# Supplementary material for: Unraveling proteomic chaos by independent component analysis—ClpX proficiency promotes the iron and oxygen limitation responses of Staphylococcus aureus and affects the intracellular bacterial behavior
Source: mSystems. 2025 Nov 17;10(12):e01092-25. doi: 10.1128/msystems.01092-25 (PMC12710355; doi:10.1128/msystems.01092-25)
Supplement: Legends — for Data S1 and Tables S1-S5. [file msystems.01092-25-s0003.docx]

**Supporting Information**

**Data S1. Bar plots of all quantified proteins. Rescaled MaxLFQ protein level values are shown. Values were rescaled based on relative iBAQ-values.**

**Table S1. Rescaled MaxLFQ protein level values. Values were rescaled based on relative iBAQ-values.**

**Table S2. GSEA-based regulon analysis of principle components.**

**Table S3. Statistical results of the proteome analysis. Statistics were calculated using the ROPECA approach.**

**Table S4. (A) Overview of the ClpX modulon under control conditions, iron limitation and oxygen limitation. (B) Evaluation of the effect ClpX-deficiency under control conditions in the exponential and in the stationary growth phase. (C) Evaluation of the effect ClpX-deficiency under iron limitation in the exponential and in the stationary growth phase. (D) Evaluation of the effect ClpX-deficiency under oxygen limitation in the exponential and in the stationary growth phase.**

**Table S5. (A) Overview of i-modulon members for all i-modulons according to the i-modulon-specific loading threshold. (B) Weight/Loading matrix if the ICA. (C) Activity matrix of the ICA. (D) Kruskal-Wallis-based analyses of functional associations of the i-modulons. (E) GSEA-based regulon analysis of the i-modulons. (F) Overview of associated regulons and functional experimental parameters of the i-modulons.**
