# Supplementary material for: Unraveling proteomic chaos by independent component analysis—ClpX proficiency promotes the iron and oxygen limitation responses of Staphylococcus aureus and affects the intracellular bacterial behavior
Source: mSystems. 2025 Nov 17;10(12):e01092-25. doi: 10.1128/msystems.01092-25 (PMC12710355; doi:10.1128/msystems.01092-25)
Supplement: Data S1 — Protein abundance barplots. [file msystems.01092-25-s0001.pdf]

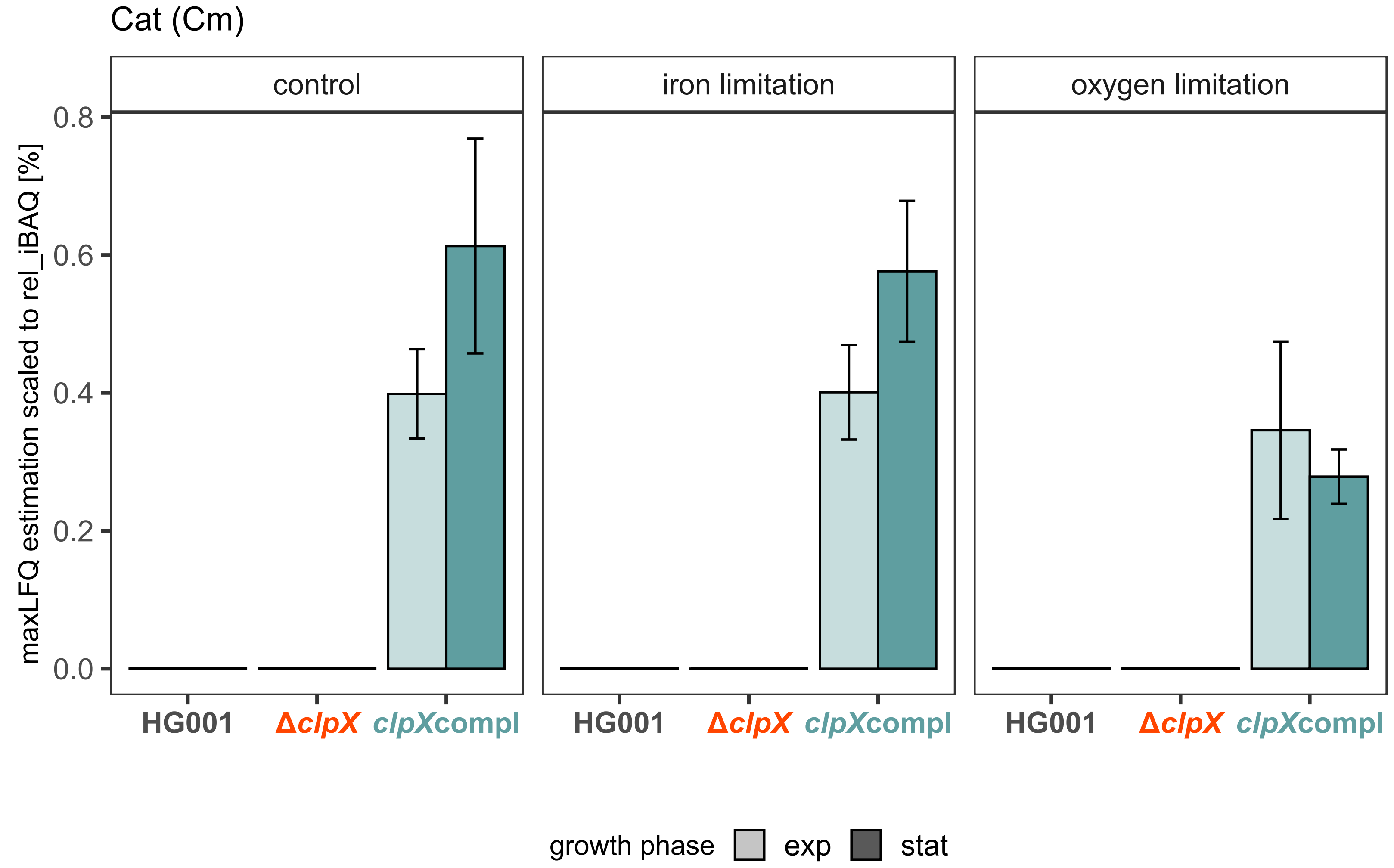

Cat (Cm)
control
iron limitation
oxygen limitation
maxLFQ estimation scaled to rel_iBAQ [%]
0.0
0.2
0.4
0.6
0.8
HG001
ΔclpX
clpXcompl
growth phase
exp
stat

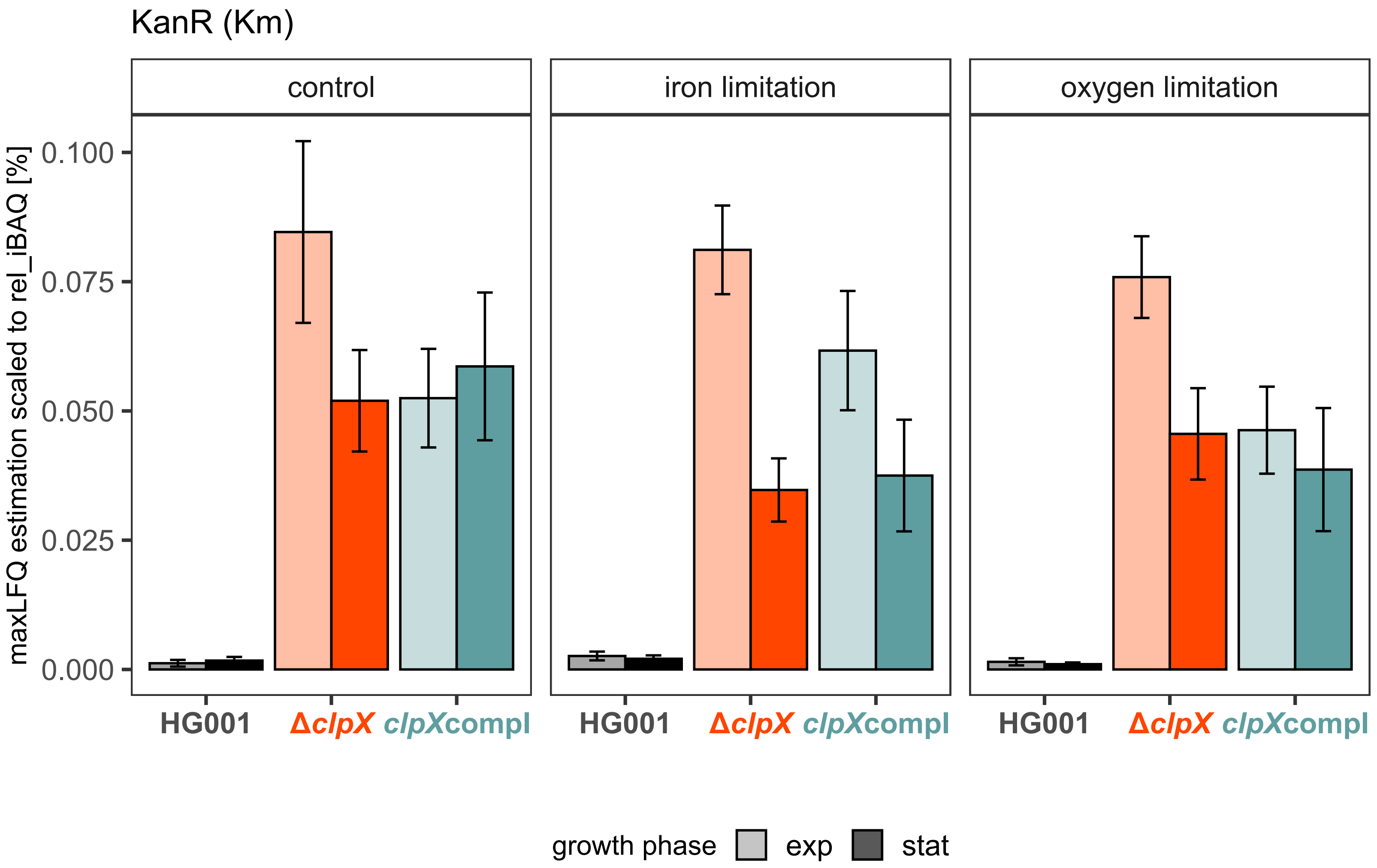
KanR (Km)
control
iron limitation
oxygen limitation
maxLFQ estimation scaled to rel_iBAQ [%]
0.100
0.075
0.050
0.025
0.000
HG001
ΔclpX
clpXcompl
growth phase
exp
stat

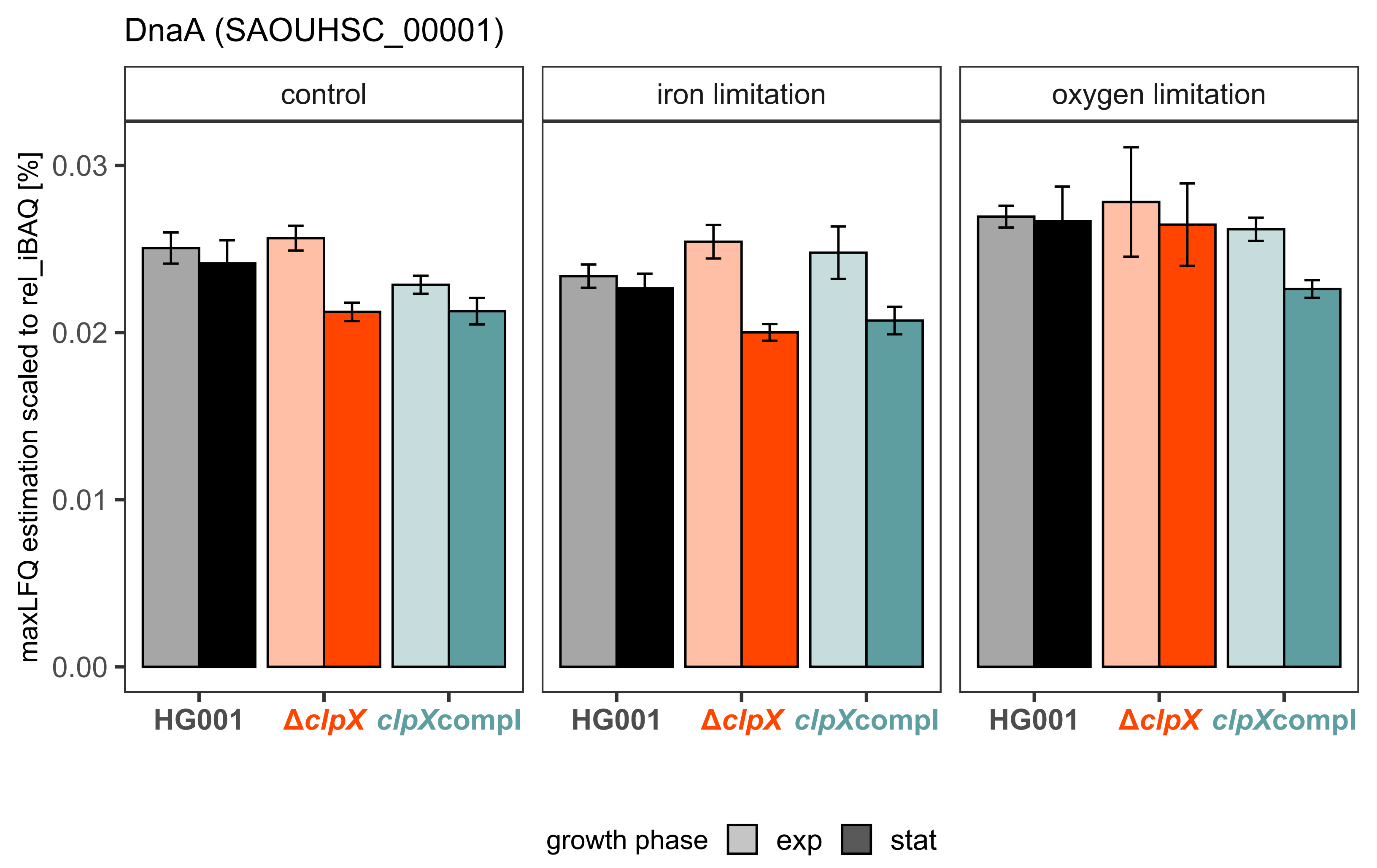

DnaA (SAOUHSC_00001)
control
iron limitation
oxygen limitation
maxLFQ estimation scaled to rel_iBAQ [%]
0.03
0.02
0.01
0.00
HG001
ΔclpX
clpXcompl
growth phase
exp
stat

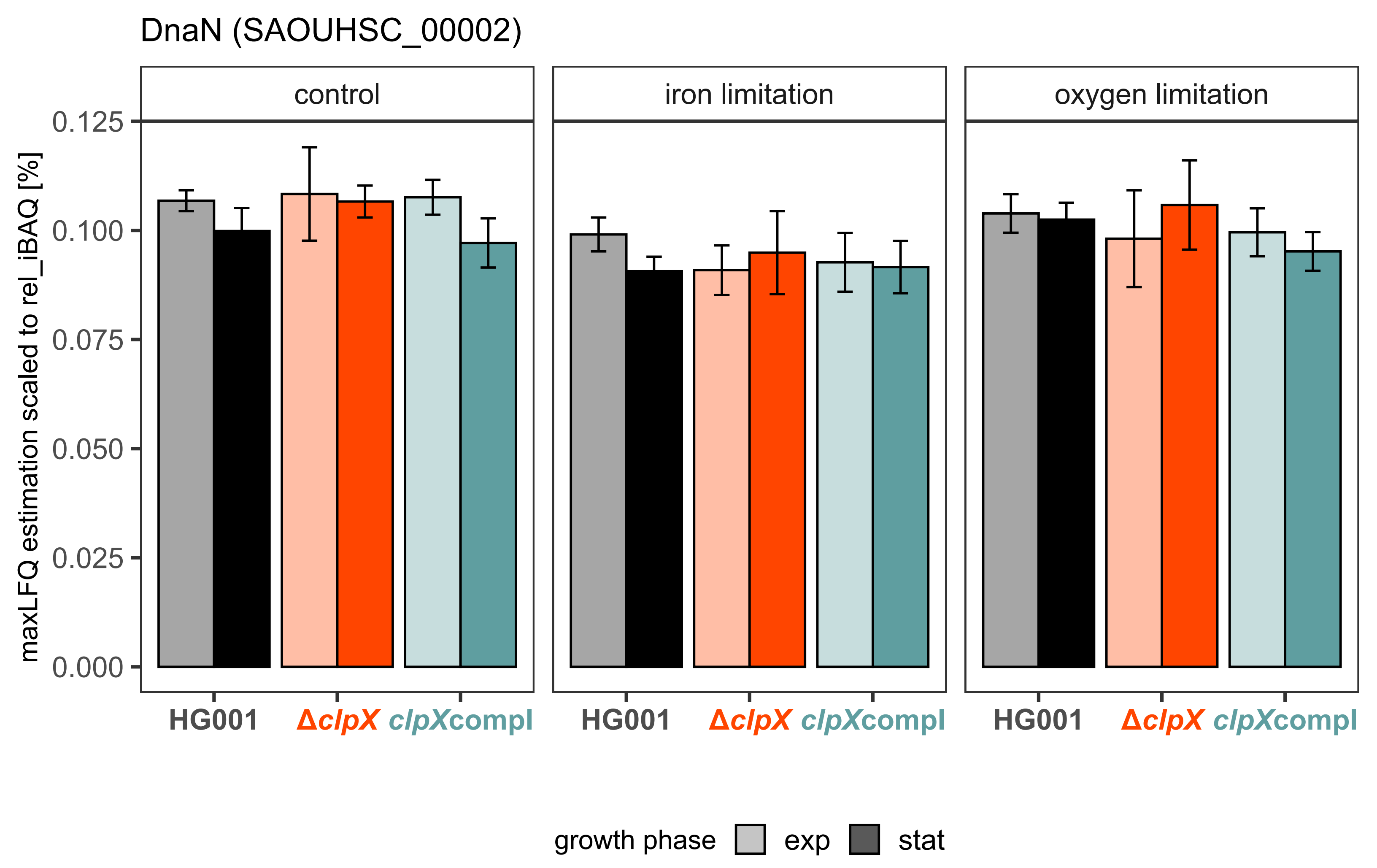

DnaN (SAOUHSC_00002)
control
iron limitation
oxygen limitation
maxLFQ estimation scaled to rel_iBAQ [%]
0.125
0.100
0.075
0.050
0.025
0.000
HG001
ΔclpX
clpXcompl
growth phase
exp
stat

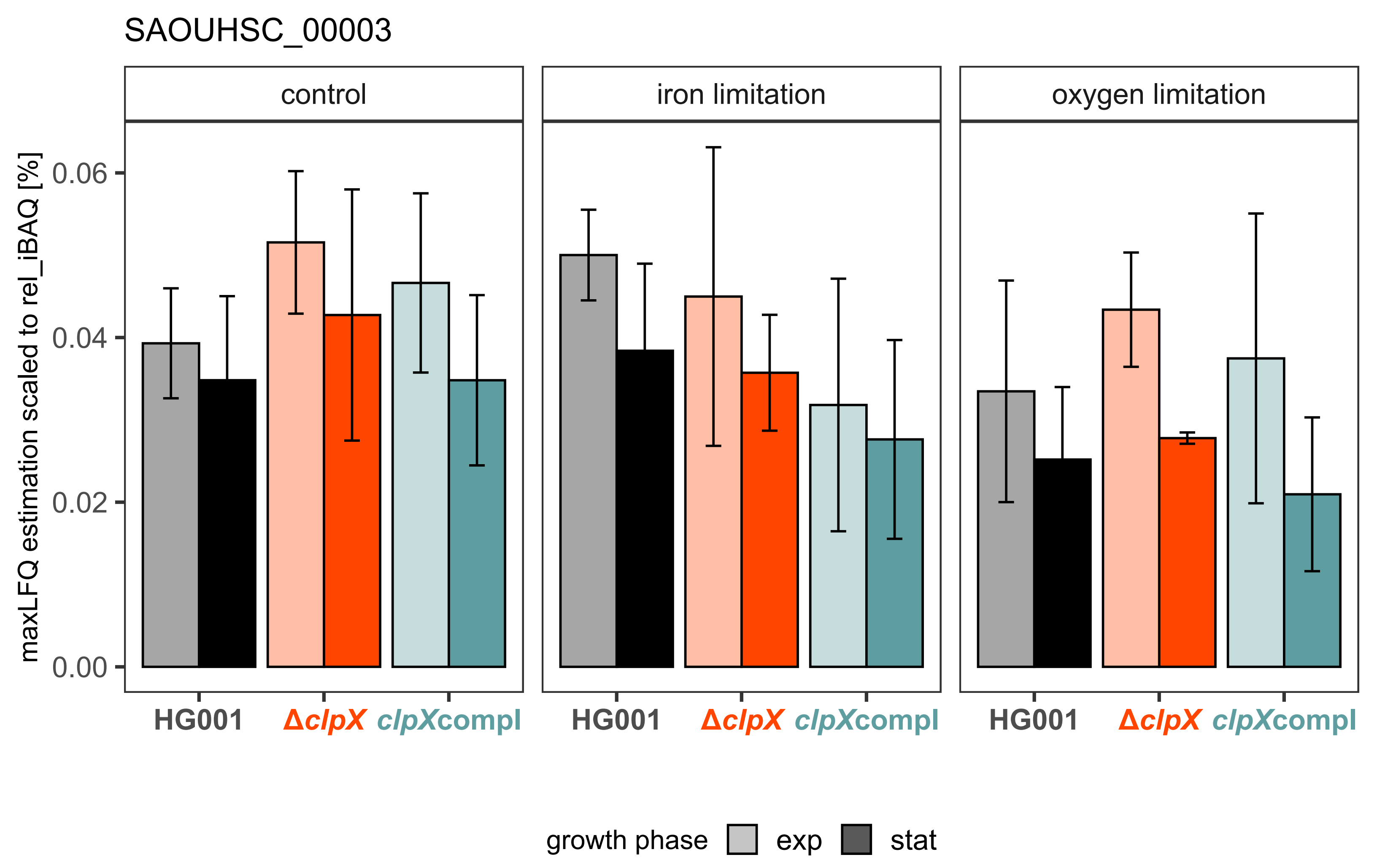
SAOUHSC_00003
control
iron limitation
oxygen limitation
maxLFQ estimation scaled to rel_iBAQ [%]
0.06
0.04
0.02
0.00
HG001
ΔclpX
clpXcompl
growth phase
exp
stat

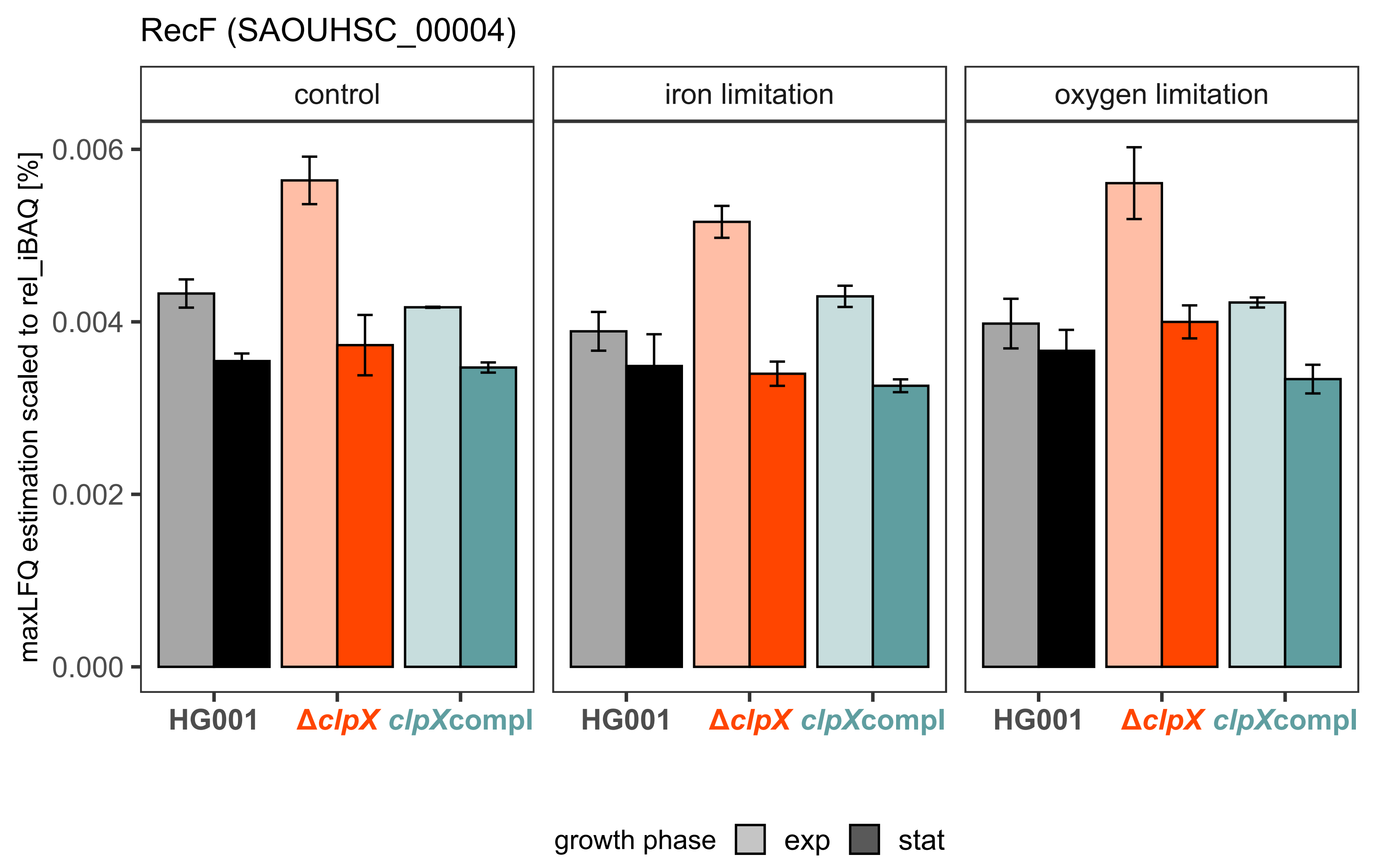
RecF (SAOUHSC_00004)
control
iron limitation
oxygen limitation
maxLFQ estimation scaled to rel_iBAQ [%]
0.006
0.004
0.002
0.000
HG001
ΔclpX
clpXcompl
growth phase
exp
stat

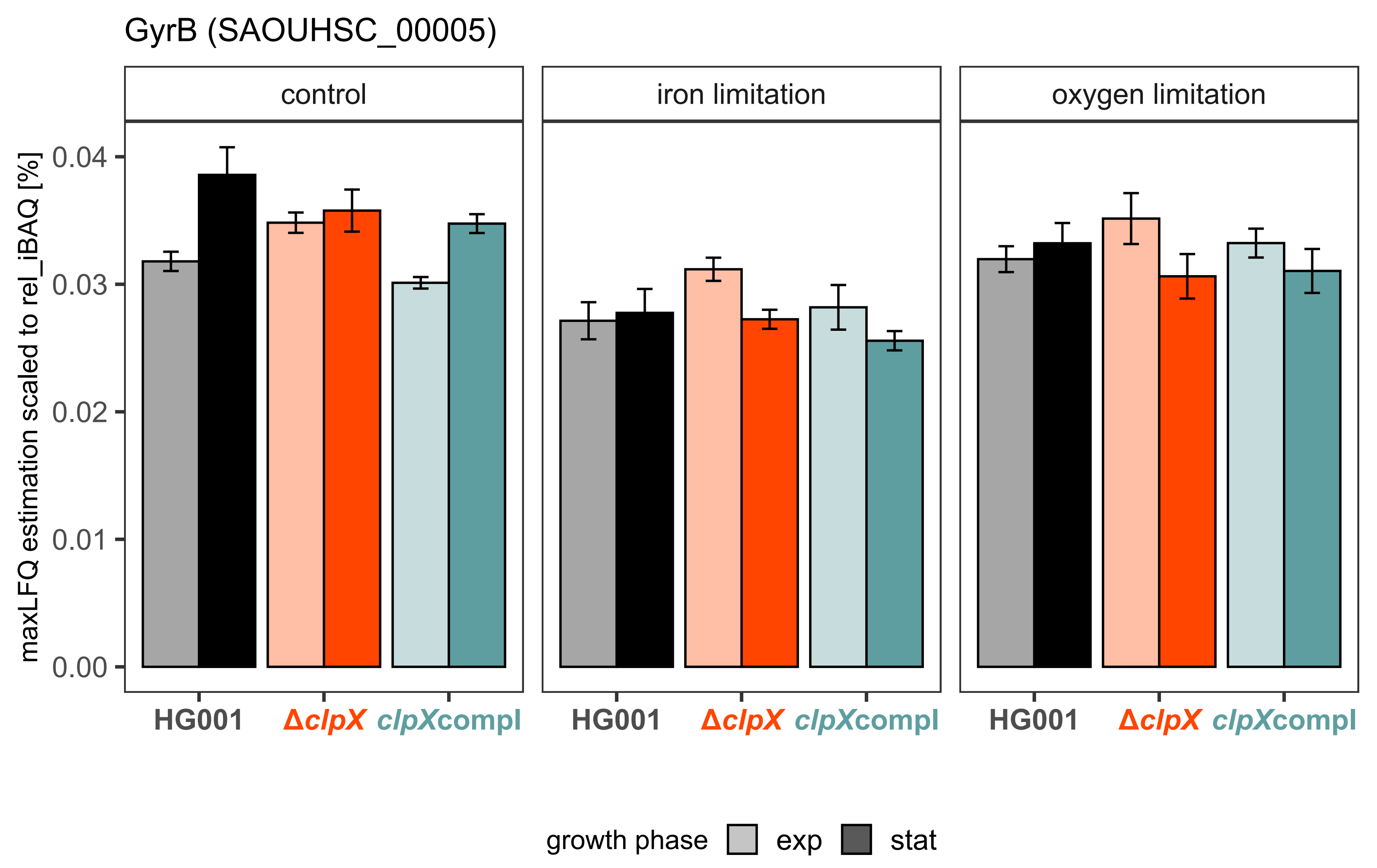
GyrB (SAOUHSC_00005)
control
iron limitation
oxygen limitation
maxLFQ estimation scaled to rel_iBAQ [%]
0.04
0.03
0.02
0.01
0.00
HG001
ΔclpX
clpXcompl
growth phase
exp
stat

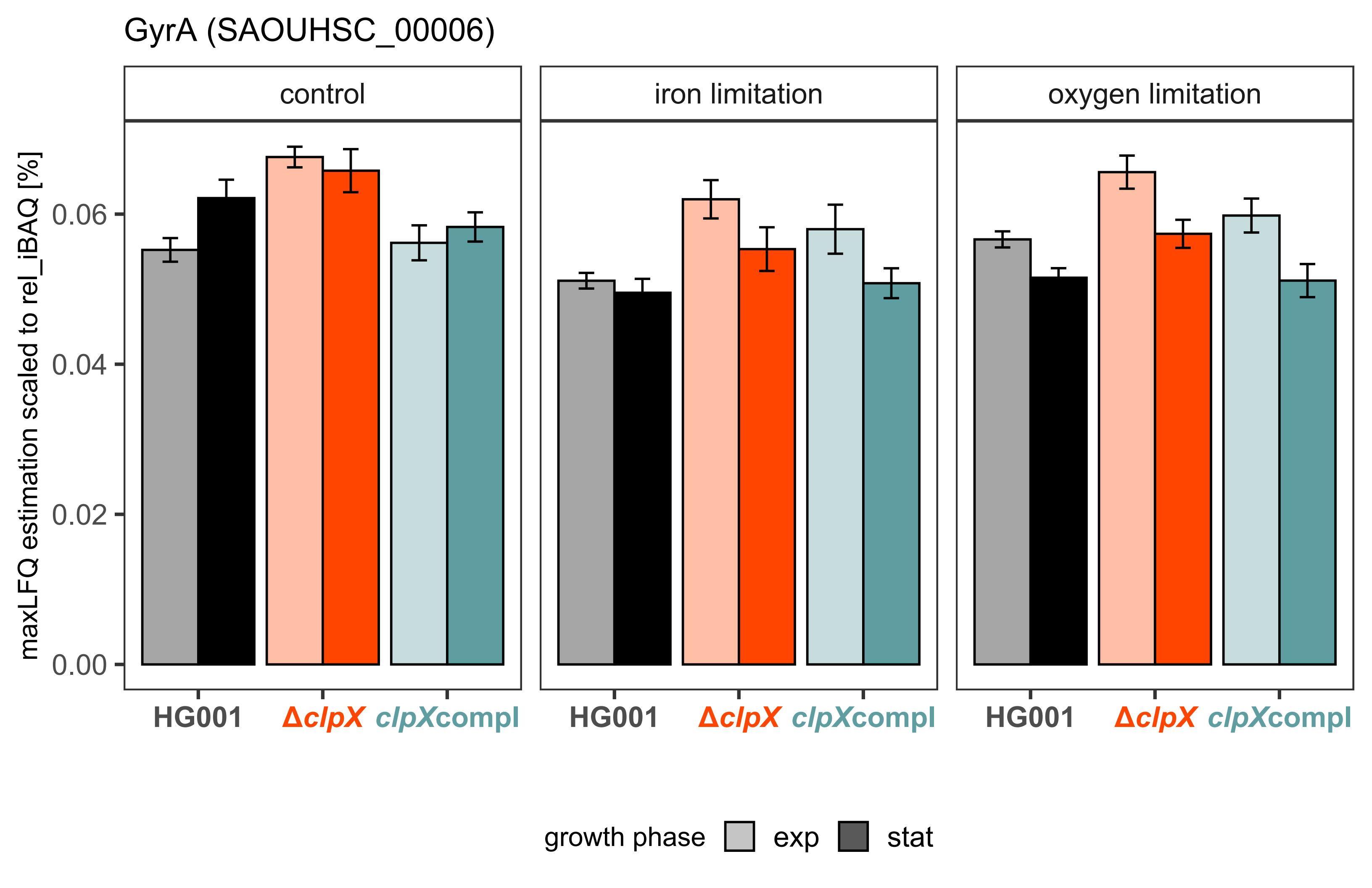
GyrA (SAOUHSC_00006)
control
iron limitation
oxygen limitation
maxLFQ estimation scaled to rel_iBAQ [%]
0.06
0.04
0.02
0.00
HG001
ΔclpX
clpXcompl
growth phase
exp
stat

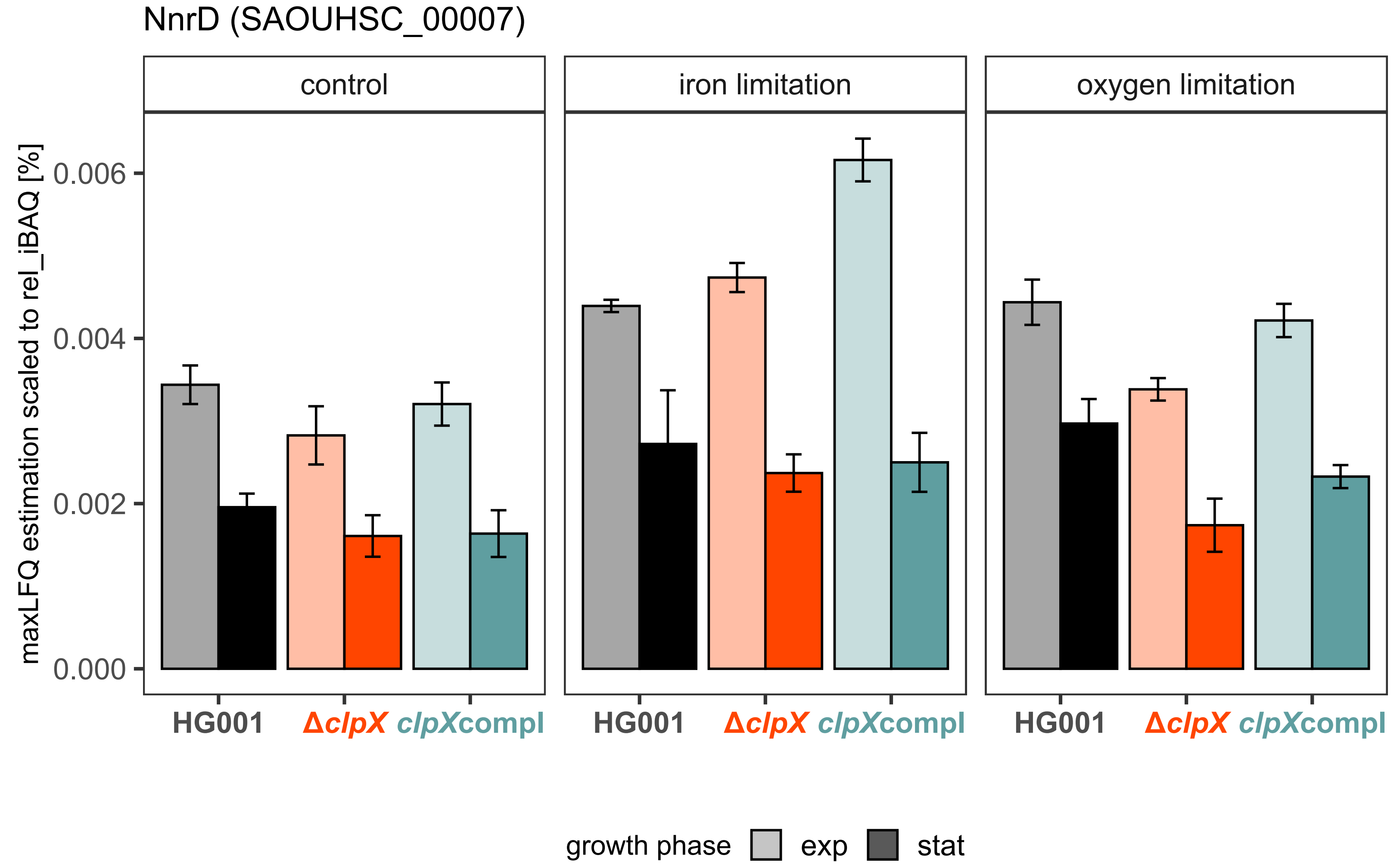

NnrD (SAOUHSC_00007)
control
iron limitation
oxygen limitation
maxLFQ estimation scaled to rel_iBAQ [%]
0.006
0.004
0.002
0.000
HG001
ΔclpX
clpXcompl
growth phase
exp
stat

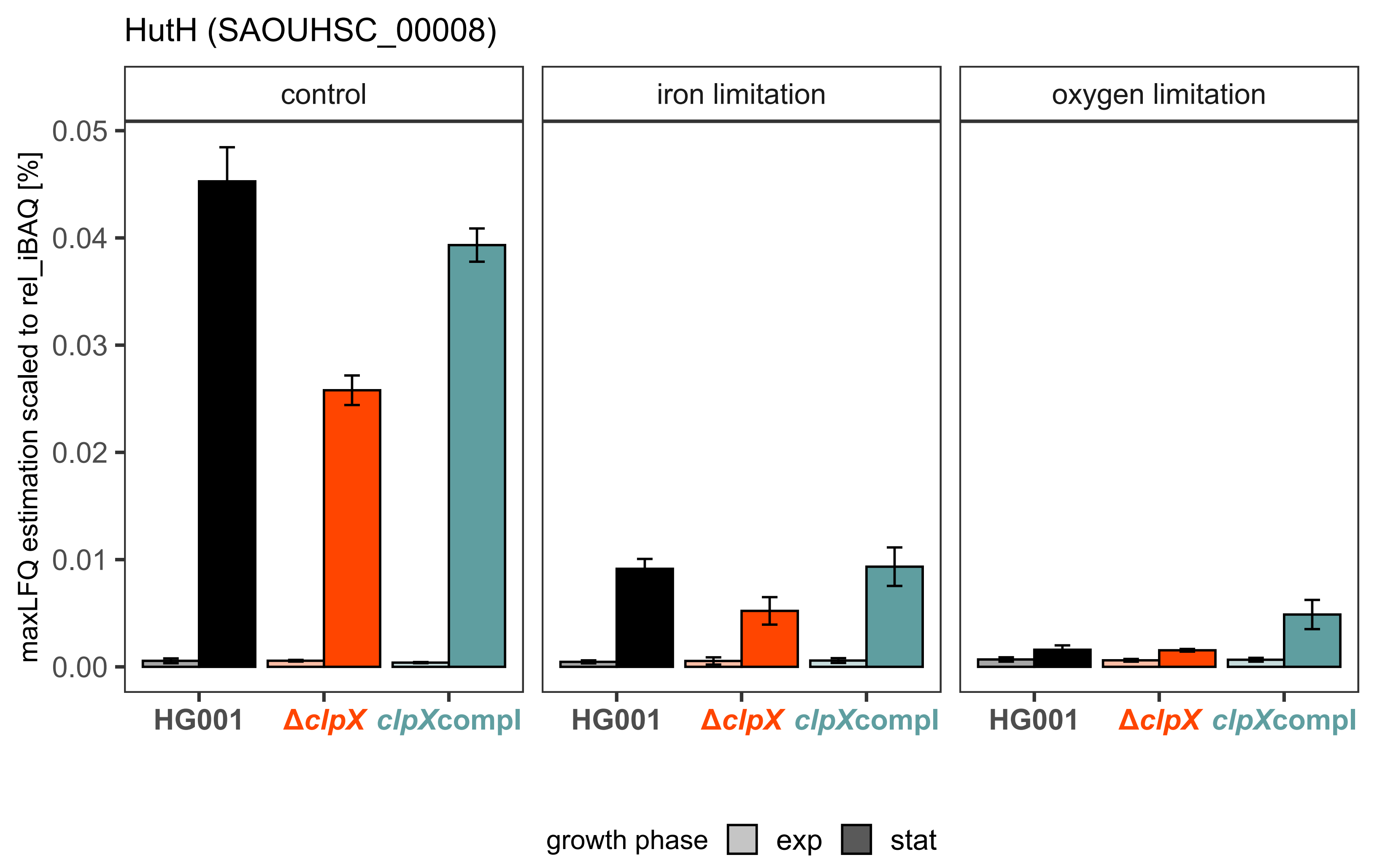

HutH (SAOUHSC_00008)
control
iron limitation
oxygen limitation
maxLFQ estimation scaled to rel_iBAQ [%]
0.05
0.04
0.03
0.02
0.01
0.00
HG001
ΔclpX
clpXcompl
growth phase
exp
stat

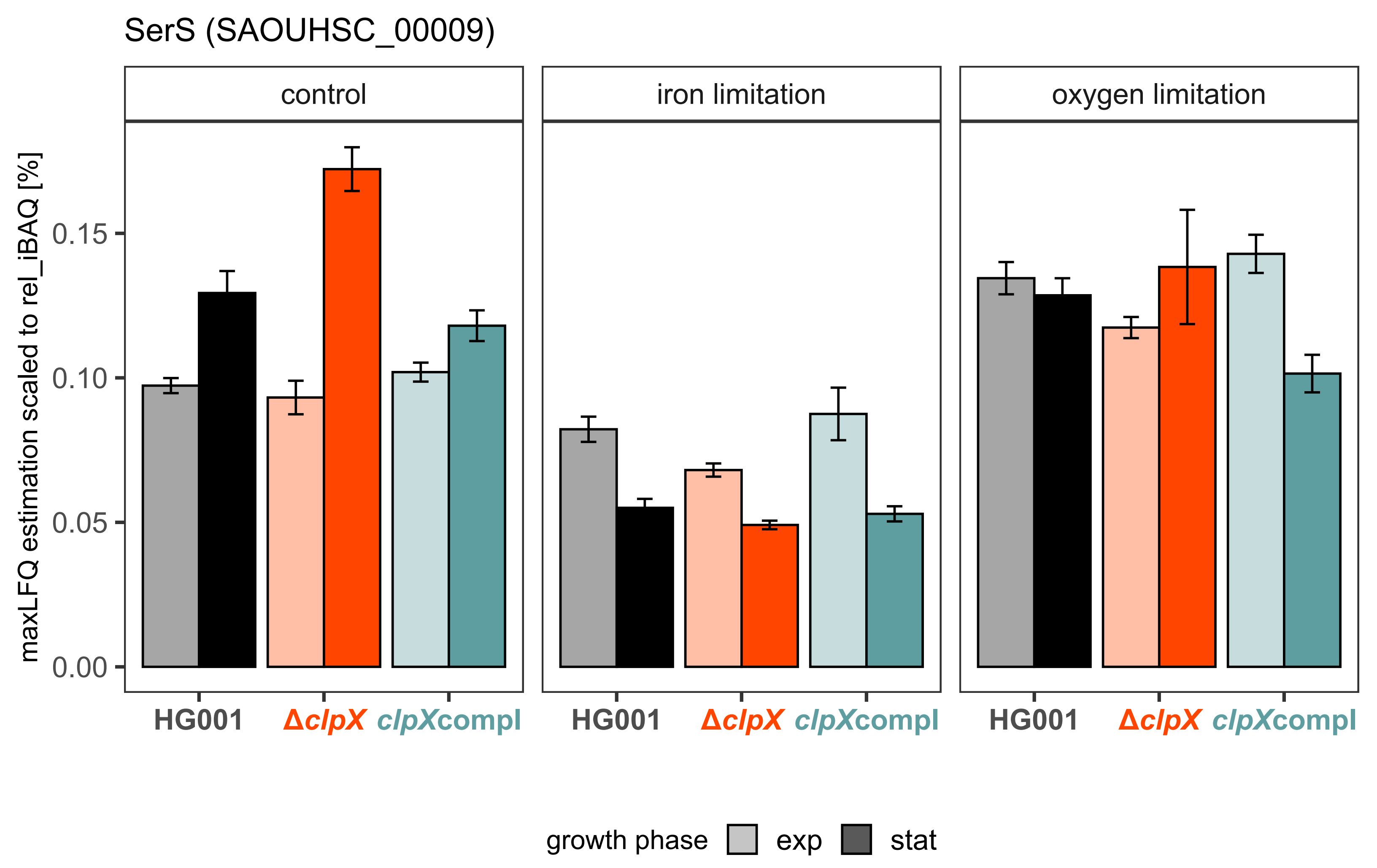

SerS (SAOUHSC_00009)
control
iron limitation
oxygen limitation
maxLFQ estimation scaled to rel_iBAQ [%]
0.15
0.10
0.05
0.00
HG001
ΔclpX
clpXcompl
growth phase
exp
stat

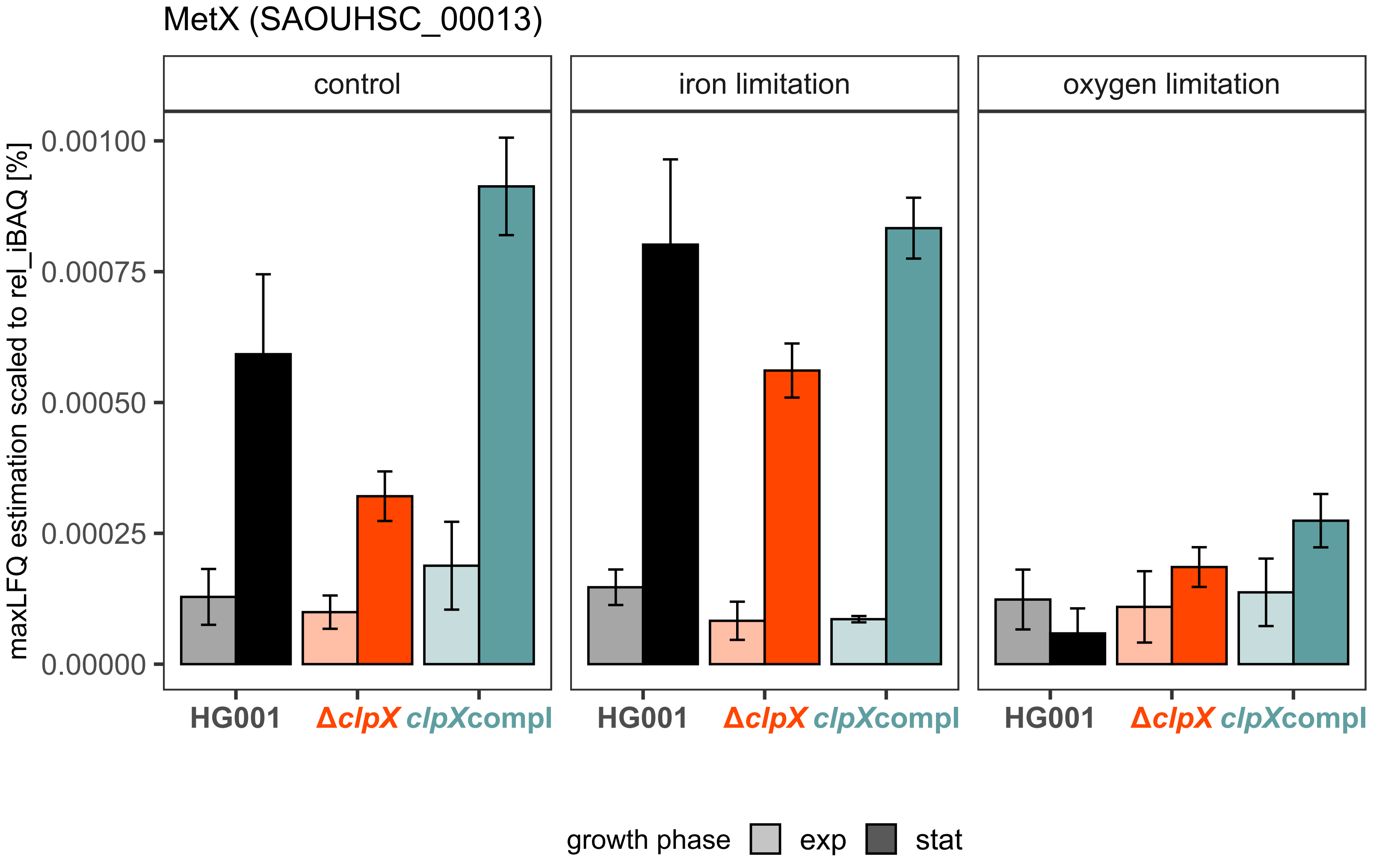

MetX (SAOUHSC_00013)
control
iron limitation
oxygen limitation
maxLFQ estimation scaled to rel_iBAQ [%]
0.00100
0.00075
0.00050
0.00025
0.00000
HG001
ΔclpX
clpXcompl
growth phase
exp
stat

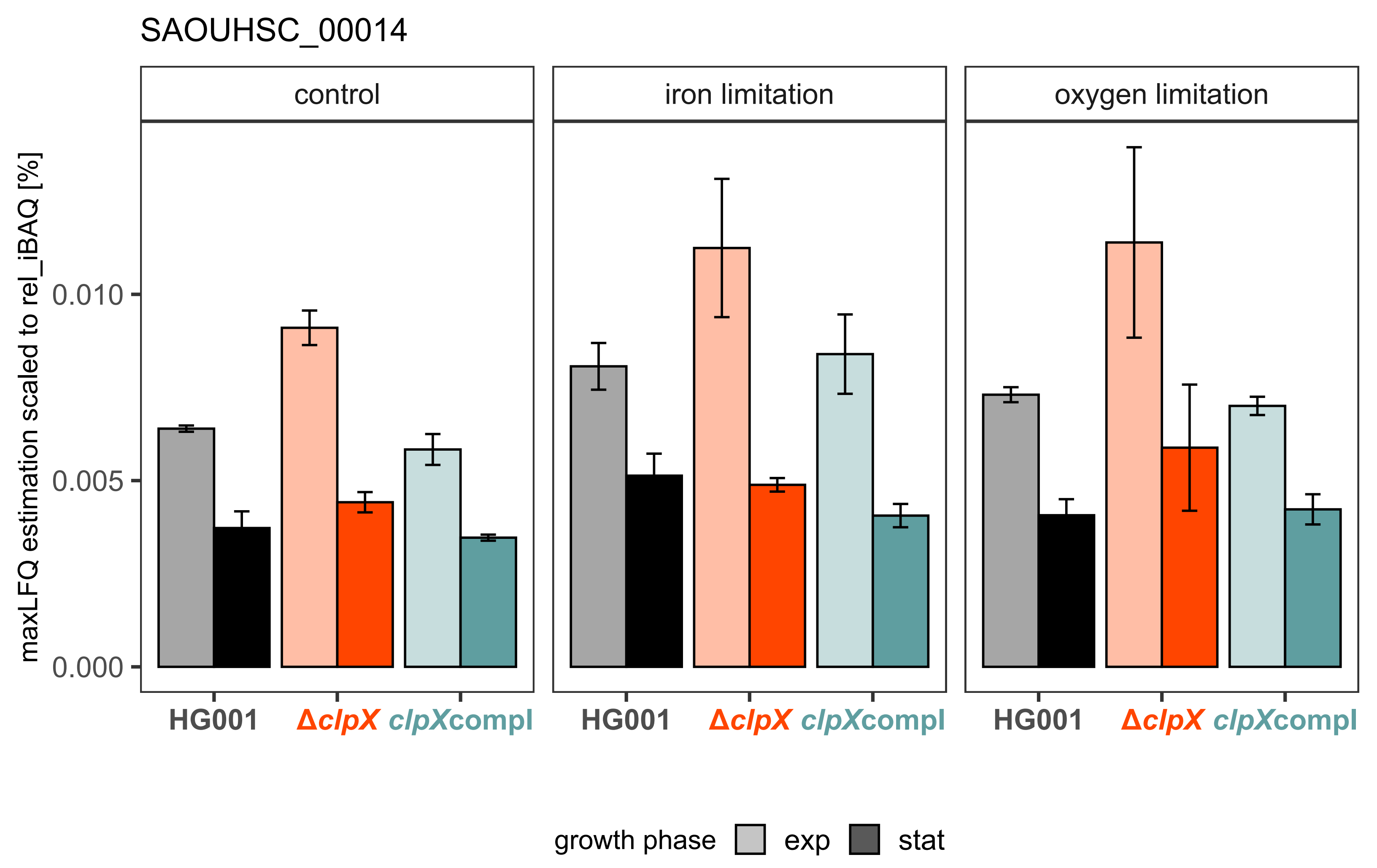
SAOUHSC_00014
control
iron limitation
oxygen limitation
maxLFQ estimation scaled to rel_iBAQ [%]
0.010
0.005
0.000
HG001
ΔclpX
clpXcompl
growth phase
exp
stat

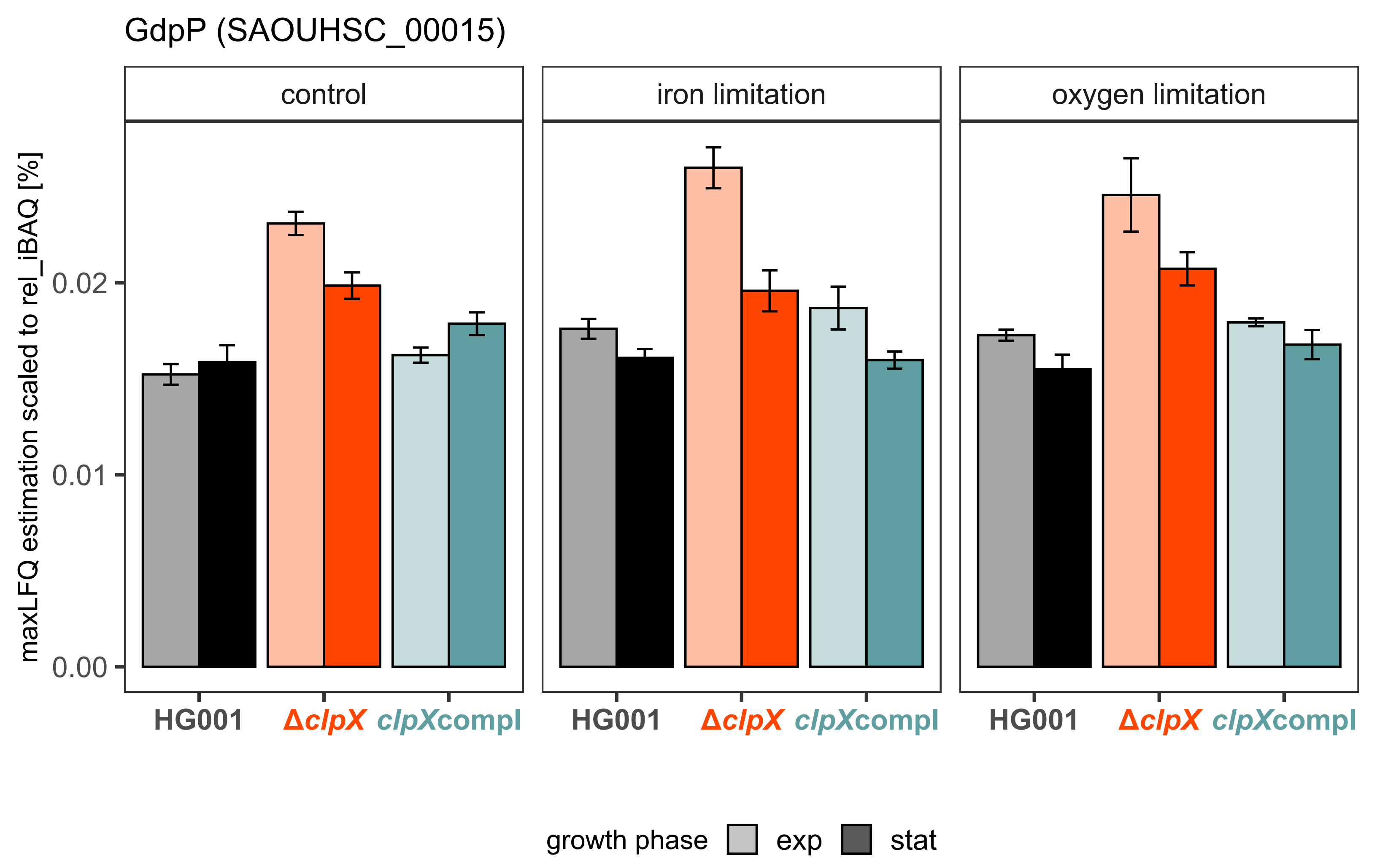
GdpP (SAOUHSC_00015)
control
iron limitation
oxygen limitation
maxLFQ estimation scaled to rel_iBAQ [%]
0.00
0.01
0.02
HG001
ΔclpX
clpXcompl
growth phase
exp
stat

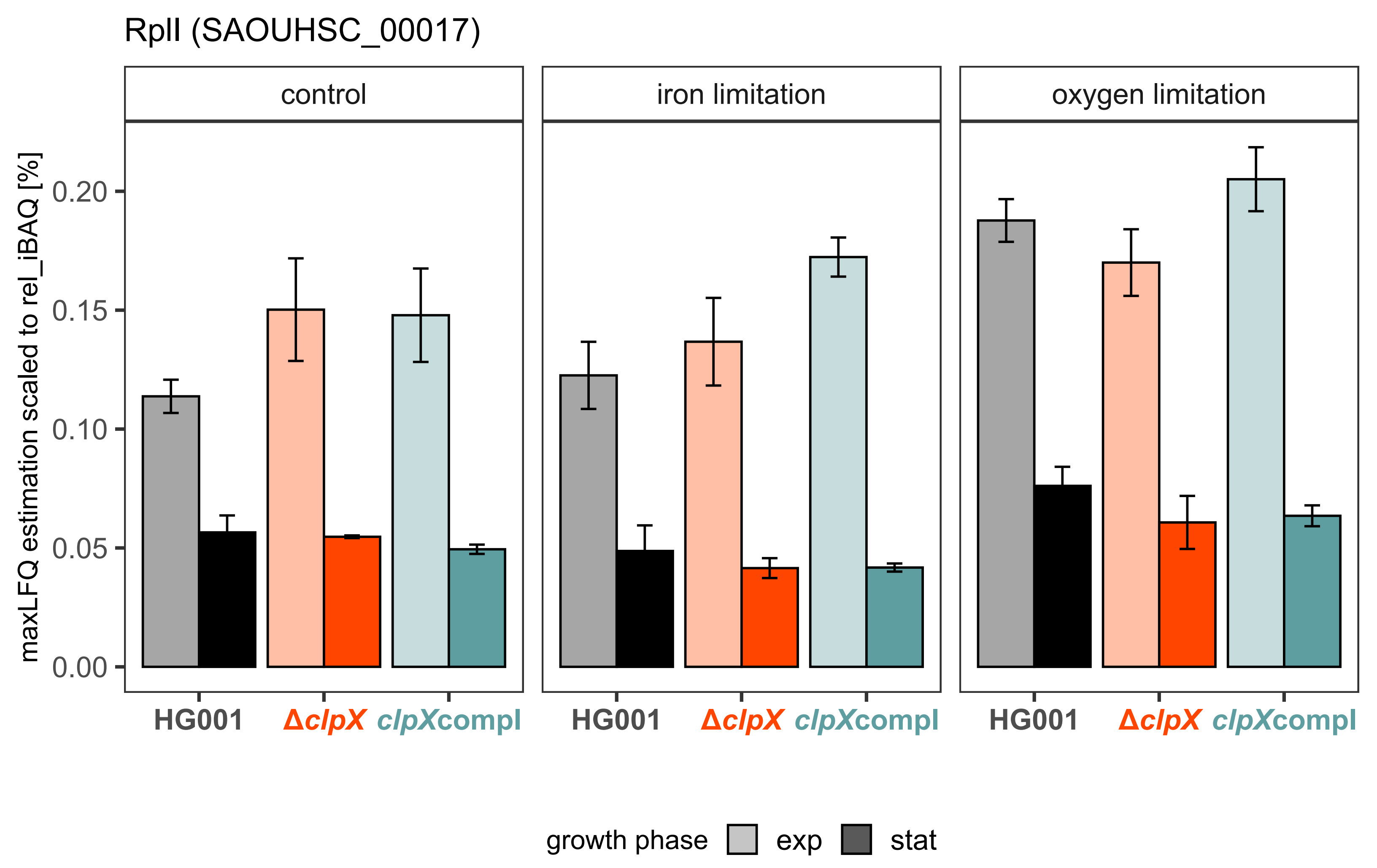

RplI (SAOUHSC_00017)
control
iron limitation
oxygen limitation
maxLFQ estimation scaled to rel_iBAQ [%]
0.20
0.15
0.10
0.05
0.00
HG001
ΔclpX
clpXcompl
growth phase
exp
stat

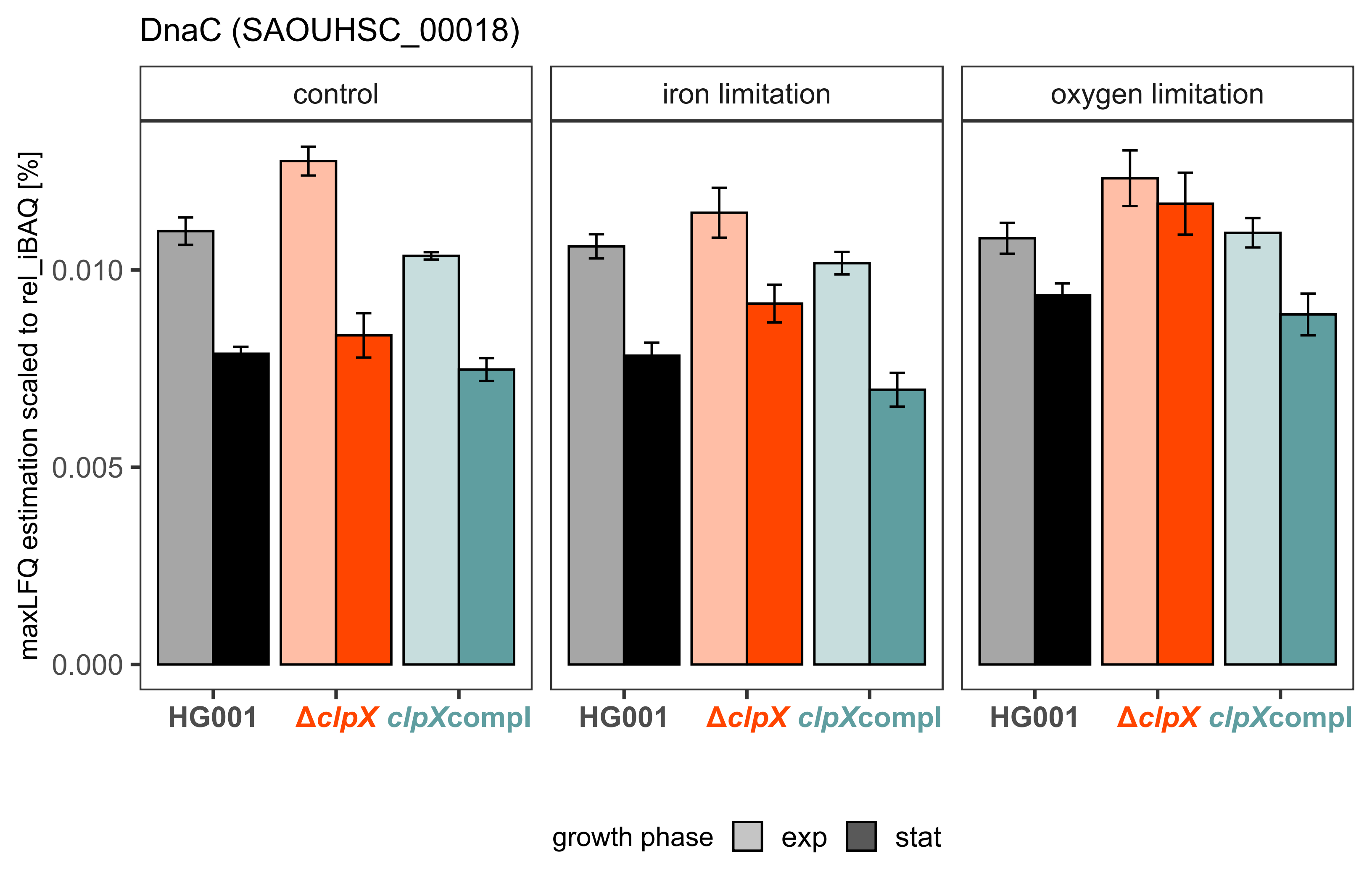

DnaC (SAOUHSC_00018)
control
iron limitation
oxygen limitation
maxLFQ estimation scaled to rel_iBAQ [%]
0.000
0.005
0.010
HG001
ΔclpX
clpXcompl
growth phase
exp
stat

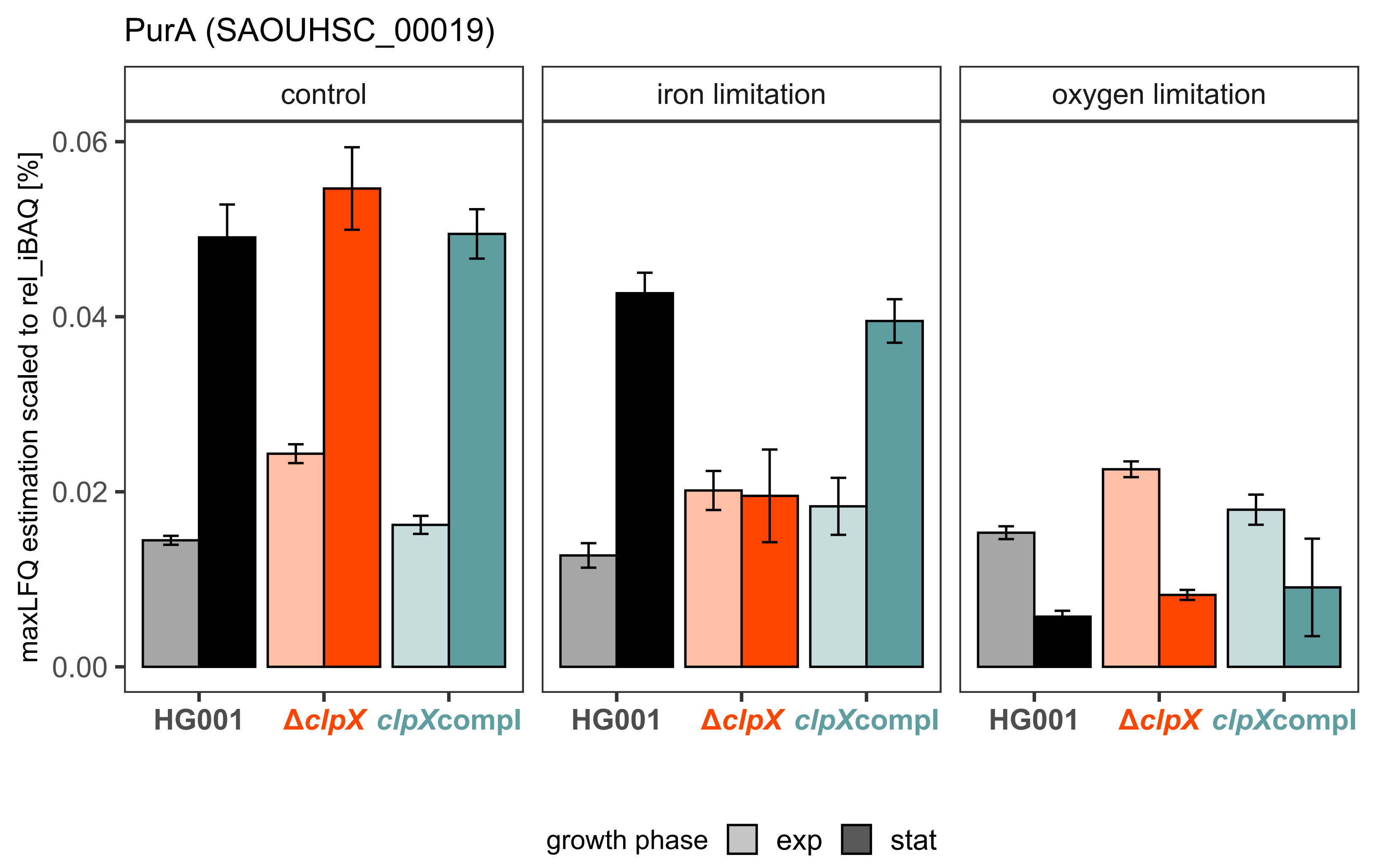

PurA (SAOUHSC_00019)
control
iron limitation
oxygen limitation
maxLFQ estimation scaled to rel_iBAQ [%]
0.06
0.04
0.02
0.00
HG001
ΔclpX
clpXcompl
growth phase
exp
stat

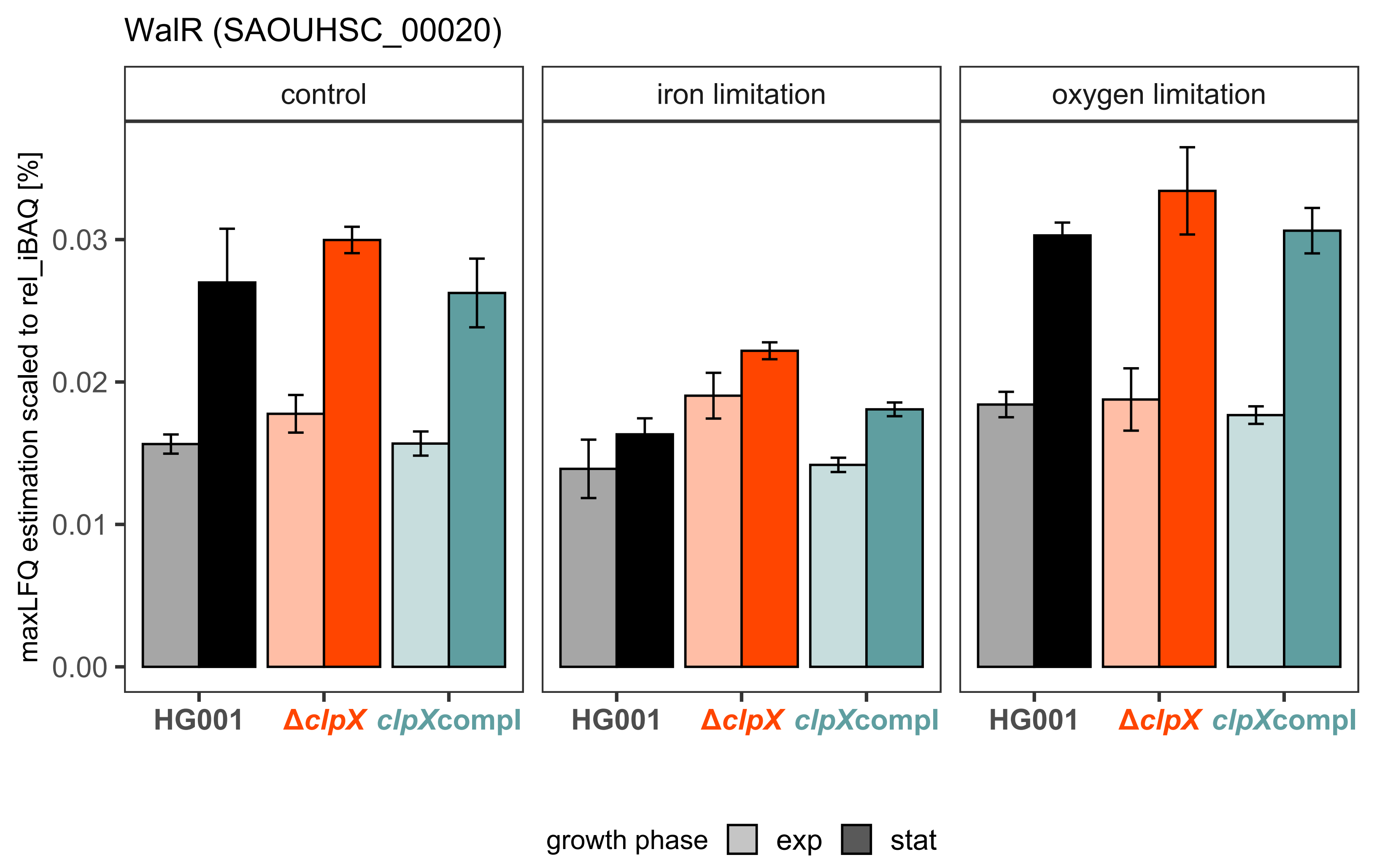

WalR (SAOUHSC_00020)
control
iron limitation
oxygen limitation
maxLFQ estimation scaled to rel_iBAQ [%]
0.00
0.01
0.02
0.03
HG001
ΔclpX
clpXcompl
growth phase
exp
stat

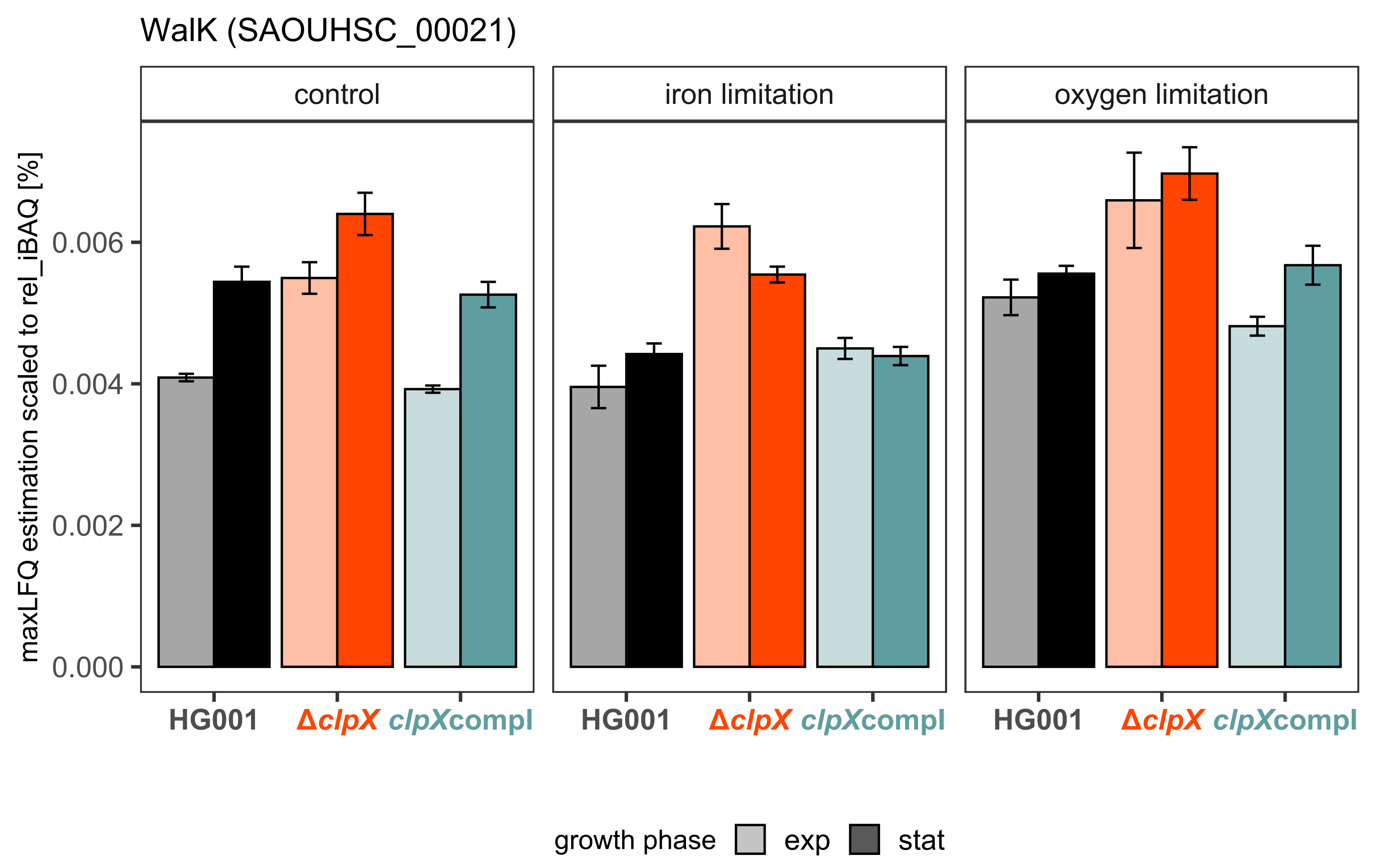

WalK (SAOUHSC_00021)
control
iron limitation
oxygen limitation
maxLFQ estimation scaled to rel_iBAQ [%]
0.006
0.004
0.002
0.000
HG001
ΔclpX
clpXcompl
growth phase
exp
stat

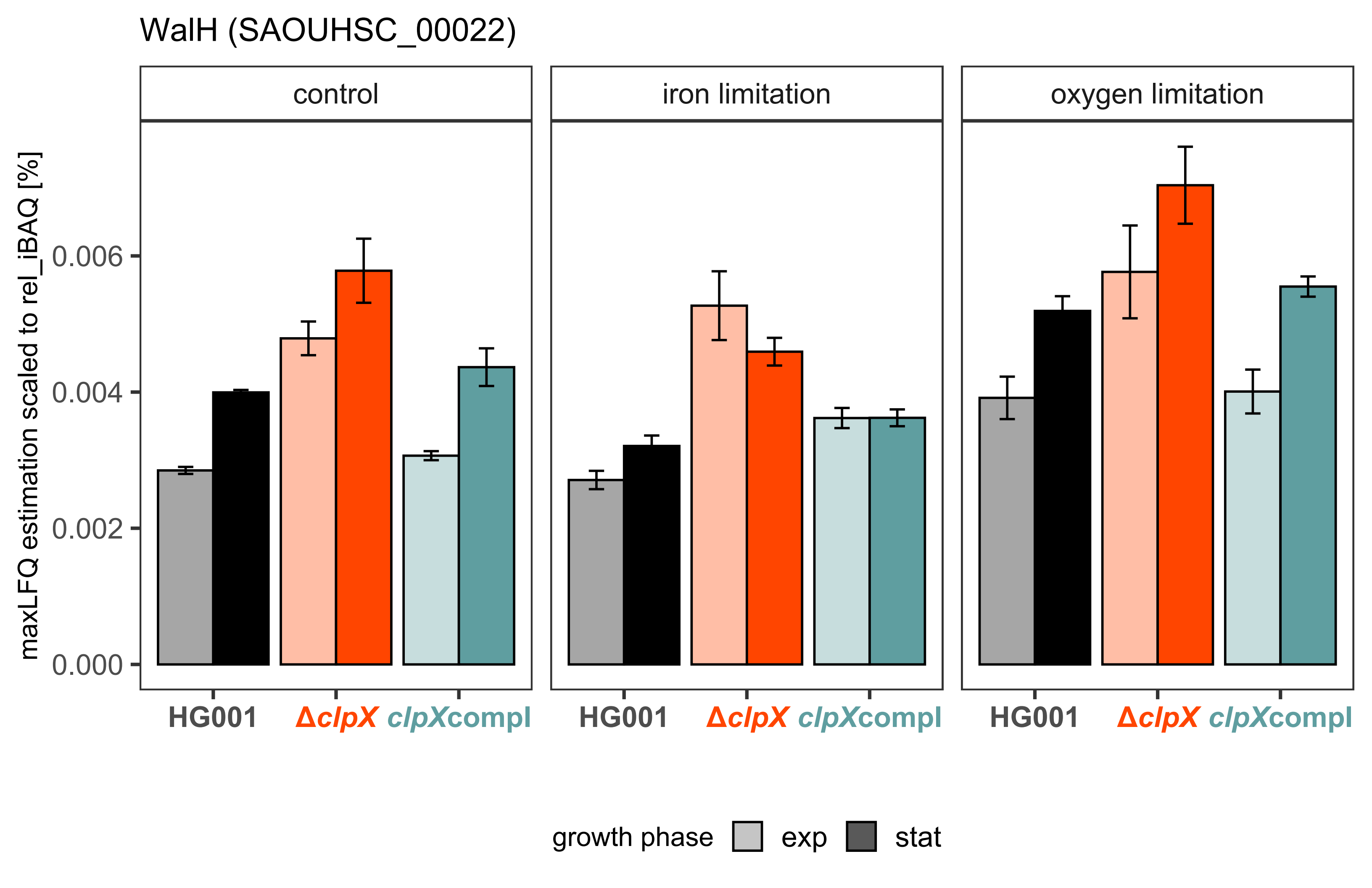
WalH (SAOUHSC_00022)
control
iron limitation
oxygen limitation
maxLFQ estimation scaled to rel_iBAQ [%]
0.006
0.004
0.002
0.000
HG001
ΔclpX
clpXcompl
growth phase
exp
stat

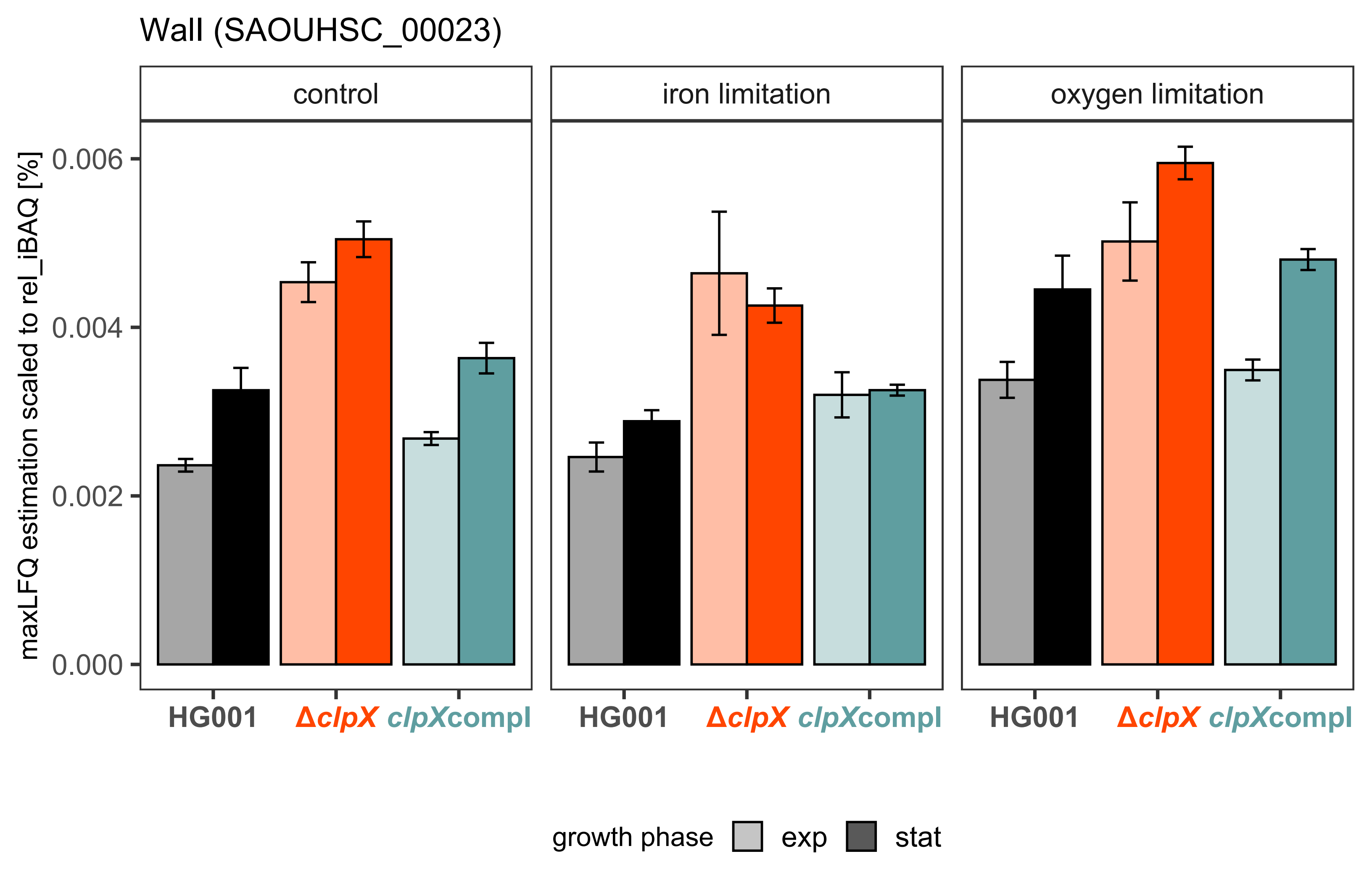

WalI (SAOUHSC_00023)
control
iron limitation
oxygen limitation
maxLFQ estimation scaled to rel_iBAQ [%]
0.006
0.004
0.002
0.000
HG001
ΔclpX
clpXcompl
growth phase
exp
stat

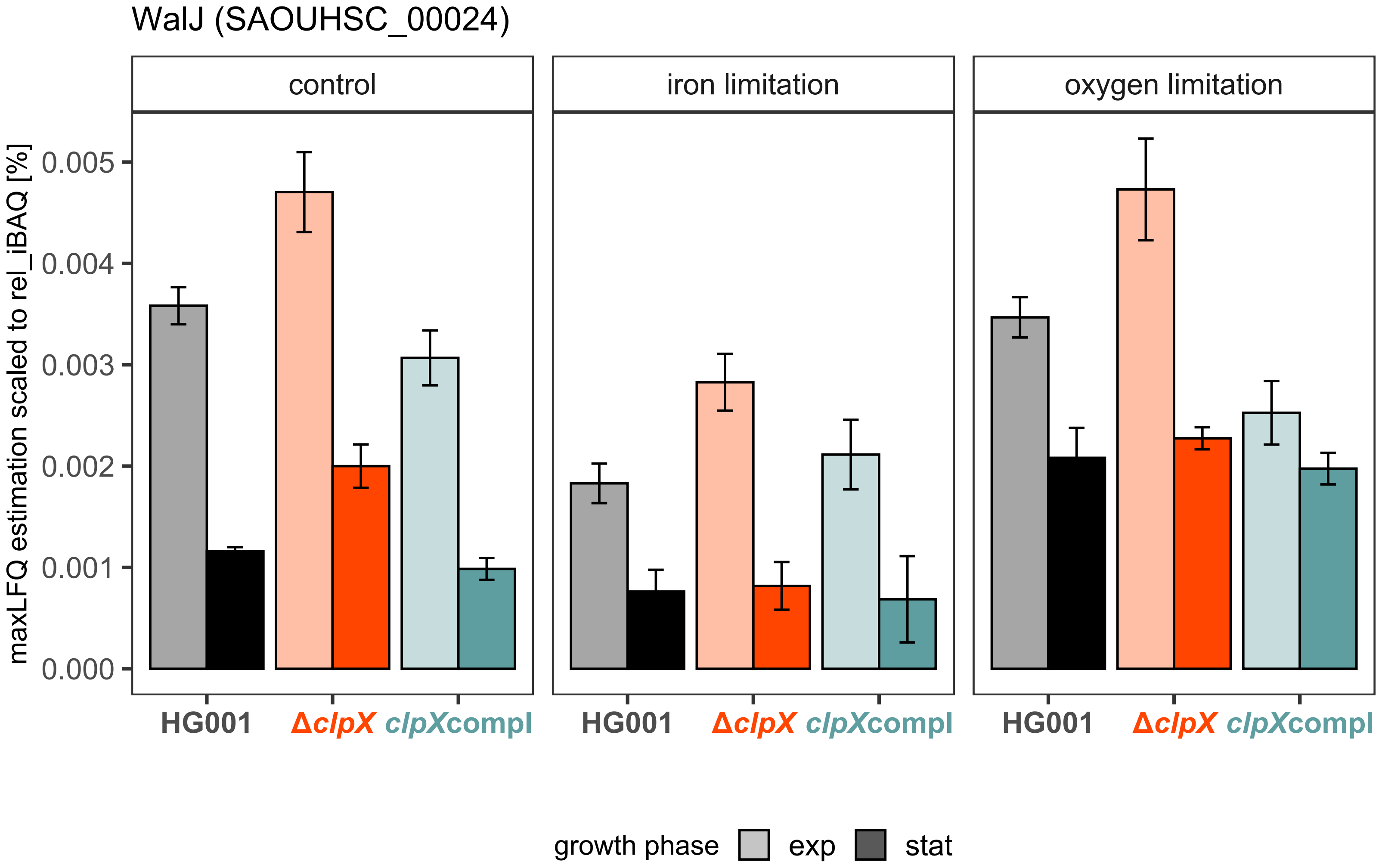
WalJ (SAOUHSC_00024)
control
iron limitation
oxygen limitation
maxLFQ estimation scaled to rel_iBAQ [%]
0.005
0.004
0.003
0.002
0.001
0.000
HG001
ΔclpX
clpXcompl
HG001
ΔclpX
clpXcompl
HG001
ΔclpX
clpXcompl
growth phase
exp
stat

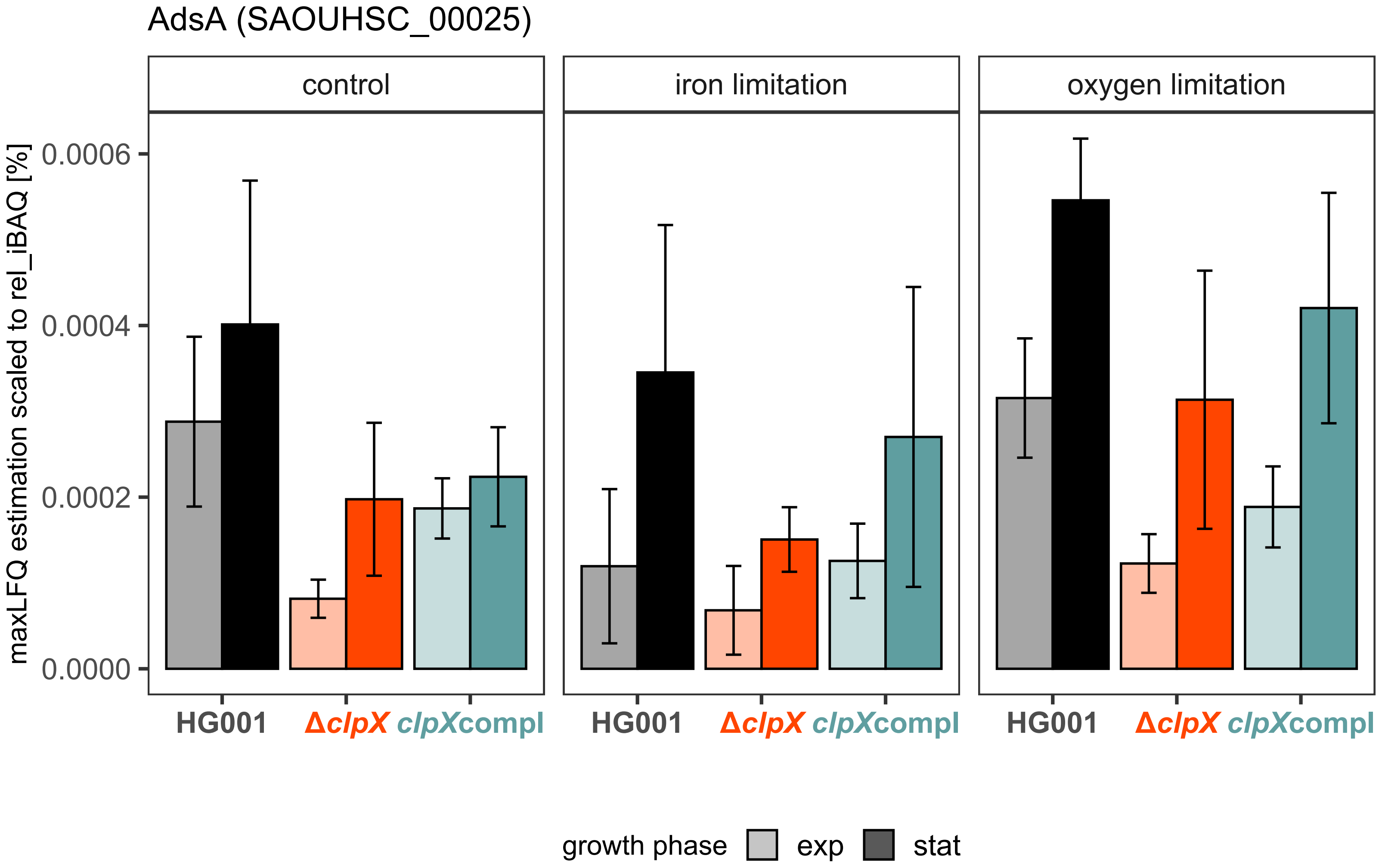
AdsA (SAOUHSC_00025)
control
iron limitation
oxygen limitation
maxLFQ estimation scaled to rel_iBAQ [%]
0.0006
0.0004
0.0002
0.0000
HG001
ΔclpX
clpXcompl
growth phase
exp
stat

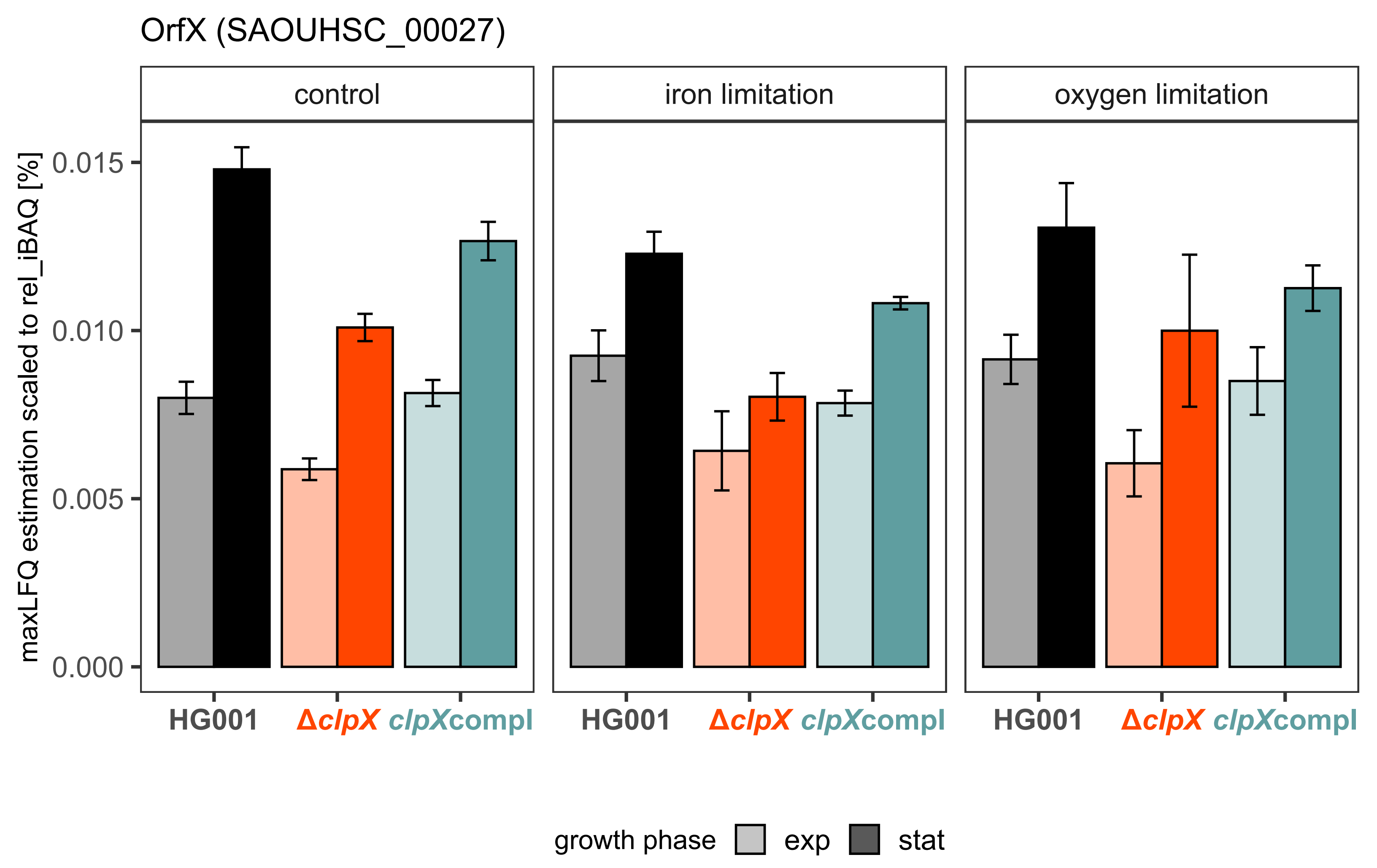
OrfX (SAOUHSC_00027)
control
iron limitation
oxygen limitation
maxLFQ estimation scaled to rel_iBAQ [%]
0.015
0.010
0.005
0.000
HG001
ΔclpX
clpXcompl
growth phase
exp
stat

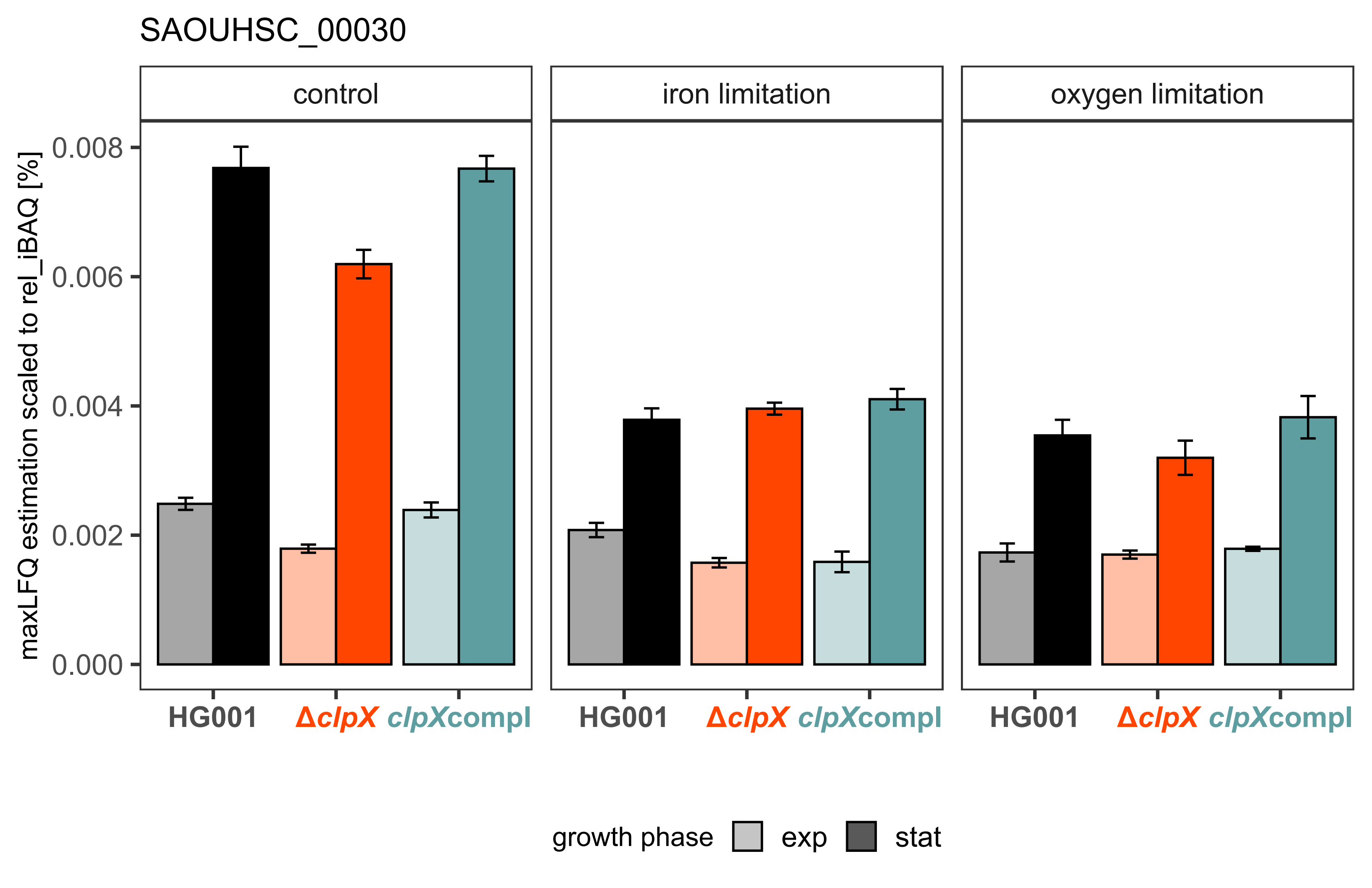
SAOUHSC_00030
control
iron limitation
oxygen limitation
maxLFQ estimation scaled to rel_iBAQ [%]
0.008
0.006
0.004
0.002
0.000
HG001
ΔclpX
clpXcompl
growth phase
exp
stat

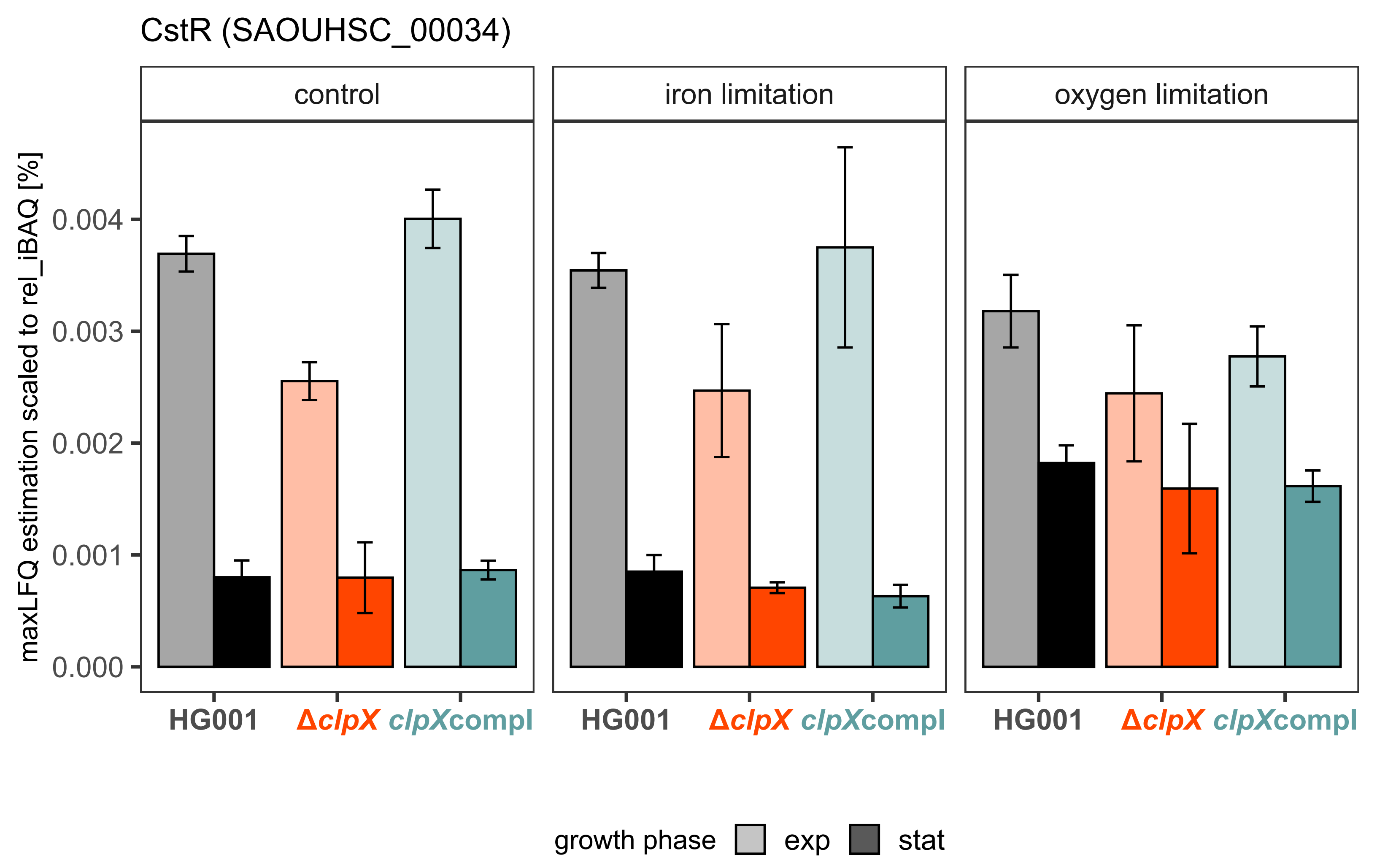
CstR (SAOUHSC_00034)
control
iron limitation
oxygen limitation
maxLFQ estimation scaled to rel_iBAQ [%]
0.004
0.003
0.002
0.001
0.000
HG001
ΔclpX
clpXcompl
growth phase
exp
stat

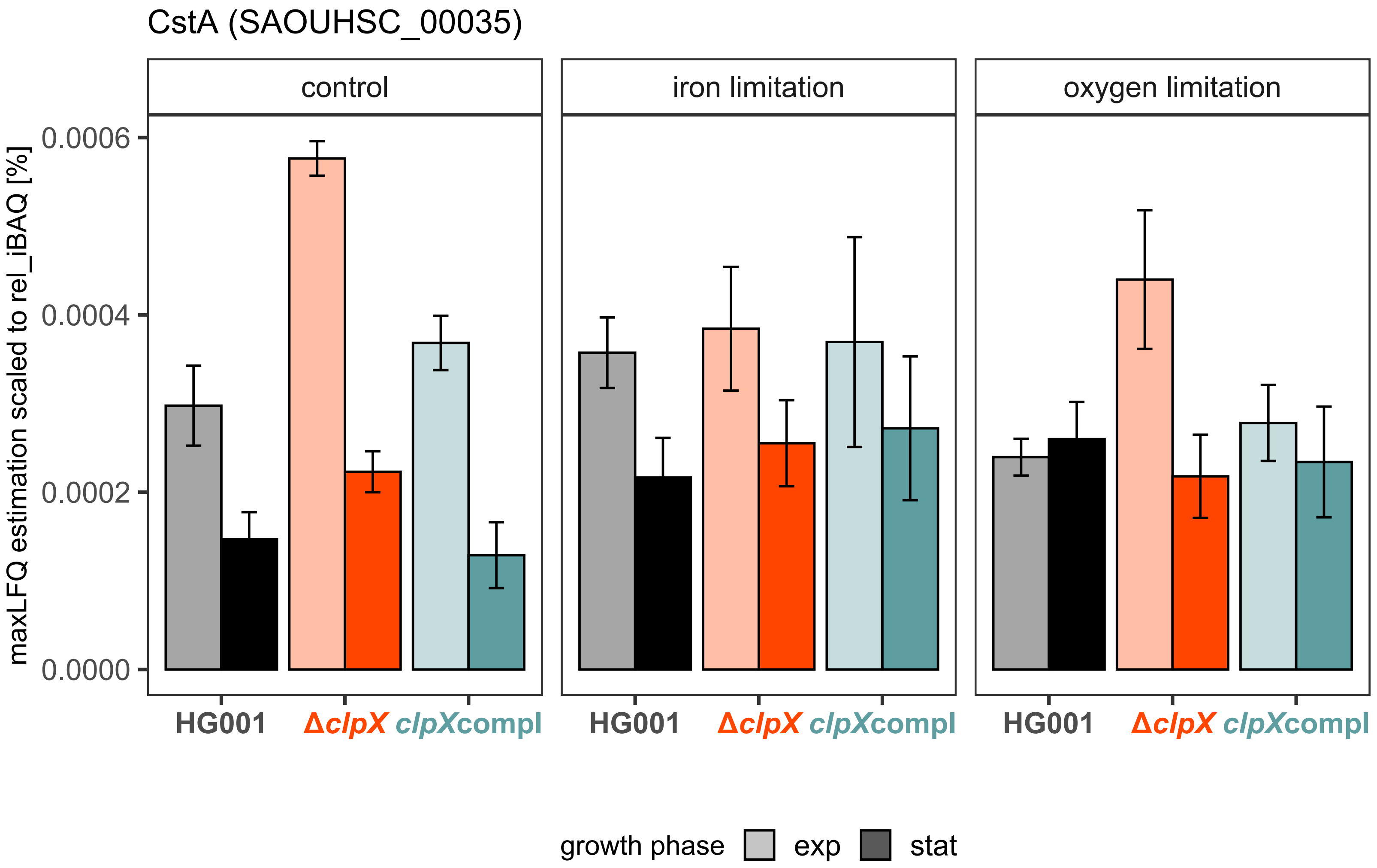
CstA (SAOUHSC_00035)
control
iron limitation
oxygen limitation
0.0006
0.0004
0.0002
0.0000
maxLFQ estimation scaled to rel_iBAQ [%]
HG001
ΔclpX
clpXcompl
growth phase
exp
stat

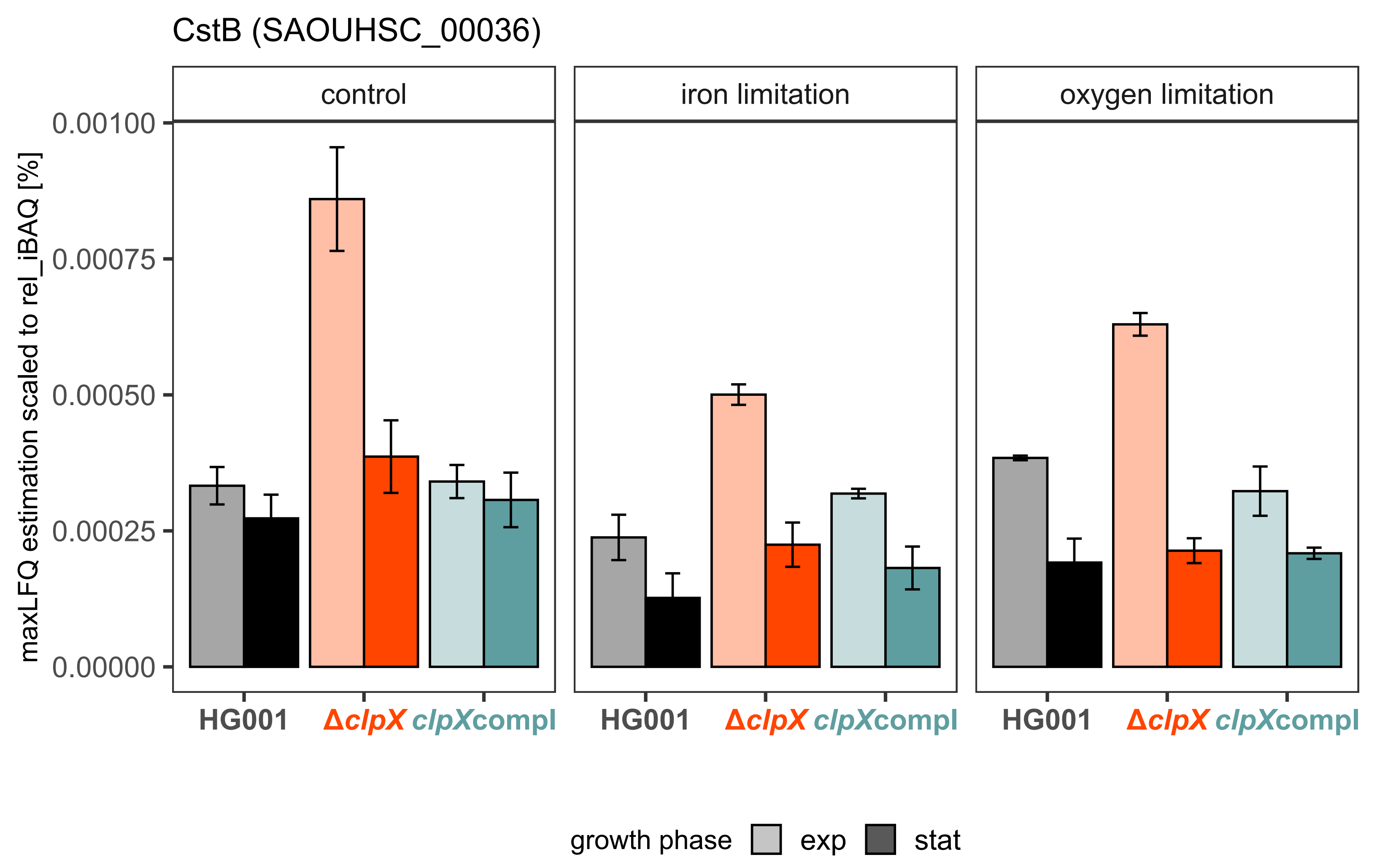

CstB (SAOUHSC_00036)
control
iron limitation
oxygen limitation
maxLFQ estimation scaled to rel_iBAQ [%]
0.00100
0.00075
0.00050
0.00025
0.00000
HG001
ΔclpX
clpXcompl
growth phase
exp
stat

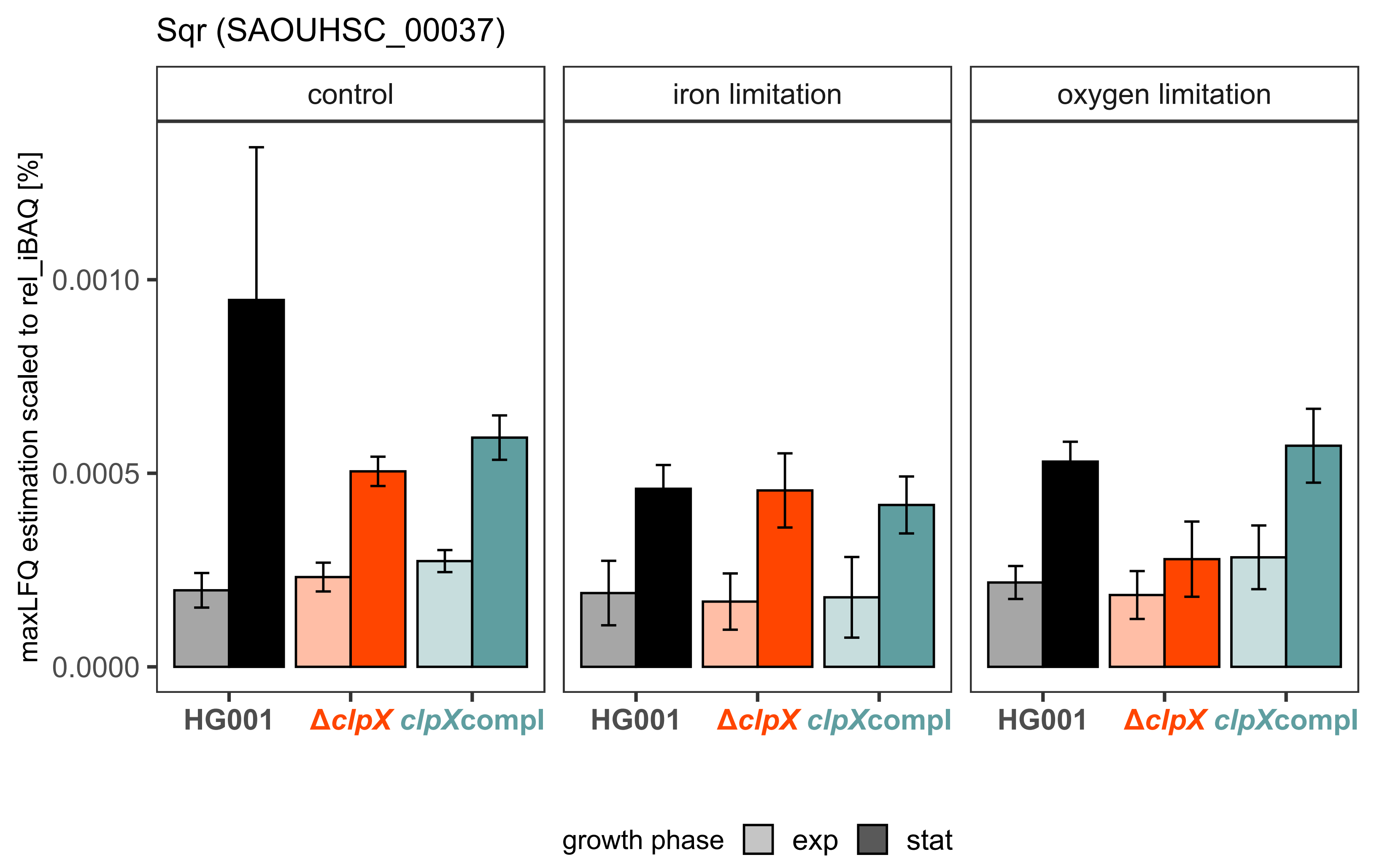

Sqr (SAOUHSC_00037)
control
iron limitation
oxygen limitation
maxLFQ estimation scaled to rel_iBAQ [%]
0.0010
0.0005
0.0000
HG001
ΔclpX
clpXcompl
growth phase
exp
stat

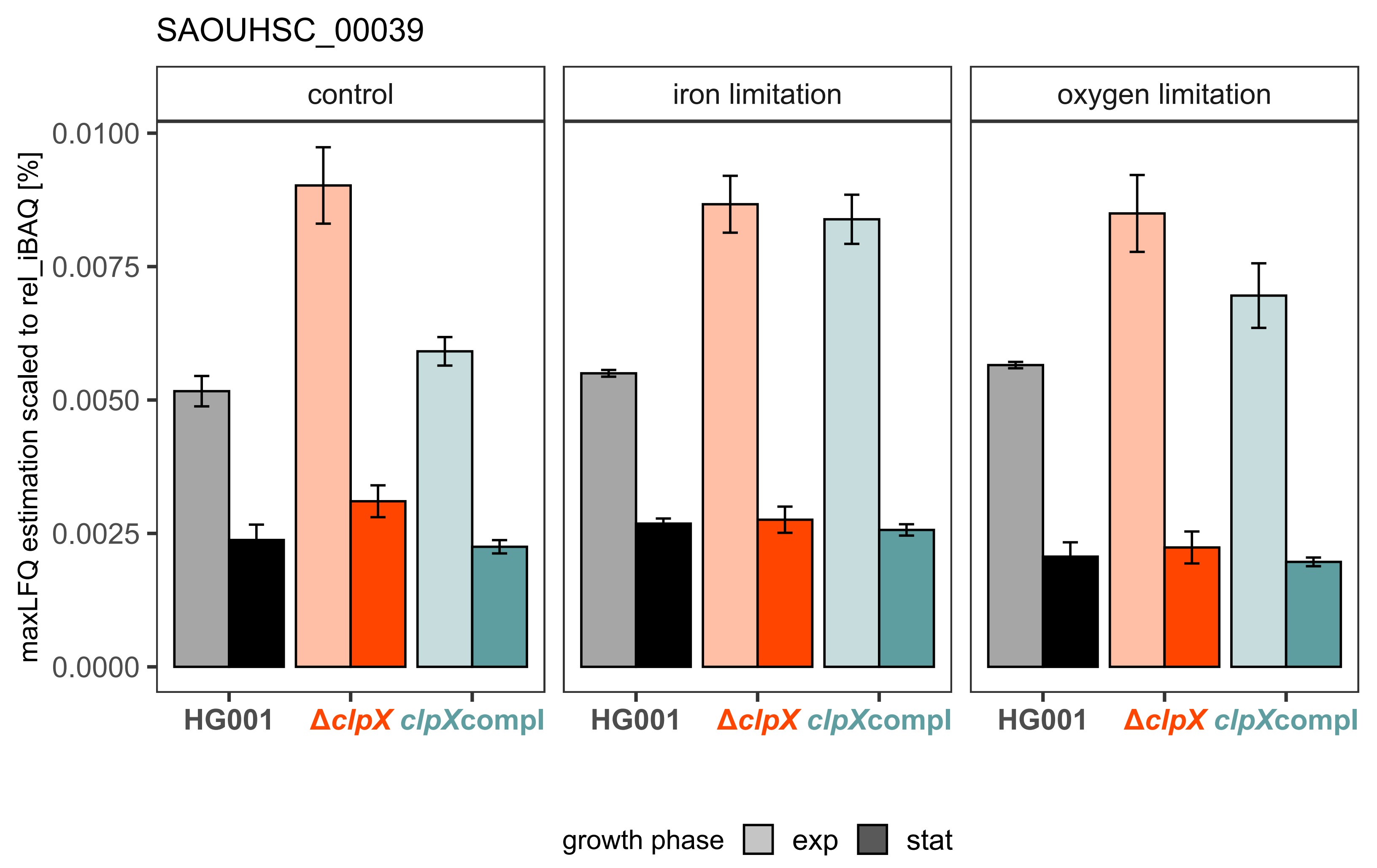
SAOUHSC_00039
control
iron limitation
oxygen limitation
maxLFQ estimation scaled to rel_iBAQ [%]
0.0100
0.0075
0.0050
0.0025
0.0000
HG001
ΔclpX
clpXcompl
growth phase
exp
stat

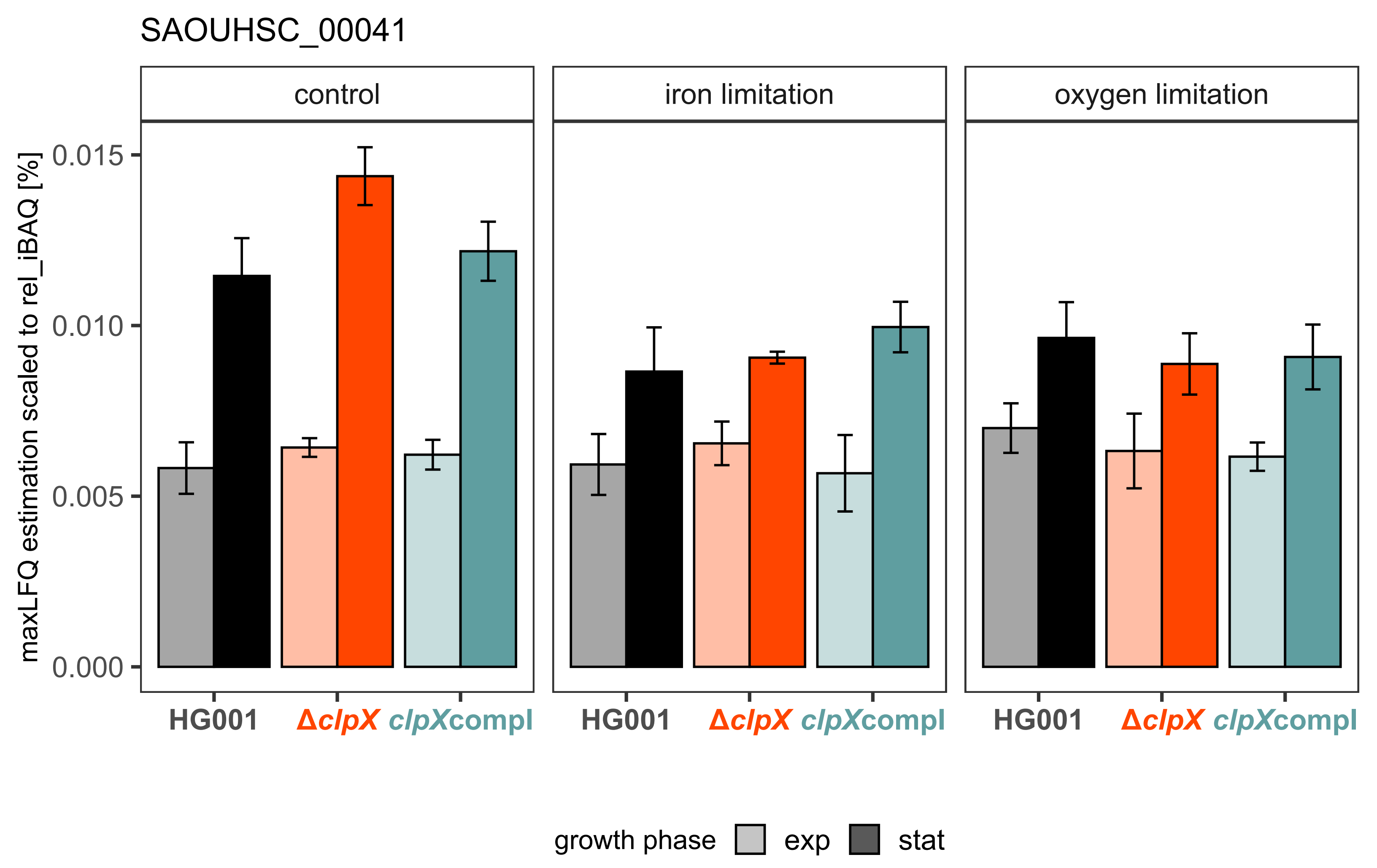
SAOUHSC_00041
control
iron limitation
oxygen limitation
maxLFQ estimation scaled to rel_iBAQ [%]
0.015
0.010
0.005
0.000
HG001
ΔclpX
clpXcompl
growth phase
exp
stat

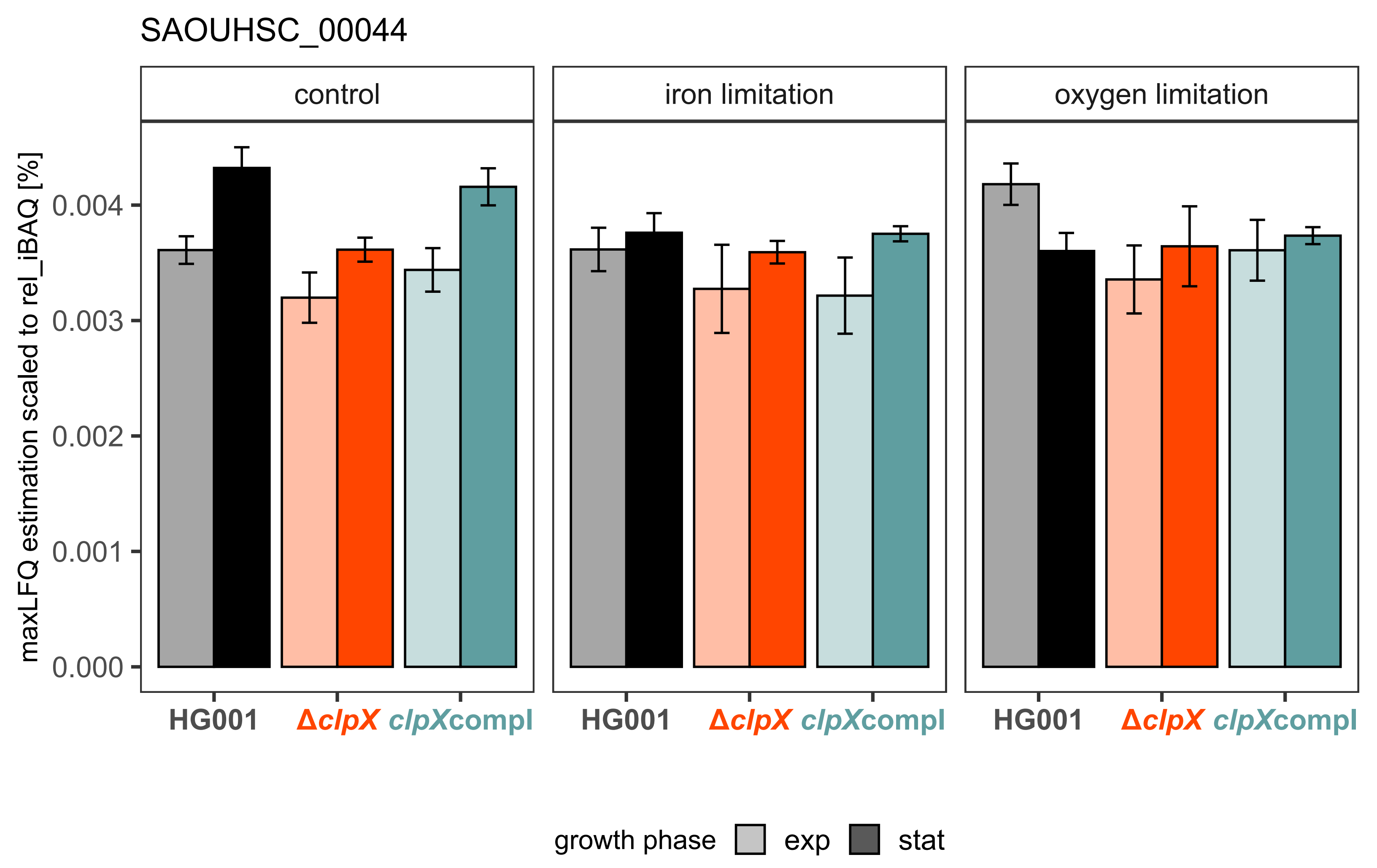

SAOUHSC_00044
control
iron limitation
oxygen limitation
maxLFQ estimation scaled to rel_iBAQ [%]
0.000
0.001
0.002
0.003
0.004
HG001
ΔclpX
clpXcompl
growth phase
exp
stat

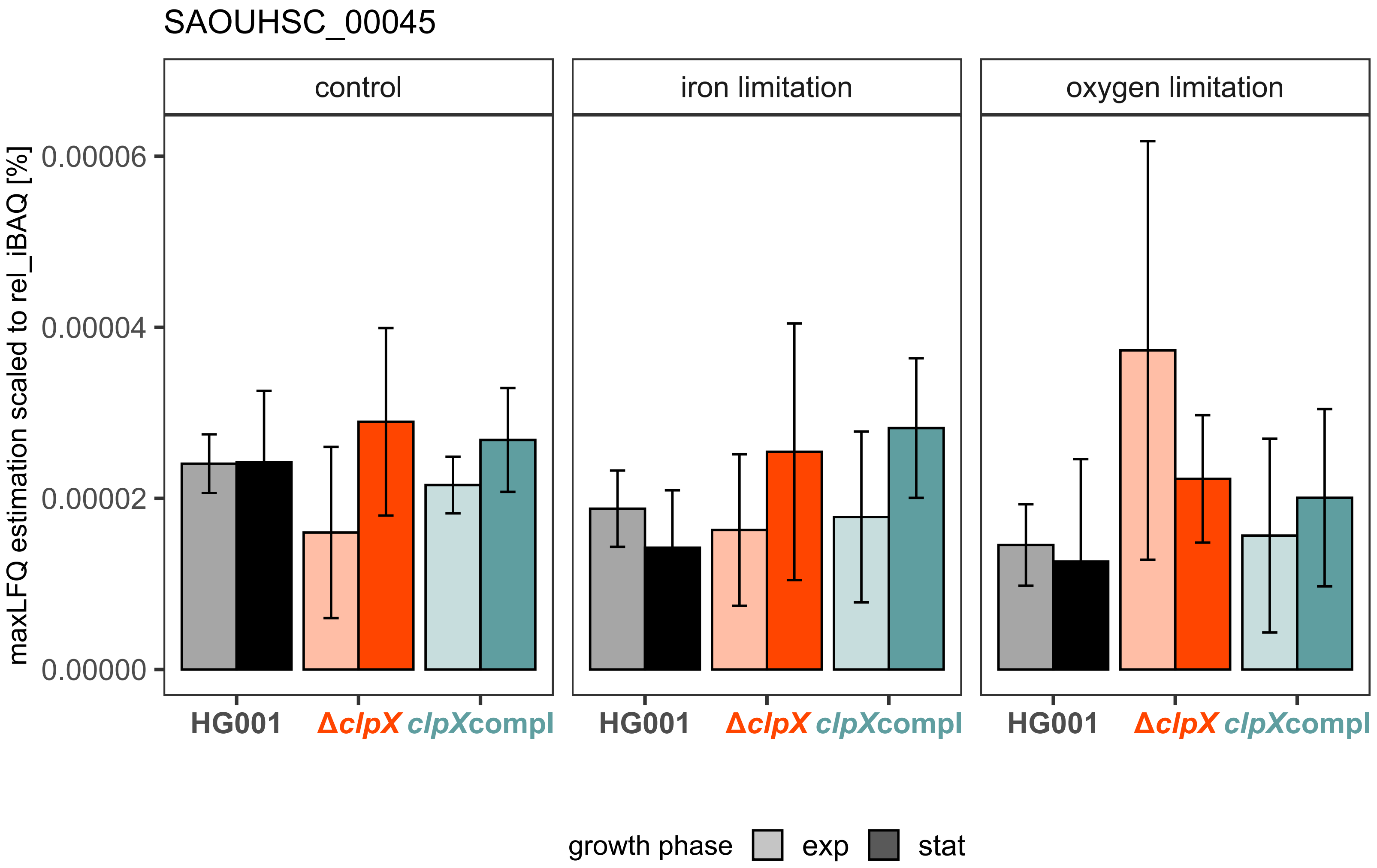

SAOUHSC_00045
control
iron limitation
oxygen limitation
maxLFQ estimation scaled to rel_iBAQ [%]
0.00006
0.00004
0.00002
0.00000
HG001
ΔclpX
clpXcompl
growth phase
exp
stat

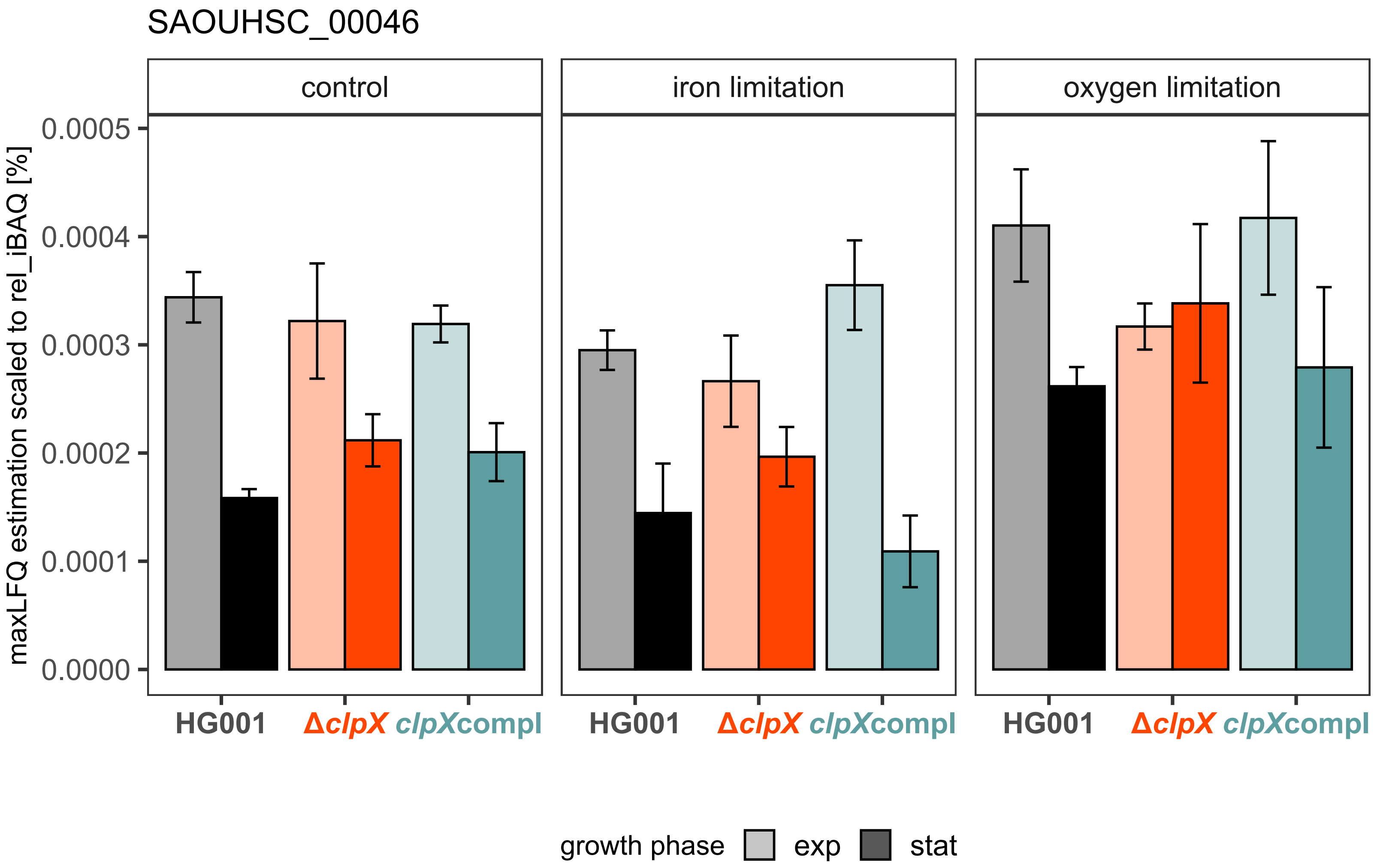

SAOUHSC_00046
control
iron limitation
oxygen limitation
maxLFQ estimation scaled to rel_iBAQ [%]
0.0005
0.0004
0.0003
0.0002
0.0001
0.0000
HG001
ΔclpX
clpXcompl
growth phase
exp
stat

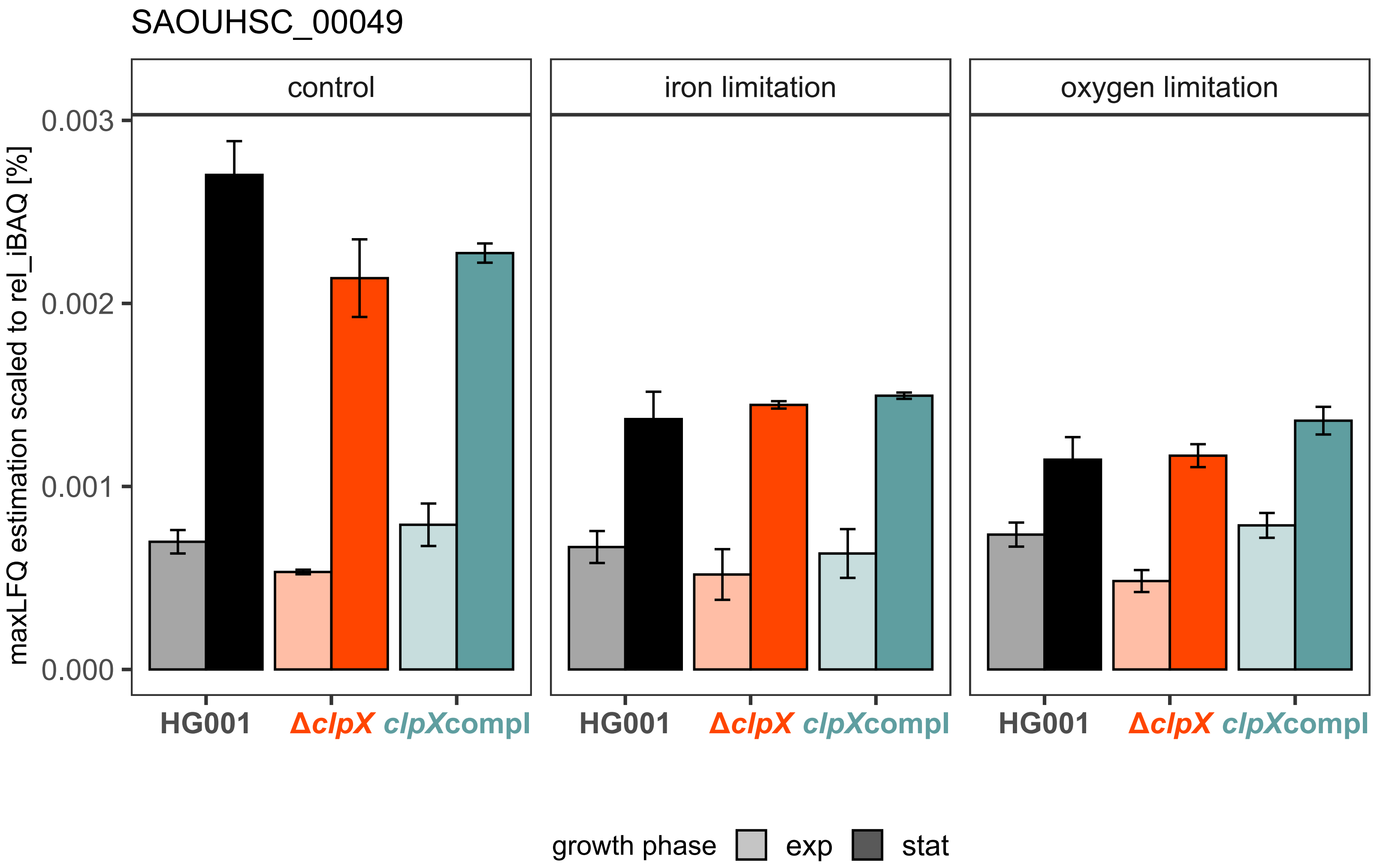
SAOUHSC_00049
control
iron limitation
oxygen limitation
maxLFQ estimation scaled to rel_iBAQ [%]
0.003
0.002
0.001
0.000
HG001
ΔclpX
clpXcompl
growth phase
exp
stat

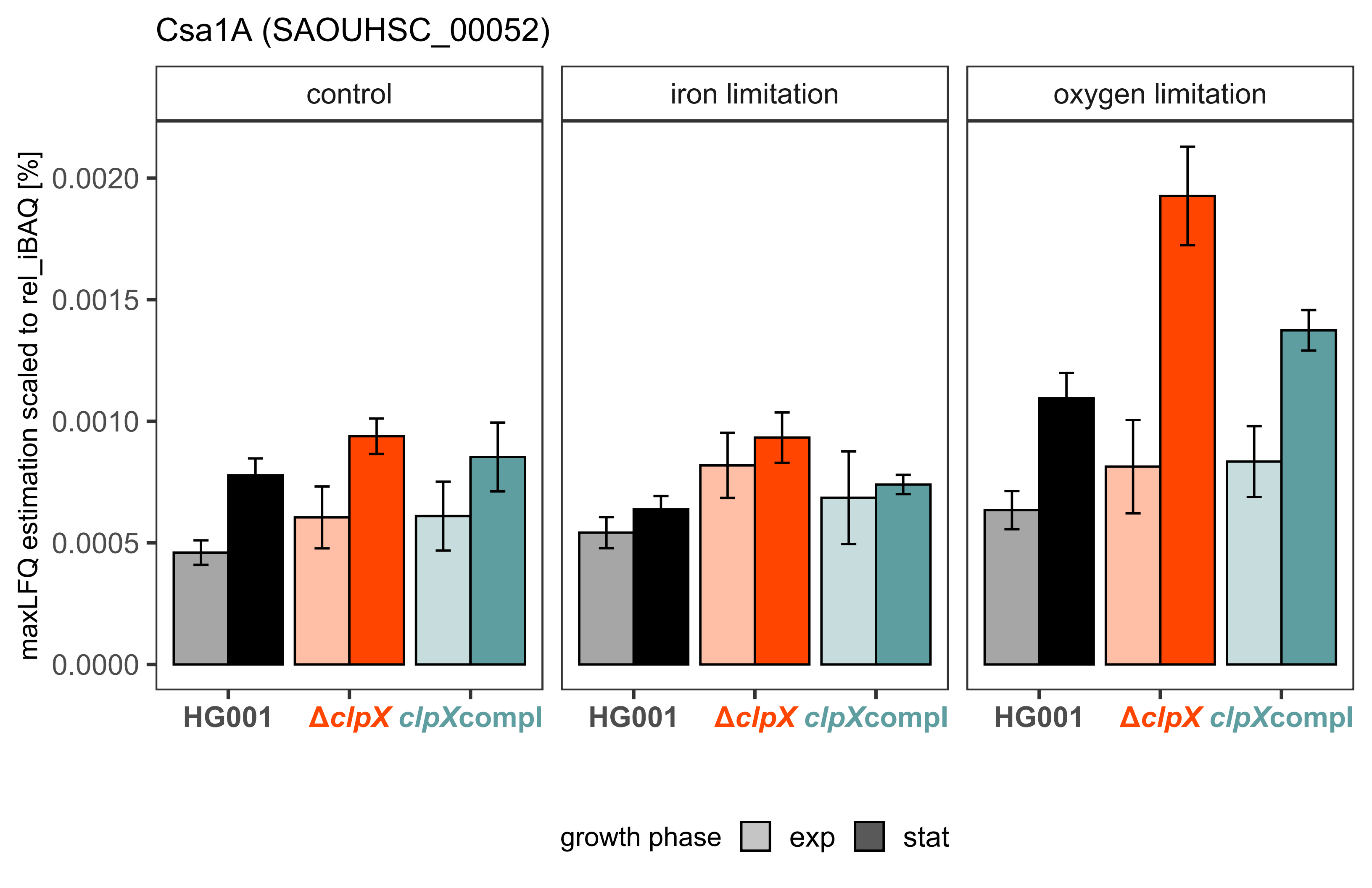
Csa1A (SAOUHSC_00052)
control
iron limitation
oxygen limitation
maxLFQ estimation scaled to rel_iBAQ [%]
0.0020
0.0015
0.0010
0.0005
0.0000
HG001
ΔclpX
clpXcompl
growth phase
exp
stat

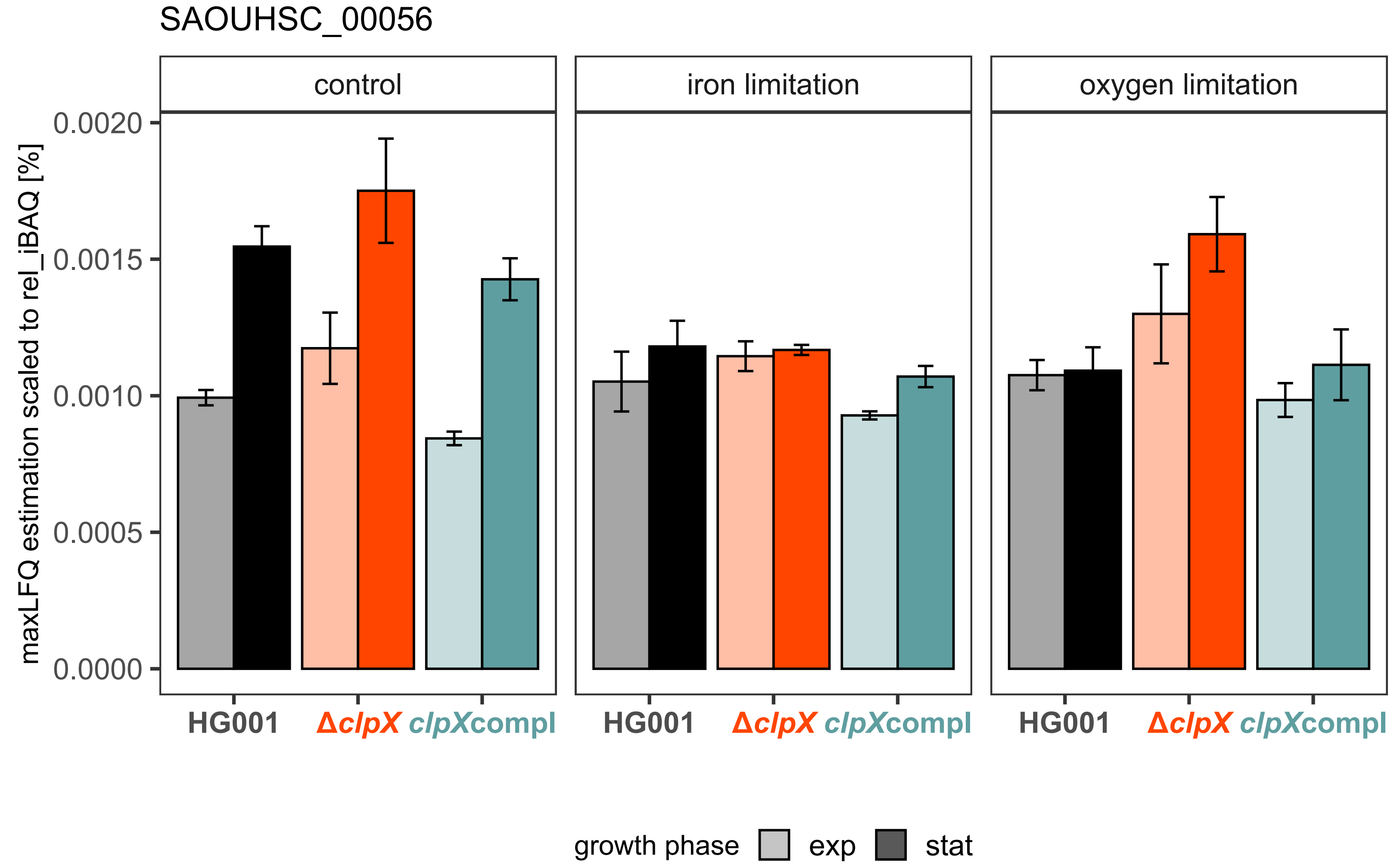

SAOUHSC_00056
control
iron limitation
oxygen limitation
maxLFQ estimation scaled to rel_iBAQ [%]
0.0020
0.0015
0.0010
0.0005
0.0000
HG001
ΔclpX
clpXcompl
growth phase
exp
stat

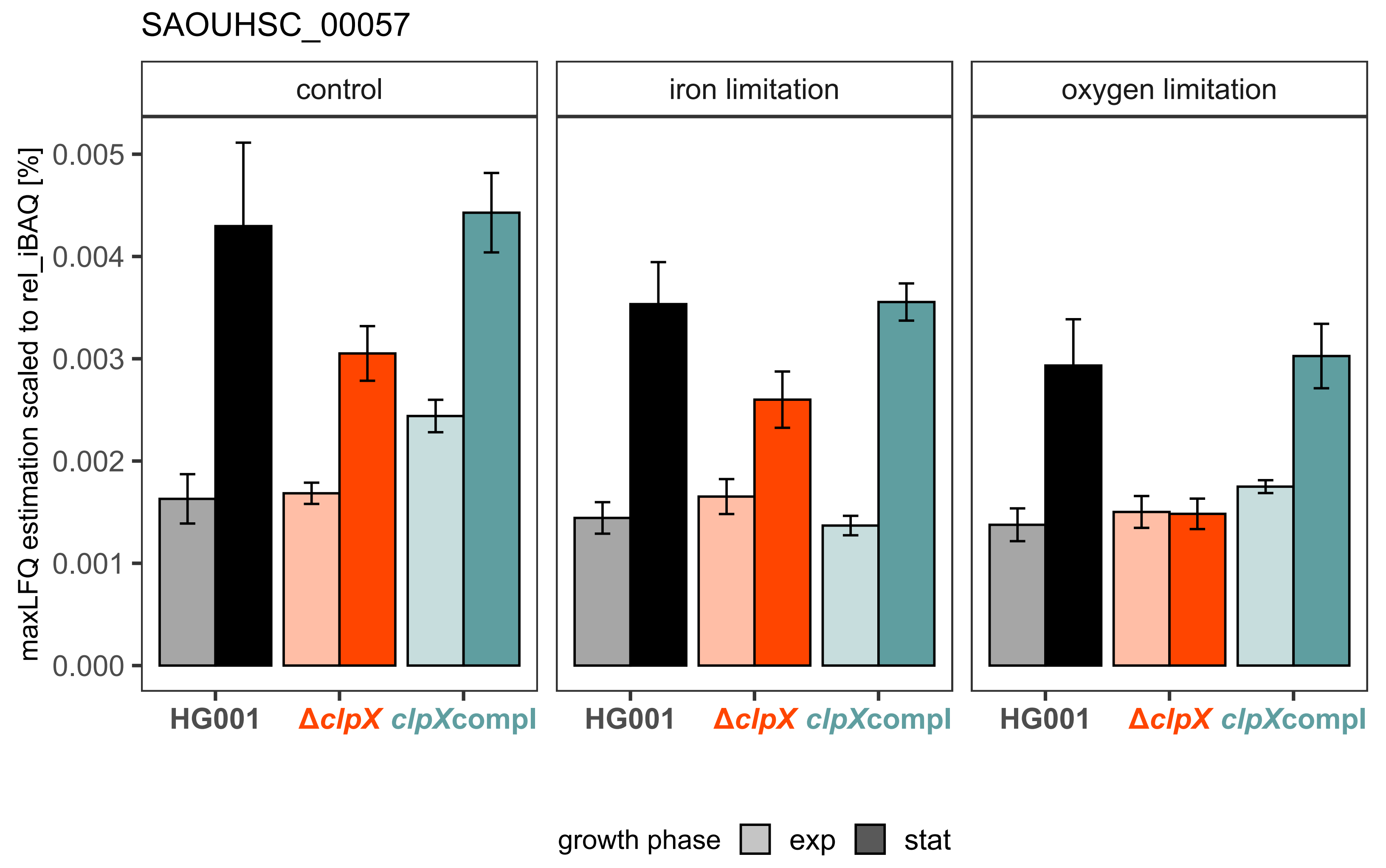
SAOUHSC_00057
control
iron limitation
oxygen limitation
maxLFQ estimation scaled to rel_iBAQ [%]
0.005
0.004
0.003
0.002
0.001
0.000
HG001
ΔclpX
clpXcompl
growth phase
exp
stat

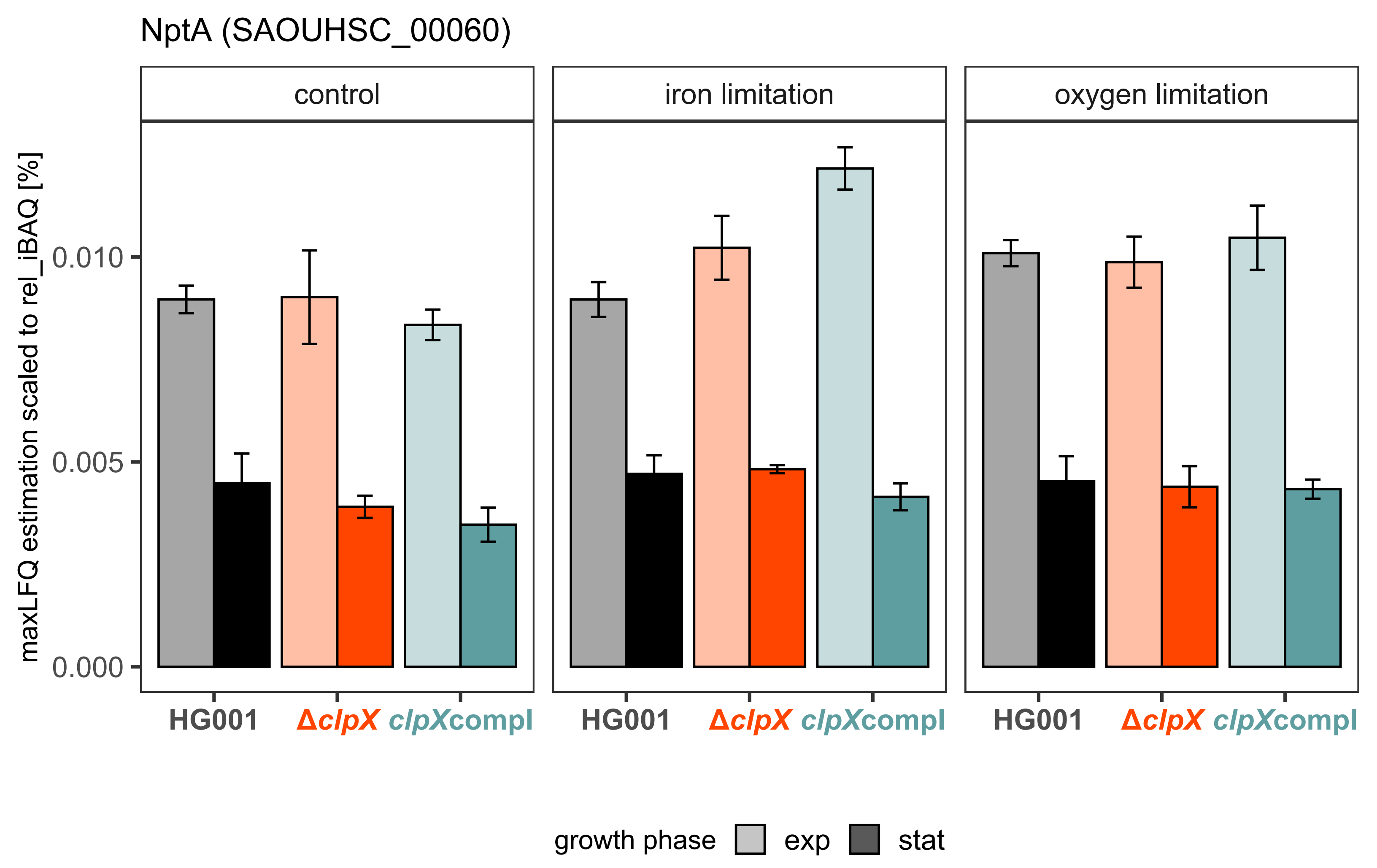
NptA (SAOUHSC_00060)
control
iron limitation
oxygen limitation
maxLFQ estimation scaled to rel_iBAQ [%]
0.010
0.005
0.000
HG001
ΔclpX
clpXcompl
growth phase
exp
stat

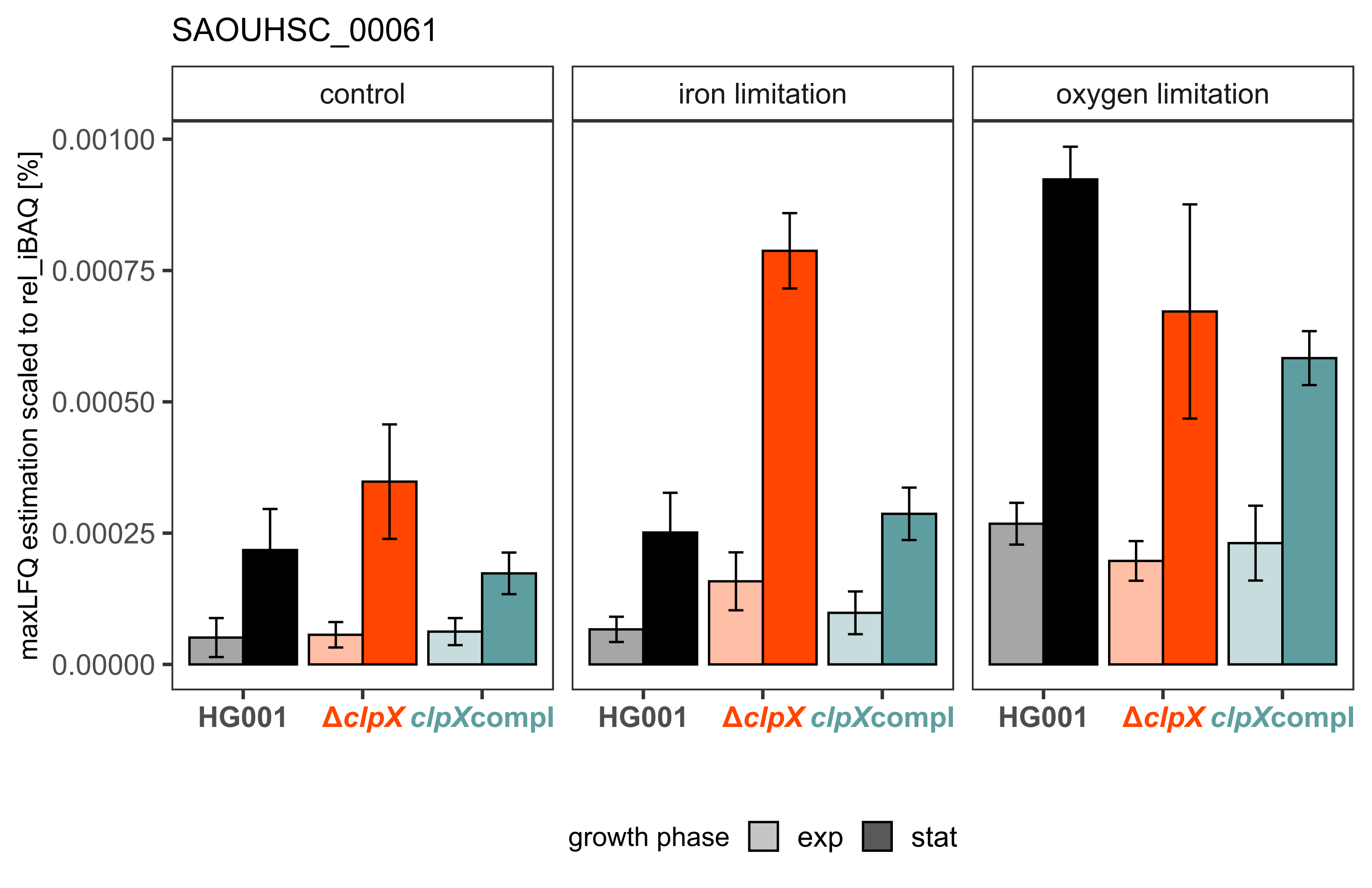
SAOUHSC_00061
control
iron limitation
oxygen limitation
maxLFQ estimation scaled to rel_iBAQ [%]
0.00100
0.00075
0.00050
0.00025
0.00000
HG001
ΔclpX
clpXcompl
growth phase
exp
stat

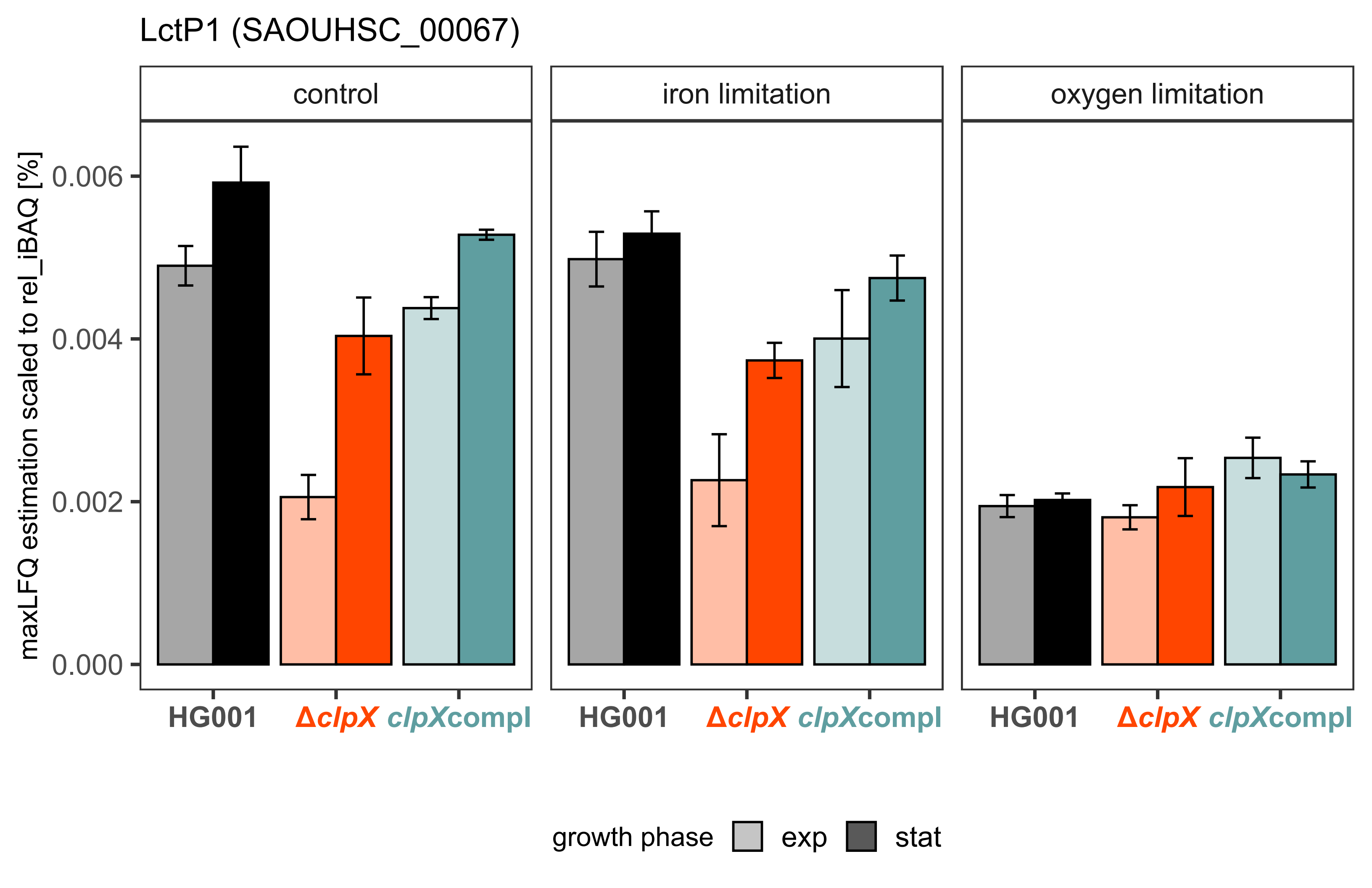

LctP1 (SAOUHSC_00067)
control
iron limitation
oxygen limitation
maxLFQ estimation scaled to rel_iBAQ [%]
0.006
0.004
0.002
0.000
HG001
ΔclpX
clpXcompl
growth phase
exp
stat

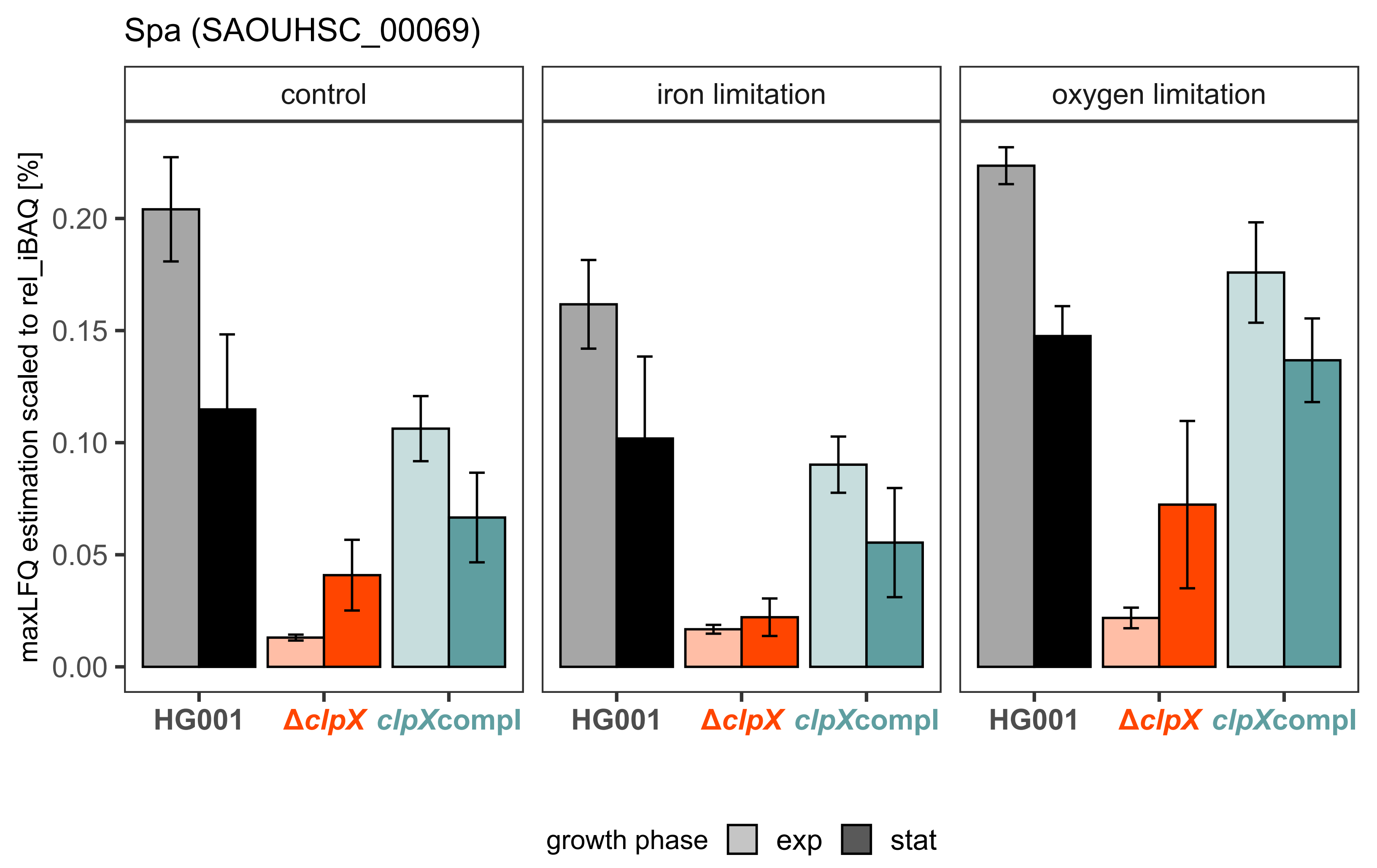
Spa (SAOUHSC_00069)
control
iron limitation
oxygen limitation
maxLFQ estimation scaled to rel_iBAQ [%]
0.00
0.05
0.10
0.15
0.20
HG001
ΔclpX
clpXcompl
growth phase
exp
stat

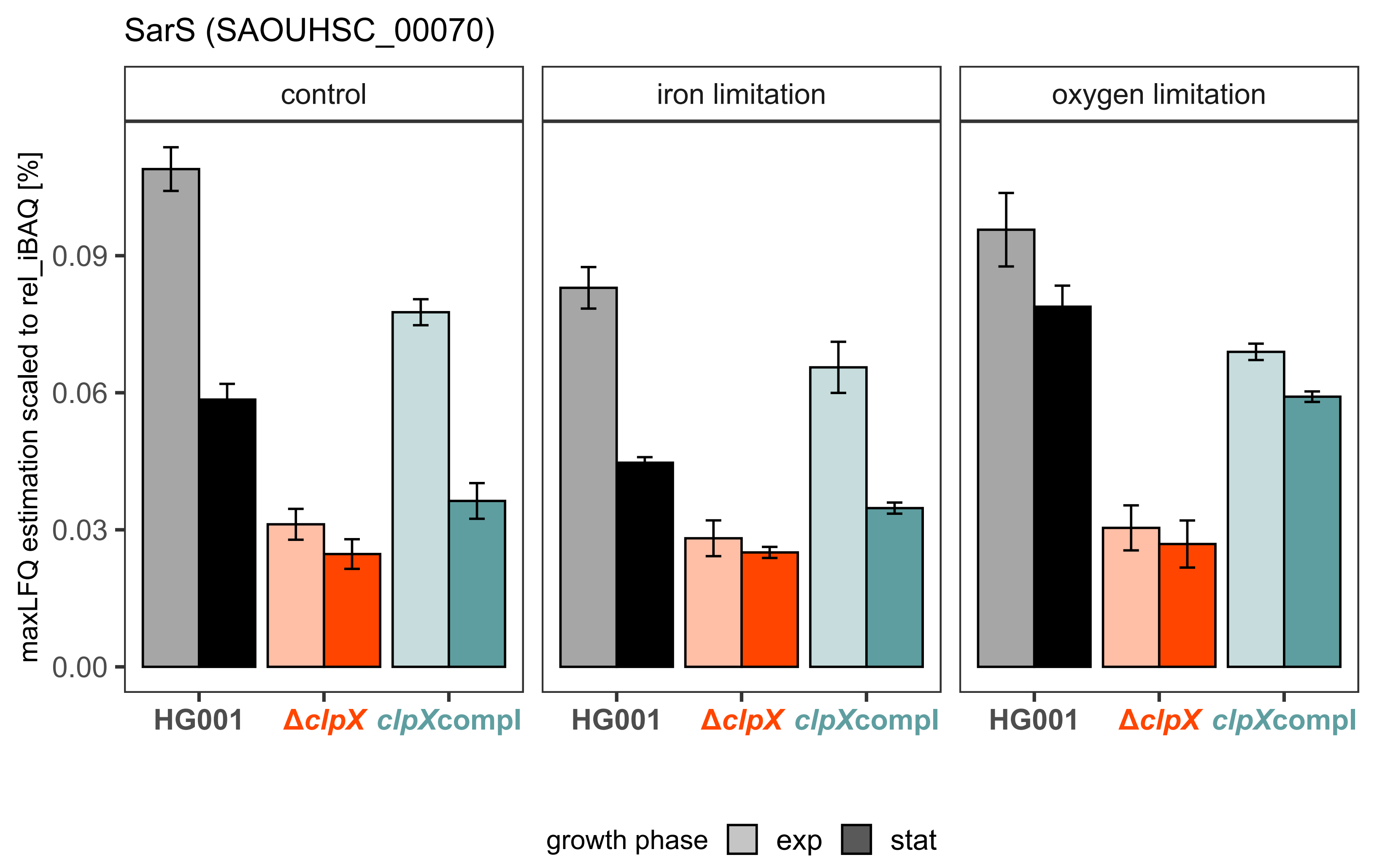

SarS (SAOUHSC_00070)
control
iron limitation
oxygen limitation
maxLFQ estimation scaled to rel_iBAQ [%]
0.00
0.03
0.06
0.09
HG001
ΔclpX
clpXcompl
growth phase
exp
stat

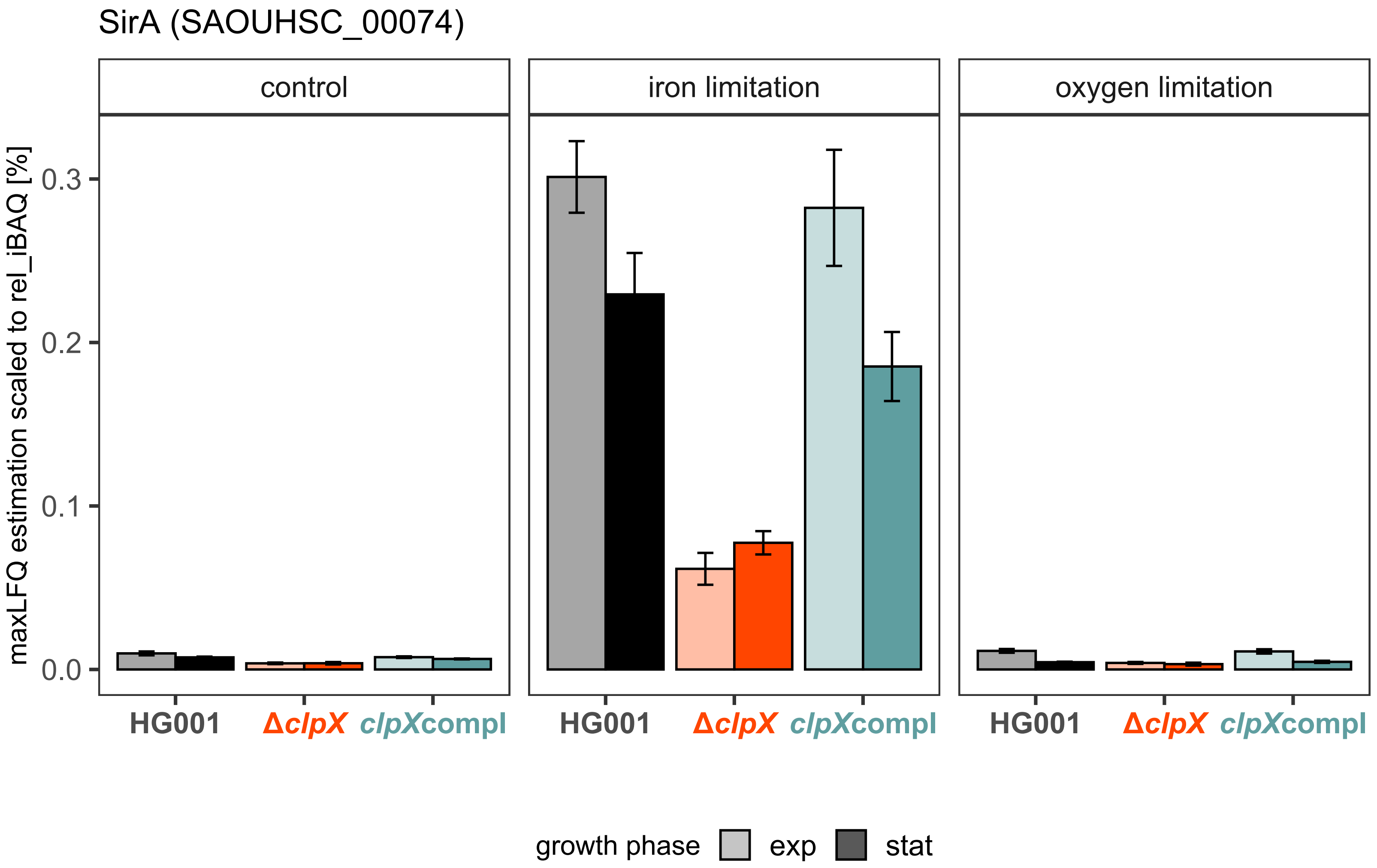
SirA (SAOUHSC_00074)
control
iron limitation
oxygen limitation
maxLFQ estimation scaled to rel_iBAQ [%]
0.0
0.1
0.2
0.3
HG001
ΔclpX
clpXcompl
growth phase
exp
stat

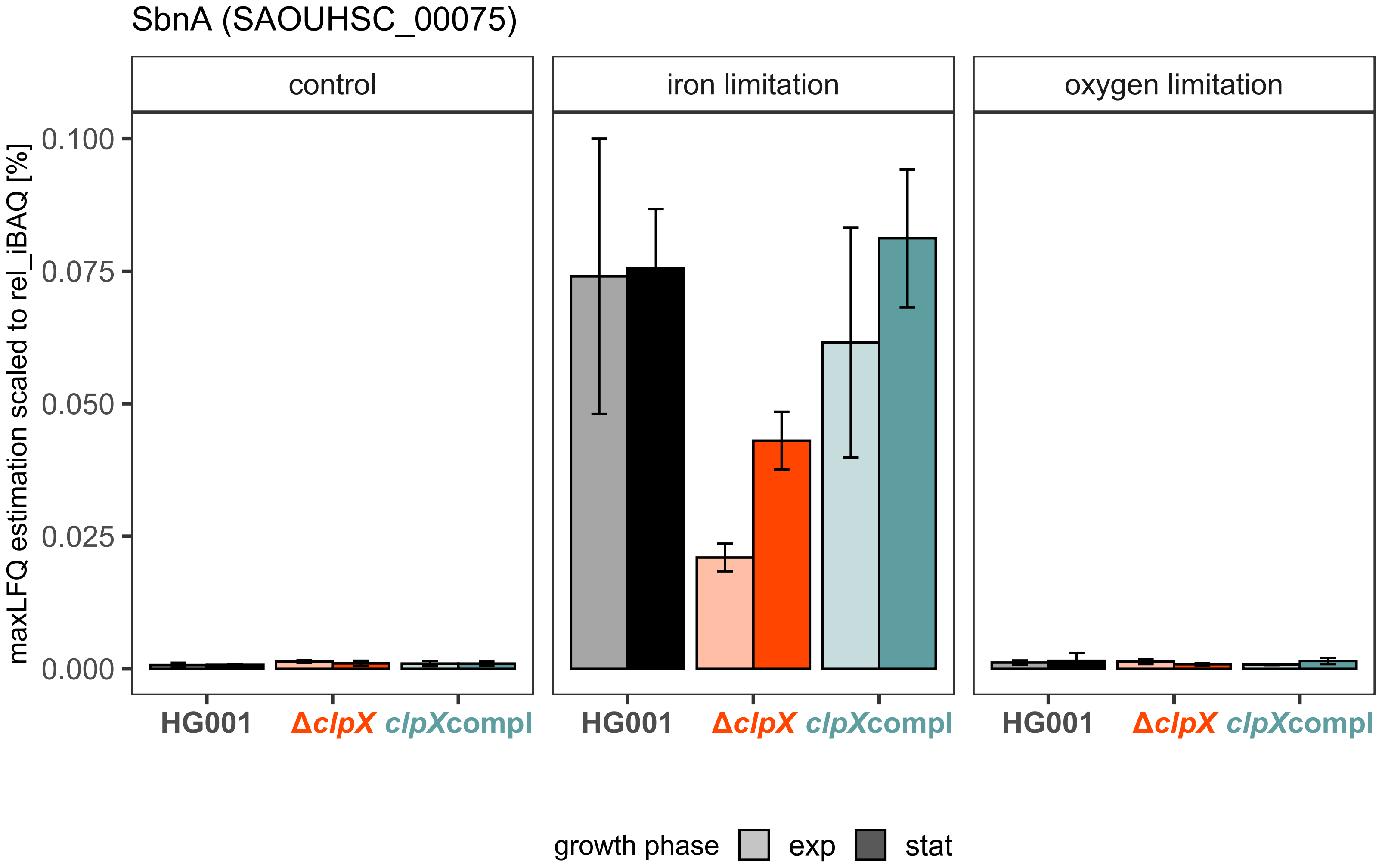
SbnA (SAOUHSC_00075)
control
iron limitation
oxygen limitation
maxLFQ estimation scaled to rel_iBAQ [%]
0.100
0.075
0.050
0.025
0.000
HG001
ΔclpX
clpXcompl
growth phase
exp
stat

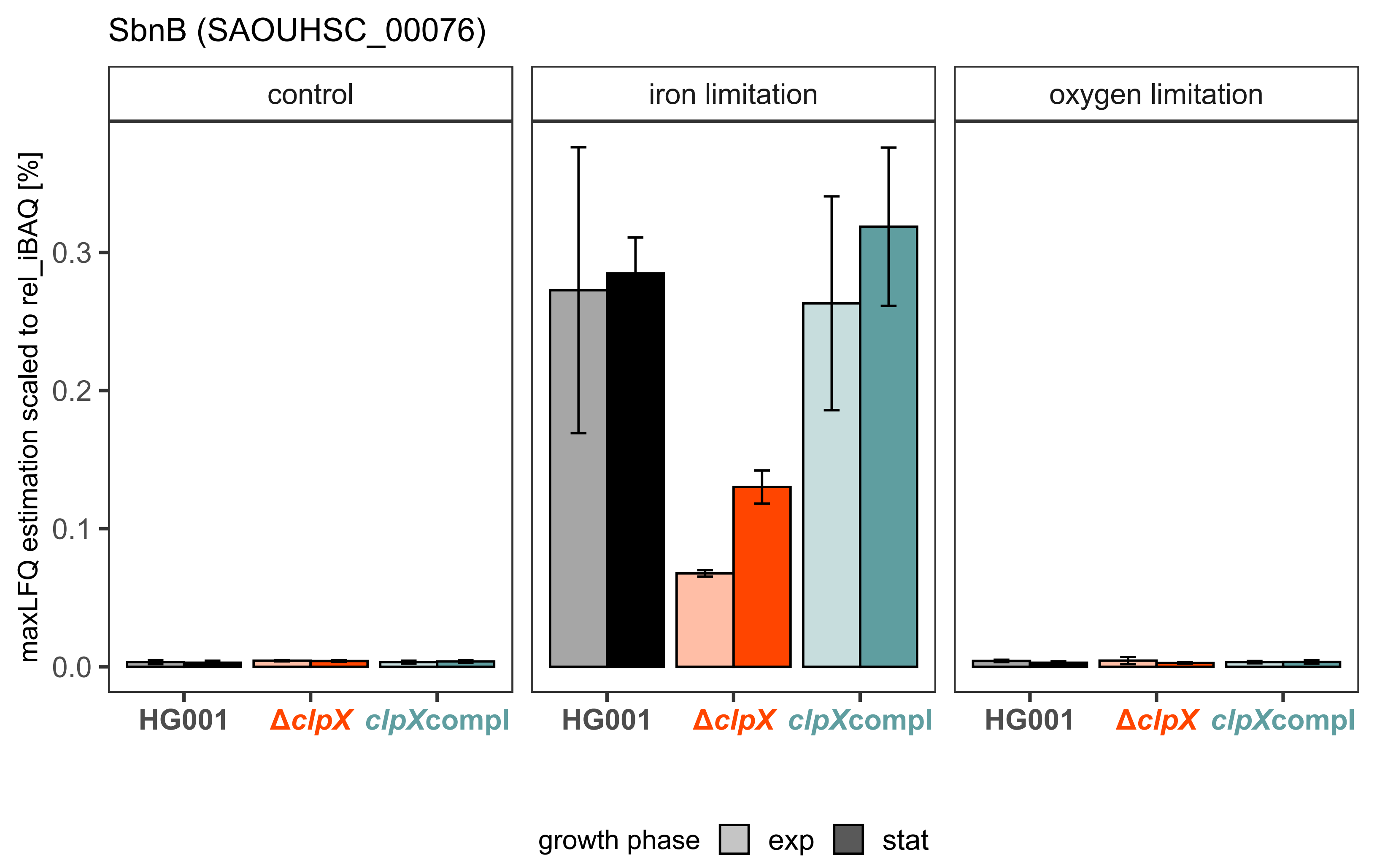

SbnB (SAOUHSC_00076)
control
iron limitation
oxygen limitation
maxLFQ estimation scaled to rel_iBAQ [%]
0.0
0.1
0.2
0.3
HG001
ΔclpX
clpXcompl
growth phase
exp
stat

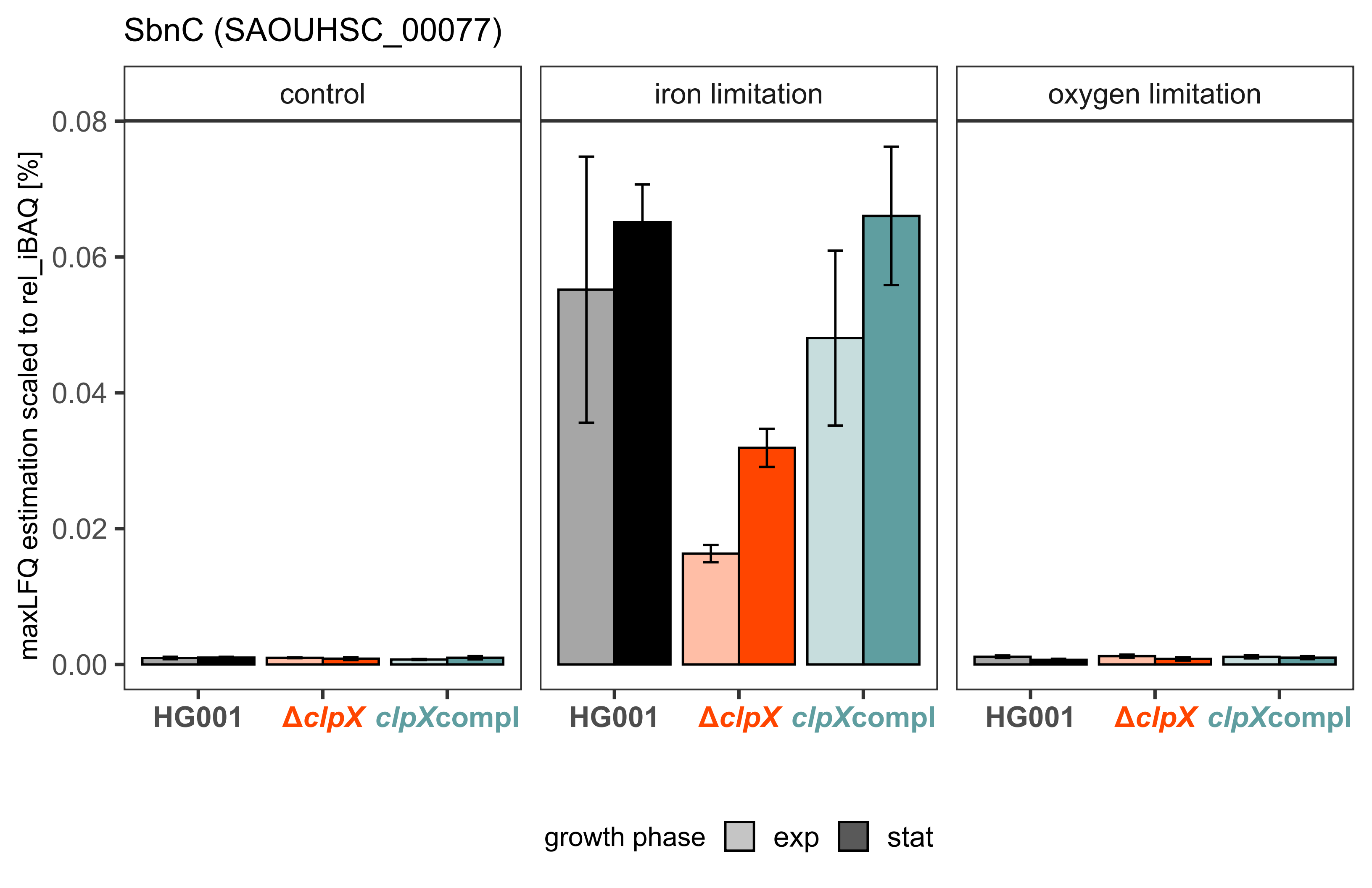

SbnC (SAOUHSC_00077)
control
iron limitation
oxygen limitation
0.08
0.06
0.04
0.02
0.00
maxLFQ estimation scaled to rel_iBAQ [%]
HG001
ΔclpX
clpXcompl
growth phase
exp
stat

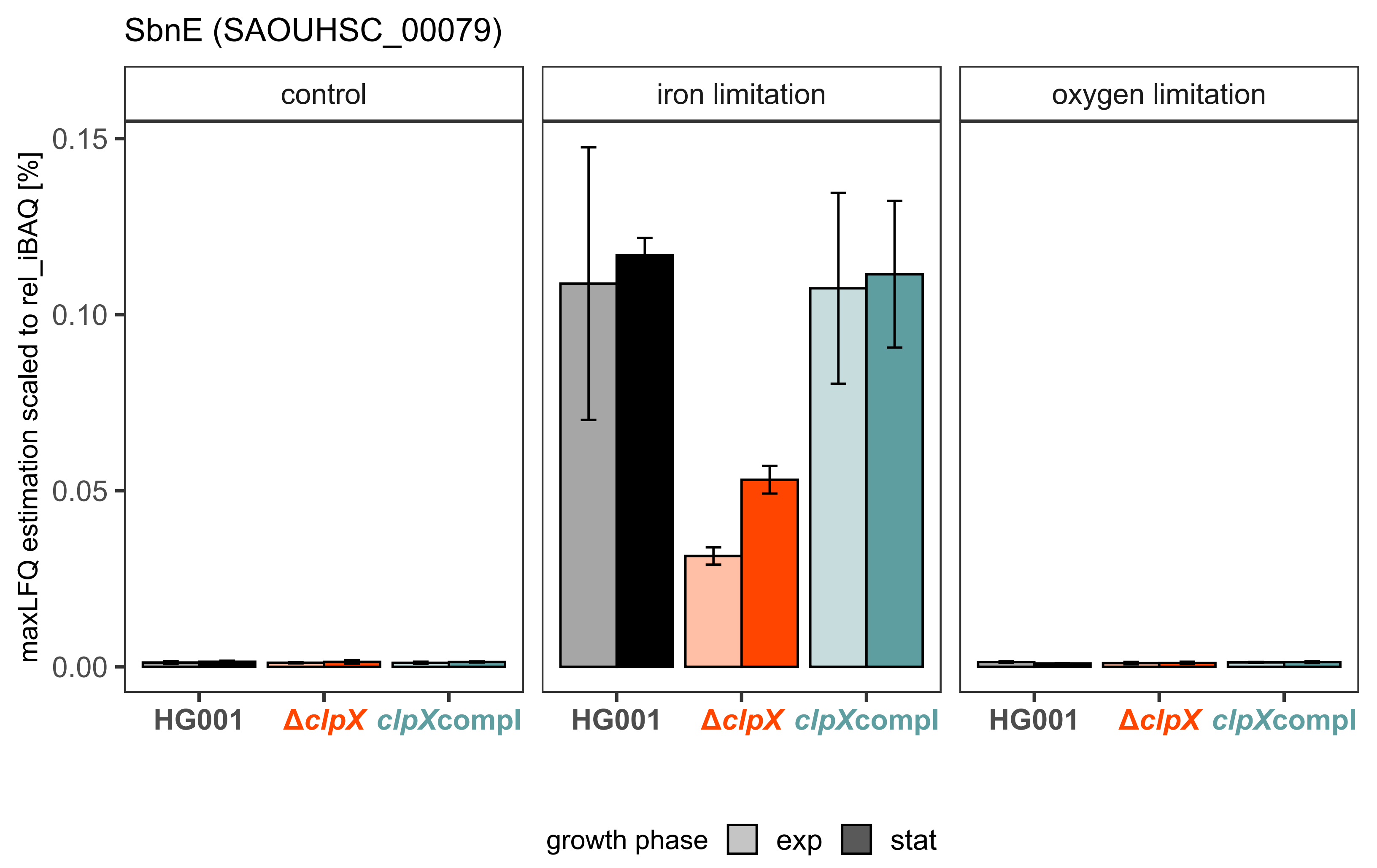
SbnE (SAOUHSC_00079)
control
iron limitation
oxygen limitation
maxLFQ estimation scaled to rel_iBAQ [%]
0.15
0.10
0.05
0.00
HG001
ΔclpX
clpXcompl
growth phase
exp
stat

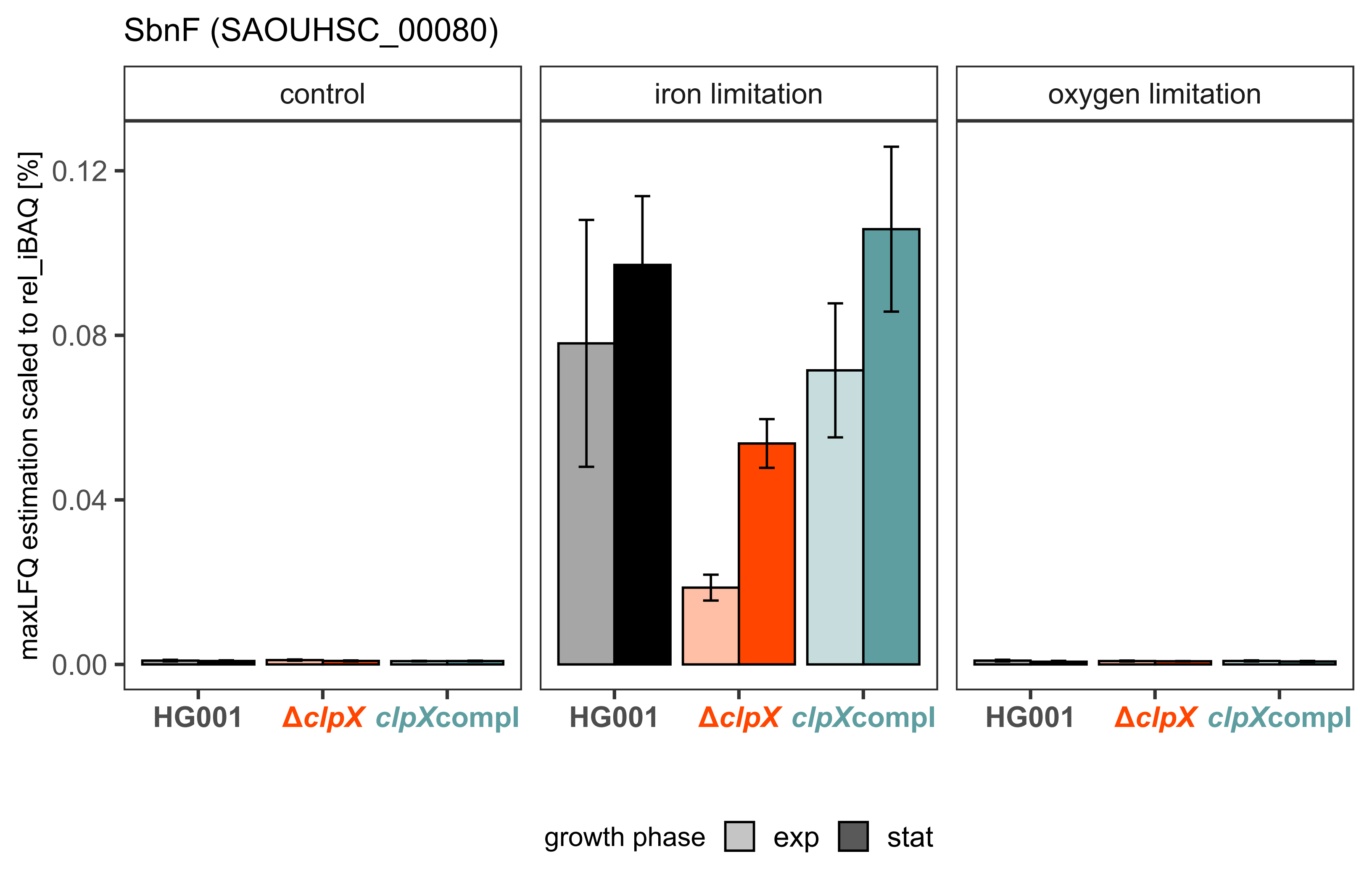
SbnF (SAOUHSC_00080)
control
iron limitation
oxygen limitation
maxLFQ estimation scaled to rel_iBAQ [%]
0.12
0.08
0.04
0.00
HG001
ΔclpX
clpXcompl
growth phase
exp
stat

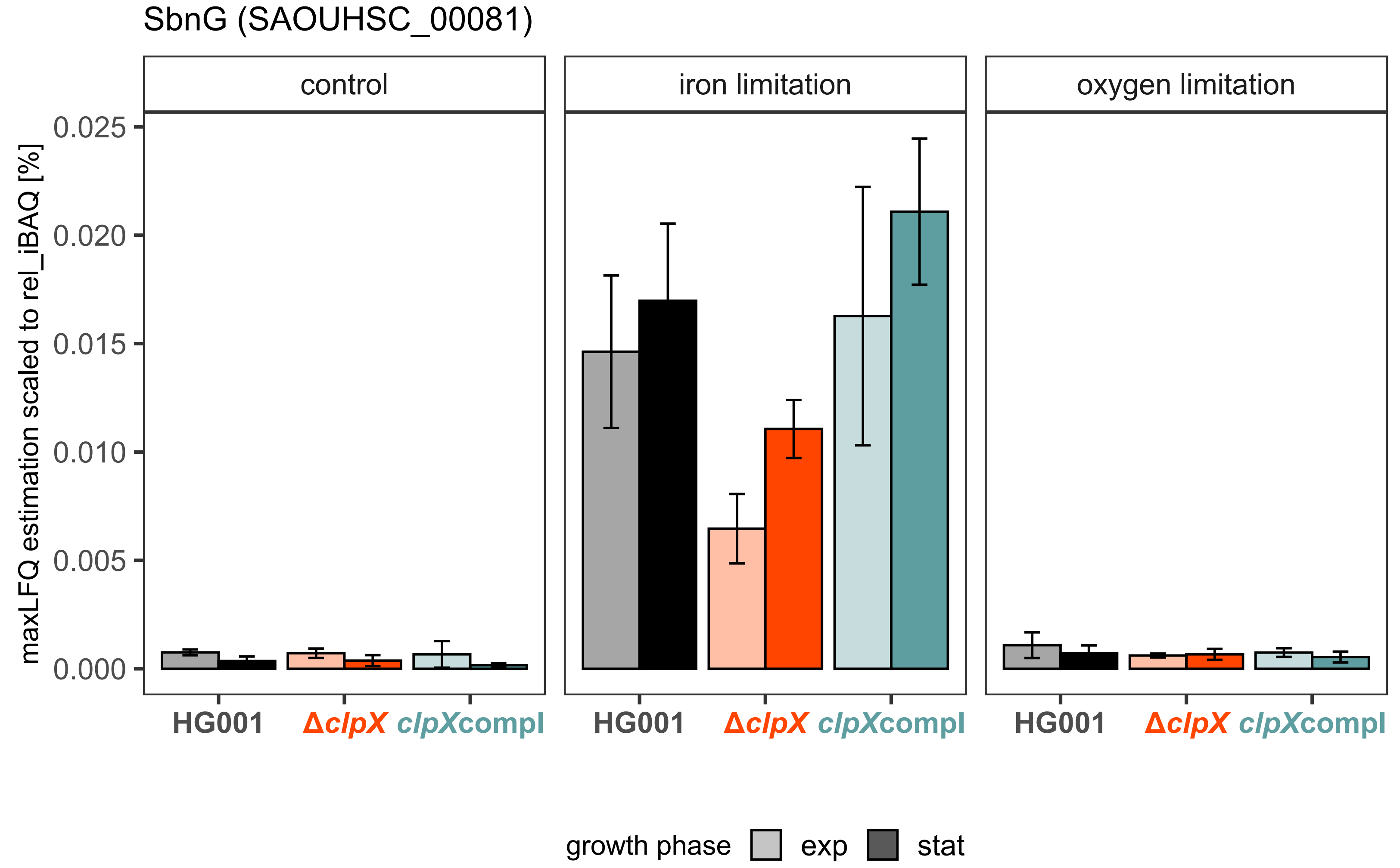

SbnG (SAOUHSC_00081)
control
iron limitation
oxygen limitation
maxLFQ estimation scaled to rel_iBAQ [%]
0.025
0.020
0.015
0.010
0.005
0.000
HG001
ΔclpX
clpXcompl
growth phase
exp
stat

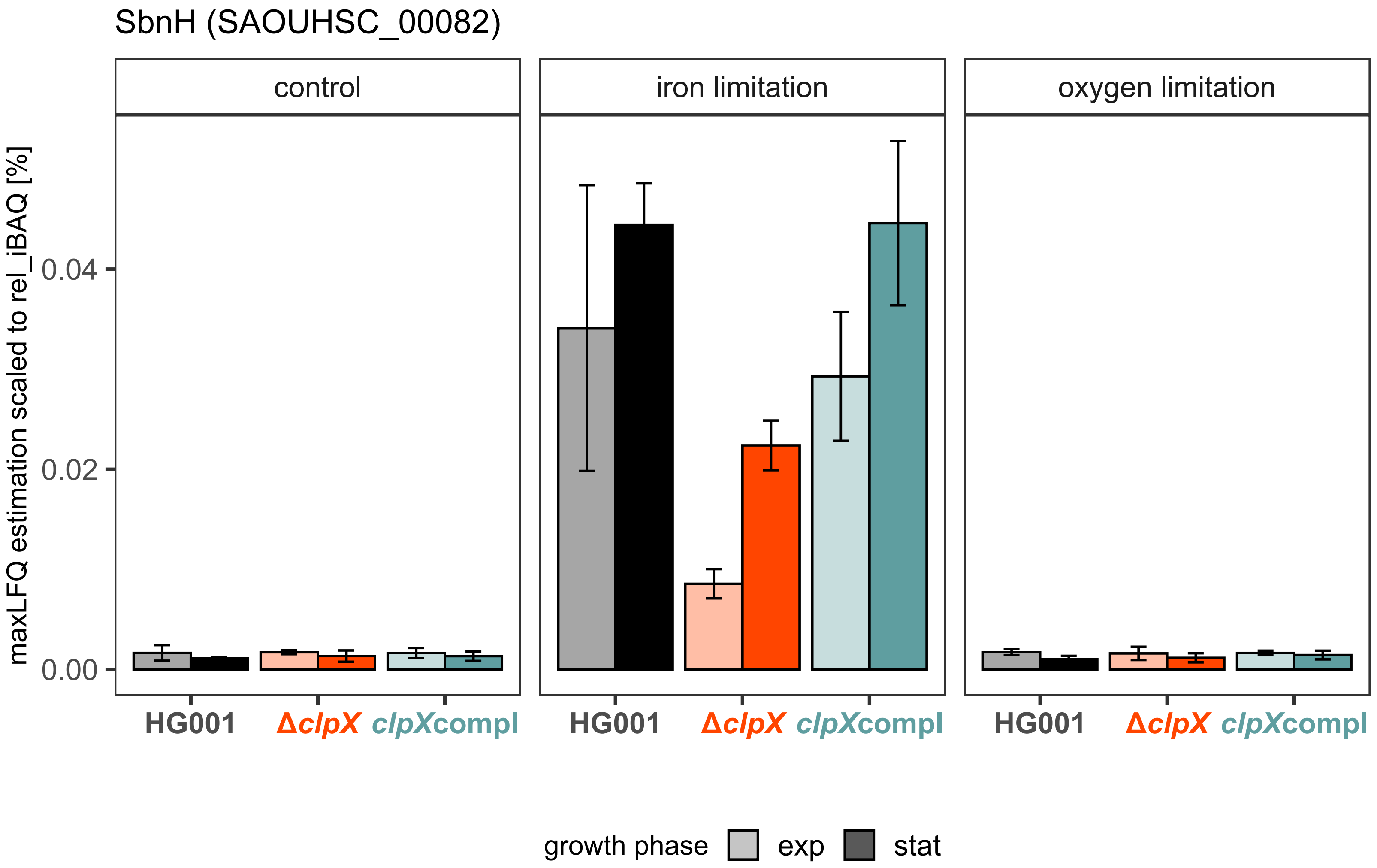
SbnH (SAOUHSC_00082)
control
iron limitation
oxygen limitation
maxLFQ estimation scaled to rel_iBAQ [%]
0.04
0.02
0.00
HG001
ΔclpX
clpXcompl
growth phase
exp
stat

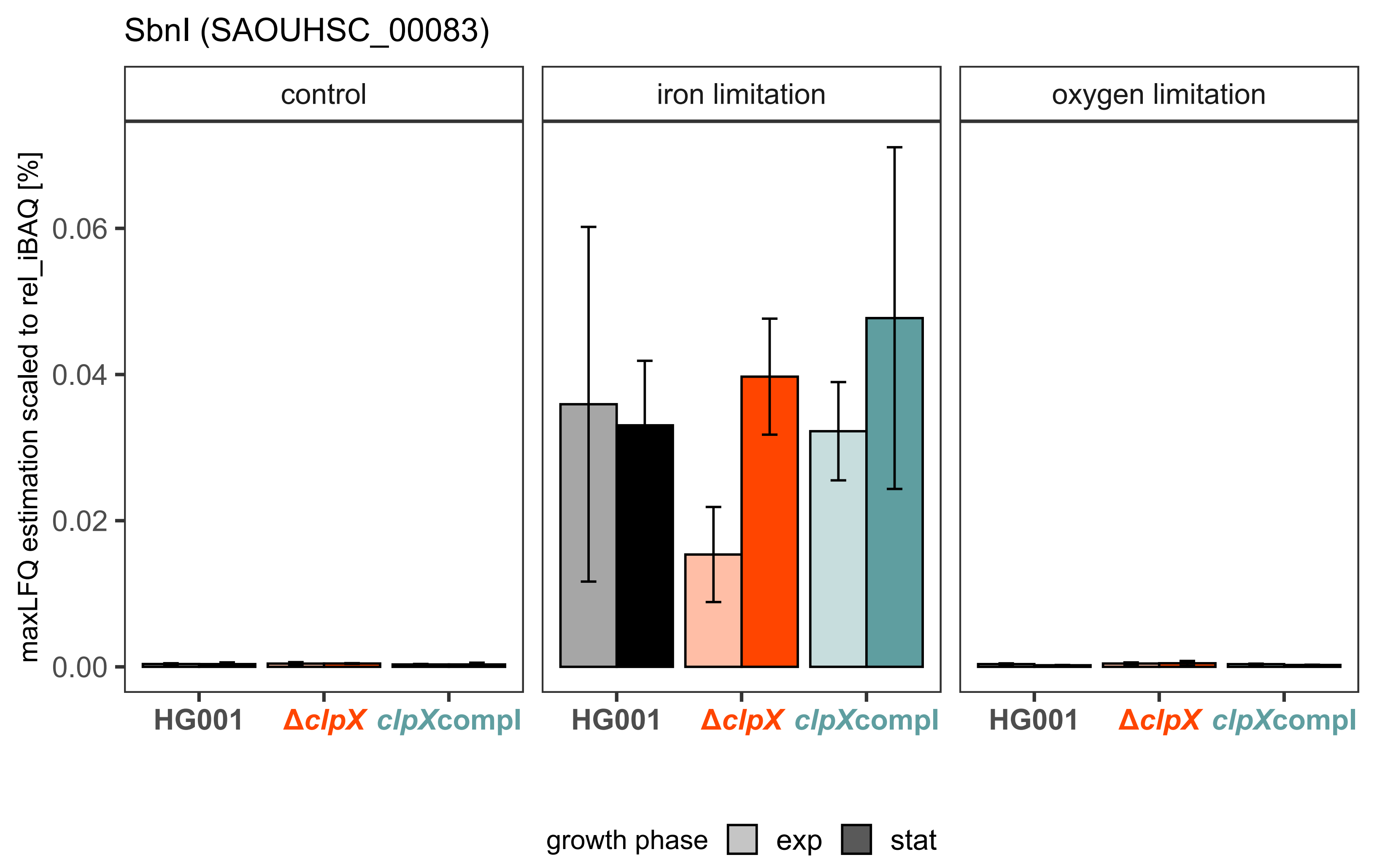
SbnI (SAOUHSC_00083)
control
iron limitation
oxygen limitation
maxLFQ estimation scaled to rel_iBAQ [%]
0.06
0.04
0.02
0.00
HG001
ΔclpX
clpXcompl
growth phase
exp
stat

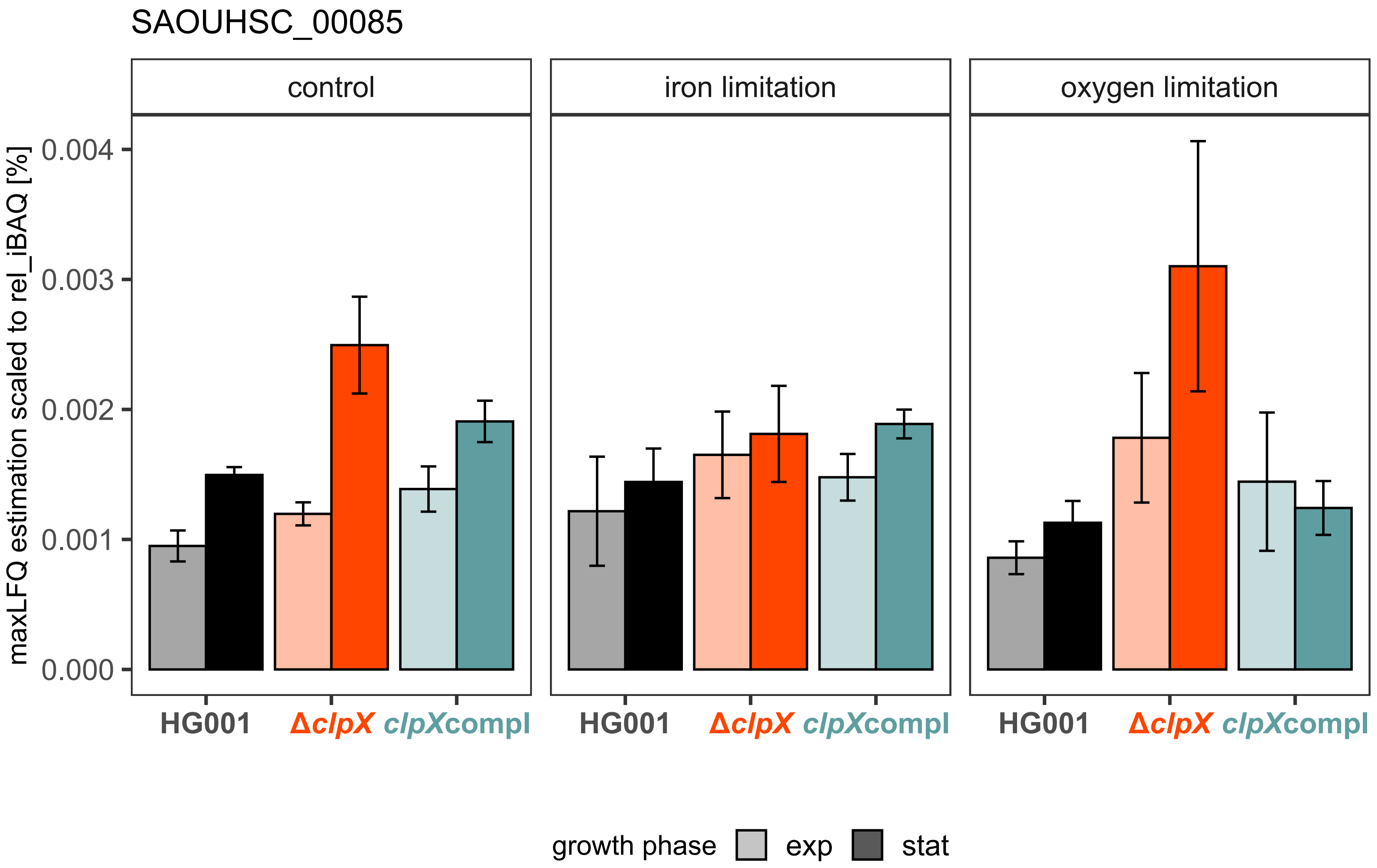

SAOUHSC_00085
control
iron limitation
oxygen limitation
maxLFQ estimation scaled to rel_iBAQ [%]
0.004
0.003
0.002
0.001
0.000
HG001
ΔclpX
clpXcompl
growth phase
exp
stat

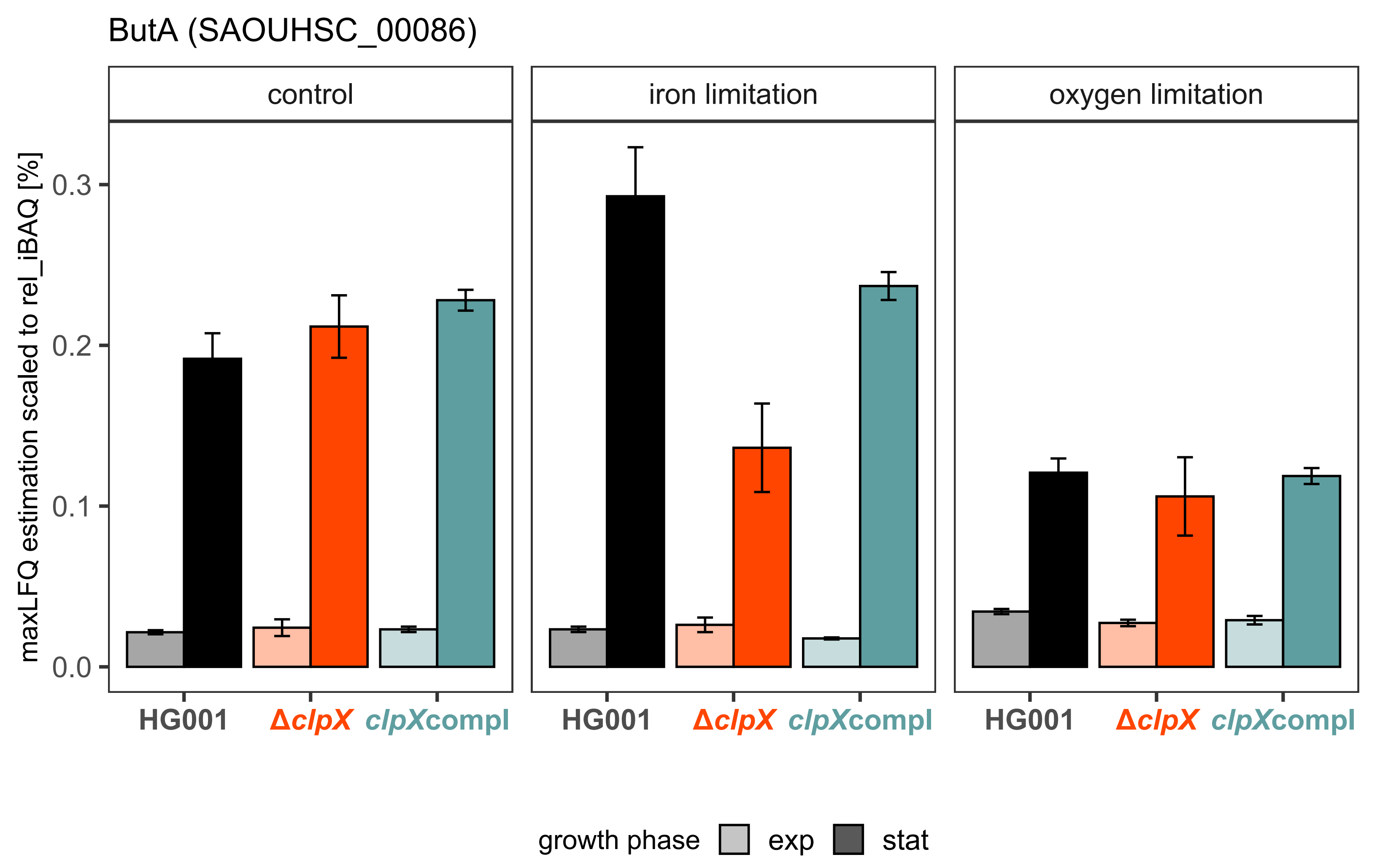

ButA (SAOUHSC_00086)
control
iron limitation
oxygen limitation
maxLFQ estimation scaled to rel_iBAQ [%]
0.0
0.1
0.2
0.3
HG001
ΔclpX
clpXcompl
growth phase
exp
stat

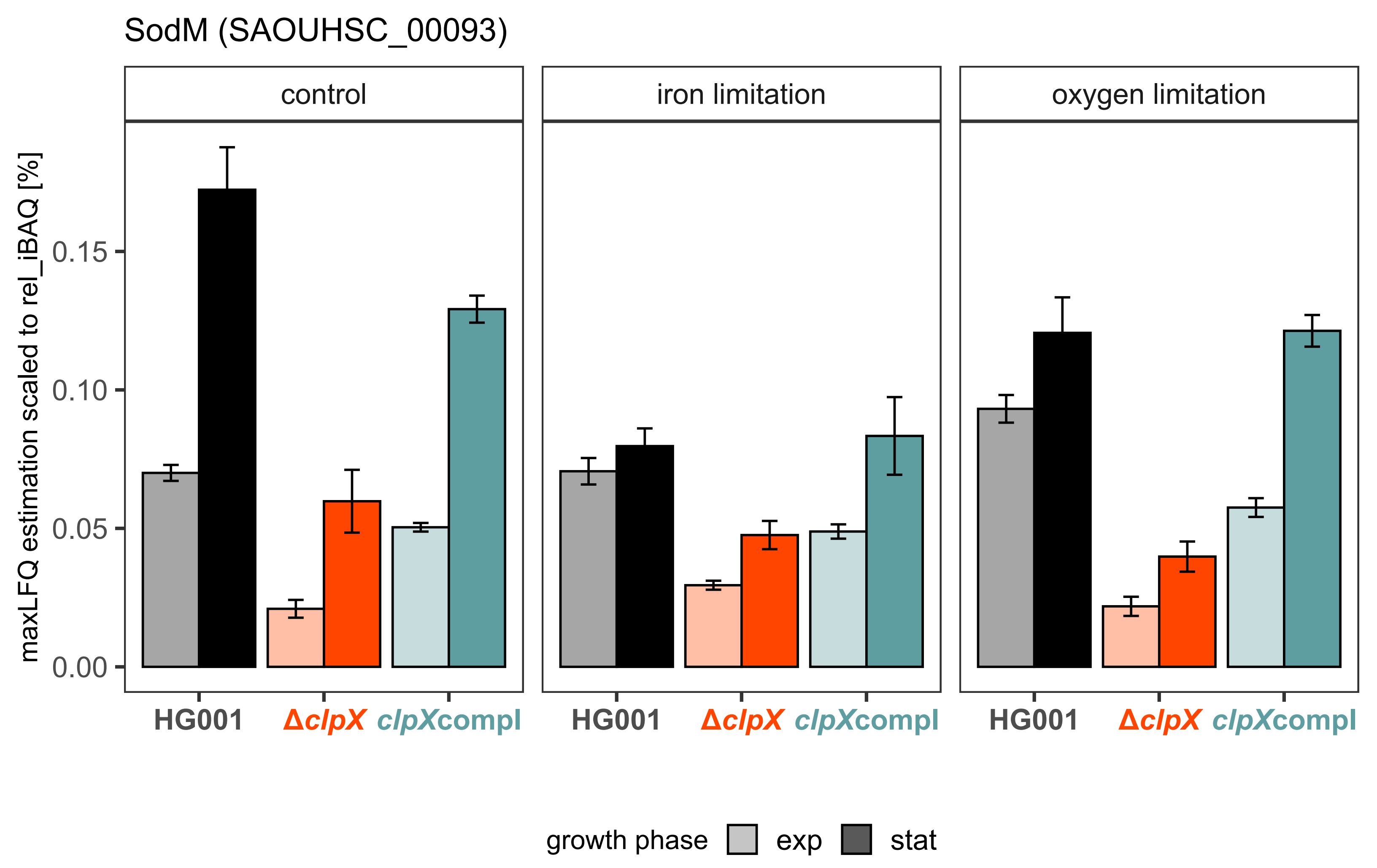

SodM (SAOUHSC_00093)
control
iron limitation
oxygen limitation
maxLFQ estimation scaled to rel_iBAQ [%]
0.15
0.10
0.05
0.00
HG001
ΔclpX
clpXcompl
growth phase
exp
stat

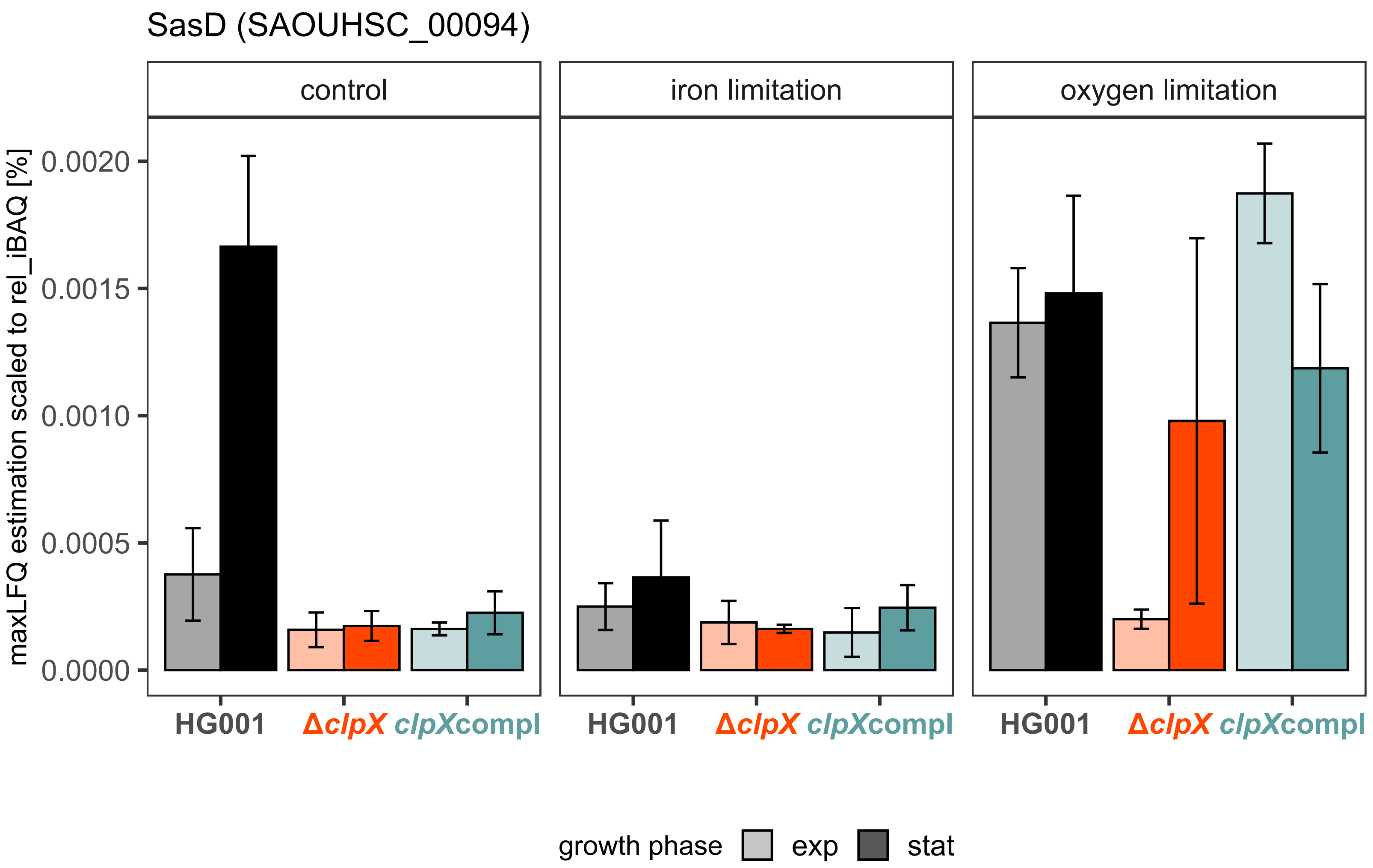

SasD (SAOUHSC_00094)
control
iron limitation
oxygen limitation
maxLFQ estimation scaled to rel_iBAQ [%]
0.0020
0.0015
0.0010
0.0005
0.0000
HG001
ΔclpX
clpXcompl
growth phase
exp
stat

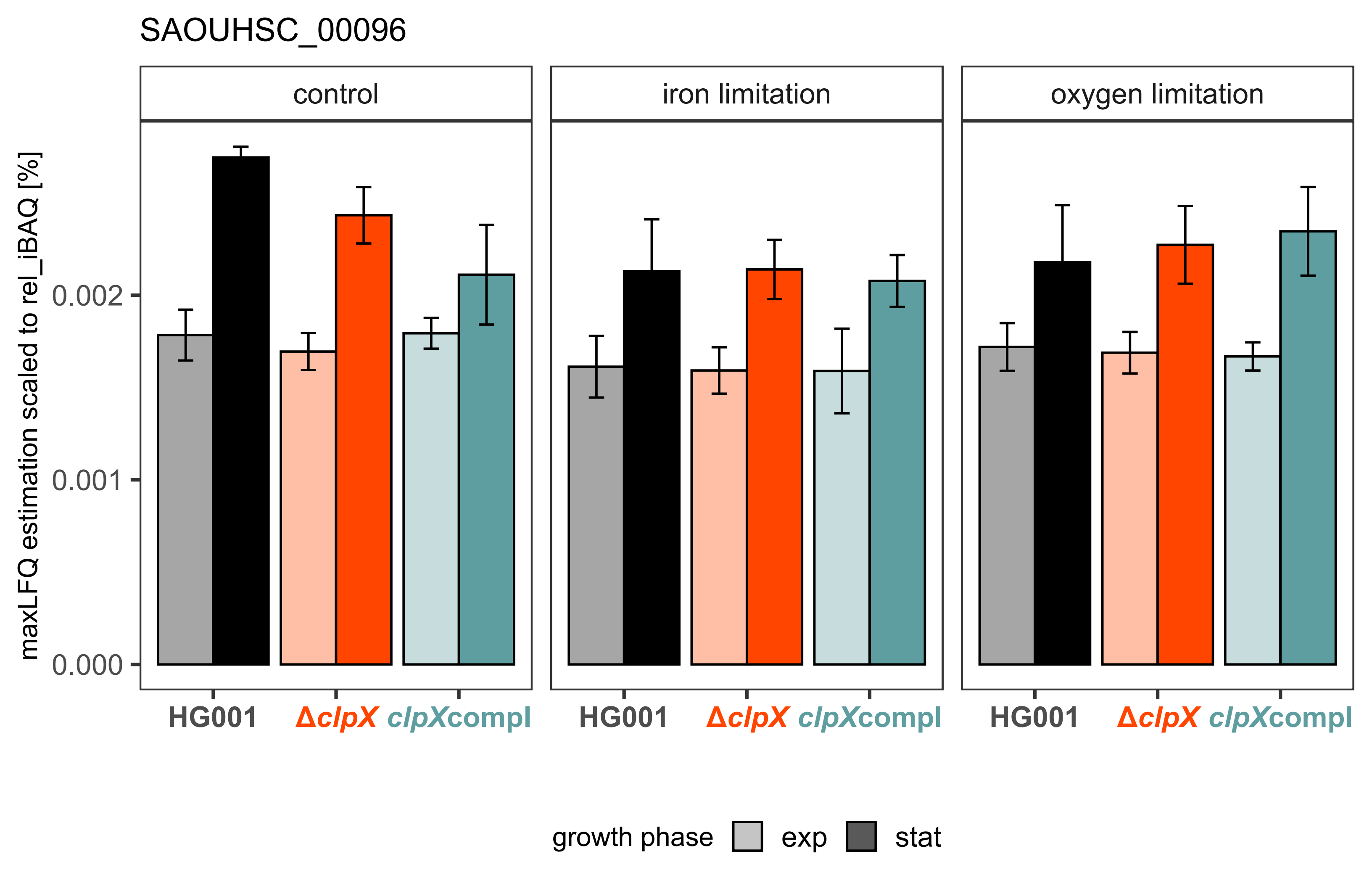
SAOUHSC_00096
control
iron limitation
oxygen limitation
maxLFQ estimation scaled to rel_iBAQ [%]
0.000
0.001
0.002
HG001
ΔclpX
clpXcompl
growth phase
exp
stat

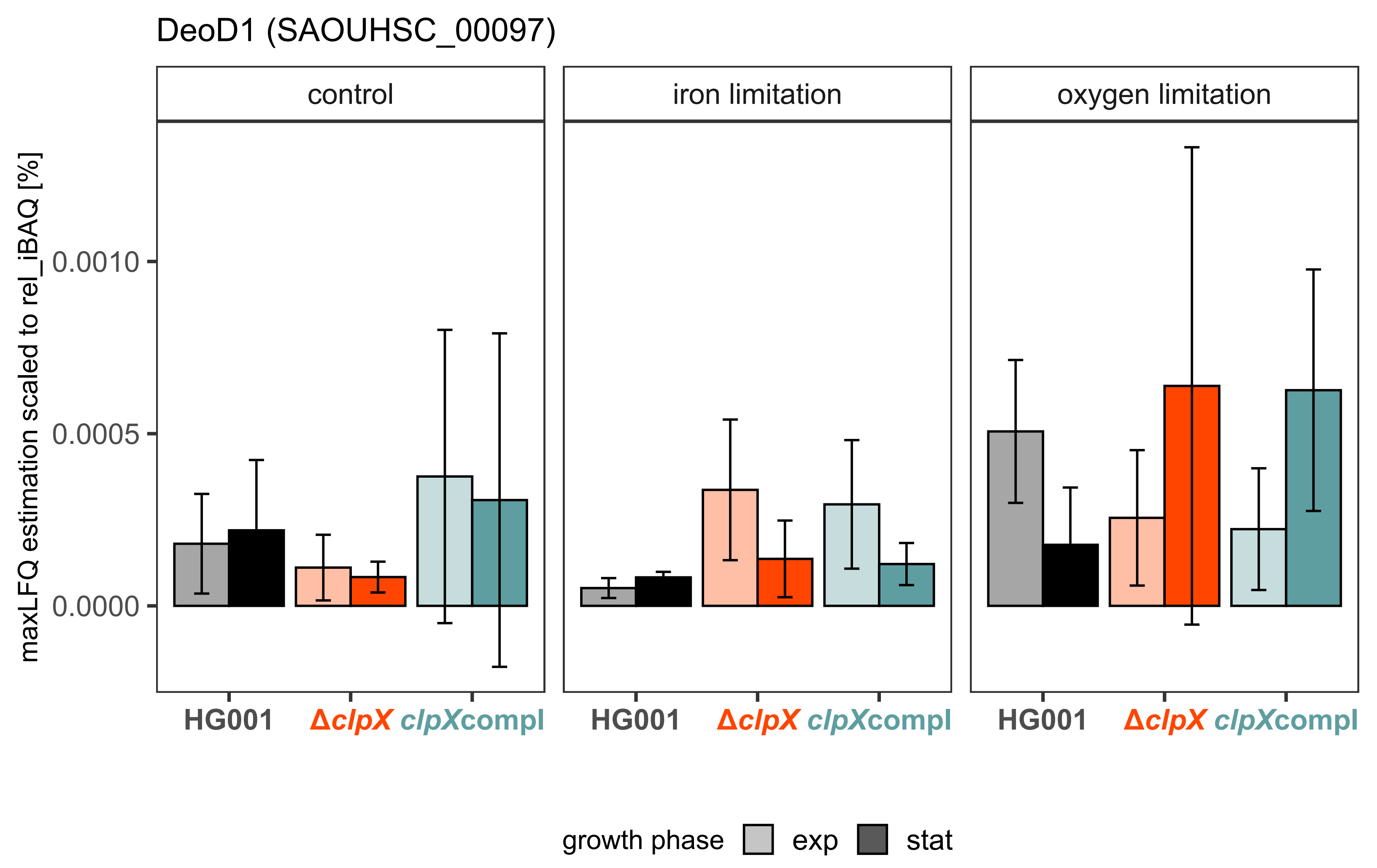
DeoD1 (SAOUHSC_00097)
control
iron limitation
oxygen limitation
maxLFQ estimation scaled to rel_iBAQ [%]
0.0010
0.0005
0.0000
HG001
ΔclpX
clpXcompl
growth phase
exp
stat

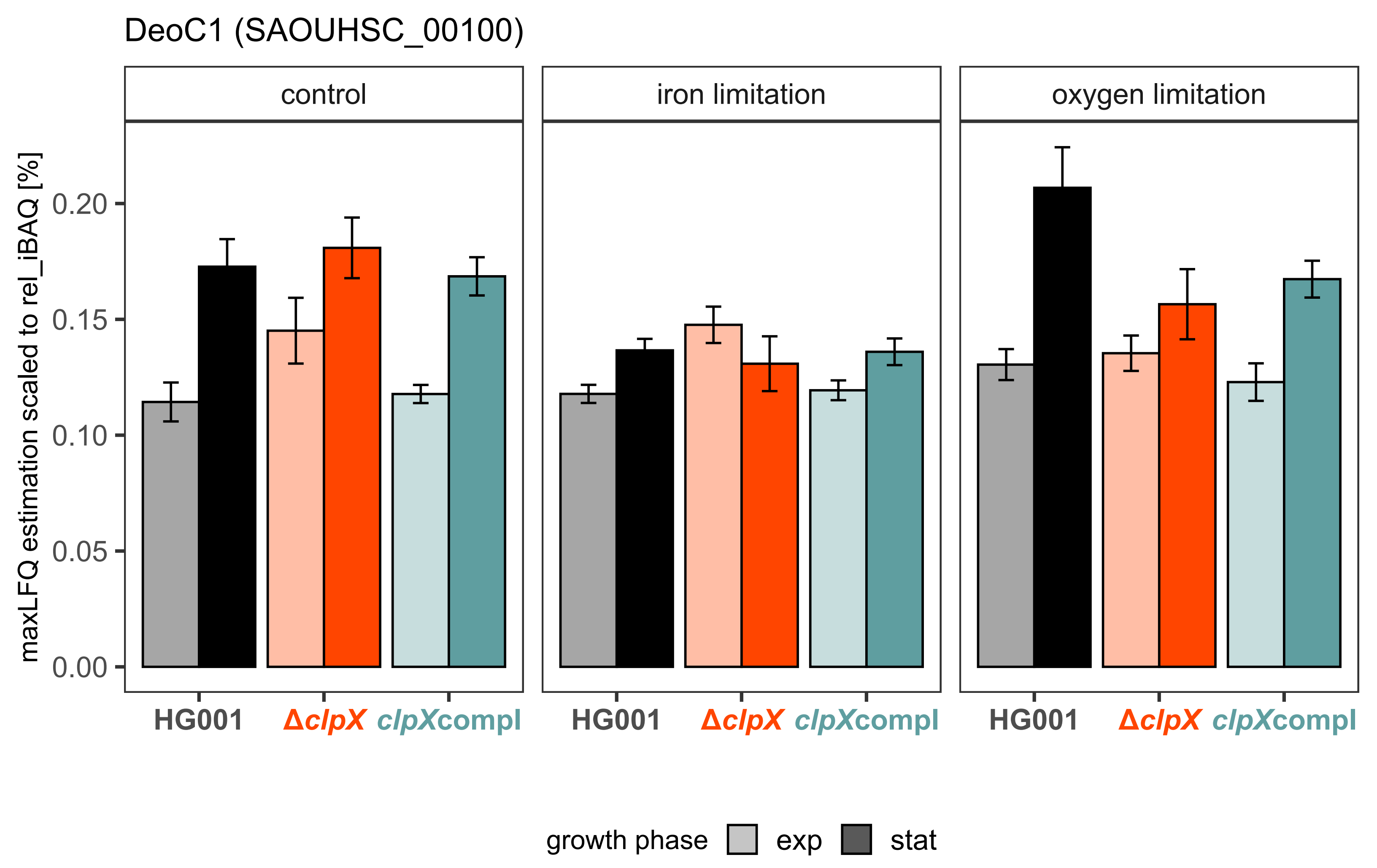
DeoC1 (SAOUHSC_00100)
control
iron limitation
oxygen limitation
maxLFQ estimation scaled to rel_iBAQ [%]
0.00
0.05
0.10
0.15
0.20
HG001
ΔclpX
clpXcompl
growth phase
exp
stat

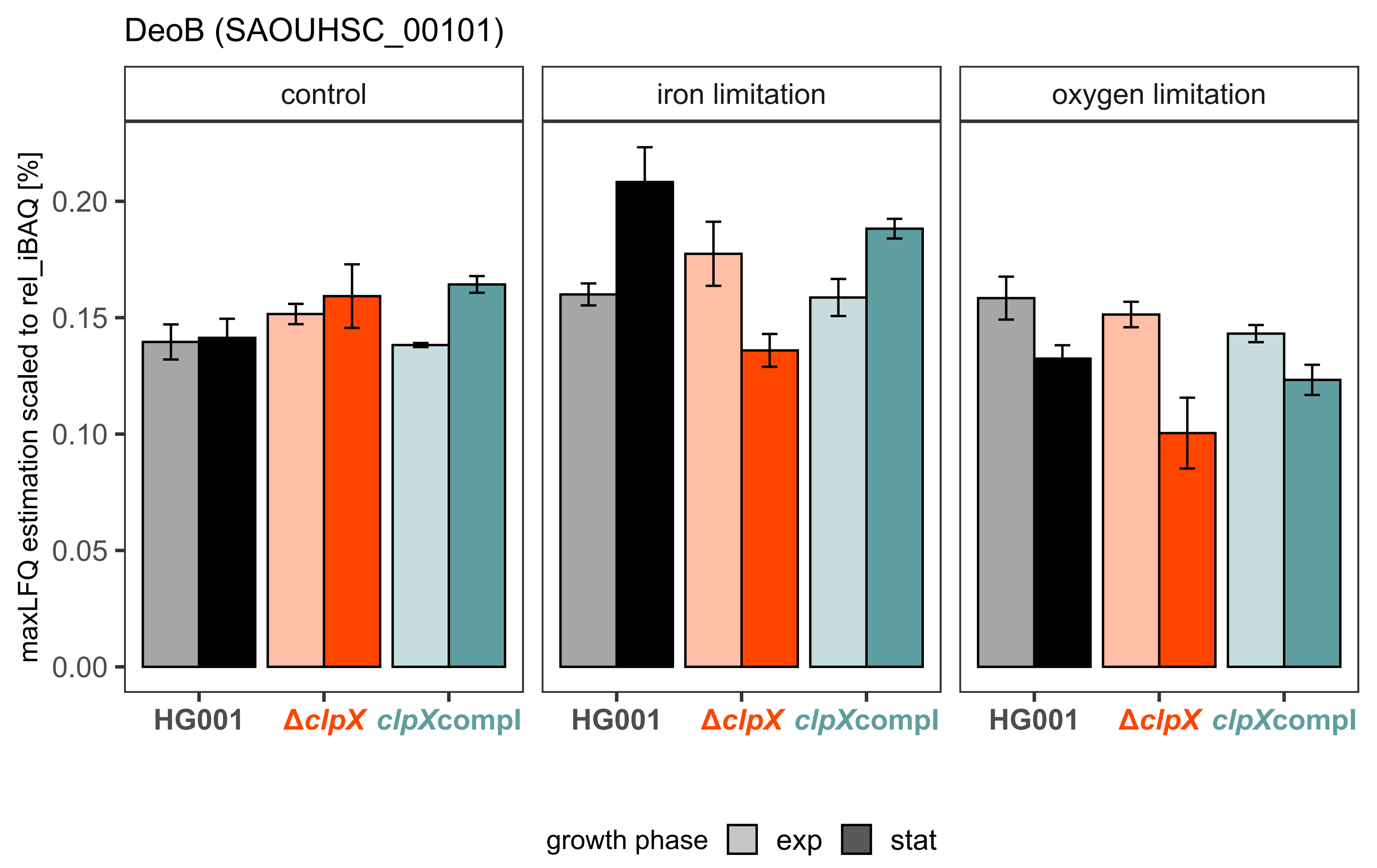
DeoB (SAOUHSC_00101)
control
iron limitation
oxygen limitation
maxLFQ estimation scaled to rel_iBAQ [%]
0.00
0.05
0.10
0.15
0.20
HG001
ΔclpX
clpXcompl
growth phase
exp
stat

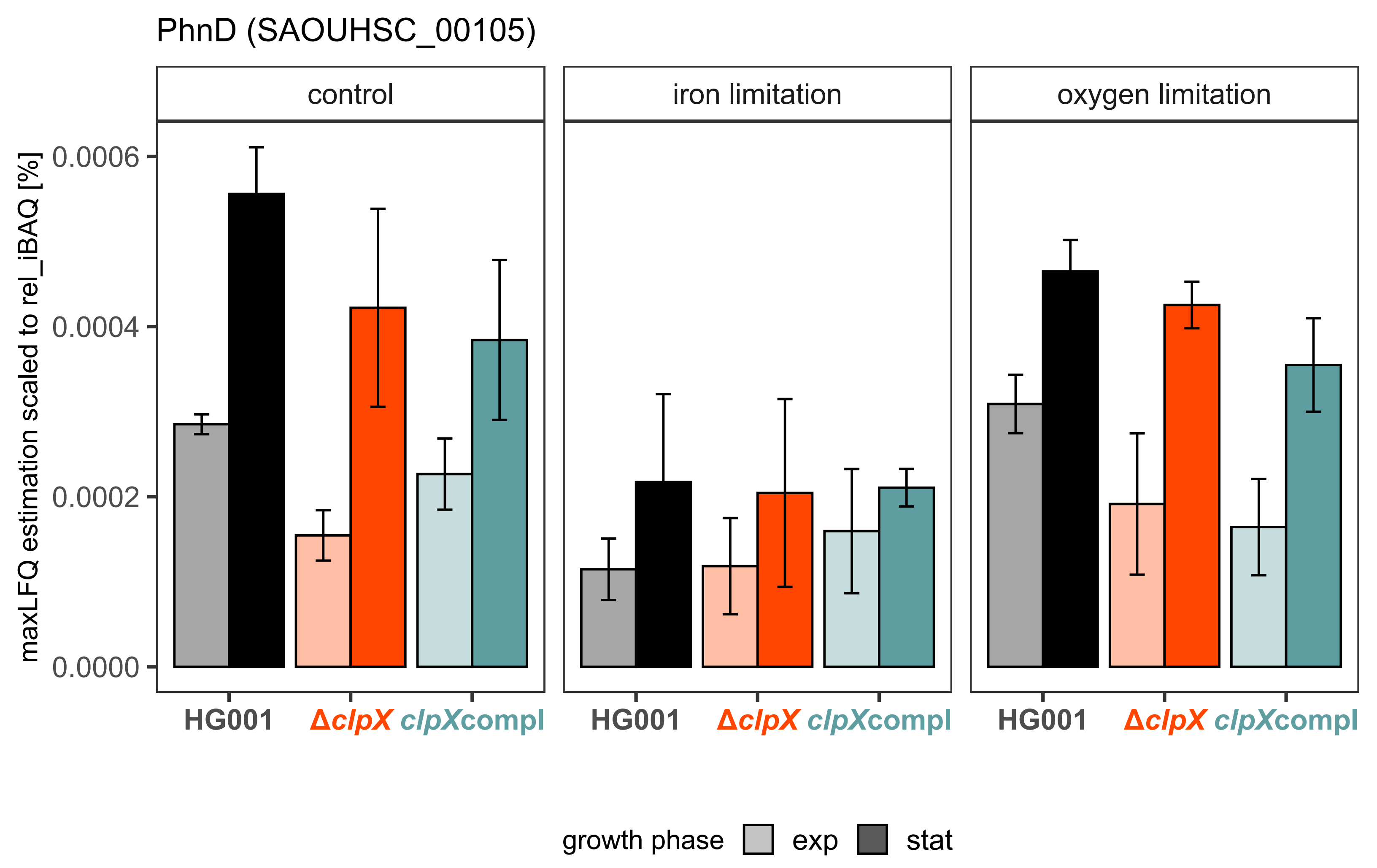

PhnD (SAOUHSC_00105)
control
iron limitation
oxygen limitation
maxLFQ estimation scaled to rel_iBAQ [%]
0.0006
0.0004
0.0002
0.0000
HG001
ΔclpX
clpXcompl
growth phase
exp
stat

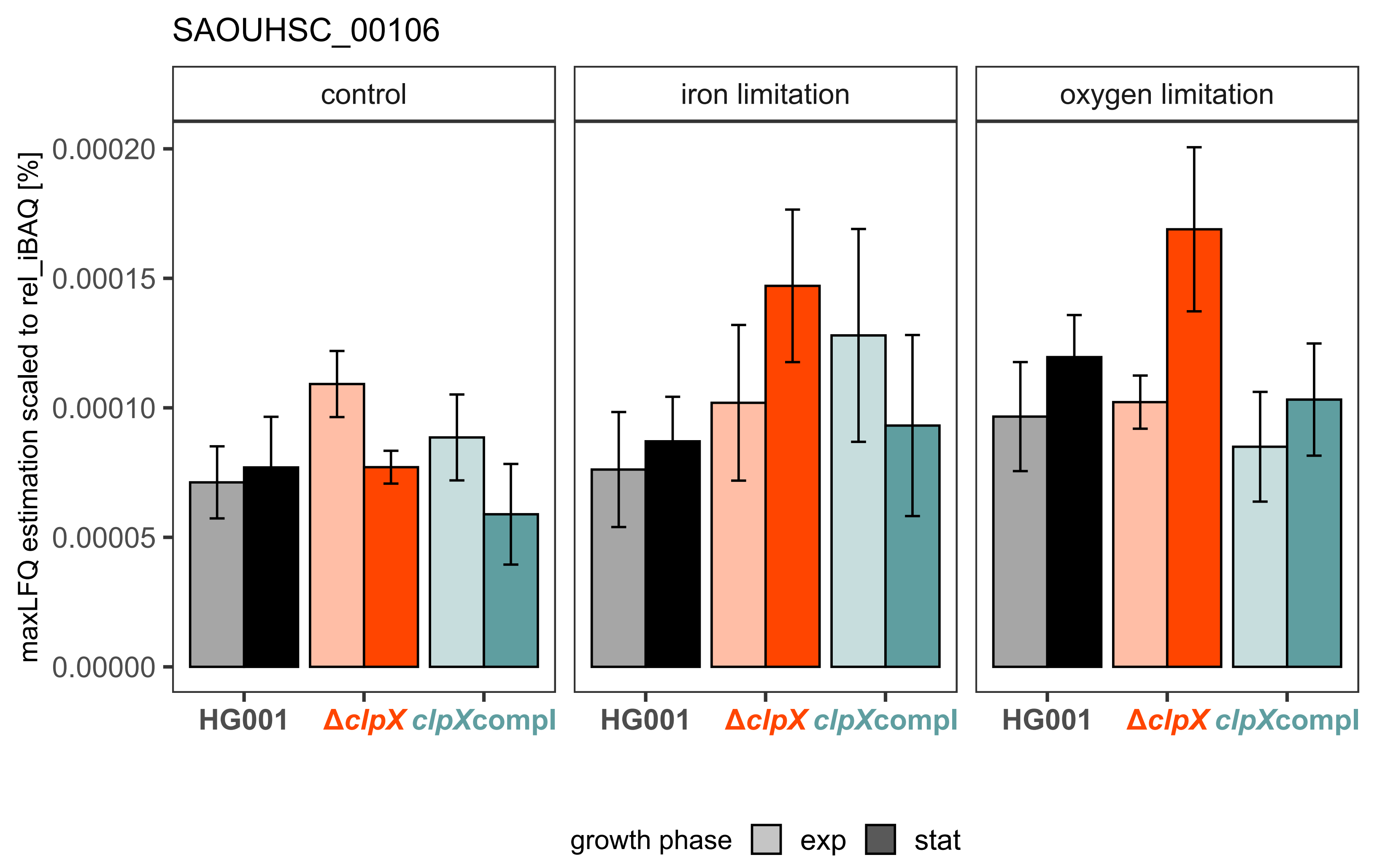

SAOUHSC_00106
control
iron limitation
oxygen limitation
maxLFQ estimation scaled to rel_iBAQ [%]
0.00020
0.00015
0.00010
0.00005
0.00000
HG001
ΔclpX
clpXcompl
growth phase
exp
stat

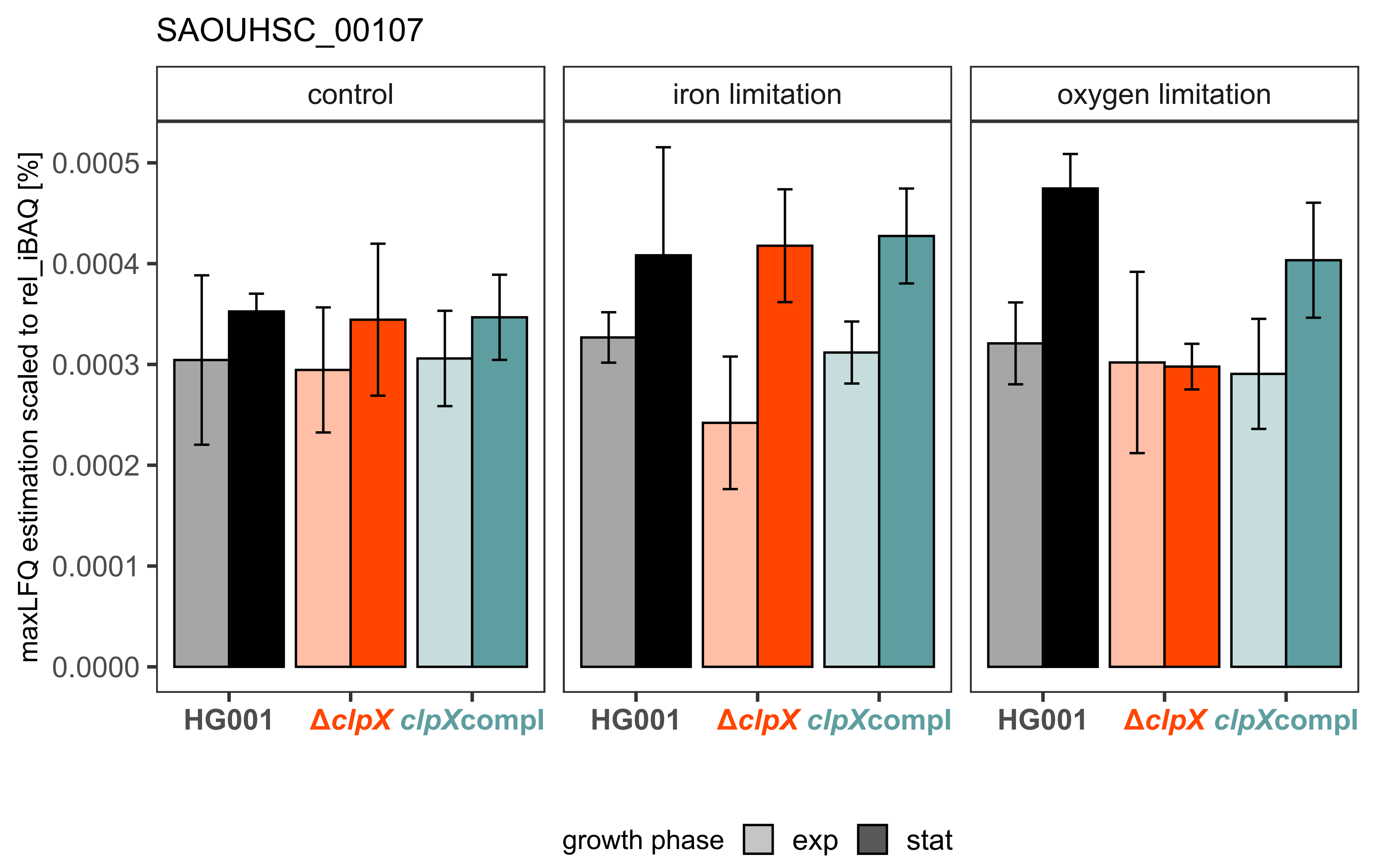

SAOUHSC_00107
control
iron limitation
oxygen limitation
maxLFQ estimation scaled to rel_iBAQ [%]
0.0005
0.0004
0.0003
0.0002
0.0001
0.0000
HG001
ΔclpX
clpXcompl
growth phase
exp
stat

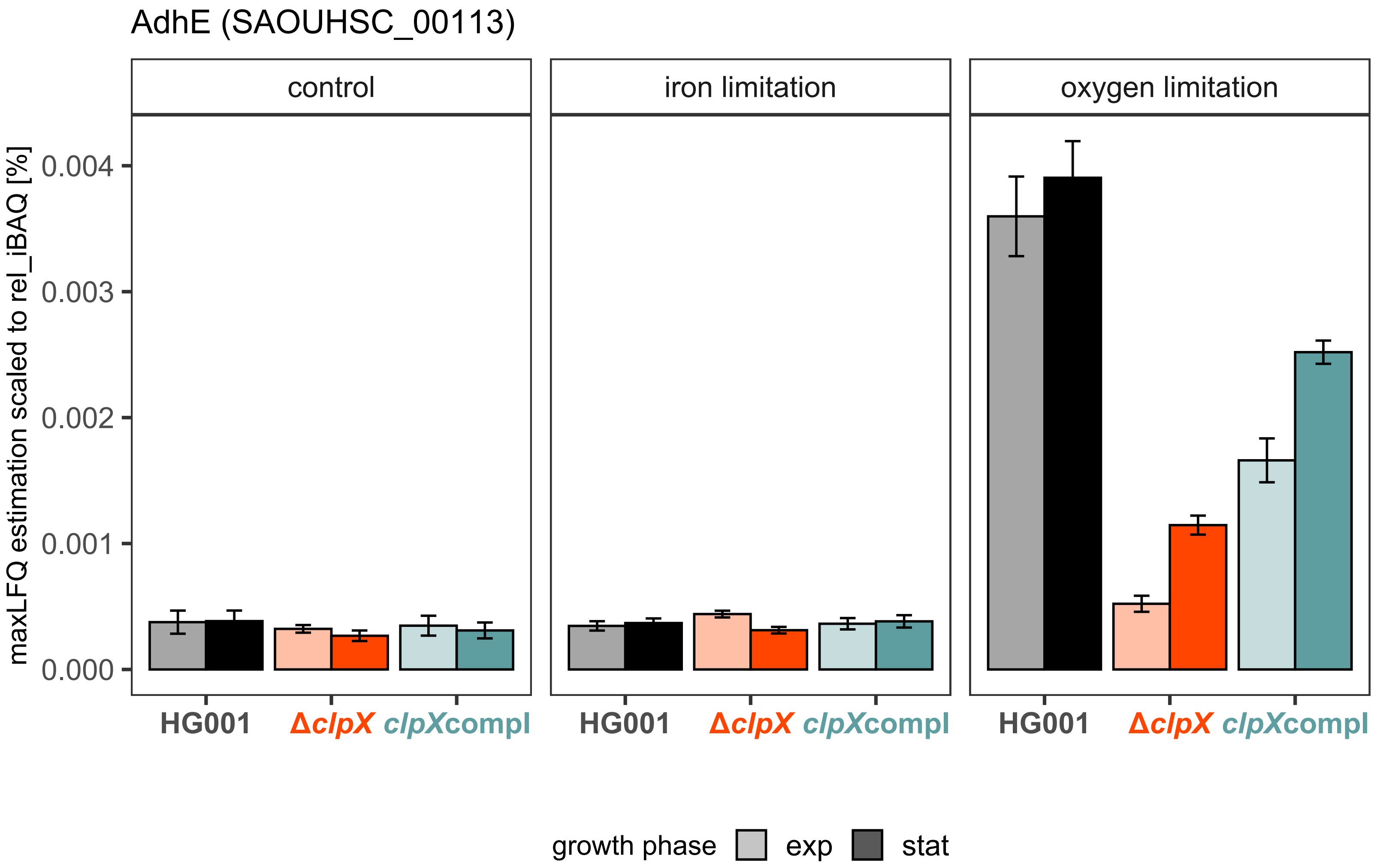
AdhE (SAOUHSC_00113)
control
iron limitation
oxygen limitation
maxLFQ estimation scaled to rel_iBAQ [%]
0.004
0.003
0.002
0.001
0.000
HG001
ΔclpX
clpXcompl
growth phase
exp
stat

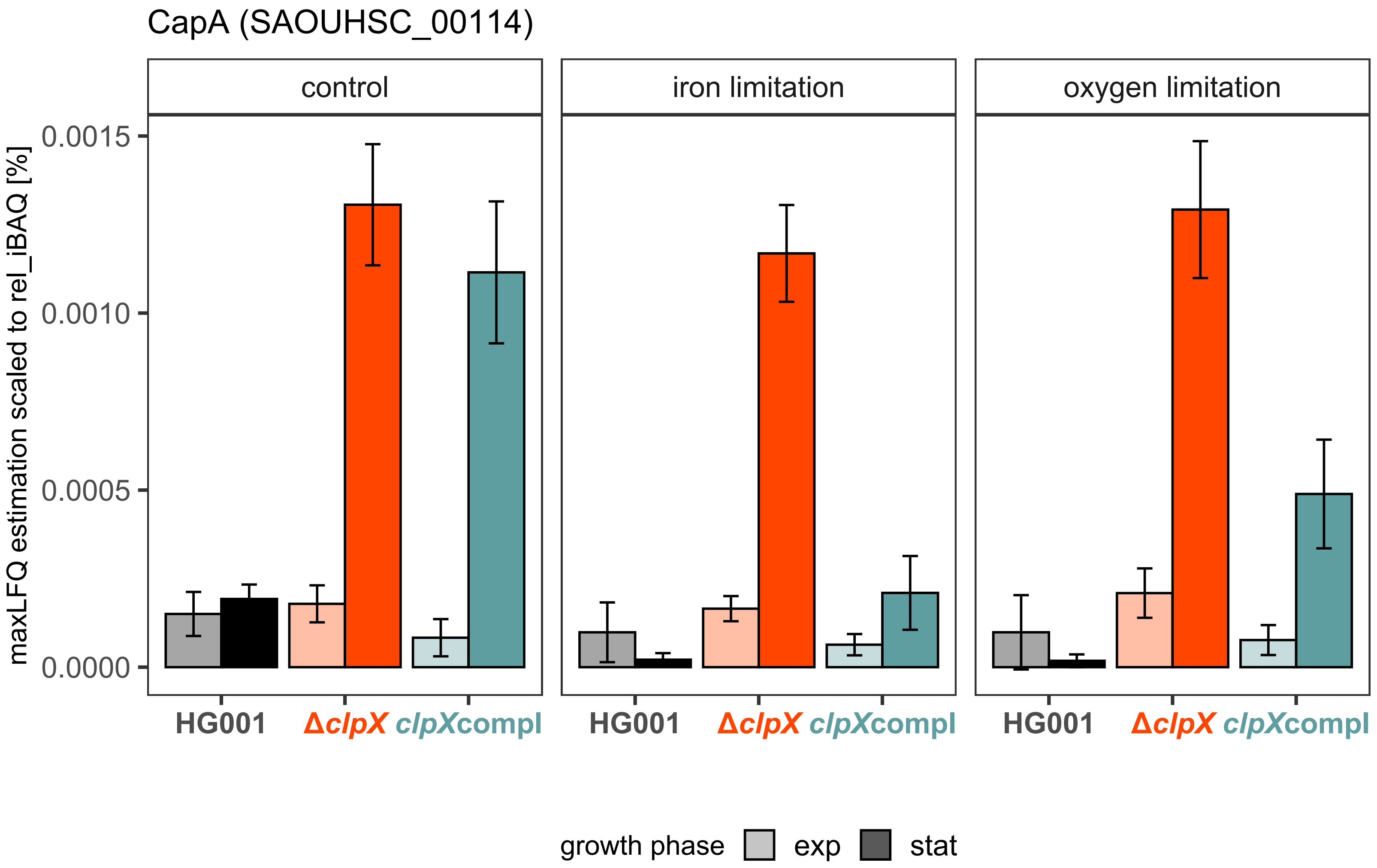

CapA (SAOUHSC_00114)
control
iron limitation
oxygen limitation
maxLFQ estimation scaled to rel_iBAQ [%]
0.0015
0.0010
0.0005
0.0000
HG001
ΔclpX
clpXcompl
growth phase
exp
stat

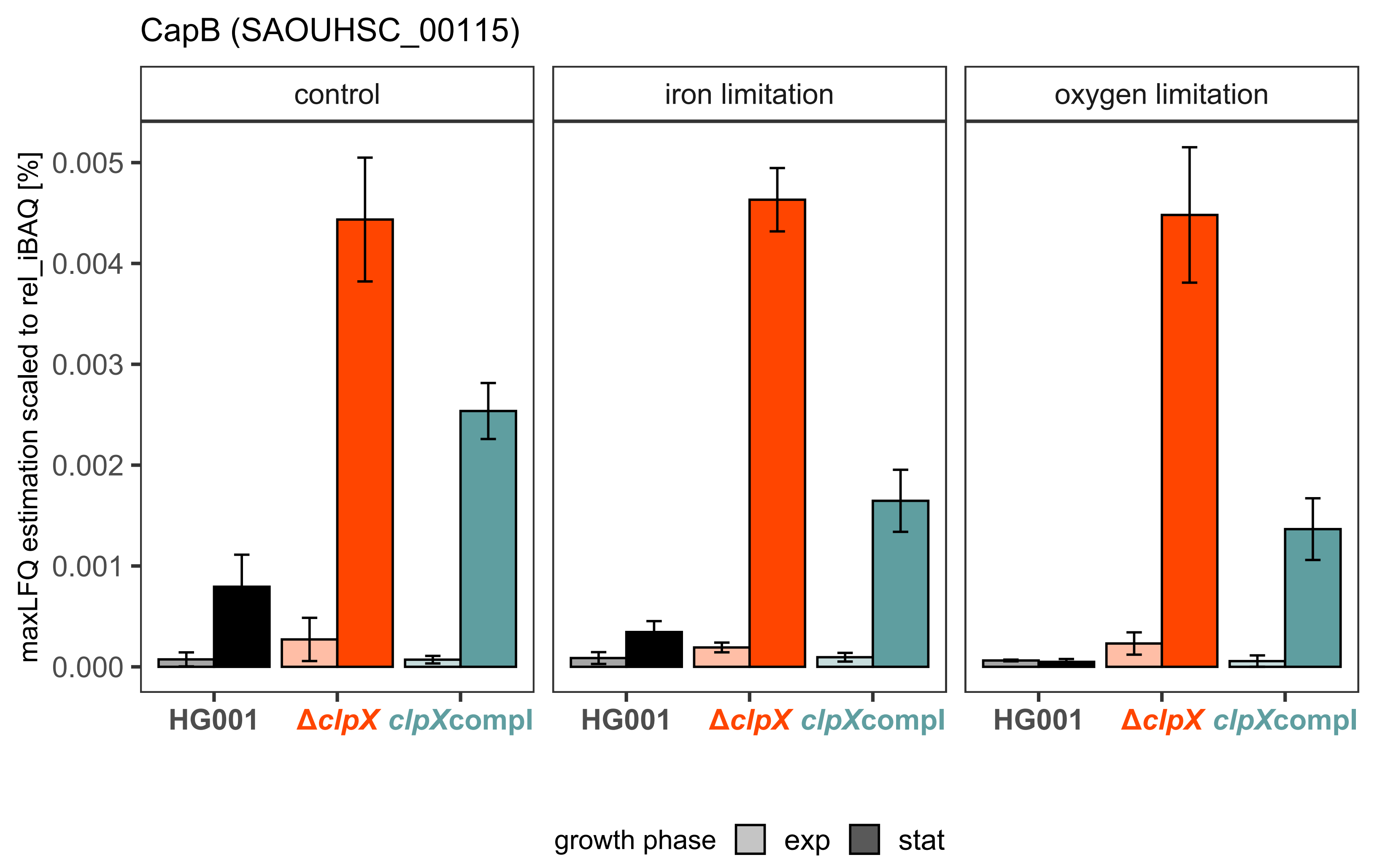

CapB (SAOUHSC_00115)
control
iron limitation
oxygen limitation
maxLFQ estimation scaled to rel_iBAQ [%]
0.005
0.004
0.003
0.002
0.001
0.000
HG001
ΔclpX
clpXcompl
growth phase
exp
stat

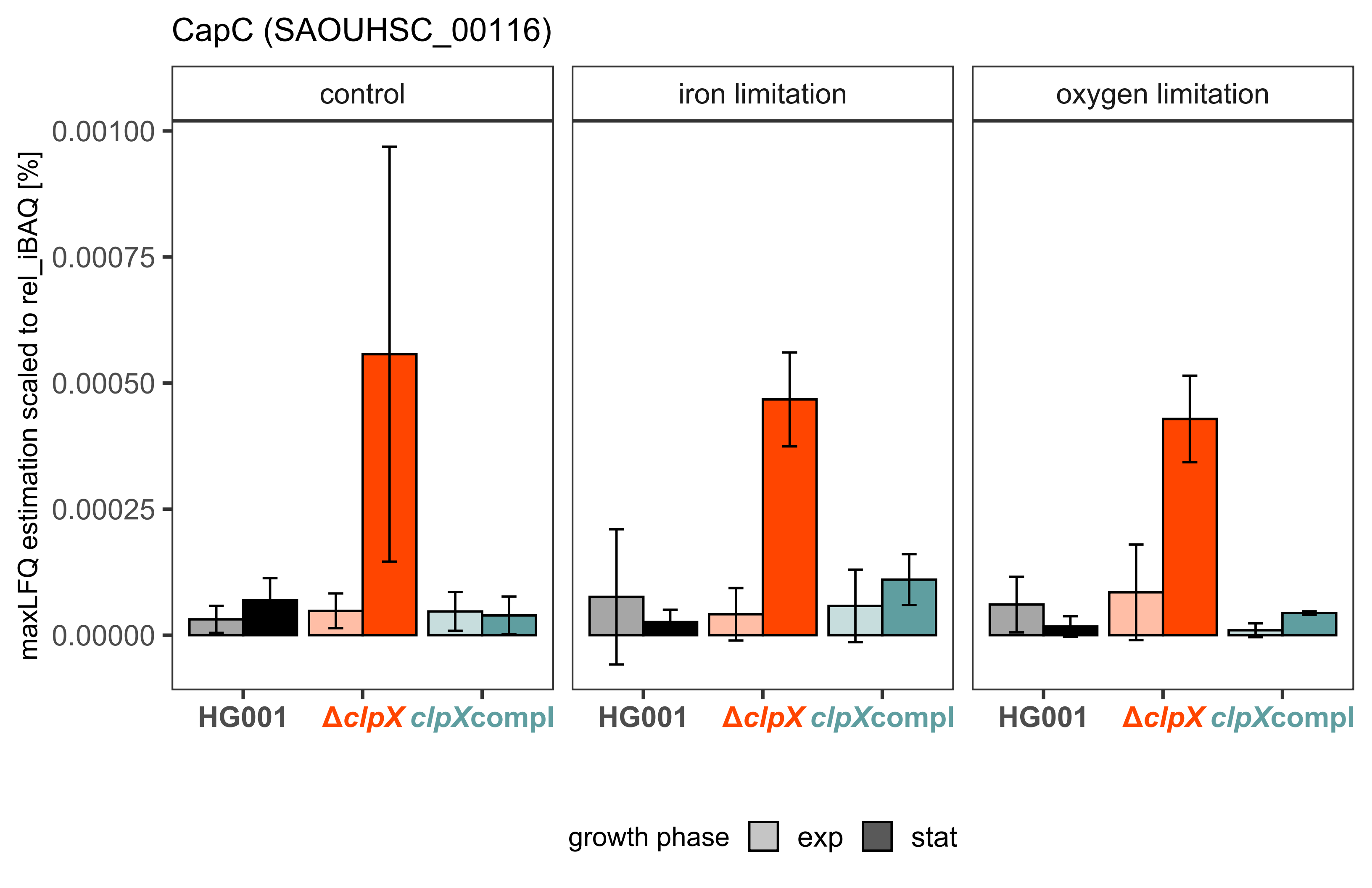
CapC (SAOUHSC_00116)
control
iron limitation
oxygen limitation
maxLFQ estimation scaled to rel_iBAQ [%]
0.00100
0.00075
0.00050
0.00025
0.00000
HG001
ΔclpX
clpXcompl
growth phase
exp
stat

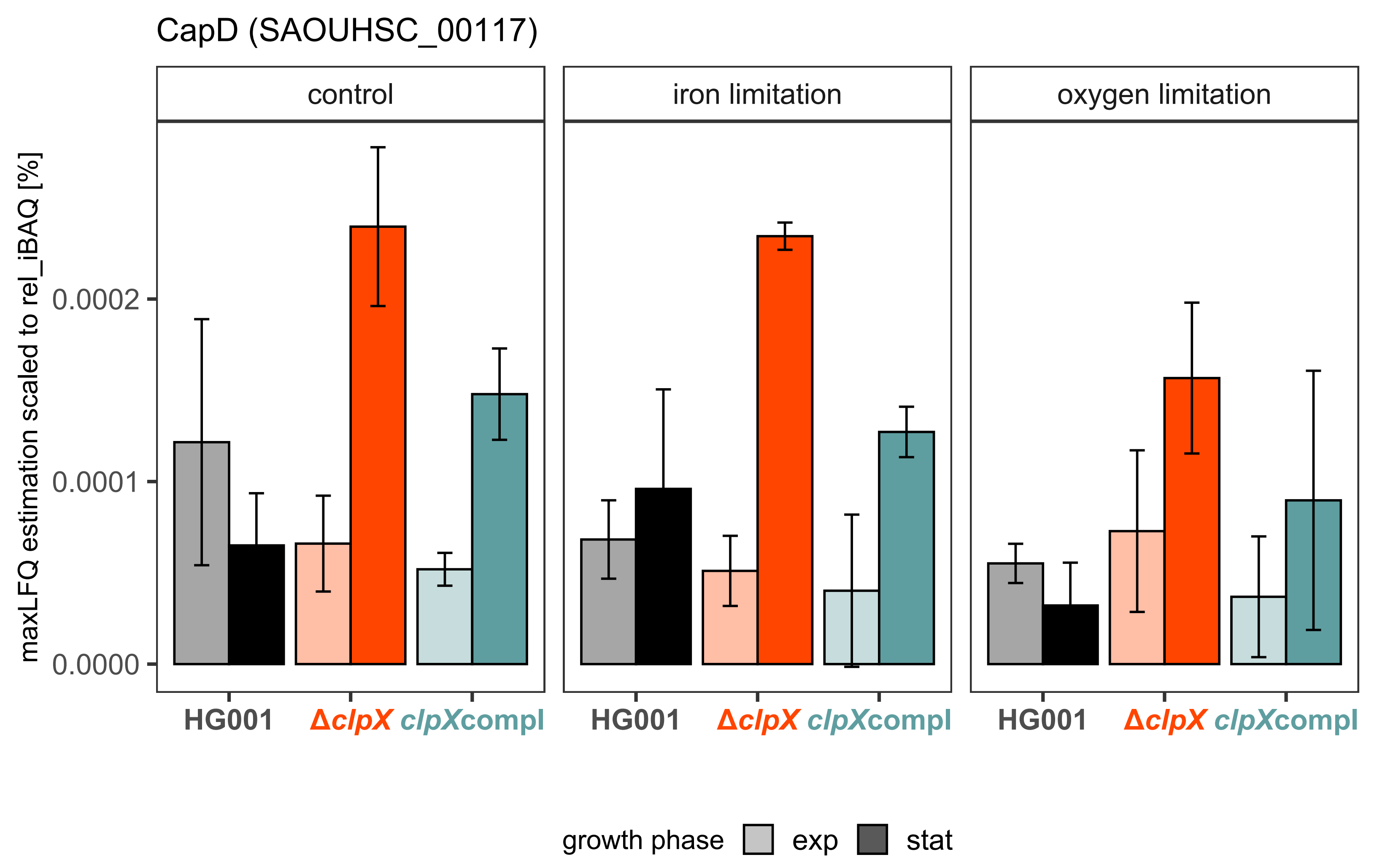
CapD (SAOUHSC_00117)
control
iron limitation
oxygen limitation
maxLFQ estimation scaled to rel_iBAQ [%]
0.0000
0.0001
0.0002
HG001
ΔclpX
clpXcompl
growth phase
exp
stat

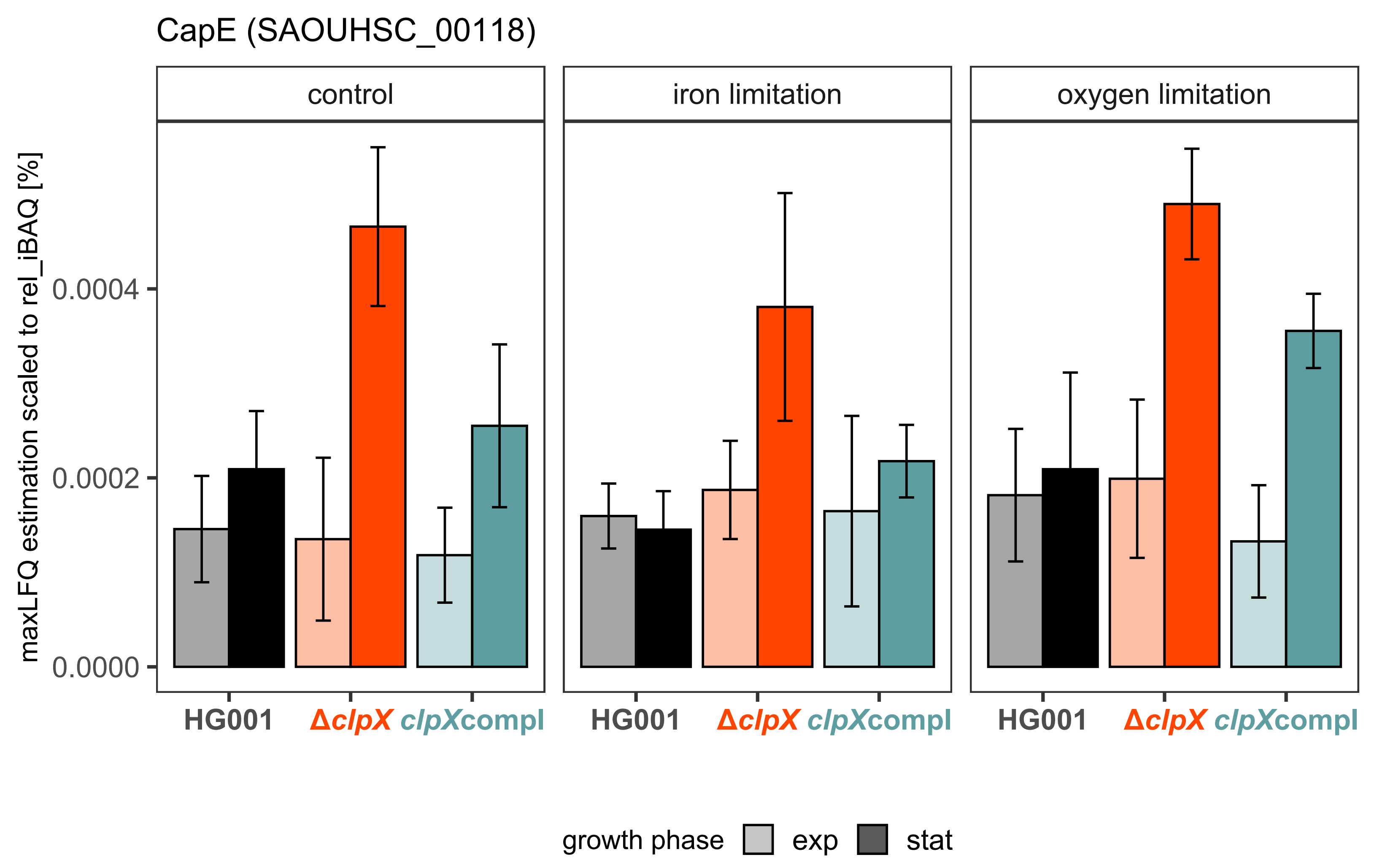
CapE (SAOUHSC_00118)
control
iron limitation
oxygen limitation
maxLFQ estimation scaled to rel_iBAQ [%]
0.0004
0.0002
0.0000
HG001
ΔclpX
clpXcompl
growth phase
exp
stat

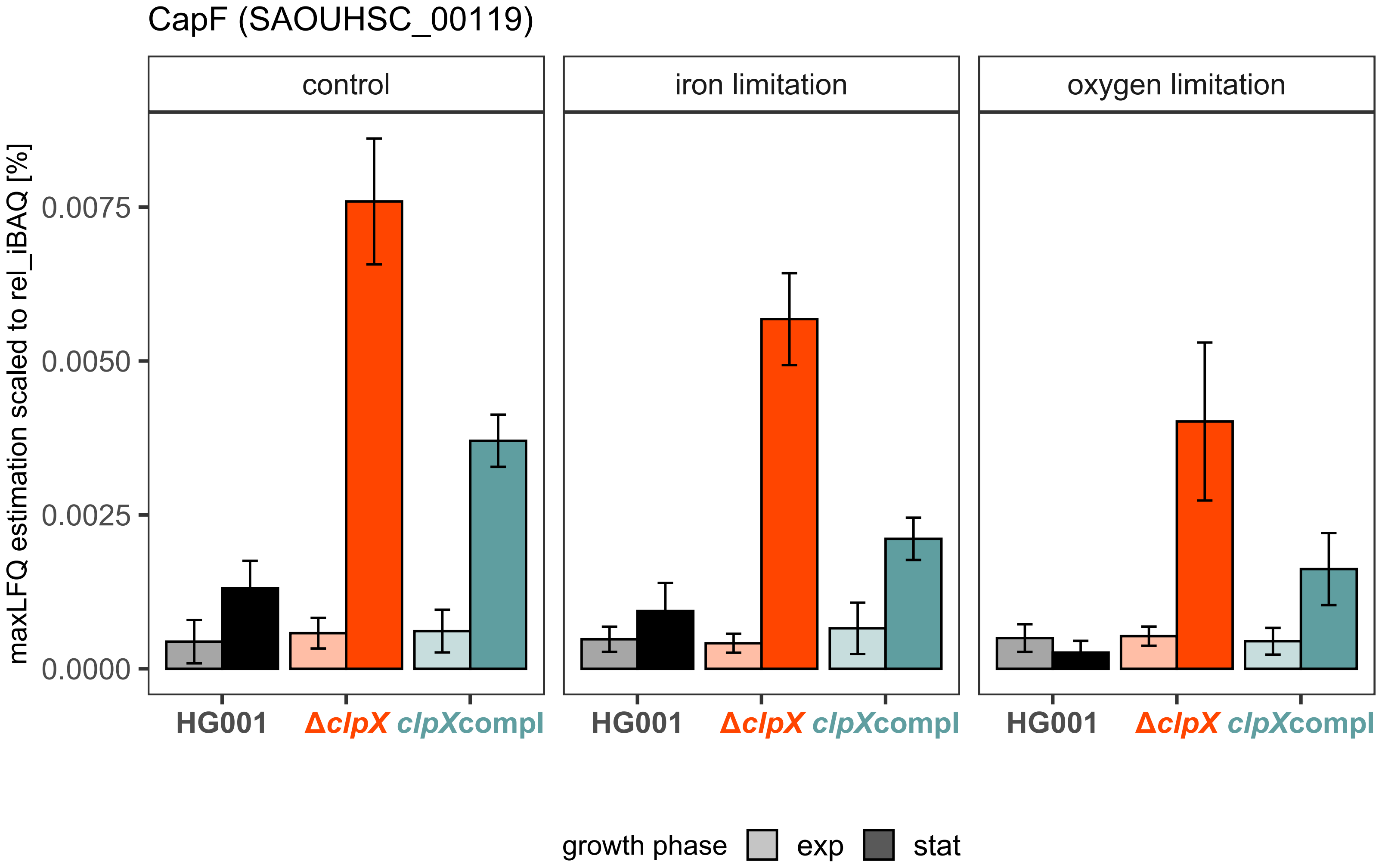
CapF (SAOUHSC_00119)
control
iron limitation
oxygen limitation
maxLFQ estimation scaled to rel_iBAQ [%]
0.0075
0.0050
0.0025
0.0000
HG001
ΔclpX
clpXcompl
growth phase
exp
stat

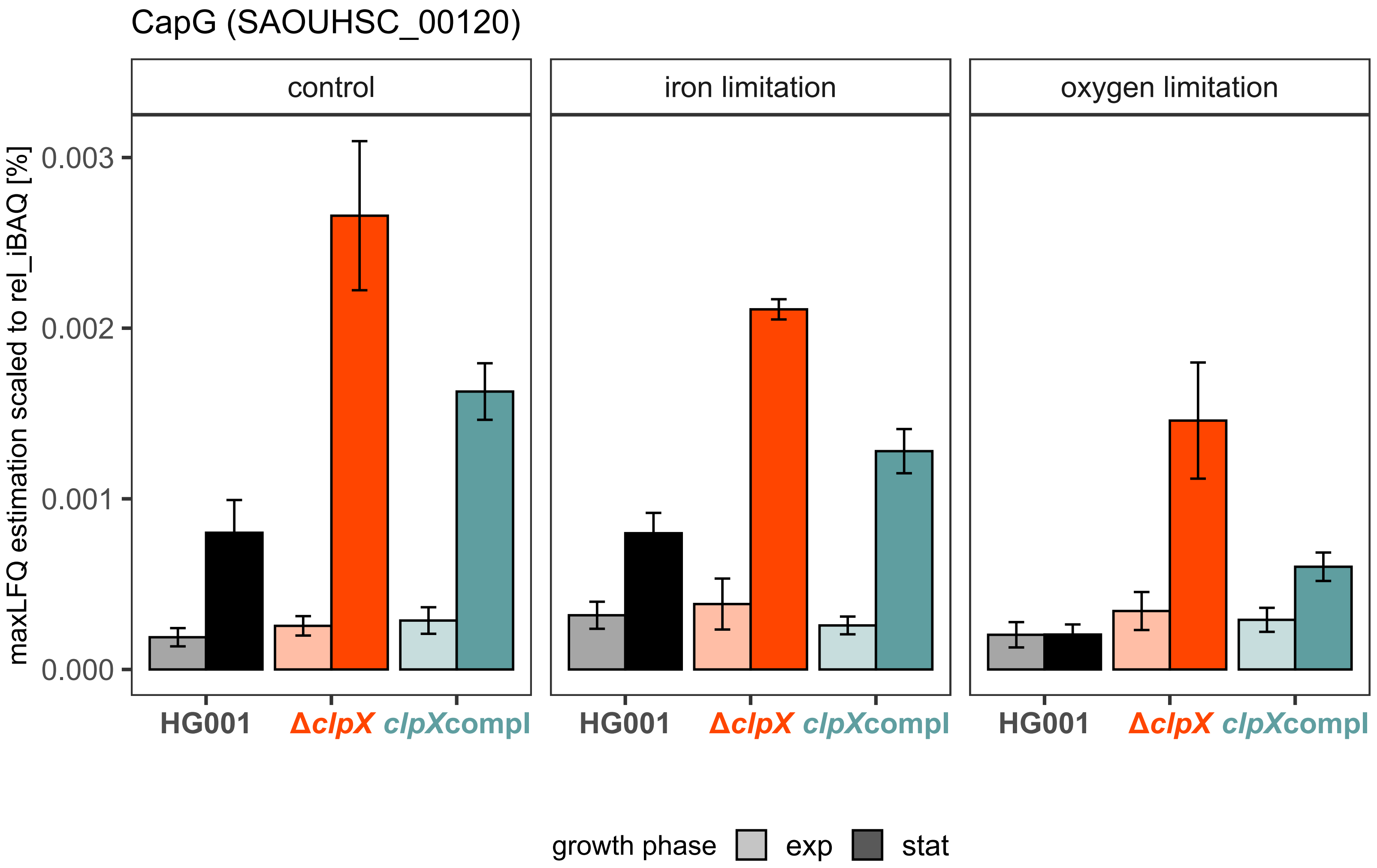
CapG (SAOUHSC_00120)
control
iron limitation
oxygen limitation
maxLFQ estimation scaled to rel_iBAQ [%]
0.003
0.002
0.001
0.000
HG001
ΔclpX
clpXcompl
growth phase
exp
stat

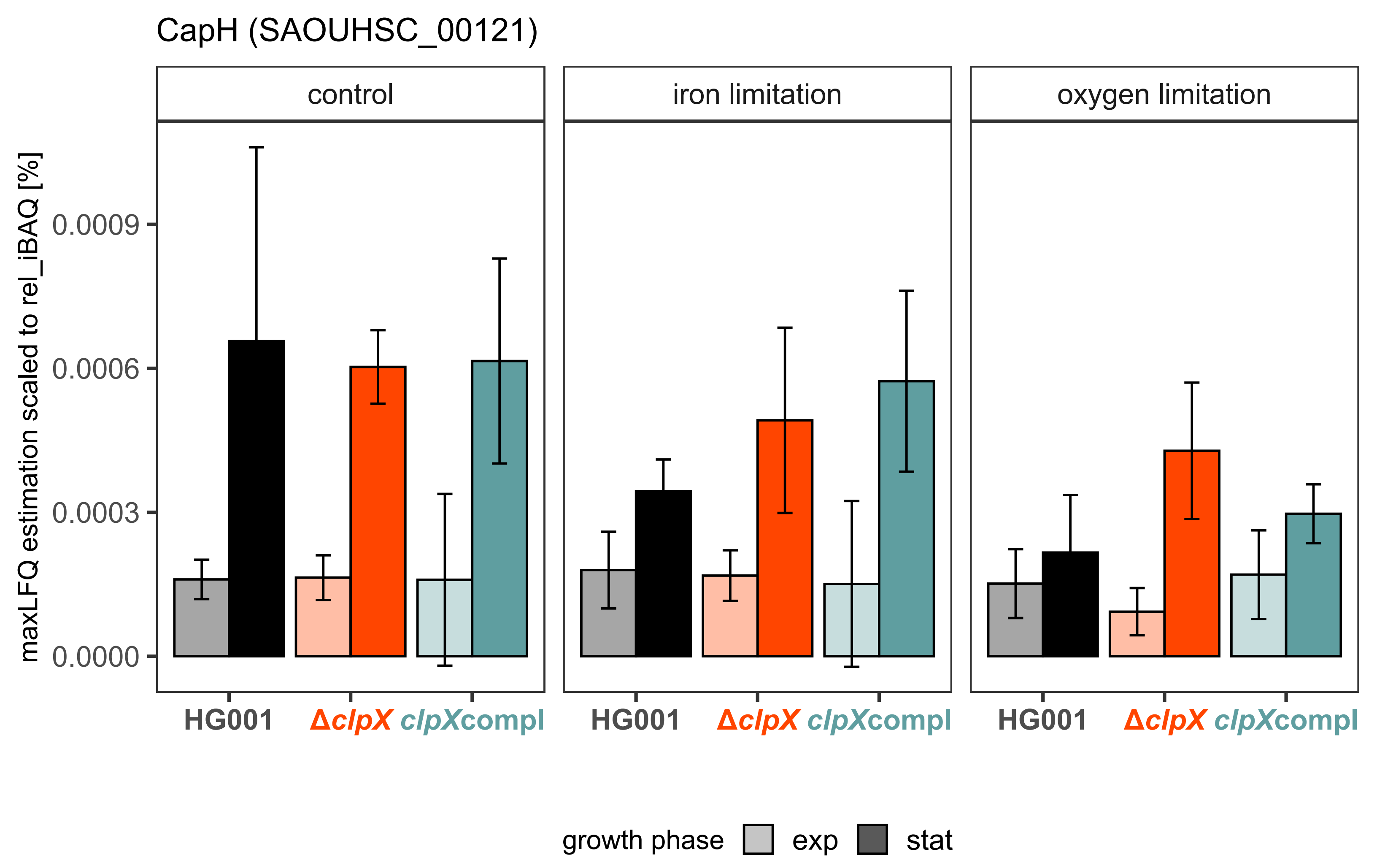

CapH (SAOUHSC_00121)
control
iron limitation
oxygen limitation
maxLFQ estimation scaled to rel_iBAQ [%]
0.0009
0.0006
0.0003
0.0000
HG001
ΔclpX
clpXcompl
growth phase
exp
stat

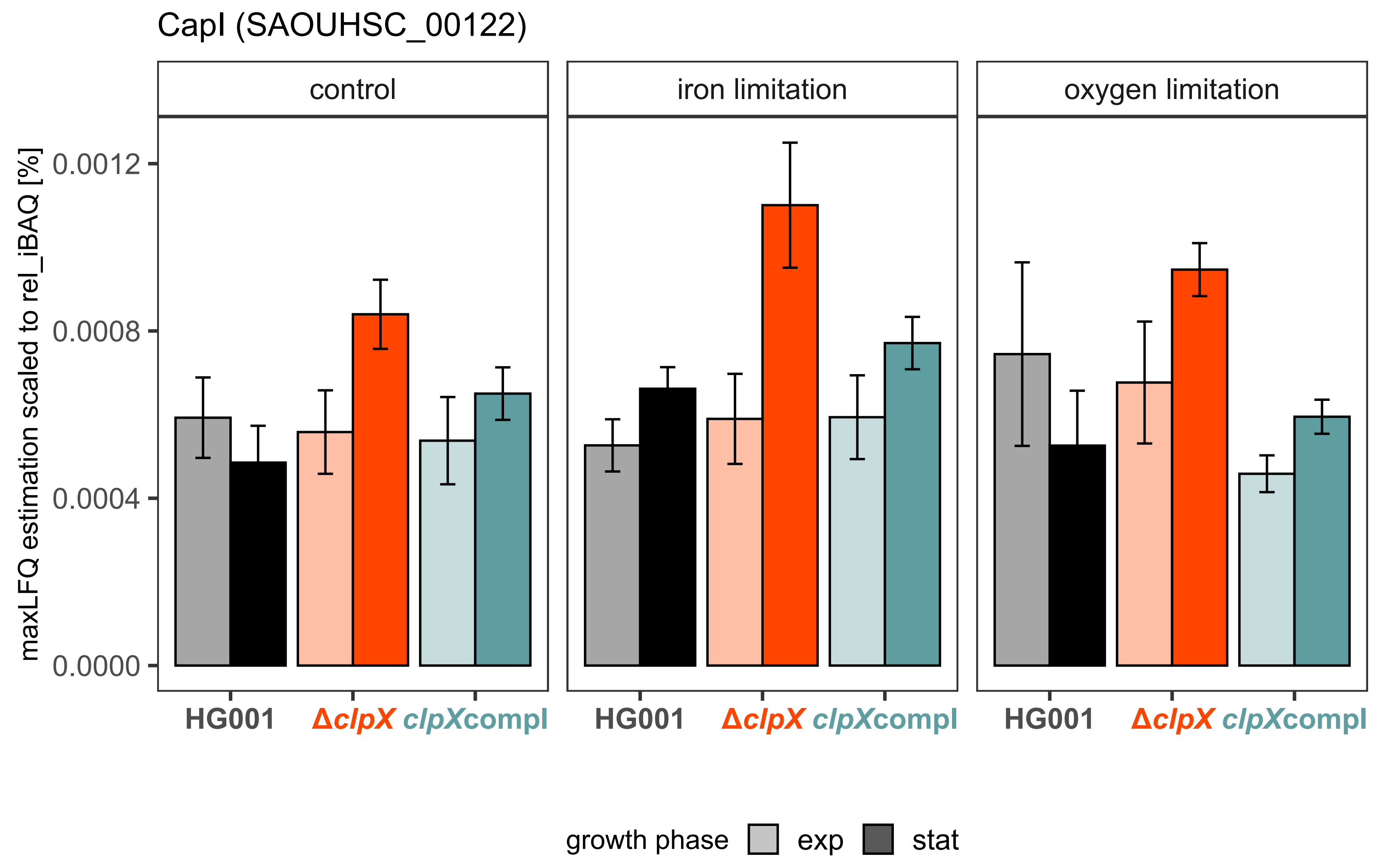
CapI (SAOUHSC_00122)
control
iron limitation
oxygen limitation
maxLFQ estimation scaled to rel_iBAQ [%]
0.0012
0.0008
0.0004
0.0000
HG001
ΔclpX
clpXcompl
growth phase
exp
stat

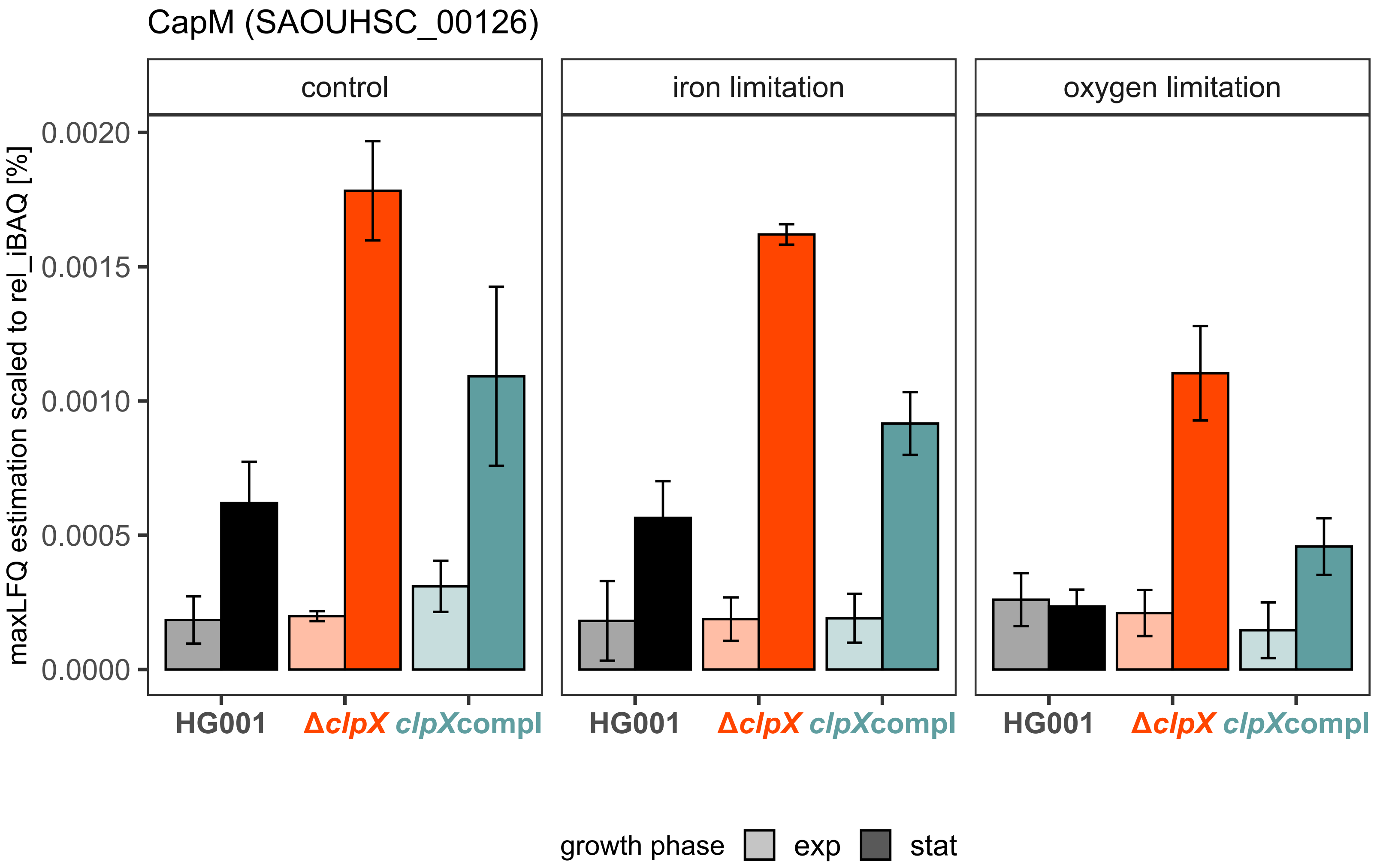

CapM (SAOUHSC_00126)
control
iron limitation
oxygen limitation
maxLFQ estimation scaled to rel_iBAQ [%]
0.0020
0.0015
0.0010
0.0005
0.0000
HG001
ΔclpX
clpXcompl
growth phase
exp
stat

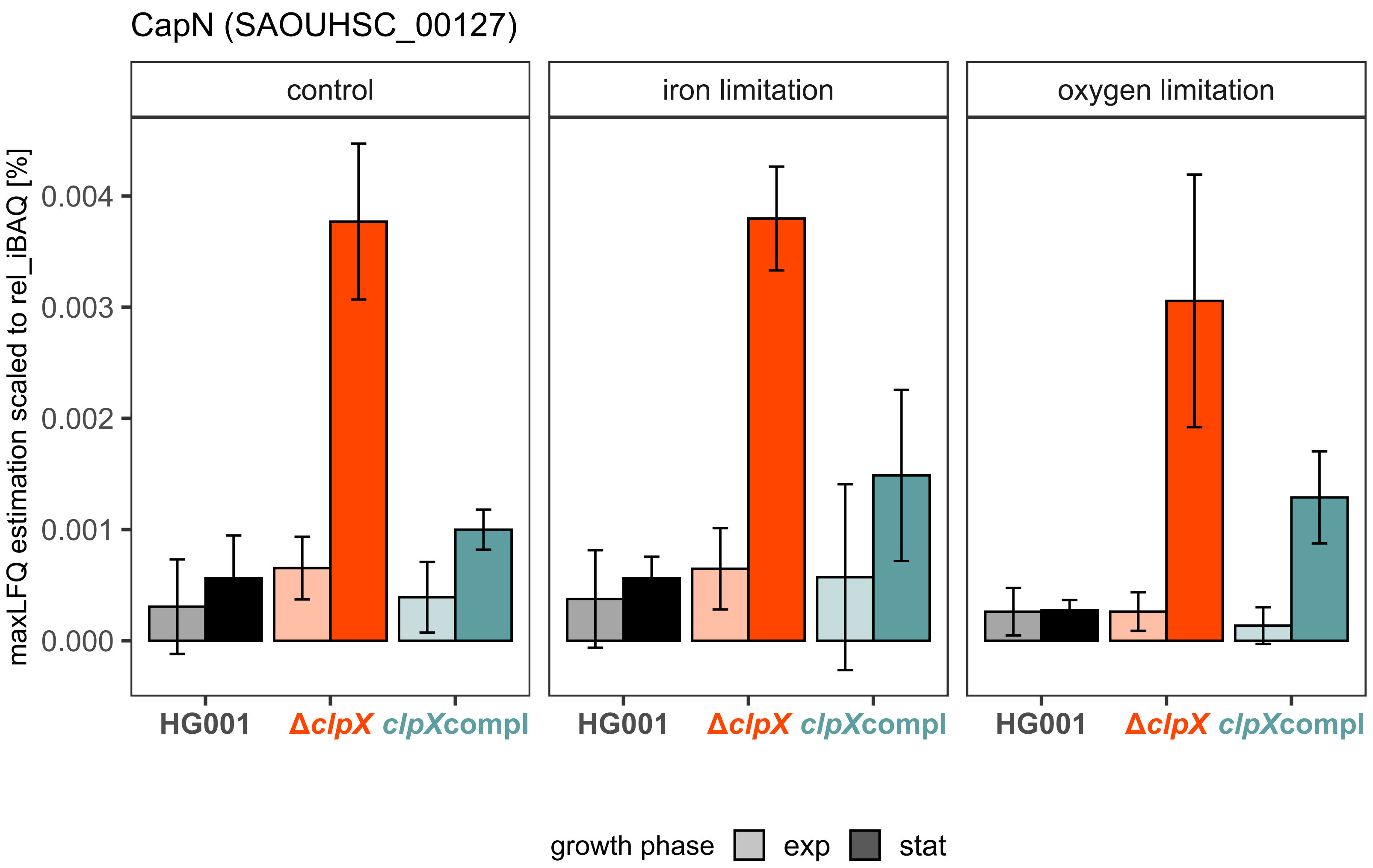
CapN (SAOUHSC_00127)
control
iron limitation
oxygen limitation
maxLFQ estimation scaled to rel_iBAQ [%]
0.004
0.003
0.002
0.001
0.000
HG001
ΔclpX
clpXcompl
growth phase
exp
stat

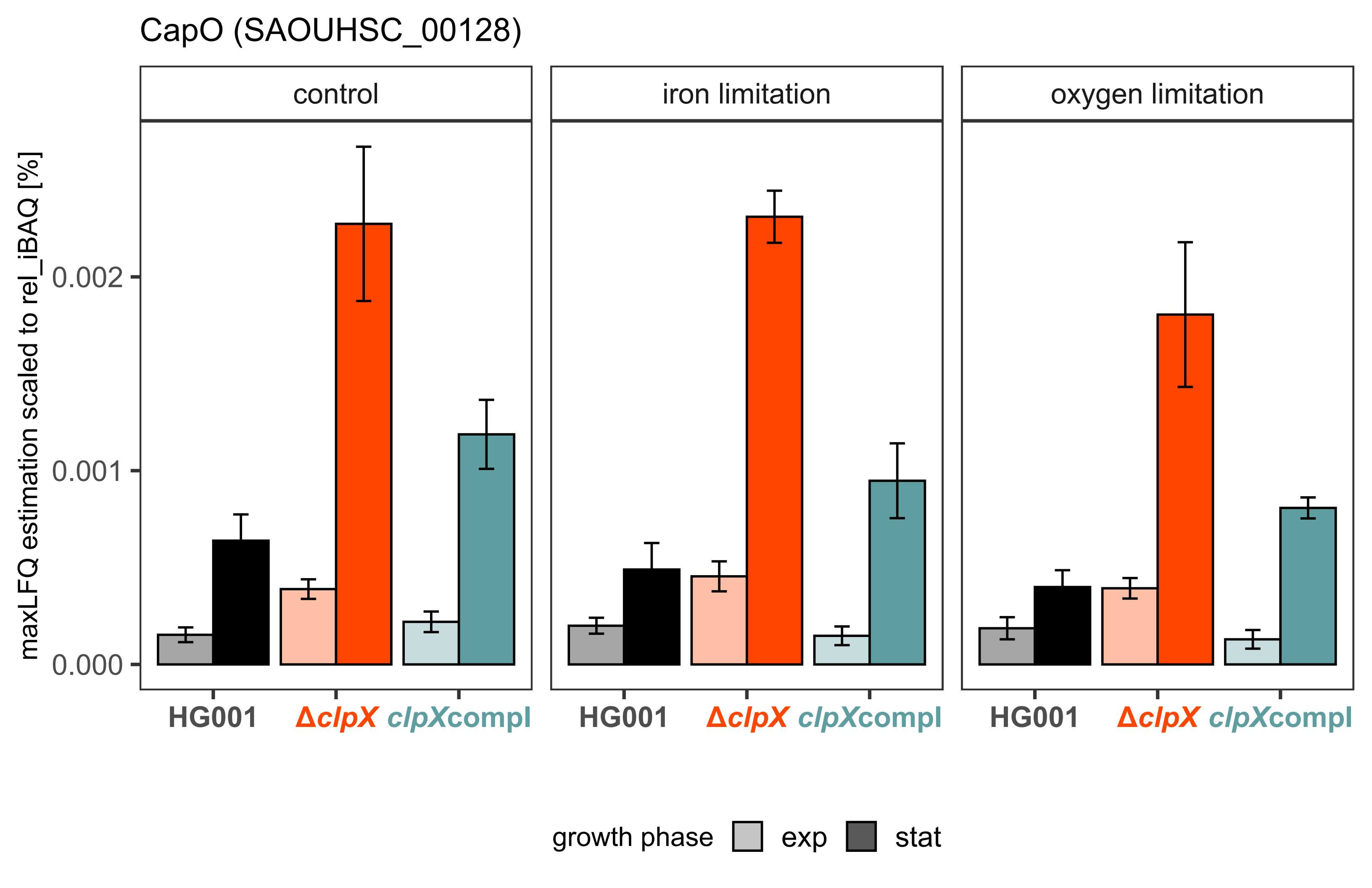

CapO (SAOUHSC_00128)
control
iron limitation
oxygen limitation
maxLFQ estimation scaled to rel_iBAQ [%]
0.000
0.001
0.002
HG001
ΔclpX
clpXcompl
growth phase
exp
stat

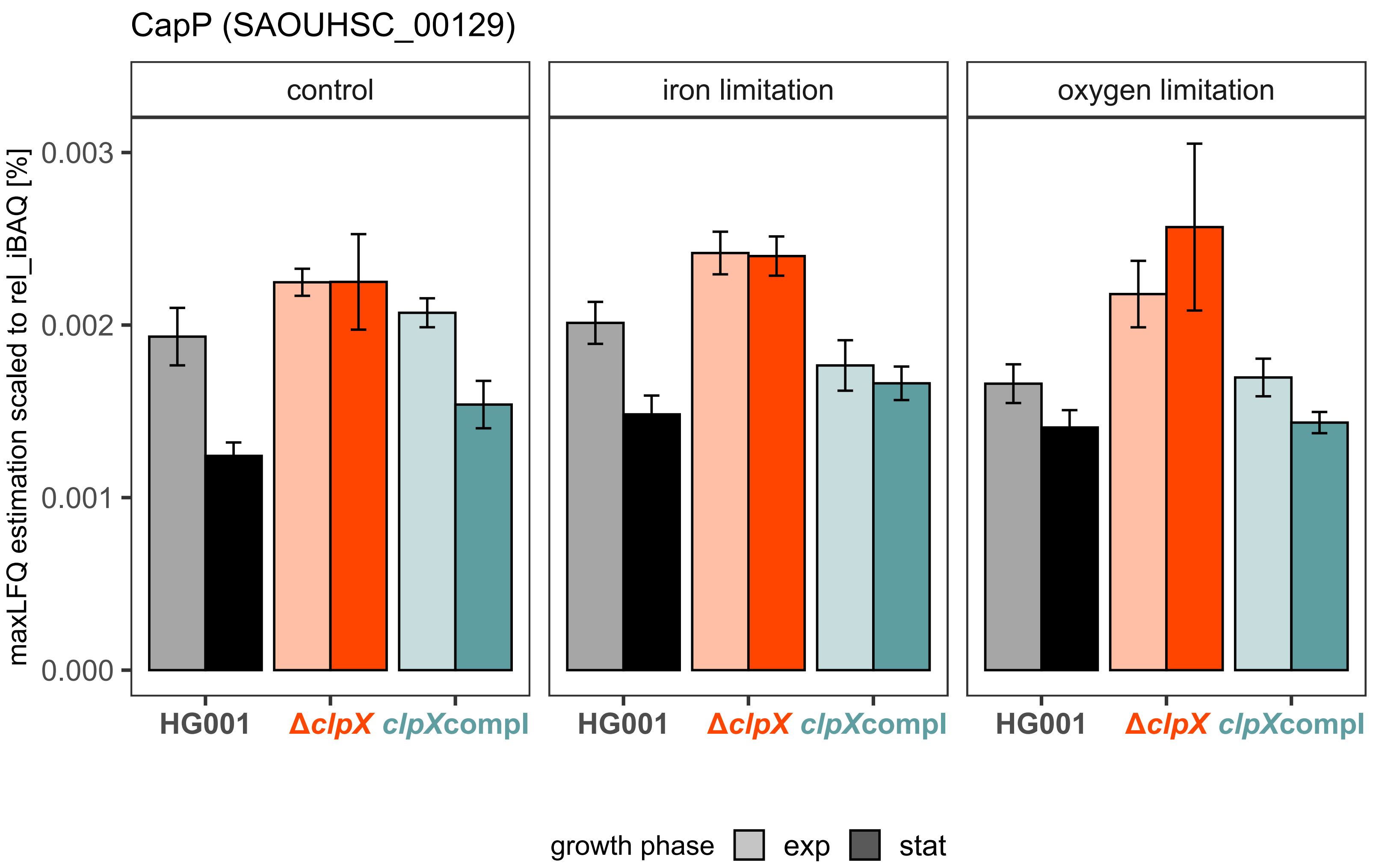

CapP (SAOUHSC_00129)
control
iron limitation
oxygen limitation
maxLFQ estimation scaled to rel_iBAQ [%]
0.003
0.002
0.001
0.000
HG001
ΔclpX
clpXcompl
growth phase
exp
stat

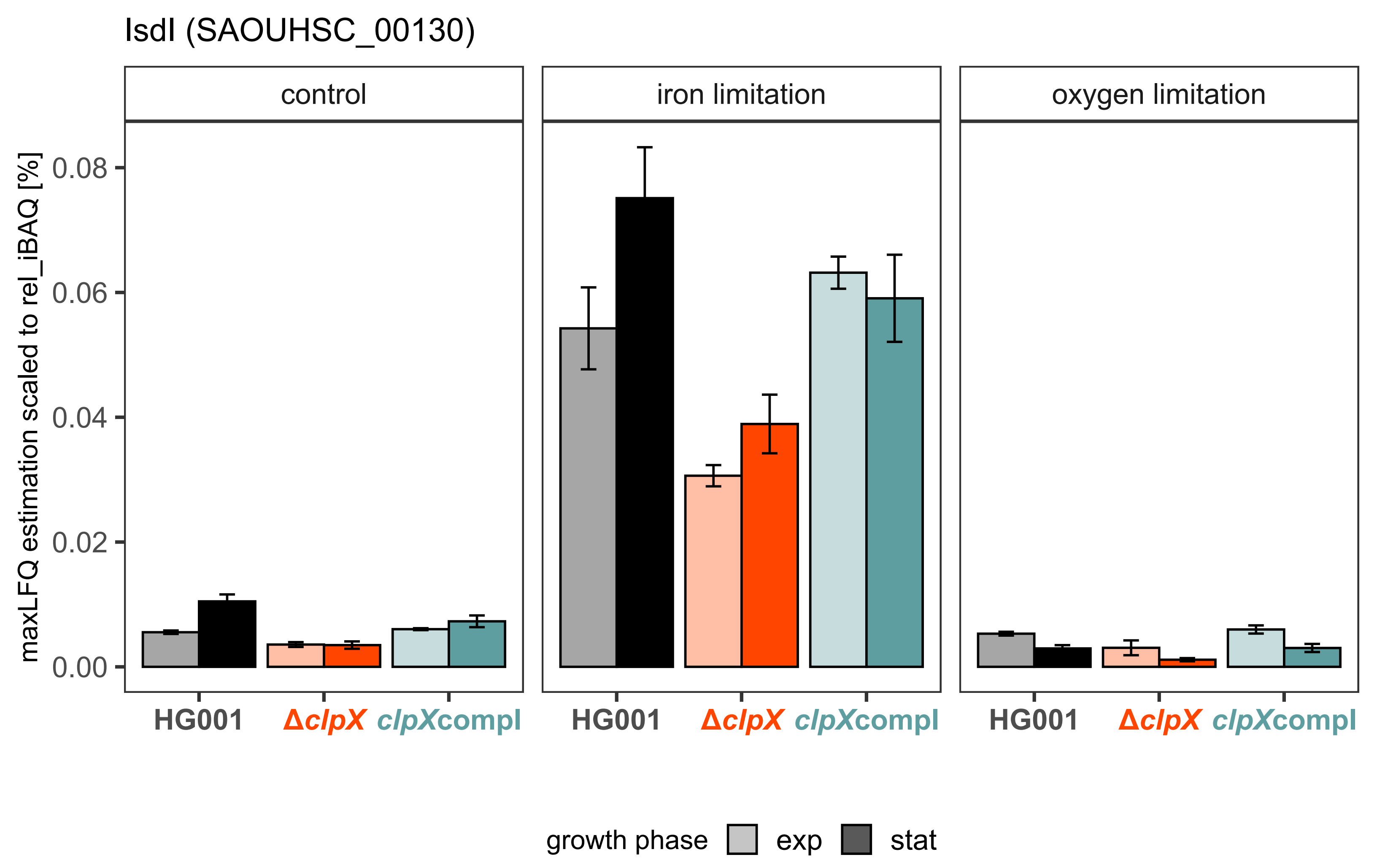
IsdI (SAOUHSC_00130)
control
iron limitation
oxygen limitation
maxLFQ estimation scaled to rel_iBAQ [%]
0.08
0.06
0.04
0.02
0.00
HG001
ΔclpX
clpXcompl
growth phase
exp
stat

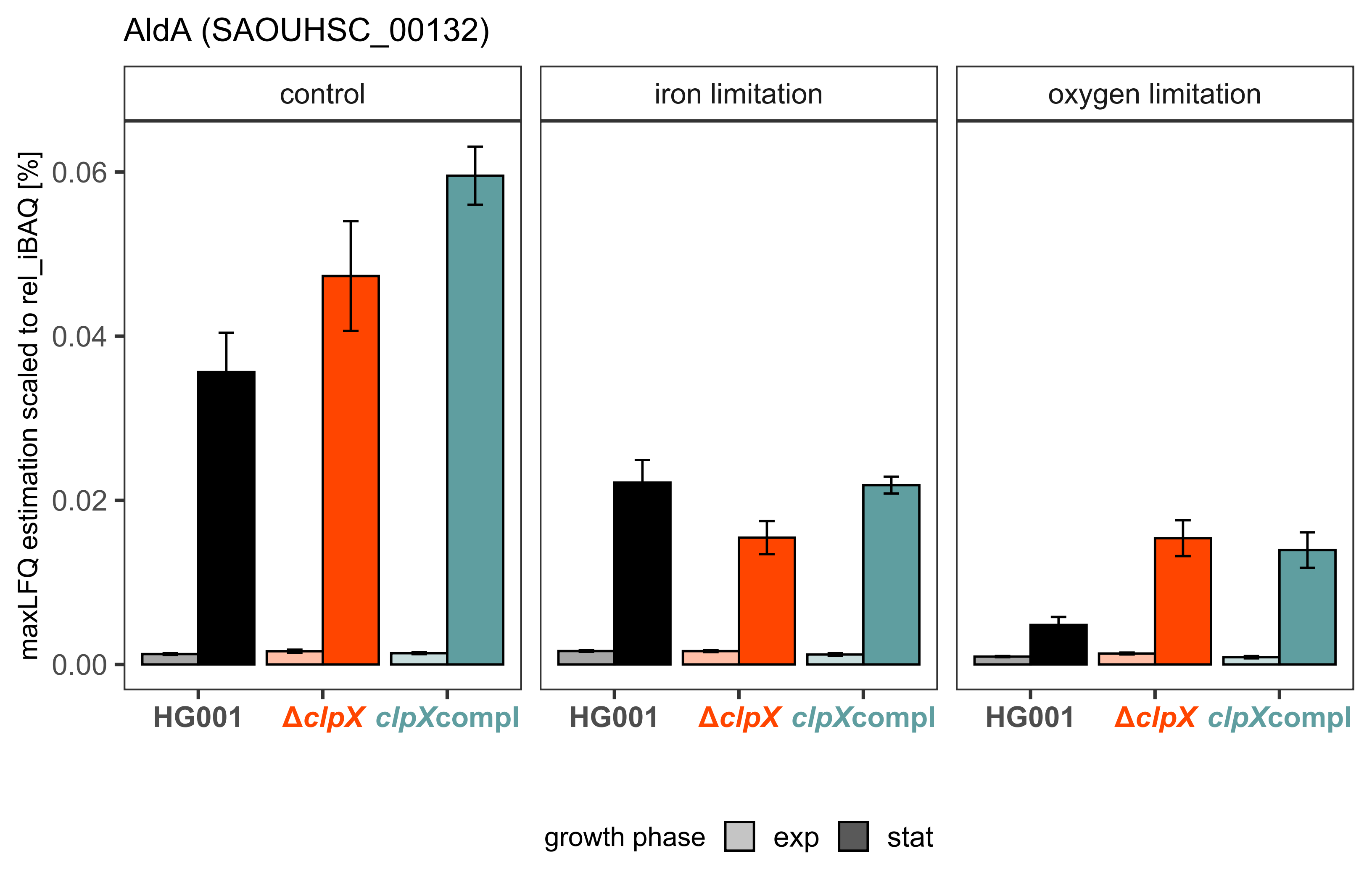
AldA (SAOUHSC_00132)
control
iron limitation
oxygen limitation
maxLFQ estimation scaled to rel_iBAQ [%]
0.06
0.04
0.02
0.00
HG001
ΔclpX
clpXcompl
growth phase
exp
stat

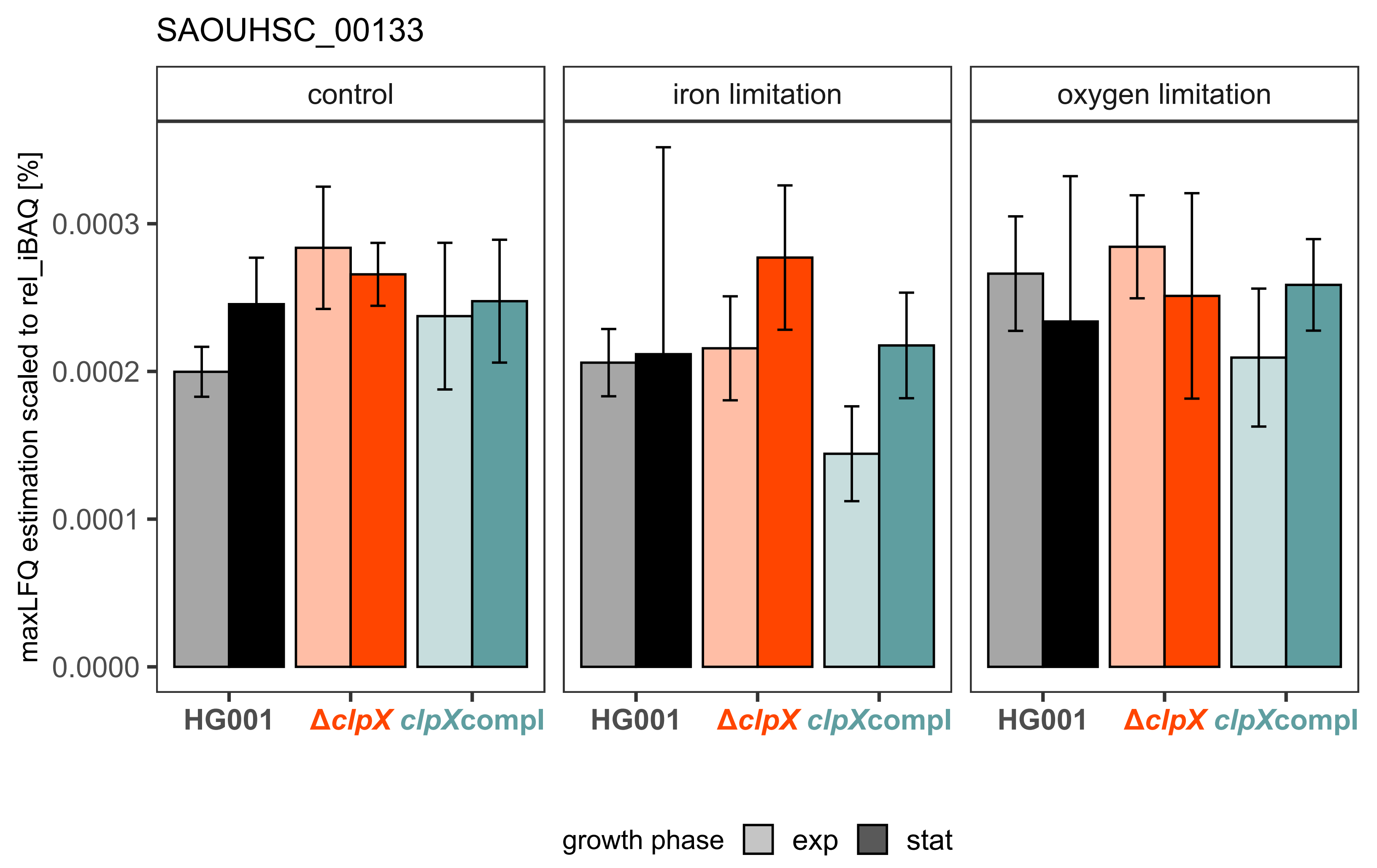

SAOUHSC_00133
control
iron limitation
oxygen limitation
maxLFQ estimation scaled to rel_iBAQ [%]
0.0003
0.0002
0.0001
0.0000
HG001
ΔclpX
clpXcompl
growth phase
exp
stat

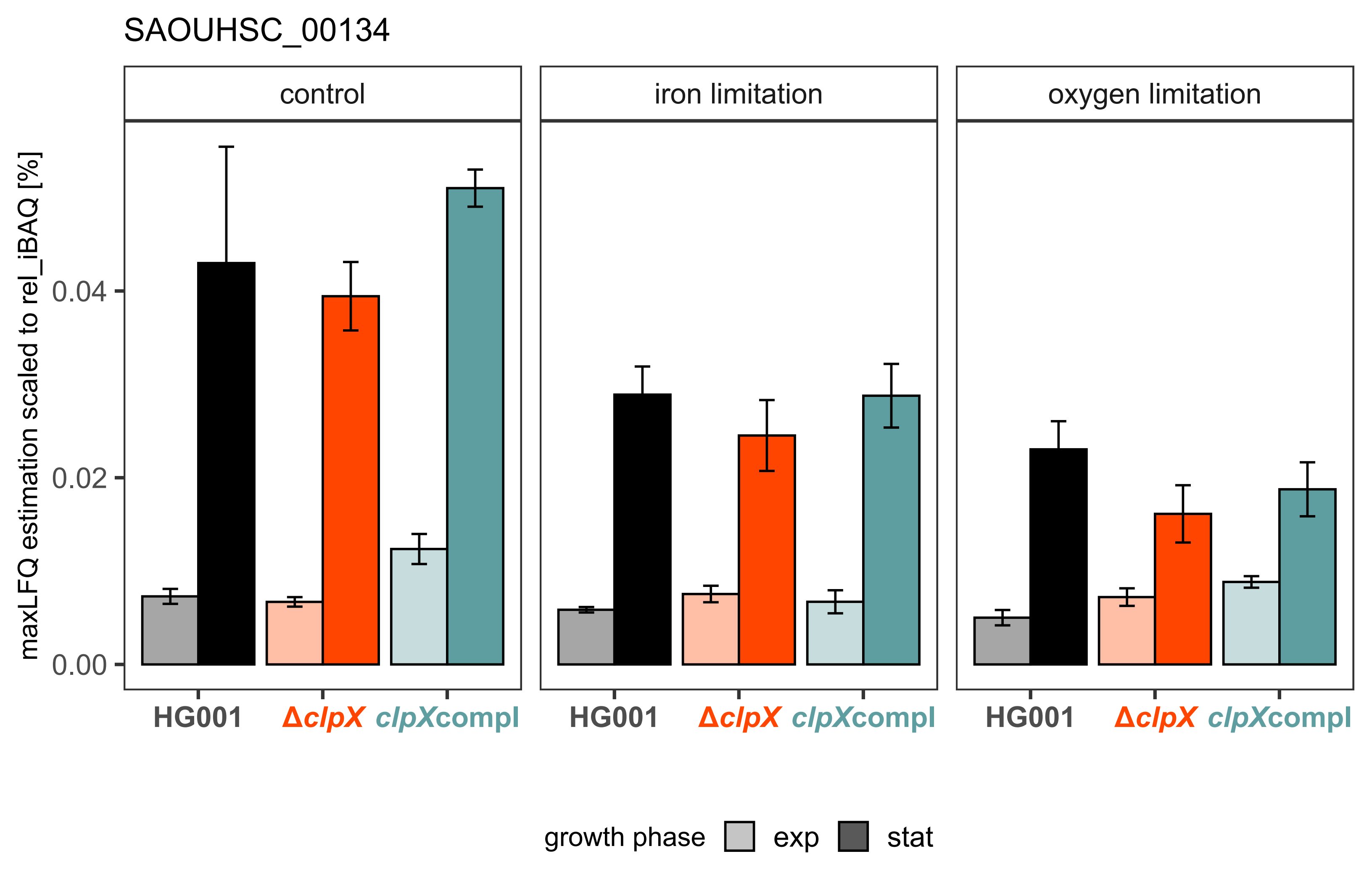
SAOUHSC_00134
control
iron limitation
oxygen limitation
maxLFQ estimation scaled to rel_iBAQ [%]
0.00
0.02
0.04
HG001
ΔclpX
clpXcompl
growth phase
exp
stat

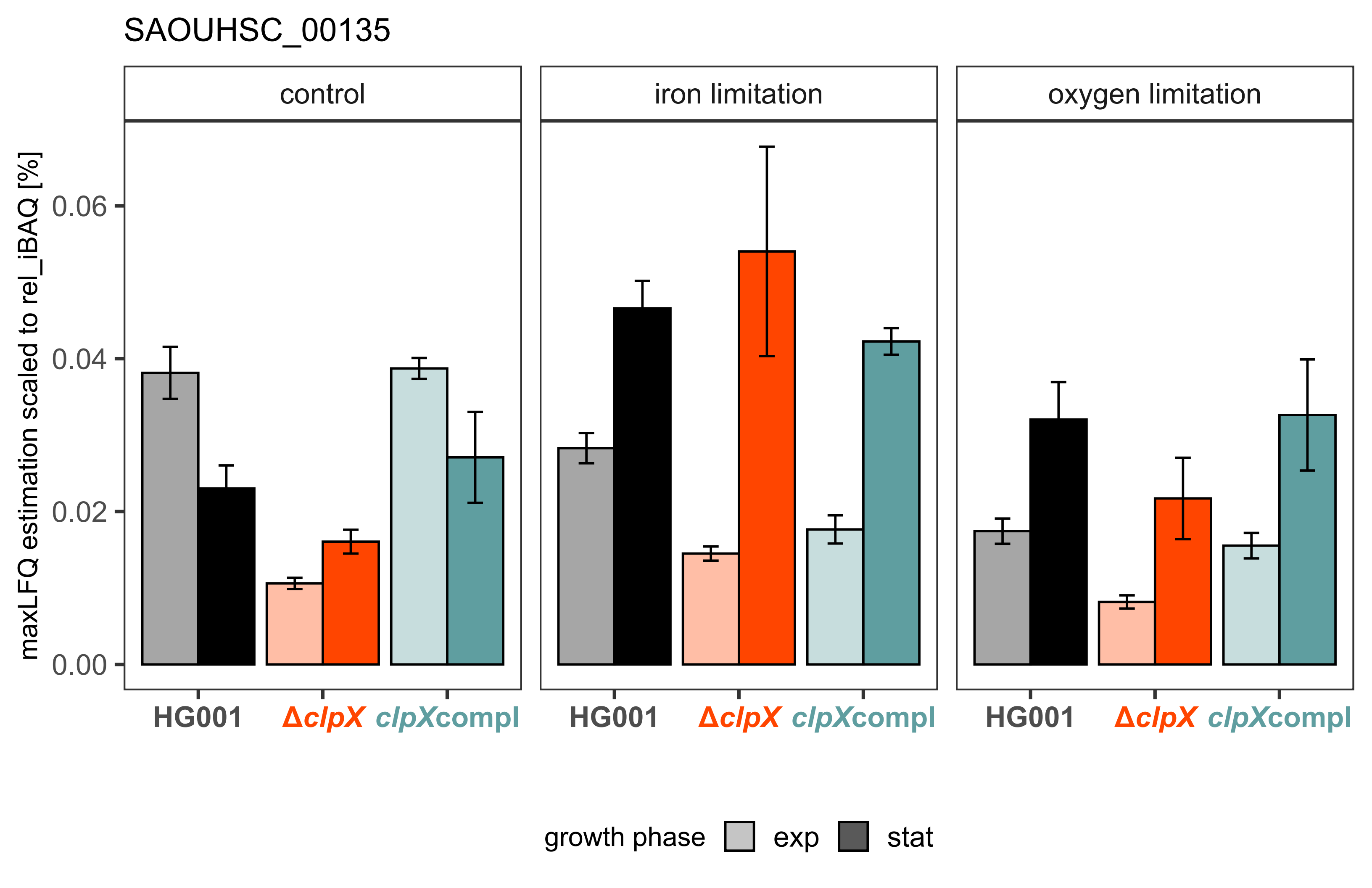

SAOUHSC_00135
control
iron limitation
oxygen limitation
maxLFQ estimation scaled to rel_iBAQ [%]
0.06
0.04
0.02
0.00
HG001
ΔclpX
clpXcompl
growth phase
exp
stat

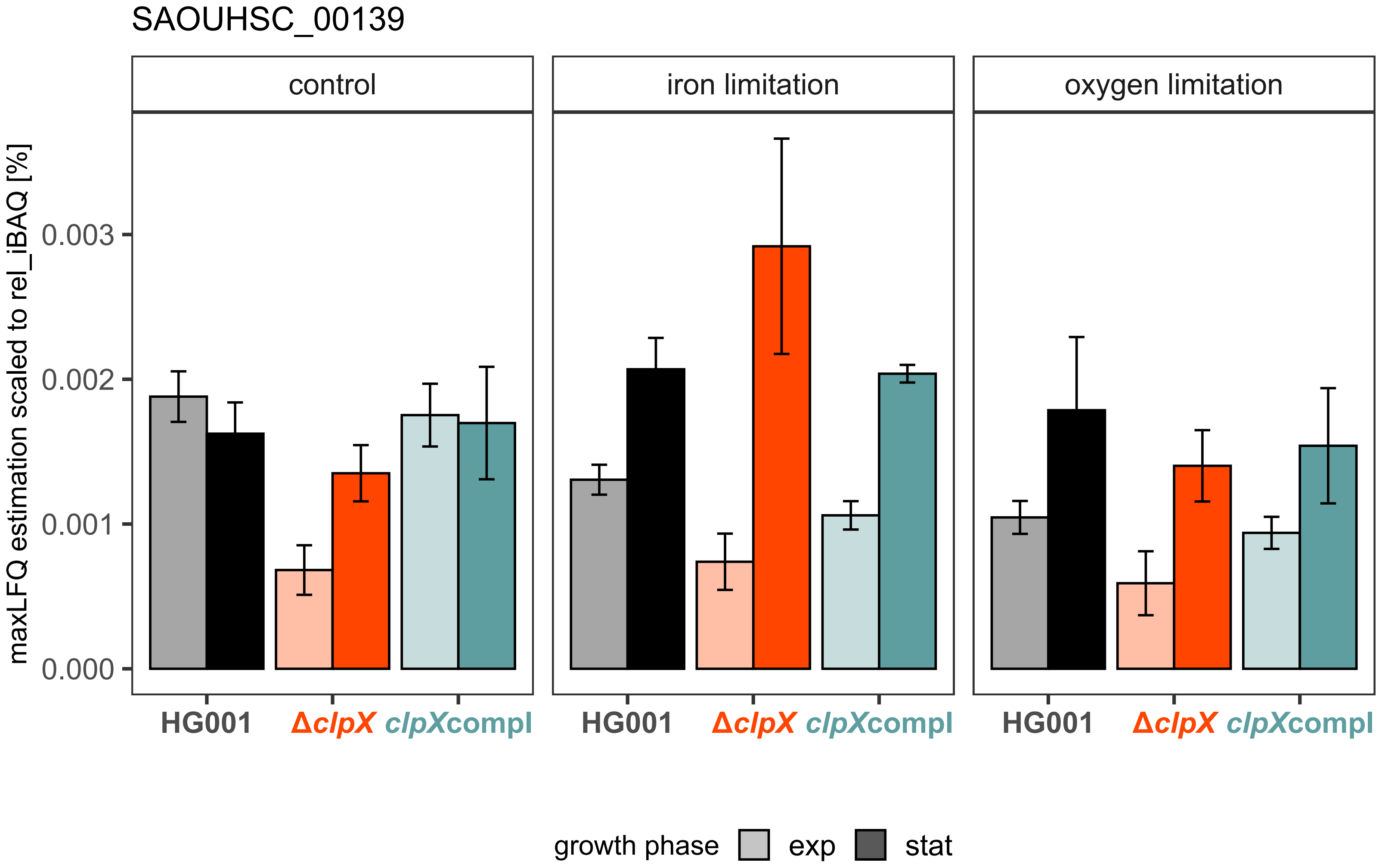

SAOUHSC_00139
control
iron limitation
oxygen limitation
maxLFQ estimation scaled to rel_iBAQ [%]
0.003
0.002
0.001
0.000
HG001
ΔclpX
clpXcompl
growth phase
exp
stat

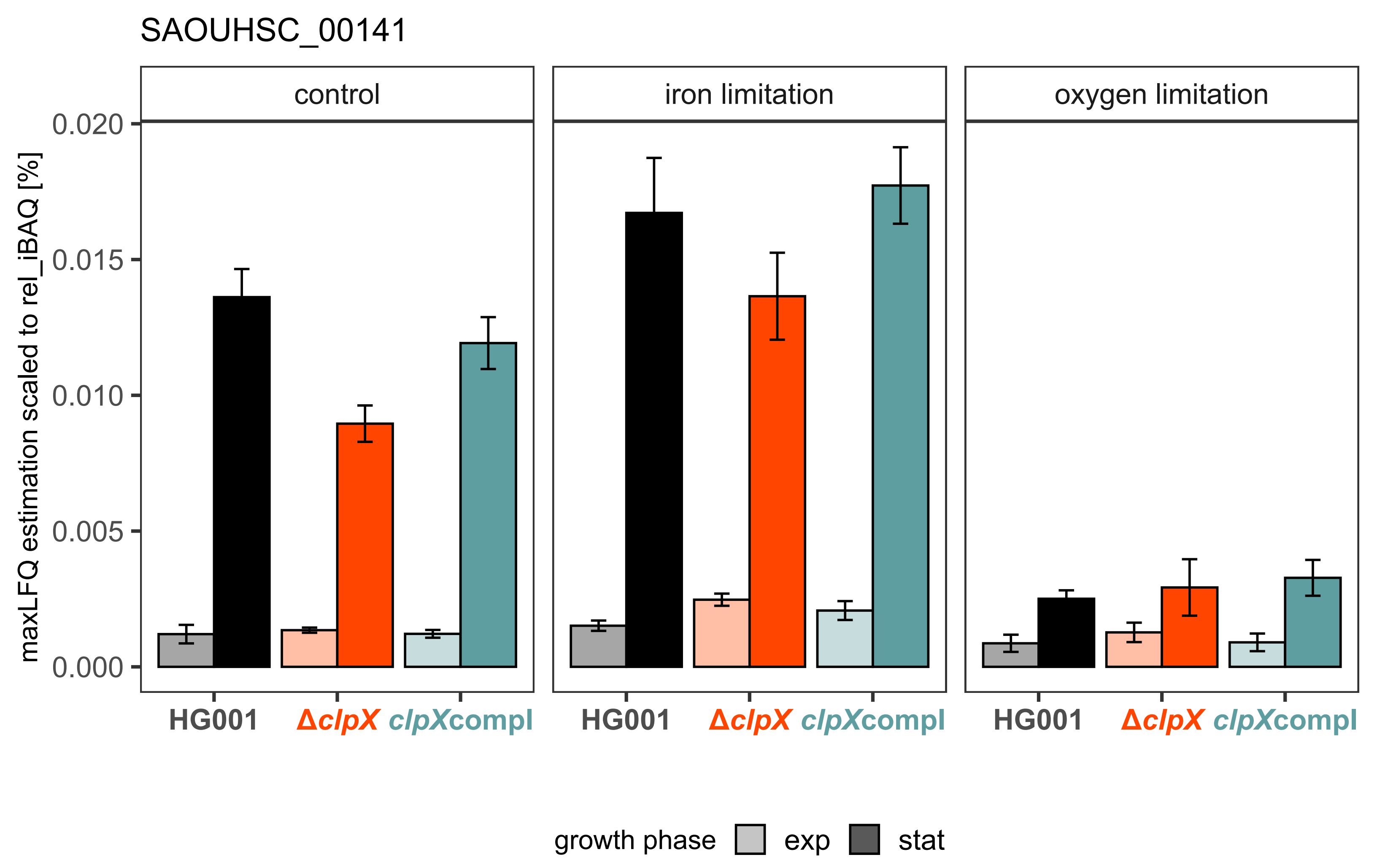

SAOUHSC_00141
control
iron limitation
oxygen limitation
maxLFQ estimation scaled to rel_iBAQ [%]
0.020
0.015
0.010
0.005
0.000
HG001
ΔclpX
clpXcompl
growth phase
exp
stat

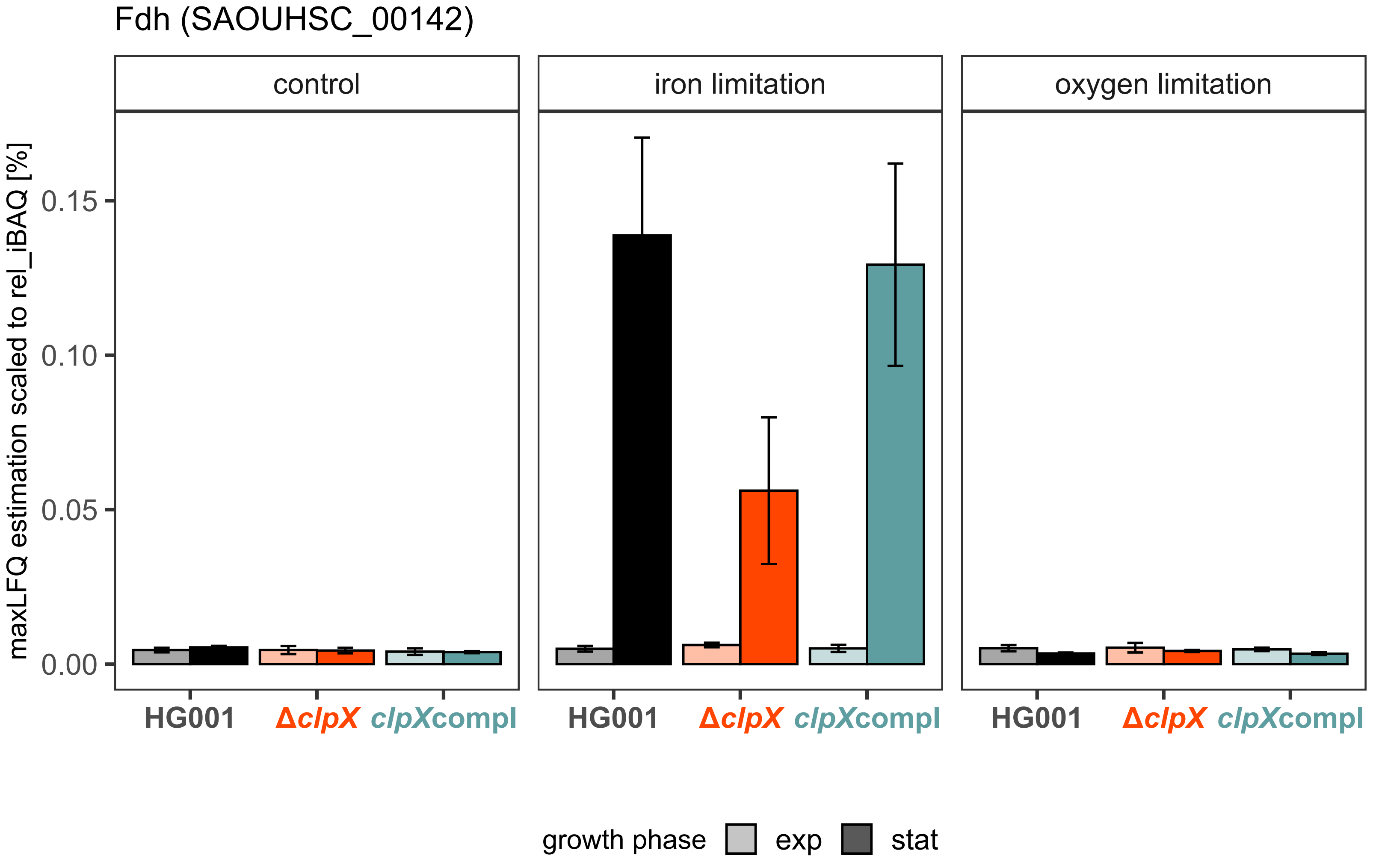

Fdh (SAOUHSC_00142)
control
iron limitation
oxygen limitation
maxLFQ estimation scaled to rel_iBAQ [%]
0.15
0.10
0.05
0.00
HG001
ΔclpX
clpXcompl
growth phase
exp
stat

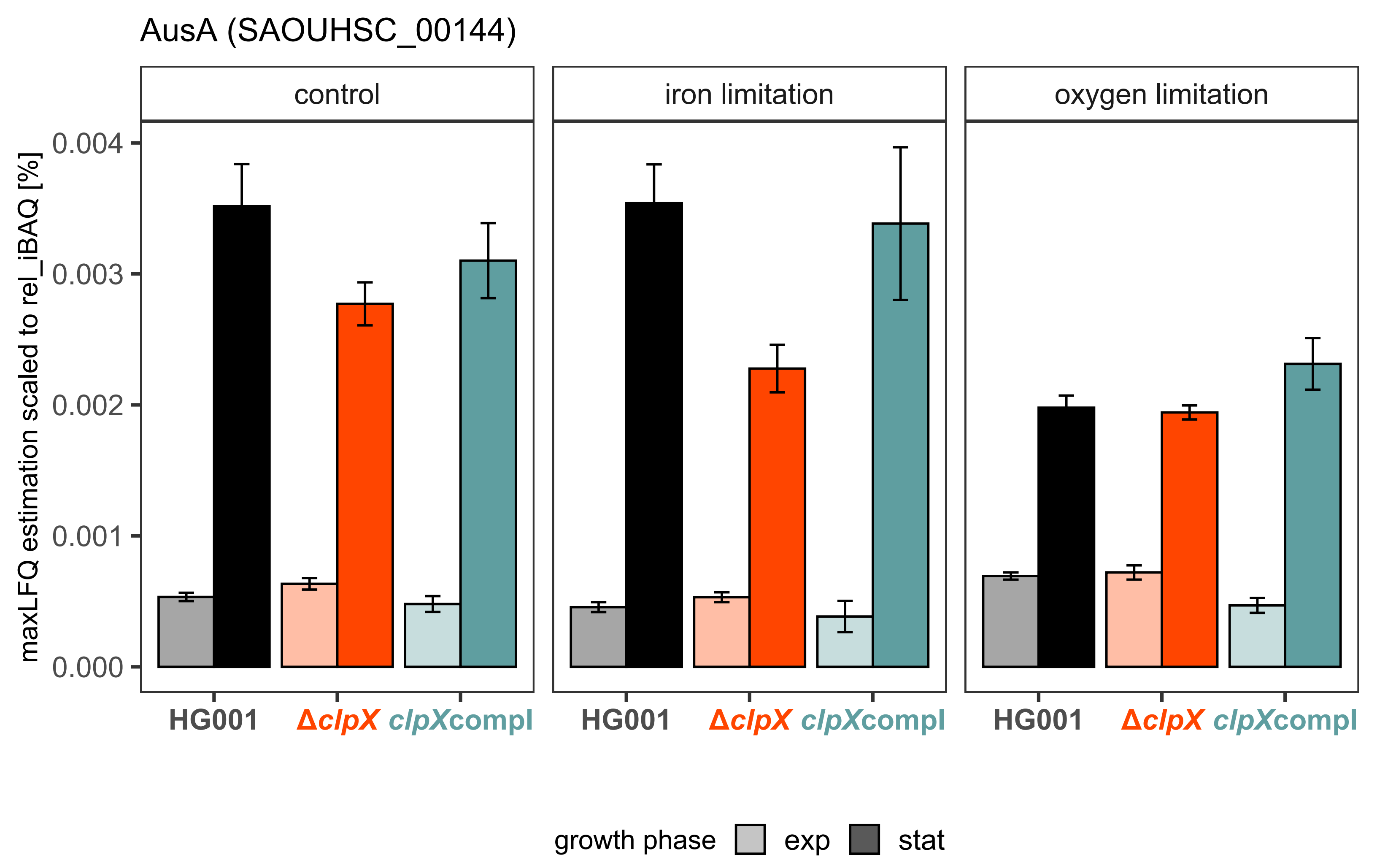

AusA (SAOUHSC_00144)
control
iron limitation
oxygen limitation
maxLFQ estimation scaled to rel_iBAQ [%]
0.004
0.003
0.002
0.001
0.000
HG001
ΔclpX
clpXcompl
growth phase
exp
stat

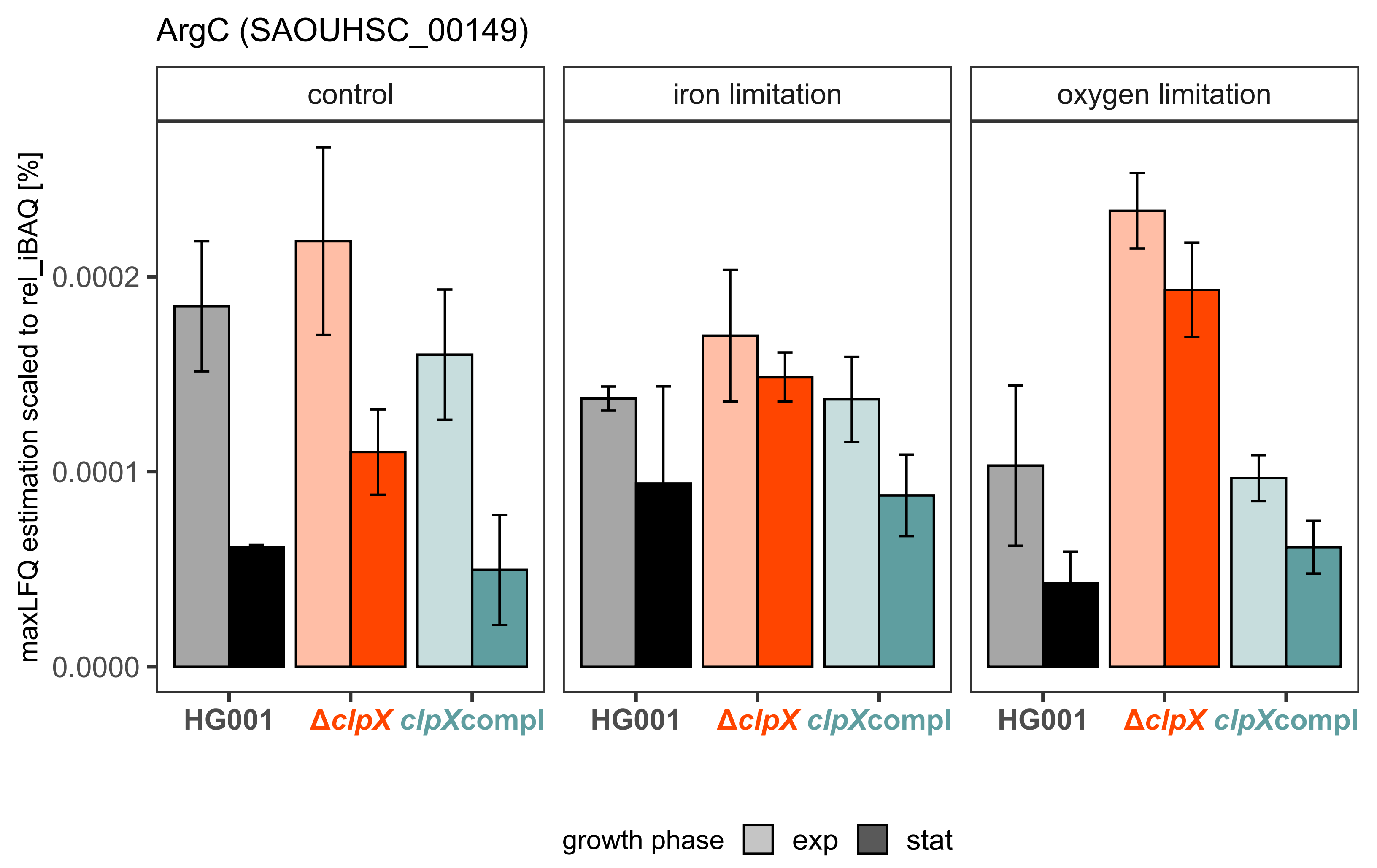

ArgC (SAOUHSC_00149)
control
iron limitation
oxygen limitation
maxLFQ estimation scaled to rel_iBAQ [%]
0.0002
0.0001
0.0000
HG001
ΔclpX
clpXcompl
growth phase
exp
stat

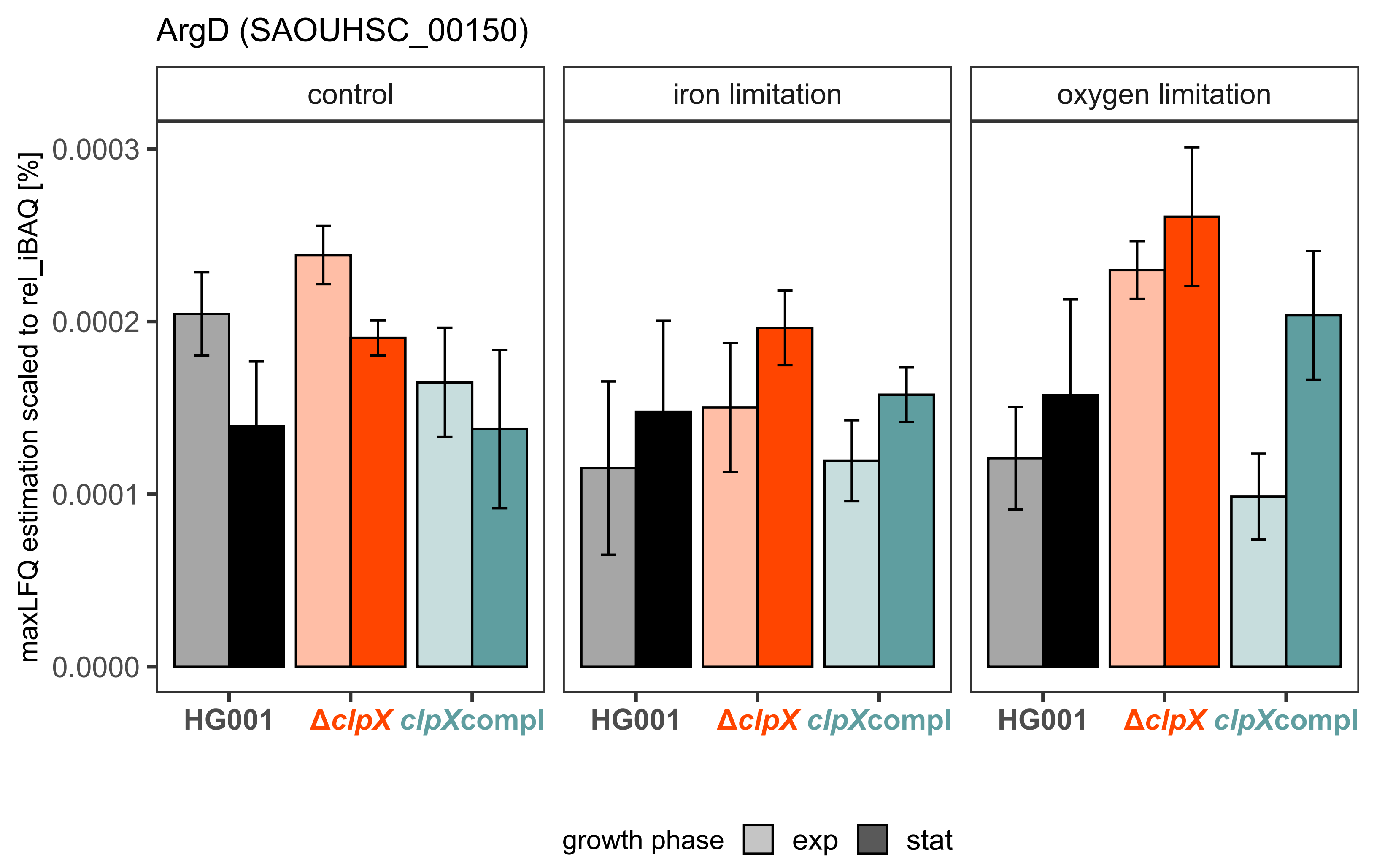
ArgD (SAOUHSC_00150)
control
iron limitation
oxygen limitation
maxLFQ estimation scaled to rel_iBAQ [%]
0.0003
0.0002
0.0001
0.0000
HG001
ΔclpX
clpXcompl
growth phase
exp
stat

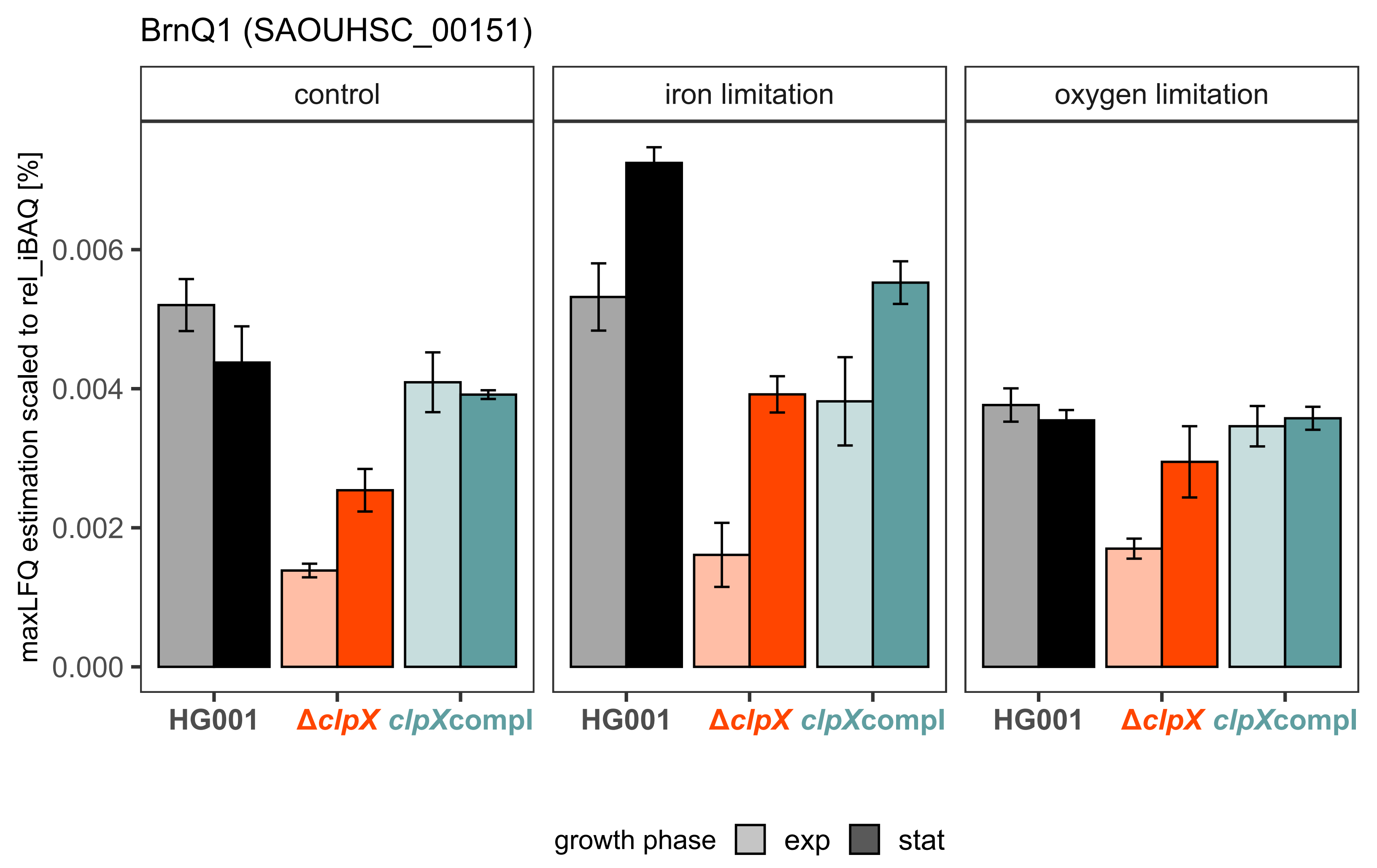
BrnQ1 (SAOUHSC_00151)
control
iron limitation
oxygen limitation
maxLFQ estimation scaled to rel_iBAQ [%]
0.006
0.004
0.002
0.000
HG001
ΔclpX
clpXcompl
growth phase
exp
stat

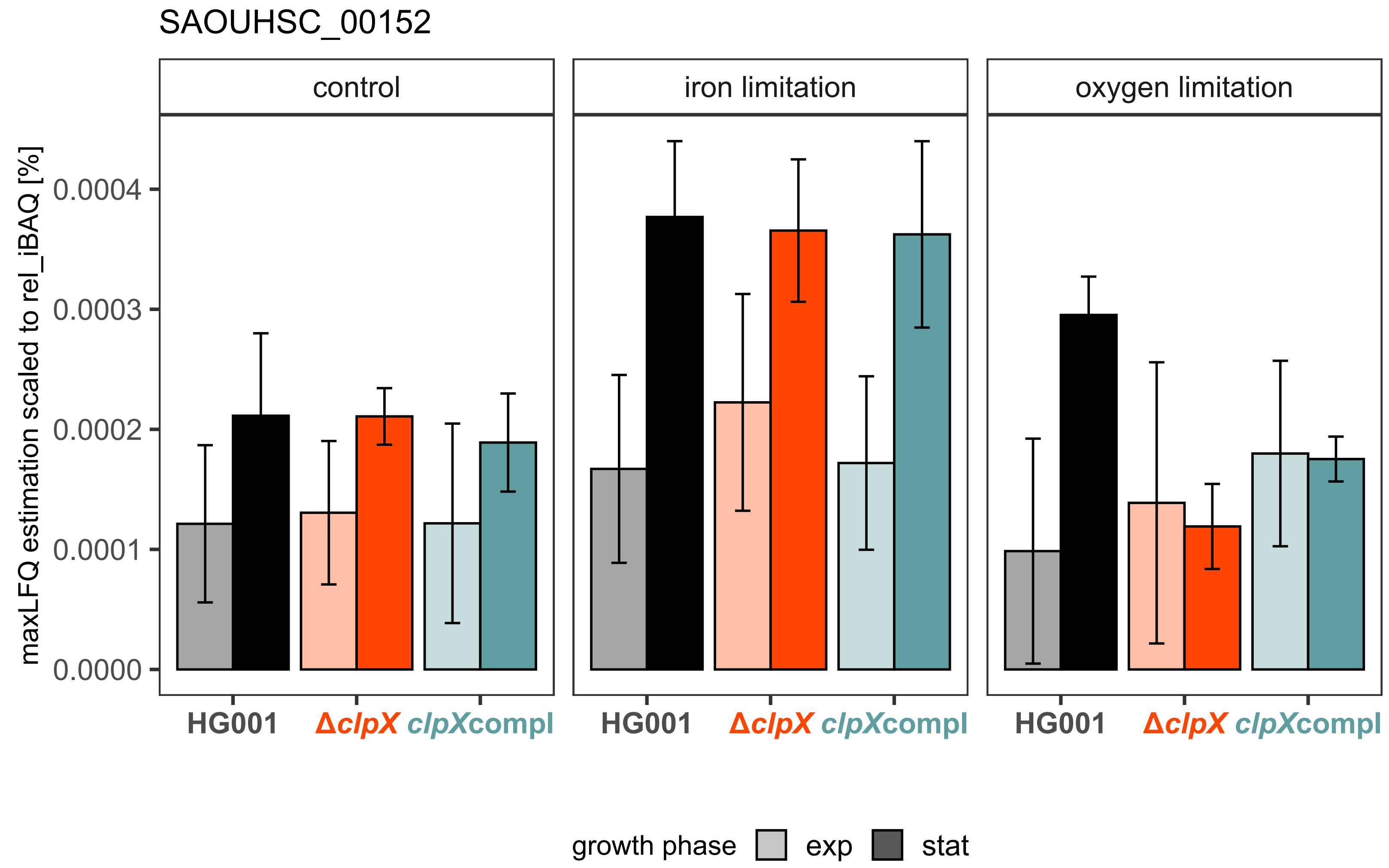
SAOUHSC_00152
control
iron limitation
oxygen limitation
maxLFQ estimation scaled to rel_iBAQ [%]
0.0004
0.0003
0.0002
0.0001
0.0000
HG001
ΔclpX
clpXcompl
growth phase
exp
stat

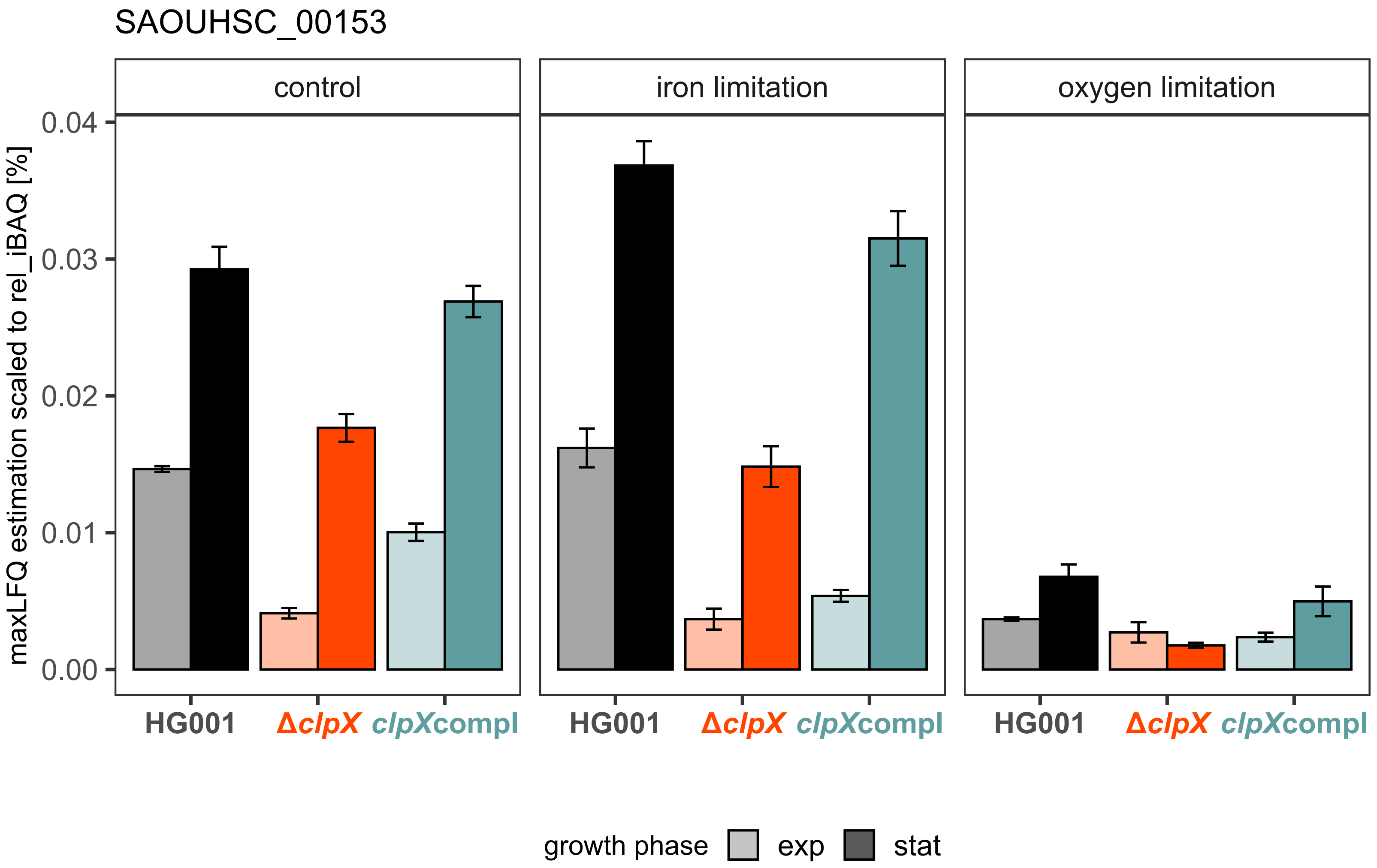
SAOUHSC_00153
control
iron limitation
oxygen limitation
maxLFQ estimation scaled to rel_iBAQ [%]
0.04
0.03
0.02
0.01
0.00
HG001
ΔclpX
clpXcompl
growth phase
exp
stat

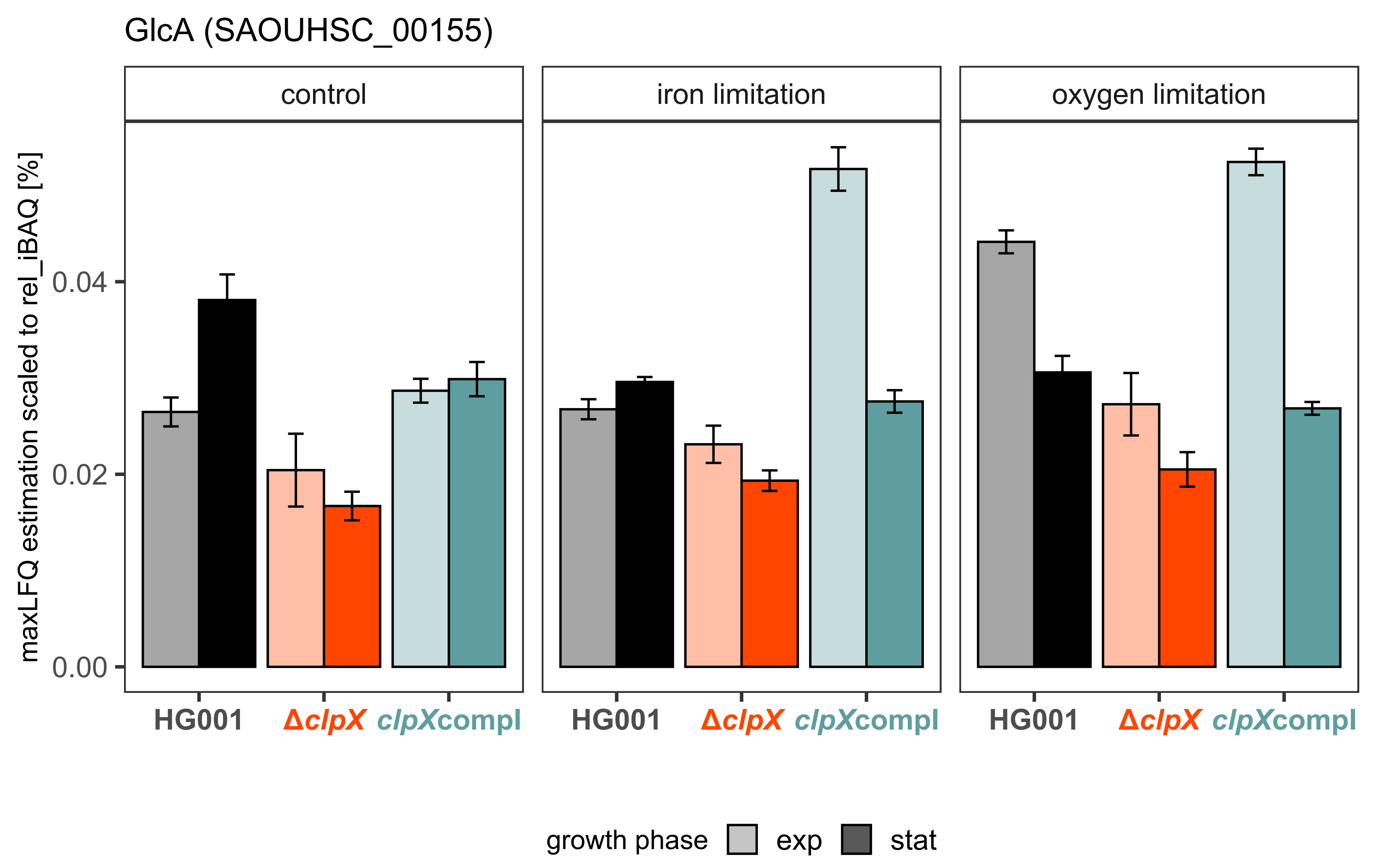
GlcA (SAOUHSC_00155)
control
iron limitation
oxygen limitation
maxLFQ estimation scaled to rel_iBAQ [%]
0.00
0.02
0.04
HG001
ΔclpX
clpXcompl
growth phase
exp
stat

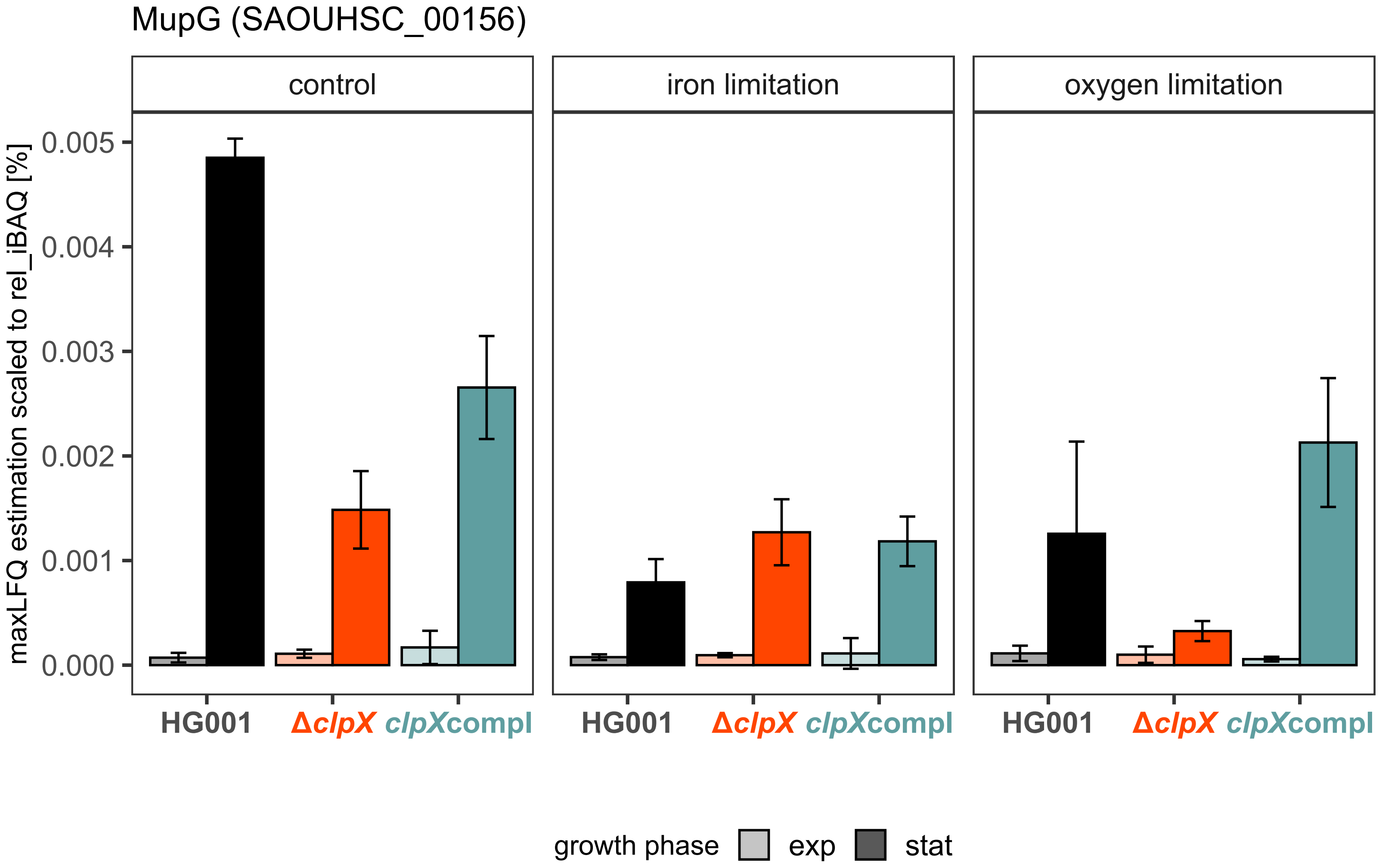

MupG (SAOUHSC_00156)
control
iron limitation
oxygen limitation
maxLFQ estimation scaled to rel_iBAQ [%]
0.005
0.004
0.003
0.002
0.001
0.000
HG001
ΔclpX
clpXcompl
growth phase
exp
stat

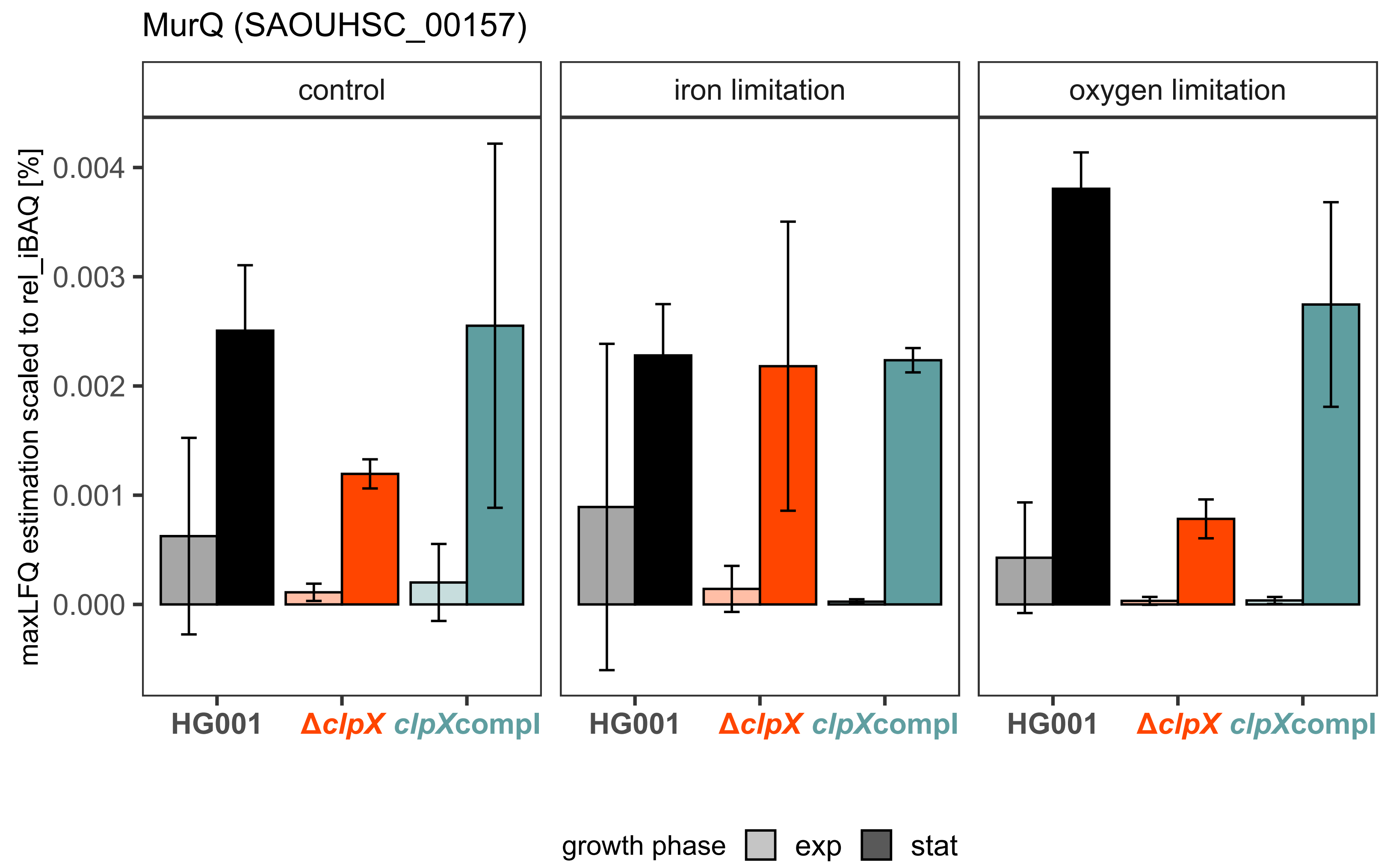

MurQ (SAOUHSC_00157)
control
iron limitation
oxygen limitation
maxLFQ estimation scaled to rel_iBAQ [%]
0.004
0.003
0.002
0.001
0.000
HG001
ΔclpX
clpXcompl
growth phase
exp
stat

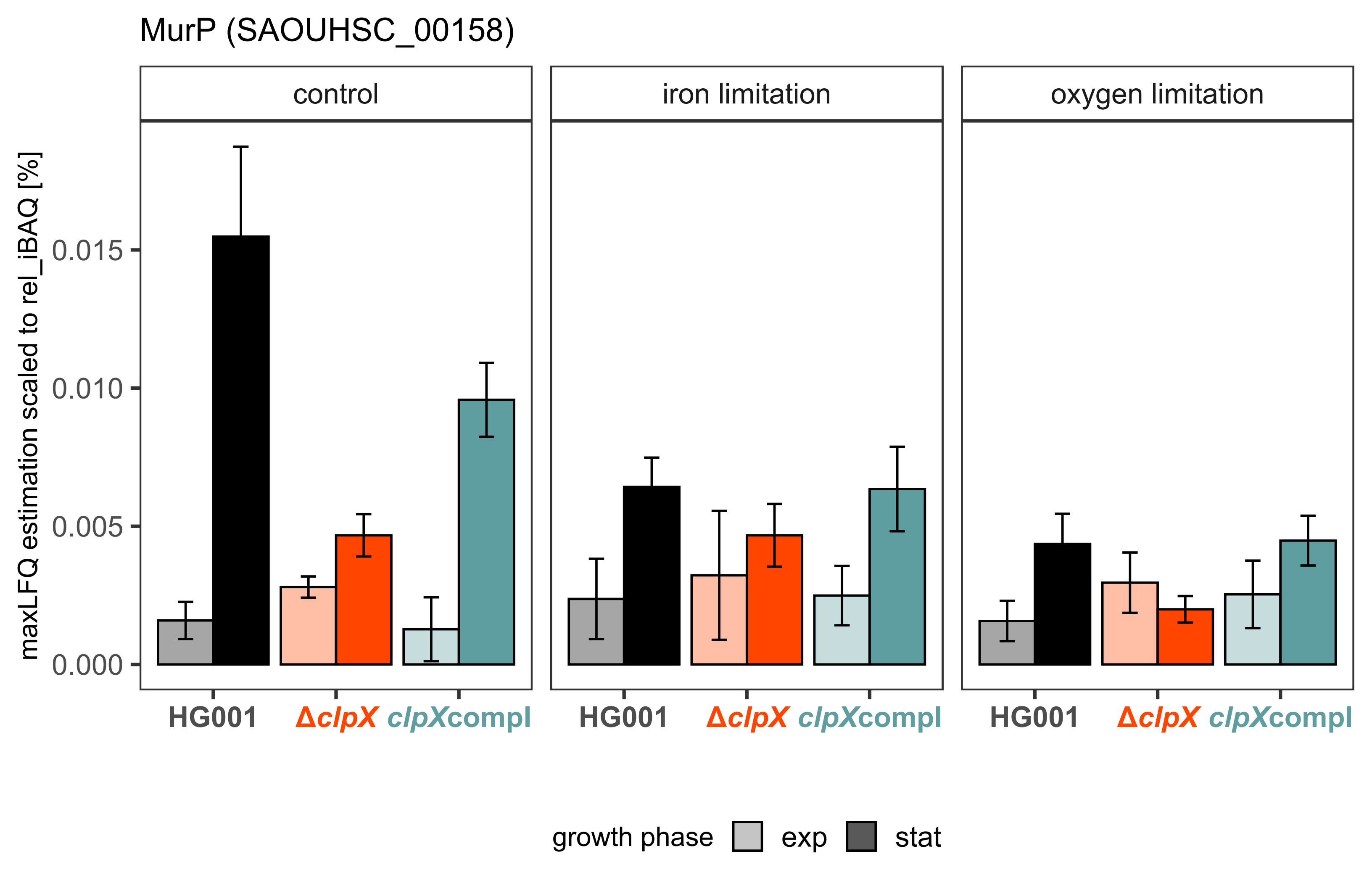
MurP (SAOUHSC_00158)
control
iron limitation
oxygen limitation
maxLFQ estimation scaled to rel_iBAQ [%]
0.015
0.010
0.005
0.000
HG001
ΔclpX
clpXcompl
growth phase
exp
stat

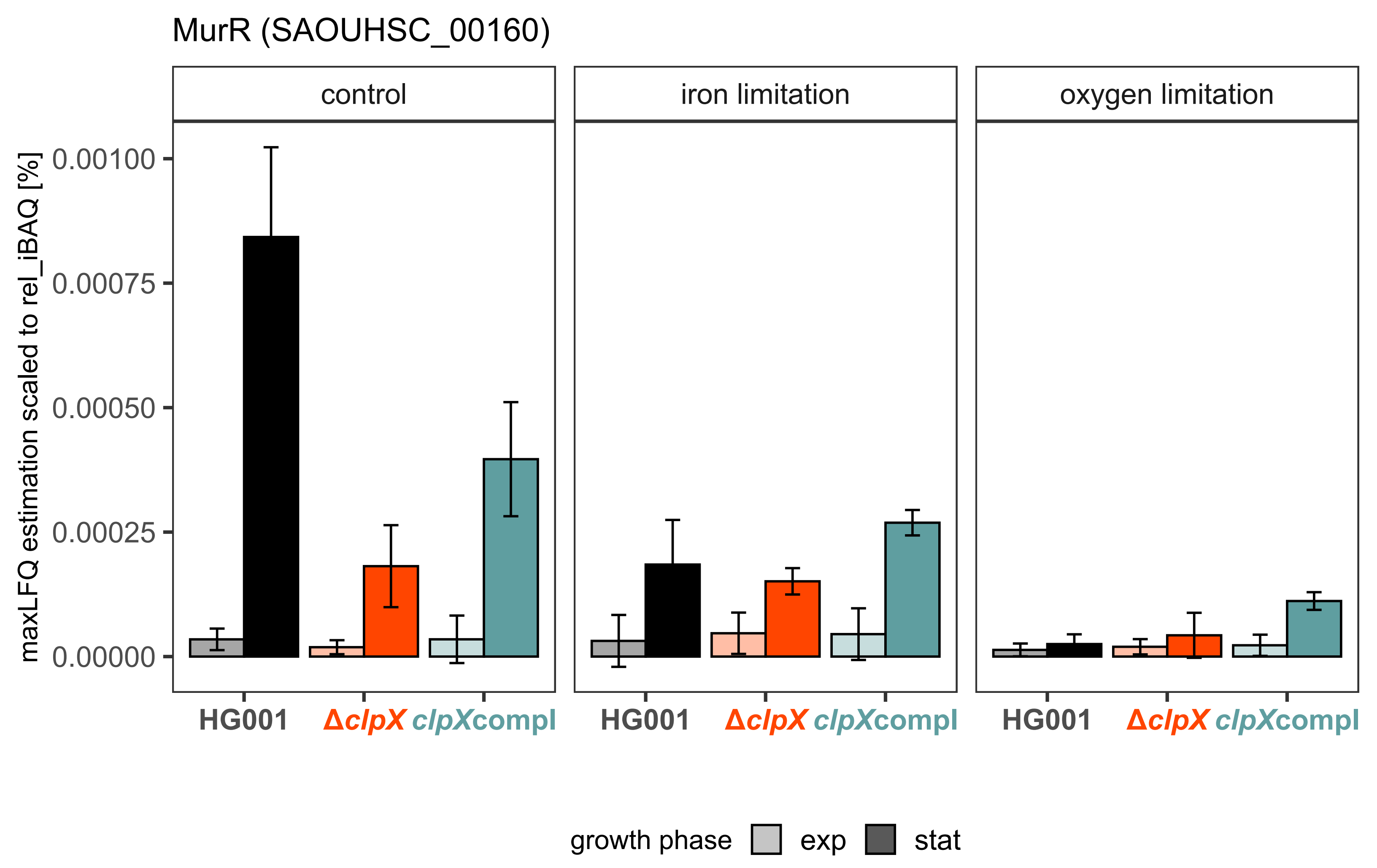

MurR (SAOUHSC_00160)
control
iron limitation
oxygen limitation
maxLFQ estimation scaled to rel_iBAQ [%]
0.00100
0.00075
0.00050
0.00025
0.00000
HG001
ΔclpX
clpXcompl
growth phase
exp
stat

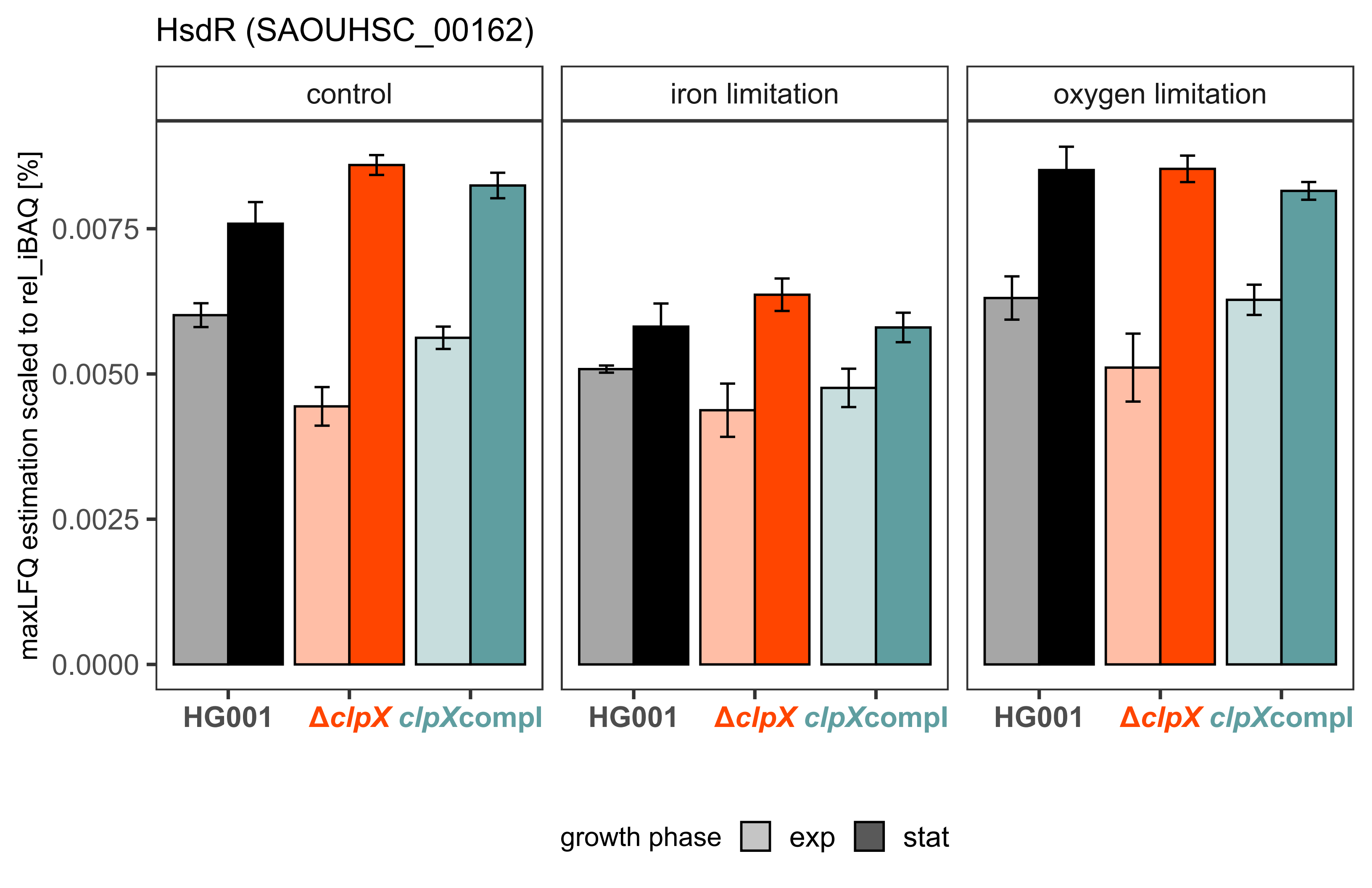
HsdR (SAOUHSC_00162)
control
iron limitation
oxygen limitation
maxLFQ estimation scaled to rel_iBAQ [%]
0.0000
0.0025
0.0050
0.0075
HG001
ΔclpX
clpXcompl
growth phase
exp
stat

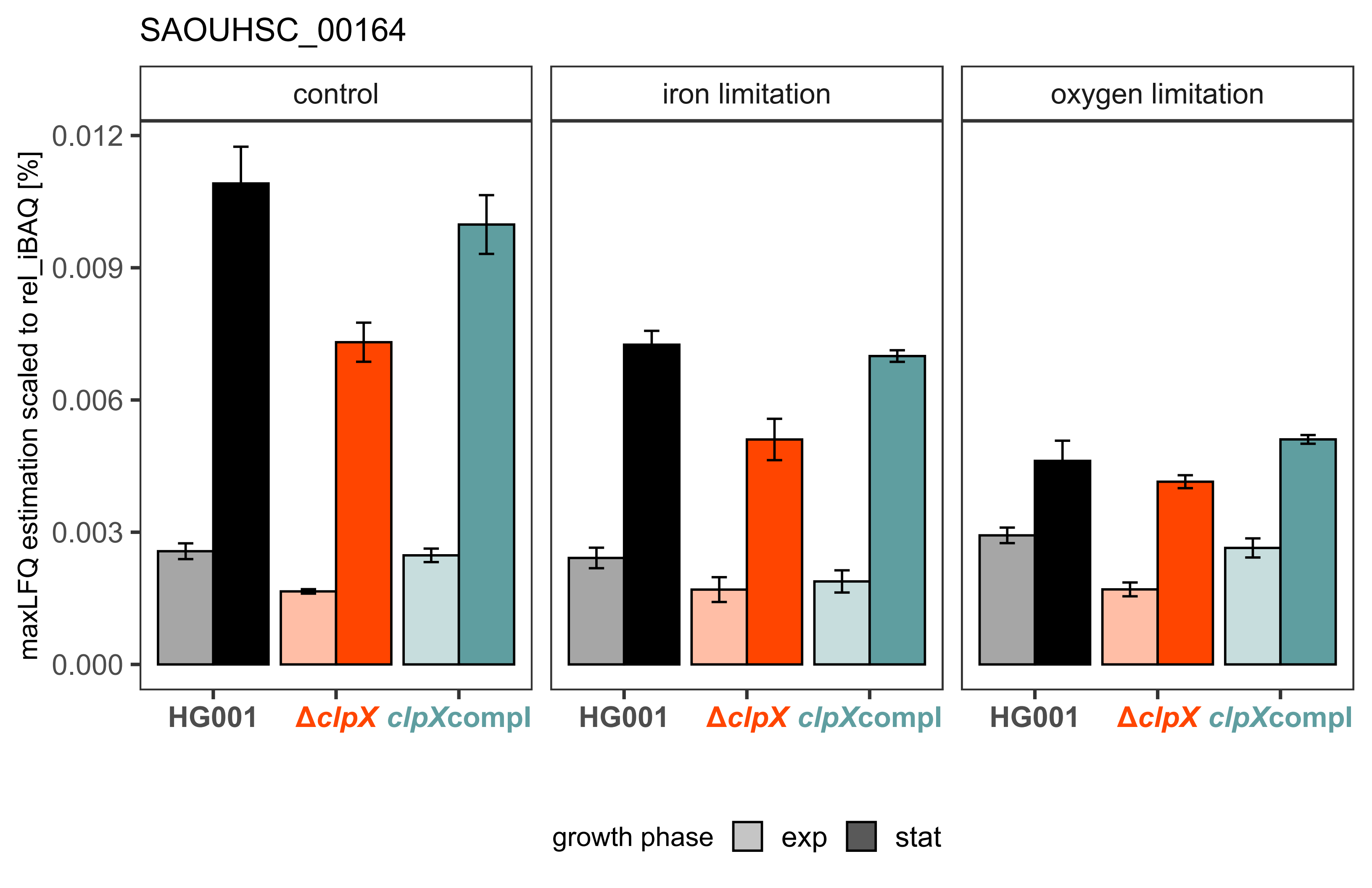
SAOUHSC_00164
control
iron limitation
oxygen limitation
maxLFQ estimation scaled to rel_iBAQ [%]
0.012
0.009
0.006
0.003
0.000
HG001
ΔclpX
clpXcompl
growth phase
exp
stat

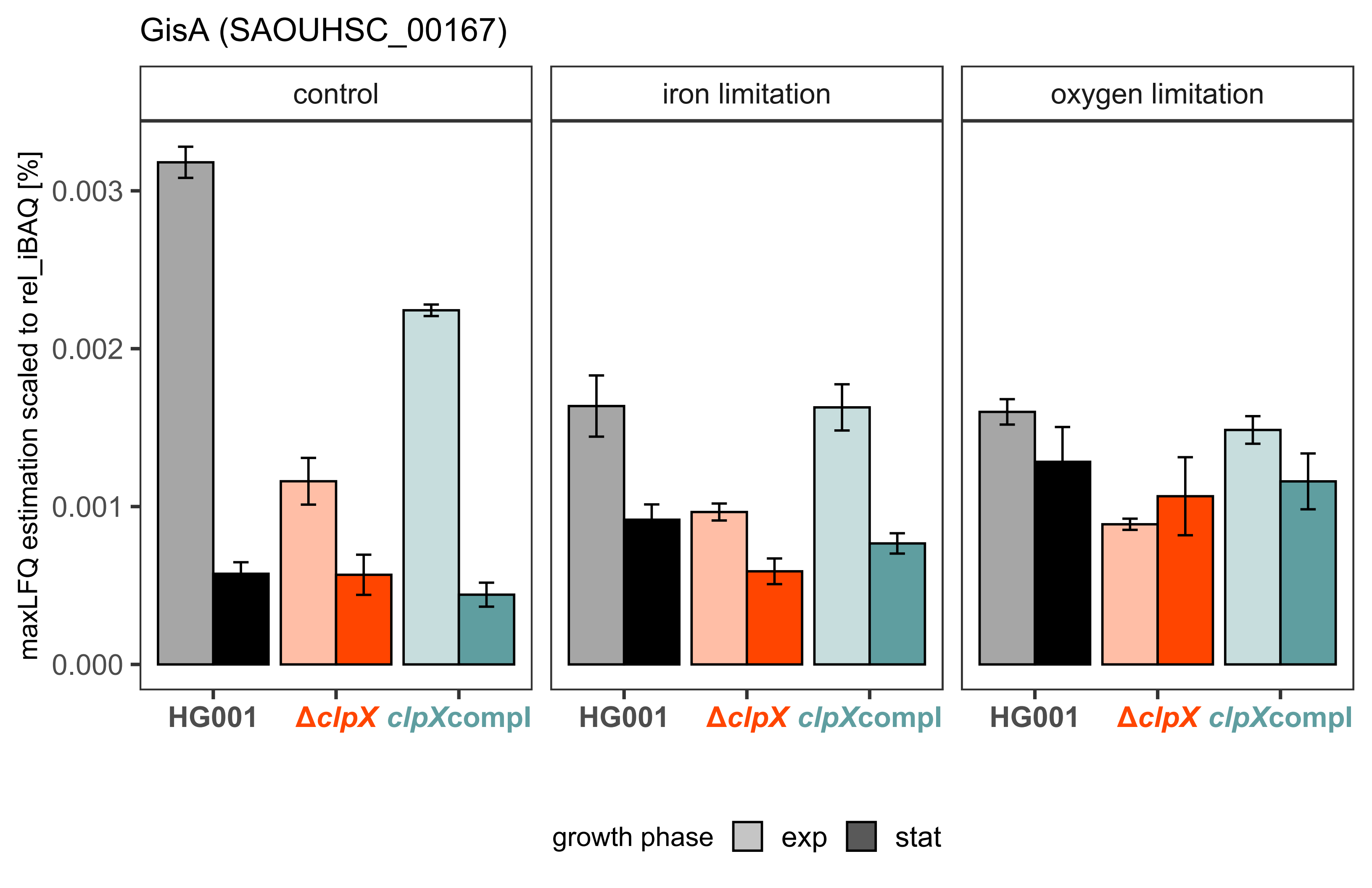

GisA (SAOUHSC_00167)
control
iron limitation
oxygen limitation
maxLFQ estimation scaled to rel_iBAQ [%]
0.003
0.002
0.001
0.000
HG001
ΔclpX
clpXcompl
growth phase
exp
stat

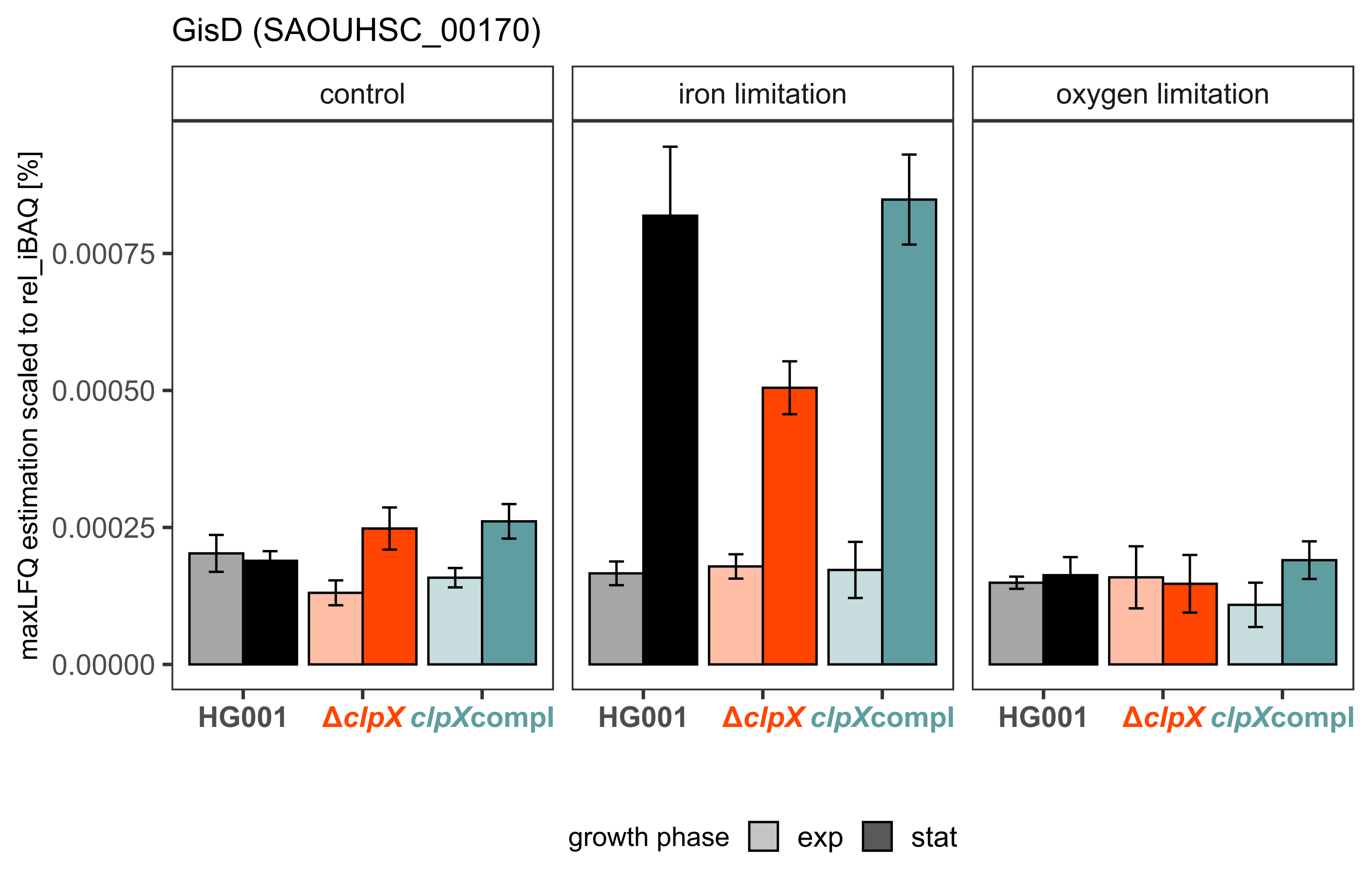

GisD (SAOUHSC_00170)
control
iron limitation
oxygen limitation
maxLFQ estimation scaled to rel_iBAQ [%]
0.00075
0.00050
0.00025
0.00000
HG001
ΔclpX
clpXcompl
growth phase
exp
stat

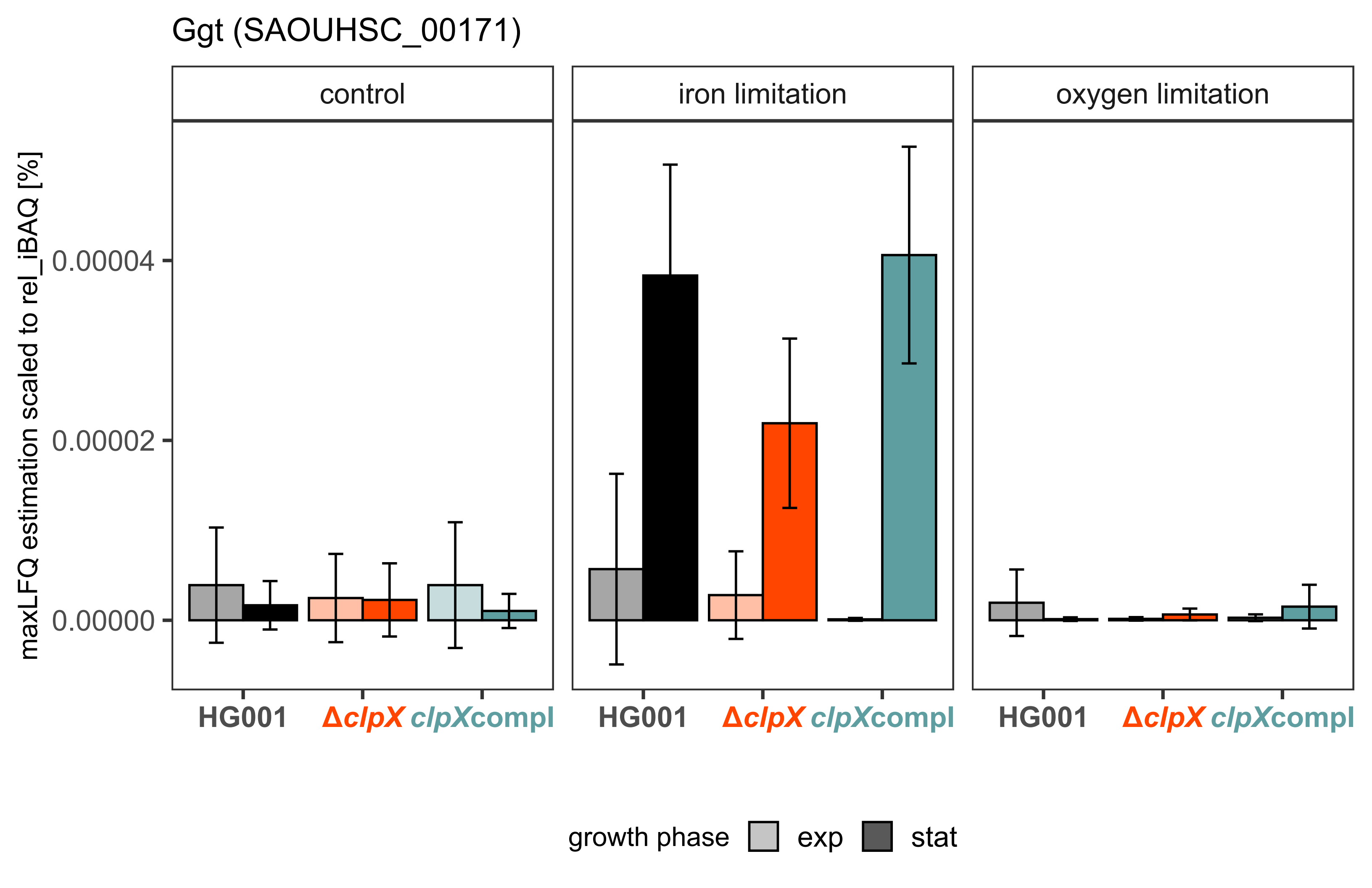

Ggt (SAOUHSC_00171)
control
iron limitation
oxygen limitation
maxLFQ estimation scaled to rel_iBAQ [%]
0.00004
0.00002
0.00000
HG001
ΔclpX
clpXcompl
HG001
ΔclpX
clpXcompl
HG001
ΔclpX
clpXcompl
growth phase
exp
stat

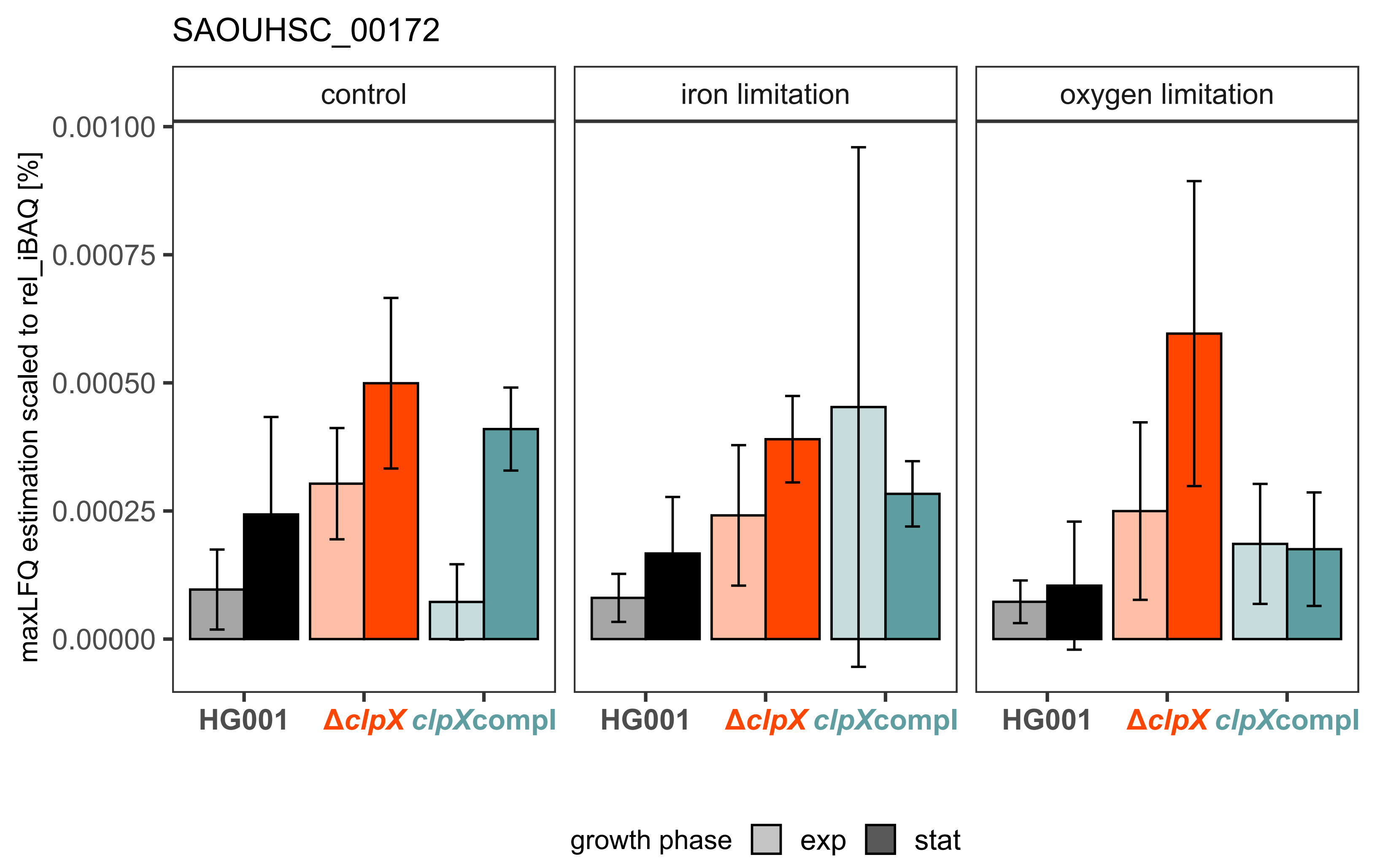
SAOUHSC_00172
control
iron limitation
oxygen limitation
maxLFQ estimation scaled to rel_iBAQ [%]
0.00100
0.00075
0.00050
0.00025
0.00000
HG001
ΔclpX
clpXcompl
growth phase
exp
stat

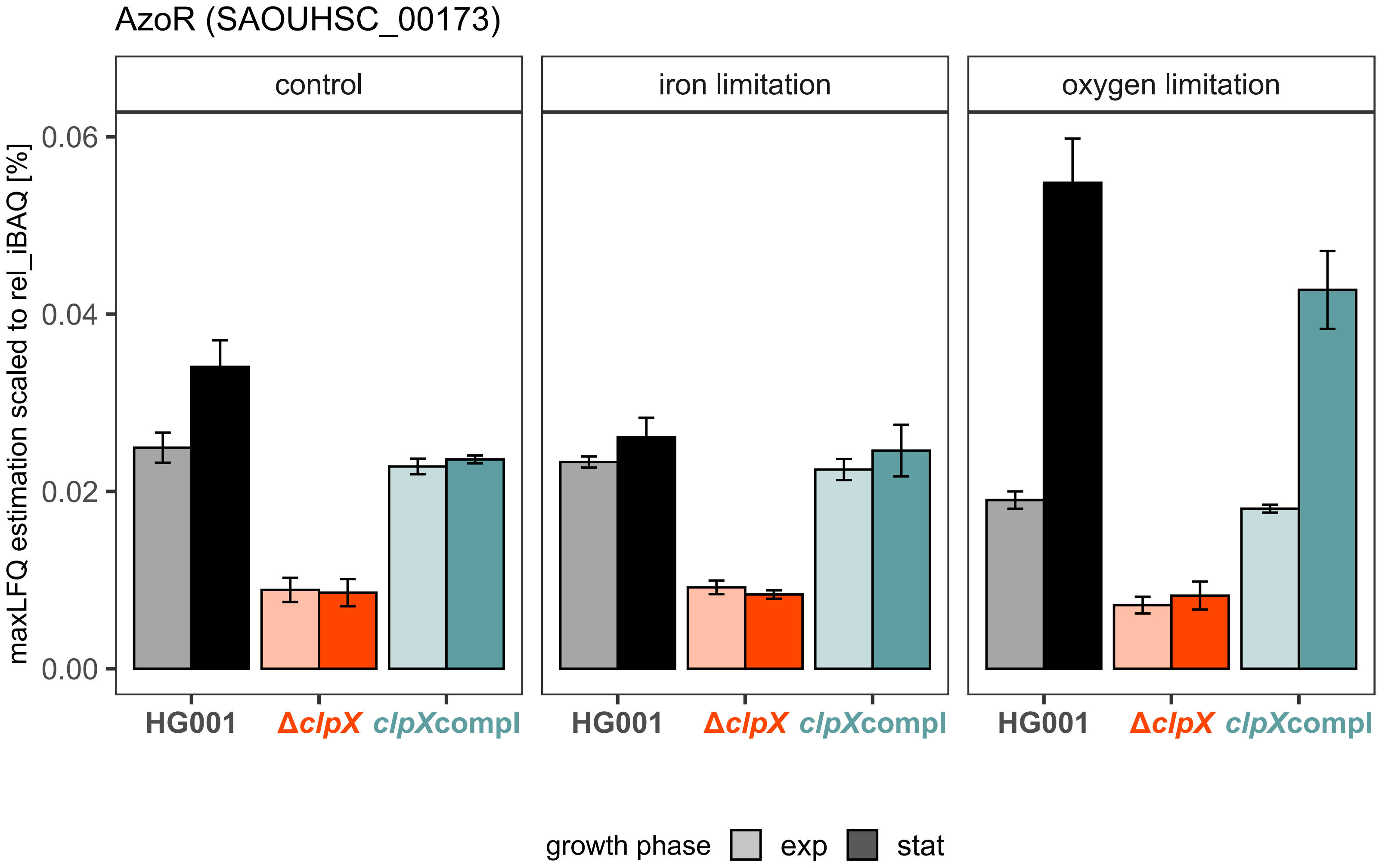

AzoR (SAOUHSC_00173)
control
iron limitation
oxygen limitation
maxLFQ estimation scaled to rel_iBAQ [%]
0.06
0.04
0.02
0.00
HG001
ΔclpX
clpXcompl
growth phase
exp
stat

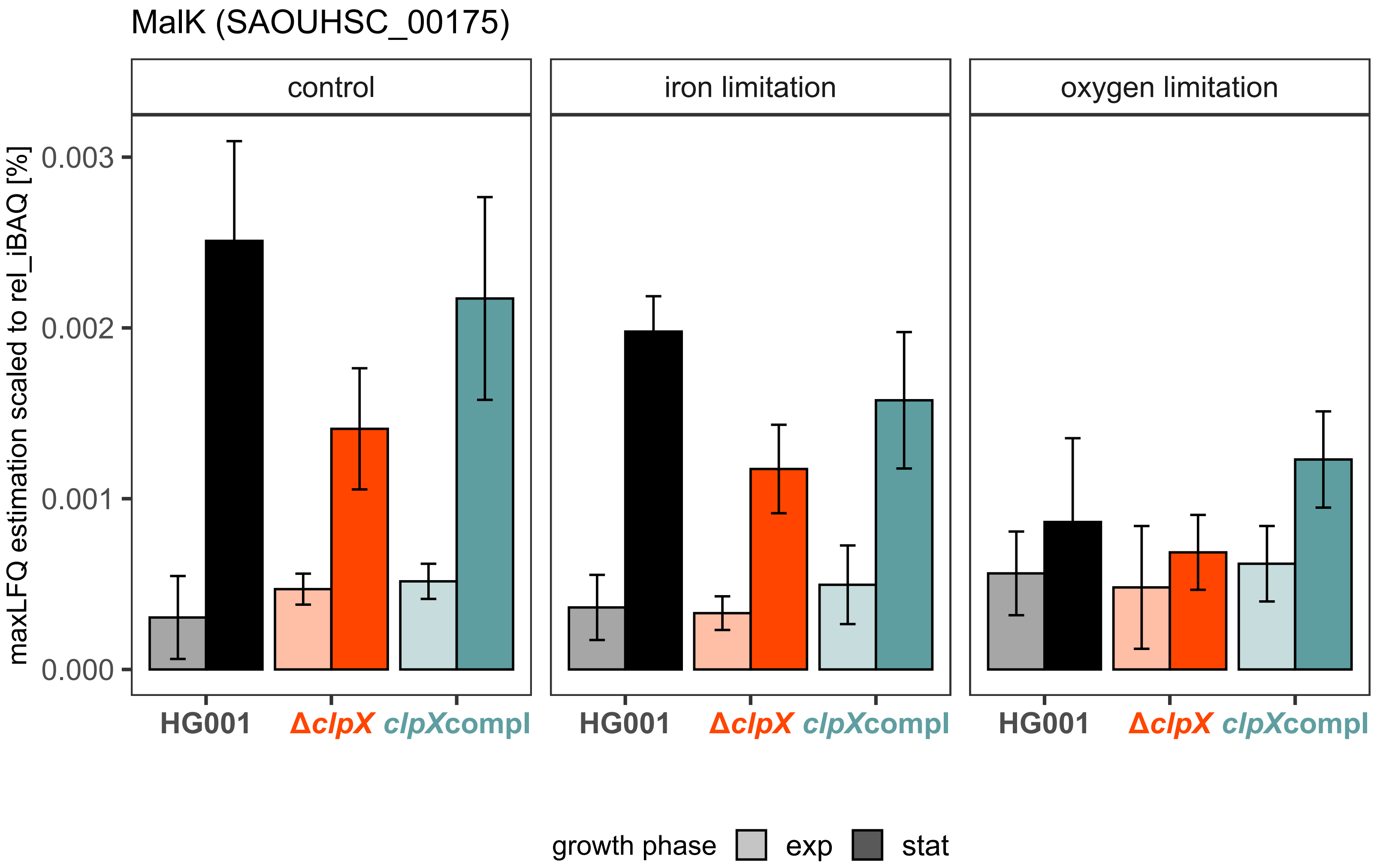
MalK (SAOUHSC_00175)
control
iron limitation
oxygen limitation
maxLFQ estimation scaled to rel_iBAQ [%]
0.003
0.002
0.001
0.000
HG001
ΔclpX
clpXcompl
growth phase
exp
stat

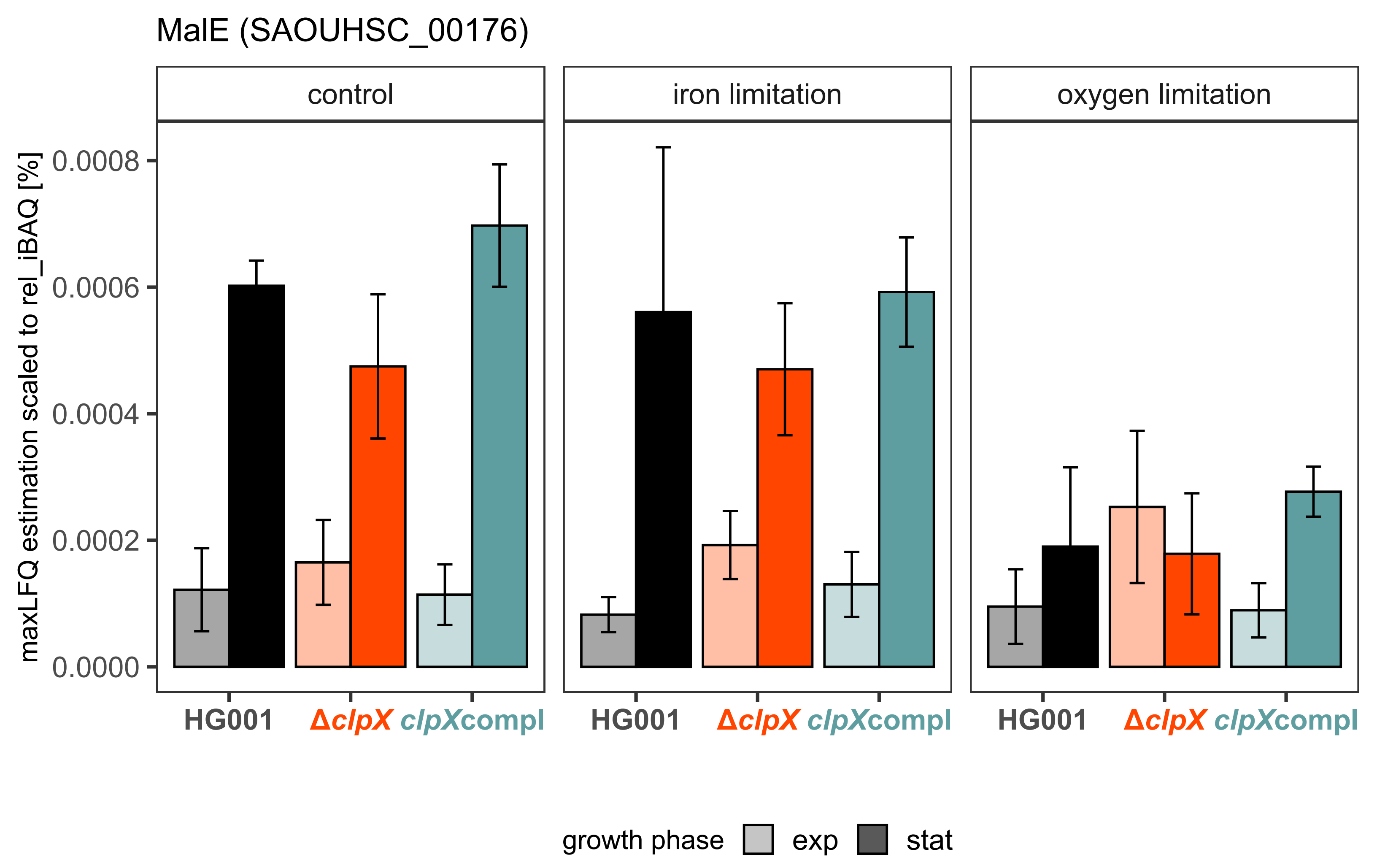

MalE (SAOUHSC_00176)
control
iron limitation
oxygen limitation
maxLFQ estimation scaled to rel_iBAQ [%]
0.0008
0.0006
0.0004
0.0002
0.0000
HG001
ΔclpX
clpXcompl
growth phase
exp
stat

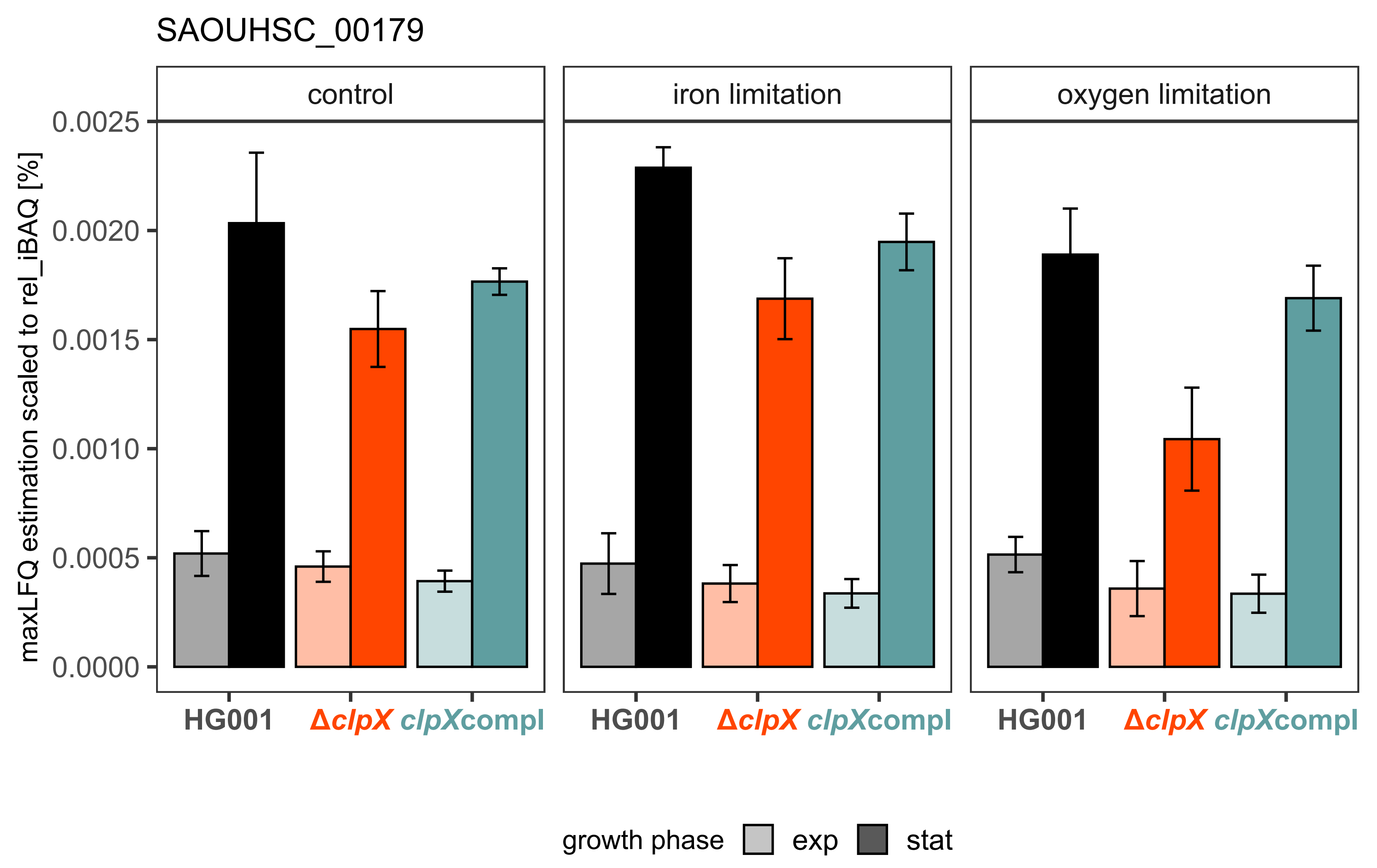
SAOUHSC_00179
control
iron limitation
oxygen limitation
maxLFQ estimation scaled to rel_iBAQ [%]
0.0025
0.0020
0.0015
0.0010
0.0005
0.0000
HG001
ΔclpX
clpXcompl
growth phase
exp
stat

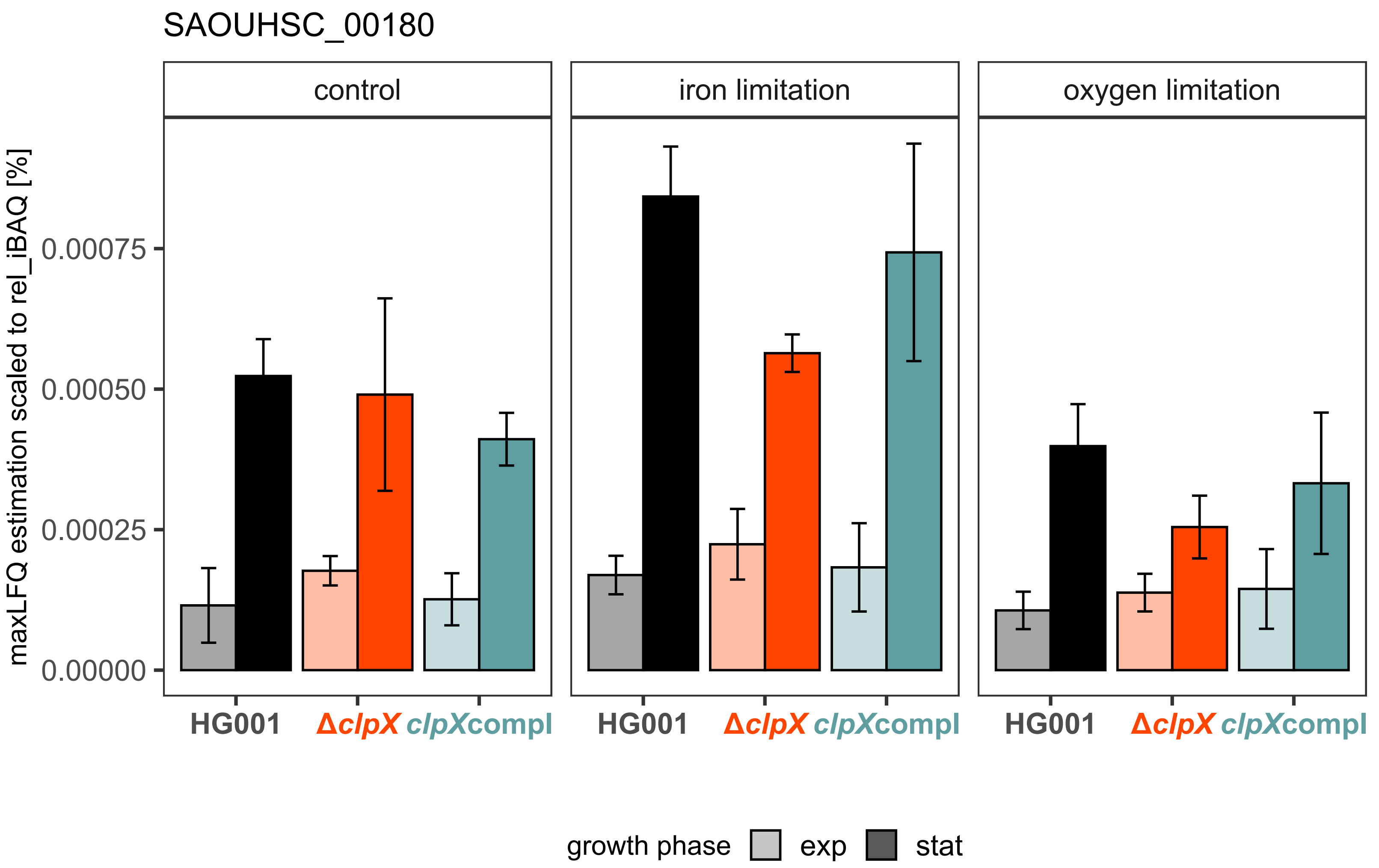
SAOUHSC_00180
control
iron limitation
oxygen limitation
maxLFQ estimation scaled to rel_iBAQ [%]
0.00075
0.00050
0.00025
0.00000
HG001
ΔclpX
clpXcompl
growth phase
exp
stat

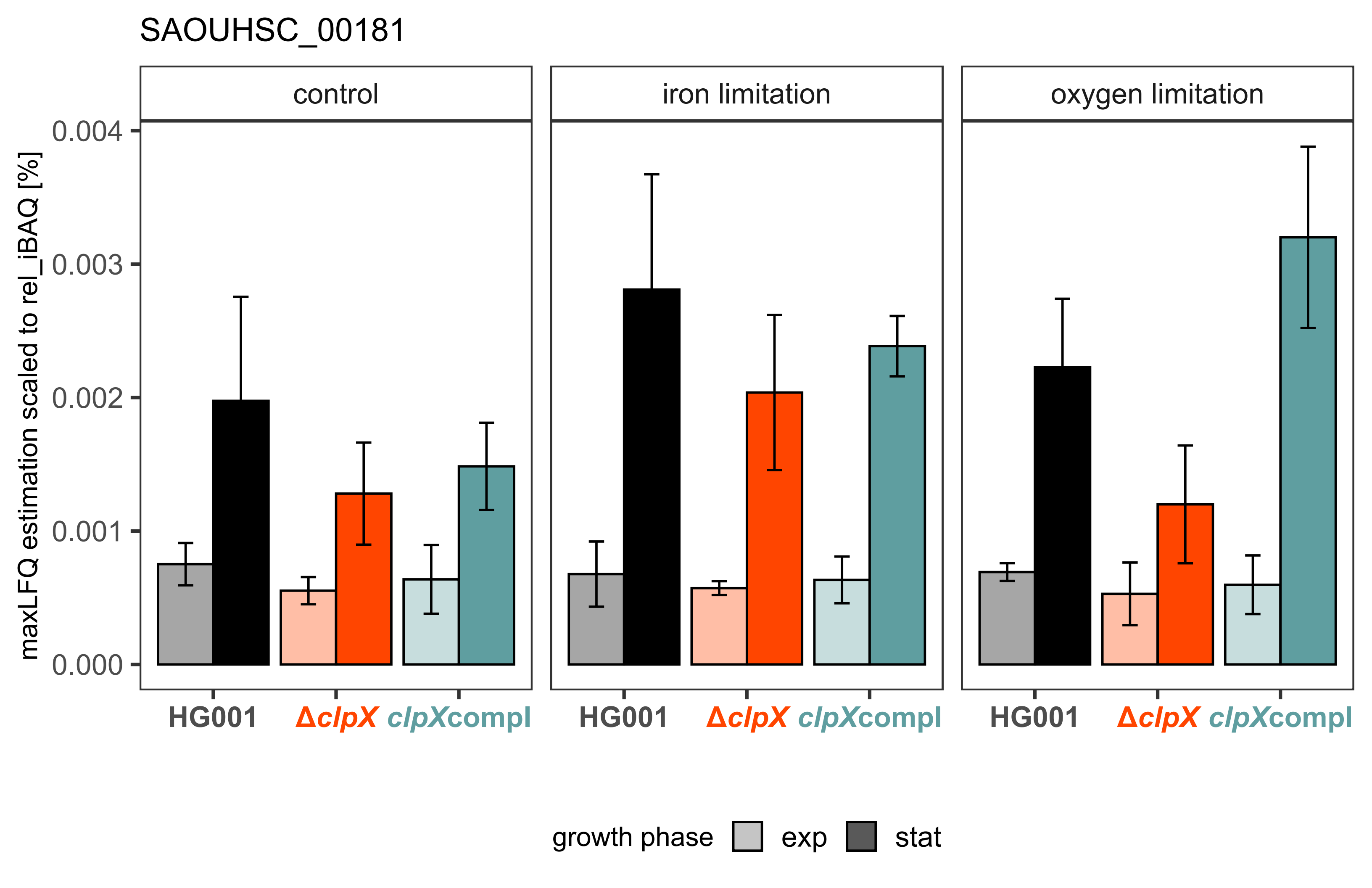

SAOUHSC_00181
control
iron limitation
oxygen limitation
maxLFQ estimation scaled to rel_iBAQ [%]
0.004
0.003
0.002
0.001
0.000
HG001
ΔclpX
clpXcompl
growth phase
exp
stat

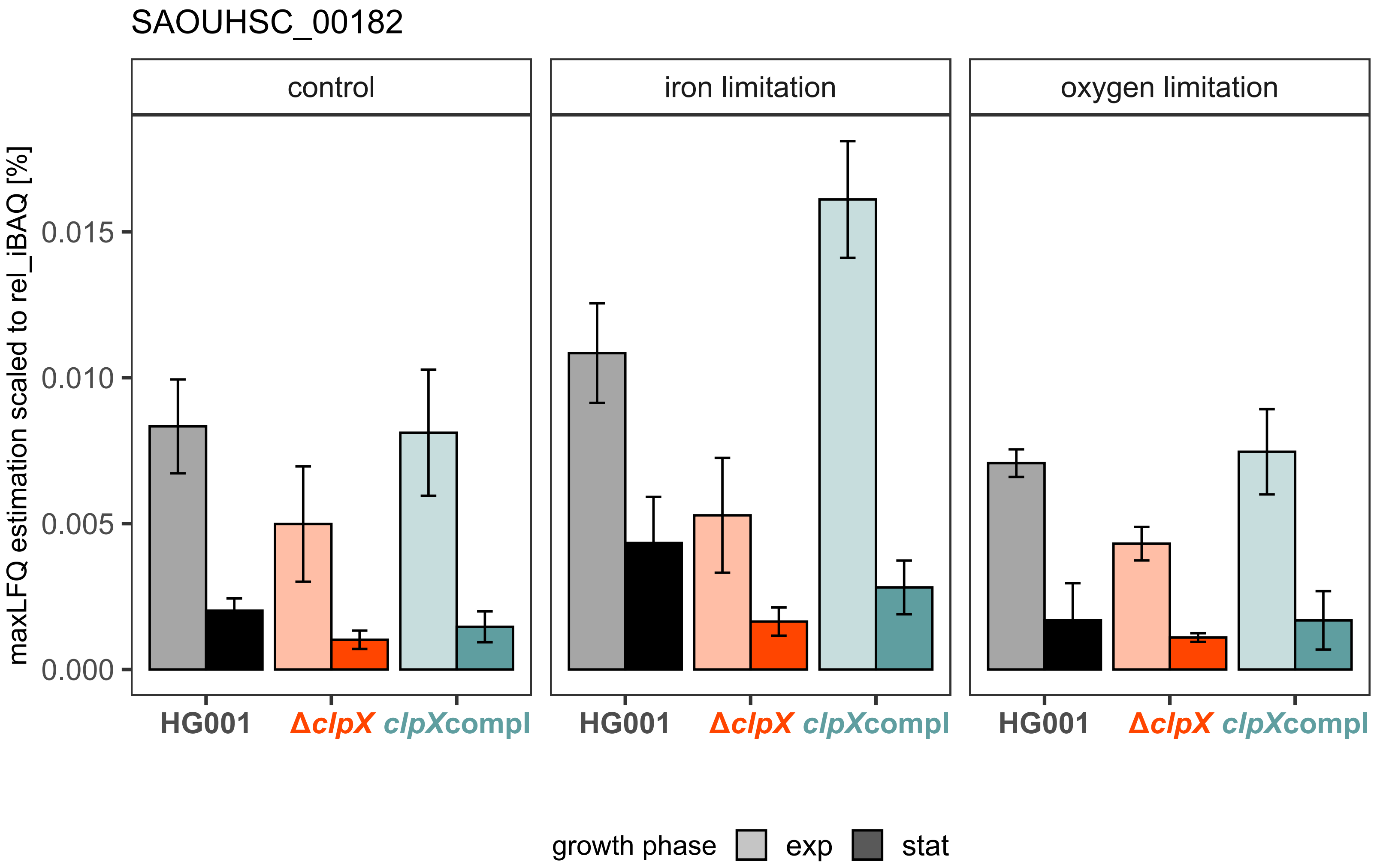

SAOUHSC_00182
control
iron limitation
oxygen limitation
maxLFQ estimation scaled to rel_iBAQ [%]
0.015
0.010
0.005
0.000
HG001
ΔclpX
clpXcompl
growth phase
exp
stat

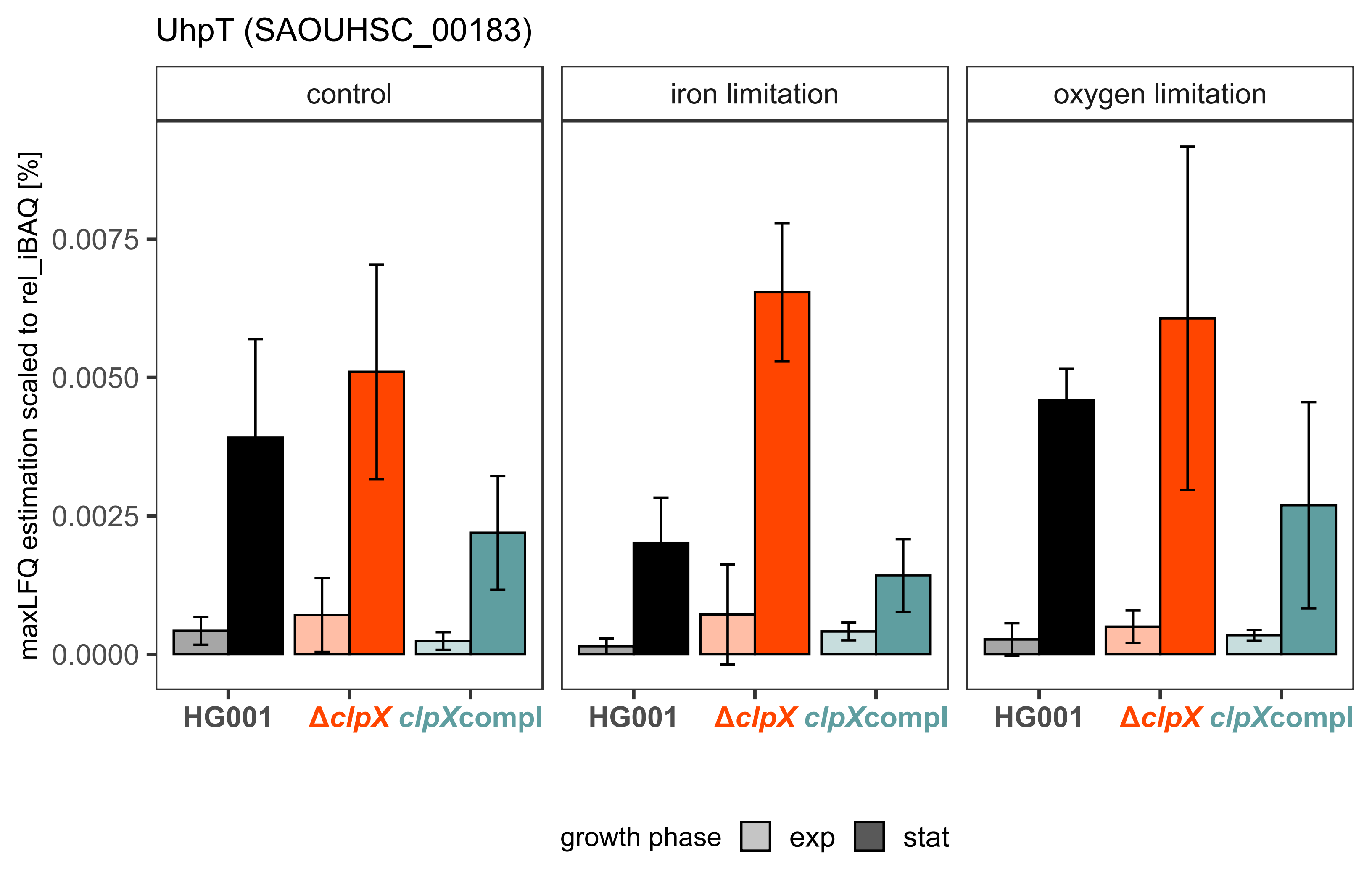
UhpT (SAOUHSC_00183)
control
iron limitation
oxygen limitation
maxLFQ estimation scaled to rel_iBAQ [%]
0.0075
0.0050
0.0025
0.0000
HG001
ΔclpX
clpXcompl
growth phase
exp
stat

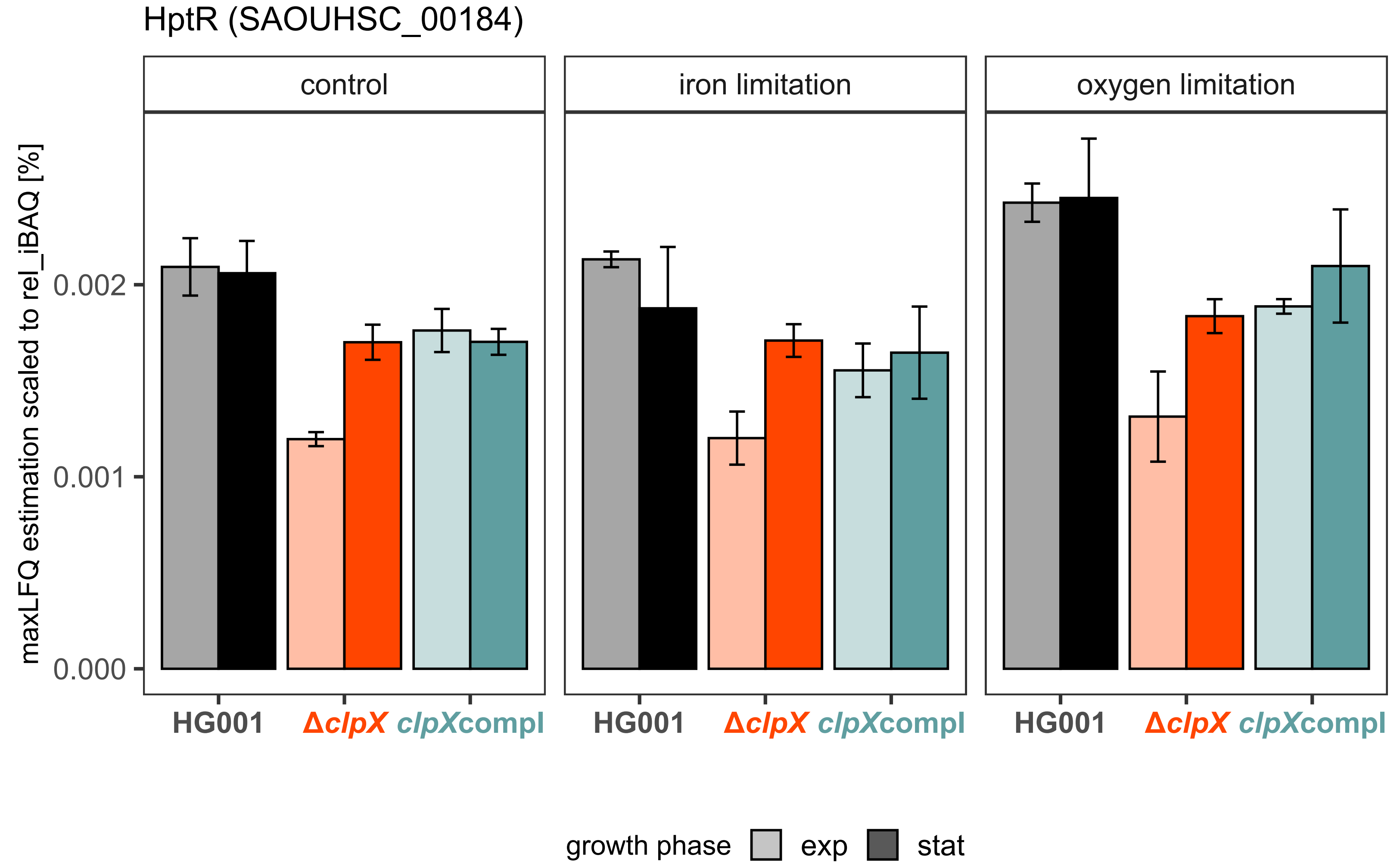
HptR (SAOUHSC_00184)
control
iron limitation
oxygen limitation
maxLFQ estimation scaled to rel_iBAQ [%]
0.002
0.001
0.000
HG001
ΔclpX
clpXcompl
growth phase
exp
stat

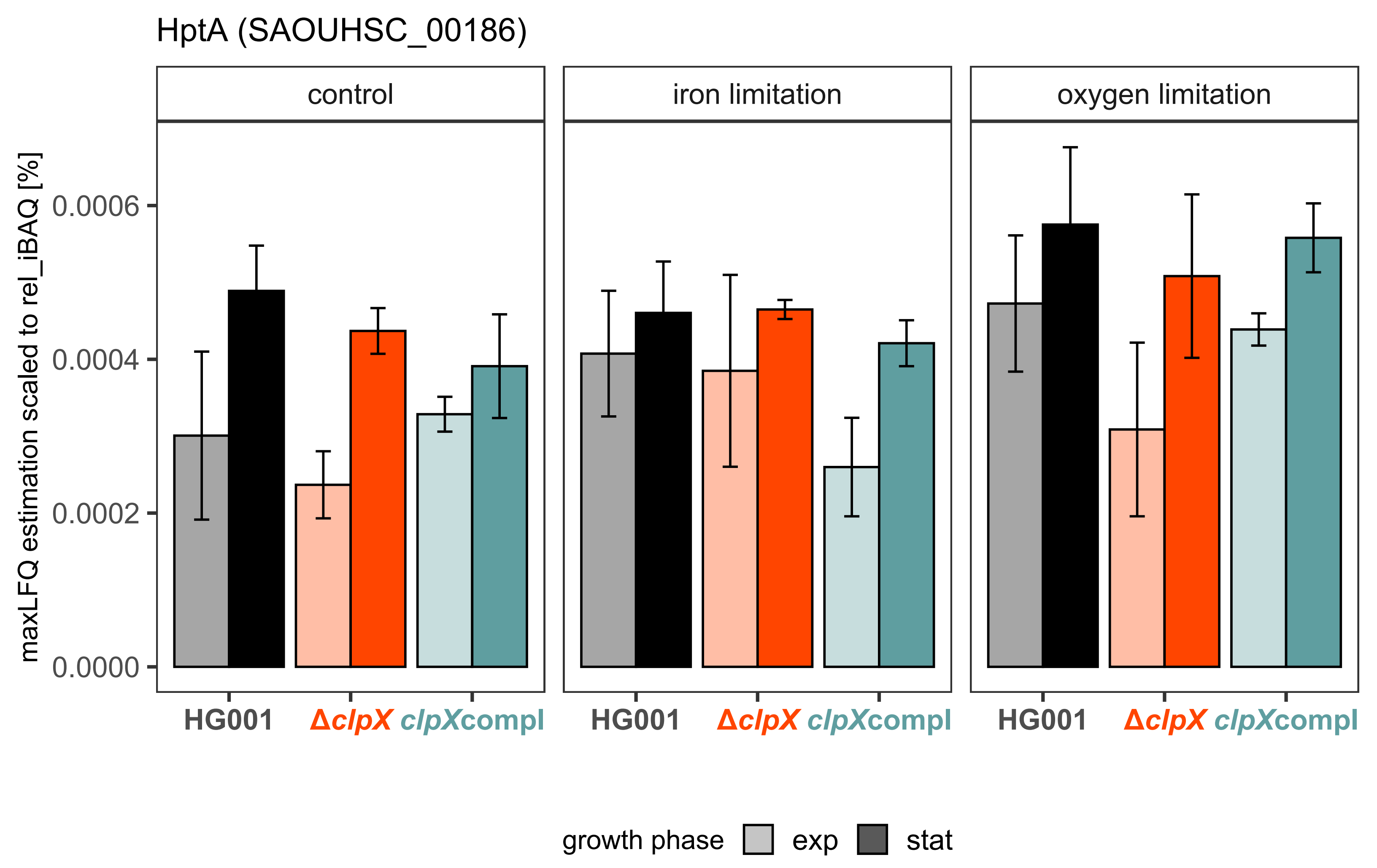

HptA (SAOUHSC_00186)
control
iron limitation
oxygen limitation
maxLFQ estimation scaled to rel_iBAQ [%]
0.0006
0.0004
0.0002
0.0000
HG001
ΔclpX
clpXcompl
growth phase
exp
stat

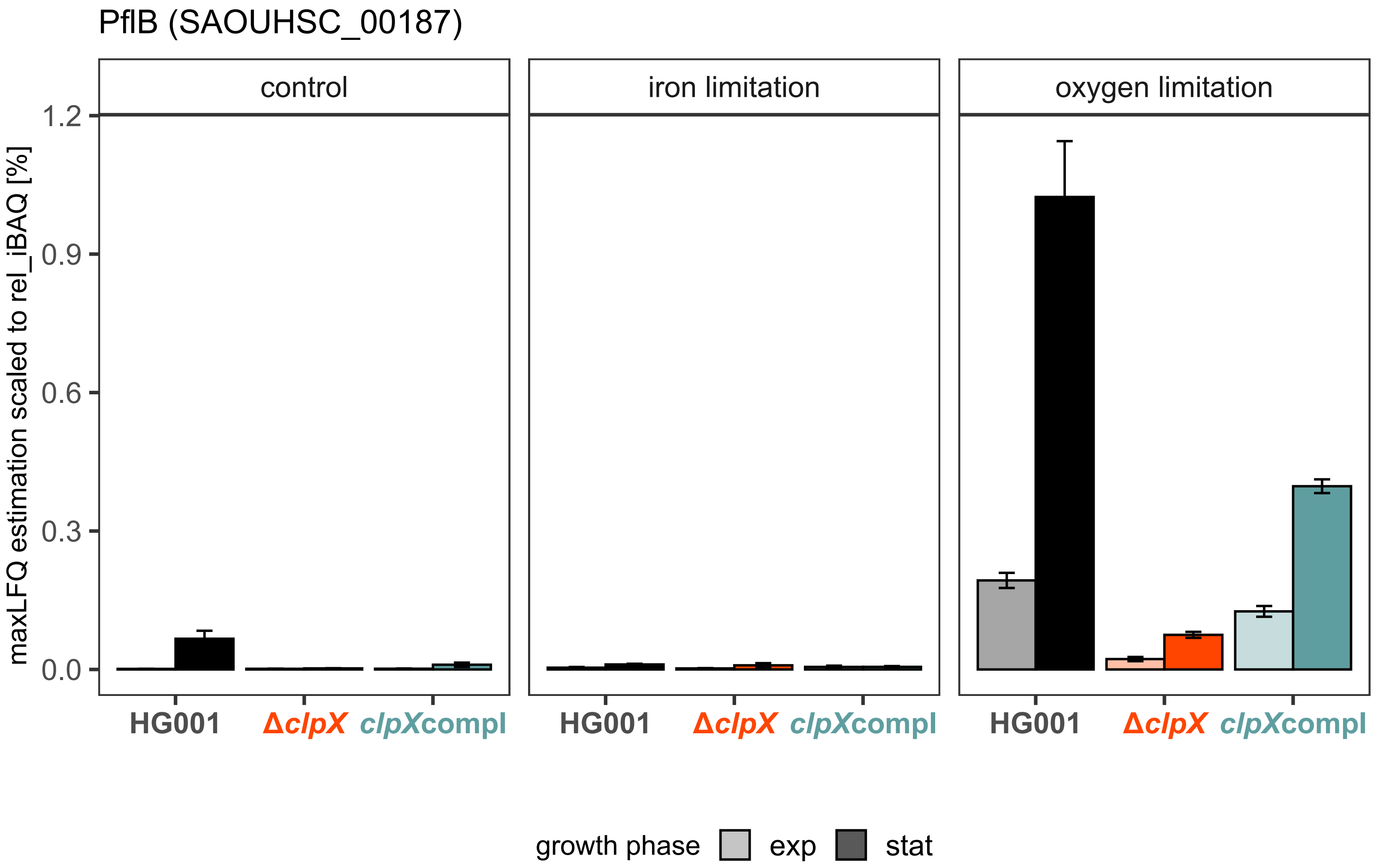
PflB (SAOUHSC_00187)
control
iron limitation
oxygen limitation
maxLFQ estimation scaled to rel_iBAQ [%]
1.2
0.9
0.6
0.3
0.0
HG001
ΔclpX
clpXcompl
HG001
ΔclpX
clpXcompl
HG001
ΔclpX
clpXcompl
growth phase
exp
stat

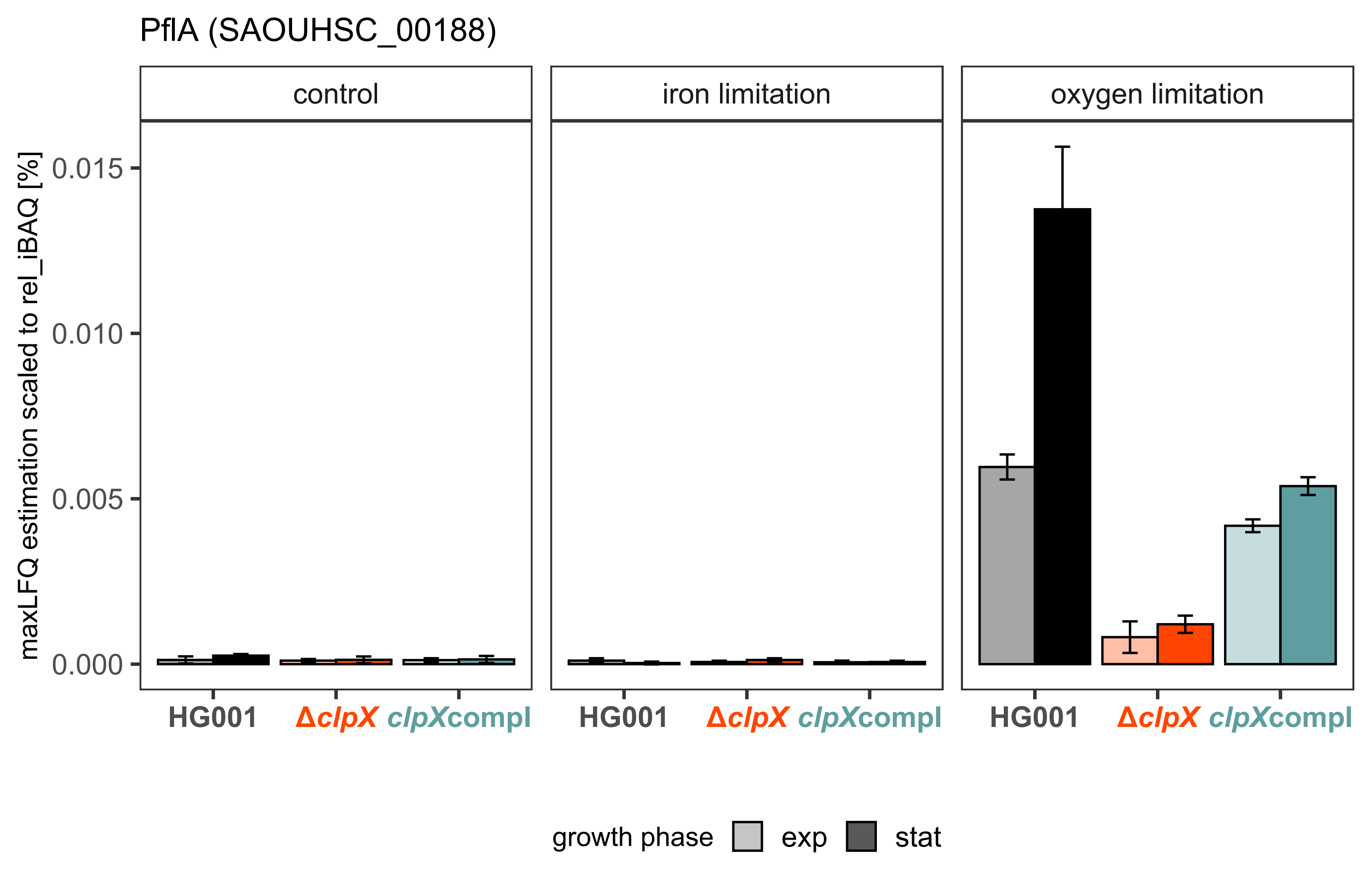

PflA (SAOUHSC_00188)
control
iron limitation
oxygen limitation
maxLFQ estimation scaled to rel_iBAQ [%]
0.015
0.010
0.005
0.000
HG001
ΔclpX
clpXcompl
growth phase
exp
stat

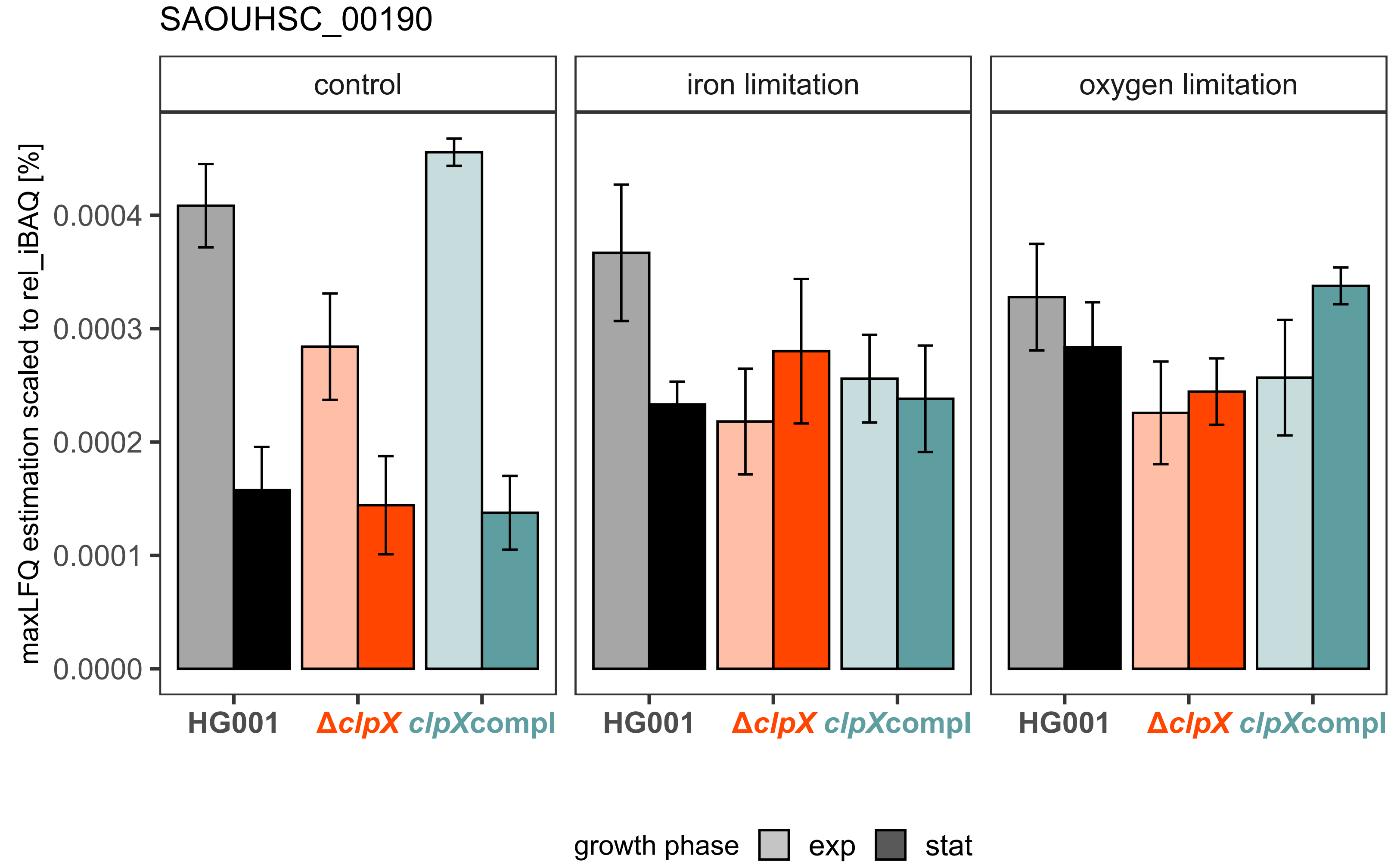

SAOUHSC_00190
control
iron limitation
oxygen limitation
maxLFQ estimation scaled to rel_iBAQ [%]
0.0000
0.0001
0.0002
0.0003
0.0004
HG001
ΔclpX
clpXcompl
growth phase
exp
stat

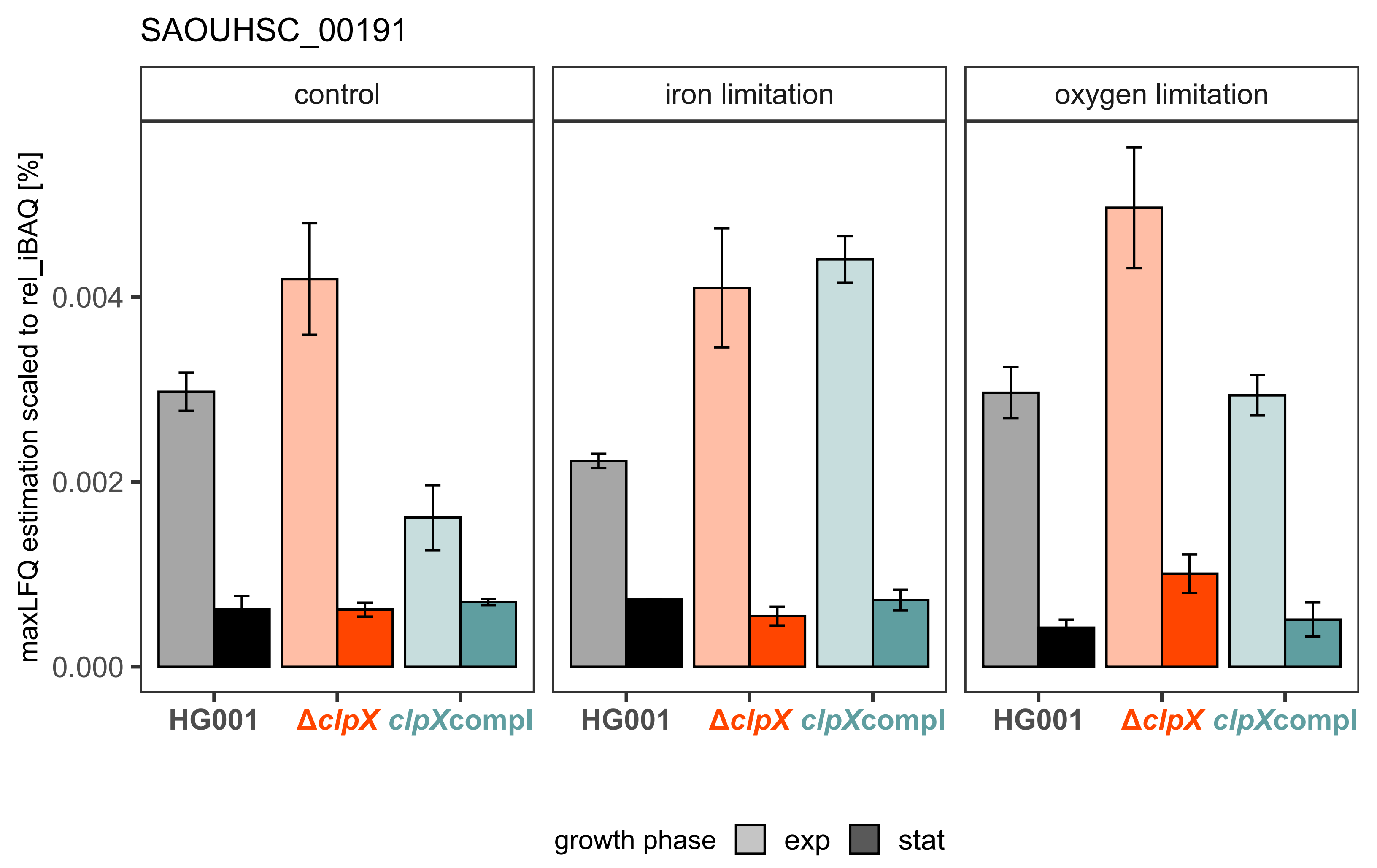
SAOUHSC_00191
control
iron limitation
oxygen limitation
maxLFQ estimation scaled to rel_iBAQ [%]
0.004
0.002
0.000
HG001
ΔclpX
clpXcompl
growth phase
exp
stat

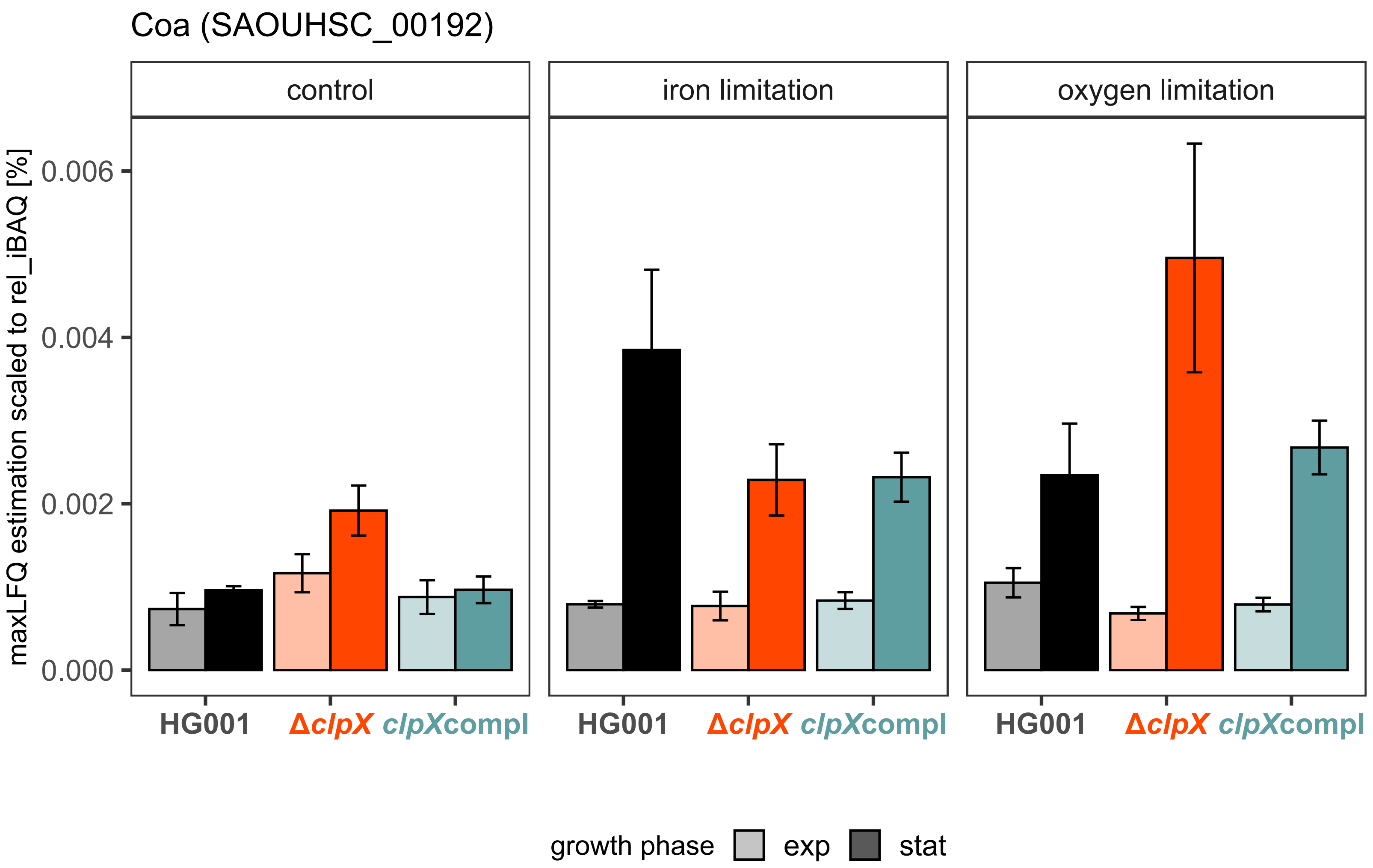

Coa (SAOUHSC_00192)
control
iron limitation
oxygen limitation
maxLFQ estimation scaled to rel_iBAQ [%]
0.006
0.004
0.002
0.000
HG001
ΔclpX
clpXcompl
growth phase
exp
stat

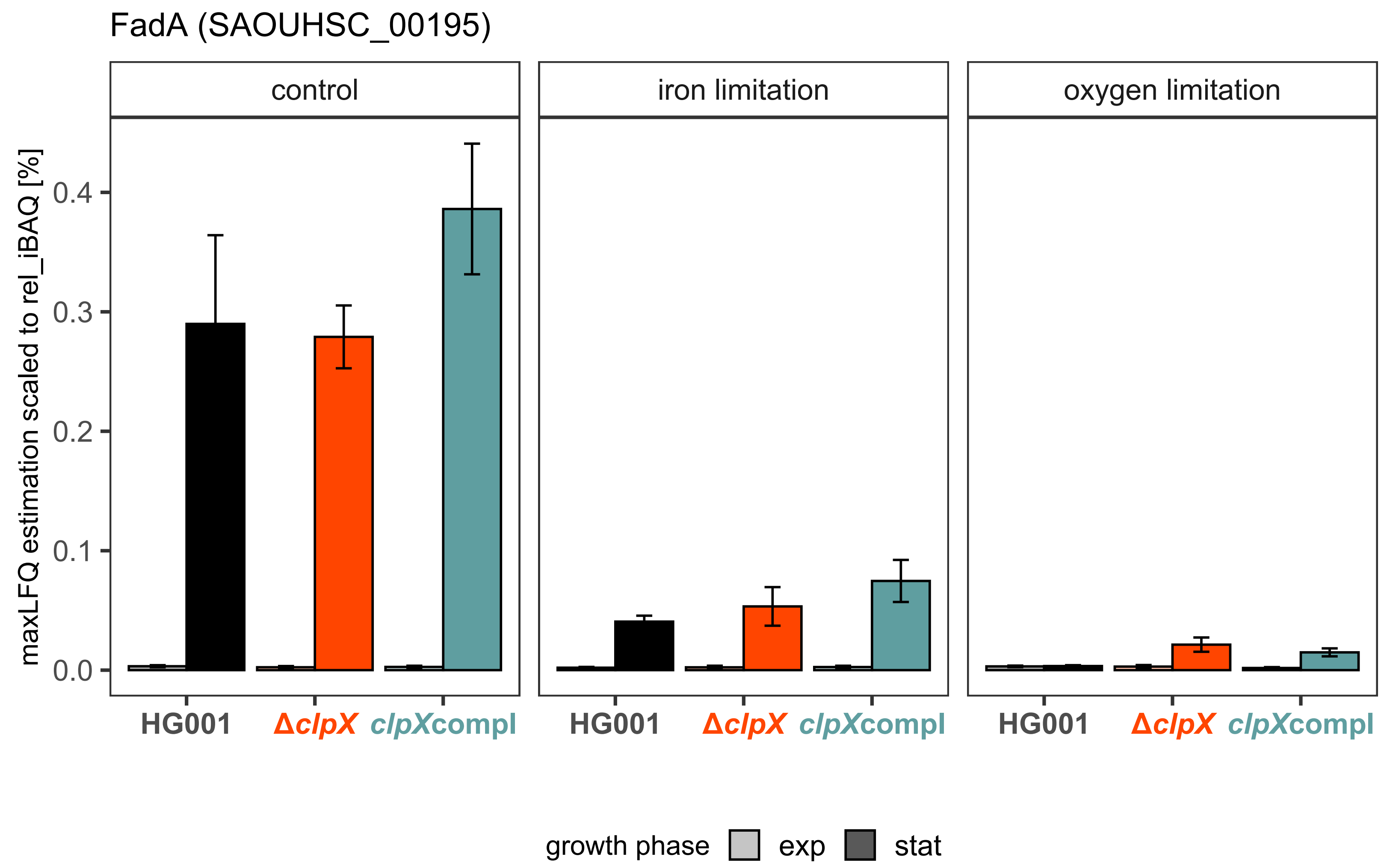

FadA (SAOUHSC_00195)
control
iron limitation
oxygen limitation
maxLFQ estimation scaled to rel_iBAQ [%]
0.0
0.1
0.2
0.3
0.4
HG001
ΔclpX
clpXcompl
growth phase
exp
stat

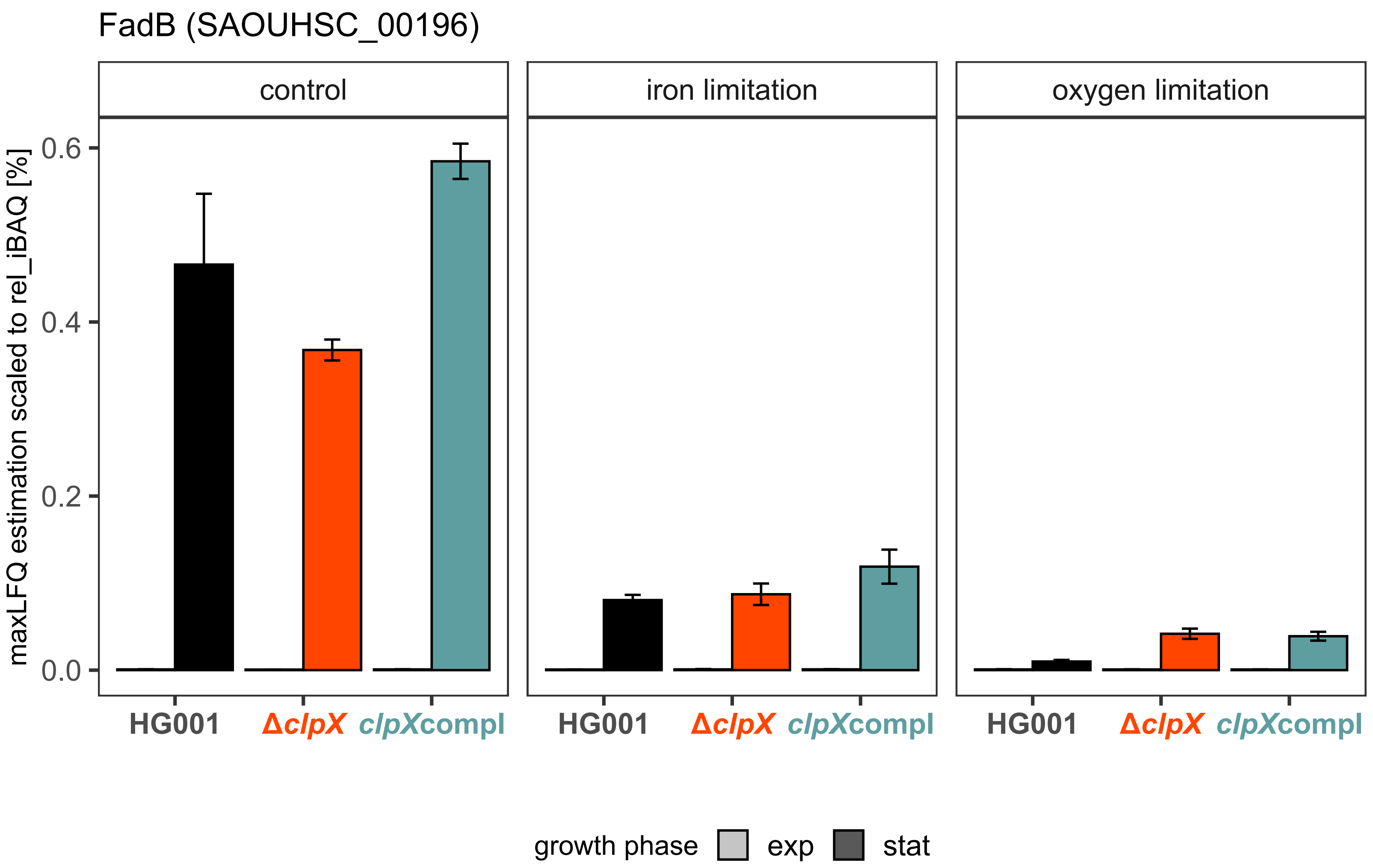

FadB (SAOUHSC_00196)
control
iron limitation
oxygen limitation
maxLFQ estimation scaled to rel_iBAQ [%]
0.6
0.4
0.2
0.0
HG001
ΔclpX
clpXcompl
growth phase
exp
stat

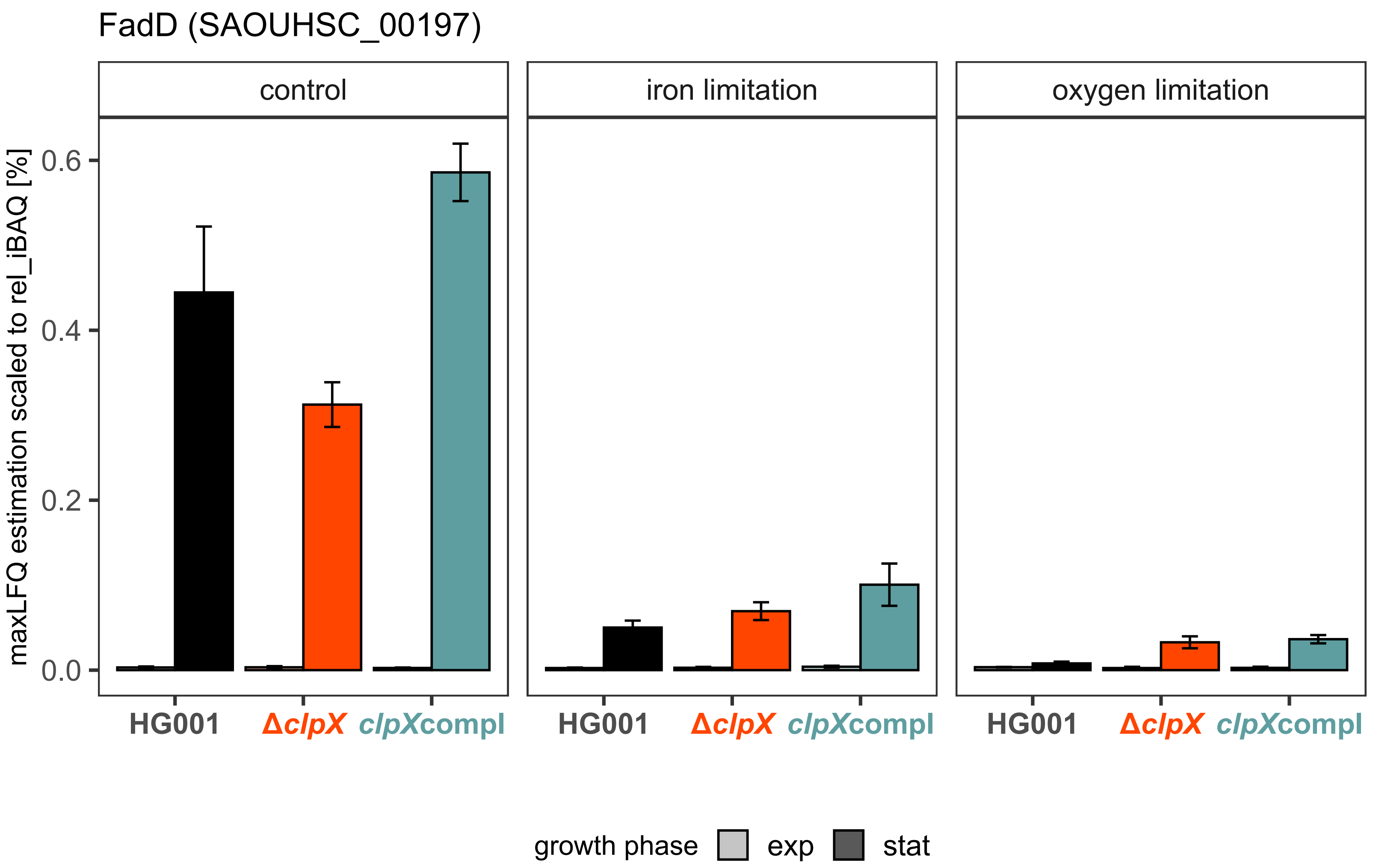
FadD (SAOUHSC_00197)
control
iron limitation
oxygen limitation
maxLFQ estimation scaled to rel_iBAQ [%]
0.0
0.2
0.4
0.6
HG001
ΔclpX
clpXcompl
growth phase
exp
stat

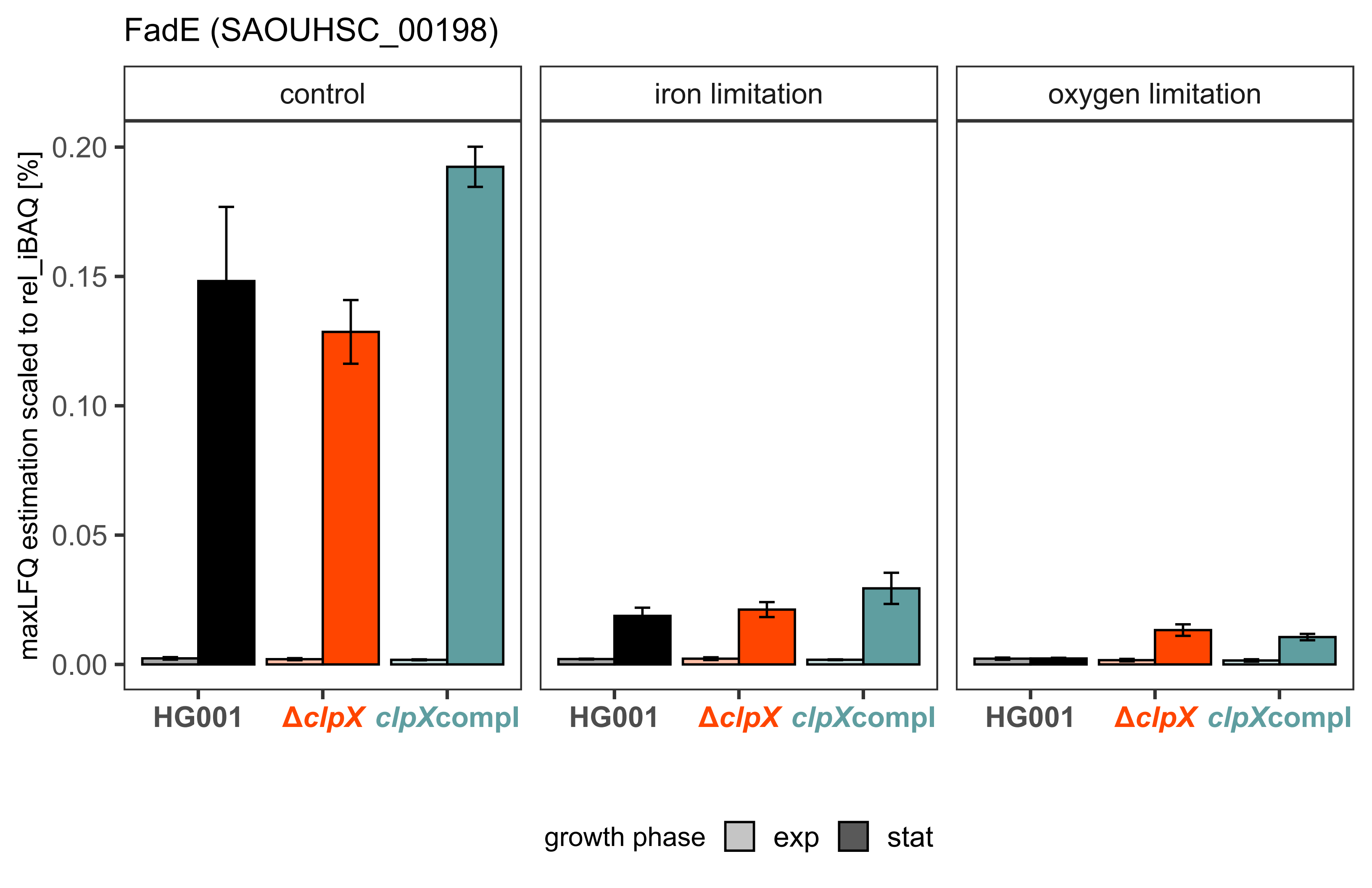

FadE (SAOUHSC_00198)
control
iron limitation
oxygen limitation
maxLFQ estimation scaled to rel_iBAQ [%]
0.20
0.15
0.10
0.05
0.00
HG001
ΔclpX
clpXcompl
HG001
ΔclpX
clpXcompl
HG001
ΔclpX
clpXcompl
growth phase
exp
stat

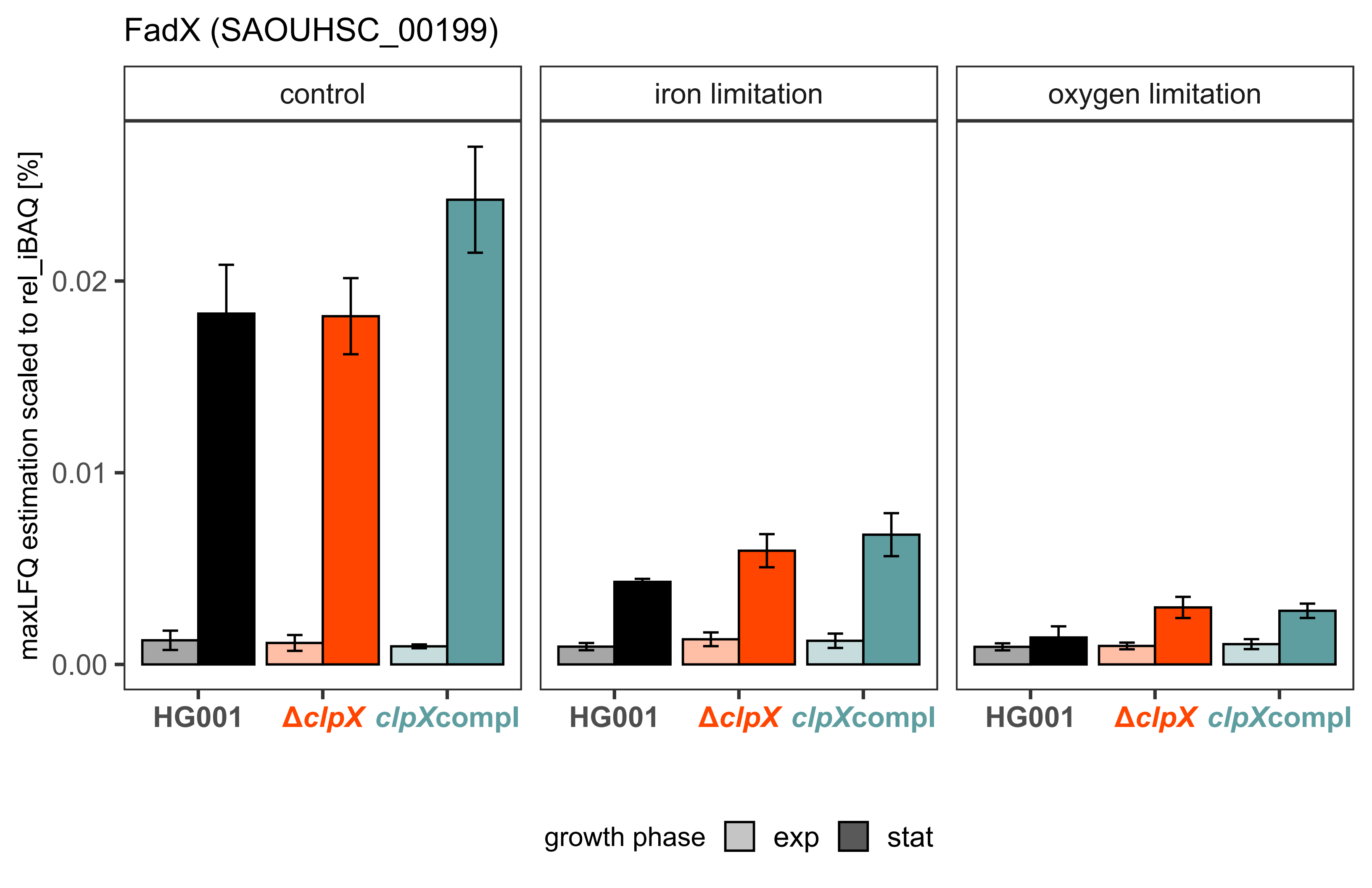
FadX (SAOUHSC_00199)
control
iron limitation
oxygen limitation
maxLFQ estimation scaled to rel_iBAQ [%]
0.02
0.01
0.00
HG001
ΔclpX
clpXcompl
growth phase
exp
stat

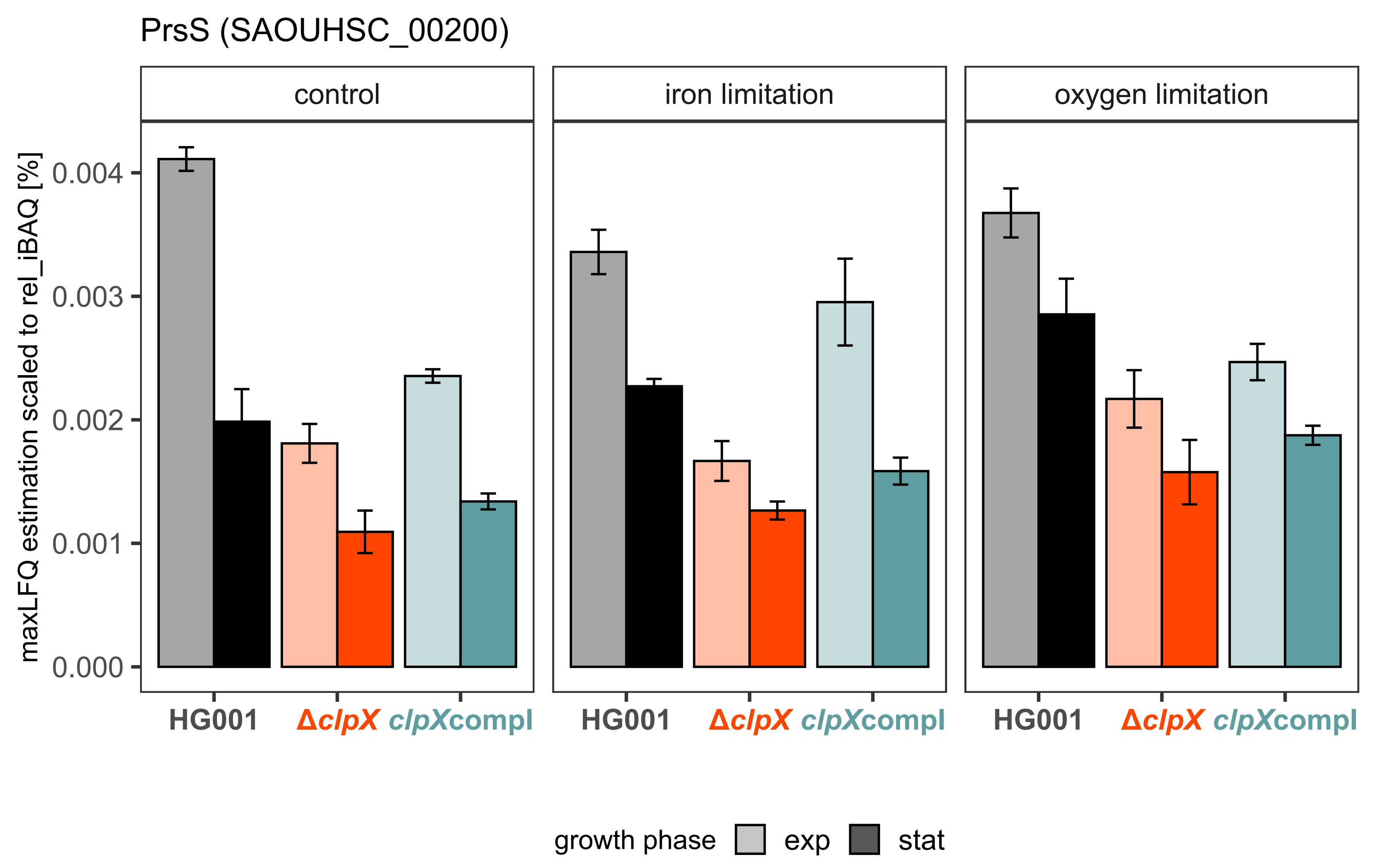

PrsS (SAOUHSC_00200)
control
iron limitation
oxygen limitation
maxLFQ estimation scaled to rel_iBAQ [%]
0.004
0.003
0.002
0.001
0.000
HG001
ΔclpX
clpXcompl
growth phase
exp
stat

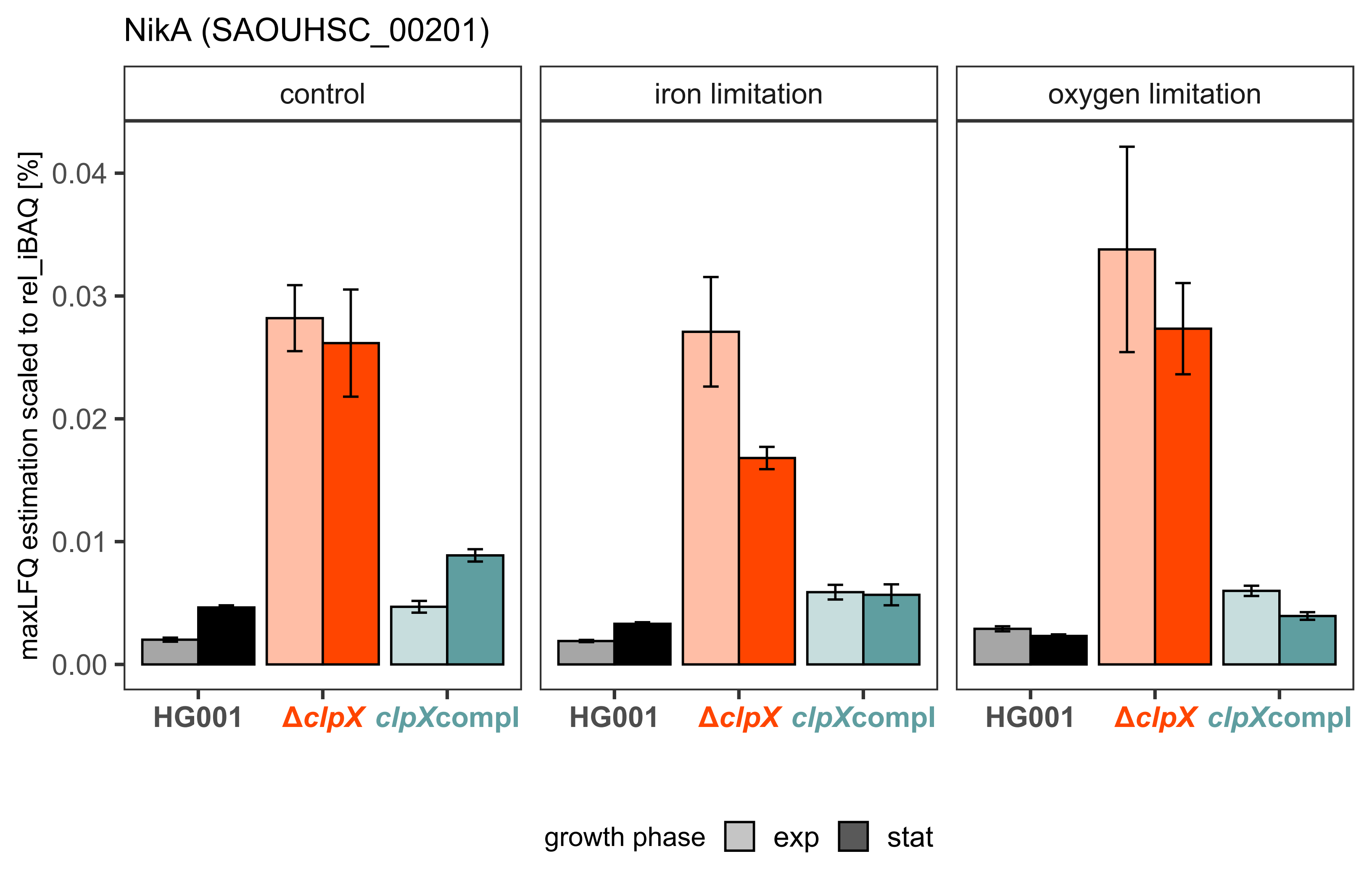
NikA (SAOUHSC_00201)
control
iron limitation
oxygen limitation
maxLFQ estimation scaled to rel_iBAQ [%]
0.04
0.03
0.02
0.01
0.00
HG001
ΔclpX
clpXcompl
growth phase
exp
stat

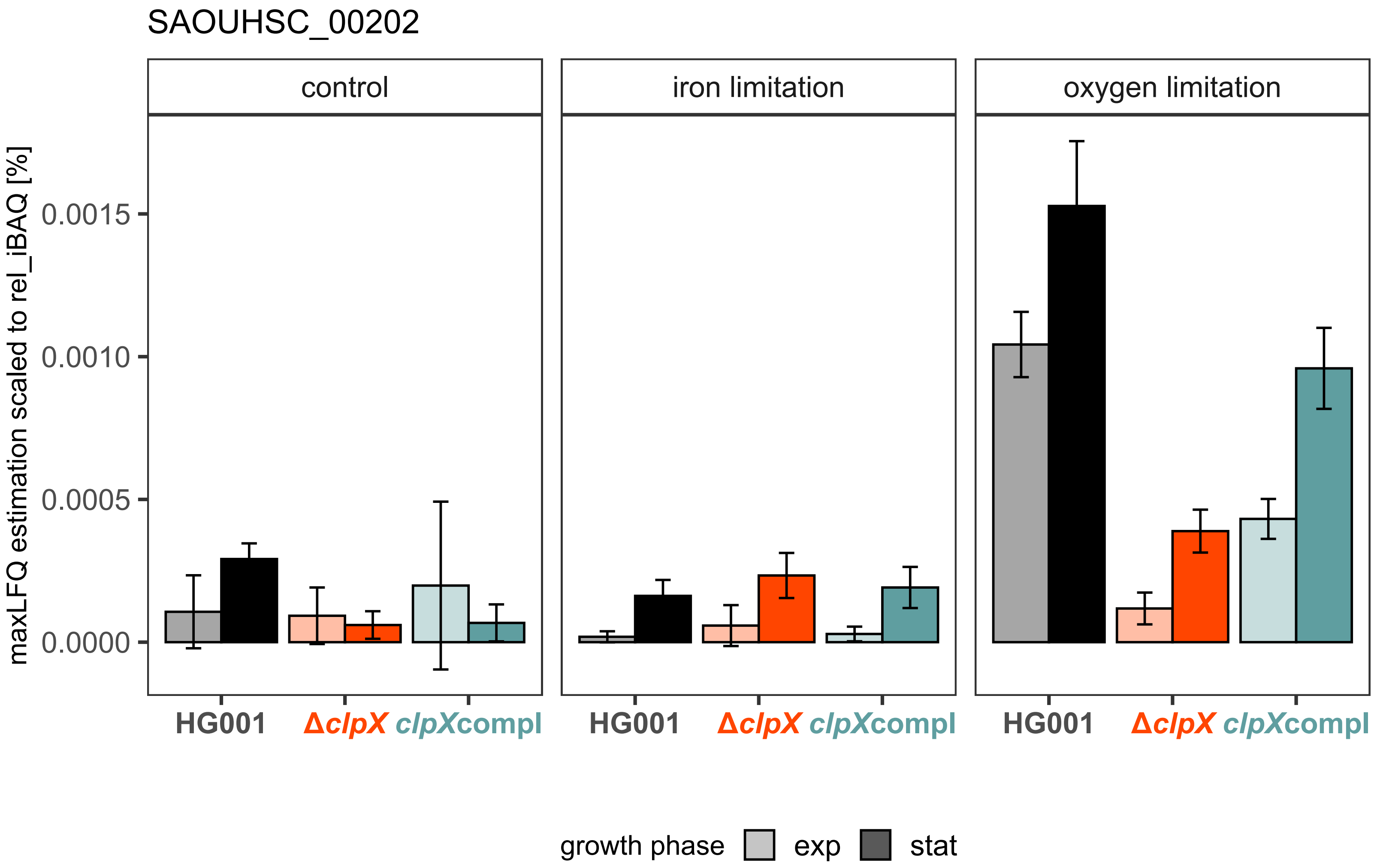

SAOUHSC_00202
control
iron limitation
oxygen limitation
maxLFQ estimation scaled to rel_iBAQ [%]
0.0015
0.0010
0.0005
0.0000
HG001
ΔclpX
clpXcompl
growth phase
exp
stat

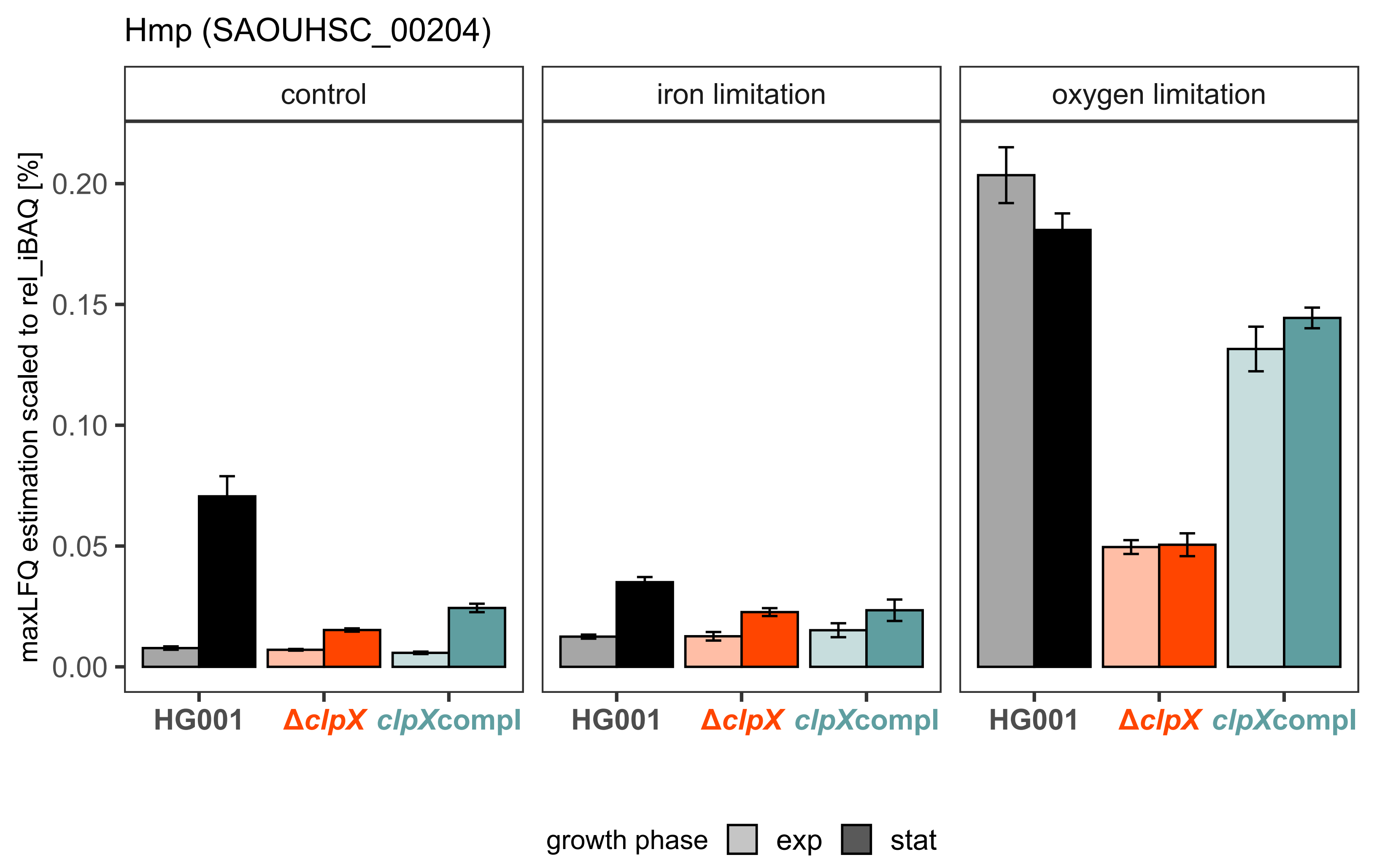
Hmp (SAOUHSC_00204)
control
iron limitation
oxygen limitation
maxLFQ estimation scaled to rel_iBAQ [%]
0.20
0.15
0.10
0.05
0.00
HG001
ΔclpX
clpXcompl
growth phase
exp
stat

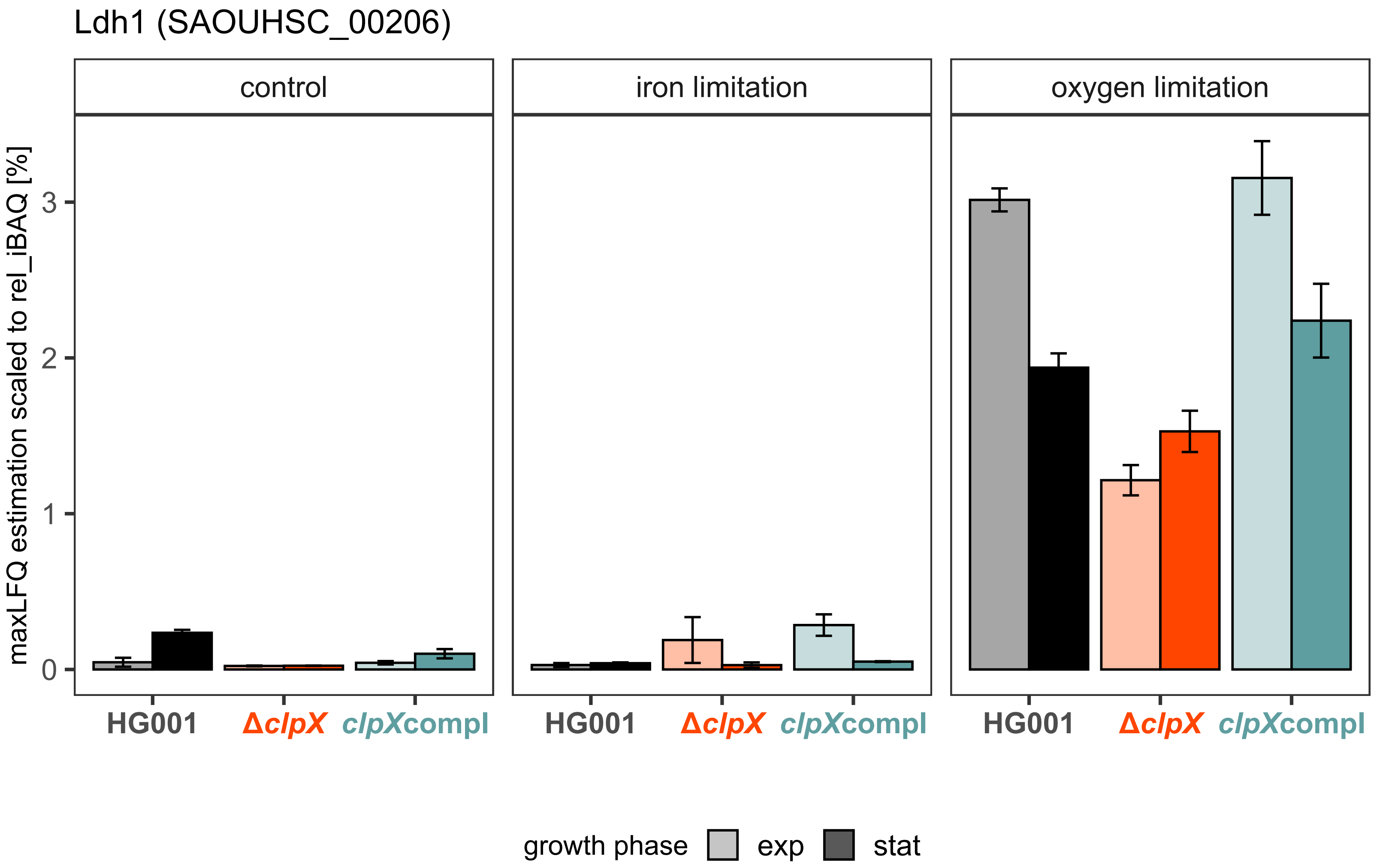

Ldh1 (SAOUHSC_00206)
control
iron limitation
oxygen limitation
maxLFQ estimation scaled to rel_iBAQ [%]
0
1
2
3
HG001
ΔclpX
clpXcompl
growth phase
exp
stat

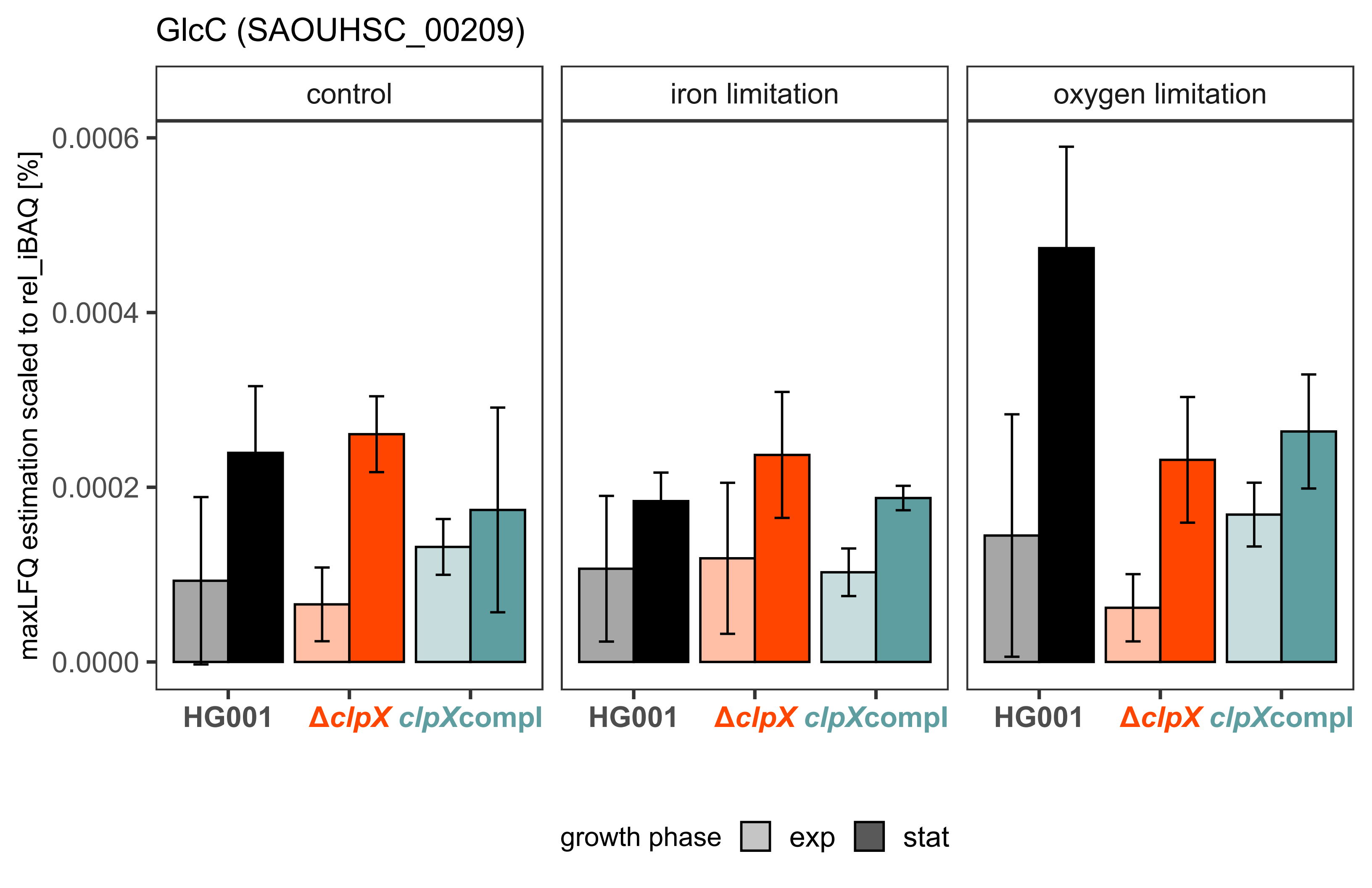

GlcC (SAOUHSC_00209)
control
iron limitation
oxygen limitation
maxLFQ estimation scaled to rel_iBAQ [%]
0.0006
0.0004
0.0002
0.0000
HG001
ΔclpX
clpXcompl
growth phase
exp
stat

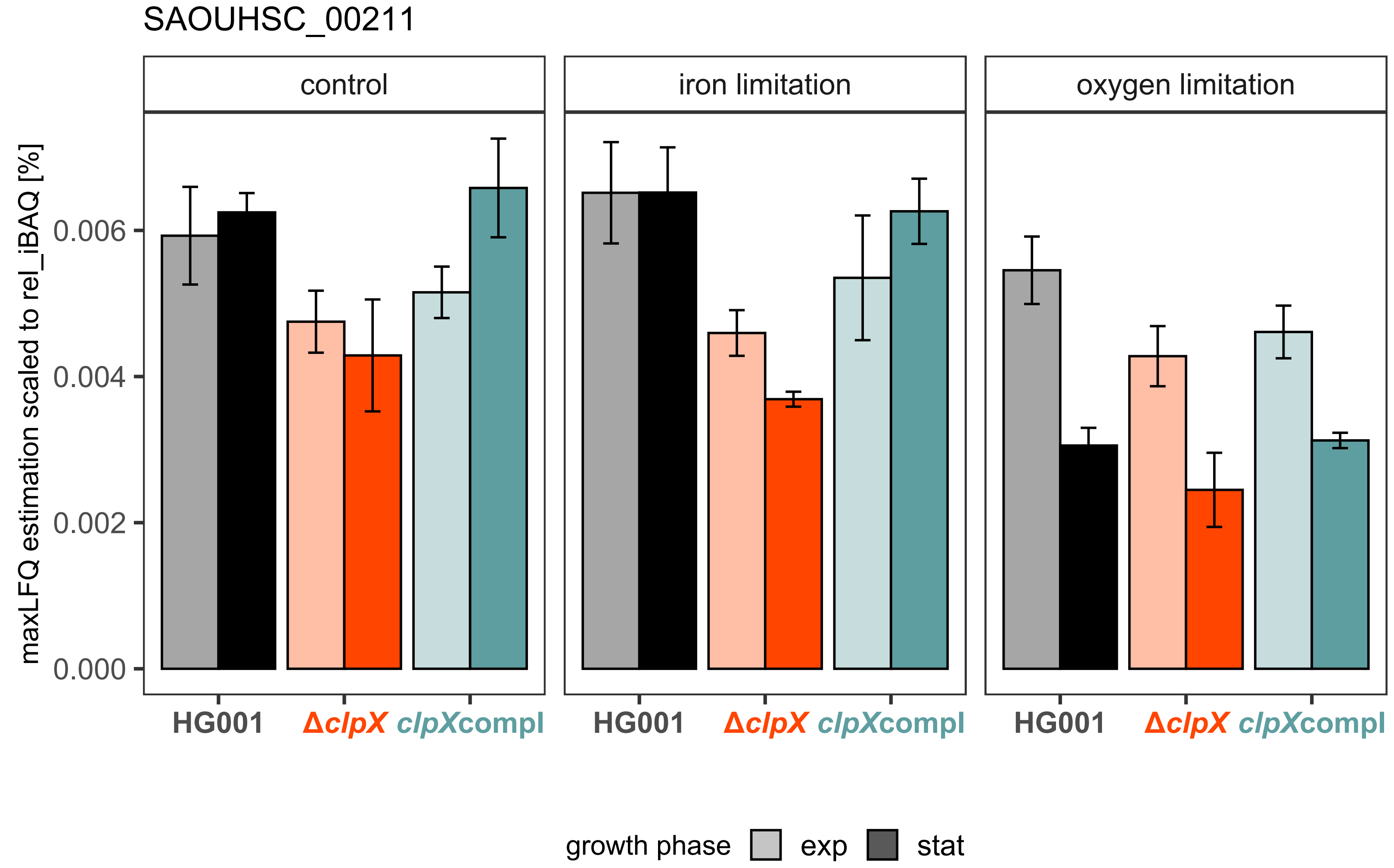

SAOUHSC_00211
control
iron limitation
oxygen limitation
maxLFQ estimation scaled to rel_iBAQ [%]
0.000
0.002
0.004
0.006
HG001
ΔclpX
clpXcompl
growth phase
exp
stat

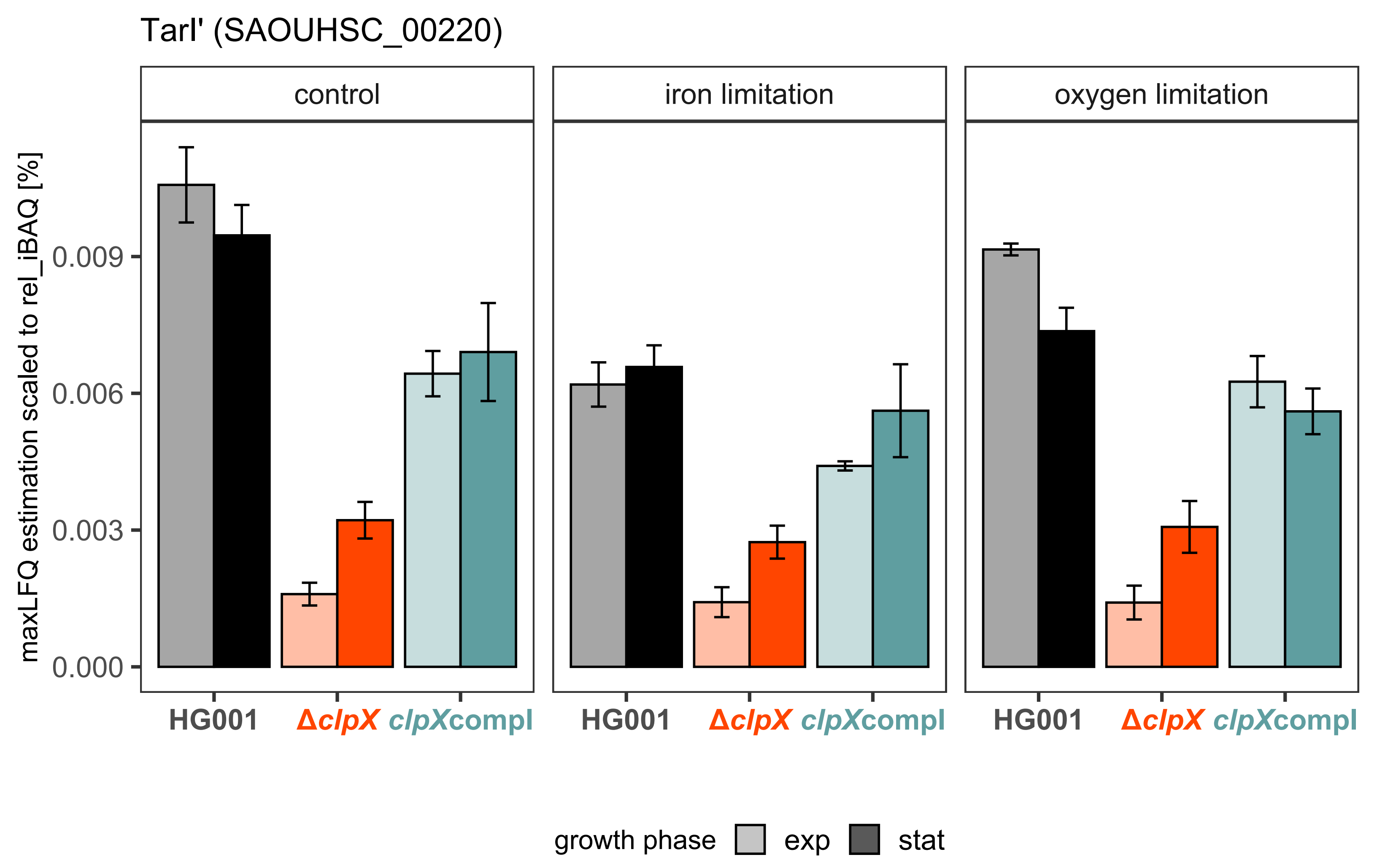
TarI' (SAOUHSC_00220)
control
iron limitation
oxygen limitation
maxLFQ estimation scaled to rel_iBAQ [%]
0.009
0.006
0.003
0.000
HG001
ΔclpX
clpXcompl
growth phase
exp
stat

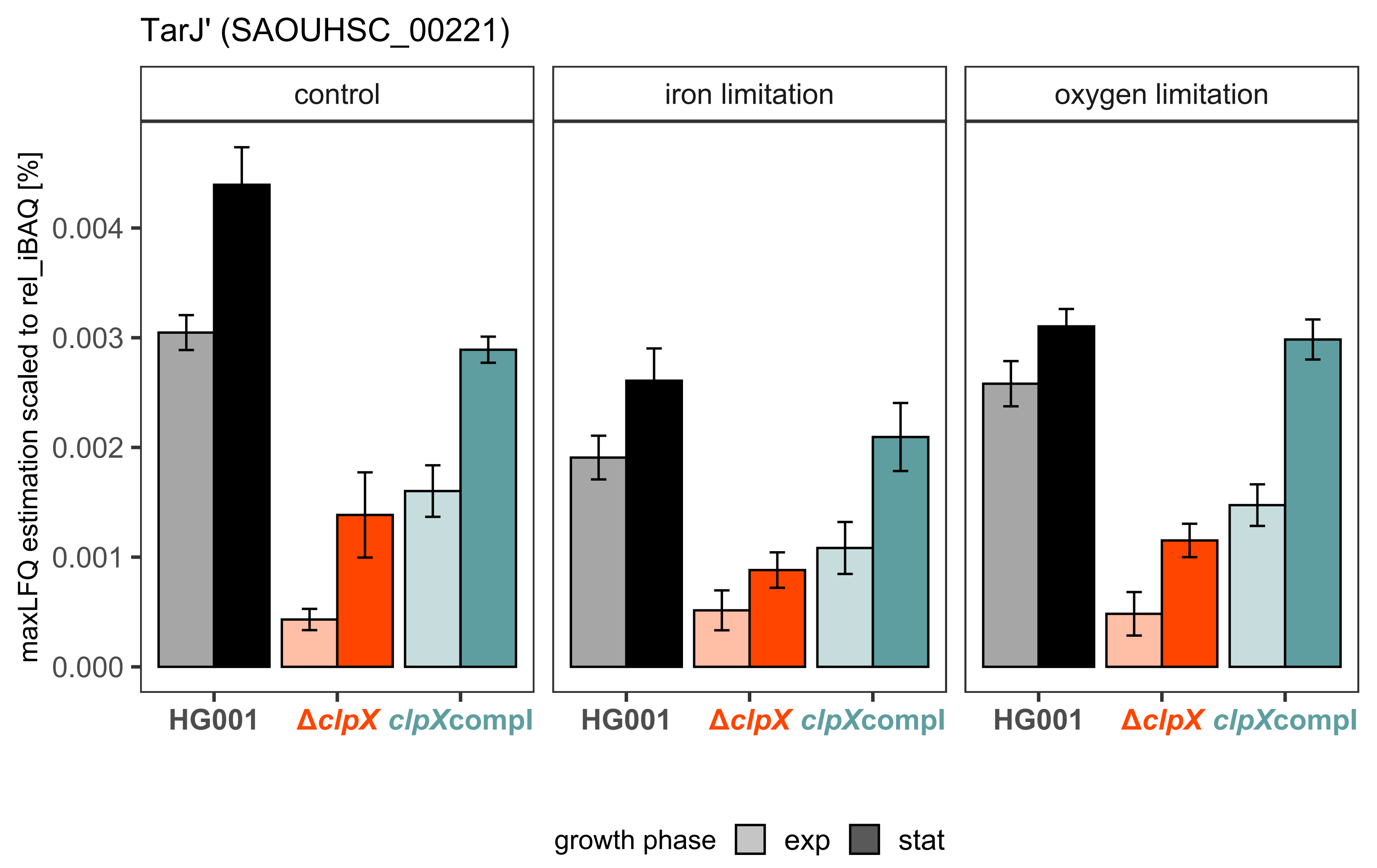

TarJ' (SAOUHSC_00221)
control
iron limitation
oxygen limitation
maxLFQ estimation scaled to rel_iBAQ [%]
0.004
0.003
0.002
0.001
0.000
HG001
ΔclpX
clpXcompl
growth phase
exp
stat

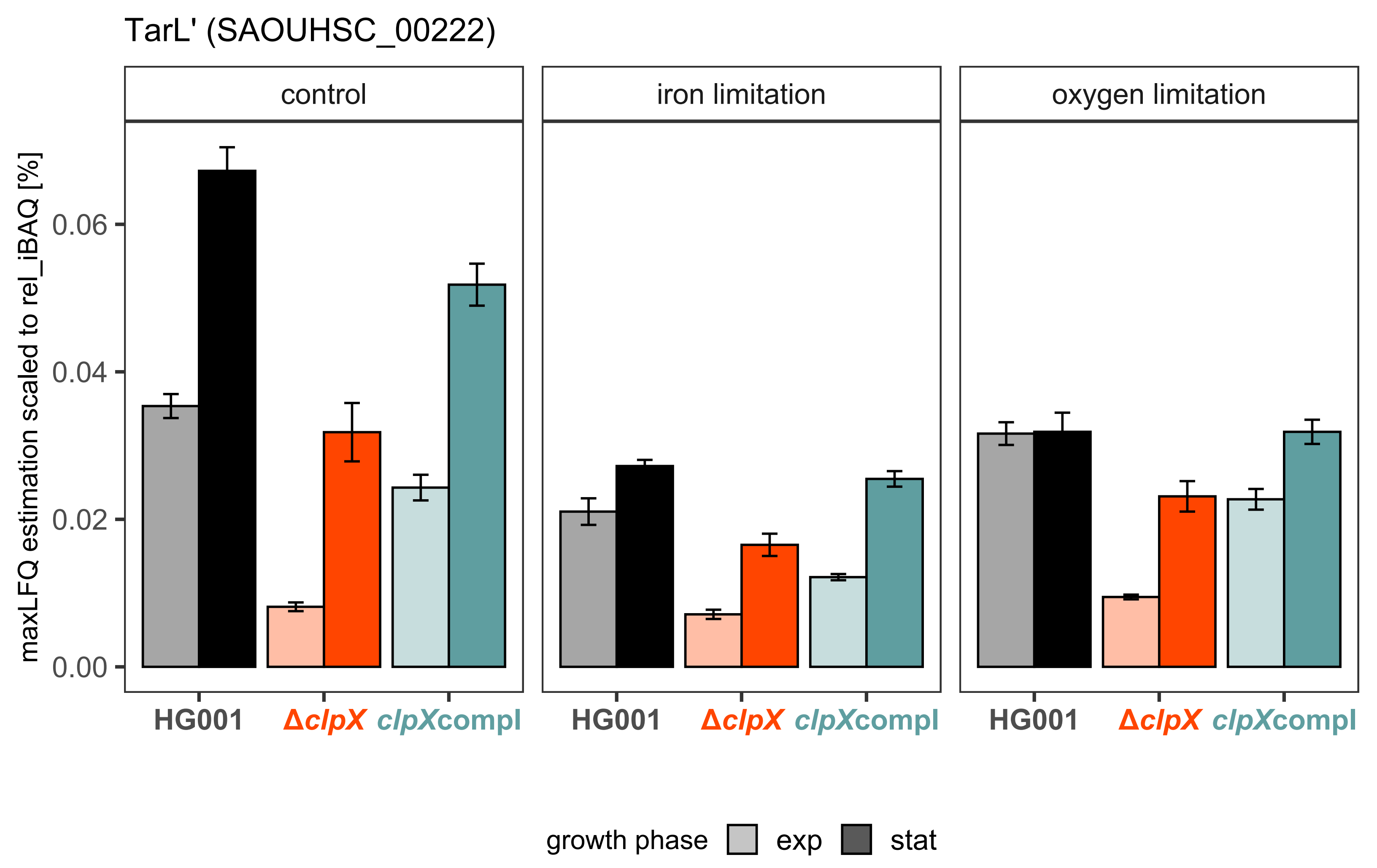

TarL' (SAOUHSC_00222)
control
iron limitation
oxygen limitation
maxLFQ estimation scaled to rel_iBAQ [%]
0.06
0.04
0.02
0.00
HG001
ΔclpX
clpXcompl
growth phase
exp
stat

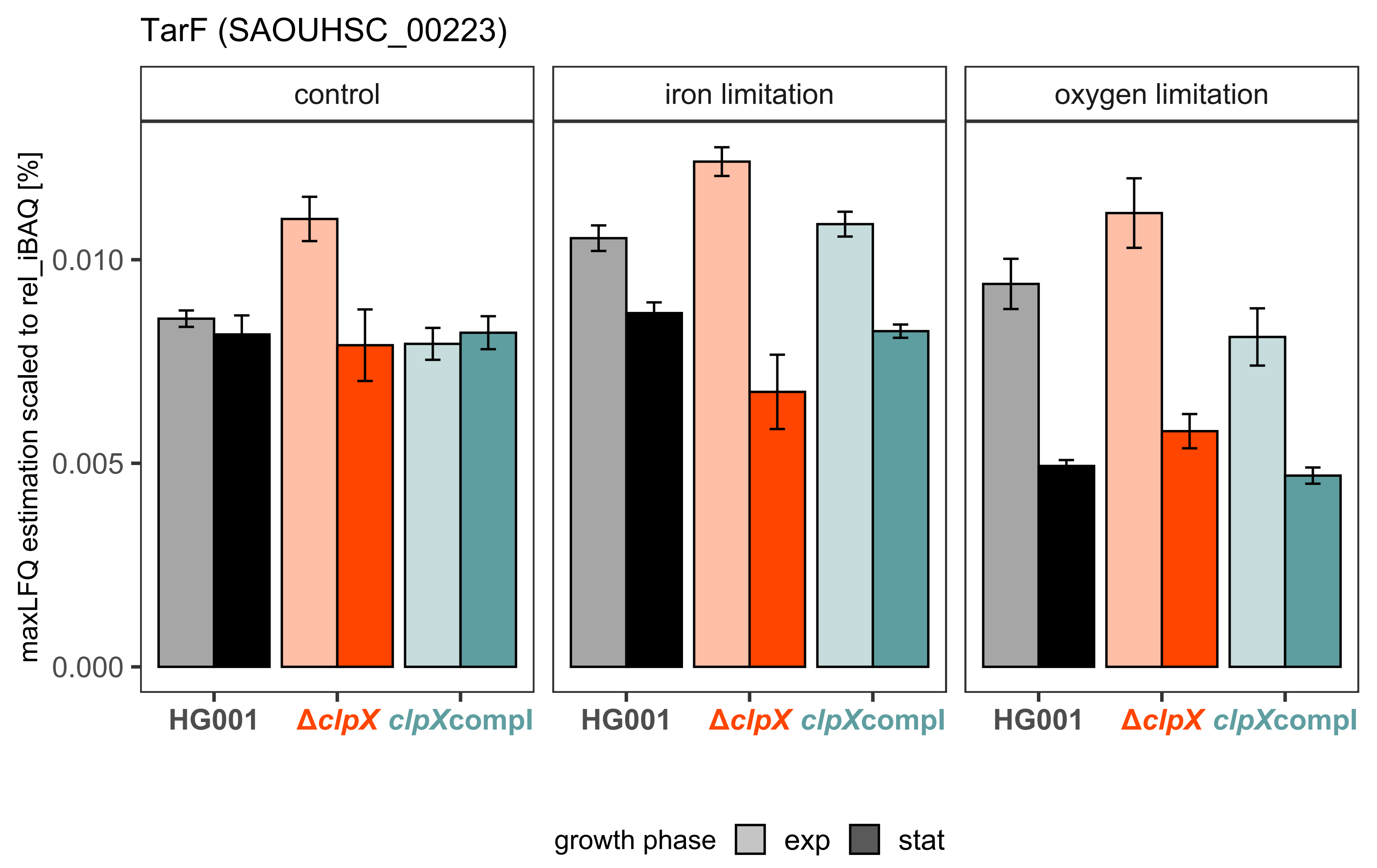
TarF (SAOUHSC_00223)
control
iron limitation
oxygen limitation
maxLFQ estimation scaled to rel_iBAQ [%]
0.010
0.005
0.000
HG001
ΔclpX
clpXcompl
growth phase
exp
stat

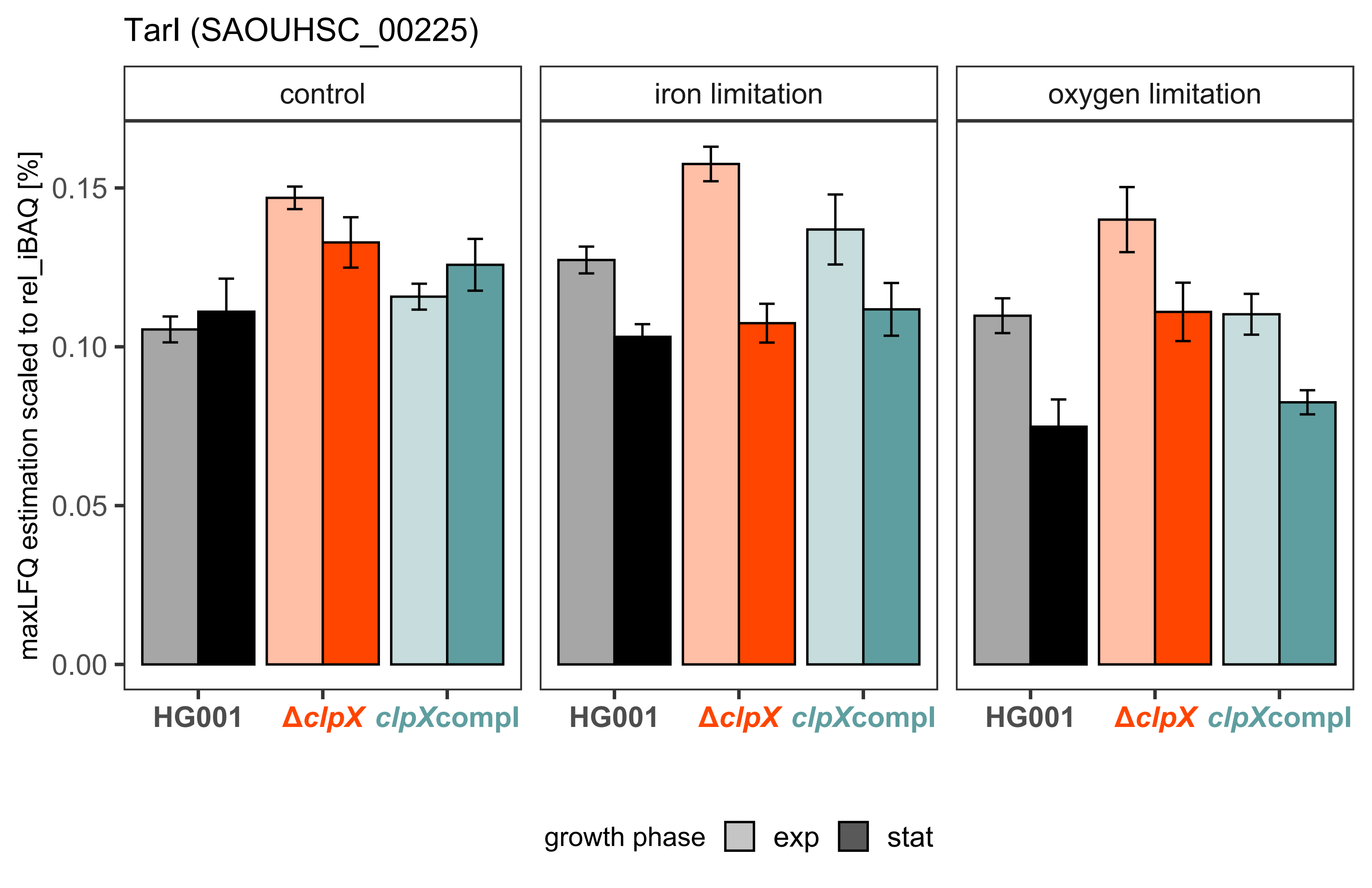
TarI (SAOUHSC_00225)
control
iron limitation
oxygen limitation
maxLFQ estimation scaled to rel_iBAQ [%]
0.15
0.10
0.05
0.00
HG001
ΔclpX
clpXcompl
growth phase
exp
stat

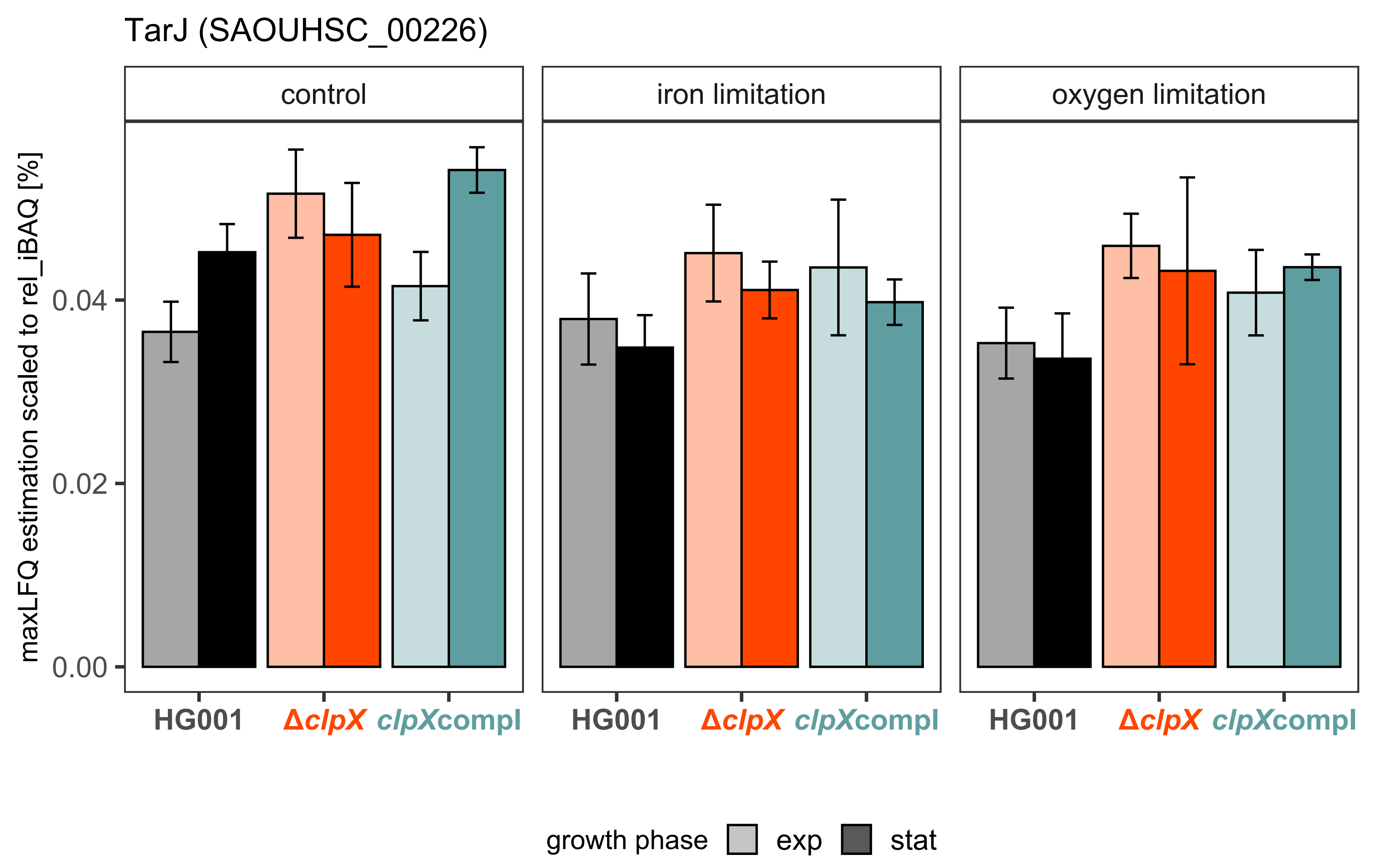
TarJ (SAOUHSC_00226)
control
iron limitation
oxygen limitation
maxLFQ estimation scaled to rel_iBAQ [%]
0.00
0.02
0.04
HG001
ΔclpX
clpXcompl
growth phase
exp
stat

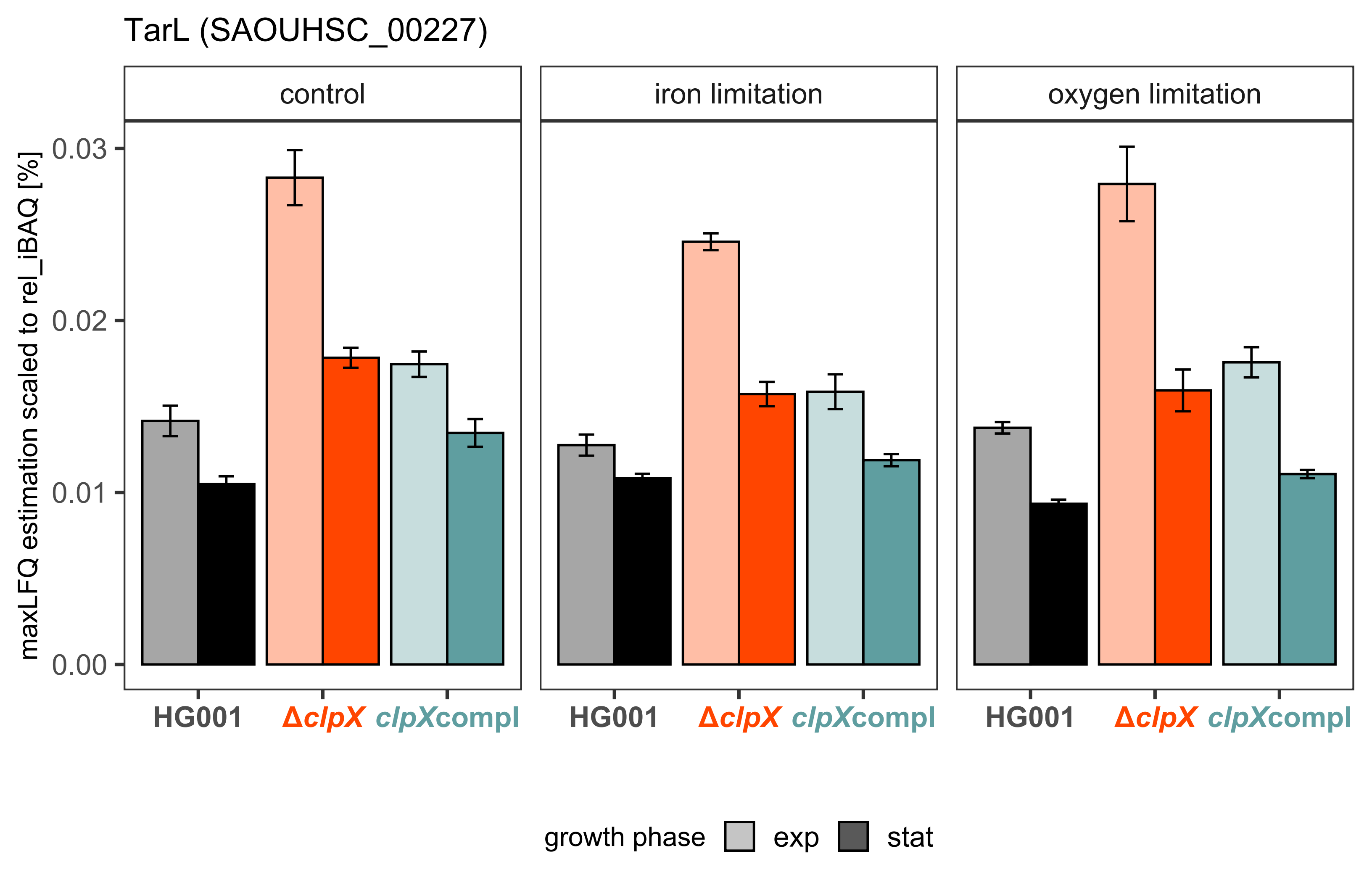
TarL (SAOUHSC_00227)
control
iron limitation
oxygen limitation
maxLFQ estimation scaled to rel_iBAQ [%]
0.03
0.02
0.01
0.00
HG001
ΔclpX
clpXcompl
growth phase
exp
stat

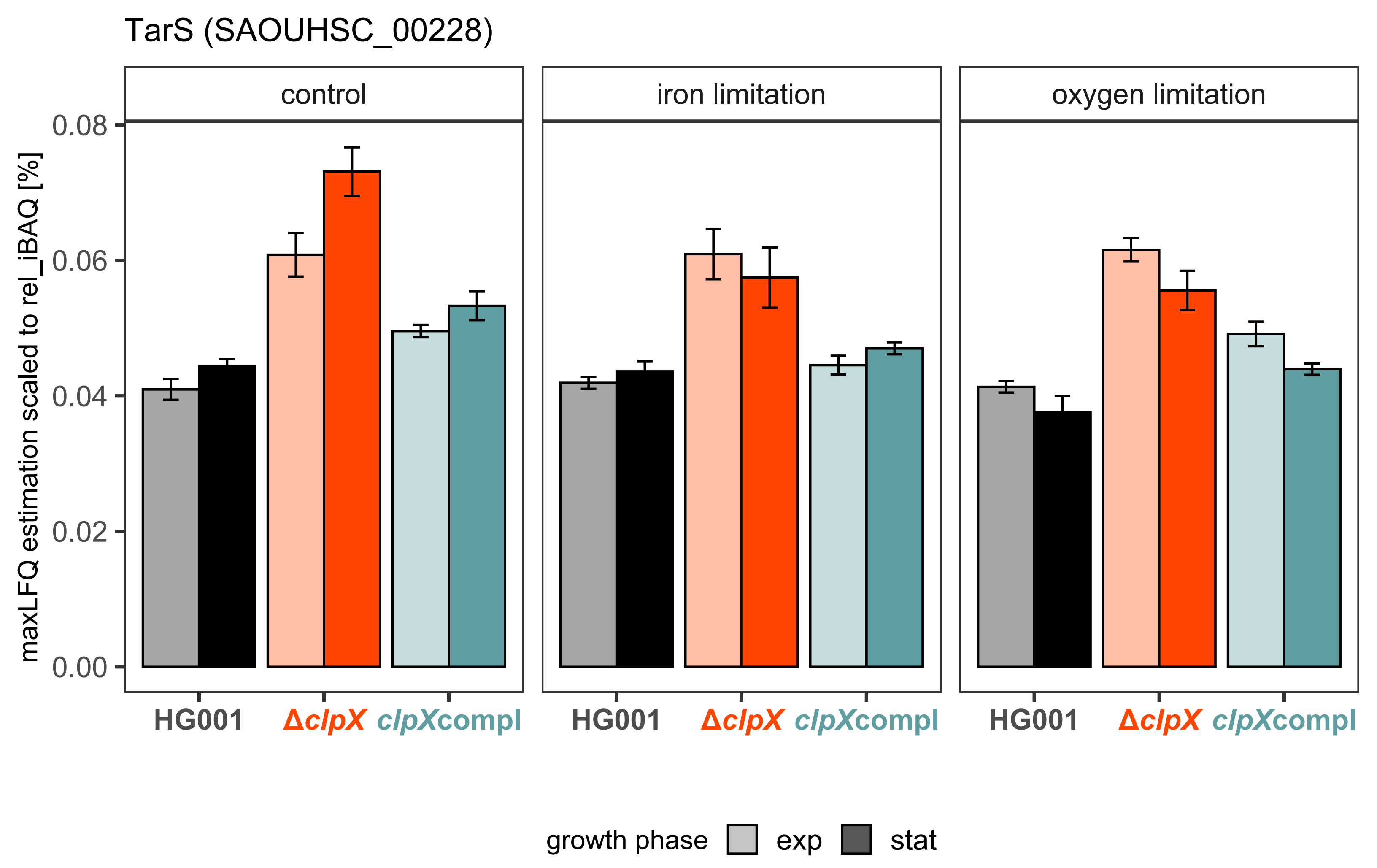

TarS (SAOUHSC_00228)
control
iron limitation
oxygen limitation
maxLFQ estimation scaled to rel_iBAQ [%]
0.08
0.06
0.04
0.02
0.00
HG001
ΔclpX
clpXcompl
growth phase
exp
stat

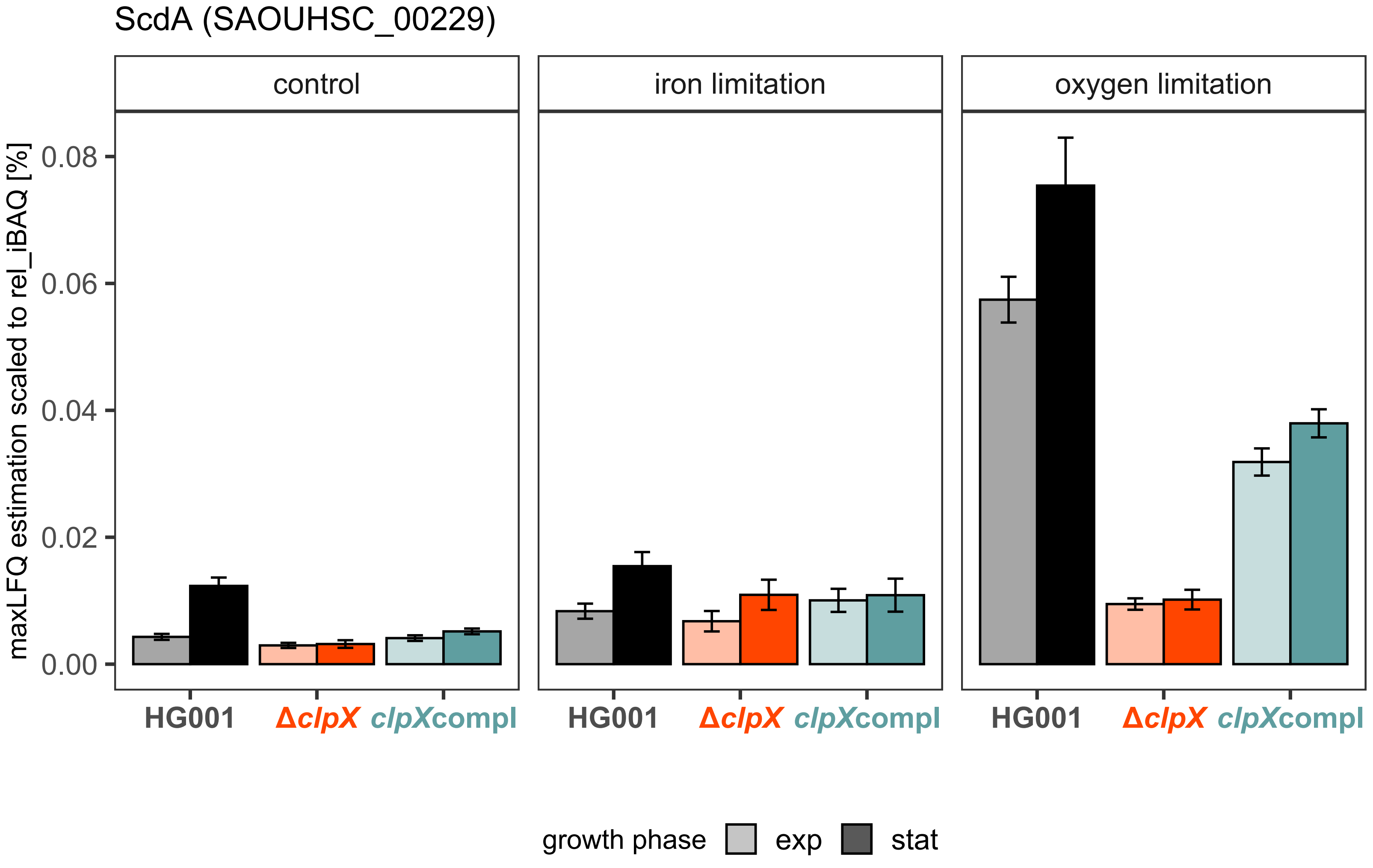
ScdA (SAOUHSC_00229)
control
iron limitation
oxygen limitation
maxLFQ estimation scaled to rel_iBAQ [%]
0.08
0.06
0.04
0.02
0.00
HG001
ΔclpX
clpXcompl
growth phase
exp
stat

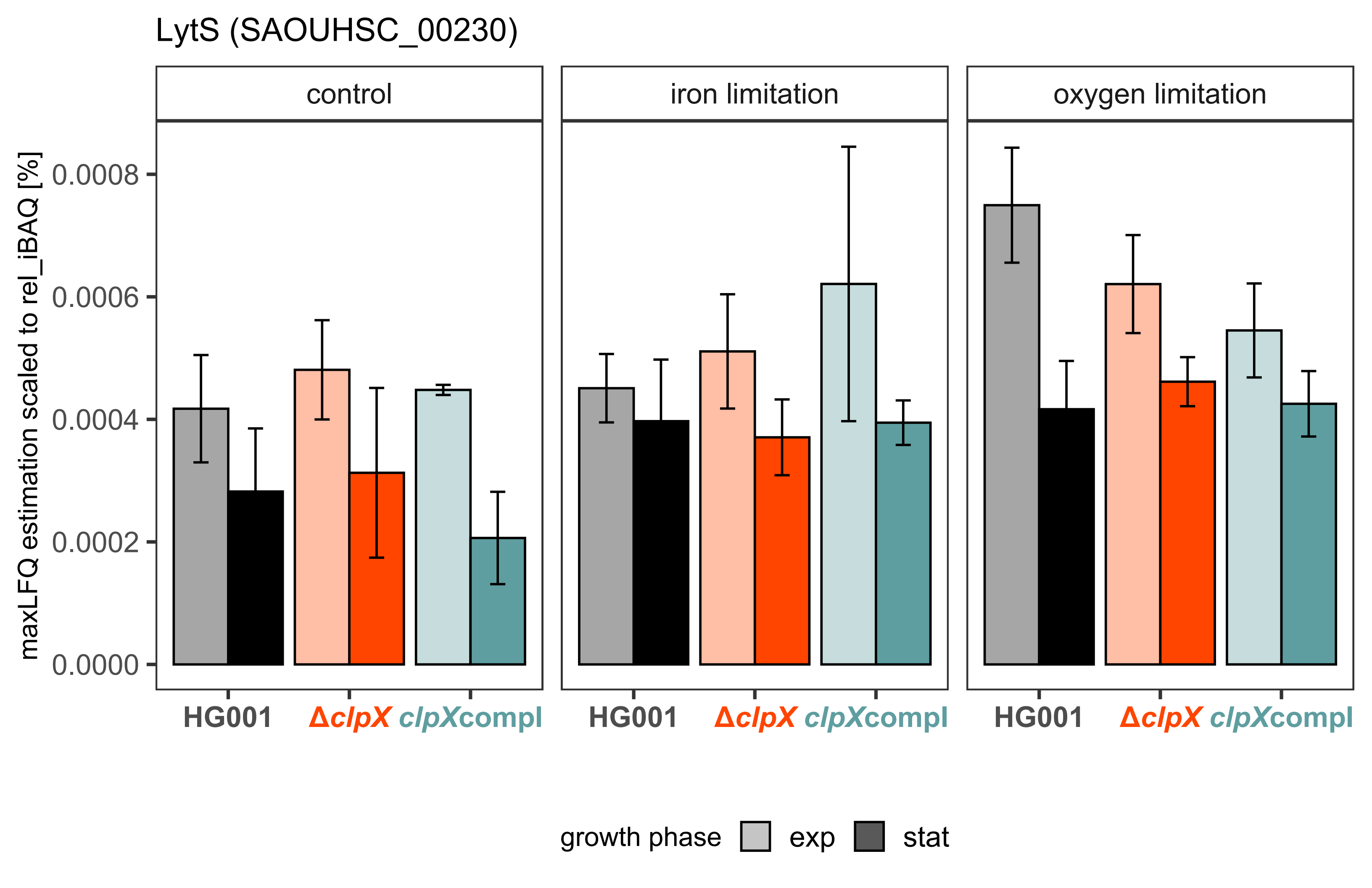

LytS (SAOUHSC_00230)
control
iron limitation
oxygen limitation
maxLFQ estimation scaled to rel_iBAQ [%]
0.0008
0.0006
0.0004
0.0002
0.0000
HG001
ΔclpX
clpXcompl
growth phase
exp
stat

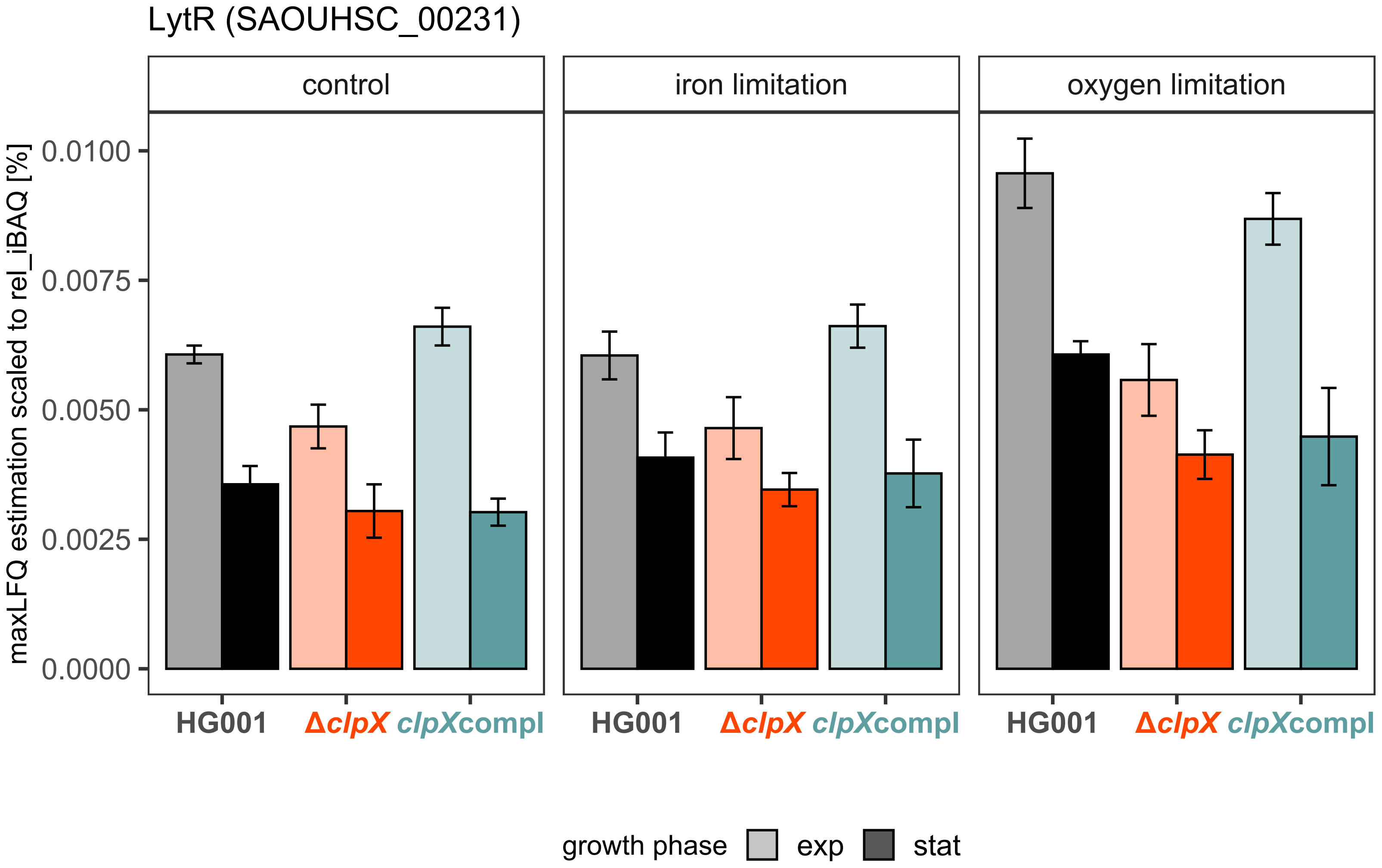
LytR (SAOUHSC_00231)
control
iron limitation
oxygen limitation
maxLFQ estimation scaled to rel_iBAQ [%]
0.0100
0.0075
0.0050
0.0025
0.0000
HG001
ΔclpX
clpXcompl
growth phase
exp
stat

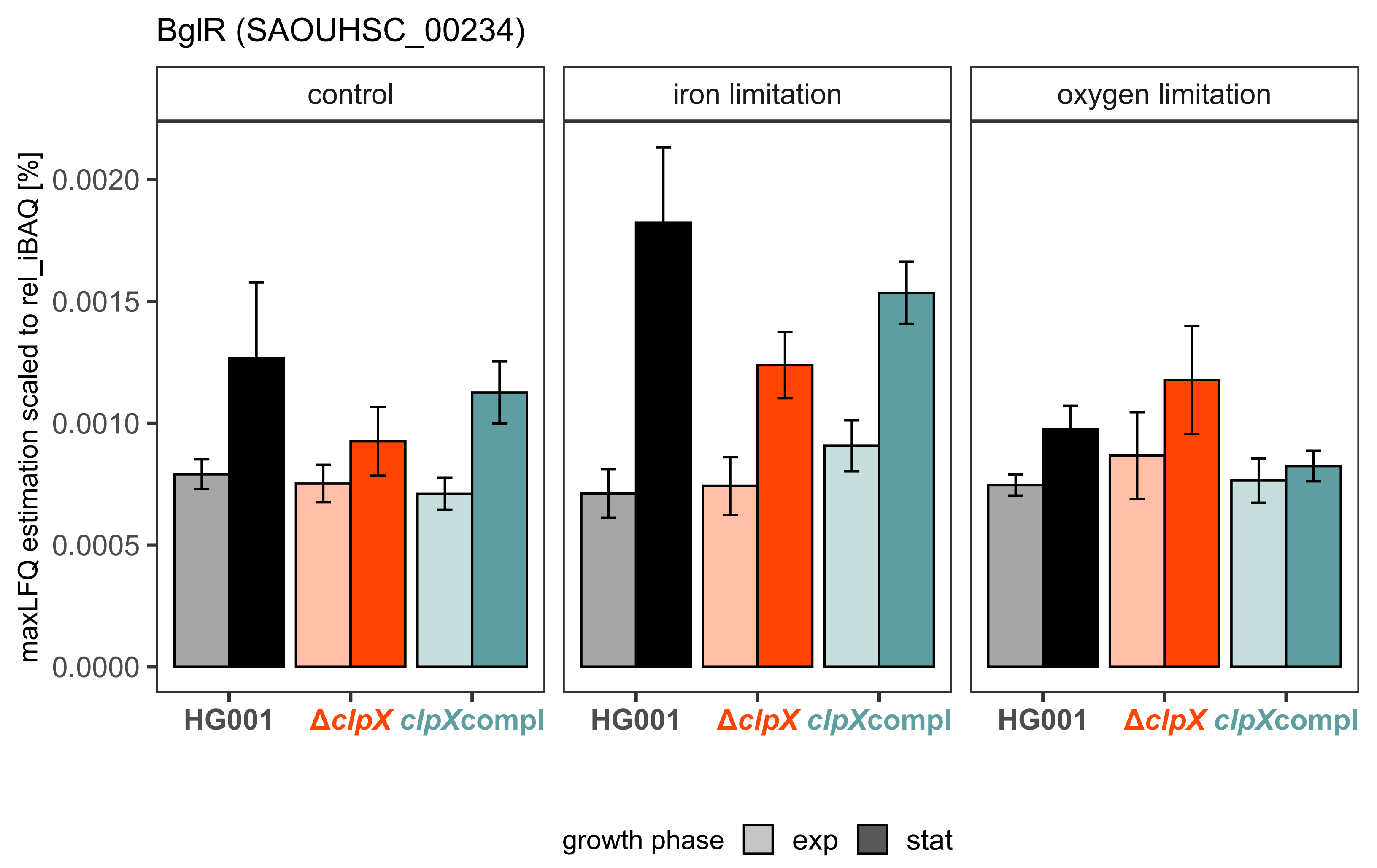

BglR (SAOUHSC_00234)
control
iron limitation
oxygen limitation
maxLFQ estimation scaled to rel_iBAQ [%]
0.0020
0.0015
0.0010
0.0005
0.0000
HG001
ΔclpX
clpXcompl
growth phase
exp
stat

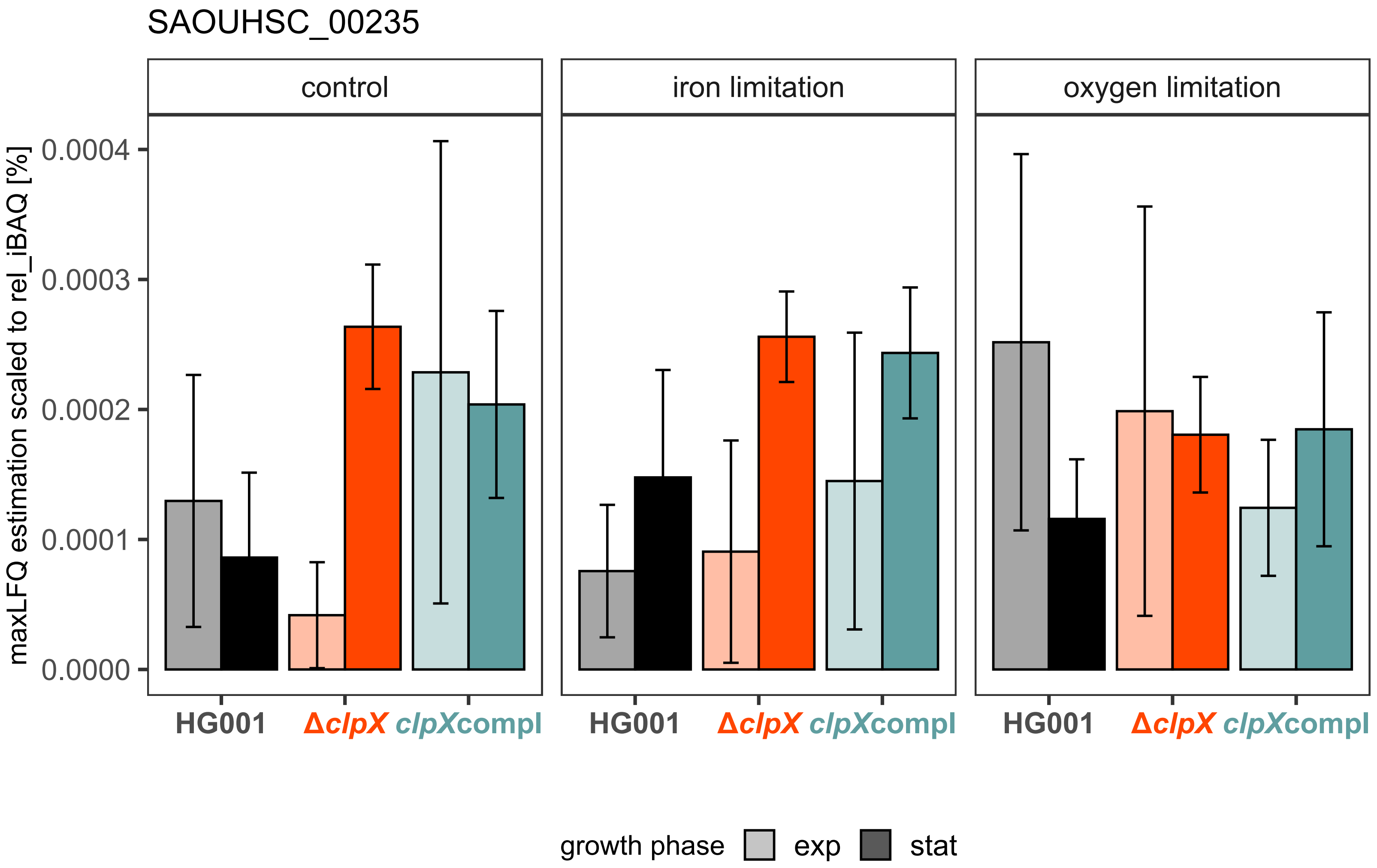
SAOUHSC_00235
control
iron limitation
oxygen limitation
maxLFQ estimation scaled to rel_iBAQ [%]
0.0004
0.0003
0.0002
0.0001
0.0000
HG001
ΔclpX
clpXcompl
growth phase
exp
stat

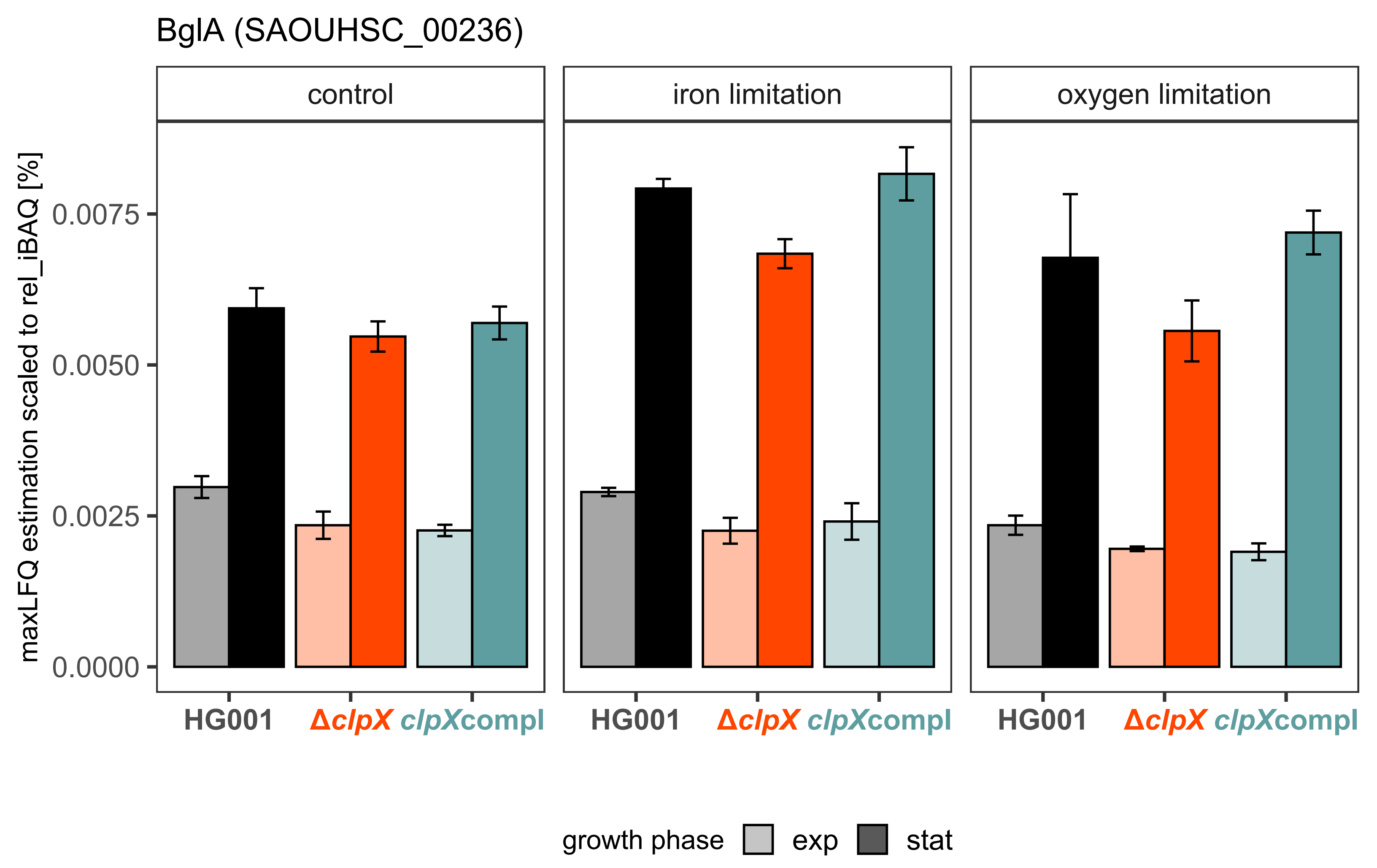

BglA (SAOUHSC_00236)
control
iron limitation
oxygen limitation
maxLFQ estimation scaled to rel_iBAQ [%]
0.0075
0.0050
0.0025
0.0000
HG001
ΔclpX
clpXcompl
growth phase
exp
stat

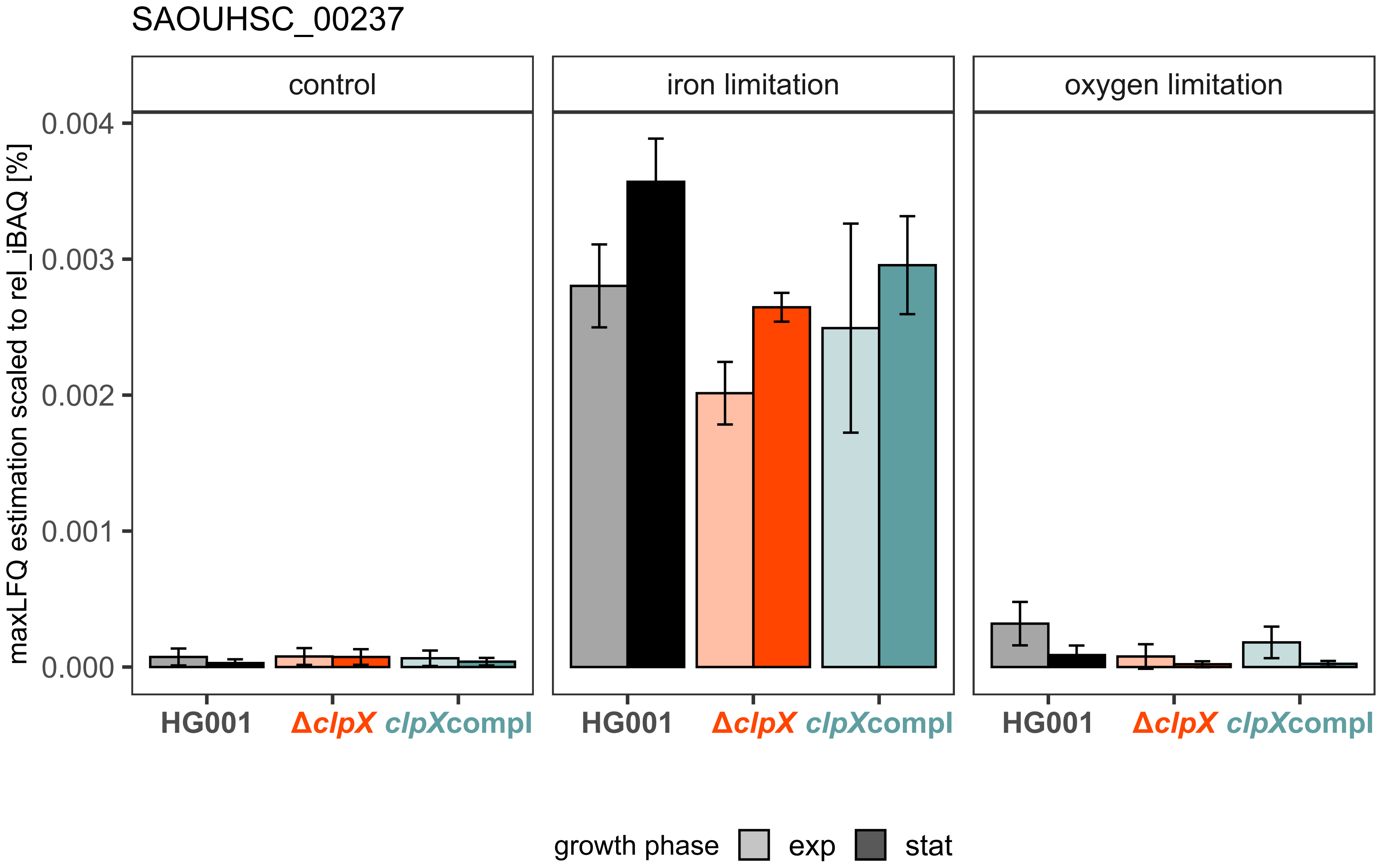
SAOUHSC_00237
control
iron limitation
oxygen limitation
maxLFQ estimation scaled to rel_iBAQ [%]
0.004
0.003
0.002
0.001
0.000
HG001
ΔclpX
clpXcompl
growth phase
exp
stat

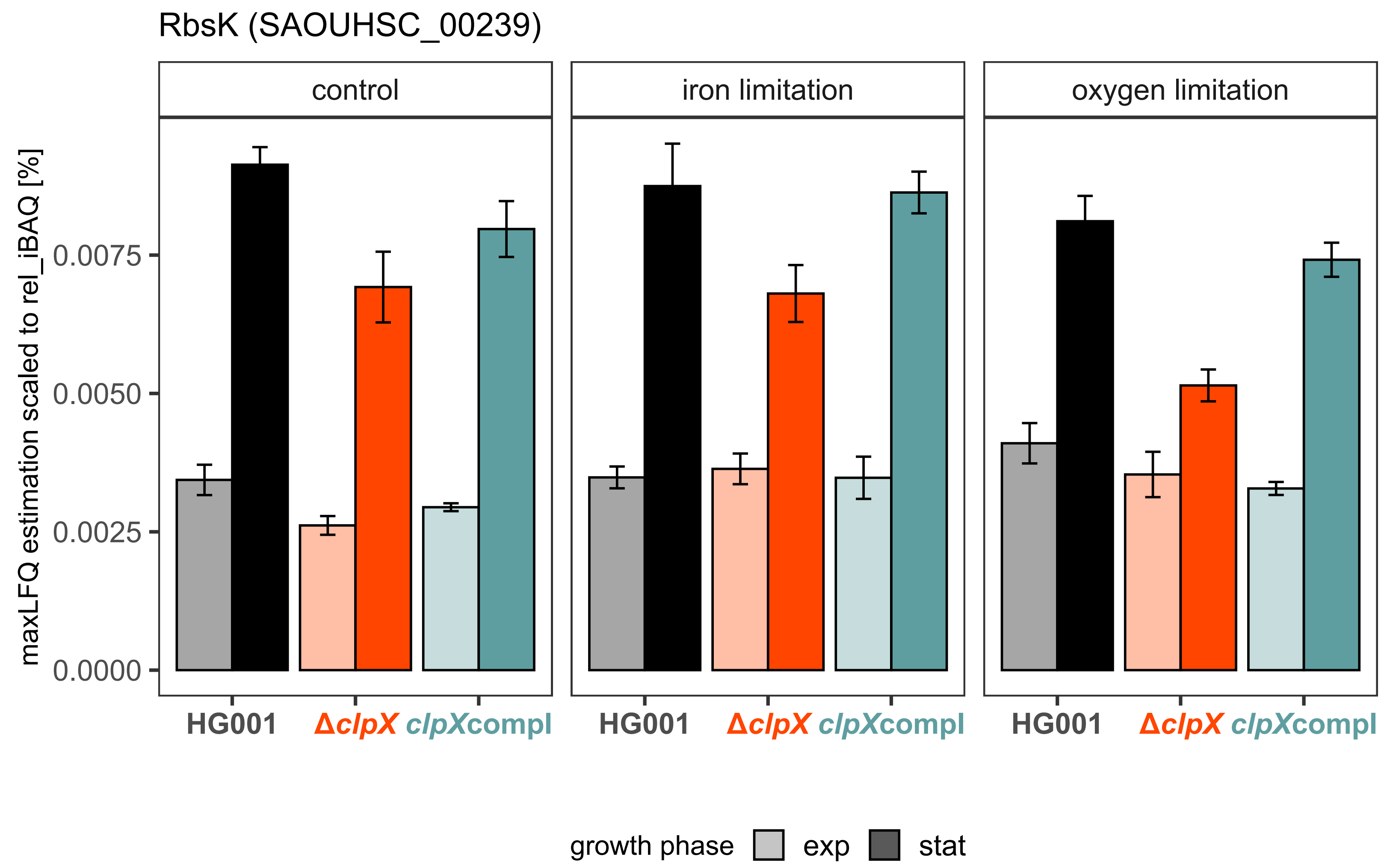

RbsK (SAOUHSC_00239)
control
iron limitation
oxygen limitation
maxLFQ estimation scaled to rel_iBAQ [%]
0.0075
0.0050
0.0025
0.0000
HG001
ΔclpX
clpXcompl
growth phase
exp
stat

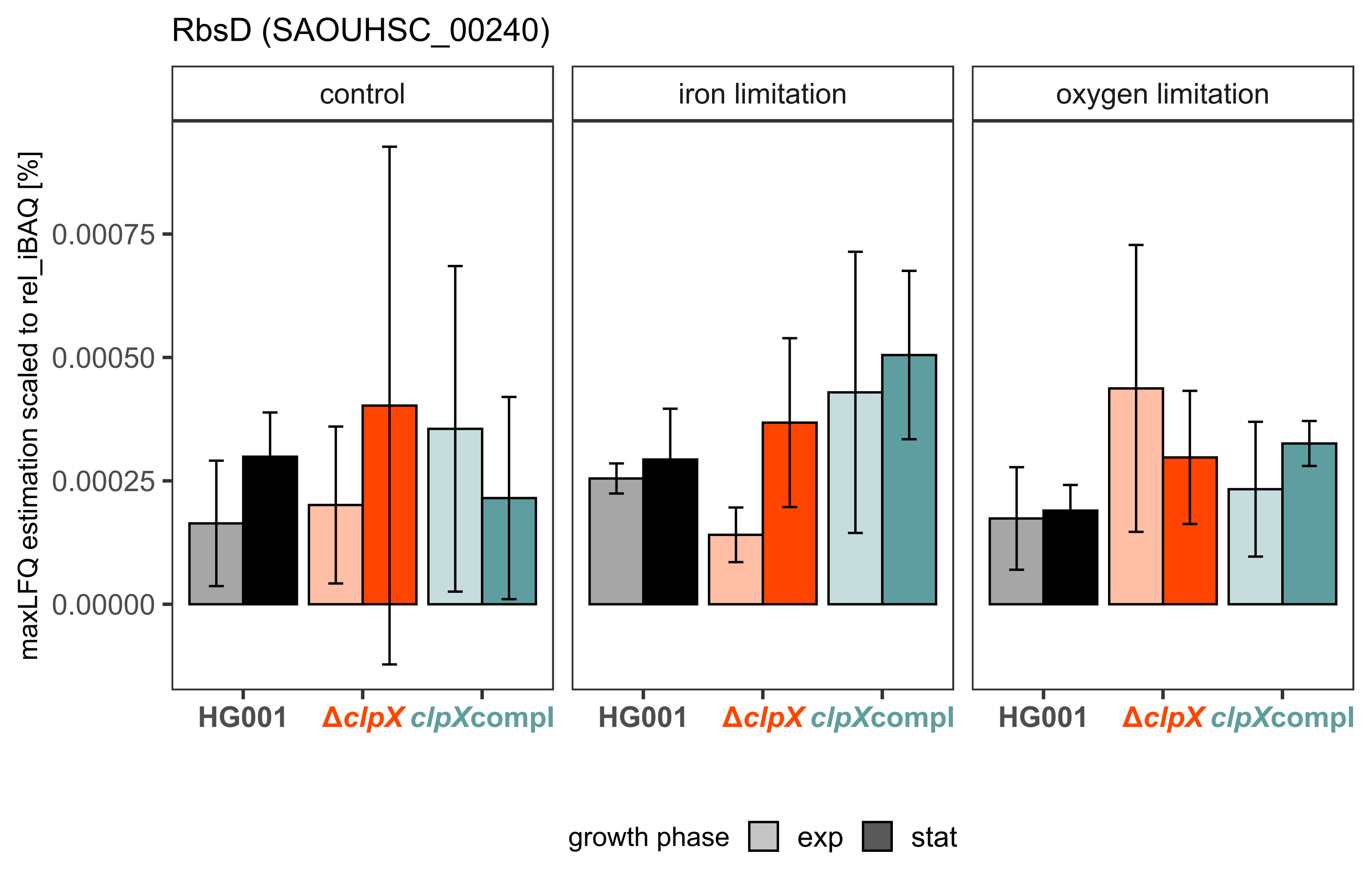

RbsD (SAOUHSC_00240)
control
iron limitation
oxygen limitation
maxLFQ estimation scaled to rel_iBAQ [%]
0.00075
0.00050
0.00025
0.00000
HG001
ΔclpX
clpXcompl
growth phase
exp
stat

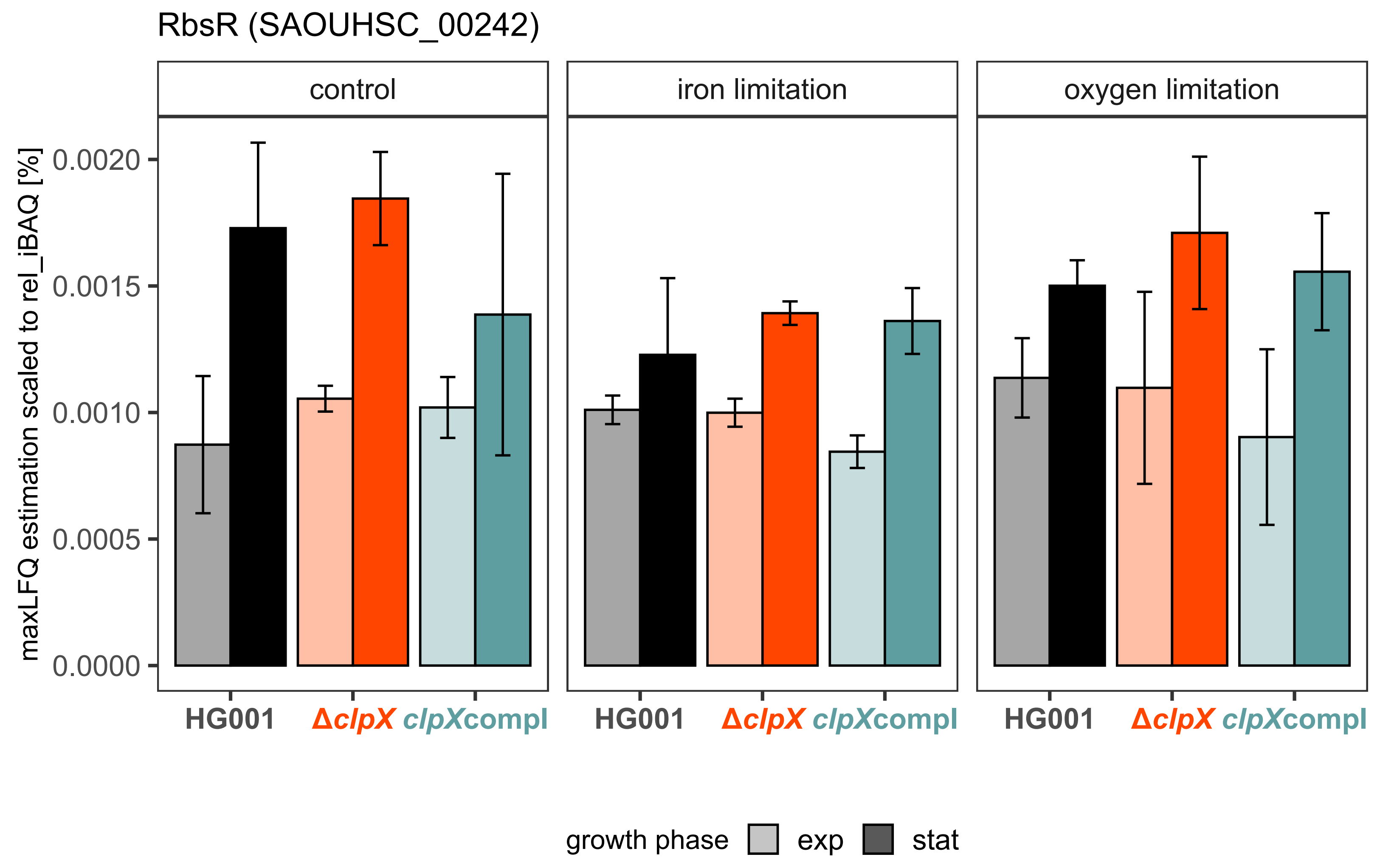

RbsR (SAOUHSC_00242)
control
iron limitation
oxygen limitation
maxLFQ estimation scaled to rel_iBAQ [%]
0.0020
0.0015
0.0010
0.0005
0.0000
HG001
ΔclpX
clpXcompl
growth phase
exp
stat

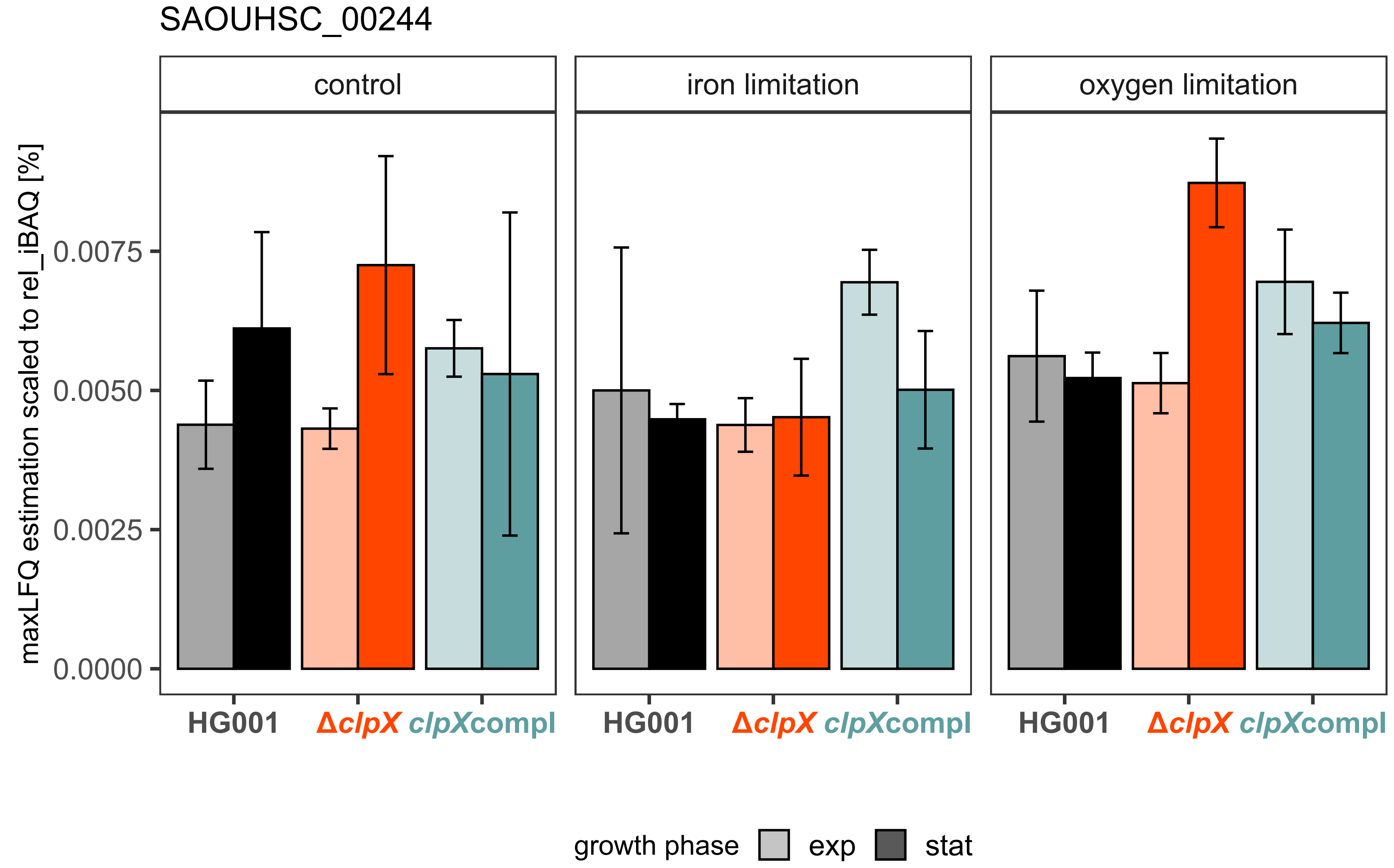

SAOUHSC_00244
control
iron limitation
oxygen limitation
maxLFQ estimation scaled to rel_iBAQ [%]
0.0075
0.0050
0.0025
0.0000
HG001
ΔclpX
clpXcompl
growth phase
exp
stat

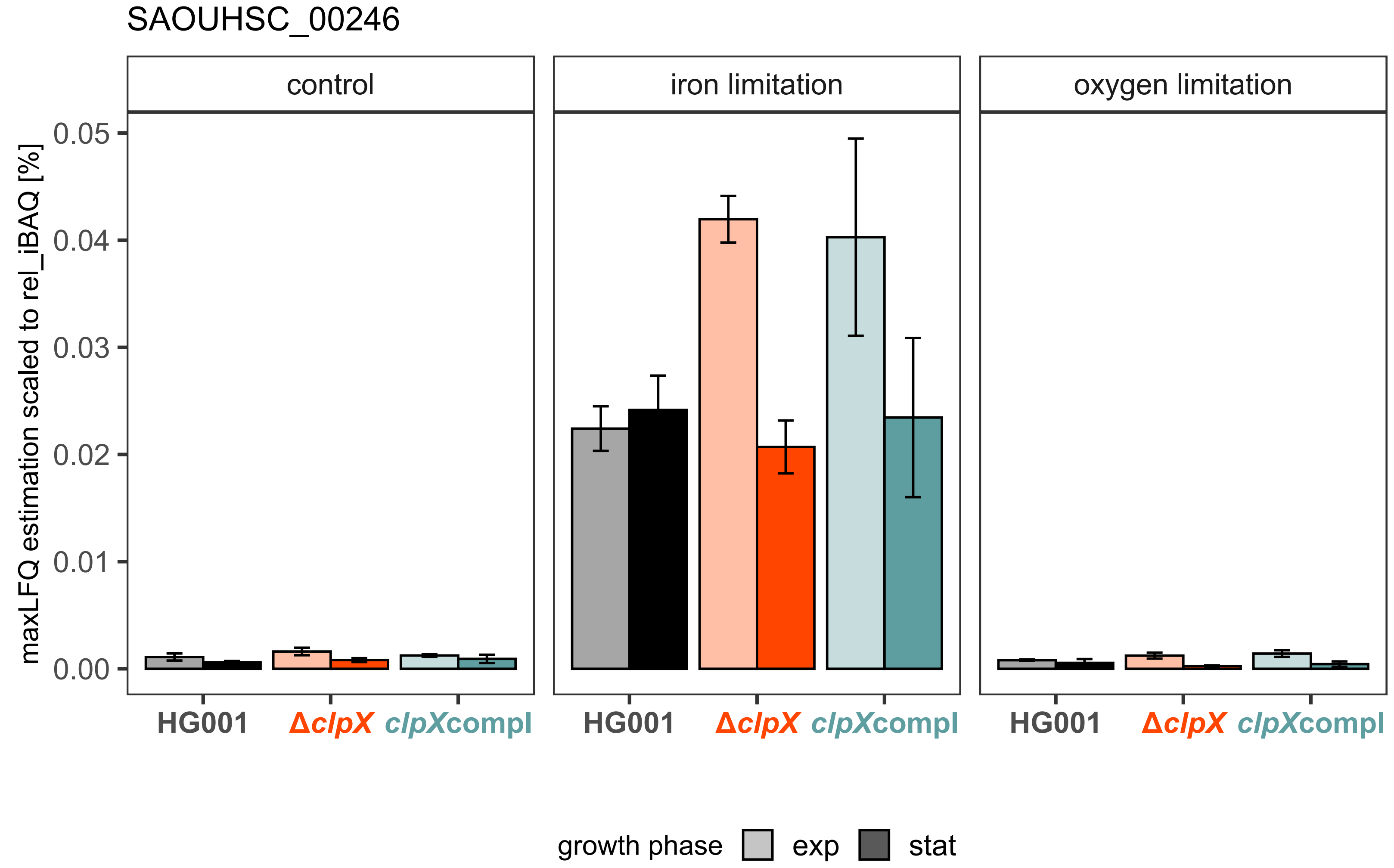
SAOUHSC_00246
control
iron limitation
oxygen limitation
maxLFQ estimation scaled to rel_iBAQ [%]
0.05
0.04
0.03
0.02
0.01
0.00
HG001
ΔclpX
clpXcompl
growth phase
exp
stat

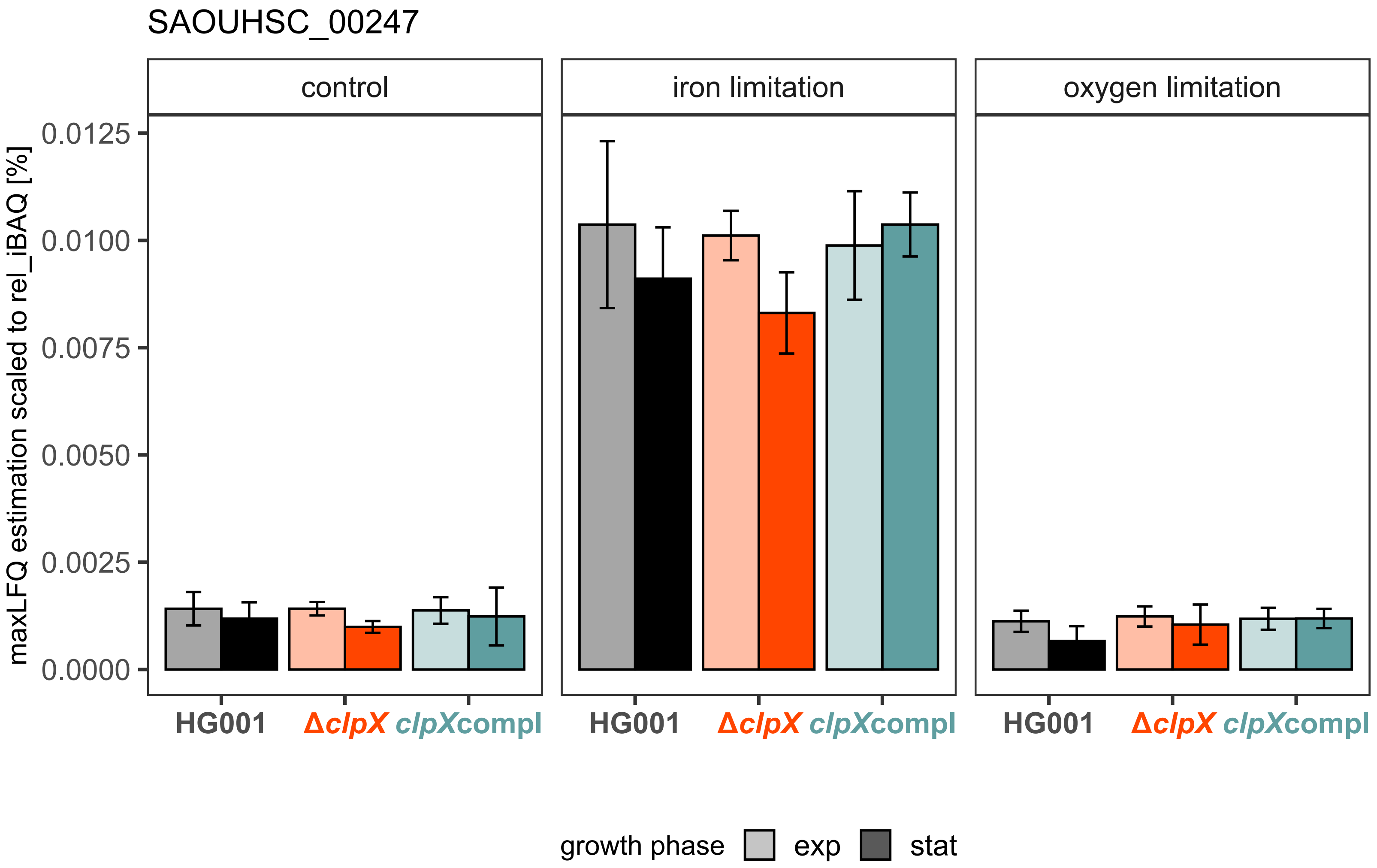

SAOUHSC_00247
control
iron limitation
oxygen limitation
maxLFQ estimation scaled to rel_iBAQ [%]
0.0125
0.0100
0.0075
0.0050
0.0025
0.0000
HG001
ΔclpX
clpXcompl
growth phase
exp
stat

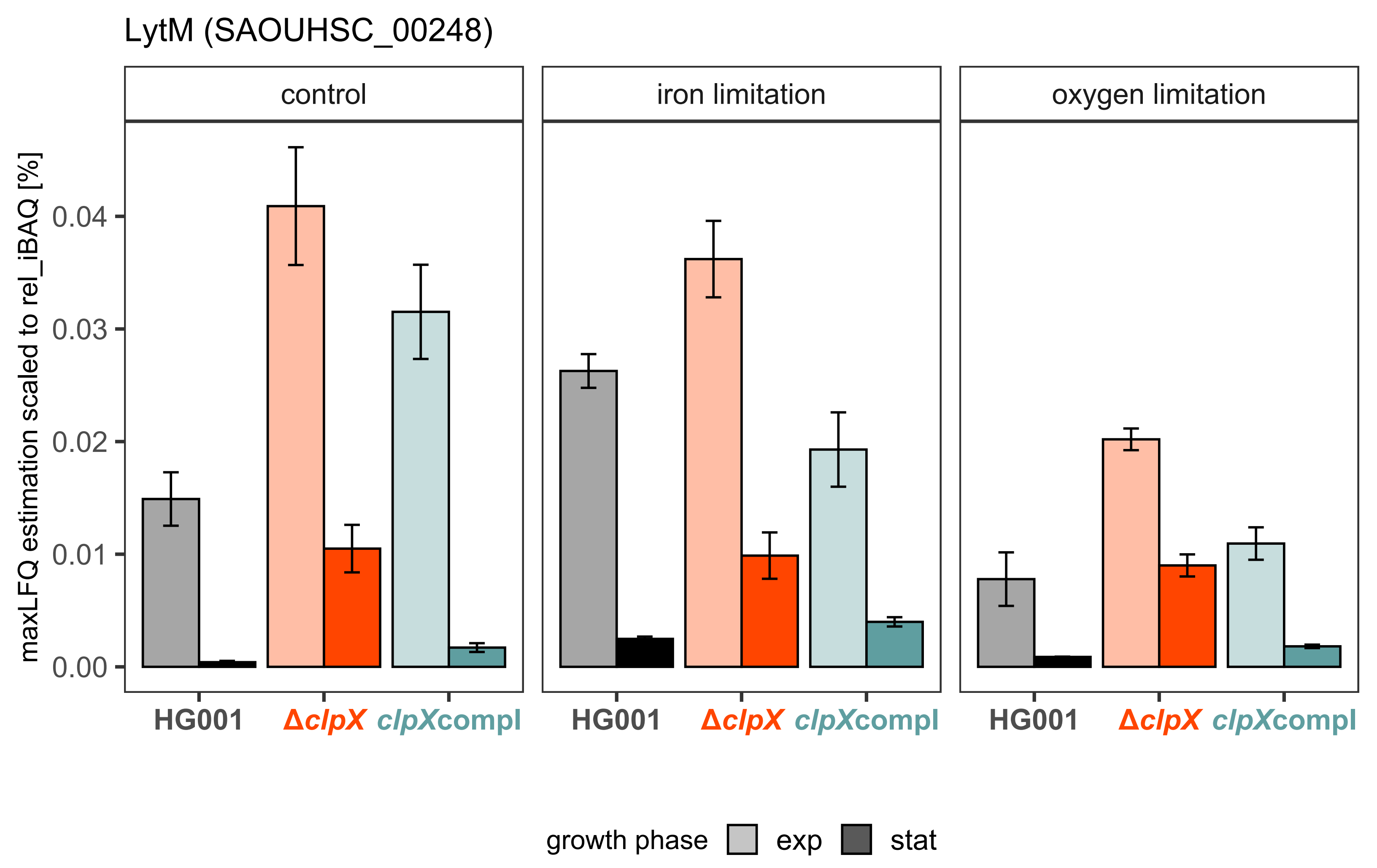
LytM (SAOUHSC_00248)
control
iron limitation
oxygen limitation
maxLFQ estimation scaled to rel_iBAQ [%]
0.04
0.03
0.02
0.01
0.00
HG001
ΔclpX
clpXcompl
growth phase
exp
stat

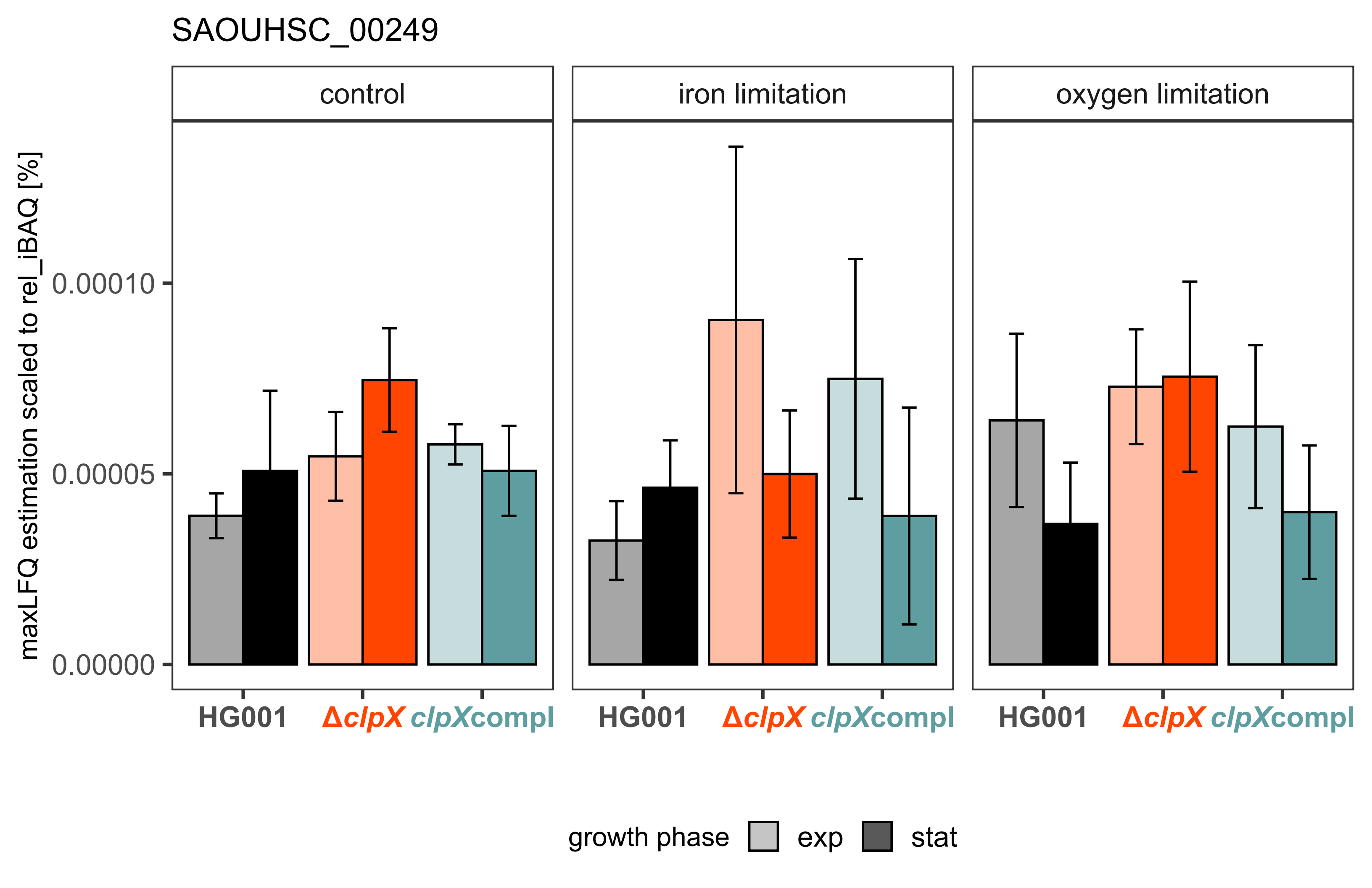
SAOUHSC_00249
control
iron limitation
oxygen limitation
maxLFQ estimation scaled to rel_iBAQ [%]
0.00010
0.00005
0.00000
HG001
ΔclpX
clpXcompl
growth phase
exp
stat

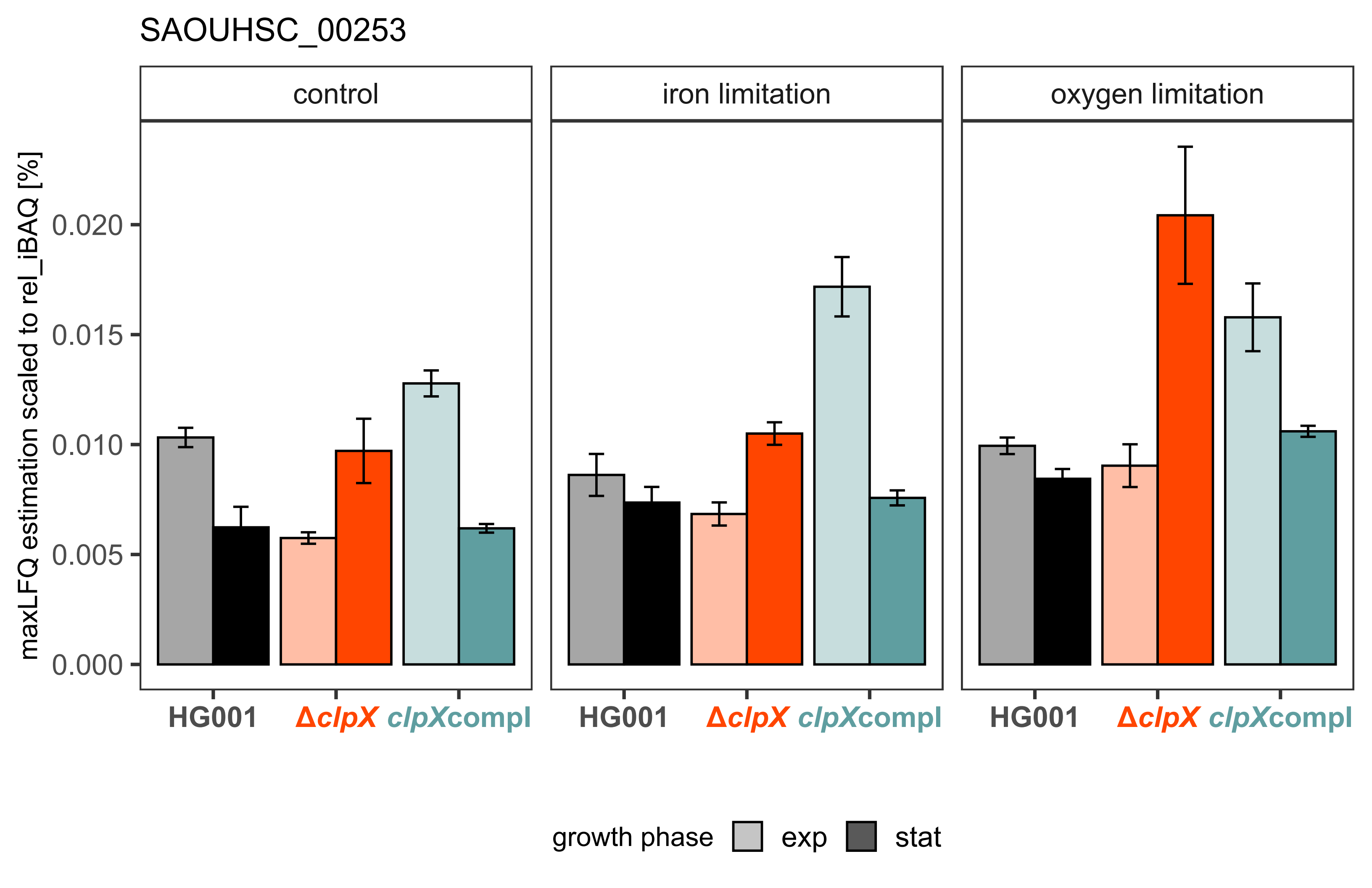
SAOUHSC_00253
control
iron limitation
oxygen limitation
maxLFQ estimation scaled to rel_iBAQ [%]
0.020
0.015
0.010
0.005
0.000
HG001
ΔclpX
clpXcompl
growth phase
exp
stat

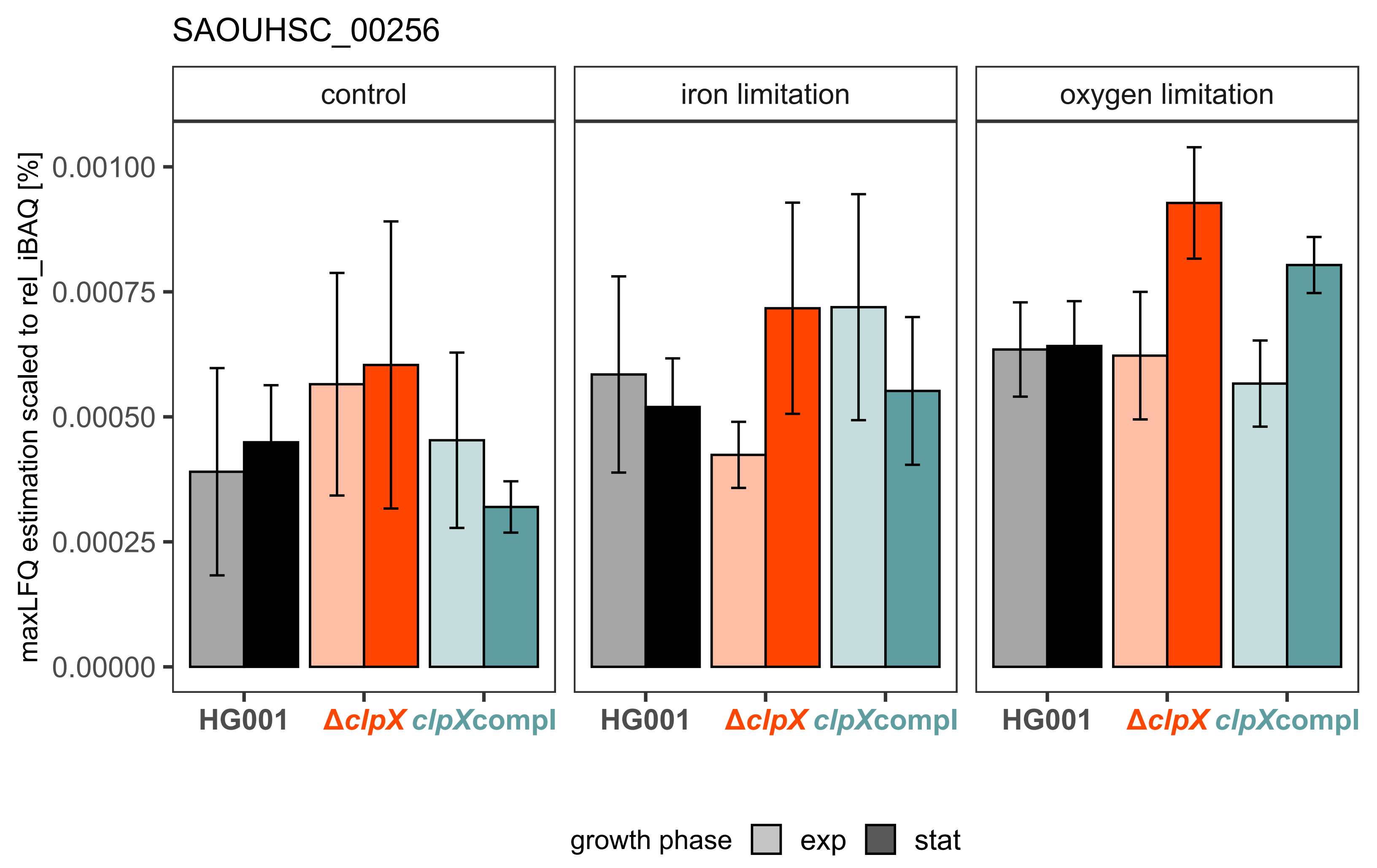
SAOUHSC_00256
control
iron limitation
oxygen limitation
maxLFQ estimation scaled to rel_iBAQ [%]
0.00100
0.00075
0.00050
0.00025
0.00000
HG001
ΔclpX
clpXcompl
growth phase
exp
stat

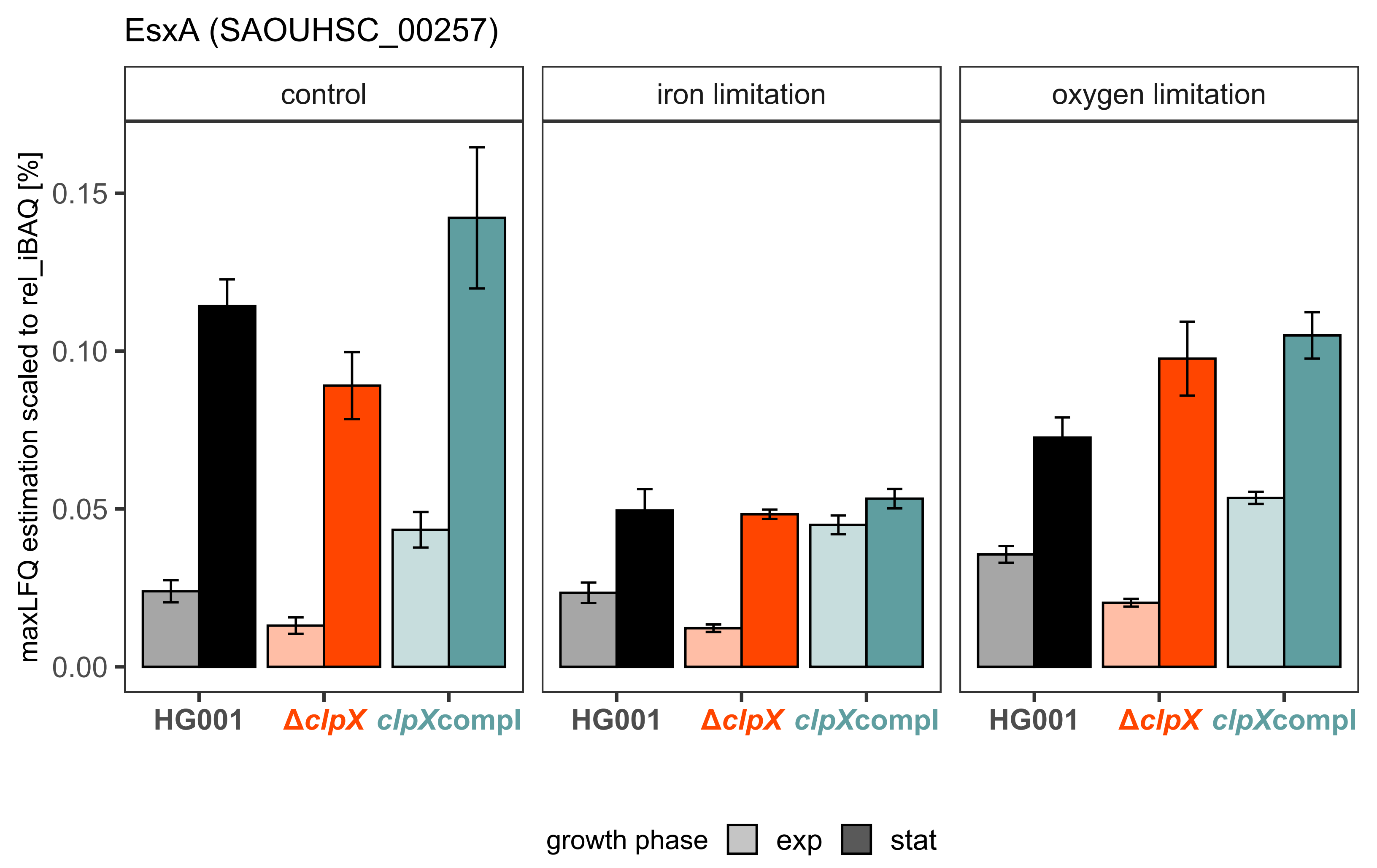

EsxA (SAOUHSC_00257)
control
iron limitation
oxygen limitation
maxLFQ estimation scaled to rel_iBAQ [%]
0.15
0.10
0.05
0.00
HG001
ΔclpX
clpXcompl
growth phase
exp
stat

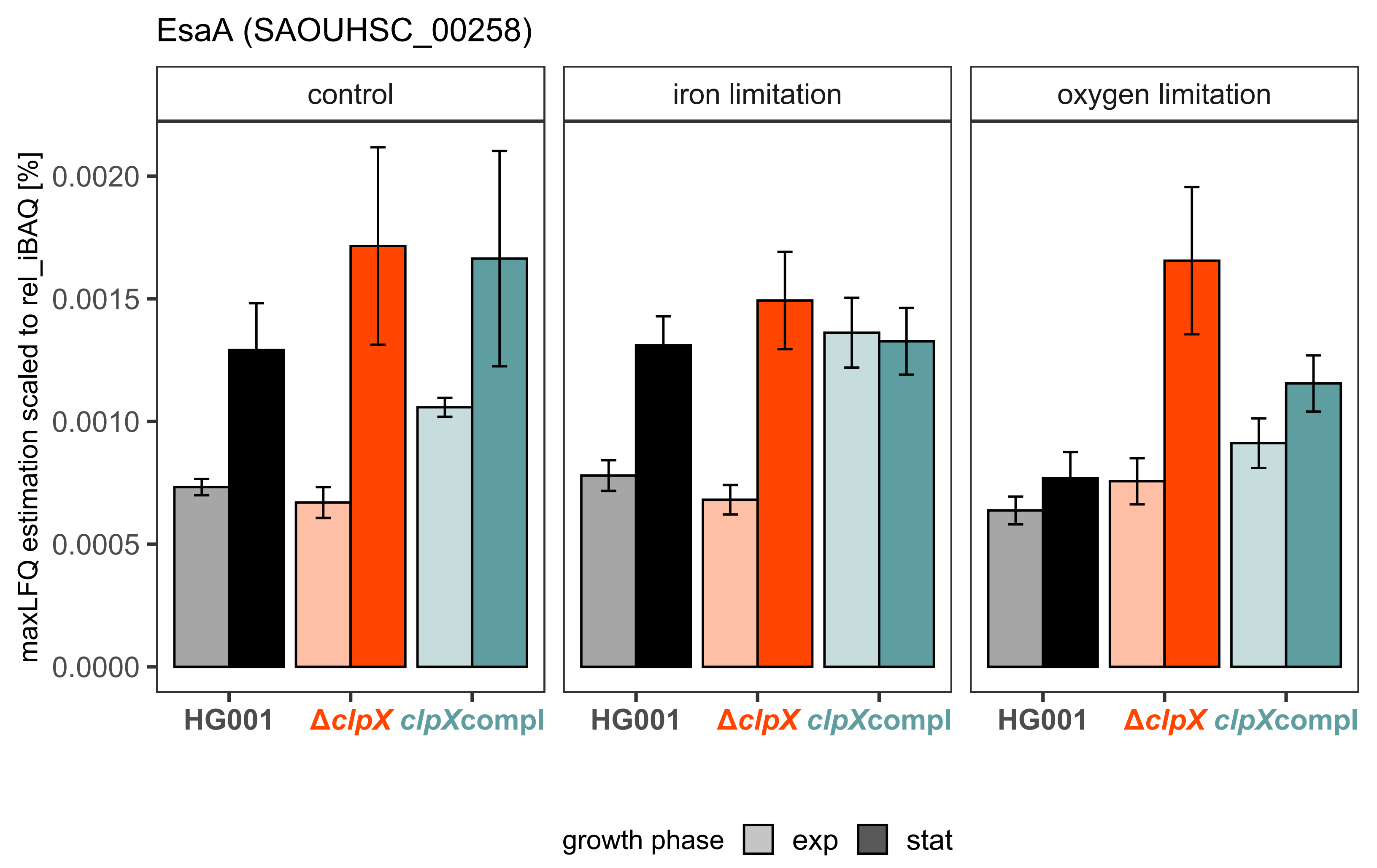

EsaA (SAOUHSC_00258)
control
iron limitation
oxygen limitation
maxLFQ estimation scaled to rel_iBAQ [%]
0.0020
0.0015
0.0010
0.0005
0.0000
HG001
ΔclpX
clpXcompl
growth phase
exp
stat

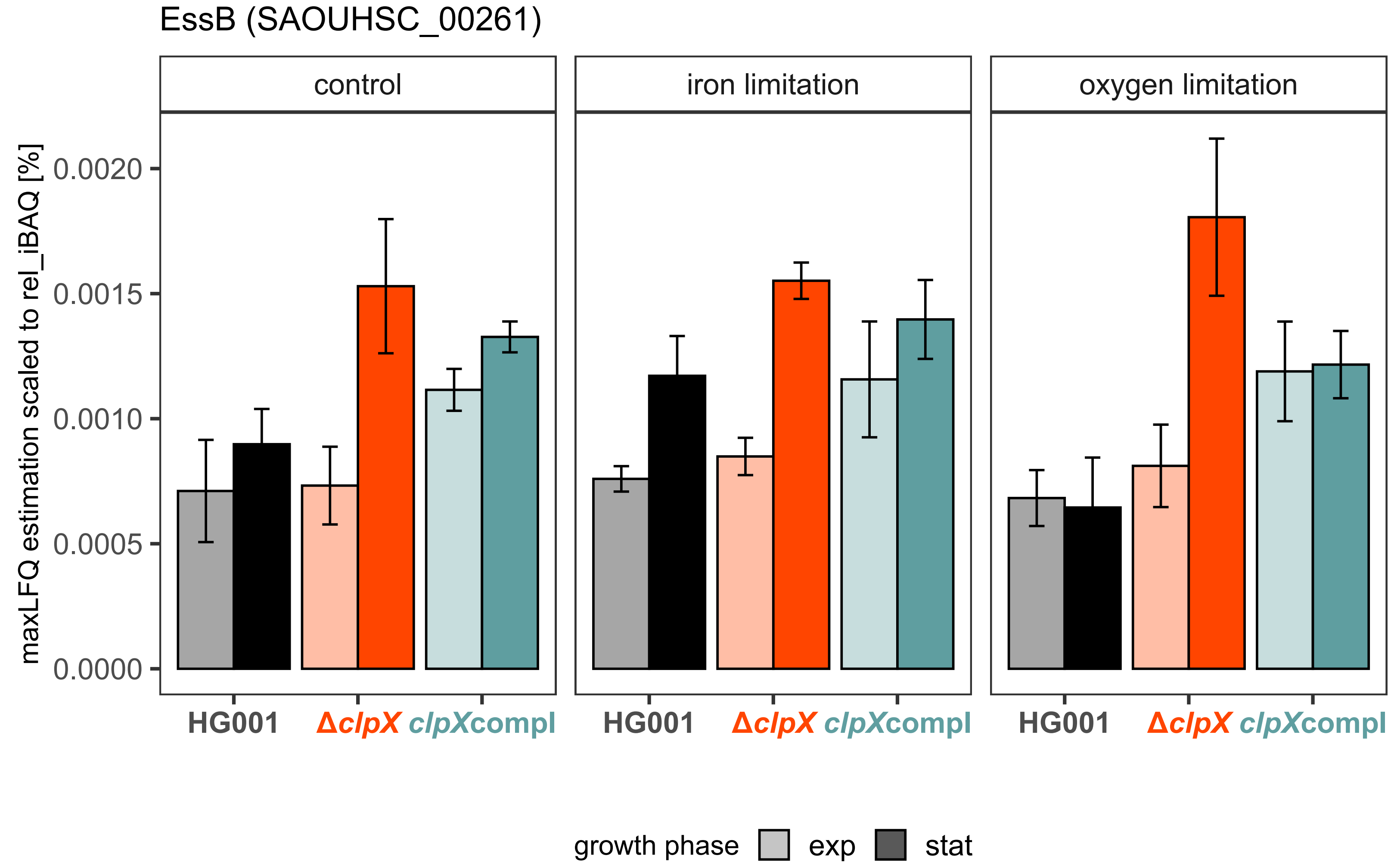
EssB (SAOUHSC_00261)
control
iron limitation
oxygen limitation
maxLFQ estimation scaled to rel_iBAQ [%]
0.0020
0.0015
0.0010
0.0005
0.0000
HG001
ΔclpX
clpXcompl
growth phase
exp
stat

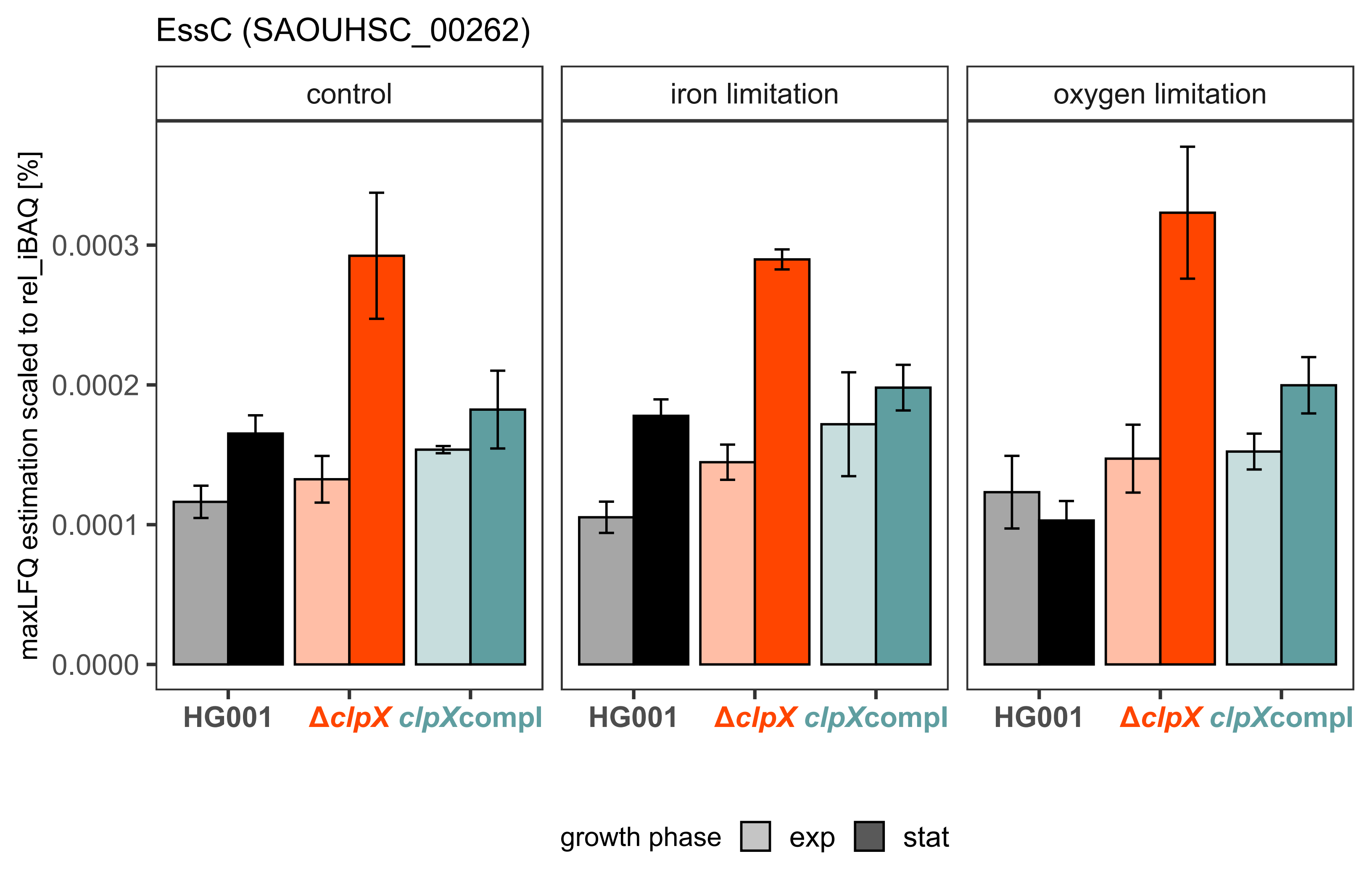
EssC (SAOUHSC_00262)
control
iron limitation
oxygen limitation
maxLFQ estimation scaled to rel_iBAQ [%]
0.0003
0.0002
0.0001
0.0000
HG001
ΔclpX
clpXcompl
growth phase
exp
stat

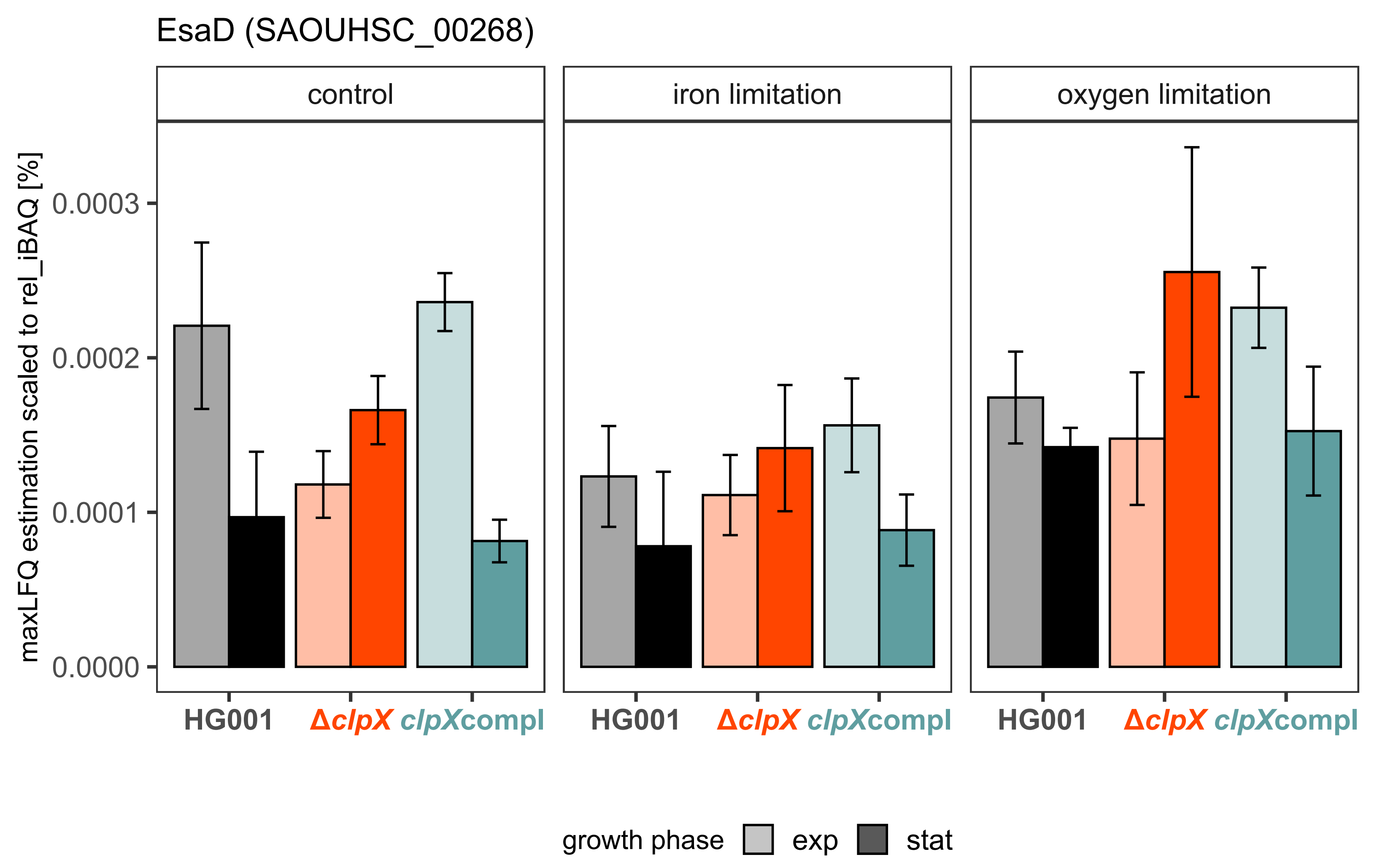
EsaD (SAOUHSC_00268)
control
iron limitation
oxygen limitation
maxLFQ estimation scaled to rel_iBAQ [%]
0.0003
0.0002
0.0001
0.0000
HG001
ΔclpX
clpXcompl
growth phase
exp
stat

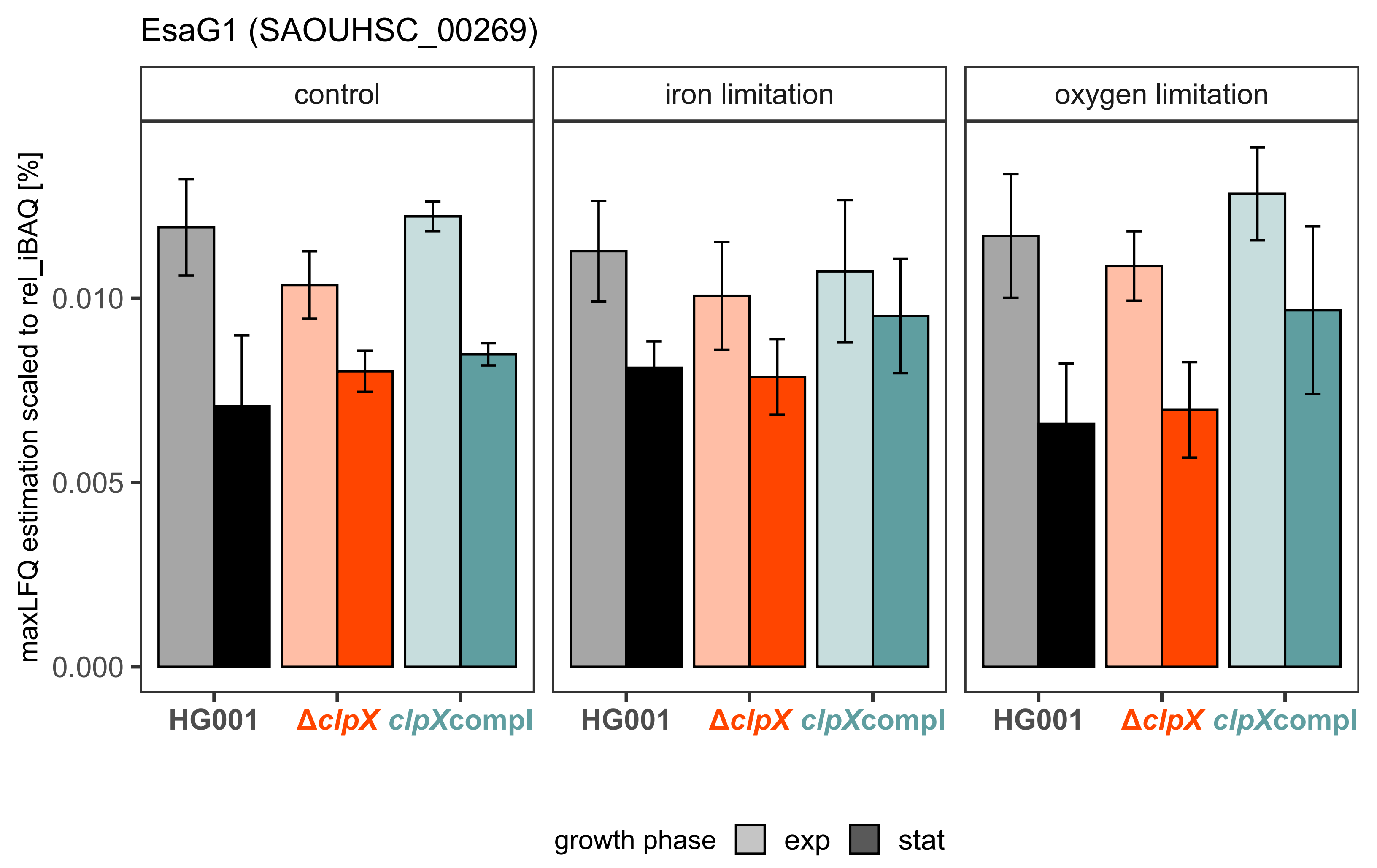
EsaG1 (SAOUHSC_00269)
control
iron limitation
oxygen limitation
maxLFQ estimation scaled to rel_iBAQ [%]
0.010
0.005
0.000
HG001
ΔclpX
clpXcompl
growth phase
exp
stat

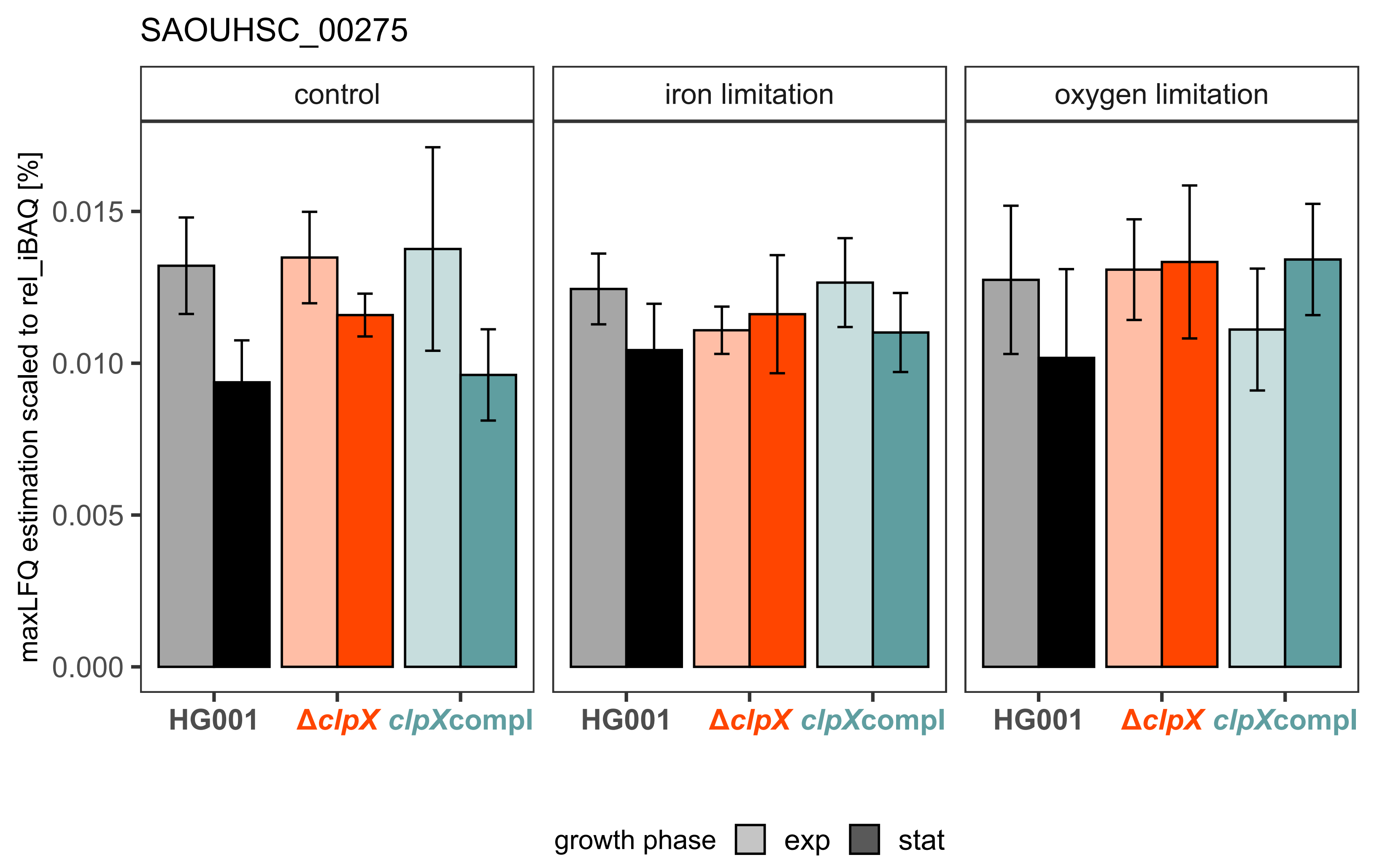

SAOUHSC_00275
control
iron limitation
oxygen limitation
maxLFQ estimation scaled to rel_iBAQ [%]
0.015
0.010
0.005
0.000
HG001
ΔclpX
clpXcompl
growth phase
exp
stat

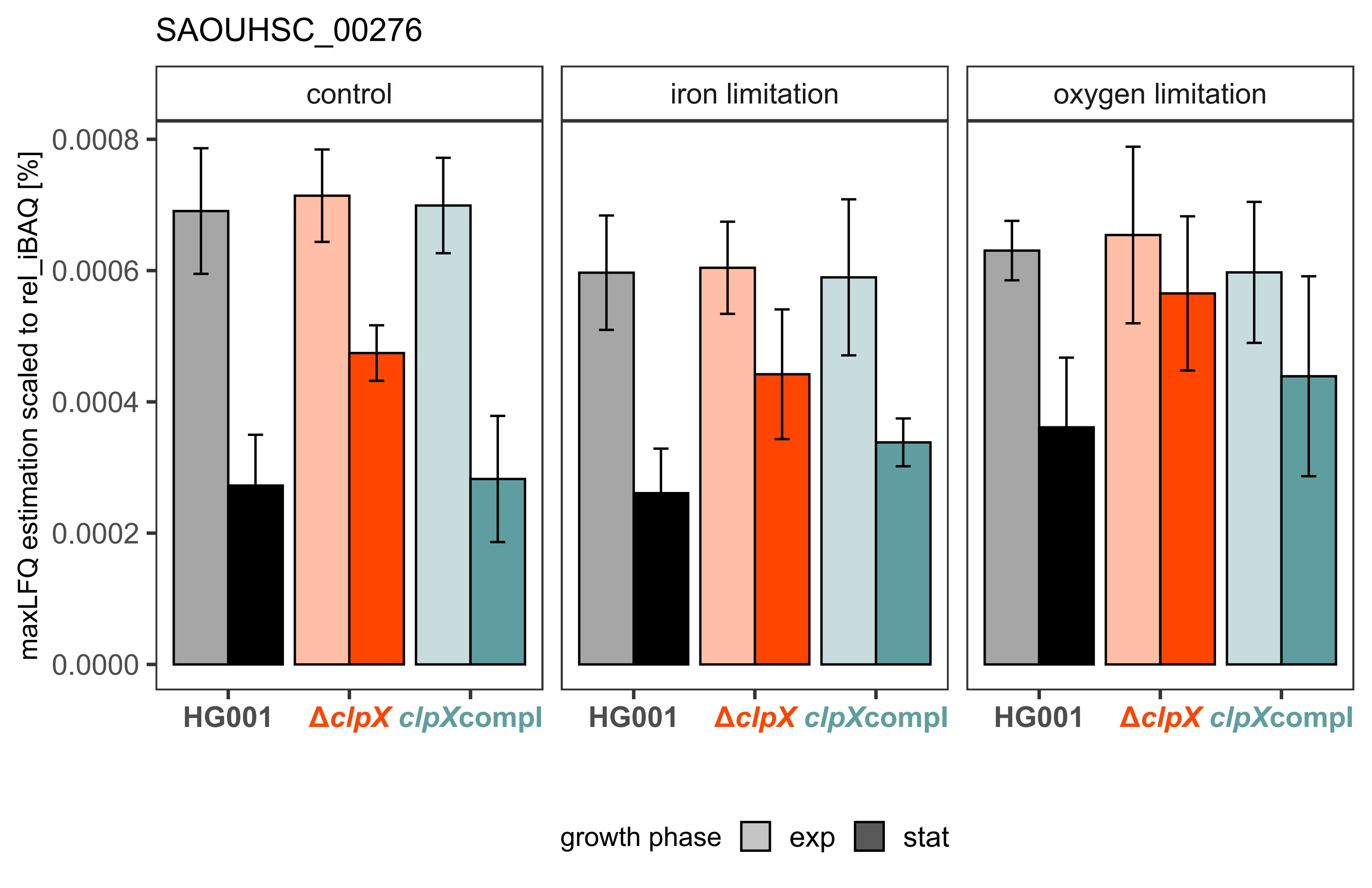

SAOUHSC_00276
control
iron limitation
oxygen limitation
maxLFQ estimation scaled to rel_iBAQ [%]
0.0008
0.0006
0.0004
0.0002
0.0000
HG001
ΔclpX
clpXcompl
growth phase
exp
stat

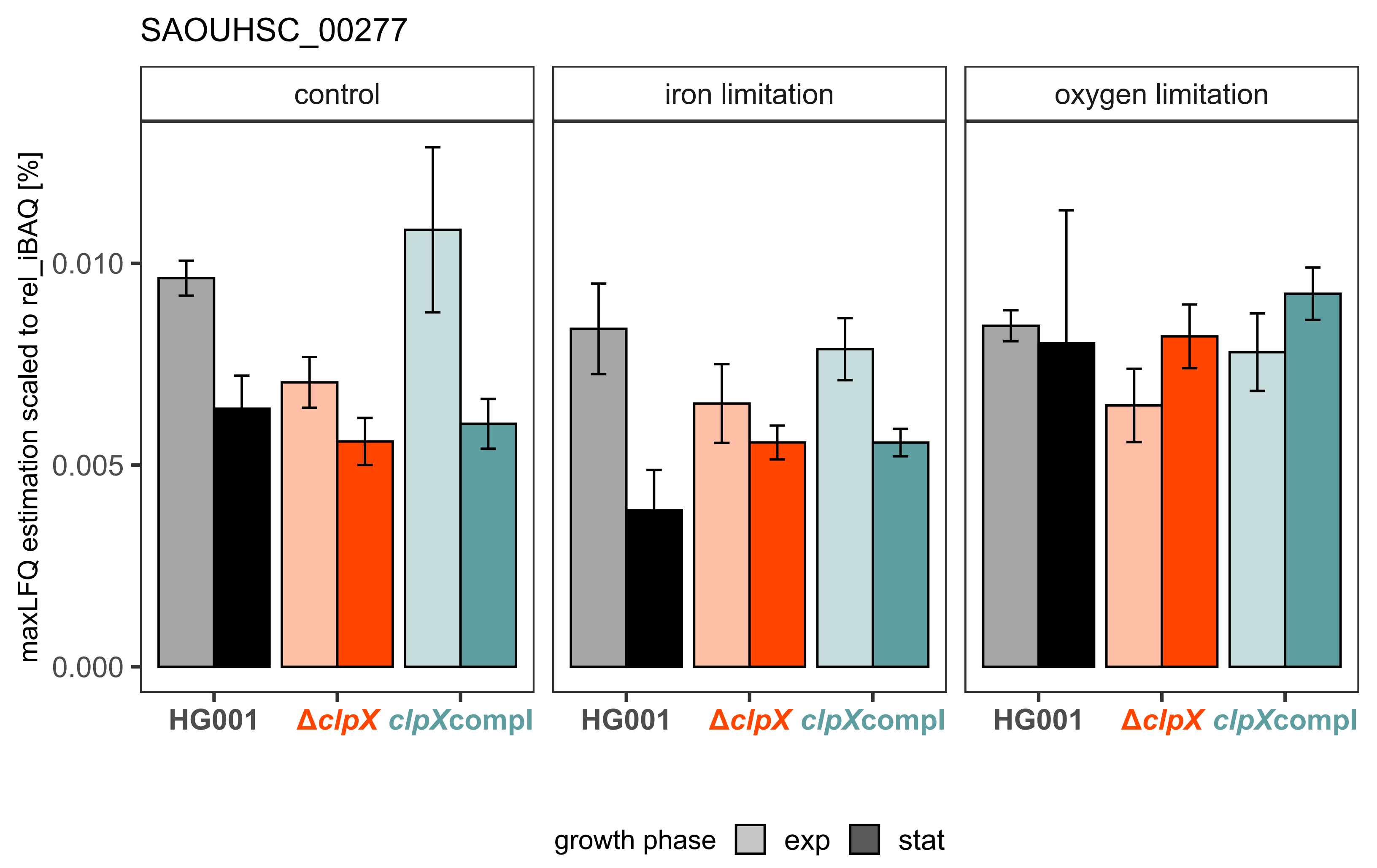

SAOUHSC_00277
control
iron limitation
oxygen limitation
maxLFQ estimation scaled to rel_iBAQ [%]
0.010
0.005
0.000
HG001
ΔclpX
clpXcompl
growth phase
exp
stat

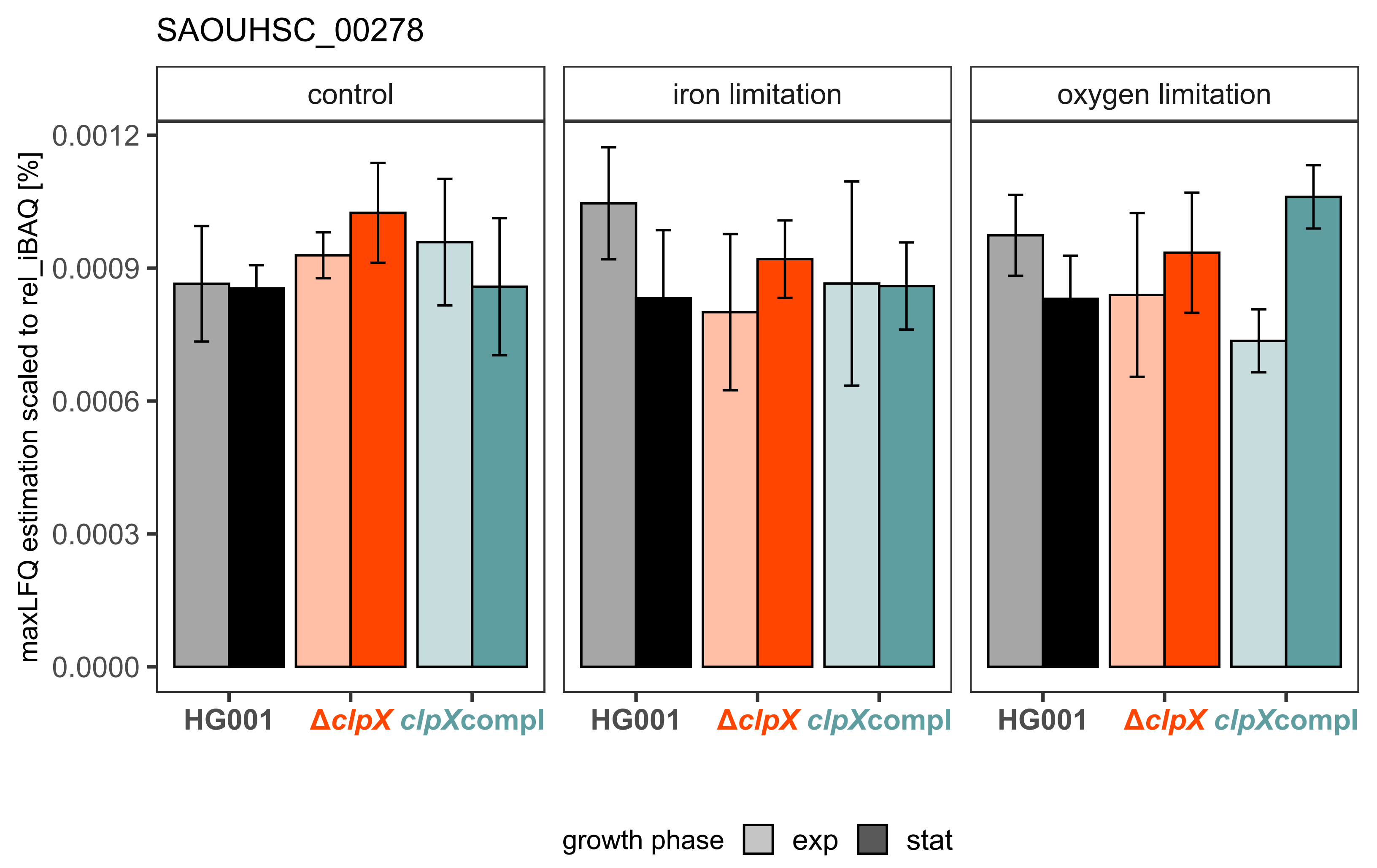
SAOUHSC_00278
control
iron limitation
oxygen limitation
maxLFQ estimation scaled to rel_iBAQ [%]
0.0012
0.0009
0.0006
0.0003
0.0000
HG001
ΔclpX
clpXcompl
growth phase
exp
stat

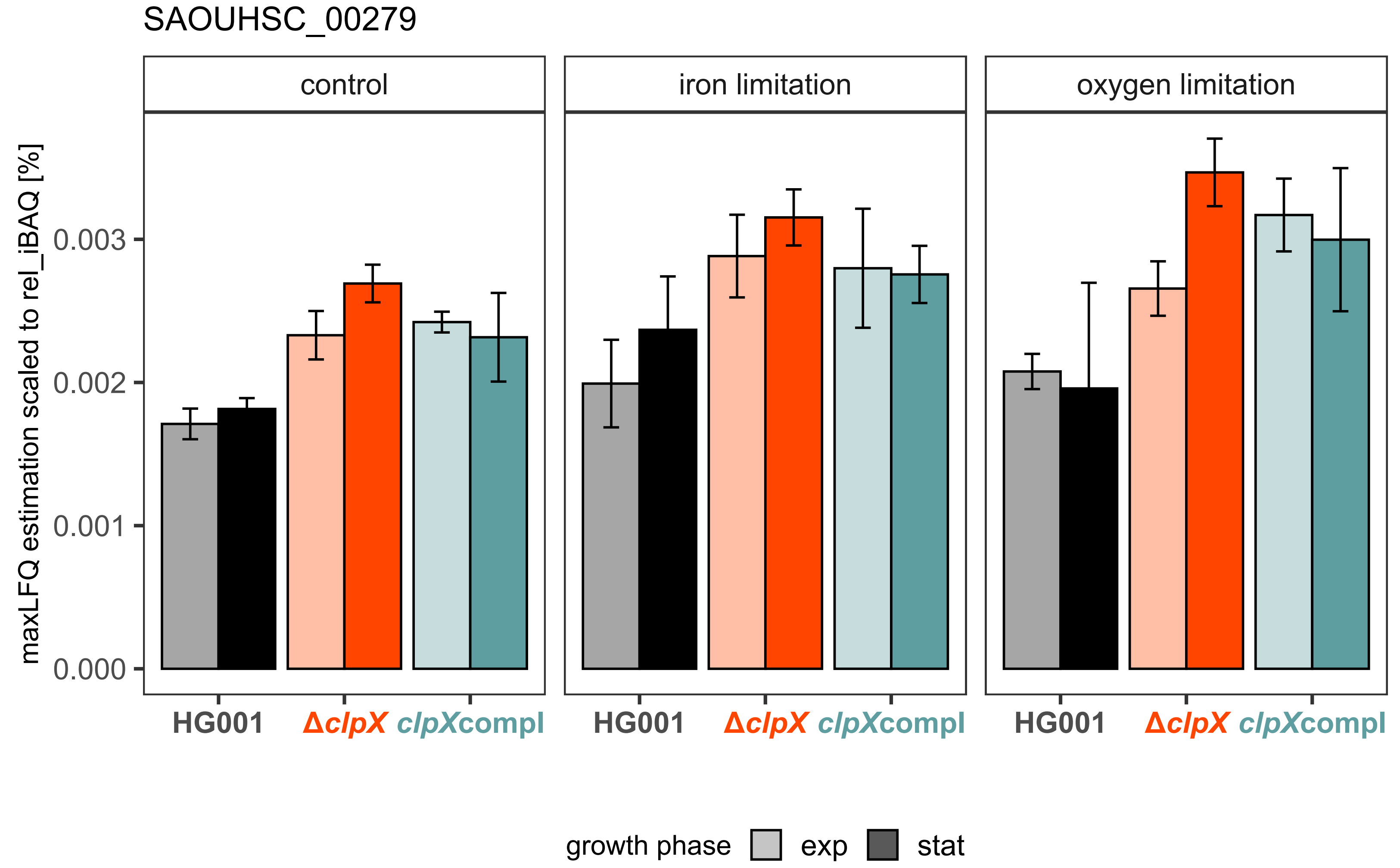

SAOUHSC_00279
control
iron limitation
oxygen limitation
maxLFQ estimation scaled to rel_iBAQ [%]
0.003
0.002
0.001
0.000
HG001
ΔclpX
clpXcompl
growth phase
exp
stat

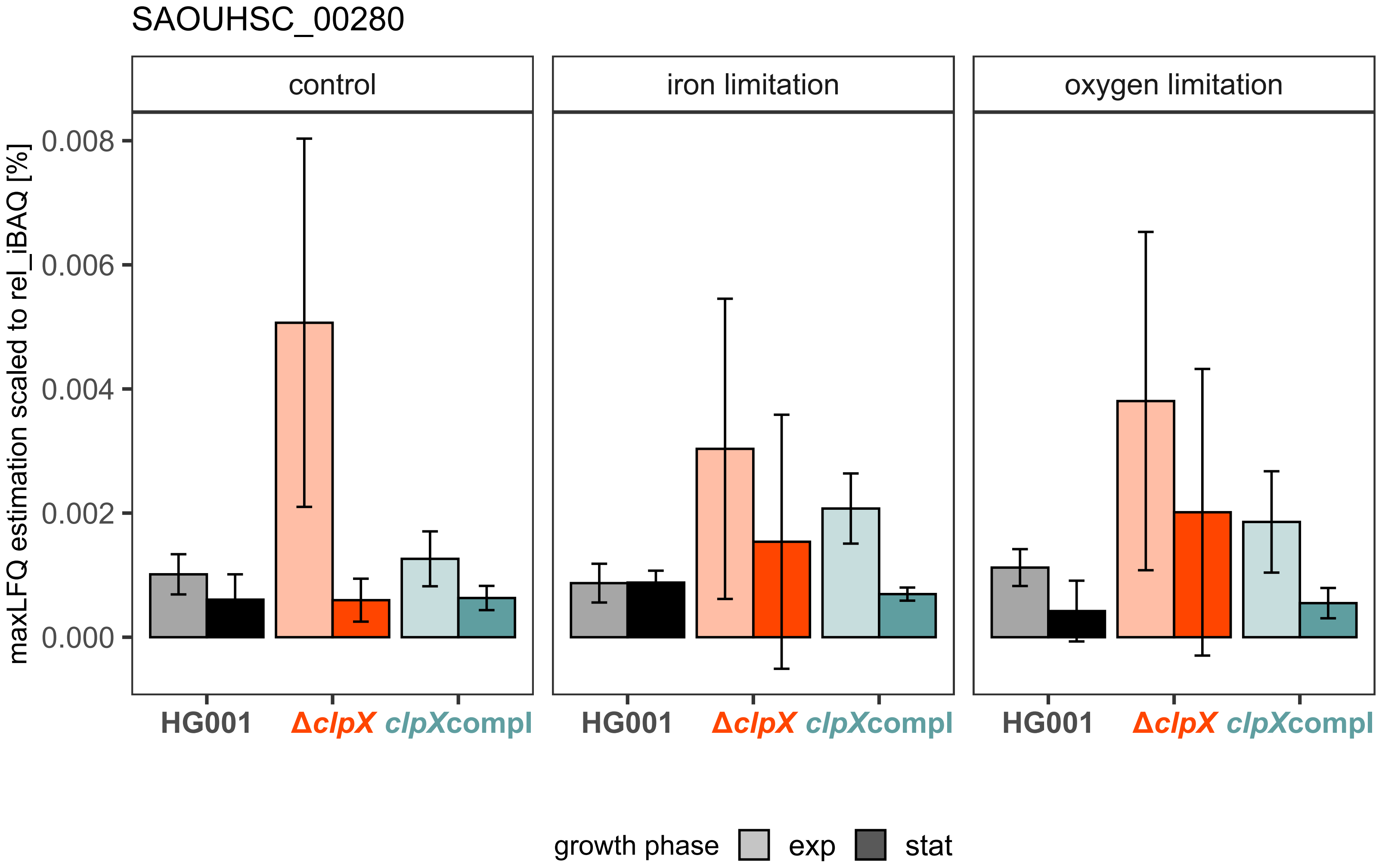
SAOUHSC_00280
control
iron limitation
oxygen limitation
maxLFQ estimation scaled to rel_iBAQ [%]
0.008
0.006
0.004
0.002
0.000
HG001
ΔclpX
clpXcompl
growth phase
exp
stat

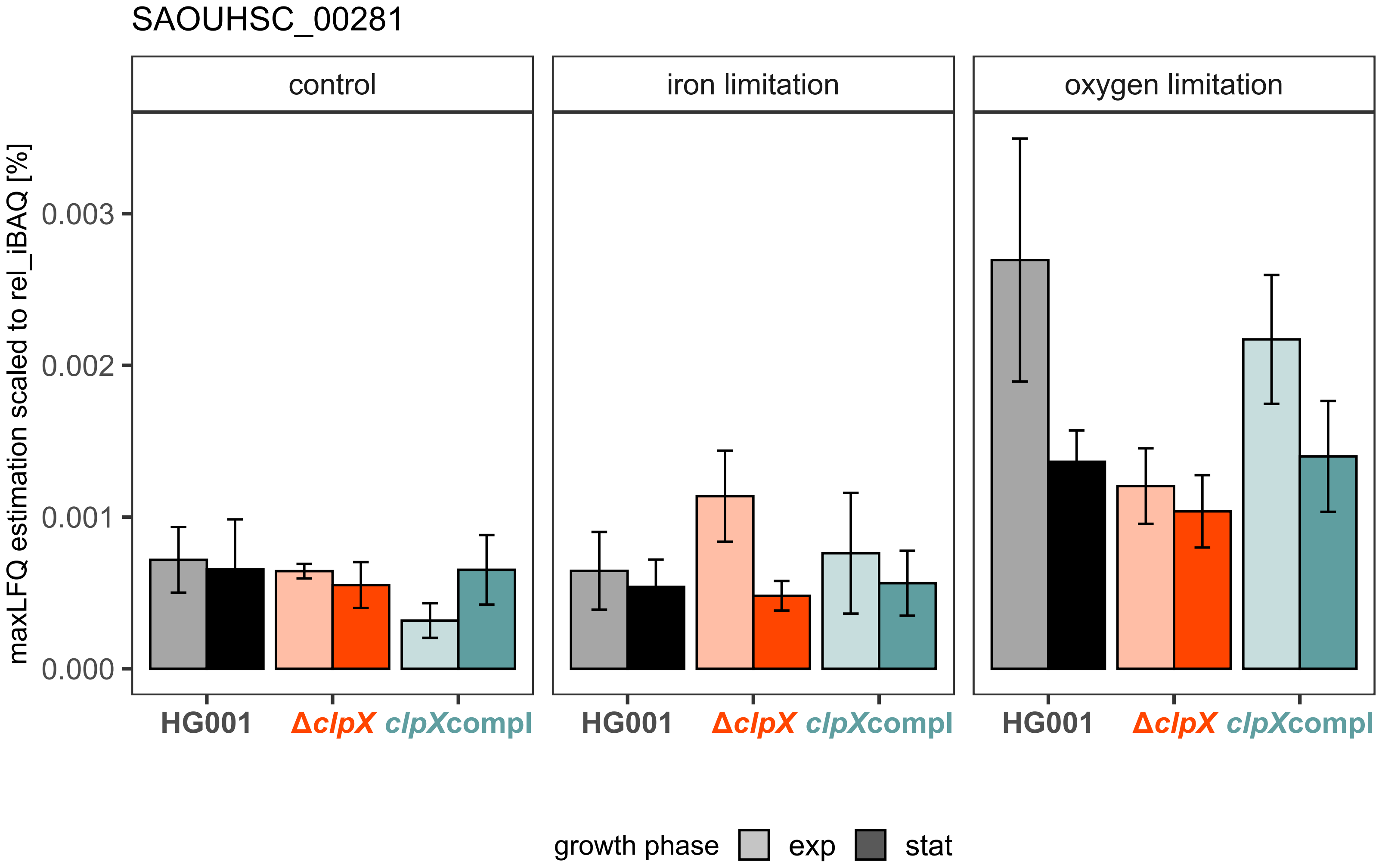
SAOUHSC_00281
control
iron limitation
oxygen limitation
maxLFQ estimation scaled to rel_iBAQ [%]
0.003
0.002
0.001
0.000
HG001
ΔclpX
clpXcompl
growth phase
exp
stat

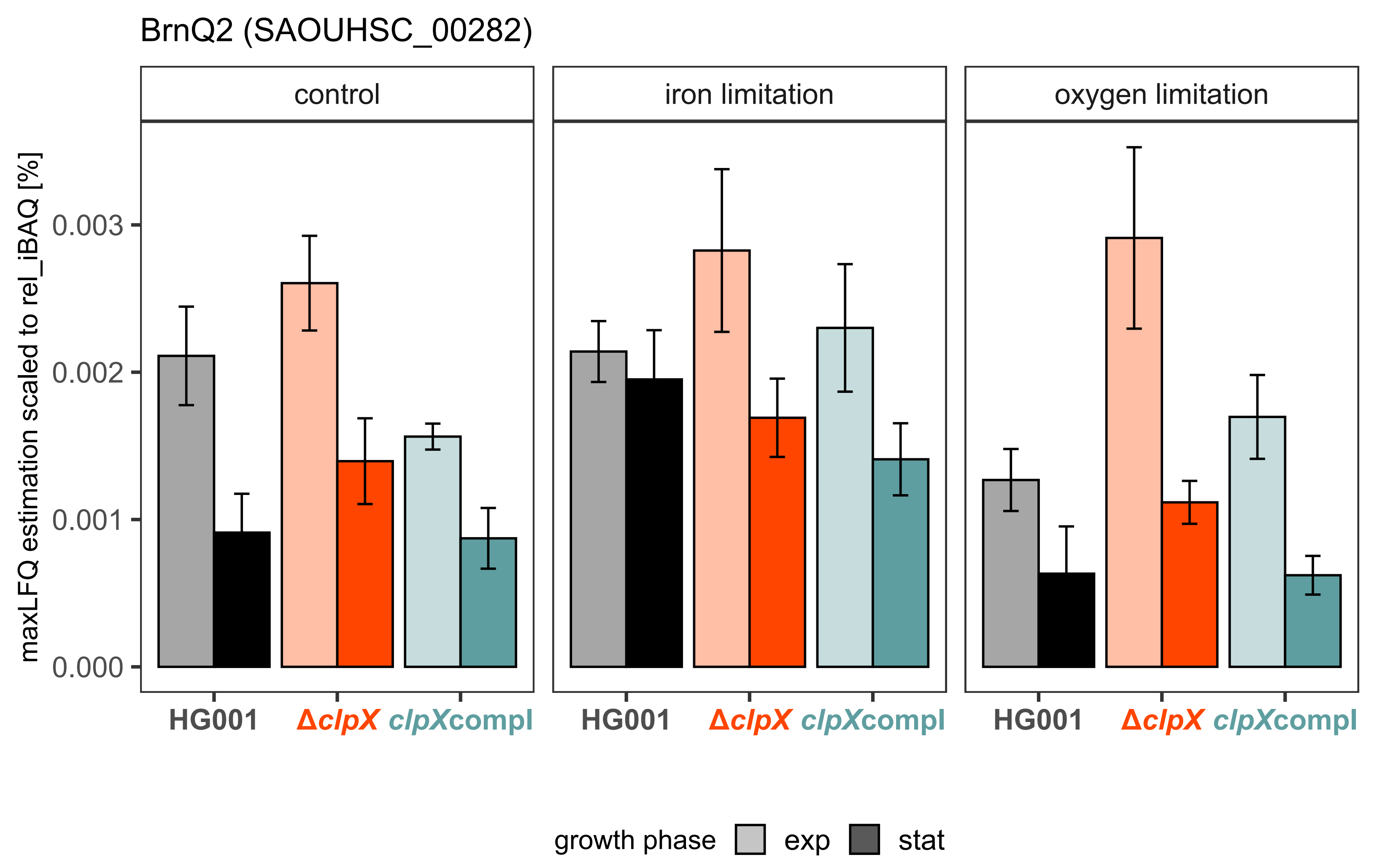

BrnQ2 (SAOUHSC_00282)
control
iron limitation
oxygen limitation
maxLFQ estimation scaled to rel_iBAQ [%]
0.003
0.002
0.001
0.000
HG001
ΔclpX
clpXcompl
growth phase
exp
stat

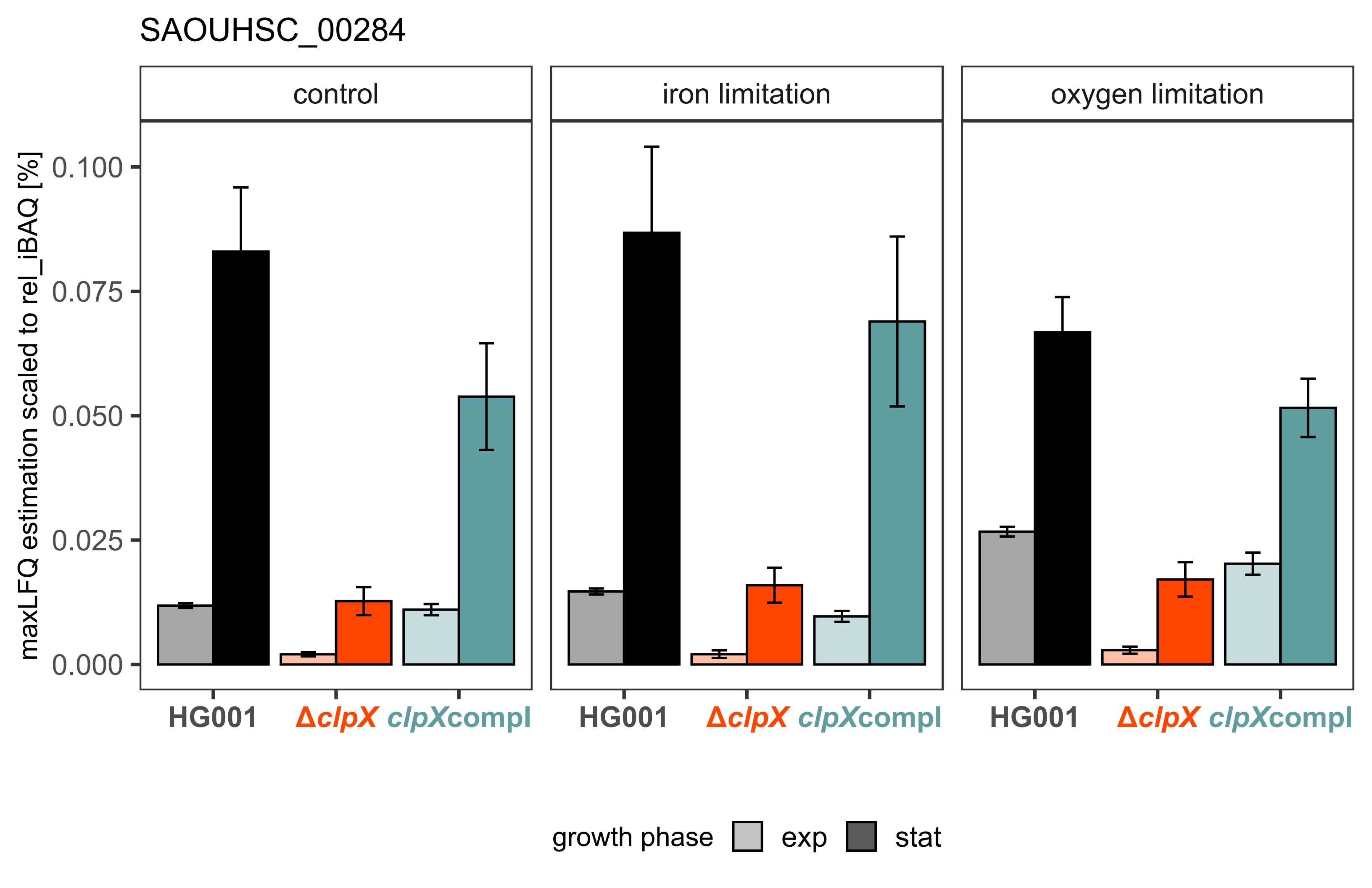
SAOUHSC_00284
control
iron limitation
oxygen limitation
maxLFQ estimation scaled to rel_iBAQ [%]
0.100
0.075
0.050
0.025
0.000
HG001
ΔclpX
clpXcompl
growth phase
exp
stat

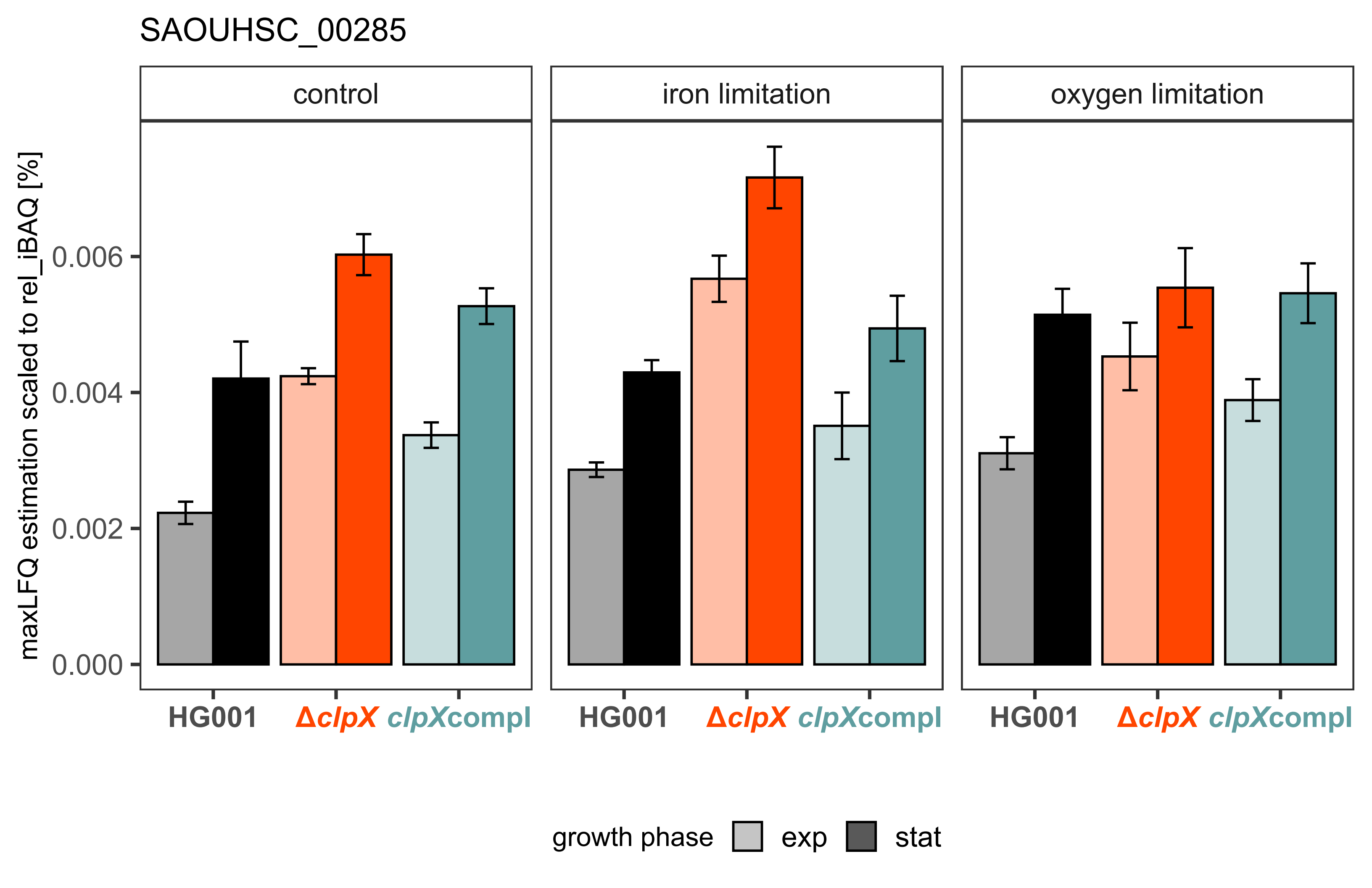
SAOUHSC_00285
control
iron limitation
oxygen limitation
maxLFQ estimation scaled to rel_iBAQ [%]
0.006
0.004
0.002
0.000
HG001
ΔclpX
clpXcompl
growth phase
exp
stat

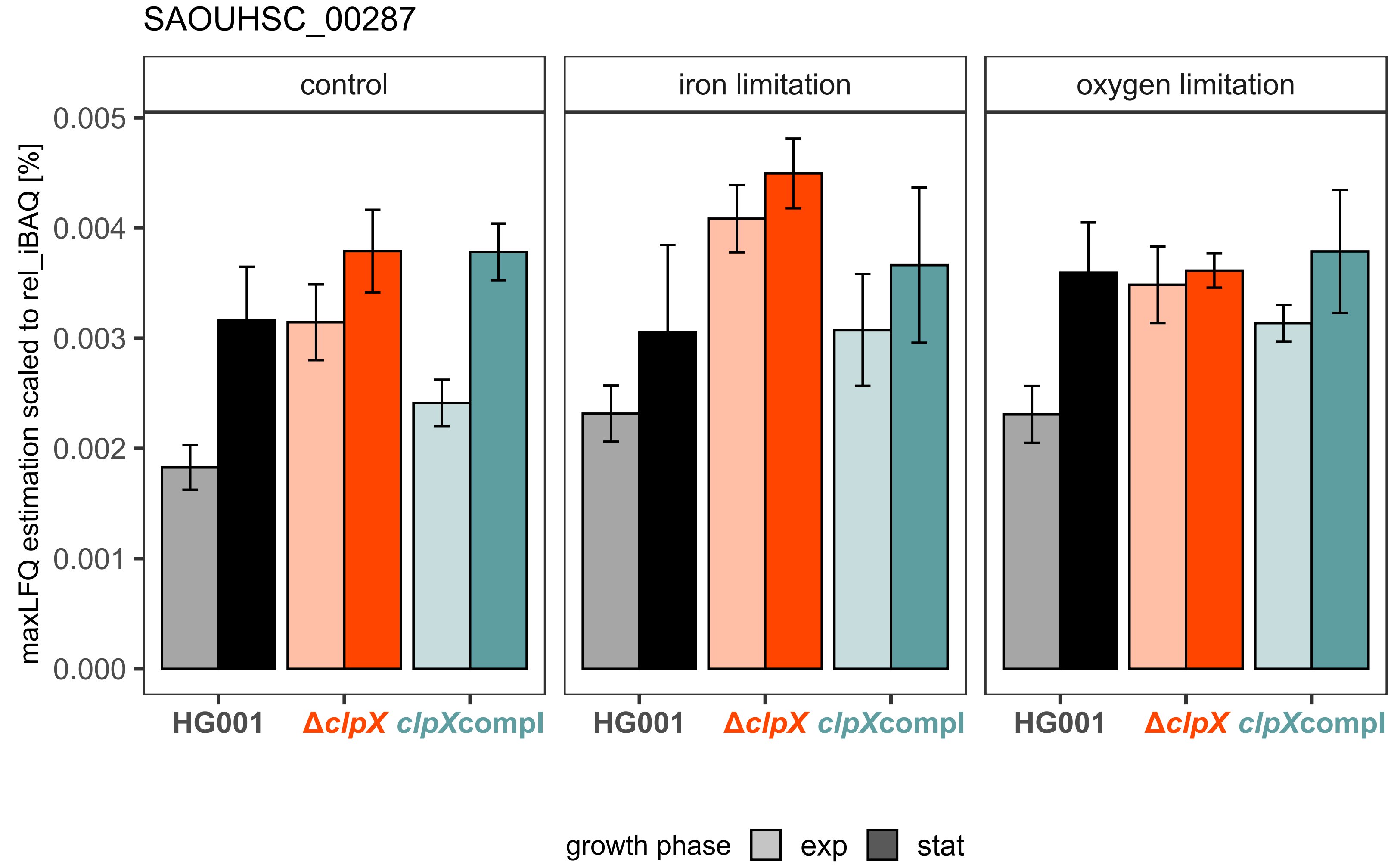
SAOUHSC_00287
control
iron limitation
oxygen limitation
maxLFQ estimation scaled to rel_iBAQ [%]
0.005
0.004
0.003
0.002
0.001
0.000
HG001
ΔclpX
clpXcompl
growth phase
exp
stat

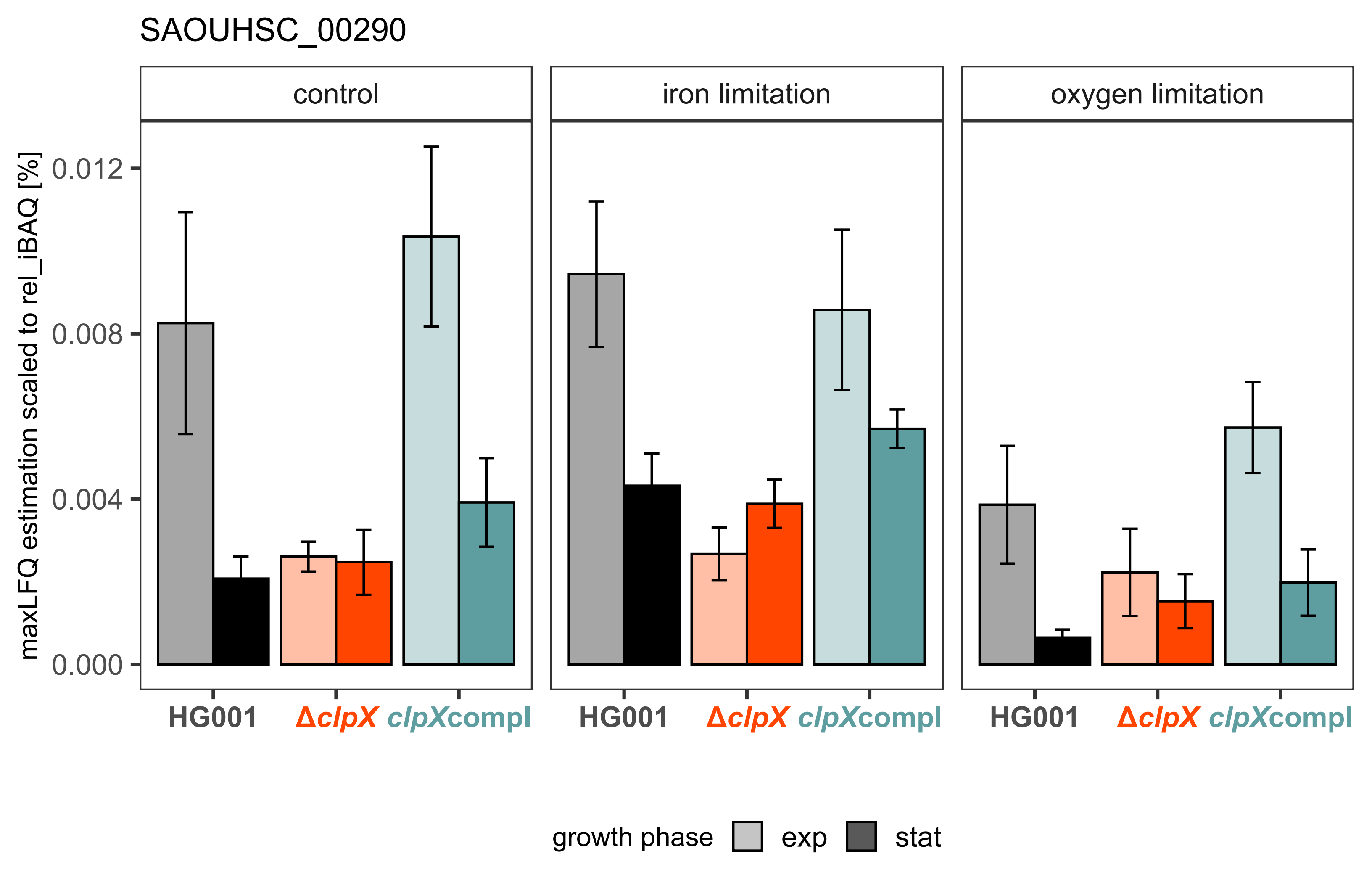
SAOUHSC_00290
control
iron limitation
oxygen limitation
maxLFQ estimation scaled to rel_iBAQ [%]
0.012
0.008
0.004
0.000
HG001
ΔclpX
clpXcompl
growth phase
exp
stat

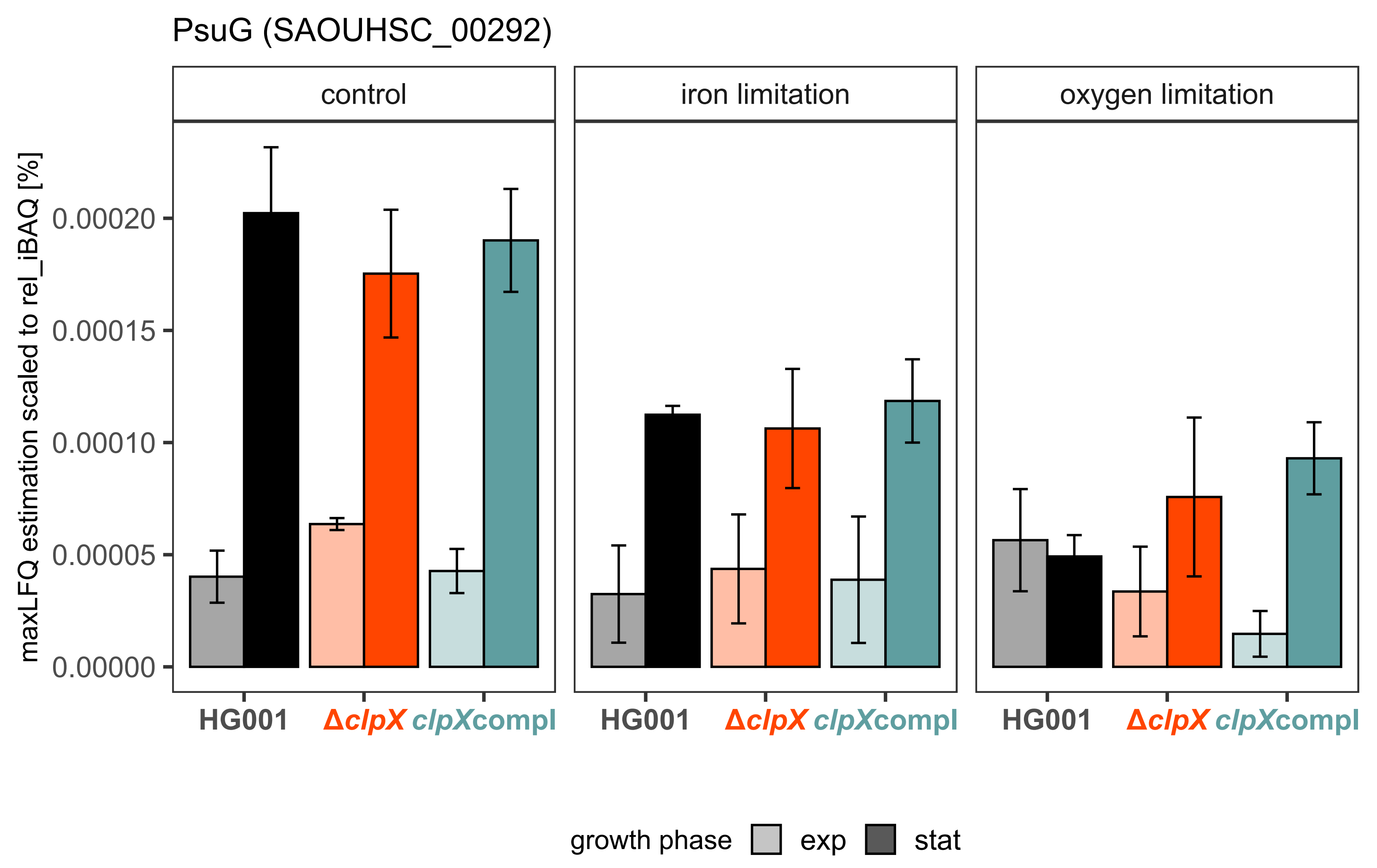

PsuG (SAOUHSC_00292)
control
iron limitation
oxygen limitation
maxLFQ estimation scaled to rel_iBAQ [%]
0.00020
0.00015
0.00010
0.00005
0.00000
HG001
ΔclpX
clpXcompl
growth phase
exp
stat

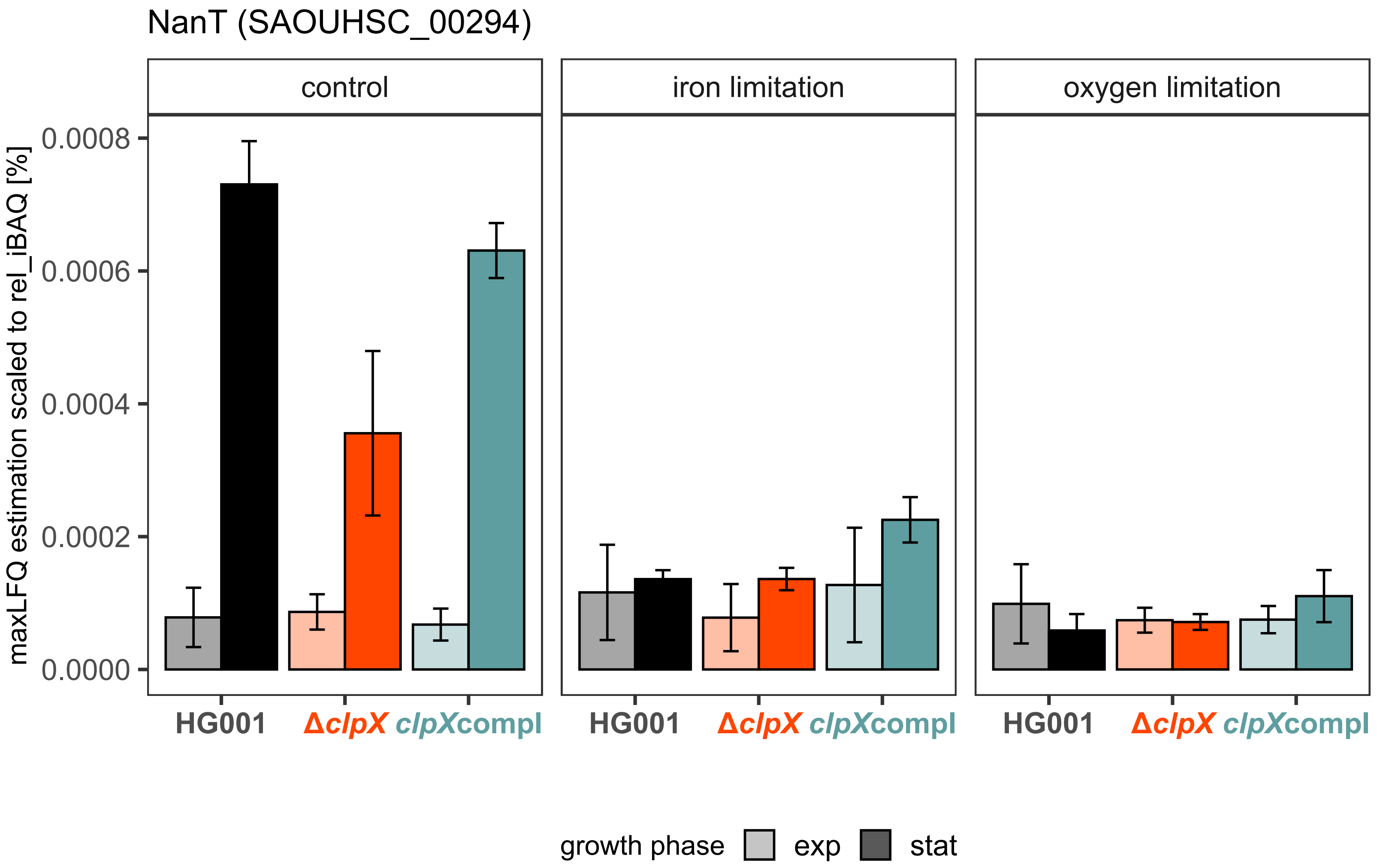
NanT (SAOUHSC_00294)
control
iron limitation
oxygen limitation
maxLFQ estimation scaled to rel_iBAQ [%]
0.0008
0.0006
0.0004
0.0002
0.0000
HG001
ΔclpX
clpXcompl
growth phase
exp
stat

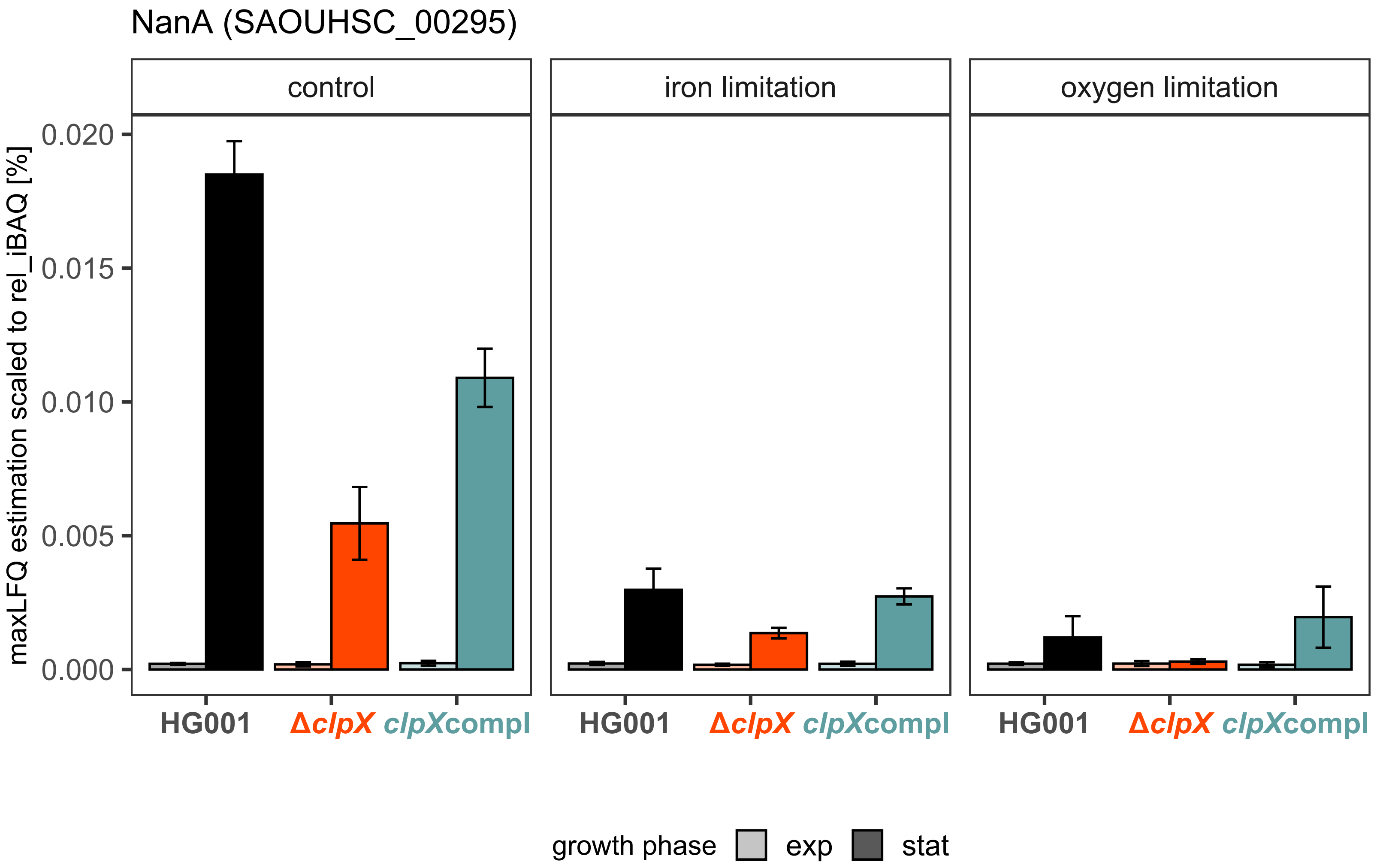

NanA (SAOUHSC_00295)
control
iron limitation
oxygen limitation
maxLFQ estimation scaled to rel_iBAQ [%]
0.020
0.015
0.010
0.005
0.000
HG001
ΔclpX
clpXcompl
growth phase
exp
stat

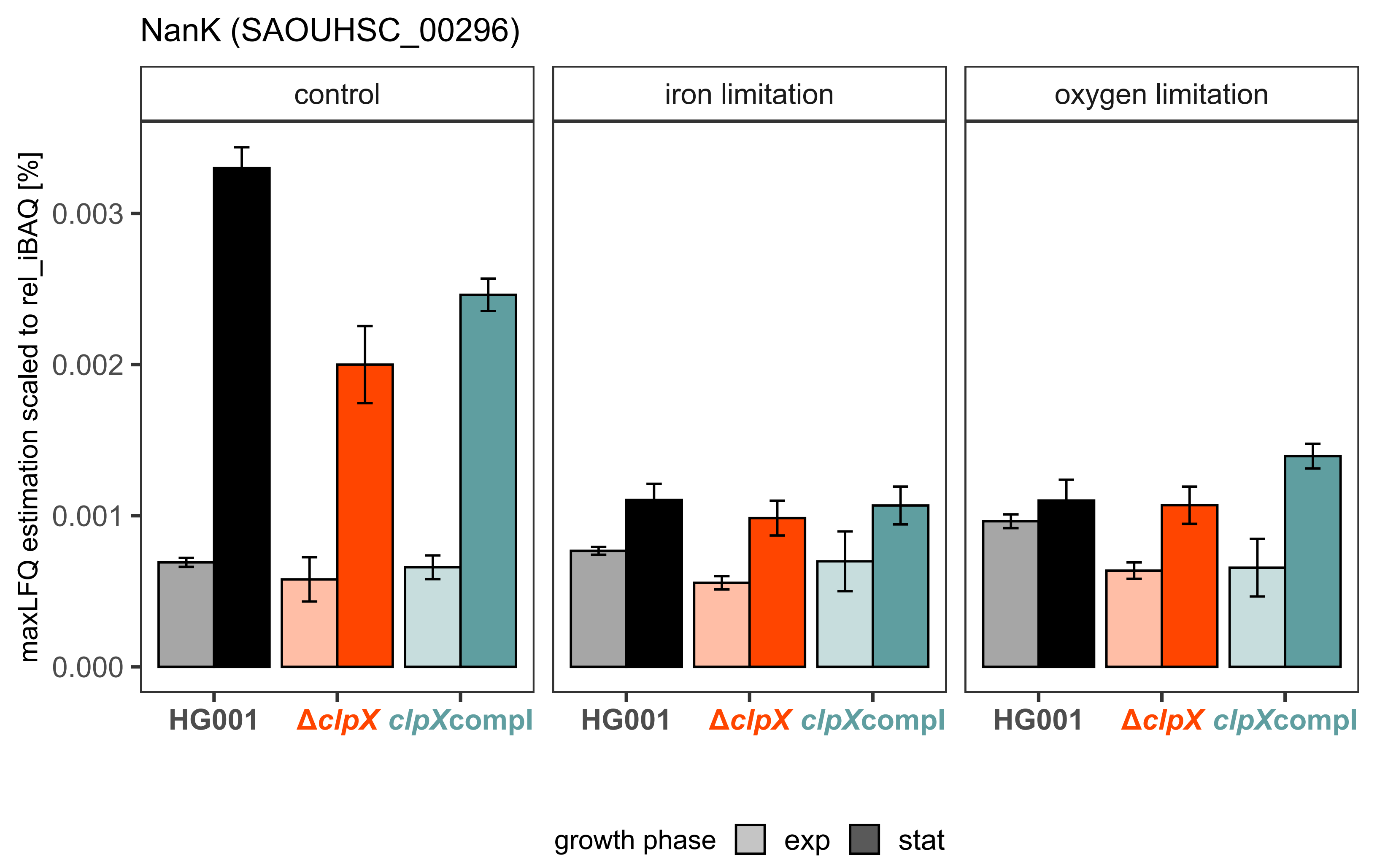

NanK (SAOUHSC_00296)
control
iron limitation
oxygen limitation
maxLFQ estimation scaled to rel_iBAQ [%]
0.003
0.002
0.001
0.000
HG001
ΔclpX
clpXcompl
growth phase
exp
stat

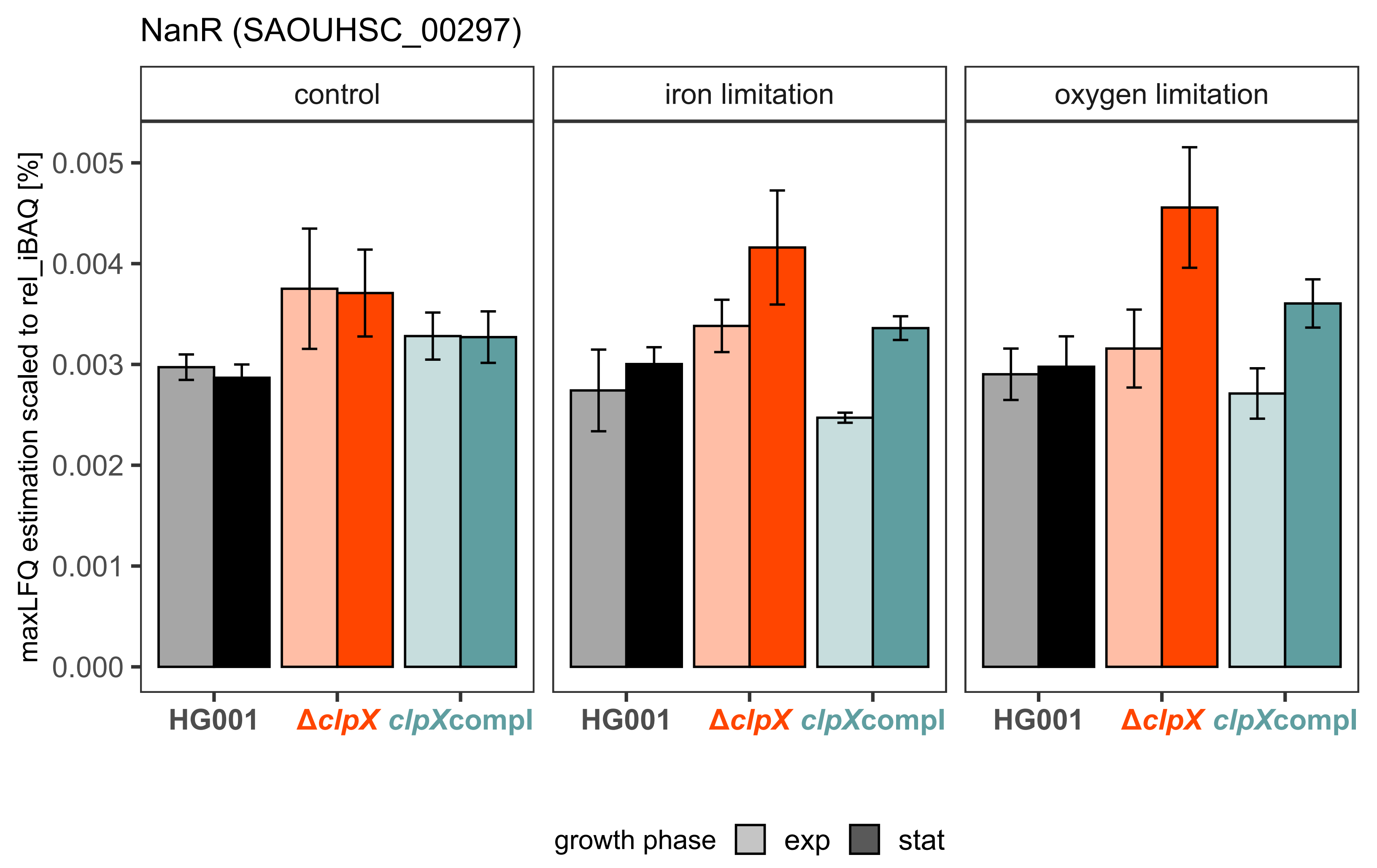
NanR (SAOUHSC_00297)
control
iron limitation
oxygen limitation
maxLFQ estimation scaled to rel_iBAQ [%]
0.005
0.004
0.003
0.002
0.001
0.000
HG001
ΔclpX
clpXcompl
growth phase
exp
stat

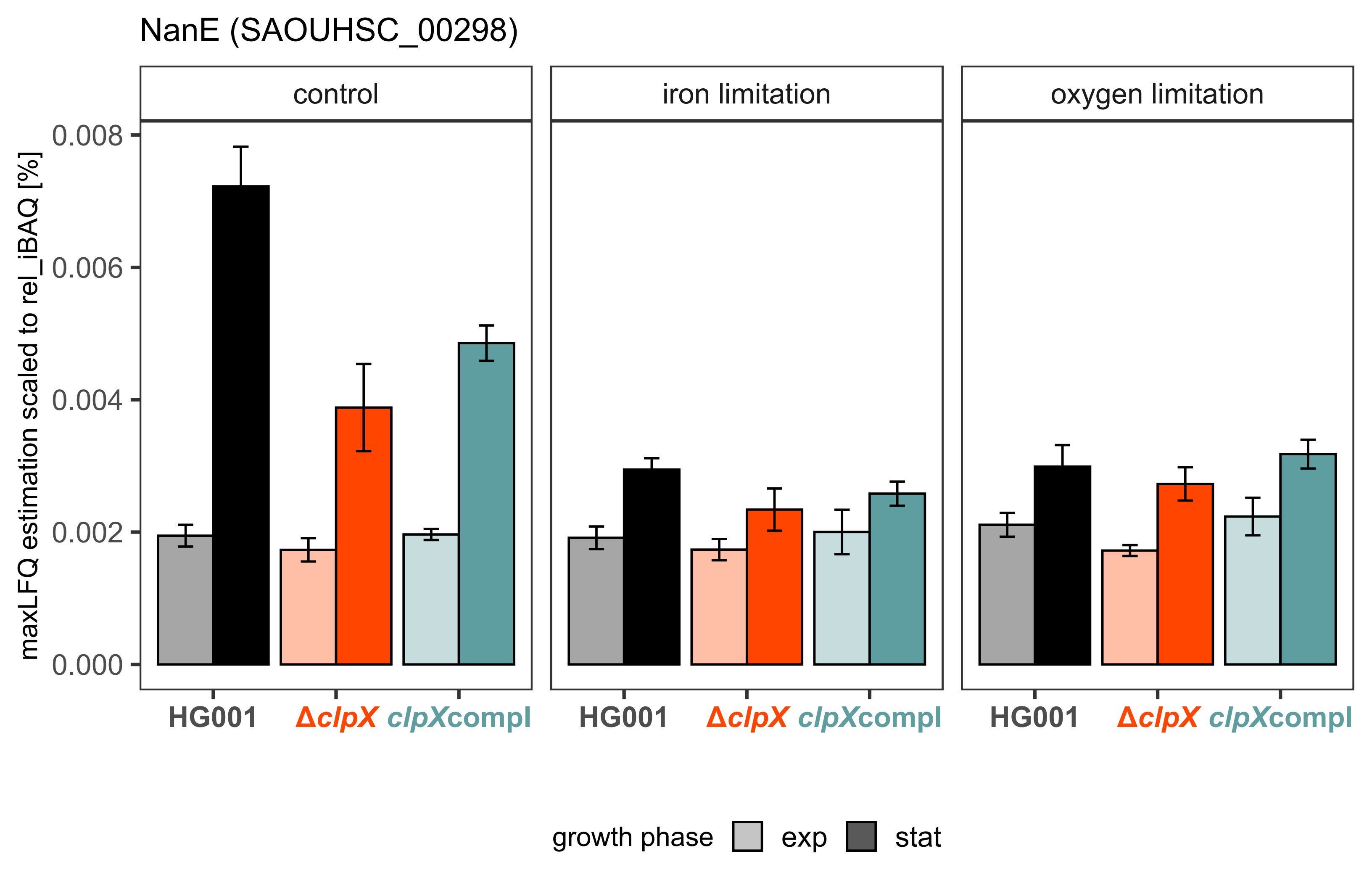

NanE (SAOUHSC_00298)
control
iron limitation
oxygen limitation
maxLFQ estimation scaled to rel_iBAQ [%]
0.008
0.006
0.004
0.002
0.000
HG001
ΔclpX
clpXcompl
growth phase
exp
stat

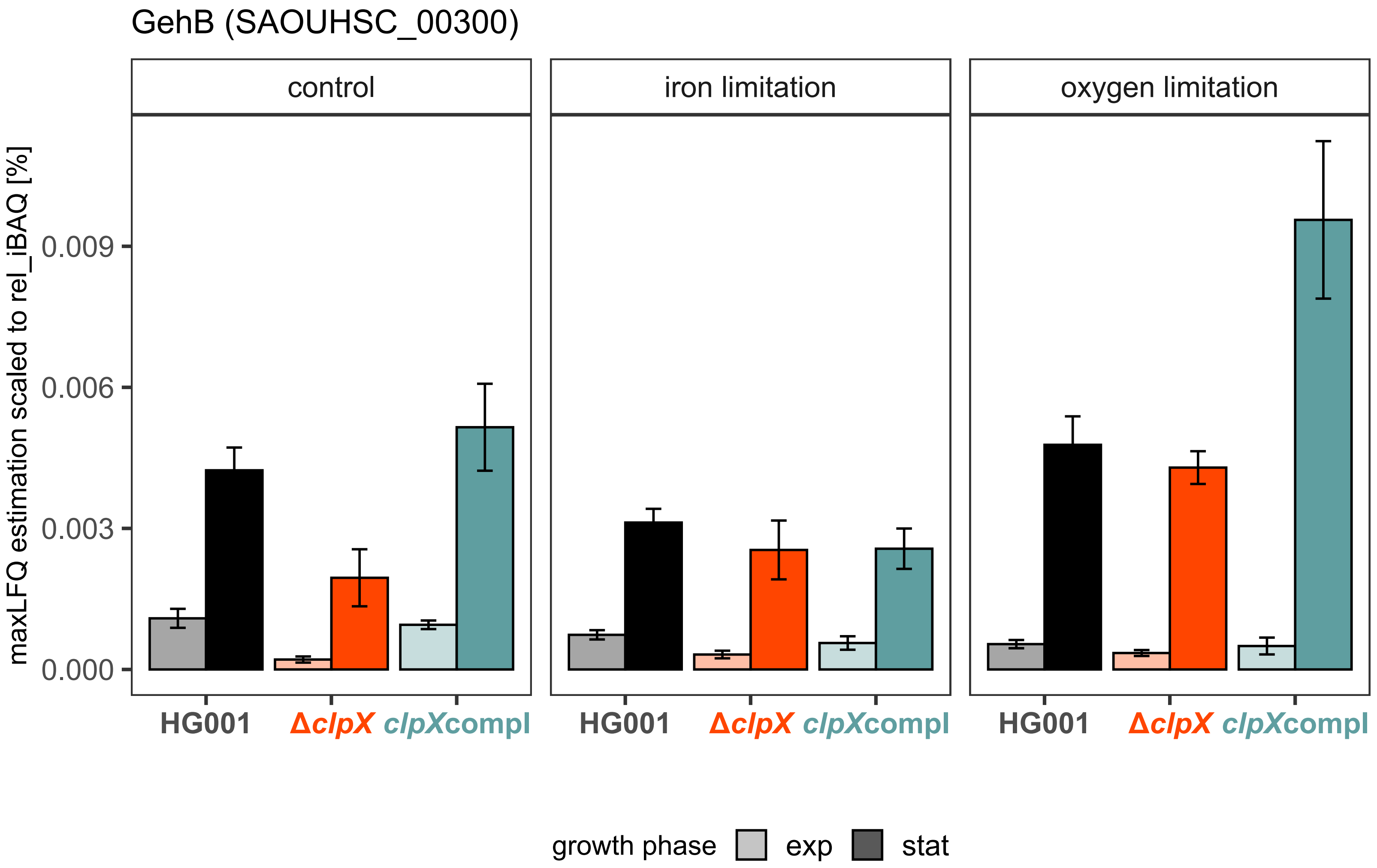
GehB (SAOUHSC_00300)
control
iron limitation
oxygen limitation
maxLFQ estimation scaled to rel_iBAQ [%]
0.009
0.006
0.003
0.000
HG001
ΔclpX
clpXcompl
growth phase
exp
stat

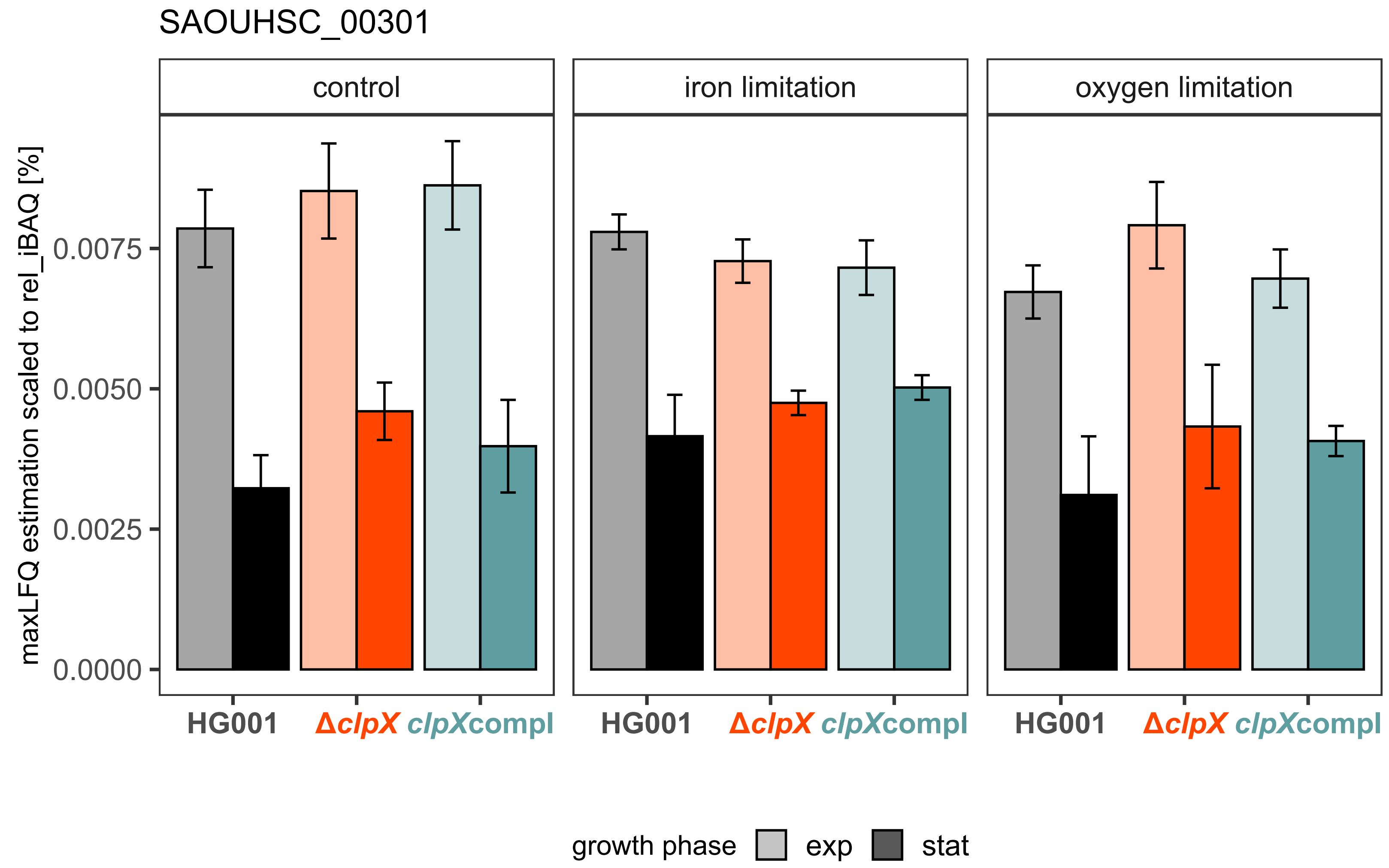

SAOUHSC_00301
control
iron limitation
oxygen limitation
maxLFQ estimation scaled to rel_iBAQ [%]
0.0075
0.0050
0.0025
0.0000
HG001
ΔclpX
clpXcompl
growth phase
exp
stat

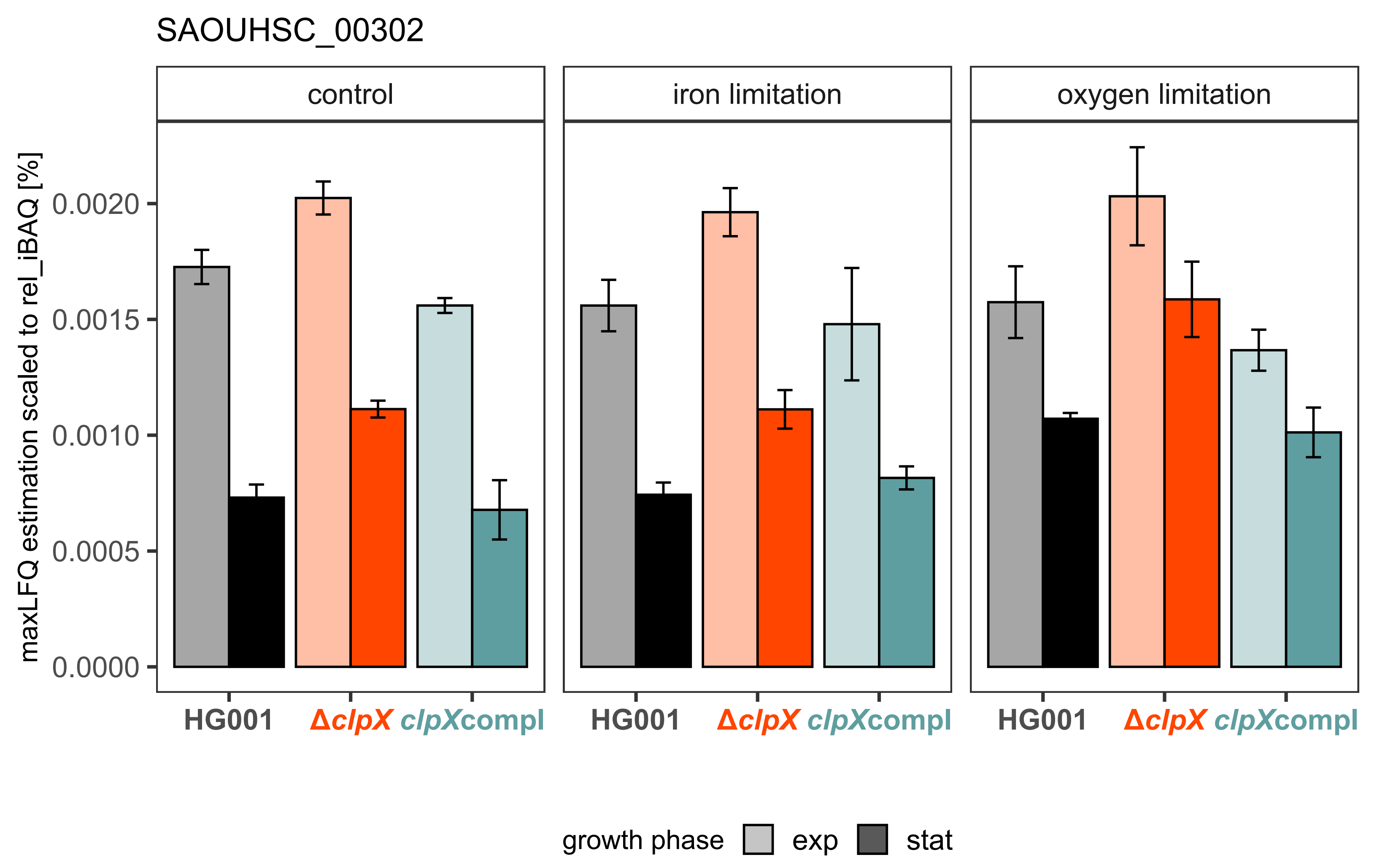

SAOUHSC_00302
control
iron limitation
oxygen limitation
maxLFQ estimation scaled to rel_iBAQ [%]
0.0020
0.0015
0.0010
0.0005
0.0000
HG001
ΔclpX
clpXcompl
growth phase
exp
stat

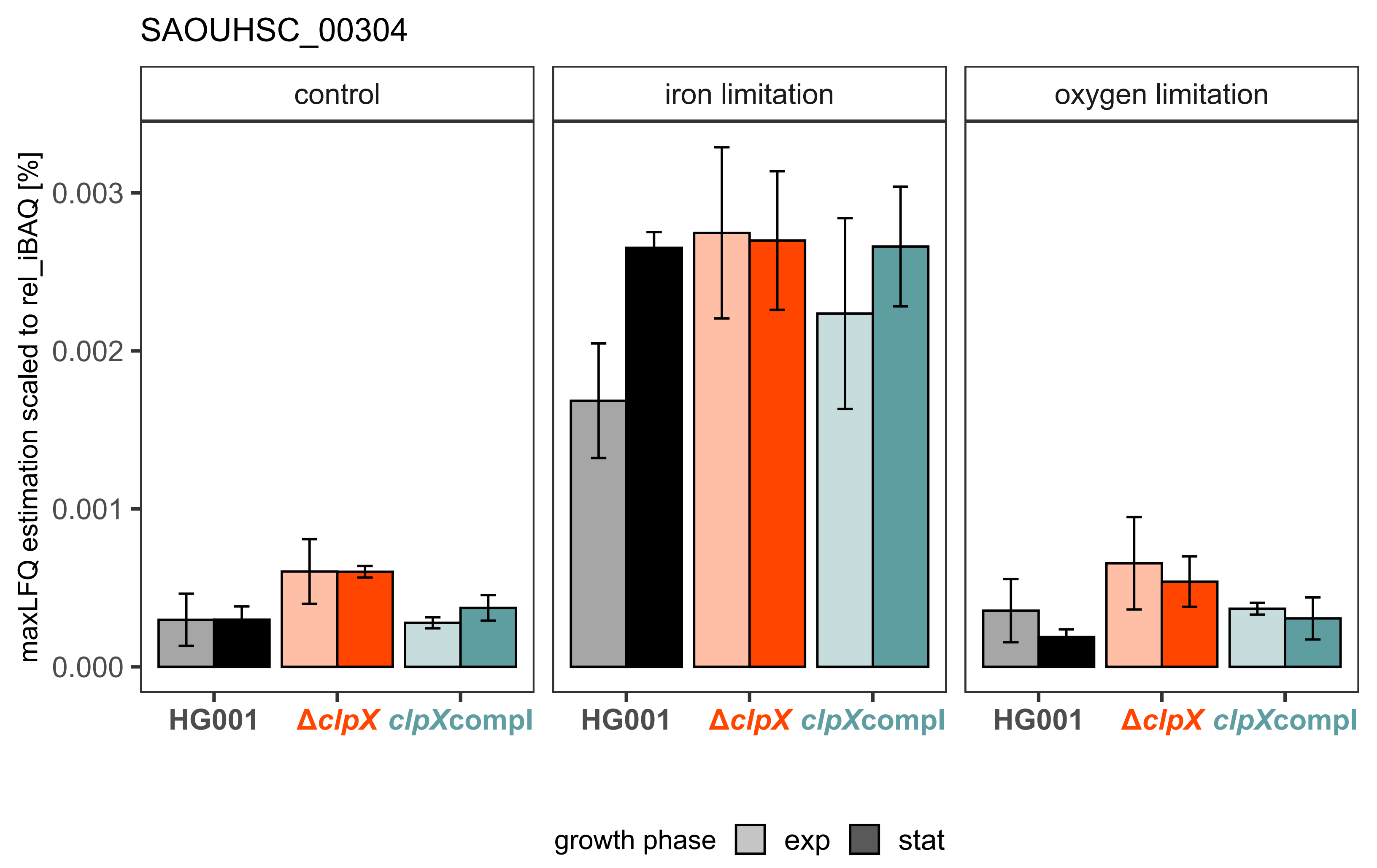
SAOUHSC_00304
control
iron limitation
oxygen limitation
maxLFQ estimation scaled to rel_iBAQ [%]
0.003
0.002
0.001
0.000
HG001
ΔclpX
clpXcompl
growth phase
exp
stat

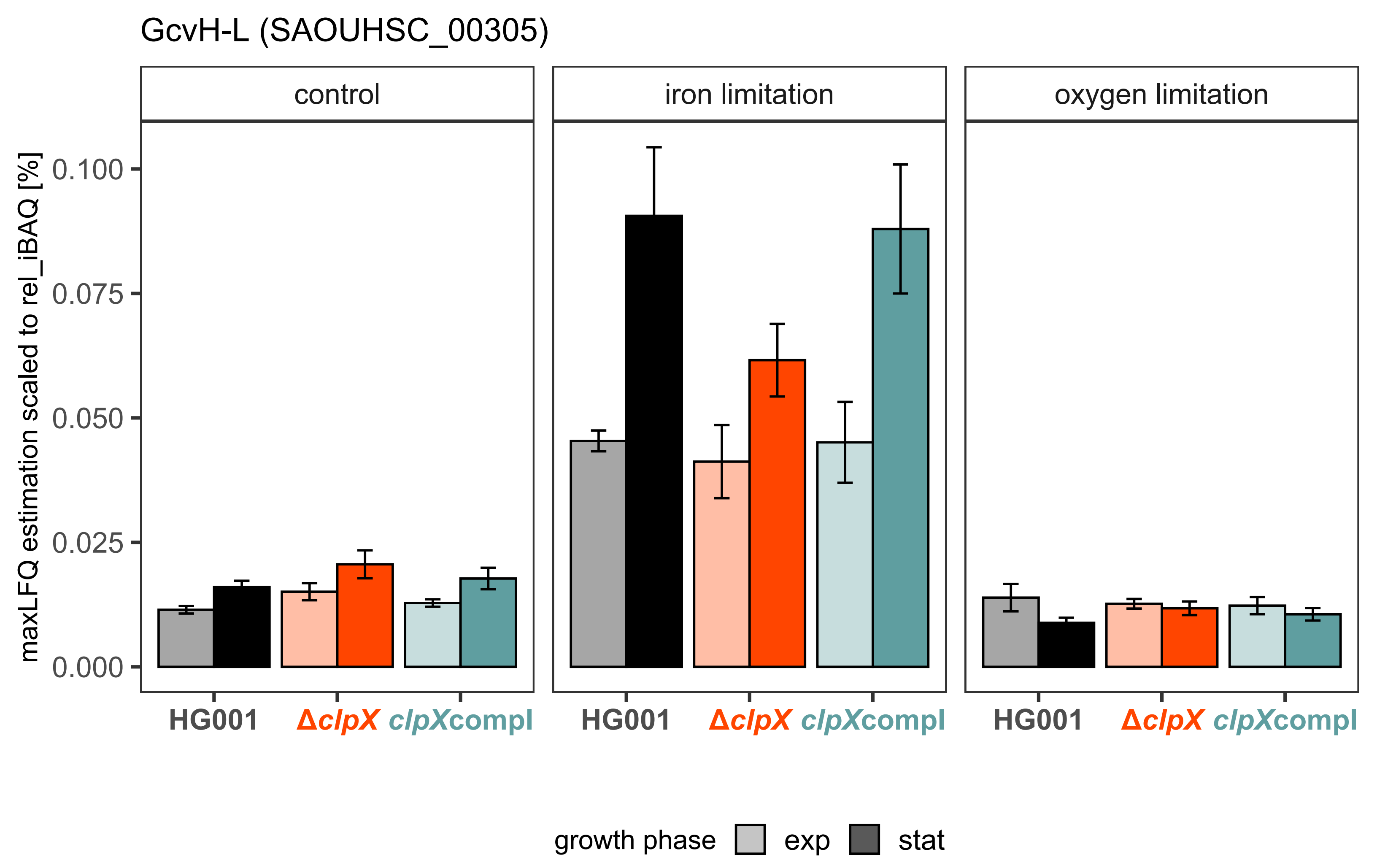
GcvH-L (SAOUHSC_00305)
control
iron limitation
oxygen limitation
maxLFQ estimation scaled to rel_iBAQ [%]
0.100
0.075
0.050
0.025
0.000
HG001
ΔclpX
clpXcompl
growth phase
exp
stat

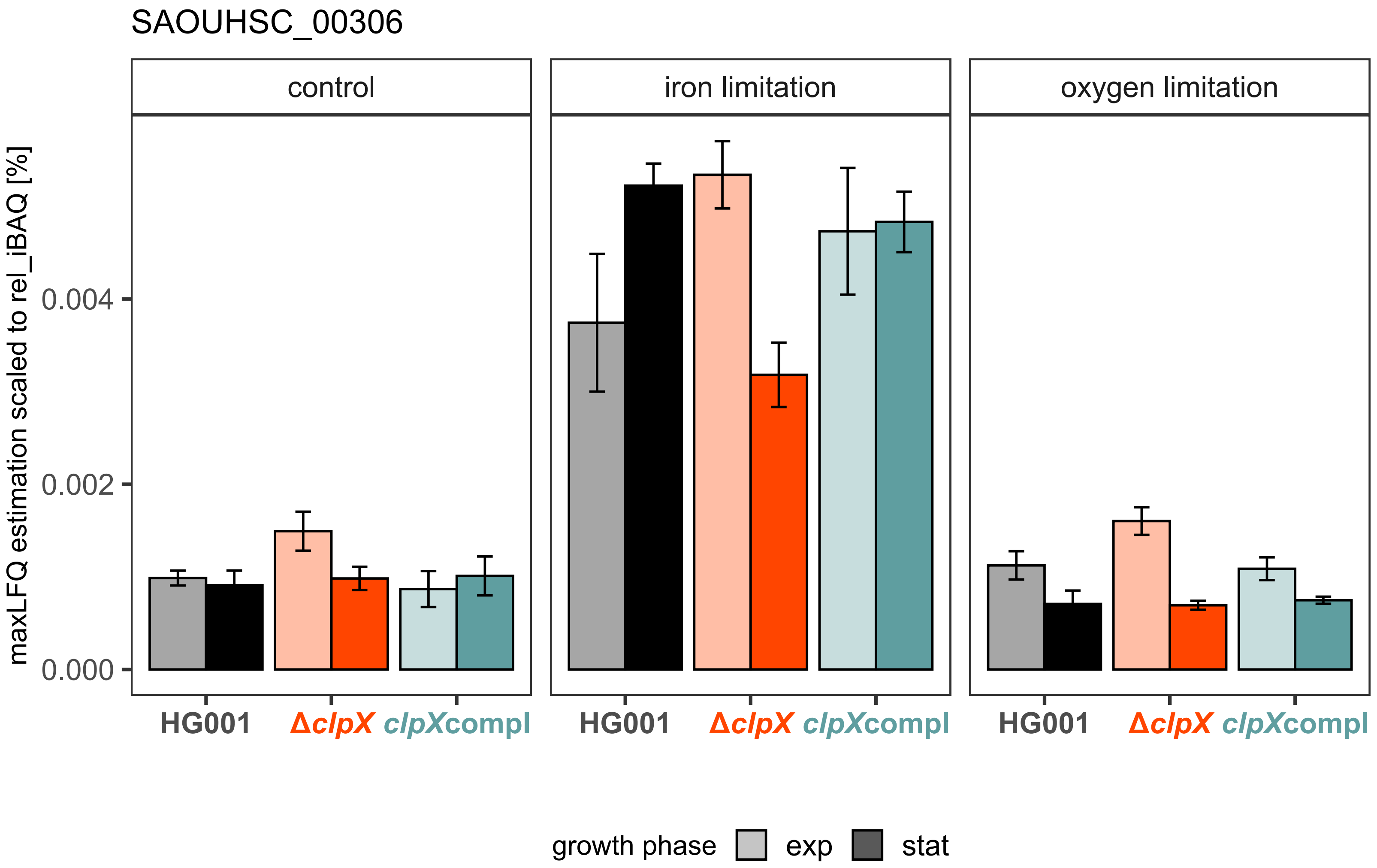

SAOUHSC_00306
control
iron limitation
oxygen limitation
maxLFQ estimation scaled to rel_iBAQ [%]
0.000
0.002
0.004
HG001
ΔclpX
clpXcompl
growth phase
exp
stat

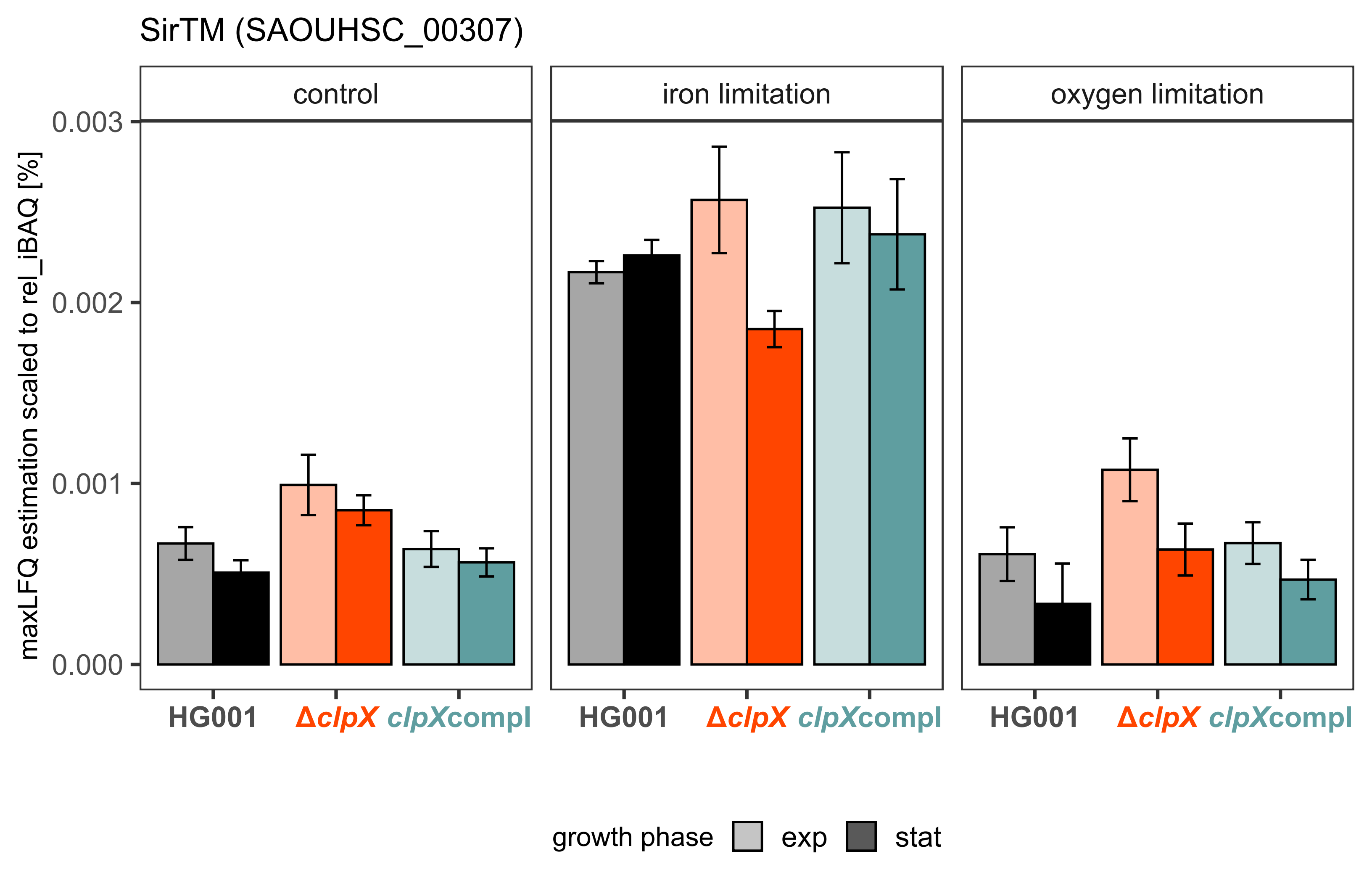

SirTM (SAOUHSC_00307)
control
iron limitation
oxygen limitation
maxLFQ estimation scaled to rel_iBAQ [%]
0.003
0.002
0.001
0.000
HG001
ΔclpX
clpXcompl
growth phase
exp
stat

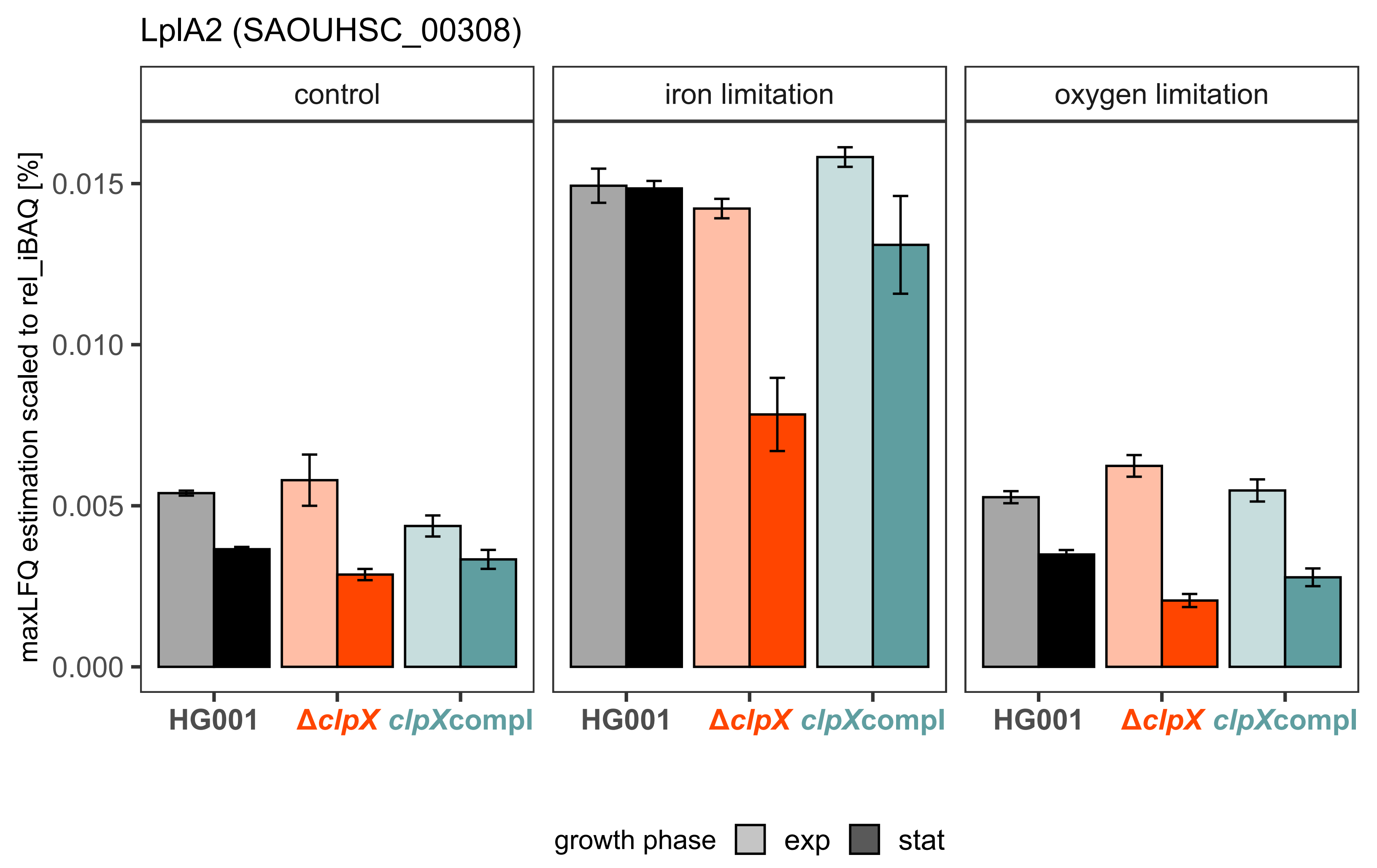

LplA2 (SAOUHSC_00308)
control
iron limitation
oxygen limitation
maxLFQ estimation scaled to rel_iBAQ [%]
0.015
0.010
0.005
0.000
HG001
ΔclpX
clpXcompl
growth phase
exp
stat

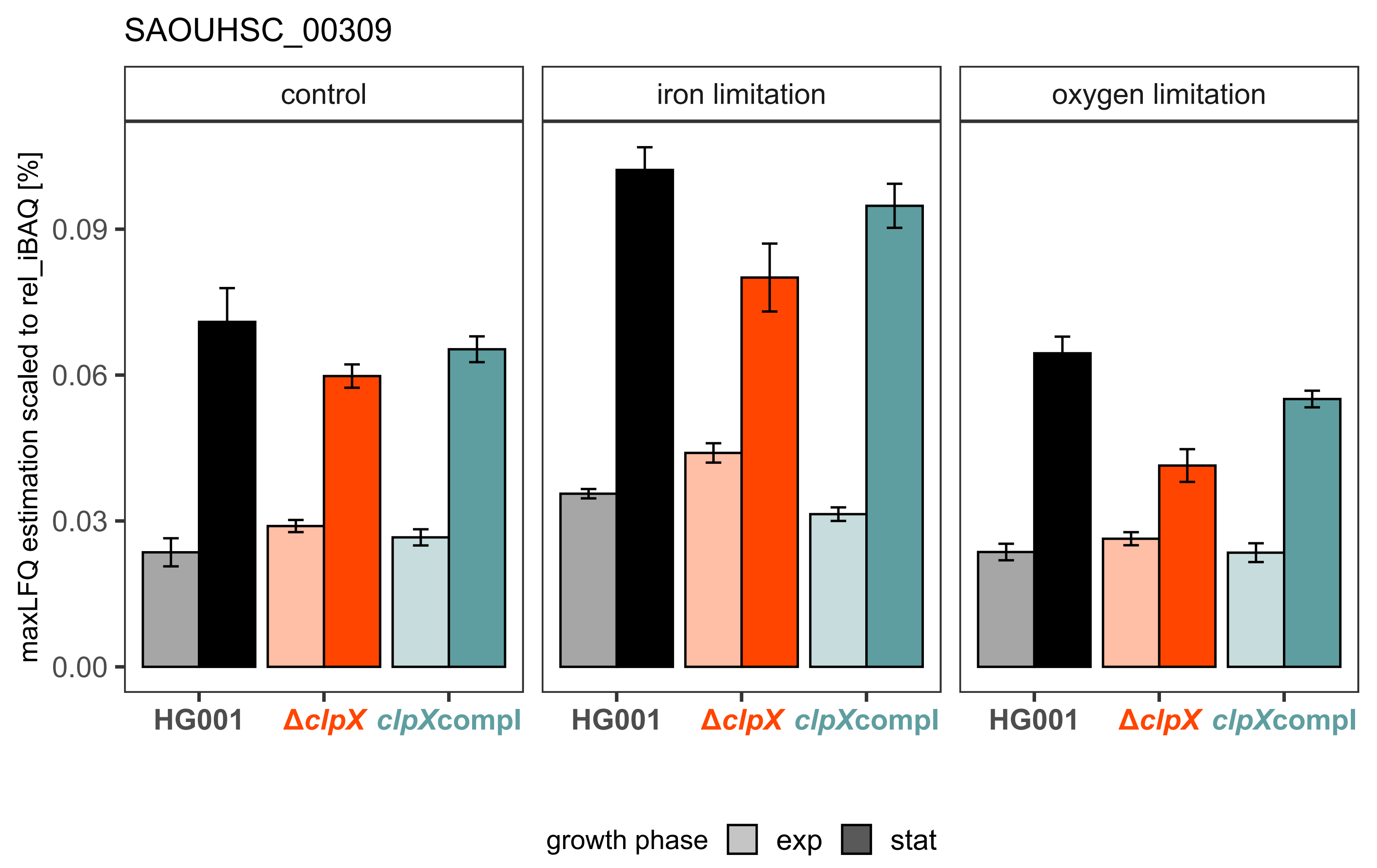
SAOUHSC_00309
control
iron limitation
oxygen limitation
maxLFQ estimation scaled to rel_iBAQ [%]
0.09
0.06
0.03
0.00
HG001
ΔclpX
clpXcompl
growth phase
exp
stat

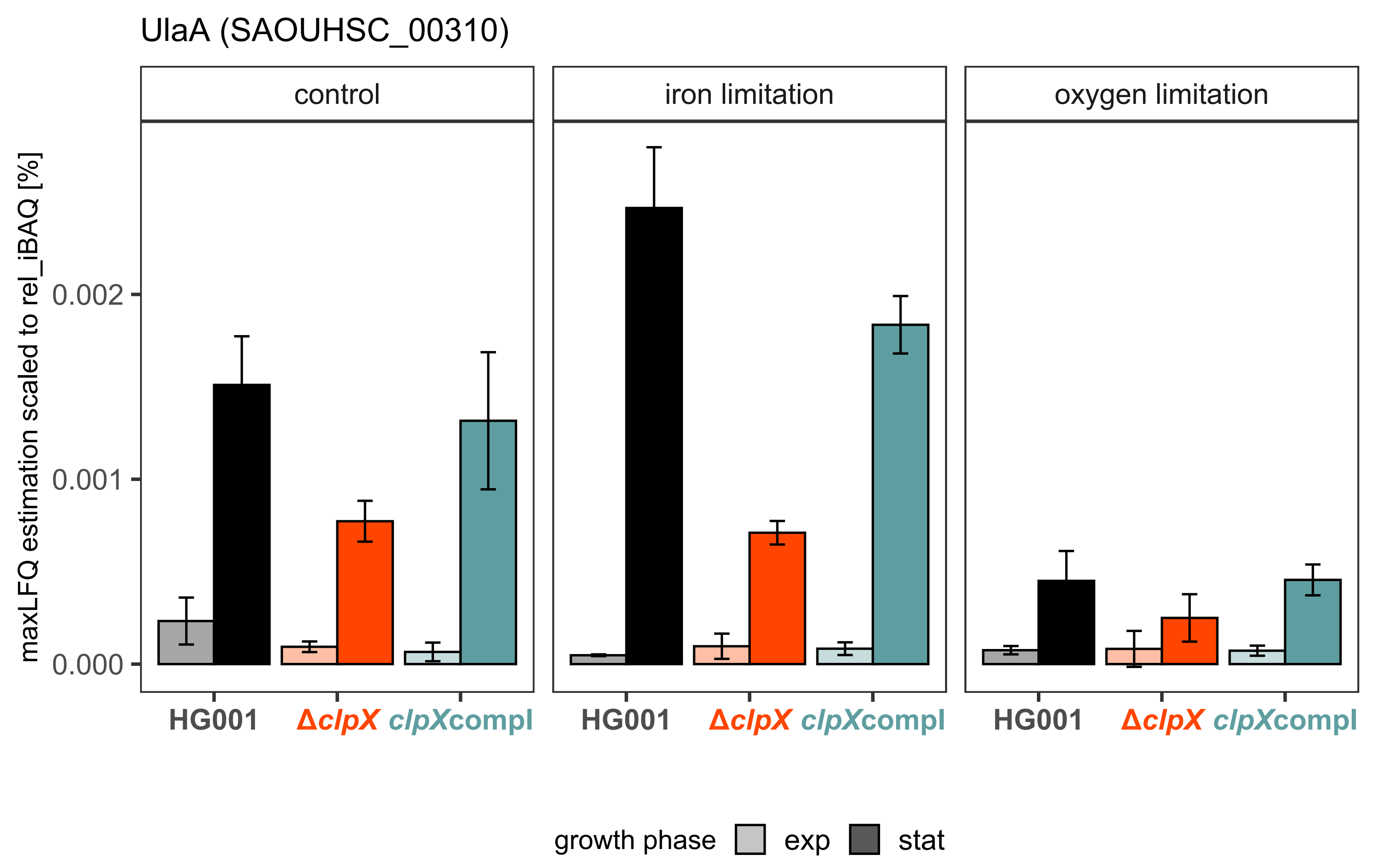
UlaA (SAOUHSC_00310)
control
iron limitation
oxygen limitation
maxLFQ estimation scaled to rel_iBAQ [%]
0.000
0.001
0.002
HG001
ΔclpX
clpXcompl
growth phase
exp
stat

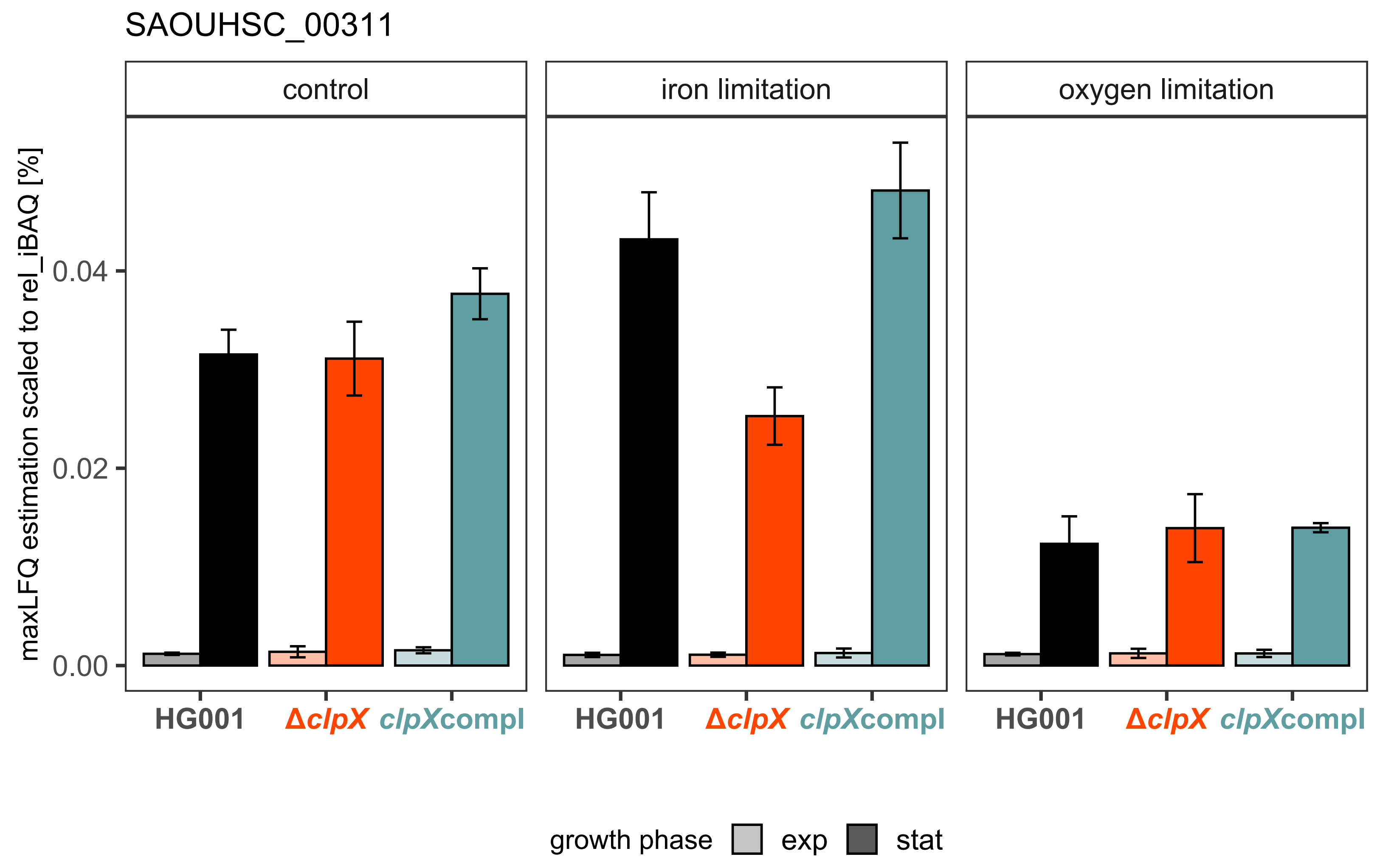

SAOUHSC_00311
control
iron limitation
oxygen limitation
maxLFQ estimation scaled to rel_iBAQ [%]
0.04
0.02
0.00
HG001
ΔclpX
clpXcompl
growth phase
exp
stat

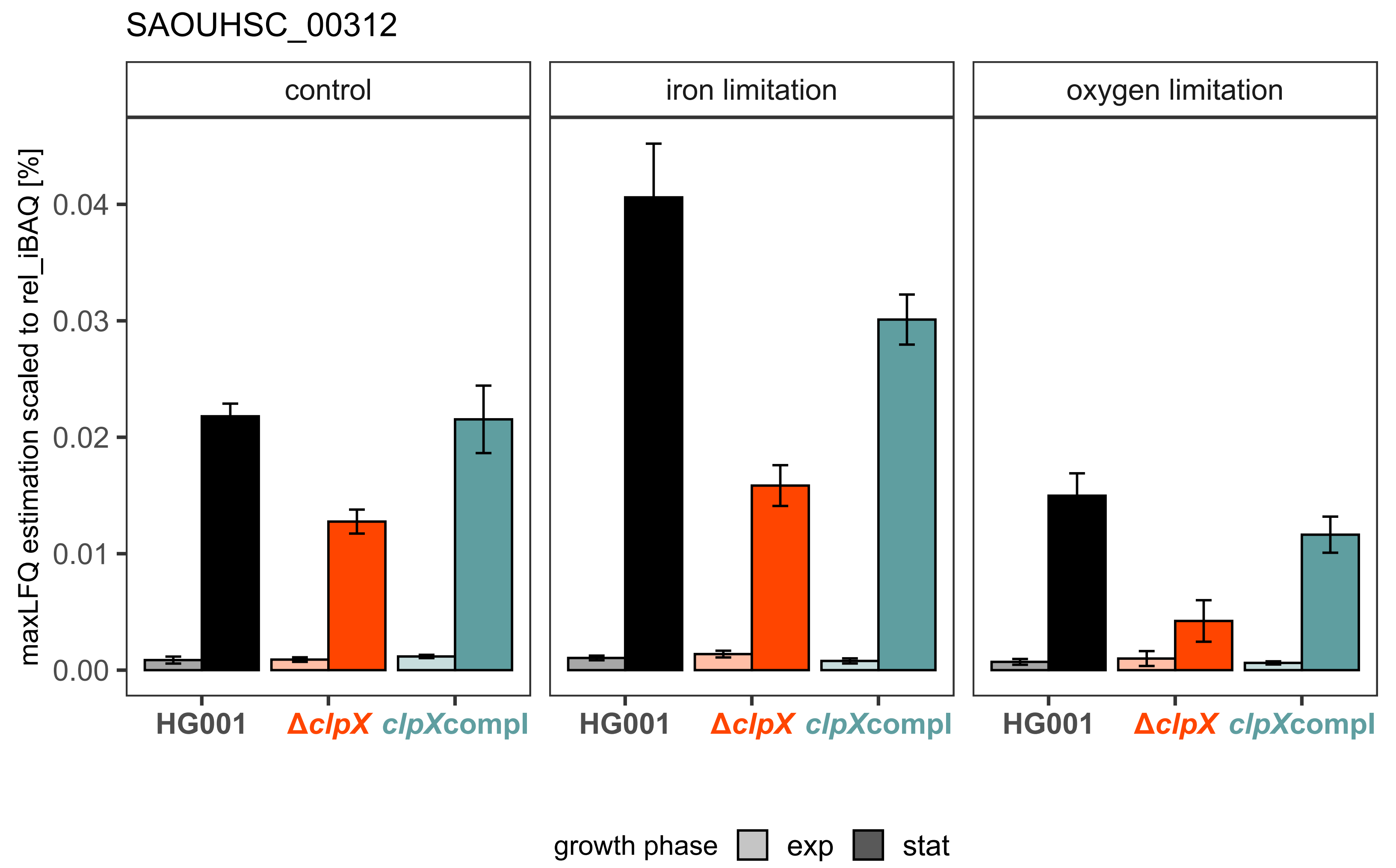

SAOUHSC_00312
control
iron limitation
oxygen limitation
maxLFQ estimation scaled to rel_iBAQ [%]
0.04
0.03
0.02
0.01
0.00
HG001
ΔclpX
clpXcompl
growth phase
exp
stat

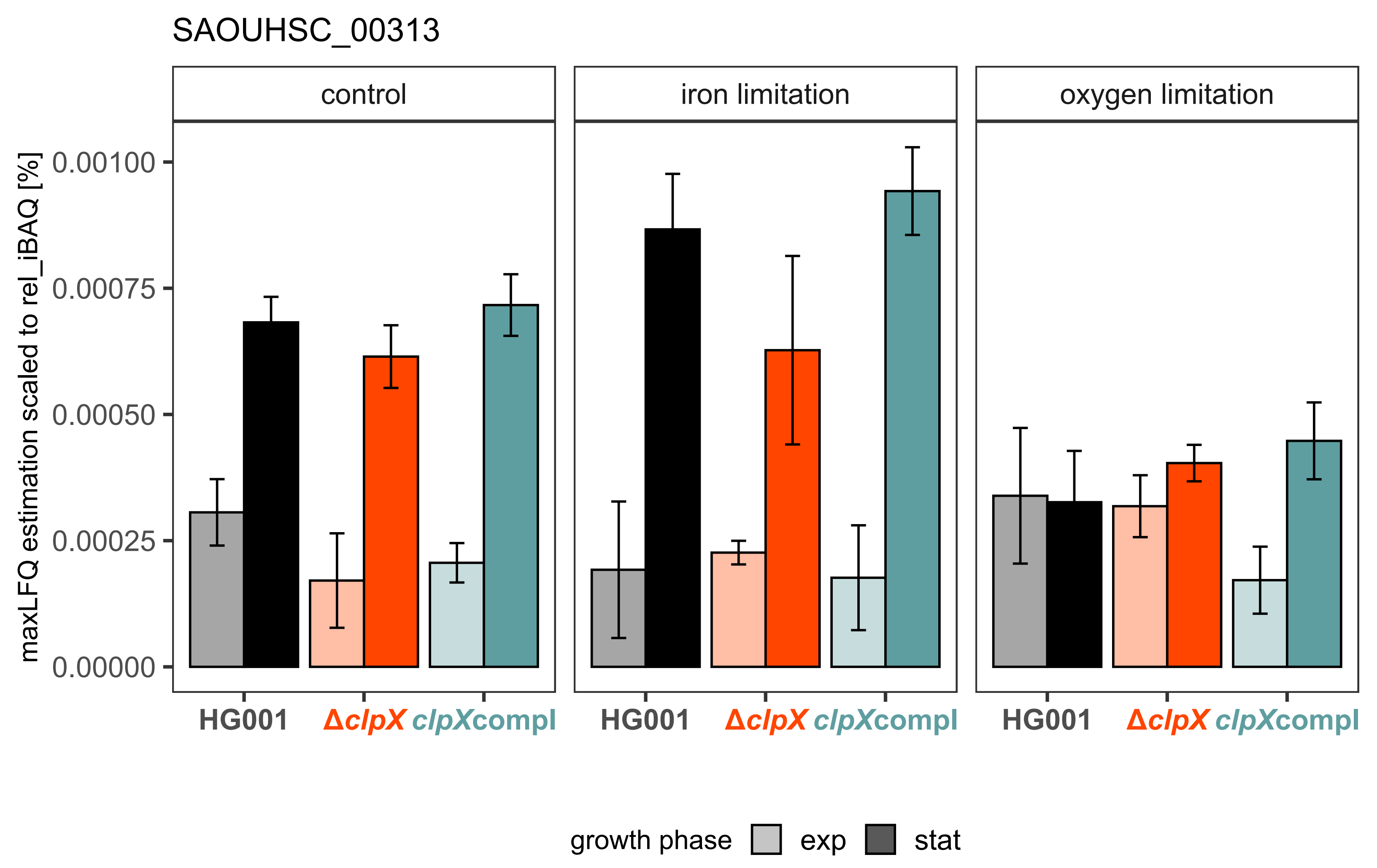

SAOUHSC_00313
control
iron limitation
oxygen limitation
maxLFQ estimation scaled to rel_iBAQ [%]
0.00100
0.00075
0.00050
0.00025
0.00000
HG001
ΔclpX
clpXcompl
growth phase
exp
stat

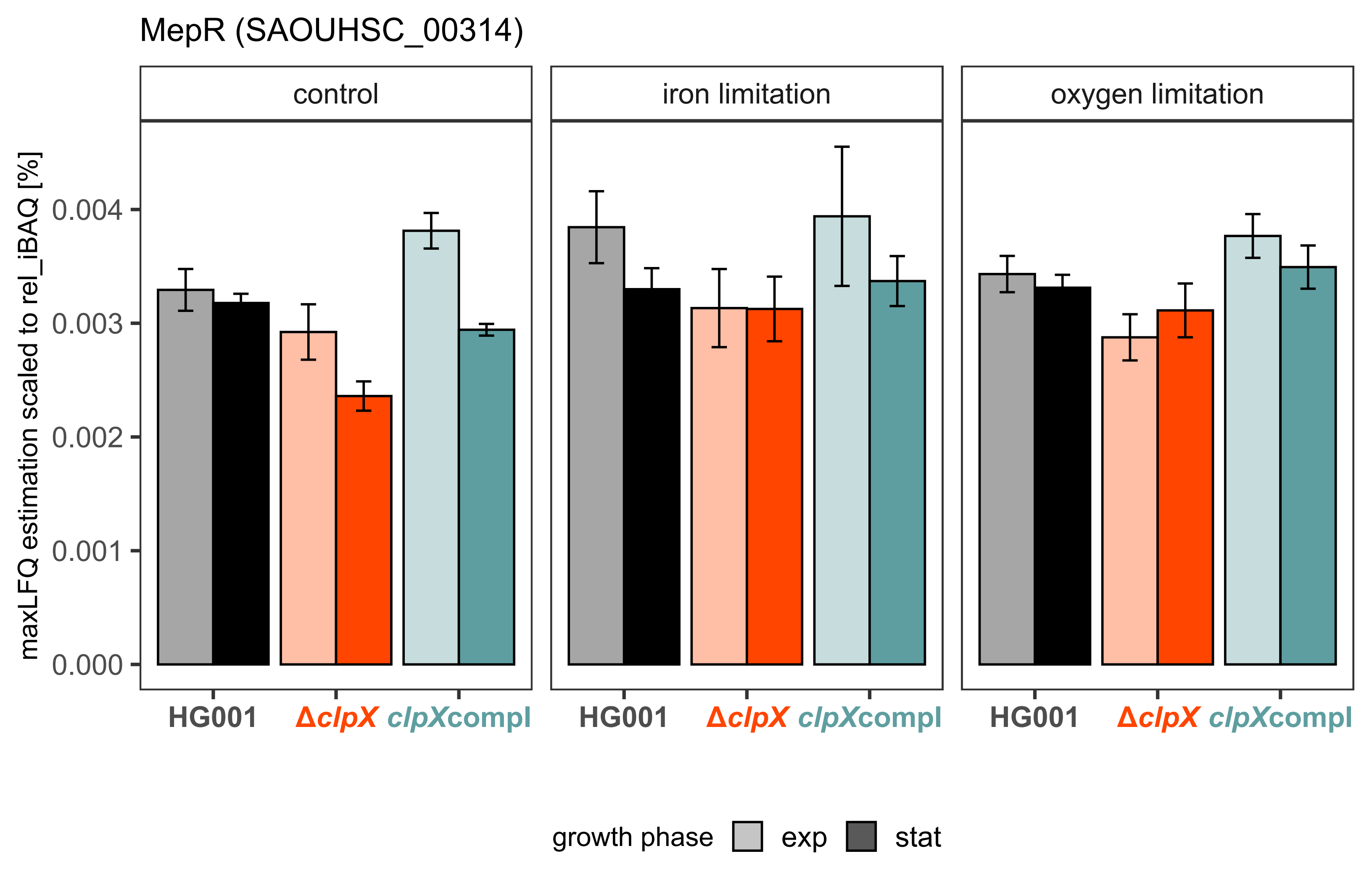

MepR (SAOUHSC_00314)
control
iron limitation
oxygen limitation
maxLFQ estimation scaled to rel_iBAQ [%]
0.004
0.003
0.002
0.001
0.000
HG001
ΔclpX
clpXcompl
growth phase
exp
stat

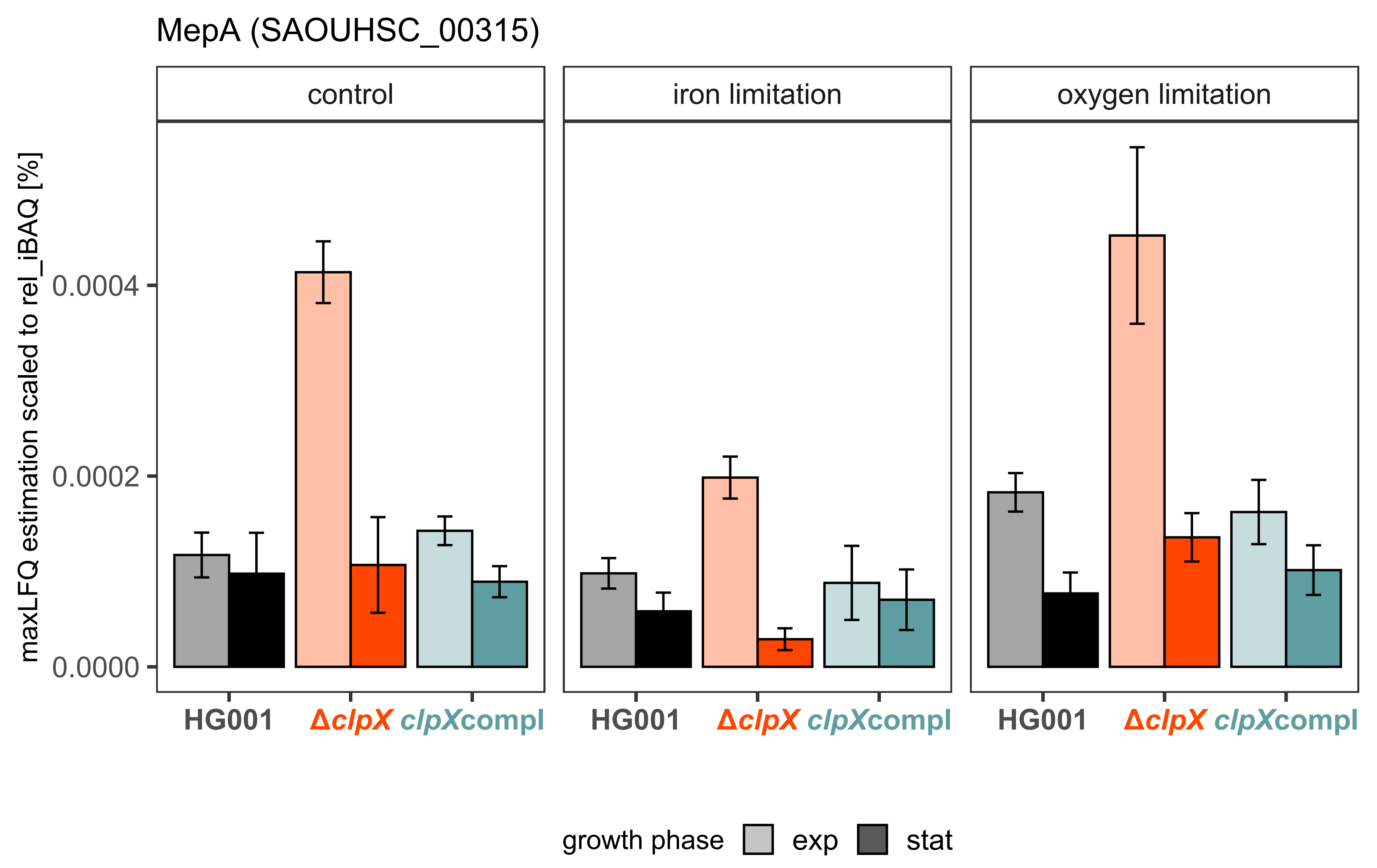

MepA (SAOUHSC_00315)
control
iron limitation
oxygen limitation
maxLFQ estimation scaled to rel_iBAQ [%]
0.0004
0.0002
0.0000
HG001
ΔclpX
clpXcompl
HG001
ΔclpX
clpXcompl
HG001
ΔclpX
clpXcompl
growth phase
exp
stat

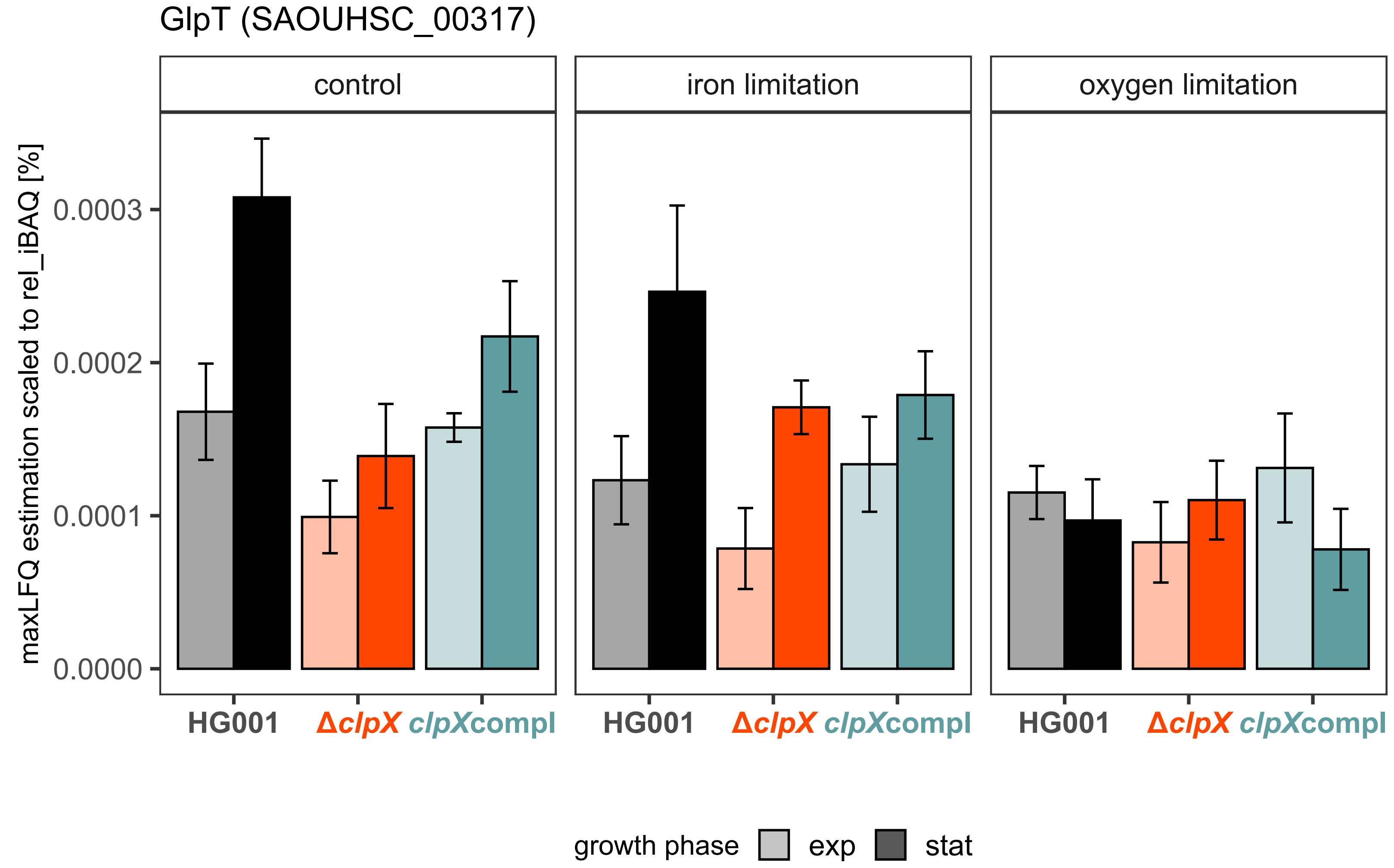
GlpT (SAOUHSC_00317)
control
iron limitation
oxygen limitation
maxLFQ estimation scaled to rel_iBAQ [%]
0.0003
0.0002
0.0001
0.0000
HG001
ΔclpX
clpXcompl
growth phase
exp
stat

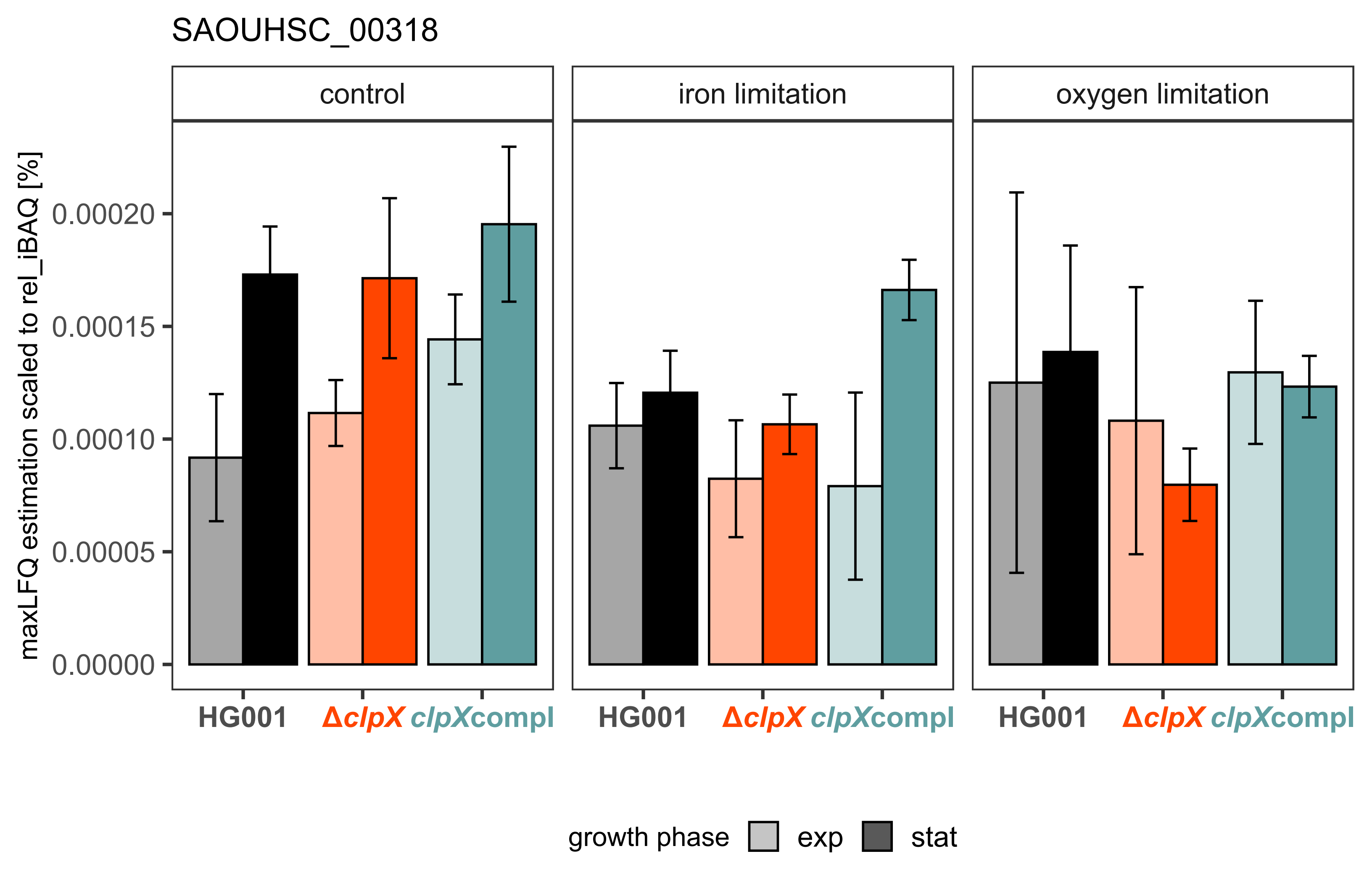

SAOUHSC_00318
control
iron limitation
oxygen limitation
maxLFQ estimation scaled to rel_iBAQ [%]
0.00020
0.00015
0.00010
0.00005
0.00000
HG001
ΔclpX
clpXcompl
growth phase
exp
stat

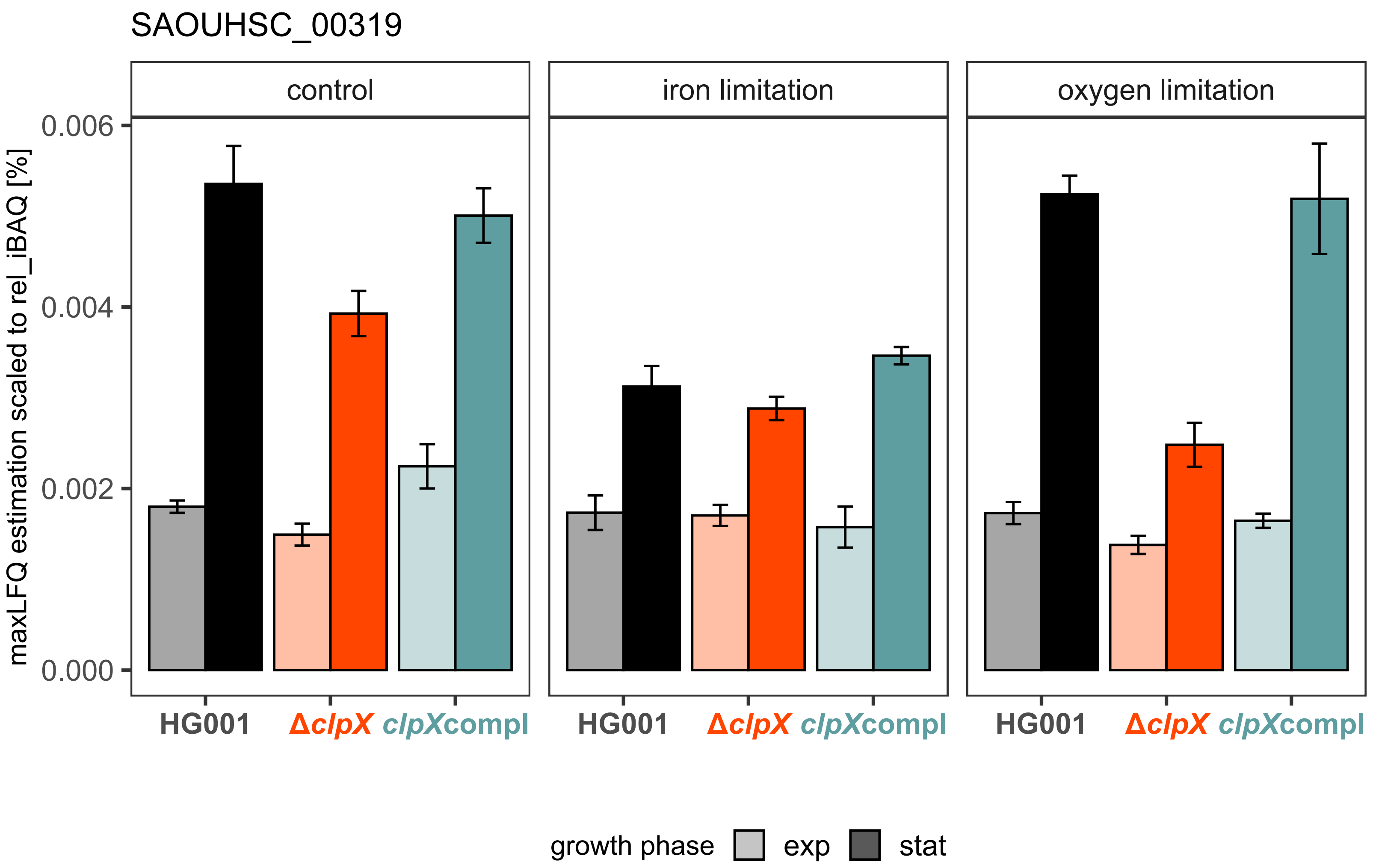
SAOUHSC_00319
control
iron limitation
oxygen limitation
maxLFQ estimation scaled to rel_iBAQ [%]
0.006
0.004
0.002
0.000
HG001
ΔclpX
clpXcompl
growth phase
exp
stat

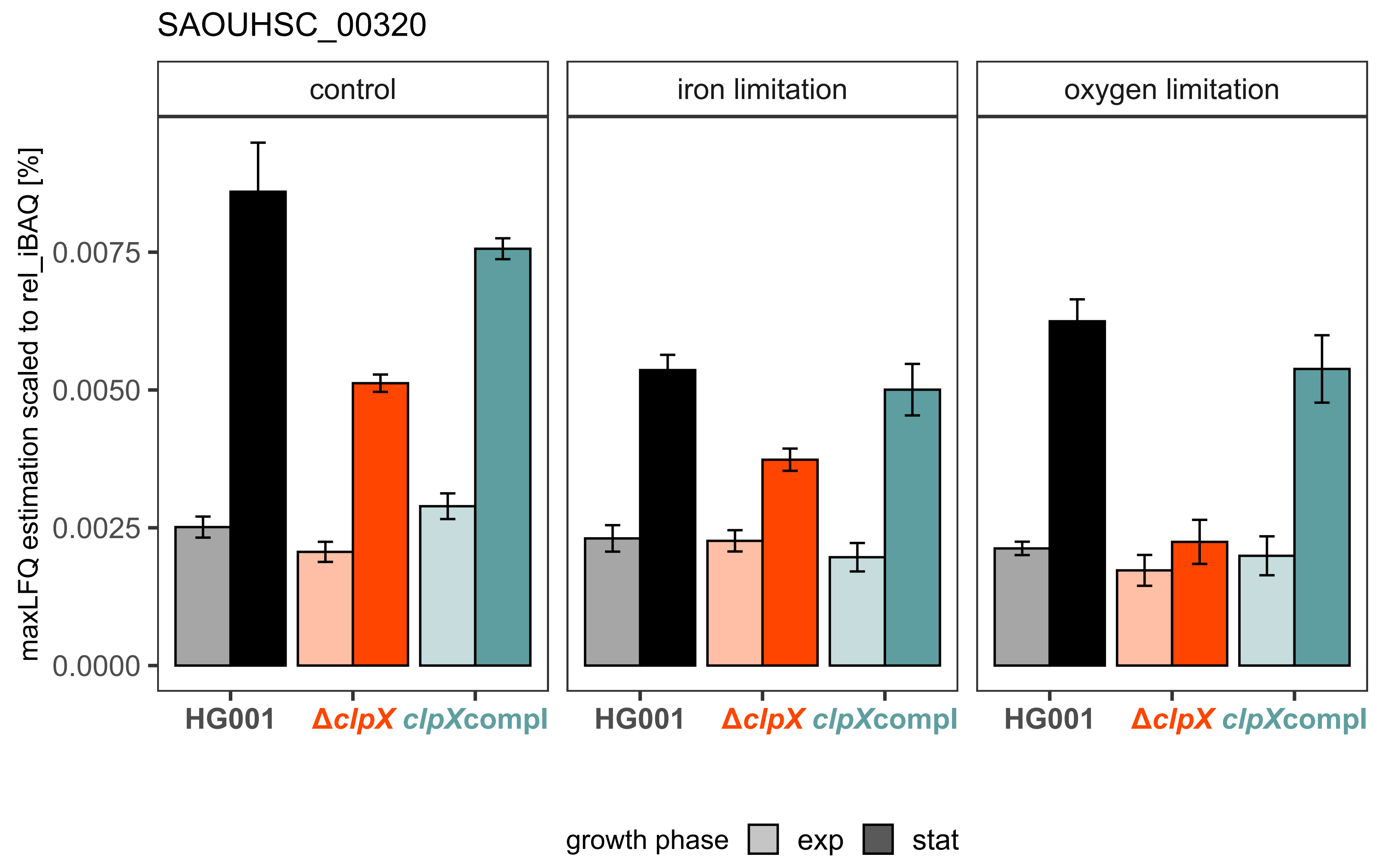

SAOUHSC_00320
control
iron limitation
oxygen limitation
maxLFQ estimation scaled to rel_iBAQ [%]
0.0075
0.0050
0.0025
0.0000
HG001
ΔclpX
clpXcompl
growth phase
exp
stat

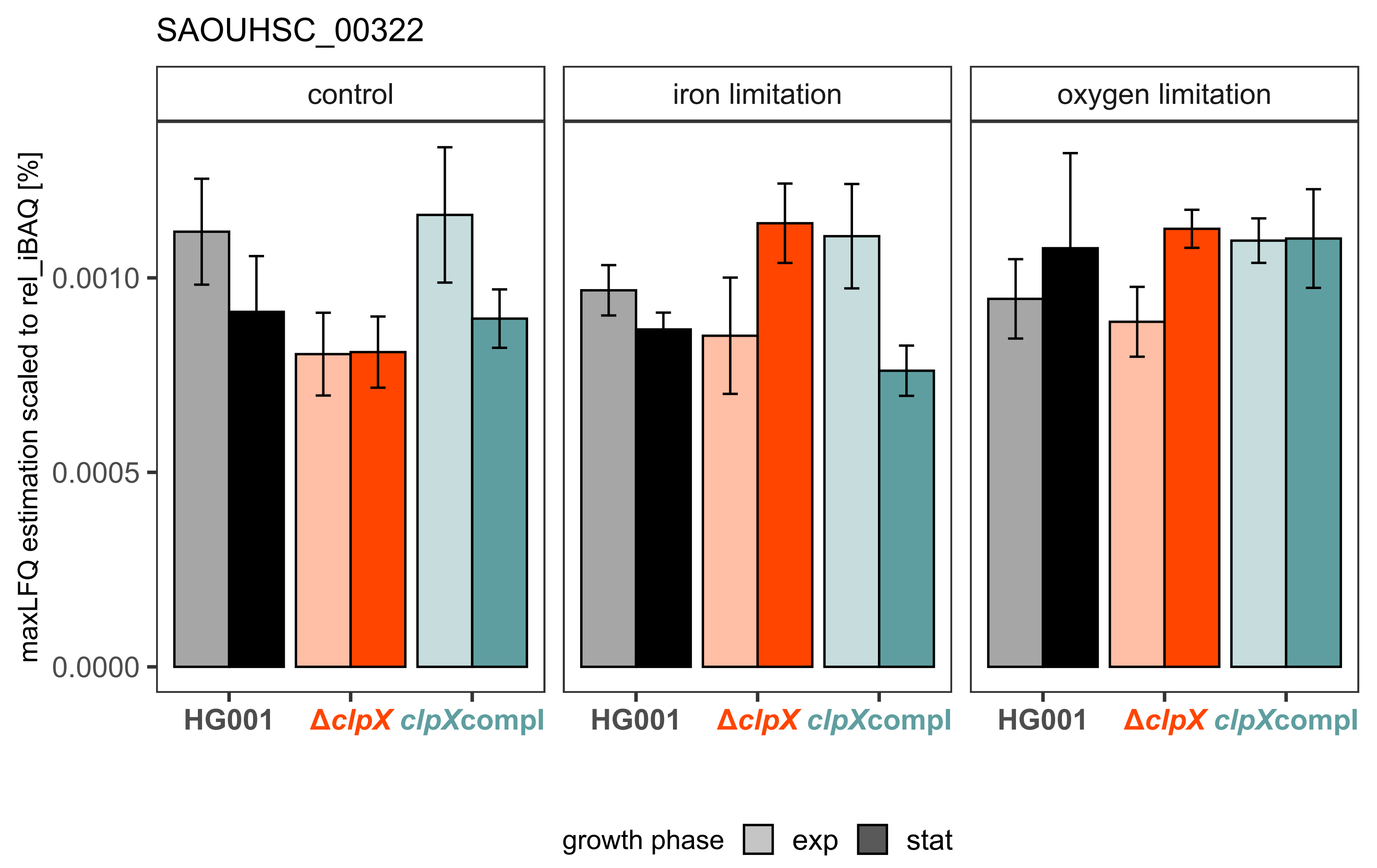

SAOUHSC_00322
control
iron limitation
oxygen limitation
maxLFQ estimation scaled to rel_iBAQ [%]
0.0010
0.0005
0.0000
HG001
ΔclpX
clpXcompl
growth phase
exp
stat

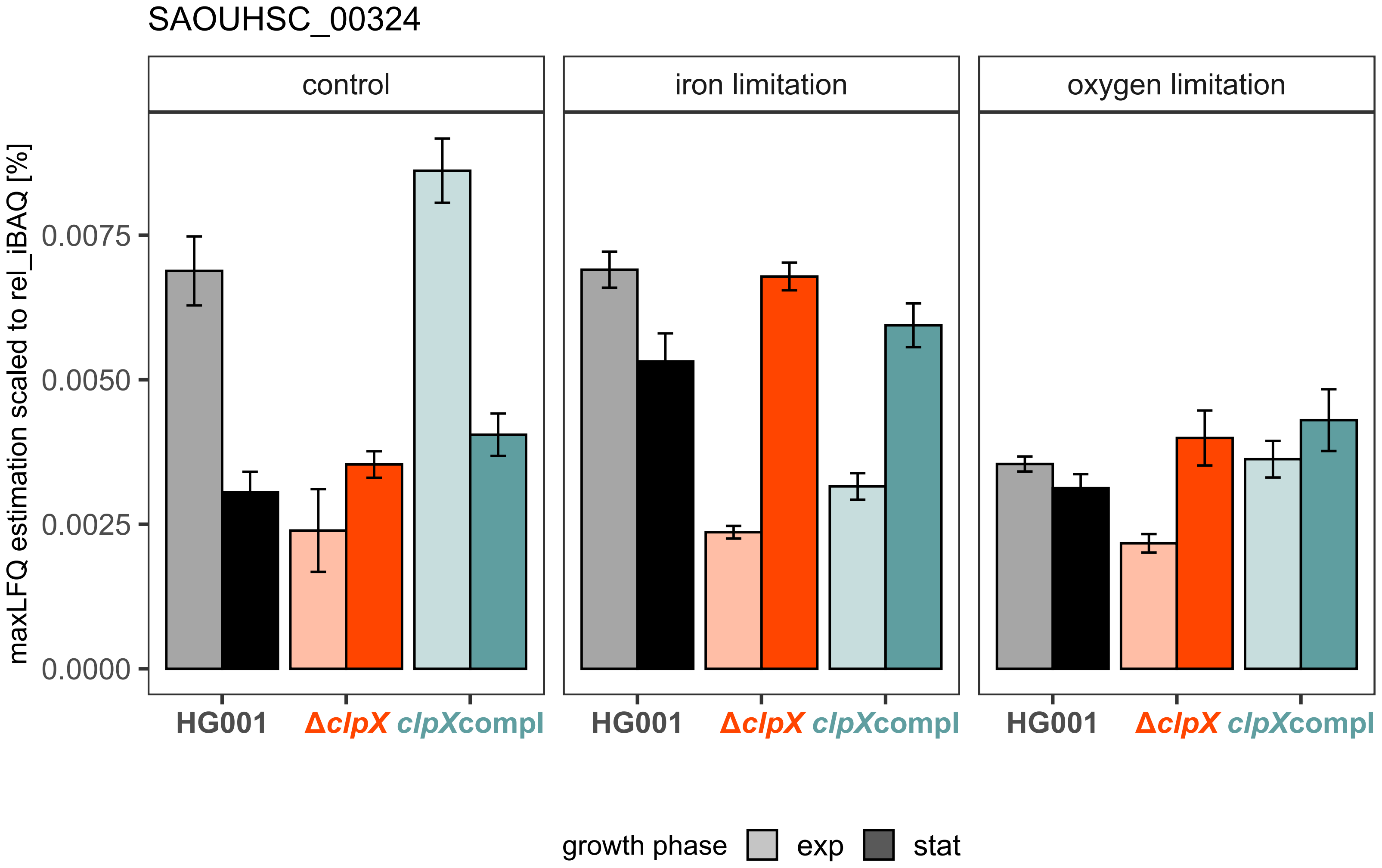
SAOUHSC_00324
control
iron limitation
oxygen limitation
maxLFQ estimation scaled to rel_iBAQ [%]
0.0075
0.0050
0.0025
0.0000
HG001
ΔclpX
clpXcompl
growth phase
exp
stat

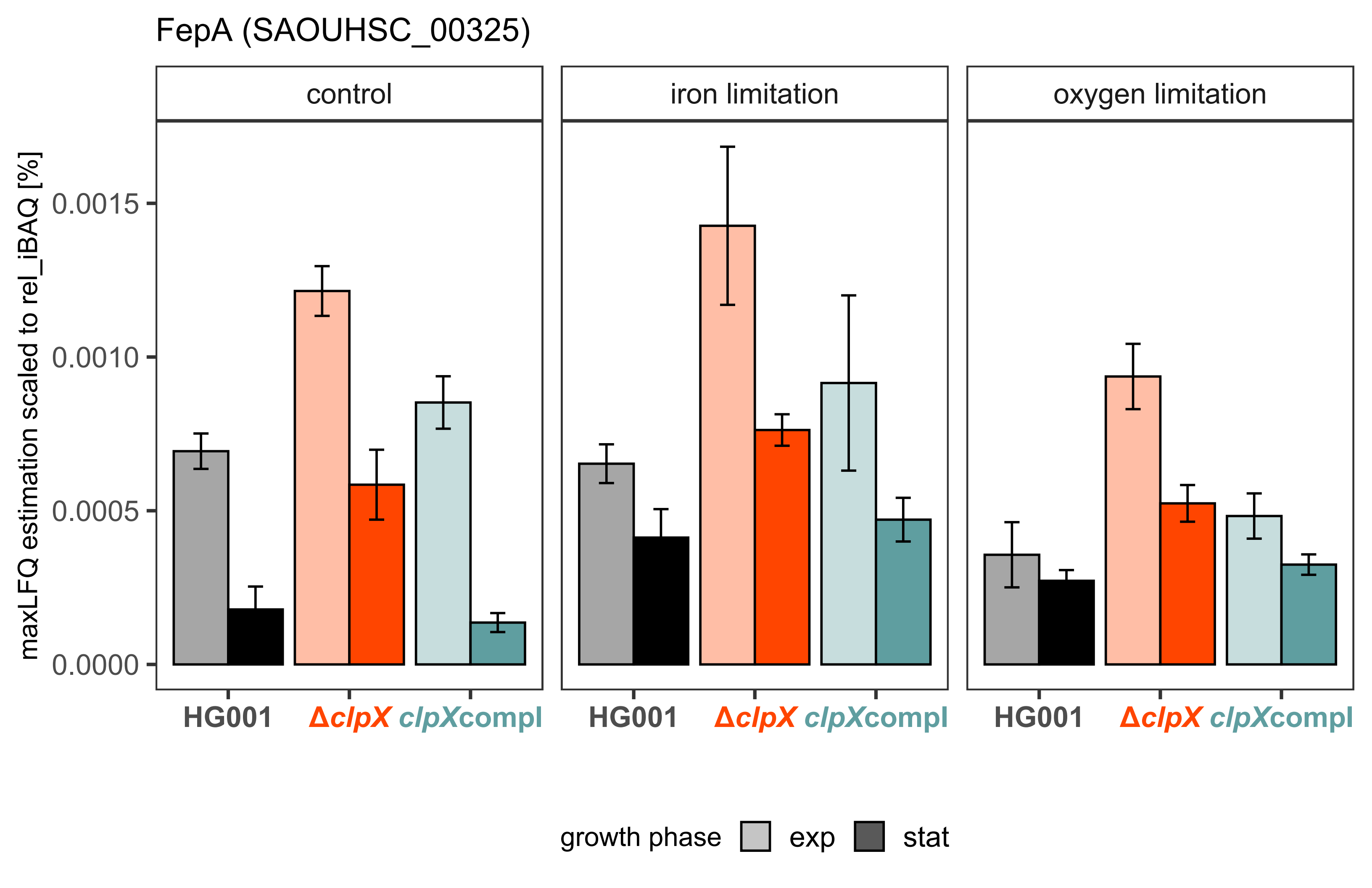

FepA (SAOUHSC_00325)
control
iron limitation
oxygen limitation
maxLFQ estimation scaled to rel_iBAQ [%]
0.0015
0.0010
0.0005
0.0000
HG001
ΔclpX
clpXcompl
growth phase
exp
stat

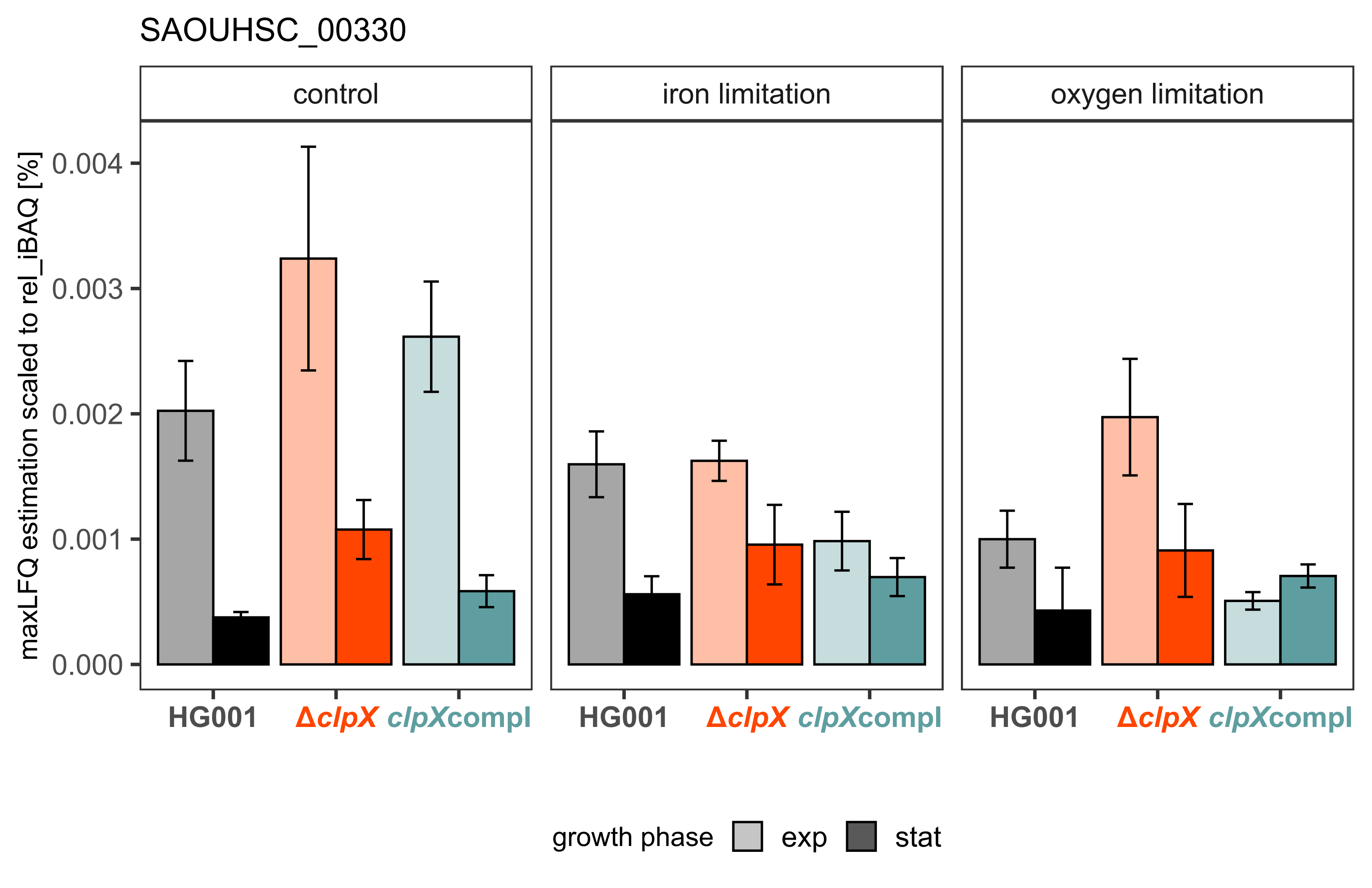

SAOUHSC_00330
control
iron limitation
oxygen limitation
maxLFQ estimation scaled to rel_iBAQ [%]
0.004
0.003
0.002
0.001
0.000
HG001
ΔclpX
clpXcompl
growth phase
exp
stat

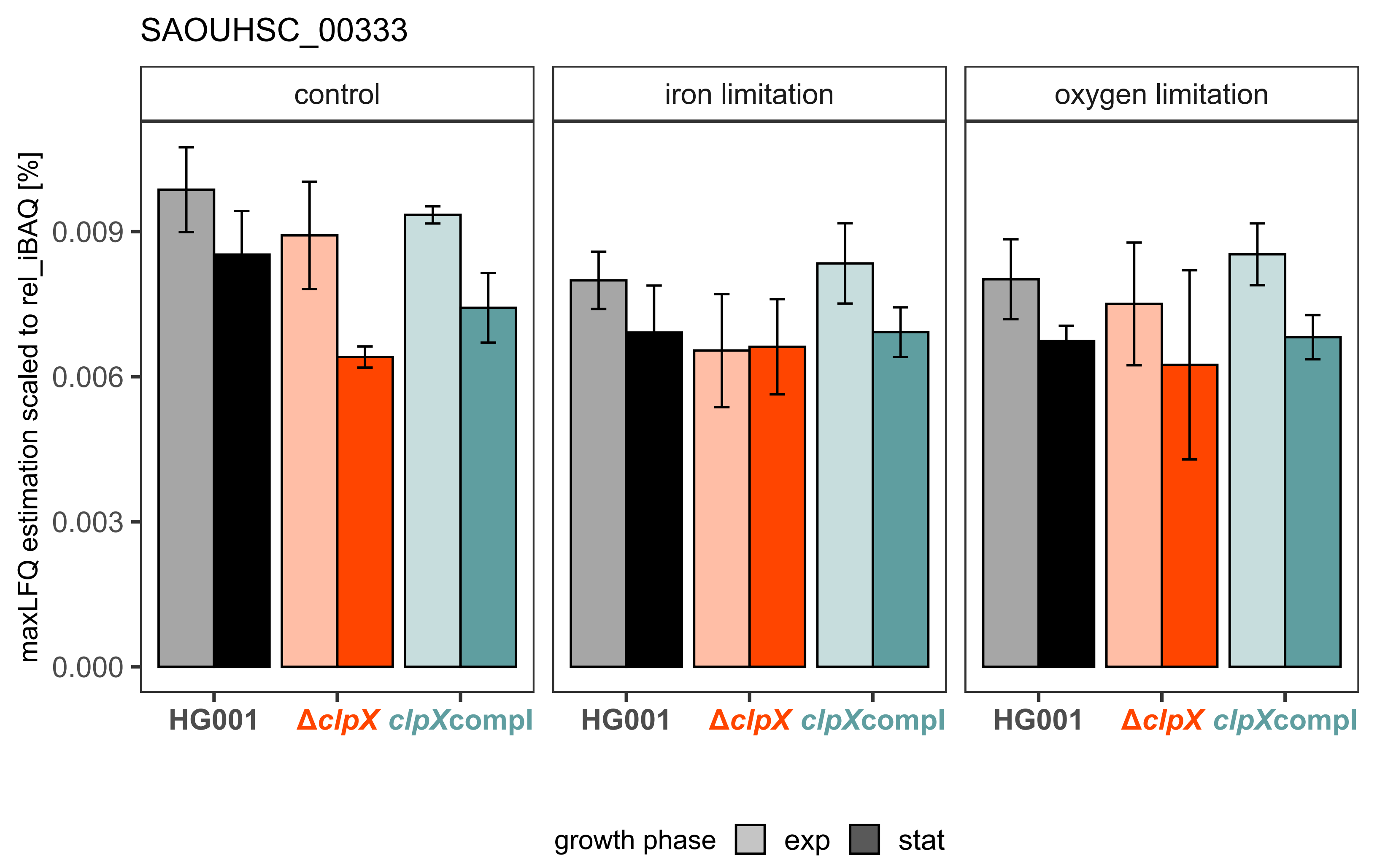

SAOUHSC_00333
control
iron limitation
oxygen limitation
maxLFQ estimation scaled to rel_iBAQ [%]
0.009
0.006
0.003
0.000
HG001
ΔclpX
clpXcompl
growth phase
exp
stat

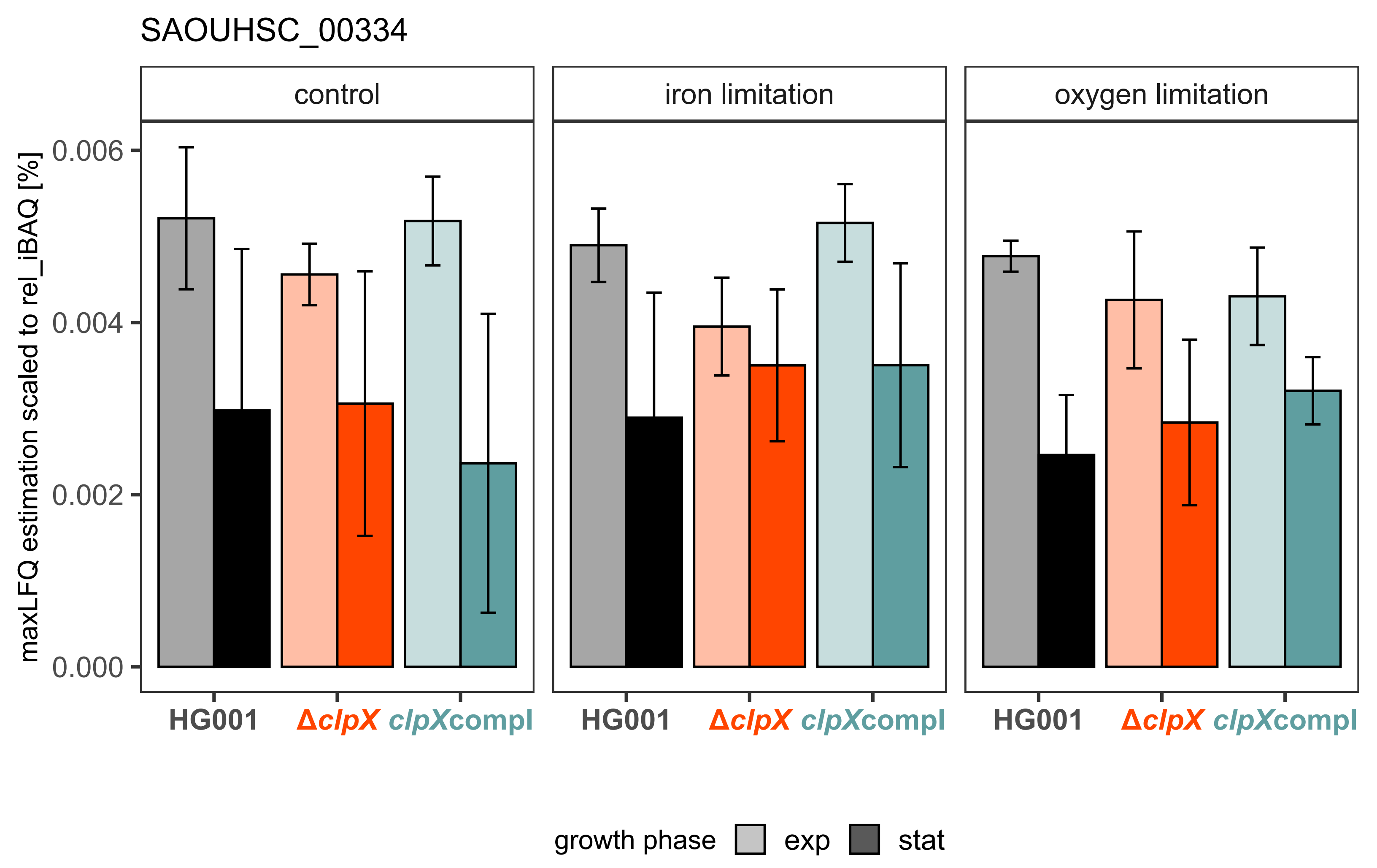
SAOUHSC_00334
control
iron limitation
oxygen limitation
maxLFQ estimation scaled to rel_iBAQ [%]
0.006
0.004
0.002
0.000
HG001
ΔclpX
clpXcompl
growth phase
exp
stat

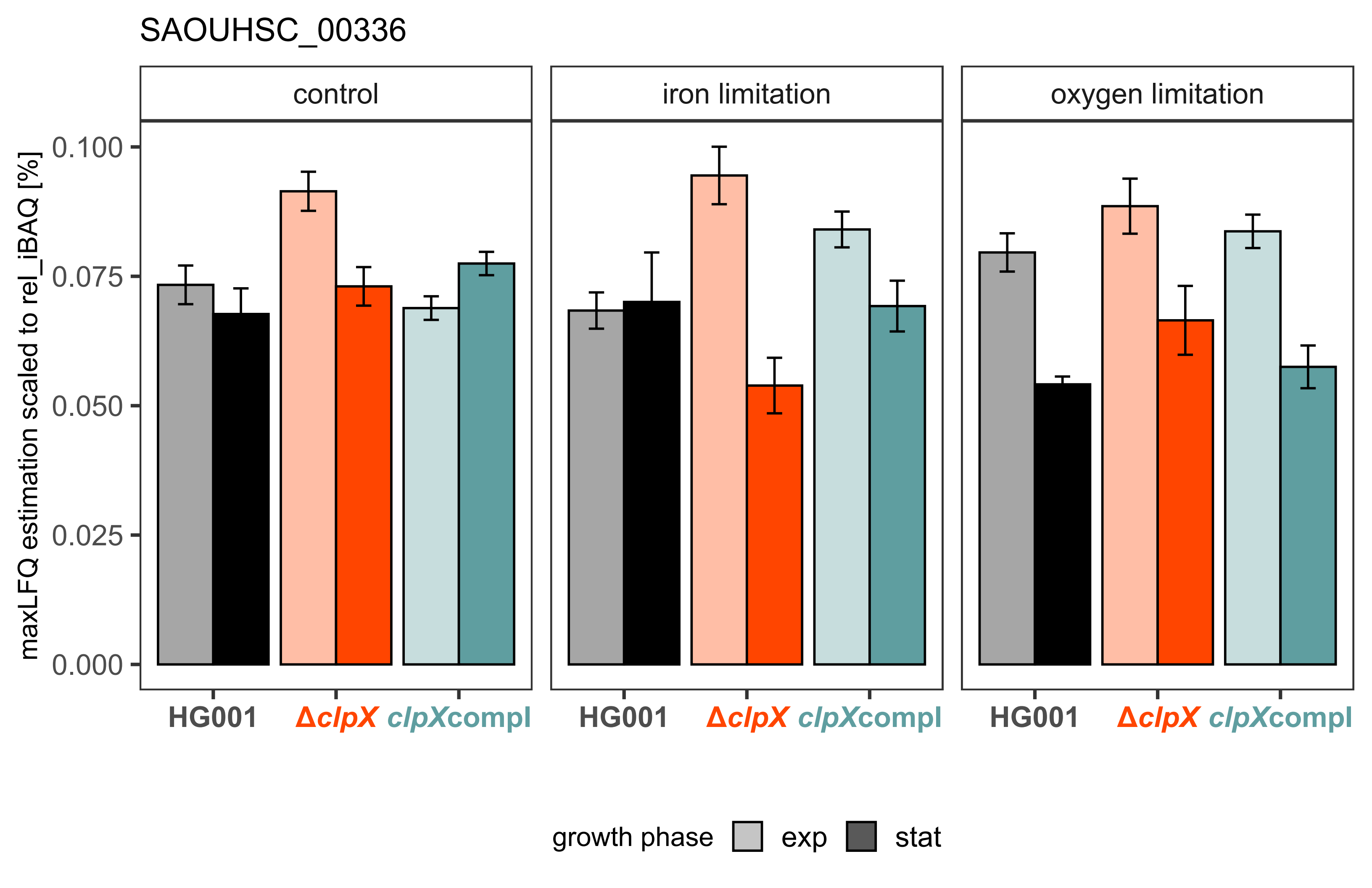
SAOUHSC_00336
control
iron limitation
oxygen limitation
maxLFQ estimation scaled to rel_iBAQ [%]
0.100
0.075
0.050
0.025
0.000
HG001
ΔclpX
clpXcompl
growth phase
exp
stat

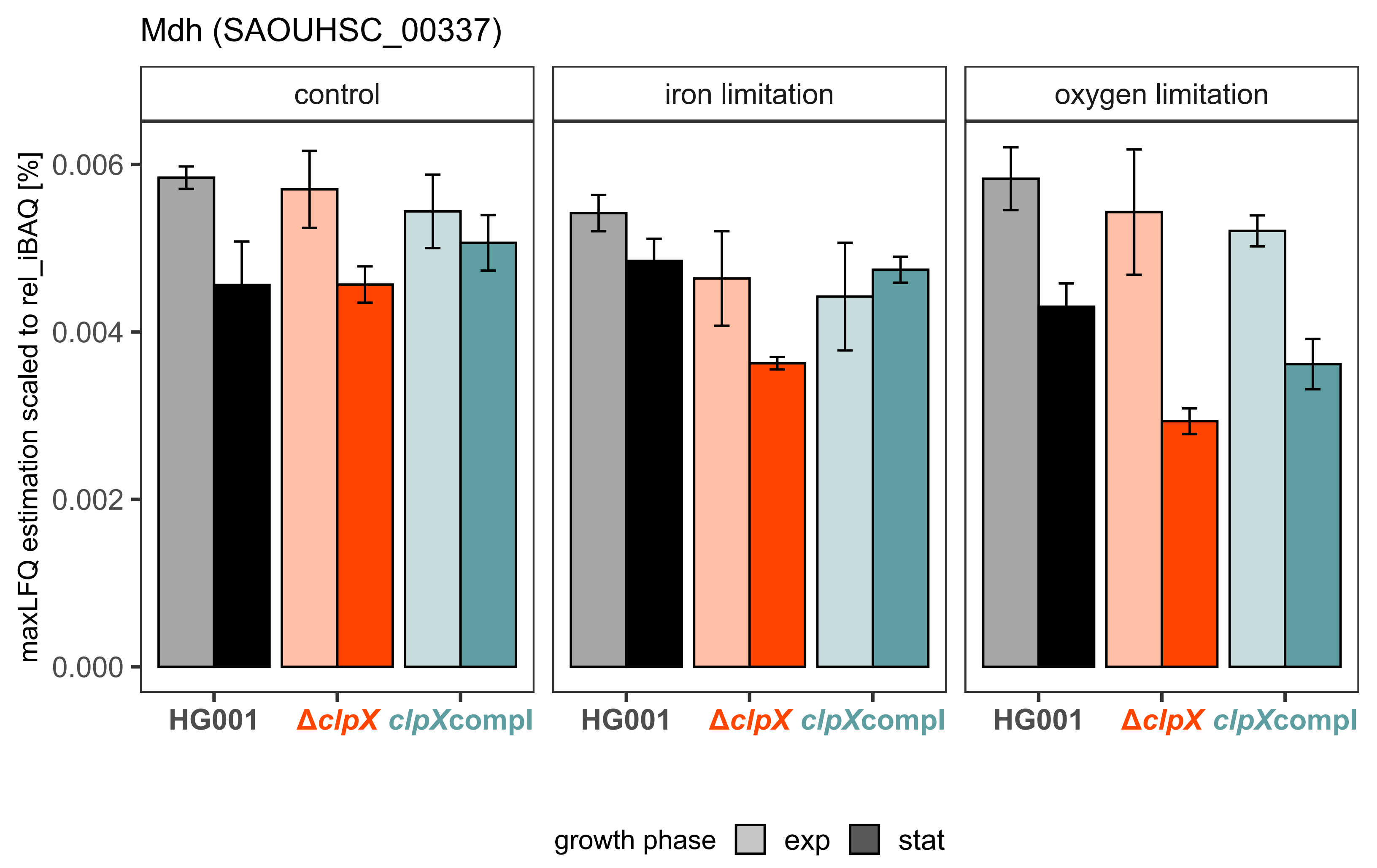
Mdh (SAOUHSC_00337)
control
iron limitation
oxygen limitation
maxLFQ estimation scaled to rel_iBAQ [%]
0.006
0.004
0.002
0.000
HG001
ΔclpX
clpXcompl
growth phase
exp
stat

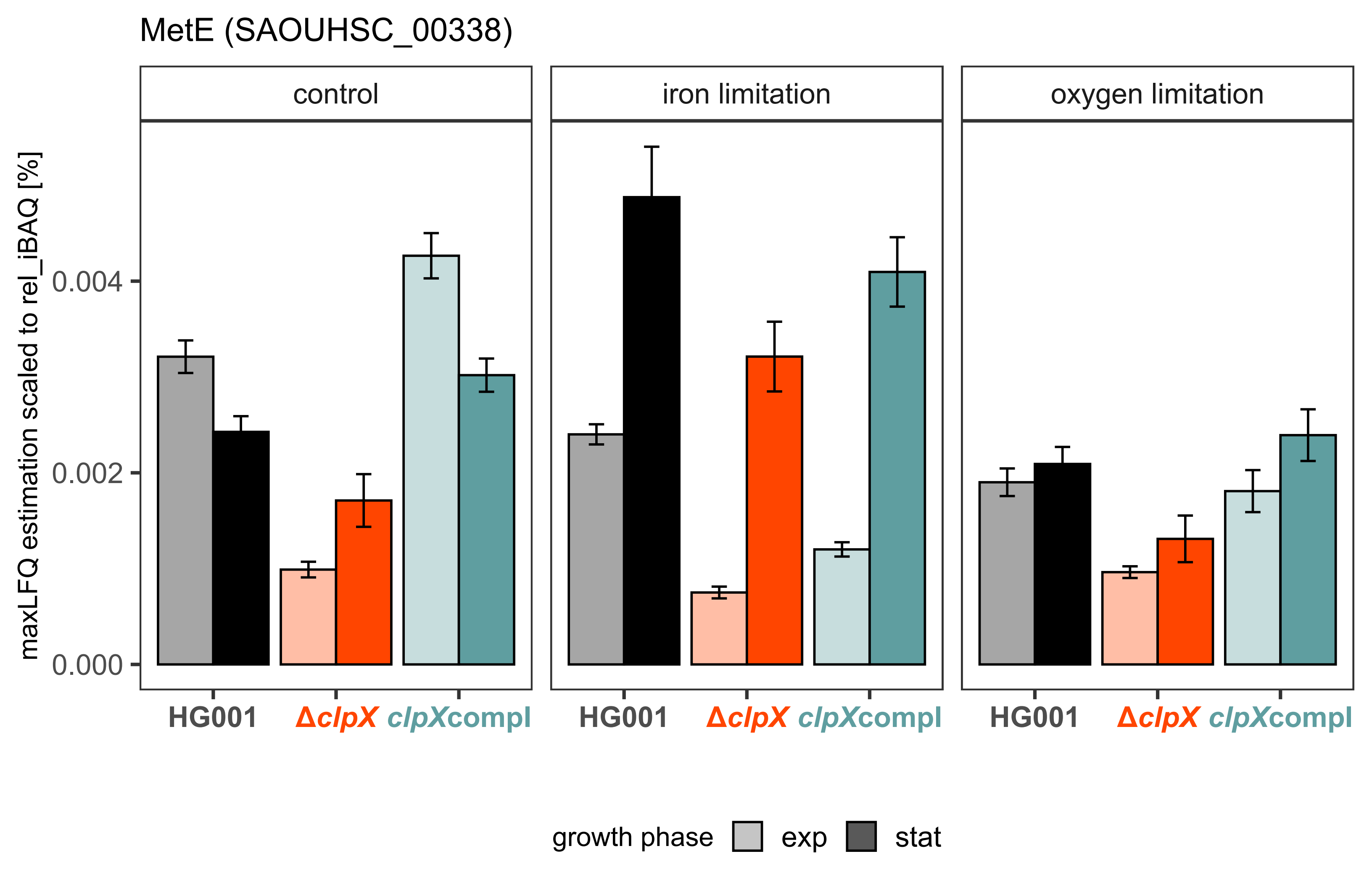
MetE (SAOUHSC_00338)
control
iron limitation
oxygen limitation
maxLFQ estimation scaled to rel_iBAQ [%]
0.004
0.002
0.000
HG001
ΔclpX
clpXcompl
growth phase
exp
stat

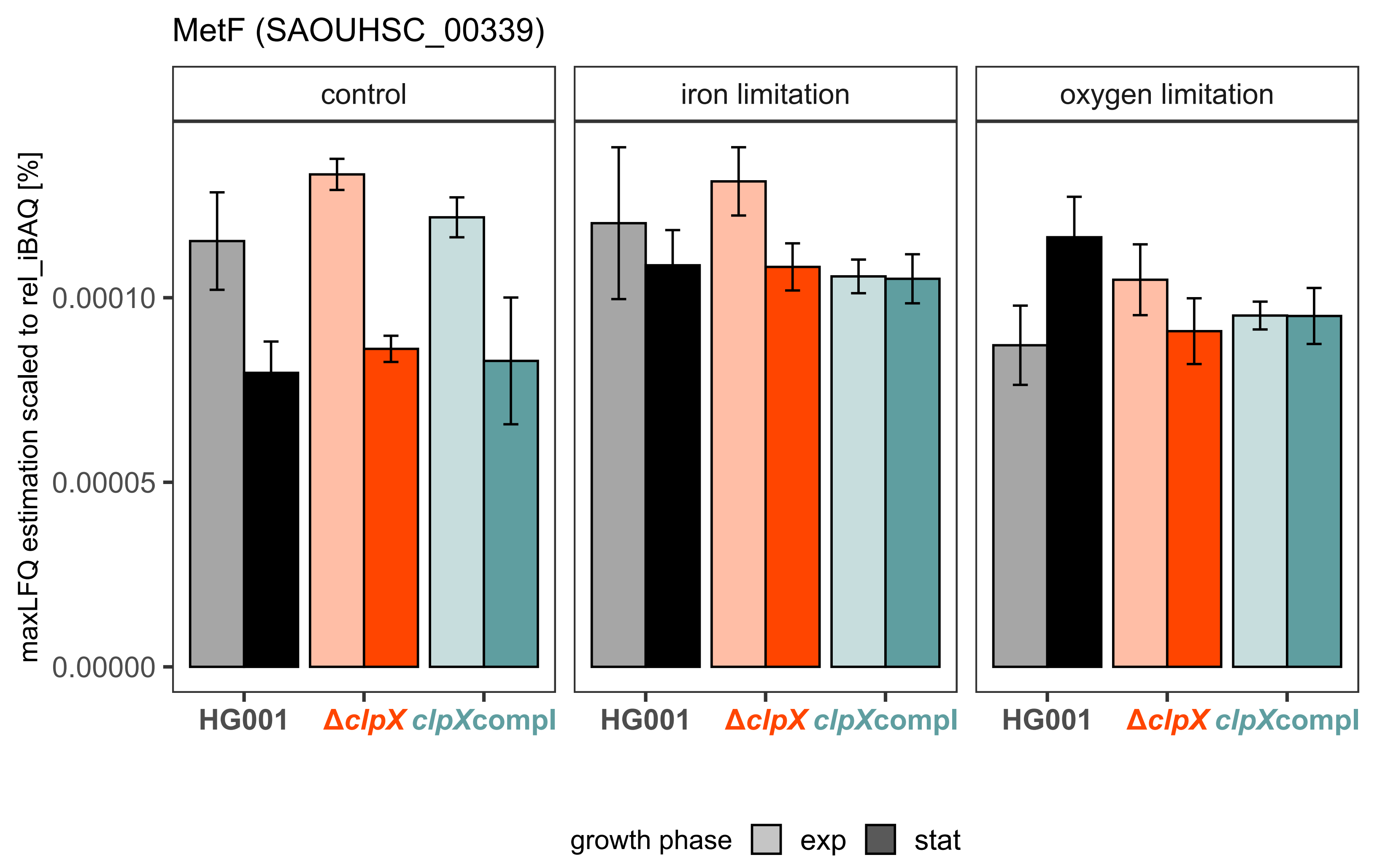
MetF (SAOUHSC_00339)
control
iron limitation
oxygen limitation
maxLFQ estimation scaled to rel_iBAQ [%]
0.00010
0.00005
0.00000
HG001
ΔclpX
clpXcompl
growth phase
exp
stat

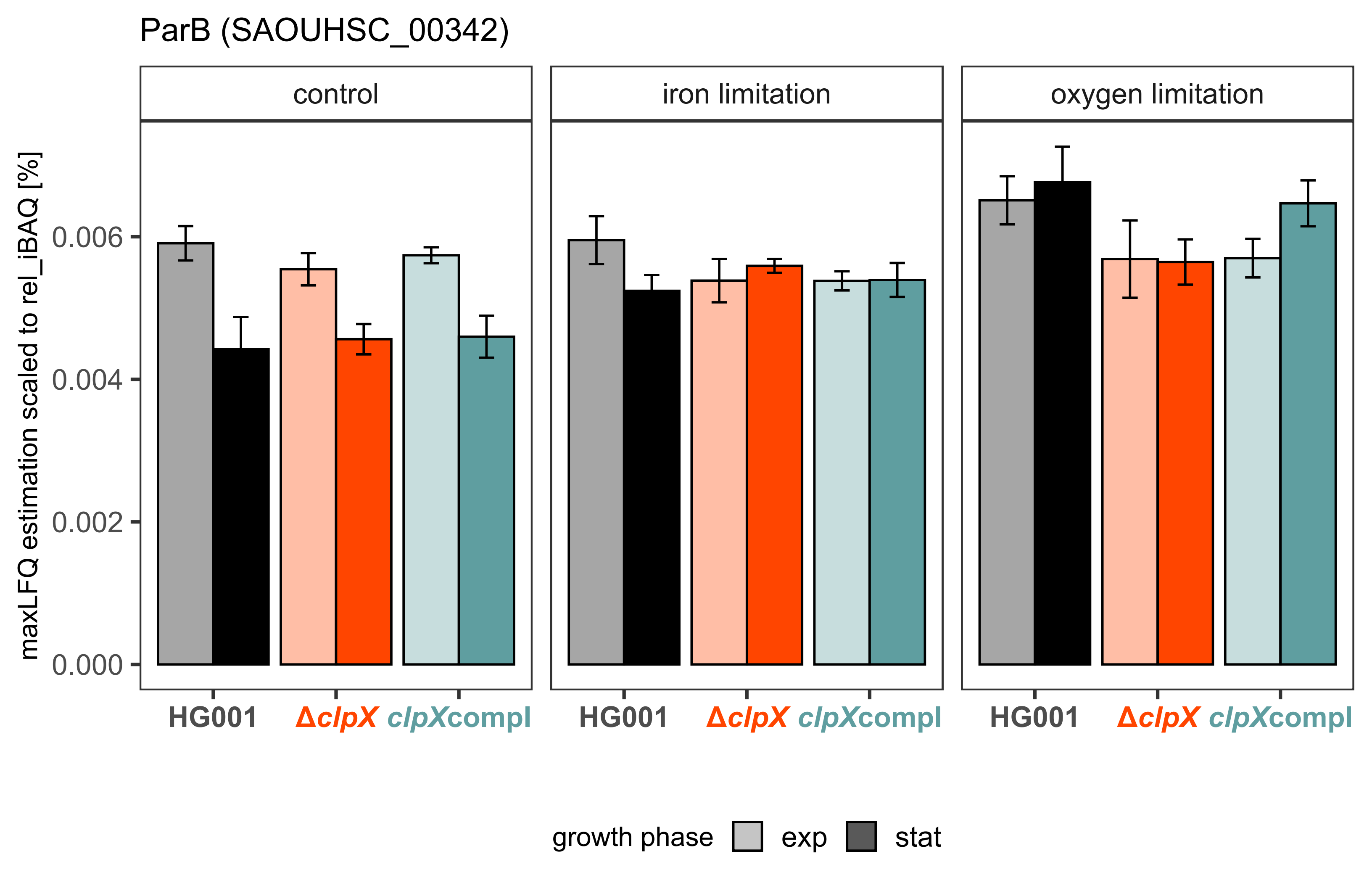
ParB (SAOUHSC_00342)
control
iron limitation
oxygen limitation
maxLFQ estimation scaled to rel_iBAQ [%]
0.000
0.002
0.004
0.006
HG001
ΔclpX
clpXcompl
growth phase
exp
stat

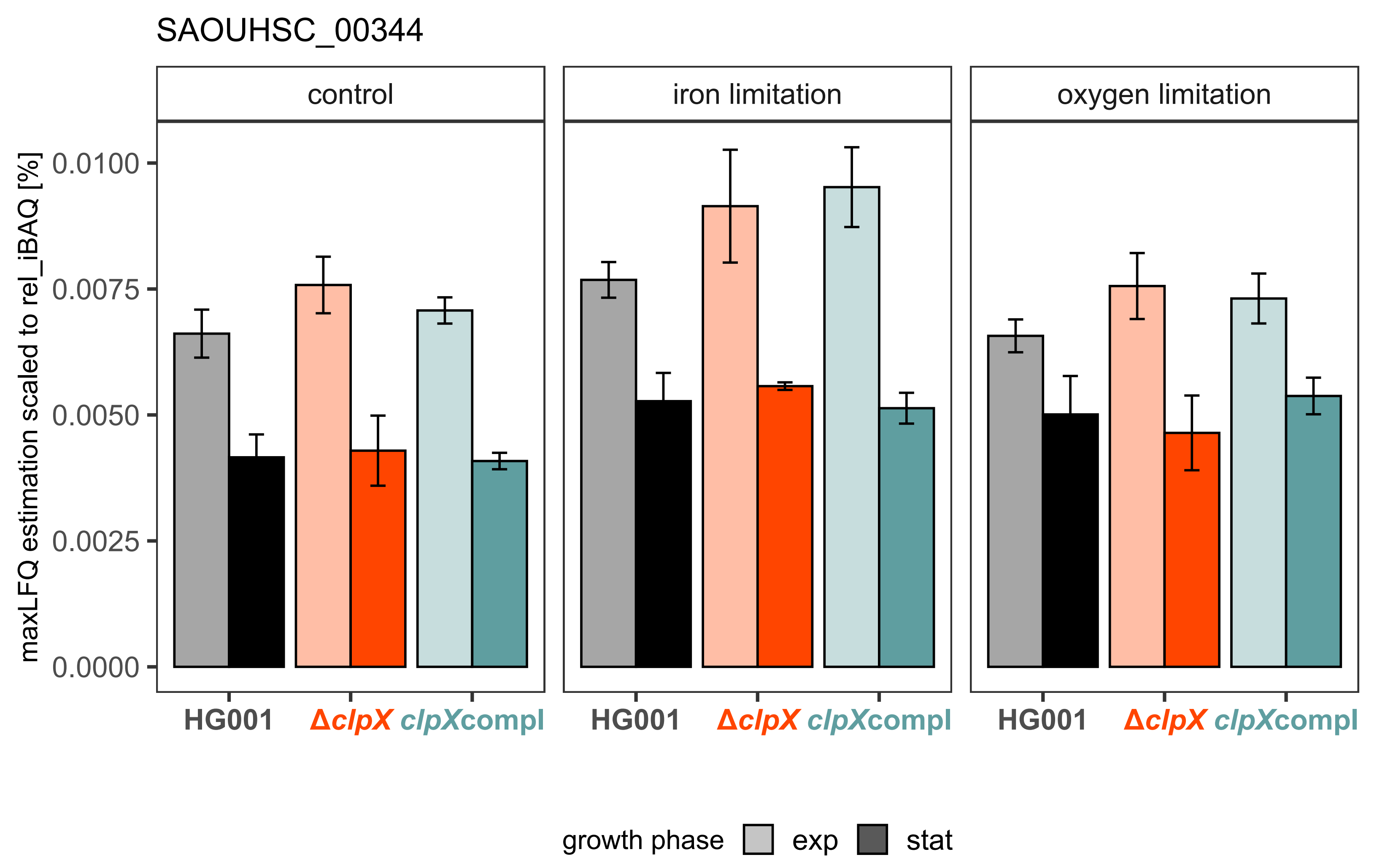
SAOUHSC_00344
control
iron limitation
oxygen limitation
maxLFQ estimation scaled to rel_iBAQ [%]
0.0100
0.0075
0.0050
0.0025
0.0000
HG001
ΔclpX
clpXcompl
growth phase
exp
stat

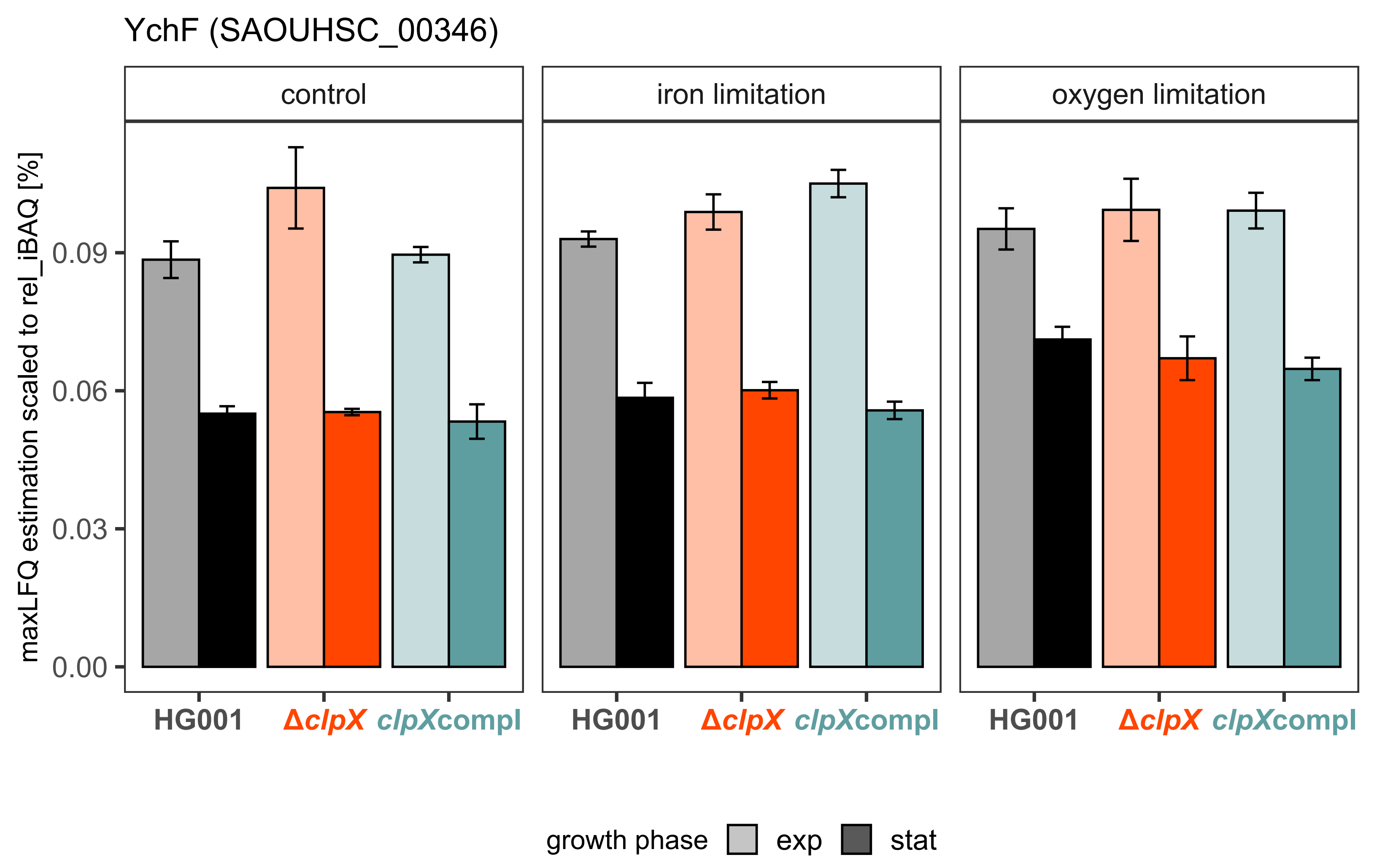

YchF (SAOUHSC_00346)
control
iron limitation
oxygen limitation
maxLFQ estimation scaled to rel_iBAQ [%]
0.09
0.06
0.03
0.00
HG001
ΔclpX
clpXcompl
growth phase
exp
stat

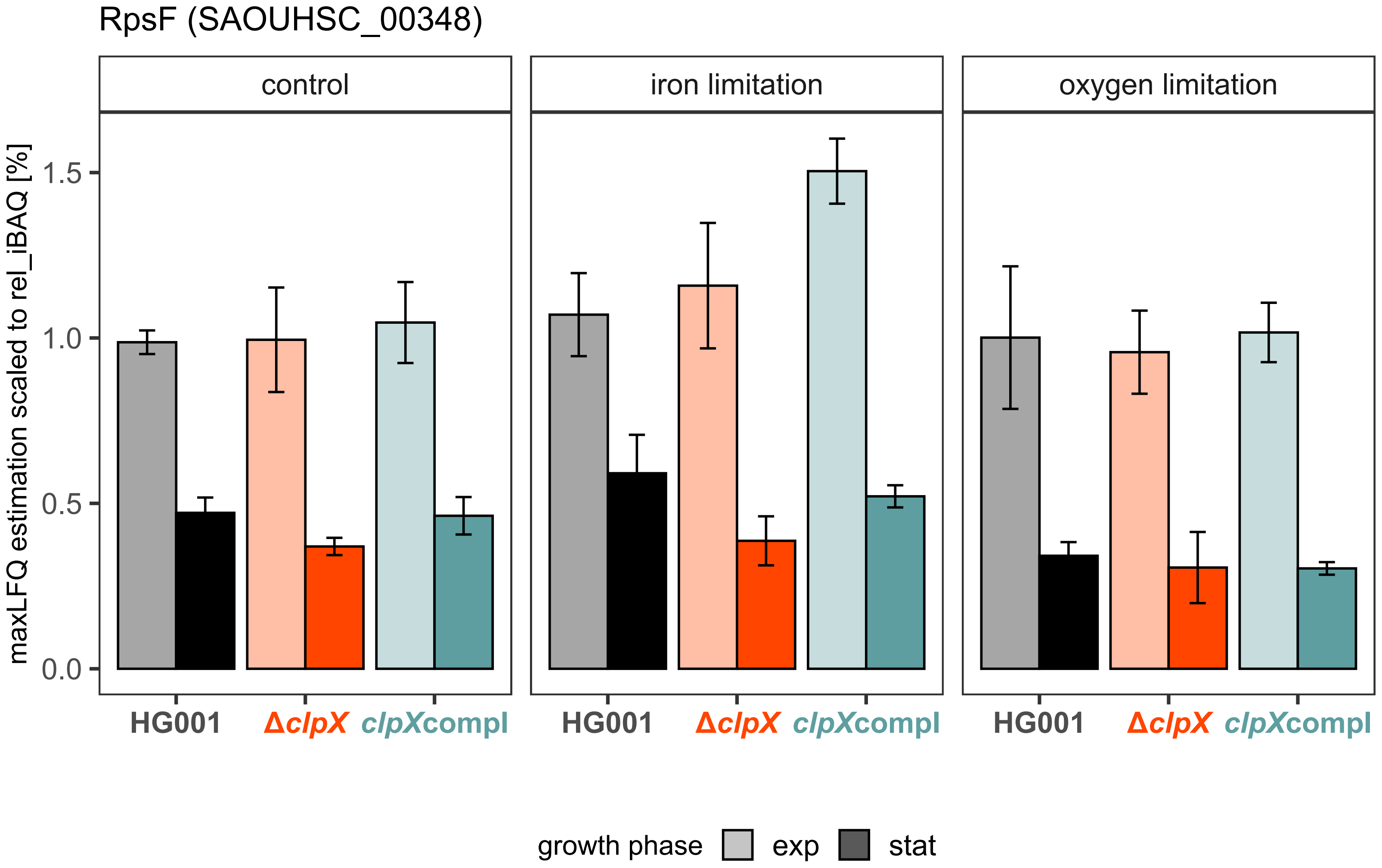
RpsF (SAOUHSC_00348)
control
iron limitation
oxygen limitation
maxLFQ estimation scaled to rel_iBAQ [%]
0.0
0.5
1.0
1.5
HG001
ΔclpX
clpXcompl
growth phase
exp
stat

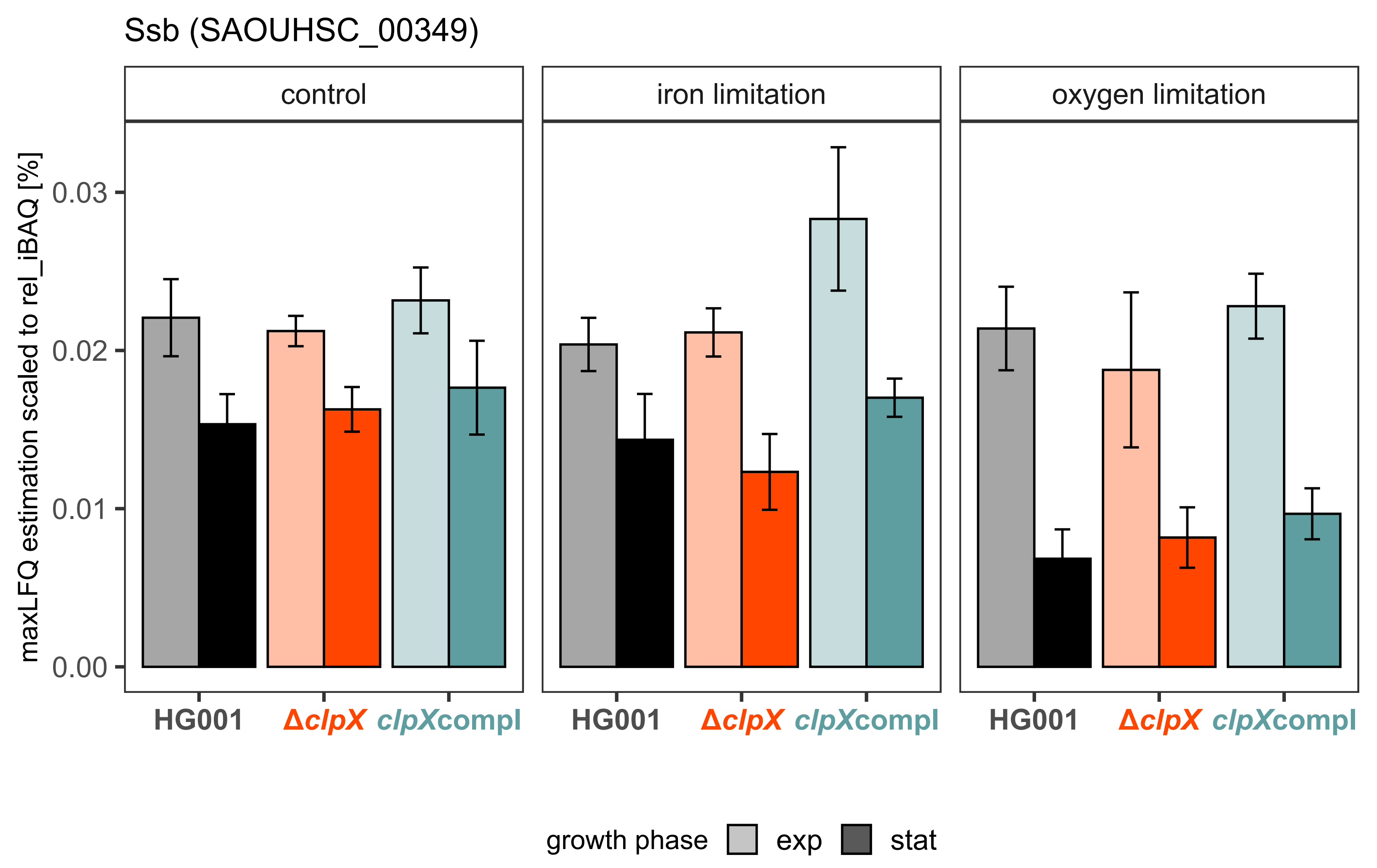
Ssb (SAOUHSC_00349)
control
iron limitation
oxygen limitation
maxLFQ estimation scaled to rel_iBAQ [%]
0.00
0.01
0.02
0.03
HG001
ΔclpX
clpXcompl
growth phase
exp
stat

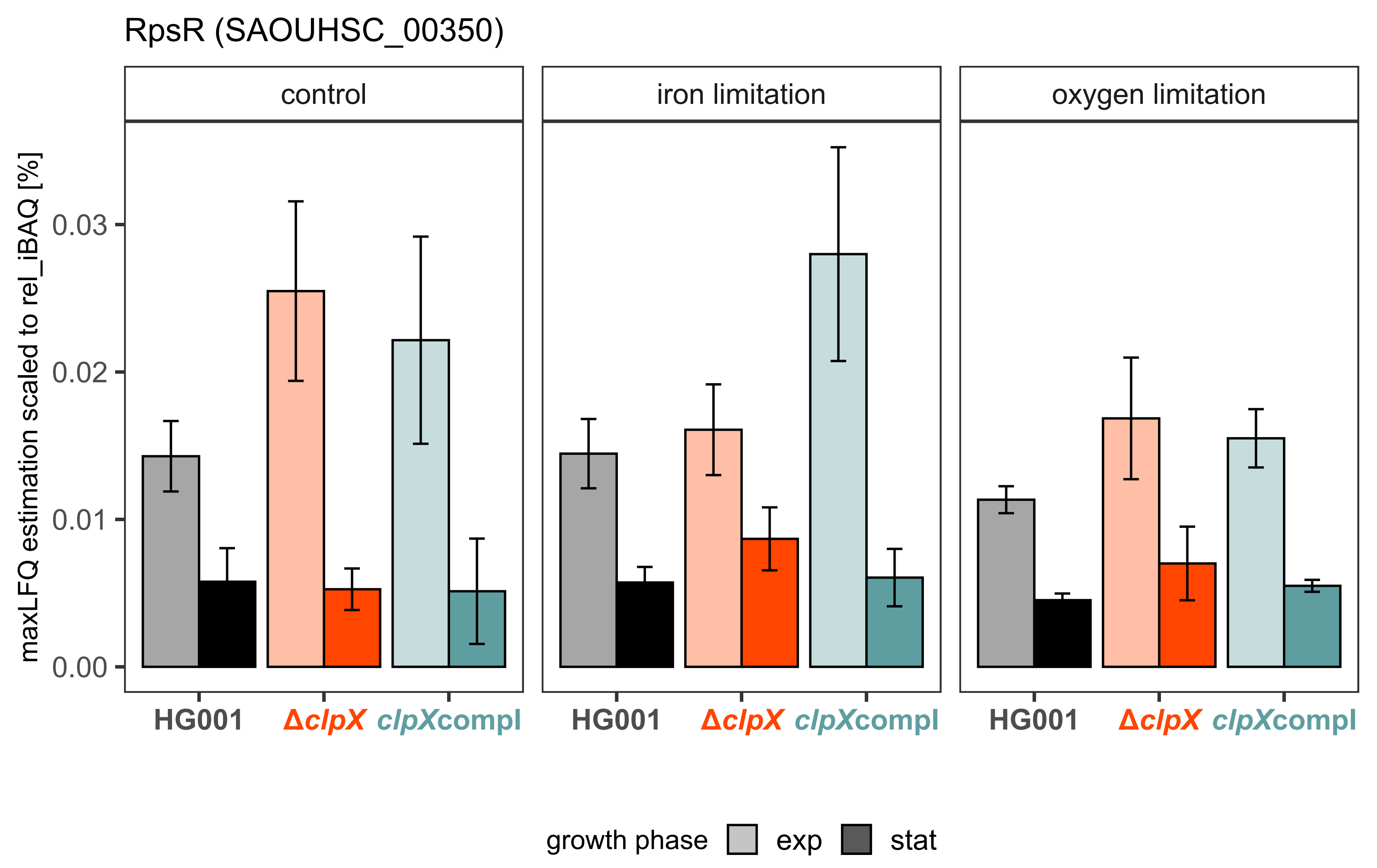
RpsR (SAOUHSC_00350)
control
iron limitation
oxygen limitation
maxLFQ estimation scaled to rel_iBAQ [%]
0.00
0.01
0.02
0.03
HG001
ΔclpX
clpXcompl
growth phase
exp
stat

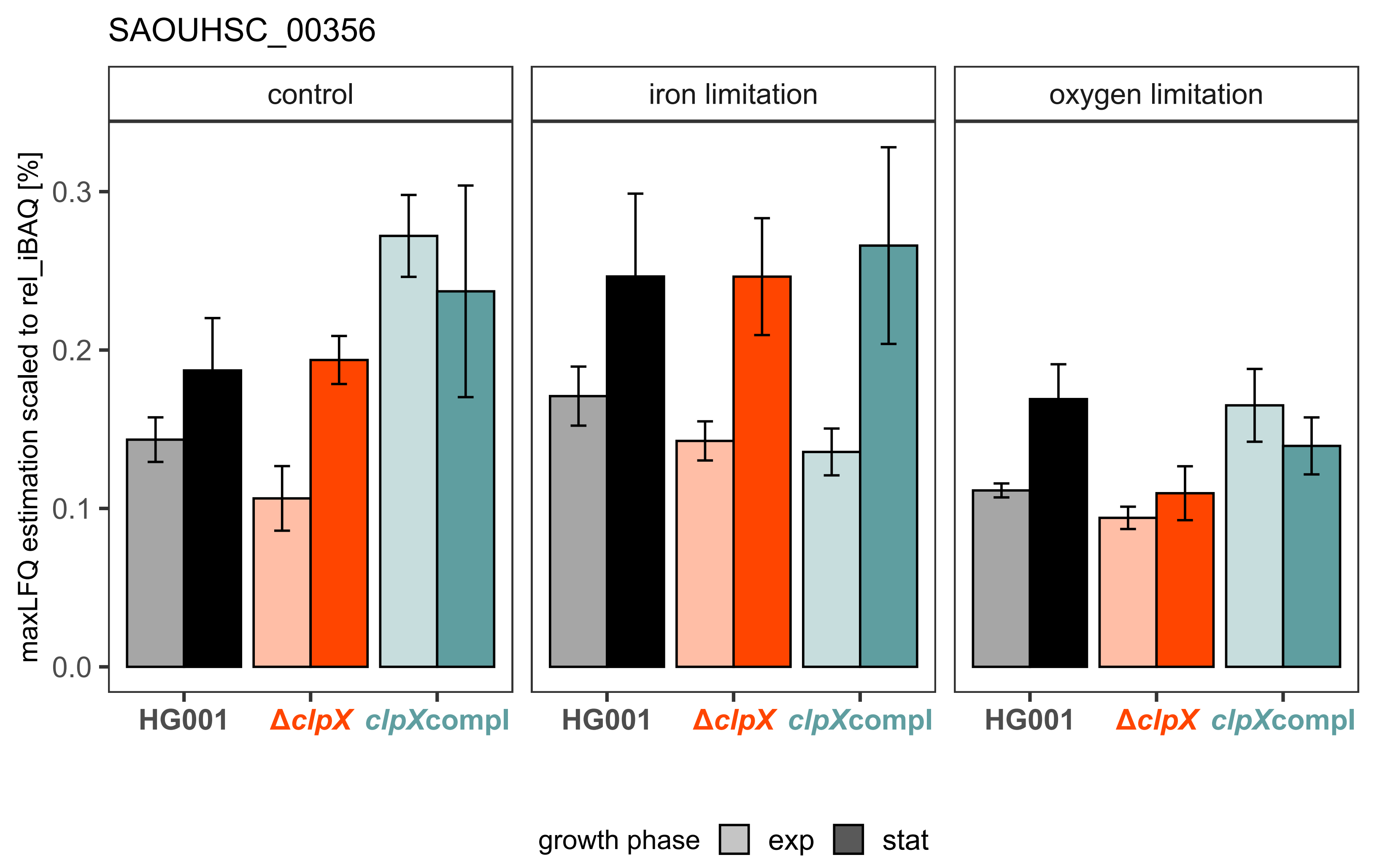

SAOUHSC_00356
control
iron limitation
oxygen limitation
maxLFQ estimation scaled to rel_iBAQ [%]
0.0
0.1
0.2
0.3
HG001
ΔclpX
clpXcompl
growth phase
exp
stat

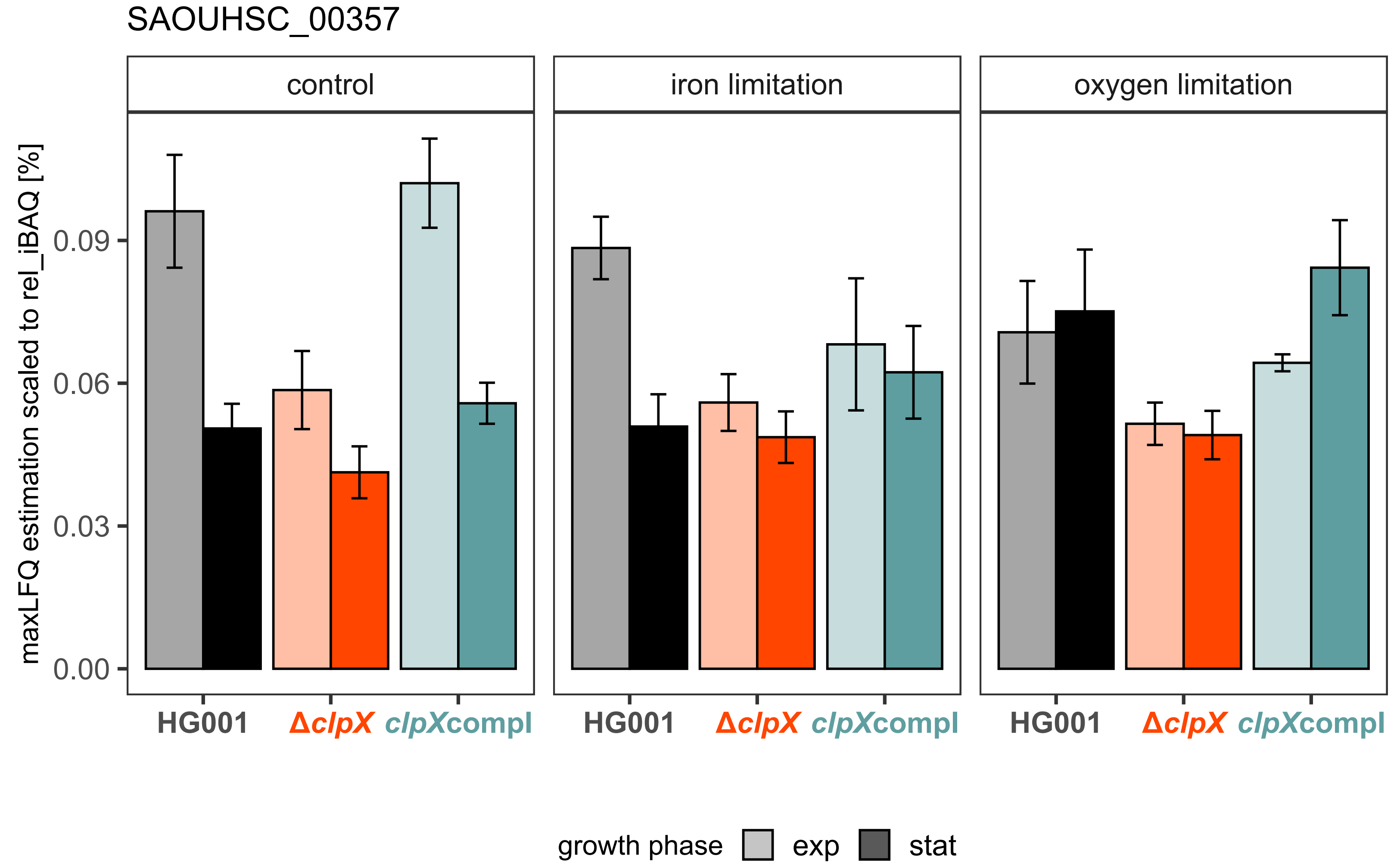
SAOUHSC_00357
control
iron limitation
oxygen limitation
maxLFQ estimation scaled to rel_iBAQ [%]
0.00
0.03
0.06
0.09
HG001
ΔclpX
clpXcompl
growth phase
exp
stat

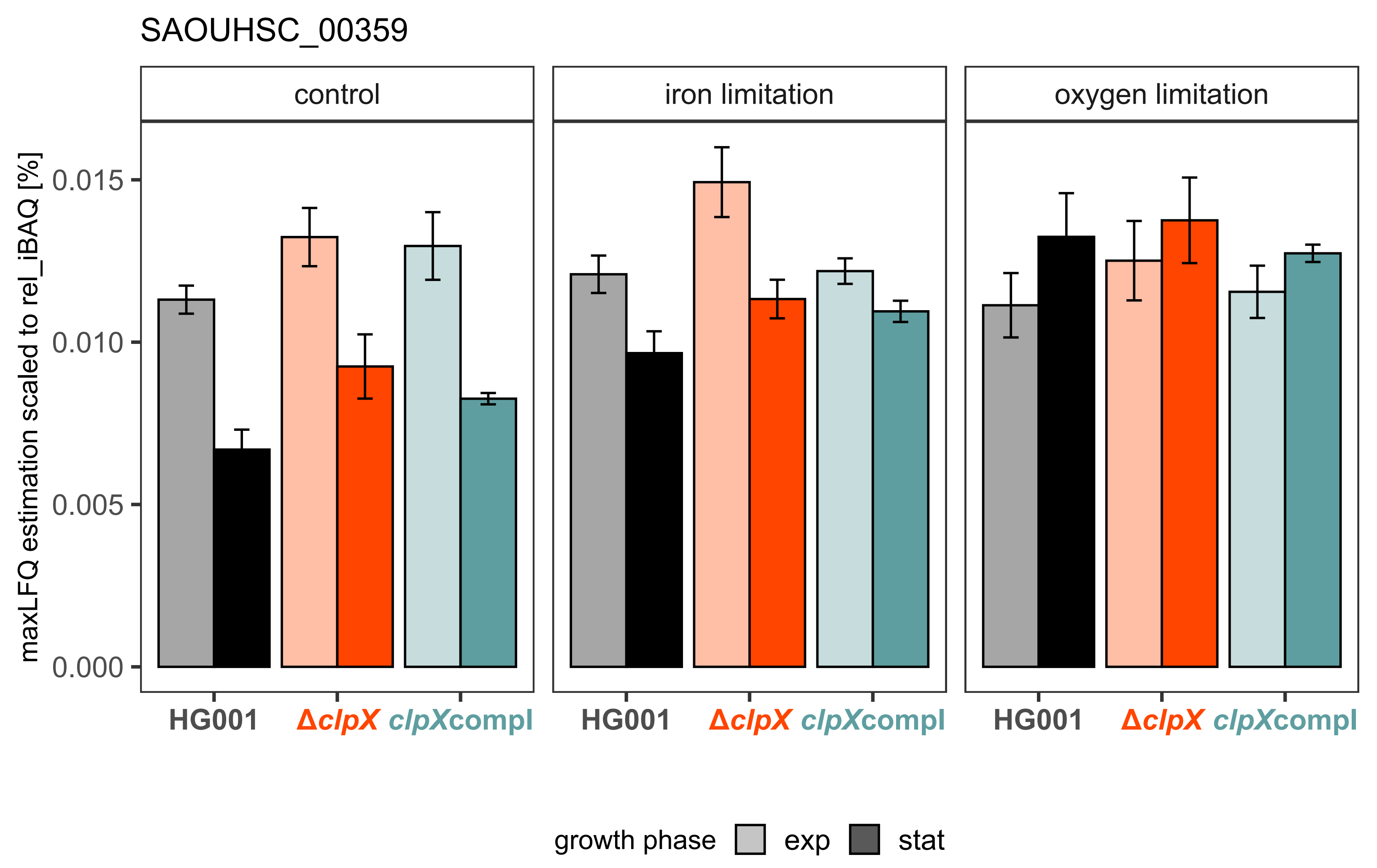

SAOUHSC_00359
control
iron limitation
oxygen limitation
maxLFQ estimation scaled to rel_iBAQ [%]
0.015
0.010
0.005
0.000
HG001
ΔclpX
clpXcompl
growth phase
exp
stat

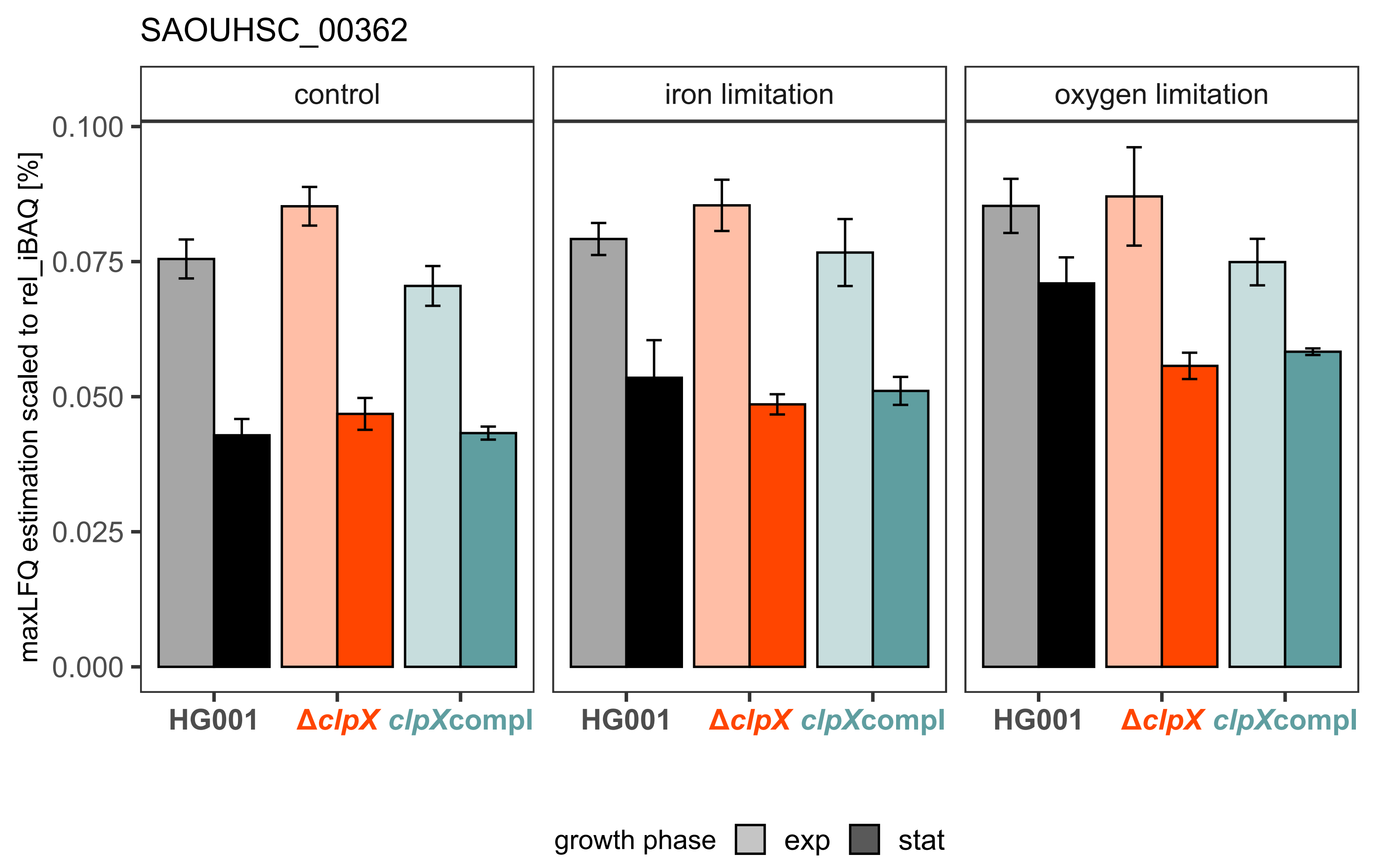

SAOUHSC_00362
control
iron limitation
oxygen limitation
maxLFQ estimation scaled to rel_iBAQ [%]
0.100
0.075
0.050
0.025
0.000
HG001
ΔclpX
clpXcompl
growth phase
exp
stat

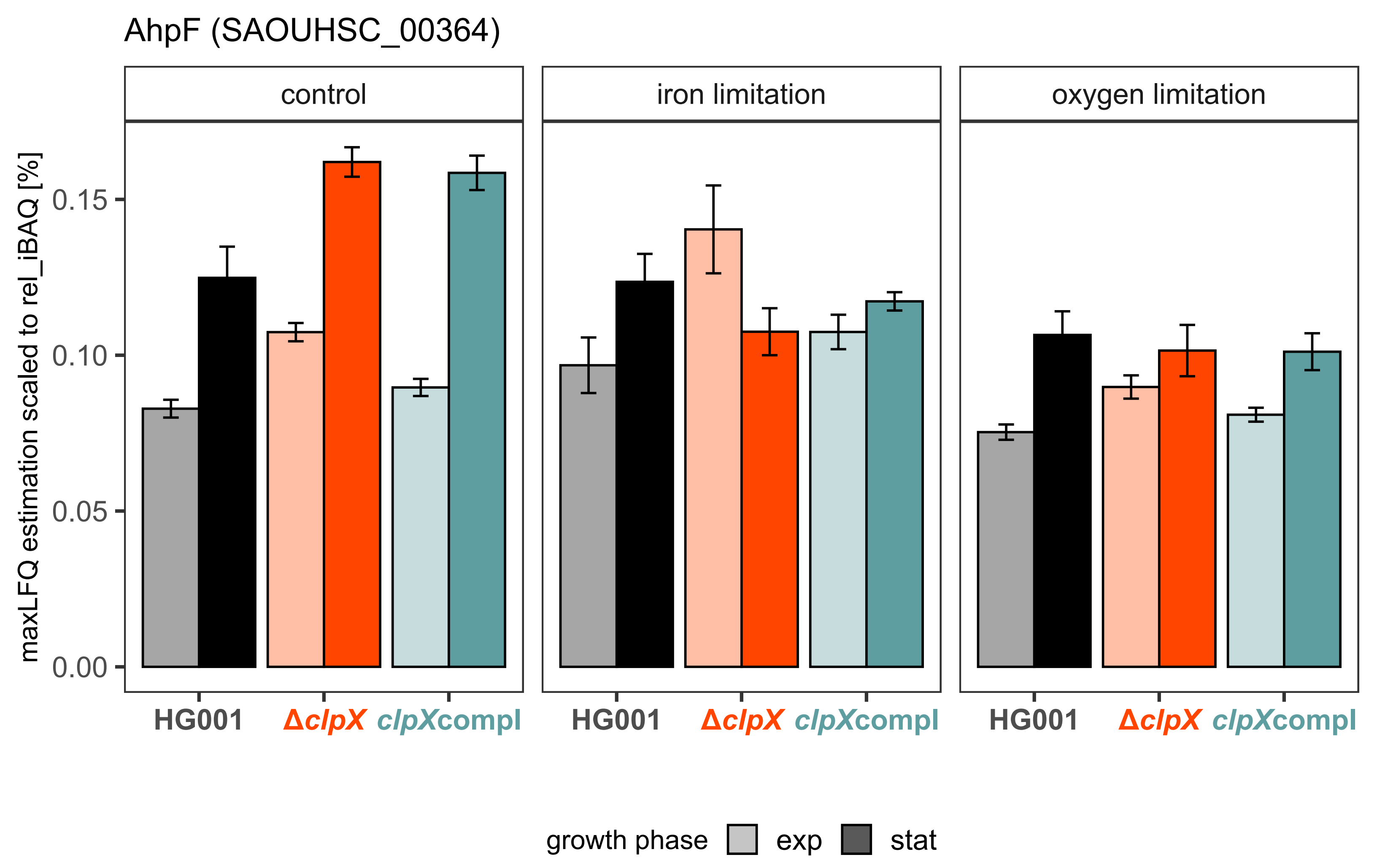
AhpF (SAOUHSC_00364)
control
iron limitation
oxygen limitation
maxLFQ estimation scaled to rel_iBAQ [%]
0.00
0.05
0.10
0.15
HG001
ΔclpX
clpXcompl
growth phase
exp
stat

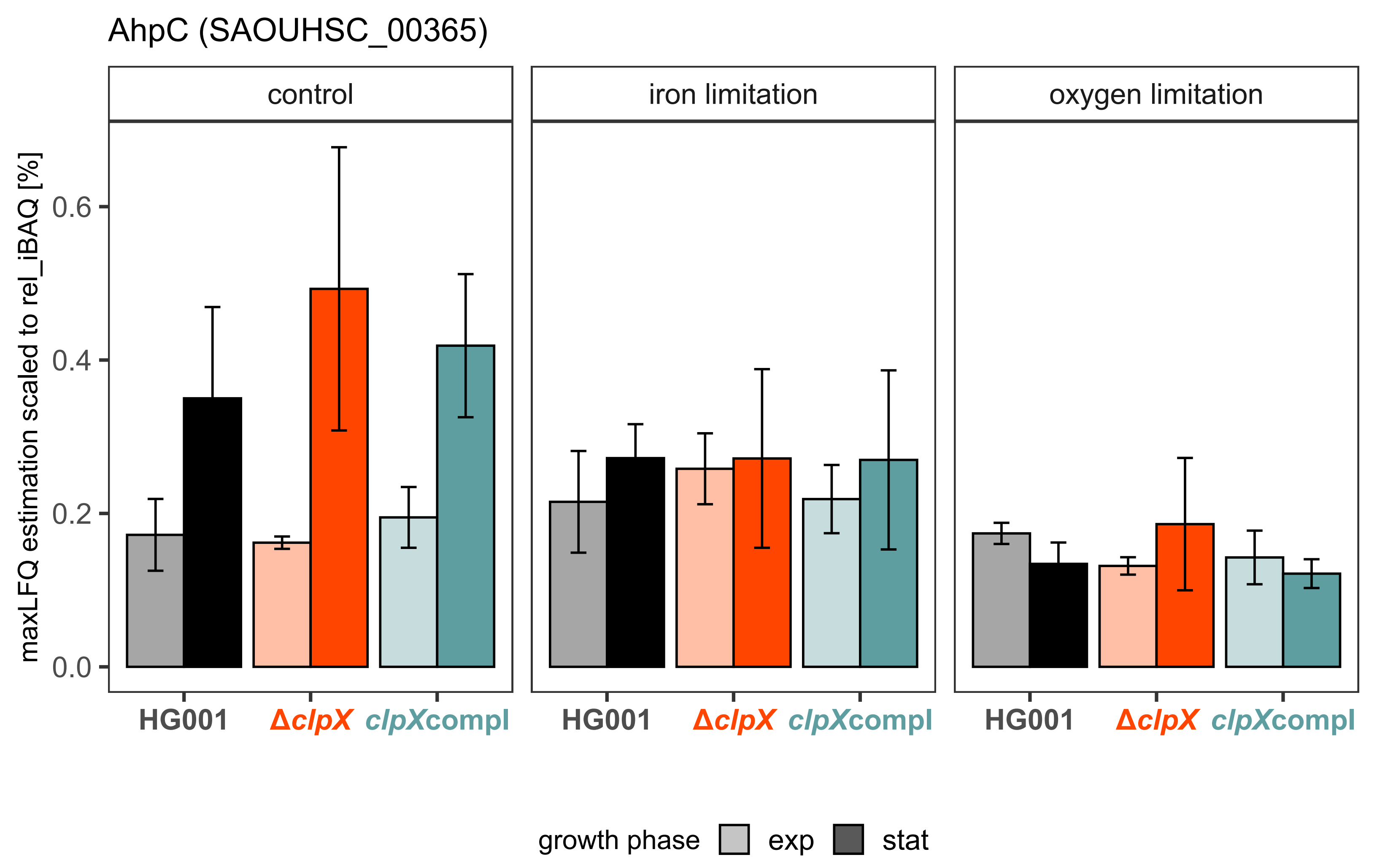
AhpC (SAOUHSC_00365)
control
iron limitation
oxygen limitation
maxLFQ estimation scaled to rel_iBAQ [%]
0.0
0.2
0.4
0.6
HG001
ΔclpX
clpXcompl
growth phase
exp
stat

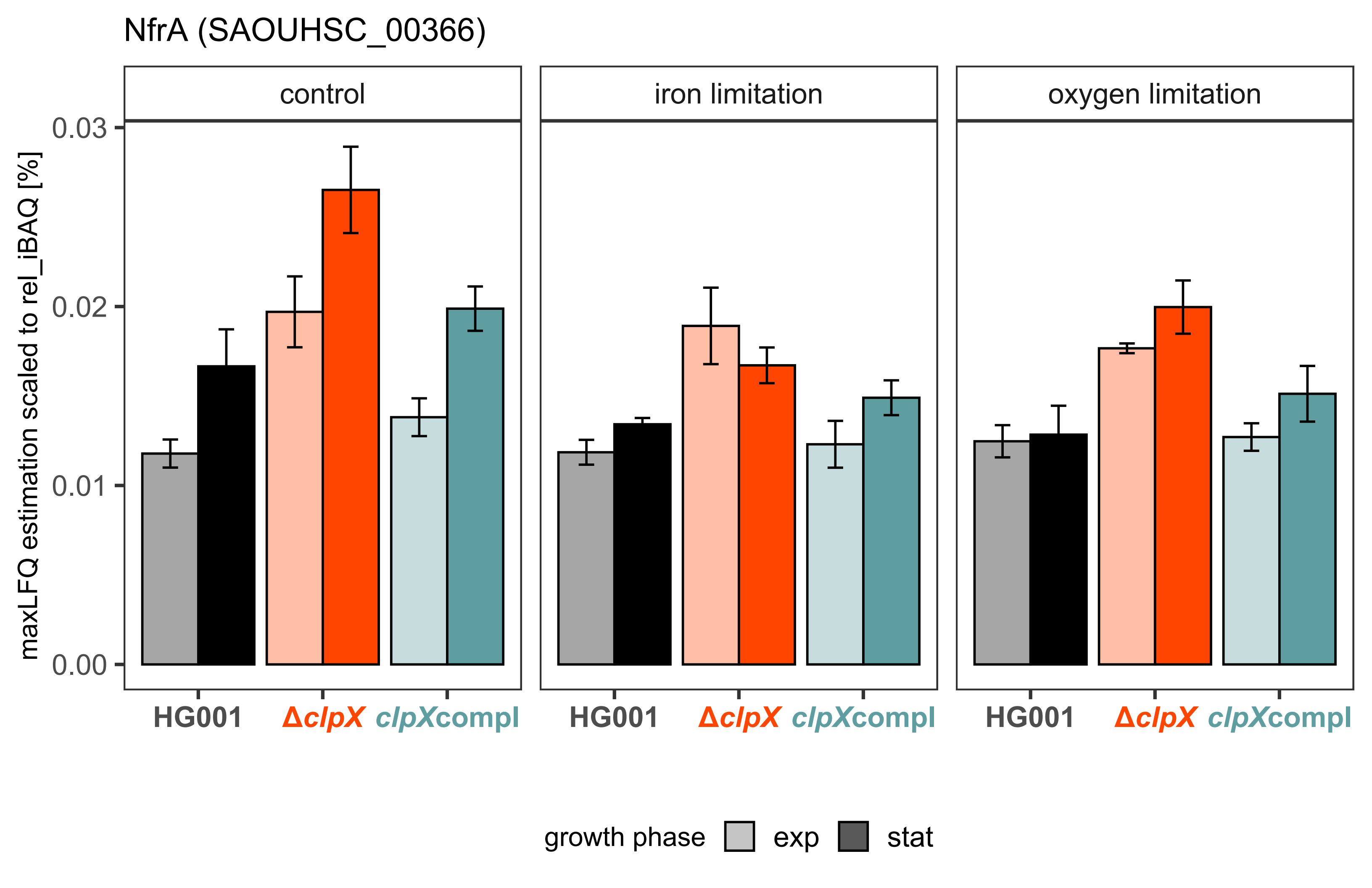
NfrA (SAOUHSC_00366)
control
iron limitation
oxygen limitation
maxLFQ estimation scaled to rel_iBAQ [%]
0.03
0.02
0.01
0.00
HG001
ΔclpX
clpXcompl
growth phase
exp
stat

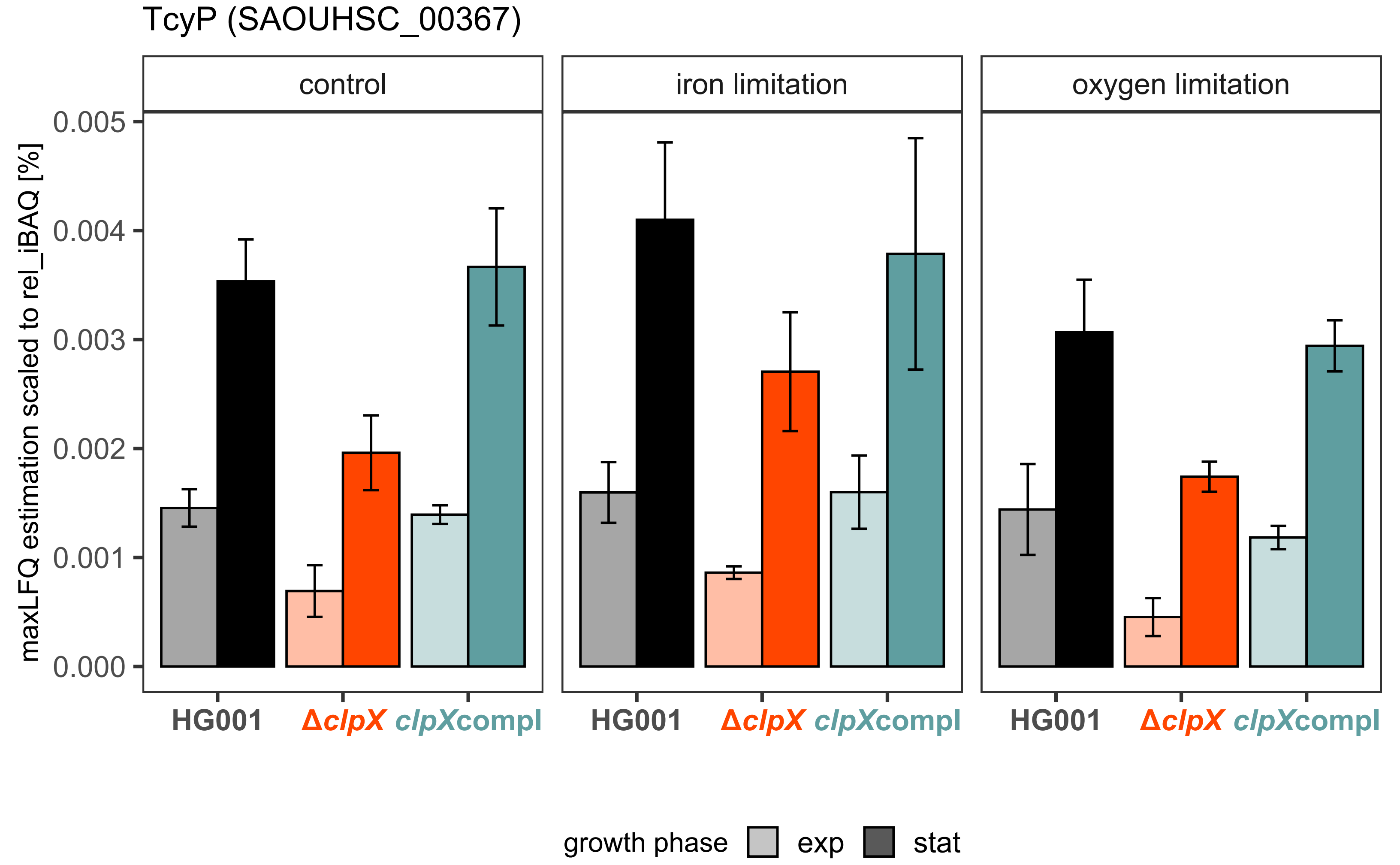

TcyP (SAOUHSC_00367)
control
iron limitation
oxygen limitation
maxLFQ estimation scaled to rel_iBAQ [%]
0.000
0.001
0.002
0.003
0.004
0.005
HG001
ΔclpX
clpXcompl
growth phase
exp
stat

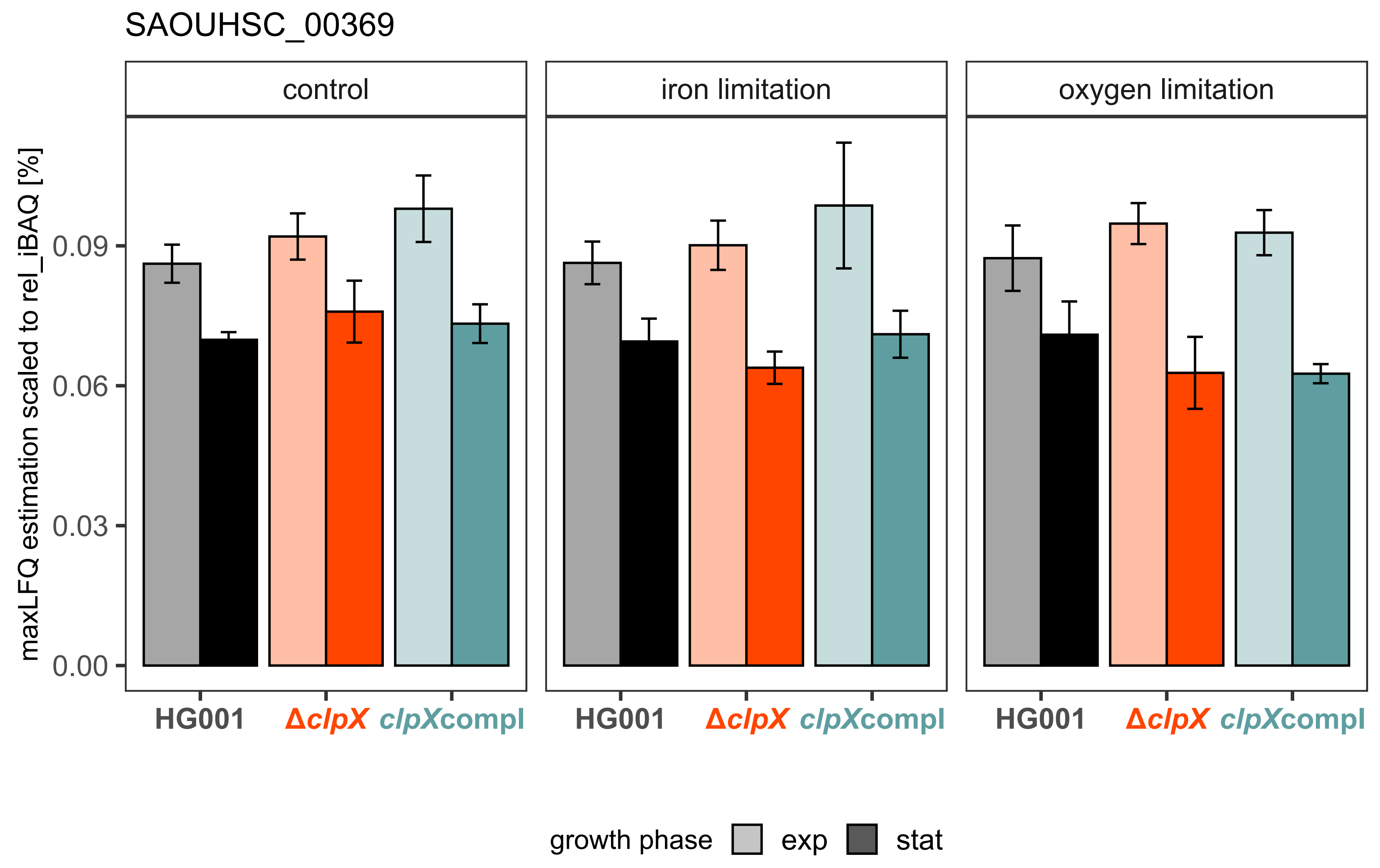
SAOUHSC_00369
control
iron limitation
oxygen limitation
maxLFQ estimation scaled to rel_iBAQ [%]
0.09
0.06
0.03
0.00
HG001
ΔclpX
clpXcompl
growth phase
exp
stat

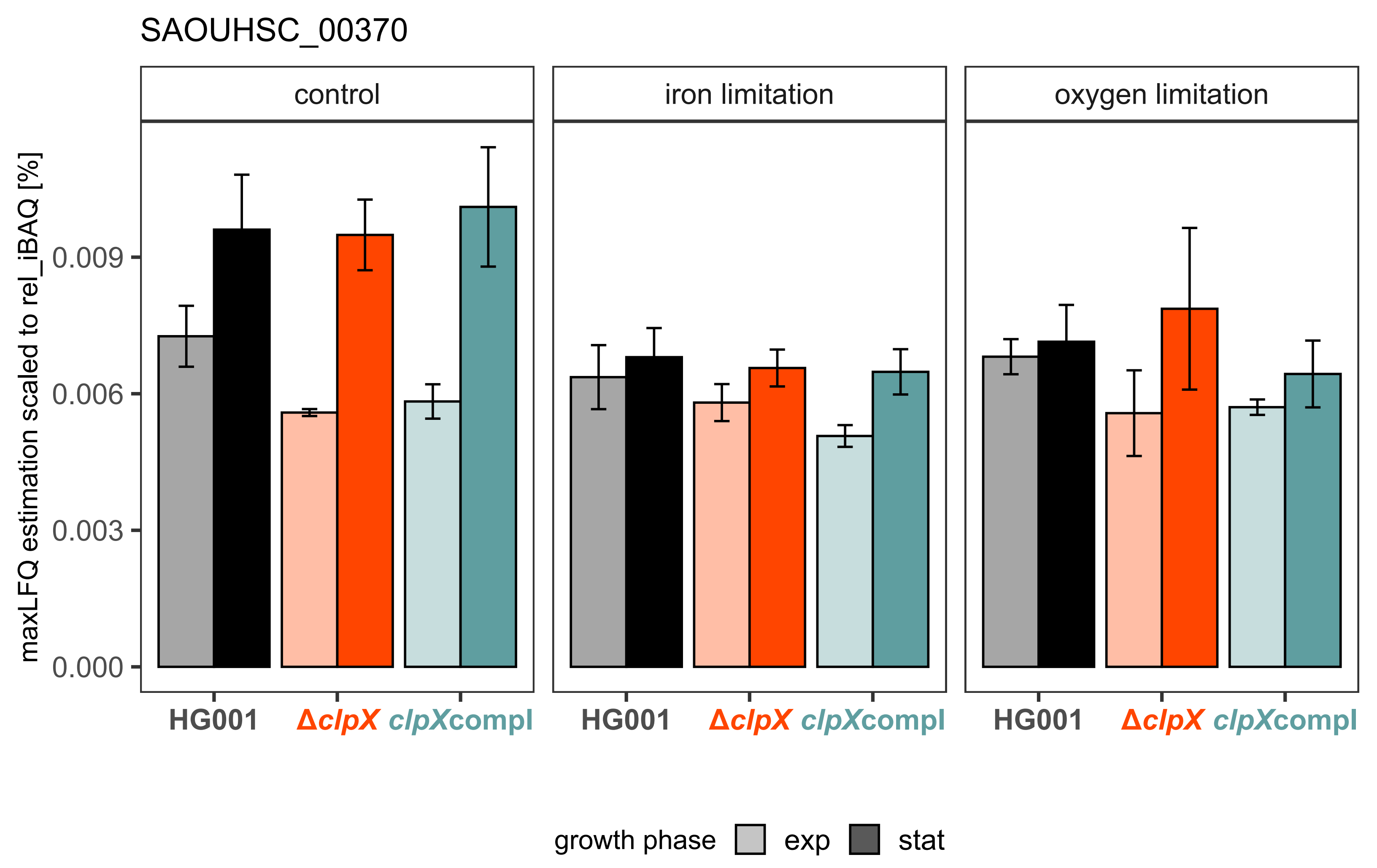

SAOUHSC_00370
control
iron limitation
oxygen limitation
maxLFQ estimation scaled to rel_iBAQ [%]
0.009
0.006
0.003
0.000
HG001
ΔclpX
clpXcompl
growth phase
exp
stat

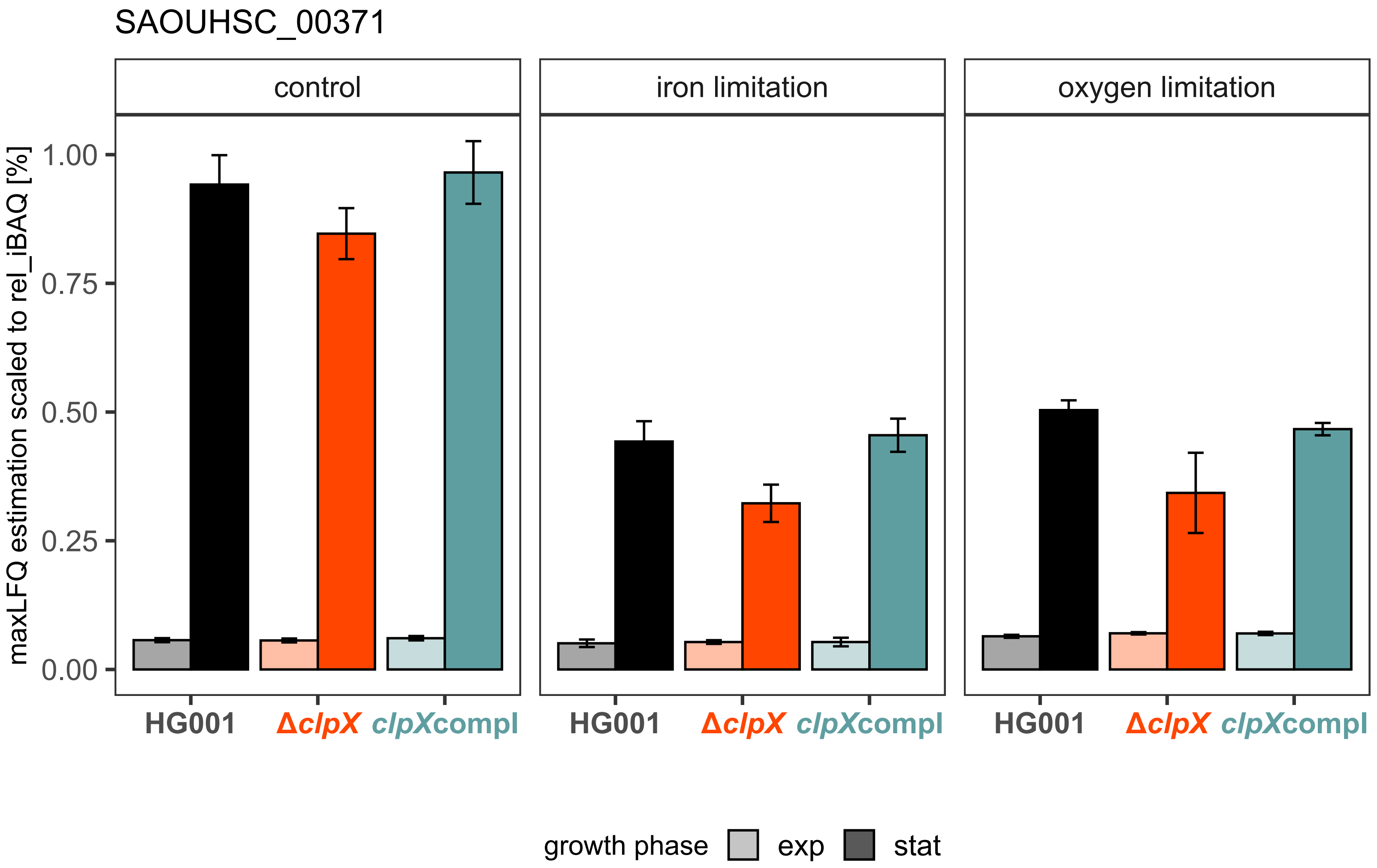

SAOUHSC_00371
control
iron limitation
oxygen limitation
maxLFQ estimation scaled to rel_iBAQ [%]
1.00
0.75
0.50
0.25
0.00
HG001
ΔclpX
clpXcompl
growth phase
exp
stat

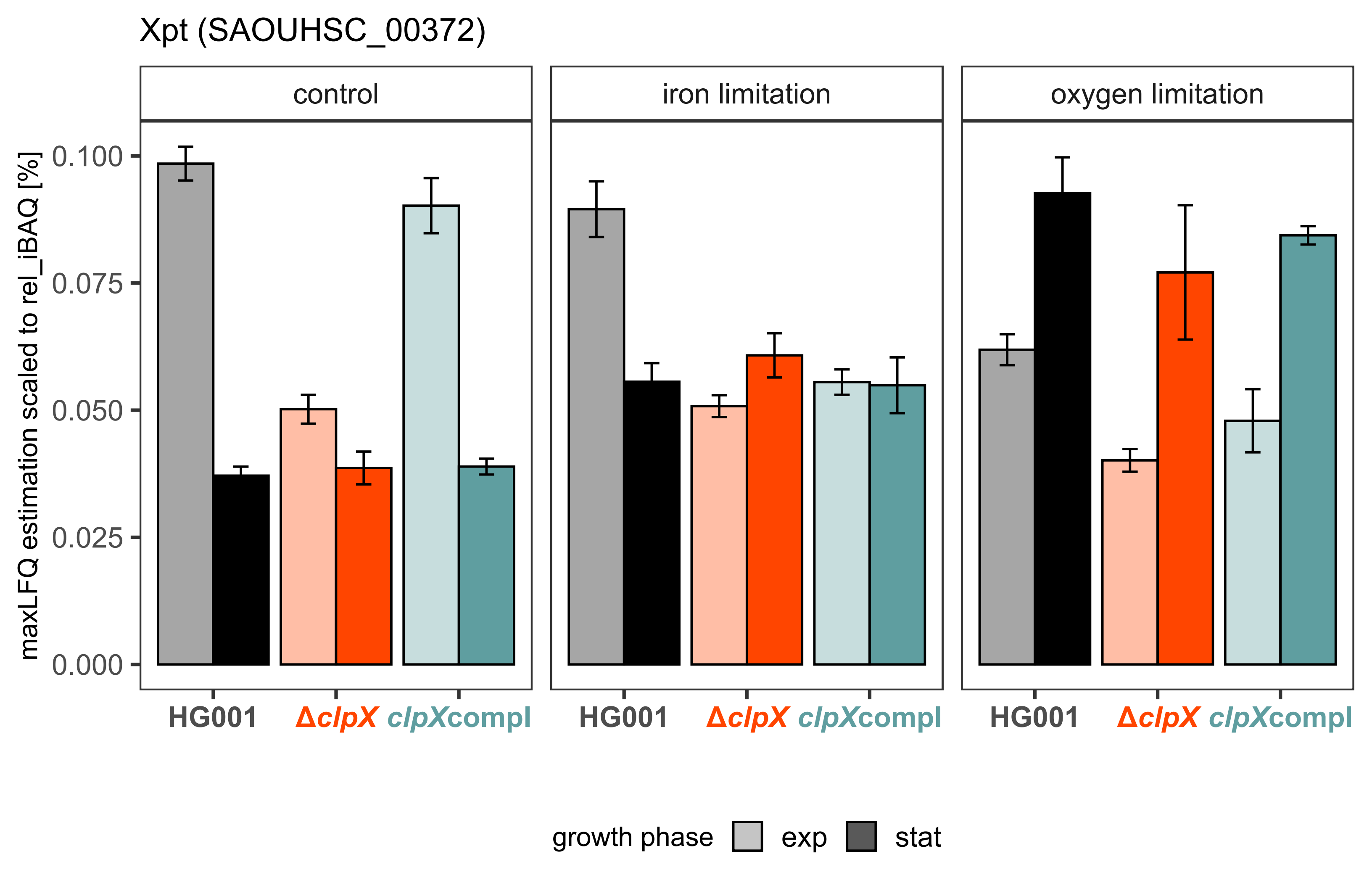

Xpt (SAOUHSC_00372)
control
iron limitation
oxygen limitation
maxLFQ estimation scaled to rel_iBAQ [%]
0.100
0.075
0.050
0.025
0.000
HG001
ΔclpX
clpXcompl
growth phase
exp
stat

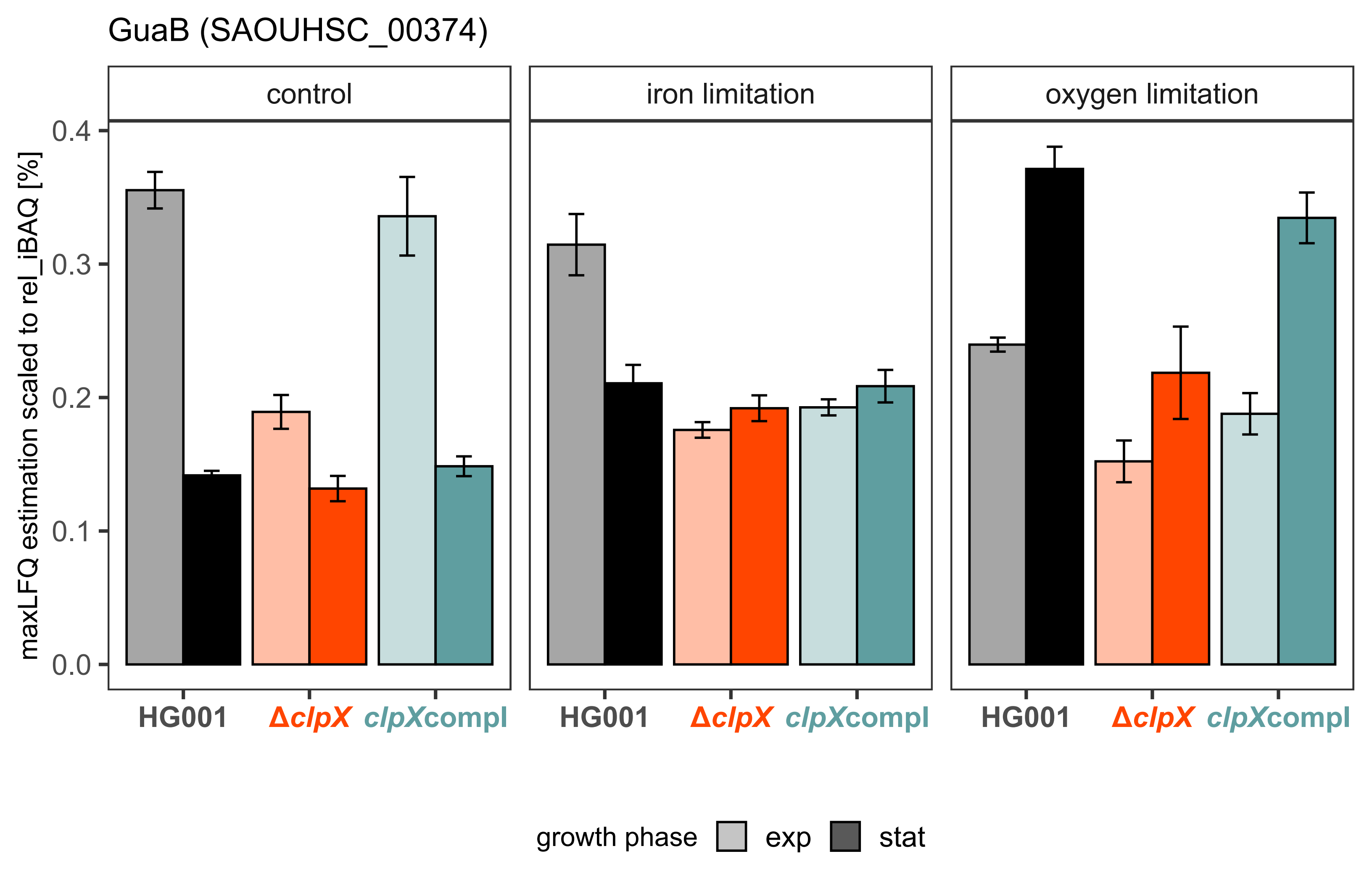
GuaB (SAOUHSC_00374)
control
iron limitation
oxygen limitation
maxLFQ estimation scaled to rel_iBAQ [%]
0.0
0.1
0.2
0.3
0.4
HG001
ΔclpX
clpXcompl
growth phase
exp
stat

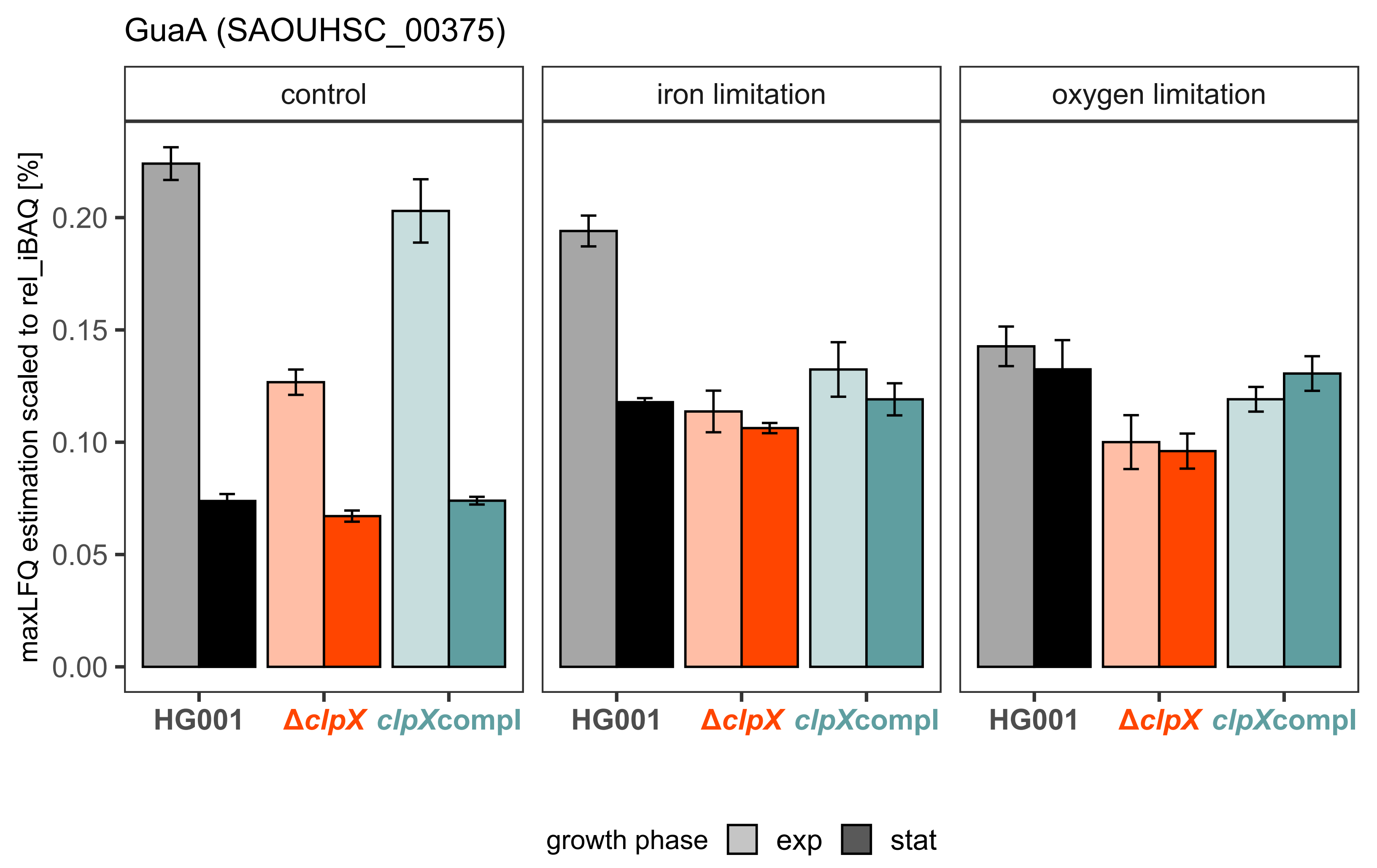
GuaA (SAOUHSC_00375)
control
iron limitation
oxygen limitation
maxLFQ estimation scaled to rel_iBAQ [%]
0.20
0.15
0.10
0.05
0.00
HG001
ΔclpX
clpXcompl
growth phase
exp
stat

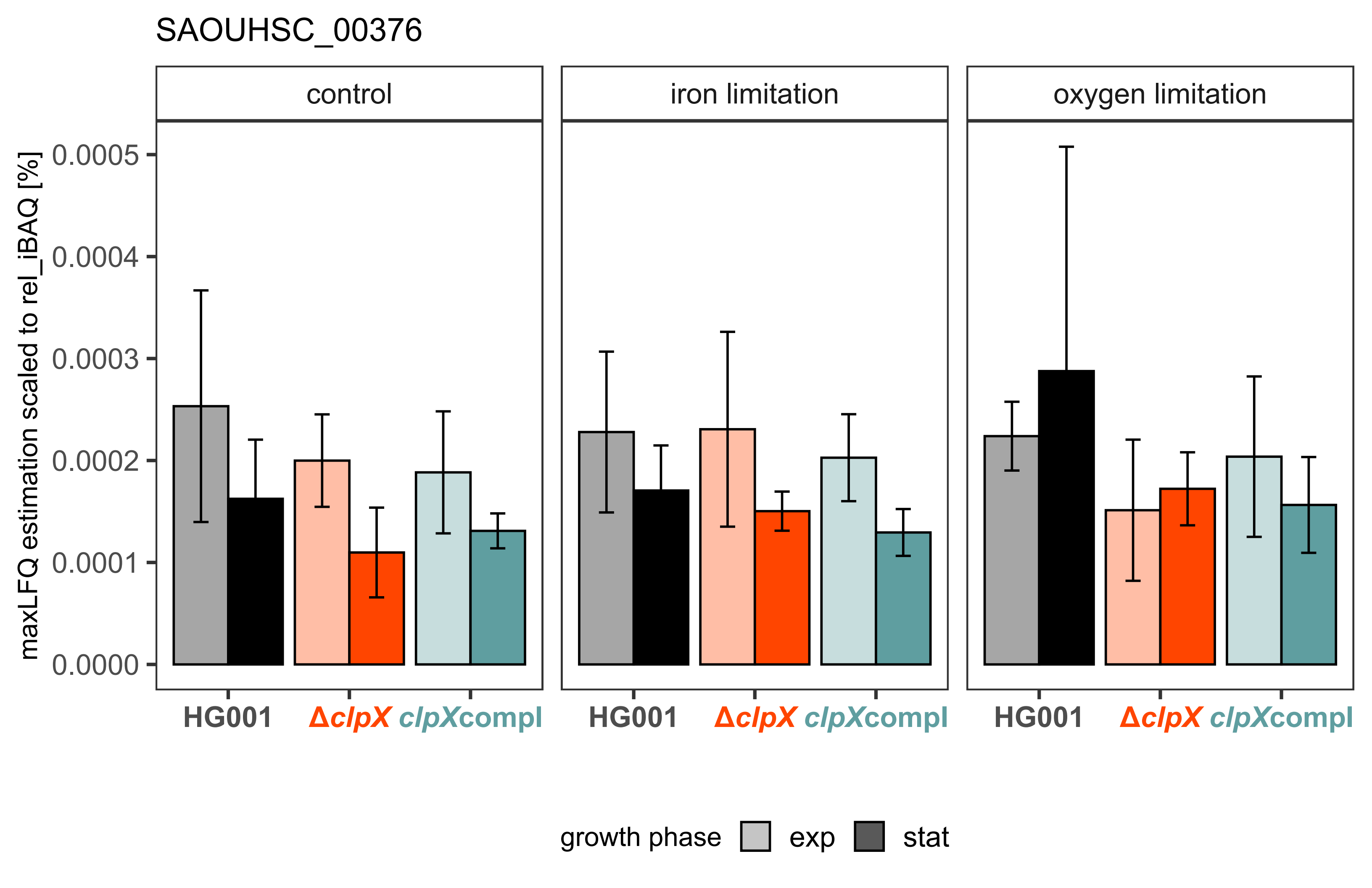
SAOUHSC_00376
control
iron limitation
oxygen limitation
maxLFQ estimation scaled to rel_iBAQ [%]
0.0005
0.0004
0.0003
0.0002
0.0001
0.0000
HG001
ΔclpX
clpXcompl
growth phase
exp
stat

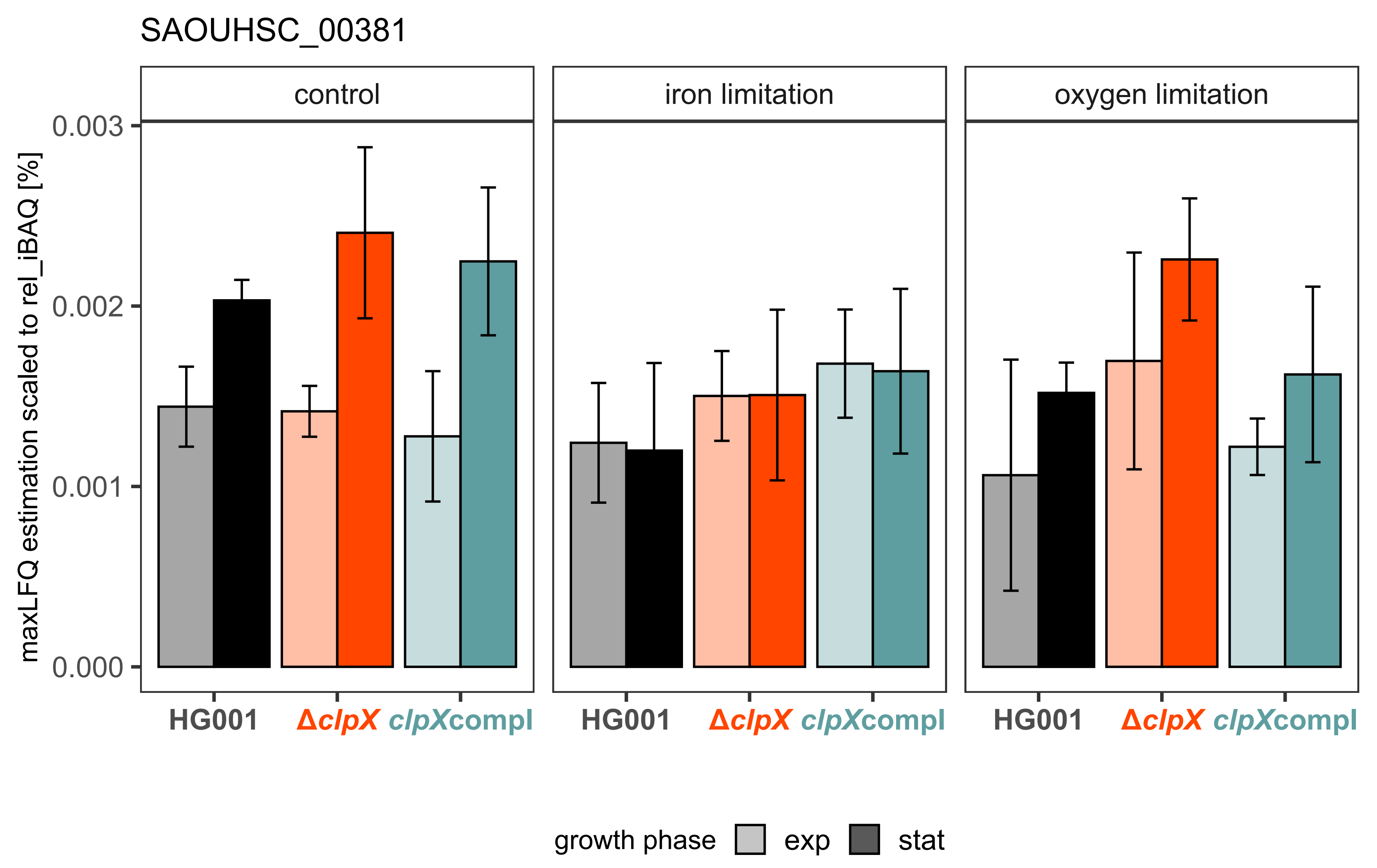

SAOUHSC_00381
control
iron limitation
oxygen limitation
maxLFQ estimation scaled to rel_iBAQ [%]
0.003
0.002
0.001
0.000
HG001
ΔclpX
clpXcompl
growth phase
exp
stat

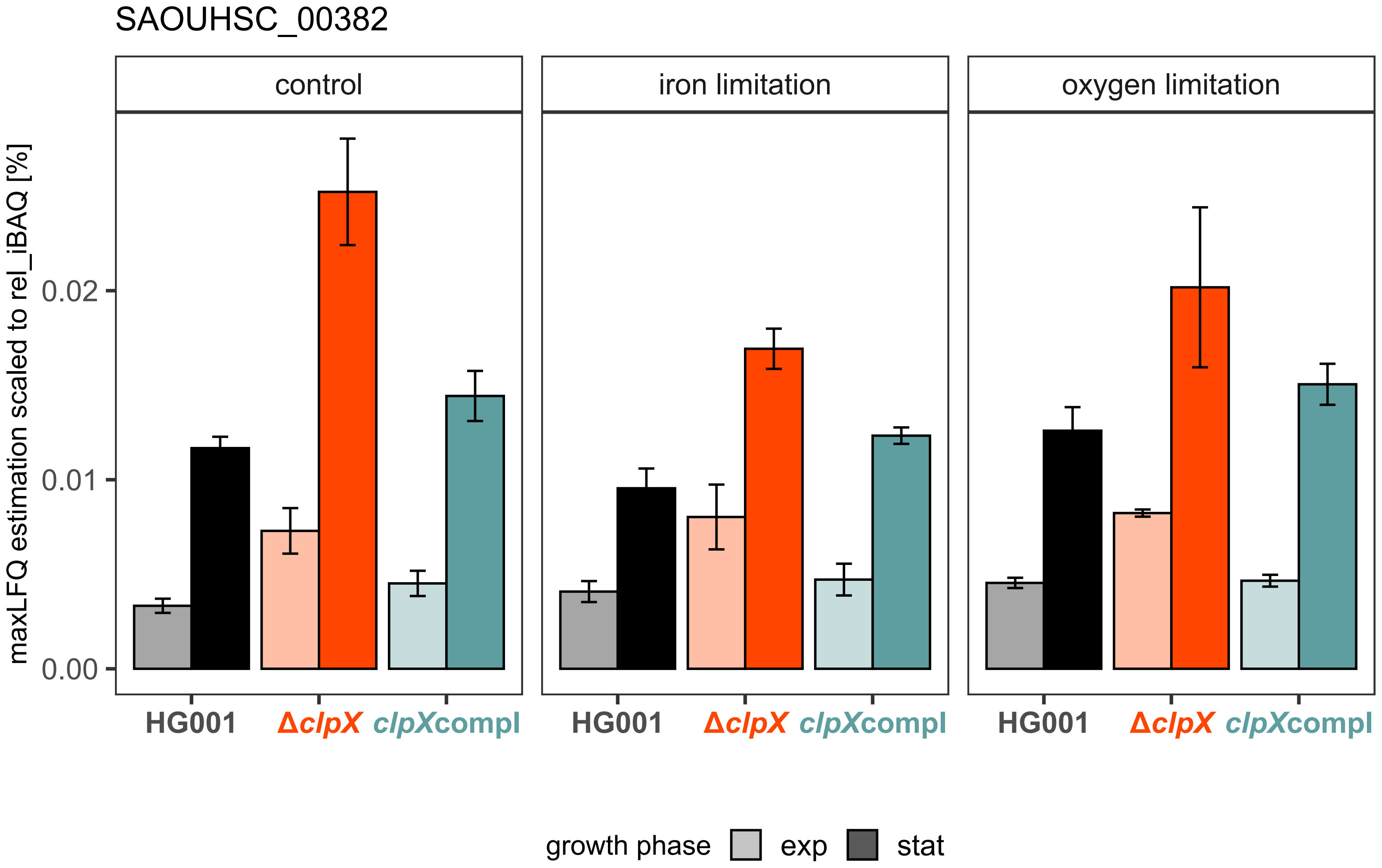

SAOUHSC_00382
control
iron limitation
oxygen limitation
maxLFQ estimation scaled to rel_iBAQ [%]
0.00
0.01
0.02
HG001
ΔclpX
clpXcompl
growth phase
exp
stat

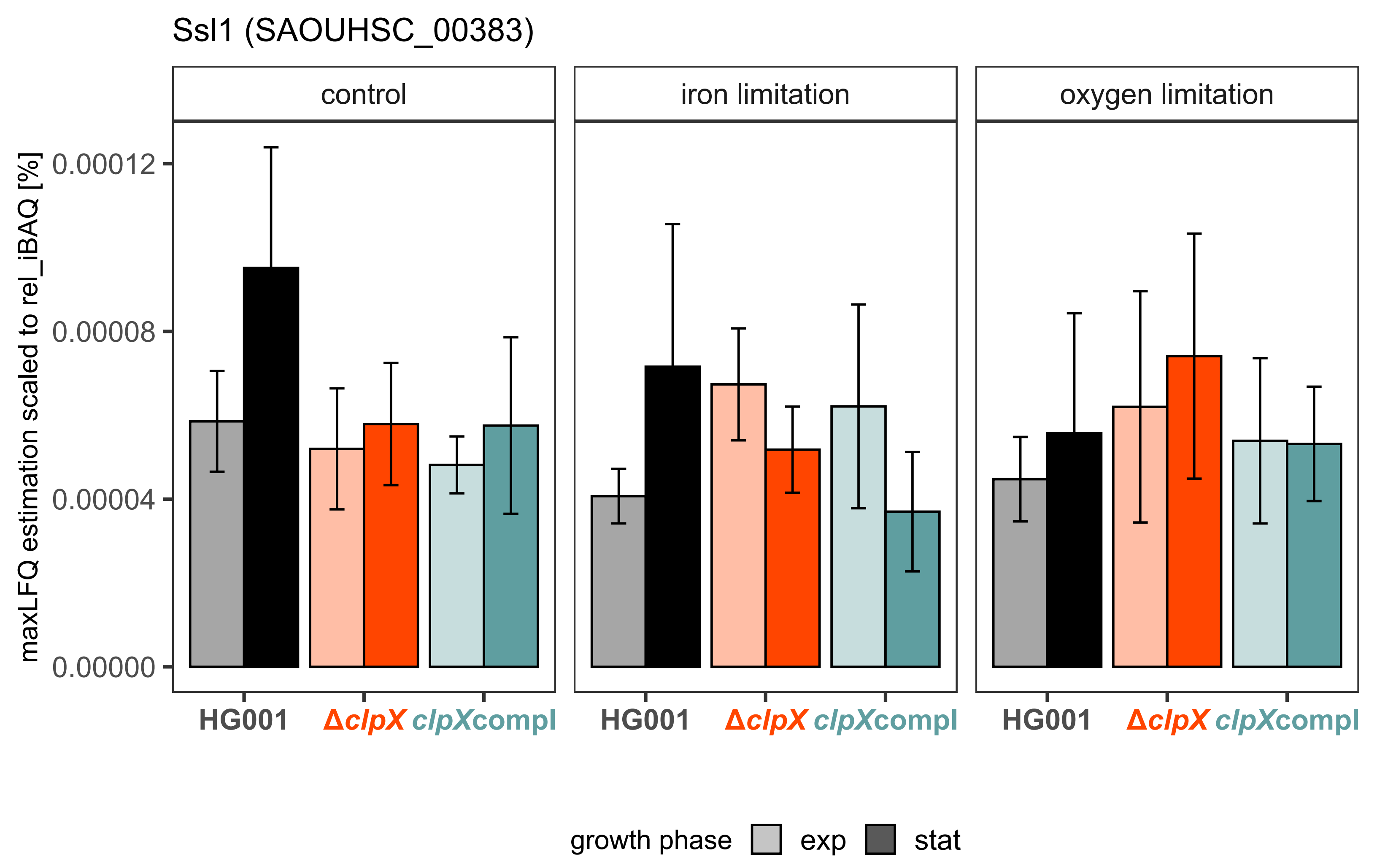
Ssl1 (SAOUHSC_00383)
control
iron limitation
oxygen limitation
maxLFQ estimation scaled to rel_iBAQ [%]
0.00012
0.00008
0.00004
0.00000
HG001
ΔclpX
clpXcompl
growth phase
exp
stat

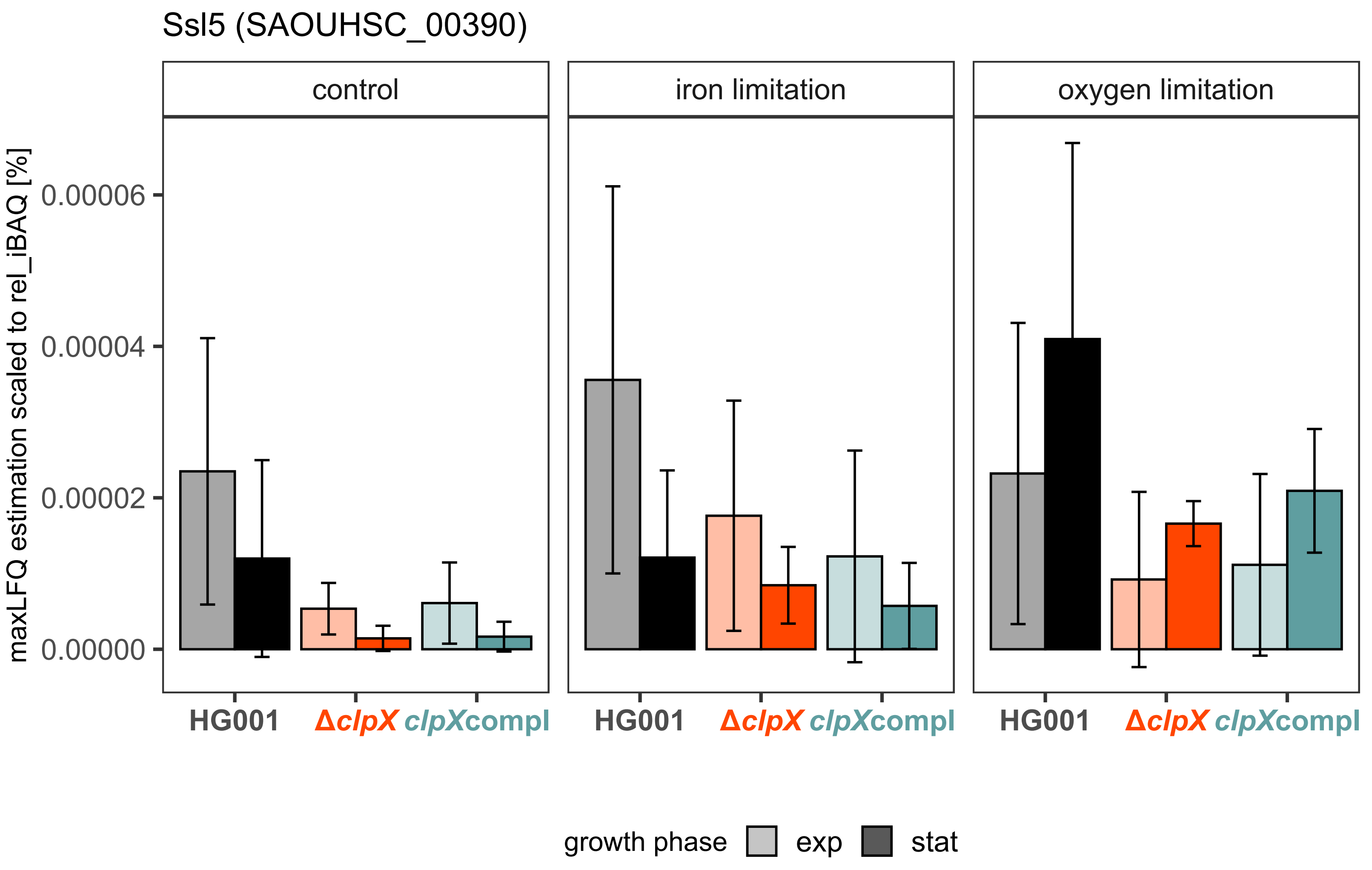

Ssl5 (SAOUHSC_00390)
control
iron limitation
oxygen limitation
maxLFQ estimation scaled to rel_iBAQ [%]
0.00006
0.00004
0.00002
0.00000
HG001
ΔclpX
clpXcompl
growth phase
exp
stat

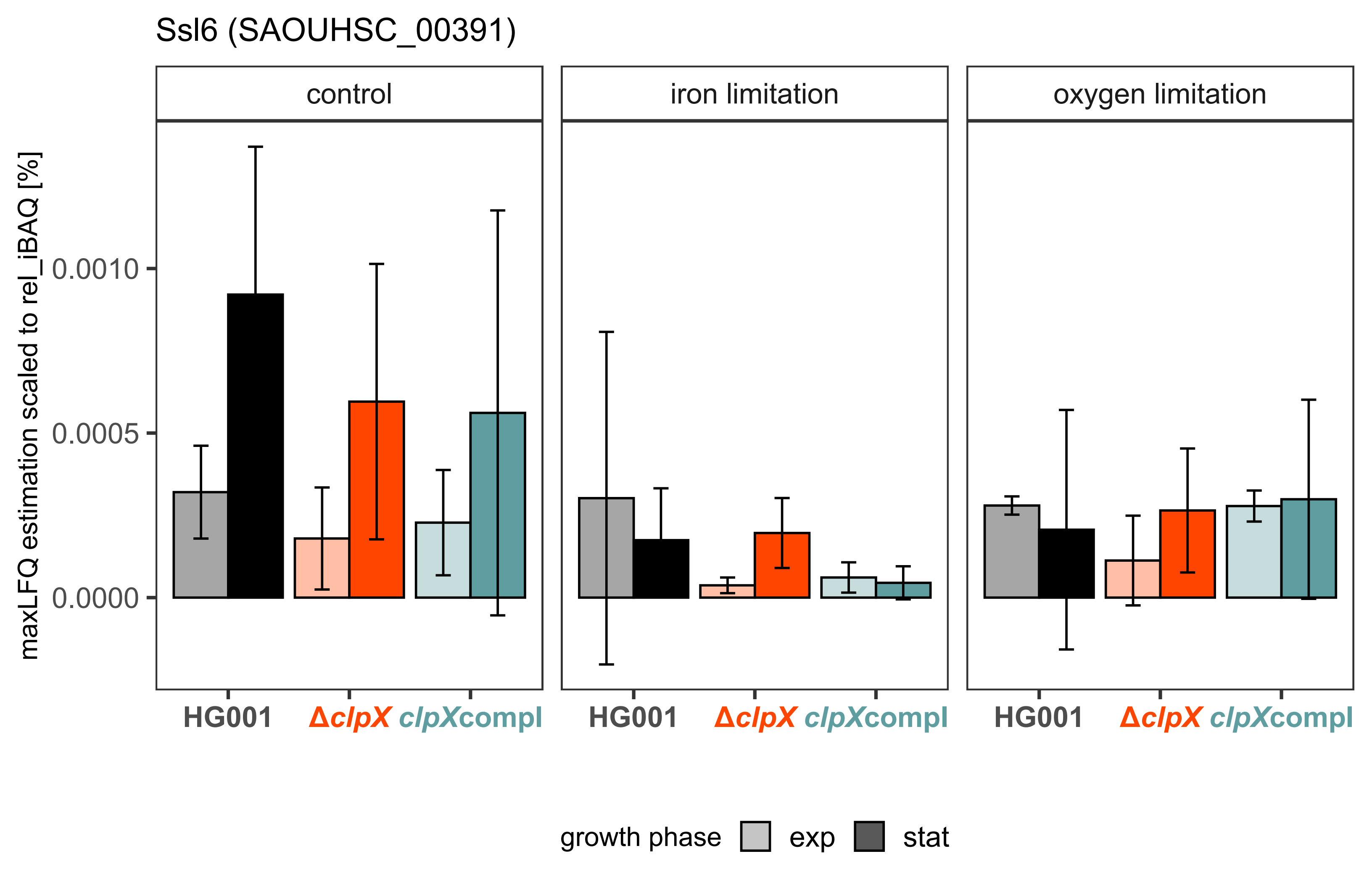

Ssl6 (SAOUHSC_00391)
control
iron limitation
oxygen limitation
maxLFQ estimation scaled to rel_iBAQ [%]
0.0010
0.0005
0.0000
HG001
ΔclpX
clpXcompl
growth phase
exp
stat

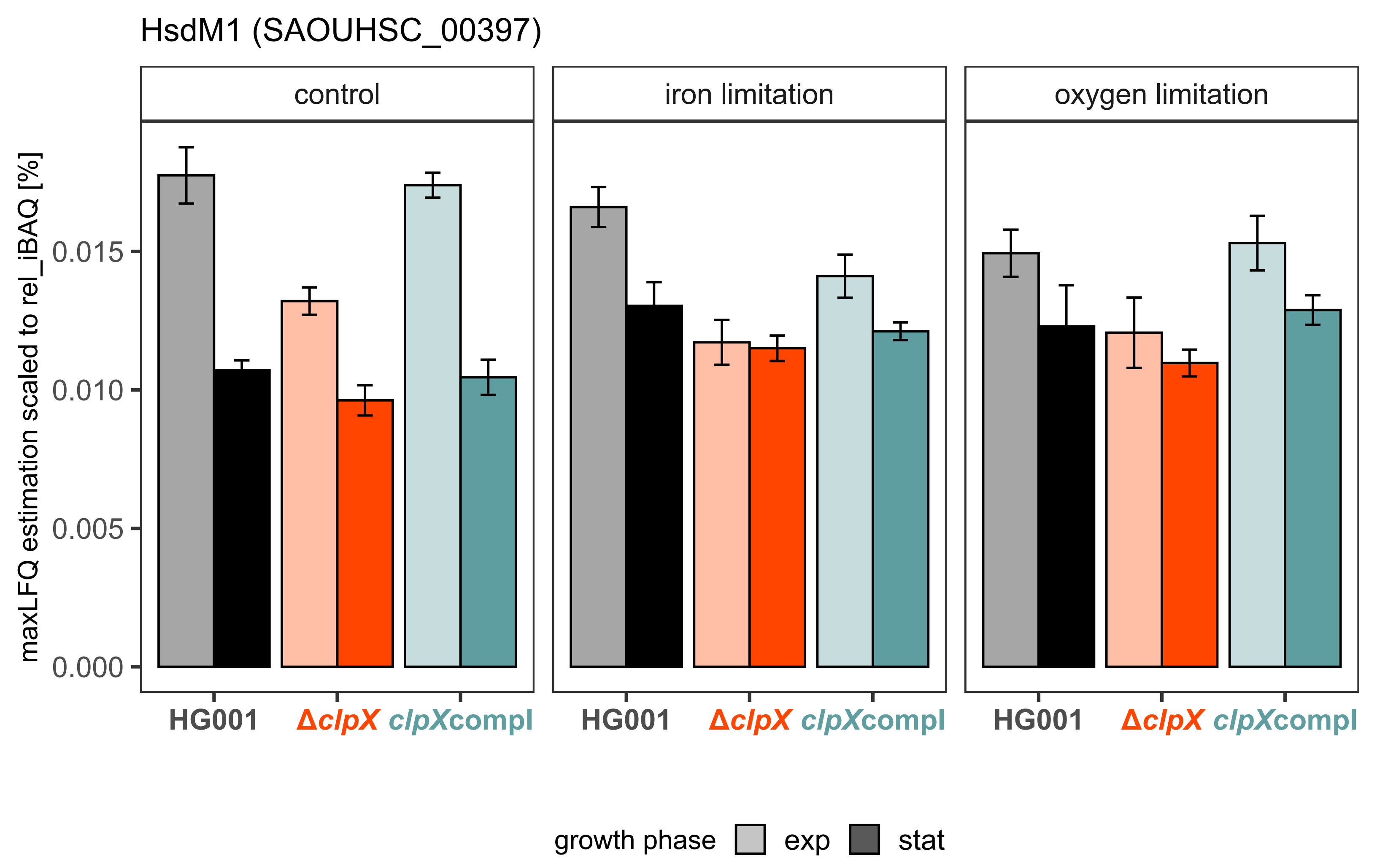

HsdM1 (SAOUHSC_00397)
control
iron limitation
oxygen limitation
maxLFQ estimation scaled to rel_iBAQ [%]
0.015
0.010
0.005
0.000
HG001
ΔclpX
clpXcompl
growth phase
exp
stat

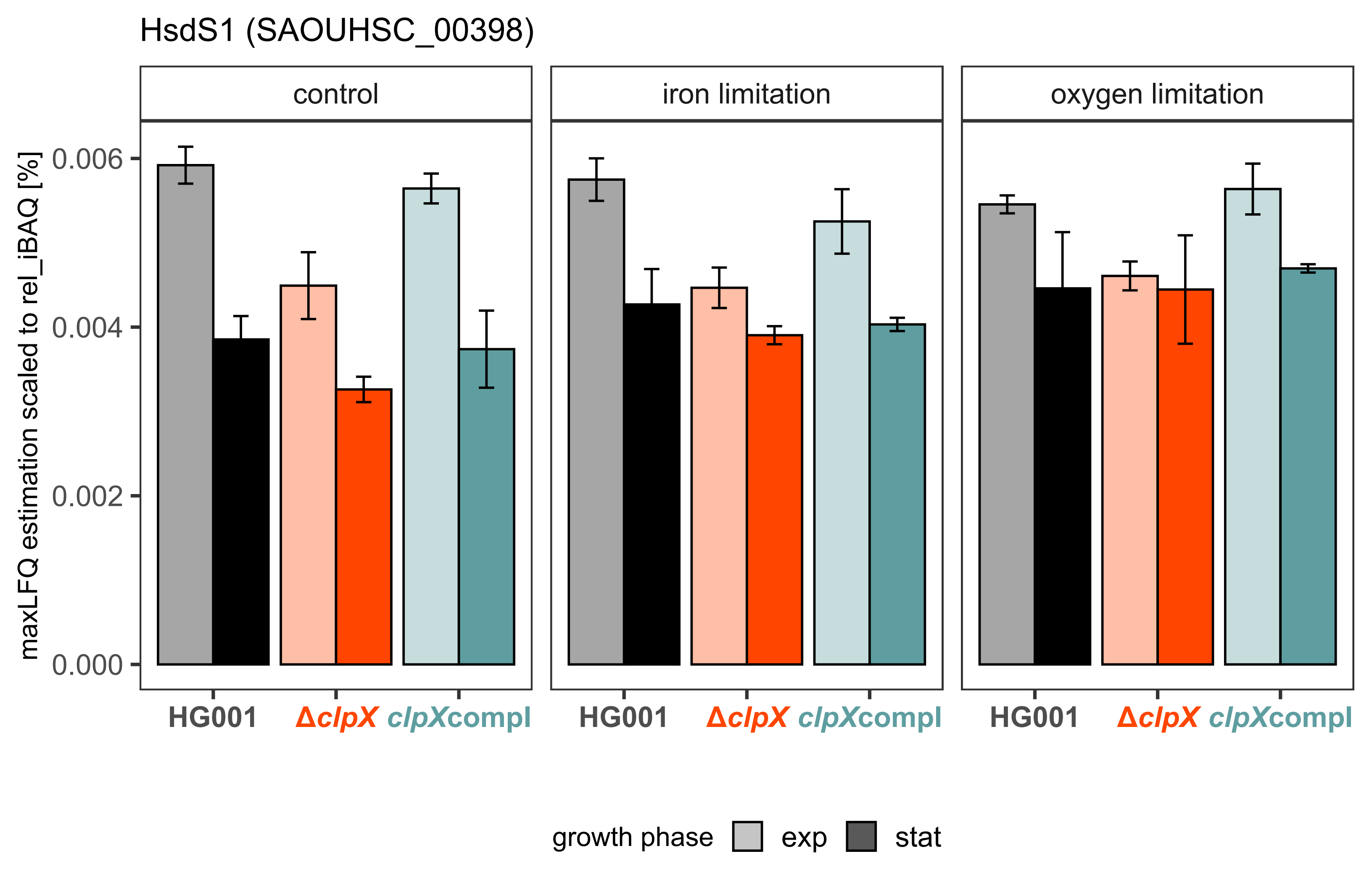

HsdS1 (SAOUHSC_00398)
control
iron limitation
oxygen limitation
maxLFQ estimation scaled to rel_iBAQ [%]
0.006
0.004
0.002
0.000
HG001
ΔclpX
clpXcompl
growth phase
exp
stat

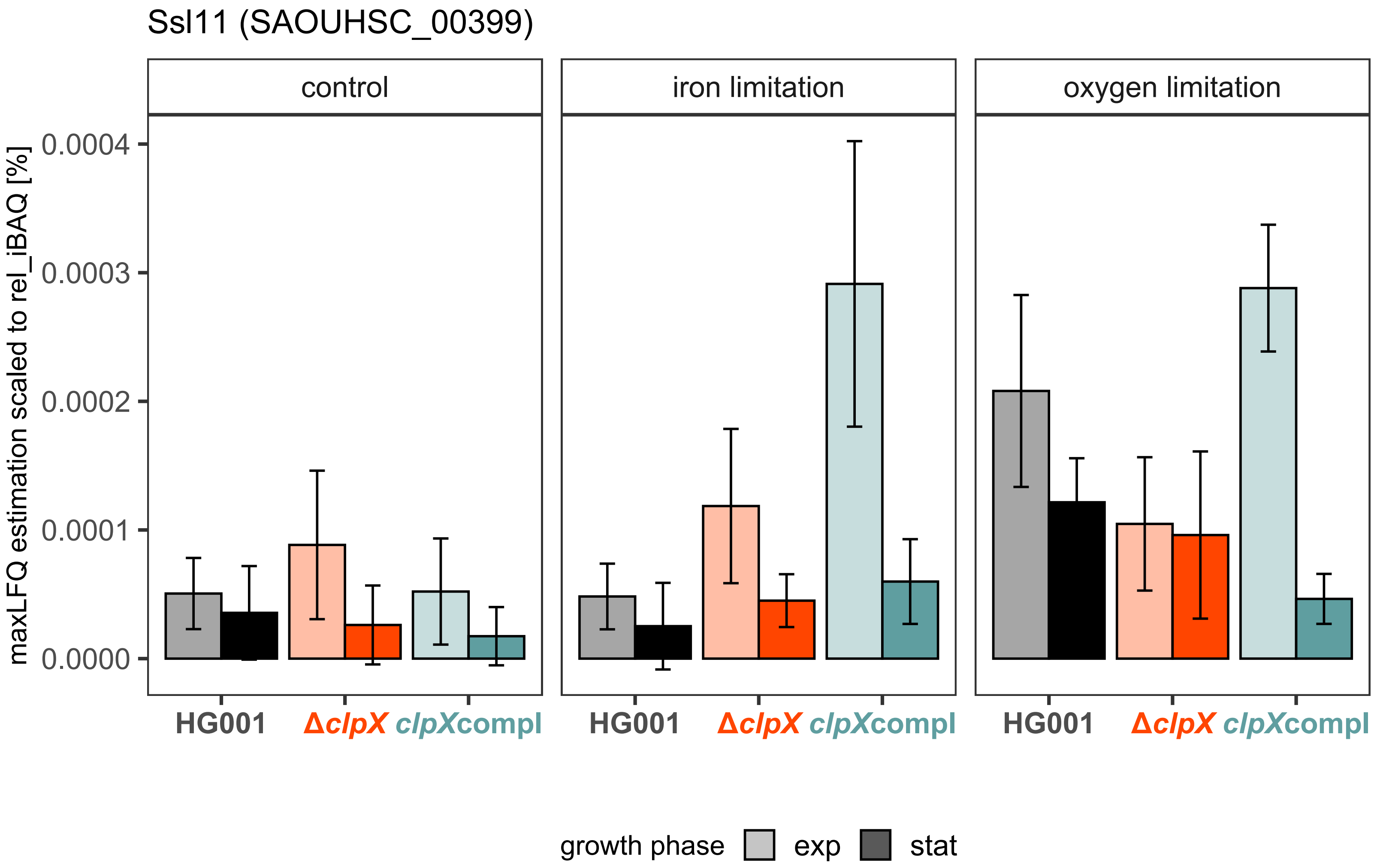

Ssl11 (SAOUHSC_00399)
control
iron limitation
oxygen limitation
maxLFQ estimation scaled to rel_iBAQ [%]
0.0004
0.0003
0.0002
0.0001
0.0000
HG001
ΔclpX
clpXcompl
growth phase
exp
stat

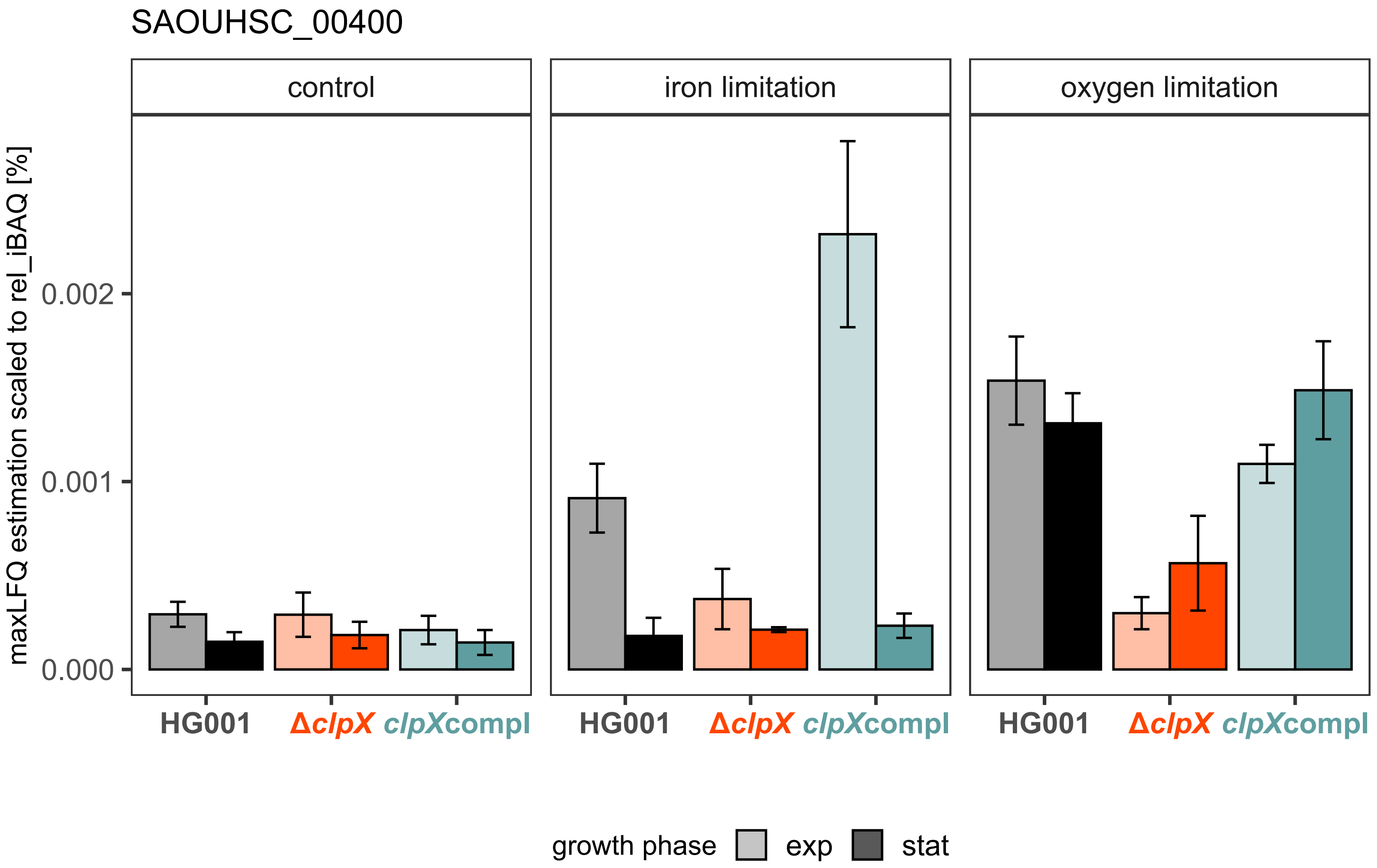
SAOUHSC_00400
control
iron limitation
oxygen limitation
maxLFQ estimation scaled to rel_iBAQ [%]
0.000
0.001
0.002
HG001
ΔclpX
clpXcompl
growth phase
exp
stat

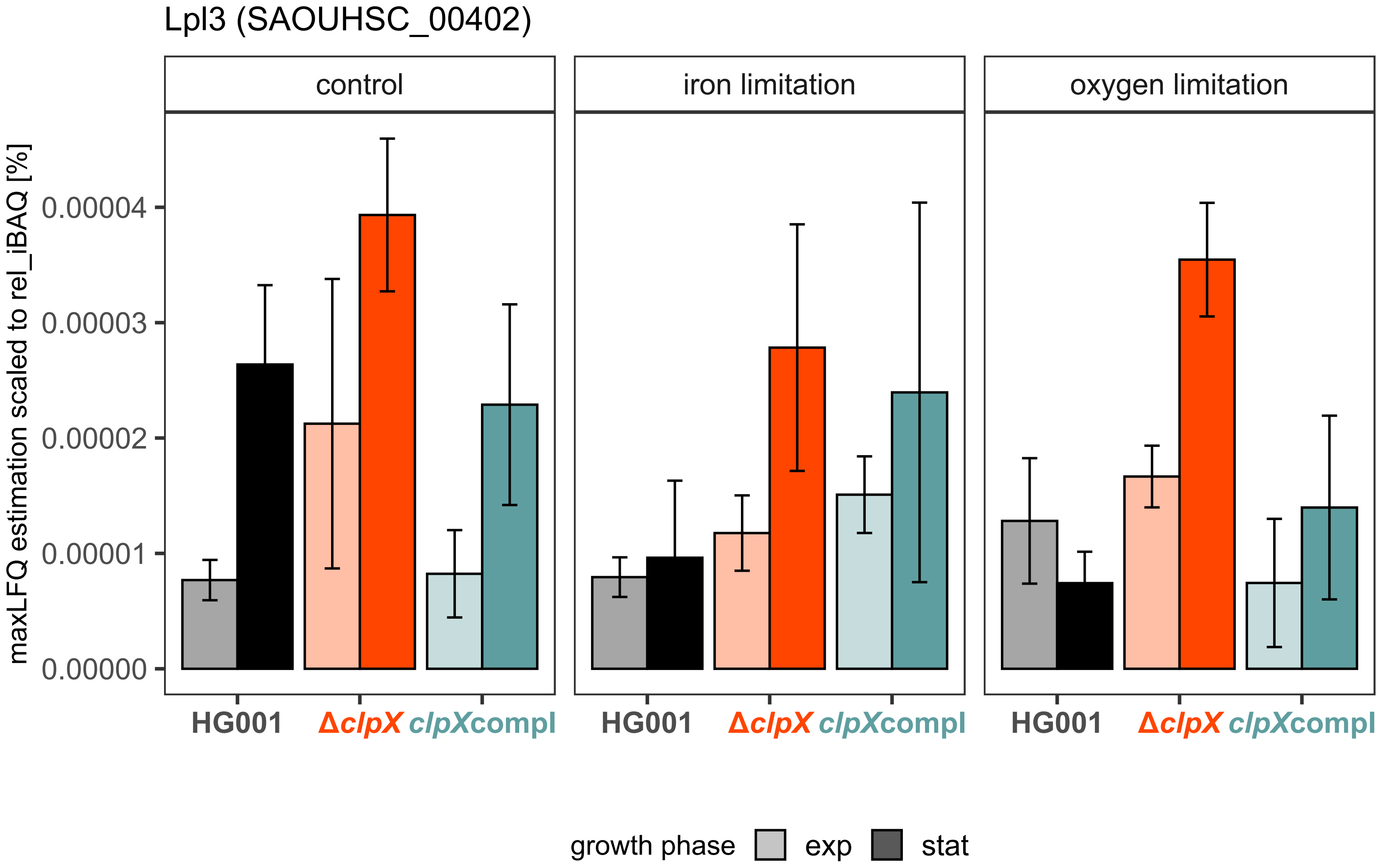
Lpl3 (SAOUHSC_00402)
control
iron limitation
oxygen limitation
maxLFQ estimation scaled to rel_iBAQ [%]
0.00004
0.00003
0.00002
0.00001
0.00000
HG001
ΔclpX
clpXcompl
growth phase
exp
stat

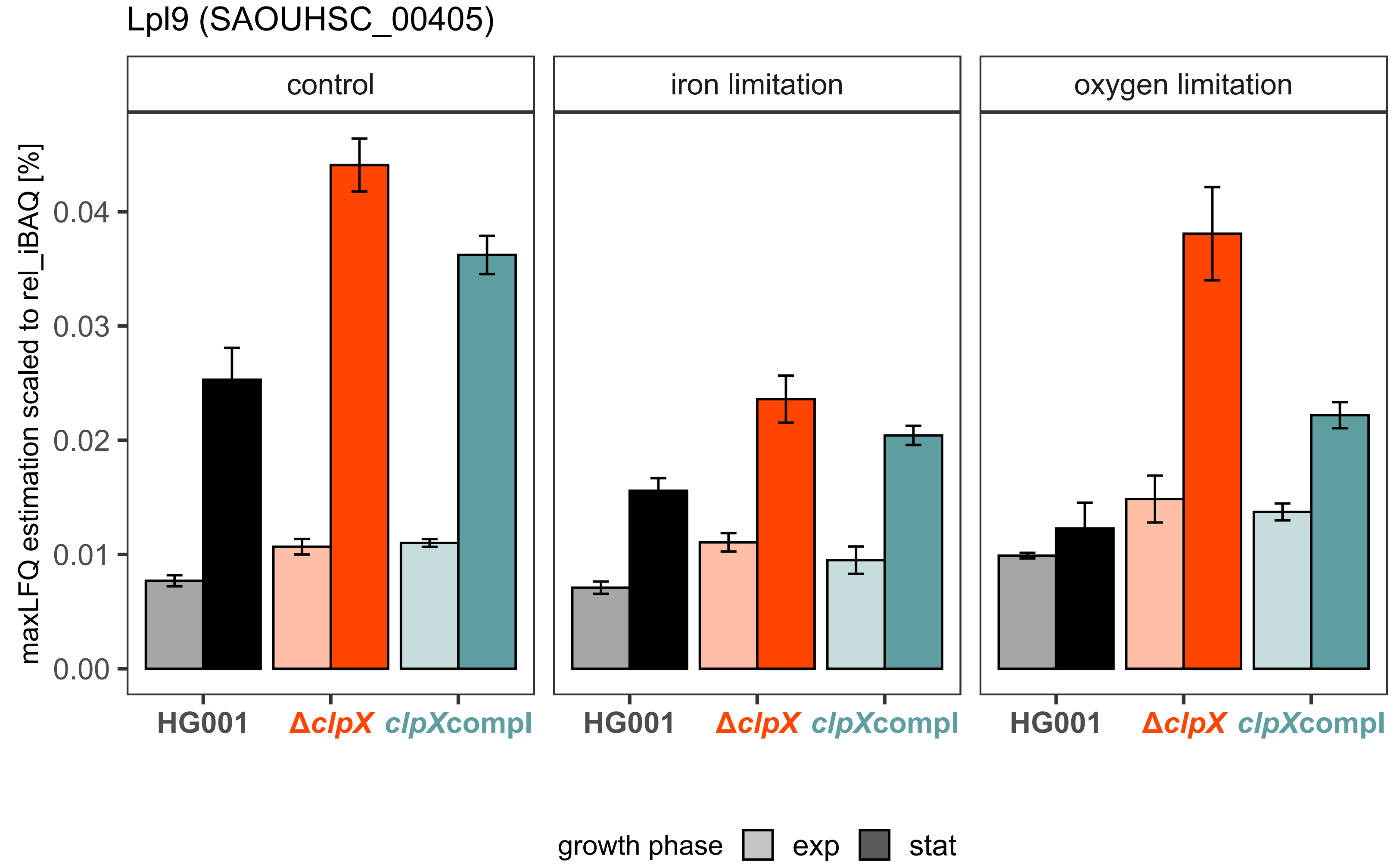
Lpl9 (SAOUHSC_00405)
control
iron limitation
oxygen limitation
maxLFQ estimation scaled to rel_iBAQ [%]
0.00
0.01
0.02
0.03
0.04
HG001
ΔclpX
clpXcompl
growth phase
exp
stat

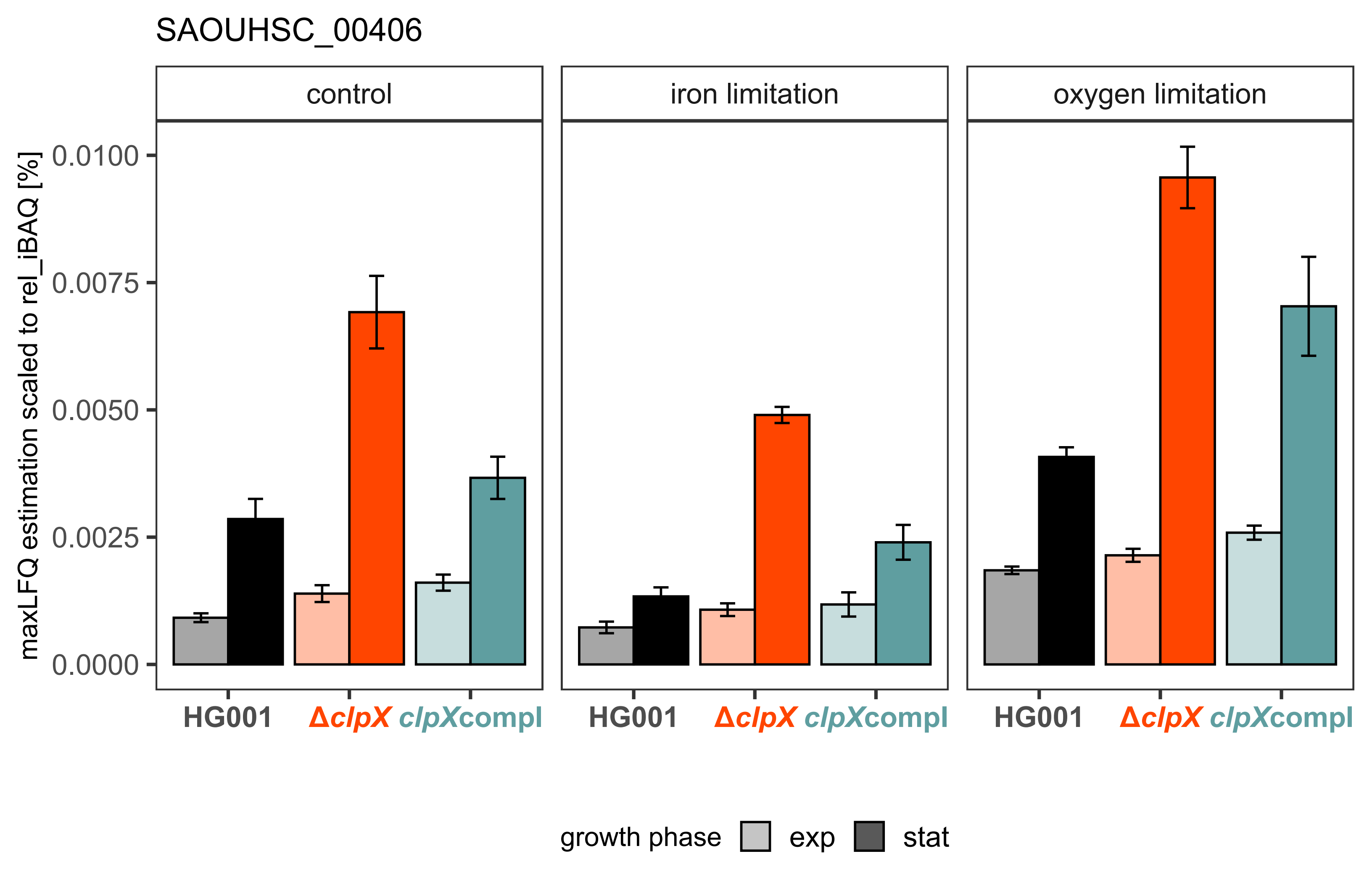

SAOUHSC_00406
control
iron limitation
oxygen limitation
maxLFQ estimation scaled to rel_iBAQ [%]
0.0100
0.0075
0.0050
0.0025
0.0000
HG001
ΔclpX
clpXcompl
growth phase
exp
stat

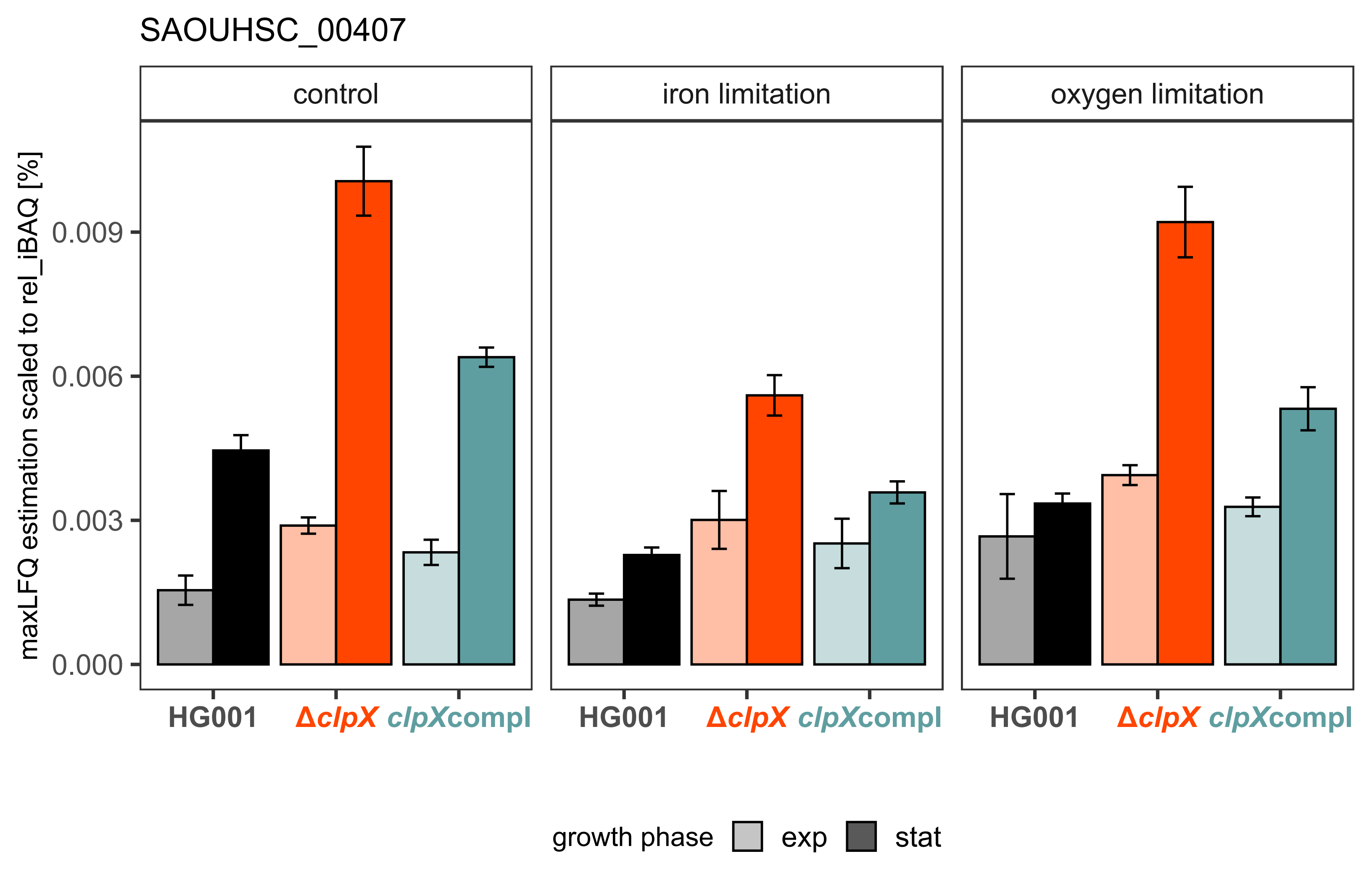

SAOUHSC_00407
control
iron limitation
oxygen limitation
maxLFQ estimation scaled to rel_iBAQ [%]
0.009
0.006
0.003
0.000
HG001
ΔclpX
clpXcompl
growth phase
exp
stat

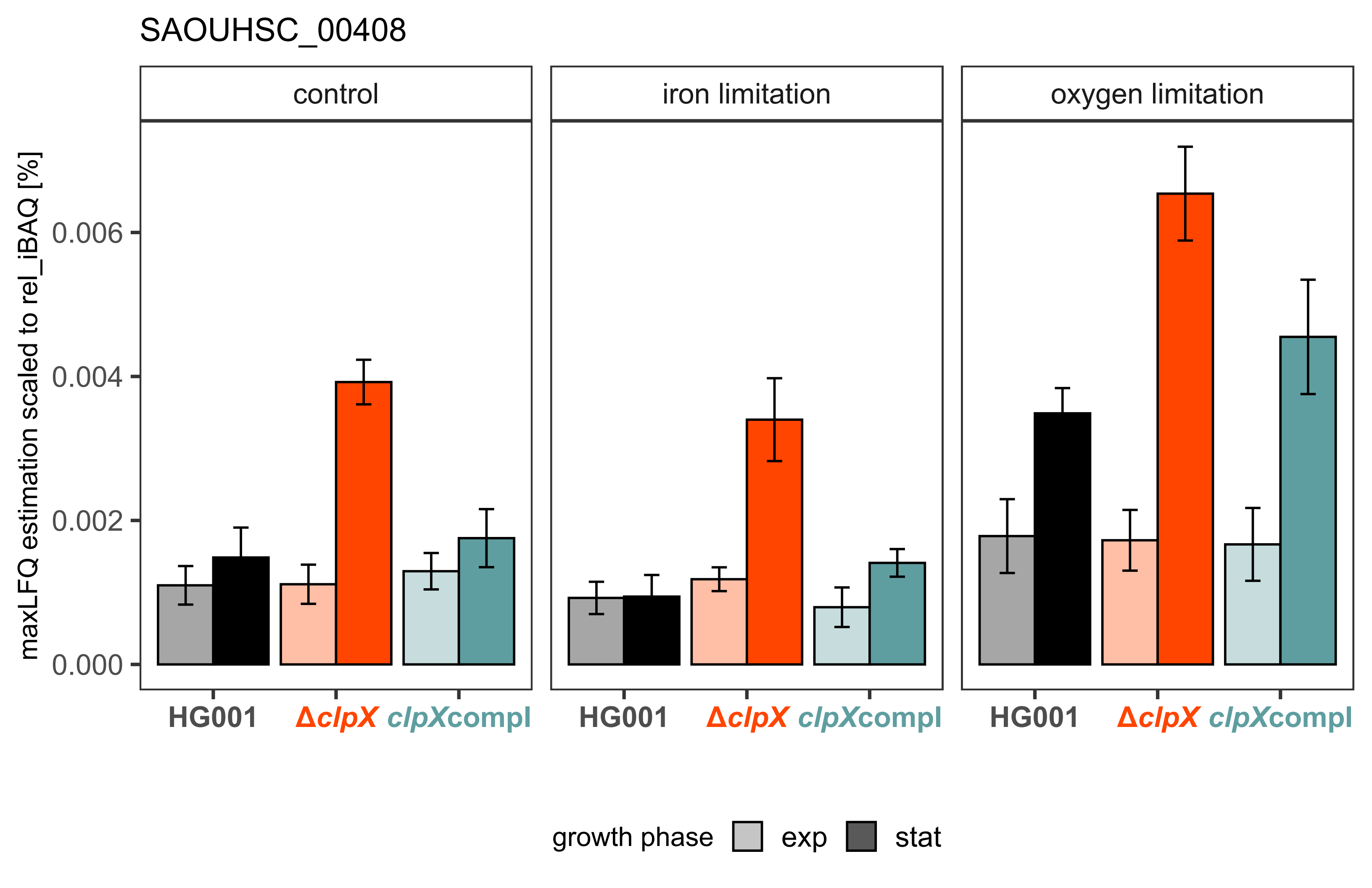
SAOUHSC_00408
control
iron limitation
oxygen limitation
maxLFQ estimation scaled to rel_iBAQ [%]
0.006
0.004
0.002
0.000
HG001
ΔclpX
clpXcompl
growth phase
exp
stat

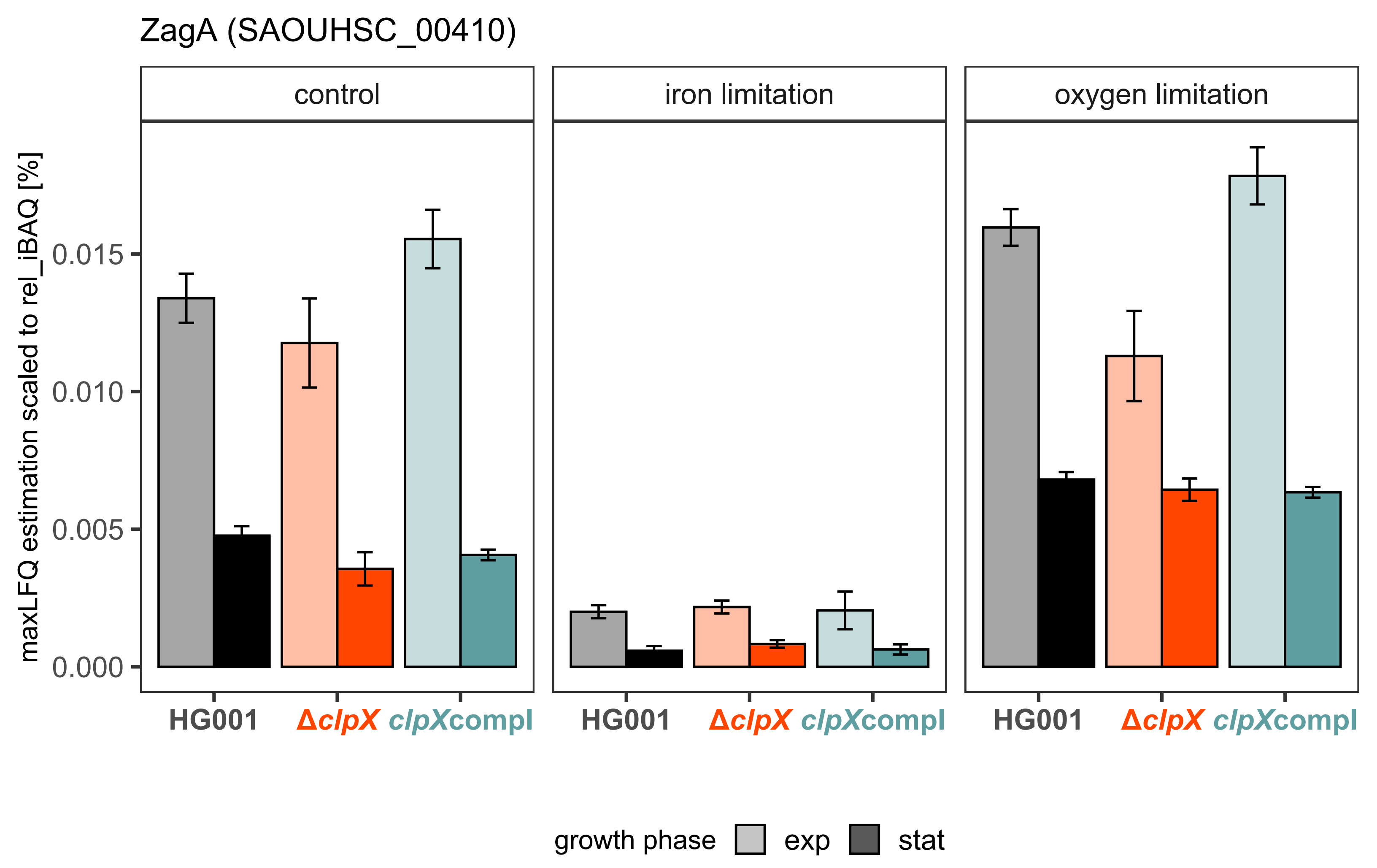
ZagA (SAOUHSC_00410)
control
iron limitation
oxygen limitation
maxLFQ estimation scaled to rel_iBAQ [%]
0.015
0.010
0.005
0.000
HG001
ΔclpX
clpXcompl
growth phase
exp
stat

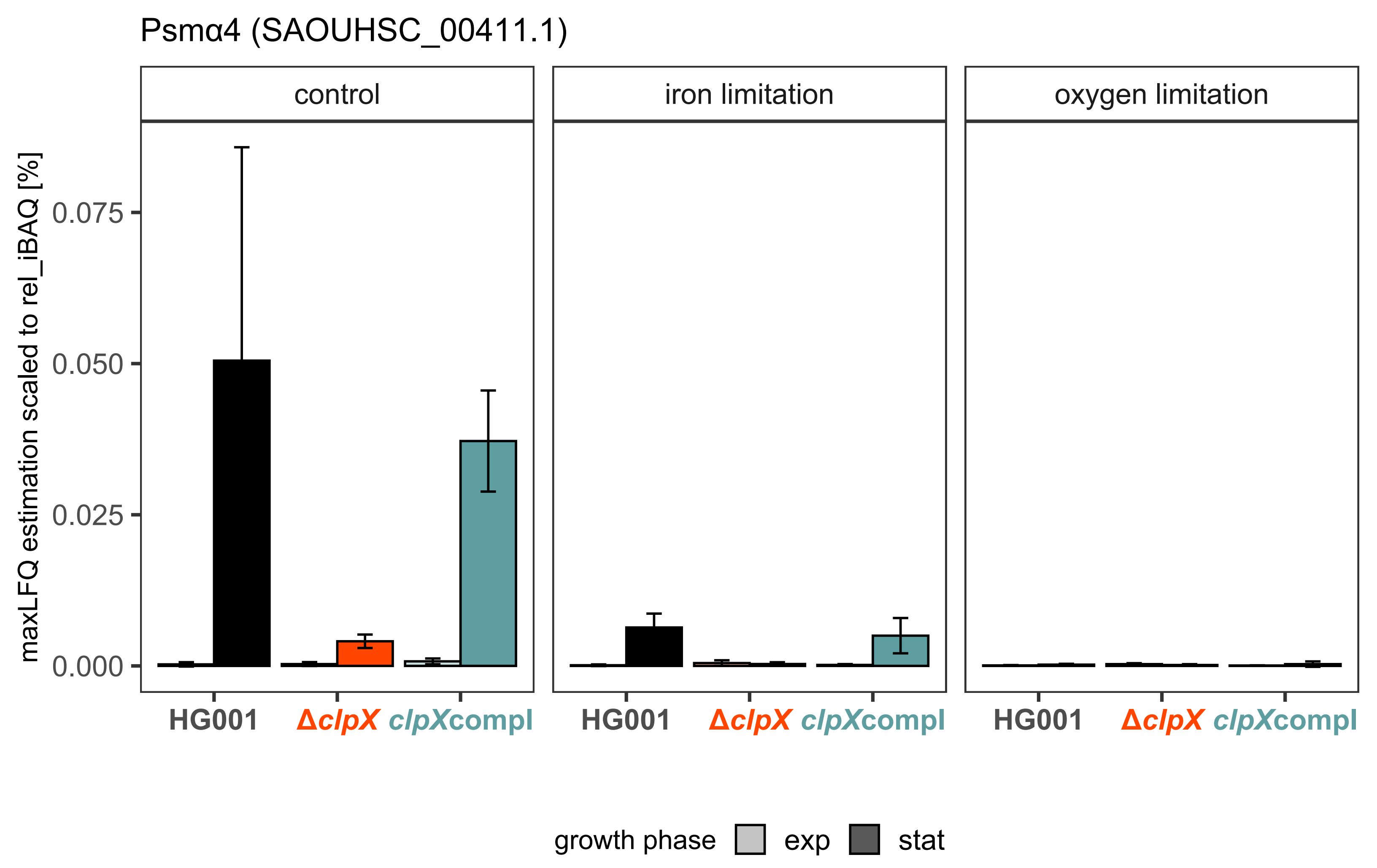
Psmα4 (SAOUHSC_00411.1)
control
iron limitation
oxygen limitation
maxLFQ estimation scaled to rel_iBAQ [%]
0.075
0.050
0.025
0.000
HG001
ΔclpX
clpXcompl
growth phase
exp
stat

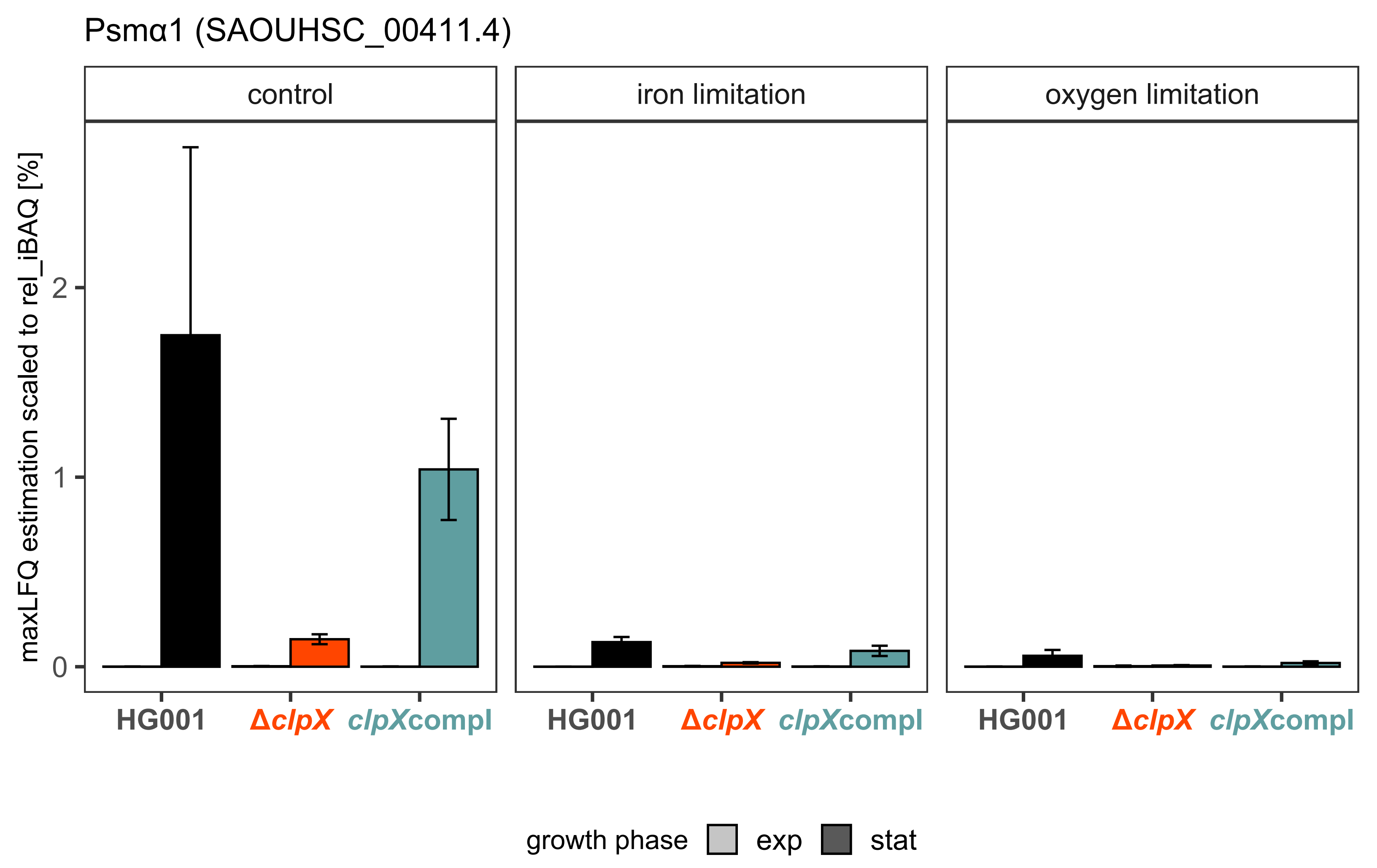
Psmα1 (SAOUHSC_00411.4)
control
iron limitation
oxygen limitation
maxLFQ estimation scaled to rel_iBAQ [%]
0
1
2
HG001
ΔclpX
clpXcompl
growth phase
exp
stat

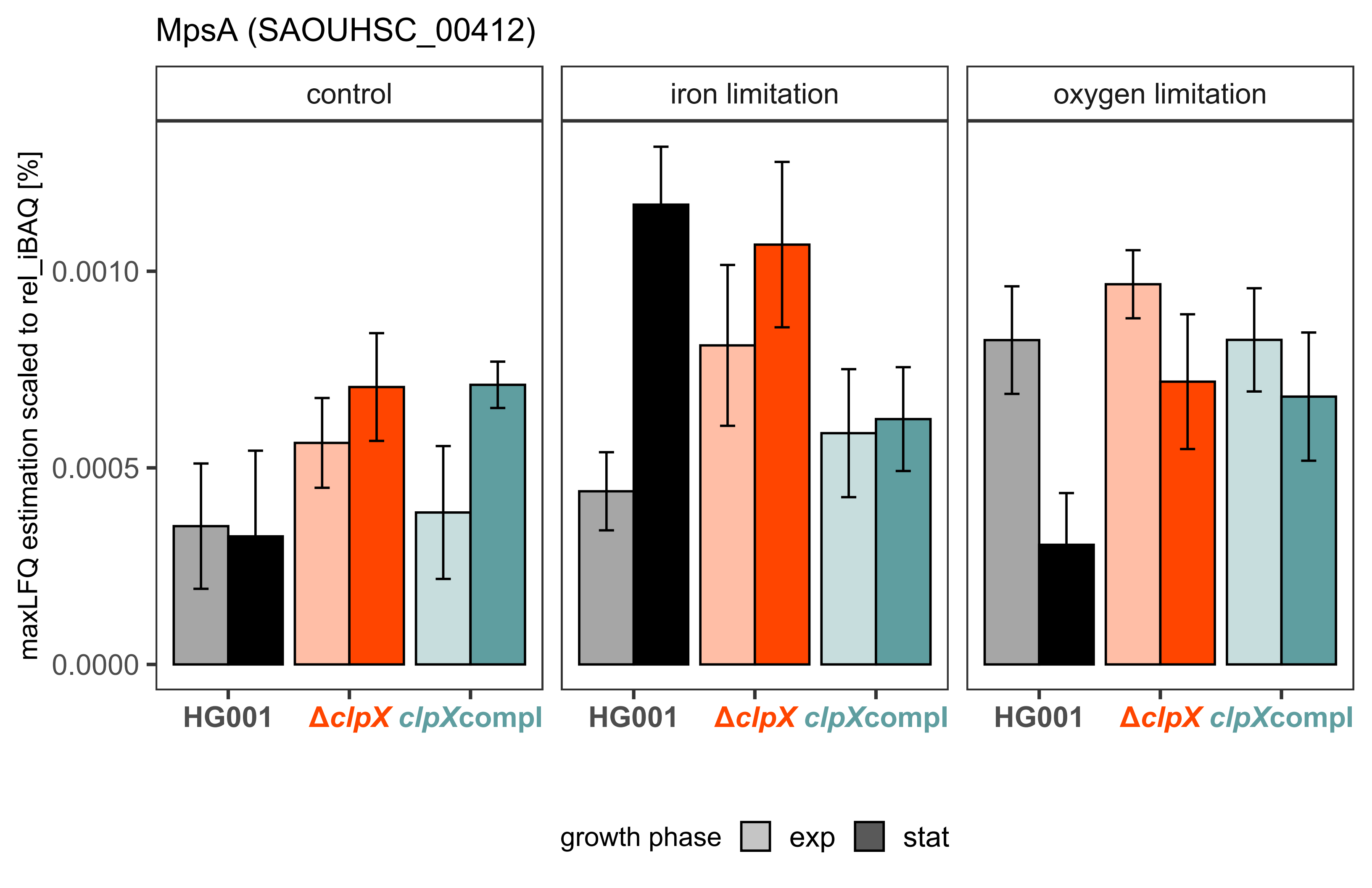

MpsA (SAOUHSC_00412)
control
iron limitation
oxygen limitation
maxLFQ estimation scaled to rel_iBAQ [%]
0.0010
0.0005
0.0000
HG001
ΔclpX
clpXcompl
growth phase
exp
stat

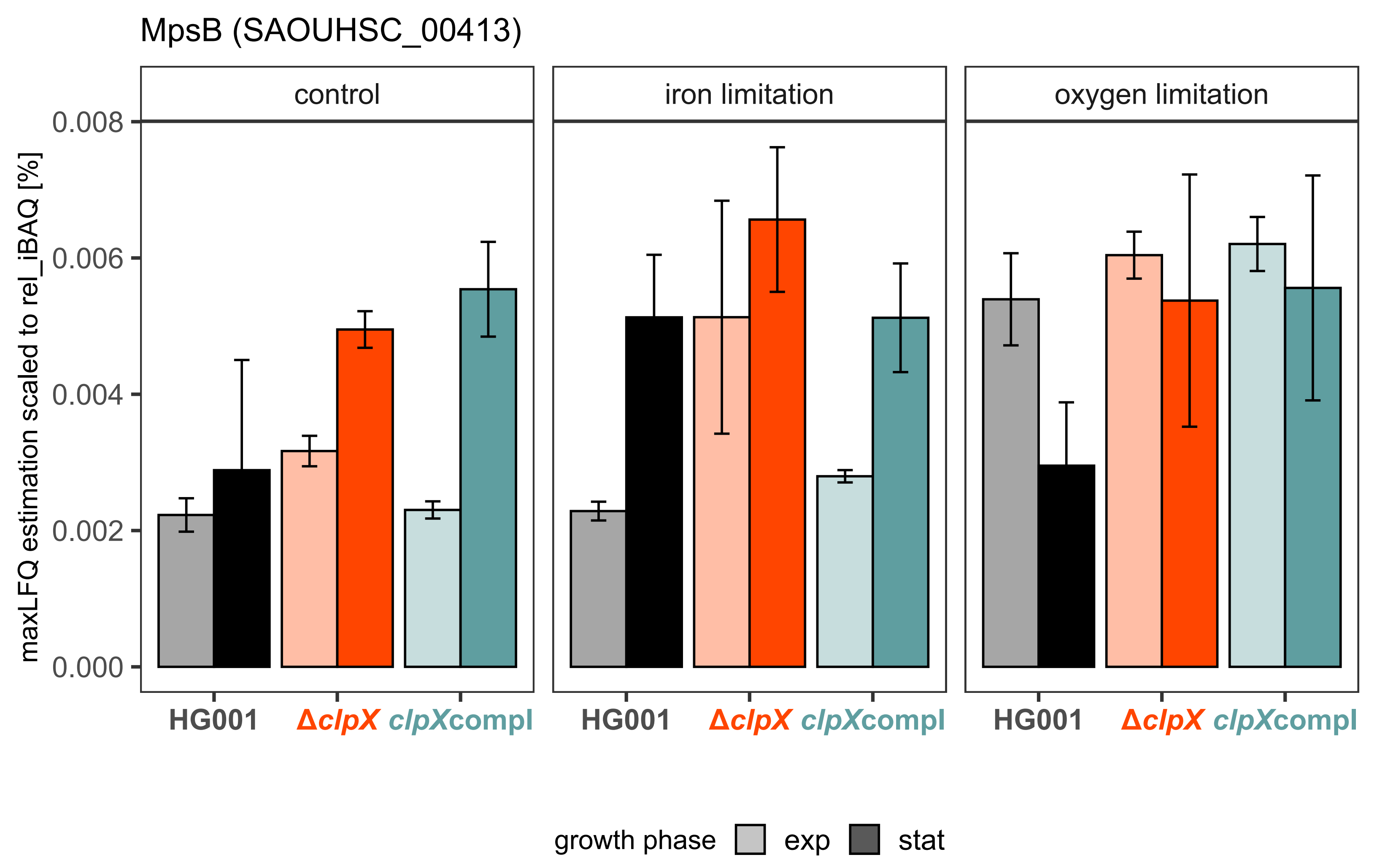

MpsB (SAOUHSC_00413)
control
iron limitation
oxygen limitation
maxLFQ estimation scaled to rel_iBAQ [%]
0.008
0.006
0.004
0.002
0.000
HG001
ΔclpX
clpXcompl
growth phase
exp
stat

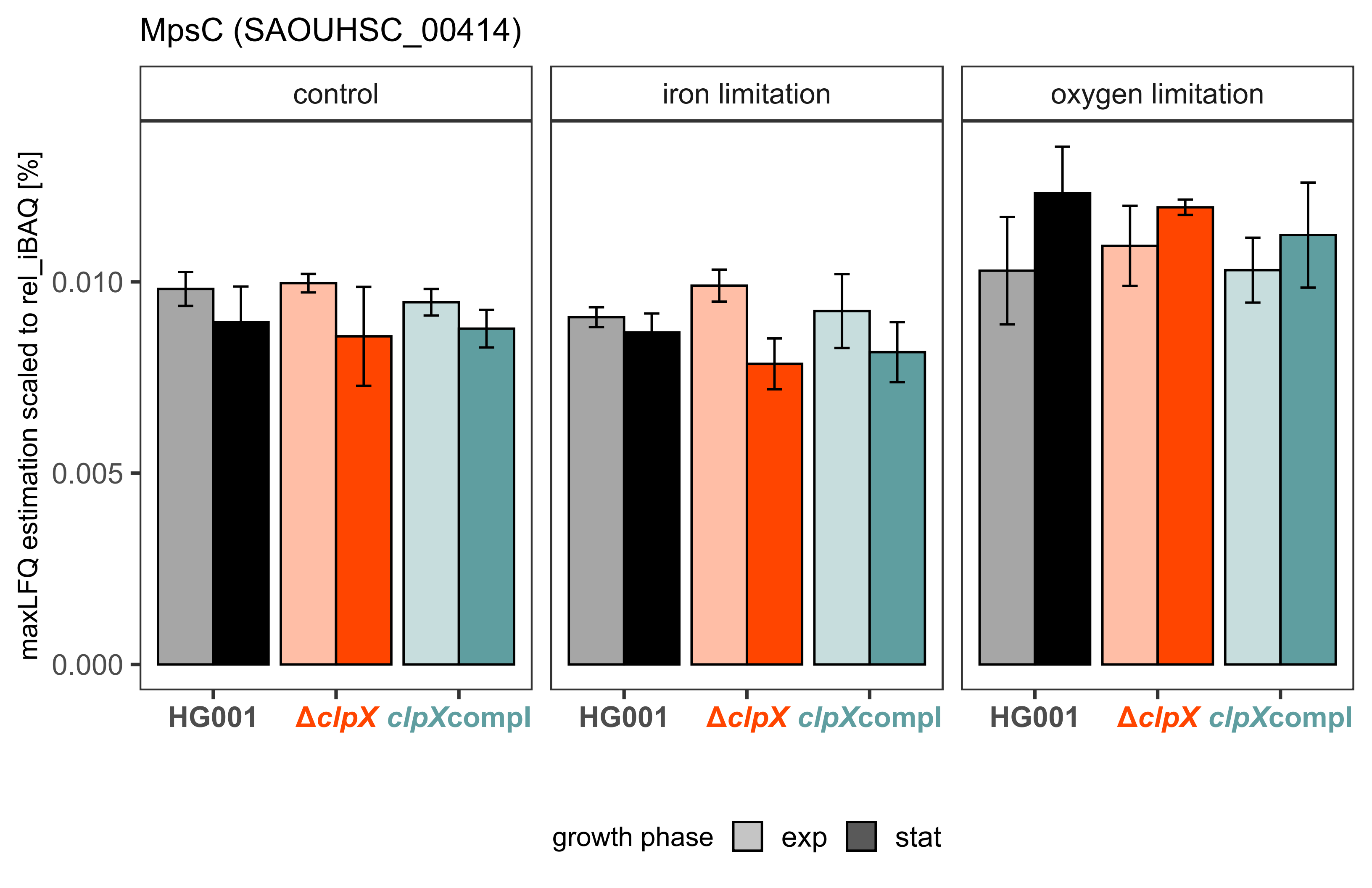

MpsC (SAOUHSC_00414)
control
iron limitation
oxygen limitation
maxLFQ estimation scaled to rel_iBAQ [%]
0.010
0.005
0.000
HG001
ΔclpX
clpXcompl
growth phase
exp
stat

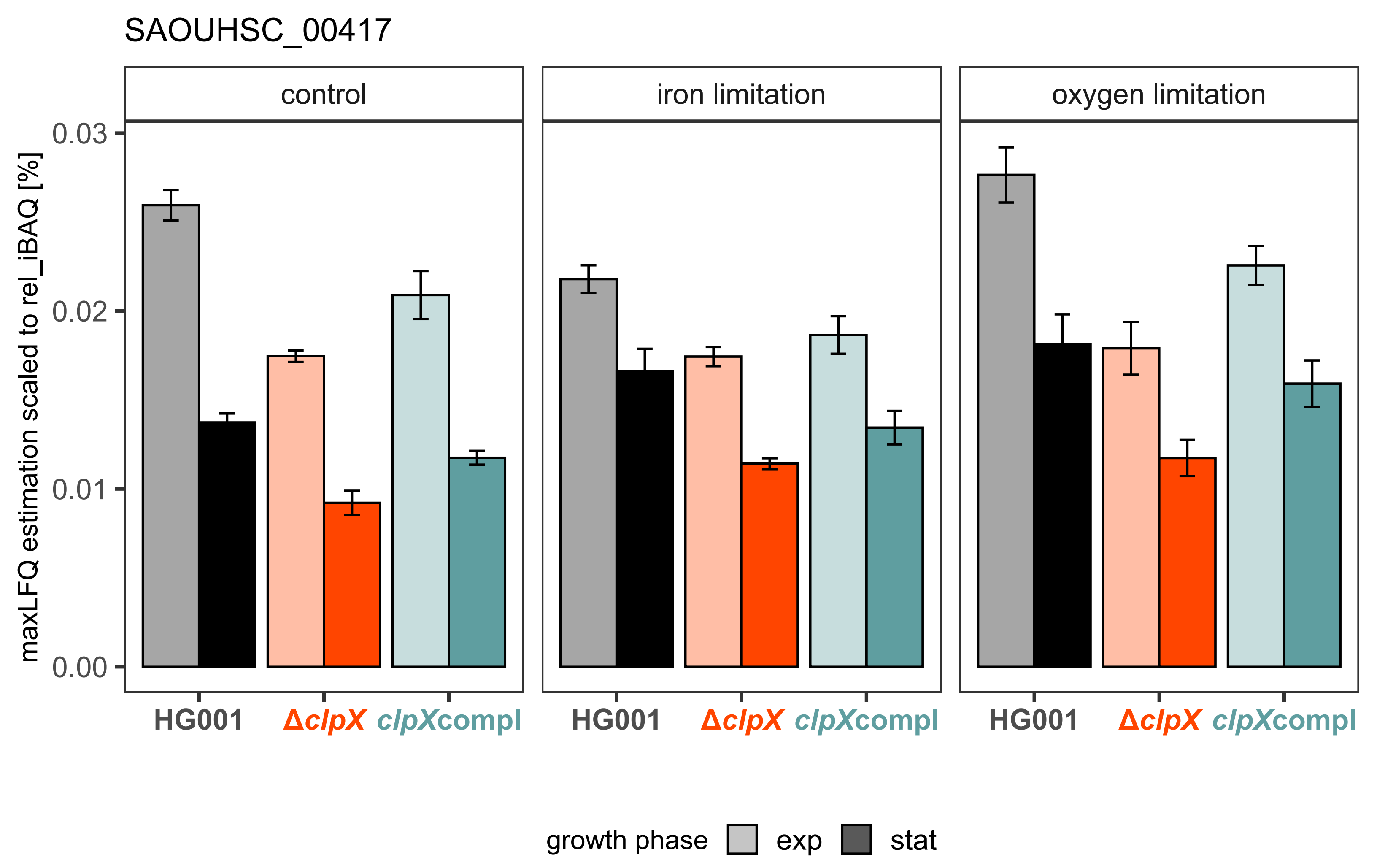
SAOUHSC_00417
control
iron limitation
oxygen limitation
maxLFQ estimation scaled to rel_iBAQ [%]
0.00
0.01
0.02
0.03
HG001
ΔclpX
clpXcompl
growth phase
exp
stat

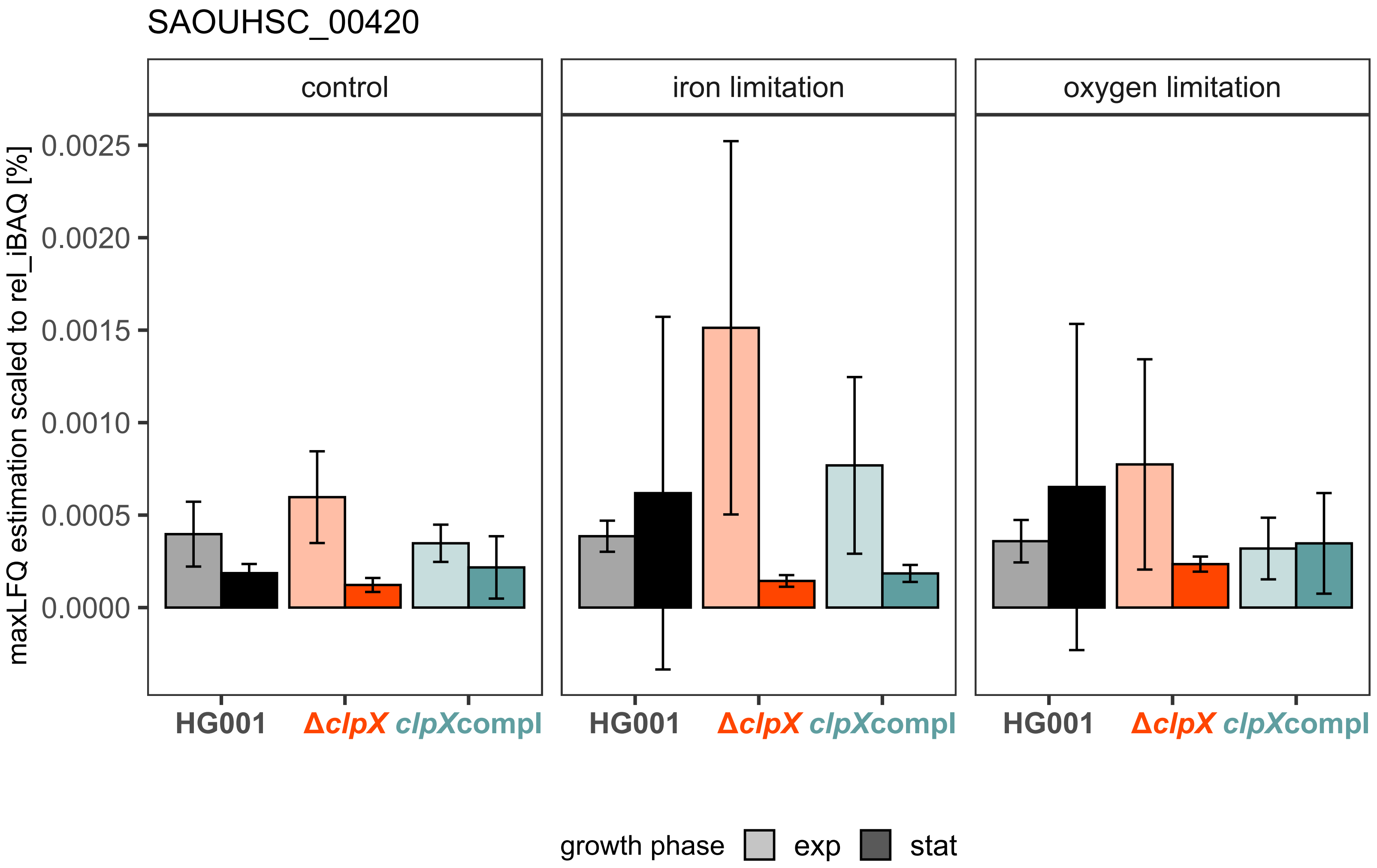
SAOUHSC_00420
control
iron limitation
oxygen limitation
maxLFQ estimation scaled to rel_iBAQ [%]
0.0025
0.0020
0.0015
0.0010
0.0005
0.0000
HG001
ΔclpX
clpXcompl
growth phase
exp
stat

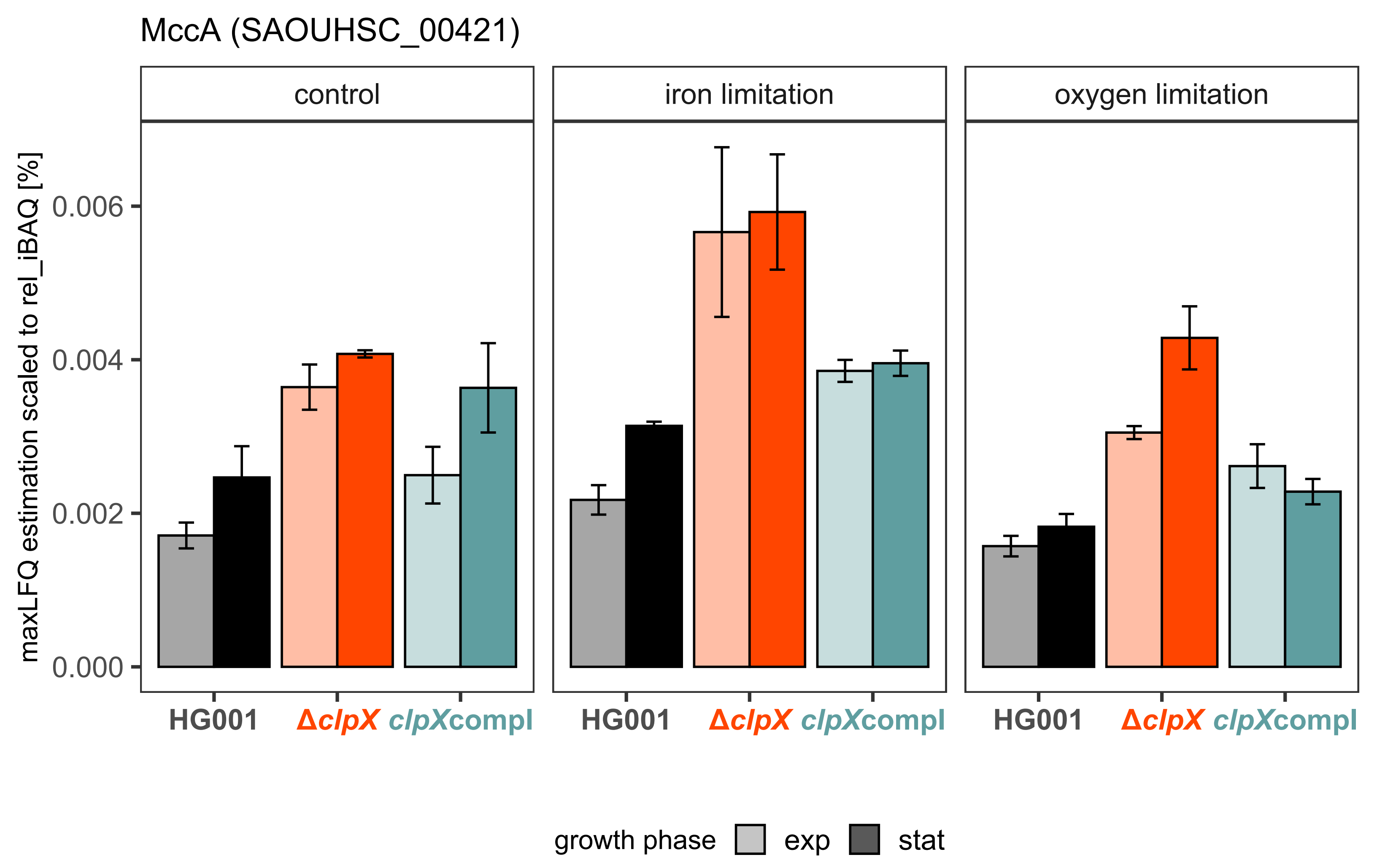

MccA (SAOUHSC_00421)
control
iron limitation
oxygen limitation
maxLFQ estimation scaled to rel_iBAQ [%]
0.006
0.004
0.002
0.000
HG001
ΔclpX
clpXcompl
growth phase
exp
stat

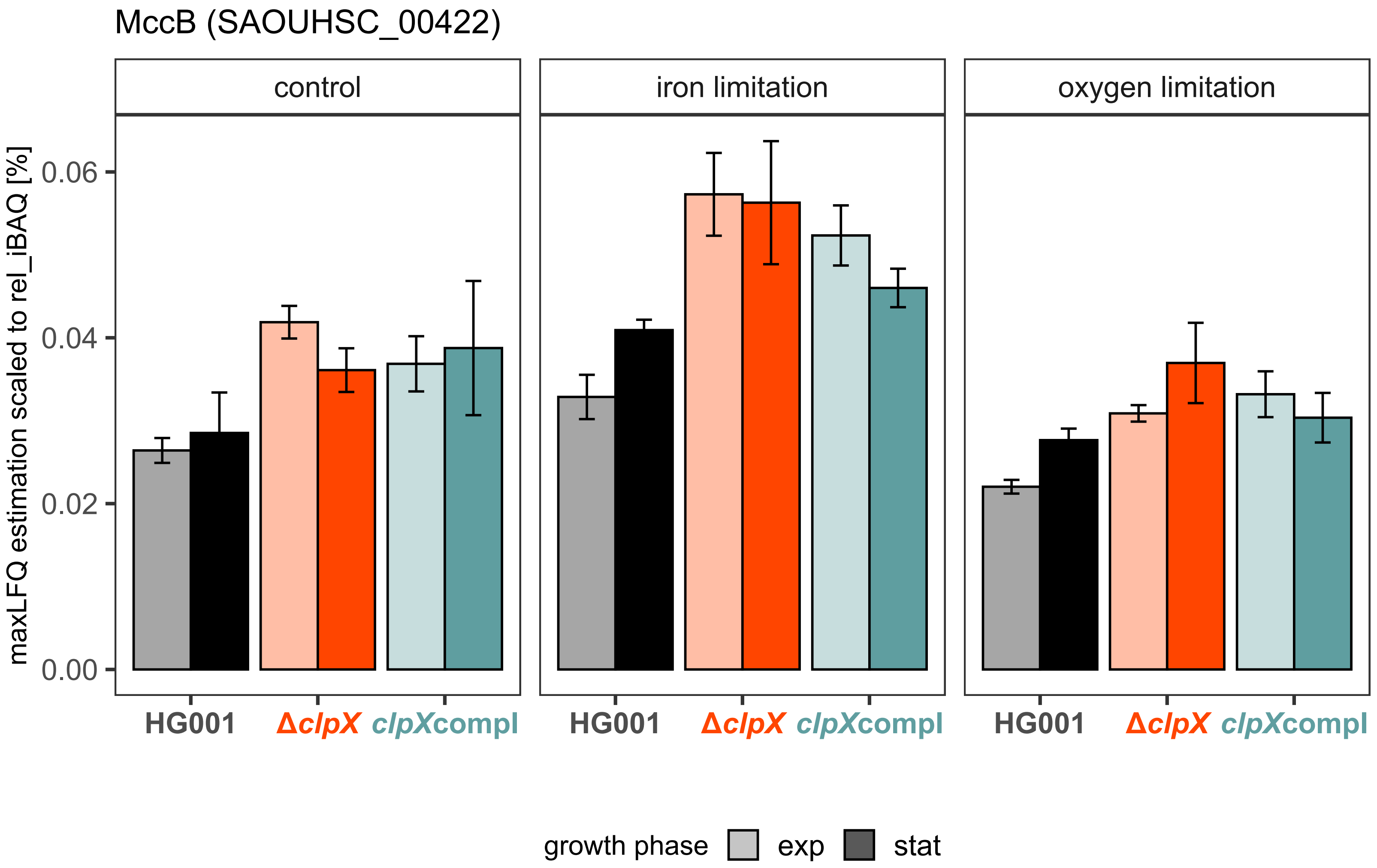
MccB (SAOUHSC_00422)
control
iron limitation
oxygen limitation
maxLFQ estimation scaled to rel_iBAQ [%]
0.06
0.04
0.02
0.00
HG001
ΔclpX
clpXcompl
growth phase
exp
stat

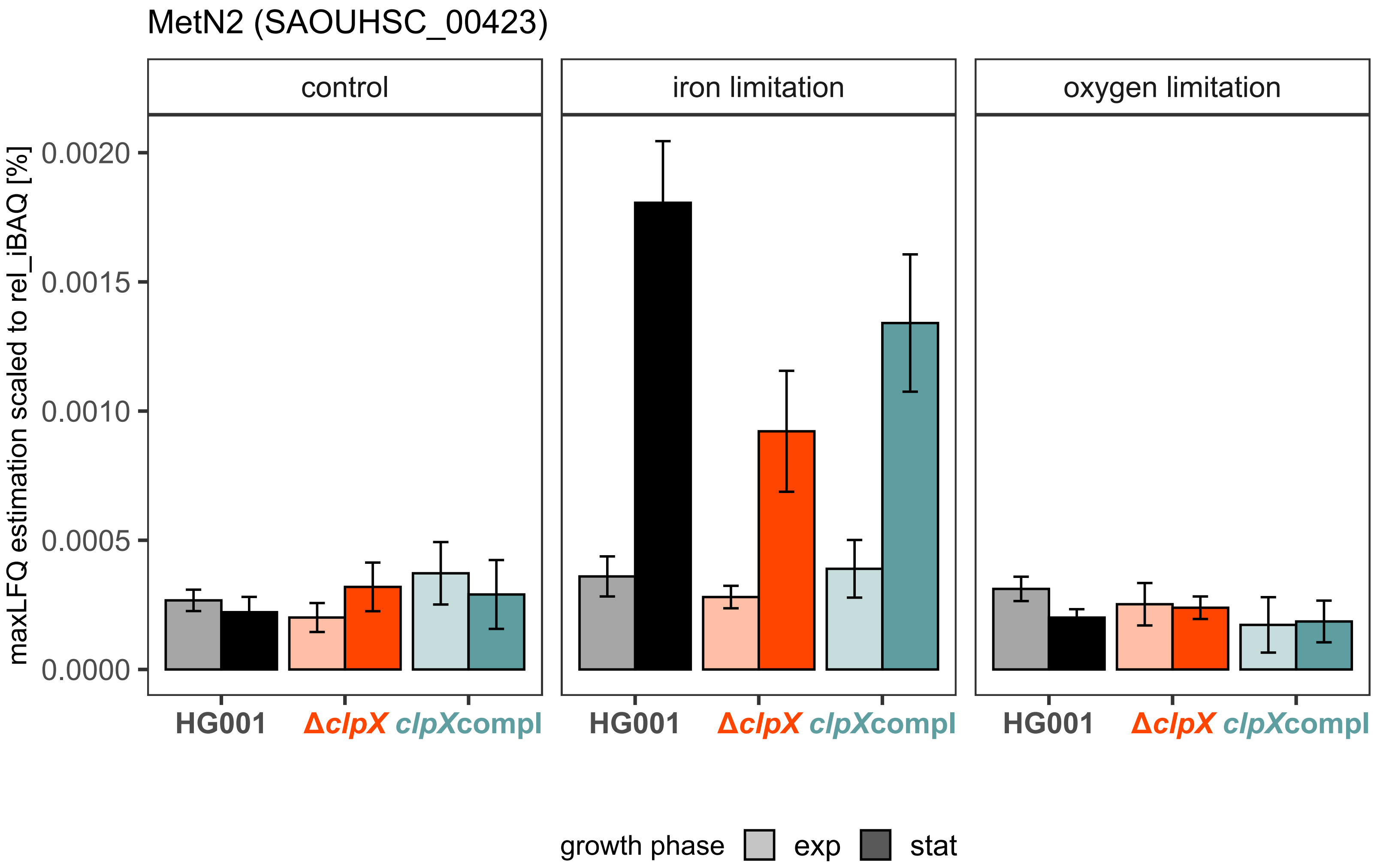

MetN2 (SAOUHSC_00423)
control
iron limitation
oxygen limitation
maxLFQ estimation scaled to rel_iBAQ [%]
0.0020
0.0015
0.0010
0.0005
0.0000
HG001
ΔclpX
clpXcompl
growth phase
exp
stat

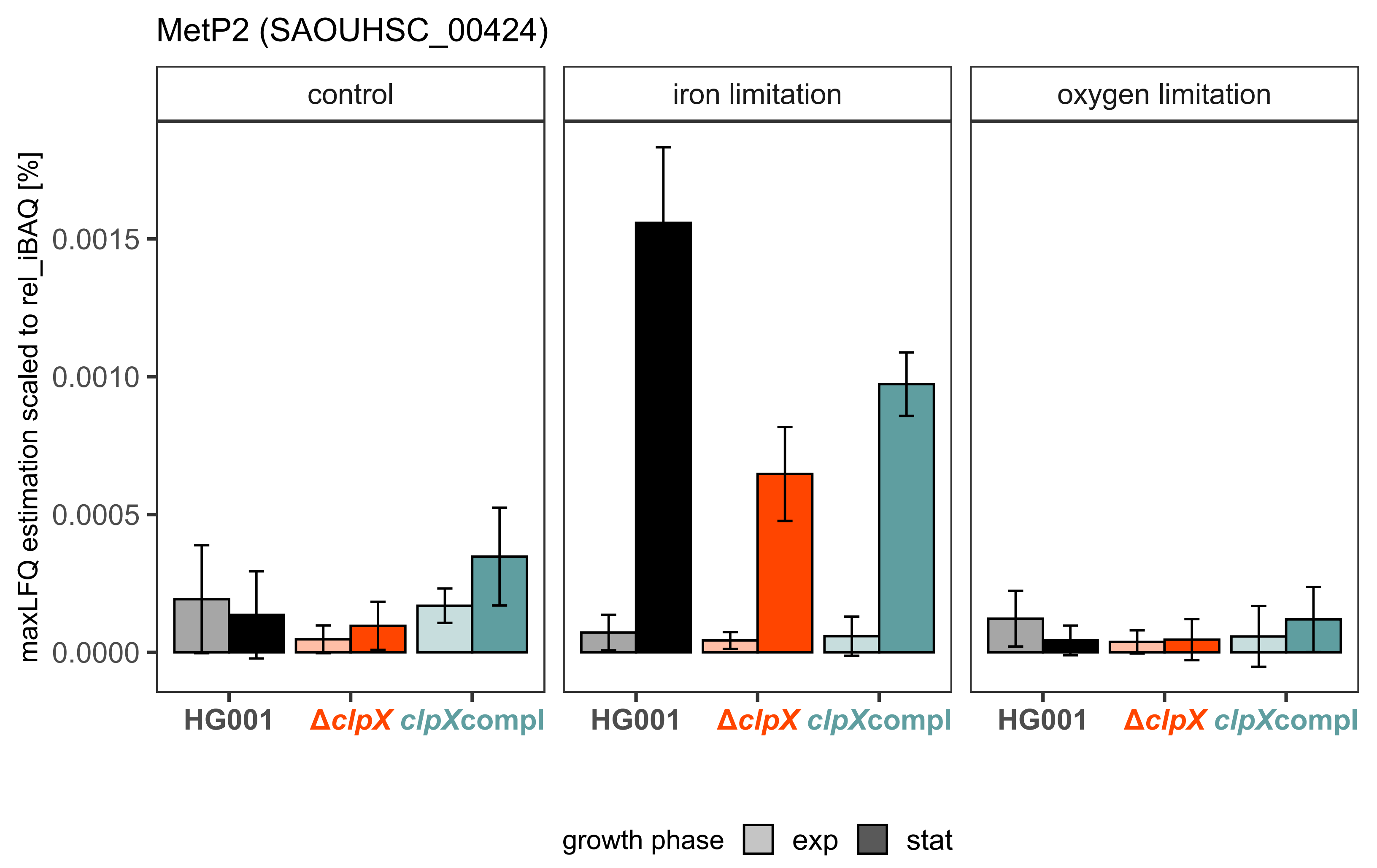
MetP2 (SAOUHSC_00424)
control
iron limitation
oxygen limitation
maxLFQ estimation scaled to rel_iBAQ [%]
0.0015
0.0010
0.0005
0.0000
HG001
ΔclpX
clpXcompl
growth phase
exp
stat

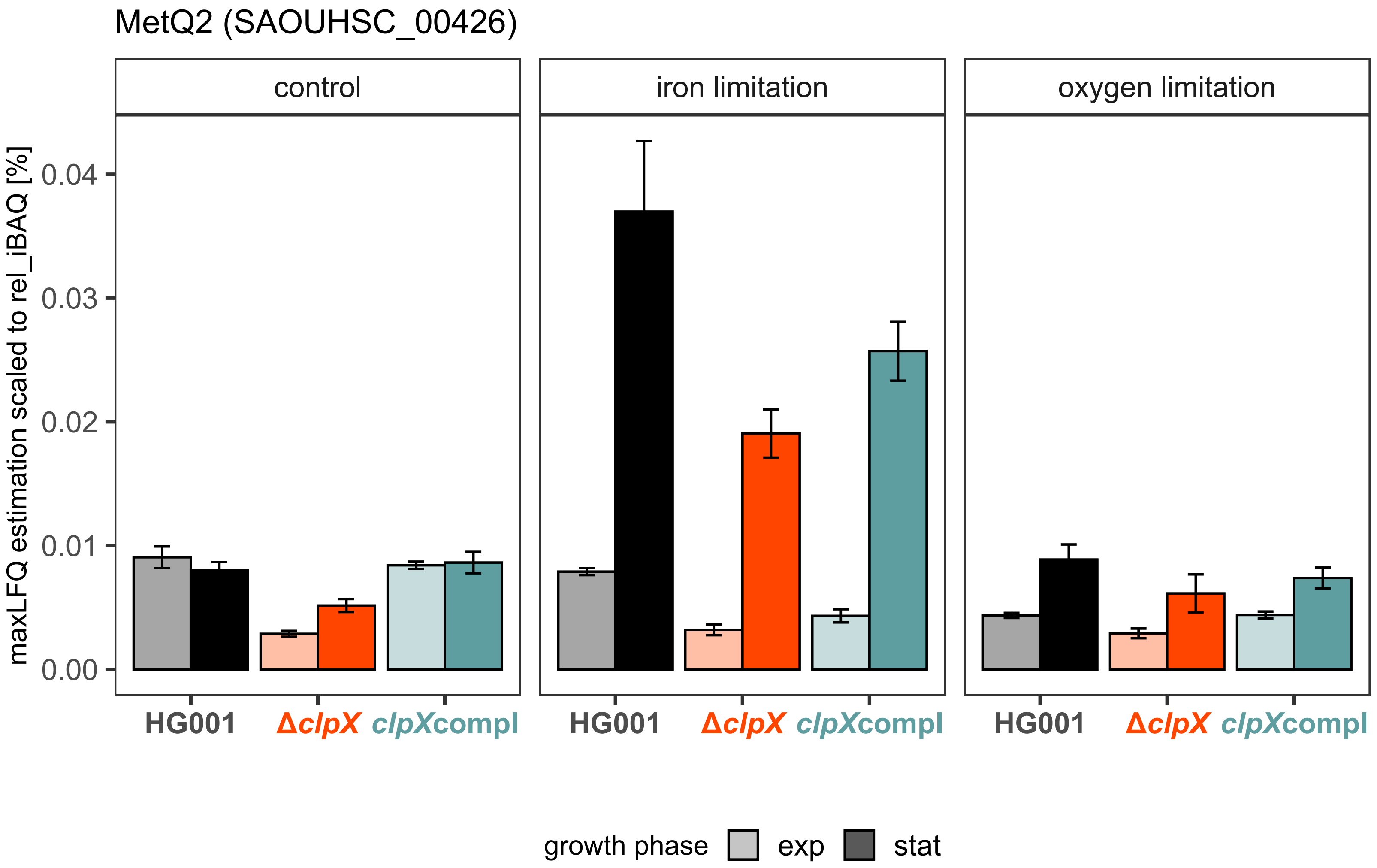
MetQ2 (SAOUHSC_00426)
control
iron limitation
oxygen limitation
maxLFQ estimation scaled to rel_iBAQ [%]
0.04
0.03
0.02
0.01
0.00
HG001
ΔclpX
clpXcompl
growth phase
exp
stat

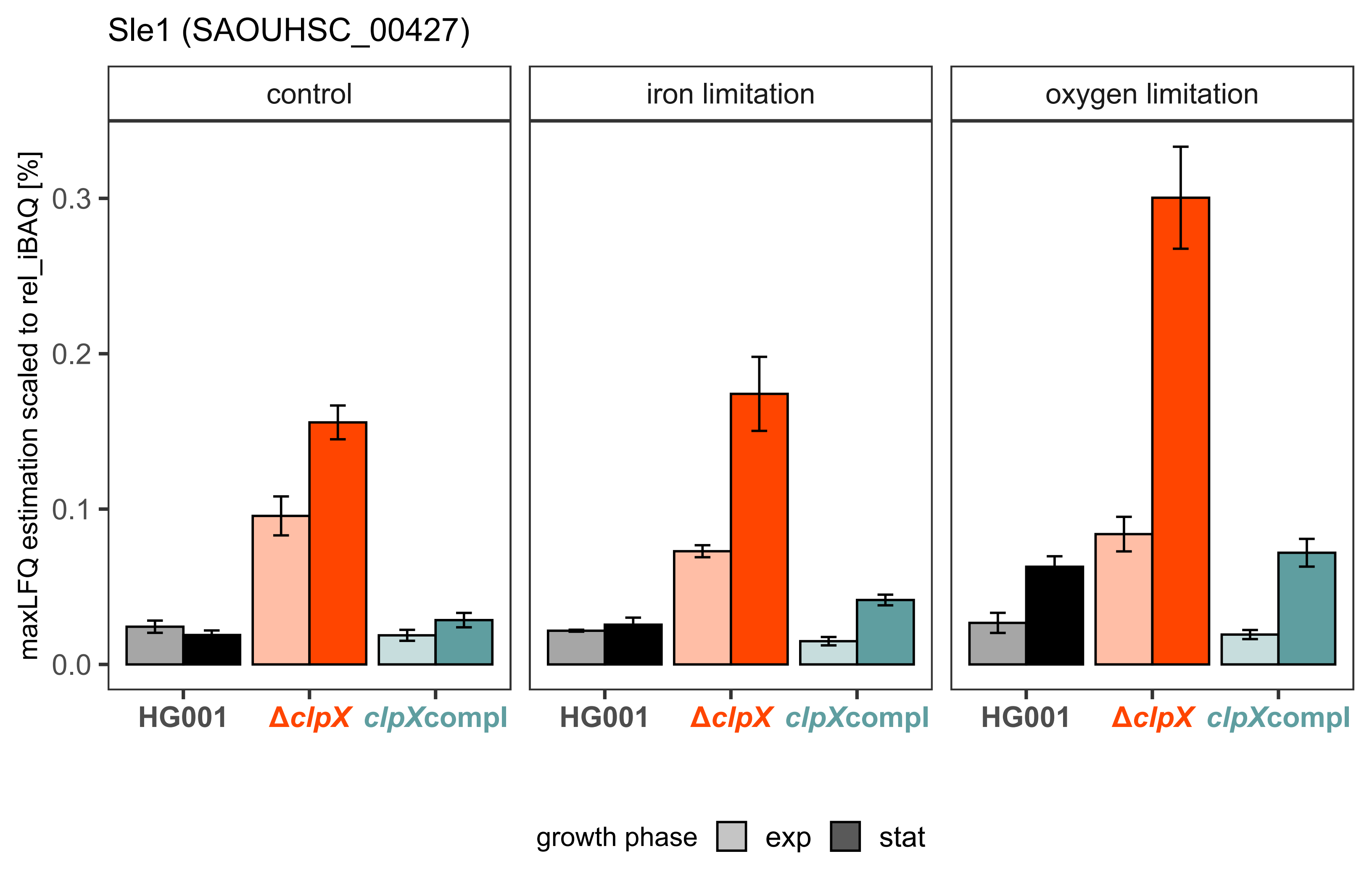

Sle1 (SAOUHSC_00427)
control
iron limitation
oxygen limitation
maxLFQ estimation scaled to rel_iBAQ [%]
0.0
0.1
0.2
0.3
HG001
ΔclpX
clpXcompl
growth phase
exp
stat

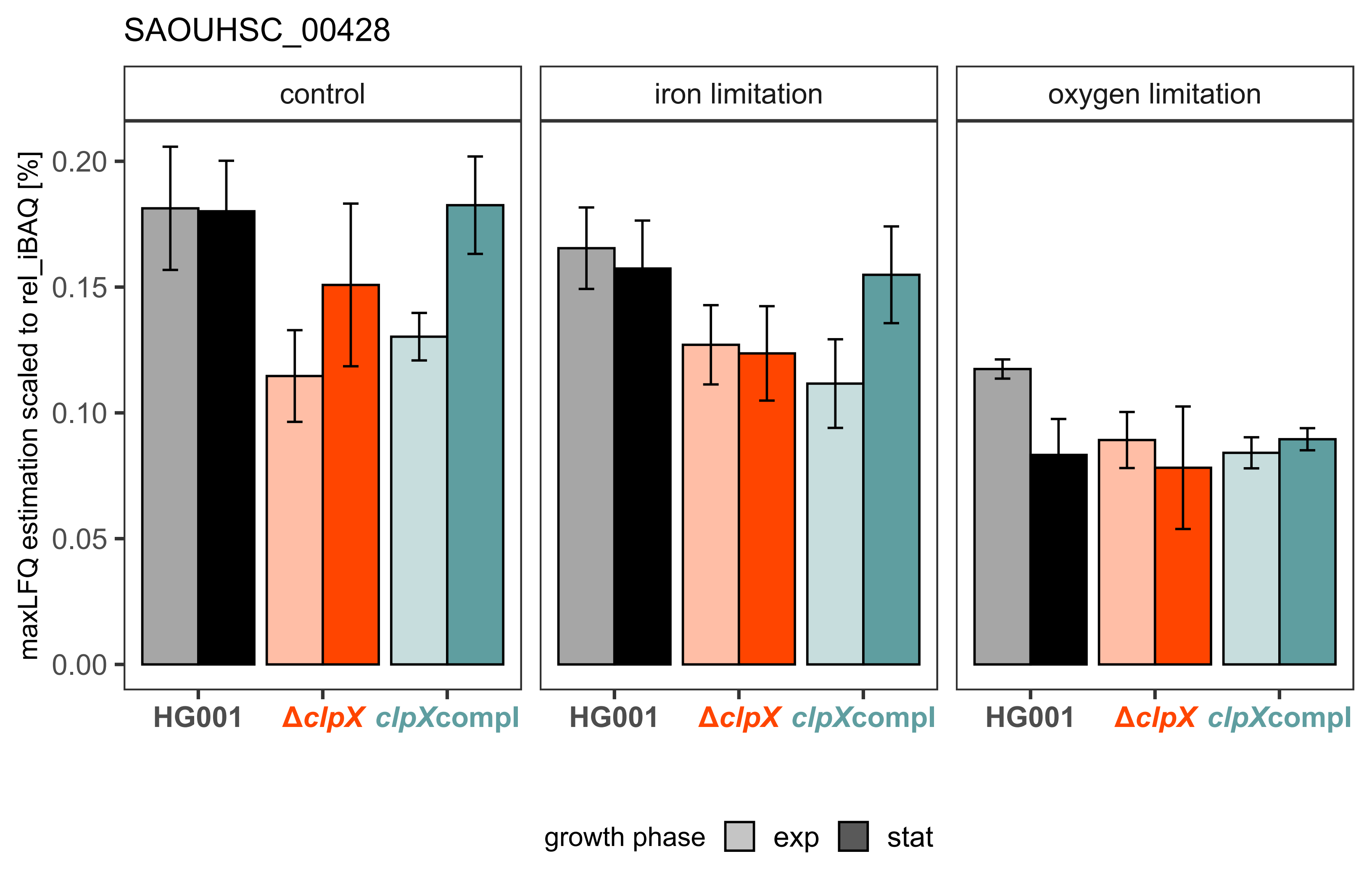
SAOUHSC_00428
control
iron limitation
oxygen limitation
maxLFQ estimation scaled to rel_iBAQ [%]
0.20
0.15
0.10
0.05
0.00
HG001
ΔclpX
clpXcompl
growth phase
exp
stat

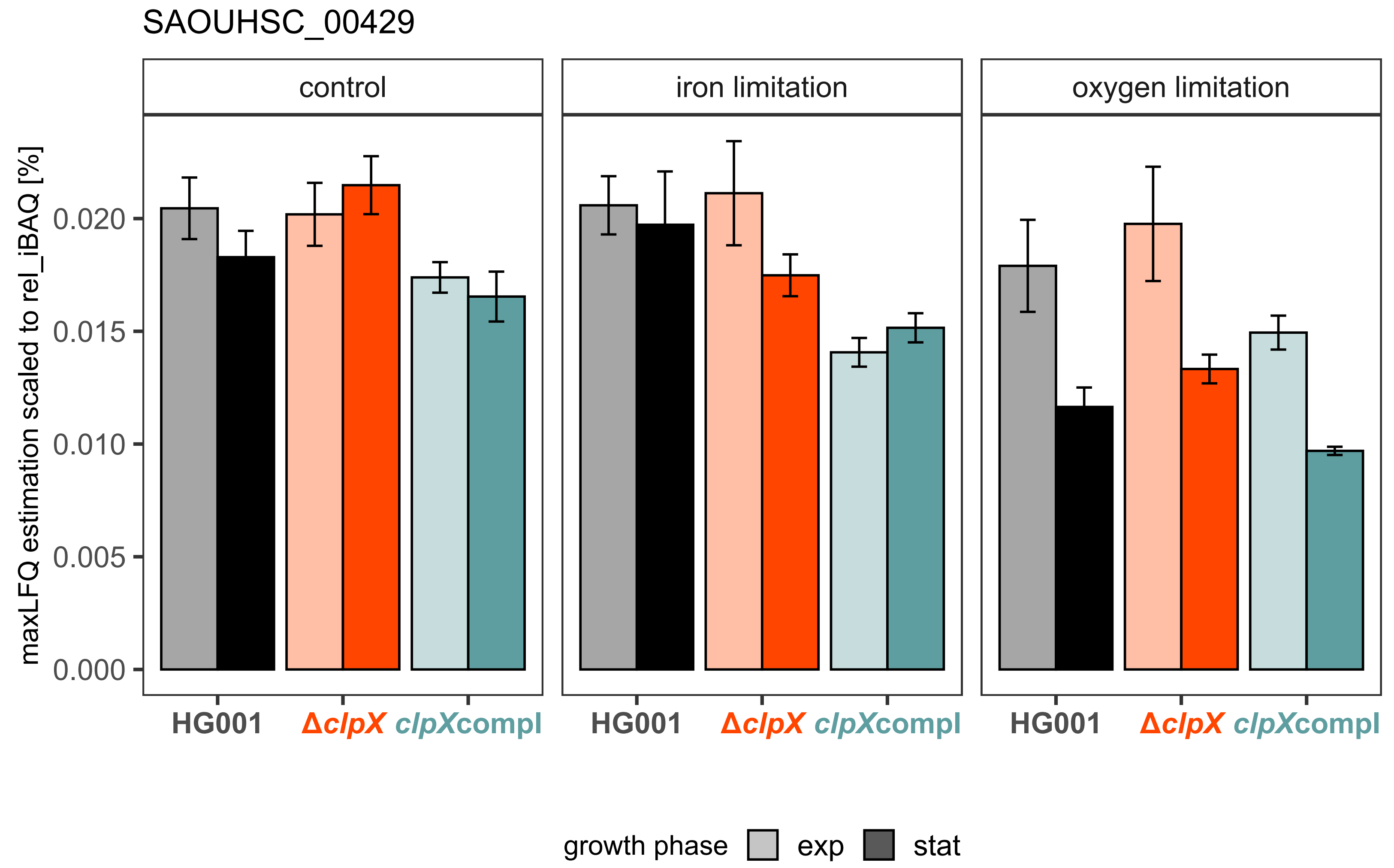
SAOUHSC_00429
control
iron limitation
oxygen limitation
maxLFQ estimation scaled to rel_iBAQ [%]
0.000
0.005
0.010
0.015
0.020
HG001
ΔclpX
clpXcompl
growth phase
exp
stat

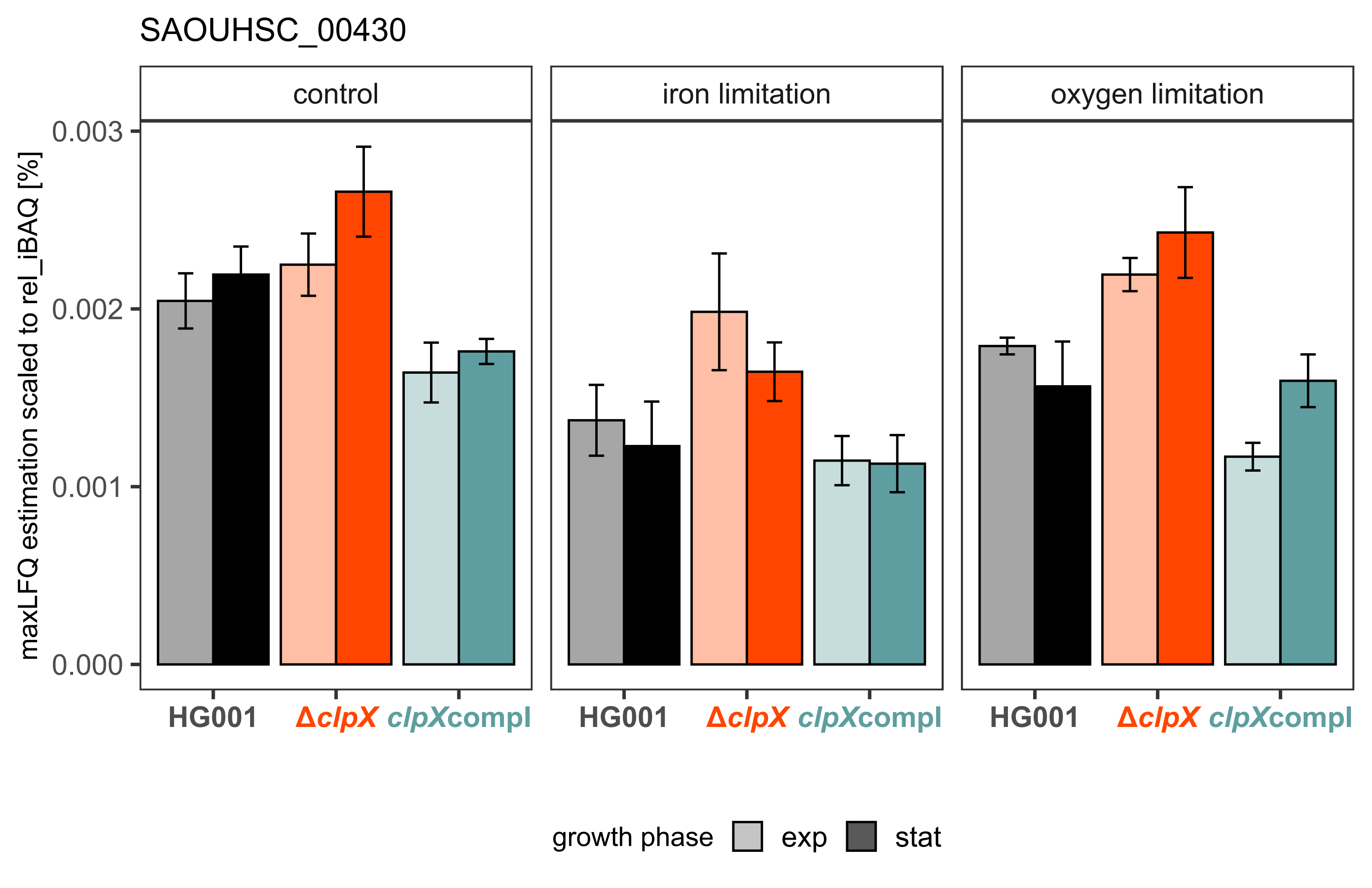
SAOUHSC_00430
control
iron limitation
oxygen limitation
maxLFQ estimation scaled to rel_iBAQ [%]
0.003
0.002
0.001
0.000
HG001
ΔclpX
clpXcompl
growth phase
exp
stat

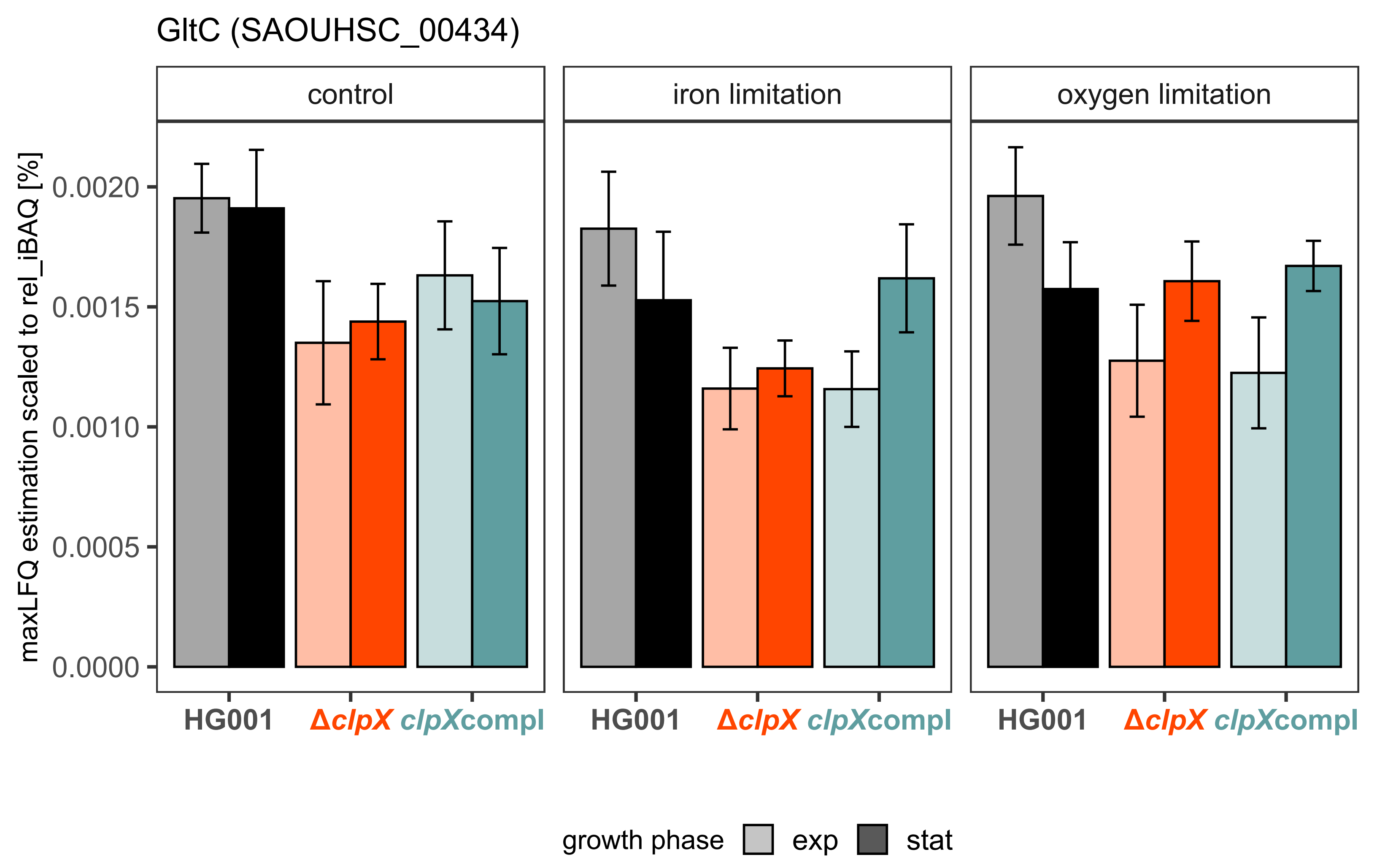

GltC (SAOUHSC_00434)
control
iron limitation
oxygen limitation
maxLFQ estimation scaled to rel_iBAQ [%]
0.0020
0.0015
0.0010
0.0005
0.0000
HG001
ΔclpX
clpXcompl
growth phase
exp
stat

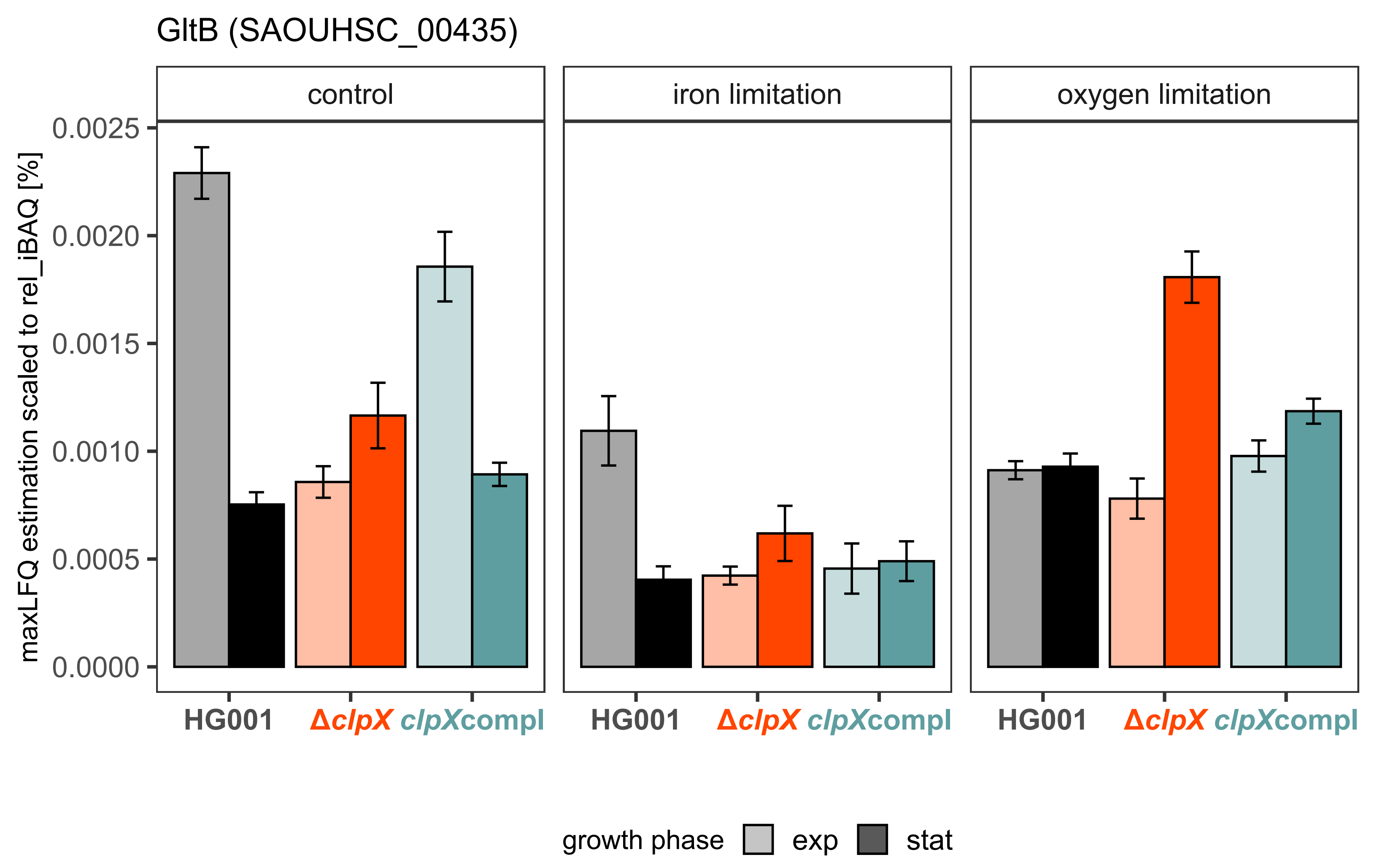

GltB (SAOUHSC_00435)
control
iron limitation
oxygen limitation
maxLFQ estimation scaled to rel_iBAQ [%]
0.0025
0.0020
0.0015
0.0010
0.0005
0.0000
HG001
ΔclpX
clpXcompl
growth phase
exp
stat

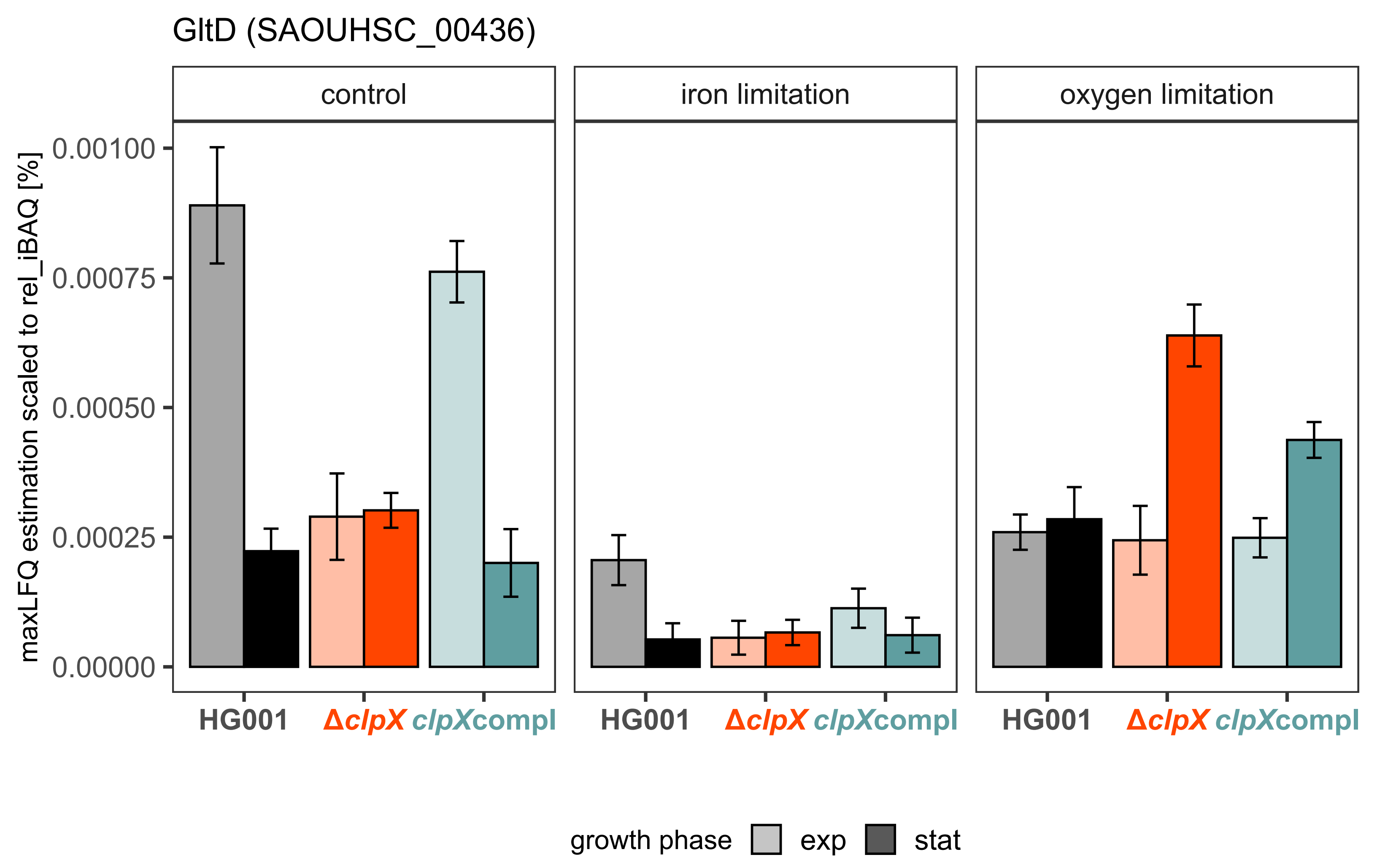
GltD (SAOUHSC_00436)
control
iron limitation
oxygen limitation
maxLFQ estimation scaled to rel_iBAQ [%]
0.00100
0.00075
0.00050
0.00025
0.00000
HG001
ΔclpX
clpXcompl
growth phase
exp
stat

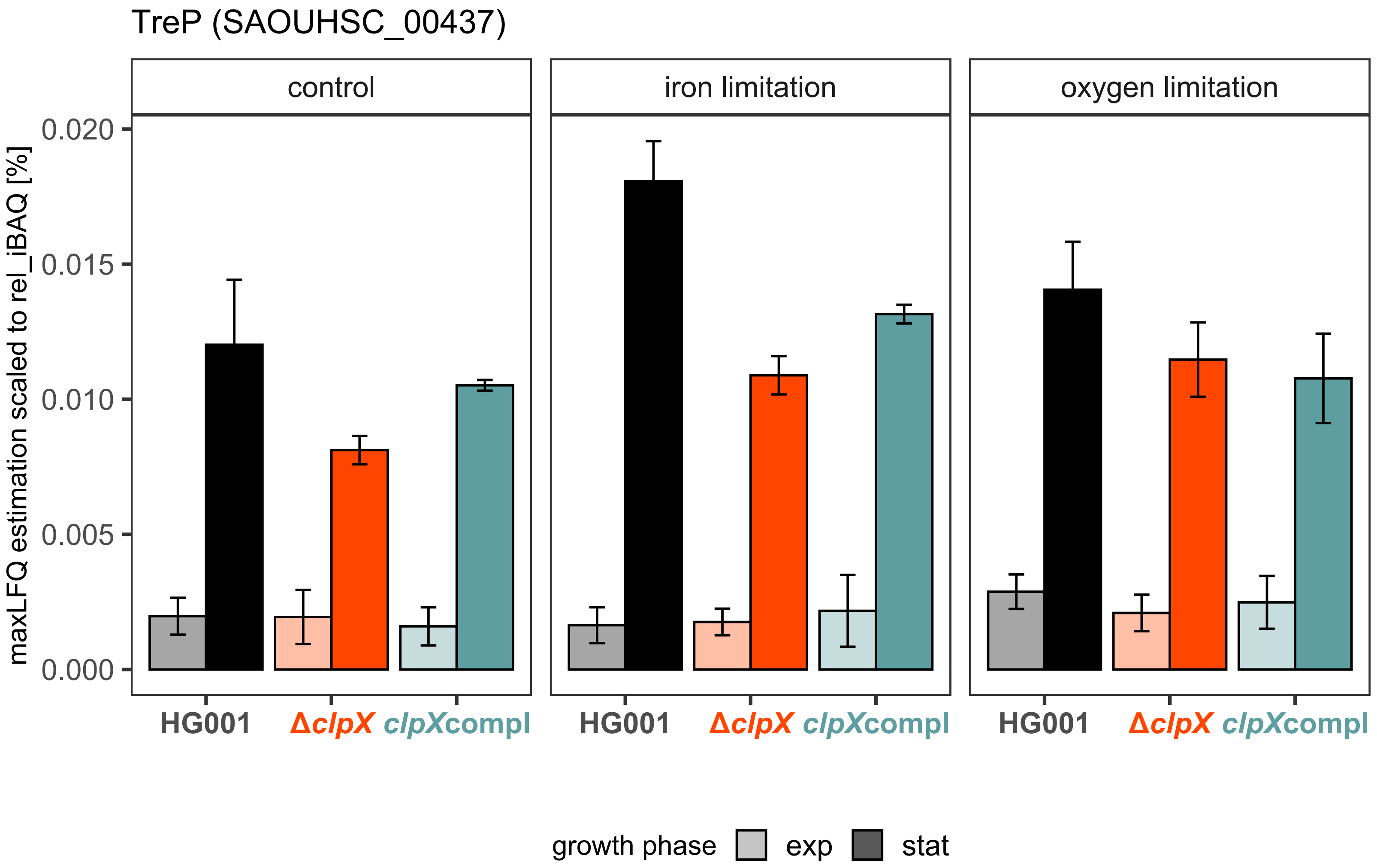

TreP (SAOUHSC_00437)
control
iron limitation
oxygen limitation
maxLFQ estimation scaled to rel_iBAQ [%]
0.020
0.015
0.010
0.005
0.000
HG001
ΔclpX
clpXcompl
growth phase
exp
stat

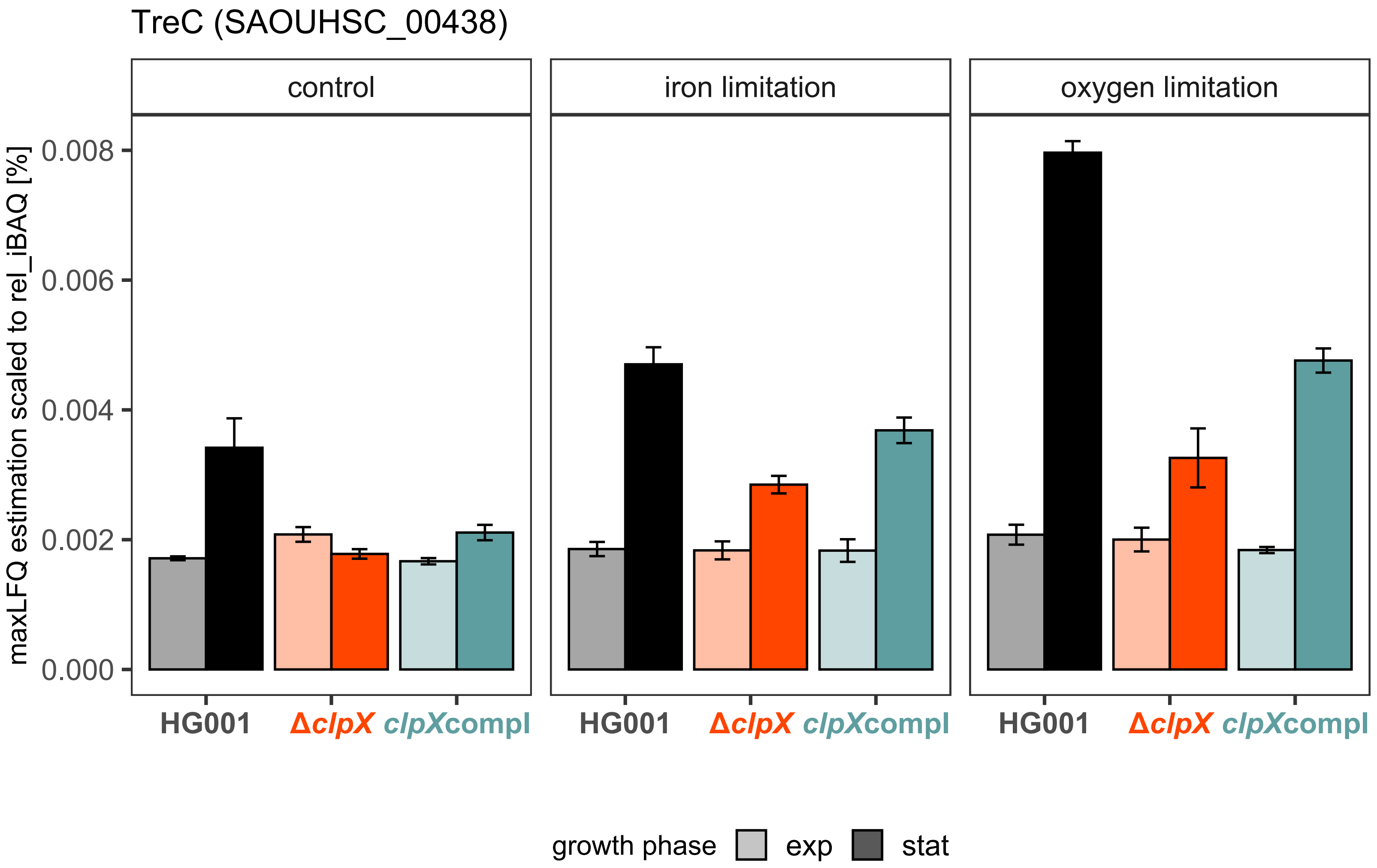

TreC (SAOUHSC_00438)
control
iron limitation
oxygen limitation
maxLFQ estimation scaled to rel_iBAQ [%]
0.008
0.006
0.004
0.002
0.000
HG001
ΔclpX
clpXcompl
growth phase
exp
stat

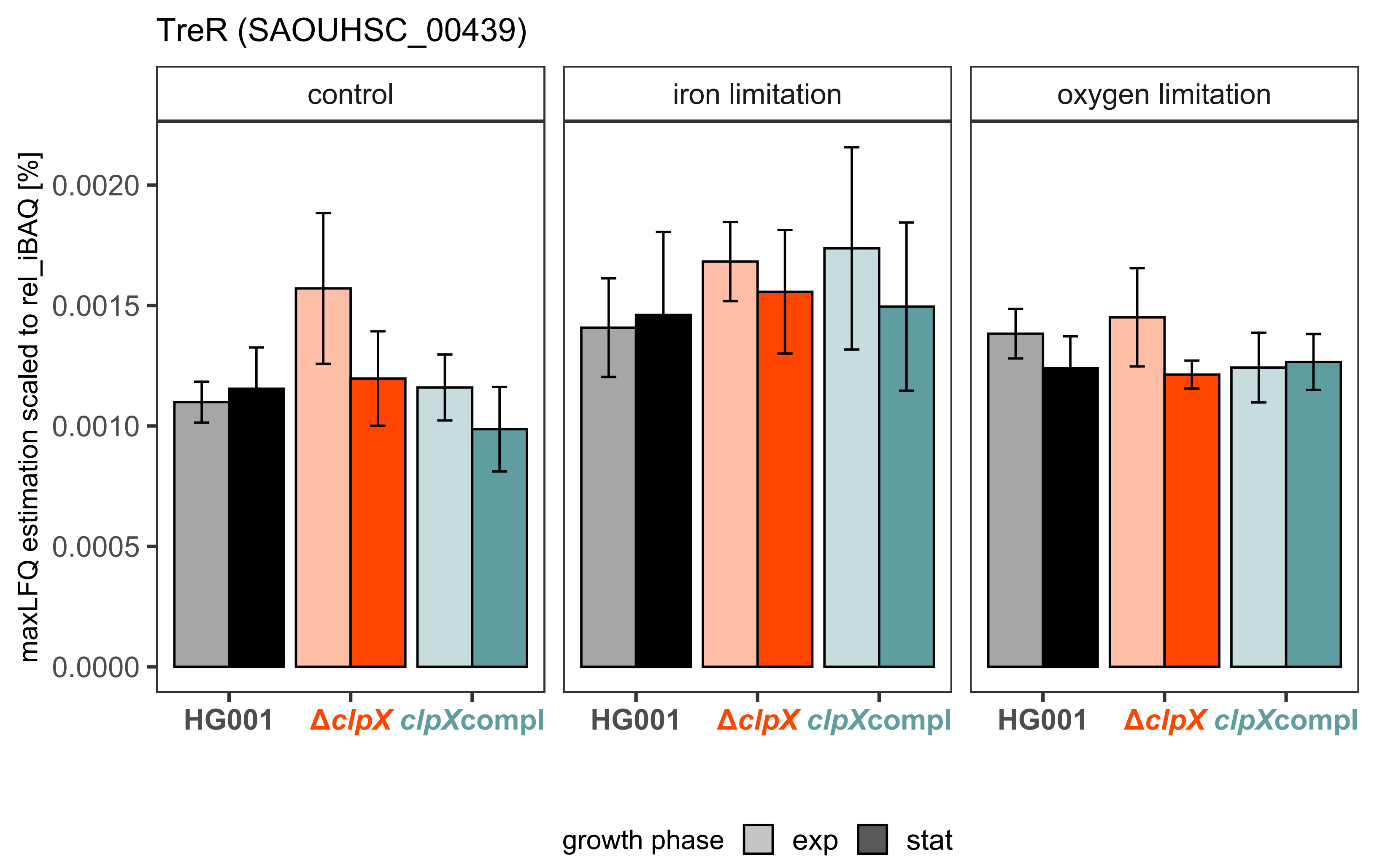
TreR (SAOUHSC_00439)
control
iron limitation
oxygen limitation
maxLFQ estimation scaled to rel_iBAQ [%]
0.0020
0.0015
0.0010
0.0005
0.0000
HG001
ΔclpX
clpXcompl
growth phase
exp
stat

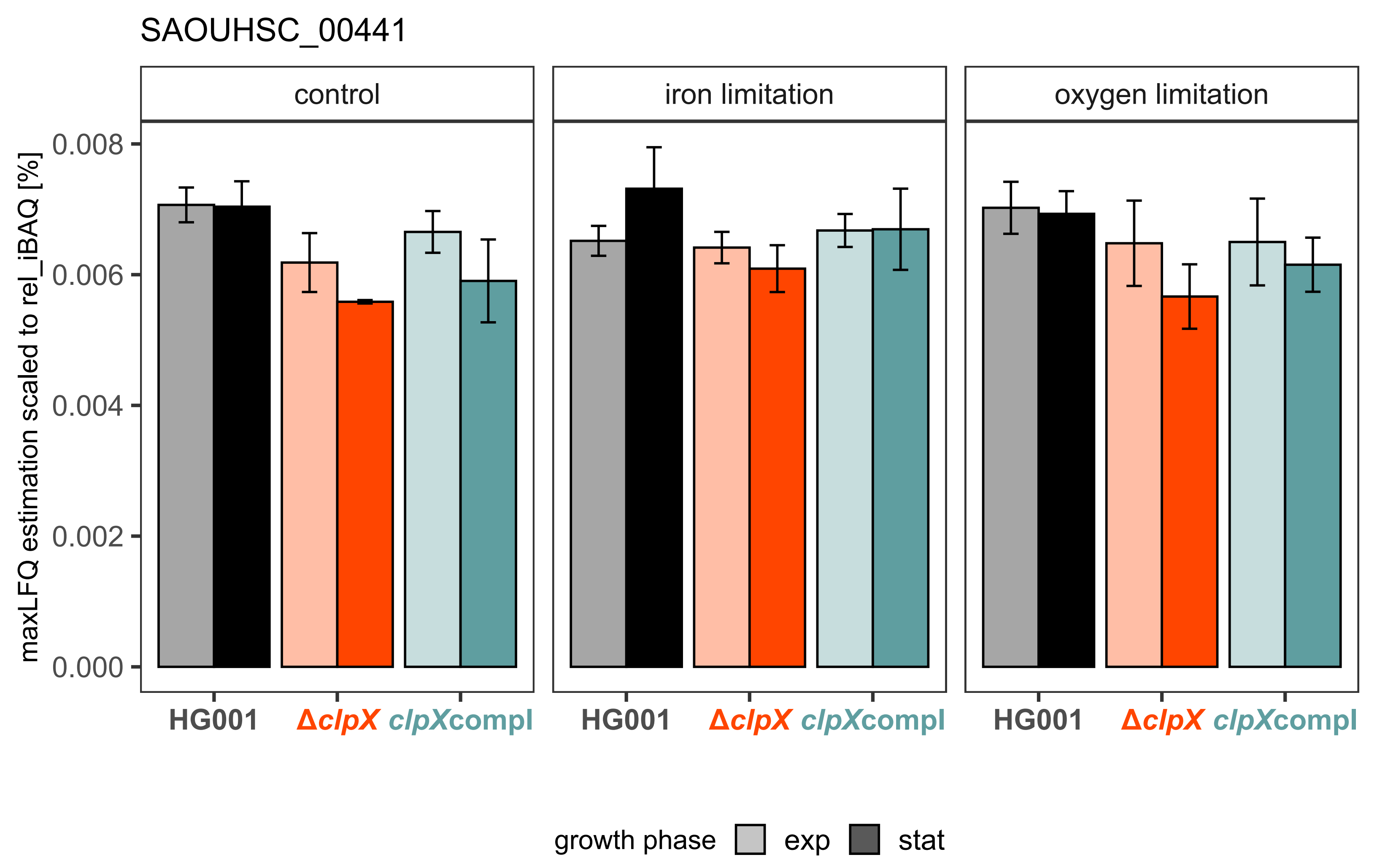
SAOUHSC_00441
control
iron limitation
oxygen limitation
maxLFQ estimation scaled to rel_iBAQ [%]
0.008
0.006
0.004
0.002
0.000
HG001
ΔclpX
clpXcompl
growth phase
exp
stat

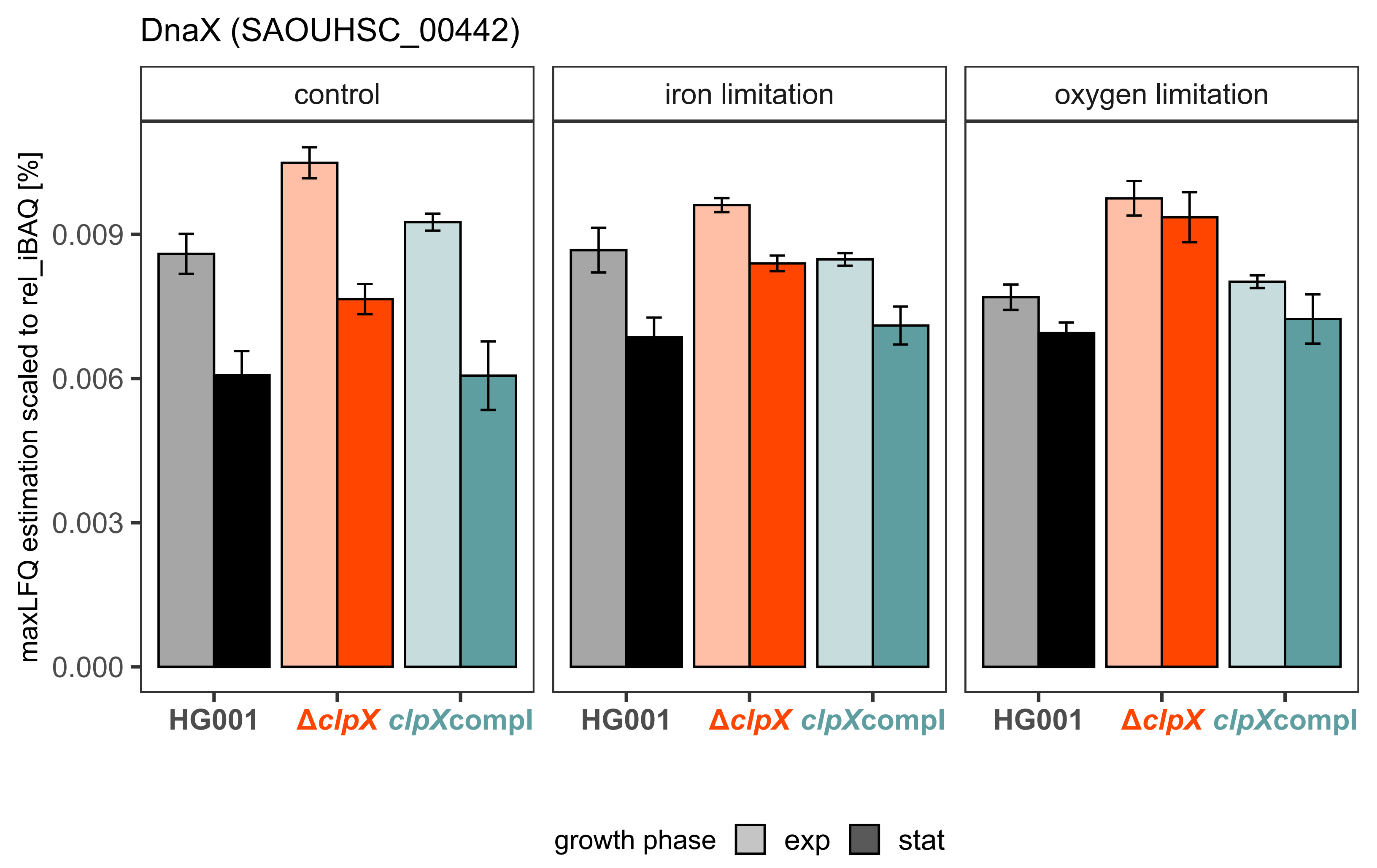
DnaX (SAOUHSC_00442)
control
iron limitation
oxygen limitation
maxLFQ estimation scaled to rel_iBAQ [%]
0.009
0.006
0.003
0.000
HG001
ΔclpX
clpXcompl
growth phase
exp
stat

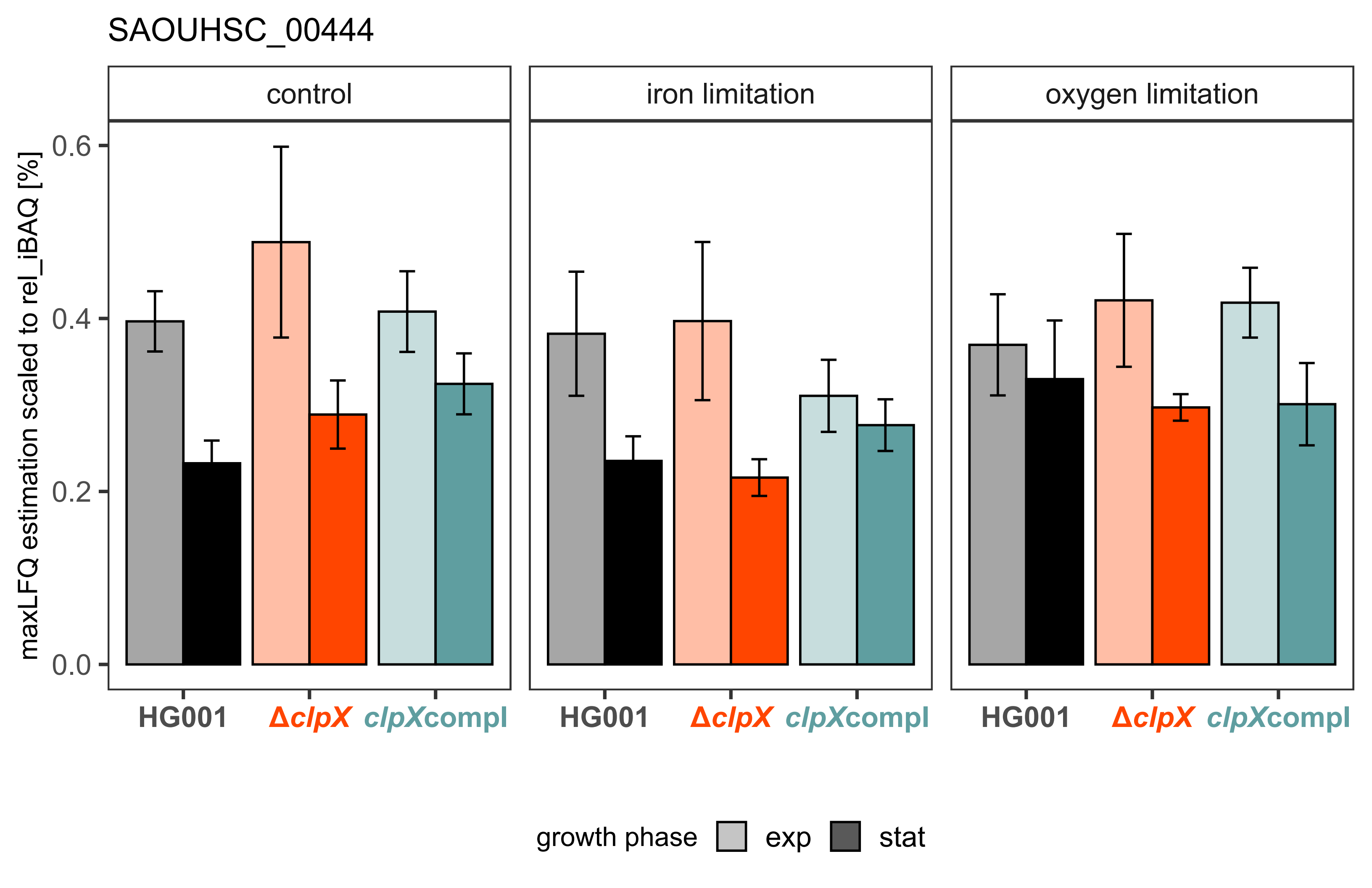

SAOUHSC_00444
control
iron limitation
oxygen limitation
maxLFQ estimation scaled to rel_iBAQ [%]
0.0
0.2
0.4
0.6
HG001
ΔclpX
clpXcompl
growth phase
exp
stat

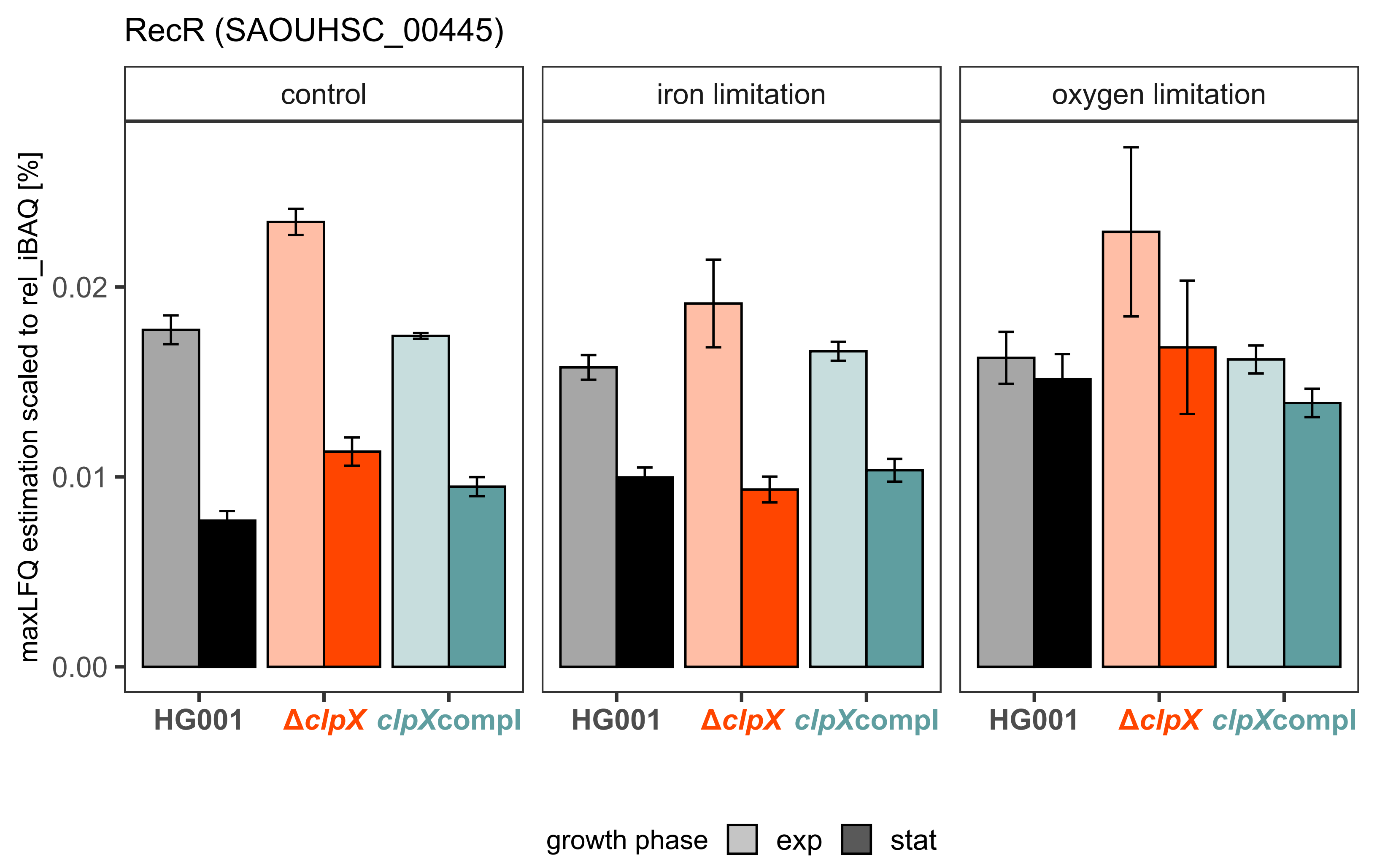

RecR (SAOUHSC_00445)
control
iron limitation
oxygen limitation
maxLFQ estimation scaled to rel_iBAQ [%]
0.00
0.01
0.02
HG001
ΔclpX
clpXcompl
growth phase
exp
stat

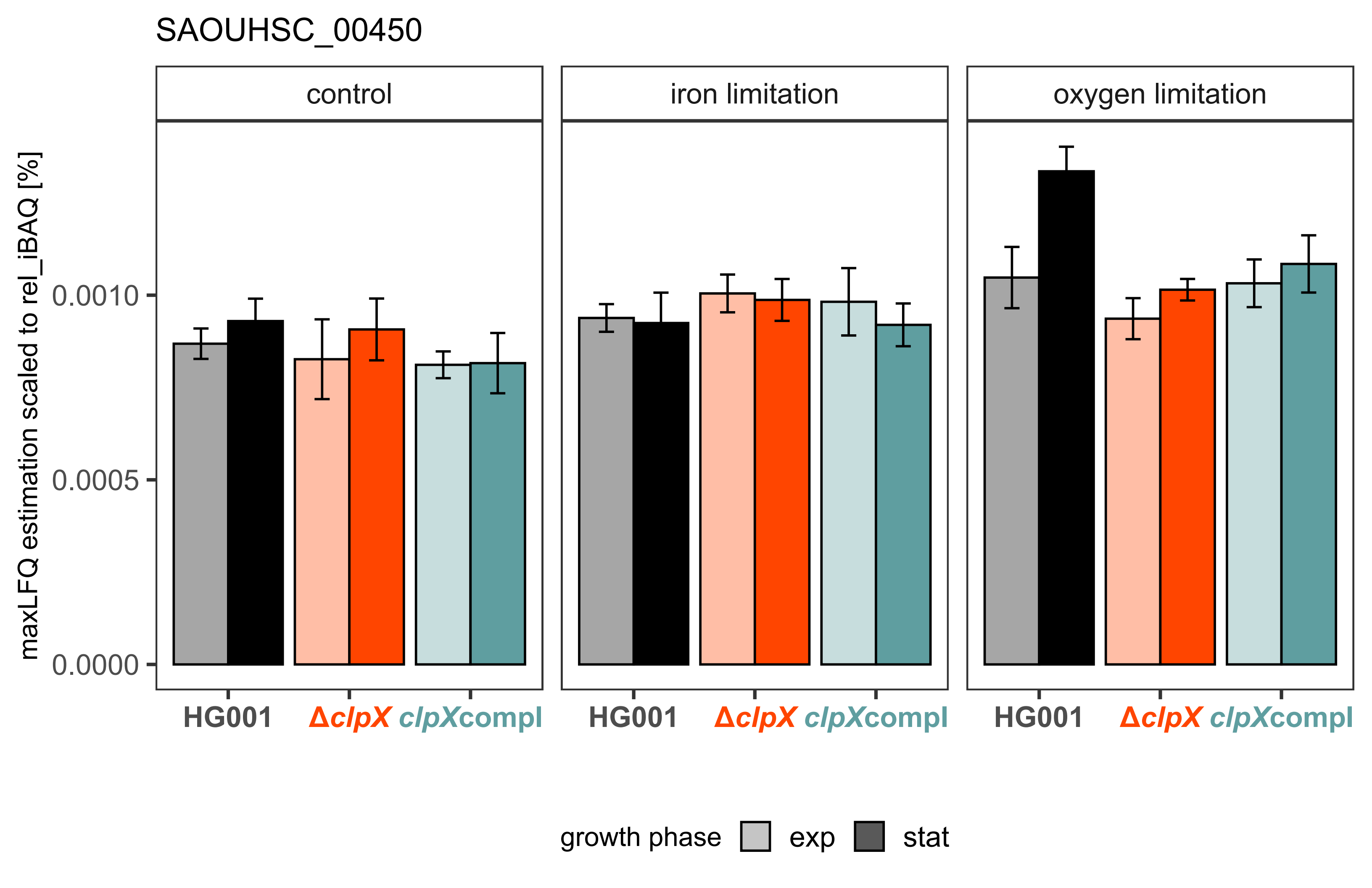

SAOUHSC_00450
control
iron limitation
oxygen limitation
maxLFQ estimation scaled to rel_iBAQ [%]
0.0010
0.0005
0.0000
HG001
ΔclpX
clpXcompl
growth phase
exp
stat

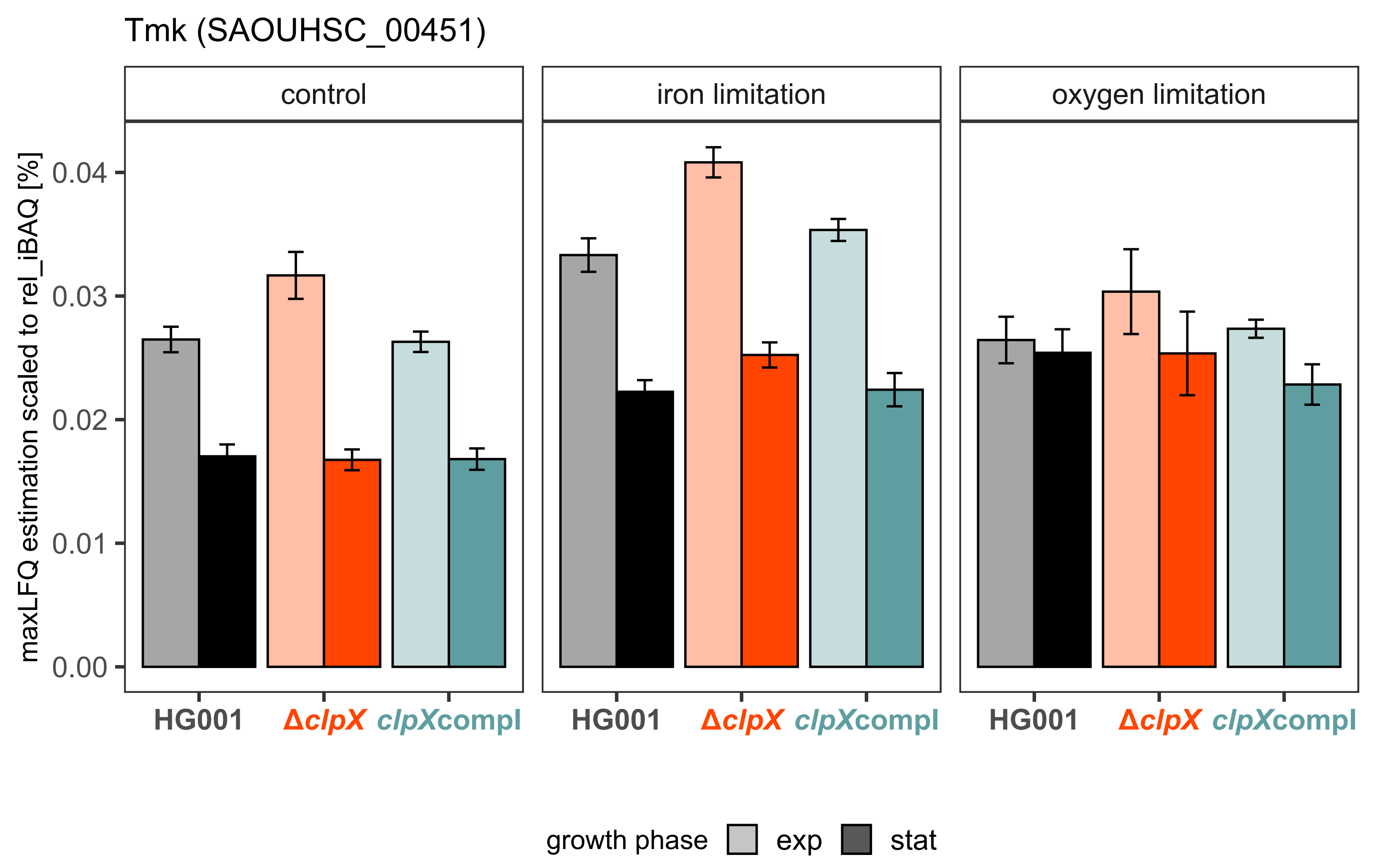

Tmk (SAOUHSC_00451)
control
iron limitation
oxygen limitation
maxLFQ estimation scaled to rel_iBAQ [%]
0.00
0.01
0.02
0.03
0.04
HG001
ΔclpX
clpXcompl
growth phase
exp
stat

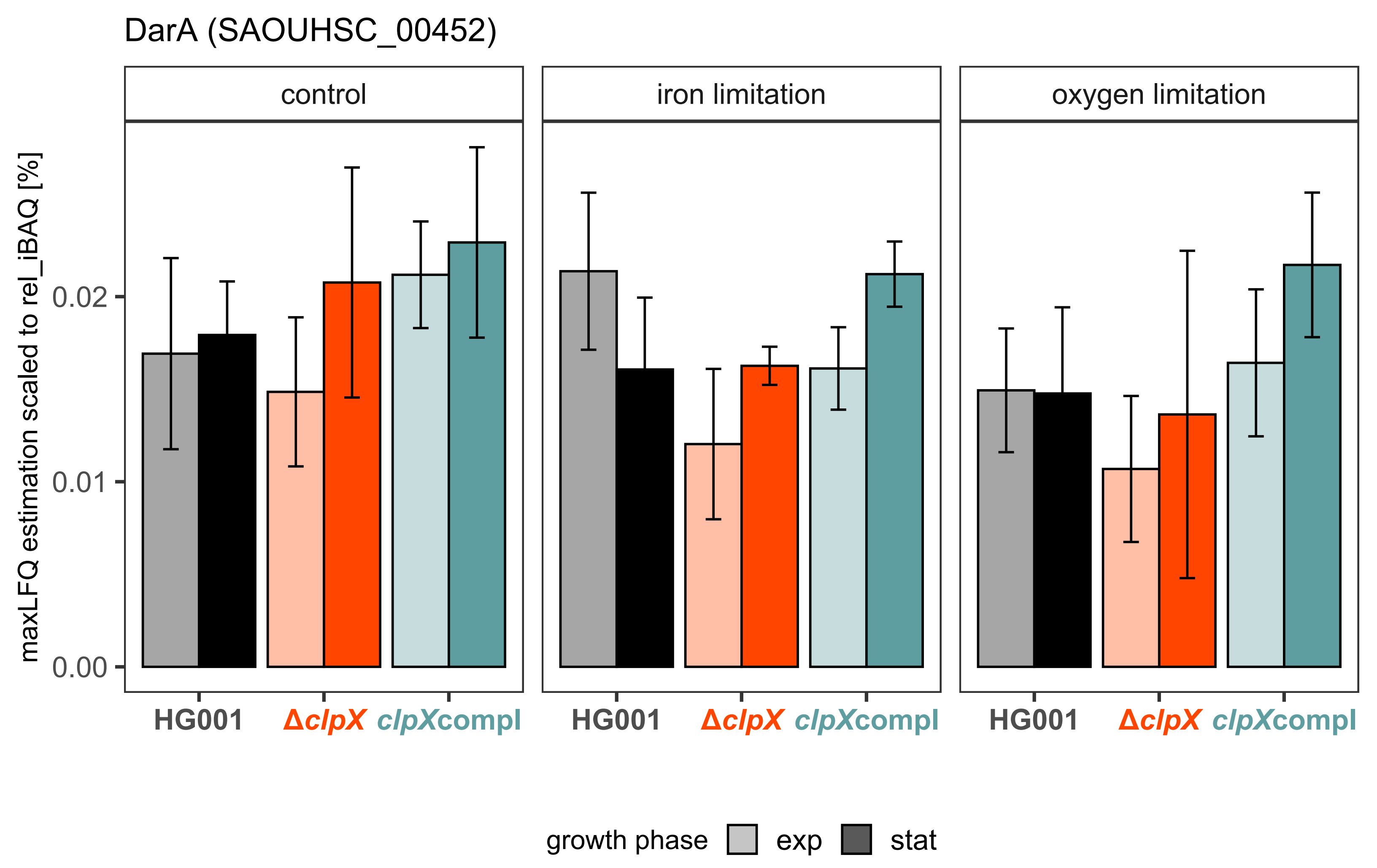
DarA (SAOUHSC_00452)
control
iron limitation
oxygen limitation
maxLFQ estimation scaled to rel_iBAQ [%]
0.00
0.01
0.02
HG001
ΔclpX
clpXcompl
growth phase
exp
stat

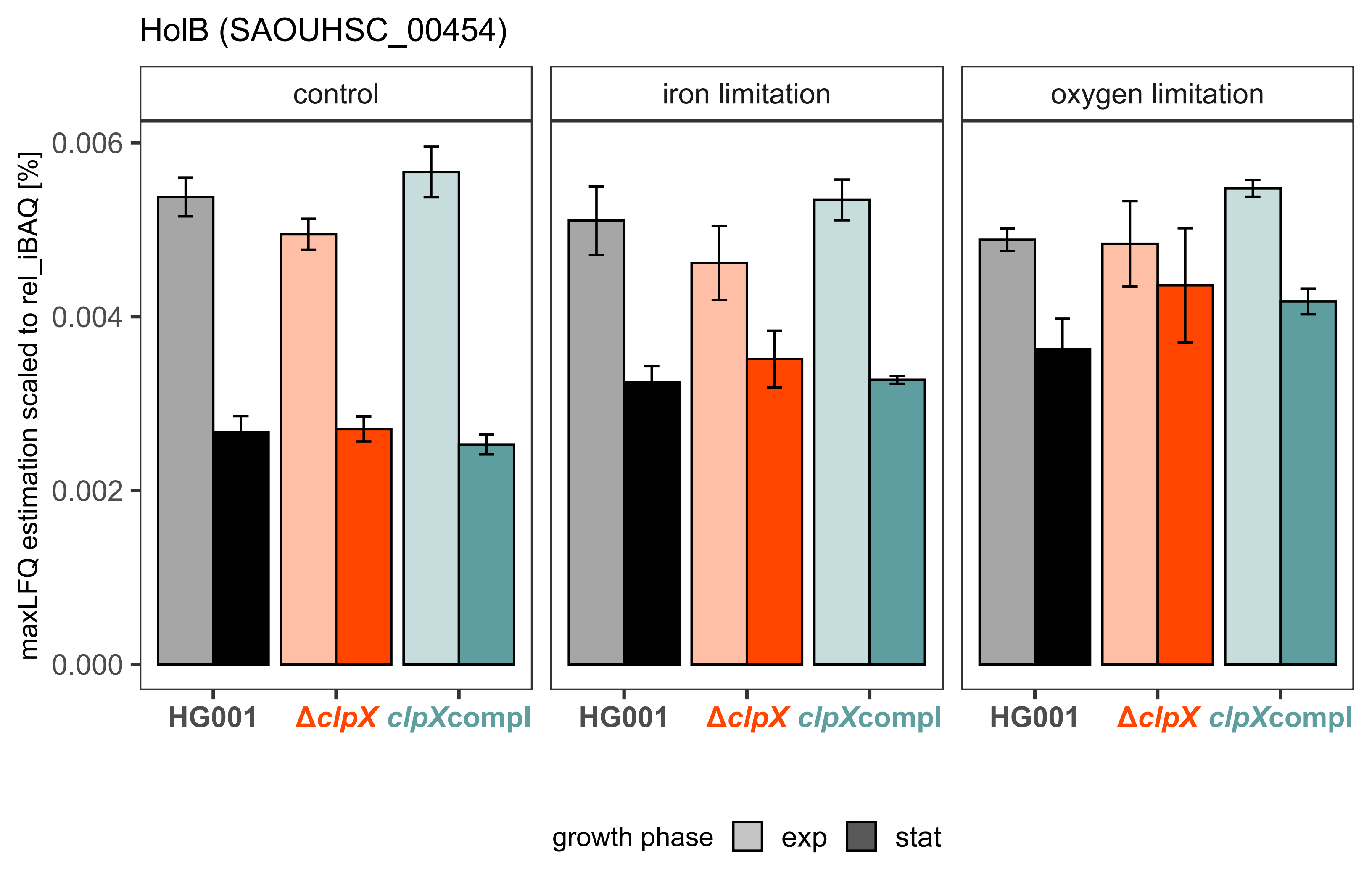
HolB (SAOUHSC_00454)
control
iron limitation
oxygen limitation
maxLFQ estimation scaled to rel_iBAQ [%]
0.006
0.004
0.002
0.000
HG001
ΔclpX
clpXcompl
growth phase
exp
stat

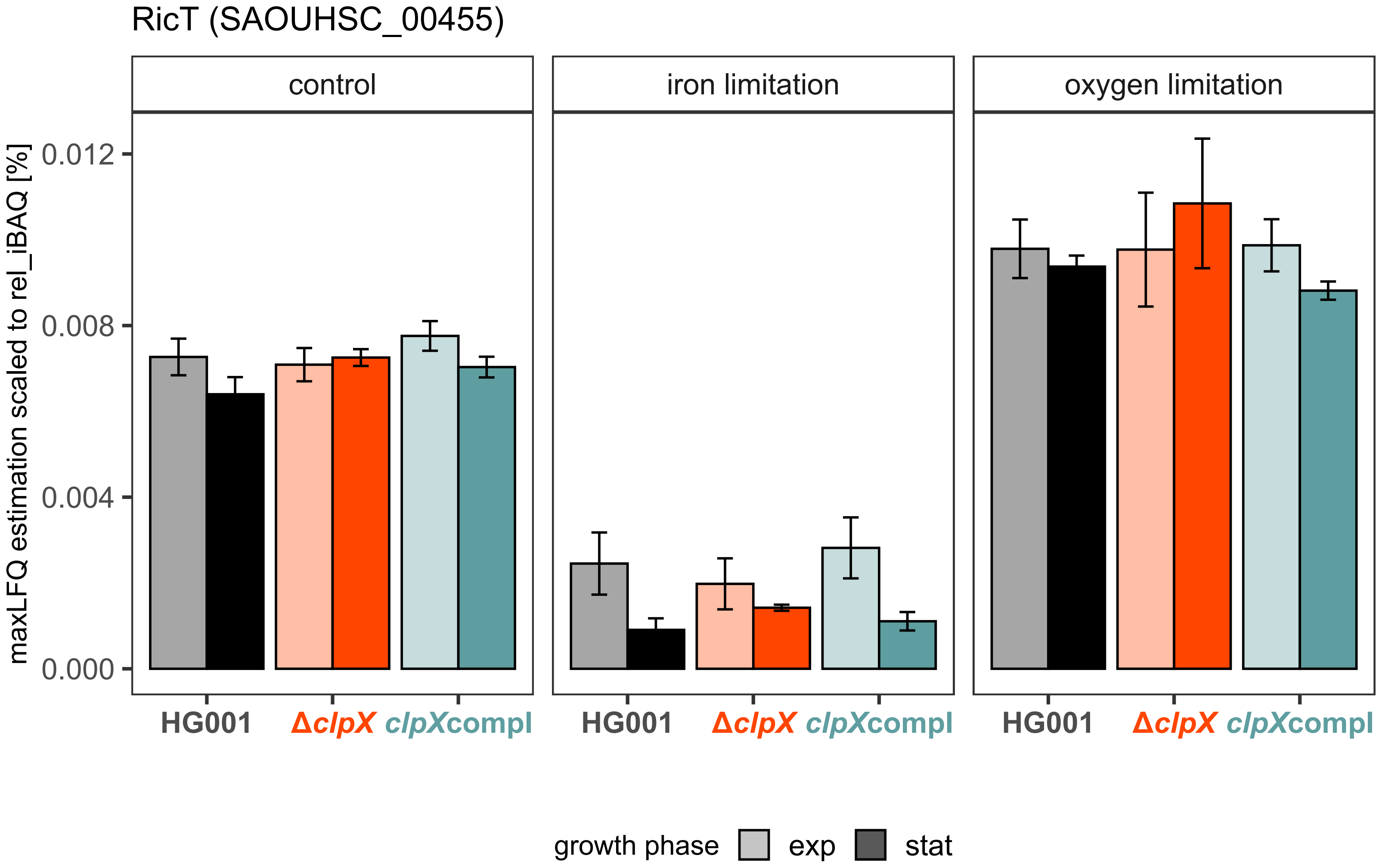

RicT (SAOUHSC_00455)
control
iron limitation
oxygen limitation
maxLFQ estimation scaled to rel_iBAQ [%]
0.012
0.008
0.004
0.000
HG001
ΔclpX
clpXcompl
growth phase
exp
stat

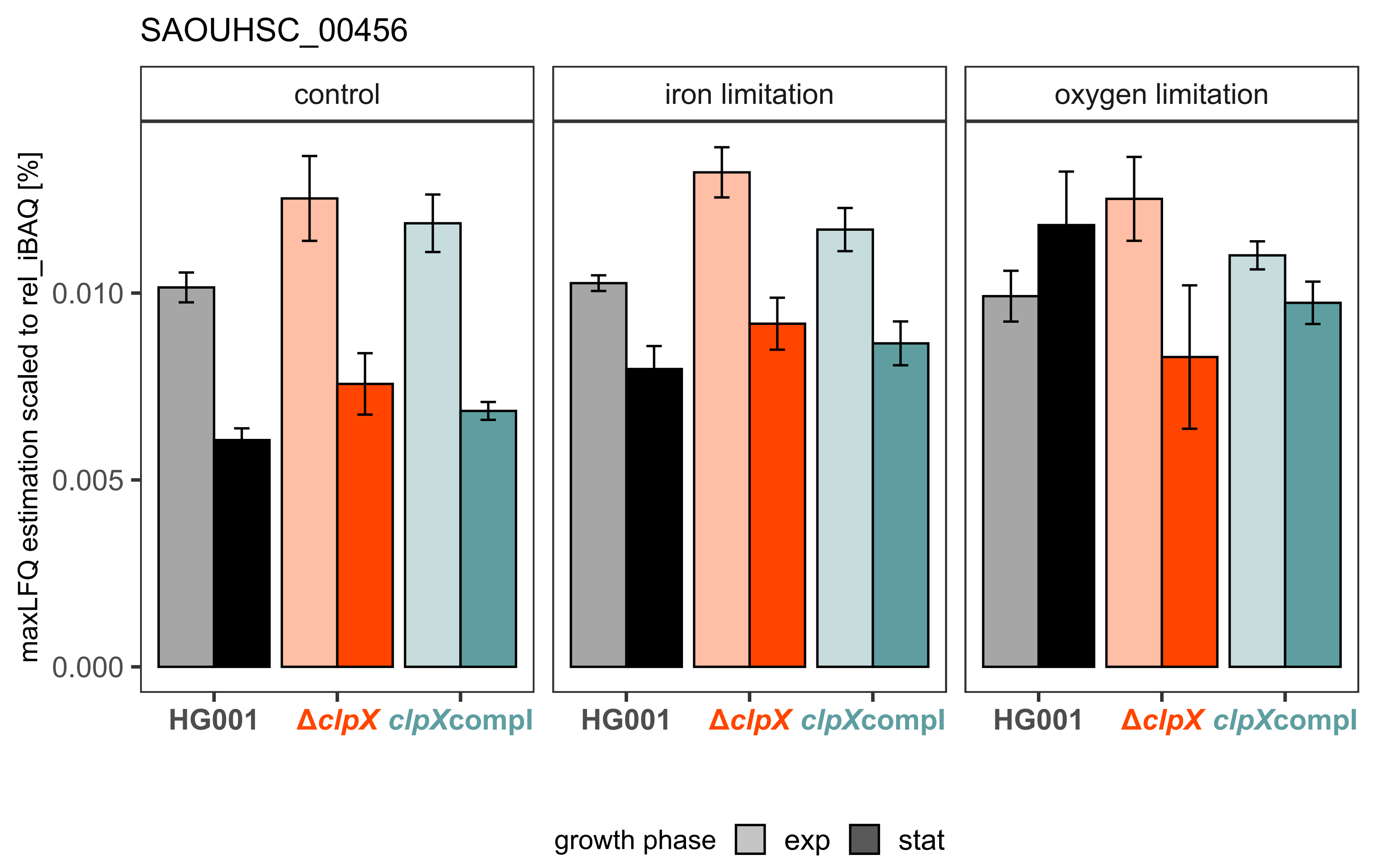

SAOUHSC_00456
control
iron limitation
oxygen limitation
maxLFQ estimation scaled to rel_iBAQ [%]
0.010
0.005
0.000
HG001
ΔclpX
clpXcompl
growth phase
exp
stat

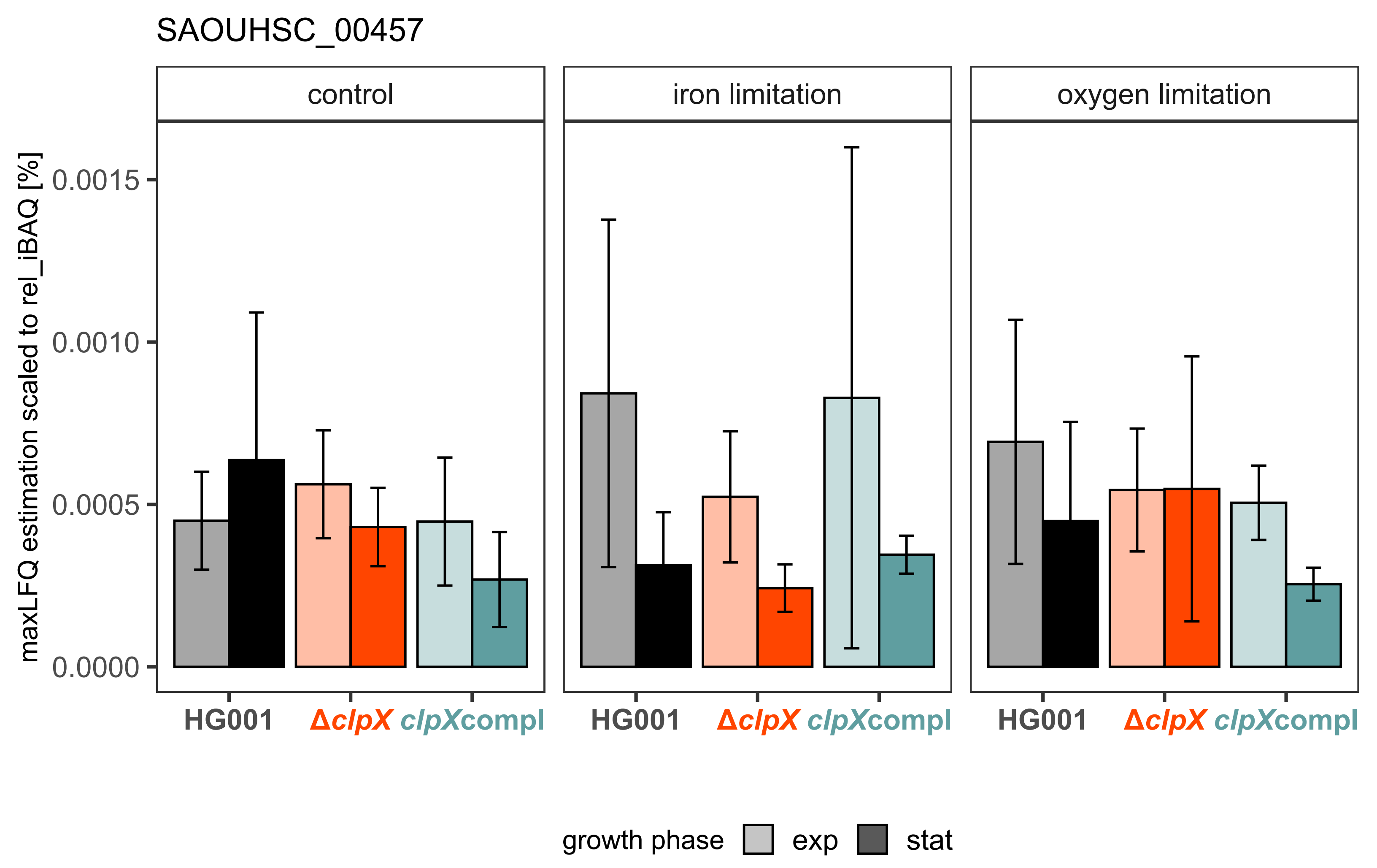

SAOUHSC_00457
control
iron limitation
oxygen limitation
maxLFQ estimation scaled to rel_iBAQ [%]
0.0015
0.0010
0.0005
0.0000
HG001
ΔclpX
clpXcompl
growth phase
exp
stat

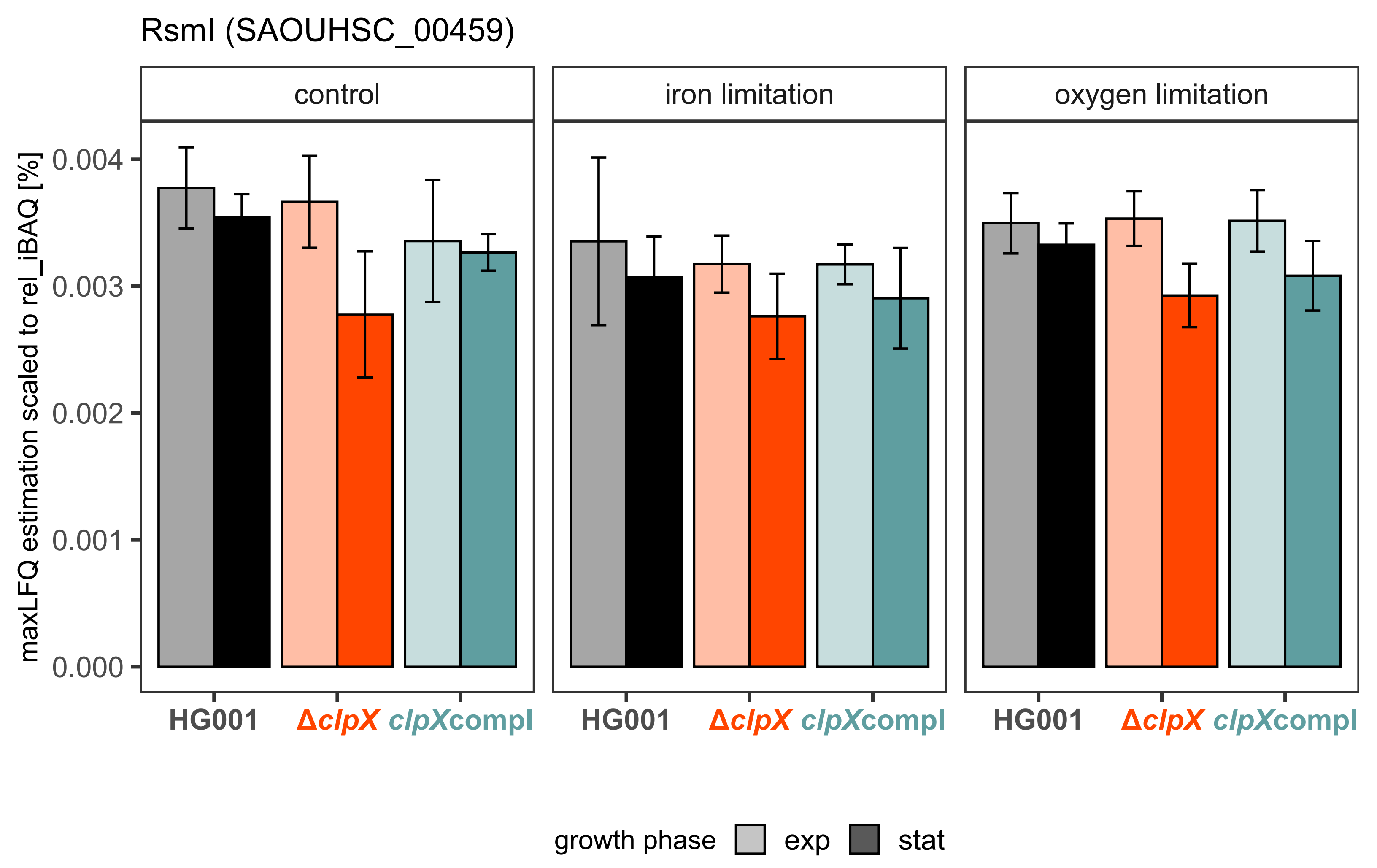

RsmI (SAOUHSC_00459)
control
iron limitation
oxygen limitation
maxLFQ estimation scaled to rel_iBAQ [%]
0.004
0.003
0.002
0.001
0.000
HG001
ΔclpX
clpXcompl
growth phase
exp
stat

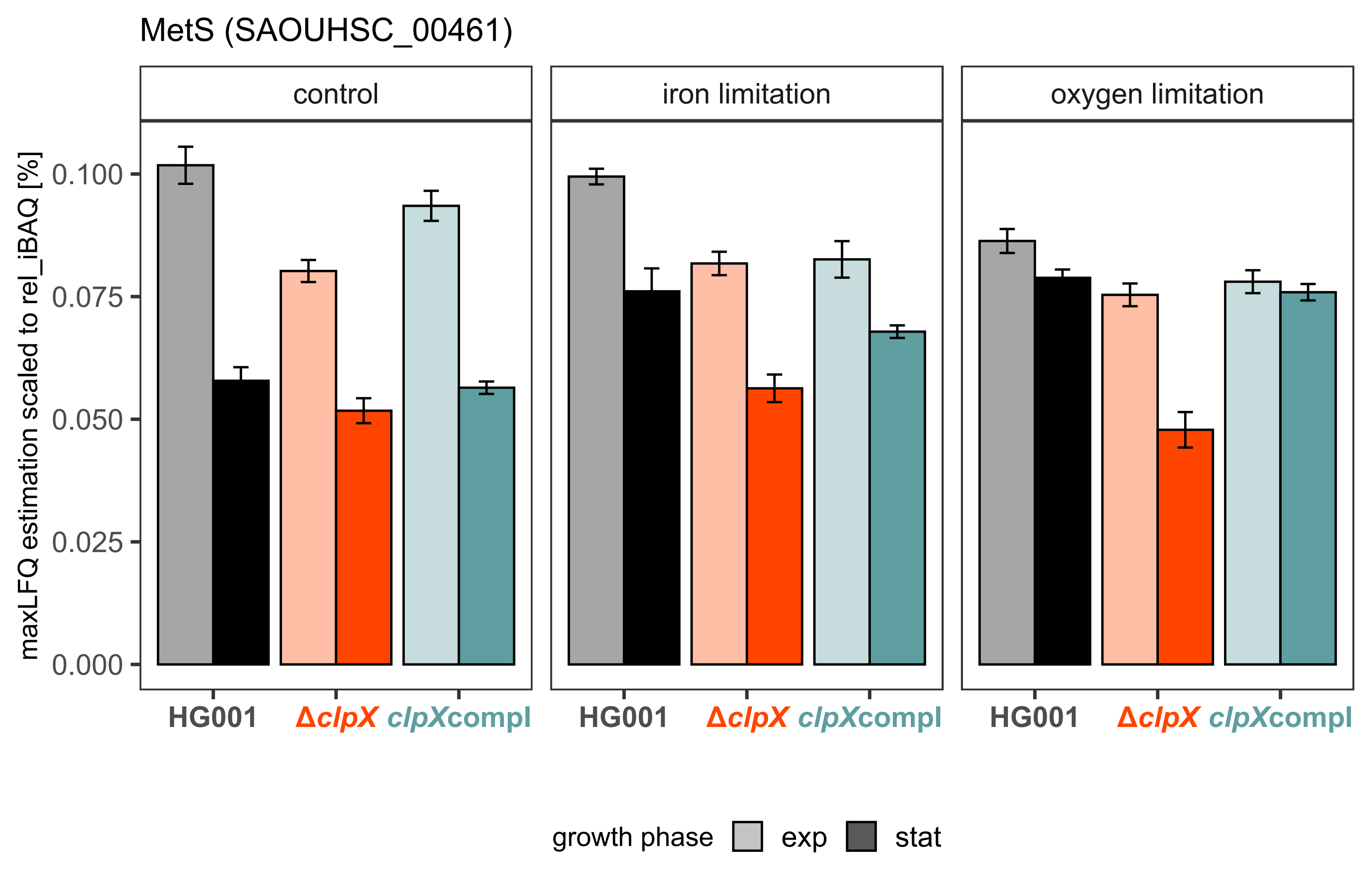

MetS (SAOUHSC_00461)
control
iron limitation
oxygen limitation
maxLFQ estimation scaled to rel_iBAQ [%]
0.100
0.075
0.050
0.025
0.000
HG001
ΔclpX
clpXcompl
growth phase
exp
stat

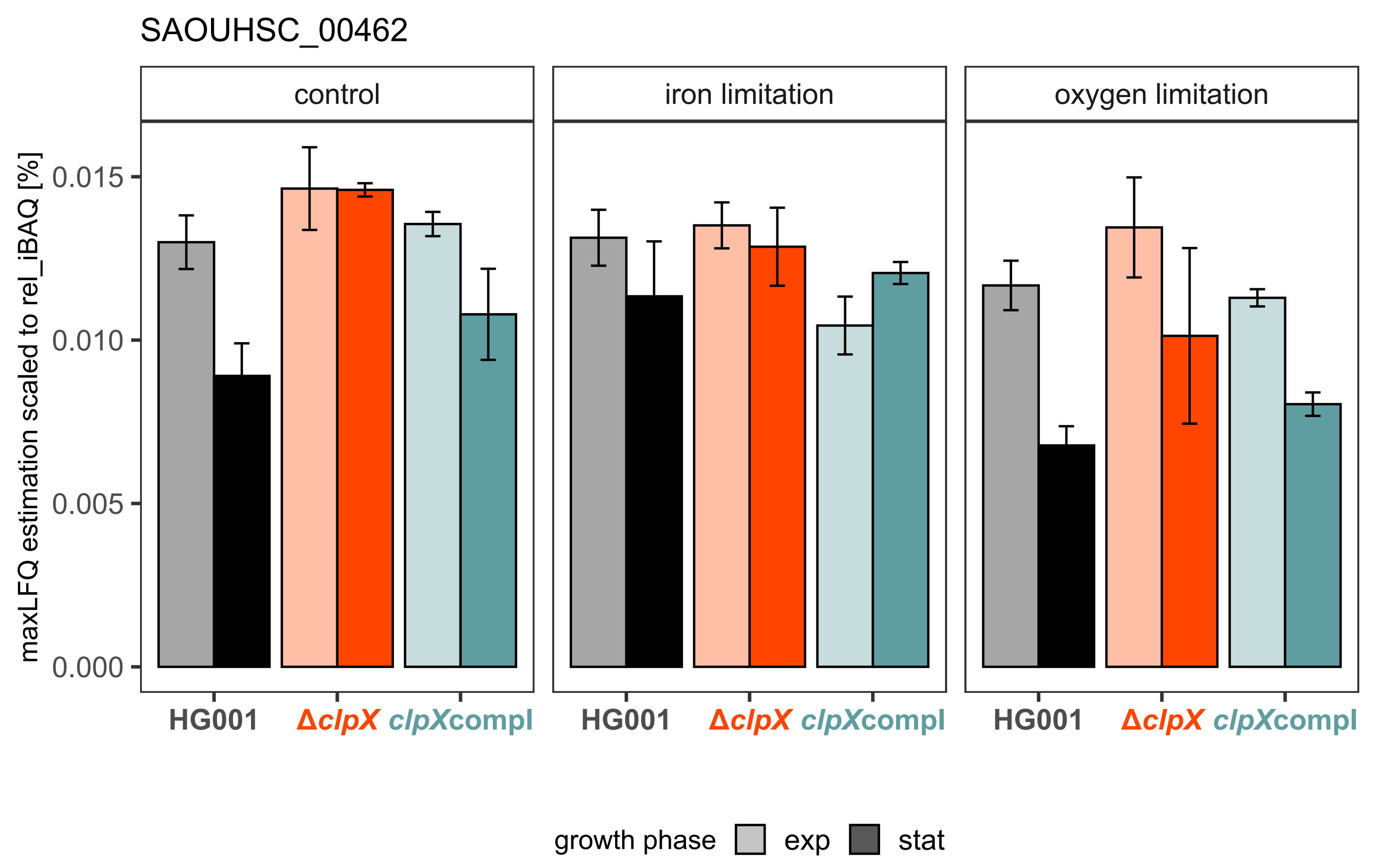

SAOUHSC_00462
control
iron limitation
oxygen limitation
maxLFQ estimation scaled to rel_iBAQ [%]
0.015
0.010
0.005
0.000
HG001
ΔclpX
clpXcompl
growth phase
exp
stat

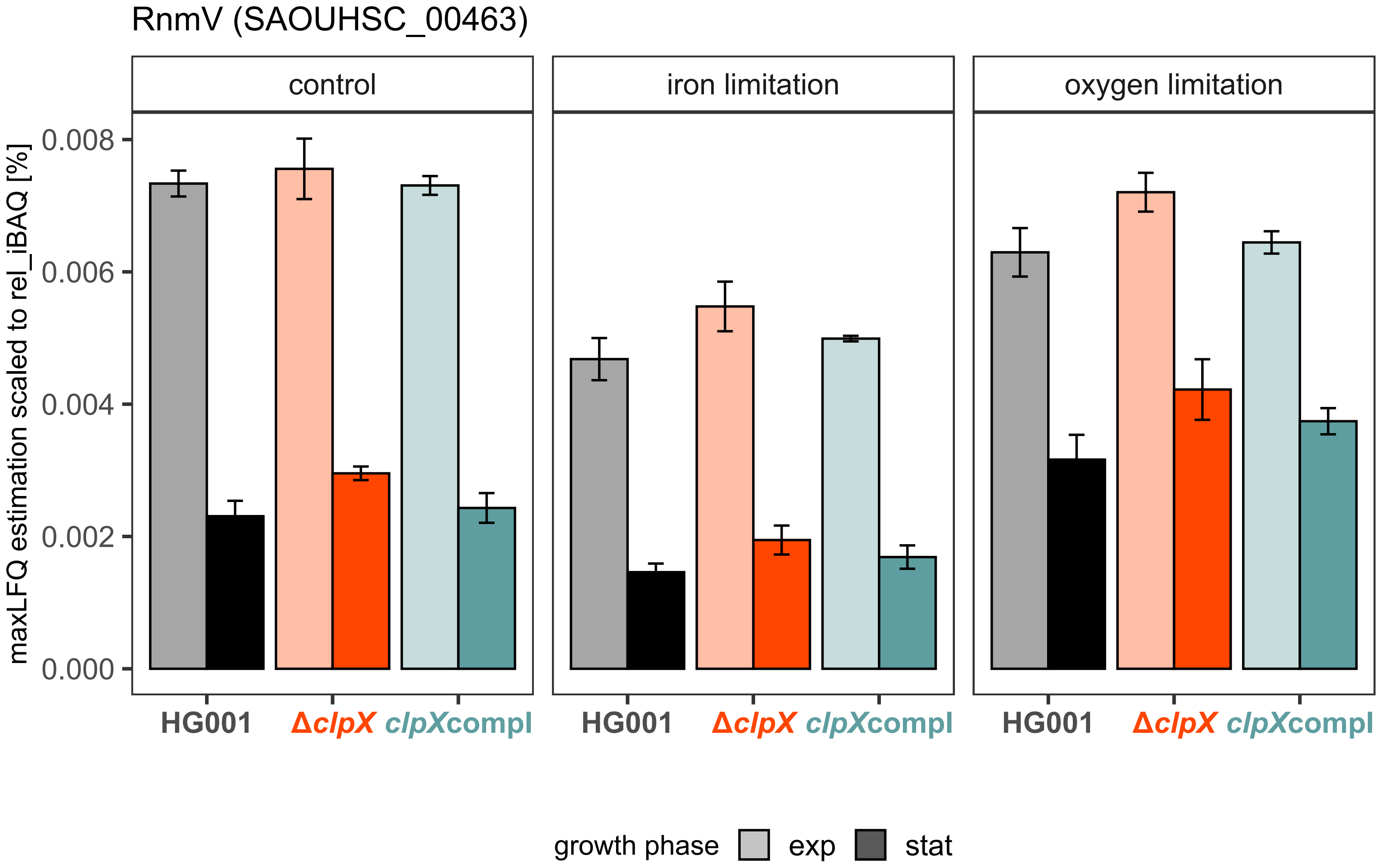

RnmV (SAOUHSC_00463)
control
iron limitation
oxygen limitation
maxLFQ estimation scaled to rel_iBAQ [%]
0.008
0.006
0.004
0.002
0.000
HG001
ΔclpX
clpXcompl
growth phase
exp
stat

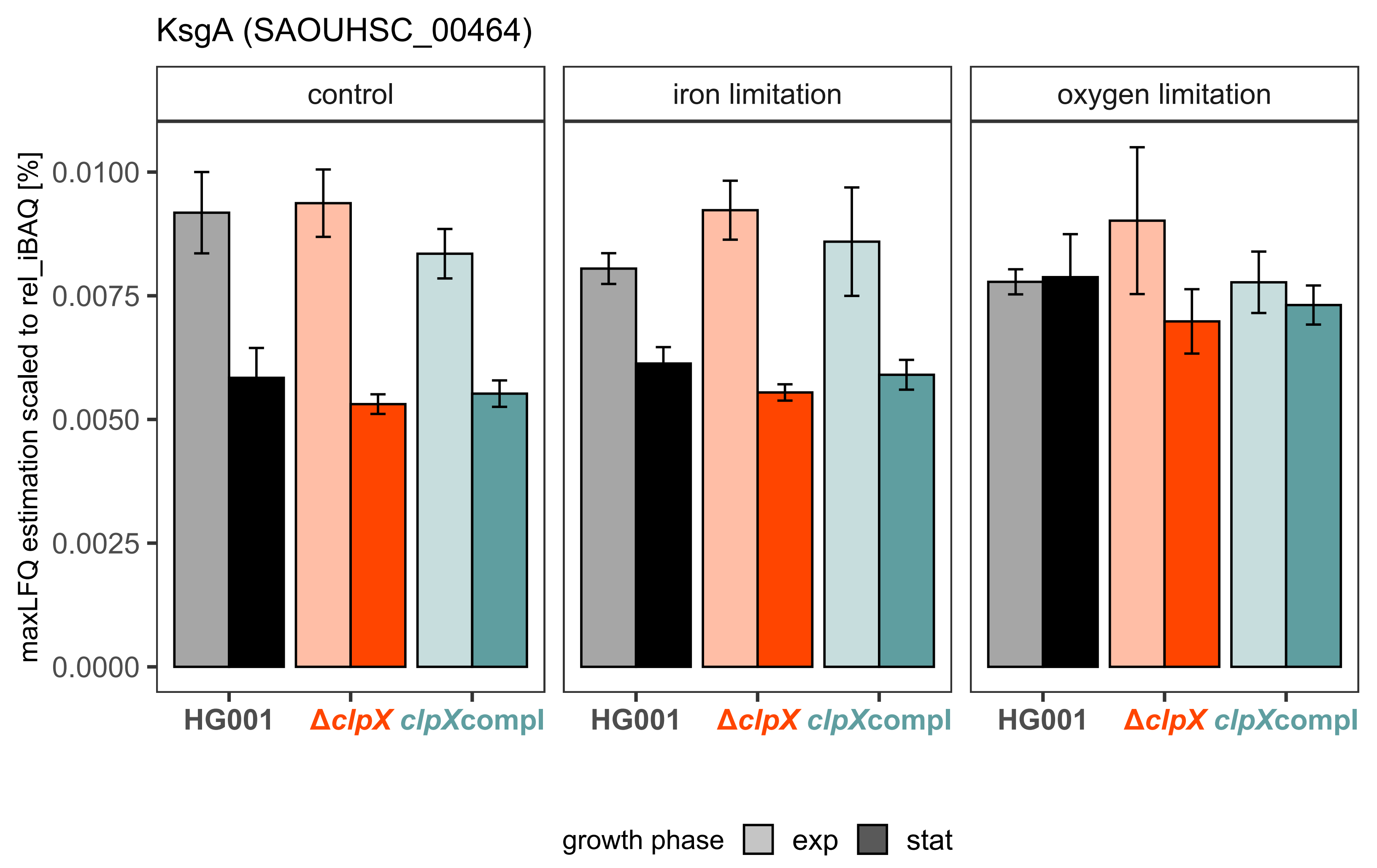

KsgA (SAOUHSC_00464)
control
iron limitation
oxygen limitation
maxLFQ estimation scaled to rel_iBAQ [%]
0.0100
0.0075
0.0050
0.0025
0.0000
HG001
ΔclpX
clpXcompl
growth phase
exp
stat

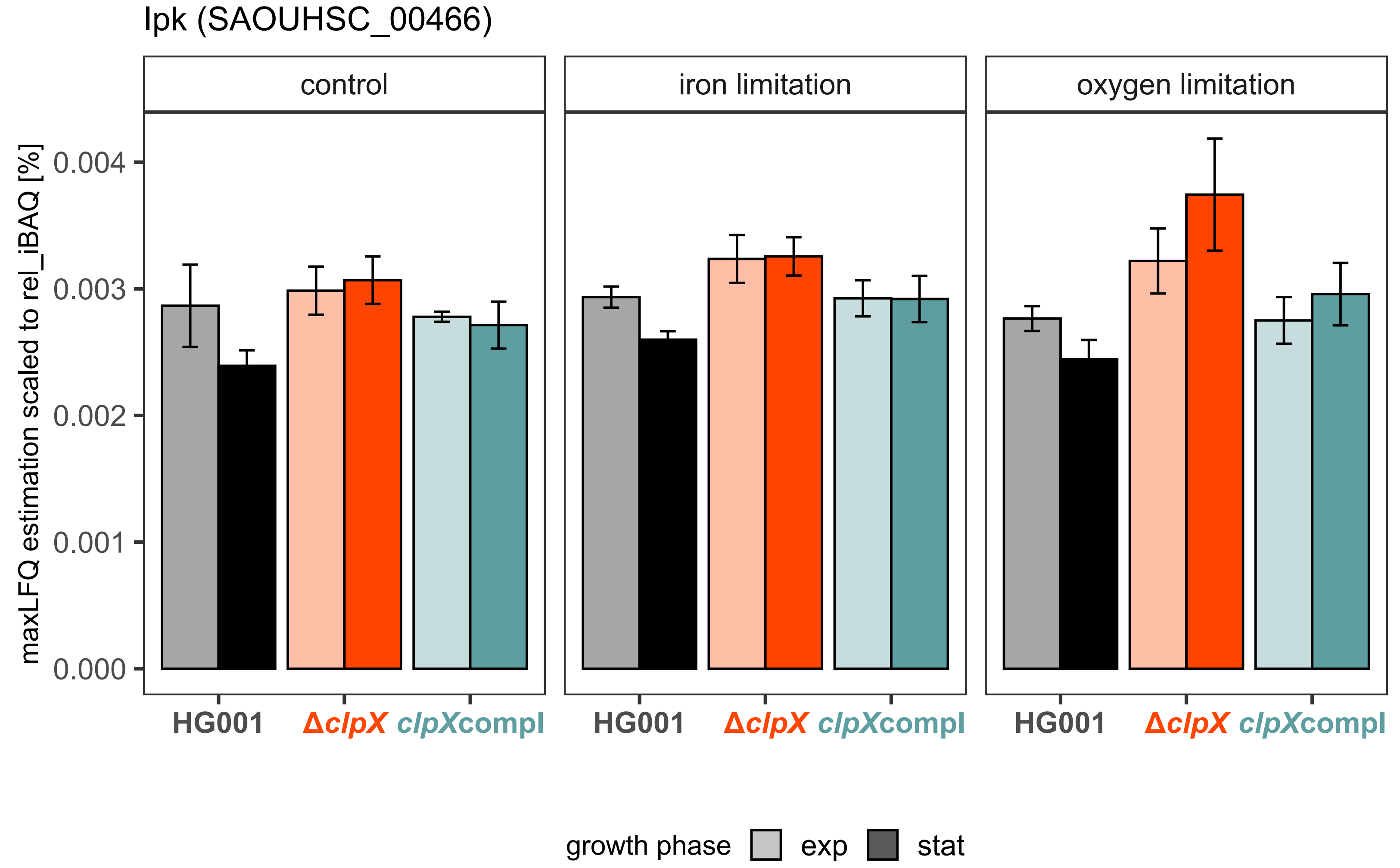
lpk (SAOUHSC_00466)
control
iron limitation
oxygen limitation
maxLFQ estimation scaled to rel_iBAQ [%]
0.004
0.003
0.002
0.001
0.000
HG001
ΔclpX
clpXcompl
growth phase
exp
stat

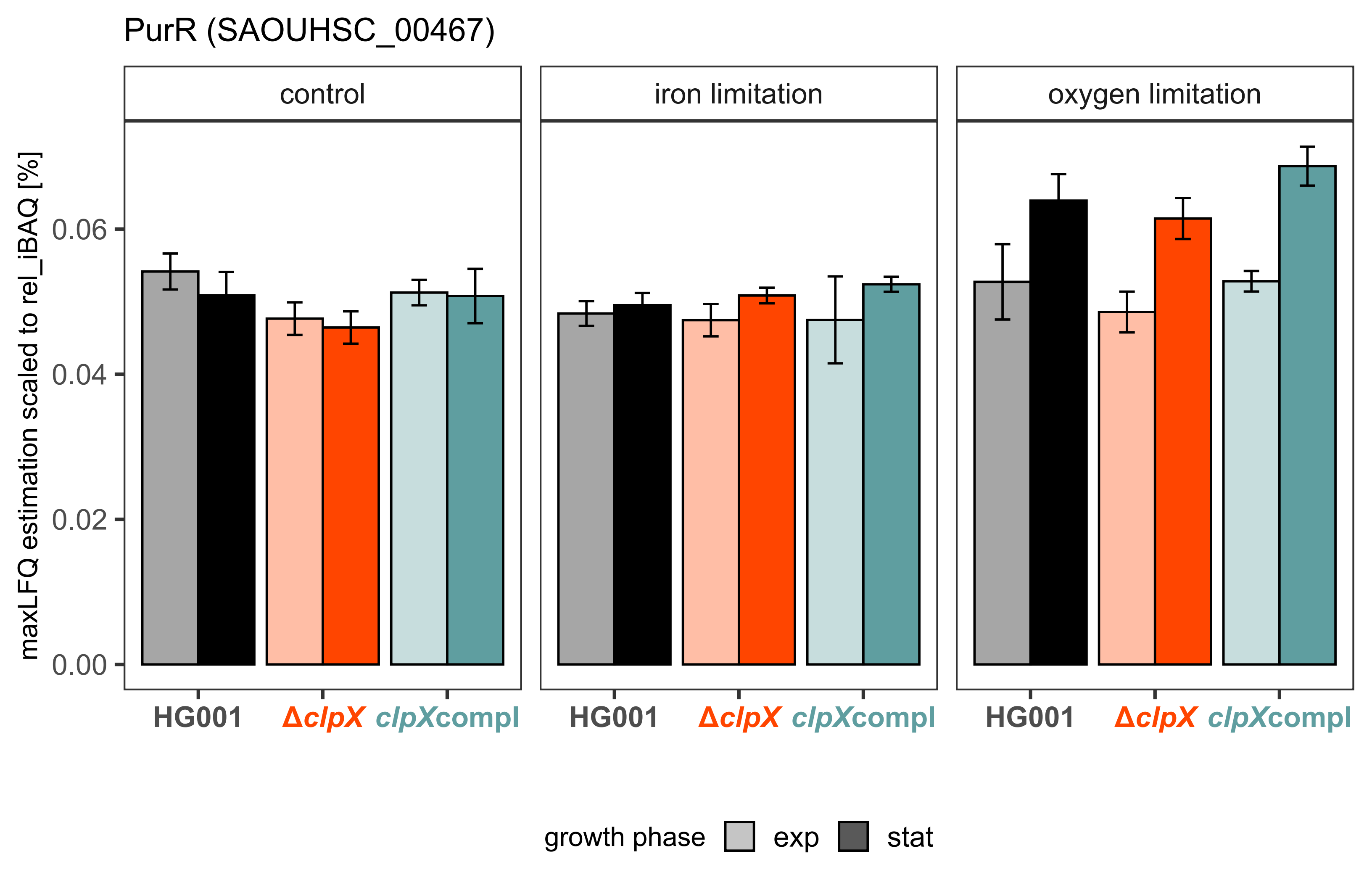

PurR (SAOUHSC_00467)
control
iron limitation
oxygen limitation
maxLFQ estimation scaled to rel_iBAQ [%]
0.00
0.02
0.04
0.06
HG001
ΔclpX
clpXcompl
growth phase
exp
stat

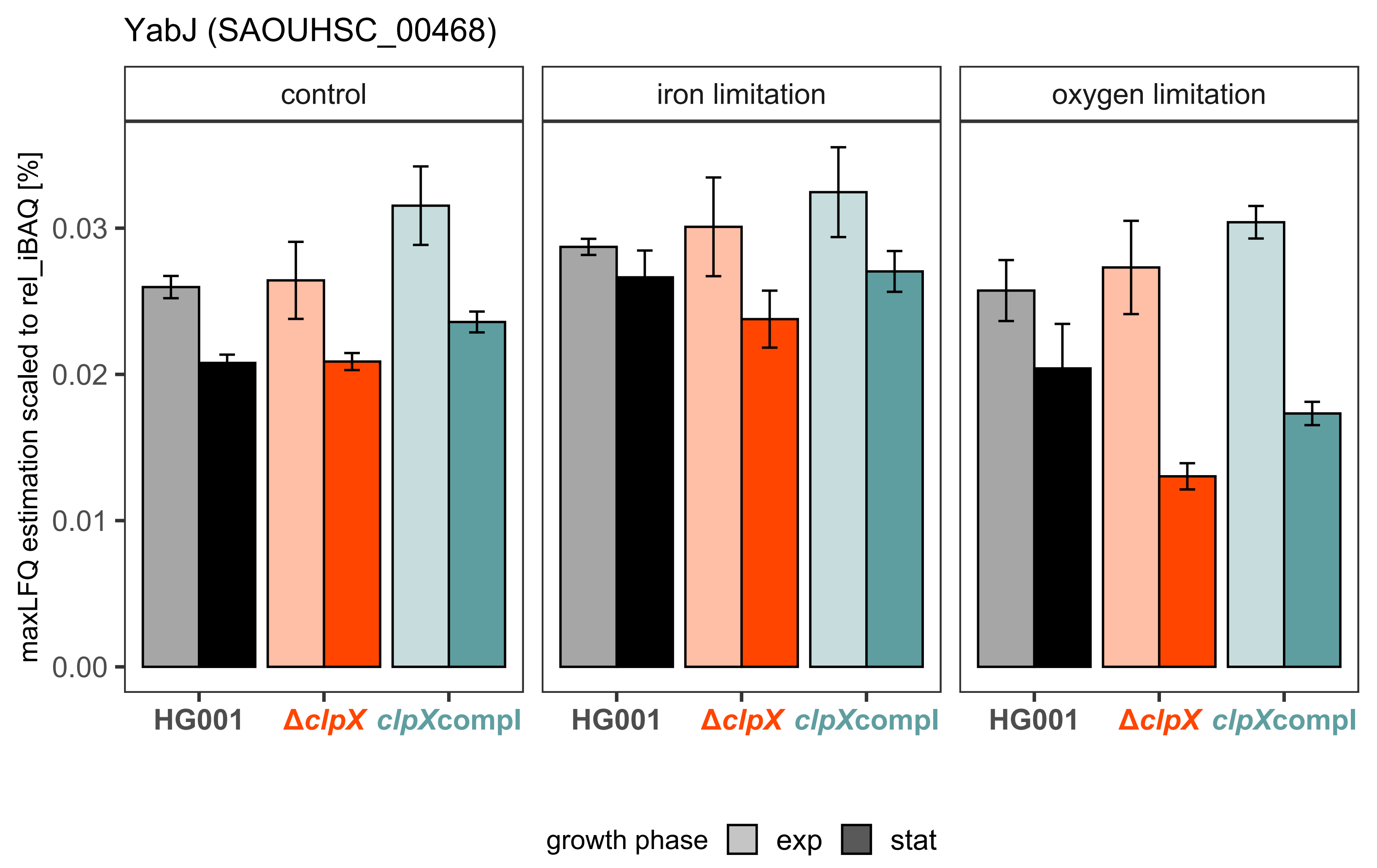

YabJ (SAOUHSC_00468)
control
iron limitation
oxygen limitation
maxLFQ estimation scaled to rel_iBAQ [%]
0.03
0.02
0.01
0.00
HG001
ΔclpX
clpXcompl
growth phase
exp
stat

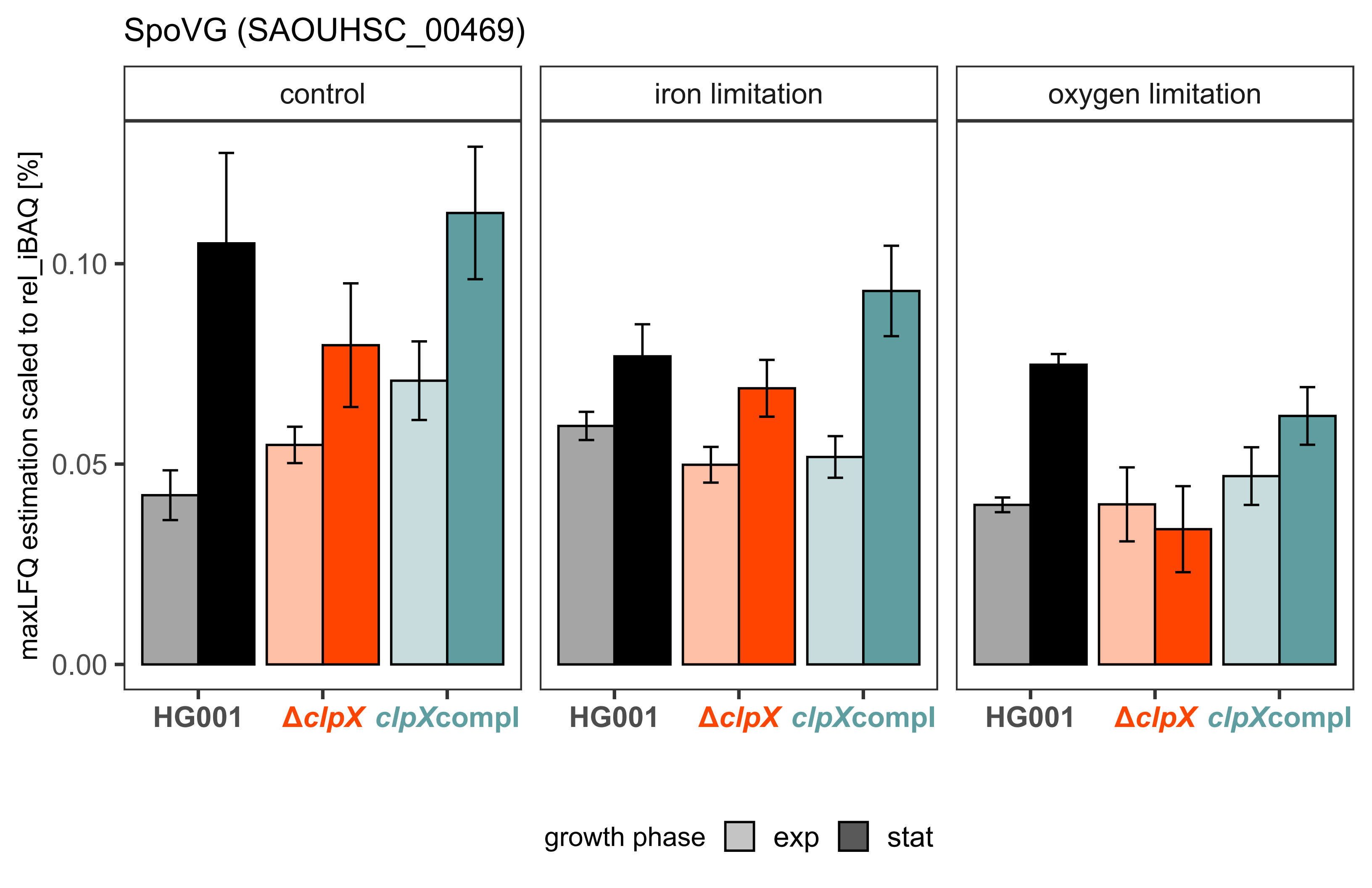

SpoVG (SAOUHSC_00469)
control
iron limitation
oxygen limitation
maxLFQ estimation scaled to rel_iBAQ [%]
0.10
0.05
0.00
HG001
ΔclpX
clpXcompl
growth phase
exp
stat

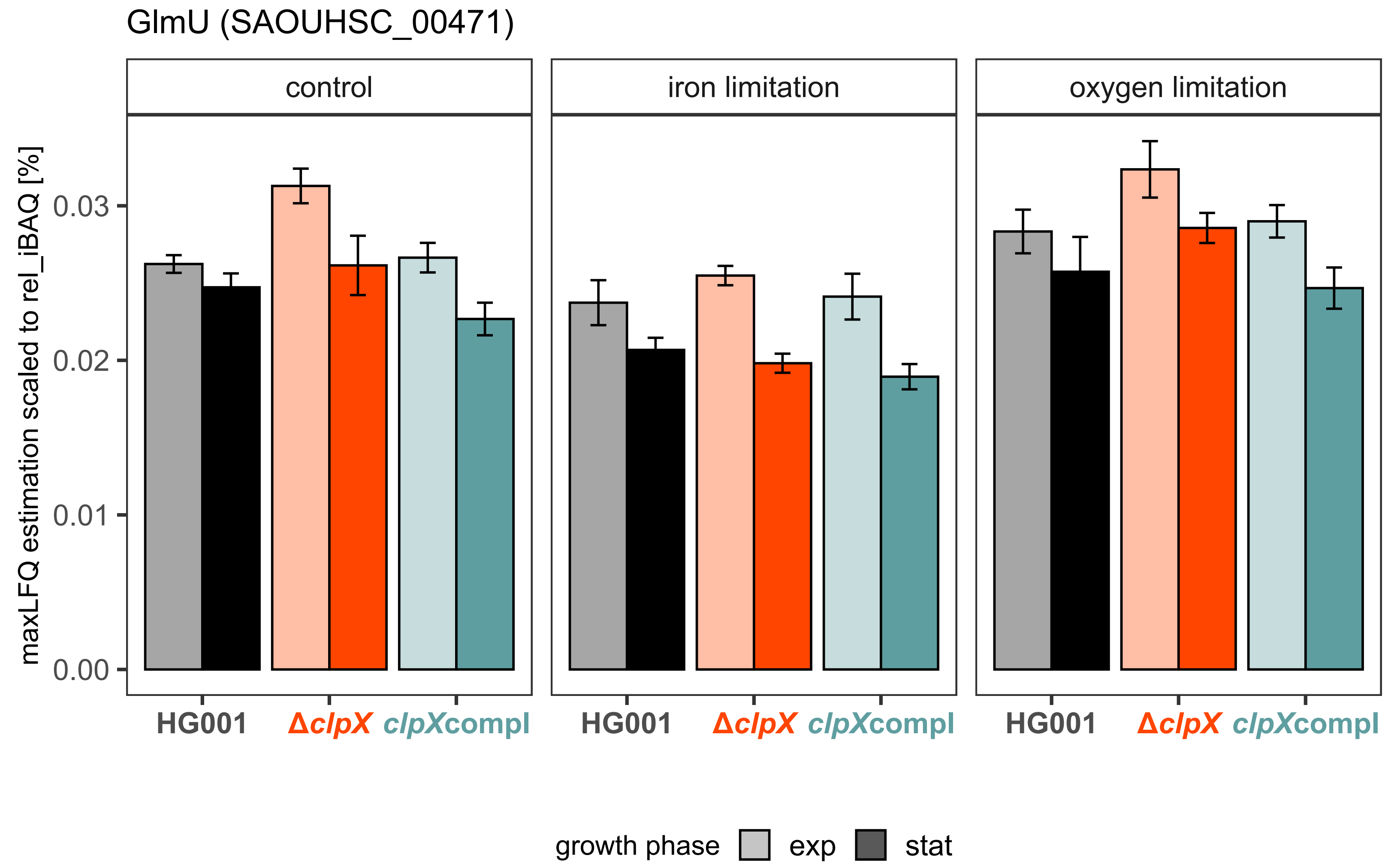
GlmU (SAOUHSC_00471)
control
iron limitation
oxygen limitation
maxLFQ estimation scaled to rel_iBAQ [%]
0.03
0.02
0.01
0.00
HG001
ΔclpX
clpXcompl
growth phase
exp
stat

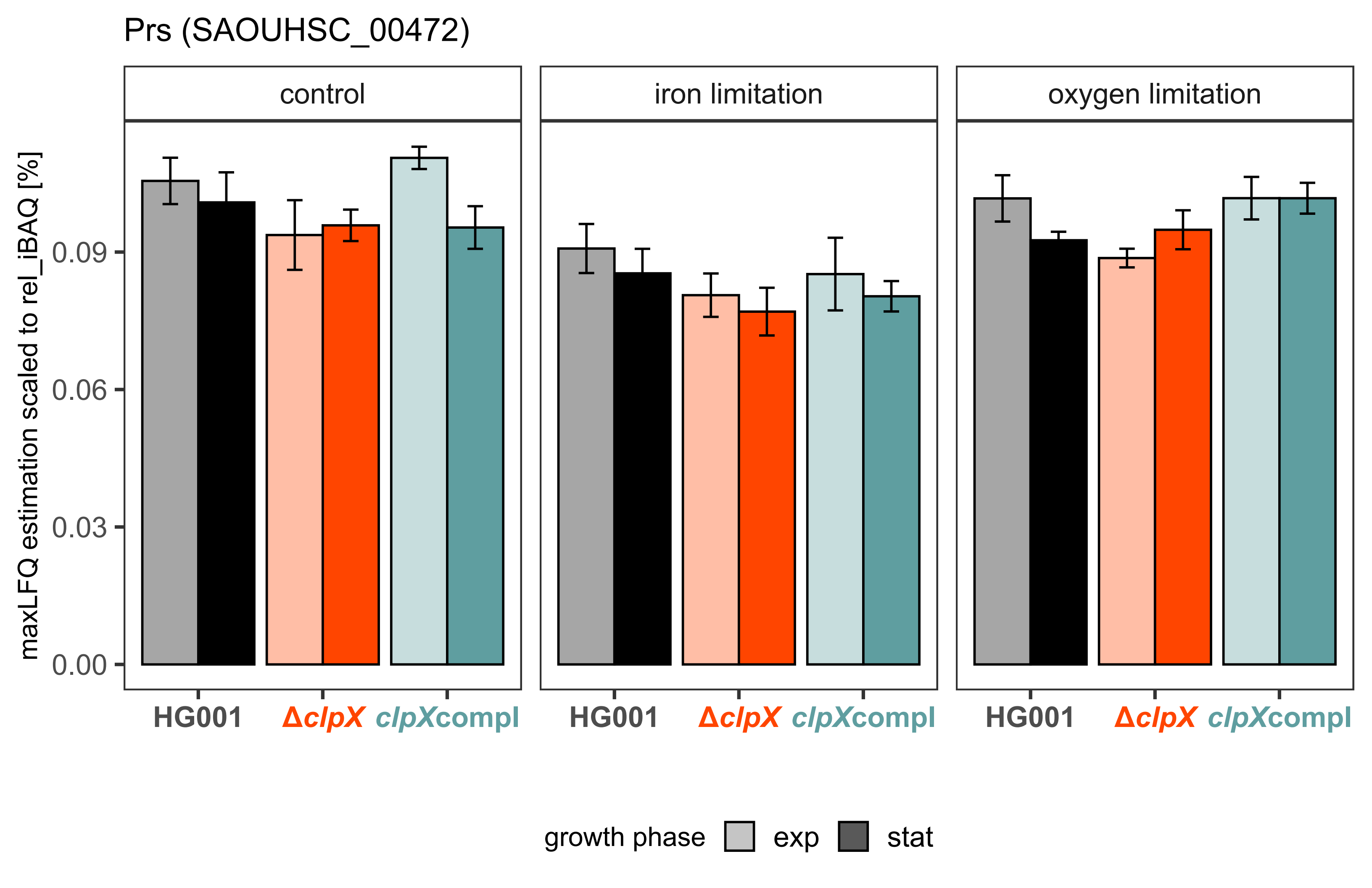
Prs (SAOUHSC_00472)
control
iron limitation
oxygen limitation
maxLFQ estimation scaled to rel_iBAQ [%]
0.00
0.03
0.06
0.09
HG001
ΔclpX
clpXcompl
growth phase
exp
stat

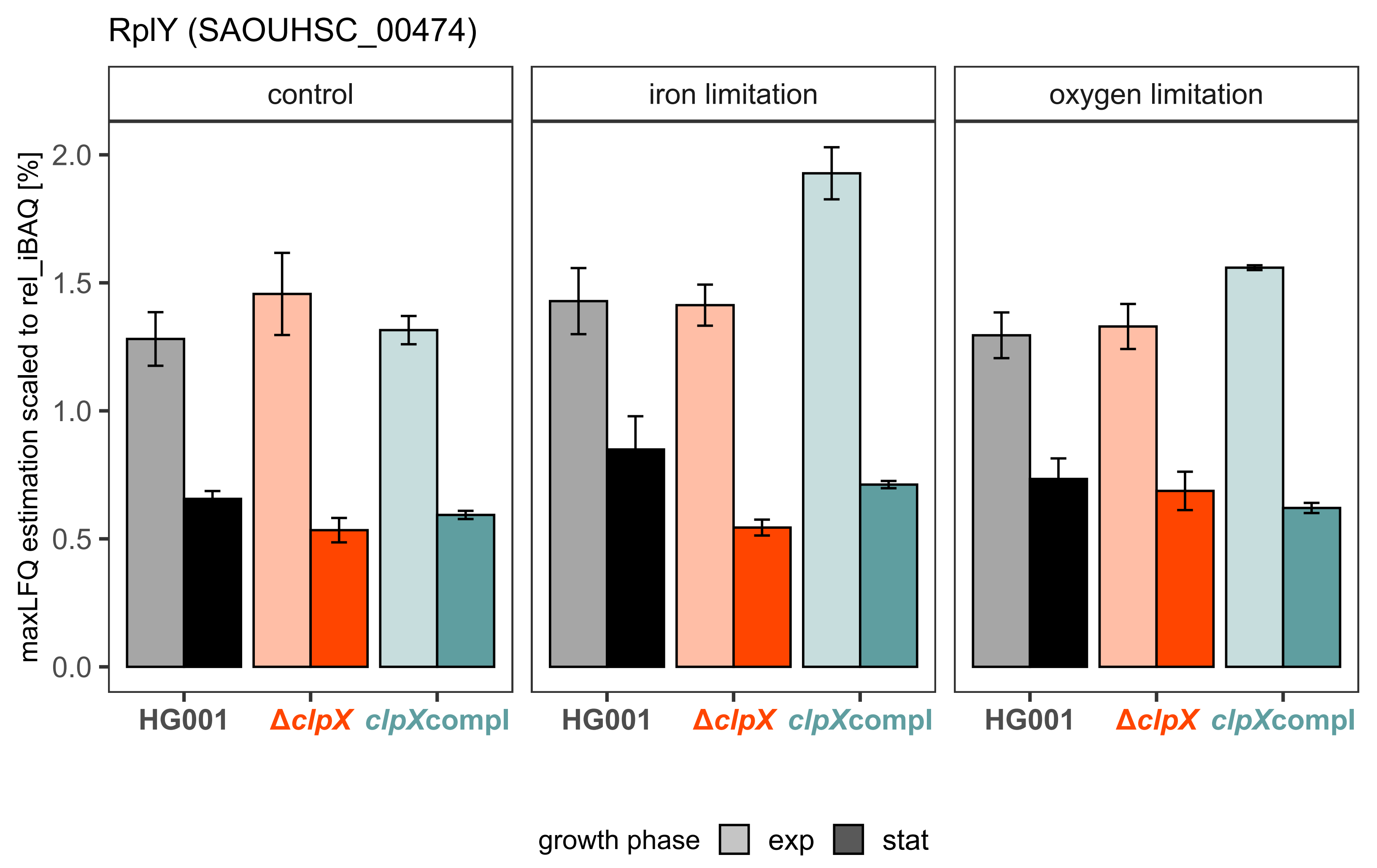
RplY (SAOUHSC_00474)
control
iron limitation
oxygen limitation
maxLFQ estimation scaled to rel_iBAQ [%]
2.0
1.5
1.0
0.5
0.0
HG001
ΔclpX
clpXcompl
growth phase
exp
stat

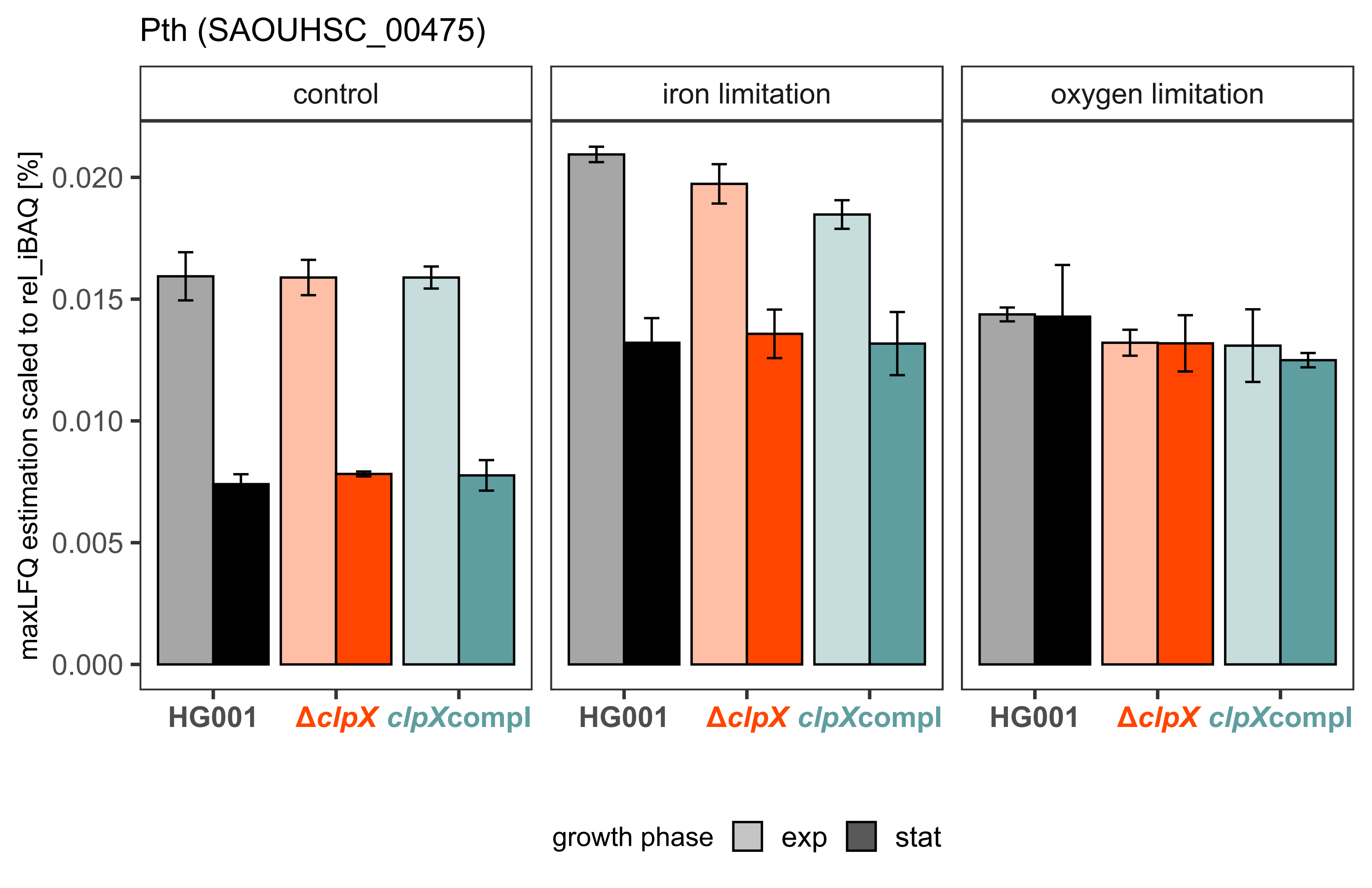

Pth (SAOUHSC_00475)
control
iron limitation
oxygen limitation
maxLFQ estimation scaled to rel_iBAQ [%]
0.020
0.015
0.010
0.005
0.000
HG001
ΔclpX
clpXcompl
growth phase
exp
stat

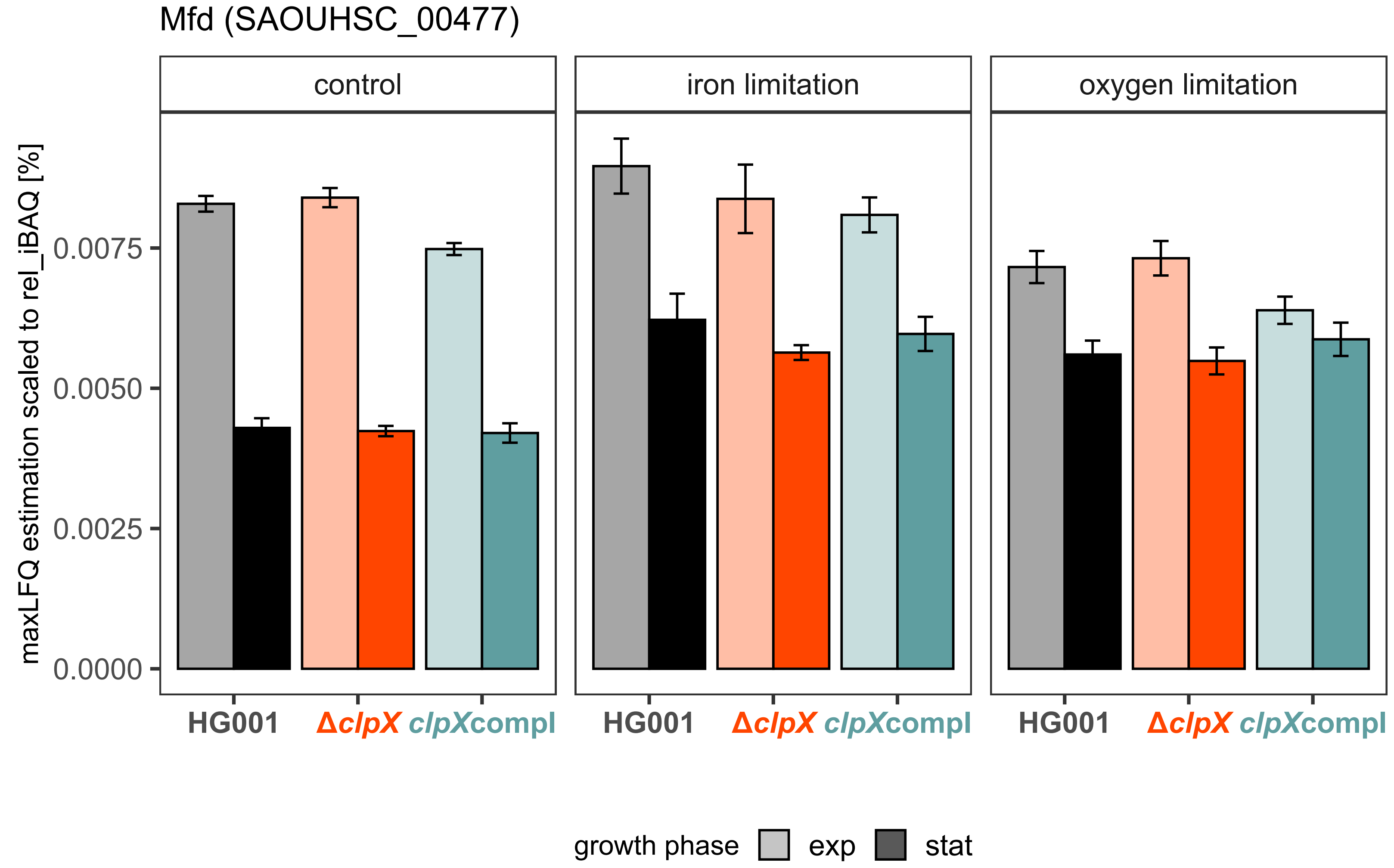

Mfd (SAOUHSC_00477)
control
iron limitation
oxygen limitation
maxLFQ estimation scaled to rel_iBAQ [%]
0.0075
0.0050
0.0025
0.0000
HG001
ΔclpX
clpXcompl
growth phase
exp
stat

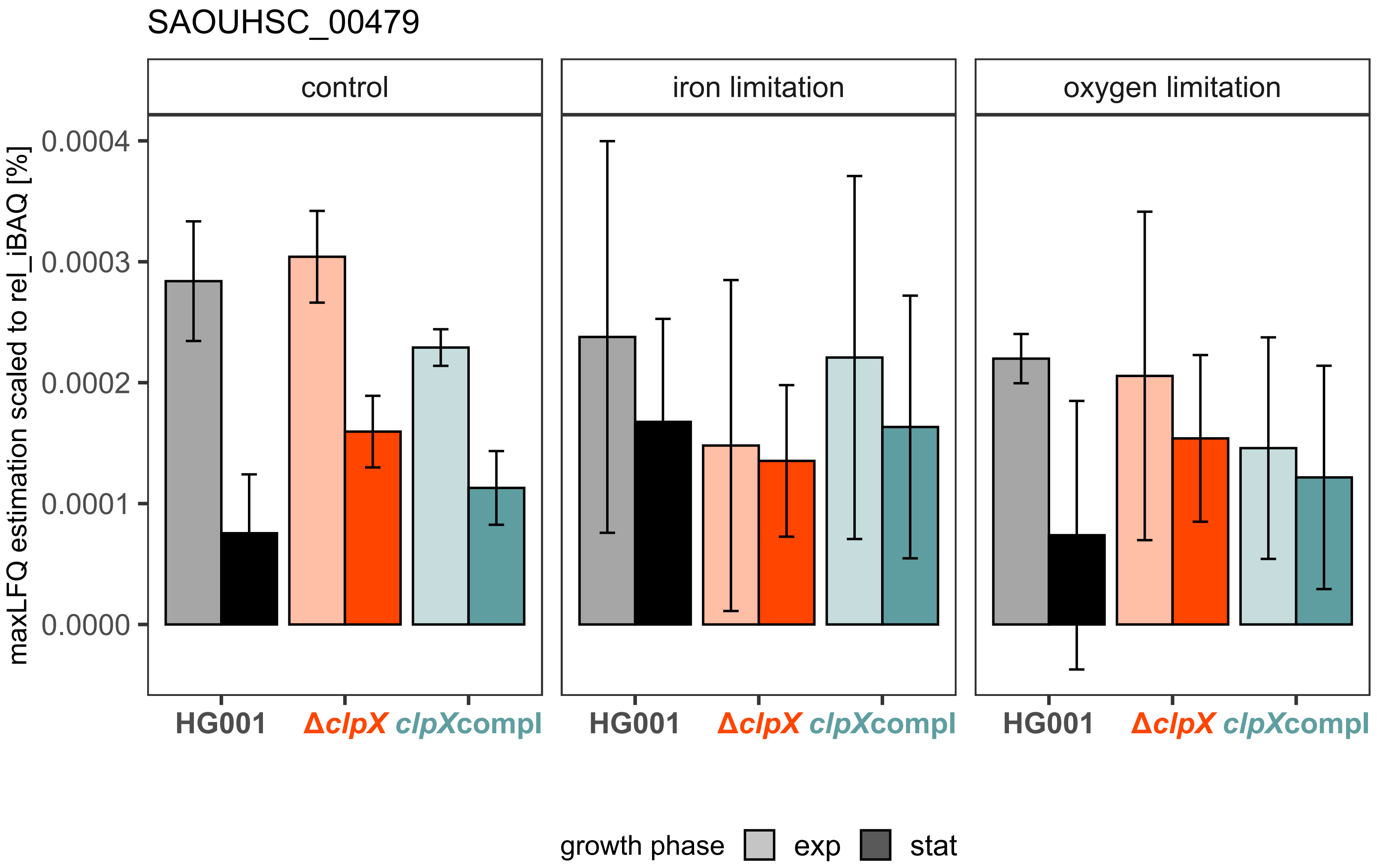

SAOUHSC_00479
control
iron limitation
oxygen limitation
maxLFQ estimation scaled to rel_iBAQ [%]
0.0004
0.0003
0.0002
0.0001
0.0000
HG001
ΔclpX
clpXcompl
growth phase
exp
stat

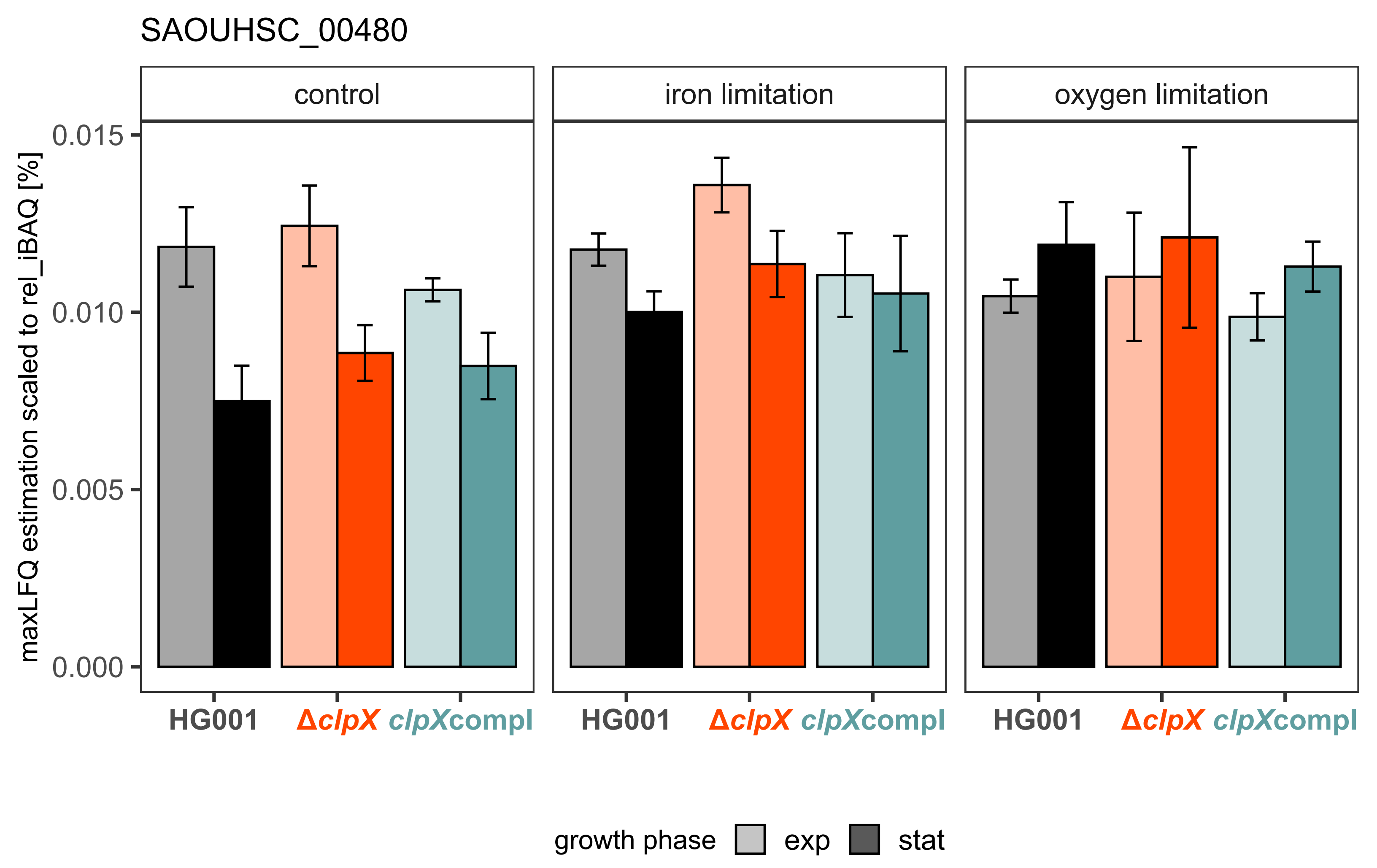
SAOUHSC_00480
control
iron limitation
oxygen limitation
maxLFQ estimation scaled to rel_iBAQ [%]
0.015
0.010
0.005
0.000
HG001
ΔclpX
clpXcompl
growth phase
exp
stat

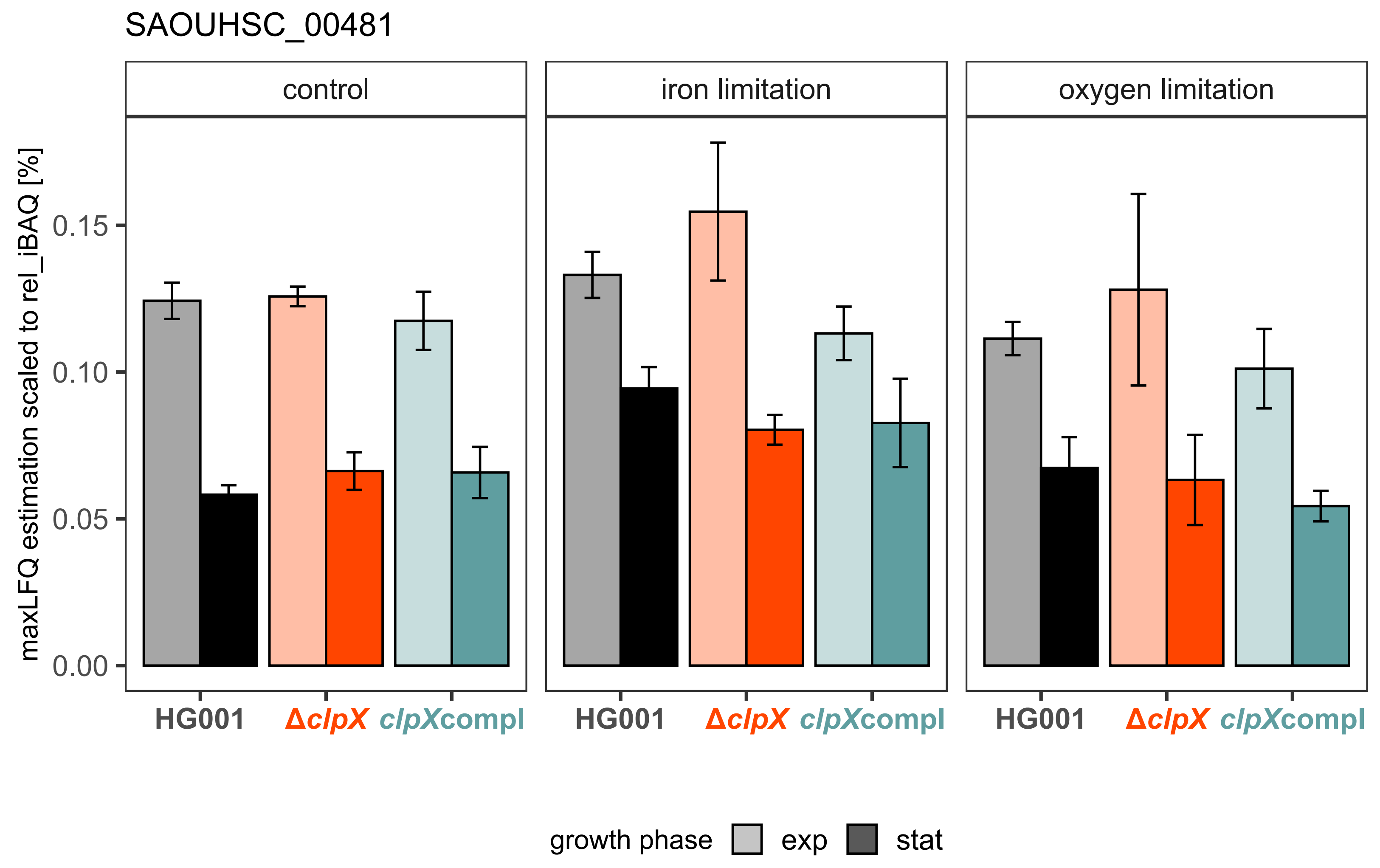
SAOUHSC_00481
control
iron limitation
oxygen limitation
maxLFQ estimation scaled to rel_iBAQ [%]
0.15
0.10
0.05
0.00
HG001
ΔclpX
clpXcompl
growth phase
exp
stat

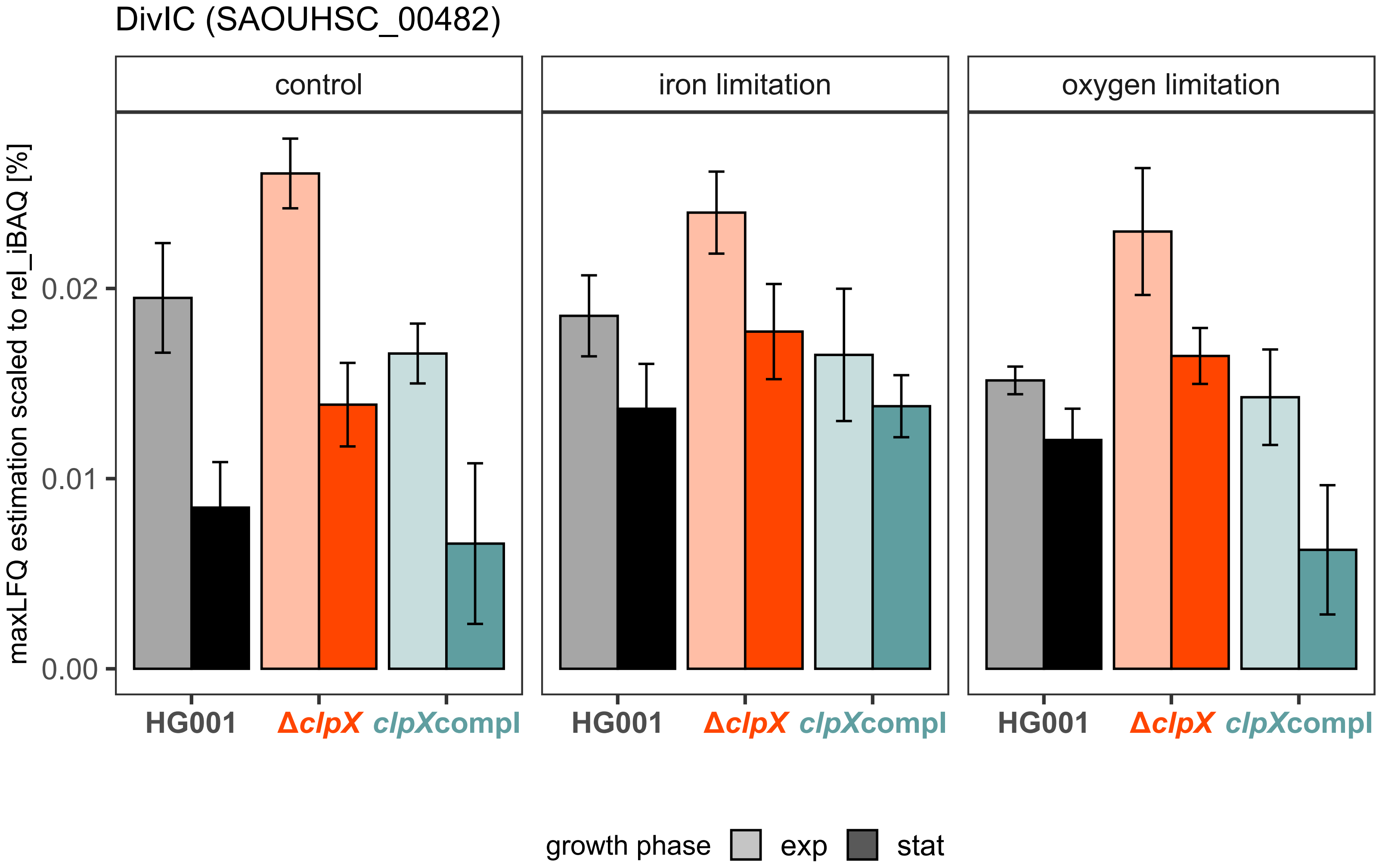

DivIC (SAOUHSC_00482)
control
iron limitation
oxygen limitation
maxLFQ estimation scaled to rel_iBAQ [%]
0.02
0.01
0.00
HG001
ΔclpX
clpXcompl
growth phase
exp
stat

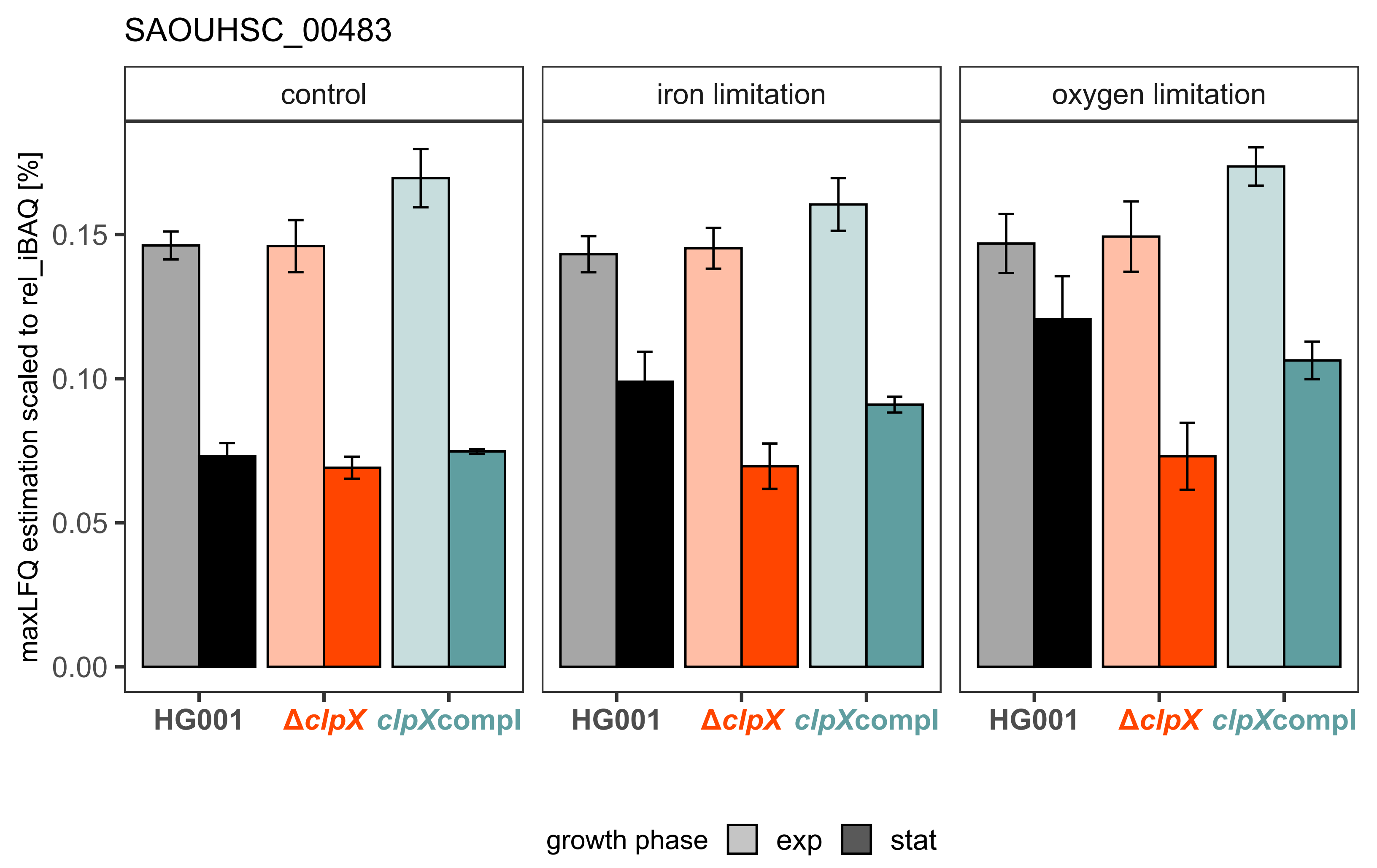
SAOUHSC_00483
control
iron limitation
oxygen limitation
maxLFQ estimation scaled to rel_iBAQ [%]
0.15
0.10
0.05
0.00
HG001
ΔclpX
clpXcompl
growth phase
exp
stat

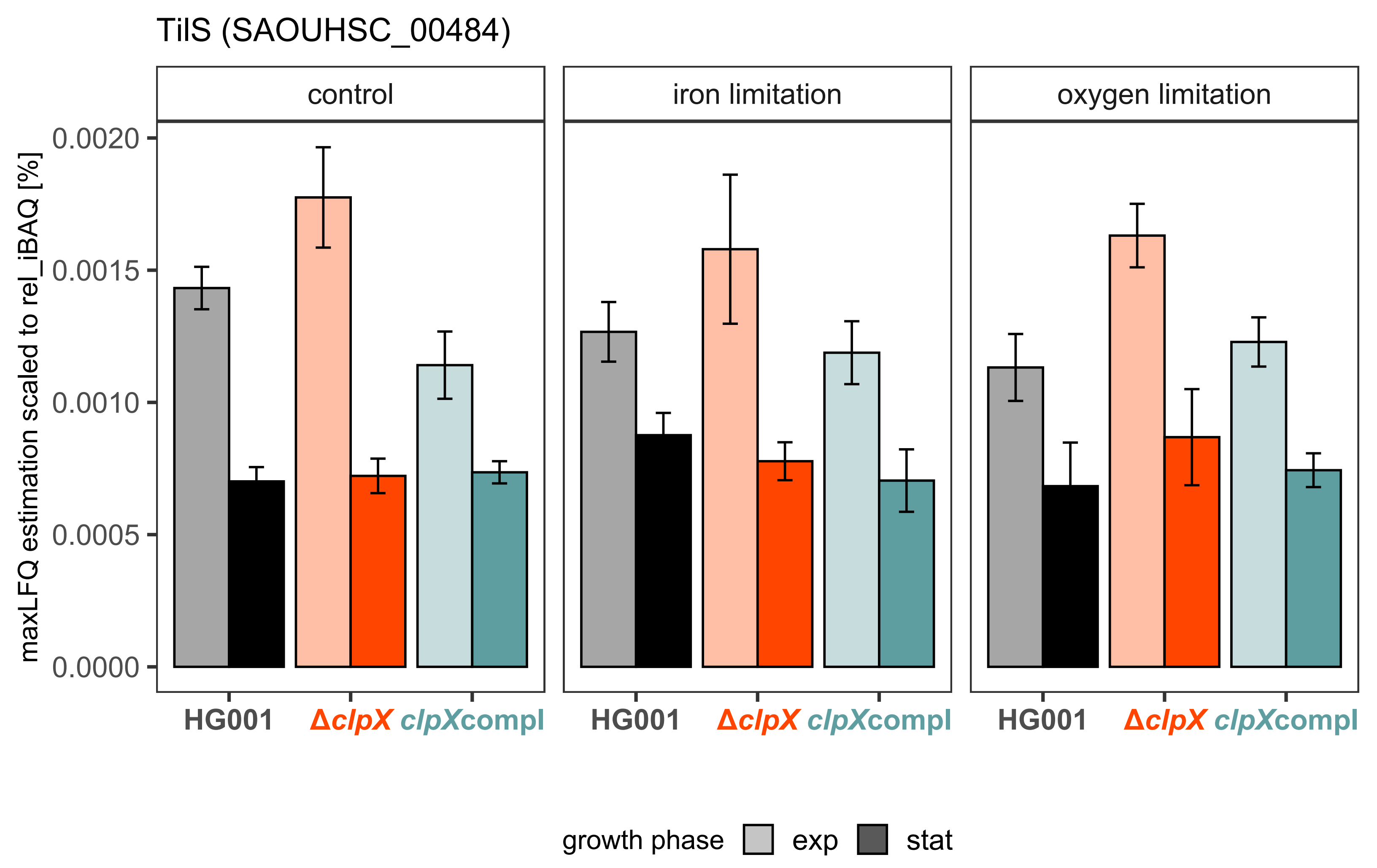

TilS (SAOUHSC_00484)
control
iron limitation
oxygen limitation
maxLFQ estimation scaled to rel_iBAQ [%]
0.0020
0.0015
0.0010
0.0005
0.0000
HG001
ΔclpX
clpXcompl
growth phase
exp
stat

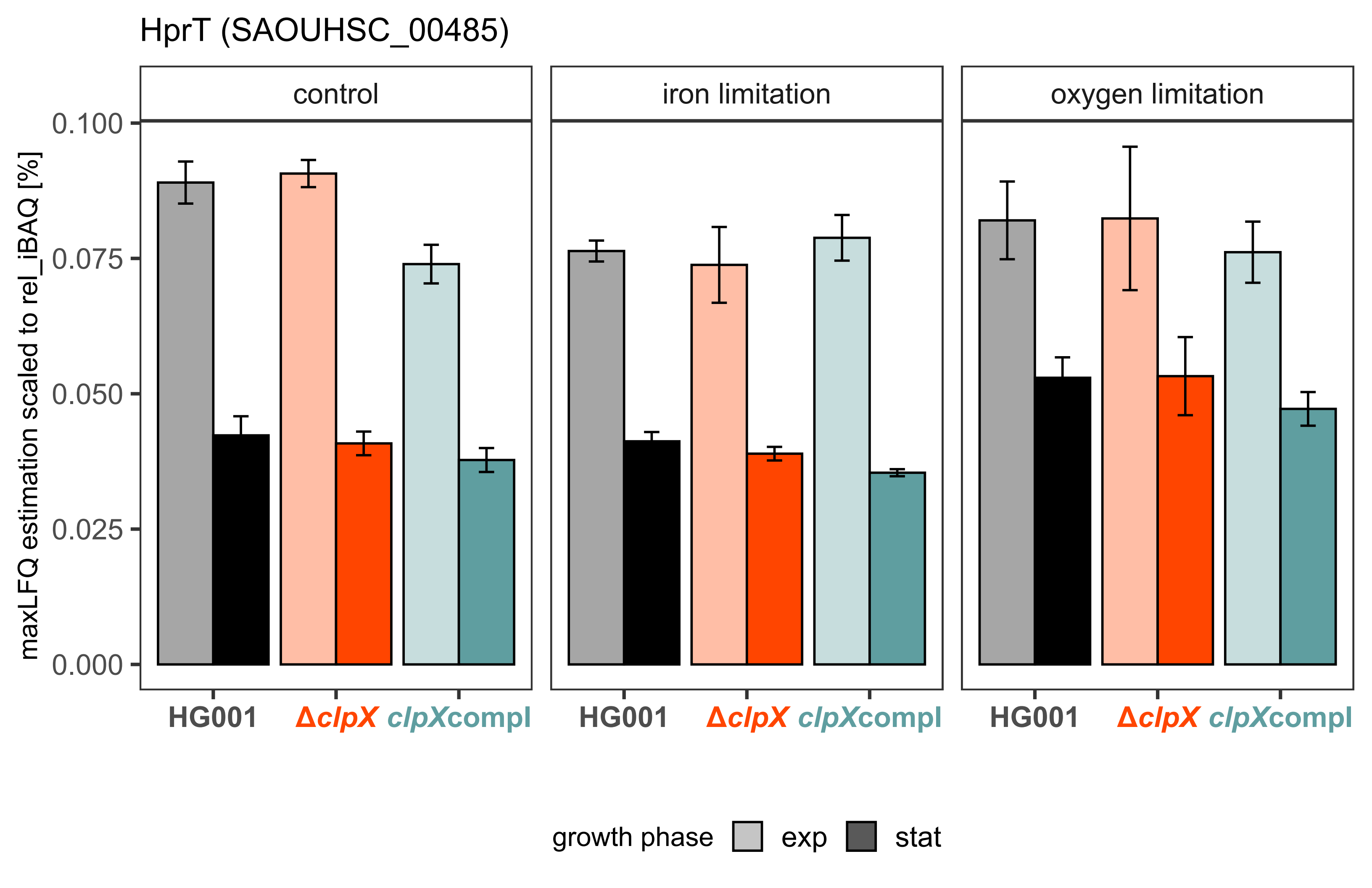
HprT (SAOUHSC_00485)
control
iron limitation
oxygen limitation
maxLFQ estimation scaled to rel_iBAQ [%]
0.100
0.075
0.050
0.025
0.000
HG001
ΔclpX
clpXcompl
growth phase
exp
stat

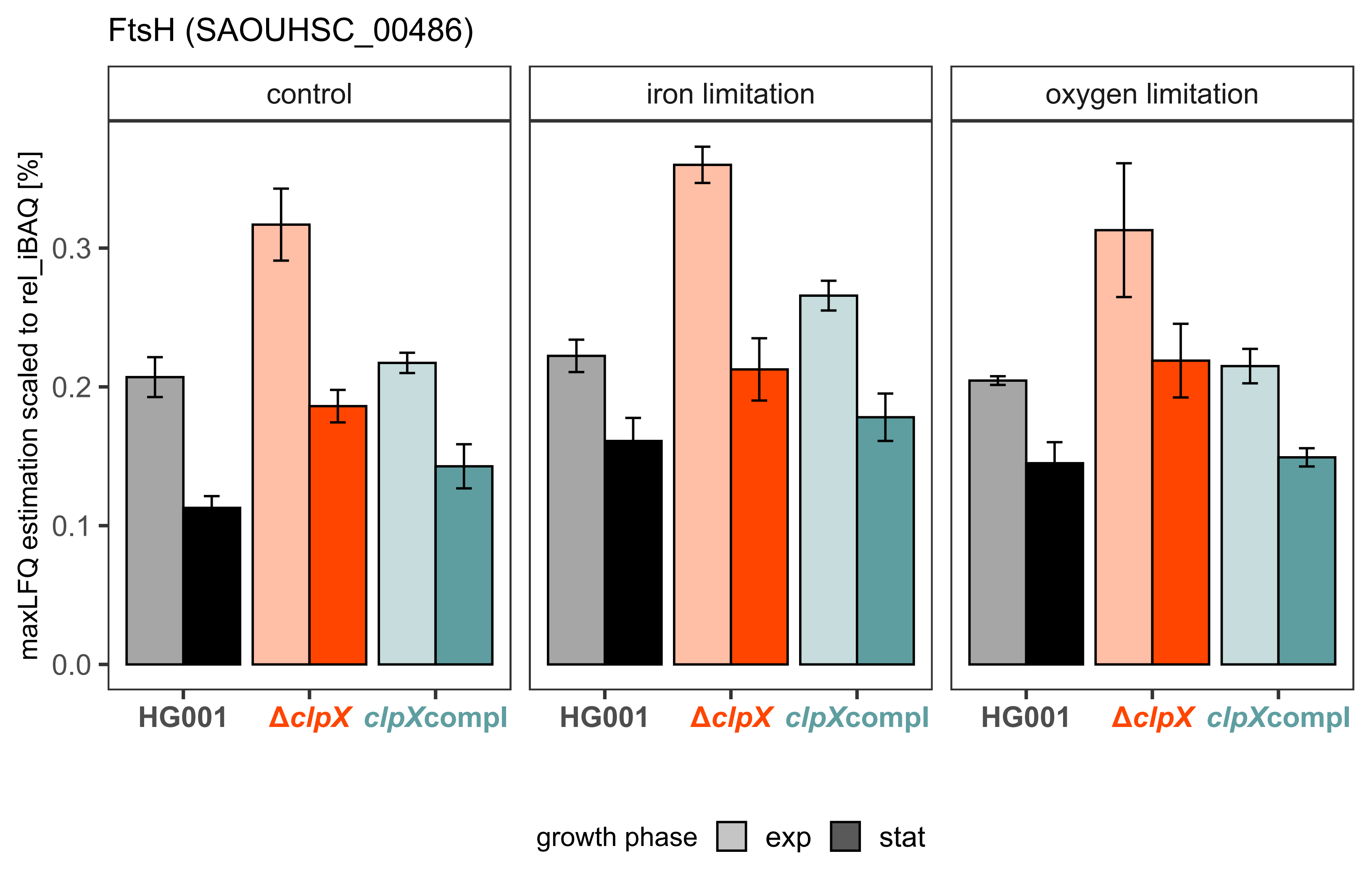
FtsH (SAOUHSC_00486)
control
iron limitation
oxygen limitation
maxLFQ estimation scaled to rel_iBAQ [%]
0.0
0.1
0.2
0.3
HG001
ΔclpX
clpXcompl
growth phase
exp
stat

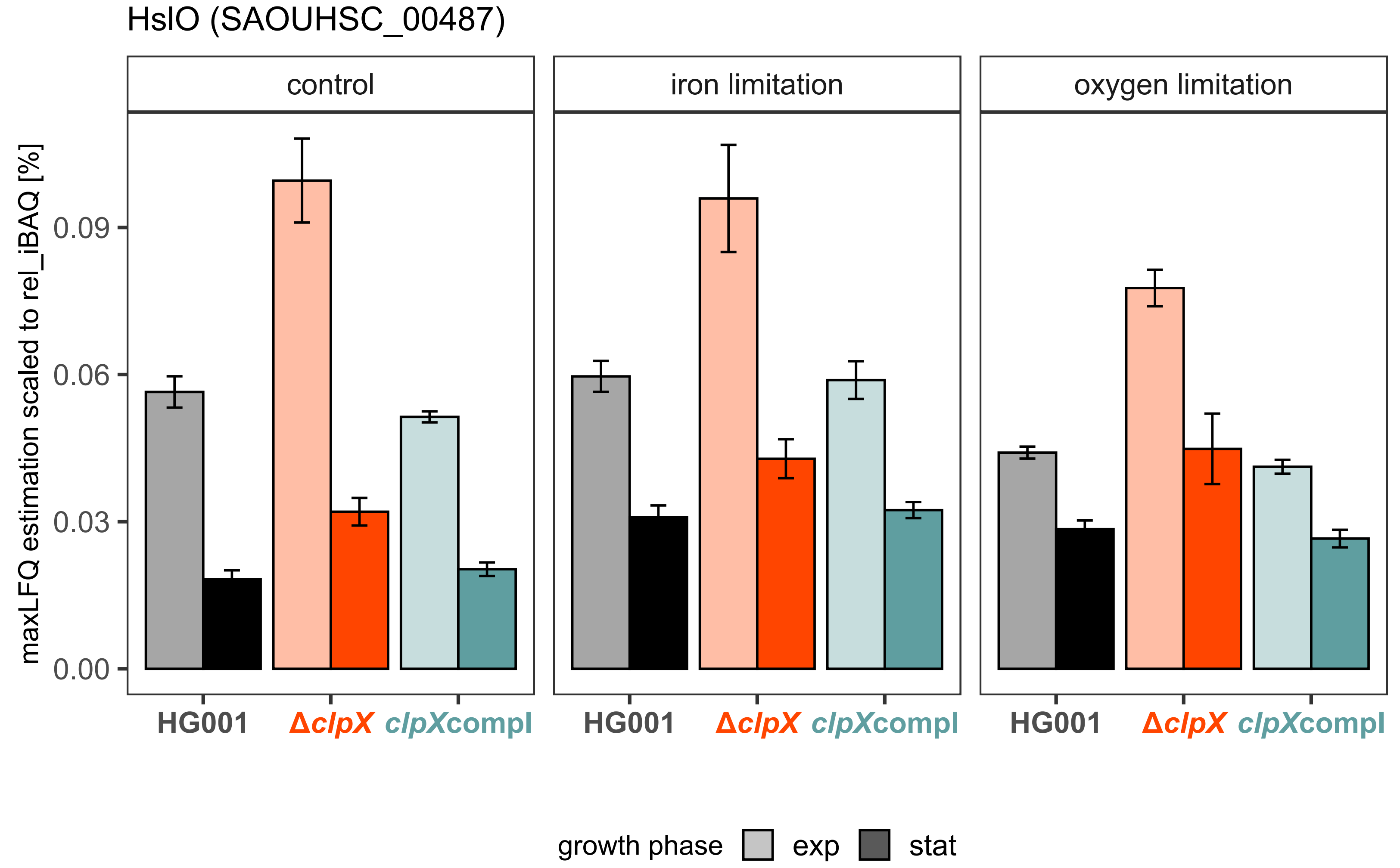

HslO (SAOUHSC_00487)
control
iron limitation
oxygen limitation
maxLFQ estimation scaled to rel_iBAQ [%]
0.00
0.03
0.06
0.09
HG001
ΔclpX
clpXcompl
growth phase
exp
stat

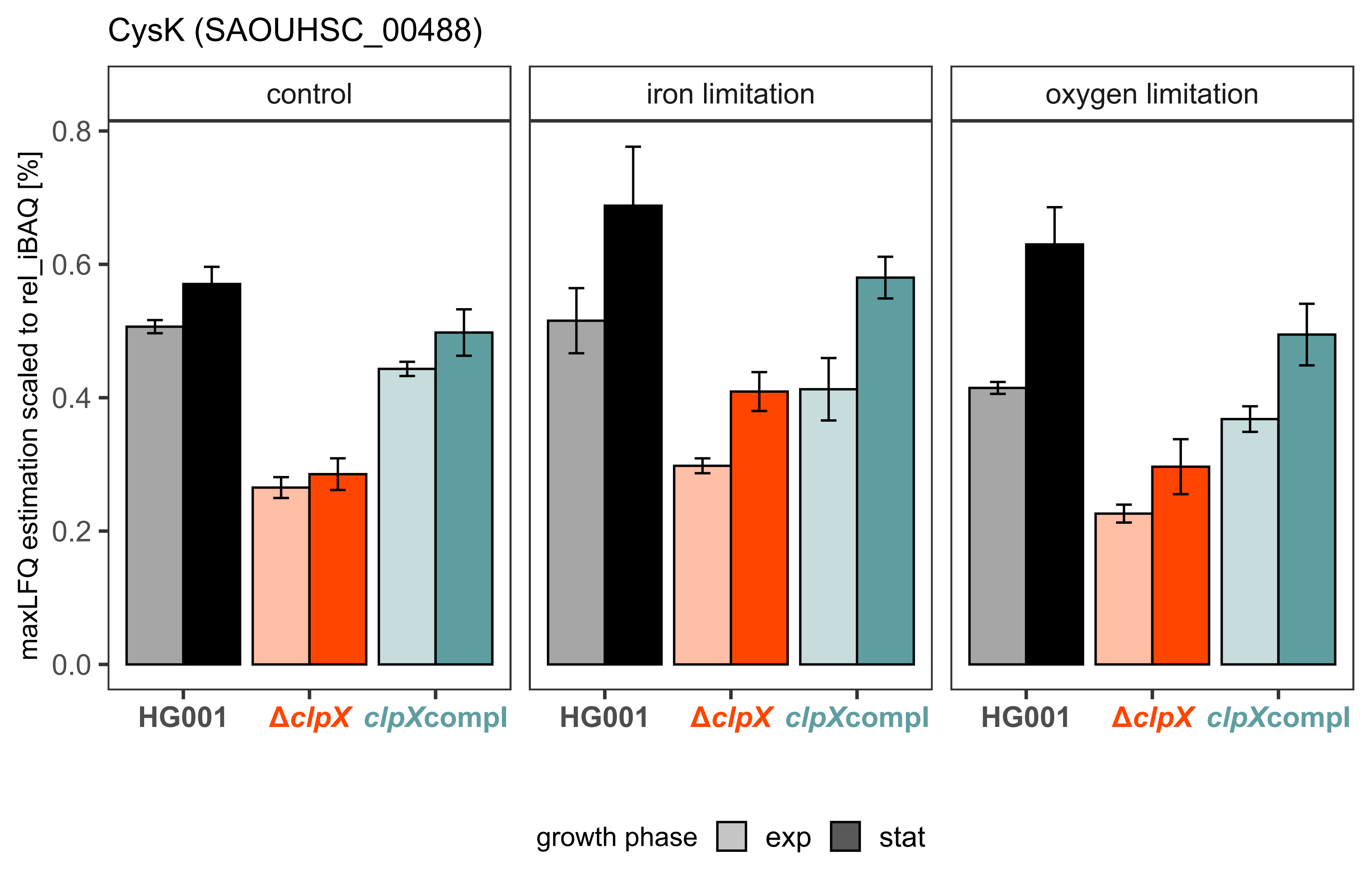

CysK (SAOUHSC_00488)
control
iron limitation
oxygen limitation
maxLFQ estimation scaled to rel_iBAQ [%]
0.0
0.2
0.4
0.6
0.8
HG001
ΔclpX
clpXcompl
growth phase
exp
stat

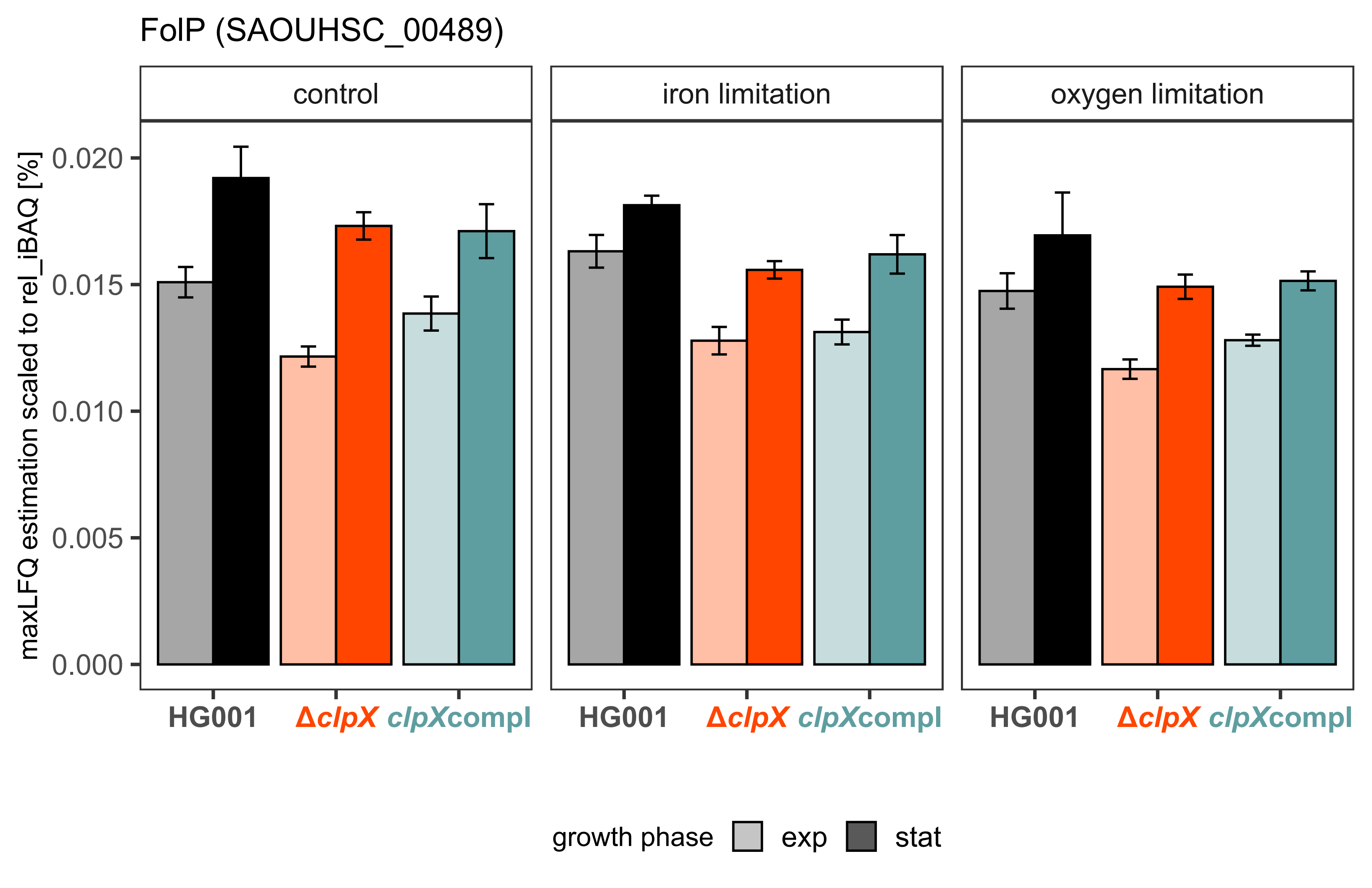
FolP (SAOUHSC_00489)
control
iron limitation
oxygen limitation
maxLFQ estimation scaled to rel_iBAQ [%]
0.020
0.015
0.010
0.005
0.000
HG001
ΔclpX
clpXcompl
growth phase
exp
stat

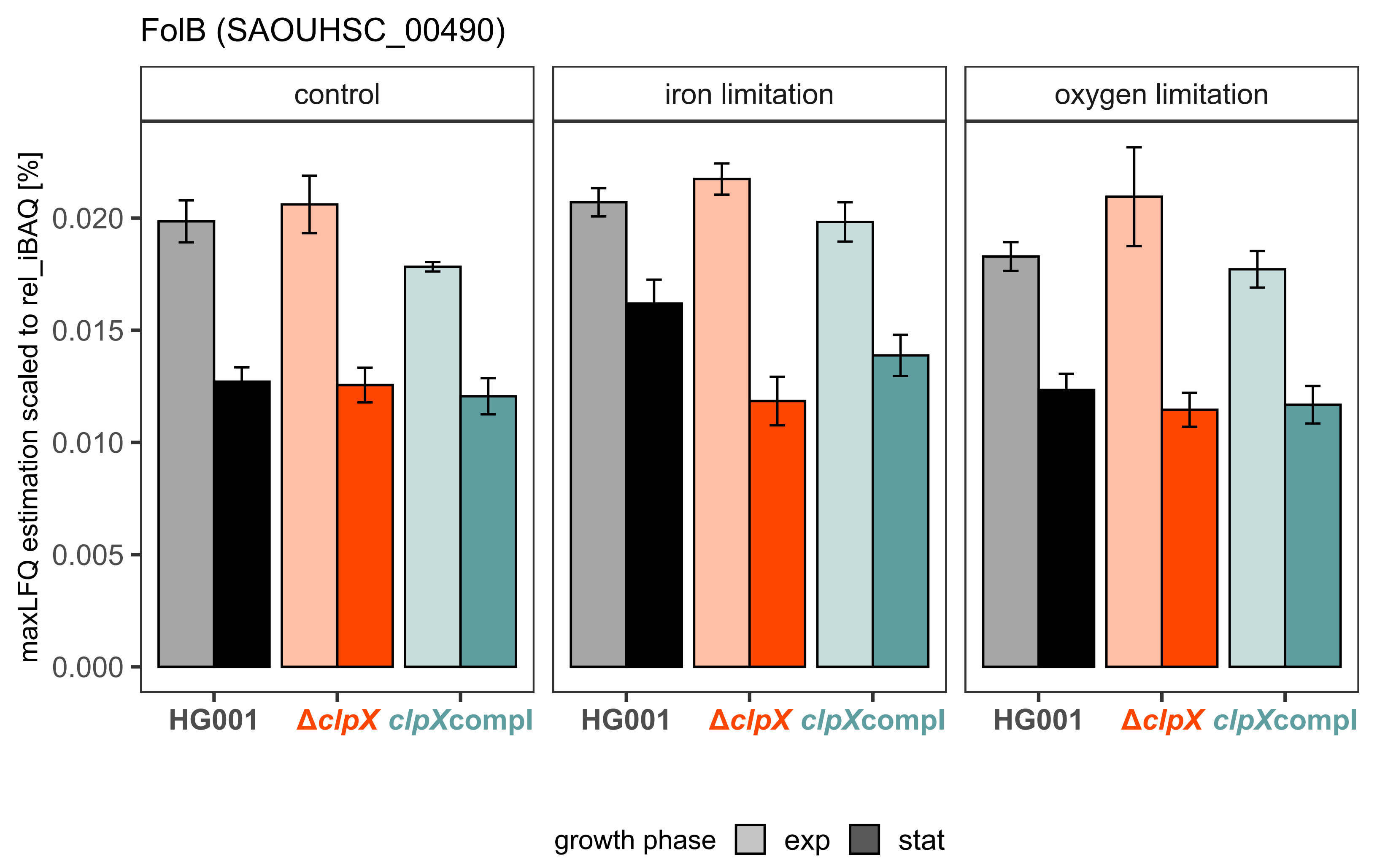
FolB (SAOUHSC_00490)
control
iron limitation
oxygen limitation
maxLFQ estimation scaled to rel_iBAQ [%]
0.020
0.015
0.010
0.005
0.000
HG001
ΔclpX
clpXcompl
growth phase
exp
stat

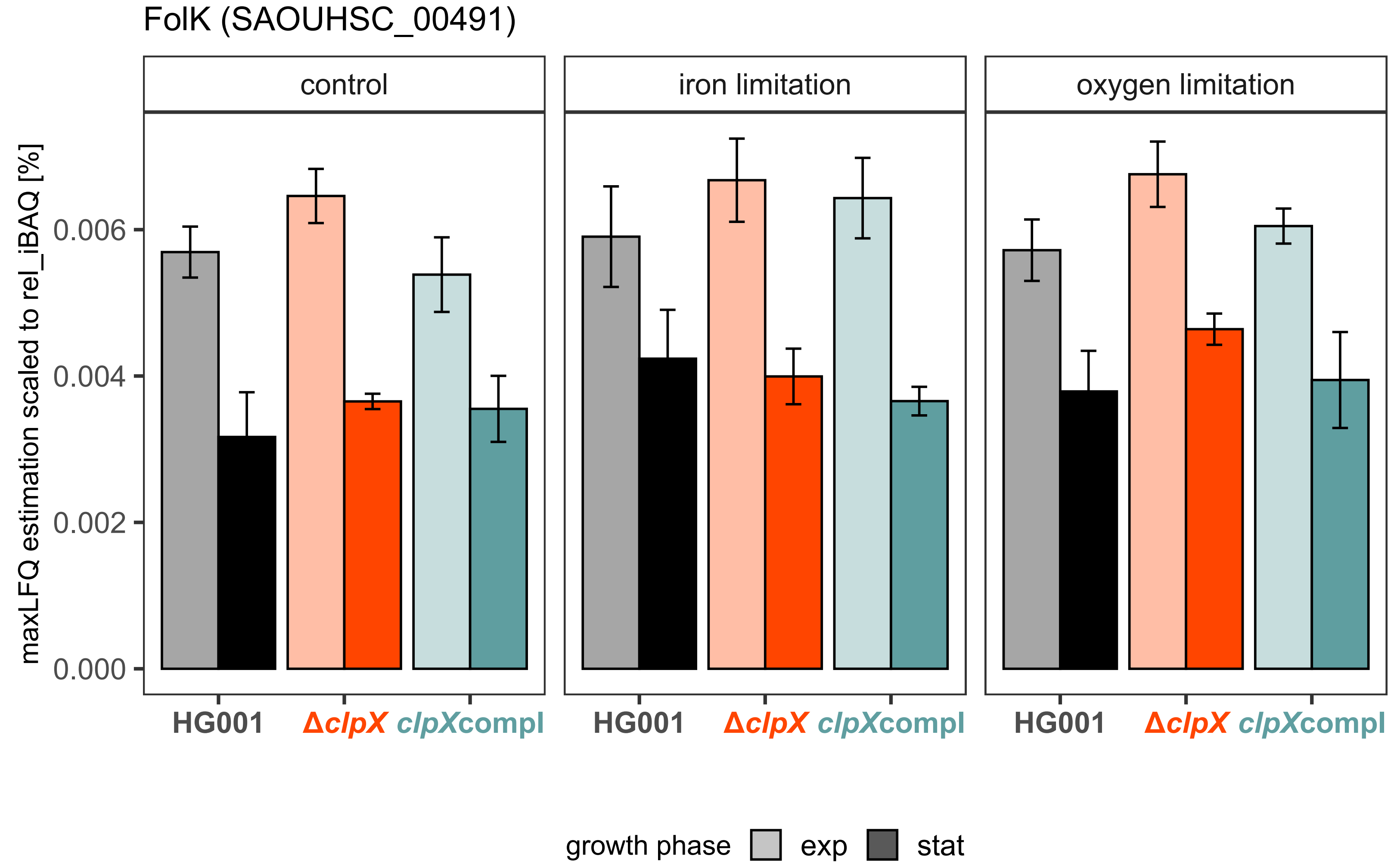
FolK (SAOUHSC_00491)
control
iron limitation
oxygen limitation
maxLFQ estimation scaled to rel_iBAQ [%]
0.006
0.004
0.002
0.000
HG001
ΔclpX
clpXcompl
growth phase
exp
stat

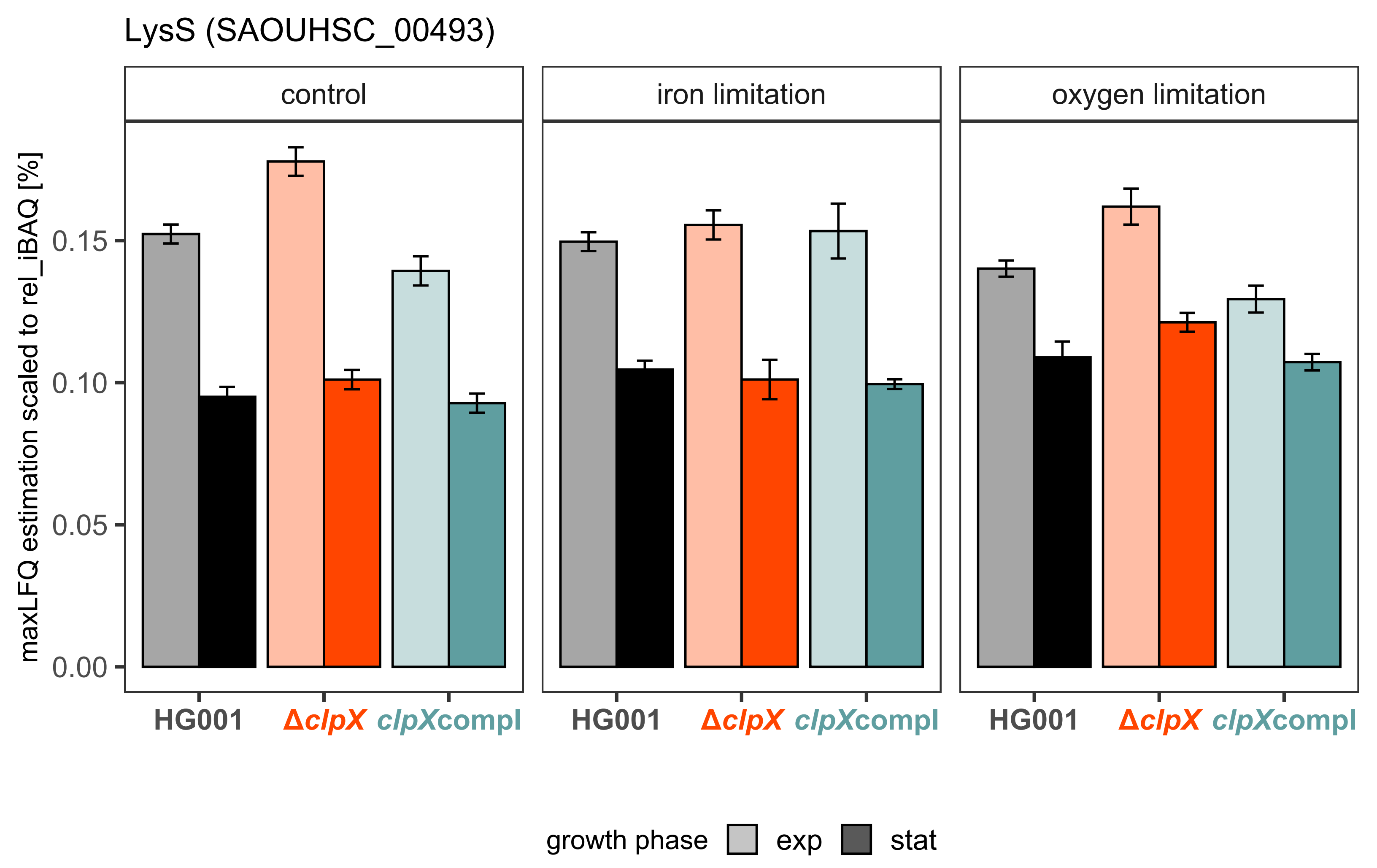
LysS (SAOUHSC_00493)
control
iron limitation
oxygen limitation
maxLFQ estimation scaled to rel_iBAQ [%]
0.15
0.10
0.05
0.00
HG001
ΔclpX
clpXcompl
growth phase
exp
stat

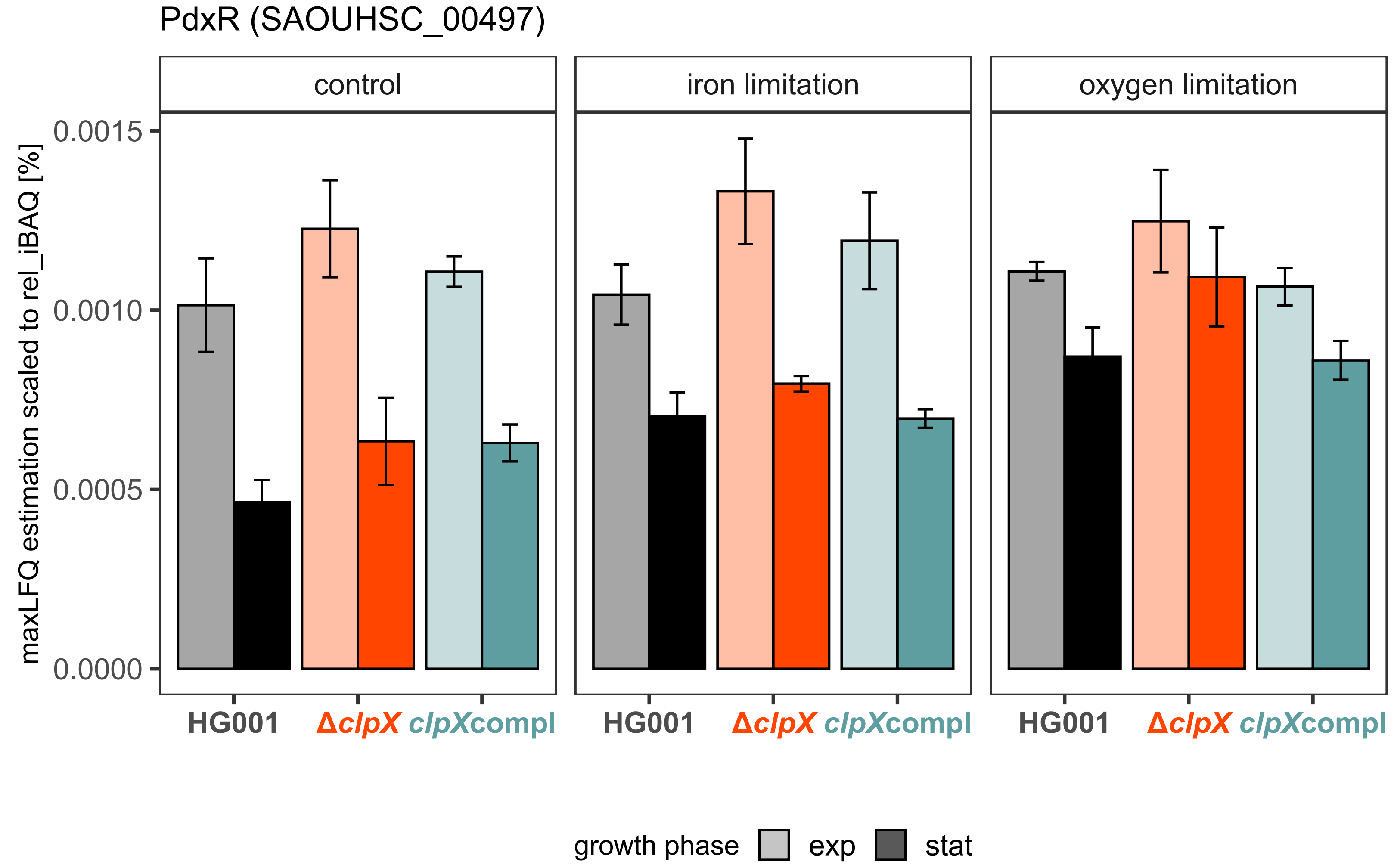

PdxR (SAOUHSC_00497)
control
iron limitation
oxygen limitation
maxLFQ estimation scaled to rel_iBAQ [%]
0.0015
0.0010
0.0005
0.0000
HG001
ΔclpX
clpXcompl
growth phase
exp
stat

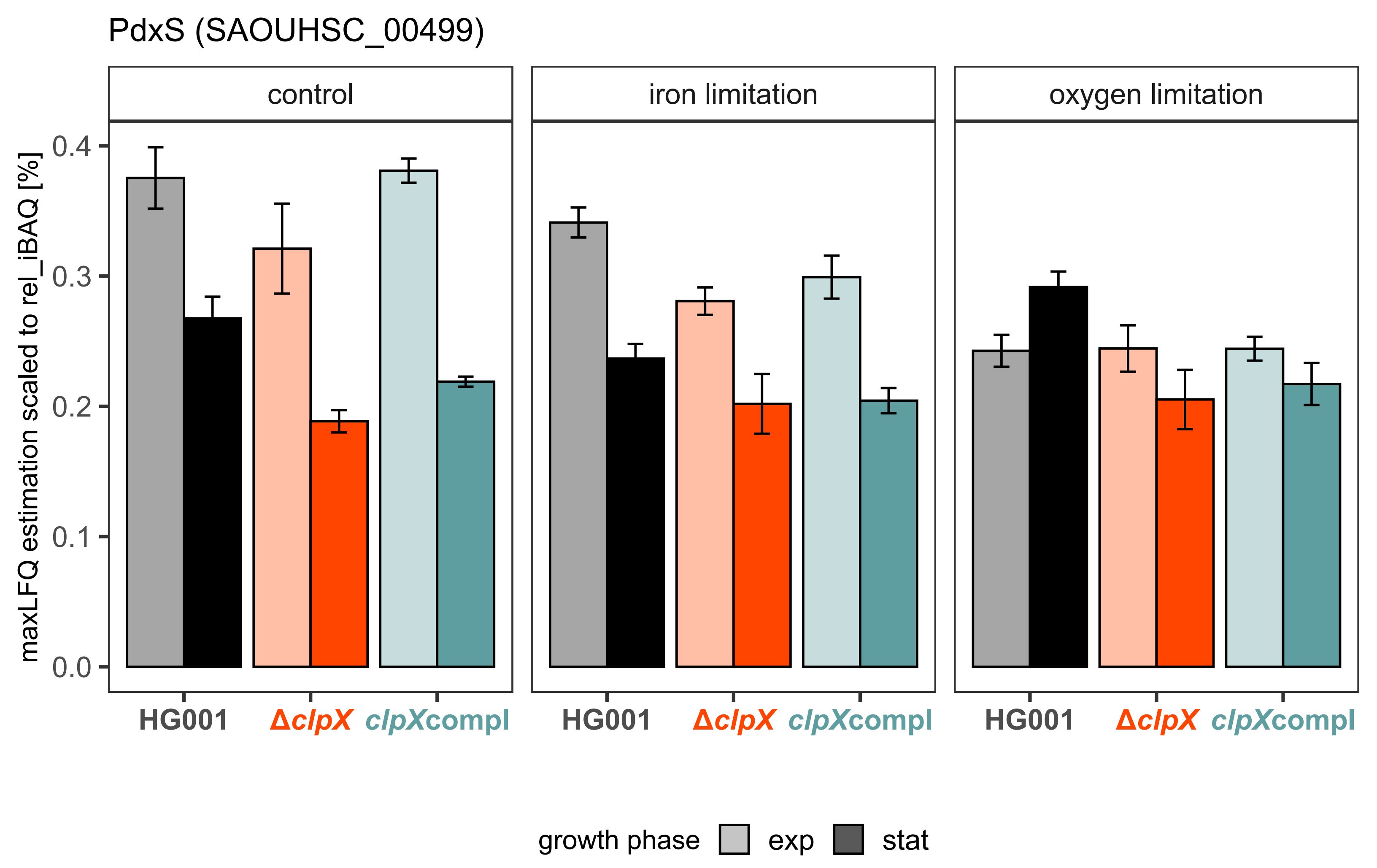

PdxS (SAOUHSC_00499)
control
iron limitation
oxygen limitation
maxLFQ estimation scaled to rel_iBAQ [%]
0.4
0.3
0.2
0.1
0.0
HG001
ΔclpX
clpXcompl
growth phase
exp
stat

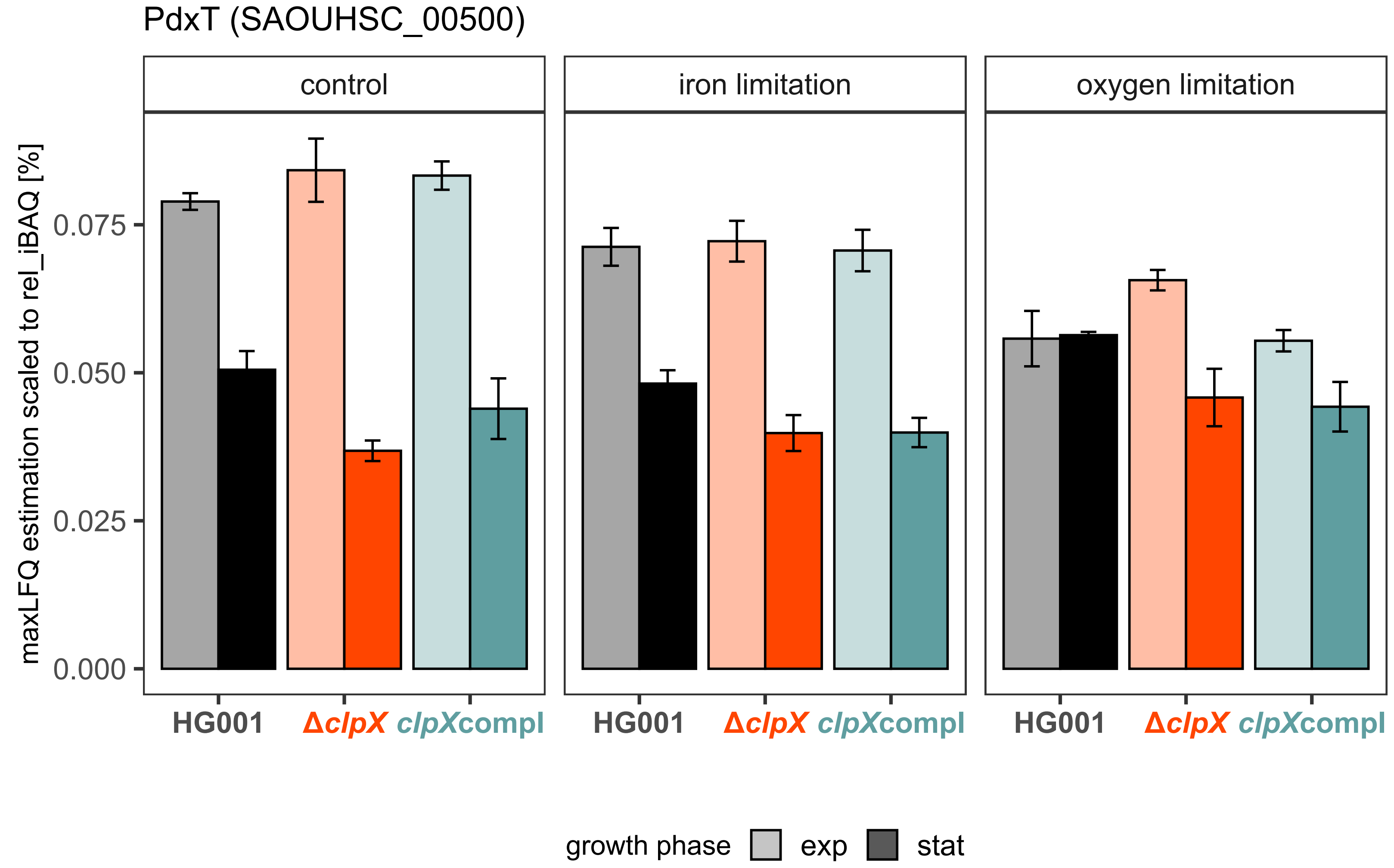
PdxT (SAOUHSC_00500)
control
iron limitation
oxygen limitation
maxLFQ estimation scaled to rel_iBAQ [%]
0.075
0.050
0.025
0.000
HG001
ΔclpX
clpXcompl
HG001
ΔclpX
clpXcompl
HG001
ΔclpX
clpXcompl
growth phase
exp
stat

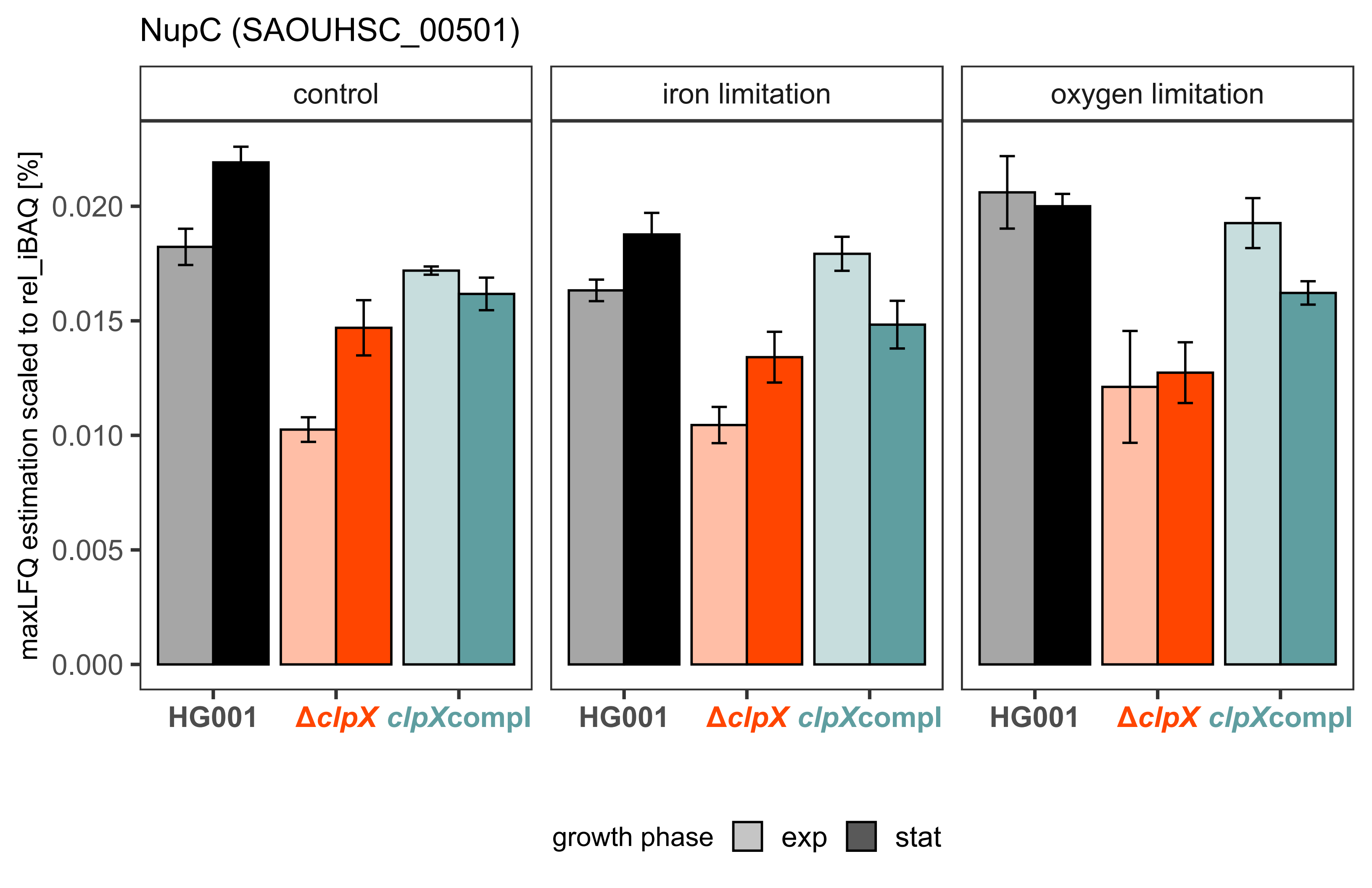

NupC (SAOUHSC_00501)
control
iron limitation
oxygen limitation
maxLFQ estimation scaled to rel_iBAQ [%]
0.020
0.015
0.010
0.005
0.000
HG001
ΔclpX
clpXcompl
growth phase
exp
stat

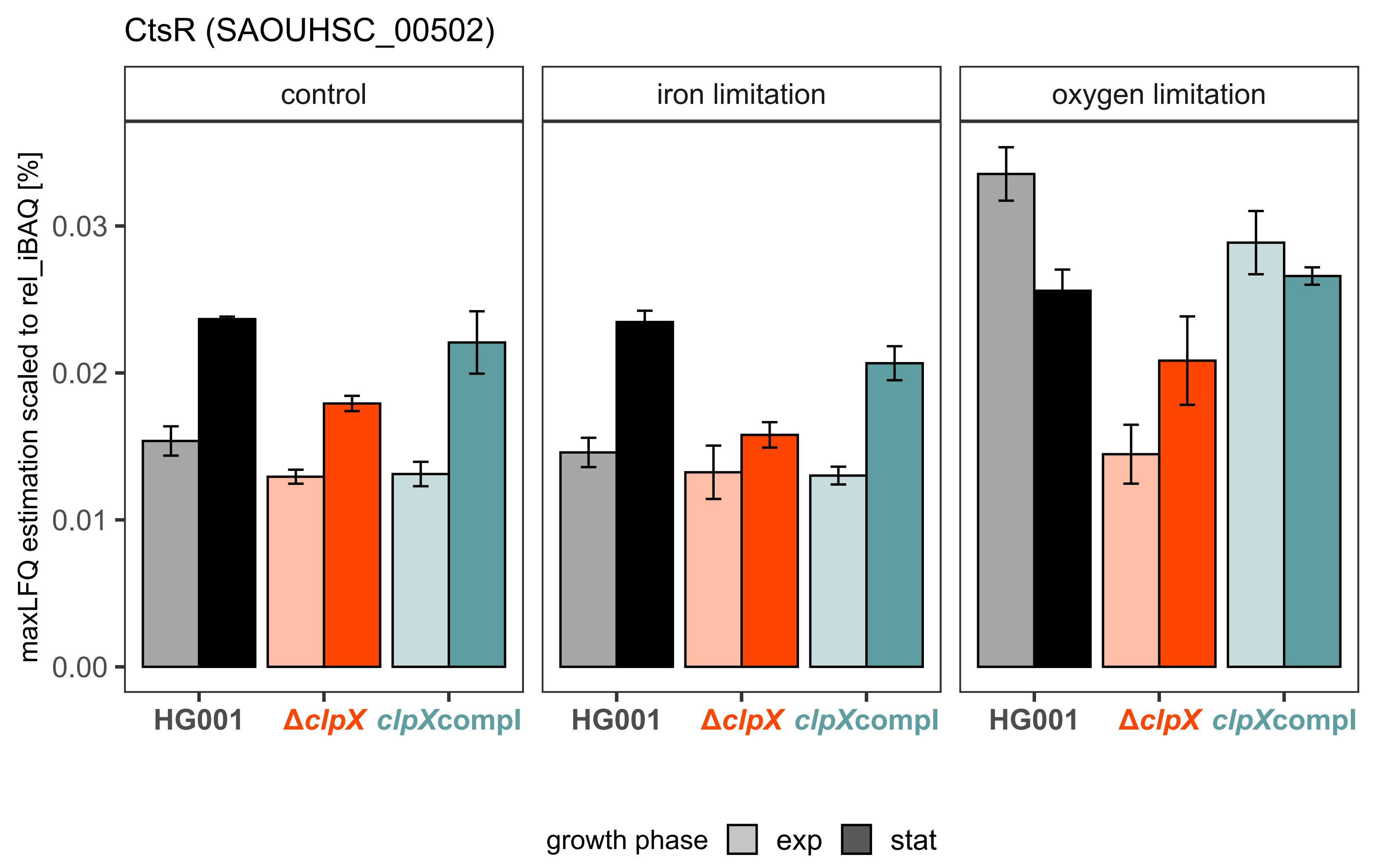

CtsR (SAOUHSC_00502)
control
iron limitation
oxygen limitation
maxLFQ estimation scaled to rel_iBAQ [%]
0.03
0.02
0.01
0.00
HG001
ΔclpX
clpXcompl
growth phase
exp
stat

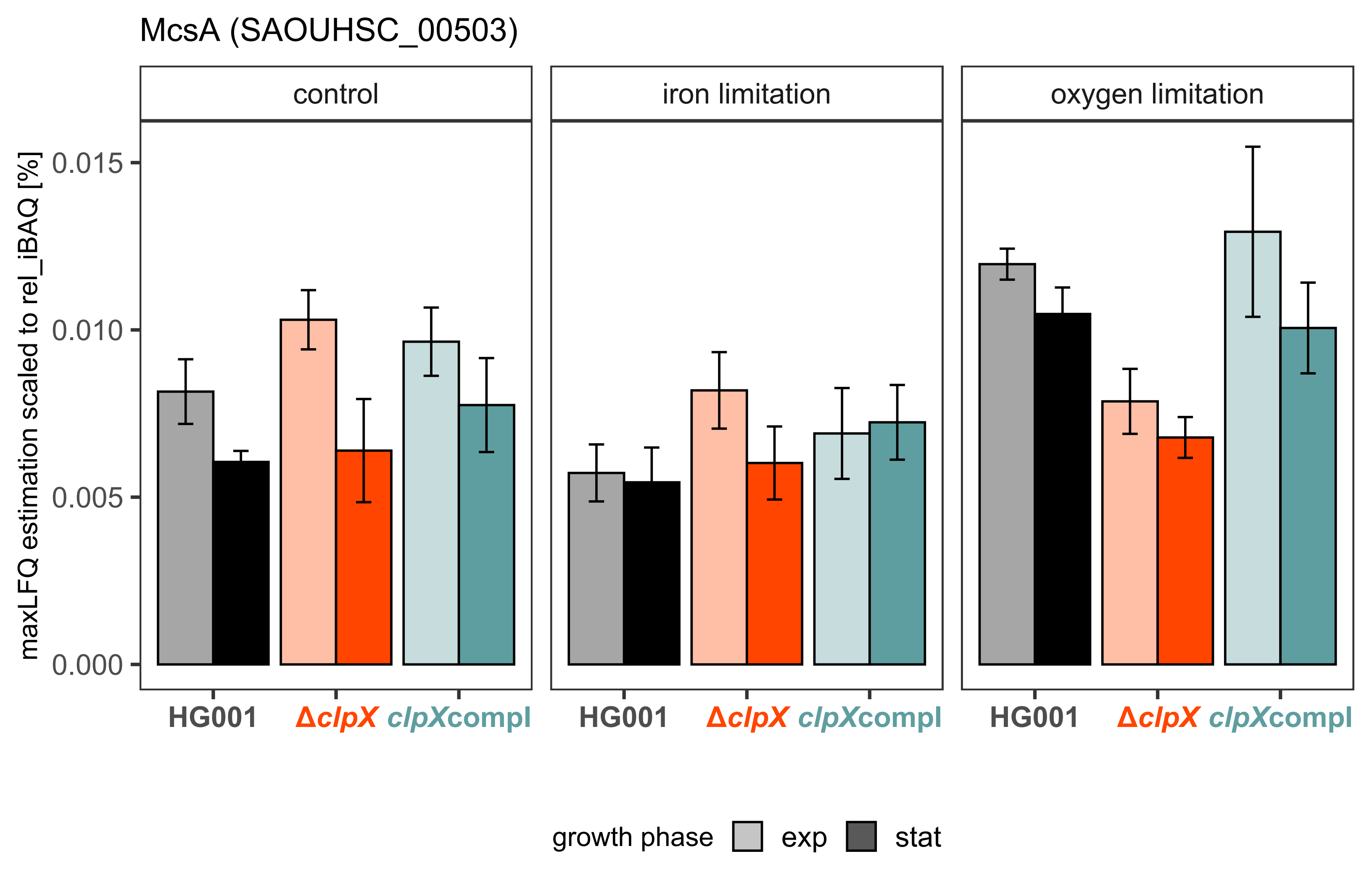
McsA (SAOUHSC_00503)
control
iron limitation
oxygen limitation
maxLFQ estimation scaled to rel_iBAQ [%]
0.015
0.010
0.005
0.000
HG001
ΔclpX
clpXcompl
growth phase
exp
stat

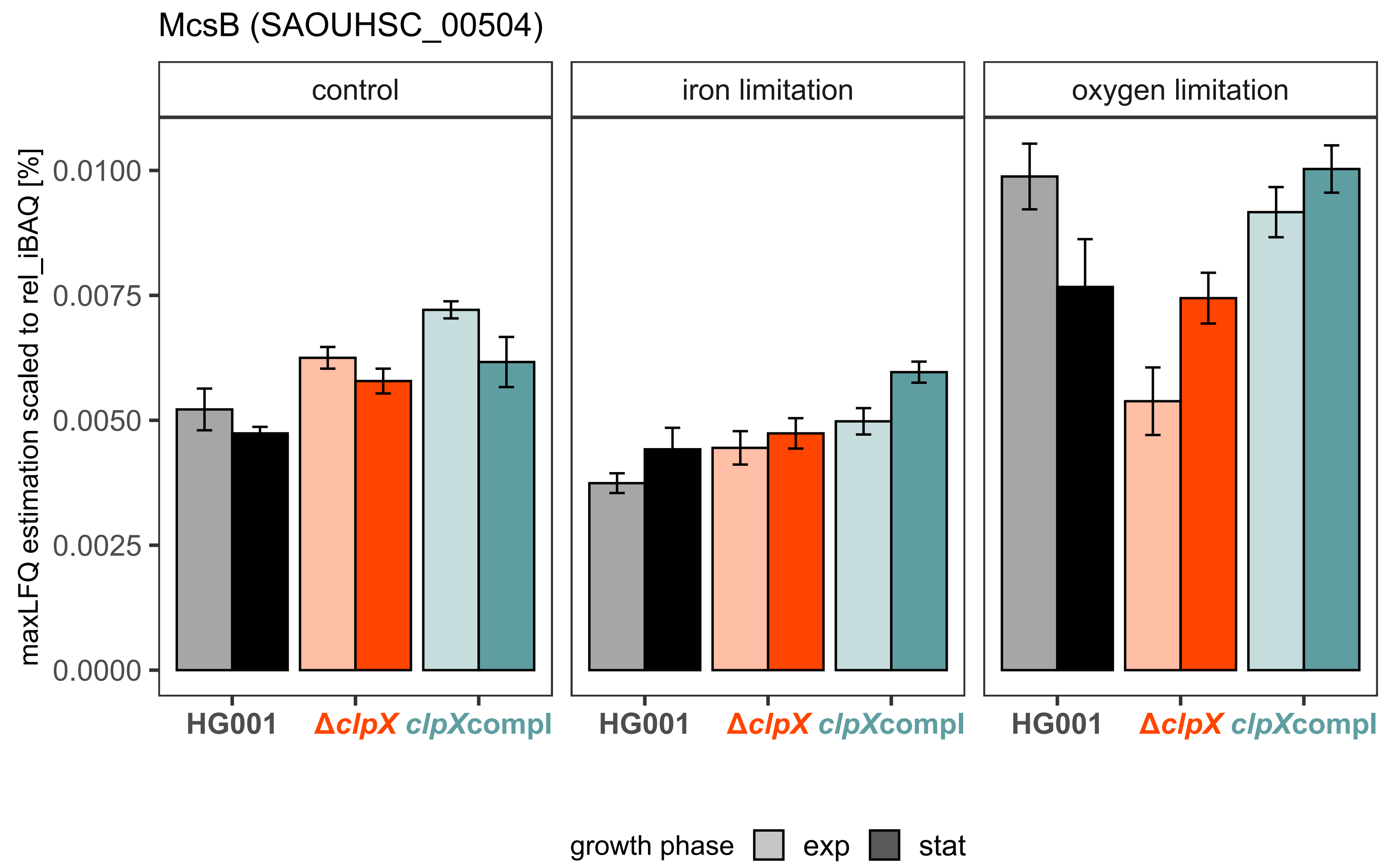
McsB (SAOUHSC_00504)
control
iron limitation
oxygen limitation
maxLFQ estimation scaled to rel_iBAQ [%]
0.0100
0.0075
0.0050
0.0025
0.0000
HG001
ΔclpX
clpXcompl
growth phase
exp
stat

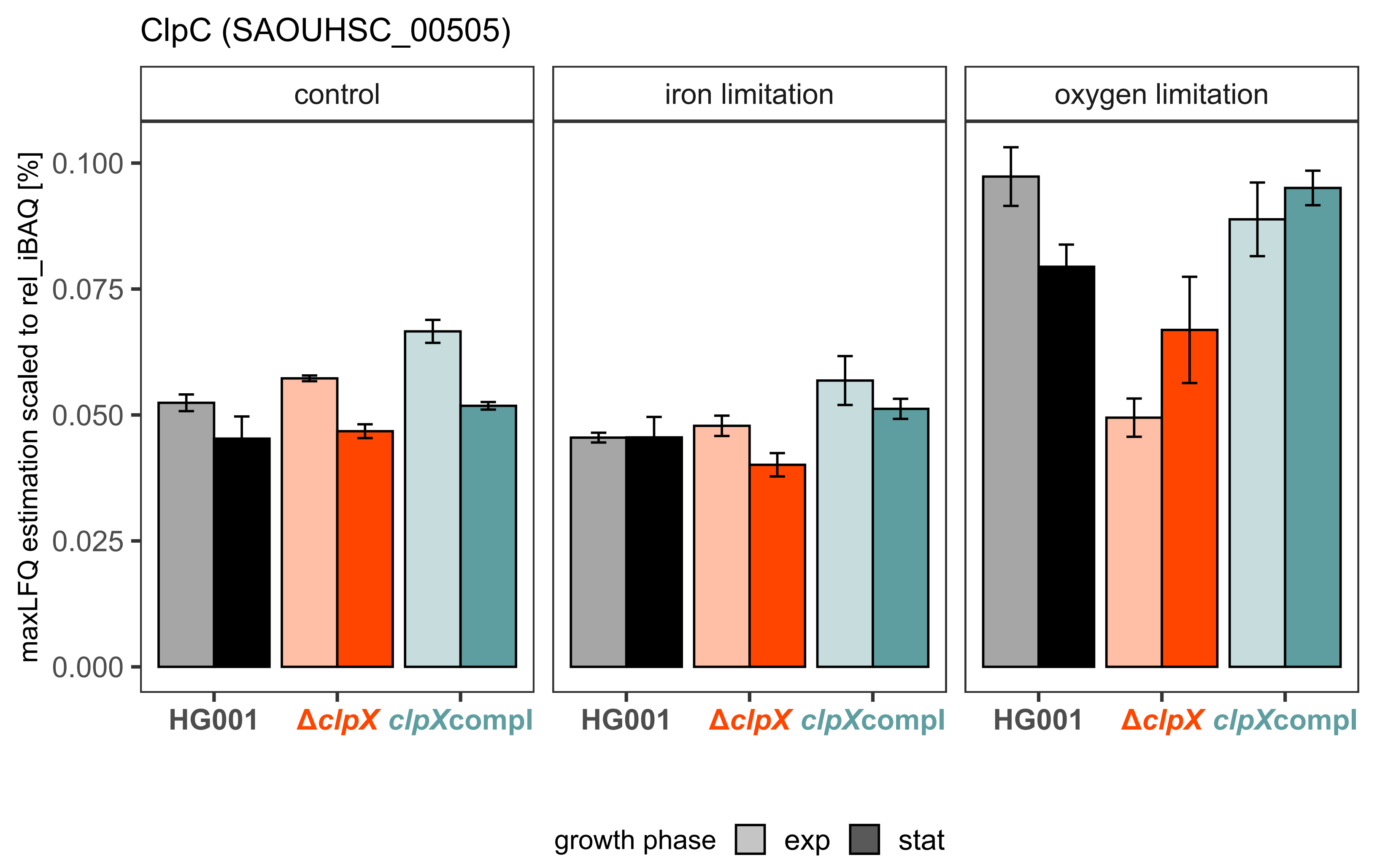
ClpC (SAOUHSC_00505)
control
iron limitation
oxygen limitation
maxLFQ estimation scaled to rel_iBAQ [%]
0.100
0.075
0.050
0.025
0.000
HG001
ΔclpX
clpXcompl
growth phase
exp
stat

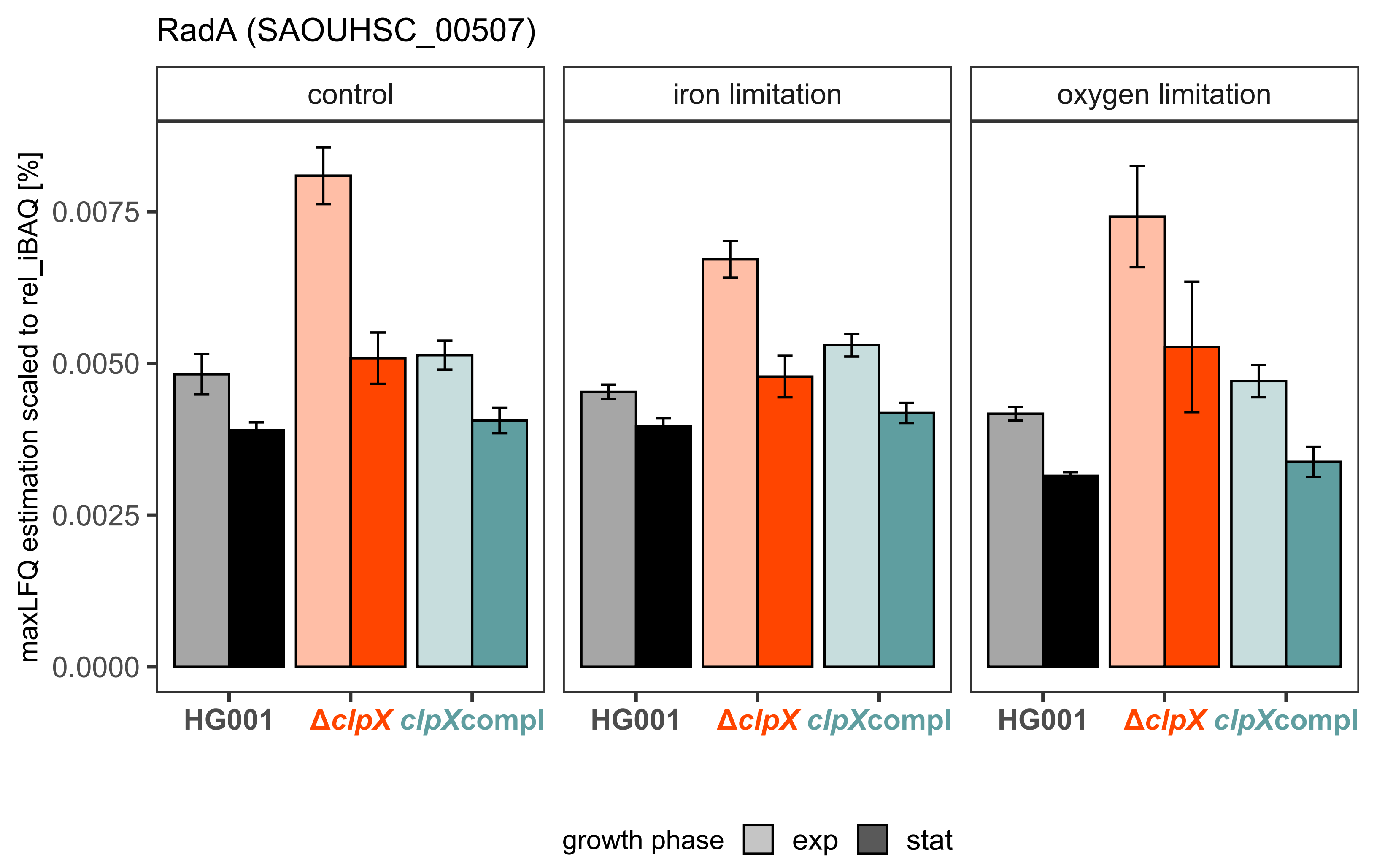

RadA (SAOUHSC_00507)
control
iron limitation
oxygen limitation
maxLFQ estimation scaled to rel_iBAQ [%]
0.0075
0.0050
0.0025
0.0000
HG001
ΔclpX
clpXcompl
growth phase
exp
stat

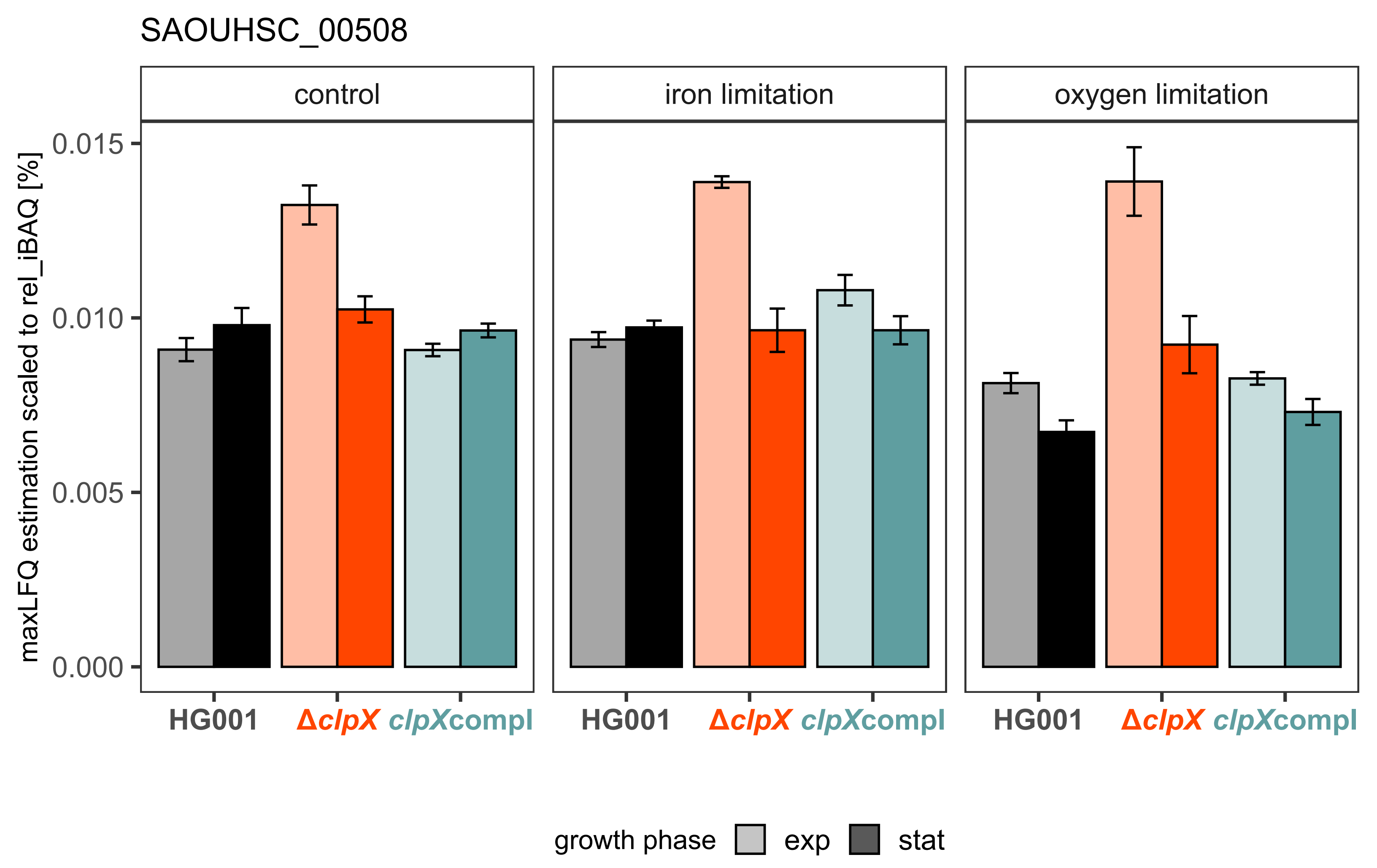
SAOUHSC_00508
control
iron limitation
oxygen limitation
maxLFQ estimation scaled to rel_iBAQ [%]
0.015
0.010
0.005
0.000
HG001
ΔclpX
clpXcompl
growth phase
exp
stat

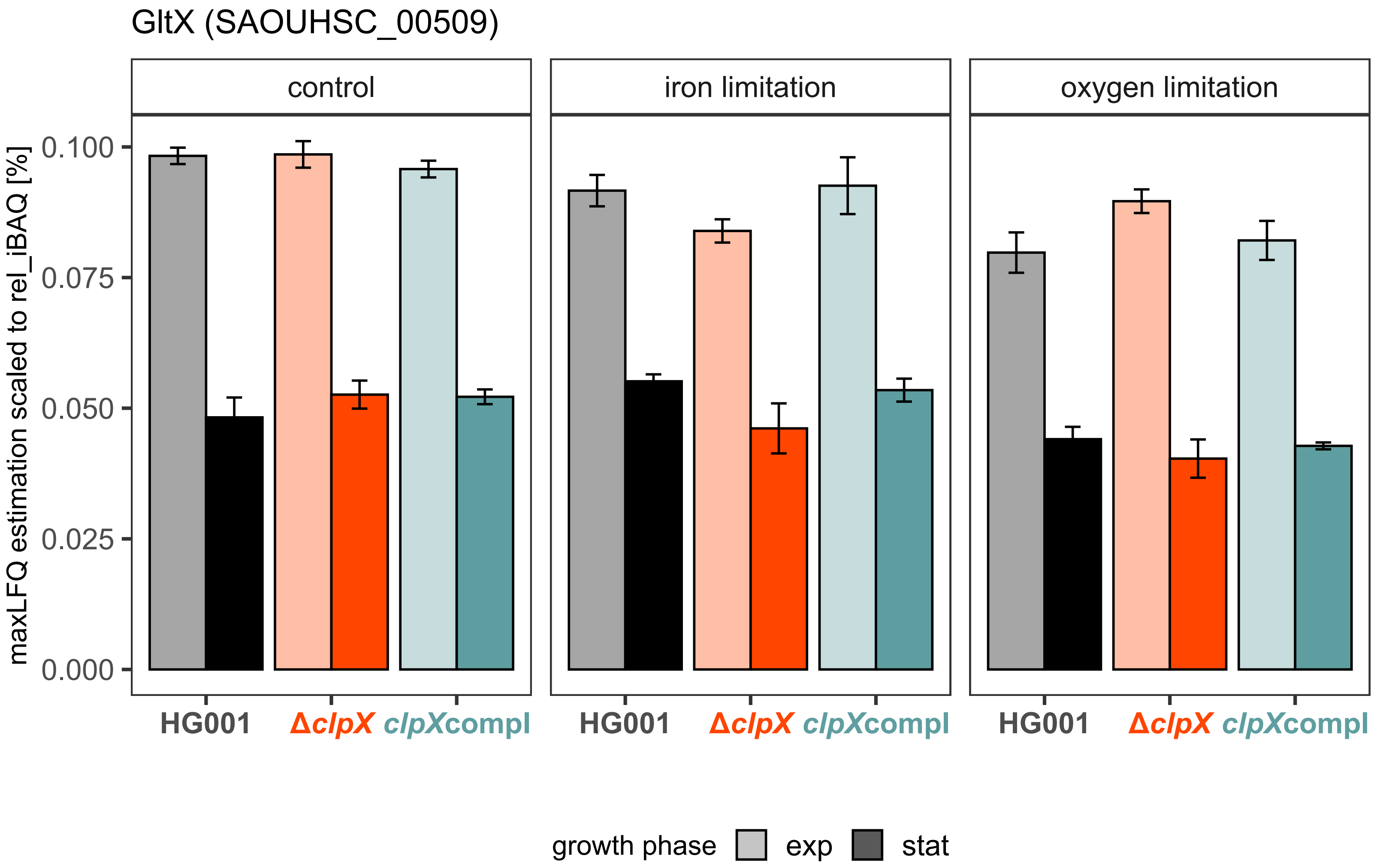

GltX (SAOUHSC_00509)
control
iron limitation
oxygen limitation
maxLFQ estimation scaled to rel_iBAQ [%]
0.100
0.075
0.050
0.025
0.000
HG001
ΔclpX
clpXcompl
growth phase
exp
stat

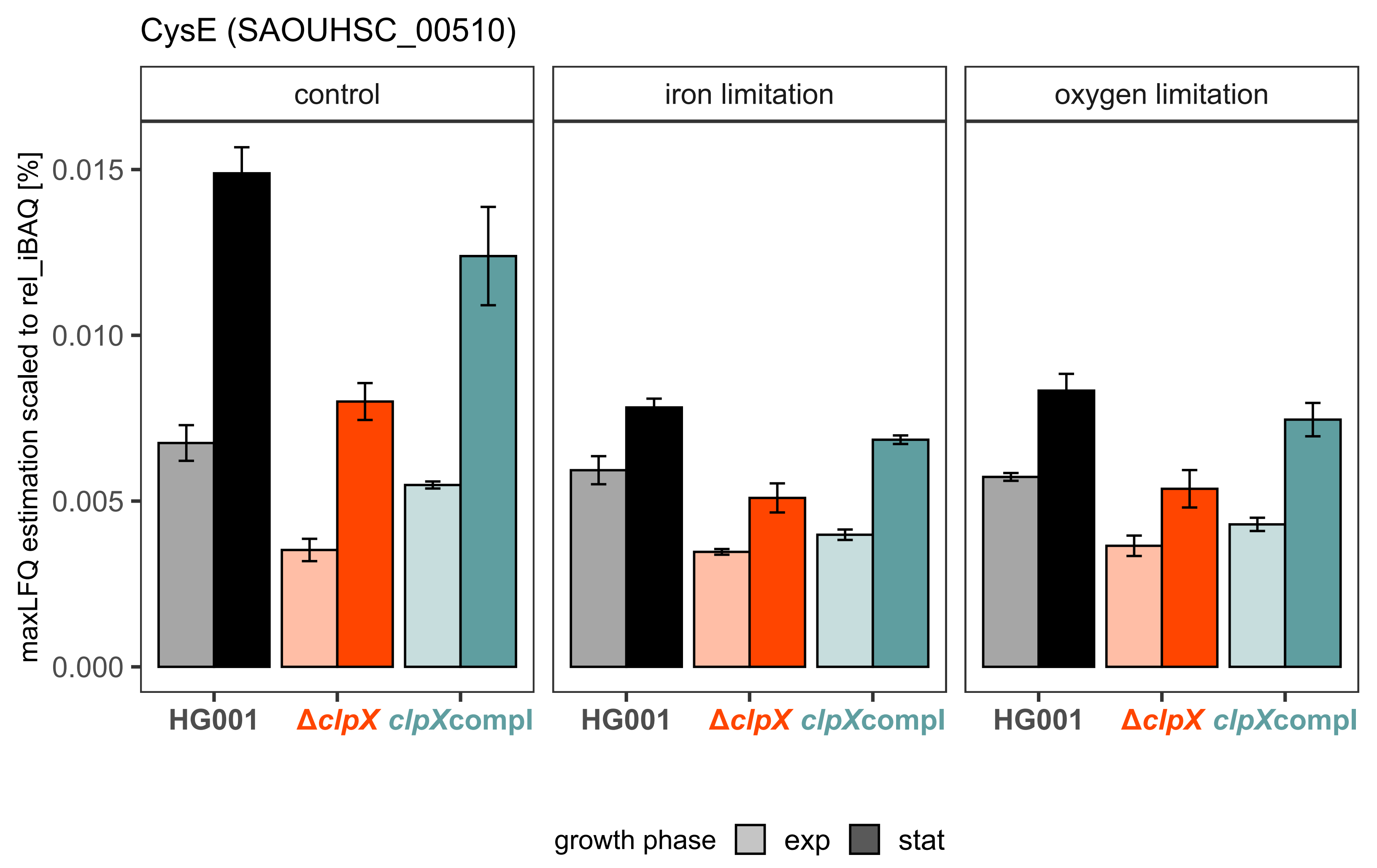

CysE (SAOUHSC_00510)
control
iron limitation
oxygen limitation
maxLFQ estimation scaled to rel_iBAQ [%]
0.015
0.010
0.005
0.000
HG001
ΔclpX
clpXcompl
growth phase
exp
stat

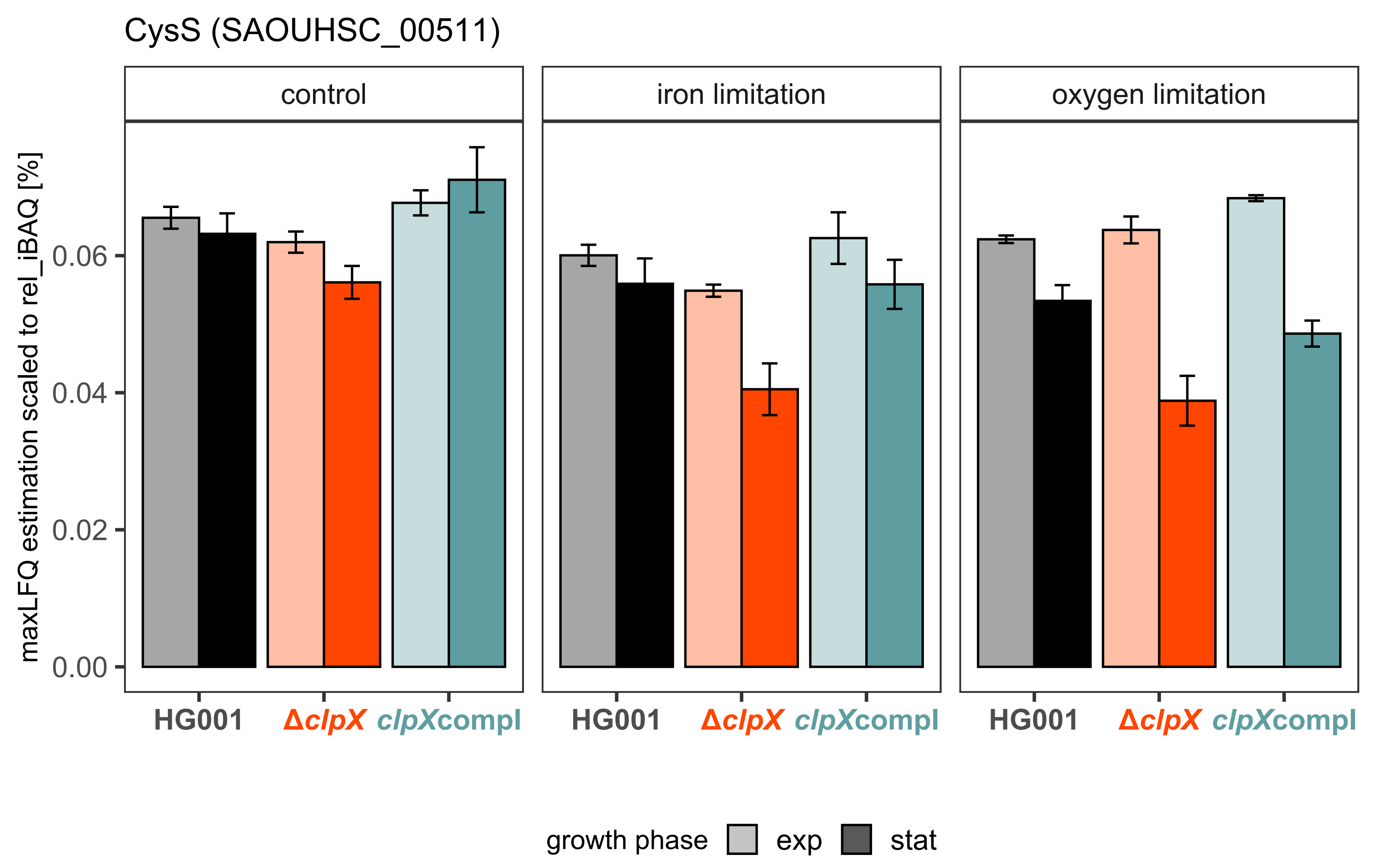
CysS (SAOUHSC_00511)
control
iron limitation
oxygen limitation
maxLFQ estimation scaled to rel_iBAQ [%]
0.00
0.02
0.04
0.06
HG001
ΔclpX
clpXcompl
growth phase
exp
stat

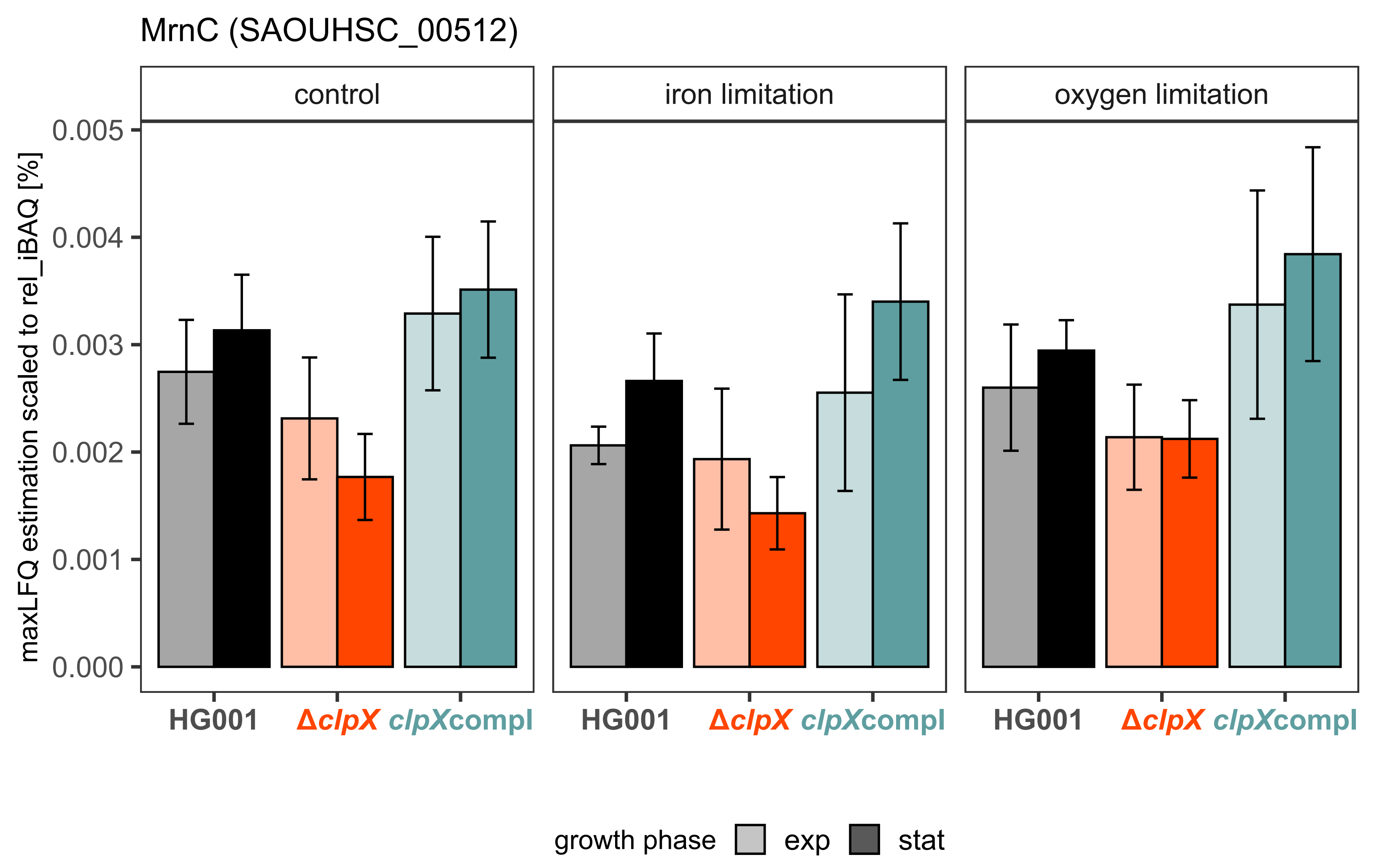
MrnC (SAOUHSC_00512)
control
iron limitation
oxygen limitation
maxLFQ estimation scaled to rel_iBAQ [%]
0.005
0.004
0.003
0.002
0.001
0.000
HG001
ΔclpX
clpXcompl
growth phase
exp
stat

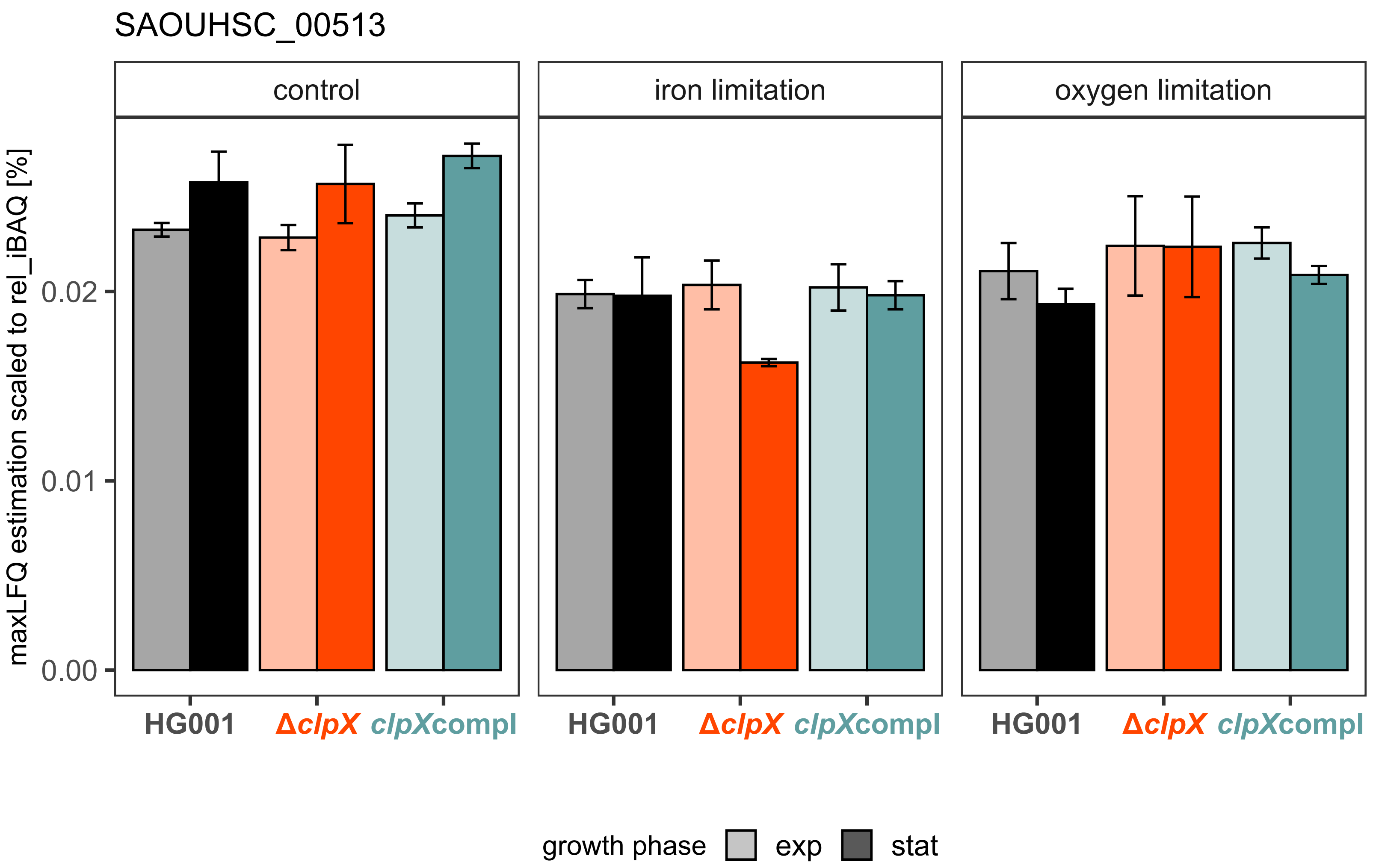
SAOUHSC_00513
control
iron limitation
oxygen limitation
maxLFQ estimation scaled to rel_iBAQ [%]
0.00
0.01
0.02
HG001
ΔclpX
clpXcompl
growth phase
exp
stat

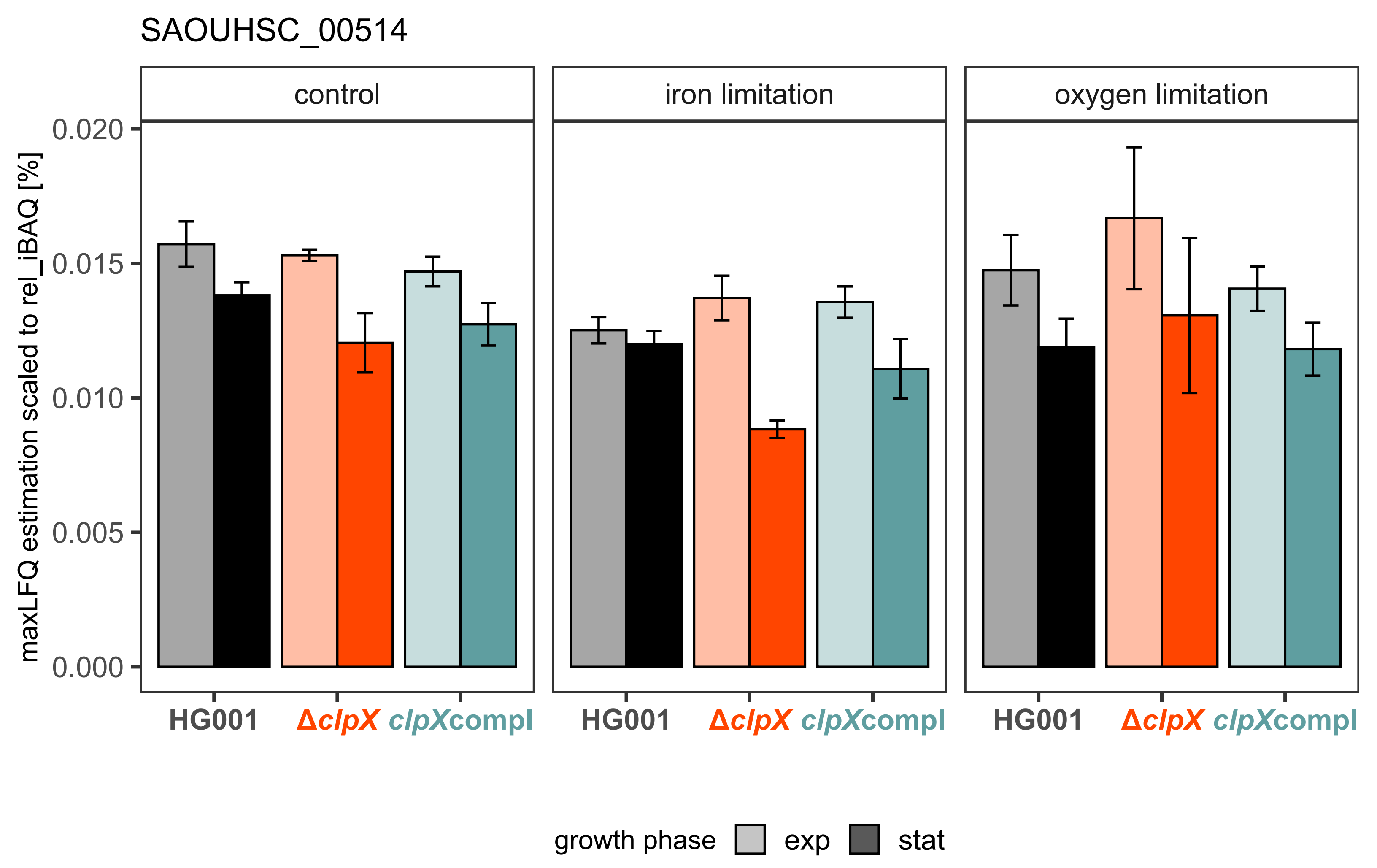

SAOUHSC_00514
control
iron limitation
oxygen limitation
maxLFQ estimation scaled to rel_iBAQ [%]
0.020
0.015
0.010
0.005
0.000
HG001
ΔclpX
clpXcompl
growth phase
exp
stat

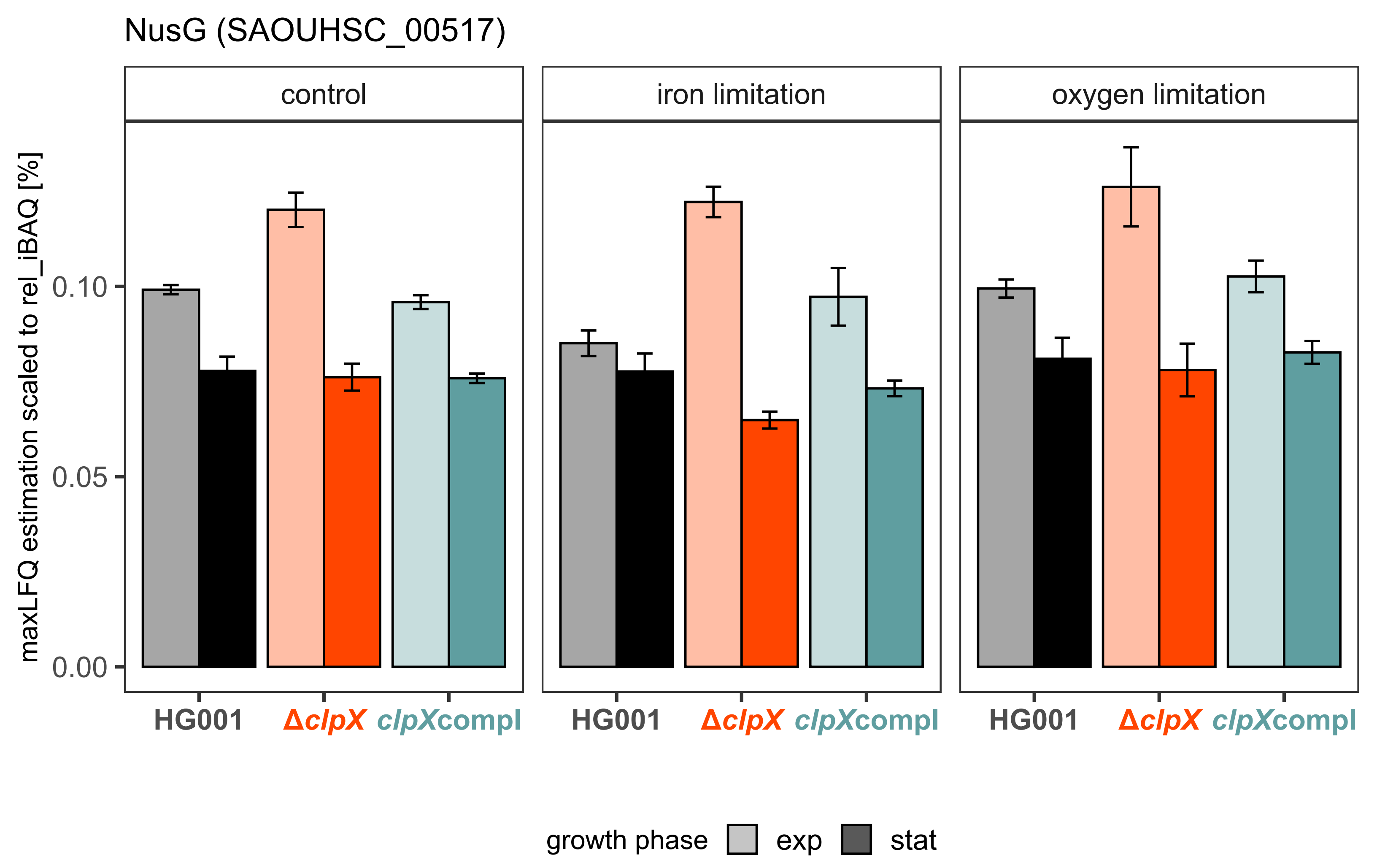
NusG (SAOUHSC_00517)
control
iron limitation
oxygen limitation
maxLFQ estimation scaled to rel_iBAQ [%]
0.10
0.05
0.00
HG001
ΔclpX
clpXcompl
growth phase
exp
stat

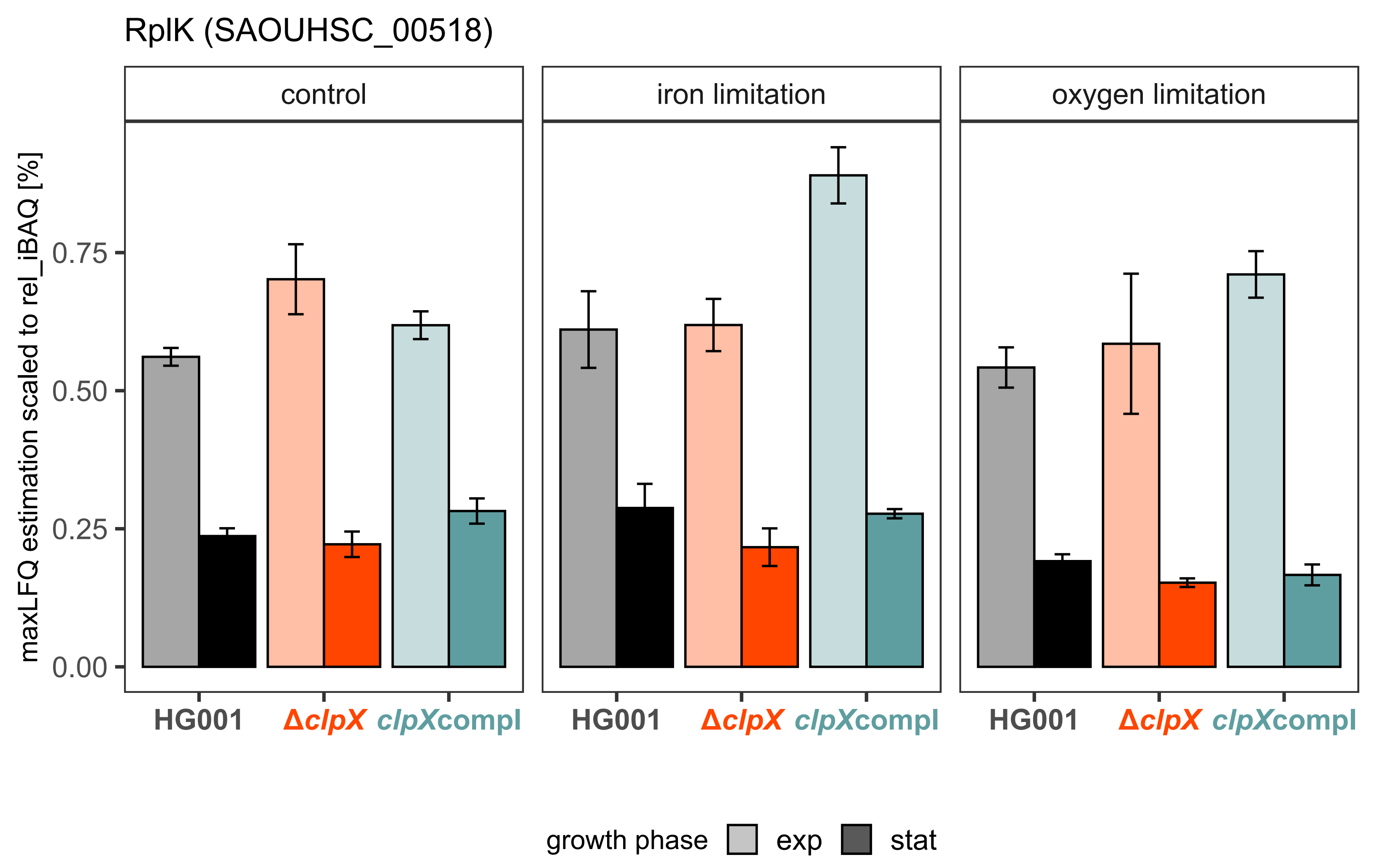
RplK (SAOUHSC_00518)
control
iron limitation
oxygen limitation
maxLFQ estimation scaled to rel_iBAQ [%]
0.00
0.25
0.50
0.75
HG001
ΔclpX
clpXcompl
growth phase
exp
stat

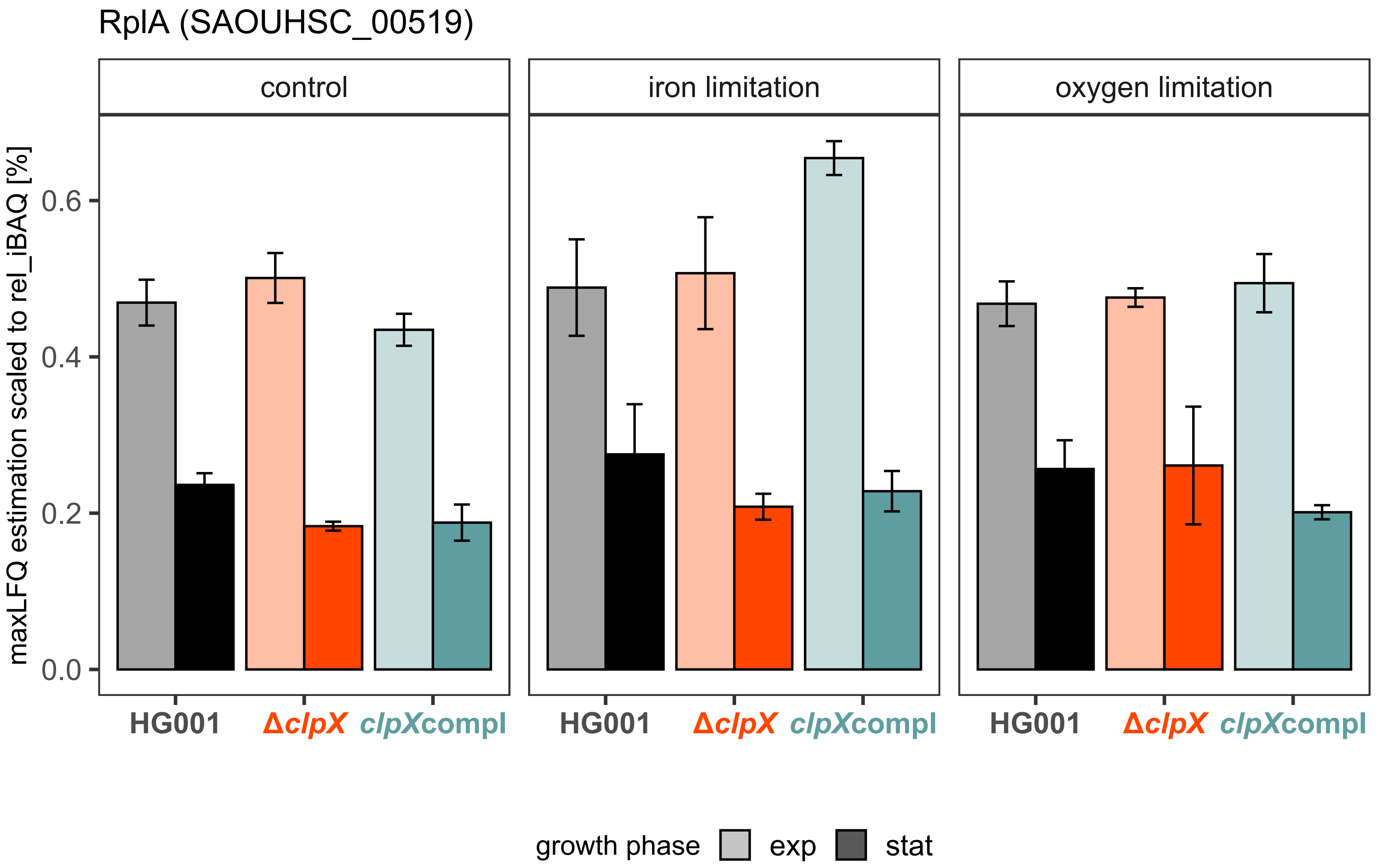
RplA (SAOUHSC_00519)
control
iron limitation
oxygen limitation
maxLFQ estimation scaled to rel_iBAQ [%]
0.6
0.4
0.2
0.0
HG001
ΔclpX
clpXcompl
growth phase
exp
stat

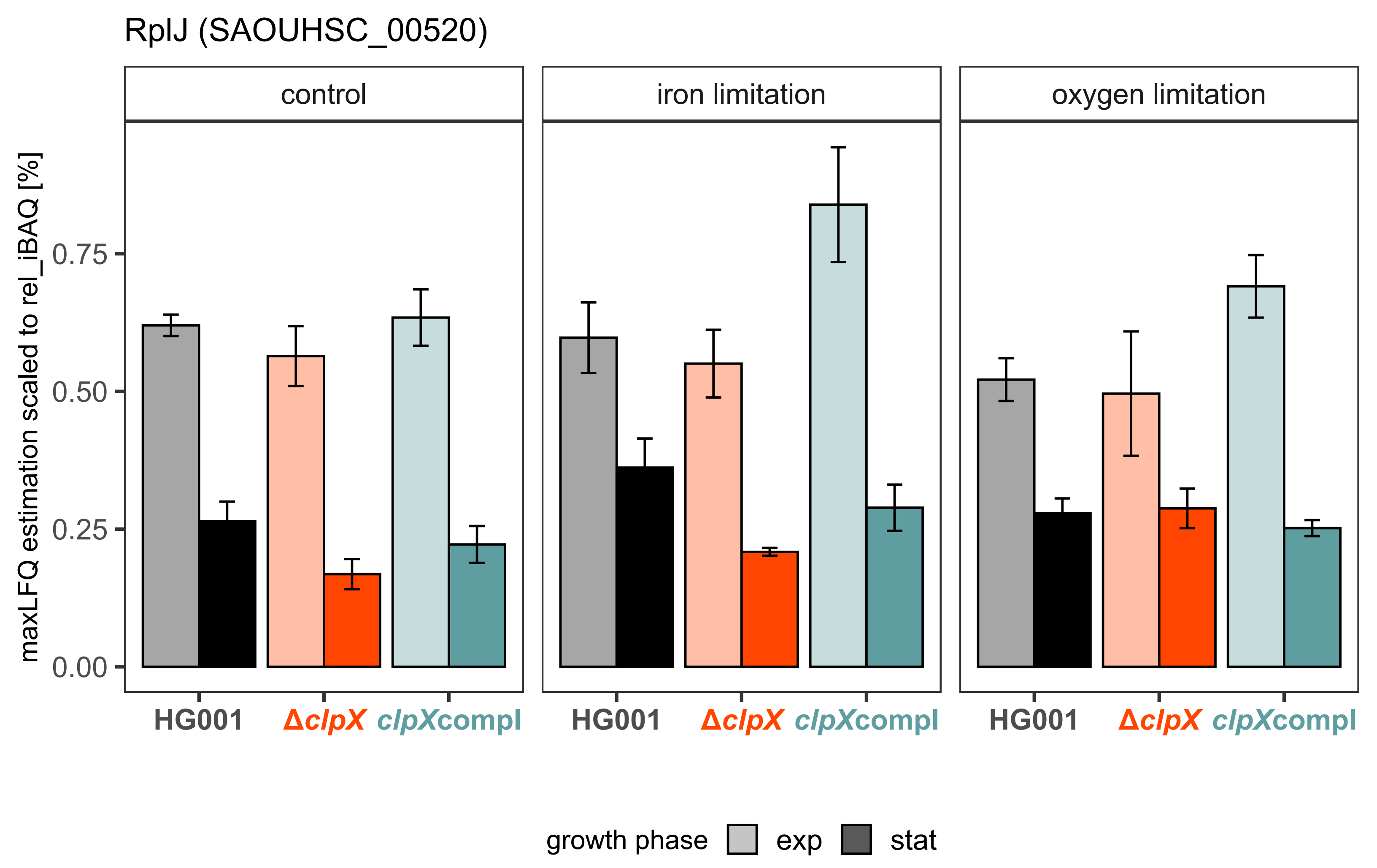

RplJ (SAOUHSC_00520)
control
iron limitation
oxygen limitation
maxLFQ estimation scaled to rel_iBAQ [%]
0.75
0.50
0.25
0.00
HG001
ΔclpX
clpXcompl
growth phase
exp
stat

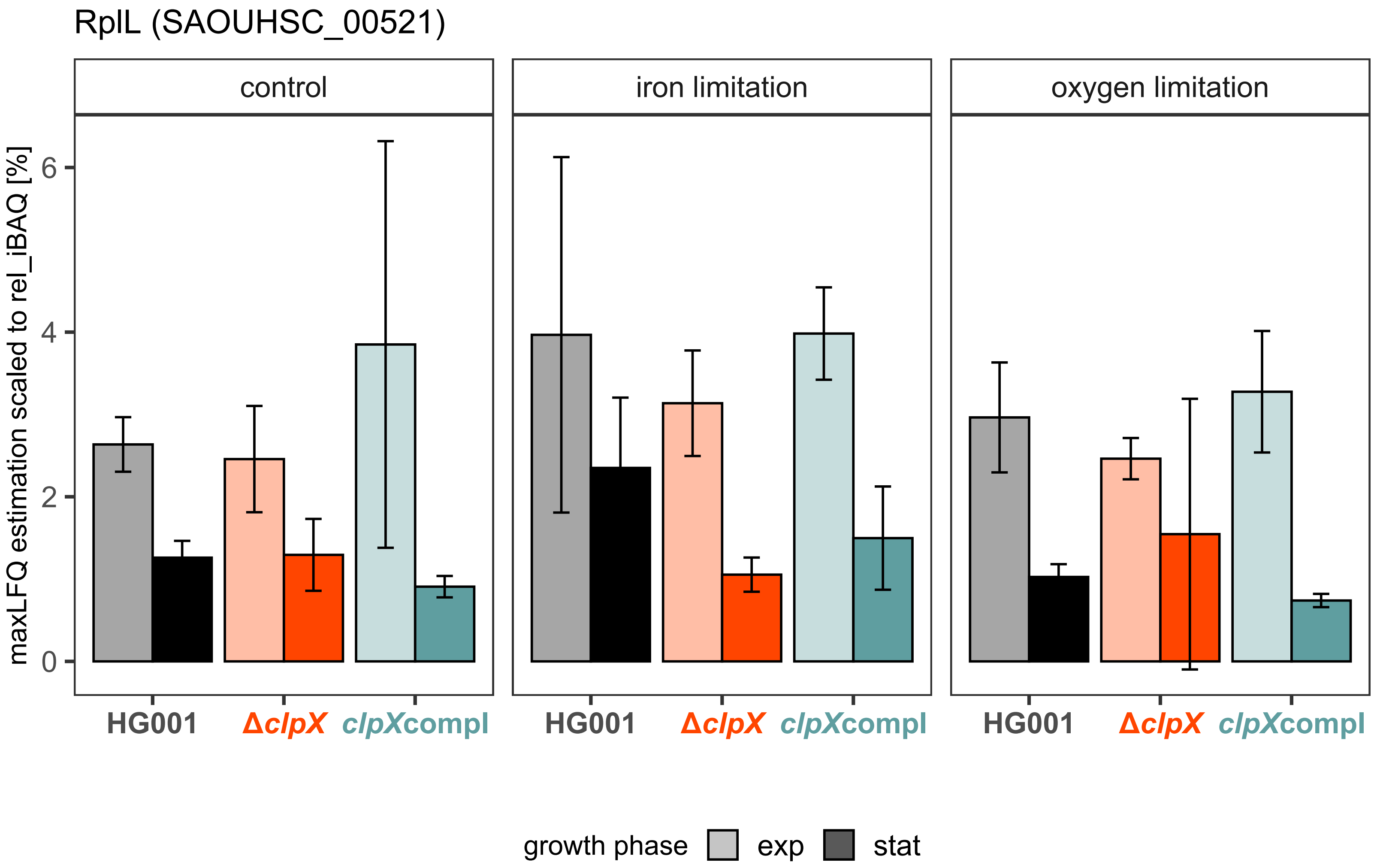

RplL (SAOUHSC_00521)
control
iron limitation
oxygen limitation
maxLFQ estimation scaled to rel_iBAQ [%]
0
2
4
6
HG001
ΔclpX
clpXcompl
growth phase
exp
stat

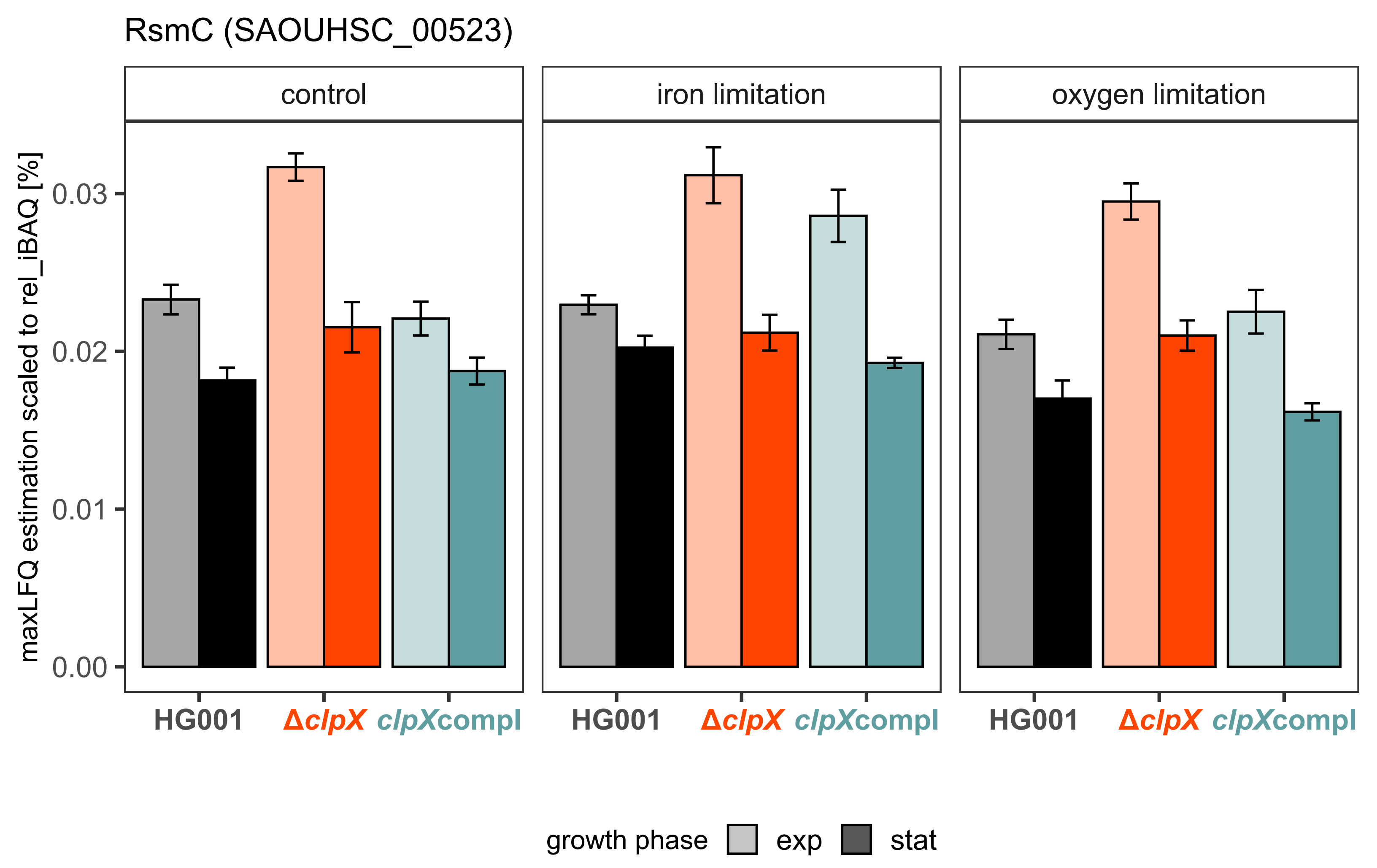
RsmC (SAOUHSC_00523)
control
iron limitation
oxygen limitation
maxLFQ estimation scaled to rel_iBAQ [%]
0.00
0.01
0.02
0.03
HG001
ΔclpX
clpXcompl
growth phase
exp
stat

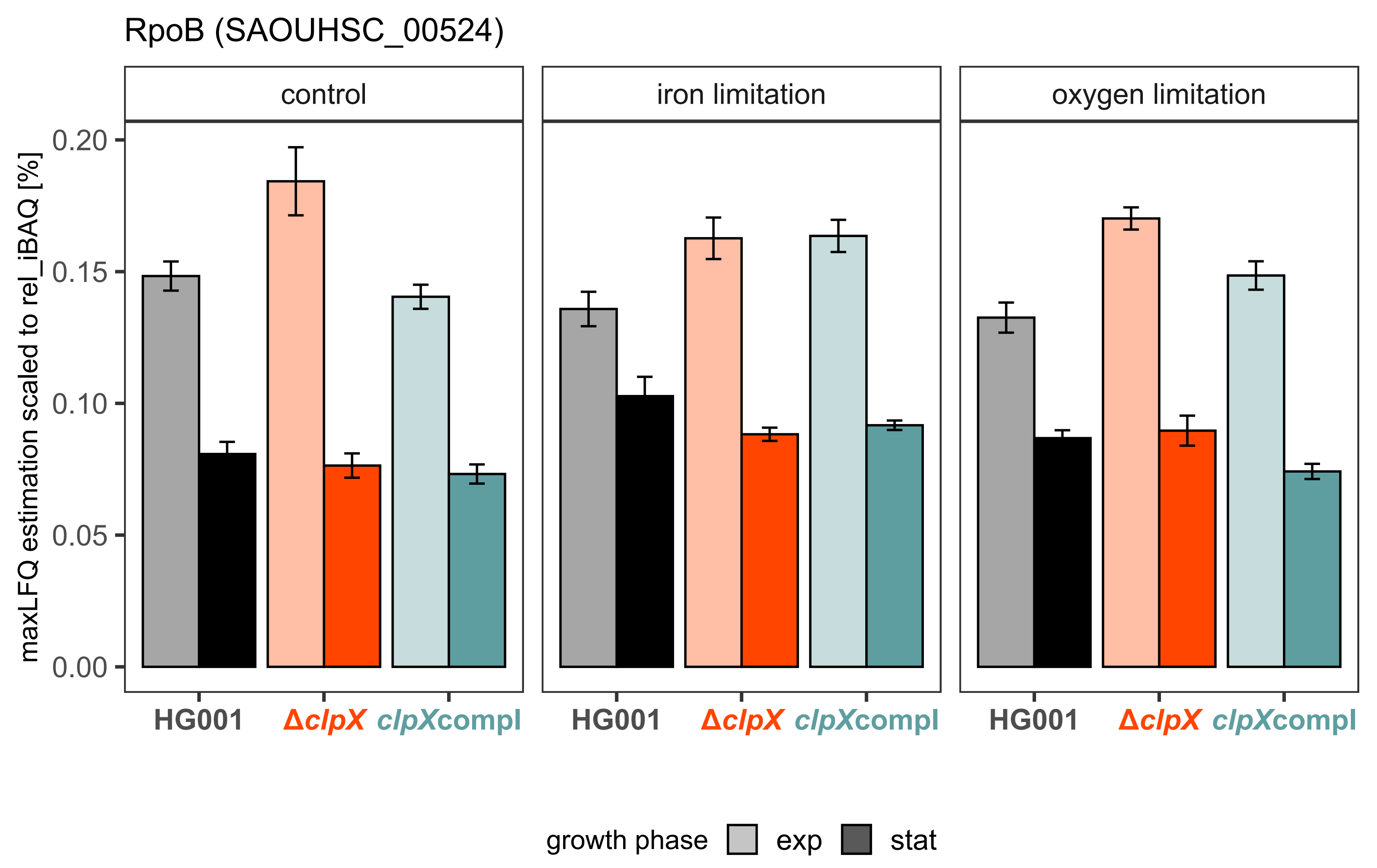

RpoB (SAOUHSC_00524)
control
iron limitation
oxygen limitation
maxLFQ estimation scaled to rel_iBAQ [%]
0.20
0.15
0.10
0.05
0.00
HG001
ΔclpX
clpXcompl
growth phase
exp
stat

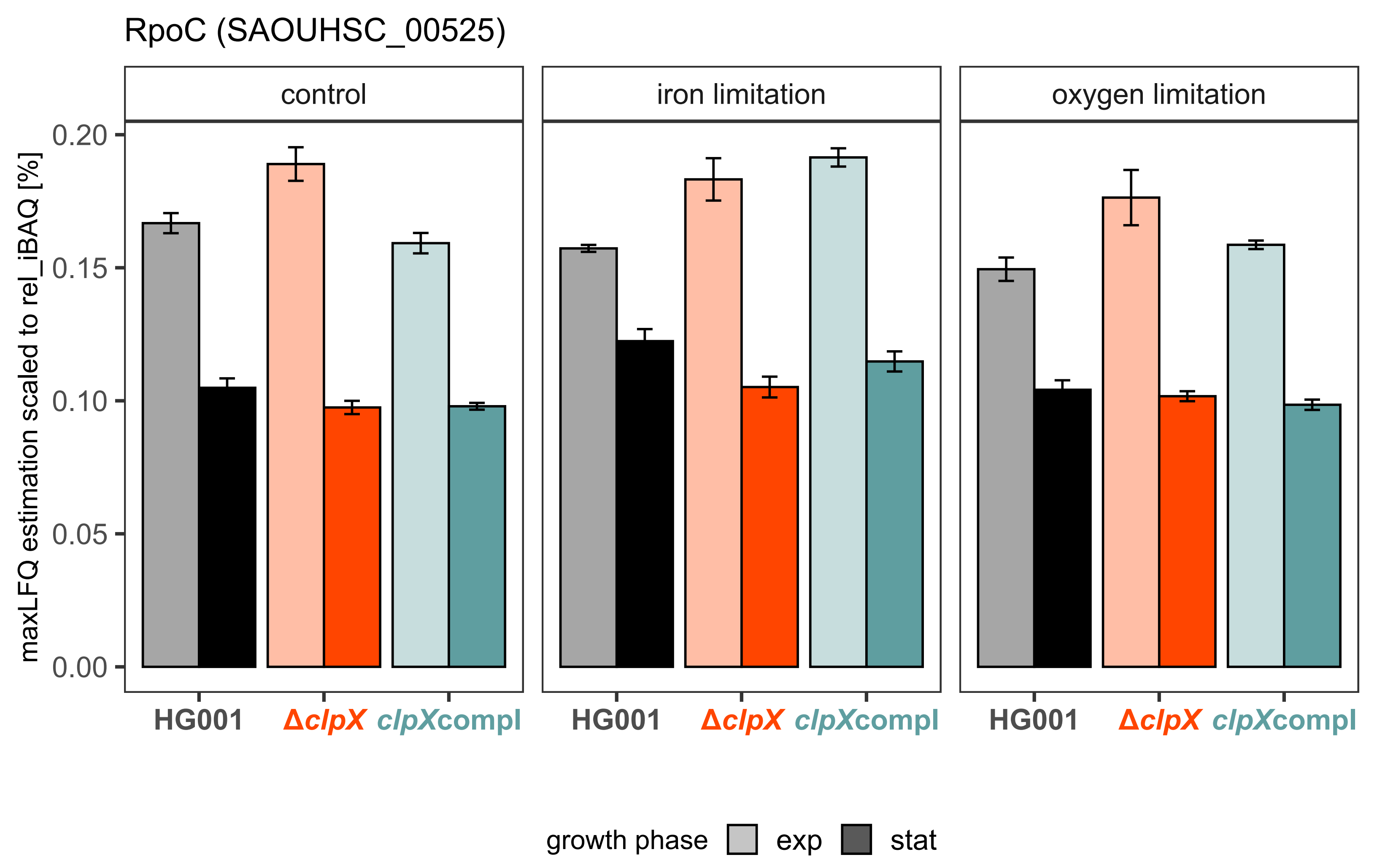

RpoC (SAOUHSC_00525)
control
iron limitation
oxygen limitation
maxLFQ estimation scaled to rel_iBAQ [%]
0.20
0.15
0.10
0.05
0.00
HG001
ΔclpX
clpXcompl
growth phase
exp
stat

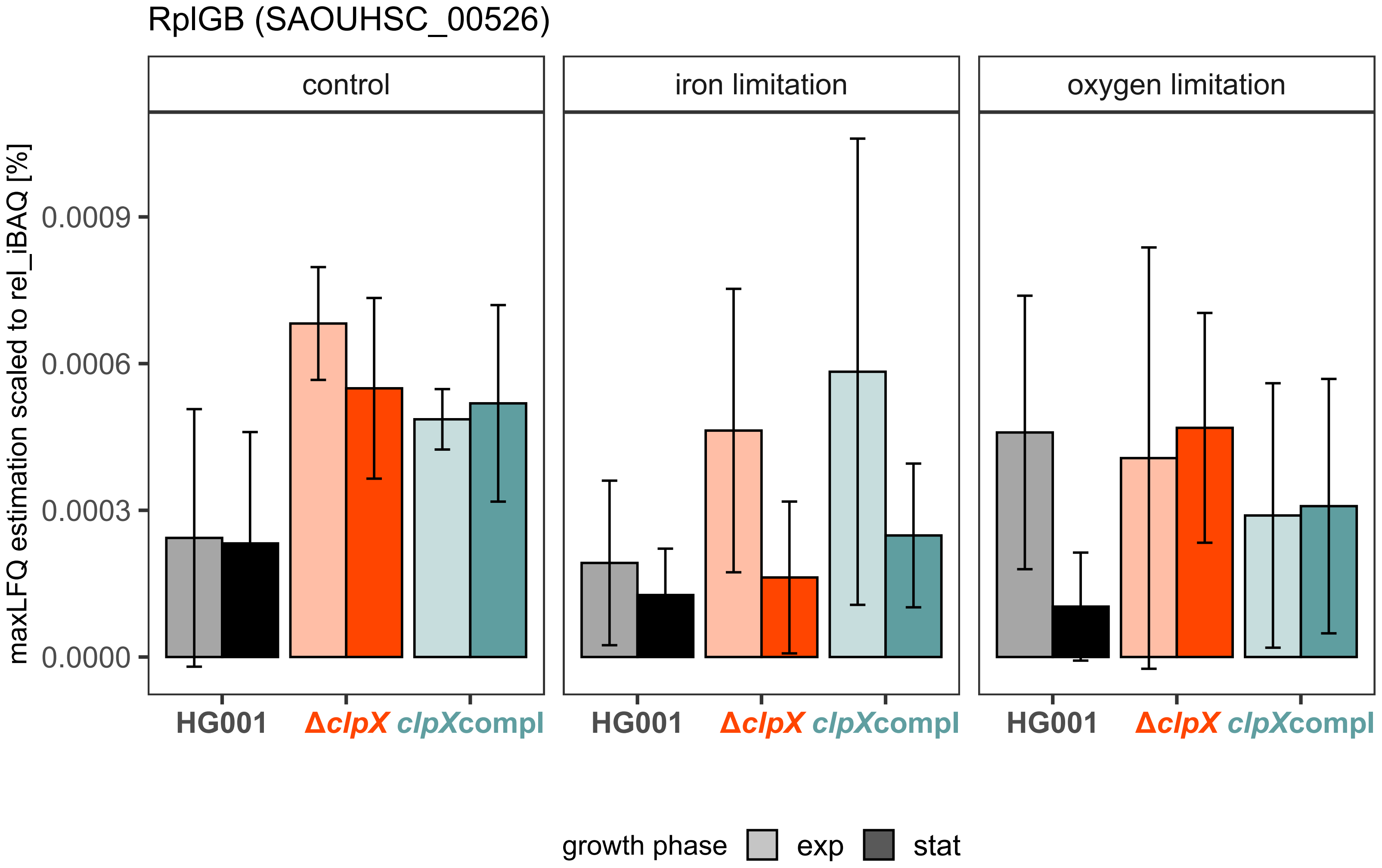

RplGB (SAOUHSC_00526)
control
iron limitation
oxygen limitation
maxLFQ estimation scaled to rel_iBAQ [%]
0.0000
0.0003
0.0006
0.0009
HG001
ΔclpX
clpXcompl
growth phase
exp
stat

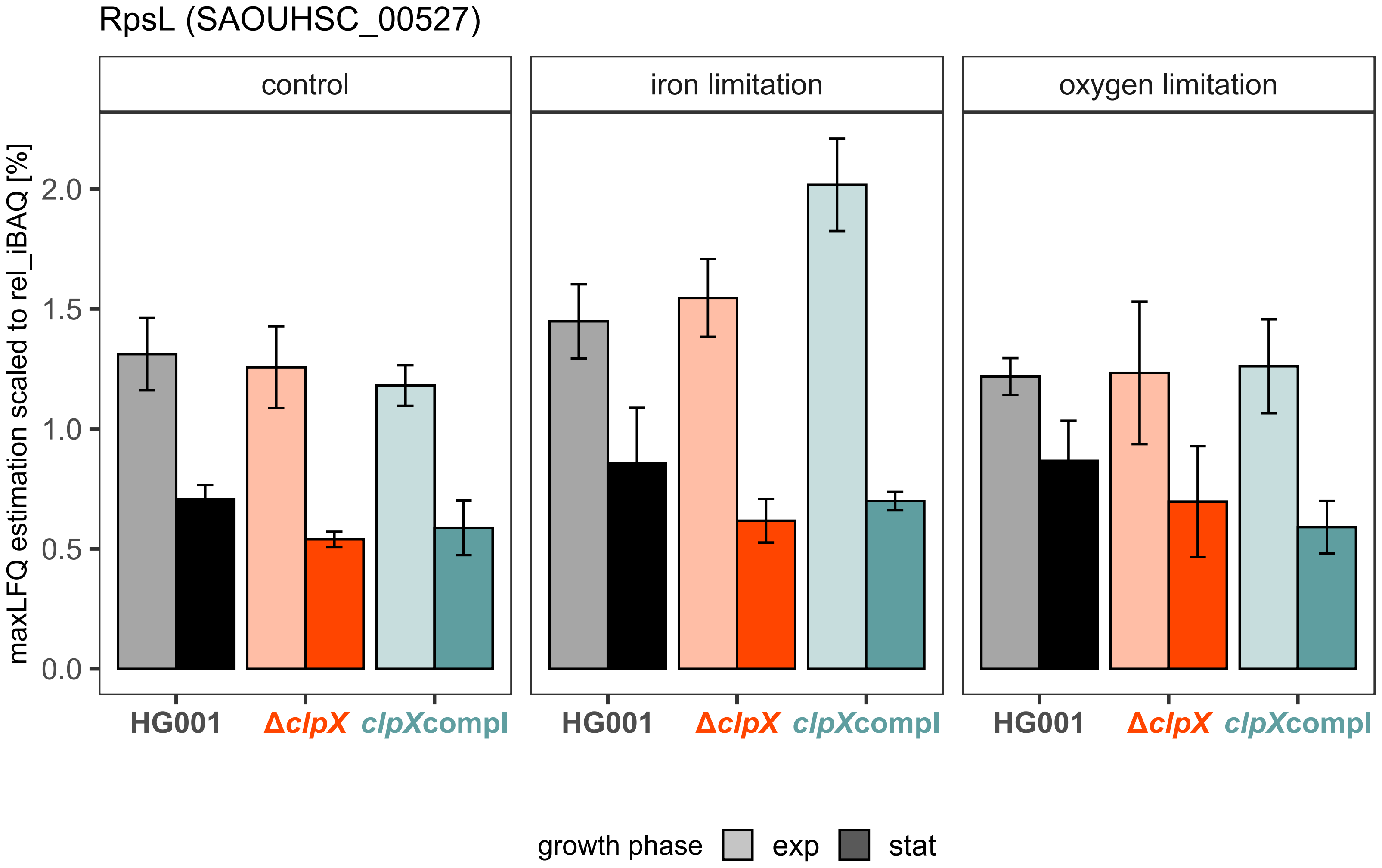
RpsL (SAOUHSC_00527)
control
iron limitation
oxygen limitation
maxLFQ estimation scaled to rel_iBAQ [%]
2.0
1.5
1.0
0.5
0.0
HG001
ΔclpX
clpXcompl
growth phase
exp
stat

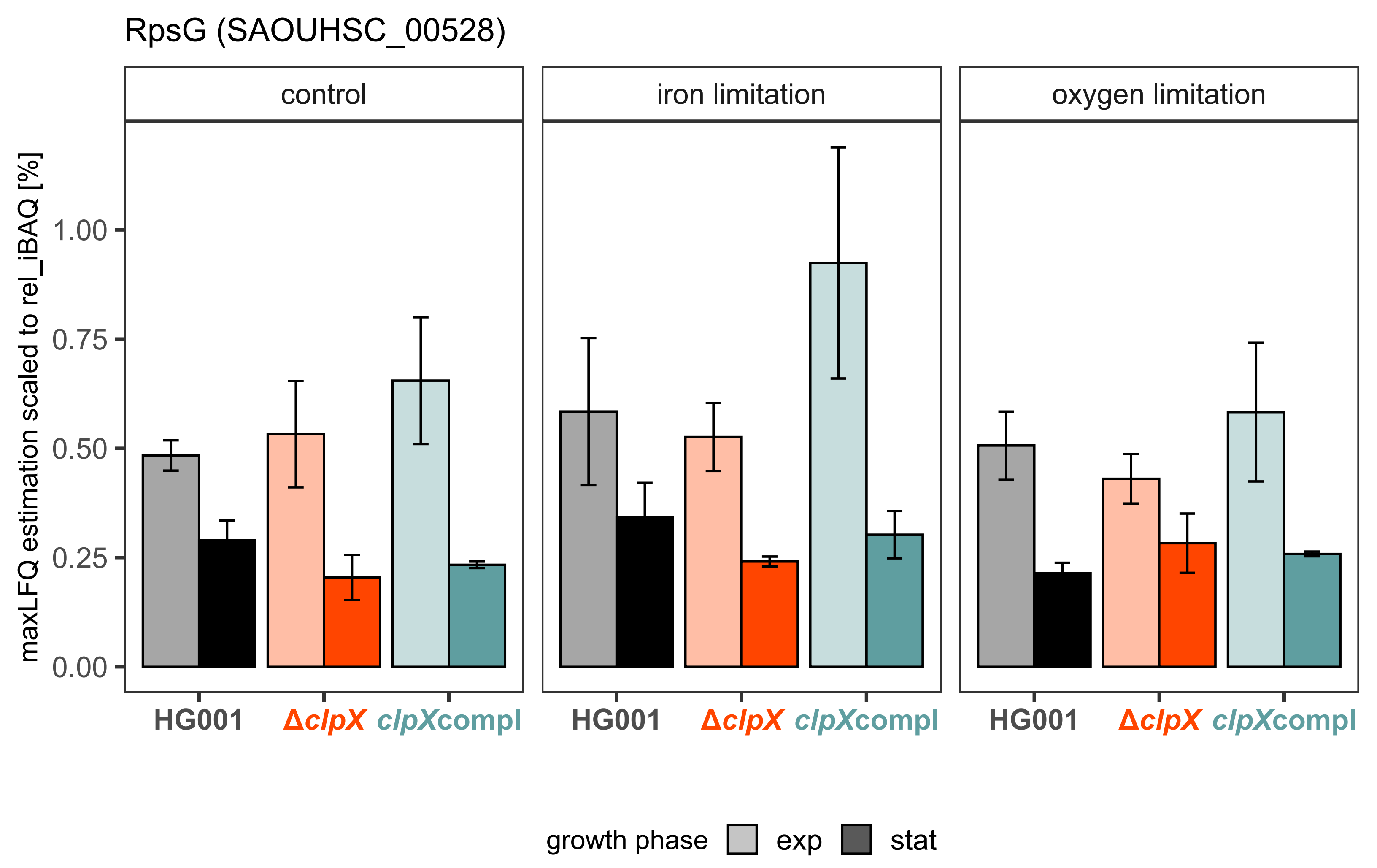
RpsG (SAOUHSC_00528)
control
iron limitation
oxygen limitation
maxLFQ estimation scaled to rel_iBAQ [%]
1.00
0.75
0.50
0.25
0.00
HG001
ΔclpX
clpXcompl
growth phase
exp
stat

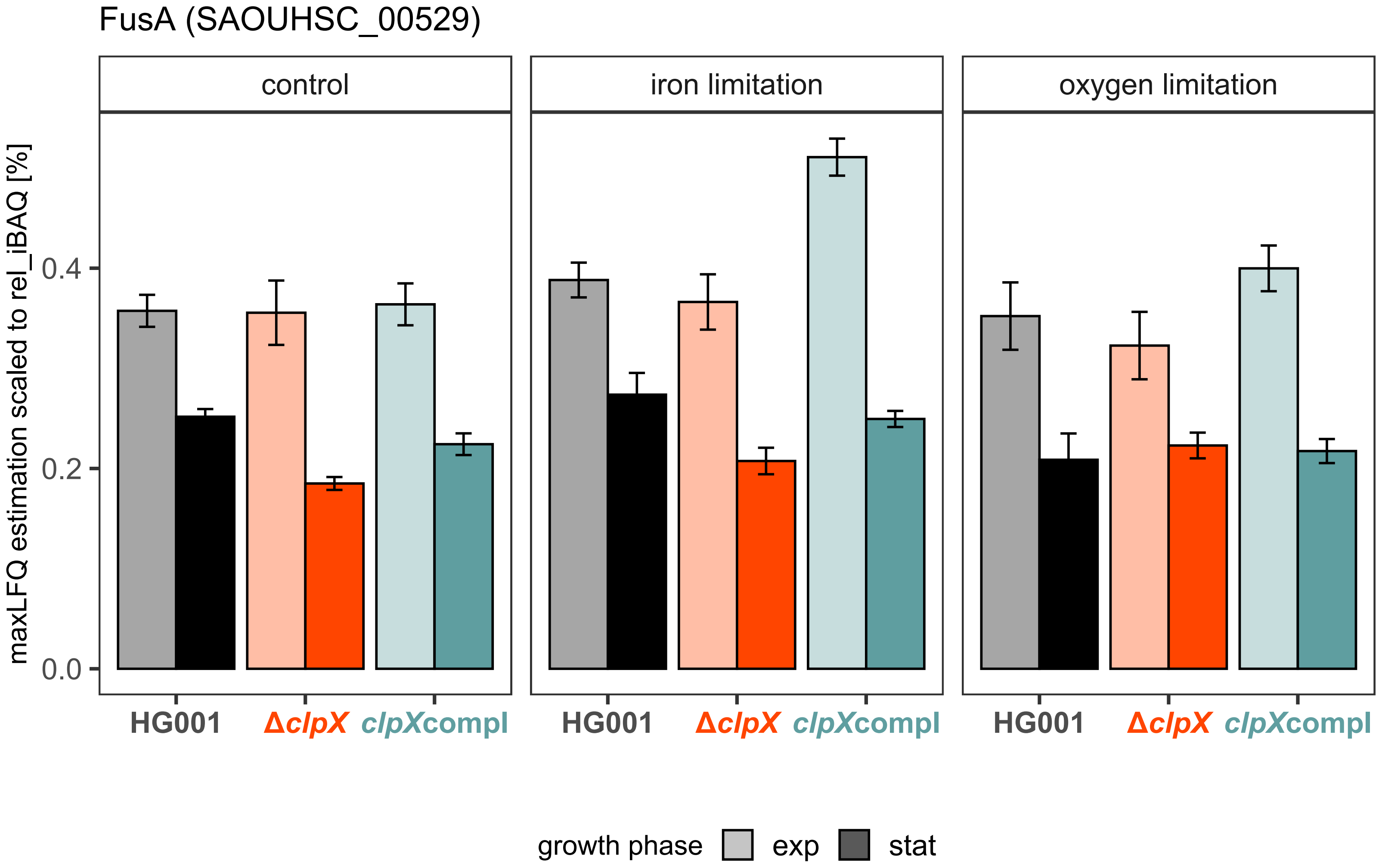
FusA (SAOUHSC_00529)
control
iron limitation
oxygen limitation
maxLFQ estimation scaled to rel_iBAQ [%]
0.0
0.2
0.4
HG001
ΔclpX
clpXcompl
growth phase
exp
stat

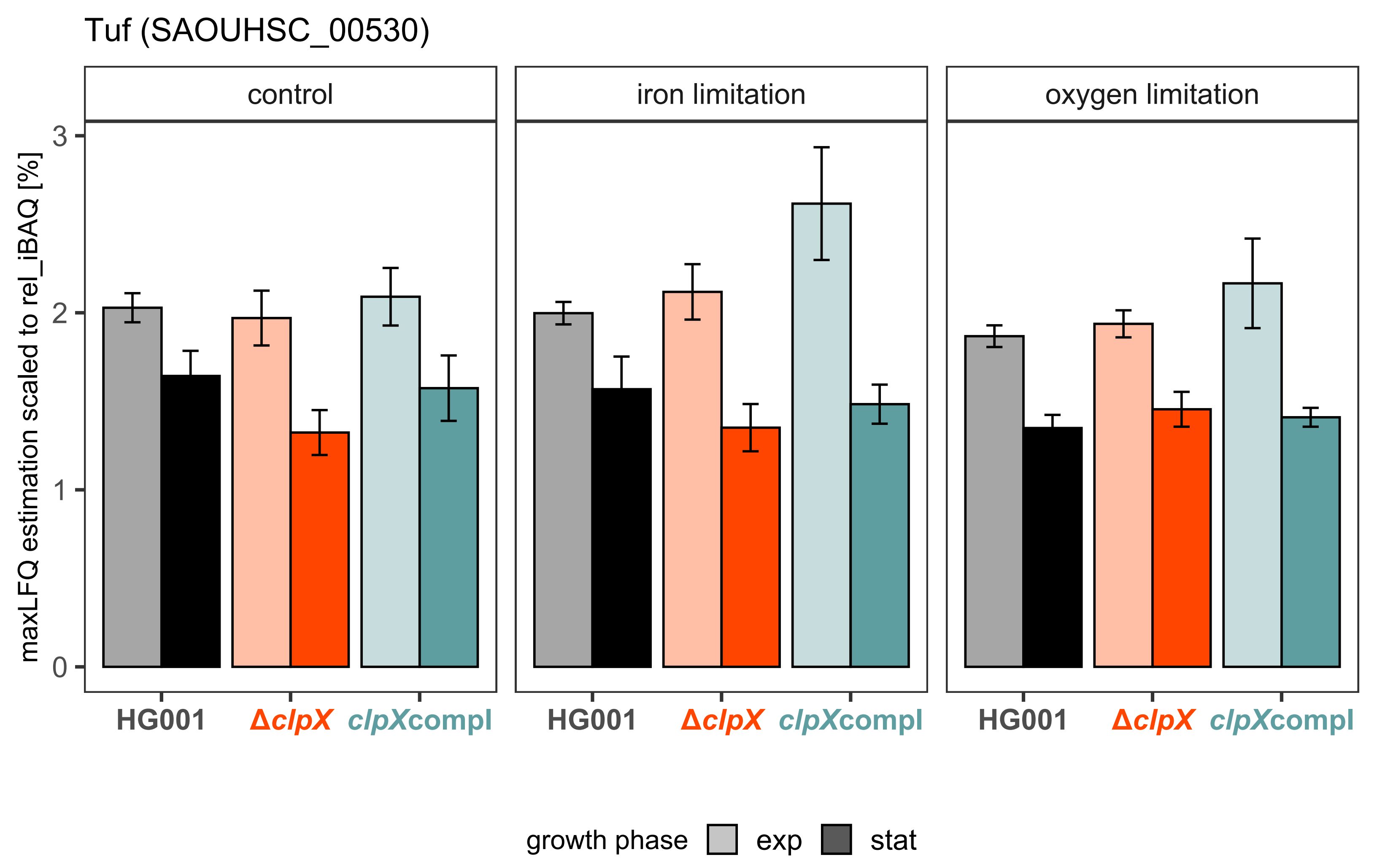

Tuf (SAOUHSC_00530)
control
iron limitation
oxygen limitation
maxLFQ estimation scaled to rel_iBAQ [%]
0
1
2
3
HG001
ΔclpX
clpXcompl
growth phase
exp
stat

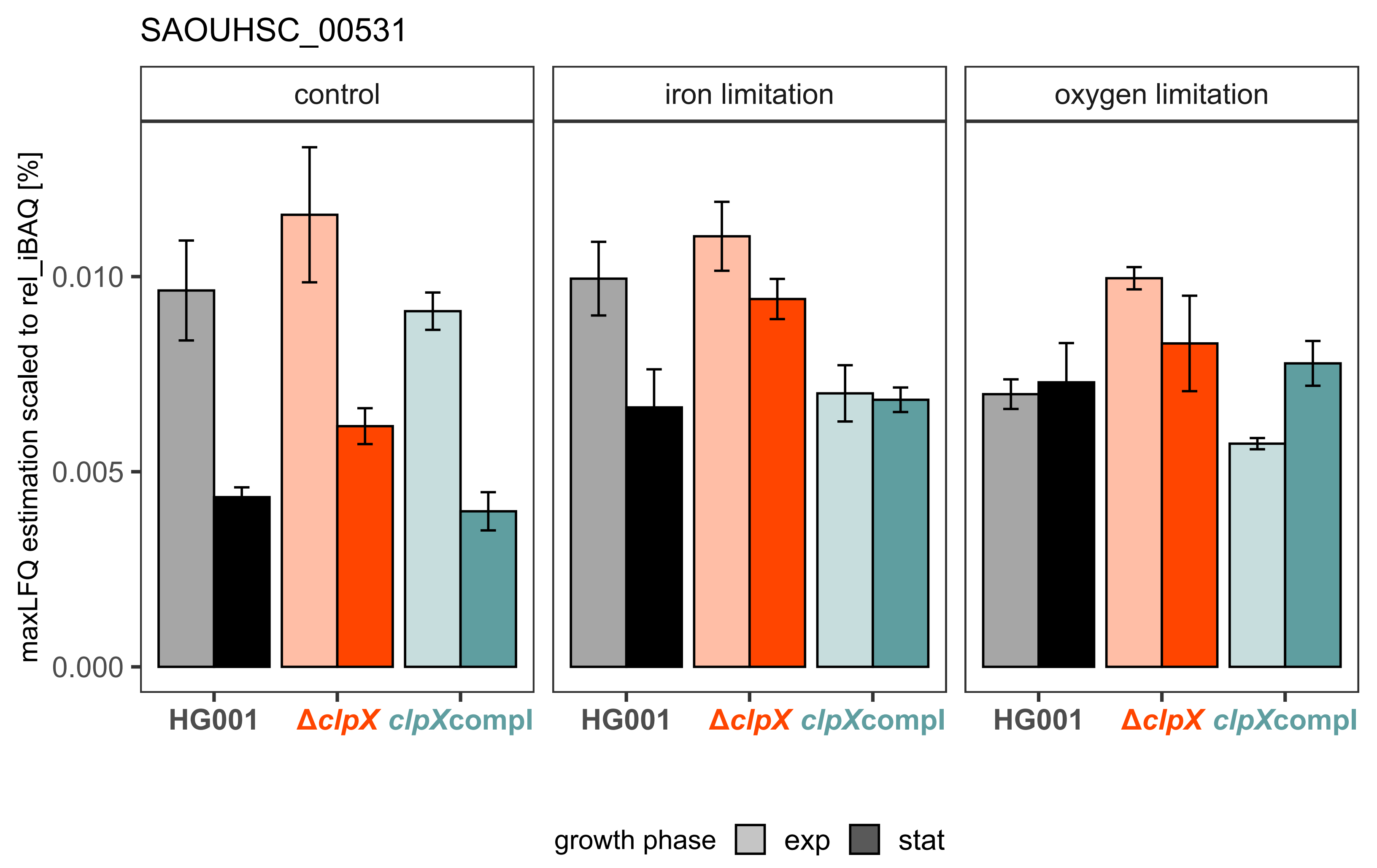
SAOUHSC_00531
control
iron limitation
oxygen limitation
maxLFQ estimation scaled to rel_iBAQ [%]
0.000
0.005
0.010
HG001
ΔclpX
clpXcompl
growth phase
exp
stat

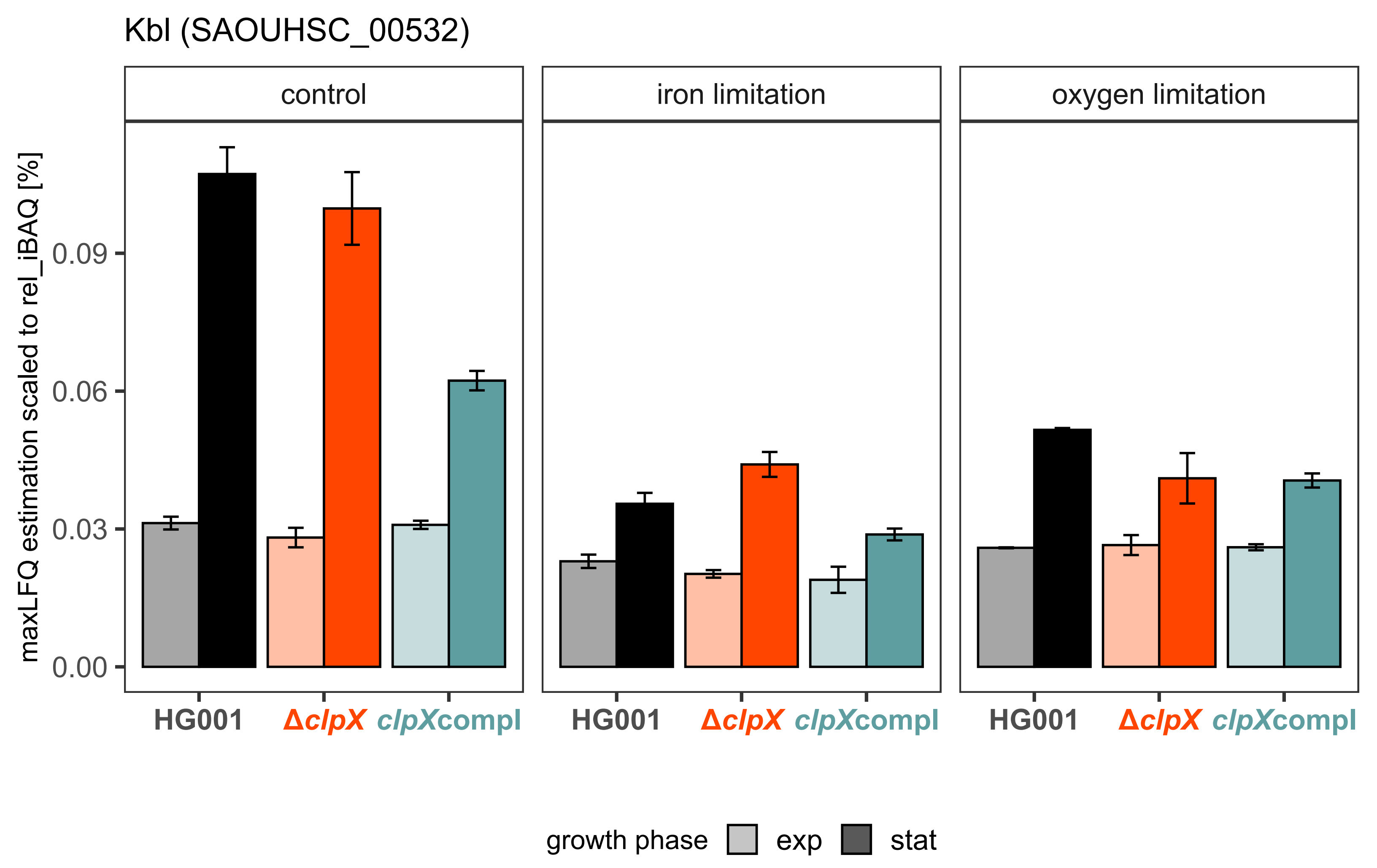

Kbl (SAOUHSC_00532)
control
iron limitation
oxygen limitation
maxLFQ estimation scaled to rel_iBAQ [%]
0.00
0.03
0.06
0.09
HG001
ΔclpX
clpXcompl
growth phase
exp
stat

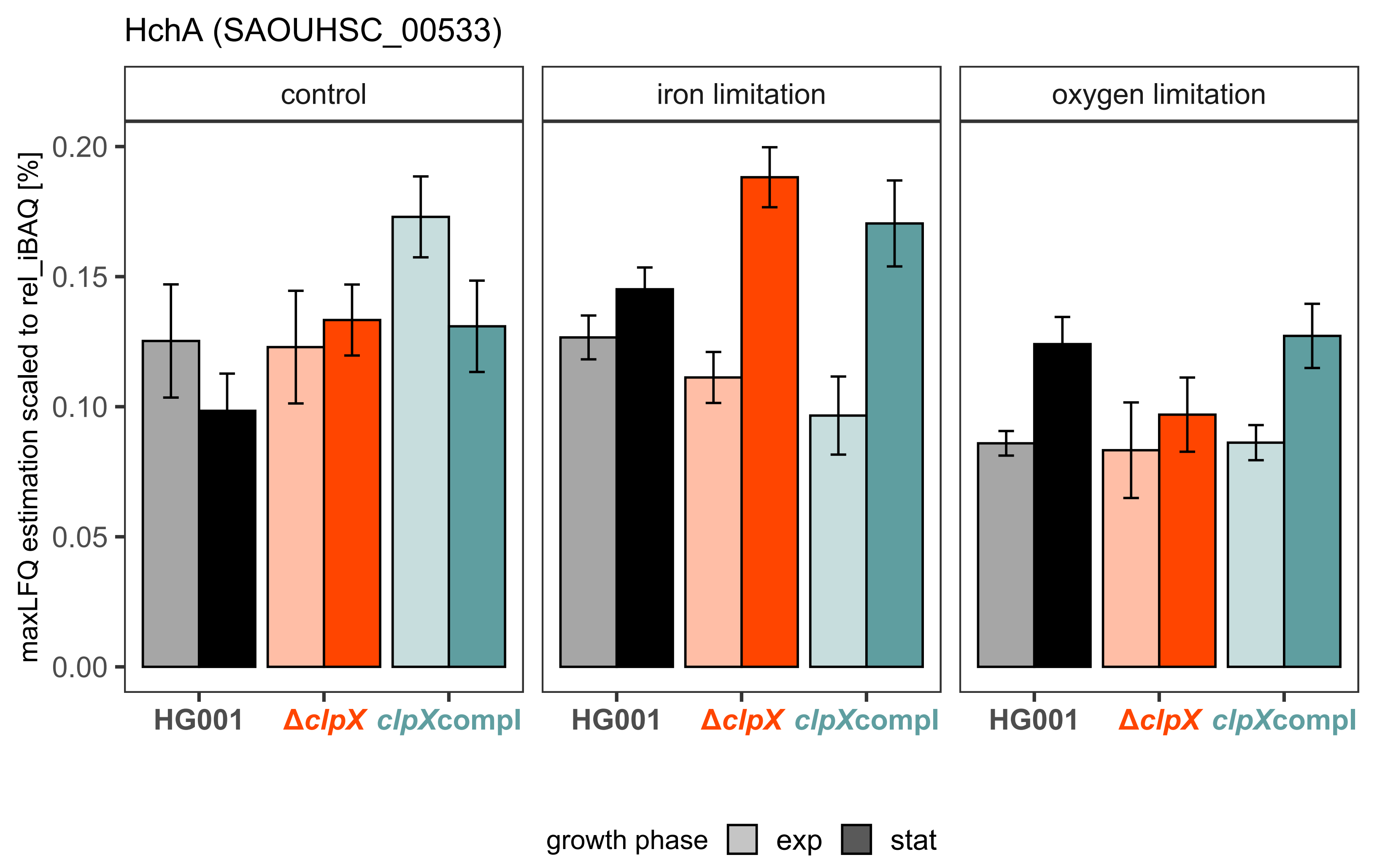
HchA (SAOUHSC_00533)
control
iron limitation
oxygen limitation
maxLFQ estimation scaled to rel_iBAQ [%]
0.20
0.15
0.10
0.05
0.00
HG001
ΔclpX
clpXcompl
growth phase
exp
stat

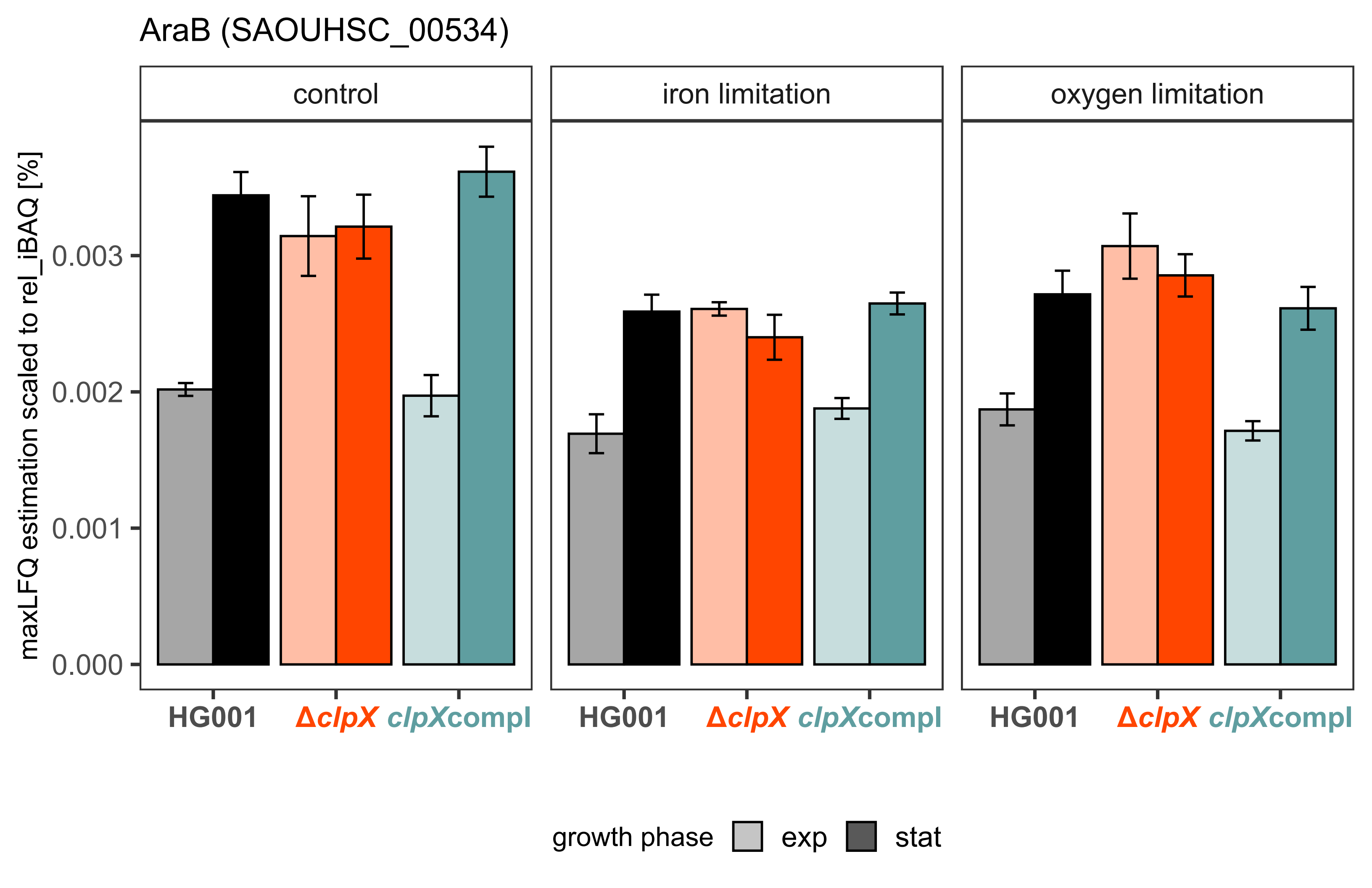
AraB (SAOUHSC_00534)
control
iron limitation
oxygen limitation
maxLFQ estimation scaled to rel_iBAQ [%]
0.000
0.001
0.002
0.003
HG001
ΔclpX
clpXcompl
growth phase
exp
stat

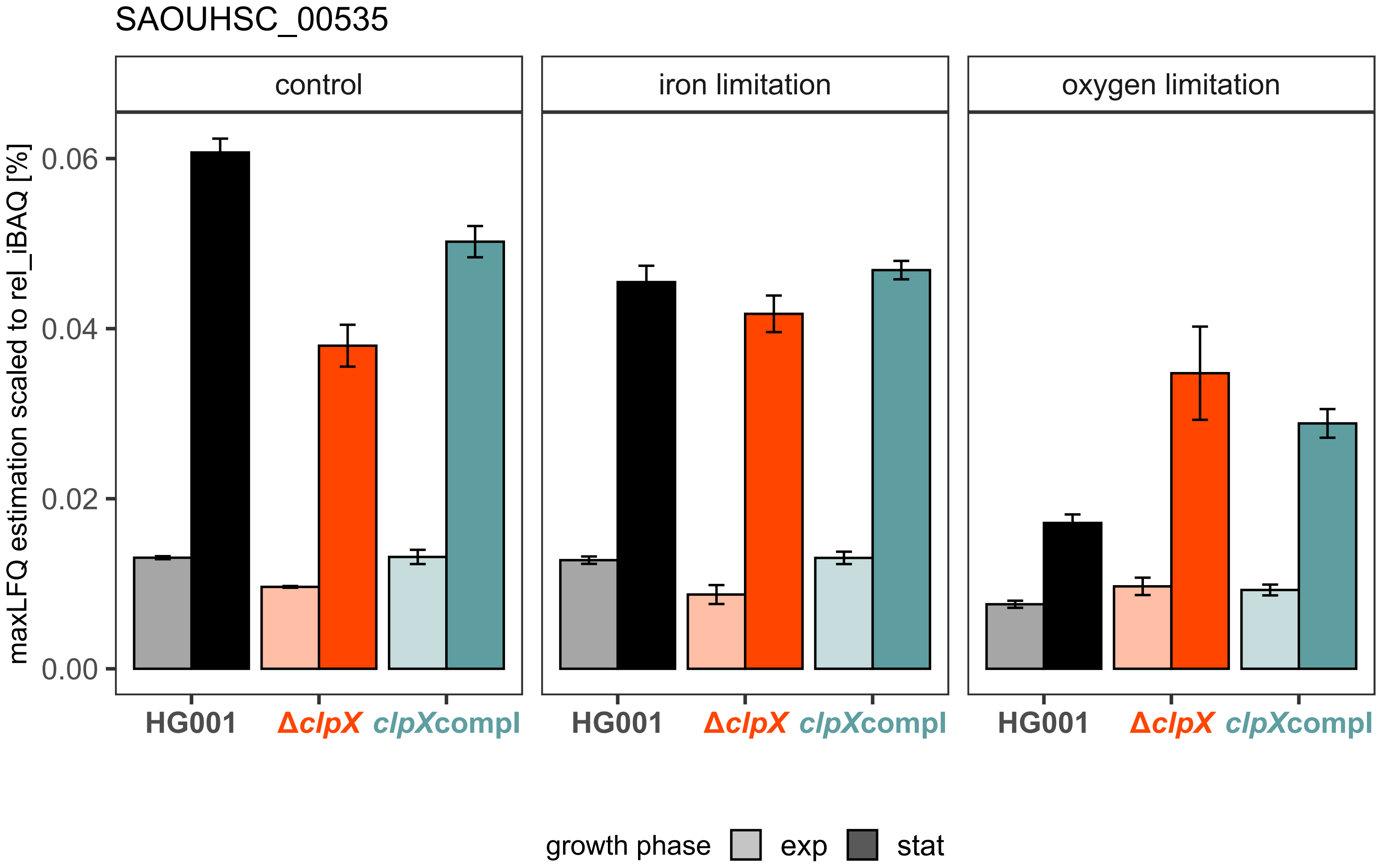

SAOUHSC_00535
control
iron limitation
oxygen limitation
maxLFQ estimation scaled to rel_iBAQ [%]
0.06
0.04
0.02
0.00
HG001
ΔclpX
clpXcompl
growth phase
exp
stat

IlvE (SAOUHSC_00536)

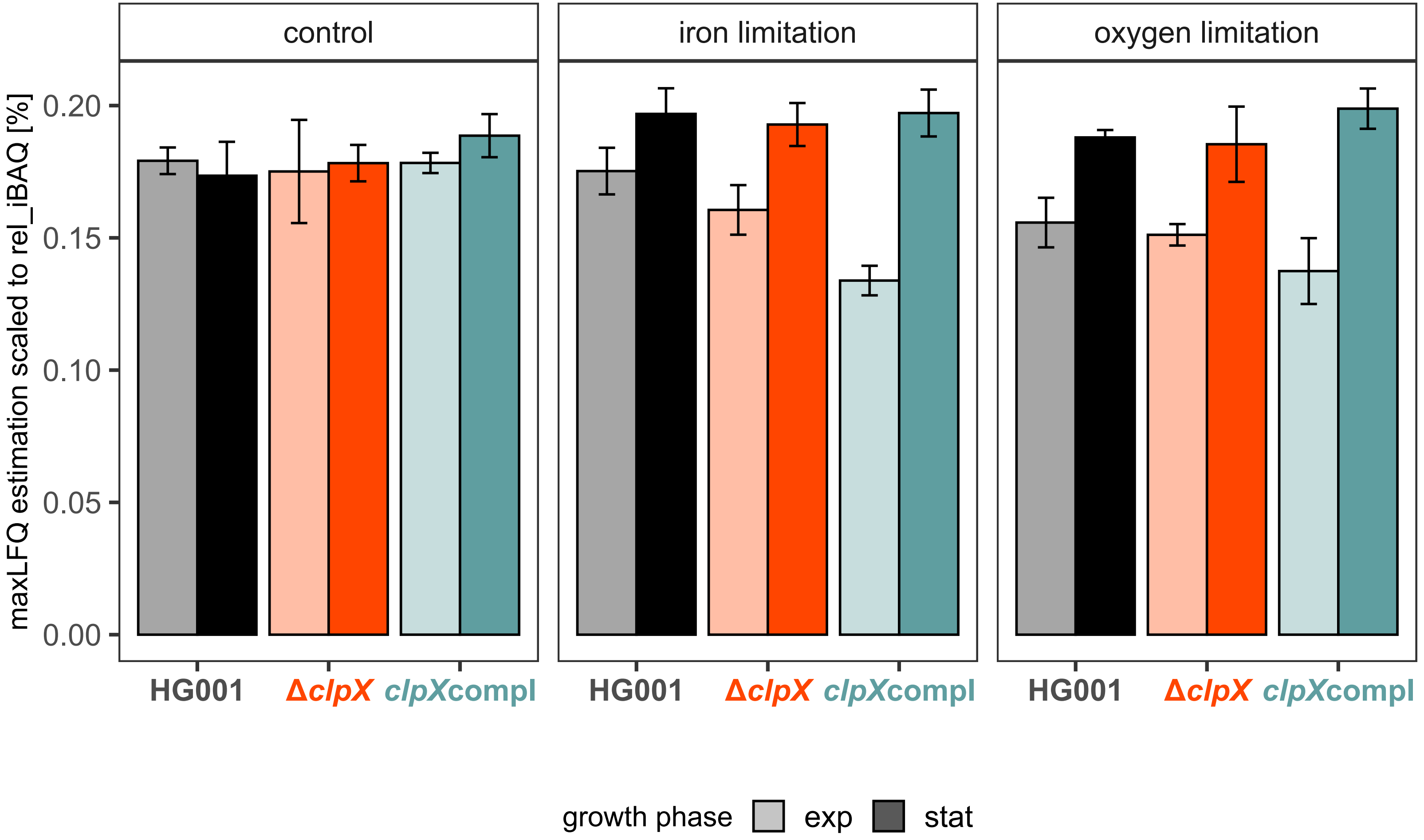

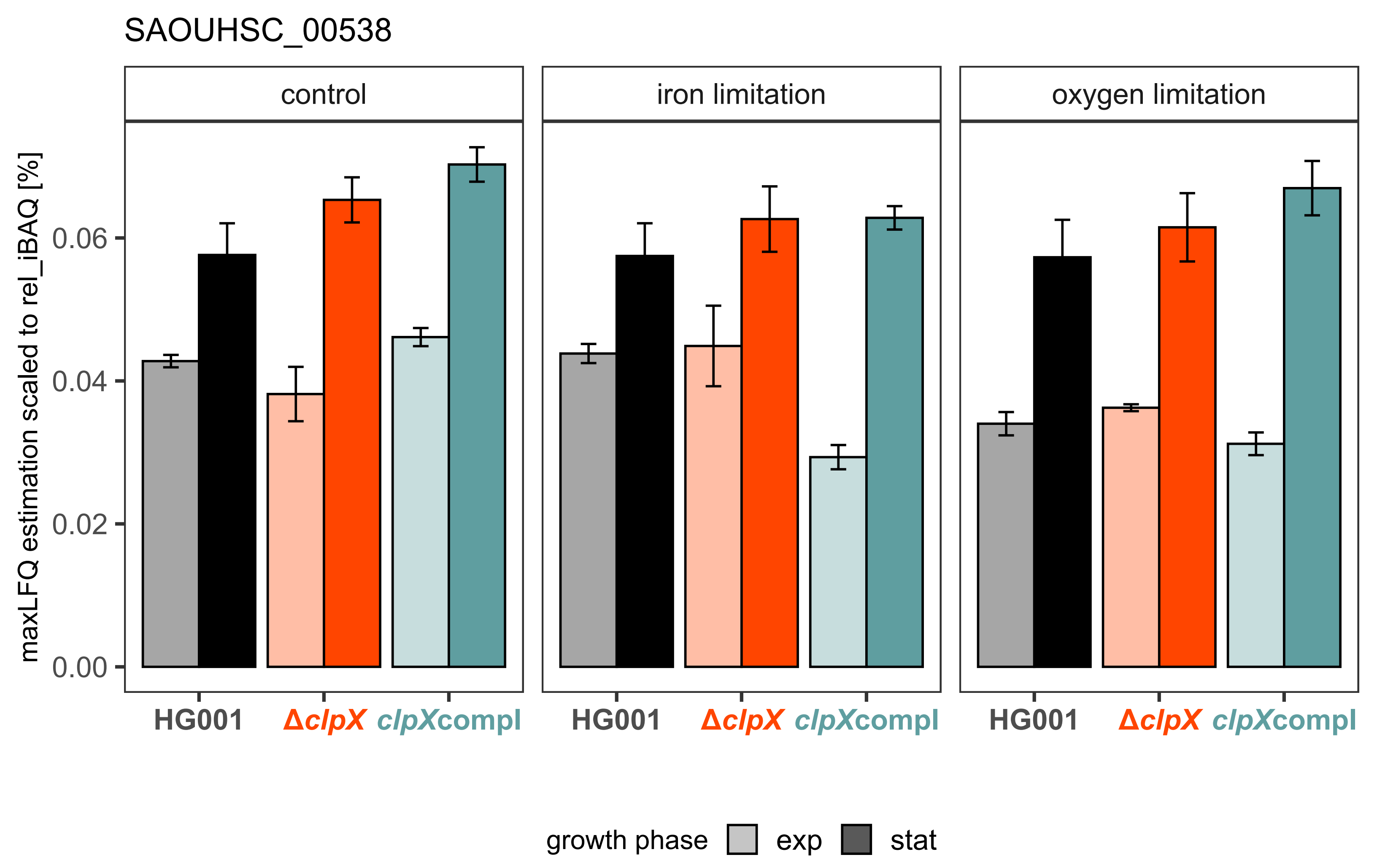

SAOUHSC_00538
control
iron limitation
oxygen limitation
maxLFQ estimation scaled to rel_iBAQ [%]
0.00
0.02
0.04
0.06
HG001
ΔclpX
clpXcompl
growth phase
exp
stat

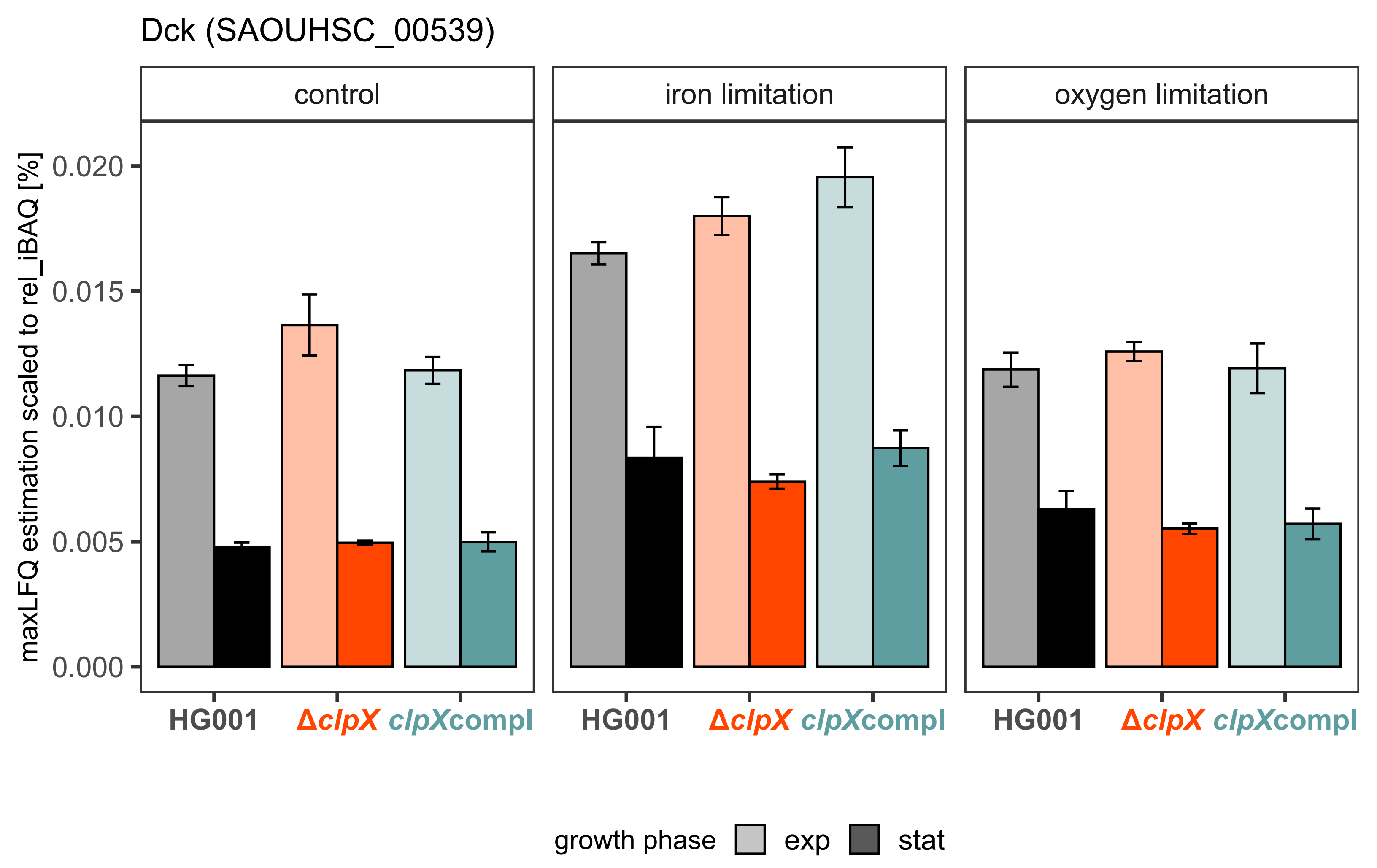

Dck (SAOUHSC_00539)
control
iron limitation
oxygen limitation
maxLFQ estimation scaled to rel_iBAQ [%]
0.020
0.015
0.010
0.005
0.000
HG001
ΔclpX
clpXcompl
growth phase
exp
stat

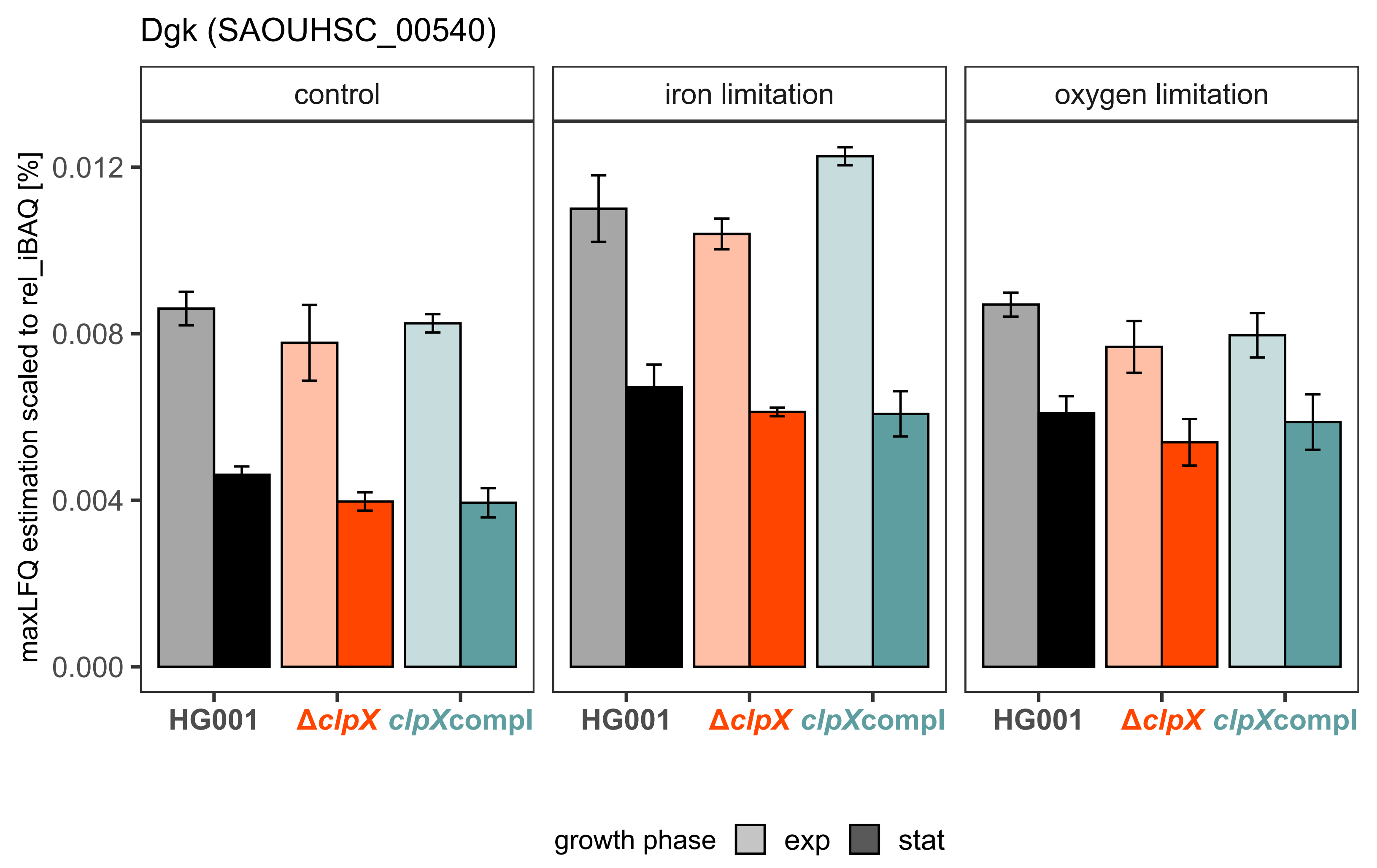

Dgk (SAOUHSC_00540)
control
iron limitation
oxygen limitation
maxLFQ estimation scaled to rel_iBAQ [%]
0.000
0.004
0.008
0.012
HG001
ΔclpX
clpXcompl
growth phase
exp
stat

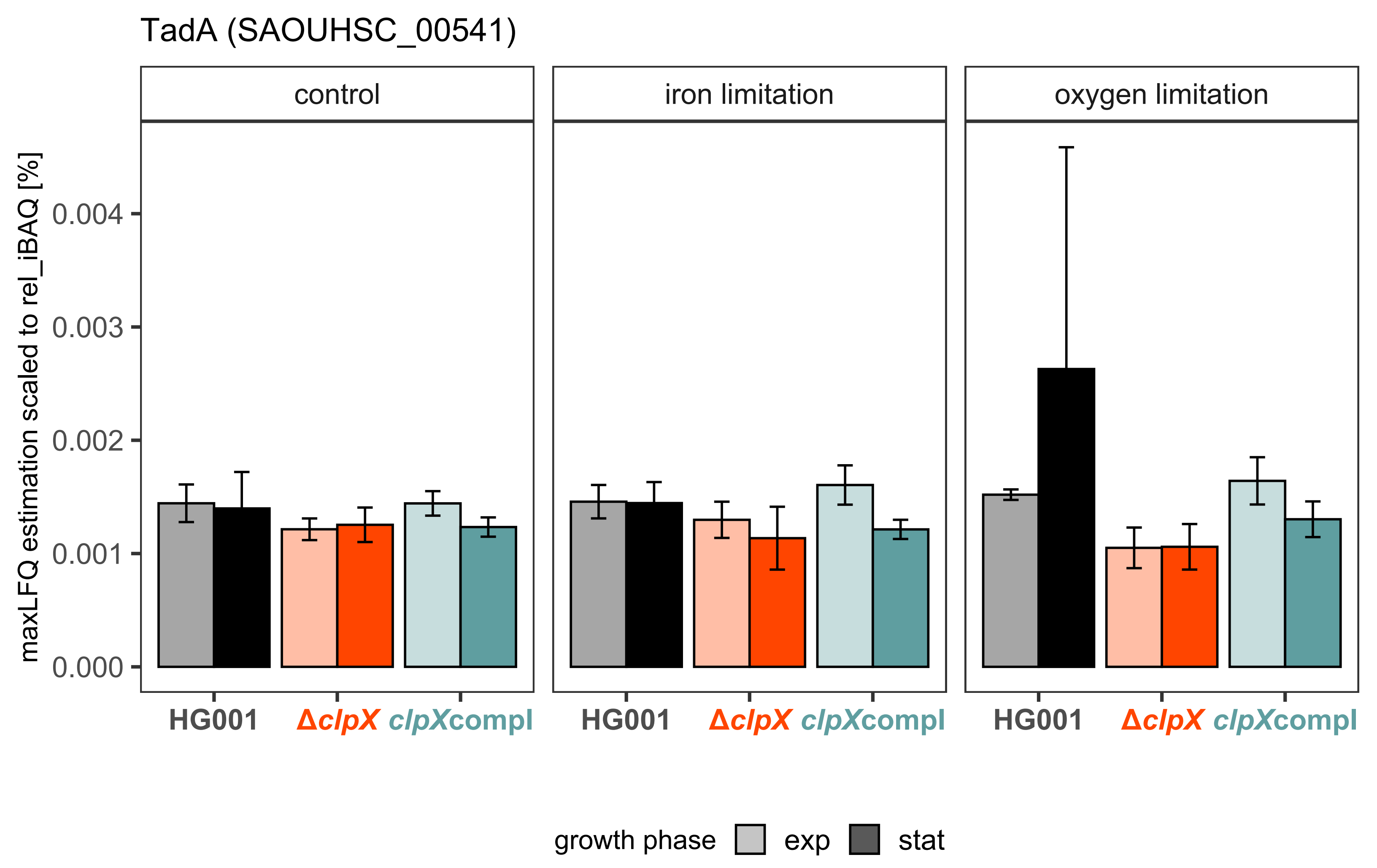
TadA (SAOUHSC_00541)
control
iron limitation
oxygen limitation
maxLFQ estimation scaled to rel_iBAQ [%]
0.004
0.003
0.002
0.001
0.000
HG001
ΔclpX
clpXcompl
growth phase
exp
stat

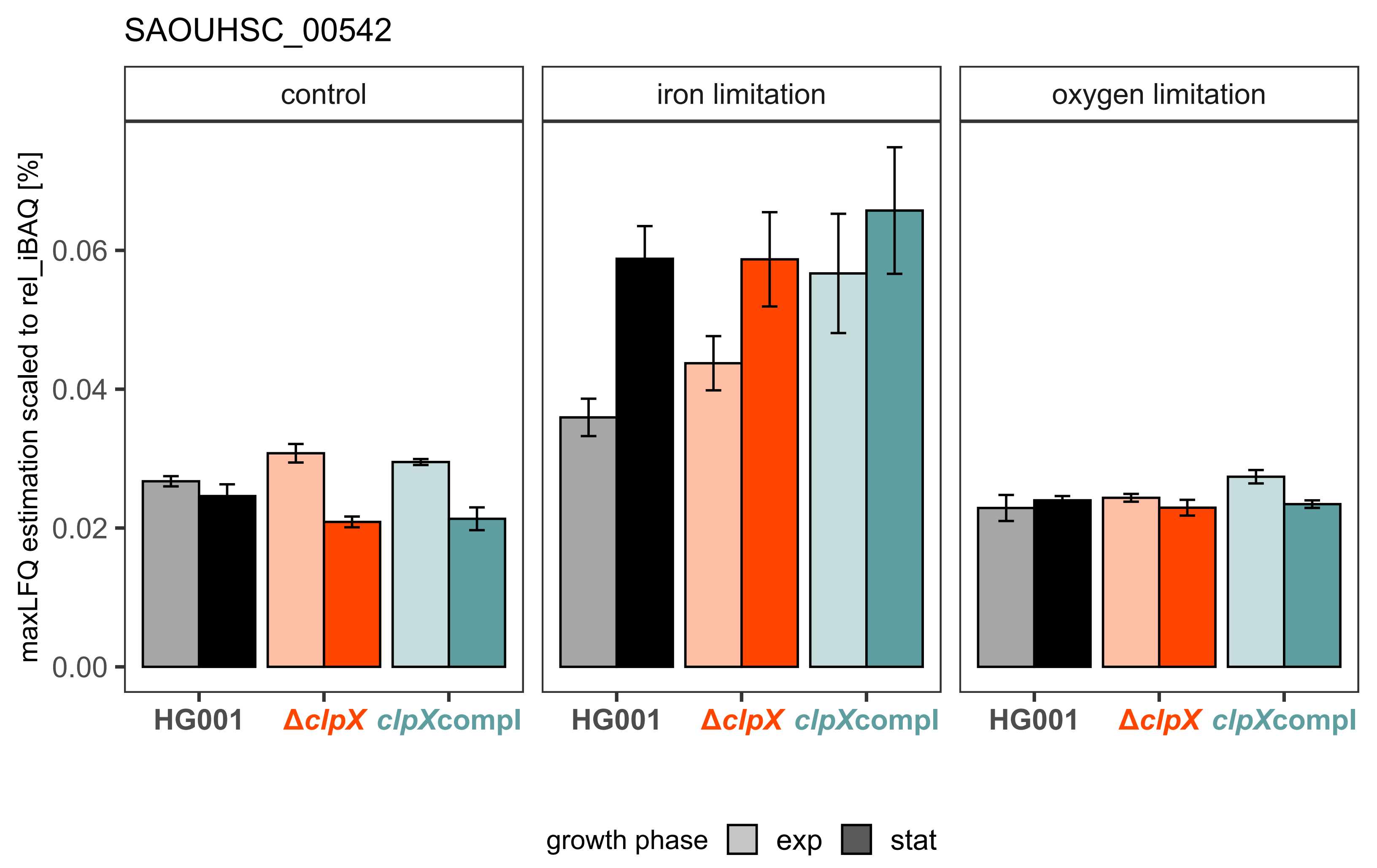

SAOUHSC_00542
control
iron limitation
oxygen limitation
maxLFQ estimation scaled to rel_iBAQ [%]
0.06
0.04
0.02
0.00
HG001
ΔclpX
clpXcompl
growth phase
exp
stat

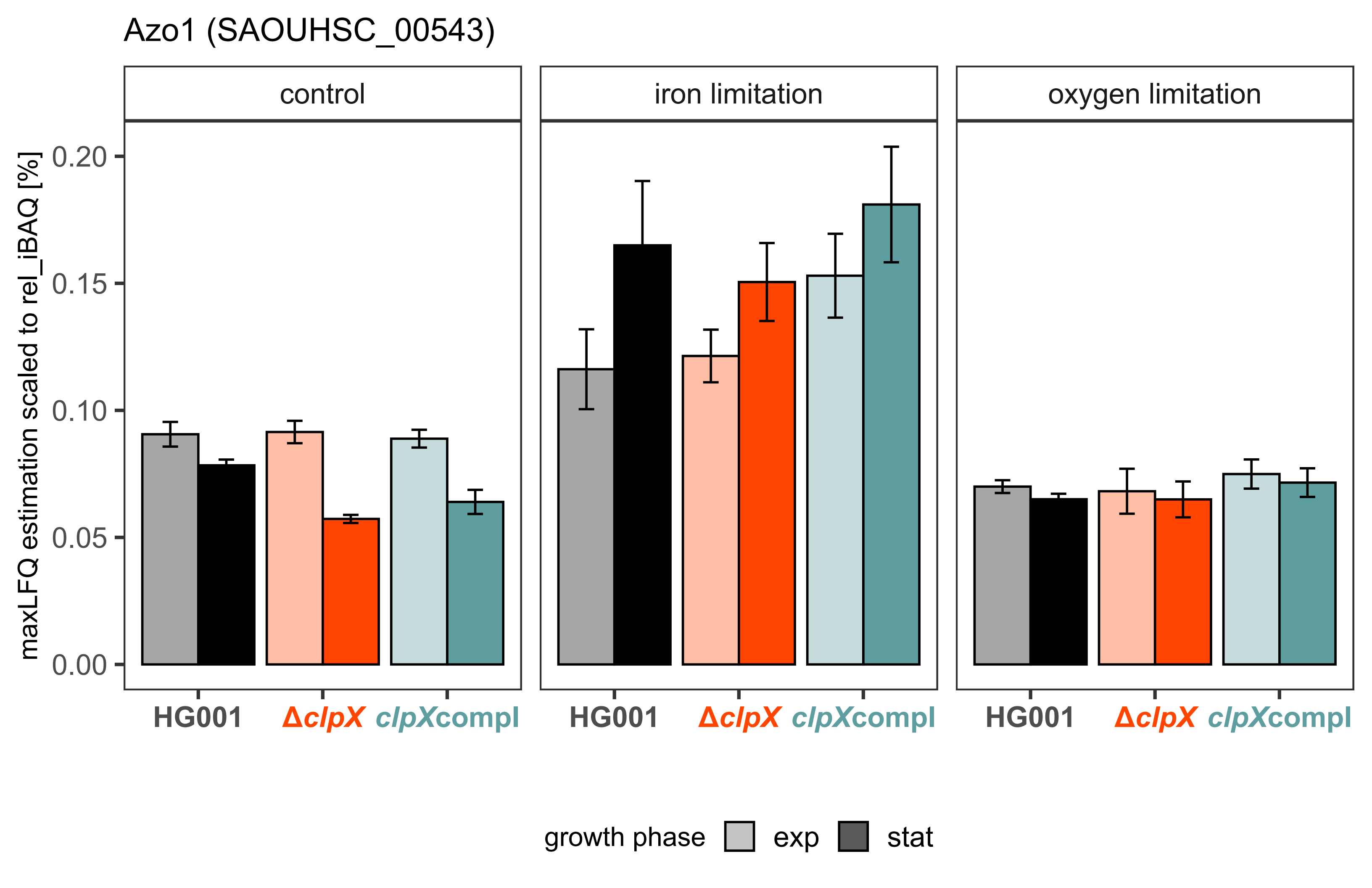

Azo1 (SAOUHSC_00543)
control
iron limitation
oxygen limitation
maxLFQ estimation scaled to rel_iBAQ [%]
0.20
0.15
0.10
0.05
0.00
HG001
ΔclpX
clpXcompl
growth phase
exp
stat

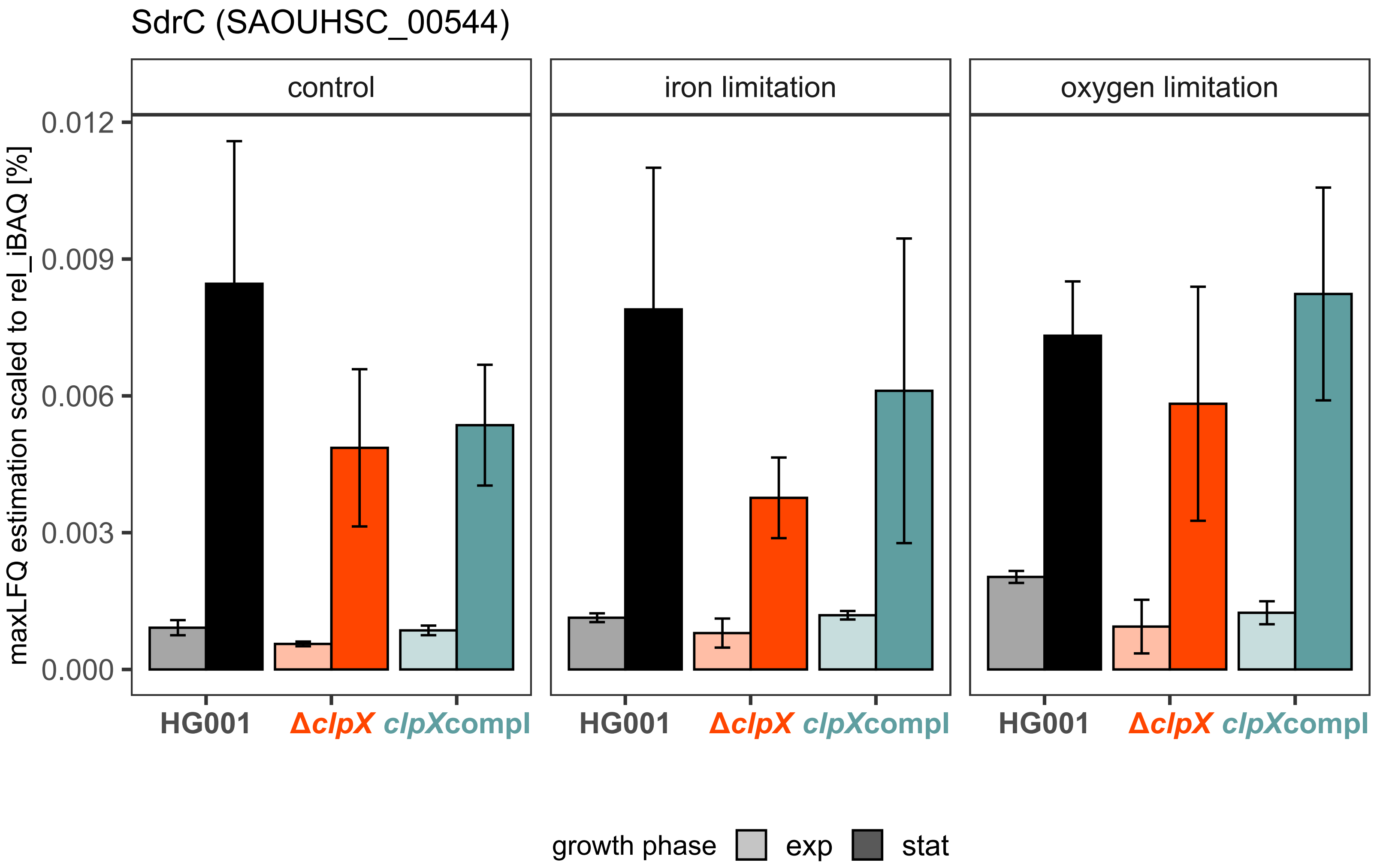
SdrC (SAOUHSC_00544)
control
iron limitation
oxygen limitation
maxLFQ estimation scaled to rel_iBAQ [%]
0.012
0.009
0.006
0.003
0.000
HG001
ΔclpX
clpXcompl
growth phase
exp
stat

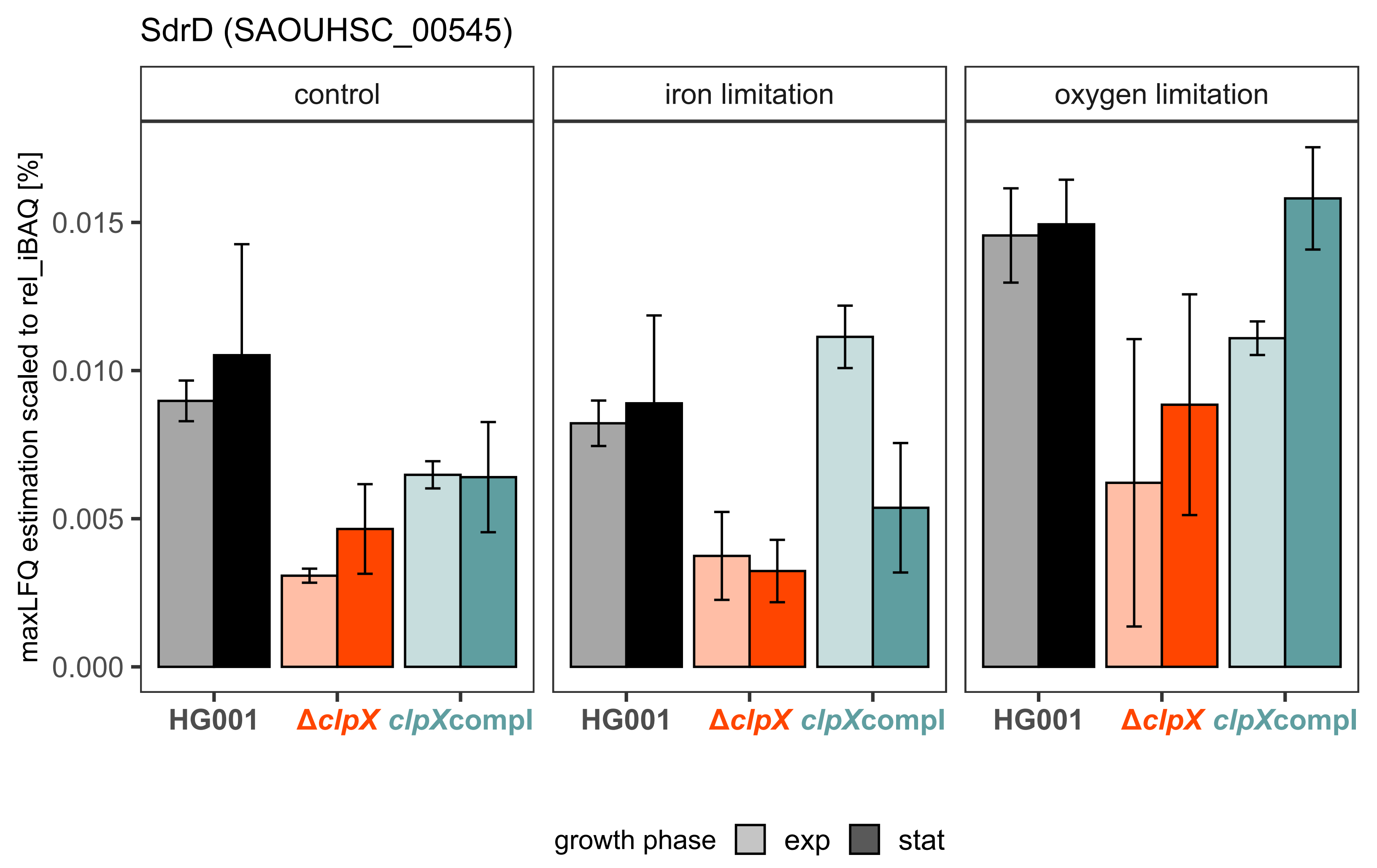
SdrD (SAOUHSC_00545)
control
iron limitation
oxygen limitation
maxLFQ estimation scaled to rel_iBAQ [%]
0.015
0.010
0.005
0.000
HG001
ΔclpX
clpXcompl
growth phase
exp
stat

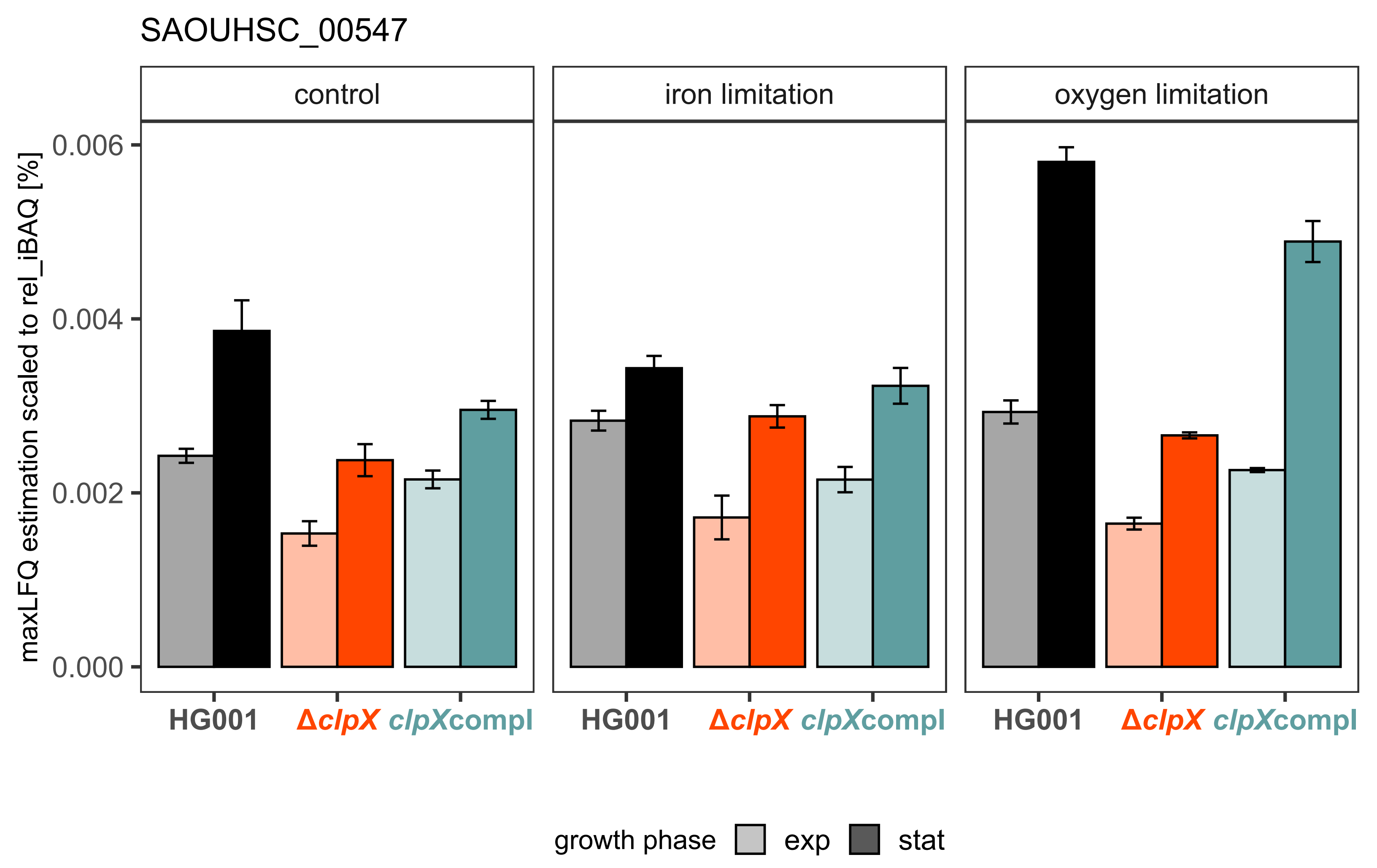
SAOUHSC_00547
control
iron limitation
oxygen limitation
maxLFQ estimation scaled to rel_iBAQ [%]
0.006
0.004
0.002
0.000
HG001
ΔclpX
clpXcompl
growth phase
exp
stat

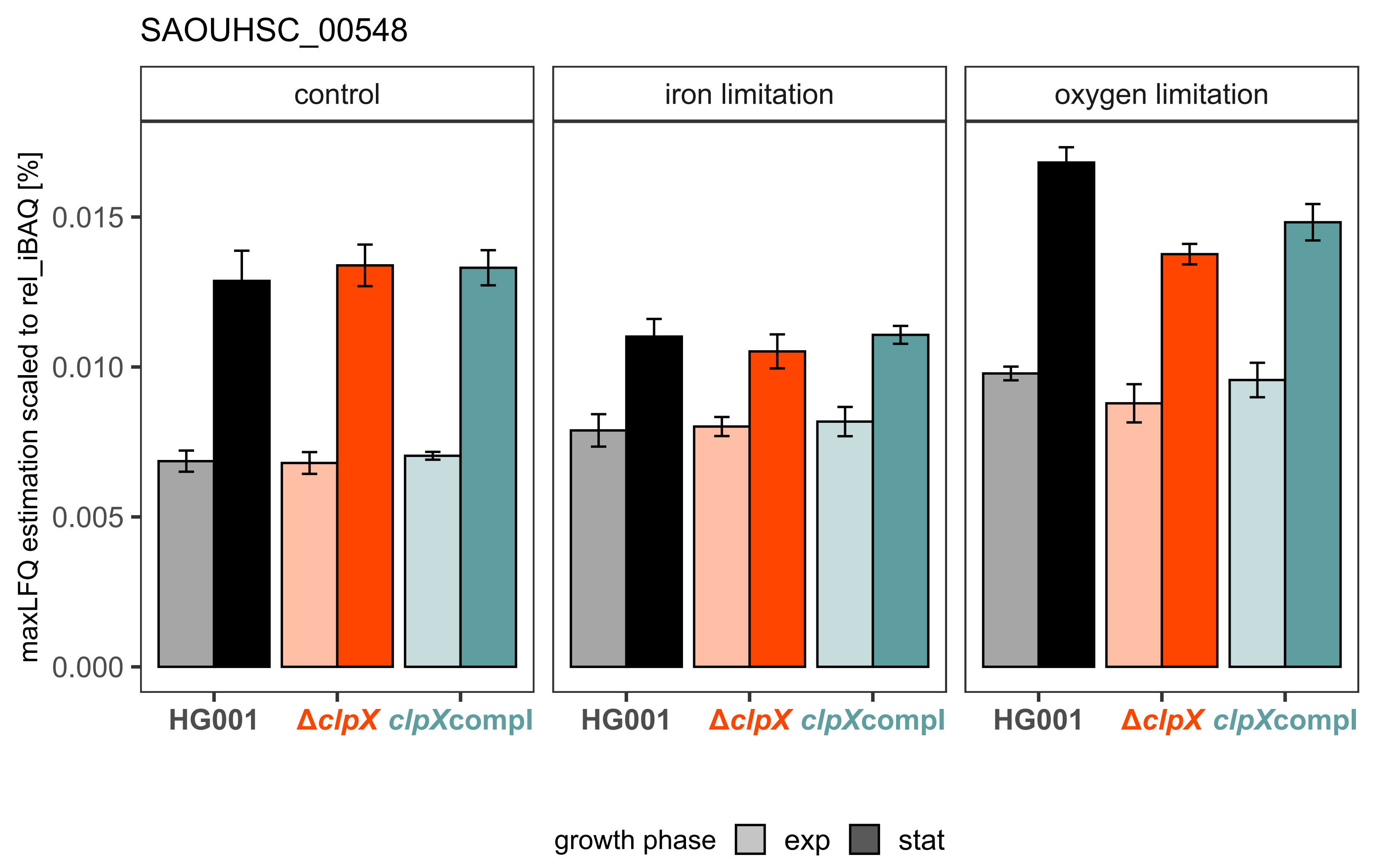

SAOUHSC_00548
control
iron limitation
oxygen limitation
maxLFQ estimation scaled to rel_iBAQ [%]
0.015
0.010
0.005
0.000
HG001
ΔclpX
clpXcompl
growth phase
exp
stat

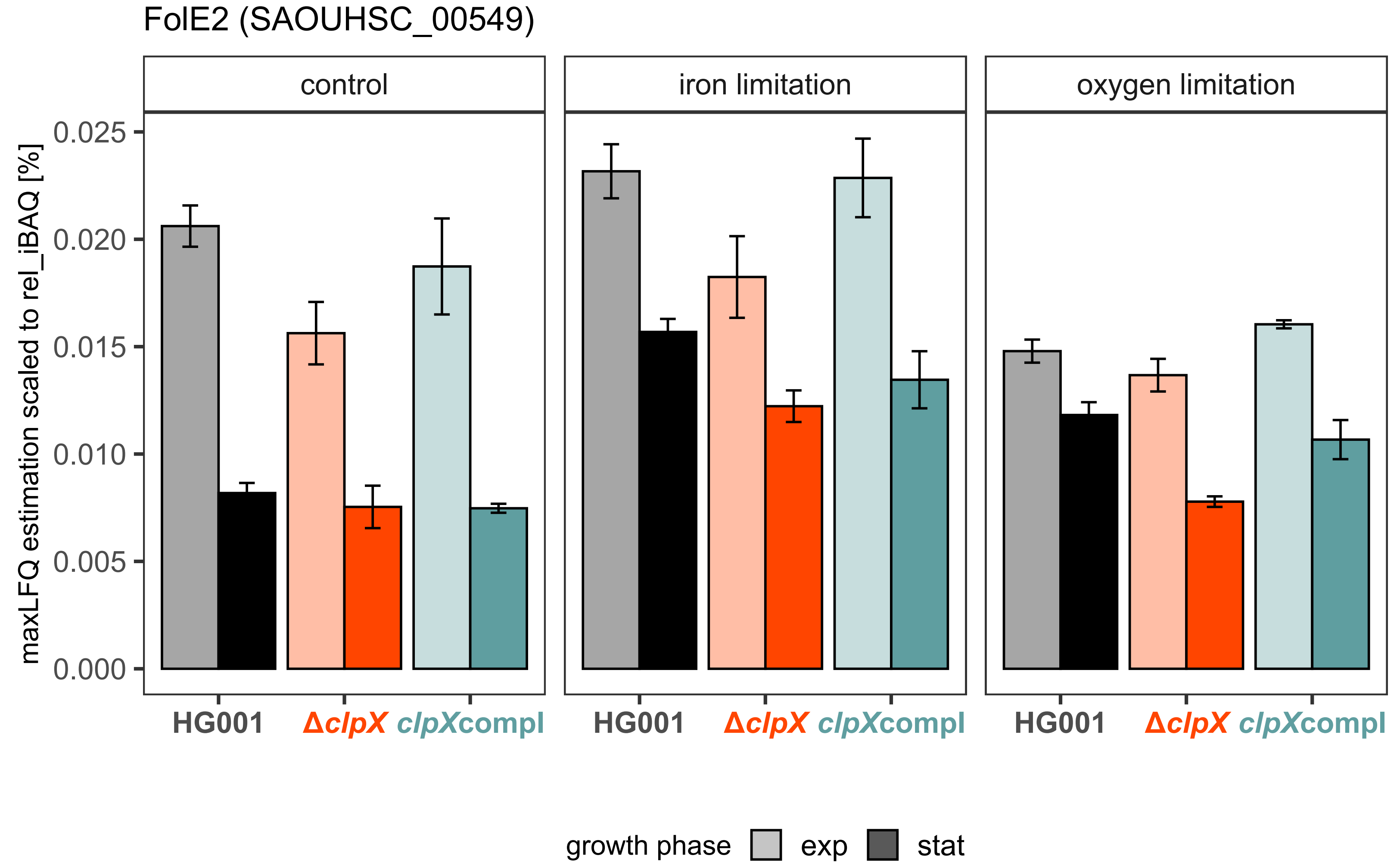
FolE2 (SAOUHSC_00549)
control
iron limitation
oxygen limitation
maxLFQ estimation scaled to rel_iBAQ [%]
0.025
0.020
0.015
0.010
0.005
0.000
HG001
ΔclpX
clpXcompl
growth phase
exp
stat

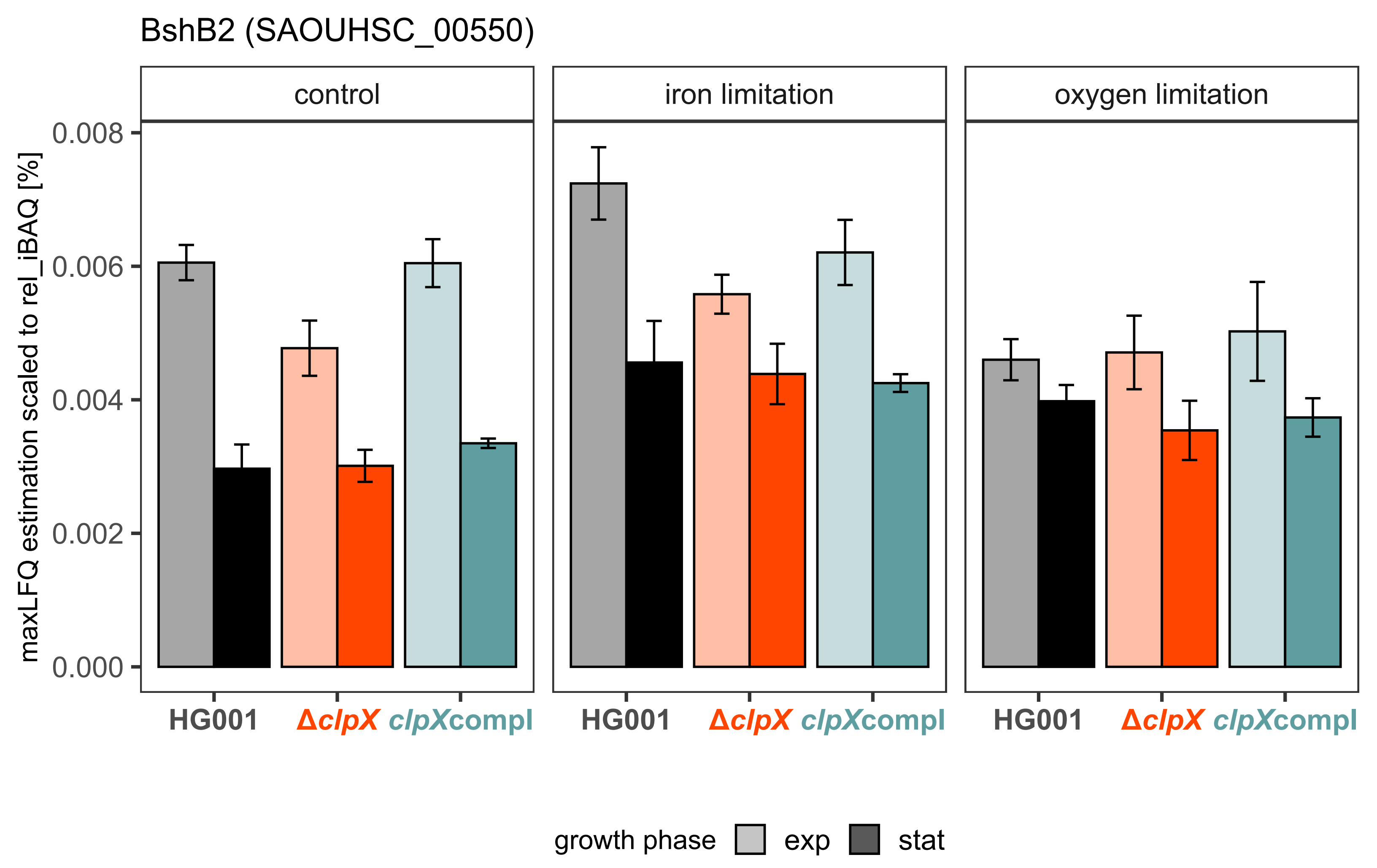

BshB2 (SAOUHSC_00550)
control
iron limitation
oxygen limitation
maxLFQ estimation scaled to rel_iBAQ [%]
0.008
0.006
0.004
0.002
0.000
HG001
ΔclpX
clpXcompl
growth phase
exp
stat

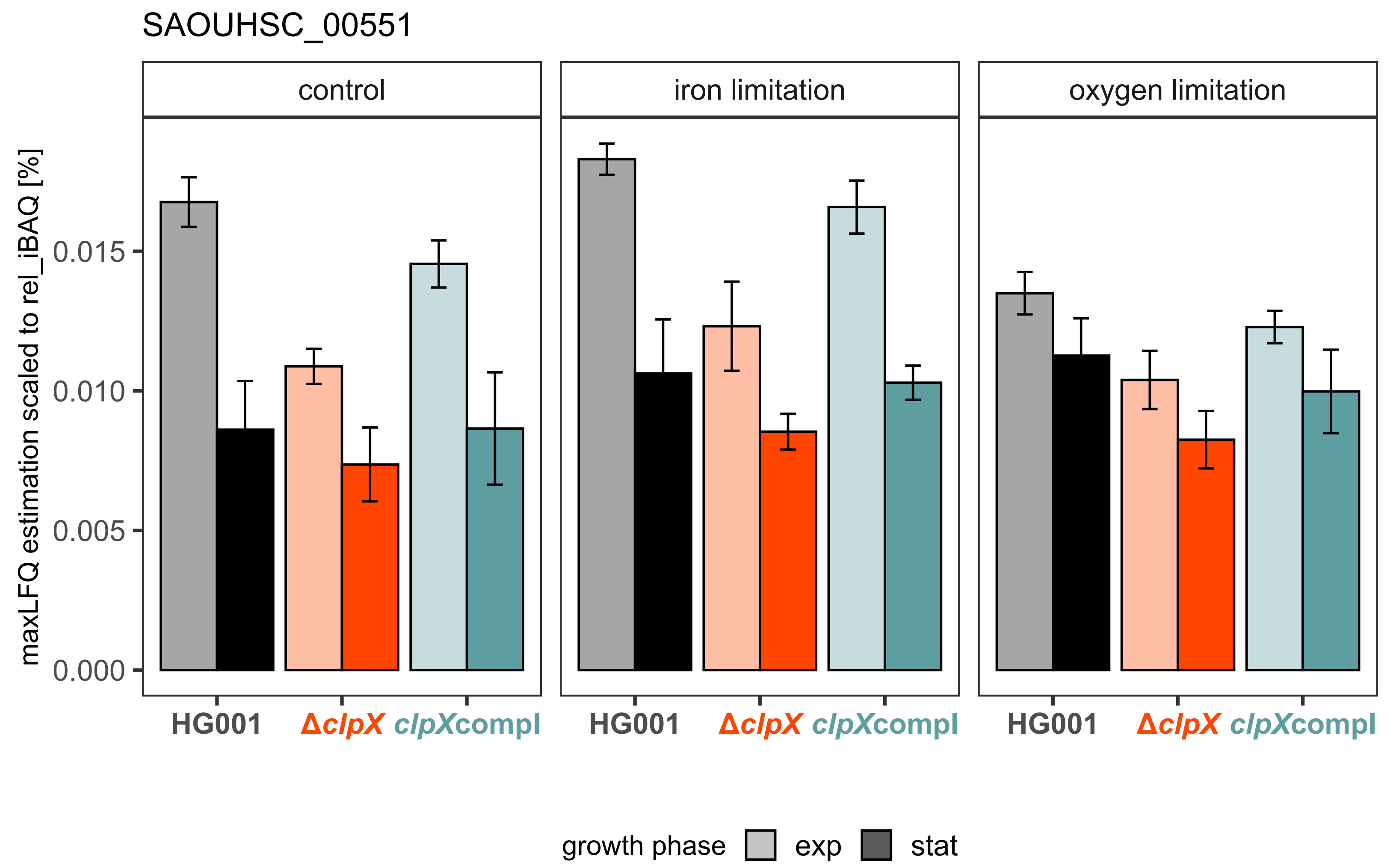

SAOUHSC_00551
control
iron limitation
oxygen limitation
maxLFQ estimation scaled to rel_iBAQ [%]
0.000
0.005
0.010
0.015
HG001
ΔclpX
clpXcompl
growth phase
exp
stat

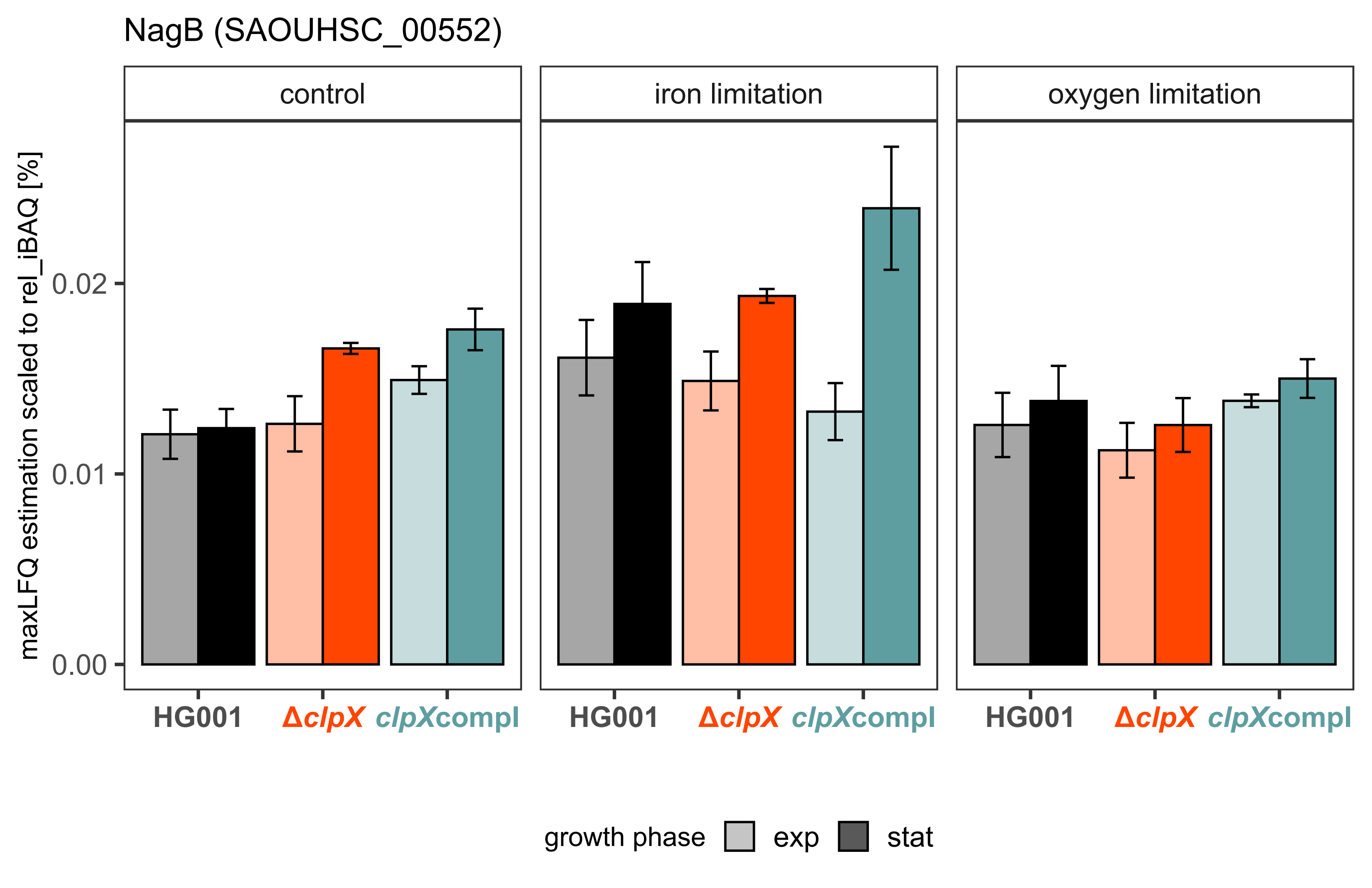
NagB (SAOUHSC_00552)
control
iron limitation
oxygen limitation
maxLFQ estimation scaled to rel_iBAQ [%]
0.02
0.01
0.00
HG001
ΔclpX
clpXcompl
growth phase
exp
stat

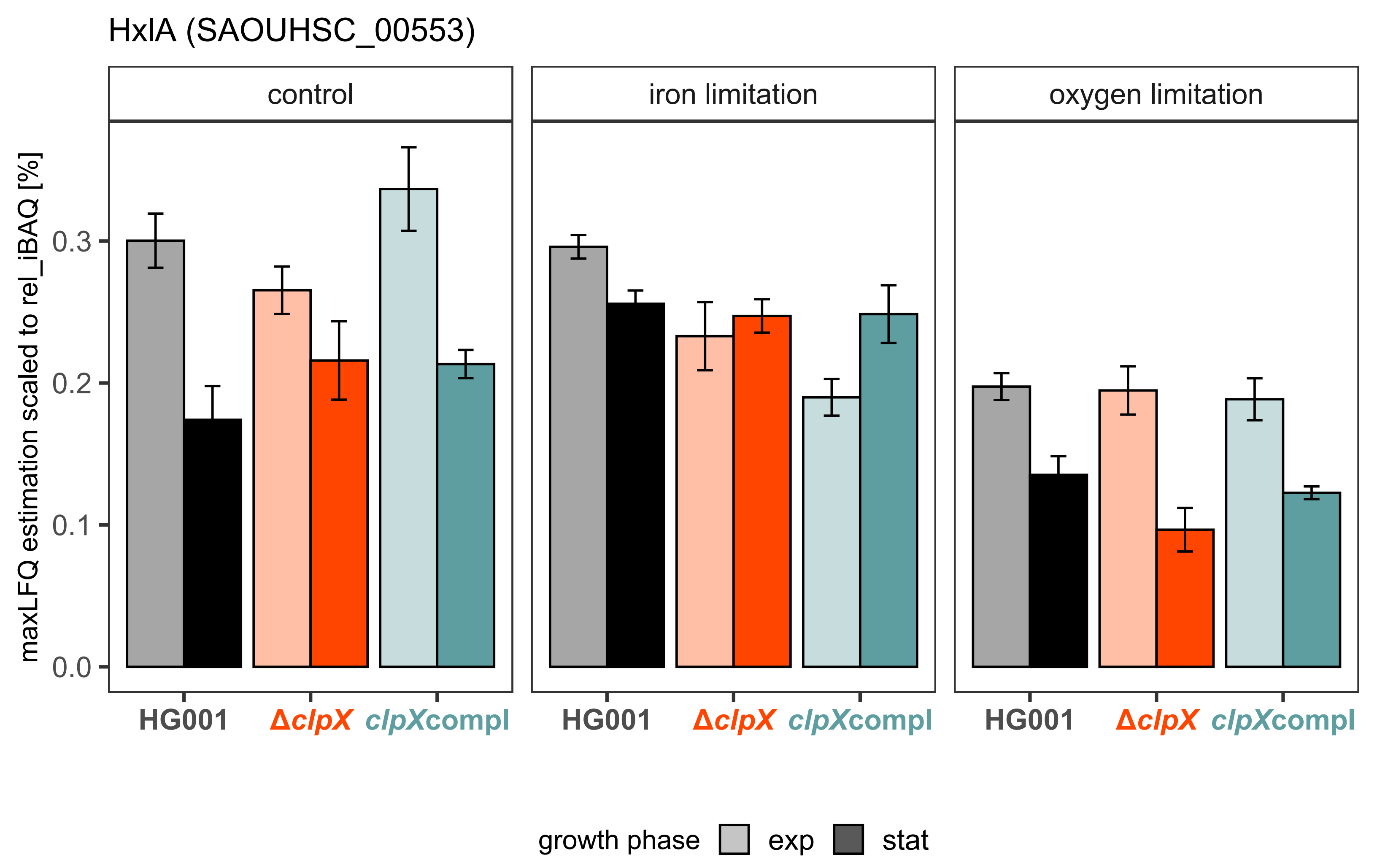

HxlA (SAOUHSC_00553)
control
iron limitation
oxygen limitation
maxLFQ estimation scaled to rel_iBAQ [%]
0.0
0.1
0.2
0.3
HG001
ΔclpX
clpXcompl
growth phase
exp
stat

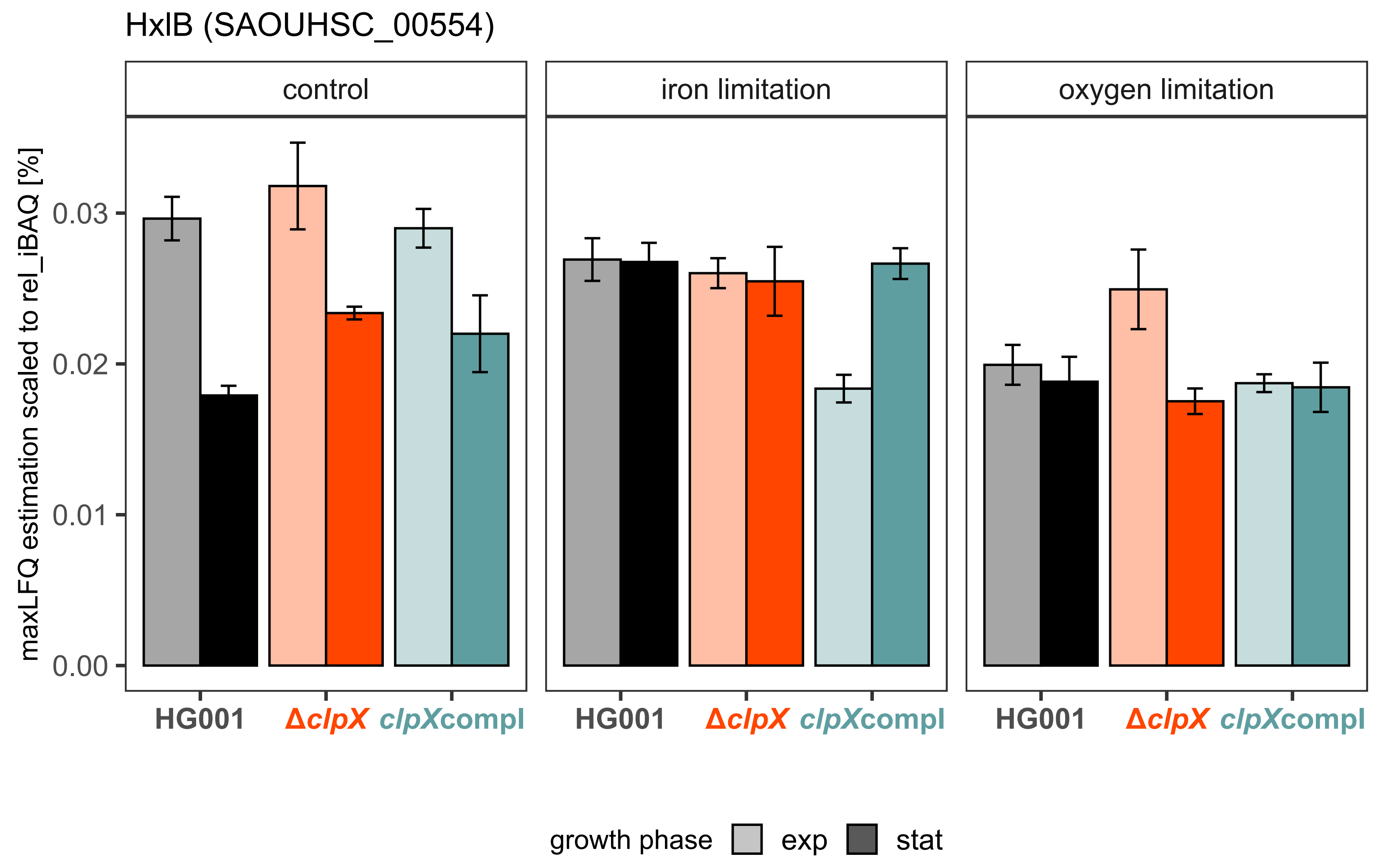

HxlB (SAOUHSC_00554)
control
iron limitation
oxygen limitation
maxLFQ estimation scaled to rel_iBAQ [%]
0.00
0.01
0.02
0.03
HG001
ΔclpX
clpXcompl
growth phase
exp
stat

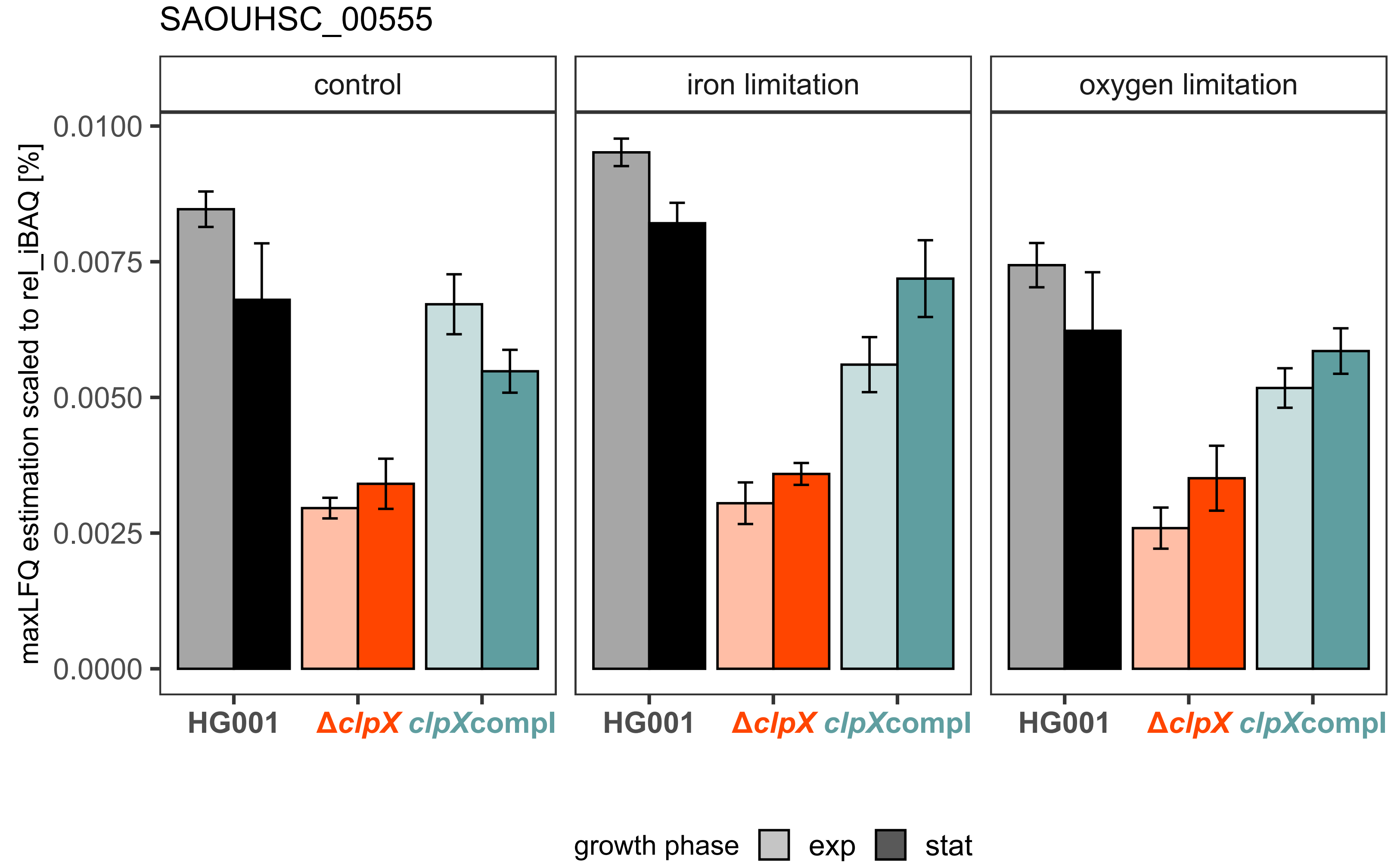

SAOUHSC_00555
control
iron limitation
oxygen limitation
maxLFQ estimation scaled to rel_iBAQ [%]
0.0100
0.0075
0.0050
0.0025
0.0000
HG001
ΔclpX
clpXcompl
growth phase
exp
stat

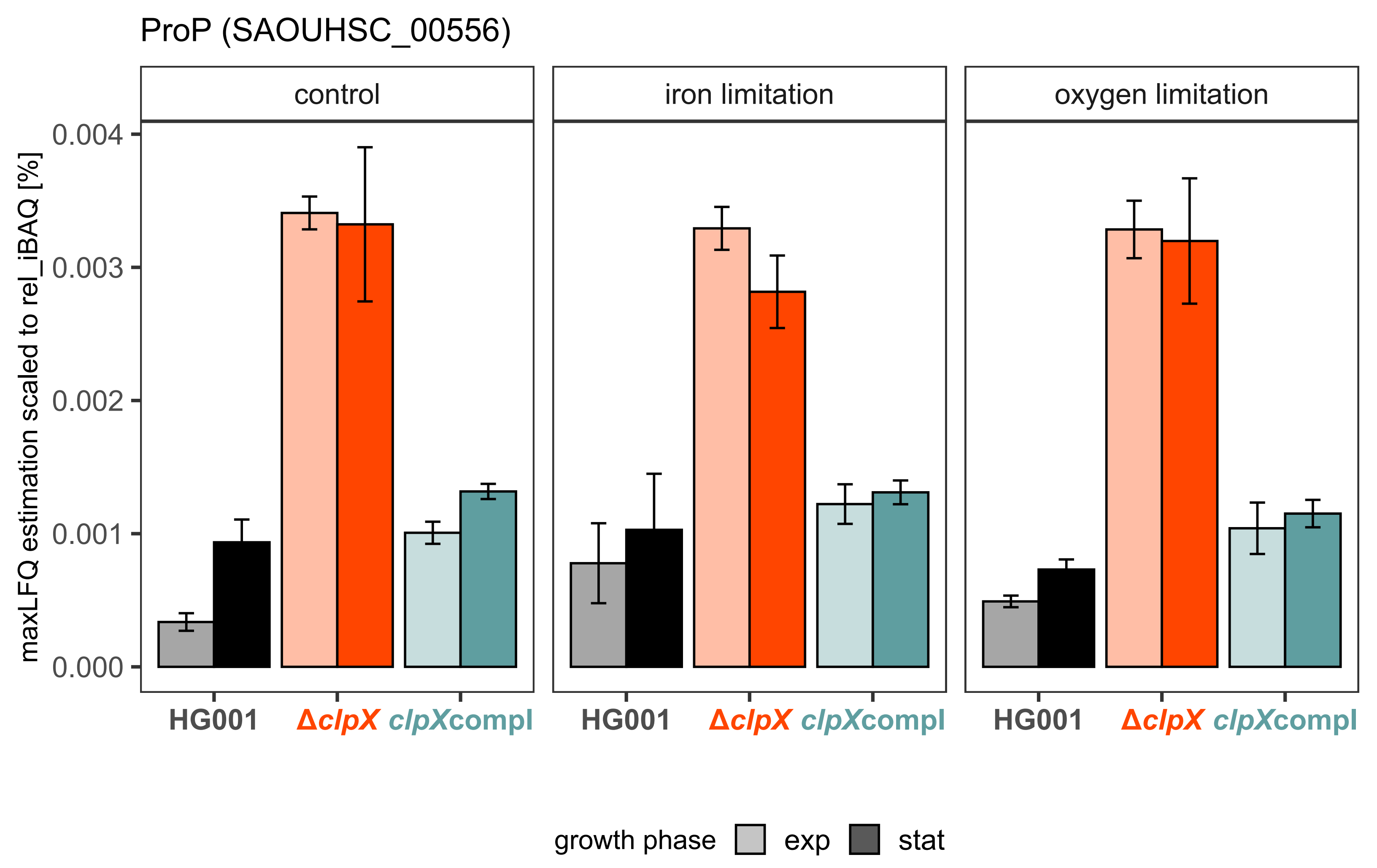
ProP (SAOUHSC_00556)
control
iron limitation
oxygen limitation
maxLFQ estimation scaled to rel_iBAQ [%]
0.004
0.003
0.002
0.001
0.000
HG001
ΔclpX
clpXcompl
growth phase
exp
stat

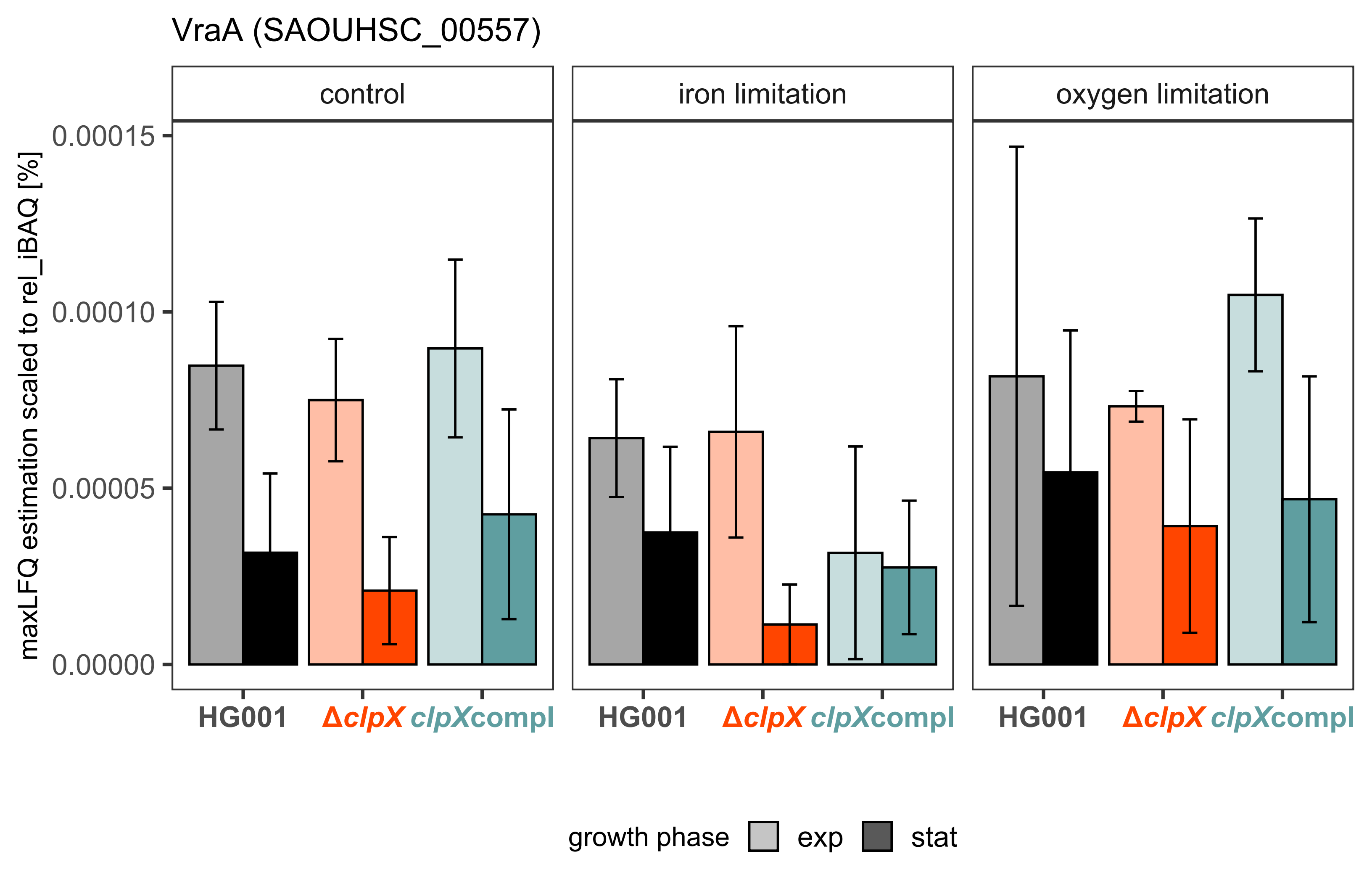

VraA (SAOUHSC_00557)
control
iron limitation
oxygen limitation
maxLFQ estimation scaled to rel_iBAQ [%]
0.00015
0.00010
0.00005
0.00000
HG001
ΔclpX
clpXcompl
growth phase
exp
stat

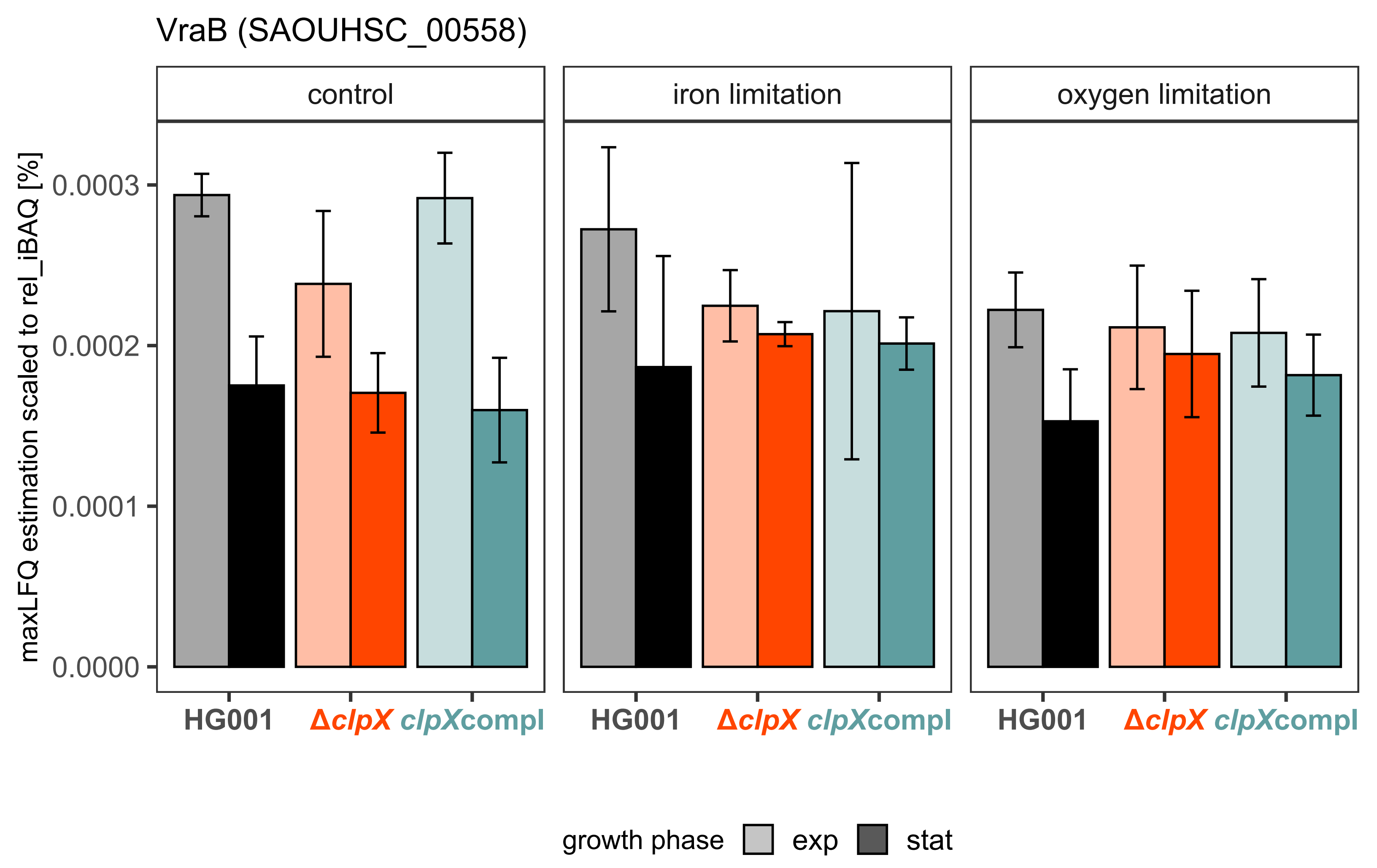
VraB (SAOUHSC_00558)
control
iron limitation
oxygen limitation
maxLFQ estimation scaled to rel_iBAQ [%]
0.0003
0.0002
0.0001
0.0000
HG001
ΔclpX
clpXcompl
growth phase
exp
stat

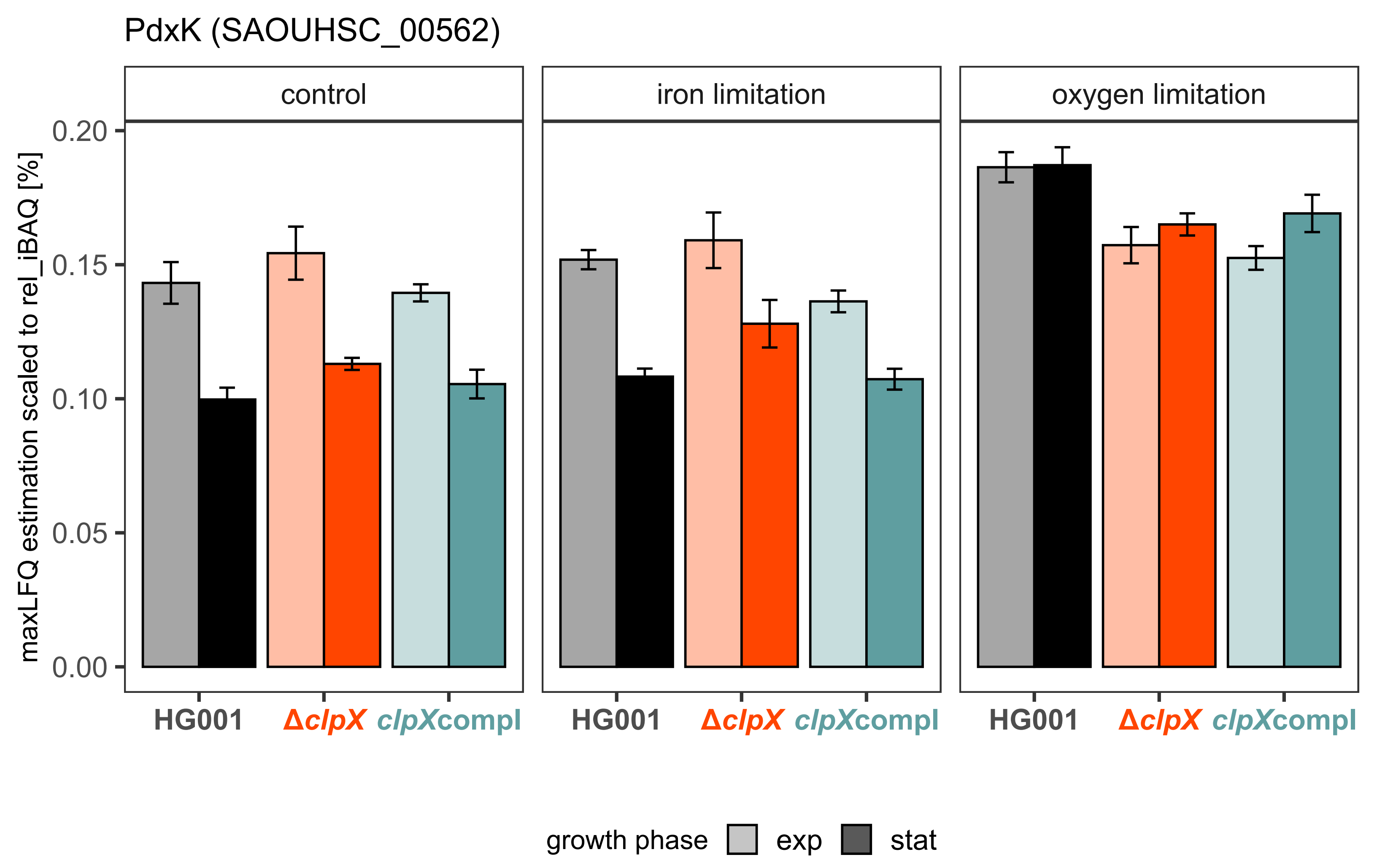
PdxK (SAOUHSC_00562)
control
iron limitation
oxygen limitation
maxLFQ estimation scaled to rel_iBAQ [%]
0.20
0.15
0.10
0.05
0.00
HG001
ΔclpX
clpXcompl
growth phase
exp
stat

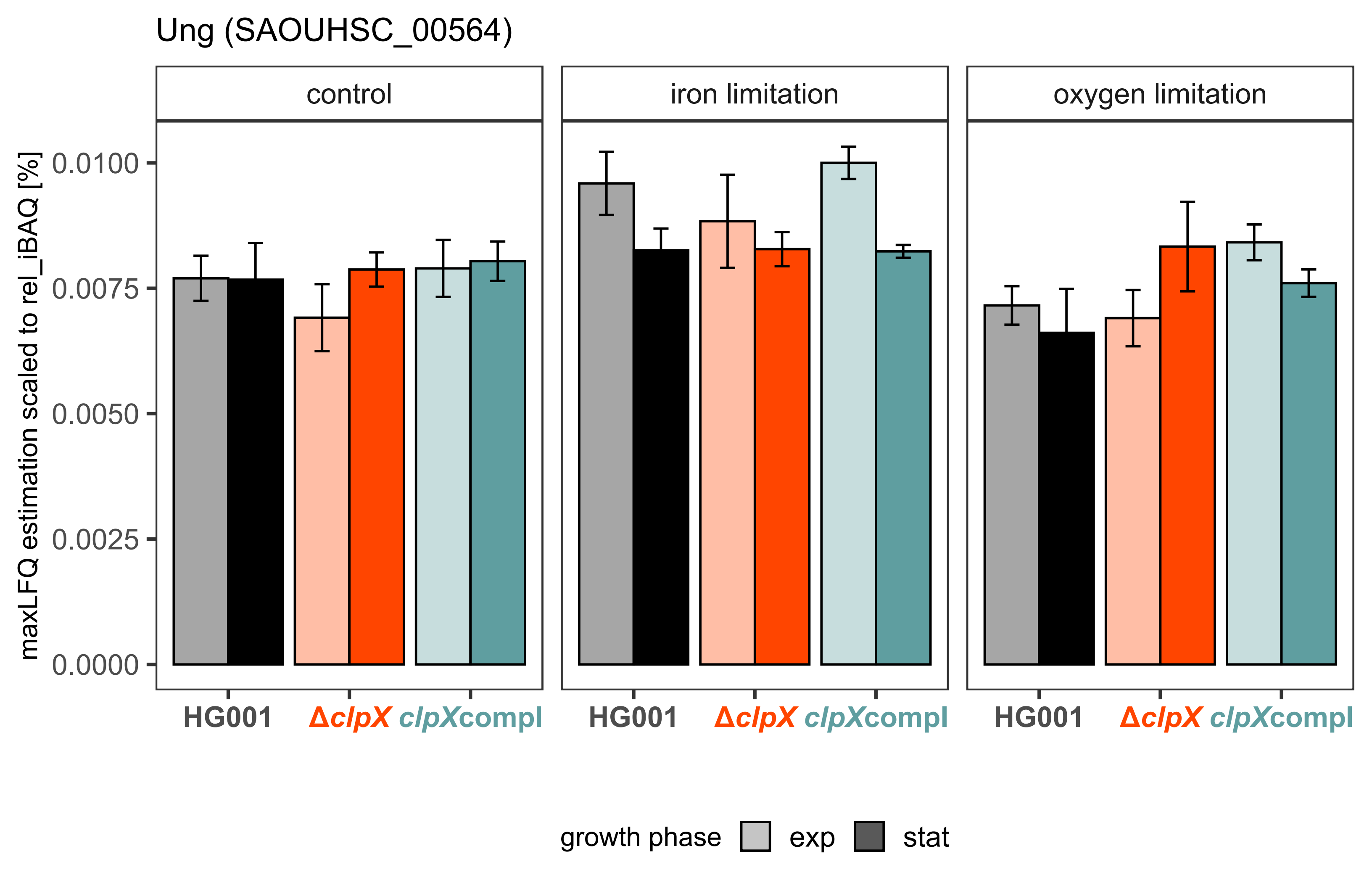

Ung (SAOUHSC_00564)
control
iron limitation
oxygen limitation
maxLFQ estimation scaled to rel_iBAQ [%]
0.0100
0.0075
0.0050
0.0025
0.0000
HG001
ΔclpX
clpXcompl
growth phase
exp
stat

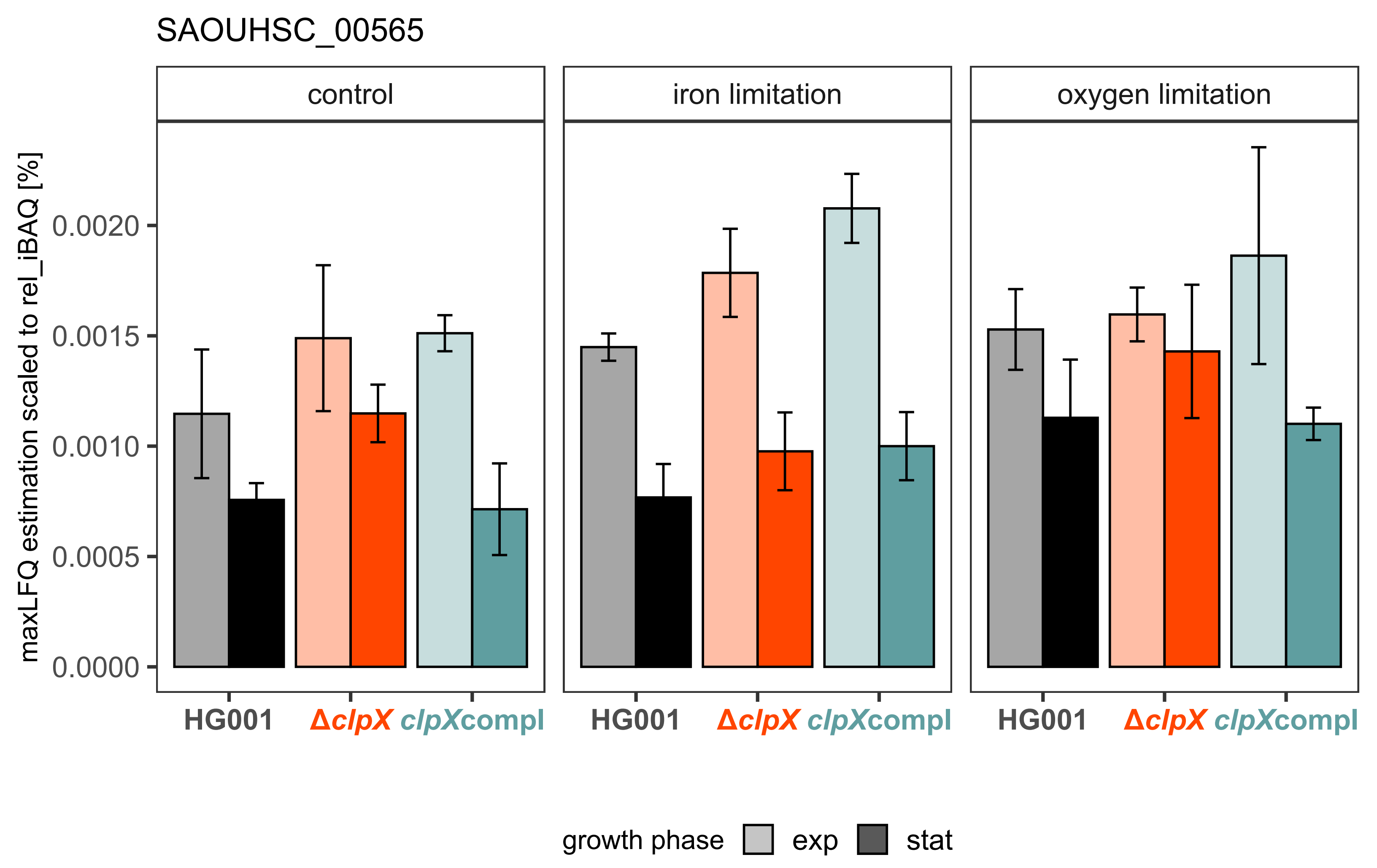
SAOUHSC_00565
control
iron limitation
oxygen limitation
maxLFQ estimation scaled to rel_iBAQ [%]
0.0020
0.0015
0.0010
0.0005
0.0000
HG001
ΔclpX
clpXcompl
growth phase
exp
stat

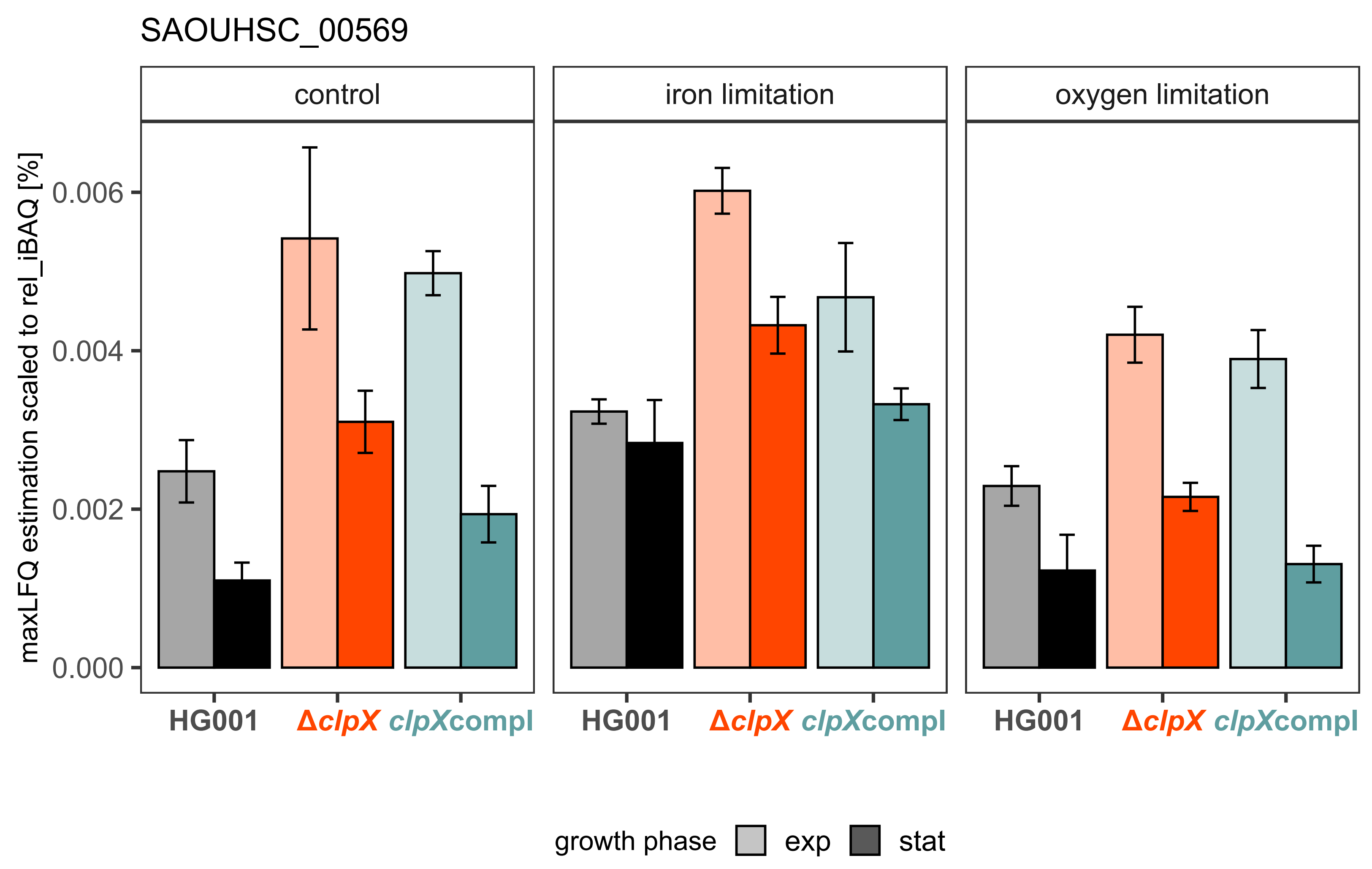
SAOUHSC_00569
control
iron limitation
oxygen limitation
maxLFQ estimation scaled to rel_iBAQ [%]
0.006
0.004
0.002
0.000
HG001
ΔclpX
clpXcompl
growth phase
exp
stat

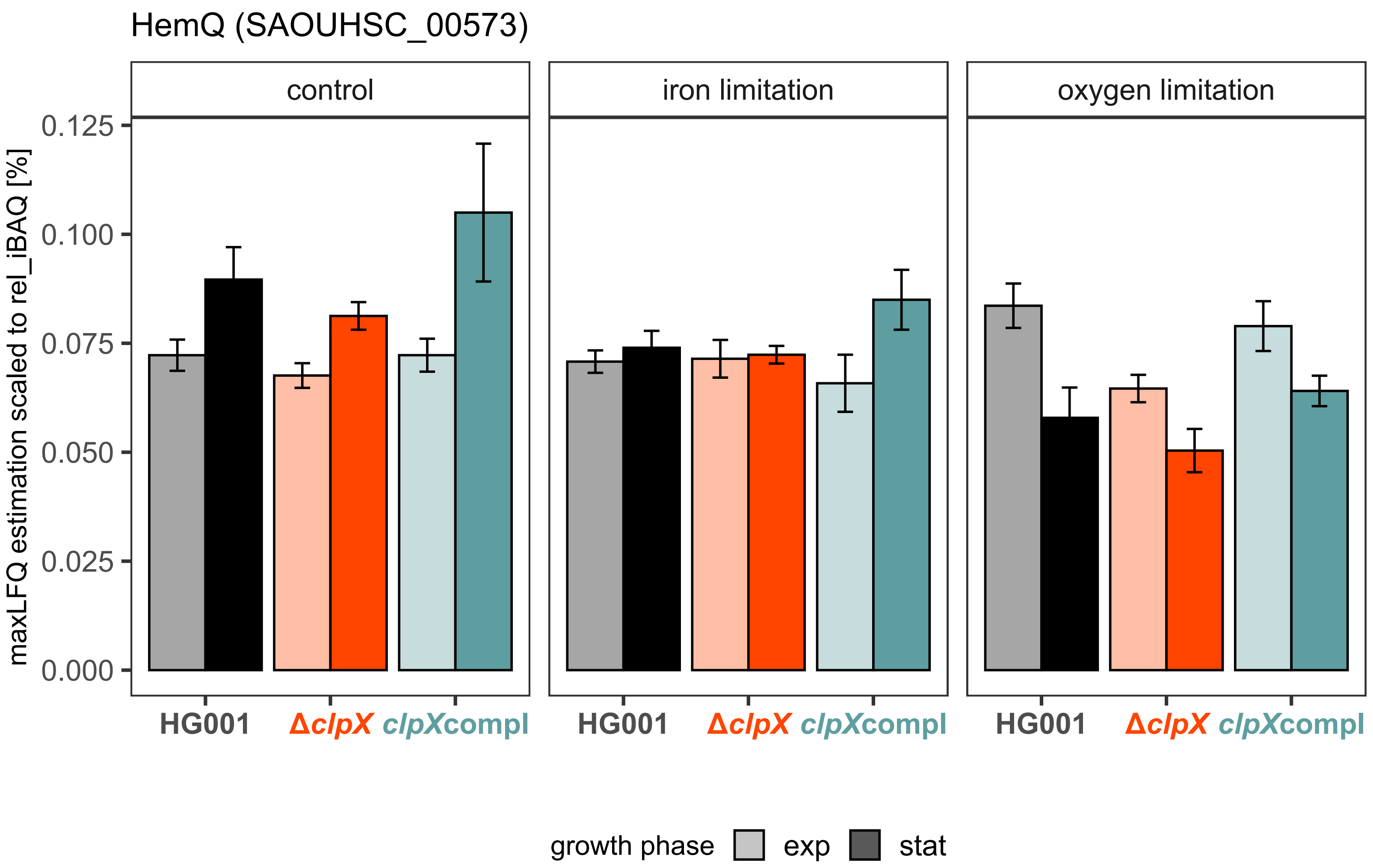
HemQ (SAOUHSC_00573)
control
iron limitation
oxygen limitation
maxLFQ estimation scaled to rel_iBAQ [%]
0.125
0.100
0.075
0.050
0.025
0.000
HG001
ΔclpX
clpXcompl
growth phase
exp
stat

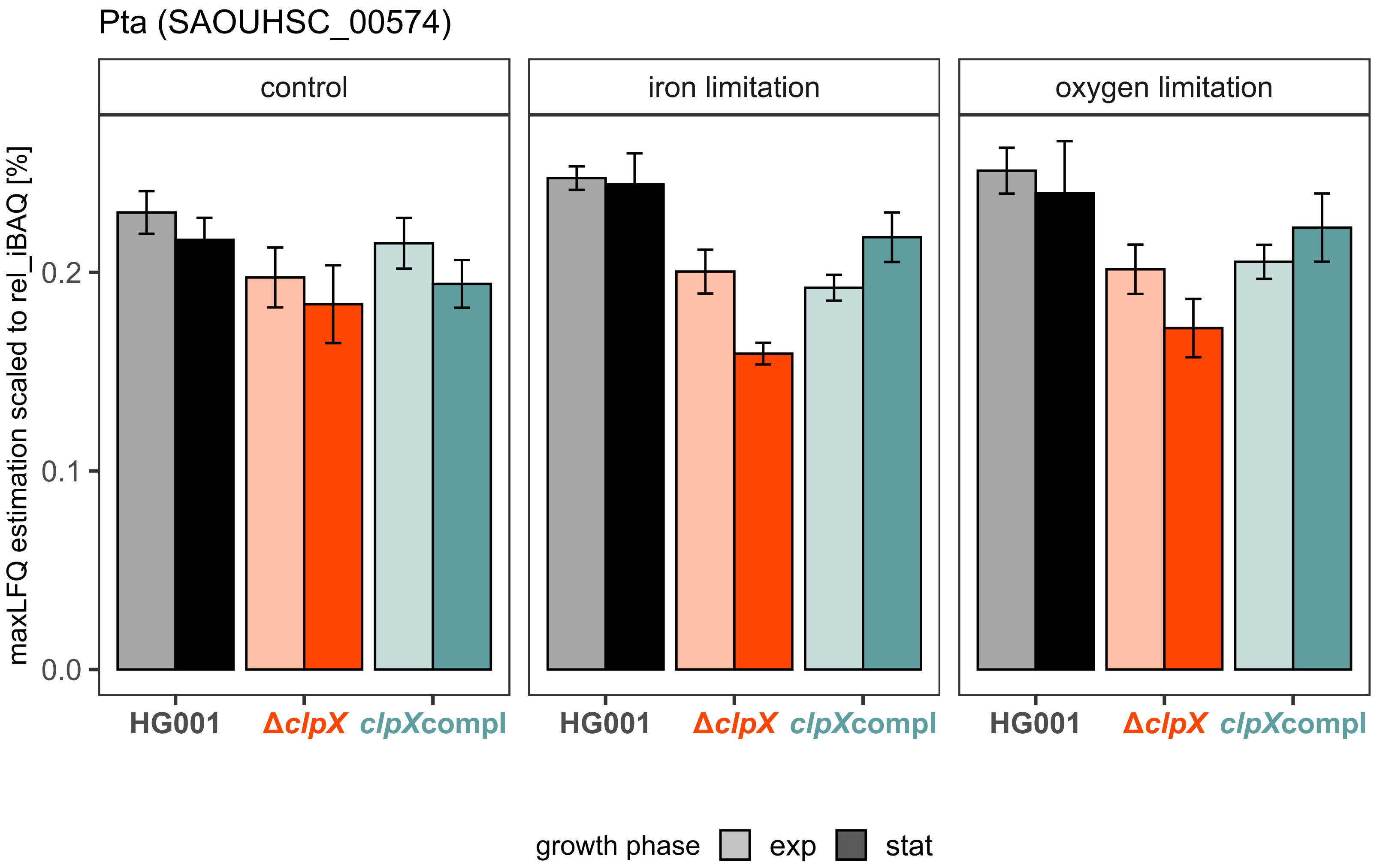

Pta (SAOUHSC_00574)
control
iron limitation
oxygen limitation
maxLFQ estimation scaled to rel_iBAQ [%]
0.0
0.1
0.2
HG001
ΔclpX
clpXcompl
growth phase
exp
stat

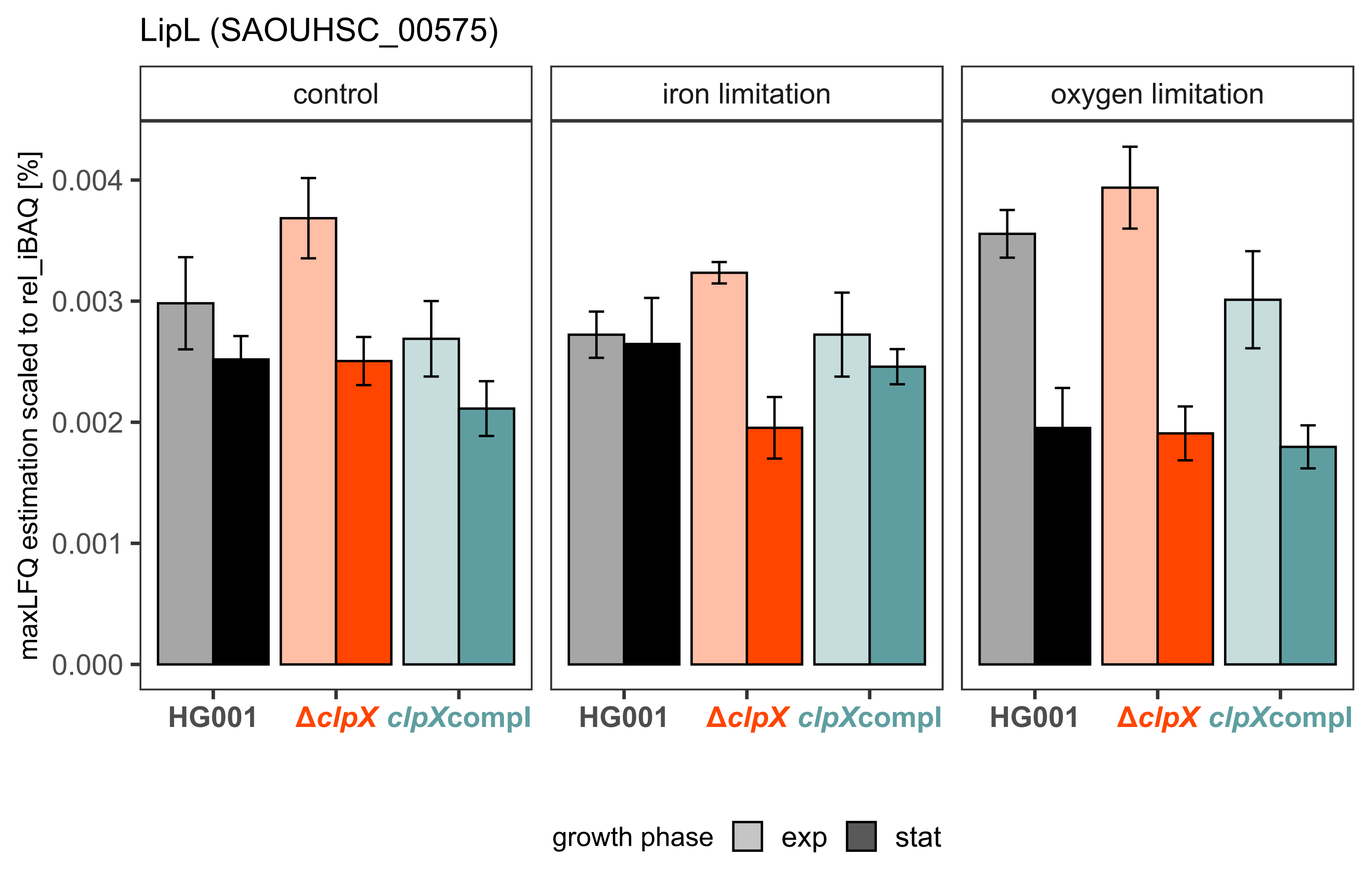

LipL (SAOUHSC_00575)
control
iron limitation
oxygen limitation
maxLFQ estimation scaled to rel_iBAQ [%]
0.004
0.003
0.002
0.001
0.000
HG001
ΔclpX
clpXcompl
growth phase
exp
stat

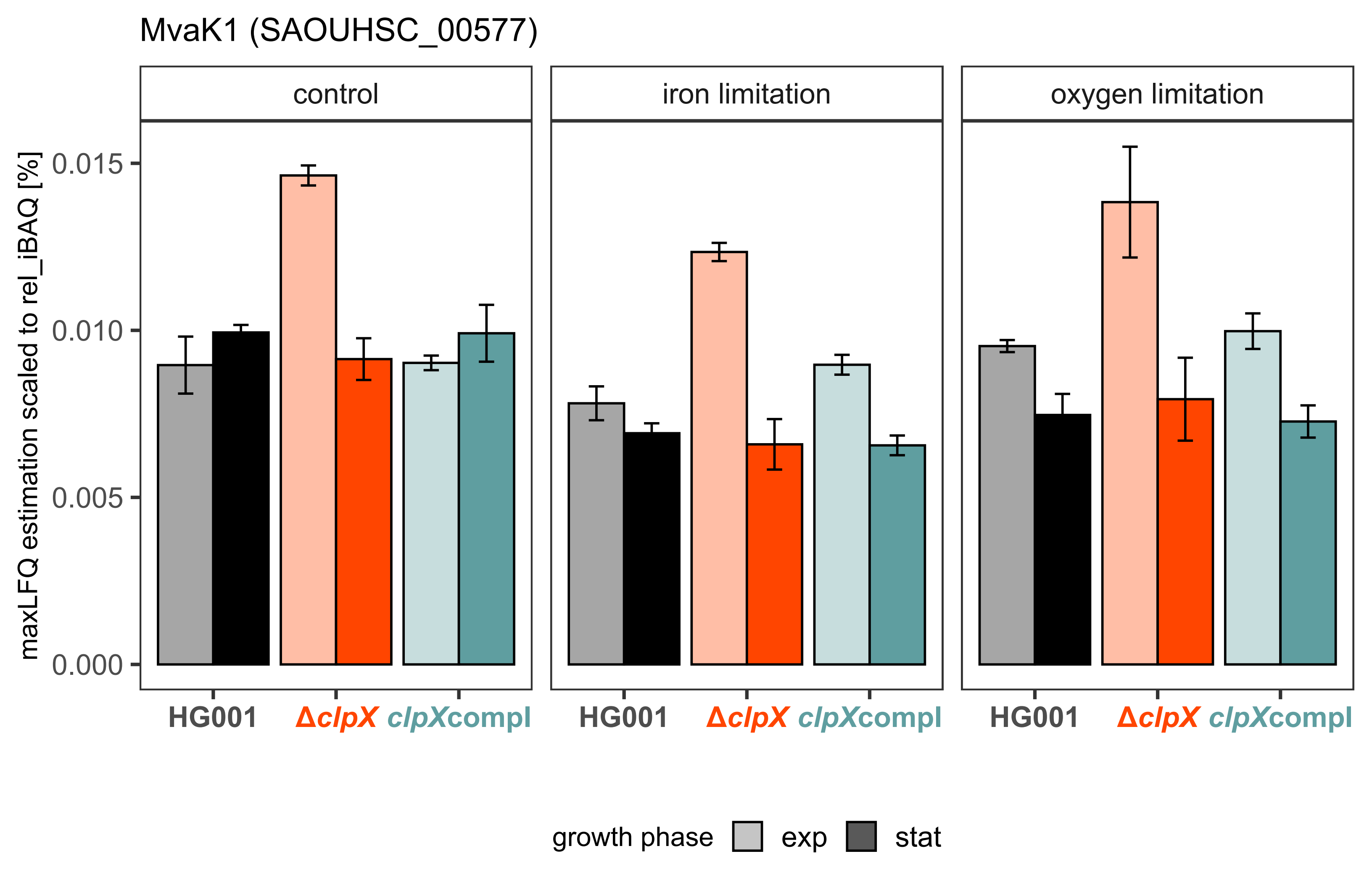

MvaK1 (SAOUHSC_00577)
control
iron limitation
oxygen limitation
maxLFQ estimation scaled to rel_iBAQ [%]
0.015
0.010
0.005
0.000
HG001
ΔclpX
clpXcompl
growth phase
exp
stat

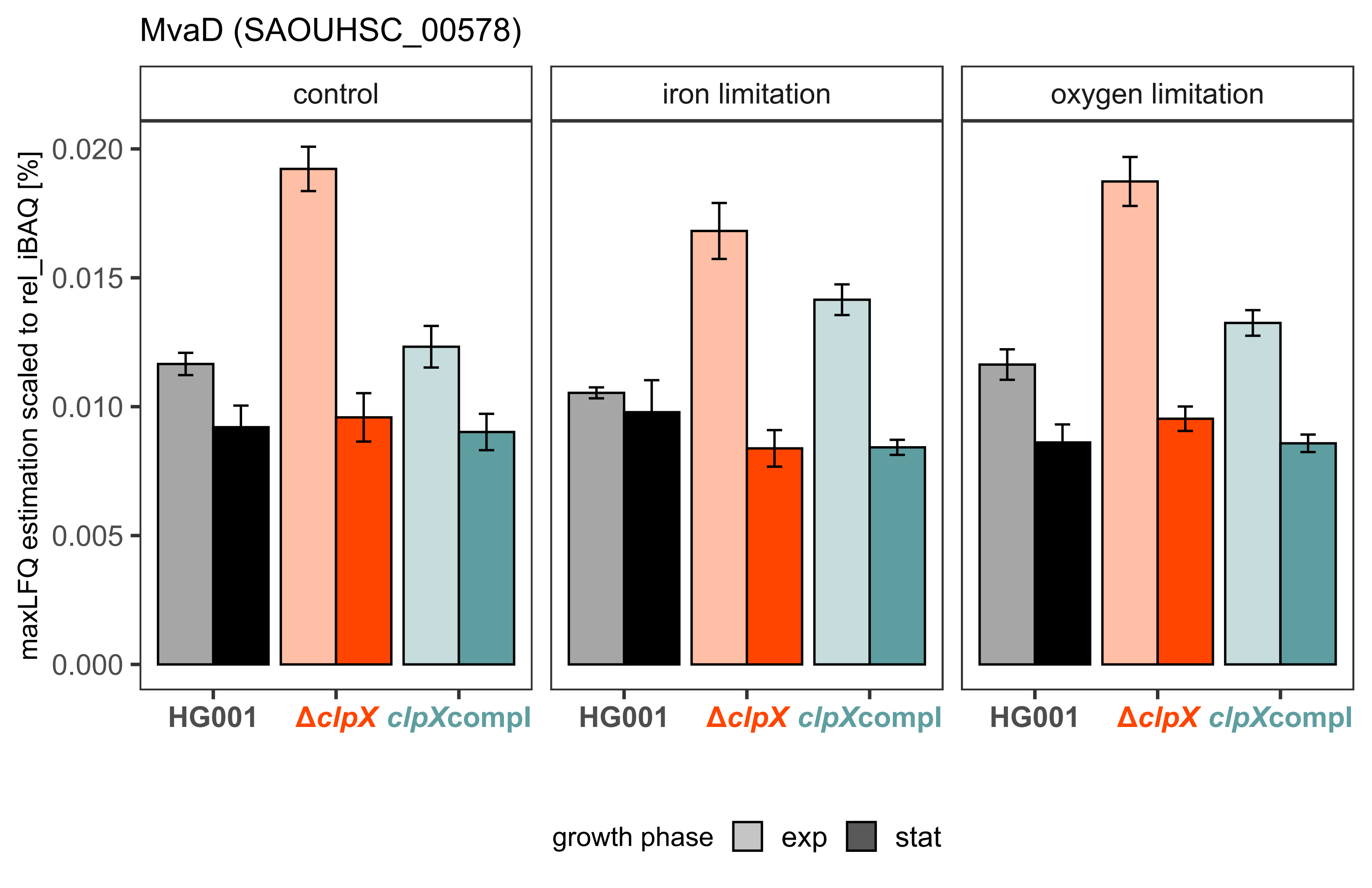
MvaD (SAOUHSC_00578)
control
iron limitation
oxygen limitation
maxLFQ estimation scaled to rel_iBAQ [%]
0.020
0.015
0.010
0.005
0.000
HG001
ΔclpX
clpXcompl
growth phase
exp
stat

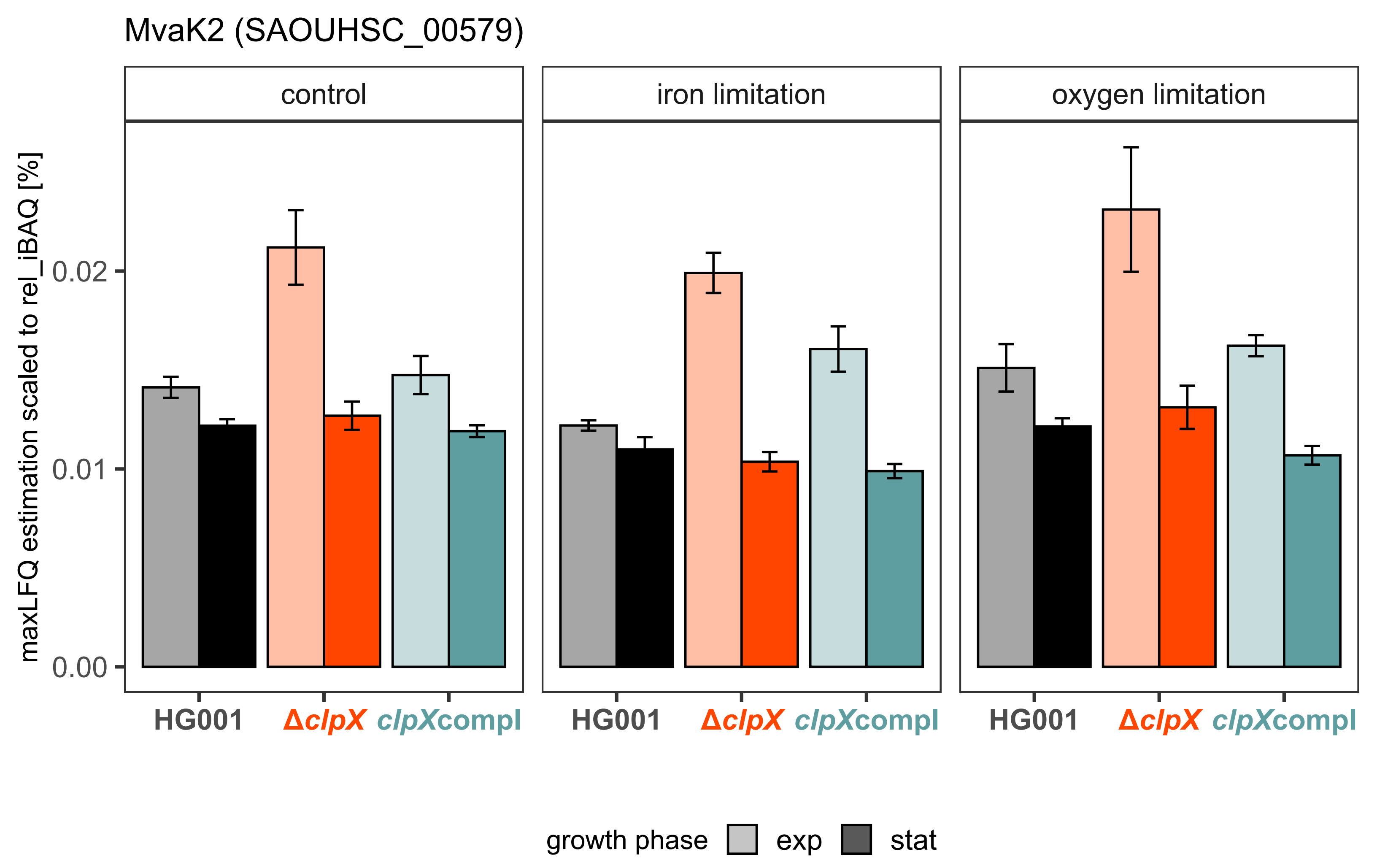
MvaK2 (SAOUHSC_00579)
control
iron limitation
oxygen limitation
maxLFQ estimation scaled to rel_iBAQ [%]
0.02
0.01
0.00
HG001
ΔclpX
clpXcompl
growth phase
exp
stat

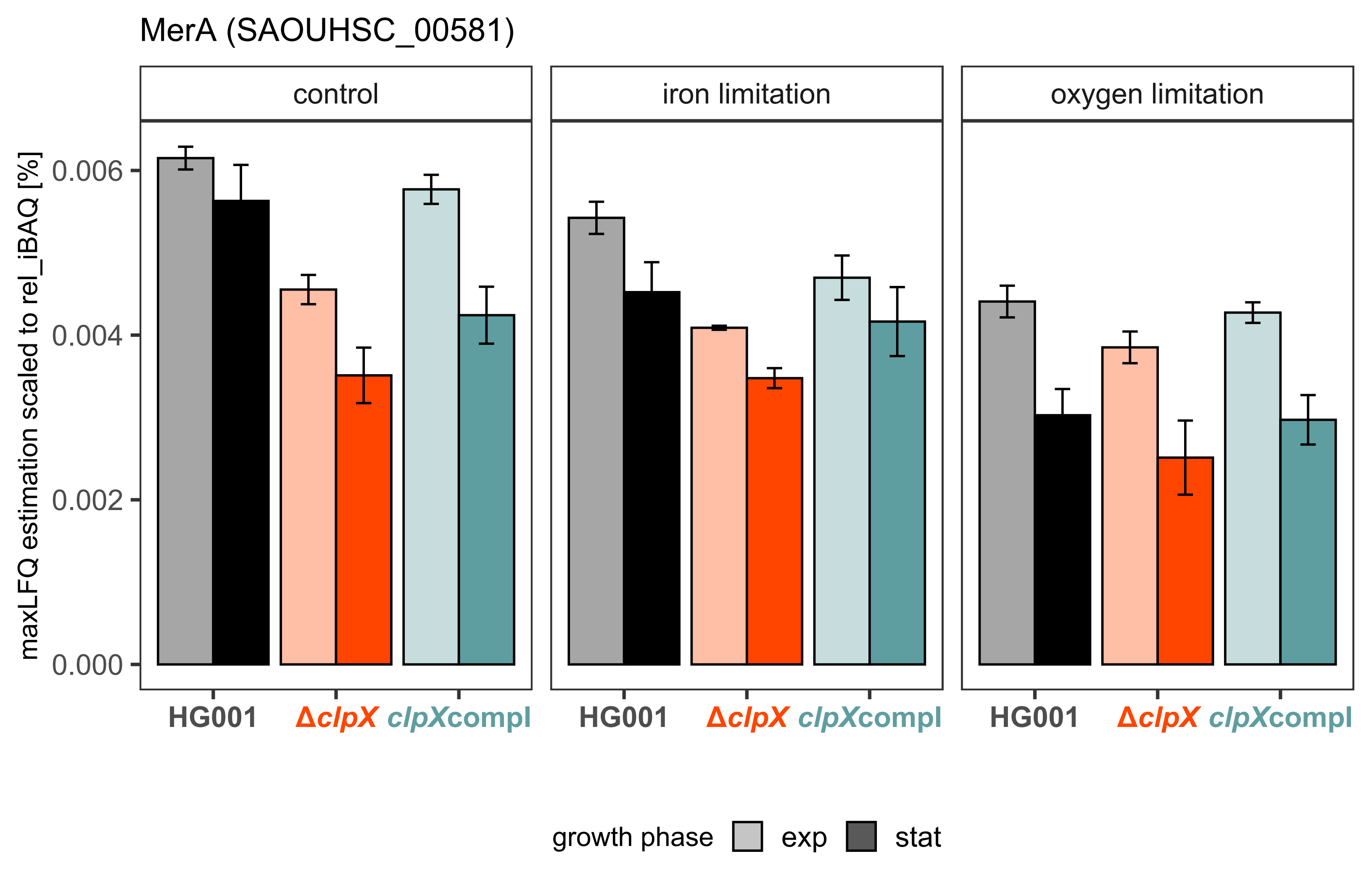

MerA (SAOUHSC_00581)
control
iron limitation
oxygen limitation
maxLFQ estimation scaled to rel_iBAQ [%]
0.006
0.004
0.002
0.000
HG001
ΔclpX
clpXcompl
growth phase
exp
stat

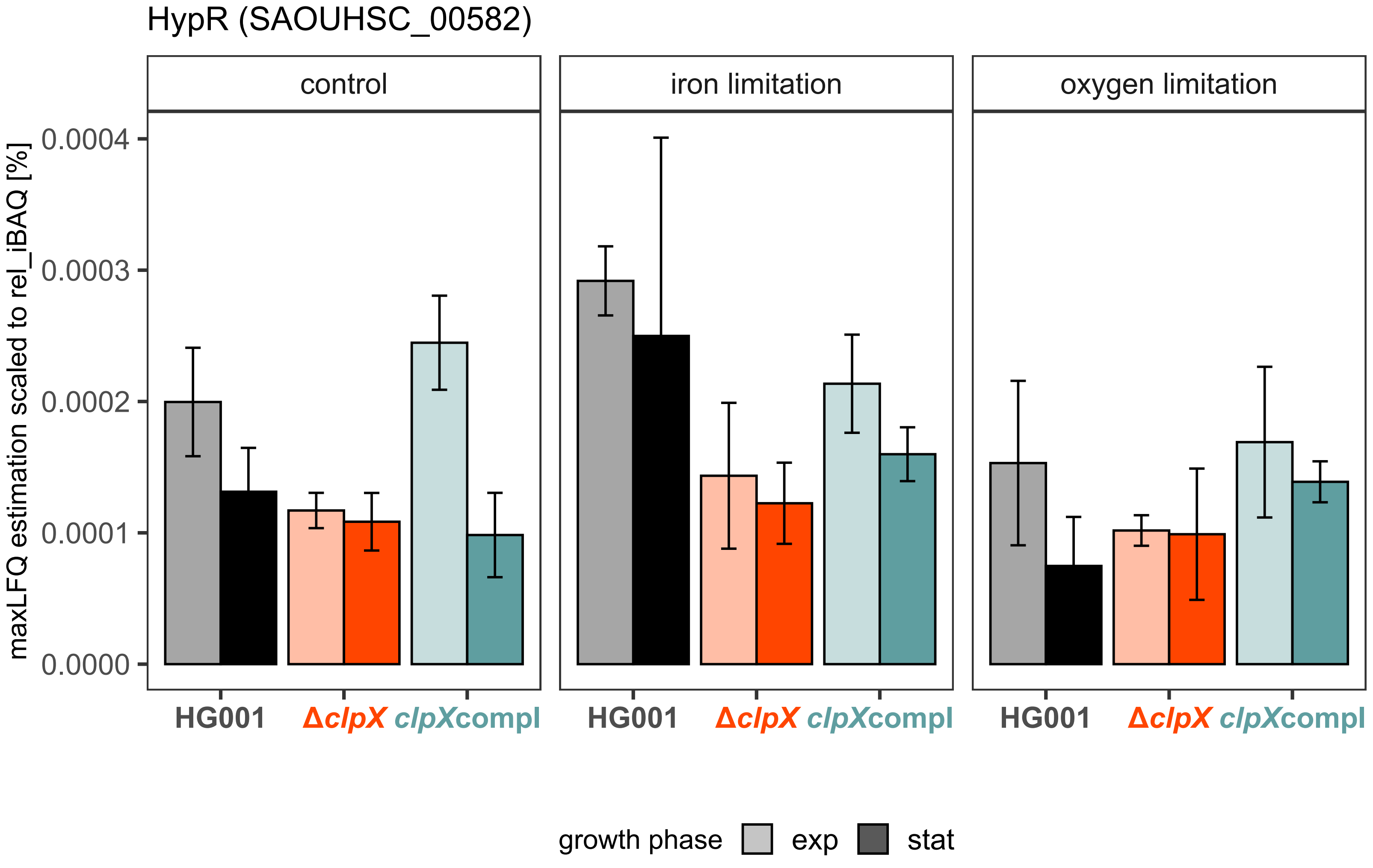

HypR (SAOUHSC_00582)
control
iron limitation
oxygen limitation
maxLFQ estimation scaled to rel_iBAQ [%]
0.0004
0.0003
0.0002
0.0001
0.0000
HG001
ΔclpX
clpXcompl
growth phase
exp
stat

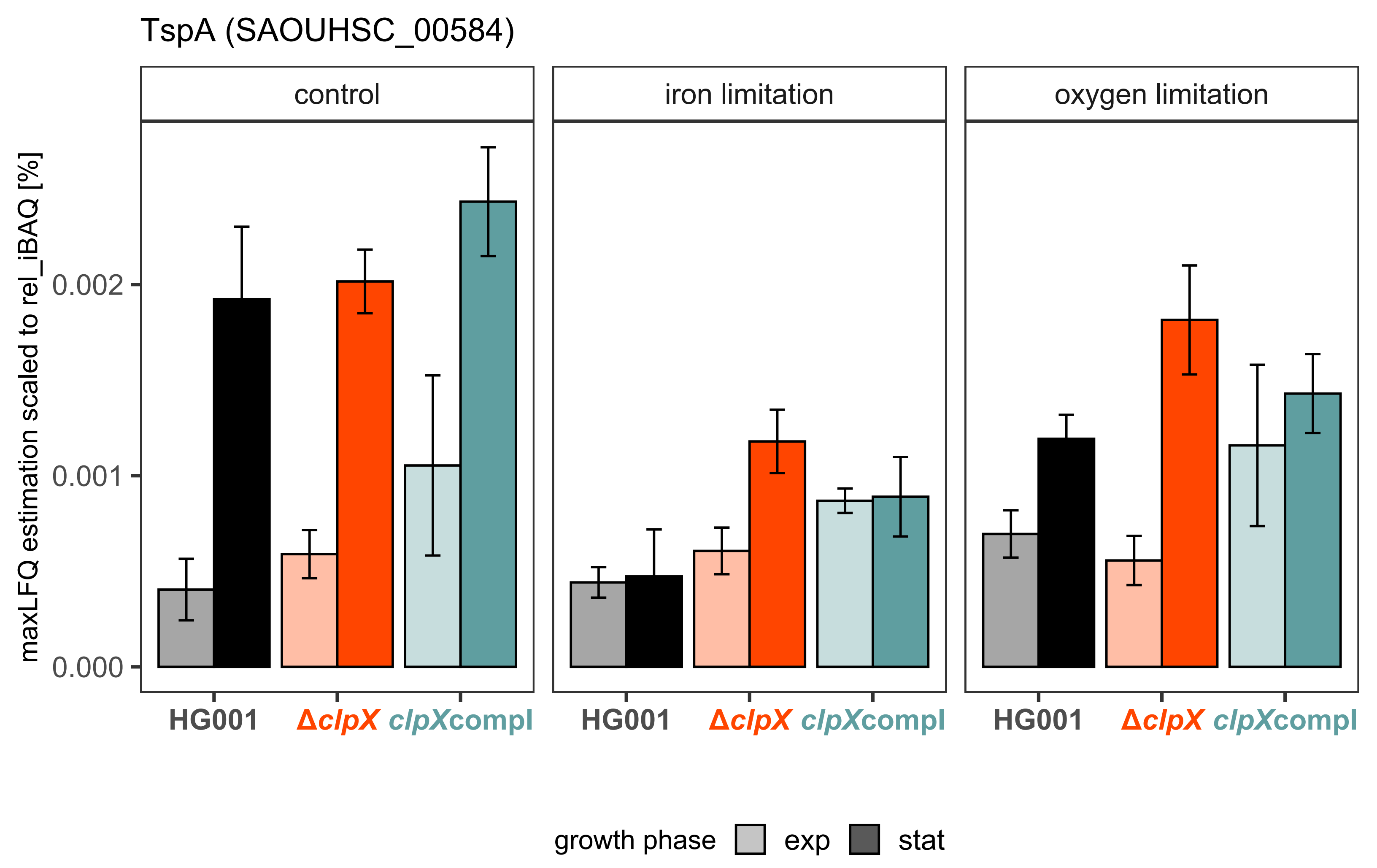

TspA (SAOUHSC_00584)
control
iron limitation
oxygen limitation
maxLFQ estimation scaled to rel_iBAQ [%]
0.000
0.001
0.002
HG001
ΔclpX
clpXcompl
growth phase
exp
stat

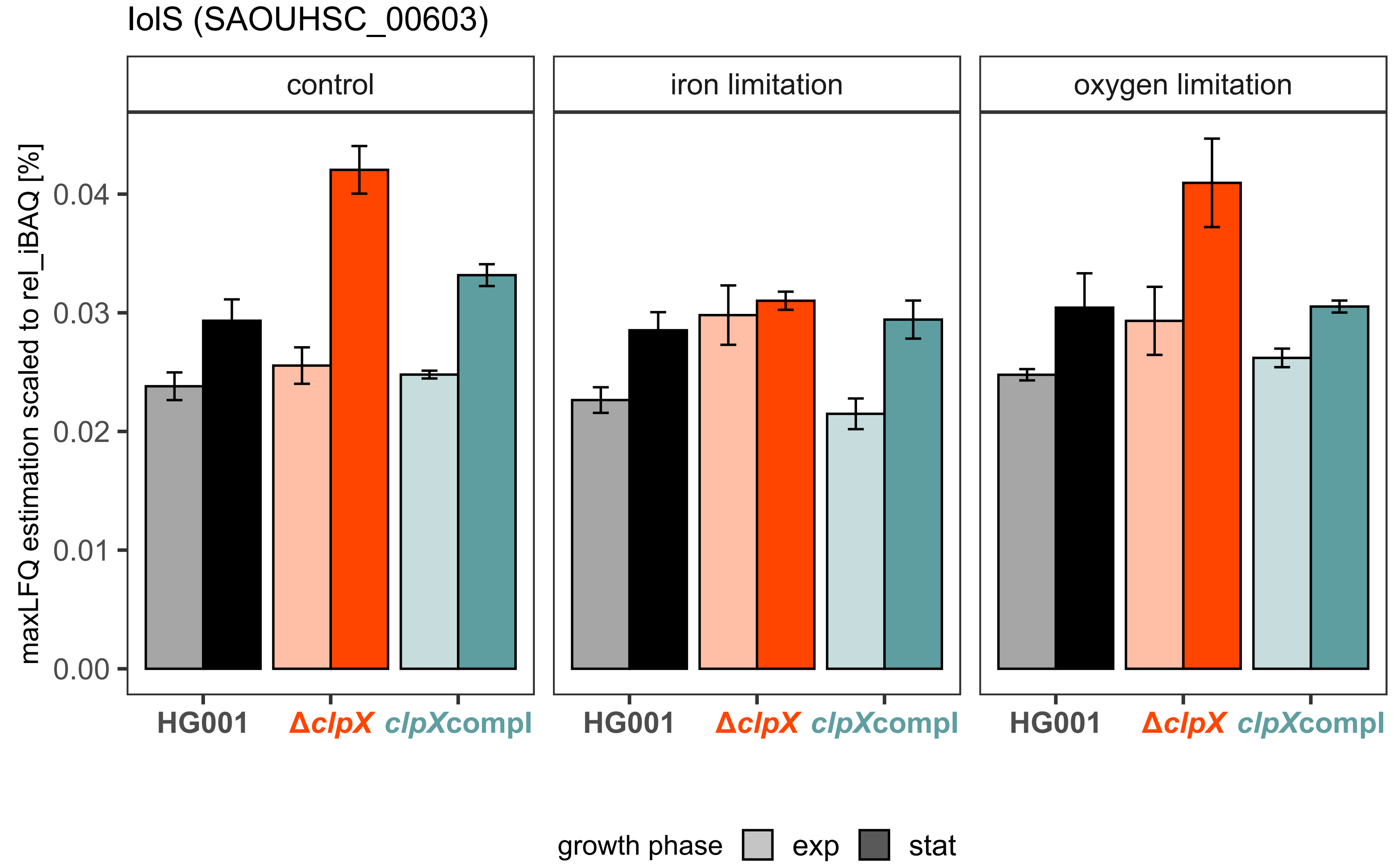
IolS (SAOUHSC_00603)
control
iron limitation
oxygen limitation
maxLFQ estimation scaled to rel_iBAQ [%]
0.00
0.01
0.02
0.03
0.04
HG001
ΔclpX
clpXcompl
growth phase
exp
stat

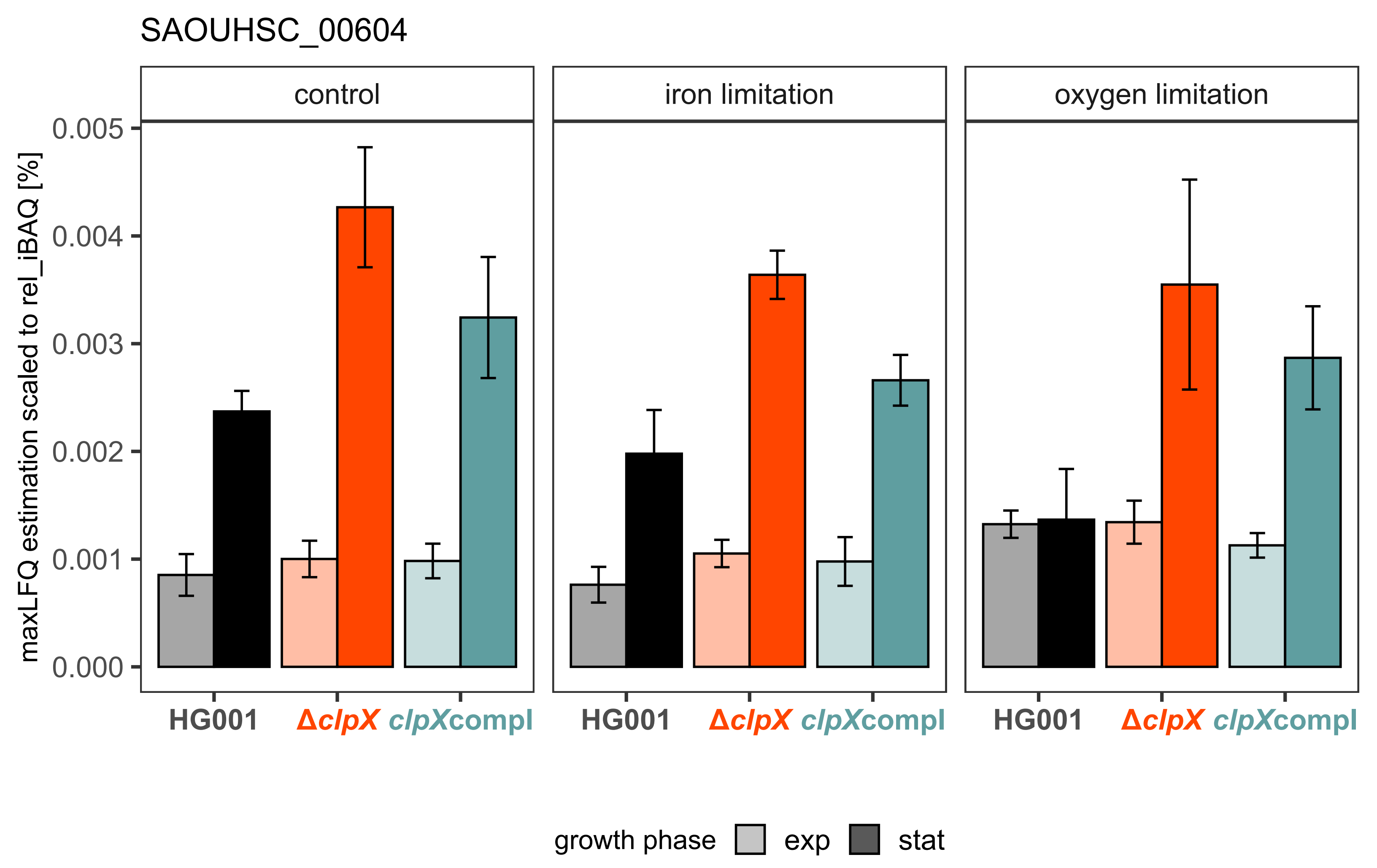
SAOUHSC_00604
control
iron limitation
oxygen limitation
maxLFQ estimation scaled to rel_iBAQ [%]
0.005
0.004
0.003
0.002
0.001
0.000
HG001
ΔclpX
clpXcompl
growth phase
exp
stat

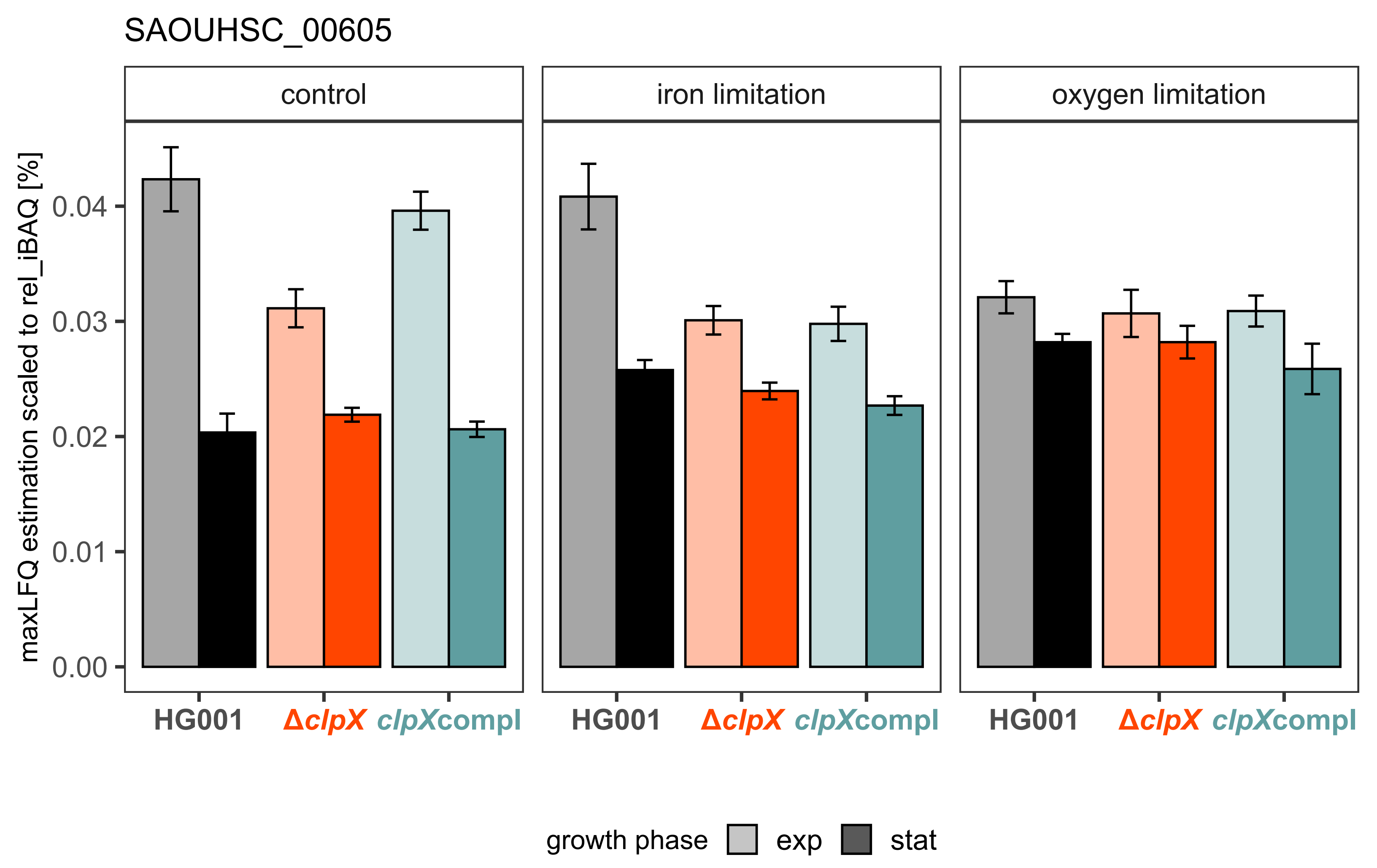

SAOUHSC_00605
control
iron limitation
oxygen limitation
maxLFQ estimation scaled to rel_iBAQ [%]
0.04
0.03
0.02
0.01
0.00
HG001
ΔclpX
clpXcompl
growth phase
exp
stat

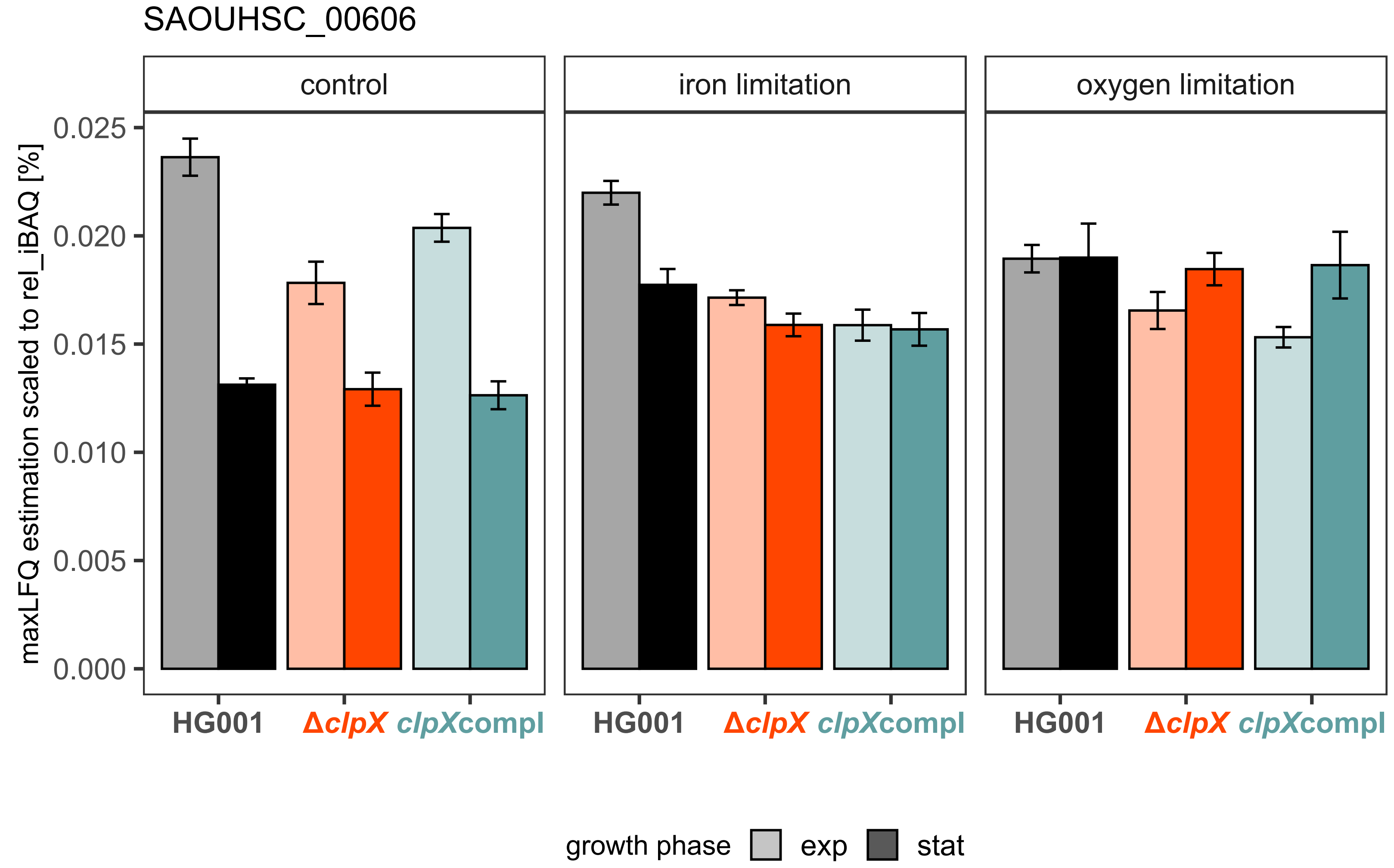
SAOUHSC_00606
control
iron limitation
oxygen limitation
maxLFQ estimation scaled to rel_iBAQ [%]
0.025
0.020
0.015
0.010
0.005
0.000
HG001
ΔclpX
clpXcompl
growth phase
exp
stat

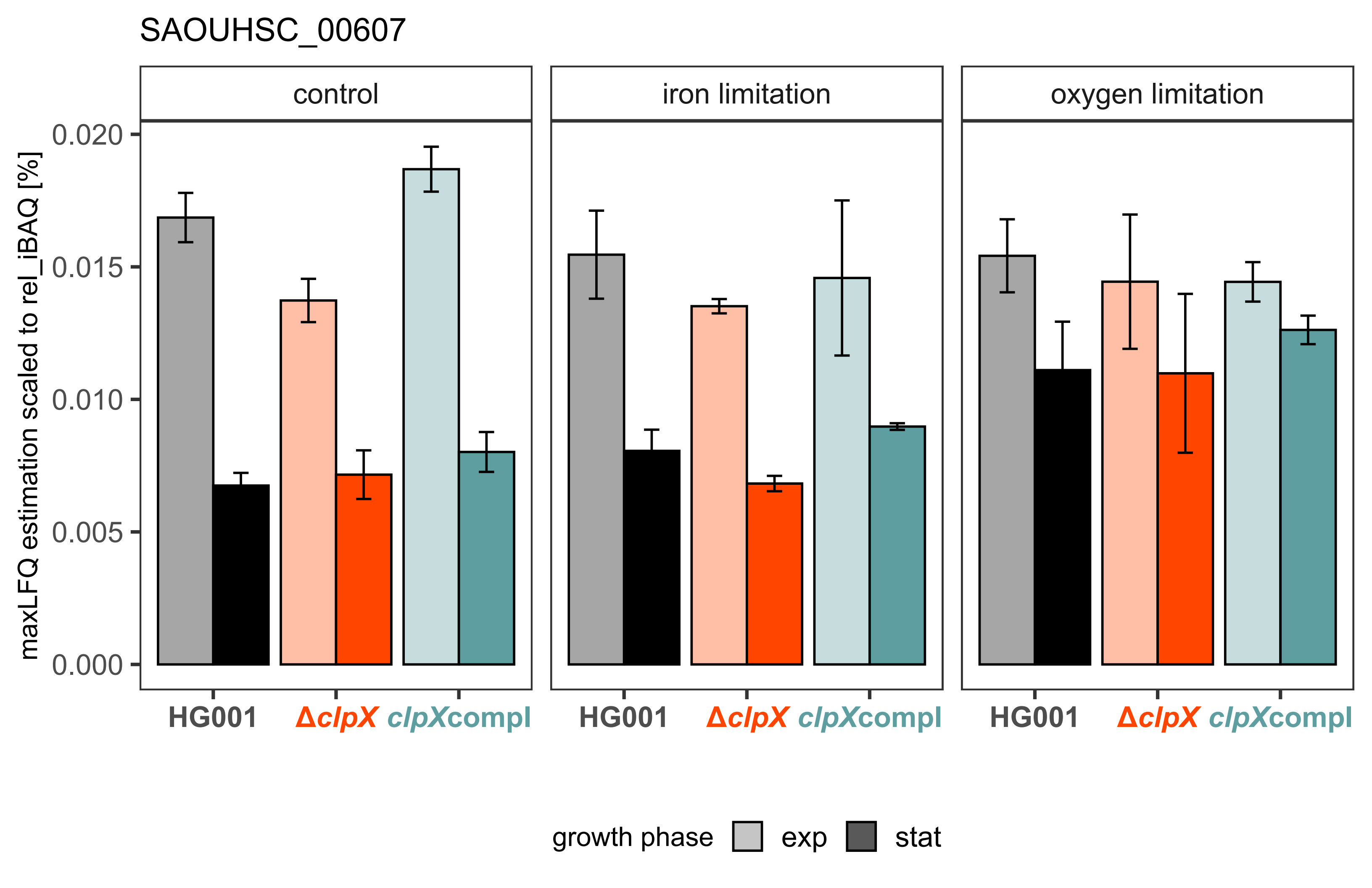

SAOUHSC_00607
control
iron limitation
oxygen limitation
maxLFQ estimation scaled to rel_iBAQ [%]
0.020
0.015
0.010
0.005
0.000
HG001
ΔclpX
clpXcompl
growth phase
exp
stat

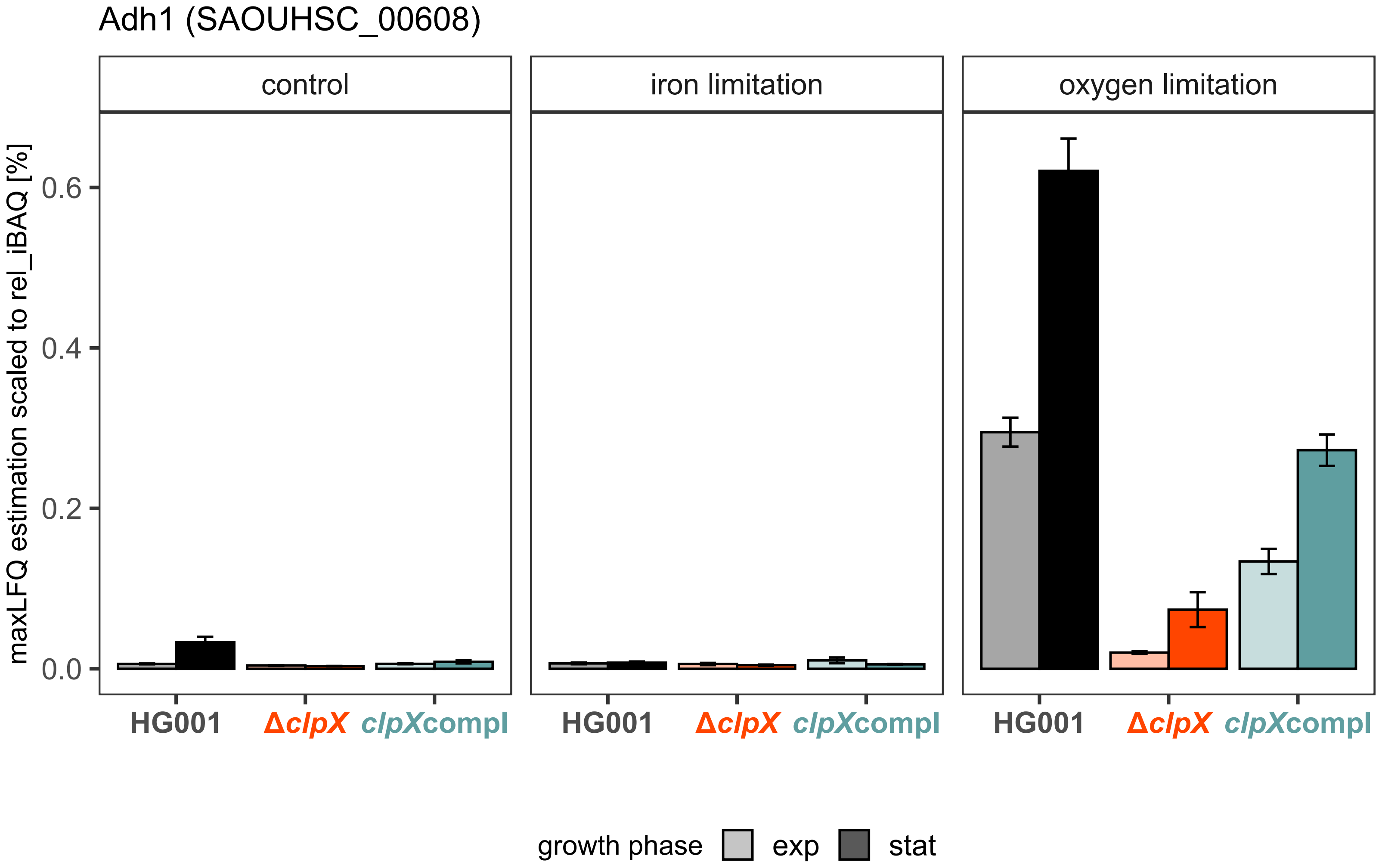

Adh1 (SAOUHSC_00608)
control
iron limitation
oxygen limitation
maxLFQ estimation scaled to rel_iBAQ [%]
0.6
0.4
0.2
0.0
HG001
ΔclpX
clpXcompl
growth phase
exp
stat

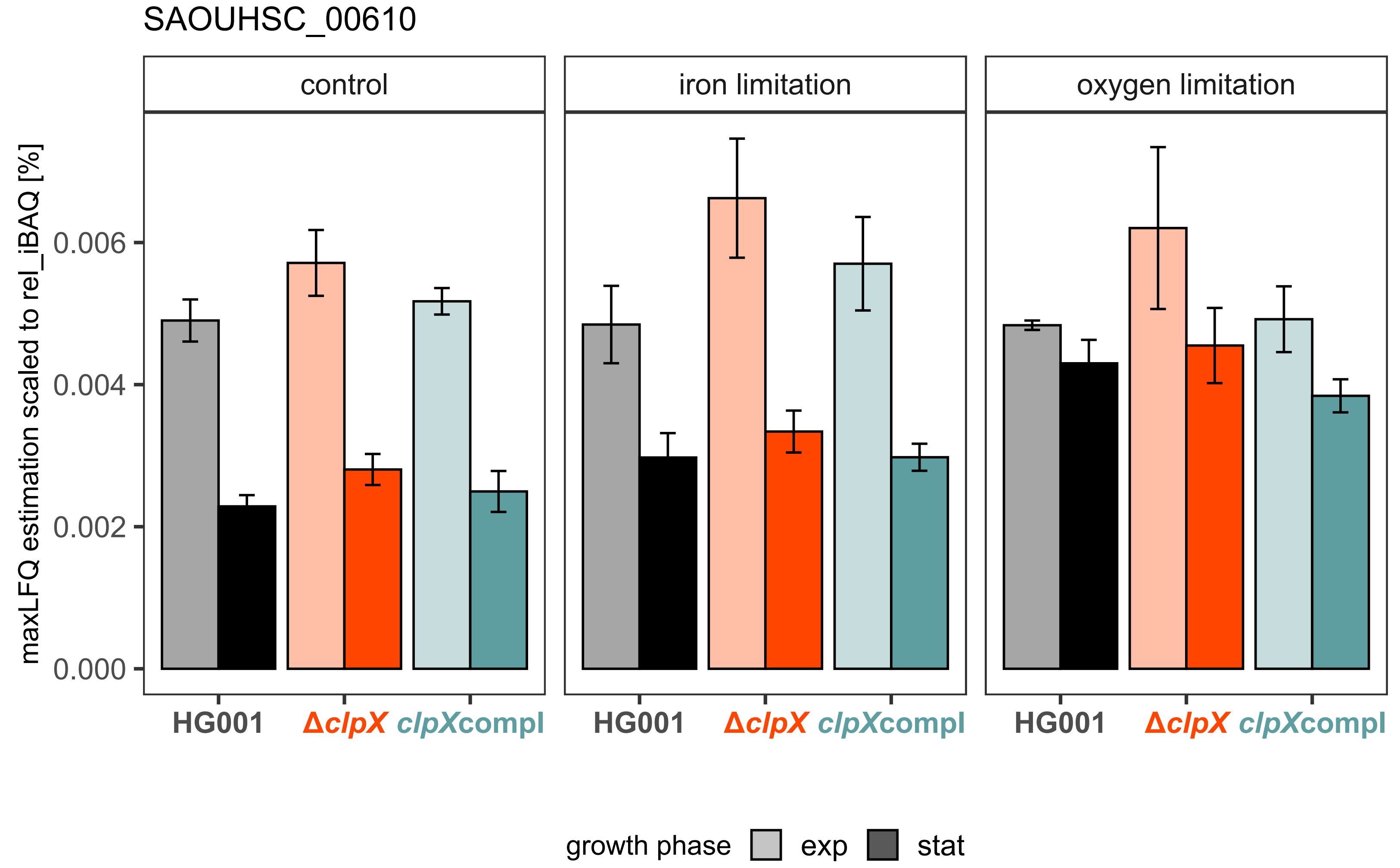
SAOUHSC_00610
control
iron limitation
oxygen limitation
maxLFQ estimation scaled to rel_iBAQ [%]
0.006
0.004
0.002
0.000
HG001
ΔclpX
clpXcompl
growth phase
exp
stat

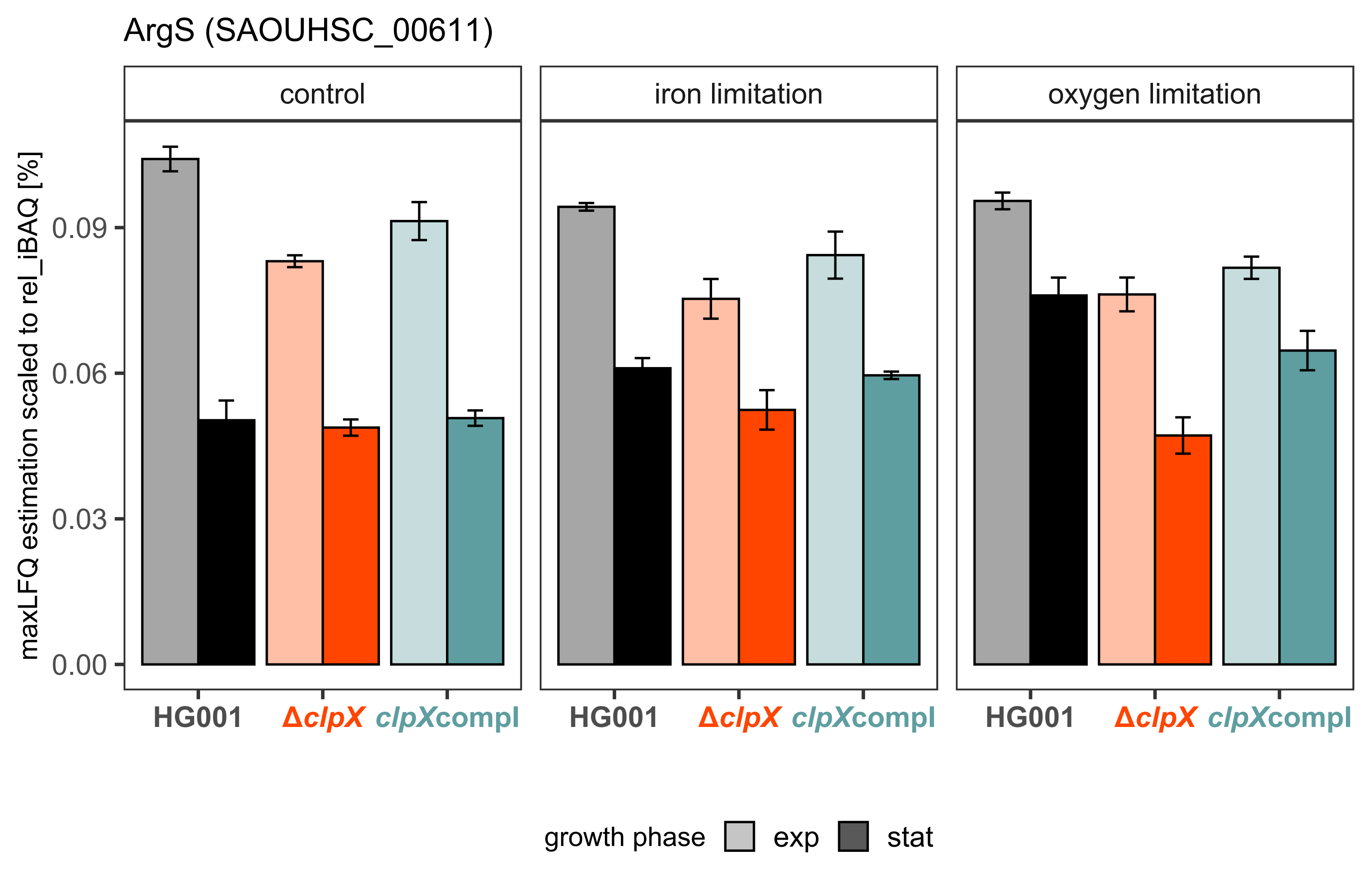

ArgS (SAOUHSC_00611)
control
iron limitation
oxygen limitation
maxLFQ estimation scaled to rel_iBAQ [%]
0.09
0.06
0.03
0.00
HG001
ΔclpX
clpXcompl
growth phase
exp
stat

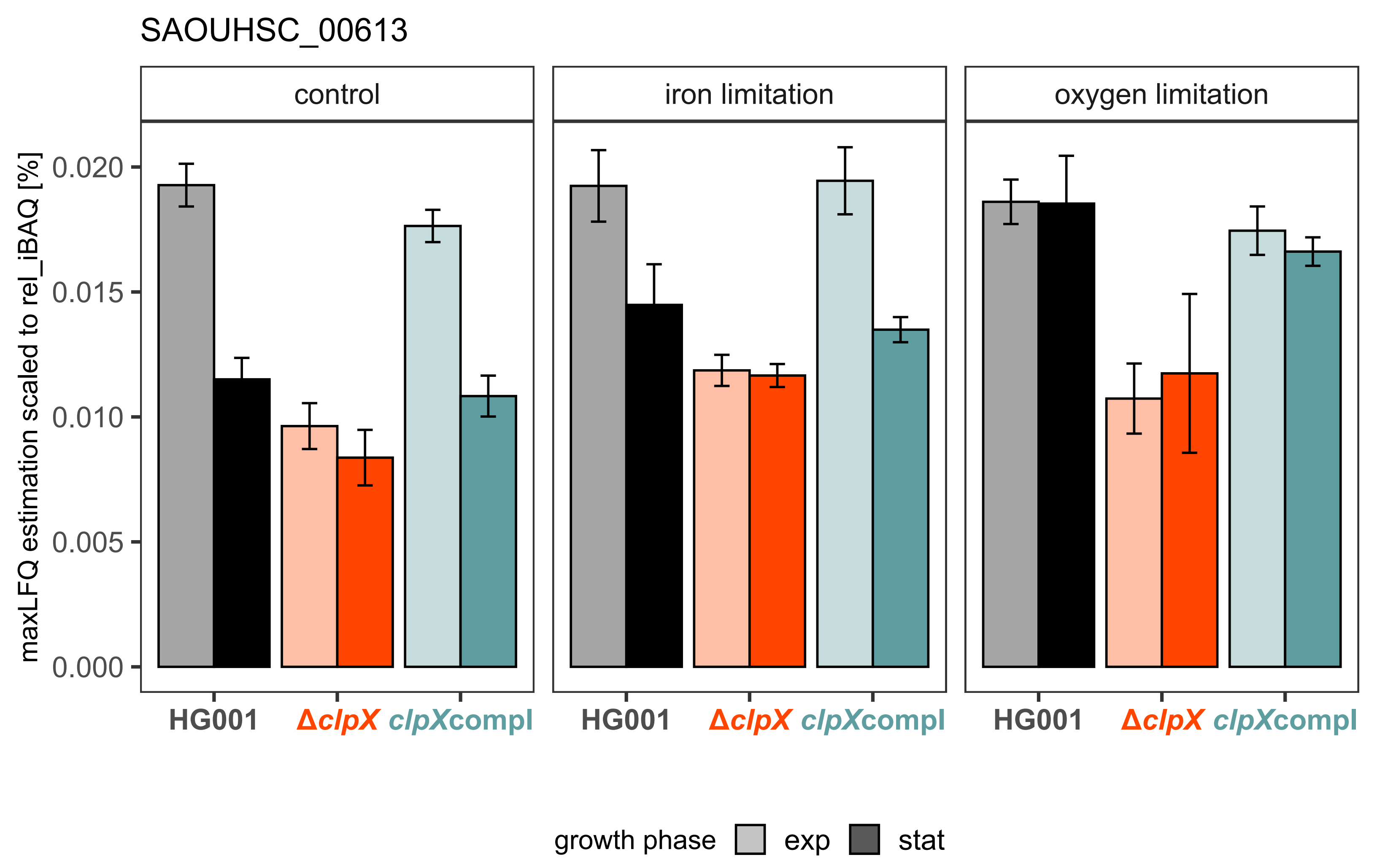
SAOUHSC_00613
control
iron limitation
oxygen limitation
maxLFQ estimation scaled to rel_iBAQ [%]
0.020
0.015
0.010
0.005
0.000
HG001
ΔclpX
clpXcompl
growth phase
exp
stat

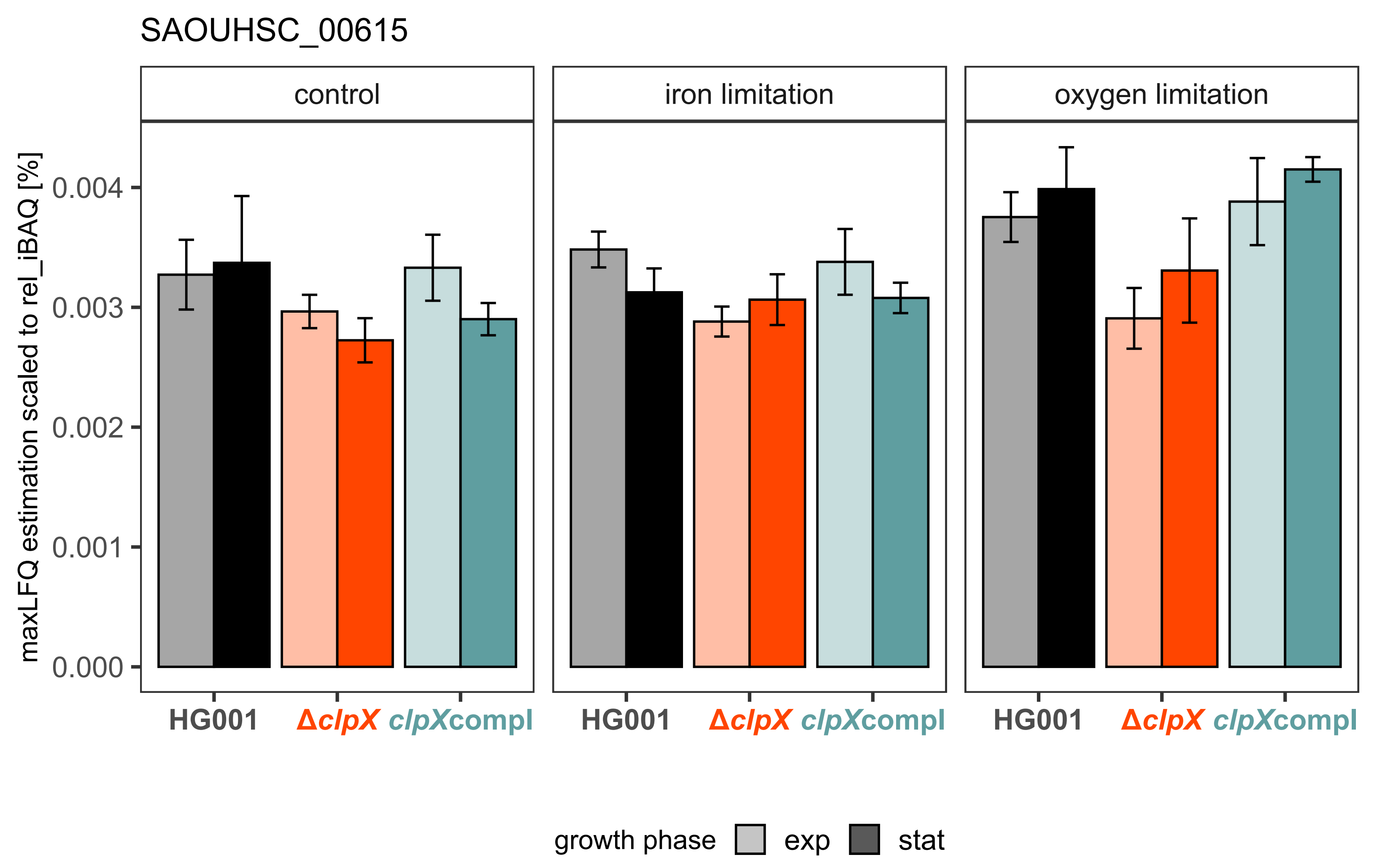

SAOUHSC_00615
control
iron limitation
oxygen limitation
maxLFQ estimation scaled to rel_iBAQ [%]
0.004
0.003
0.002
0.001
0.000
HG001
ΔclpX
clpXcompl
growth phase
exp
stat

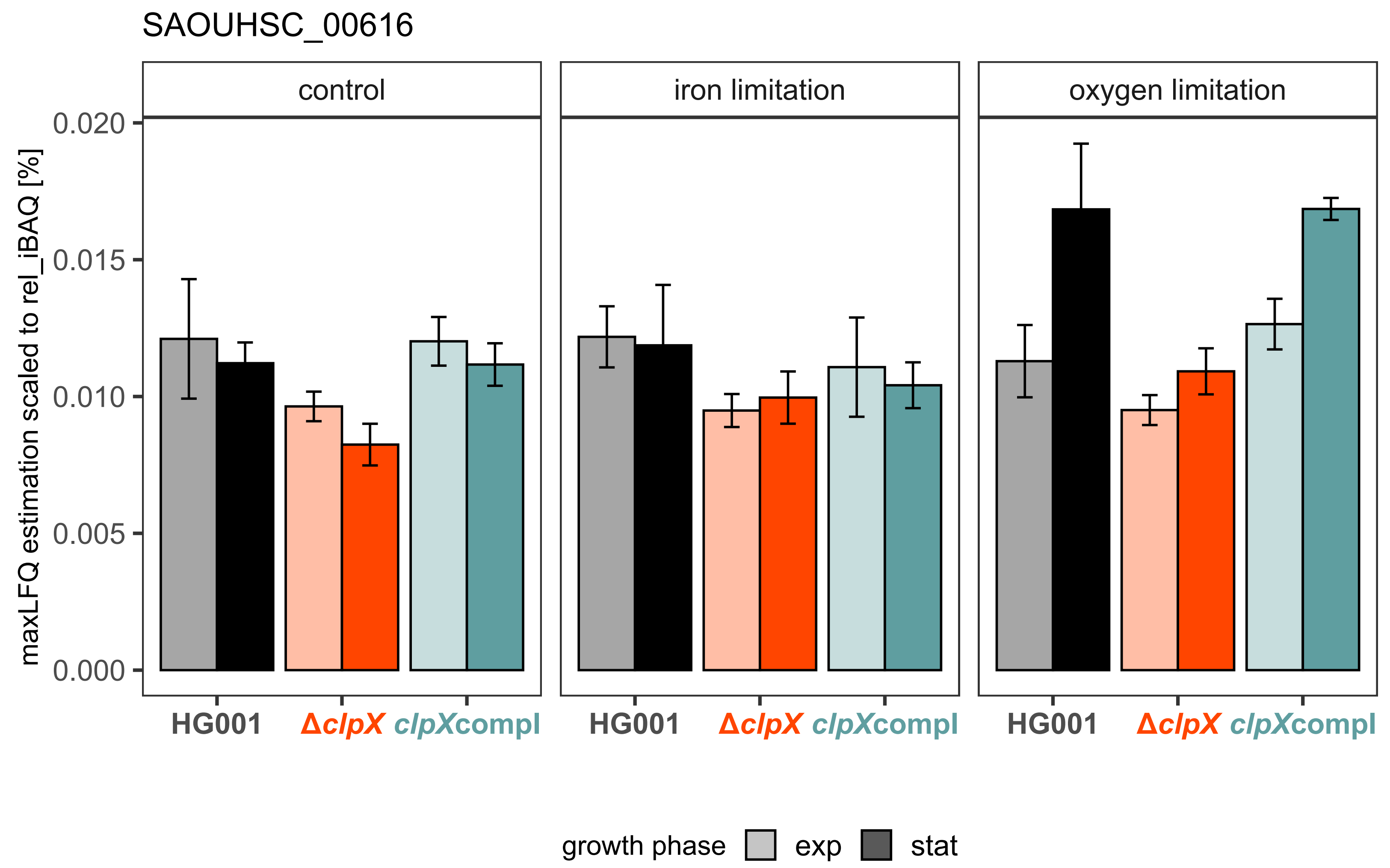

SAOUHSC_00616
control
iron limitation
oxygen limitation
maxLFQ estimation scaled to rel_iBAQ [%]
0.000
0.005
0.010
0.015
0.020
HG001
ΔclpX
clpXcompl
growth phase
exp
stat

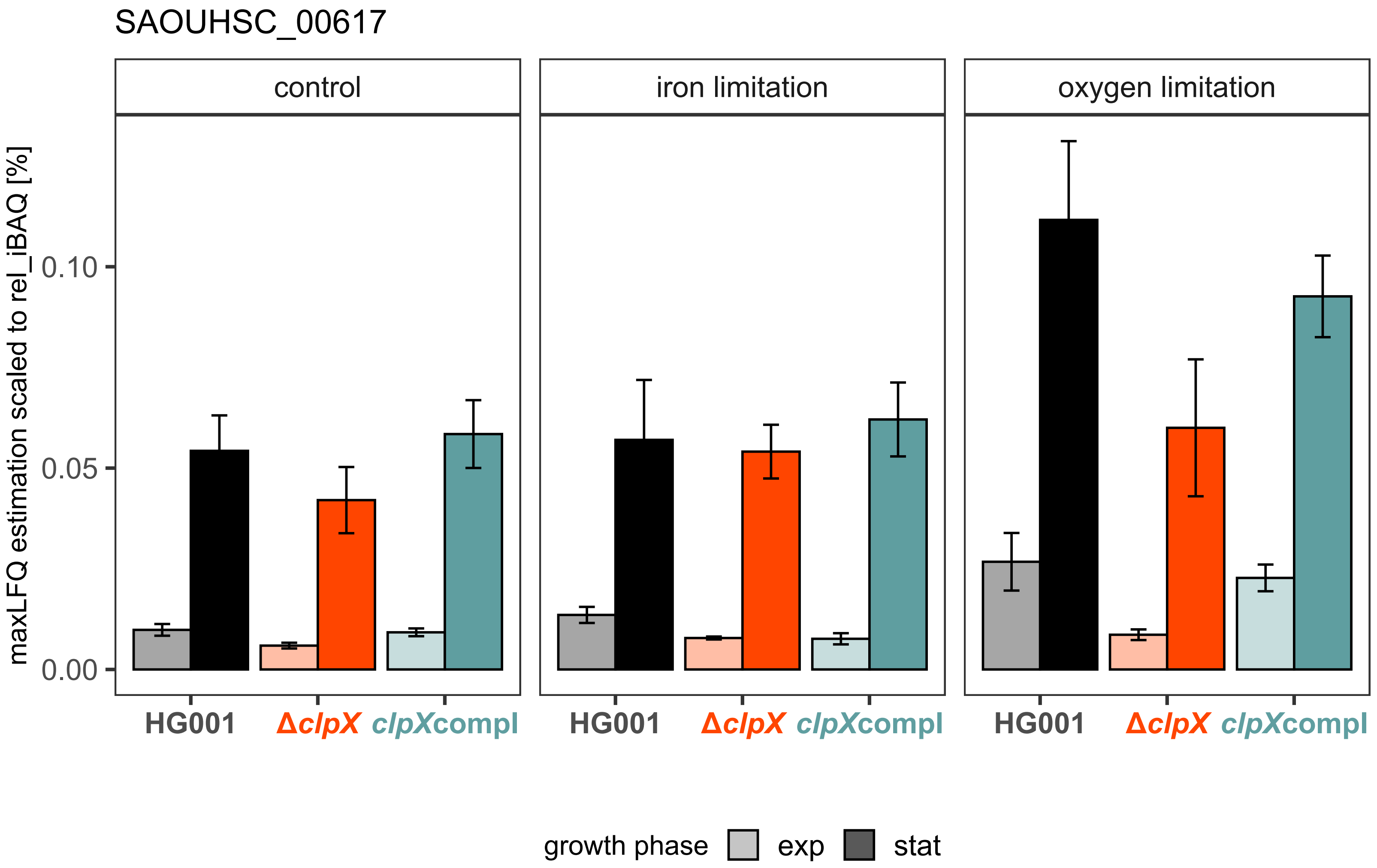

SAOUHSC_00617
control
iron limitation
oxygen limitation
maxLFQ estimation scaled to rel_iBAQ [%]
0.10
0.05
0.00
HG001
ΔclpX
clpXcompl
growth phase
exp
stat

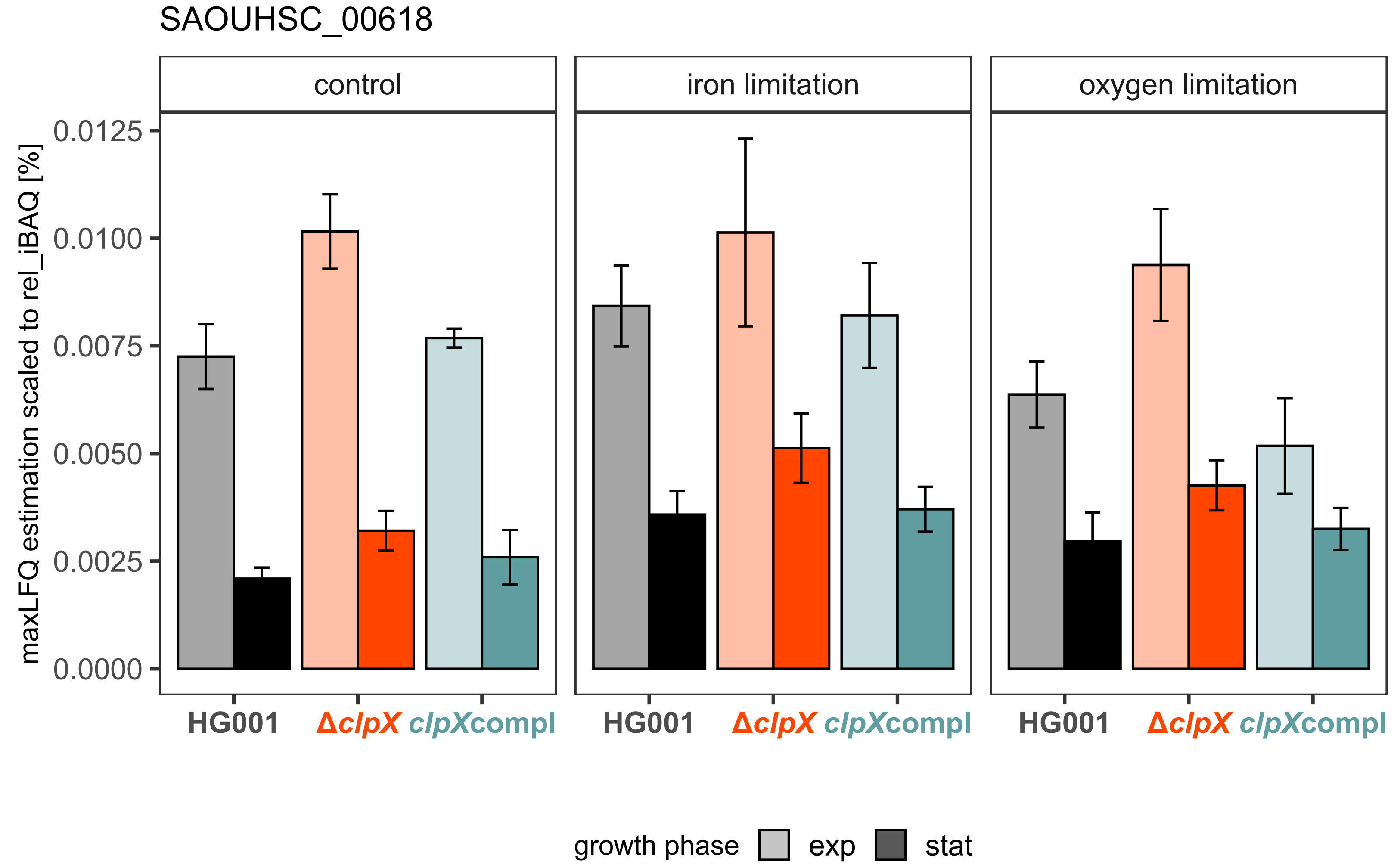

SAOUHSC_00618
control
iron limitation
oxygen limitation
maxLFQ estimation scaled to rel_iBAQ [%]
0.0125
0.0100
0.0075
0.0050
0.0025
0.0000
HG001
ΔclpX
clpXcompl
growth phase
exp
stat

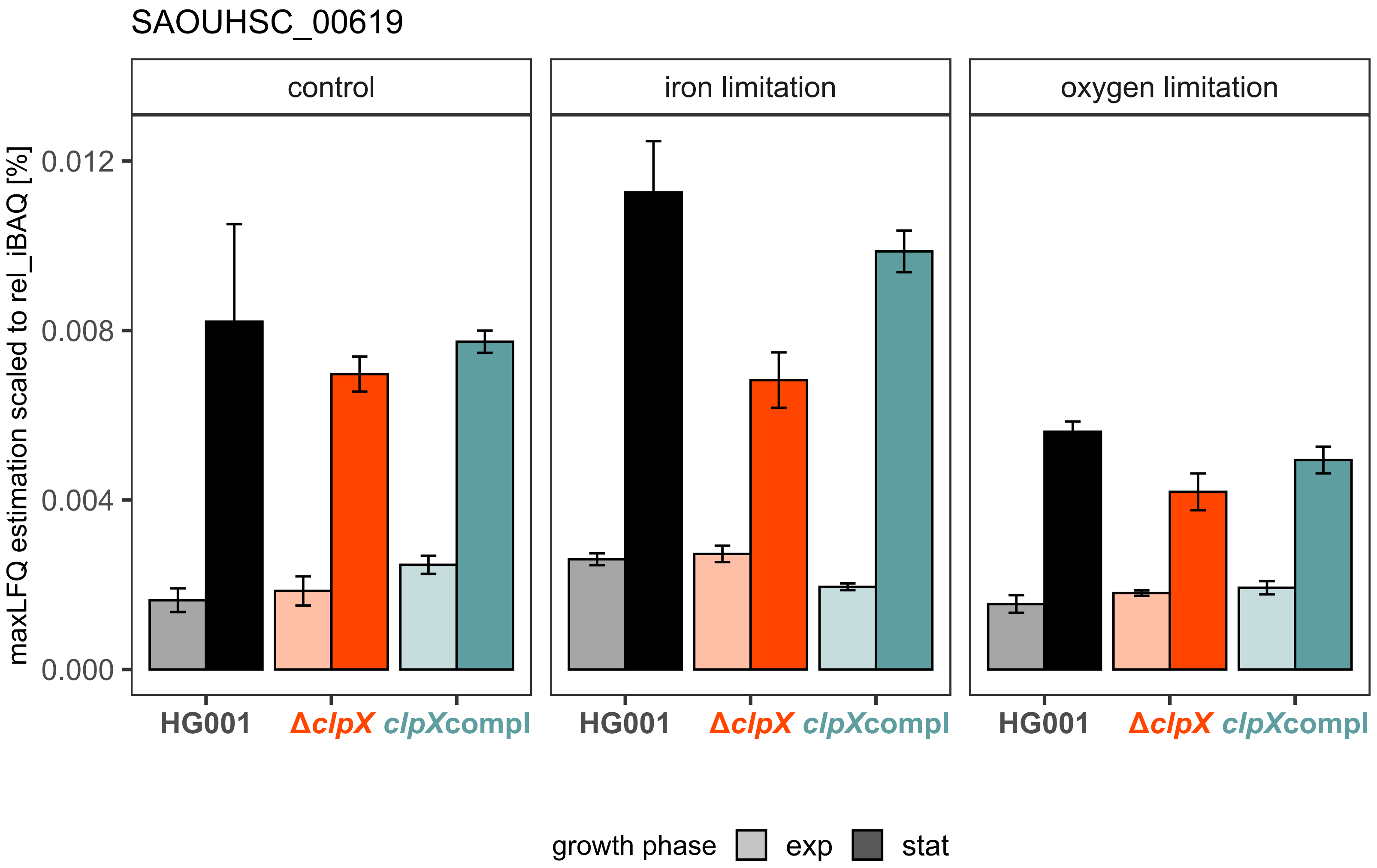
SAOUHSC_00619
control
iron limitation
oxygen limitation
maxLFQ estimation scaled to rel_iBAQ [%]
0.012
0.008
0.004
0.000
HG001
ΔclpX
clpXcompl
growth phase
exp
stat

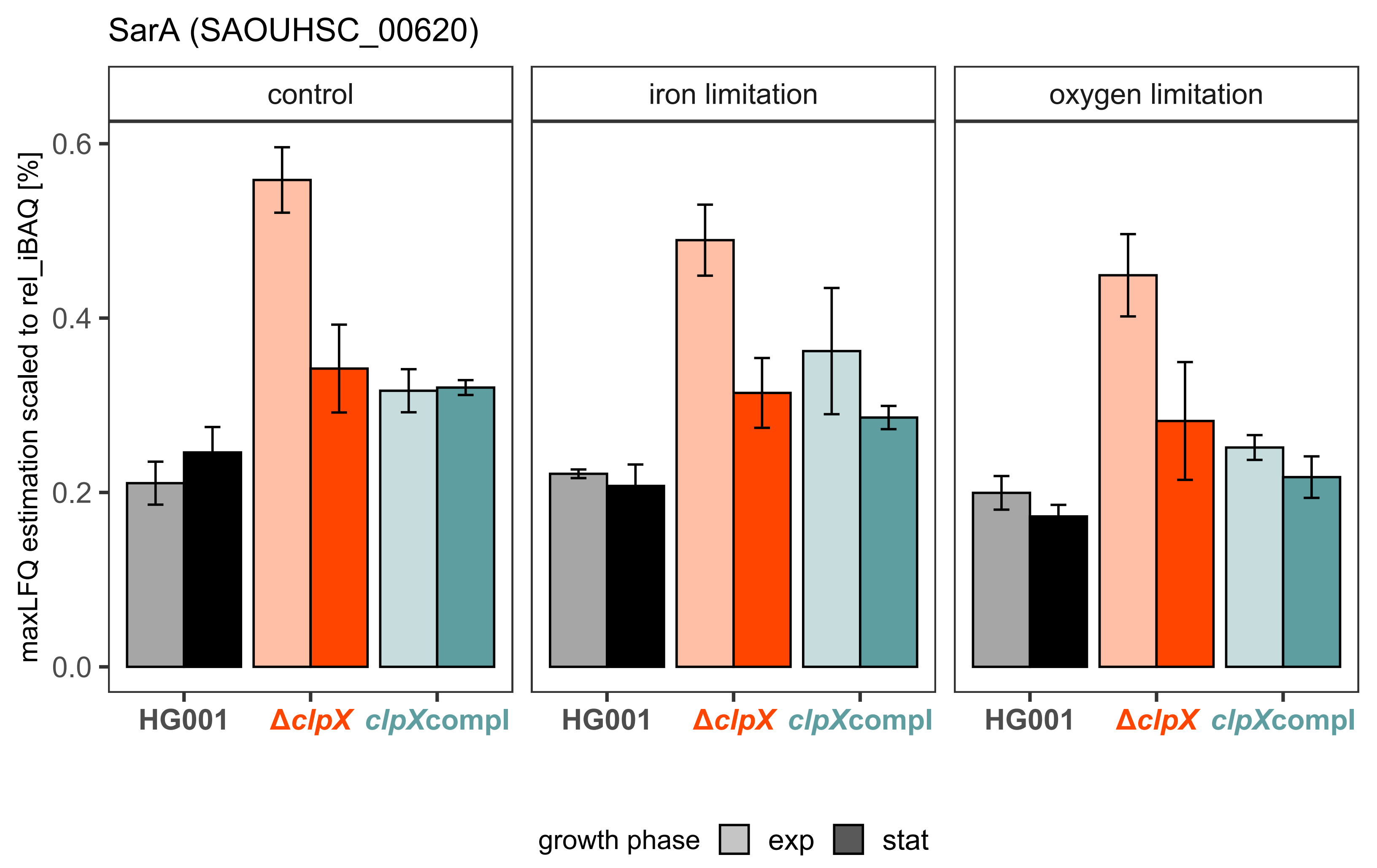
SarA (SAOUHSC_00620)
control
iron limitation
oxygen limitation
maxLFQ estimation scaled to rel_iBAQ [%]
0.0
0.2
0.4
0.6
HG001
ΔclpX
clpXcompl
growth phase
exp
stat

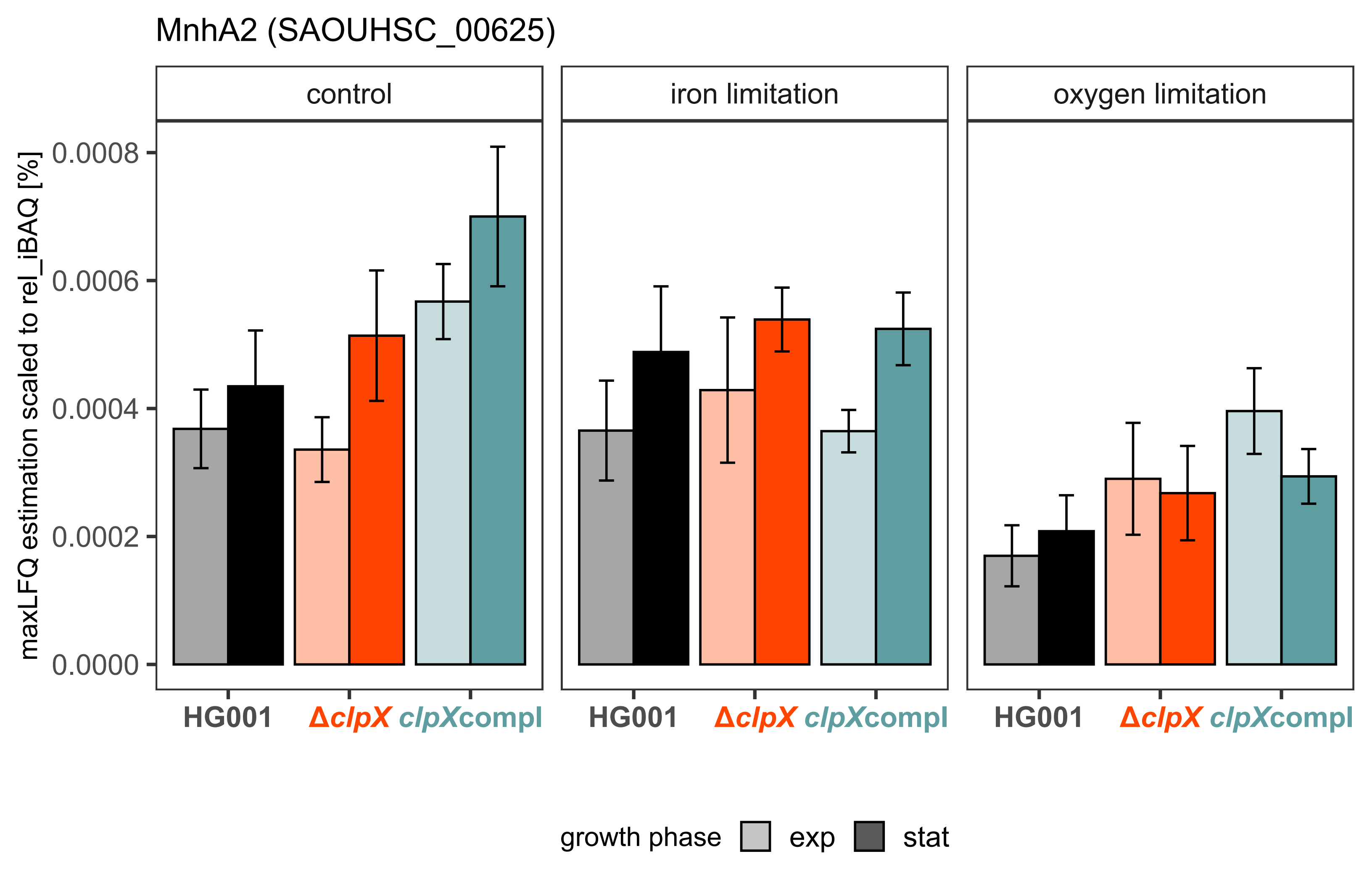
MnhA2 (SAOUHSC_00625)
control
iron limitation
oxygen limitation
maxLFQ estimation scaled to rel_iBAQ [%]
0.0008
0.0006
0.0004
0.0002
0.0000
HG001
ΔclpX
clpXcompl
growth phase
exp
stat

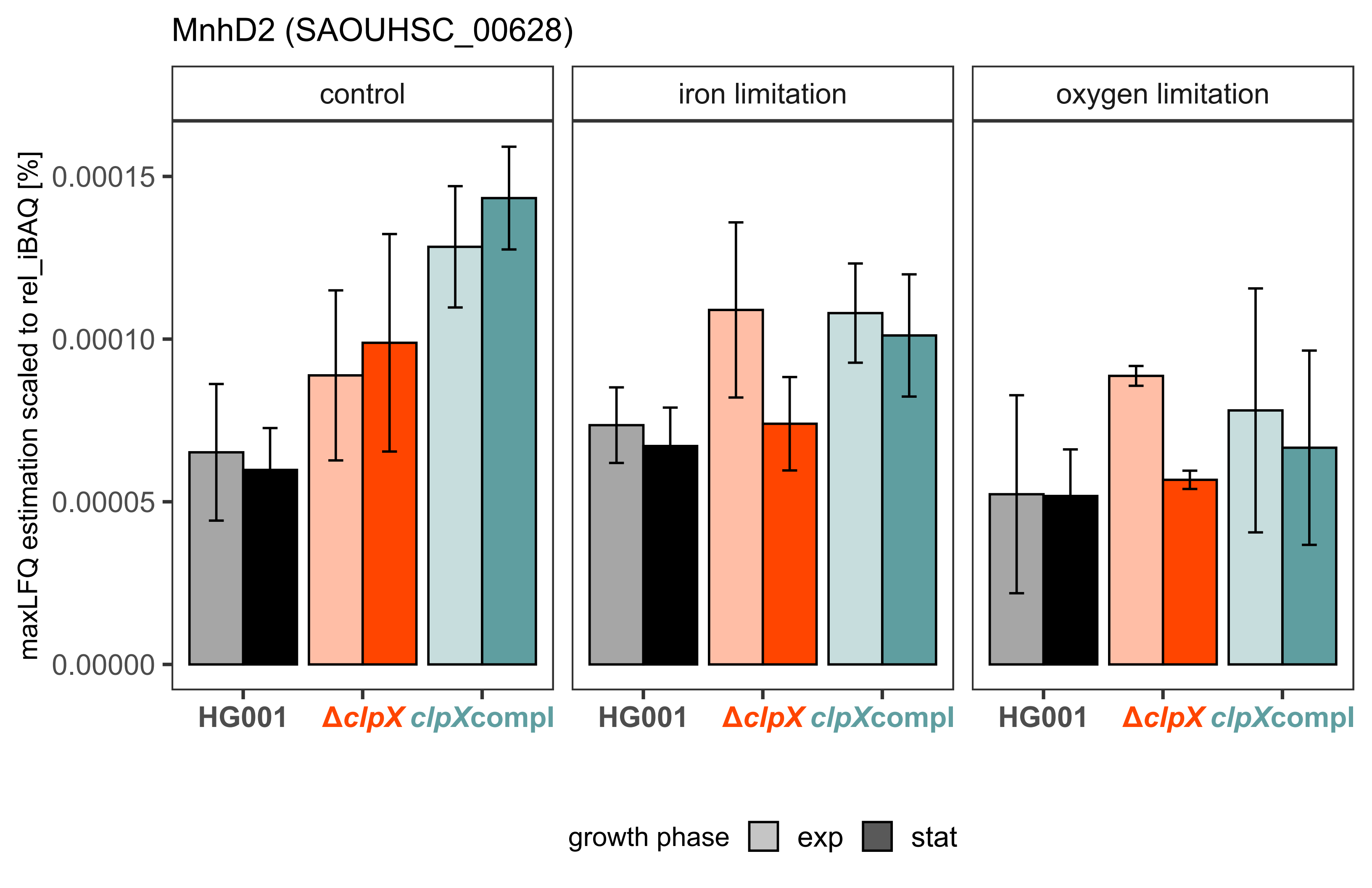

MnhD2 (SAOUHSC_00628)
control
iron limitation
oxygen limitation
maxLFQ estimation scaled to rel_iBAQ [%]
0.00015
0.00010
0.00005
0.00000
HG001
ΔclpX
clpXcompl
growth phase
exp
stat

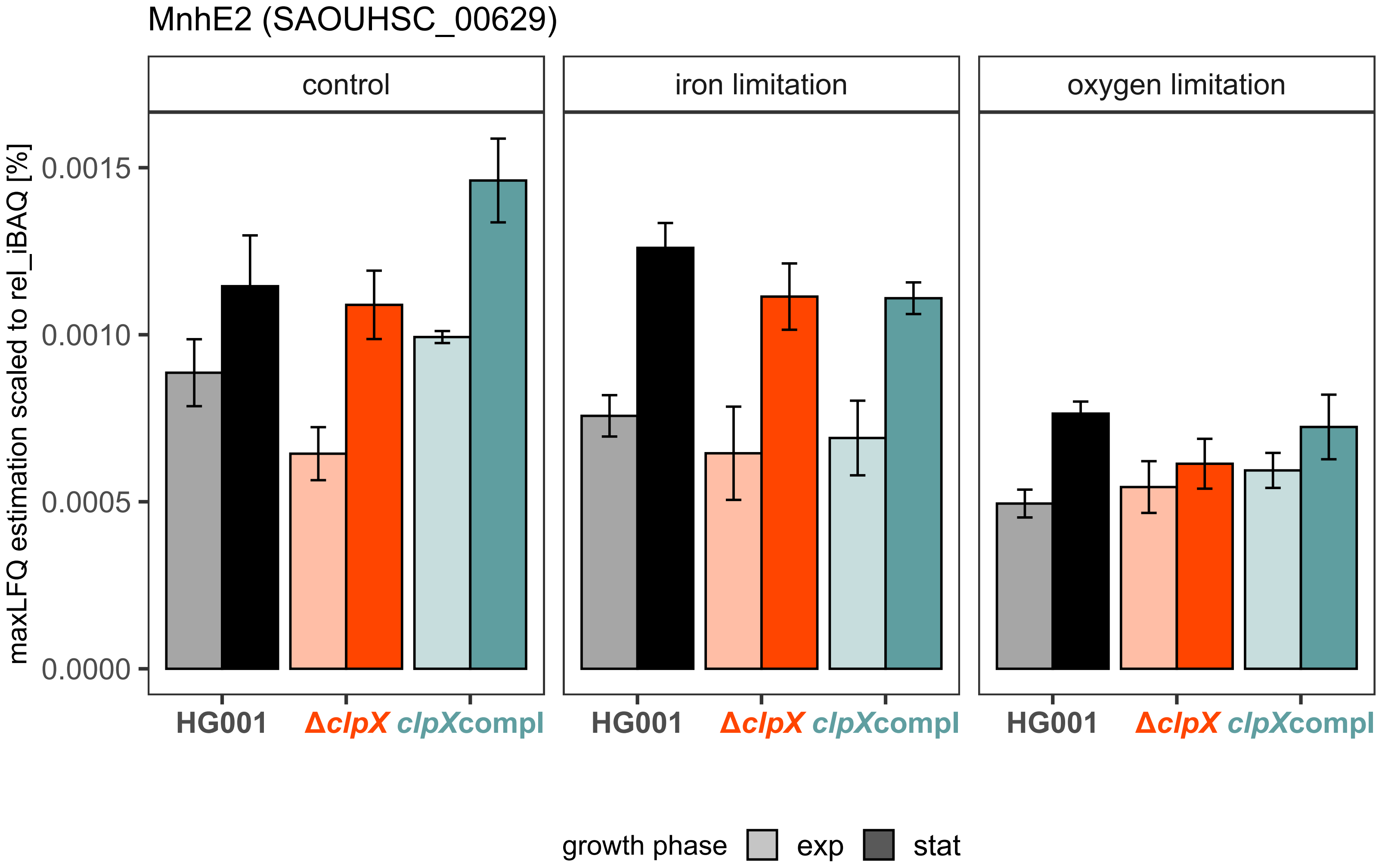
MnhE2 (SAOUHSC_00629)
control
iron limitation
oxygen limitation
maxLFQ estimation scaled to rel_iBAQ [%]
0.0015
0.0010
0.0005
0.0000
HG001
ΔclpX
clpXcompl
growth phase
exp
stat

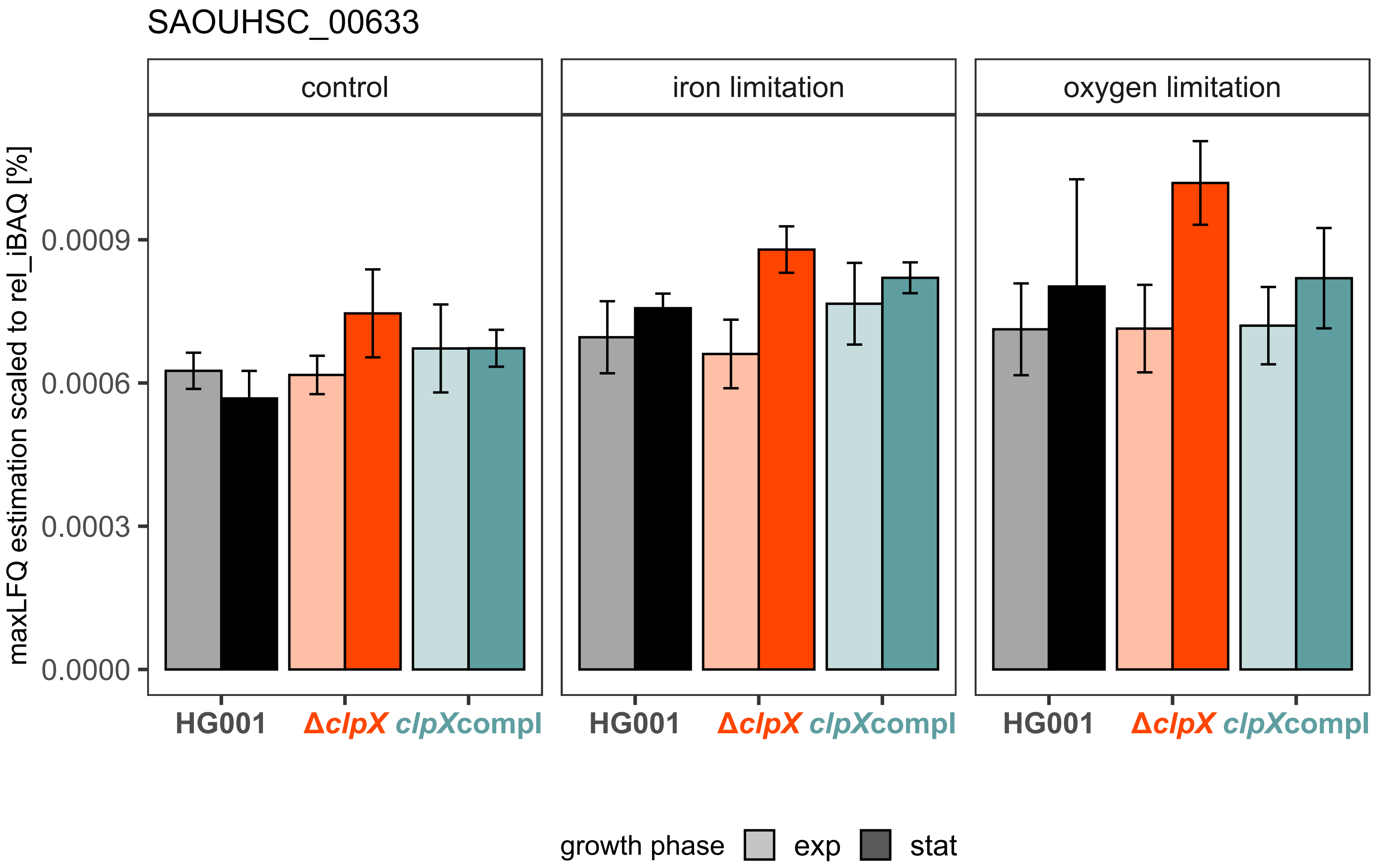

SAOUHSC_00633
control
iron limitation
oxygen limitation
maxLFQ estimation scaled to rel_iBAQ [%]
0.0009
0.0006
0.0003
0.0000
HG001
ΔclpX
clpXcompl
growth phase
exp
stat

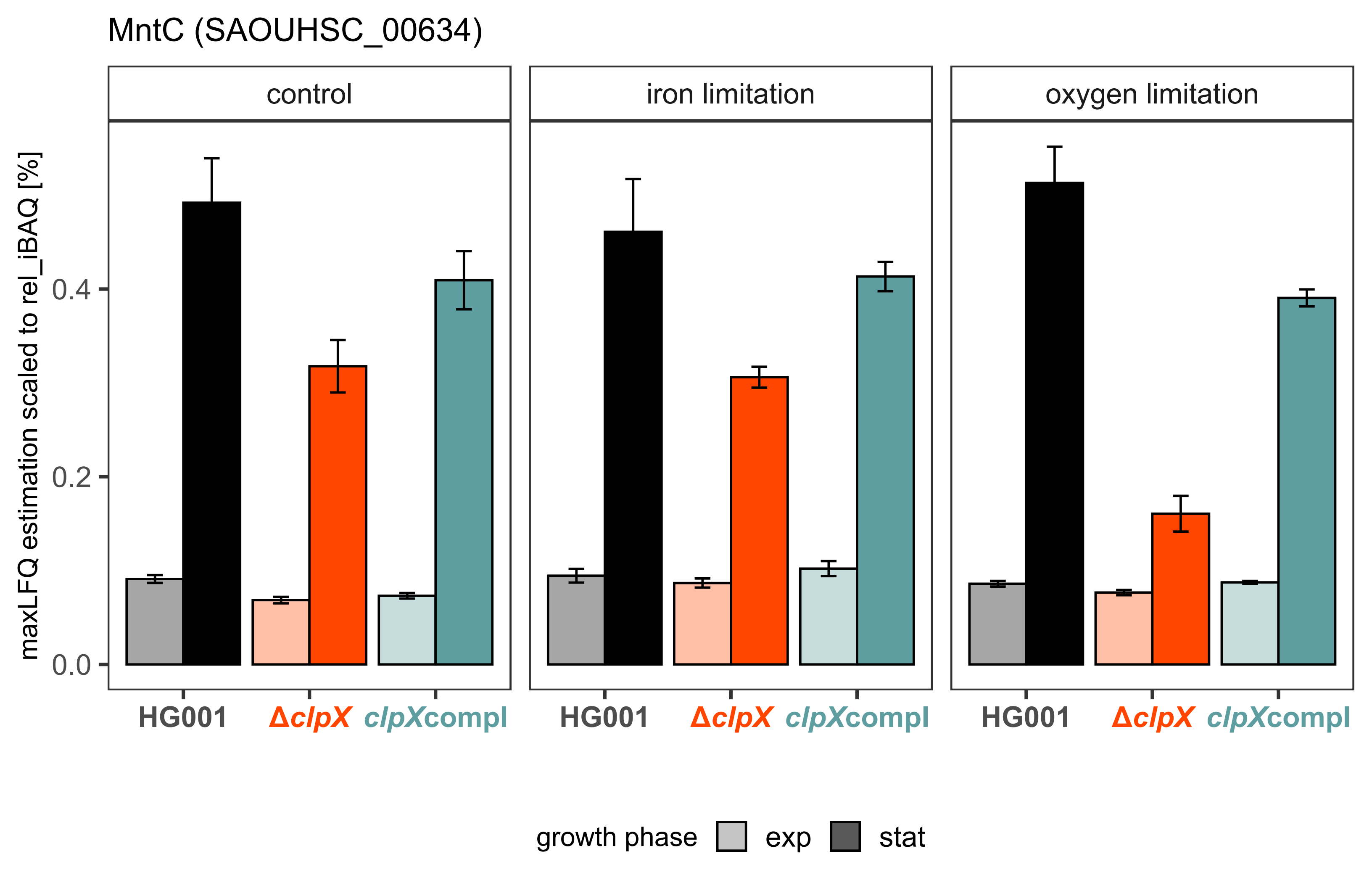

MntC (SAOUHSC_00634)
control
iron limitation
oxygen limitation
maxLFQ estimation scaled to rel_iBAQ [%]
0.0
0.2
0.4
HG001
ΔclpX
clpXcompl
growth phase
exp
stat

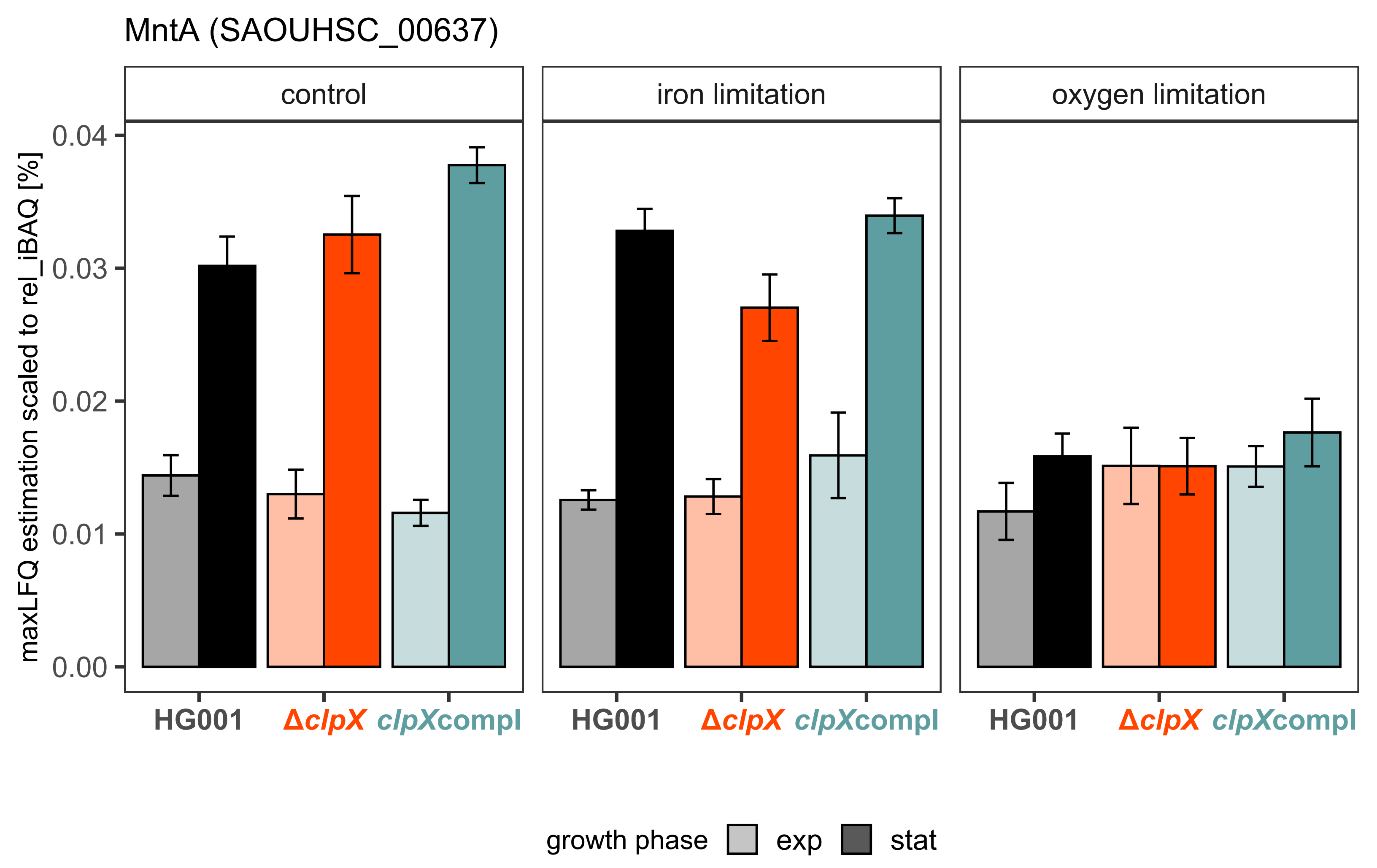
MntA (SAOUHSC_00637)
control
iron limitation
oxygen limitation
maxLFQ estimation scaled to rel_iBAQ [%]
0.00
0.01
0.02
0.03
0.04
HG001
ΔclpX
clpXcompl
growth phase
exp
stat

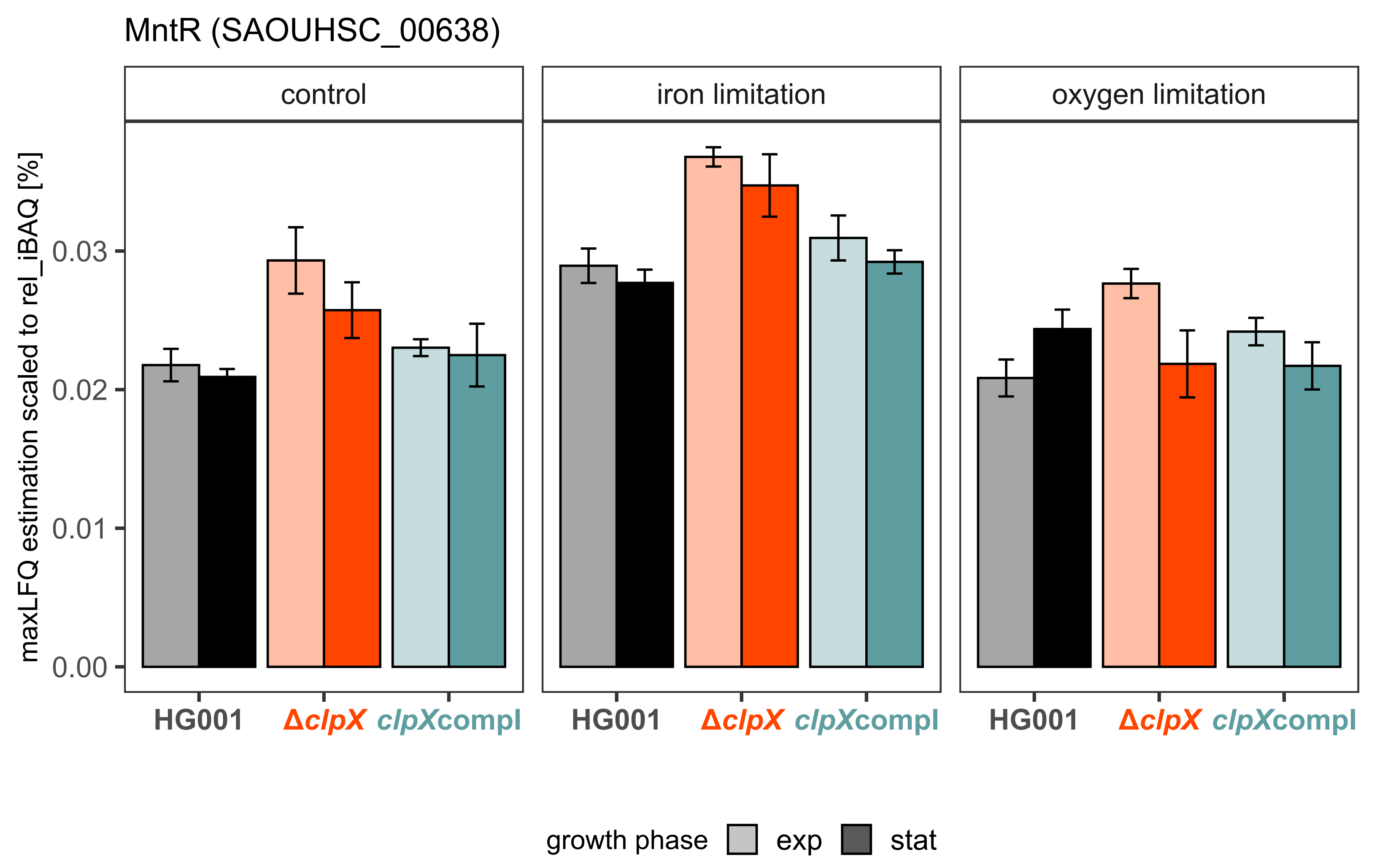
MntR (SAOUHSC_00638)
control
iron limitation
oxygen limitation
maxLFQ estimation scaled to rel_iBAQ [%]
0.03
0.02
0.01
0.00
HG001
ΔclpX
clpXcompl
growth phase
exp
stat

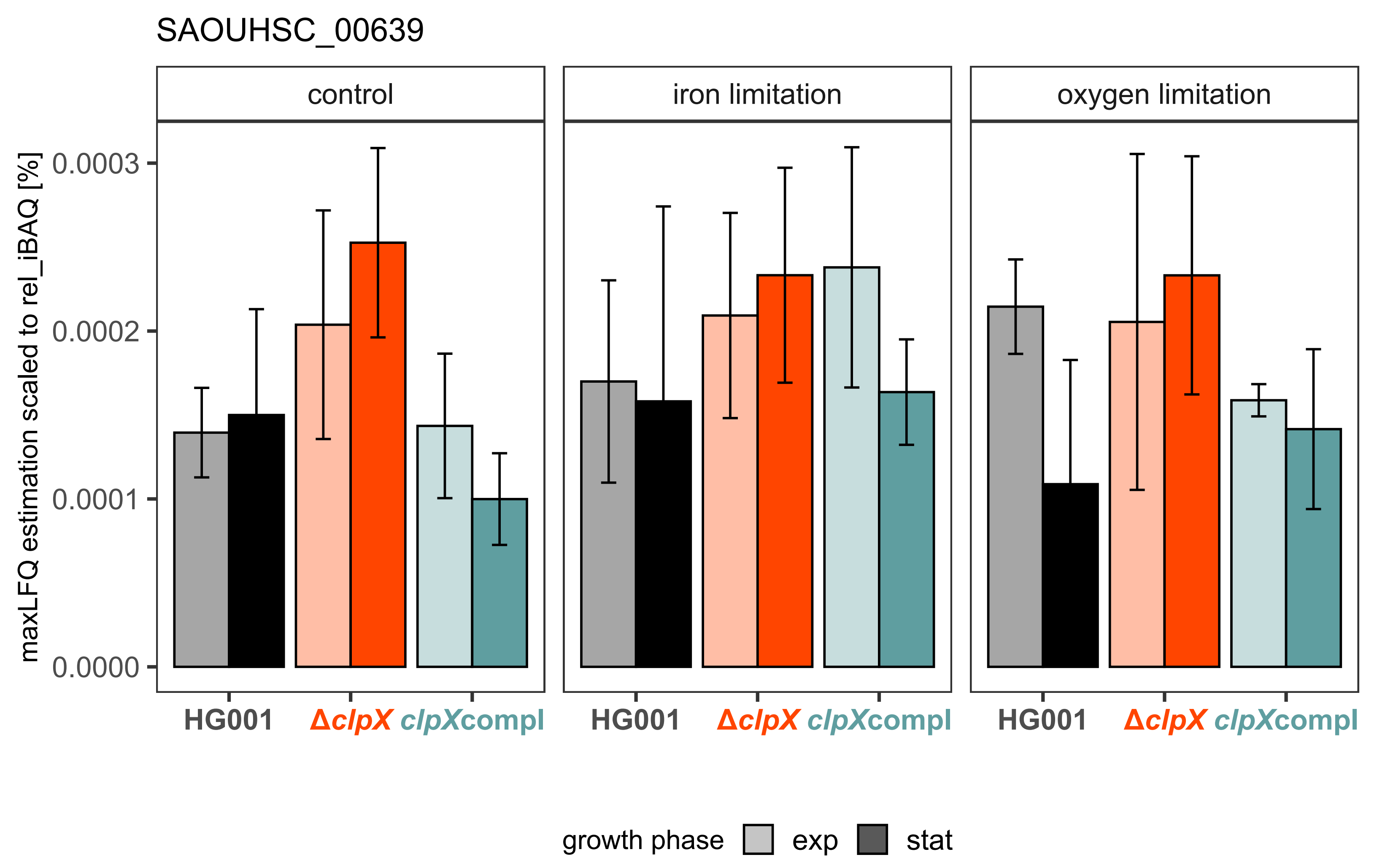

SAOUHSC_00639
control
iron limitation
oxygen limitation
maxLFQ estimation scaled to rel_iBAQ [%]
0.0003
0.0002
0.0001
0.0000
HG001
ΔclpX
clpXcompl
growth phase
exp
stat

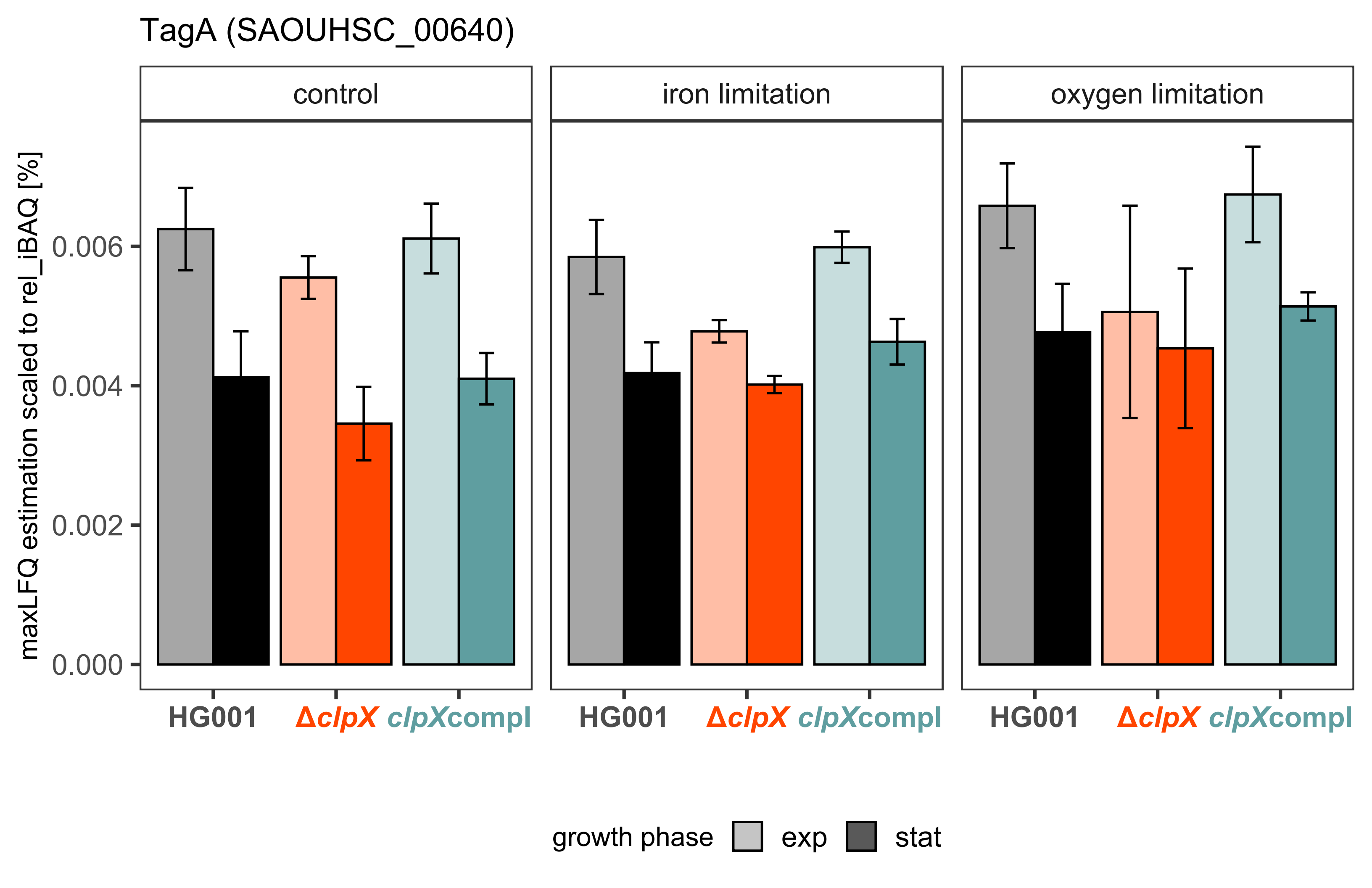
TagA (SAOUHSC_00640)
control
iron limitation
oxygen limitation
maxLFQ estimation scaled to rel_iBAQ [%]
0.006
0.004
0.002
0.000
HG001
ΔclpX
clpXcompl
growth phase
exp
stat

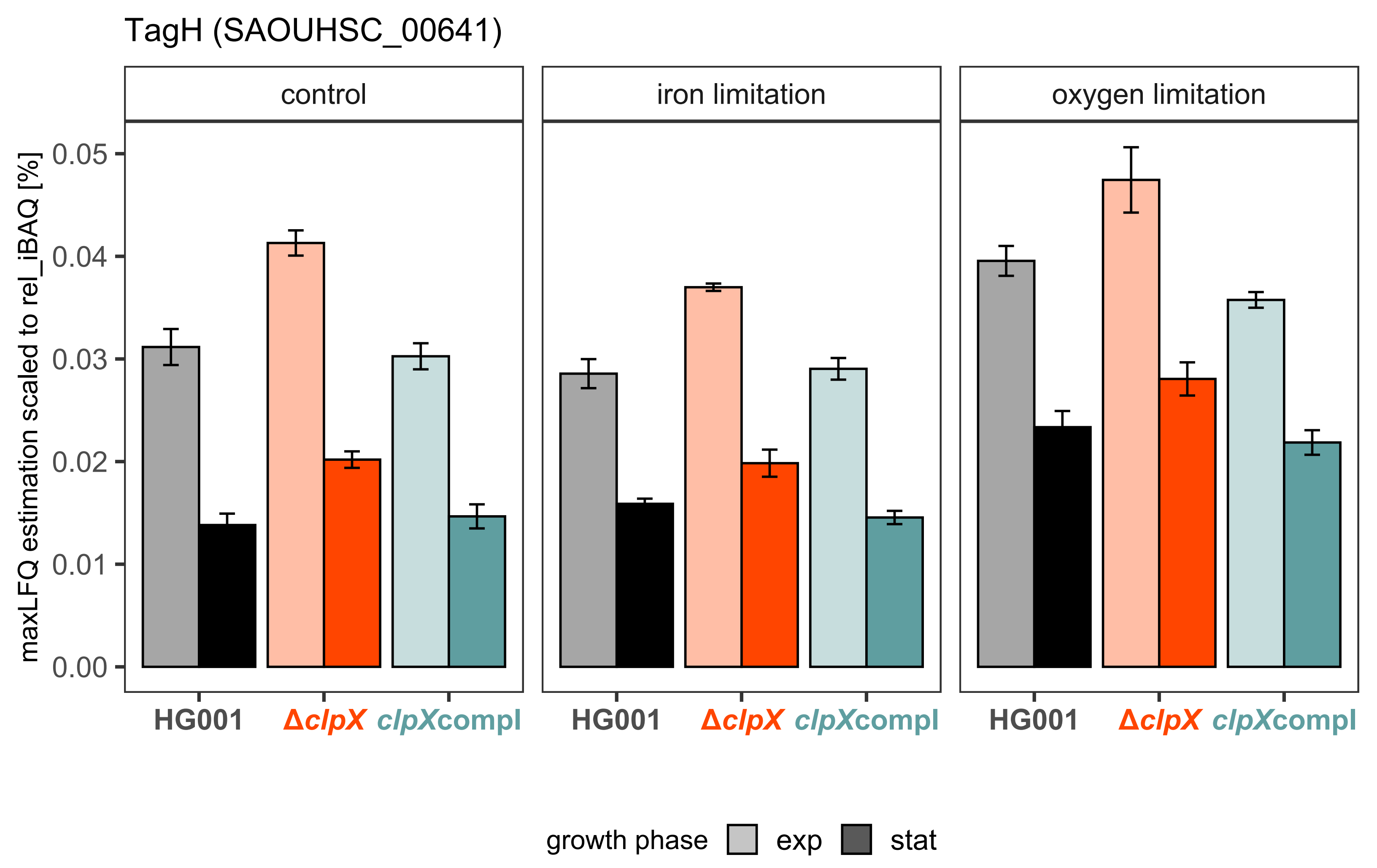

TagH (SAOUHSC_00641)
control
iron limitation
oxygen limitation
maxLFQ estimation scaled to rel_iBAQ [%]
0.05
0.04
0.03
0.02
0.01
0.00
HG001
ΔclpX
clpXcompl
growth phase
exp
stat

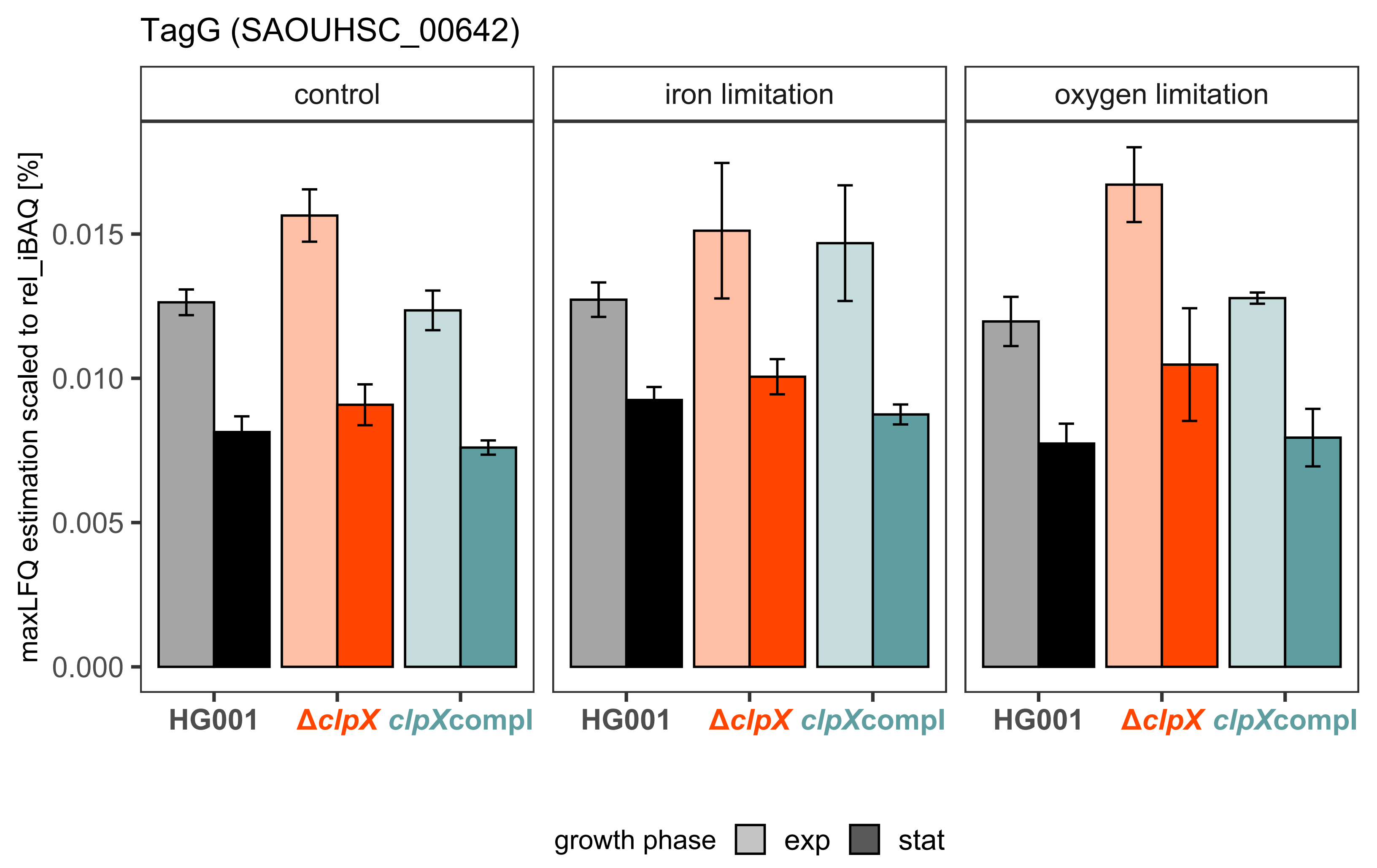

TagG (SAOUHSC_00642)
control
iron limitation
oxygen limitation
maxLFQ estimation scaled to rel_iBAQ [%]
0.015
0.010
0.005
0.000
HG001
ΔclpX
clpXcompl
growth phase
exp
stat

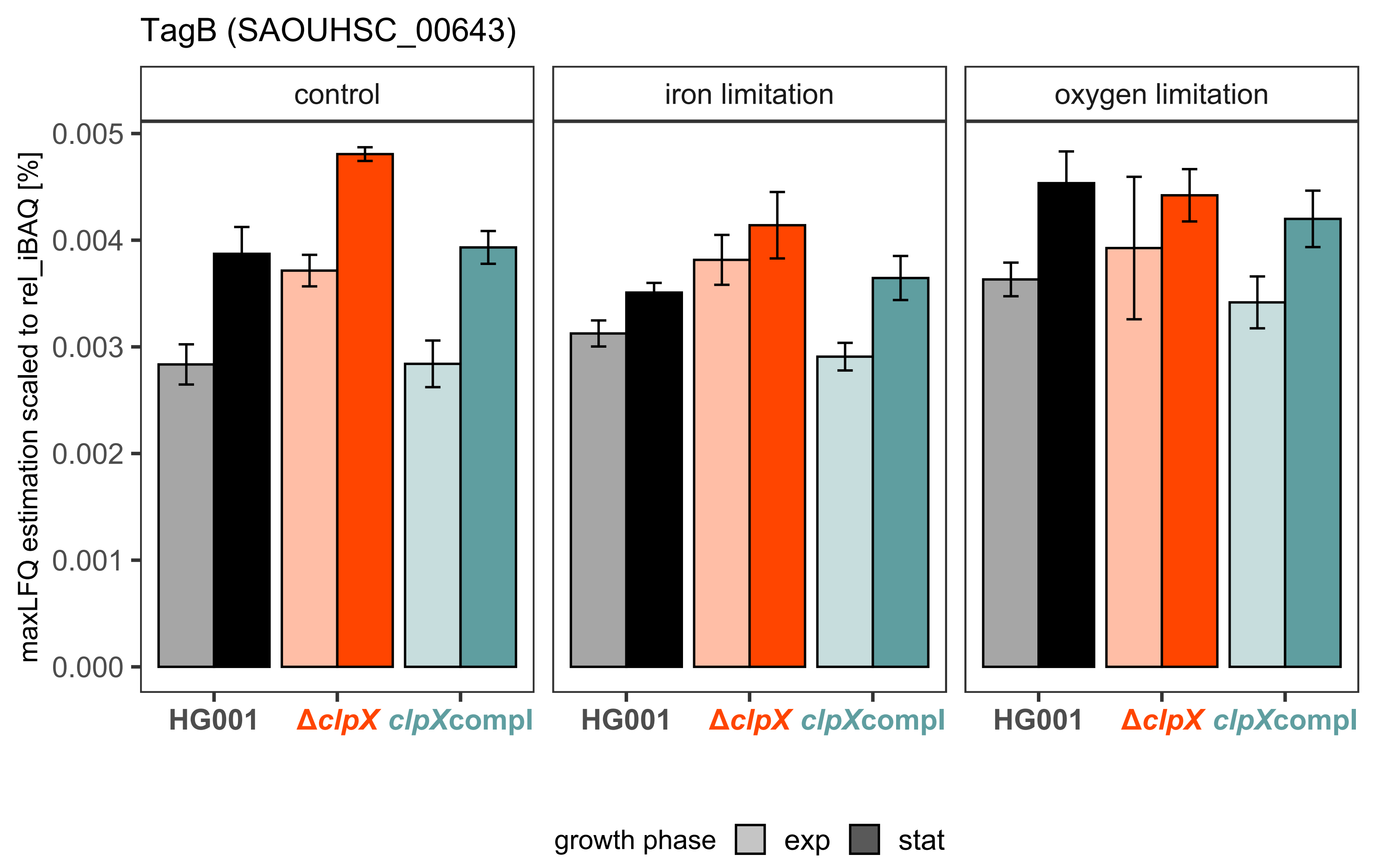

TagB (SAOUHSC_00643)
control
iron limitation
oxygen limitation
maxLFQ estimation scaled to rel_iBAQ [%]
0.005
0.004
0.003
0.002
0.001
0.000
HG001
ΔclpX
clpXcompl
growth phase
exp
stat

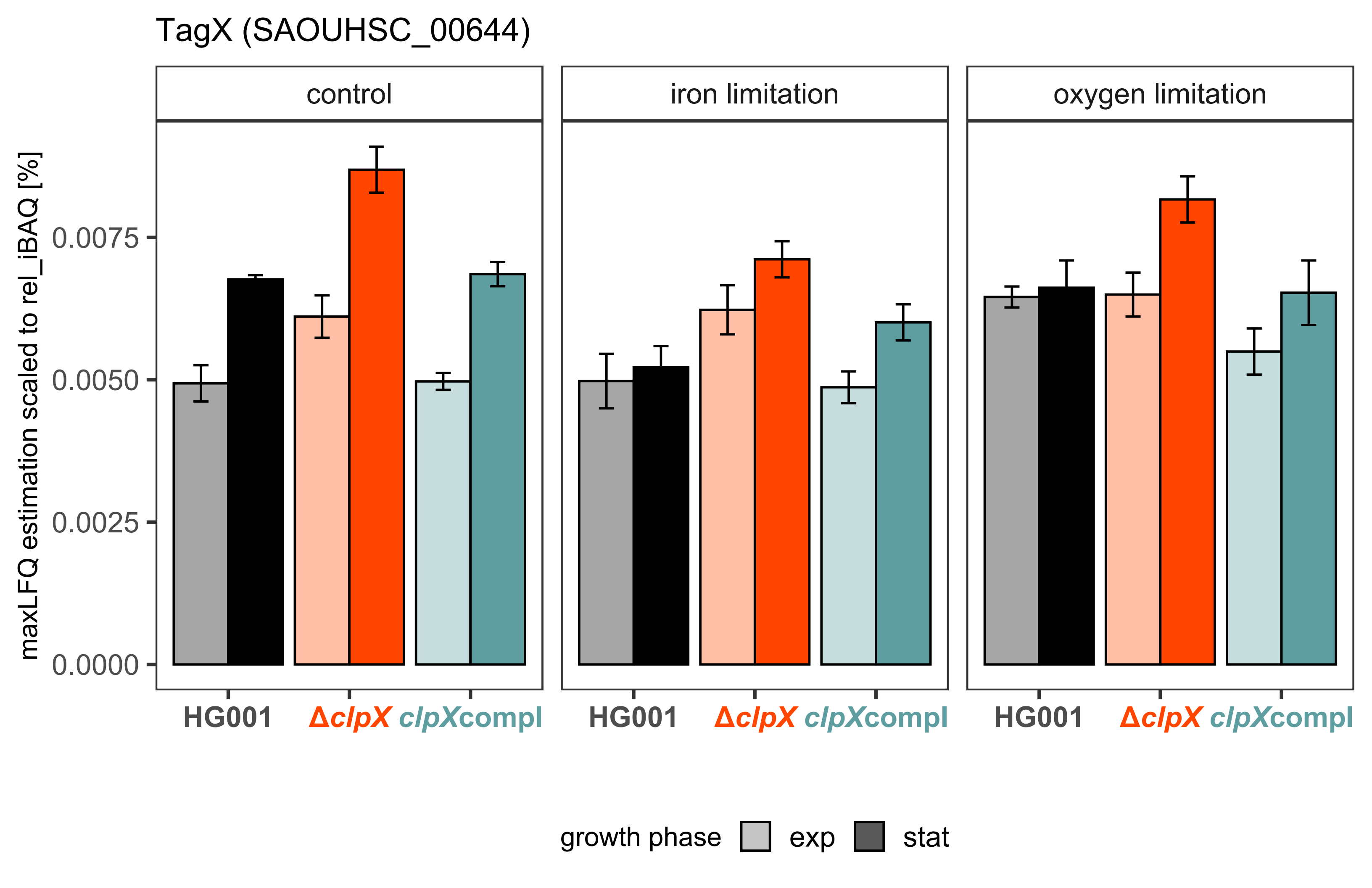

TagX (SAOUHSC_00644)
control
iron limitation
oxygen limitation
maxLFQ estimation scaled to rel_iBAQ [%]
0.0075
0.0050
0.0025
0.0000
HG001
ΔclpX
clpXcompl
growth phase
exp
stat

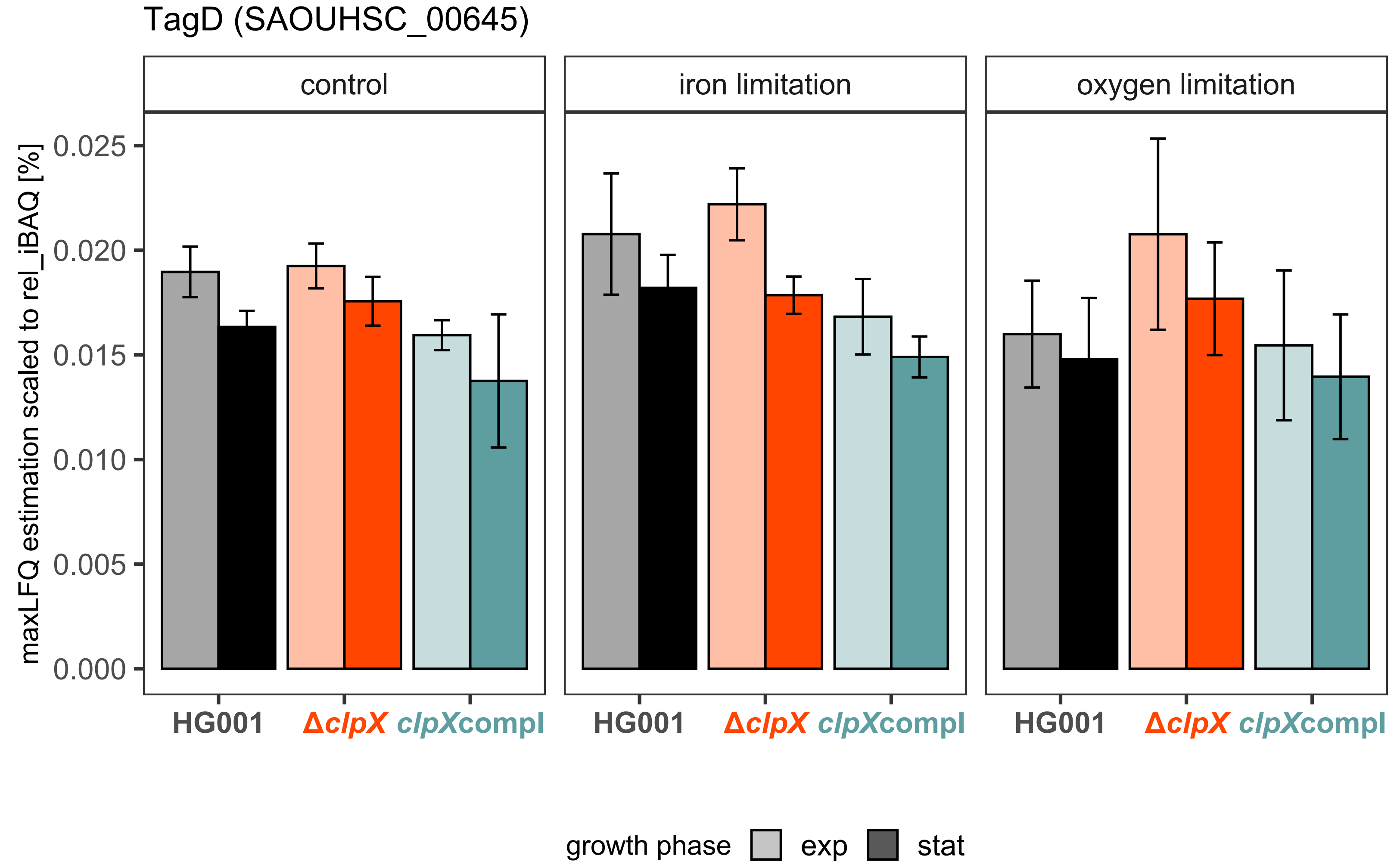
TagD (SAOUHSC_00645)
control
iron limitation
oxygen limitation
maxLFQ estimation scaled to rel_iBAQ [%]
0.025
0.020
0.015
0.010
0.005
0.000
HG001
ΔclpX
clpXcompl
growth phase
exp
stat

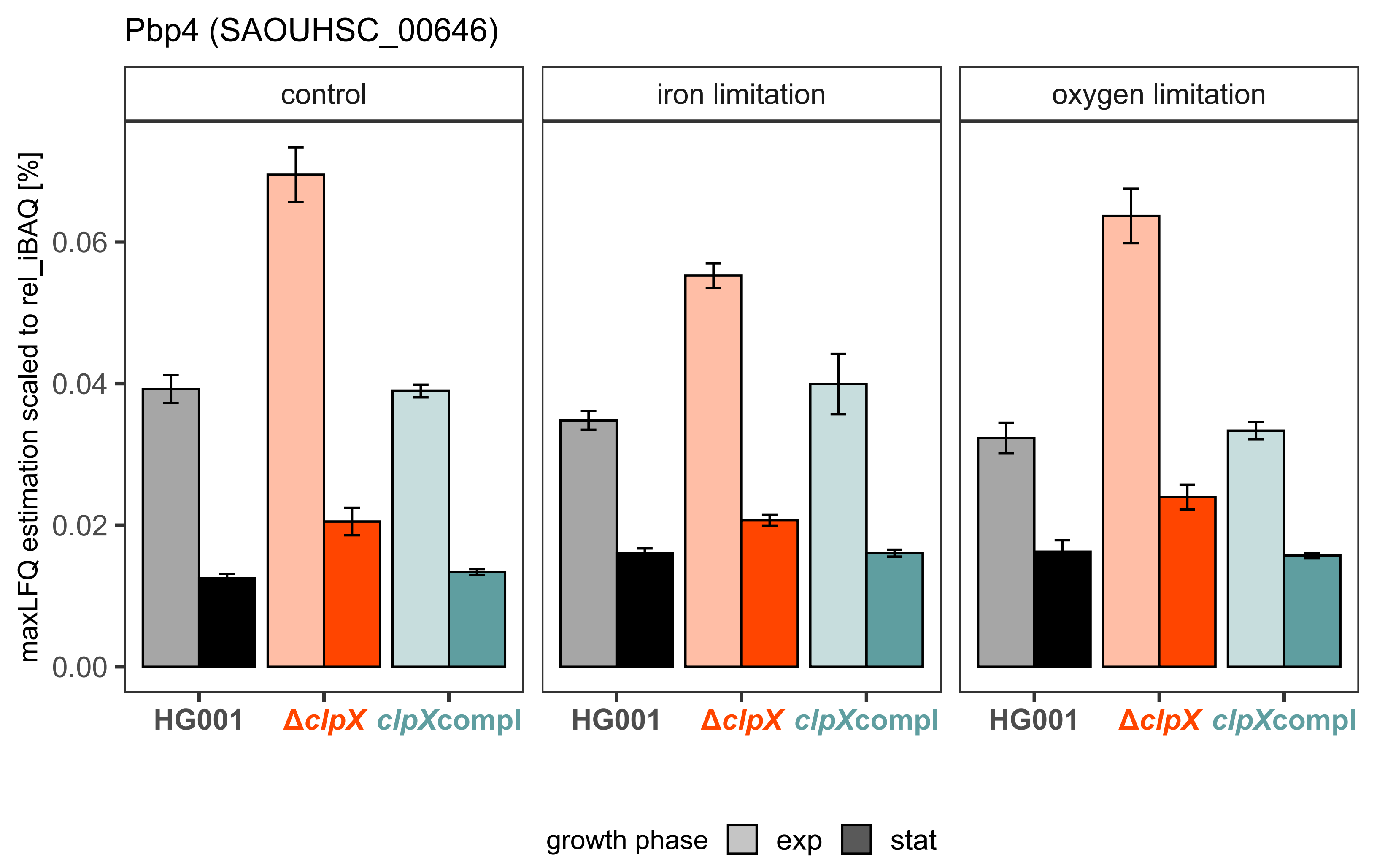
Pbp4 (SAOUHSC_00646)
control
iron limitation
oxygen limitation
maxLFQ estimation scaled to rel_iBAQ [%]
0.00
0.02
0.04
0.06
HG001
ΔclpX
clpXcompl
growth phase
exp
stat

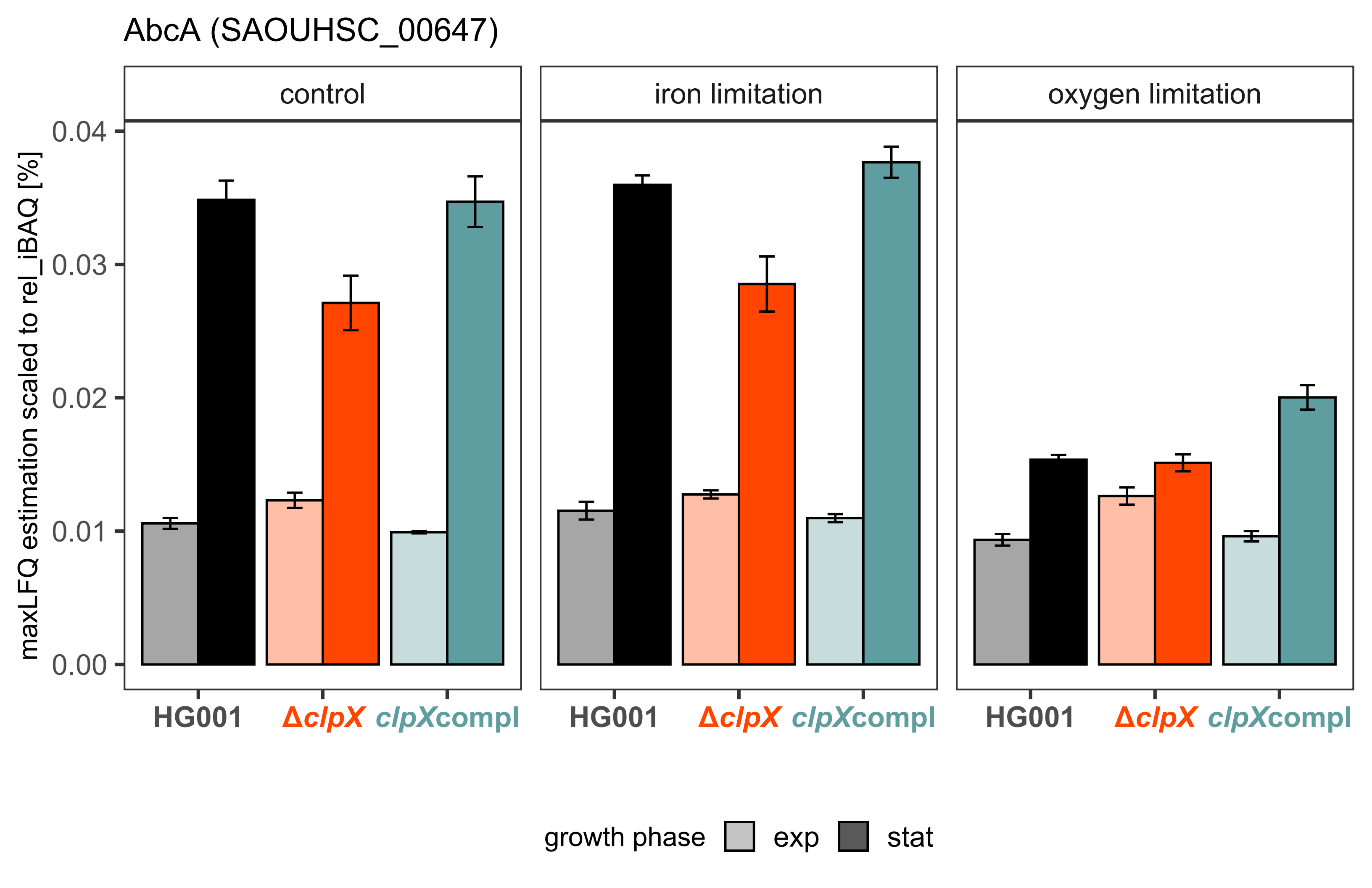
AbcA (SAOUHSC_00647)
control
iron limitation
oxygen limitation
maxLFQ estimation scaled to rel_iBAQ [%]
0.04
0.03
0.02
0.01
0.00
HG001
ΔclpX
clpXcompl
growth phase
exp
stat

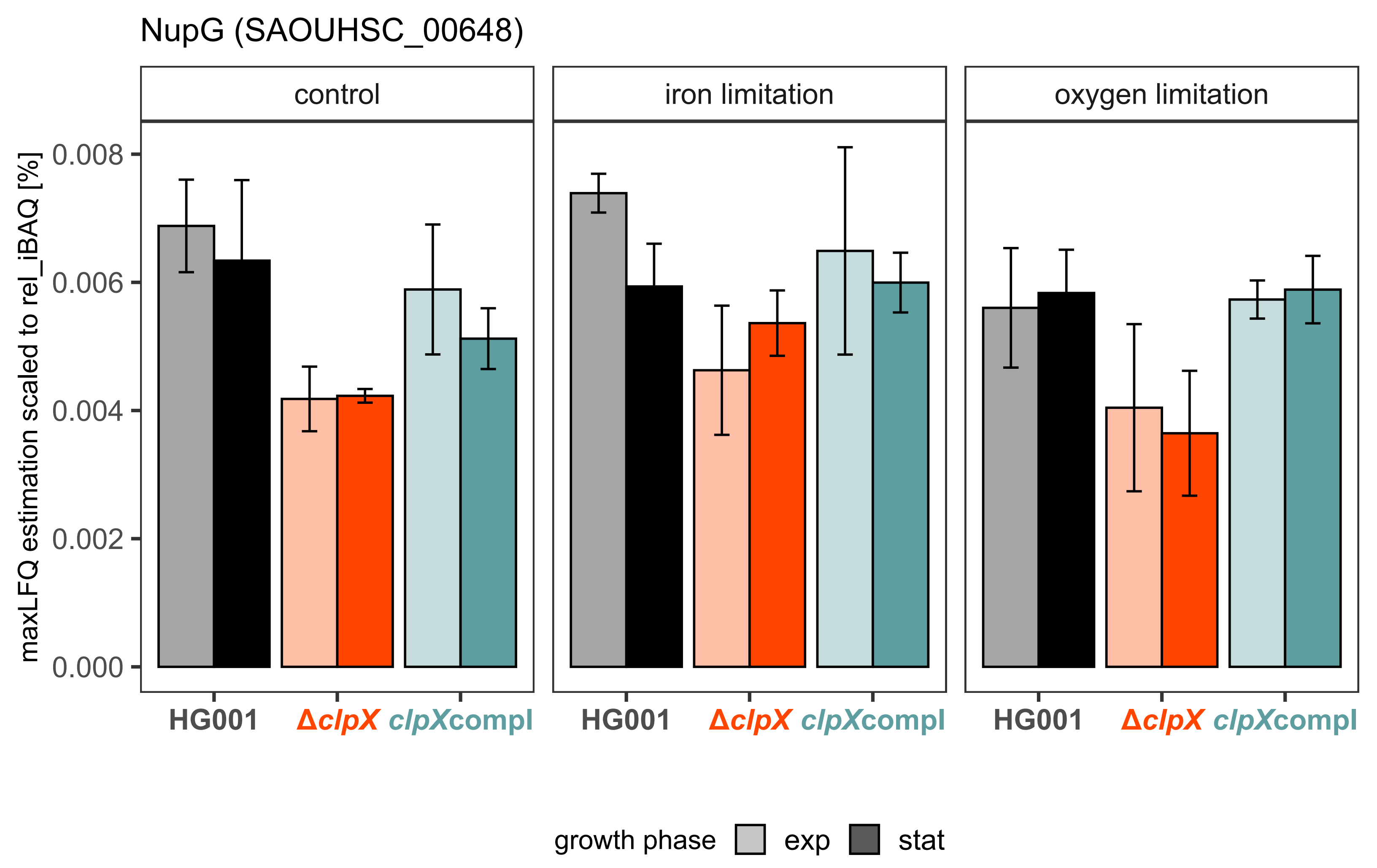
NupG (SAOUHSC_00648)
control
iron limitation
oxygen limitation
maxLFQ estimation scaled to rel_iBAQ [%]
0.008
0.006
0.004
0.002
0.000
HG001
ΔclpX
clpXcompl
growth phase
exp
stat

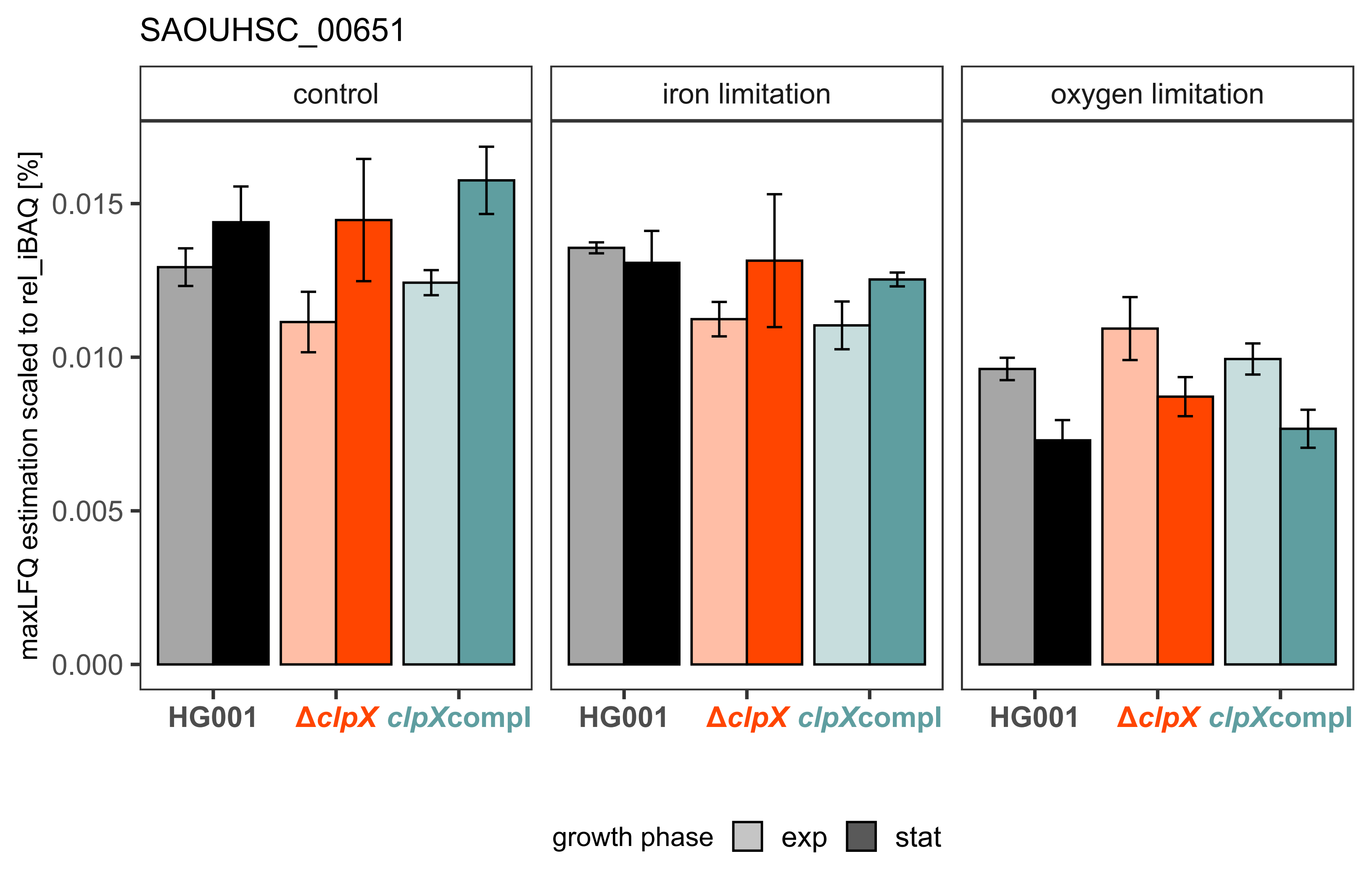

SAOUHSC_00651
control
iron limitation
oxygen limitation
maxLFQ estimation scaled to rel_iBAQ [%]
0.015
0.010
0.005
0.000
HG001
ΔclpX
clpXcompl
growth phase
exp
stat

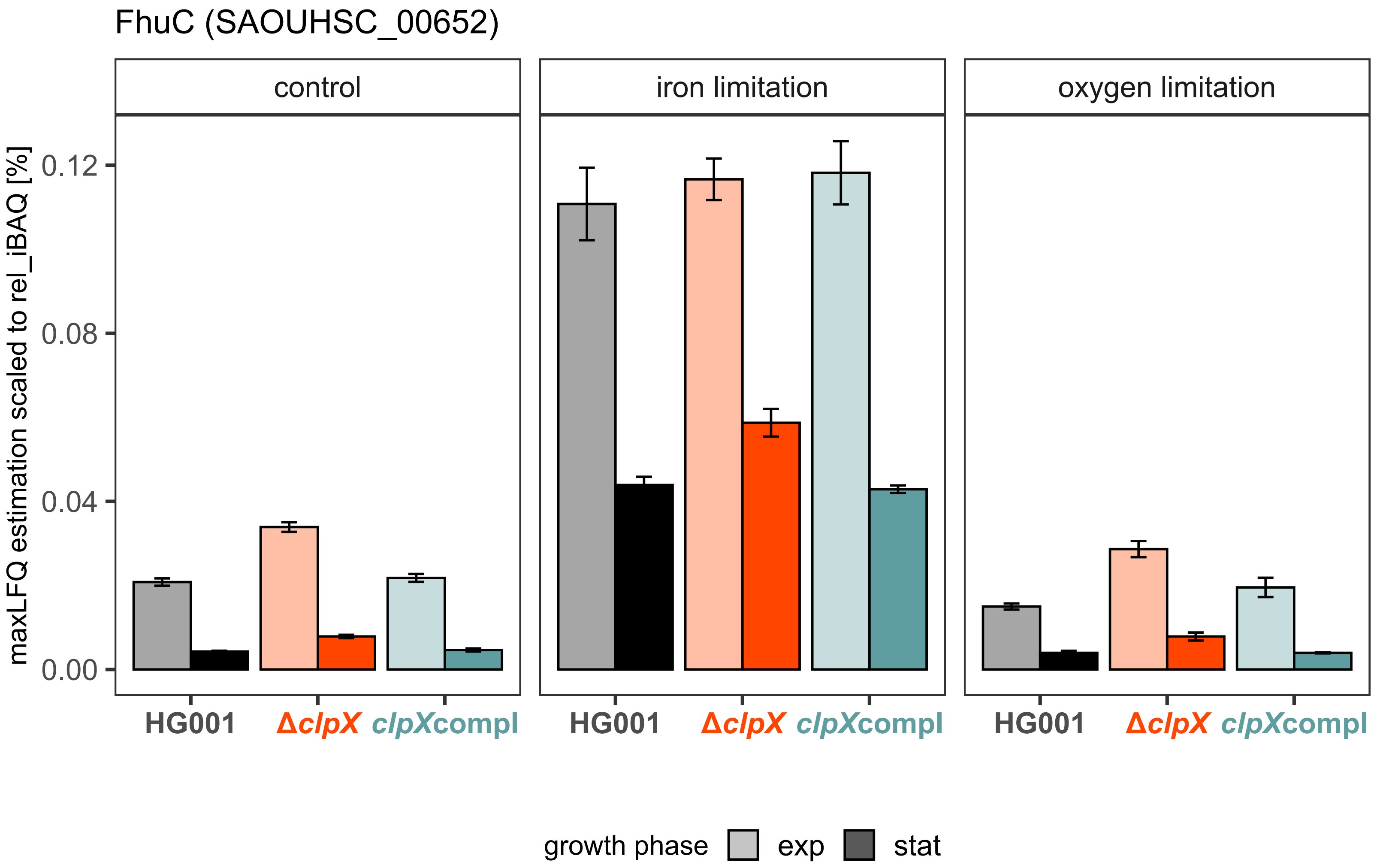

FhuC (SAOUHSC_00652)
control
iron limitation
oxygen limitation
maxLFQ estimation scaled to rel_iBAQ [%]
0.12
0.08
0.04
0.00
HG001
ΔclpX
clpXcompl
HG001
ΔclpX
clpXcompl
HG001
ΔclpX
clpXcompl
growth phase
exp
stat

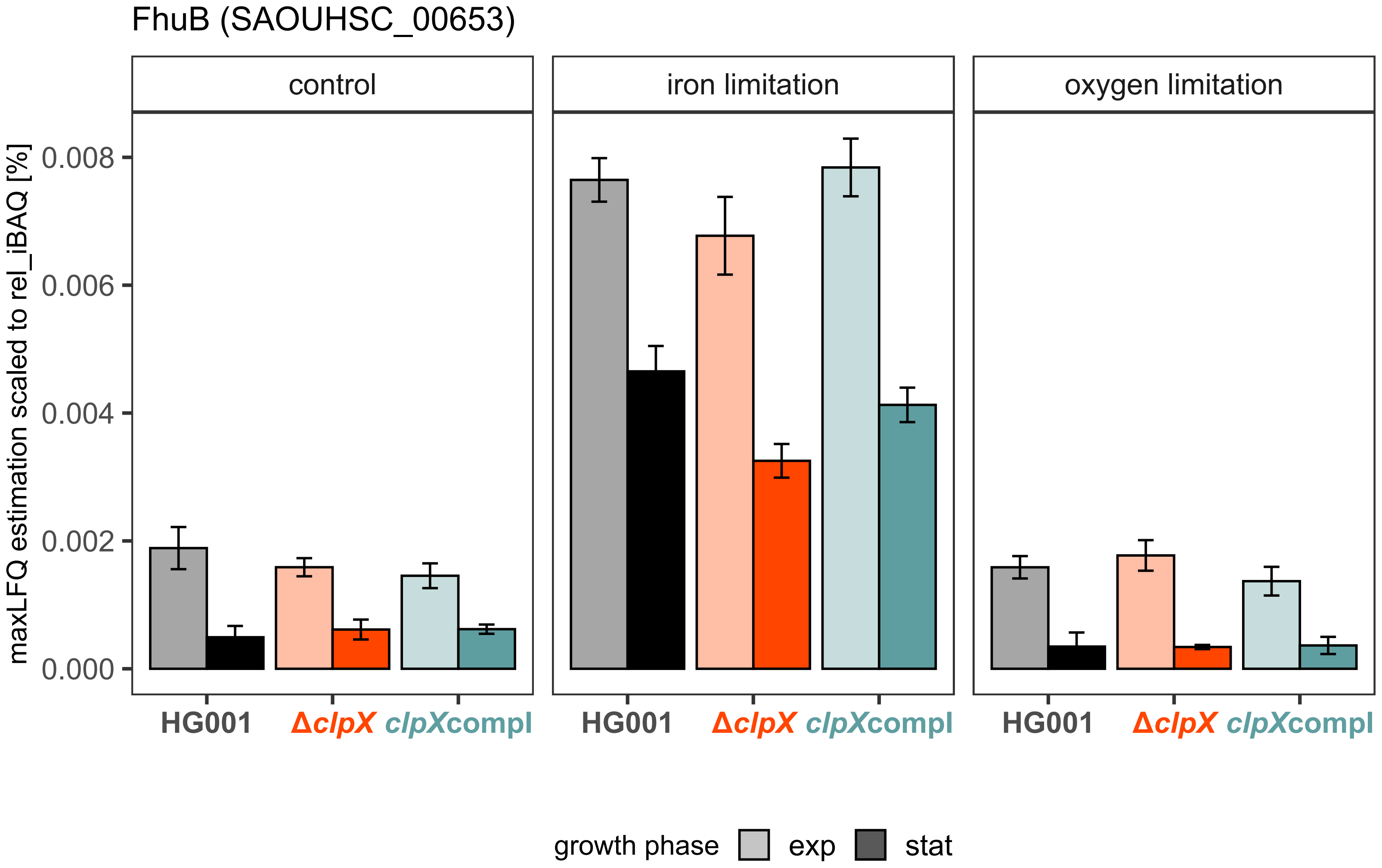

FhuB (SAOUHSC_00653)
control
iron limitation
oxygen limitation
maxLFQ estimation scaled to rel_iBAQ [%]
0.008
0.006
0.004
0.002
0.000
HG001
ΔclpX
clpXcompl
growth phase
exp
stat

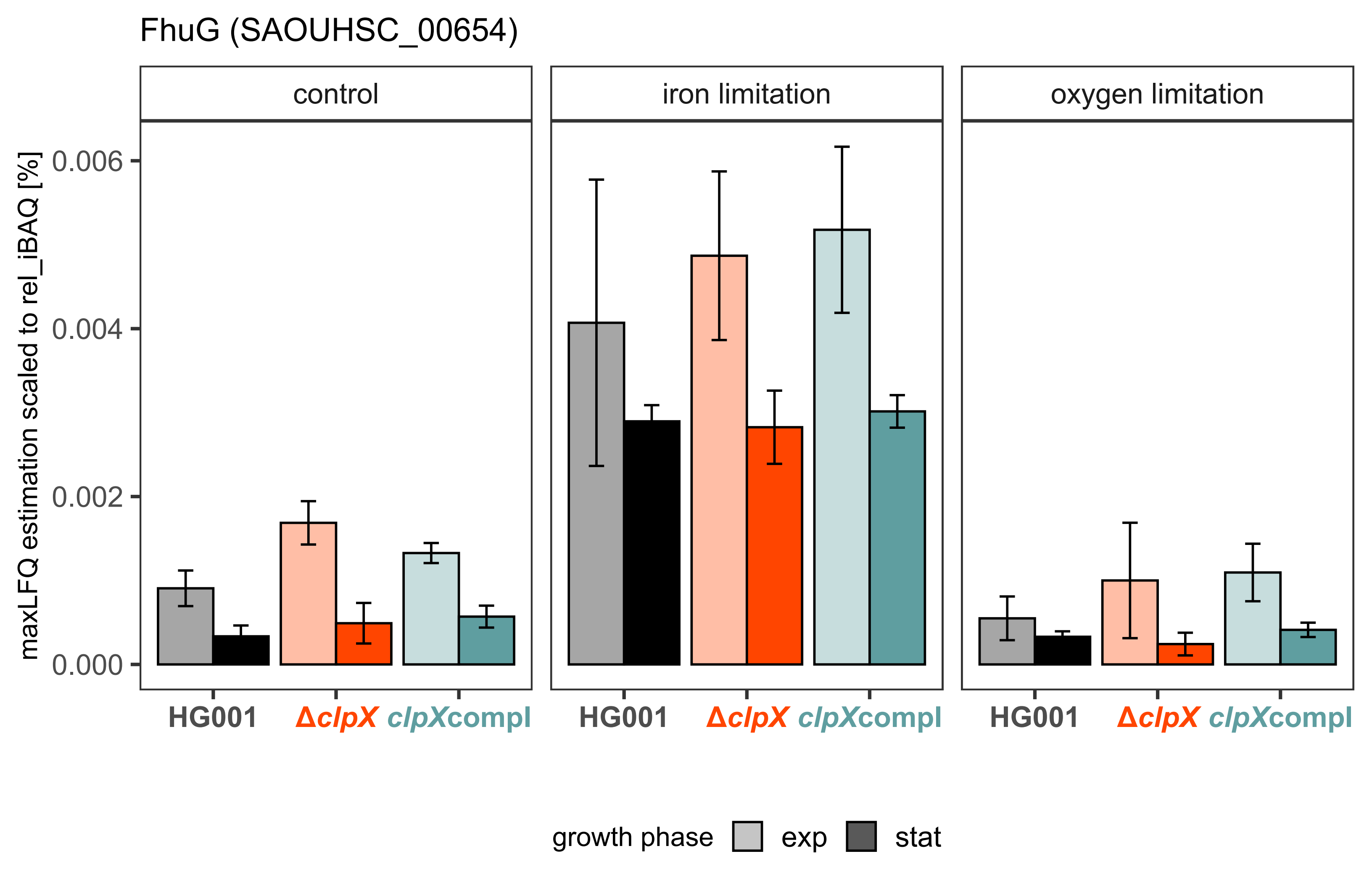
FhuG (SAOUHSC_00654)
control
iron limitation
oxygen limitation
maxLFQ estimation scaled to rel_iBAQ [%]
0.006
0.004
0.002
0.000
HG001
ΔclpX
clpXcompl
growth phase
exp
stat

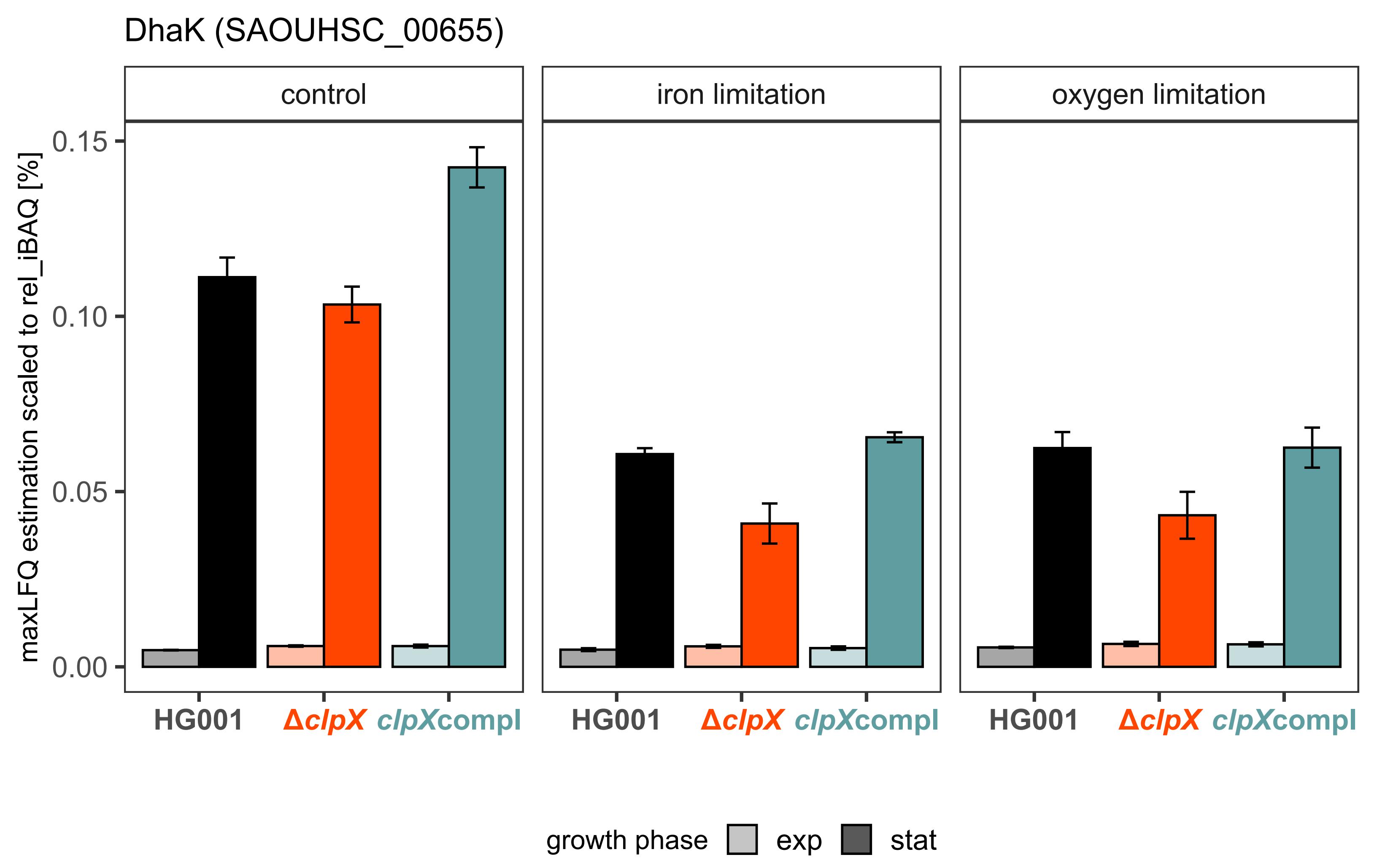

DhaK (SAOUHSC_00655)
control
iron limitation
oxygen limitation
maxLFQ estimation scaled to rel_iBAQ [%]
0.15
0.10
0.05
0.00
HG001
ΔclpX
clpXcompl
growth phase
exp
stat

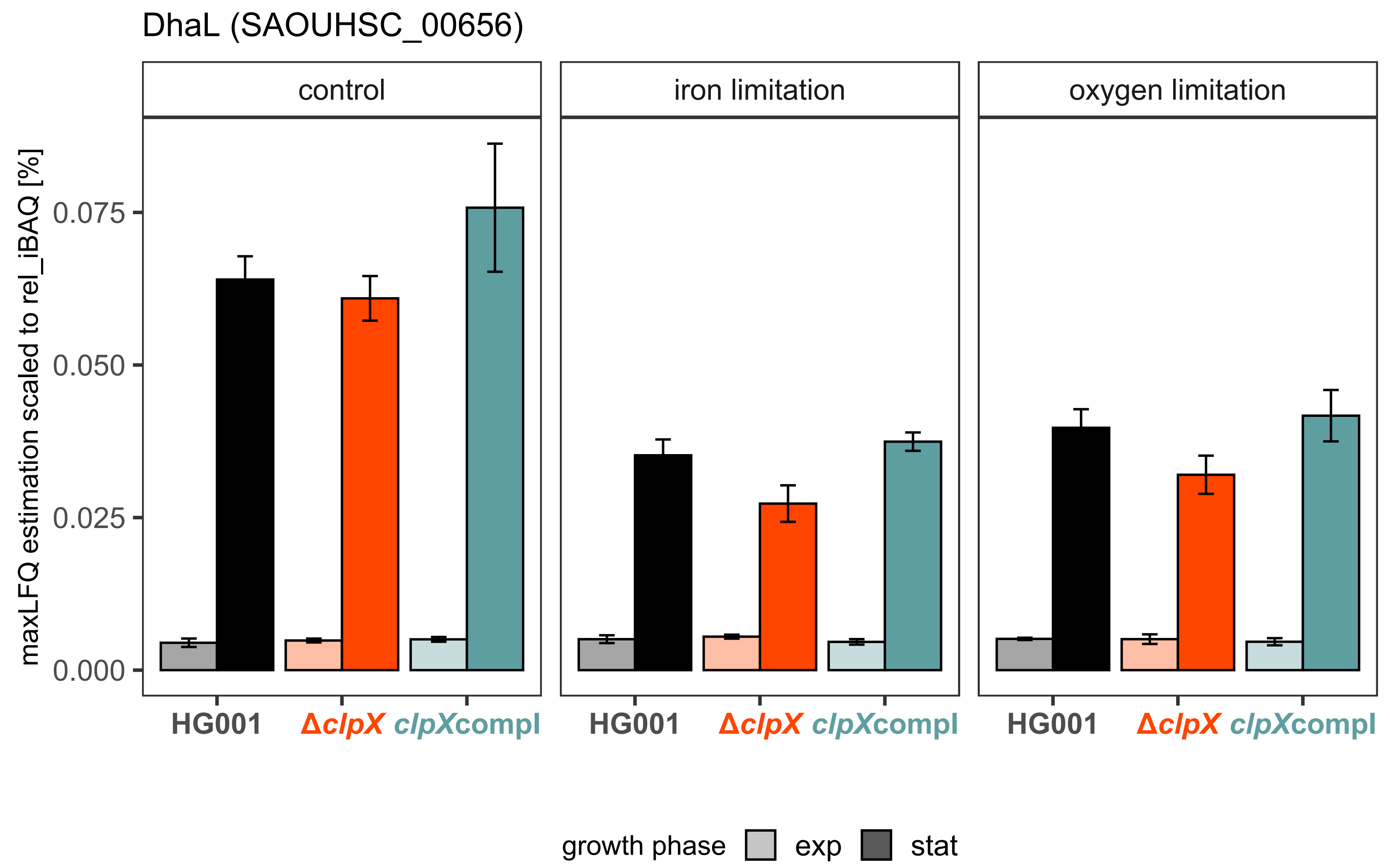

DhaL (SAOUHSC_00656)
control
iron limitation
oxygen limitation
maxLFQ estimation scaled to rel_iBAQ [%]
0.075
0.050
0.025
0.000
HG001
ΔclpX
clpXcompl
growth phase
exp
stat

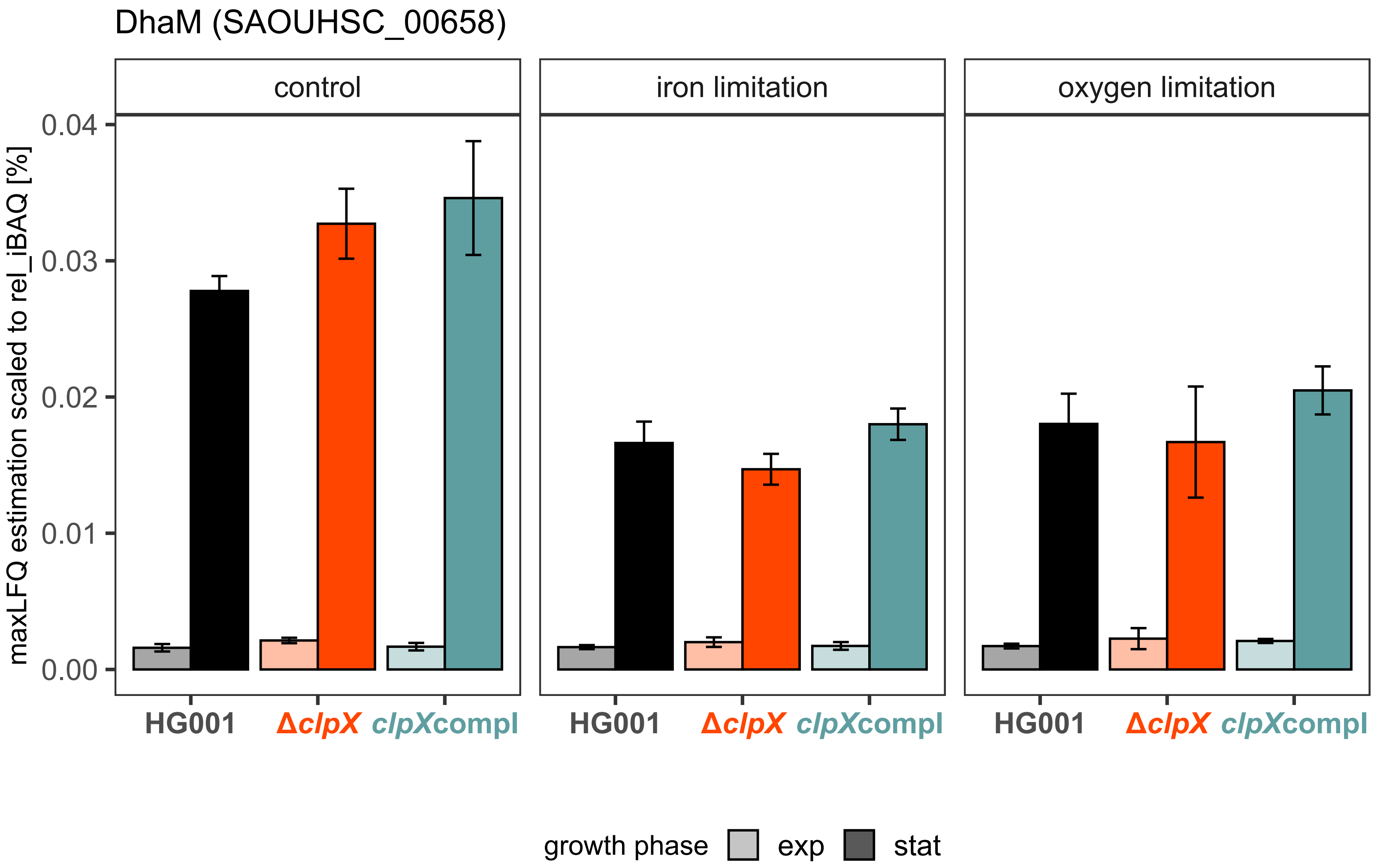

DhaM (SAOUHSC_00658)
control
iron limitation
oxygen limitation
maxLFQ estimation scaled to rel_iBAQ [%]
0.04
0.03
0.02
0.01
0.00
HG001
ΔclpX
clpXcompl
growth phase
exp
stat

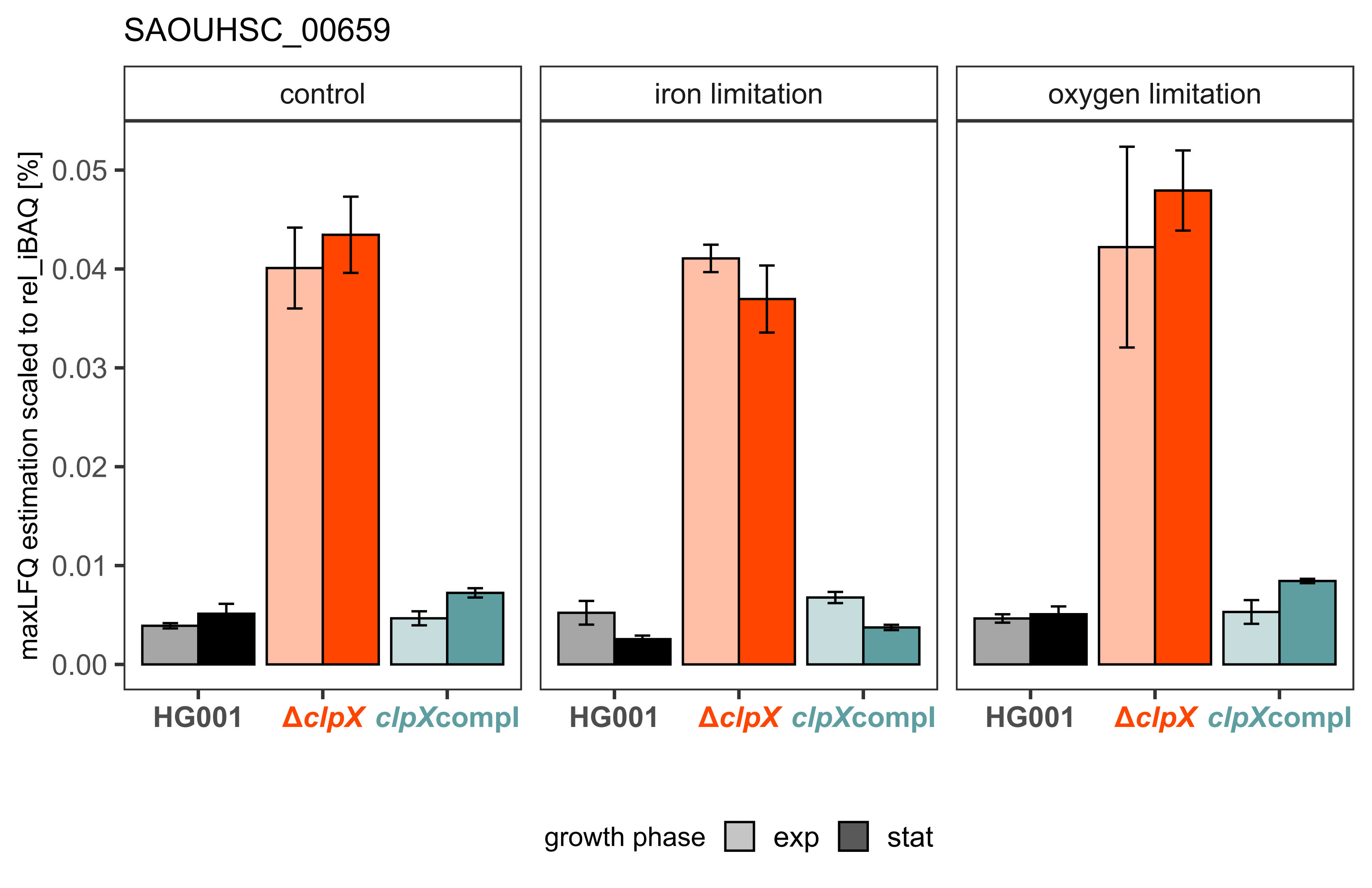
SAOUHSC_00659
control
iron limitation
oxygen limitation
maxLFQ estimation scaled to rel_iBAQ [%]
0.05
0.04
0.03
0.02
0.01
0.00
HG001
ΔclpX
clpXcompl
growth phase
exp
stat

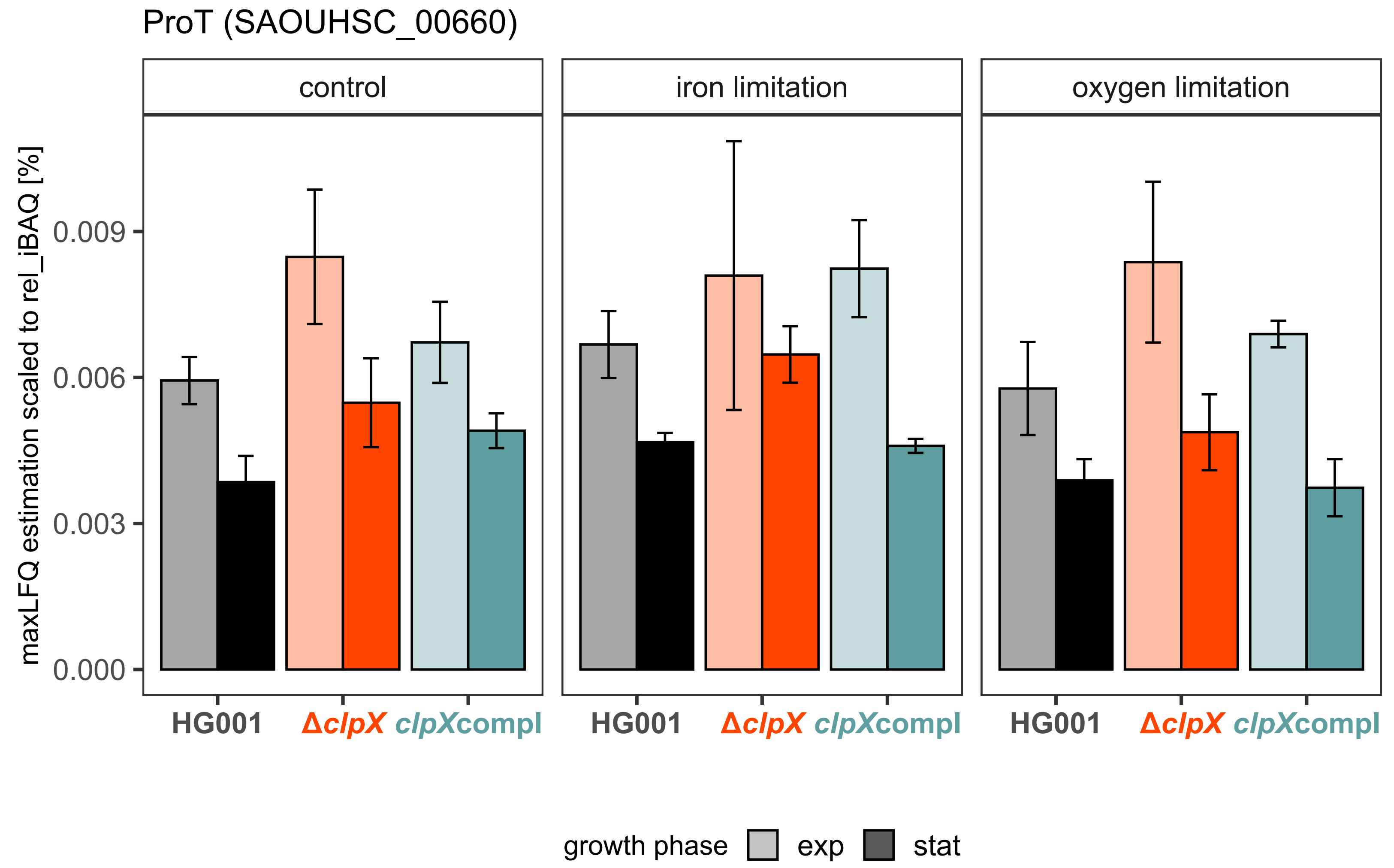
ProT (SAOUHSC_00660)
control
iron limitation
oxygen limitation
maxLFQ estimation scaled to rel_iBAQ [%]
0.009
0.006
0.003
0.000
HG001
ΔclpX
clpXcompl
growth phase
exp
stat

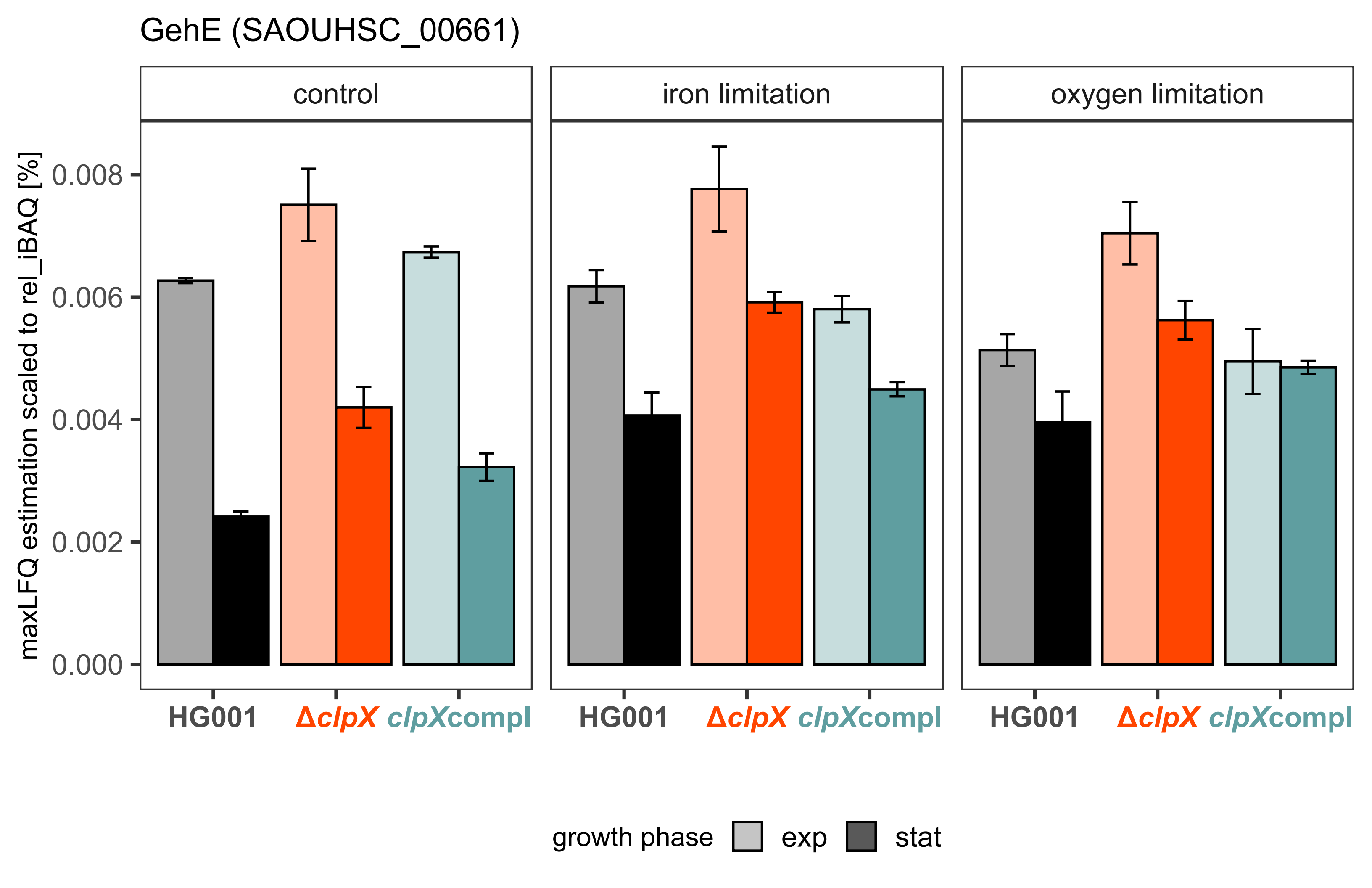

GehE (SAOUHSC_00661)
control
iron limitation
oxygen limitation
maxLFQ estimation scaled to rel_iBAQ [%]
0.008
0.006
0.004
0.002
0.000
HG001
ΔclpX
clpXcompl
growth phase
exp
stat

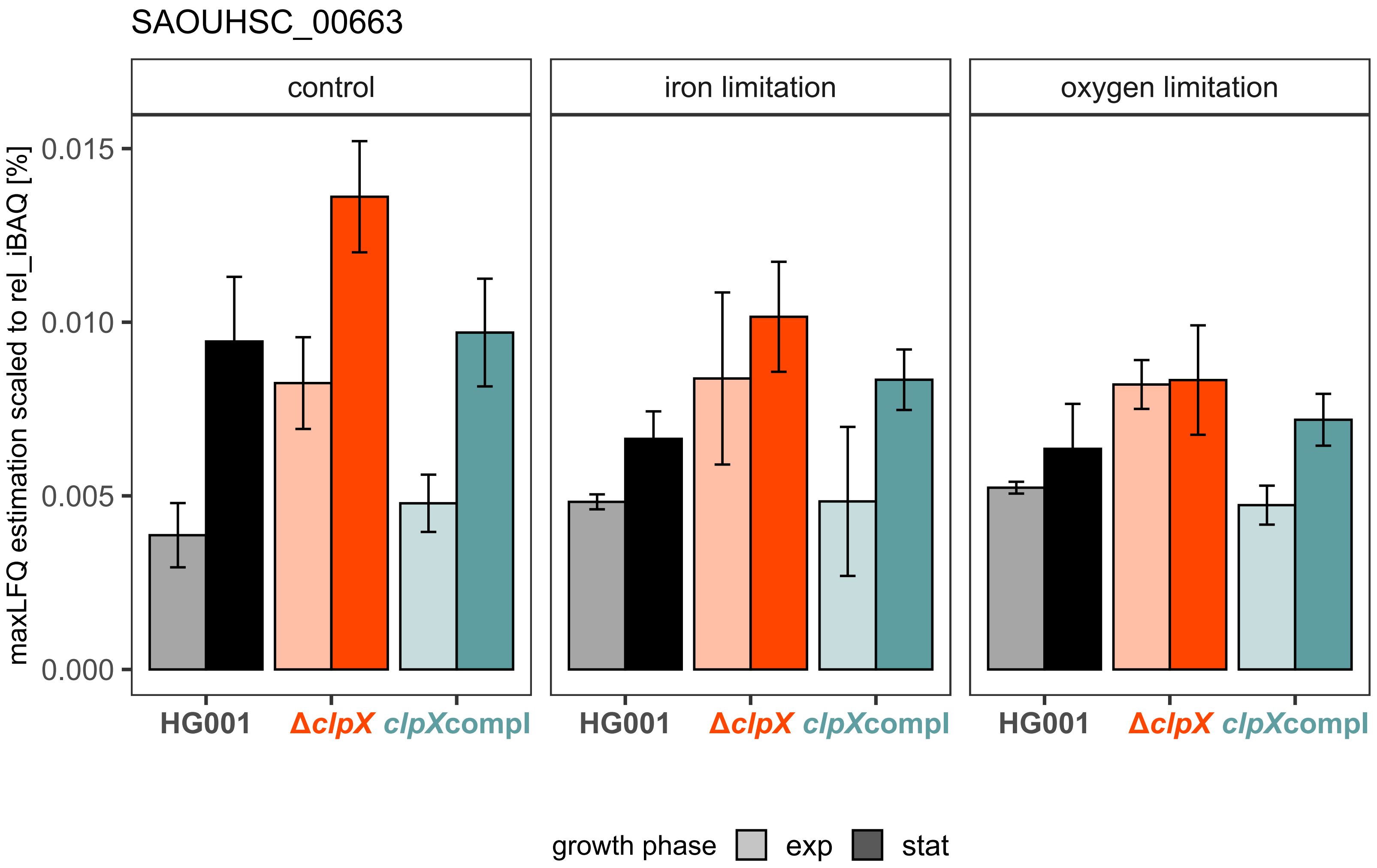
SAOUHSC_00663
control
iron limitation
oxygen limitation
maxLFQ estimation scaled to rel_iBAQ [%]
0.015
0.010
0.005
0.000
HG001
ΔclpX
clpXcompl
growth phase
exp
stat

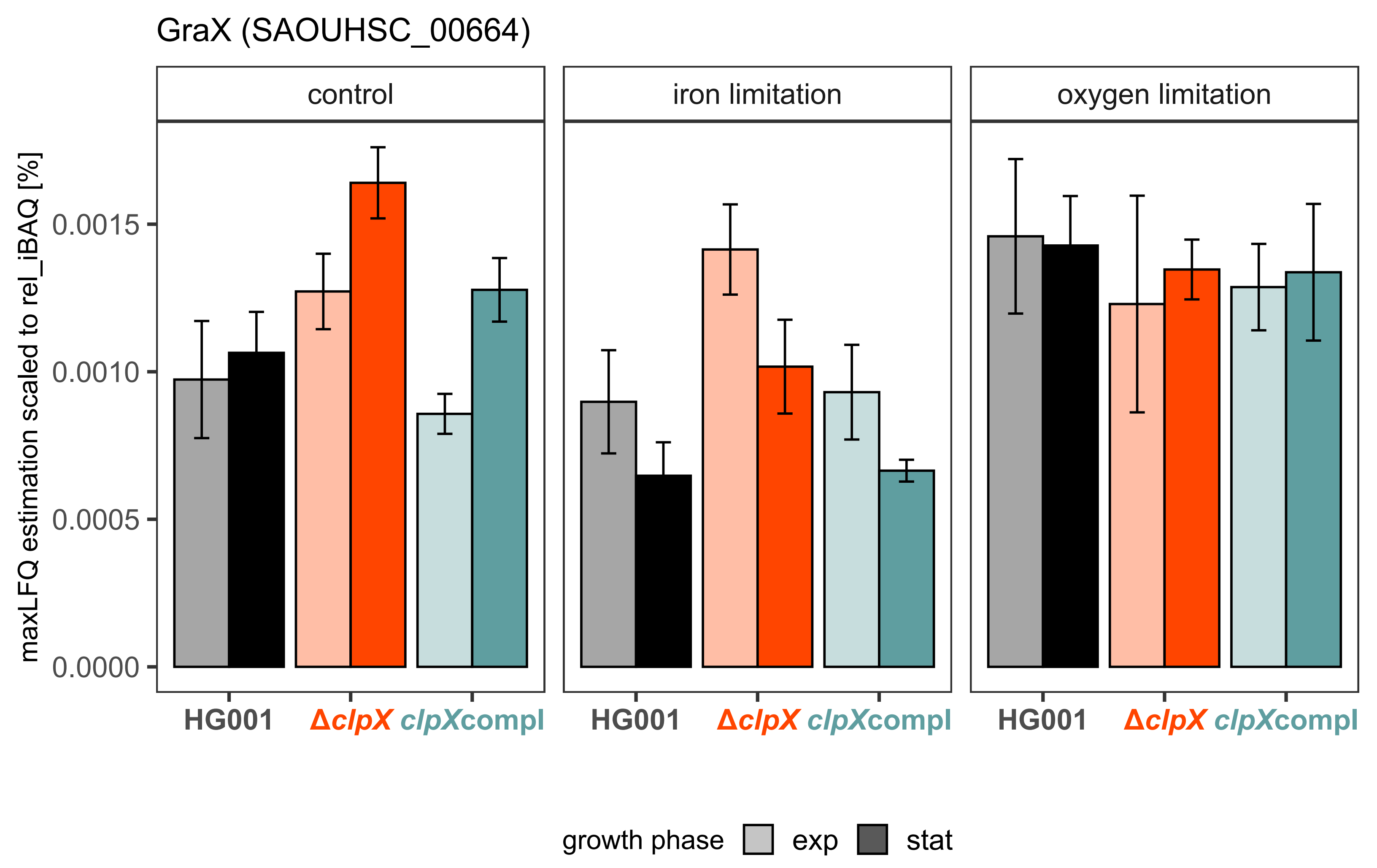

GraX (SAOUHSC_00664)
control
iron limitation
oxygen limitation
maxLFQ estimation scaled to rel_iBAQ [%]
0.0015
0.0010
0.0005
0.0000
HG001
ΔclpX
clpXcompl
growth phase
exp
stat

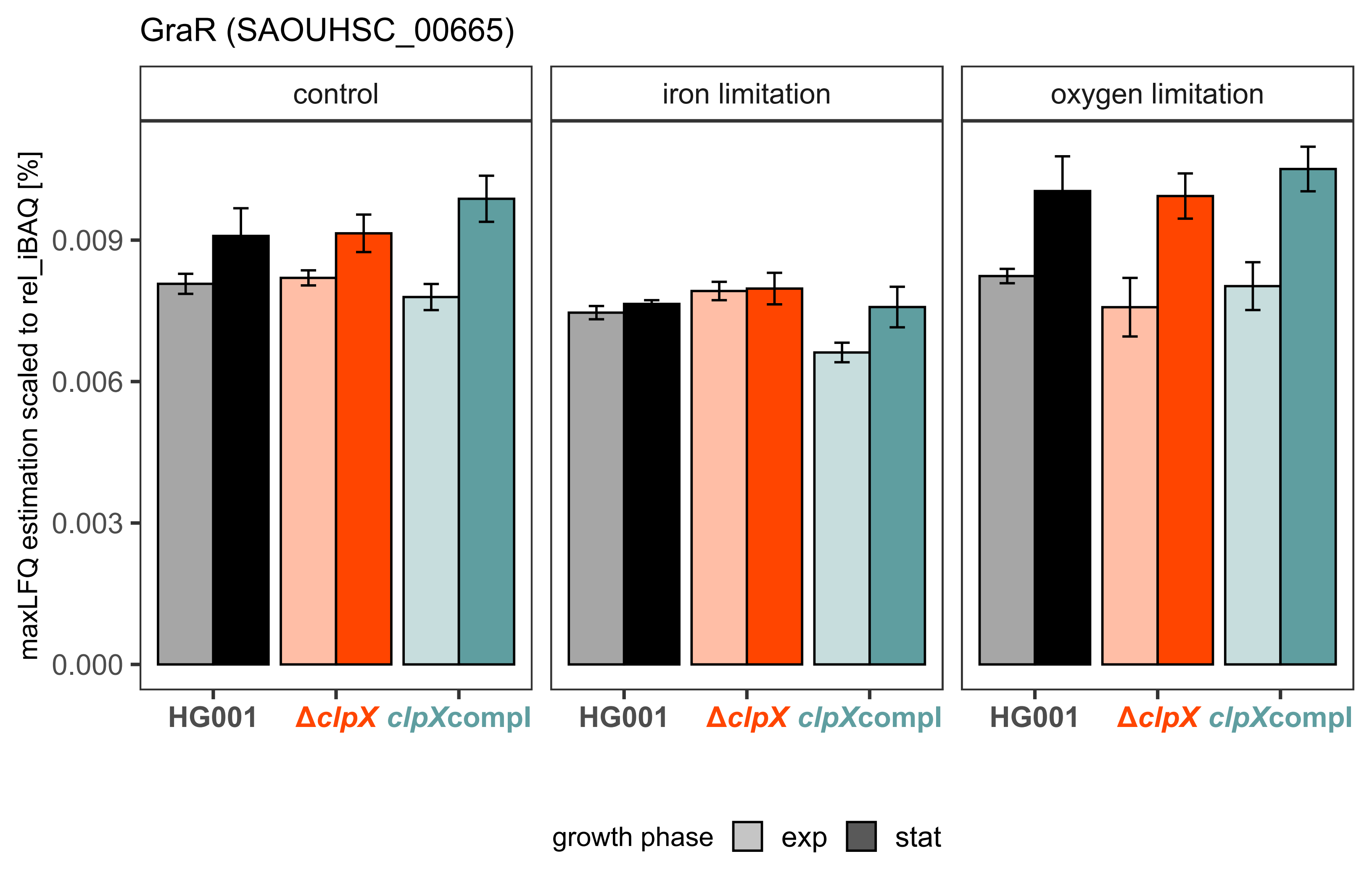
GraR (SAOUHSC_00665)
control
iron limitation
oxygen limitation
maxLFQ estimation scaled to rel_iBAQ [%]
0.009
0.006
0.003
0.000
HG001
ΔclpX
clpXcompl
growth phase
exp
stat

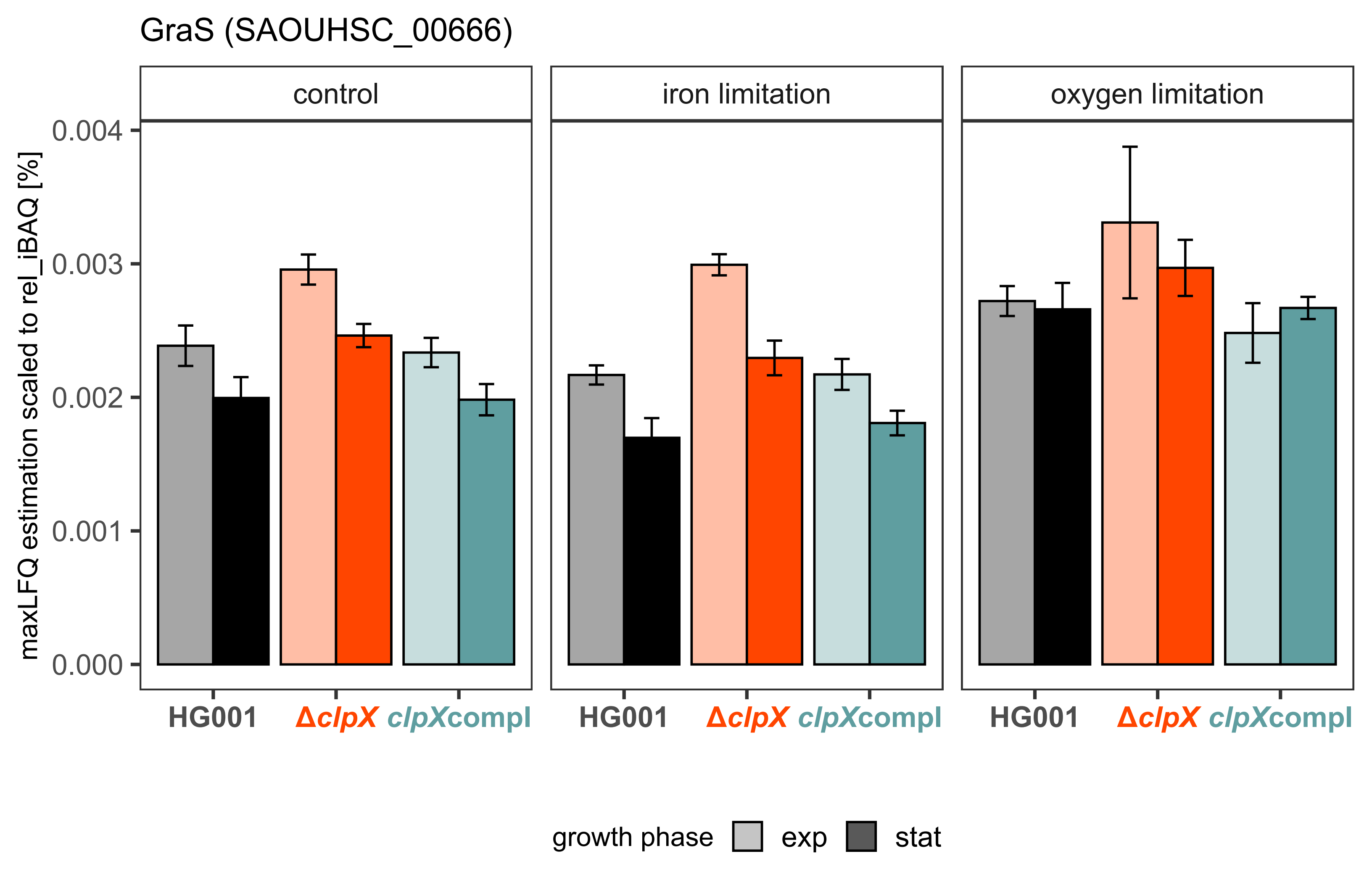
GraS (SAOUHSC_00666)
control
iron limitation
oxygen limitation
maxLFQ estimation scaled to rel_iBAQ [%]
0.004
0.003
0.002
0.001
0.000
HG001
ΔclpX
clpXcompl
growth phase
exp
stat

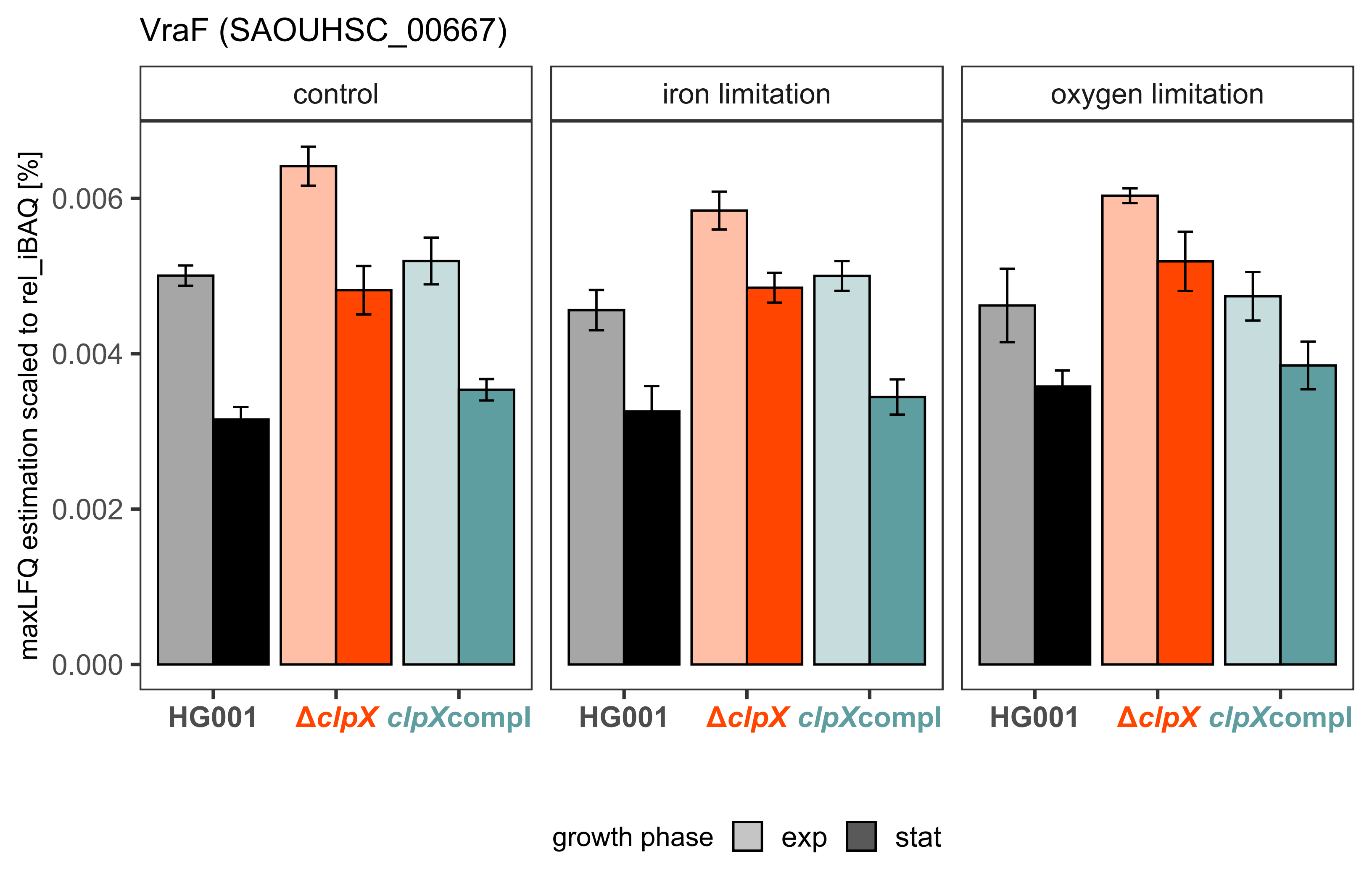

VraF (SAOUHSC_00667)
control
iron limitation
oxygen limitation
maxLFQ estimation scaled to rel_iBAQ [%]
0.000
0.002
0.004
0.006
HG001
ΔclpX
clpXcompl
growth phase
exp
stat

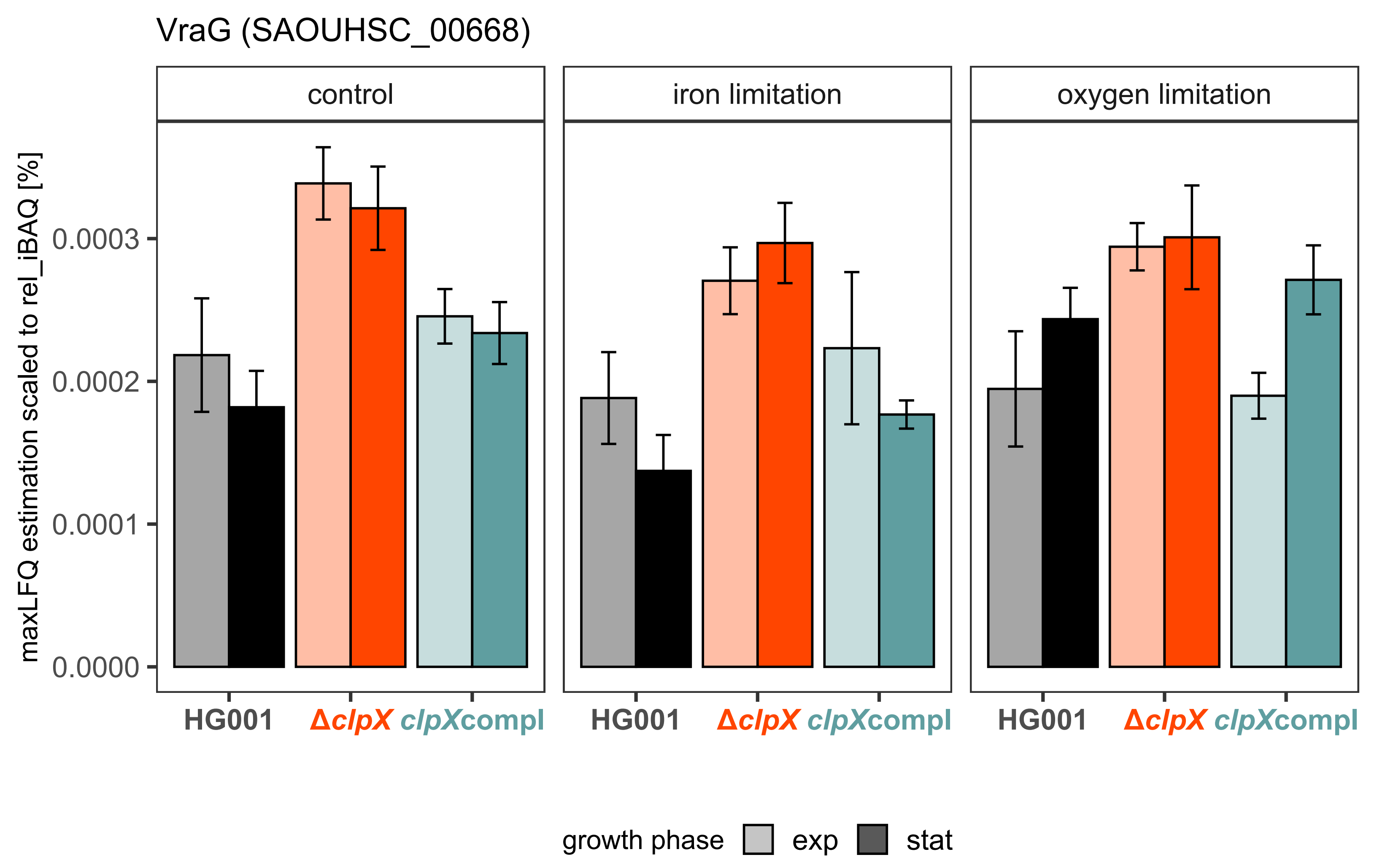

VraG (SAOUHSC_00668)
control
iron limitation
oxygen limitation
maxLFQ estimation scaled to rel_iBAQ [%]
0.0003
0.0002
0.0001
0.0000
HG001
ΔclpX
clpXcompl
growth phase
exp
stat

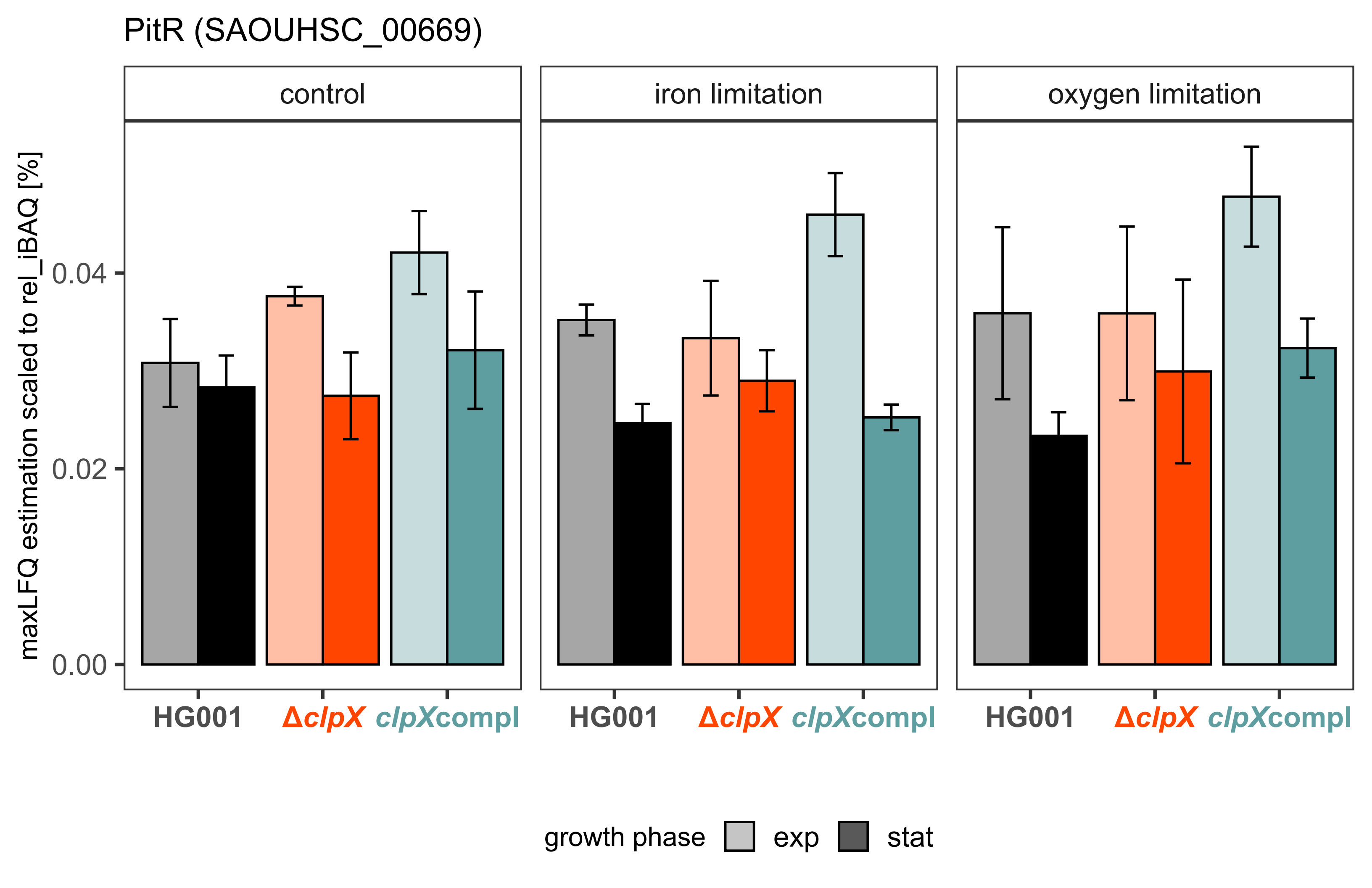
PitR (SAOUHSC_00669)
control
iron limitation
oxygen limitation
maxLFQ estimation scaled to rel_iBAQ [%]
0.04
0.02
0.00
HG001
ΔclpX
clpXcompl
growth phase
exp
stat

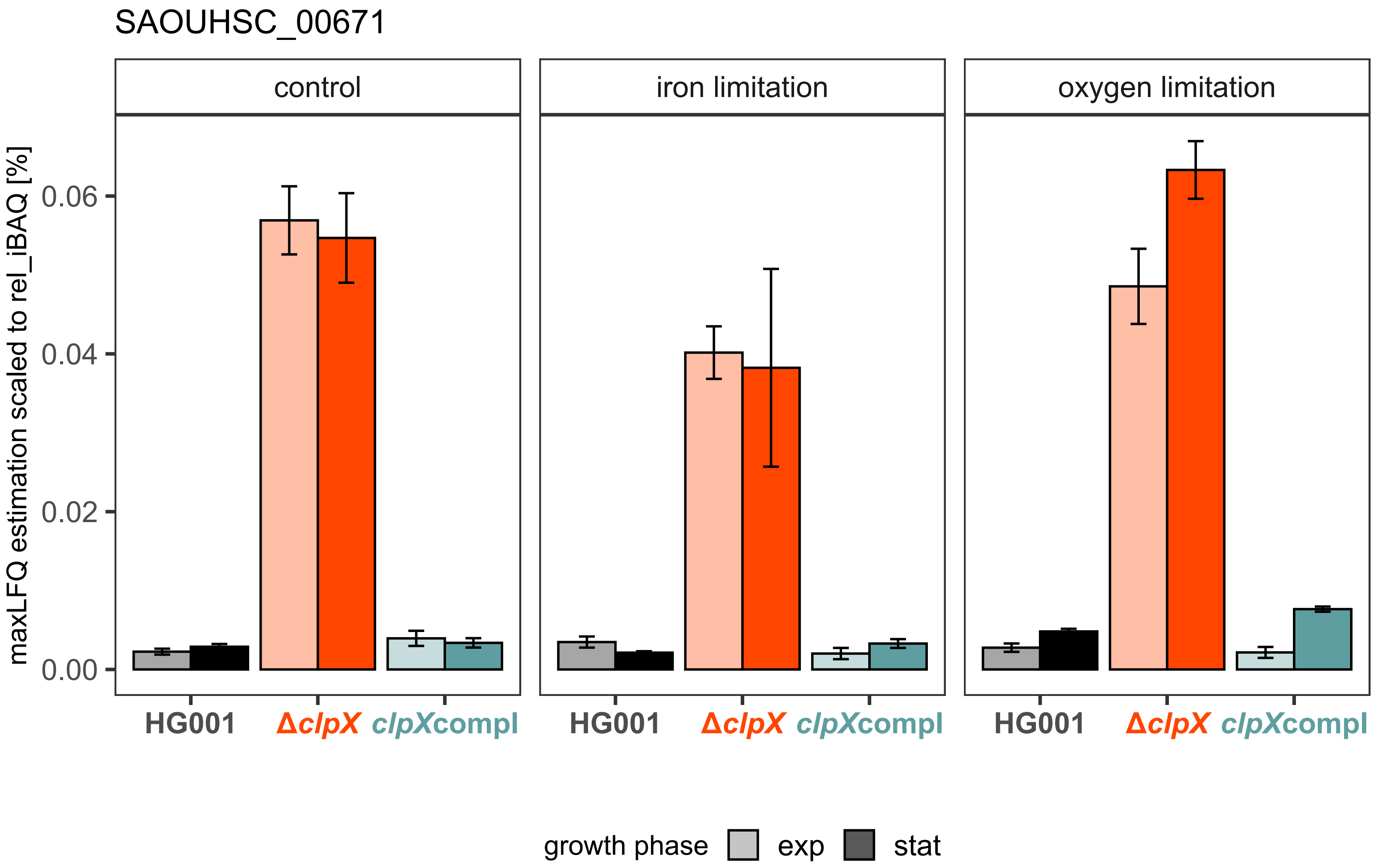
SAOUHSC_00671
control
iron limitation
oxygen limitation
maxLFQ estimation scaled to rel_iBAQ [%]
0.06
0.04
0.02
0.00
HG001
ΔclpX
clpXcompl
growth phase
exp
stat

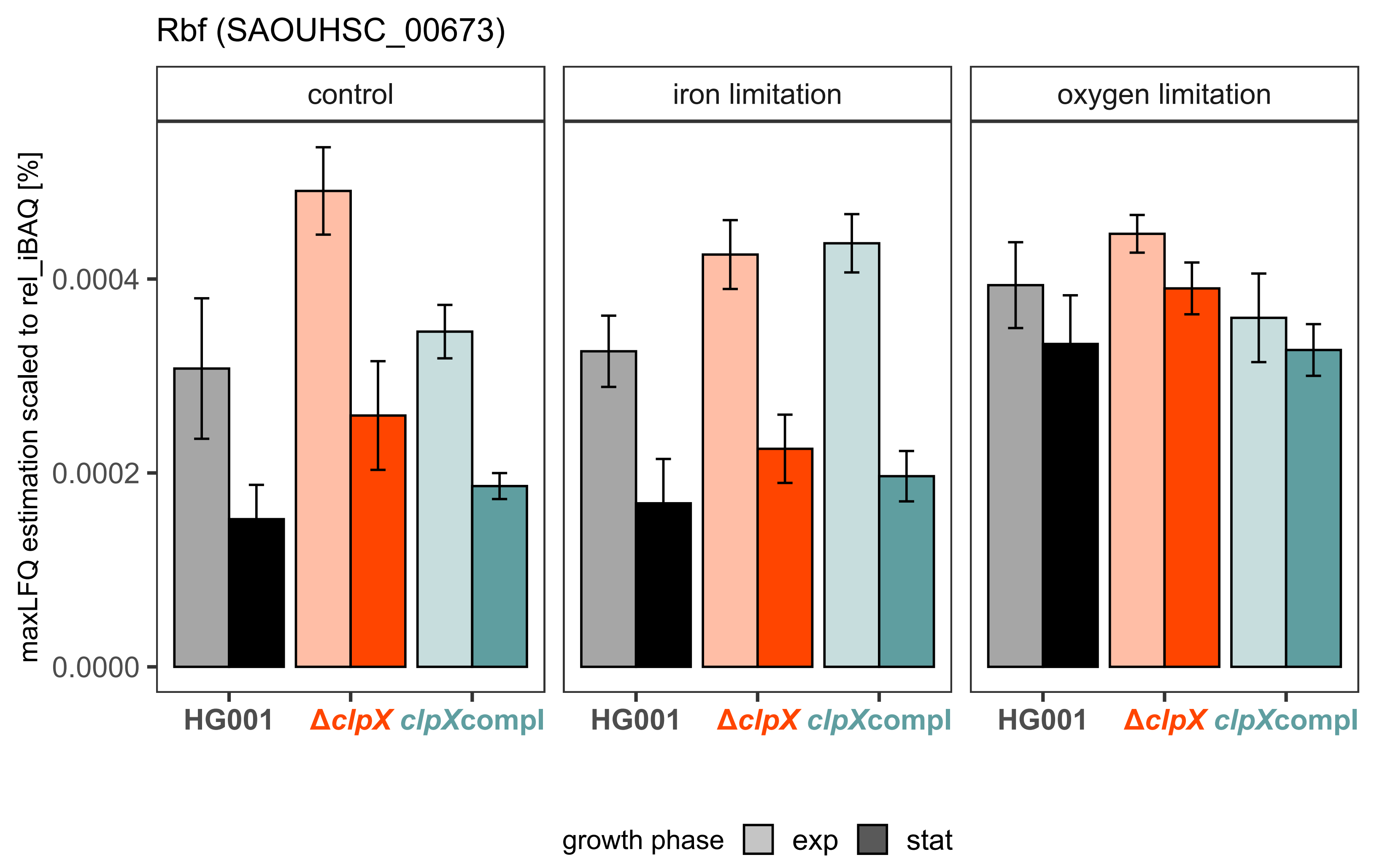

Rbf (SAOUHSC_00673)
control
iron limitation
oxygen limitation
maxLFQ estimation scaled to rel_iBAQ [%]
0.0004
0.0002
0.0000
HG001
ΔclpX
clpXcompl
growth phase
exp
stat

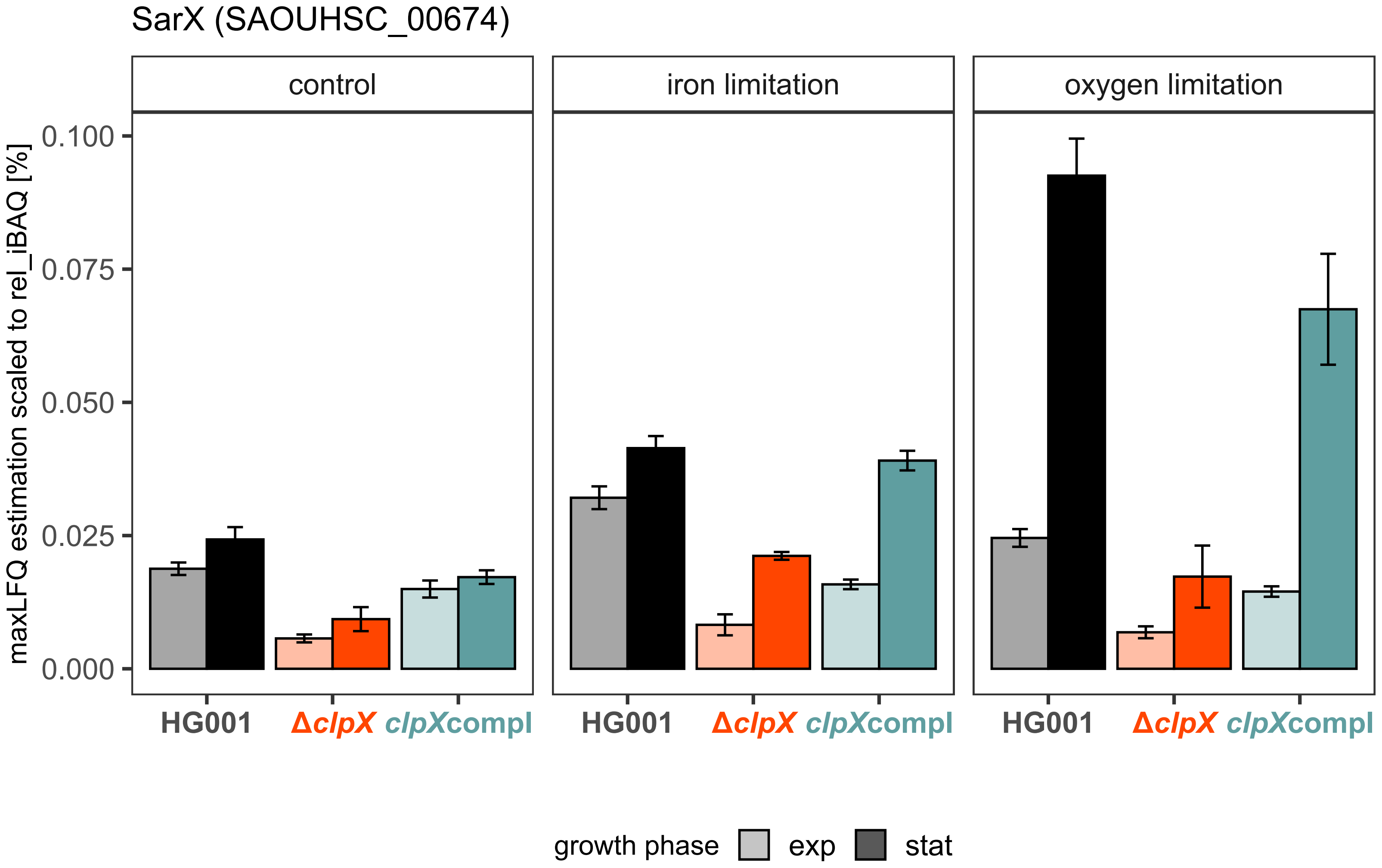
SarX (SAOUHSC_00674)
control
iron limitation
oxygen limitation
maxLFQ estimation scaled to rel_iBAQ [%]
0.100
0.075
0.050
0.025
0.000
HG001
ΔclpX
clpXcompl
growth phase
exp
stat

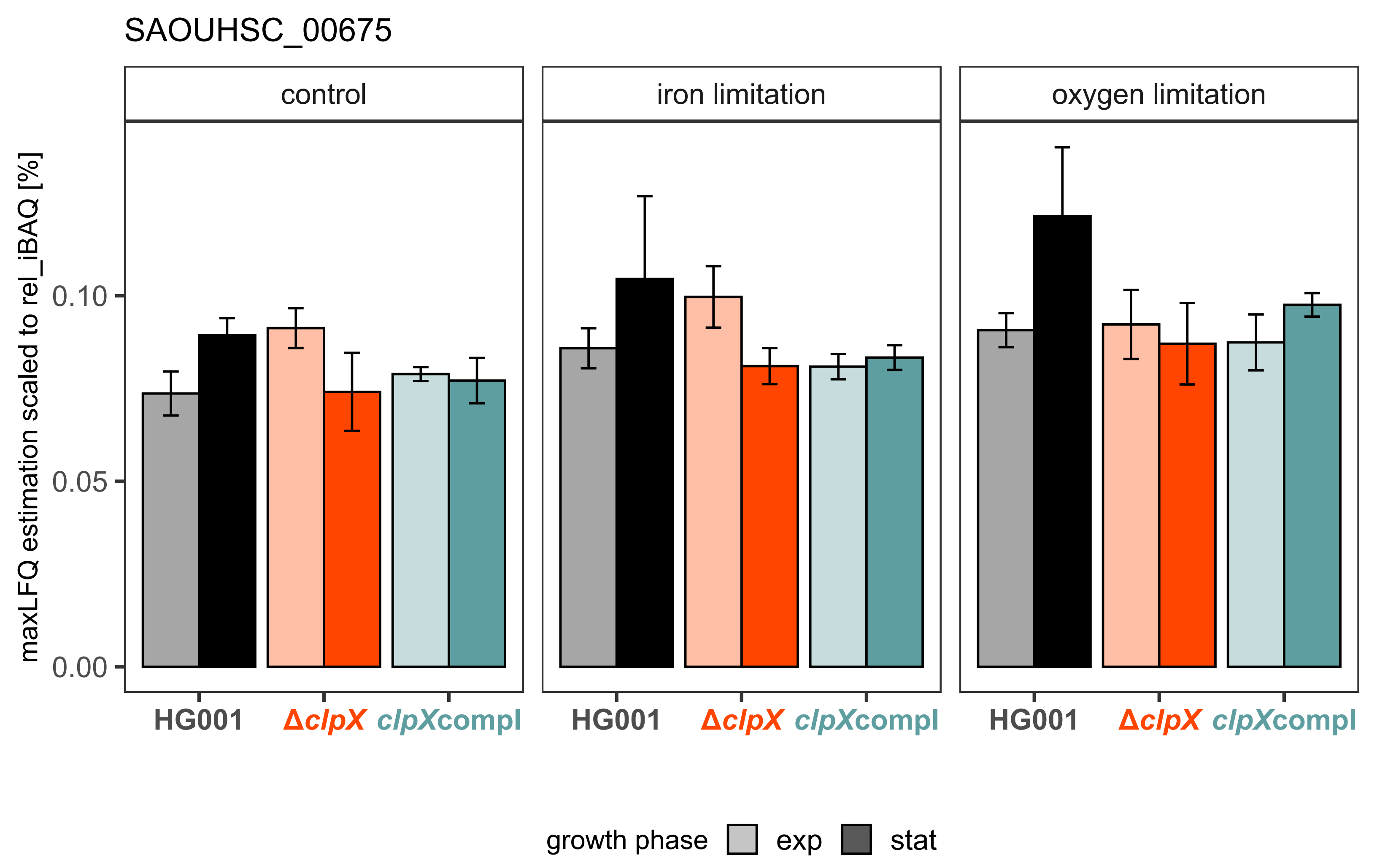

SAOUHSC_00675
control
iron limitation
oxygen limitation
maxLFQ estimation scaled to rel_iBAQ [%]
0.00
0.05
0.10
HG001
ΔclpX
clpXcompl
growth phase
exp
stat

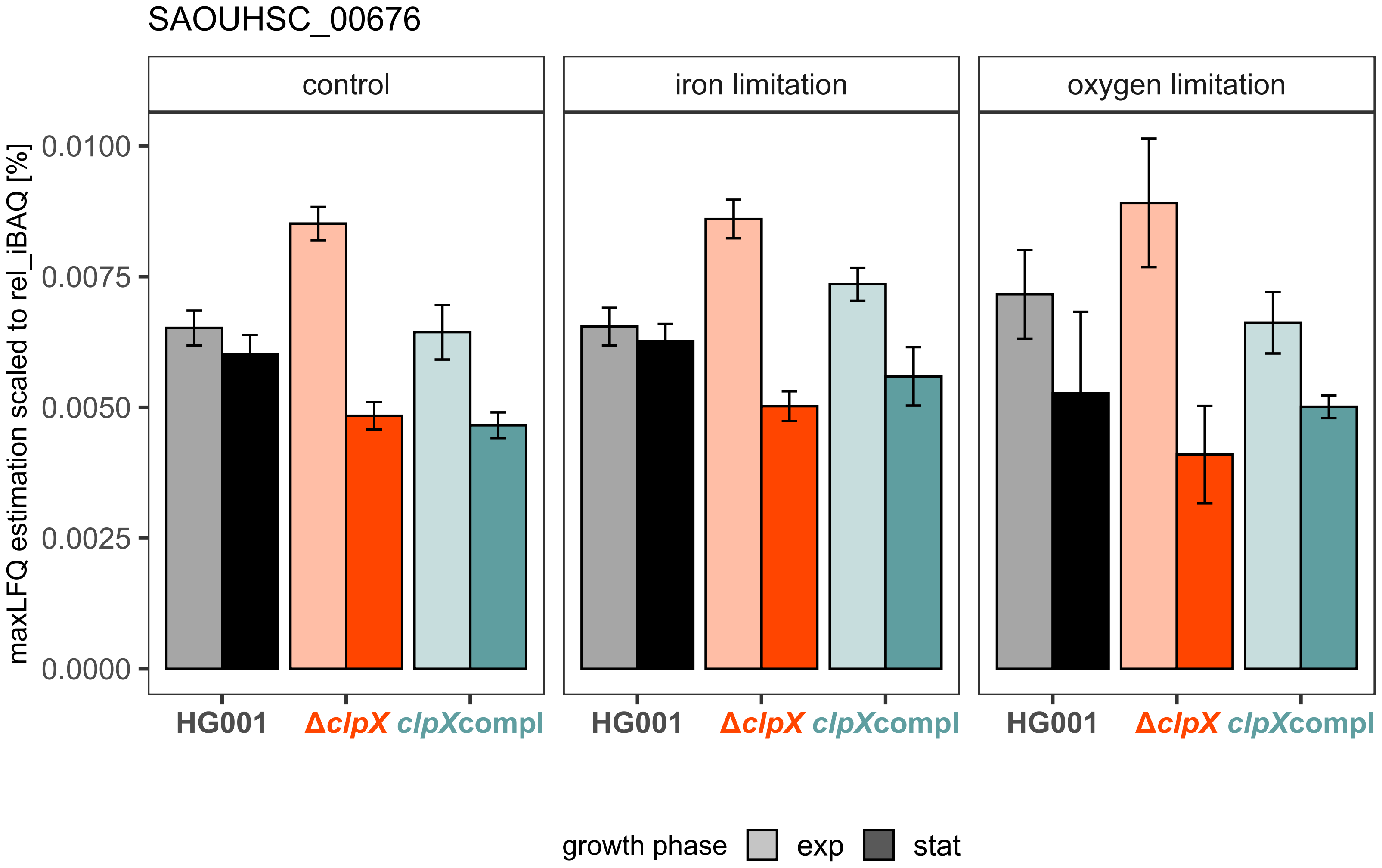
SAOUHSC_00676
control
iron limitation
oxygen limitation
maxLFQ estimation scaled to rel_iBAQ [%]
0.0100
0.0075
0.0050
0.0025
0.0000
HG001
ΔclpX
clpXcompl
HG001
ΔclpX
clpXcompl
HG001
ΔclpX
clpXcompl
growth phase
exp
stat

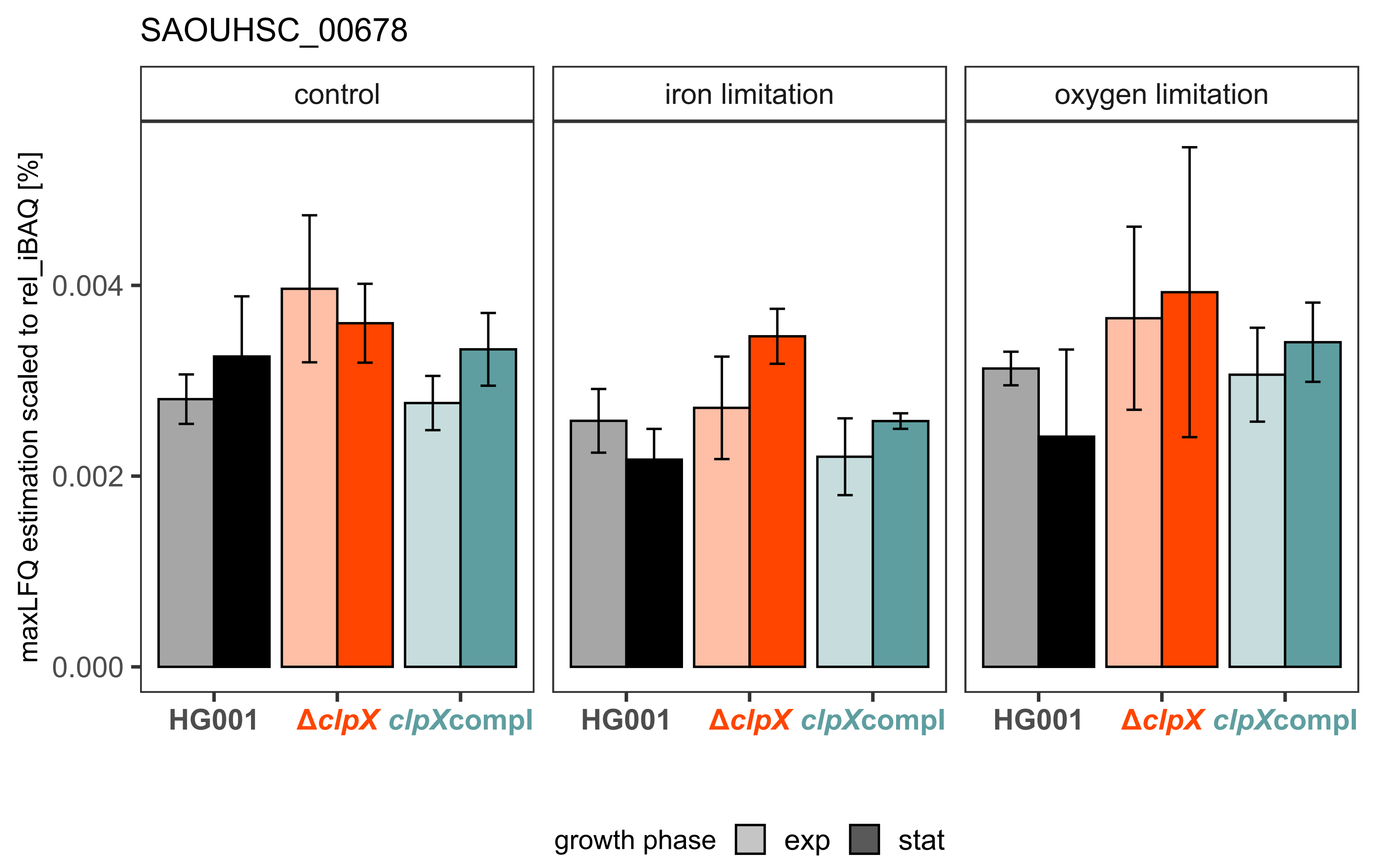

SAOUHSC_00678
control
iron limitation
oxygen limitation
maxLFQ estimation scaled to rel_iBAQ [%]
0.004
0.002
0.000
HG001
ΔclpX
clpXcompl
growth phase
exp
stat

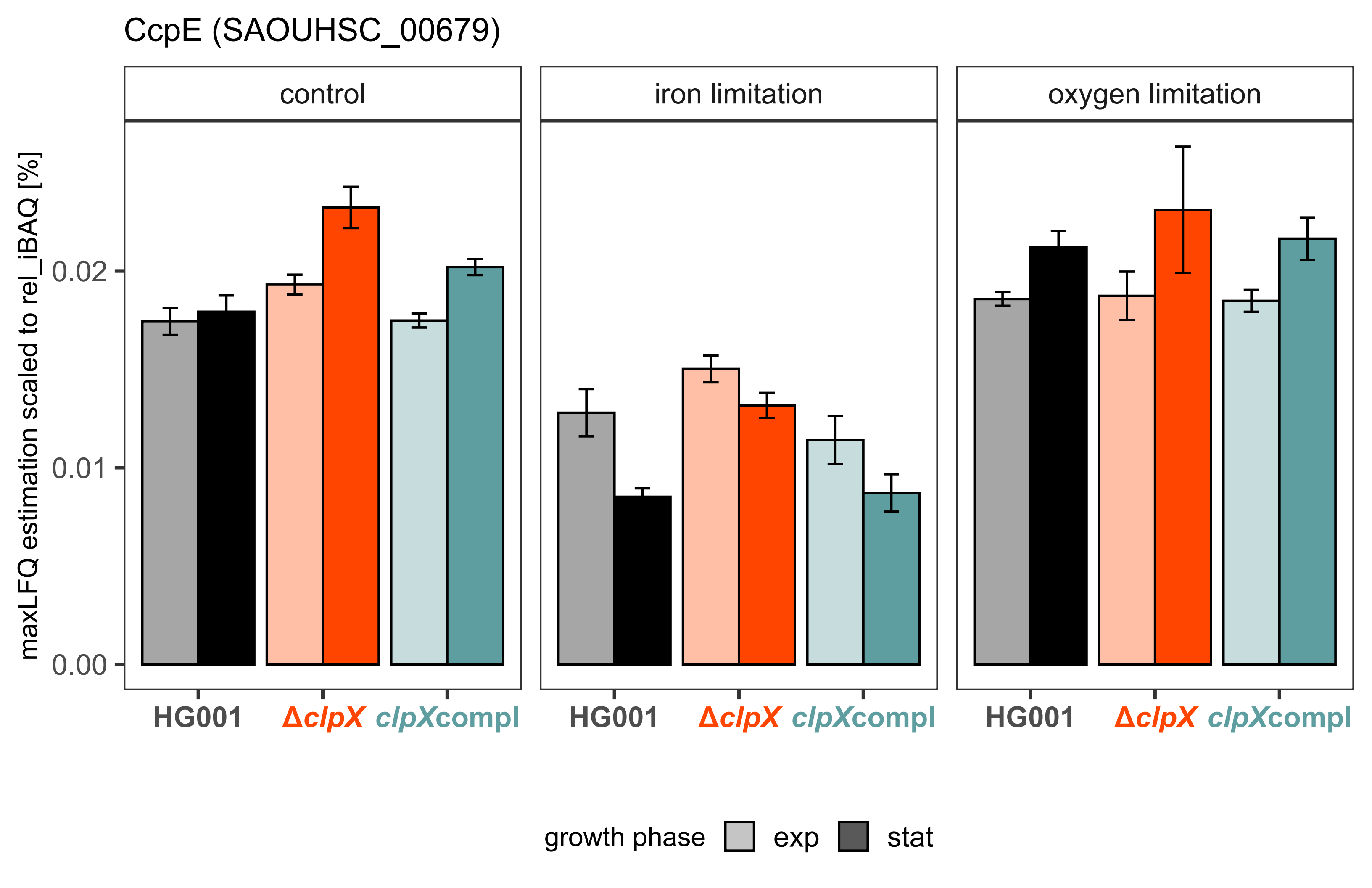

CcpE (SAOUHSC_00679)
control
iron limitation
oxygen limitation
maxLFQ estimation scaled to rel_iBAQ [%]
0.00
0.01
0.02
HG001
ΔclpX
clpXcompl
growth phase
exp
stat

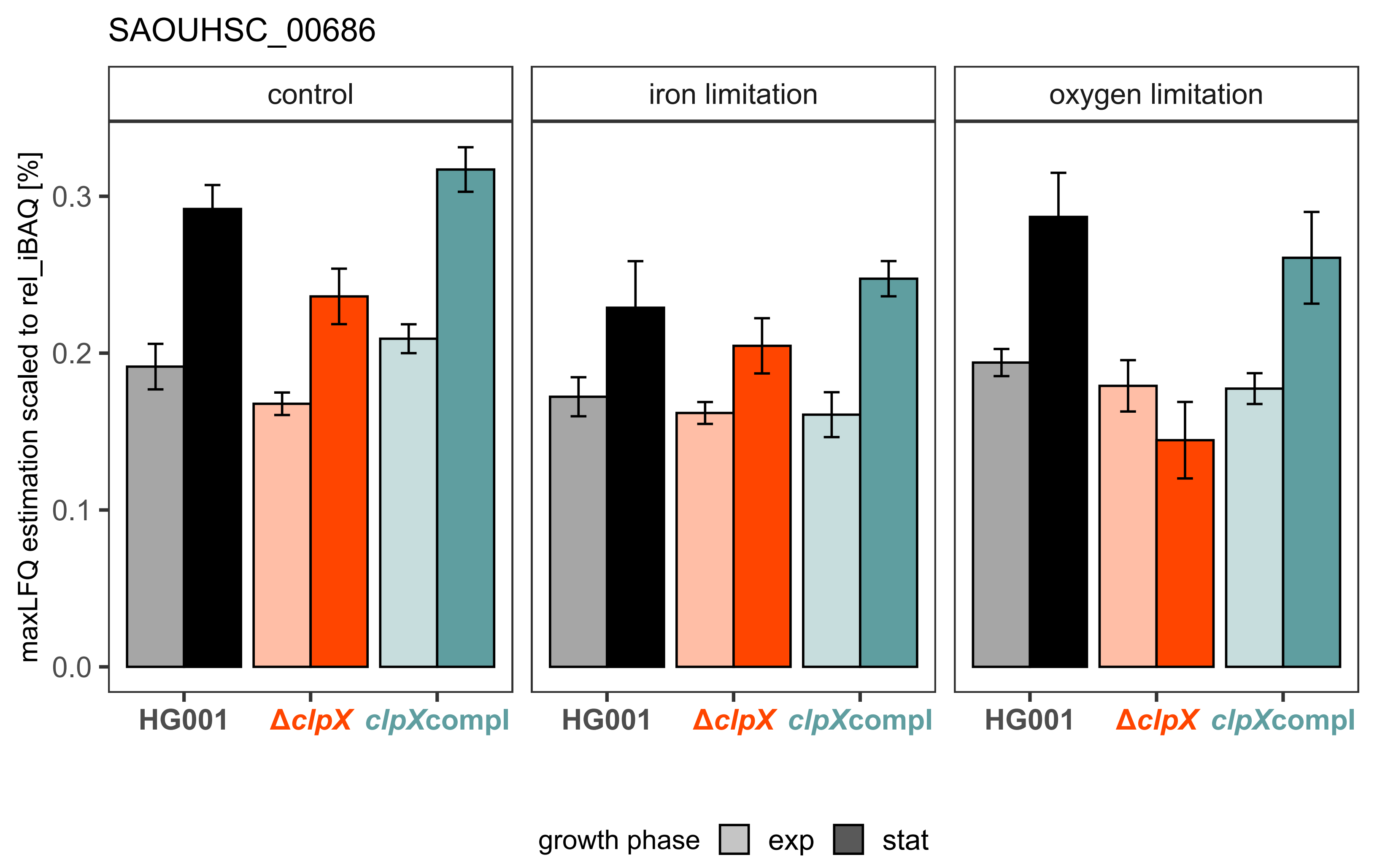

SAOUHSC_00686
control
iron limitation
oxygen limitation
maxLFQ estimation scaled to rel_iBAQ [%]
0.3
0.2
0.1
0.0
HG001
ΔclpX
clpXcompl
growth phase
exp
stat

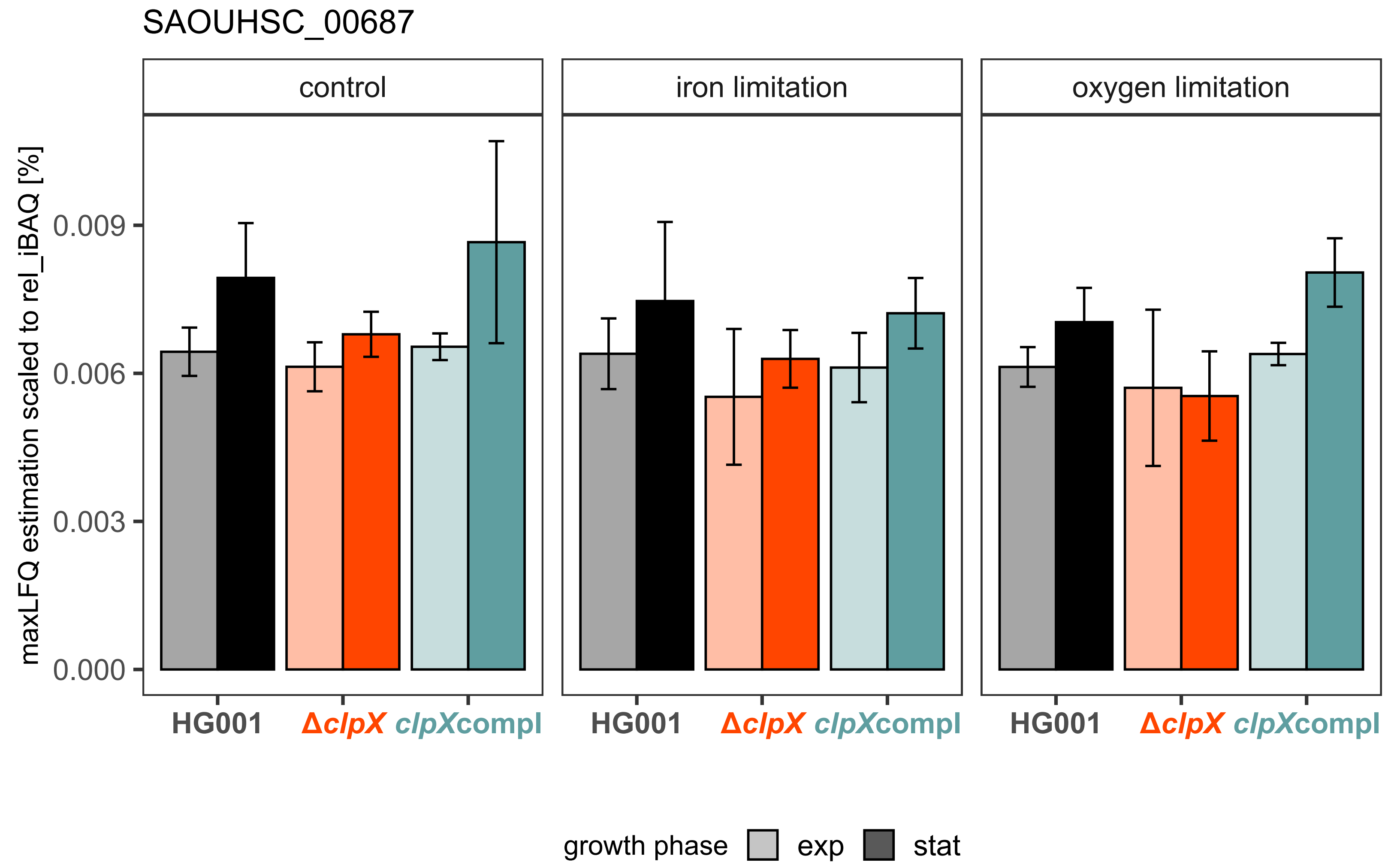
SAOUHSC_00687
control
iron limitation
oxygen limitation
maxLFQ estimation scaled to rel_iBAQ [%]
0.009
0.006
0.003
0.000
HG001
ΔclpX
clpXcompl
growth phase
exp
stat

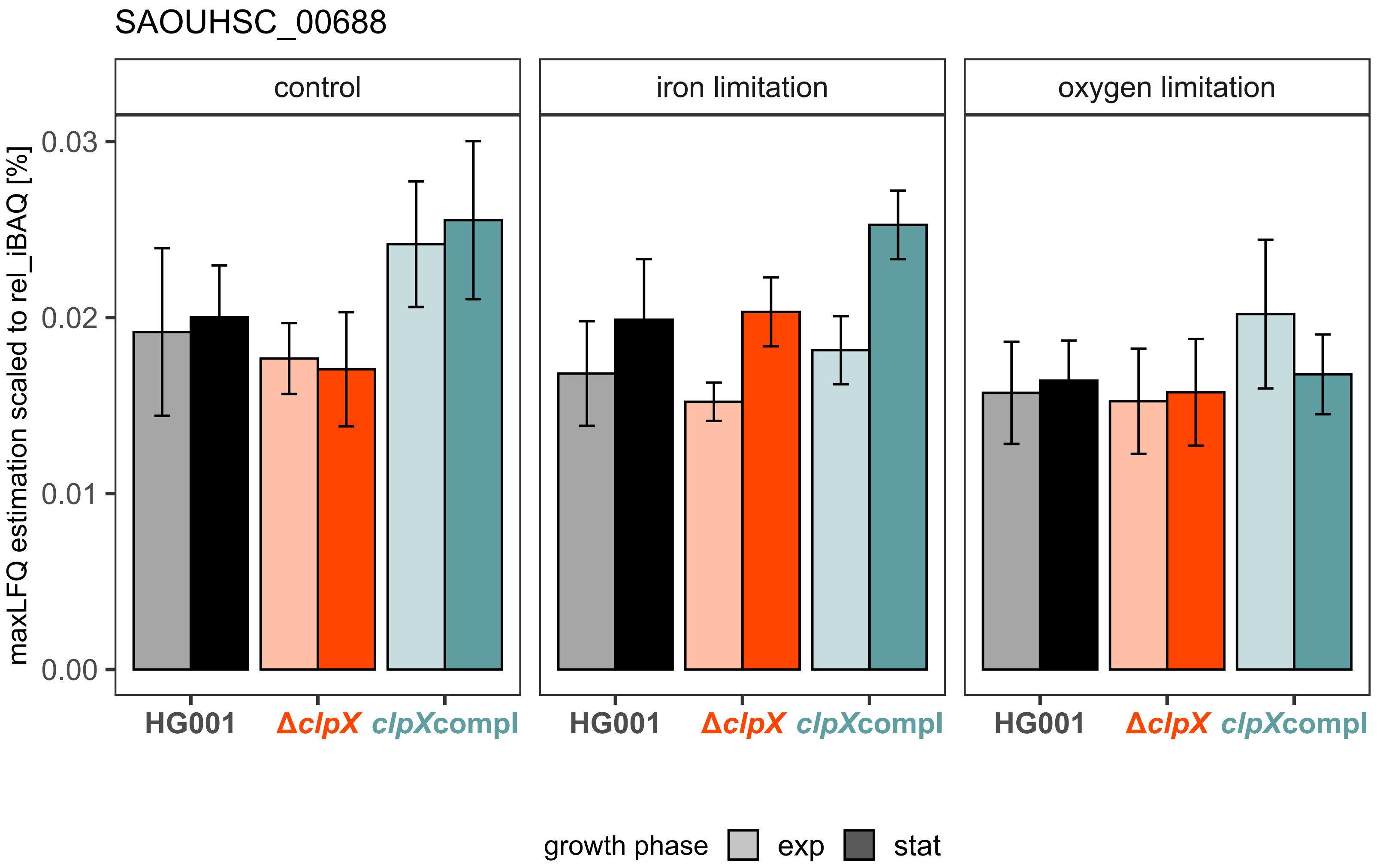

SAOUHSC_00688
control
iron limitation
oxygen limitation
maxLFQ estimation scaled to rel_iBAQ [%]
0.03
0.02
0.01
0.00
HG001
ΔclpX
clpXcompl
HG001
ΔclpX
clpXcompl
HG001
ΔclpX
clpXcompl
growth phase
exp
stat

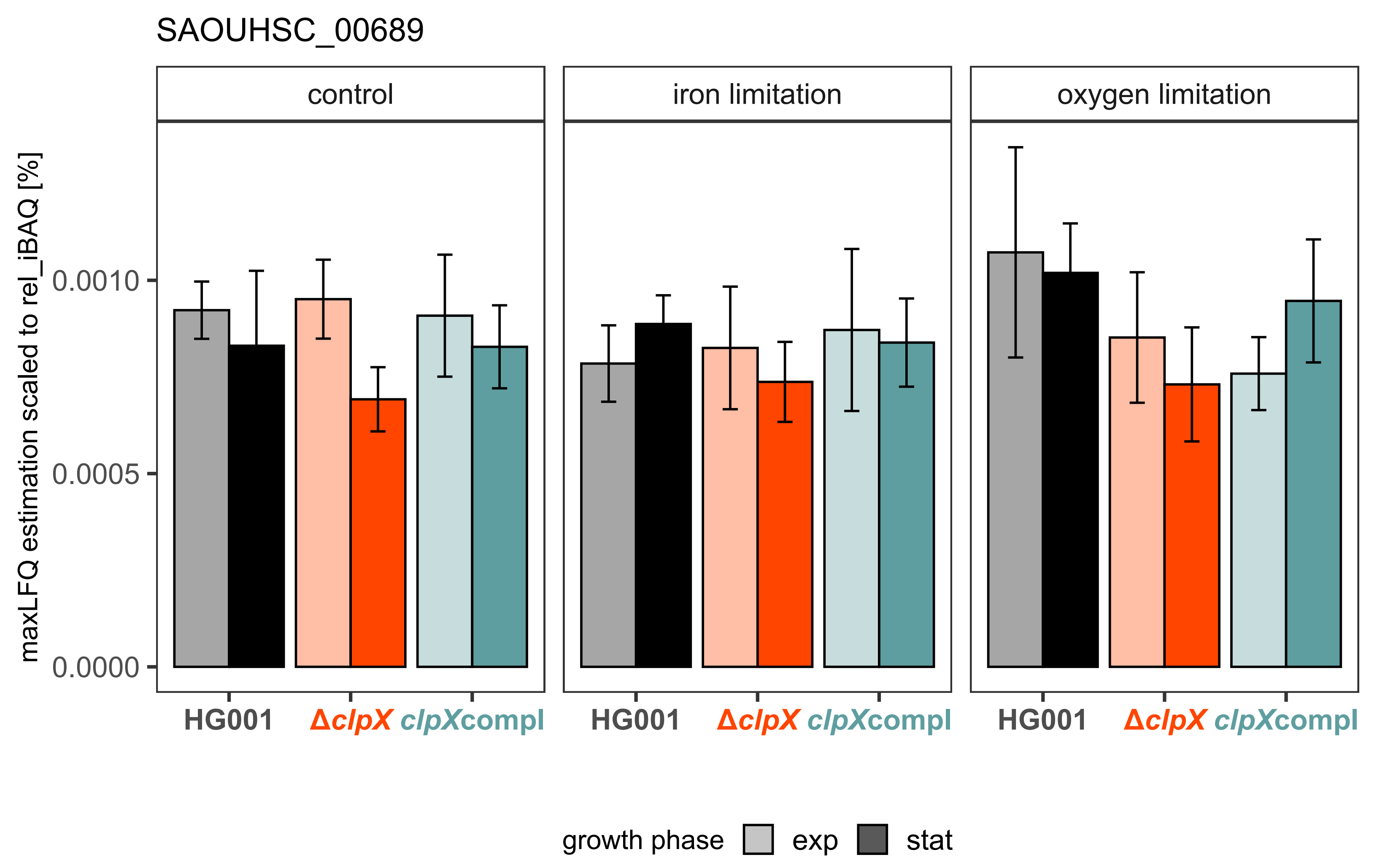

SAOUHSC_00689
control
iron limitation
oxygen limitation
maxLFQ estimation scaled to rel_iBAQ [%]
0.0010
0.0005
0.0000
HG001
ΔclpX
clpXcompl
growth phase
exp
stat

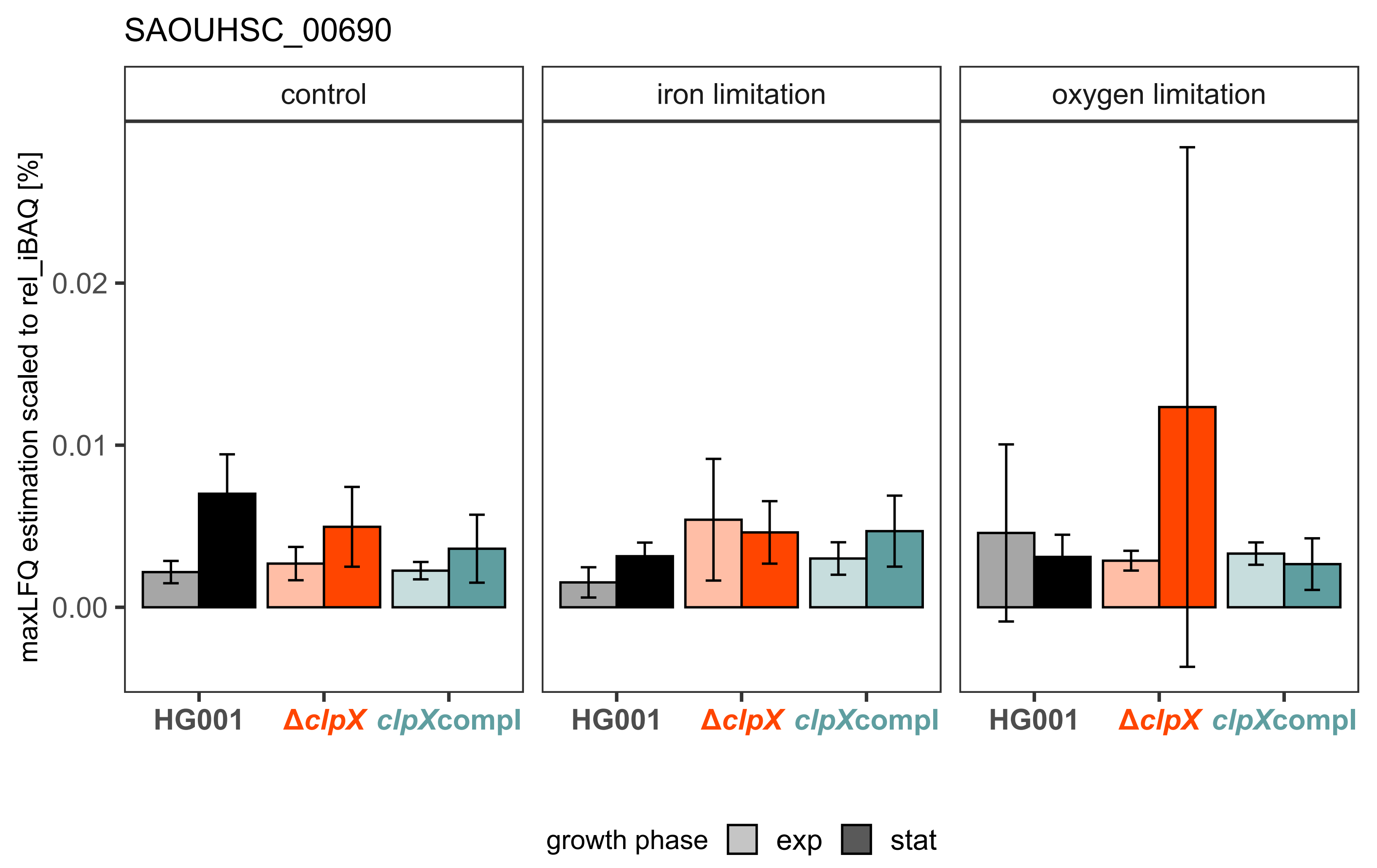
SAOUHSC_00690
control
iron limitation
oxygen limitation
maxLFQ estimation scaled to rel_iBAQ [%]
0.02
0.01
0.00
HG001
ΔclpX
clpXcompl
growth phase
exp
stat

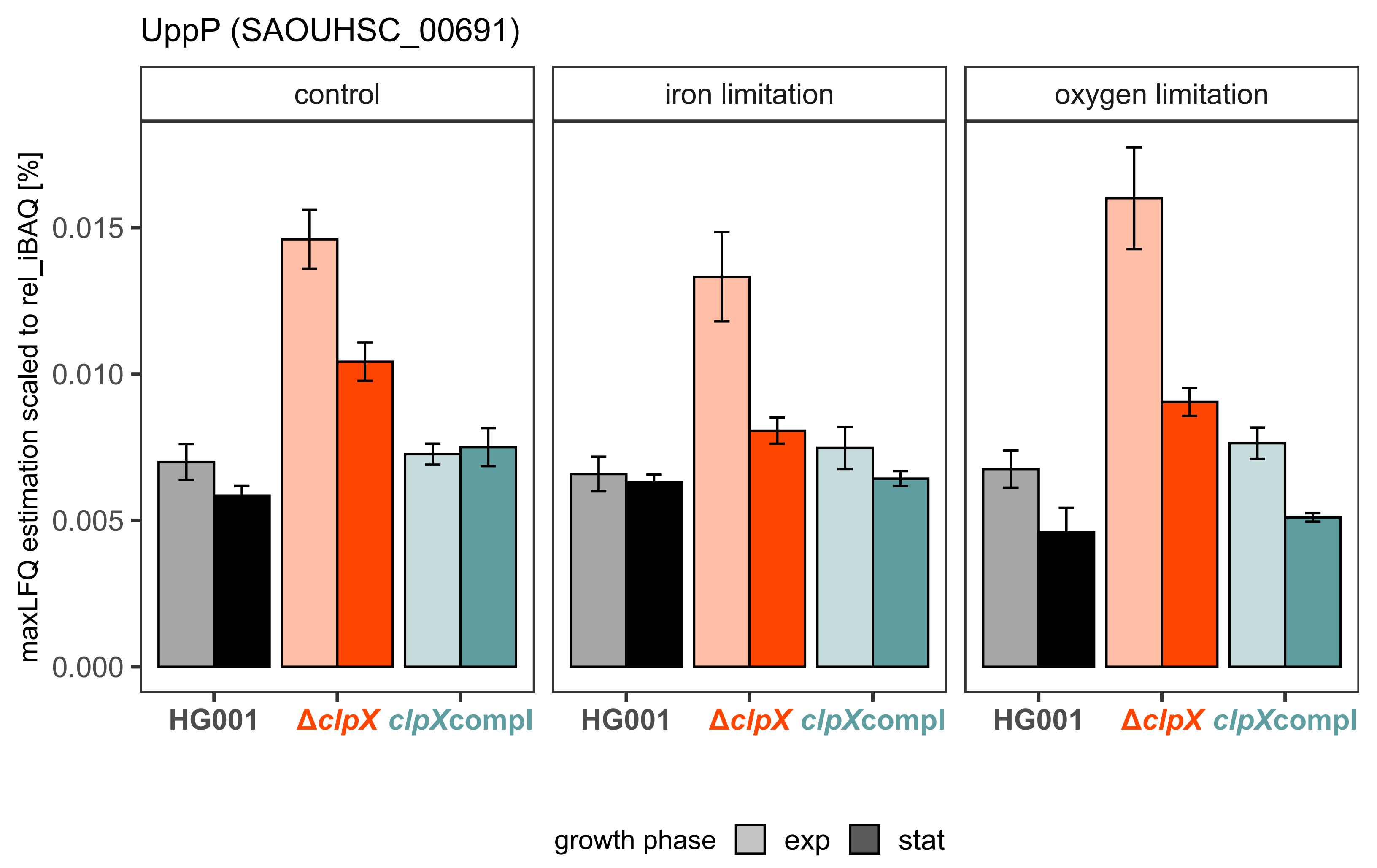
UppP (SAOUHSC_00691)
control
iron limitation
oxygen limitation
maxLFQ estimation scaled to rel_iBAQ [%]
0.015
0.010
0.005
0.000
HG001
ΔclpX
clpXcompl
growth phase
exp
stat

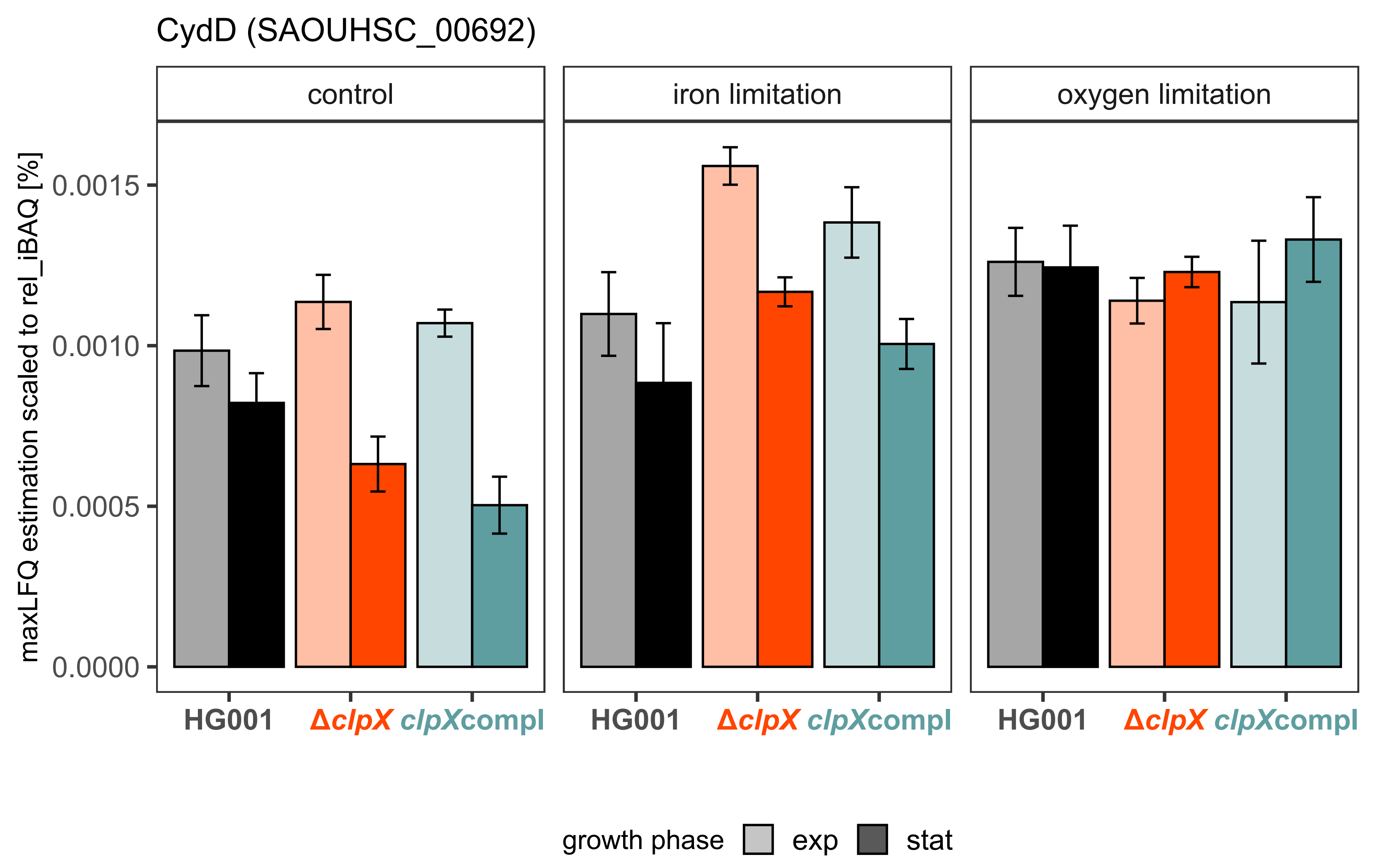

CydD (SAOUHSC_00692)
control
iron limitation
oxygen limitation
maxLFQ estimation scaled to rel_iBAQ [%]
0.0015
0.0010
0.0005
0.0000
HG001
ΔclpX
clpXcompl
growth phase
exp
stat

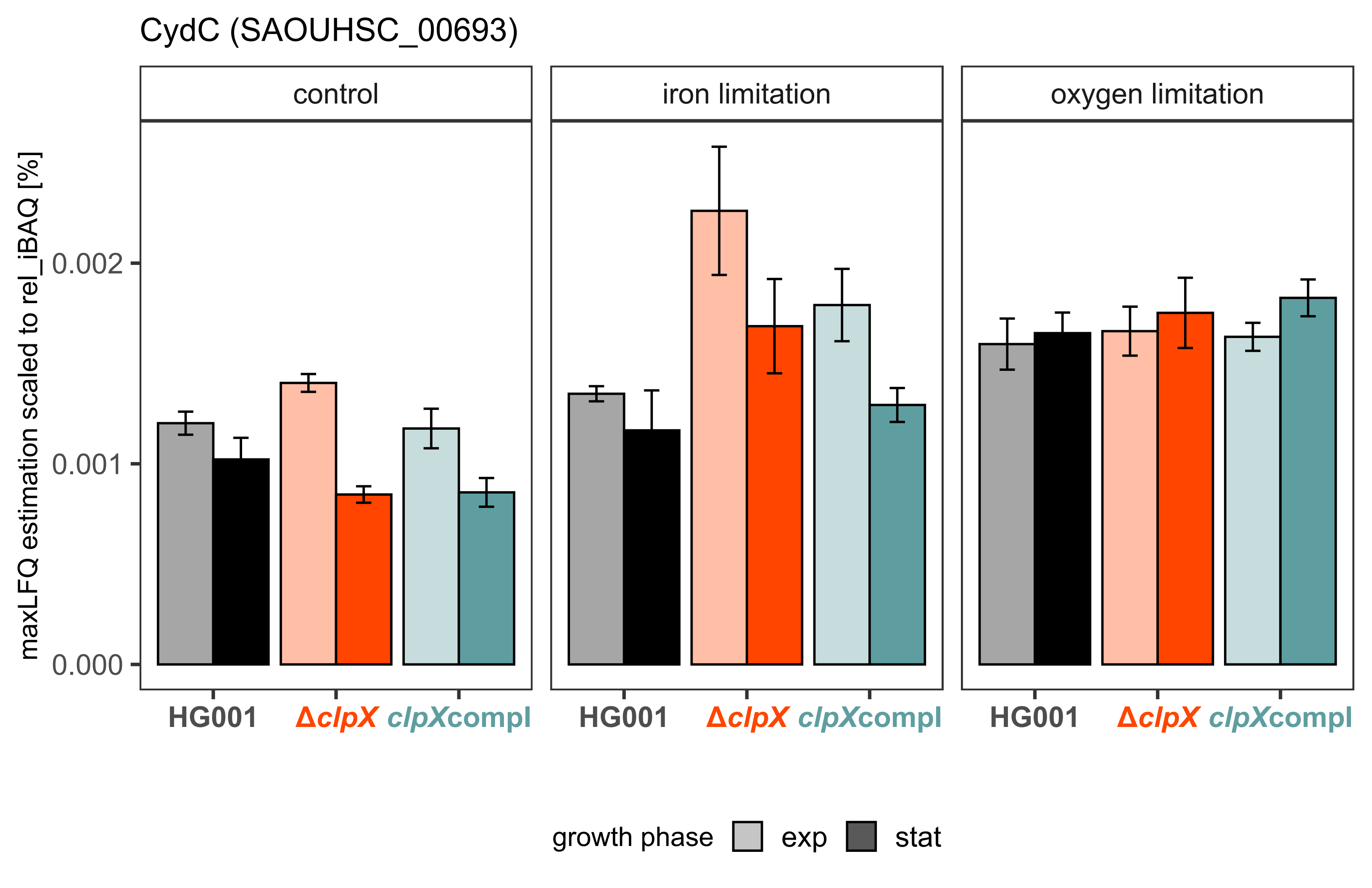
CydC (SAOUHSC_00693)
control
iron limitation
oxygen limitation
maxLFQ estimation scaled to rel_iBAQ [%]
0.000
0.001
0.002
HG001
ΔclpX
clpXcompl
growth phase
exp
stat

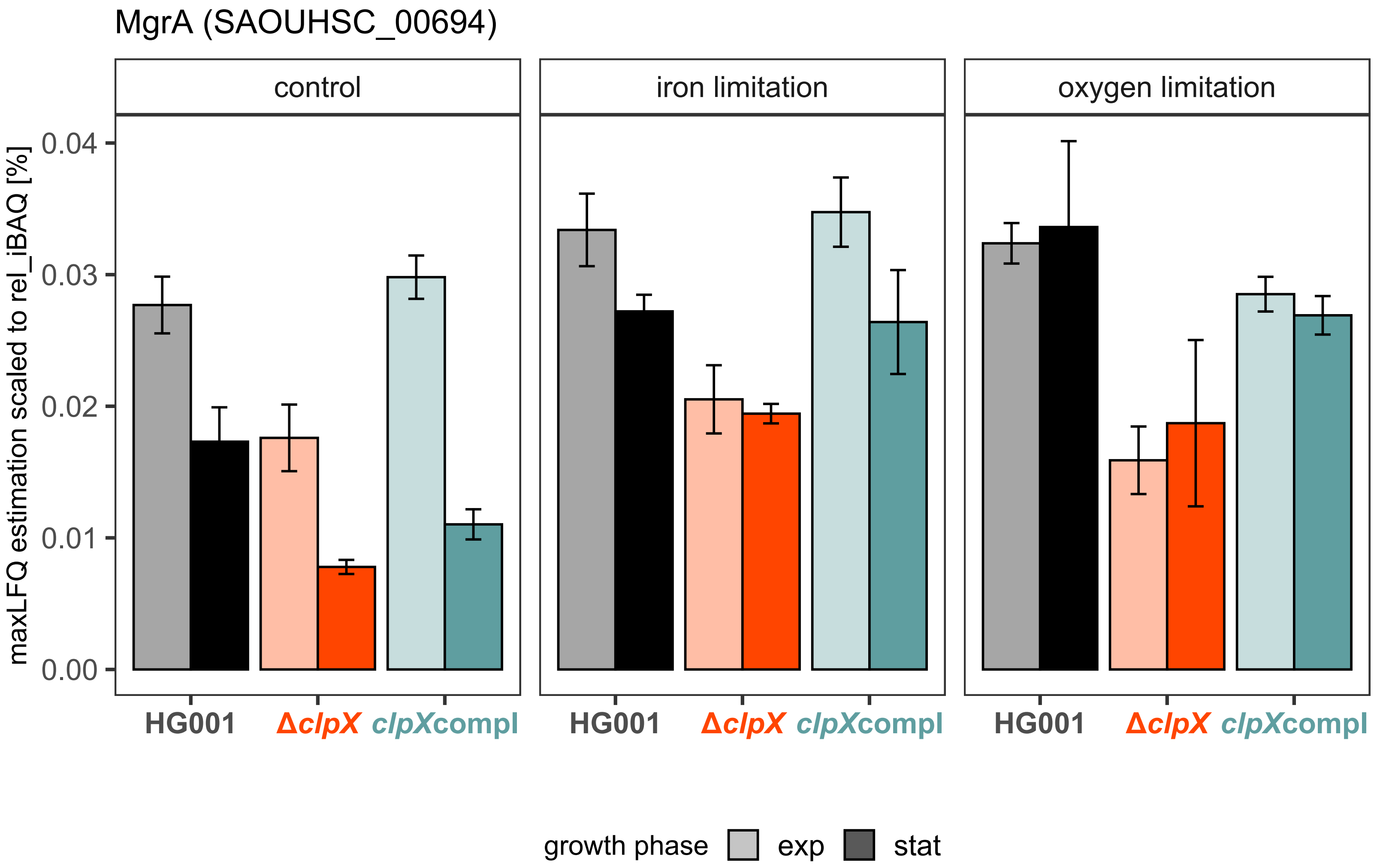
MgrA (SAOUHSC_00694)
control
iron limitation
oxygen limitation
maxLFQ estimation scaled to rel_iBAQ [%]
0.04
0.03
0.02
0.01
0.00
HG001
ΔclpX
clpXcompl
growth phase
exp
stat

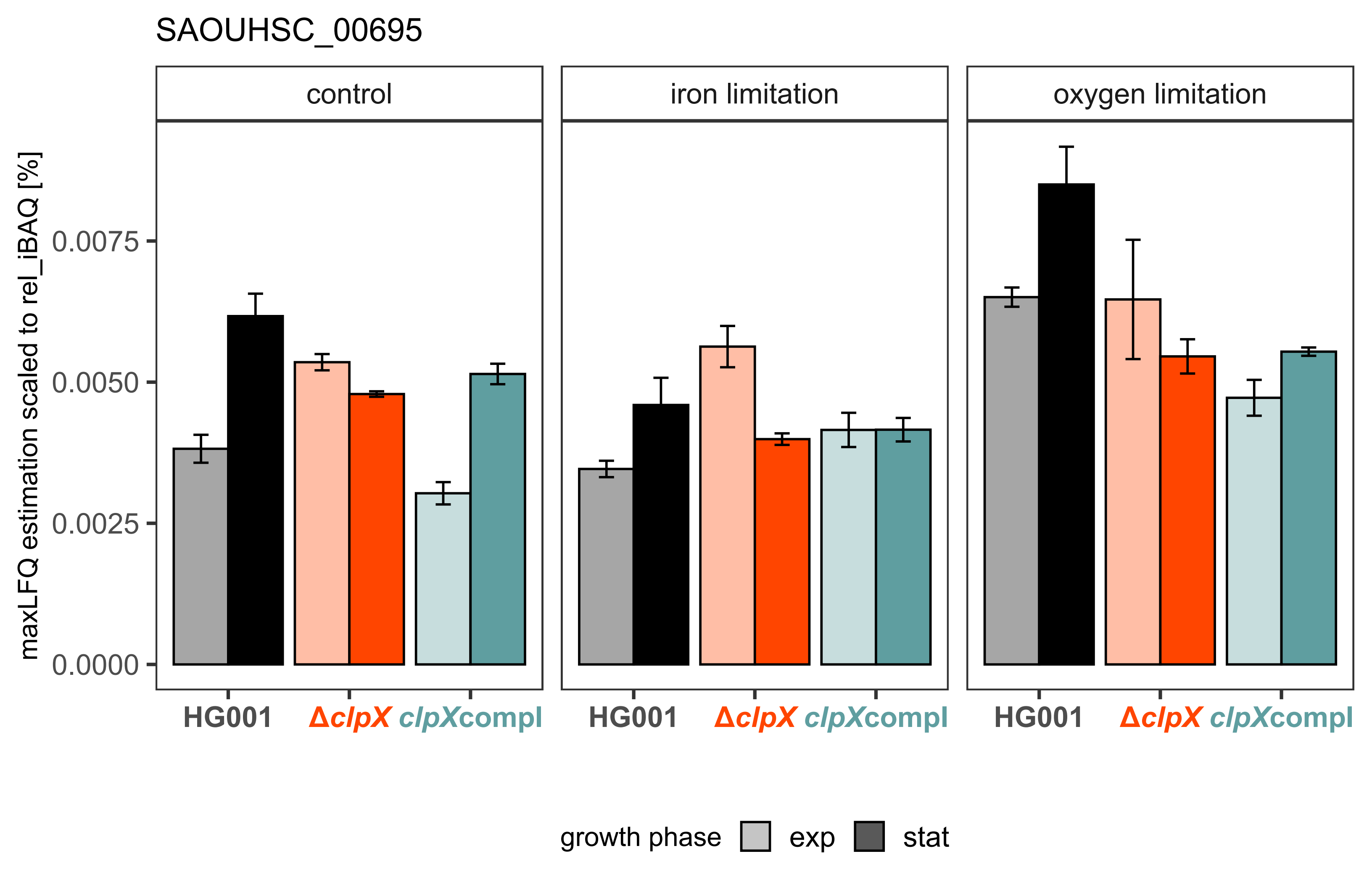

SAOUHSC_00695
control
iron limitation
oxygen limitation
maxLFQ estimation scaled to rel_iBAQ [%]
0.0075
0.0050
0.0025
0.0000
HG001
ΔclpX
clpXcompl
growth phase
exp
stat

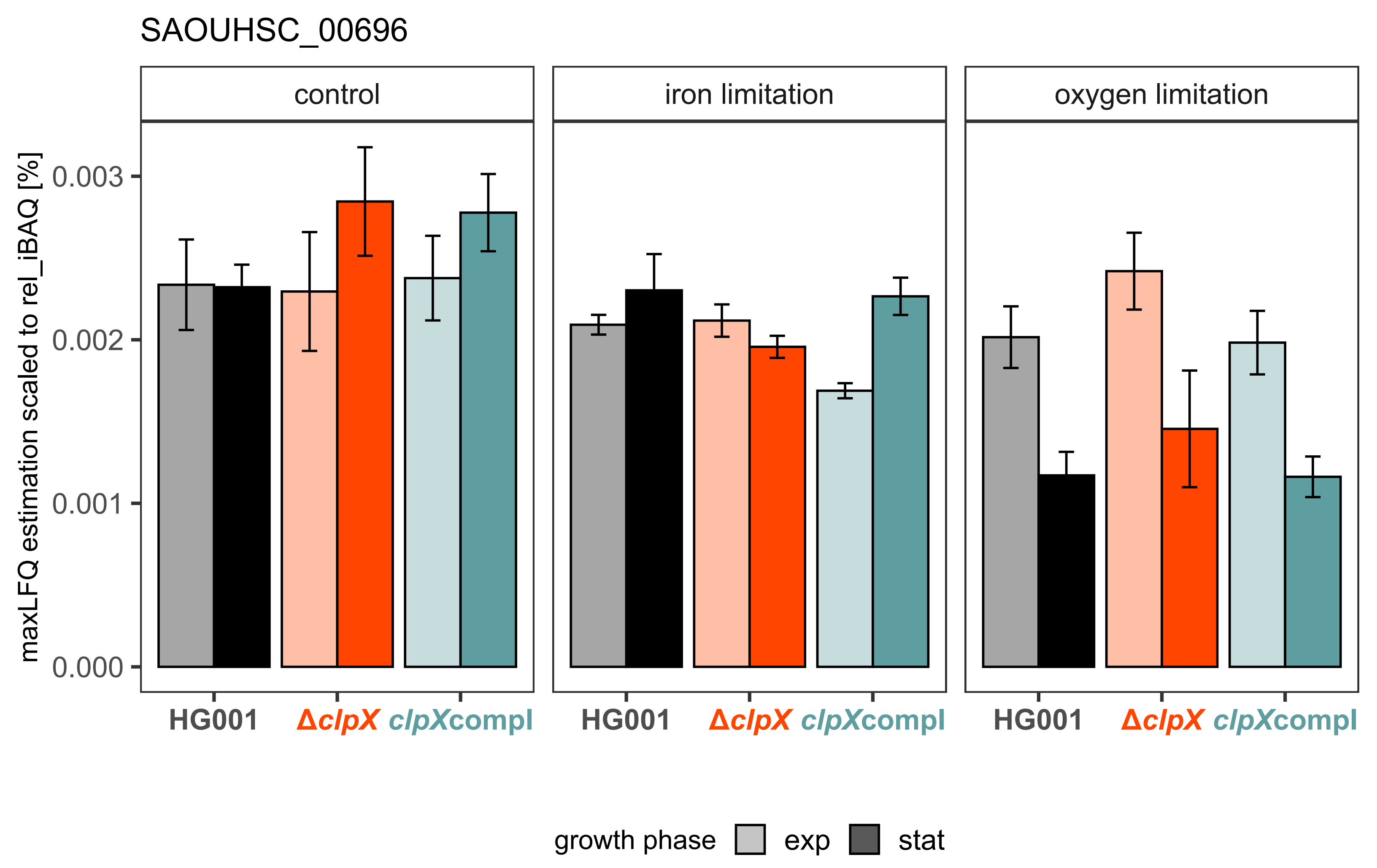
SAOUHSC_00696
control
iron limitation
oxygen limitation
maxLFQ estimation scaled to rel_iBAQ [%]
0.003
0.002
0.001
0.000
HG001
ΔclpX
clpXcompl
growth phase
exp
stat

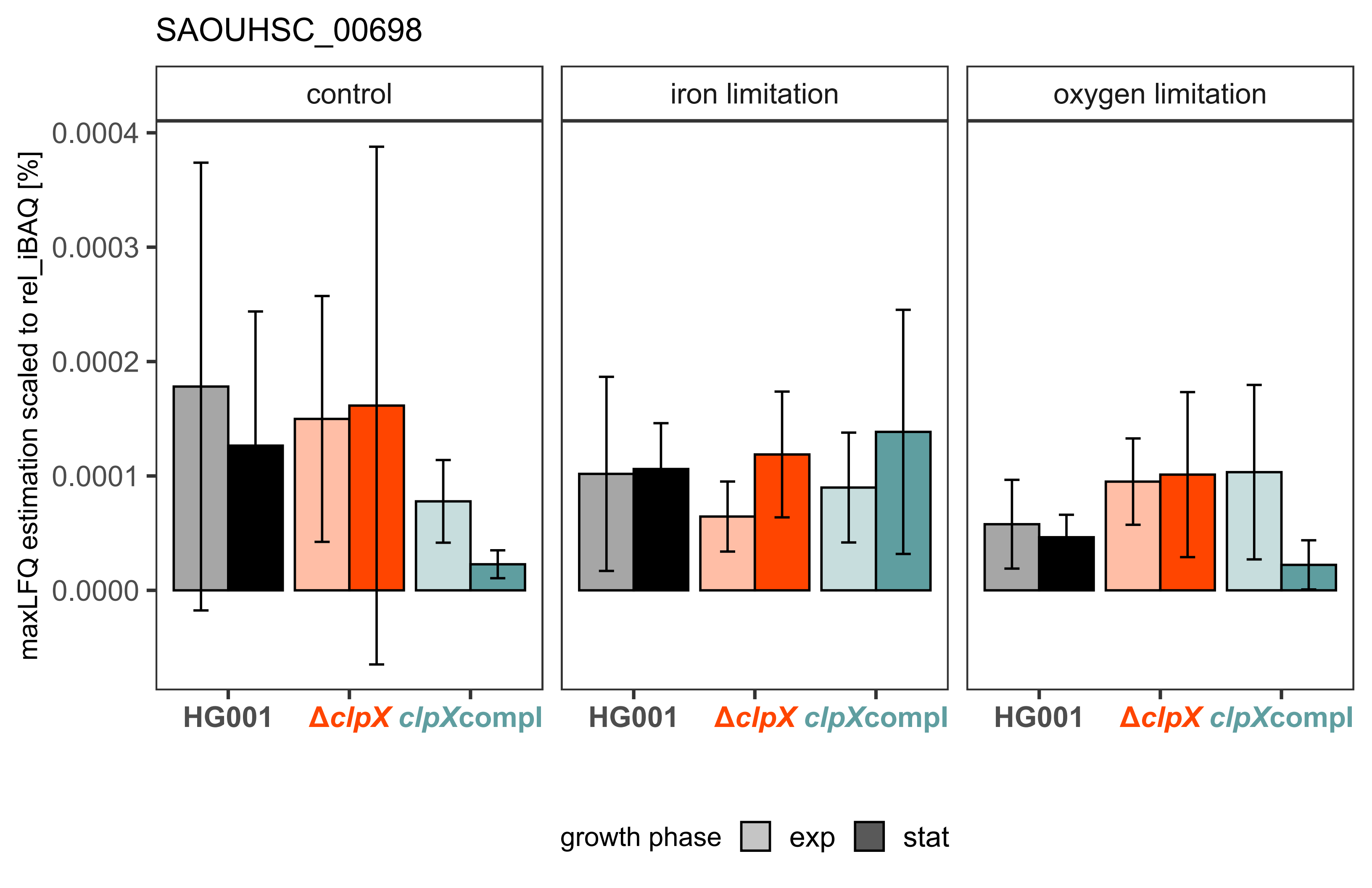

SAOUHSC_00698
control
iron limitation
oxygen limitation
maxLFQ estimation scaled to rel_iBAQ [%]
0.0004
0.0003
0.0002
0.0001
0.0000
HG001
ΔclpX
clpXcompl
growth phase
exp
stat

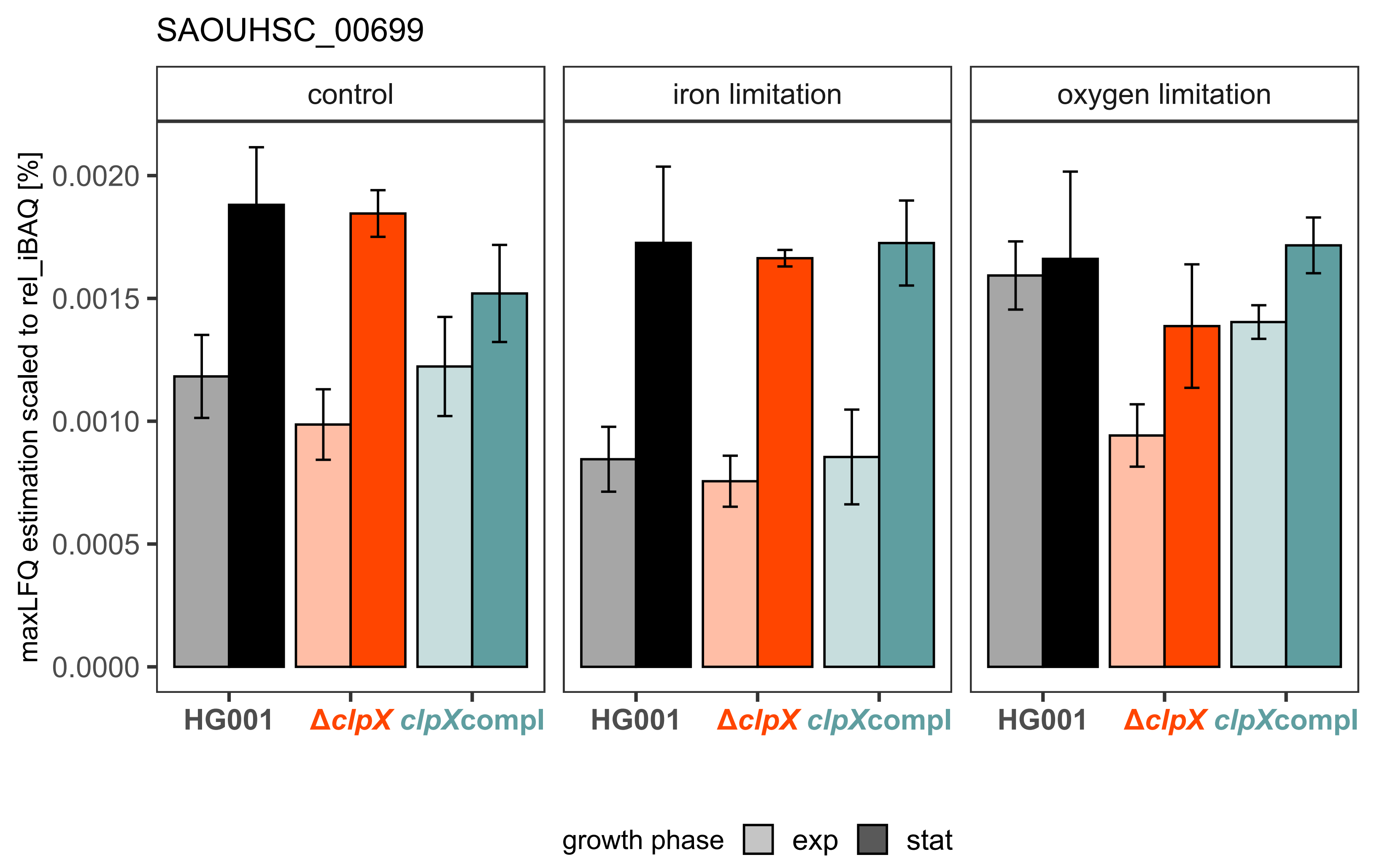

SAOUHSC_00699
control
iron limitation
oxygen limitation
maxLFQ estimation scaled to rel_iBAQ [%]
0.0020
0.0015
0.0010
0.0005
0.0000
HG001
ΔclpX
clpXcompl
growth phase
exp
stat

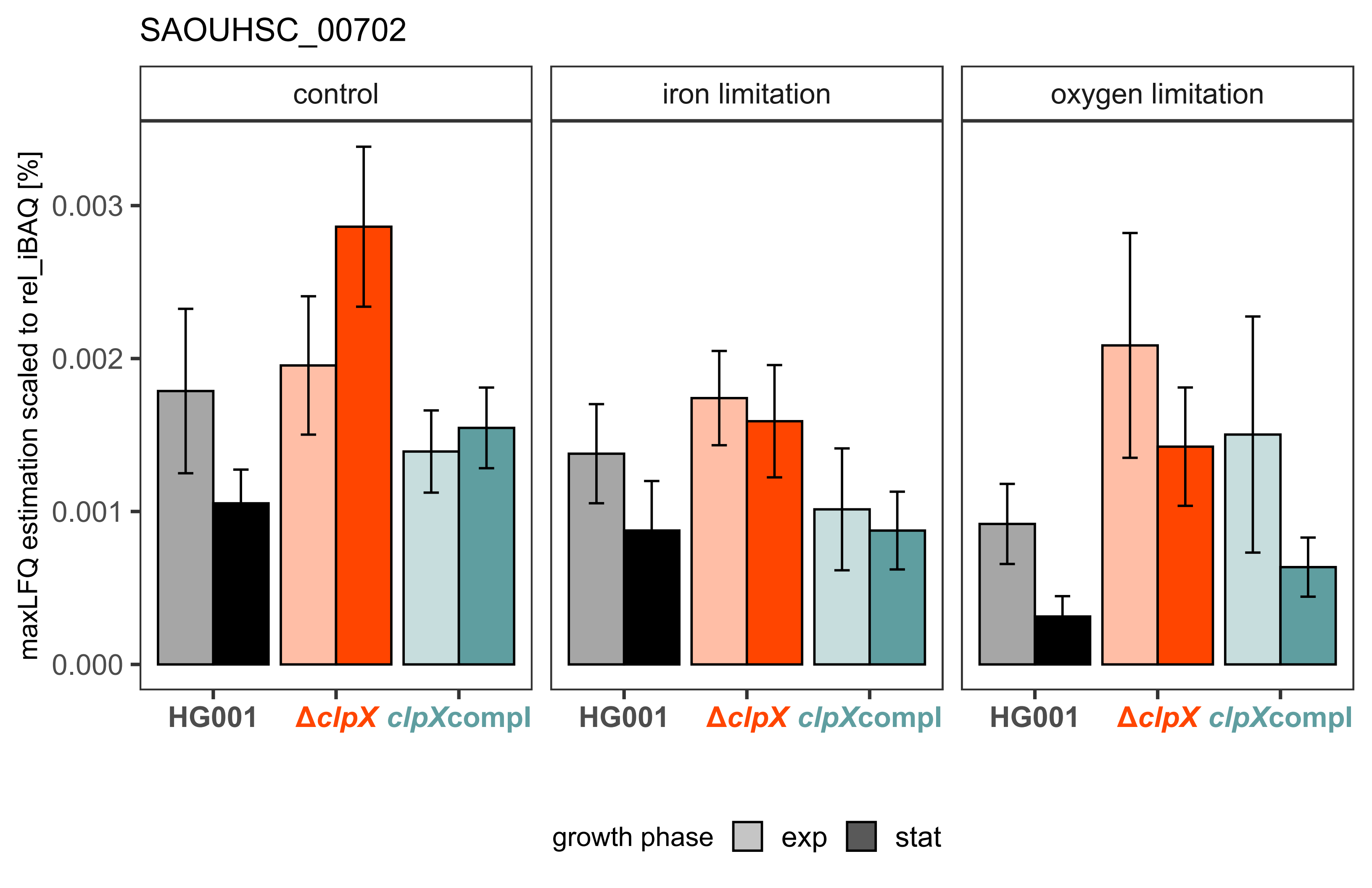

SAOUHSC_00702
control
iron limitation
oxygen limitation
maxLFQ estimation scaled to rel_iBAQ [%]
0.003
0.002
0.001
0.000
HG001
ΔclpX
clpXcompl
growth phase
exp
stat

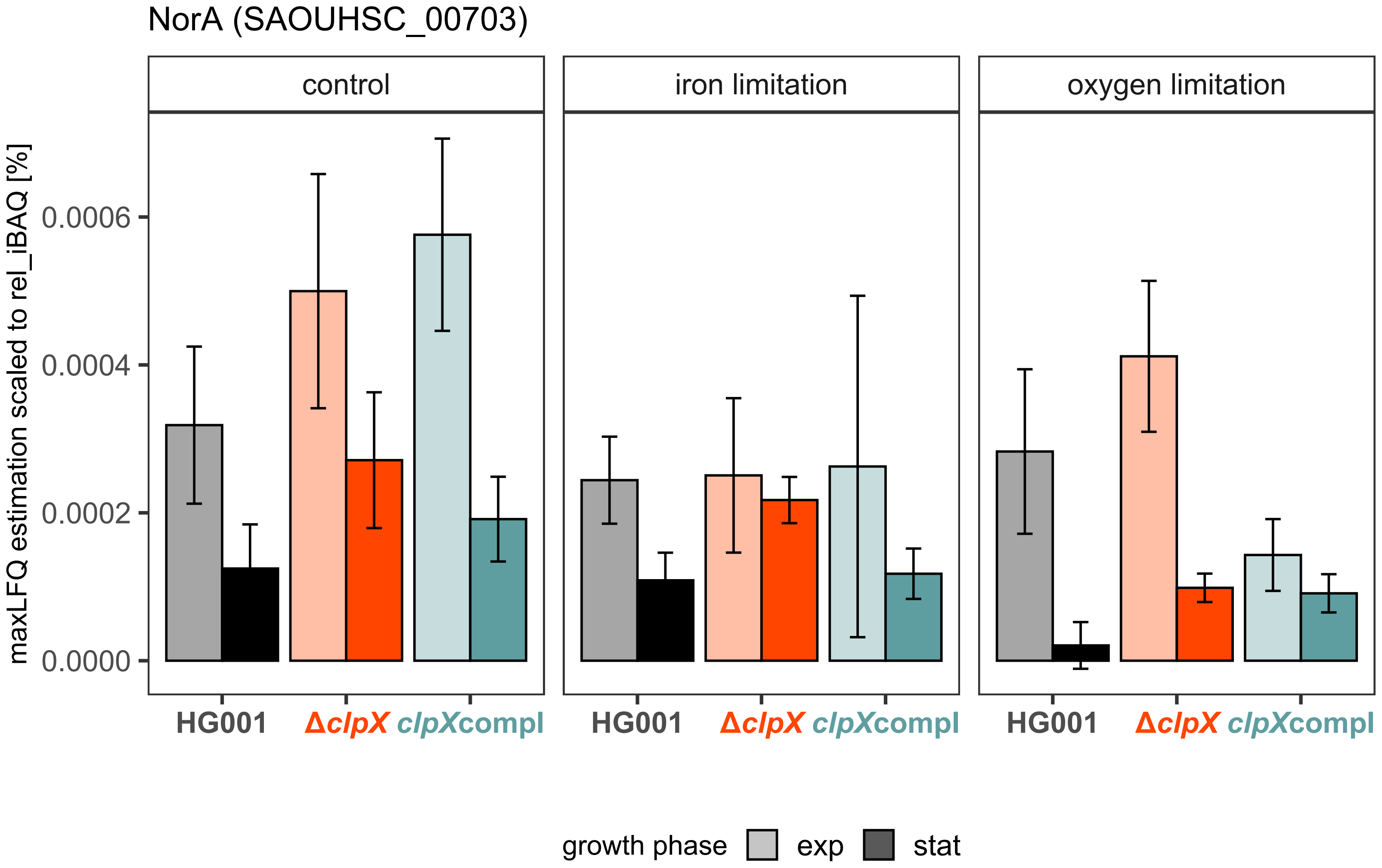

NorA (SAOUHSC_00703)
control
iron limitation
oxygen limitation
maxLFQ estimation scaled to rel_iBAQ [%]
0.0006
0.0004
0.0002
0.0000
HG001
ΔclpX
clpXcompl
growth phase
exp
stat

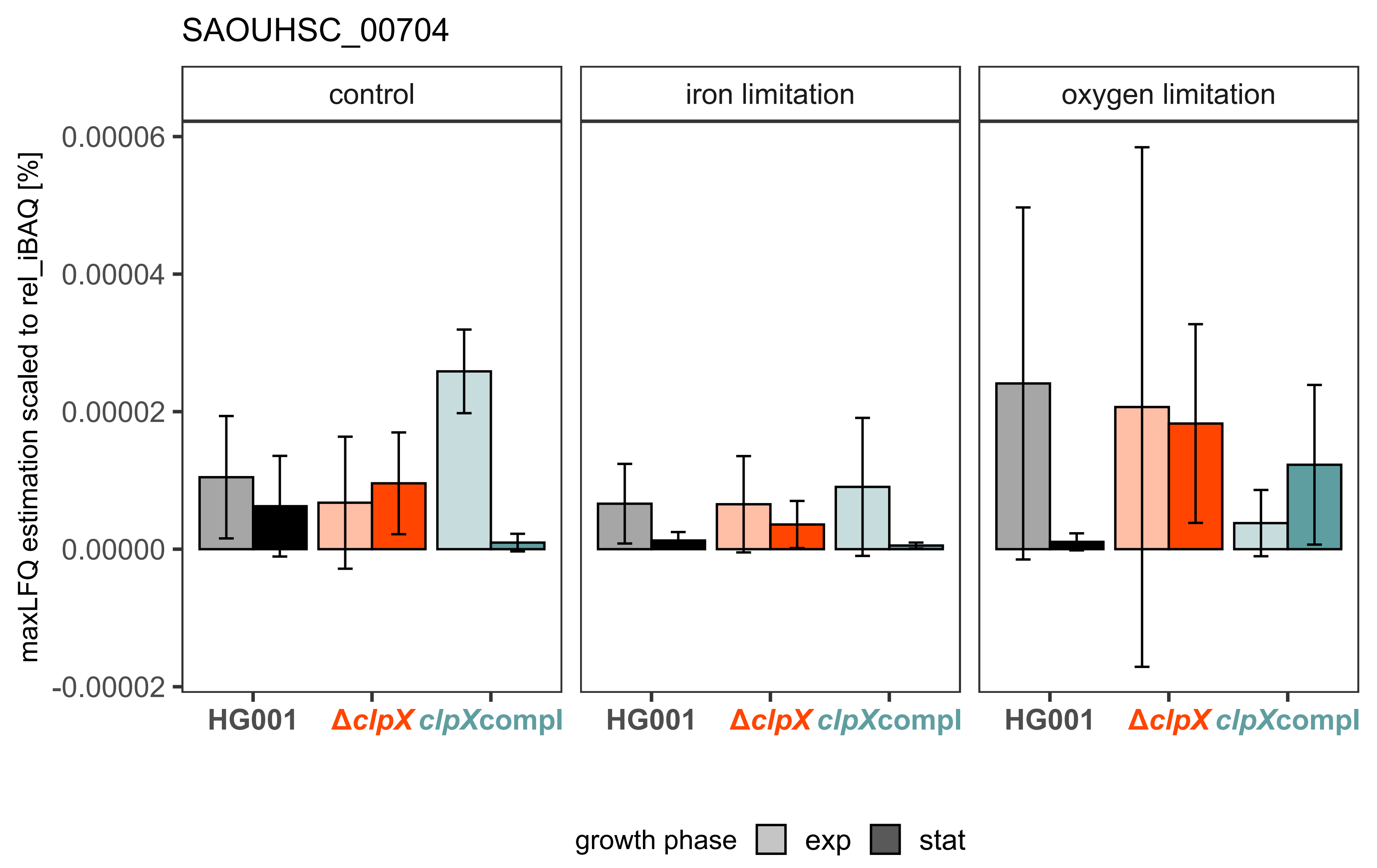
SAOUHSC_00704
control
iron limitation
oxygen limitation
maxLFQ estimation scaled to rel_iBAQ [%]
0.00006
0.00004
0.00002
0.00000
-0.00002
HG001
ΔclpX
clpXcompl
growth phase
exp
stat

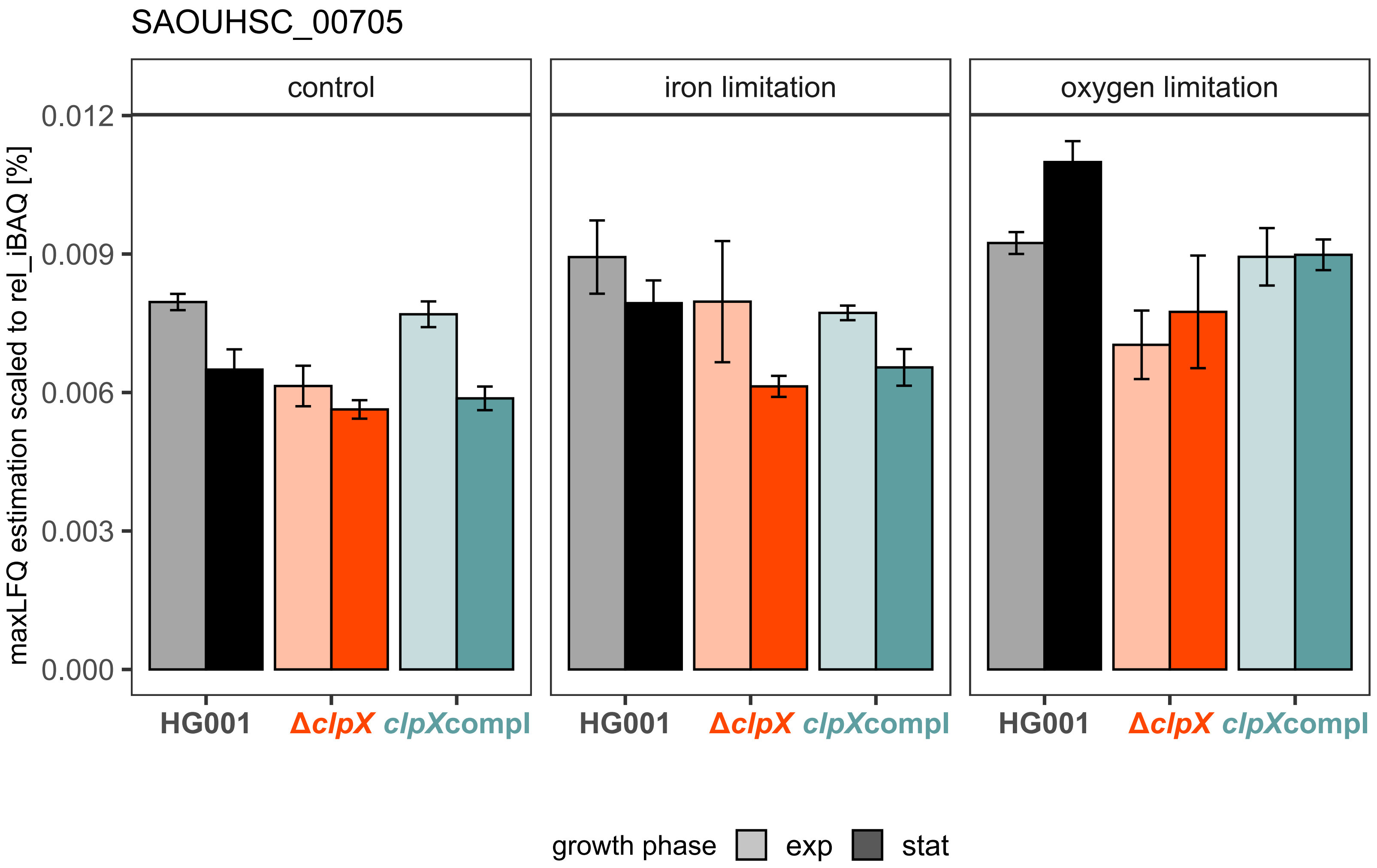

SAOUHSC_00705
control
iron limitation
oxygen limitation
maxLFQ estimation scaled to rel_iBAQ [%]
0.012
0.009
0.006
0.003
0.000
HG001
ΔclpX
clpXcompl
growth phase
exp
stat

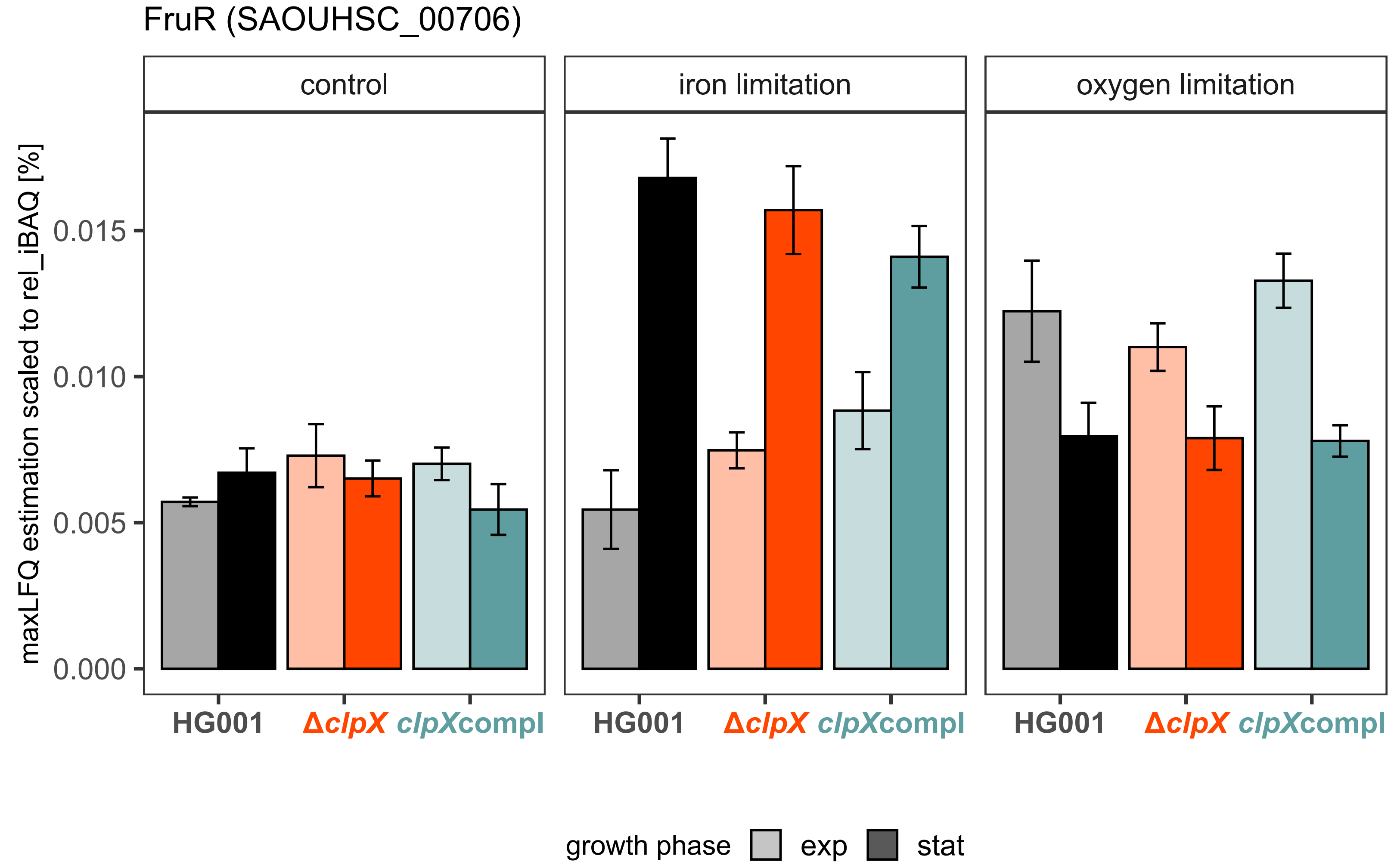
FruR (SAOUHSC_00706)
control
iron limitation
oxygen limitation
maxLFQ estimation scaled to rel_iBAQ [%]
0.015
0.010
0.005
0.000
HG001
ΔclpX
clpXcompl
growth phase
exp
stat

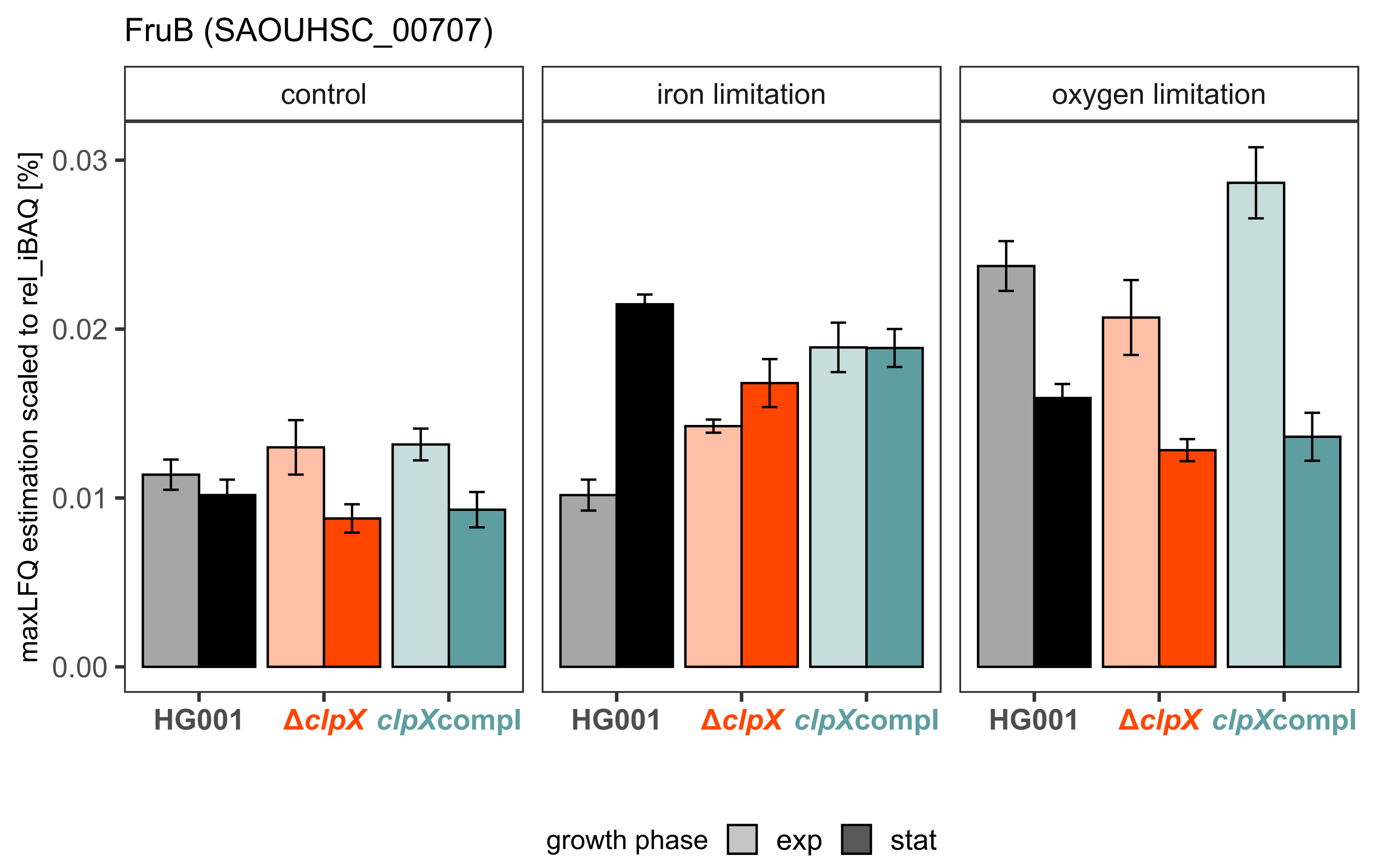

FruB (SAOUHSC_00707)
control
iron limitation
oxygen limitation
maxLFQ estimation scaled to rel_iBAQ [%]
0.03
0.02
0.01
0.00
HG001
ΔclpX
clpXcompl
growth phase
exp
stat

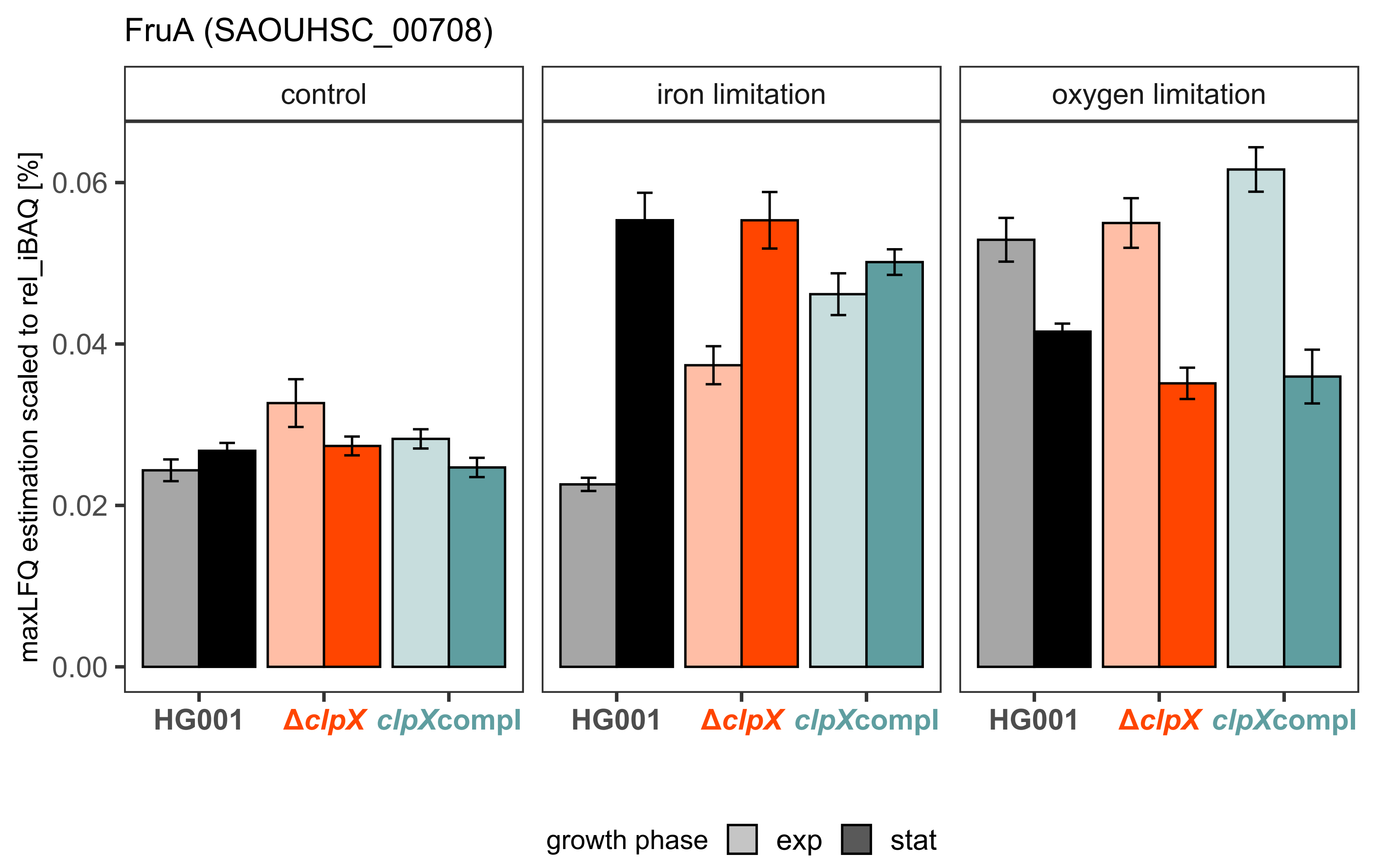
FruA (SAOUHSC_00708)
control
iron limitation
oxygen limitation
maxLFQ estimation scaled to rel_iBAQ [%]
0.06
0.04
0.02
0.00
HG001
ΔclpX
clpXcompl
HG001
ΔclpX
clpXcompl
HG001
ΔclpX
clpXcompl
growth phase
exp
stat

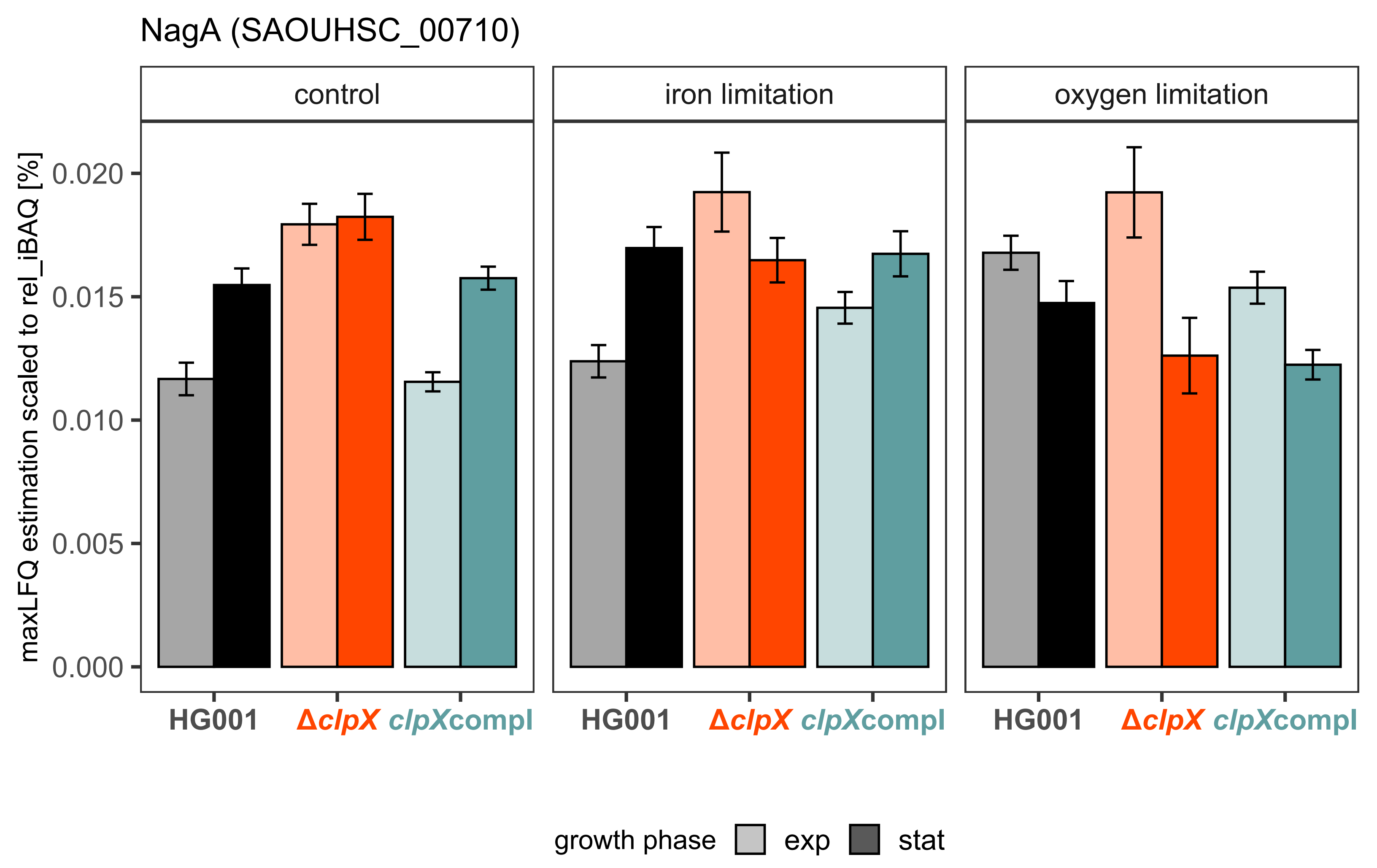
NagA (SAOUHSC_00710)
control
iron limitation
oxygen limitation
maxLFQ estimation scaled to rel_iBAQ [%]
0.020
0.015
0.010
0.005
0.000
HG001
ΔclpX
clpXcompl
growth phase
exp
stat

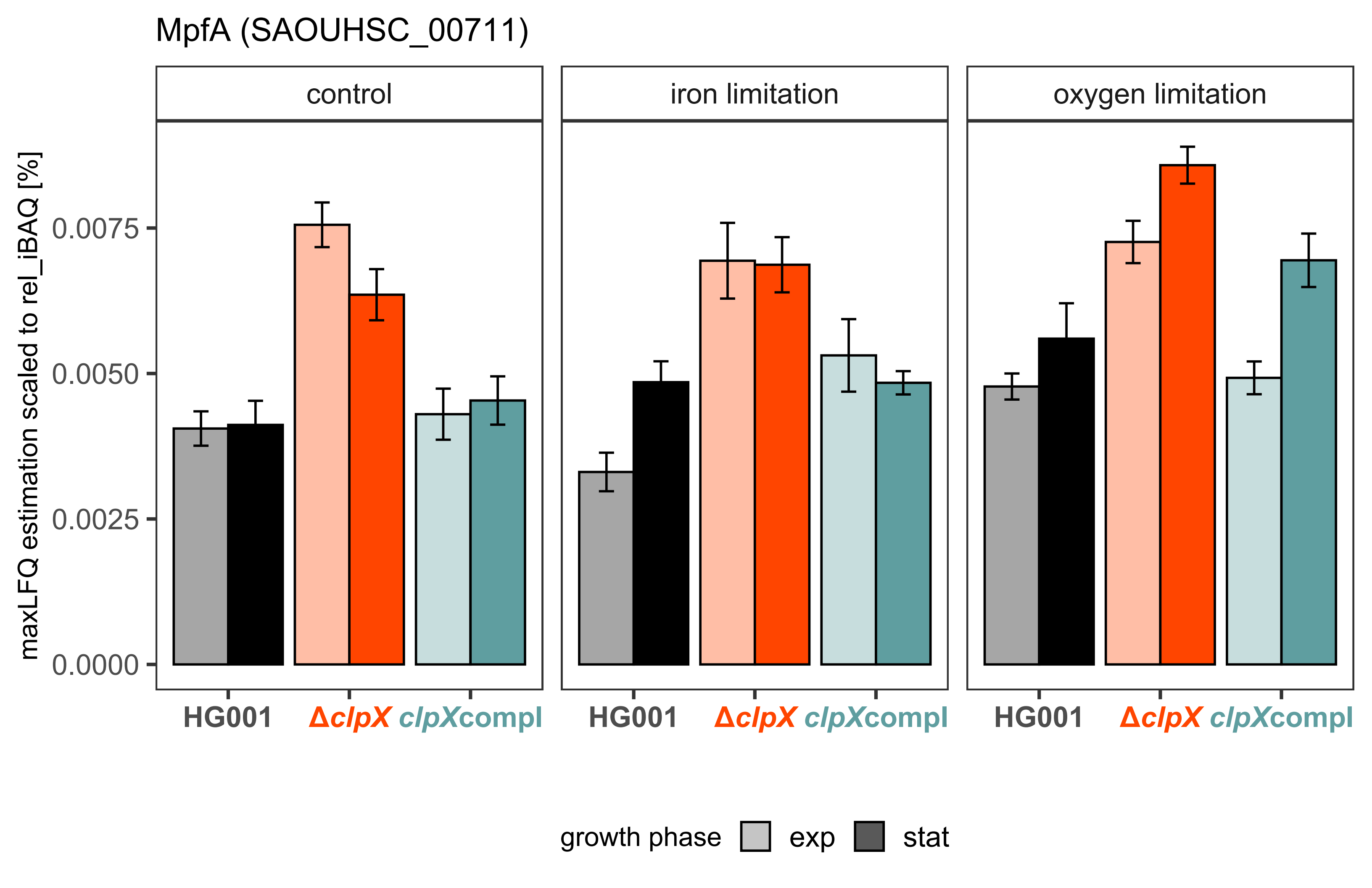

MpfA (SAOUHSC_00711)
control
iron limitation
oxygen limitation
maxLFQ estimation scaled to rel_iBAQ [%]
0.0075
0.0050
0.0025
0.0000
HG001
ΔclpX
clpXcompl
growth phase
exp
stat

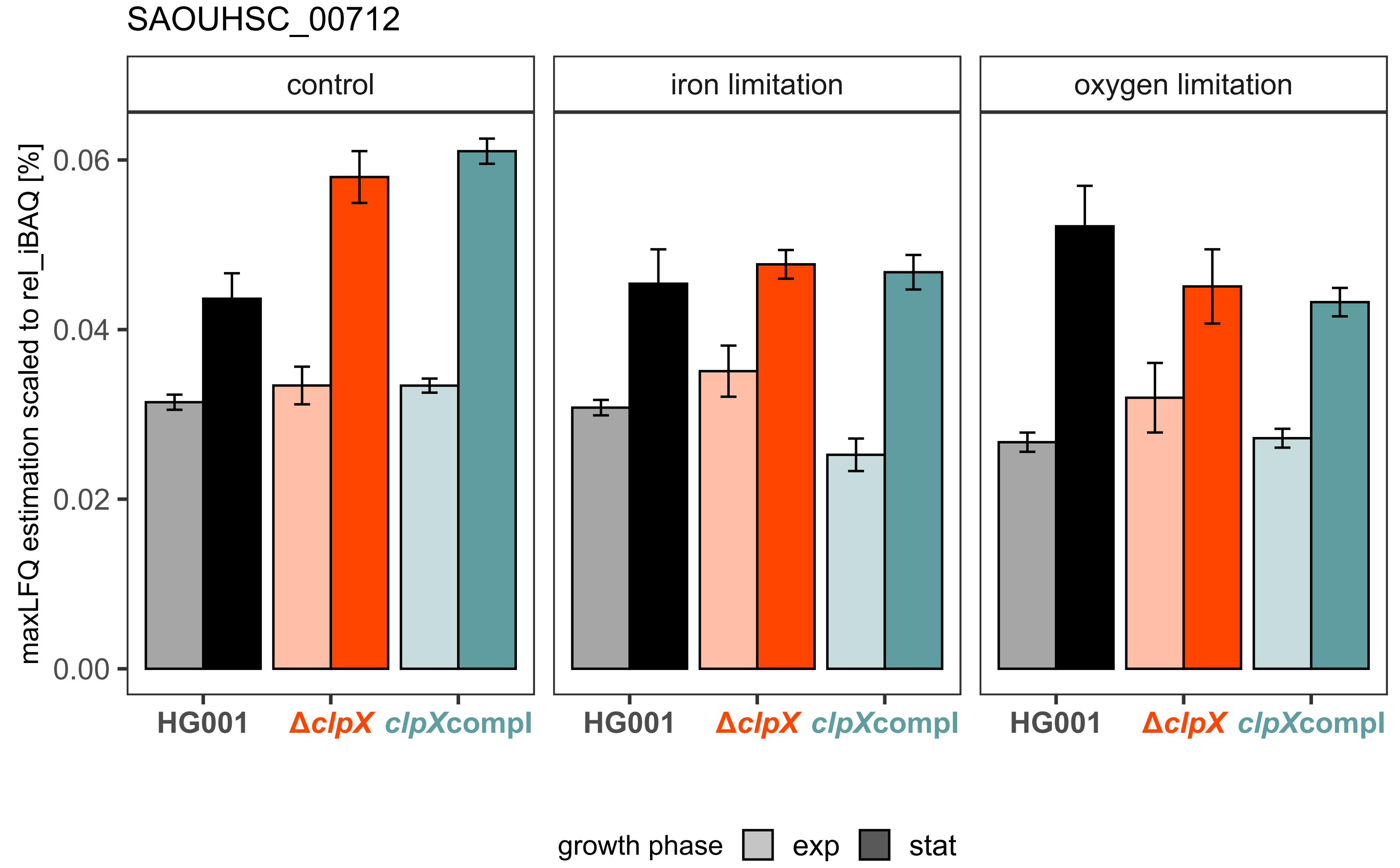
SAOUHSC_00712
control
iron limitation
oxygen limitation
maxLFQ estimation scaled to rel_iBAQ [%]
0.06
0.04
0.02
0.00
HG001
ΔclpX
clpXcompl
growth phase
exp
stat

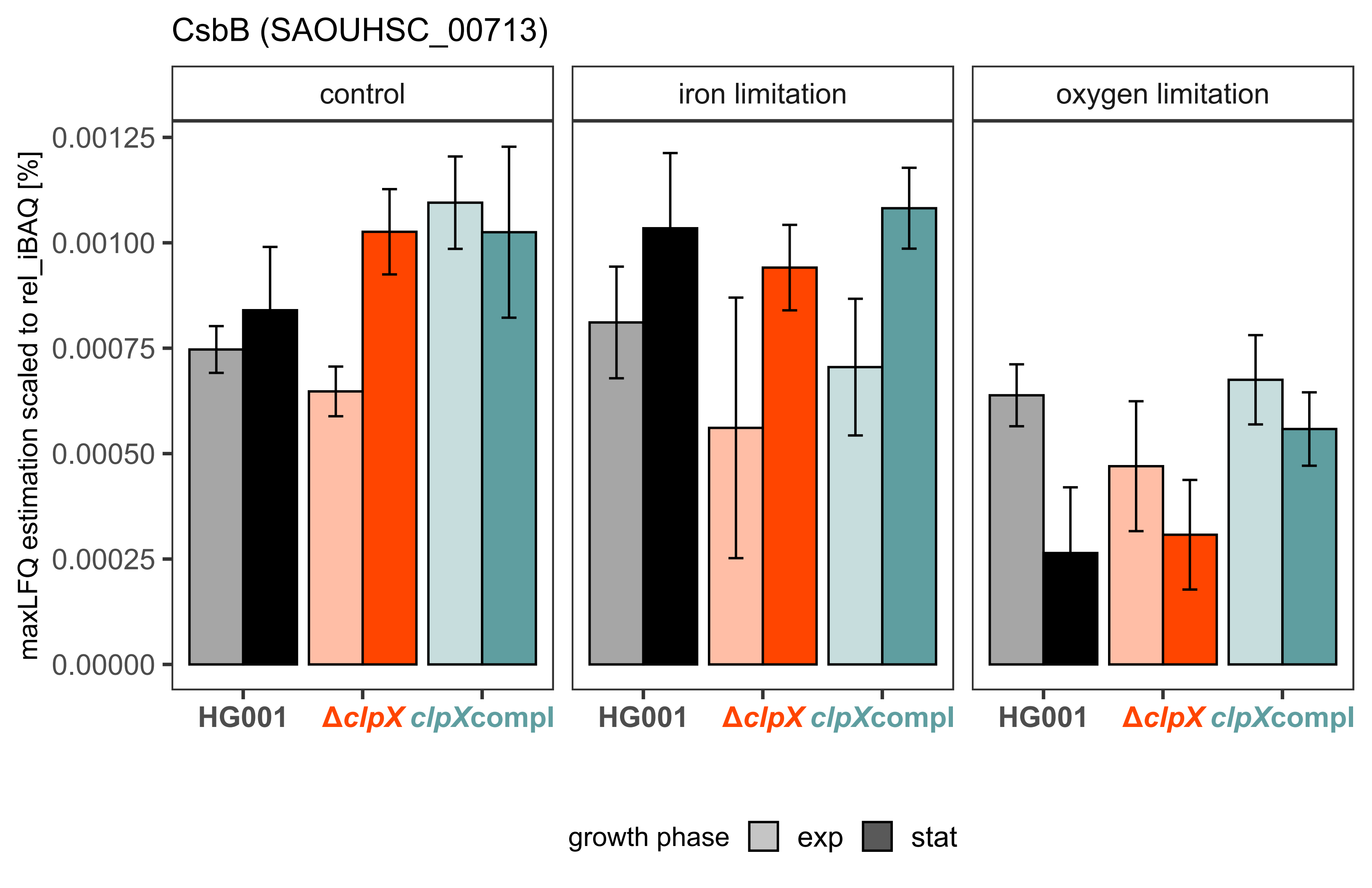

CsbB (SAOUHSC_00713)
control
iron limitation
oxygen limitation
maxLFQ estimation scaled to rel_iBAQ [%]
0.00125
0.00100
0.00075
0.00050
0.00025
0.00000
HG001
ΔclpX
clpXcompl
growth phase
exp
stat

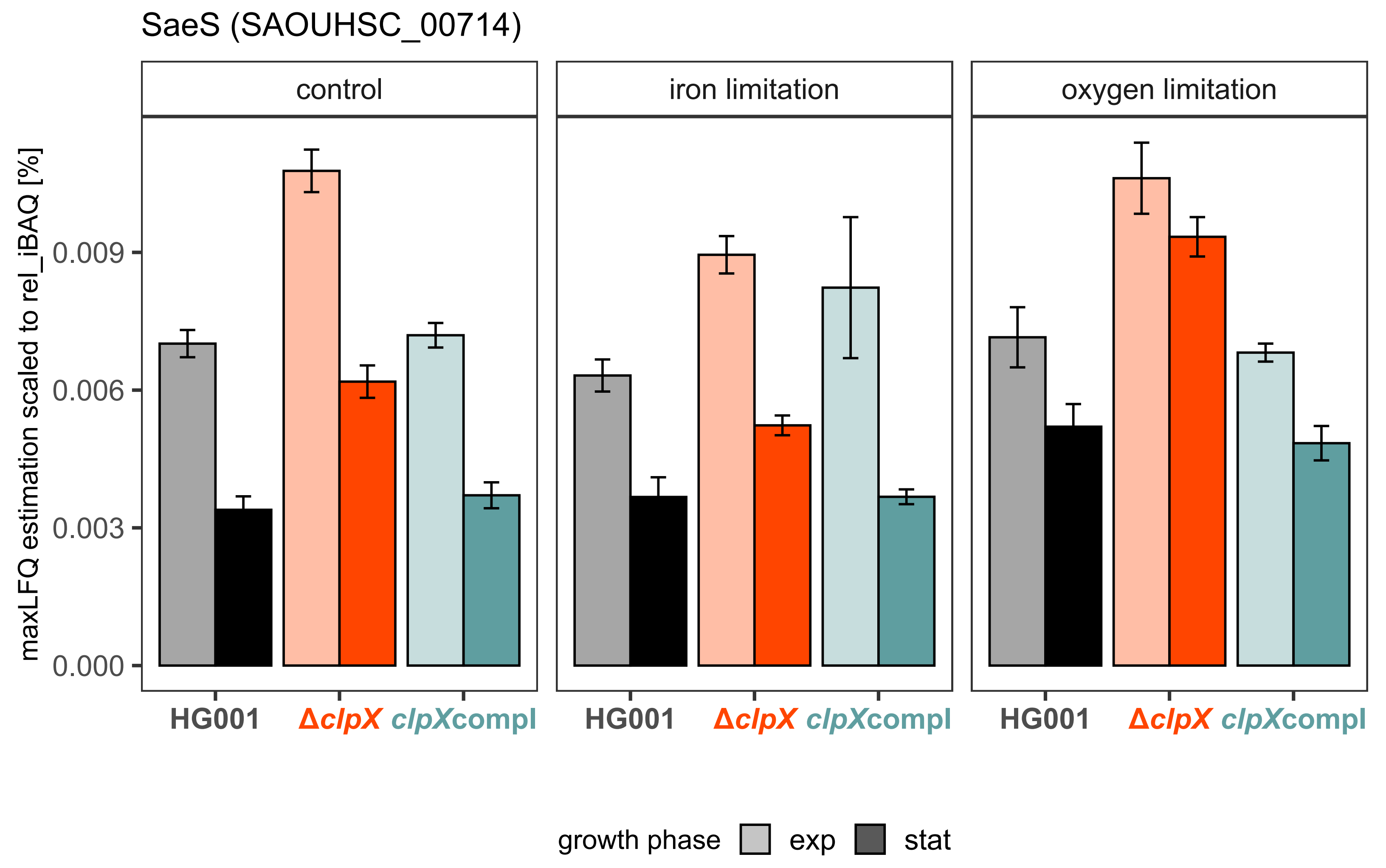
SaeS (SAOUHSC_00714)
control
iron limitation
oxygen limitation
maxLFQ estimation scaled to rel_iBAQ [%]
0.009
0.006
0.003
0.000
HG001
ΔclpX
clpXcompl
growth phase
exp
stat

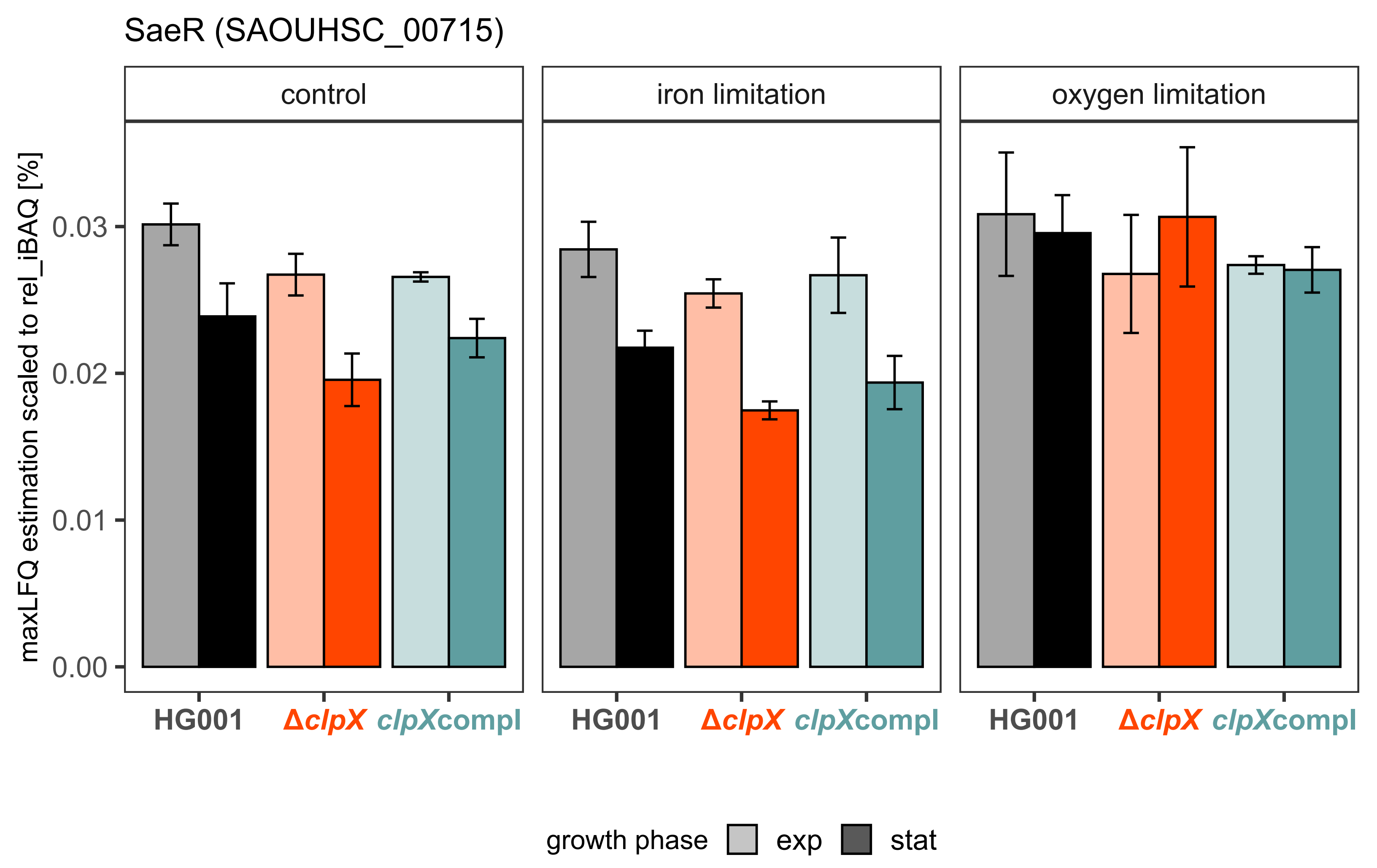

SaeR (SAOUHSC_00715)
control
iron limitation
oxygen limitation
maxLFQ estimation scaled to rel_iBAQ [%]
0.00
0.01
0.02
0.03
HG001
ΔclpX
clpXcompl
growth phase
exp
stat

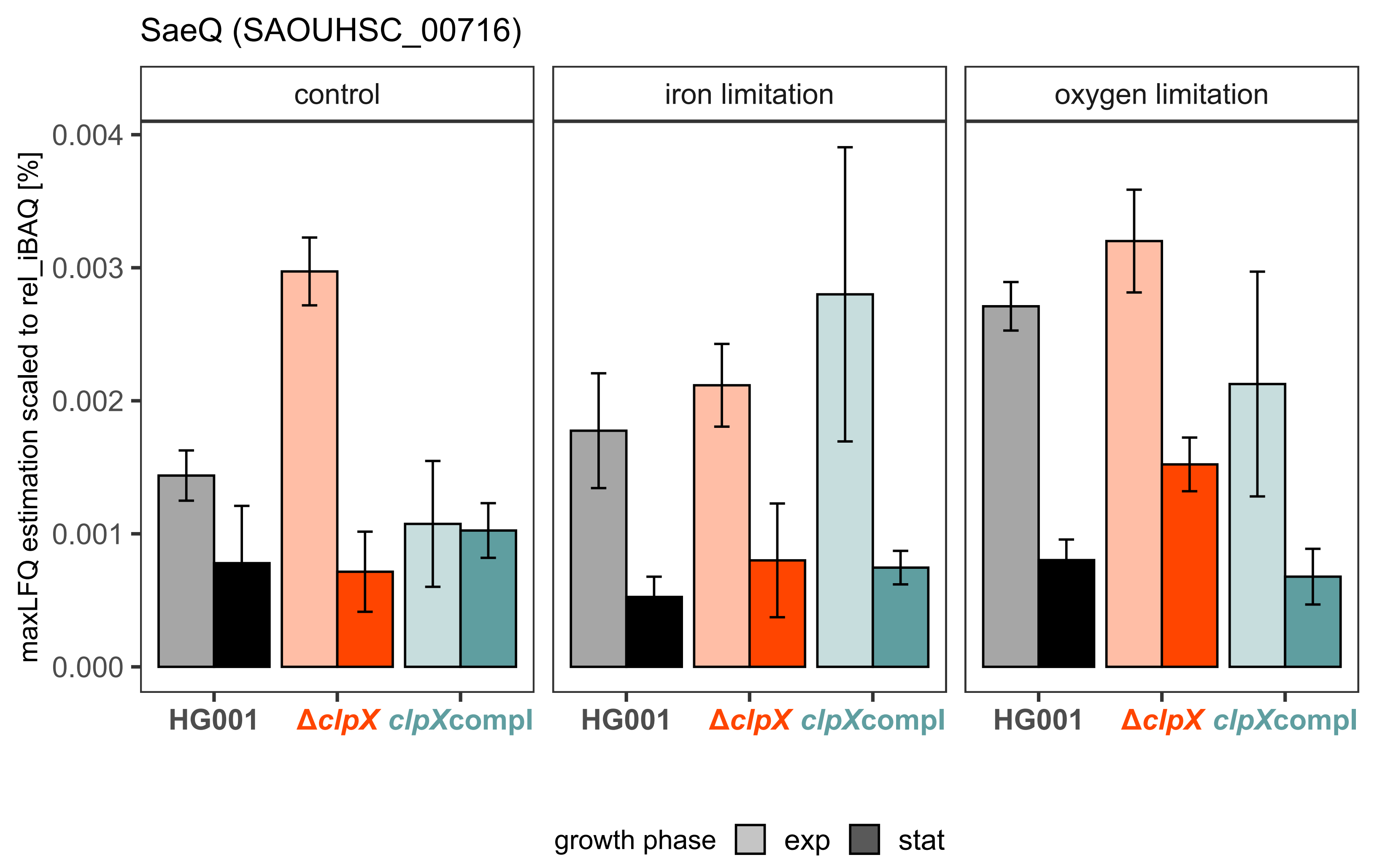

SaeQ (SAOUHSC_00716)
control
iron limitation
oxygen limitation
maxLFQ estimation scaled to rel_iBAQ [%]
0.004
0.003
0.002
0.001
0.000
HG001
ΔclpX
clpXcompl
growth phase
exp
stat

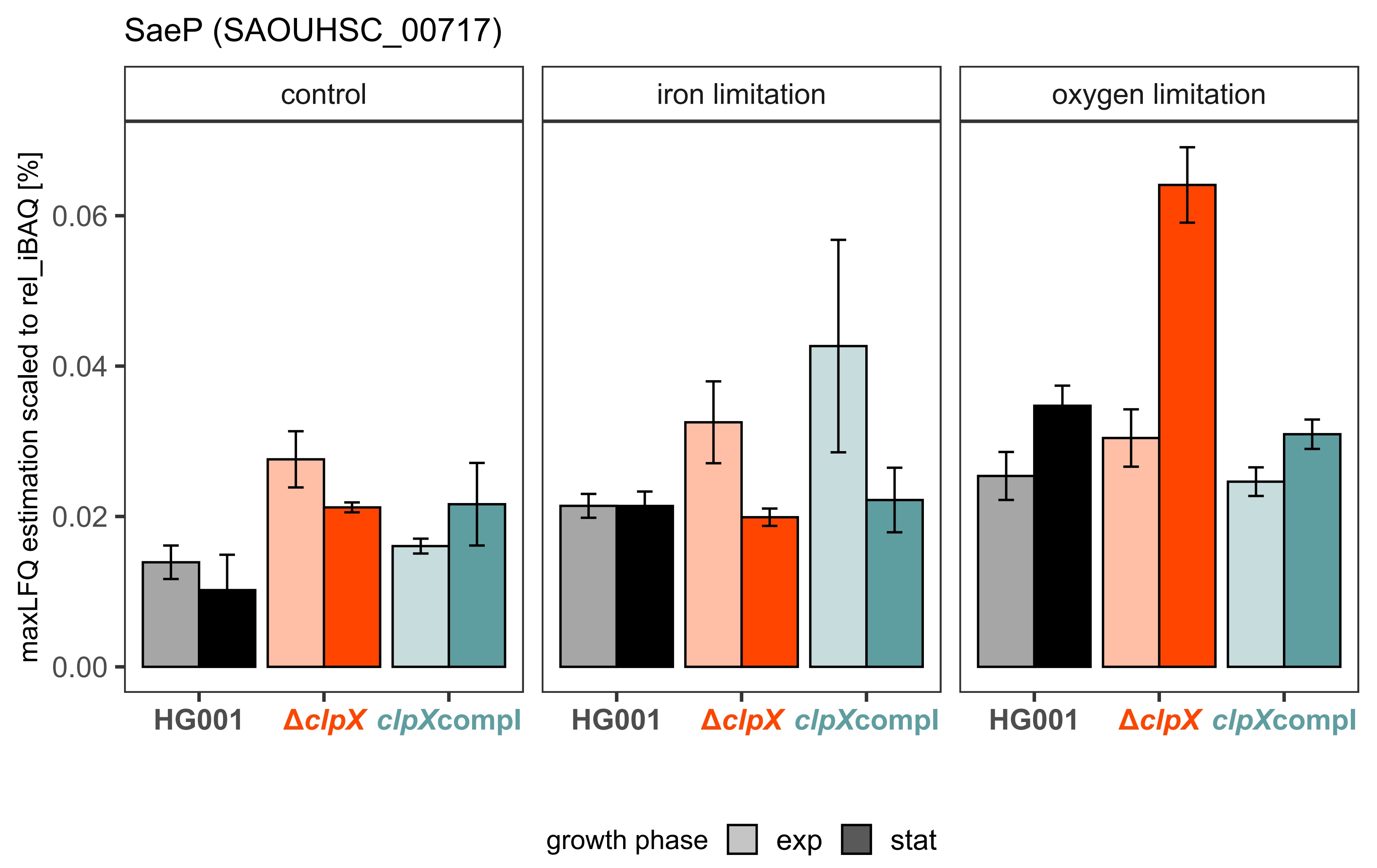

SaeP (SAOUHSC_00717)
control
iron limitation
oxygen limitation
maxLFQ estimation scaled to rel_iBAQ [%]
0.06
0.04
0.02
0.00
HG001
ΔclpX
clpXcompl
growth phase
exp
stat

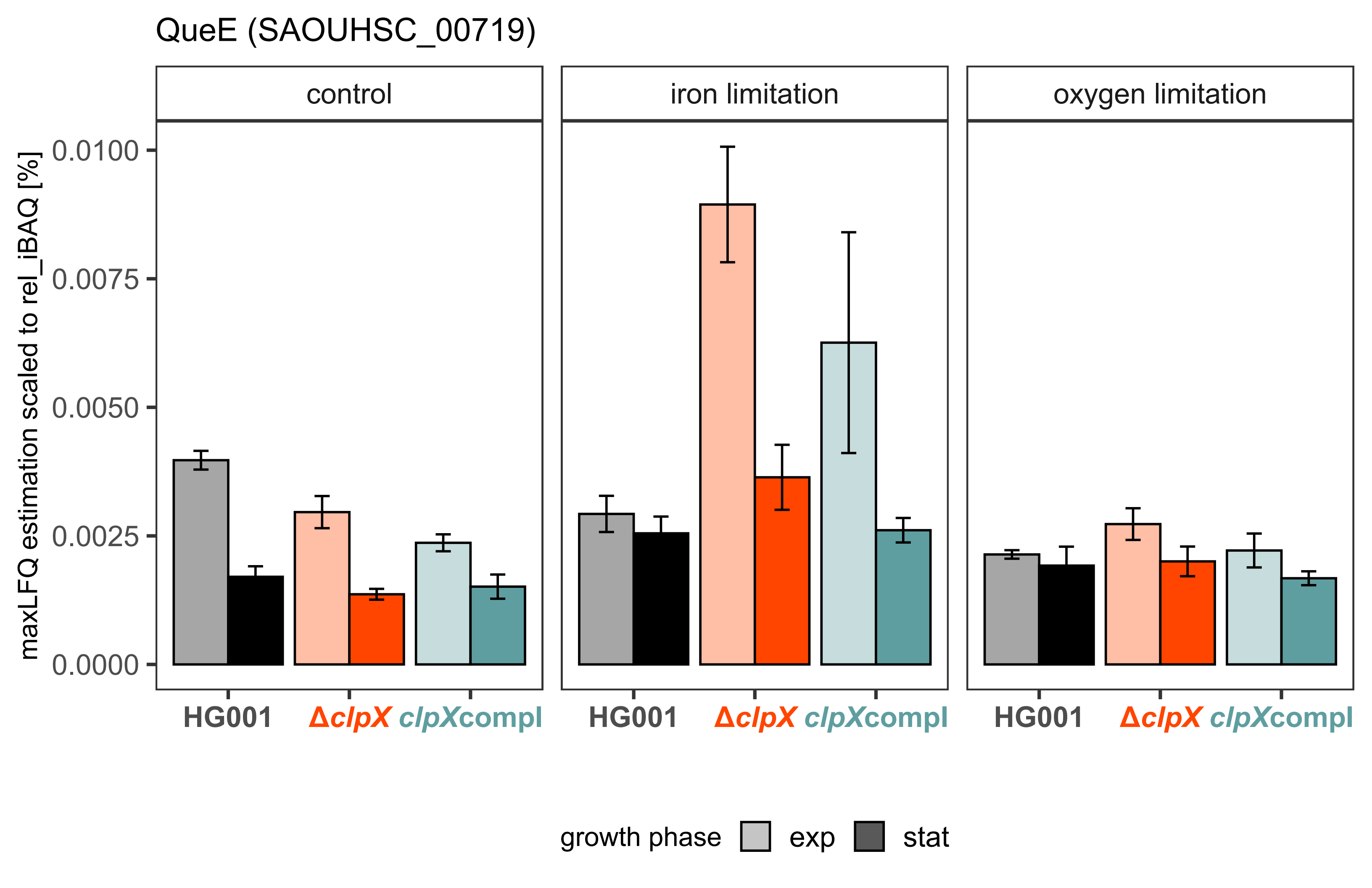
QueE (SAOUHSC_00719)
control
iron limitation
oxygen limitation
maxLFQ estimation scaled to rel_iBAQ [%]
0.0100
0.0075
0.0050
0.0025
0.0000
HG001
ΔclpX
clpXcompl
growth phase
exp
stat

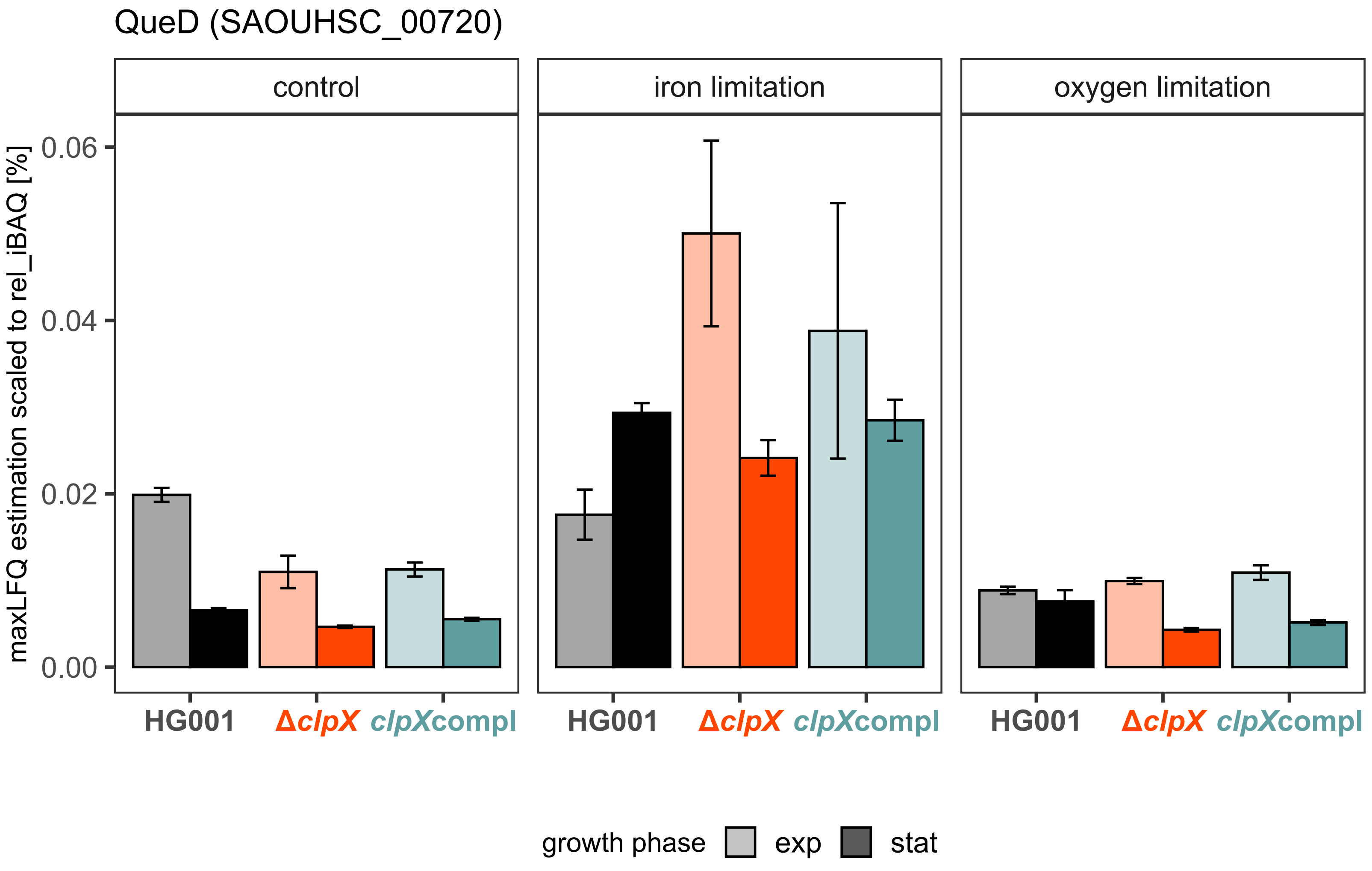

QueD (SAOUHSC_00720)
control
iron limitation
oxygen limitation
maxLFQ estimation scaled to rel_iBAQ [%]
0.06
0.04
0.02
0.00
HG001
ΔclpX
clpXcompl
growth phase
exp
stat

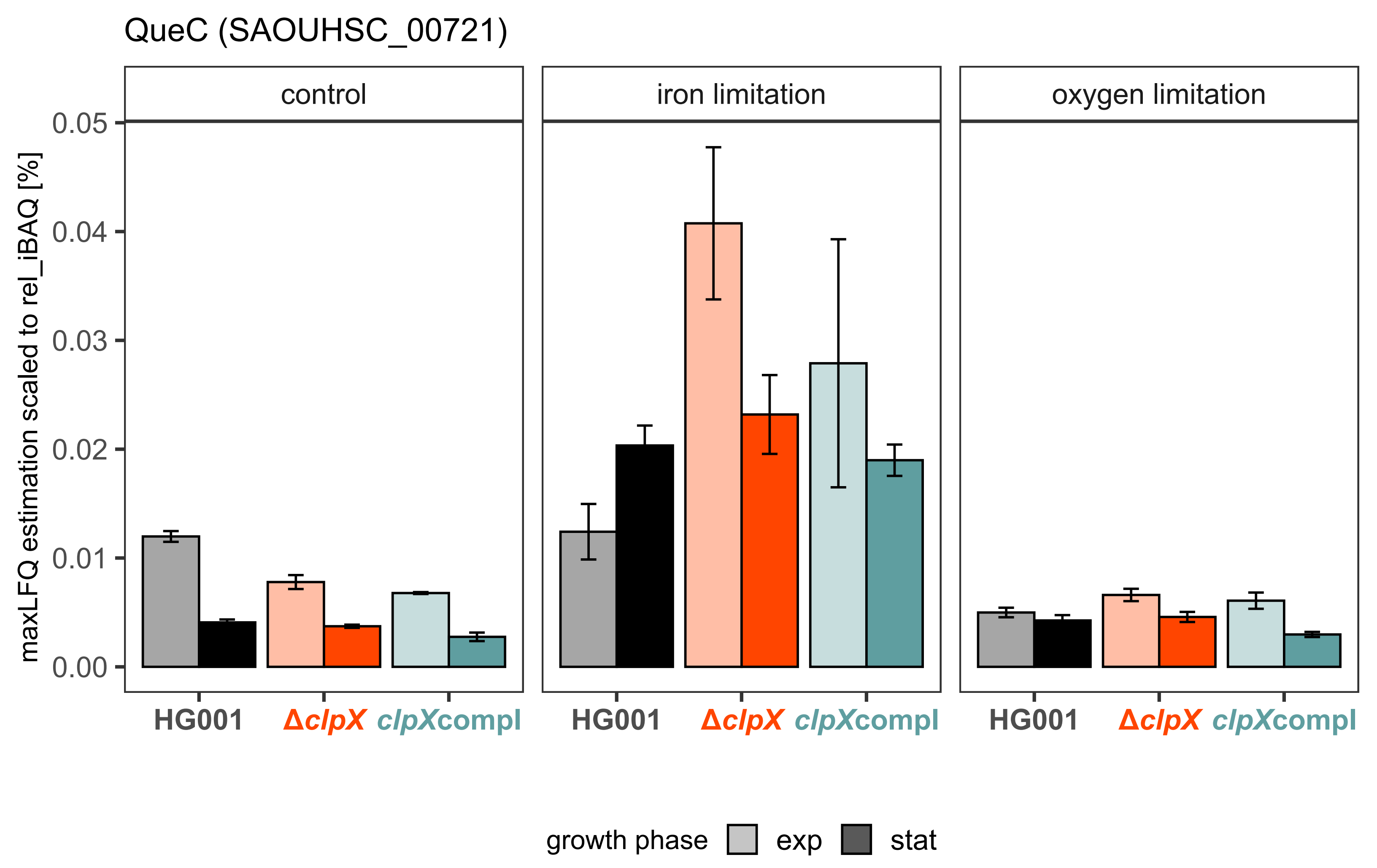
QueC (SAOUHSC_00721)
control
iron limitation
oxygen limitation
maxLFQ estimation scaled to rel_iBAQ [%]
0.05
0.04
0.03
0.02
0.01
0.00
HG001
ΔclpX
clpXcompl
growth phase
exp
stat

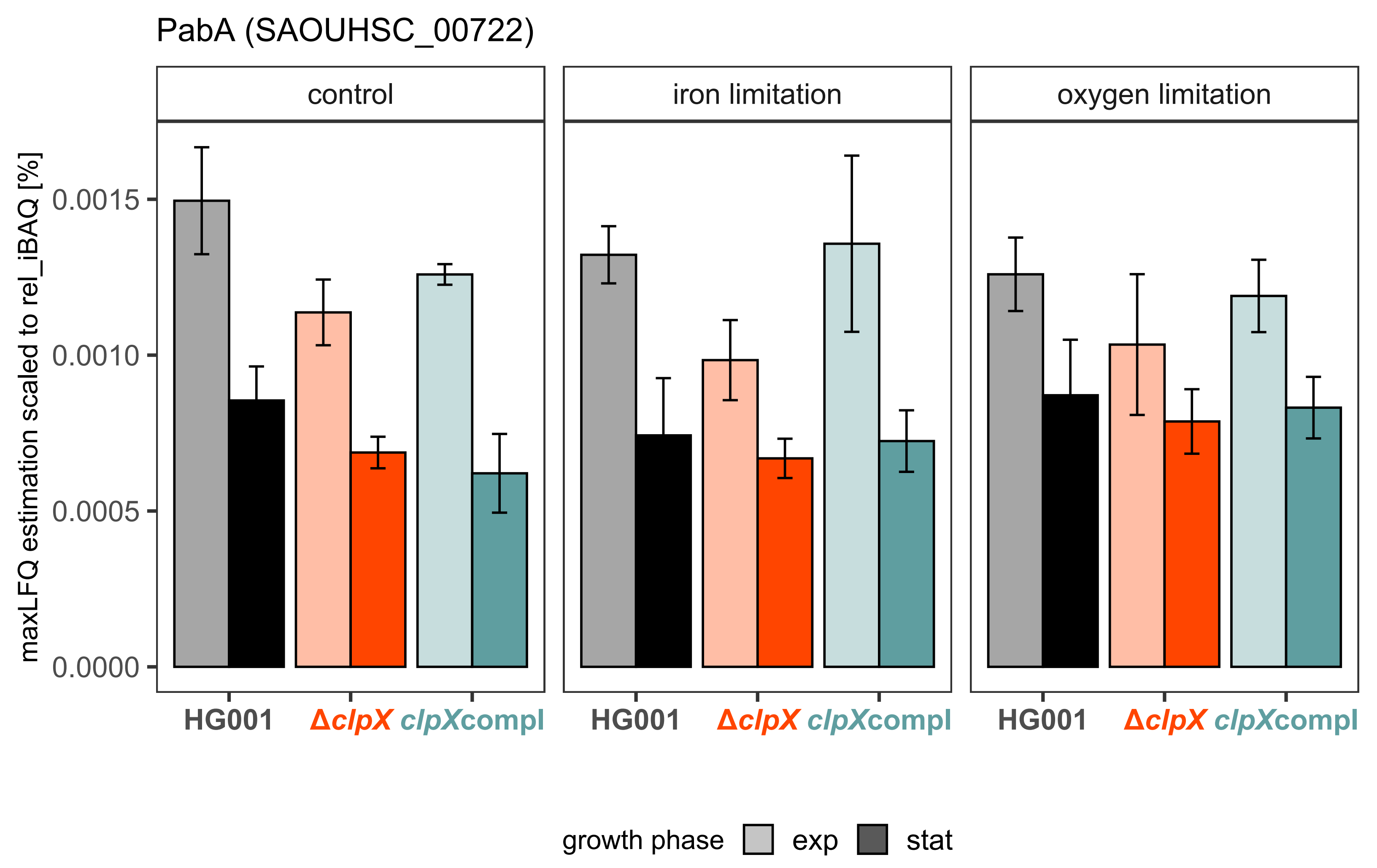

PabA (SAOUHSC_00722)
control
iron limitation
oxygen limitation
maxLFQ estimation scaled to rel_iBAQ [%]
0.0015
0.0010
0.0005
0.0000
HG001
ΔclpX
clpXcompl
growth phase
exp
stat

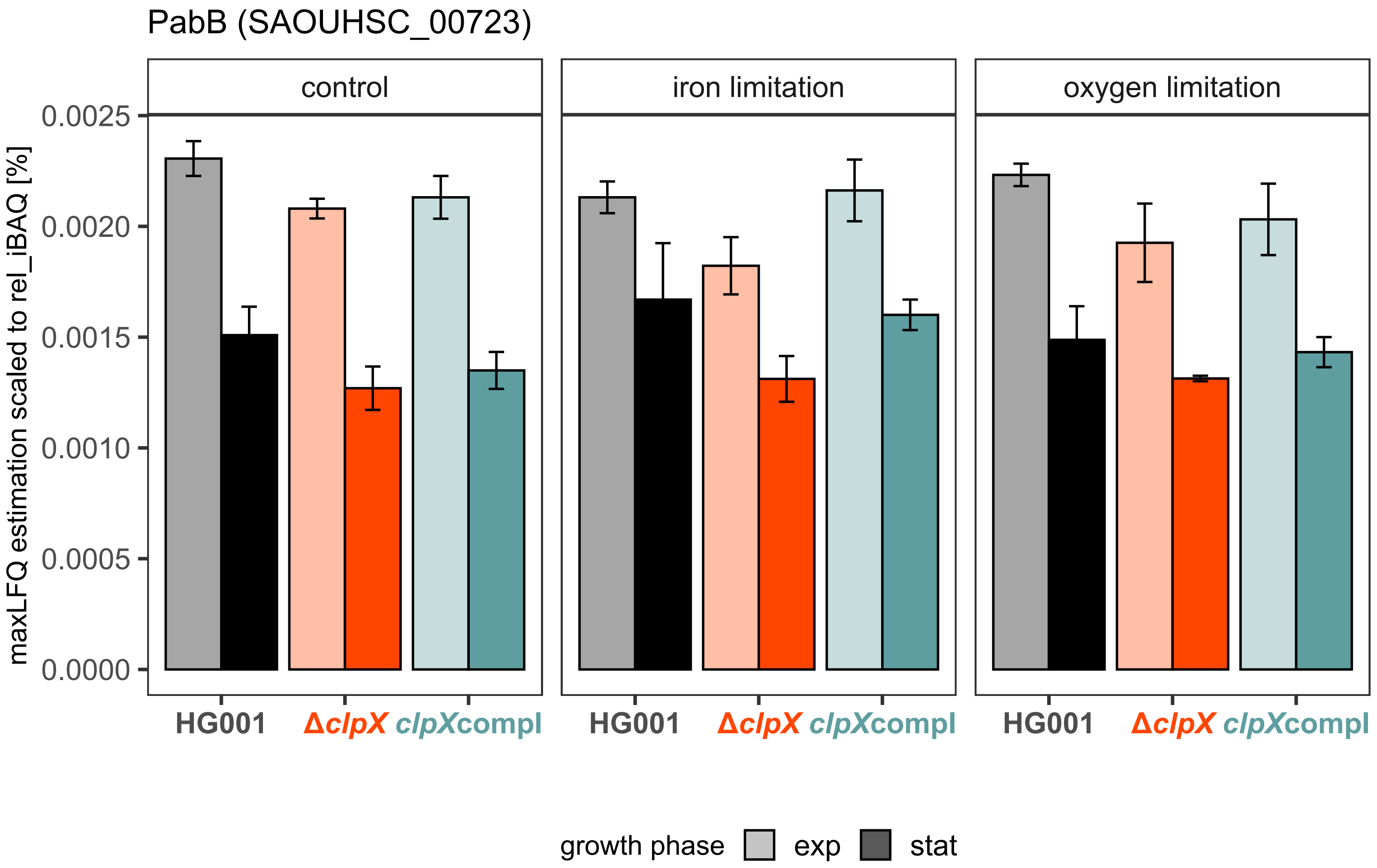
PabB (SAOUHSC_00723)
control
iron limitation
oxygen limitation
maxLFQ estimation scaled to rel_iBAQ [%]
0.0025
0.0020
0.0015
0.0010
0.0005
0.0000
HG001
ΔclpX
clpXcompl
growth phase
exp
stat

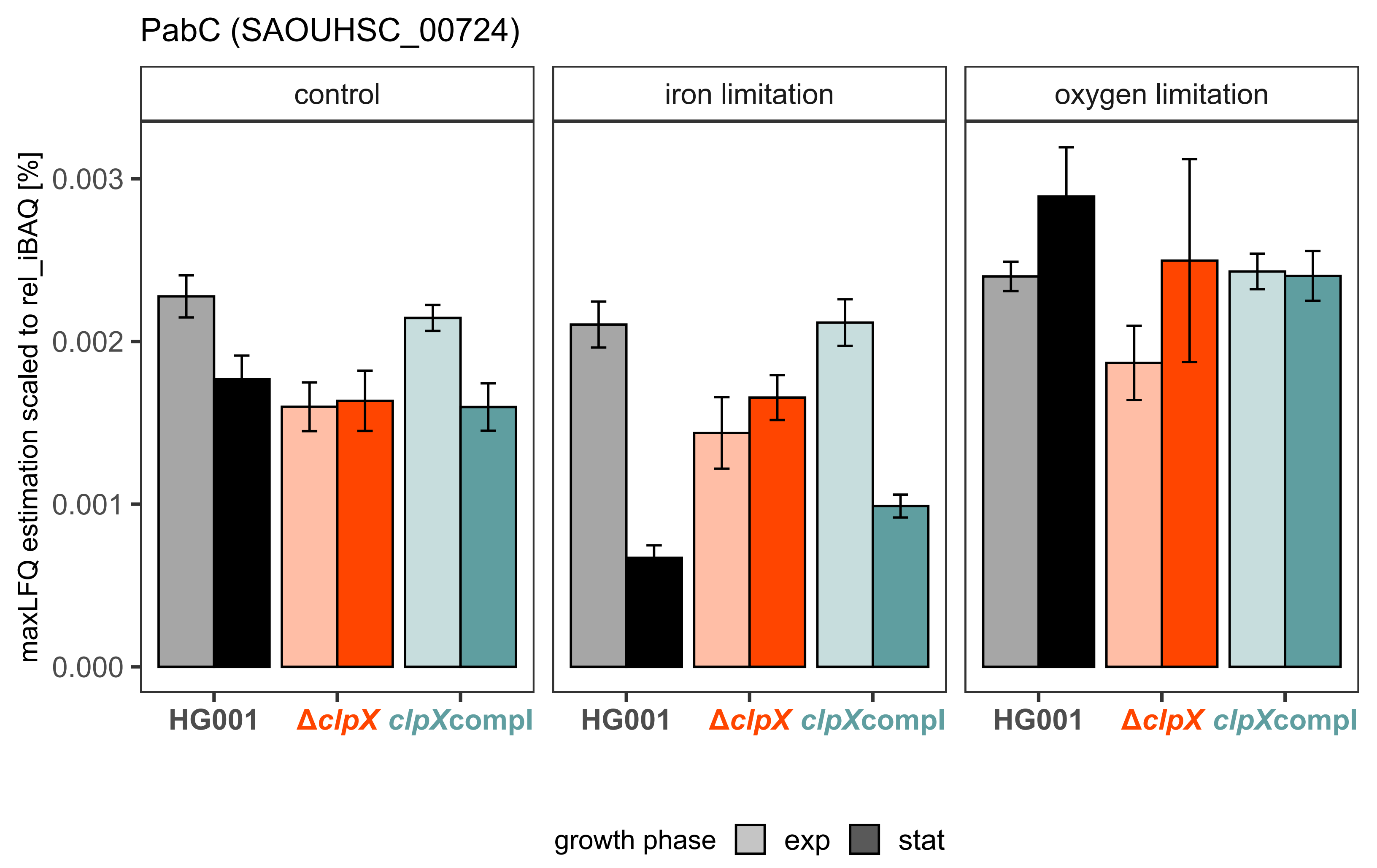
PabC (SAOUHSC_00724)
control
iron limitation
oxygen limitation
maxLFQ estimation scaled to rel_iBAQ [%]
0.003
0.002
0.001
0.000
HG001
ΔclpX
clpXcompl
growth phase
exp
stat

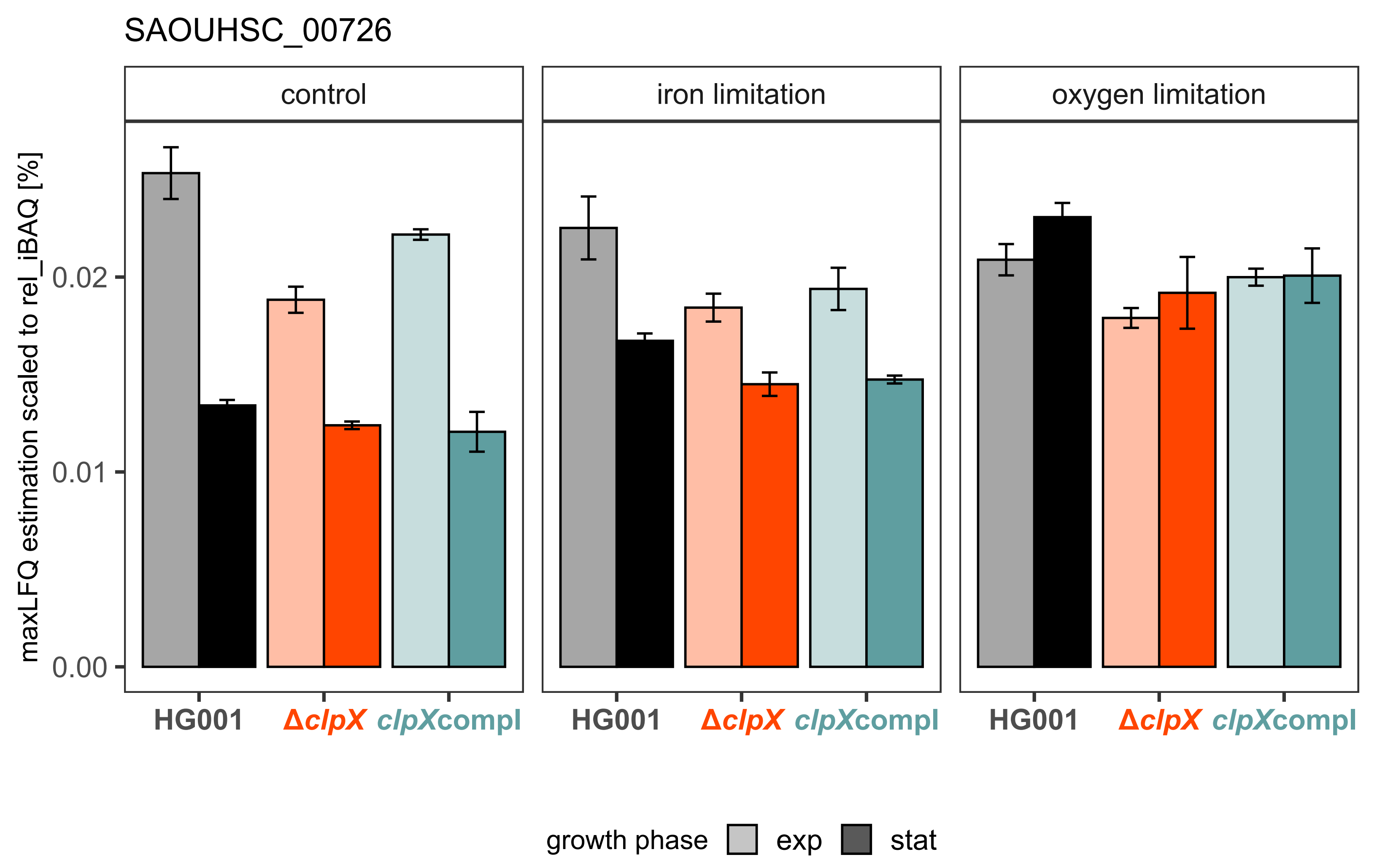
SAOUHSC_00726
control
iron limitation
oxygen limitation
maxLFQ estimation scaled to rel_iBAQ [%]
0.00
0.01
0.02
HG001
ΔclpX
clpXcompl
growth phase
exp
stat

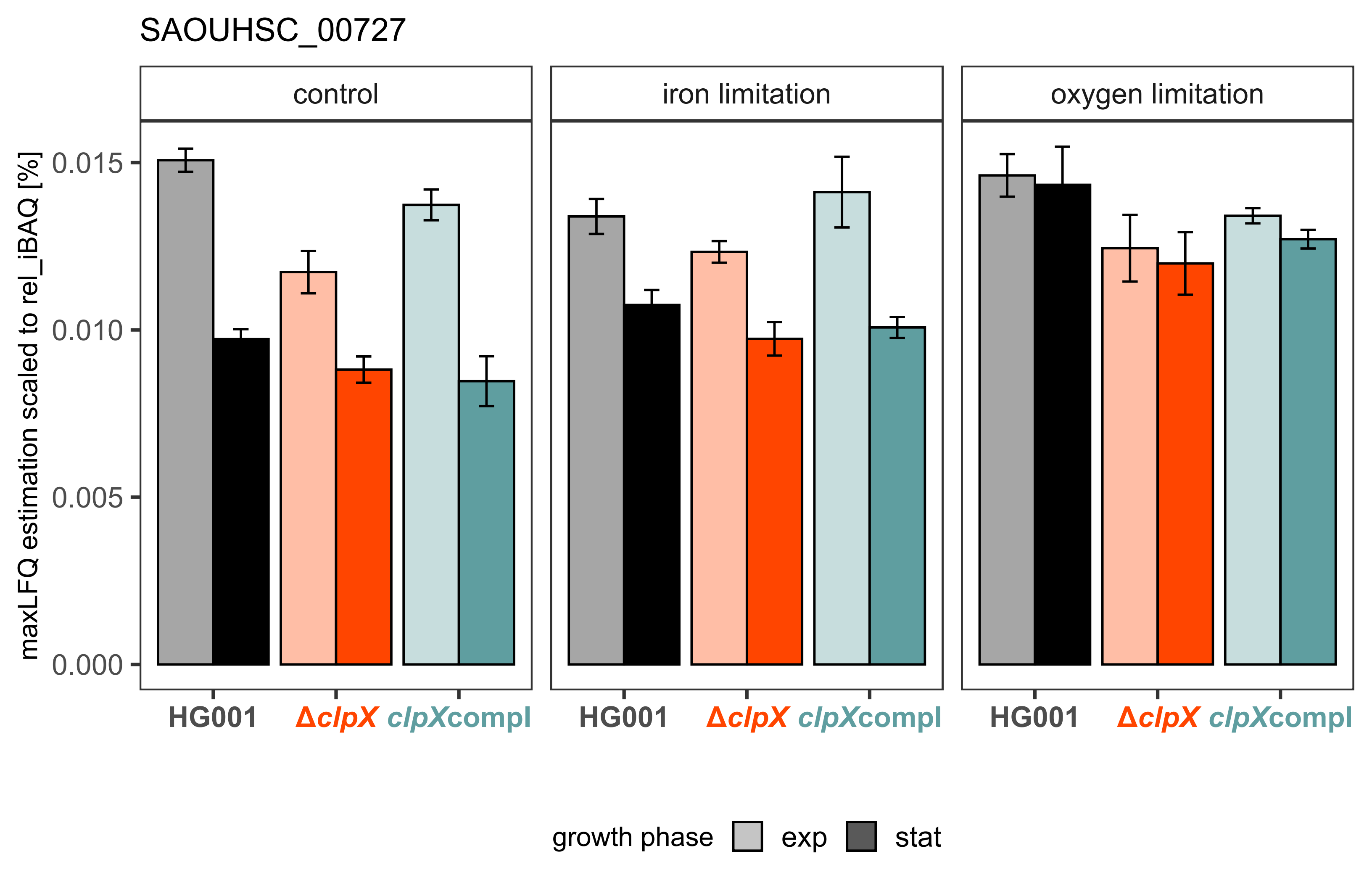
SAOUHSC_00727
control
iron limitation
oxygen limitation
maxLFQ estimation scaled to rel_iBAQ [%]
0.015
0.010
0.005
0.000
HG001
ΔclpX
clpXcompl
growth phase
exp
stat

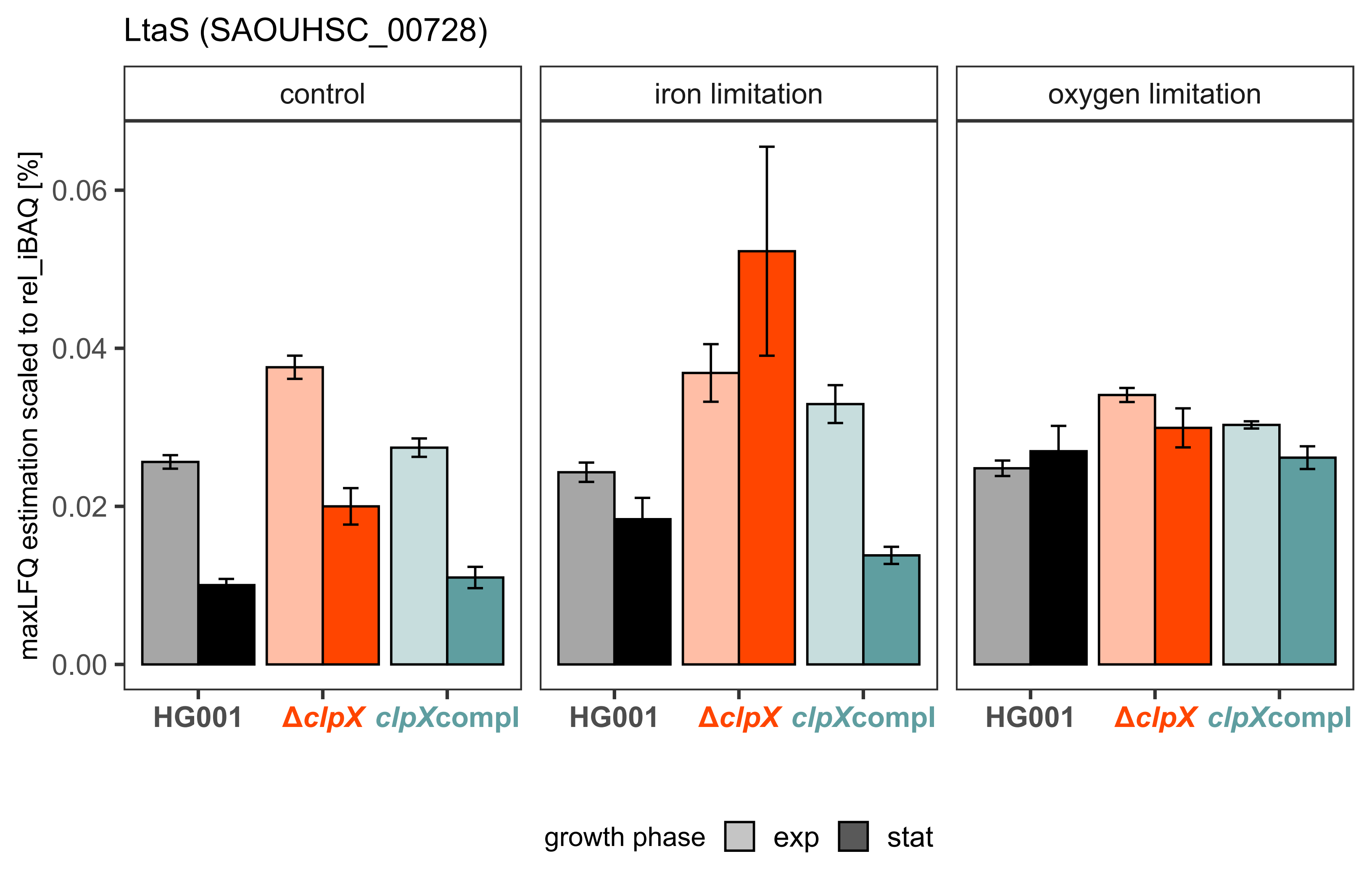
LtaS (SAOUHSC_00728)
control
iron limitation
oxygen limitation
maxLFQ estimation scaled to rel_iBAQ [%]
0.06
0.04
0.02
0.00
HG001
ΔclpX
clpXcompl
growth phase
exp
stat

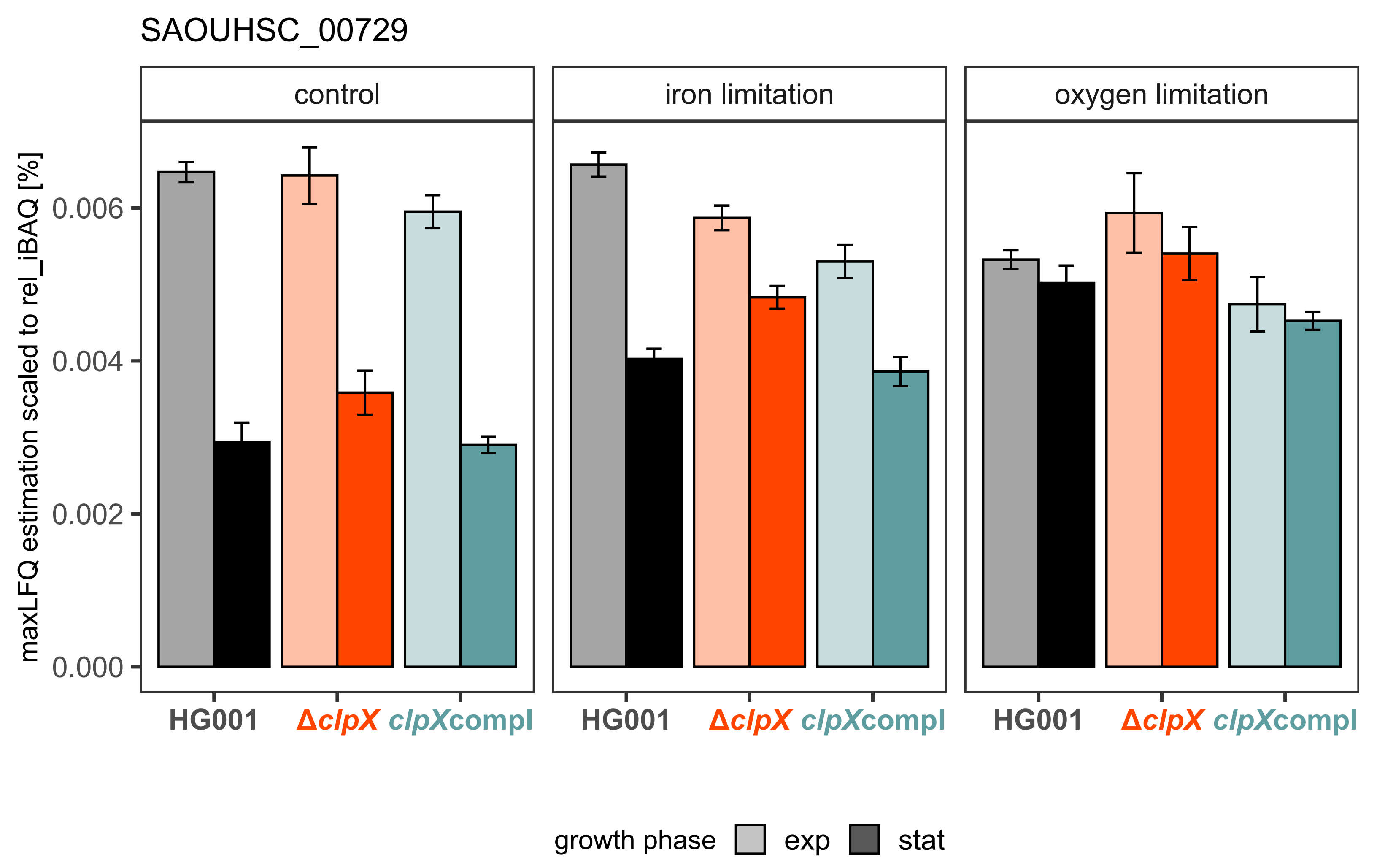
SAOUHSC_00729
control
iron limitation
oxygen limitation
maxLFQ estimation scaled to rel_iBAQ [%]
0.006
0.004
0.002
0.000
HG001
ΔclpX
clpXcompl
growth phase
exp
stat

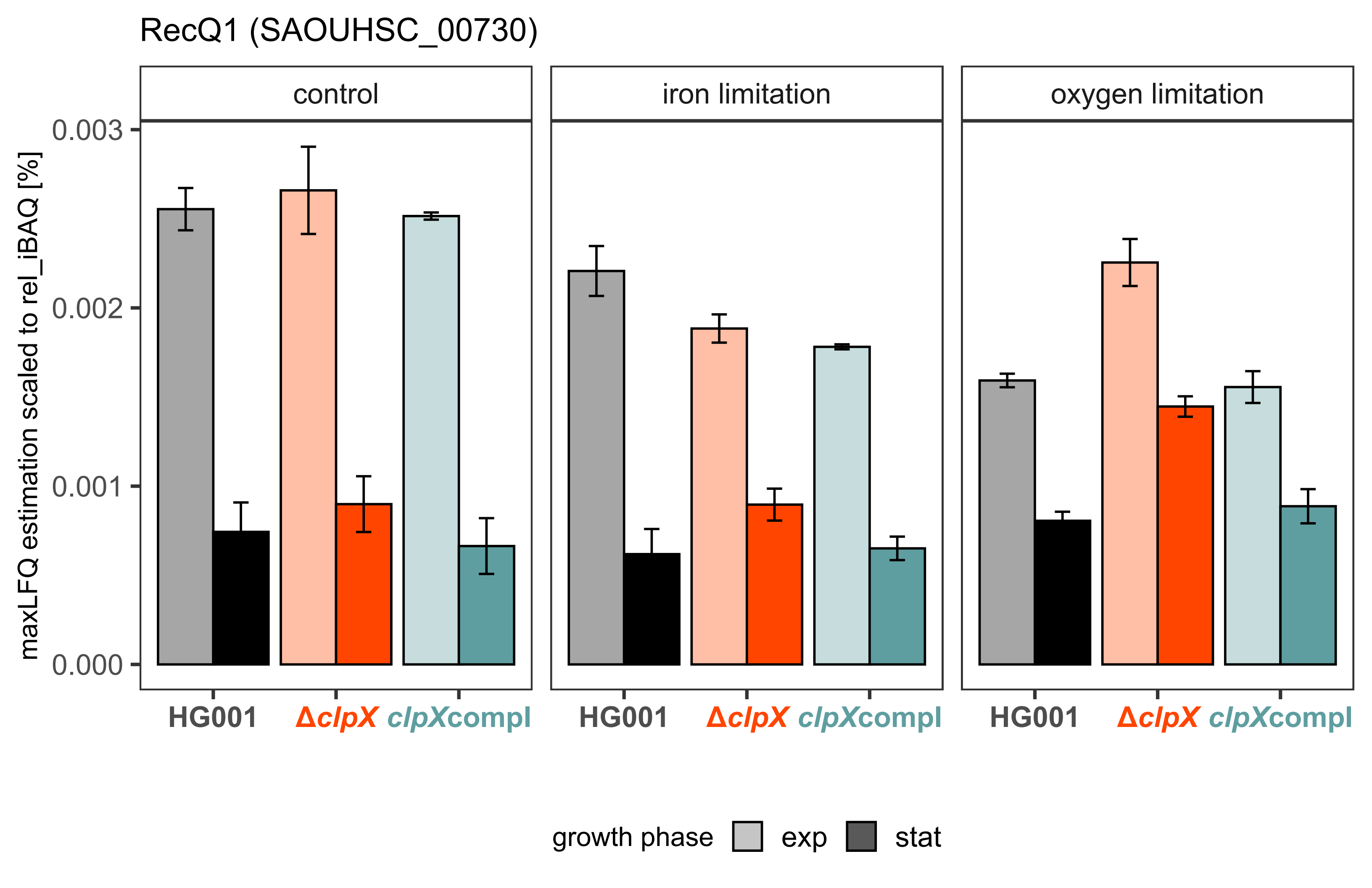

RecQ1 (SAOUHSC_00730)
control
iron limitation
oxygen limitation
maxLFQ estimation scaled to rel_iBAQ [%]
0.003
0.002
0.001
0.000
HG001
ΔclpX
clpXcompl
growth phase
exp
stat

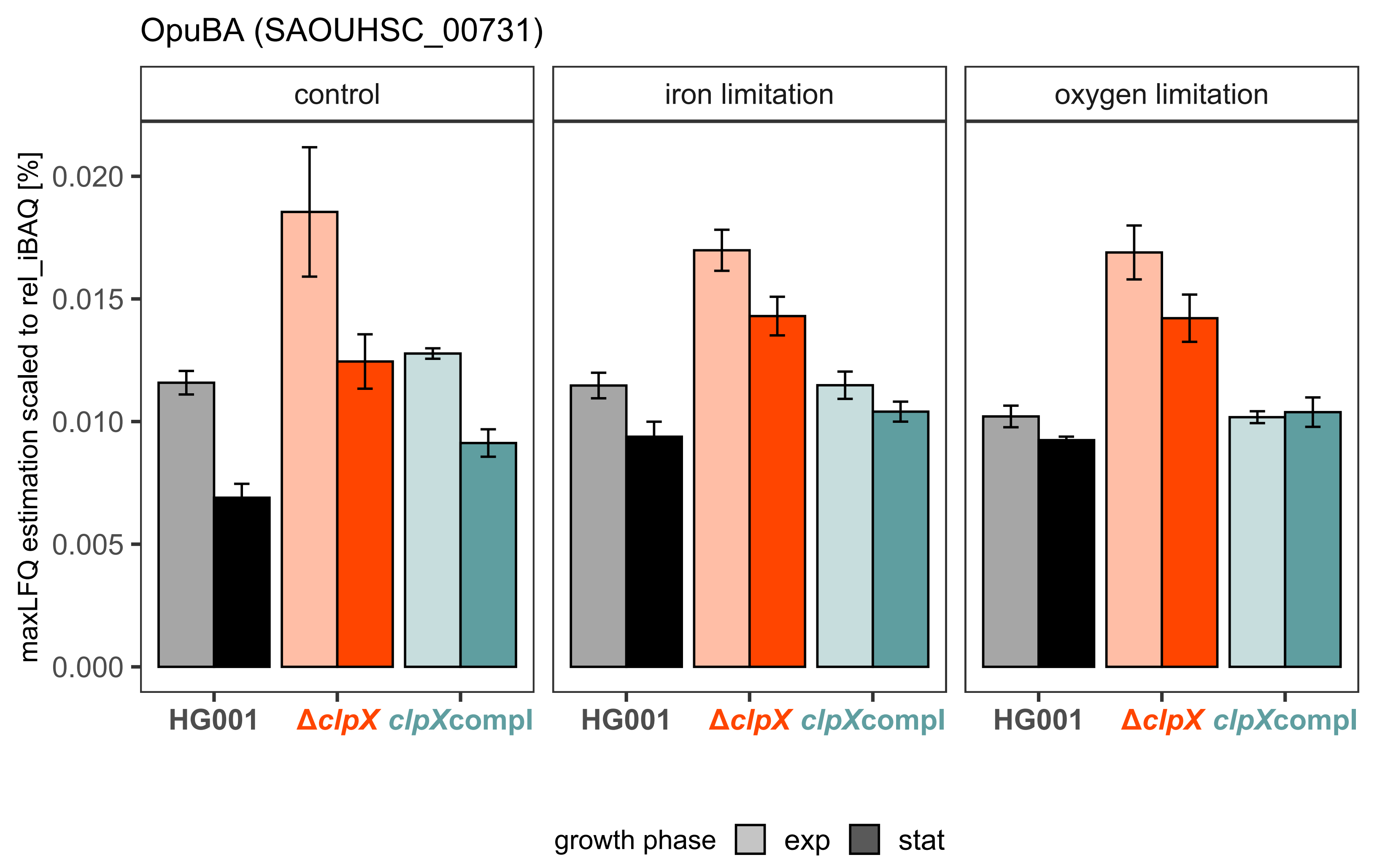
OpuBA (SAOUHSC_00731)
control
iron limitation
oxygen limitation
maxLFQ estimation scaled to rel_iBAQ [%]
0.020
0.015
0.010
0.005
0.000
HG001
ΔclpX
clpXcompl
growth phase
exp
stat

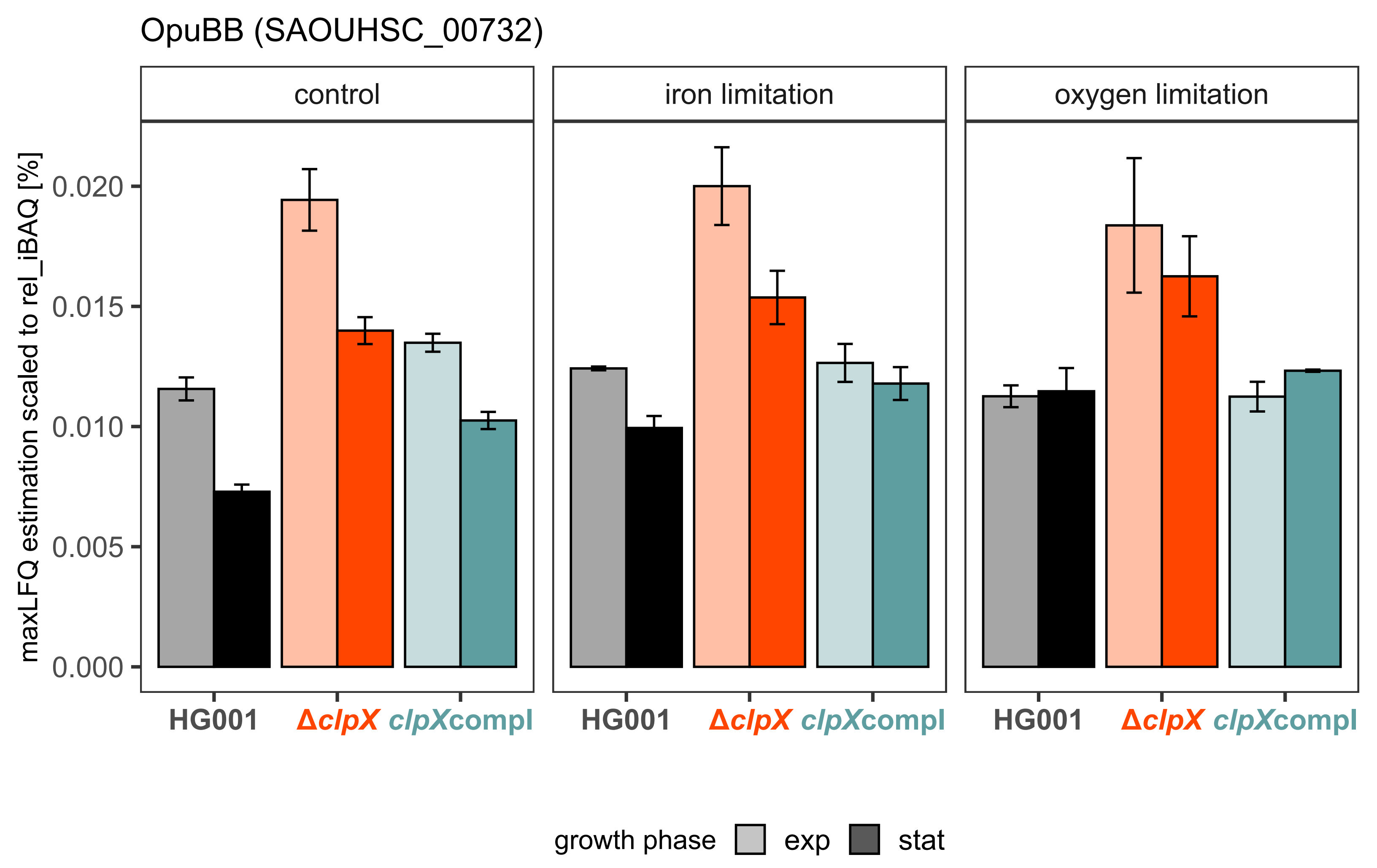
OpuBB (SAOUHSC_00732)
control
iron limitation
oxygen limitation
maxLFQ estimation scaled to rel_iBAQ [%]
0.020
0.015
0.010
0.005
0.000
HG001
ΔclpX
clpXcompl
growth phase
exp
stat

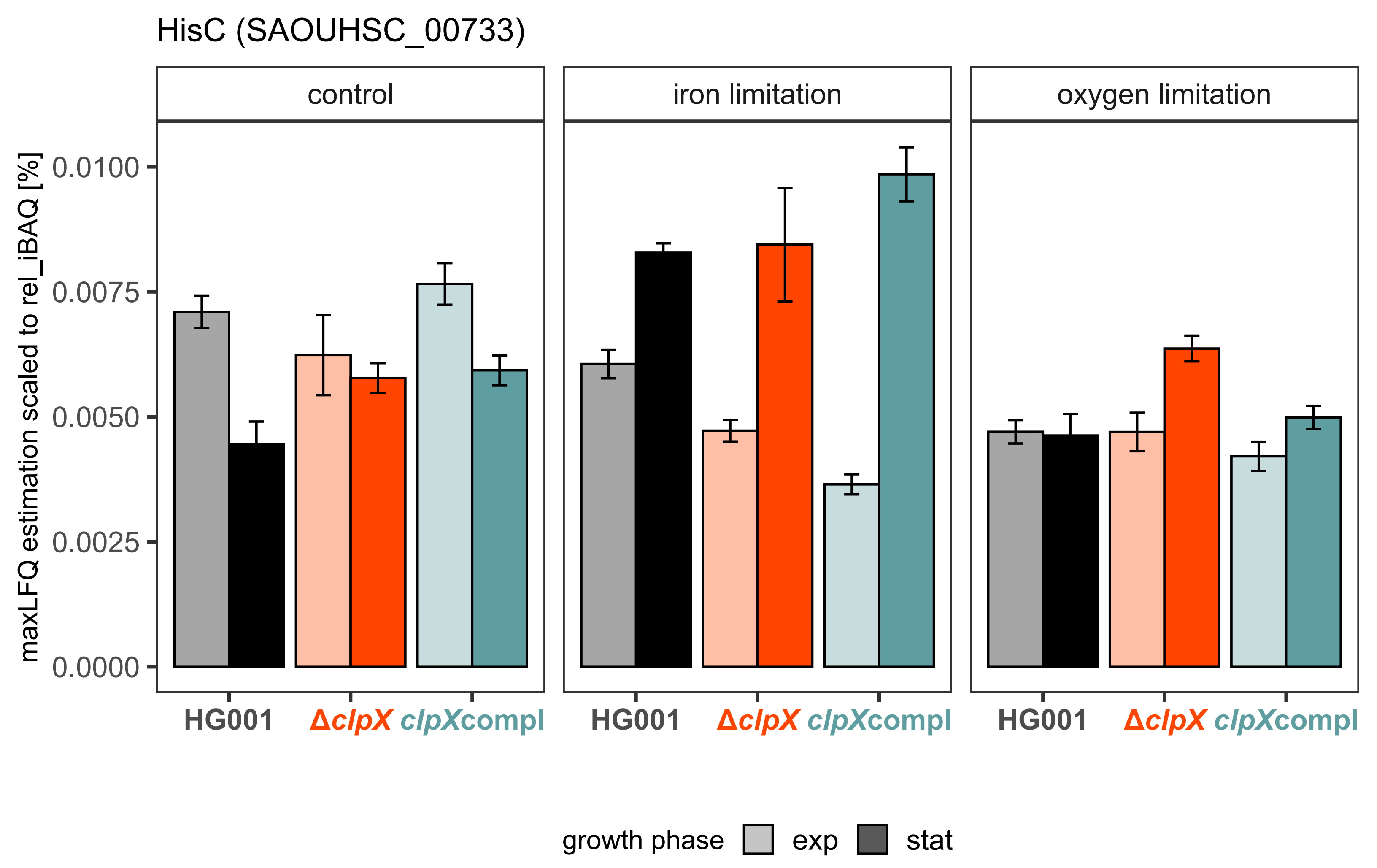

HisC (SAOUHSC_00733)
control
iron limitation
oxygen limitation
maxLFQ estimation scaled to rel_iBAQ [%]
0.0100
0.0075
0.0050
0.0025
0.0000
HG001
ΔclpX
clpXcompl
growth phase
exp
stat

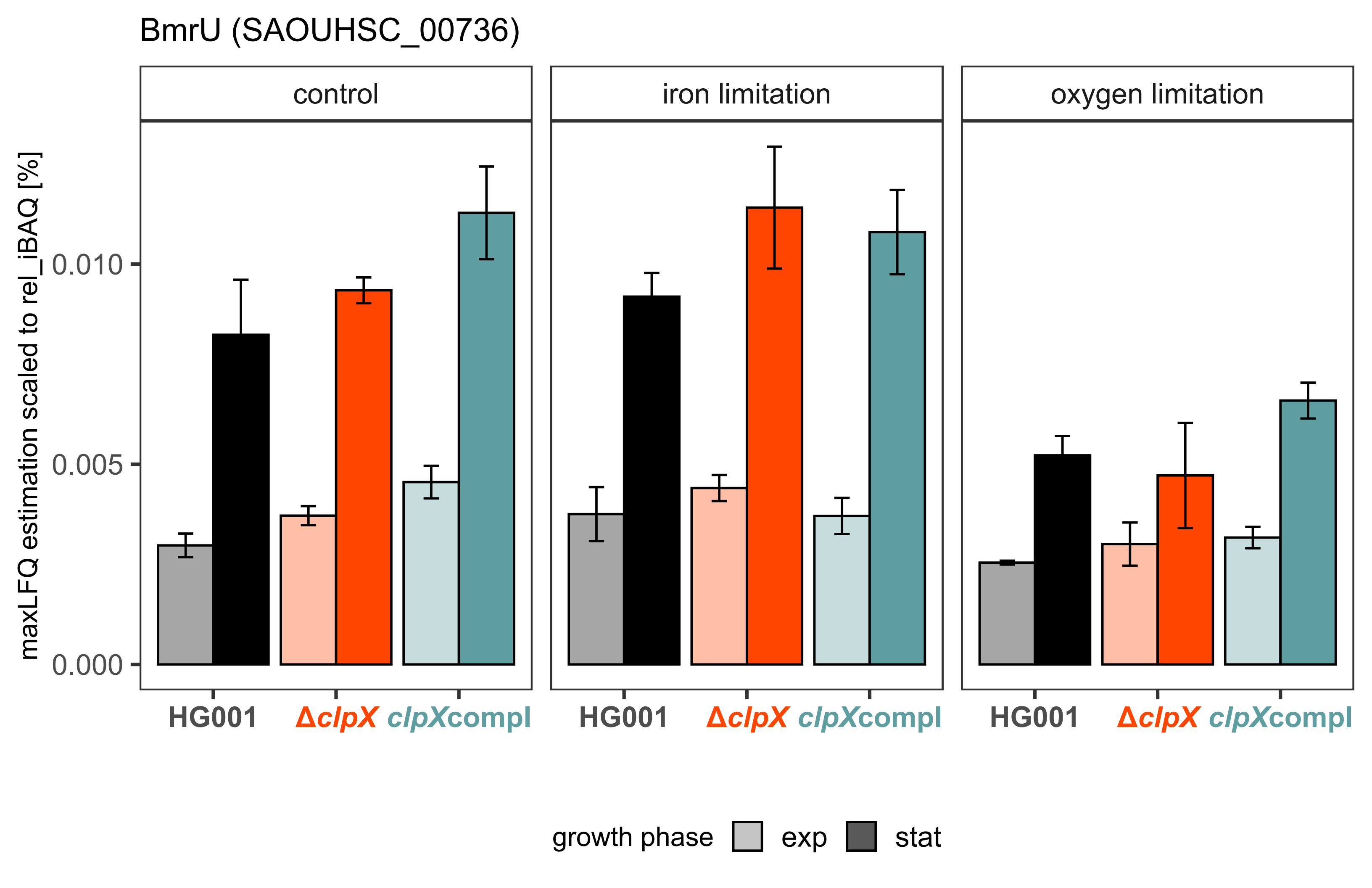
BmrU (SAOUHSC_00736)
control
iron limitation
oxygen limitation
maxLFQ estimation scaled to rel_iBAQ [%]
0.000
0.005
0.010
HG001
ΔclpX
clpXcompl
growth phase
exp
stat

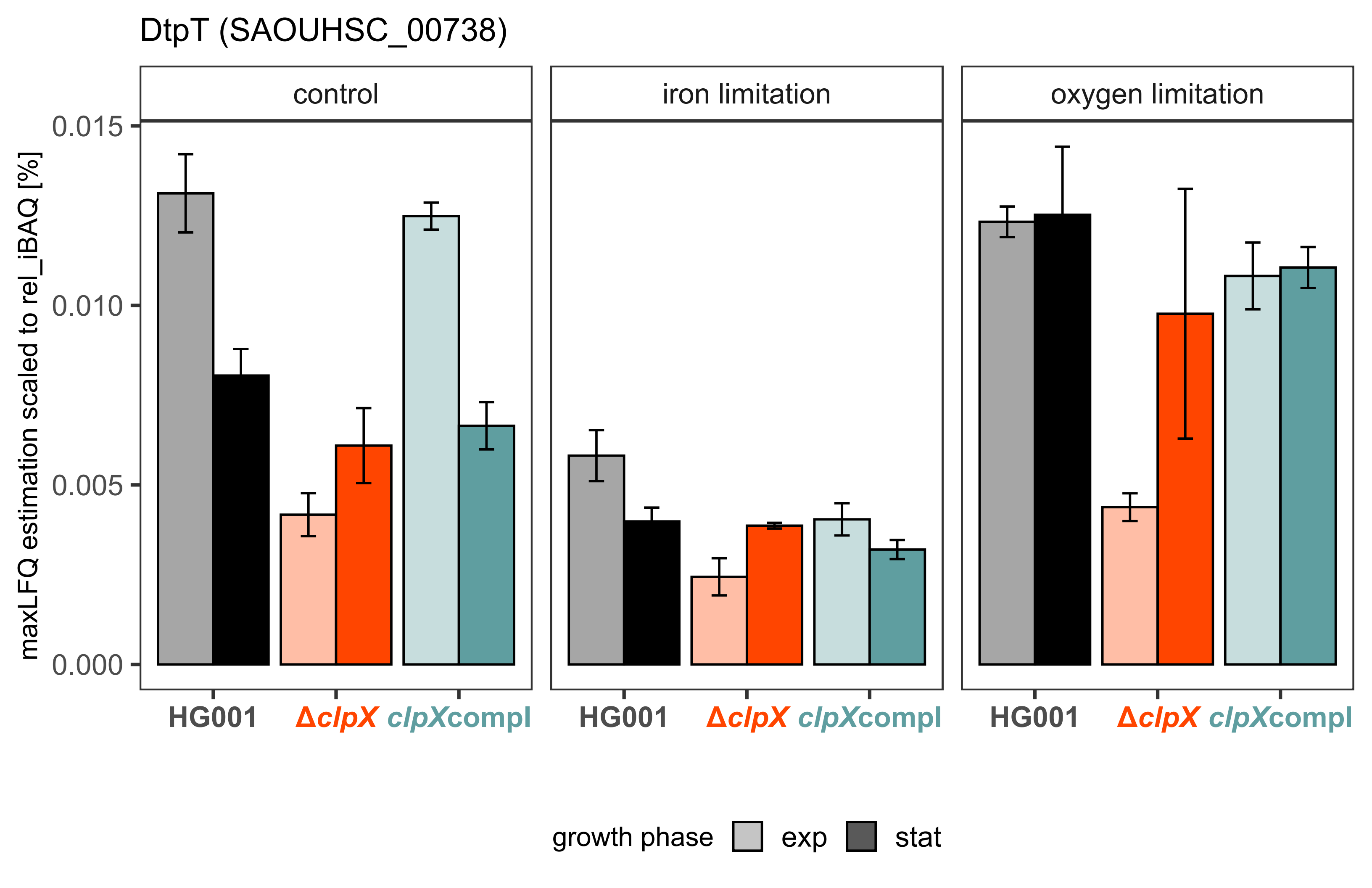
DtpT (SAOUHSC_00738)
control
iron limitation
oxygen limitation
maxLFQ estimation scaled to rel_iBAQ [%]
0.015
0.010
0.005
0.000
HG001
ΔclpX
clpXcompl
growth phase
exp
stat

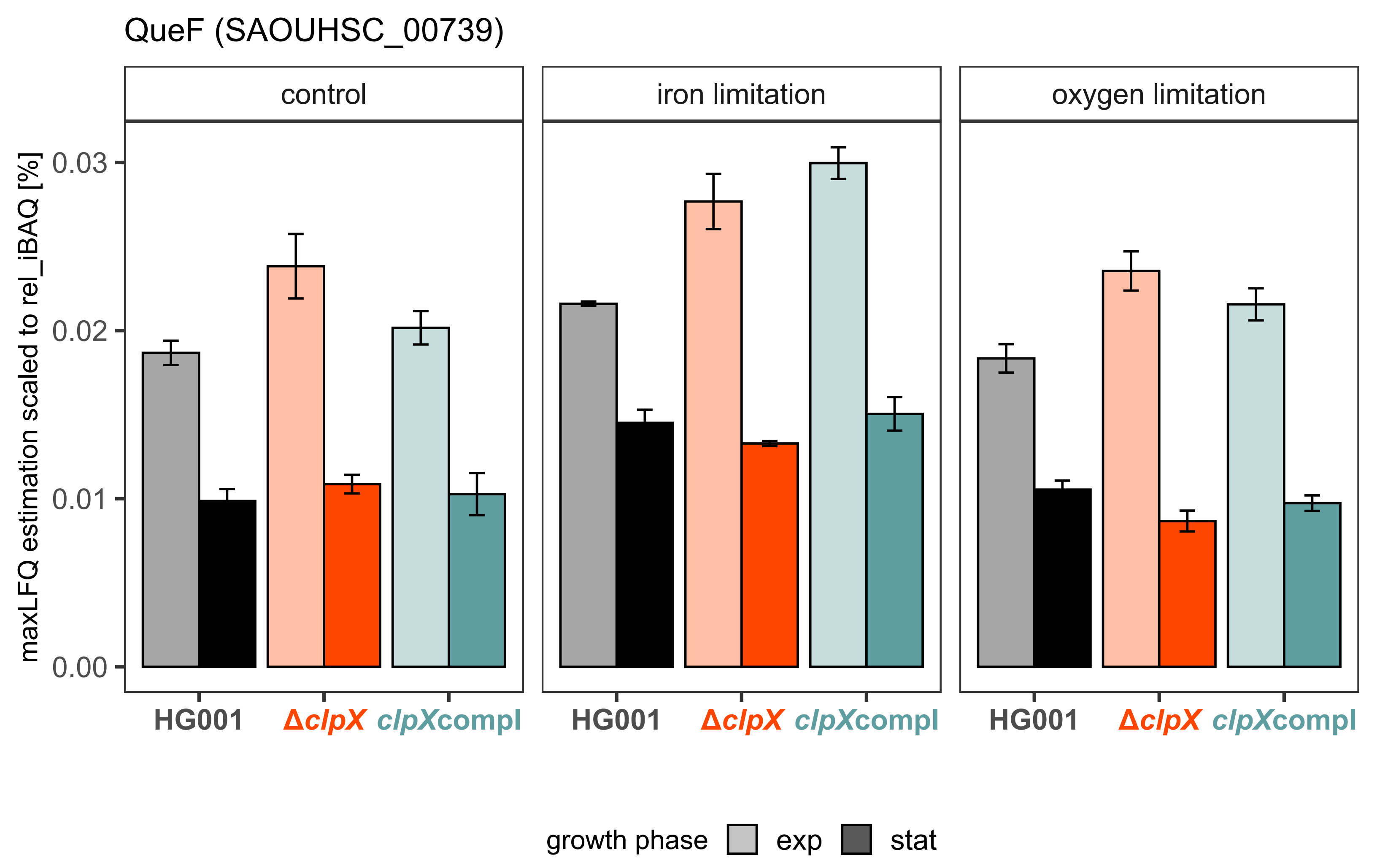

QueF (SAOUHSC_00739)
control
iron limitation
oxygen limitation
maxLFQ estimation scaled to rel_iBAQ [%]
0.03
0.02
0.01
0.00
HG001
ΔclpX
clpXcompl
growth phase
exp
stat

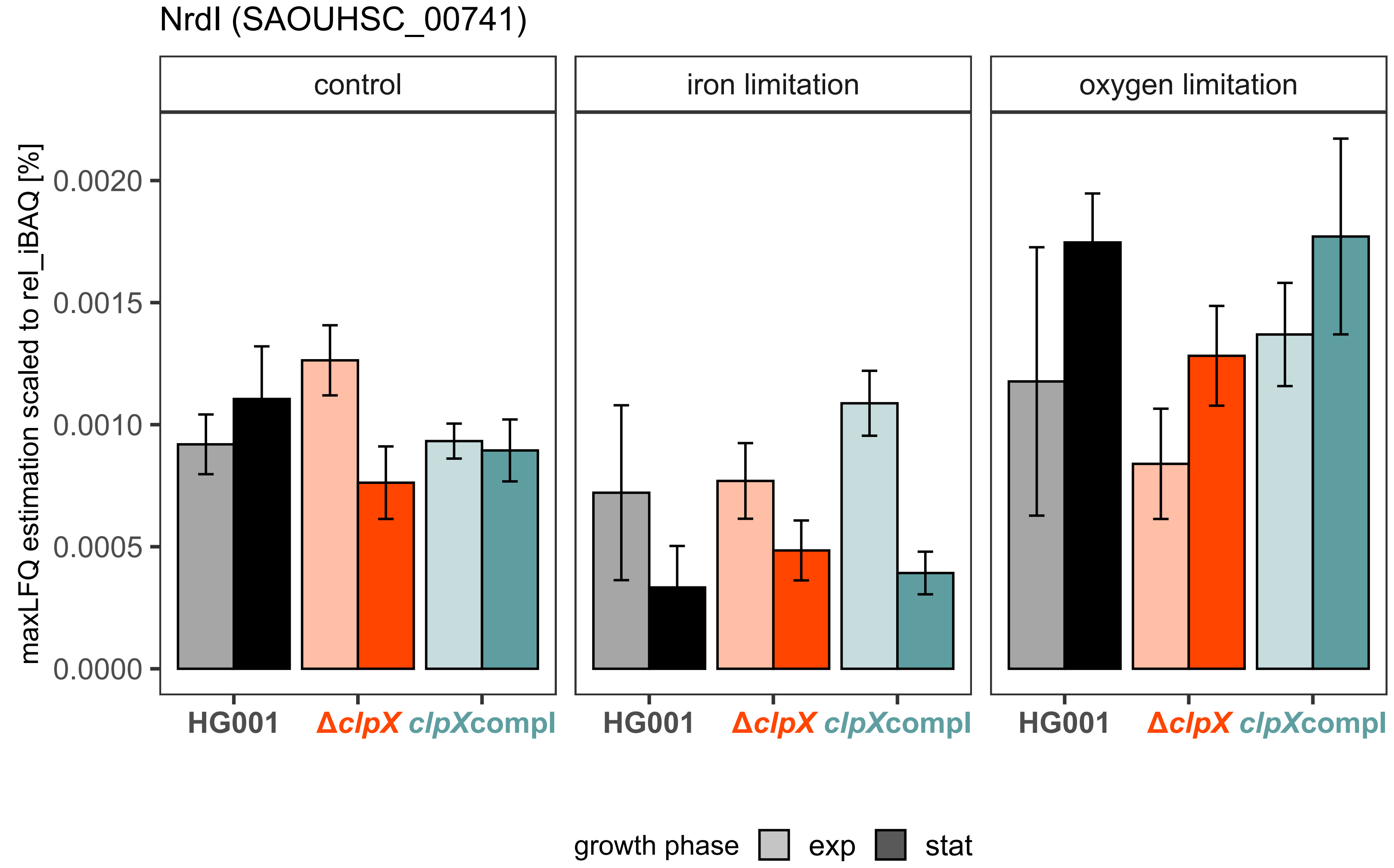
NrdI (SAOUHSC_00741)
control
iron limitation
oxygen limitation
maxLFQ estimation scaled to rel_iBAQ [%]
0.0020
0.0015
0.0010
0.0005
0.0000
HG001
ΔclpX
clpXcompl
growth phase
exp
stat

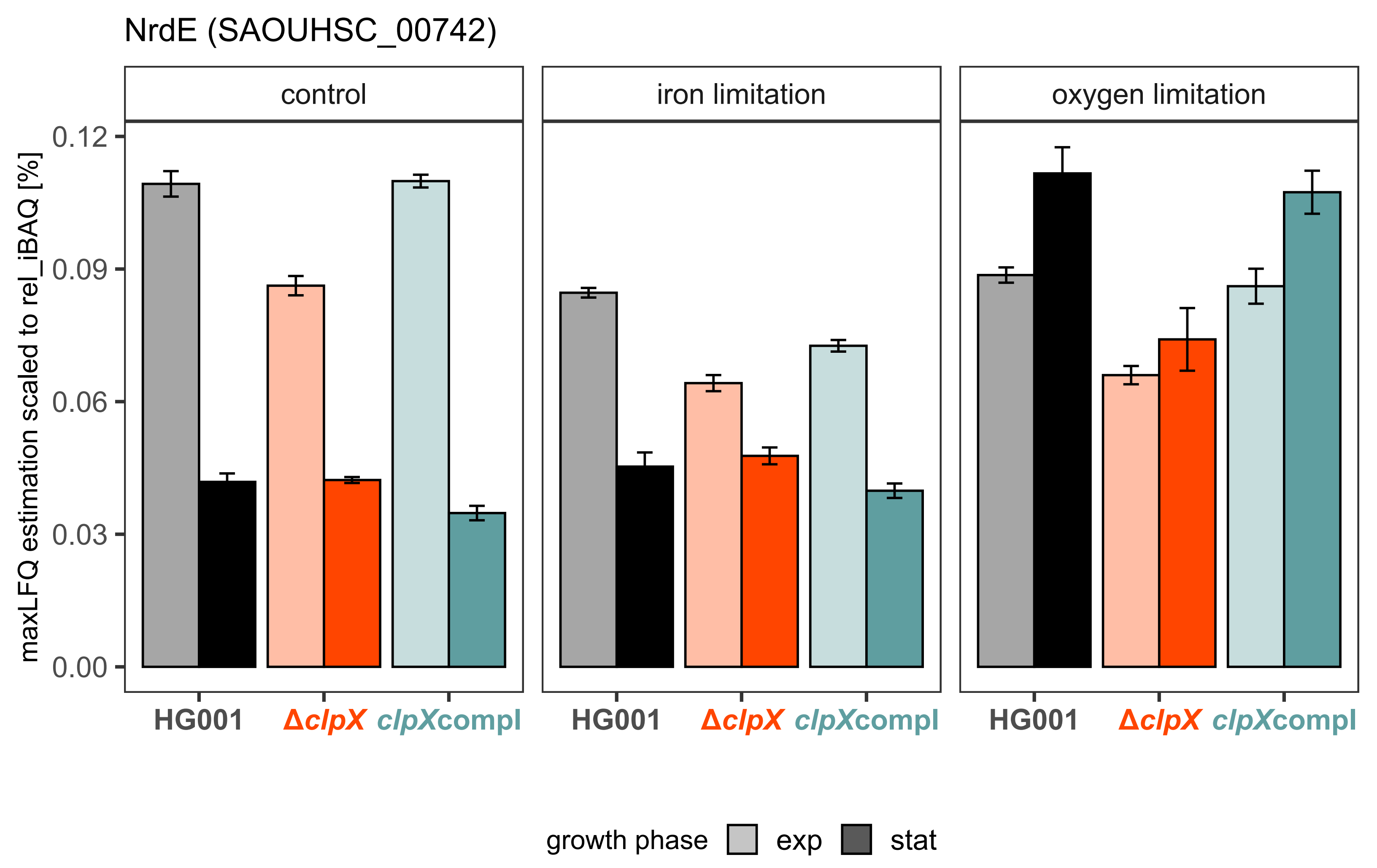
NrdE (SAOUHSC_00742)
control
iron limitation
oxygen limitation
maxLFQ estimation scaled to rel_iBAQ [%]
0.00
0.03
0.06
0.09
0.12
HG001
ΔclpX
clpXcompl
growth phase
exp
stat

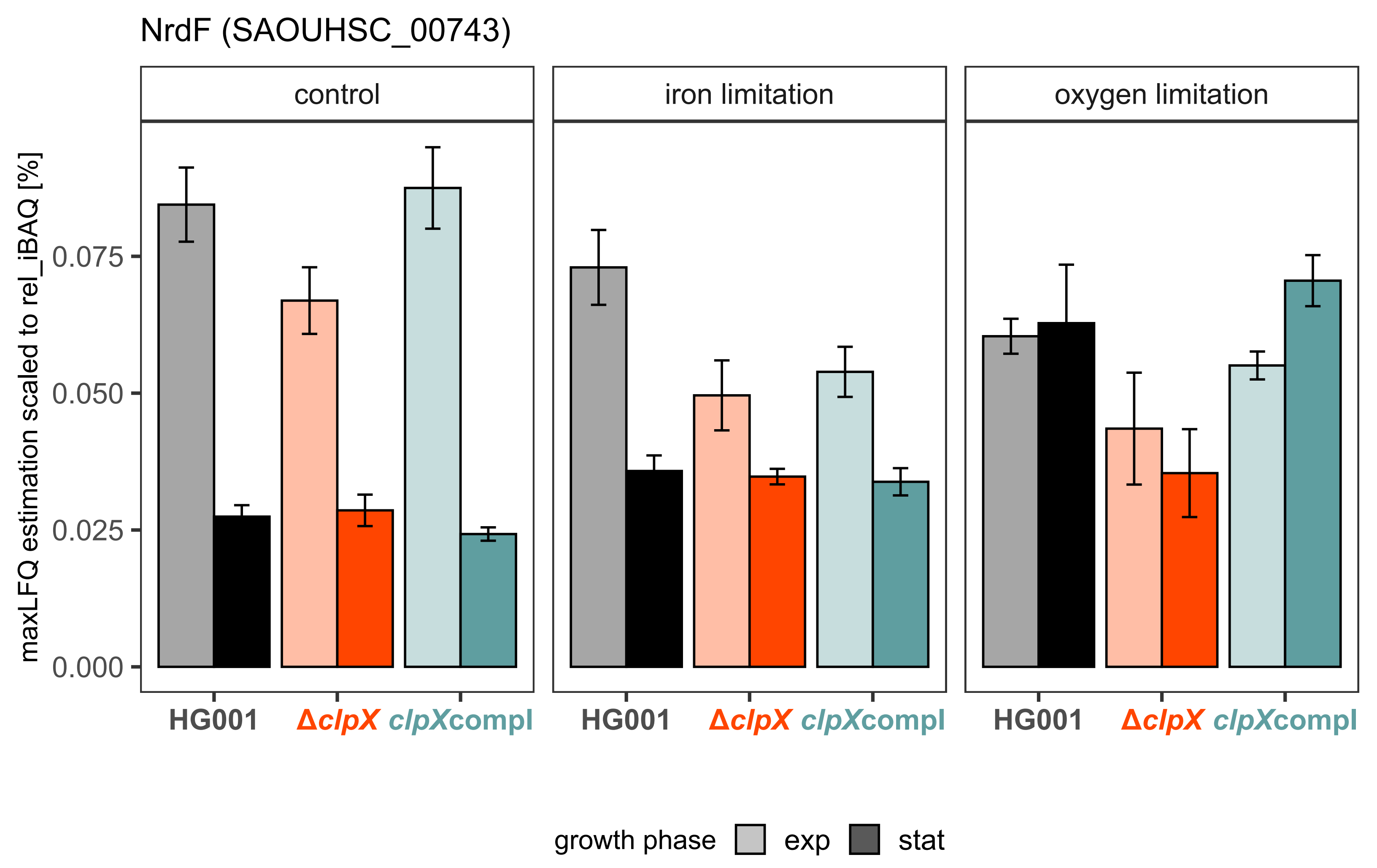

NrdF (SAOUHSC_00743)
control
iron limitation
oxygen limitation
maxLFQ estimation scaled to rel_iBAQ [%]
0.075
0.050
0.025
0.000
HG001
ΔclpX
clpXcompl
growth phase
exp
stat

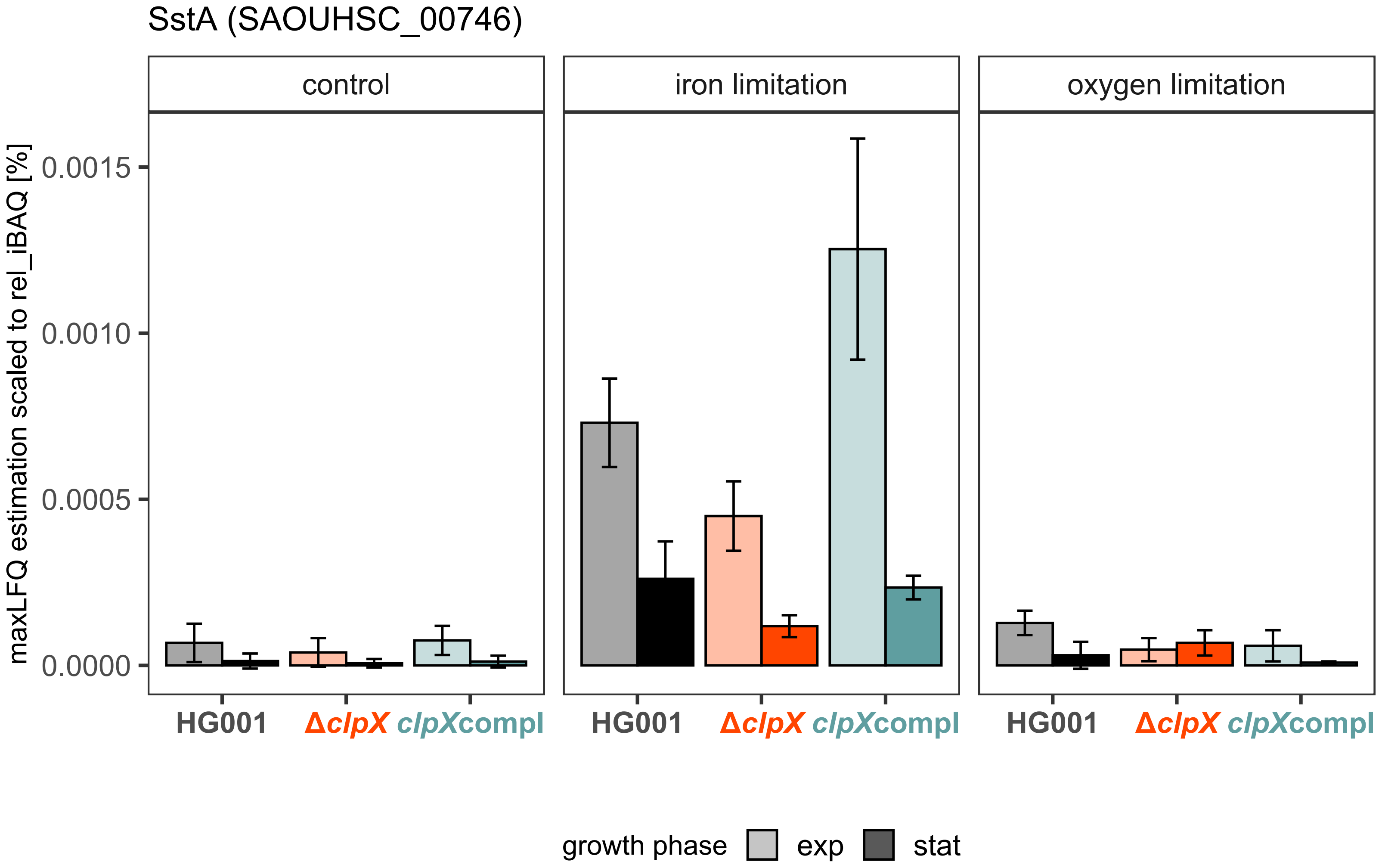
SstA (SAOUHSC_00746)
control
iron limitation
oxygen limitation
maxLFQ estimation scaled to rel_iBAQ [%]
0.0015
0.0010
0.0005
0.0000
HG001
ΔclpX
clpXcompl
growth phase
exp
stat

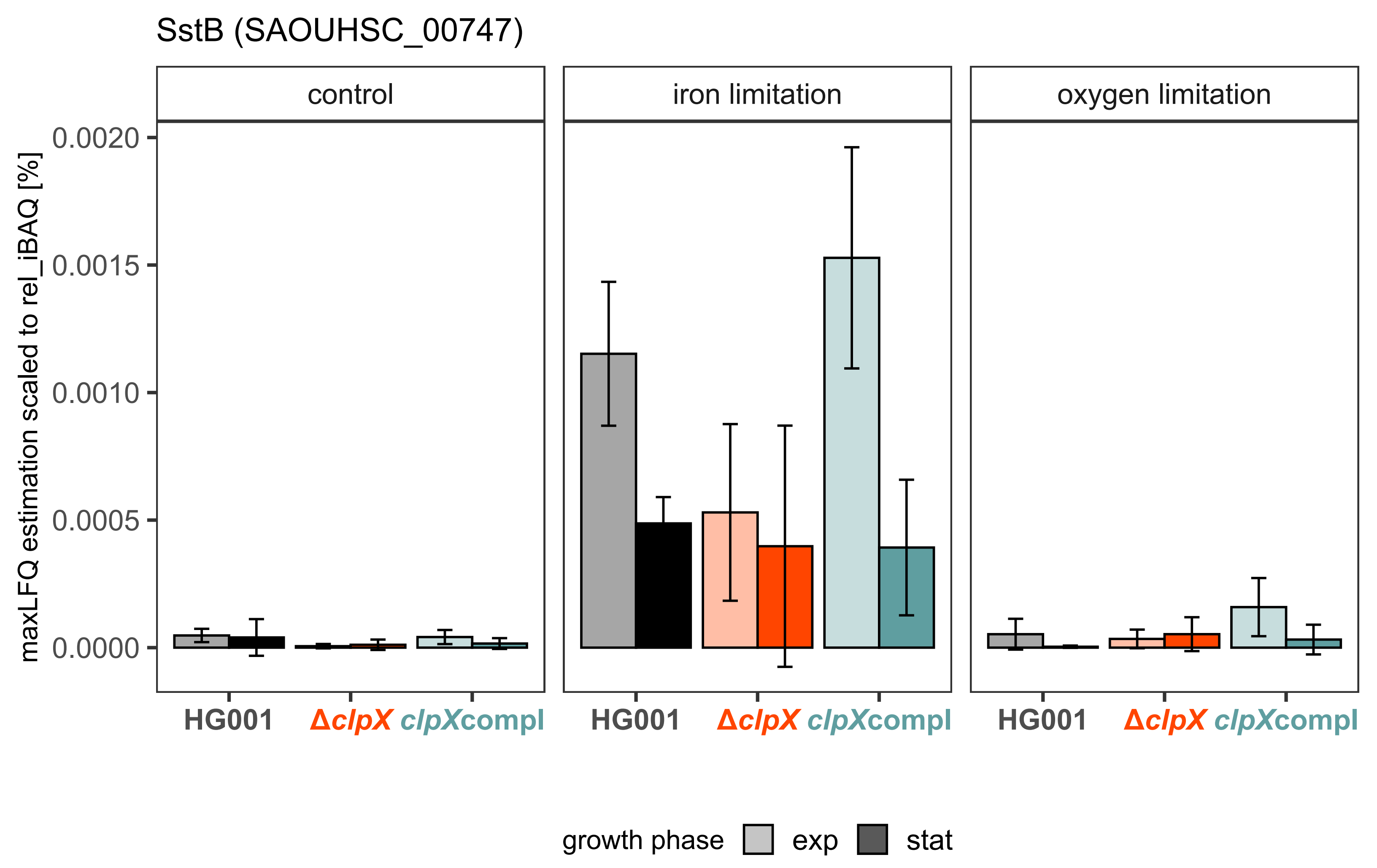
SstB (SAOUHSC_00747)
control
iron limitation
oxygen limitation
maxLFQ estimation scaled to rel_iBAQ [%]
0.0020
0.0015
0.0010
0.0005
0.0000
HG001
ΔclpX
clpXcompl
growth phase
exp
stat

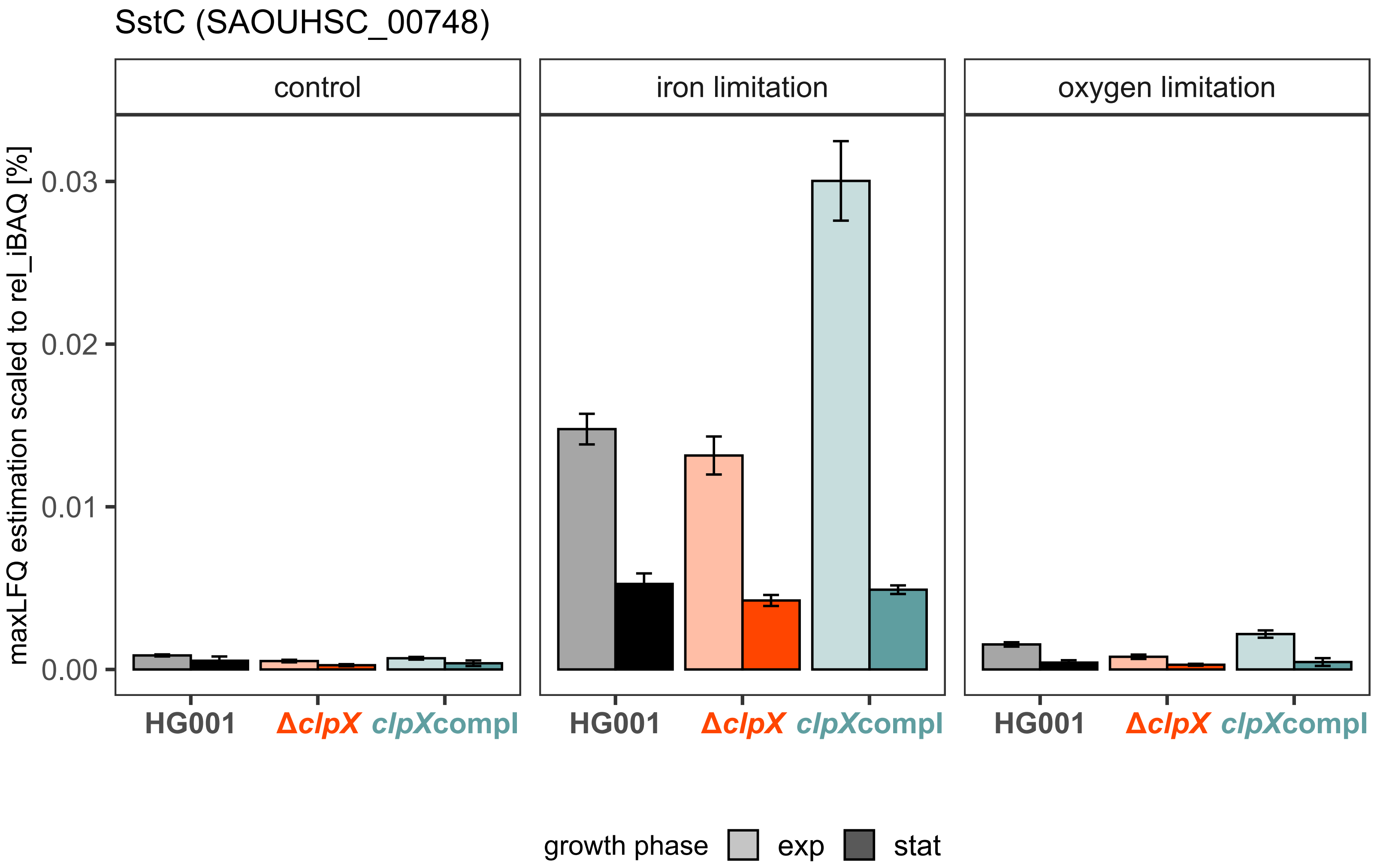
SstC (SAOUHSC_00748)
control
iron limitation
oxygen limitation
maxLFQ estimation scaled to rel_iBAQ [%]
0.03
0.02
0.01
0.00
HG001
ΔclpX
clpXcompl
growth phase
exp
stat

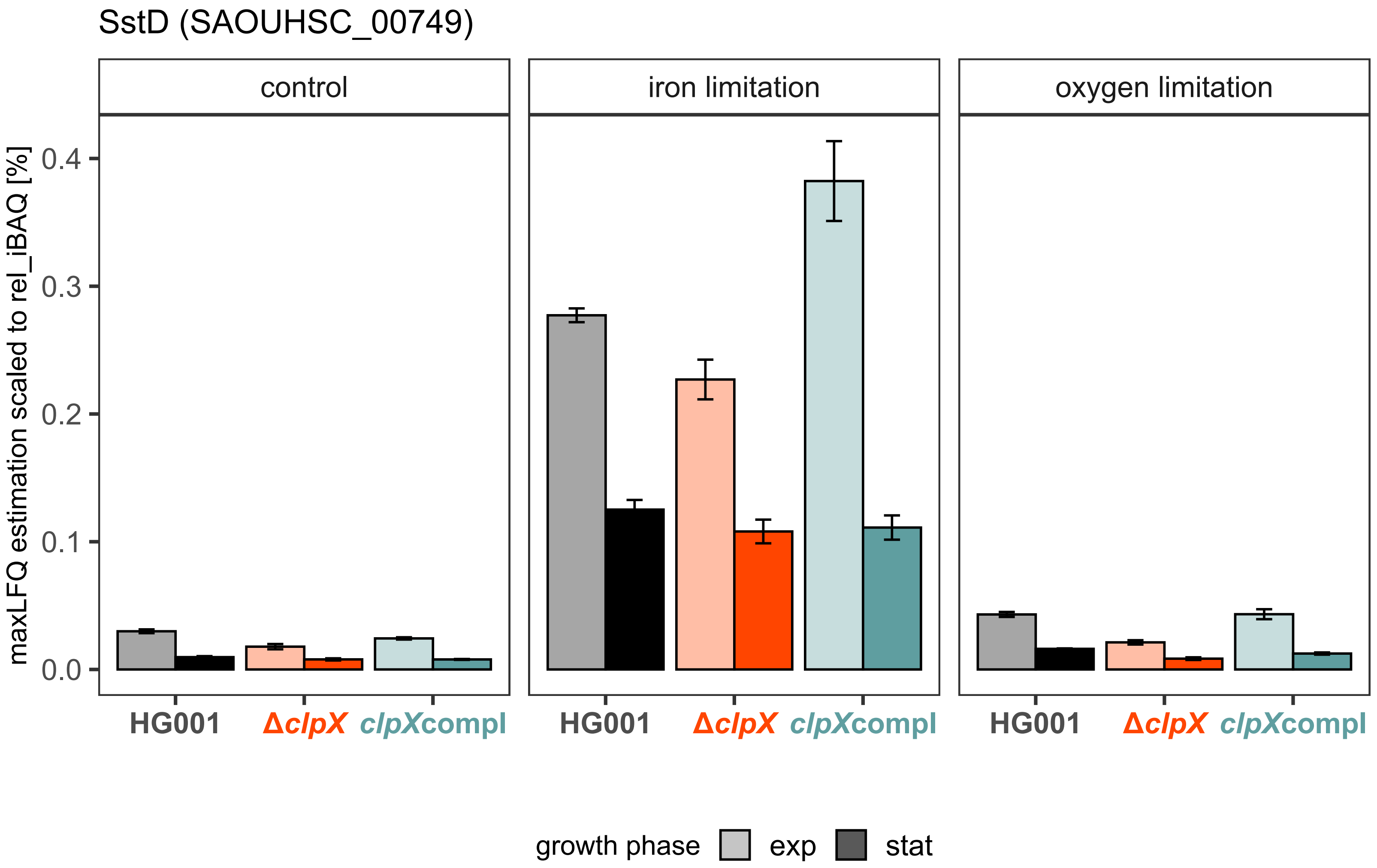
SstD (SAOUHSC_00749)
control
iron limitation
oxygen limitation
maxLFQ estimation scaled to rel_iBAQ [%]
0.0
0.1
0.2
0.3
0.4
HG001
ΔclpX
clpXcompl
growth phase
exp
stat

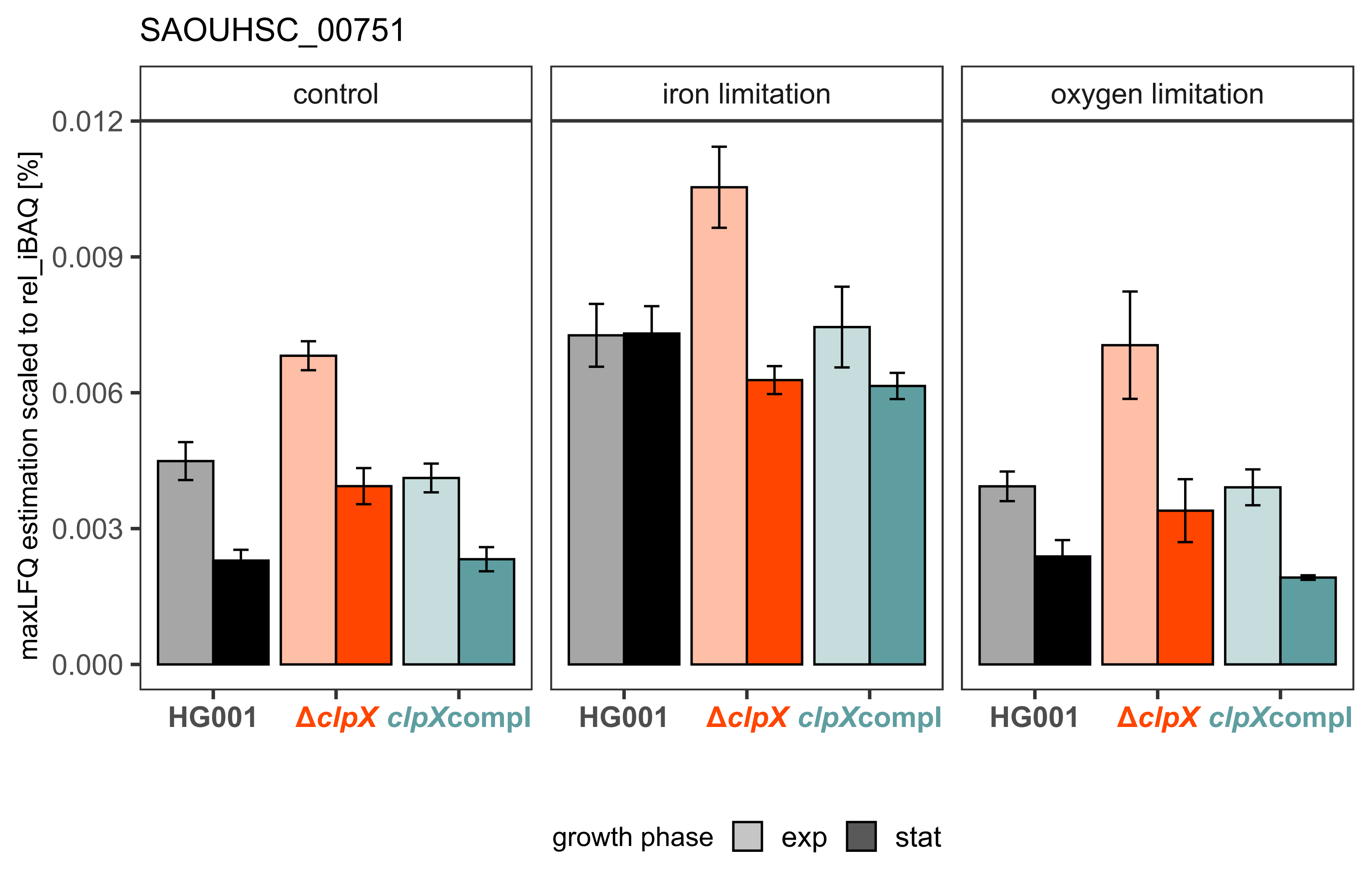
SAOUHSC_00751
control
iron limitation
oxygen limitation
maxLFQ estimation scaled to rel_iBAQ [%]
0.012
0.009
0.006
0.003
0.000
HG001
ΔclpX
clpXcompl
growth phase
exp
stat

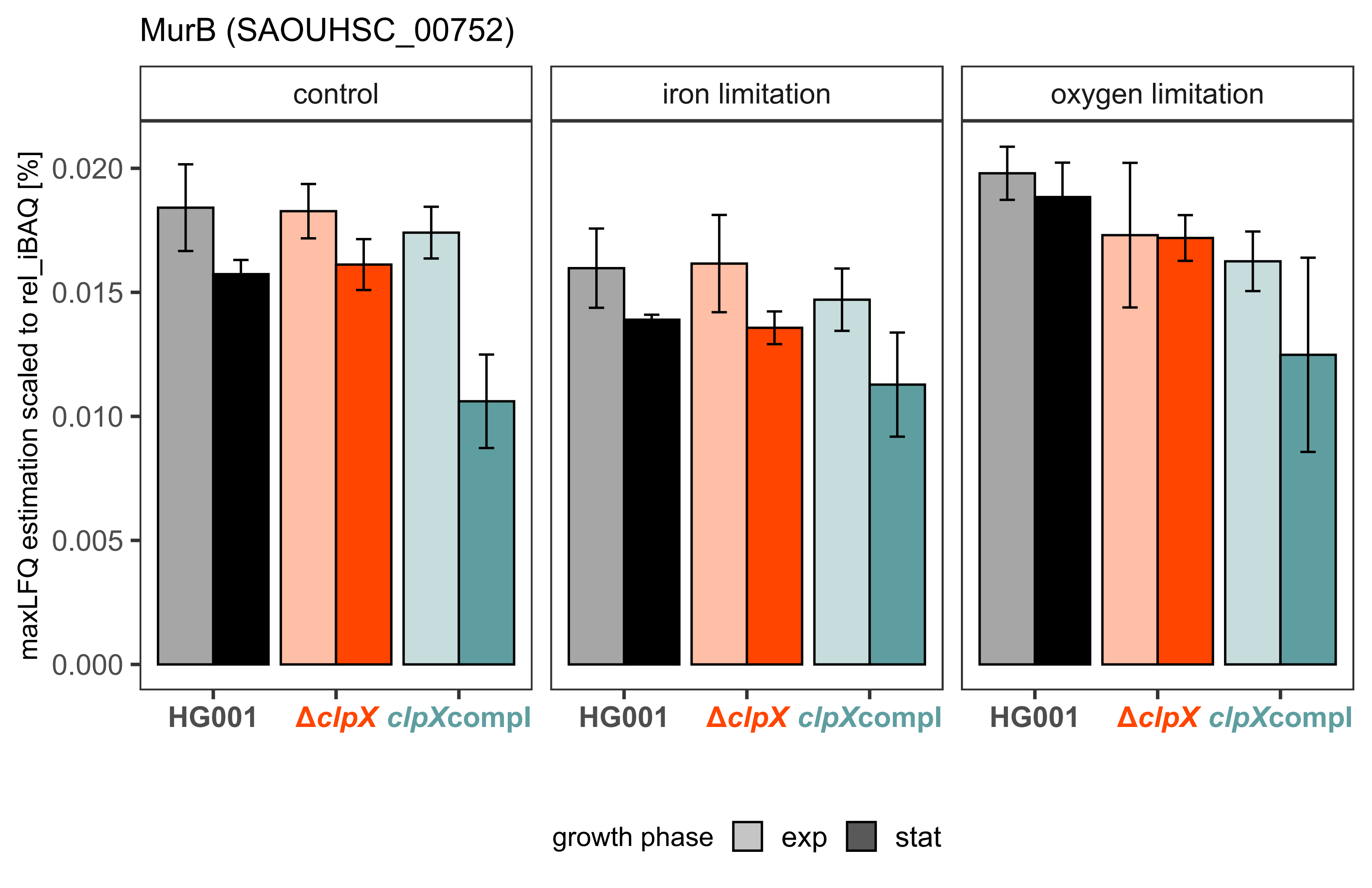

MurB (SAOUHSC_00752)
control
iron limitation
oxygen limitation
maxLFQ estimation scaled to rel_iBAQ [%]
0.020
0.015
0.010
0.005
0.000
HG001
ΔclpX
clpXcompl
growth phase
exp
stat

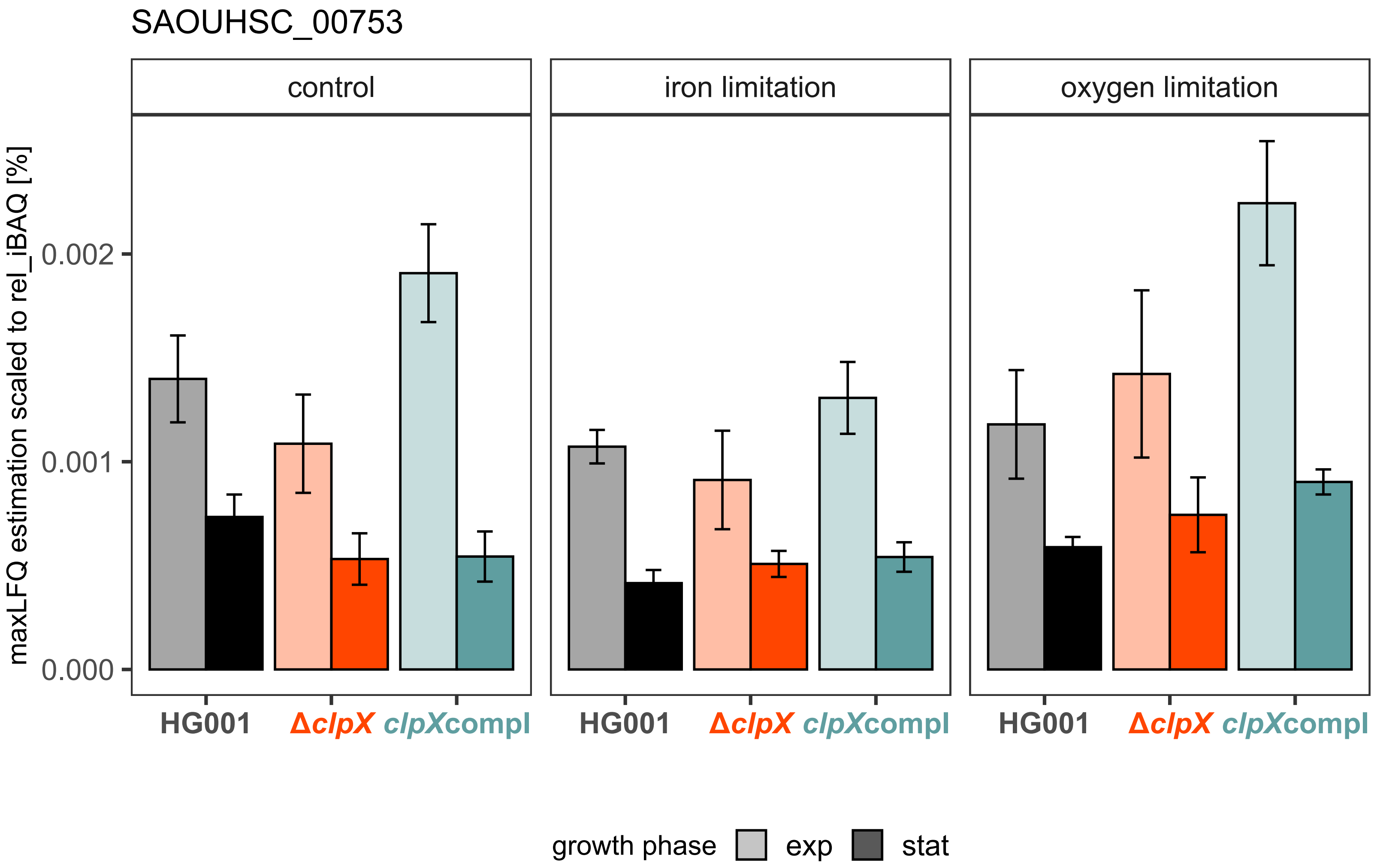
SAOUHSC_00753
control
iron limitation
oxygen limitation
maxLFQ estimation scaled to rel_iBAQ [%]
0.002
0.001
0.000
HG001
ΔclpX
clpXcompl
growth phase
exp
stat

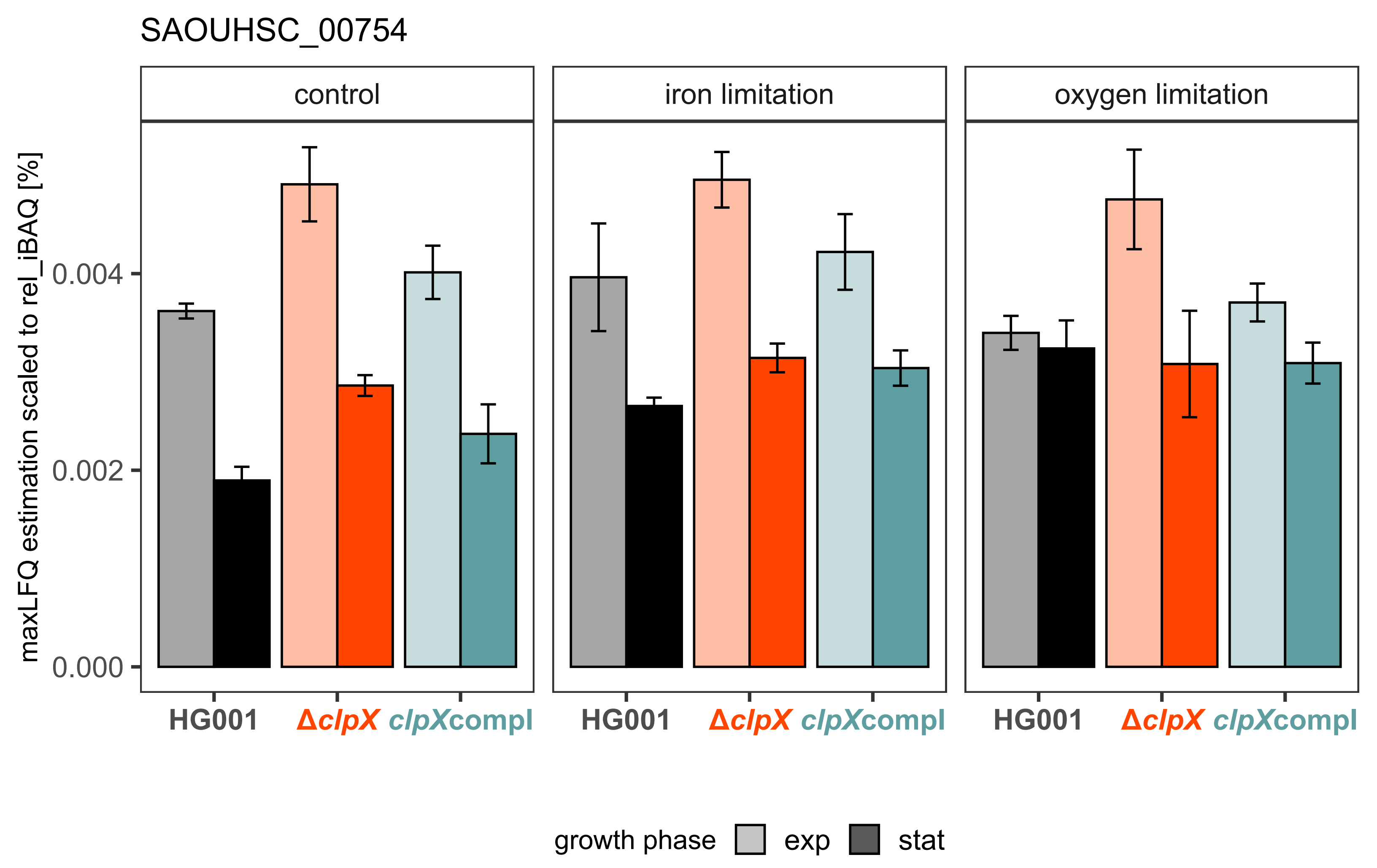

SAOUHSC_00754
control
iron limitation
oxygen limitation
maxLFQ estimation scaled to rel_iBAQ [%]
0.004
0.002
0.000
HG001
ΔclpX
clpXcompl
growth phase
exp
stat

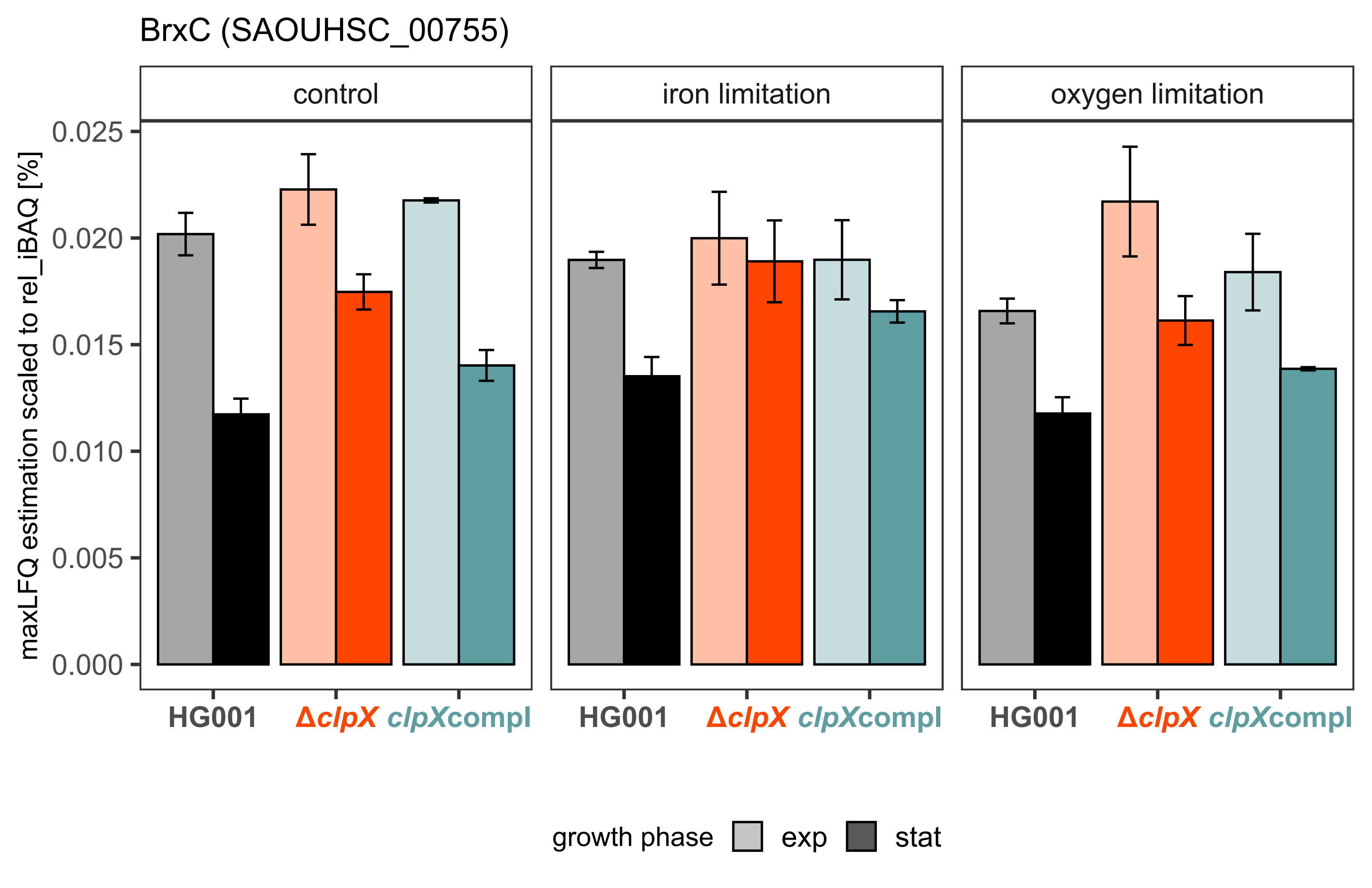

BrxC (SAOUHSC_00755)
control
iron limitation
oxygen limitation
maxLFQ estimation scaled to rel_iBAQ [%]
0.025
0.020
0.015
0.010
0.005
0.000
HG001
ΔclpX
clpXcompl
growth phase
exp
stat

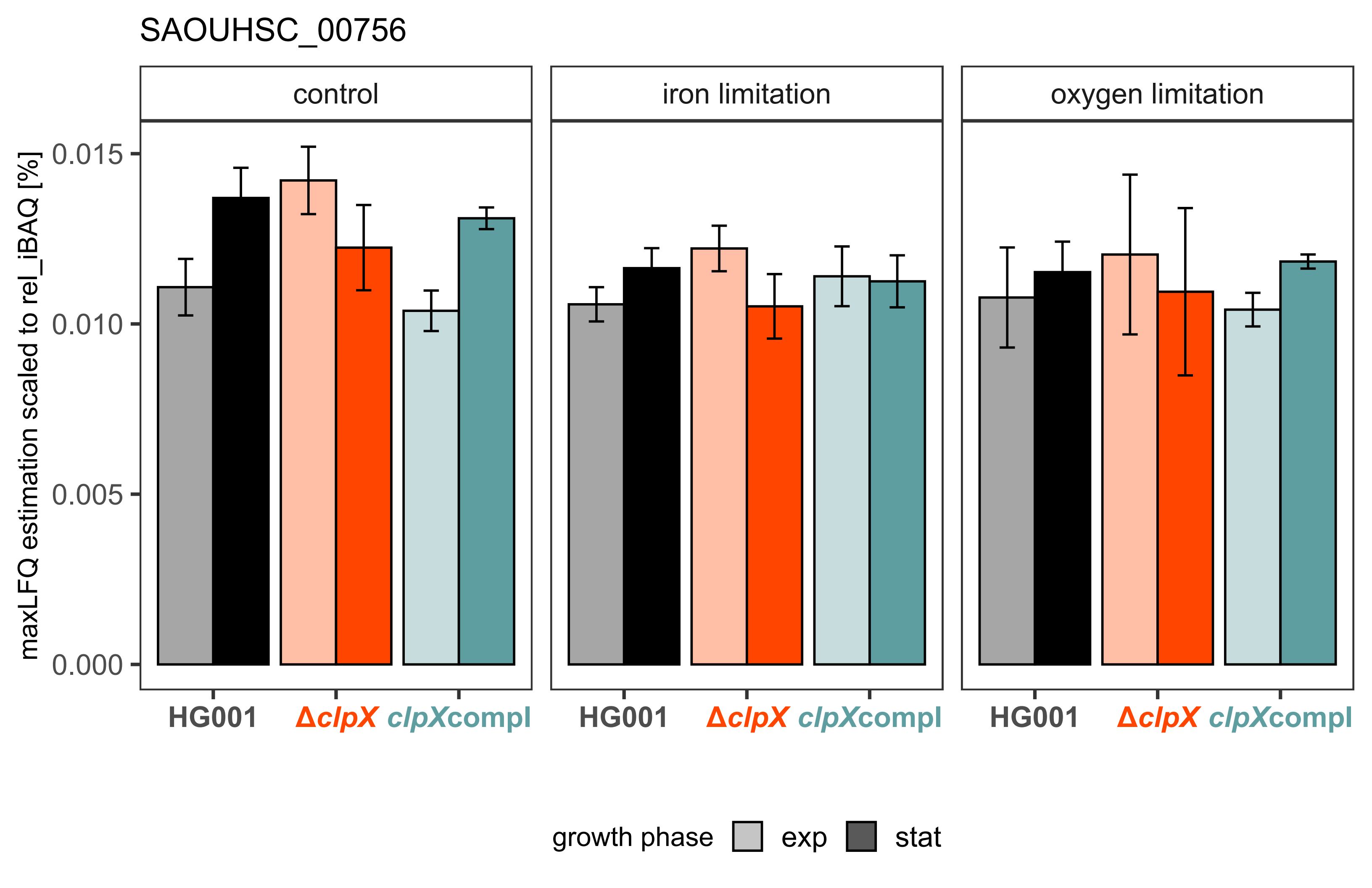

SAOUHSC_00756
control
iron limitation
oxygen limitation
maxLFQ estimation scaled to rel_iBAQ [%]
0.015
0.010
0.005
0.000
HG001
ΔclpX
clpXcompl
growth phase
exp
stat

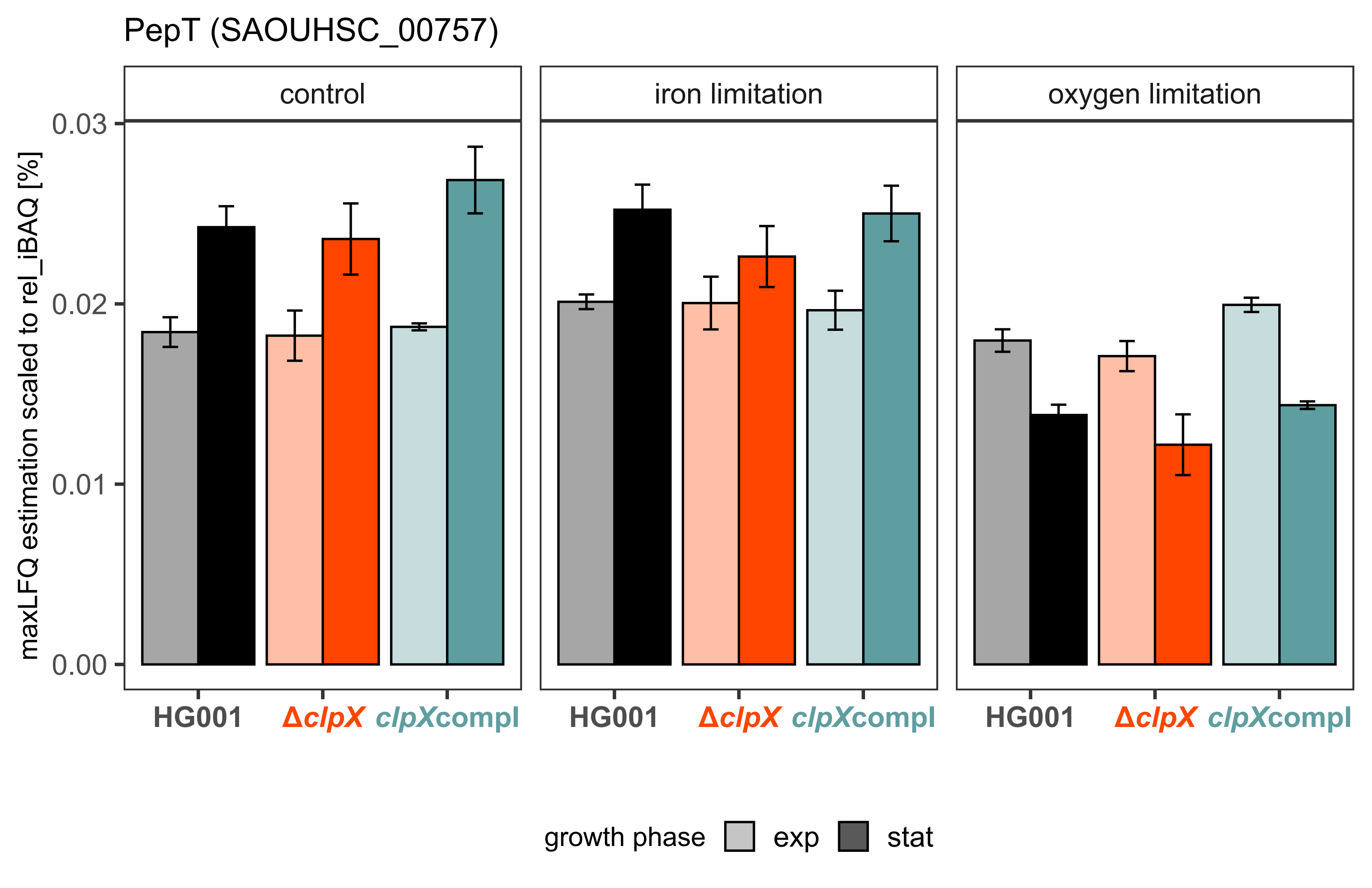

PepT (SAOUHSC_00757)
control
iron limitation
oxygen limitation
maxLFQ estimation scaled to rel_iBAQ [%]
0.03
0.02
0.01
0.00
HG001
ΔclpX
clpXcompl
growth phase
exp
stat

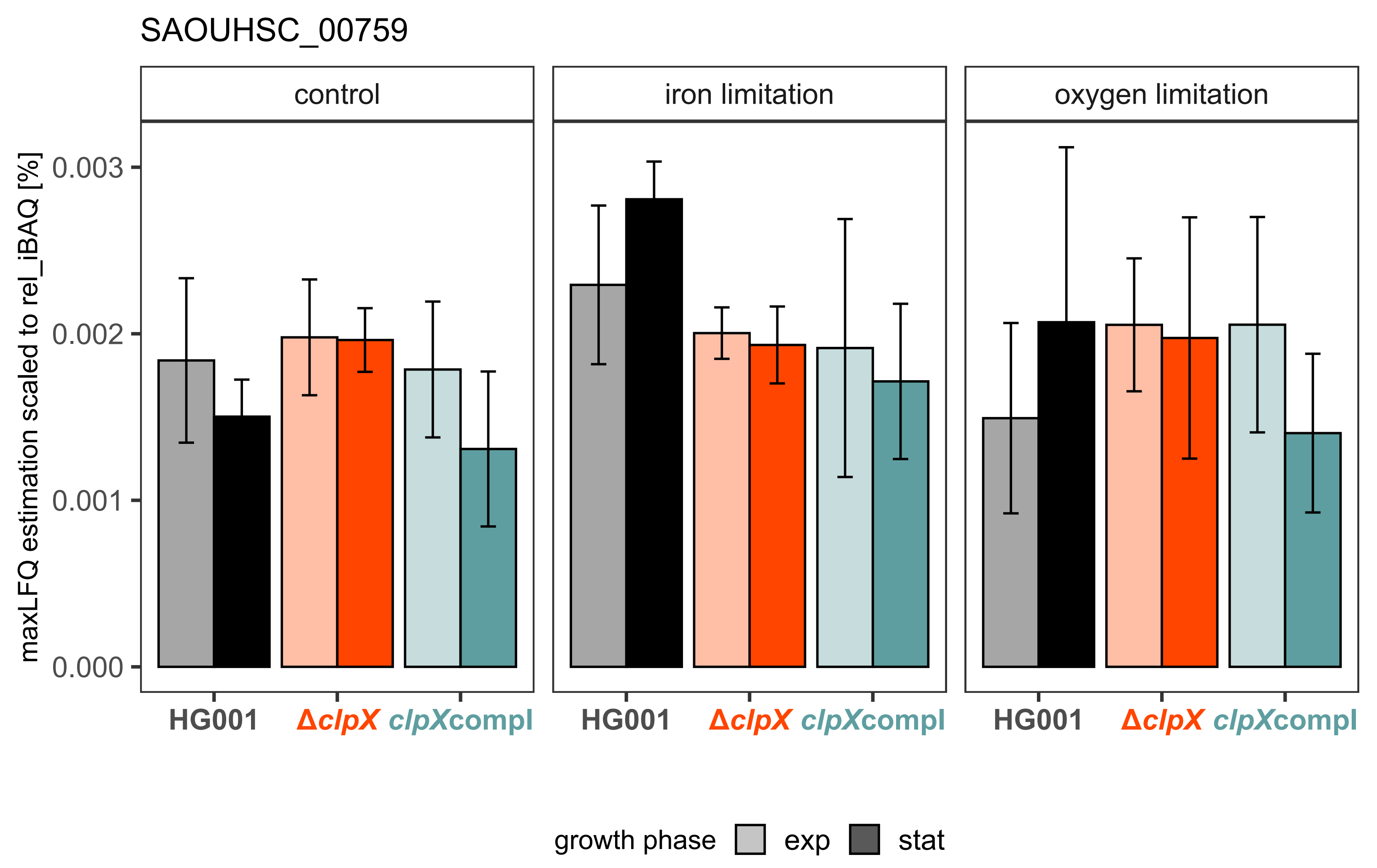
SAOUHSC_00759
control
iron limitation
oxygen limitation
maxLFQ estimation scaled to rel_iBAQ [%]
0.003
0.002
0.001
0.000
HG001
ΔclpX
clpXcompl
growth phase
exp
stat

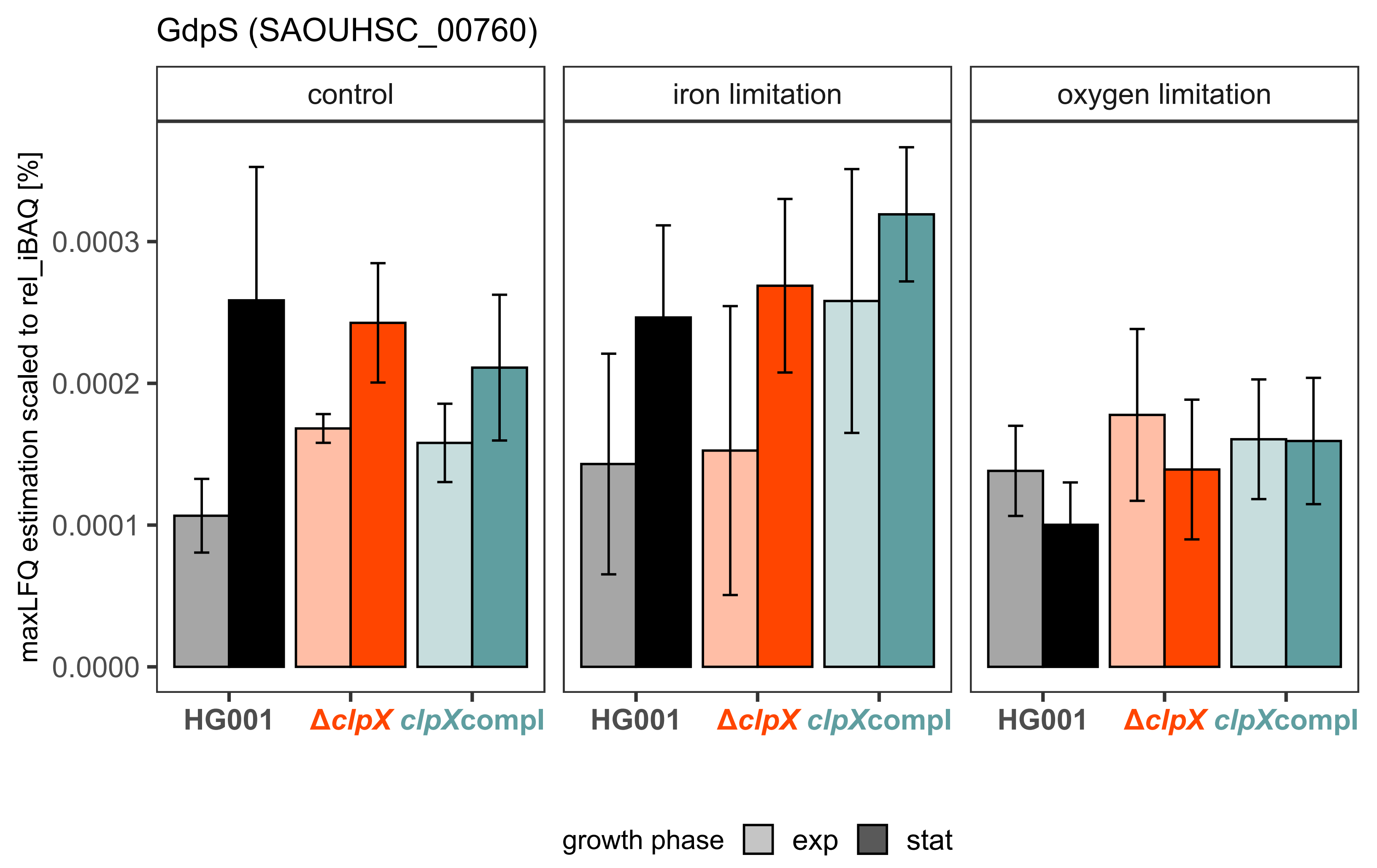
GdpS (SAOUHSC_00760)
control
iron limitation
oxygen limitation
maxLFQ estimation scaled to rel_iBAQ [%]
0.0003
0.0002
0.0001
0.0000
HG001
ΔclpX
clpXcompl
growth phase
exp
stat

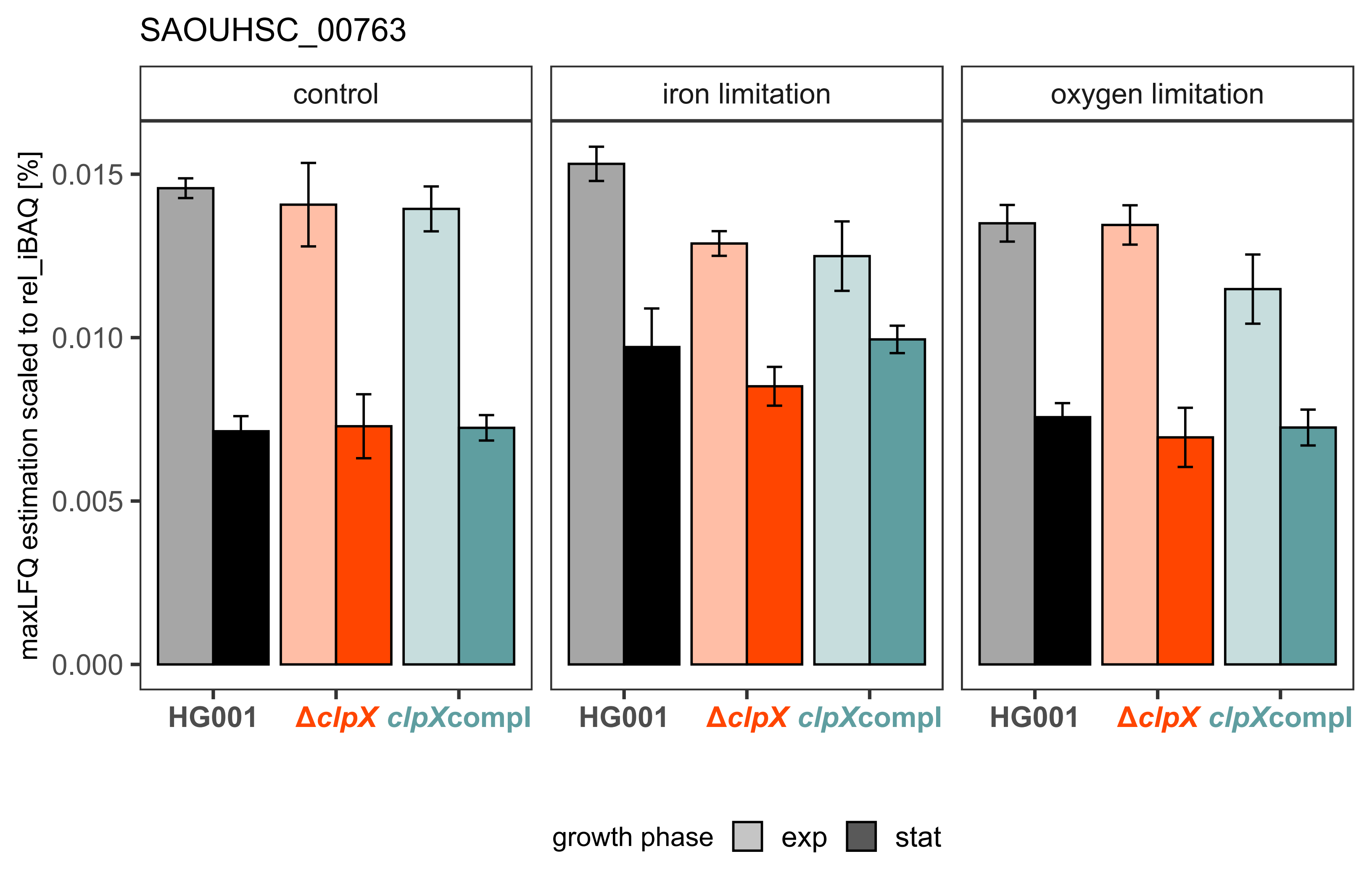
SAOUHSC_00763
control
iron limitation
oxygen limitation
maxLFQ estimation scaled to rel_iBAQ [%]
0.015
0.010
0.005
0.000
HG001
ΔclpX
clpXcompl
growth phase
exp
stat

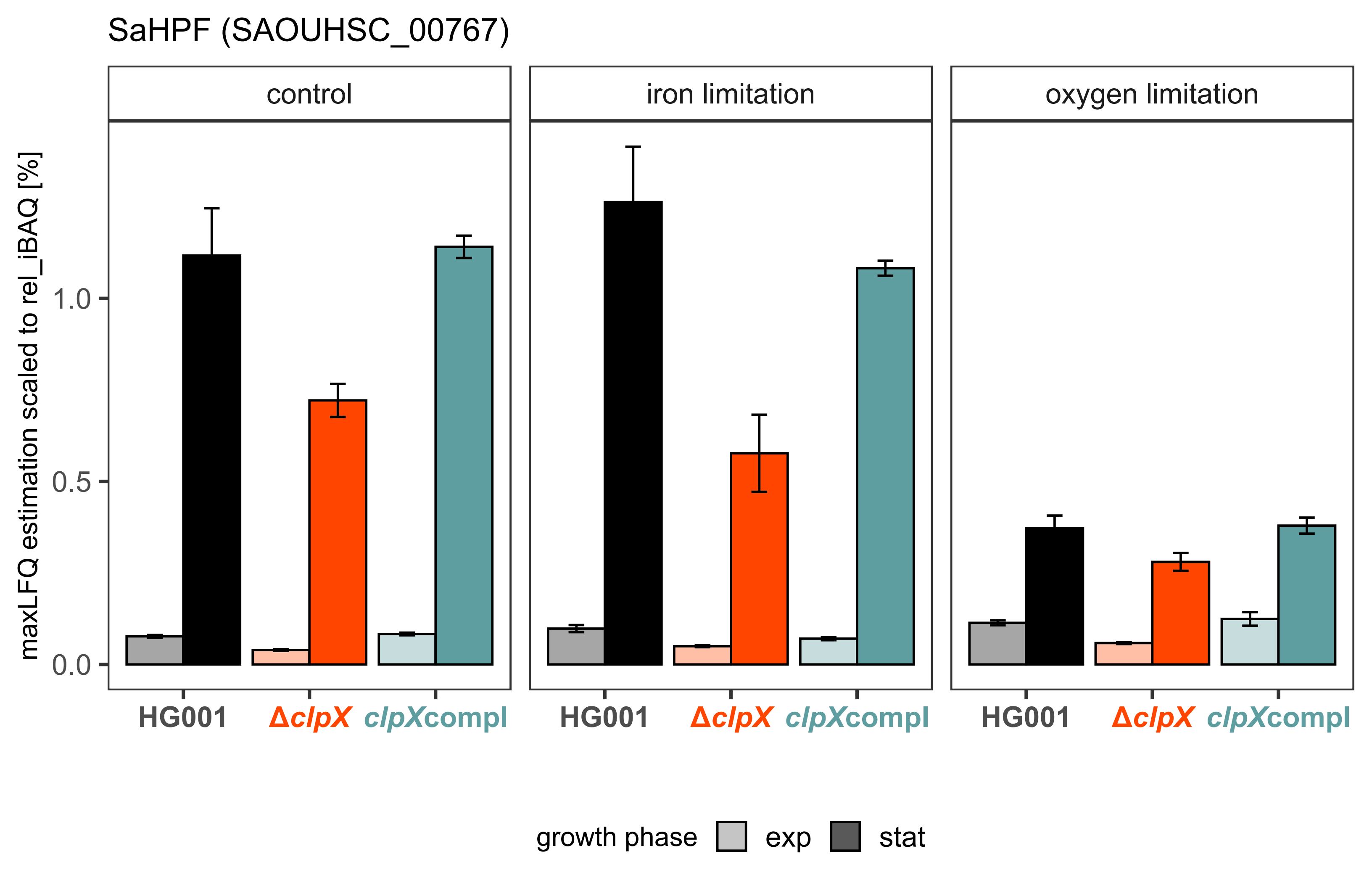

SaHPF (SAOUHSC_00767)
control
iron limitation
oxygen limitation
maxLFQ estimation scaled to rel_iBAQ [%]
0.0
0.5
1.0
HG001
ΔclpX
clpXcompl
growth phase
exp
stat

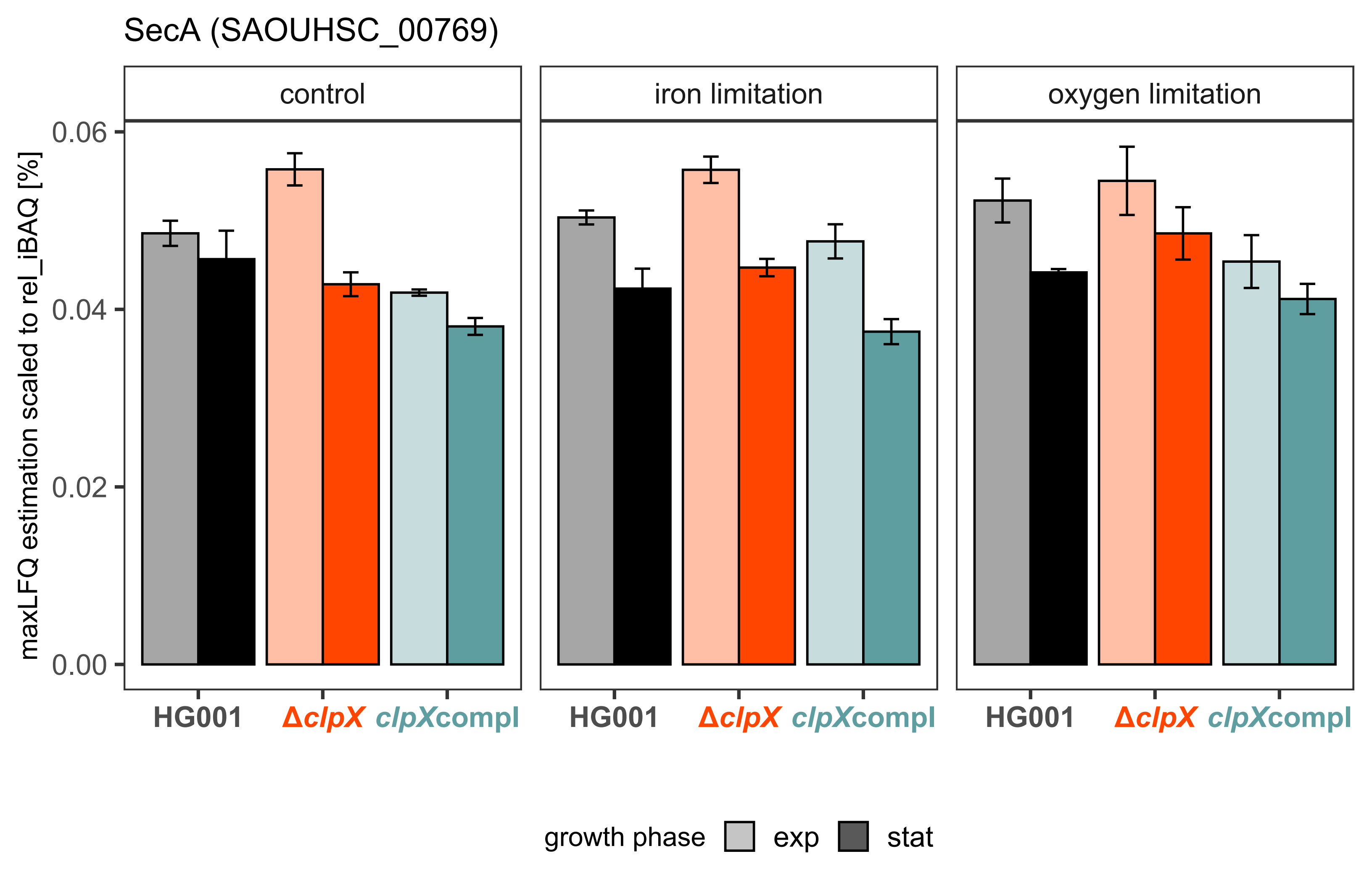

SecA (SAOUHSC_00769)
control
iron limitation
oxygen limitation
maxLFQ estimation scaled to rel_iBAQ [%]
0.06
0.04
0.02
0.00
HG001
ΔclpX
clpXcompl
growth phase
exp
stat

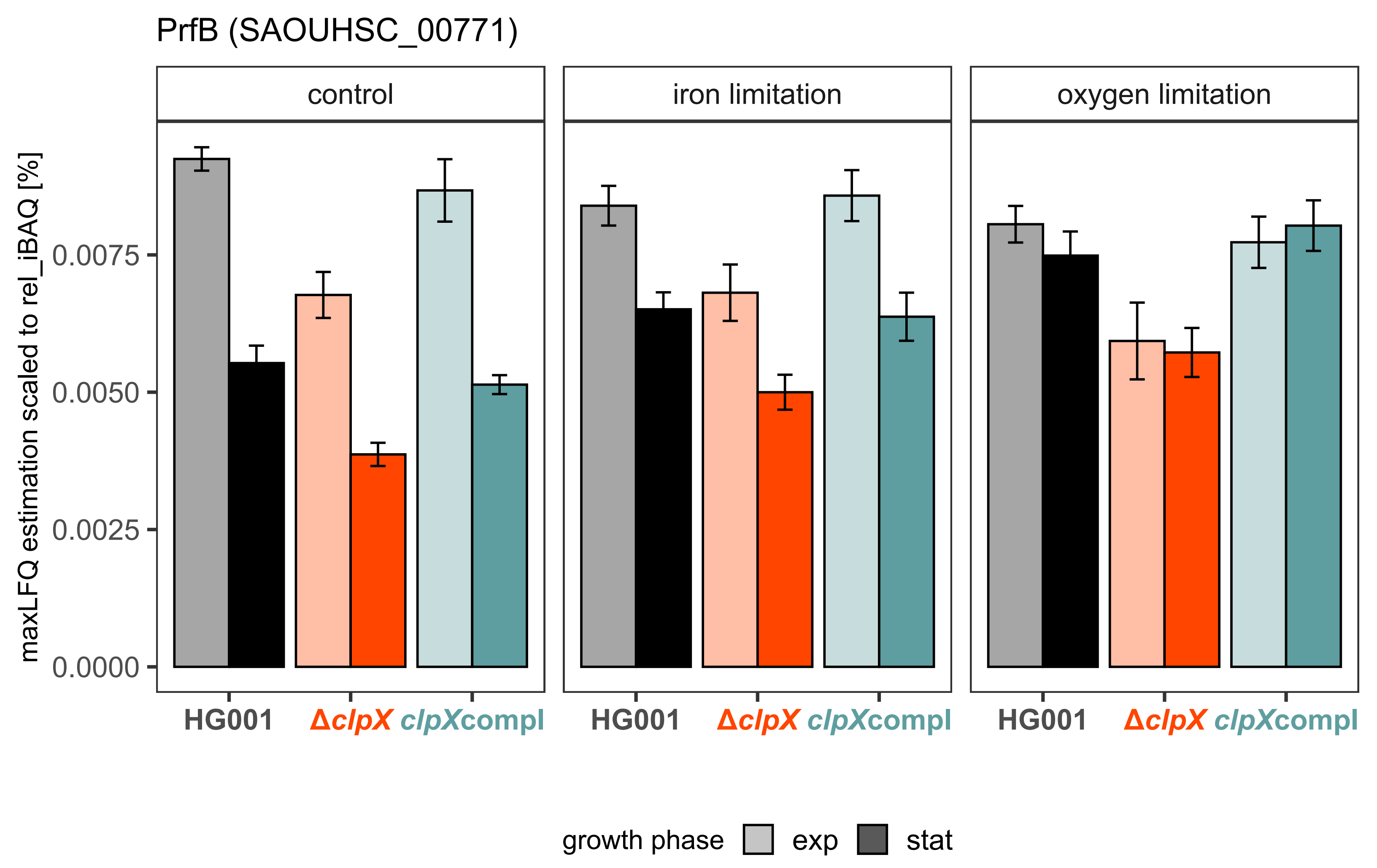
PrfB (SAOUHSC_00771)
control
iron limitation
oxygen limitation
maxLFQ estimation scaled to rel_iBAQ [%]
0.0000
0.0025
0.0050
0.0075
HG001
ΔclpX
clpXcompl
growth phase
exp
stat

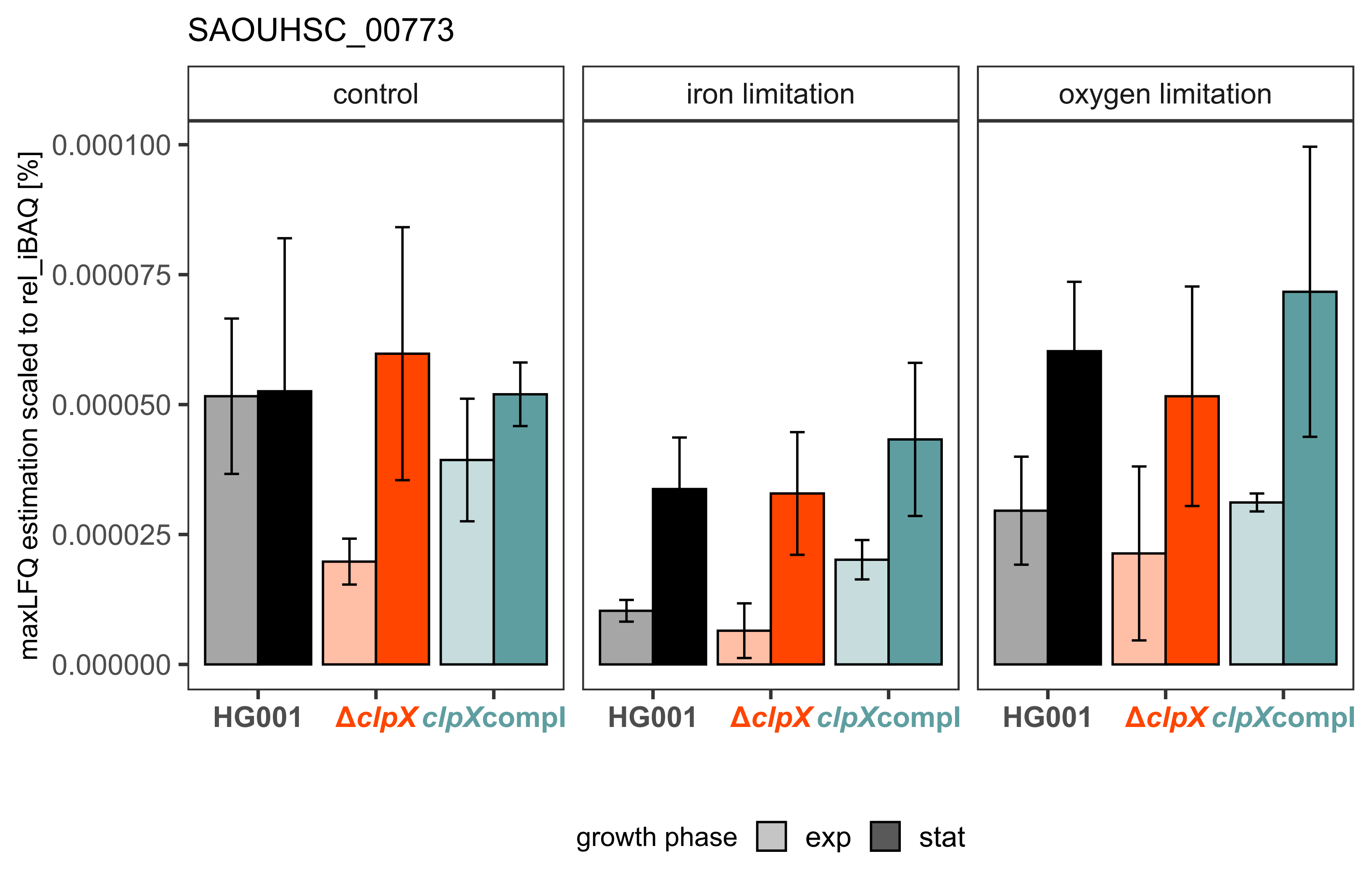

SAOUHSC_00773
control
iron limitation
oxygen limitation
maxLFQ estimation scaled to rel_iBAQ [%]
0.000100
0.000075
0.000050
0.000025
0.000000
HG001
ΔclpX
clpXcompl
growth phase
exp
stat

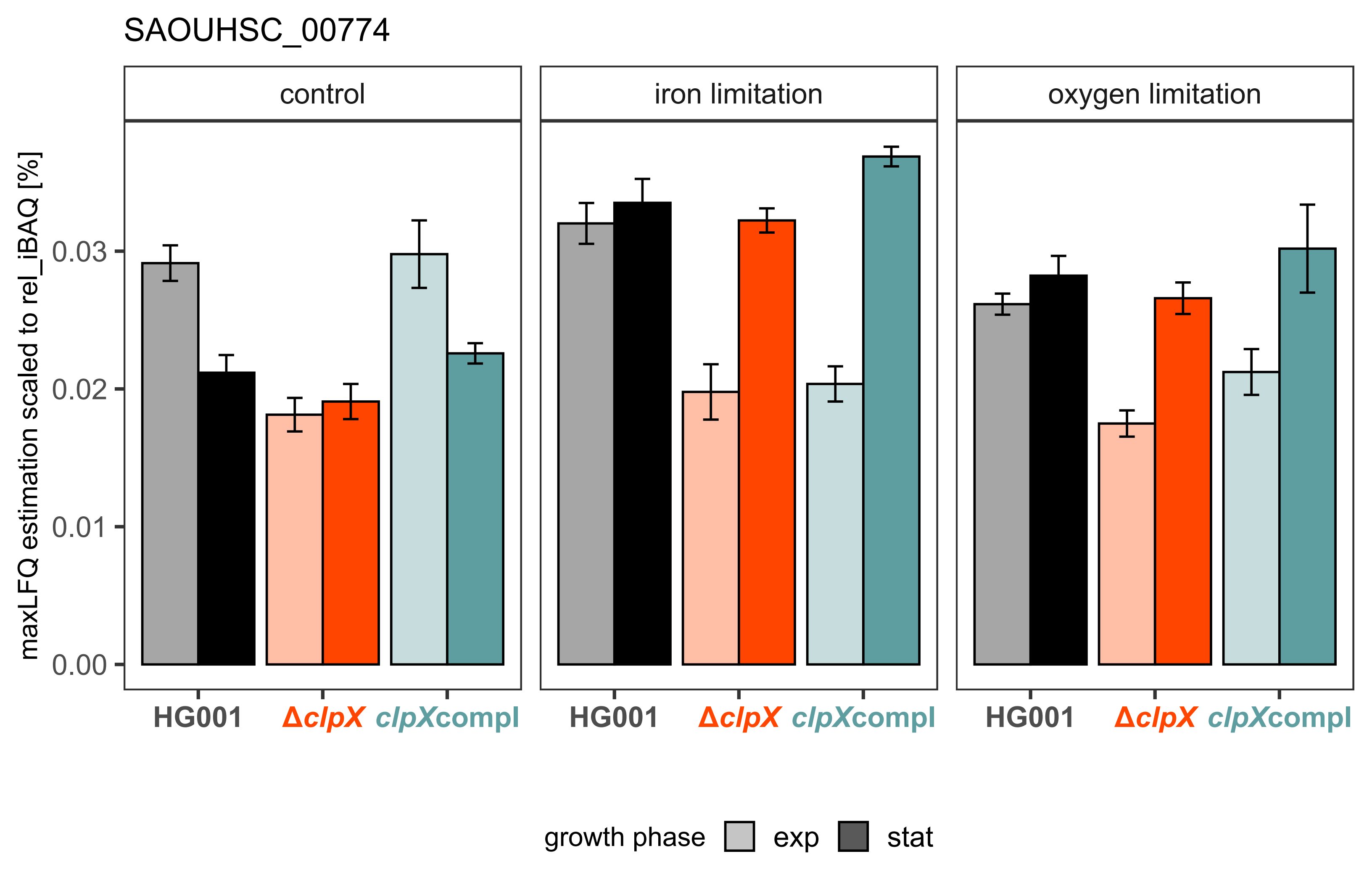
SAOUHSC_00774
control
iron limitation
oxygen limitation
maxLFQ estimation scaled to rel_iBAQ [%]
0.03
0.02
0.01
0.00
HG001
ΔclpX
clpXcompl
growth phase
exp
stat

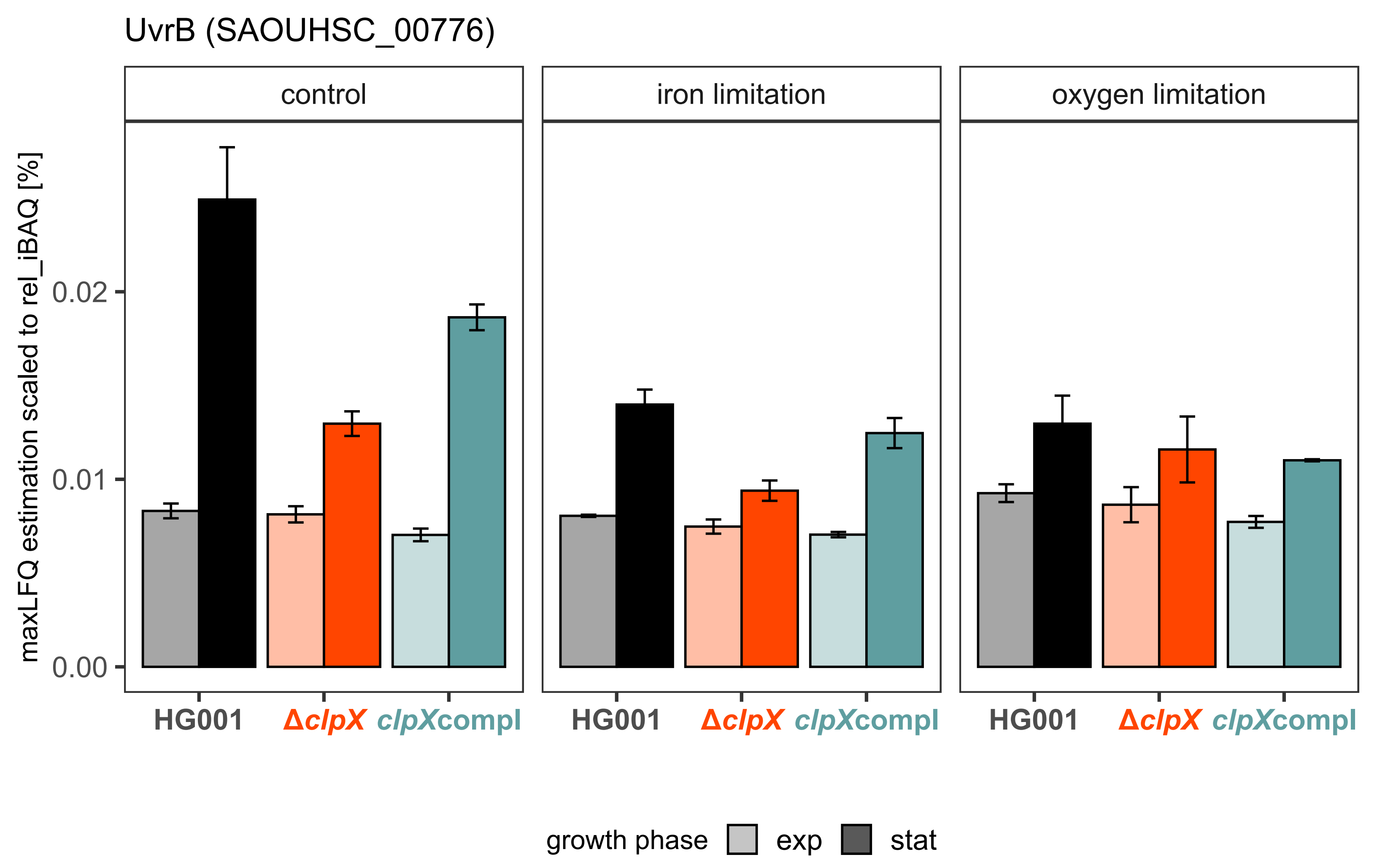
UvrB (SAOUHSC_00776)
control
iron limitation
oxygen limitation
maxLFQ estimation scaled to rel_iBAQ [%]
0.02
0.01
0.00
HG001
ΔclpX
clpXcompl
growth phase
exp
stat

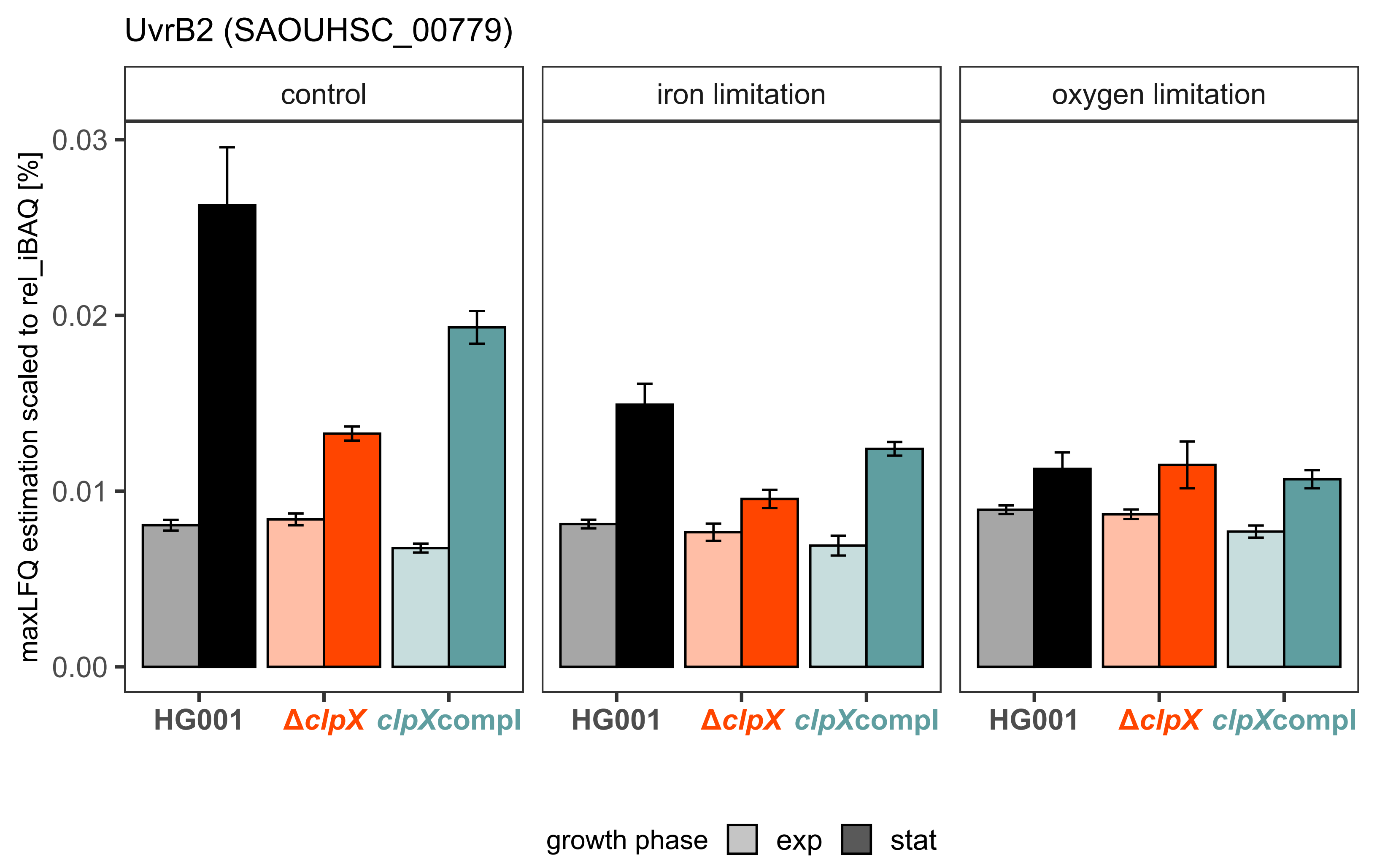
UvrB2 (SAOUHSC_00779)
control
iron limitation
oxygen limitation
maxLFQ estimation scaled to rel_iBAQ [%]
0.03
0.02
0.01
0.00
HG001
ΔclpX
clpXcompl
growth phase
exp
stat

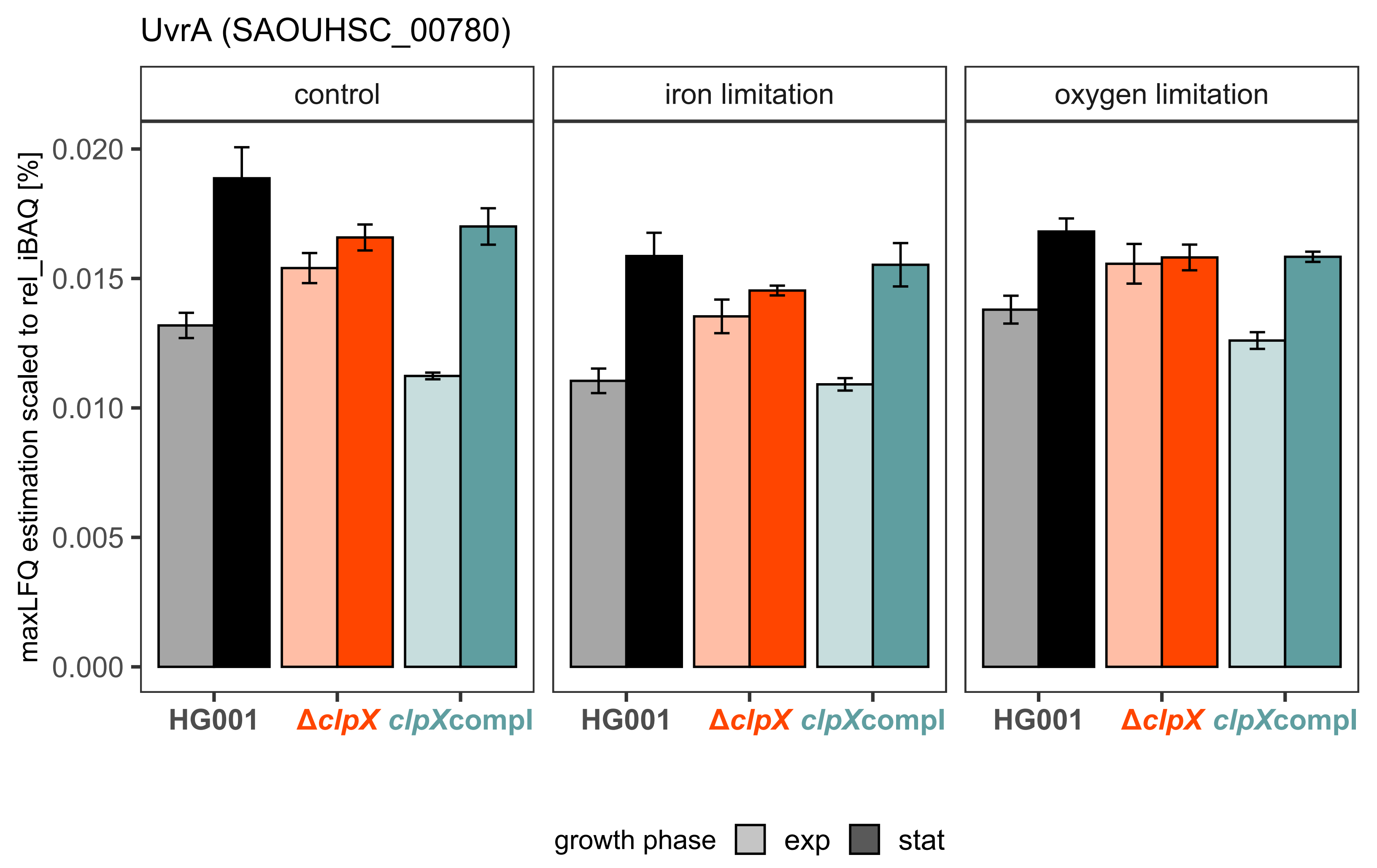
UvrA (SAOUHSC_00780)
control
iron limitation
oxygen limitation
maxLFQ estimation scaled to rel_iBAQ [%]
0.020
0.015
0.010
0.005
0.000
HG001
ΔclpX
clpXcompl
growth phase
exp
stat

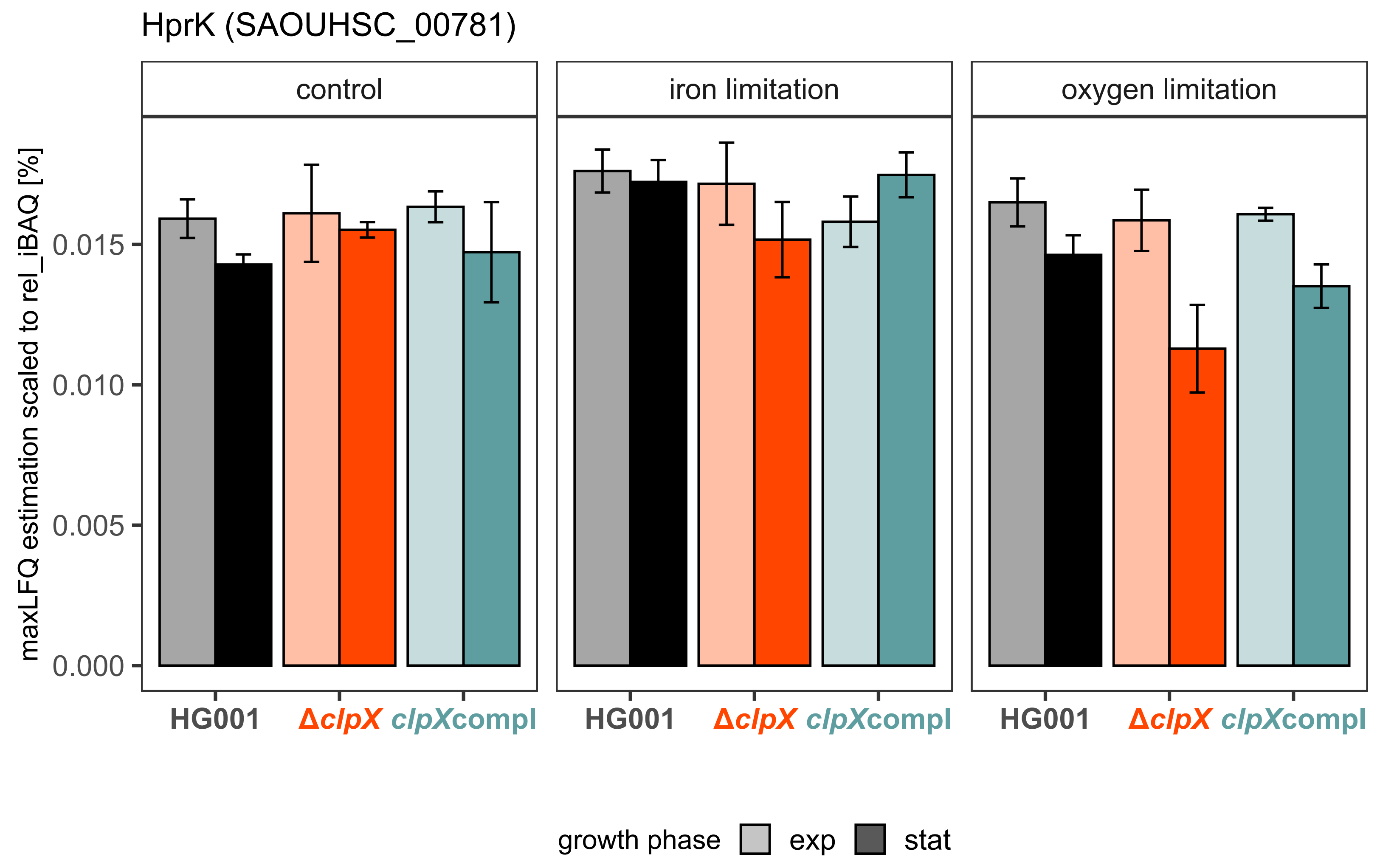
HprK (SAOUHSC_00781)
control
iron limitation
oxygen limitation
maxLFQ estimation scaled to rel_iBAQ [%]
0.015
0.010
0.005
0.000
HG001
ΔclpX
clpXcompl
growth phase
exp
stat

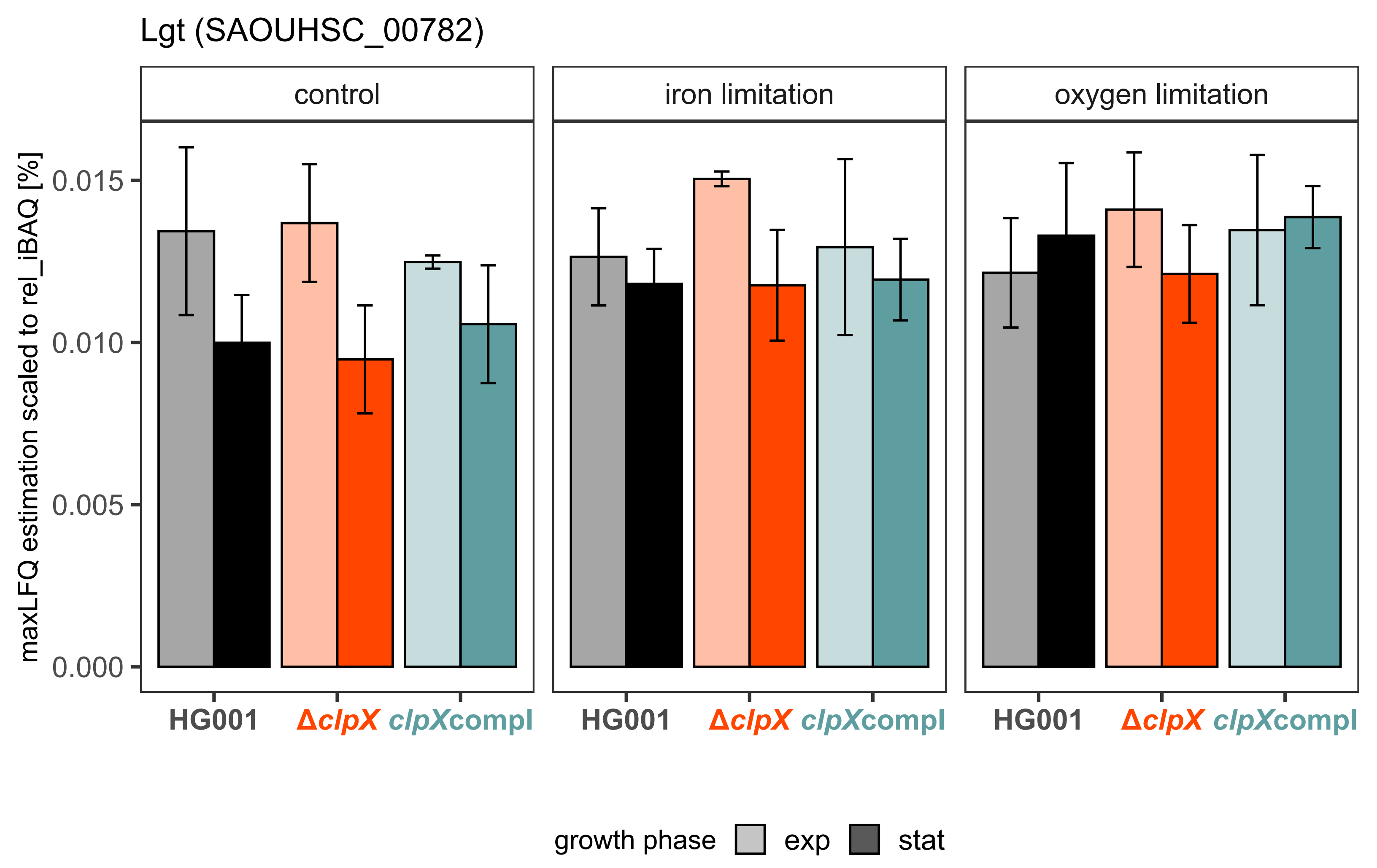

Lgt (SAOUHSC_00782)
control
iron limitation
oxygen limitation
maxLFQ estimation scaled to rel_iBAQ [%]
0.015
0.010
0.005
0.000
HG001
ΔclpX
clpXcompl
growth phase
exp
stat

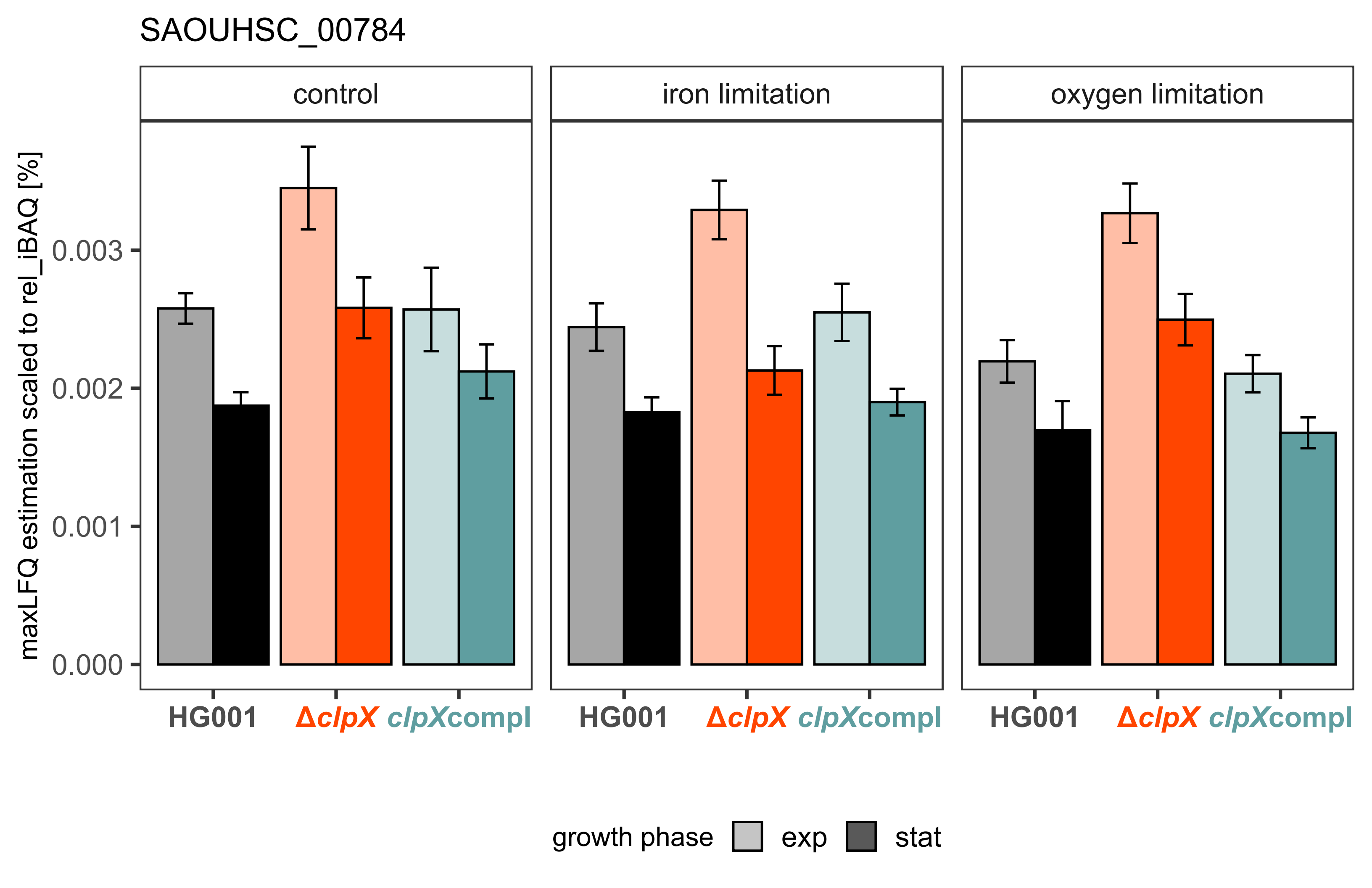

SAOUHSC_00784
control
iron limitation
oxygen limitation
maxLFQ estimation scaled to rel_iBAQ [%]
0.000
0.001
0.002
0.003
HG001
ΔclpX
clpXcompl
growth phase
exp
stat

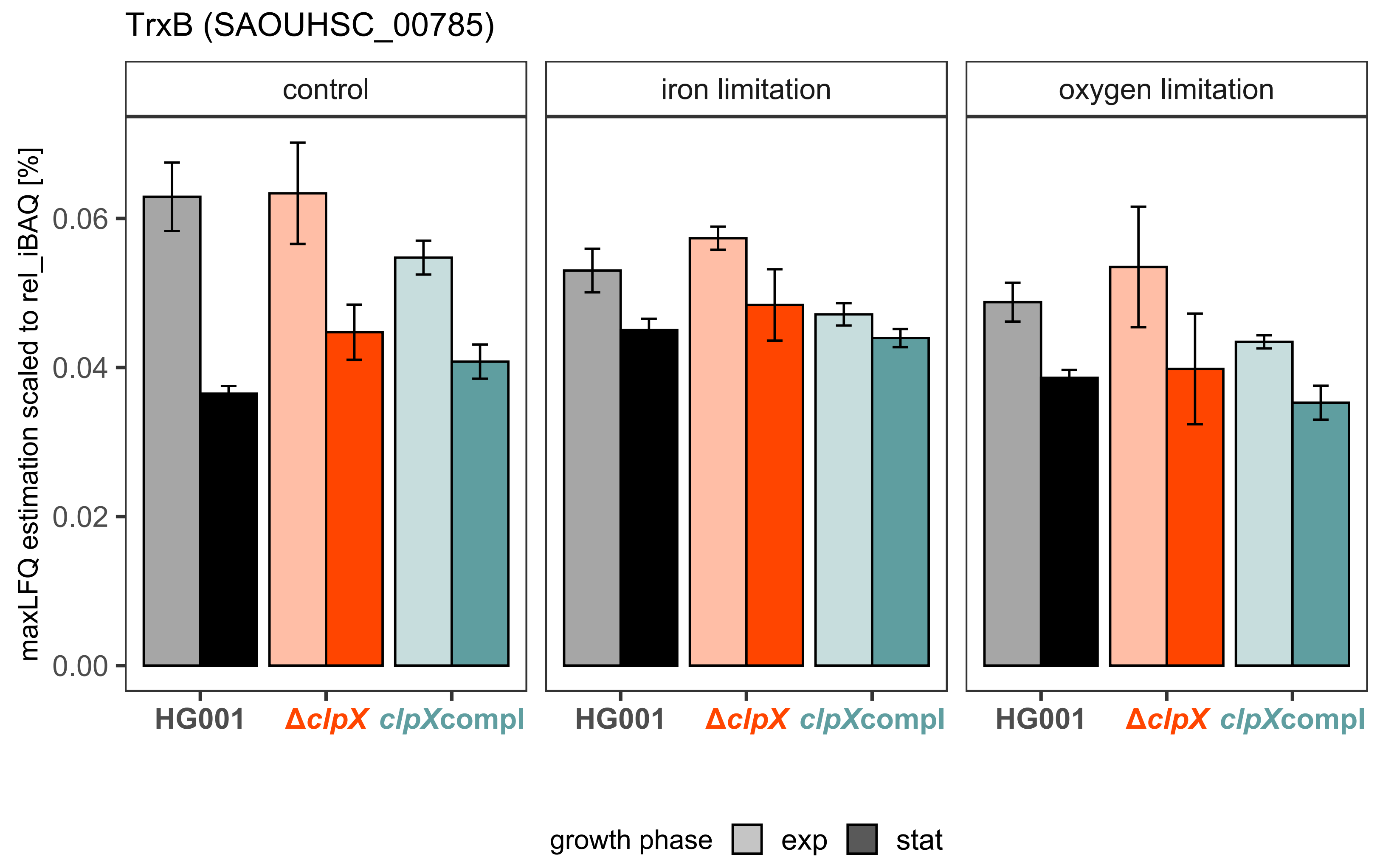
TrxB (SAOUHSC_00785)
control
iron limitation
oxygen limitation
maxLFQ estimation scaled to rel_iBAQ [%]
0.06
0.04
0.02
0.00
HG001
ΔclpX
clpXcompl
growth phase
exp
stat

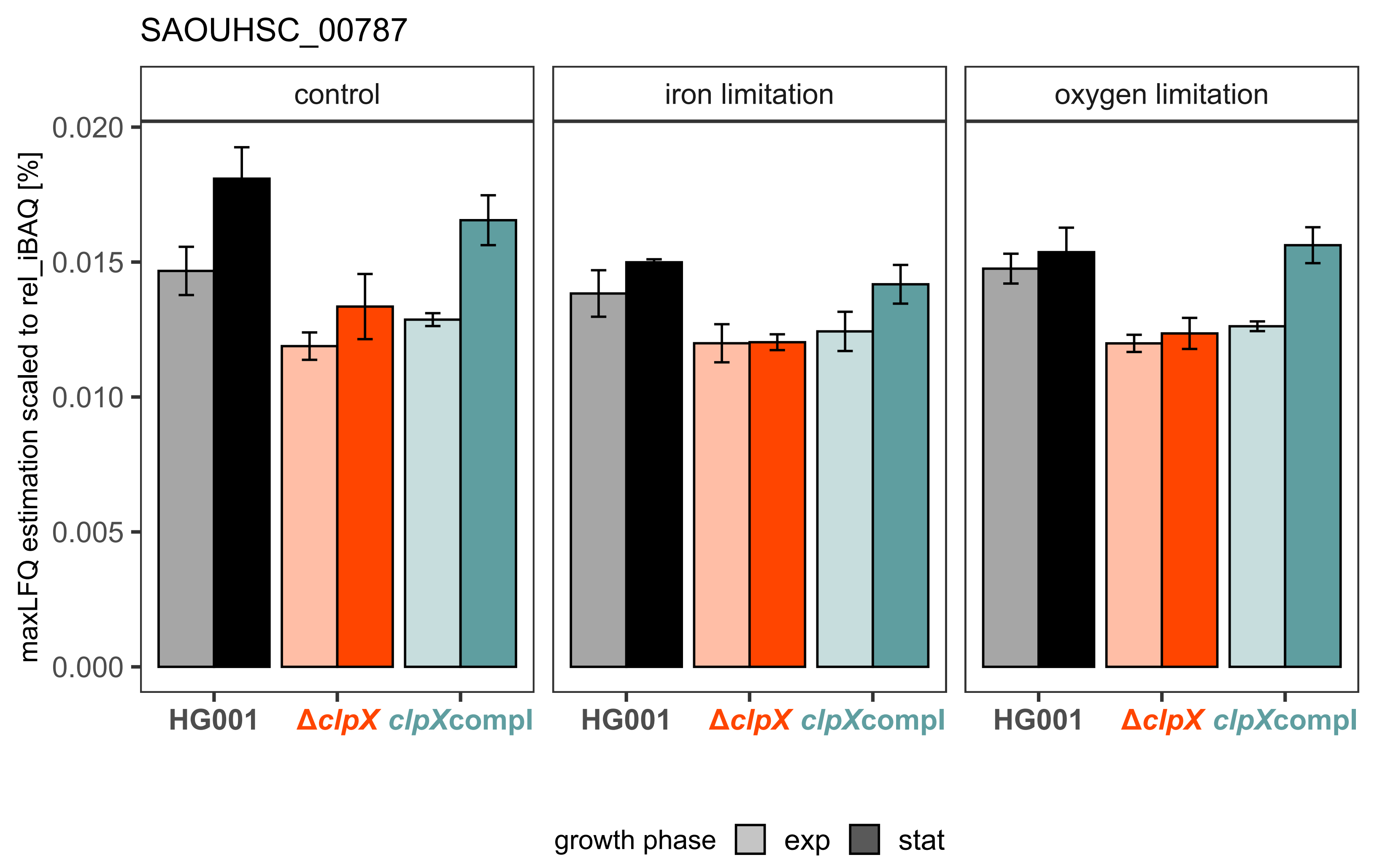

SAOUHSC_00787
control
iron limitation
oxygen limitation
maxLFQ estimation scaled to rel_iBAQ [%]
0.020
0.015
0.010
0.005
0.000
HG001
ΔclpX
clpXcompl
growth phase
exp
stat

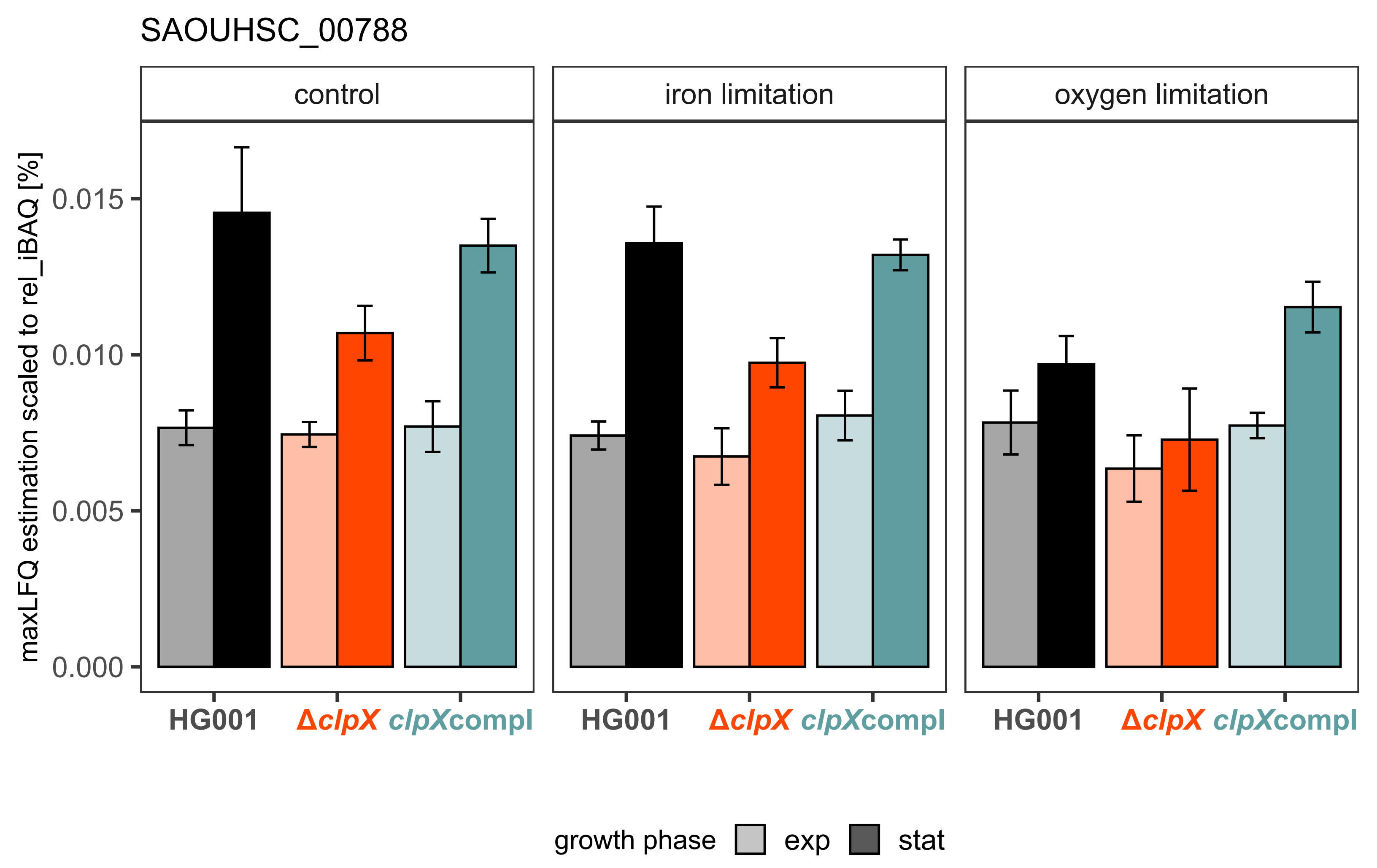
SAOUHSC_00788
control
iron limitation
oxygen limitation
maxLFQ estimation scaled to rel_iBAQ [%]
0.015
0.010
0.005
0.000
HG001
ΔclpX
clpXcompl
growth phase
exp
stat

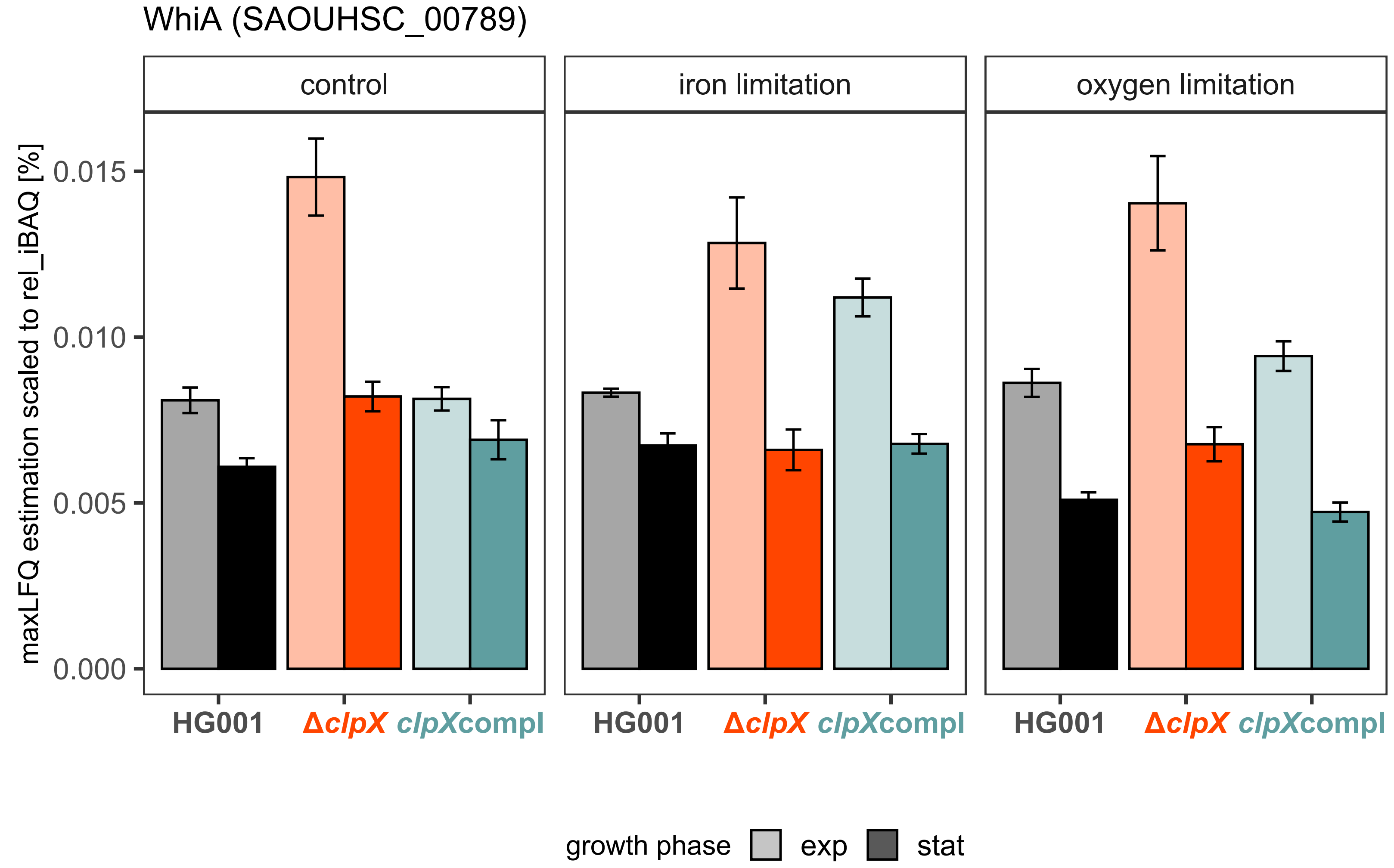
WhiA (SAOUHSC_00789)
control
iron limitation
oxygen limitation
maxLFQ estimation scaled to rel_iBAQ [%]
0.015
0.010
0.005
0.000
HG001
ΔclpX
clpXcompl
growth phase
exp
stat

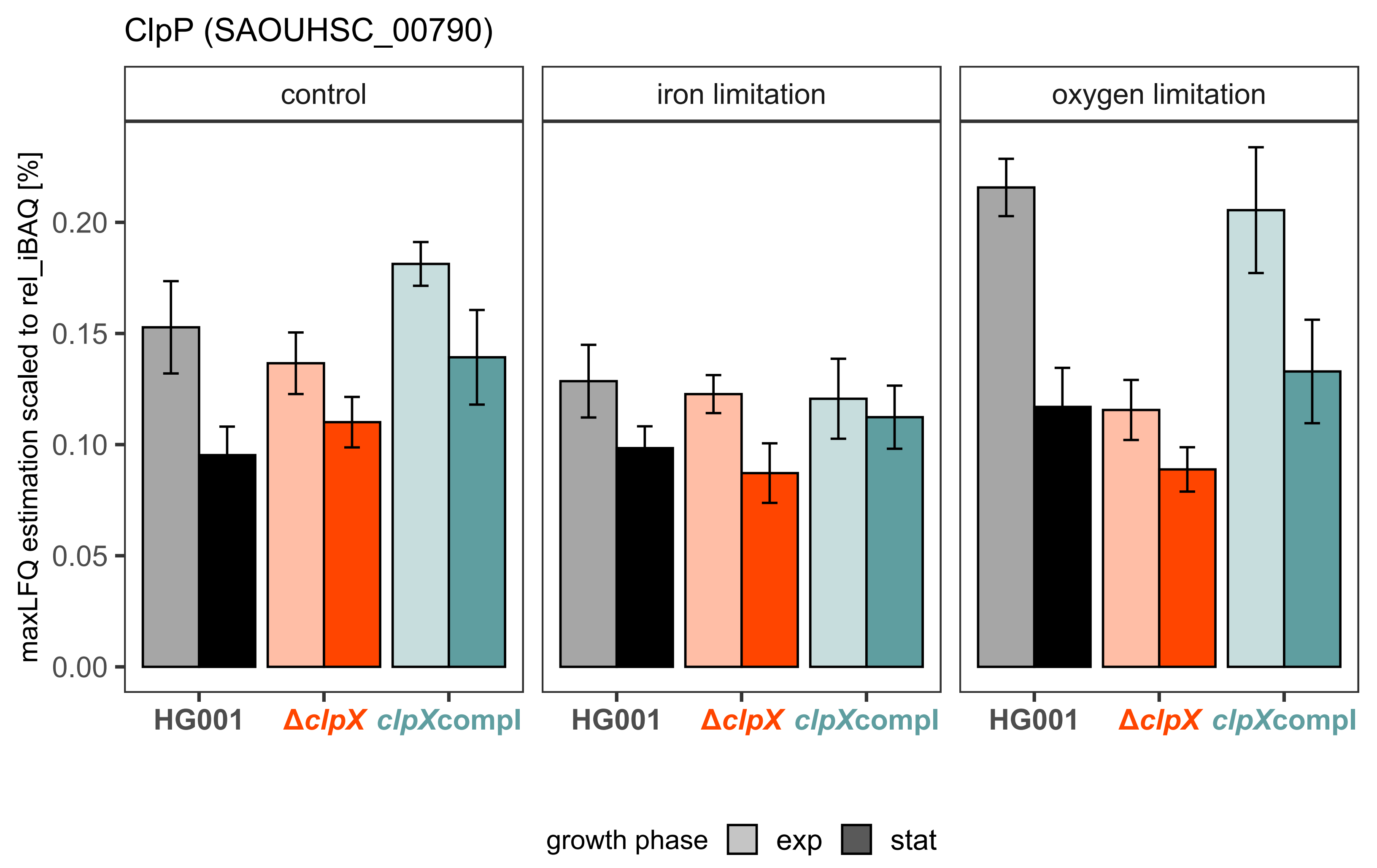

ClpP (SAOUHSC_00790)
control
iron limitation
oxygen limitation
maxLFQ estimation scaled to rel_iBAQ [%]
0.00
0.05
0.10
0.15
0.20
HG001
ΔclpX
clpXcompl
growth phase
exp
stat

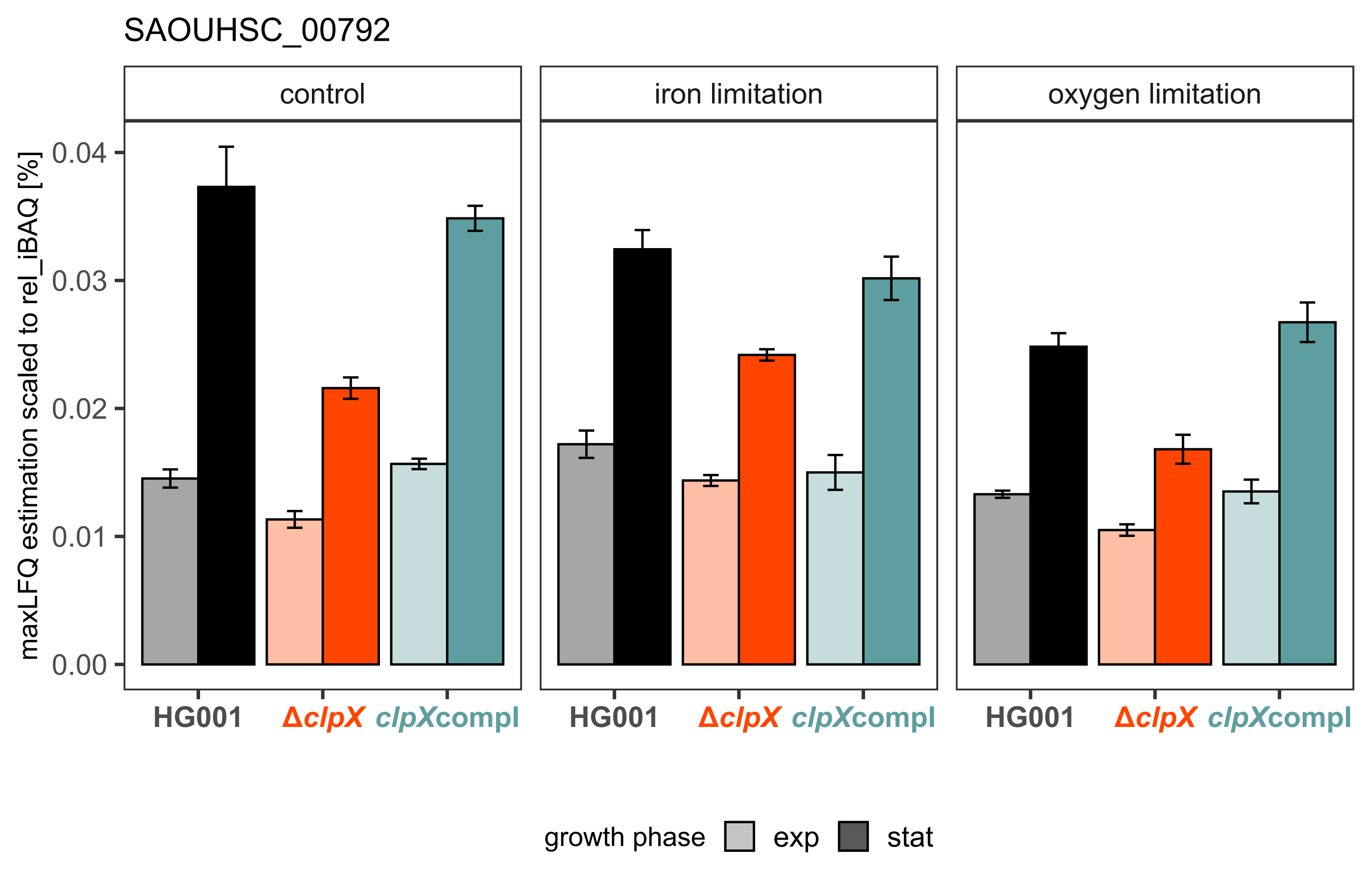

SAOUHSC_00792
control
iron limitation
oxygen limitation
maxLFQ estimation scaled to rel_iBAQ [%]
0.04
0.03
0.02
0.01
0.00
HG001
ΔclpX
clpXcompl
growth phase
exp
stat

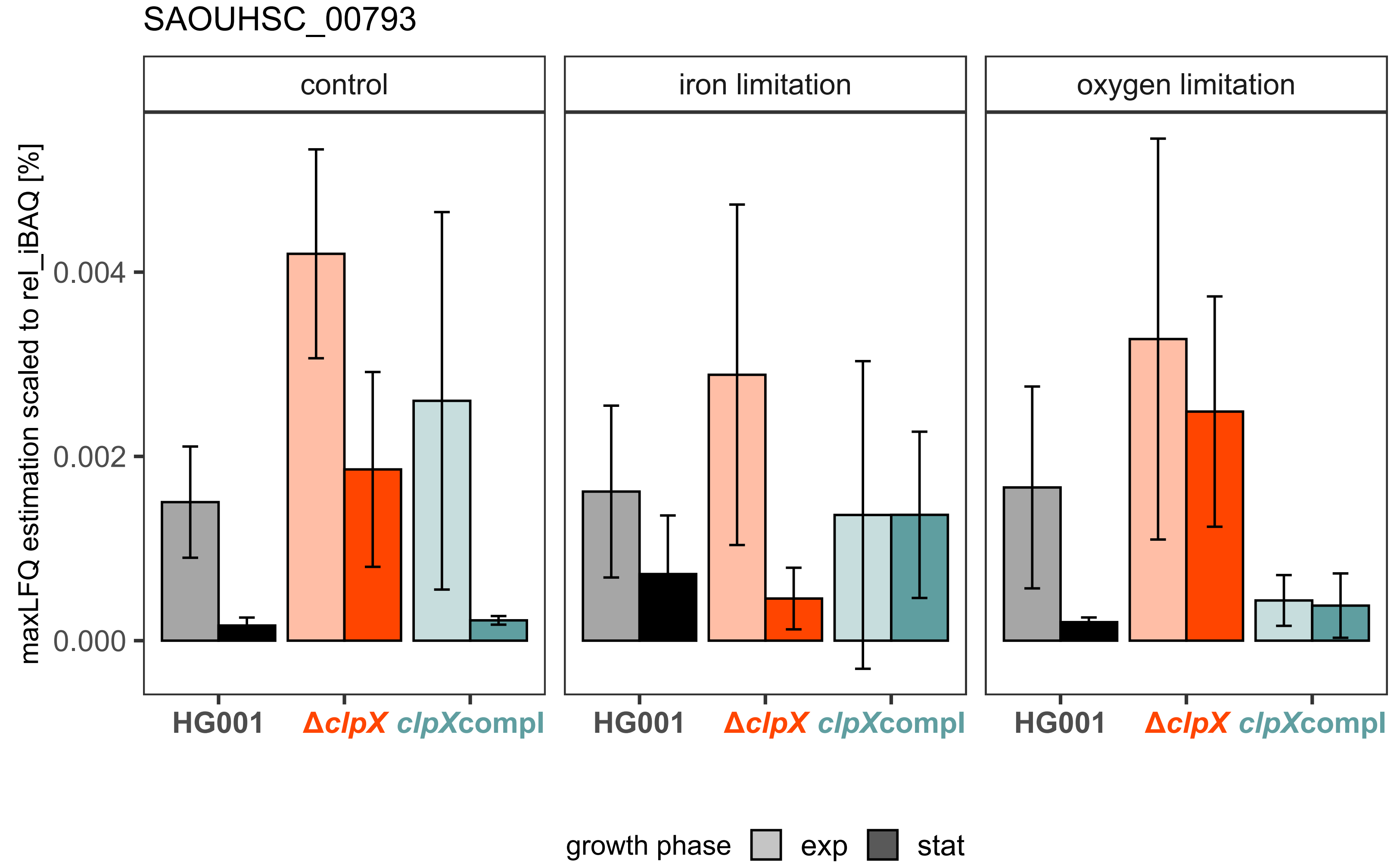
SAOUHSC_00793
control
iron limitation
oxygen limitation
maxLFQ estimation scaled to rel_iBAQ [%]
0.004
0.002
0.000
HG001
ΔclpX
clpXcompl
growth phase
exp
stat

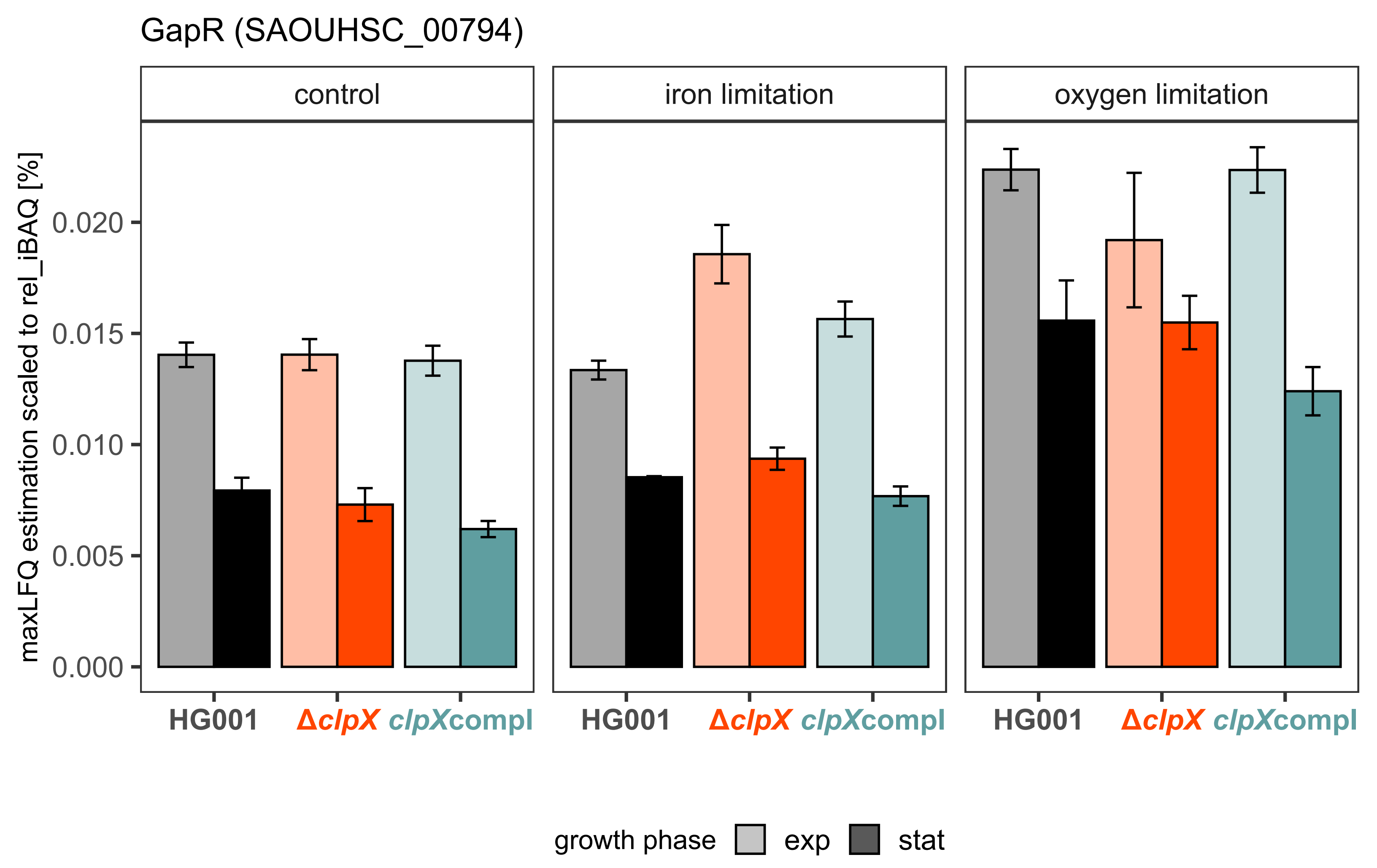

GapR (SAOUHSC_00794)
control
iron limitation
oxygen limitation
maxLFQ estimation scaled to rel_iBAQ [%]
0.000
0.005
0.010
0.015
0.020
HG001
ΔclpX
clpXcompl
growth phase
exp
stat

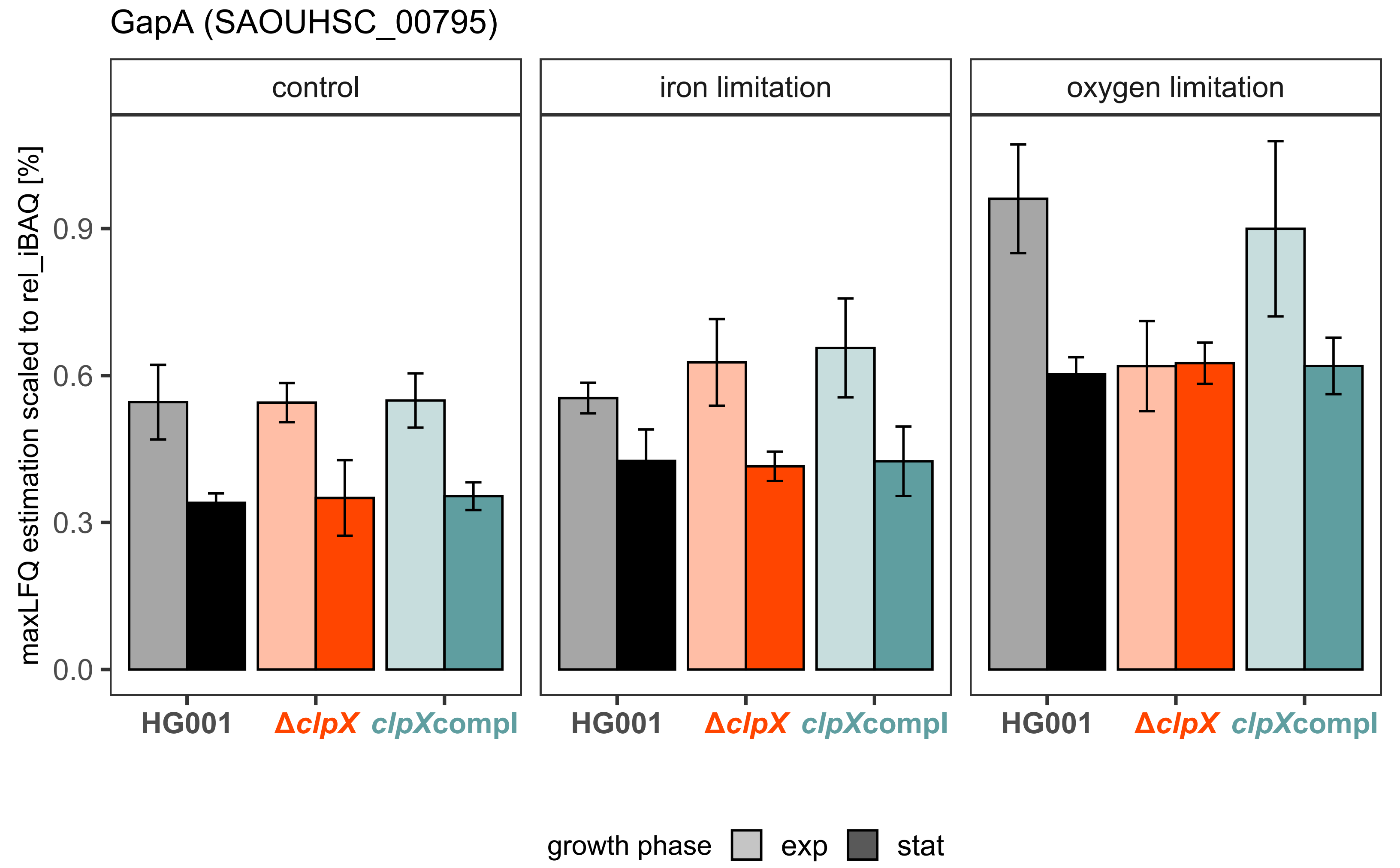
GapA (SAOUHSC_00795)
control
iron limitation
oxygen limitation
maxLFQ estimation scaled to rel_iBAQ [%]
0.0
0.3
0.6
0.9
HG001
ΔclpX
clpXcompl
growth phase
exp
stat

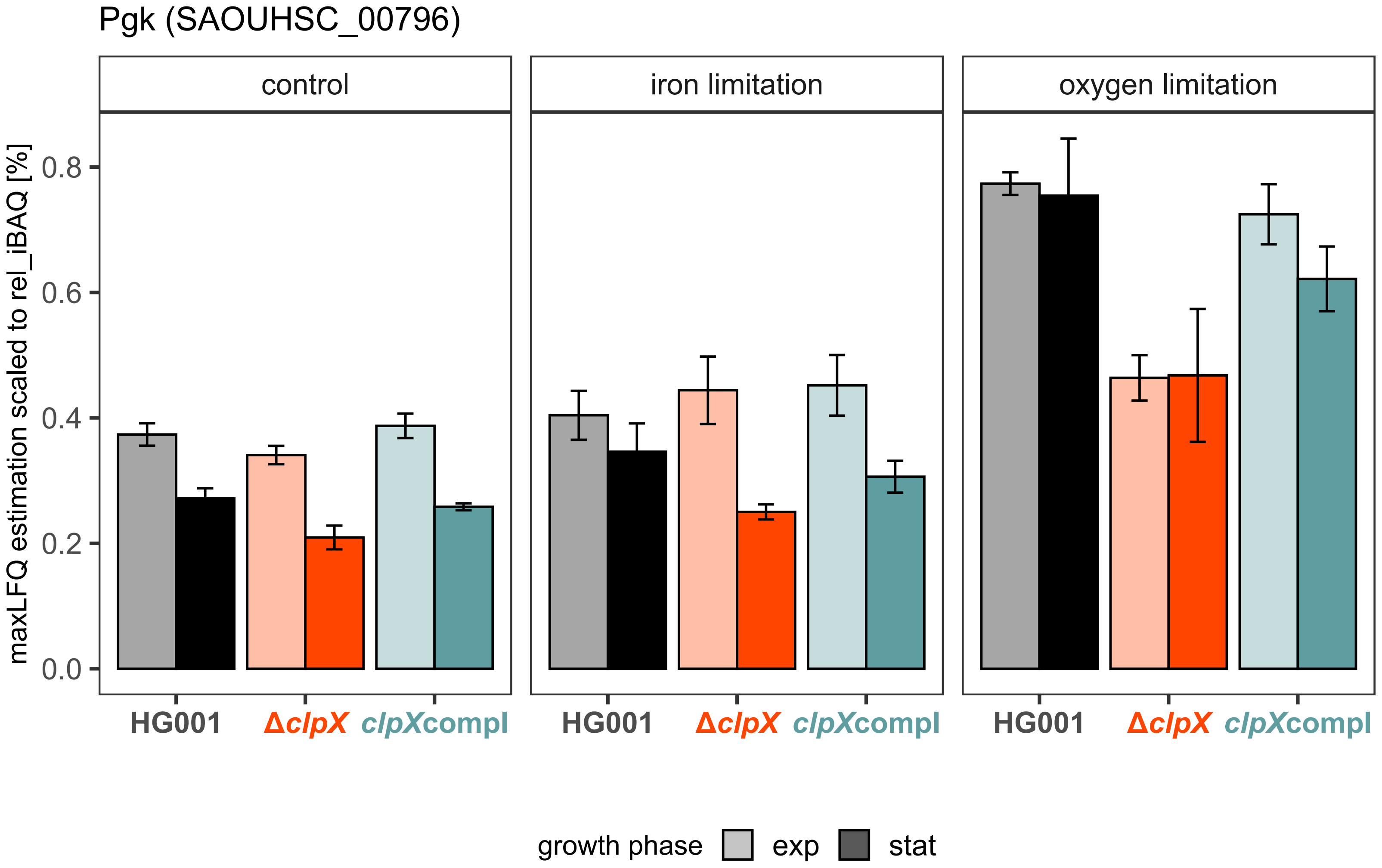

Pgk (SAOUHSC_00796)
control
iron limitation
oxygen limitation
maxLFQ estimation scaled to rel_iBAQ [%]
0.0
0.2
0.4
0.6
0.8
HG001
ΔclpX
clpXcompl
growth phase
exp
stat

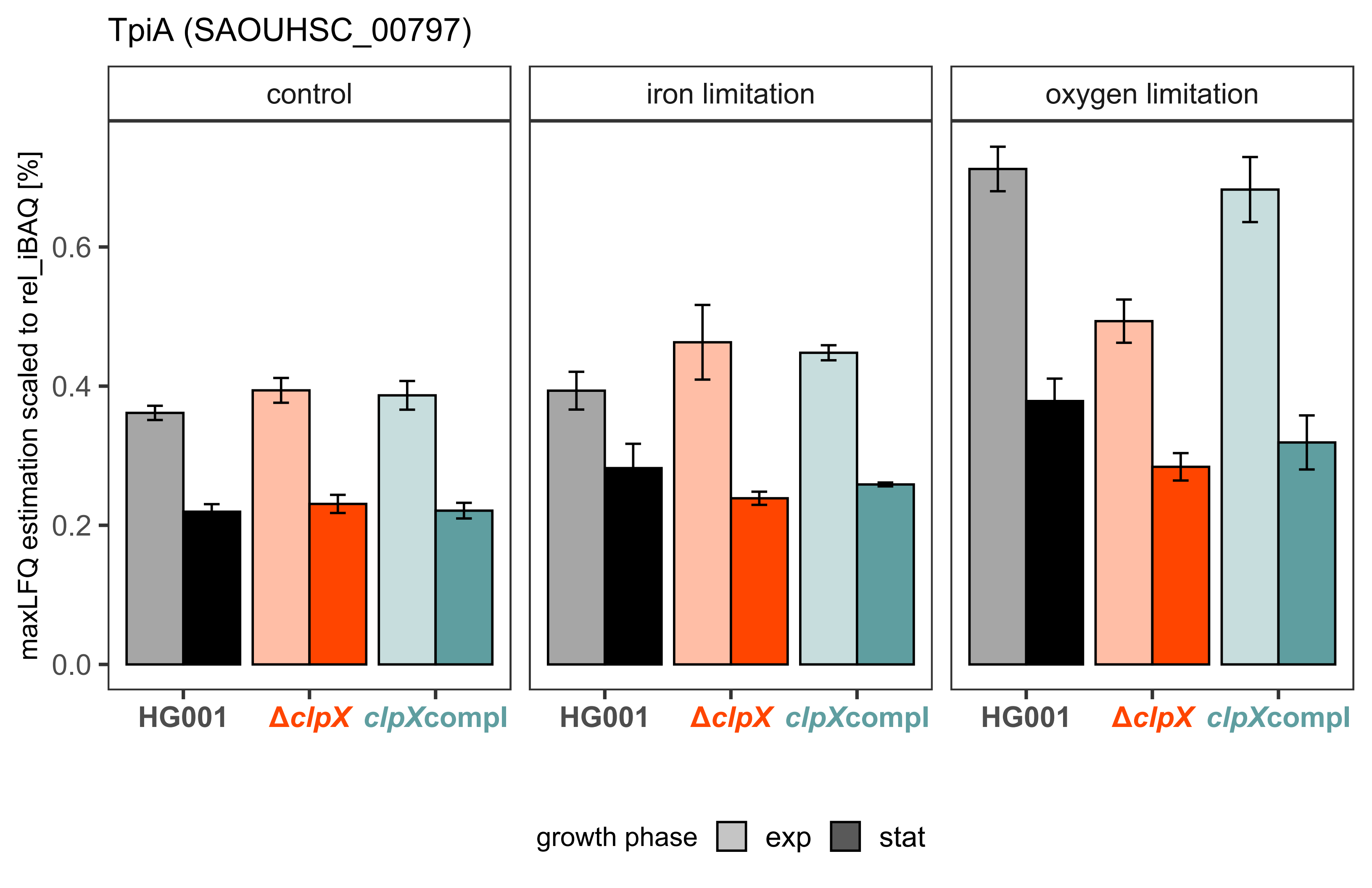

TpiA (SAOUHSC_00797)
control
iron limitation
oxygen limitation
maxLFQ estimation scaled to rel_iBAQ [%]
0.0
0.2
0.4
0.6
HG001
ΔclpX
clpXcompl
growth phase
exp
stat

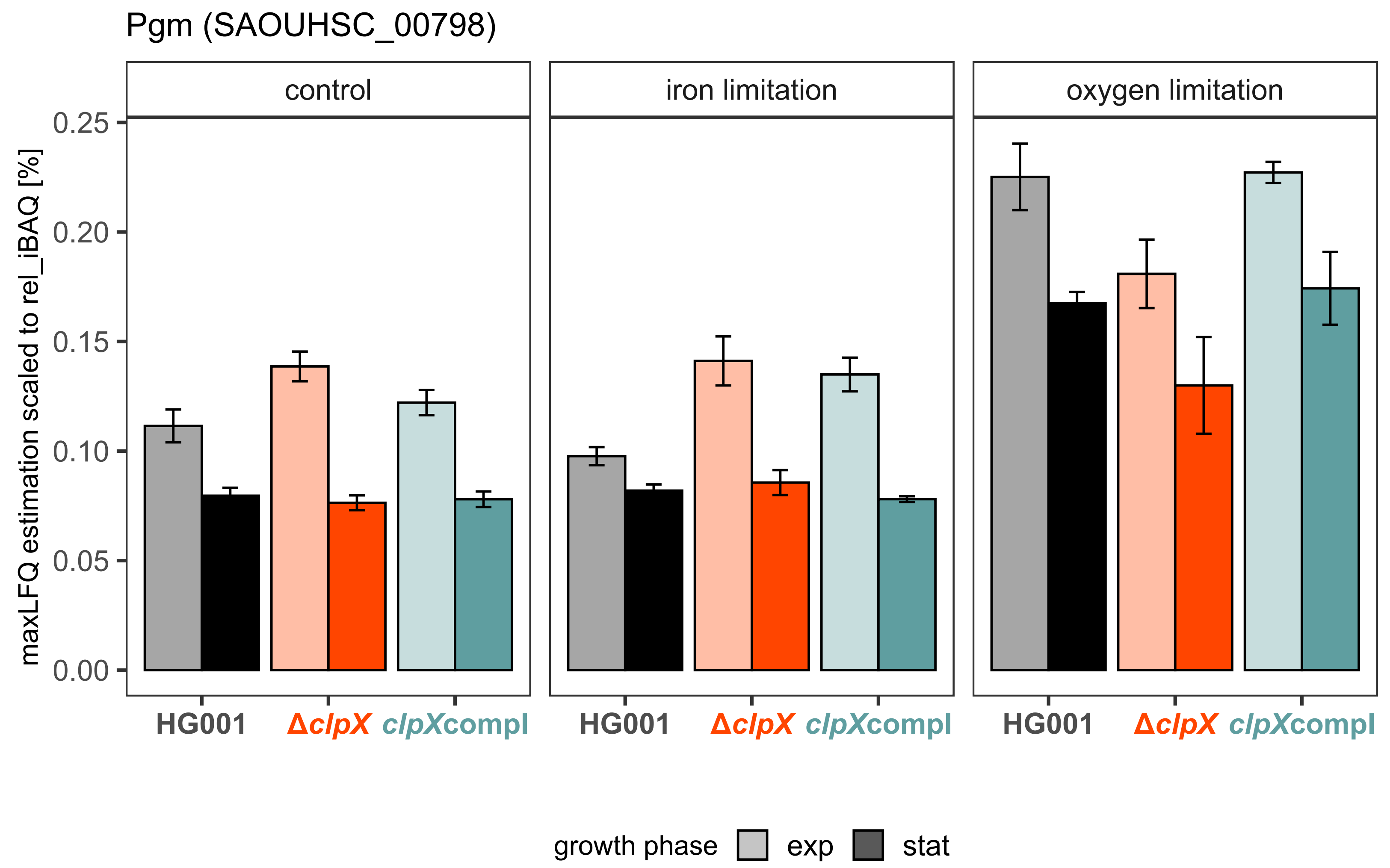
Pgm (SAOUHSC_00798)
control
iron limitation
oxygen limitation
maxLFQ estimation scaled to rel_iBAQ [%]
0.25
0.20
0.15
0.10
0.05
0.00
HG001
ΔclpX
clpXcompl
growth phase
exp
stat

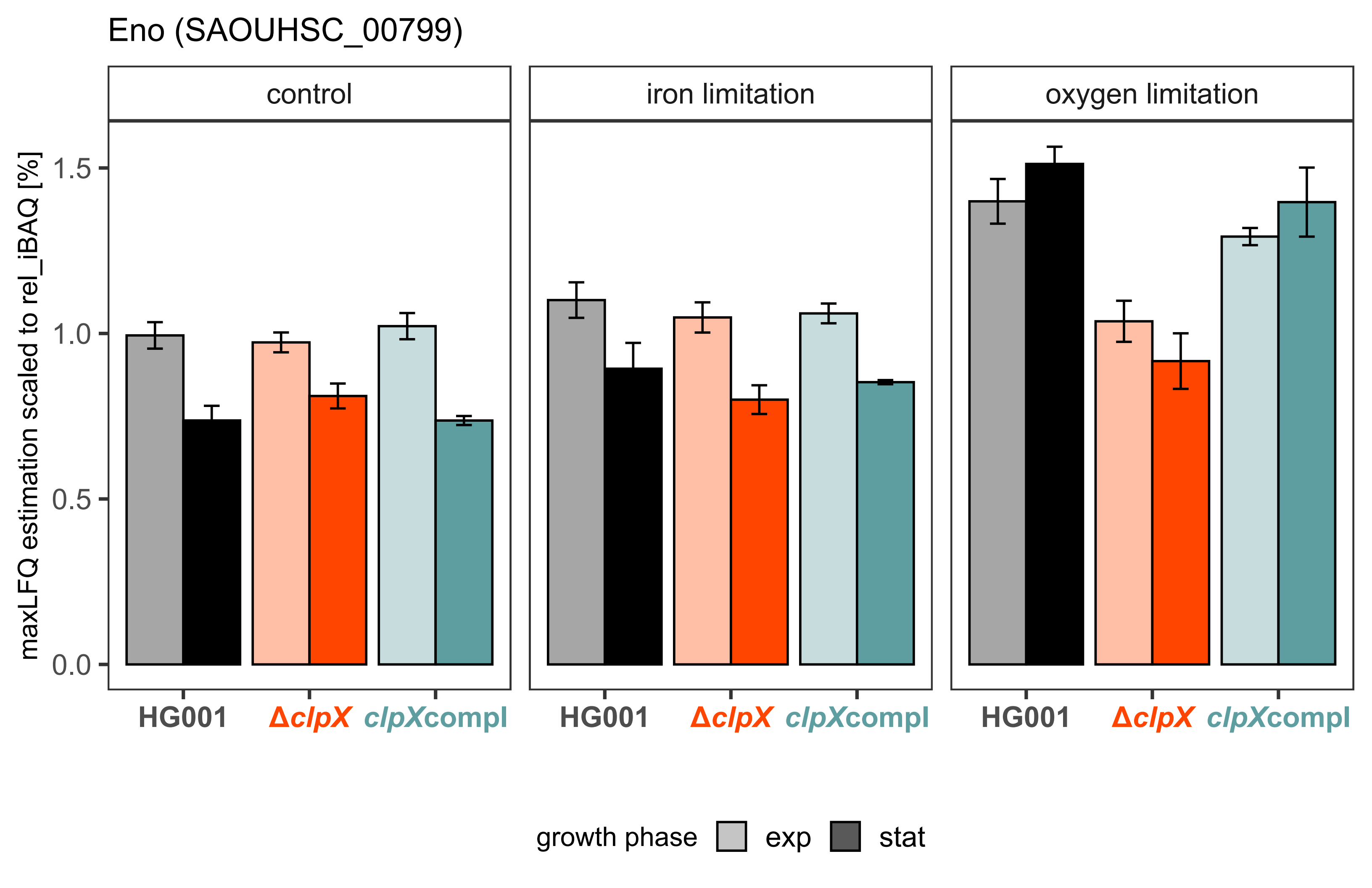

Eno (SAOUHSC_00799)
control
iron limitation
oxygen limitation
maxLFQ estimation scaled to rel_iBAQ [%]
0.0
0.5
1.0
1.5
HG001
ΔclpX
clpXcompl
growth phase
exp
stat

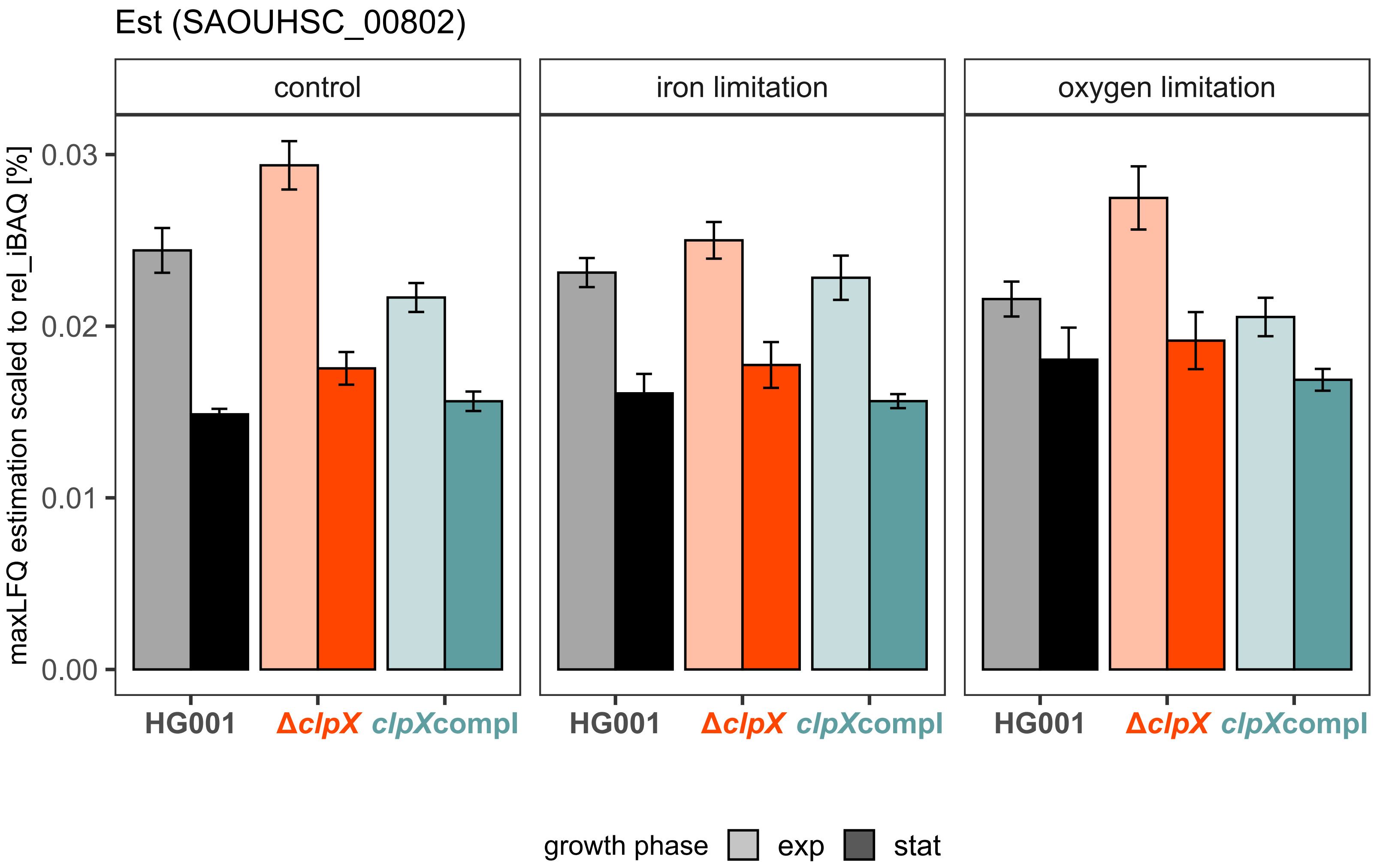

Est (SAOUHSC_00802)
control
iron limitation
oxygen limitation
maxLFQ estimation scaled to rel_iBAQ [%]
0.03
0.02
0.01
0.00
HG001
ΔclpX
clpXcompl
growth phase
exp
stat

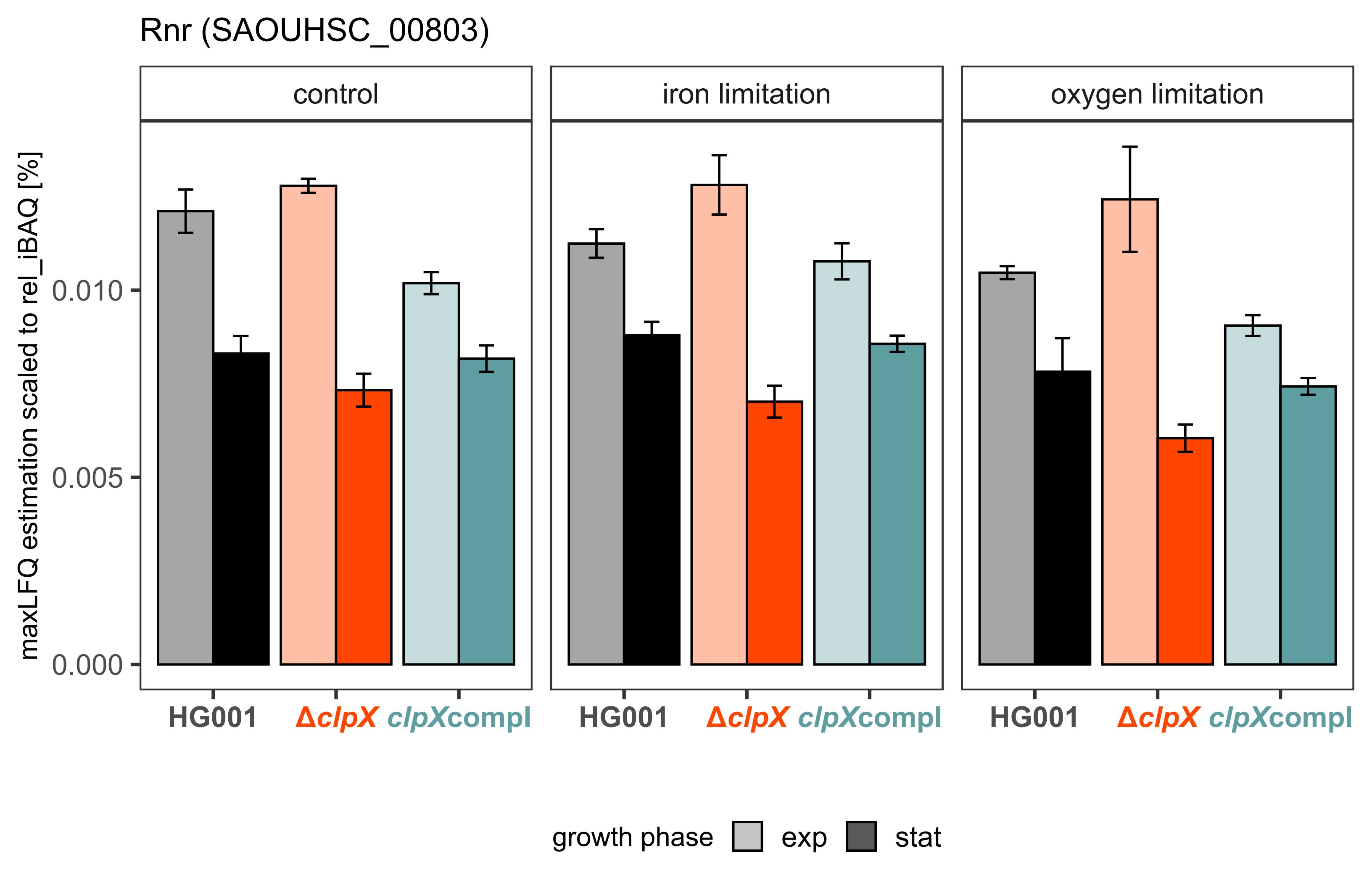
Rnr (SAOUHSC_00803)
control
iron limitation
oxygen limitation
maxLFQ estimation scaled to rel_iBAQ [%]
0.000
0.005
0.010
HG001
ΔclpX
clpXcompl
growth phase
exp
stat

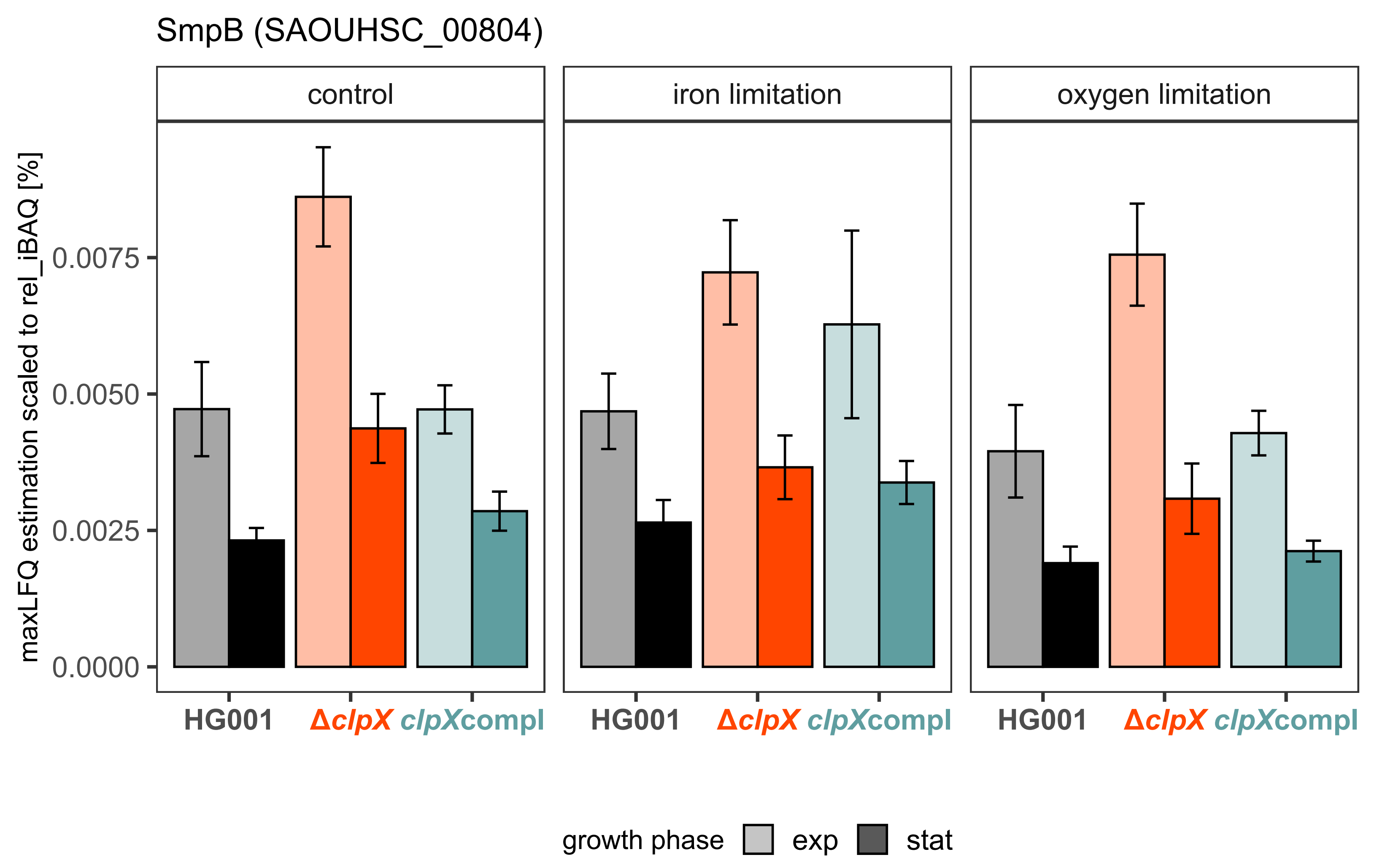

SmpB (SAOUHSC_00804)
control
iron limitation
oxygen limitation
maxLFQ estimation scaled to rel_iBAQ [%]
0.0000
0.0025
0.0050
0.0075
HG001
ΔclpX
clpXcompl
growth phase
exp
stat

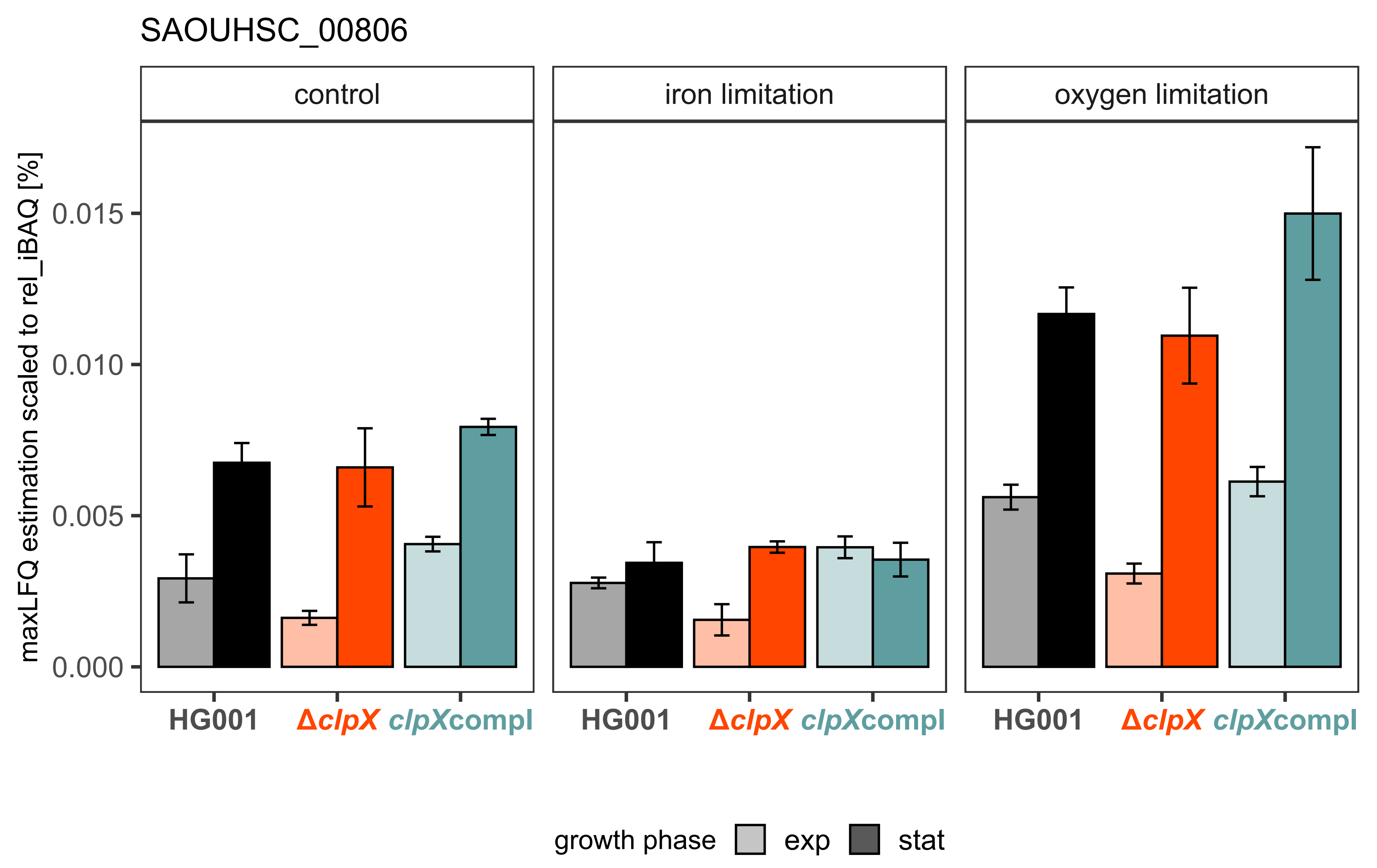

SAOUHSC_00806
control
iron limitation
oxygen limitation
maxLFQ estimation scaled to rel_iBAQ [%]
0.015
0.010
0.005
0.000
HG001
ΔclpX
clpXcompl
growth phase
exp
stat

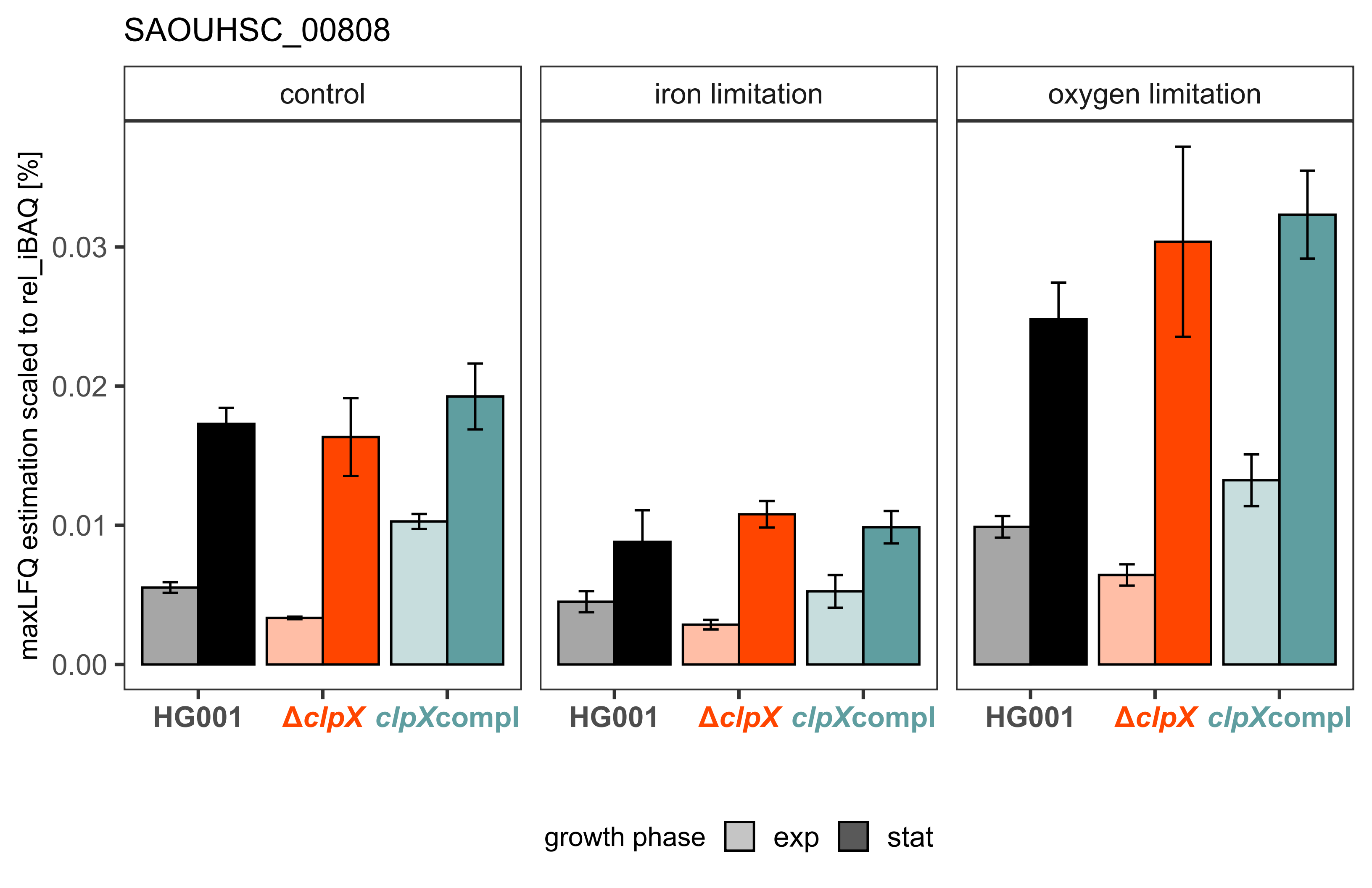

SAOUHSC_00808
control
iron limitation
oxygen limitation
maxLFQ estimation scaled to rel_iBAQ [%]
0.03
0.02
0.01
0.00
HG001
ΔclpX
clpXcompl
growth phase
exp
stat

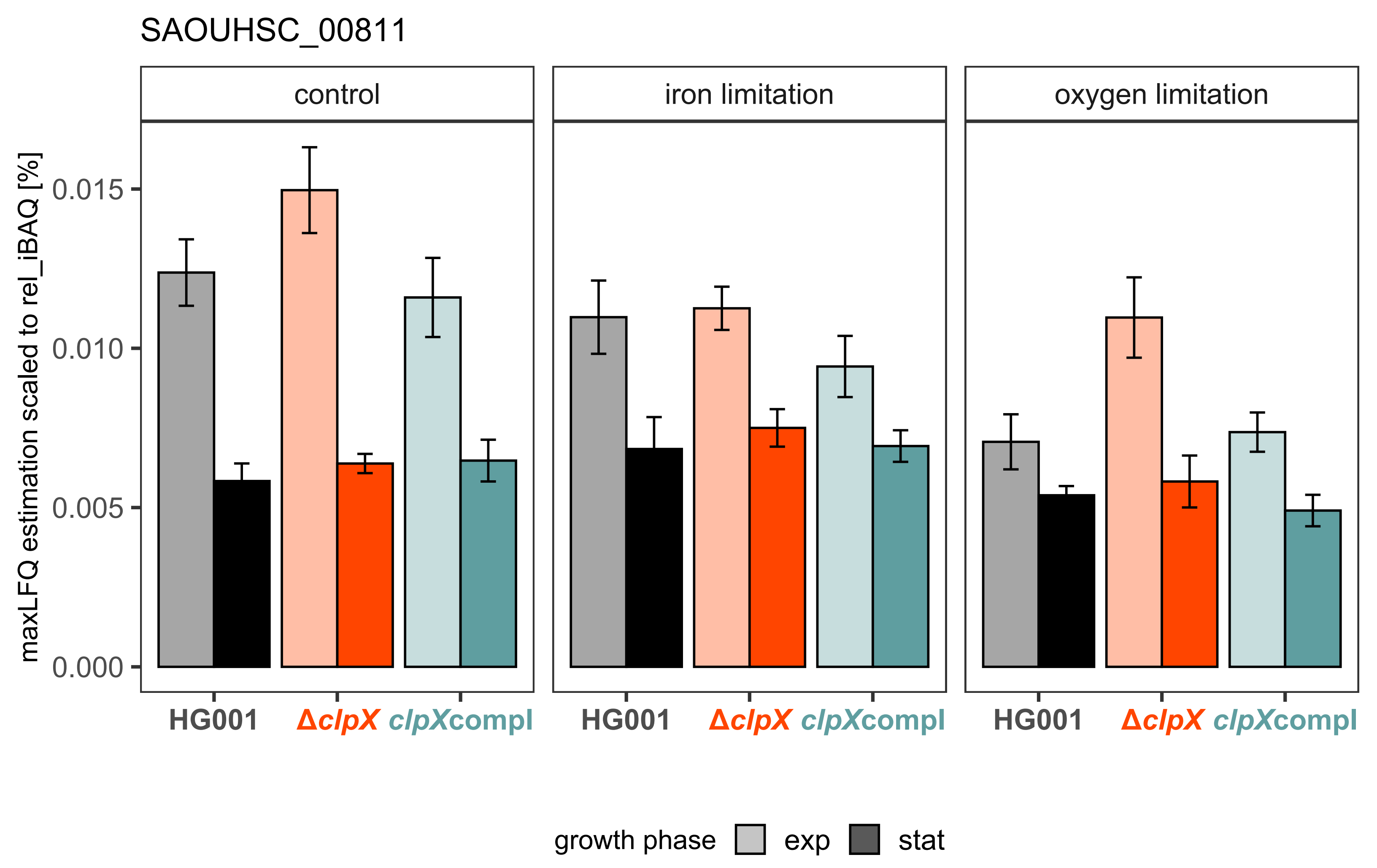
SAOUHSC_00811
control
iron limitation
oxygen limitation
maxLFQ estimation scaled to rel_iBAQ [%]
0.015
0.010
0.005
0.000
HG001
ΔclpX
clpXcompl
growth phase
exp
stat

ClfA (SAOUHSC_00812)

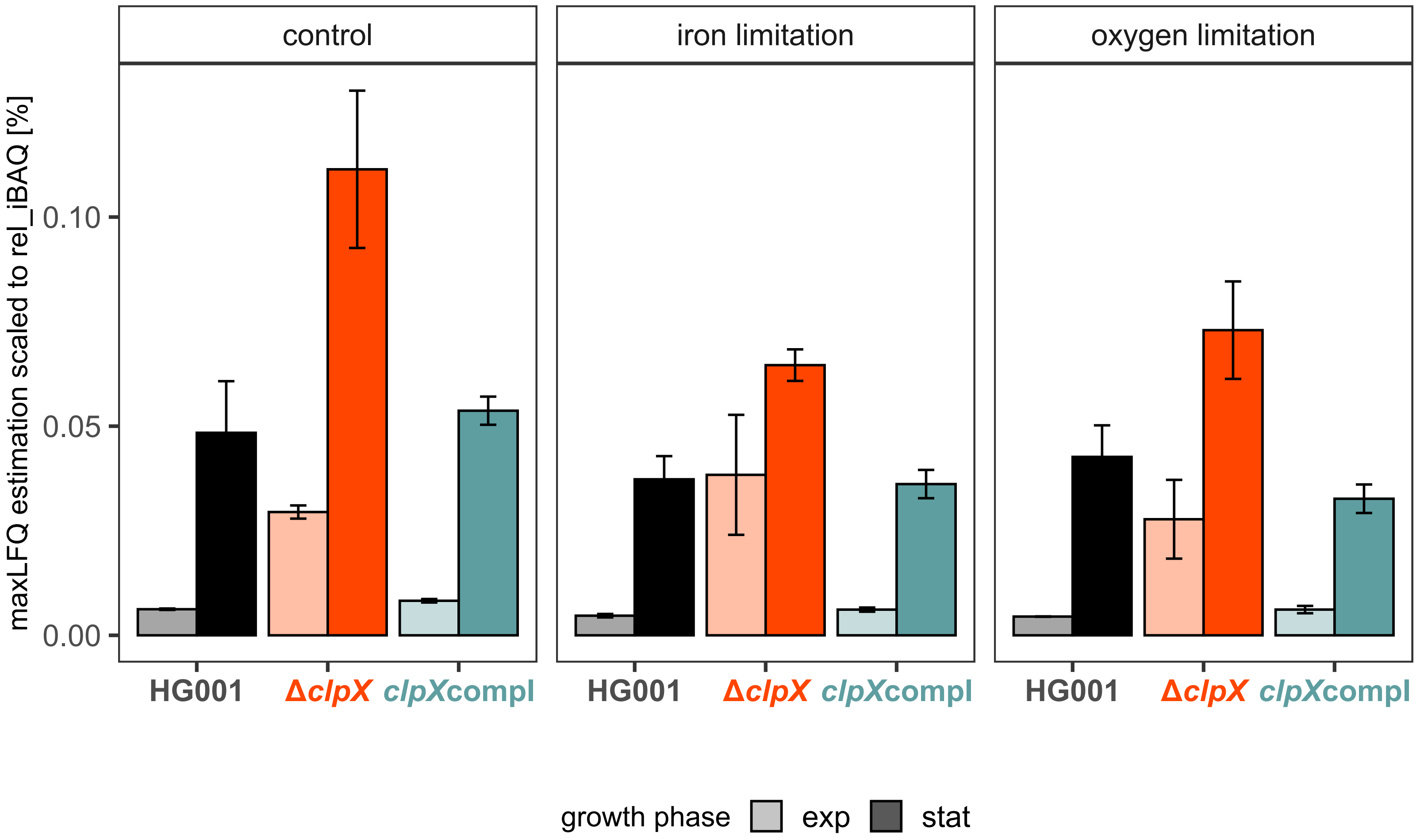

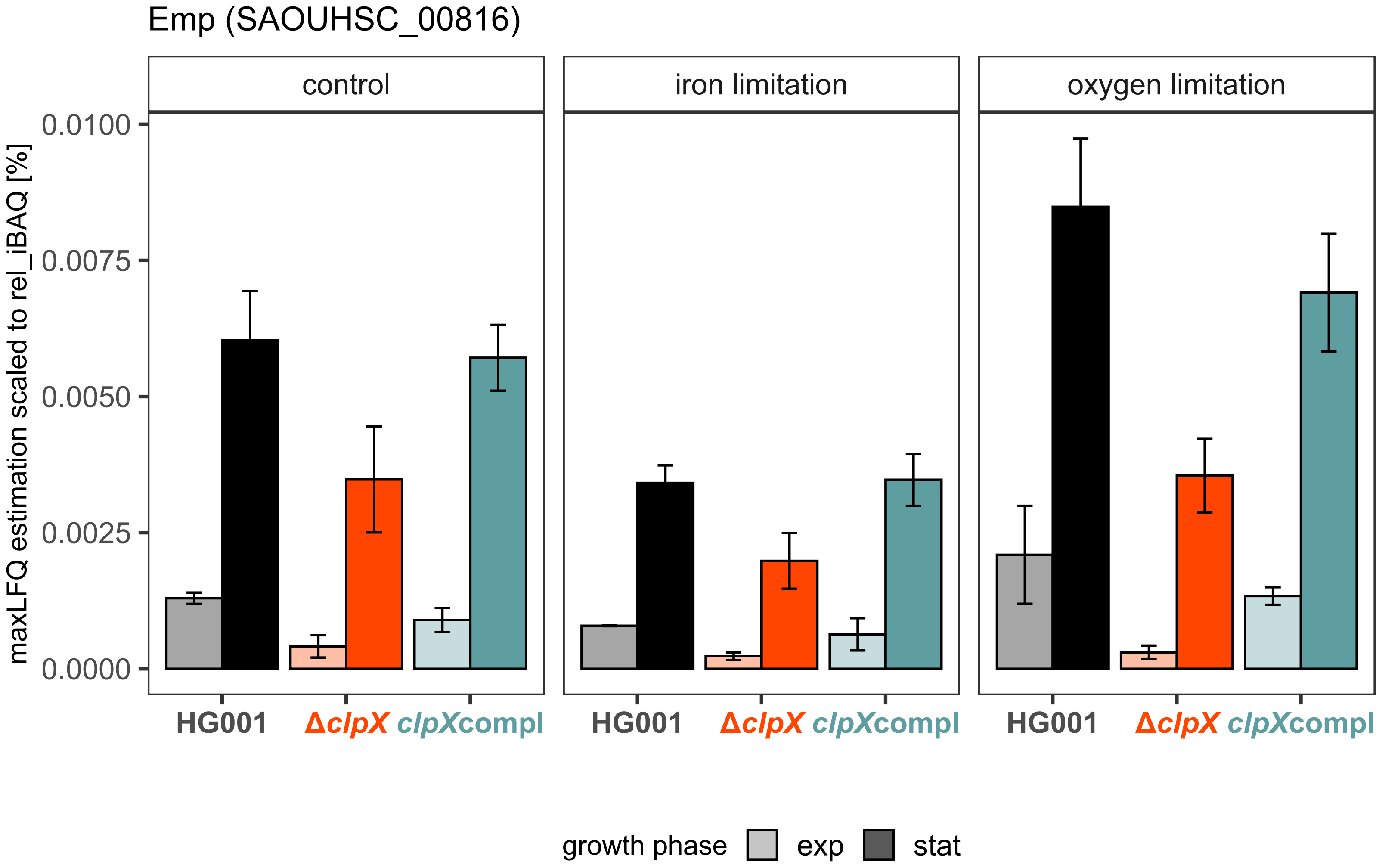
Emp (SAOUHSC_00816)
control
iron limitation
oxygen limitation
maxLFQ estimation scaled to rel_iBAQ [%]
0.0100
0.0075
0.0050
0.0025
0.0000
HG001
ΔclpX
clpXcompl
growth phase
exp
stat

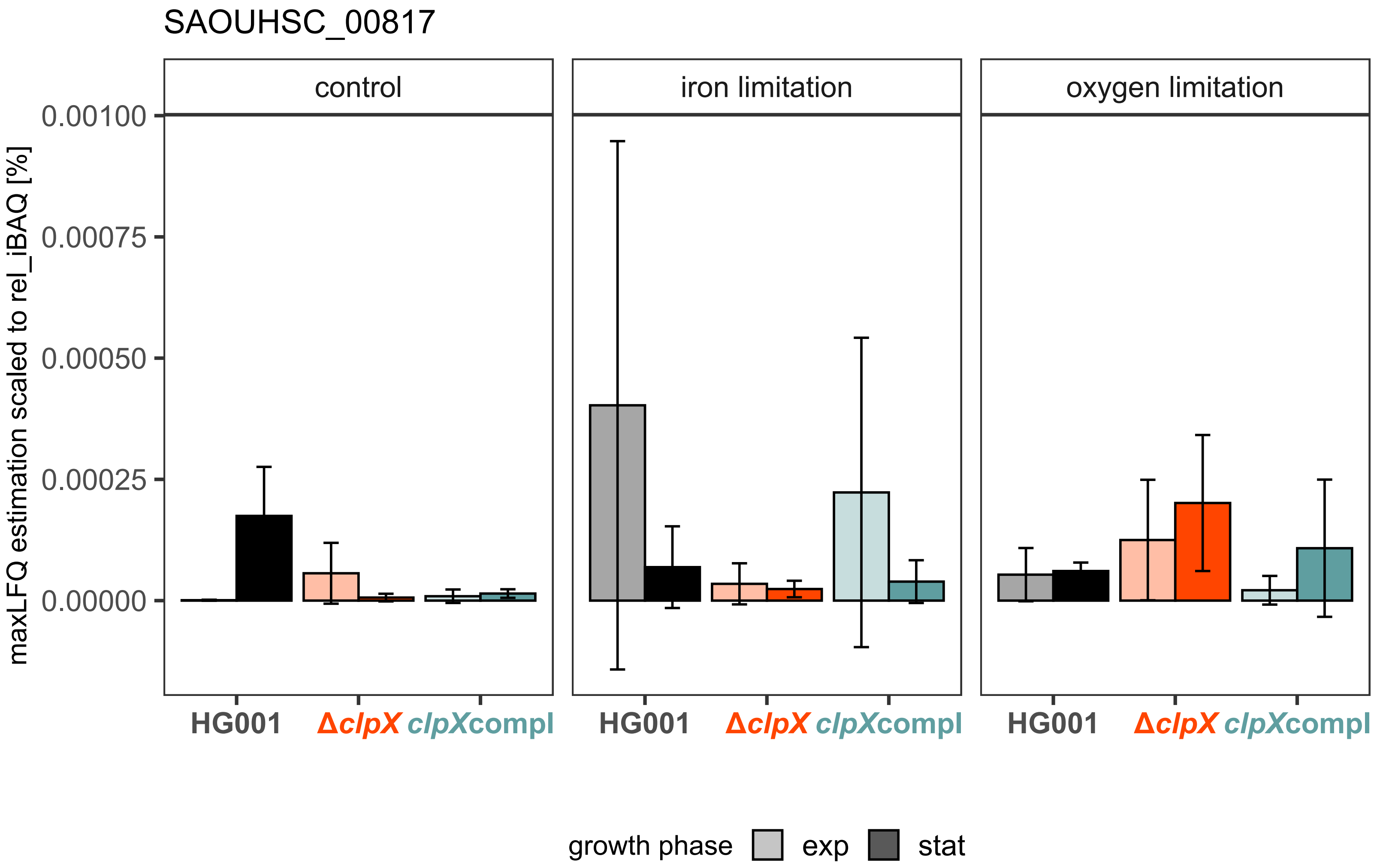
SAOUHSC_00817
control
iron limitation
oxygen limitation
0.00100
0.00075
0.00050
0.00025
0.00000
maxLFQ estimation scaled to rel_iBAQ [%]
HG001
ΔclpX
clpXcompl
growth phase
exp
stat

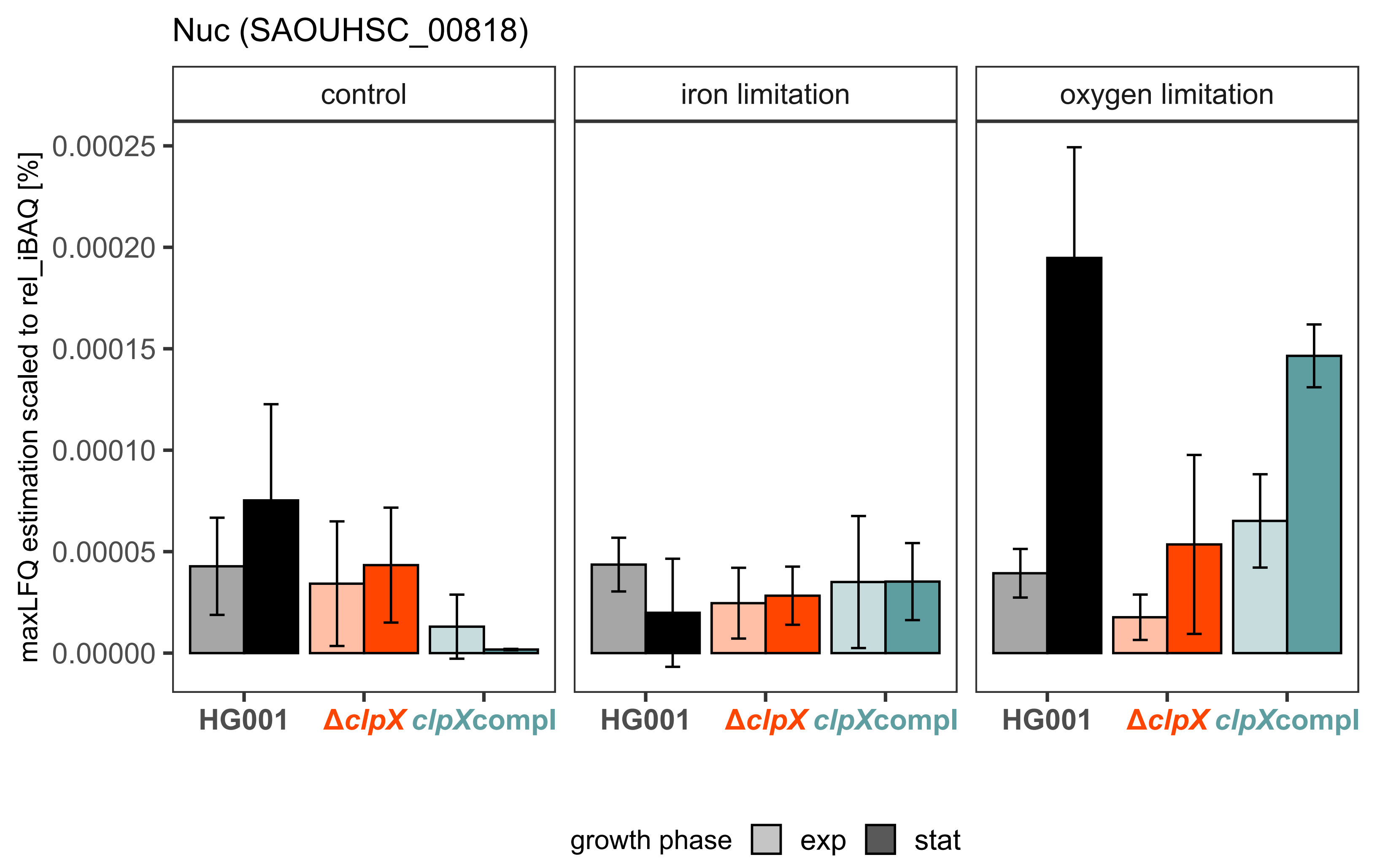
Nuc (SAOUHSC_00818)
control
iron limitation
oxygen limitation
maxLFQ estimation scaled to rel_iBAQ [%]
0.00025
0.00020
0.00015
0.00010
0.00005
0.00000
HG001
ΔclpX
clpXcompl
growth phase
exp
stat

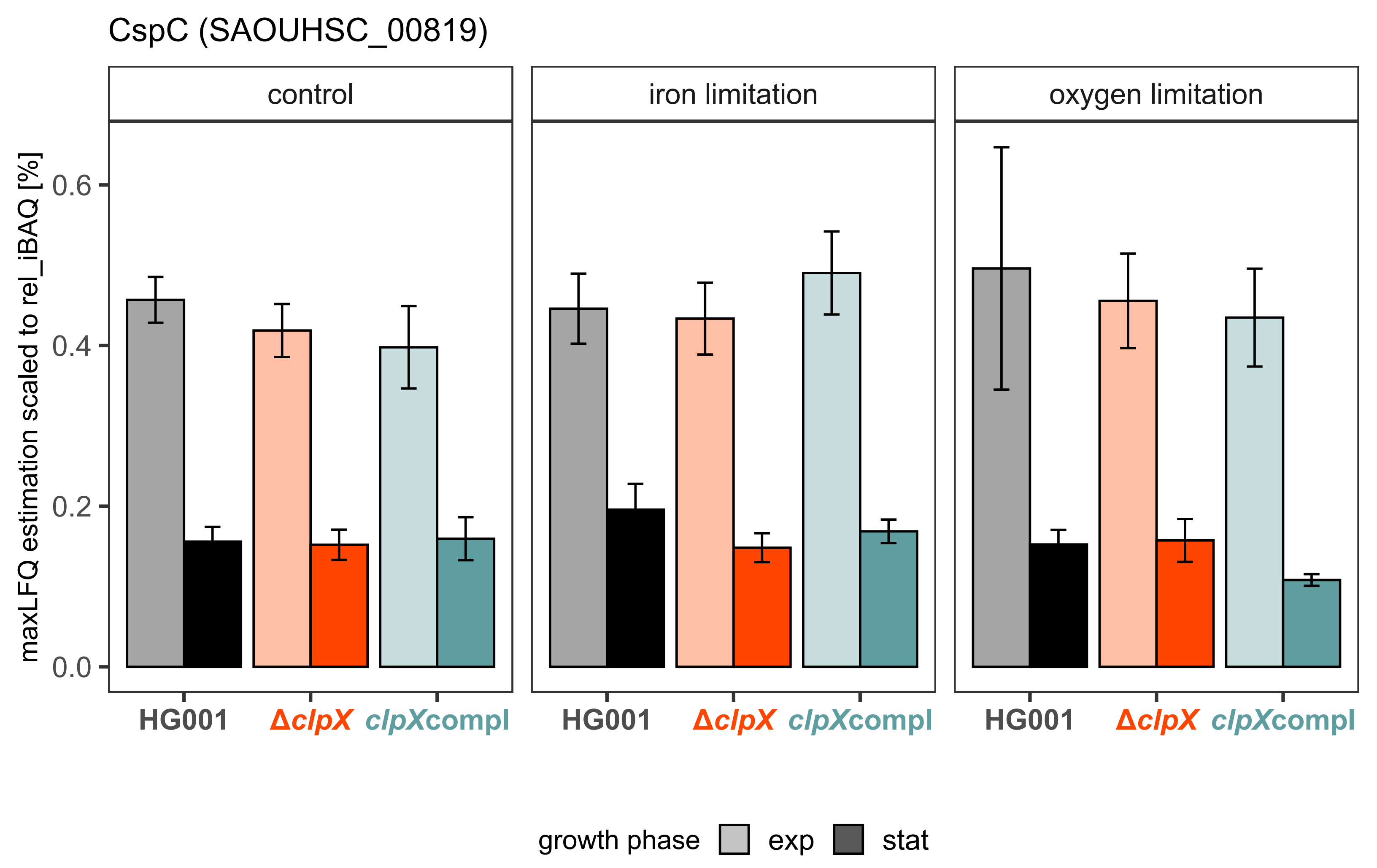
CspC (SAOUHSC_00819)
control
iron limitation
oxygen limitation
maxLFQ estimation scaled to rel_iBAQ [%]
0.0
0.2
0.4
0.6
HG001
ΔclpX
clpXcompl
growth phase
exp
stat

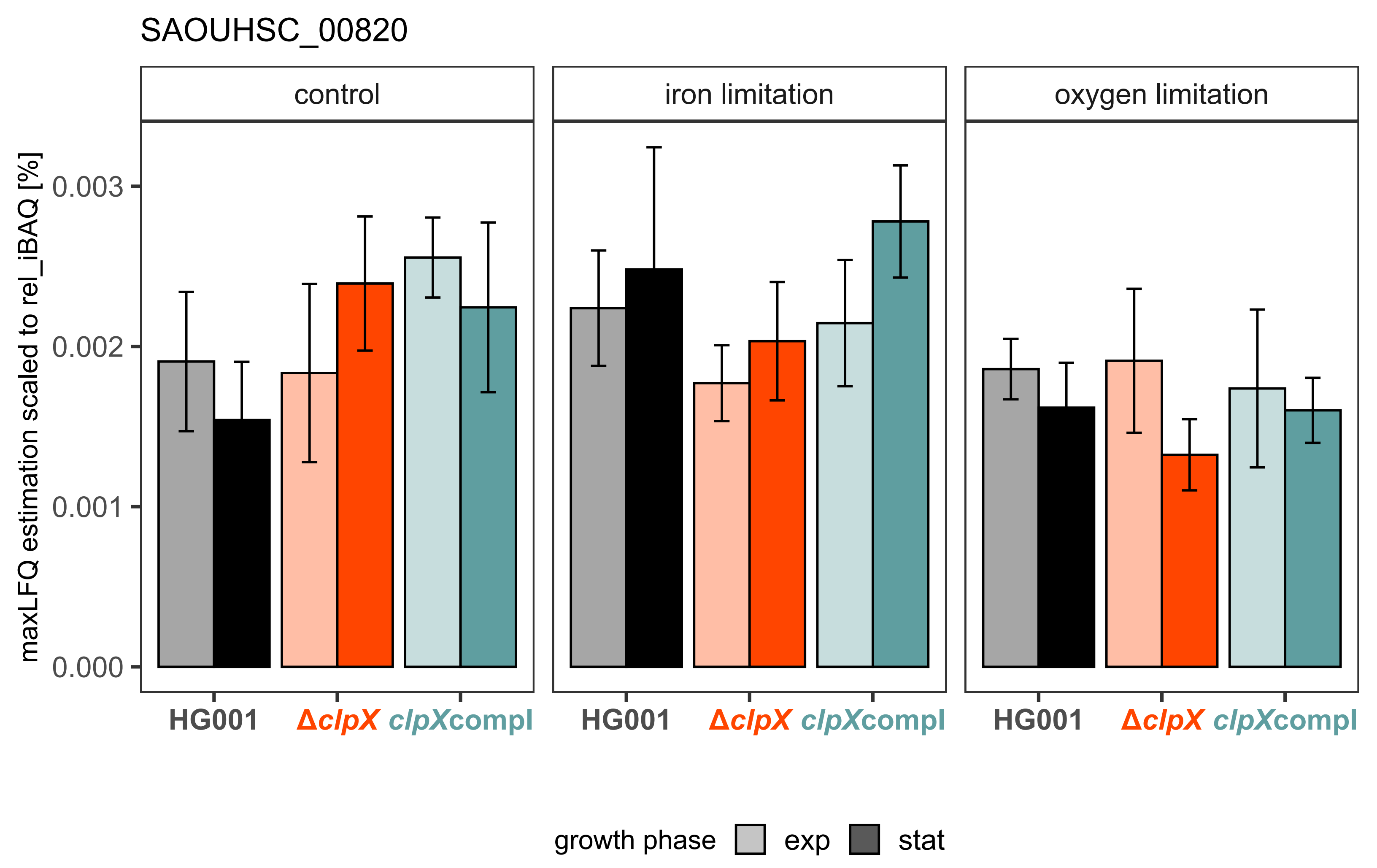

SAOUHSC_00820
control
iron limitation
oxygen limitation
maxLFQ estimation scaled to rel_iBAQ [%]
0.003
0.002
0.001
0.000
HG001
ΔclpX
clpXcompl
growth phase
exp
stat

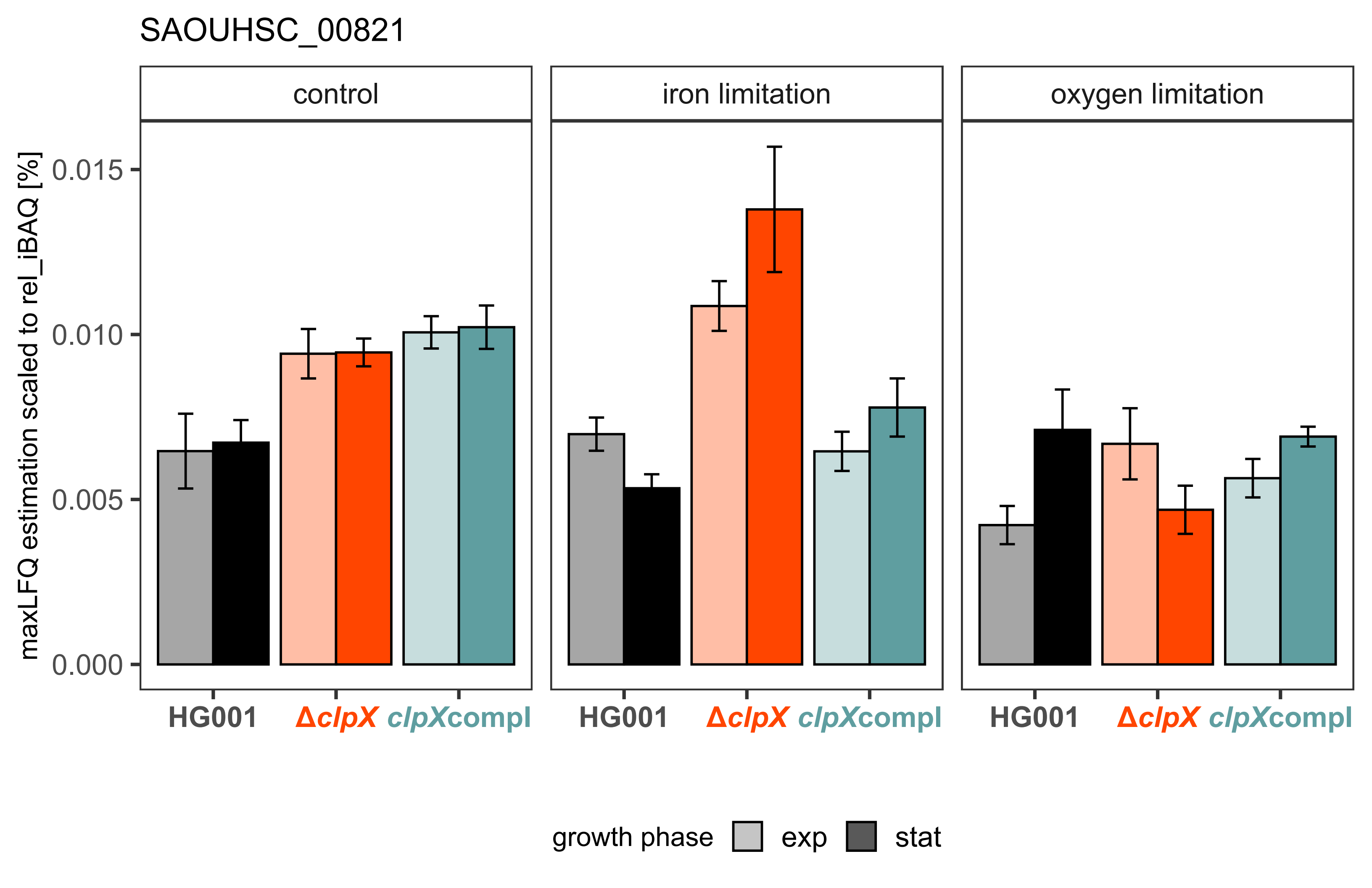
SAOUHSC_00821
control
iron limitation
oxygen limitation
maxLFQ estimation scaled to rel_iBAQ [%]
0.015
0.010
0.005
0.000
HG001
ΔclpX
clpXcompl
growth phase
exp
stat

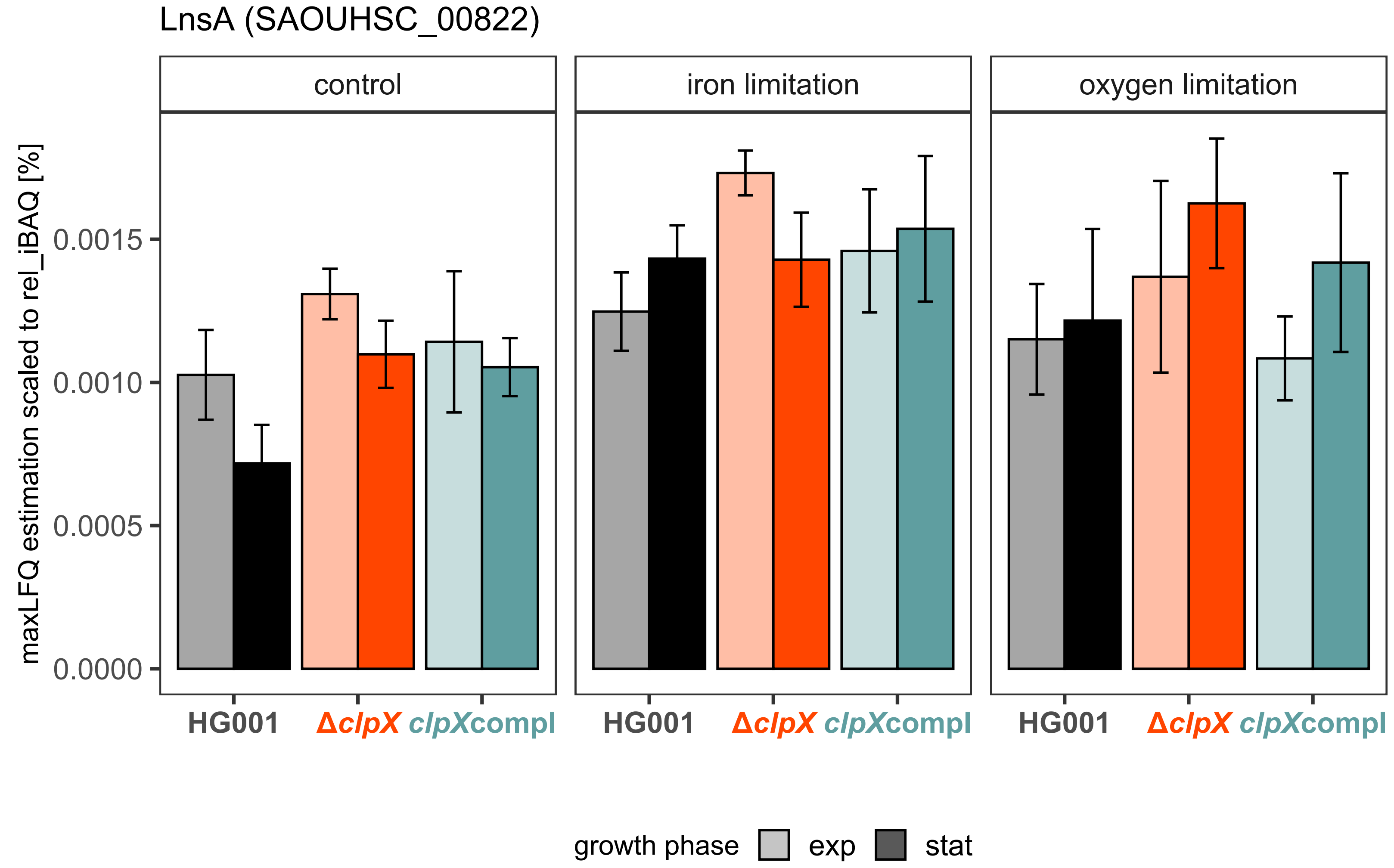
LnsA (SAOUHSC_00822)
control
iron limitation
oxygen limitation
maxLFQ estimation scaled to rel_iBAQ [%]
0.0015
0.0010
0.0005
0.0000
HG001
ΔclpX
clpXcompl
growth phase
exp
stat

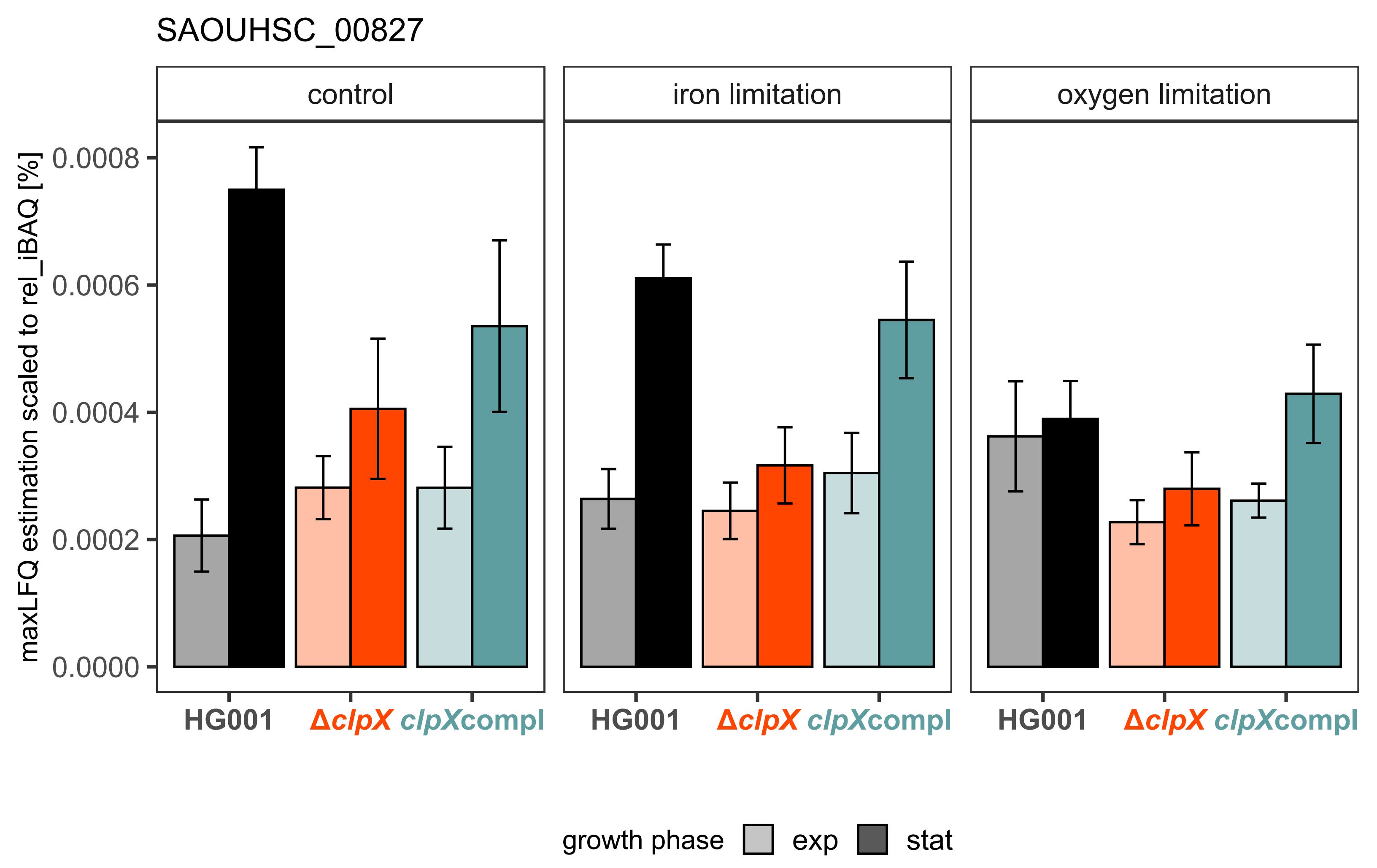

SAOUHSC_00827
control
iron limitation
oxygen limitation
maxLFQ estimation scaled to rel_iBAQ [%]
0.0008
0.0006
0.0004
0.0002
0.0000
HG001
ΔclpX
clpXcompl
growth phase
exp
stat

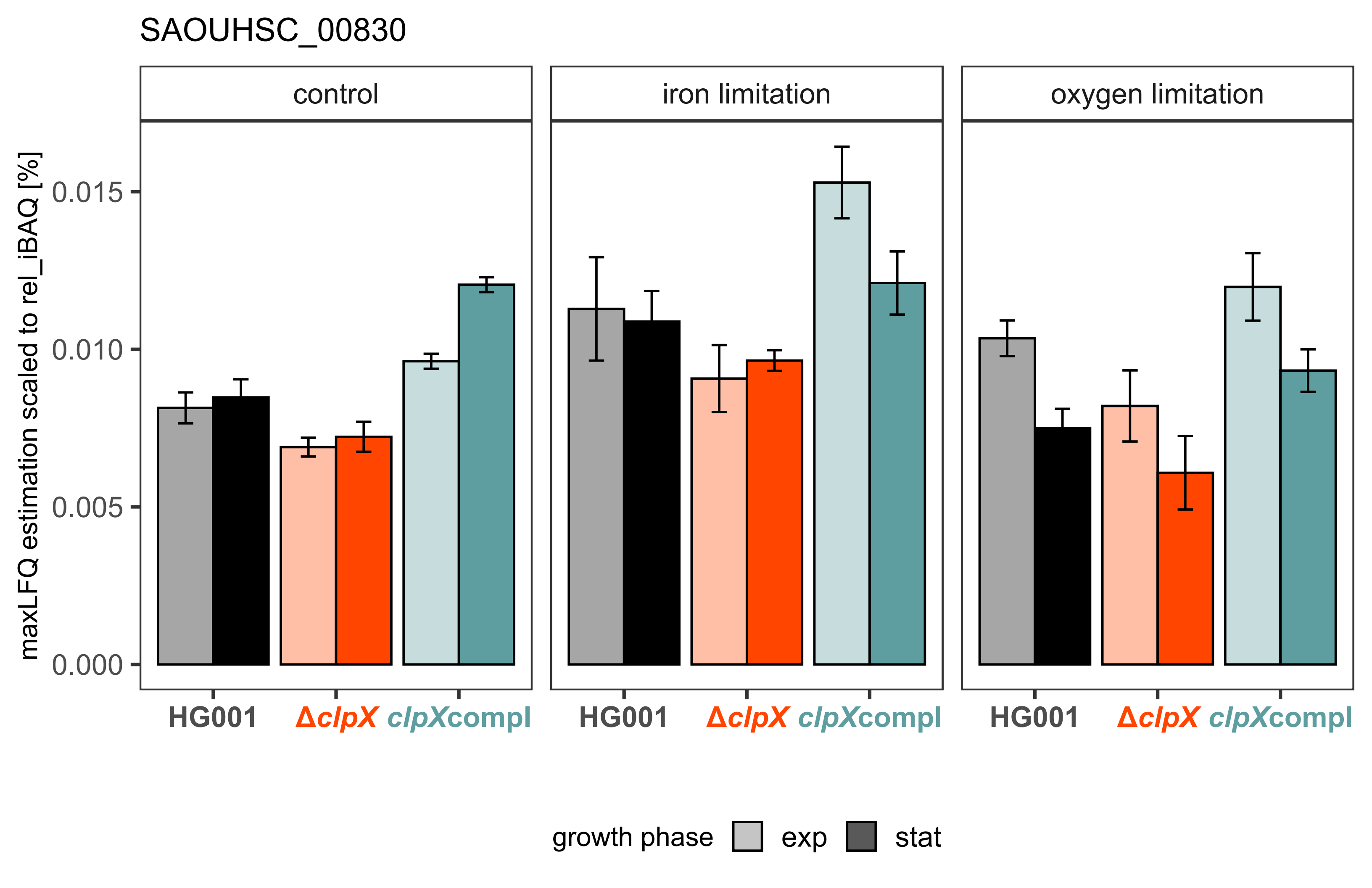

SAOUHSC_00830
control
iron limitation
oxygen limitation
maxLFQ estimation scaled to rel_iBAQ [%]
0.015
0.010
0.005
0.000
HG001
ΔclpX
clpXcompl
growth phase
exp
stat

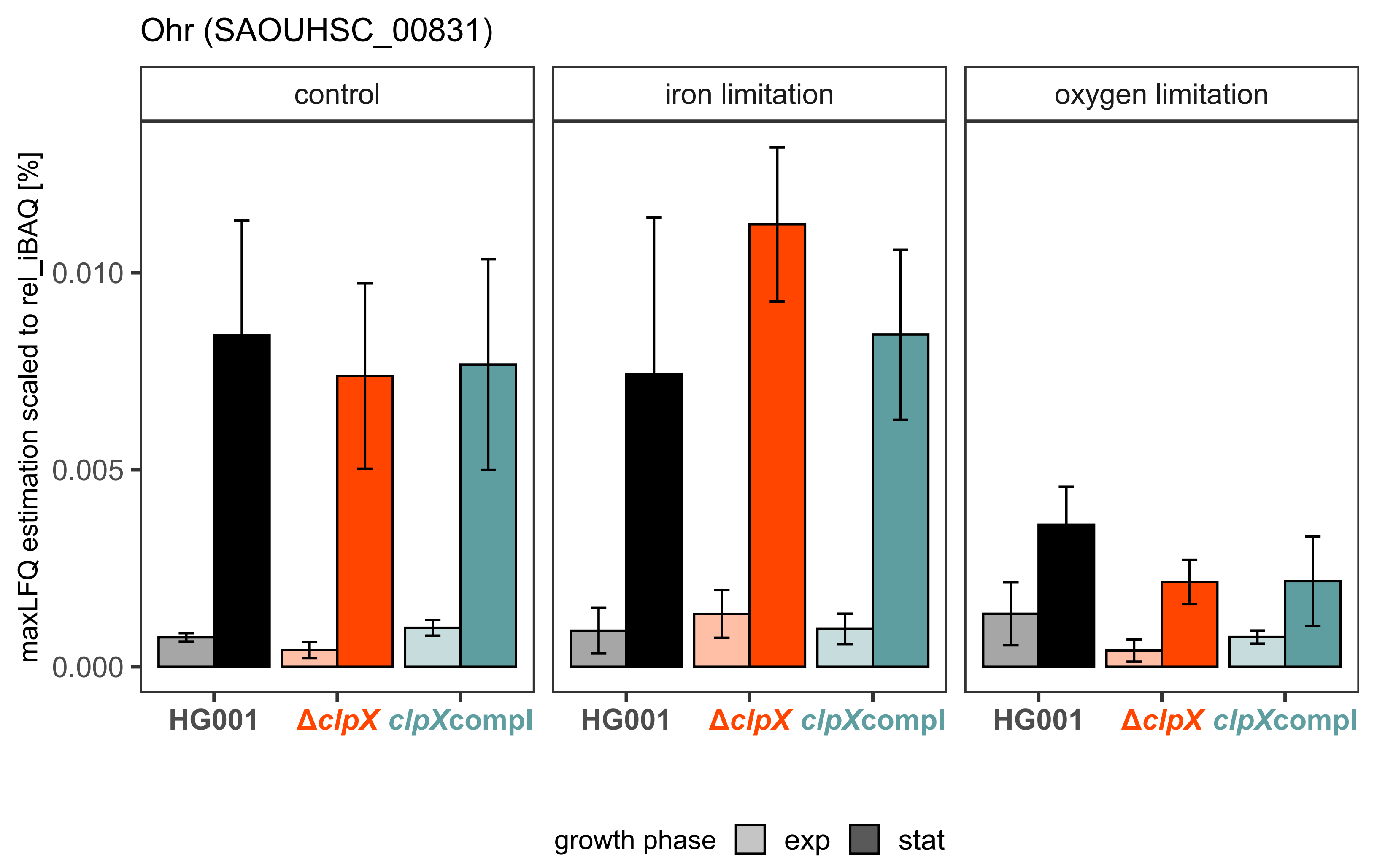
Ohr (SAOUHSC_00831)
control
iron limitation
oxygen limitation
maxLFQ estimation scaled to rel_iBAQ [%]
0.010
0.005
0.000
HG001
ΔclpX
clpXcompl
growth phase
exp
stat

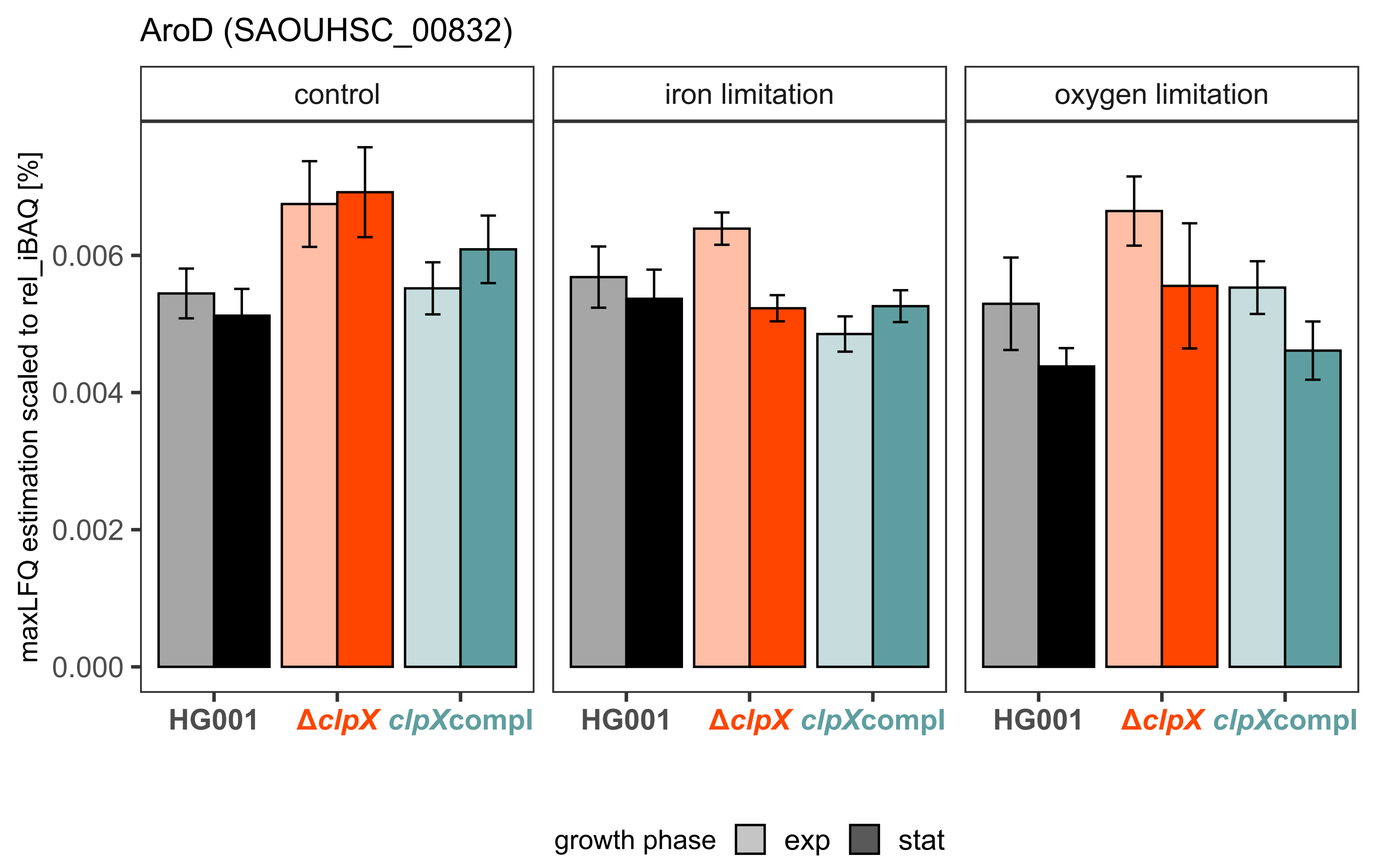
AroD (SAOUHSC_00832)
control
iron limitation
oxygen limitation
maxLFQ estimation scaled to rel_iBAQ [%]
0.006
0.004
0.002
0.000
HG001
ΔclpX
clpXcompl
growth phase
exp
stat

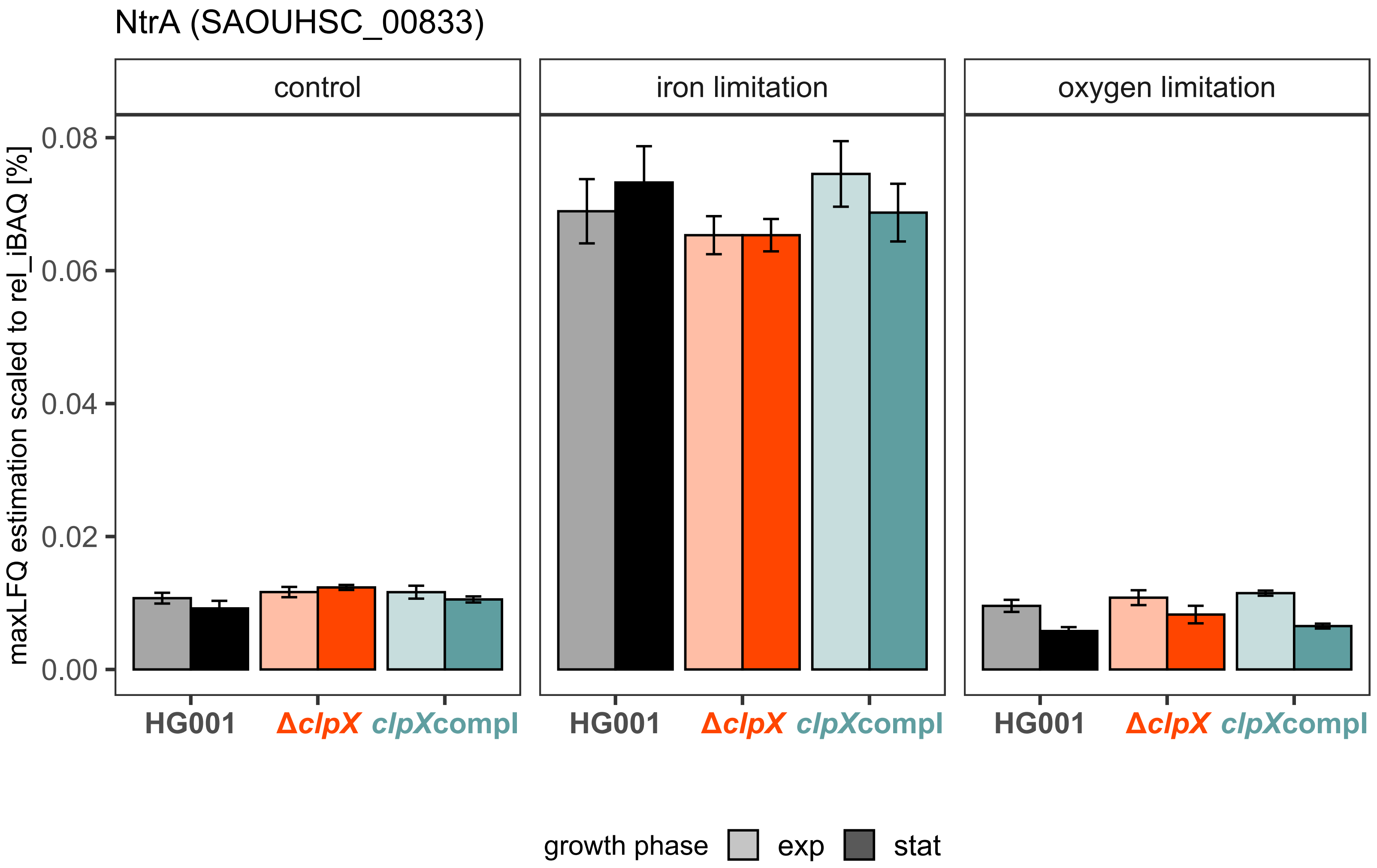

NtrA (SAOUHSC_00833)
control
iron limitation
oxygen limitation
maxLFQ estimation scaled to rel_iBAQ [%]
0.08
0.06
0.04
0.02
0.00
HG001
ΔclpX
clpXcompl
growth phase
exp
stat

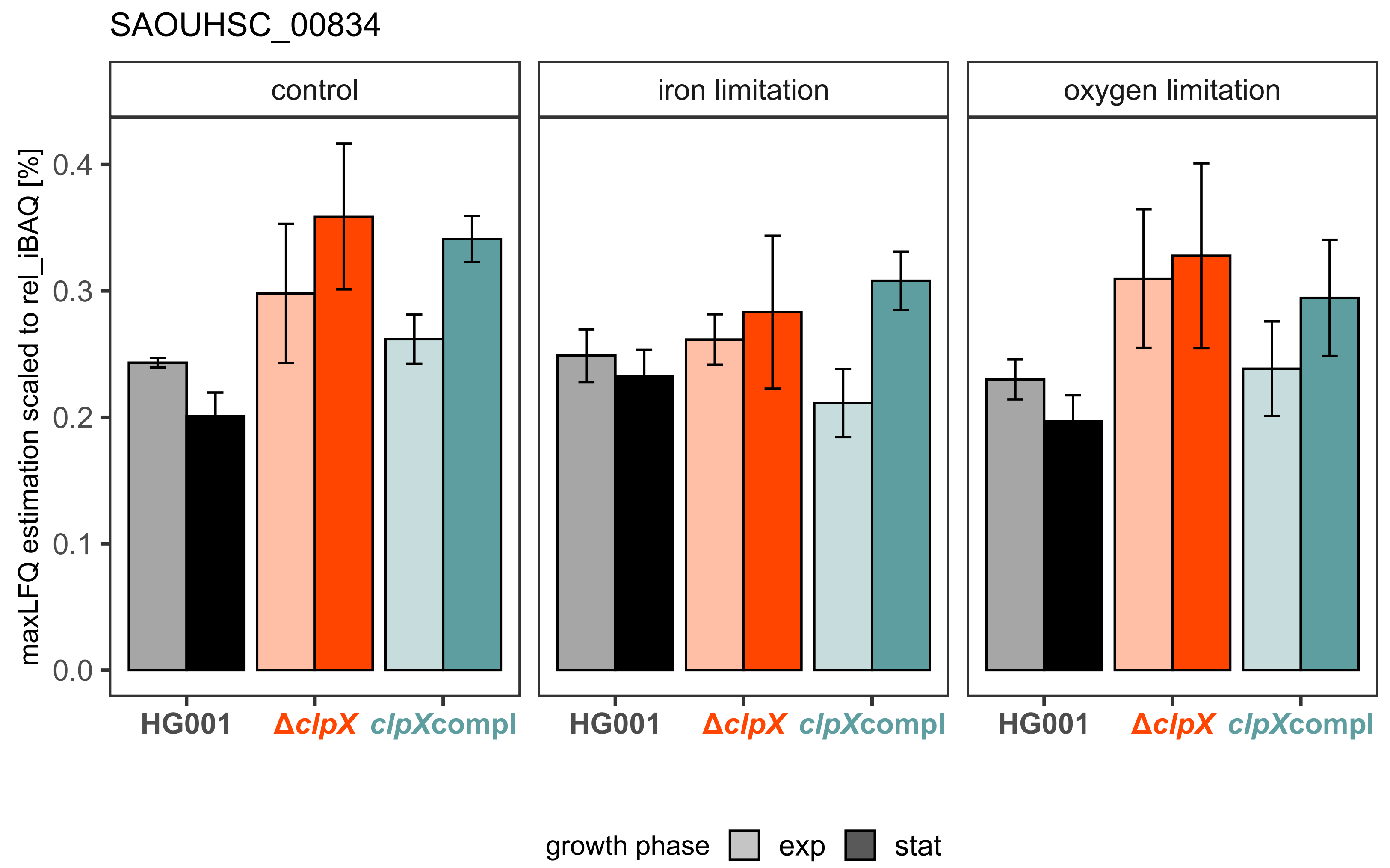

SAOUHSC_00834
control
iron limitation
oxygen limitation
maxLFQ estimation scaled to rel_iBAQ [%]
0.0
0.1
0.2
0.3
0.4
HG001
ΔclpX
clpXcompl
growth phase
exp
stat

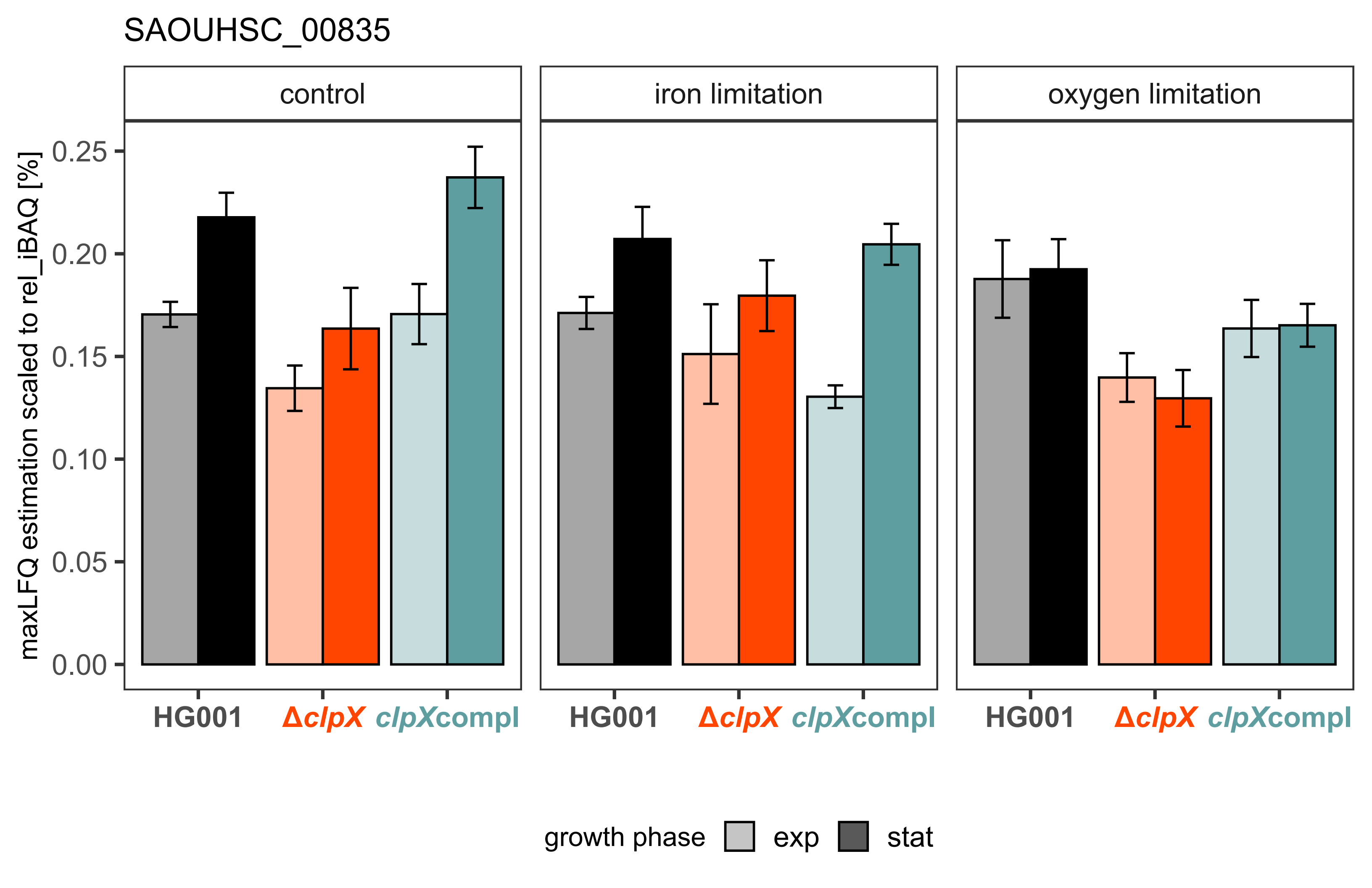

SAOUHSC_00835
control
iron limitation
oxygen limitation
maxLFQ estimation scaled to rel_iBAQ [%]
0.25
0.20
0.15
0.10
0.05
0.00
HG001
ΔclpX
clpXcompl
growth phase
exp
stat

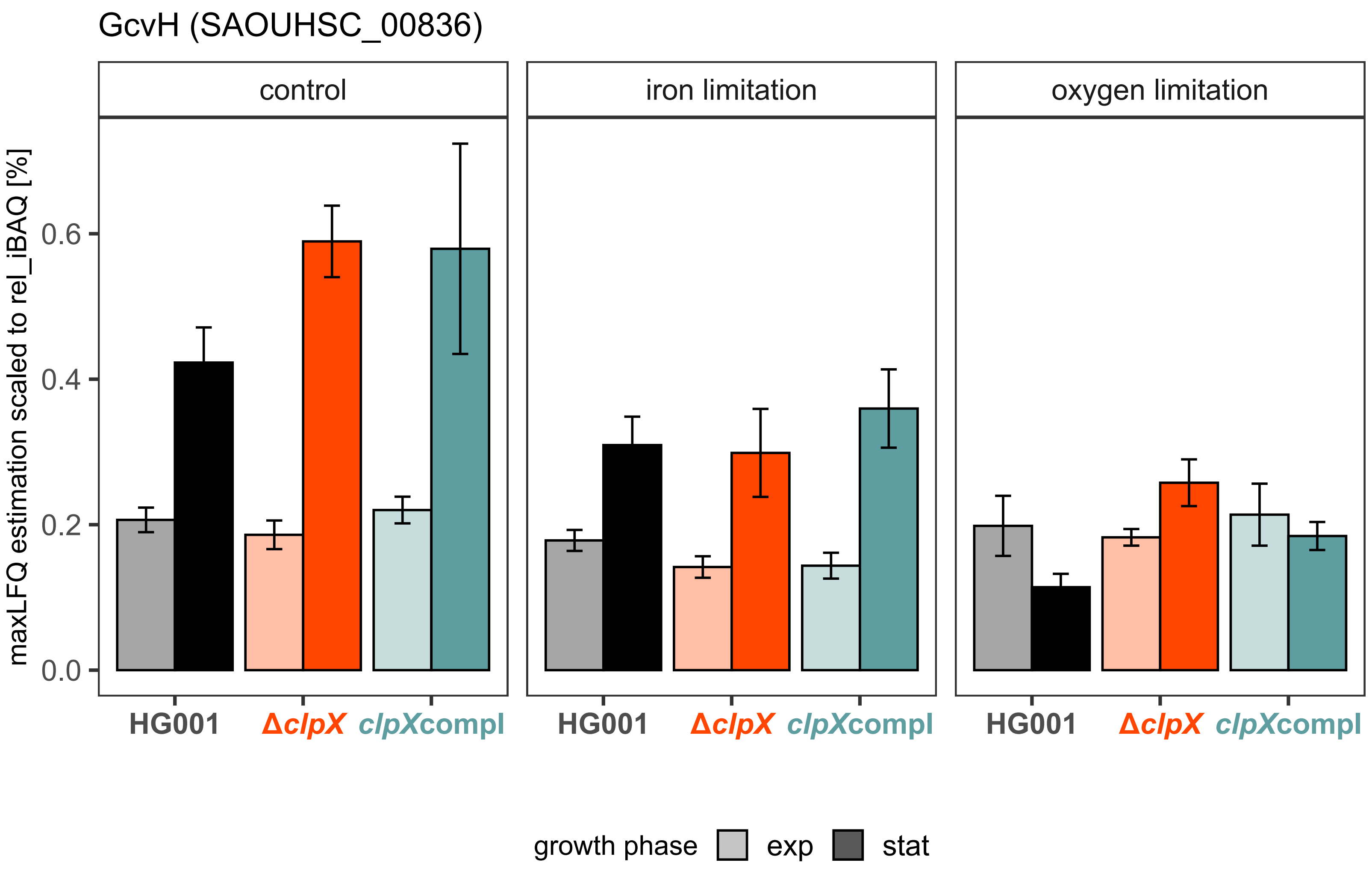
GcvH (SAOUHSC_00836)
control
iron limitation
oxygen limitation
maxLFQ estimation scaled to rel_iBAQ [%]
0.0
0.2
0.4
0.6
HG001
ΔclpX
clpXcompl
growth phase
exp
stat

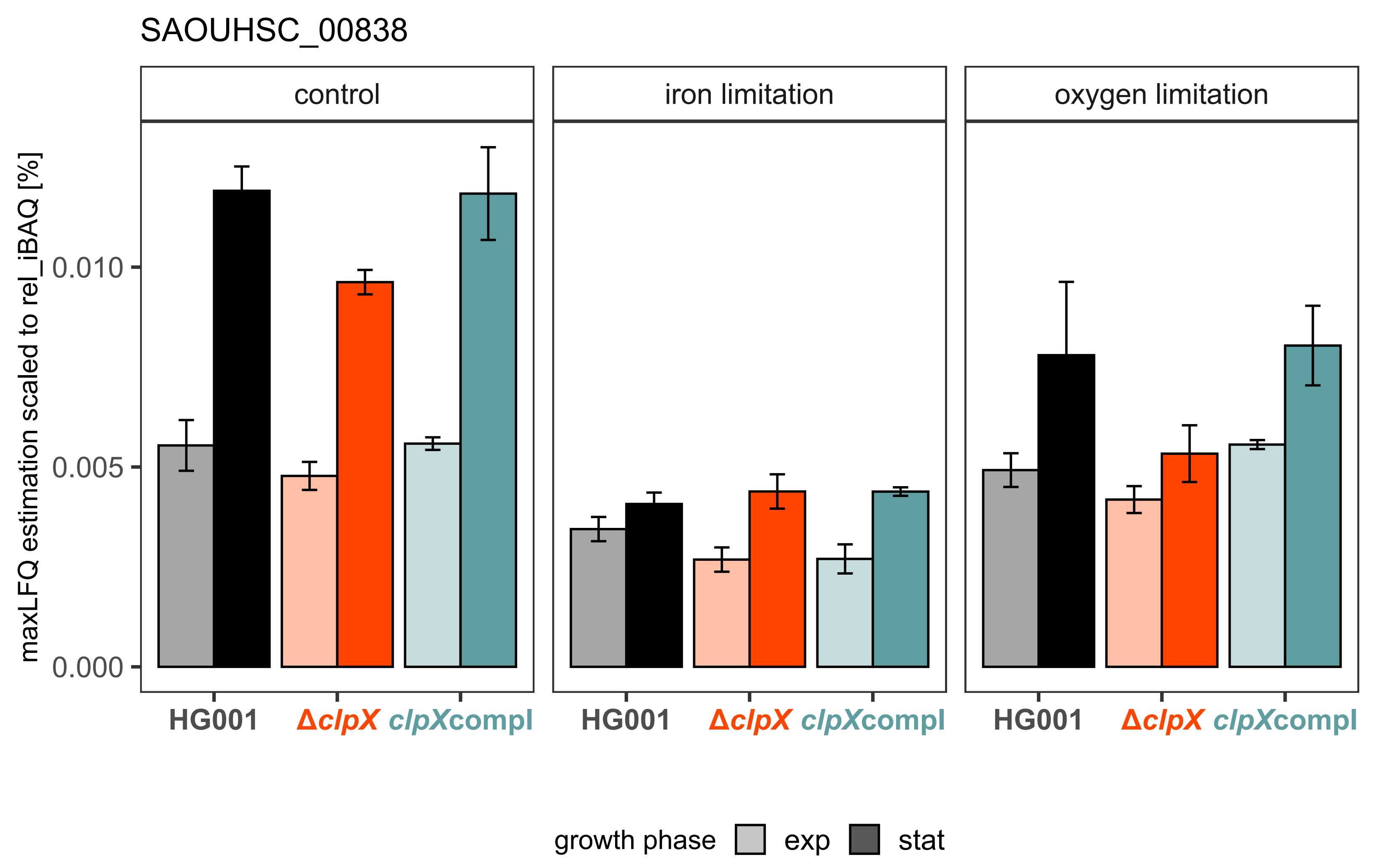
SAOUHSC_00838
control
iron limitation
oxygen limitation
maxLFQ estimation scaled to rel_iBAQ [%]
0.010
0.005
0.000
HG001
ΔclpX
clpXcompl
growth phase
exp
stat

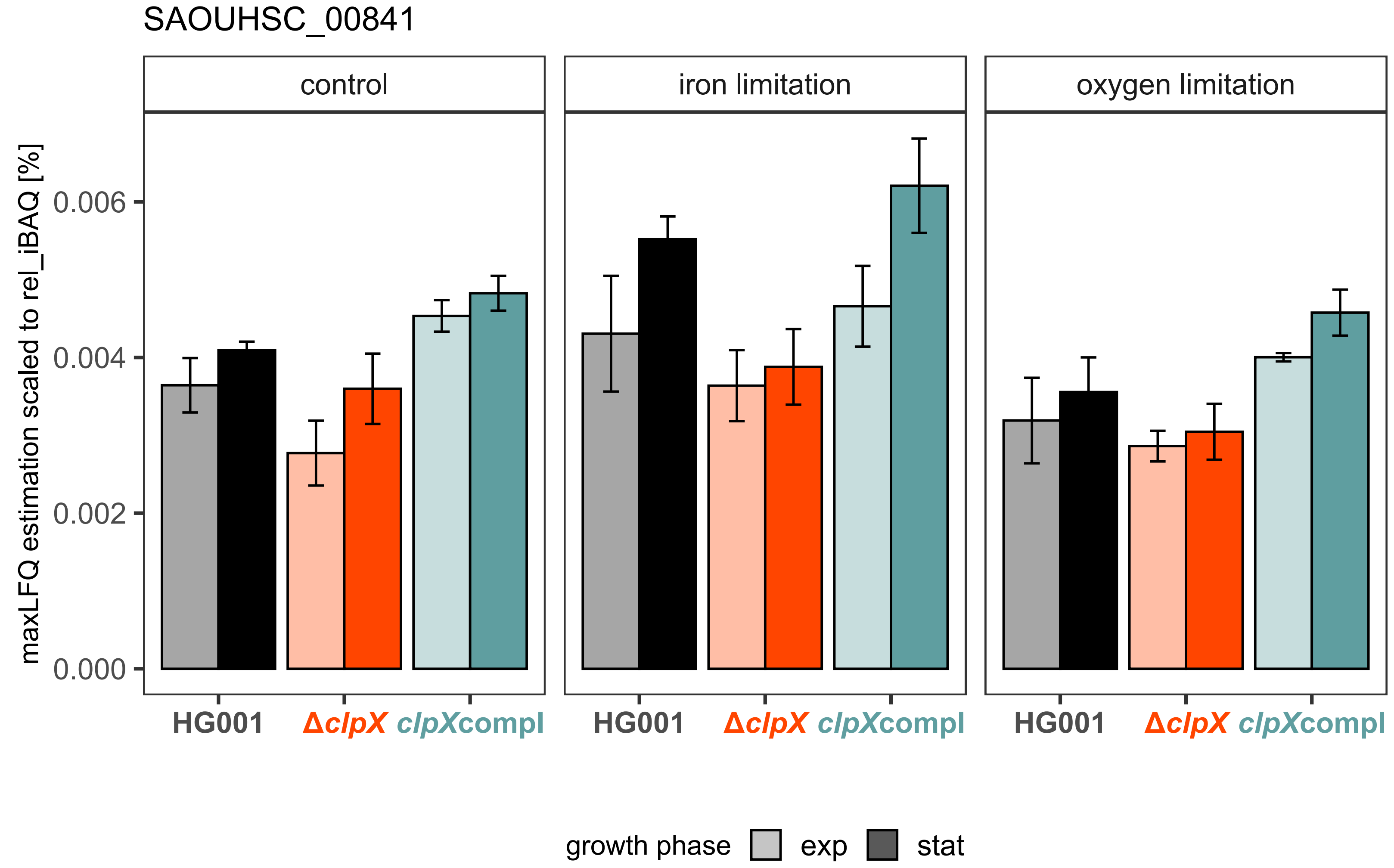
SAOUHSC_00841
control
iron limitation
oxygen limitation
maxLFQ estimation scaled to rel_iBAQ [%]
0.006
0.004
0.002
0.000
HG001
ΔclpX
clpXcompl
growth phase
exp
stat

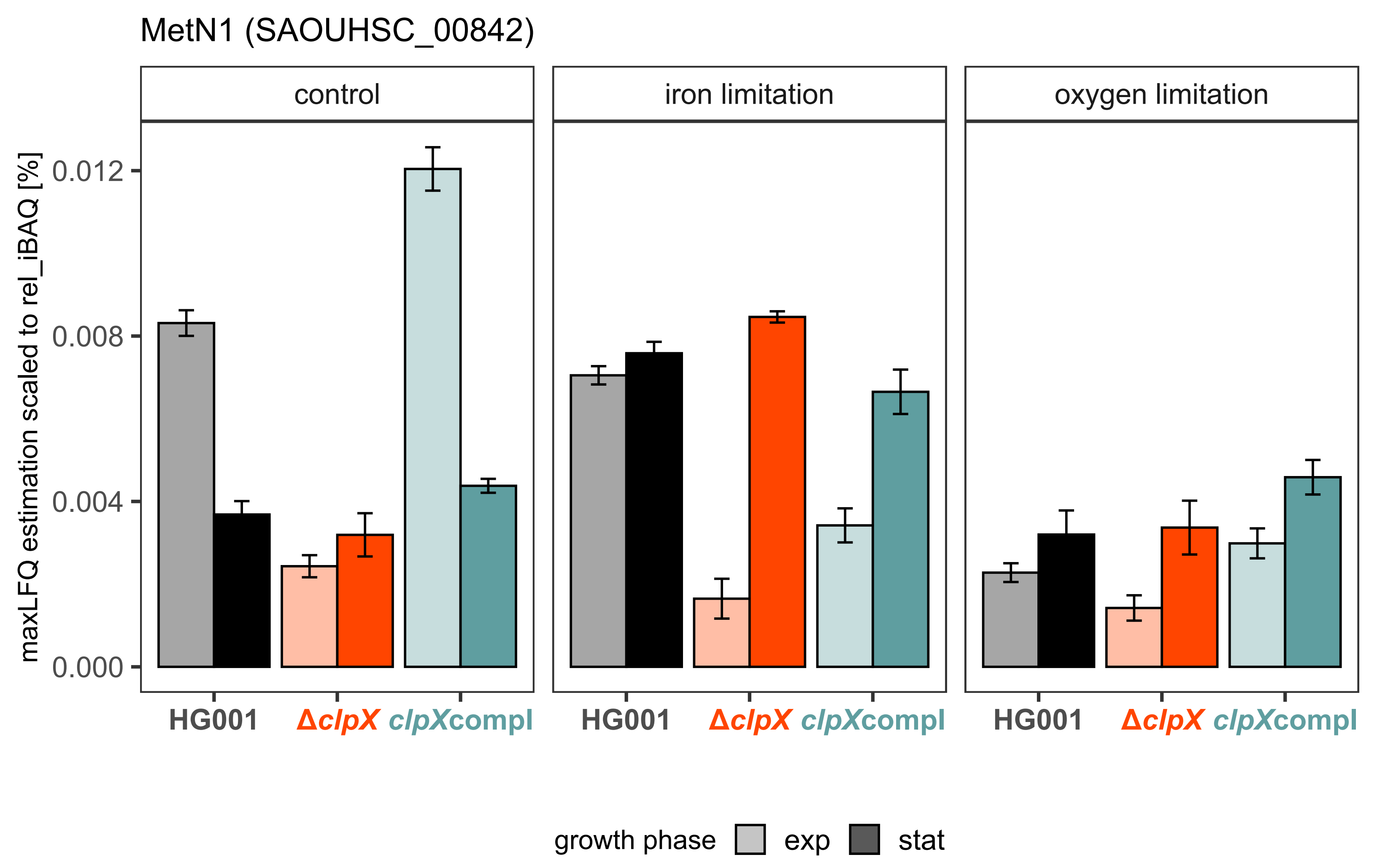
MetN1 (SAOUHSC_00842)
control
iron limitation
oxygen limitation
maxLFQ estimation scaled to rel_iBAQ [%]
0.012
0.008
0.004
0.000
HG001
ΔclpX
clpXcompl
growth phase
exp
stat

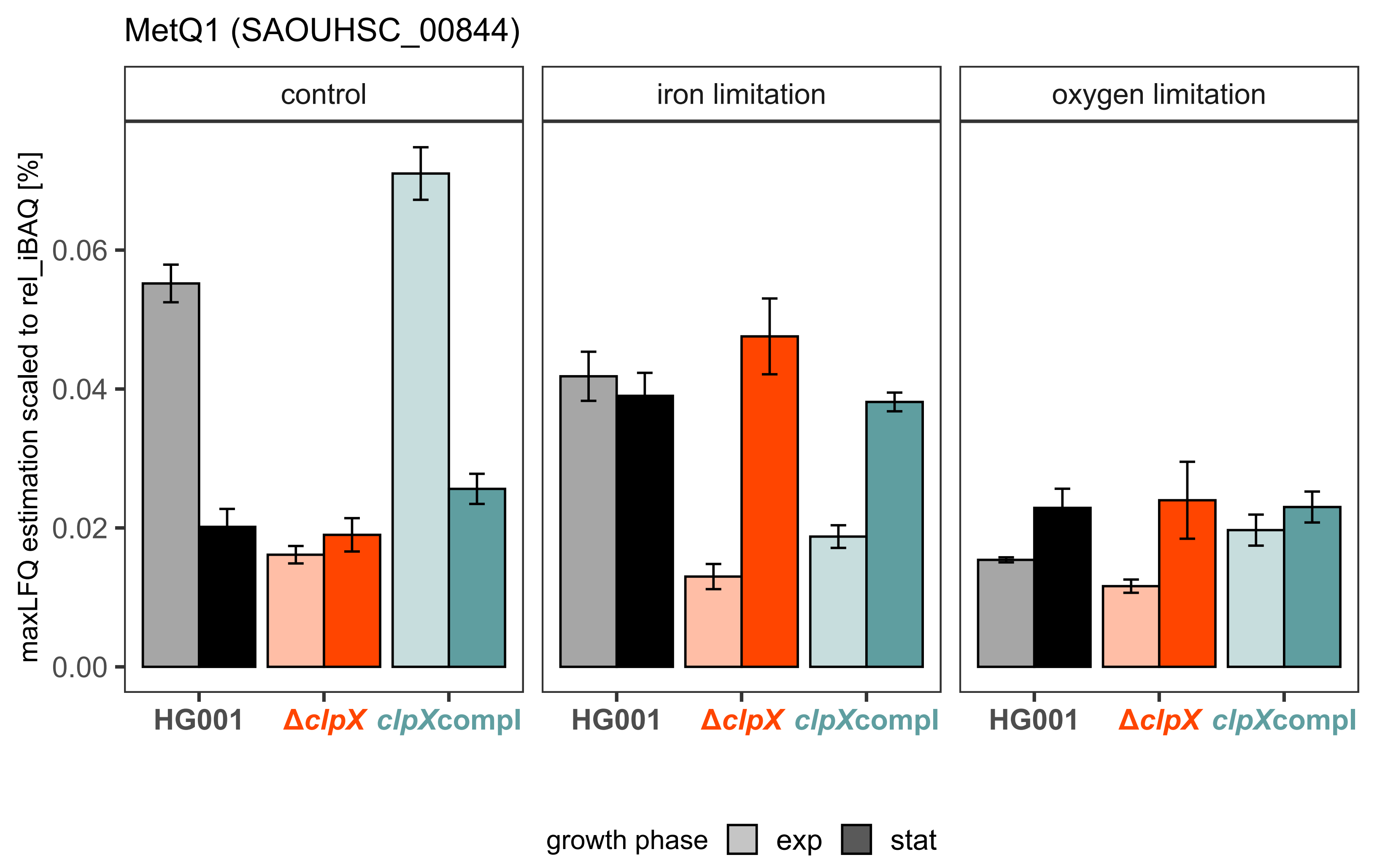
MetQ1 (SAOUHSC_00844)
control
iron limitation
oxygen limitation
maxLFQ estimation scaled to rel_iBAQ [%]
0.00
0.02
0.04
0.06
HG001
ΔclpX
clpXcompl
growth phase
exp
stat

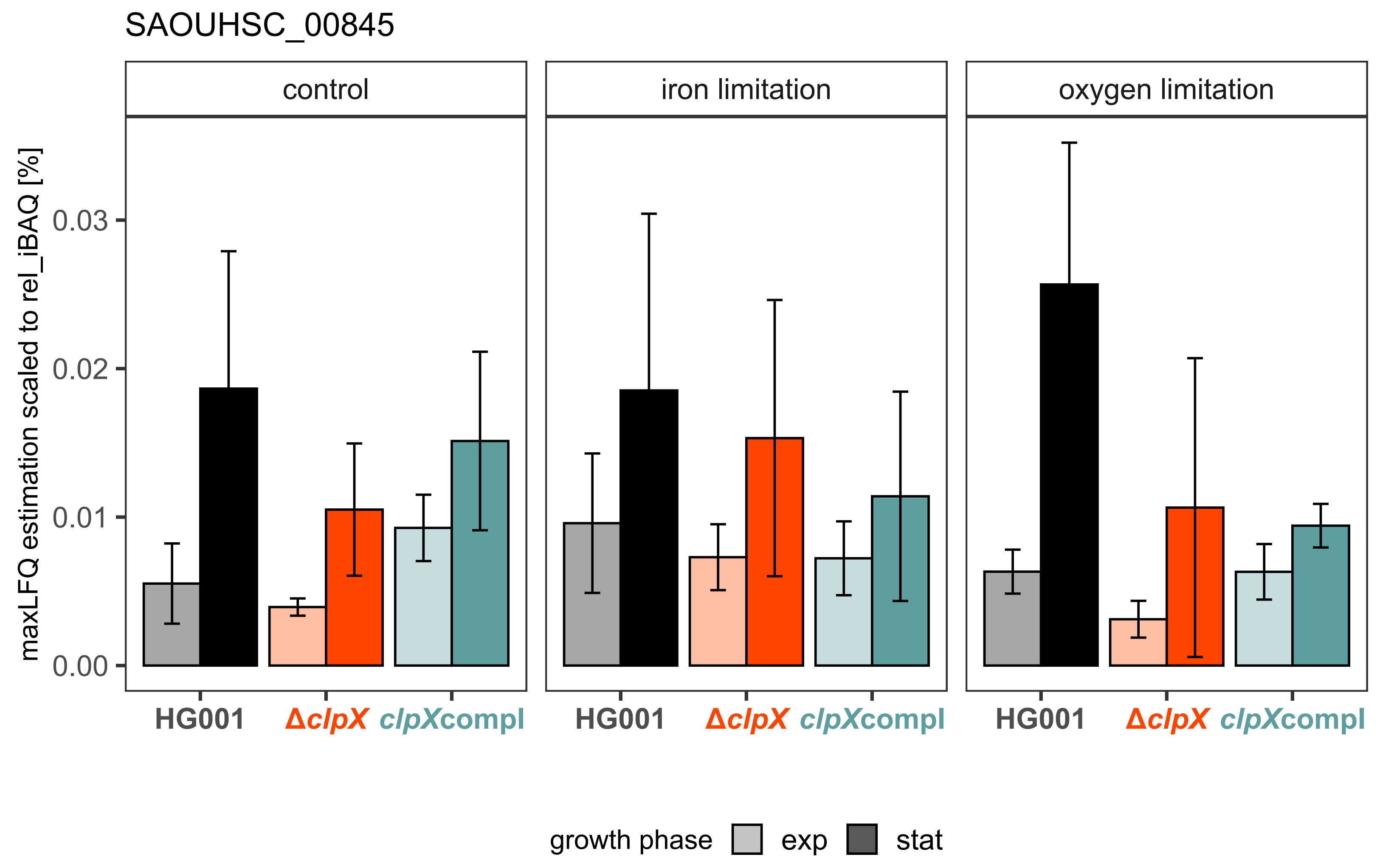

SAOUHSC_00845
control
iron limitation
oxygen limitation
maxLFQ estimation scaled to rel_iBAQ [%]
0.00
0.01
0.02
0.03
HG001
ΔclpX
clpXcompl
growth phase
exp
stat

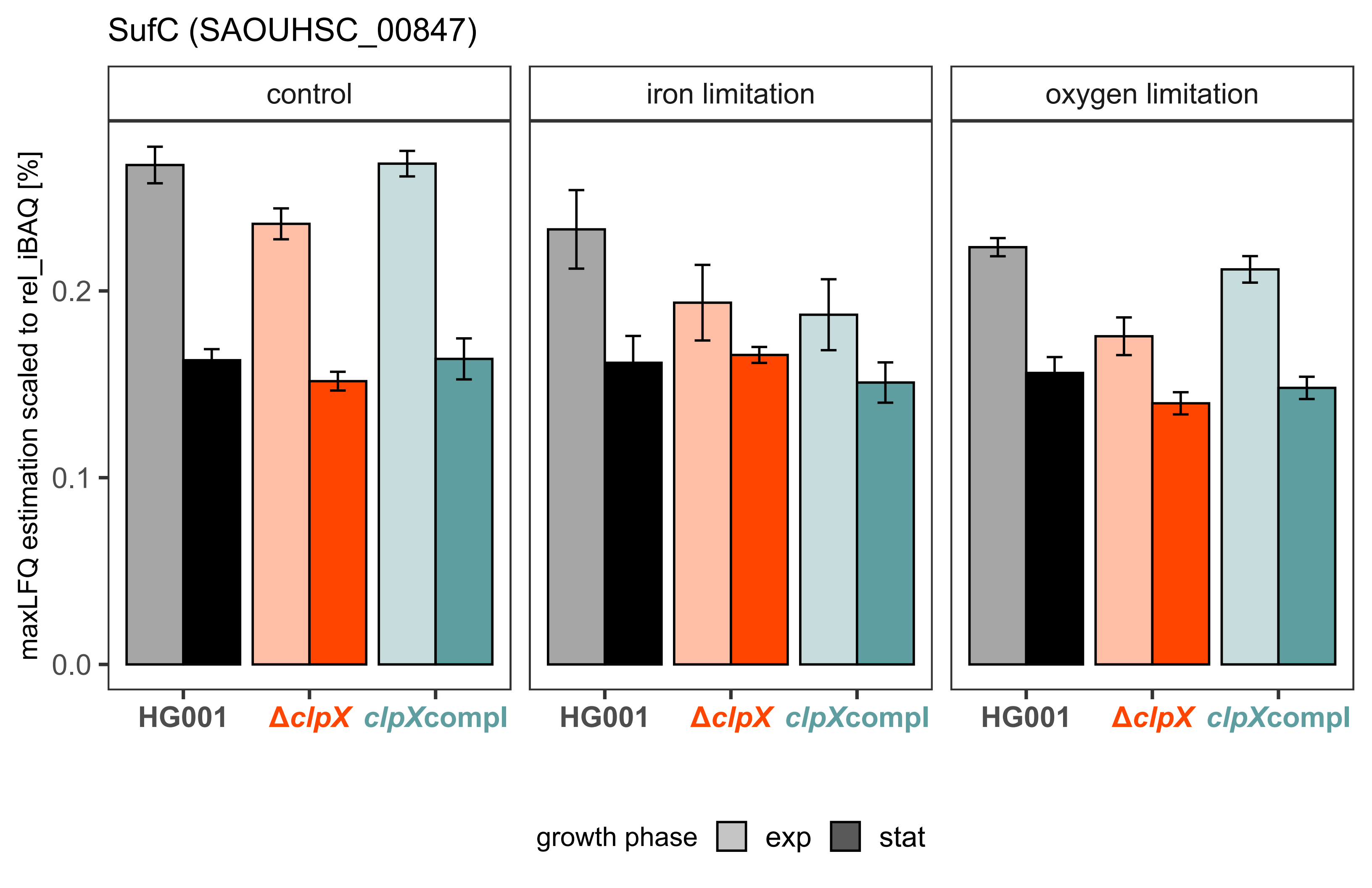
SufC (SAOUHSC_00847)
control
iron limitation
oxygen limitation
maxLFQ estimation scaled to rel_iBAQ [%]
0.0
0.1
0.2
HG001
ΔclpX
clpXcompl
growth phase
exp
stat

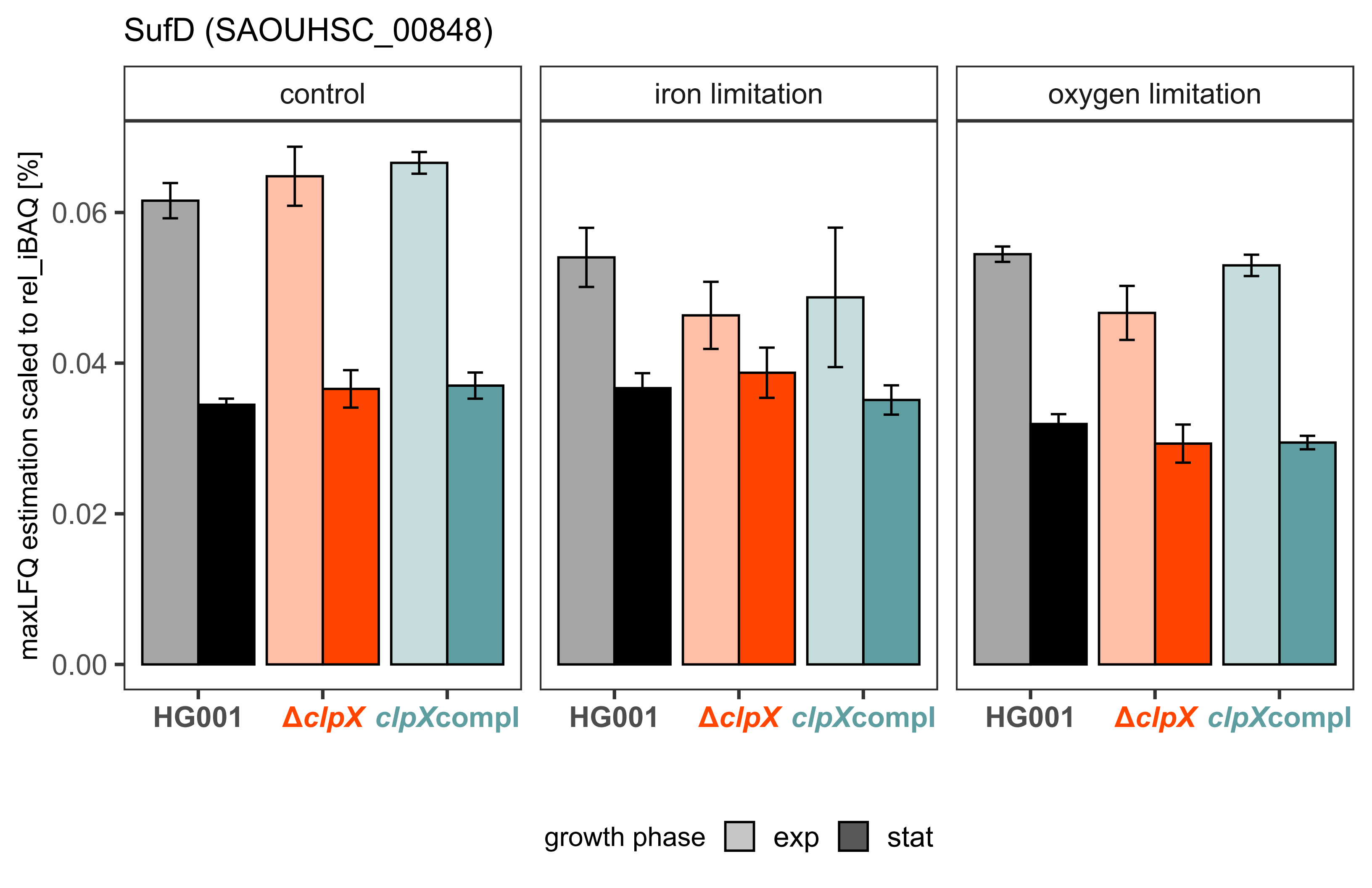

SufD (SAOUHSC_00848)
control
iron limitation
oxygen limitation
maxLFQ estimation scaled to rel_iBAQ [%]
0.06
0.04
0.02
0.00
HG001
ΔclpX
clpXcompl
HG001
ΔclpX
clpXcompl
HG001
ΔclpX
clpXcompl
growth phase
exp
stat

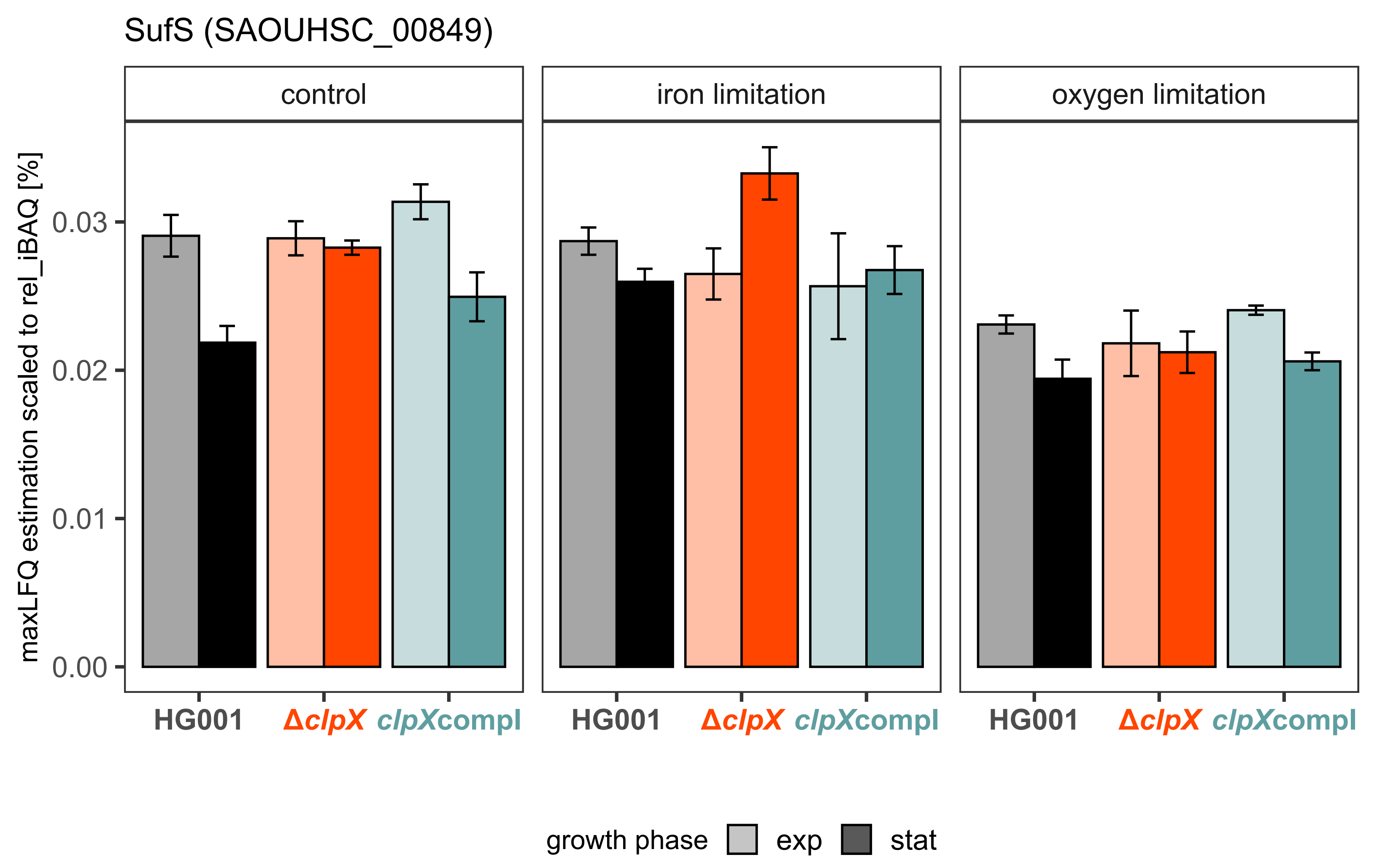
SufS (SAOUHSC_00849)
control
iron limitation
oxygen limitation
maxLFQ estimation scaled to rel_iBAQ [%]
0.03
0.02
0.01
0.00
HG001
ΔclpX
clpXcompl
growth phase
exp
stat

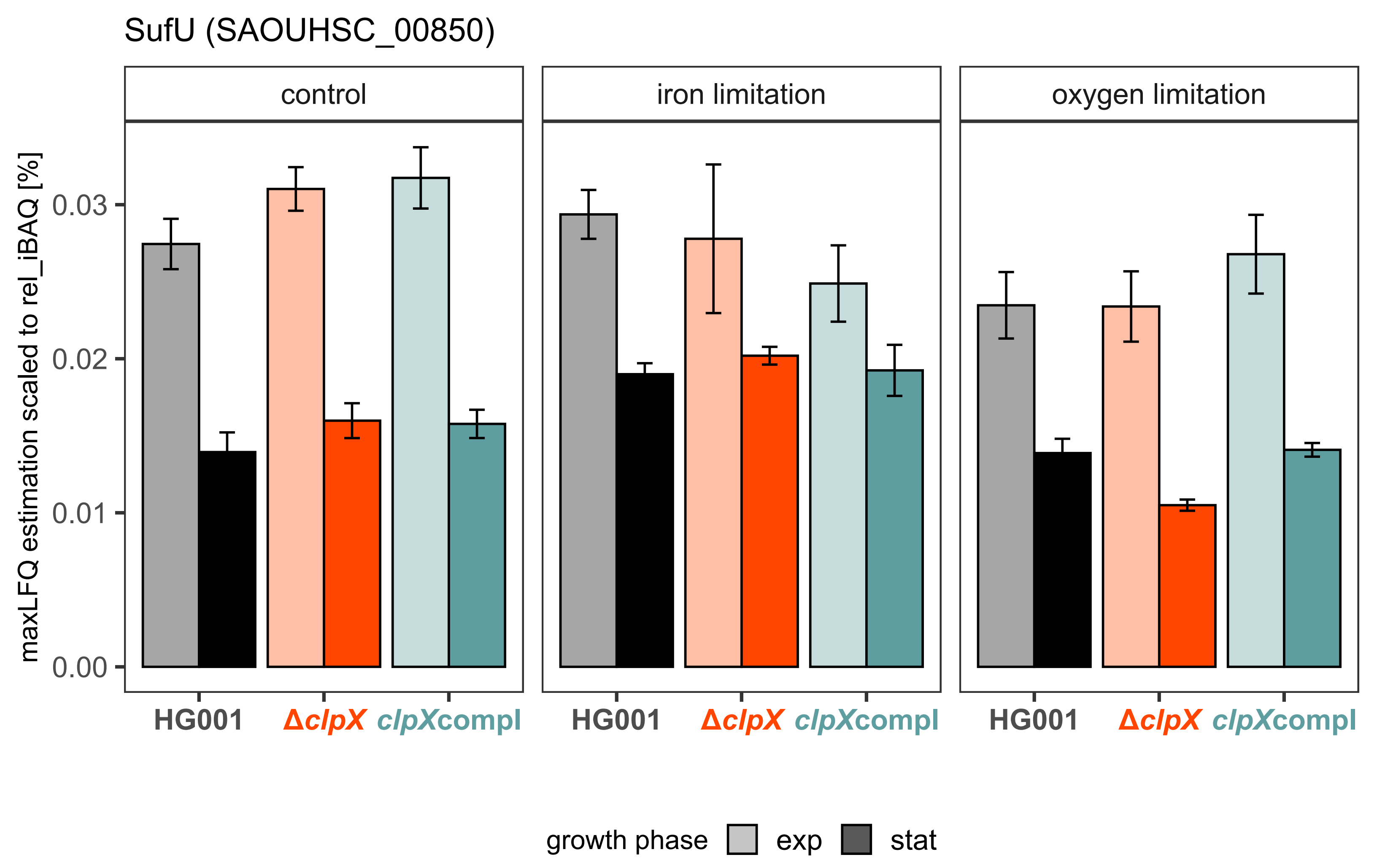
SufU (SAOUHSC_00850)
control
iron limitation
oxygen limitation
maxLFQ estimation scaled to rel_iBAQ [%]
0.03
0.02
0.01
0.00
HG001
ΔclpX
clpXcompl
growth phase
exp
stat

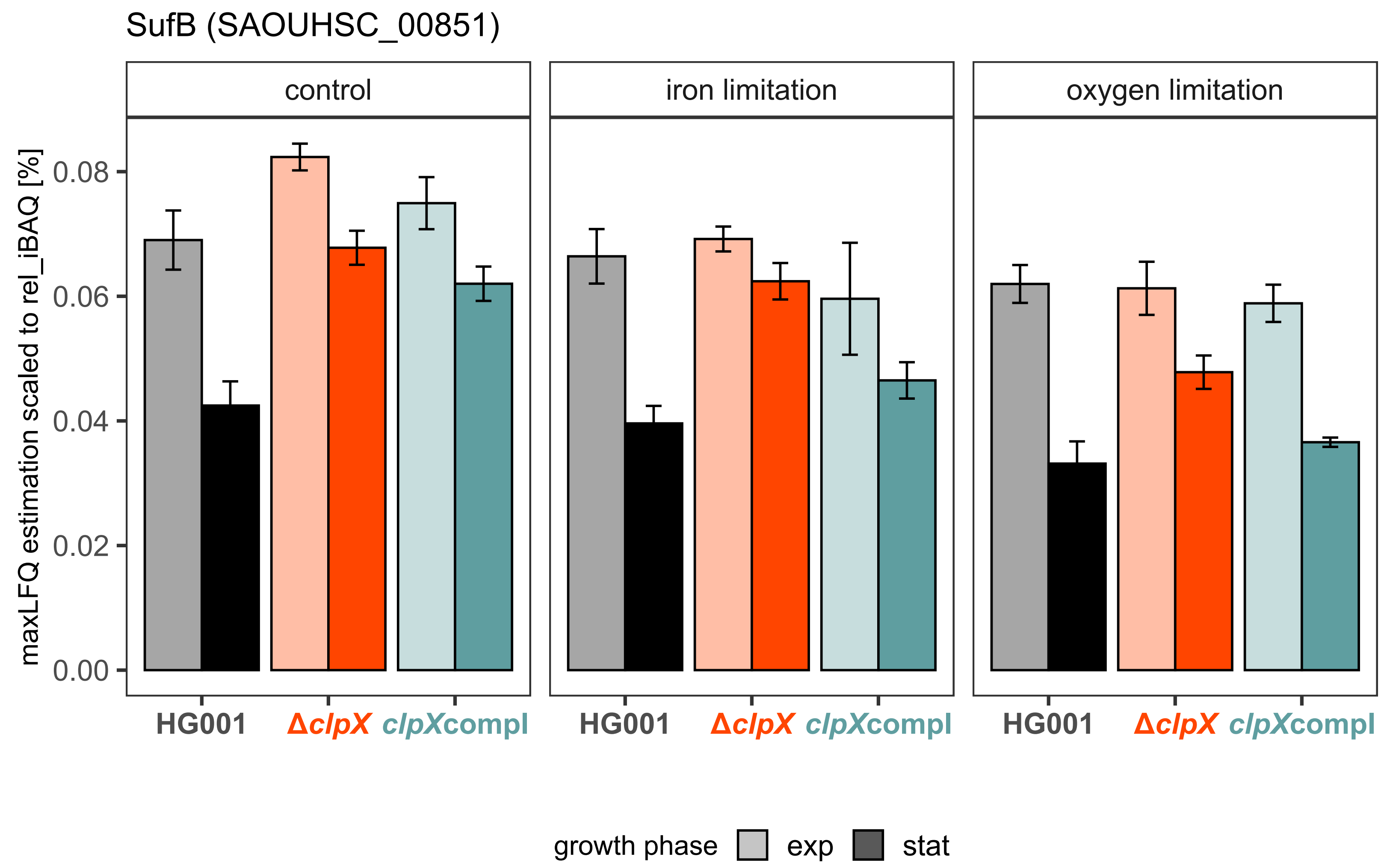
SufB (SAOUHSC_00851)
control
iron limitation
oxygen limitation
maxLFQ estimation scaled to rel_iBAQ [%]
0.00
0.02
0.04
0.06
0.08
HG001
ΔclpX
clpXcompl
growth phase
exp
stat

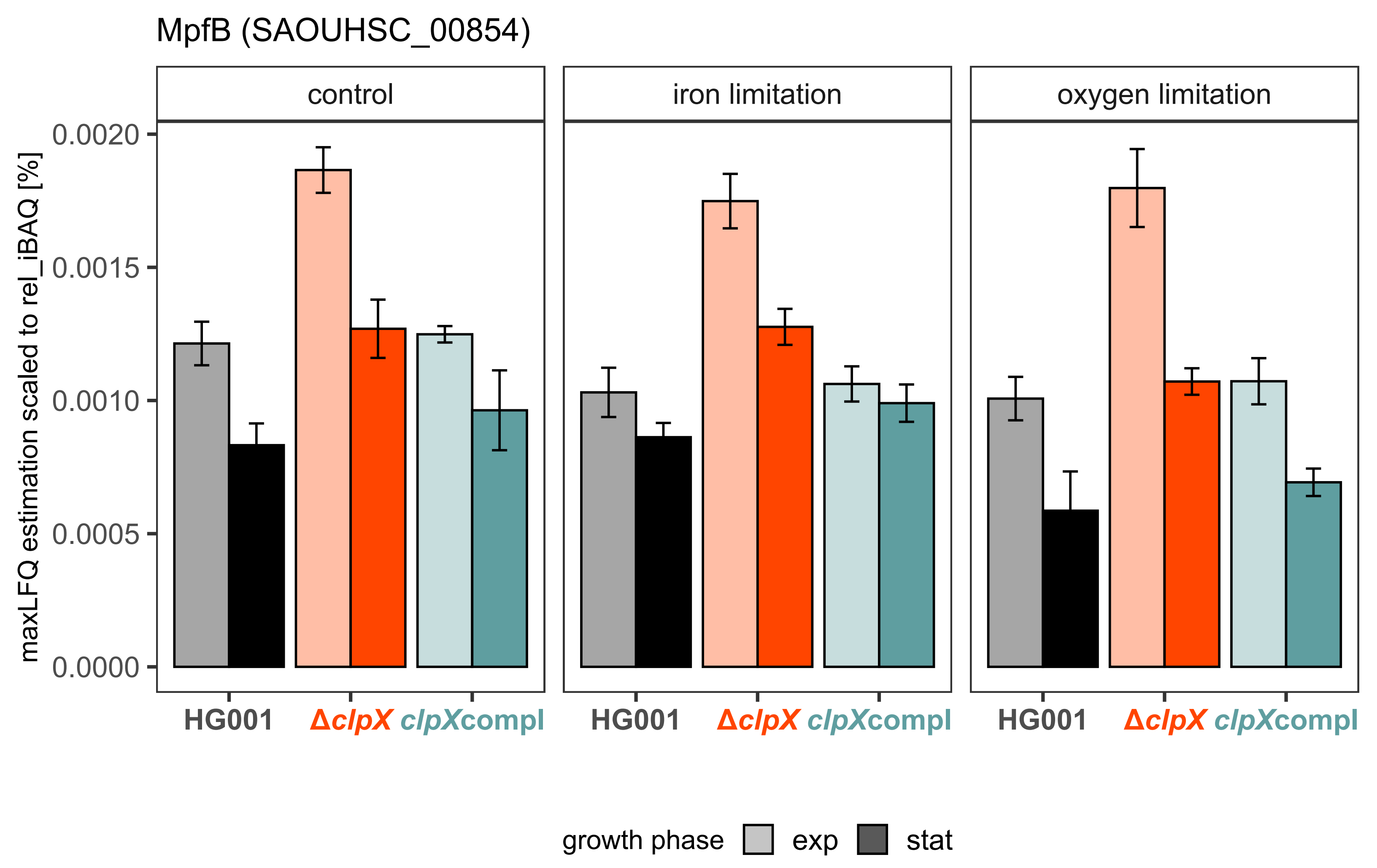

MpfB (SAOUHSC_00854)
control
iron limitation
oxygen limitation
maxLFQ estimation scaled to rel_iBAQ [%]
0.0020
0.0015
0.0010
0.0005
0.0000
HG001
ΔclpX
clpXcompl
growth phase
exp
stat

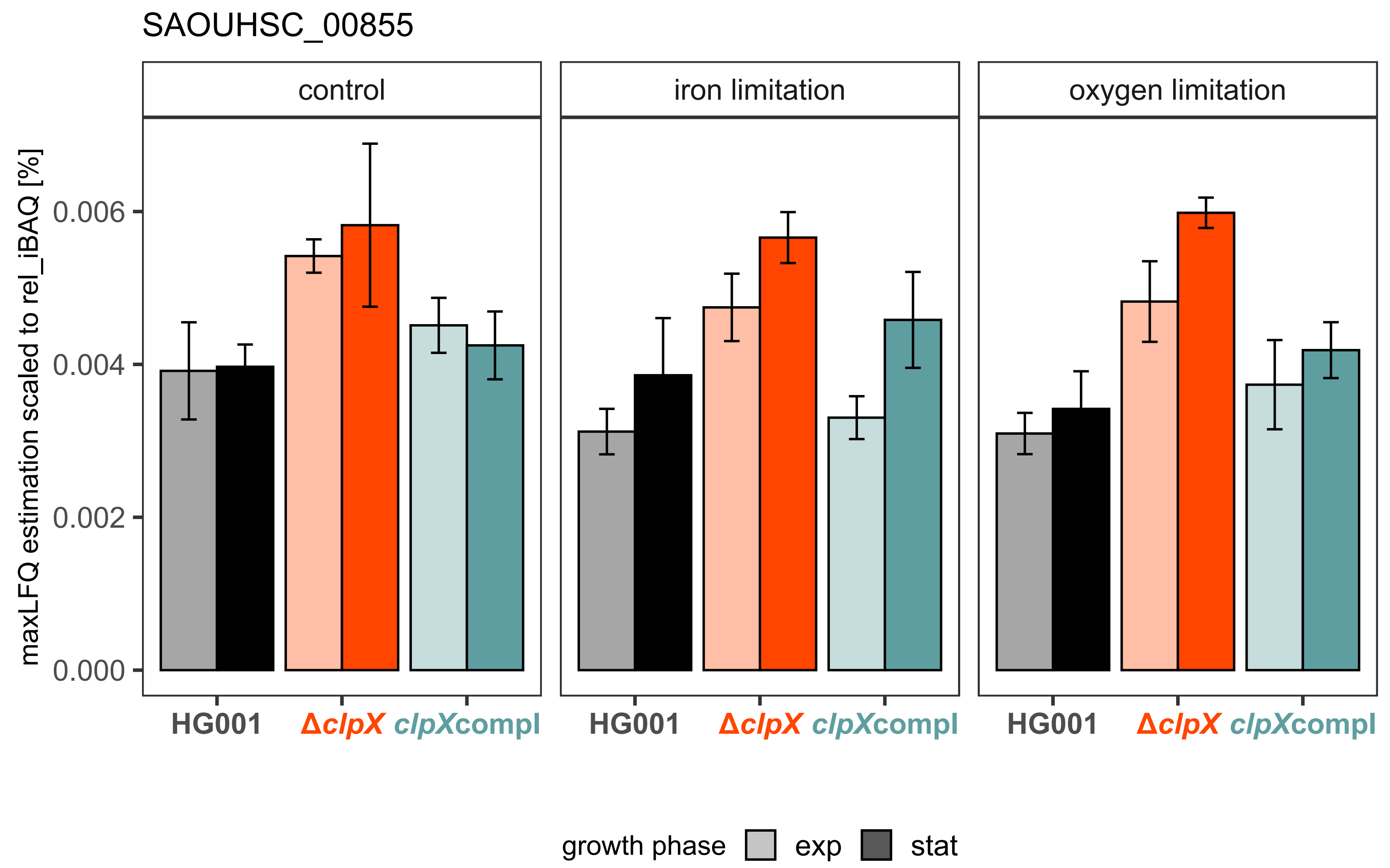
SAOUHSC_00855
control
iron limitation
oxygen limitation
maxLFQ estimation scaled to rel_iBAQ [%]
0.006
0.004
0.002
0.000
HG001
ΔclpX
clpXcompl
growth phase
exp
stat

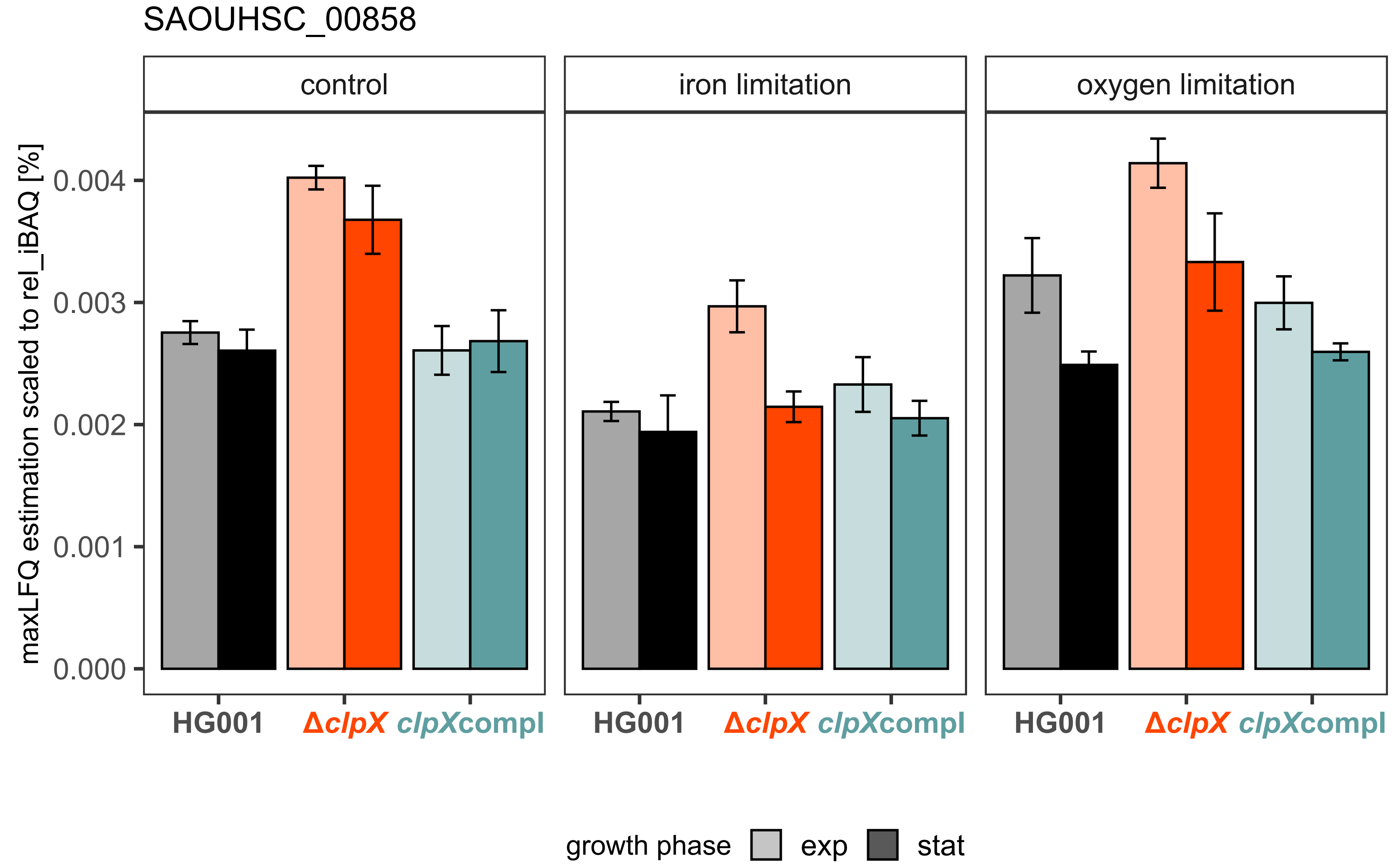

SAOUHSC_00858
control
iron limitation
oxygen limitation
maxLFQ estimation scaled to rel_iBAQ [%]
0.004
0.003
0.002
0.001
0.000
HG001
ΔclpX
clpXcompl
growth phase
exp
stat

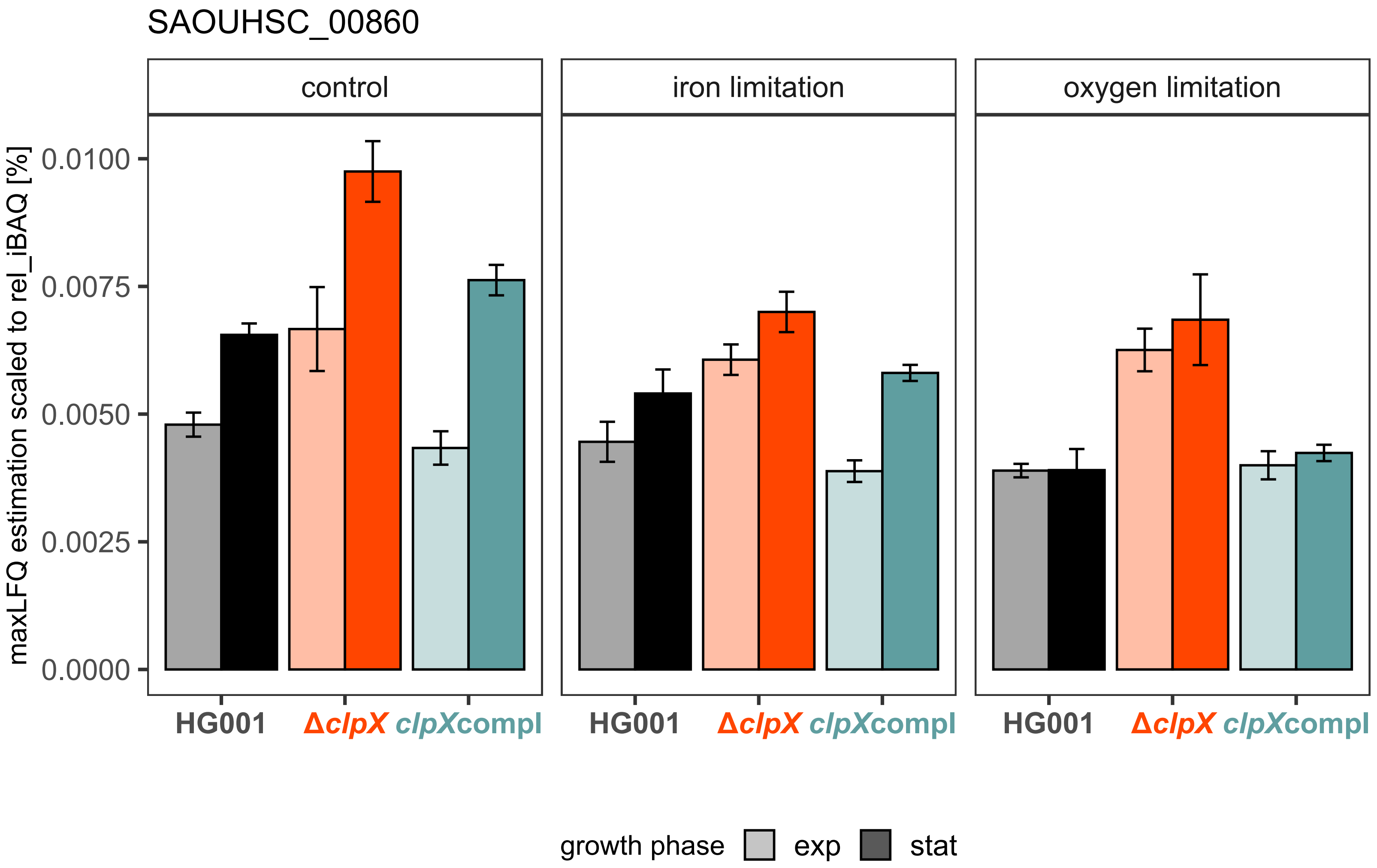
SAOUHSC_00860
control
iron limitation
oxygen limitation
maxLFQ estimation scaled to rel_iBAQ [%]
0.0100
0.0075
0.0050
0.0025
0.0000
HG001
ΔclpX
clpXcompl
growth phase
exp
stat

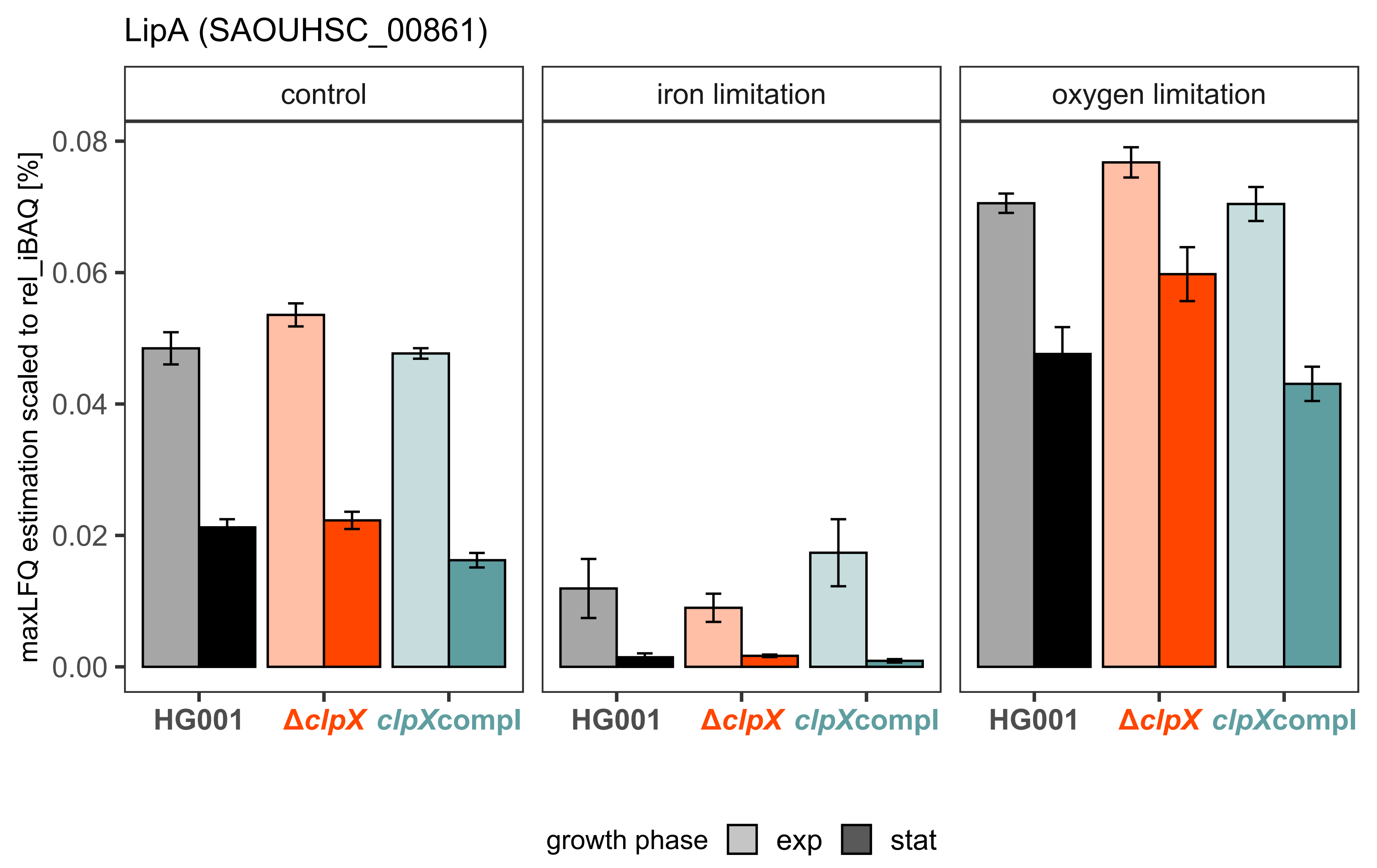
LipA (SAOUHSC_00861)
control
iron limitation
oxygen limitation
maxLFQ estimation scaled to rel_iBAQ [%]
0.08
0.06
0.04
0.02
0.00
HG001
ΔclpX
clpXcompl
growth phase
exp
stat

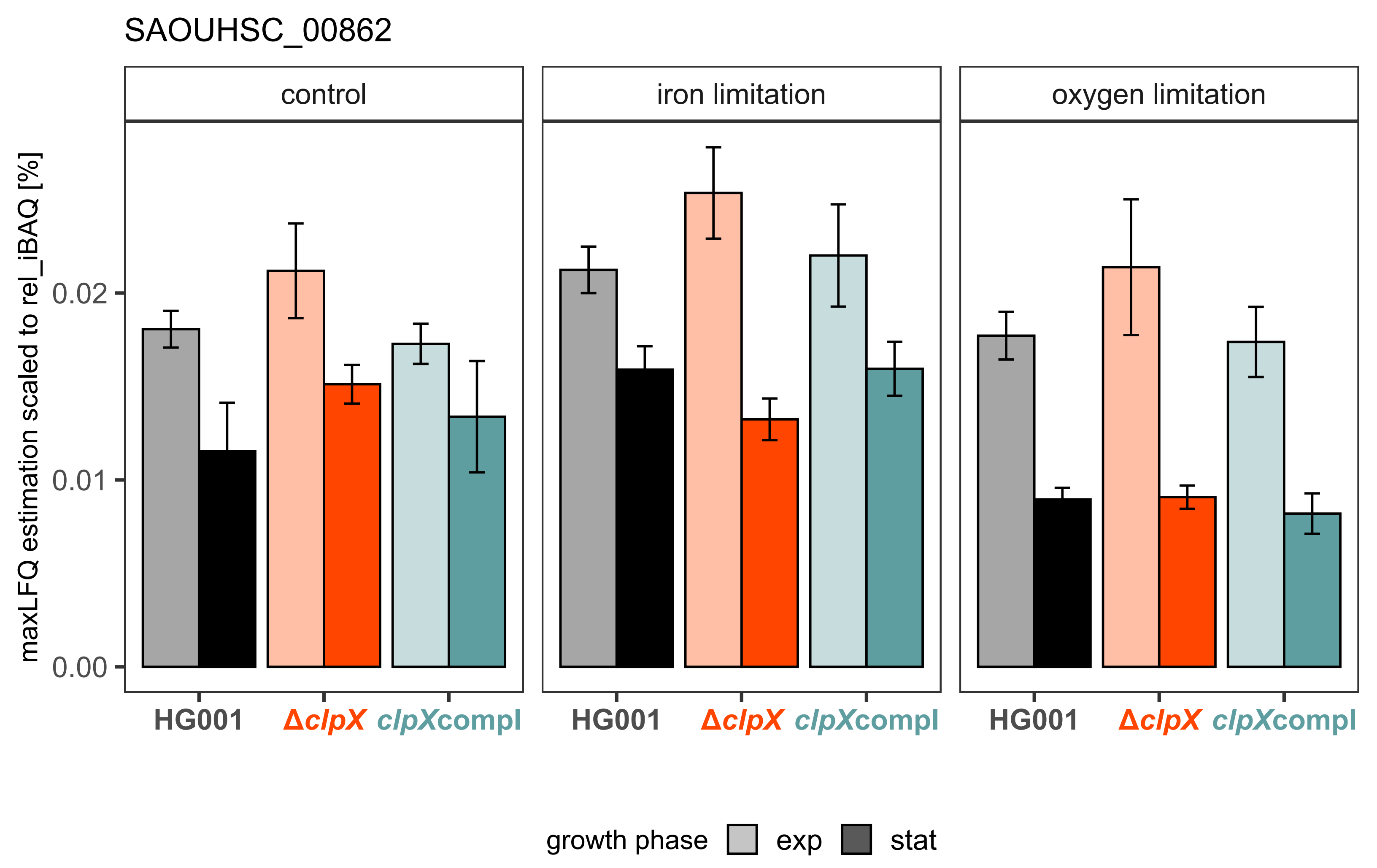
SAOUHSC_00862
control
iron limitation
oxygen limitation
maxLFQ estimation scaled to rel_iBAQ [%]
0.00
0.01
0.02
HG001
ΔclpX
clpXcompl
growth phase
exp
stat

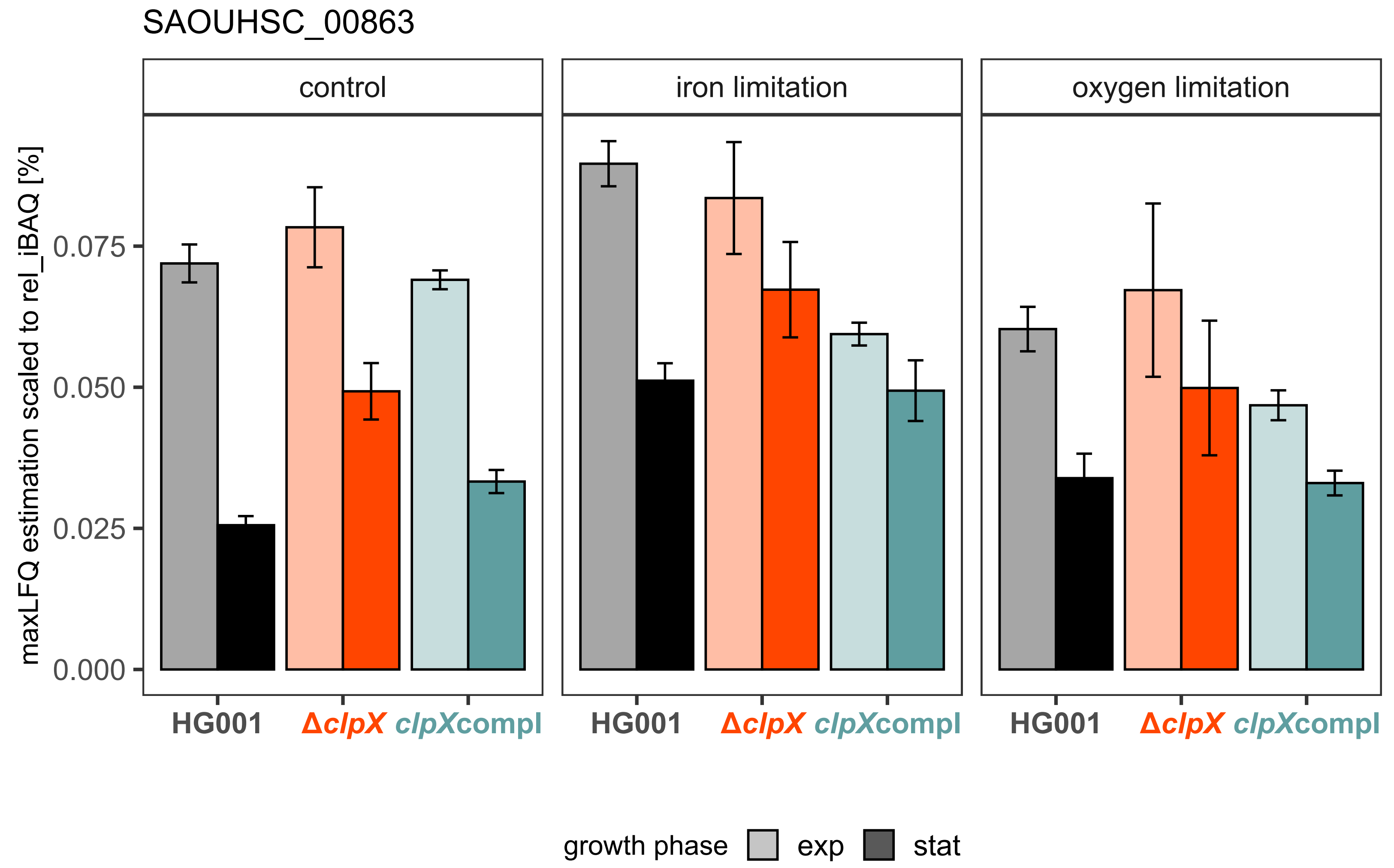

SAOUHSC_00863
control
iron limitation
oxygen limitation
maxLFQ estimation scaled to rel_iBAQ [%]
0.000
0.025
0.050
0.075
HG001
ΔclpX
clpXcompl
growth phase
exp
stat

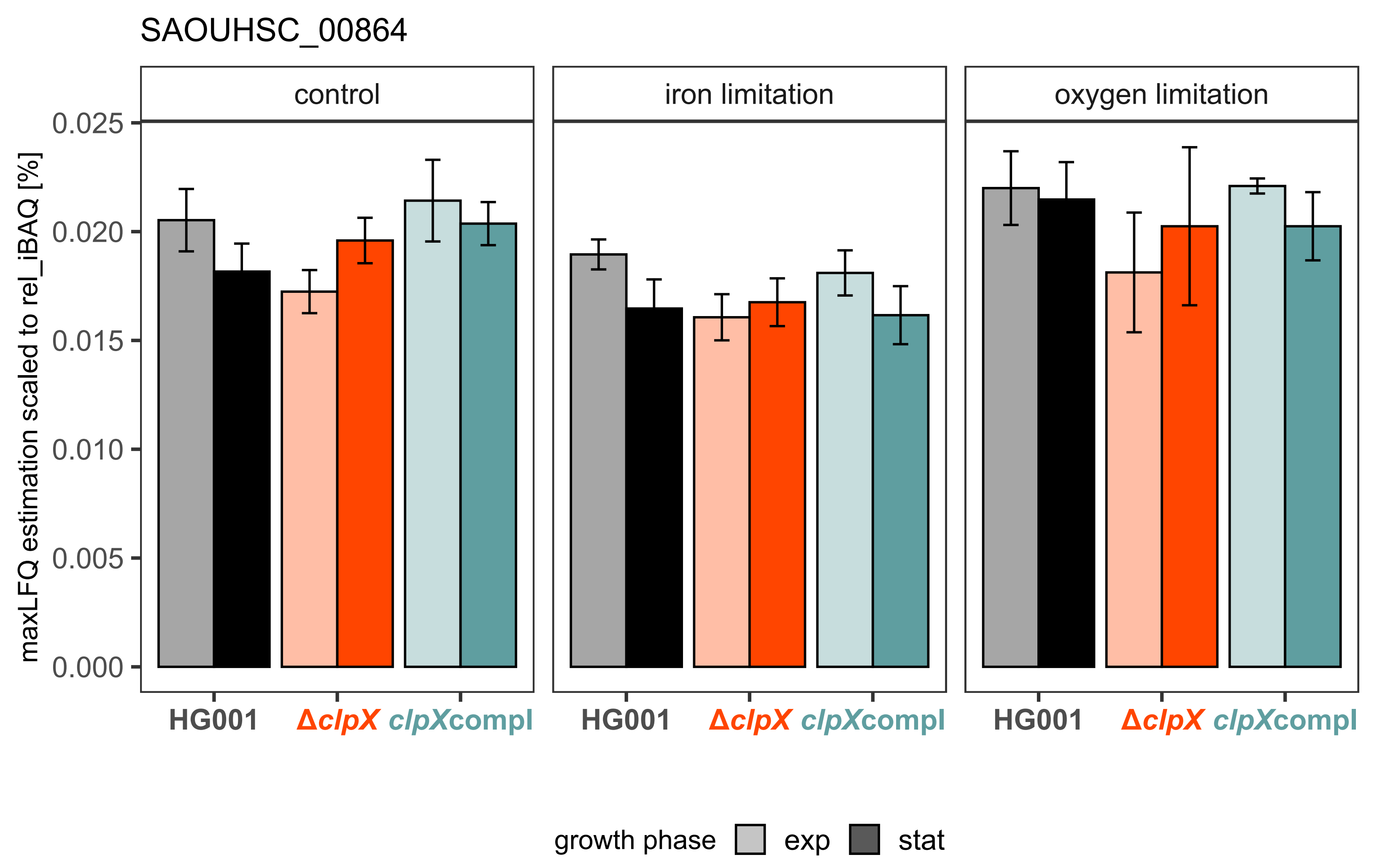

SAOUHSC_00864
control
iron limitation
oxygen limitation
maxLFQ estimation scaled to rel_iBAQ [%]
0.025
0.020
0.015
0.010
0.005
0.000
HG001
ΔclpX
clpXcompl
growth phase
exp
stat

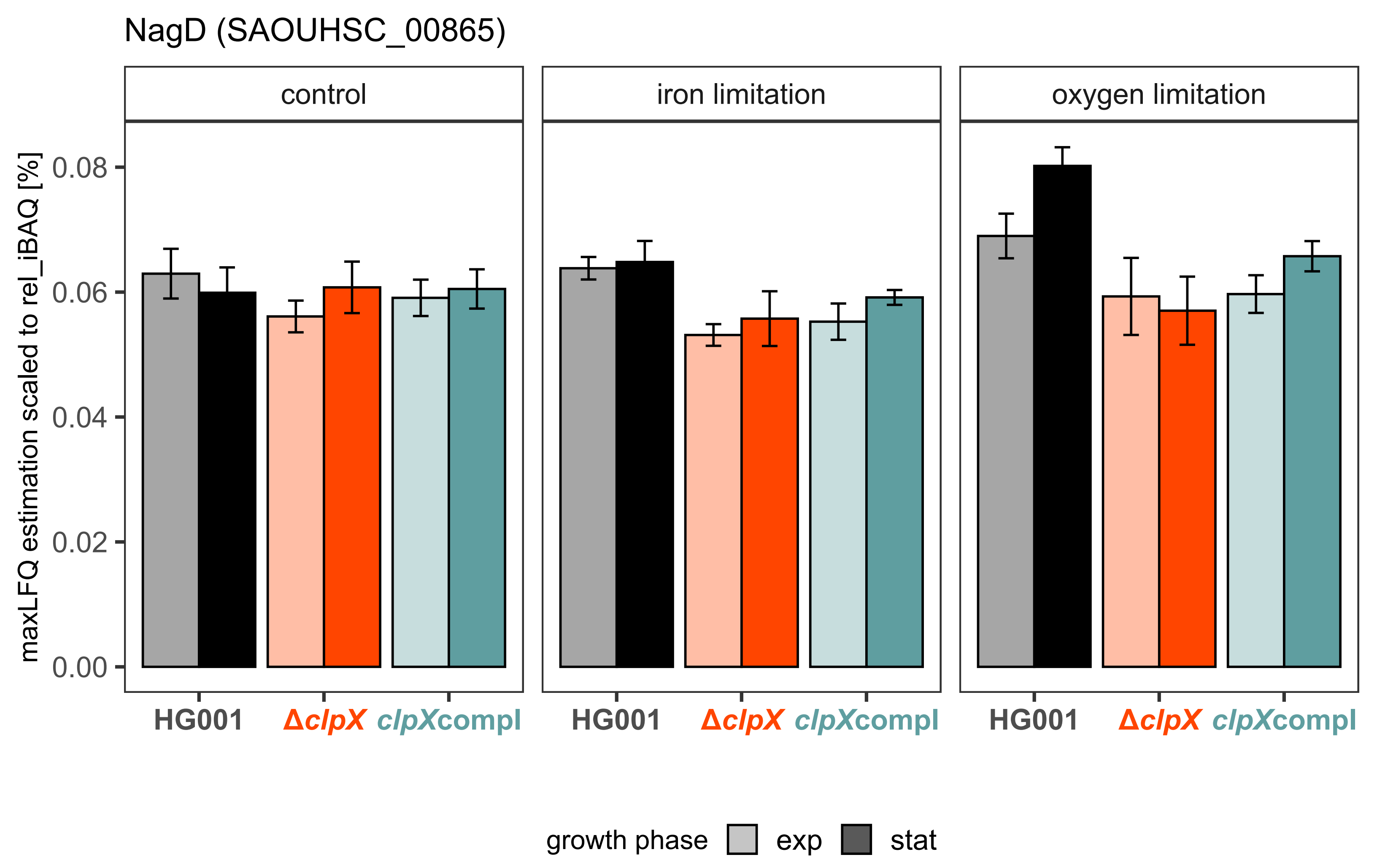
NagD (SAOUHSC_00865)
control
iron limitation
oxygen limitation
maxLFQ estimation scaled to rel_iBAQ [%]
0.00
0.02
0.04
0.06
0.08
HG001
ΔclpX
clpXcompl
growth phase
exp
stat

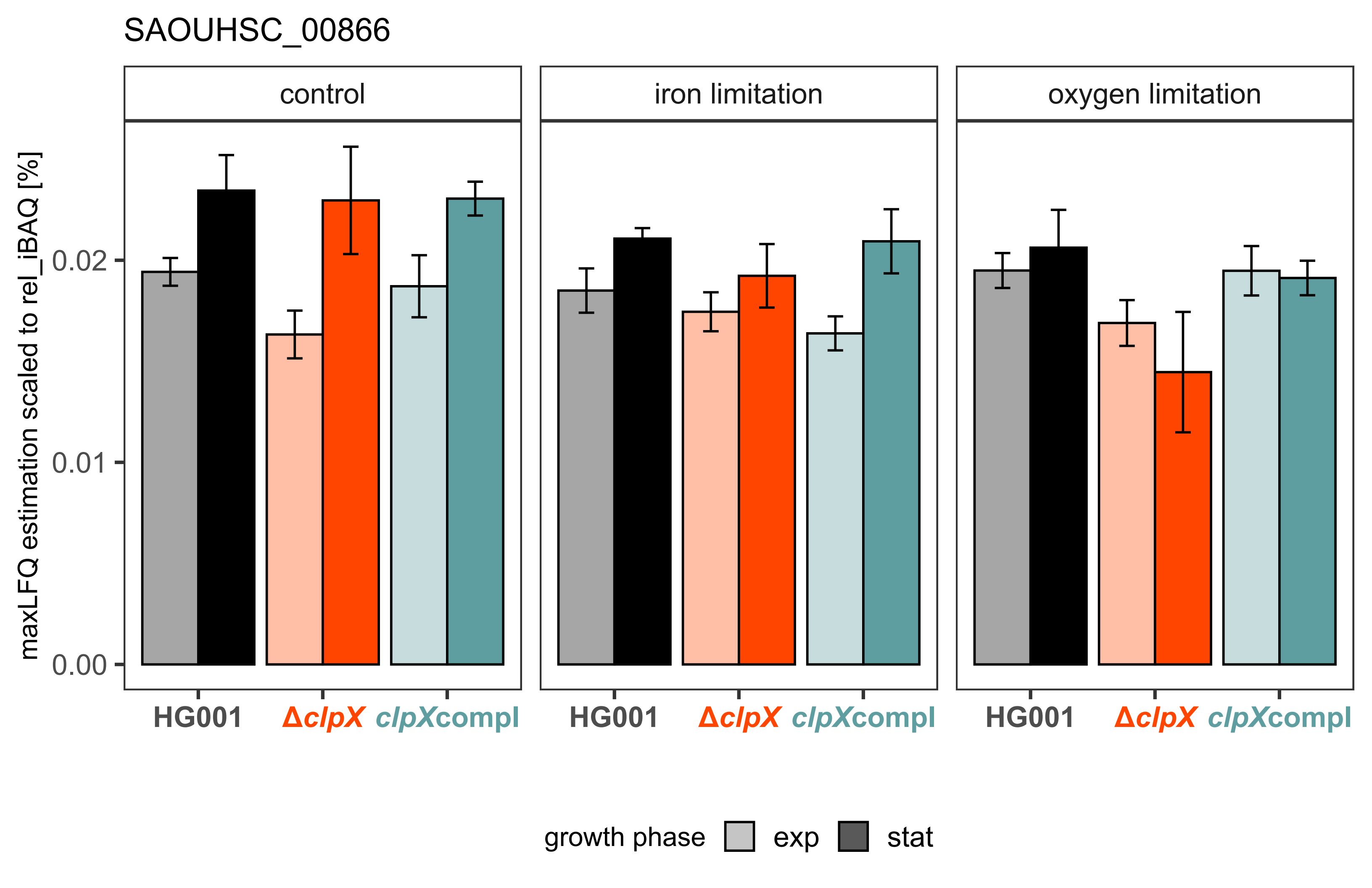

SAOUHSC_00866
control
iron limitation
oxygen limitation
maxLFQ estimation scaled to rel_iBAQ [%]
0.00
0.01
0.02
HG001
ΔclpX
clpXcompl
growth phase
exp
stat

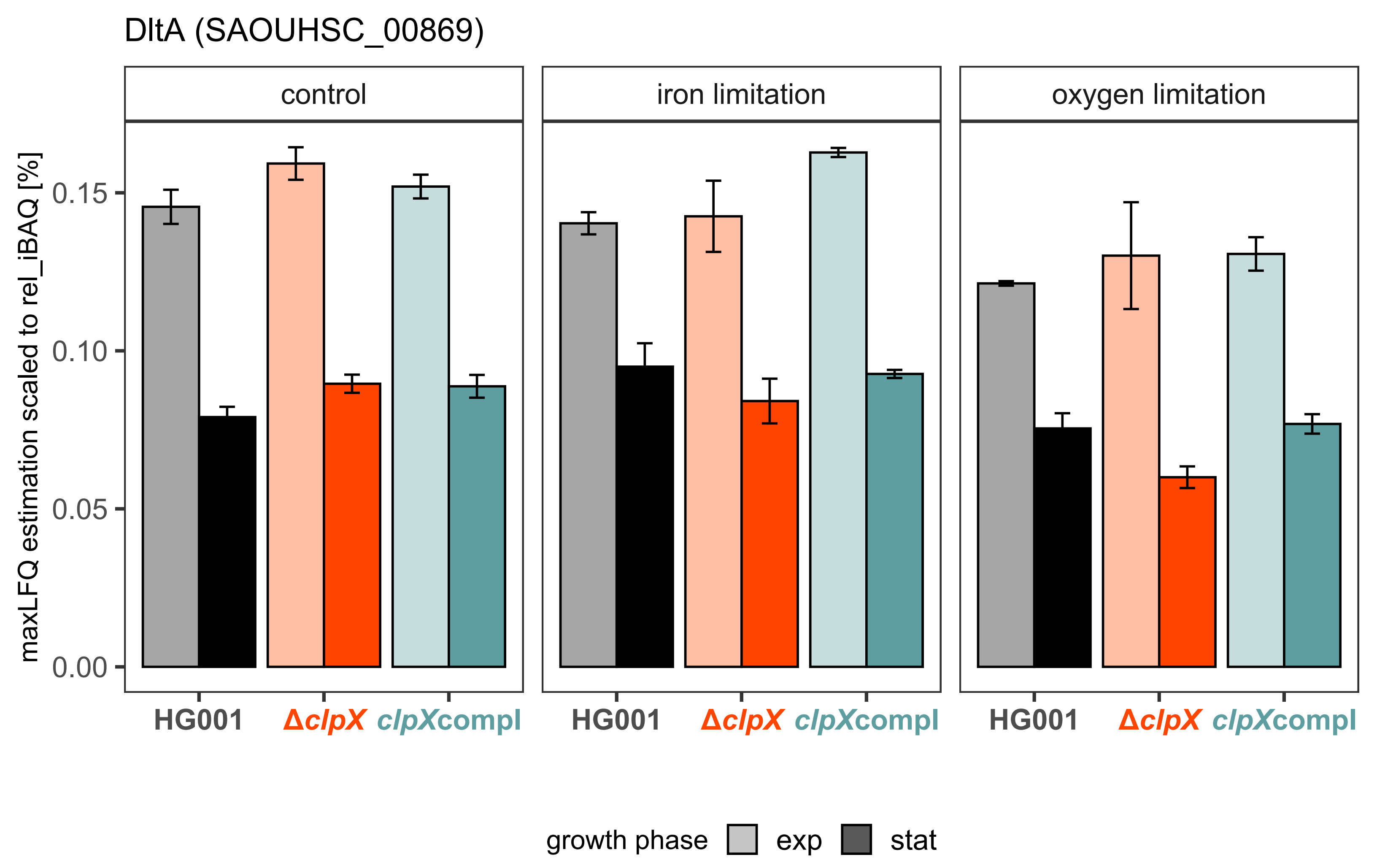

DltA (SAOUHSC_00869)
control
iron limitation
oxygen limitation
maxLFQ estimation scaled to rel_iBAQ [%]
0.15
0.10
0.05
0.00
HG001
ΔclpX
clpXcompl
growth phase
exp
stat

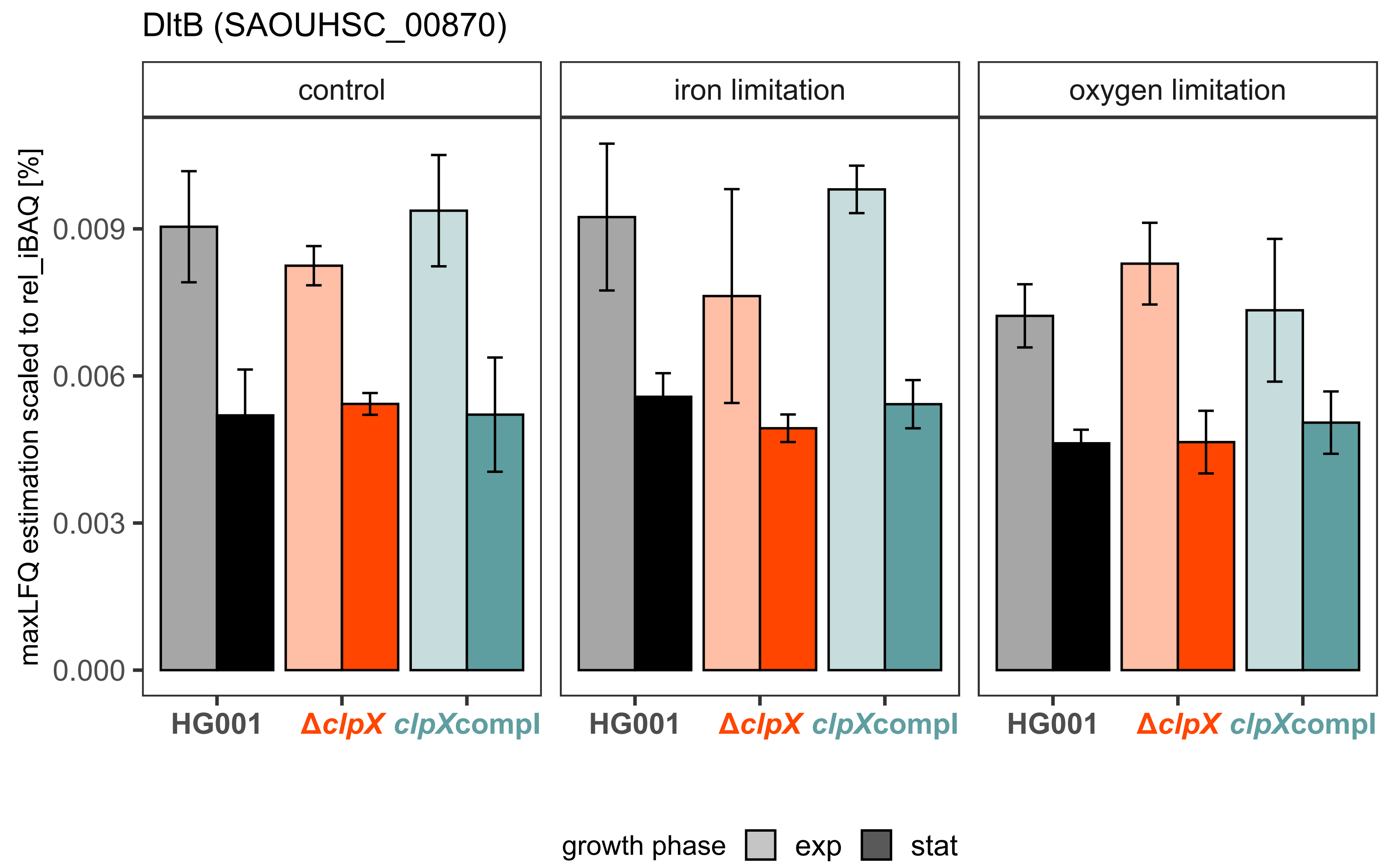

DltB (SAOUHSC_00870)
control
iron limitation
oxygen limitation
maxLFQ estimation scaled to rel_iBAQ [%]
0.009
0.006
0.003
0.000
HG001
ΔclpX
clpXcompl
growth phase
exp
stat

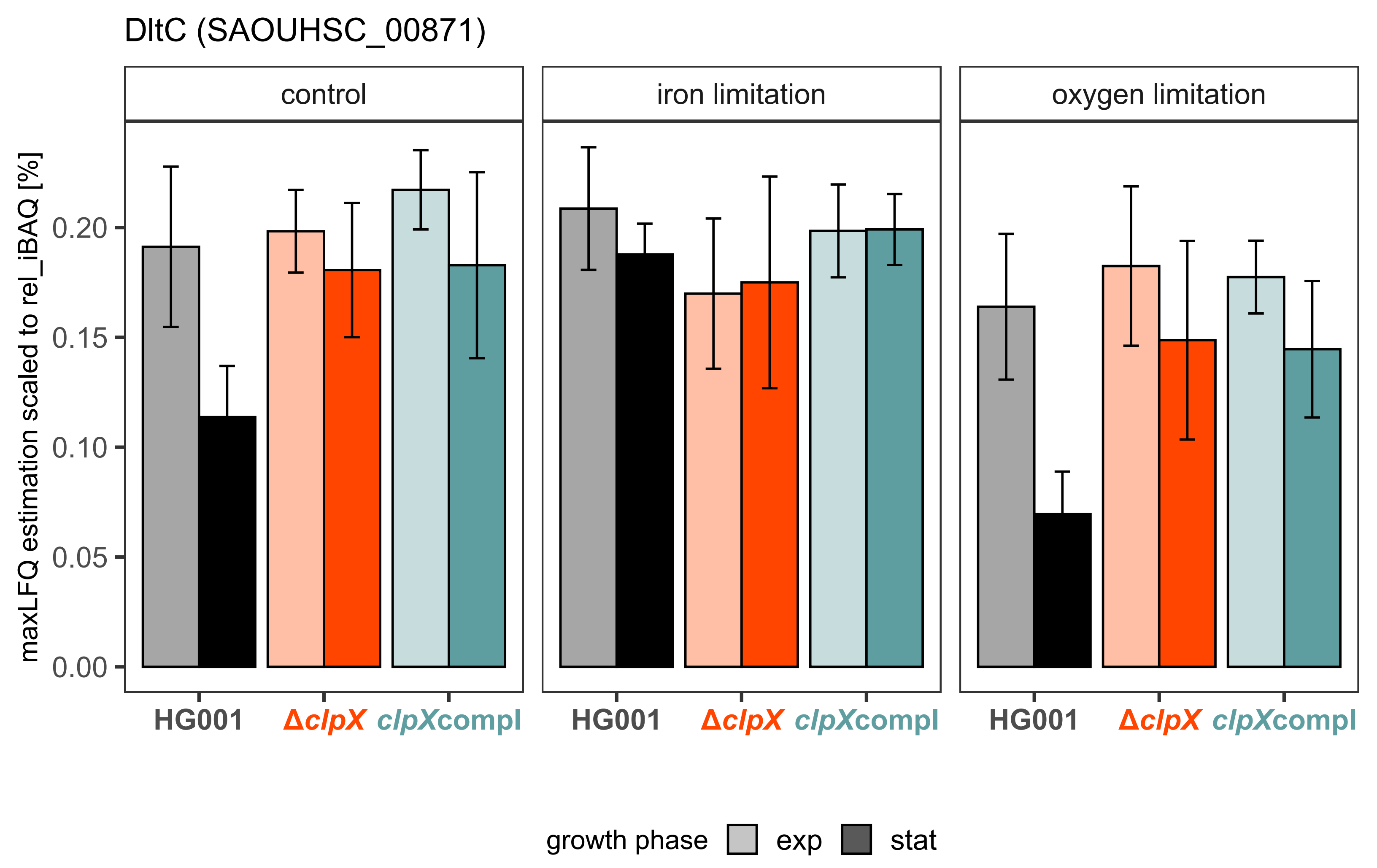
DltC (SAOUHSC_00871)
control
iron limitation
oxygen limitation
maxLFQ estimation scaled to rel_iBAQ [%]
0.20
0.15
0.10
0.05
0.00
HG001
ΔclpX
clpXcompl
growth phase
exp
stat

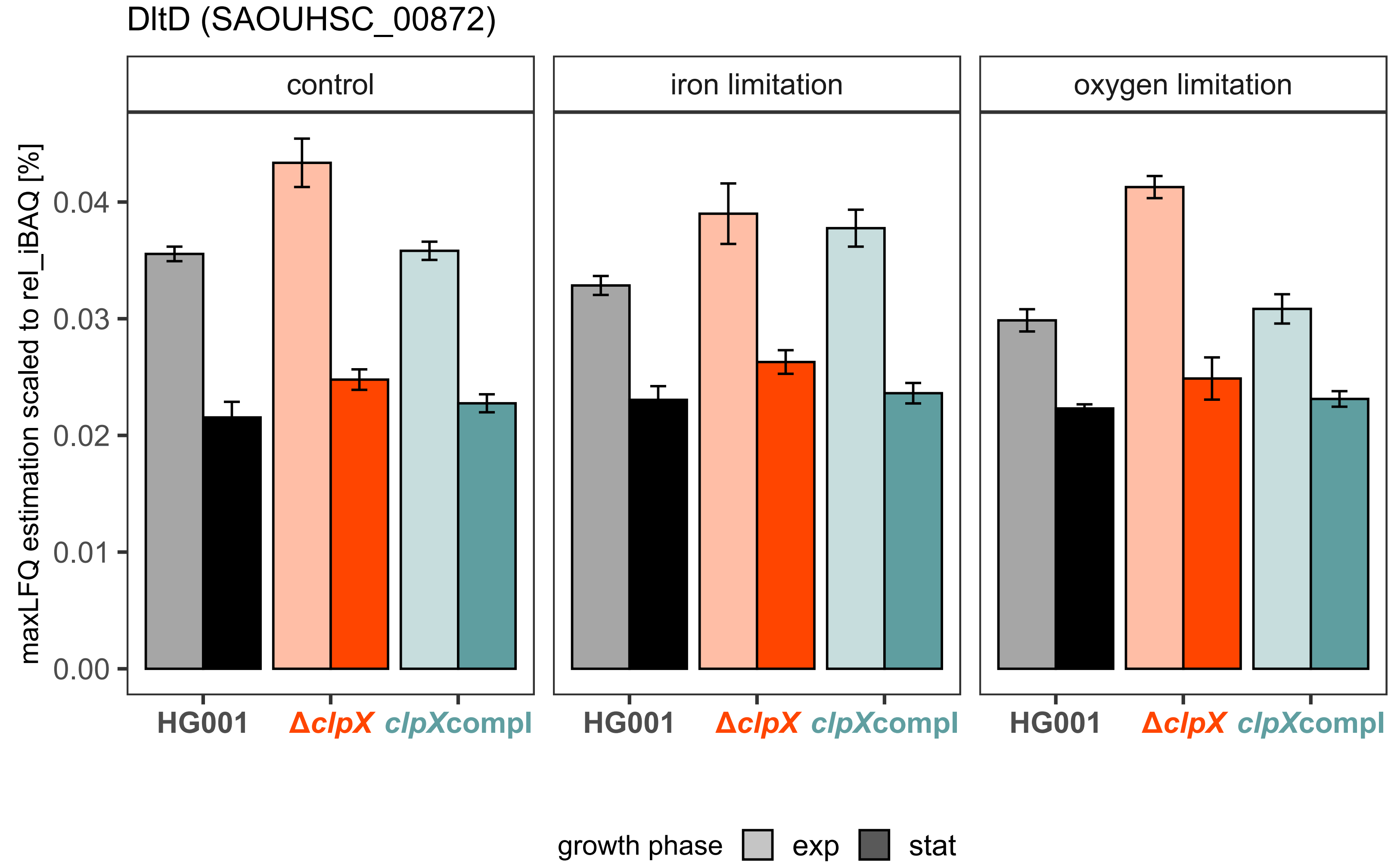

DltD (SAOUHSC_00872)
control
iron limitation
oxygen limitation
maxLFQ estimation scaled to rel_iBAQ [%]
0.00
0.01
0.02
0.03
0.04
HG001
ΔclpX
clpXcompl
growth phase
exp
stat

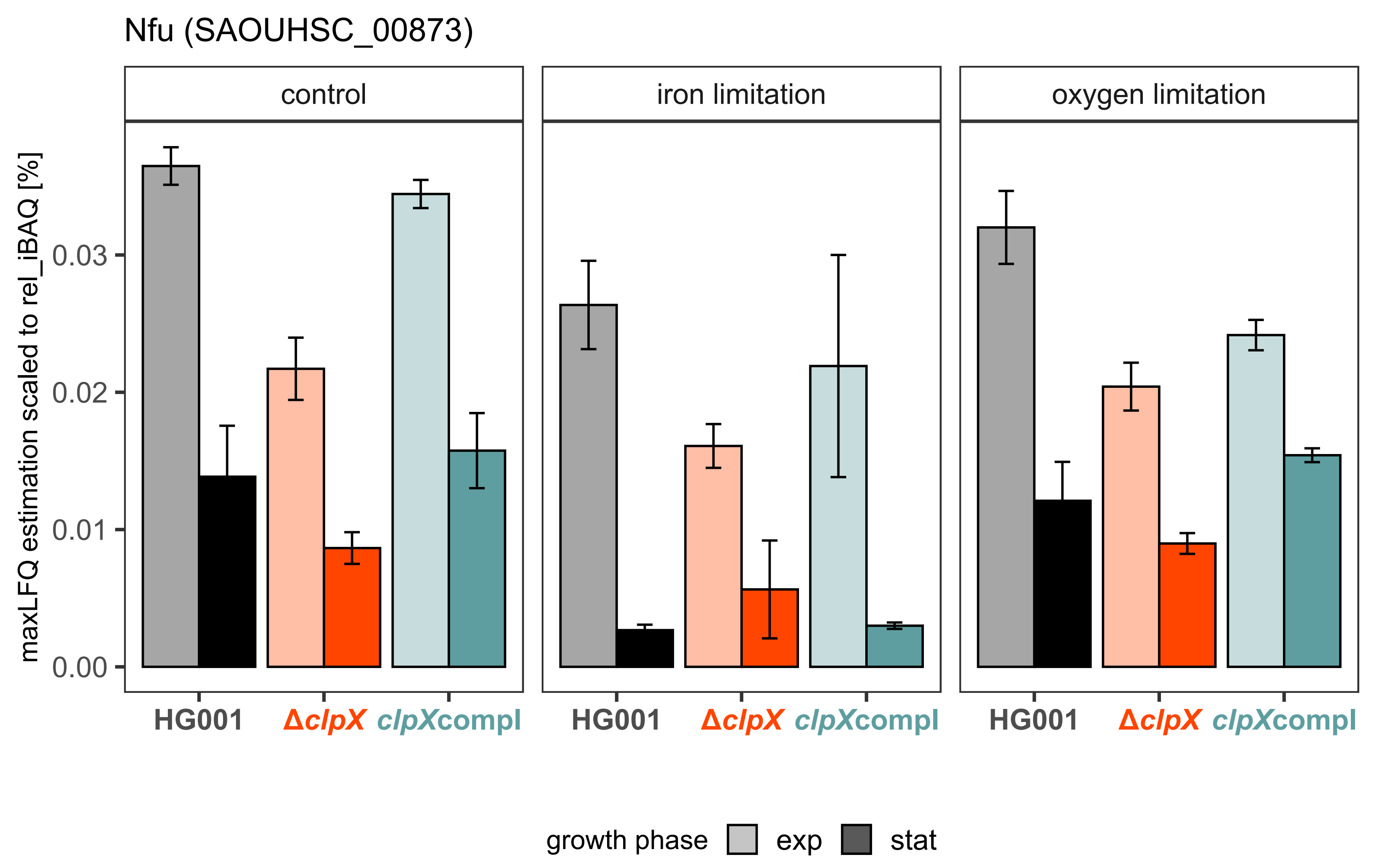

Nfu (SAOUHSC_00873)
control
iron limitation
oxygen limitation
maxLFQ estimation scaled to rel_iBAQ [%]
0.00
0.01
0.02
0.03
HG001
ΔclpX
clpXcompl
growth phase
exp
stat

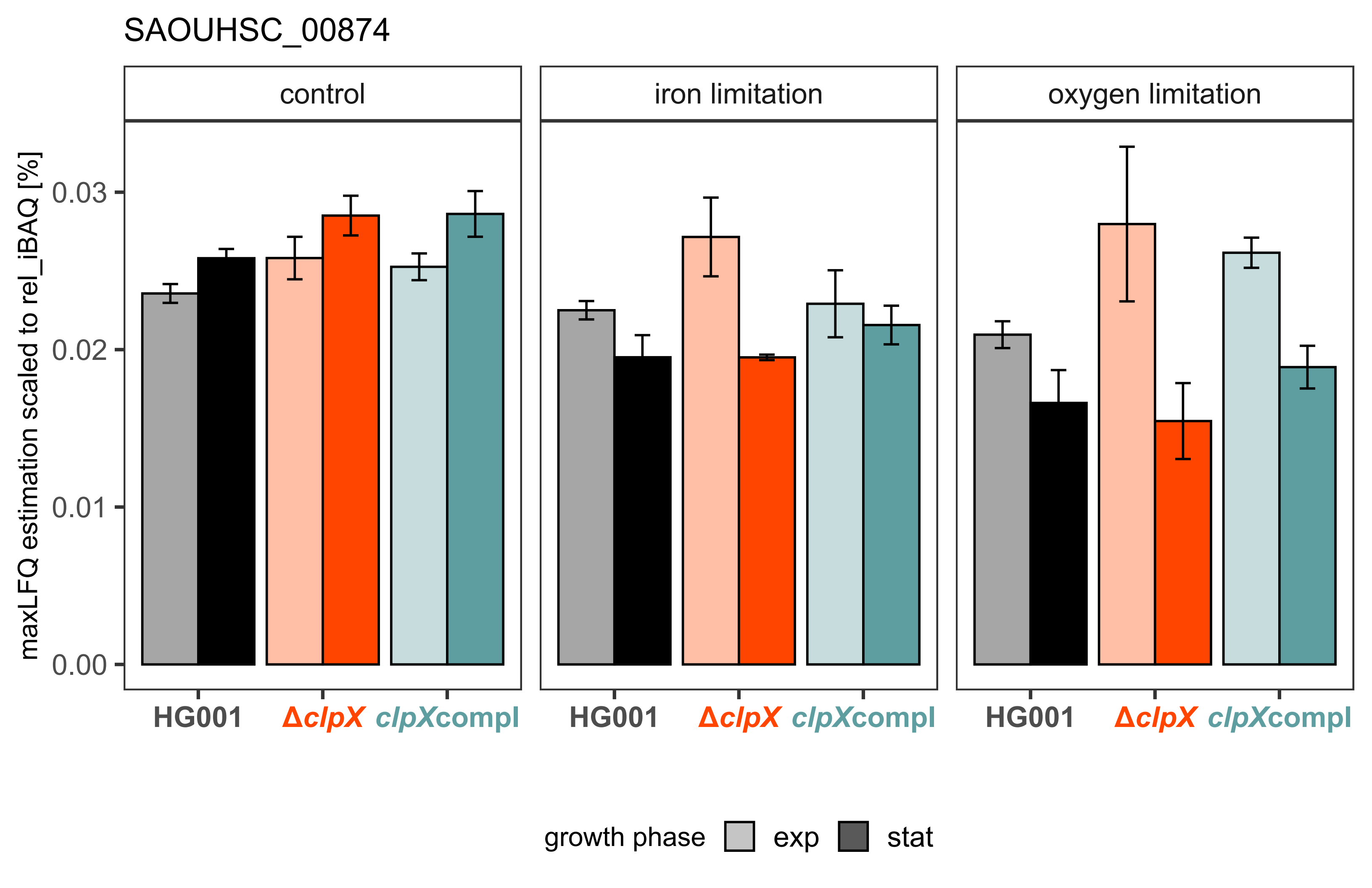

SAOUHSC_00874
control
iron limitation
oxygen limitation
maxLFQ estimation scaled to rel_iBAQ [%]
0.00
0.01
0.02
0.03
HG001
ΔclpX
clpXcompl
growth phase
exp
stat

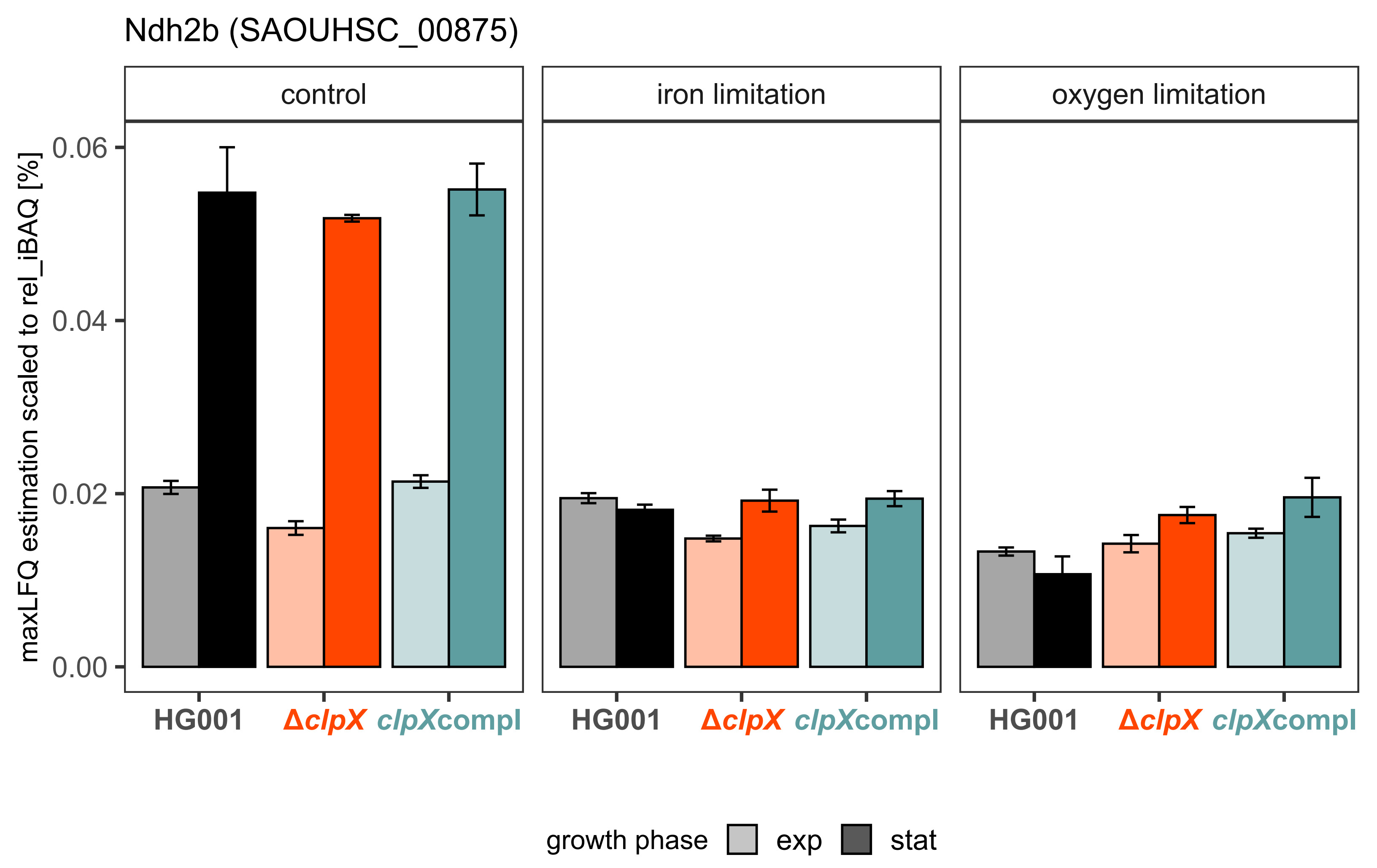

Ndh2b (SAOUHSC_00875)
control
iron limitation
oxygen limitation
maxLFQ estimation scaled to rel_iBAQ [%]
0.06
0.04
0.02
0.00
HG001
ΔclpX
clpXcompl
growth phase
exp
stat

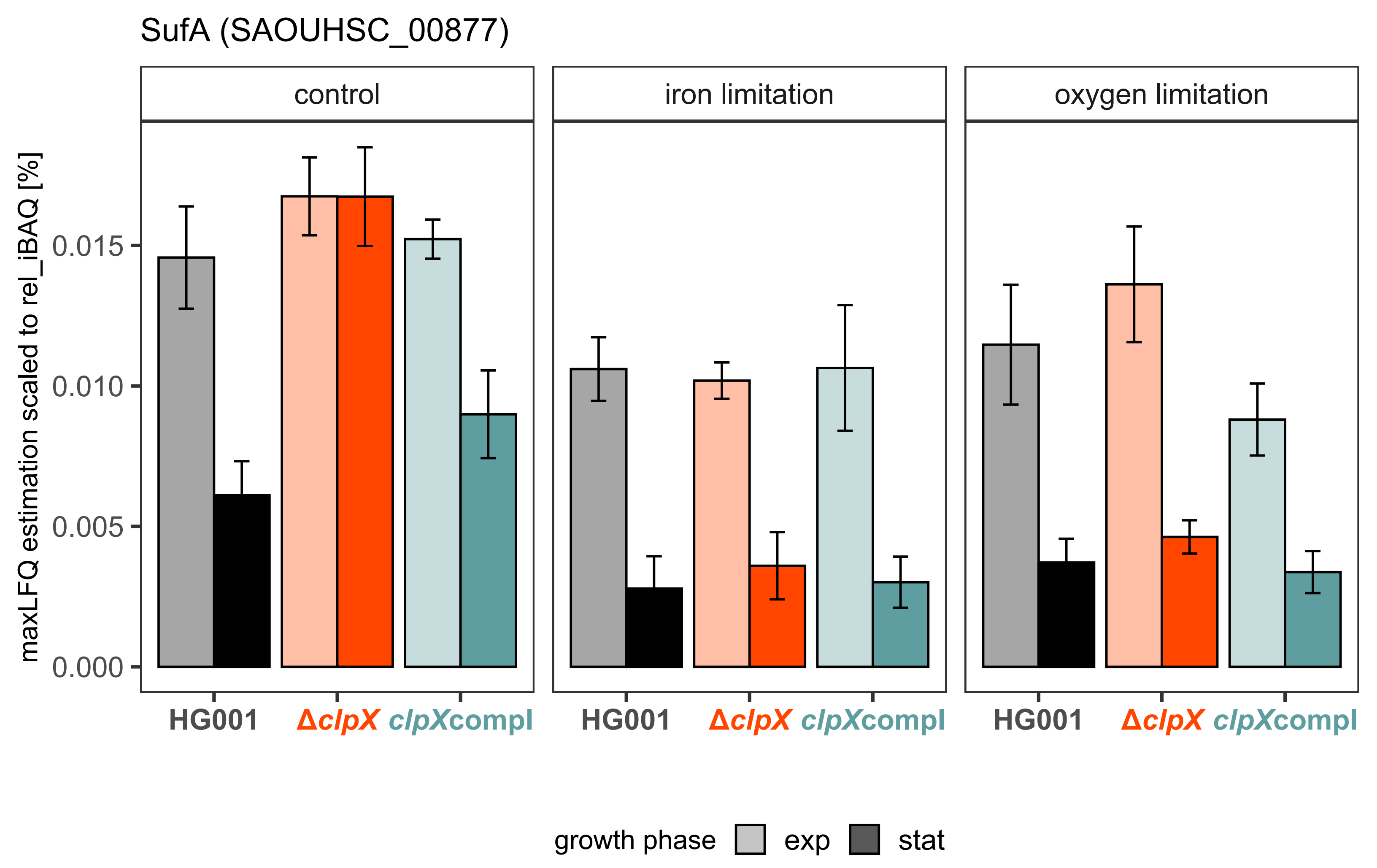

SufA (SAOUHSC_00877)
control
iron limitation
oxygen limitation
maxLFQ estimation scaled to rel_iBAQ [%]
0.015
0.010
0.005
0.000
HG001
ΔclpX
clpXcompl
growth phase
exp
stat

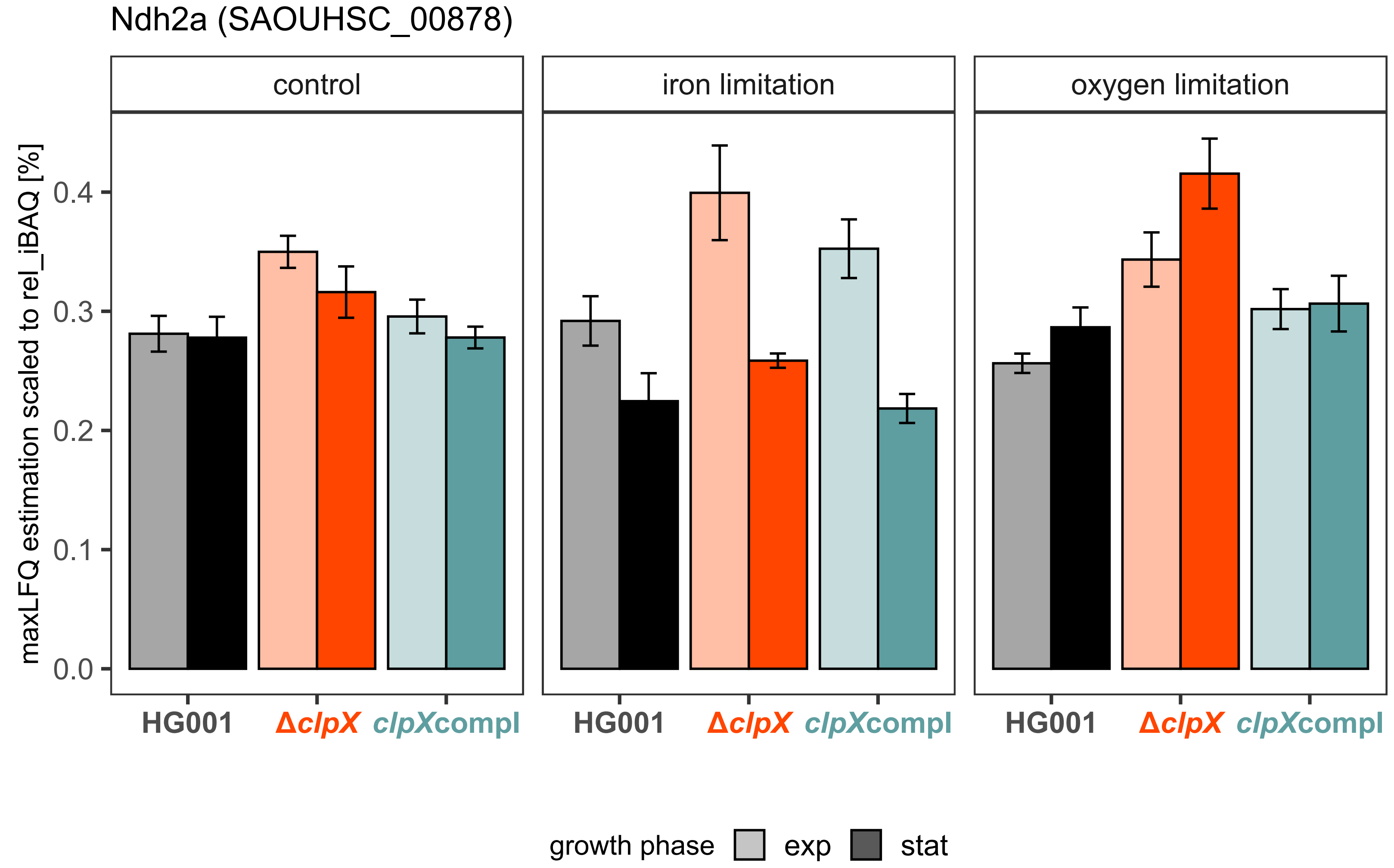
Ndh2a (SAOUHSC_00878)
control
iron limitation
oxygen limitation
maxLFQ estimation scaled to rel_iBAQ [%]
0.0
0.1
0.2
0.3
0.4
HG001
ΔclpX
clpXcompl
growth phase
exp
stat

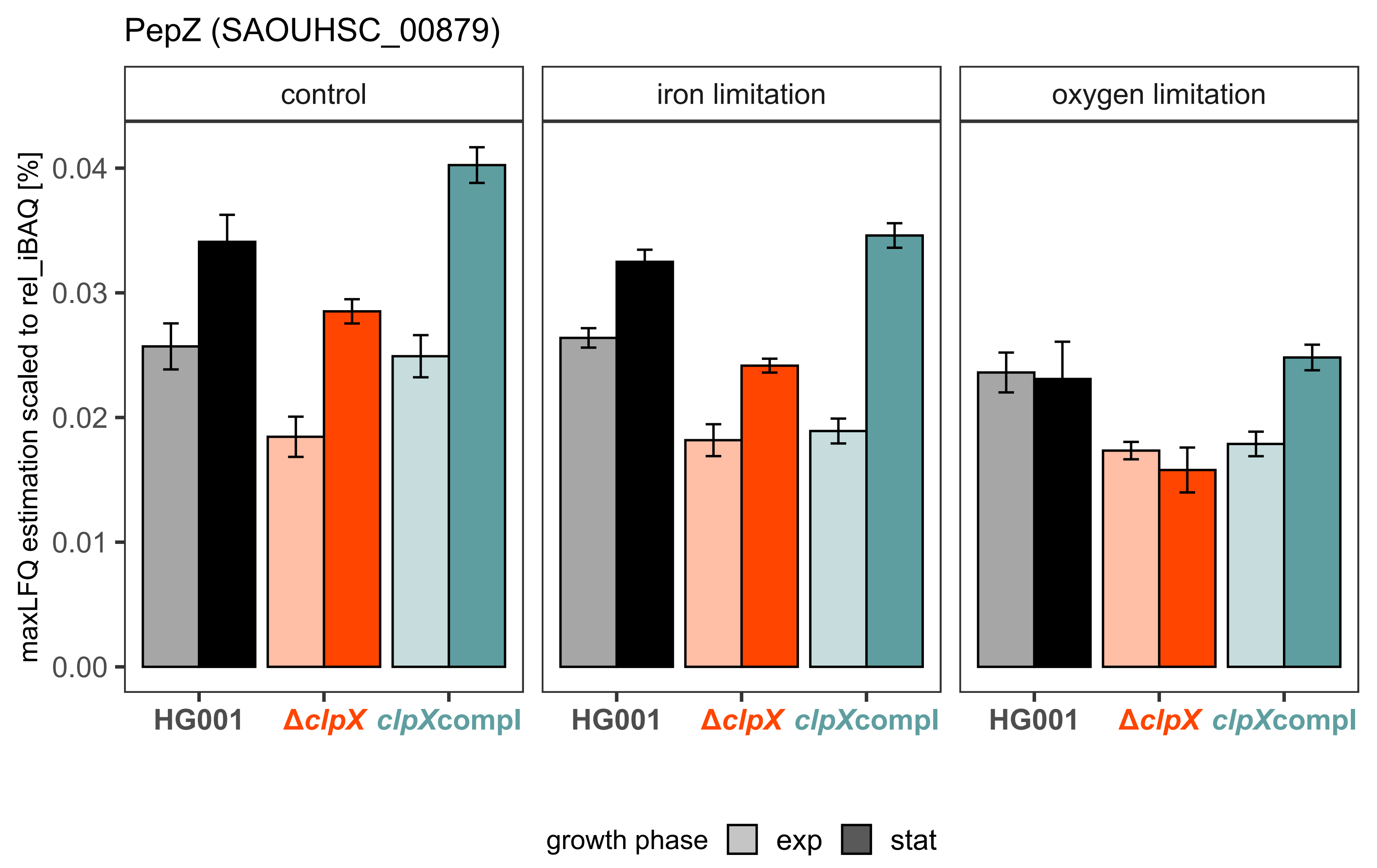
PepZ (SAOUHSC_00879)
control
iron limitation
oxygen limitation
maxLFQ estimation scaled to rel_iBAQ [%]
0.04
0.03
0.02
0.01
0.00
HG001
ΔclpX
clpXcompl
growth phase
exp
stat

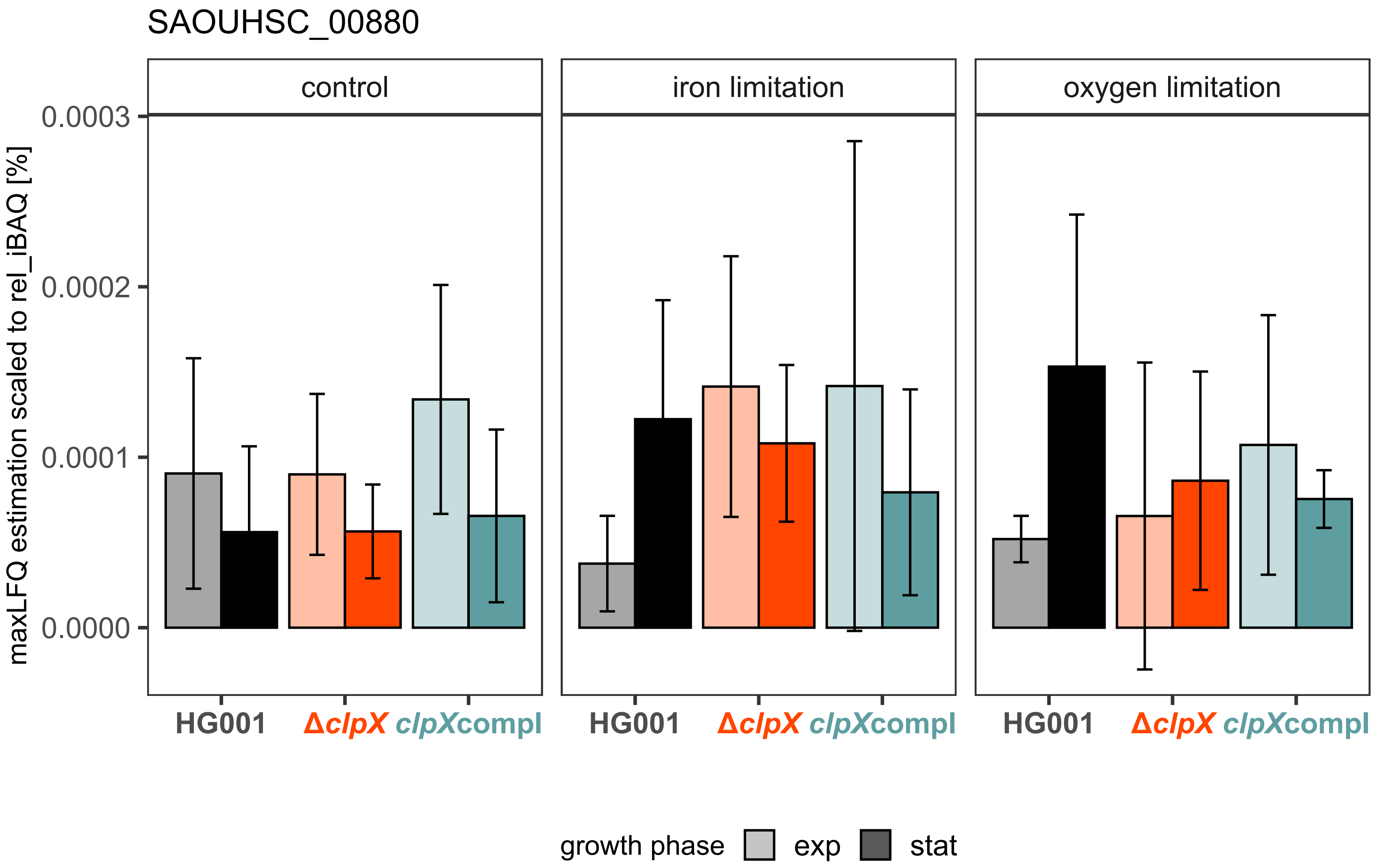
SAOUHSC_00880
control
iron limitation
oxygen limitation
0.0003
0.0002
0.0001
0.0000
maxLFQ estimation scaled to rel_iBAQ [%]
HG001
ΔclpX
clpXcompl
growth phase
exp
stat

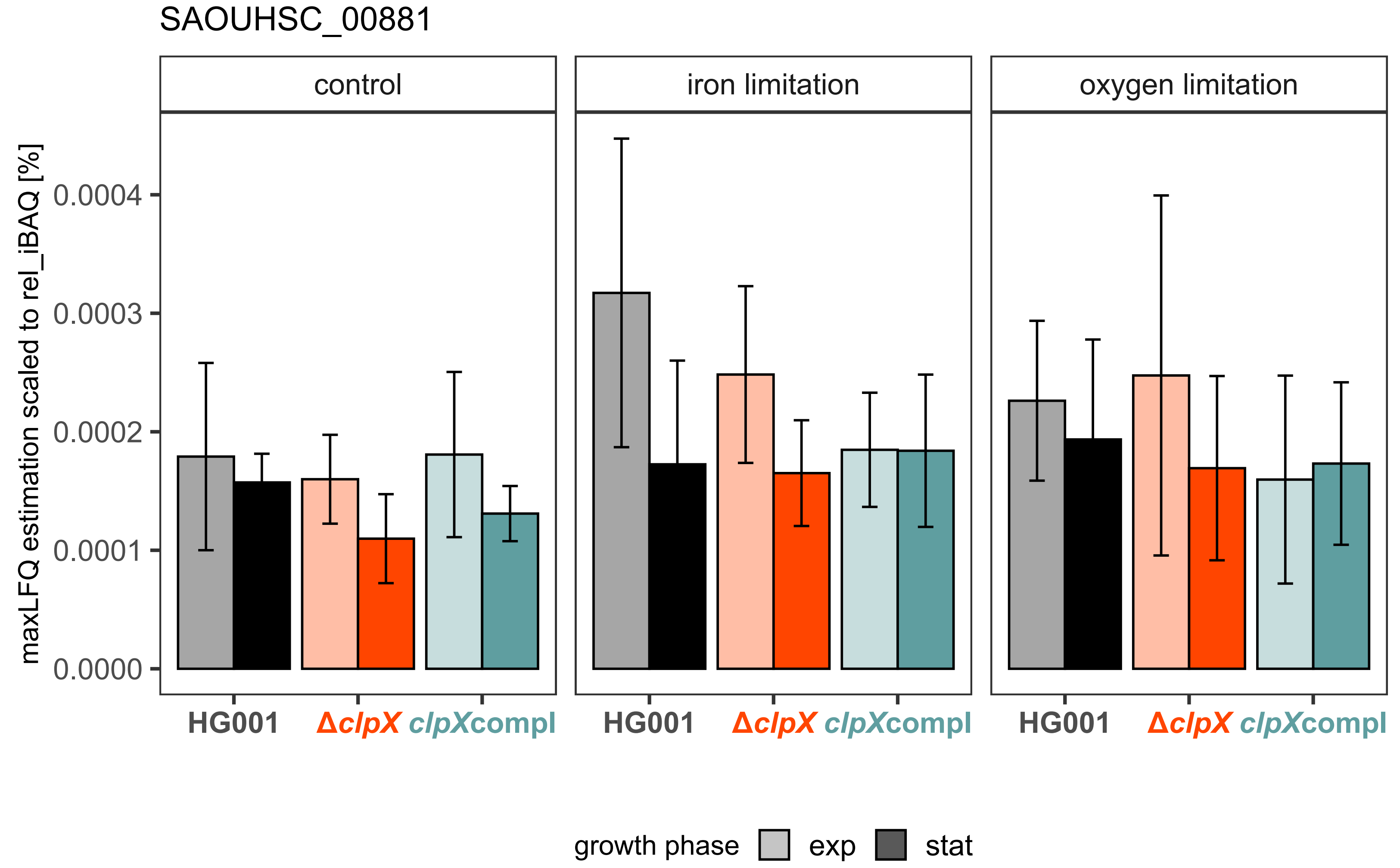

SAOUHSC_00881
control
iron limitation
oxygen limitation
maxLFQ estimation scaled to rel_iBAQ [%]
0.0004
0.0003
0.0002
0.0001
0.0000
HG001
ΔclpX
clpXcompl
growth phase
exp
stat

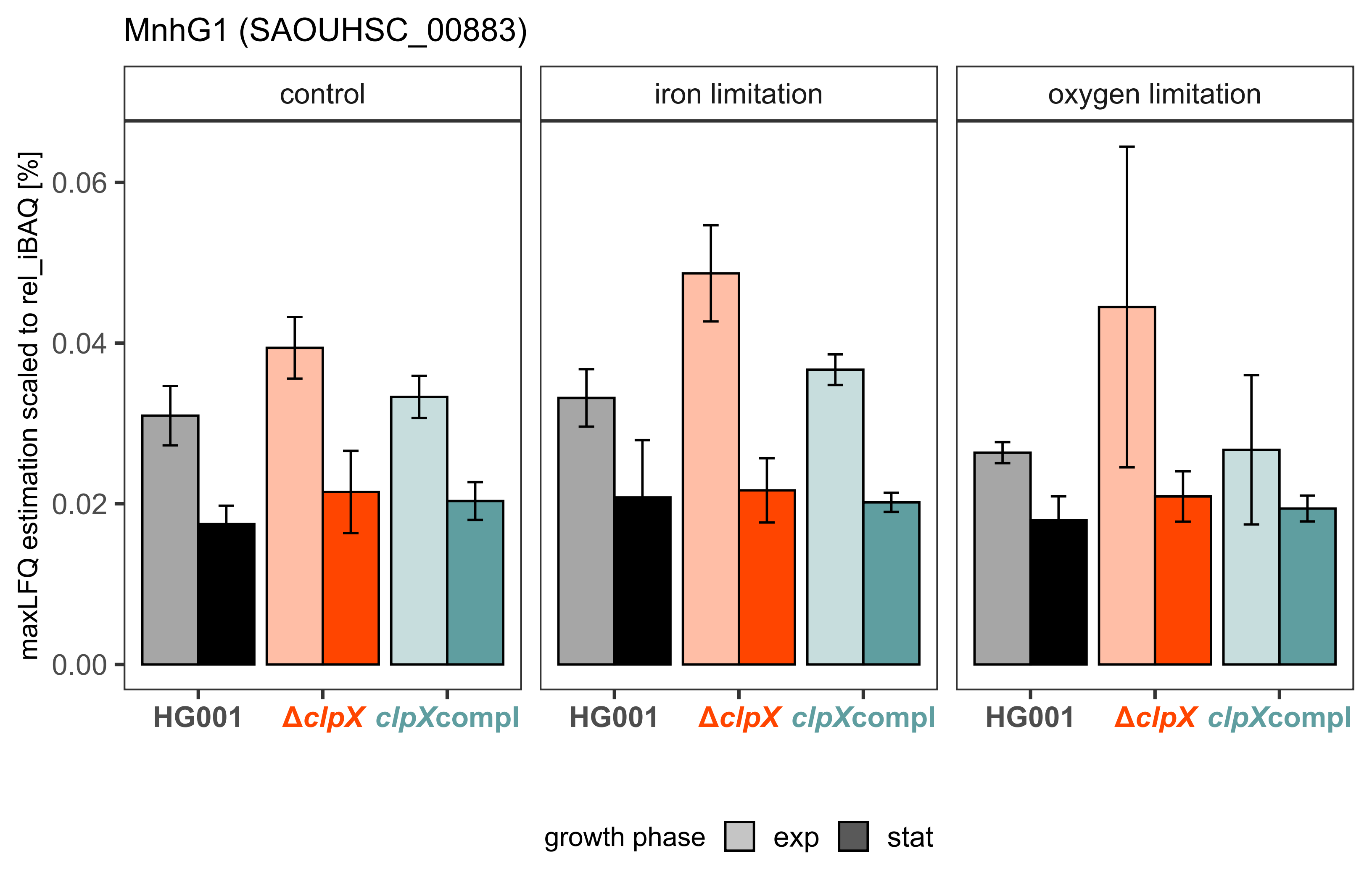
MnhG1 (SAOUHSC_00883)
control
iron limitation
oxygen limitation
maxLFQ estimation scaled to rel_iBAQ [%]
0.06
0.04
0.02
0.00
HG001
ΔclpX
clpXcompl
growth phase
exp
stat

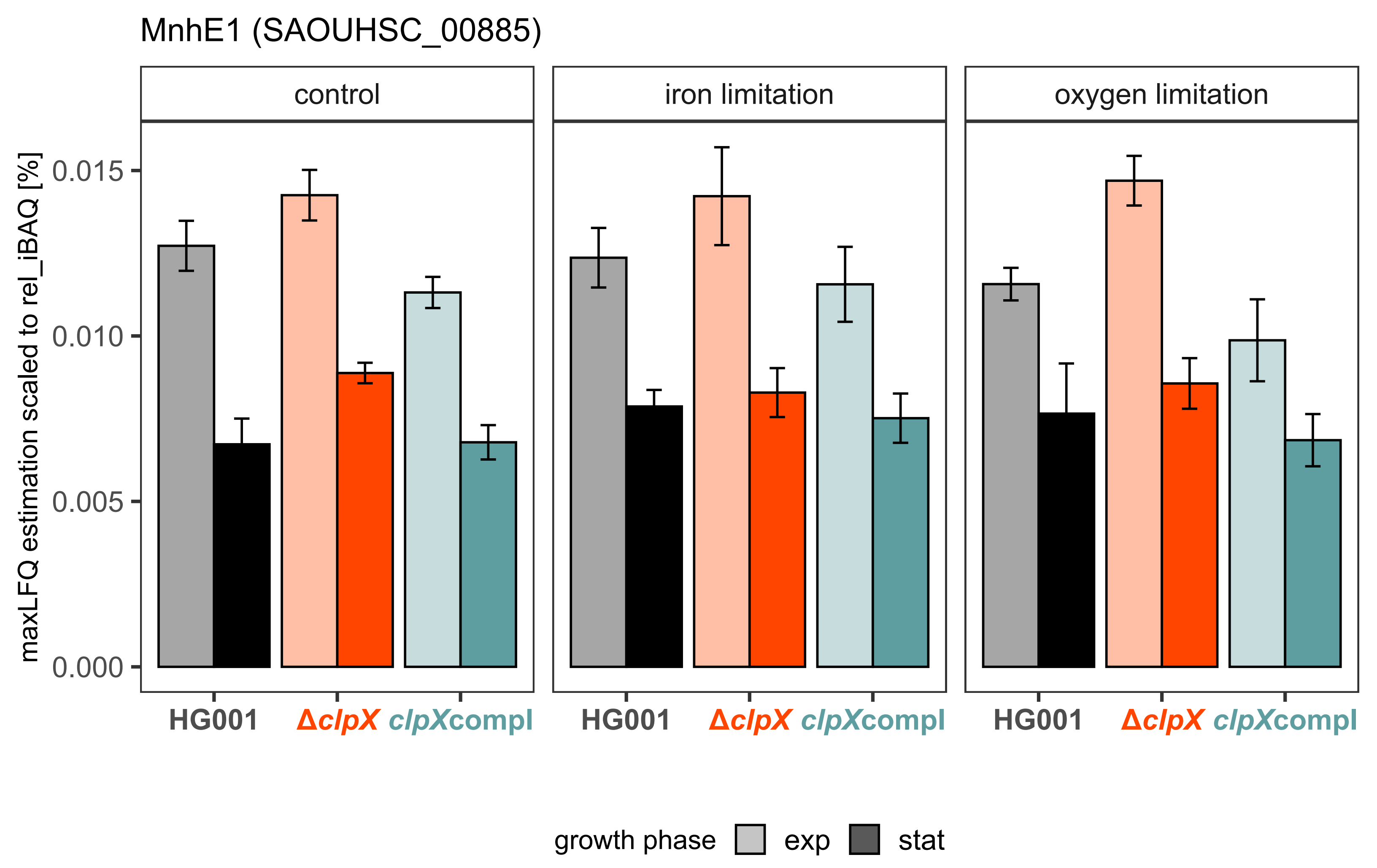

MnhE1 (SAOUHSC_00885)
control
iron limitation
oxygen limitation
maxLFQ estimation scaled to rel_iBAQ [%]
0.015
0.010
0.005
0.000
HG001
ΔclpX
clpXcompl
growth phase
exp
stat

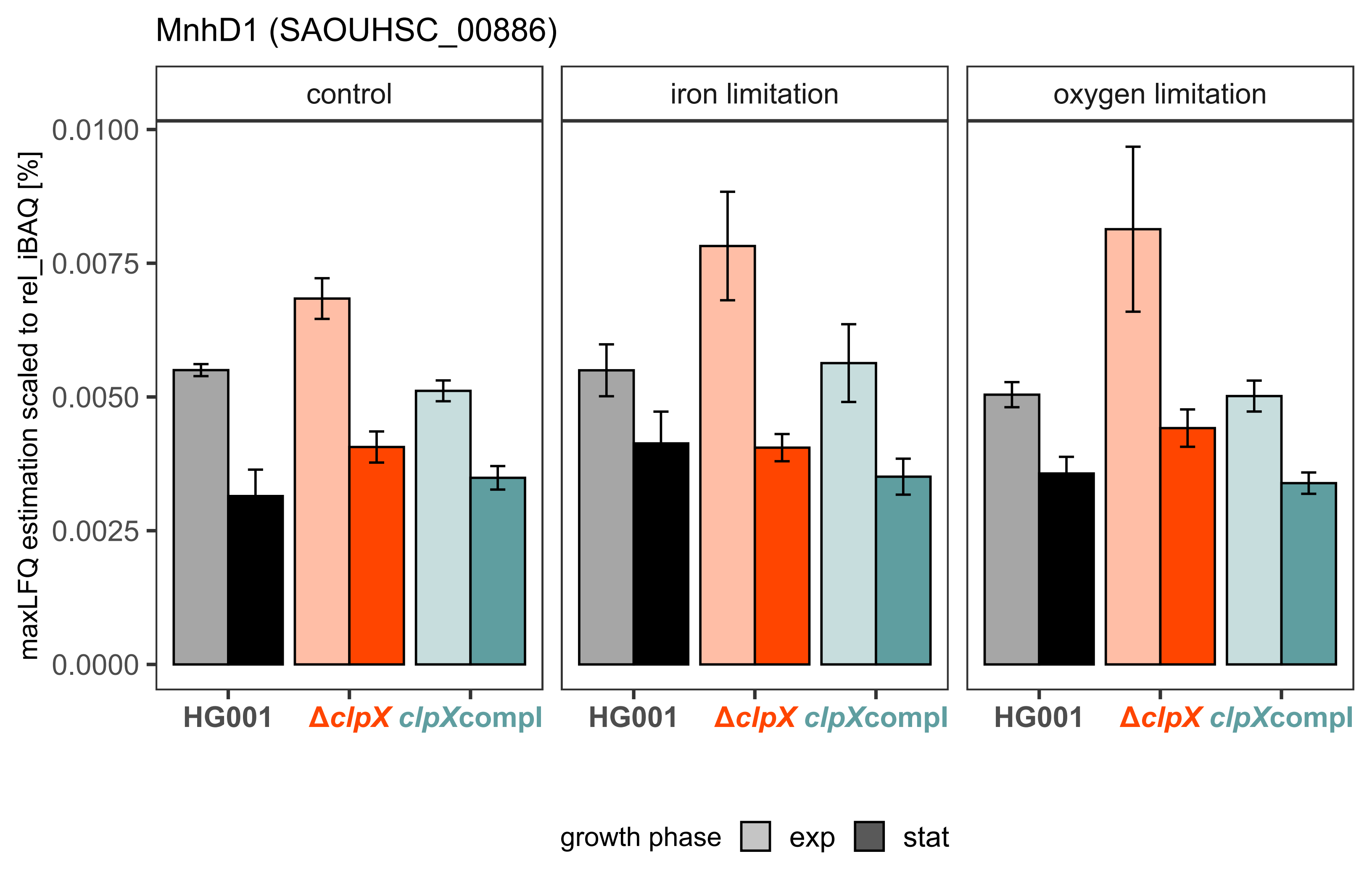

MnhD1 (SAOUHSC_00886)
control
iron limitation
oxygen limitation
maxLFQ estimation scaled to rel_iBAQ [%]
0.0100
0.0075
0.0050
0.0025
0.0000
HG001
ΔclpX
clpXcompl
growth phase
exp
stat

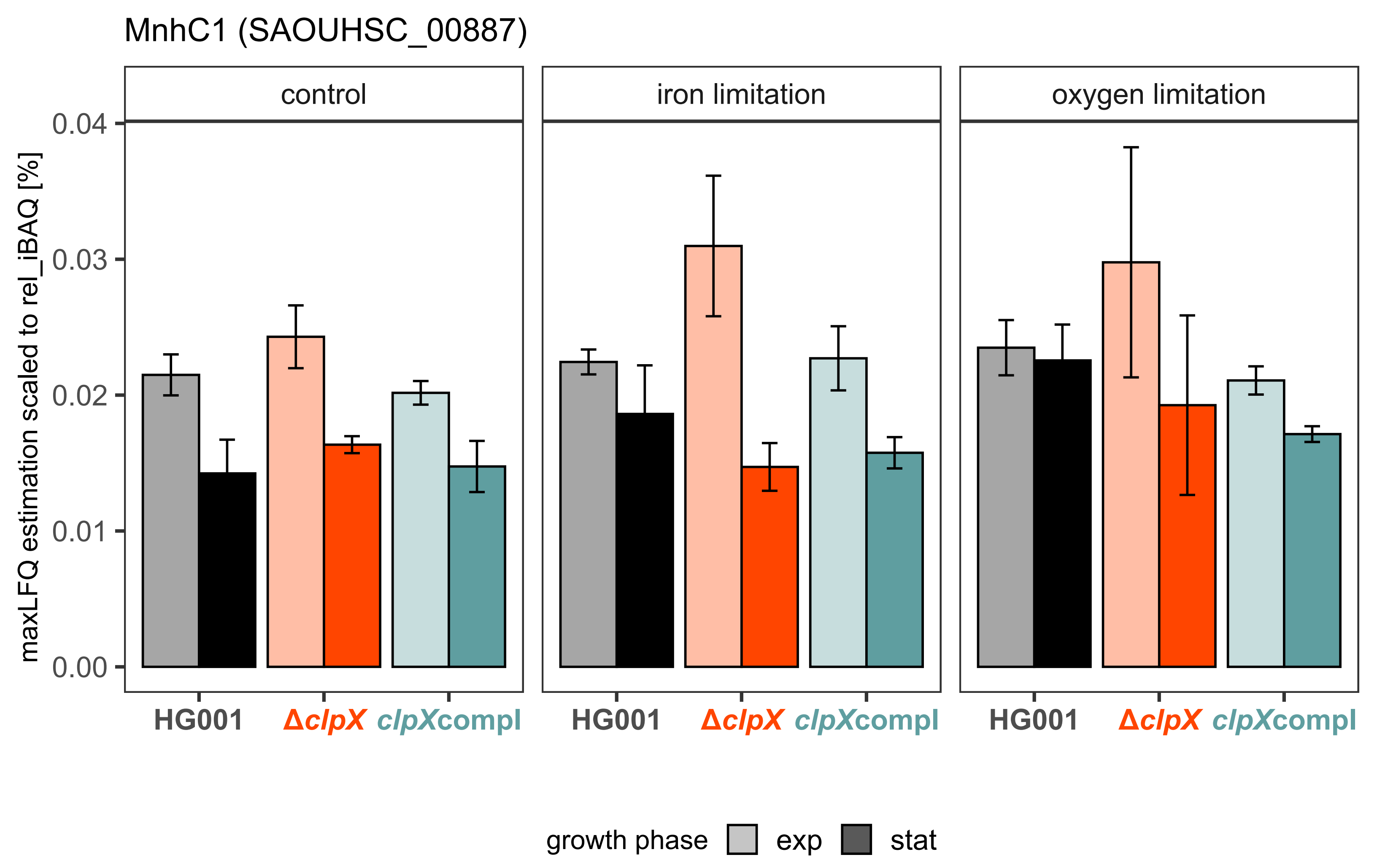

MnhC1 (SAOUHSC_00887)
control
iron limitation
oxygen limitation
maxLFQ estimation scaled to rel_iBAQ [%]
0.00
0.01
0.02
0.03
0.04
HG001
ΔclpX
clpXcompl
growth phase
exp
stat

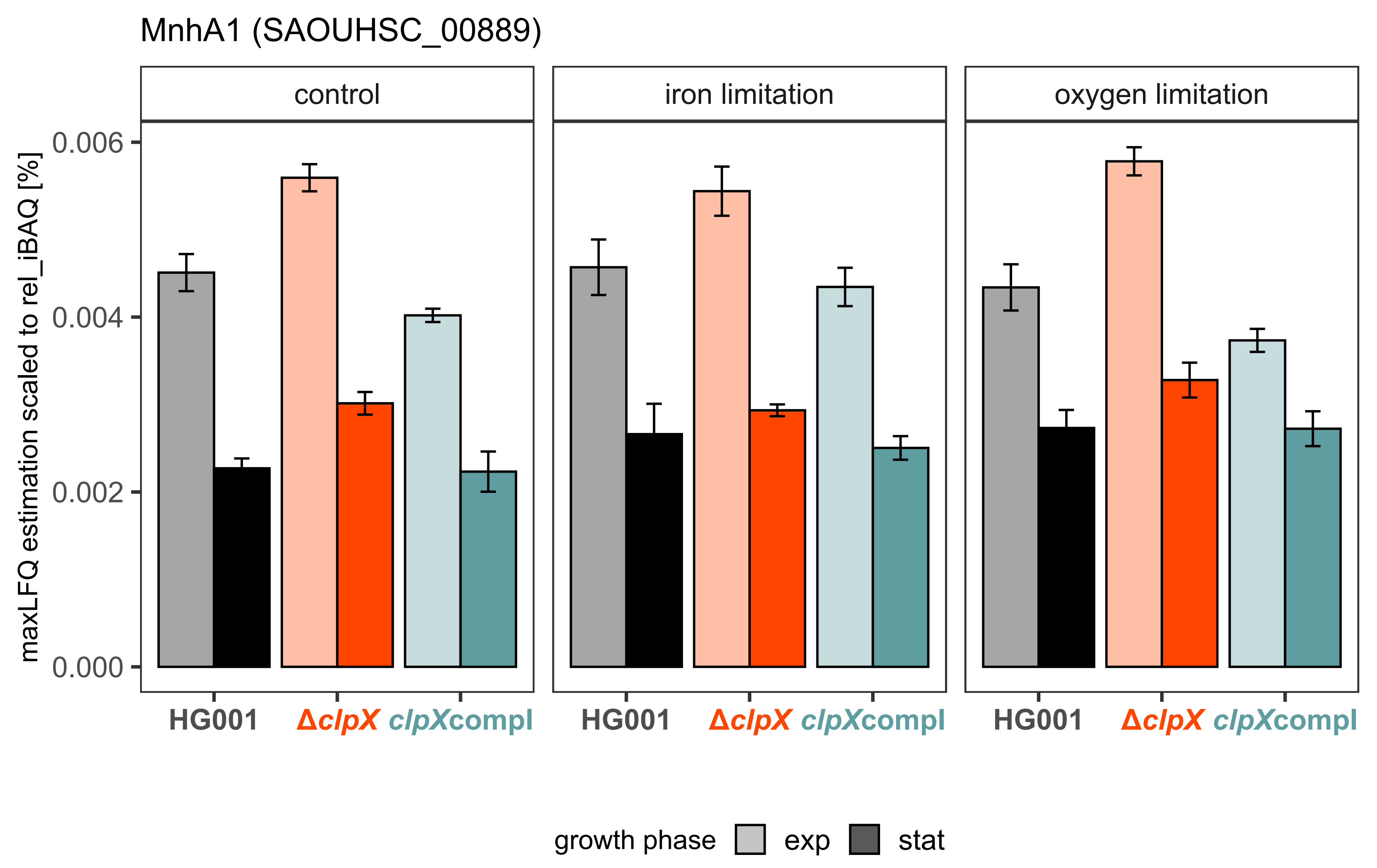
MnhA1 (SAOUHSC_00889)
control
iron limitation
oxygen limitation
0.006
0.004
0.002
0.000
maxLFQ estimation scaled to rel_iBAQ [%]
HG001
ΔclpX
clpXcompl
growth phase
exp
stat

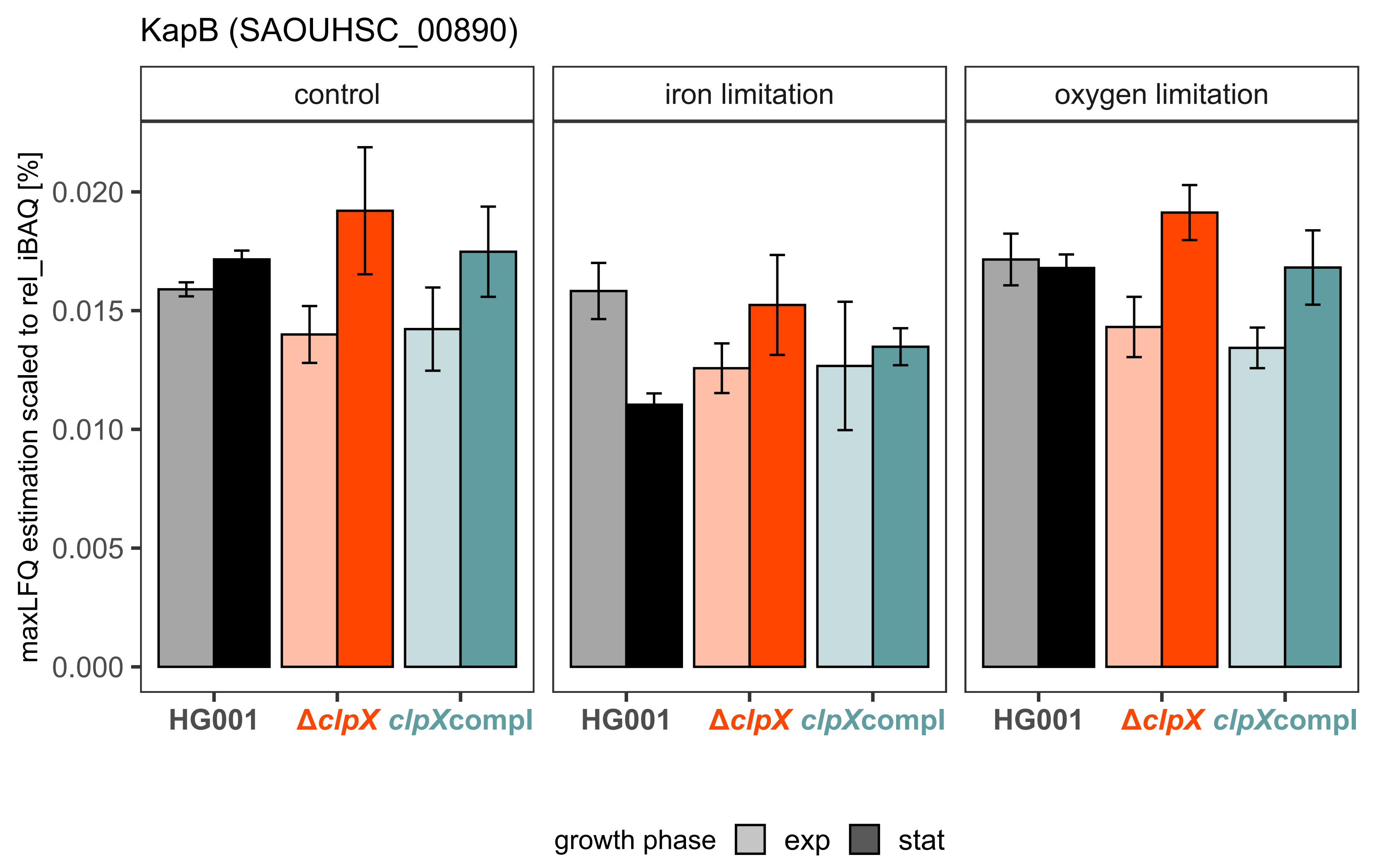

KapB (SAOUHSC_00890)
control
iron limitation
oxygen limitation
maxLFQ estimation scaled to rel_iBAQ [%]
0.020
0.015
0.010
0.005
0.000
HG001
ΔclpX
clpXcompl
growth phase
exp
stat

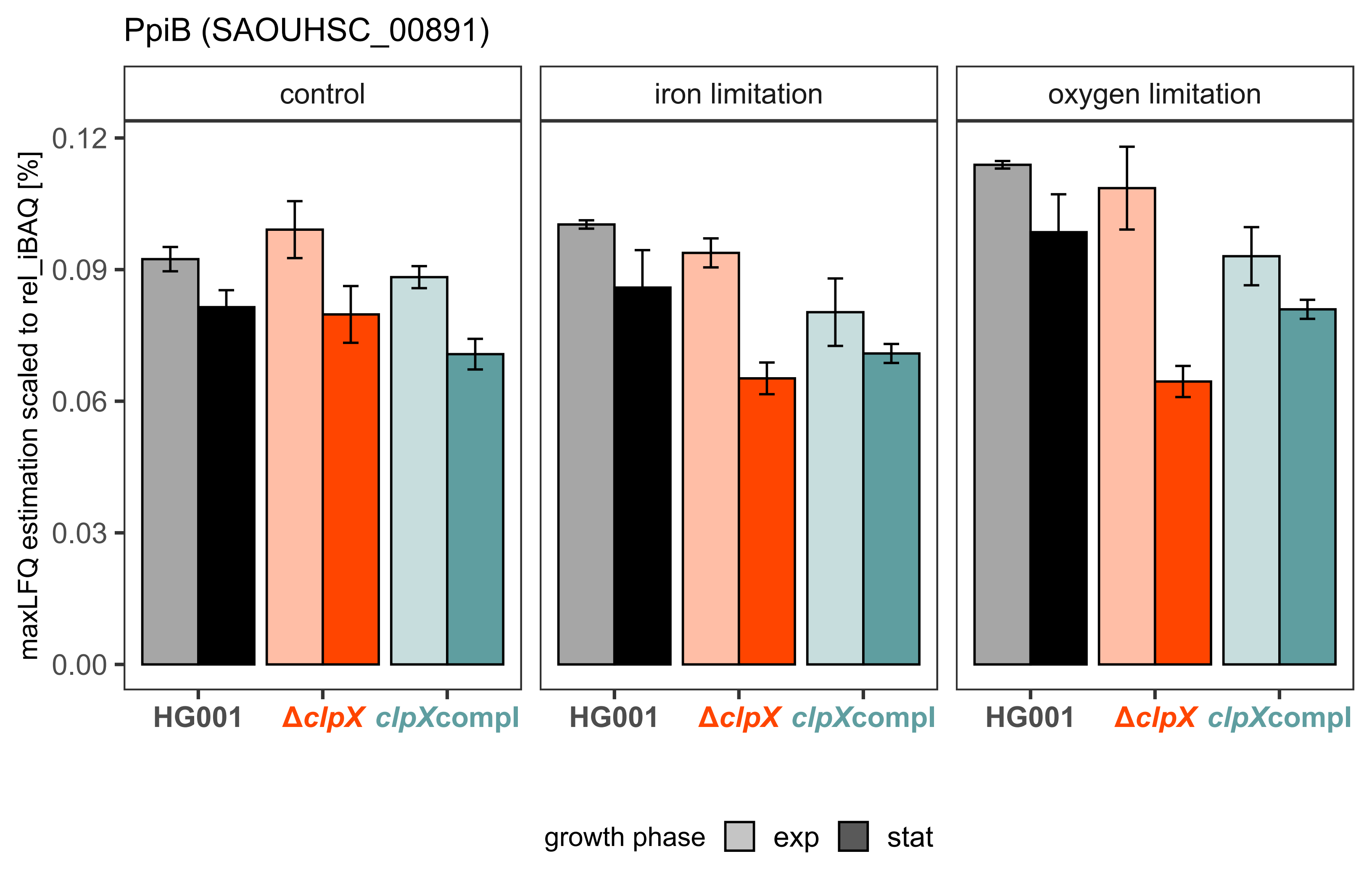

PpiB (SAOUHSC_00891)
control
iron limitation
oxygen limitation
maxLFQ estimation scaled to rel_iBAQ [%]
0.12
0.09
0.06
0.03
0.00
HG001
ΔclpX
clpXcompl
growth phase
exp
stat

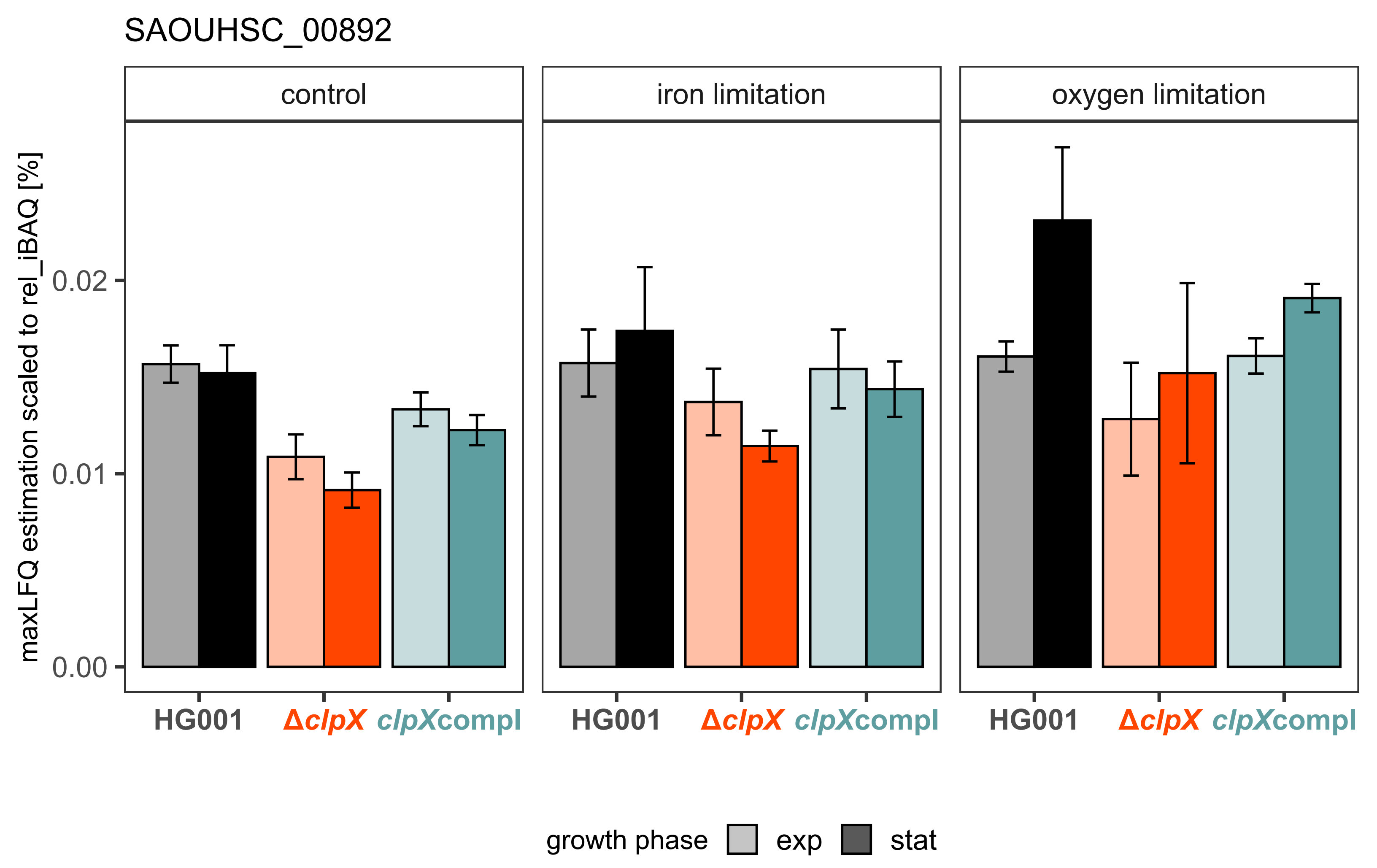

SAOUHSC_00892
control
iron limitation
oxygen limitation
maxLFQ estimation scaled to rel_iBAQ [%]
0.00
0.01
0.02
HG001
ΔclpX
clpXcompl
growth phase
exp
stat

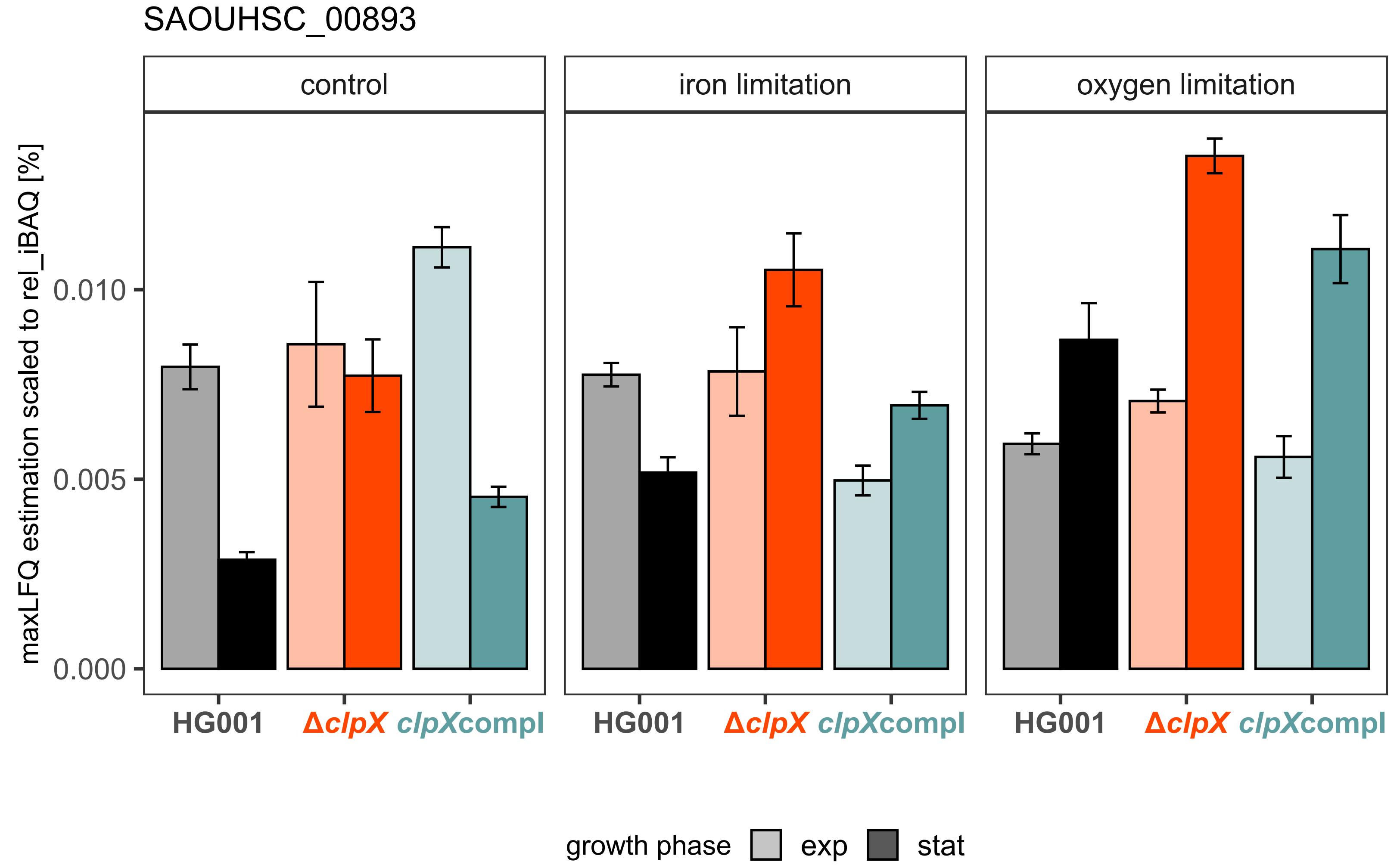
SAOUHSC_00893
control
iron limitation
oxygen limitation
maxLFQ estimation scaled to rel_iBAQ [%]
0.010
0.005
0.000
HG001
ΔclpX
clpXcompl
growth phase
exp
stat

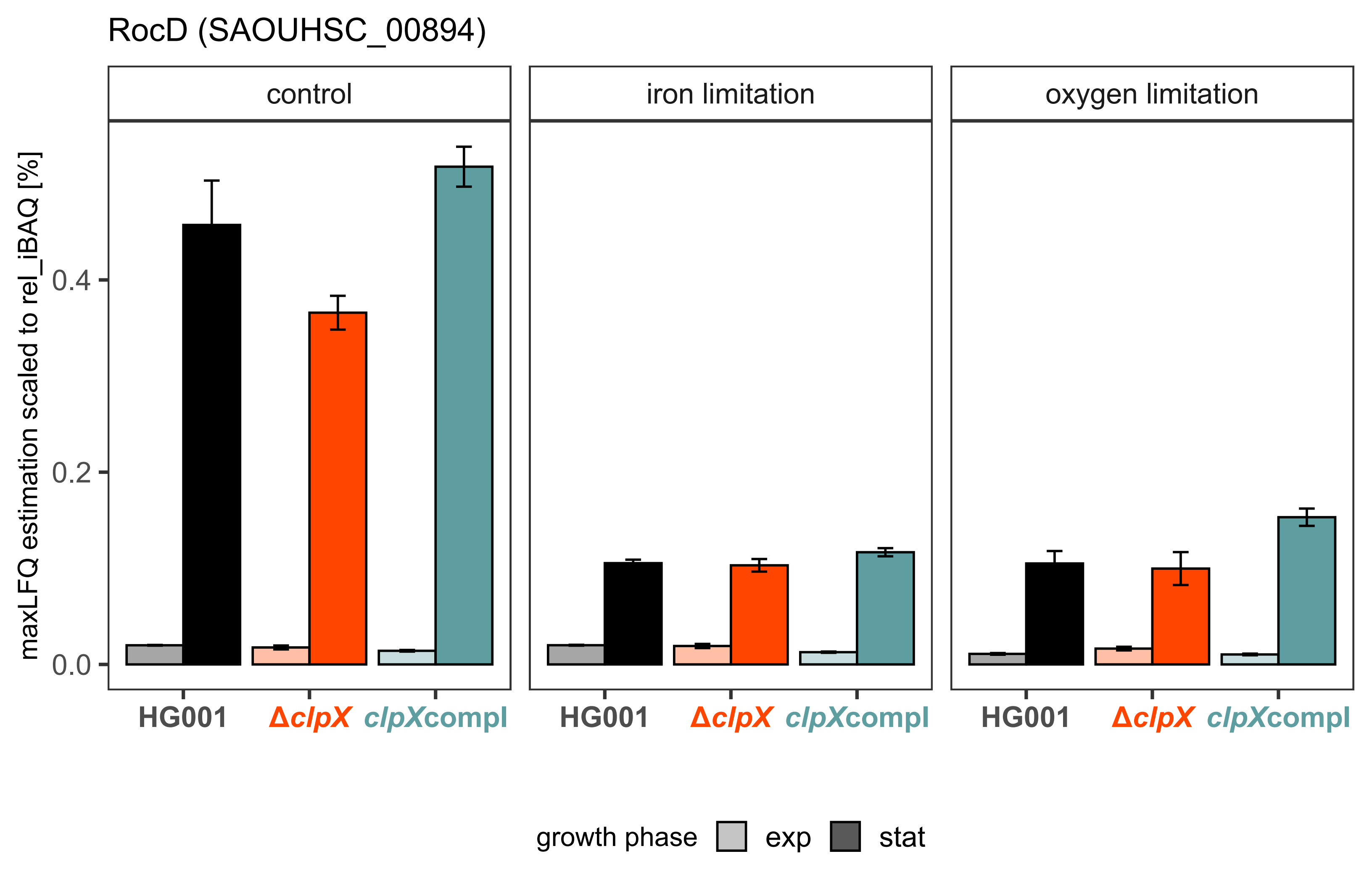

RocD (SAOUHSC_00894)
control
iron limitation
oxygen limitation
maxLFQ estimation scaled to rel_iBAQ [%]
0.0
0.2
0.4
HG001
ΔclpX
clpXcompl
growth phase
exp
stat

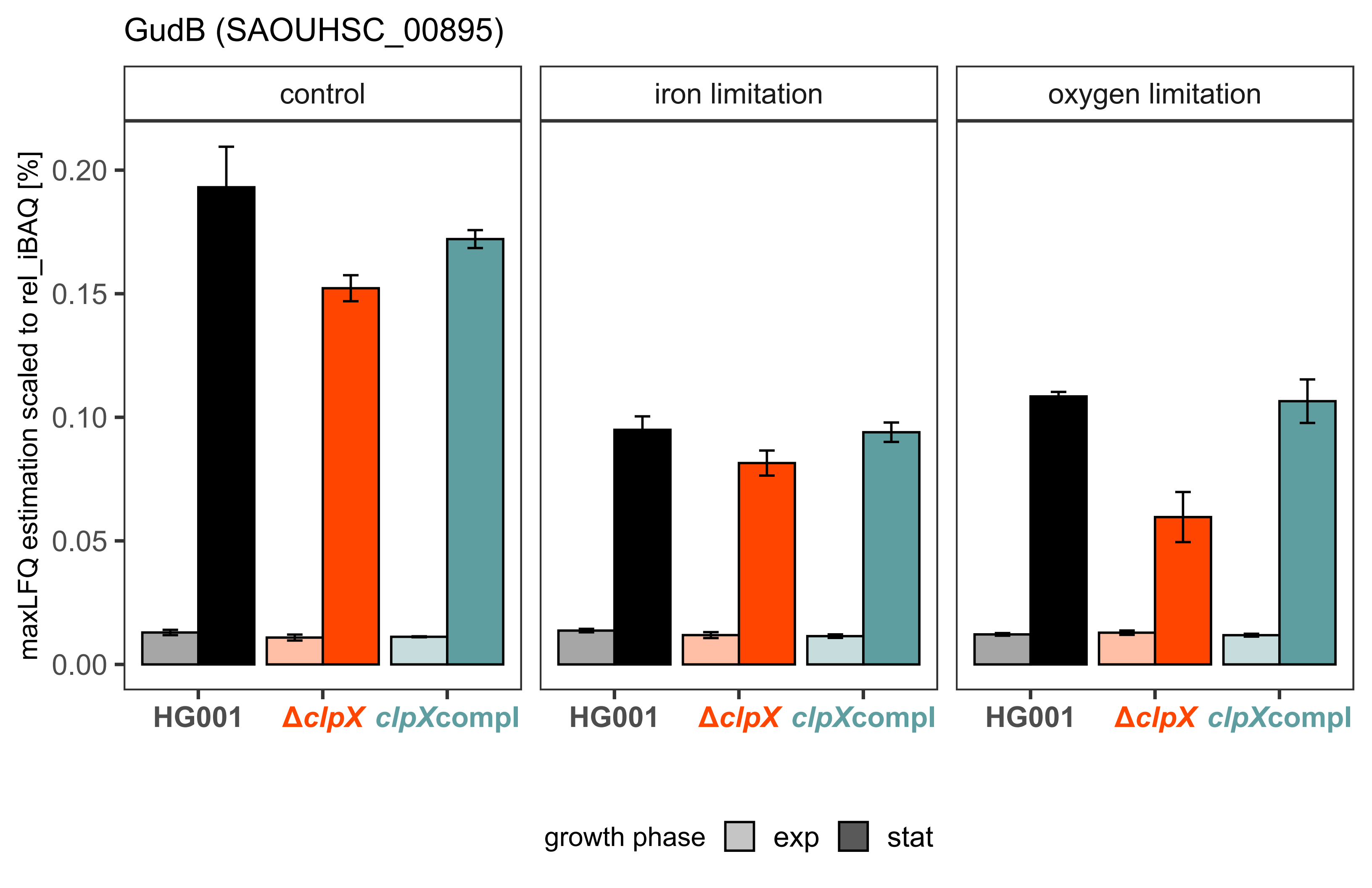
GudB (SAOUHSC_00895)
control
iron limitation
oxygen limitation
maxLFQ estimation scaled to rel_iBAQ [%]
0.20
0.15
0.10
0.05
0.00
HG001
ΔclpX
clpXcompl
growth phase
exp
stat

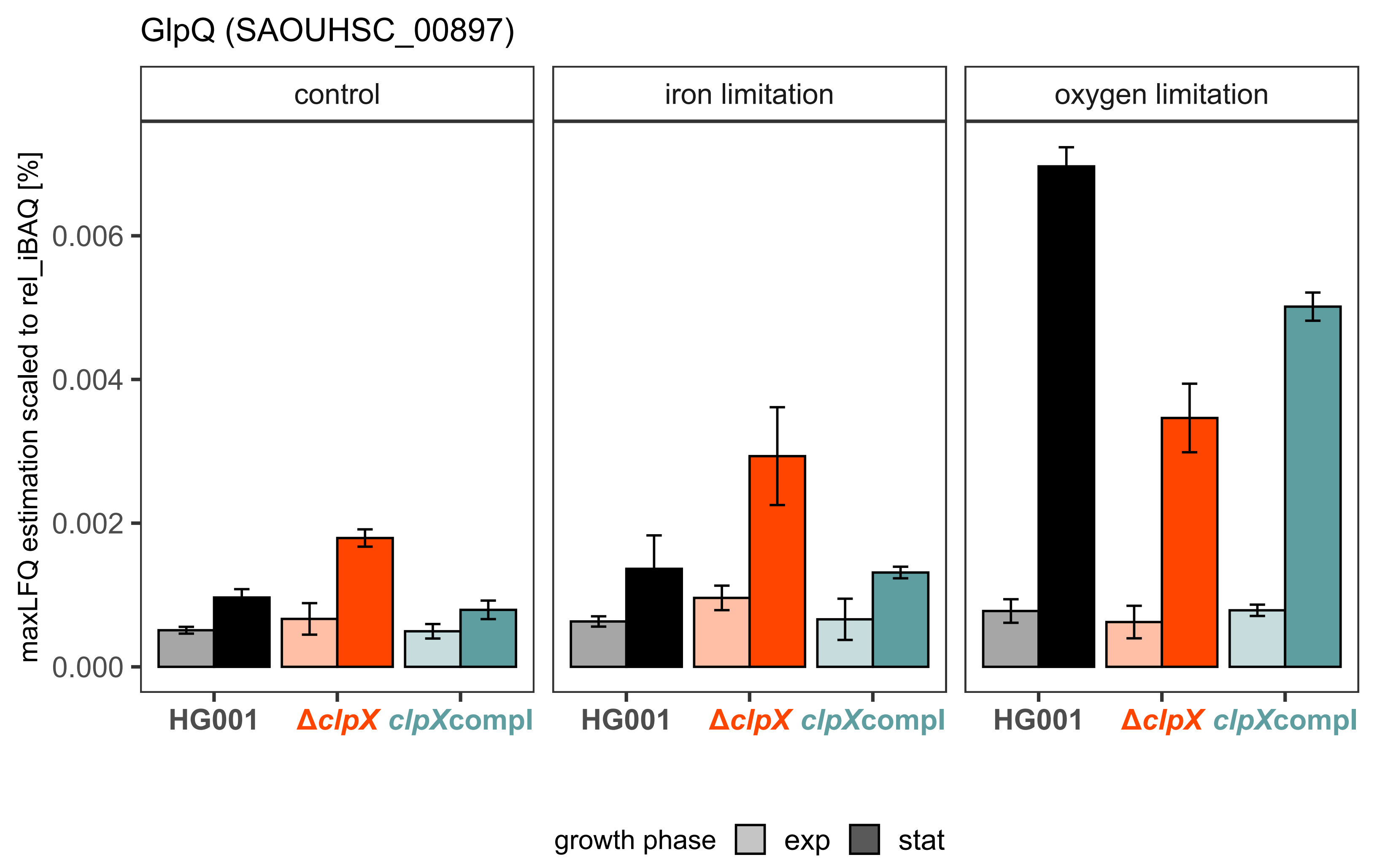
GlpQ (SAOUHSC_00897)
control
iron limitation
oxygen limitation
maxLFQ estimation scaled to rel_iBAQ [%]
0.006
0.004
0.002
0.000
HG001
ΔclpX
clpXcompl
growth phase
exp
stat

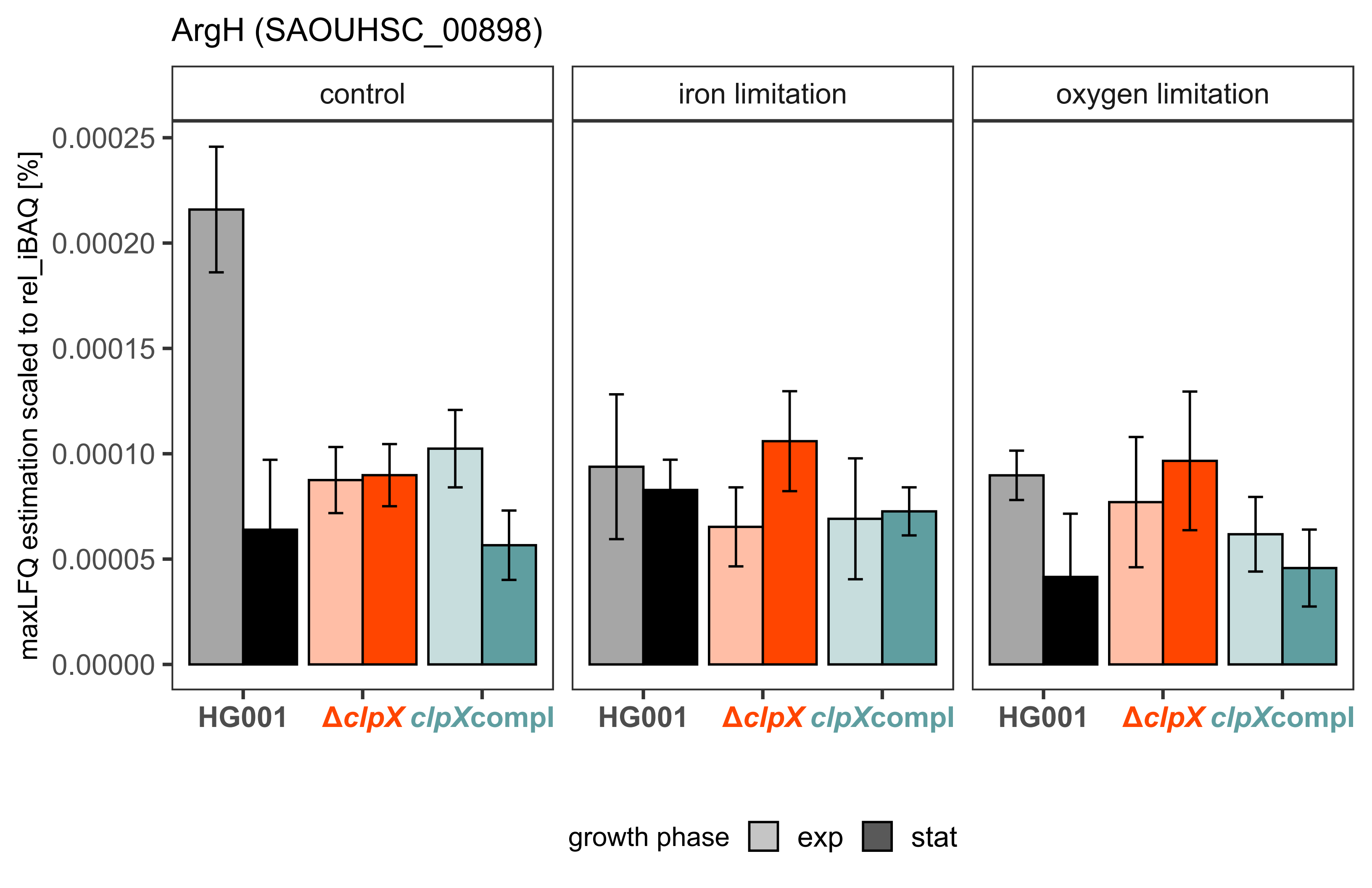

ArgH (SAOUHSC_00898)
control
iron limitation
oxygen limitation
maxLFQ estimation scaled to rel_iBAQ [%]
0.00025
0.00020
0.00015
0.00010
0.00005
0.00000
HG001
ΔclpX
clpXcompl
growth phase
exp
stat

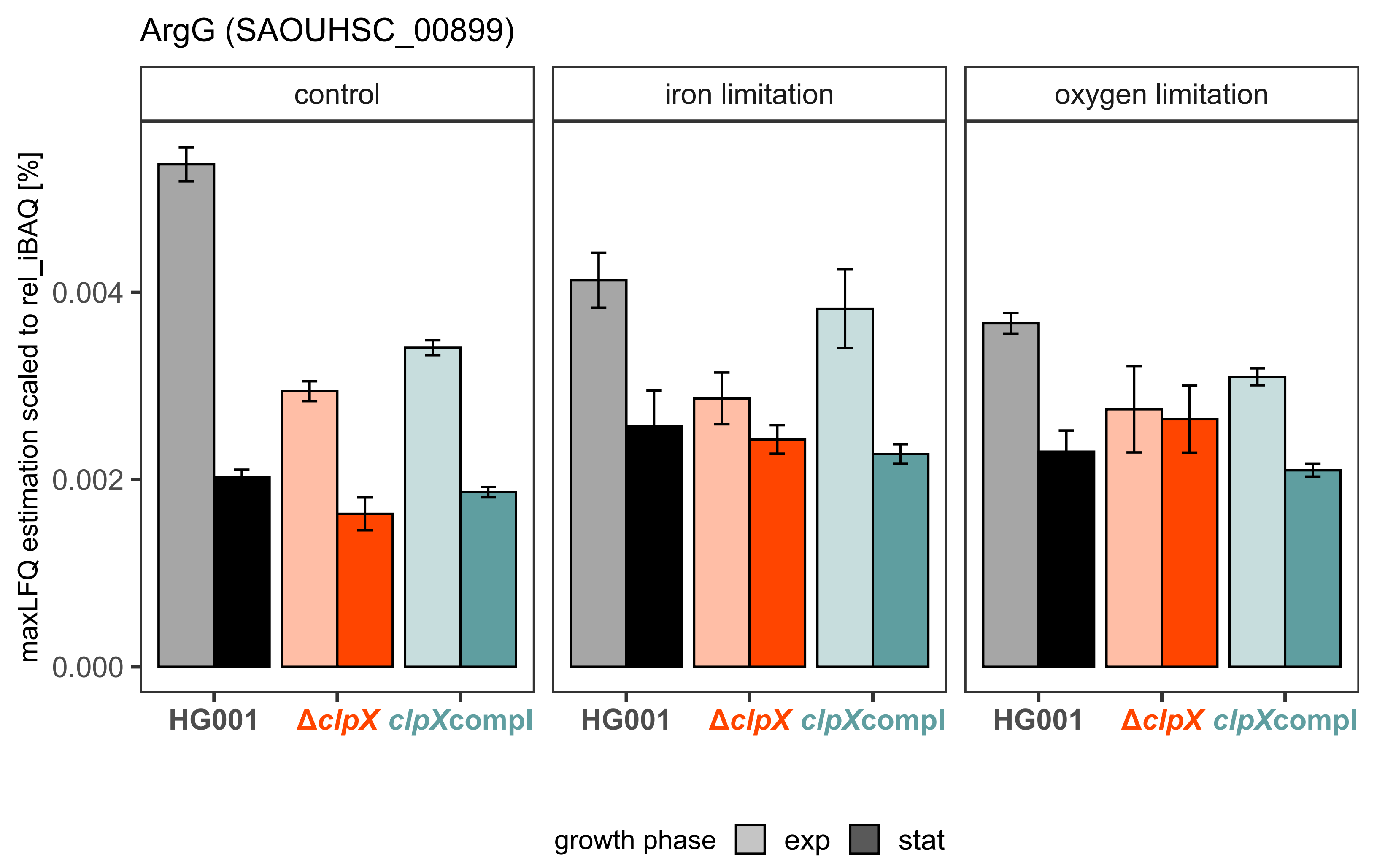

ArgG (SAOUHSC_00899)
control
iron limitation
oxygen limitation
maxLFQ estimation scaled to rel_iBAQ [%]
0.004
0.002
0.000
HG001
ΔclpX
clpXcompl
growth phase
exp
stat

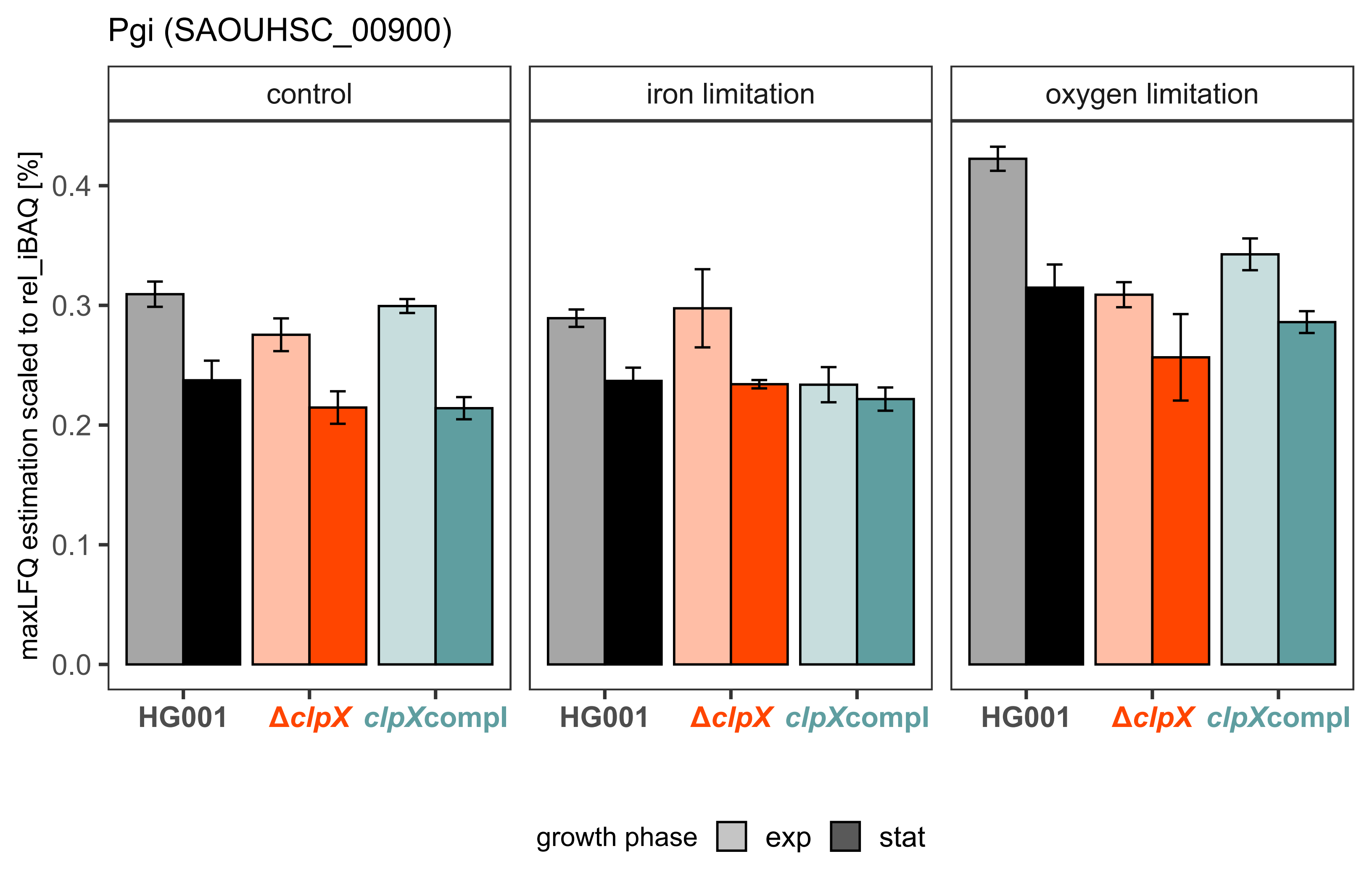

Pgi (SAOUHSC_00900)
control
iron limitation
oxygen limitation
maxLFQ estimation scaled to rel_iBAQ [%]
0.0
0.1
0.2
0.3
0.4
HG001
ΔclpX
clpXcompl
growth phase
exp
stat

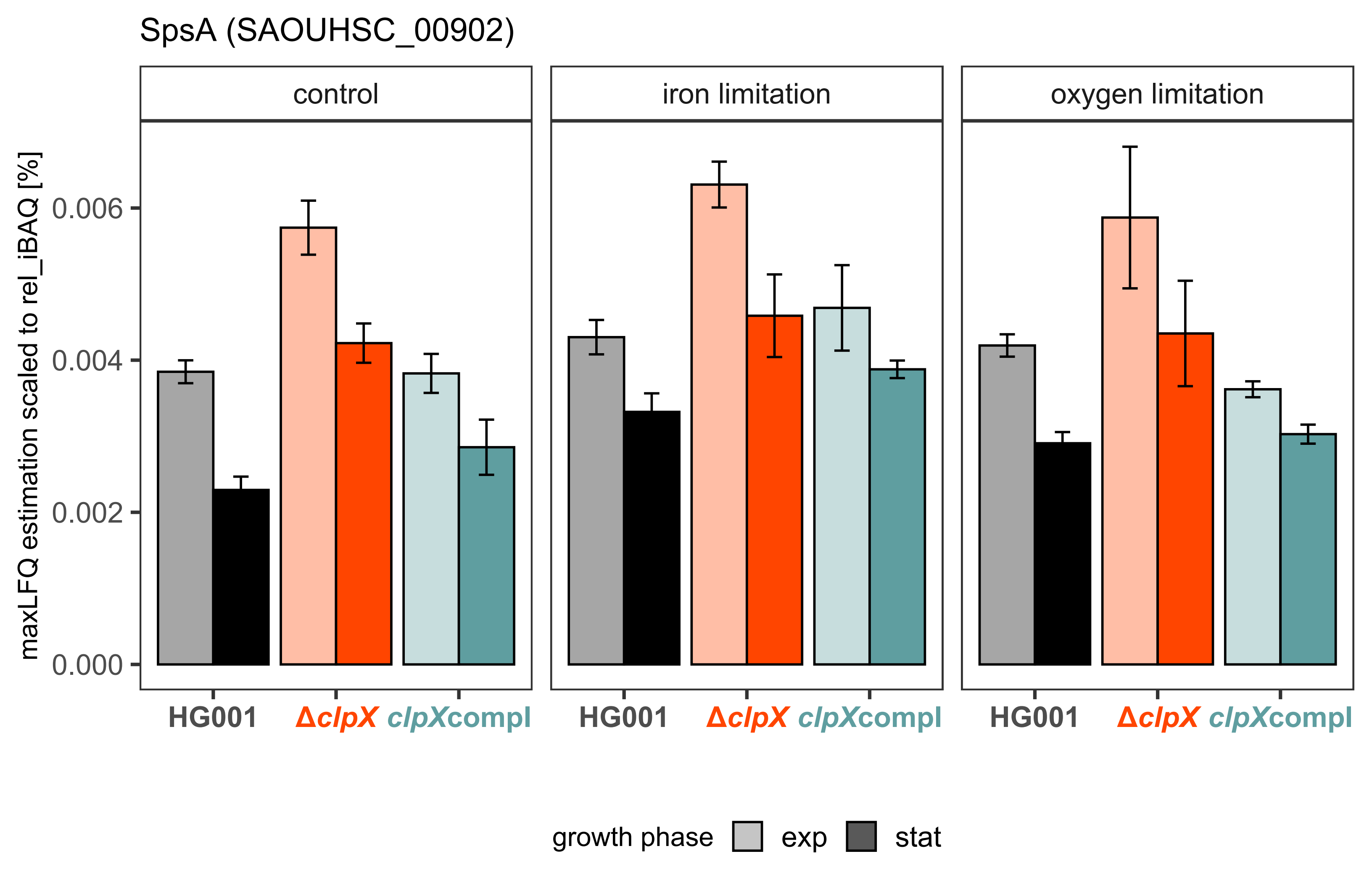
SpsA (SAOUHSC_00902)
control
iron limitation
oxygen limitation
maxLFQ estimation scaled to rel_iBAQ [%]
0.006
0.004
0.002
0.000
HG001
ΔclpX
clpXcompl
growth phase
exp
stat

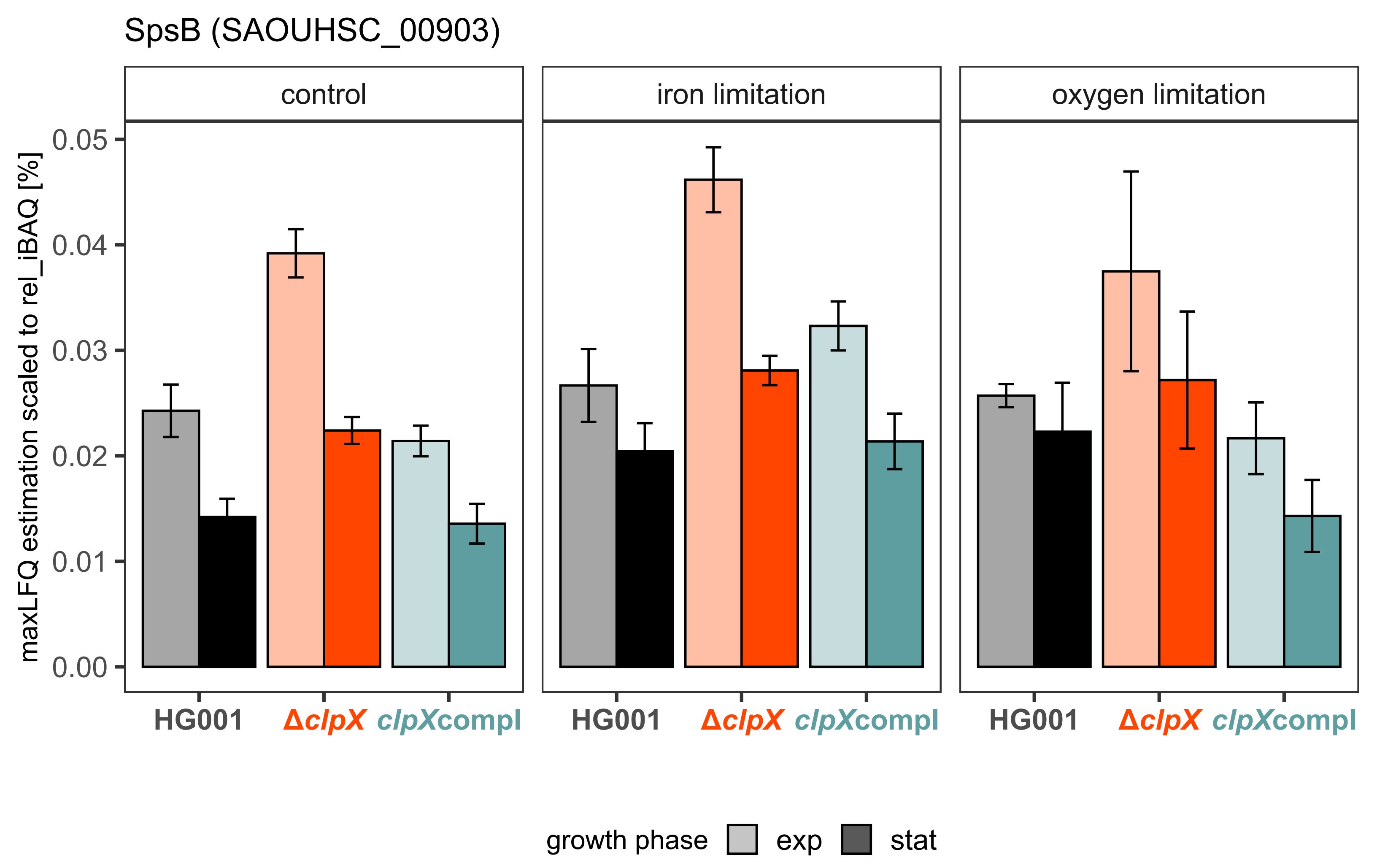

SpsB (SAOUHSC_00903)
control
iron limitation
oxygen limitation
maxLFQ estimation scaled to rel_iBAQ [%]
0.05
0.04
0.03
0.02
0.01
0.00
HG001
ΔclpX
clpXcompl
growth phase
exp
stat

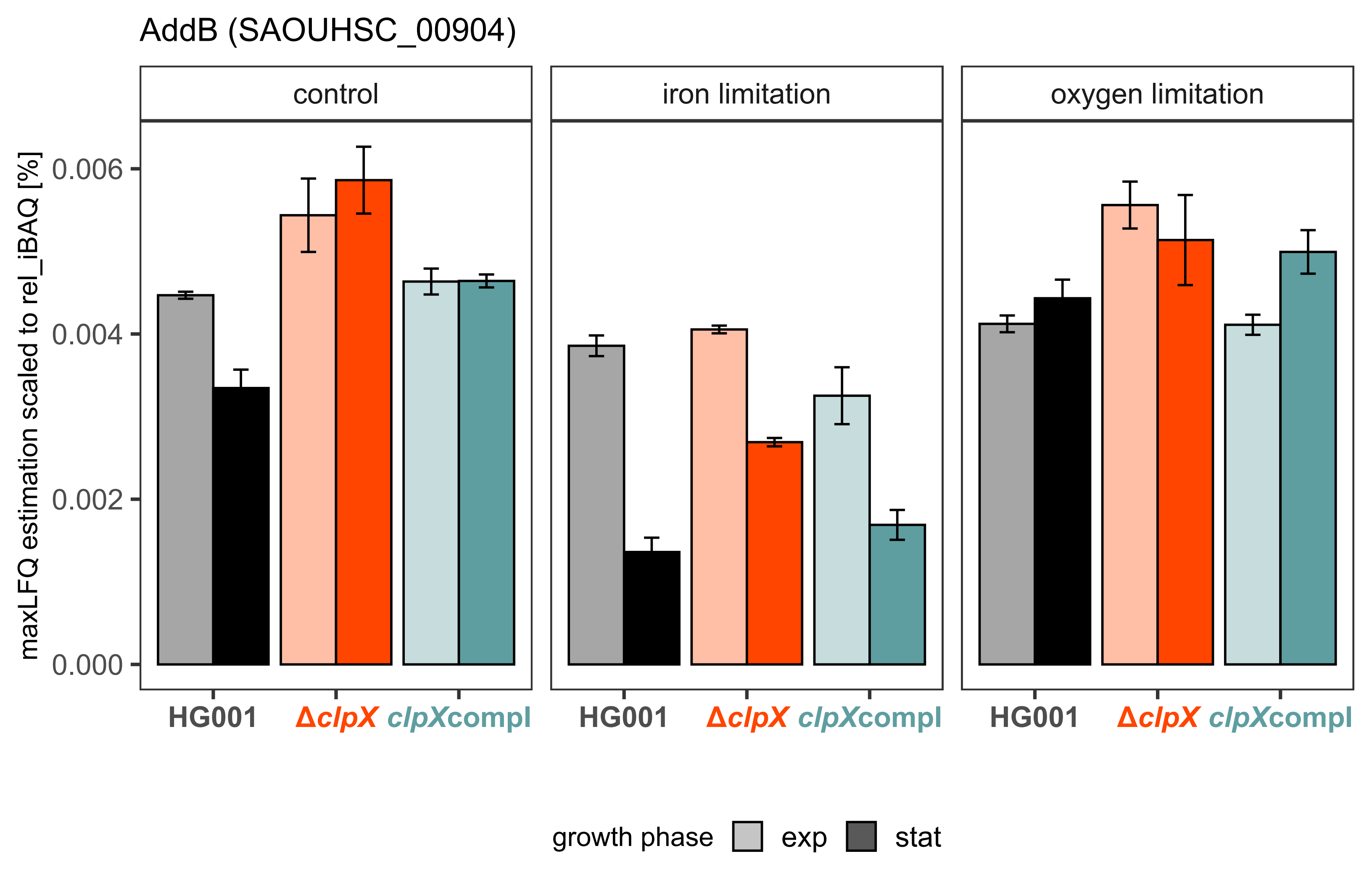
AddB (SAOUHSC_00904)
control
iron limitation
oxygen limitation
maxLFQ estimation scaled to rel_iBAQ [%]
0.006
0.004
0.002
0.000
HG001
ΔclpX
clpXcompl
growth phase
exp
stat

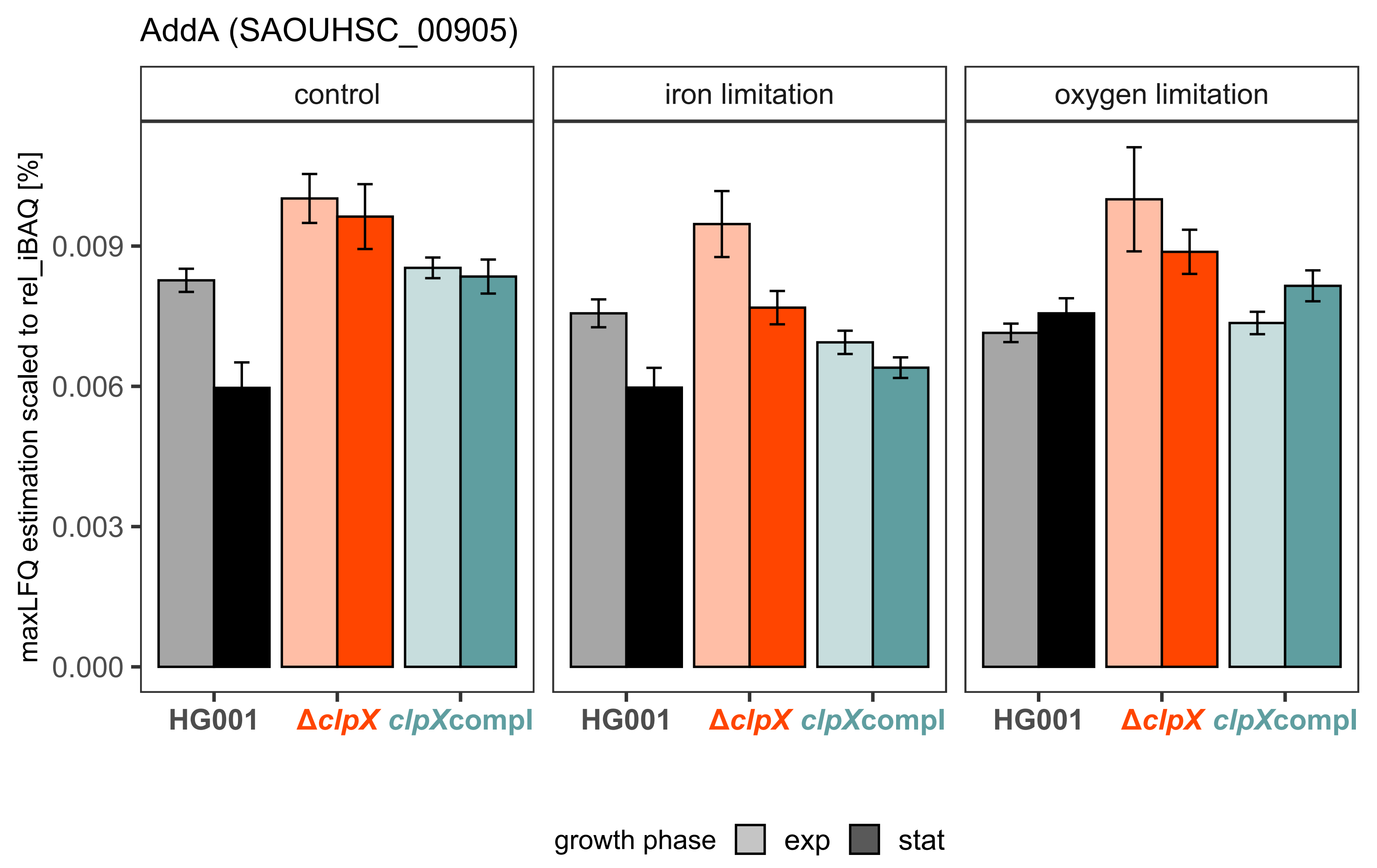
AddA (SAOUHSC_00905)
control
iron limitation
oxygen limitation
maxLFQ estimation scaled to rel_iBAQ [%]
0.009
0.006
0.003
0.000
HG001
ΔclpX
clpXcompl
growth phase
exp
stat

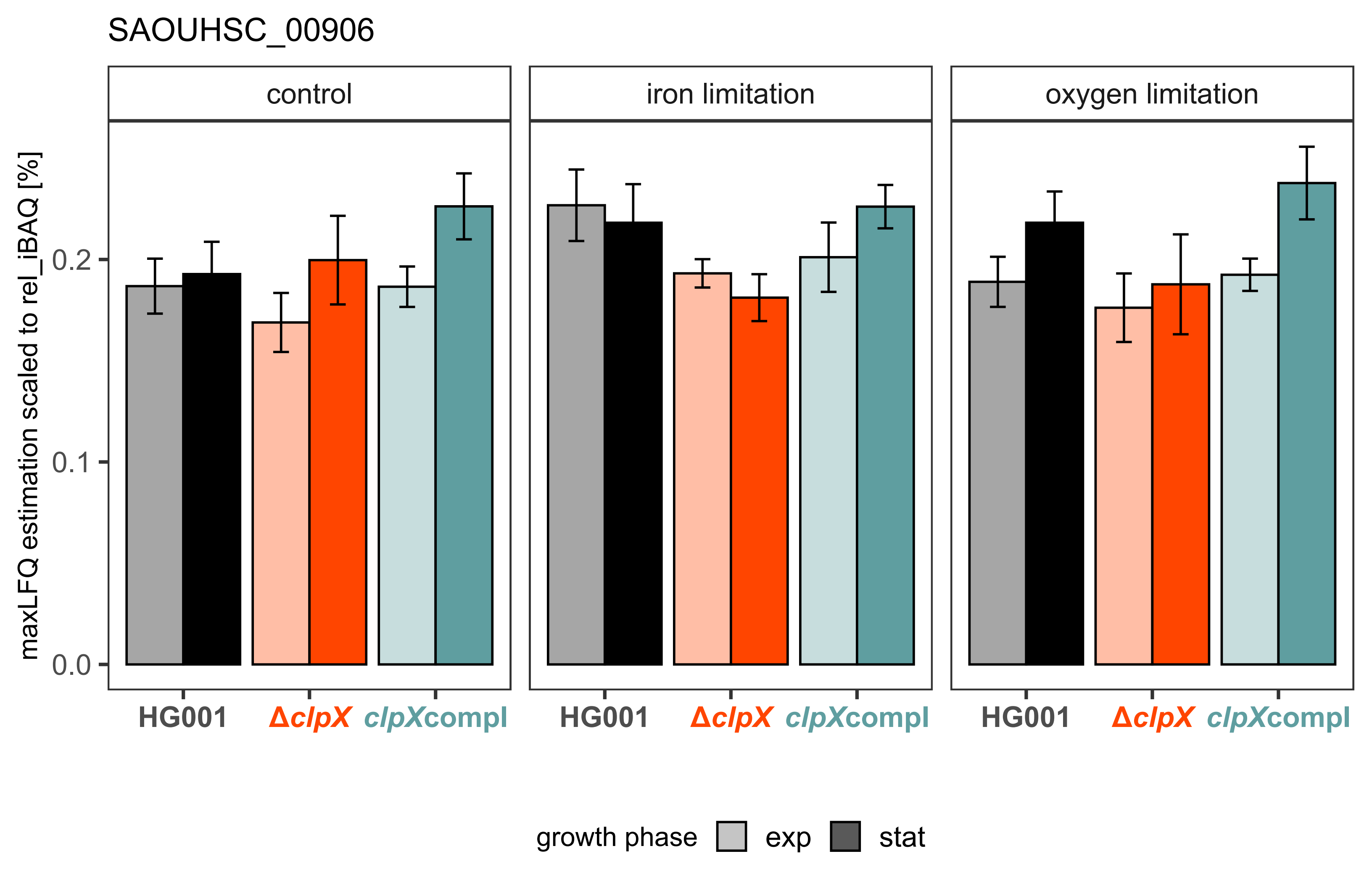
SAOUHSC_00906
control
iron limitation
oxygen limitation
maxLFQ estimation scaled to rel_iBAQ [%]
0.0
0.1
0.2
HG001
ΔclpX
clpXcompl
HG001
ΔclpX
clpXcompl
HG001
ΔclpX
clpXcompl
growth phase
exp
stat

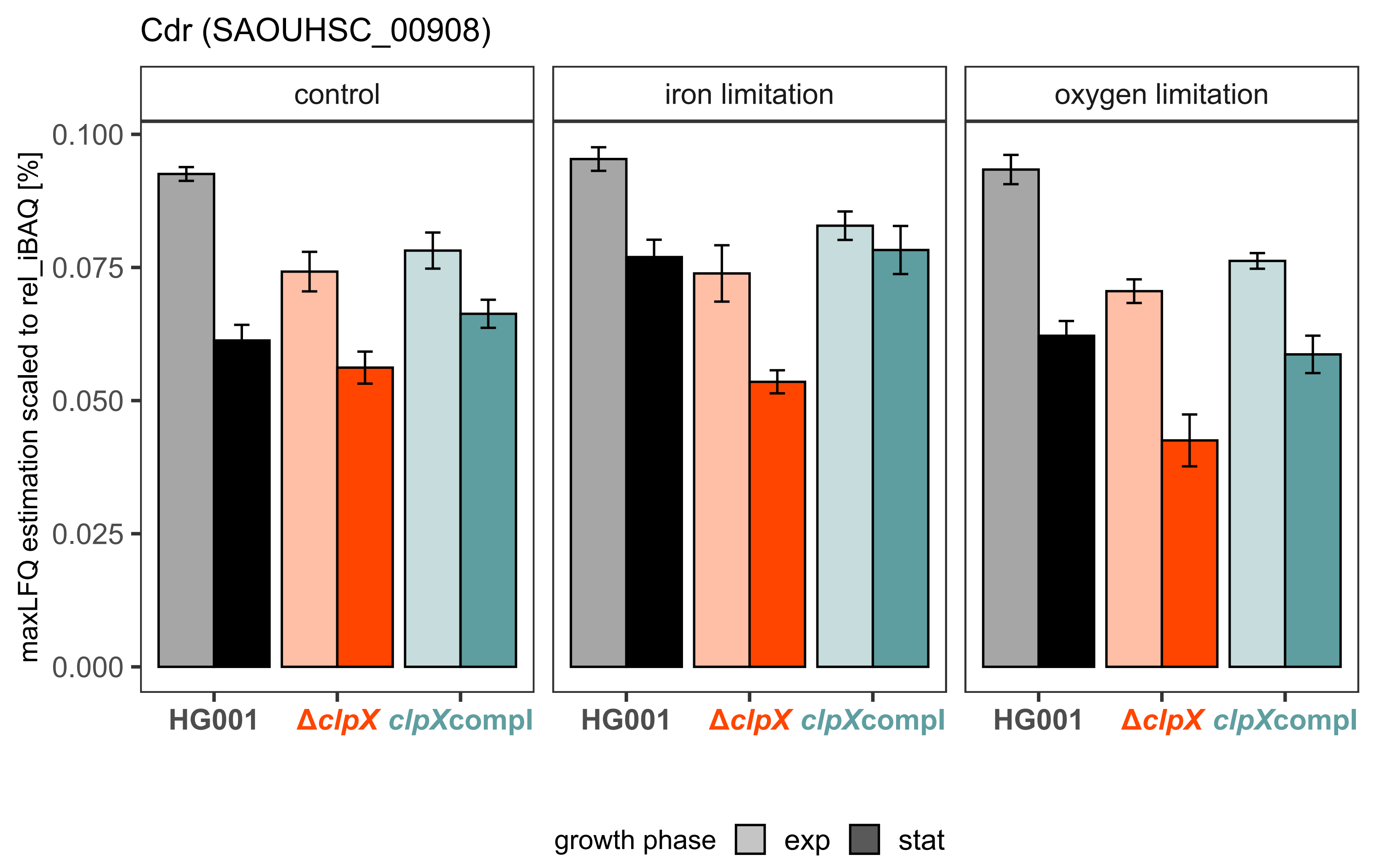

Cdr (SAOUHSC_00908)
control
iron limitation
oxygen limitation
maxLFQ estimation scaled to rel_iBAQ [%]
0.100
0.075
0.050
0.025
0.000
HG001
ΔclpX
clpXcompl
growth phase
exp
stat

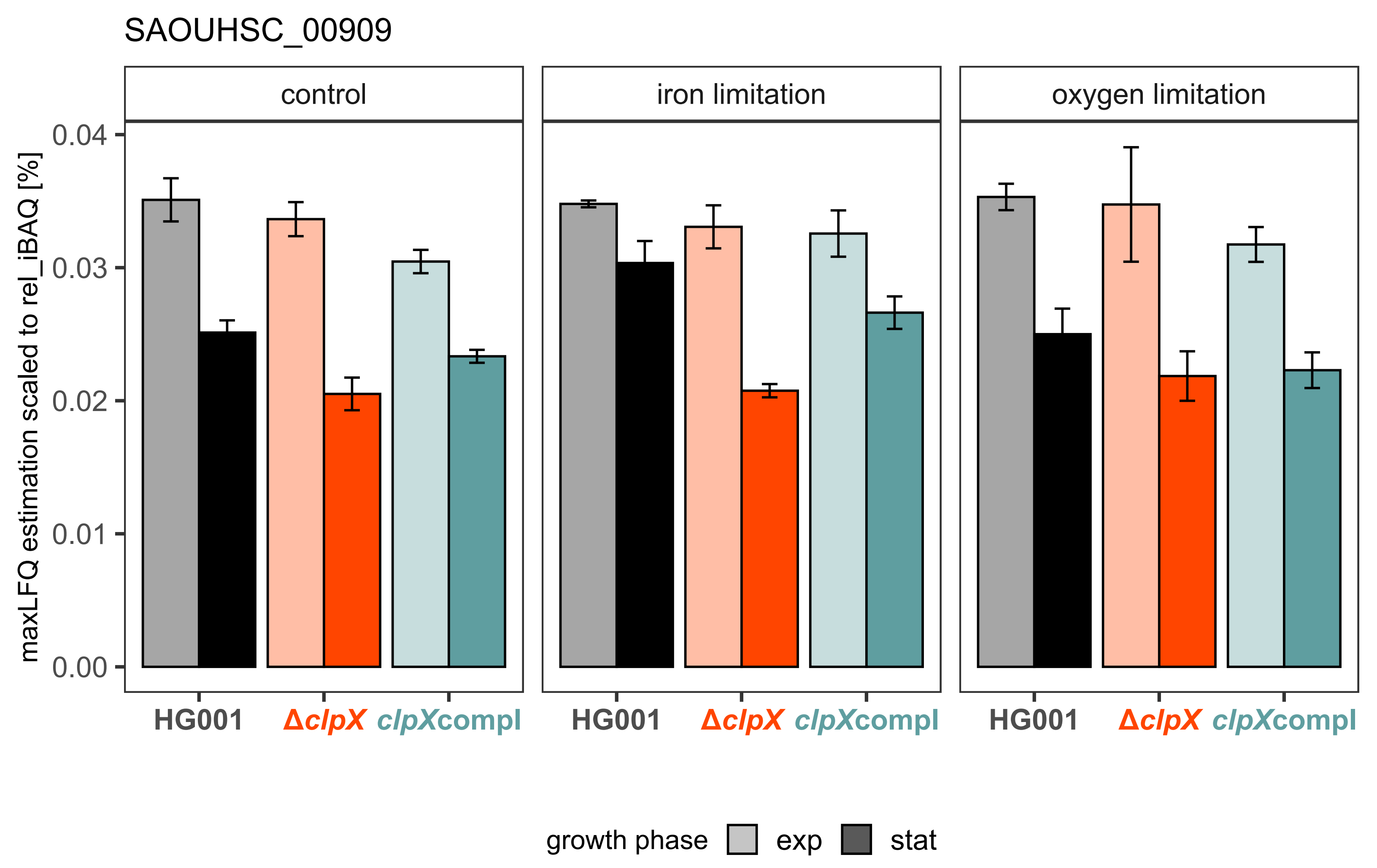
SAOUHSC_00909
control
iron limitation
oxygen limitation
maxLFQ estimation scaled to rel_iBAQ [%]
0.04
0.03
0.02
0.01
0.00
HG001
ΔclpX
clpXcompl
growth phase
exp
stat

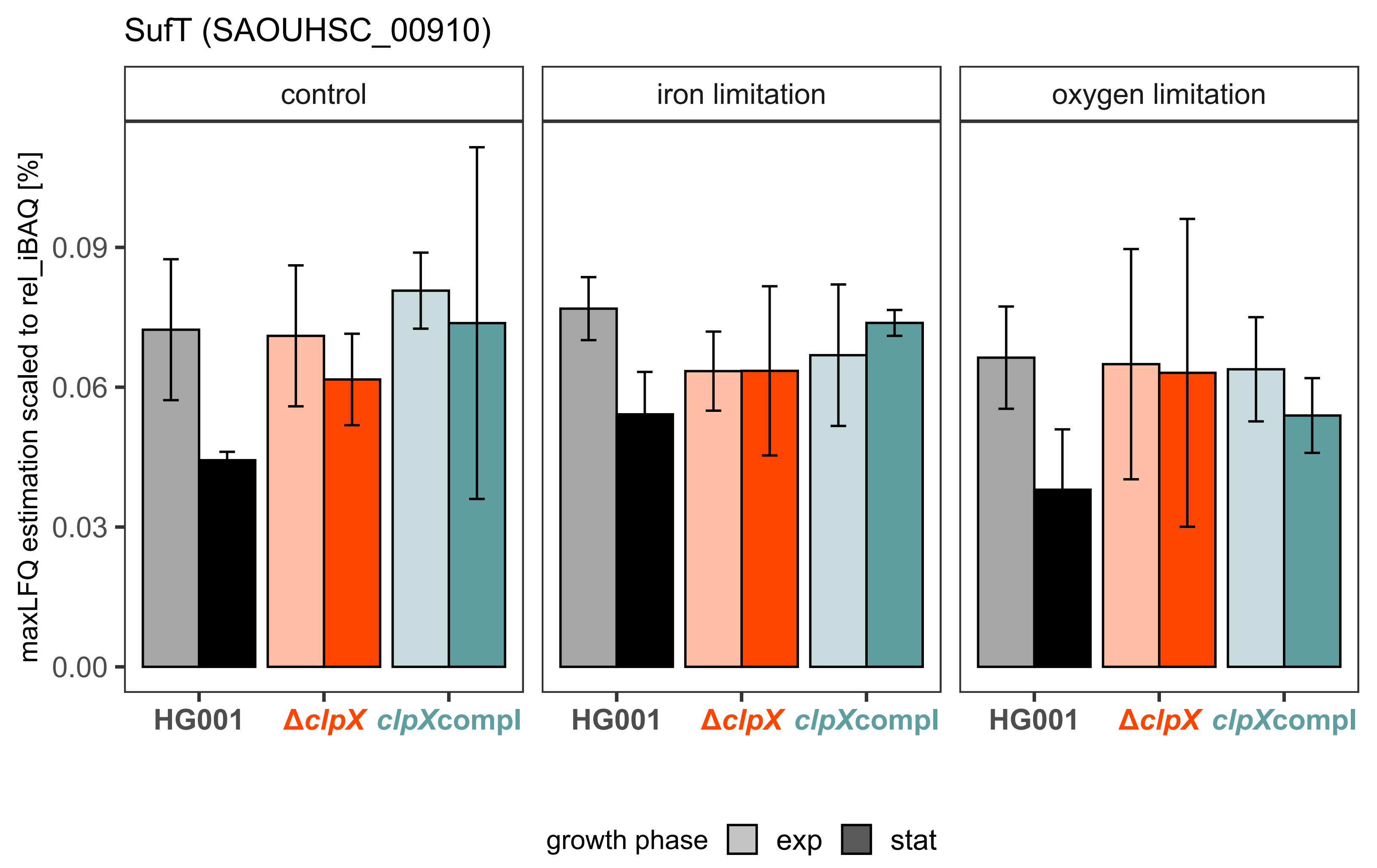

SufT (SAOUHSC_00910)
control
iron limitation
oxygen limitation
maxLFQ estimation scaled to rel_iBAQ [%]
0.00
0.03
0.06
0.09
HG001
ΔclpX
clpXcompl
growth phase
exp
stat

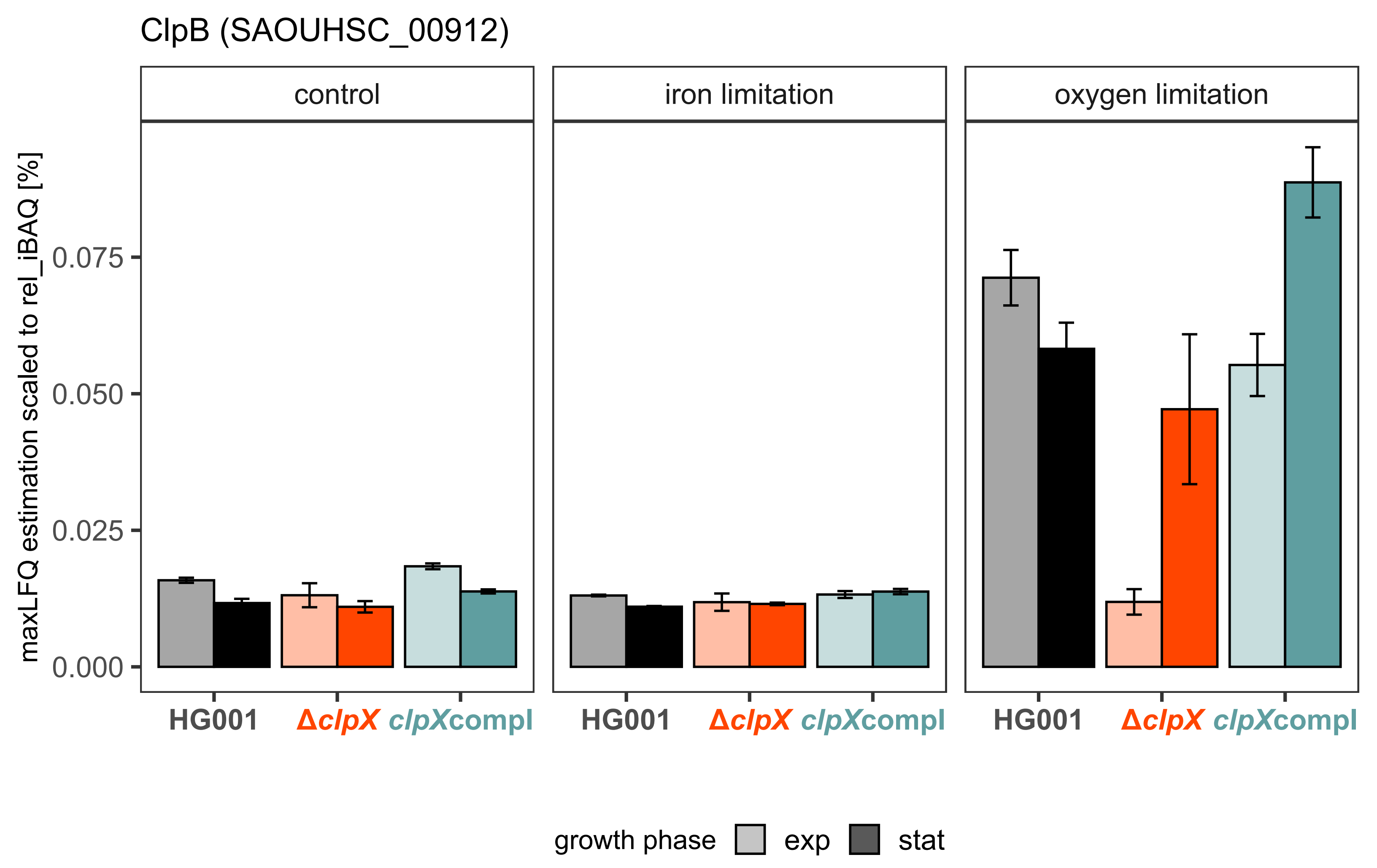

ClpB (SAOUHSC_00912)
control
iron limitation
oxygen limitation
maxLFQ estimation scaled to rel_iBAQ [%]
0.000
0.025
0.050
0.075
HG001
ΔclpX
clpXcompl
growth phase
exp
stat

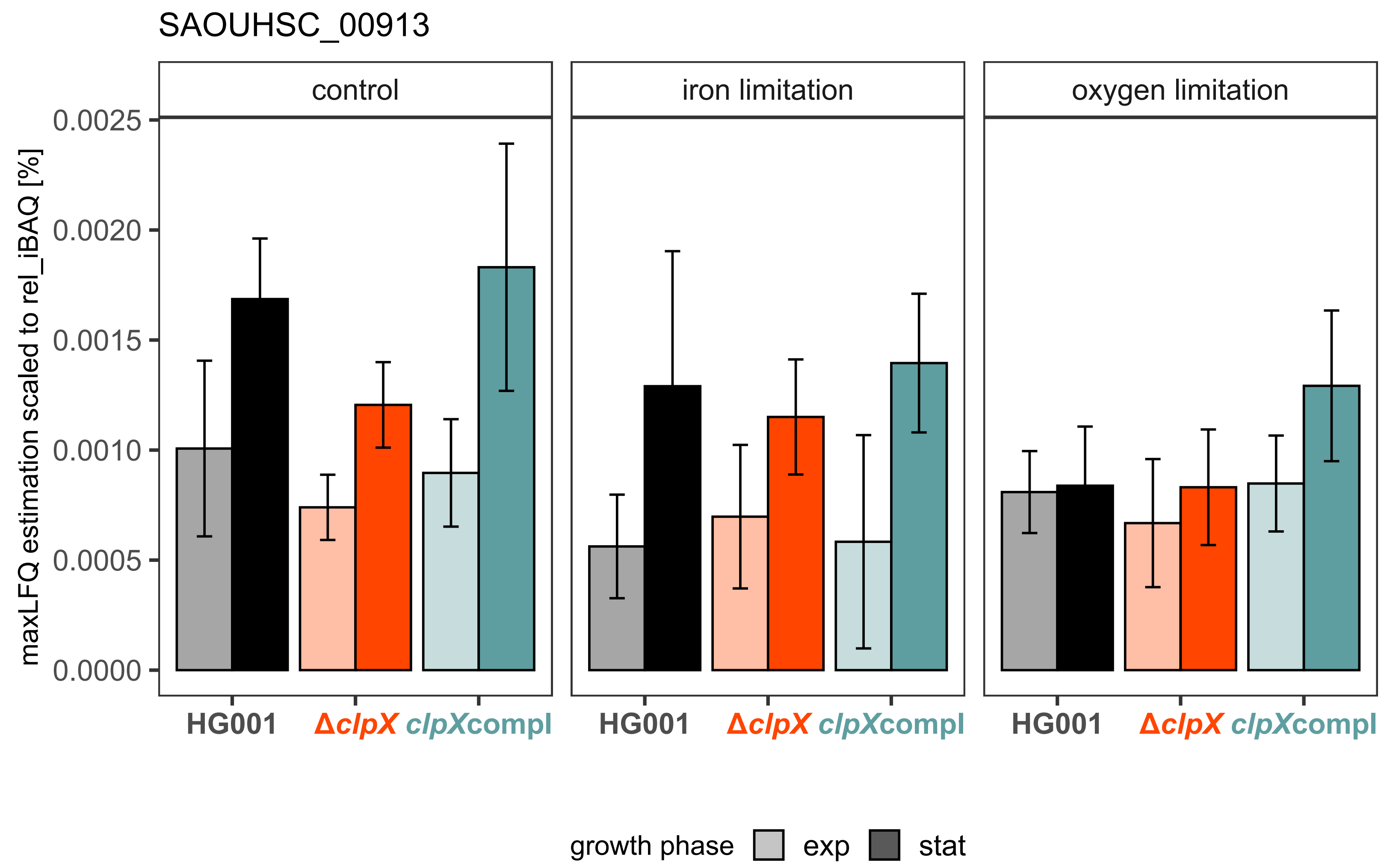

SAOUHSC_00913
control
iron limitation
oxygen limitation
maxLFQ estimation scaled to rel_iBAQ [%]
0.0025
0.0020
0.0015
0.0010
0.0005
0.0000
HG001
ΔclpX
clpXcompl
growth phase
exp
stat

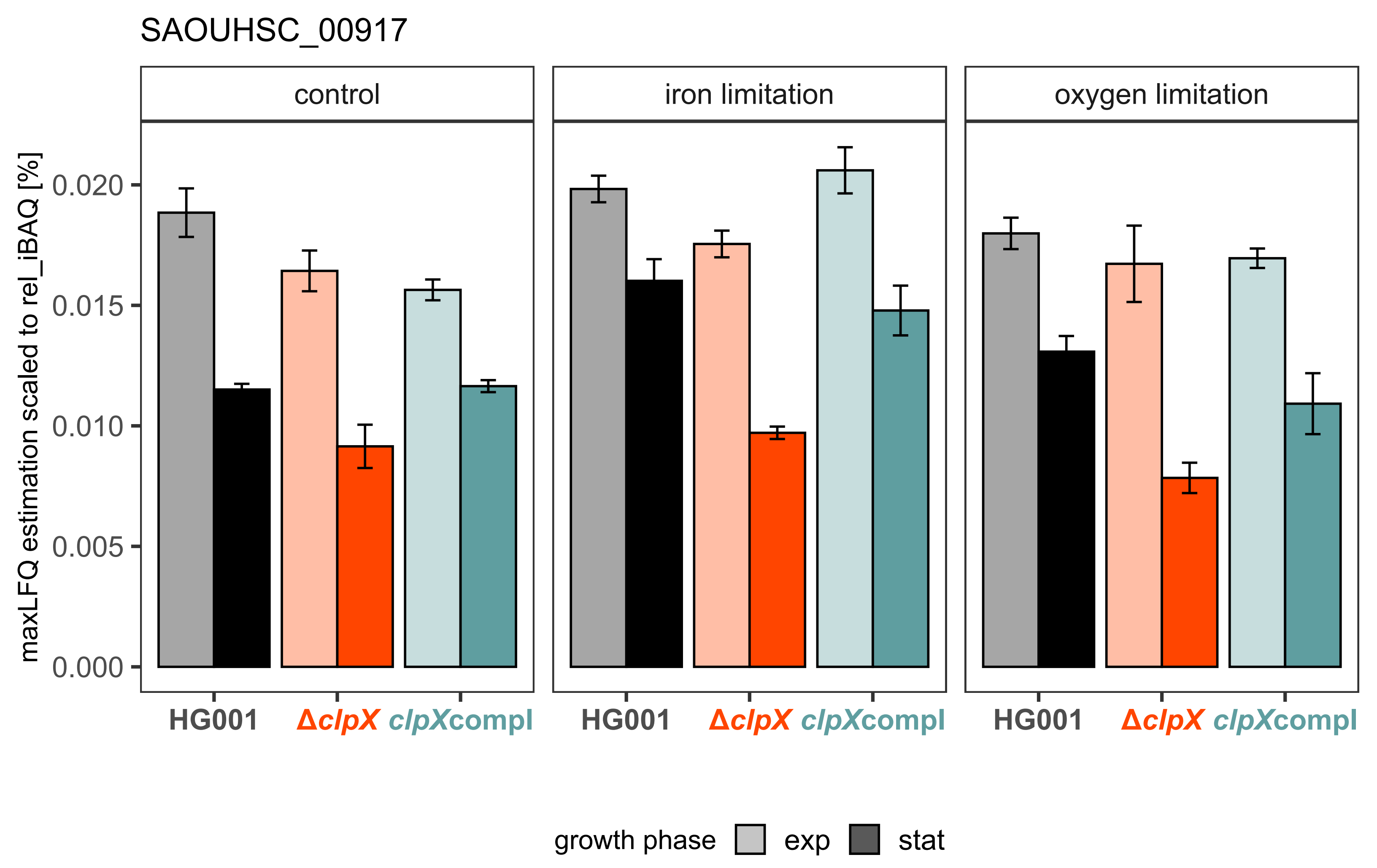
SAOUHSC_00917
control
iron limitation
oxygen limitation
maxLFQ estimation scaled to rel_iBAQ [%]
0.020
0.015
0.010
0.005
0.000
HG001
ΔclpX
clpXcompl
growth phase
exp
stat

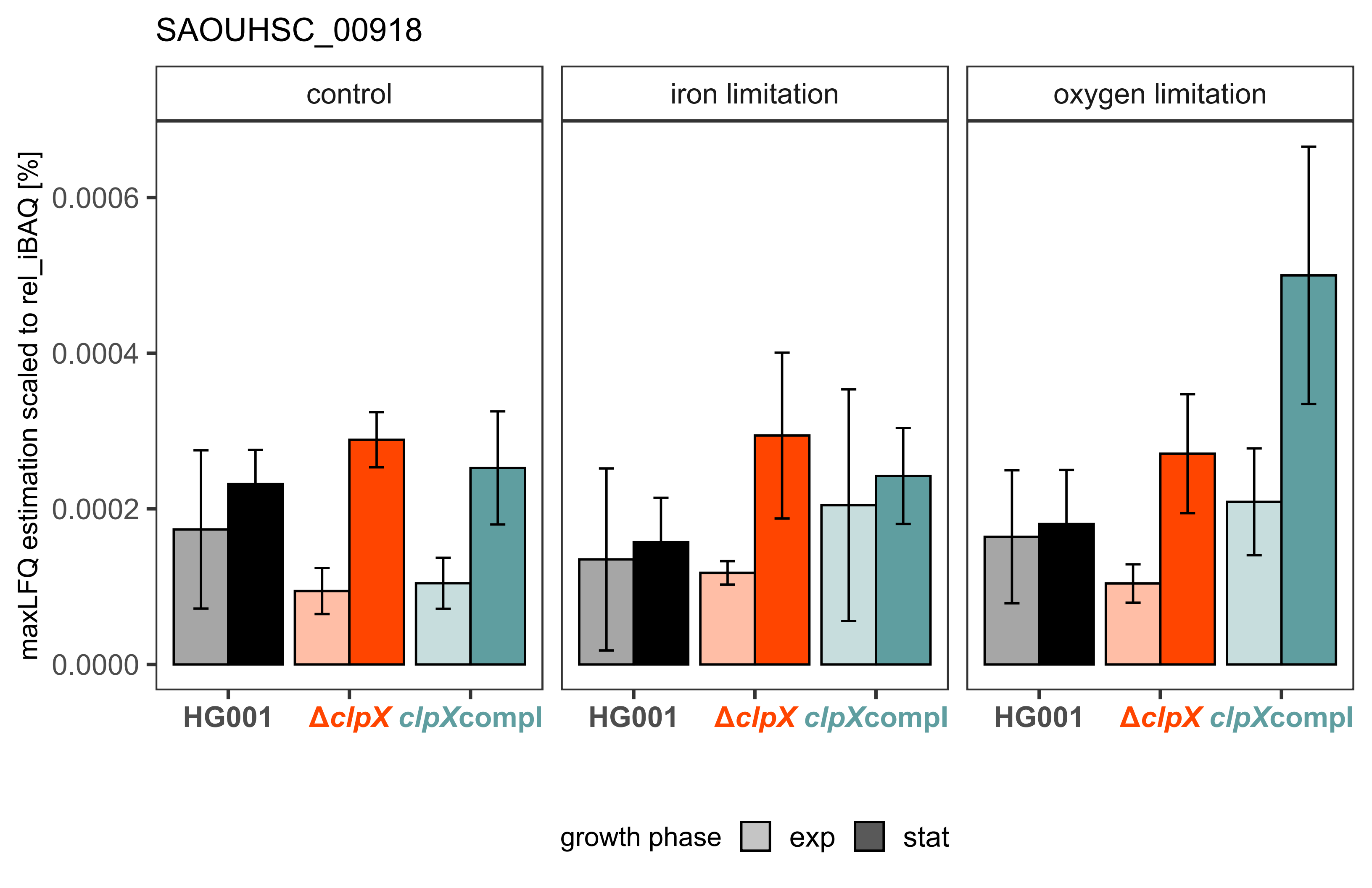
SAOUHSC_00918
control
iron limitation
oxygen limitation
maxLFQ estimation scaled to rel_iBAQ [%]
0.0006
0.0004
0.0002
0.0000
HG001
ΔclpX
clpXcompl
growth phase
exp
stat

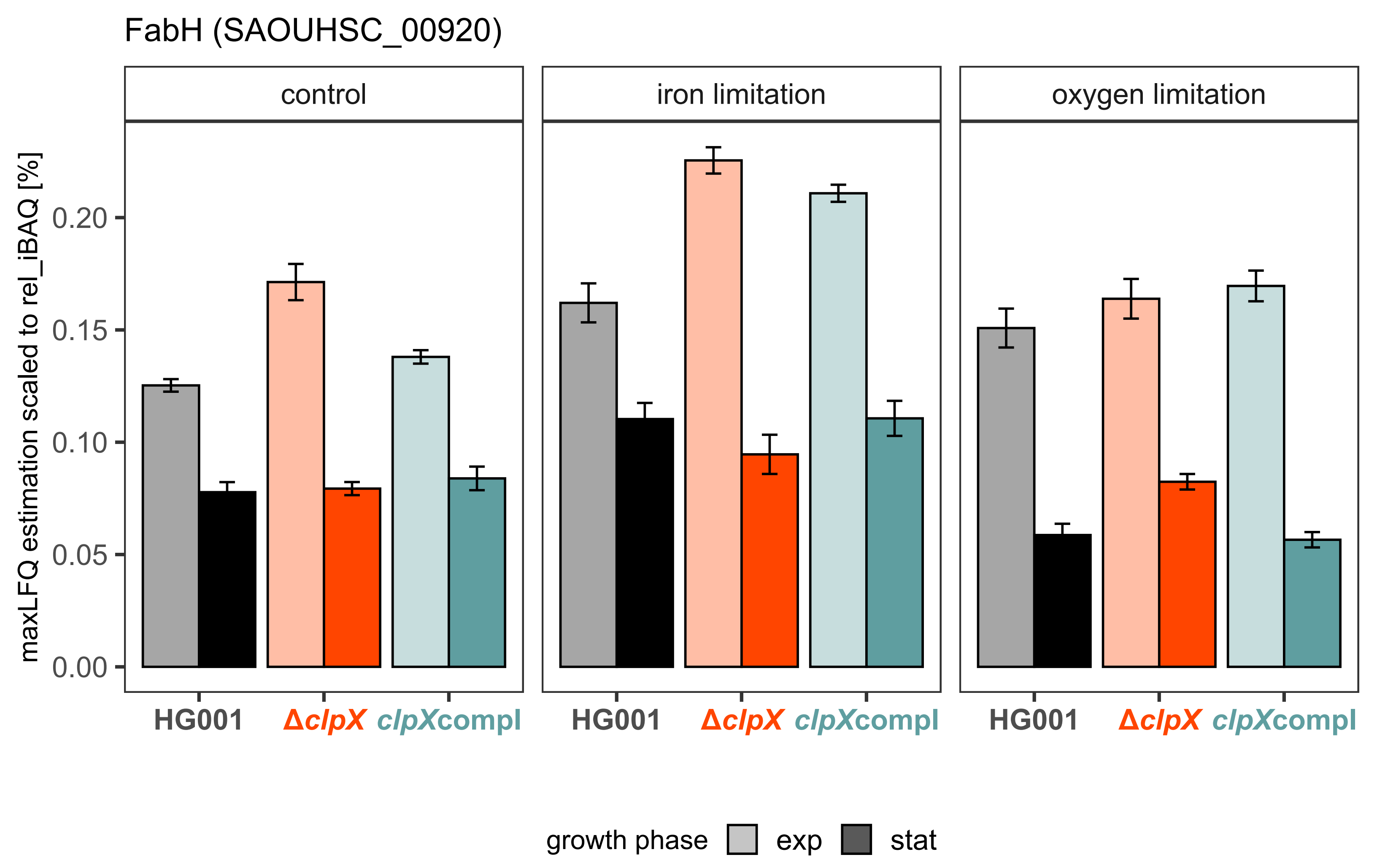
FabH (SAOUHSC_00920)
control
iron limitation
oxygen limitation
maxLFQ estimation scaled to rel_iBAQ [%]
0.00
0.05
0.10
0.15
0.20
HG001
ΔclpX
clpXcompl
growth phase
exp
stat

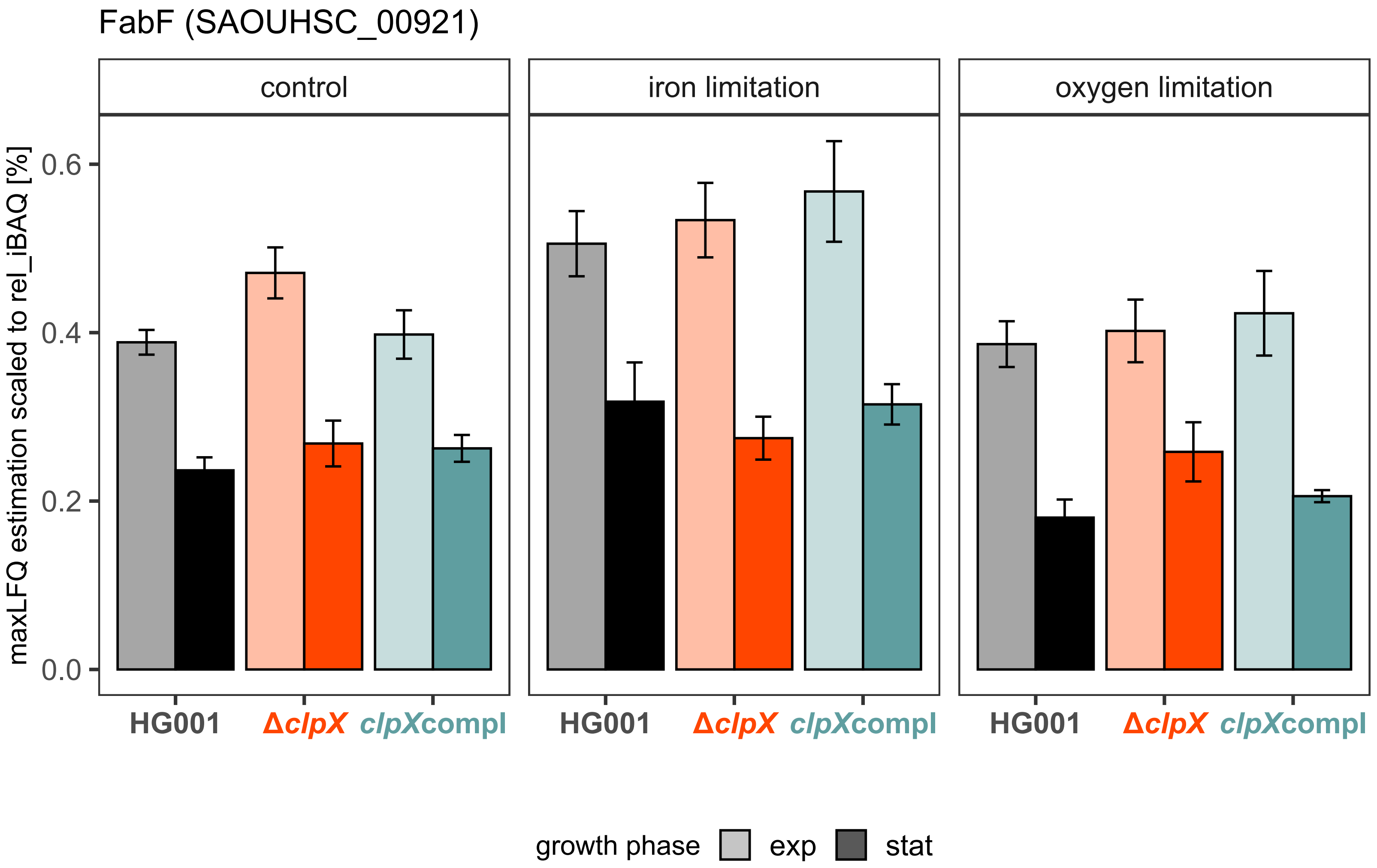
FabF (SAOUHSC_00921)
control
iron limitation
oxygen limitation
maxLFQ estimation scaled to rel_iBAQ [%]
0.0
0.2
0.4
0.6
HG001
ΔclpX
clpXcompl
growth phase
exp
stat

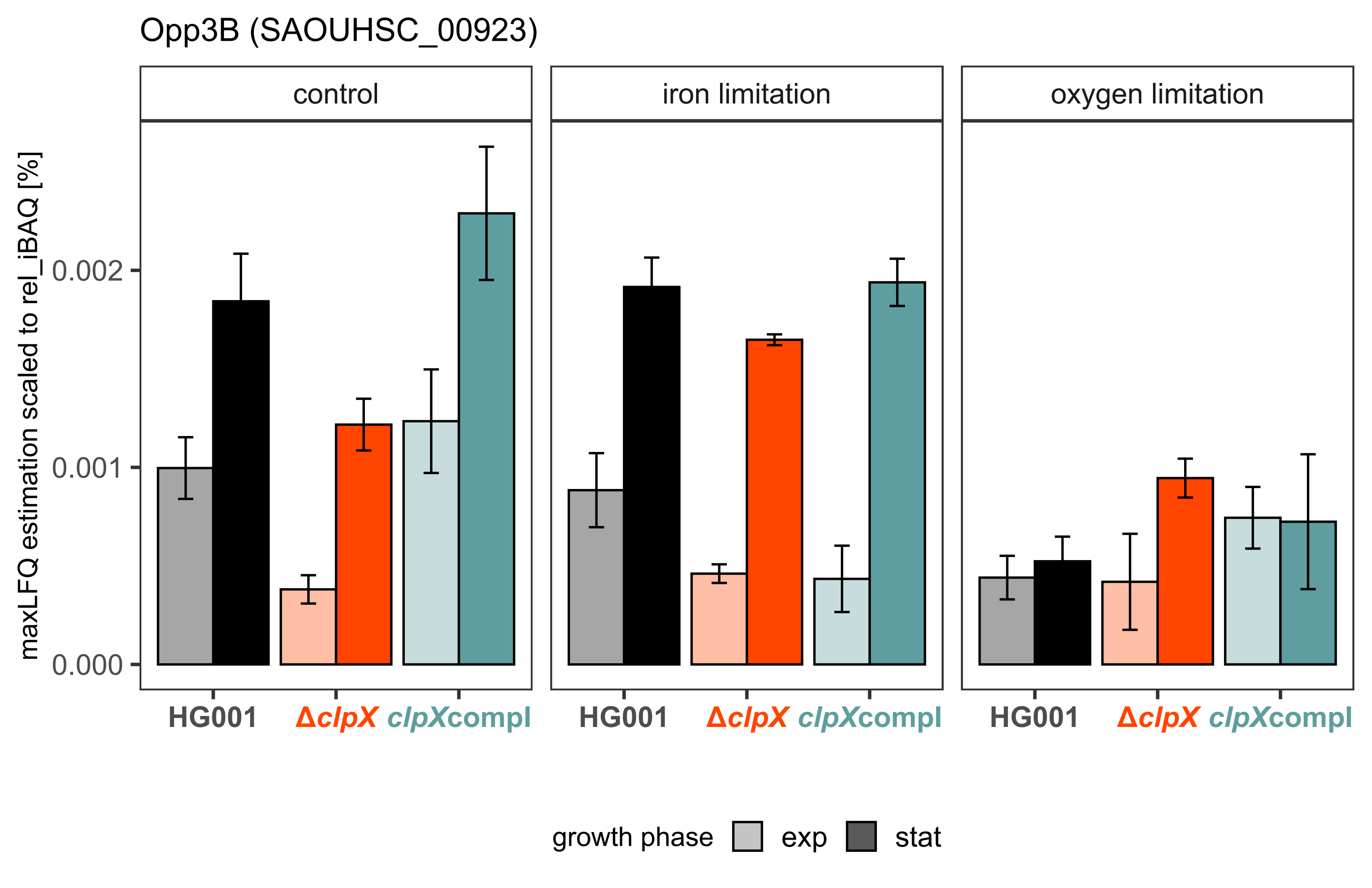
Opp3B (SAOUHSC_00923)
control
iron limitation
oxygen limitation
maxLFQ estimation scaled to rel_iBAQ [%]
0.002
0.001
0.000
HG001
ΔclpX
clpXcompl
growth phase
exp
stat

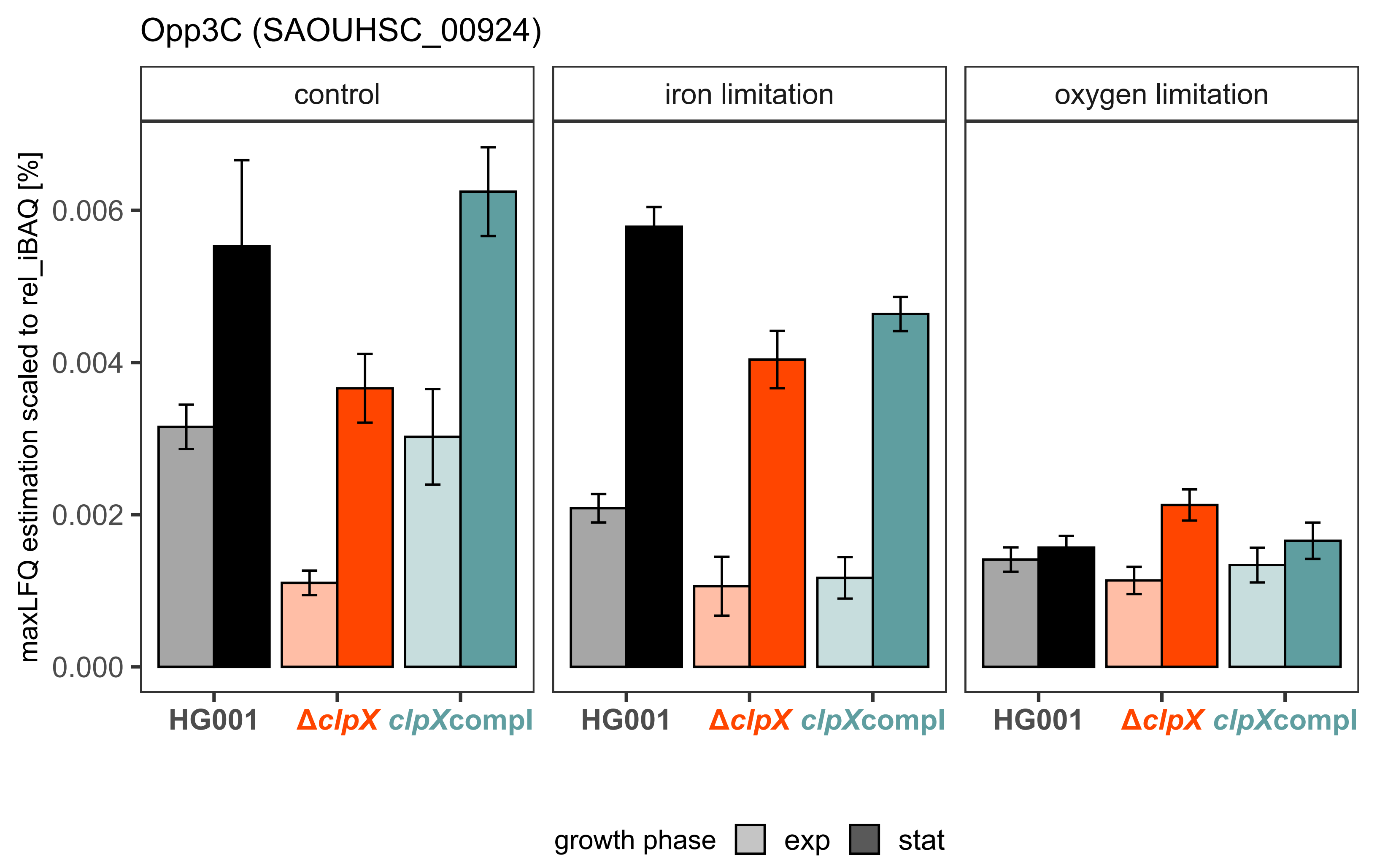

Opp3C (SAOUHSC_00924)
control
iron limitation
oxygen limitation
maxLFQ estimation scaled to rel_iBAQ [%]
0.006
0.004
0.002
0.000
HG001
ΔclpX
clpXcompl
growth phase
exp
stat

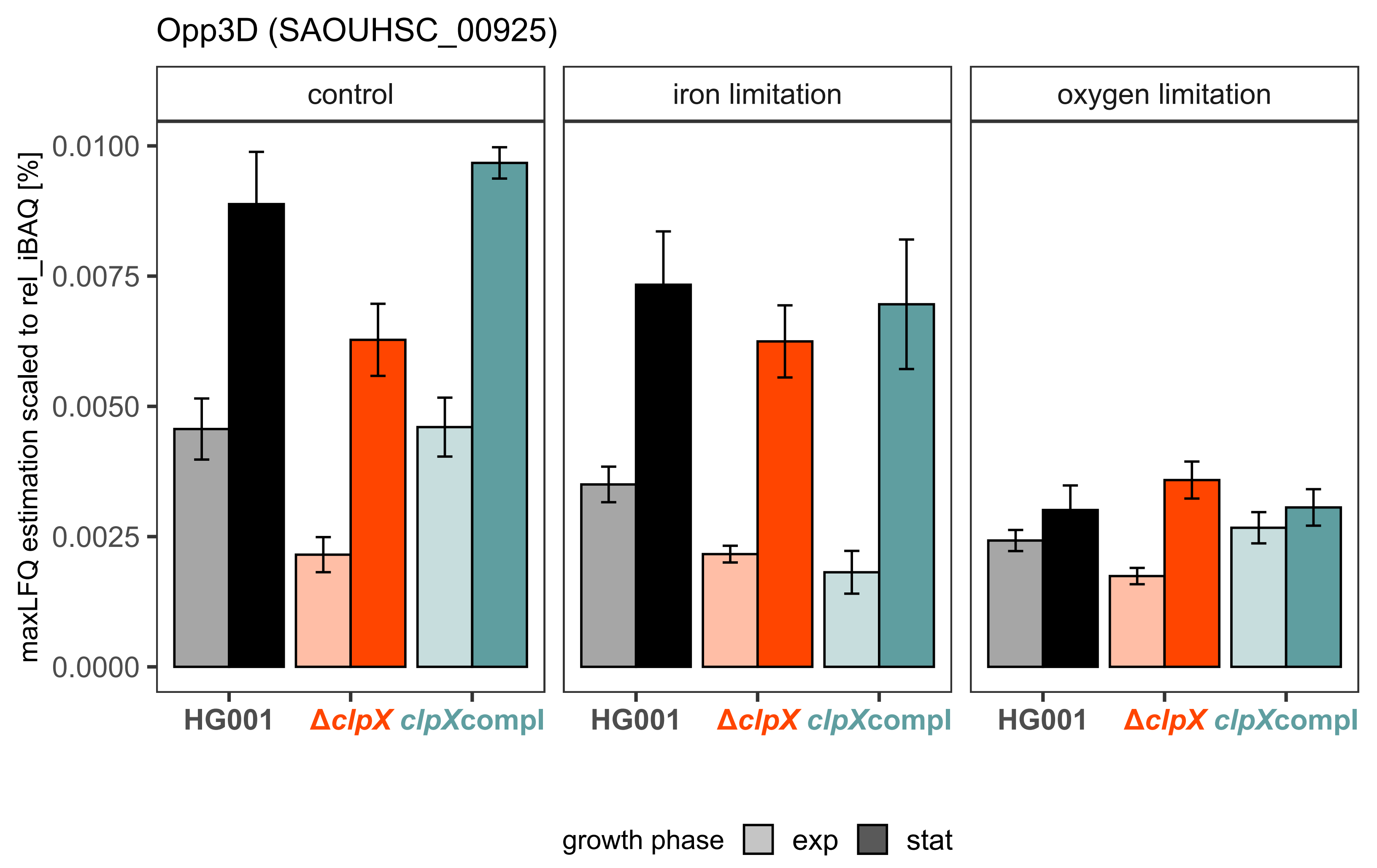

Opp3D (SAOUHSC_00925)
control
iron limitation
oxygen limitation
maxLFQ estimation scaled to rel_iBAQ [%]
0.0100
0.0075
0.0050
0.0025
0.0000
HG001
ΔclpX
clpXcompl
growth phase
exp
stat

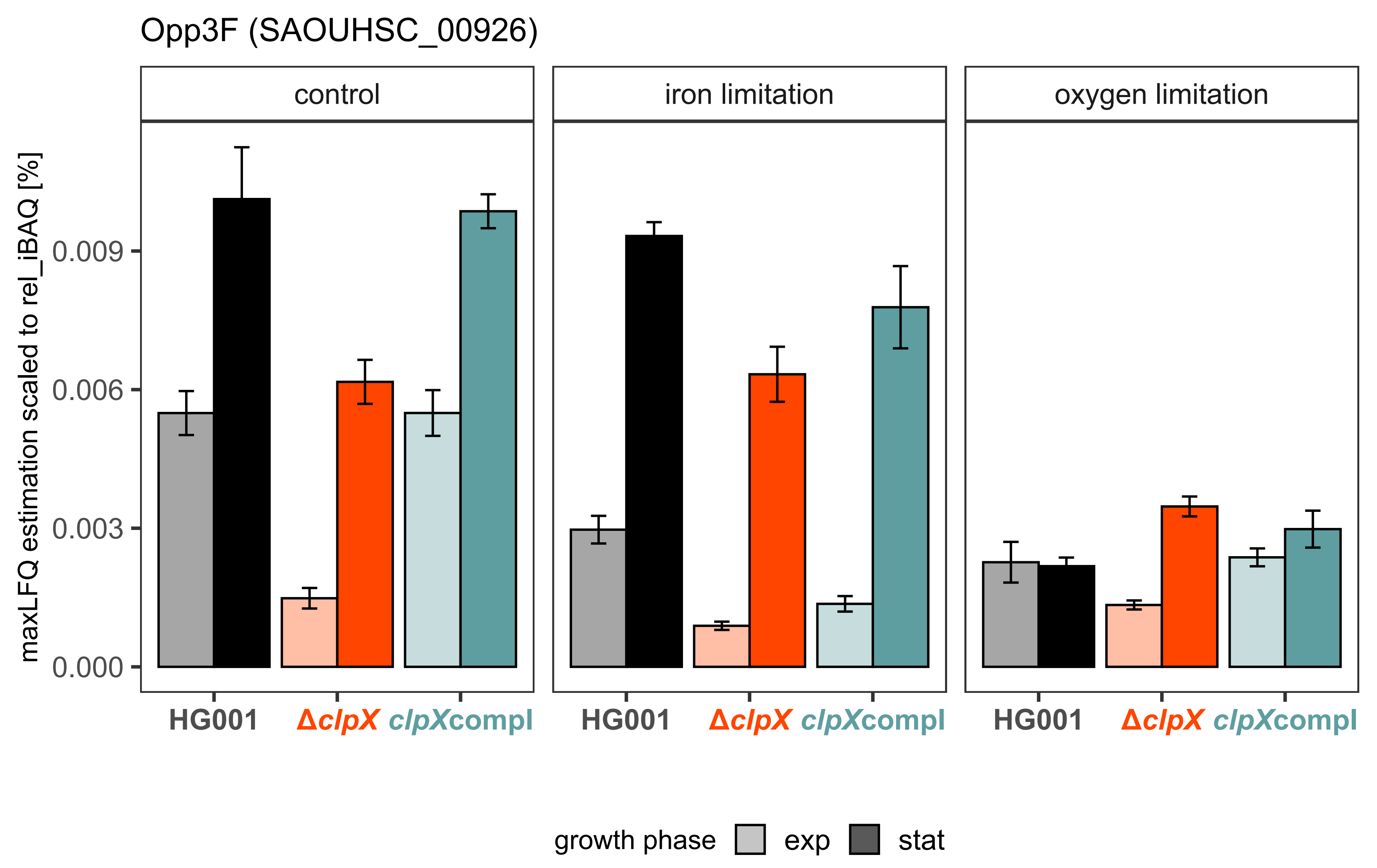
Opp3F (SAOUHSC_00926)
control
iron limitation
oxygen limitation
maxLFQ estimation scaled to rel_iBAQ [%]
0.009
0.006
0.003
0.000
HG001
ΔclpX
clpXcompl
growth phase
exp
stat

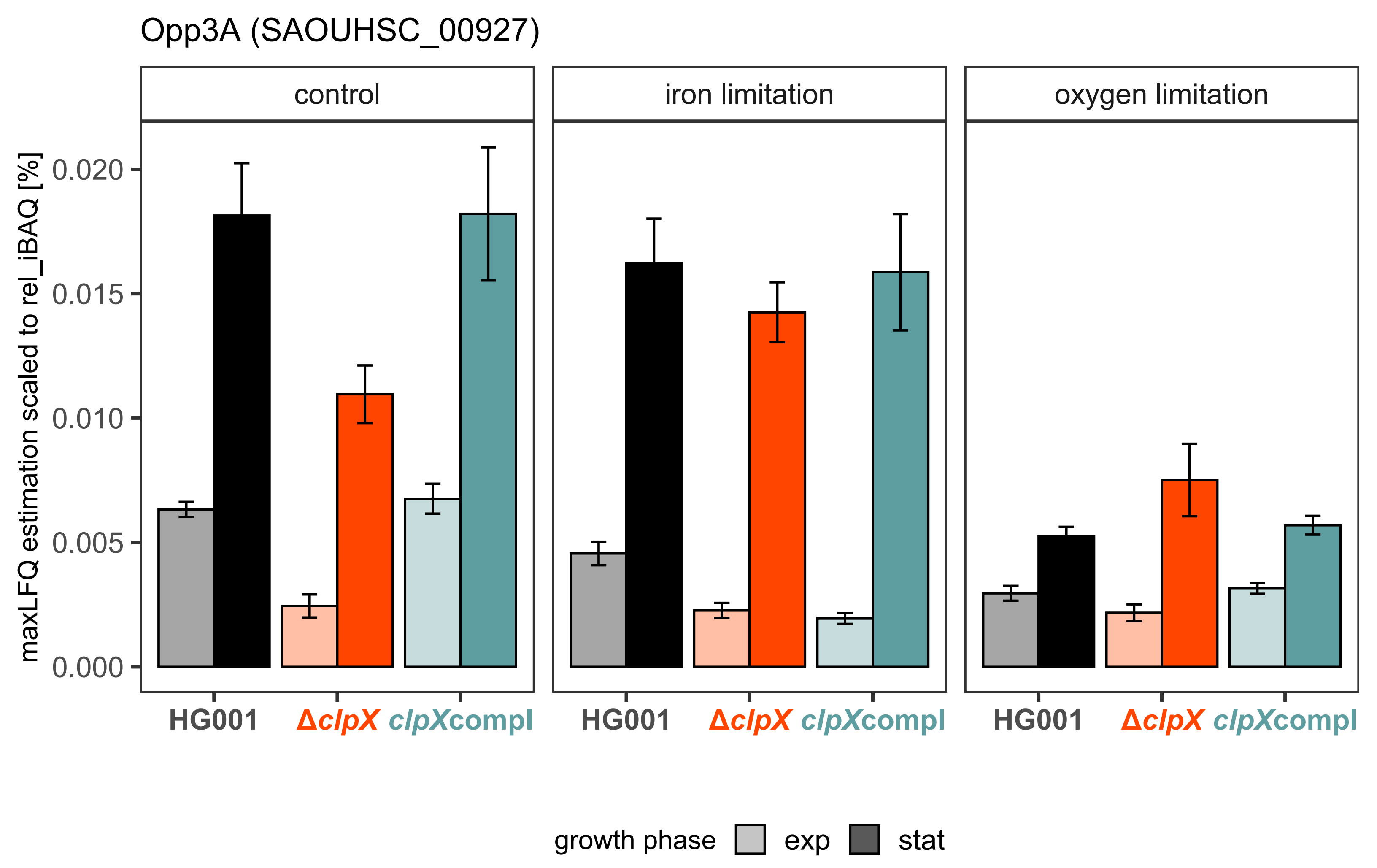
Opp3A (SAOUHSC_00927)
control
iron limitation
oxygen limitation
maxLFQ estimation scaled to rel_iBAQ [%]
0.020
0.015
0.010
0.005
0.000
HG001
ΔclpX
clpXcompl
growth phase
exp
stat

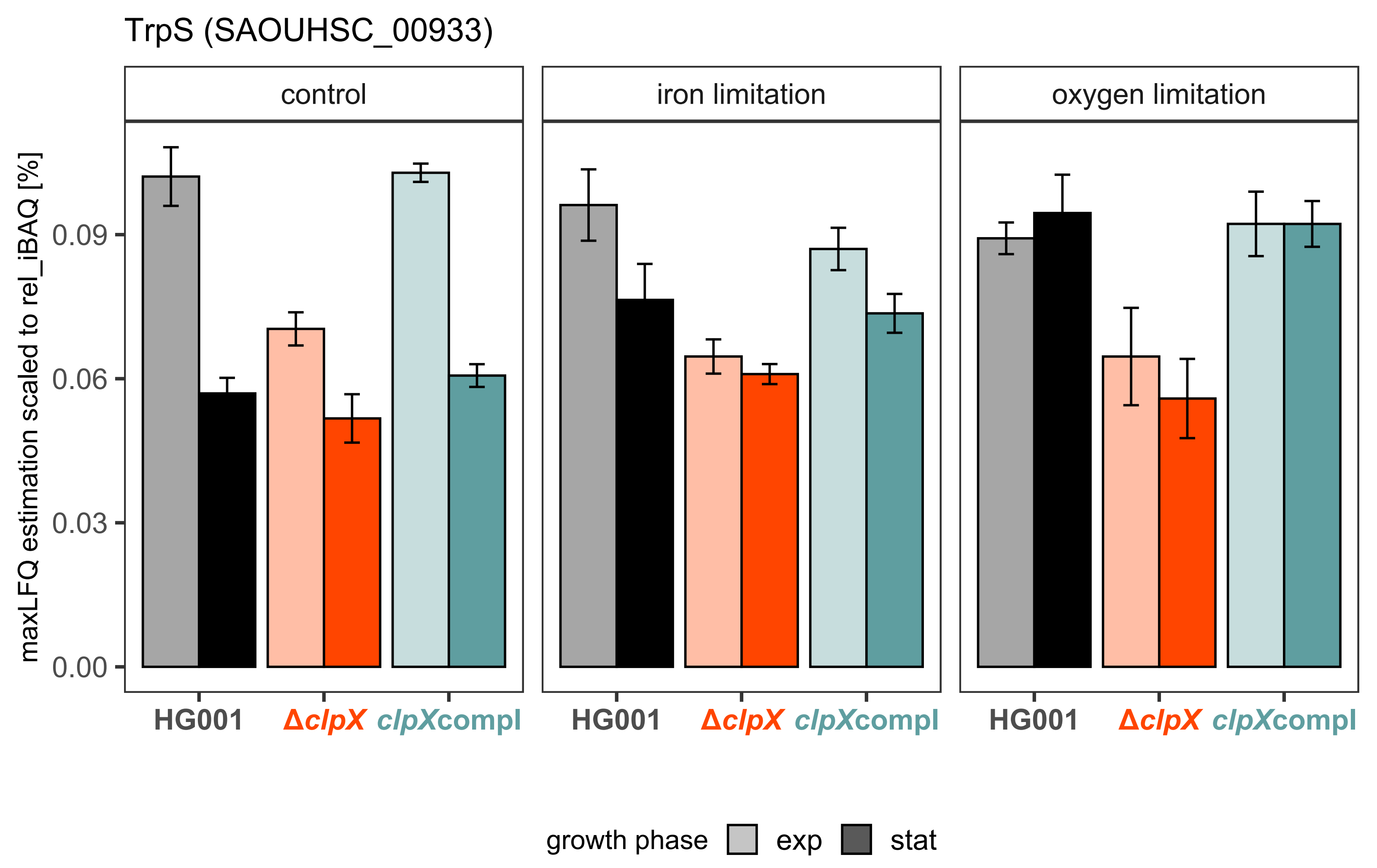
TrpS (SAOUHSC_00933)
control
iron limitation
oxygen limitation
maxLFQ estimation scaled to rel_iBAQ [%]
0.09
0.06
0.03
0.00
HG001
ΔclpX
clpXcompl
growth phase
exp
stat

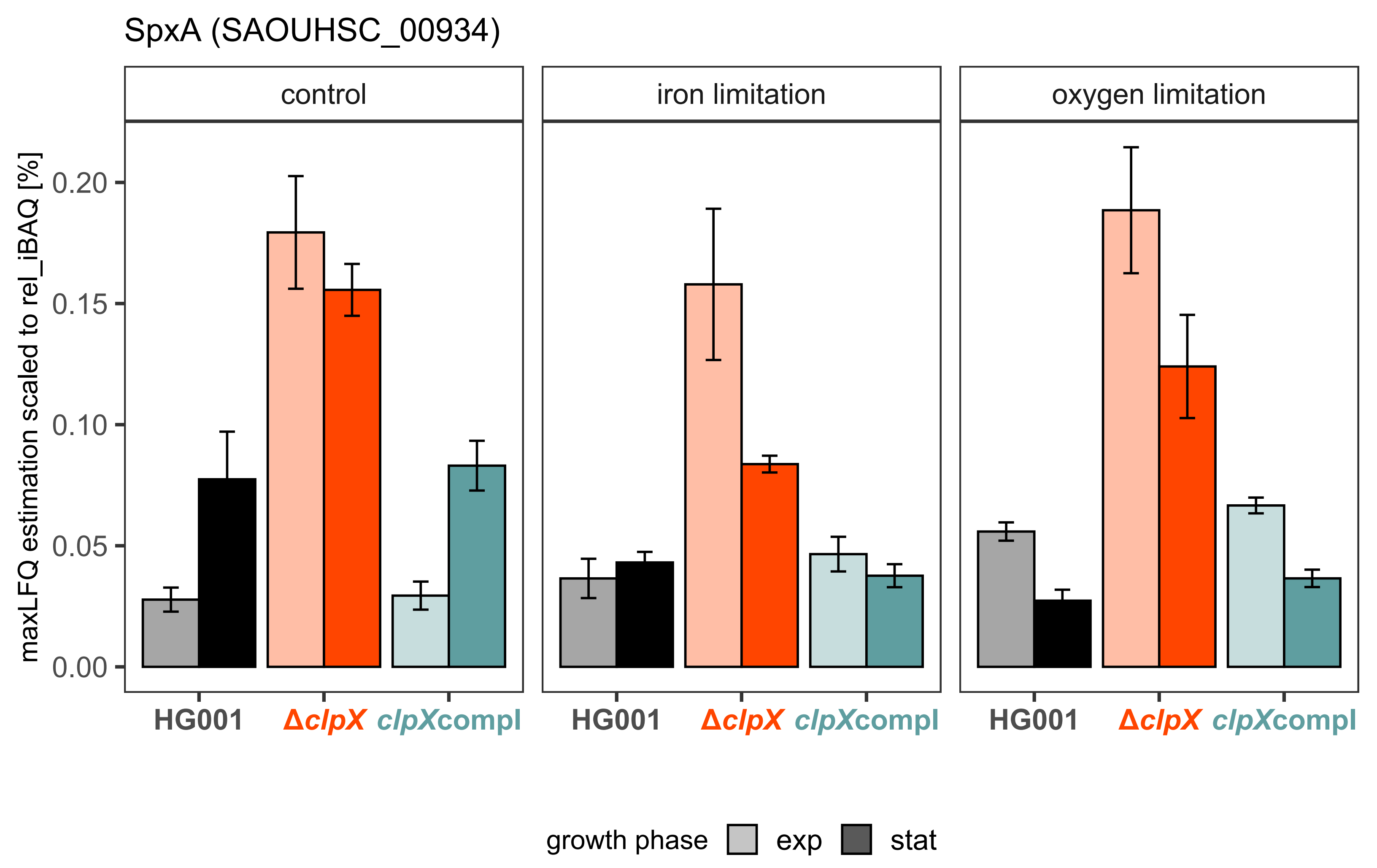

SpxA (SAOUHSC_00934)
control
iron limitation
oxygen limitation
maxLFQ estimation scaled to rel_iBAQ [%]
0.00
0.05
0.10
0.15
0.20
HG001
ΔclpX
clpXcompl
growth phase
exp
stat

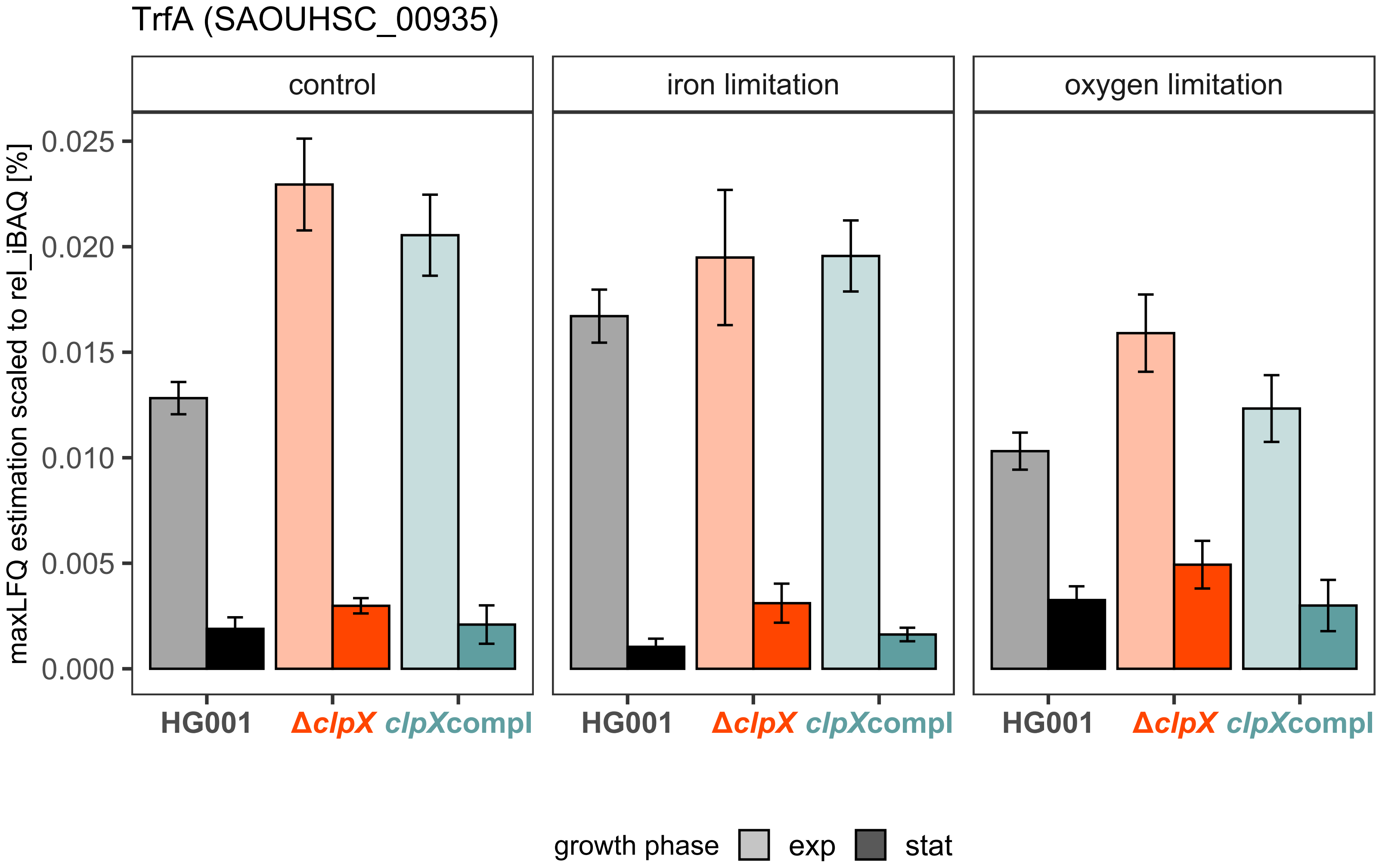
TrfA (SAOUHSC_00935)
control
iron limitation
oxygen limitation
maxLFQ estimation scaled to rel_iBAQ [%]
0.025
0.020
0.015
0.010
0.005
0.000
HG001
ΔclpX
clpXcompl
growth phase
exp
stat

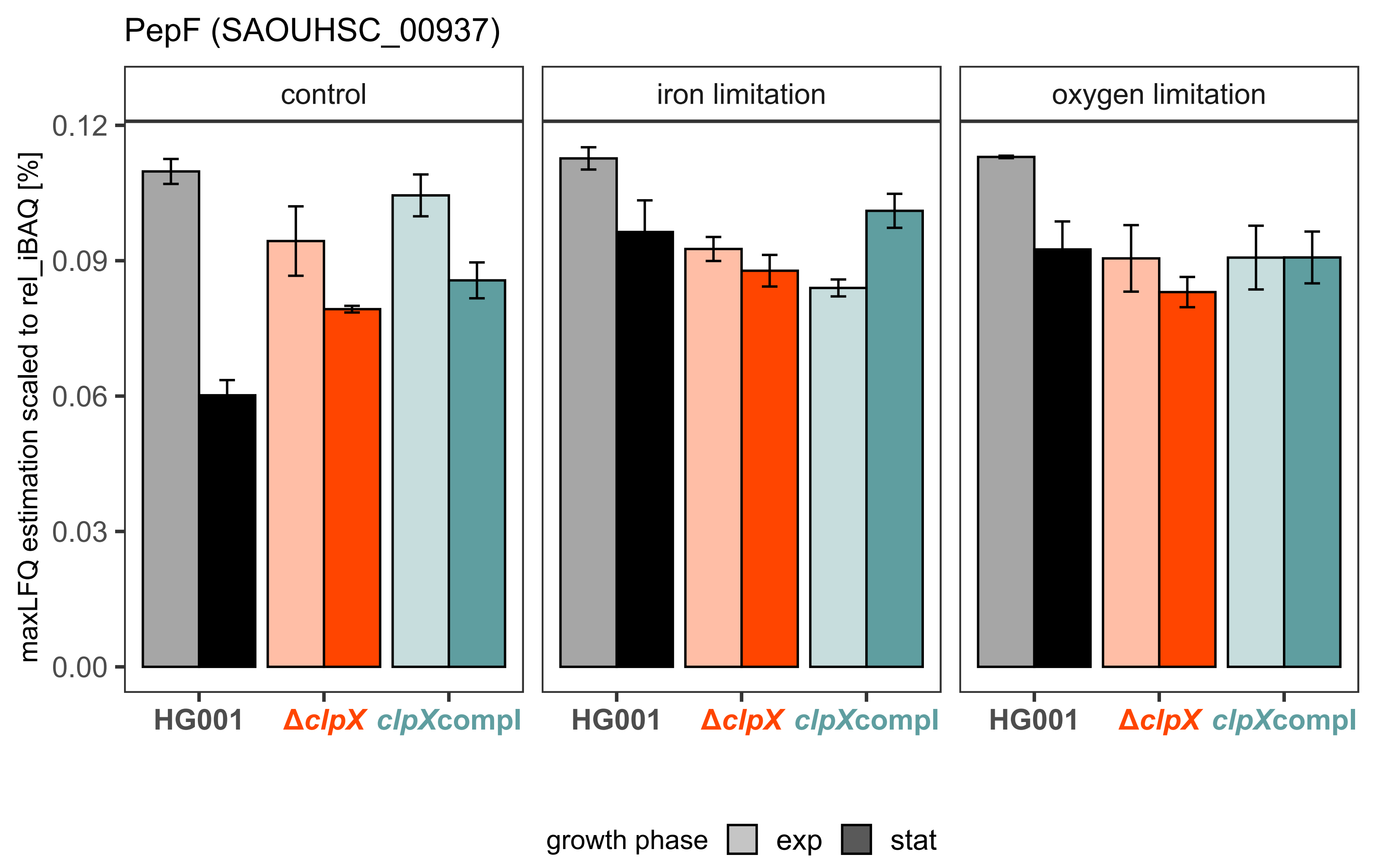
PepF (SAOUHSC_00937)
control
iron limitation
oxygen limitation
maxLFQ estimation scaled to rel_iBAQ [%]
0.12
0.09
0.06
0.03
0.00
HG001
ΔclpX
clpXcompl
growth phase
exp
stat

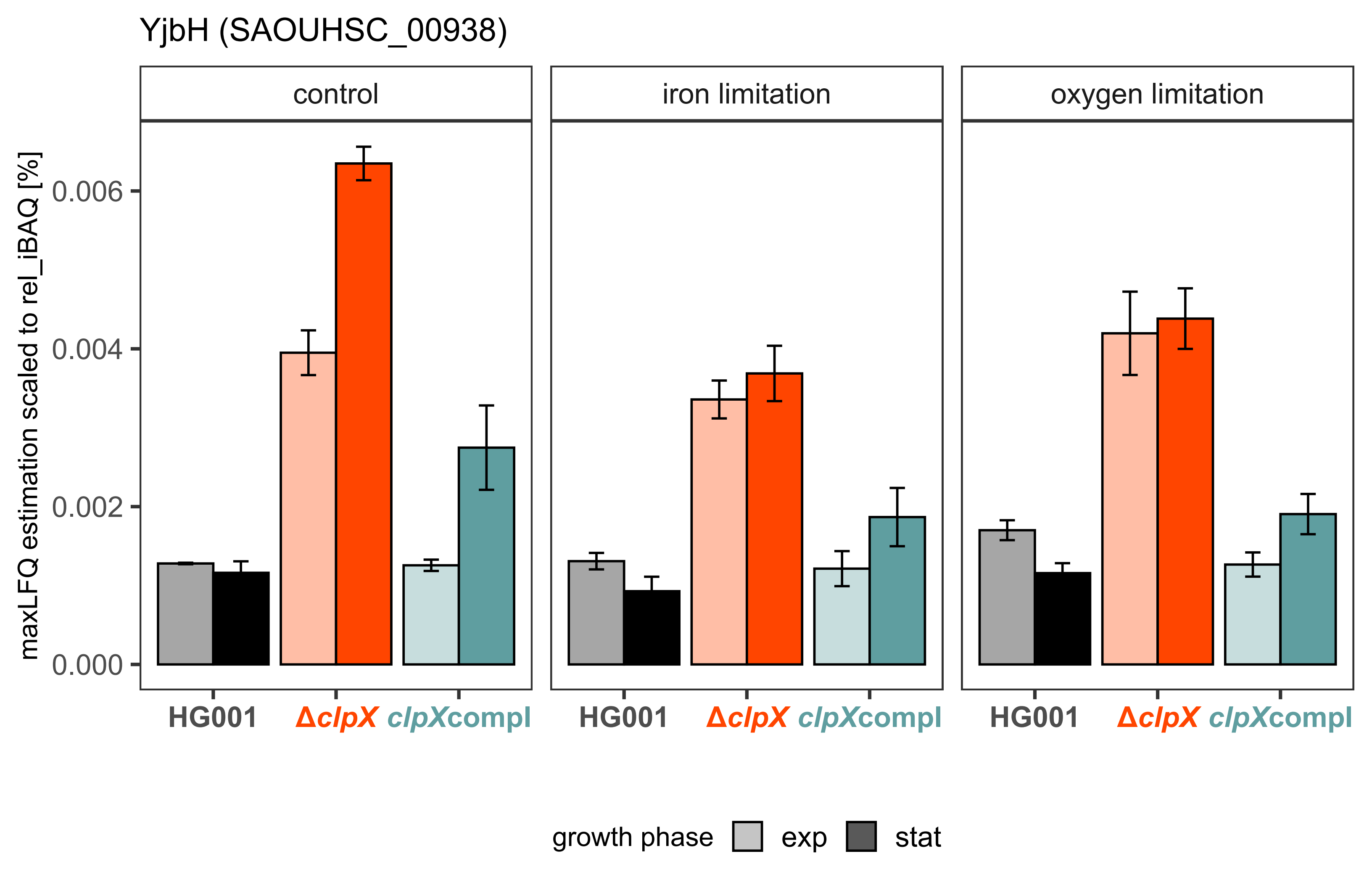

YjbH (SAOUHSC_00938)
control
iron limitation
oxygen limitation
maxLFQ estimation scaled to rel_iBAQ [%]
0.006
0.004
0.002
0.000
HG001
ΔclpX
clpXcompl
growth phase
exp
stat

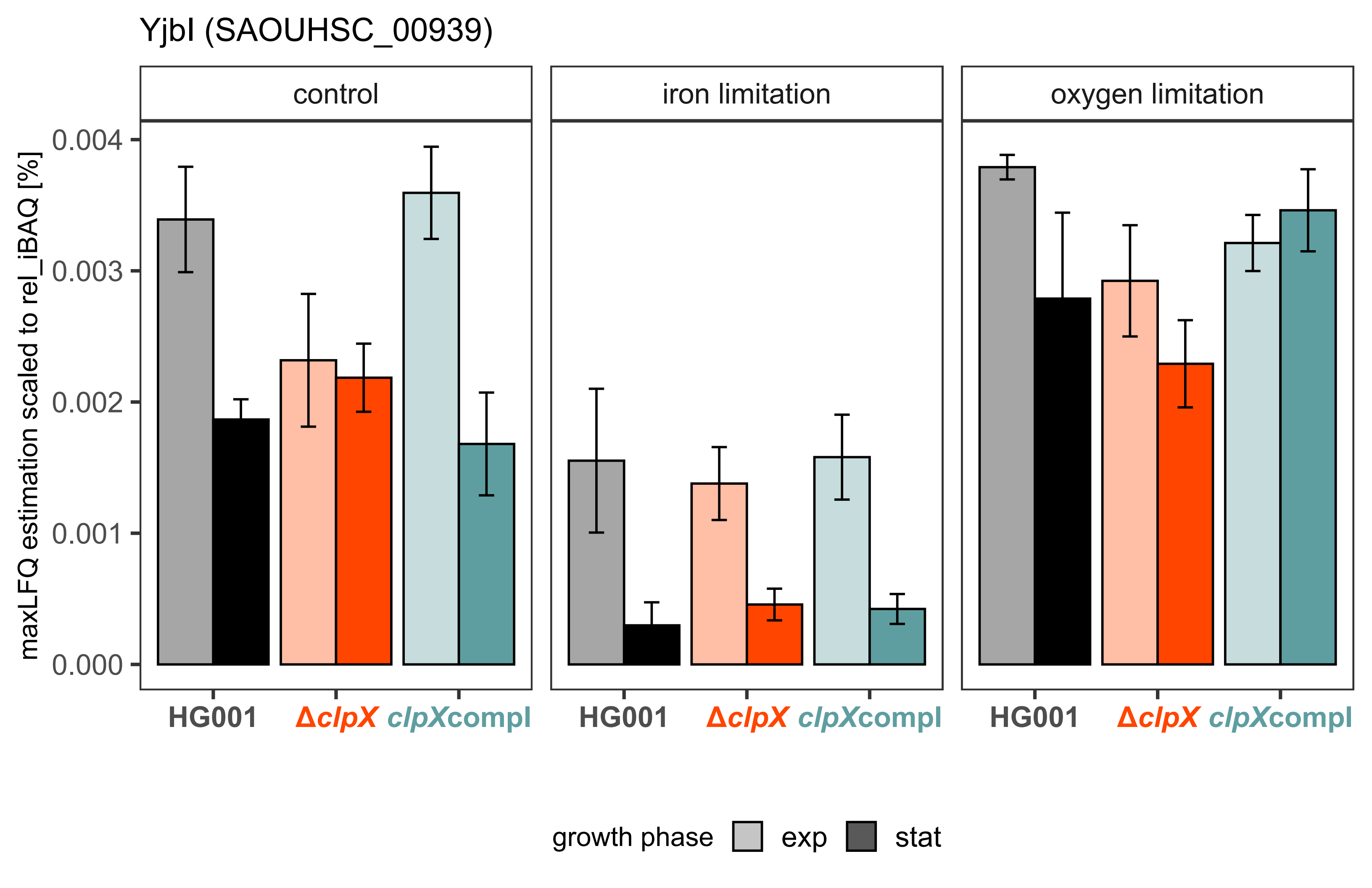

YjbI (SAOUHSC_00939)
control
iron limitation
oxygen limitation
maxLFQ estimation scaled to rel_iBAQ [%]
0.004
0.003
0.002
0.001
0.000
HG001
ΔclpX
clpXcompl
growth phase
exp
stat

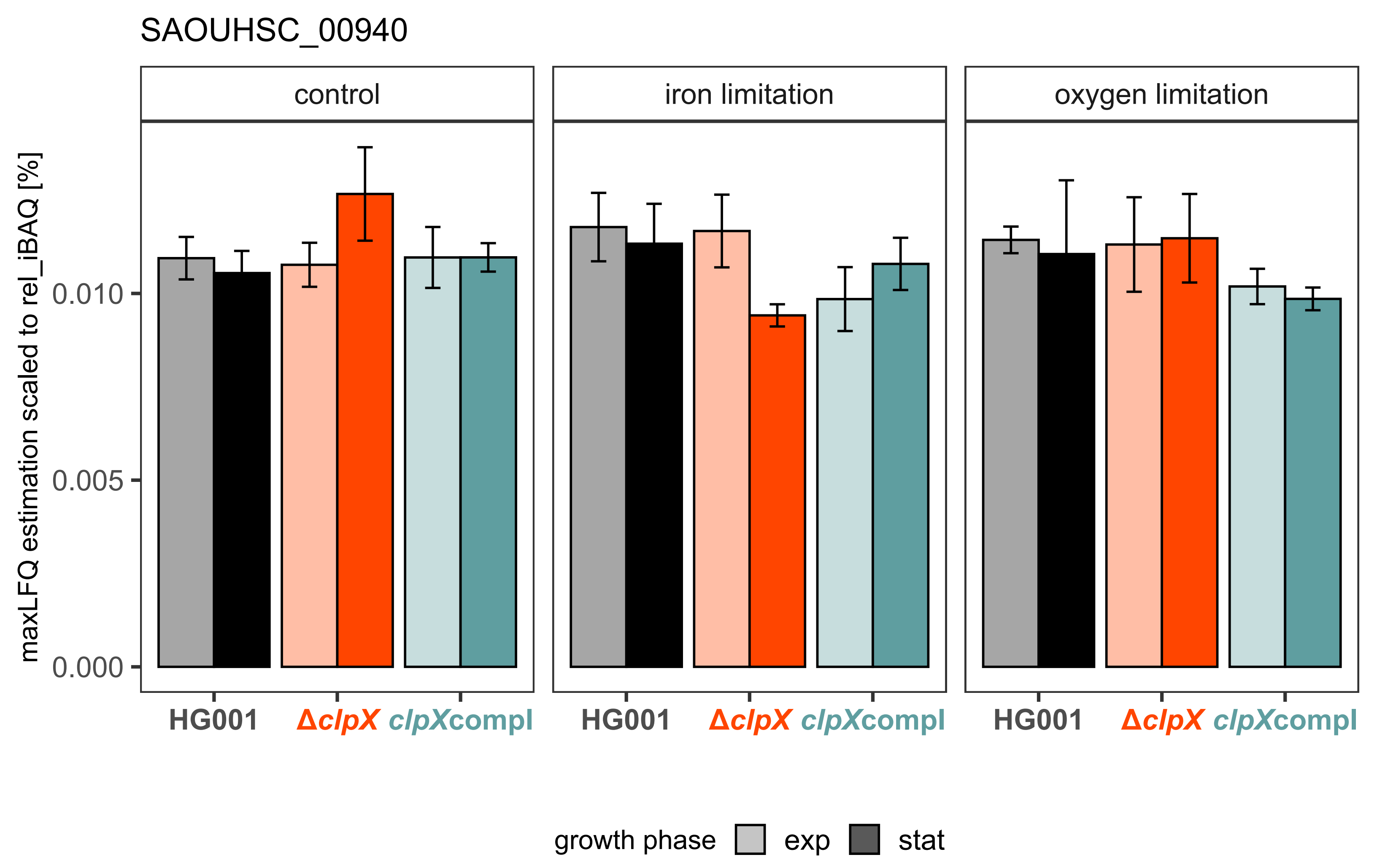

SAOUHSC_00940
control
iron limitation
oxygen limitation
maxLFQ estimation scaled to rel_iBAQ [%]
0.010
0.005
0.000
HG001
ΔclpX
clpXcompl
growth phase
exp
stat

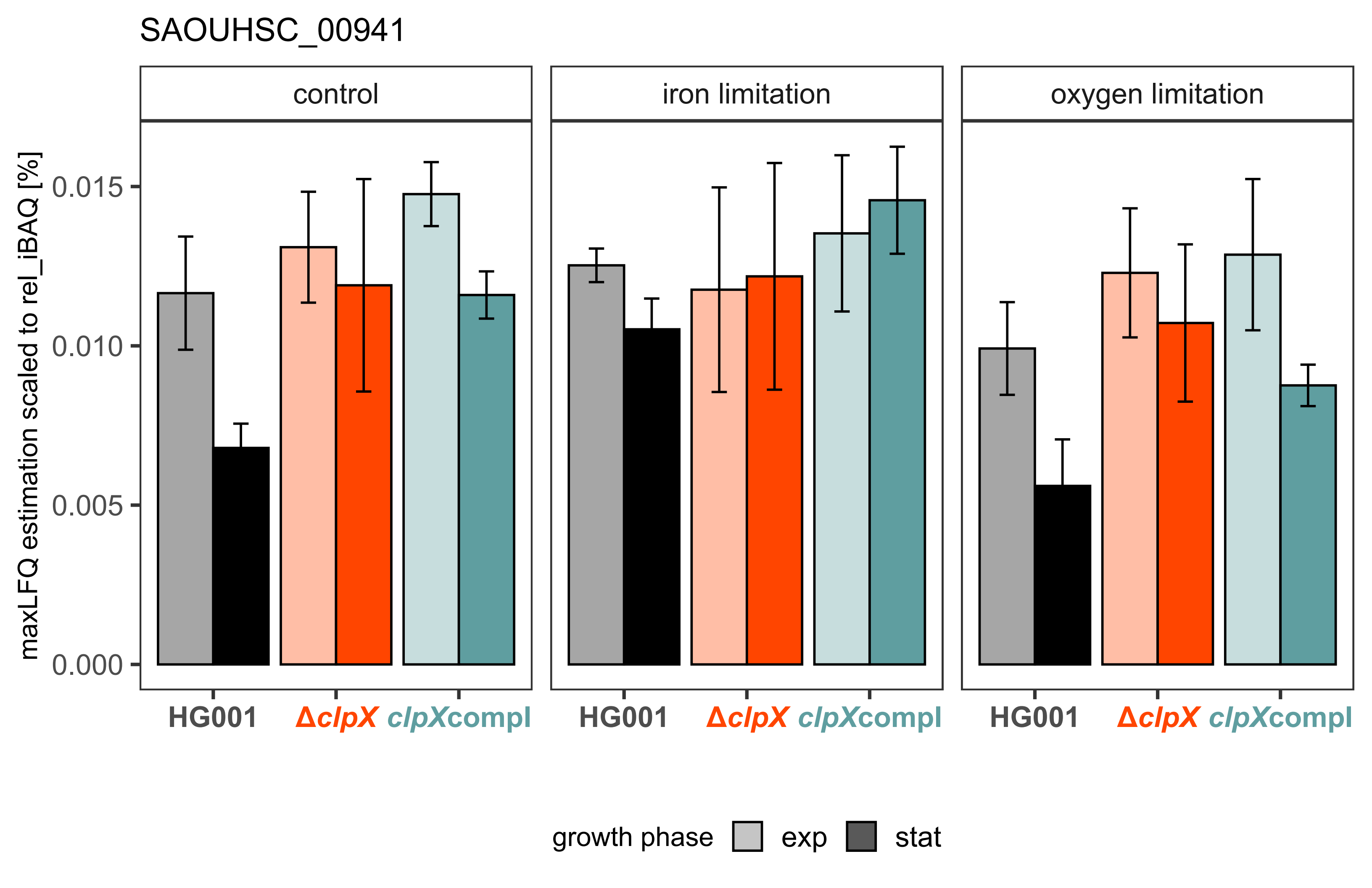
SAOUHSC_00941
control
iron limitation
oxygen limitation
maxLFQ estimation scaled to rel_iBAQ [%]
0.015
0.010
0.005
0.000
HG001
ΔclpX
clpXcompl
growth phase
exp
stat

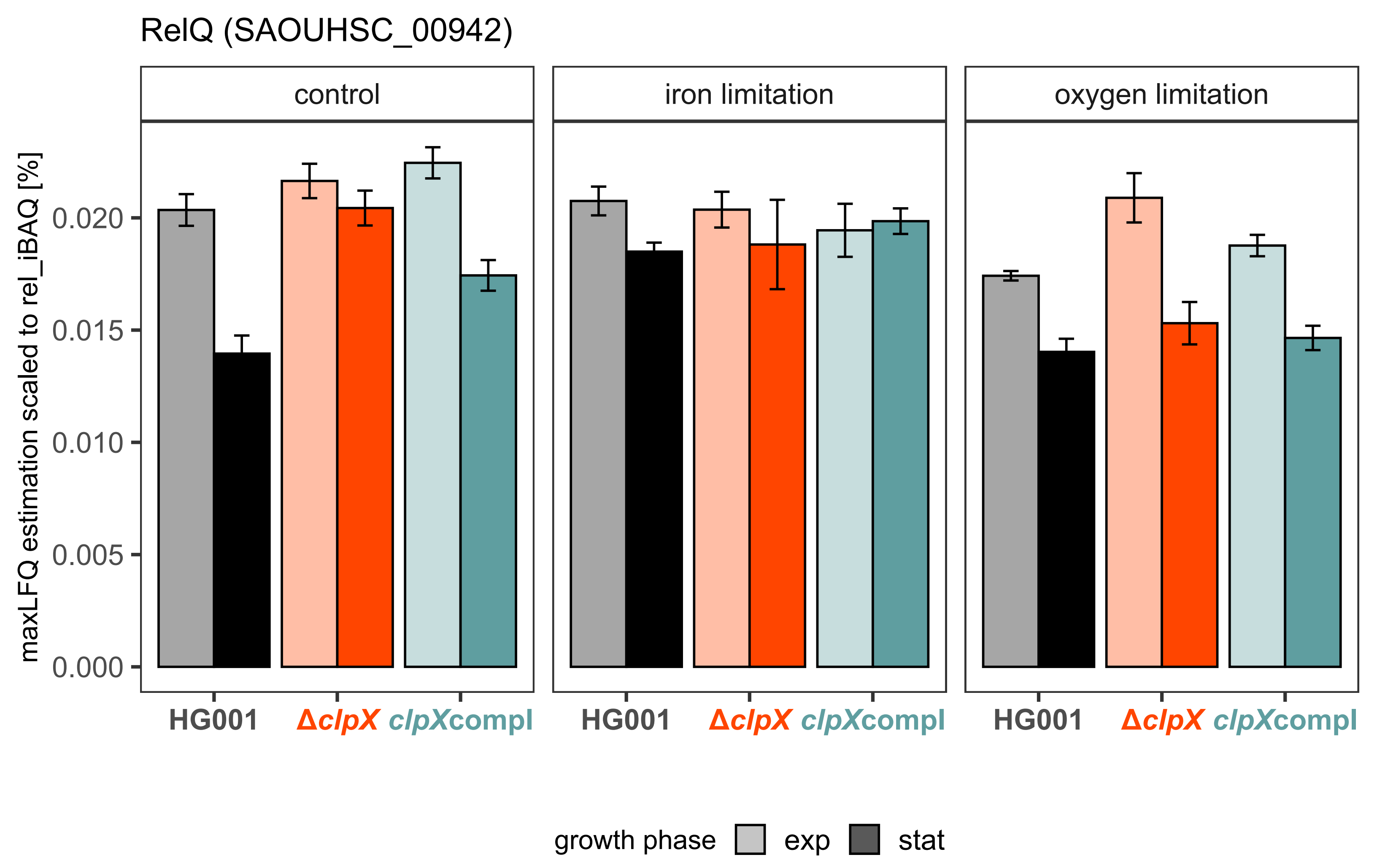
RelQ (SAOUHSC_00942)
control
iron limitation
oxygen limitation
maxLFQ estimation scaled to rel_iBAQ [%]
0.000
0.005
0.010
0.015
0.020
HG001
ΔclpX
clpXcompl
growth phase
exp
stat

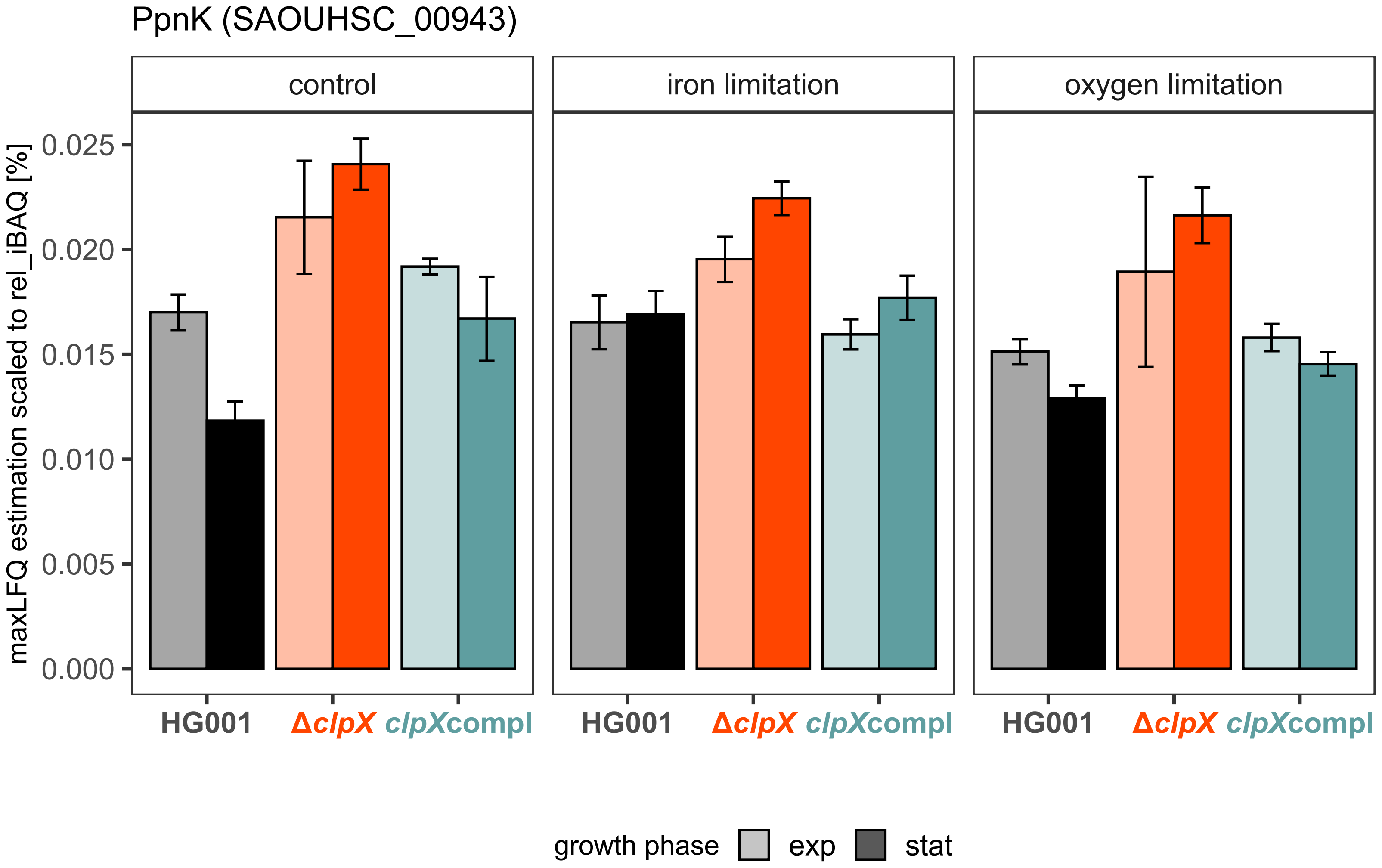

PpnK (SAOUHSC_00943)
control
iron limitation
oxygen limitation
maxLFQ estimation scaled to rel_iBAQ [%]
0.025
0.020
0.015
0.010
0.005
0.000
HG001
ΔclpX
clpXcompl
growth phase
exp
stat

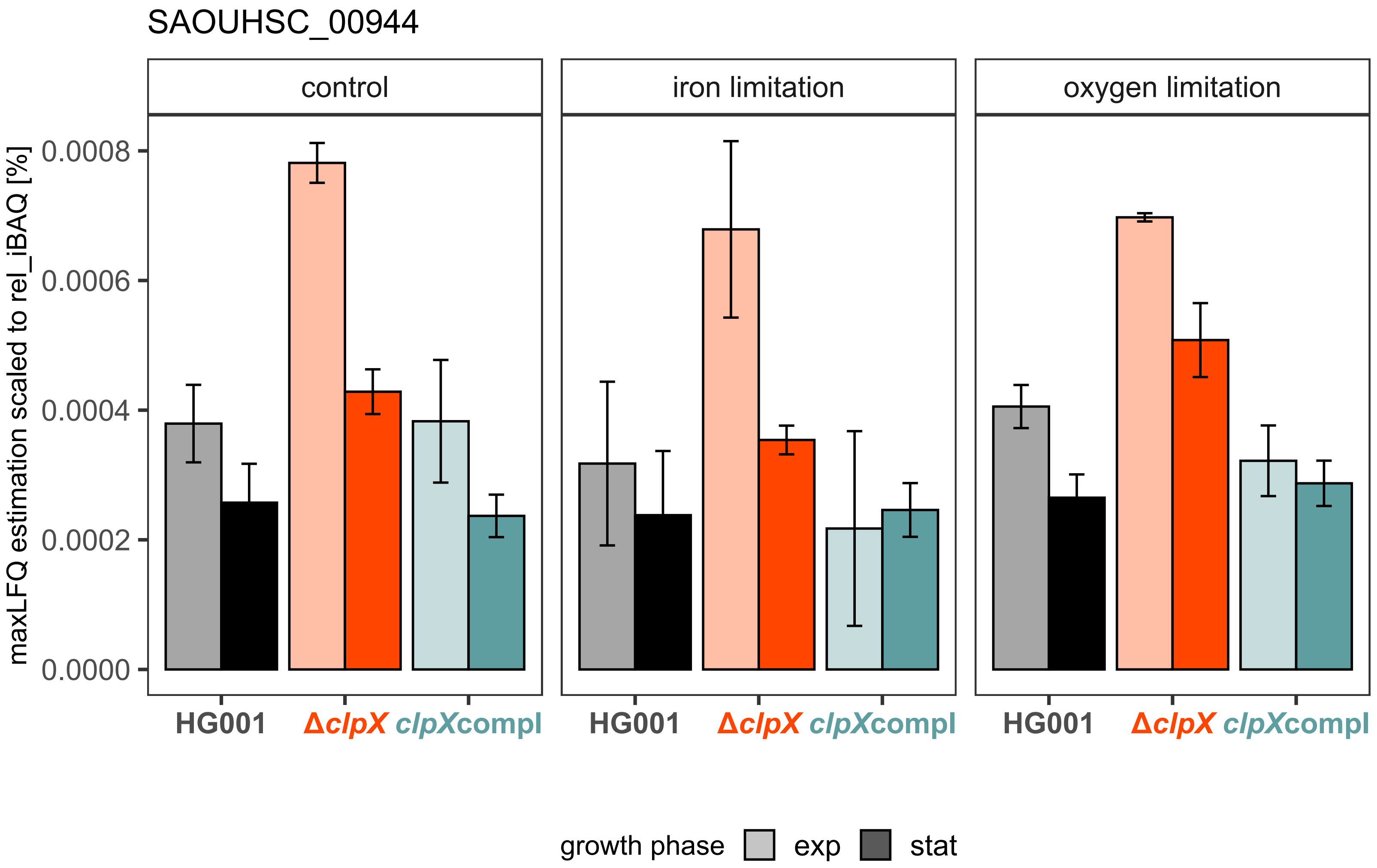
SAOUHSC_00944
control
iron limitation
oxygen limitation
maxLFQ estimation scaled to rel_iBAQ [%]
0.0008
0.0006
0.0004
0.0002
0.0000
HG001
ΔclpX
clpXcompl
growth phase
exp
stat

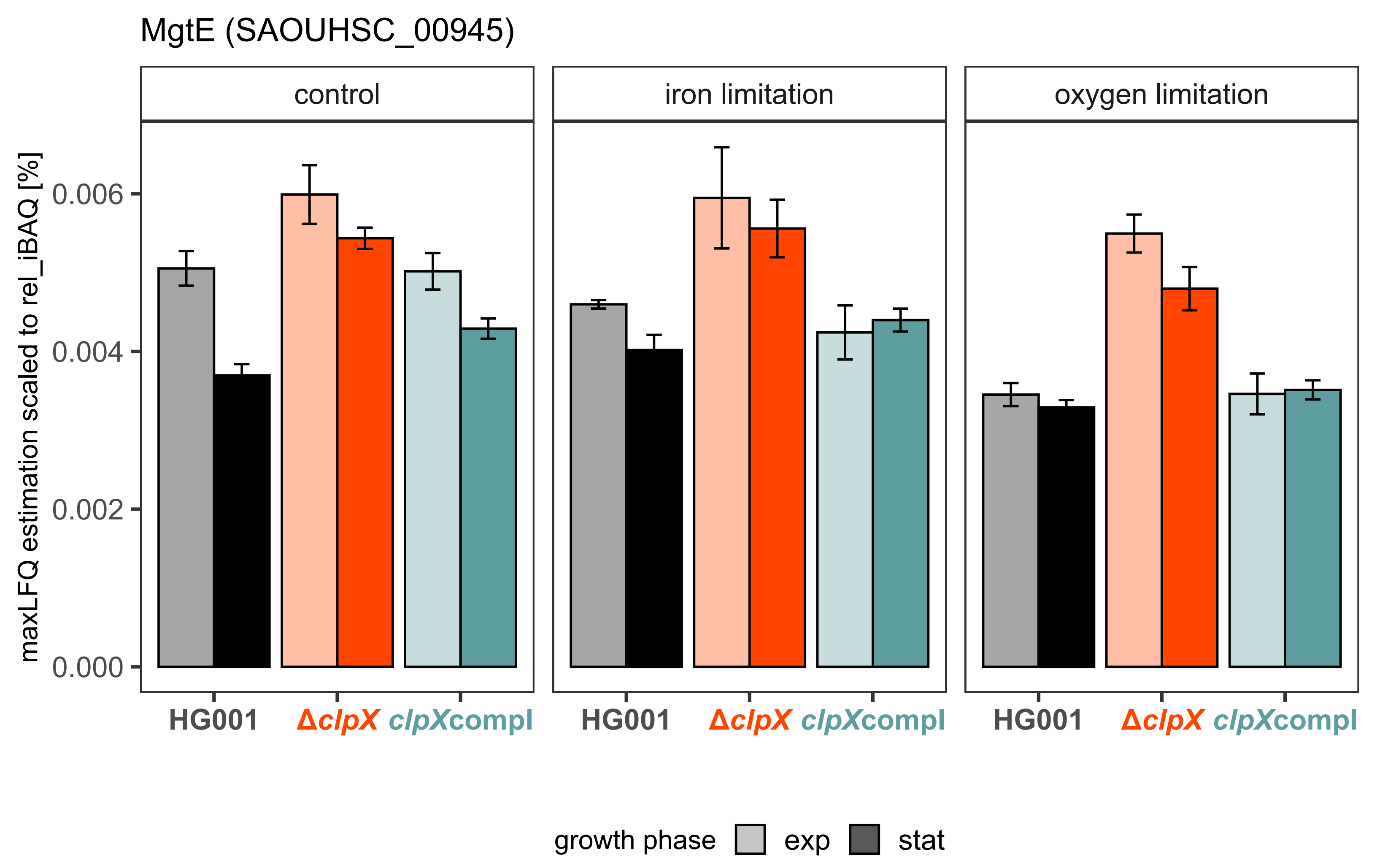
MgtE (SAOUHSC_00945)
control
iron limitation
oxygen limitation
maxLFQ estimation scaled to rel_iBAQ [%]
0.006
0.004
0.002
0.000
HG001
ΔclpX
clpXcompl
growth phase
exp
stat

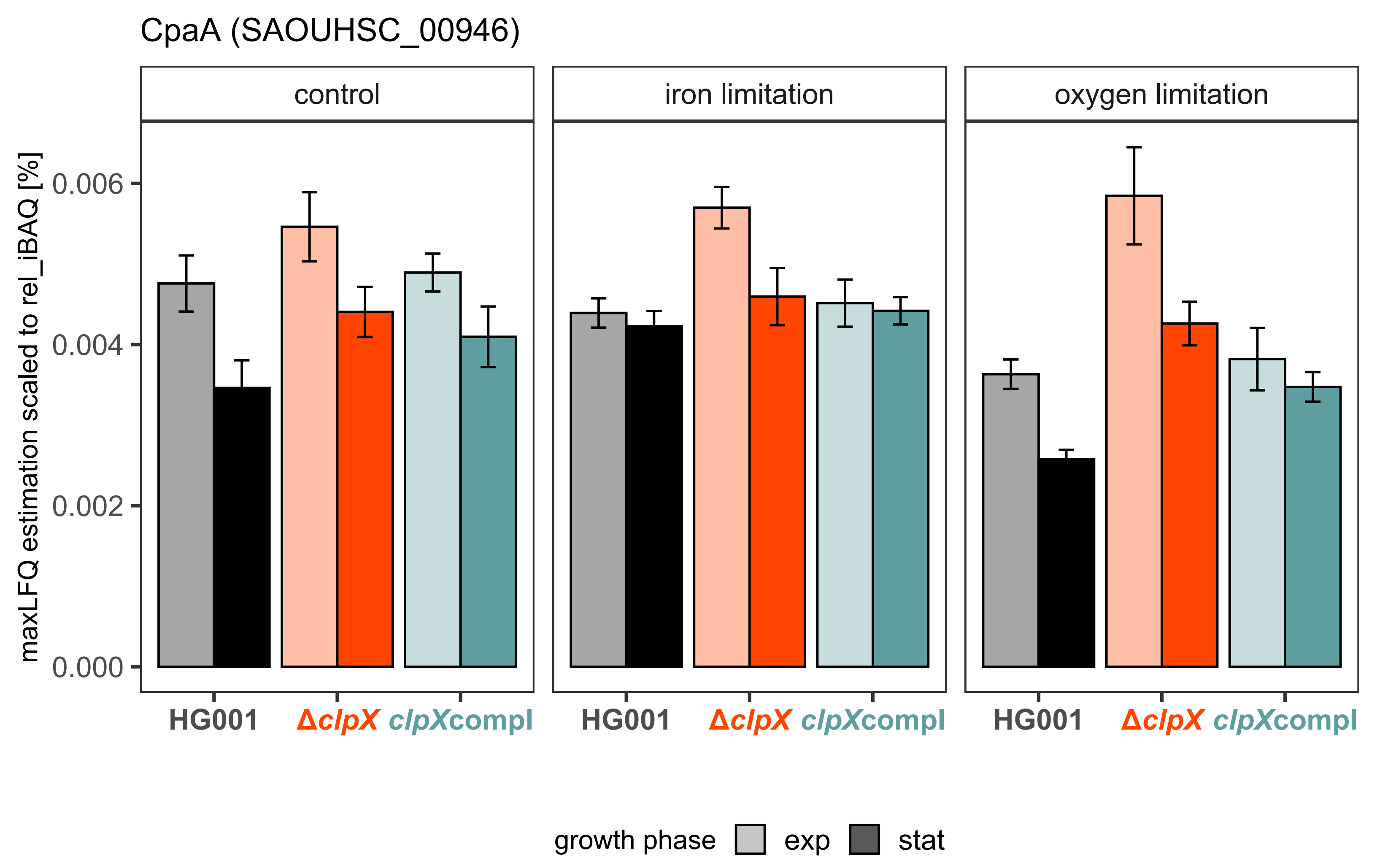
CpaA (SAOUHSC_00946)
control
iron limitation
oxygen limitation
maxLFQ estimation scaled to rel_iBAQ [%]
0.006
0.004
0.002
0.000
HG001
ΔclpX
clpXcompl
growth phase
exp
stat

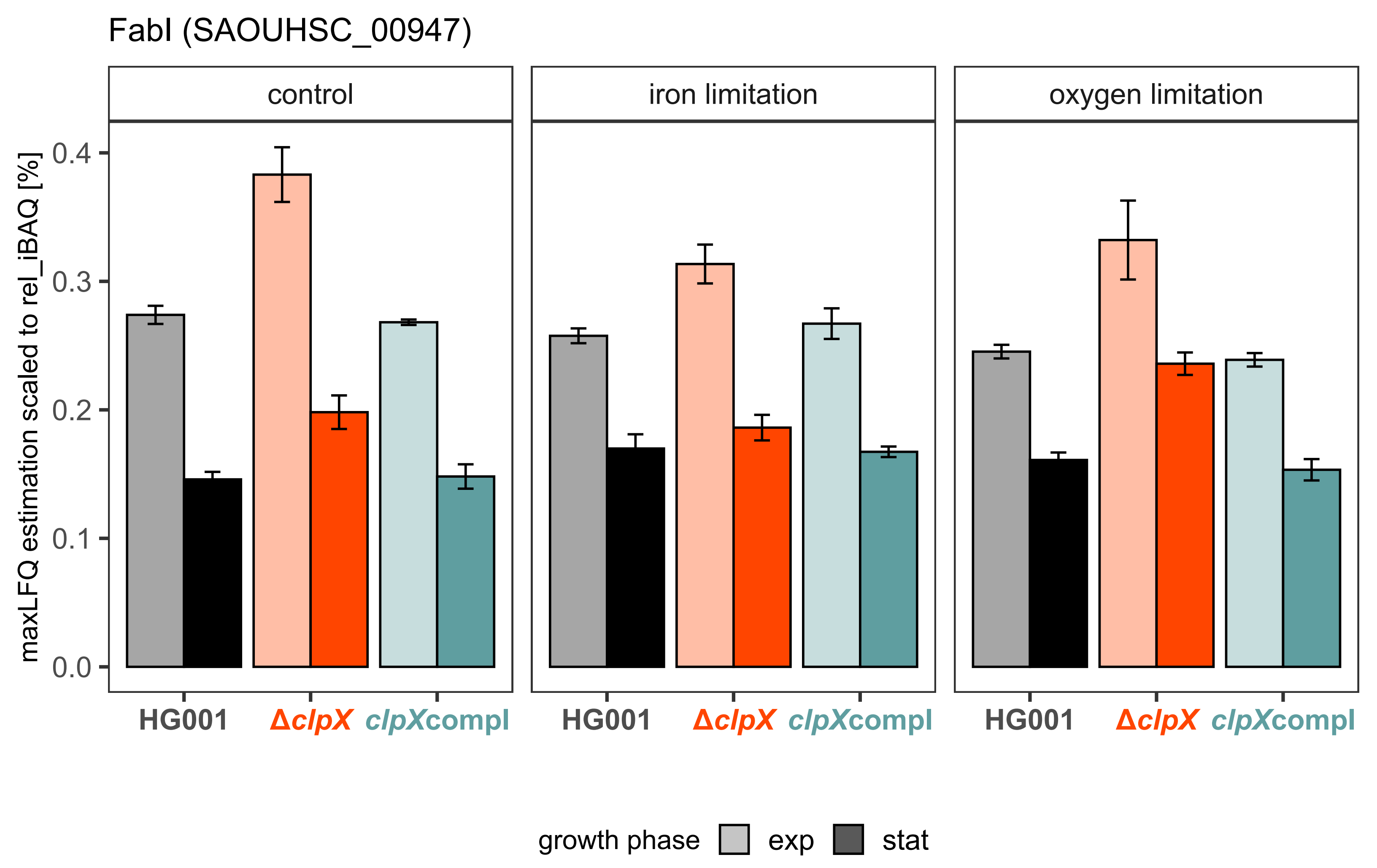
FabI (SAOUHSC_00947)
control
iron limitation
oxygen limitation
maxLFQ estimation scaled to rel_iBAQ [%]
0.0
0.1
0.2
0.3
0.4
HG001
ΔclpX
clpXcompl
growth phase
exp
stat

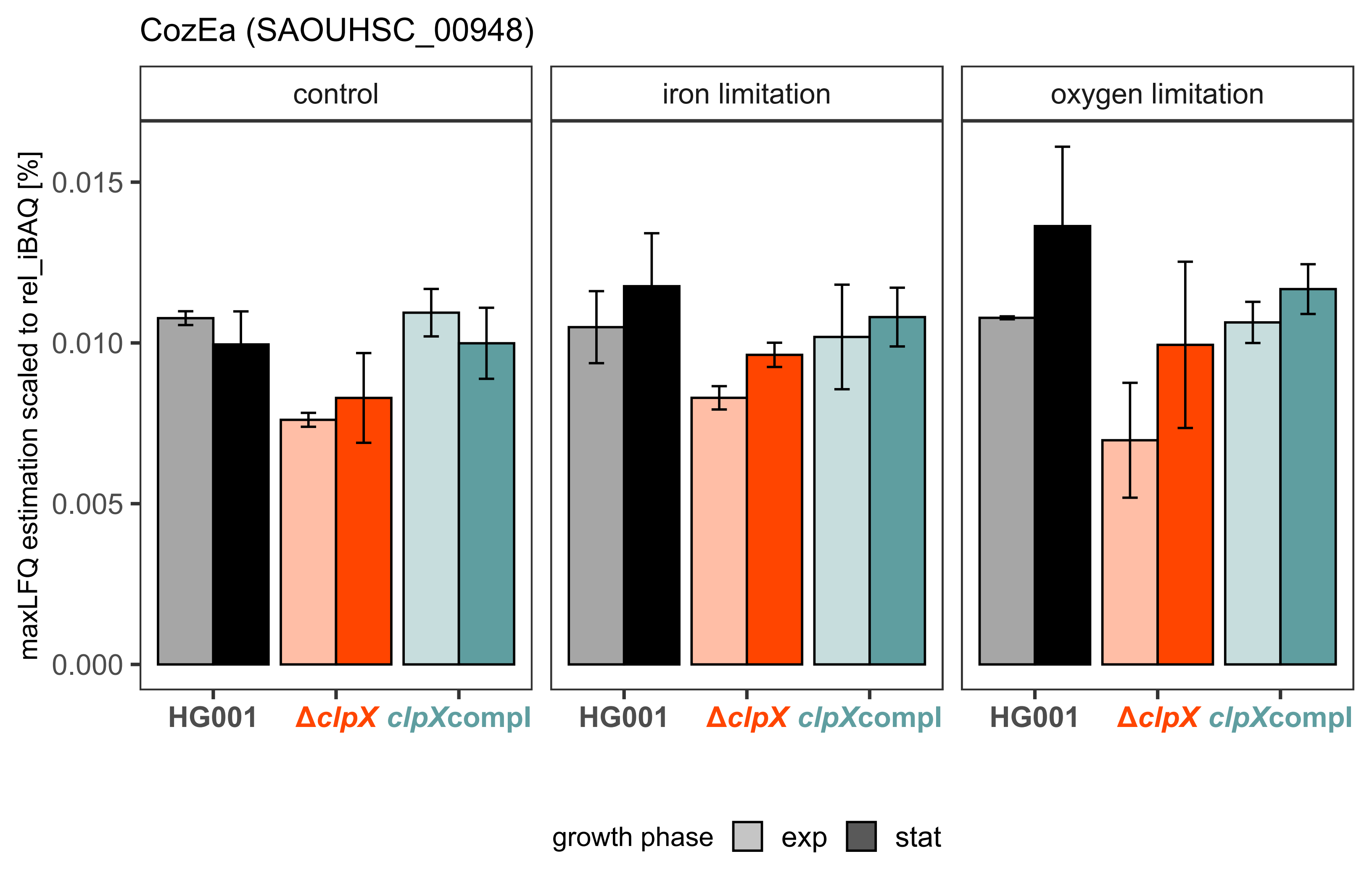

CozEa (SAOUHSC_00948)
control
iron limitation
oxygen limitation
maxLFQ estimation scaled to rel_iBAQ [%]
0.015
0.010
0.005
0.000
HG001
ΔclpX
clpXcompl
growth phase
exp
stat

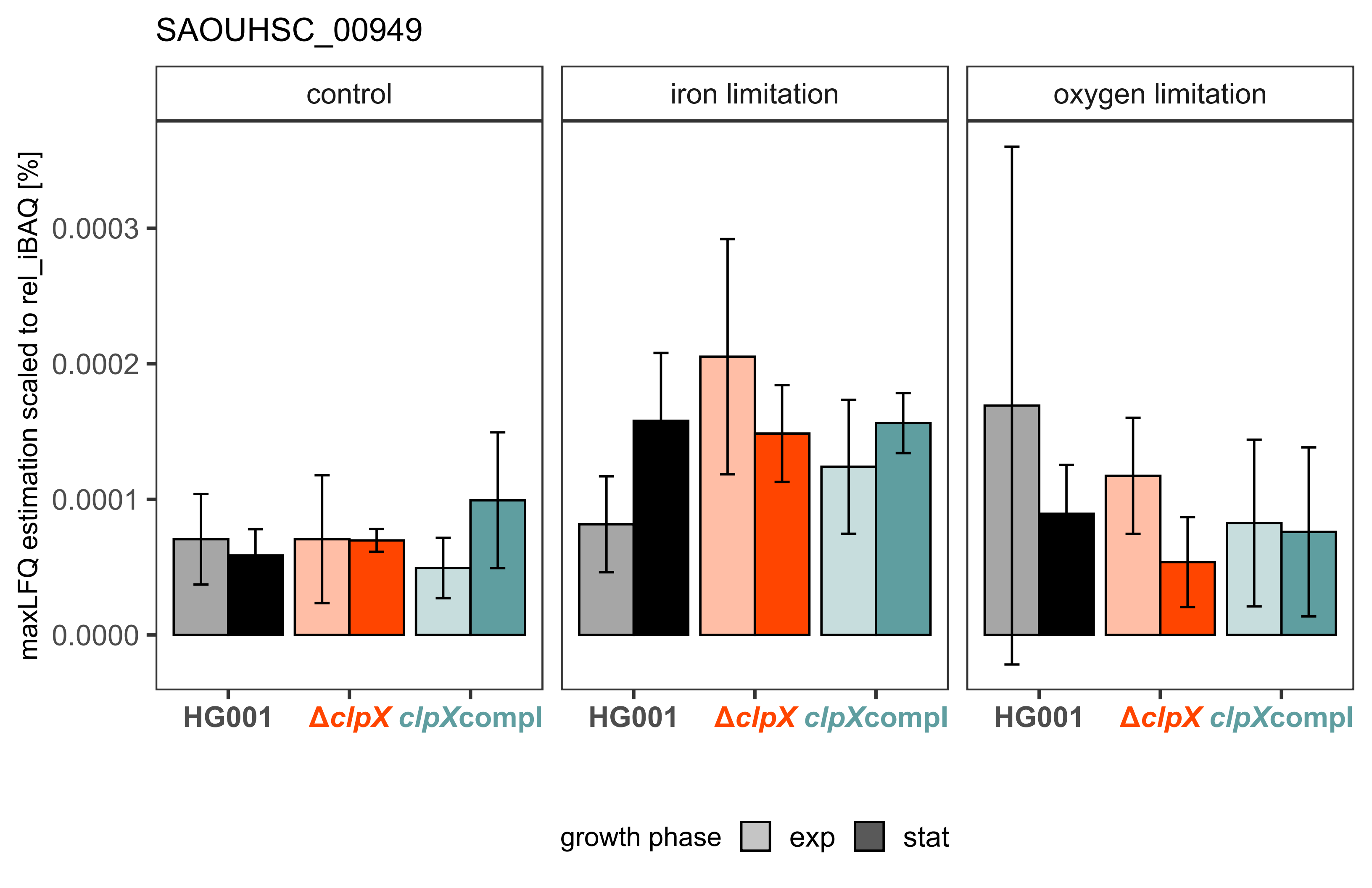

SAOUHSC_00949
control
iron limitation
oxygen limitation
maxLFQ estimation scaled to rel_iBAQ [%]
0.0003
0.0002
0.0001
0.0000
HG001
ΔclpX
clpXcompl
growth phase
exp
stat

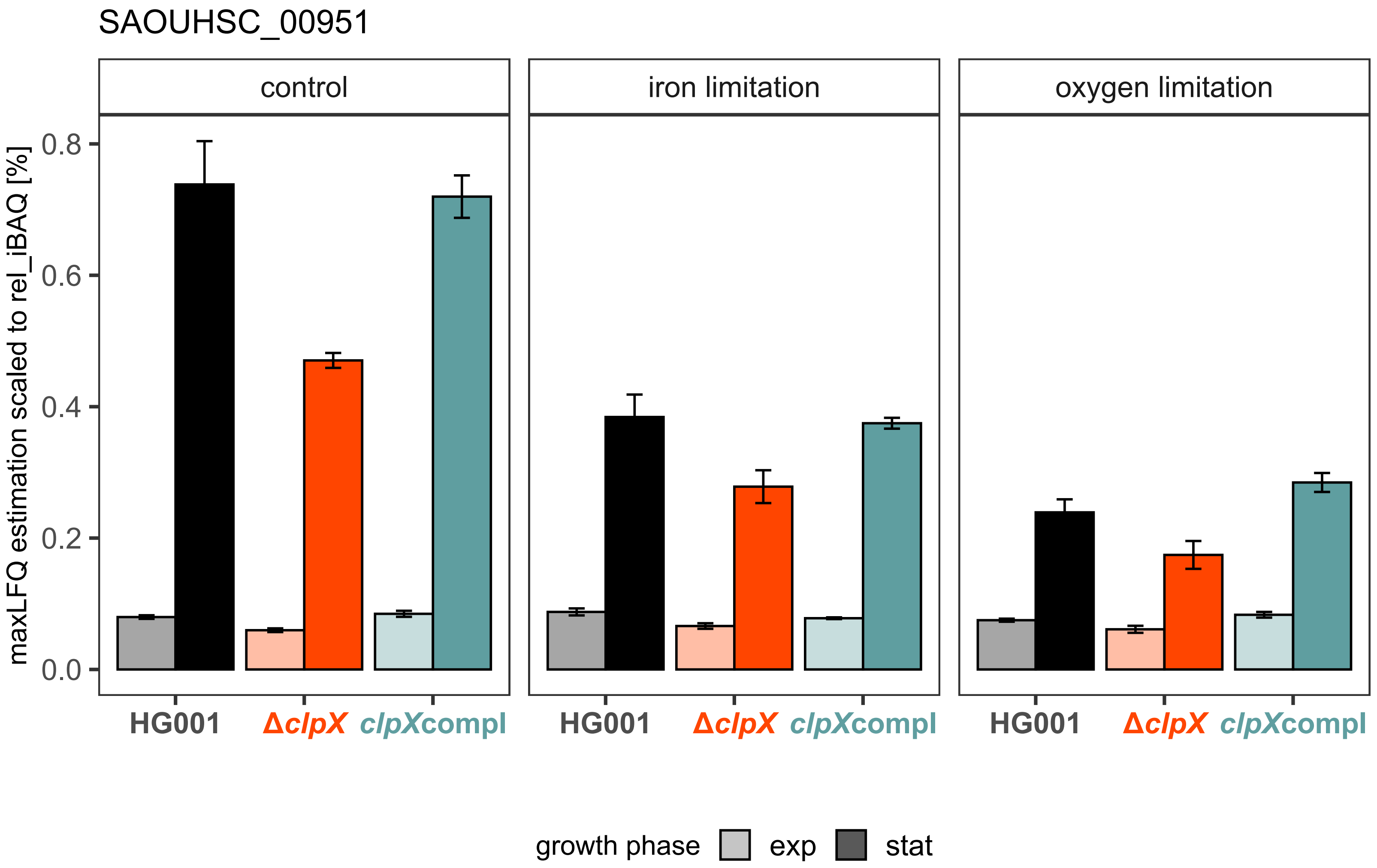
SAOUHSC_00951
control
iron limitation
oxygen limitation
maxLFQ estimation scaled to rel_iBAQ [%]
0.8
0.6
0.4
0.2
0.0
HG001
ΔclpX
clpXcompl
growth phase
exp
stat

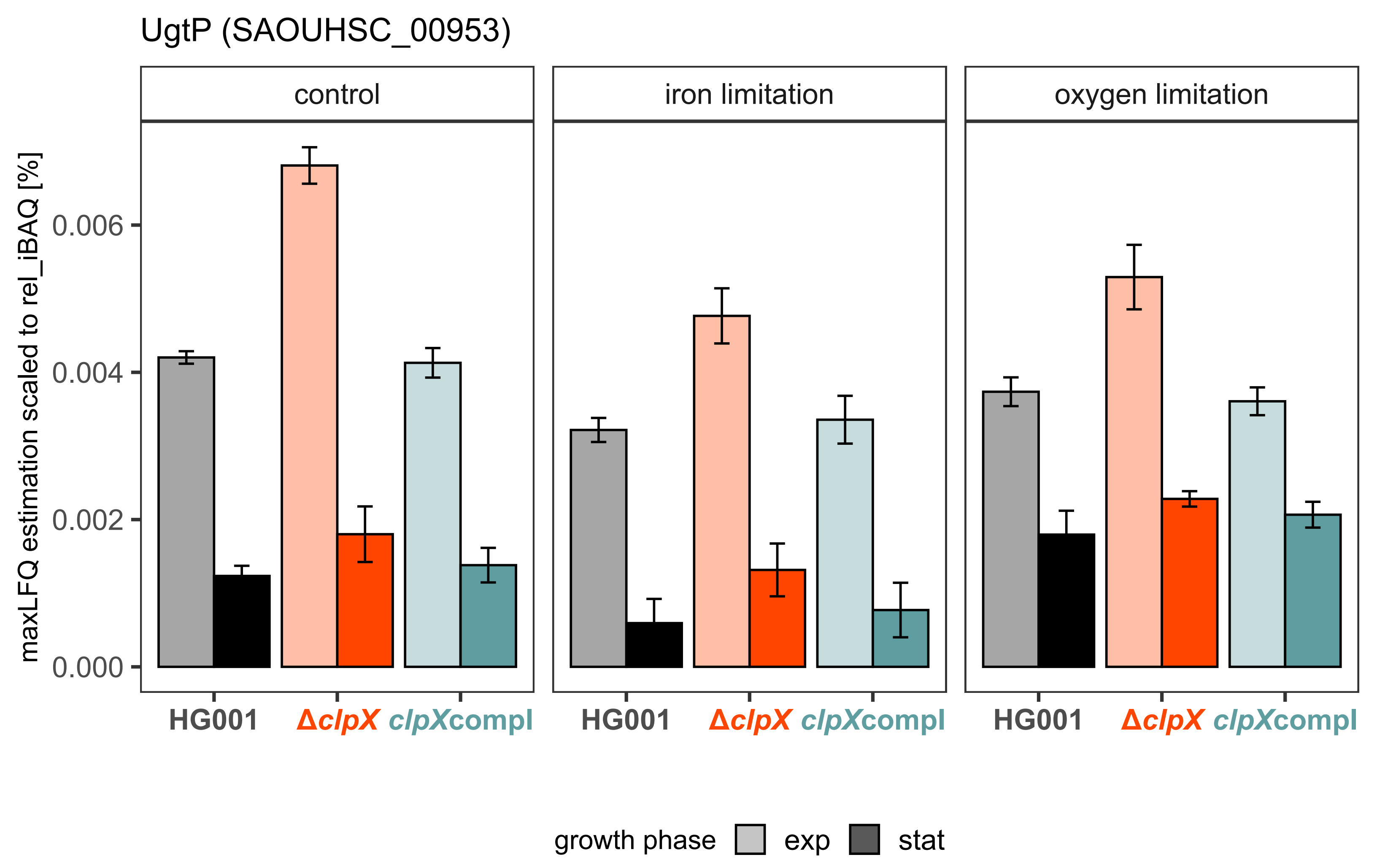
UgtP (SAOUHSC_00953)
control
iron limitation
oxygen limitation
maxLFQ estimation scaled to rel_iBAQ [%]
0.000
0.002
0.004
0.006
HG001
ΔclpX
clpXcompl
growth phase
exp
stat

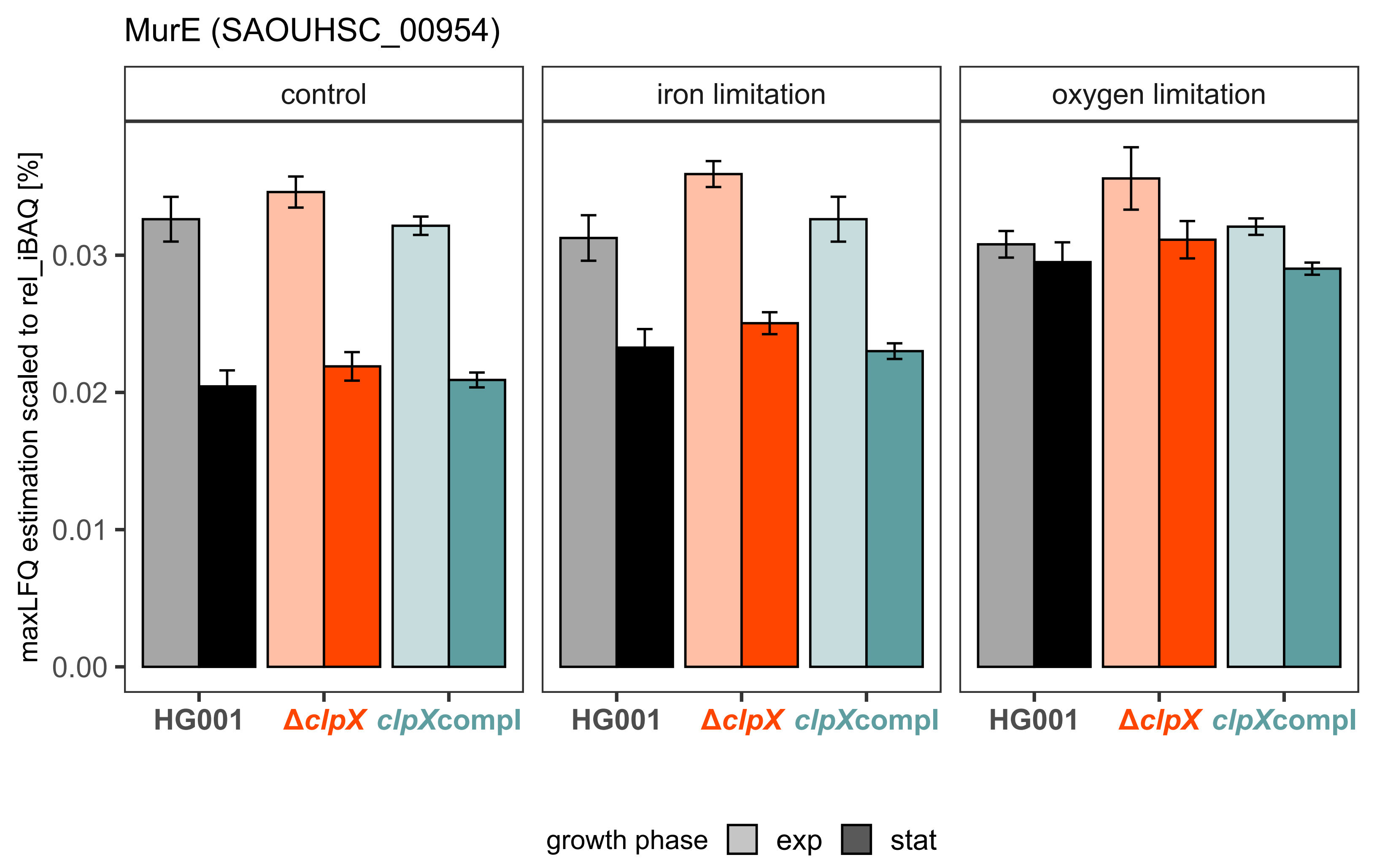
MurE (SAOUHSC_00954)
control
iron limitation
oxygen limitation
maxLFQ estimation scaled to rel_iBAQ [%]
0.00
0.01
0.02
0.03
HG001
ΔclpX
clpXcompl
growth phase
exp
stat

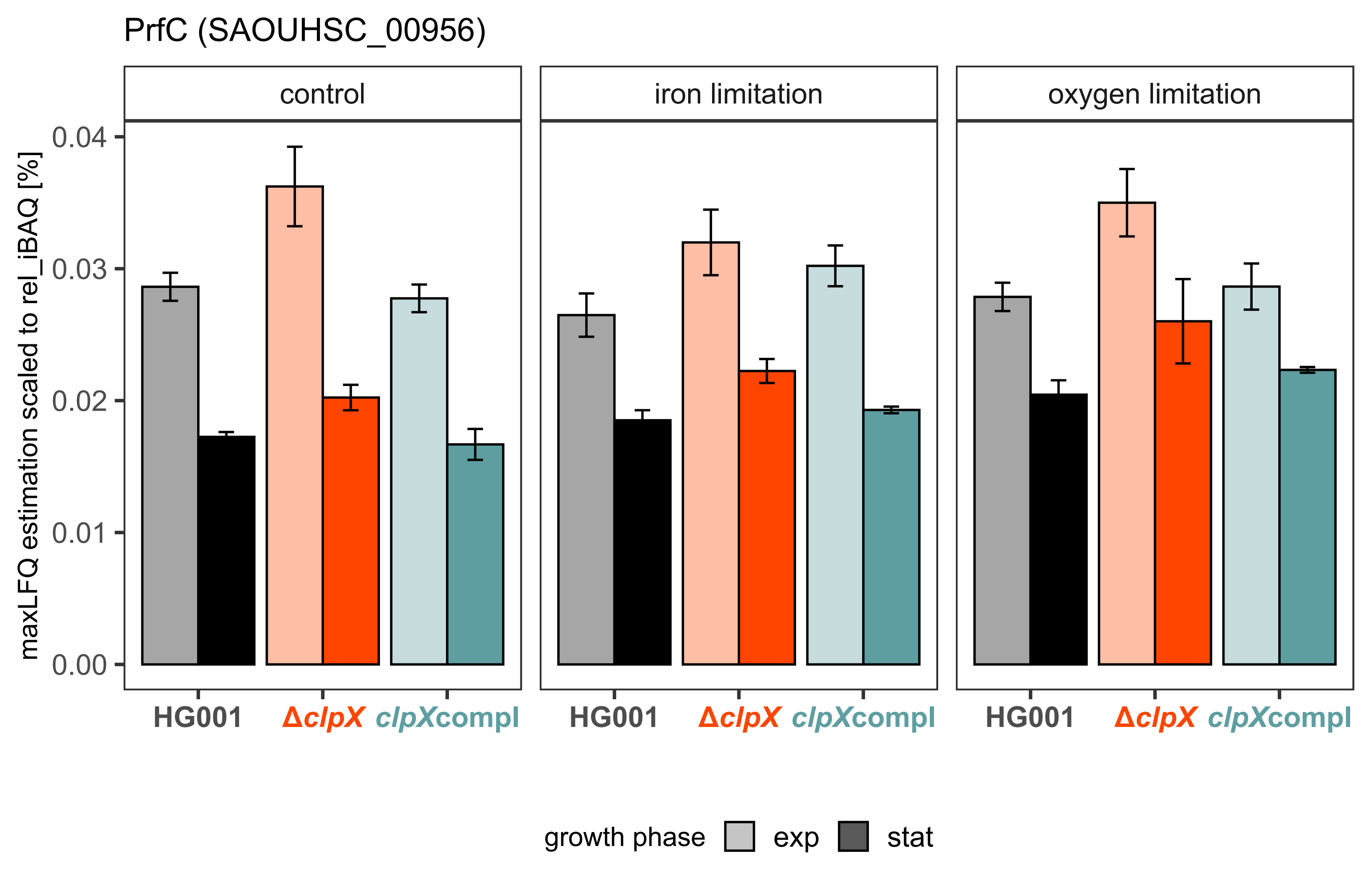

PrfC (SAOUHSC_00956)
control
iron limitation
oxygen limitation
maxLFQ estimation scaled to rel_iBAQ [%]
0.04
0.03
0.02
0.01
0.00
HG001
ΔclpX
clpXcompl
growth phase
exp
stat

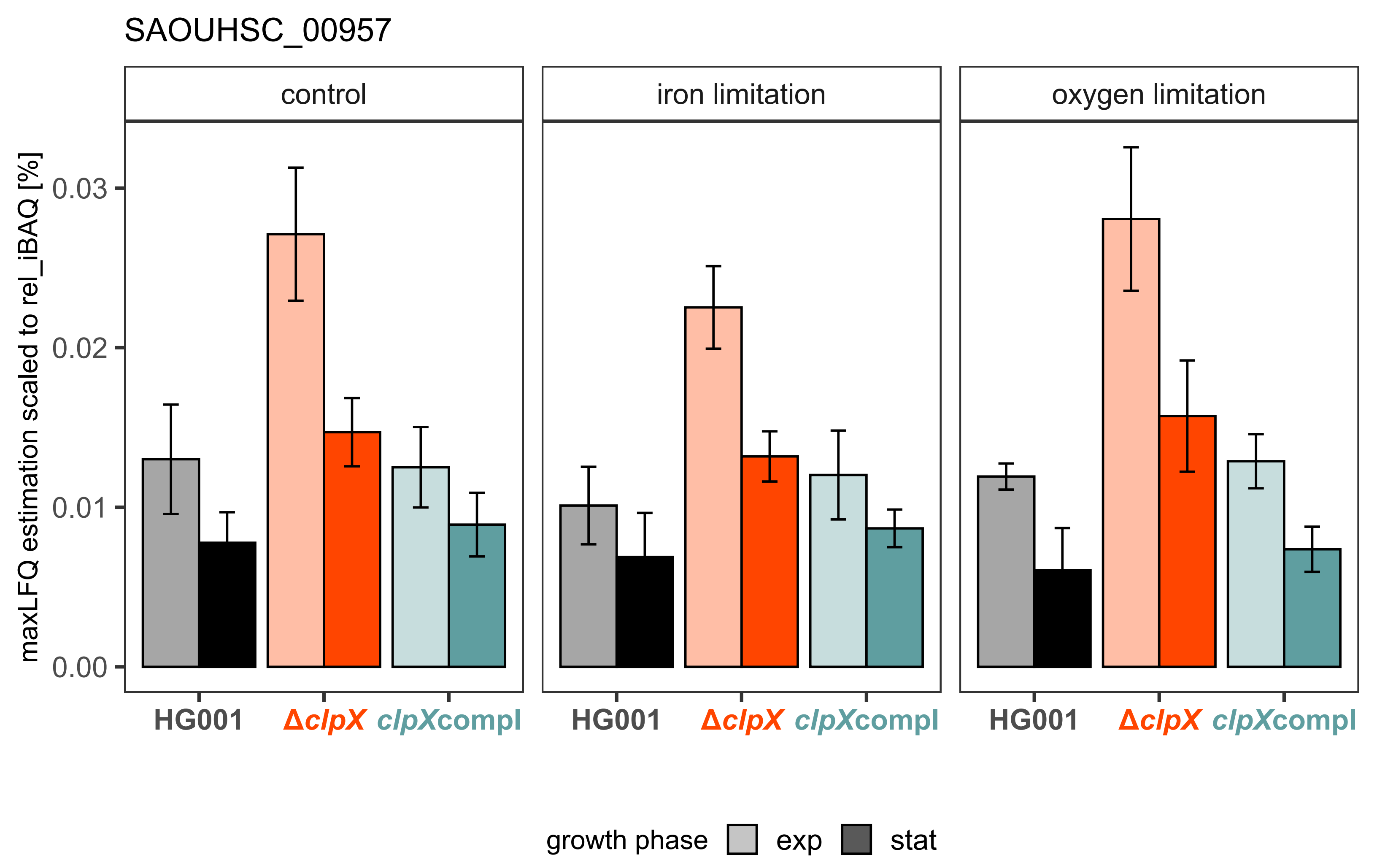
SAOUHSC_00957
control
iron limitation
oxygen limitation
maxLFQ estimation scaled to rel_iBAQ [%]
0.00
0.01
0.02
0.03
HG001
ΔclpX
clpXcompl
growth phase
exp
stat

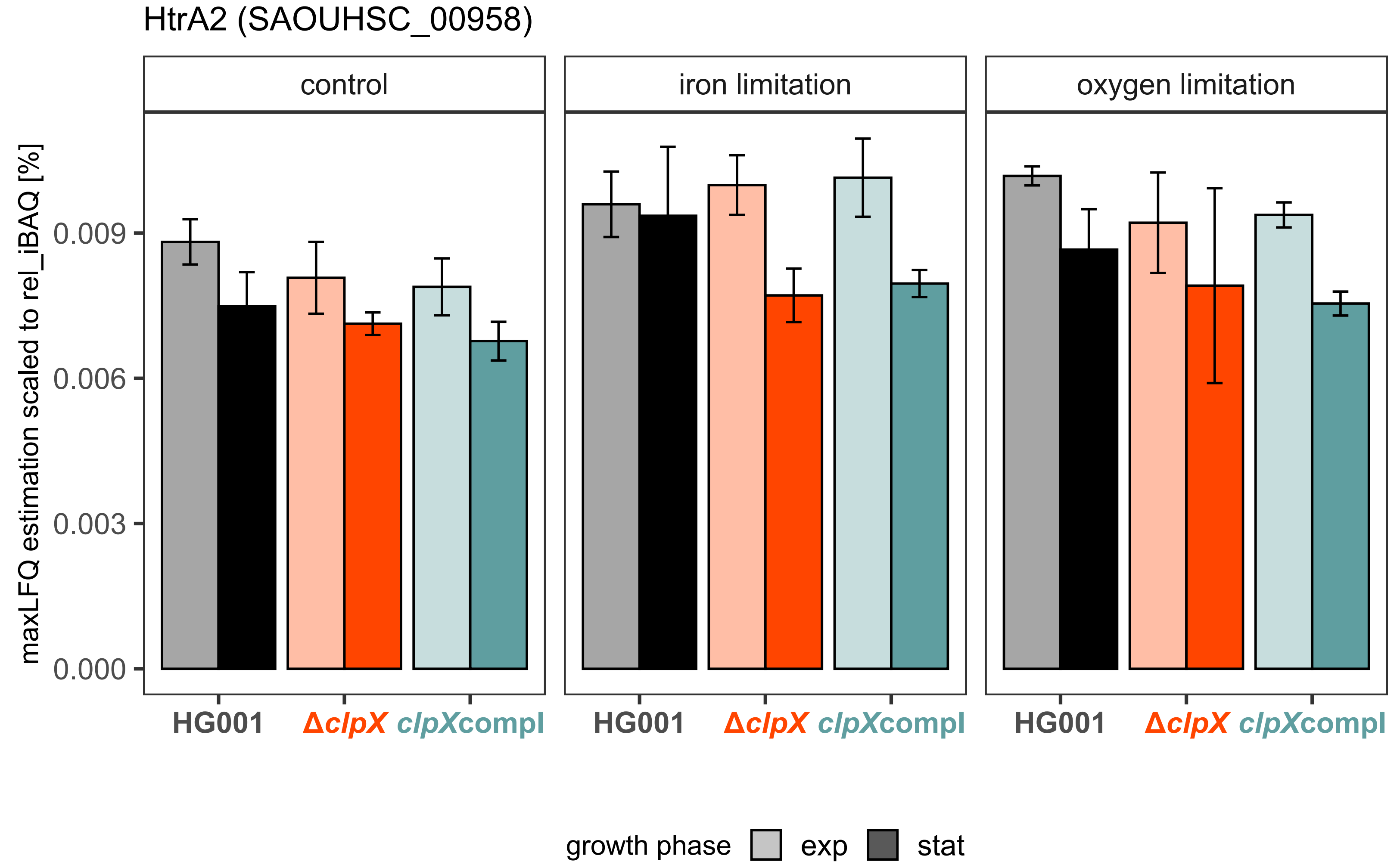

HtrA2 (SAOUHSC_00958)
control
iron limitation
oxygen limitation
maxLFQ estimation scaled to rel_iBAQ [%]
0.009
0.006
0.003
0.000
HG001
ΔclpX
clpXcompl
growth phase
exp
stat

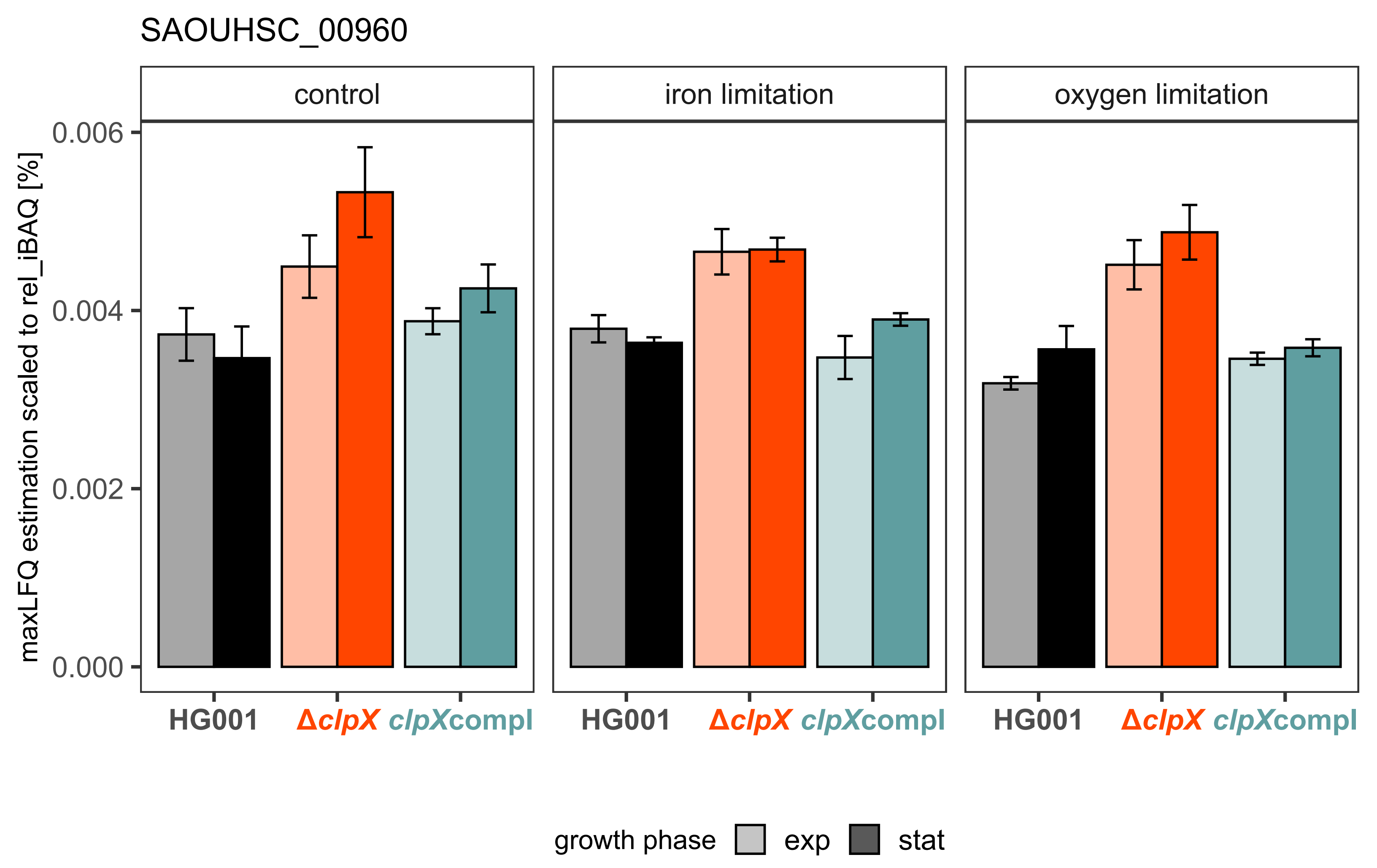

SAOUHSC_00960
control
iron limitation
oxygen limitation
maxLFQ estimation scaled to rel_iBAQ [%]
0.006
0.004
0.002
0.000
HG001
ΔclpX
clpXcompl
growth phase
exp
stat

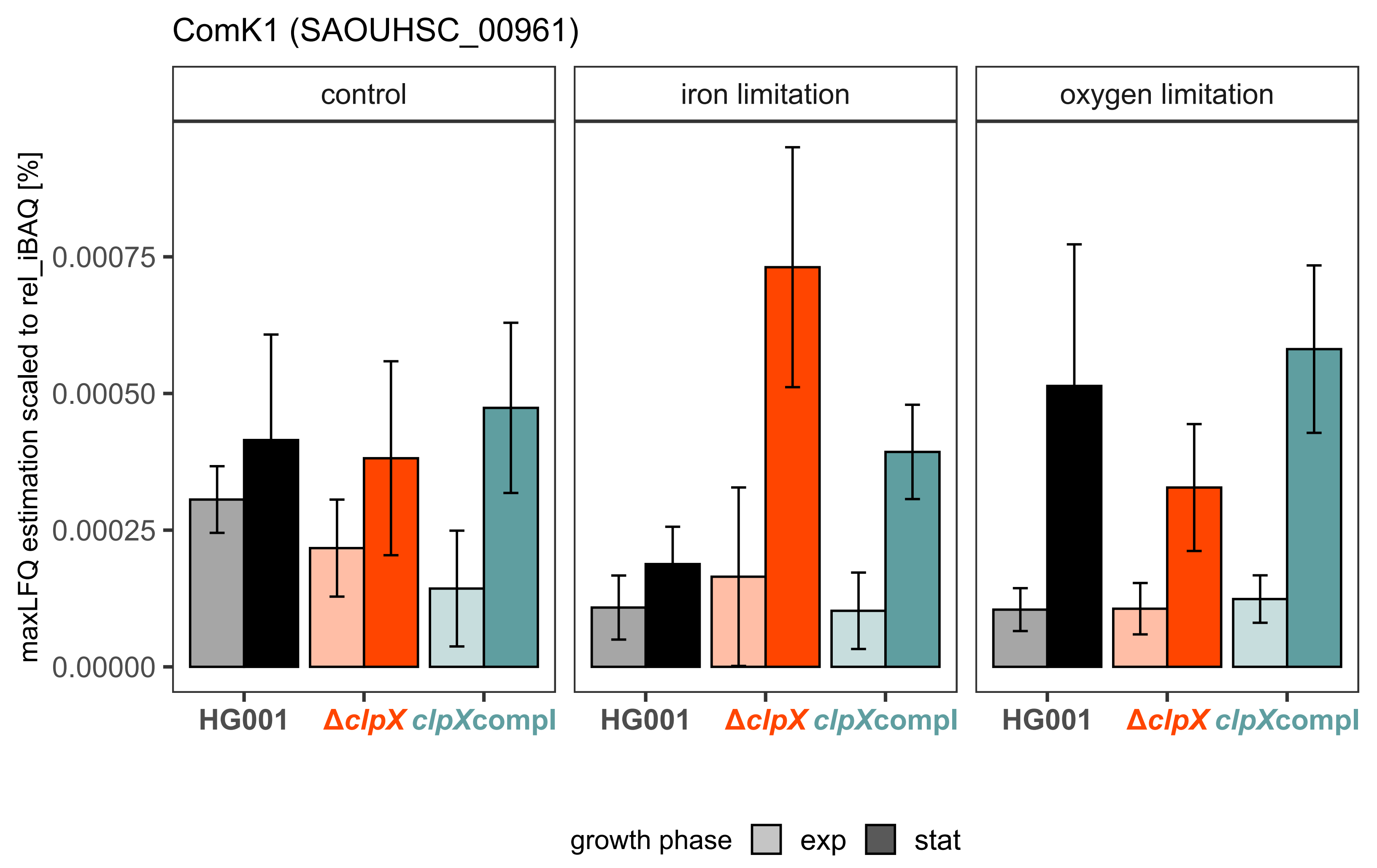

ComK1 (SAOUHSC_00961)
control
iron limitation
oxygen limitation
maxLFQ estimation scaled to rel_iBAQ [%]
0.00075
0.00050
0.00025
0.00000
HG001
ΔclpX
clpXcompl
growth phase
exp
stat

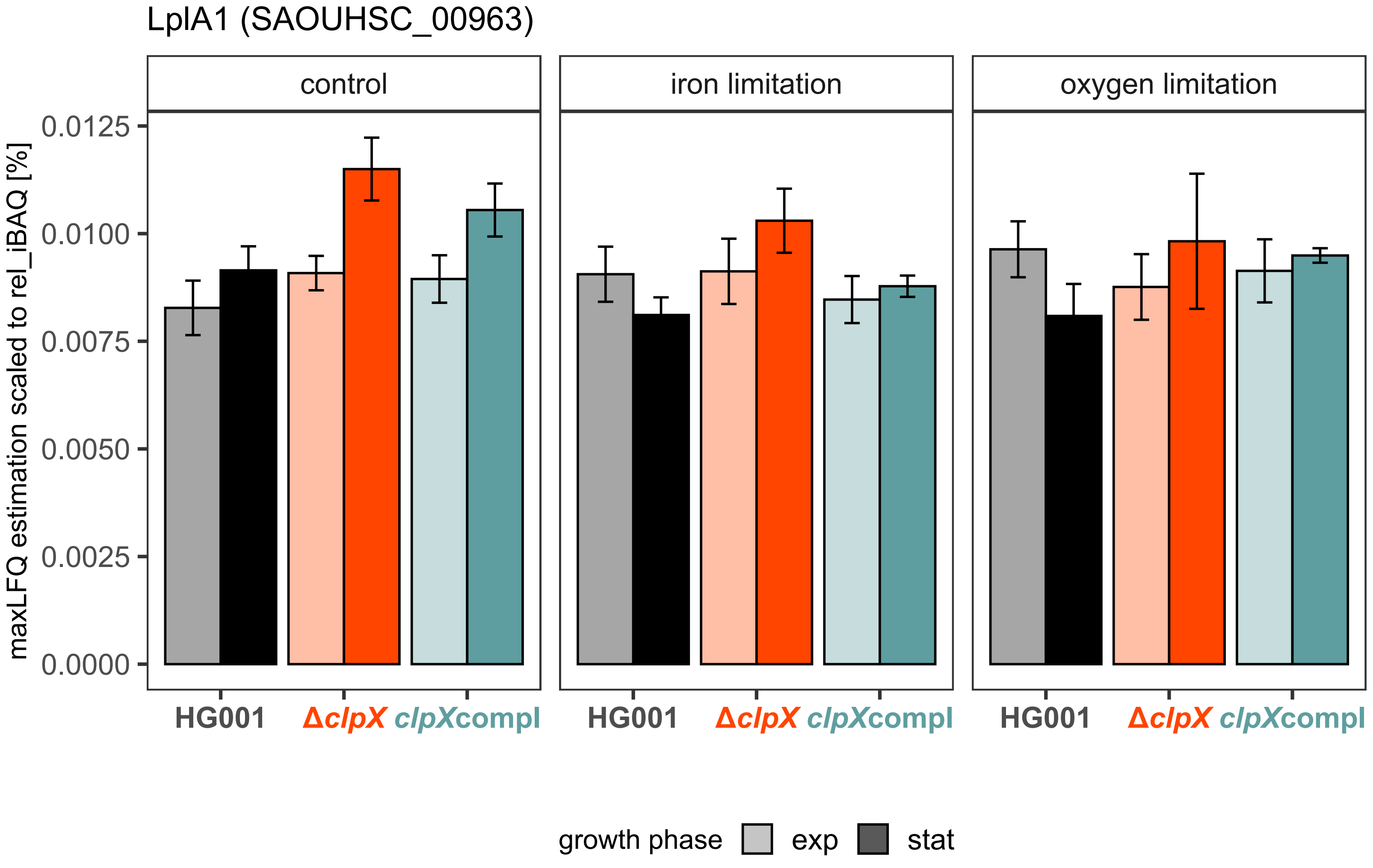

LplA1 (SAOUHSC_00963)
control
iron limitation
oxygen limitation
maxLFQ estimation scaled to rel_iBAQ [%]
0.0125
0.0100
0.0075
0.0050
0.0025
0.0000
HG001
ΔclpX
clpXcompl
growth phase
exp
stat

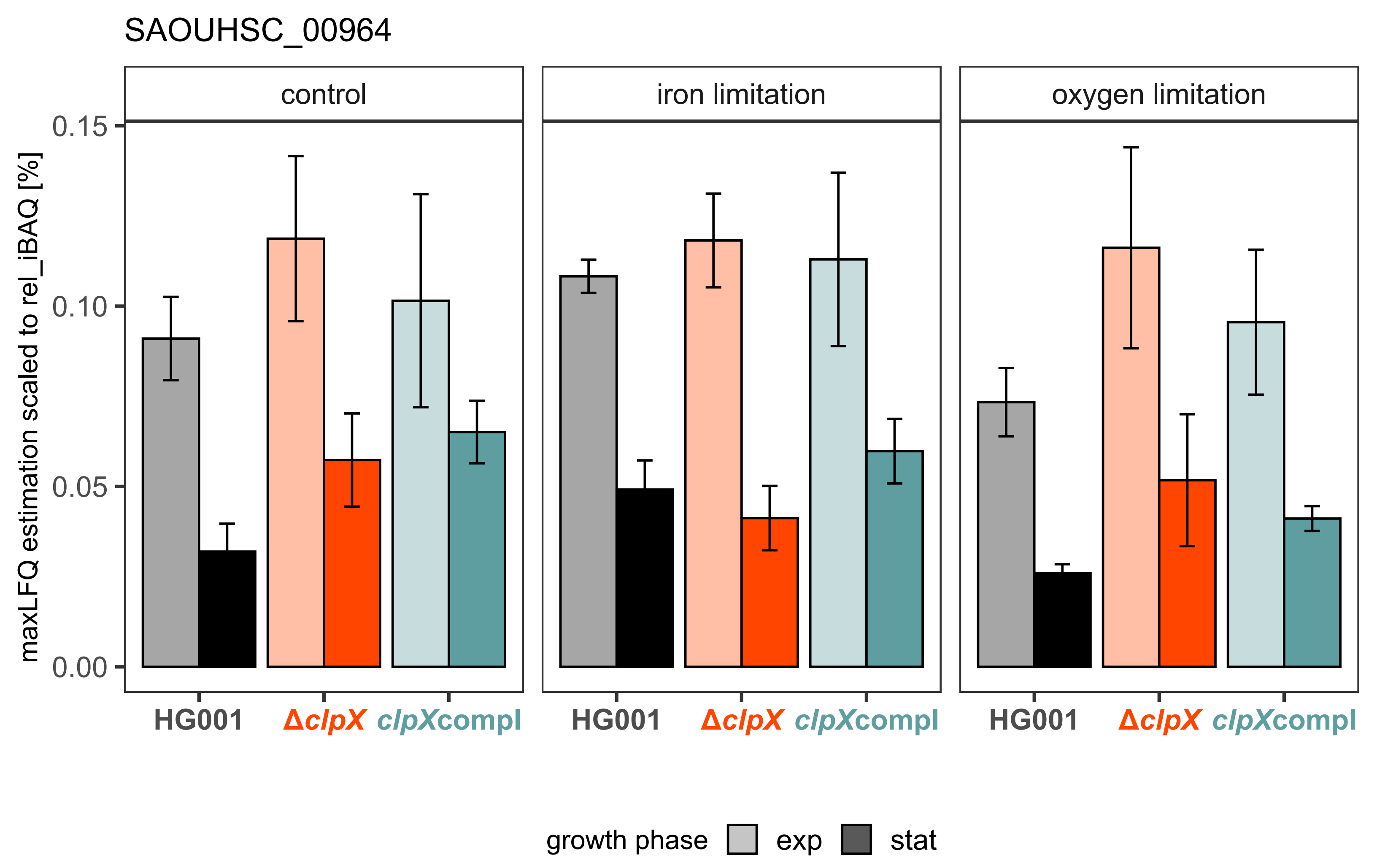

SAOUHSC_00964
control
iron limitation
oxygen limitation
maxLFQ estimation scaled to rel_iBAQ [%]
0.15
0.10
0.05
0.00
HG001
ΔclpX
clpXcompl
growth phase
exp
stat

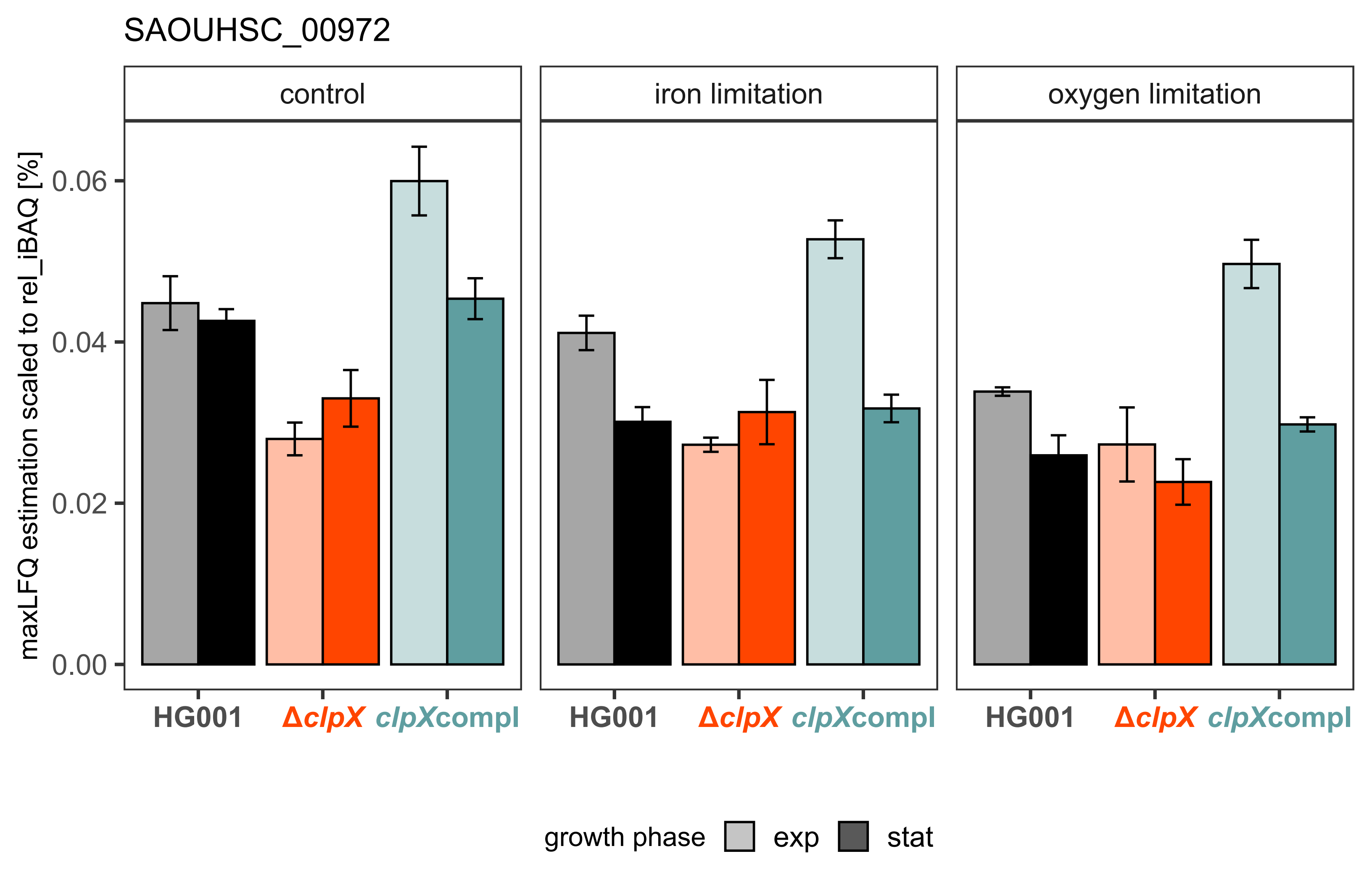
SAOUHSC_00972
control
iron limitation
oxygen limitation
maxLFQ estimation scaled to rel_iBAQ [%]
0.06
0.04
0.02
0.00
HG001
ΔclpX
clpXcompl
growth phase
exp
stat

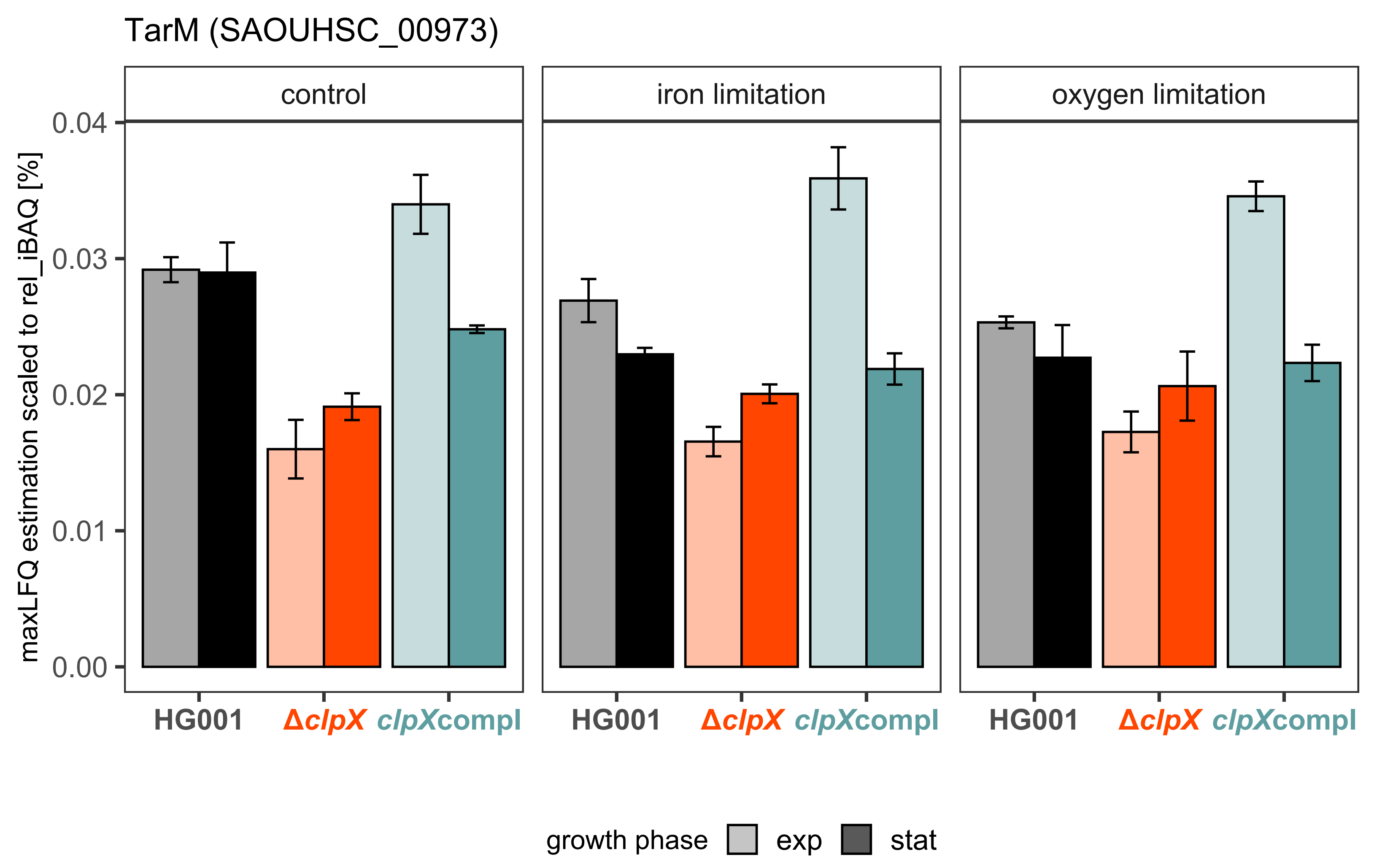
TarM (SAOUHSC_00973)
control
iron limitation
oxygen limitation
maxLFQ estimation scaled to rel_iBAQ [%]
0.04
0.03
0.02
0.01
0.00
HG001
ΔclpX
clpXcompl
growth phase
exp
stat

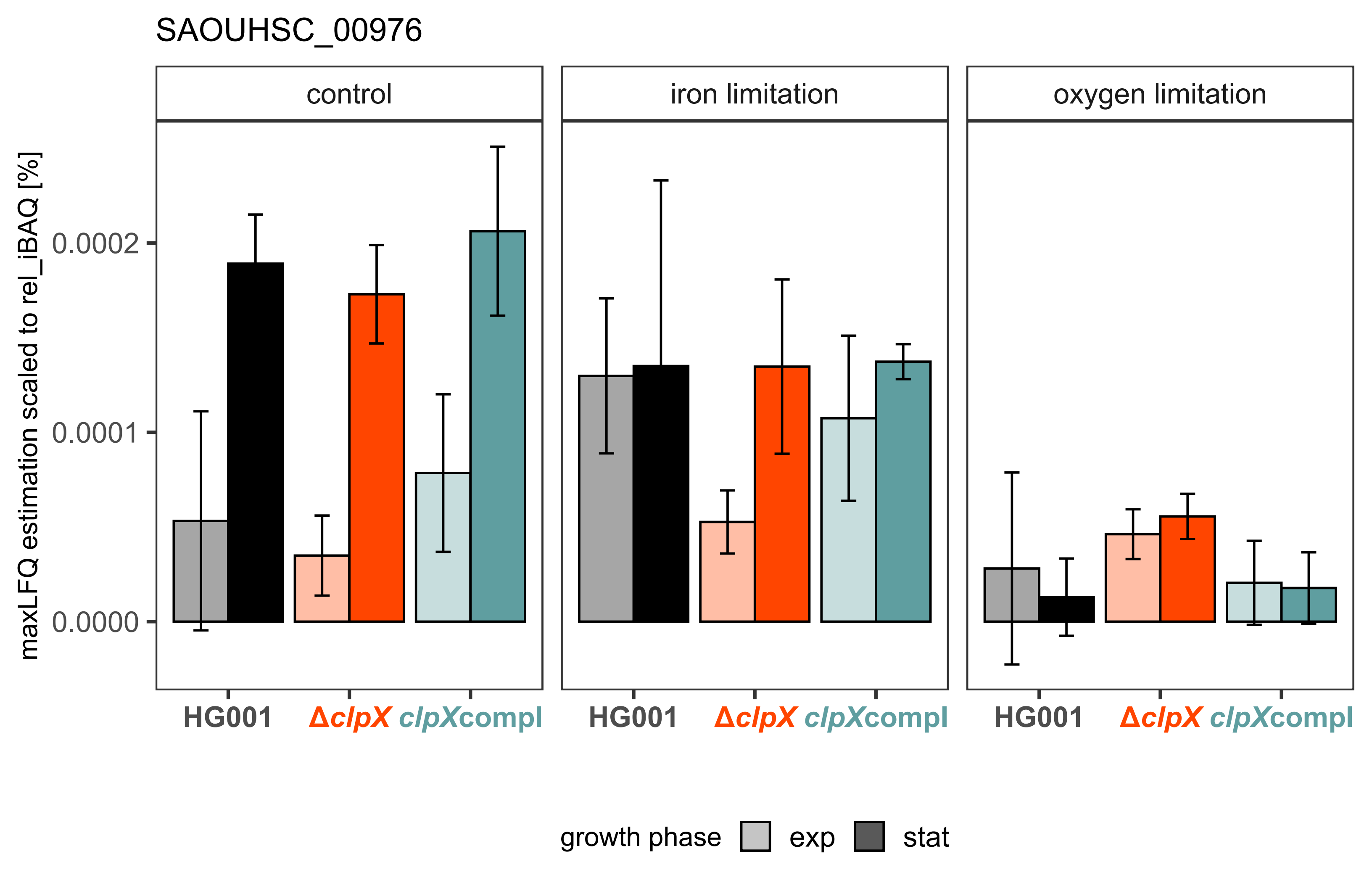

SAOUHSC_00976
control
iron limitation
oxygen limitation
maxLFQ estimation scaled to rel_iBAQ [%]
0.0002
0.0001
0.0000
HG001
ΔclpX
clpXcompl
growth phase
exp
stat

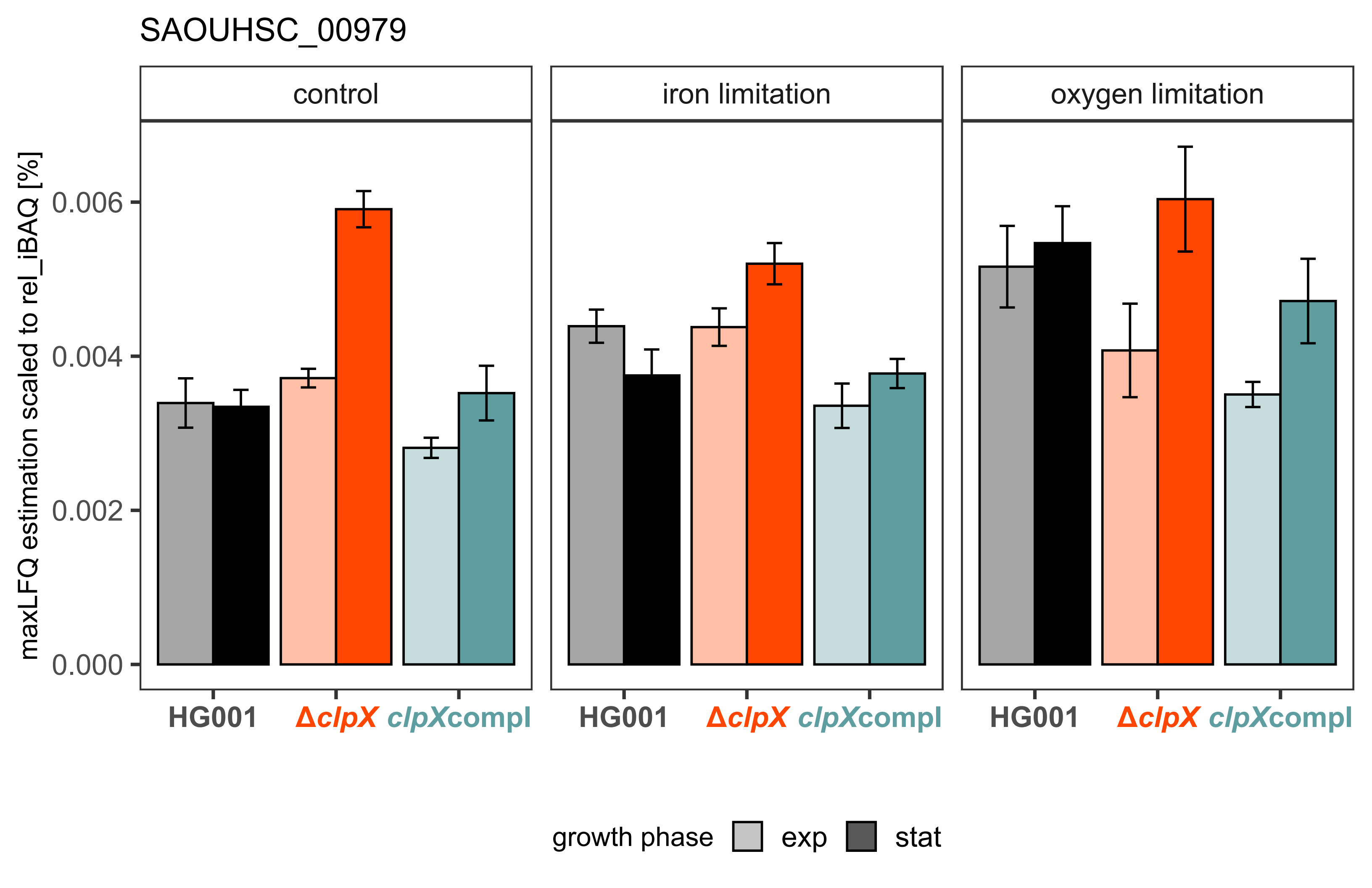
SAOUHSC_00979
control
iron limitation
oxygen limitation
maxLFQ estimation scaled to rel_iBAQ [%]
0.006
0.004
0.002
0.000
HG001
ΔclpX
clpXcompl
growth phase
exp
stat

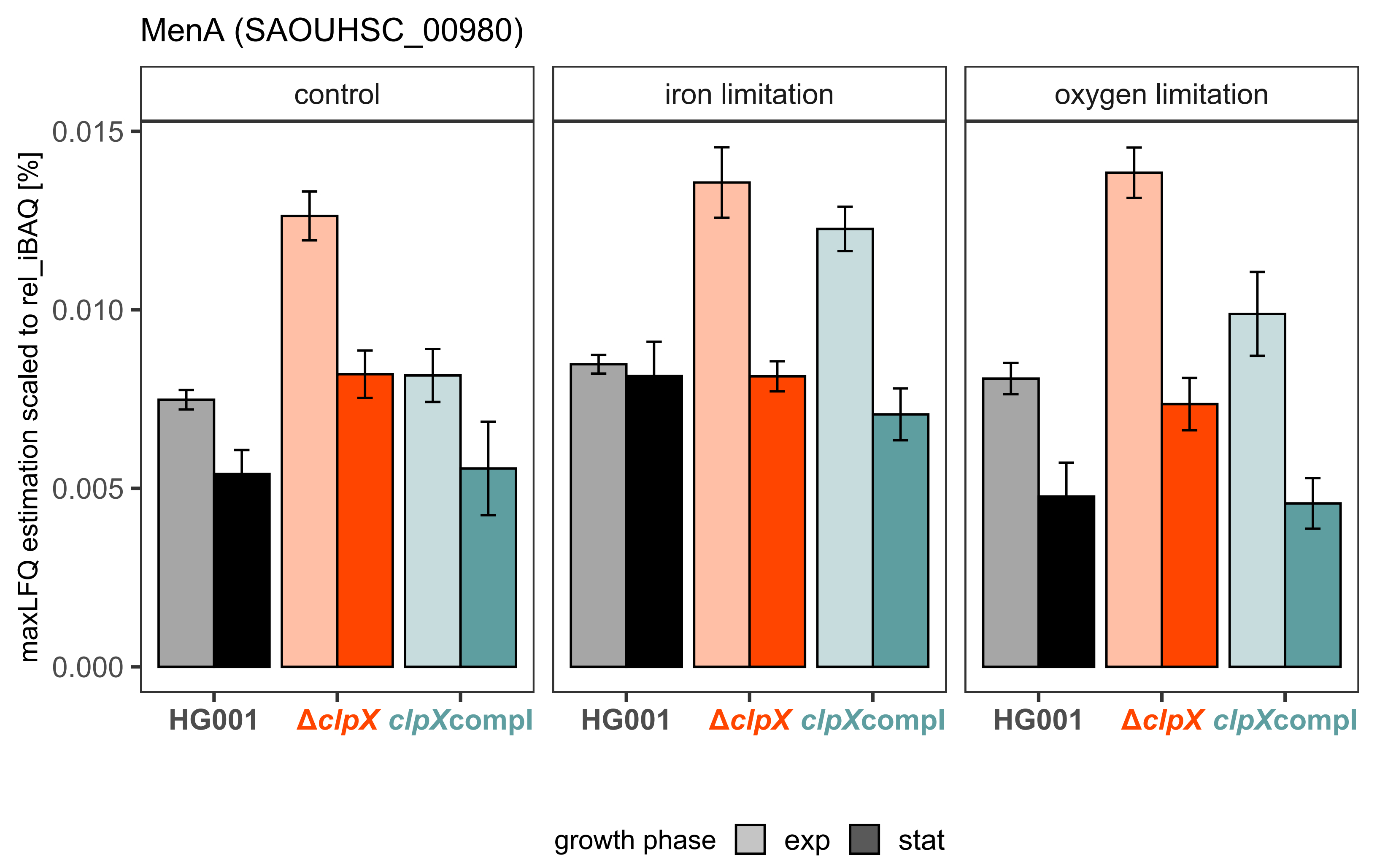

MenA (SAOUHSC_00980)
control
iron limitation
oxygen limitation
maxLFQ estimation scaled to rel_iBAQ [%]
0.015
0.010
0.005
0.000
HG001
ΔclpX
clpXcompl
growth phase
exp
stat

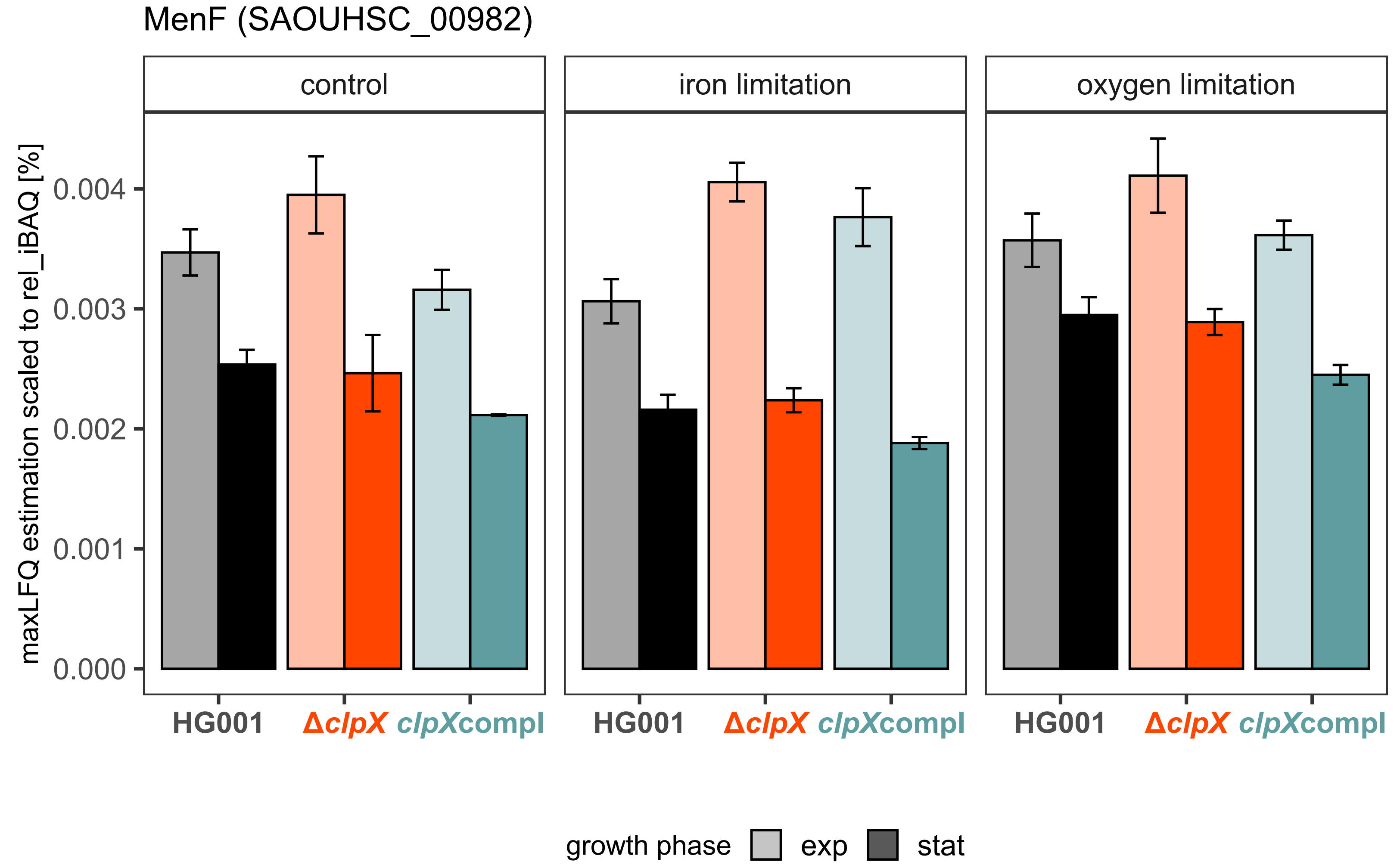
MenF (SAOUHSC_00982)
control
iron limitation
oxygen limitation
maxLFQ estimation scaled to rel_iBAQ [%]
0.004
0.003
0.002
0.001
0.000
HG001
ΔclpX
clpXcompl
growth phase
exp
stat

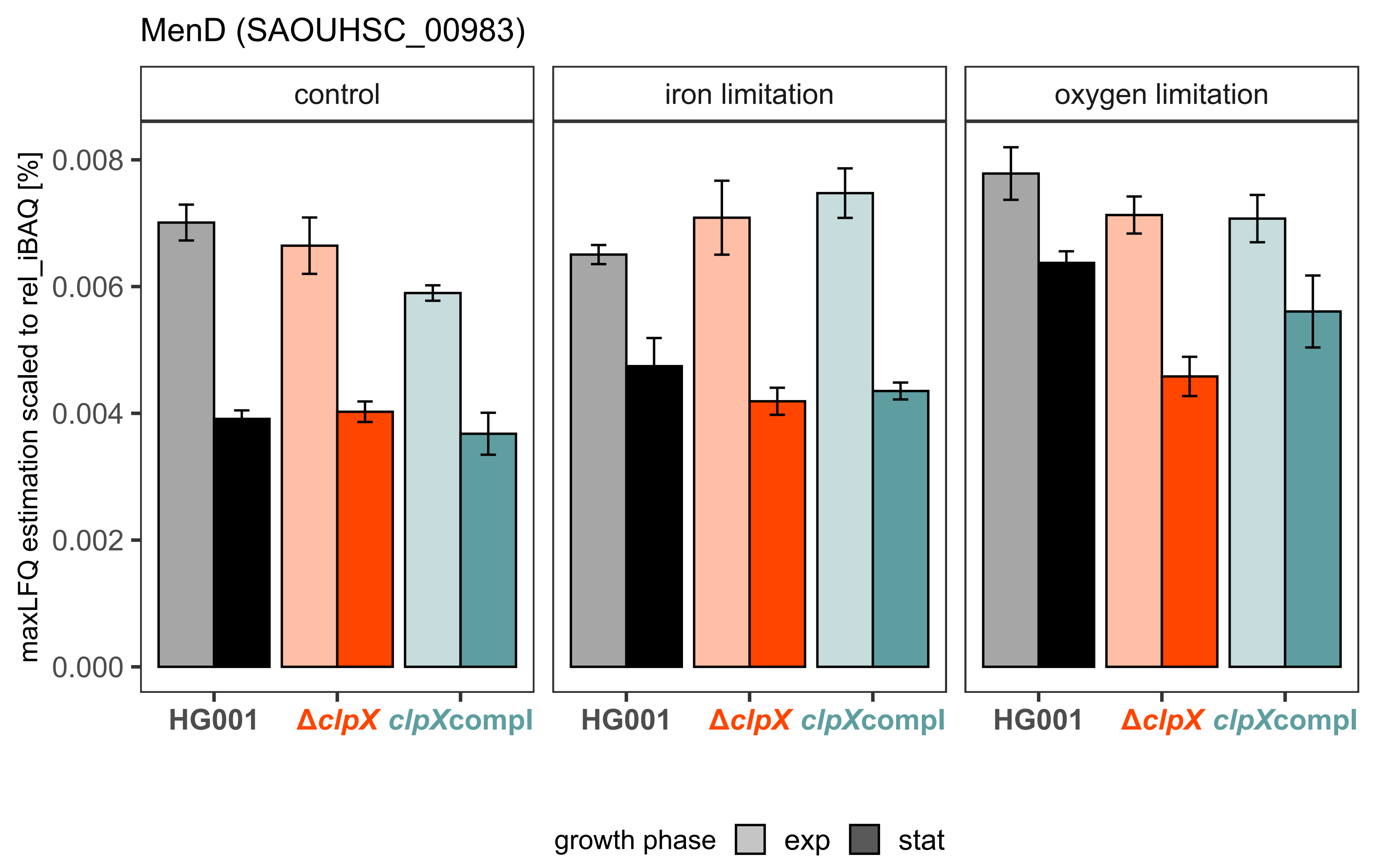
MenD (SAOUHSC_00983)
control
iron limitation
oxygen limitation
maxLFQ estimation scaled to rel_iBAQ [%]
0.008
0.006
0.004
0.002
0.000
HG001
ΔclpX
clpXcompl
growth phase
exp
stat

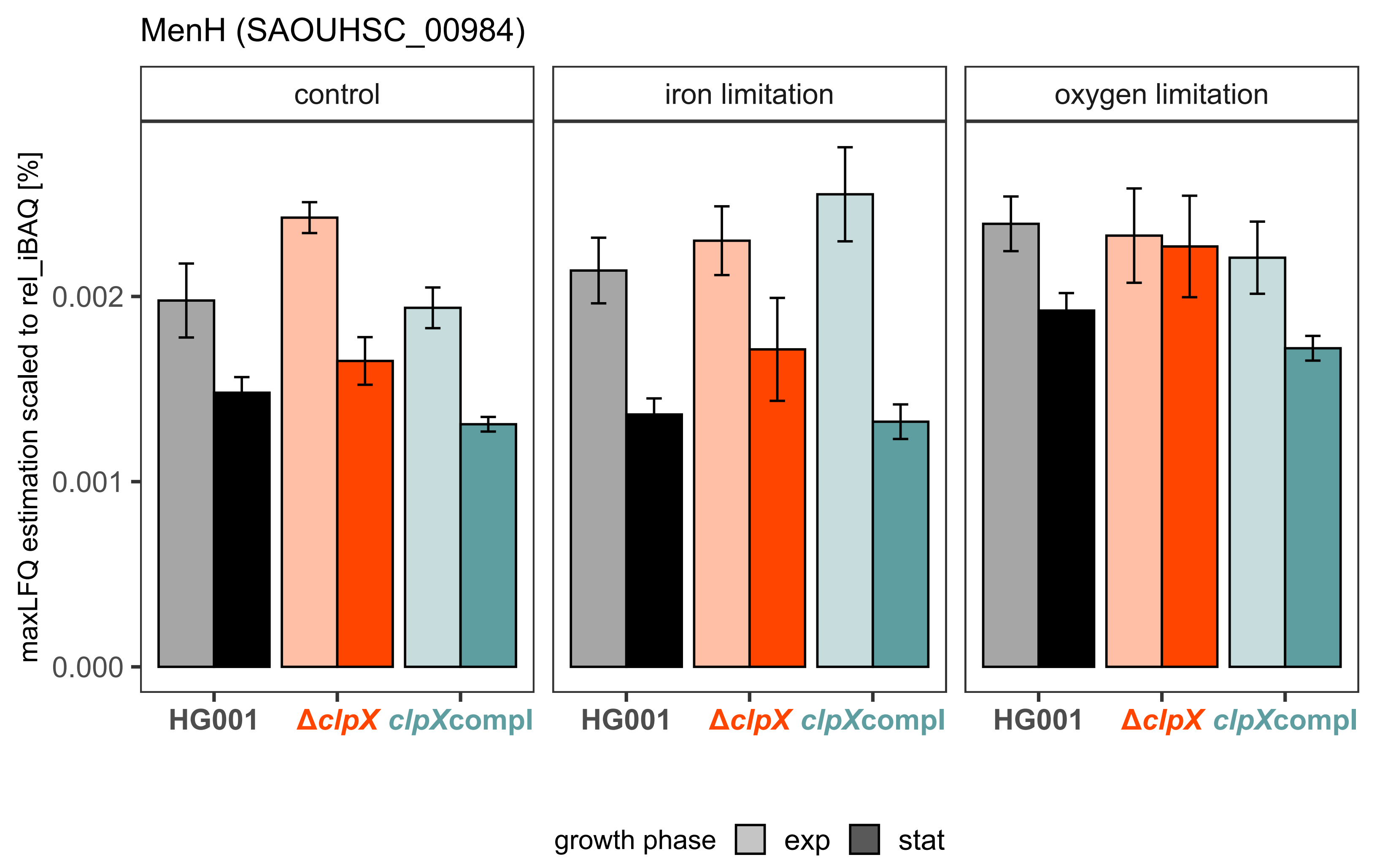
MenH (SAOUHSC_00984)
control
iron limitation
oxygen limitation
maxLFQ estimation scaled to rel_iBAQ [%]
0.002
0.001
0.000
HG001
ΔclpX
clpXcompl
growth phase
exp
stat

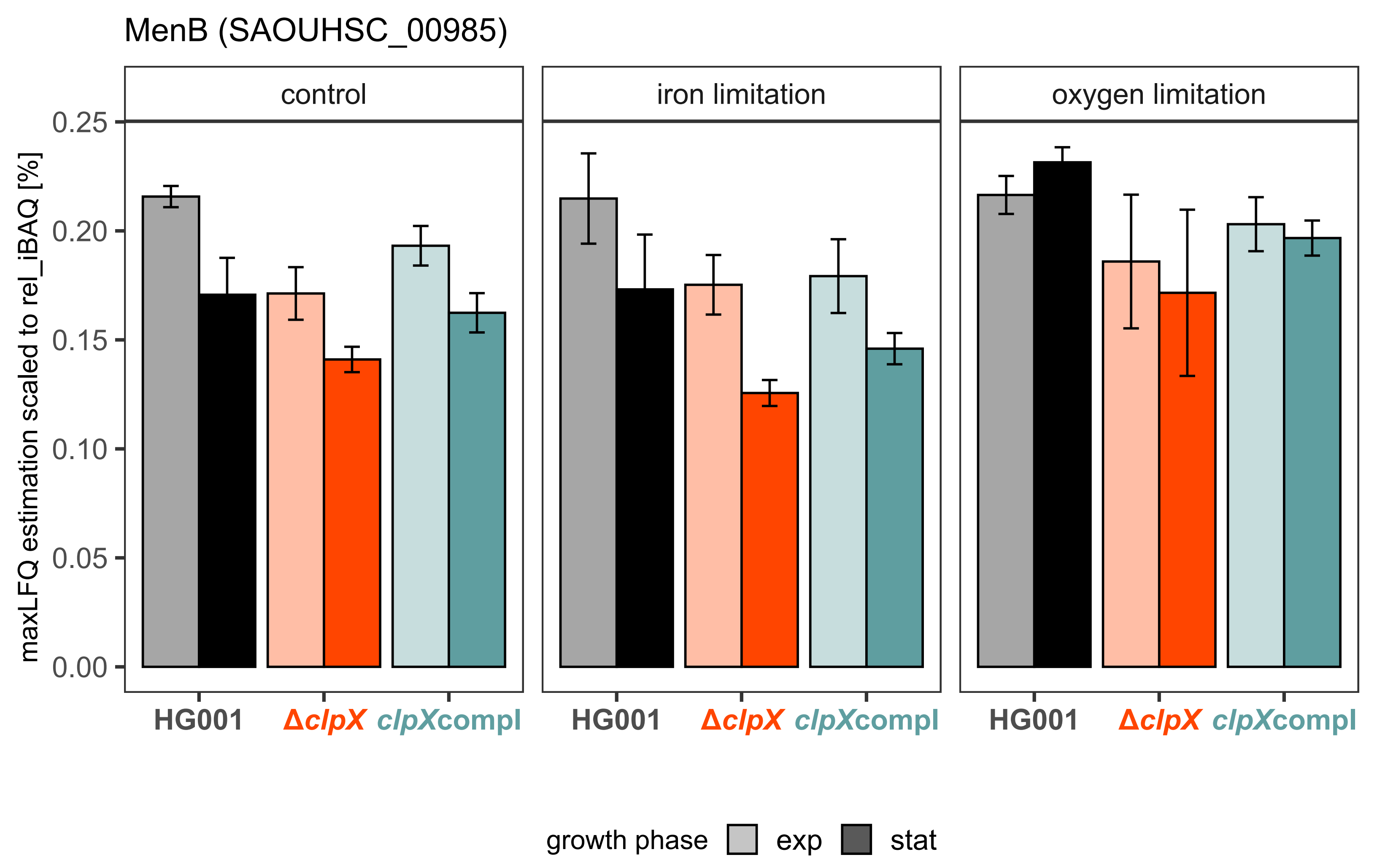

MenB (SAOUHSC_00985)
control
iron limitation
oxygen limitation
maxLFQ estimation scaled to rel_iBAQ [%]
0.25
0.20
0.15
0.10
0.05
0.00
HG001
ΔclpX
clpXcompl
growth phase
exp
stat

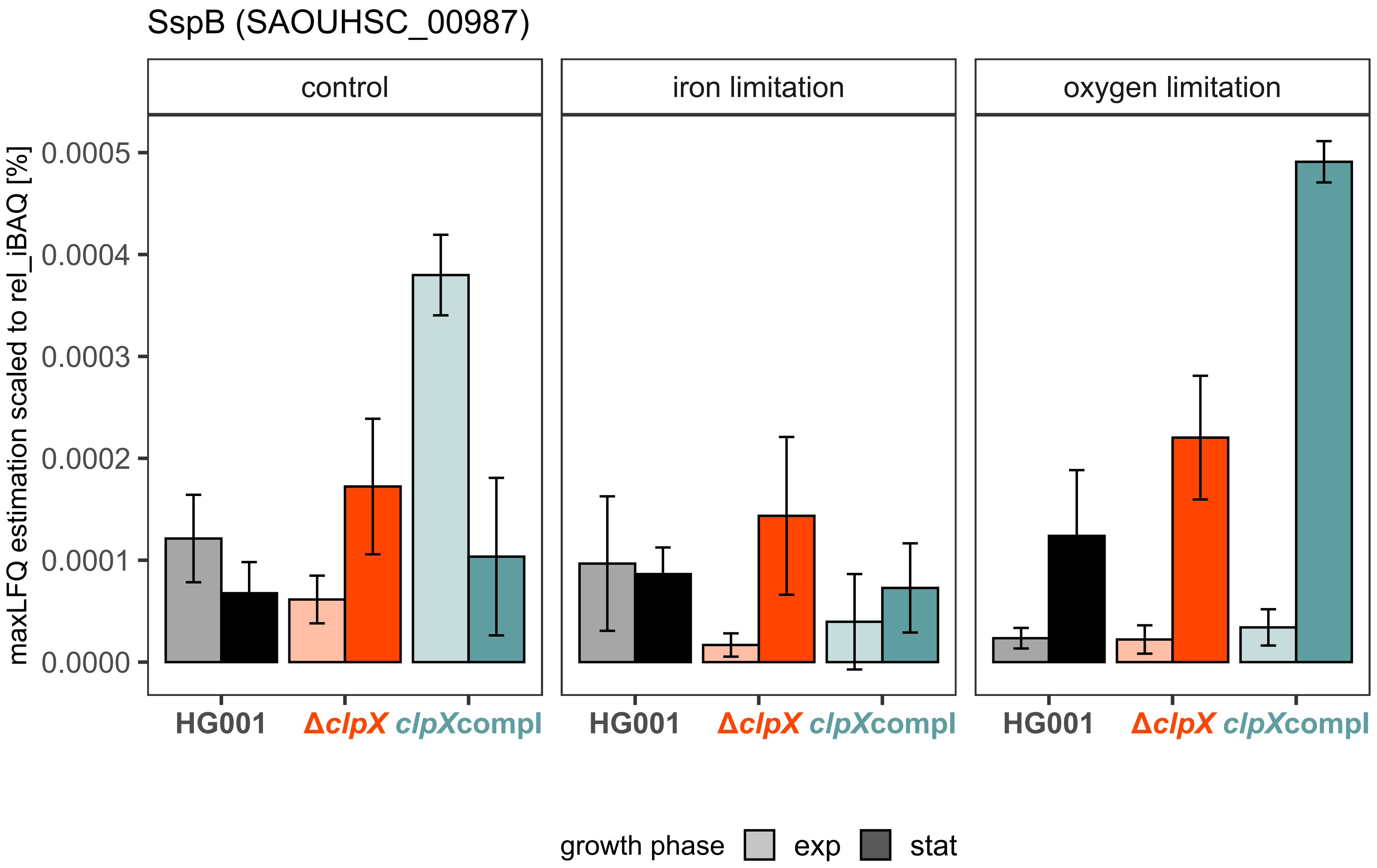

SspB (SAOUHSC_00987)
control
iron limitation
oxygen limitation
maxLFQ estimation scaled to rel_iBAQ [%]
0.0005
0.0004
0.0003
0.0002
0.0001
0.0000
HG001
ΔclpX
clpXcompl
growth phase
exp
stat

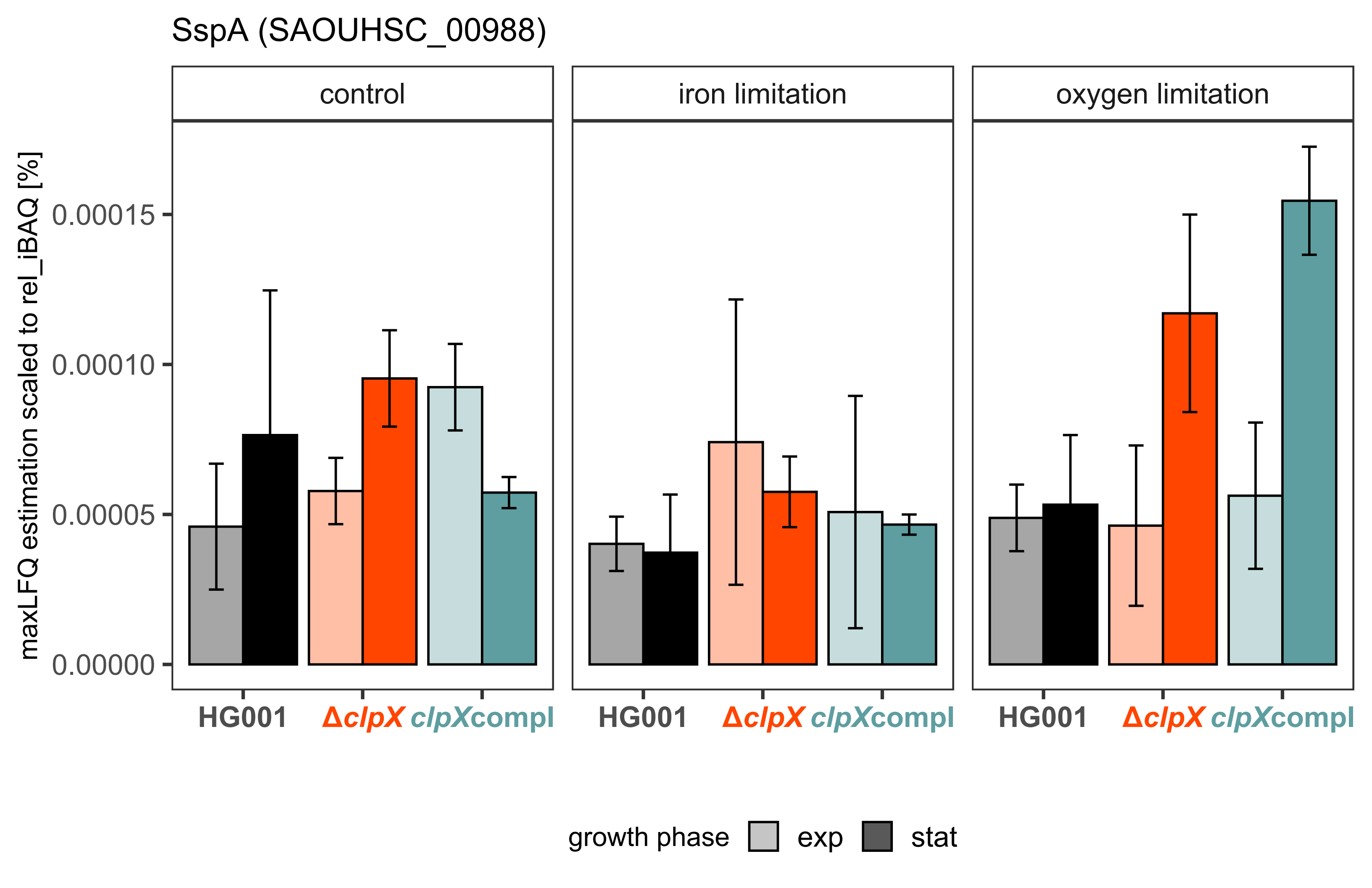

SspA (SAOUHSC_00988)
control
iron limitation
oxygen limitation
maxLFQ estimation scaled to rel_iBAQ [%]
0.00015
0.00010
0.00005
0.00000
HG001
ΔclpX
clpXcompl
growth phase
exp
stat

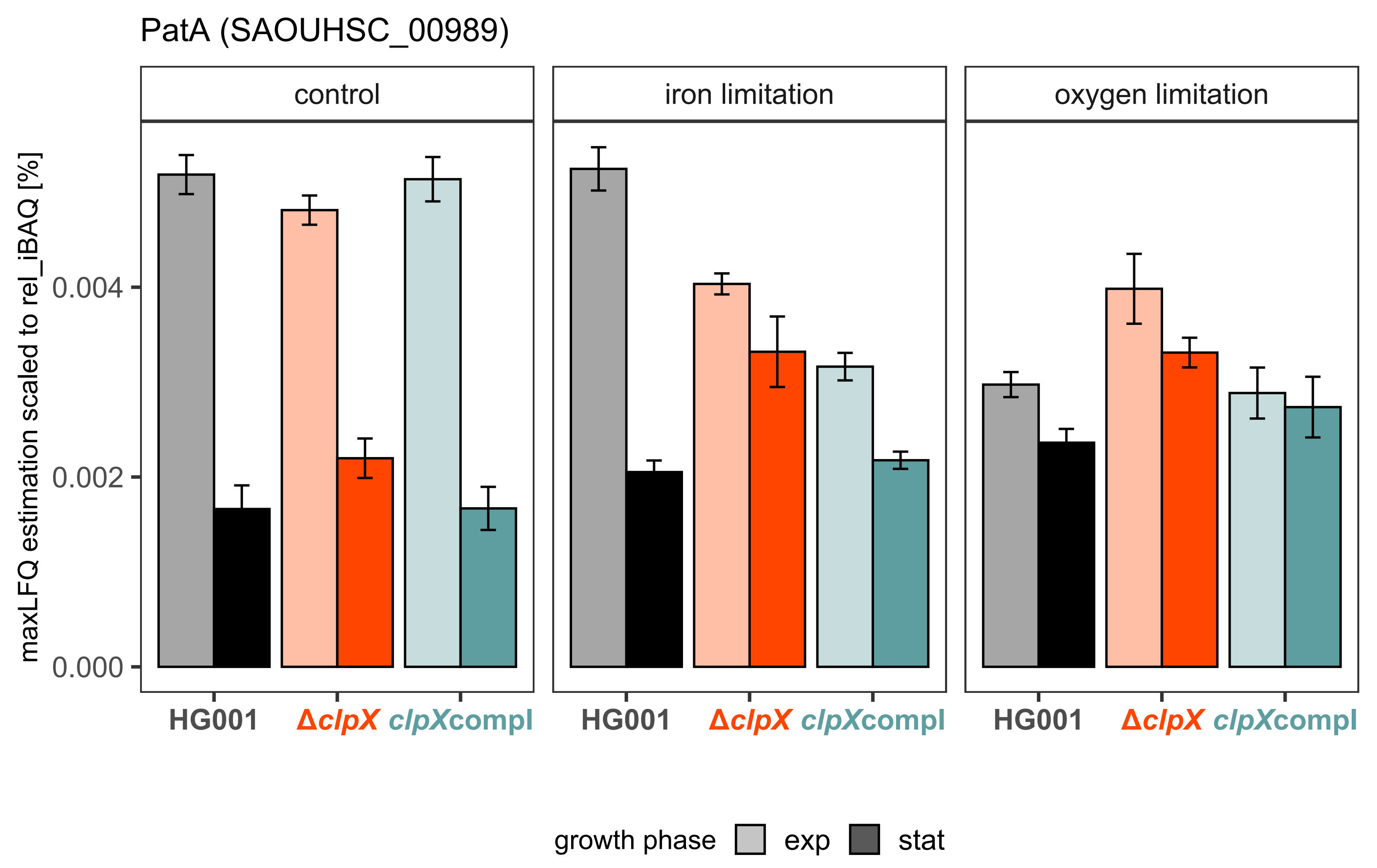

PatA (SAOUHSC_00989)
control
iron limitation
oxygen limitation
maxLFQ estimation scaled to rel_iBAQ [%]
0.004
0.002
0.000
HG001
ΔclpX
clpXcompl
growth phase
exp
stat

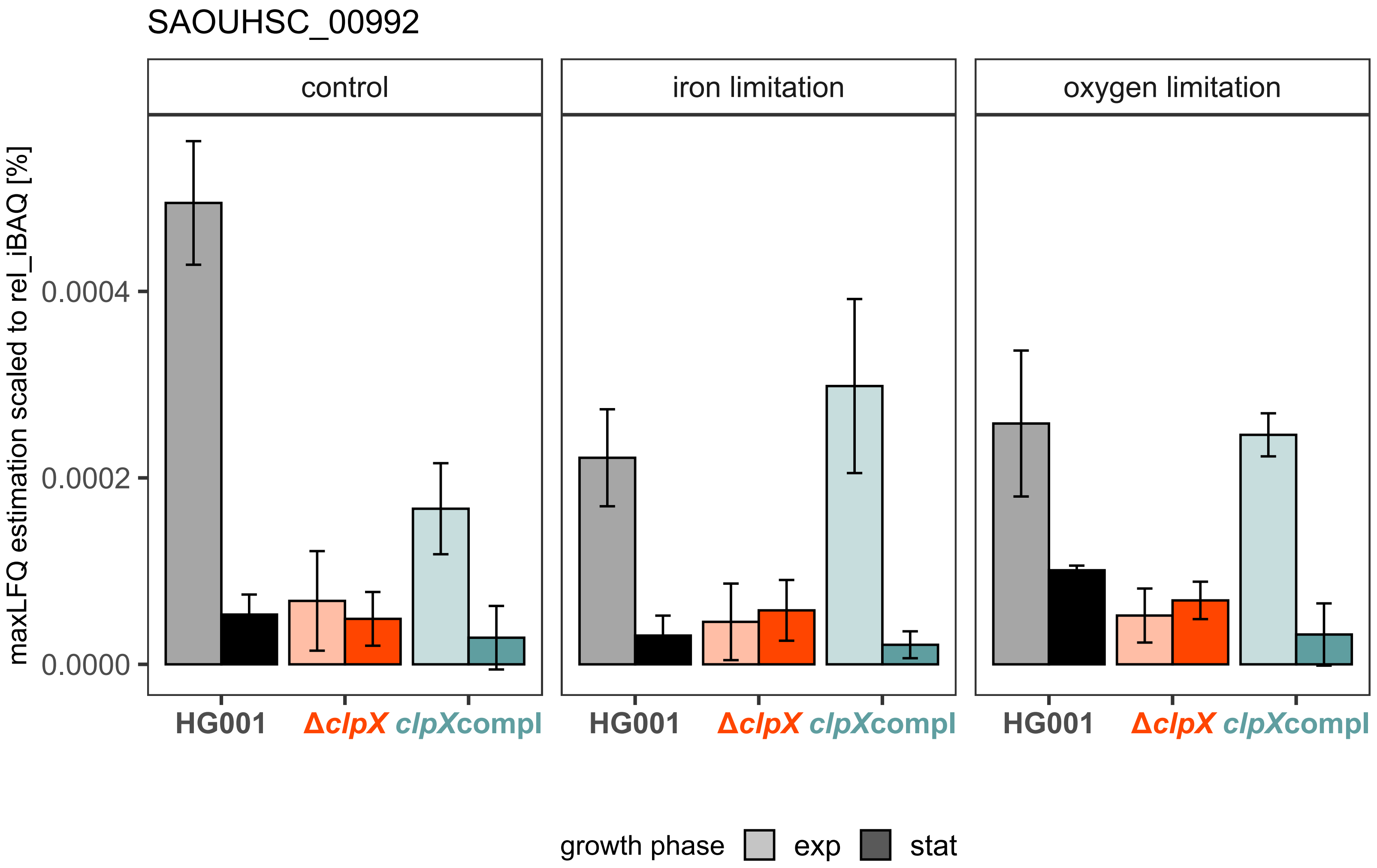
SAOUHSC_00992
control
iron limitation
oxygen limitation
maxLFQ estimation scaled to rel_iBAQ [%]
0.0004
0.0002
0.0000
HG001
ΔclpX
clpXcompl
HG001
ΔclpX
clpXcompl
HG001
ΔclpX
clpXcompl
growth phase
exp
stat

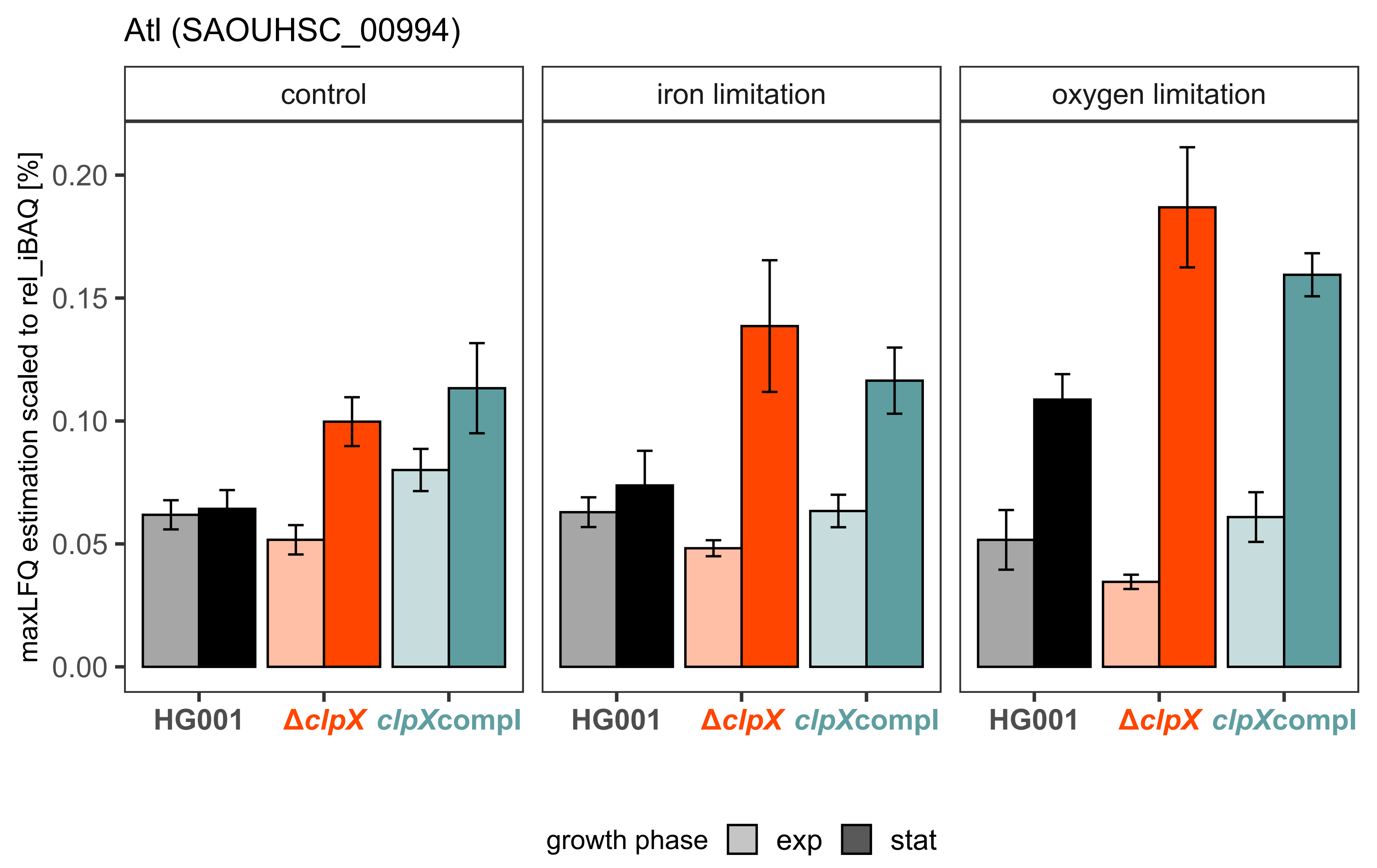
Atl (SAOUHSC_00994)
control
iron limitation
oxygen limitation
maxLFQ estimation scaled to rel_iBAQ [%]
0.20
0.15
0.10
0.05
0.00
HG001
ΔclpX
clpXcompl
growth phase
exp
stat

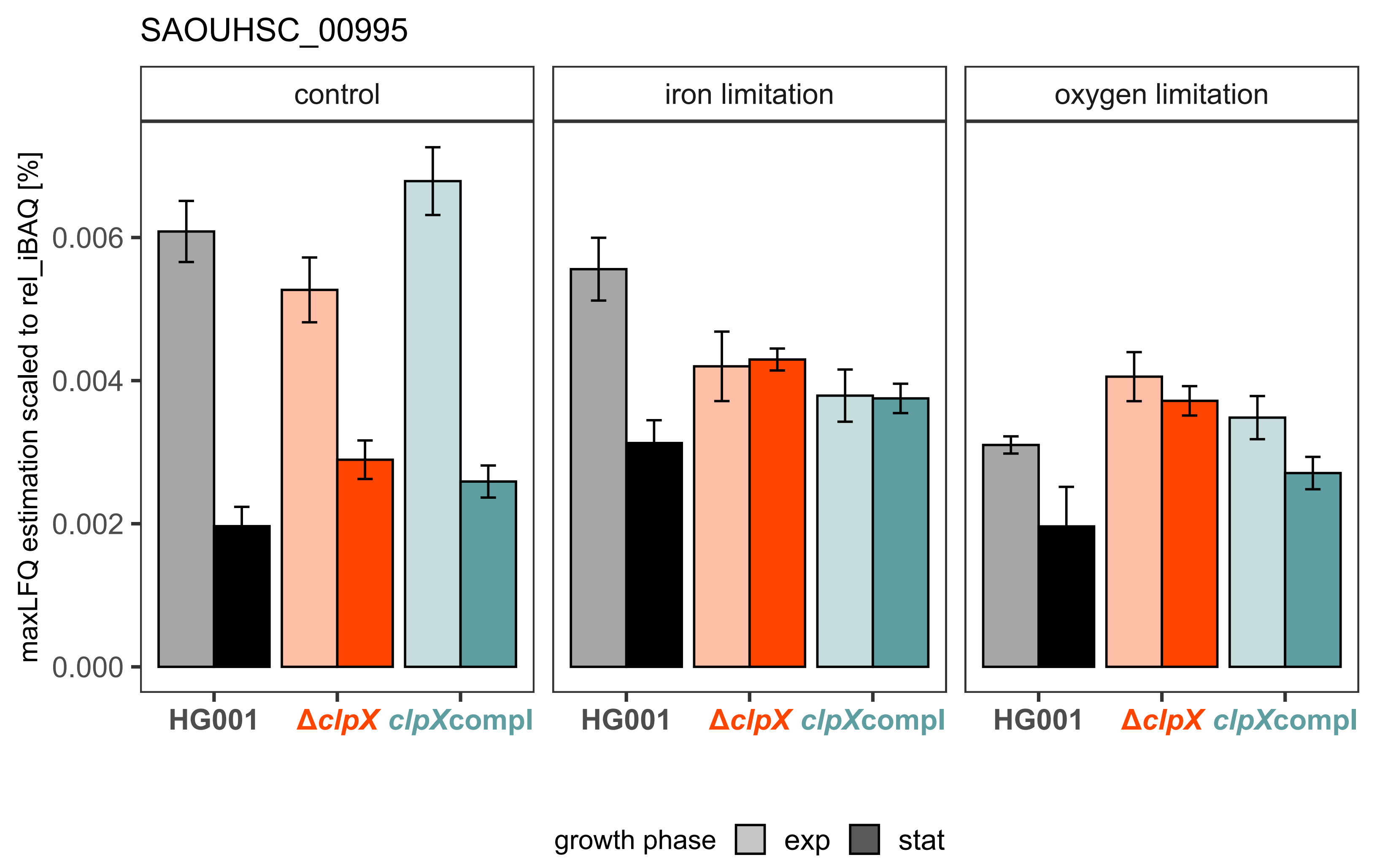

SAOUHSC_00995
control
iron limitation
oxygen limitation
maxLFQ estimation scaled to rel_iBAQ [%]
0.000
0.002
0.004
0.006
HG001
ΔclpX
clpXcompl
growth phase
exp
stat

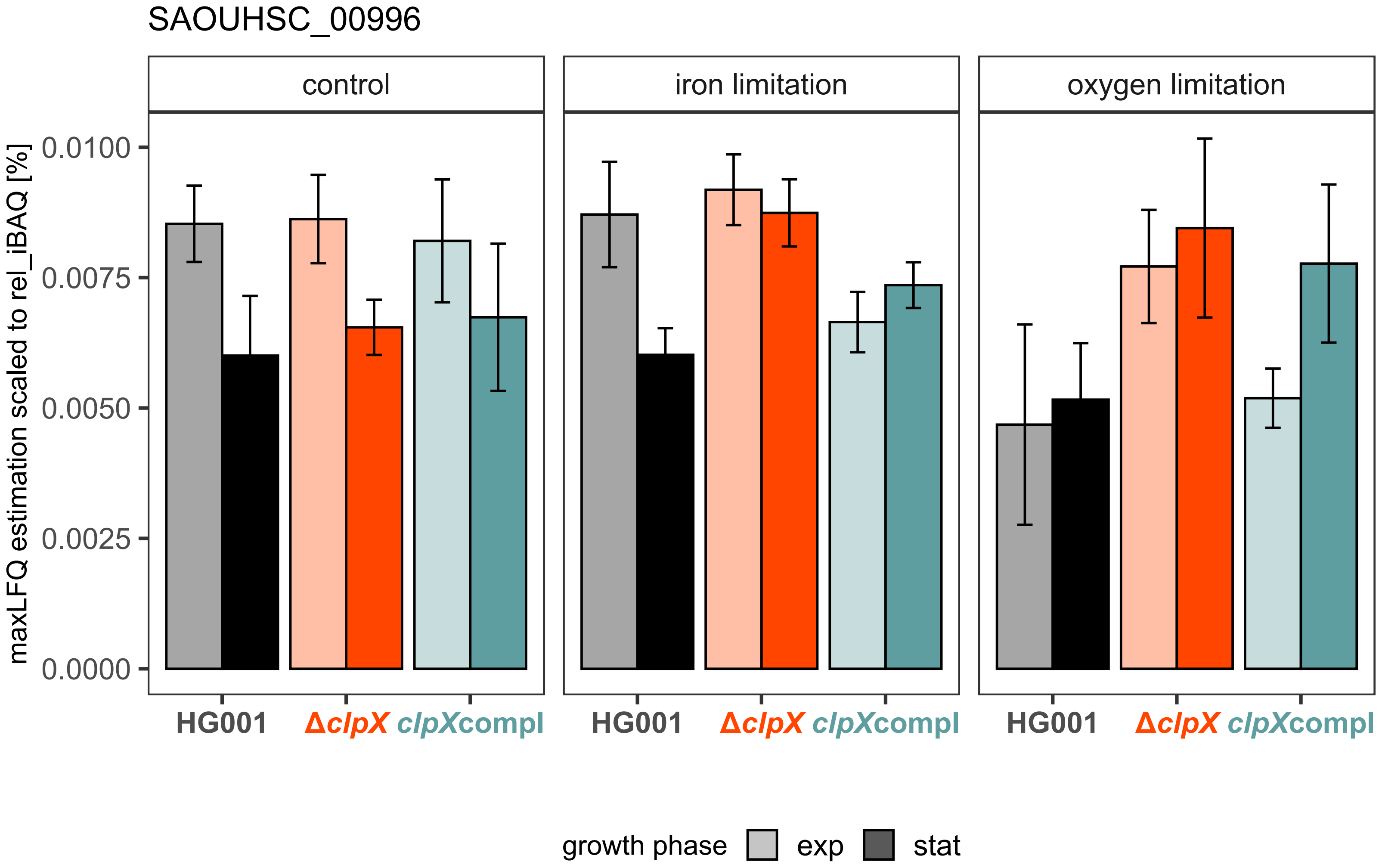
SAOUHSC_00996
control
iron limitation
oxygen limitation
maxLFQ estimation scaled to rel_iBAQ [%]
0.0100
0.0075
0.0050
0.0025
0.0000
HG001
ΔclpX
clpXcompl
growth phase
exp
stat

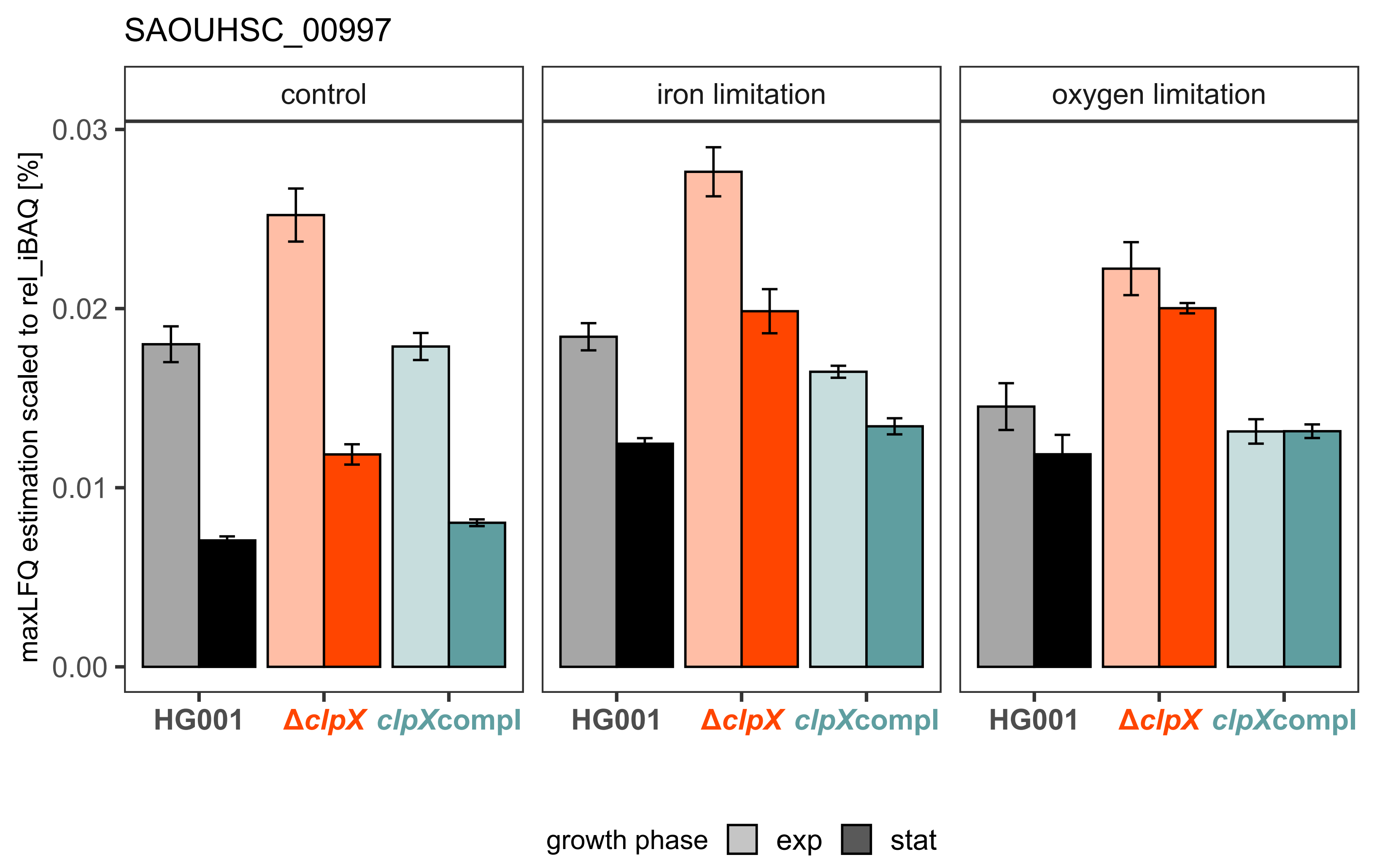

SAOUHSC_00997
control
iron limitation
oxygen limitation
maxLFQ estimation scaled to rel_iBAQ [%]
0.03
0.02
0.01
0.00
HG001
ΔclpX
clpXcompl
HG001
ΔclpX
clpXcompl
HG001
ΔclpX
clpXcompl
growth phase
exp
stat

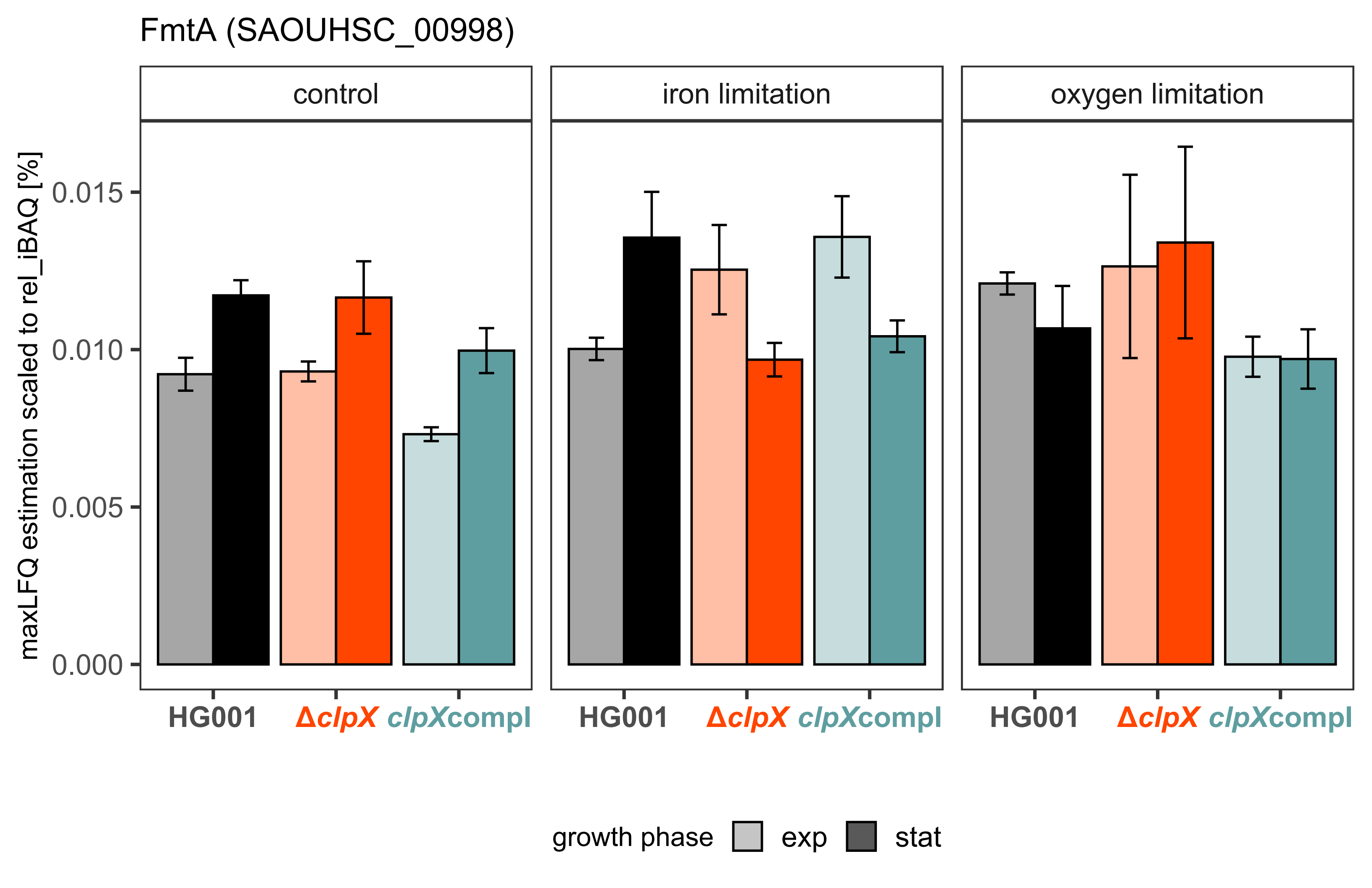
FmtA (SAOUHSC_00998)
control
iron limitation
oxygen limitation
maxLFQ estimation scaled to rel_iBAQ [%]
0.000
0.005
0.010
0.015
HG001
ΔclpX
clpXcompl
growth phase
exp
stat

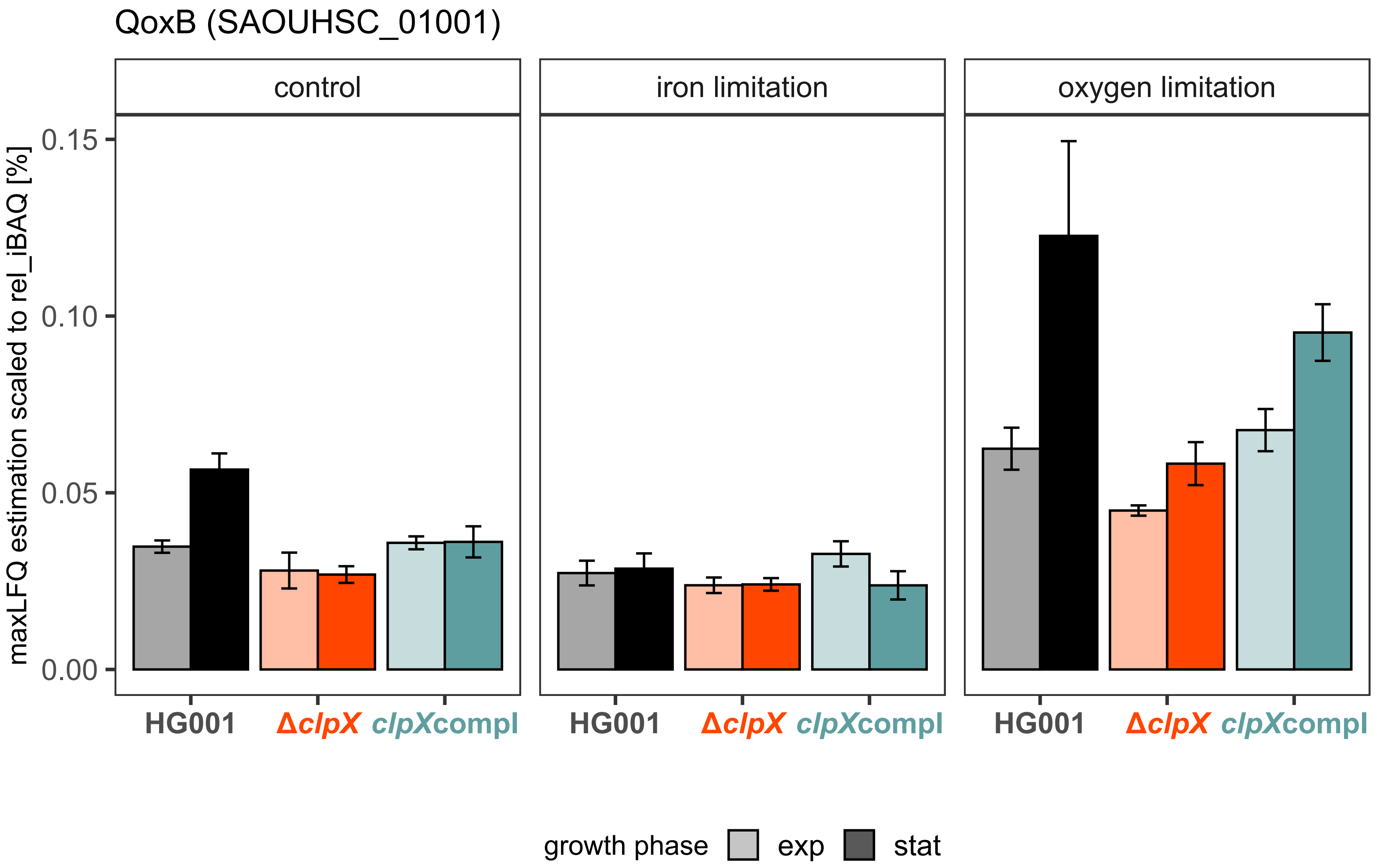

QoxB (SAOUHSC_01001)
control
iron limitation
oxygen limitation
maxLFQ estimation scaled to rel_iBAQ [%]
0.15
0.10
0.05
0.00
HG001
ΔclpX
clpXcompl
growth phase
exp
stat

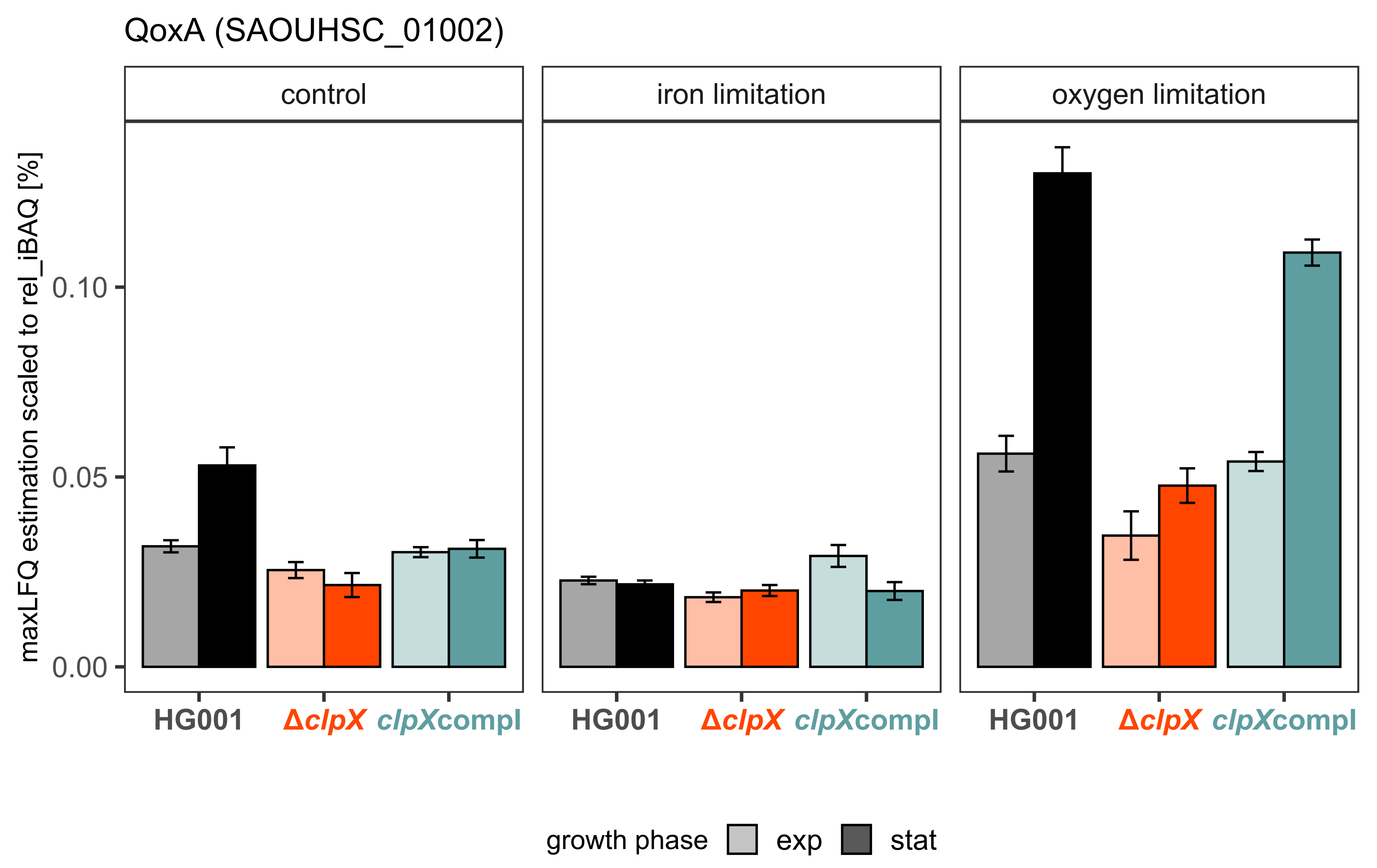

QoxA (SAOUHSC_01002)
control
iron limitation
oxygen limitation
maxLFQ estimation scaled to rel_iBAQ [%]
0.10
0.05
0.00
HG001
ΔclpX
clpXcompl
growth phase
exp
stat

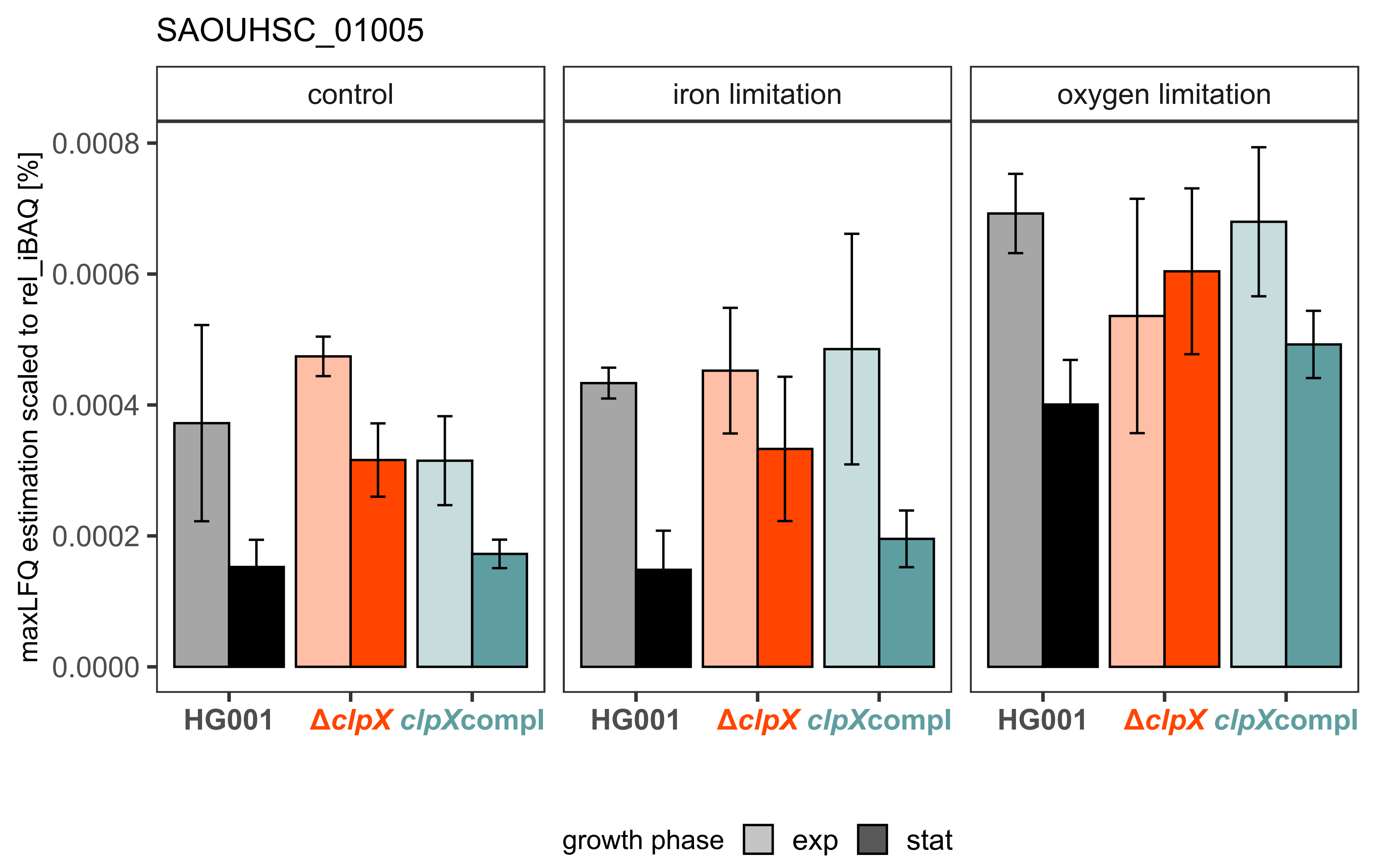
SAOUHSC_01005
control
iron limitation
oxygen limitation
0.0008
0.0006
0.0004
0.0002
0.0000
maxLFQ estimation scaled to rel_iBAQ [%]
HG001
ΔclpX
clpXcompl
growth phase
exp
stat

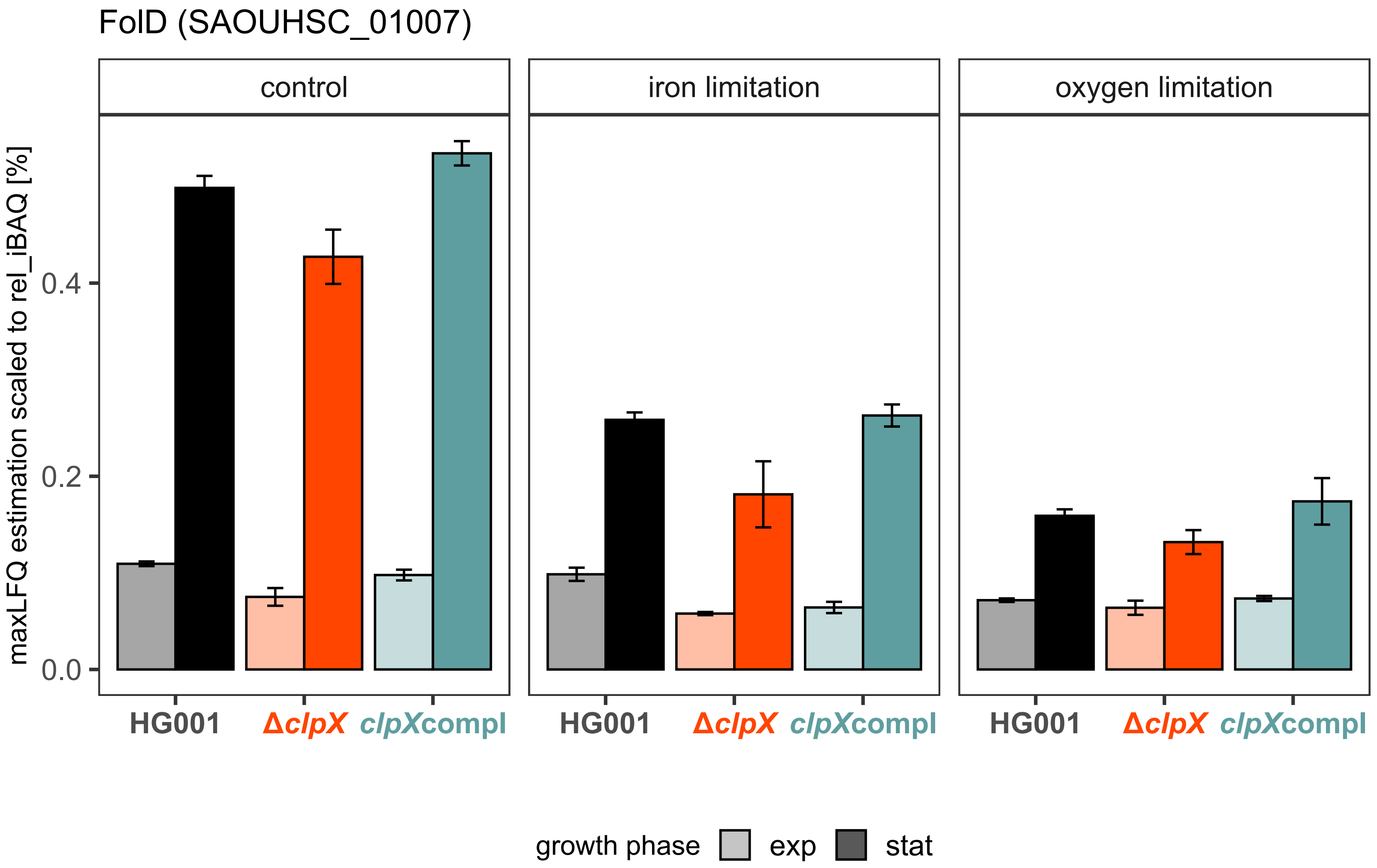
FolD (SAOUHSC_01007)
control
iron limitation
oxygen limitation
maxLFQ estimation scaled to rel_iBAQ [%]
0.0
0.2
0.4
HG001
ΔclpX
clpXcompl
growth phase
exp
stat

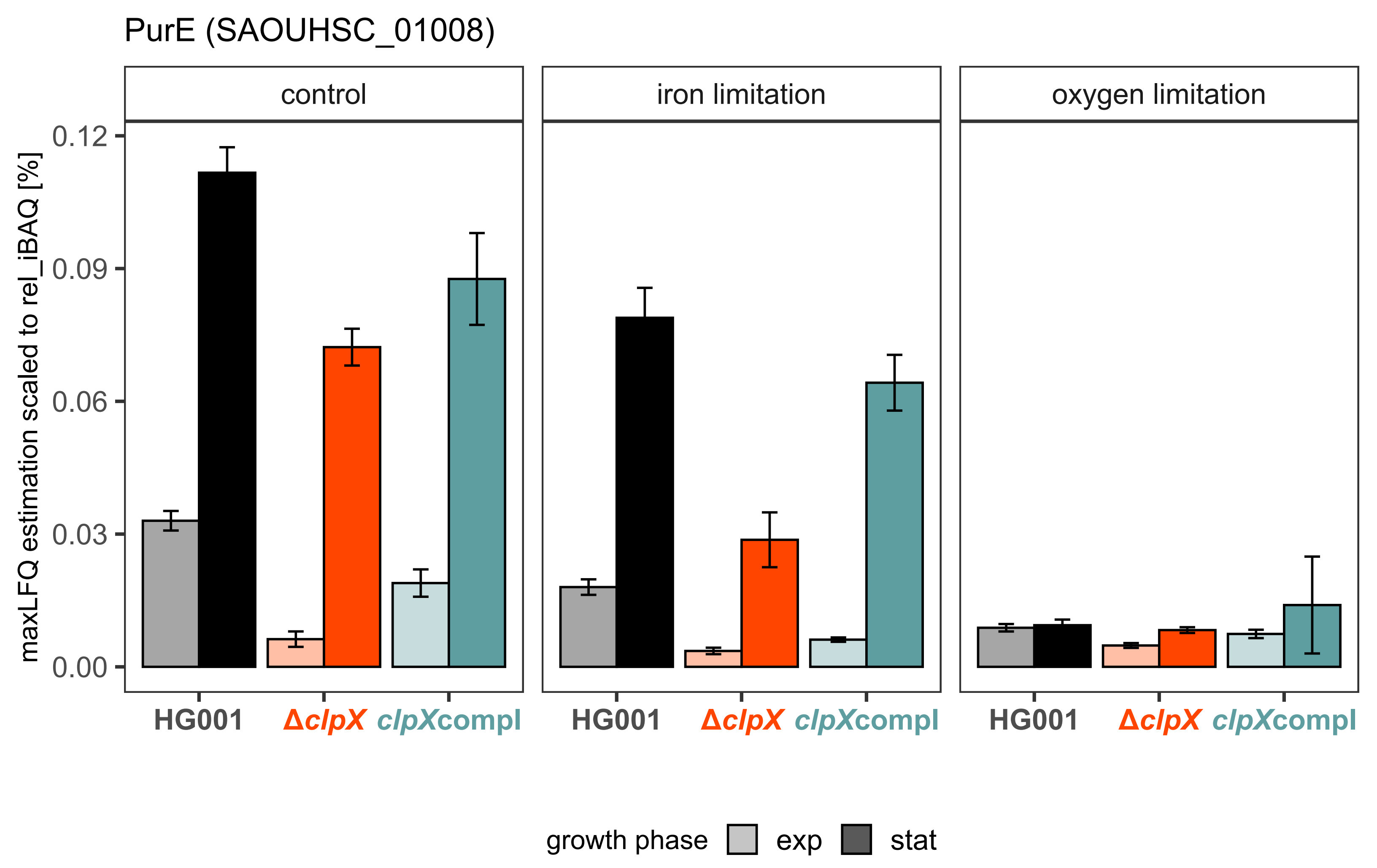

PurE (SAOUHSC_01008)
control
iron limitation
oxygen limitation
maxLFQ estimation scaled to rel_iBAQ [%]
0.12
0.09
0.06
0.03
0.00
HG001
ΔclpX
clpXcompl
growth phase
exp
stat

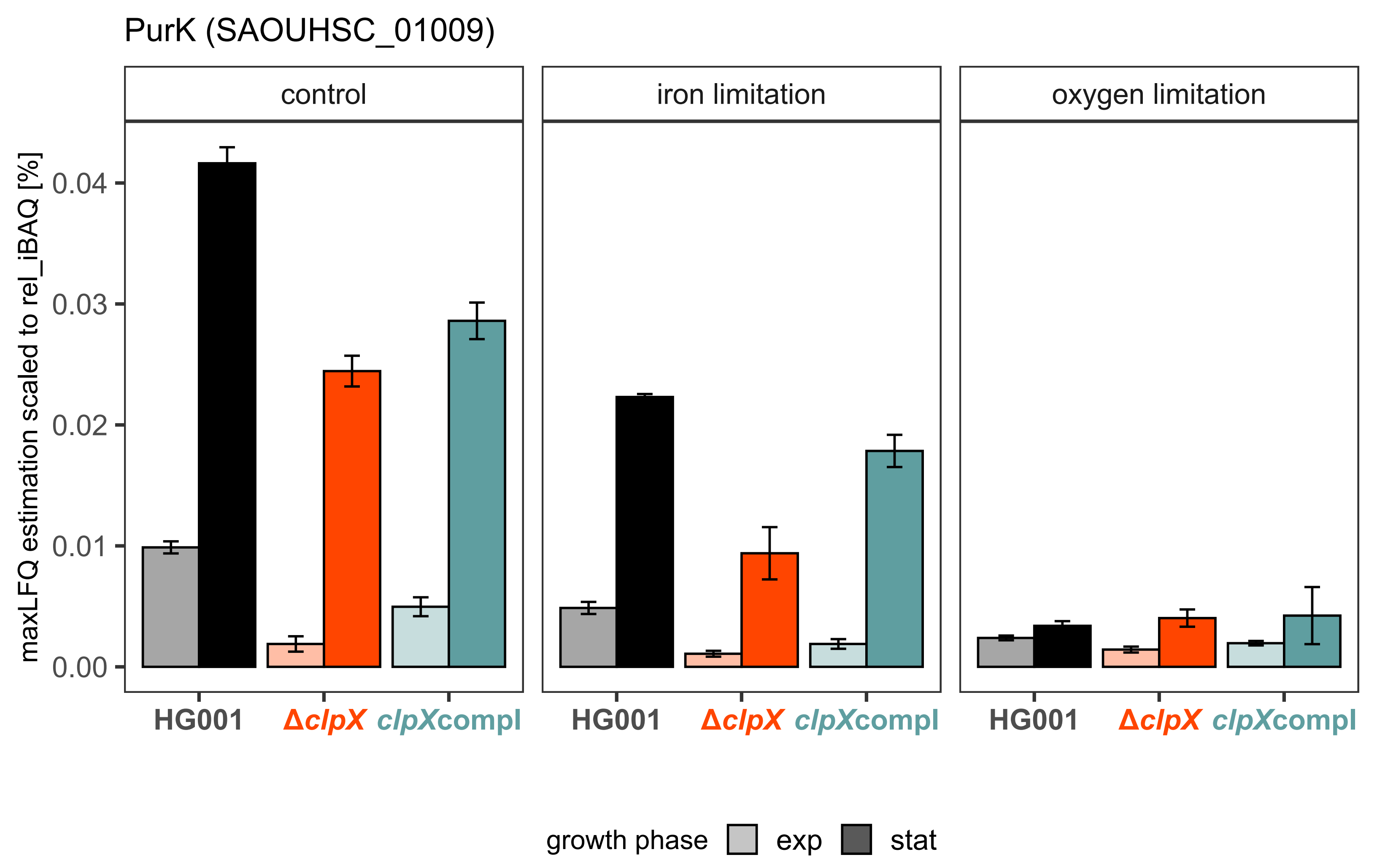
PurK (SAOUHSC_01009)
control
iron limitation
oxygen limitation
maxLFQ estimation scaled to rel_iBAQ [%]
0.00
0.01
0.02
0.03
0.04
HG001
ΔclpX
clpXcompl
growth phase
exp
stat

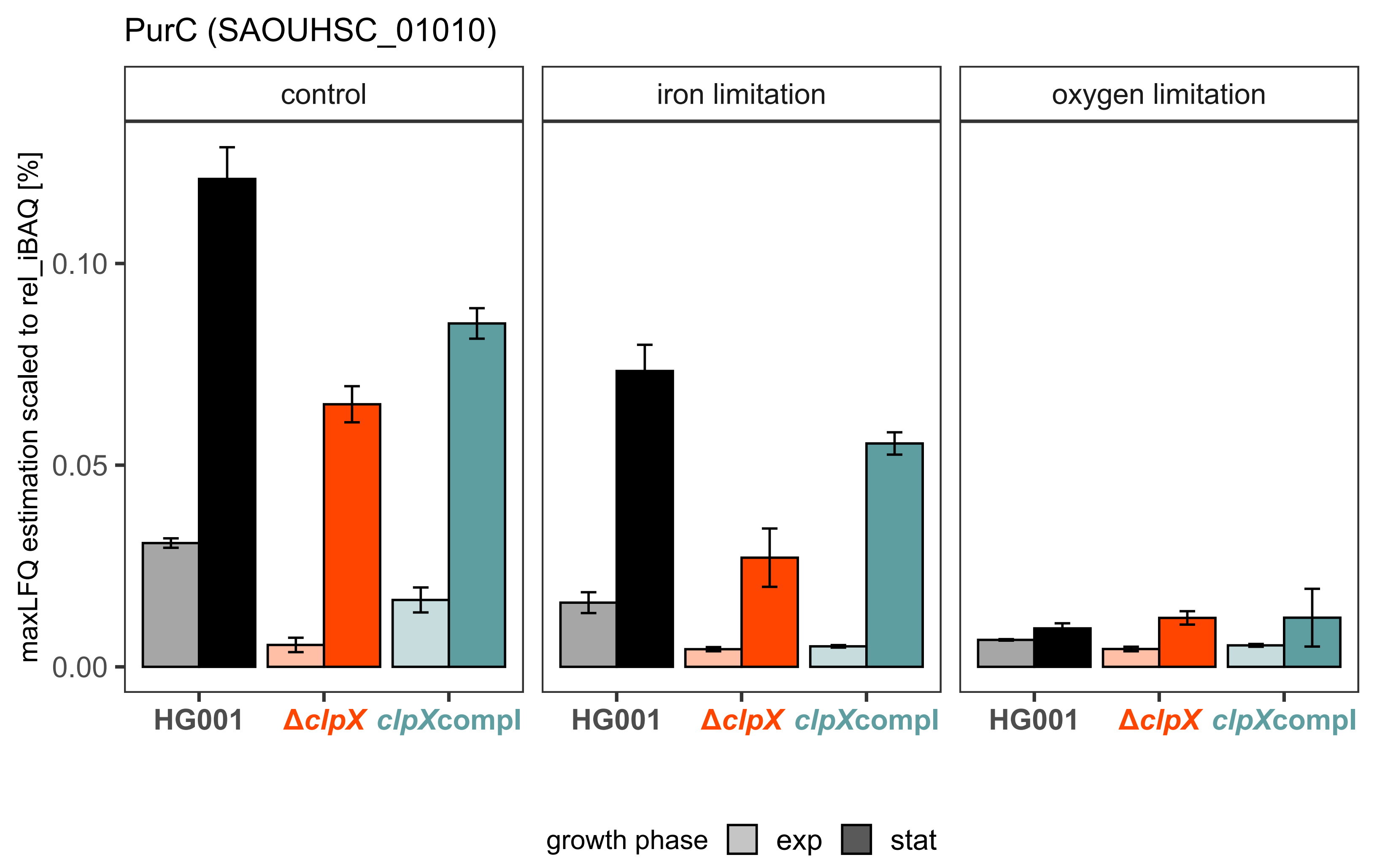

PurC (SAOUHSC_01010)
control
iron limitation
oxygen limitation
maxLFQ estimation scaled to rel_iBAQ [%]
0.10
0.05
0.00
HG001
ΔclpX
clpXcompl
growth phase
exp
stat

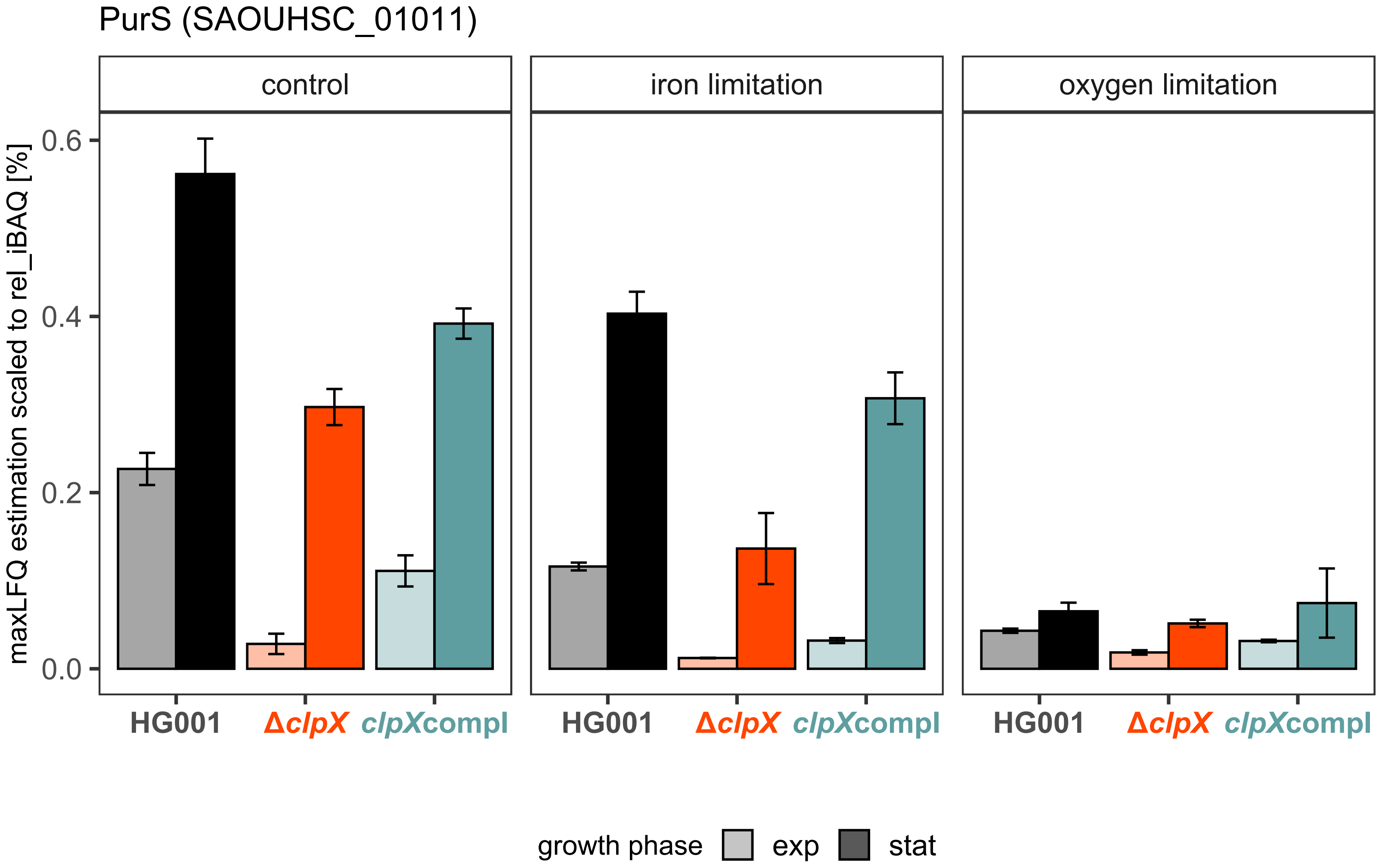
PurS (SAOUHSC_01011)
control
iron limitation
oxygen limitation
maxLFQ estimation scaled to rel_iBAQ [%]
0.0
0.2
0.4
0.6
HG001
ΔclpX
clpXcompl
growth phase
exp
stat

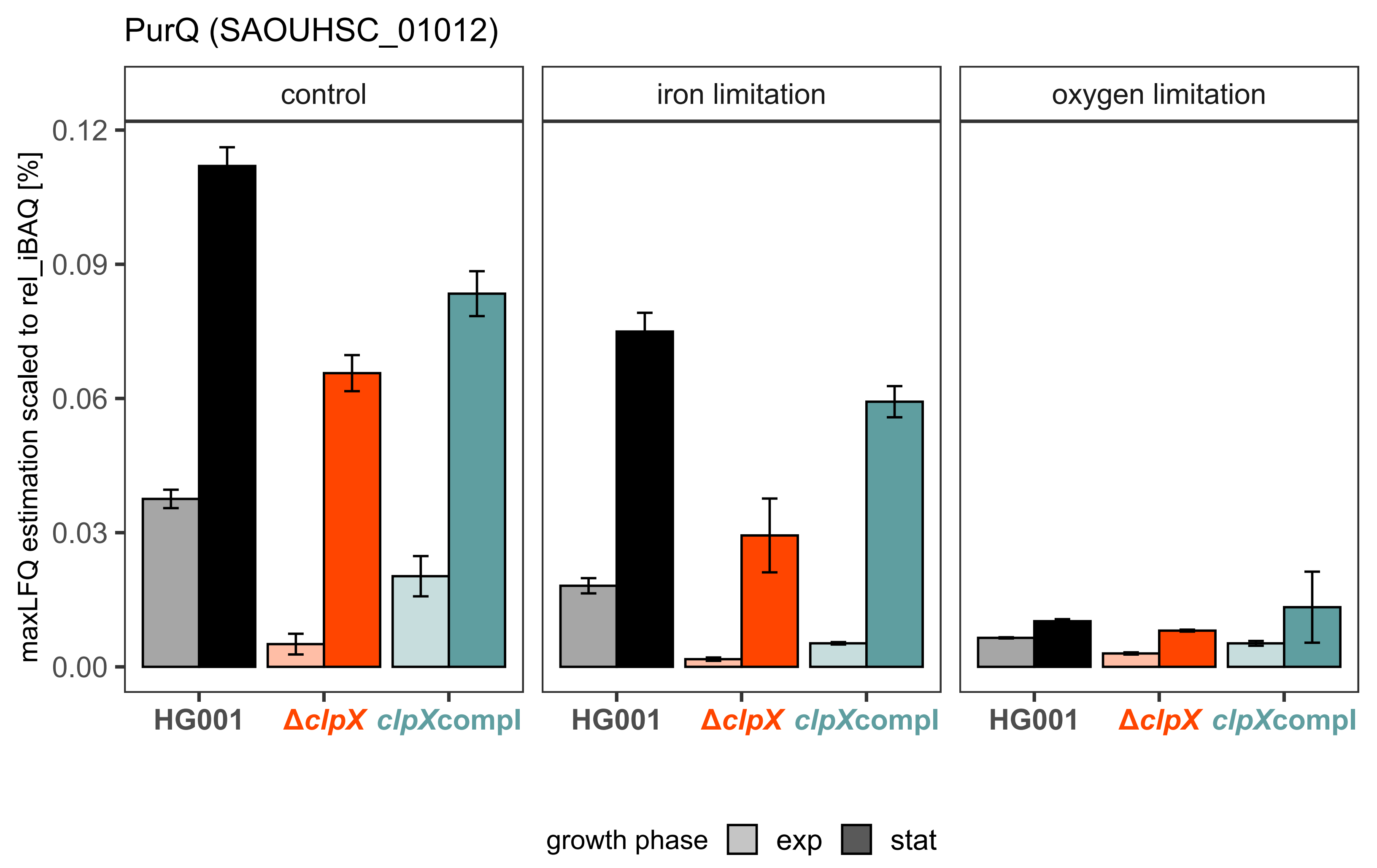
PurQ (SAOUHSC_01012)
control
iron limitation
oxygen limitation
maxLFQ estimation scaled to rel_iBAQ [%]
0.12
0.09
0.06
0.03
0.00
HG001
ΔclpX
clpXcompl
growth phase
exp
stat

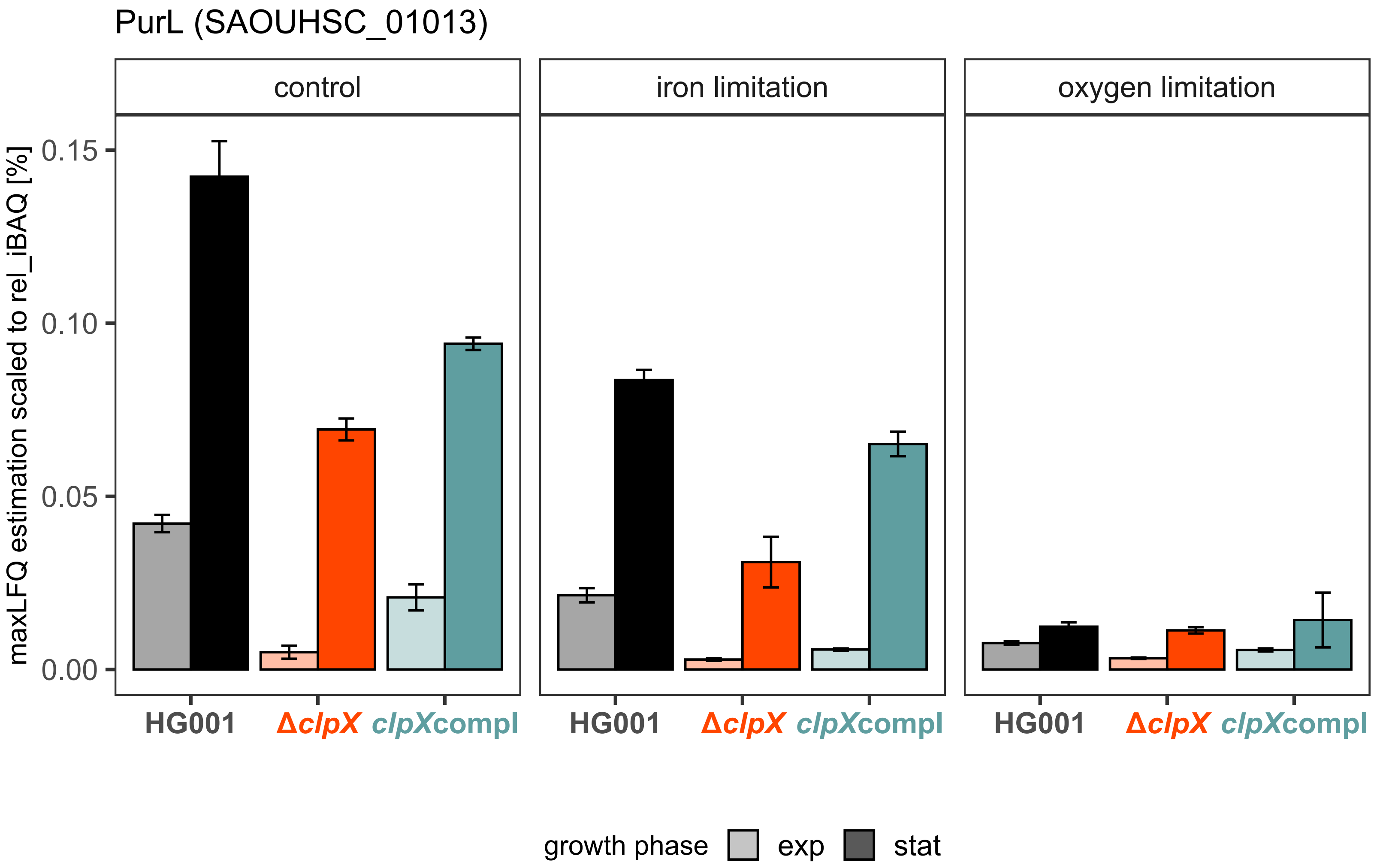
PurL (SAOUHSC_01013)
control
iron limitation
oxygen limitation
maxLFQ estimation scaled to rel_iBAQ [%]
0.15
0.10
0.05
0.00
HG001
ΔclpX
clpXcompl
growth phase
exp
stat

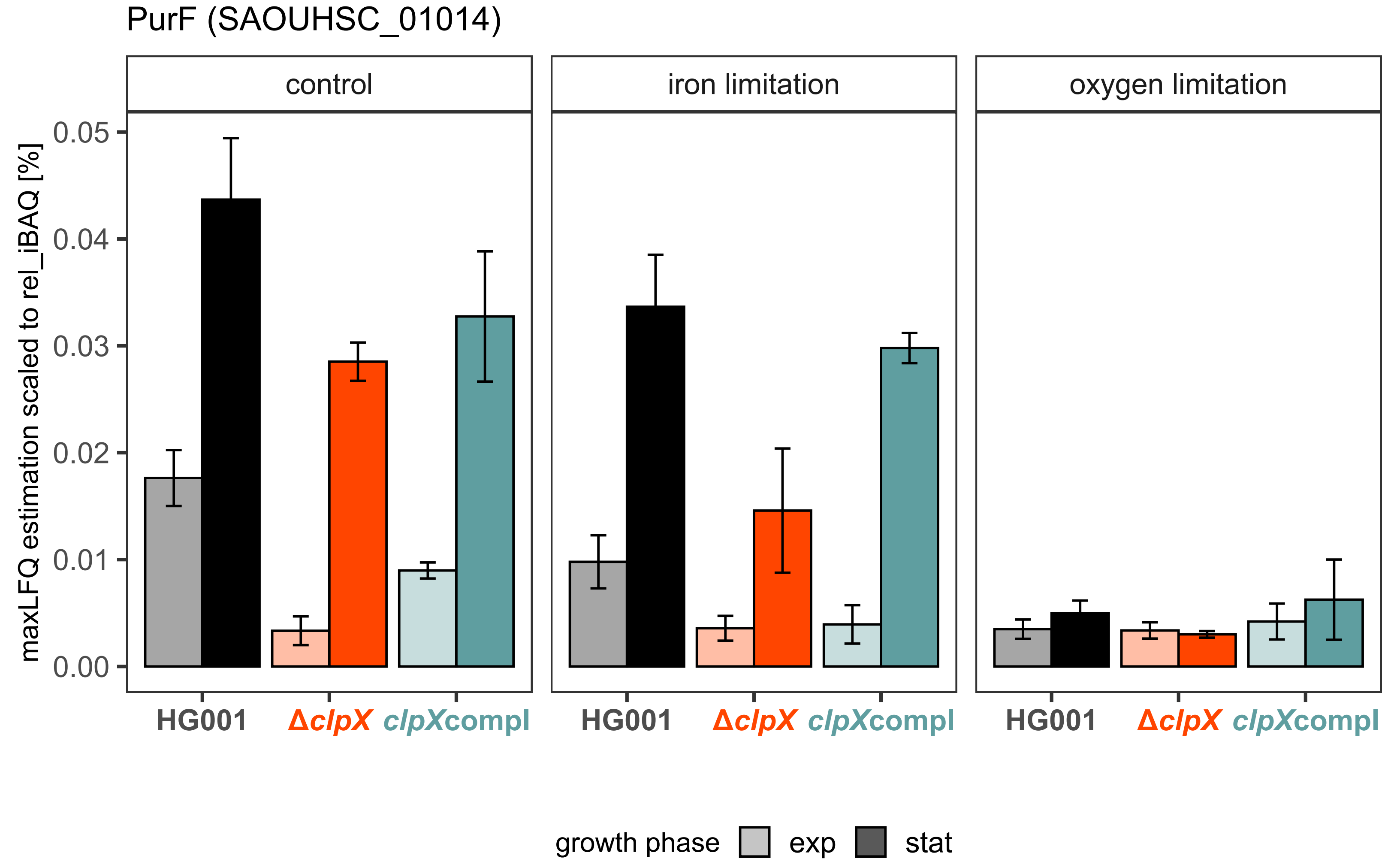
PurF (SAOUHSC_01014)
control
iron limitation
oxygen limitation
maxLFQ estimation scaled to rel_iBAQ [%]
0.05
0.04
0.03
0.02
0.01
0.00
HG001
ΔclpX
clpXcompl
growth phase
exp
stat

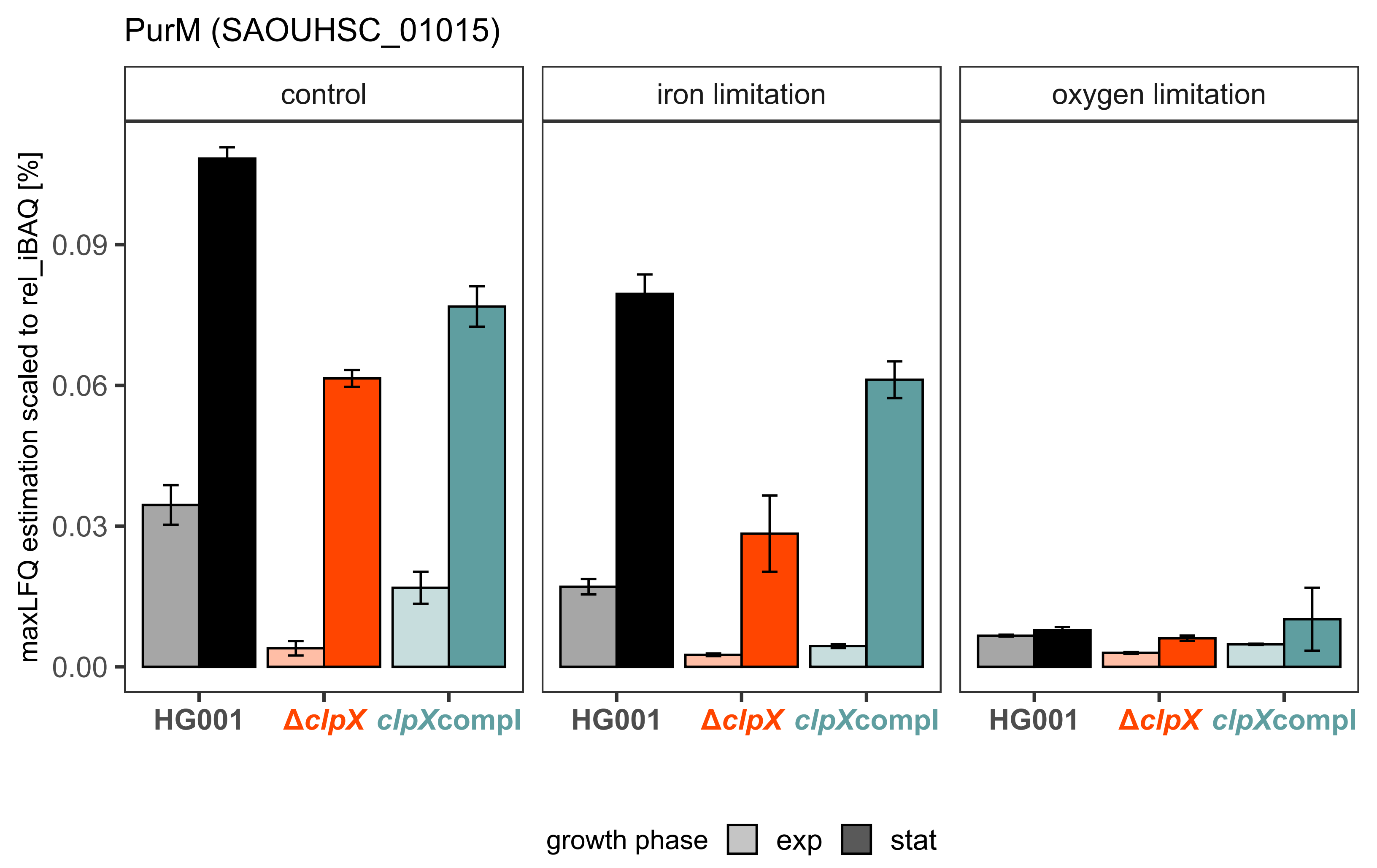
PurM (SAOUHSC_01015)
control
iron limitation
oxygen limitation
maxLFQ estimation scaled to rel_iBAQ [%]
0.09
0.06
0.03
0.00
HG001
ΔclpX
clpXcompl
growth phase
exp
stat

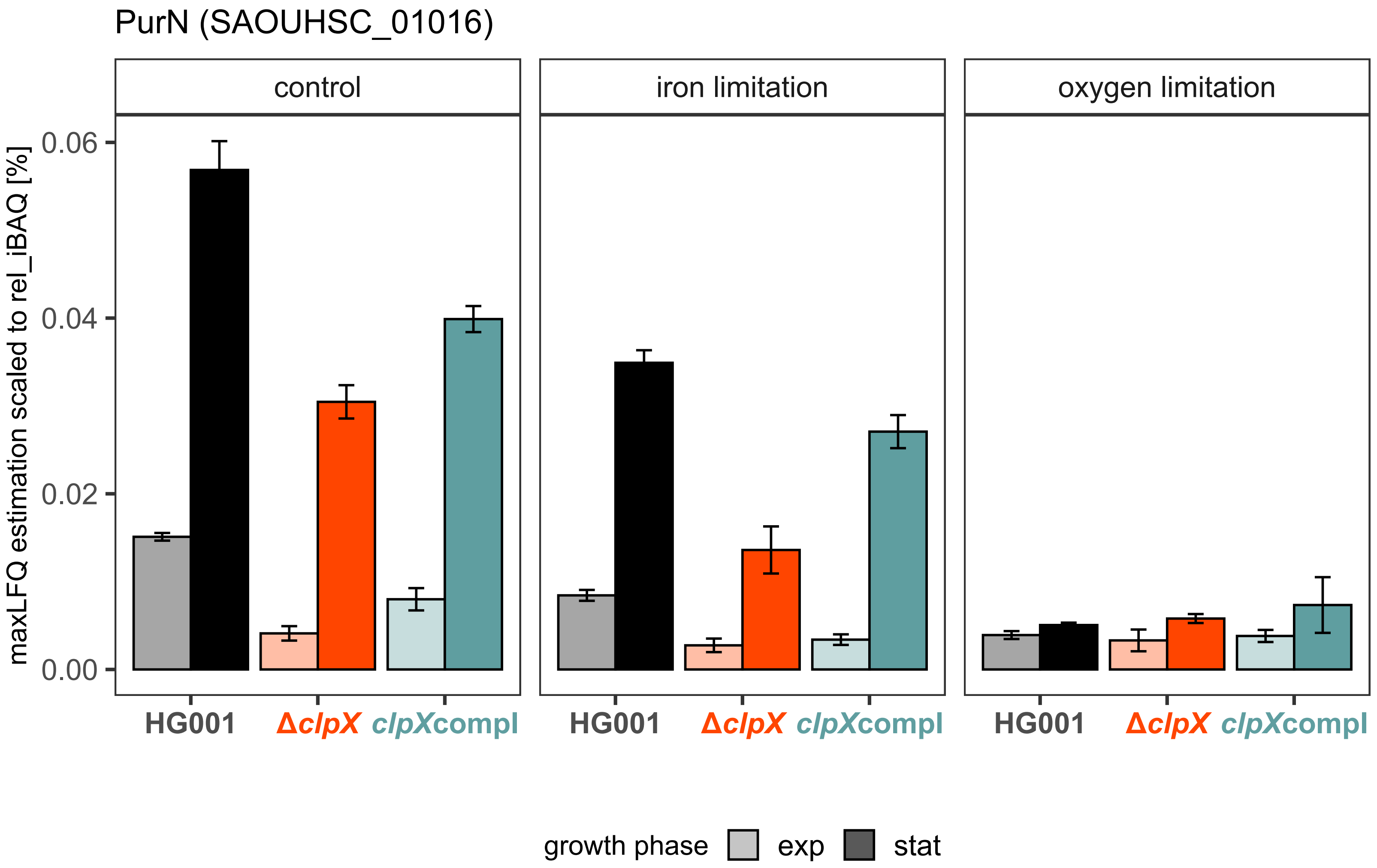
PurN (SAOUHSC_01016)
control
iron limitation
oxygen limitation
maxLFQ estimation scaled to rel_iBAQ [%]
0.06
0.04
0.02
0.00
HG001
ΔclpX
clpXcompl
growth phase
exp
stat

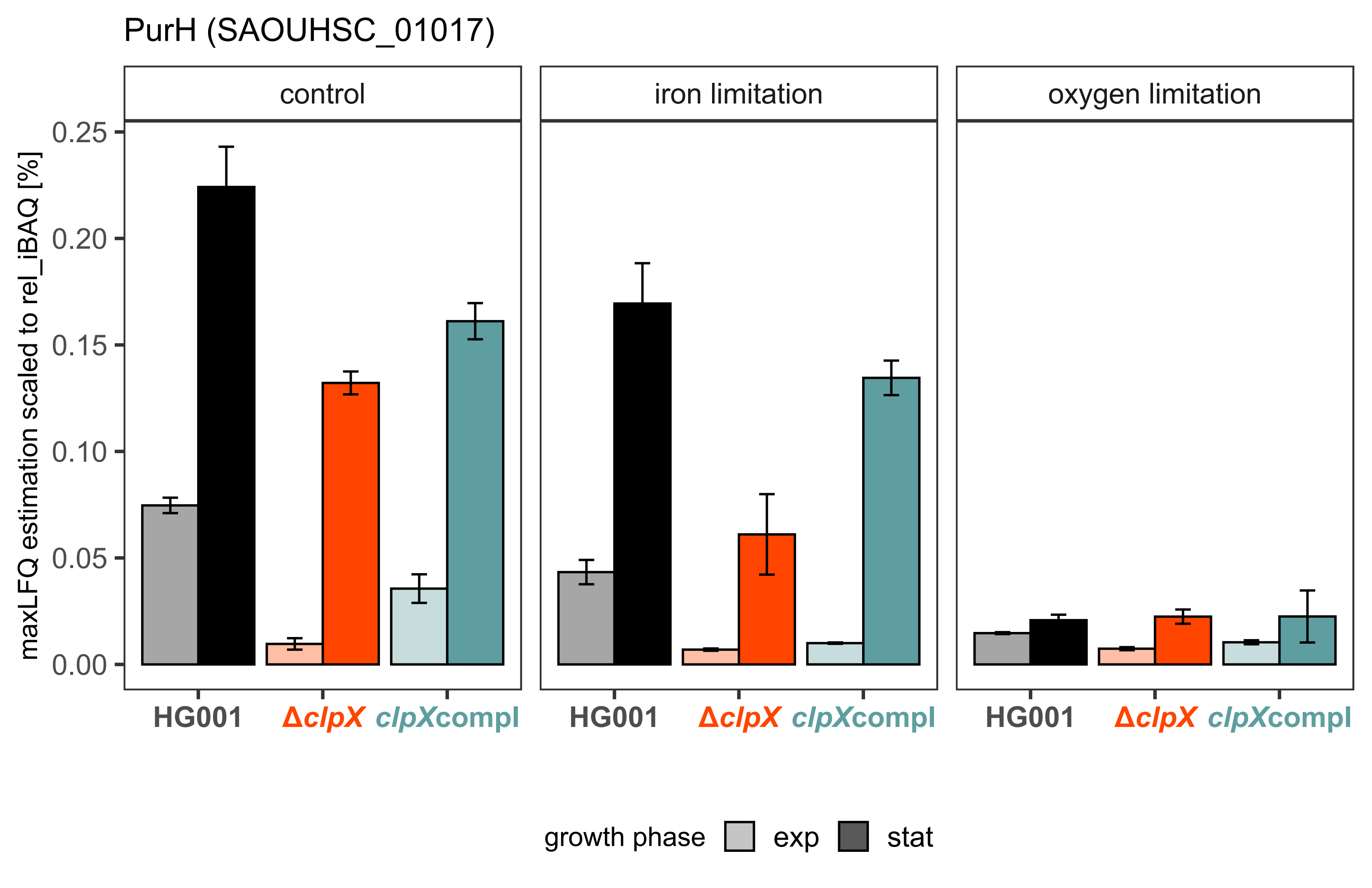
PurH (SAOUHSC_01017)
control
iron limitation
oxygen limitation
maxLFQ estimation scaled to rel_iBAQ [%]
0.25
0.20
0.15
0.10
0.05
0.00
HG001
ΔclpX
clpXcompl
growth phase
exp
stat

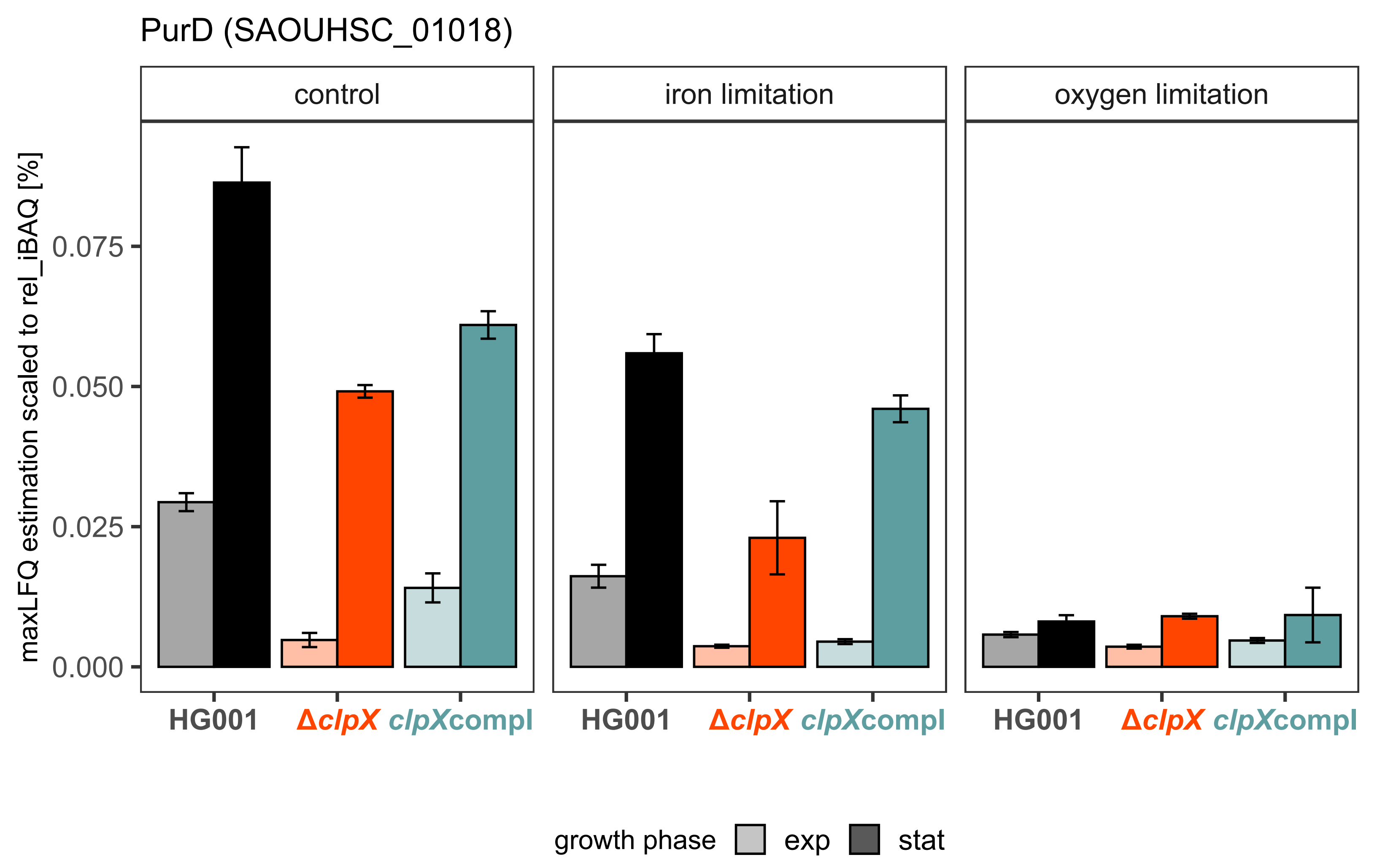
PurD (SAOUHSC_01018)
control
iron limitation
oxygen limitation
maxLFQ estimation scaled to rel_iBAQ [%]
0.075
0.050
0.025
0.000
HG001
ΔclpX
clpXcompl
growth phase
exp
stat

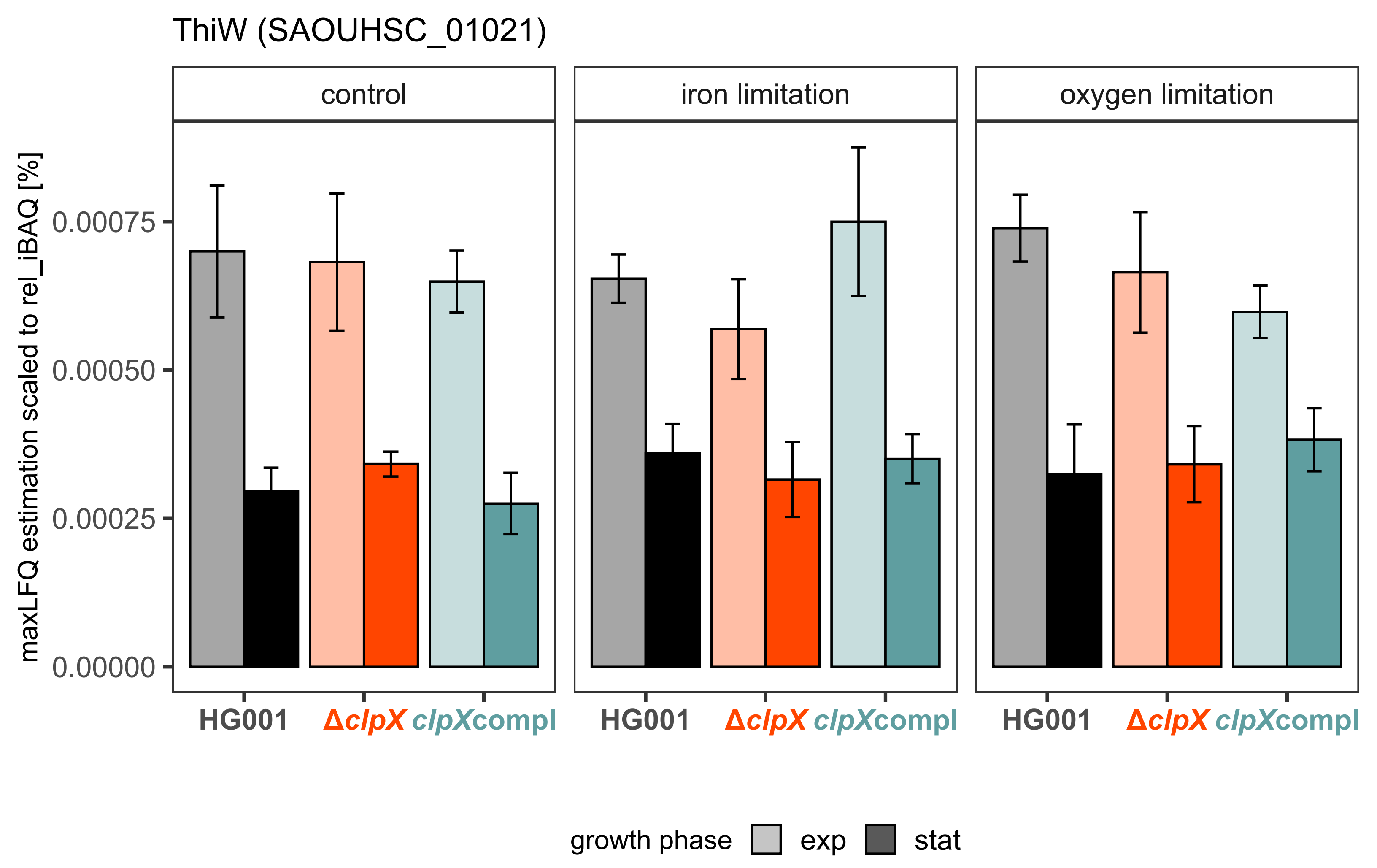
ThiW (SAOUHSC_01021)
control
iron limitation
oxygen limitation
maxLFQ estimation scaled to rel_iBAQ [%]
0.00075
0.00050
0.00025
0.00000
HG001
ΔclpX
clpXcompl
growth phase
exp
stat

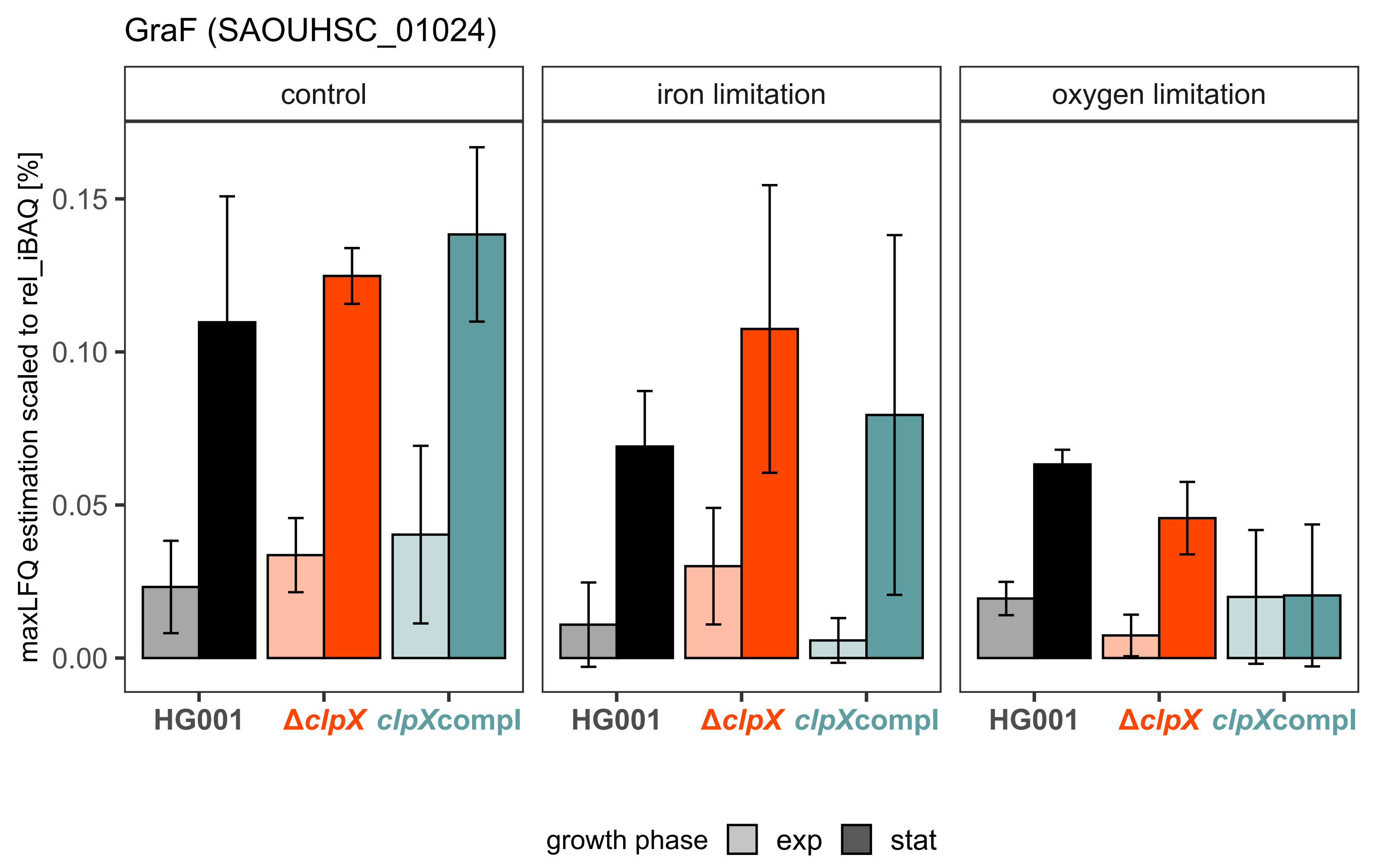

GraF (SAOUHSC_01024)
control
iron limitation
oxygen limitation
maxLFQ estimation scaled to rel_iBAQ [%]
0.15
0.10
0.05
0.00
HG001
ΔclpX
clpXcompl
growth phase
exp
stat

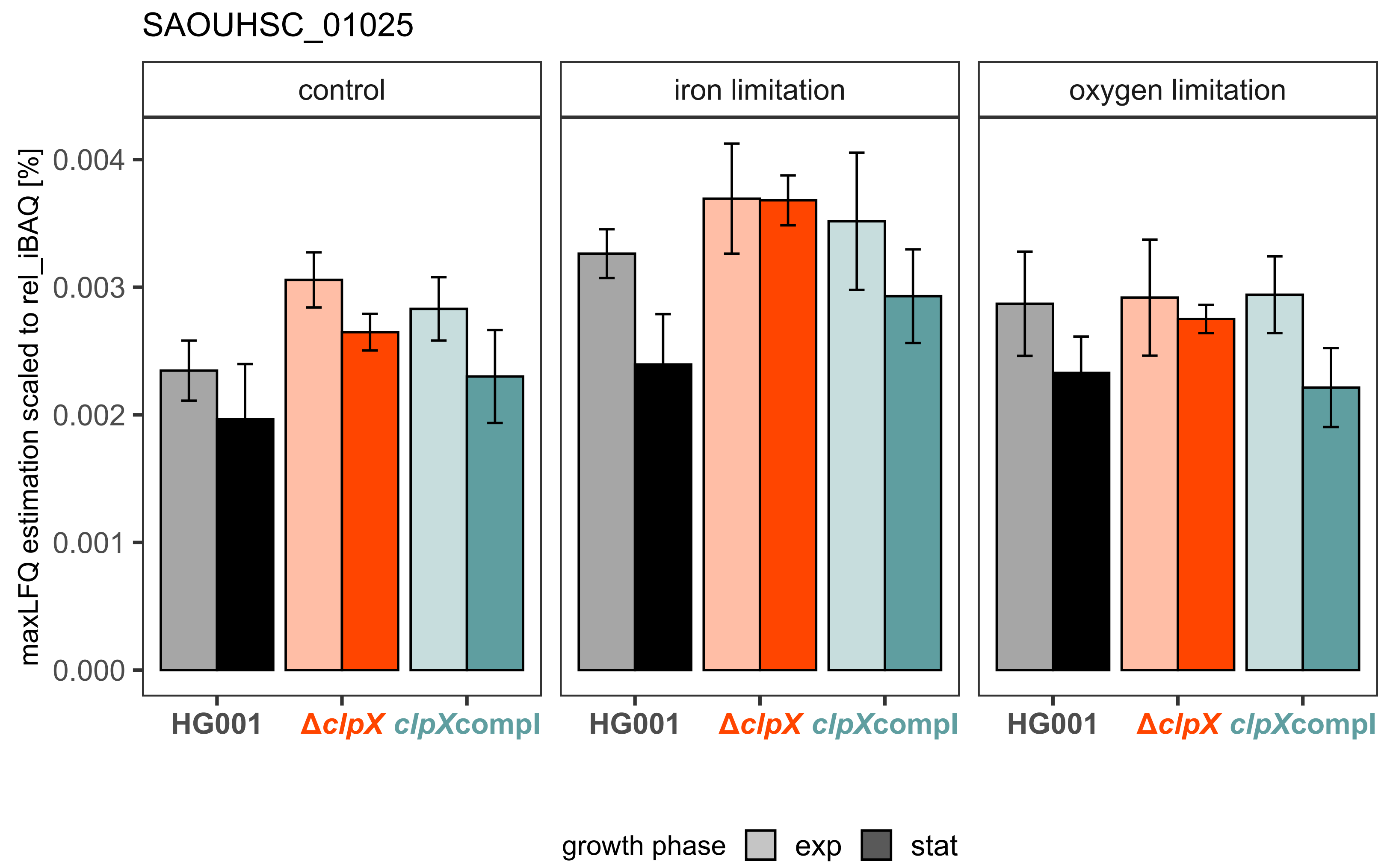

SAOUHSC_01025
control
iron limitation
oxygen limitation
maxLFQ estimation scaled to rel_iBAQ [%]
0.004
0.003
0.002
0.001
0.000
HG001
ΔclpX
clpXcompl
growth phase
exp
stat

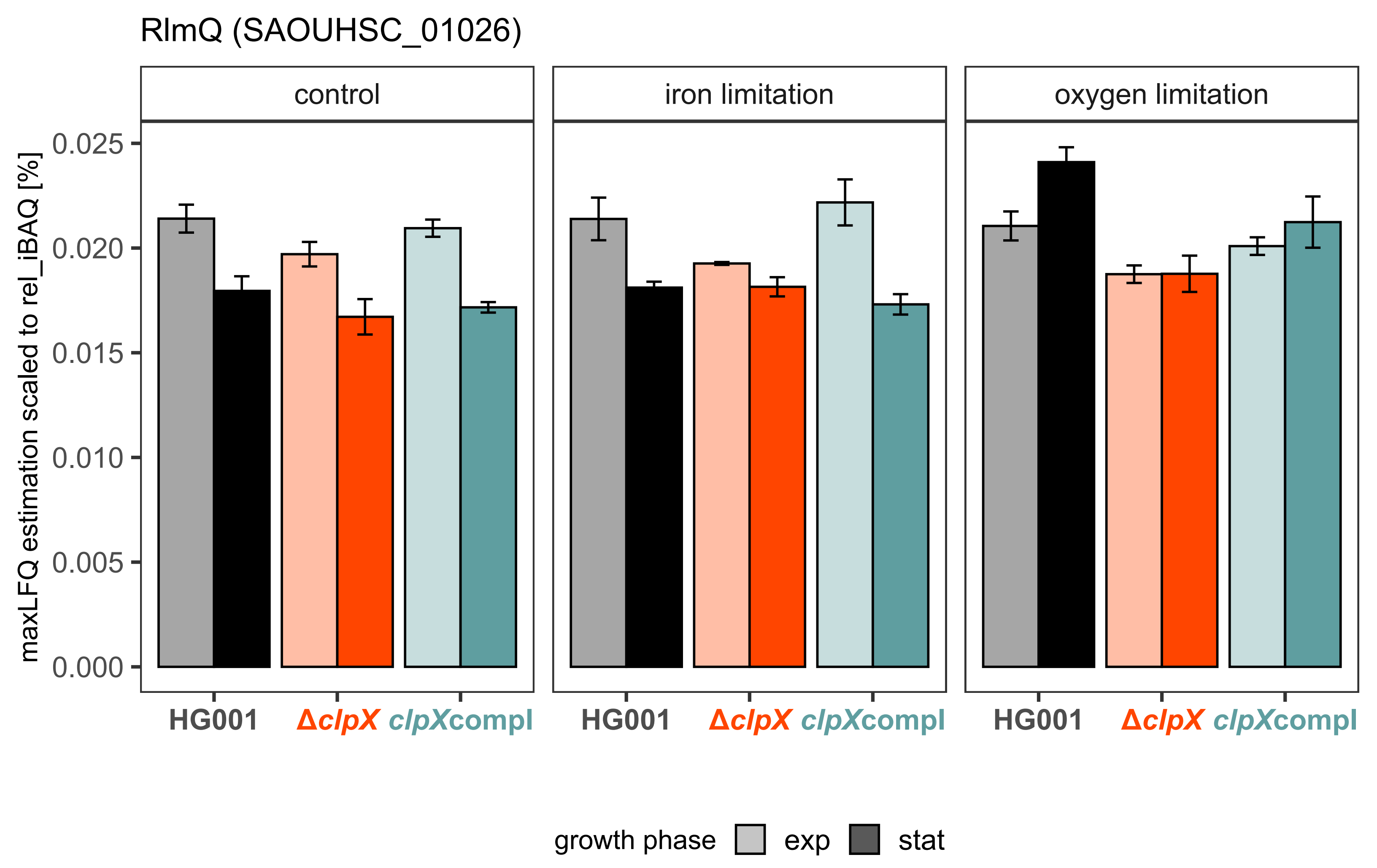

RlmQ (SAOUHSC_01026)
control
iron limitation
oxygen limitation
maxLFQ estimation scaled to rel_iBAQ [%]
0.025
0.020
0.015
0.010
0.005
0.000
HG001
ΔclpX
clpXcompl
growth phase
exp
stat

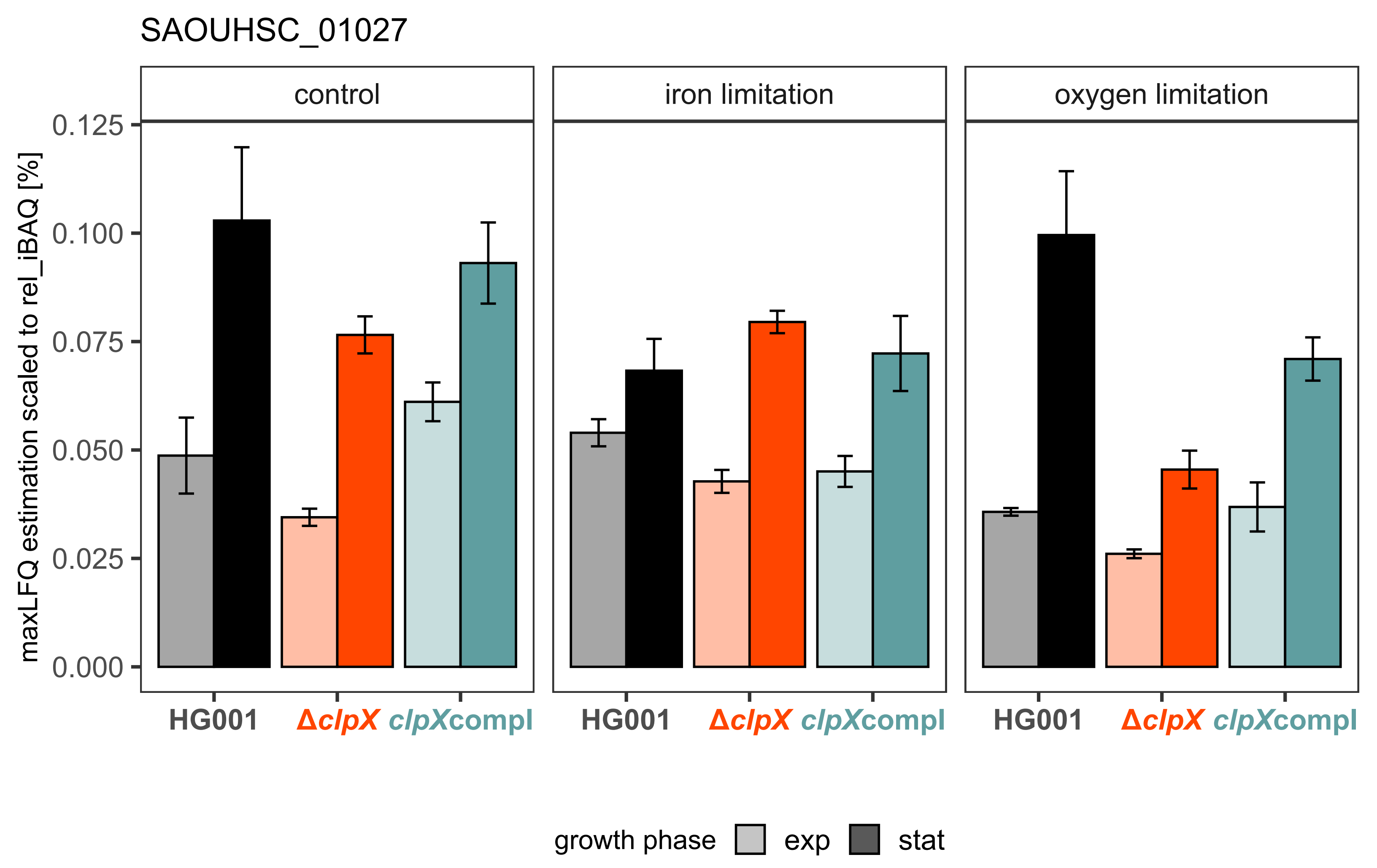
SAOUHSC_01027
control
iron limitation
oxygen limitation
maxLFQ estimation scaled to rel_iBAQ [%]
0.125
0.100
0.075
0.050
0.025
0.000
HG001
ΔclpX
clpXcompl
growth phase
exp
stat

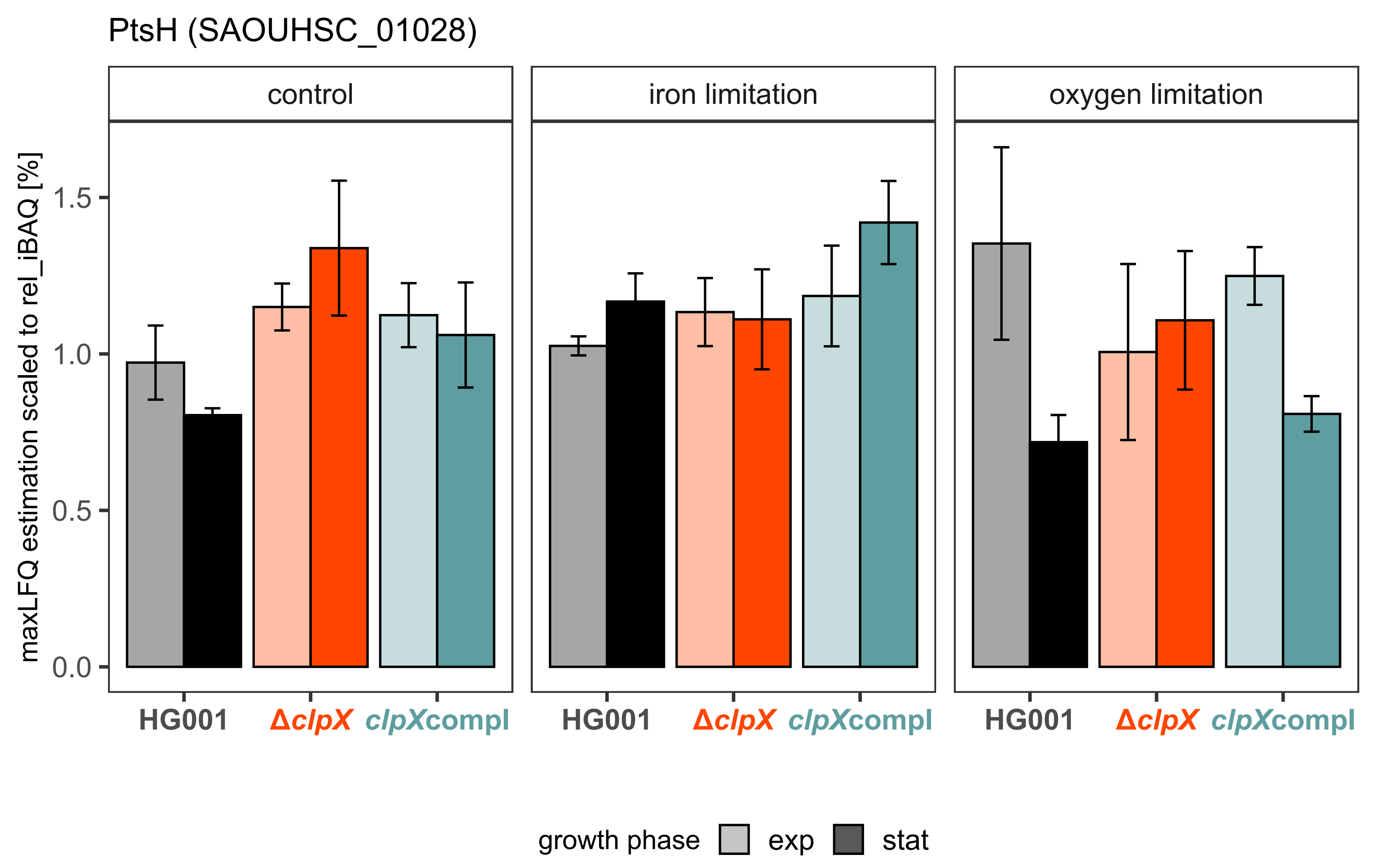
PtsH (SAOUHSC_01028)
control
iron limitation
oxygen limitation
maxLFQ estimation scaled to rel_iBAQ [%]
0.0
0.5
1.0
1.5
HG001
ΔclpX
clpXcompl
growth phase
exp
stat

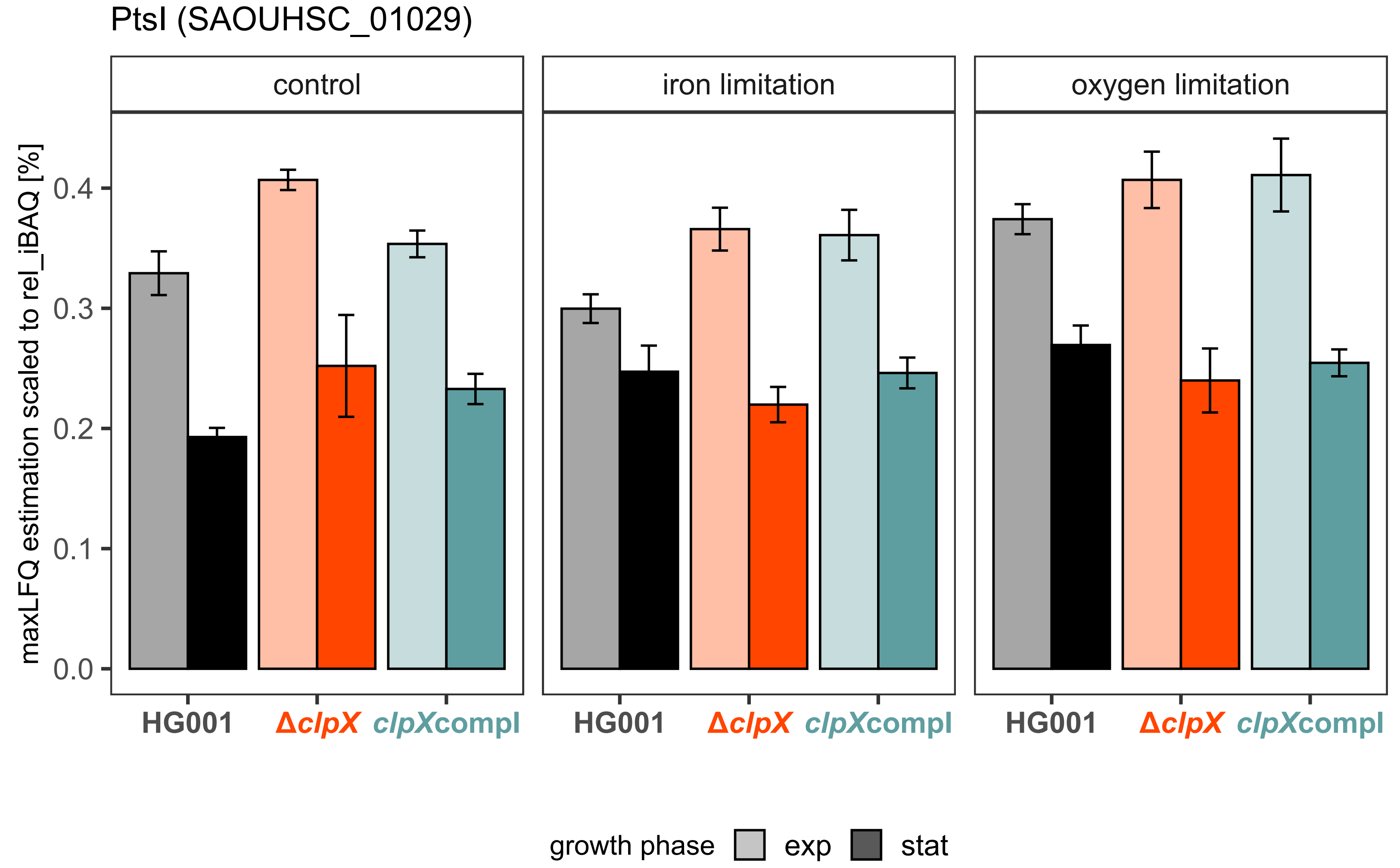
PtsI (SAOUHSC_01029)
control
iron limitation
oxygen limitation
maxLFQ estimation scaled to rel_iBAQ [%]
0.0
0.1
0.2
0.3
0.4
HG001
ΔclpX
clpXcompl
growth phase
exp
stat

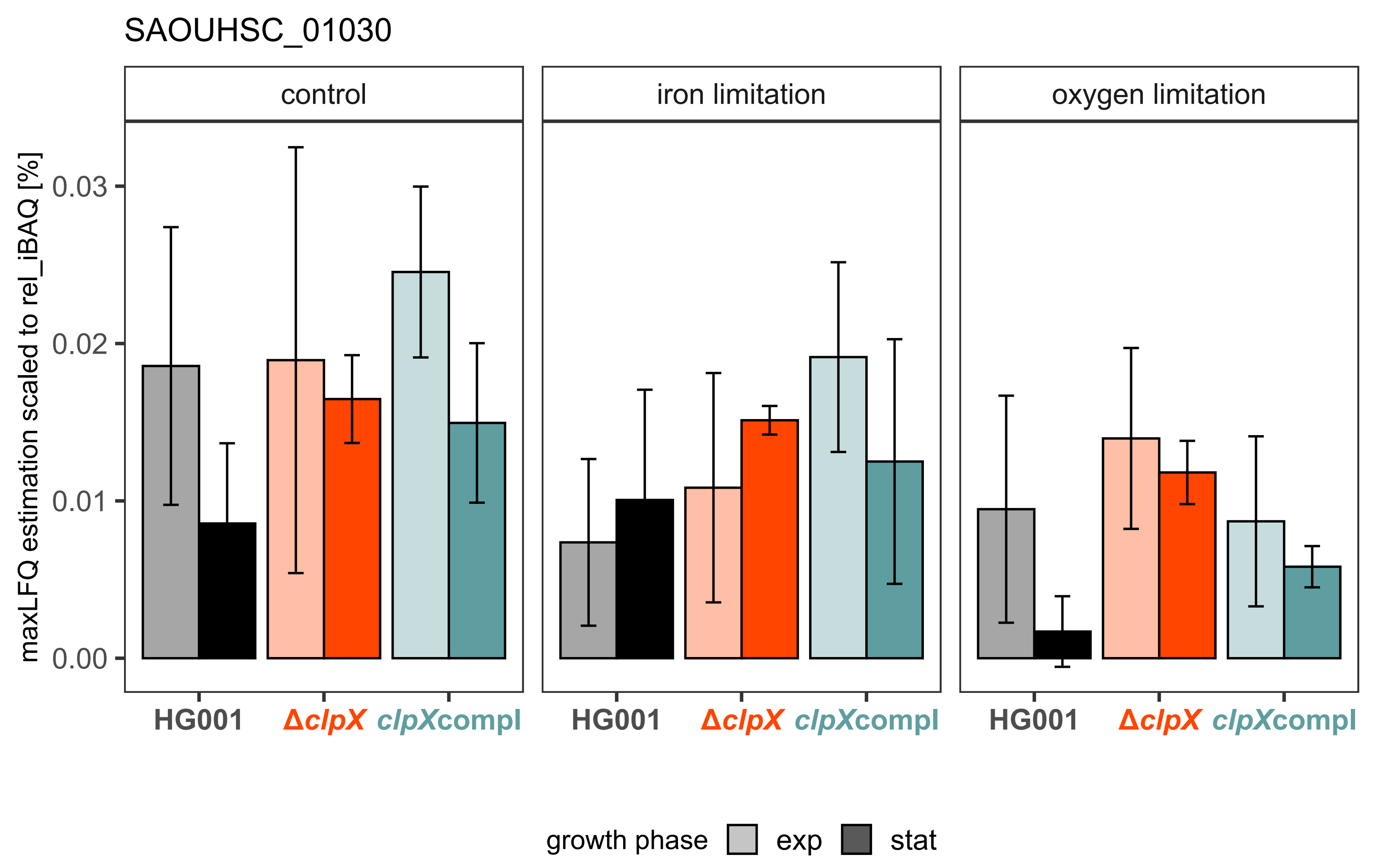
SAOUHSC_01030
control
iron limitation
oxygen limitation
maxLFQ estimation scaled to rel_iBAQ [%]
0.03
0.02
0.01
0.00
HG001
ΔclpX
clpXcompl
growth phase
exp
stat

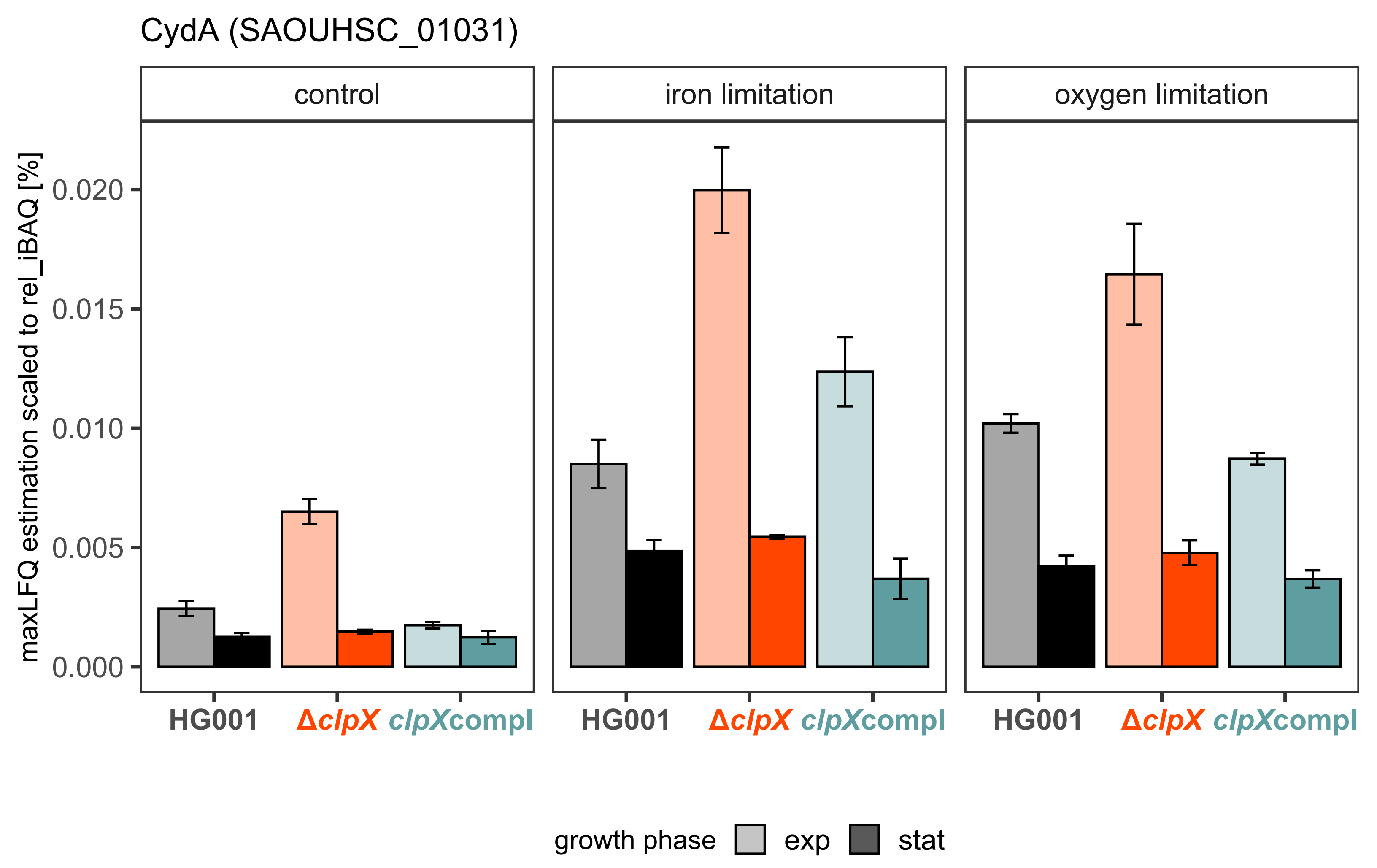
CydA (SAOUHSC_01031)
control
iron limitation
oxygen limitation
maxLFQ estimation scaled to rel_iBAQ [%]
0.020
0.015
0.010
0.005
0.000
HG001
ΔclpX
clpXcompl
growth phase
exp
stat

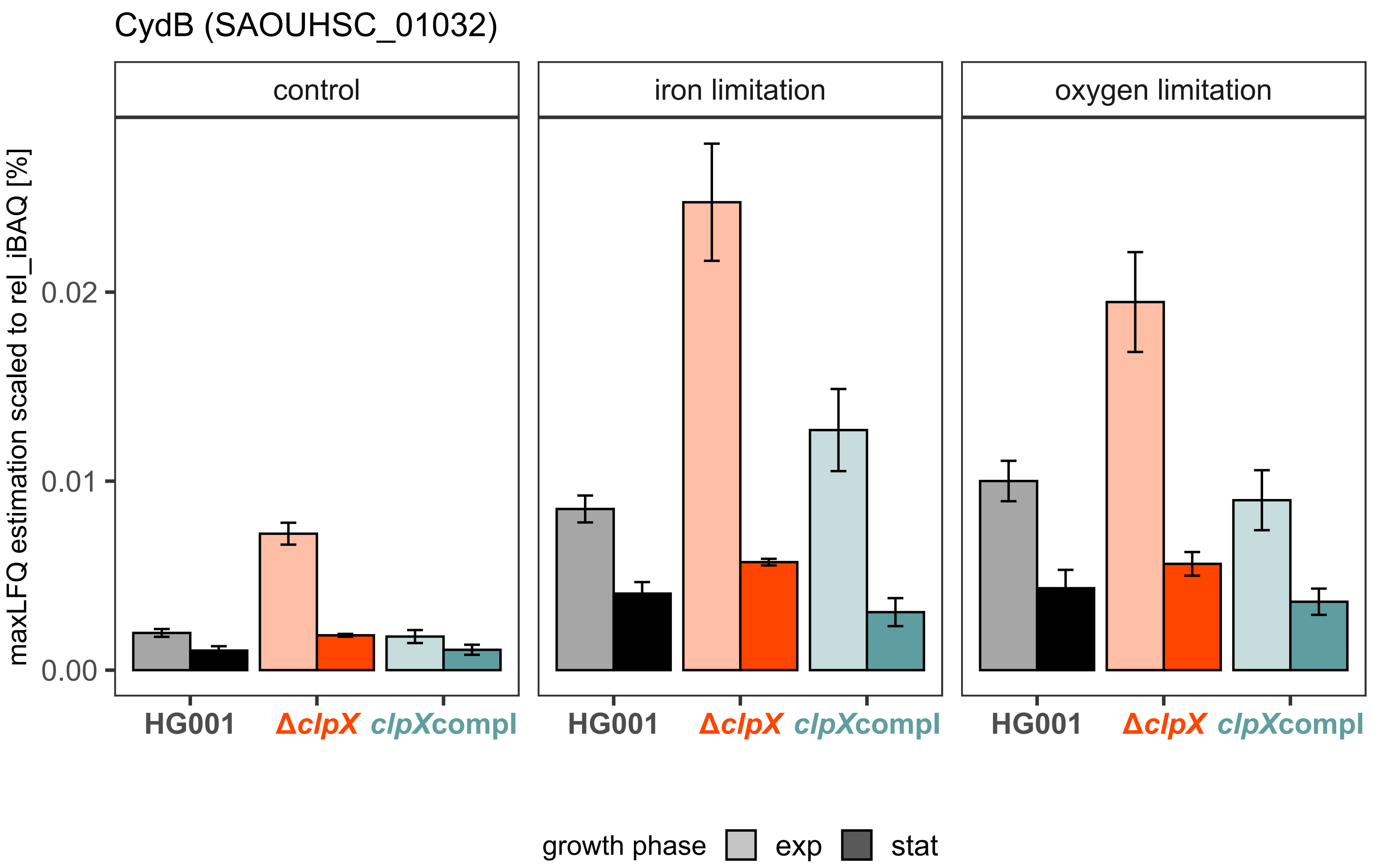

CydB (SAOUHSC_01032)
control
iron limitation
oxygen limitation
maxLFQ estimation scaled to rel_iBAQ [%]
0.00
0.01
0.02
HG001
ΔclpX
clpXcompl
growth phase
exp
stat

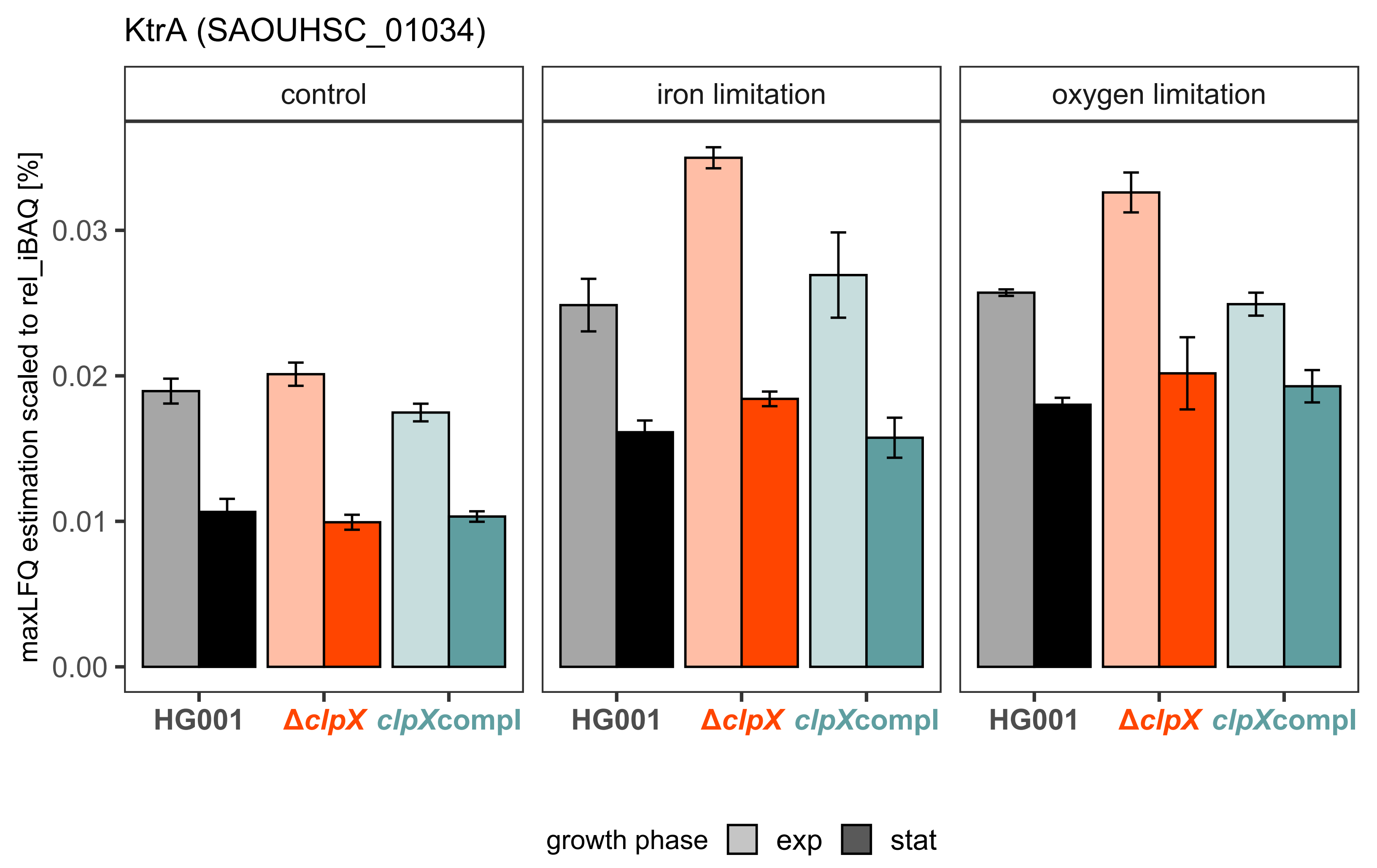

KtrA (SAOUHSC_01034)
control
iron limitation
oxygen limitation
maxLFQ estimation scaled to rel_iBAQ [%]
0.03
0.02
0.01
0.00
HG001
ΔclpX
clpXcompl
growth phase
exp
stat

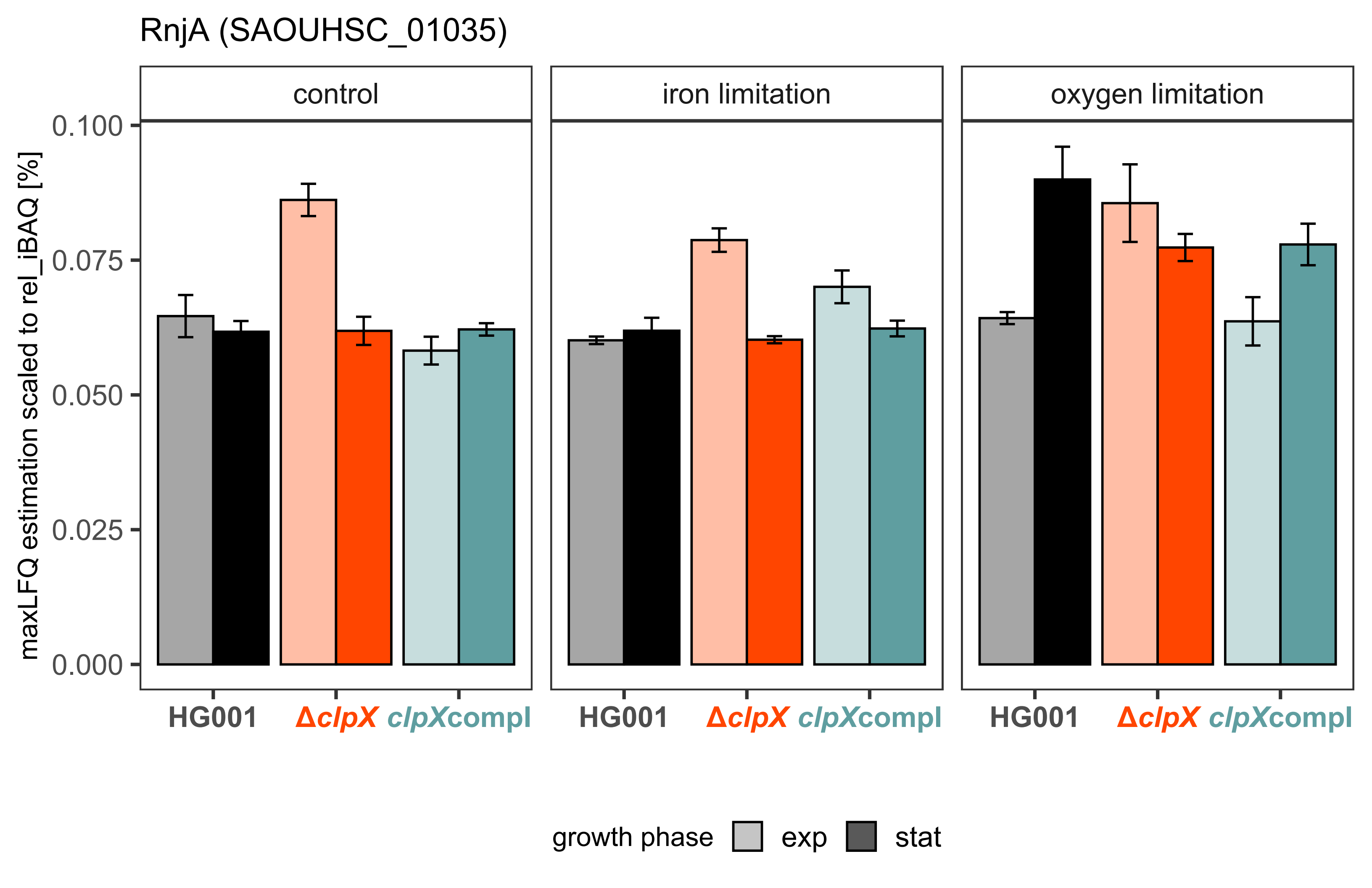
RnjA (SAOUHSC_01035)
control
iron limitation
oxygen limitation
maxLFQ estimation scaled to rel_iBAQ [%]
0.100
0.075
0.050
0.025
0.000
HG001
ΔclpX
clpXcompl
growth phase
exp
stat

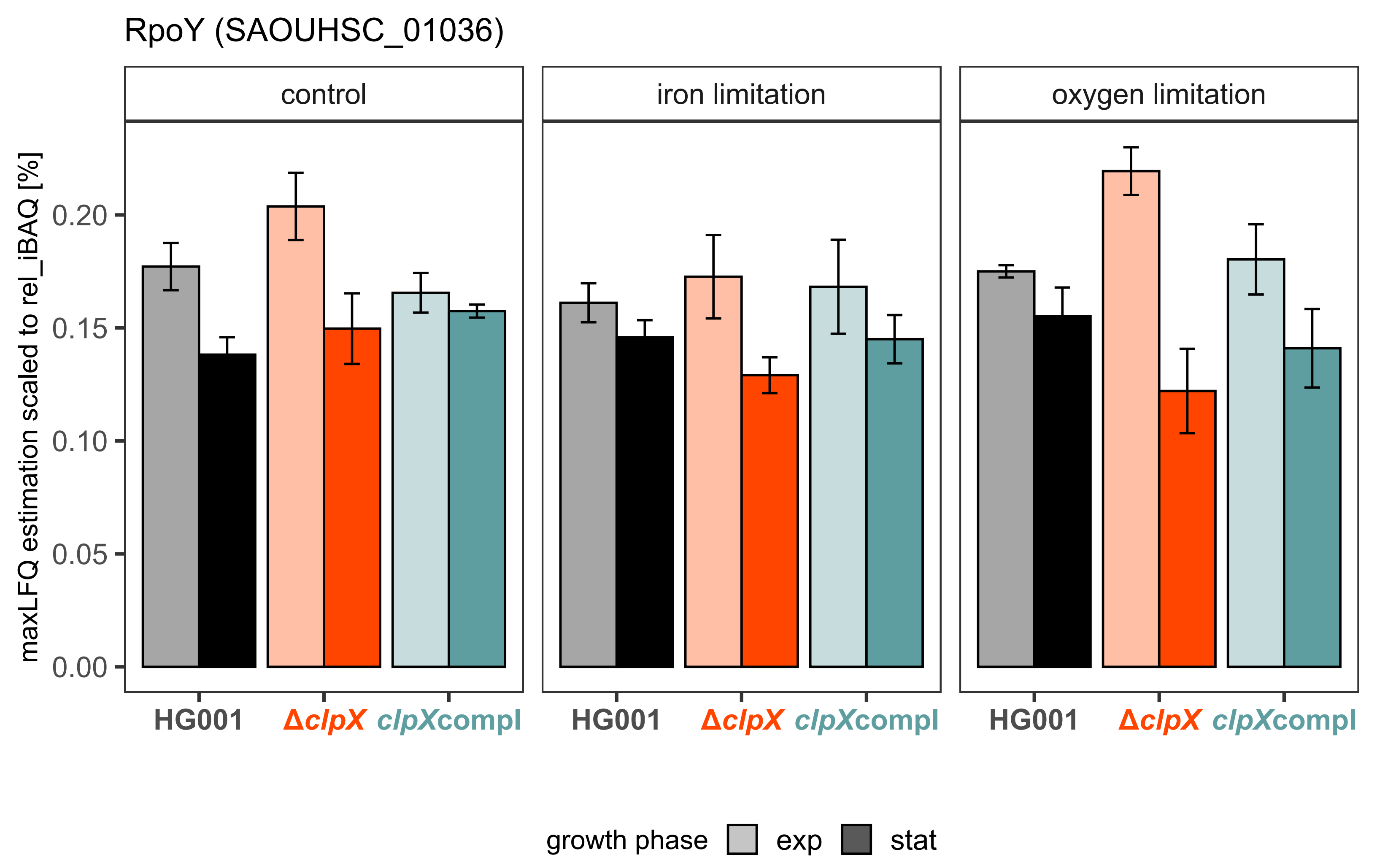

RpoY (SAOUHSC_01036)
control
iron limitation
oxygen limitation
maxLFQ estimation scaled to rel_iBAQ [%]
0.00
0.05
0.10
0.15
0.20
HG001
ΔclpX
clpXcompl
growth phase
exp
stat

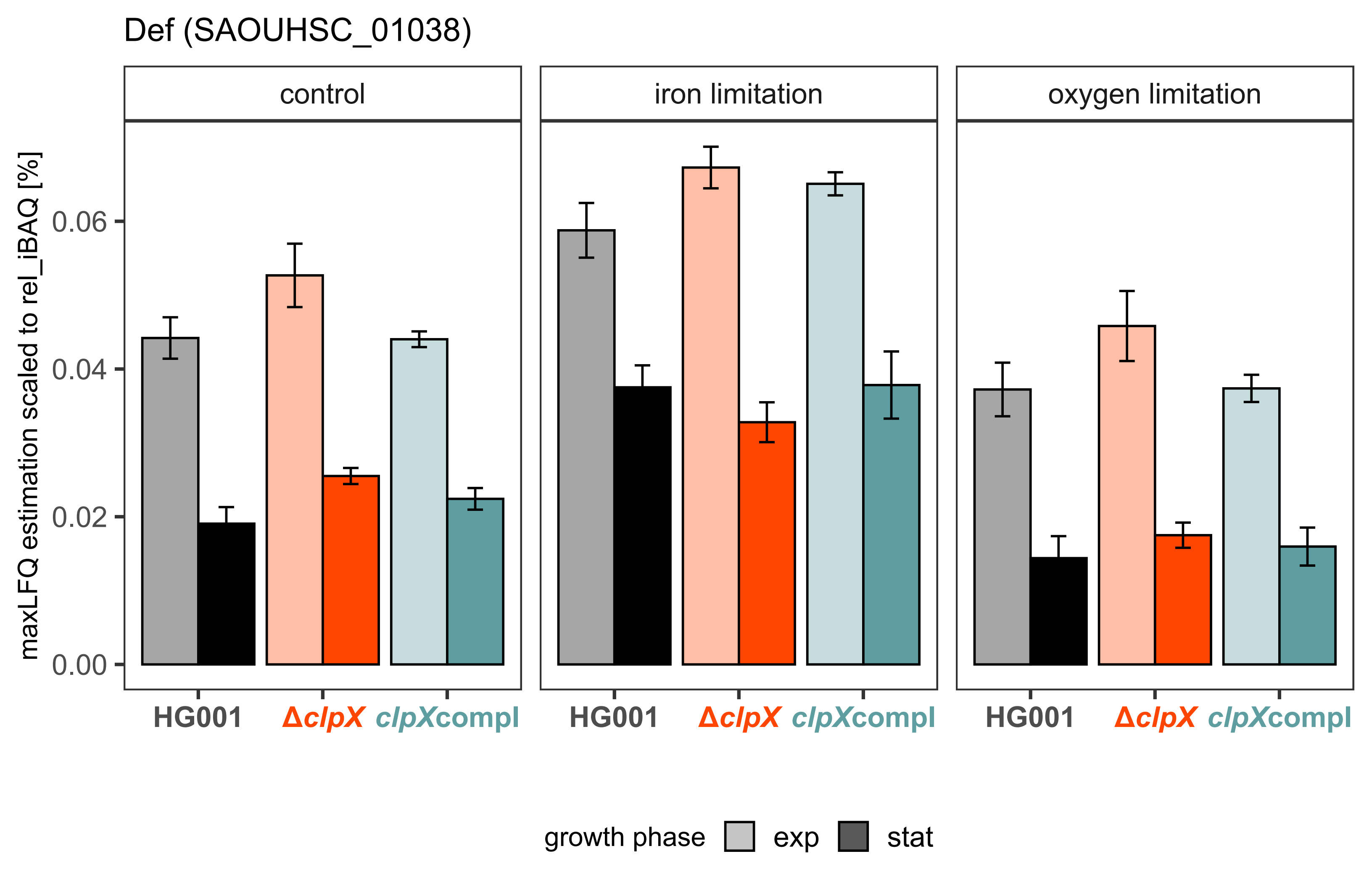

Def (SAOUHSC_01038)
control
iron limitation
oxygen limitation
maxLFQ estimation scaled to rel_iBAQ [%]
0.06
0.04
0.02
0.00
HG001
ΔclpX
clpXcompl
growth phase
exp
stat

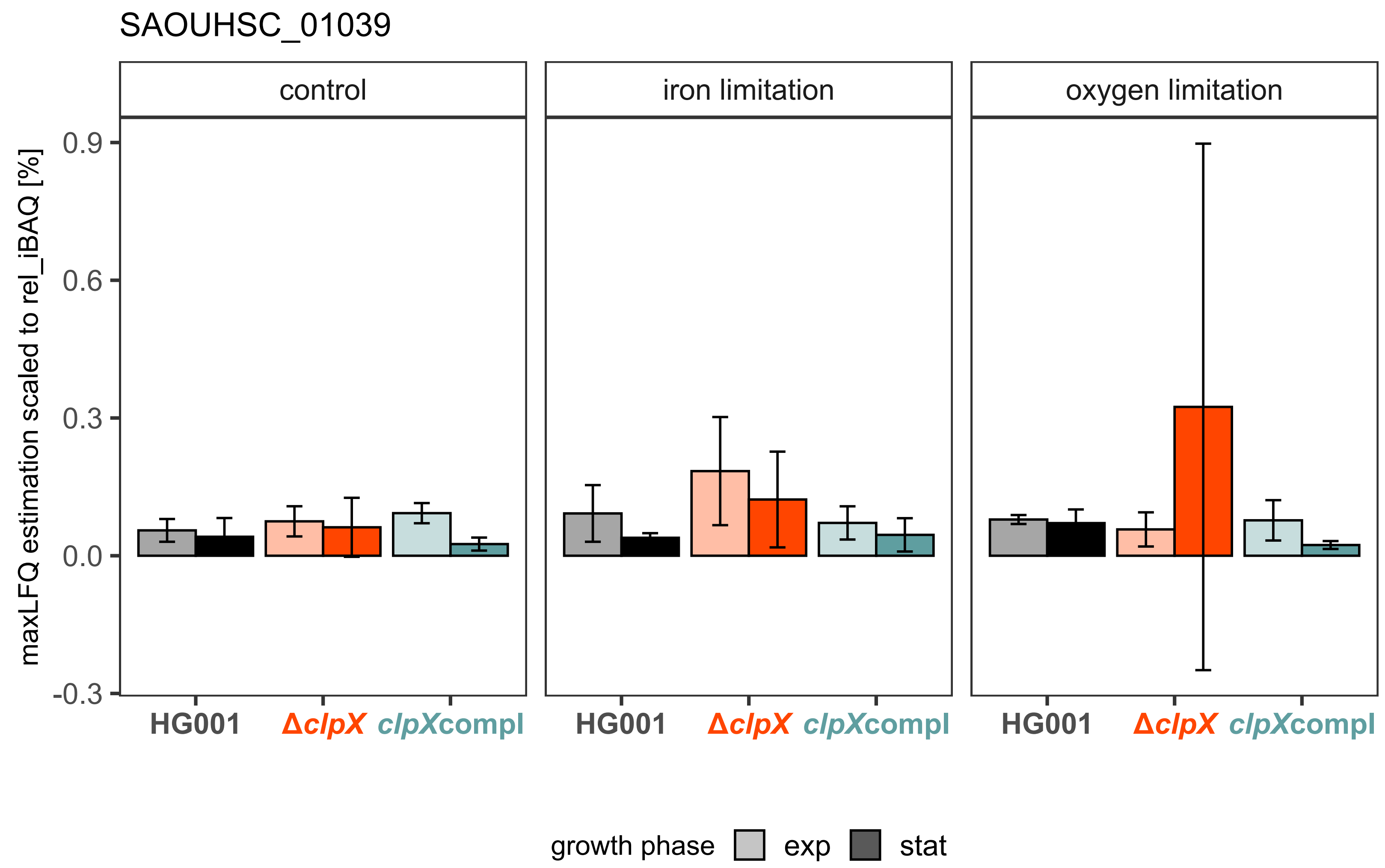

SAOUHSC_01039
control
iron limitation
oxygen limitation
maxLFQ estimation scaled to rel_iBAQ [%]
0.9
0.6
0.3
0.0
-0.3
HG001
ΔclpX
clpXcompl
growth phase
exp
stat

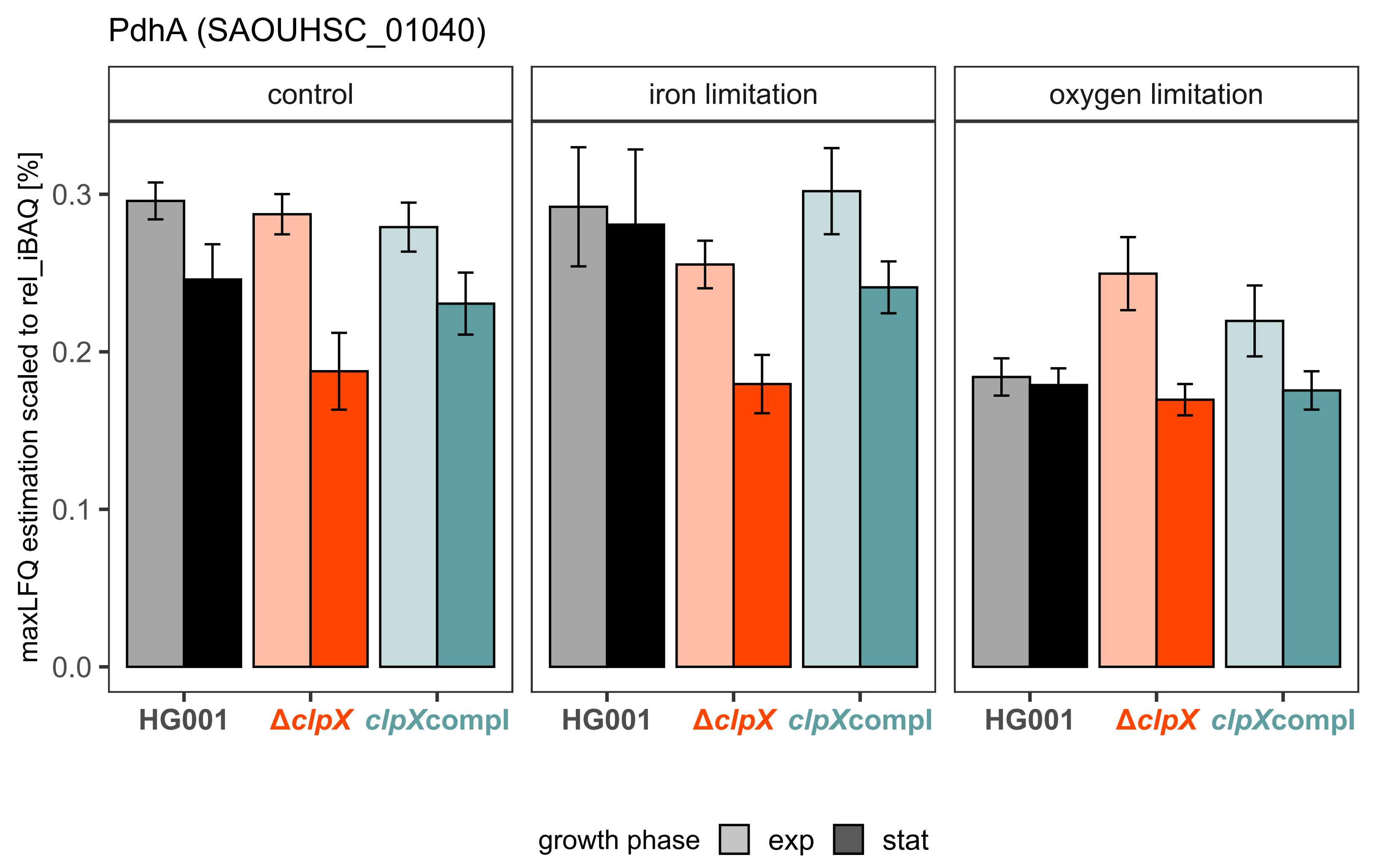

PdhA (SAOUHSC_01040)
control
iron limitation
oxygen limitation
maxLFQ estimation scaled to rel_iBAQ [%]
0.0
0.1
0.2
0.3
HG001
ΔclpX
clpXcompl
growth phase
exp
stat

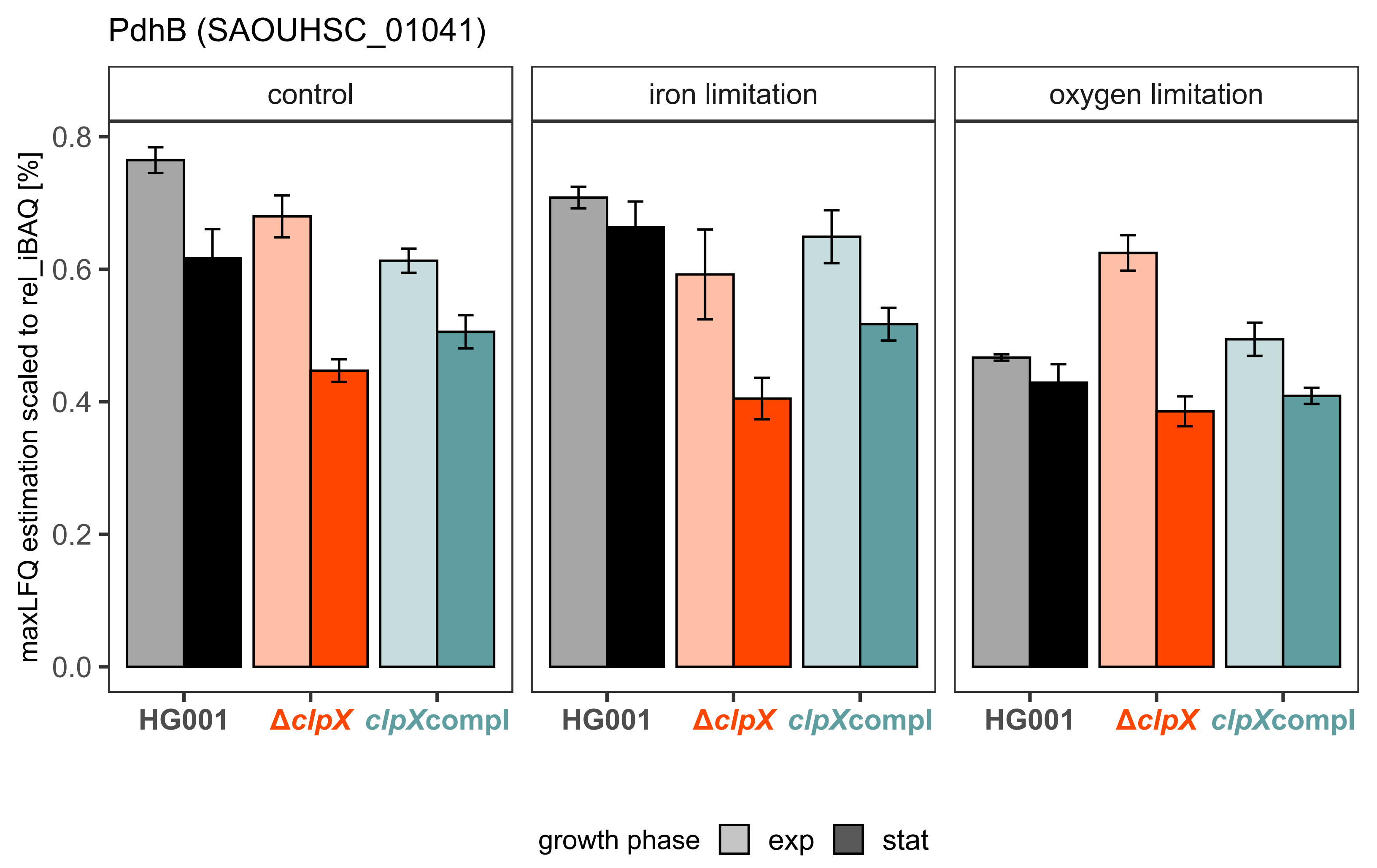
PdhB (SAOUHSC_01041)
control
iron limitation
oxygen limitation
maxLFQ estimation scaled to rel_iBAQ [%]
0.0
0.2
0.4
0.6
0.8
HG001
ΔclpX
clpXcompl
growth phase
exp
stat

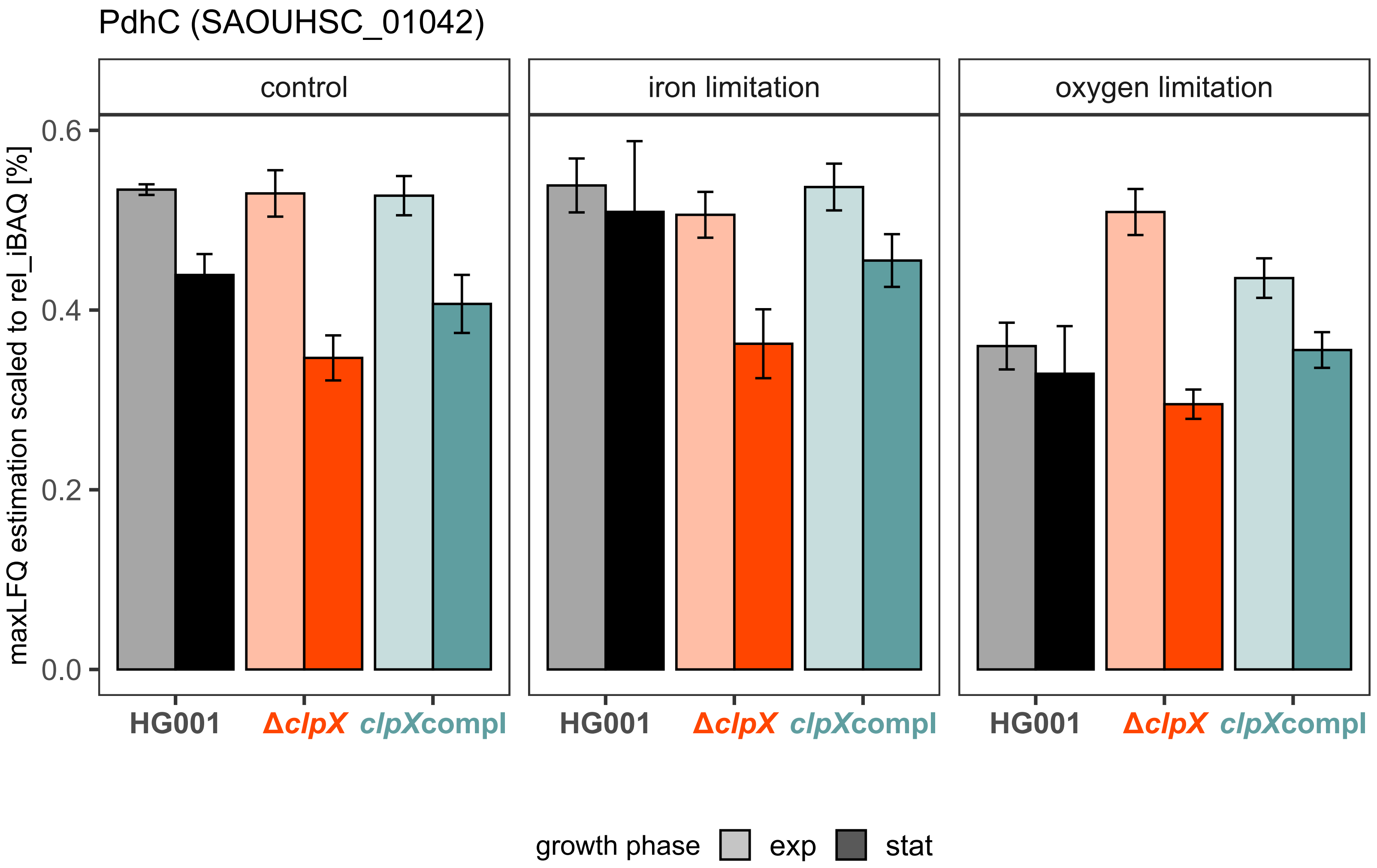
PdhC (SAOUHSC_01042)
control
iron limitation
oxygen limitation
maxLFQ estimation scaled to rel_iBAQ [%]
0.6
0.4
0.2
0.0
HG001
ΔclpX
clpXcompl
growth phase
exp
stat

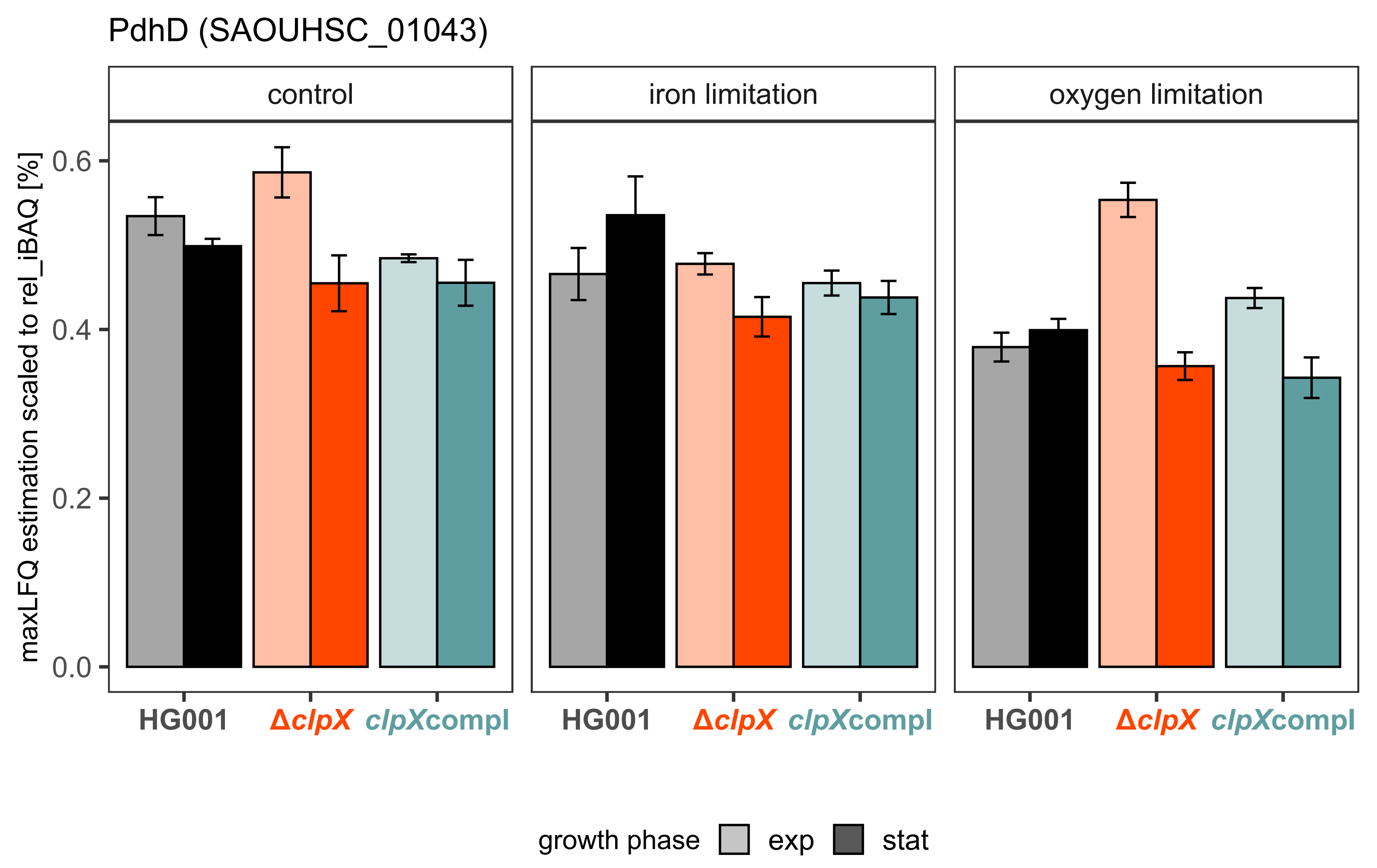
PdhD (SAOUHSC_01043)
control
iron limitation
oxygen limitation
maxLFQ estimation scaled to rel_iBAQ [%]
0.0
0.2
0.4
0.6
HG001
ΔclpX
clpXcompl
growth phase
exp
stat

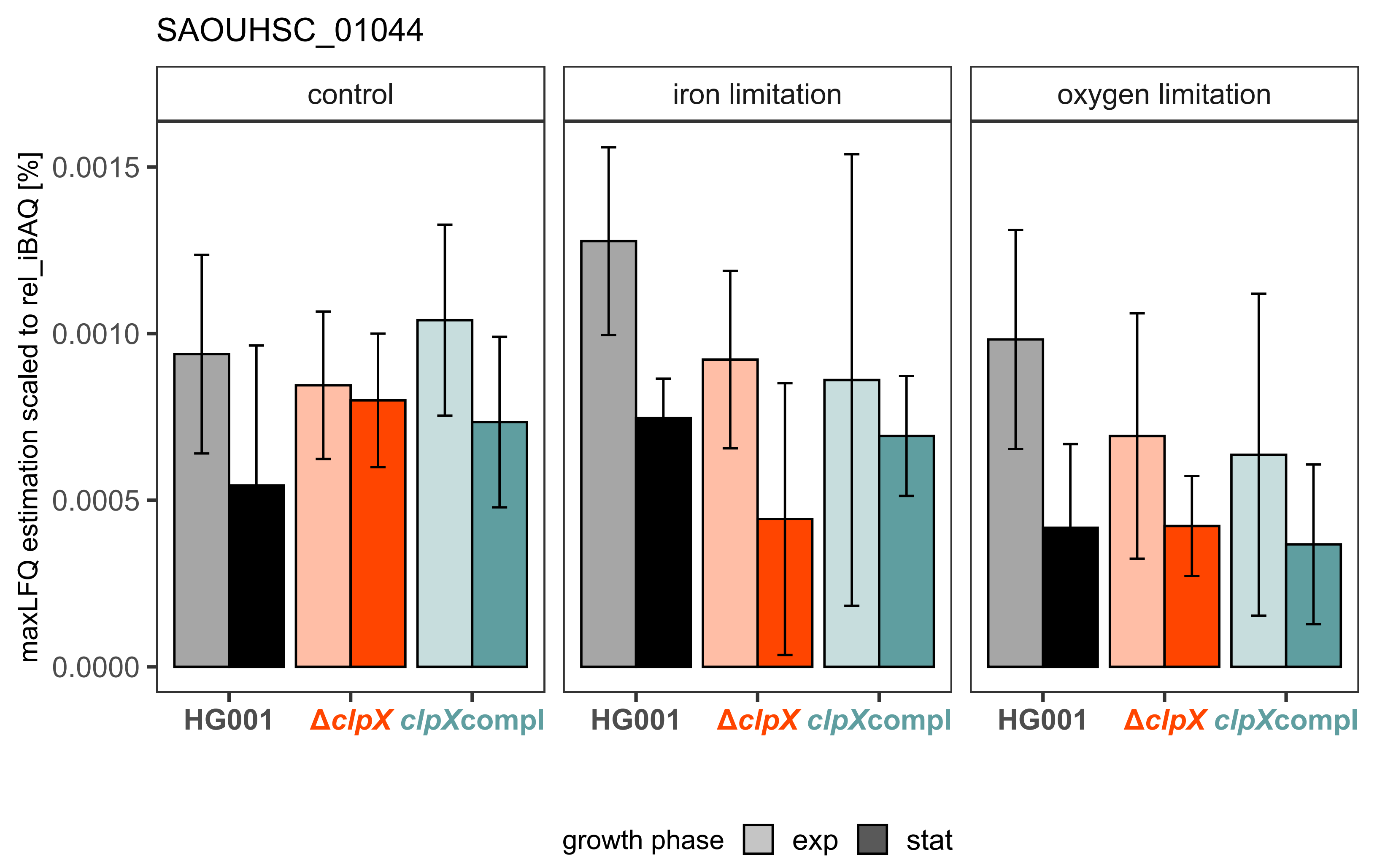
SAOUHSC_01044
control
iron limitation
oxygen limitation
maxLFQ estimation scaled to rel_iBAQ [%]
0.0015
0.0010
0.0005
0.0000
HG001
ΔclpX
clpXcompl
growth phase
exp
stat

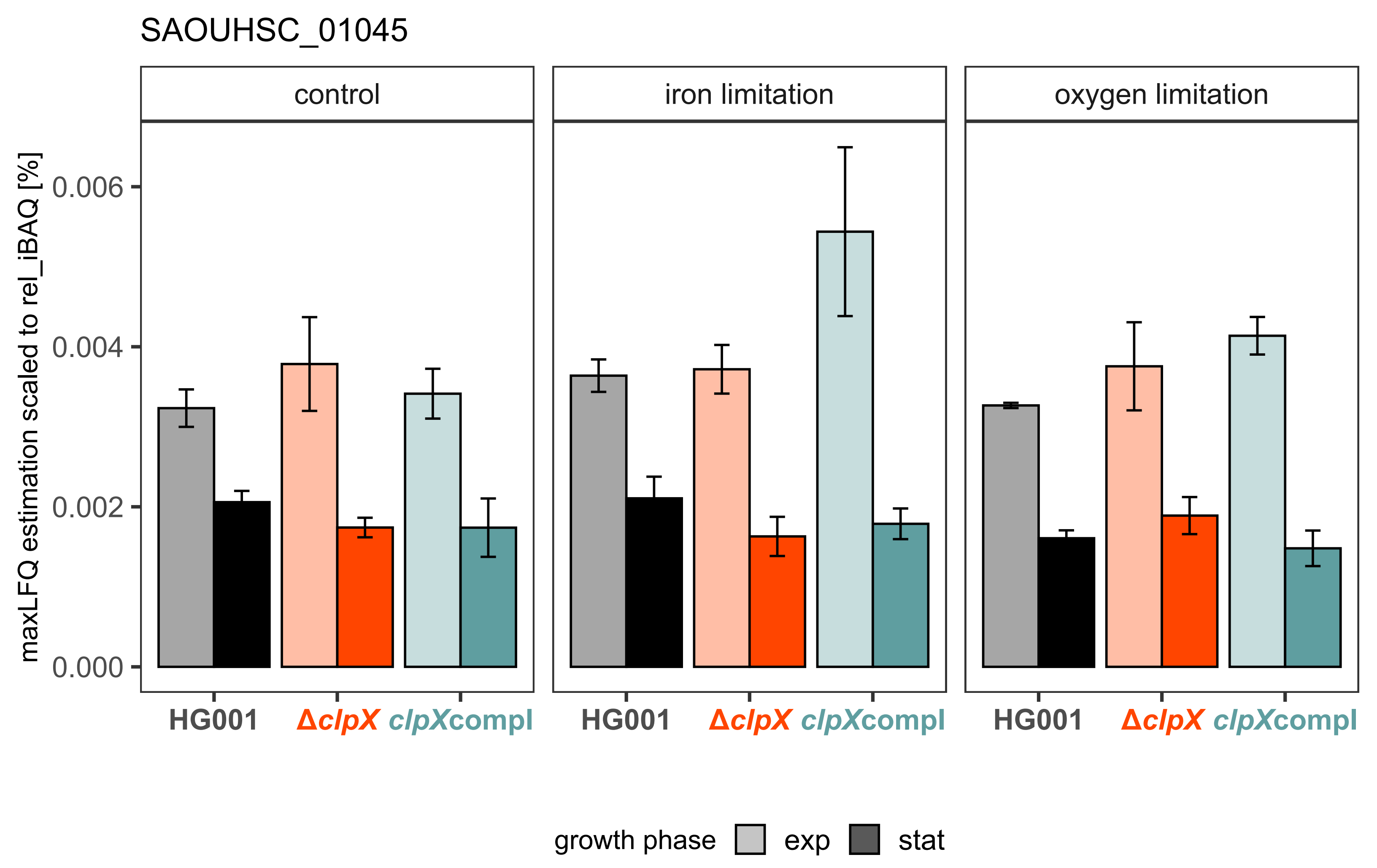
SAOUHSC_01045
control
iron limitation
oxygen limitation
maxLFQ estimation scaled to rel_iBAQ [%]
0.006
0.004
0.002
0.000
HG001
ΔclpX
clpXcompl
growth phase
exp
stat

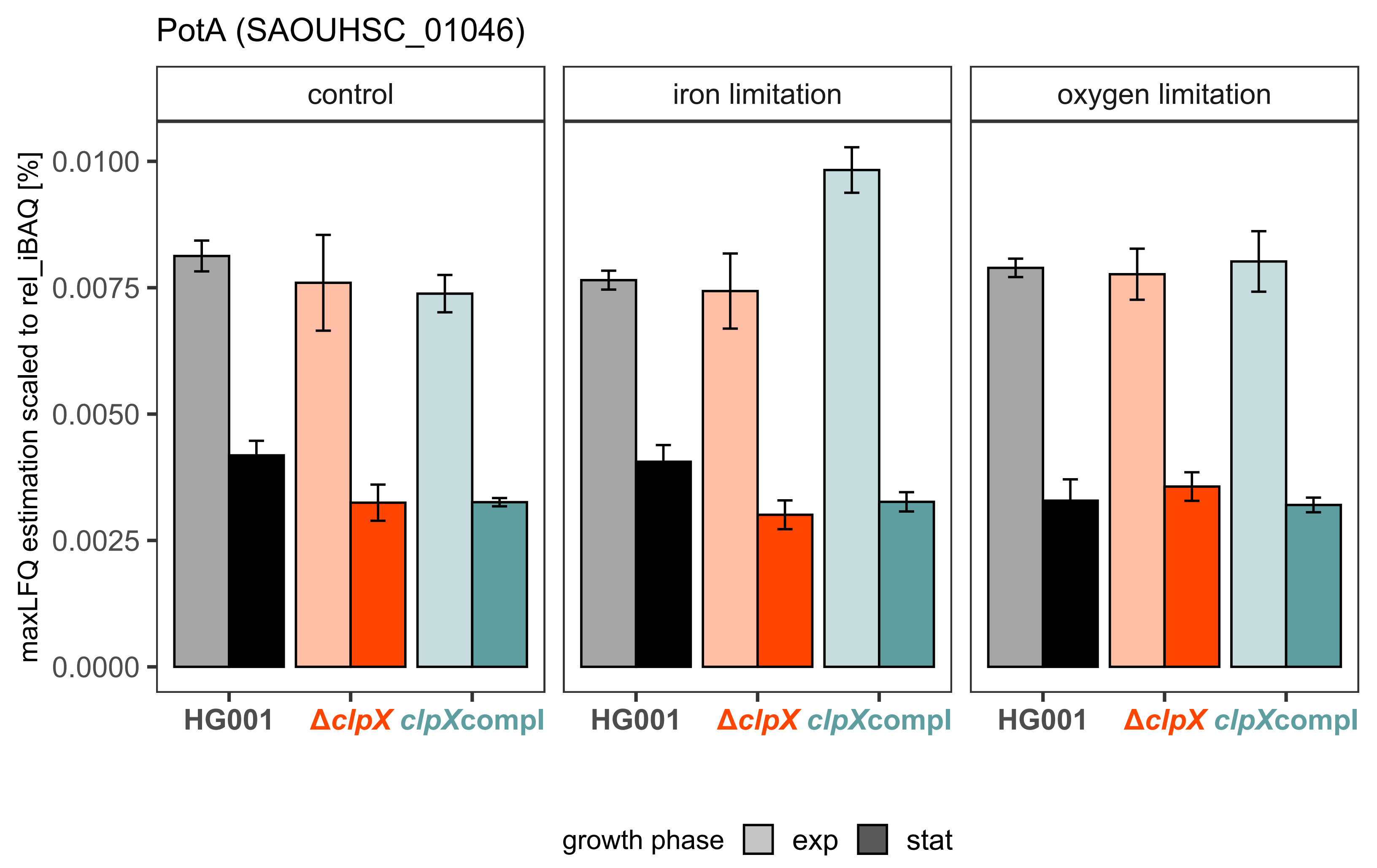
PotA (SAOUHSC_01046)
control
iron limitation
oxygen limitation
maxLFQ estimation scaled to rel_iBAQ [%]
0.0100
0.0075
0.0050
0.0025
0.0000
HG001
ΔclpX
clpXcompl
growth phase
exp
stat

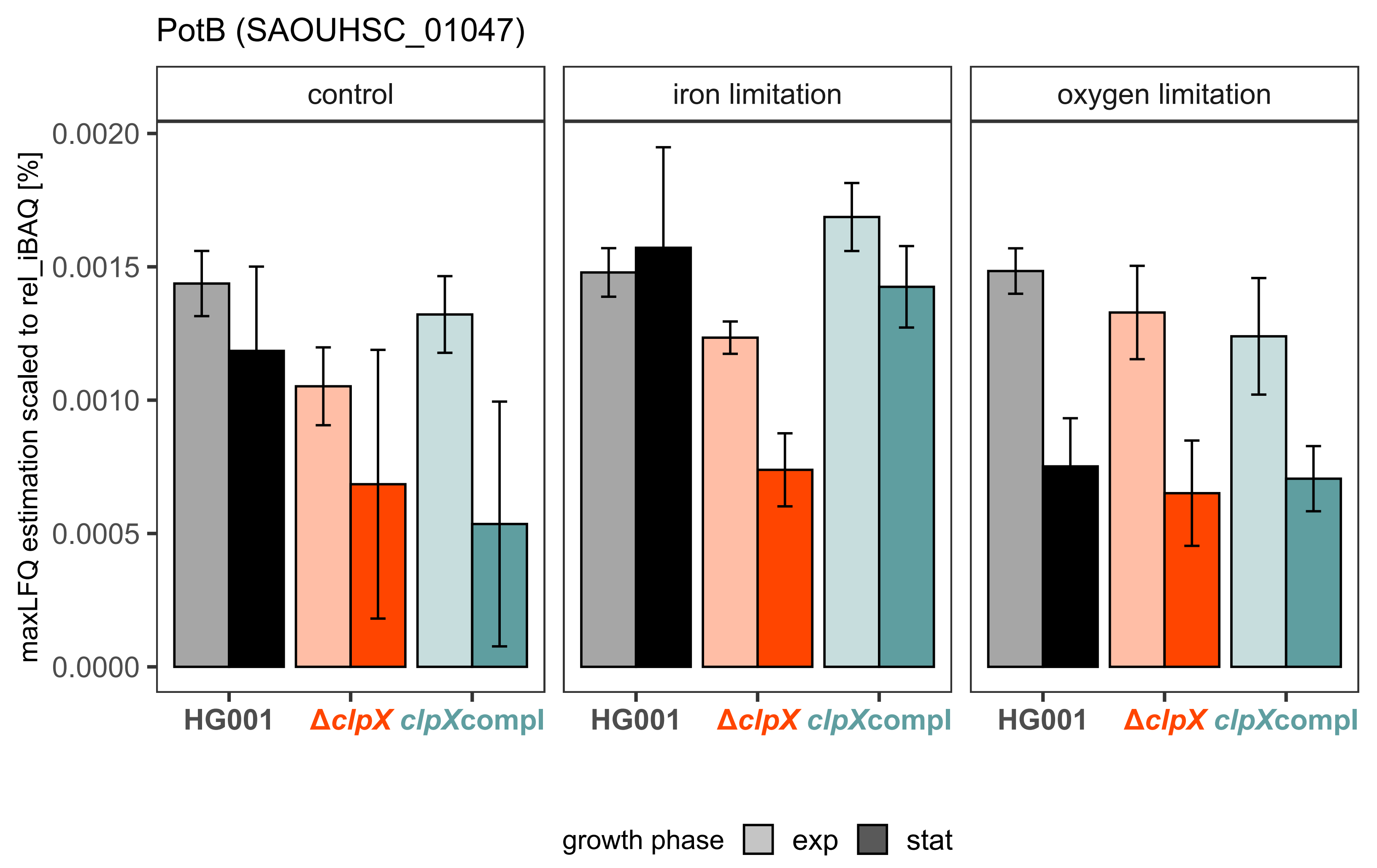
PotB (SAOUHSC_01047)
control
iron limitation
oxygen limitation
maxLFQ estimation scaled to rel_iBAQ [%]
0.0020
0.0015
0.0010
0.0005
0.0000
HG001
ΔclpX
clpXcompl
growth phase
exp
stat

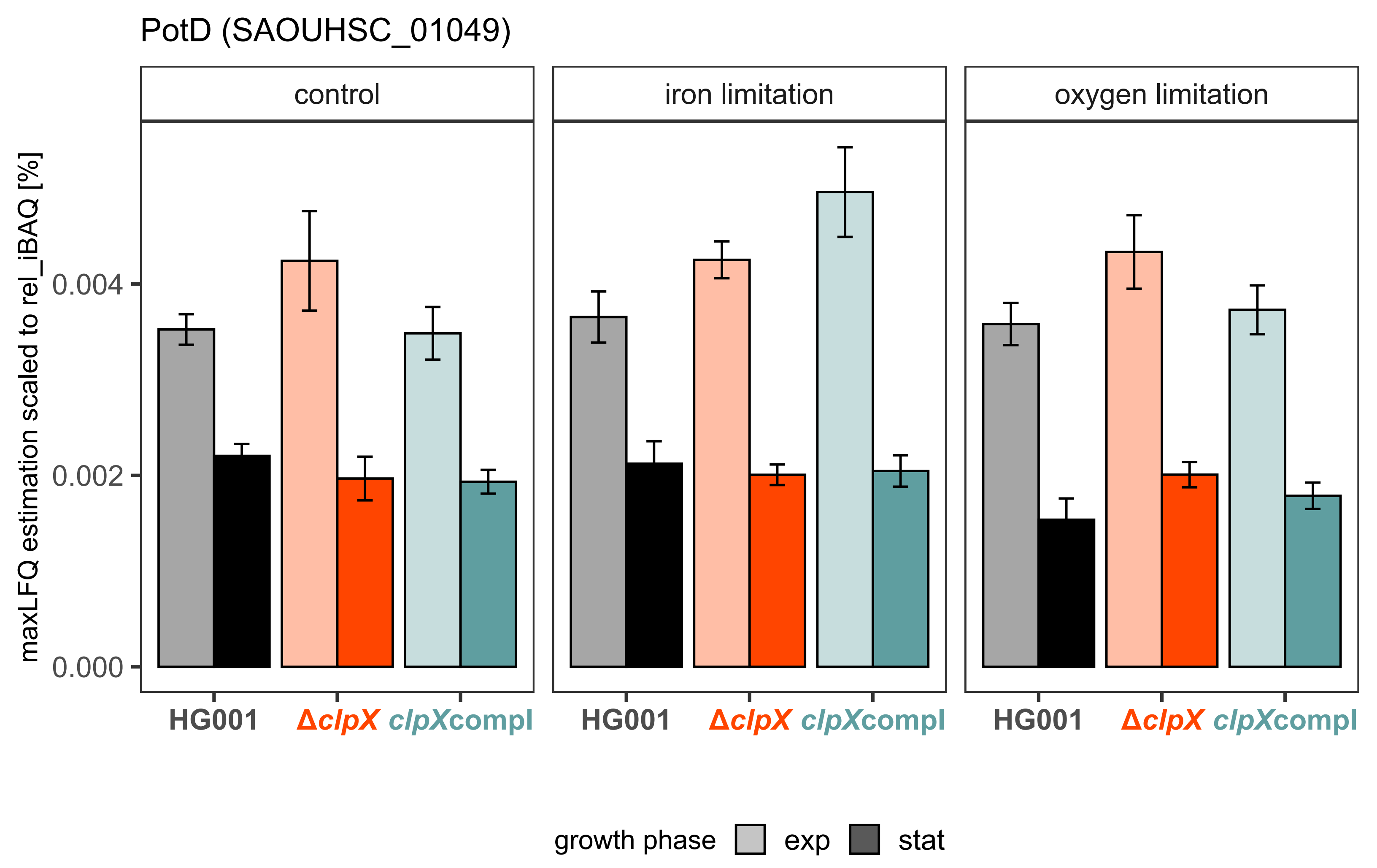

PotD (SAOUHSC_01049)
control
iron limitation
oxygen limitation
maxLFQ estimation scaled to rel_iBAQ [%]
0.004
0.002
0.000
HG001
ΔclpX
clpXcompl
HG001
ΔclpX
clpXcompl
HG001
ΔclpX
clpXcompl
growth phase
exp
stat

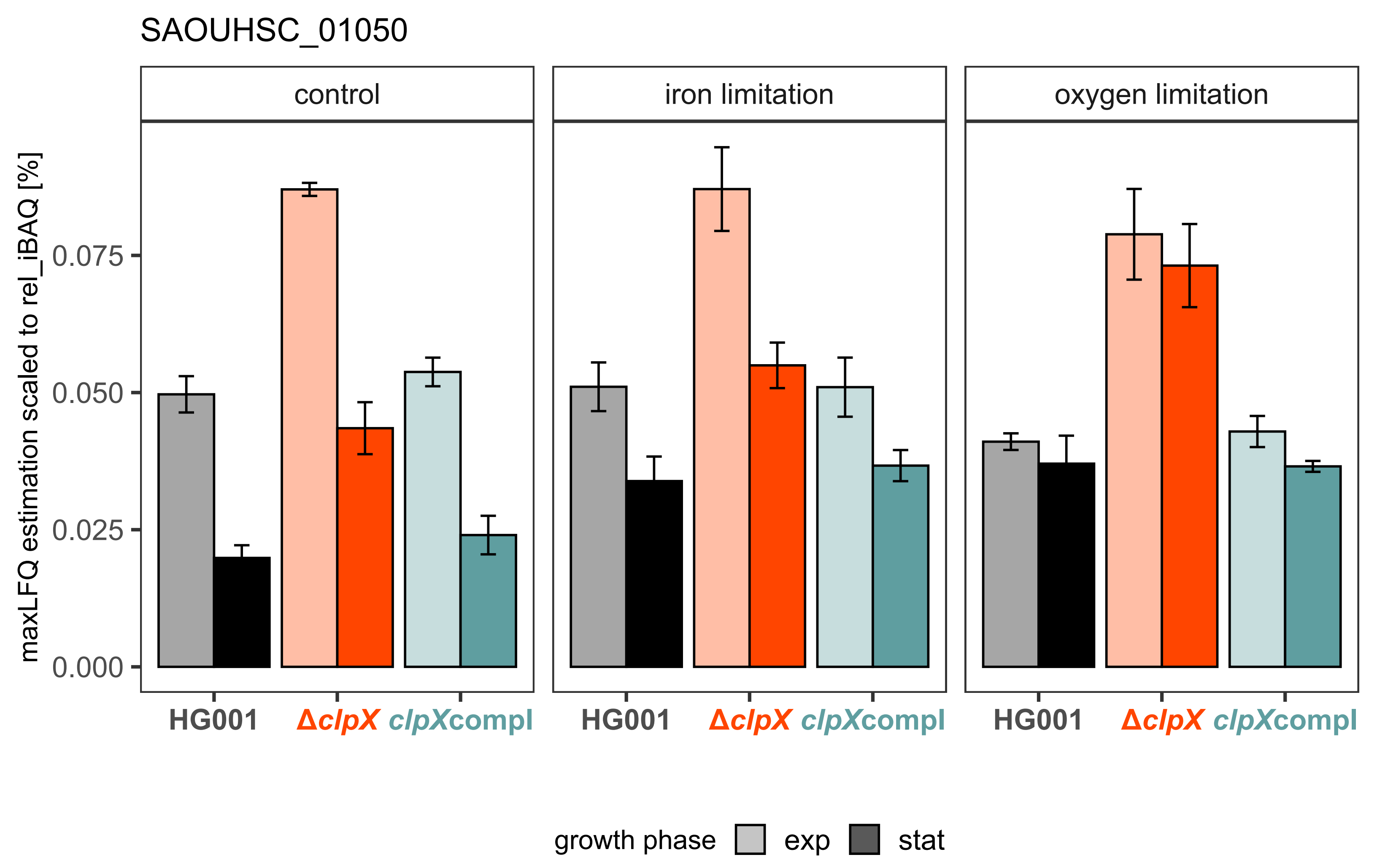

SAOUHSC_01050
control
iron limitation
oxygen limitation
maxLFQ estimation scaled to rel_iBAQ [%]
0.000
0.025
0.050
0.075
HG001
ΔclpX
clpXcompl
growth phase
exp
stat

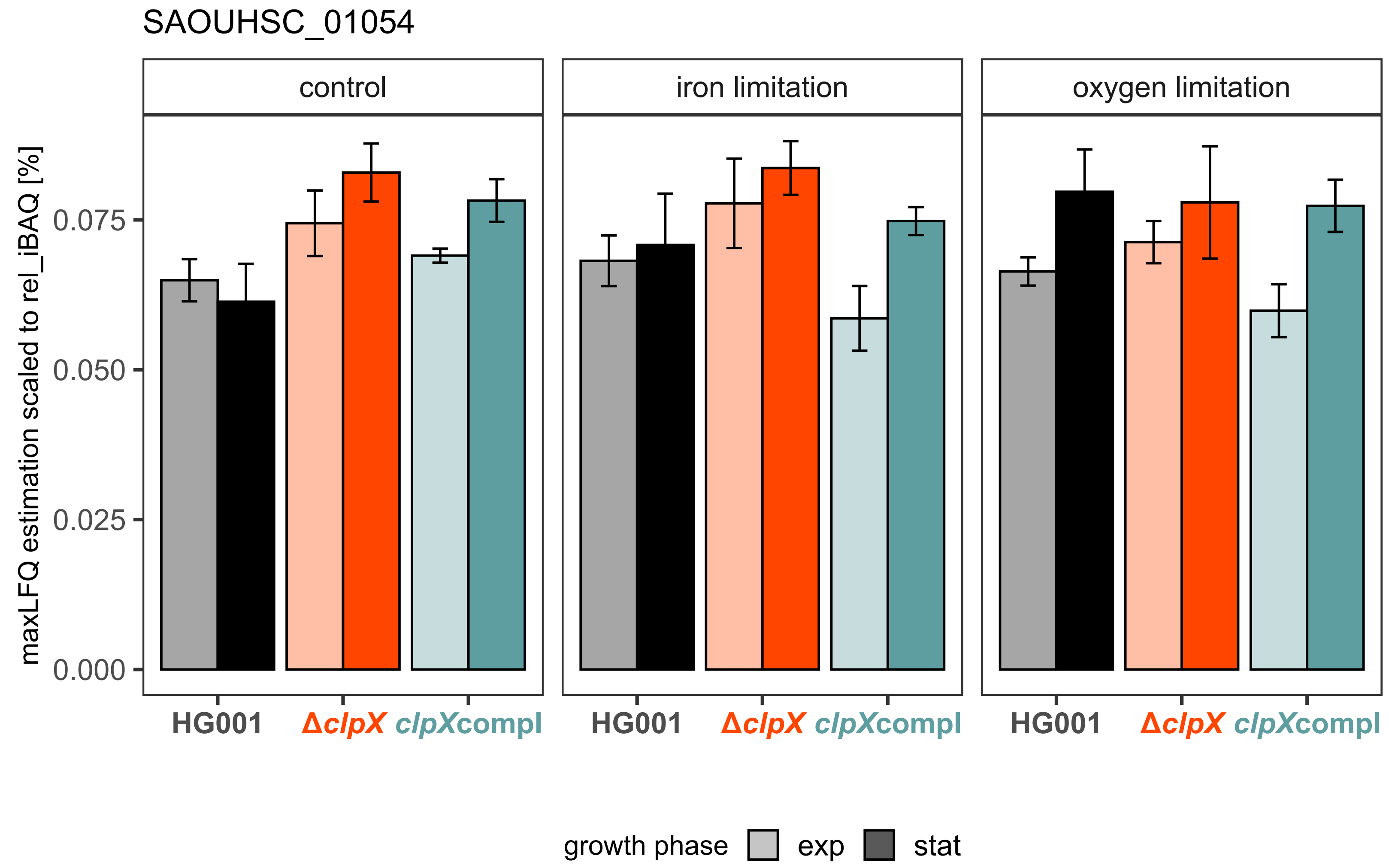
SAOUHSC_01054
control
iron limitation
oxygen limitation
maxLFQ estimation scaled to rel_iBAQ [%]
0.000
0.025
0.050
0.075
HG001
ΔclpX
clpXcompl
growth phase
exp
stat

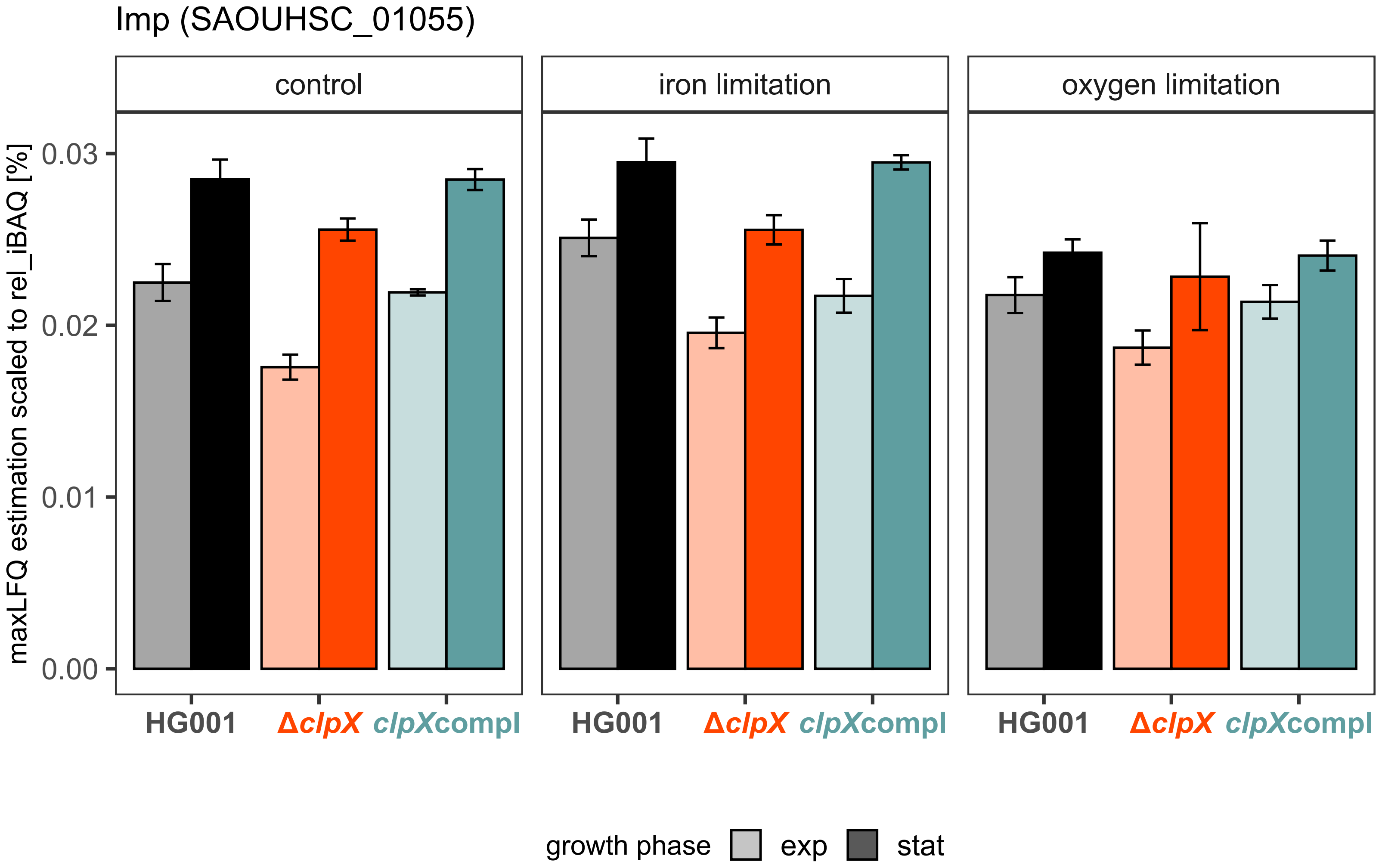
Imp (SAOUHSC_01055)
control
iron limitation
oxygen limitation
maxLFQ estimation scaled to rel_iBAQ [%]
0.03
0.02
0.01
0.00
HG001
ΔclpX
clpXcompl
growth phase
exp
stat

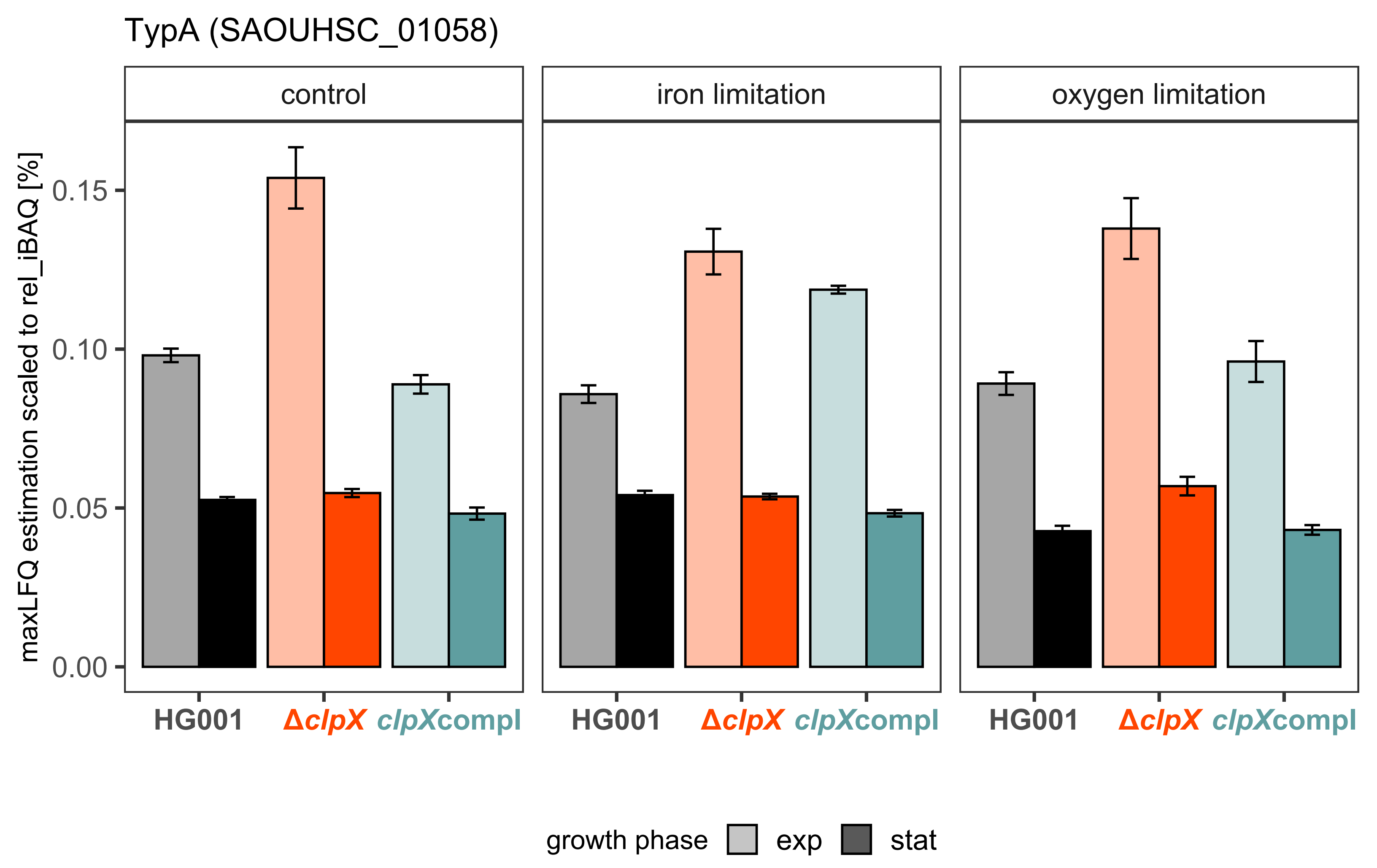
TypA (SAOUHSC_01058)
control
iron limitation
oxygen limitation
maxLFQ estimation scaled to rel_iBAQ [%]
0.15
0.10
0.05
0.00
HG001
ΔclpX
clpXcompl
growth phase
exp
stat

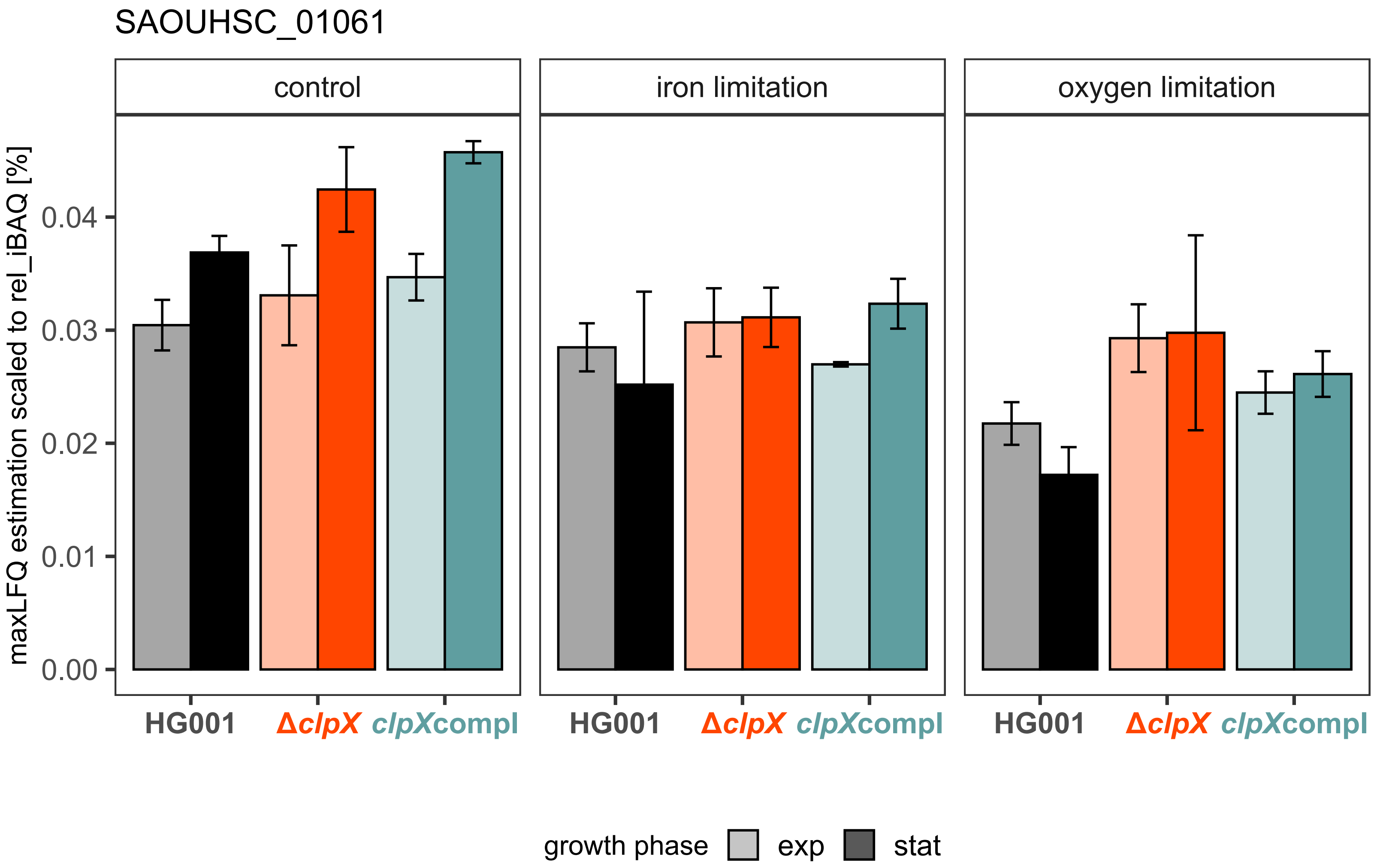
SAOUHSC_01061
control
iron limitation
oxygen limitation
maxLFQ estimation scaled to rel_iBAQ [%]
0.00
0.01
0.02
0.03
0.04
HG001
ΔclpX
clpXcompl
growth phase
exp
stat

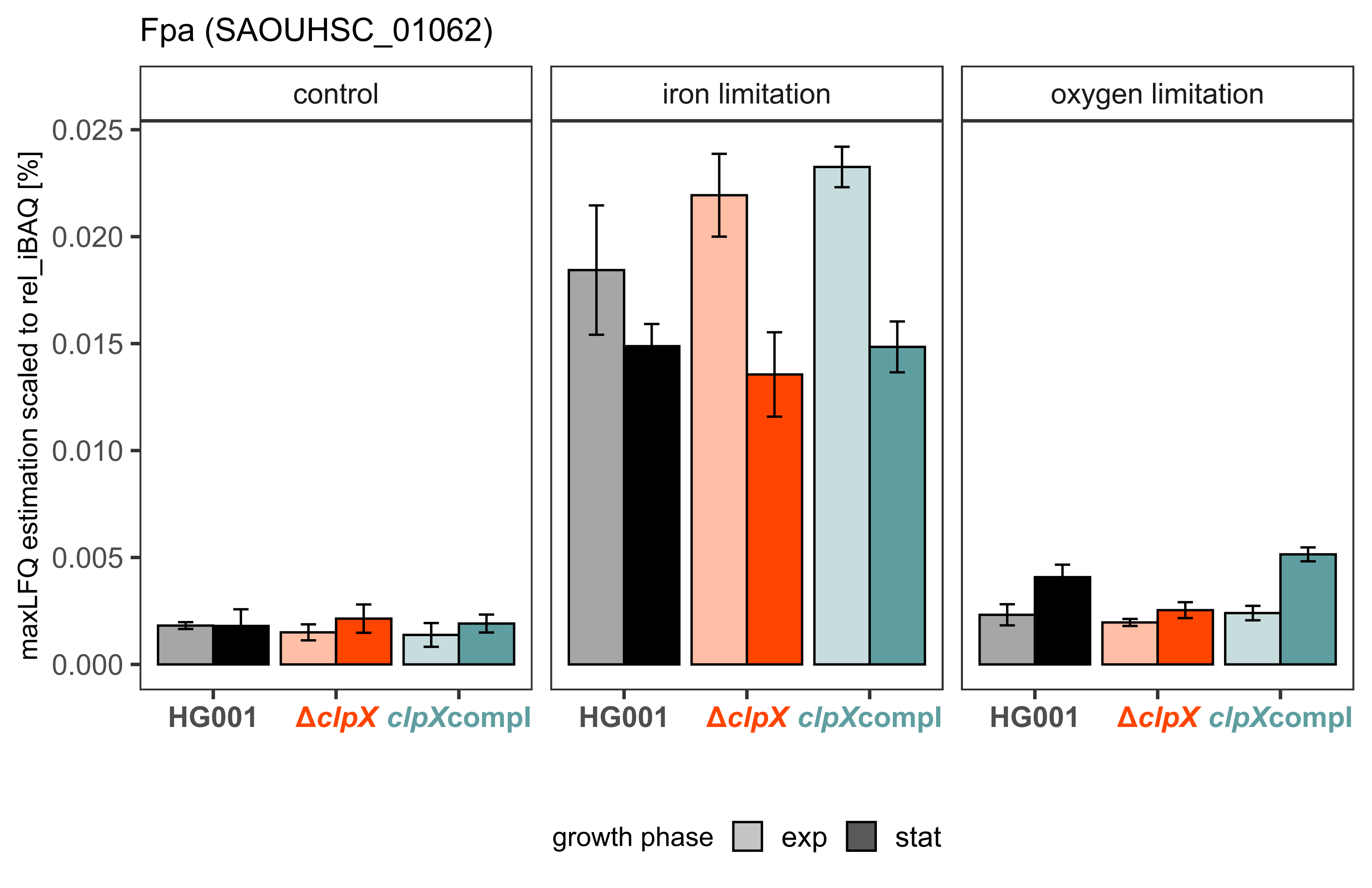
Fpa (SAOUHSC_01062)
control
iron limitation
oxygen limitation
maxLFQ estimation scaled to rel_iBAQ [%]
0.025
0.020
0.015
0.010
0.005
0.000
HG001
ΔclpX
clpXcompl
growth phase
exp
stat

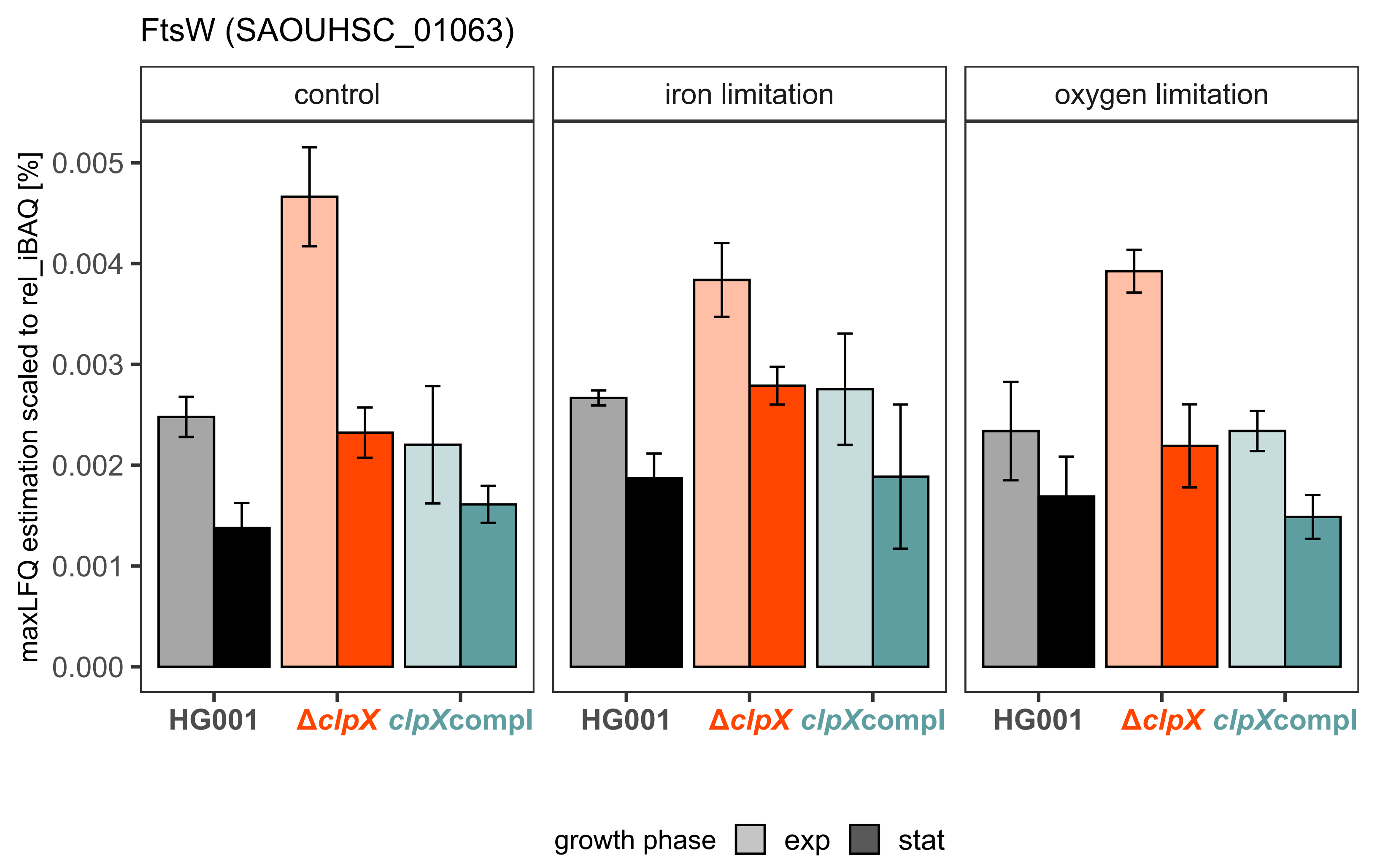

FtsW (SAOUHSC_01063)
control
iron limitation
oxygen limitation
maxLFQ estimation scaled to rel_iBAQ [%]
0.005
0.004
0.003
0.002
0.001
0.000
HG001
ΔclpX
clpXcompl
growth phase
exp
stat

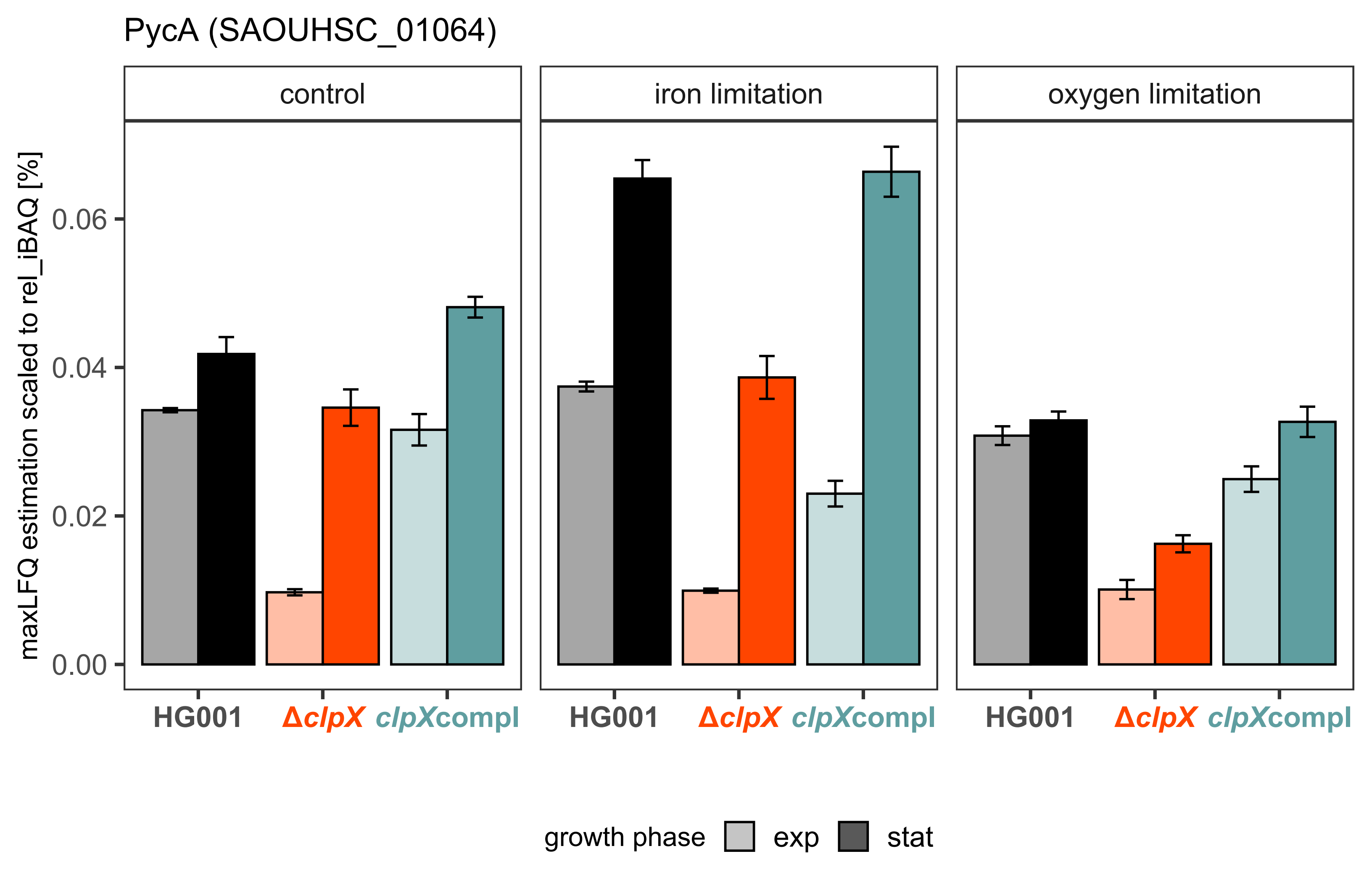

PycA (SAOUHSC_01064)
control
iron limitation
oxygen limitation
maxLFQ estimation scaled to rel_iBAQ [%]
0.06
0.04
0.02
0.00
HG001
ΔclpX
clpXcompl
growth phase
exp
stat

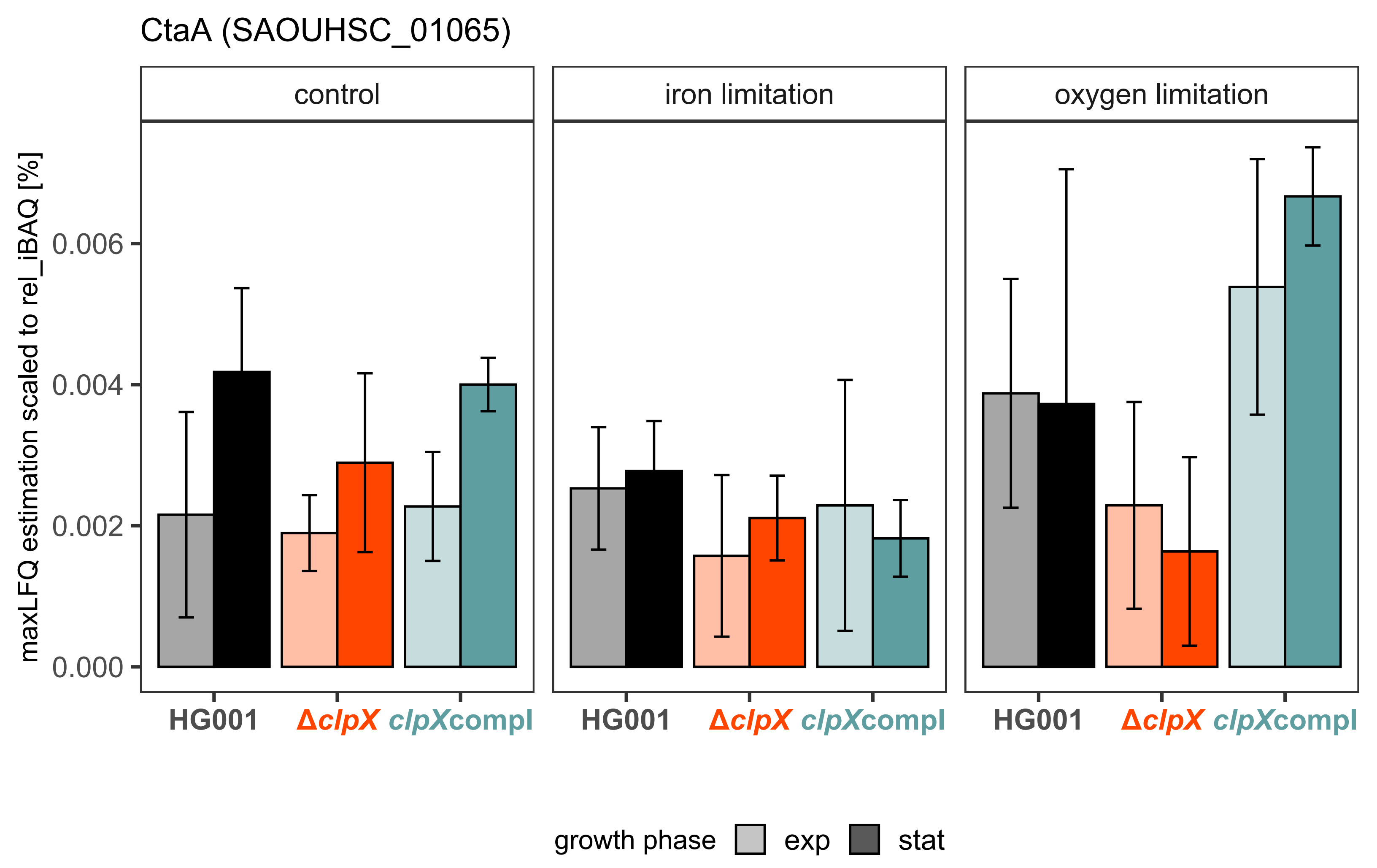
CtaA (SAOUHSC_01065)
control
iron limitation
oxygen limitation
maxLFQ estimation scaled to rel_iBAQ [%]
0.000
0.002
0.004
0.006
HG001
ΔclpX
clpXcompl
growth phase
exp
stat

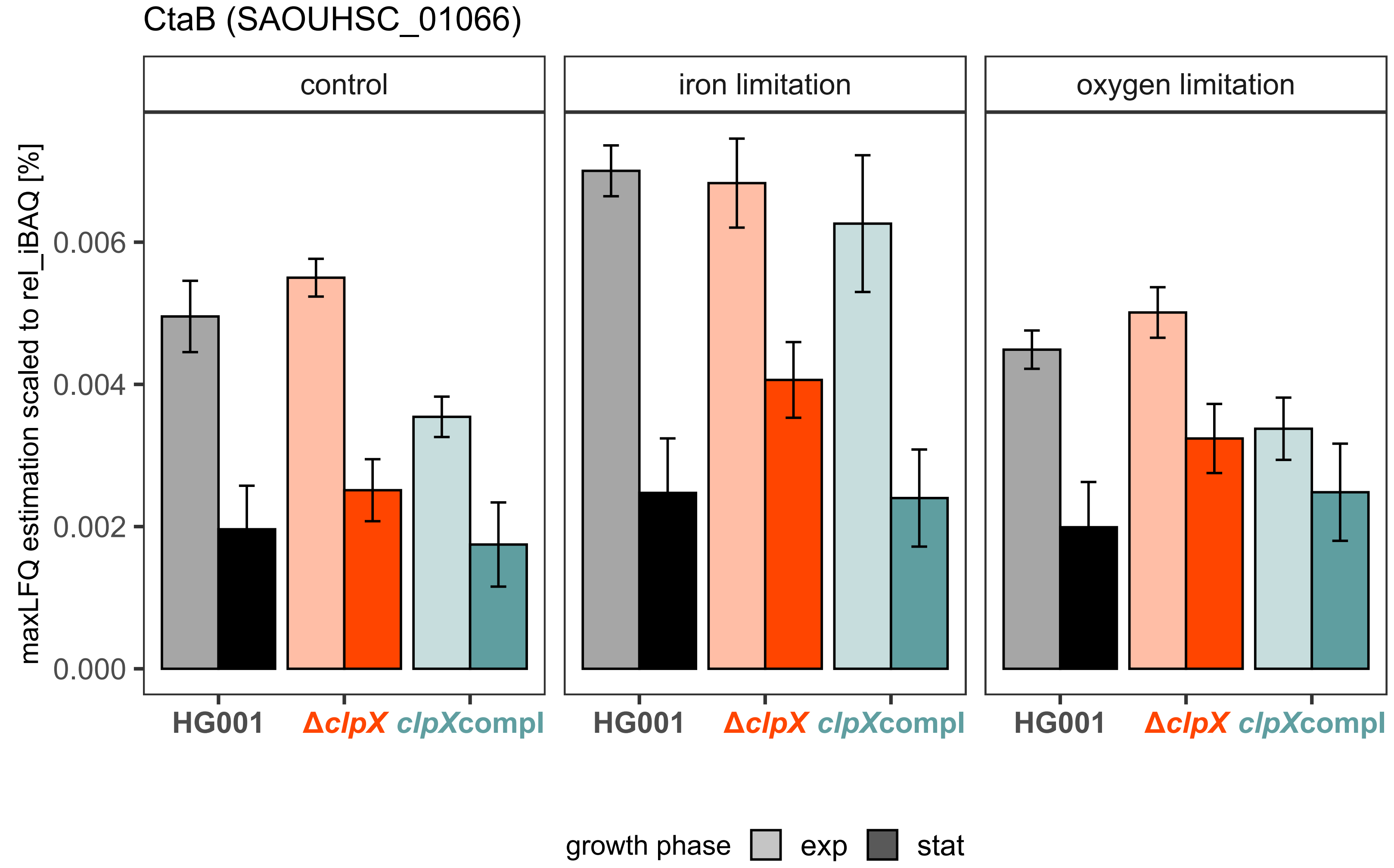

CtaB (SAOUHSC_01066)
control
iron limitation
oxygen limitation
maxLFQ estimation scaled to rel_iBAQ [%]
0.000
0.002
0.004
0.006
HG001
ΔclpX
clpXcompl
growth phase
exp
stat

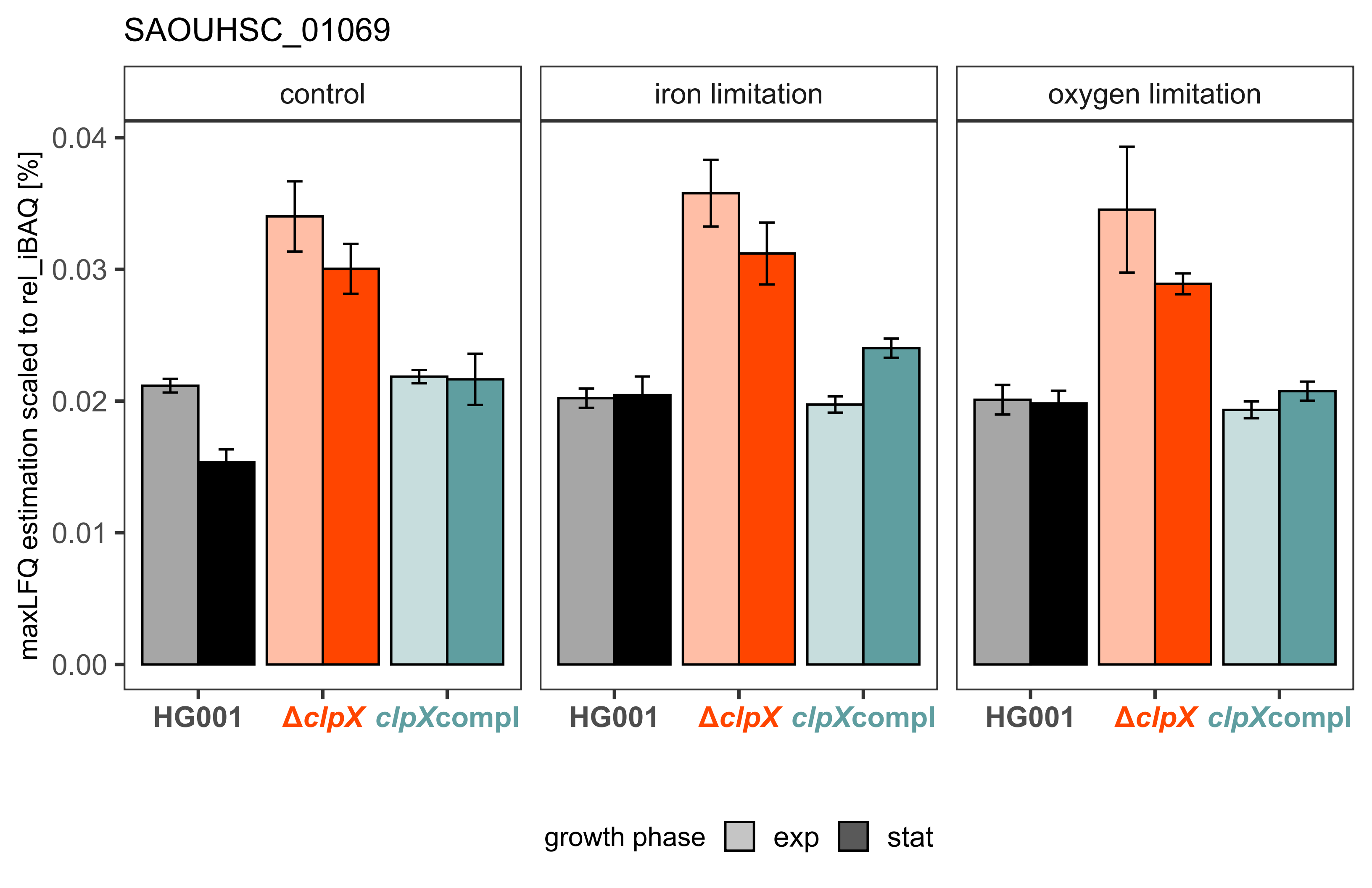
SAOUHSC_01069
control
iron limitation
oxygen limitation
maxLFQ estimation scaled to rel_iBAQ [%]
0.04
0.03
0.02
0.01
0.00
HG001
ΔclpX
clpXcompl
growth phase
exp
stat

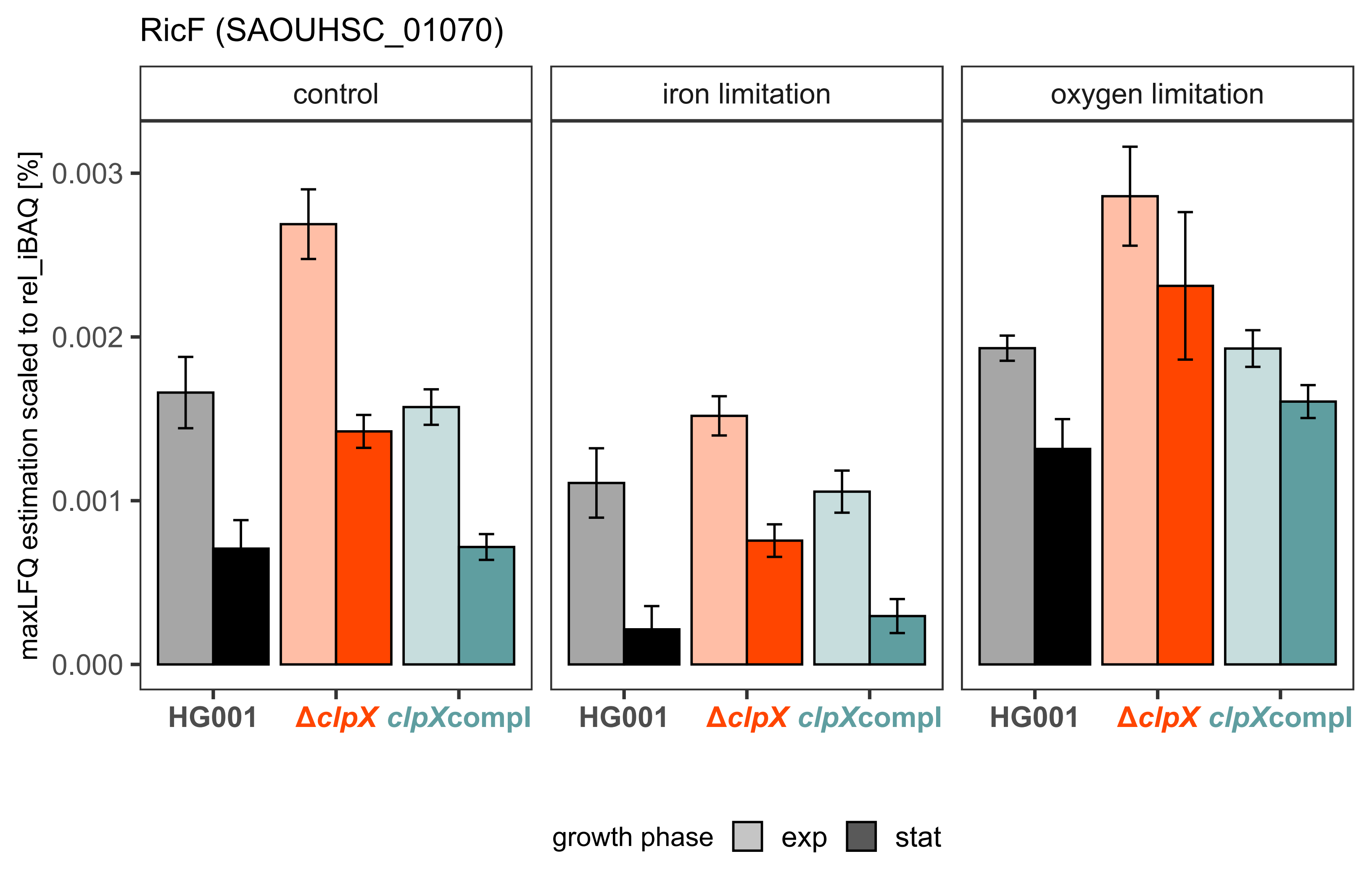

RicF (SAOUHSC_01070)
control
iron limitation
oxygen limitation
maxLFQ estimation scaled to rel_iBAQ [%]
0.003
0.002
0.001
0.000
HG001
ΔclpX
clpXcompl
growth phase
exp
stat

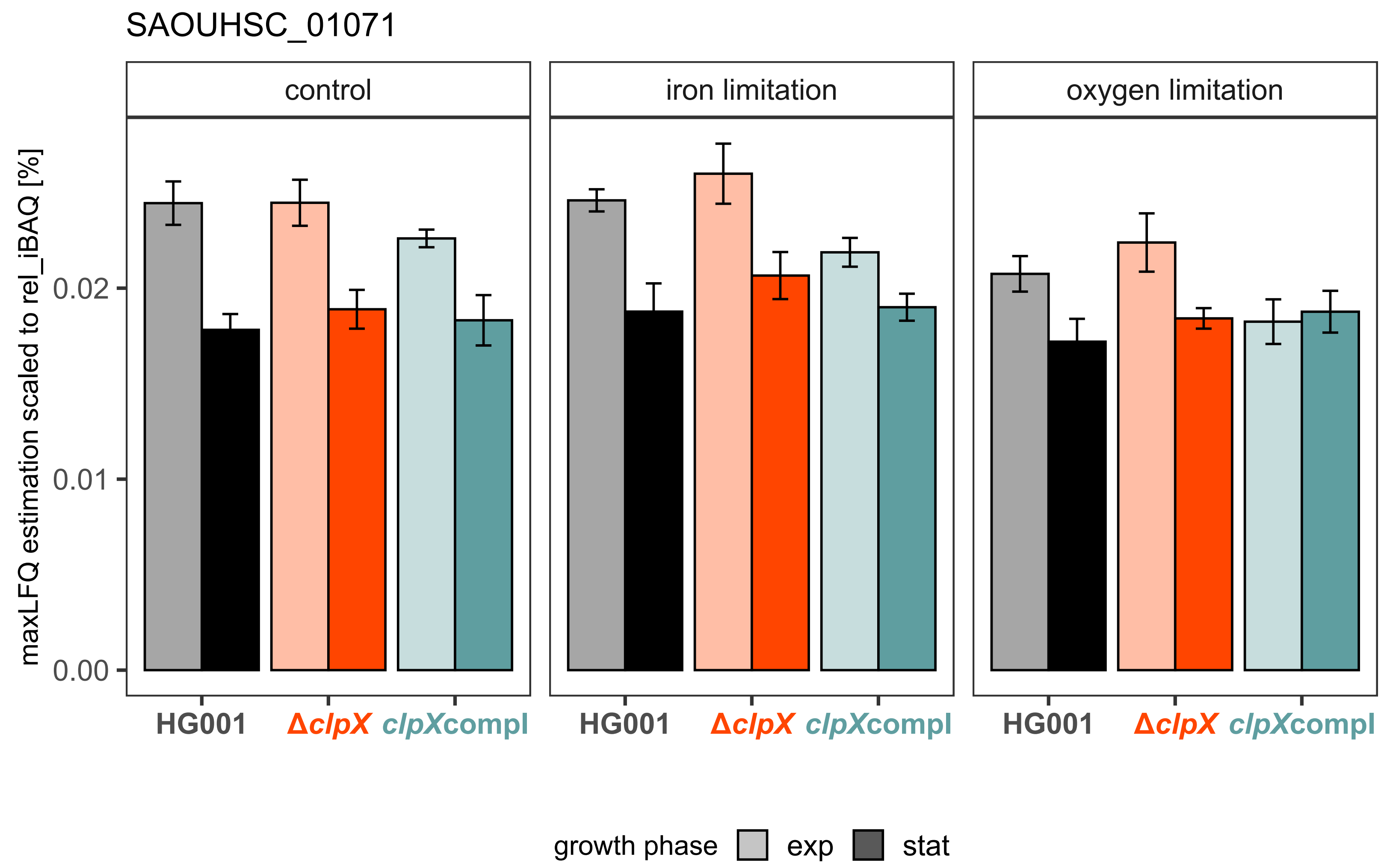
SAOUHSC_01071
control
iron limitation
oxygen limitation
maxLFQ estimation scaled to rel_iBAQ [%]
0.02
0.01
0.00
HG001
ΔclpX
clpXcompl
growth phase
exp
stat

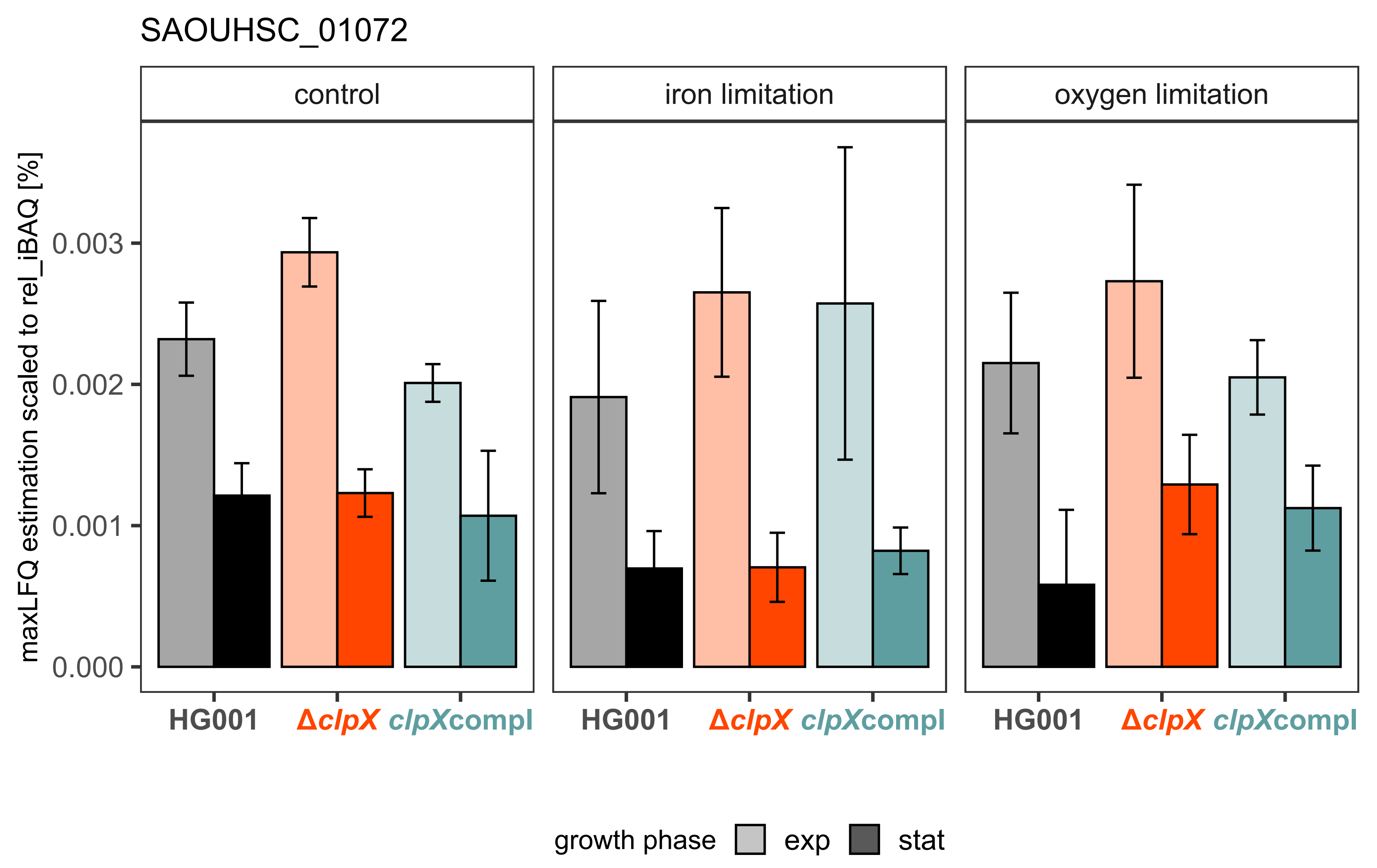

SAOUHSC_01072
control
iron limitation
oxygen limitation
maxLFQ estimation scaled to rel_iBAQ [%]
0.000
0.001
0.002
0.003
HG001
ΔclpX
clpXcompl
growth phase
exp
stat

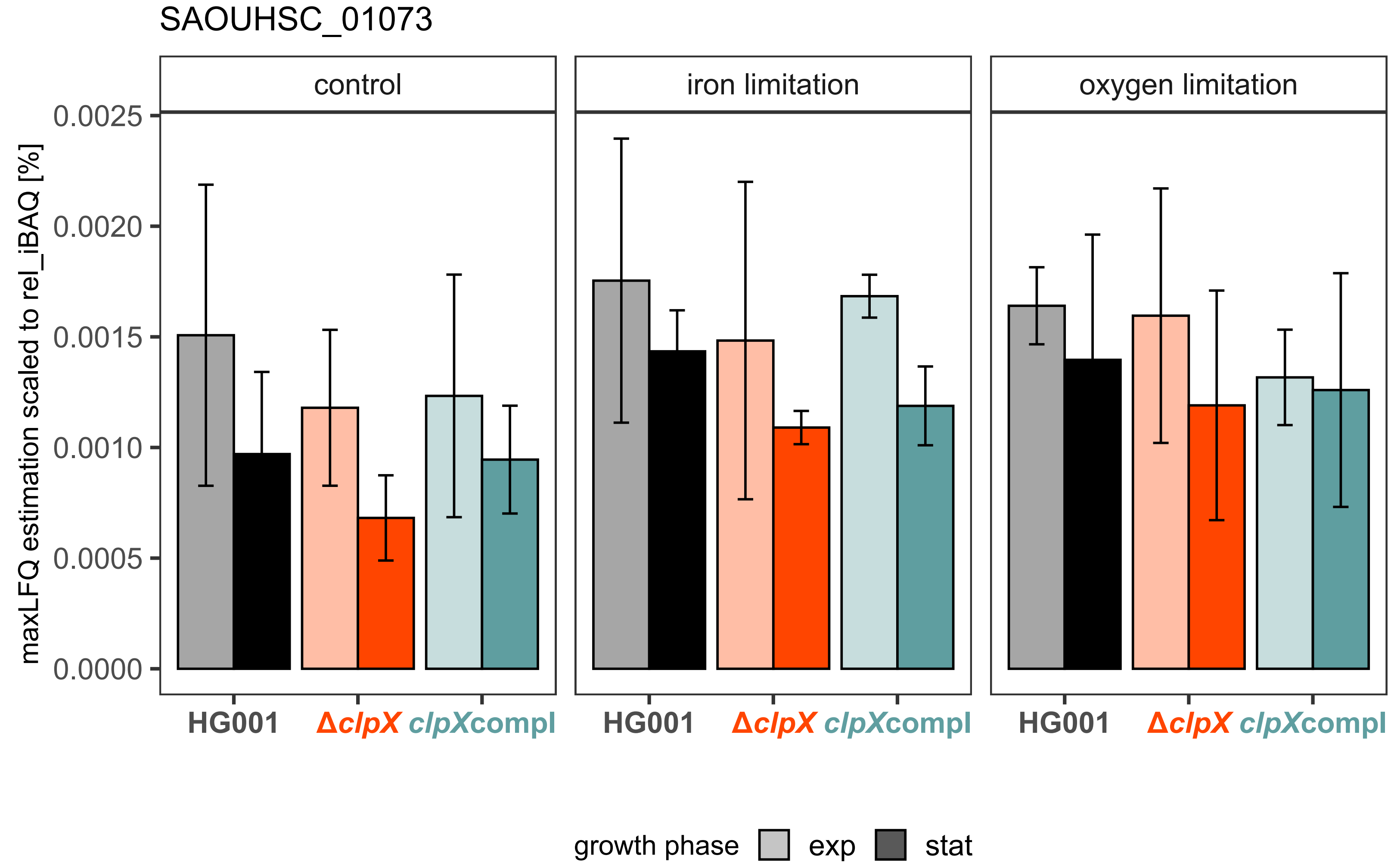

SAOUHSC_01073
control
iron limitation
oxygen limitation
maxLFQ estimation scaled to rel_iBAQ [%]
0.0025
0.0020
0.0015
0.0010
0.0005
0.0000
HG001
ΔclpX
clpXcompl
growth phase
exp
stat

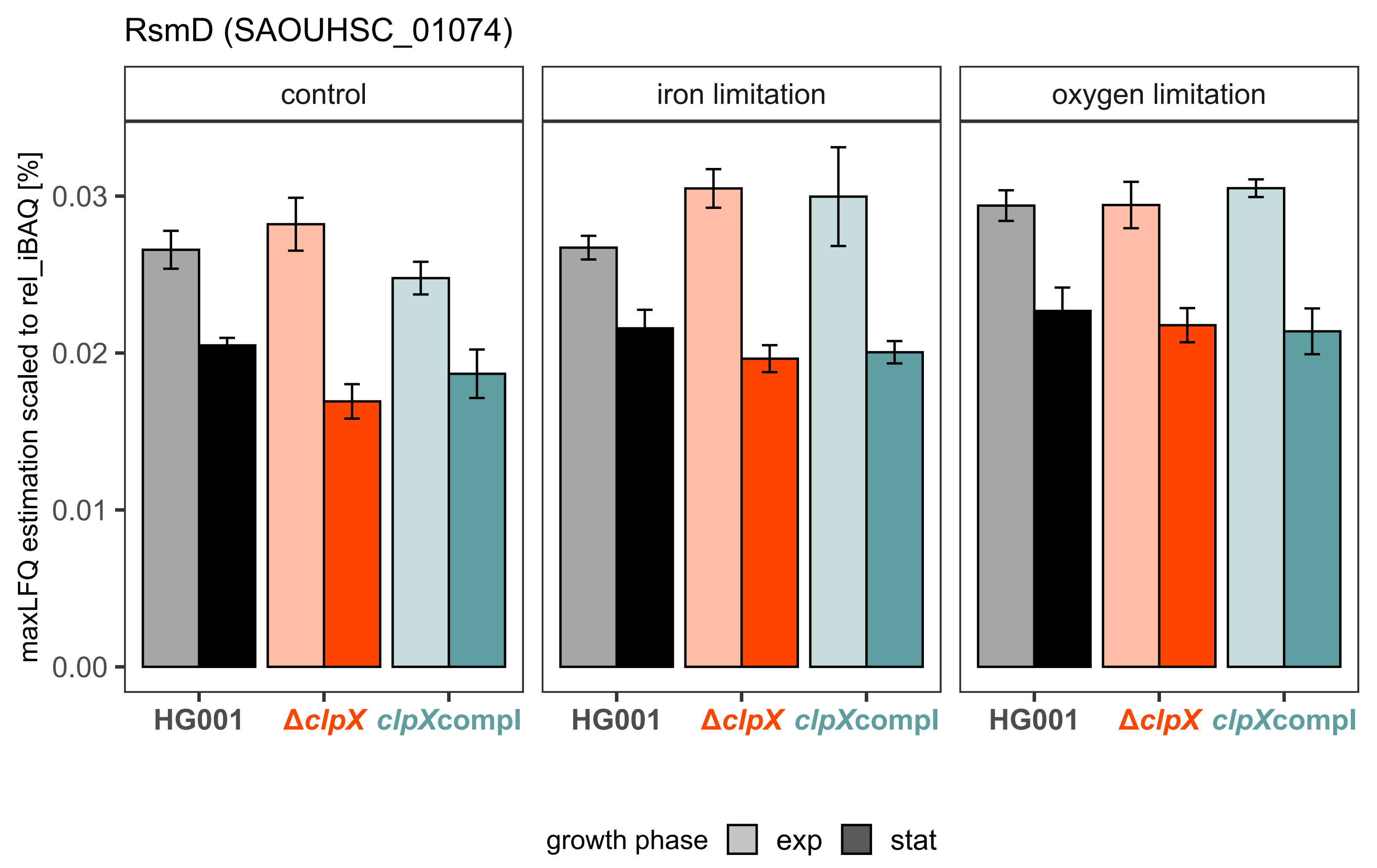

RsmD (SAOUHSC_01074)
control
iron limitation
oxygen limitation
maxLFQ estimation scaled to rel_iBAQ [%]
0.00
0.01
0.02
0.03
HG001
ΔclpX
clpXcompl
growth phase
exp
stat

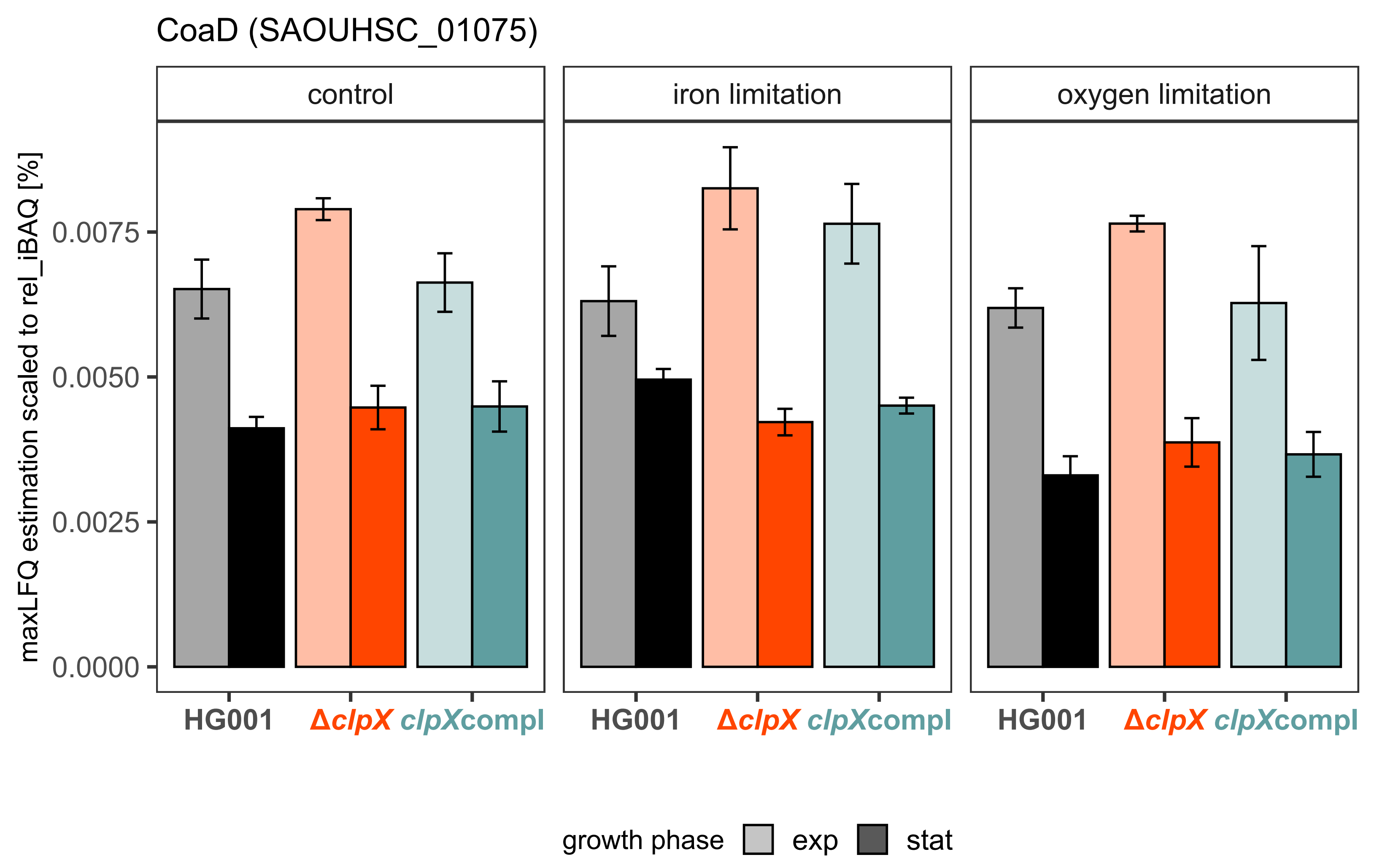
CoaD (SAOUHSC_01075)
control
iron limitation
oxygen limitation
maxLFQ estimation scaled to rel_iBAQ [%]
0.0075
0.0050
0.0025
0.0000
HG001
ΔclpX
clpXcompl
growth phase
exp
stat

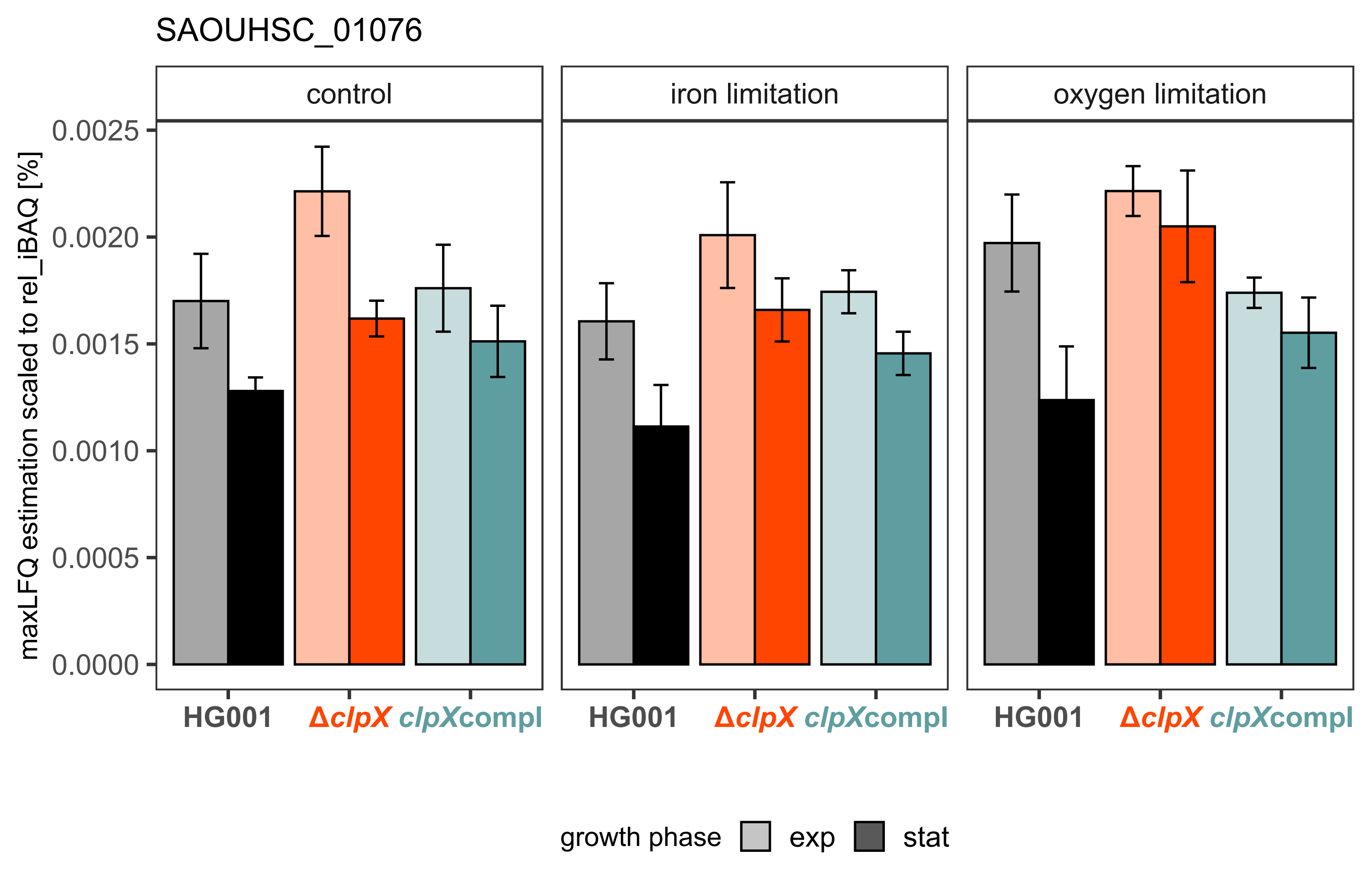
SAOUHSC_01076
control
iron limitation
oxygen limitation
maxLFQ estimation scaled to rel_iBAQ [%]
0.0025
0.0020
0.0015
0.0010
0.0005
0.0000
HG001
ΔclpX
clpXcompl
growth phase
exp
stat

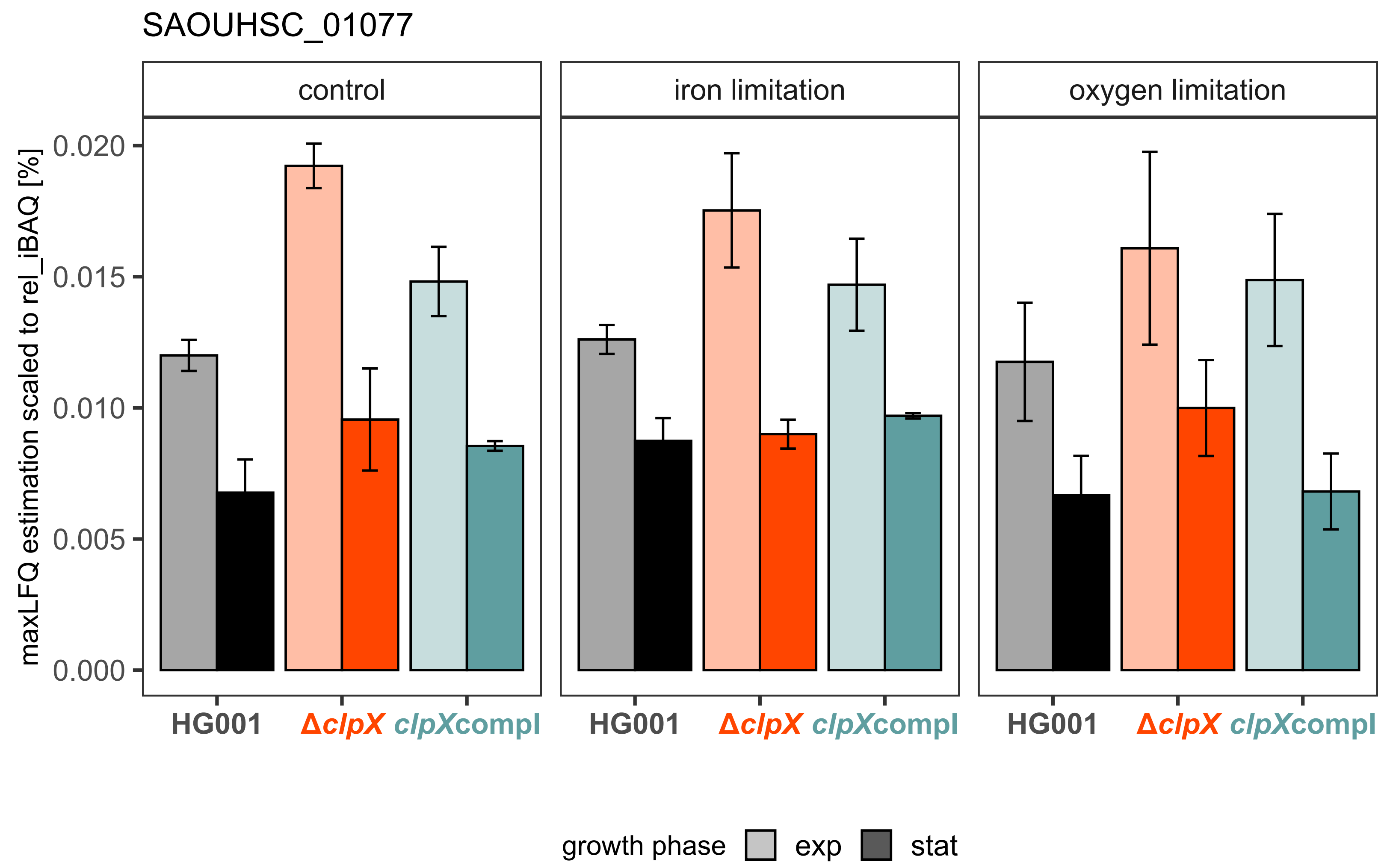

SAOUHSC_01077
control
iron limitation
oxygen limitation
maxLFQ estimation scaled to rel_iBAQ [%]
0.020
0.015
0.010
0.005
0.000
HG001
ΔclpX
clpXcompl
growth phase
exp
stat

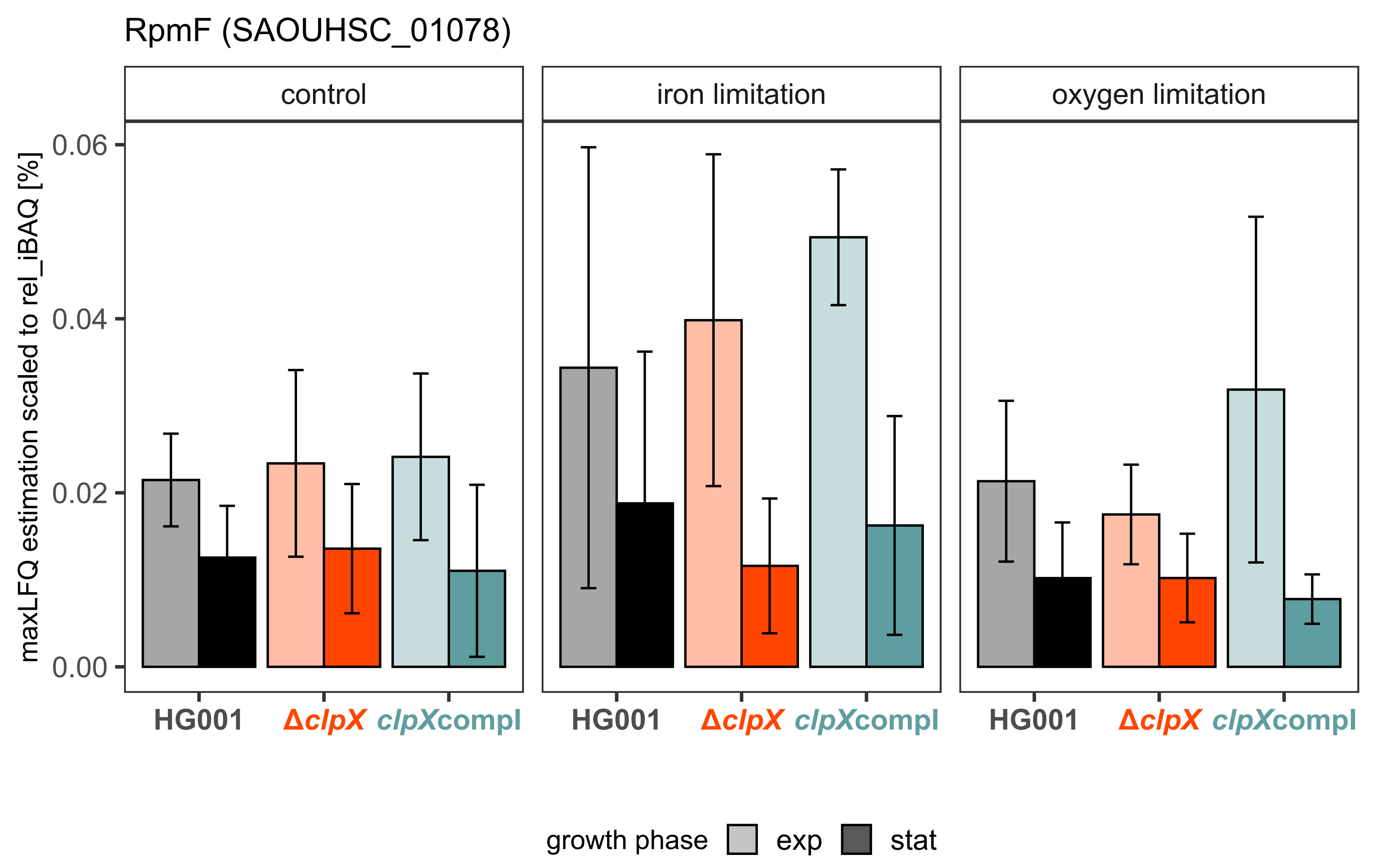

RpmF (SAOUHSC_01078)
control
iron limitation
oxygen limitation
maxLFQ estimation scaled to rel_iBAQ [%]
0.06
0.04
0.02
0.00
HG001
ΔclpX
clpXcompl
growth phase
exp
stat

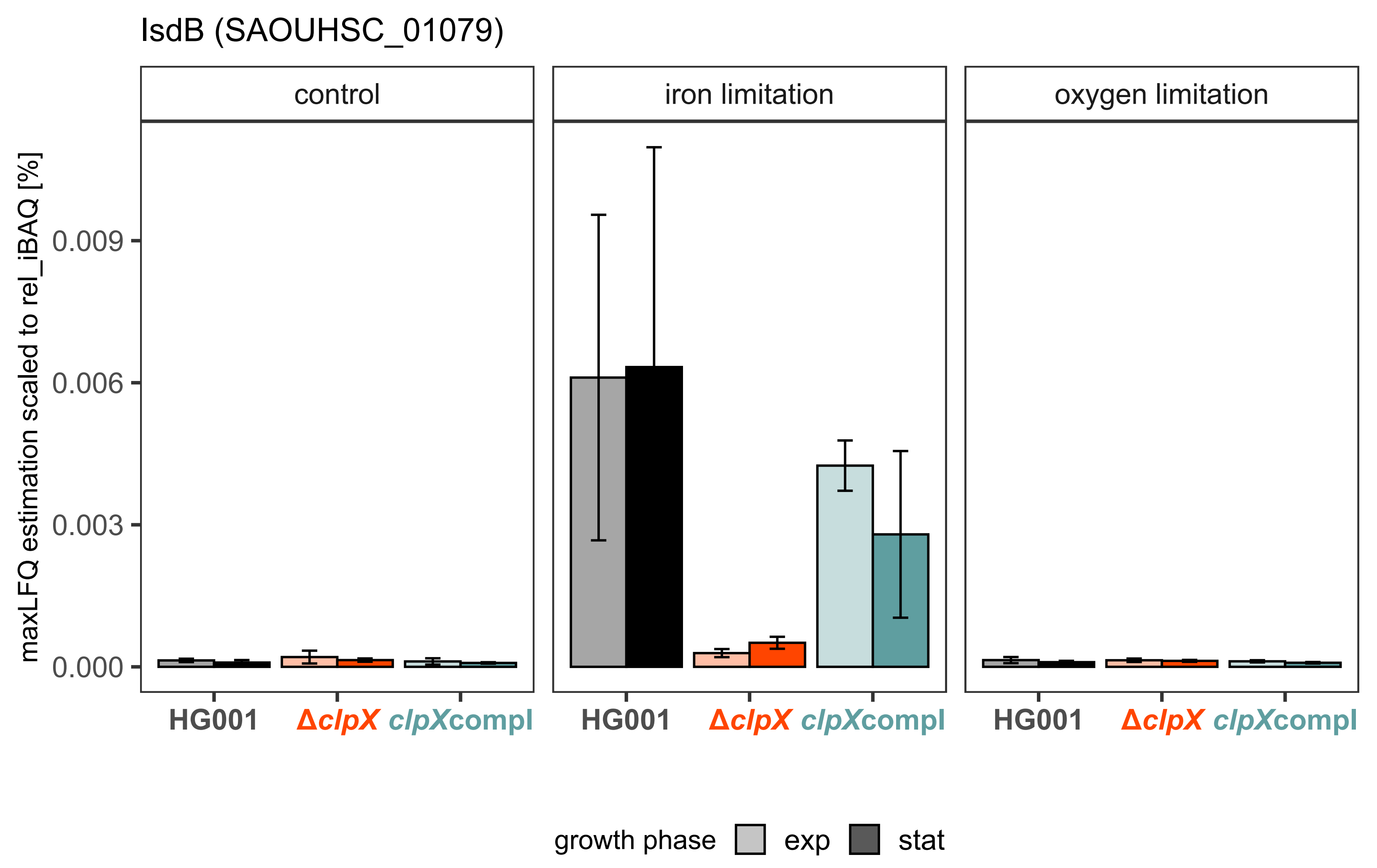
IsdB (SAOUHSC_01079)
control
iron limitation
oxygen limitation
maxLFQ estimation scaled to rel_iBAQ [%]
0.009
0.006
0.003
0.000
HG001
ΔclpX
clpXcompl
growth phase
exp
stat

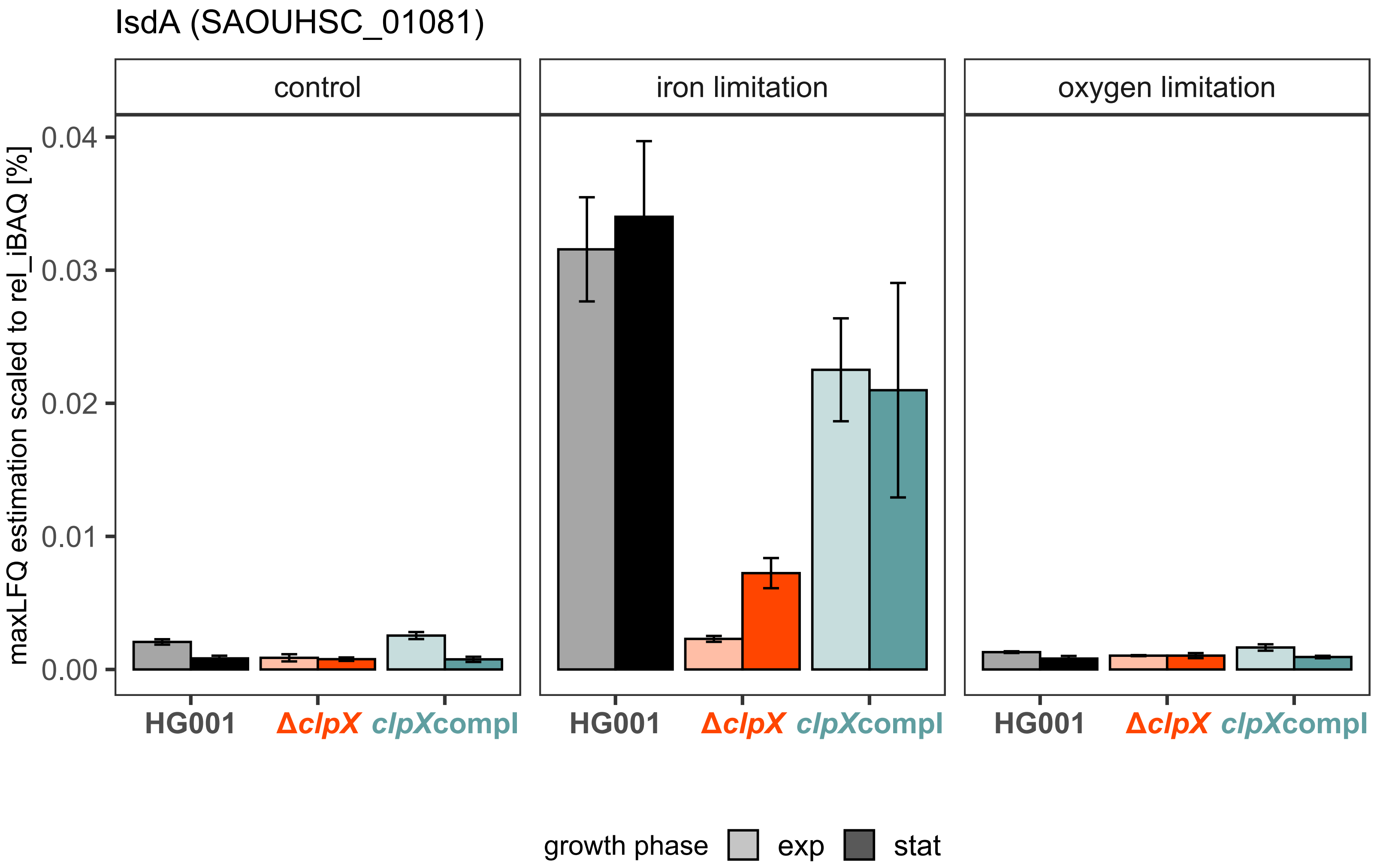

IsdA (SAOUHSC_01081)
control
iron limitation
oxygen limitation
maxLFQ estimation scaled to rel_iBAQ [%]
0.04
0.03
0.02
0.01
0.00
HG001
ΔclpX
clpXcompl
growth phase
exp
stat

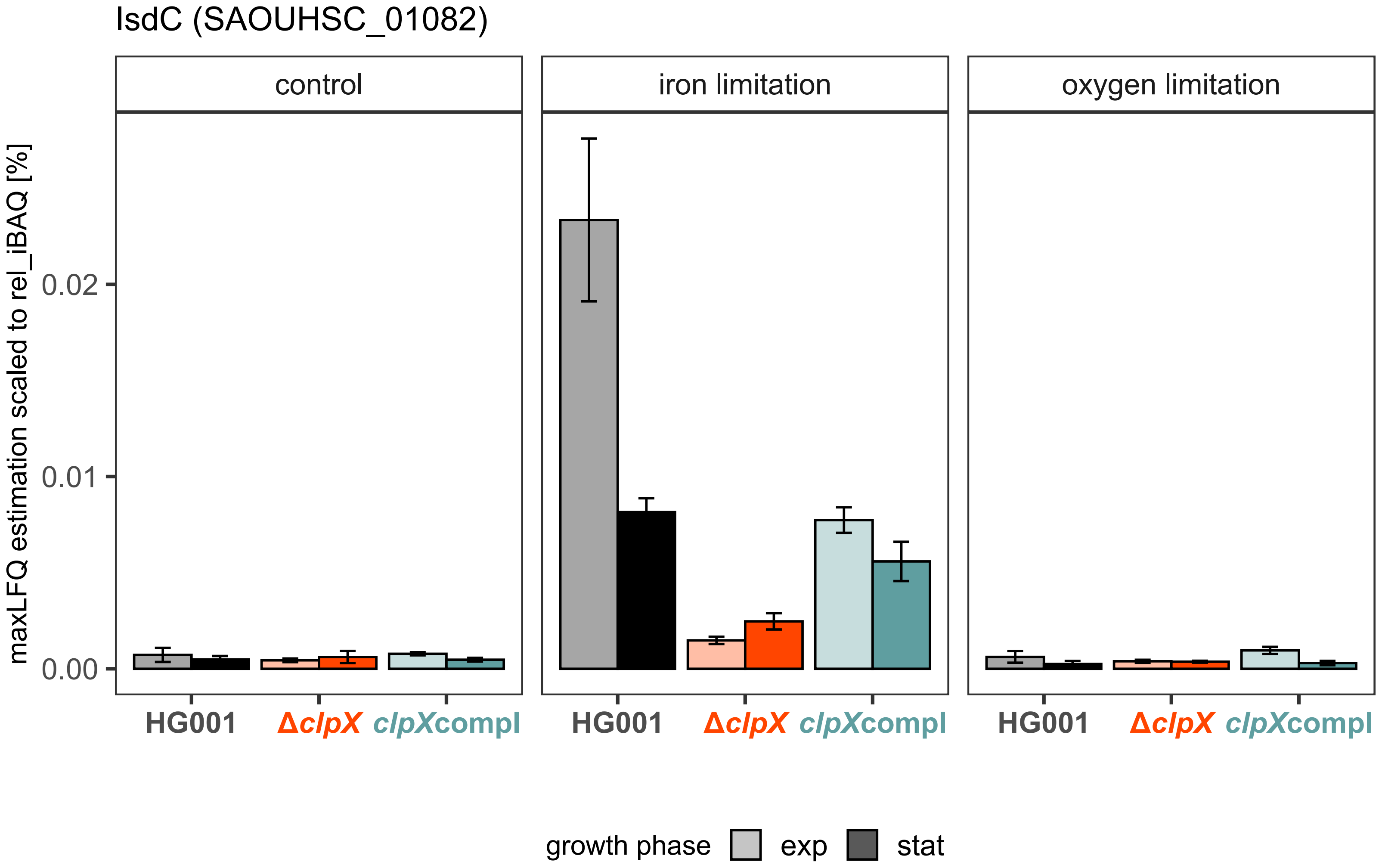
IsdC (SAOUHSC_01082)
control
iron limitation
oxygen limitation
maxLFQ estimation scaled to rel_iBAQ [%]
0.02
0.01
0.00
HG001
ΔclpX
clpXcompl
growth phase
exp
stat

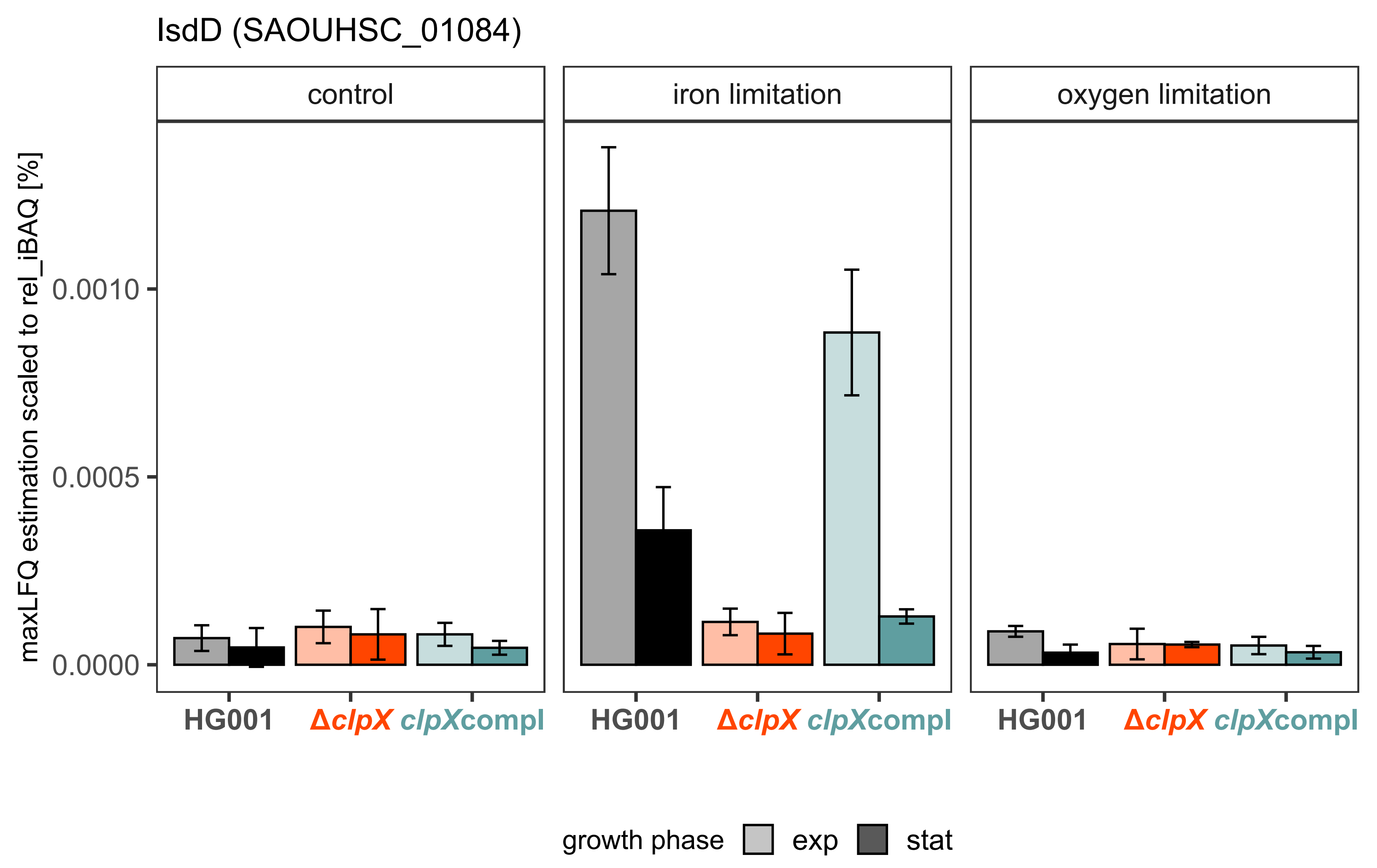
IsdD (SAOUHSC_01084)
control
iron limitation
oxygen limitation
maxLFQ estimation scaled to rel_iBAQ [%]
0.0010
0.0005
0.0000
HG001
ΔclpX
clpXcompl
growth phase
exp
stat

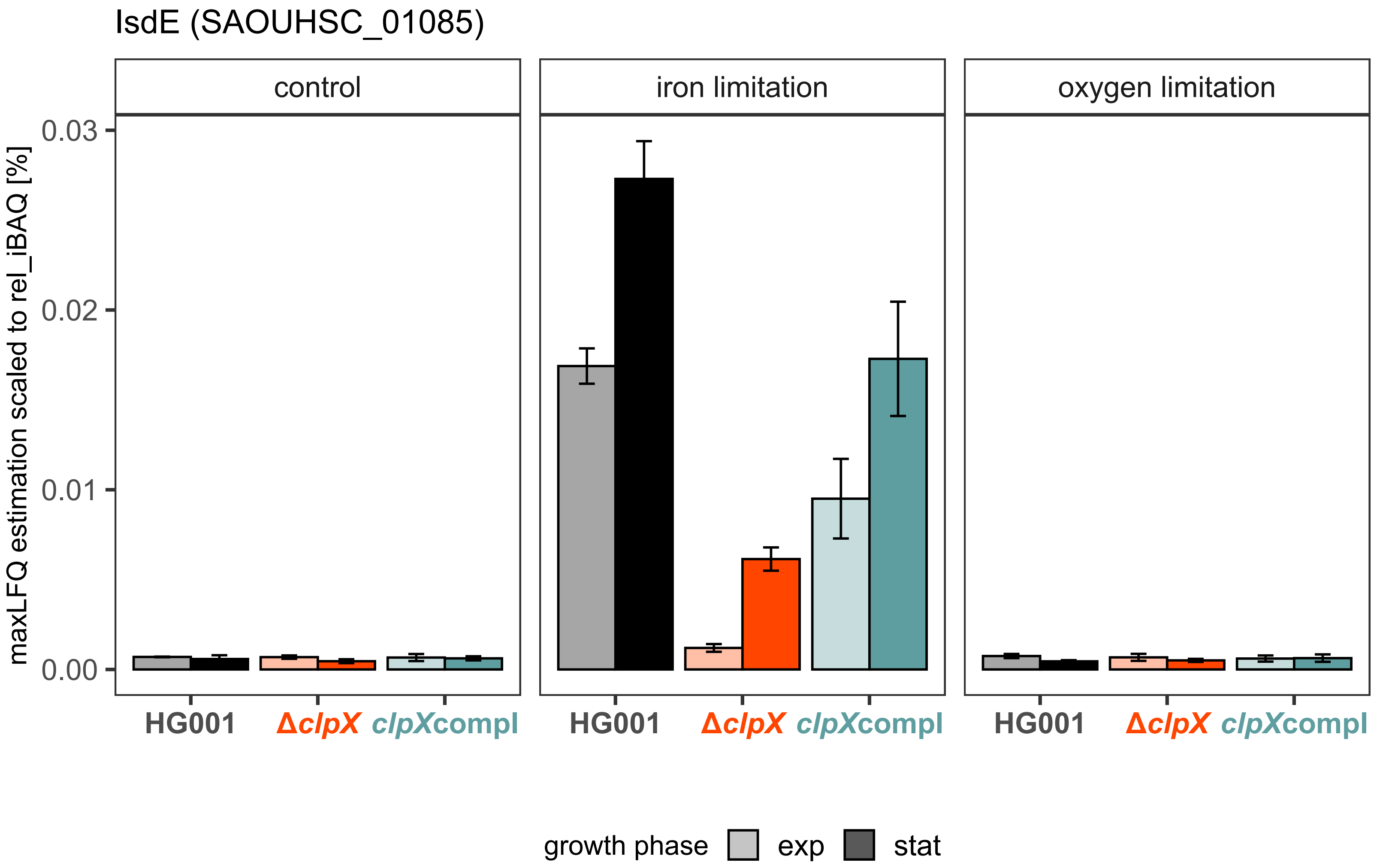

IsdE (SAOUHSC_01085)
control
iron limitation
oxygen limitation
maxLFQ estimation scaled to rel_iBAQ [%]
0.03
0.02
0.01
0.00
HG001
ΔclpX
clpXcompl
growth phase
exp
stat

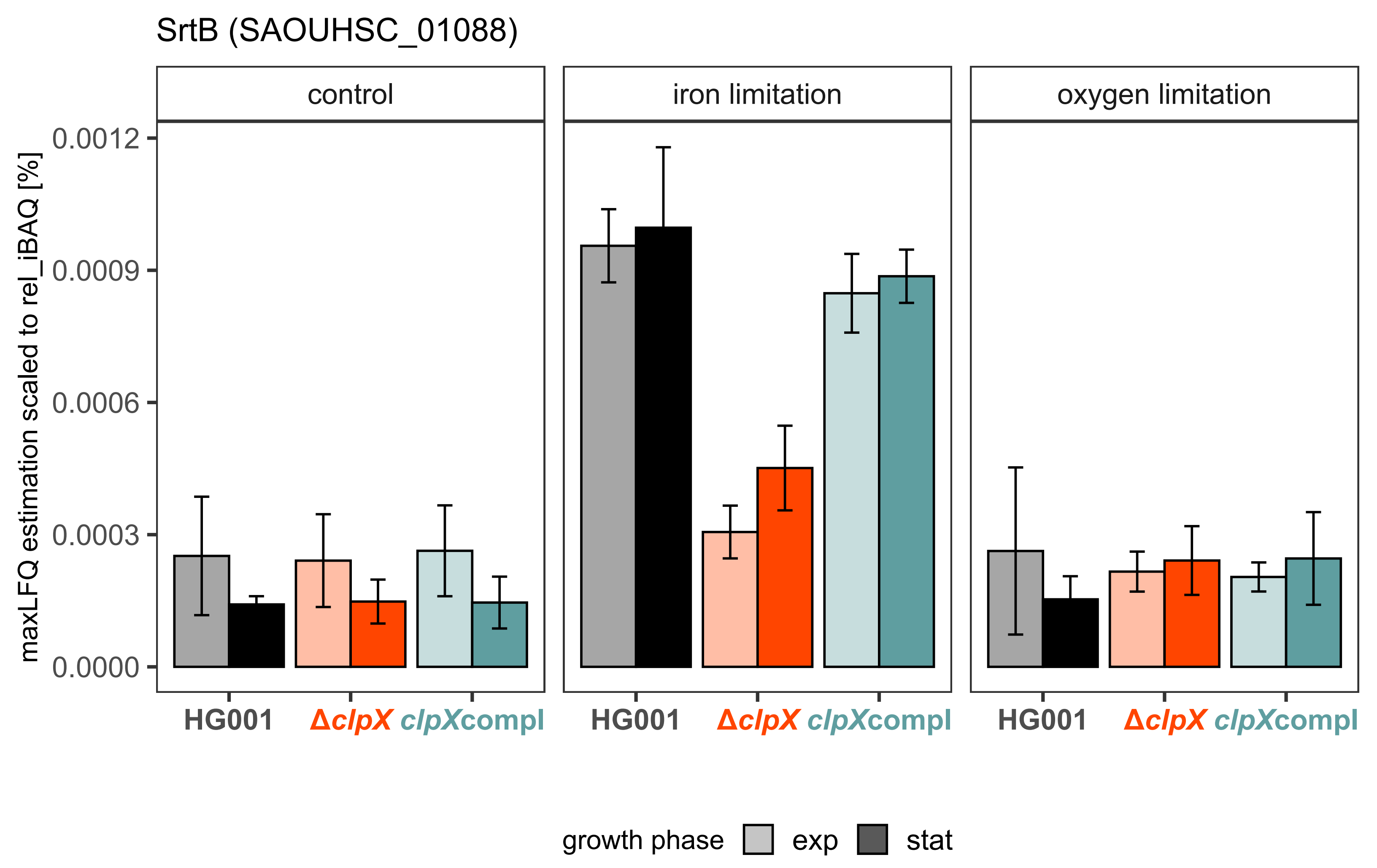

SrtB (SAOUHSC_01088)
control
iron limitation
oxygen limitation
maxLFQ estimation scaled to rel_iBAQ [%]
0.0012
0.0009
0.0006
0.0003
0.0000
HG001
ΔclpX
clpXcompl
growth phase
exp
stat

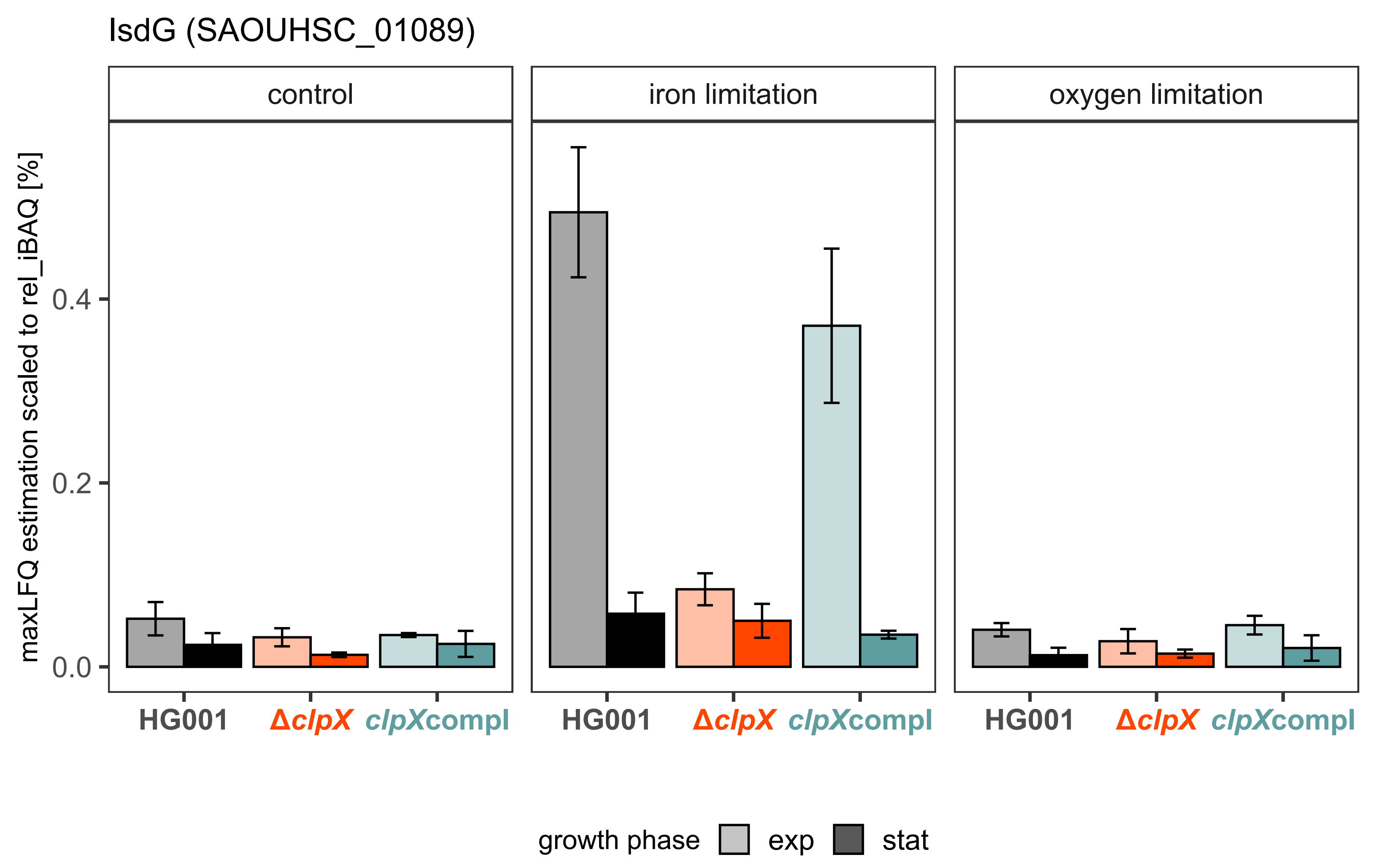
IsdG (SAOUHSC_01089)
control
iron limitation
oxygen limitation
maxLFQ estimation scaled to rel_iBAQ [%]
0.0
0.2
0.4
HG001
ΔclpX
clpXcompl
growth phase
exp
stat

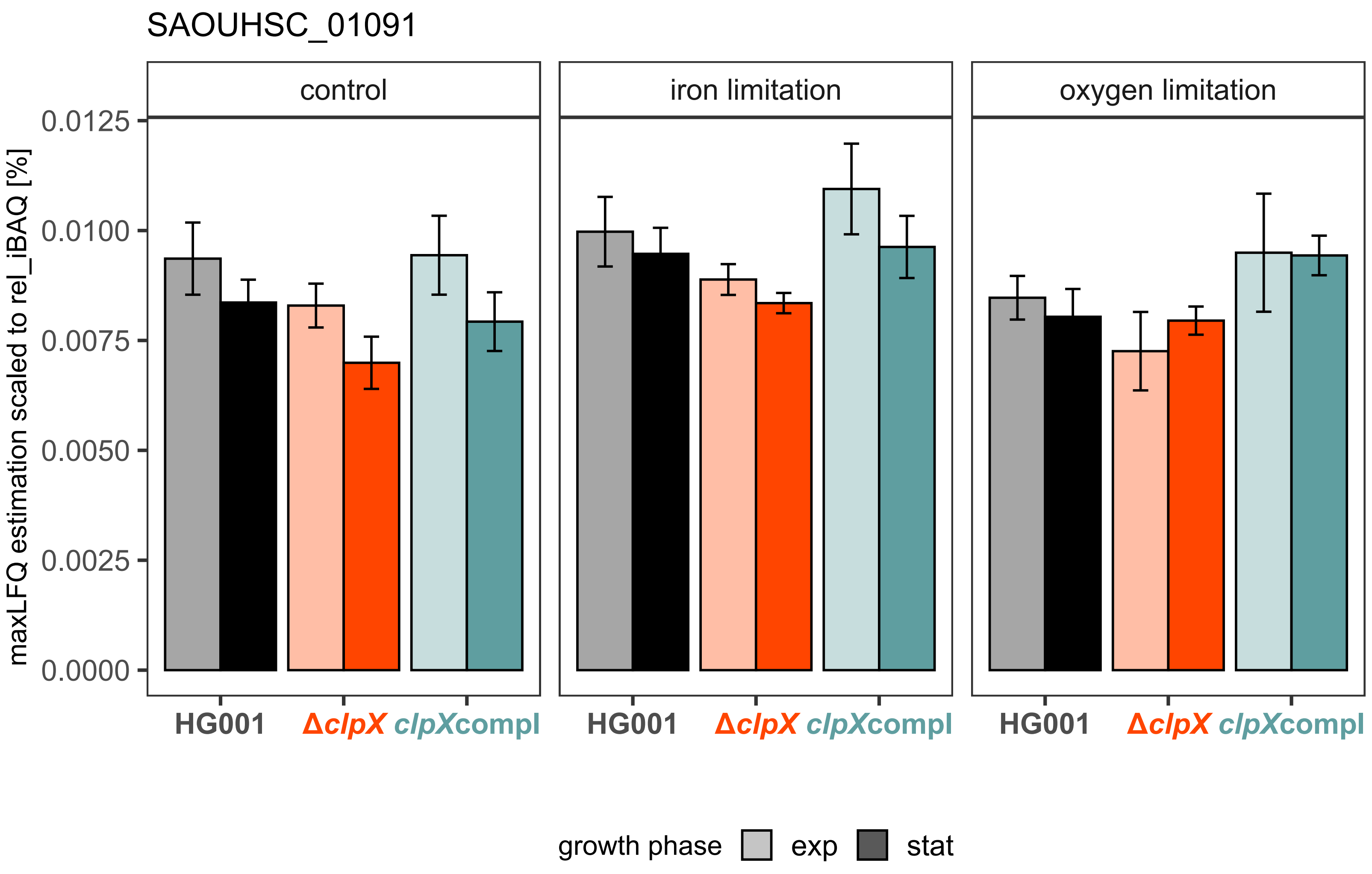
SAOUHSC_01091
control
iron limitation
oxygen limitation
maxLFQ estimation scaled to rel_iBAQ [%]
0.0125
0.0100
0.0075
0.0050
0.0025
0.0000
HG001
ΔclpX
clpXcompl
growth phase
exp
stat

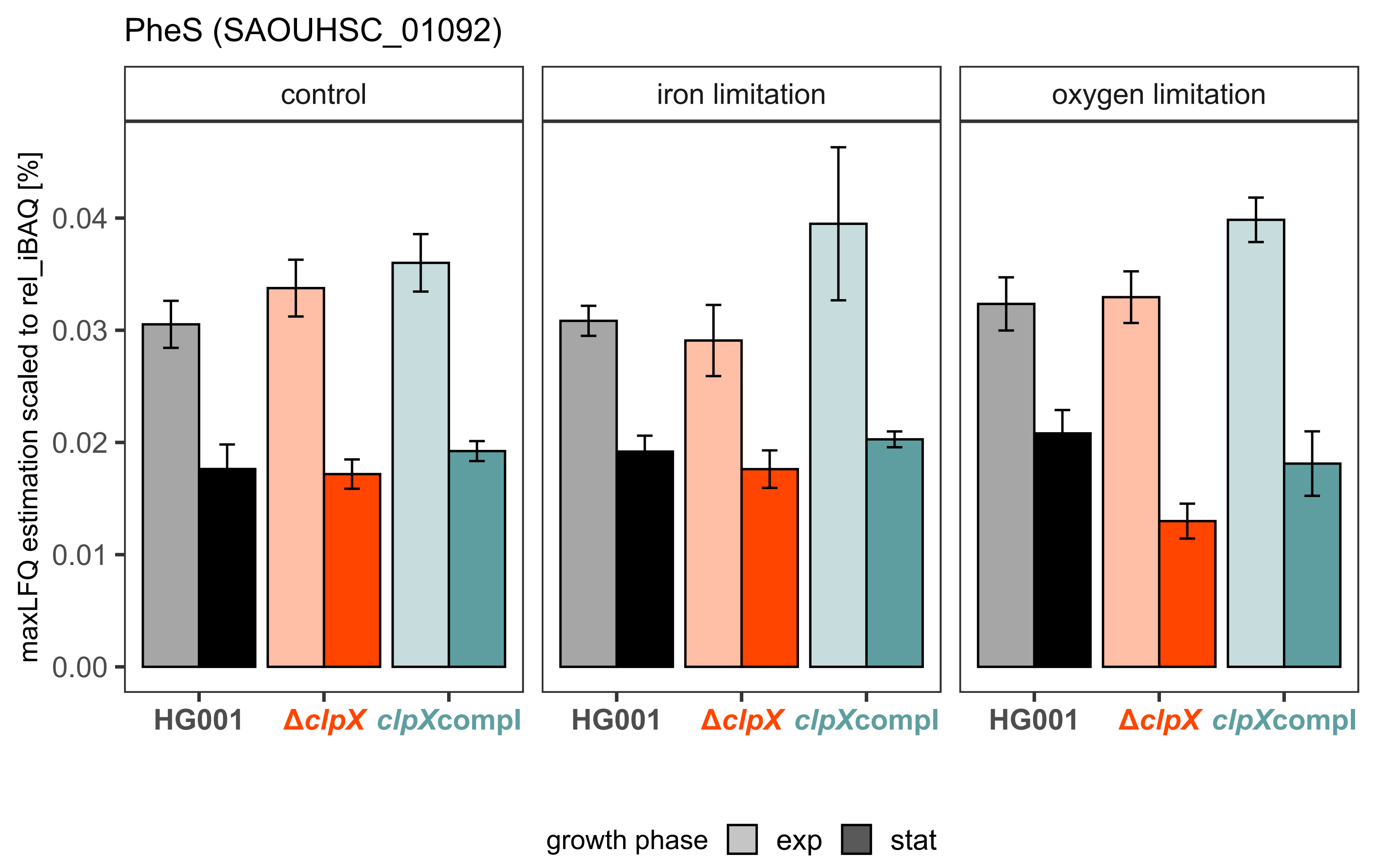
PheS (SAOUHSC_01092)
control
iron limitation
oxygen limitation
maxLFQ estimation scaled to rel_iBAQ [%]
0.00
0.01
0.02
0.03
0.04
HG001
ΔclpX
clpXcompl
growth phase
exp
stat

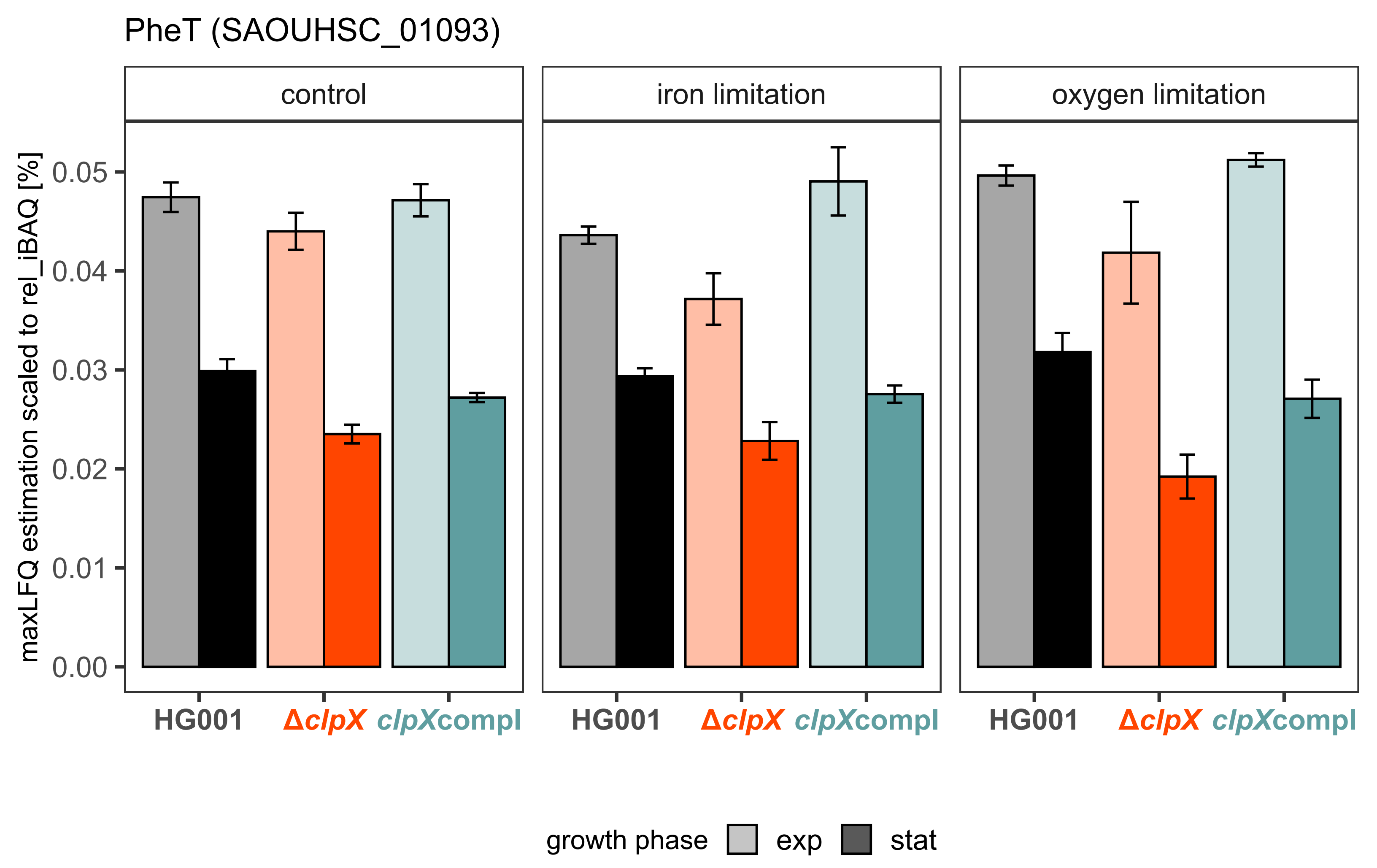
PheT (SAOUHSC_01093)
control
iron limitation
oxygen limitation
maxLFQ estimation scaled to rel_iBAQ [%]
0.05
0.04
0.03
0.02
0.01
0.00
HG001
ΔclpX
clpXcompl
growth phase
exp
stat

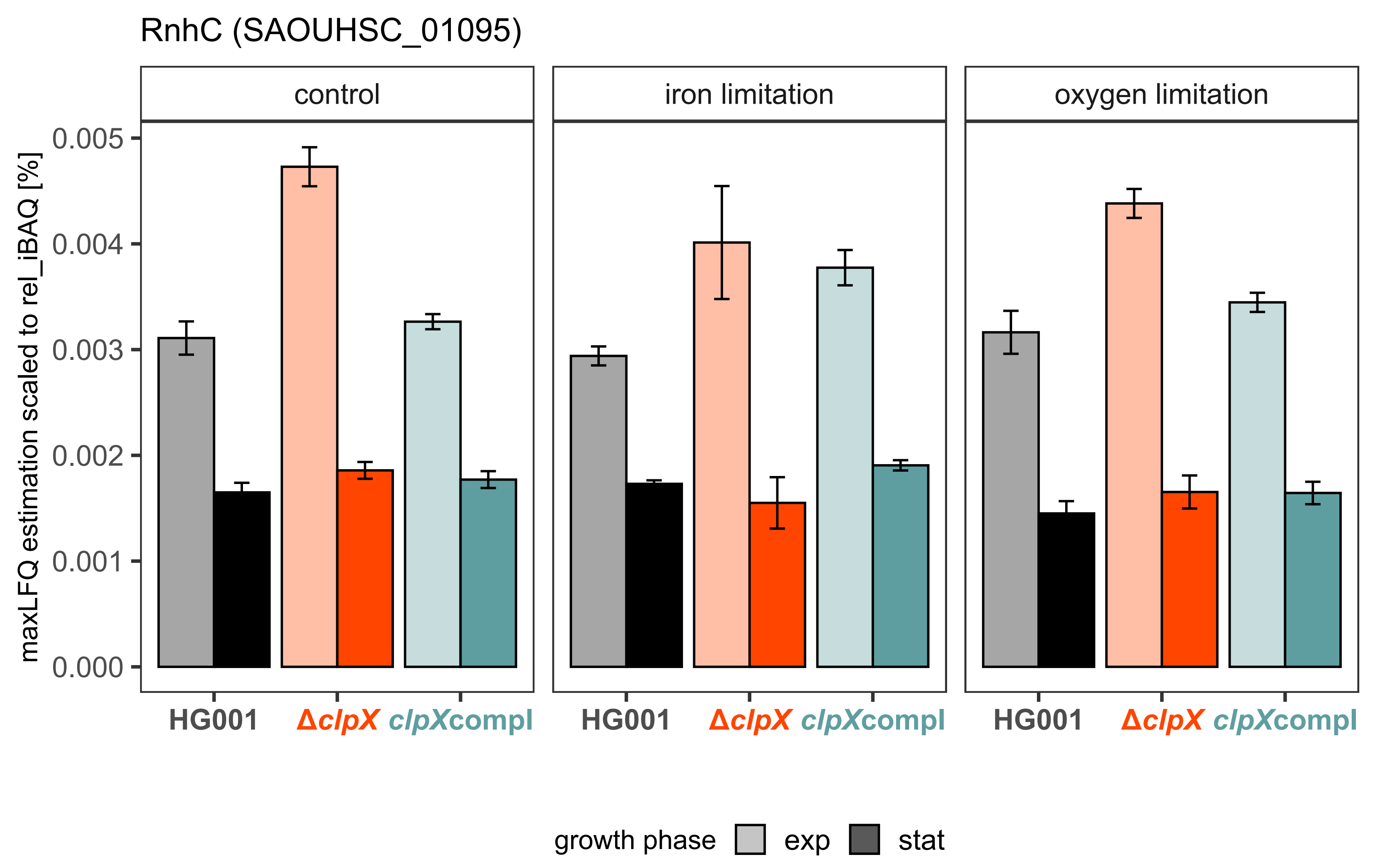
RnhC (SAOUHSC_01095)
control
iron limitation
oxygen limitation
maxLFQ estimation scaled to rel_iBAQ [%]
0.005
0.004
0.003
0.002
0.001
0.000
HG001
ΔclpX
clpXcompl
growth phase
exp
stat

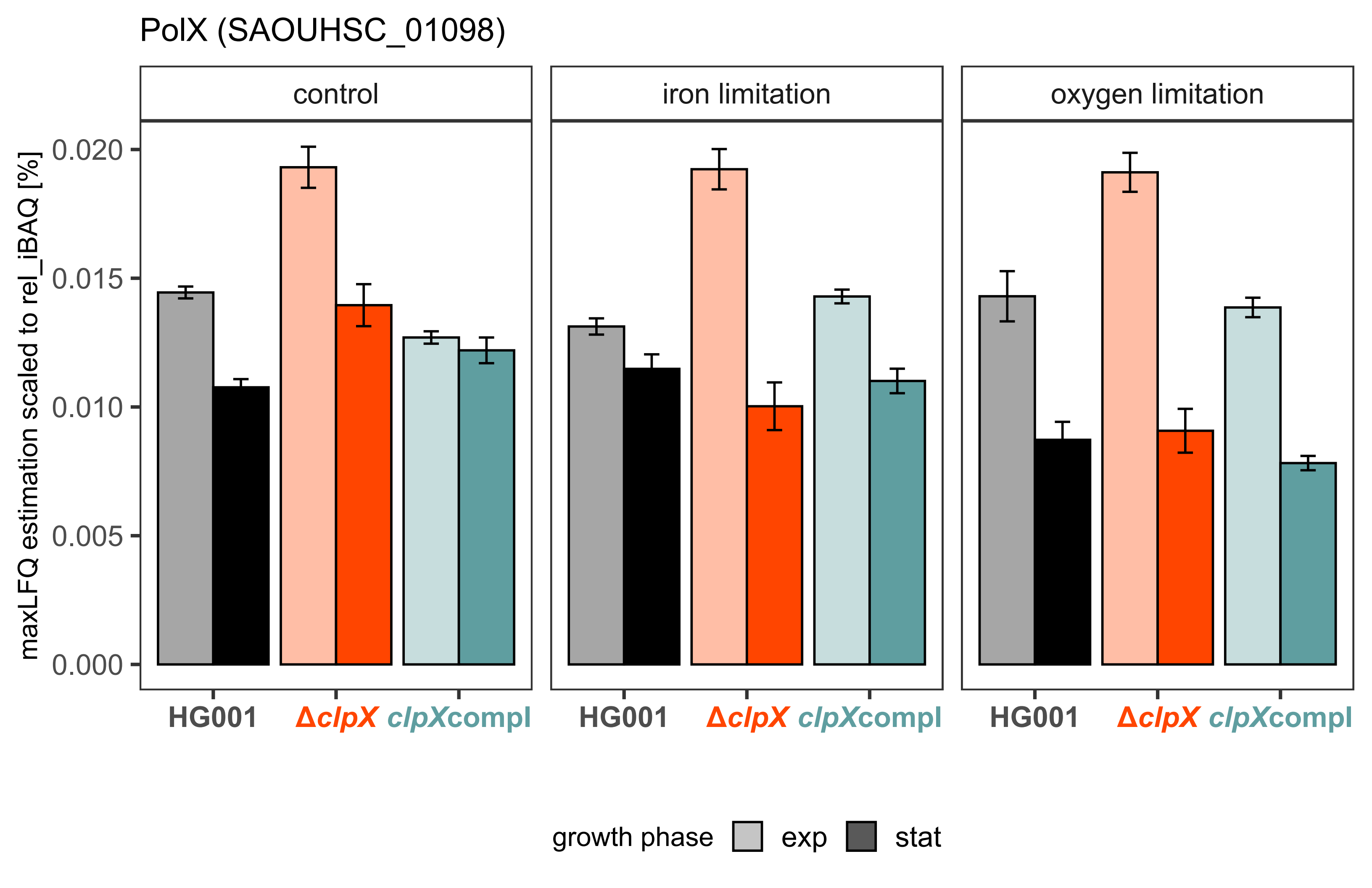

PolX (SAOUHSC_01098)
control
iron limitation
oxygen limitation
maxLFQ estimation scaled to rel_iBAQ [%]
0.020
0.015
0.010
0.005
0.000
HG001
ΔclpX
clpXcompl
growth phase
exp
stat

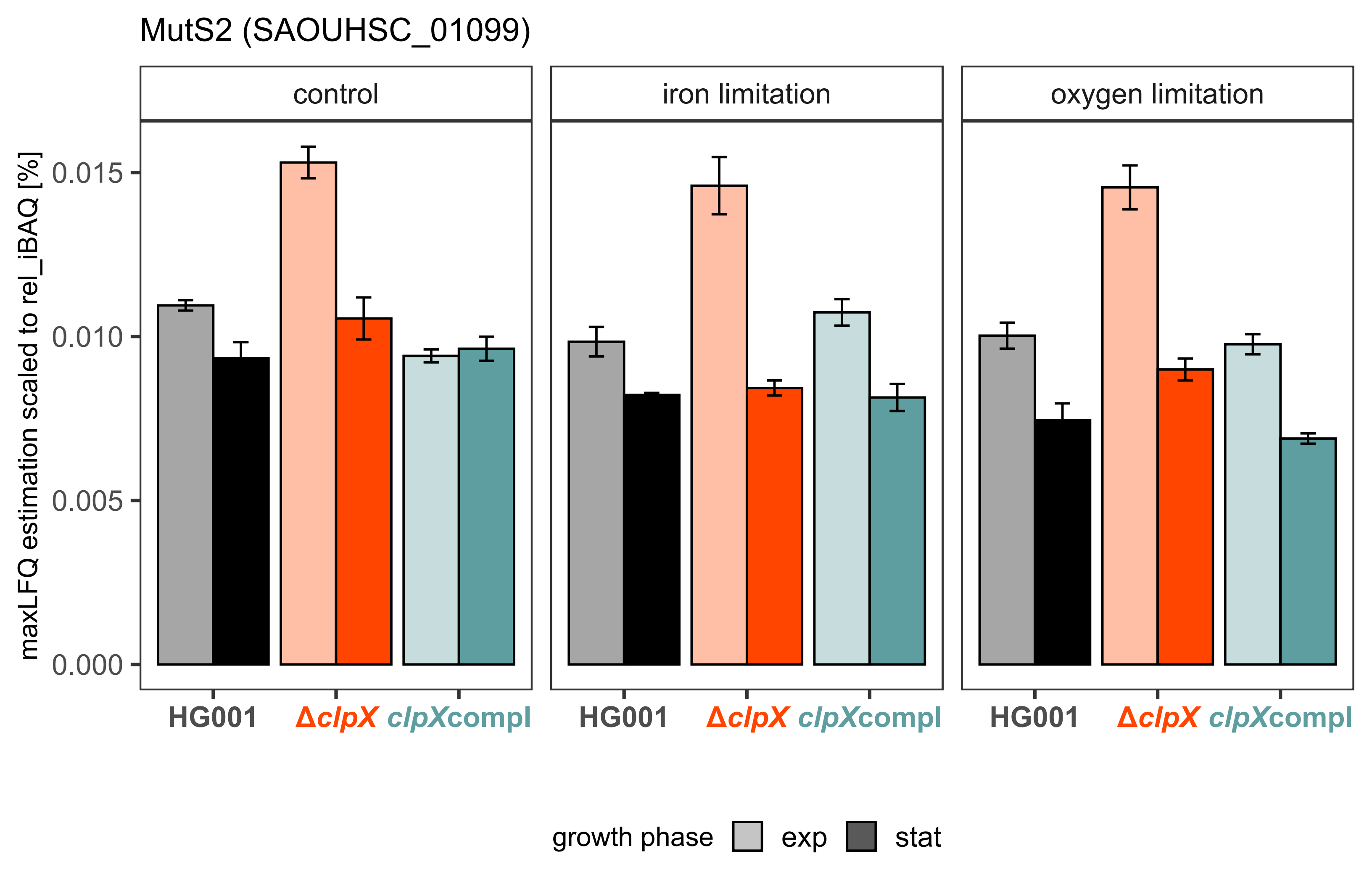

MutS2 (SAOUHSC_01099)
control
iron limitation
oxygen limitation
maxLFQ estimation scaled to rel_iBAQ [%]
0.015
0.010
0.005
0.000
HG001
ΔclpX
clpXcompl
growth phase
exp
stat

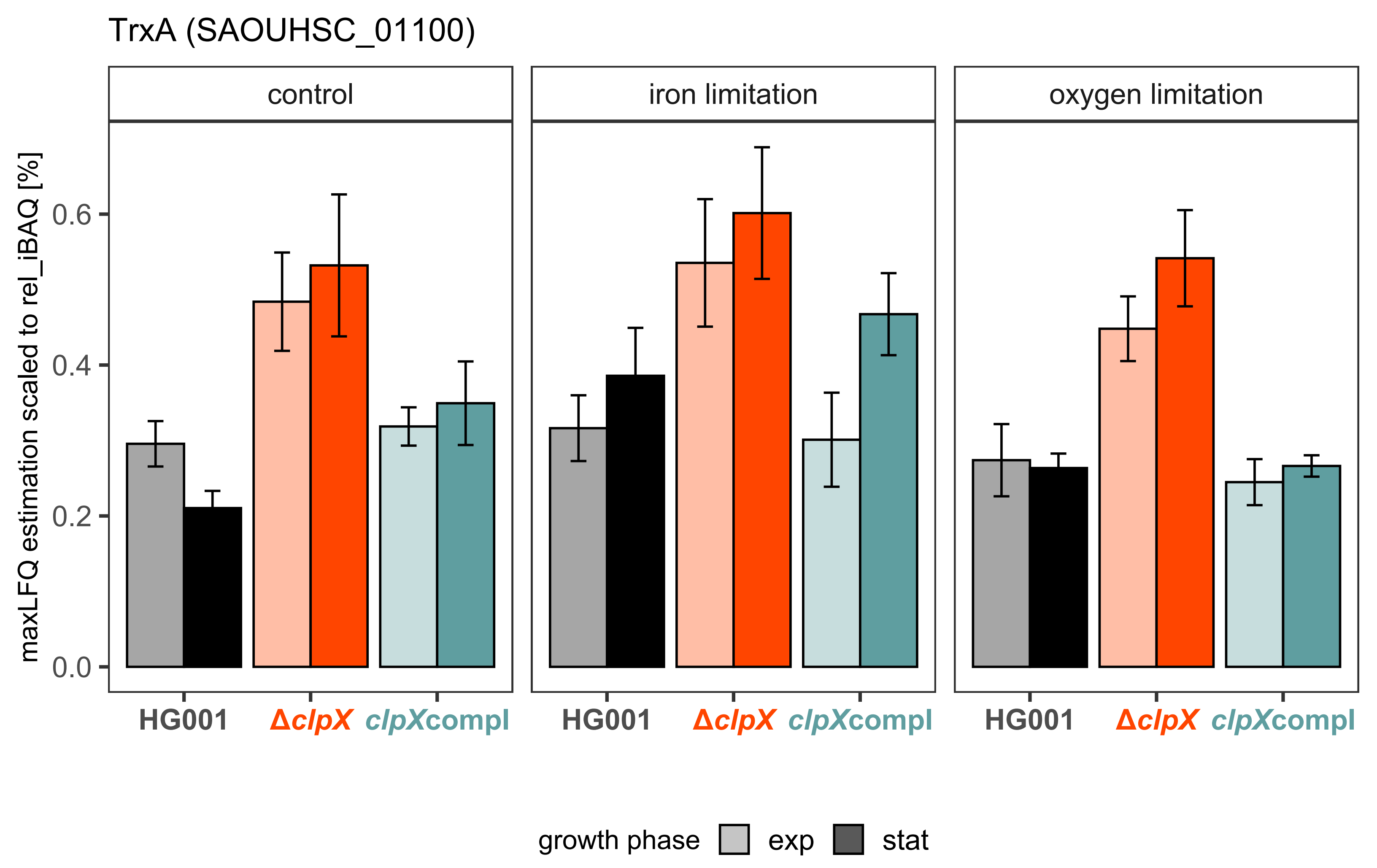
TrxA (SAOUHSC_01100)
control
iron limitation
oxygen limitation
maxLFQ estimation scaled to rel_iBAQ [%]
0.0
0.2
0.4
0.6
HG001
ΔclpX
clpXcompl
growth phase
exp
stat

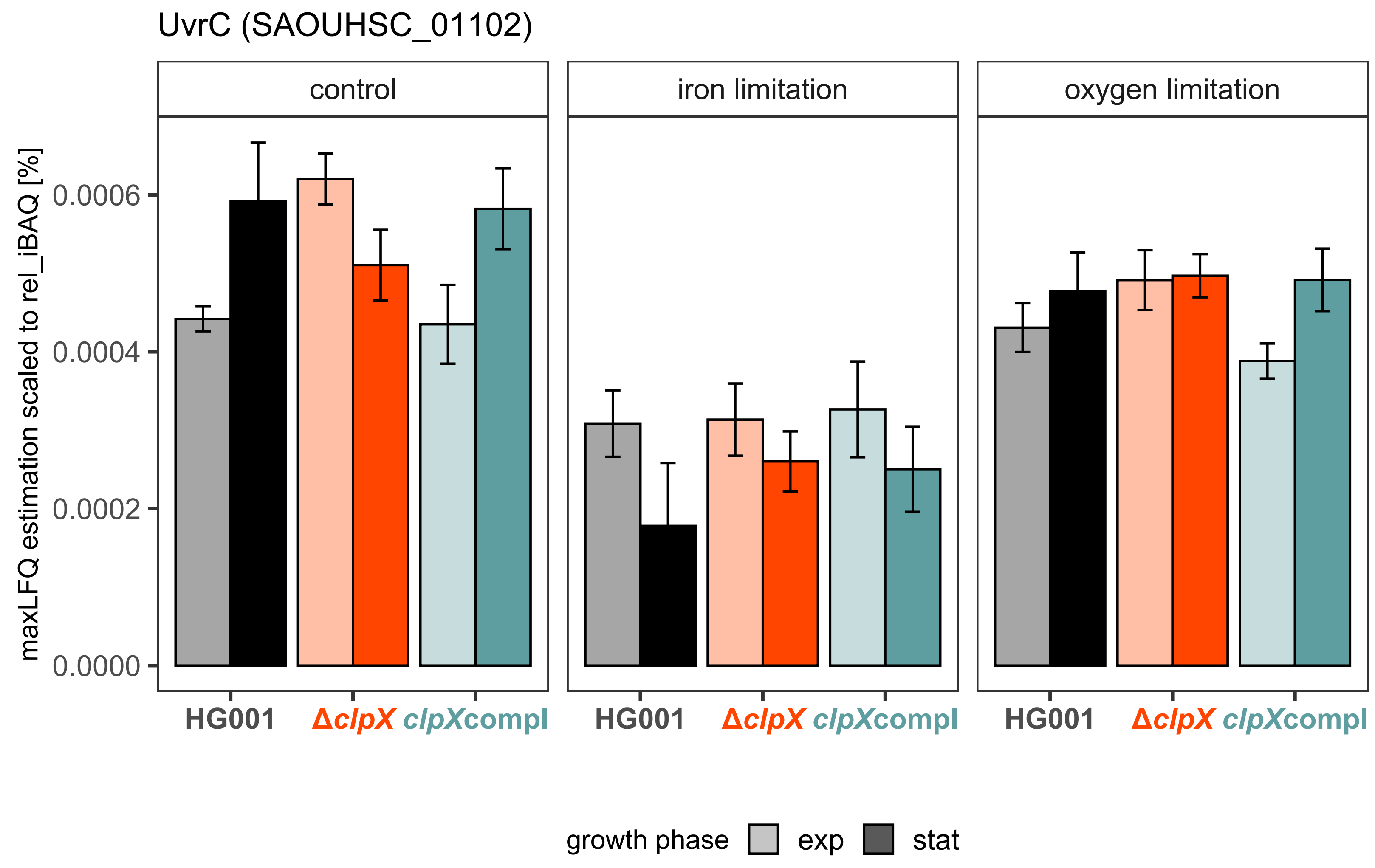

UvrC (SAOUHSC_01102)
control
iron limitation
oxygen limitation
maxLFQ estimation scaled to rel_iBAQ [%]
0.0006
0.0004
0.0002
0.0000
HG001
ΔclpX
clpXcompl
growth phase
exp
stat

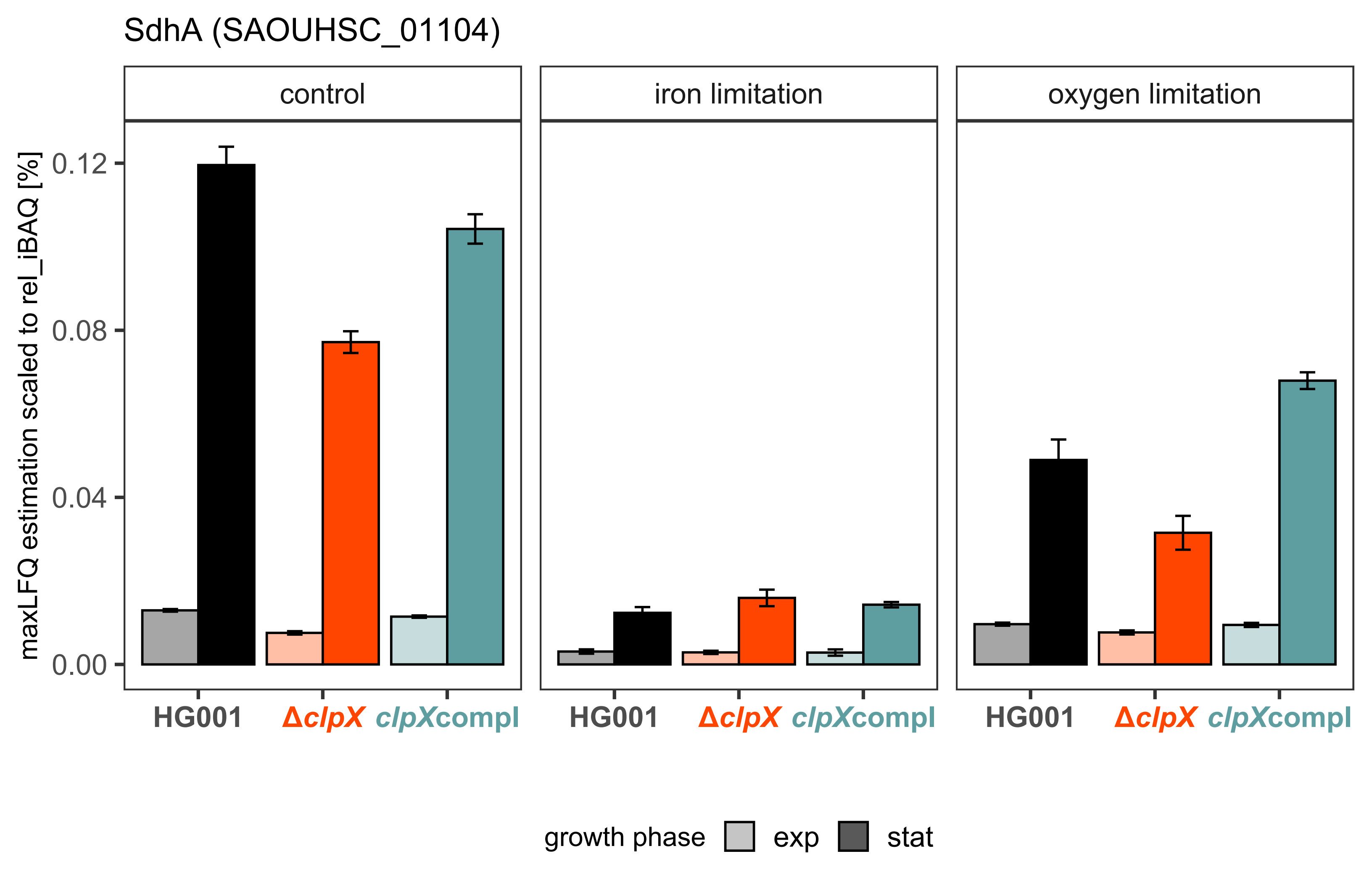

SdhA (SAOUHSC_01104)
control
iron limitation
oxygen limitation
maxLFQ estimation scaled to rel_iBAQ [%]
0.12
0.08
0.04
0.00
HG001
ΔclpX
clpXcompl
growth phase
exp
stat

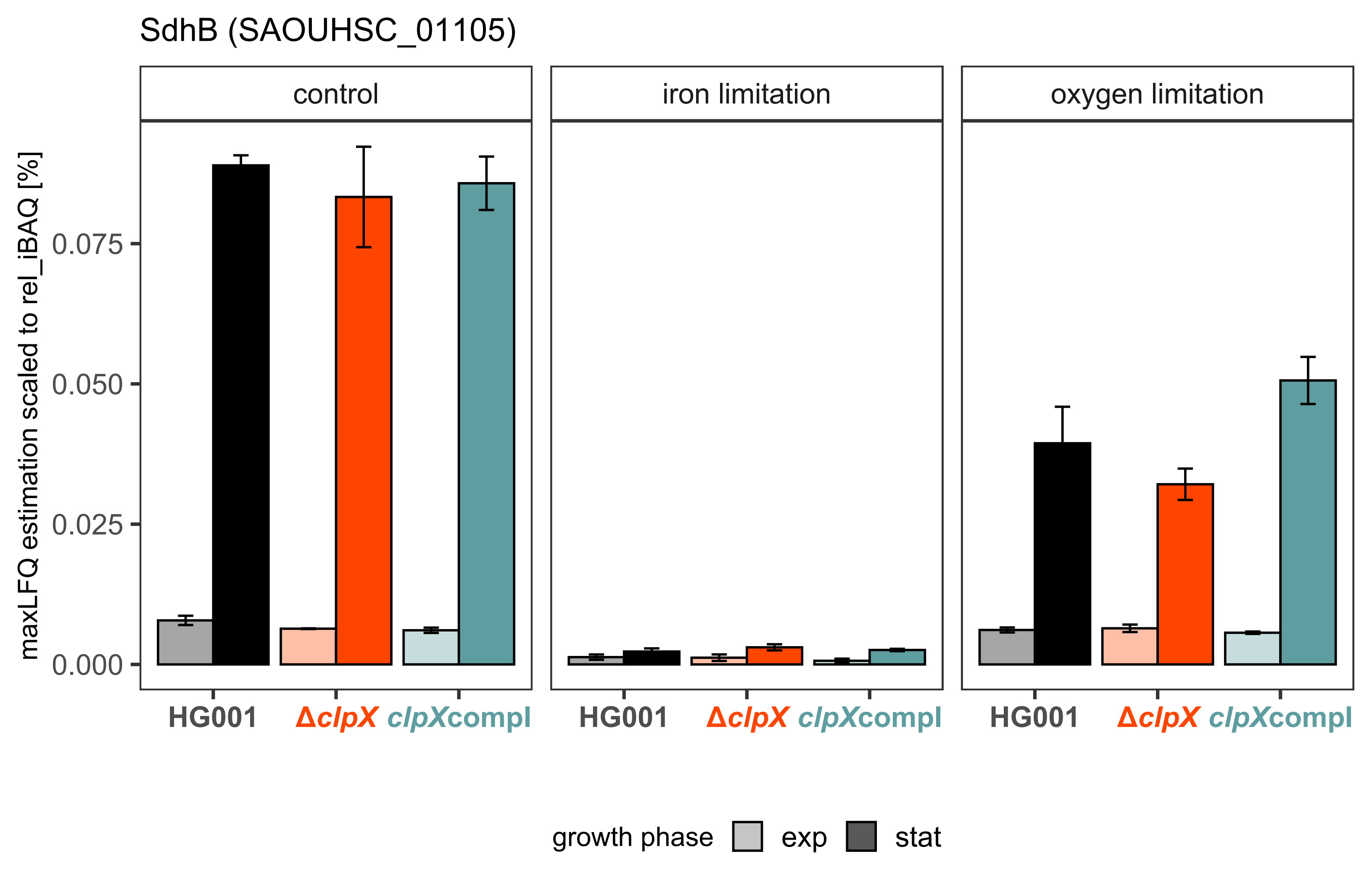
SdhB (SAOUHSC_01105)
control
iron limitation
oxygen limitation
maxLFQ estimation scaled to rel_iBAQ [%]
0.000
0.025
0.050
0.075
HG001
ΔclpX
clpXcompl
growth phase
exp
stat

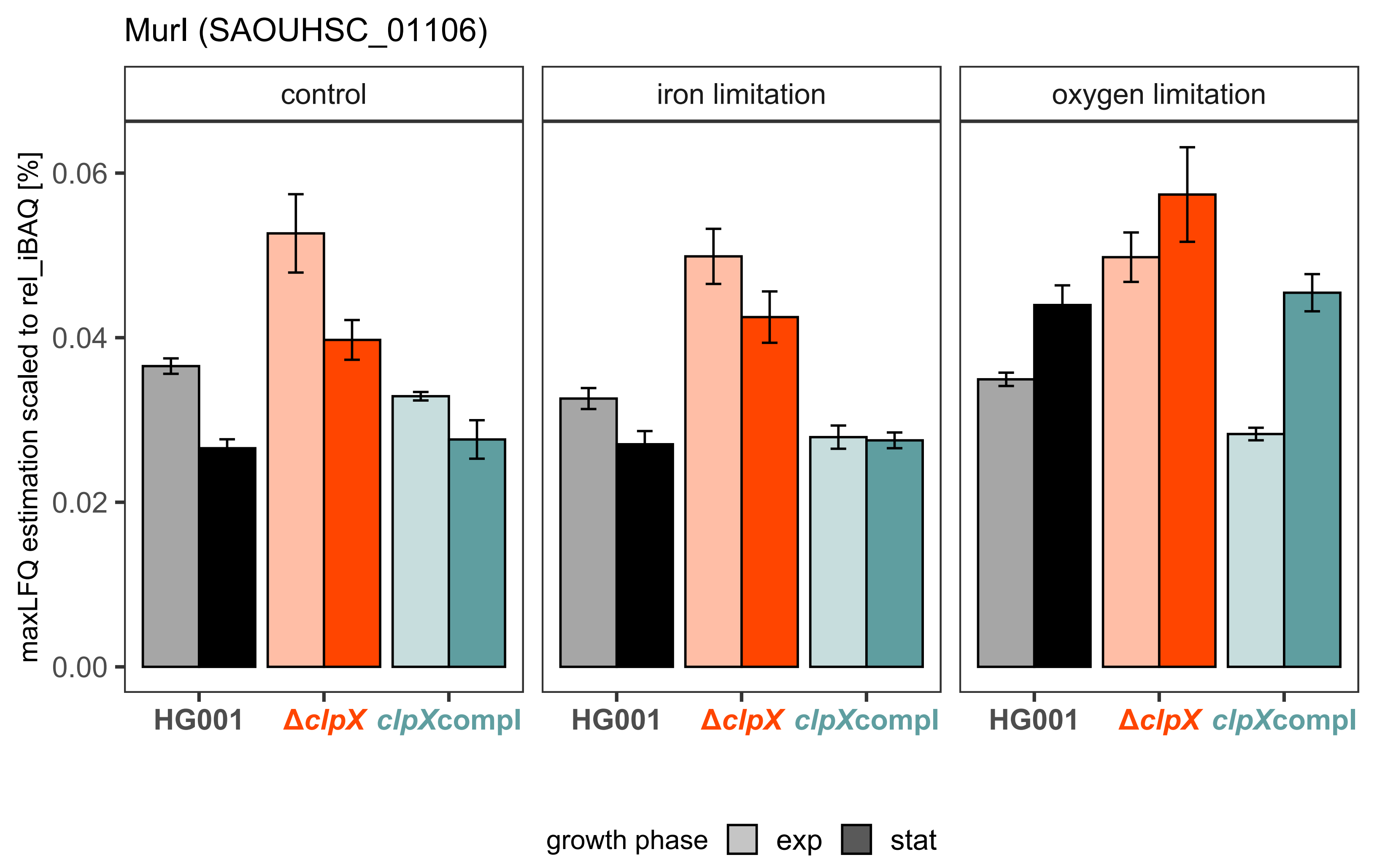
MurI (SAOUHSC_01106)
control
iron limitation
oxygen limitation
maxLFQ estimation scaled to rel_iBAQ [%]
0.06
0.04
0.02
0.00
HG001
ΔclpX
clpXcompl
growth phase
exp
stat

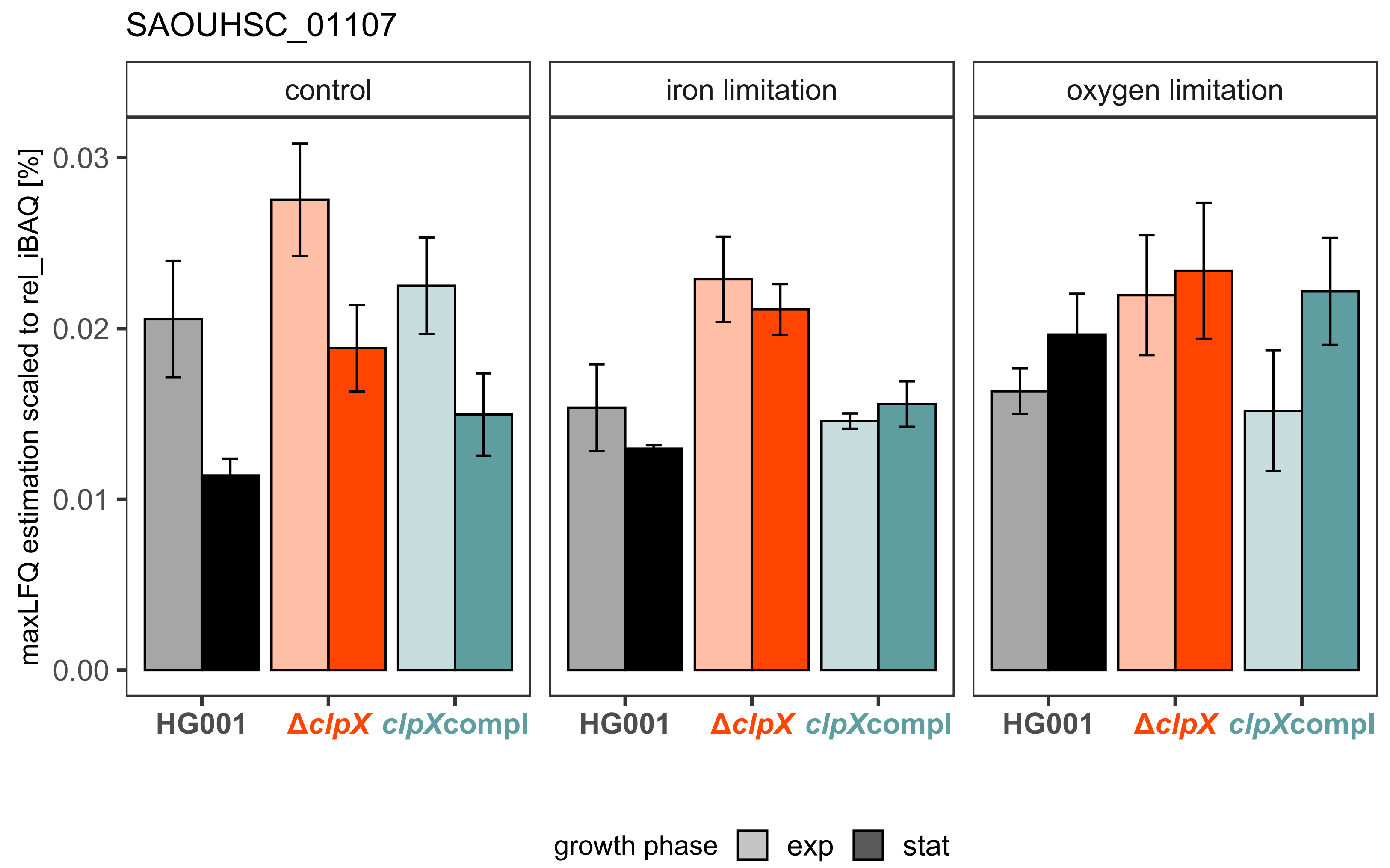
SAOUHSC_01107
control
iron limitation
oxygen limitation
maxLFQ estimation scaled to rel_iBAQ [%]
0.03
0.02
0.01
0.00
HG001
ΔclpX
clpXcompl
growth phase
exp
stat

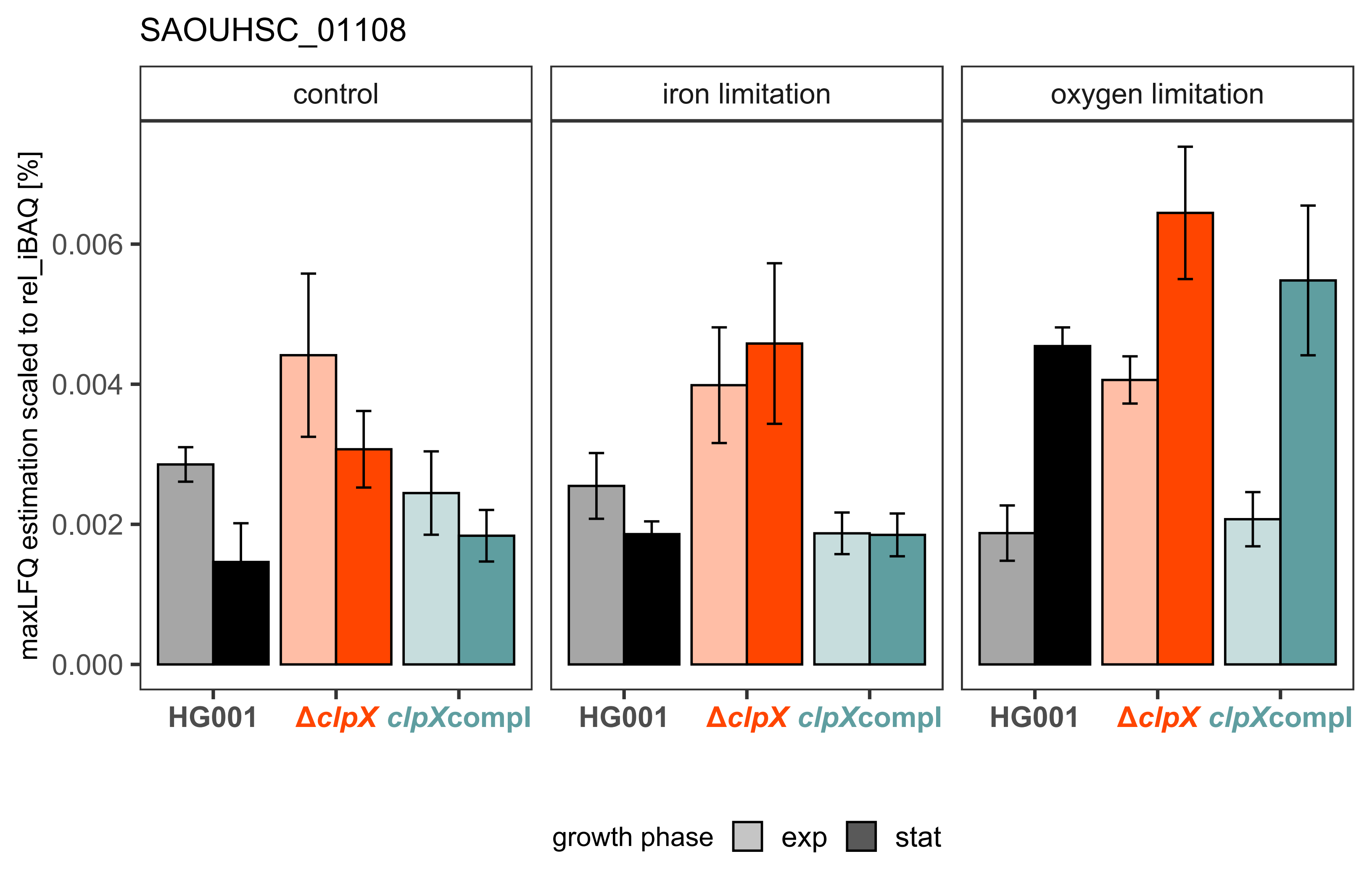
SAOUHSC_01108
control
iron limitation
oxygen limitation
maxLFQ estimation scaled to rel_iBAQ [%]
0.000
0.002
0.004
0.006
HG001
ΔclpX
clpXcompl
growth phase
exp
stat

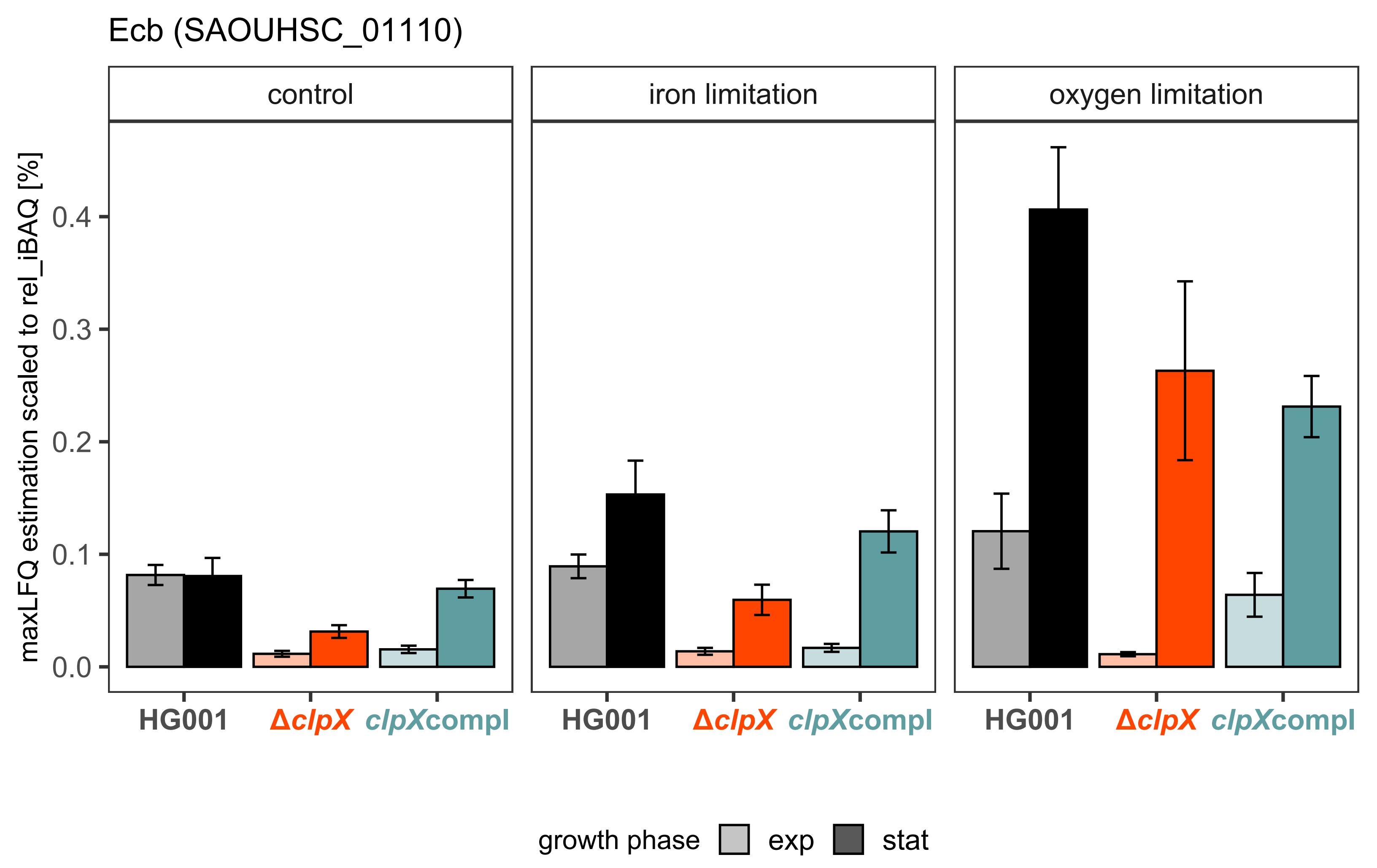

Ecb (SAOUHSC_01110)
control
iron limitation
oxygen limitation
maxLFQ estimation scaled to rel_iBAQ [%]
0.0
0.1
0.2
0.3
0.4
HG001
ΔclpX
clpXcompl
growth phase
exp
stat

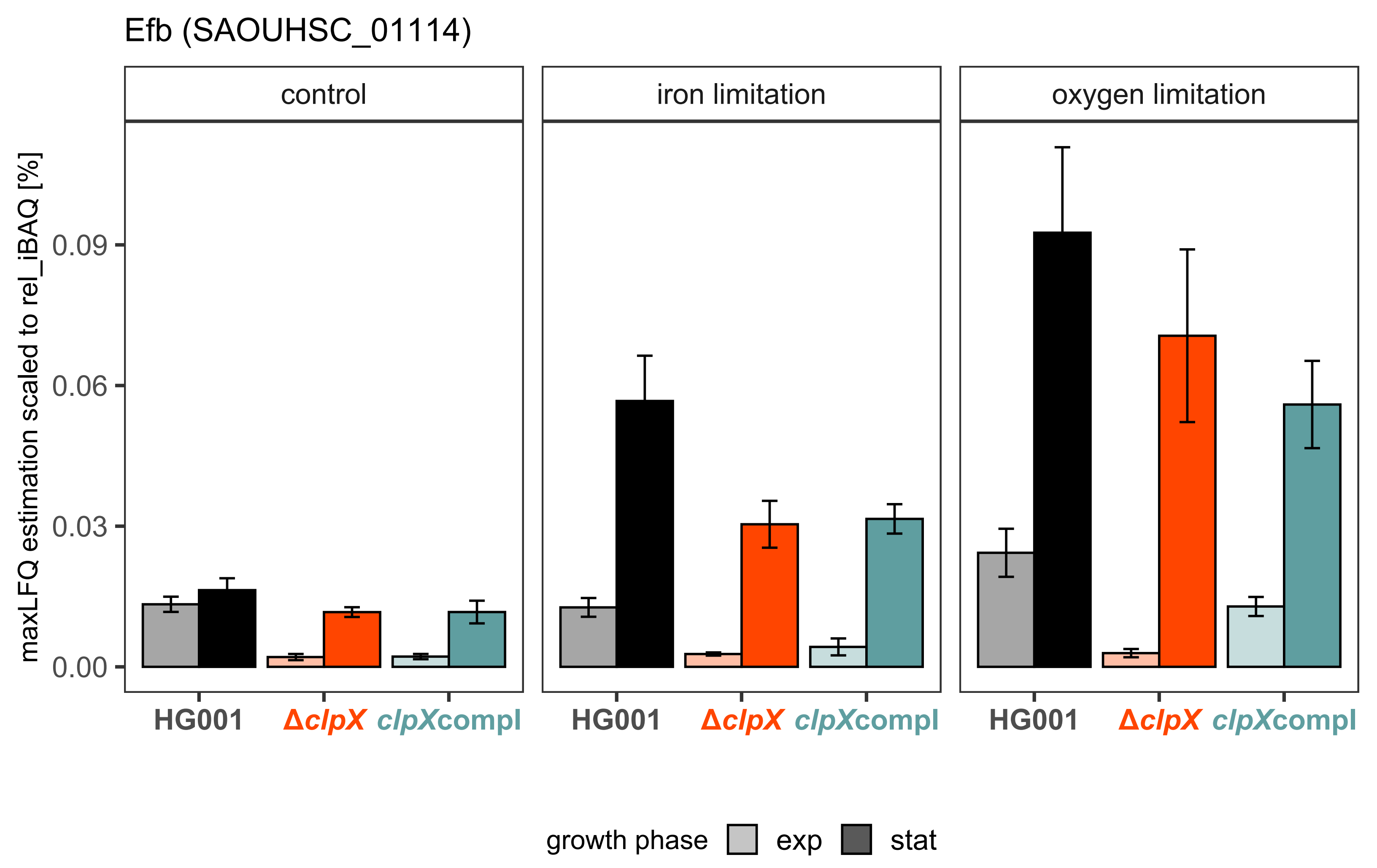

Efb (SAOUHSC_01114)
control
iron limitation
oxygen limitation
maxLFQ estimation scaled to rel_iBAQ [%]
0.09
0.06
0.03
0.00
HG001
ΔclpX
clpXcompl
HG001
ΔclpX
clpXcompl
HG001
ΔclpX
clpXcompl
growth phase
exp
stat

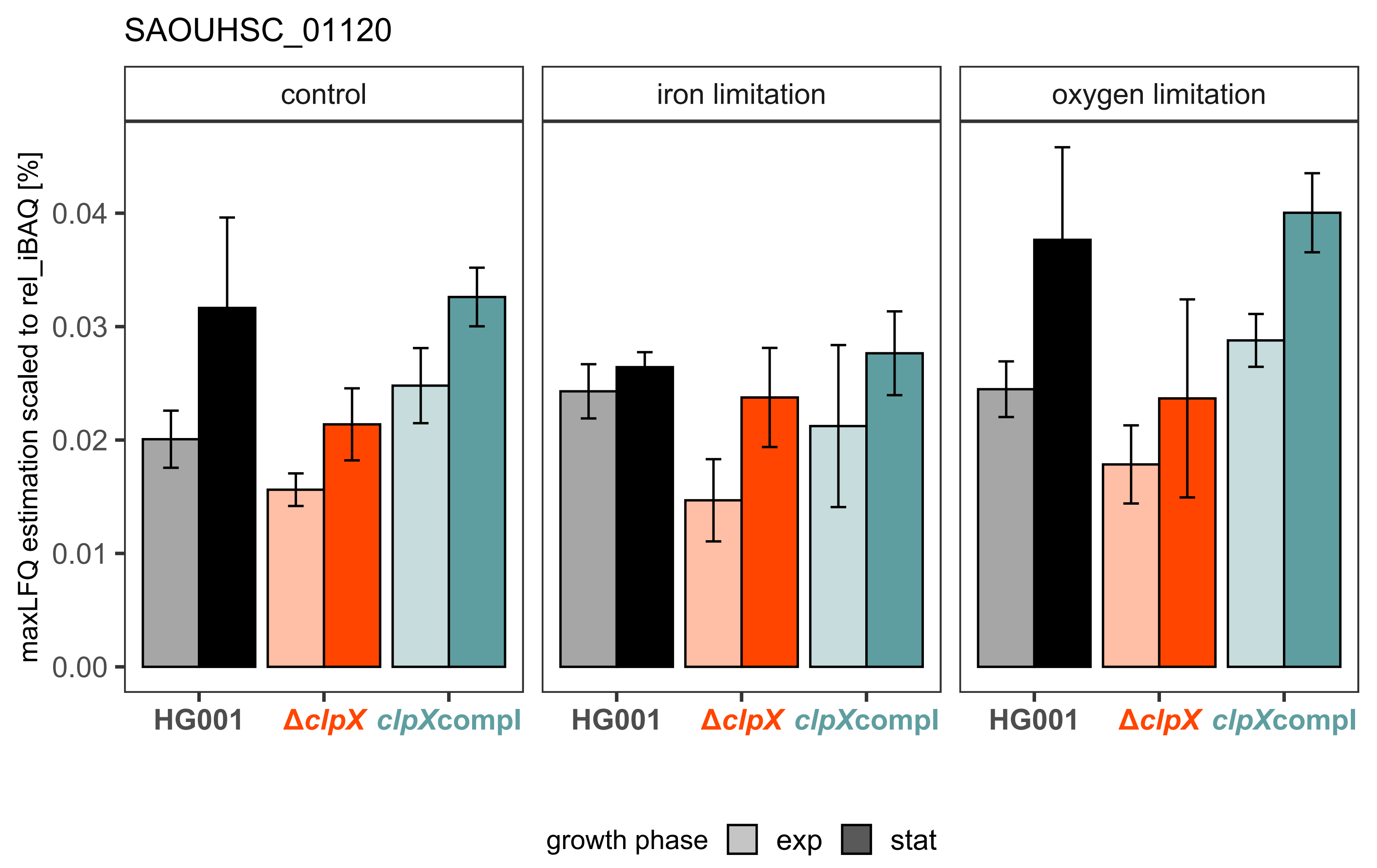
SAOUHSC_01120
control
iron limitation
oxygen limitation
maxLFQ estimation scaled to rel_iBAQ [%]
0.00
0.01
0.02
0.03
0.04
HG001
ΔclpX
clpXcompl
growth phase
exp
stat

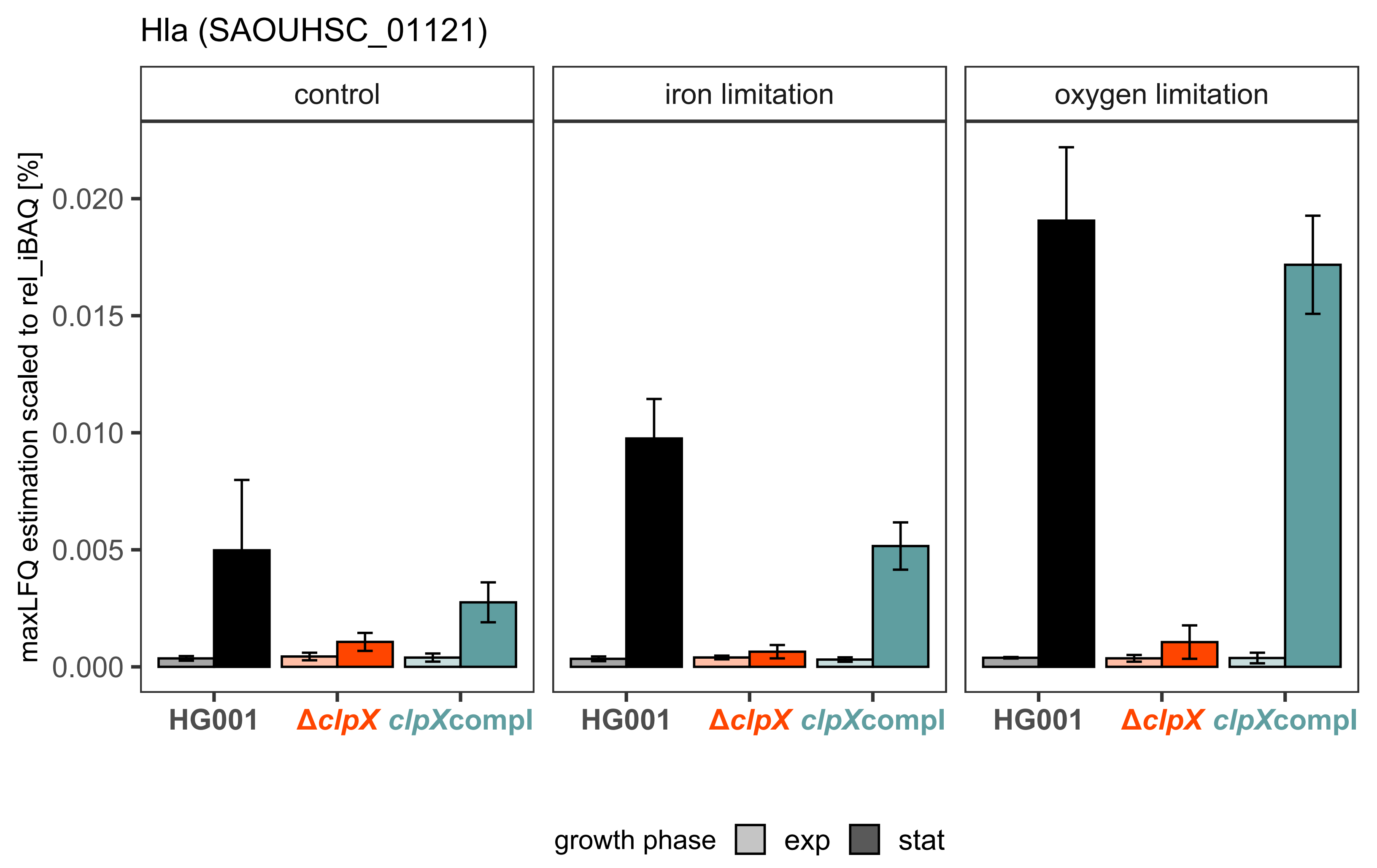

Hla (SAOUHSC_01121)
control
iron limitation
oxygen limitation
maxLFQ estimation scaled to rel_iBAQ [%]
0.000
0.005
0.010
0.015
0.020
HG001
ΔclpX
clpXcompl
growth phase
exp
stat

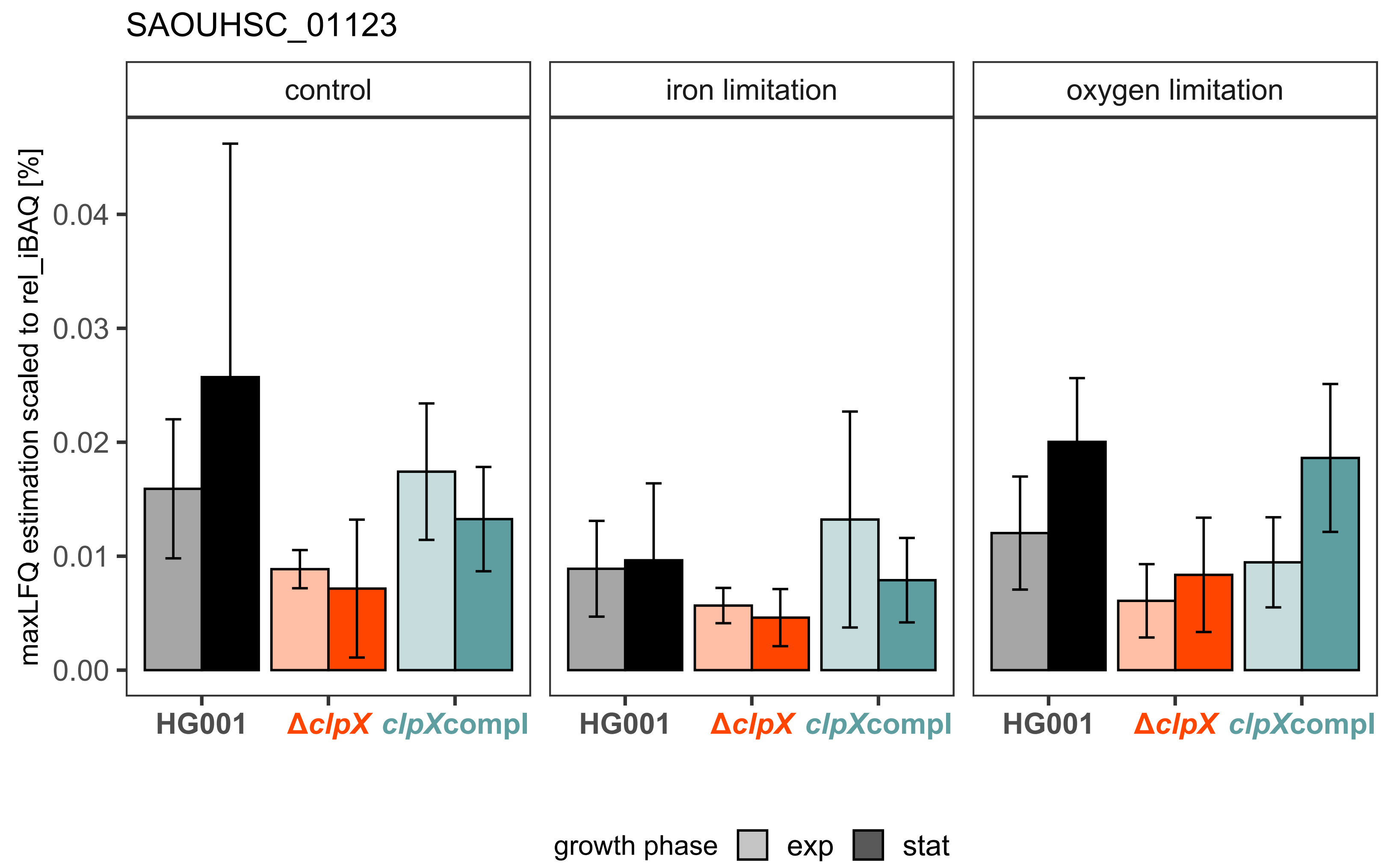
SAOUHSC_01123
control
iron limitation
oxygen limitation
maxLFQ estimation scaled to rel_iBAQ [%]
0.04
0.03
0.02
0.01
0.00
HG001
ΔclpX
clpXcompl
growth phase
exp
stat

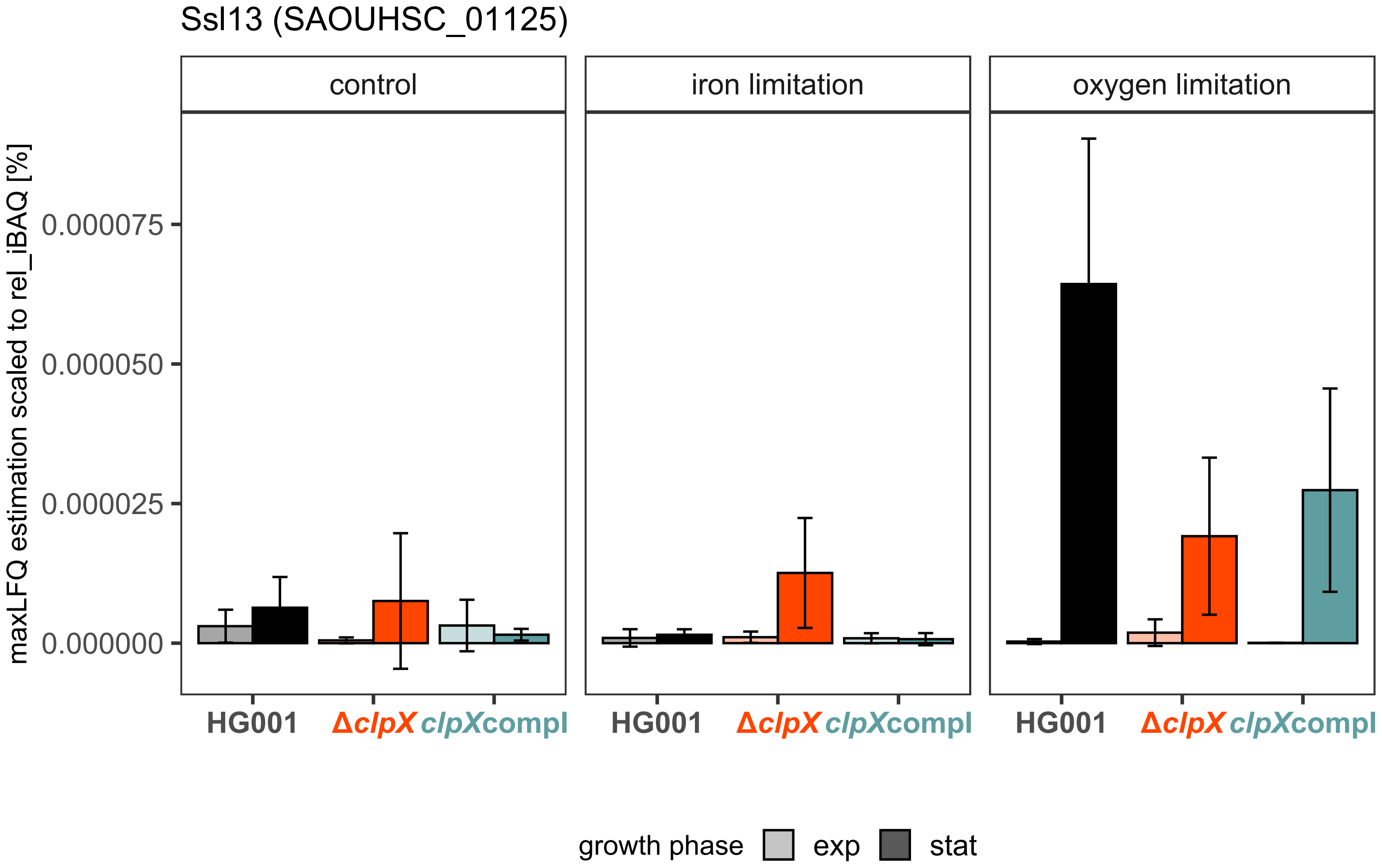

Ssl13 (SAOUHSC_01125)
control
iron limitation
oxygen limitation
maxLFQ estimation scaled to rel_iBAQ [%]
0.000075
0.000050
0.000025
0.000000
HG001
ΔclpX
clpXcompl
growth phase
exp
stat

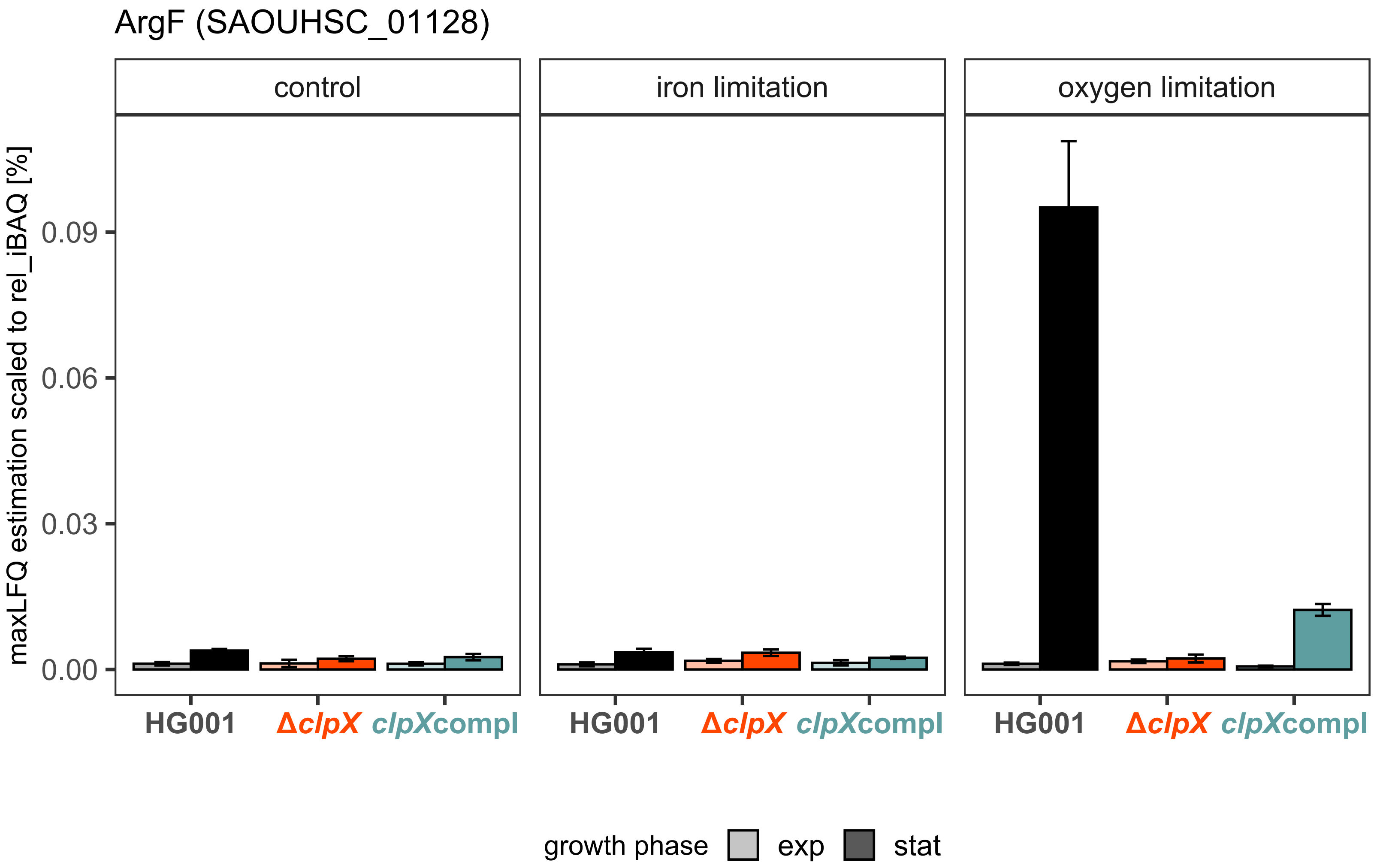

ArgF (SAOUHSC_01128)
control
iron limitation
oxygen limitation
maxLFQ estimation scaled to rel_iBAQ [%]
0.09
0.06
0.03
0.00
HG001
ΔclpX
clpXcompl
growth phase
exp
stat

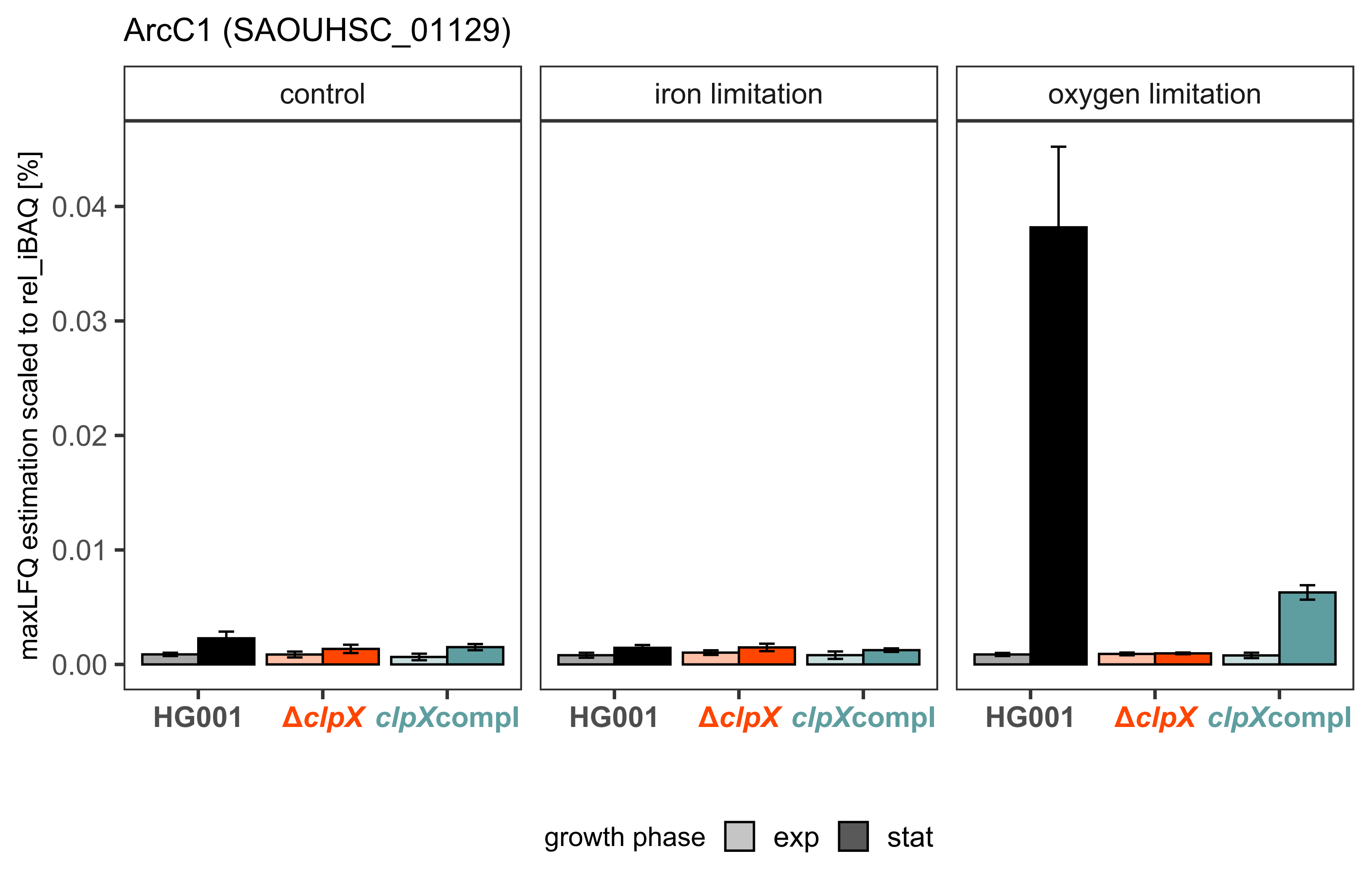
ArcC1 (SAOUHSC_01129)
control
iron limitation
oxygen limitation
maxLFQ estimation scaled to rel_iBAQ [%]
0.04
0.03
0.02
0.01
0.00
HG001
ΔclpX
clpXcompl
growth phase
exp
stat

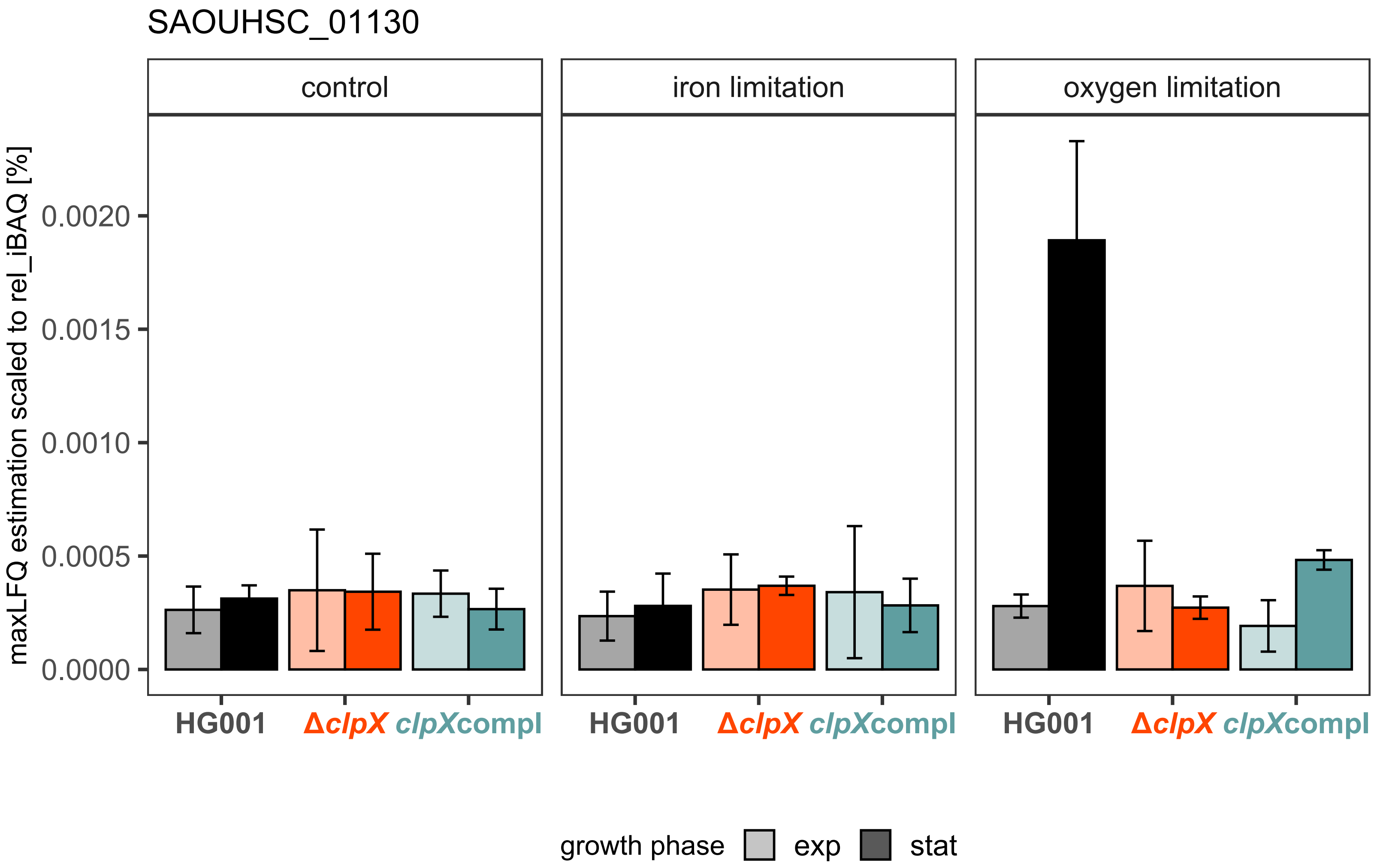

SAOUHSC_01130
control
iron limitation
oxygen limitation
maxLFQ estimation scaled to rel_iBAQ [%]
0.0020
0.0015
0.0010
0.0005
0.0000
HG001
ΔclpX
clpXcompl
growth phase
exp
stat

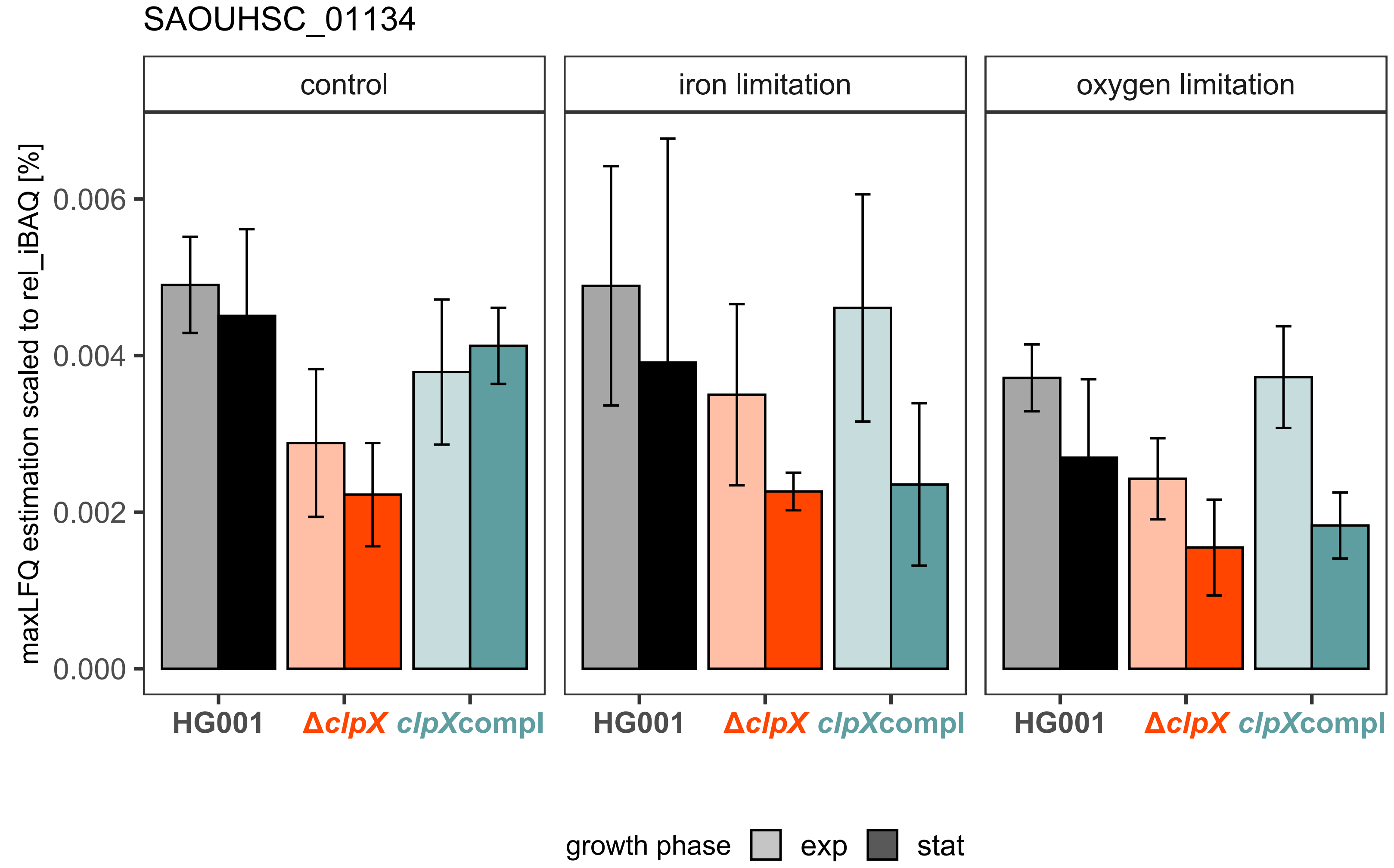
SAOUHSC_01134
control
iron limitation
oxygen limitation
maxLFQ estimation scaled to rel_iBAQ [%]
0.006
0.004
0.002
0.000
HG001
ΔclpX
clpXcompl
growth phase
exp
stat

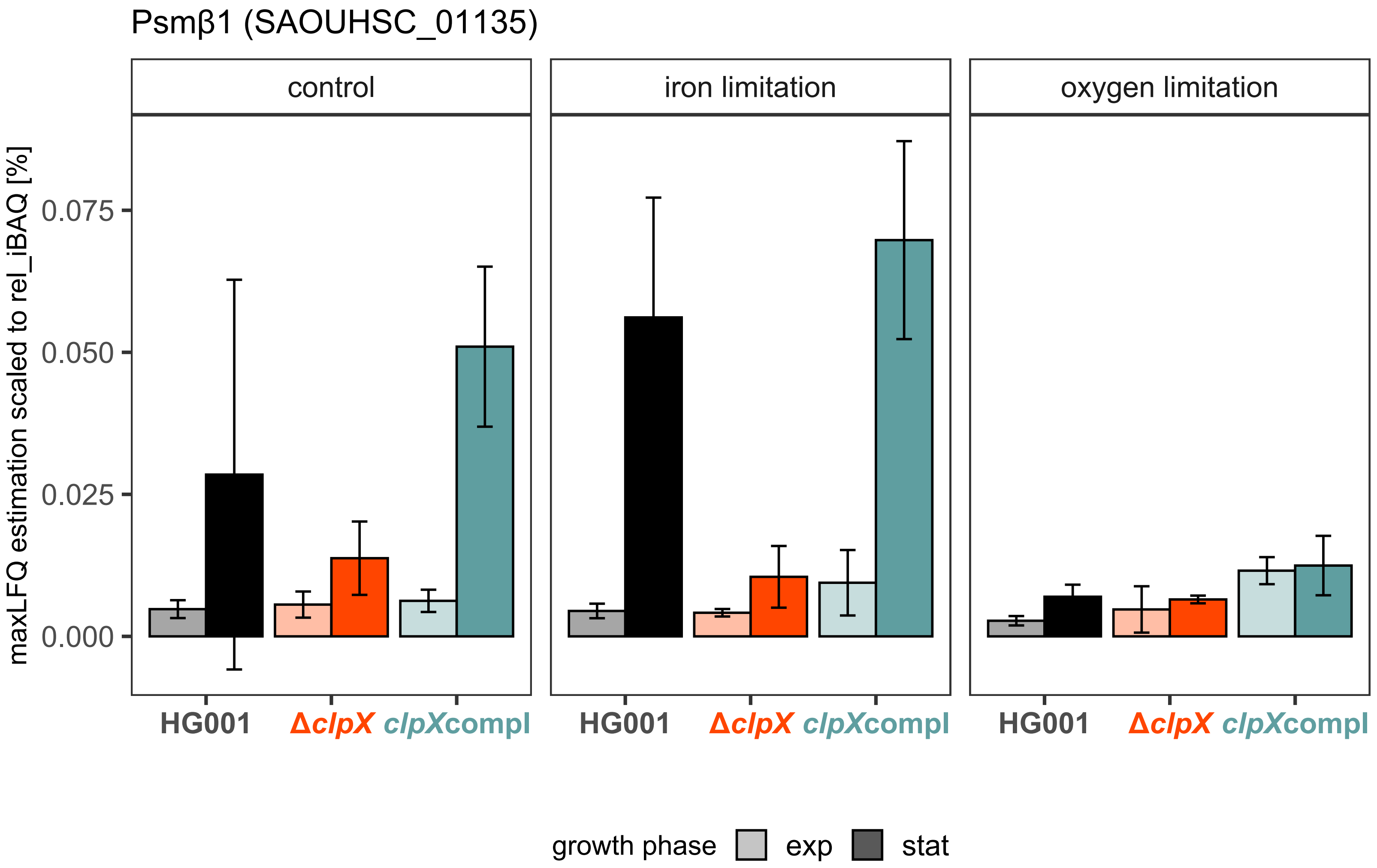
Psmβ1 (SAOUHSC_01135)
control
iron limitation
oxygen limitation
maxLFQ estimation scaled to rel_iBAQ [%]
0.075
0.050
0.025
0.000
HG001
ΔclpX
clpXcompl
growth phase
exp
stat

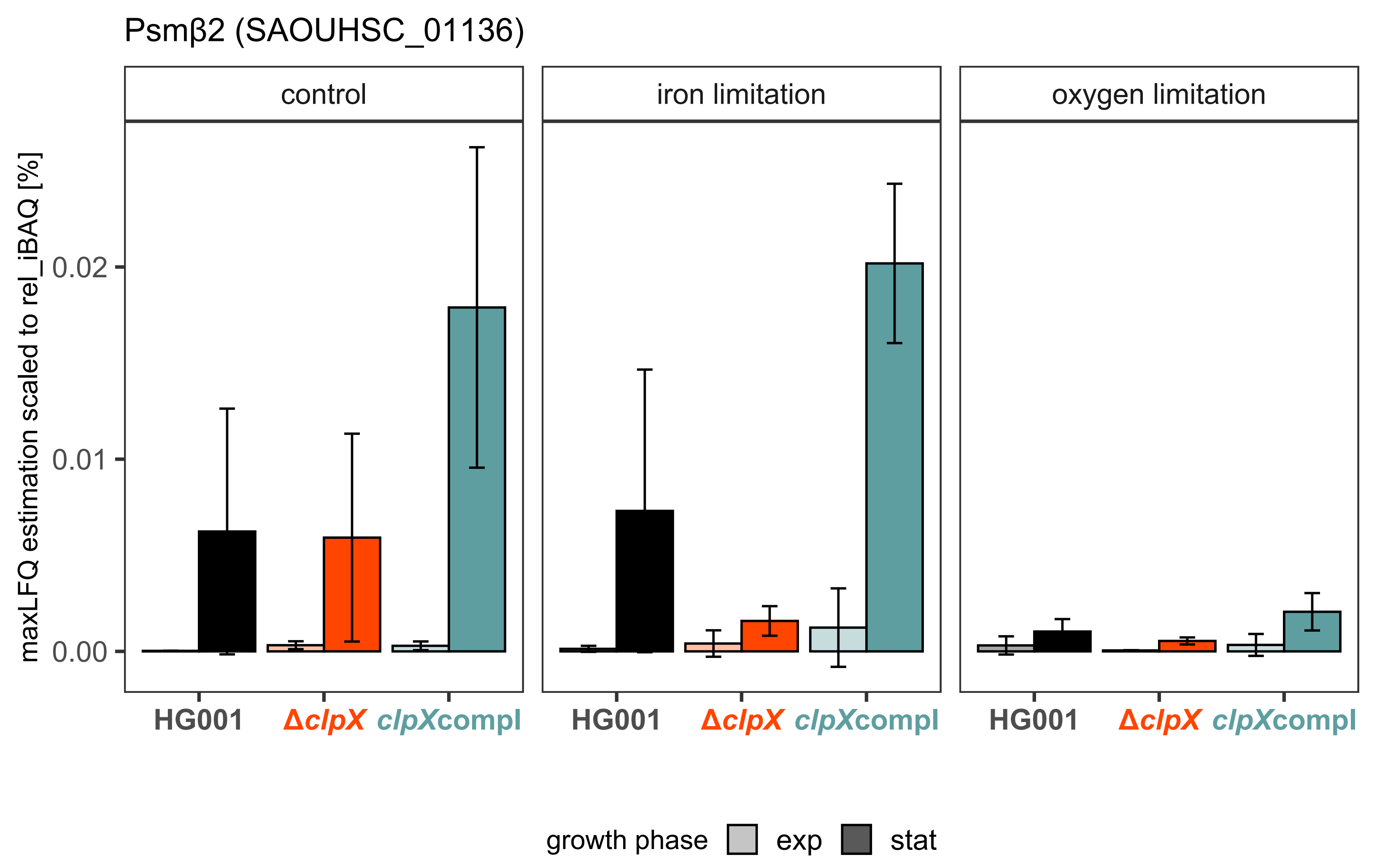

Psmβ2 (SAOUHSC_01136)
control
iron limitation
oxygen limitation
maxLFQ estimation scaled to rel_iBAQ [%]
0.02
0.01
0.00
HG001
ΔclpX
clpXcompl
HG001
ΔclpX
clpXcompl
HG001
ΔclpX
clpXcompl
growth phase
exp
stat

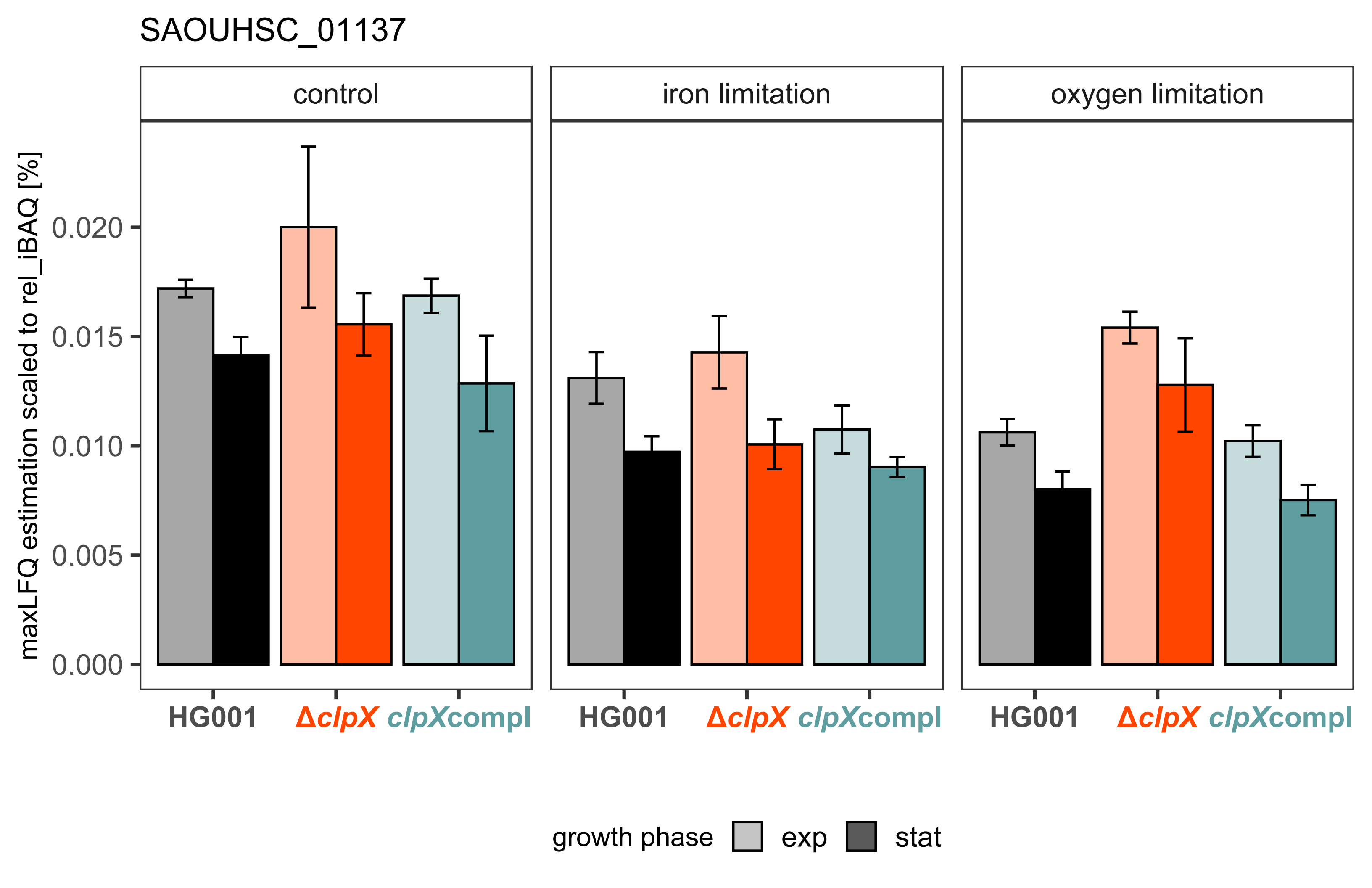

SAOUHSC_01137
control
iron limitation
oxygen limitation
maxLFQ estimation scaled to rel_iBAQ [%]
0.020
0.015
0.010
0.005
0.000
HG001
ΔclpX
clpXcompl
growth phase
exp
stat

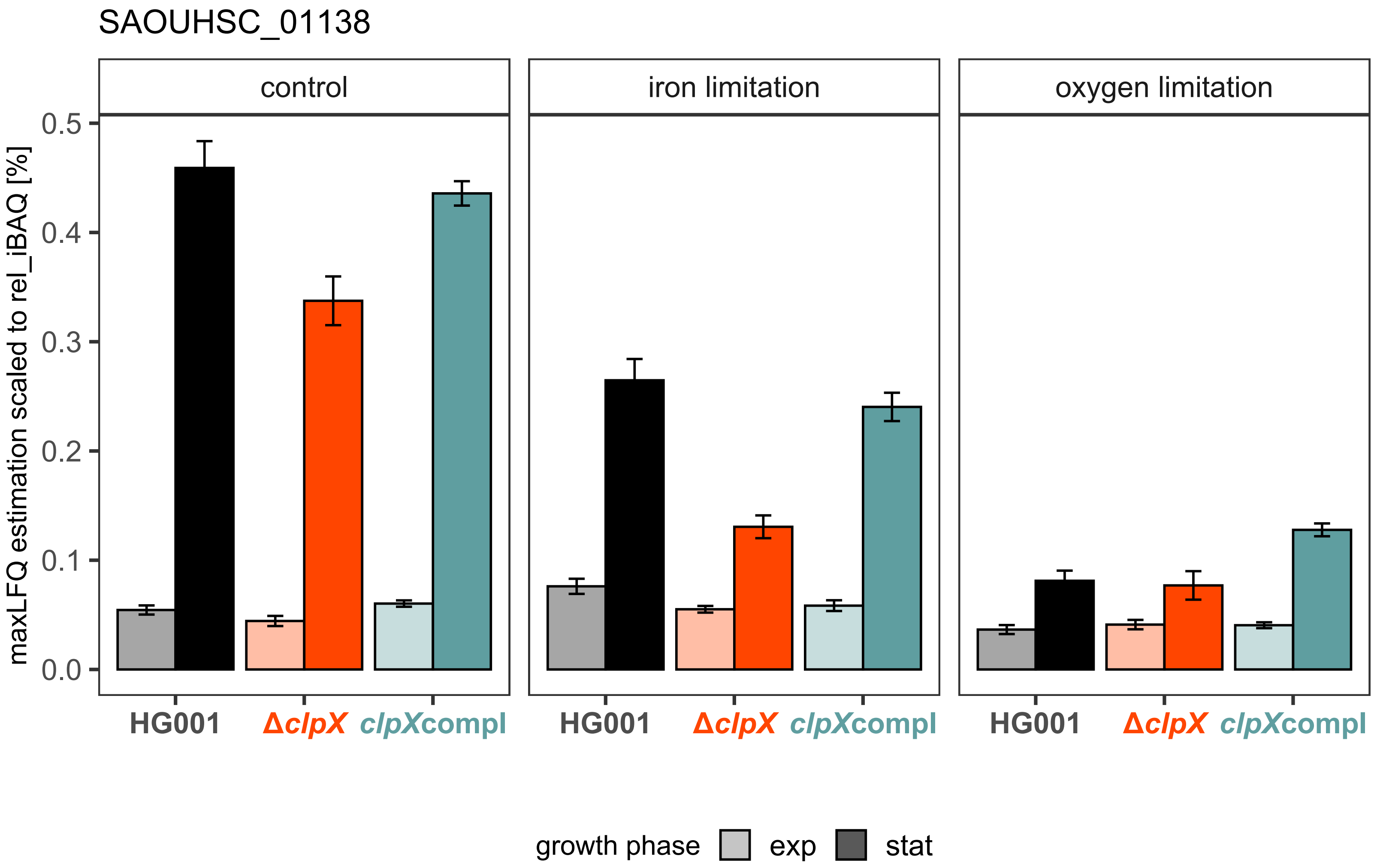
SAOUHSC_01138
control
iron limitation
oxygen limitation
maxLFQ estimation scaled to rel_iBAQ [%]
0.0
0.1
0.2
0.3
0.4
0.5
HG001
ΔclpX
clpXcompl
growth phase
exp
stat

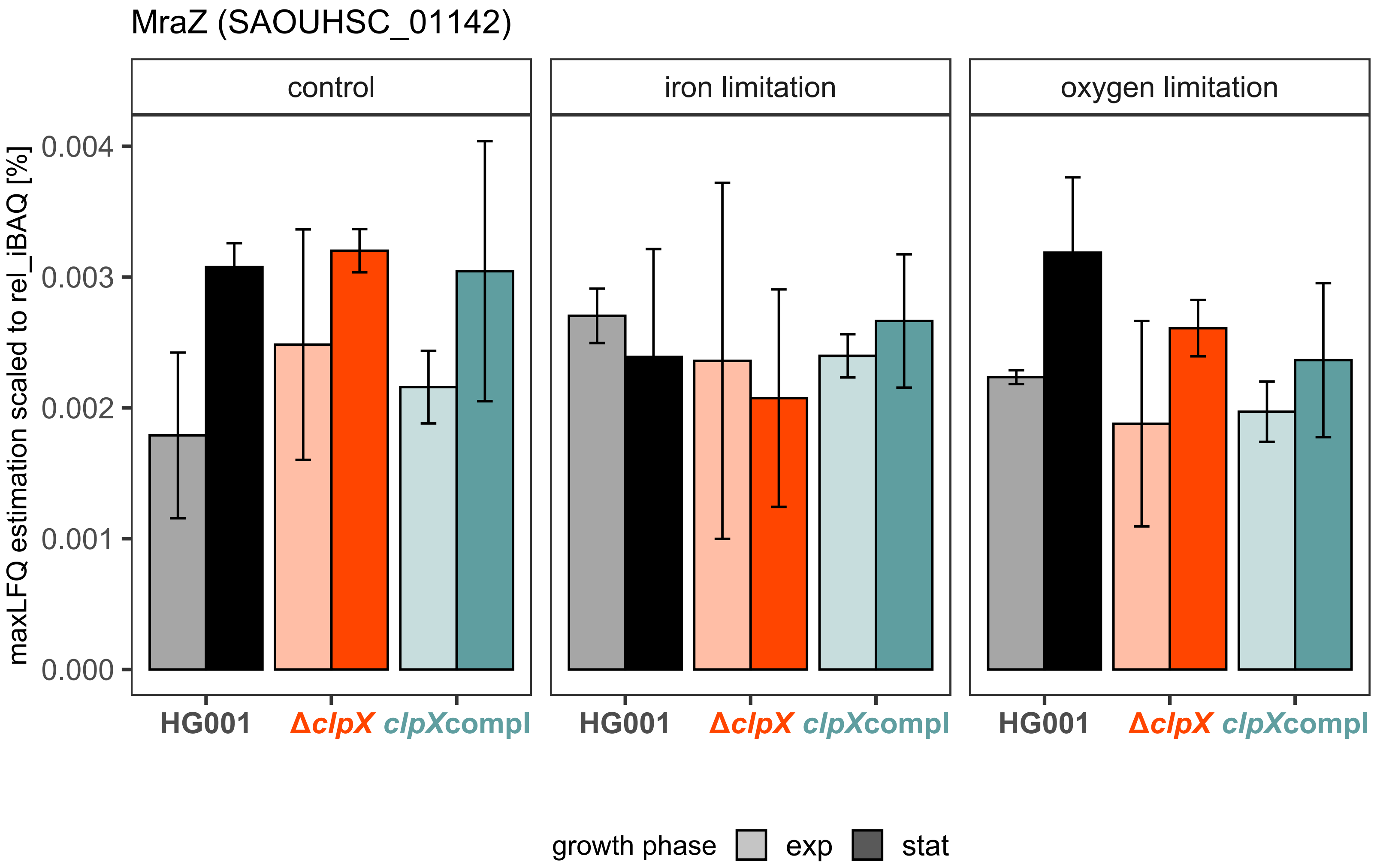

MraZ (SAOUHSC_01142)
control
iron limitation
oxygen limitation
maxLFQ estimation scaled to rel_iBAQ [%]
0.004
0.003
0.002
0.001
0.000
HG001
ΔclpX
clpXcompl
growth phase
exp
stat

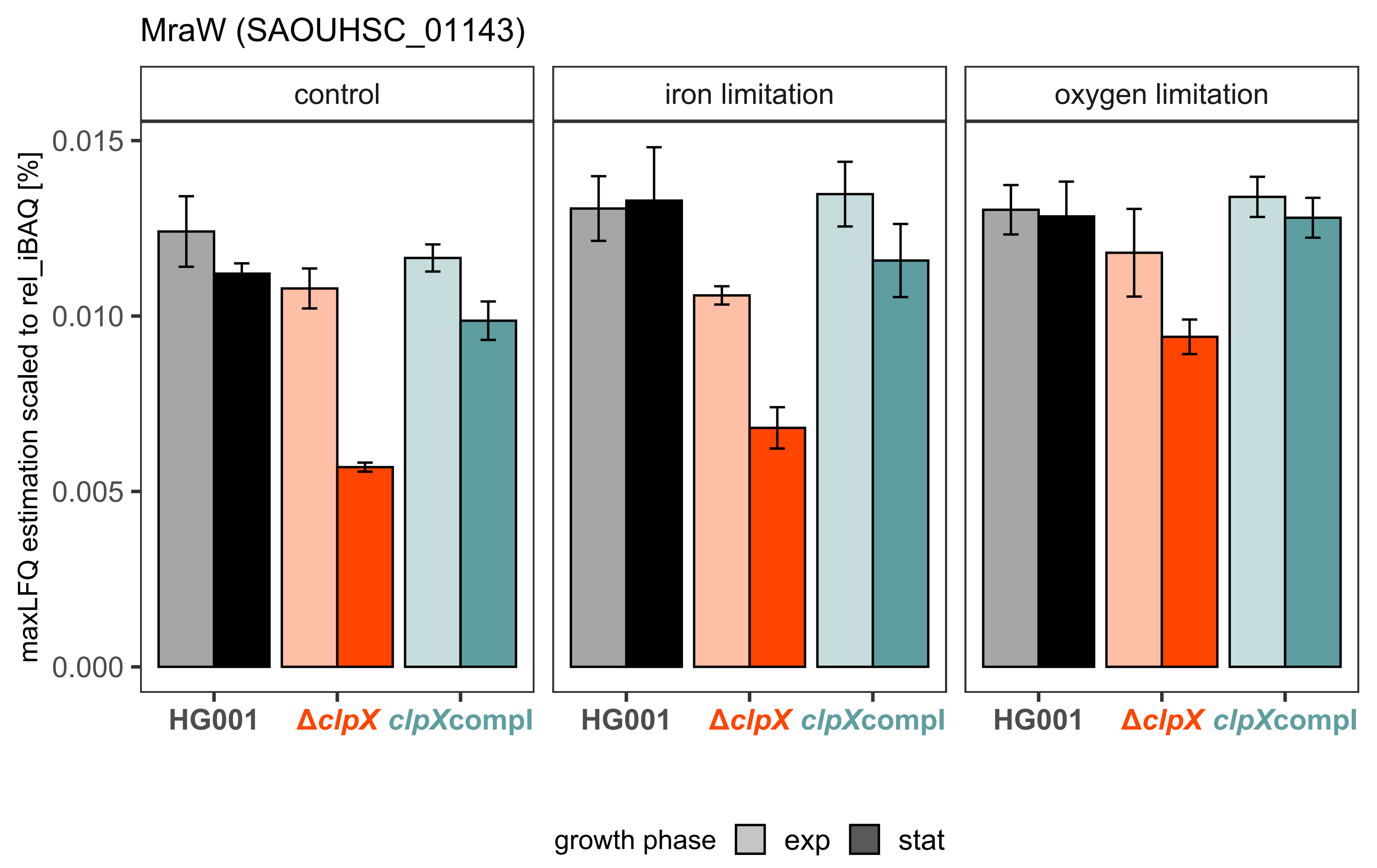

MraW (SAOUHSC_01143)
control
iron limitation
oxygen limitation
maxLFQ estimation scaled to rel_iBAQ [%]
0.015
0.010
0.005
0.000
HG001
ΔclpX
clpXcompl
growth phase
exp
stat

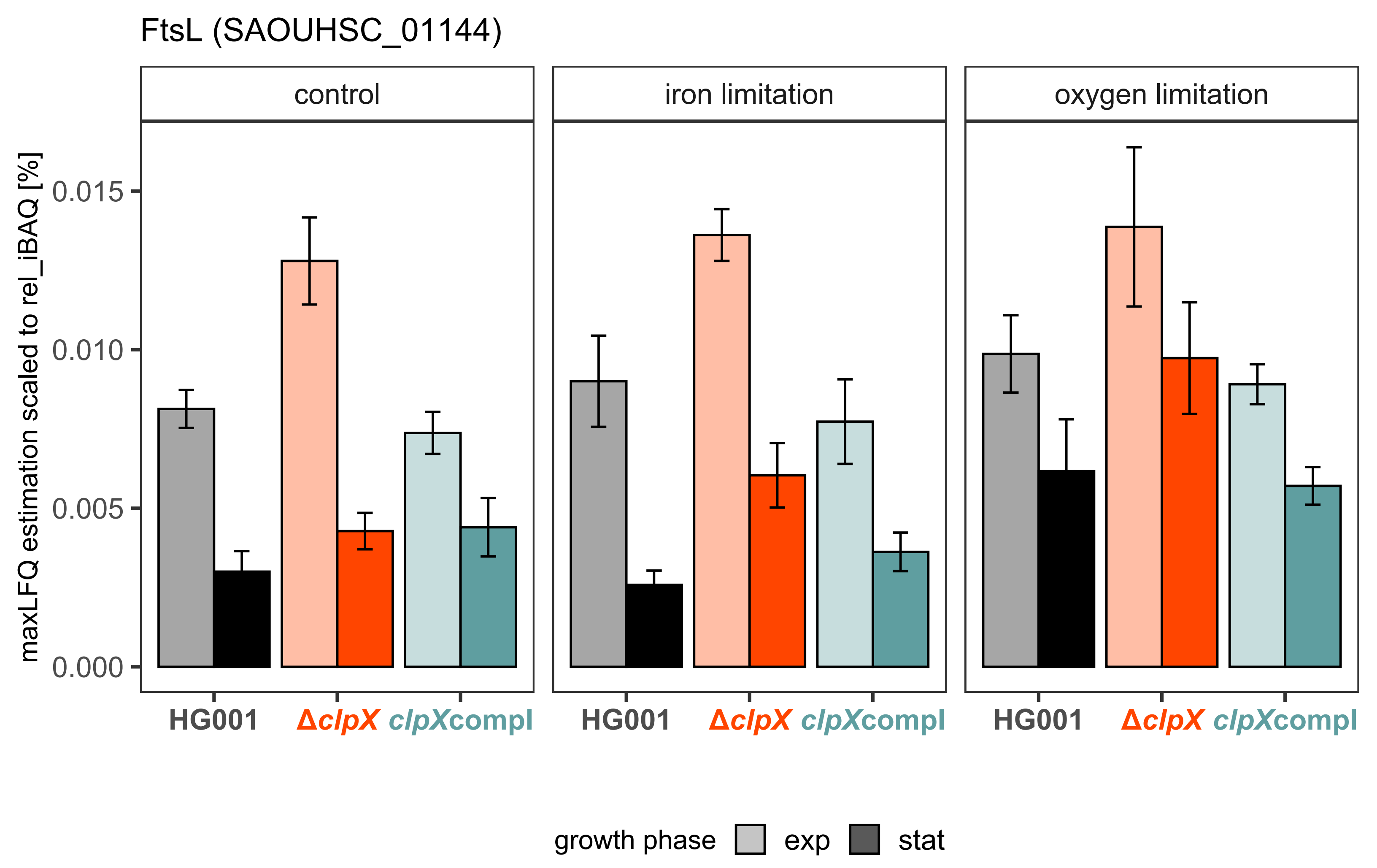
FtsL (SAOUHSC_01144)
control
iron limitation
oxygen limitation
maxLFQ estimation scaled to rel_iBAQ [%]
0.015
0.010
0.005
0.000
HG001
ΔclpX
clpXcompl
growth phase
exp
stat

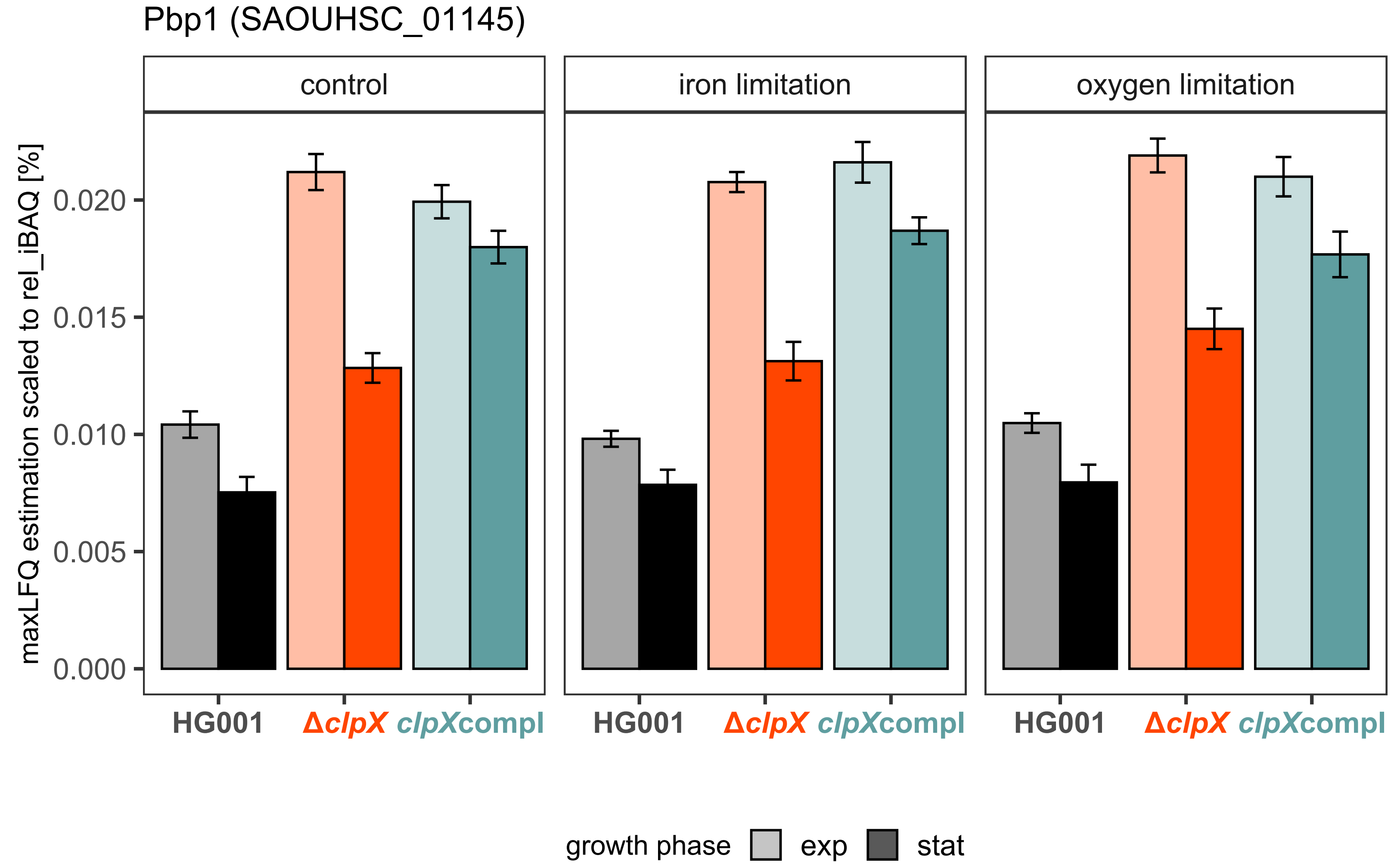
Pbp1 (SAOUHSC_01145)
control
iron limitation
oxygen limitation
maxLFQ estimation scaled to rel_iBAQ [%]
0.000
0.005
0.010
0.015
0.020
HG001
ΔclpX
clpXcompl
growth phase
exp
stat

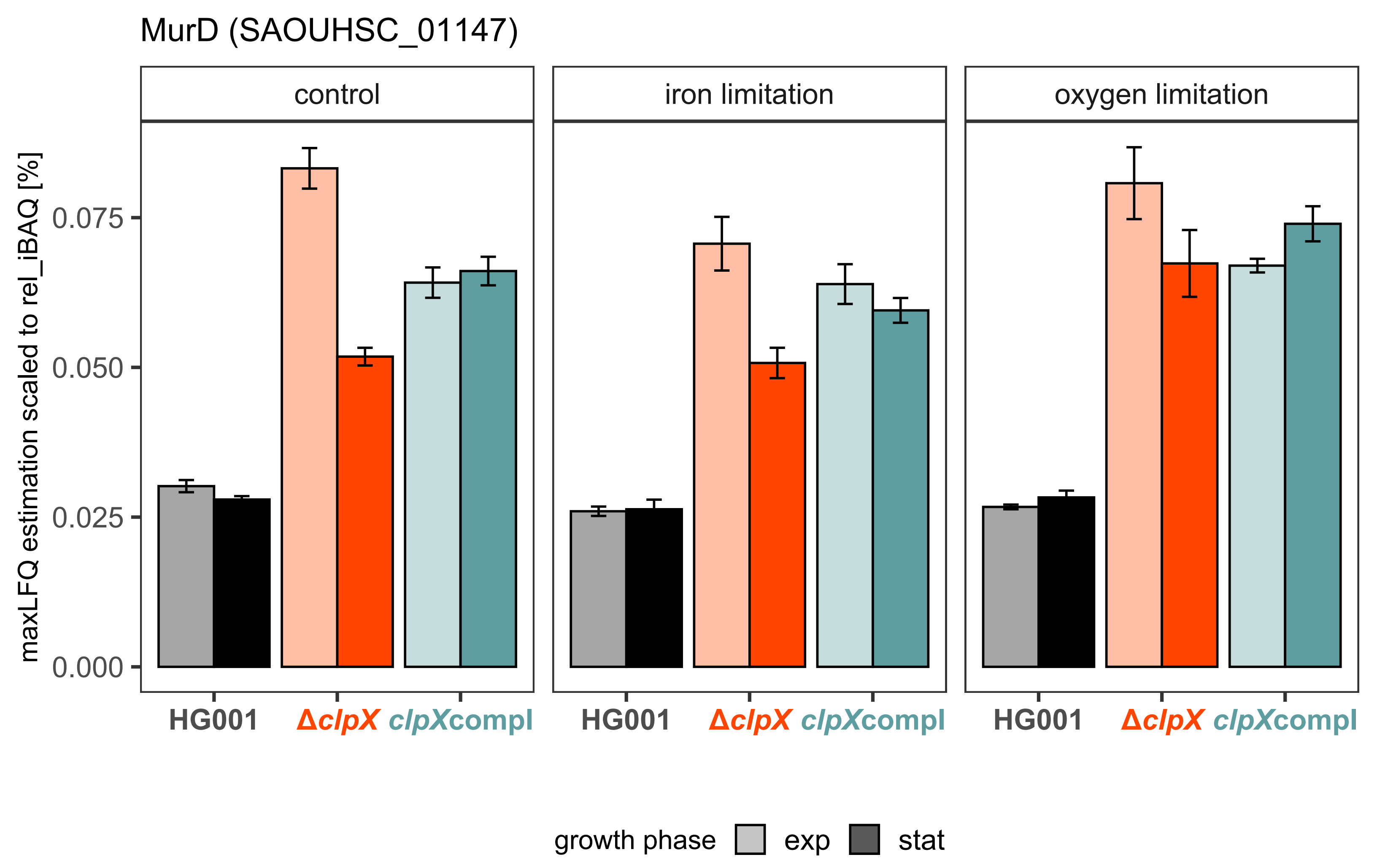
MurD (SAOUHSC_01147)
control
iron limitation
oxygen limitation
maxLFQ estimation scaled to rel_iBAQ [%]
0.000
0.025
0.050
0.075
HG001
ΔclpX
clpXcompl
growth phase
exp
stat

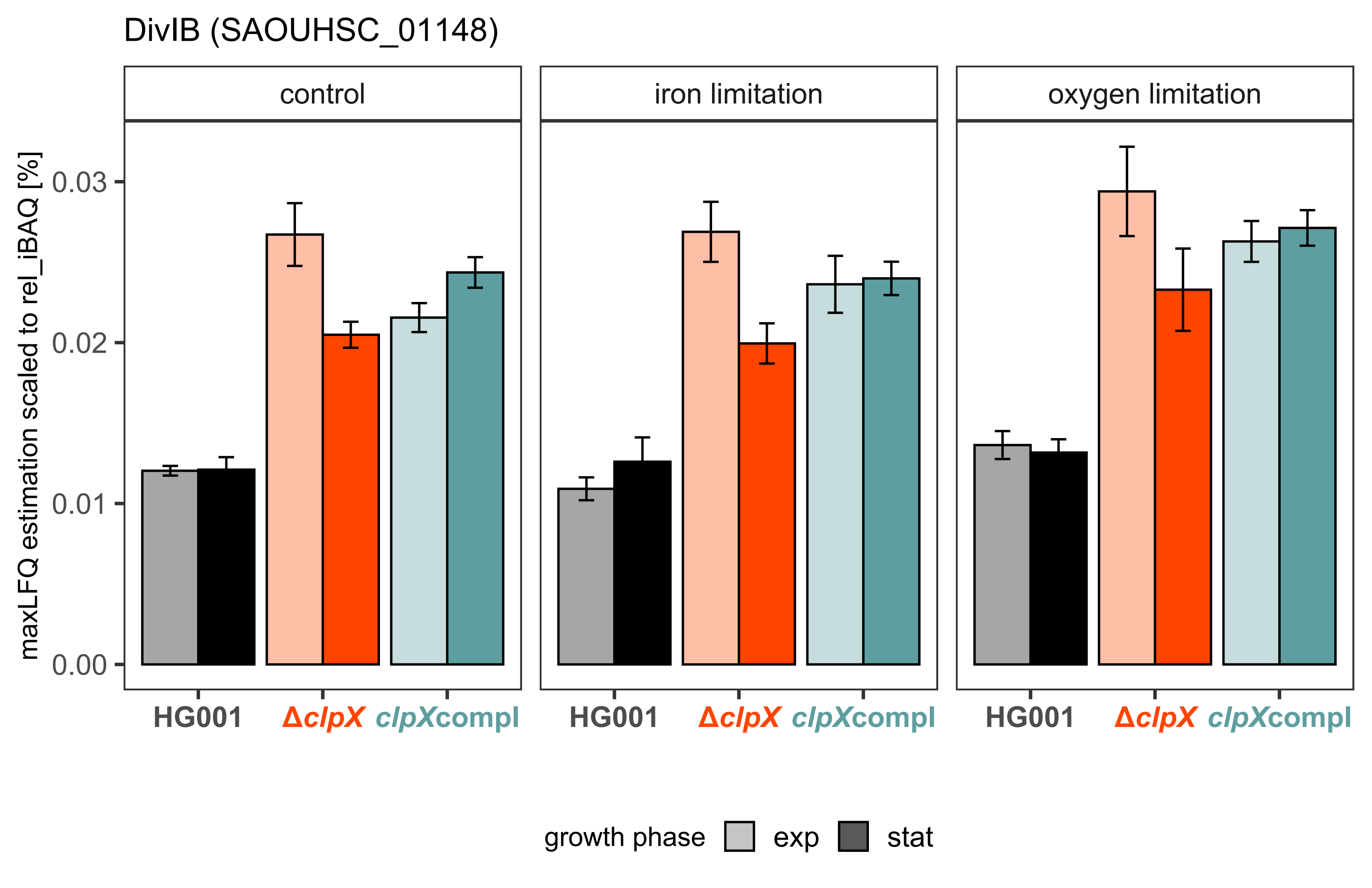
DivIB (SAOUHSC_01148)
control
iron limitation
oxygen limitation
maxLFQ estimation scaled to rel_iBAQ [%]
0.00
0.01
0.02
0.03
HG001
ΔclpX
clpXcompl
growth phase
exp
stat

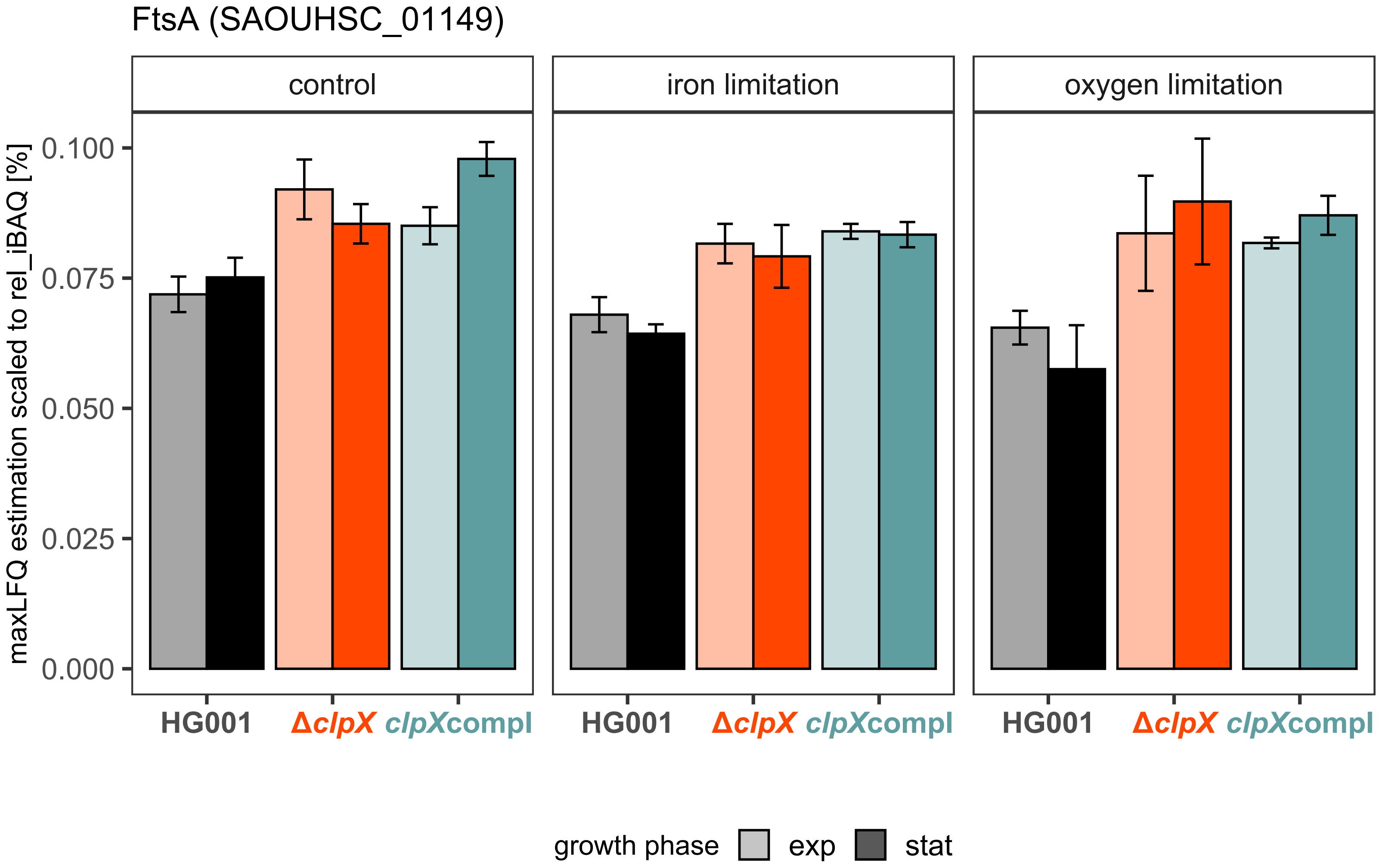
FtsA (SAOUHSC_01149)
control
iron limitation
oxygen limitation
maxLFQ estimation scaled to rel_iBAQ [%]
0.100
0.075
0.050
0.025
0.000
HG001
ΔclpX
clpXcompl
growth phase
exp
stat

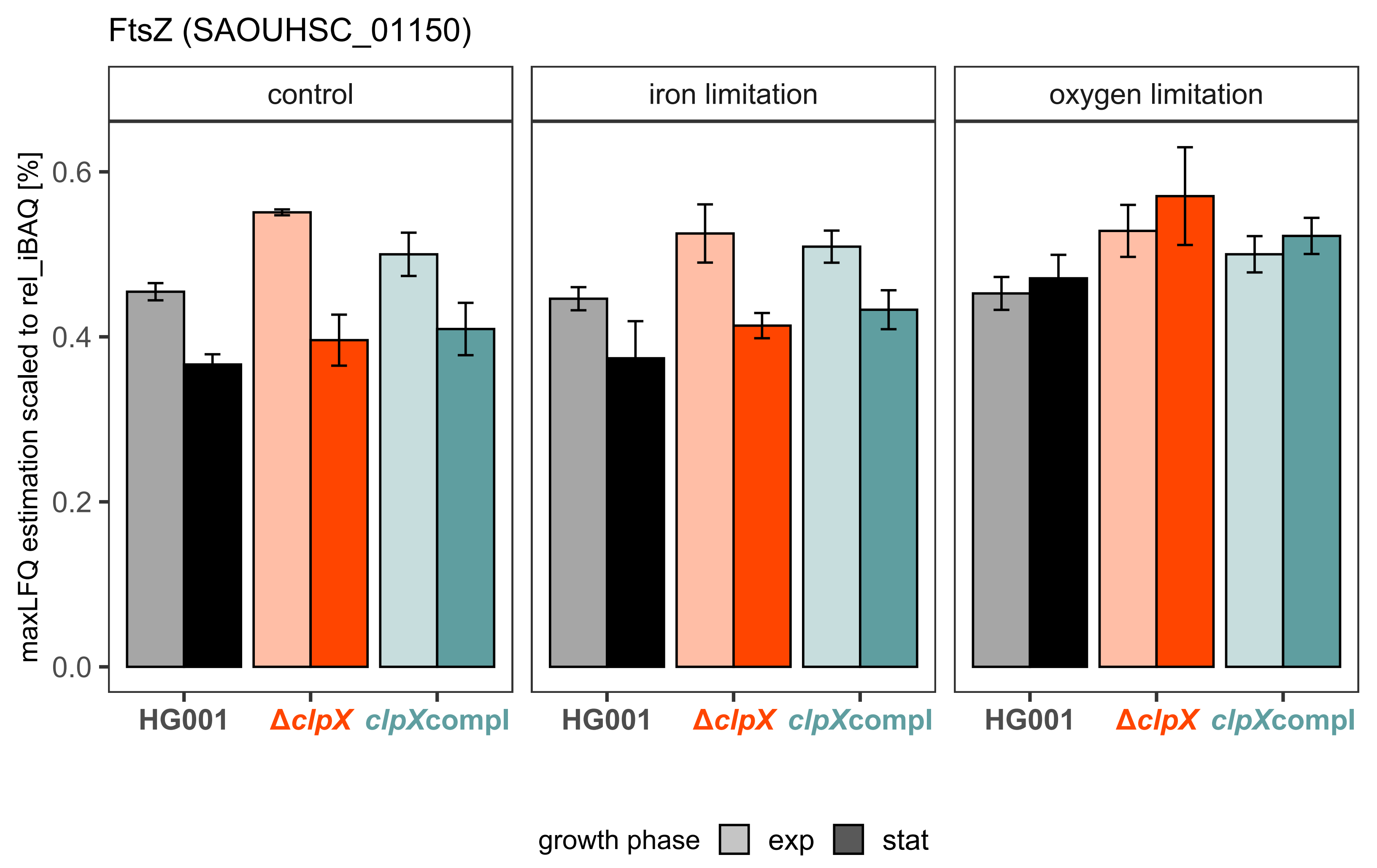
FtsZ (SAOUHSC_01150)
control
iron limitation
oxygen limitation
maxLFQ estimation scaled to rel_iBAQ [%]
0.0
0.2
0.4
0.6
HG001
ΔclpX
clpXcompl
growth phase
exp
stat

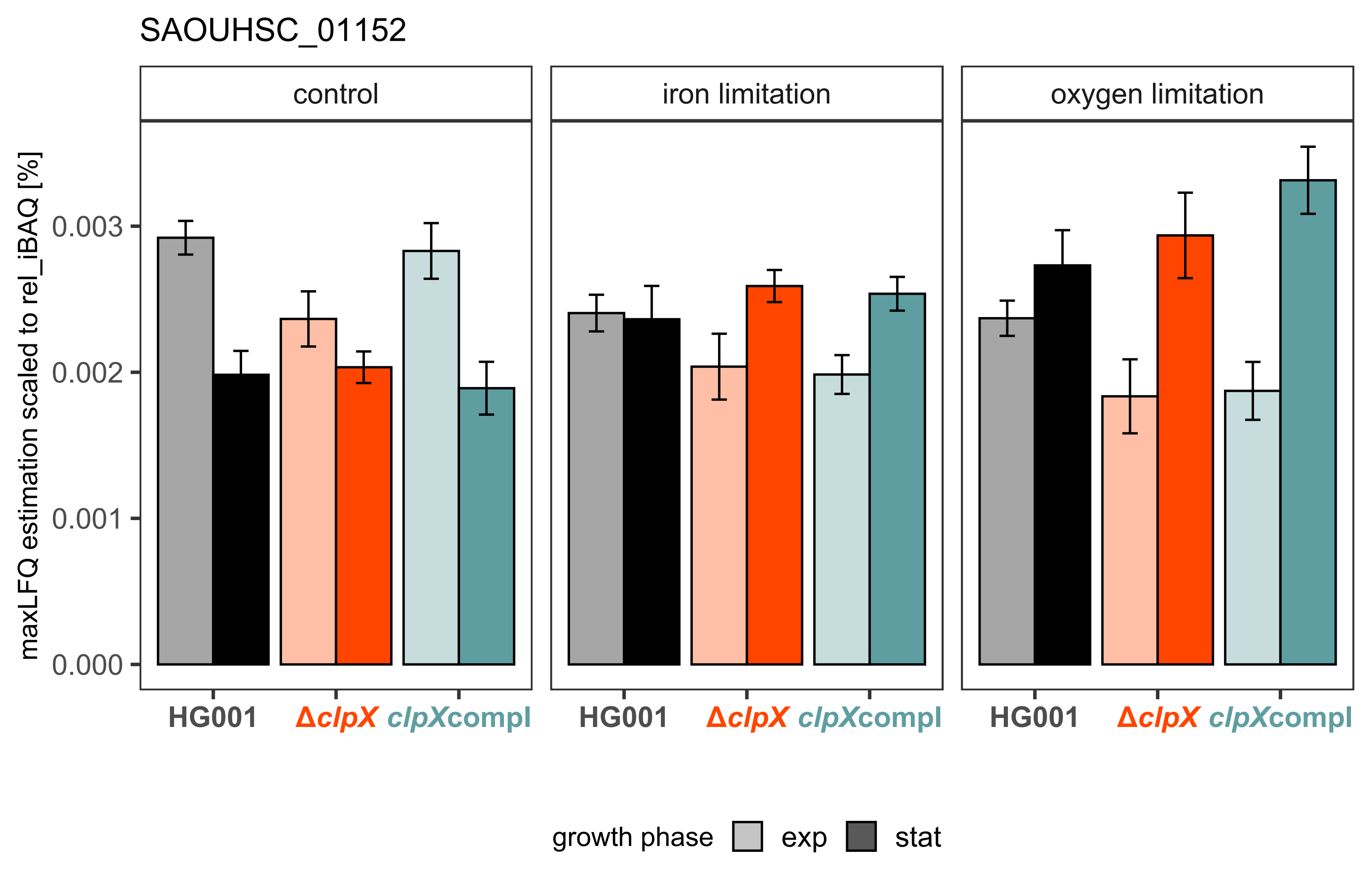

SAOUHSC_01152
control
iron limitation
oxygen limitation
maxLFQ estimation scaled to rel_iBAQ [%]
0.000
0.001
0.002
0.003
HG001
ΔclpX
clpXcompl
growth phase
exp
stat

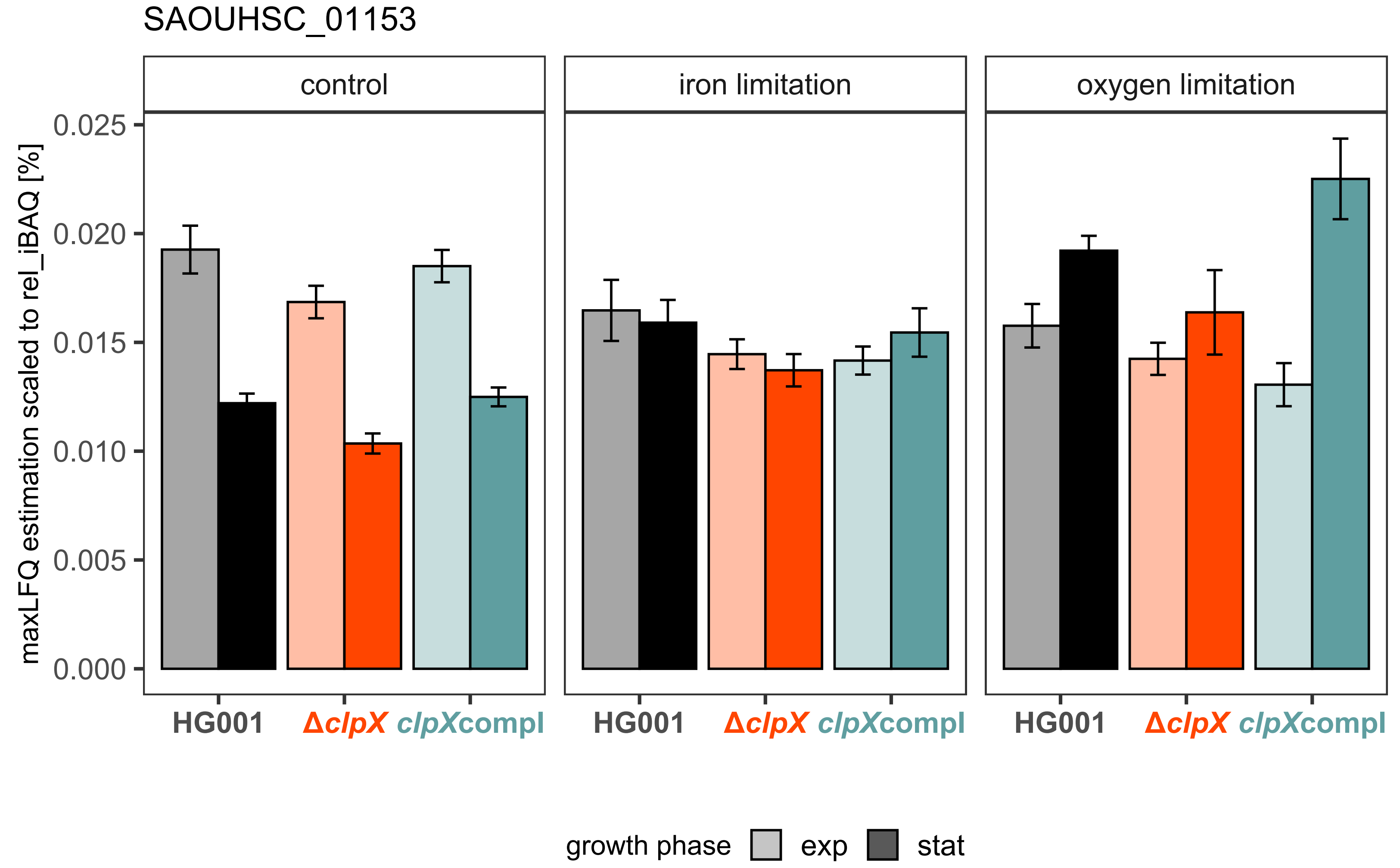

SAOUHSC_01153
control
iron limitation
oxygen limitation
maxLFQ estimation scaled to rel_iBAQ [%]
0.025
0.020
0.015
0.010
0.005
0.000
HG001
ΔclpX
clpXcompl
growth phase
exp
stat

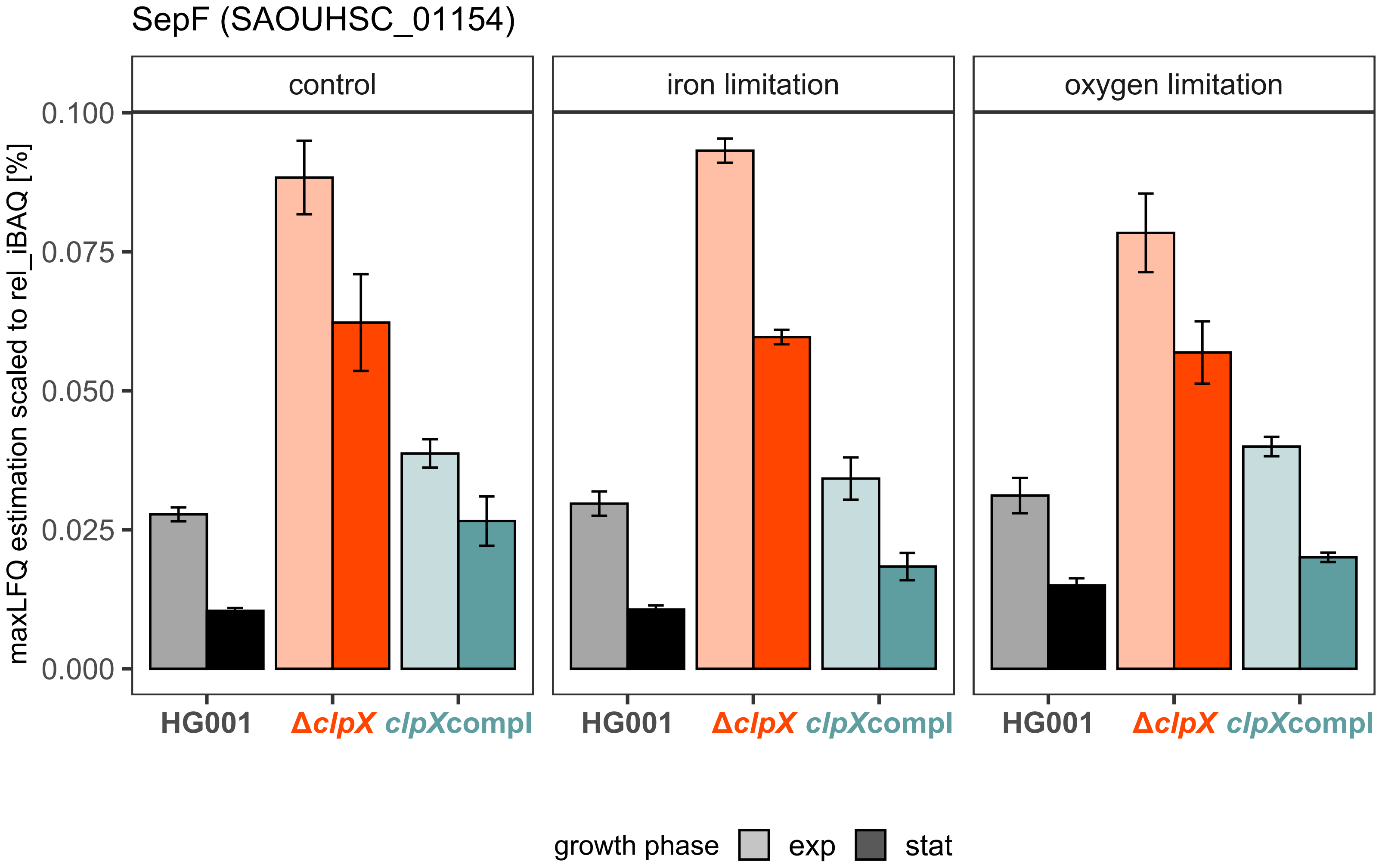
SepF (SAOUHSC_01154)
control
iron limitation
oxygen limitation
maxLFQ estimation scaled to rel_iBAQ [%]
0.100
0.075
0.050
0.025
0.000
HG001
ΔclpX
clpXcompl
growth phase
exp
stat

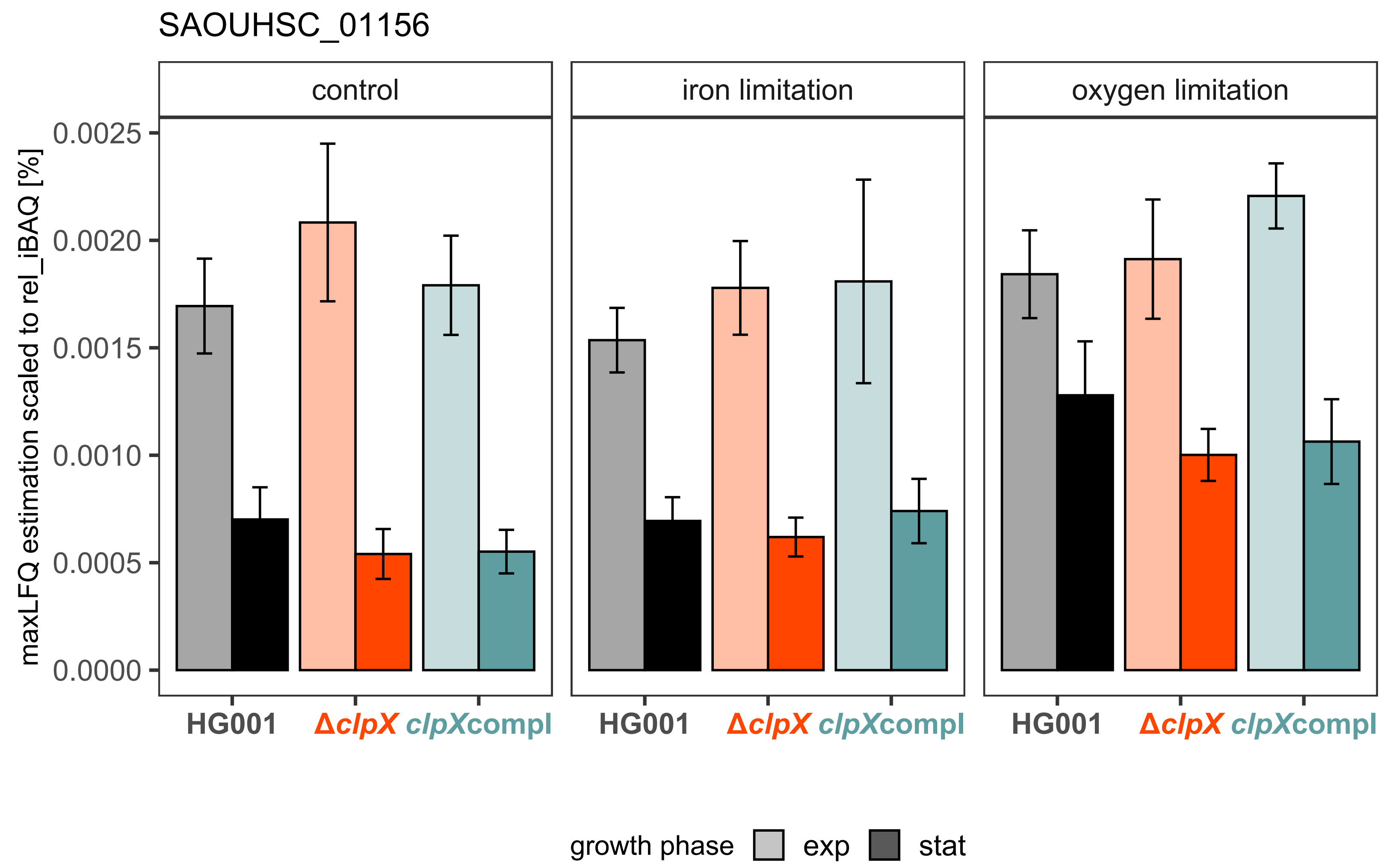

SAOUHSC_01156
control
iron limitation
oxygen limitation
maxLFQ estimation scaled to rel_iBAQ [%]
0.0025
0.0020
0.0015
0.0010
0.0005
0.0000
HG001
ΔclpX
clpXcompl
growth phase
exp
stat

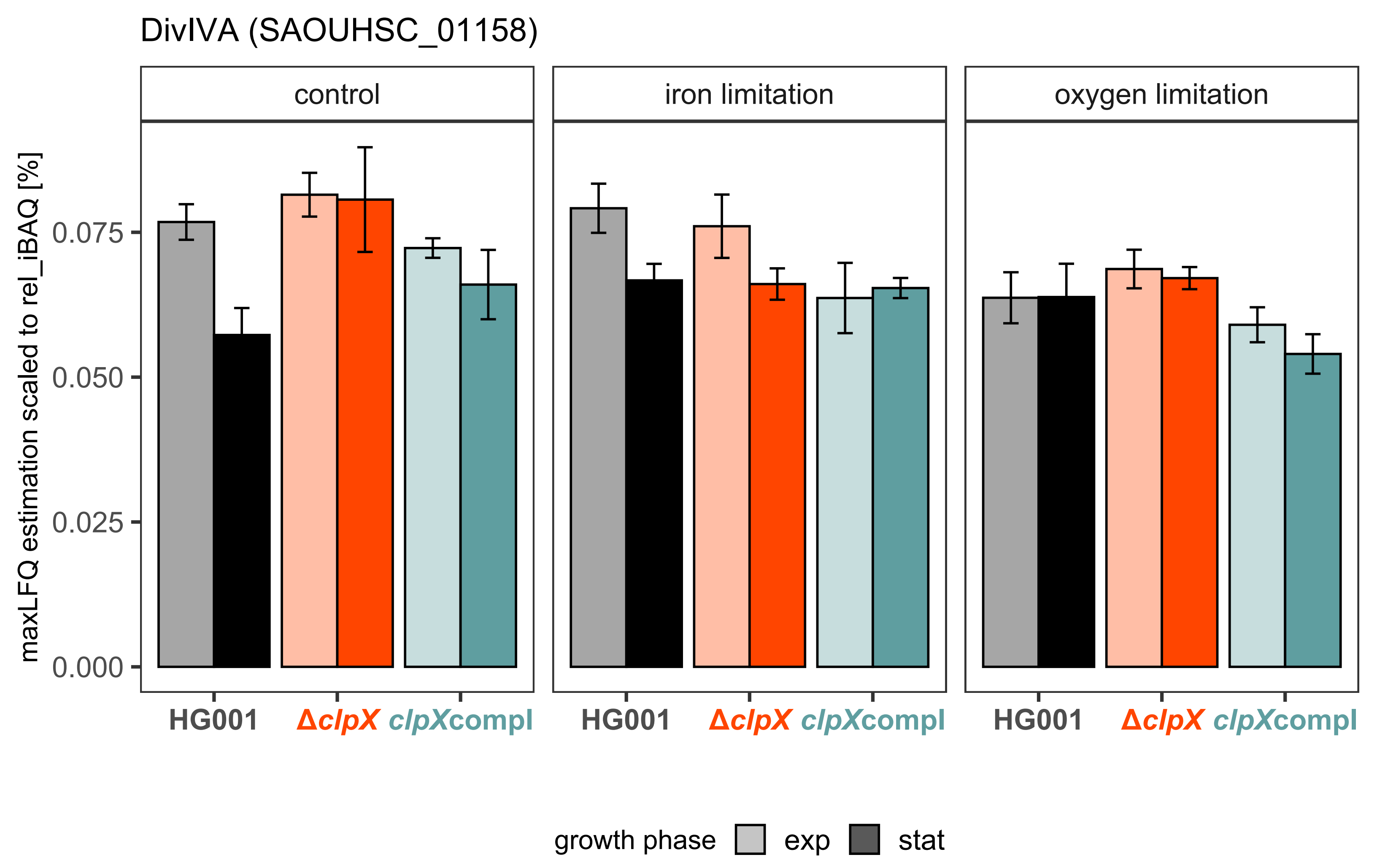
DivIVA (SAOUHSC_01158)
control
iron limitation
oxygen limitation
maxLFQ estimation scaled to rel_iBAQ [%]
0.000
0.025
0.050
0.075
HG001
ΔclpX
clpXcompl
growth phase
exp
stat

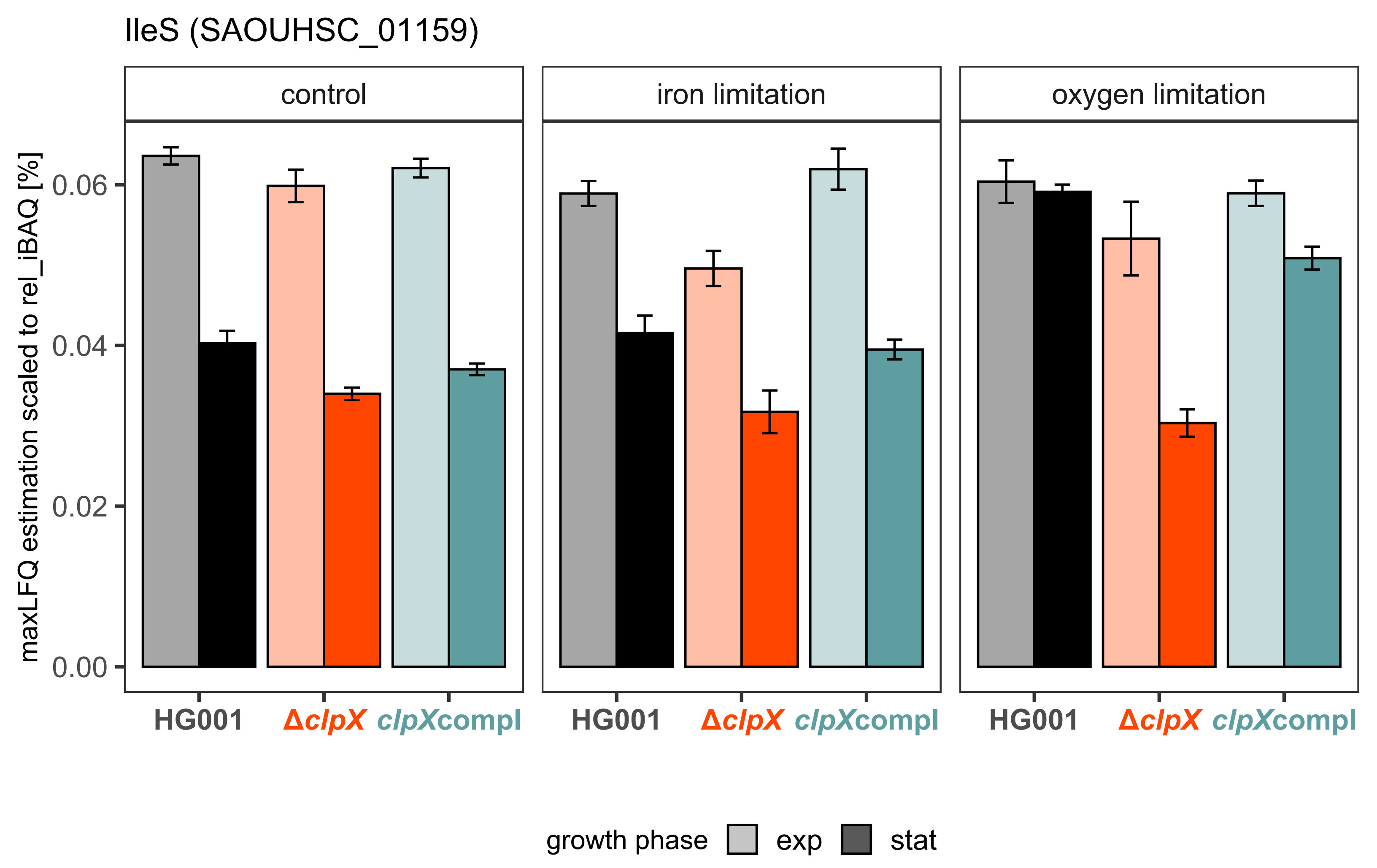
IleS (SAOUHSC_01159)
control
iron limitation
oxygen limitation
maxLFQ estimation scaled to rel_iBAQ [%]
0.06
0.04
0.02
0.00
HG001
ΔclpX
clpXcompl
growth phase
exp
stat

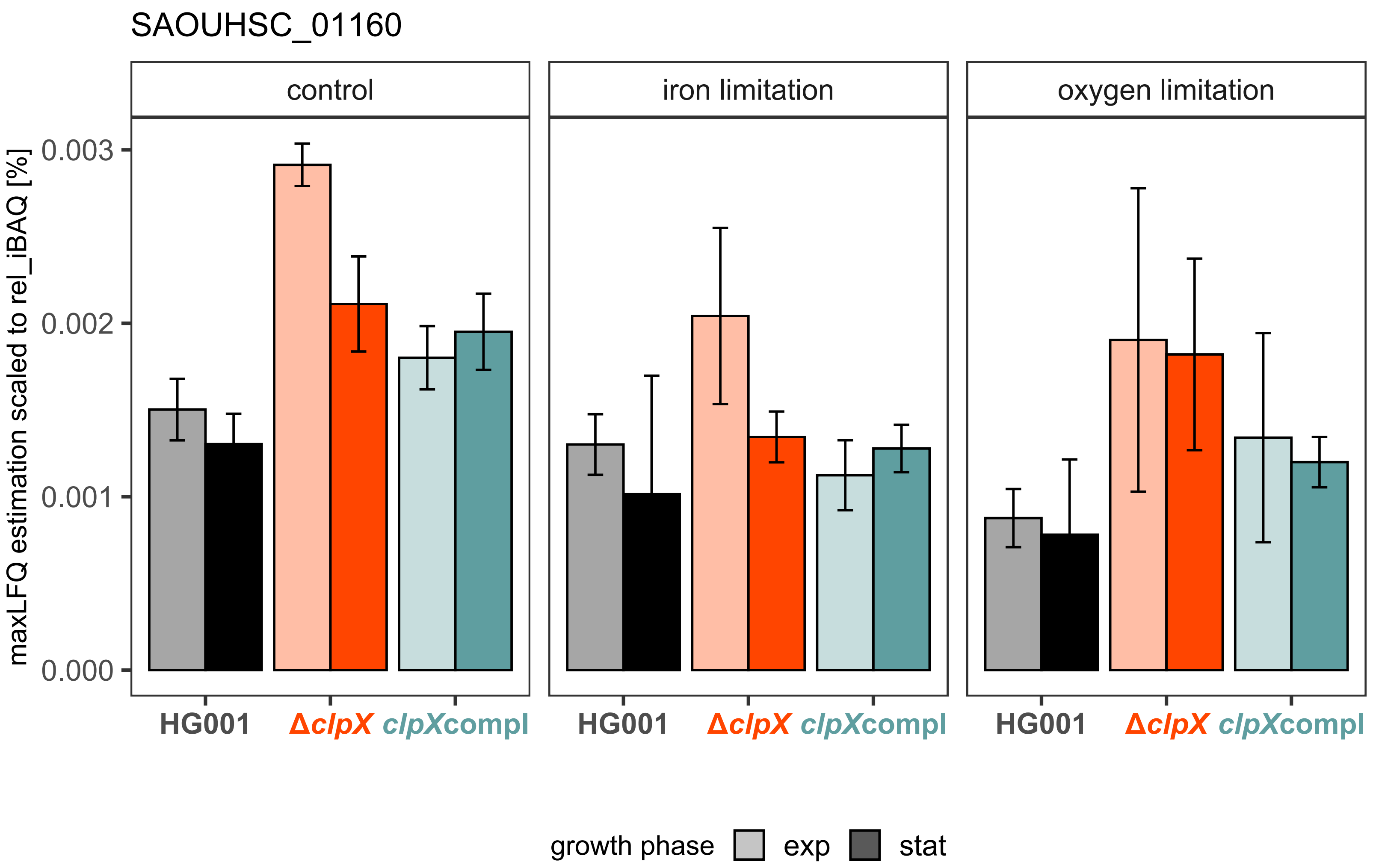
SAOUHSC_01160
control
iron limitation
oxygen limitation
maxLFQ estimation scaled to rel_iBAQ [%]
0.003
0.002
0.001
0.000
HG001
ΔclpX
clpXcompl
growth phase
exp
stat

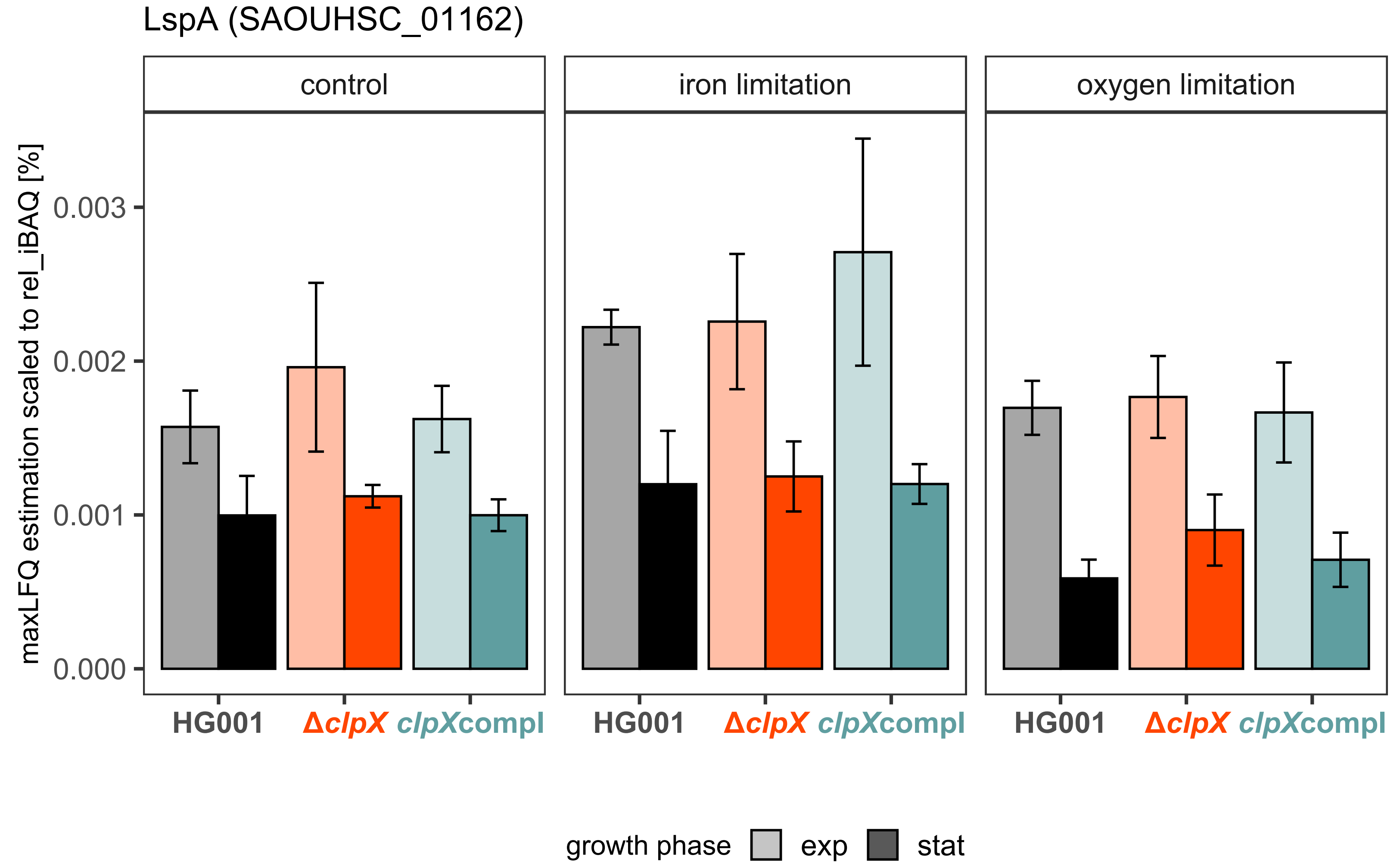

LspA (SAOUHSC_01162)
control
iron limitation
oxygen limitation
maxLFQ estimation scaled to rel_iBAQ [%]
0.003
0.002
0.001
0.000
HG001
ΔclpX
clpXcompl
growth phase
exp
stat

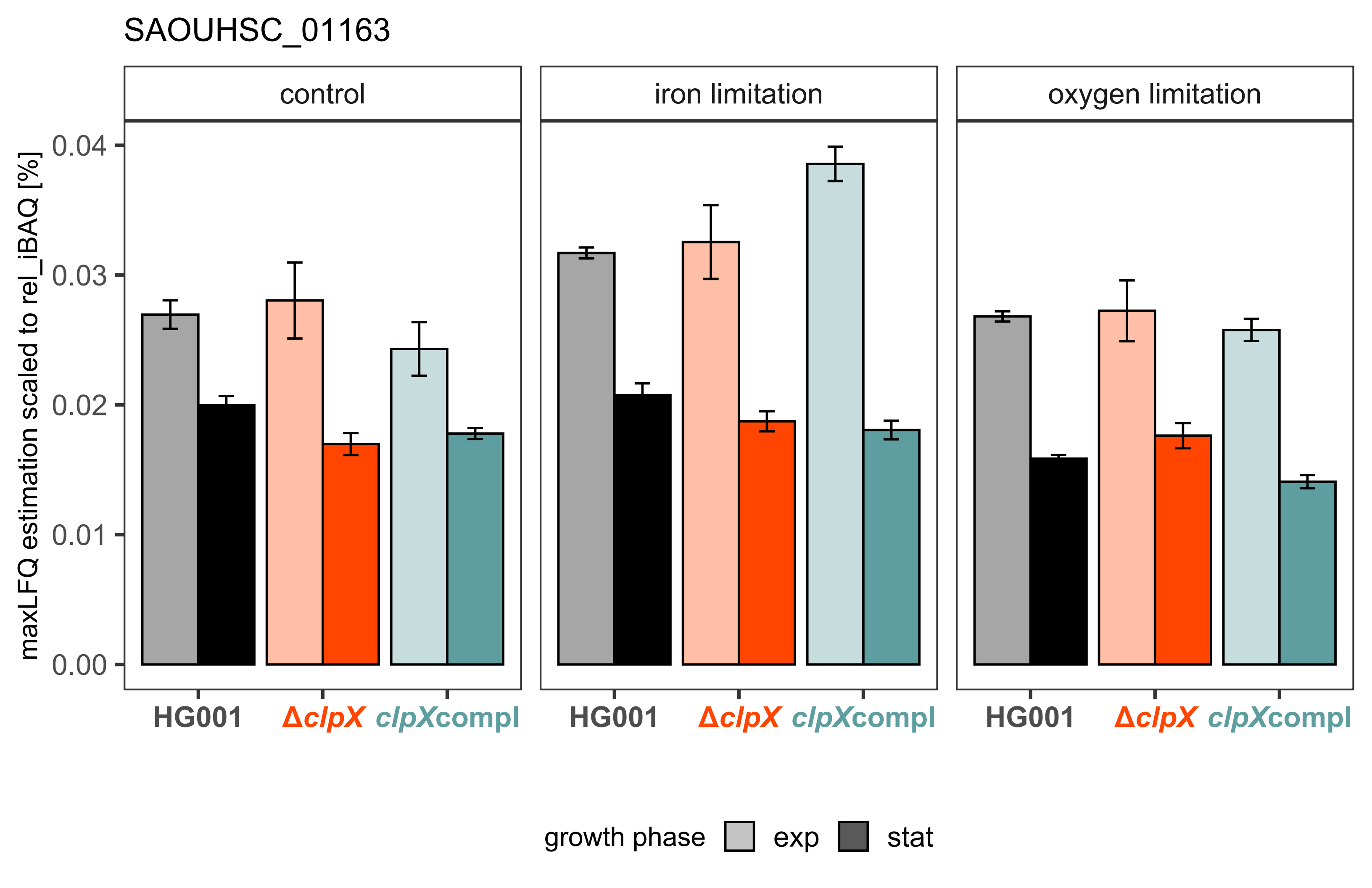
SAOUHSC_01163
control
iron limitation
oxygen limitation
maxLFQ estimation scaled to rel_iBAQ [%]
0.04
0.03
0.02
0.01
0.00
HG001
ΔclpX
clpXcompl
growth phase
exp
stat

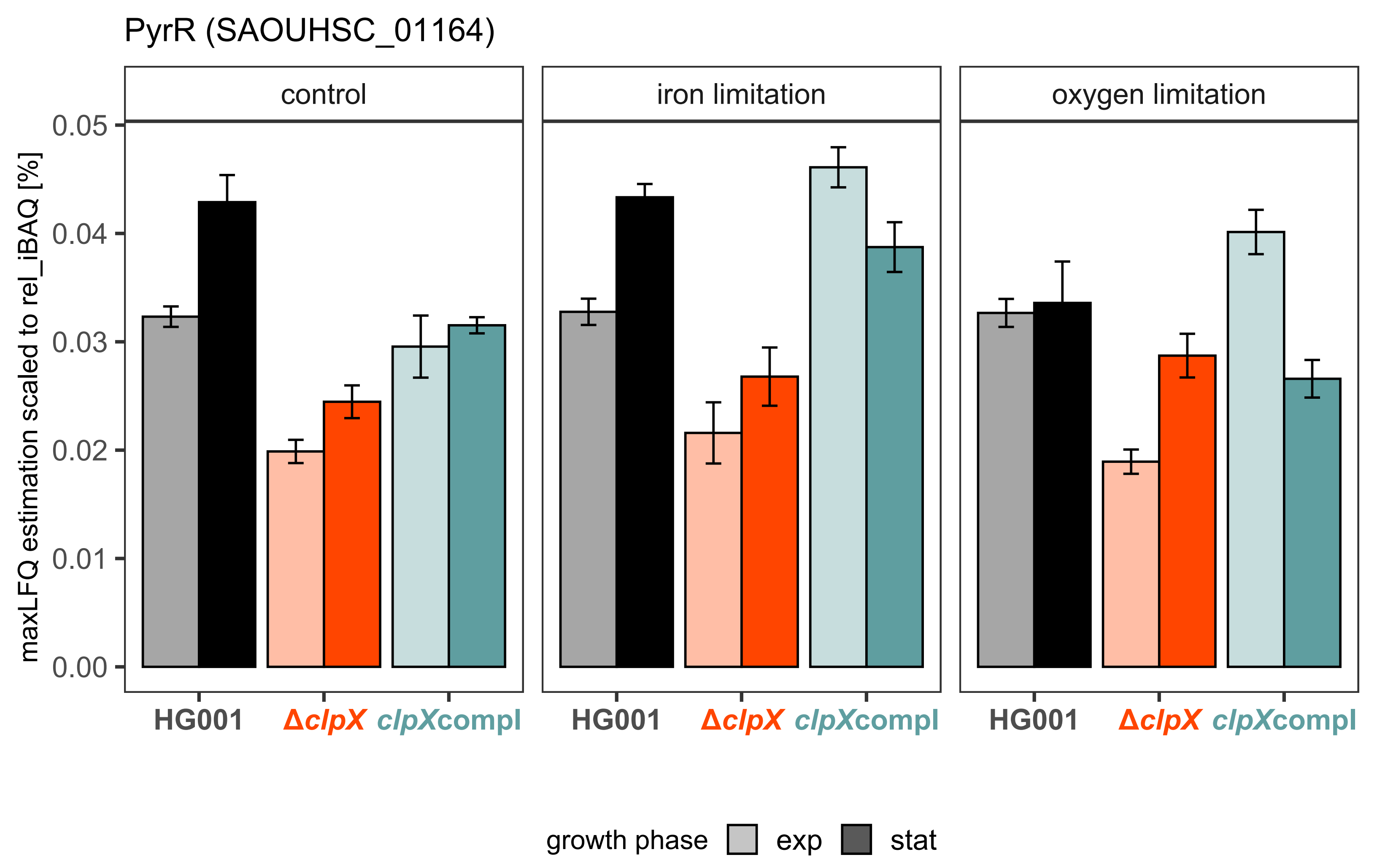
PyrR (SAOUHSC_01164)
control
iron limitation
oxygen limitation
maxLFQ estimation scaled to rel_iBAQ [%]
0.05
0.04
0.03
0.02
0.01
0.00
HG001
ΔclpX
clpXcompl
growth phase
exp
stat

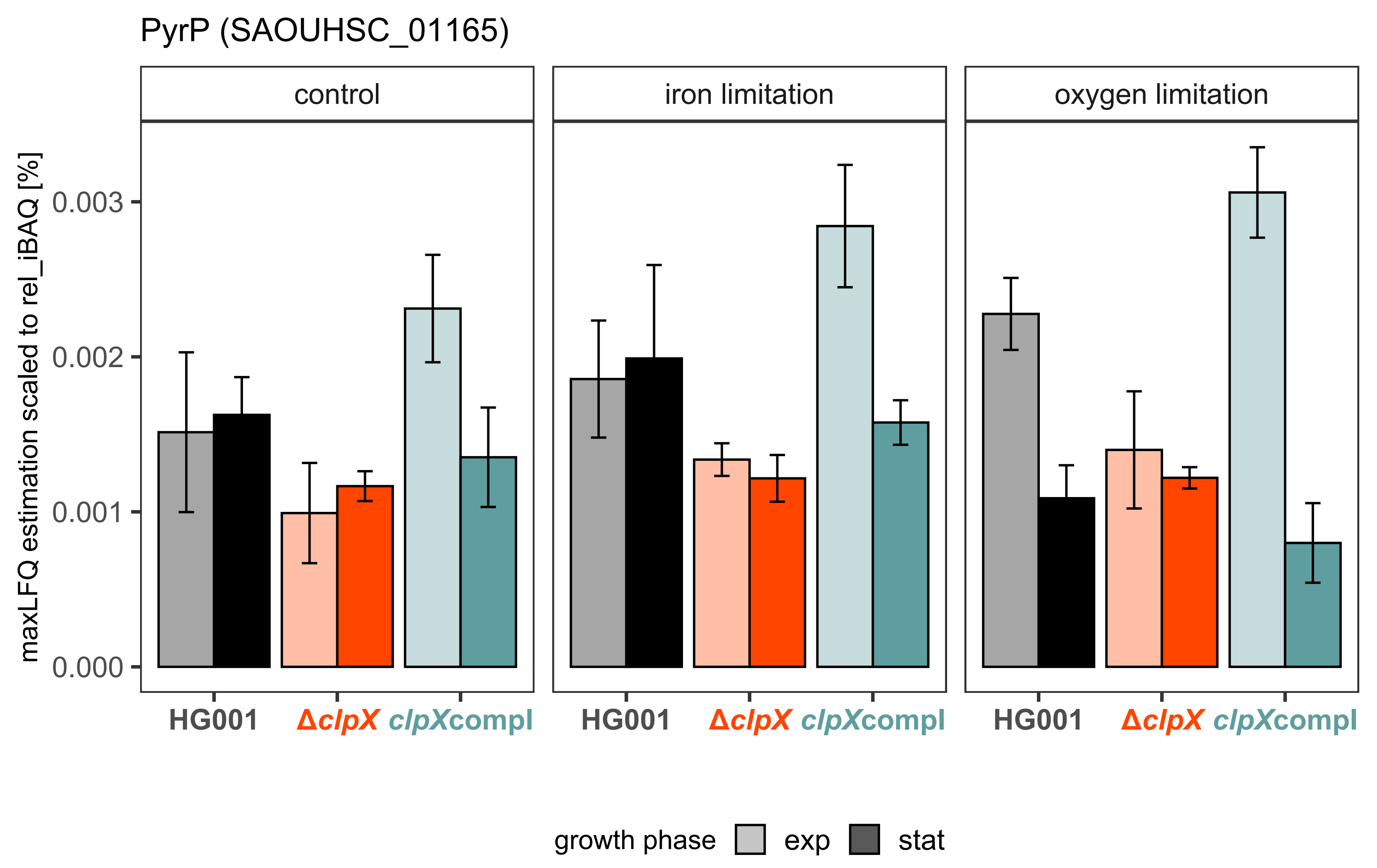
PyrP (SAOUHSC_01165)
control
iron limitation
oxygen limitation
maxLFQ estimation scaled to rel_iBAQ [%]
0.003
0.002
0.001
0.000
HG001
ΔclpX
clpXcompl
growth phase
exp
stat

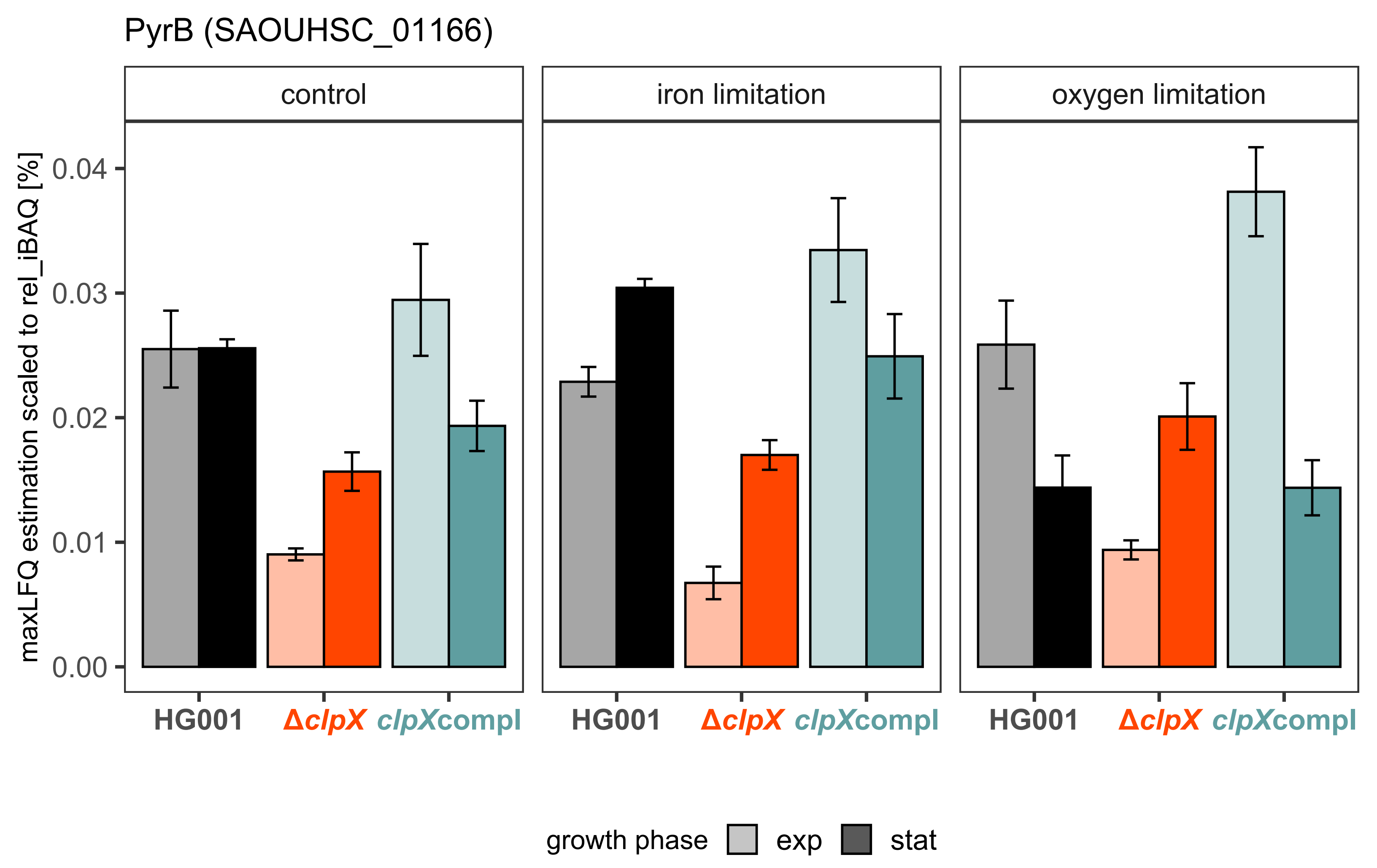

PyrB (SAOUHSC_01166)
control
iron limitation
oxygen limitation
maxLFQ estimation scaled to rel_iBAQ [%]
0.00
0.01
0.02
0.03
0.04
HG001
ΔclpX
clpXcompl
growth phase
exp
stat

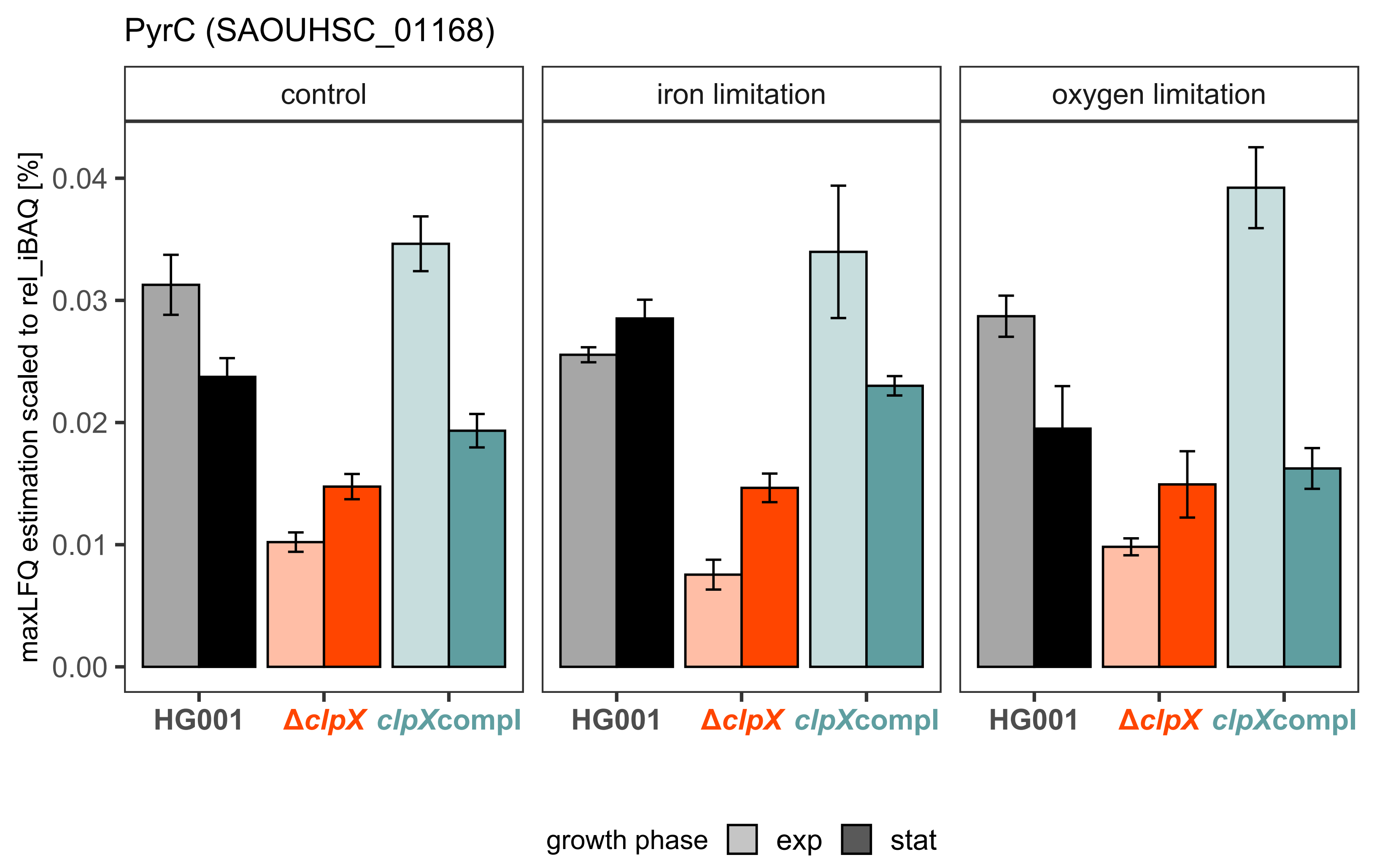
PyrC (SAOUHSC_01168)
control
iron limitation
oxygen limitation
maxLFQ estimation scaled to rel_iBAQ [%]
0.04
0.03
0.02
0.01
0.00
HG001
ΔclpX
clpXcompl
growth phase
exp
stat

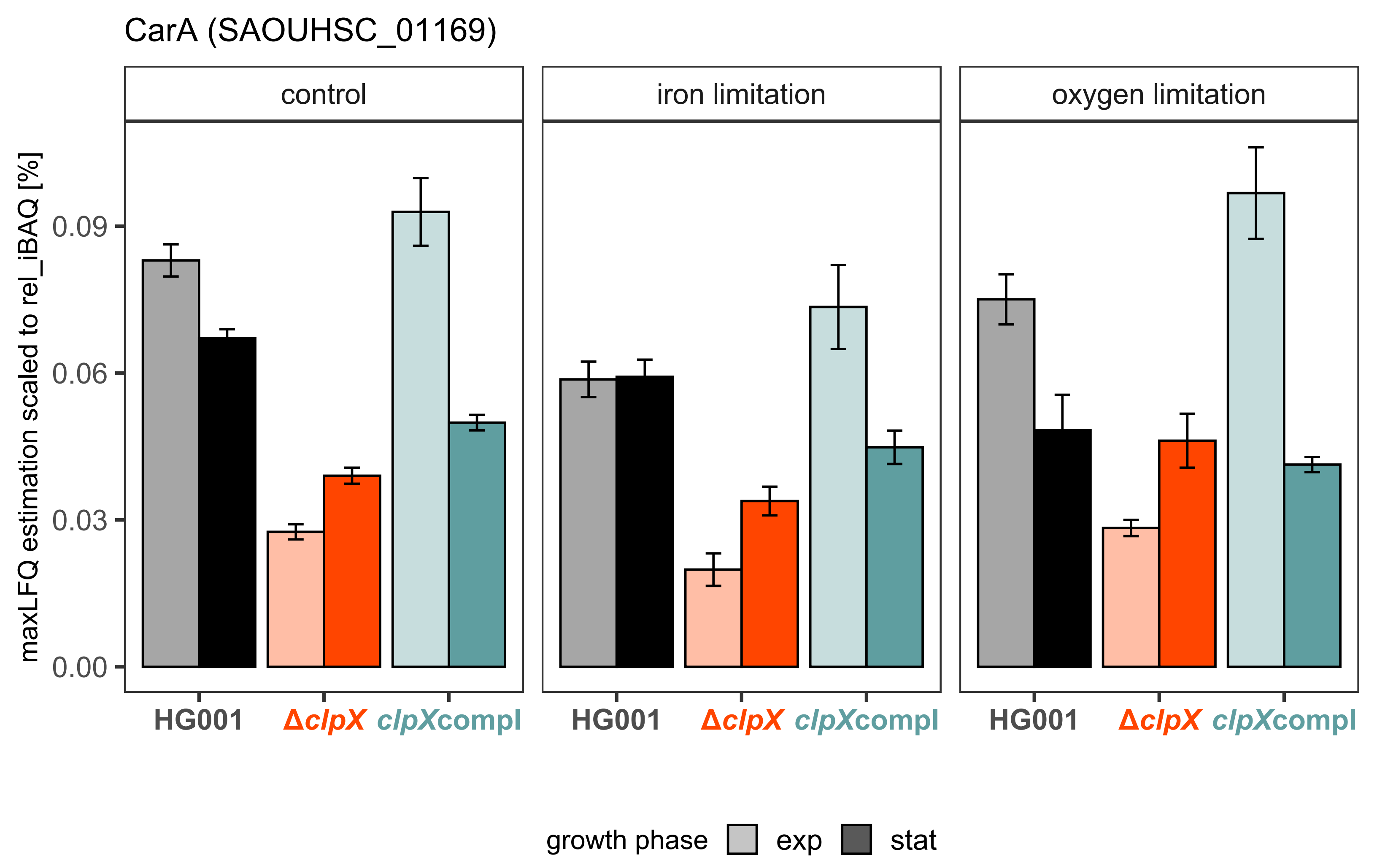
CarA (SAOUHSC_01169)
control
iron limitation
oxygen limitation
maxLFQ estimation scaled to rel_iBAQ [%]
0.09
0.06
0.03
0.00
HG001
ΔclpX
clpXcompl
growth phase
exp
stat

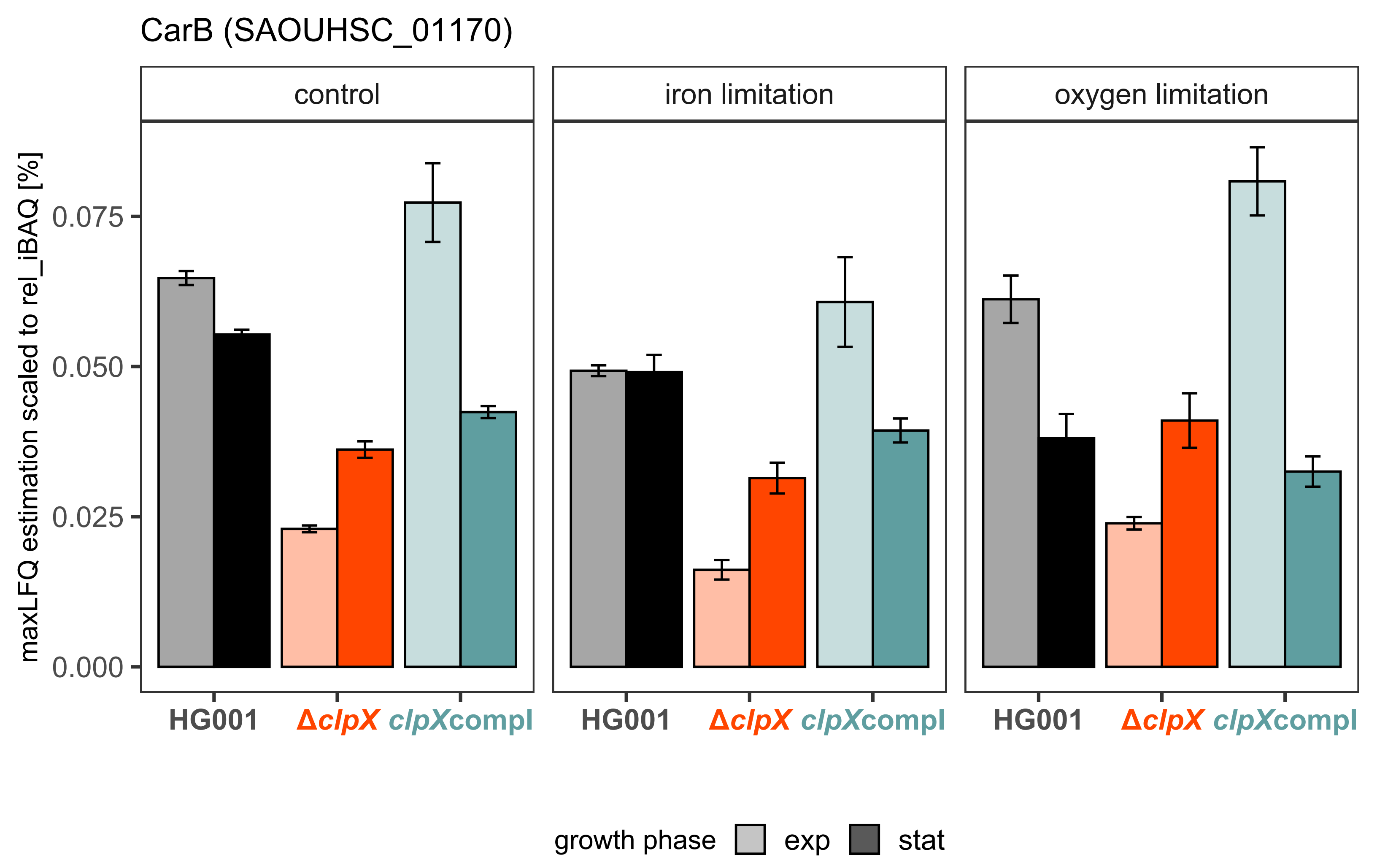
CarB (SAOUHSC_01170)
control
iron limitation
oxygen limitation
maxLFQ estimation scaled to rel_iBAQ [%]
0.075
0.050
0.025
0.000
HG001
ΔclpX
clpXcompl
growth phase
exp
stat

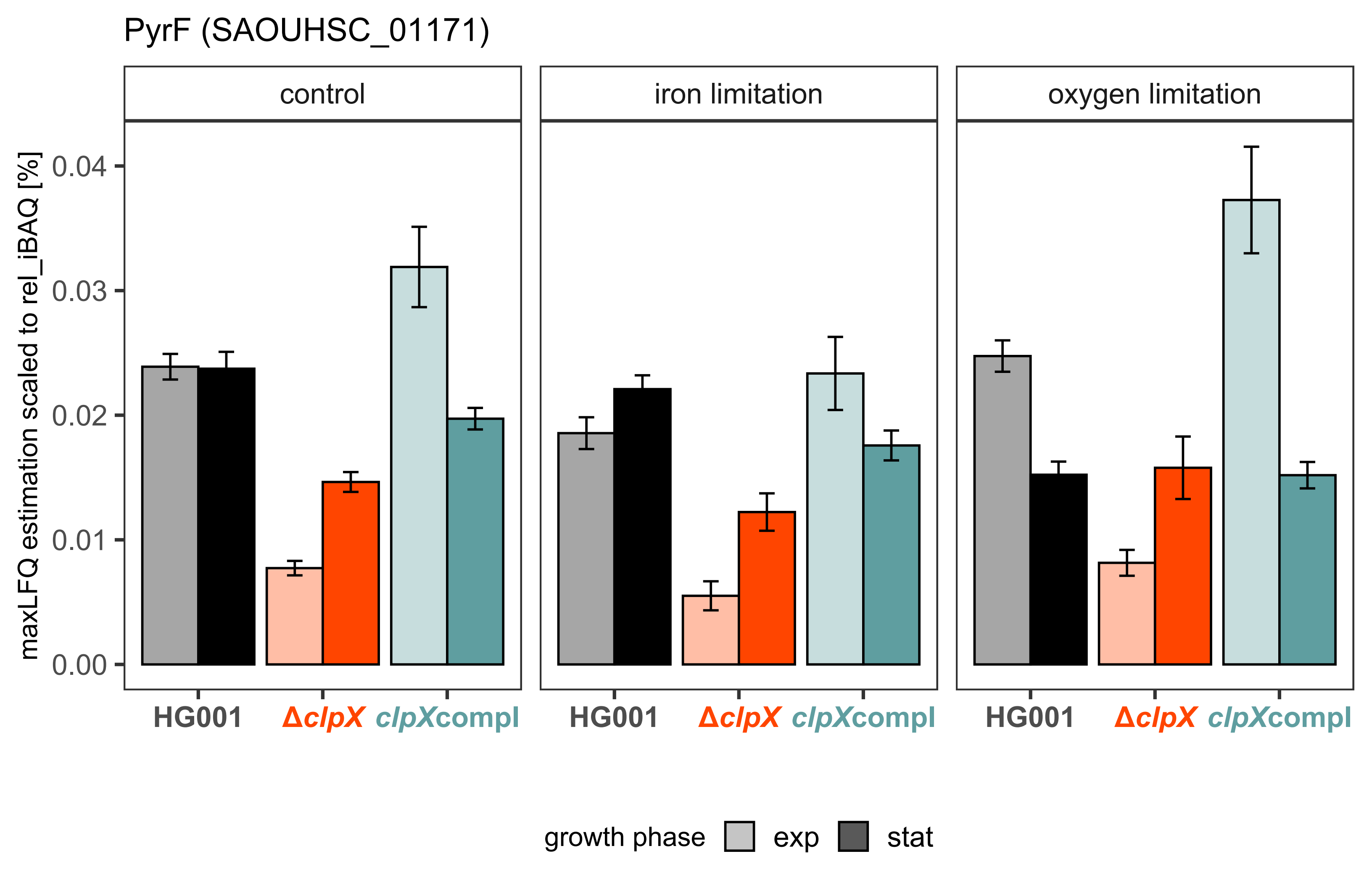
PyrF (SAOUHSC_01171)
control
iron limitation
oxygen limitation
maxLFQ estimation scaled to rel_iBAQ [%]
0.04
0.03
0.02
0.01
0.00
HG001
ΔclpX
clpXcompl
growth phase
exp
stat

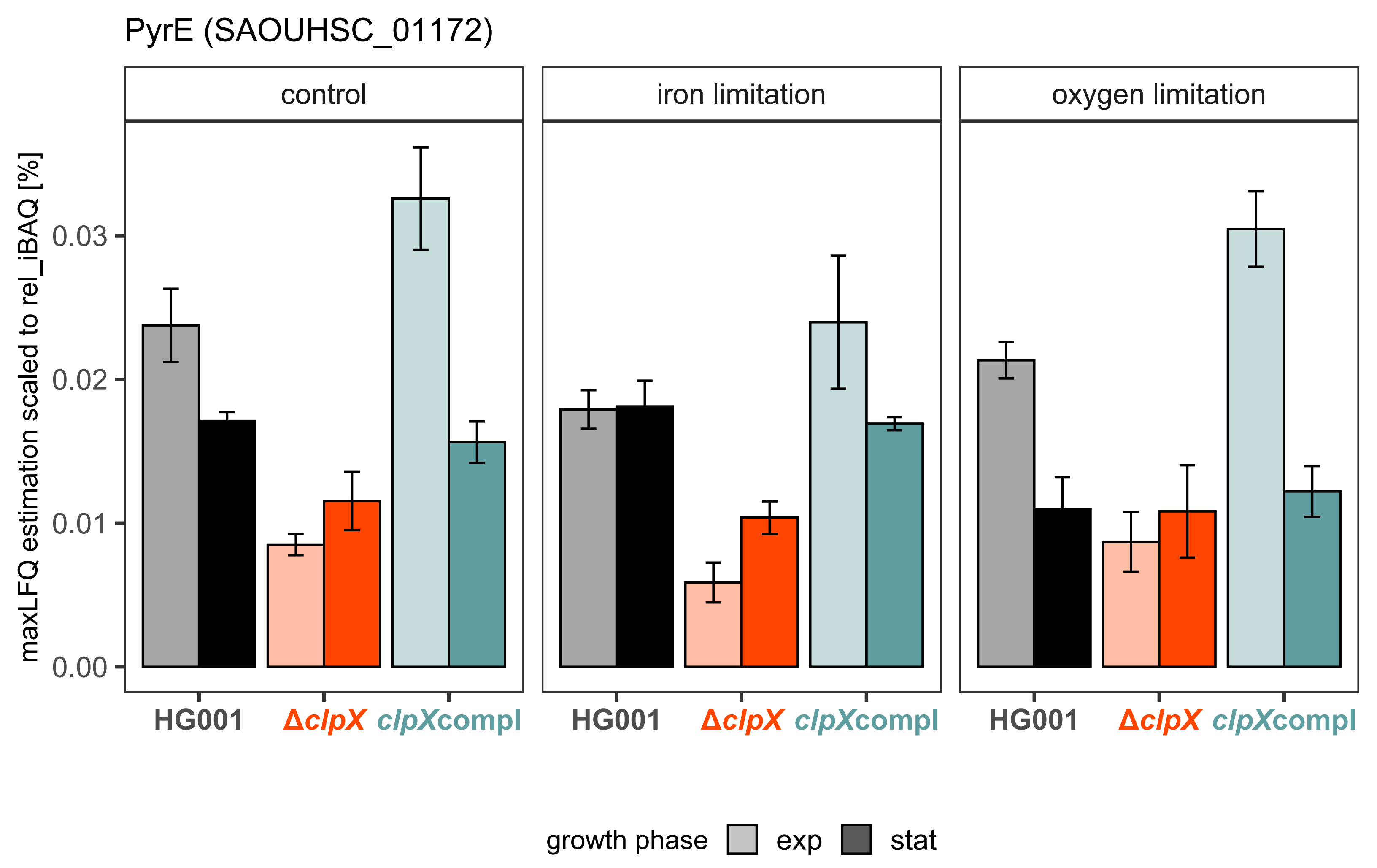

PyrE (SAOUHSC_01172)
control
iron limitation
oxygen limitation
maxLFQ estimation scaled to rel_iBAQ [%]
0.00
0.01
0.02
0.03
HG001
ΔclpX
clpXcompl
growth phase
exp
stat

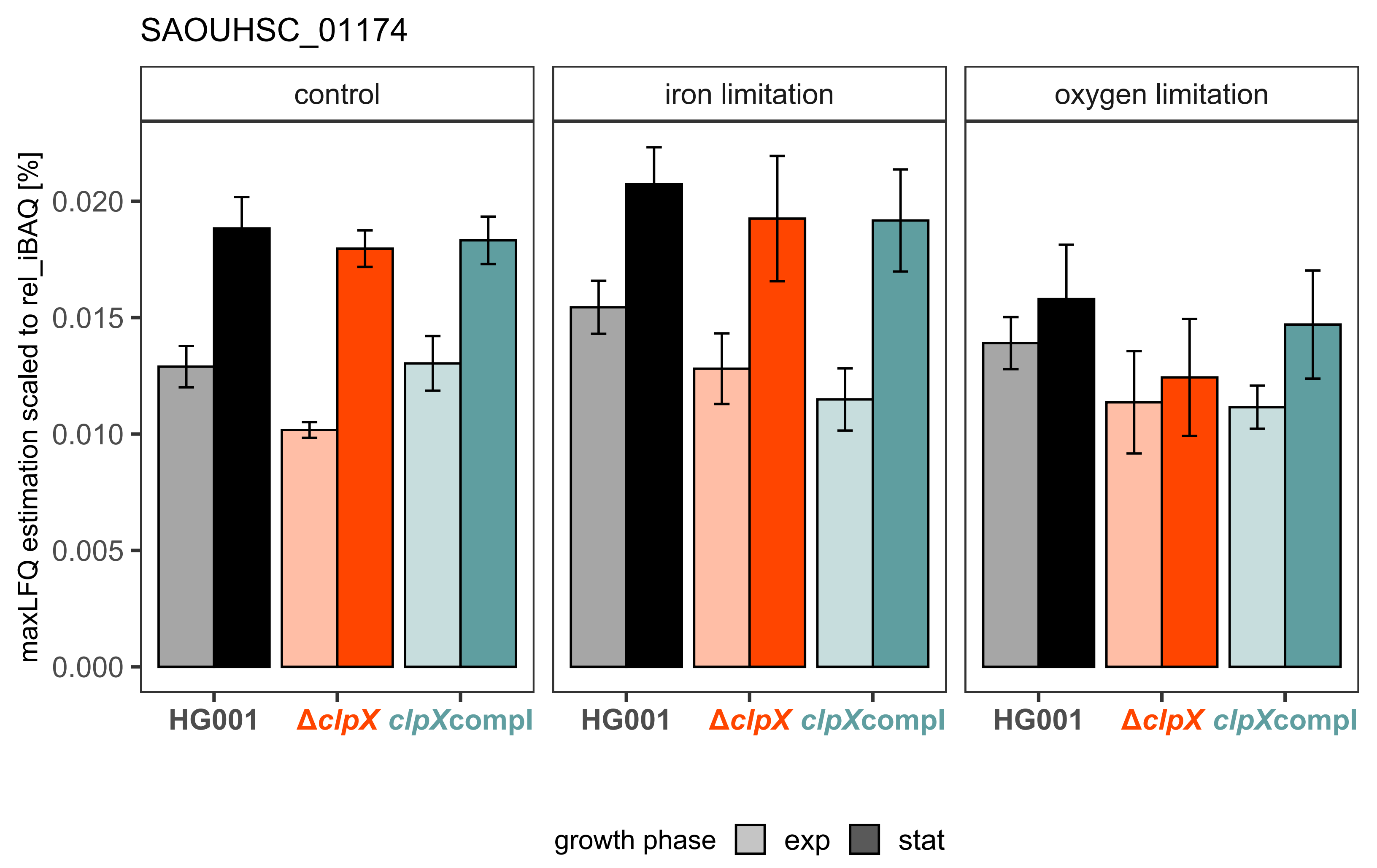

SAOUHSC_01174
control
iron limitation
oxygen limitation
maxLFQ estimation scaled to rel_iBAQ [%]
0.020
0.015
0.010
0.005
0.000
HG001
ΔclpX
clpXcompl
growth phase
exp
stat

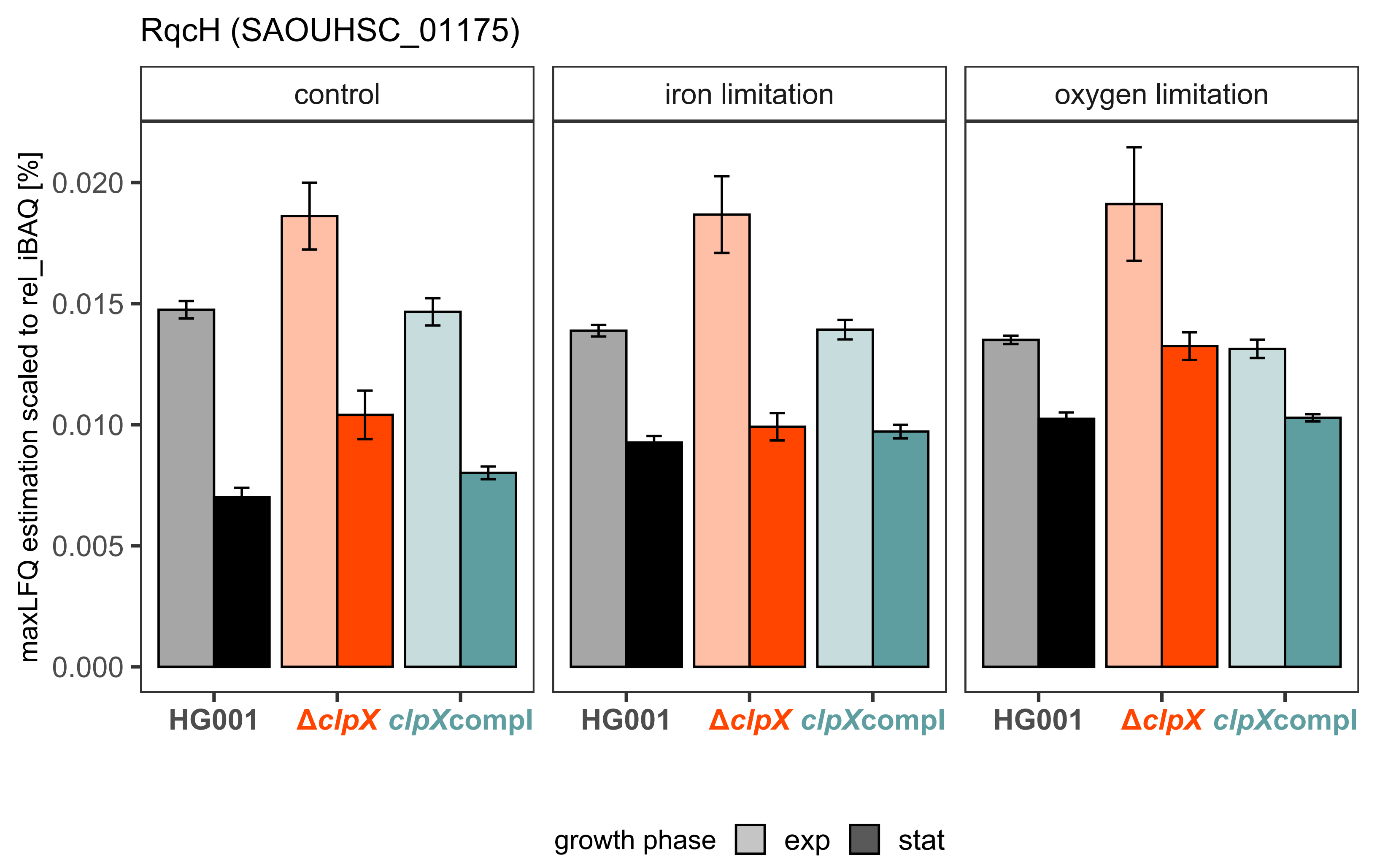

RqcH (SAOUHSC_01175)
control
iron limitation
oxygen limitation
maxLFQ estimation scaled to rel_iBAQ [%]
0.000
0.005
0.010
0.015
0.020
HG001
ΔclpX
clpXcompl
growth phase
exp
stat

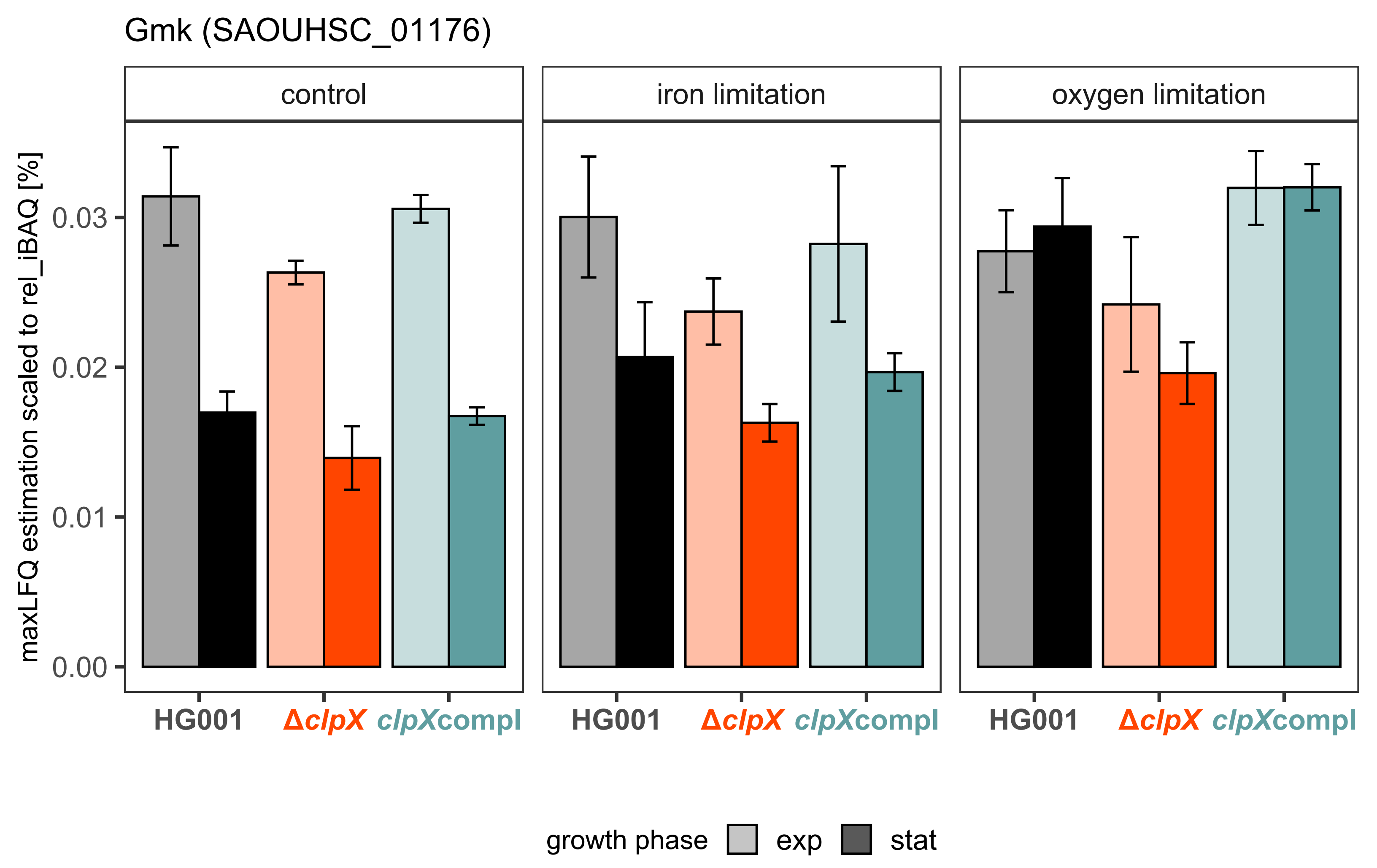

Gmk (SAOUHSC_01176)
control
iron limitation
oxygen limitation
maxLFQ estimation scaled to rel_iBAQ [%]
0.00
0.01
0.02
0.03
HG001
ΔclpX
clpXcompl
growth phase
exp
stat

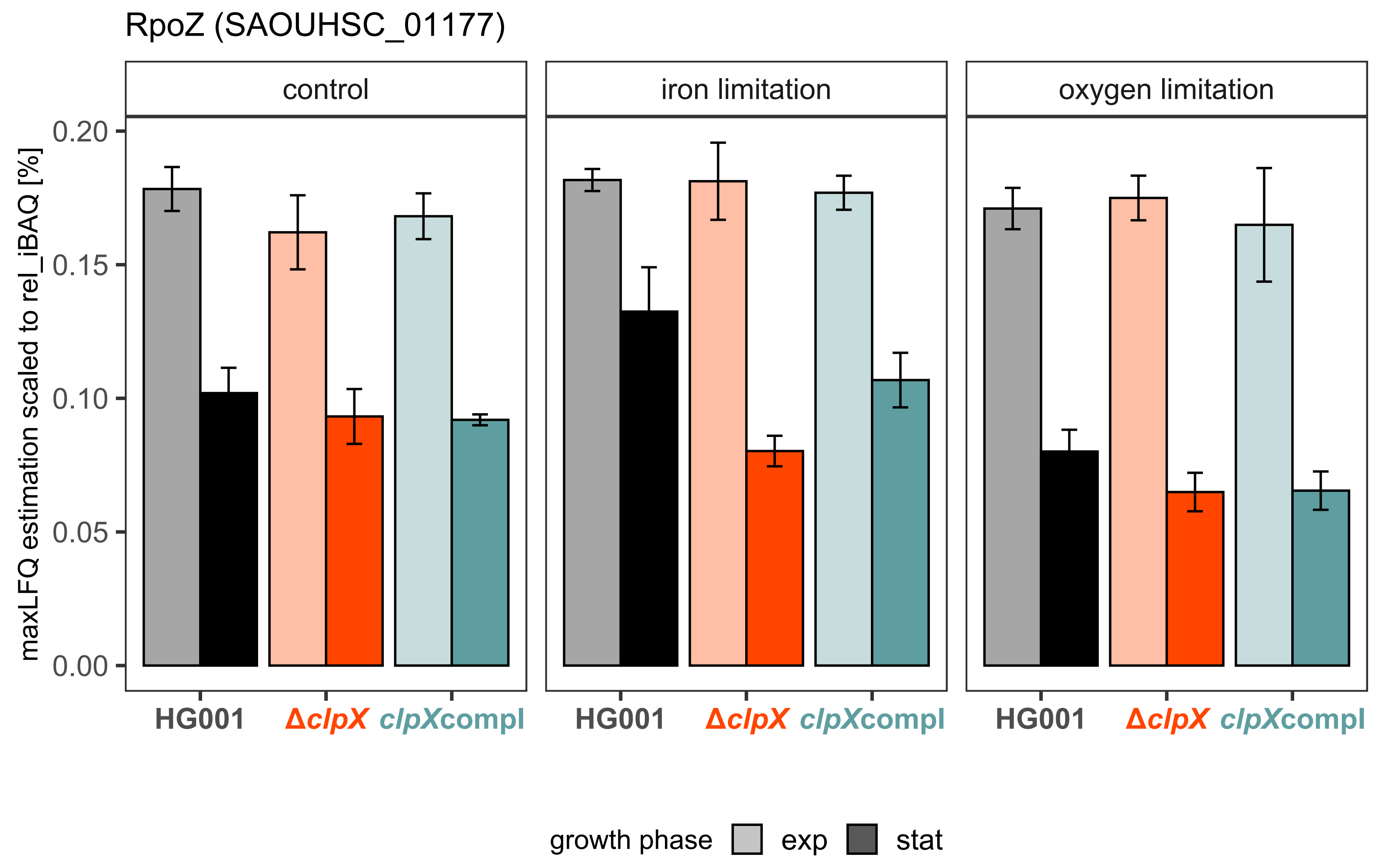
RpoZ (SAOUHSC_01177)
control
iron limitation
oxygen limitation
maxLFQ estimation scaled to rel_iBAQ [%]
0.20
0.15
0.10
0.05
0.00
HG001
ΔclpX
clpXcompl
growth phase
exp
stat

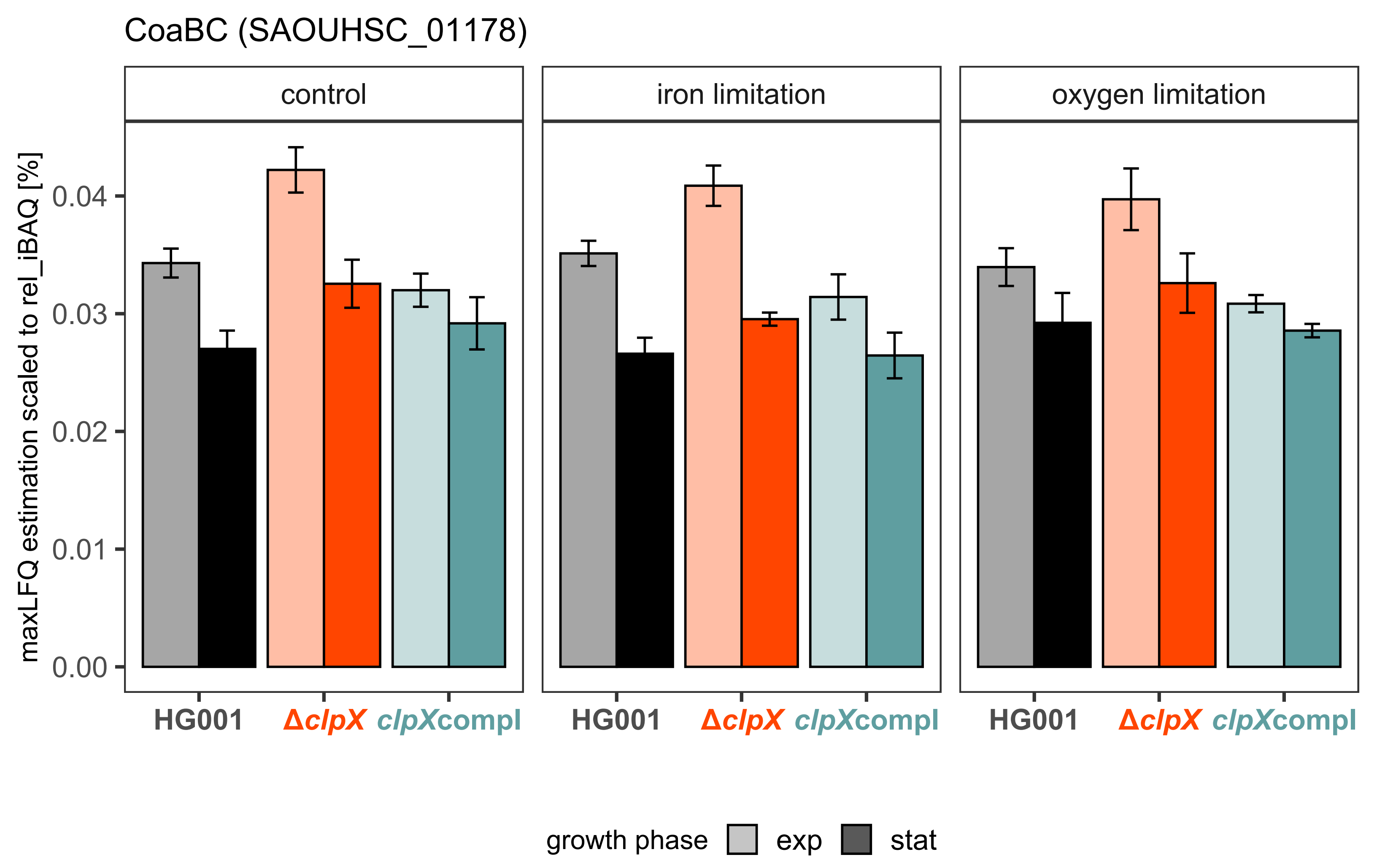

CoaBC (SAOUHSC_01178)
control
iron limitation
oxygen limitation
maxLFQ estimation scaled to rel_iBAQ [%]
0.00
0.01
0.02
0.03
0.04
HG001
ΔclpX
clpXcompl
growth phase
exp
stat

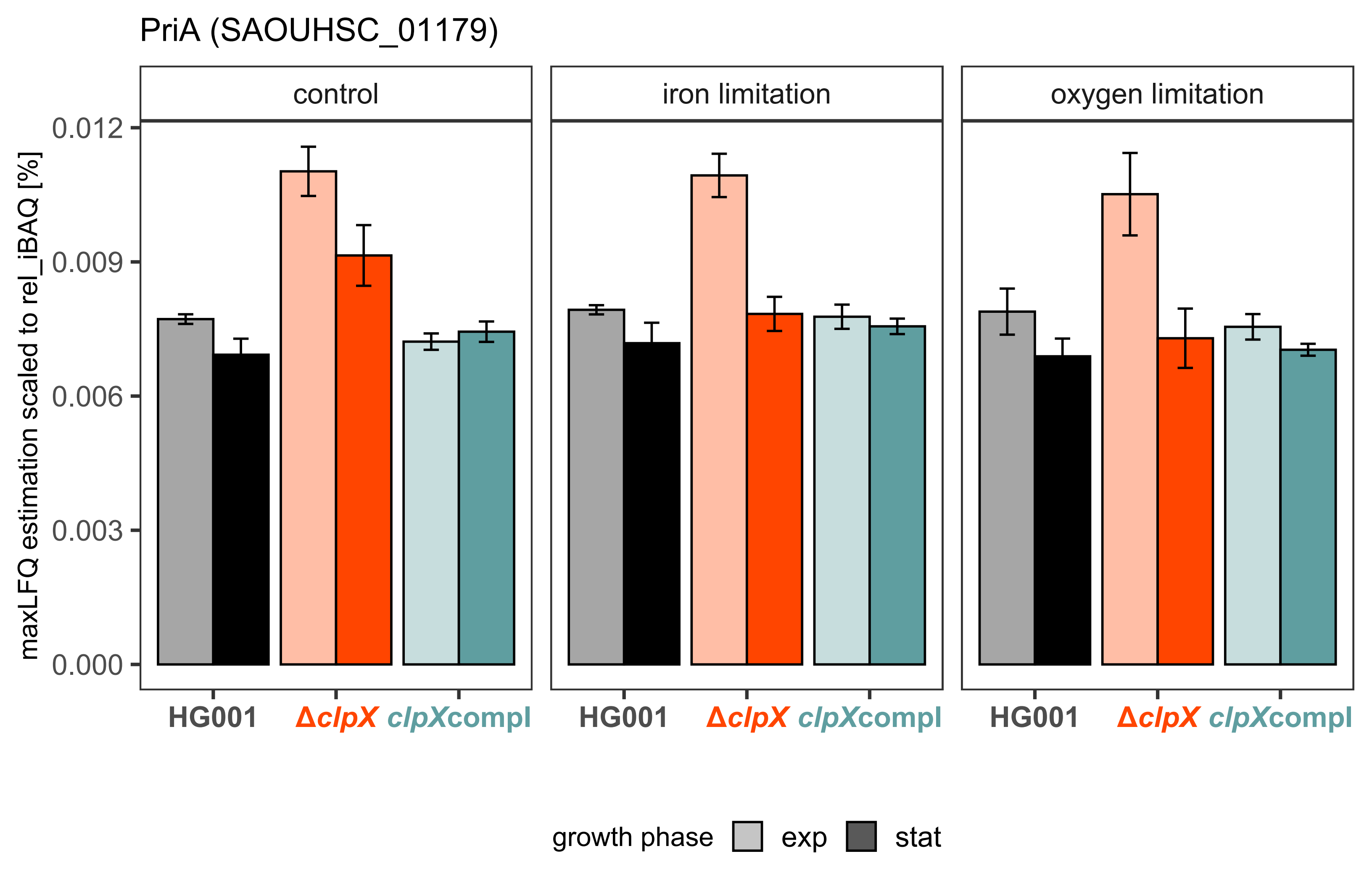

PriA (SAOUHSC_01179)
control
iron limitation
oxygen limitation
0.012
0.009
0.006
0.003
0.000
maxLFQ estimation scaled to rel_iBAQ [%]
HG001
ΔclpX
clpXcompl
growth phase
exp
stat

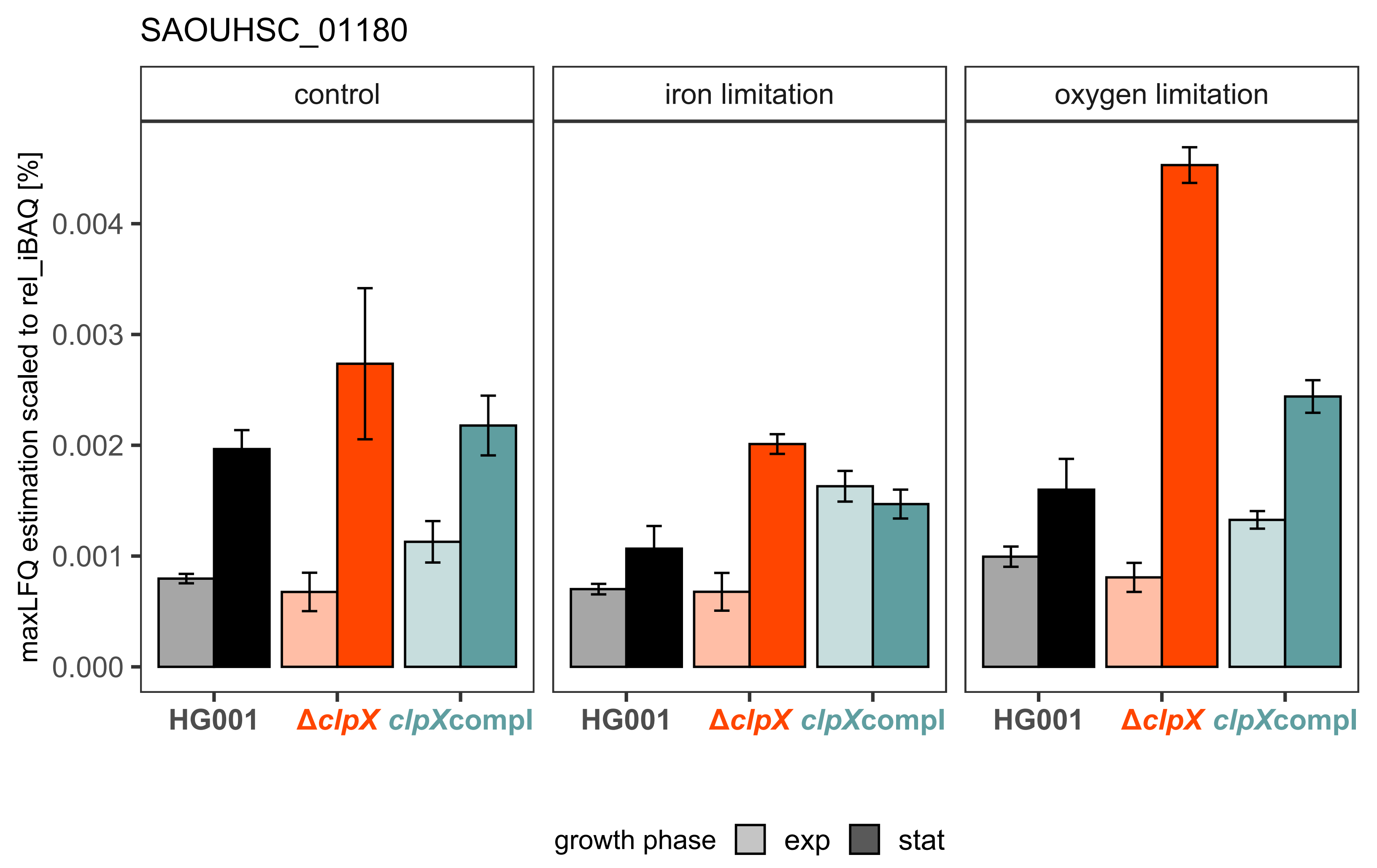

SAOUHSC_01180
control
iron limitation
oxygen limitation
maxLFQ estimation scaled to rel_iBAQ [%]
0.000
0.001
0.002
0.003
0.004
HG001
ΔclpX
clpXcompl
growth phase
exp
stat

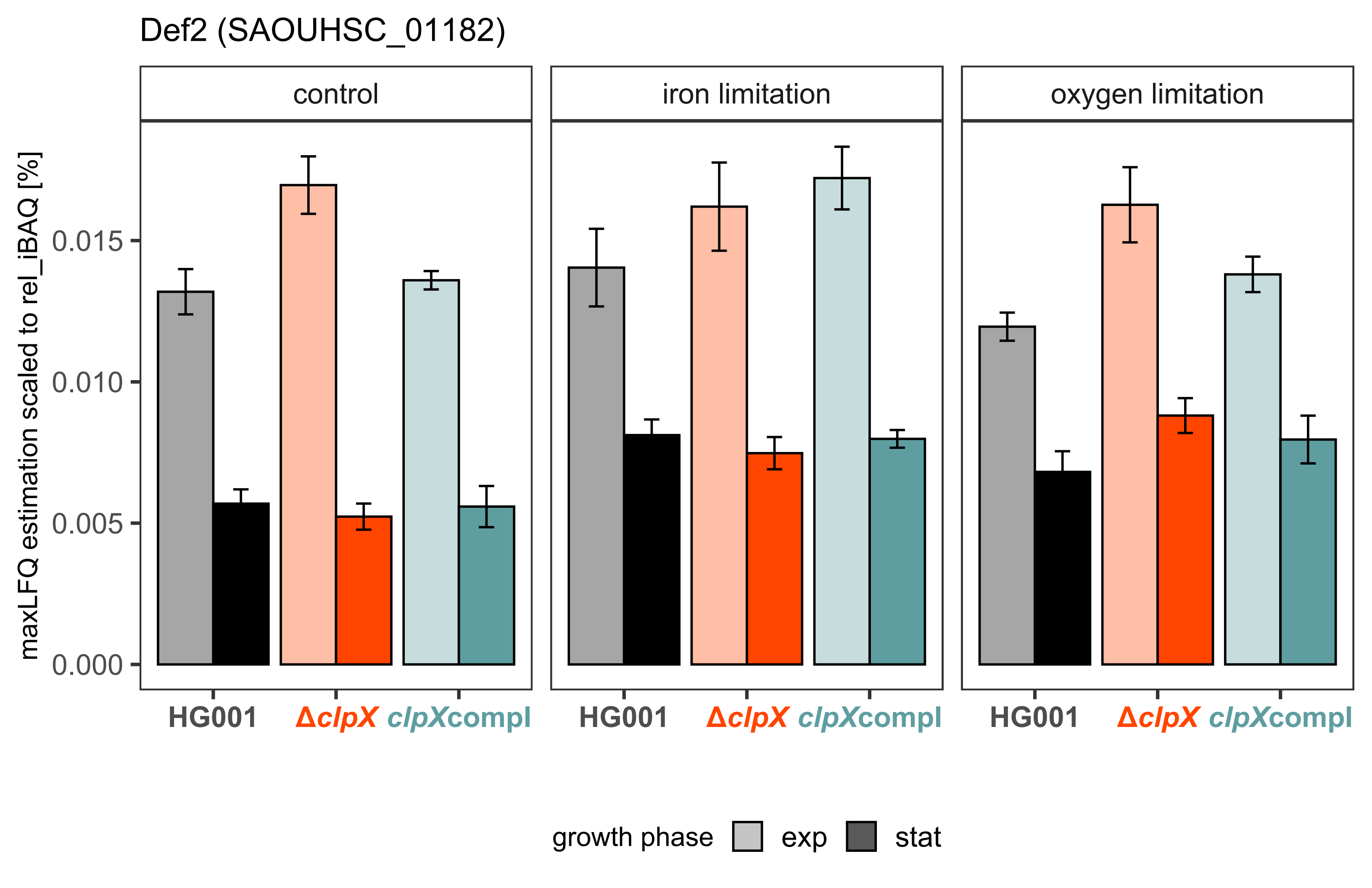

Def2 (SAOUHSC_01182)
control
iron limitation
oxygen limitation
maxLFQ estimation scaled to rel_iBAQ [%]
0.015
0.010
0.005
0.000
HG001
ΔclpX
clpXcompl
growth phase
exp
stat

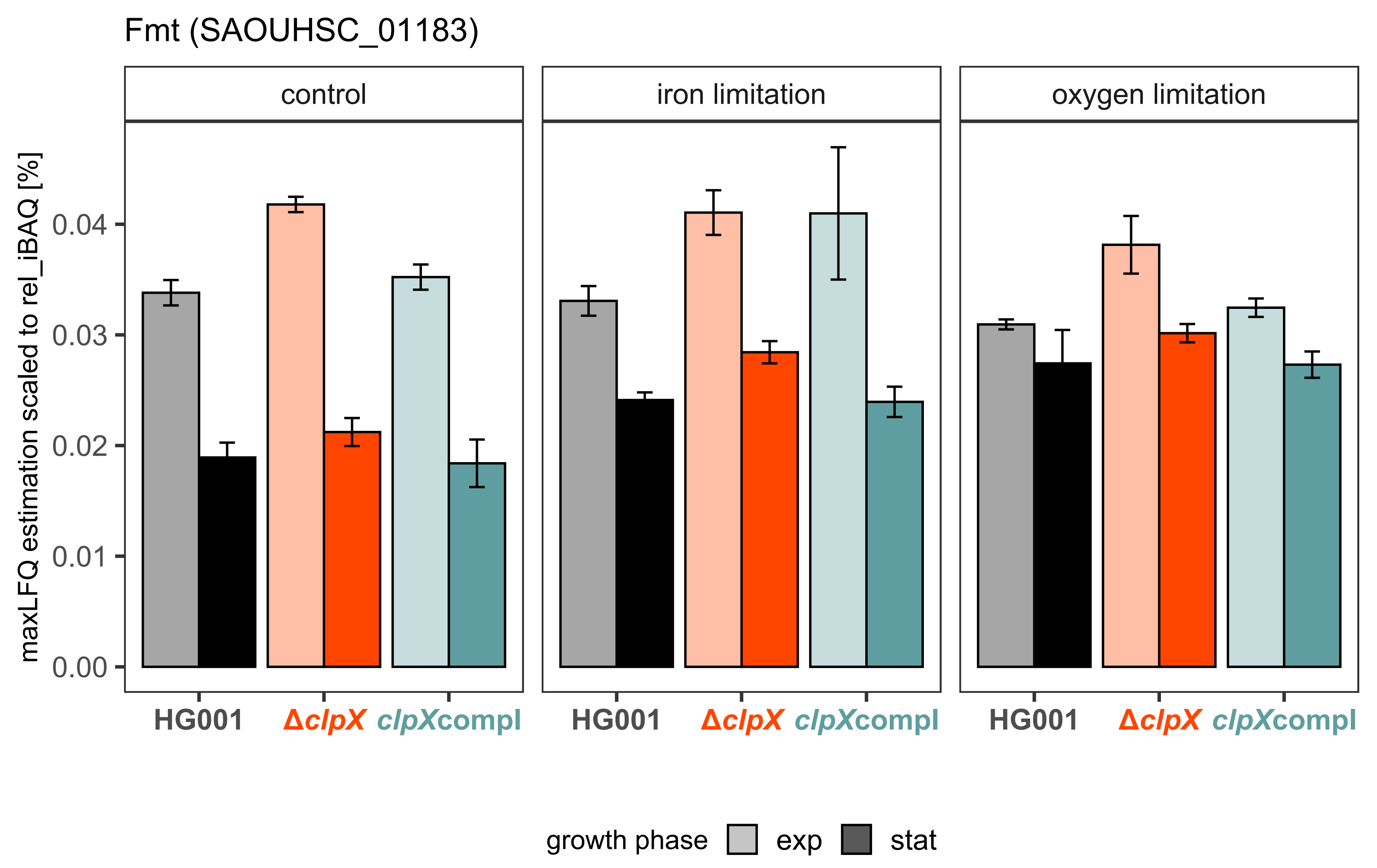

Fmt (SAOUHSC_01183)
control
iron limitation
oxygen limitation
maxLFQ estimation scaled to rel_iBAQ [%]
0.00
0.01
0.02
0.03
0.04
HG001
ΔclpX
clpXcompl
growth phase
exp
stat

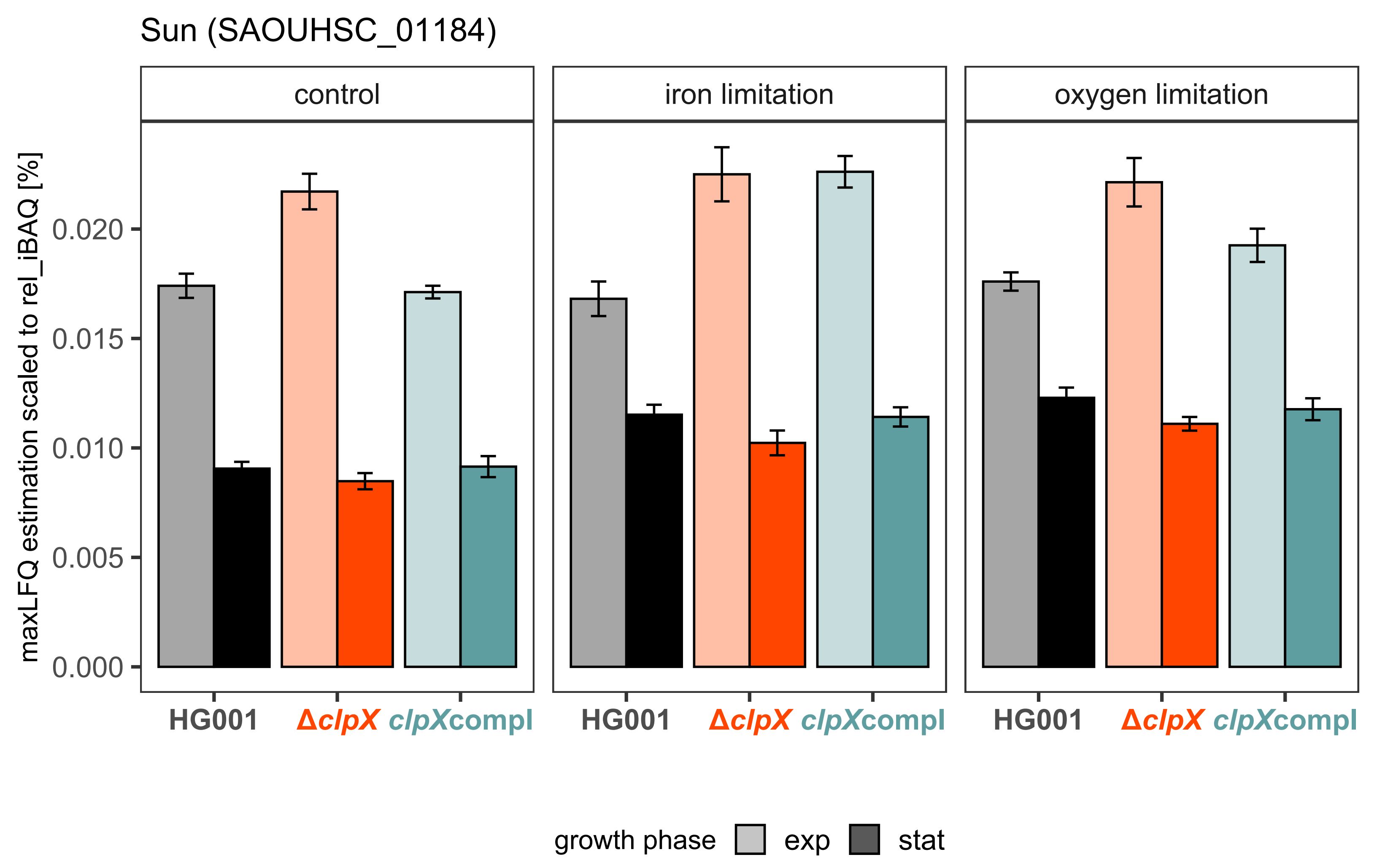

Sun (SAOUHSC_01184)
control
iron limitation
oxygen limitation
maxLFQ estimation scaled to rel_iBAQ [%]
0.000
0.005
0.010
0.015
0.020
HG001
ΔclpX
clpXcompl
growth phase
exp
stat

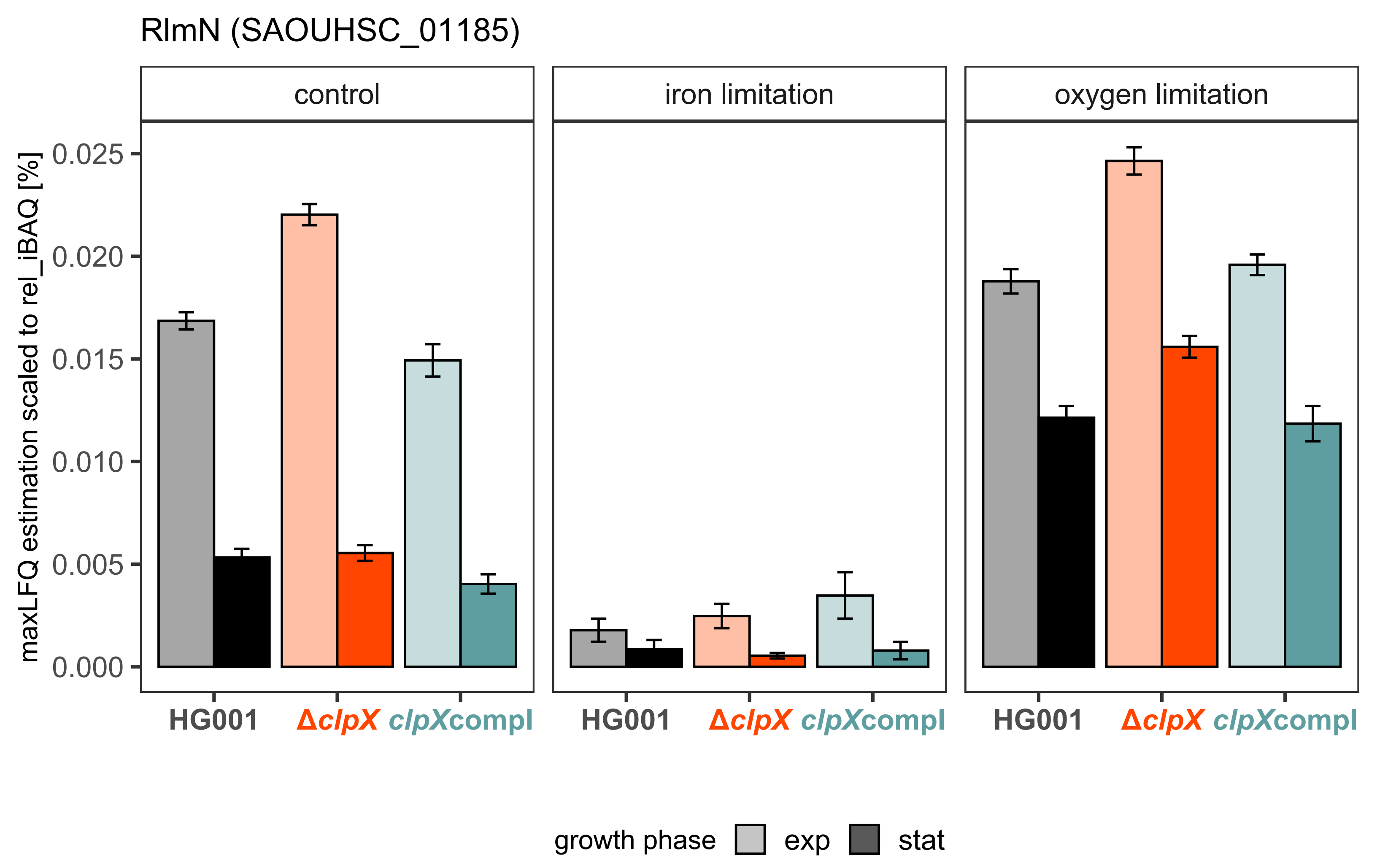

RlmN (SAOUHSC_01185)
control
iron limitation
oxygen limitation
maxLFQ estimation scaled to rel_iBAQ [%]
0.025
0.020
0.015
0.010
0.005
0.000
HG001
ΔclpX
clpXcompl
growth phase
exp
stat

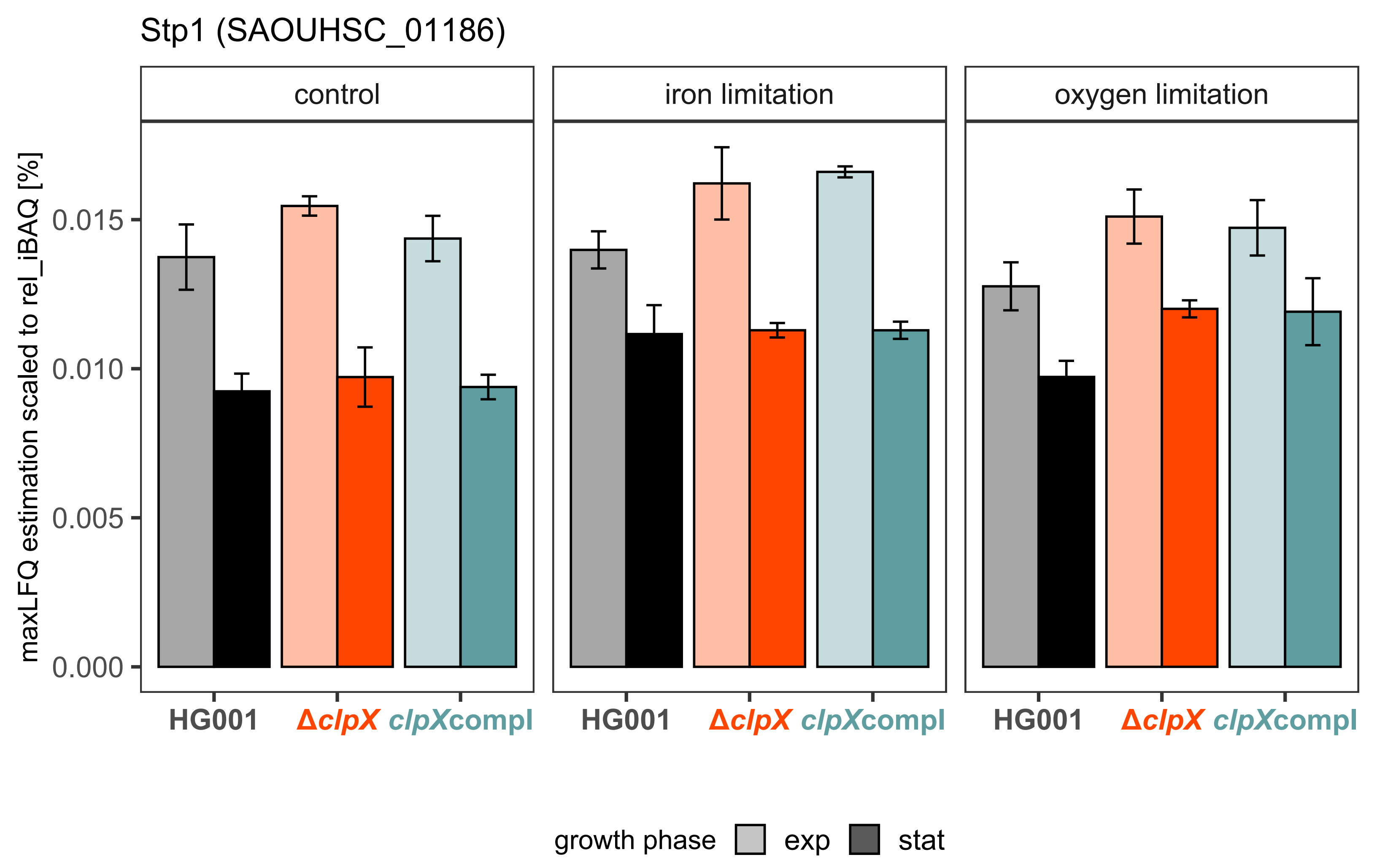

Stp1 (SAOUHSC_01186)
control
iron limitation
oxygen limitation
maxLFQ estimation scaled to rel_iBAQ [%]
0.015
0.010
0.005
0.000
HG001
ΔclpX
clpXcompl
growth phase
exp
stat

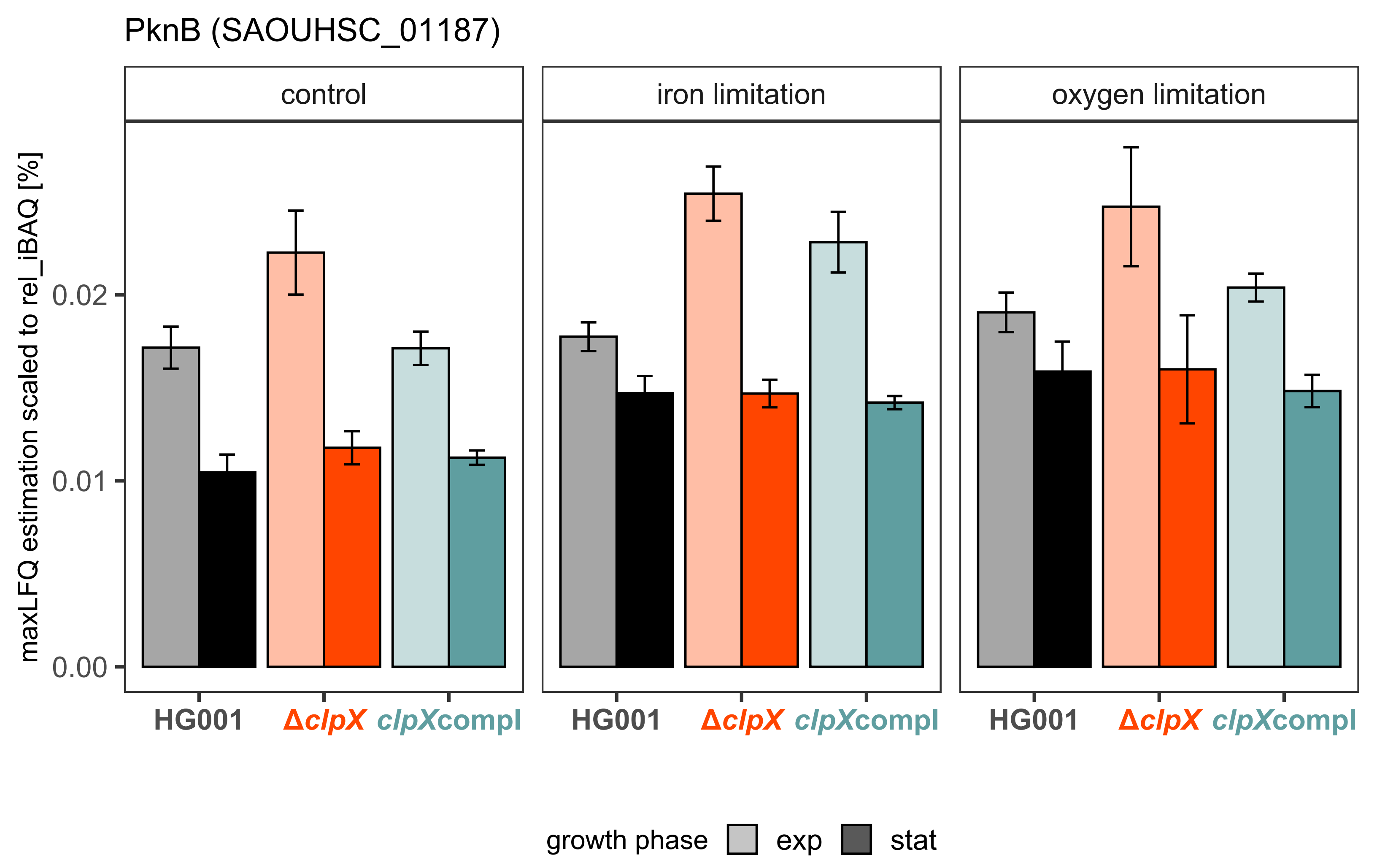

PknB (SAOUHSC_01187)
control
iron limitation
oxygen limitation
maxLFQ estimation scaled to rel_iBAQ [%]
0.00
0.01
0.02
HG001
ΔclpX
clpXcompl
growth phase
exp
stat

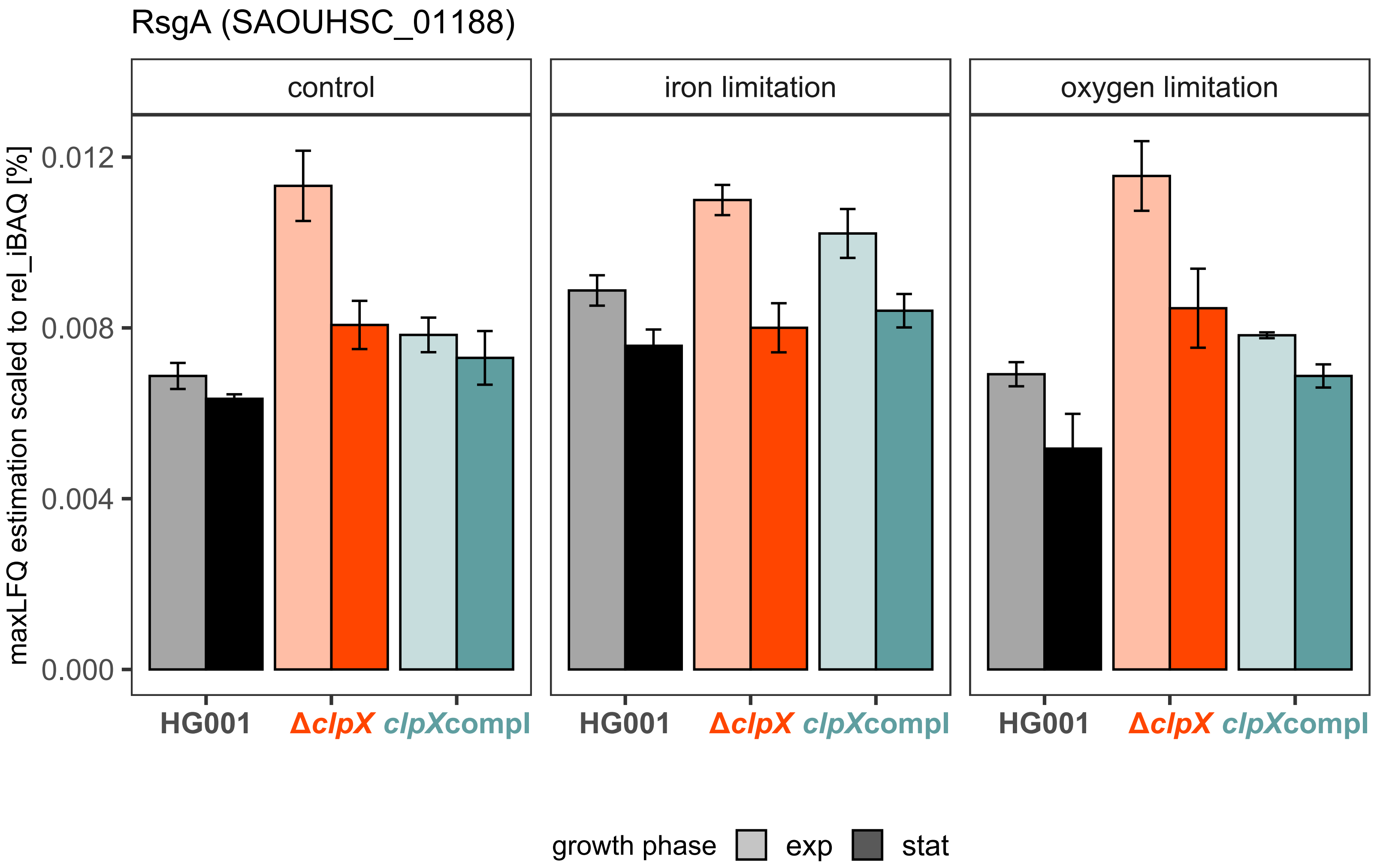
RsgA (SAOUHSC_01188)
control
iron limitation
oxygen limitation
maxLFQ estimation scaled to rel_iBAQ [%]
0.012
0.008
0.004
0.000
HG001
ΔclpX
clpXcompl
growth phase
exp
stat

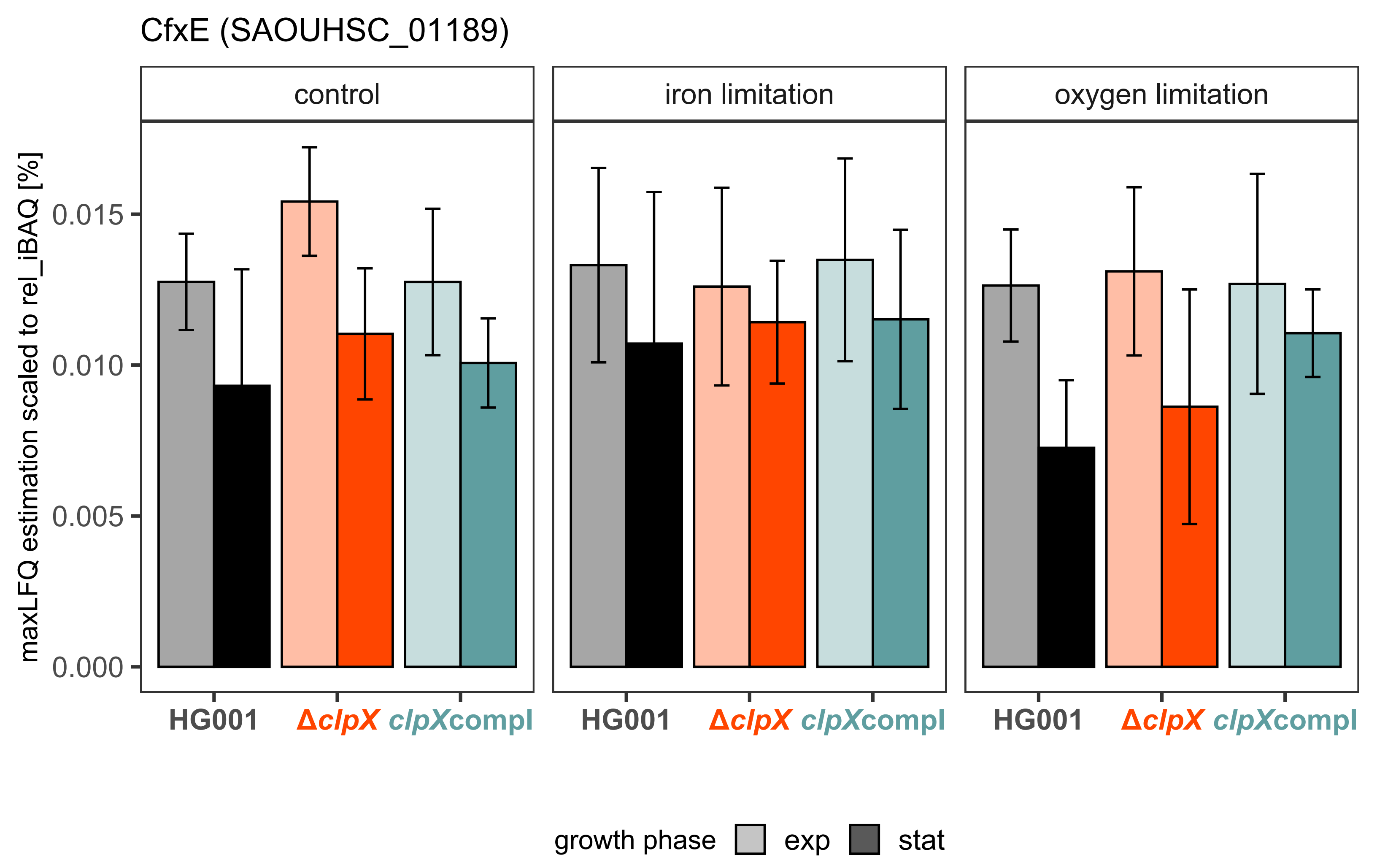
CfxE (SAOUHSC_01189)
control
iron limitation
oxygen limitation
maxLFQ estimation scaled to rel_iBAQ [%]
0.015
0.010
0.005
0.000
HG001
ΔclpX
clpXcompl
growth phase
exp
stat

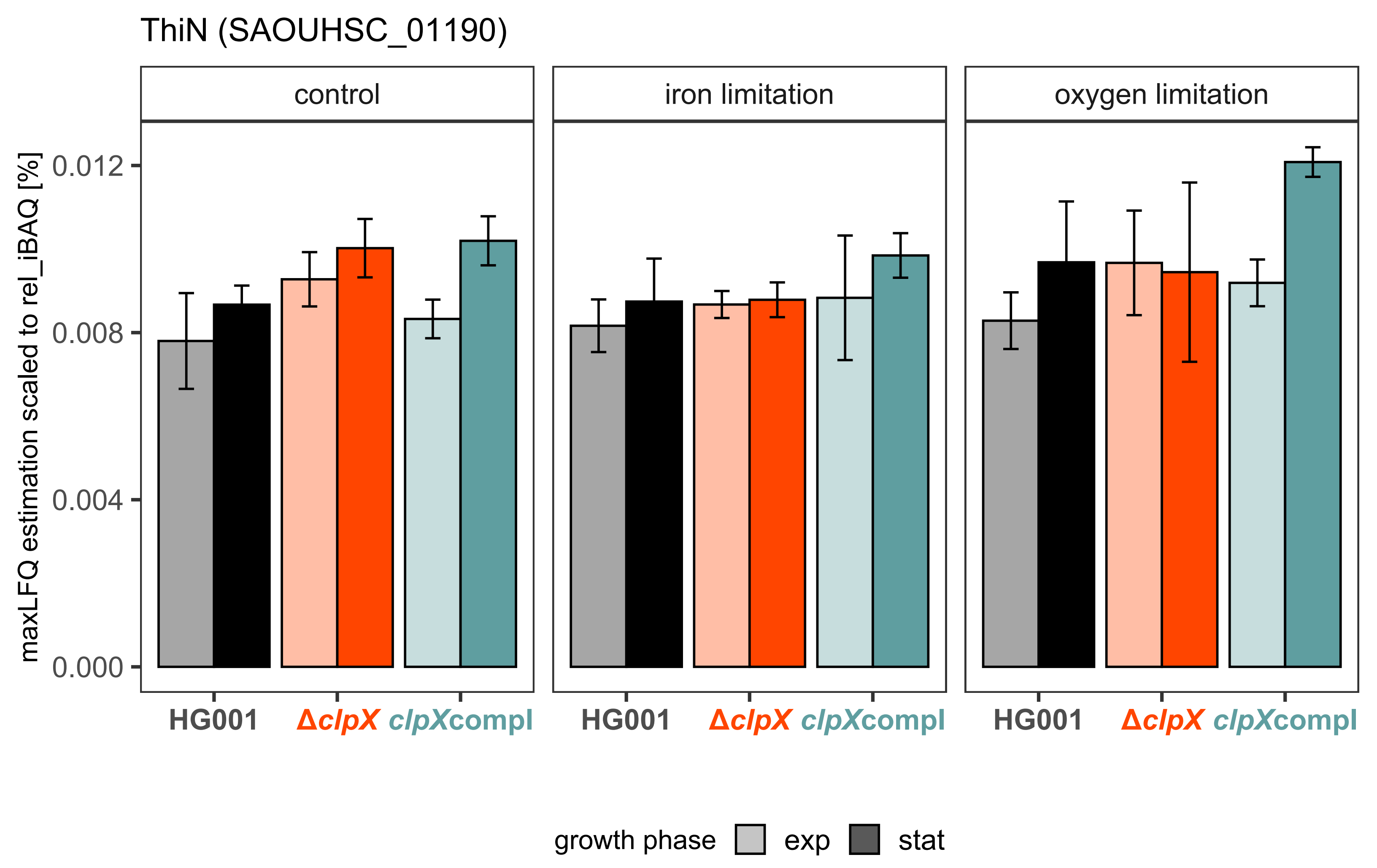
ThiN (SAOUHSC_01190)
control
iron limitation
oxygen limitation
maxLFQ estimation scaled to rel_iBAQ [%]
0.012
0.008
0.004
0.000
HG001
ΔclpX
clpXcompl
growth phase
exp
stat

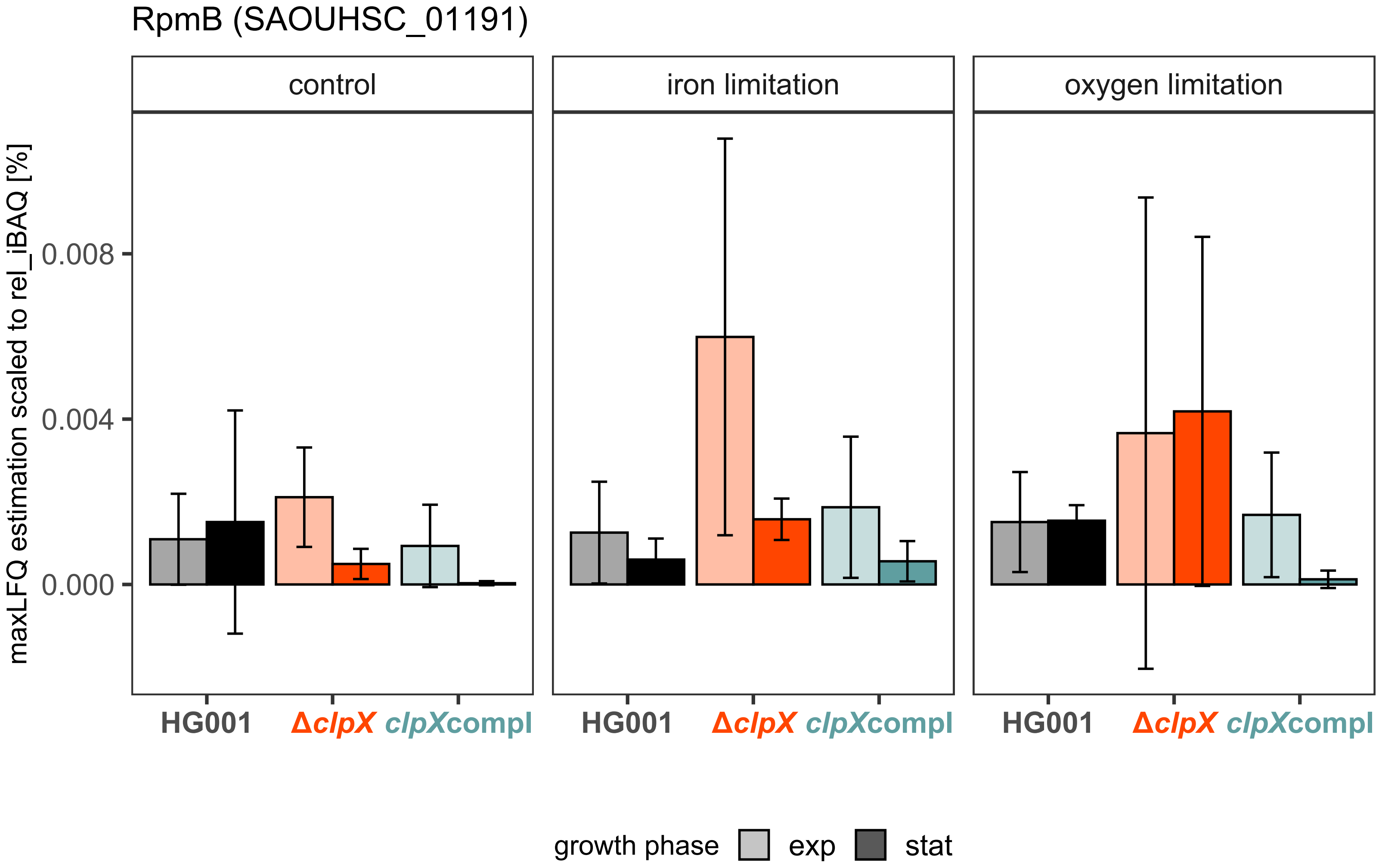
RpmB (SAOUHSC_01191)
control
iron limitation
oxygen limitation
maxLFQ estimation scaled to rel_iBAQ [%]
0.008
0.004
0.000
HG001
ΔclpX
clpXcompl
growth phase
exp
stat

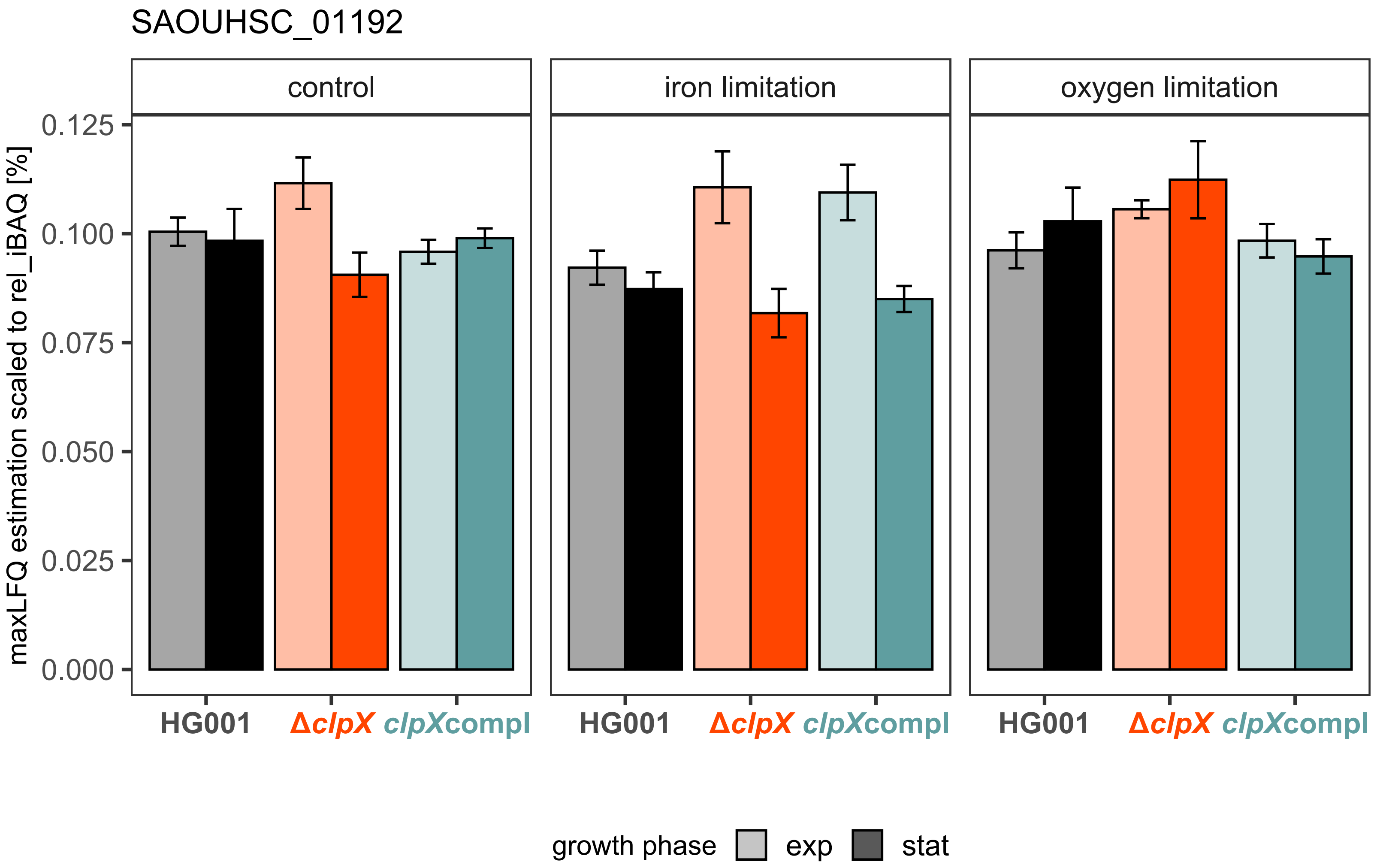

SAOUHSC_01192
control
iron limitation
oxygen limitation
maxLFQ estimation scaled to rel_iBAQ [%]
0.125
0.100
0.075
0.050
0.025
0.000
HG001
ΔclpX
clpXcompl
growth phase
exp
stat

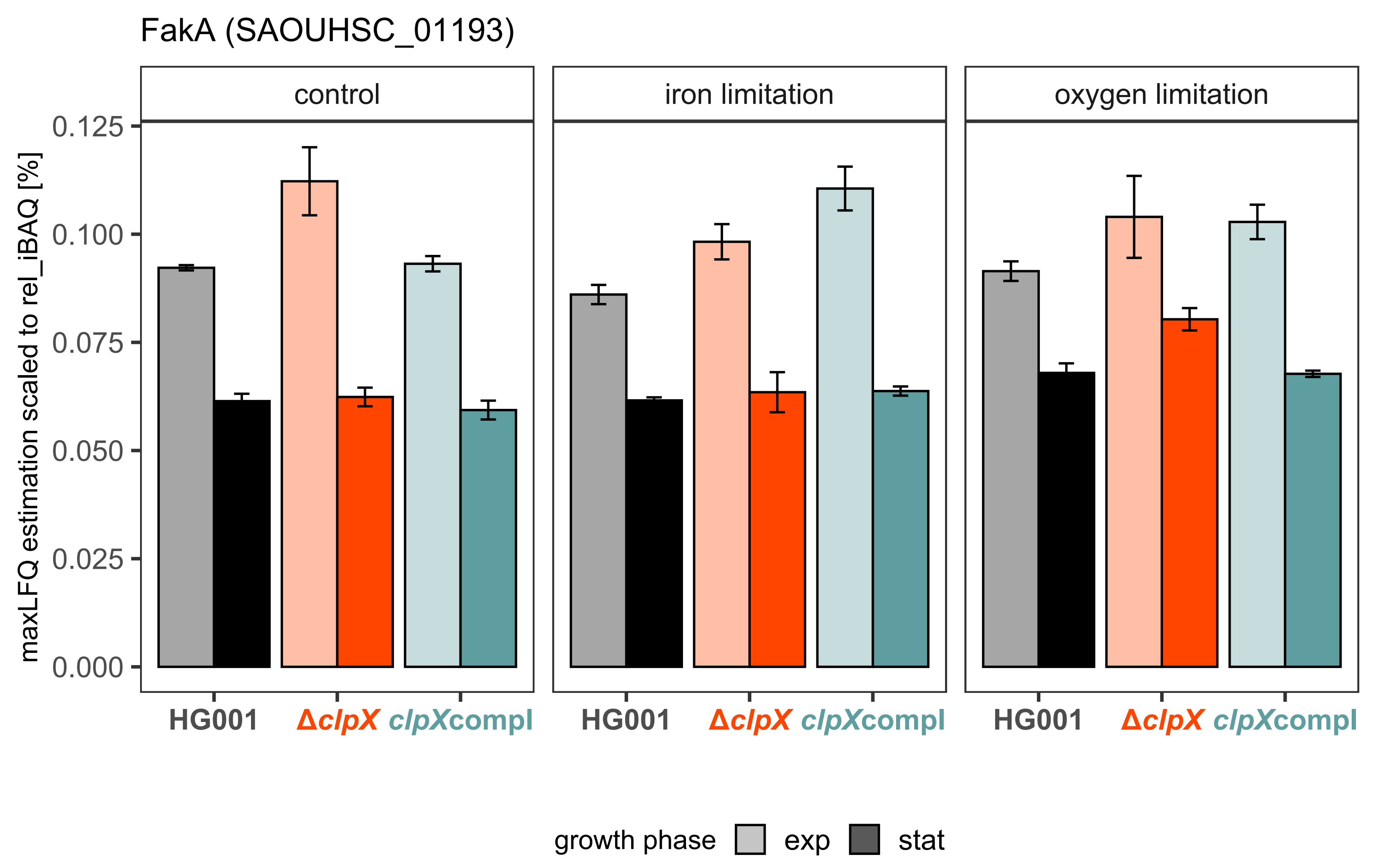
FakA (SAOUHSC_01193)
control
iron limitation
oxygen limitation
maxLFQ estimation scaled to rel_iBAQ [%]
0.125
0.100
0.075
0.050
0.025
0.000
HG001
ΔclpX
clpXcompl
growth phase
exp
stat

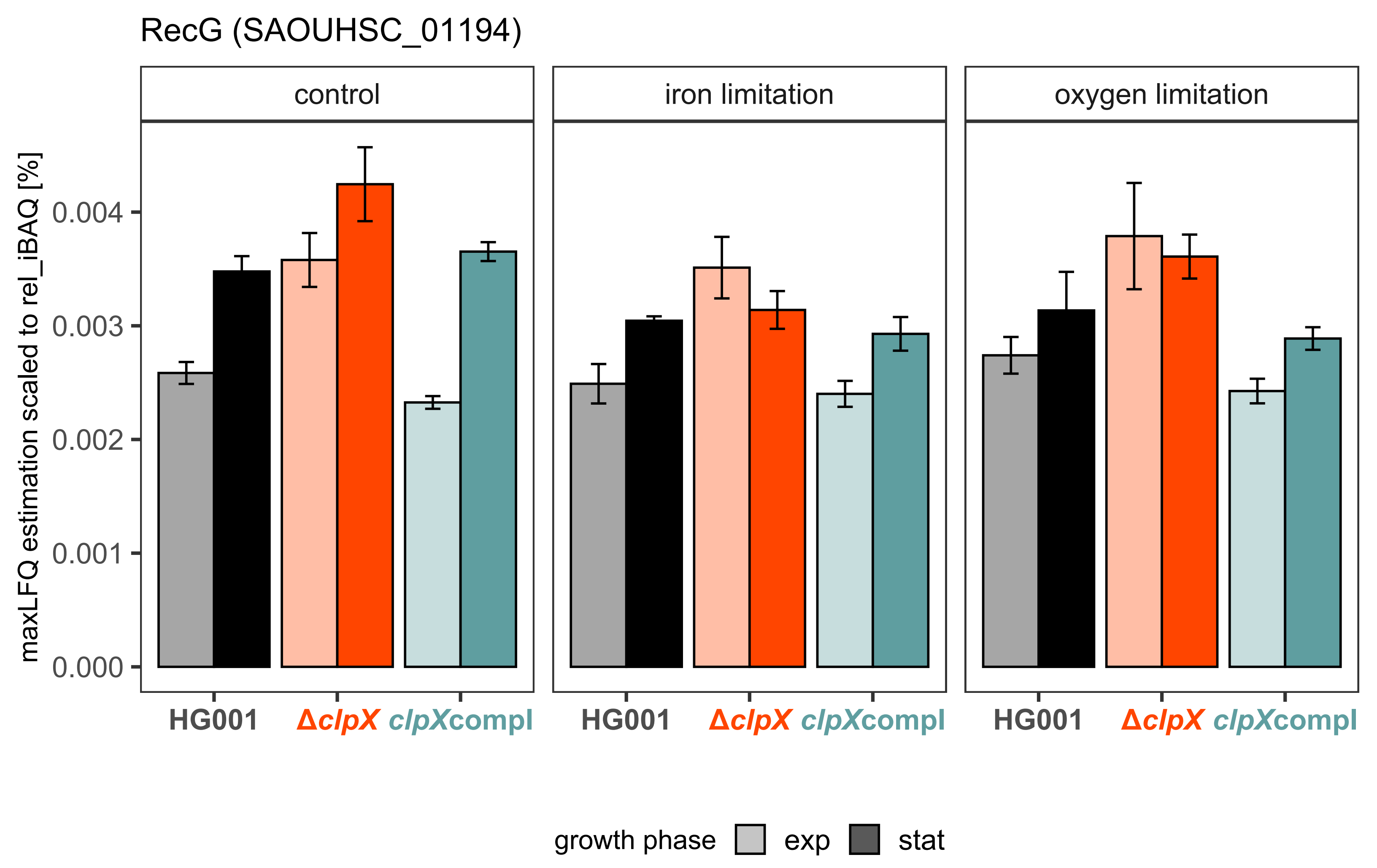
RecG (SAOUHSC_01194)
control
iron limitation
oxygen limitation
maxLFQ estimation scaled to rel_iBAQ [%]
0.004
0.003
0.002
0.001
0.000
HG001
ΔclpX
clpXcompl
growth phase
exp
stat

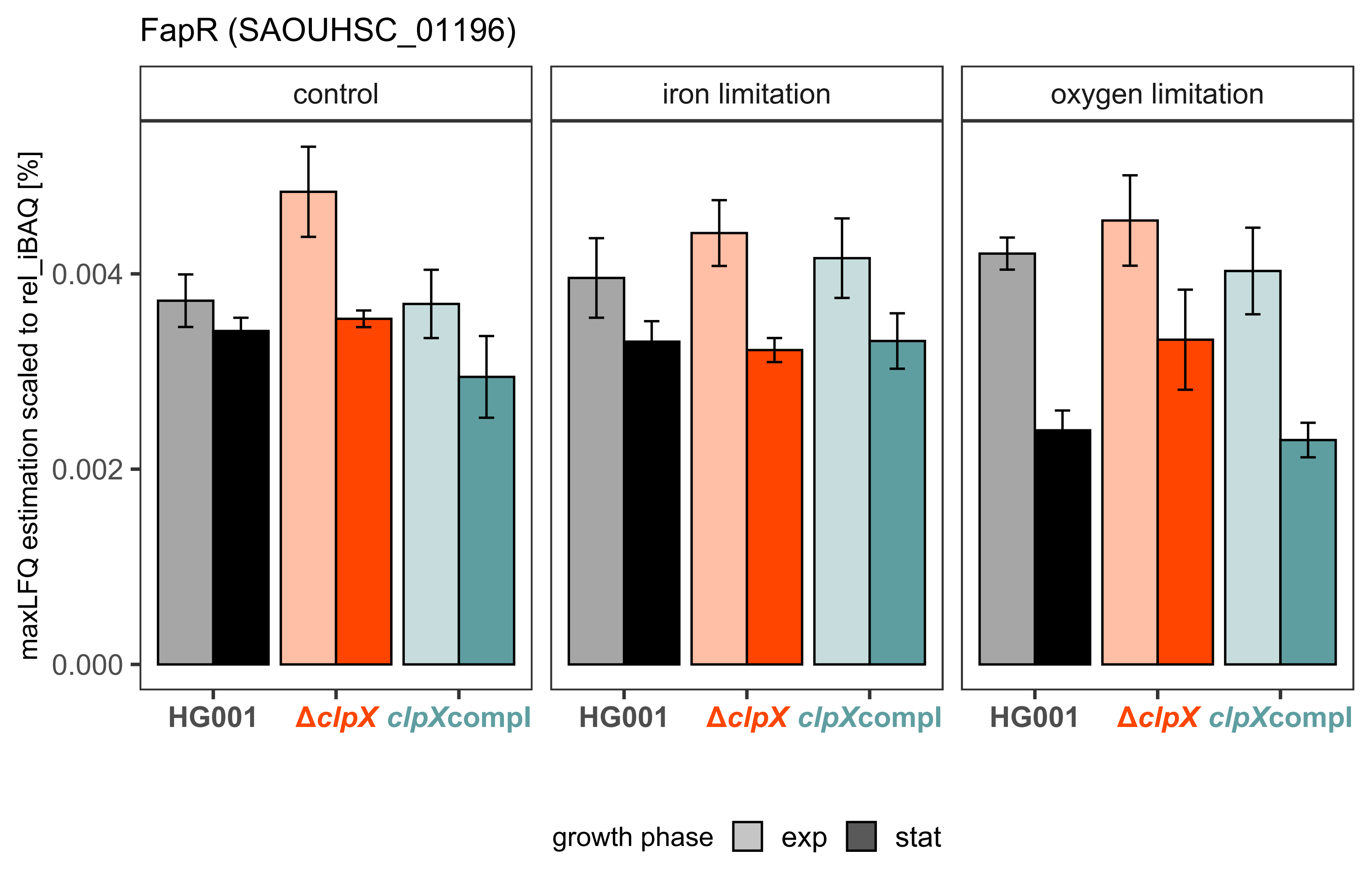

FapR (SAOUHSC_01196)
control
iron limitation
oxygen limitation
maxLFQ estimation scaled to rel_iBAQ [%]
0.000
0.002
0.004
HG001
ΔclpX
clpXcompl
growth phase
exp
stat

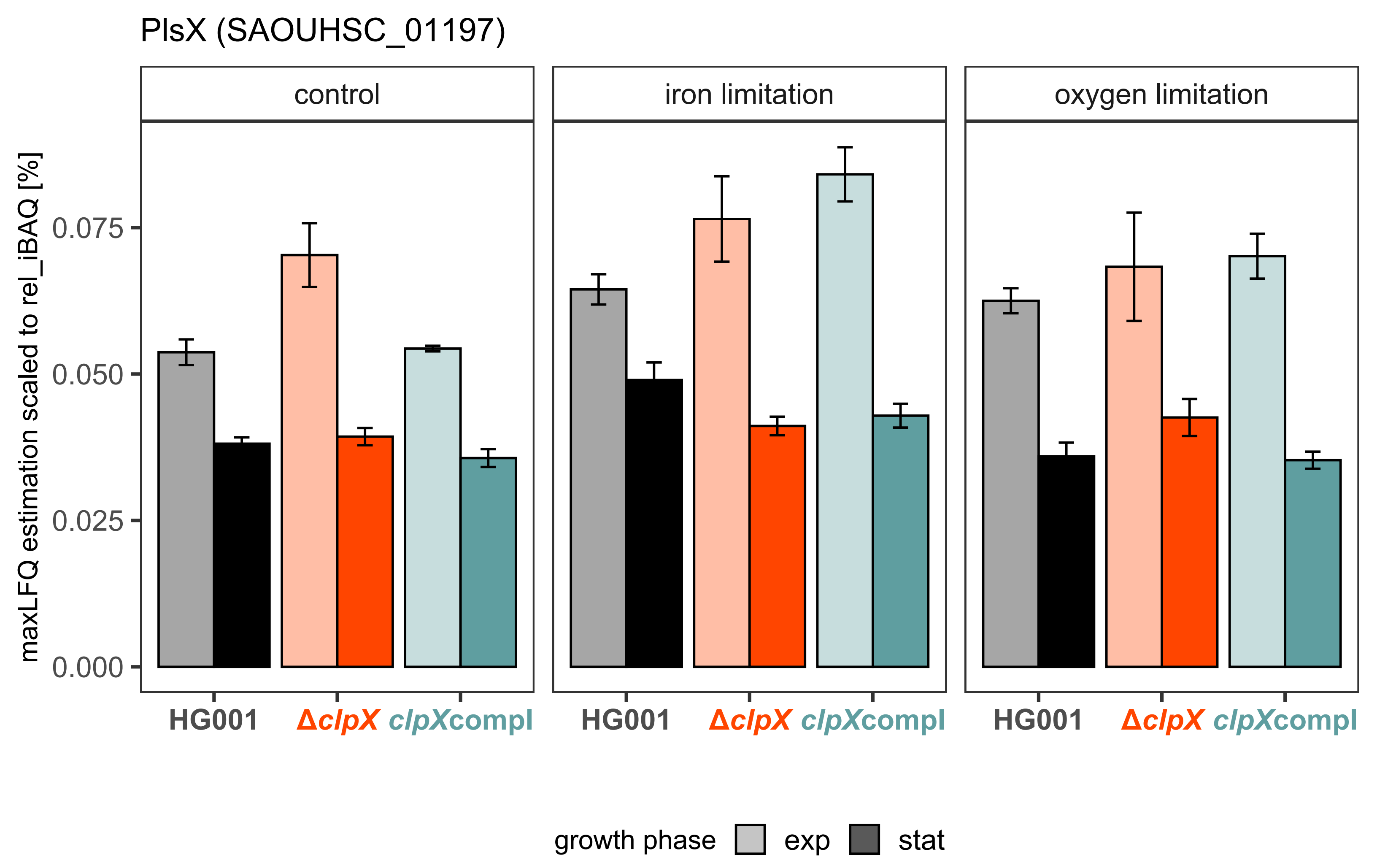
PlsX (SAOUHSC_01197)
control
iron limitation
oxygen limitation
maxLFQ estimation scaled to rel_iBAQ [%]
0.075
0.050
0.025
0.000
HG001
ΔclpX
clpXcompl
growth phase
exp
stat

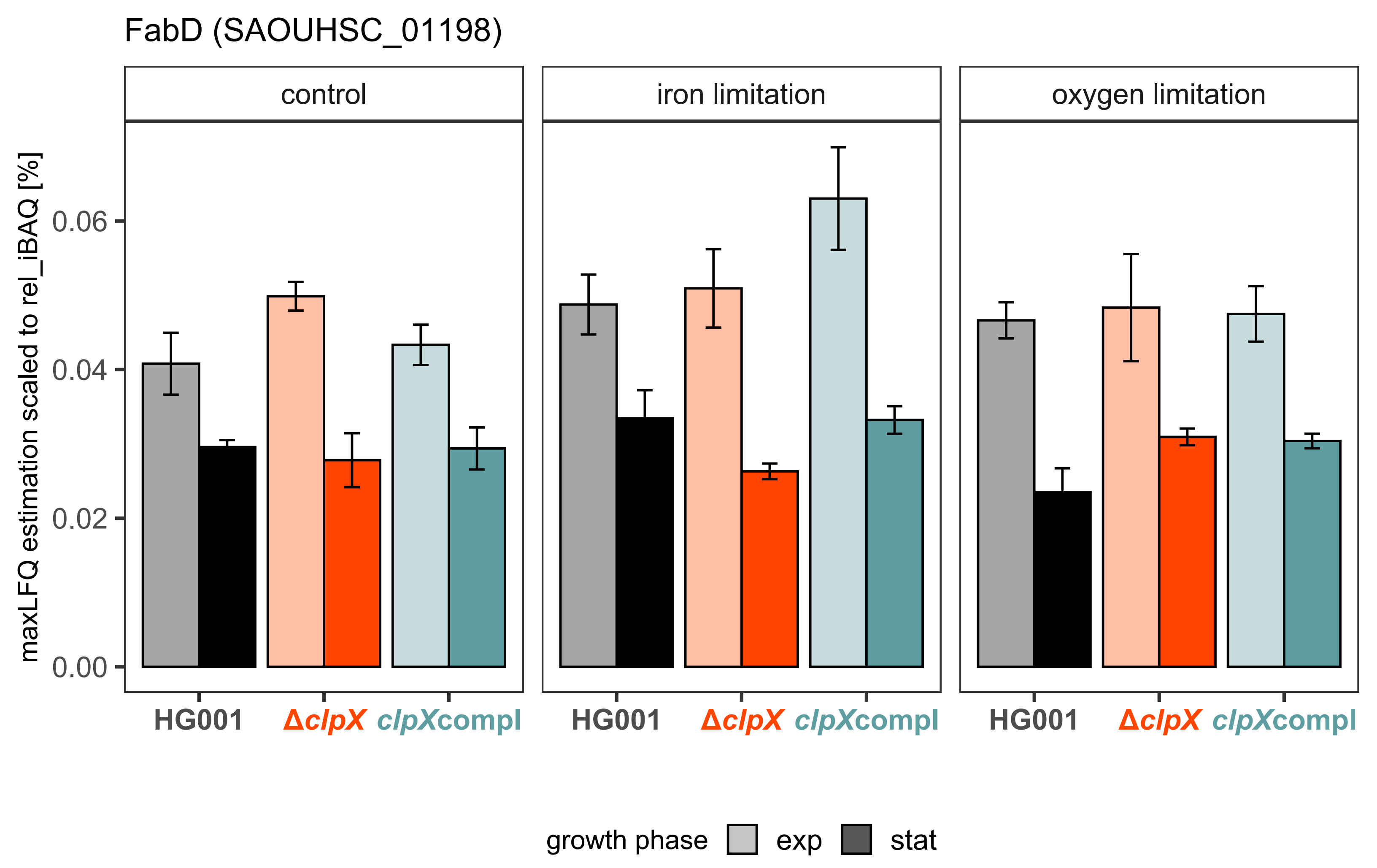
FabD (SAOUHSC_01198)
control
iron limitation
oxygen limitation
maxLFQ estimation scaled to rel_iBAQ [%]
0.00
0.02
0.04
0.06
HG001
ΔclpX
clpXcompl
growth phase
exp
stat

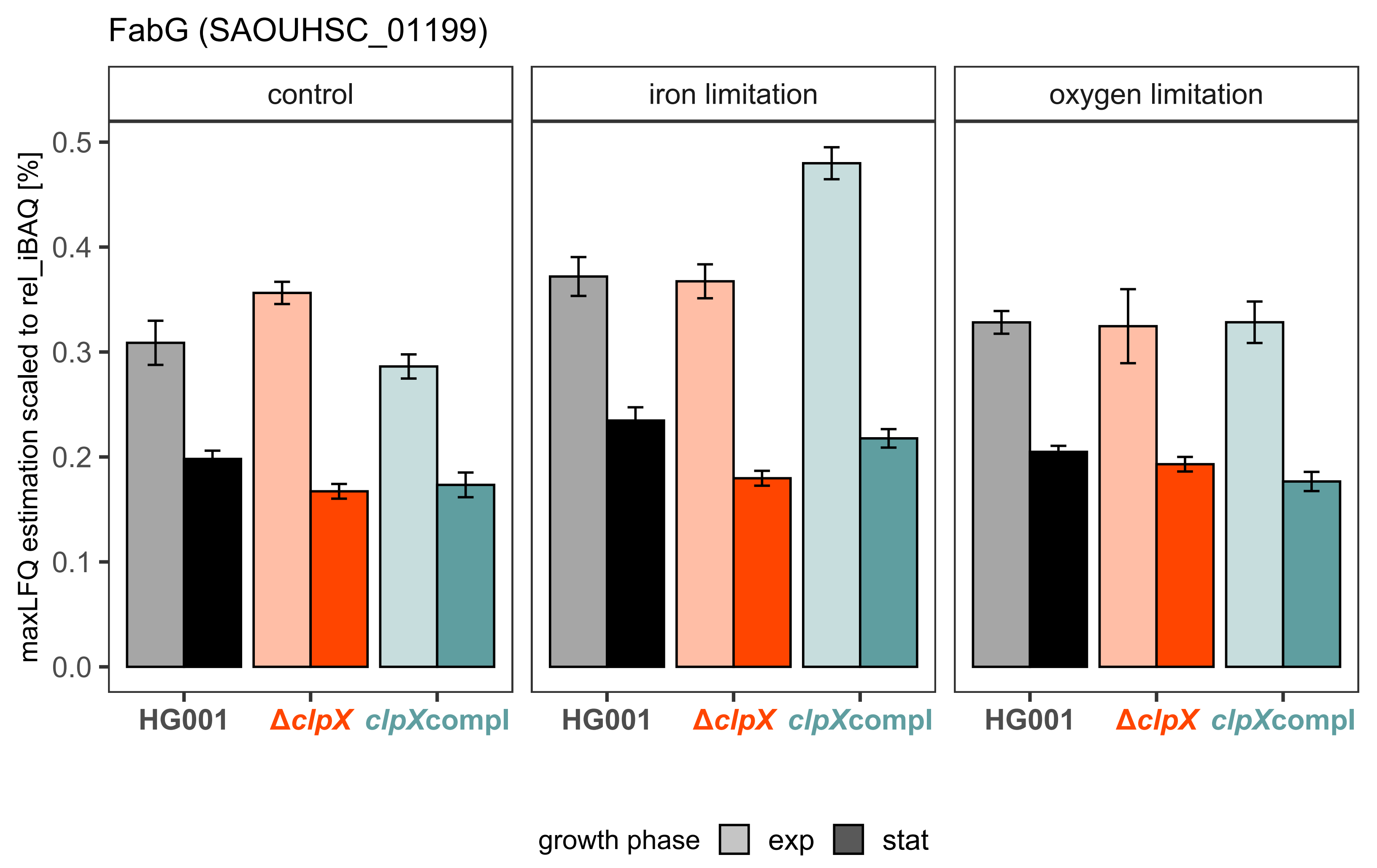
FabG (SAOUHSC_01199)
control
iron limitation
oxygen limitation
maxLFQ estimation scaled to rel_iBAQ [%]
0.0
0.1
0.2
0.3
0.4
0.5
HG001
ΔclpX
clpXcompl
growth phase
exp
stat

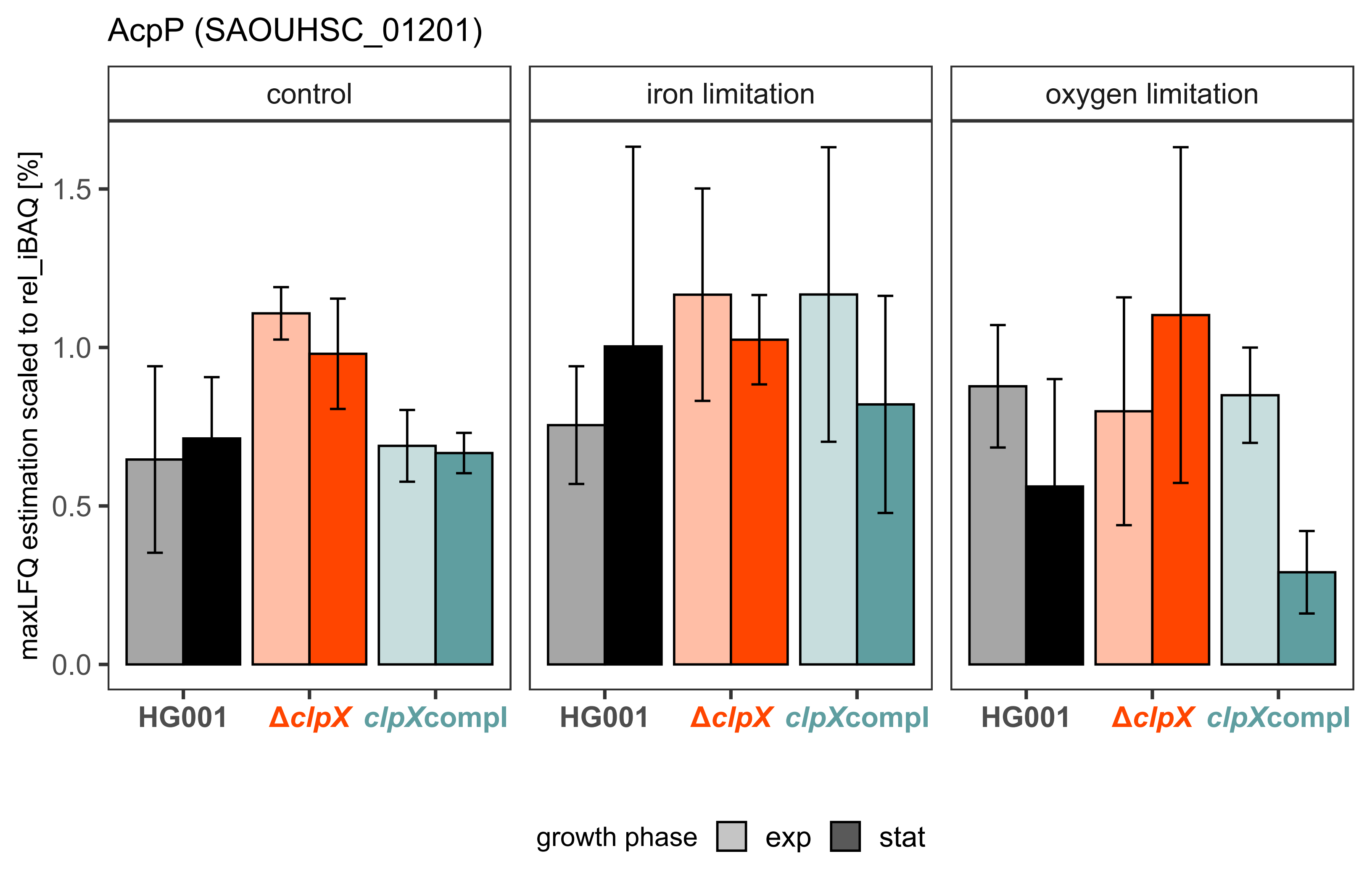
AcpP (SAOUHSC_01201)
control
iron limitation
oxygen limitation
maxLFQ estimation scaled to rel_iBAQ [%]
0.0
0.5
1.0
1.5
HG001
ΔclpX
clpXcompl
growth phase
exp
stat

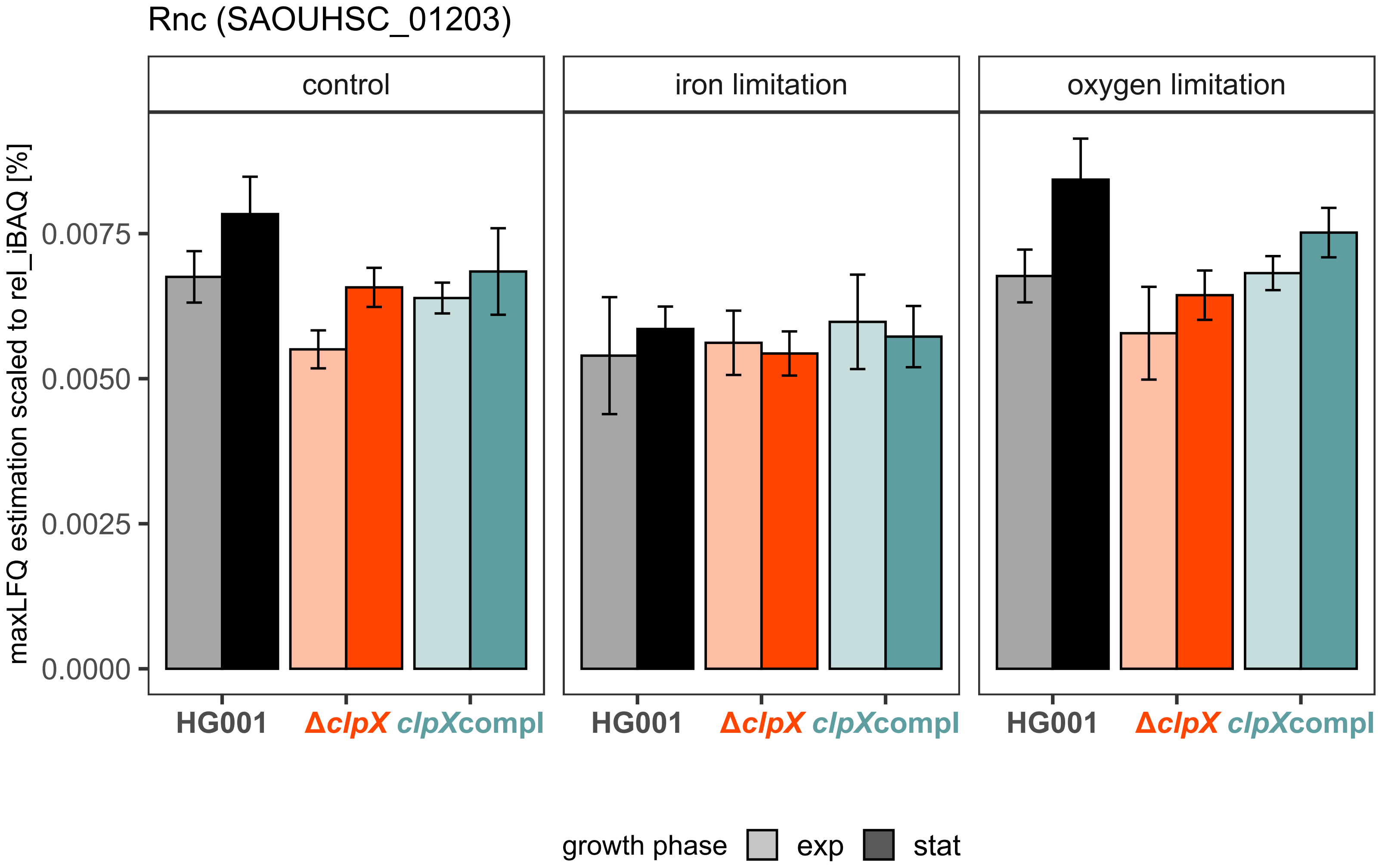

Rnc (SAOUHSC_01203)
control
iron limitation
oxygen limitation
maxLFQ estimation scaled to rel_iBAQ [%]
0.0075
0.0050
0.0025
0.0000
HG001
ΔclpX
clpXcompl
growth phase
exp
stat

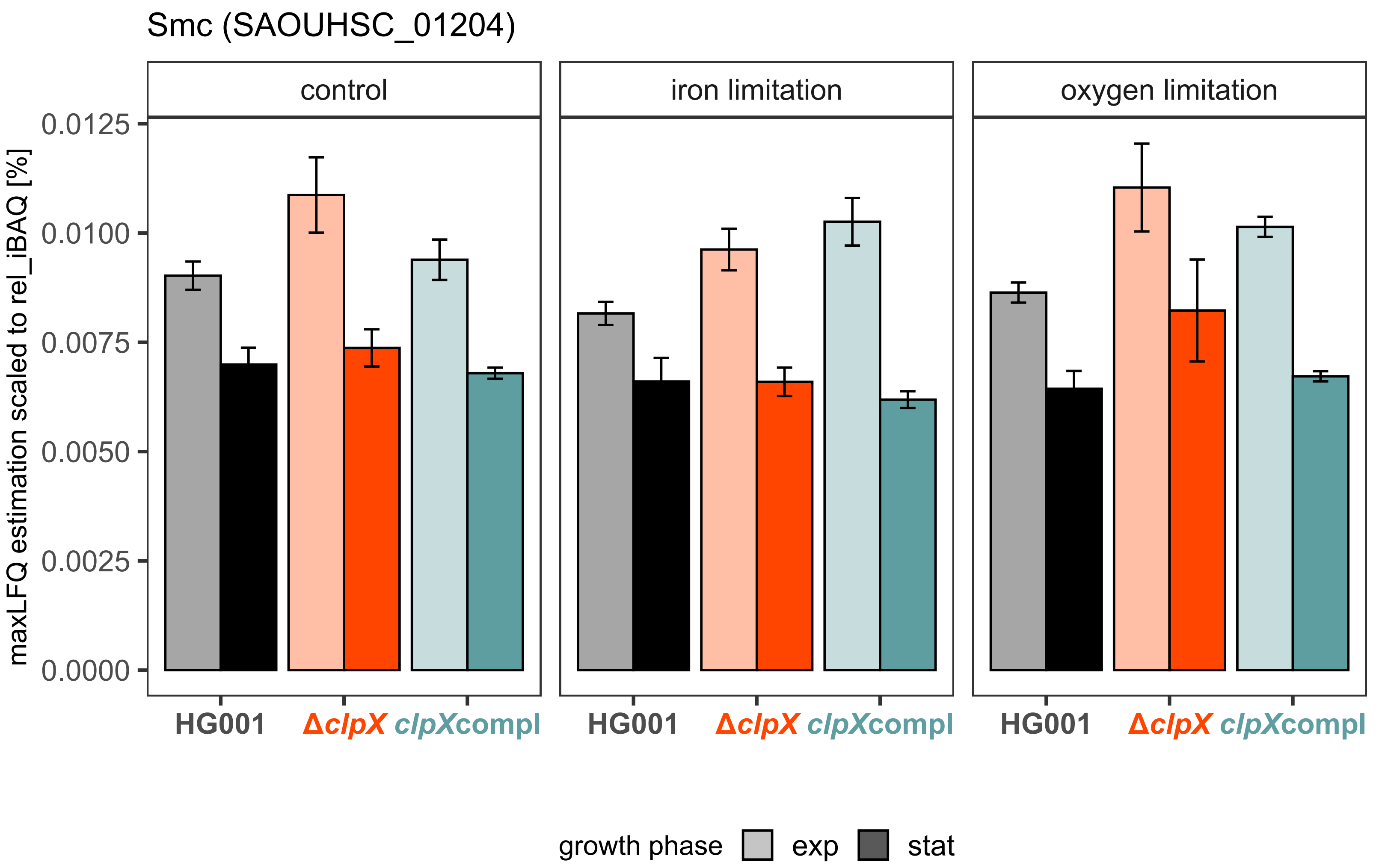

Smc (SAOUHSC_01204)
control
iron limitation
oxygen limitation
maxLFQ estimation scaled to rel_iBAQ [%]
0.0125
0.0100
0.0075
0.0050
0.0025
0.0000
HG001
ΔclpX
clpXcompl
growth phase
exp
stat

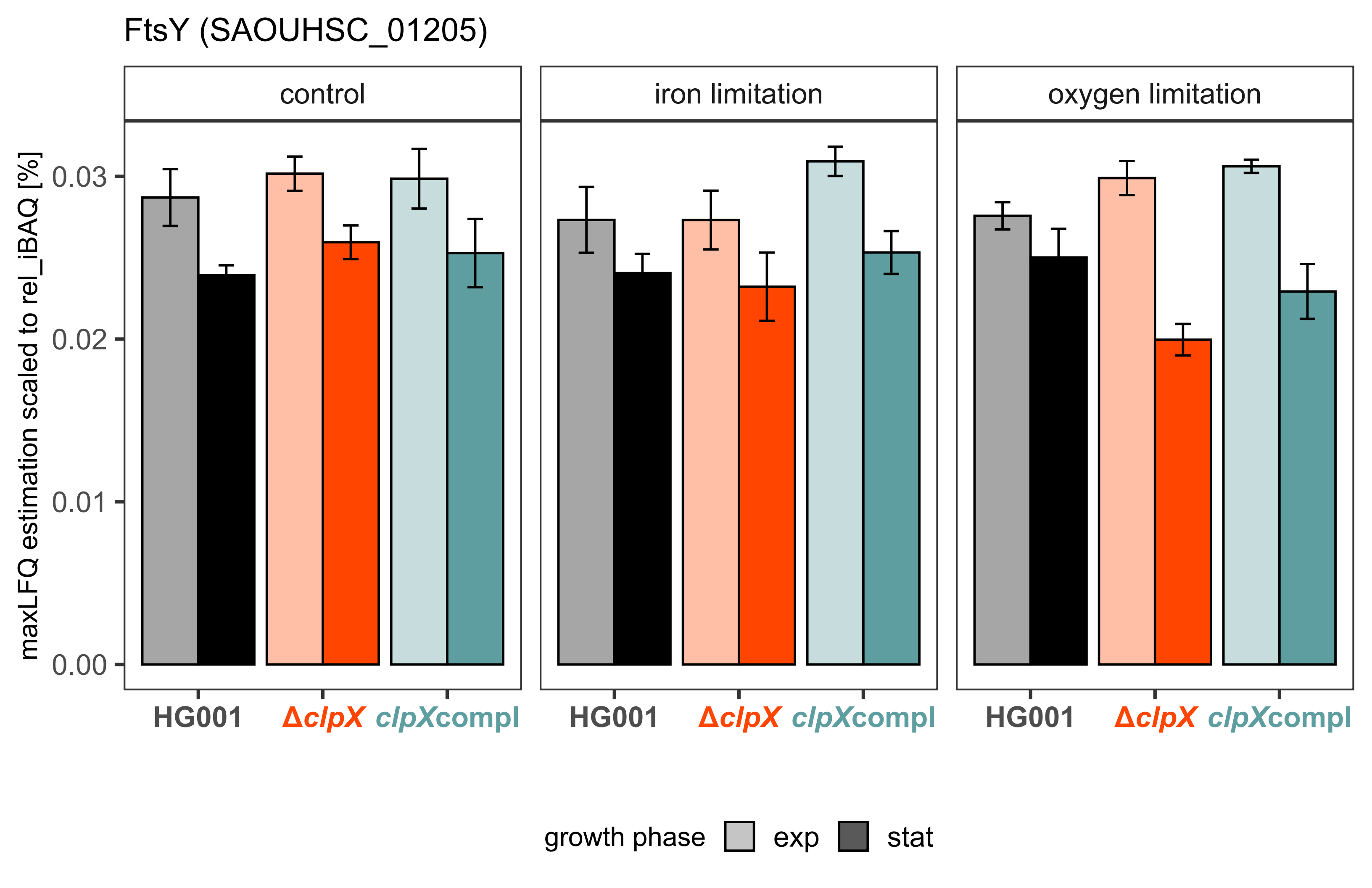
FtsY (SAOUHSC_01205)
control
iron limitation
oxygen limitation
maxLFQ estimation scaled to rel_iBAQ [%]
0.03
0.02
0.01
0.00
HG001
ΔclpX
clpXcompl
growth phase
exp
stat

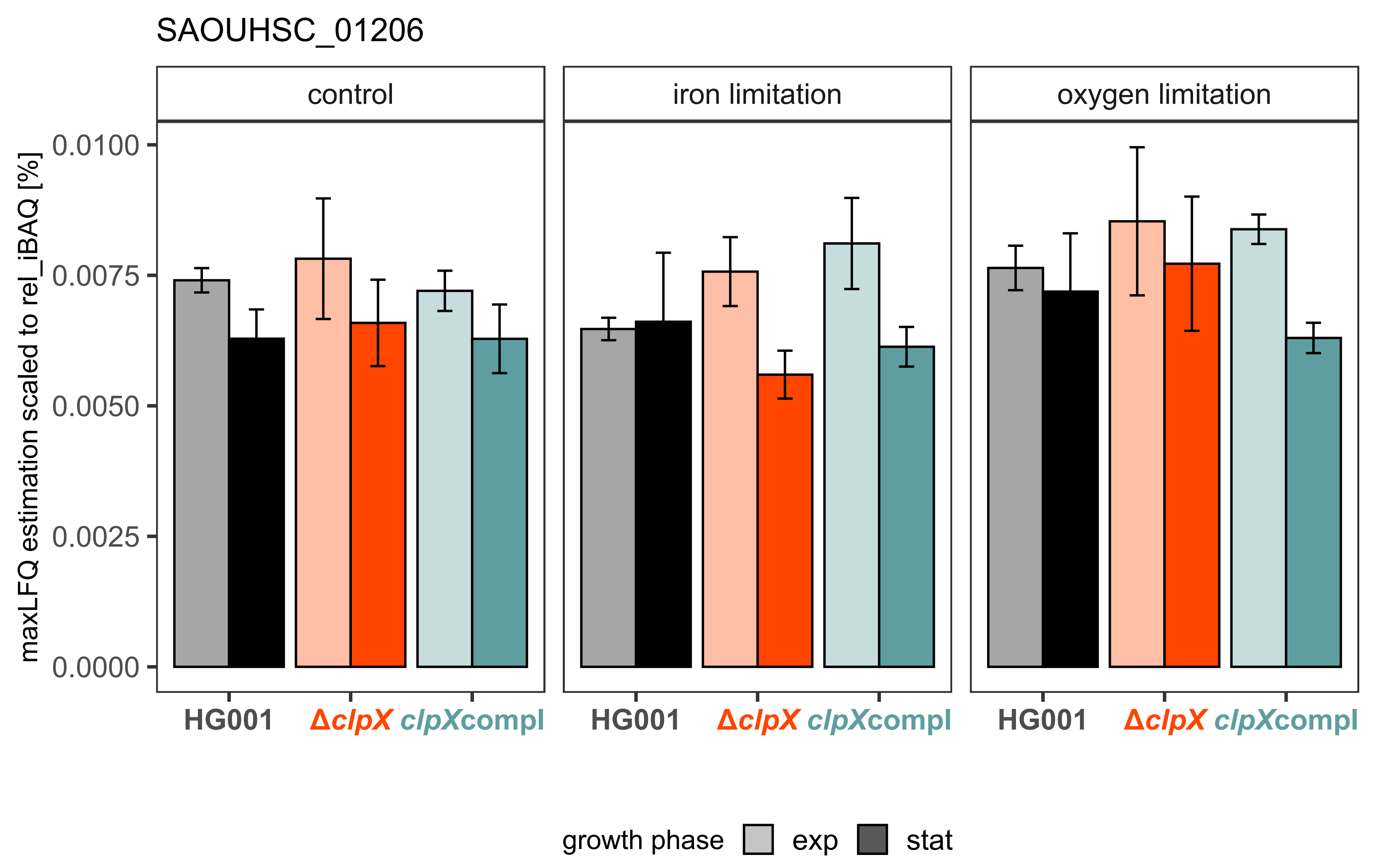
SAOUHSC_01206
control
iron limitation
oxygen limitation
maxLFQ estimation scaled to rel_iBAQ [%]
0.0100
0.0075
0.0050
0.0025
0.0000
HG001
ΔclpX
clpXcompl
growth phase
exp
stat

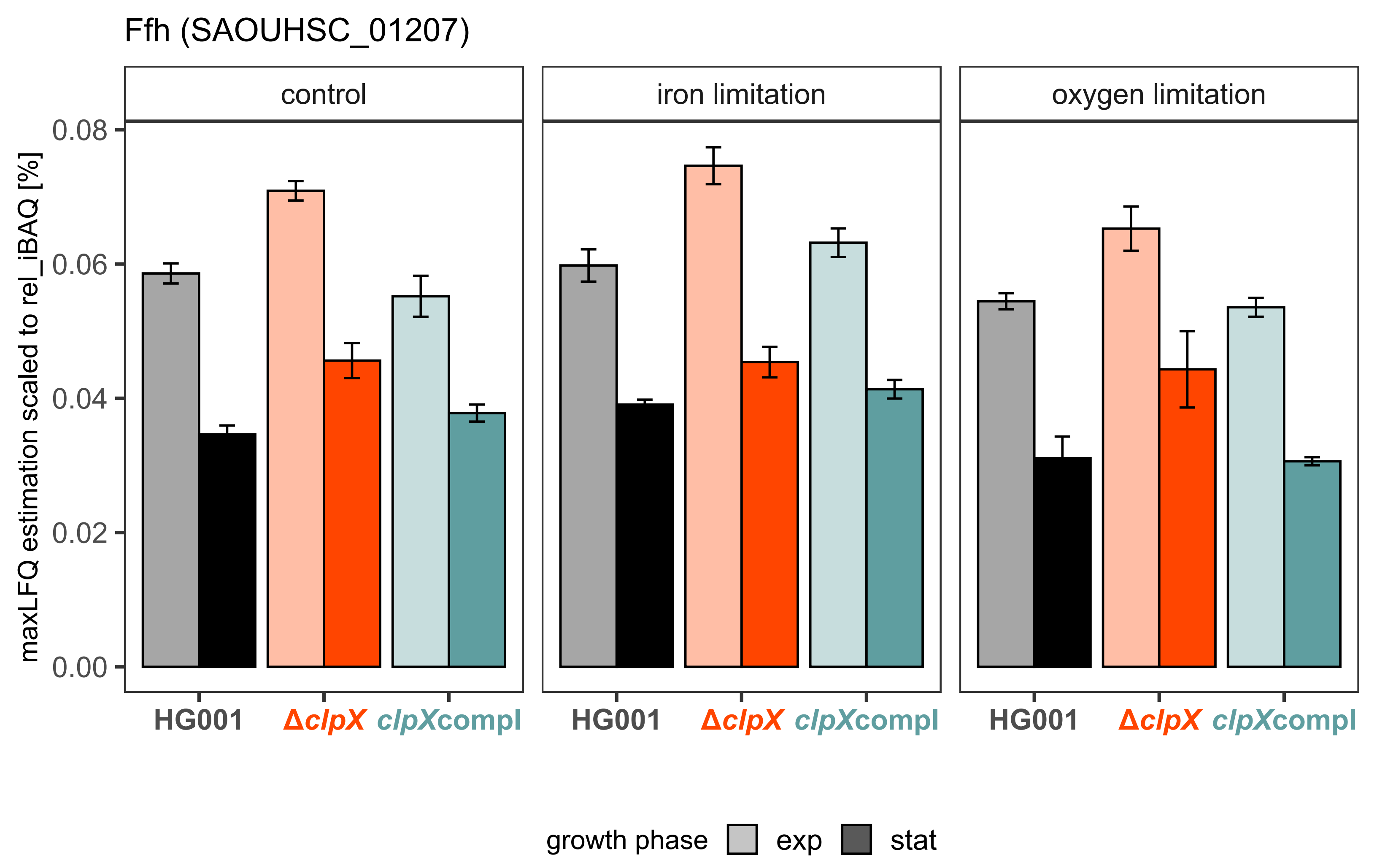
Ffh (SAOUHSC_01207)
control
iron limitation
oxygen limitation
maxLFQ estimation scaled to rel_iBAQ [%]
0.08
0.06
0.04
0.02
0.00
HG001
ΔclpX
clpXcompl
growth phase
exp
stat

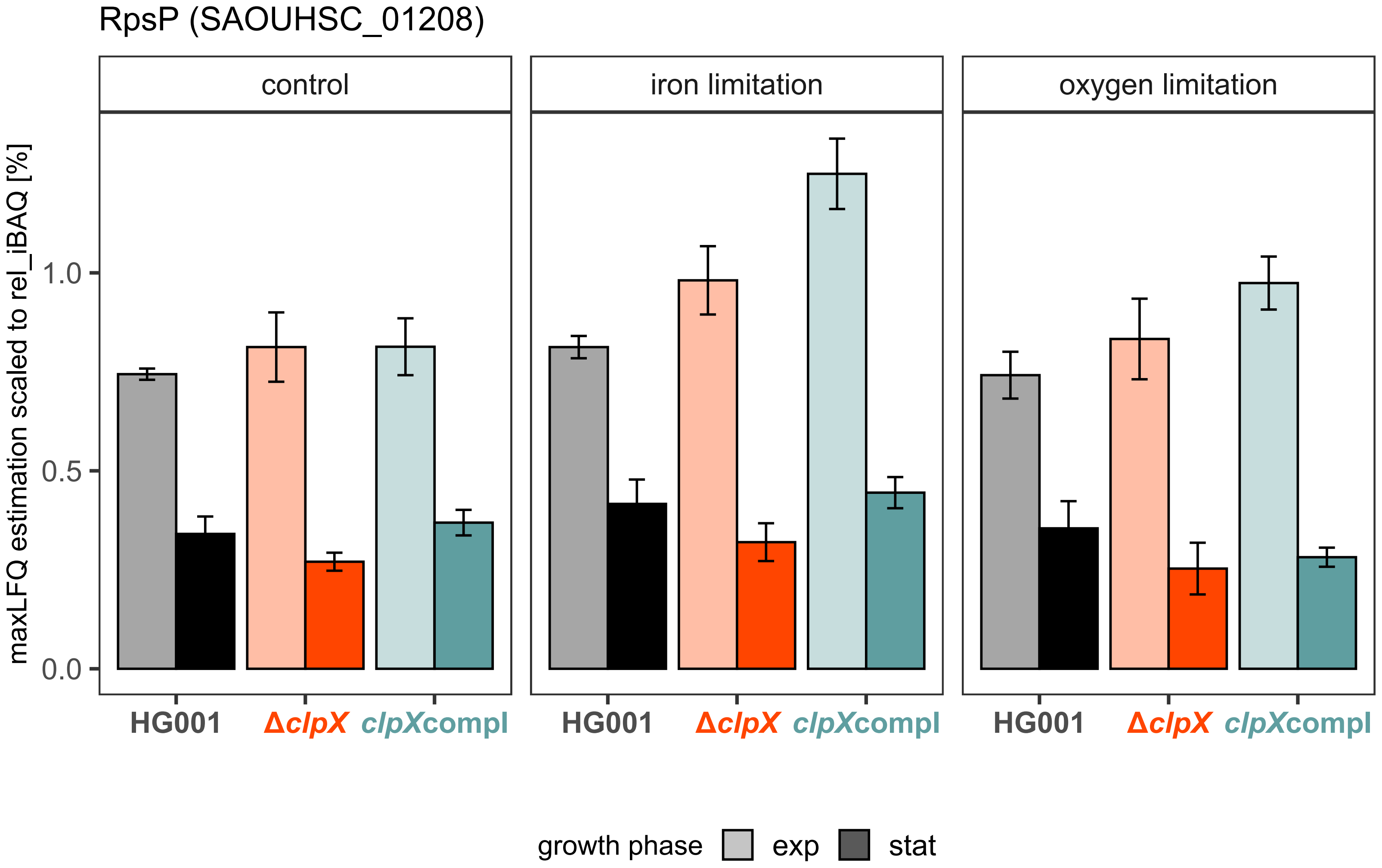

RpsP (SAOUHSC_01208)
control
iron limitation
oxygen limitation
maxLFQ estimation scaled to rel_iBAQ [%]
0.0
0.5
1.0
HG001
ΔclpX
clpXcompl
growth phase
exp
stat

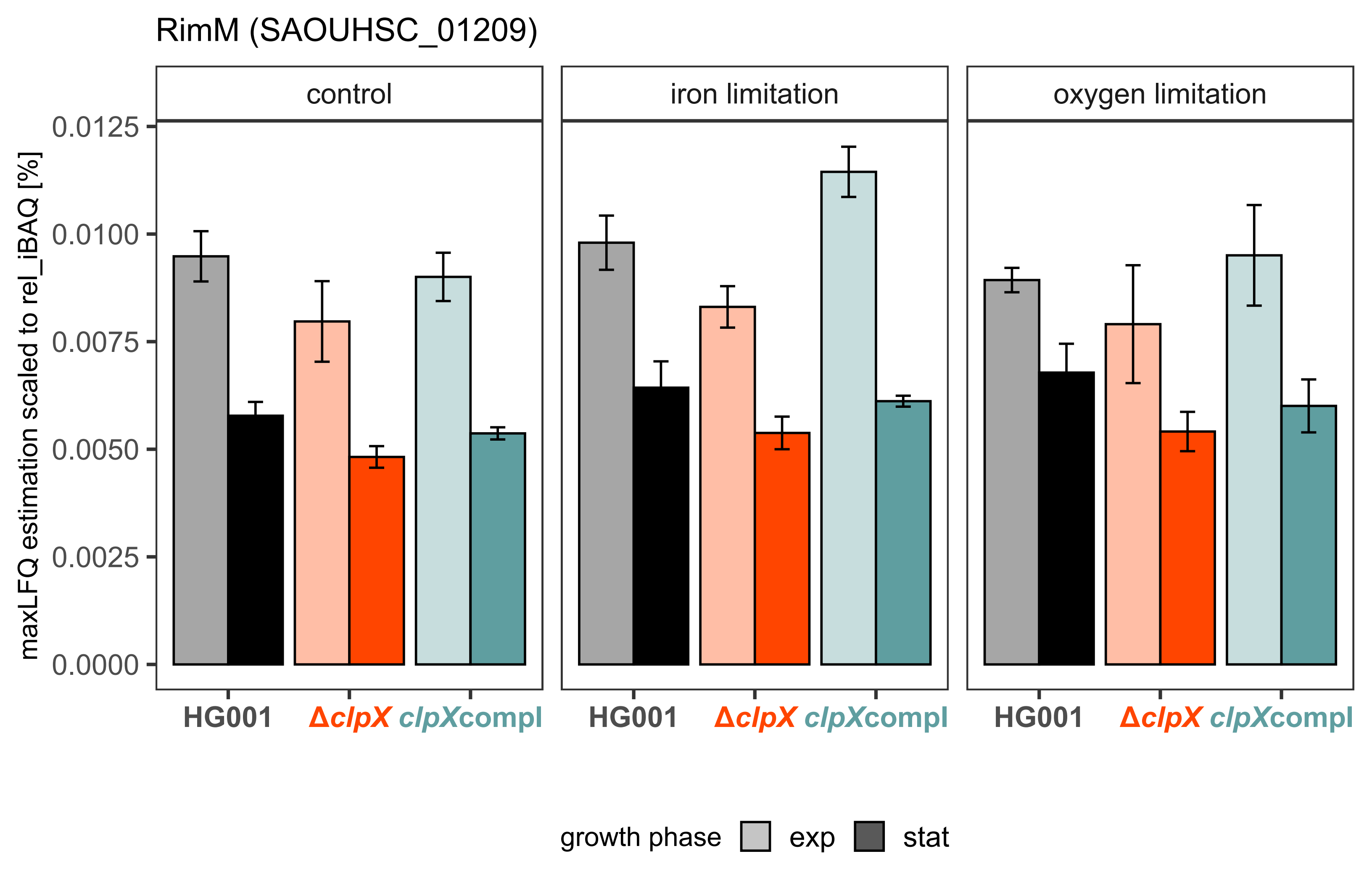
RimM (SAOUHSC_01209)
control
iron limitation
oxygen limitation
maxLFQ estimation scaled to rel_iBAQ [%]
0.0125
0.0100
0.0075
0.0050
0.0025
0.0000
HG001
ΔclpX
clpXcompl
growth phase
exp
stat

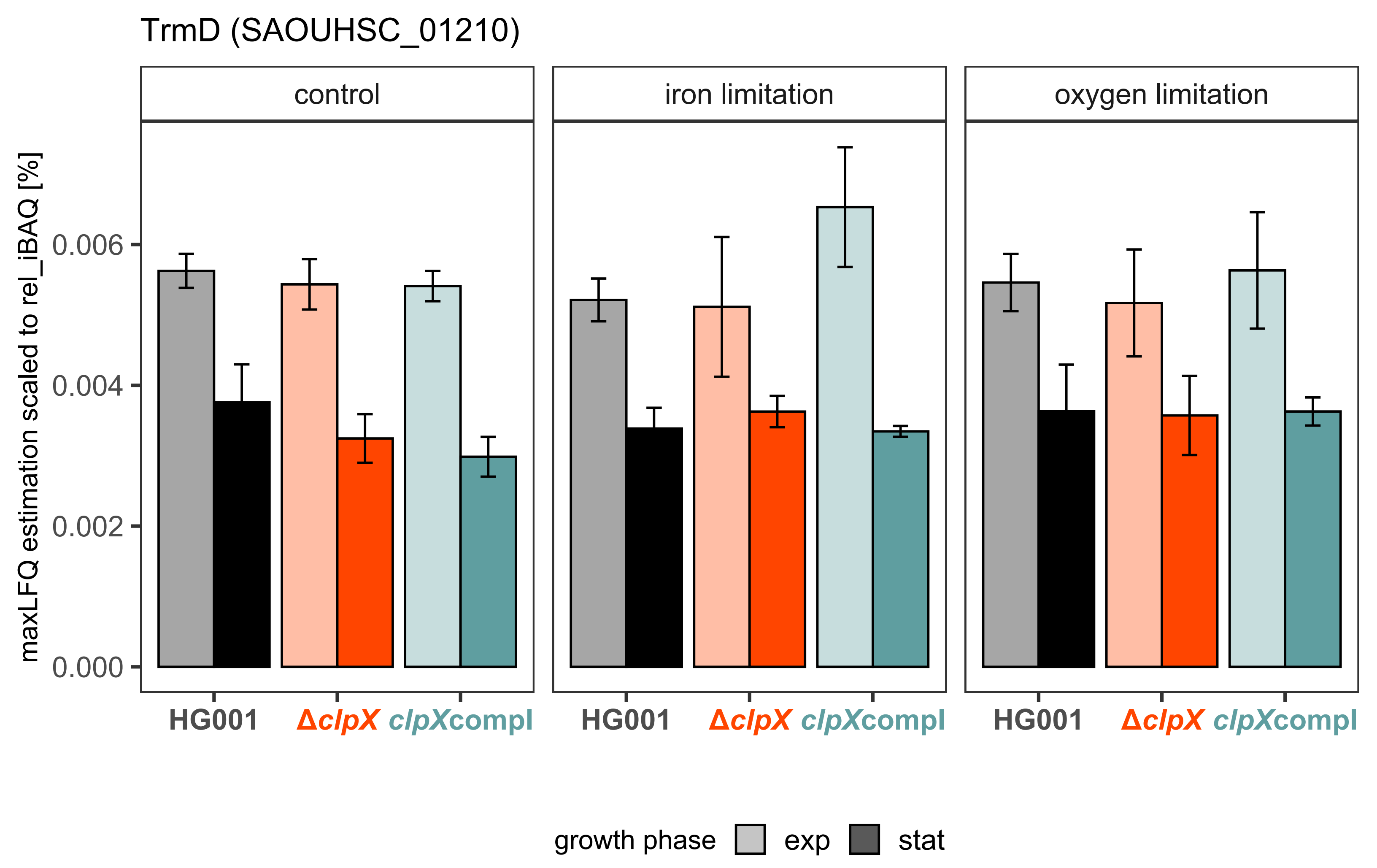
TrmD (SAOUHSC_01210)
control
iron limitation
oxygen limitation
maxLFQ estimation scaled to rel_iBAQ [%]
0.006
0.004
0.002
0.000
HG001
ΔclpX
clpXcompl
growth phase
exp
stat

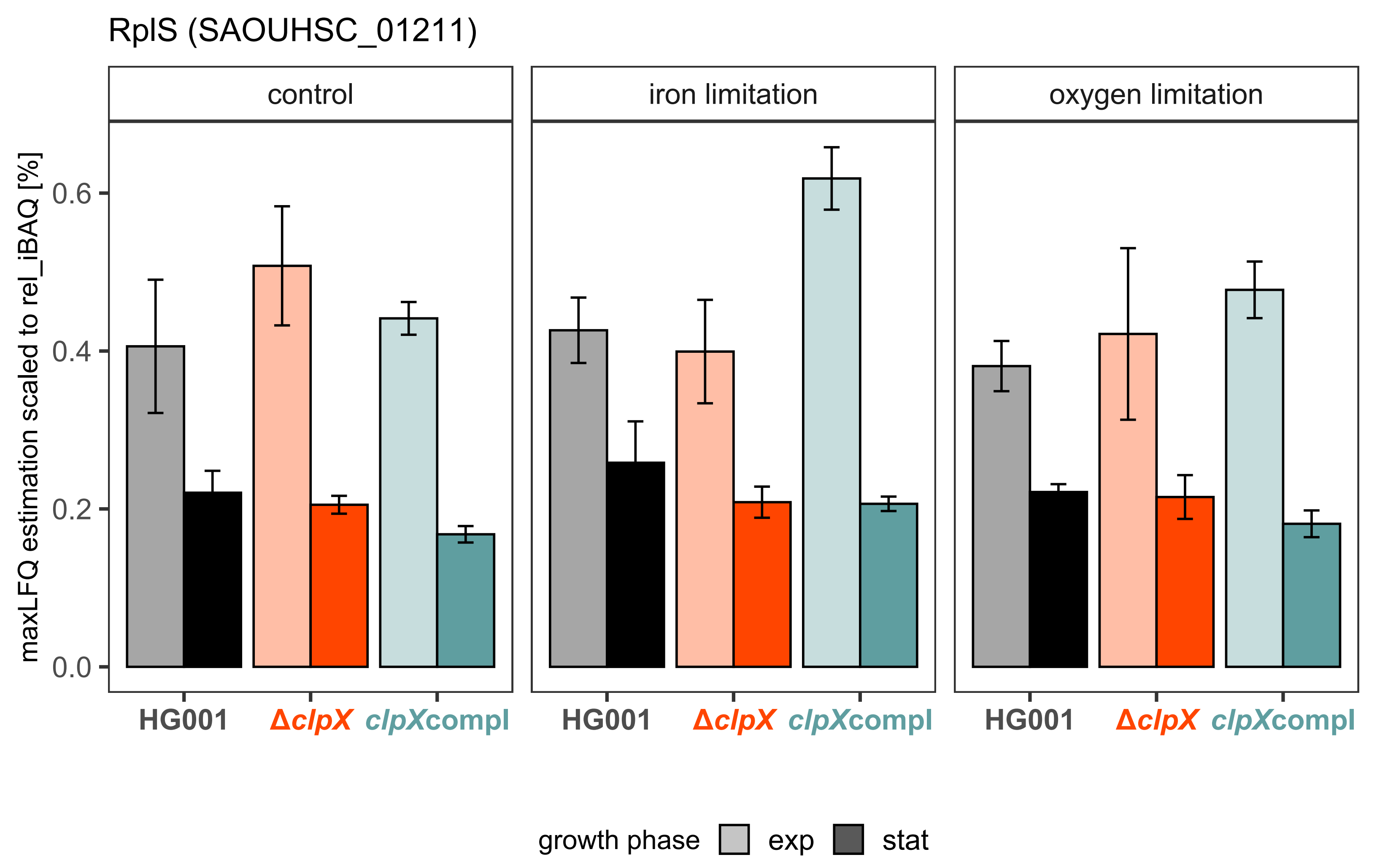
RplS (SAOUHSC_01211)
control
iron limitation
oxygen limitation
maxLFQ estimation scaled to rel_iBAQ [%]
0.0
0.2
0.4
0.6
HG001
ΔclpX
clpXcompl
growth phase
exp
stat

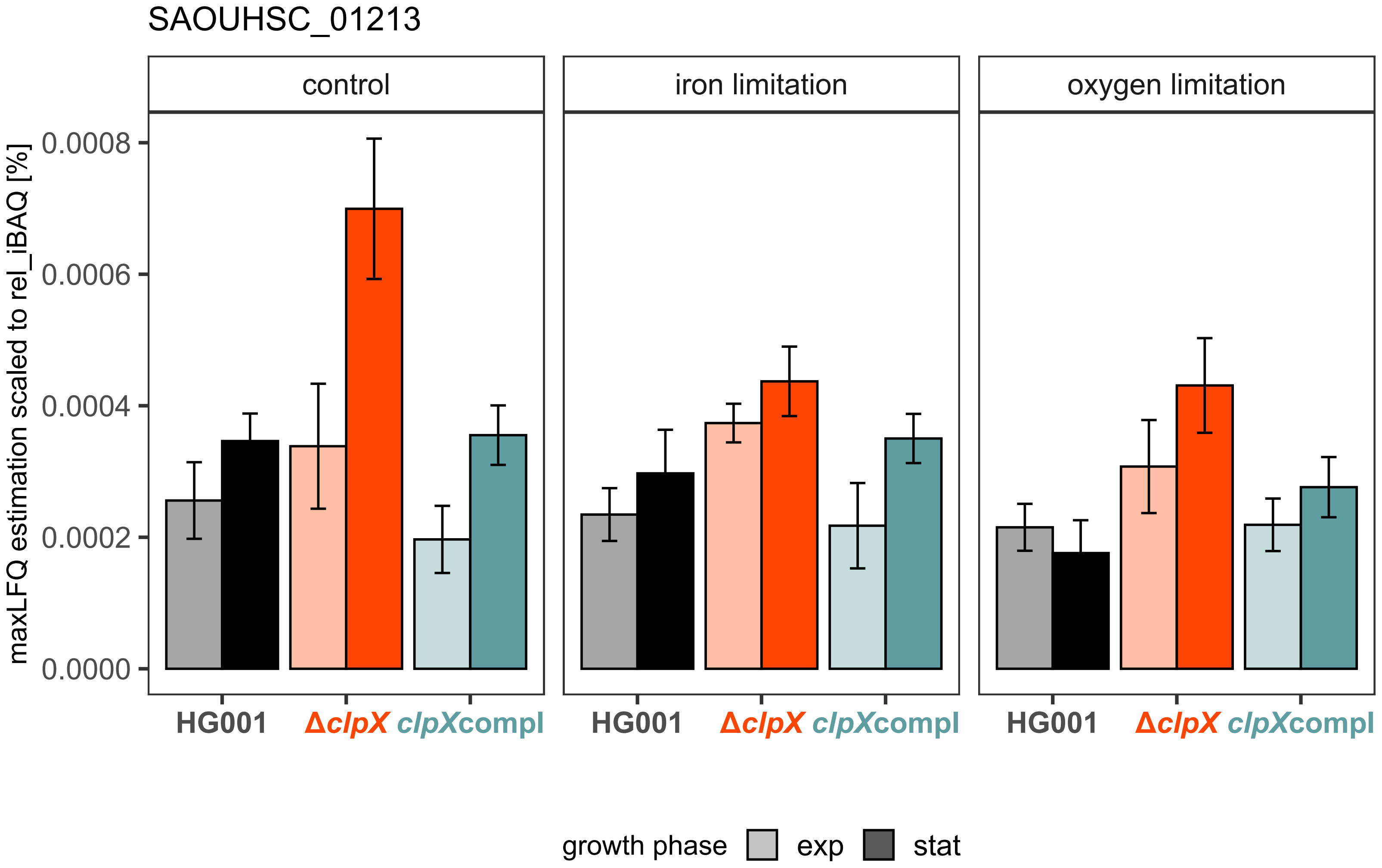

SAOUHSC_01213
control
iron limitation
oxygen limitation
maxLFQ estimation scaled to rel_iBAQ [%]
0.0008
0.0006
0.0004
0.0002
0.0000
HG001
ΔclpX
clpXcompl
growth phase
exp
stat

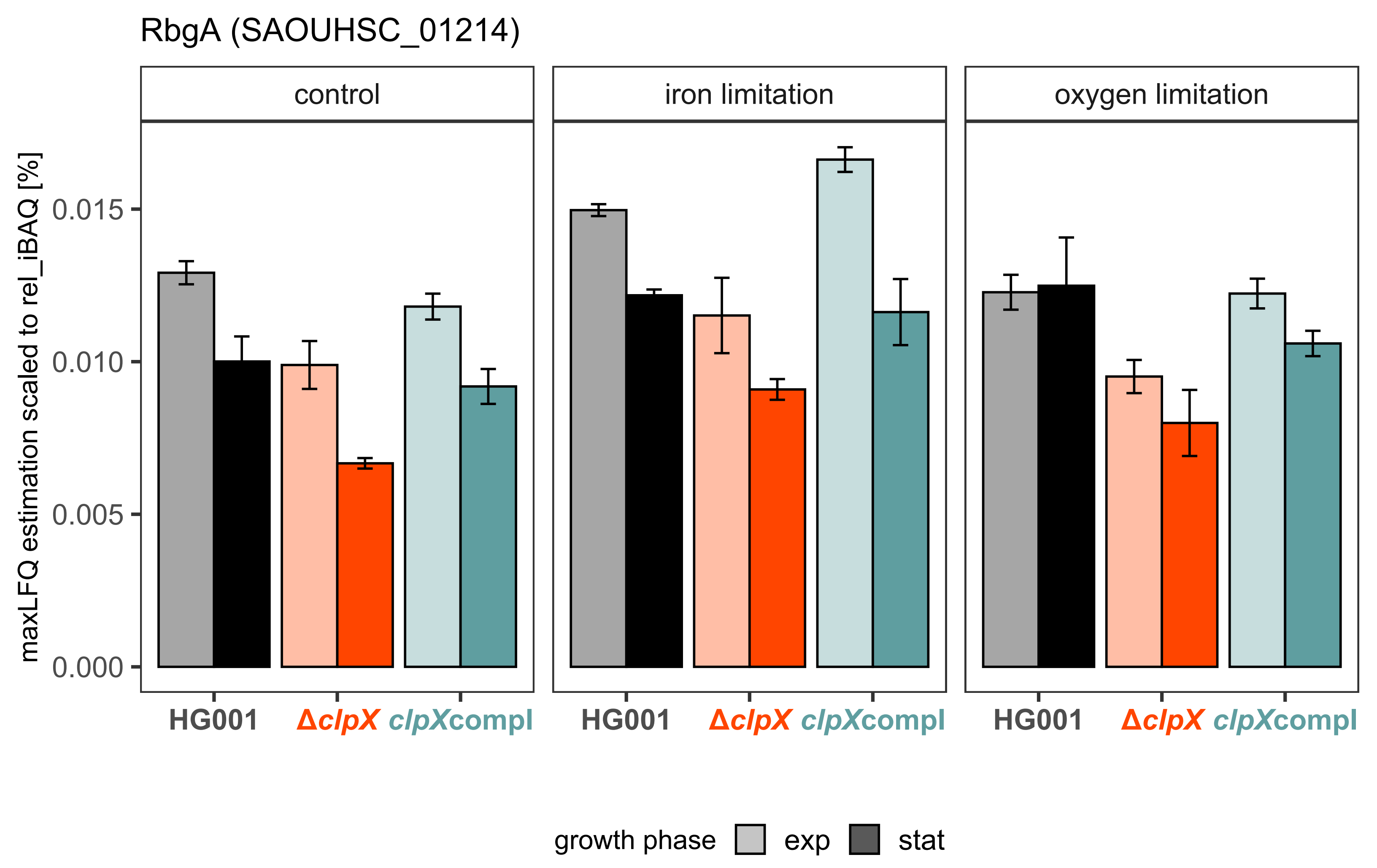

RbgA (SAOUHSC_01214)
control
iron limitation
oxygen limitation
maxLFQ estimation scaled to rel_iBAQ [%]
0.015
0.010
0.005
0.000
HG001
ΔclpX
clpXcompl
growth phase
exp
stat

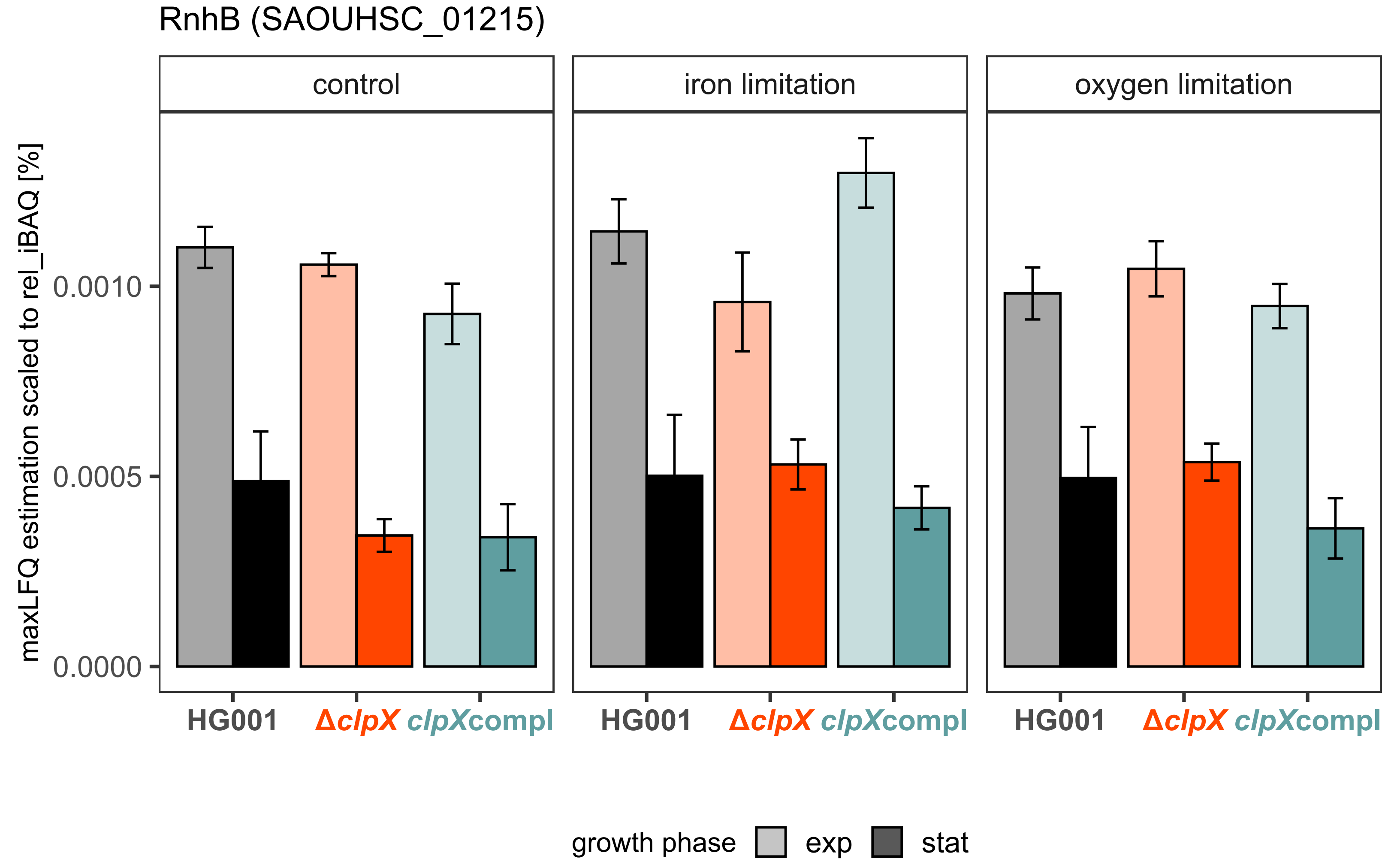
RnhB (SAOUHSC_01215)
control
iron limitation
oxygen limitation
maxLFQ estimation scaled to rel_iBAQ [%]
0.0010
0.0005
0.0000
HG001
ΔclpX
clpXcompl
growth phase
exp
stat

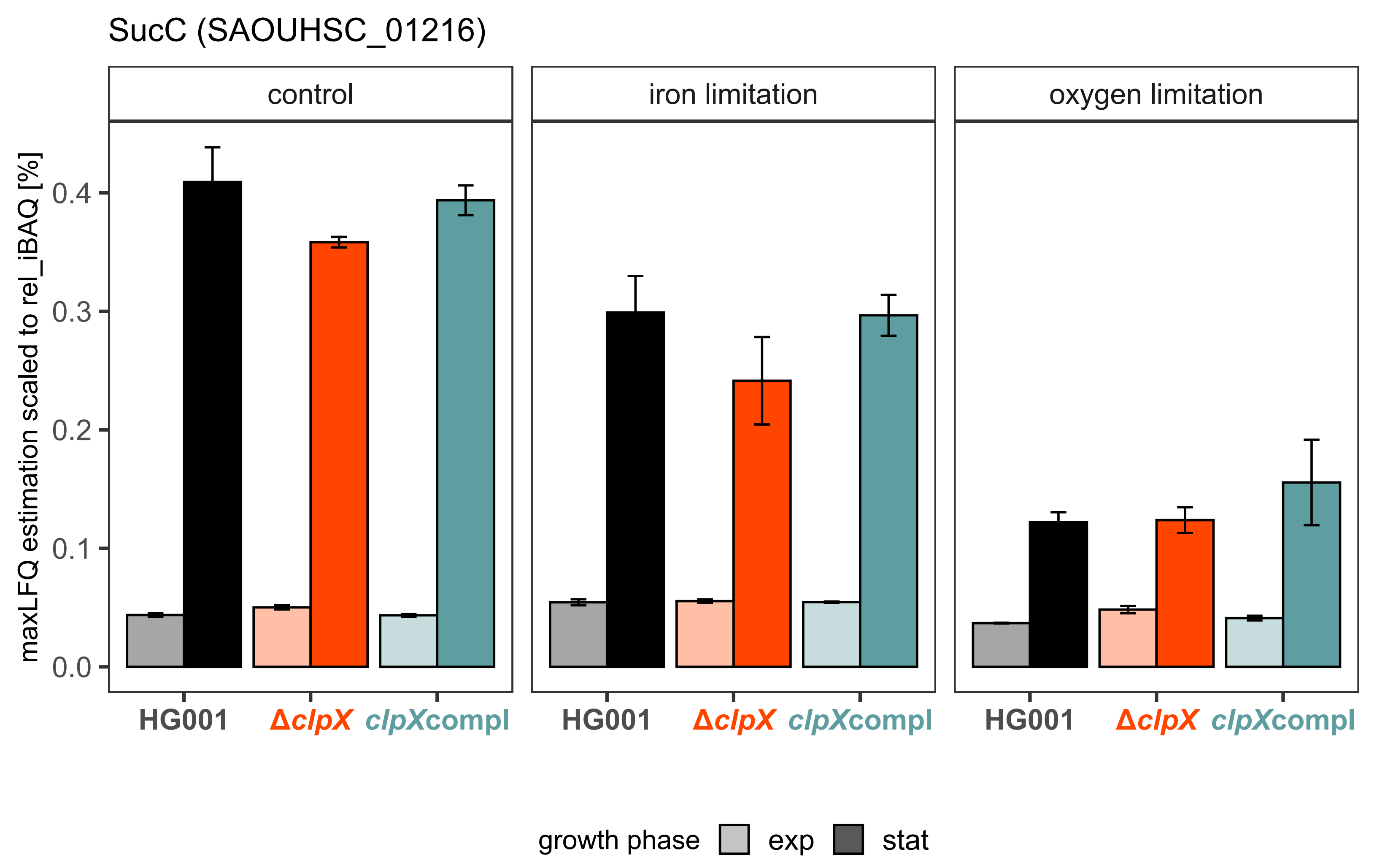
SucC (SAOUHSC_01216)
control
iron limitation
oxygen limitation
maxLFQ estimation scaled to rel_iBAQ [%]
0.0
0.1
0.2
0.3
0.4
HG001
ΔclpX
clpXcompl
growth phase
exp
stat

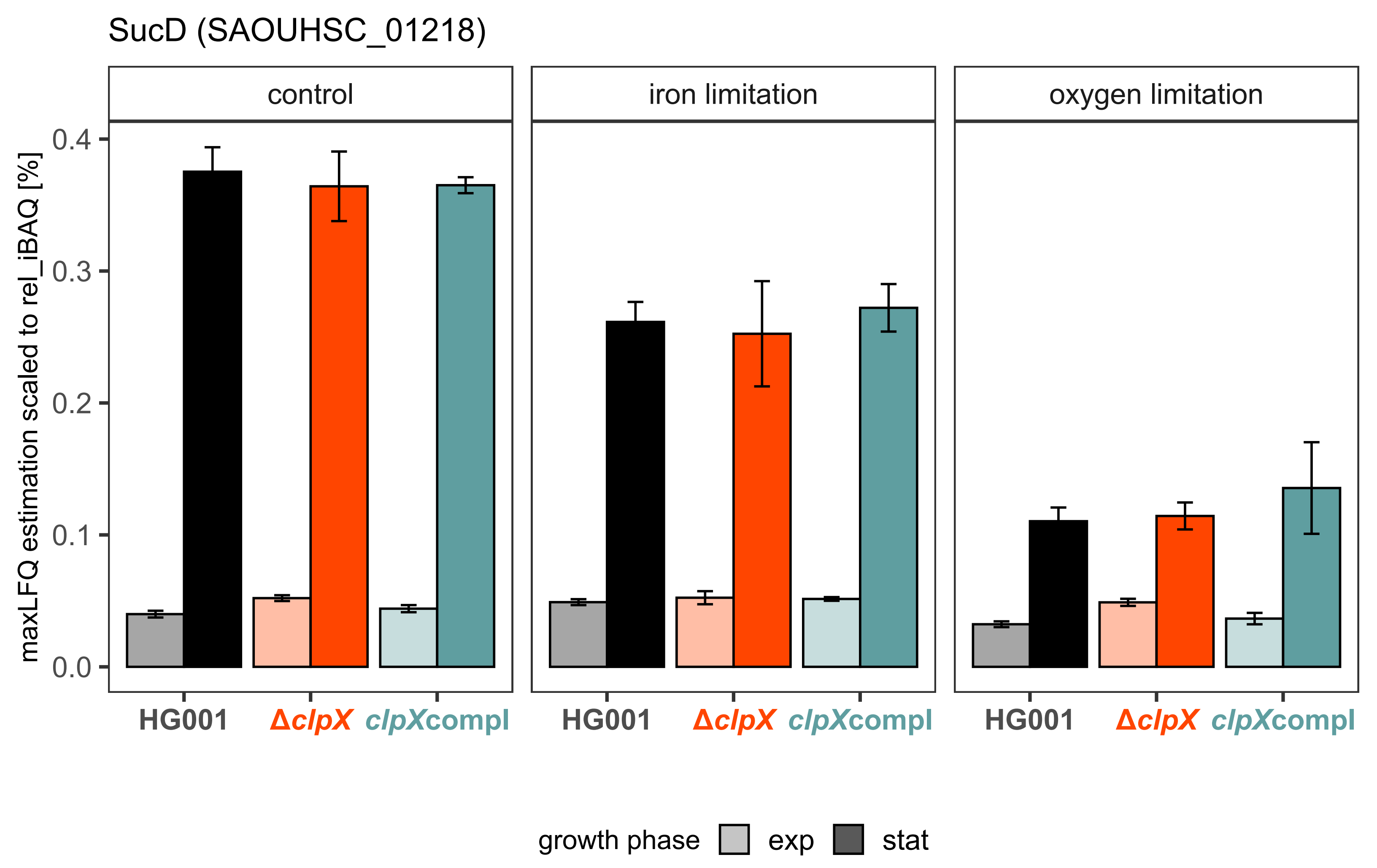

SucD (SAOUHSC_01218)
control
iron limitation
oxygen limitation
maxLFQ estimation scaled to rel_iBAQ [%]
0.4
0.3
0.2
0.1
0.0
HG001
ΔclpX
clpXcompl
growth phase
exp
stat

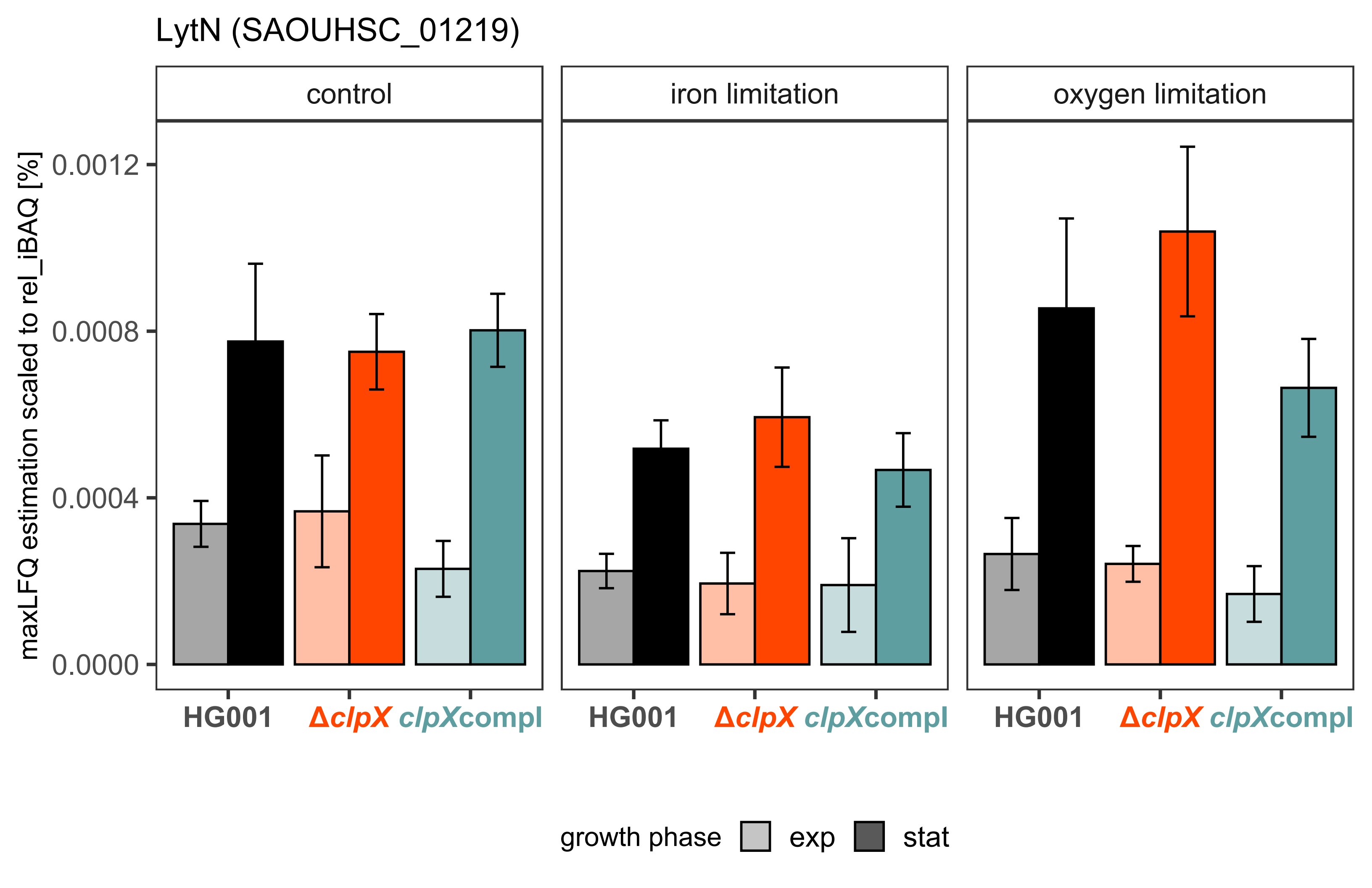

LytN (SAOUHSC_01219)
control
iron limitation
oxygen limitation
maxLFQ estimation scaled to rel_iBAQ [%]
0.0012
0.0008
0.0004
0.0000
HG001
ΔclpX
clpXcompl
growth phase
exp
stat

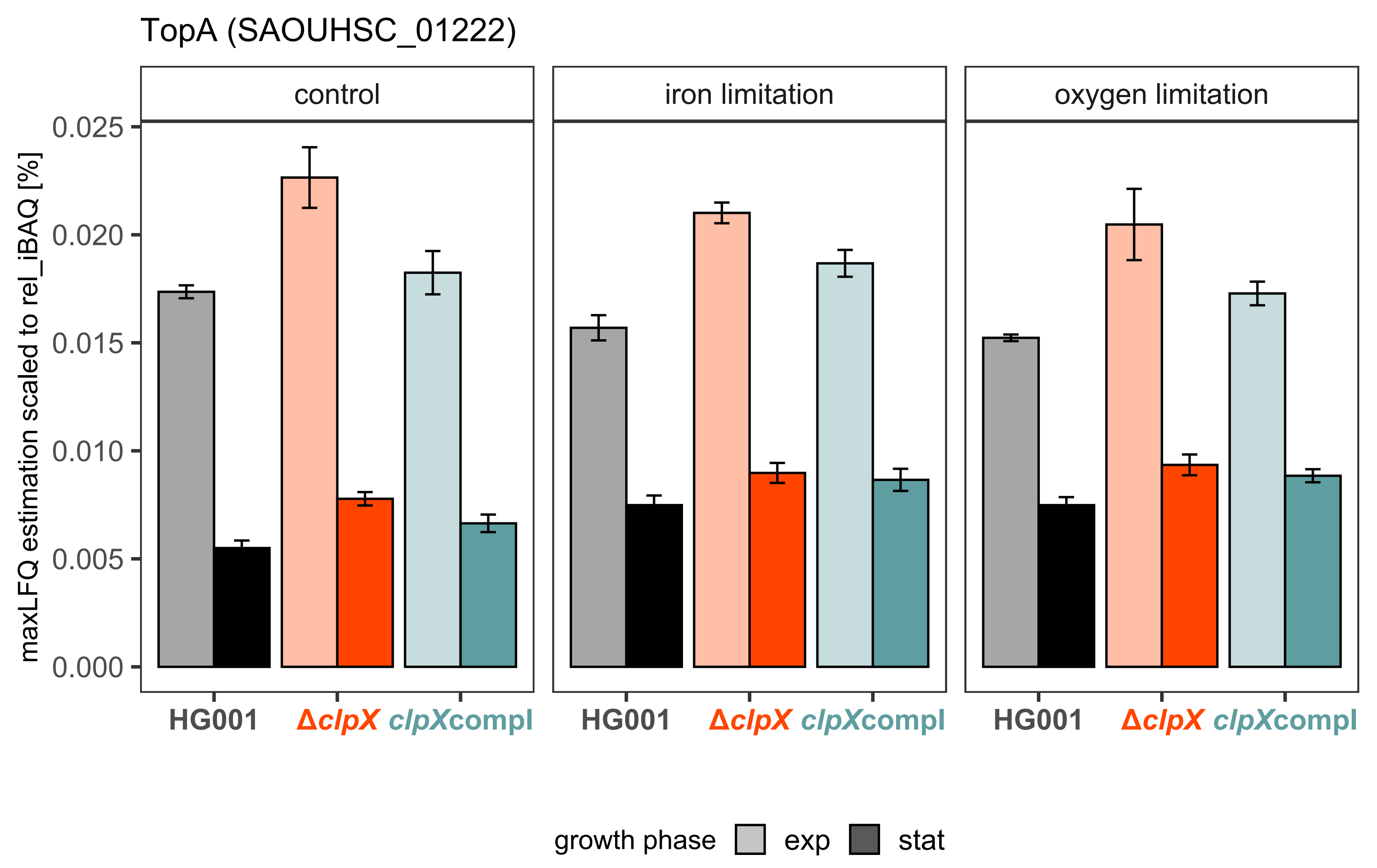
TopA (SAOUHSC_01222)
control
iron limitation
oxygen limitation
maxLFQ estimation scaled to rel_iBAQ [%]
0.025
0.020
0.015
0.010
0.005
0.000
HG001
ΔclpX
clpXcompl
growth phase
exp
stat

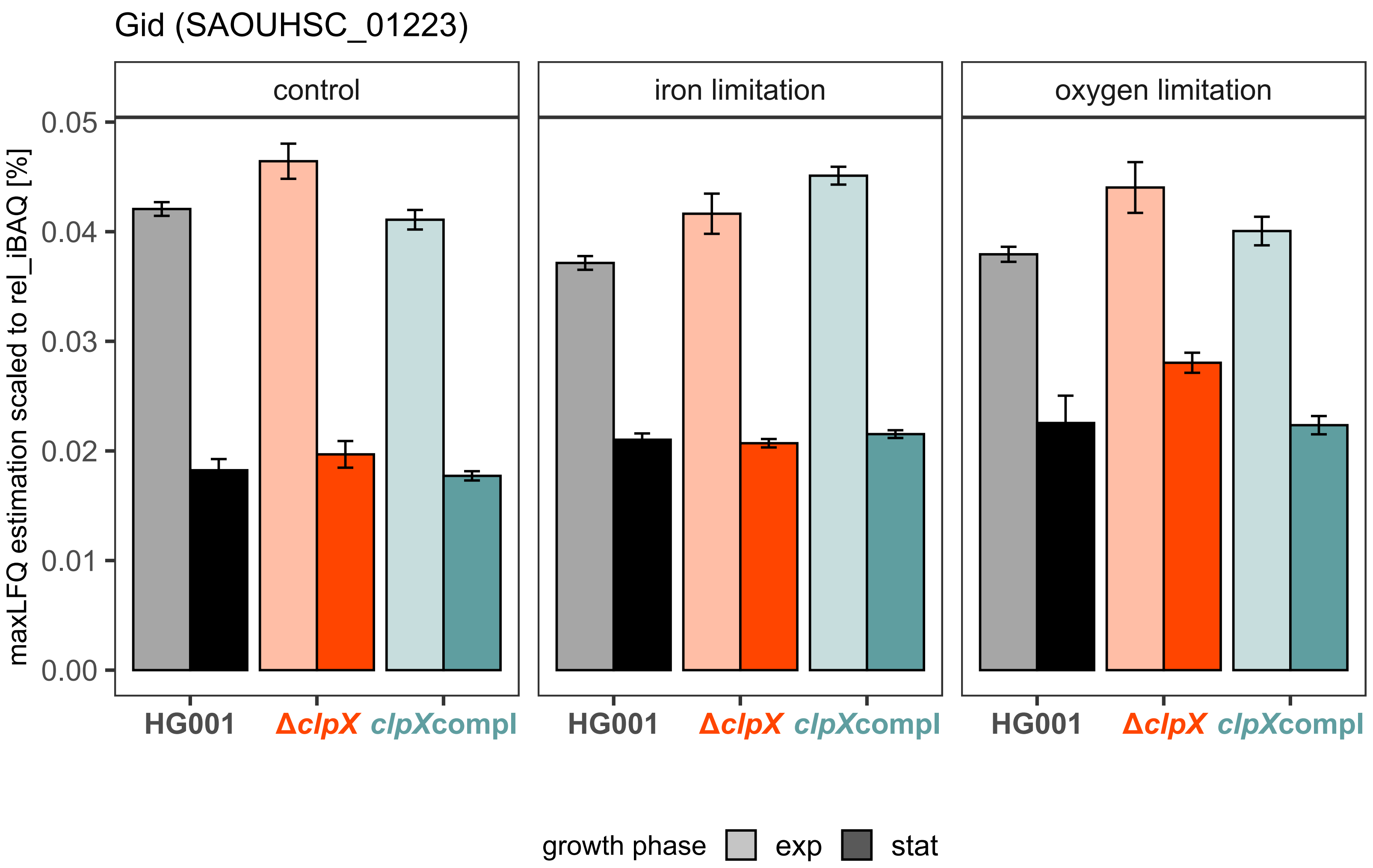

Gid (SAOUHSC_01223)
control
iron limitation
oxygen limitation
0.05
0.04
0.03
0.02
0.01
0.00
maxLFQ estimation scaled to rel_iBAQ [%]
HG001
ΔclpX
clpXcompl
growth phase
exp
stat

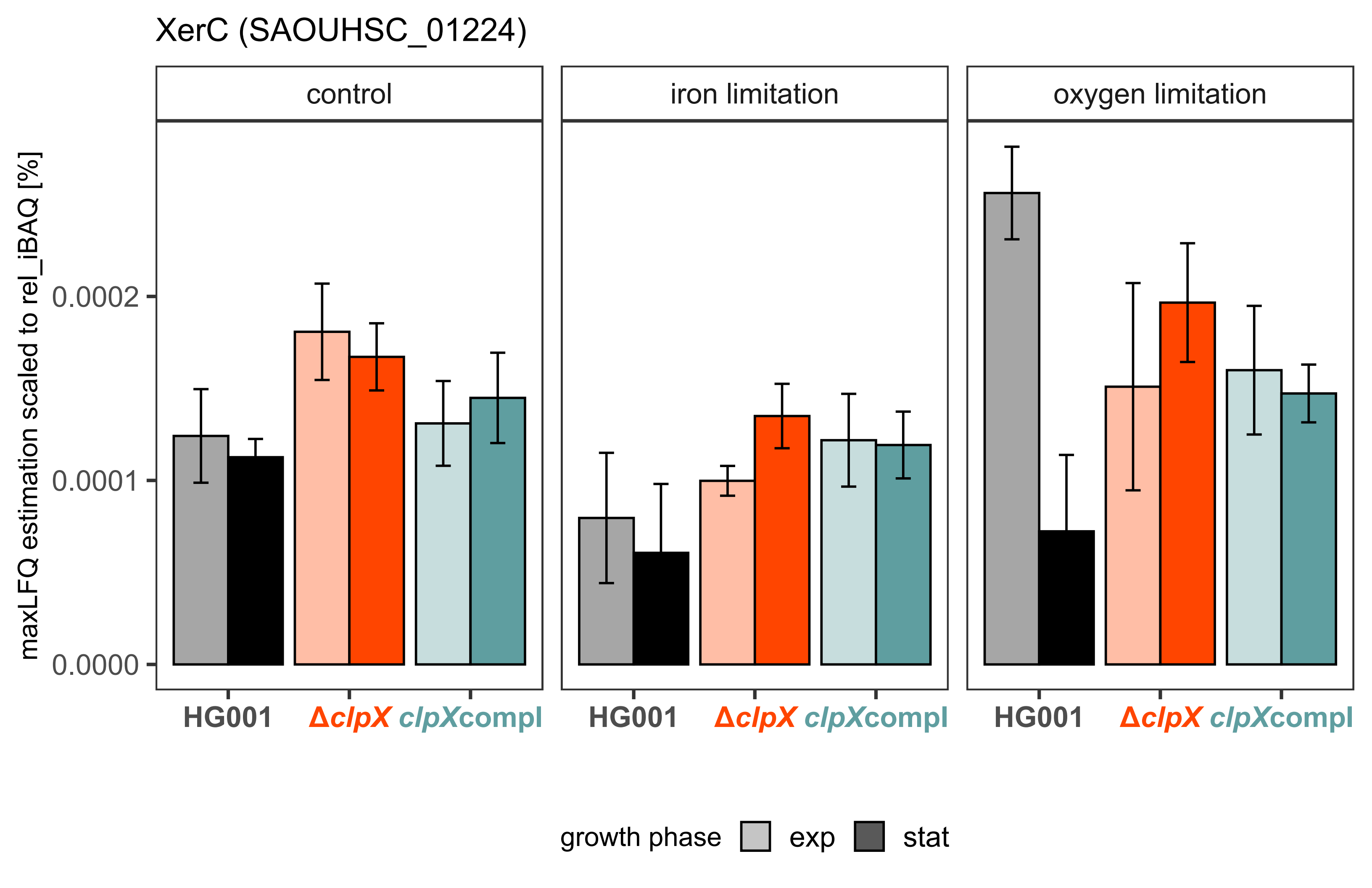

XerC (SAOUHSC_01224)
control
iron limitation
oxygen limitation
maxLFQ estimation scaled to rel_iBAQ [%]
0.0002
0.0001
0.0000
HG001
ΔclpX
clpXcompl
growth phase
exp
stat

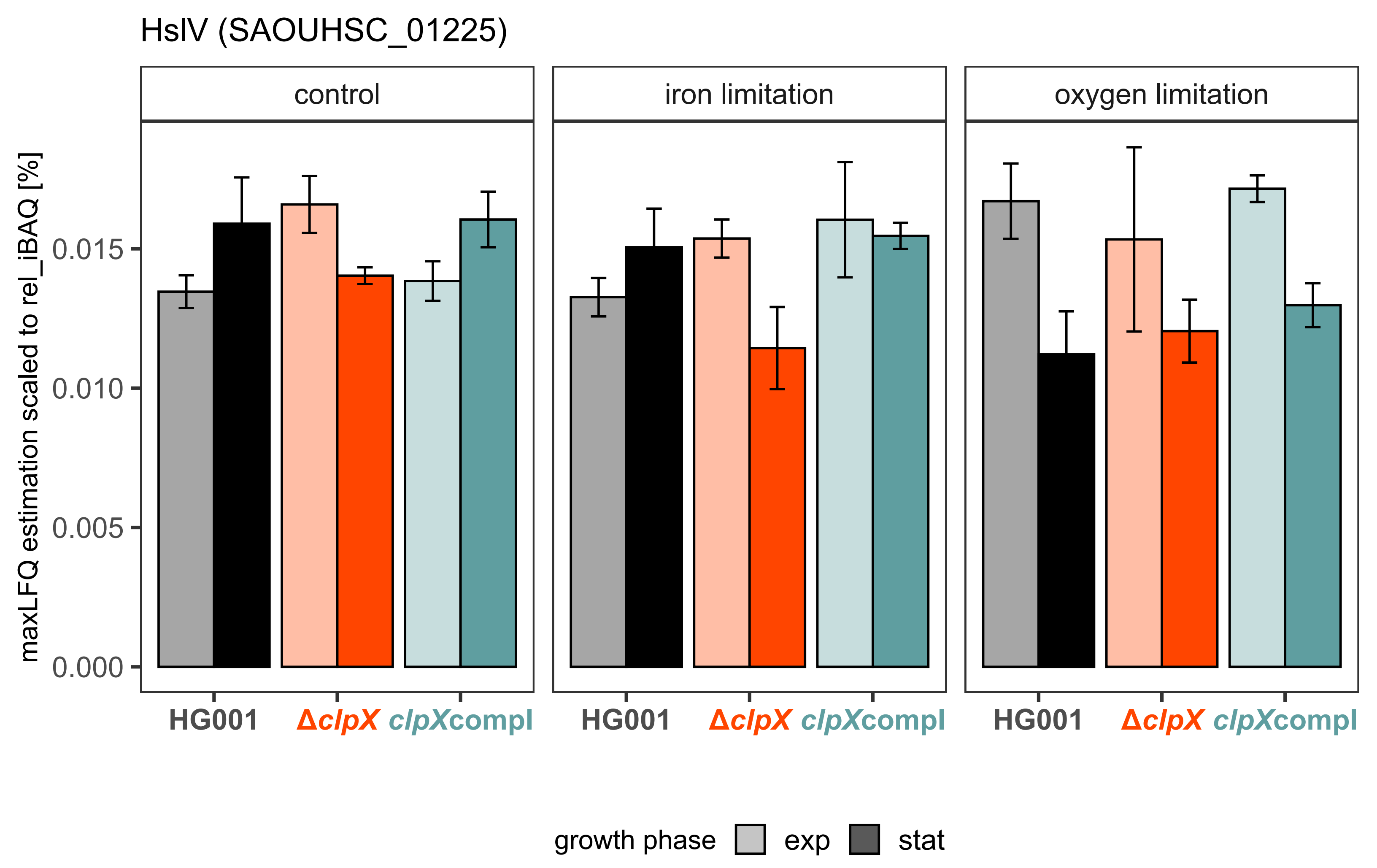
HslV (SAOUHSC_01225)
control
iron limitation
oxygen limitation
maxLFQ estimation scaled to rel_iBAQ [%]
0.000
0.005
0.010
0.015
HG001
ΔclpX
clpXcompl
growth phase
exp
stat

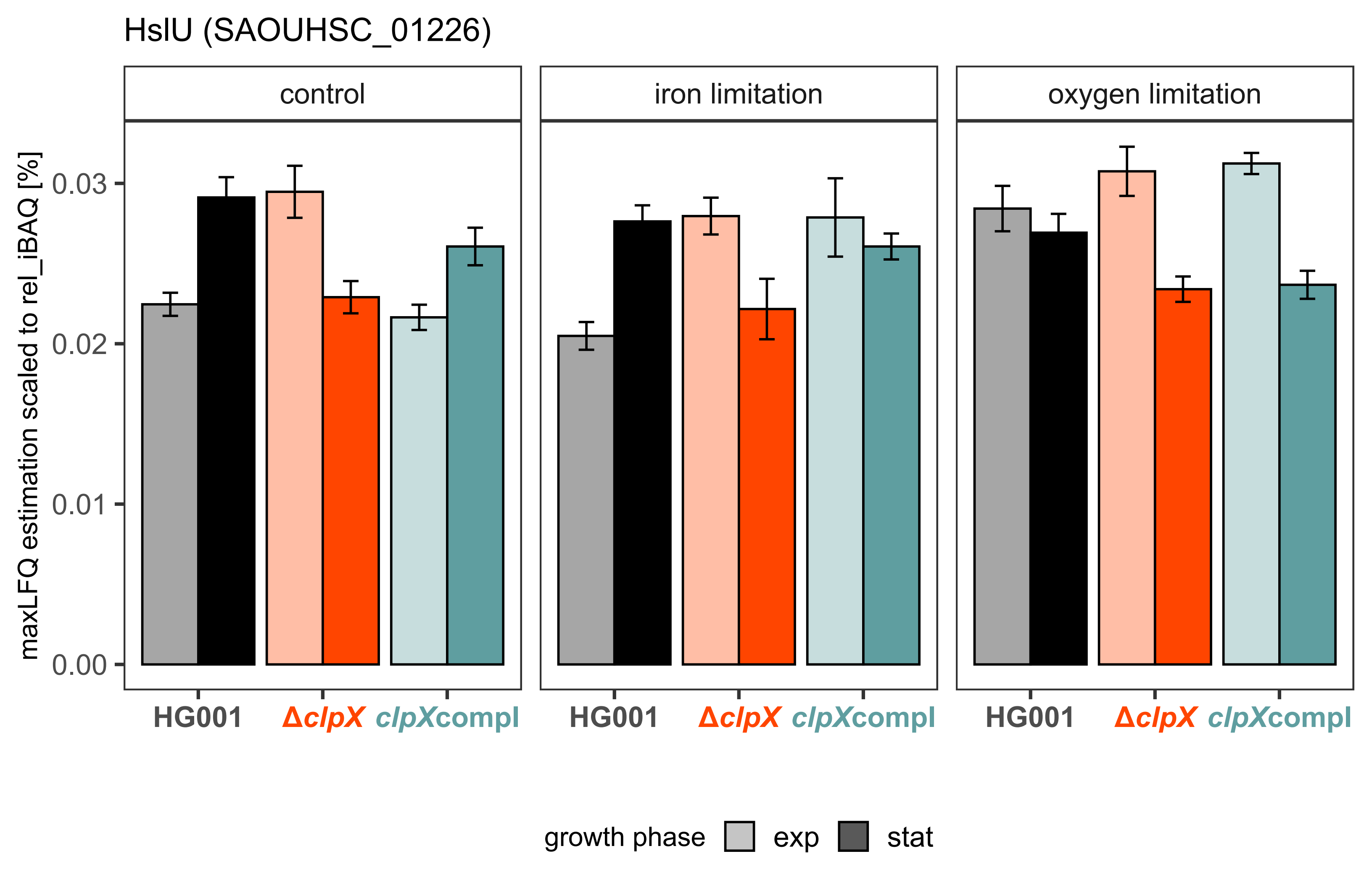

HslU (SAOUHSC_01226)
control
iron limitation
oxygen limitation
maxLFQ estimation scaled to rel_iBAQ [%]
0.00
0.01
0.02
0.03
HG001
ΔclpX
clpXcompl
growth phase
exp
stat

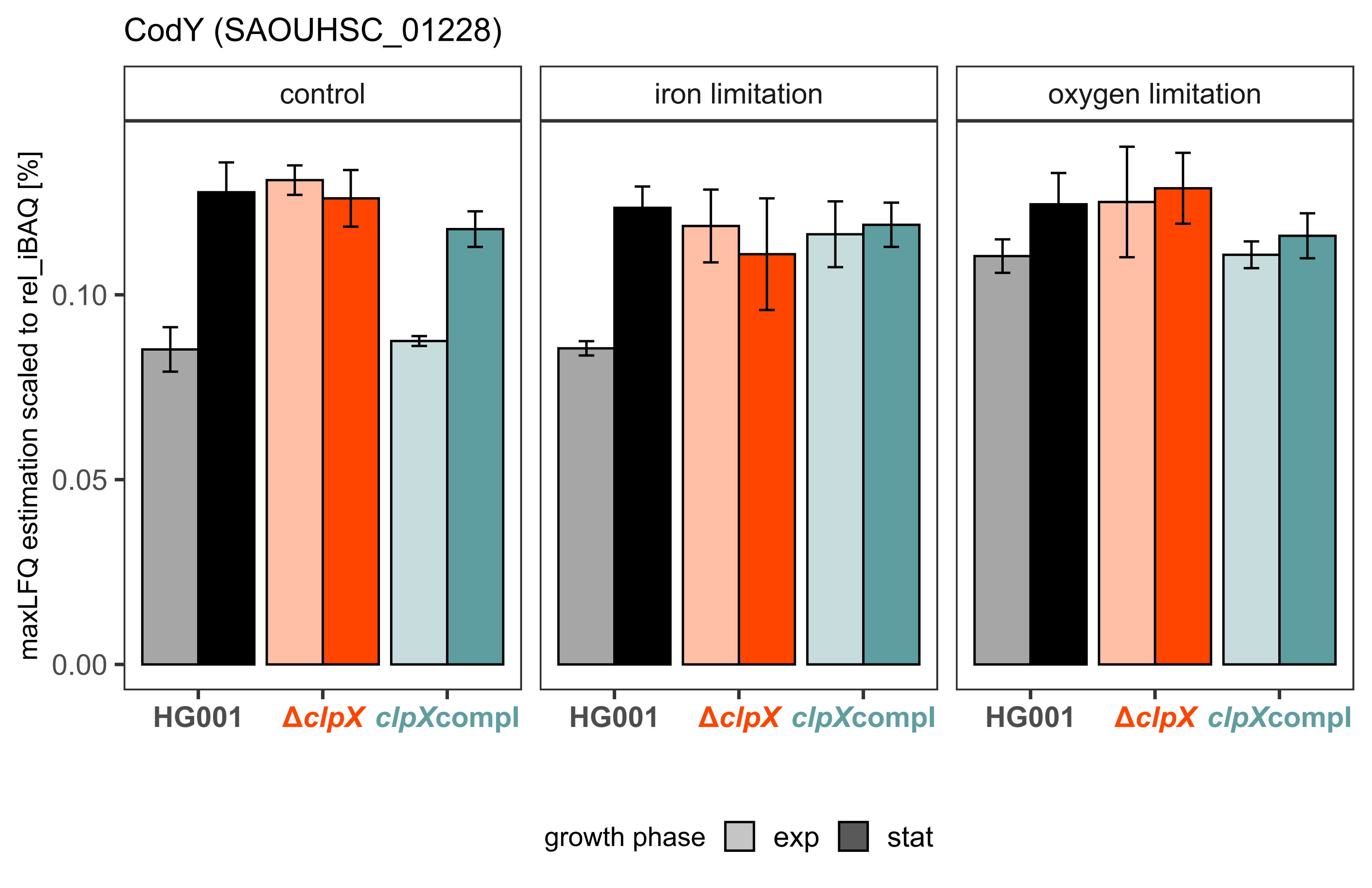
CodY (SAOUHSC_01228)
control
iron limitation
oxygen limitation
maxLFQ estimation scaled to rel_iBAQ [%]
0.10
0.05
0.00
HG001
ΔclpX
clpXcompl
growth phase
exp
stat

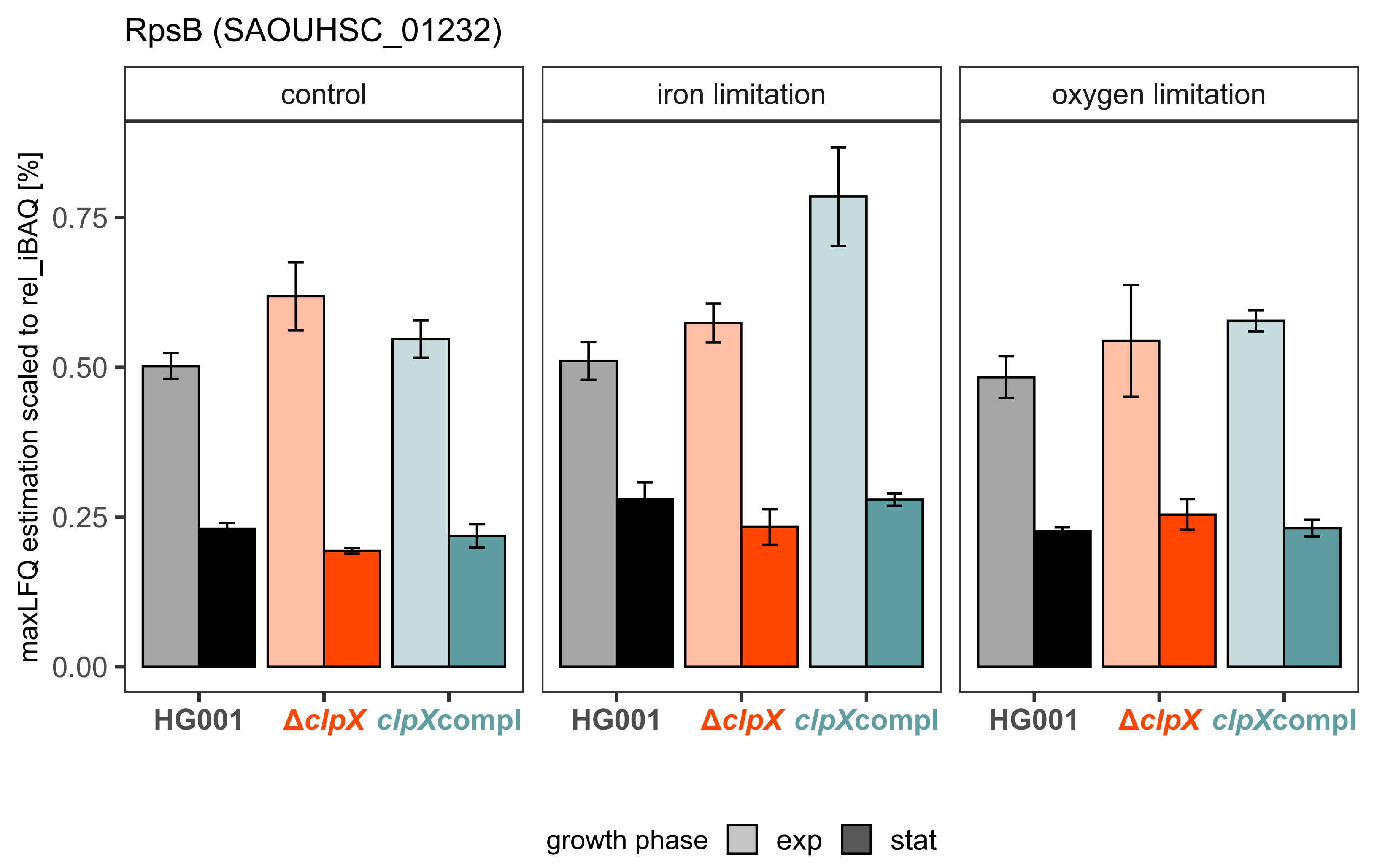
RpsB (SAOUHSC_01232)
control
iron limitation
oxygen limitation
maxLFQ estimation scaled to rel_iBAQ [%]
0.75
0.50
0.25
0.00
HG001
ΔclpX
clpXcompl
growth phase
exp
stat

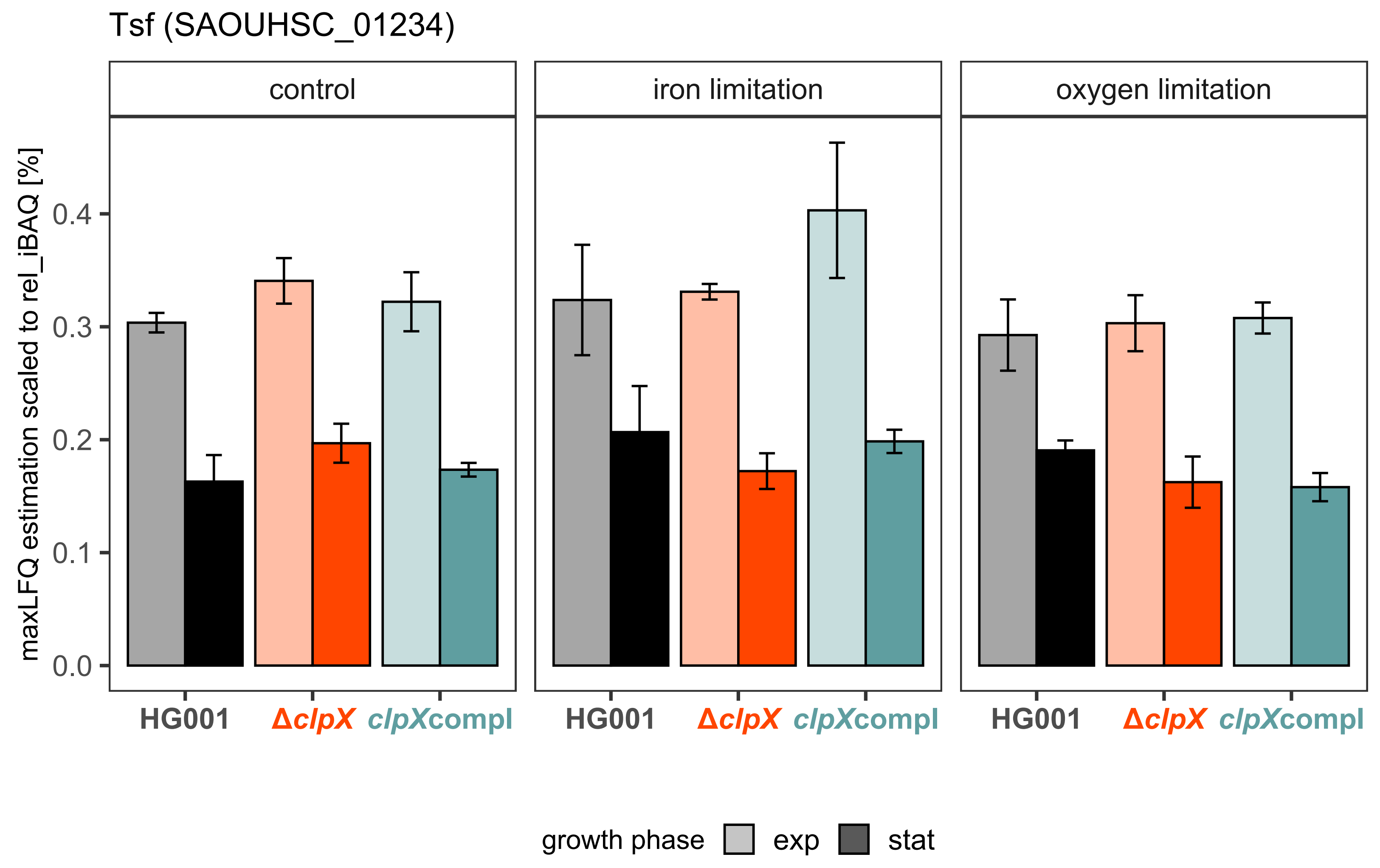
Tsf (SAOUHSC_01234)
control
iron limitation
oxygen limitation
maxLFQ estimation scaled to rel_iBAQ [%]
0.0
0.1
0.2
0.3
0.4
HG001
ΔclpX
clpXcompl
growth phase
exp
stat

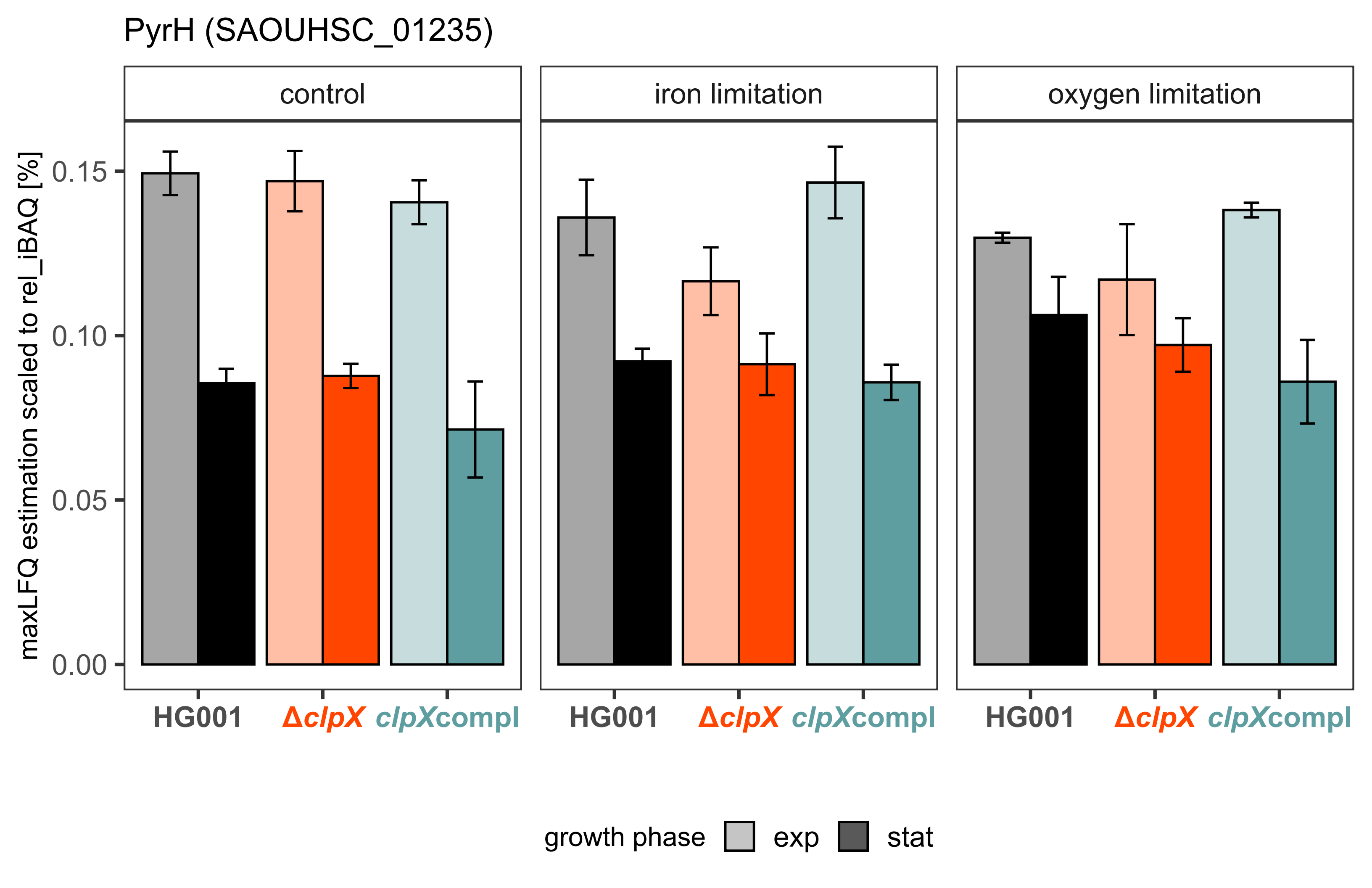

PyrH (SAOUHSC_01235)
control
iron limitation
oxygen limitation
maxLFQ estimation scaled to rel_iBAQ [%]
0.15
0.10
0.05
0.00
HG001
ΔclpX
clpXcompl
growth phase
exp
stat

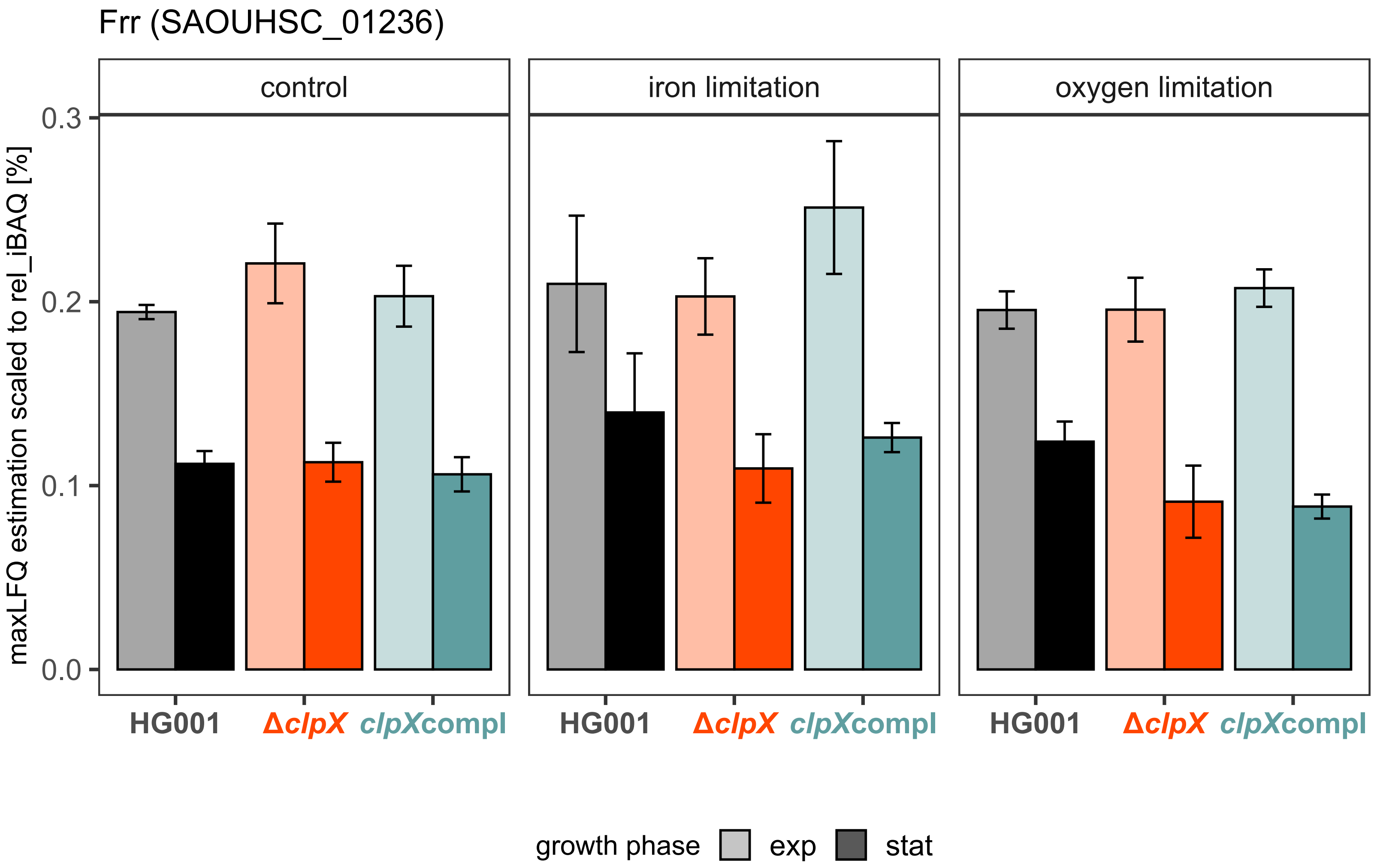

Frr (SAOUHSC_01236)
control
iron limitation
oxygen limitation
maxLFQ estimation scaled to rel_iBAQ [%]
0.0
0.1
0.2
0.3
HG001
ΔclpX
clpXcompl
growth phase
exp
stat

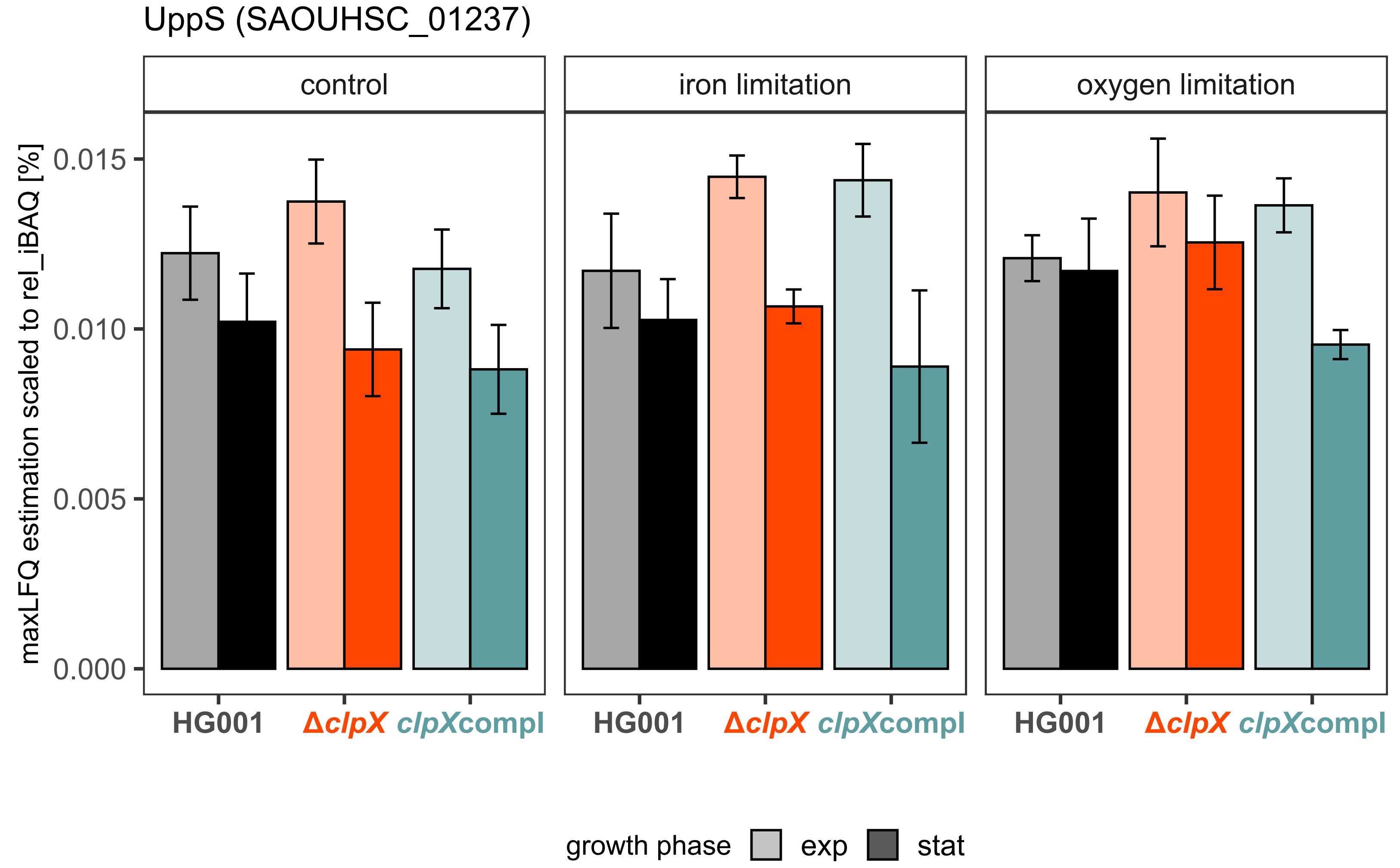
UppS (SAOUHSC_01237)
control
iron limitation
oxygen limitation
maxLFQ estimation scaled to rel_iBAQ [%]
0.015
0.010
0.005
0.000
HG001
ΔclpX
clpXcompl
growth phase
exp
stat

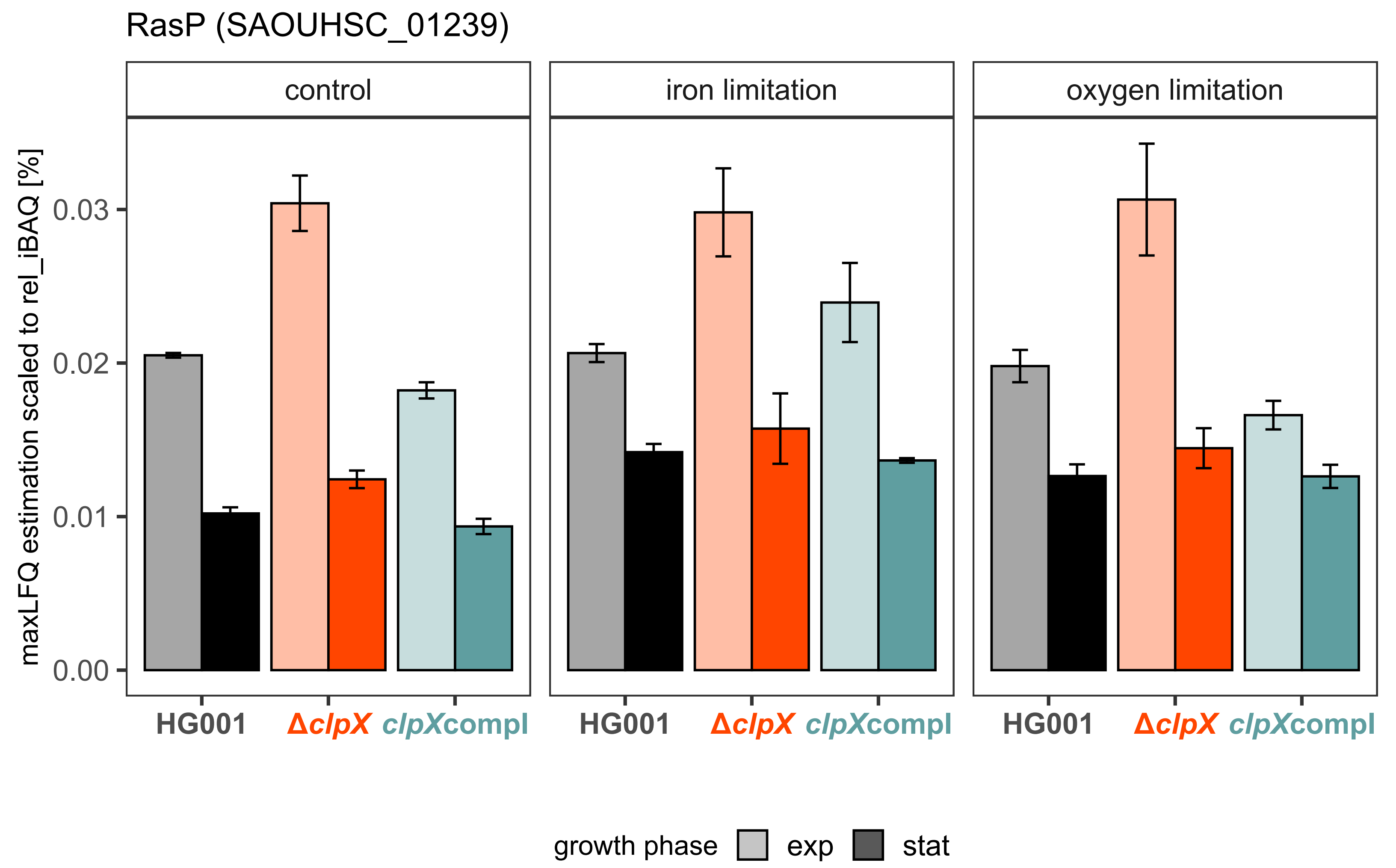

RasP (SAOUHSC_01239)
control
iron limitation
oxygen limitation
maxLFQ estimation scaled to rel_iBAQ [%]
0.00
0.01
0.02
0.03
HG001
ΔclpX
clpXcompl
growth phase
exp
stat

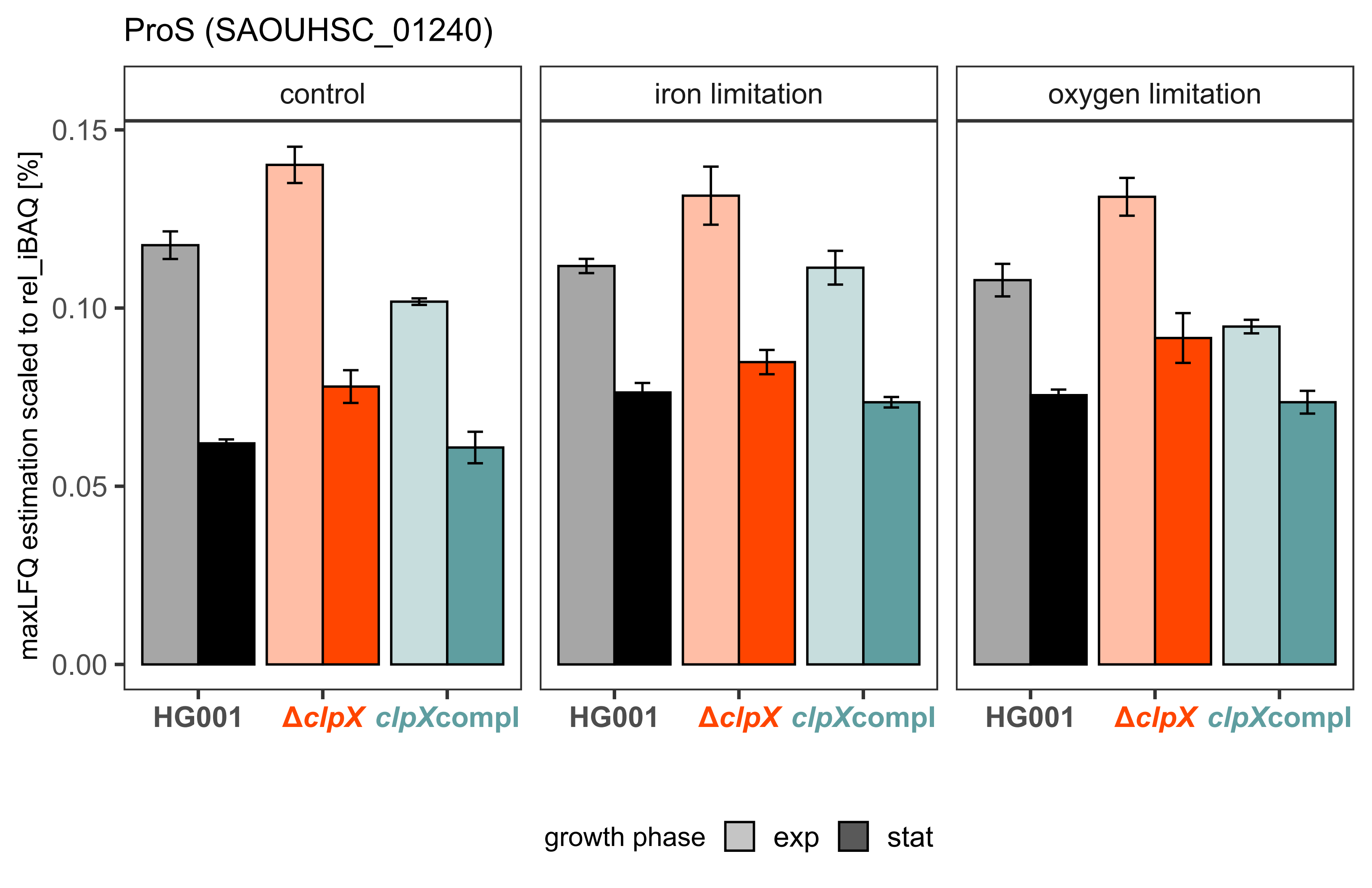

ProS (SAOUHSC_01240)
control
iron limitation
oxygen limitation
maxLFQ estimation scaled to rel_iBAQ [%]
0.15
0.10
0.05
0.00
HG001
ΔclpX
clpXcompl
growth phase
exp
stat

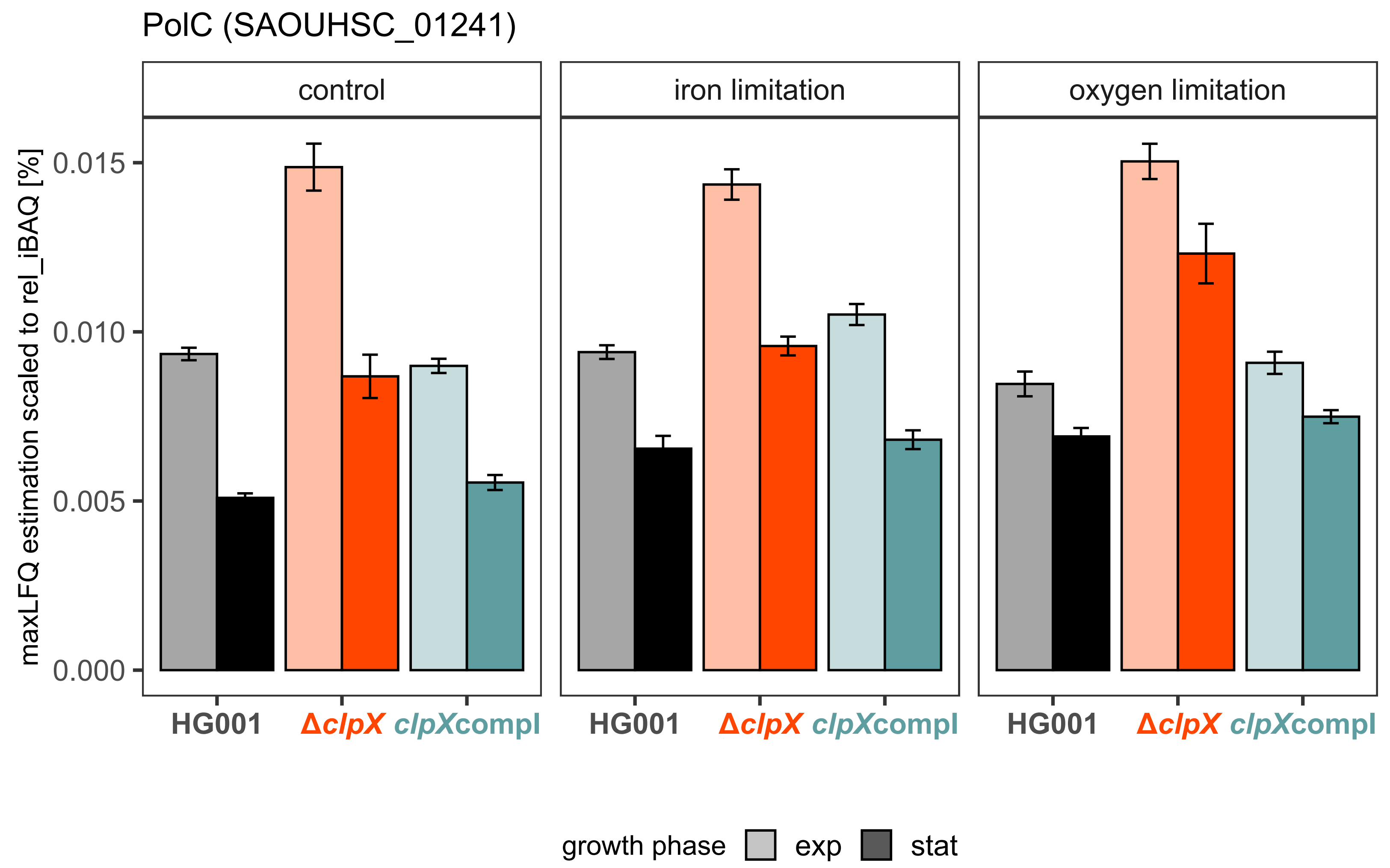

PolC (SAOUHSC_01241)
control
iron limitation
oxygen limitation
maxLFQ estimation scaled to rel_iBAQ [%]
0.015
0.010
0.005
0.000
HG001
ΔclpX
clpXcompl
growth phase
exp
stat

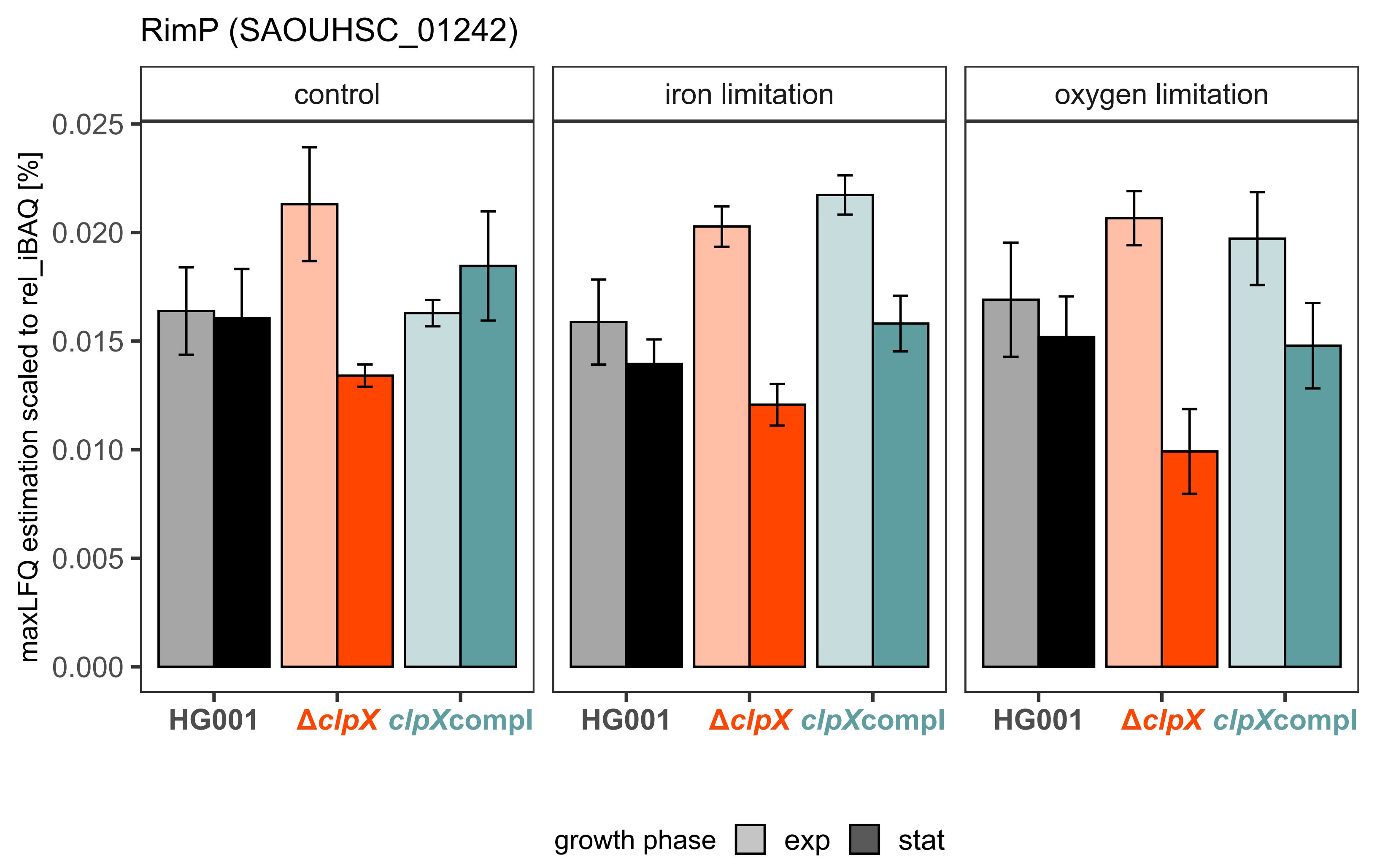

RimP (SAOUHSC_01242)
control
iron limitation
oxygen limitation
maxLFQ estimation scaled to rel_iBAQ [%]
0.025
0.020
0.015
0.010
0.005
0.000
HG001
ΔclpX
clpXcompl
growth phase
exp
stat

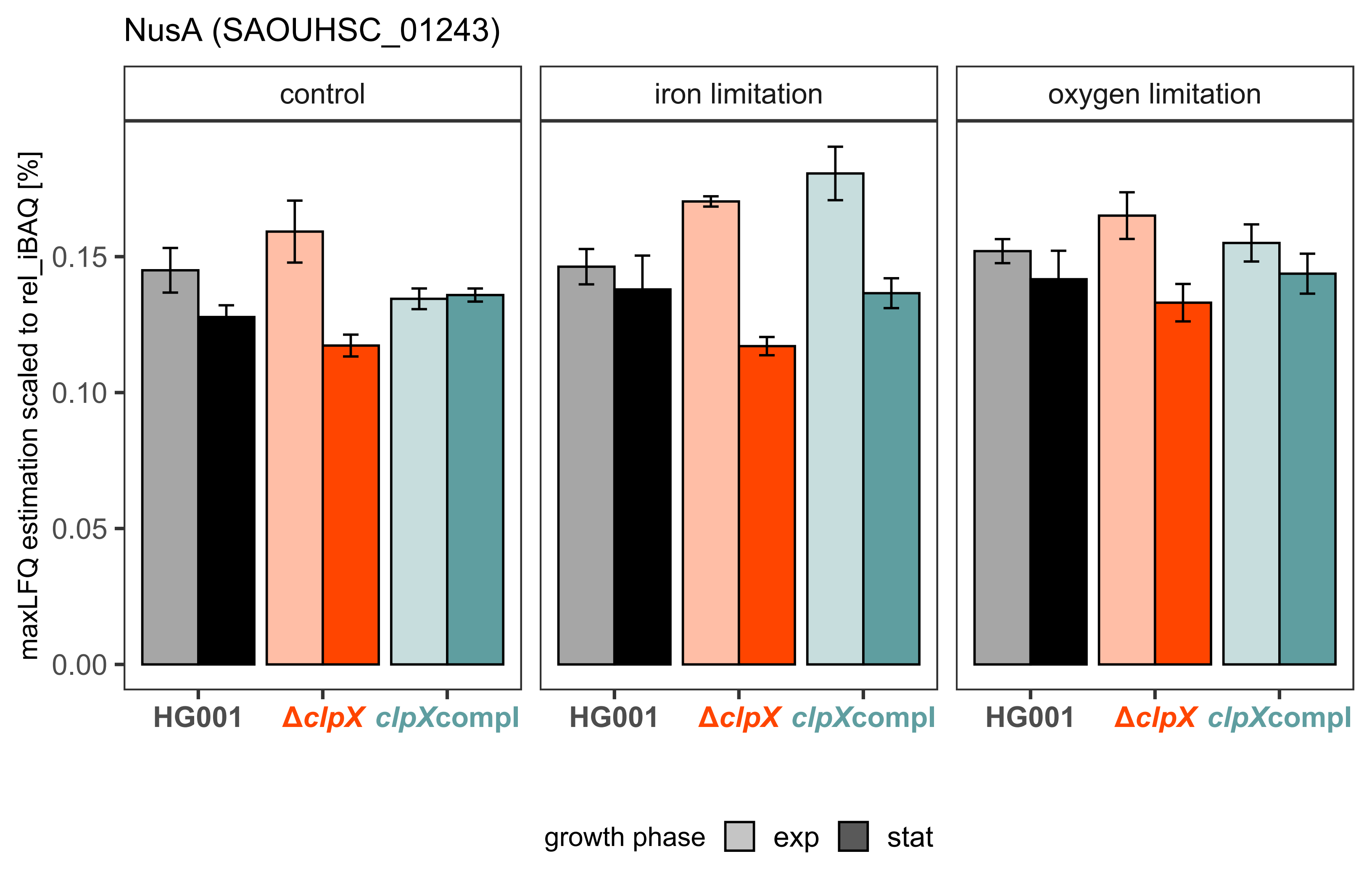

NusA (SAOUHSC_01243)
control
iron limitation
oxygen limitation
maxLFQ estimation scaled to rel_iBAQ [%]
0.00
0.05
0.10
0.15
HG001
ΔclpX
clpXcompl
growth phase
exp
stat

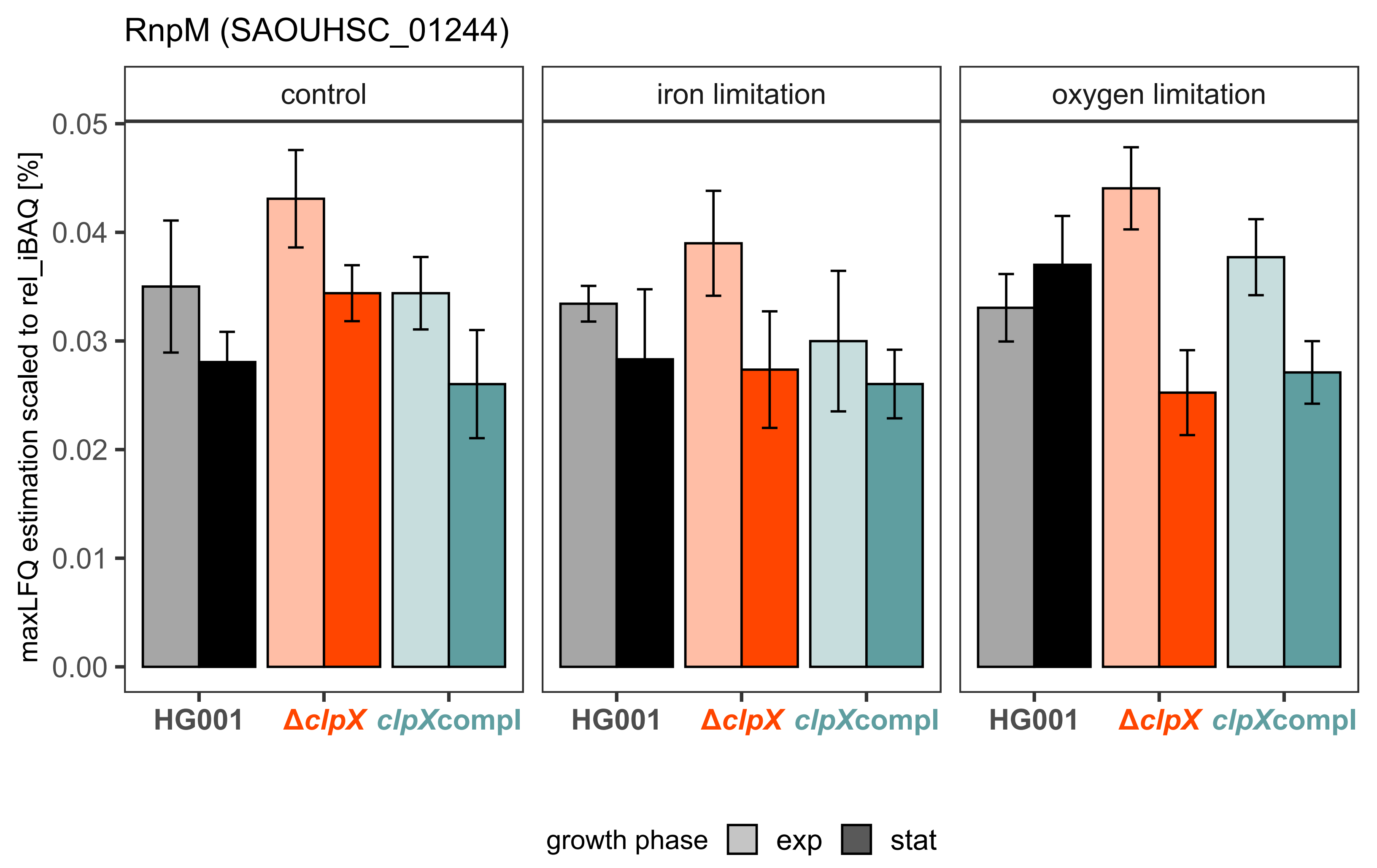
RnpM (SAOUHSC_01244)
control
iron limitation
oxygen limitation
maxLFQ estimation scaled to rel_iBAQ [%]
0.05
0.04
0.03
0.02
0.01
0.00
HG001
ΔclpX
clpXcompl
growth phase
exp
stat

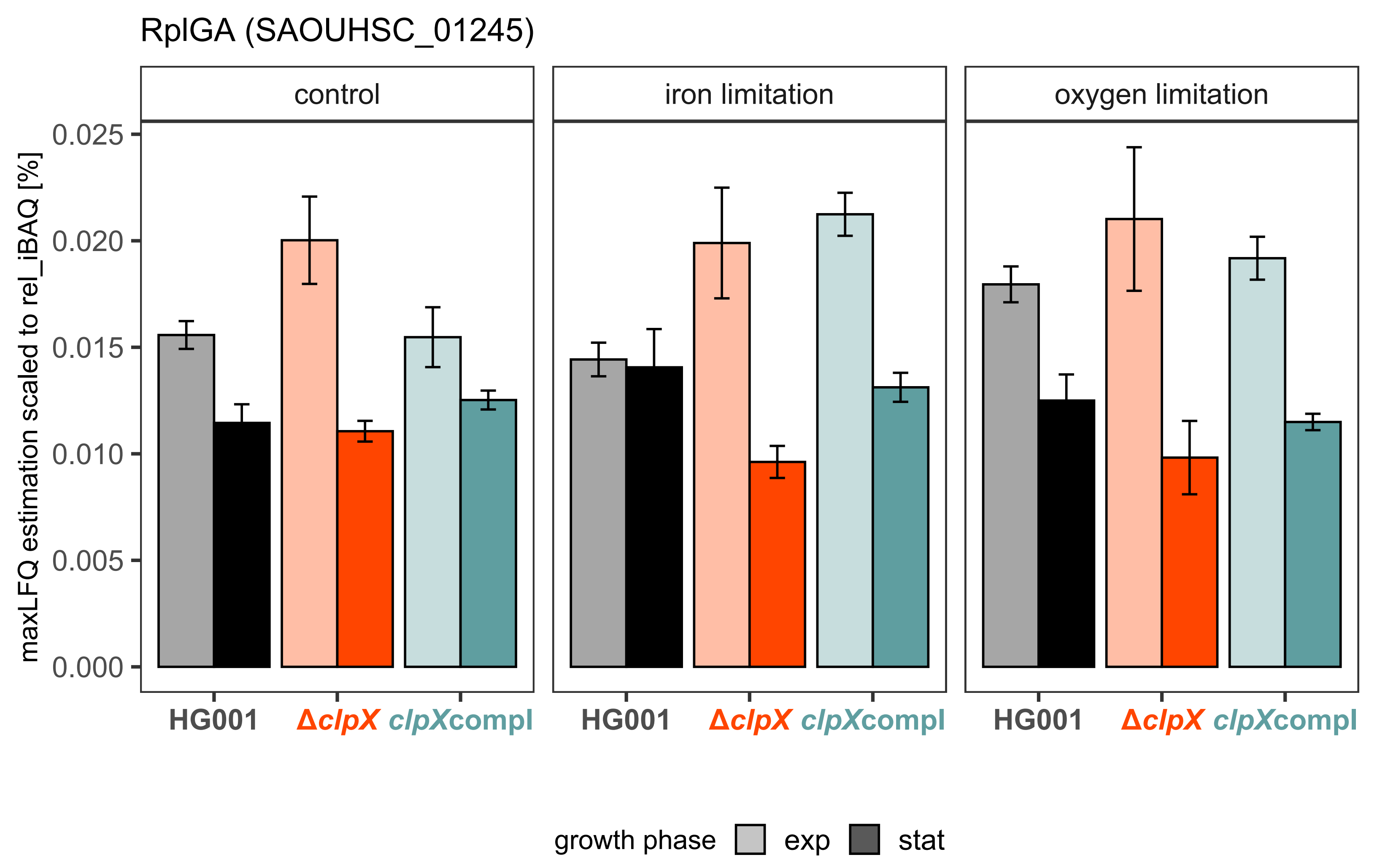
RplGA (SAOUHSC_01245)
control
iron limitation
oxygen limitation
maxLFQ estimation scaled to rel_iBAQ [%]
0.025
0.020
0.015
0.010
0.005
0.000
HG001
ΔclpX
clpXcompl
growth phase
exp
stat

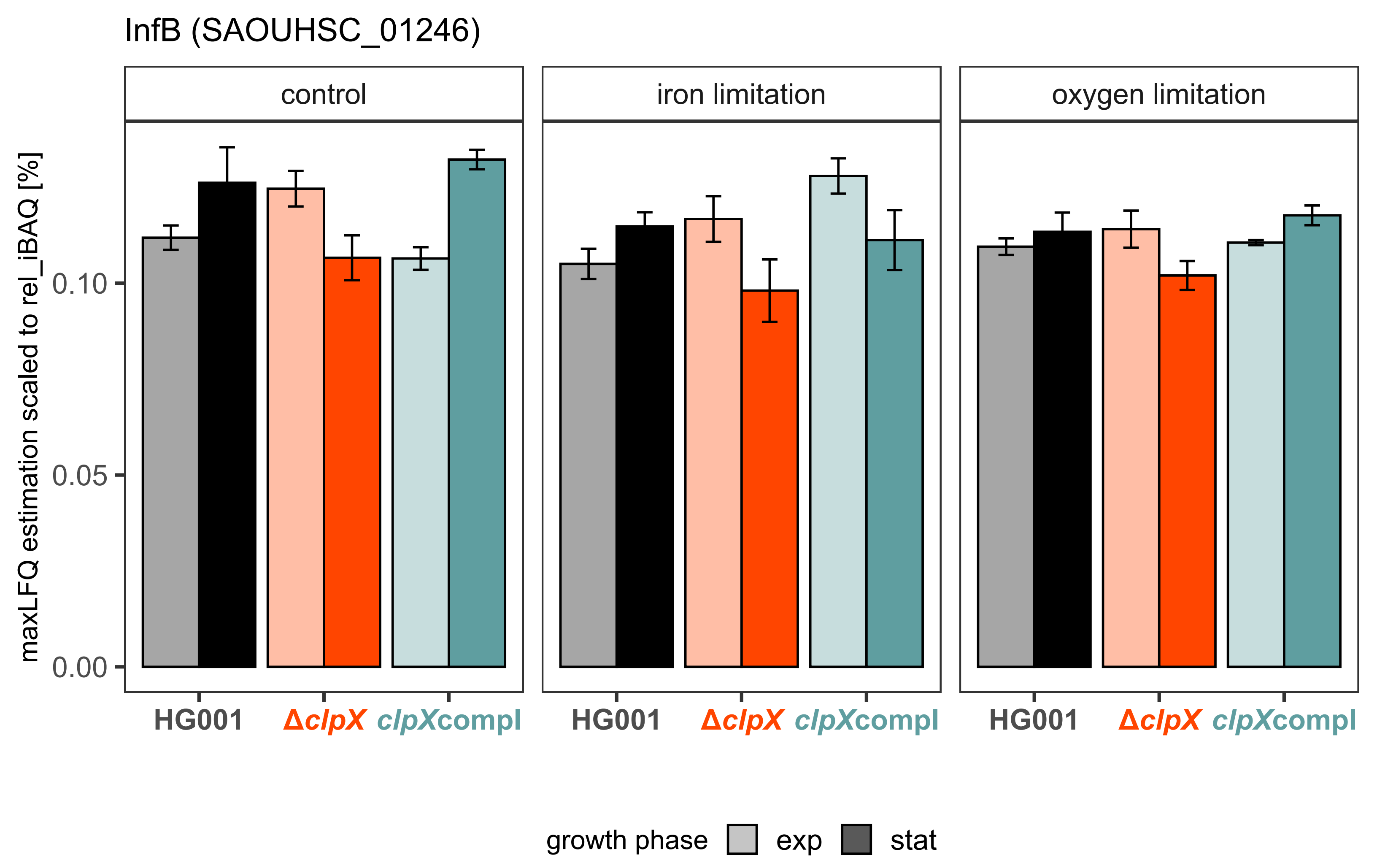

InfB (SAOUHSC_01246)
control
iron limitation
oxygen limitation
maxLFQ estimation scaled to rel_iBAQ [%]
0.10
0.05
0.00
HG001
ΔclpX
clpXcompl
growth phase
exp
stat

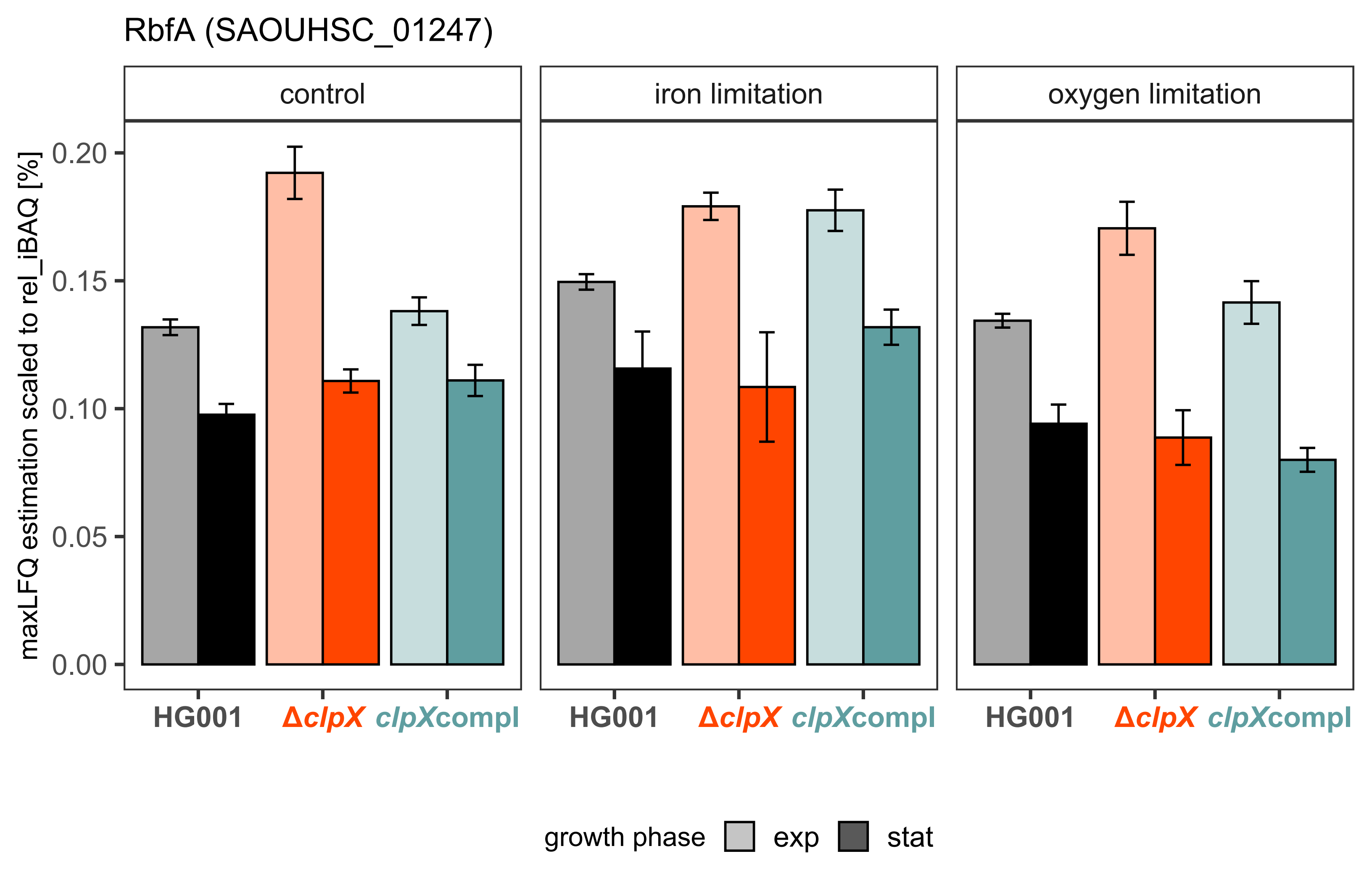

RbfA (SAOUHSC_01247)
control
iron limitation
oxygen limitation
maxLFQ estimation scaled to rel_iBAQ [%]
0.20
0.15
0.10
0.05
0.00
HG001
ΔclpX
clpXcompl
growth phase
exp
stat

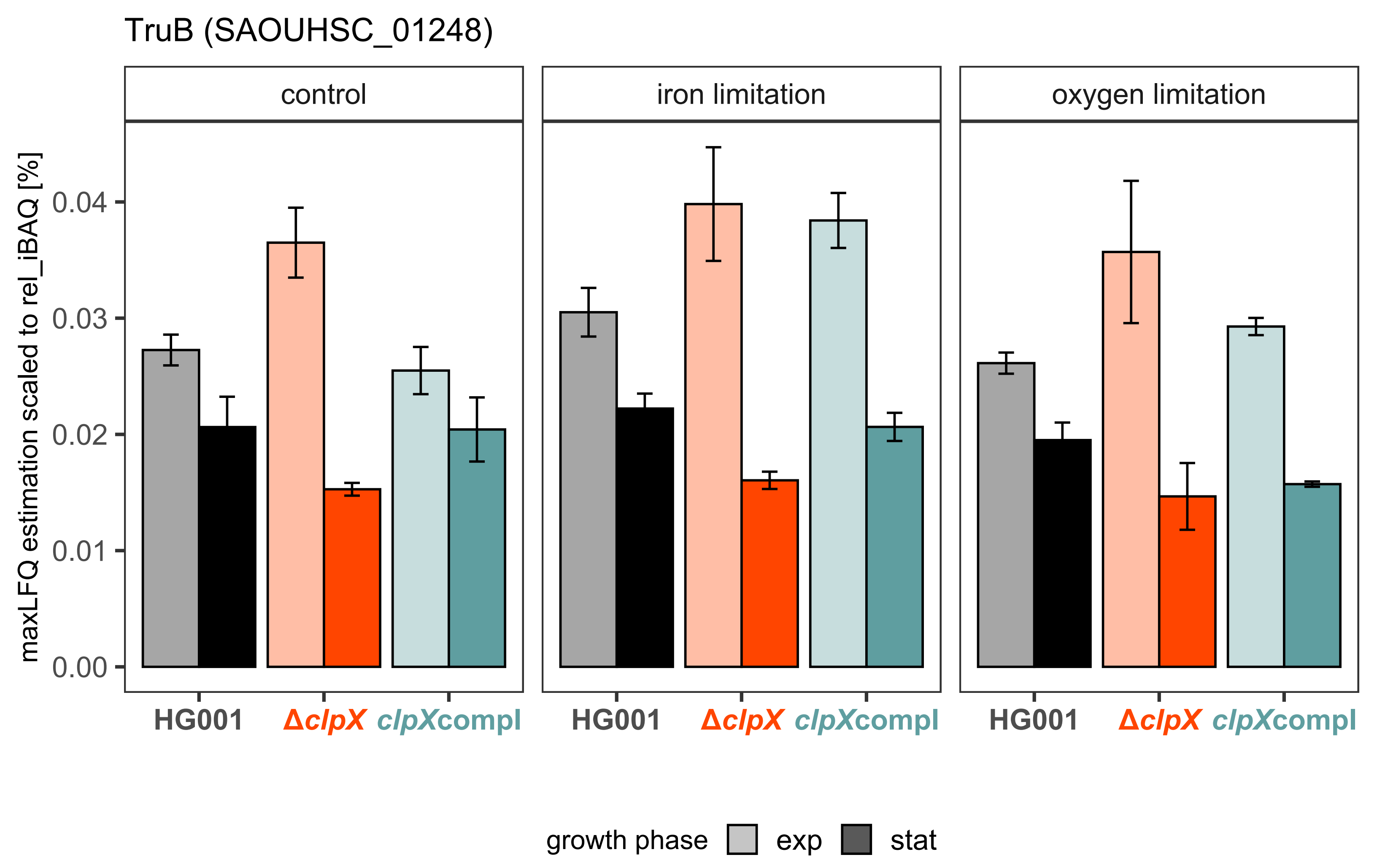

TruB (SAOUHSC_01248)
control
iron limitation
oxygen limitation
maxLFQ estimation scaled to rel_iBAQ [%]
0.00
0.01
0.02
0.03
0.04
HG001
ΔclpX
clpXcompl
growth phase
exp
stat

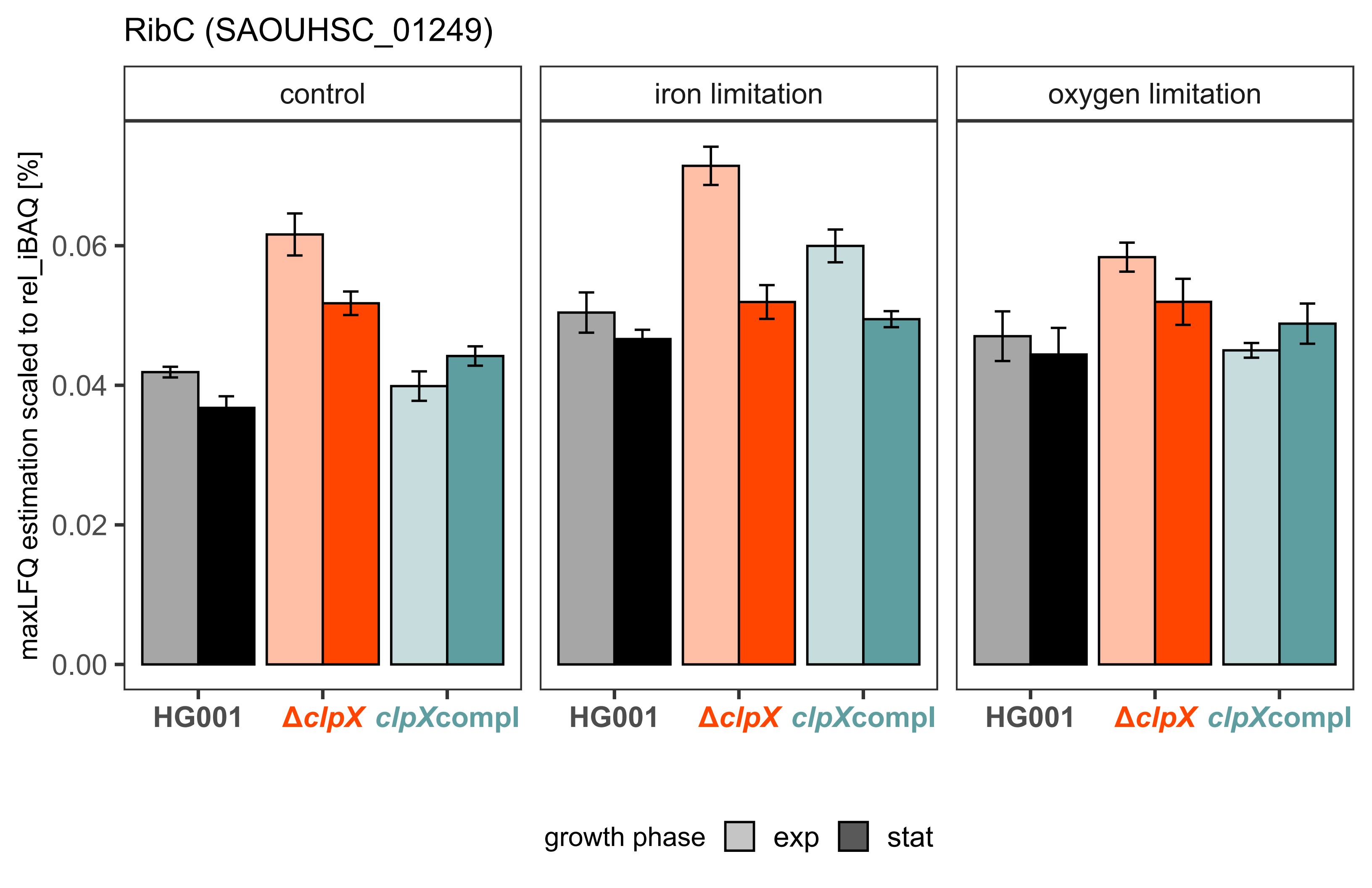

RibC (SAOUHSC_01249)
control
iron limitation
oxygen limitation
maxLFQ estimation scaled to rel_iBAQ [%]
0.00
0.02
0.04
0.06
HG001
ΔclpX
clpXcompl
growth phase
exp
stat

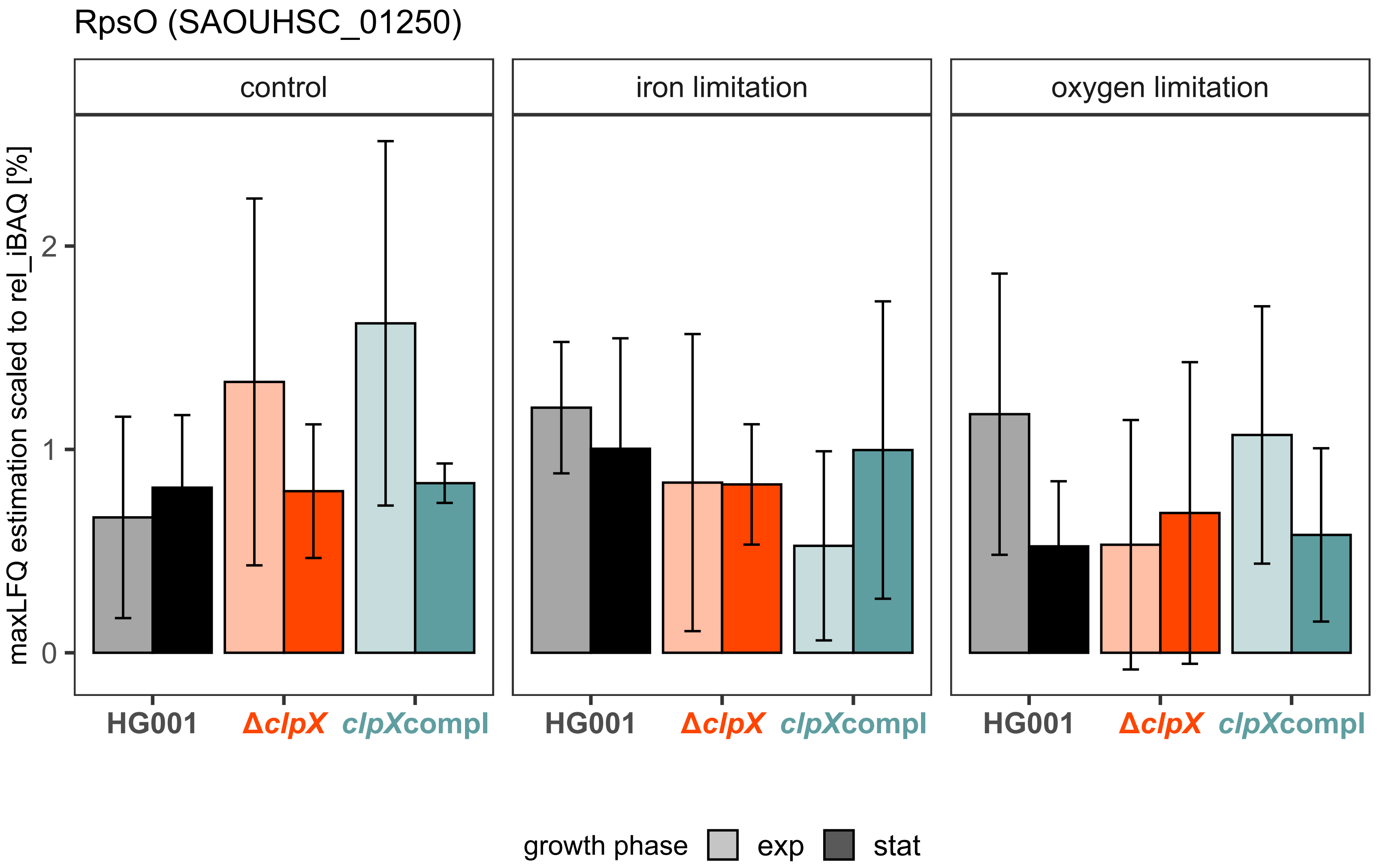
RpsO (SAOUHSC_01250)
control
iron limitation
oxygen limitation
maxLFQ estimation scaled to rel_iBAQ [%]
0
1
2
HG001
ΔclpX
clpXcompl
growth phase
exp
stat

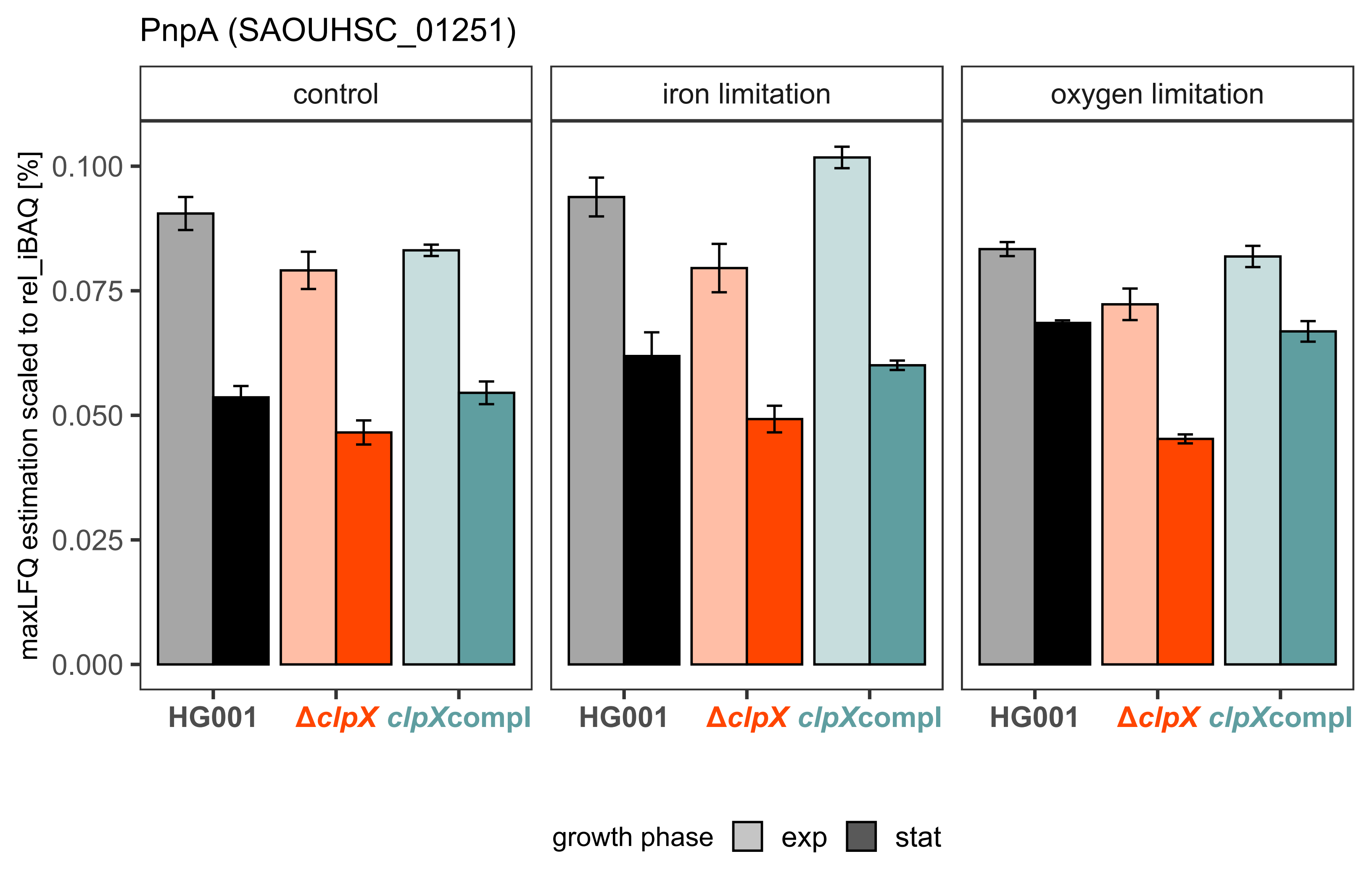
PnpA (SAOUHSC_01251)
control
iron limitation
oxygen limitation
maxLFQ estimation scaled to rel_iBAQ [%]
0.100
0.075
0.050
0.025
0.000
HG001
ΔclpX
clpXcompl
growth phase
exp
stat

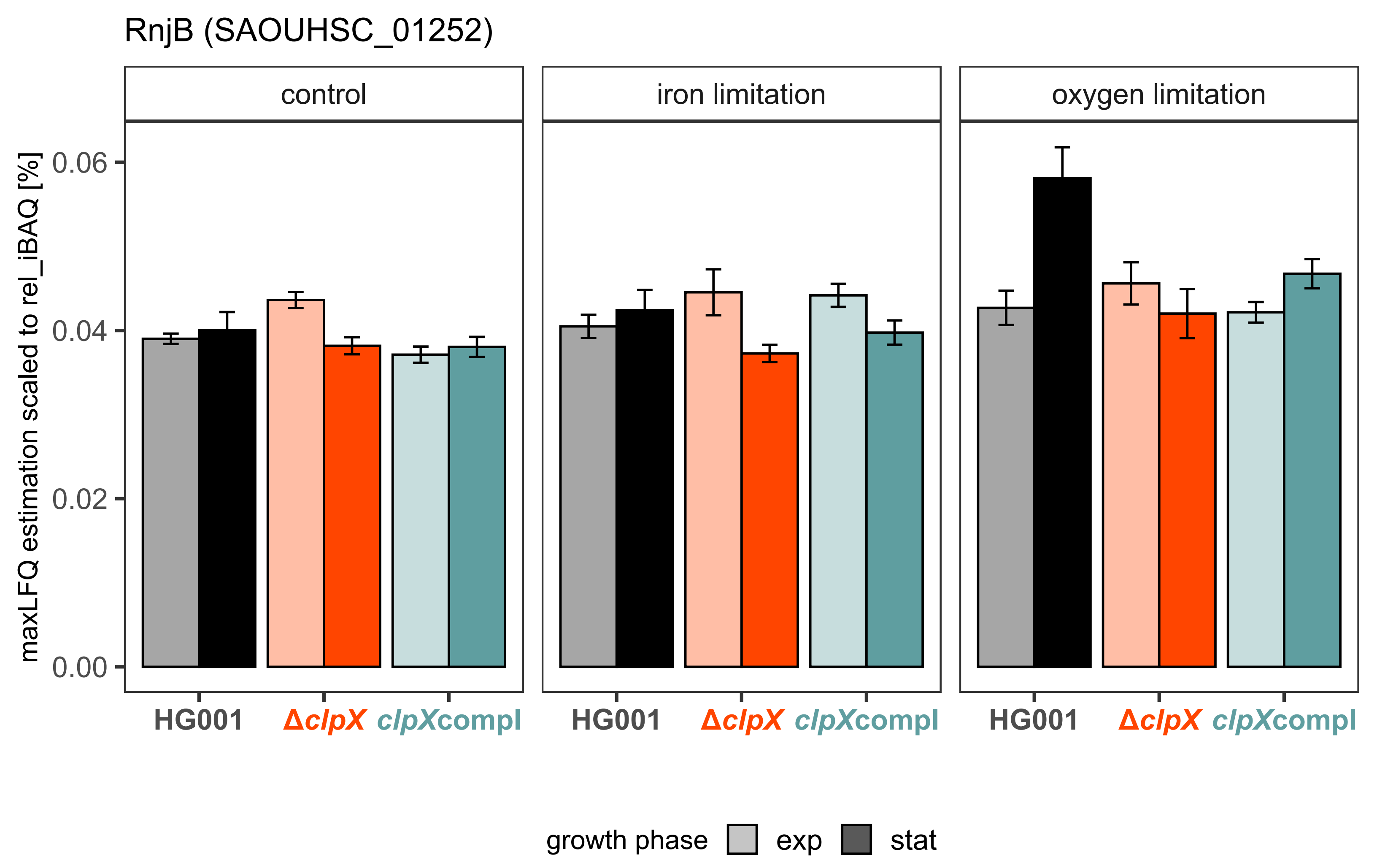

RnjB (SAOUHSC_01252)
control
iron limitation
oxygen limitation
maxLFQ estimation scaled to rel_iBAQ [%]
0.06
0.04
0.02
0.00
HG001
ΔclpX
clpXcompl
growth phase
exp
stat

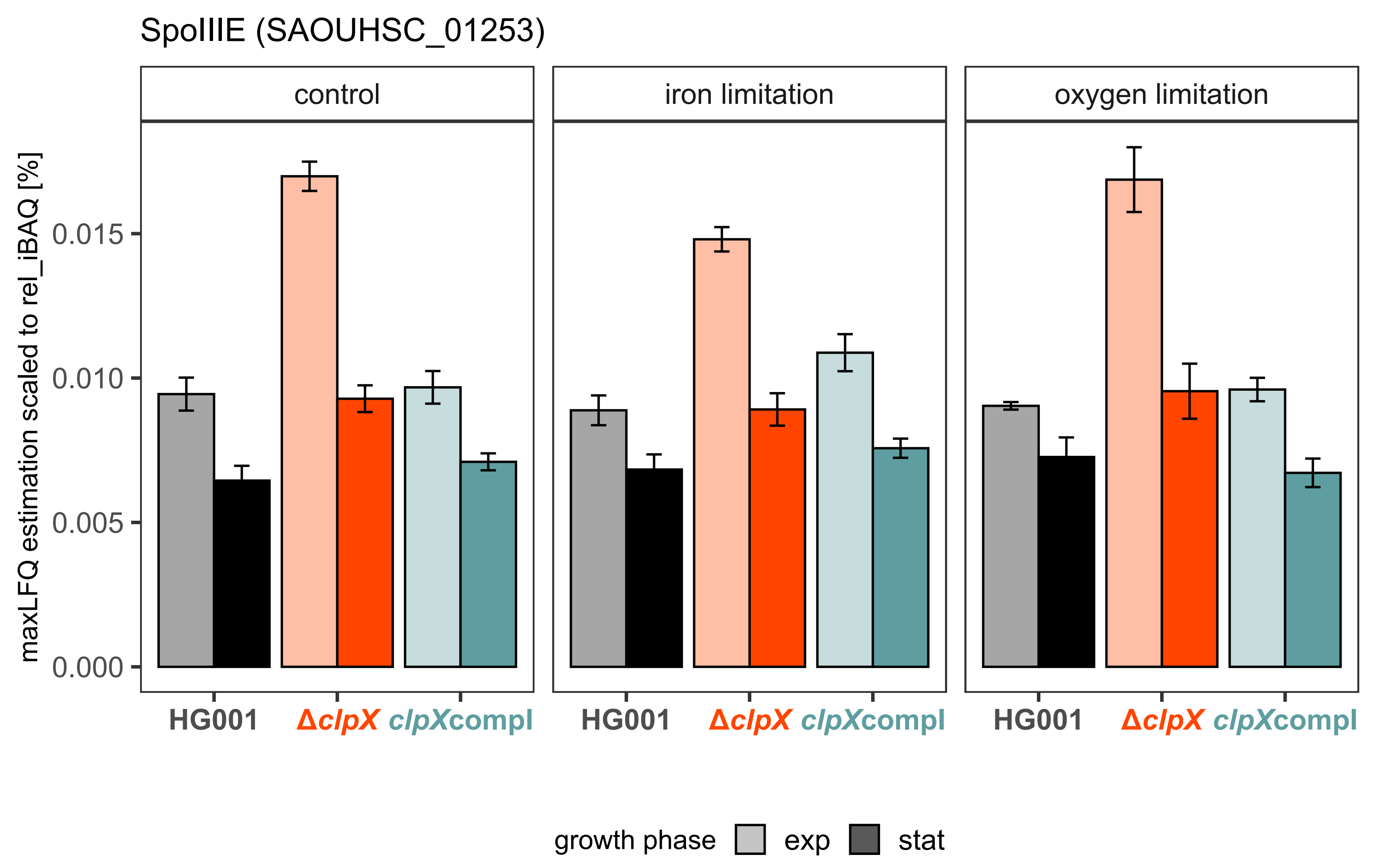

SpoIIIE (SAOUHSC_01253)
control
iron limitation
oxygen limitation
maxLFQ estimation scaled to rel_iBAQ [%]
0.015
0.010
0.005
0.000
HG001
ΔclpX
clpXcompl
growth phase
exp
stat

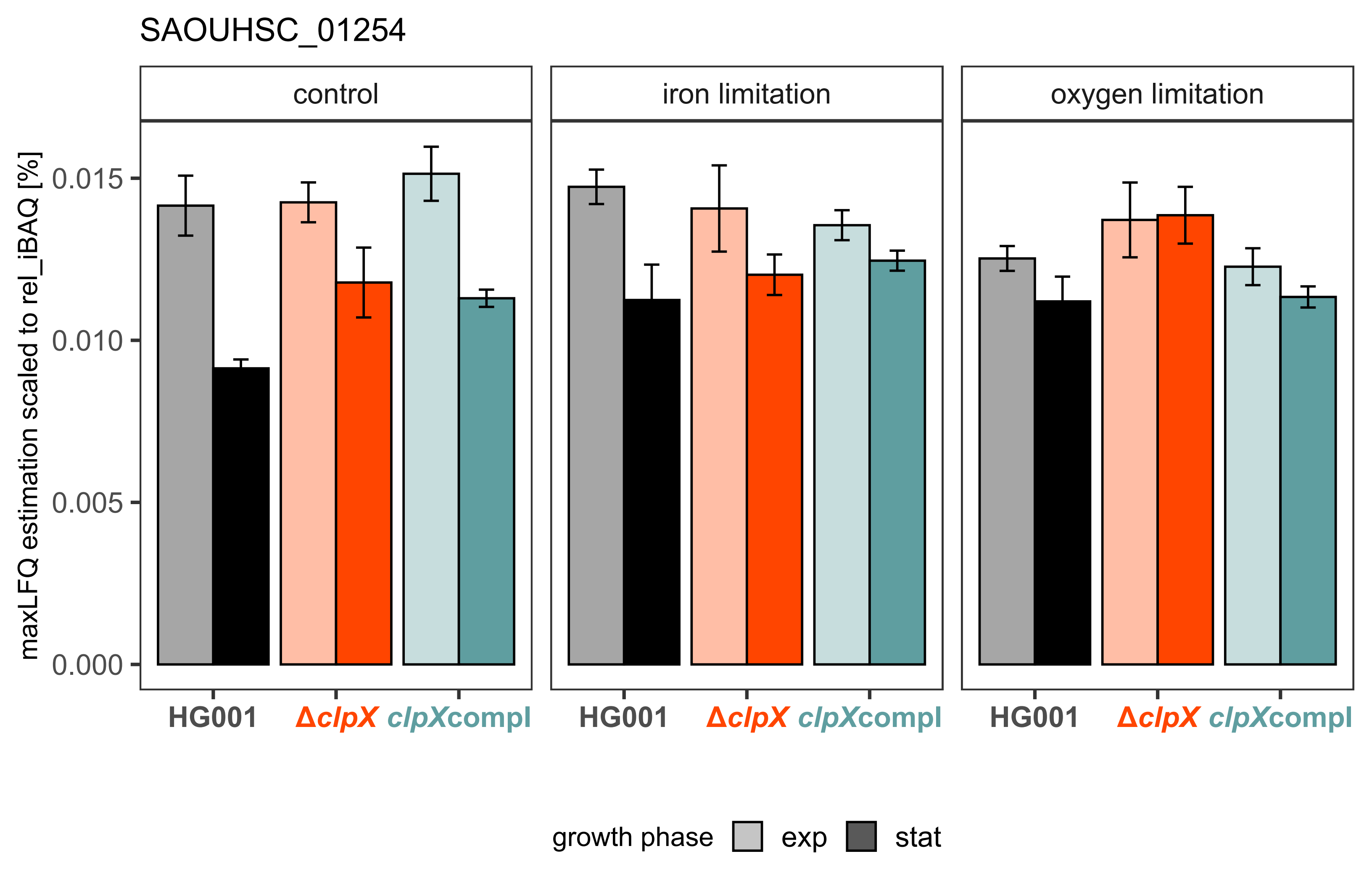
SAOUHSC_01254
control
iron limitation
oxygen limitation
maxLFQ estimation scaled to rel_iBAQ [%]
0.000
0.005
0.010
0.015
HG001
ΔclpX
clpXcompl
growth phase
exp
stat

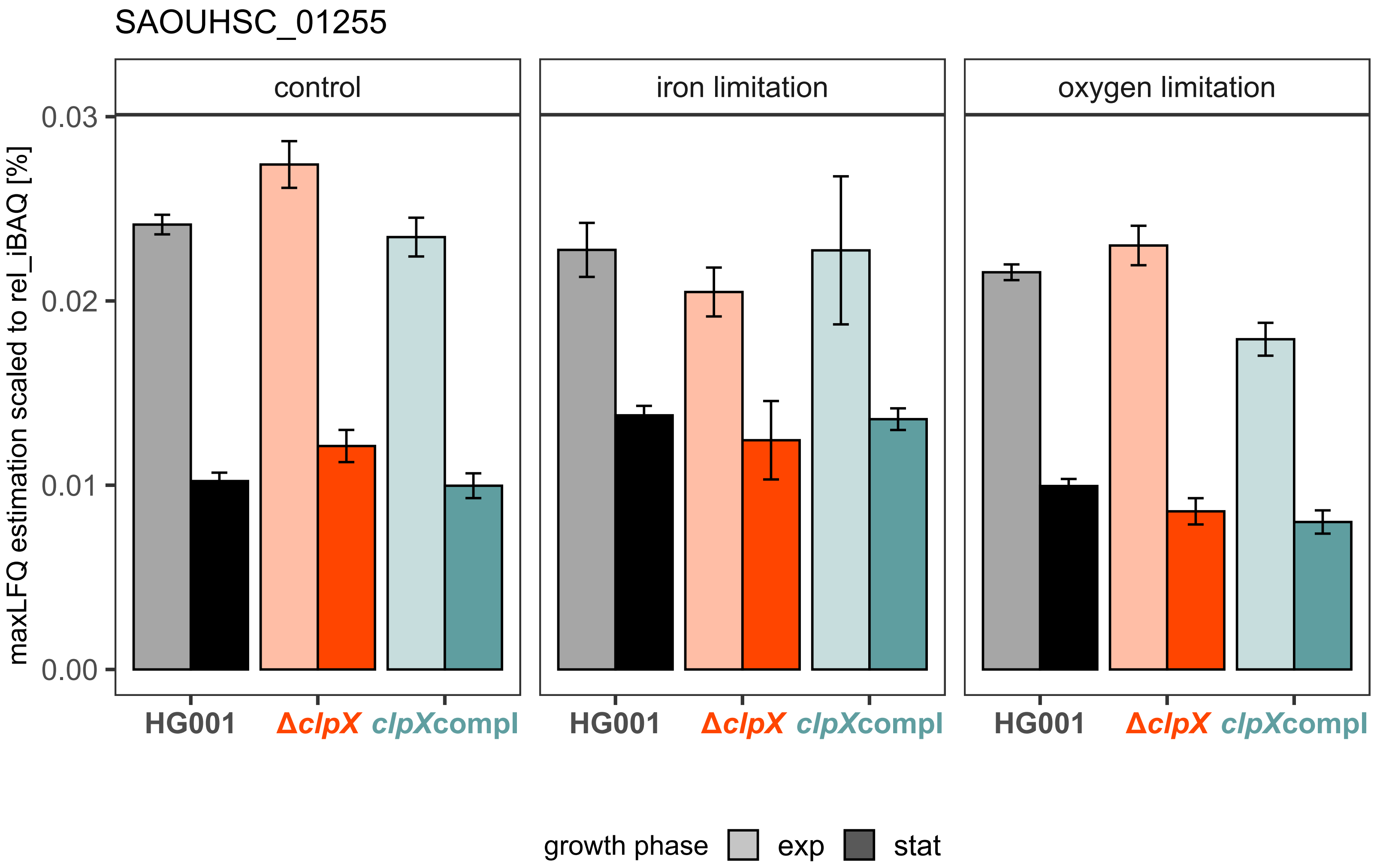

SAOUHSC_01255
control
iron limitation
oxygen limitation
maxLFQ estimation scaled to rel_iBAQ [%]
0.03
0.02
0.01
0.00
HG001
ΔclpX
clpXcompl
growth phase
exp
stat

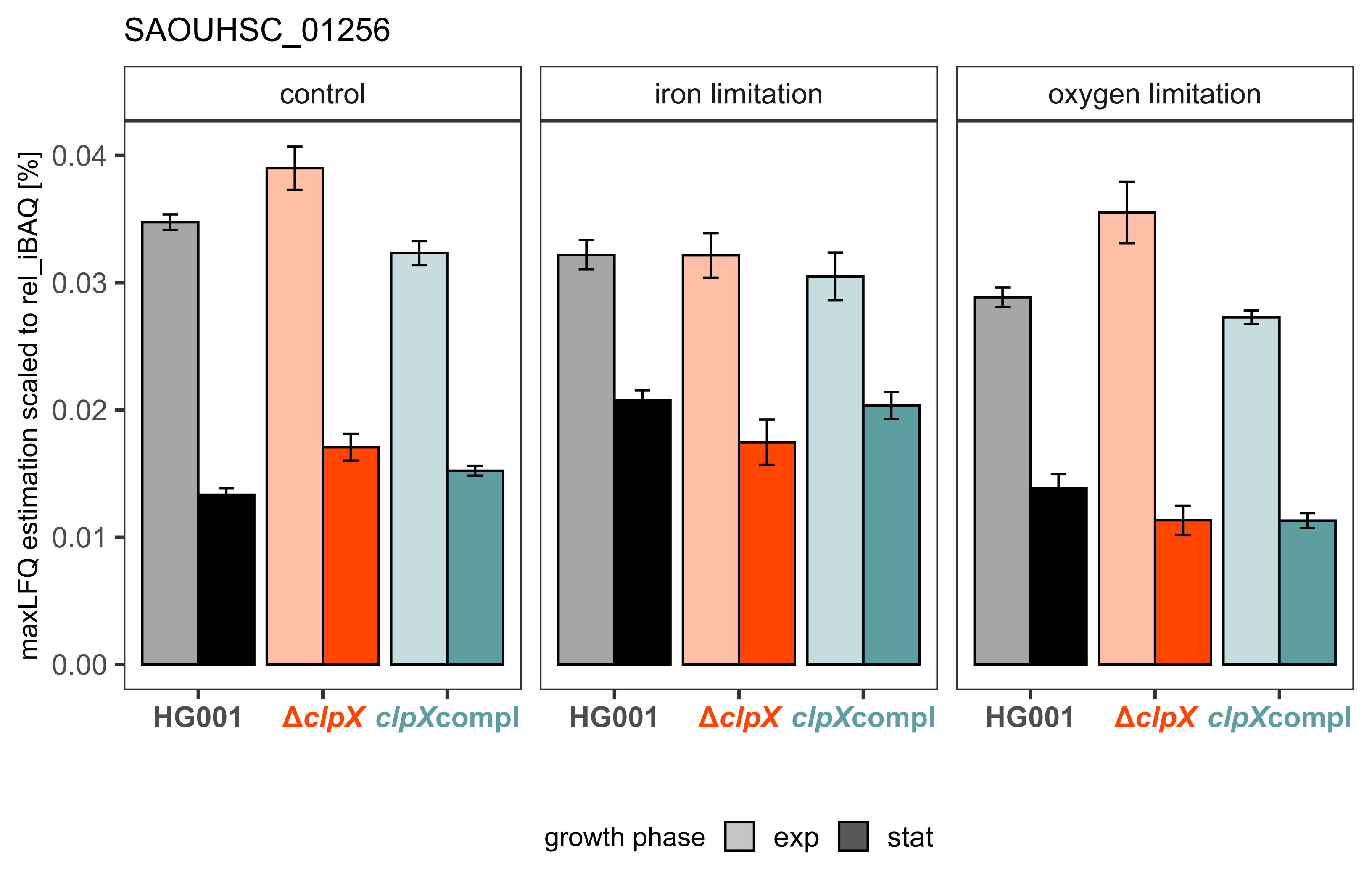

SAOUHSC_01256
control
iron limitation
oxygen limitation
maxLFQ estimation scaled to rel_iBAQ [%]
0.00
0.01
0.02
0.03
0.04
HG001
ΔclpX
clpXcompl
growth phase
exp
stat

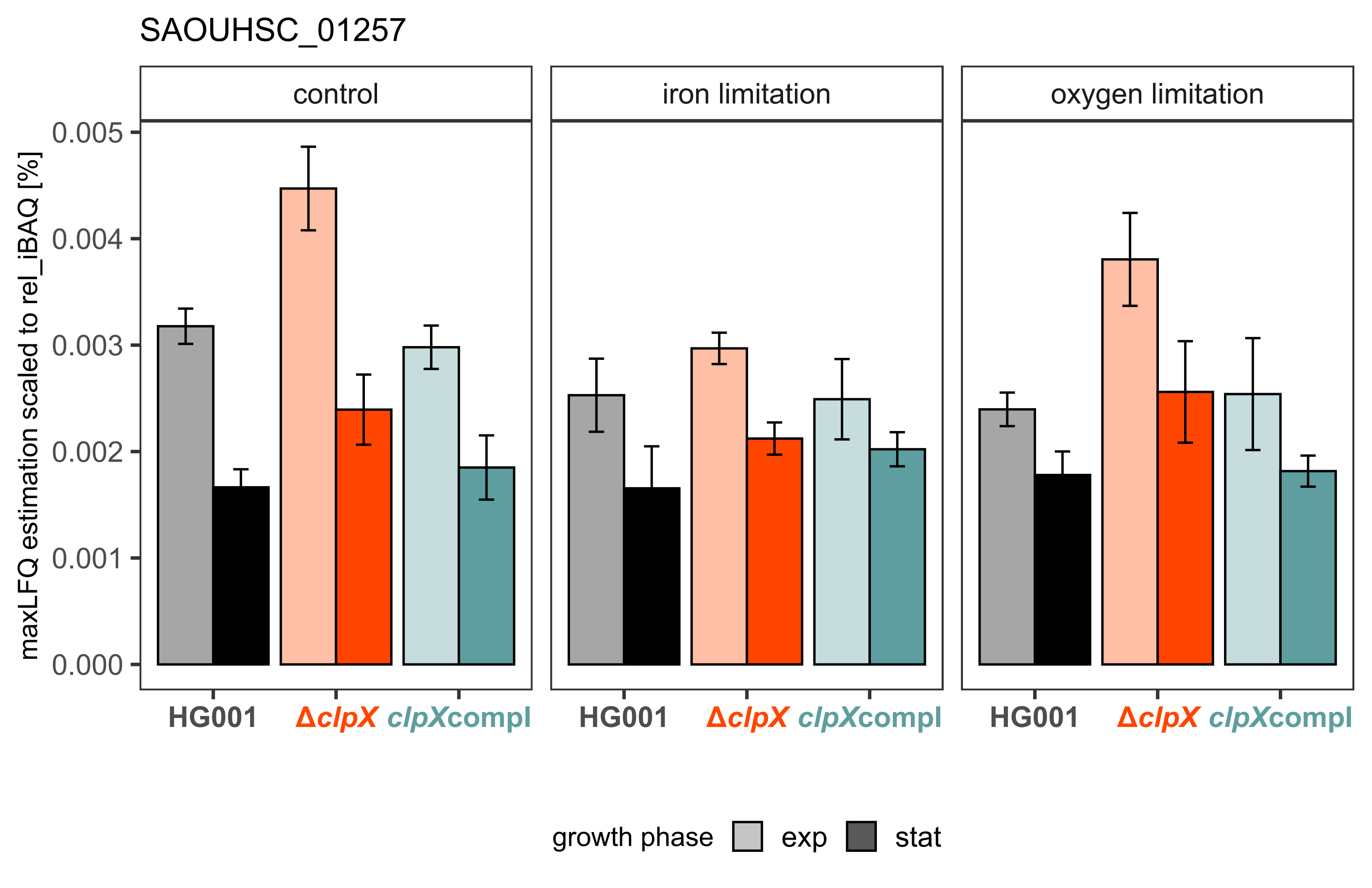
SAOUHSC_01257
control
iron limitation
oxygen limitation
maxLFQ estimation scaled to rel_iBAQ [%]
0.005
0.004
0.003
0.002
0.001
0.000
HG001
ΔclpX
clpXcompl
growth phase
exp
stat

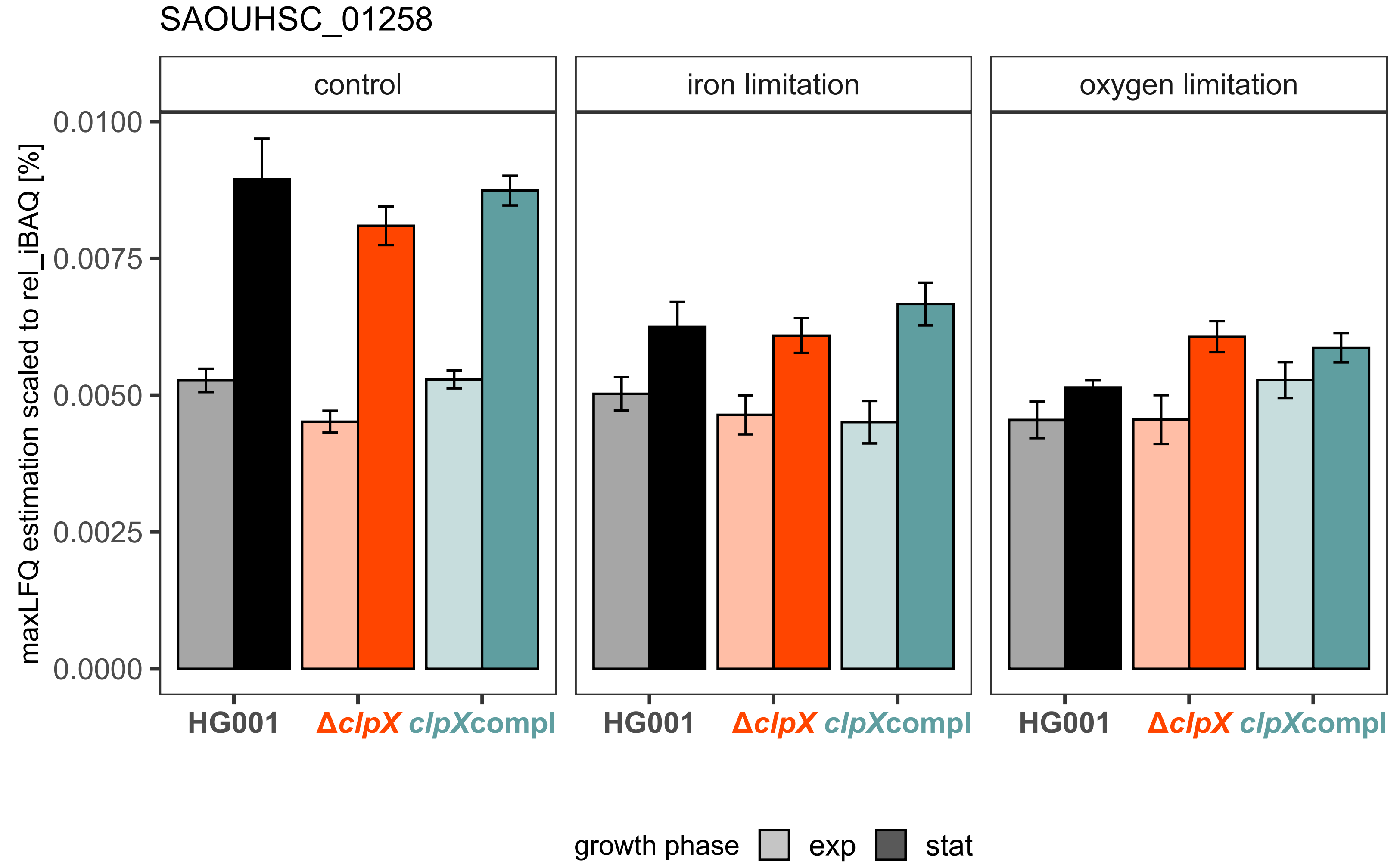

SAOUHSC_01258
control
iron limitation
oxygen limitation
maxLFQ estimation scaled to rel_iBAQ [%]
0.0100
0.0075
0.0050
0.0025
0.0000
HG001
ΔclpX
clpXcompl
growth phase
exp
stat

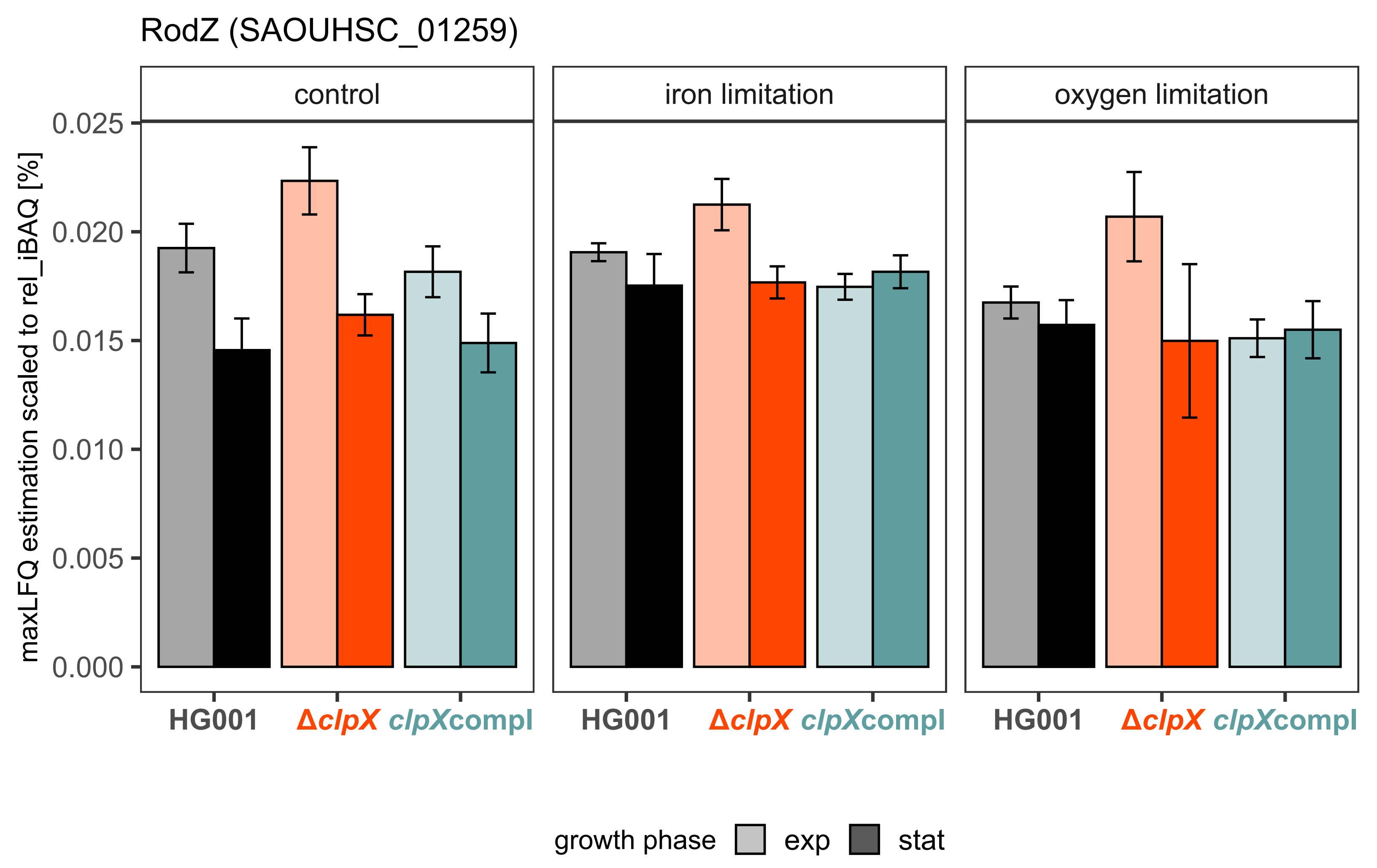
RodZ (SAOUHSC_01259)
control
iron limitation
oxygen limitation
maxLFQ estimation scaled to rel_iBAQ [%]
0.025
0.020
0.015
0.010
0.005
0.000
HG001
ΔclpX
clpXcompl
growth phase
exp
stat

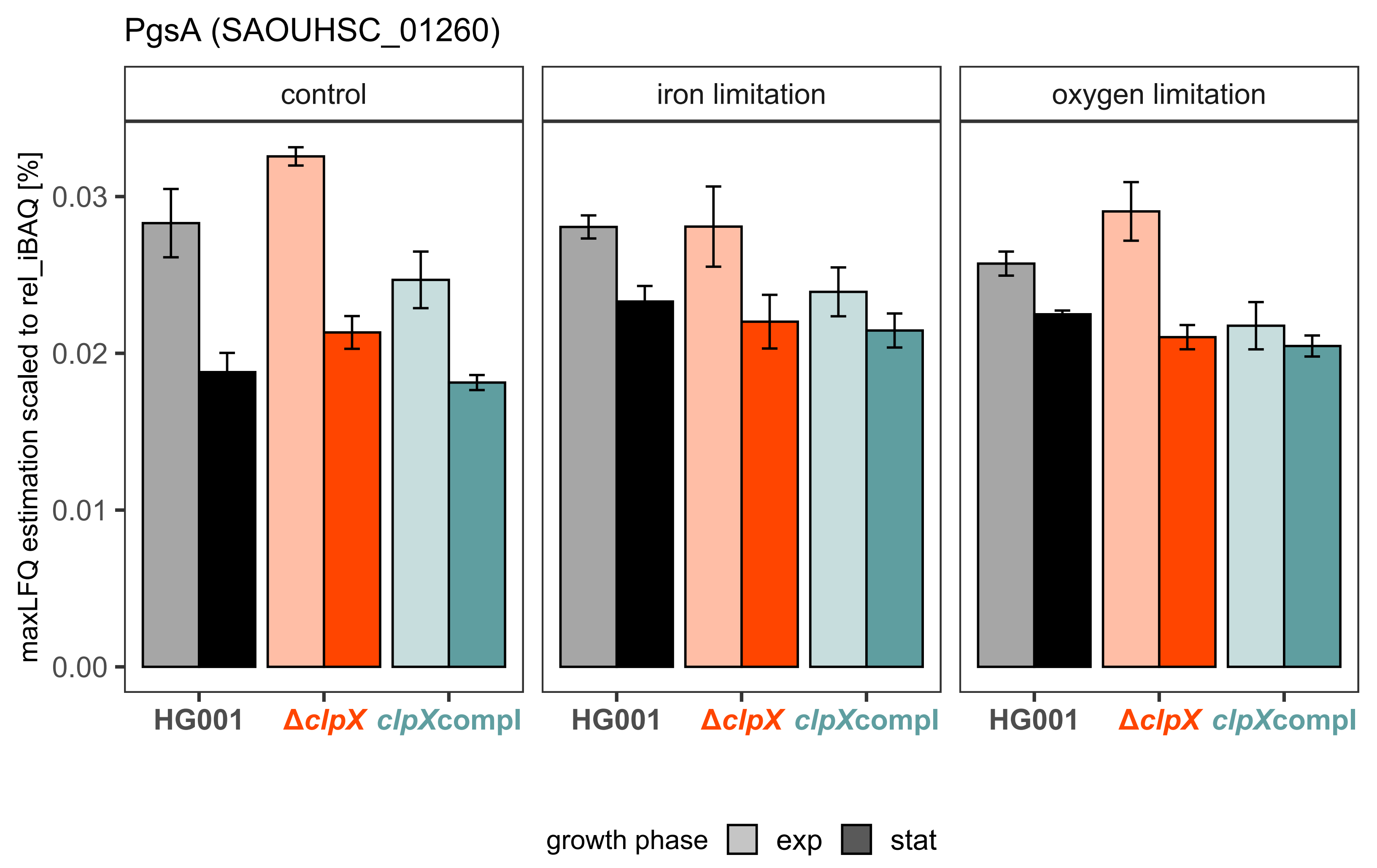
PgsA (SAOUHSC_01260)
control
iron limitation
oxygen limitation
maxLFQ estimation scaled to rel_iBAQ [%]
0.00
0.01
0.02
0.03
HG001
ΔclpX
clpXcompl
growth phase
exp
stat

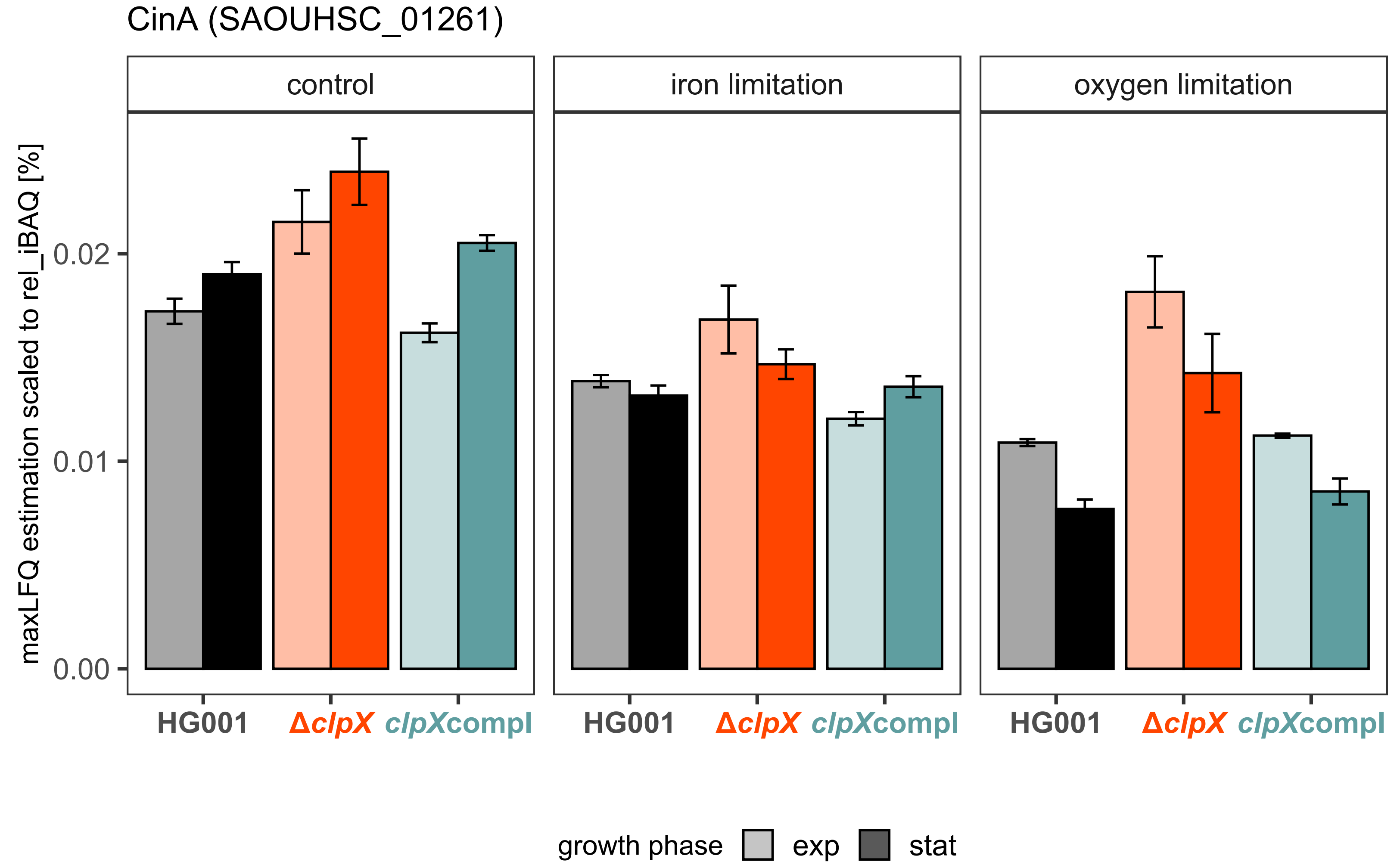

CinA (SAOUHSC_01261)
control
iron limitation
oxygen limitation
maxLFQ estimation scaled to rel_iBAQ [%]
0.00
0.01
0.02
HG001
ΔclpX
clpXcompl
growth phase
exp
stat

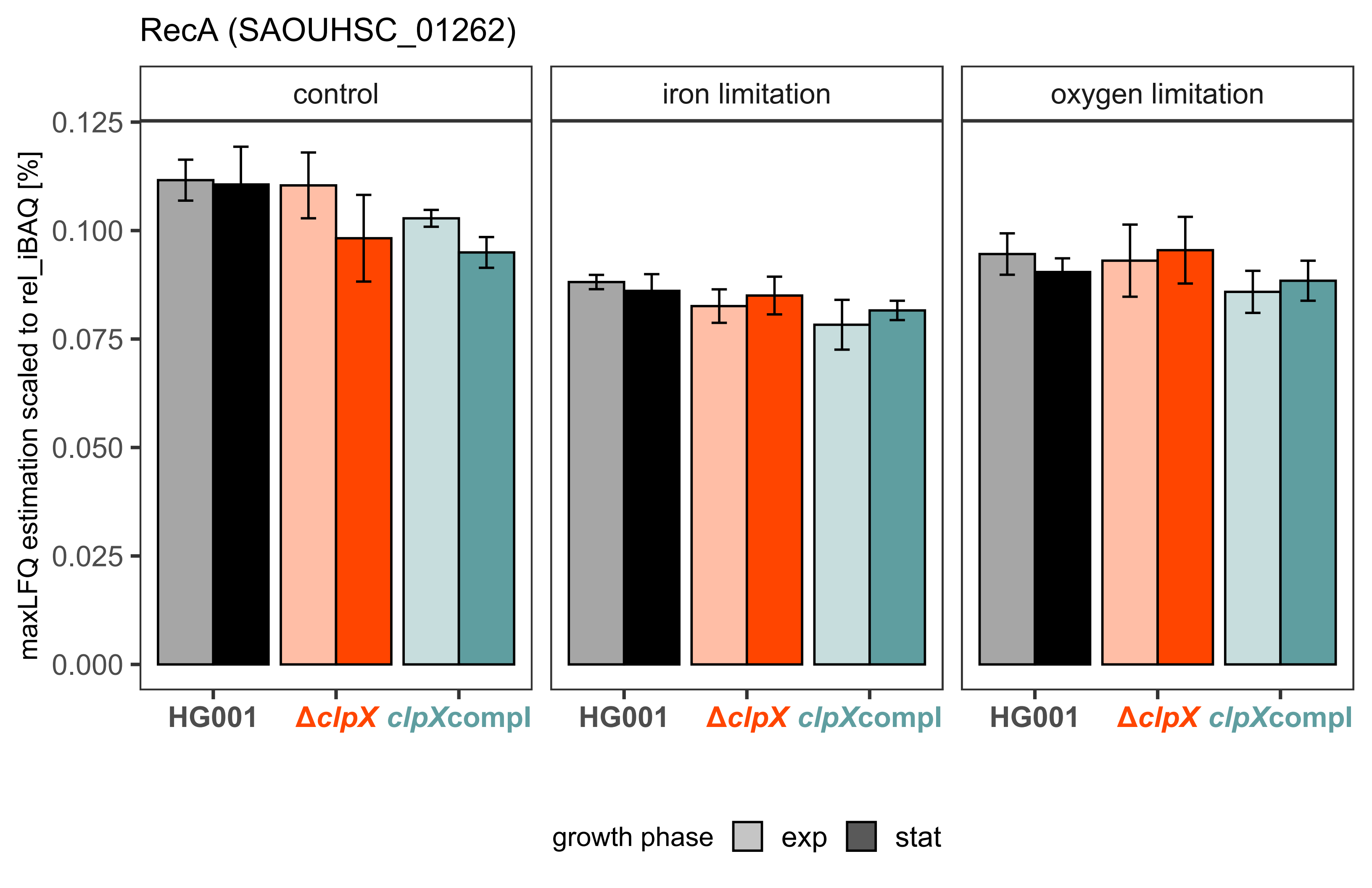

RecA (SAOUHSC_01262)
control
iron limitation
oxygen limitation
0.125
0.100
0.075
0.050
0.025
0.000
maxLFQ estimation scaled to rel_iBAQ [%]
HG001
ΔclpX
clpXcompl
growth phase
exp
stat

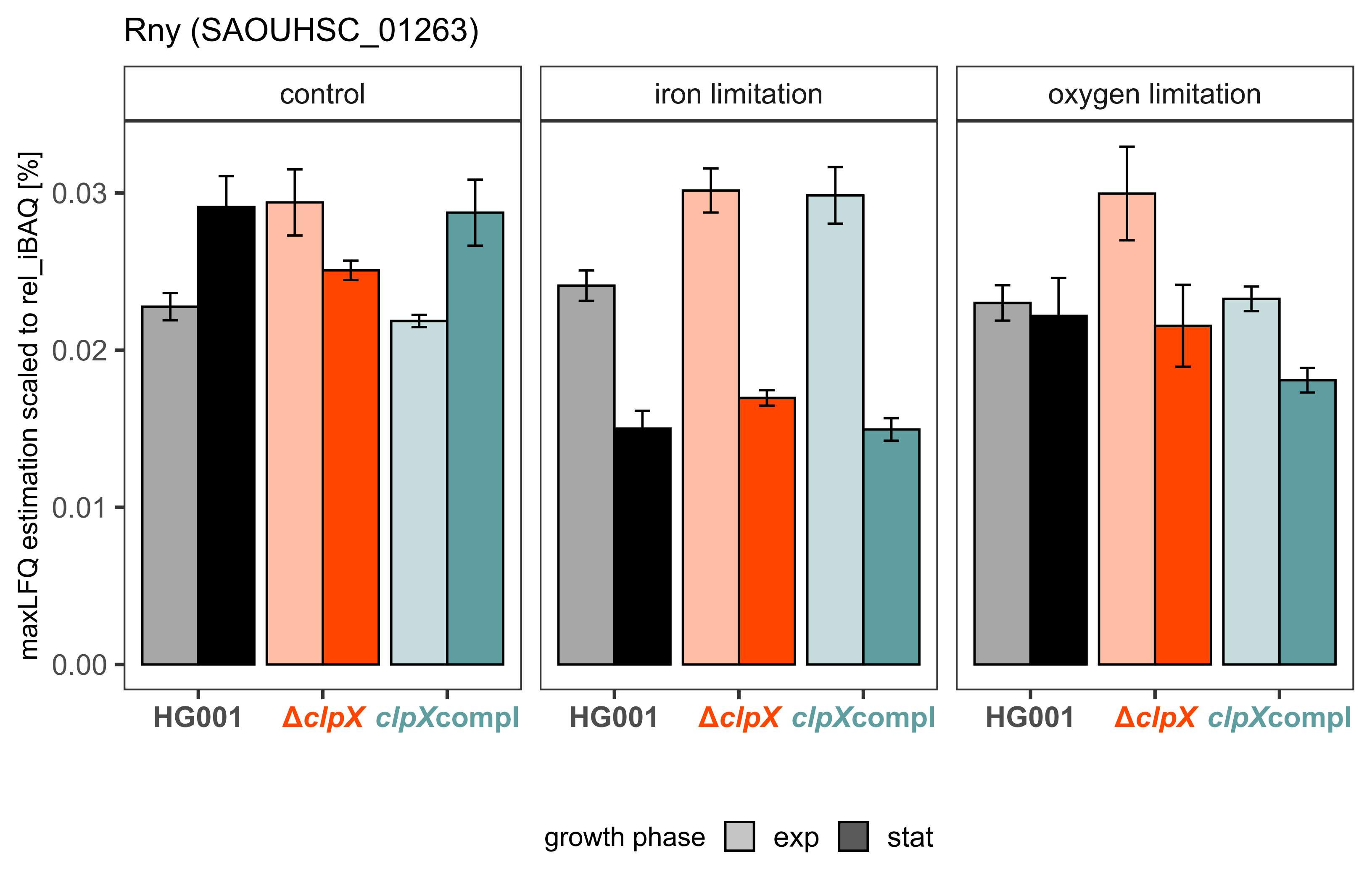

Rny (SAOUHSC_01263)
control
iron limitation
oxygen limitation
maxLFQ estimation scaled to rel_iBAQ [%]
0.00
0.01
0.02
0.03
HG001
ΔclpX
clpXcompl
growth phase
exp
stat

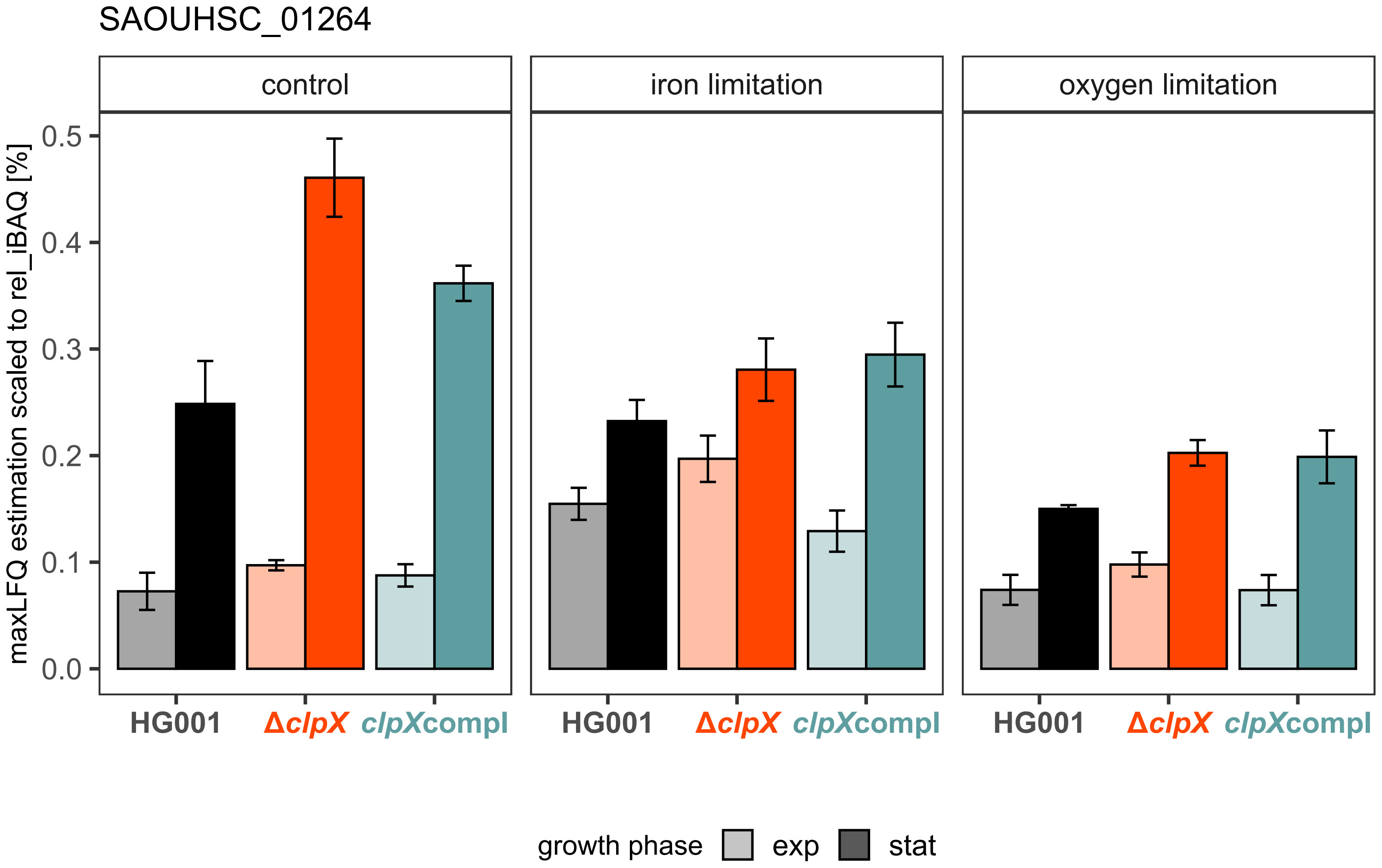
SAOUHSC_01264
control
iron limitation
oxygen limitation
maxLFQ estimation scaled to rel_iBAQ [%]
0.0
0.1
0.2
0.3
0.4
0.5
HG001
ΔclpX
clpXcompl
growth phase
exp
stat

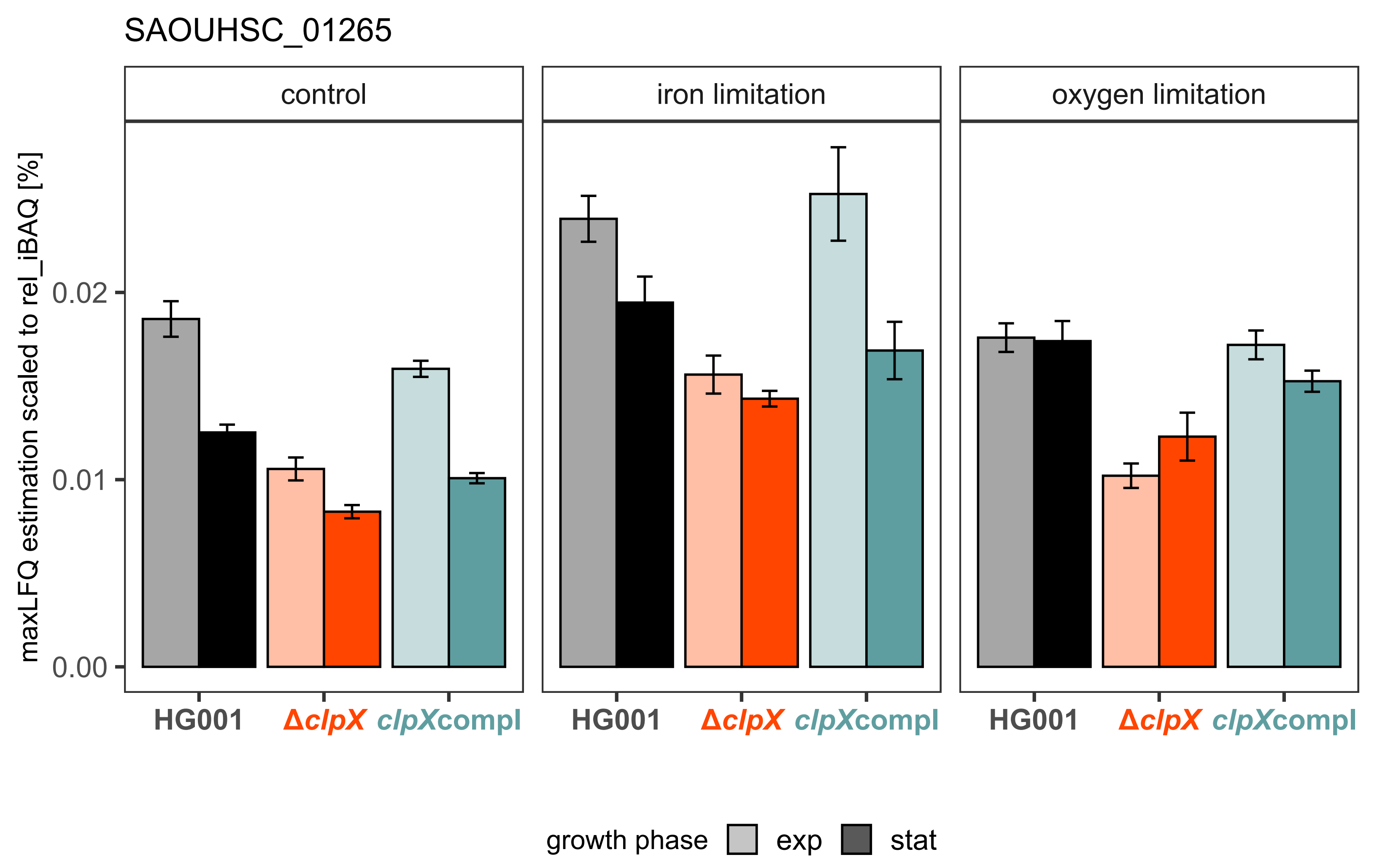
SAOUHSC_01265
control
iron limitation
oxygen limitation
maxLFQ estimation scaled to rel_iBAQ [%]
0.00
0.01
0.02
HG001
ΔclpX
clpXcompl
growth phase
exp
stat

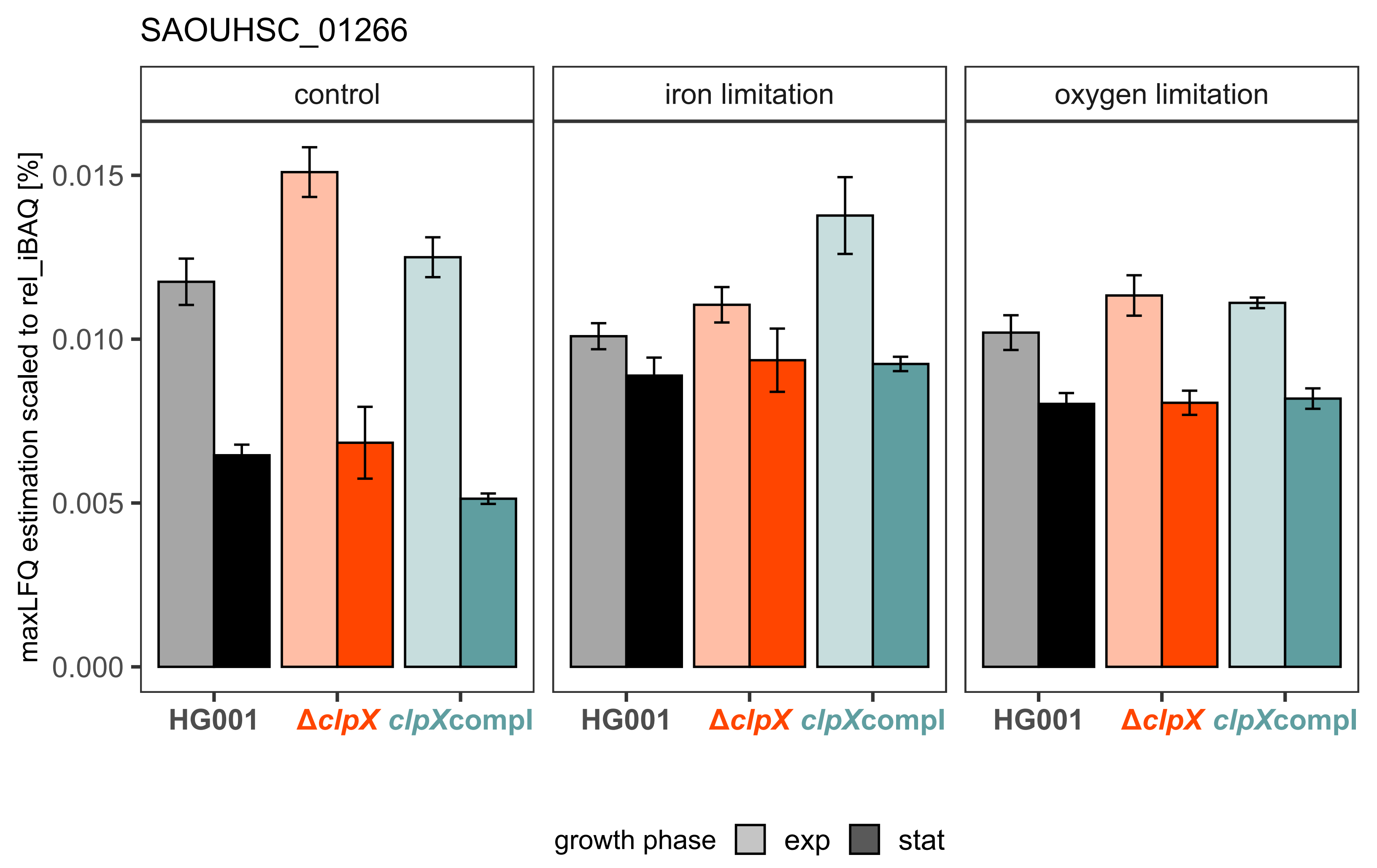

SAOUHSC_01266
control
iron limitation
oxygen limitation
maxLFQ estimation scaled to rel_iBAQ [%]
0.015
0.010
0.005
0.000
HG001
ΔclpX
clpXcompl
growth phase
exp
stat

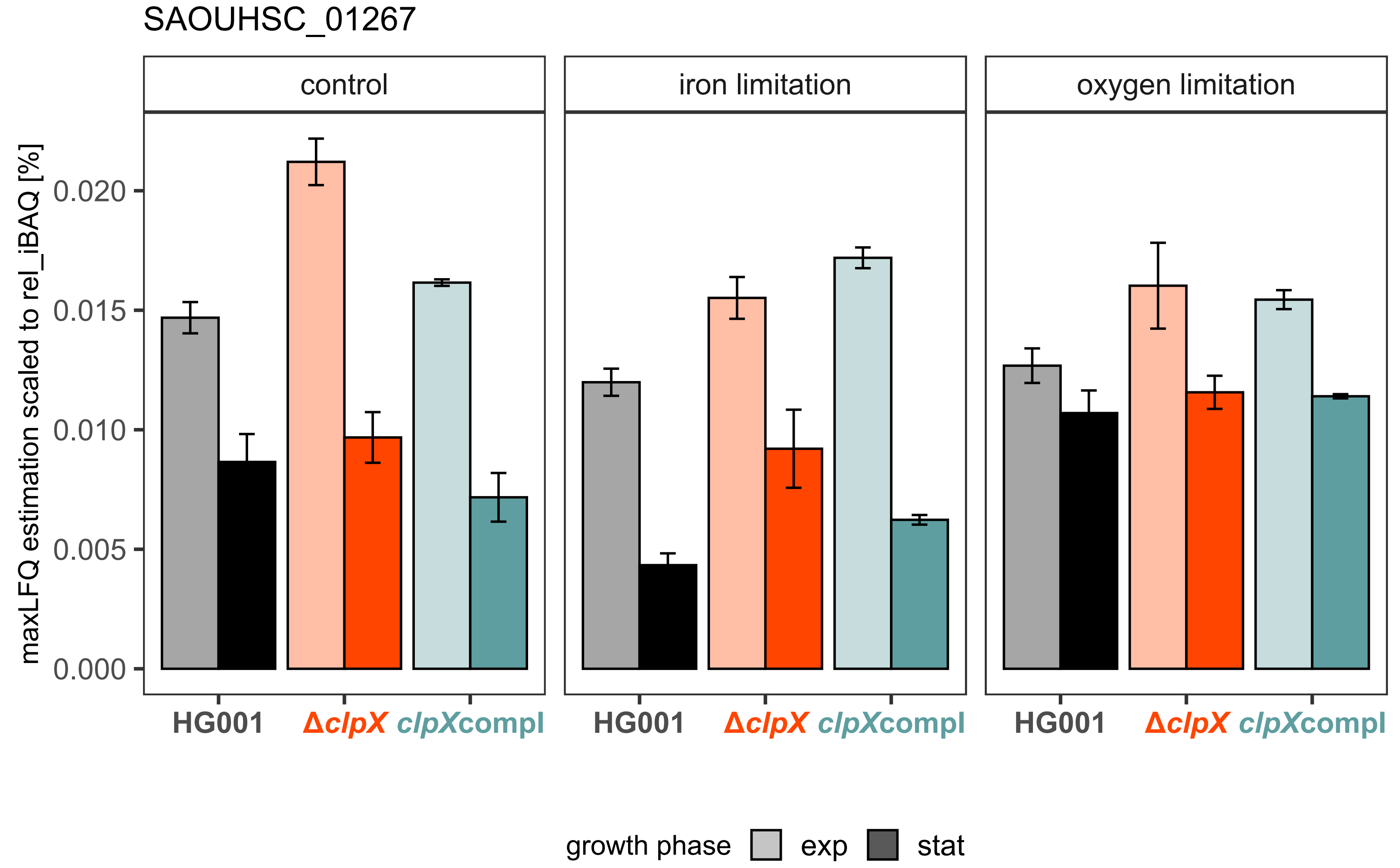
SAOUHSC_01267
control
iron limitation
oxygen limitation
maxLFQ estimation scaled to rel_iBAQ [%]
0.000
0.005
0.010
0.015
0.020
HG001
ΔclpX
clpXcompl
growth phase
exp
stat

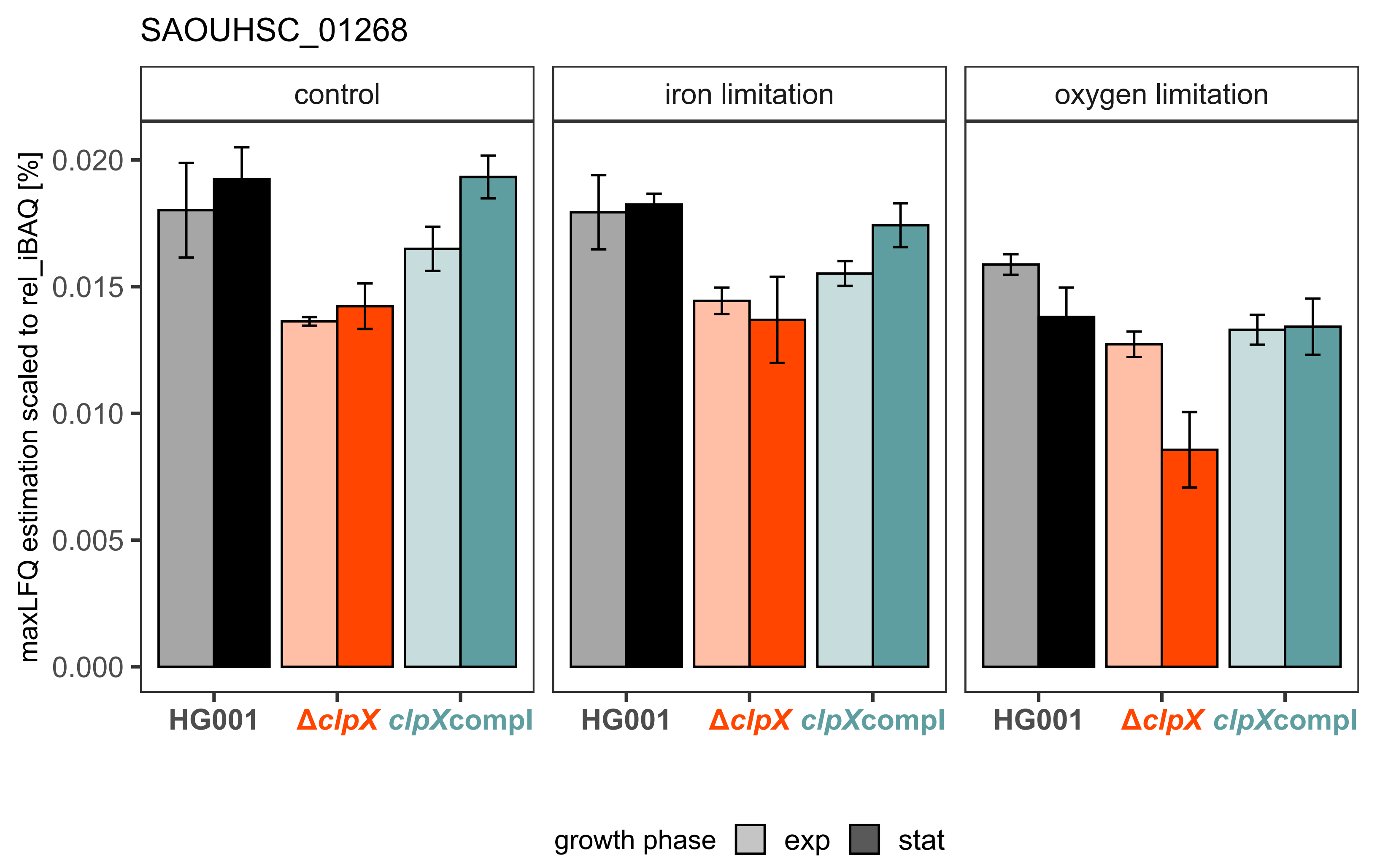

SAOUHSC_01268
control
iron limitation
oxygen limitation
maxLFQ estimation scaled to rel_iBAQ [%]
0.020
0.015
0.010
0.005
0.000
HG001
ΔclpX
clpXcompl
growth phase
exp
stat

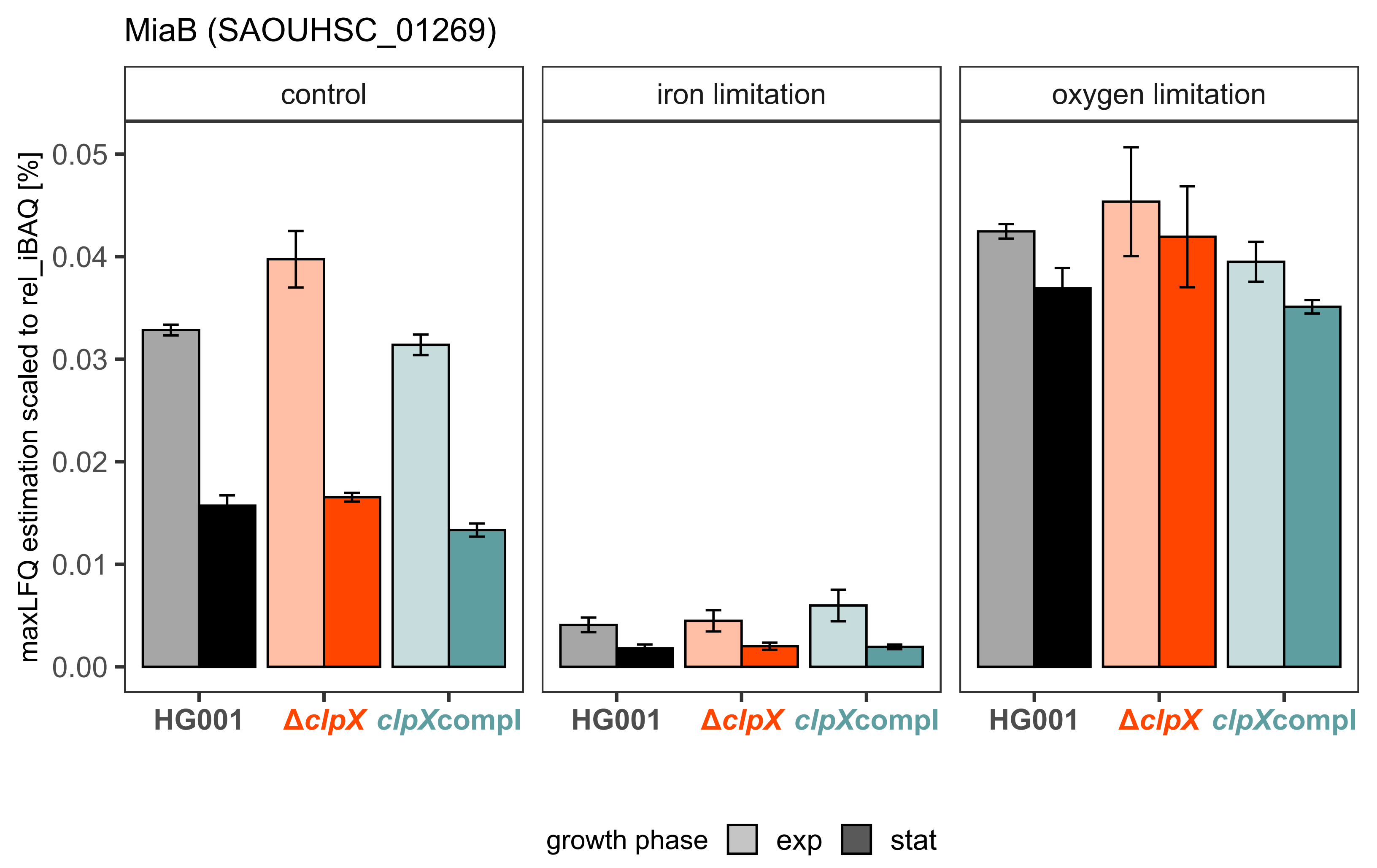
MiaB (SAOUHSC_01269)
control
iron limitation
oxygen limitation
maxLFQ estimation scaled to rel_iBAQ [%]
0.05
0.04
0.03
0.02
0.01
0.00
HG001
ΔclpX
clpXcompl
growth phase
exp
stat

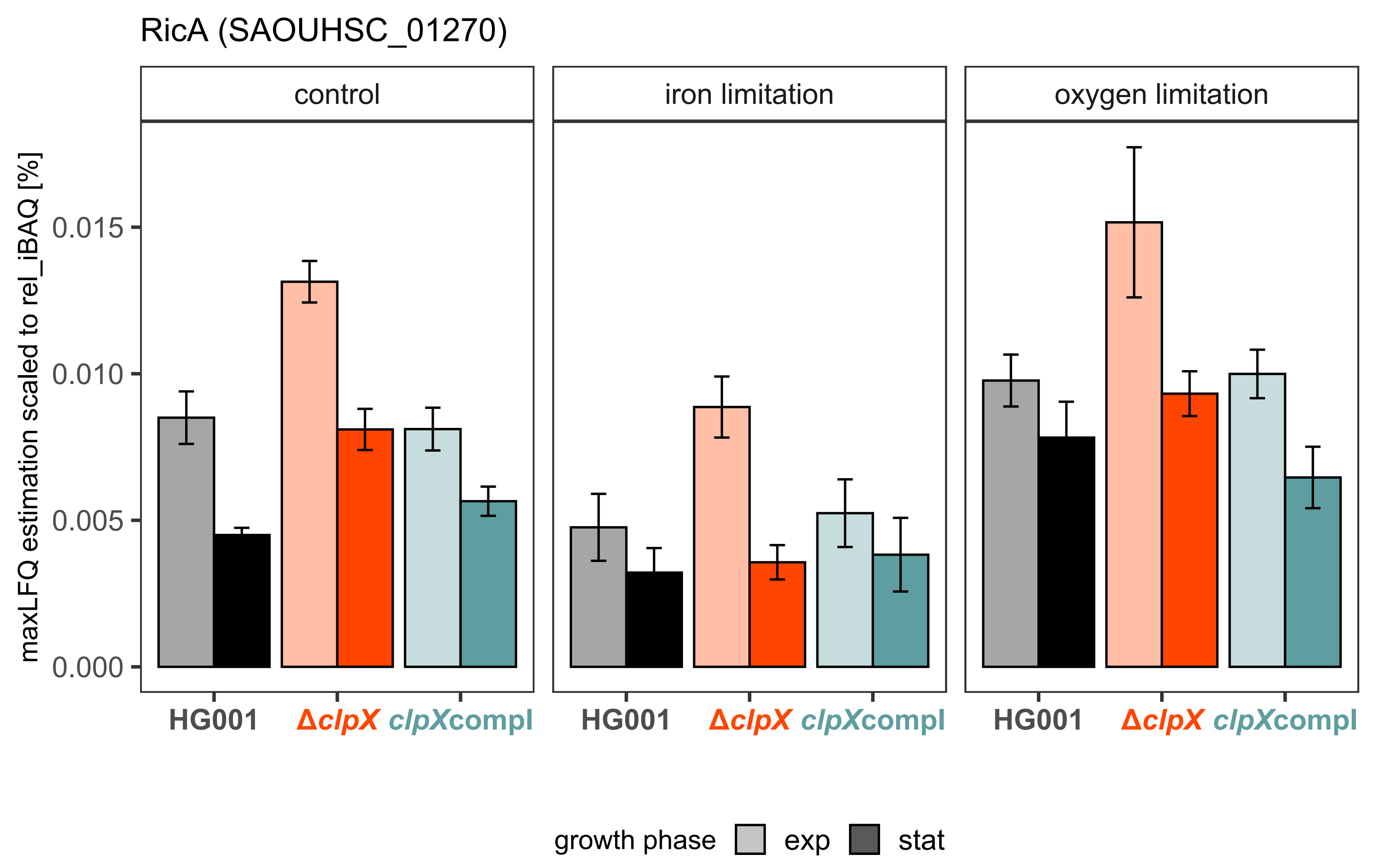

RicA (SAOUHSC_01270)
control
iron limitation
oxygen limitation
maxLFQ estimation scaled to rel_iBAQ [%]
0.015
0.010
0.005
0.000
HG001
ΔclpX
clpXcompl
growth phase
exp
stat

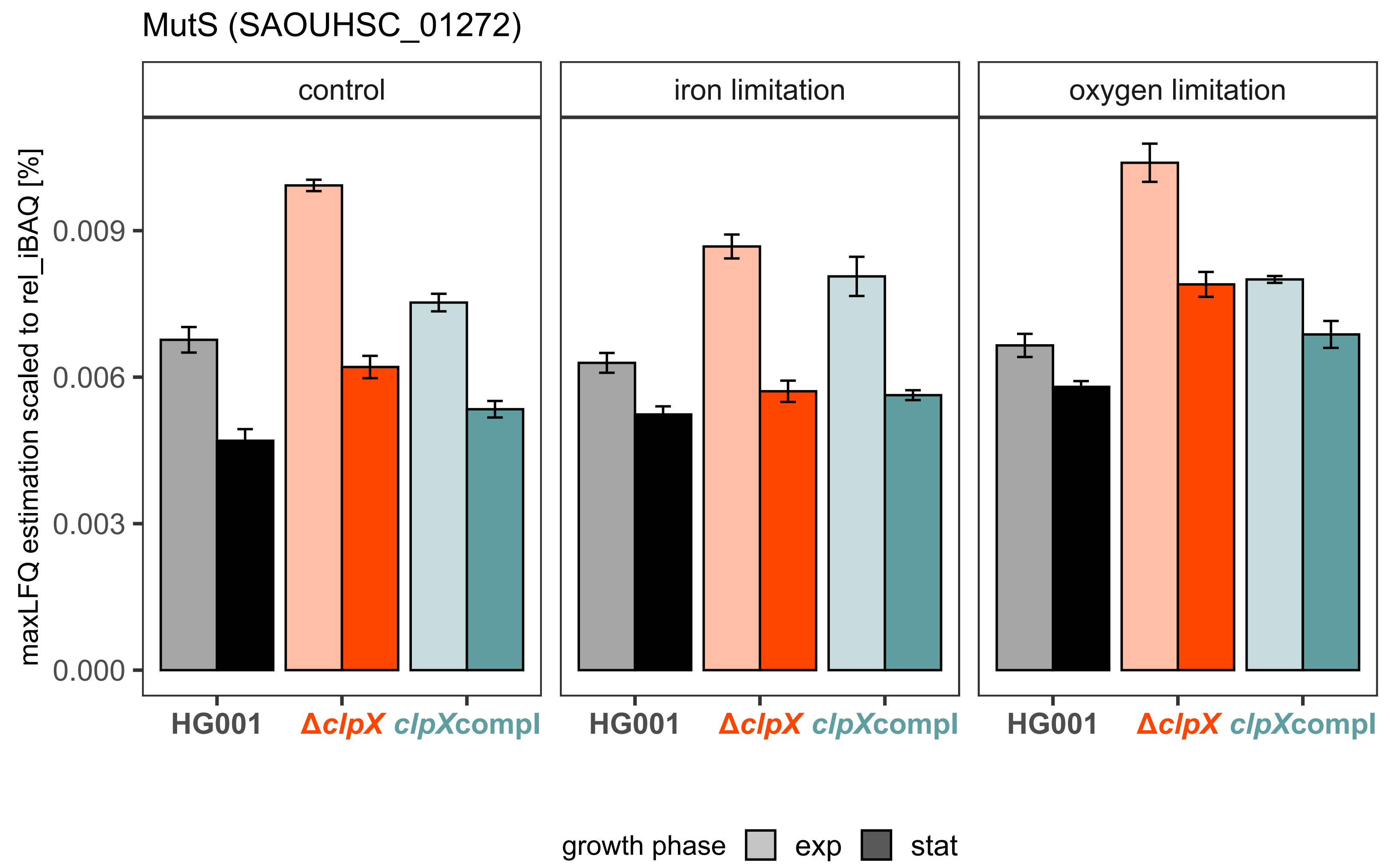

MutS (SAOUHSC_01272)
control
iron limitation
oxygen limitation
maxLFQ estimation scaled to rel_iBAQ [%]
0.009
0.006
0.003
0.000
HG001
ΔclpX
clpXcompl
growth phase
exp
stat

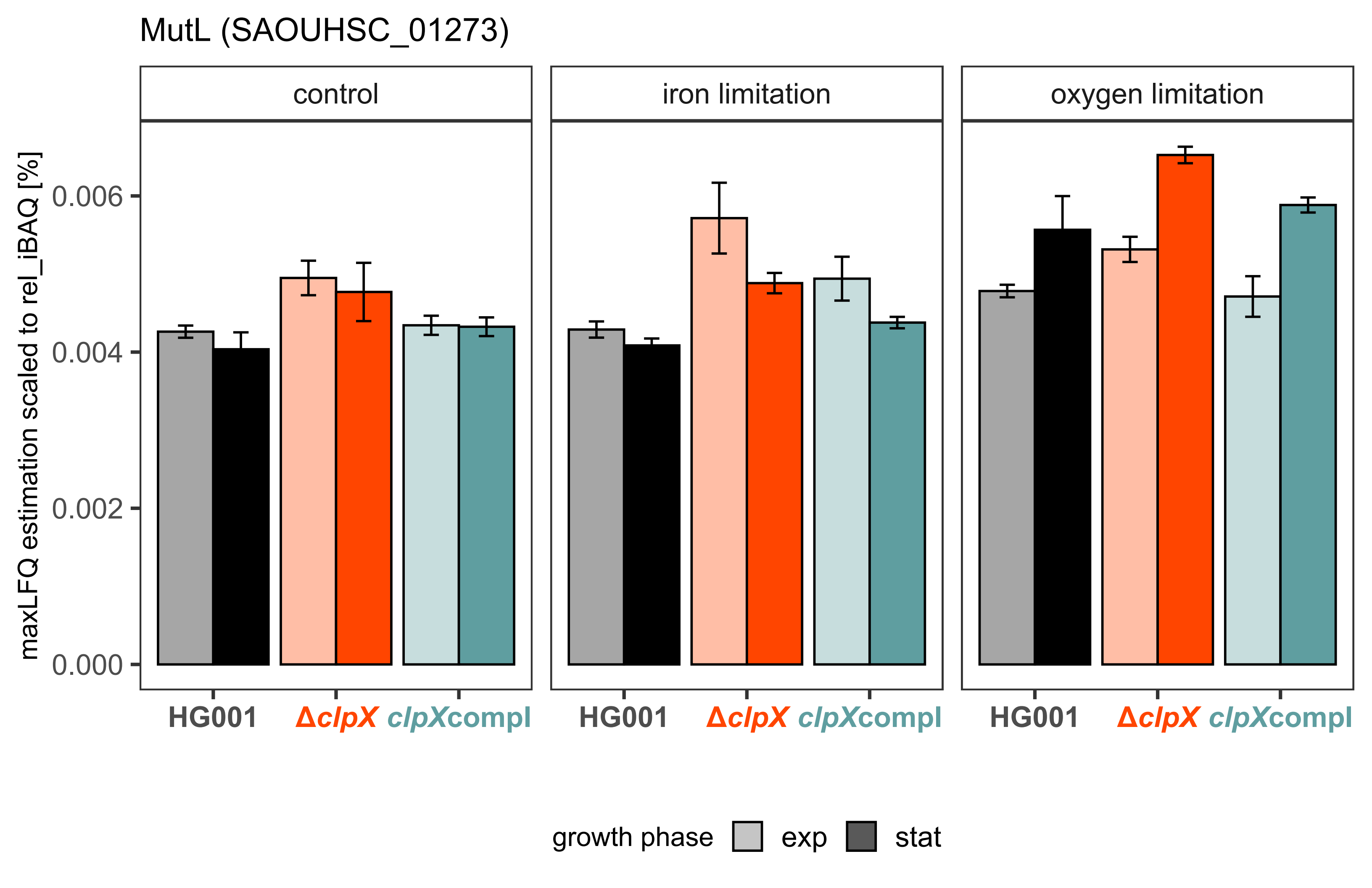
MutL (SAOUHSC_01273)
control
iron limitation
oxygen limitation
maxLFQ estimation scaled to rel_iBAQ [%]
0.000
0.002
0.004
0.006
HG001
ΔclpX
clpXcompl
growth phase
exp
stat

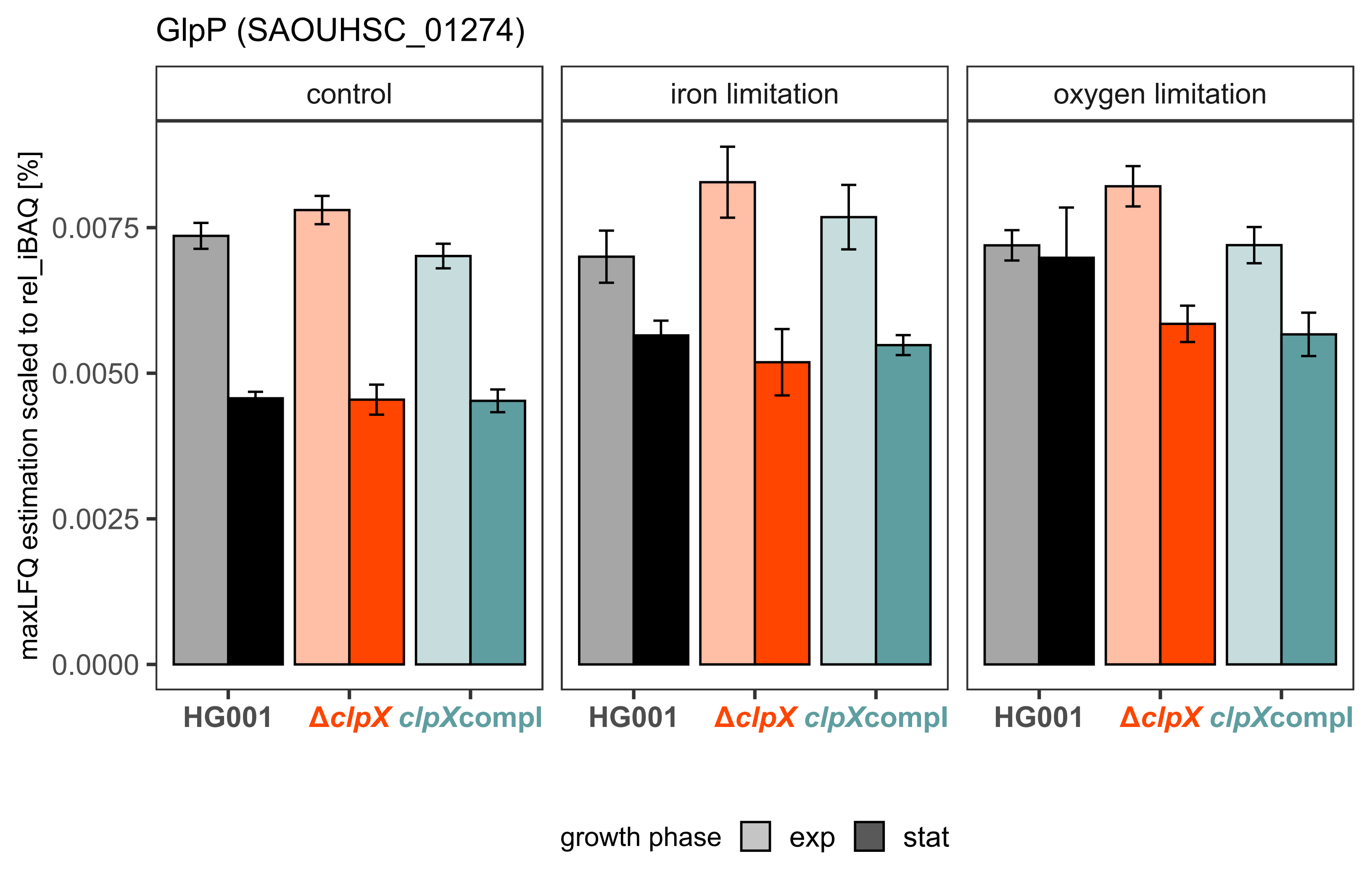

GlpP (SAOUHSC_01274)
control
iron limitation
oxygen limitation
maxLFQ estimation scaled to rel_iBAQ [%]
0.0000
0.0025
0.0050
0.0075
HG001
ΔclpX
clpXcompl
growth phase
exp
stat

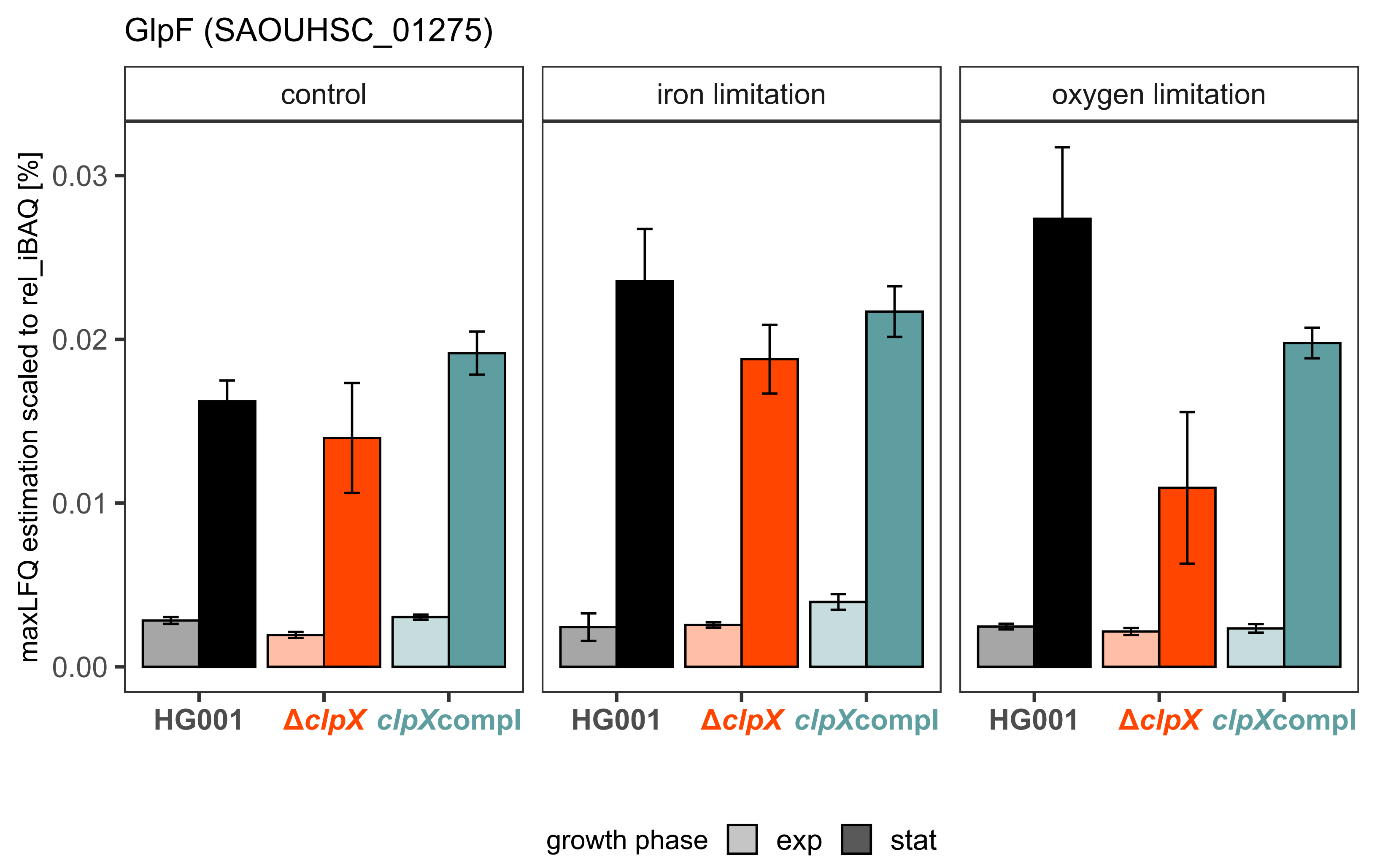
GlpF (SAOUHSC_01275)
control
iron limitation
oxygen limitation
maxLFQ estimation scaled to rel_iBAQ [%]
0.03
0.02
0.01
0.00
HG001
ΔclpX
clpXcompl
growth phase
exp
stat

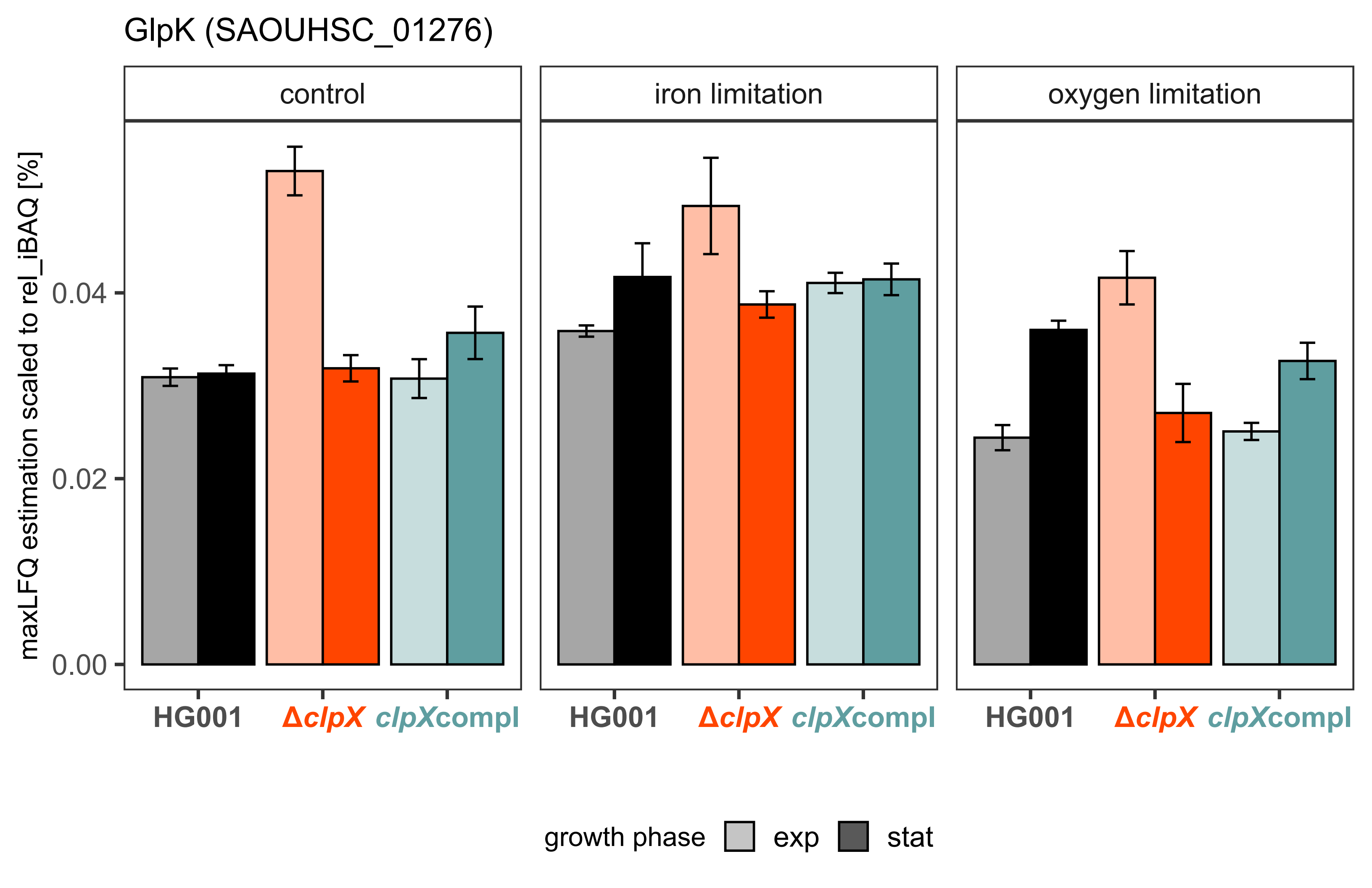
GlpK (SAOUHSC_01276)
control
iron limitation
oxygen limitation
maxLFQ estimation scaled to rel_iBAQ [%]
0.00
0.02
0.04
HG001
ΔclpX
clpXcompl
growth phase
exp
stat

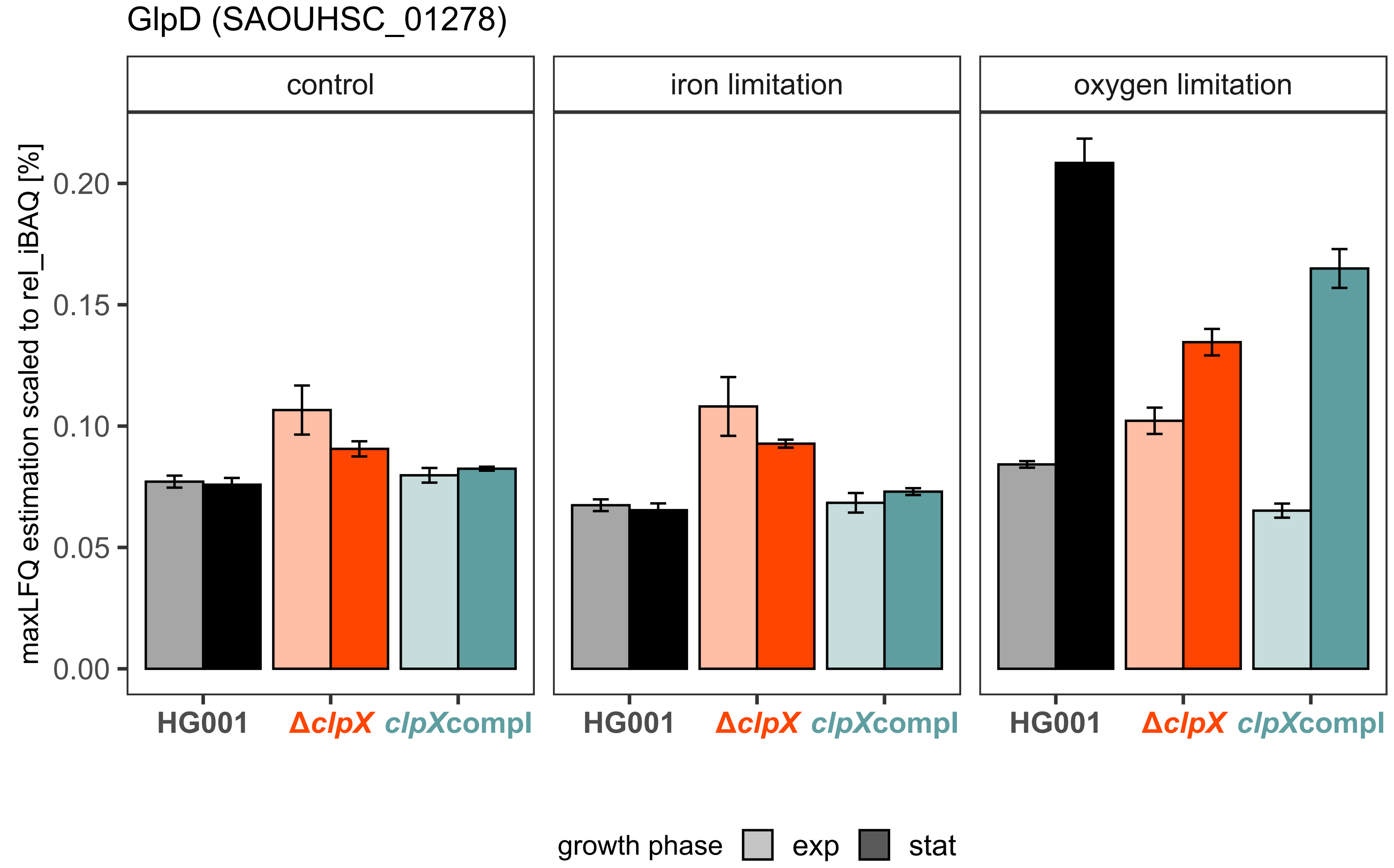
GlpD (SAOUHSC_01278)
control
iron limitation
oxygen limitation
maxLFQ estimation scaled to rel_iBAQ [%]
0.20
0.15
0.10
0.05
0.00
HG001
ΔclpX
clpXcompl
growth phase
exp
stat

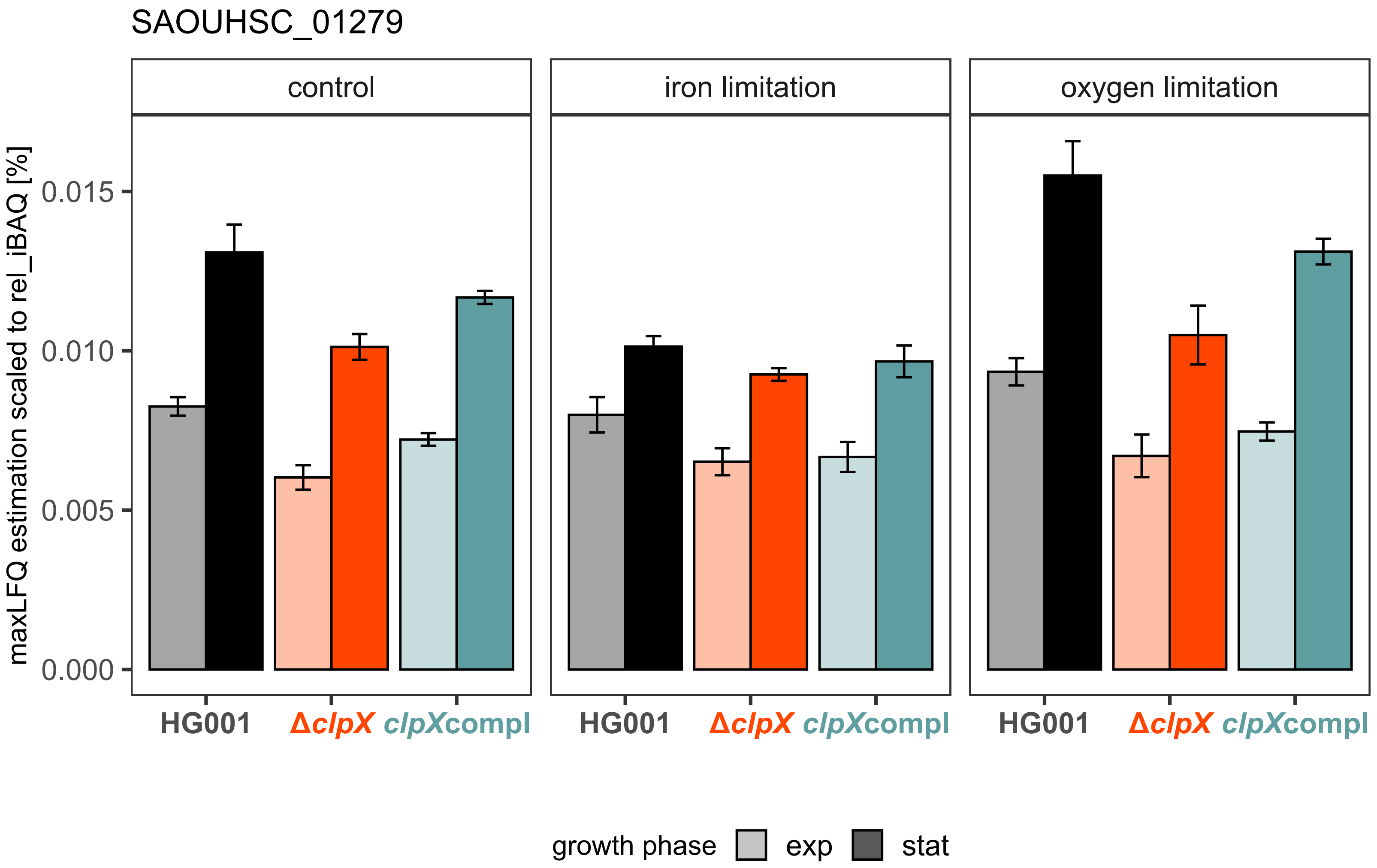

SAOUHSC_01279
control
iron limitation
oxygen limitation
maxLFQ estimation scaled to rel_iBAQ [%]
0.015
0.010
0.005
0.000
HG001
ΔclpX
clpXcompl
growth phase
exp
stat

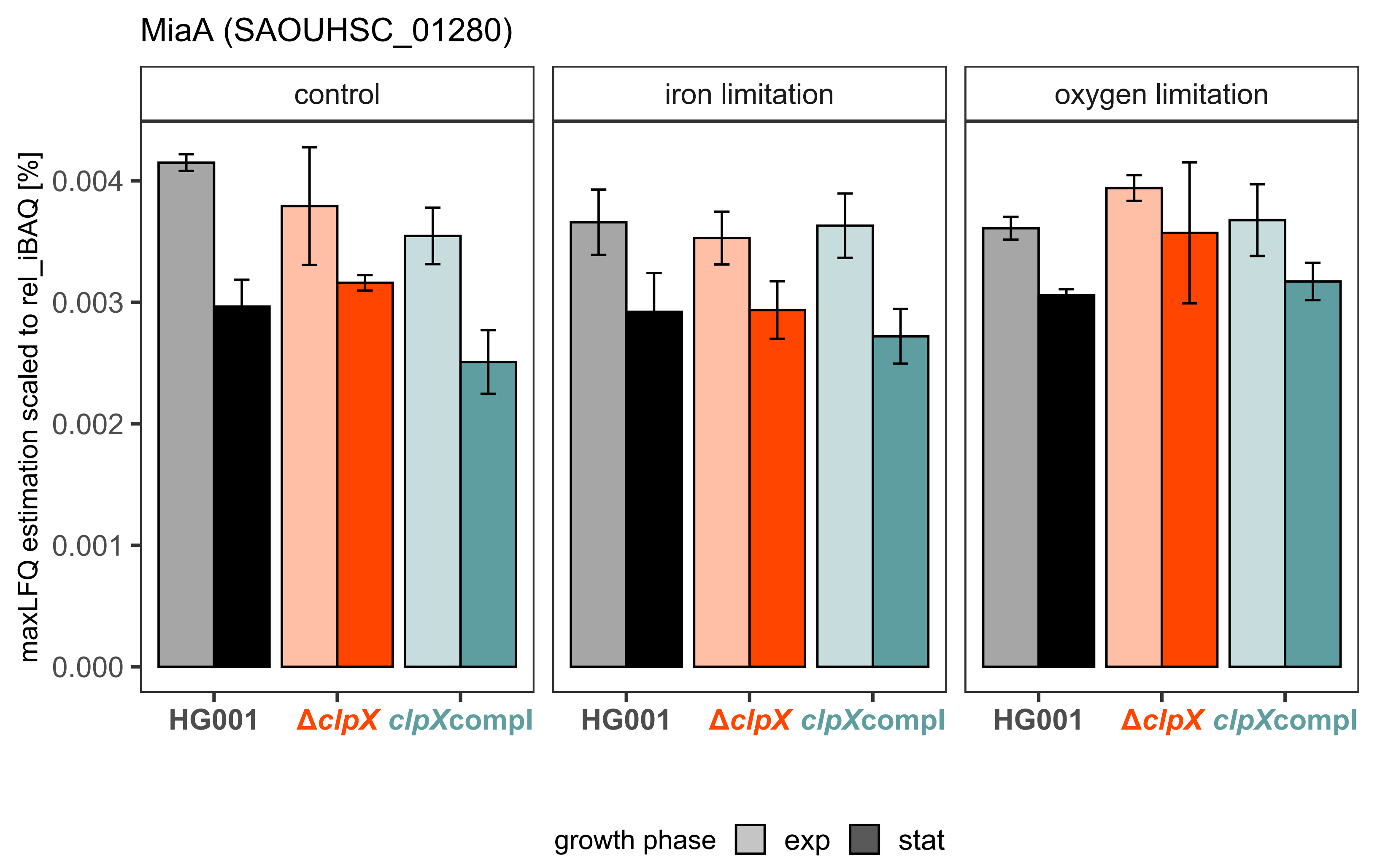
MiaA (SAOUHSC_01280)
control
iron limitation
oxygen limitation
maxLFQ estimation scaled to rel_iBAQ [%]
0.004
0.003
0.002
0.001
0.000
HG001
ΔclpX
clpXcompl
growth phase
exp
stat

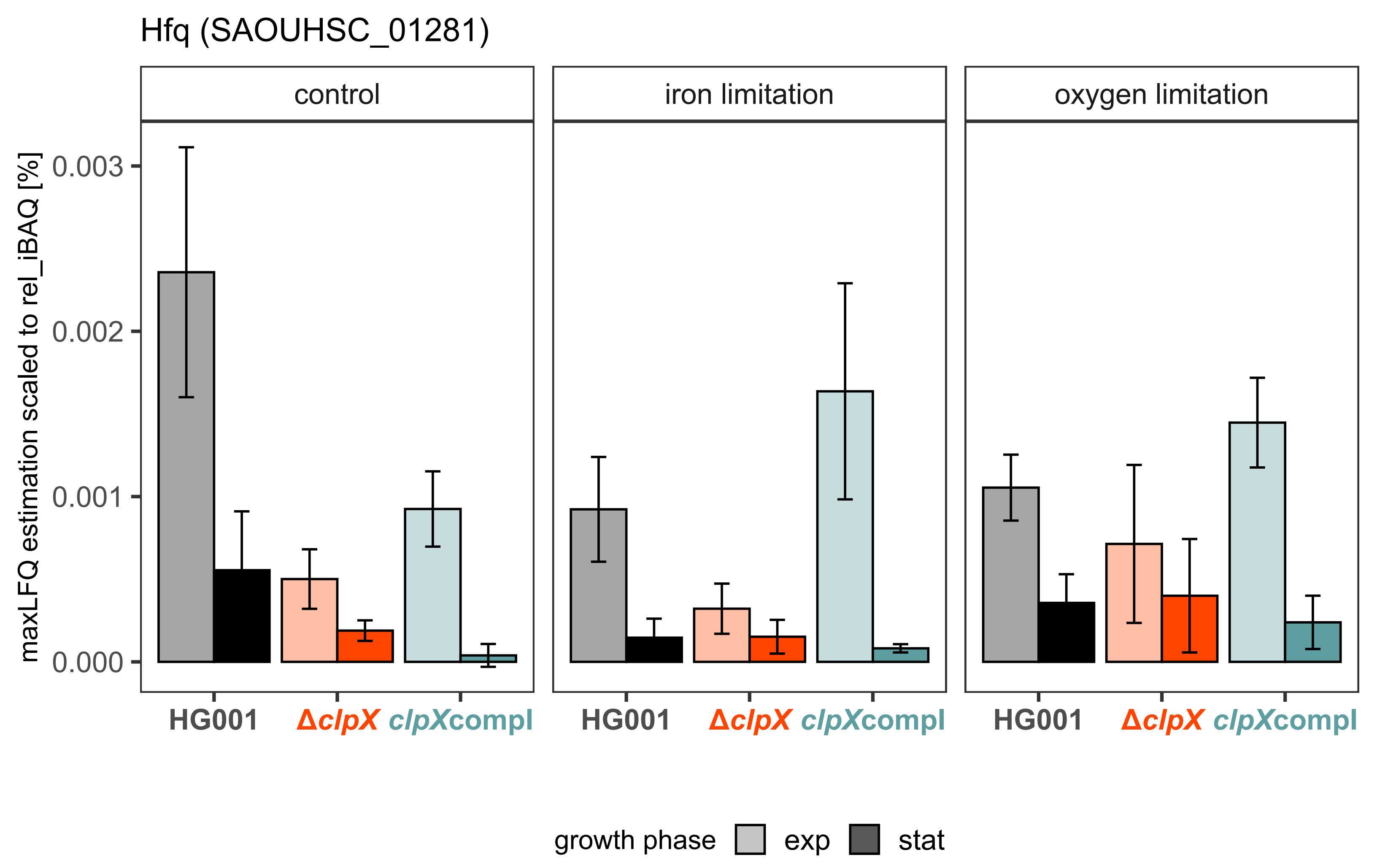

Hfq (SAOUHSC_01281)
control
iron limitation
oxygen limitation
maxLFQ estimation scaled to rel_iBAQ [%]
0.003
0.002
0.001
0.000
HG001
ΔclpX
clpXcompl
growth phase
exp
stat

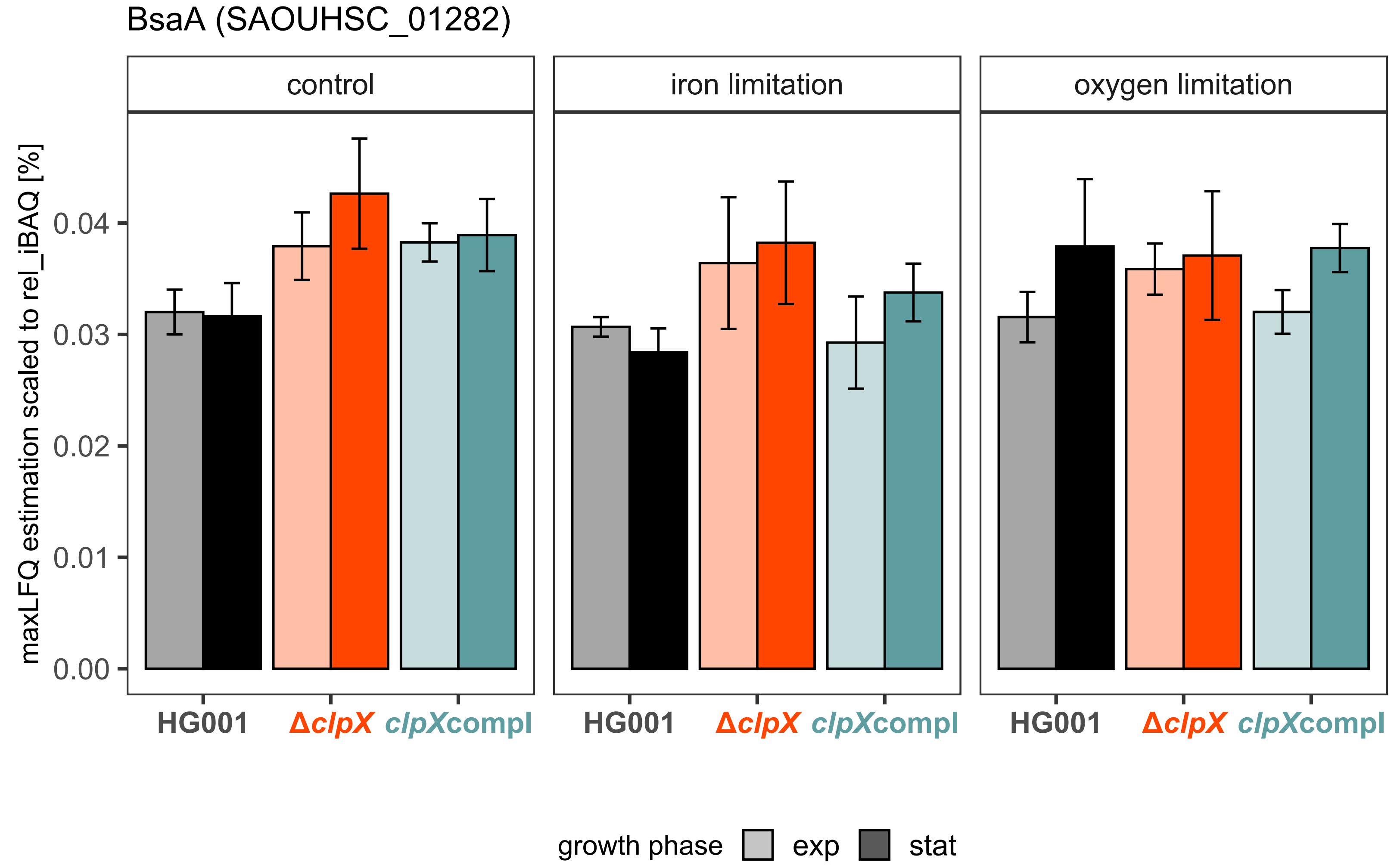

BsaA (SAOUHSC_01282)
control
iron limitation
oxygen limitation
maxLFQ estimation scaled to rel_iBAQ [%]
0.00
0.01
0.02
0.03
0.04
HG001
ΔclpX
clpXcompl
growth phase
exp
stat

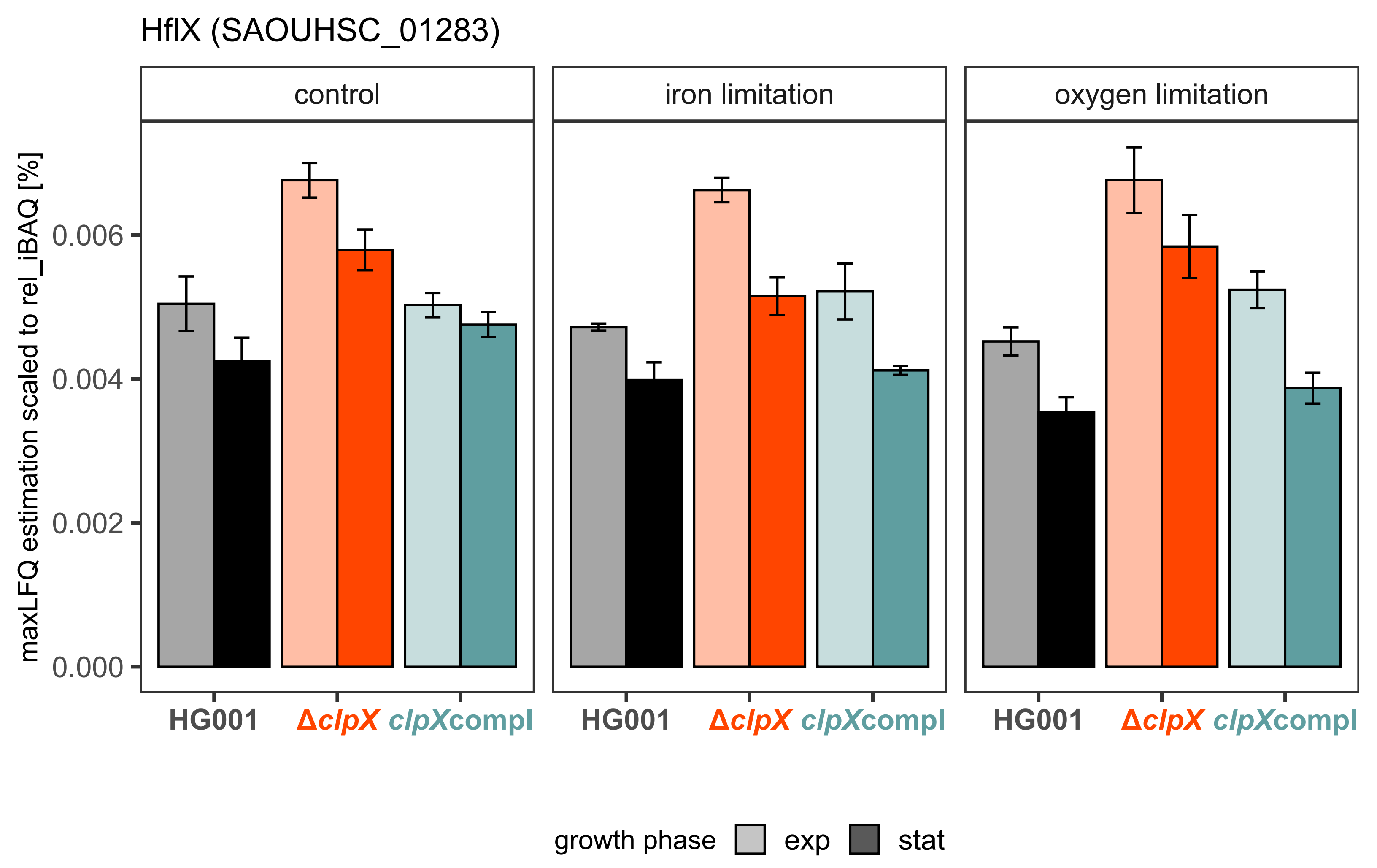
HflX (SAOUHSC_01283)
control
iron limitation
oxygen limitation
maxLFQ estimation scaled to rel_iBAQ [%]
0.000
0.002
0.004
0.006
HG001
ΔclpX
clpXcompl
growth phase
exp
stat

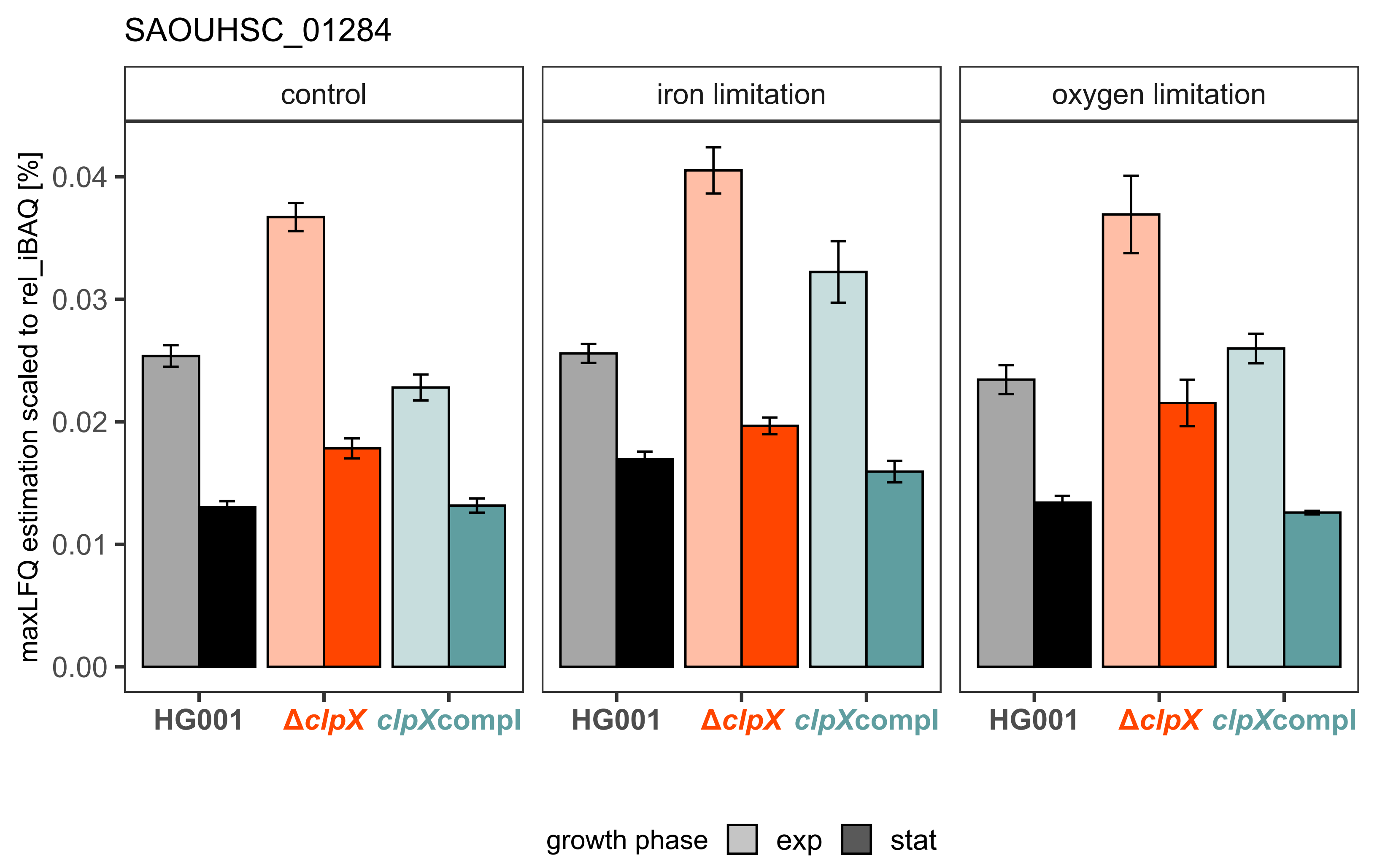
SAOUHSC_01284
control
iron limitation
oxygen limitation
maxLFQ estimation scaled to rel_iBAQ [%]
0.04
0.03
0.02
0.01
0.00
HG001
ΔclpX
clpXcompl
growth phase
exp
stat

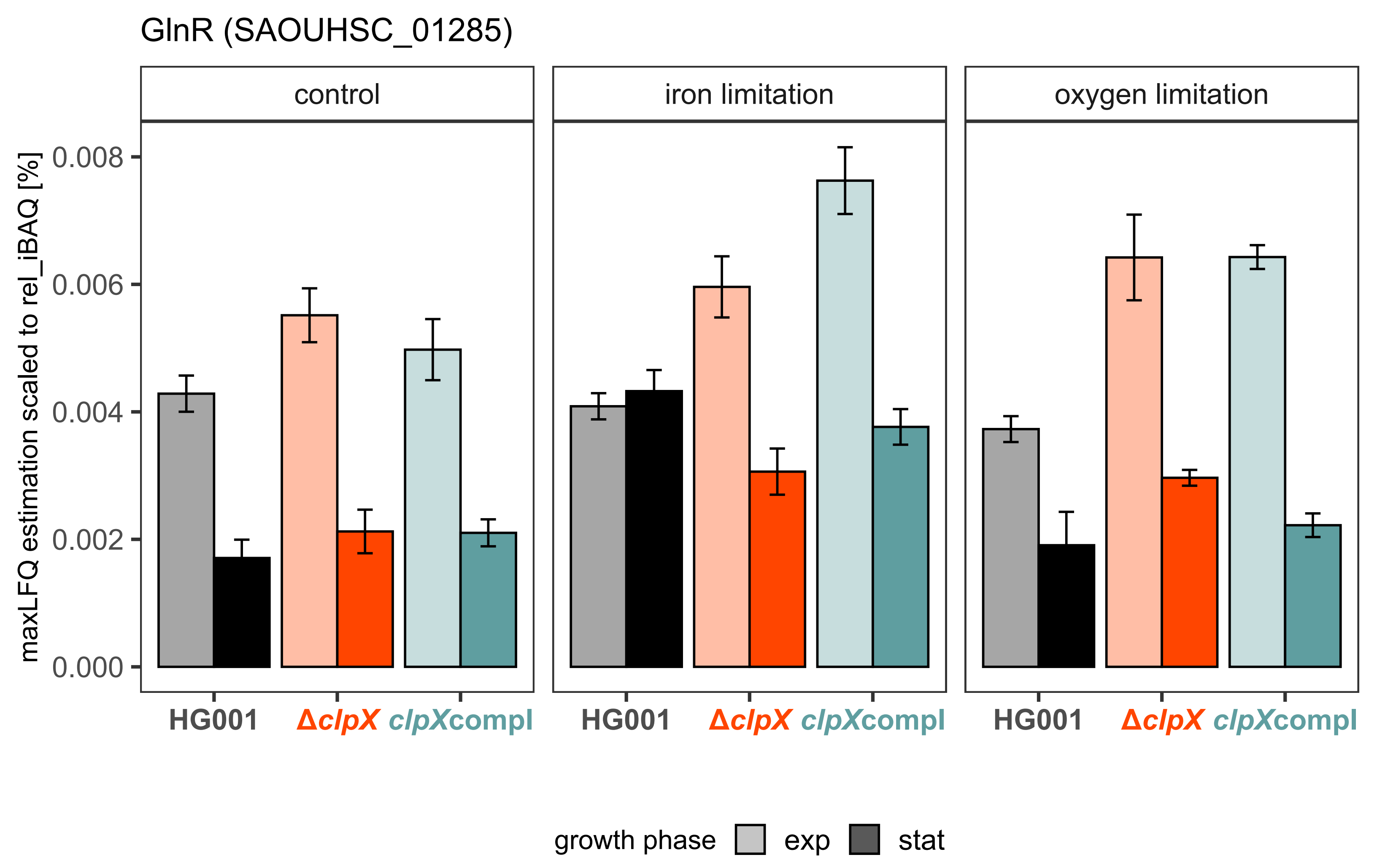
GlnR (SAOUHSC_01285)
control
iron limitation
oxygen limitation
maxLFQ estimation scaled to rel_iBAQ [%]
0.000
0.002
0.004
0.006
0.008
HG001
ΔclpX
clpXcompl
growth phase
exp
stat

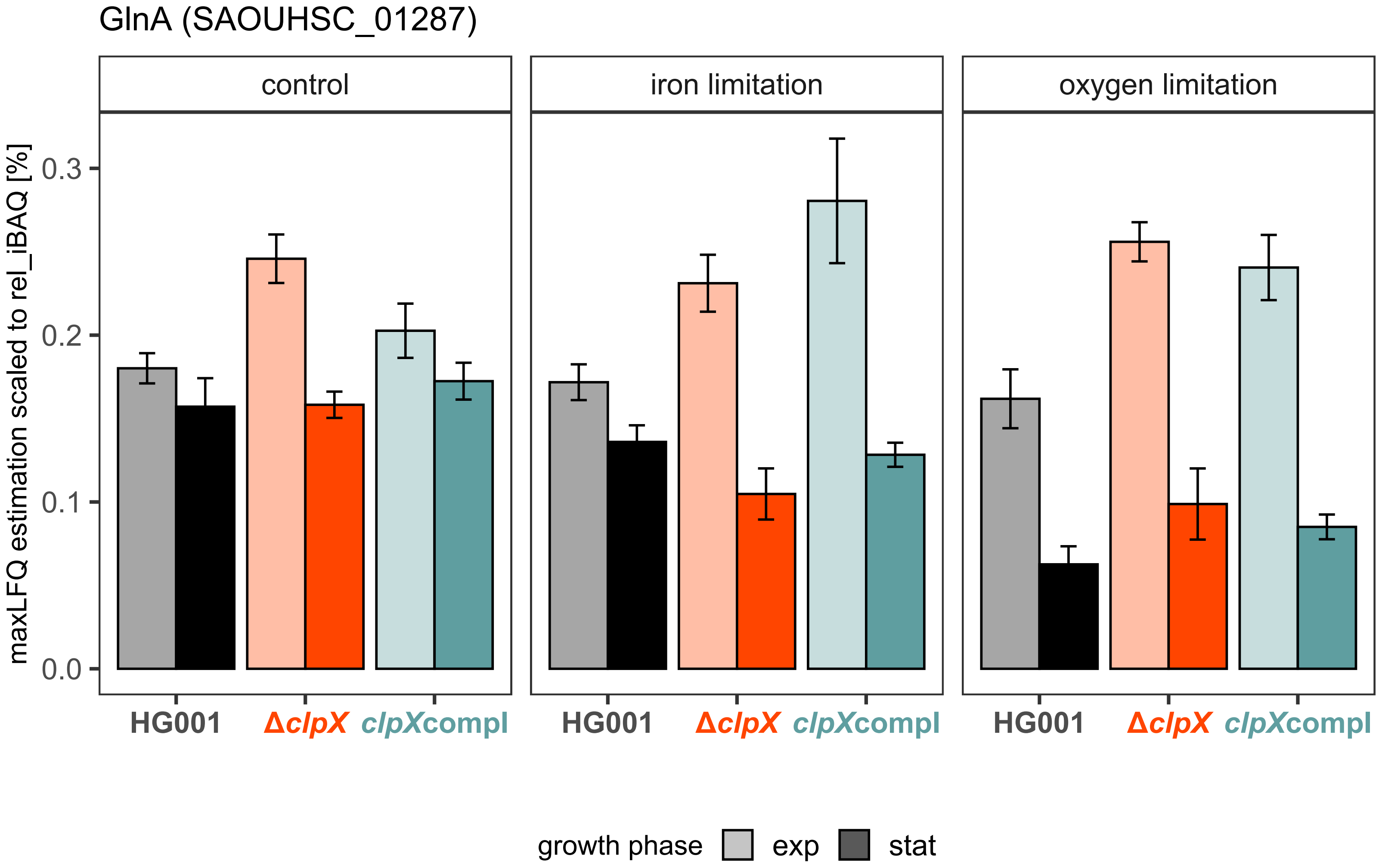

GlnA (SAOUHSC_01287)
control
iron limitation
oxygen limitation
maxLFQ estimation scaled to rel_iBAQ [%]
0.3
0.2
0.1
0.0
HG001
ΔclpX
clpXcompl
growth phase
exp
stat

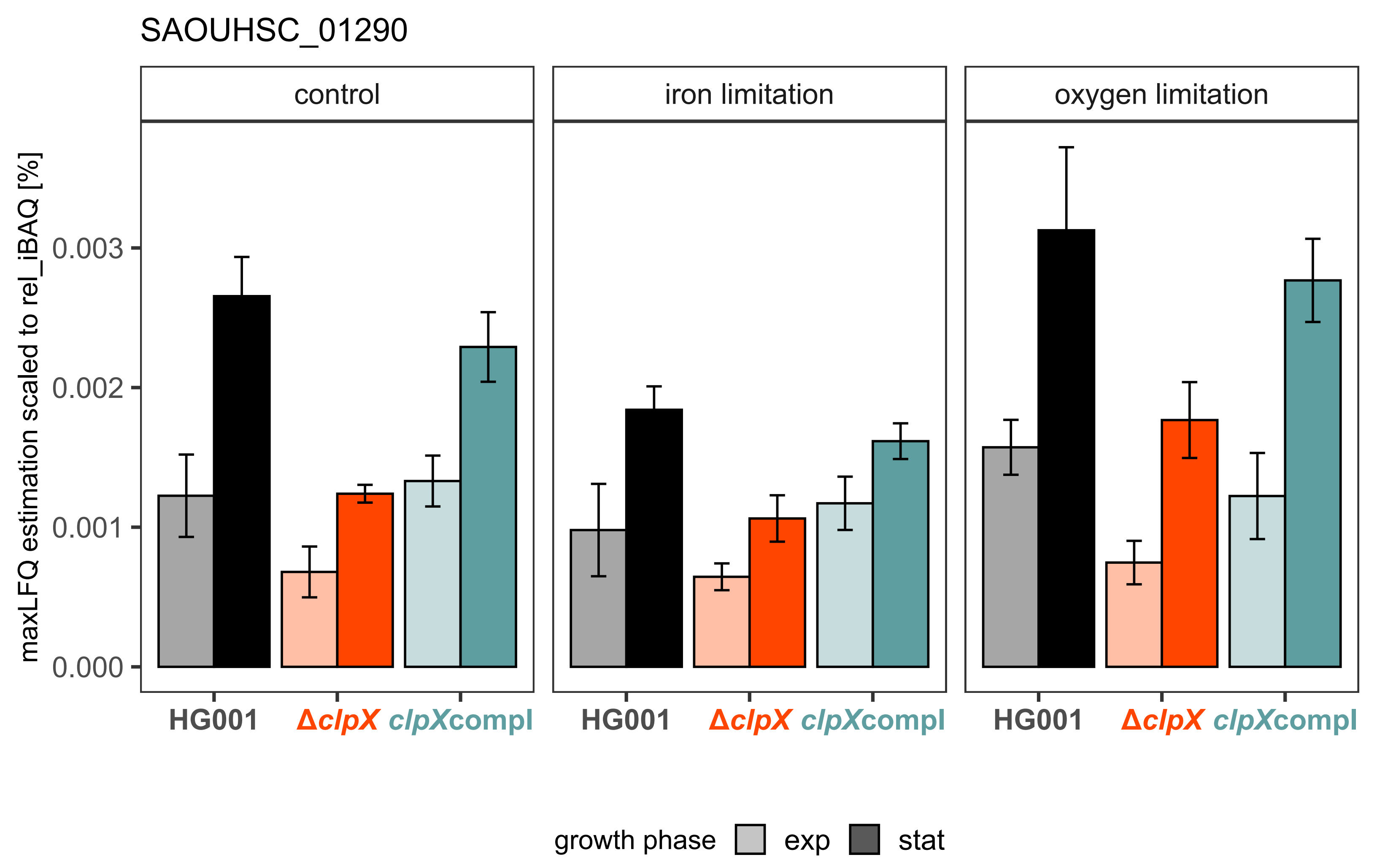

SAOUHSC_01290
control
iron limitation
oxygen limitation
maxLFQ estimation scaled to rel_iBAQ [%]
0.003
0.002
0.001
0.000
HG001
ΔclpX
clpXcompl
growth phase
exp
stat

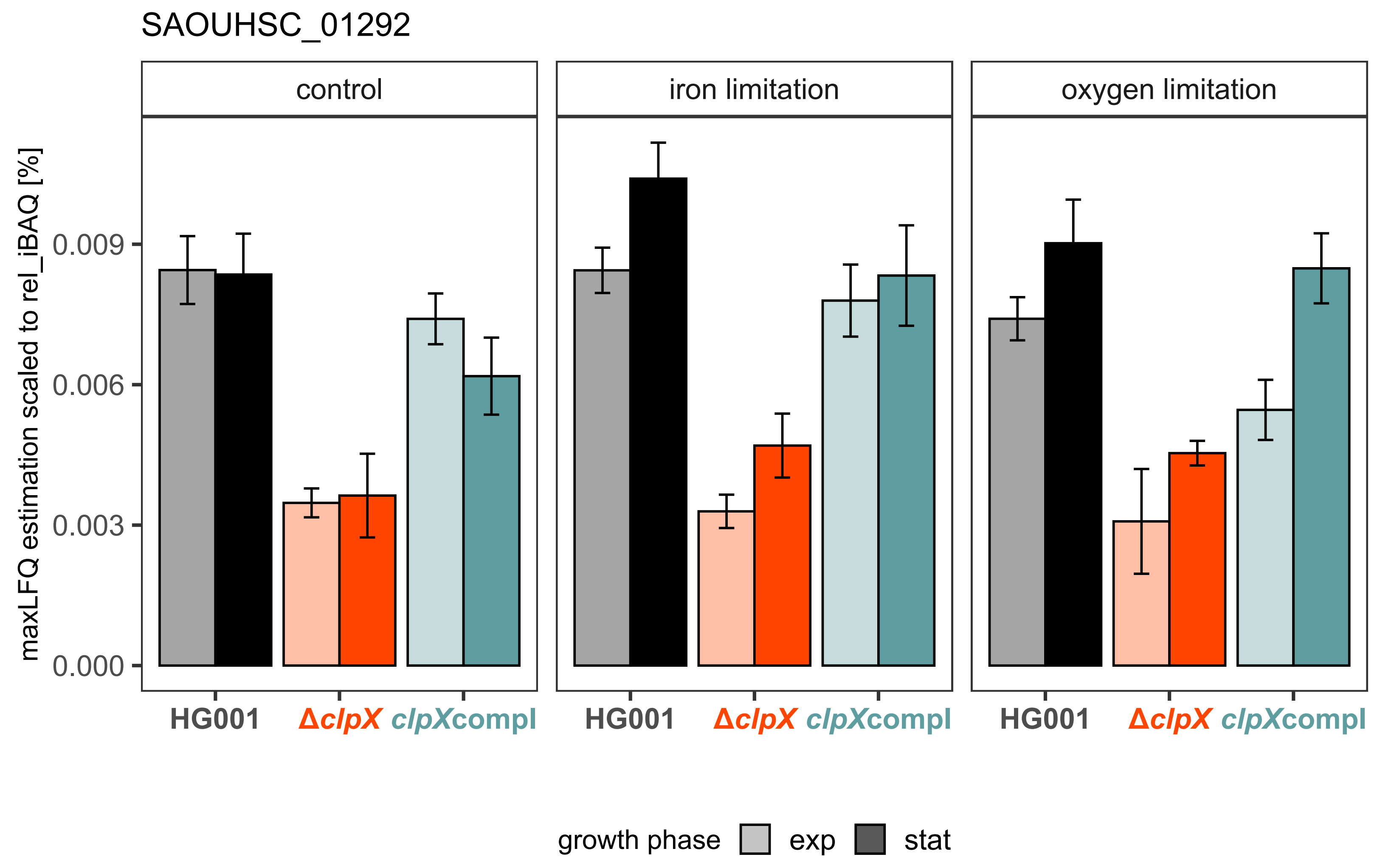
SAOUHSC_01292
control
iron limitation
oxygen limitation
maxLFQ estimation scaled to rel_iBAQ [%]
0.009
0.006
0.003
0.000
HG001
ΔclpX
clpXcompl
growth phase
exp
stat

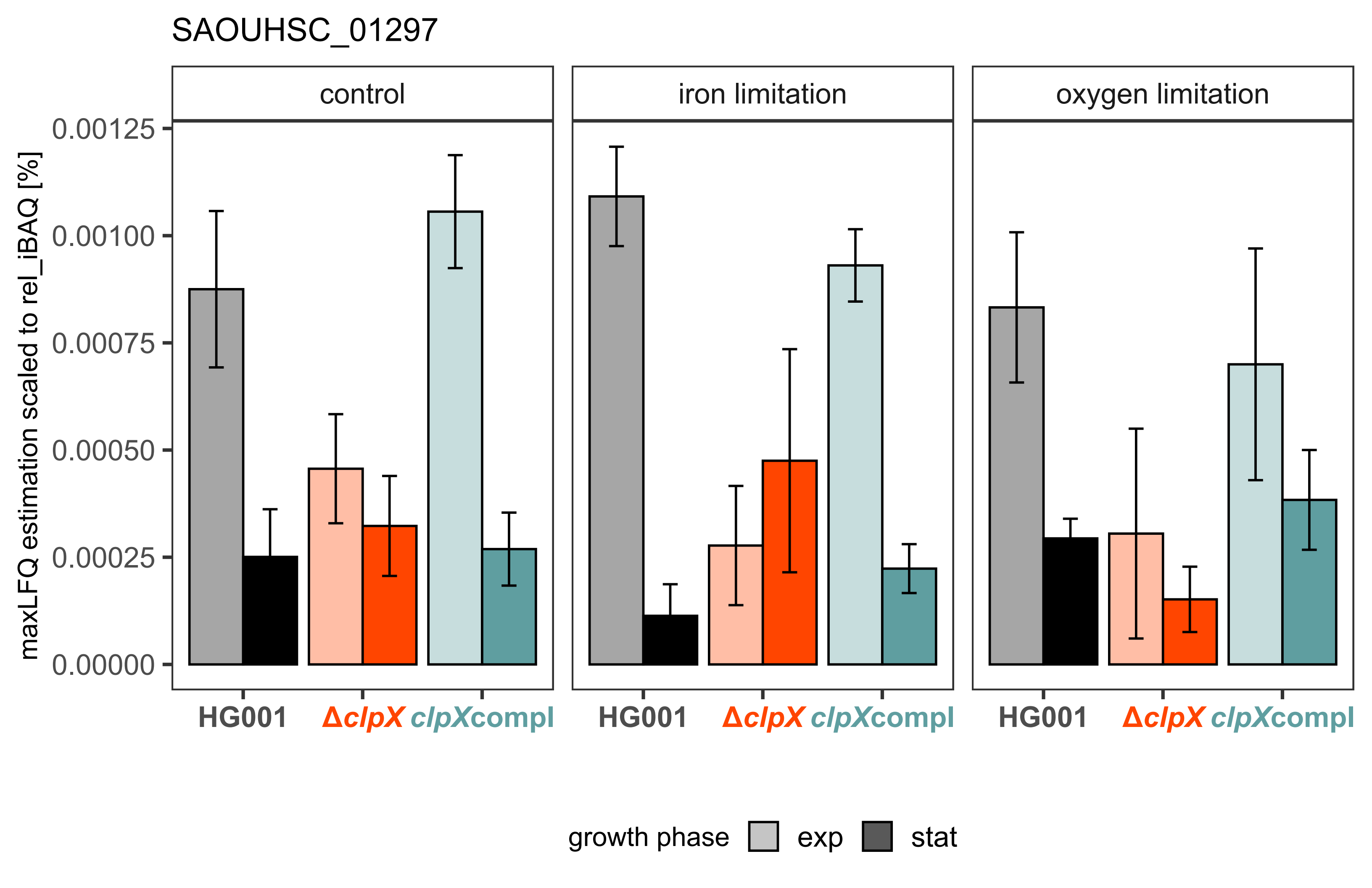
SAOUHSC_01297
control
iron limitation
oxygen limitation
maxLFQ estimation scaled to rel_iBAQ [%]
0.00125
0.00100
0.00075
0.00050
0.00025
0.00000
HG001
ΔclpX
clpXcompl
growth phase
exp
stat

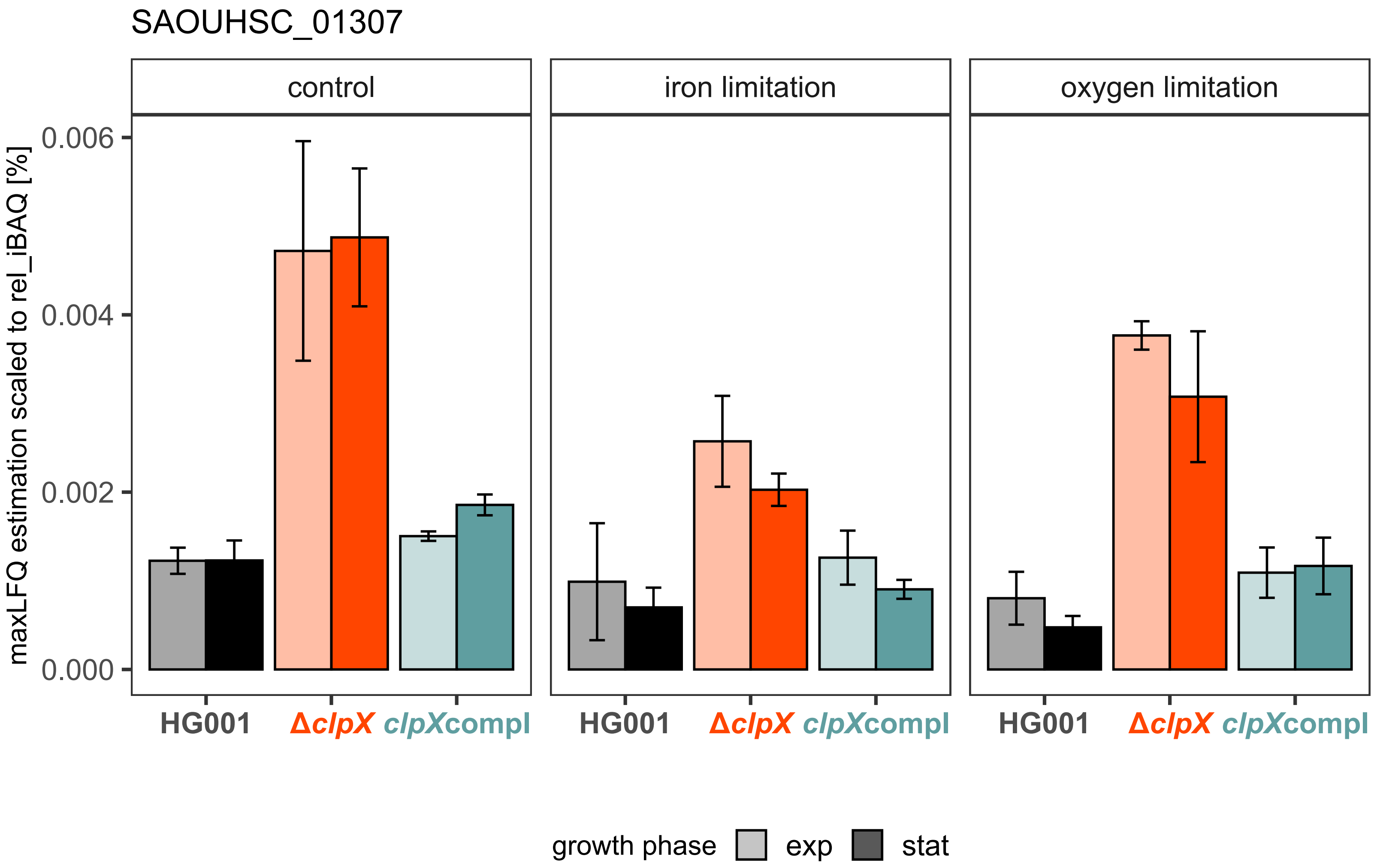

SAOUHSC_01307
control
iron limitation
oxygen limitation
maxLFQ estimation scaled to rel_iBAQ [%]
0.006
0.004
0.002
0.000
HG001
ΔclpX
clpXcompl
growth phase
exp
stat

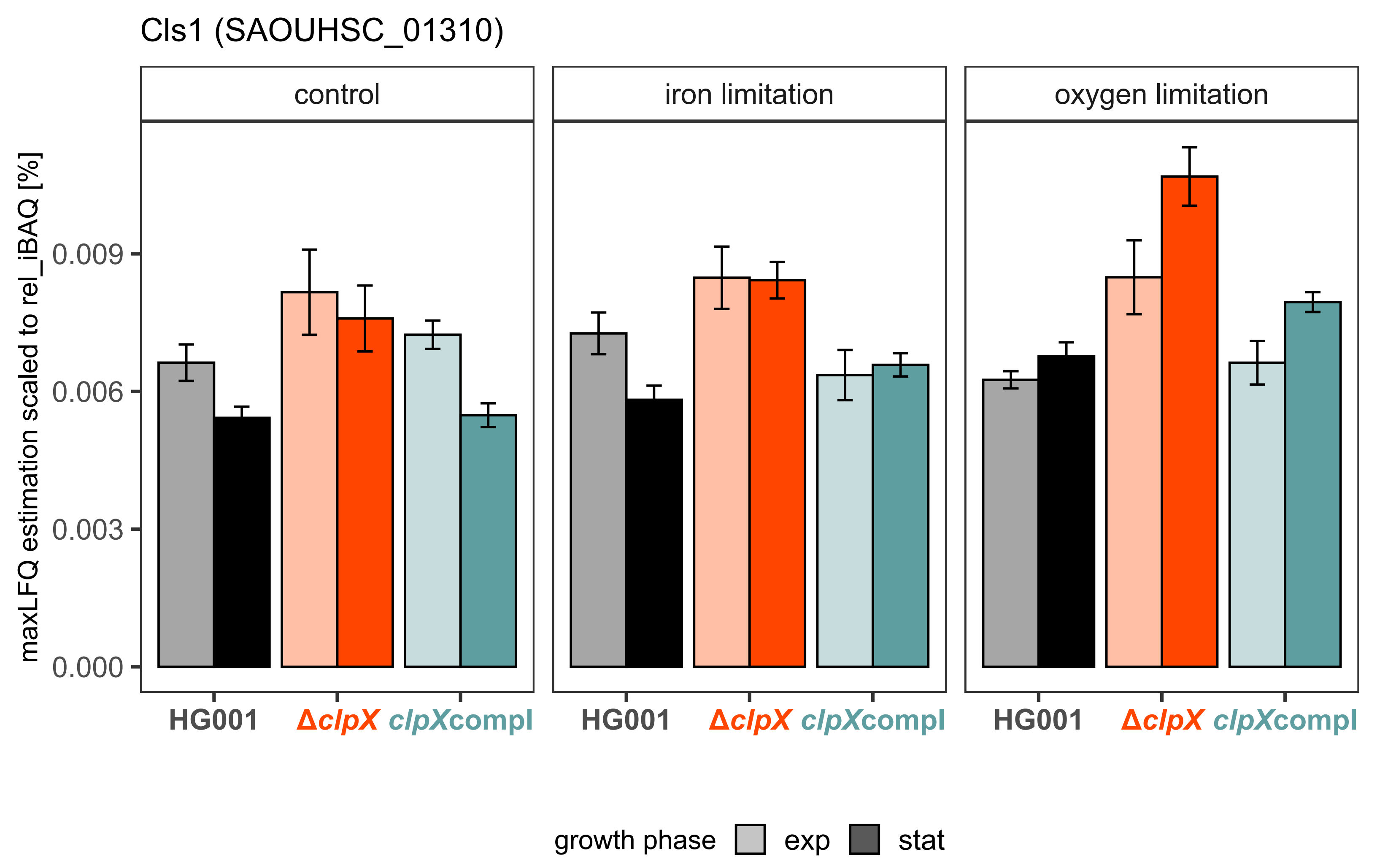
Cls1 (SAOUHSC_01310)
control
iron limitation
oxygen limitation
maxLFQ estimation scaled to rel_iBAQ [%]
0.009
0.006
0.003
0.000
HG001
ΔclpX
clpXcompl
growth phase
exp
stat

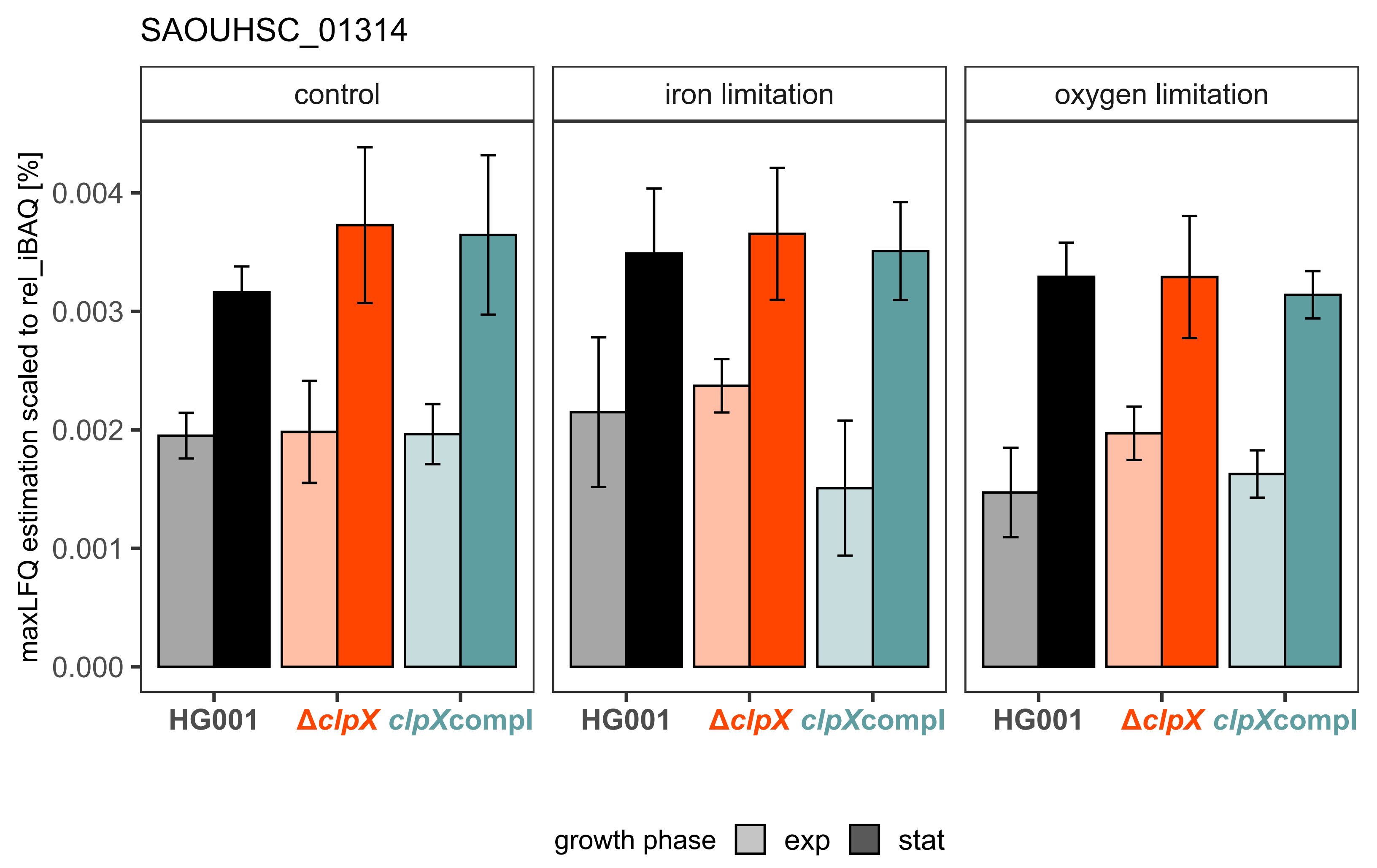
SAOUHSC_01314
control
iron limitation
oxygen limitation
maxLFQ estimation scaled to rel_iBAQ [%]
0.004
0.003
0.002
0.001
0.000
HG001
ΔclpX
clpXcompl
growth phase
exp
stat

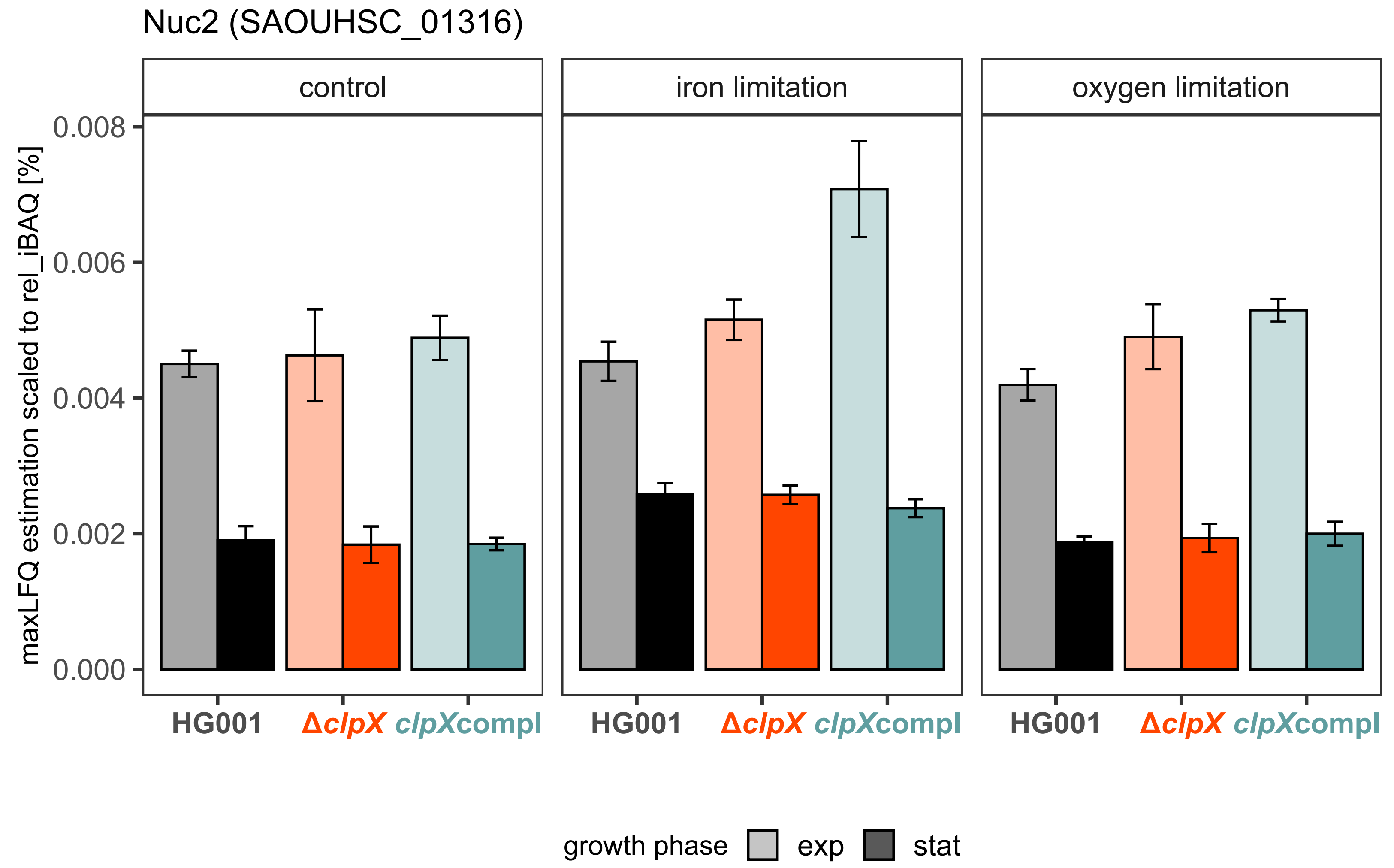
Nuc2 (SAOUHSC_01316)
control
iron limitation
oxygen limitation
maxLFQ estimation scaled to rel_iBAQ [%]
0.008
0.006
0.004
0.002
0.000
HG001
ΔclpX
clpXcompl
growth phase
exp
stat

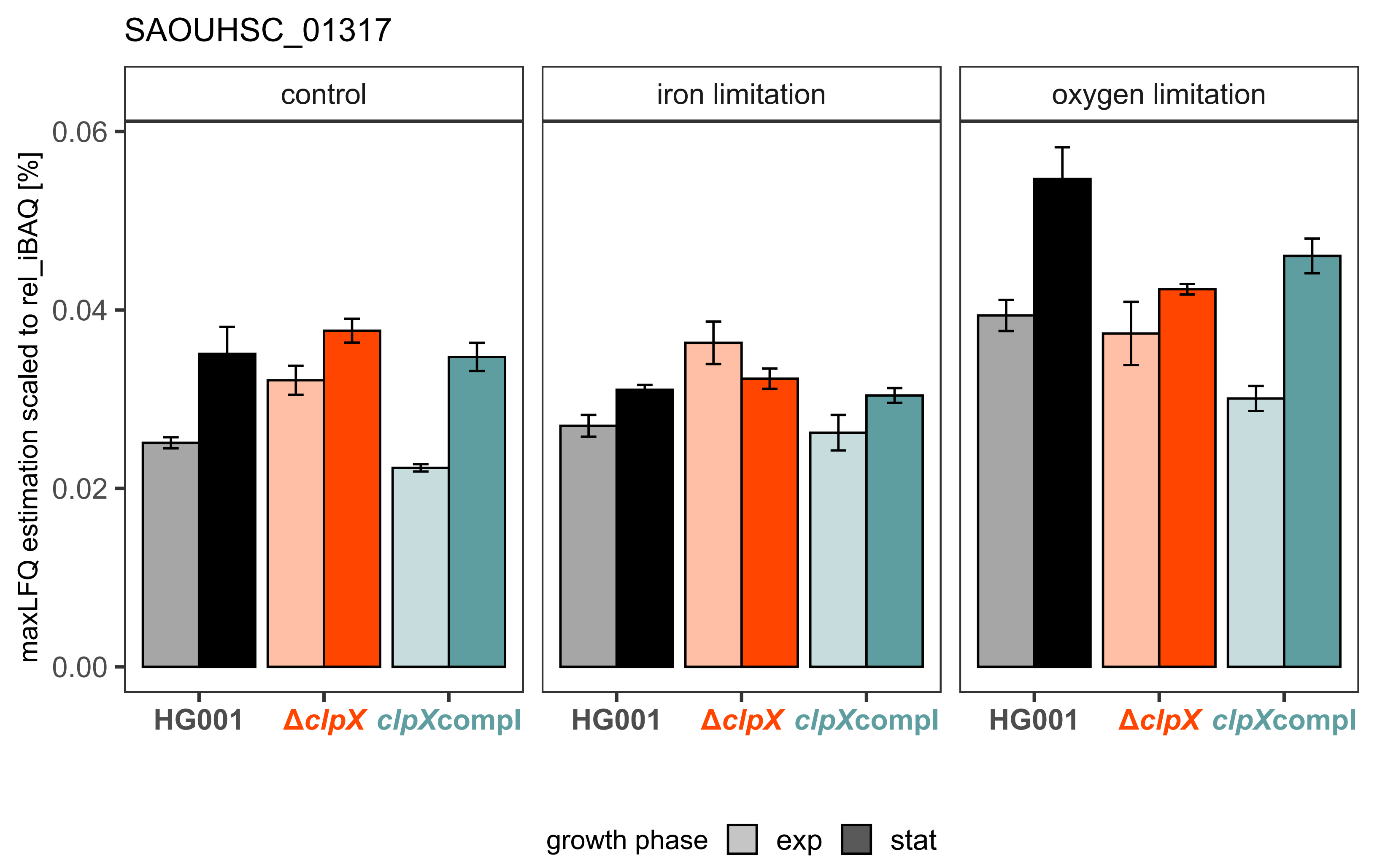
SAOUHSC_01317
control
iron limitation
oxygen limitation
maxLFQ estimation scaled to rel_iBAQ [%]
0.06
0.04
0.02
0.00
HG001
ΔclpX
clpXcompl
growth phase
exp
stat

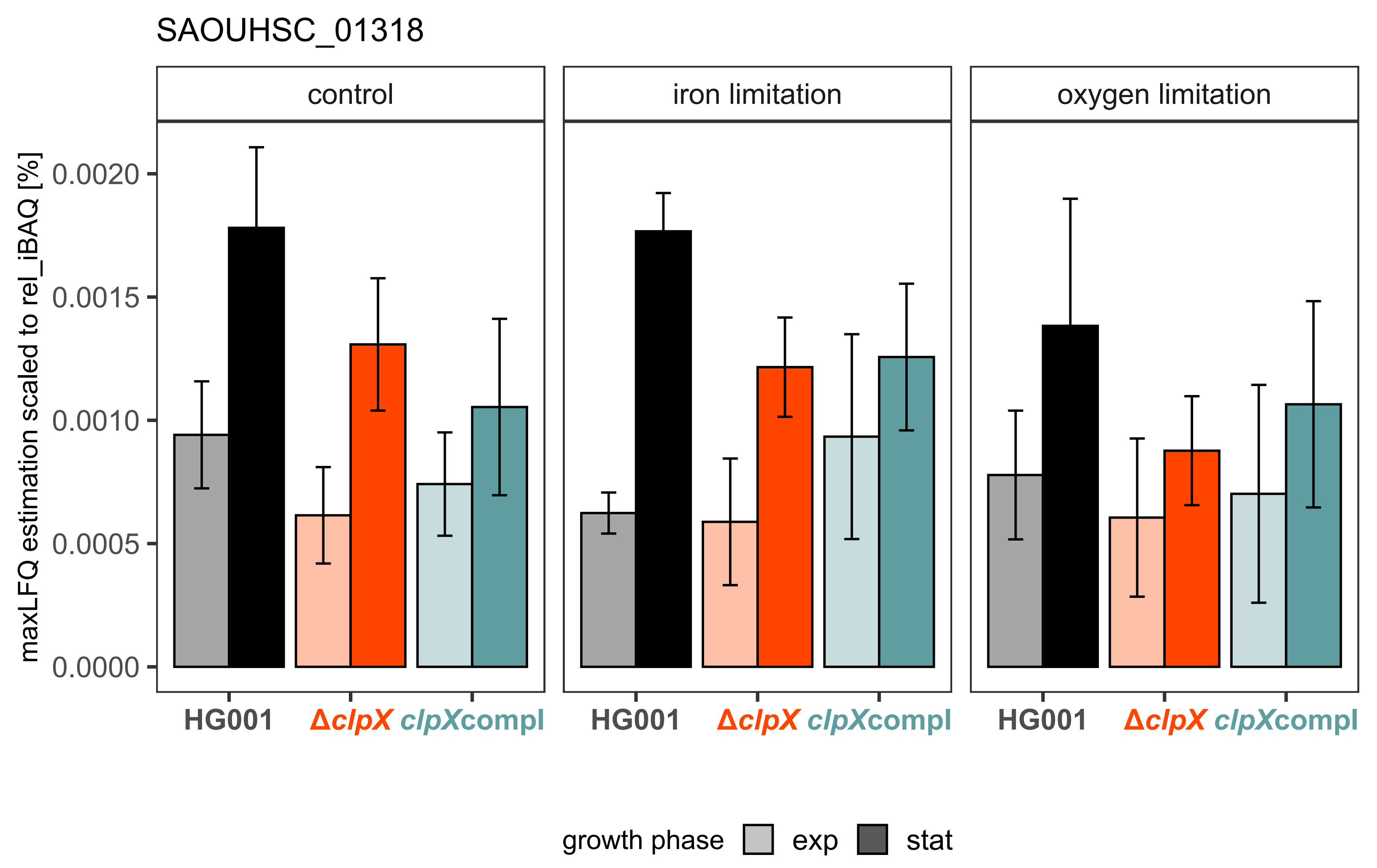

SAOUHSC_01318
control
iron limitation
oxygen limitation
maxLFQ estimation scaled to rel_iBAQ [%]
0.0020
0.0015
0.0010
0.0005
0.0000
HG001
ΔclpX
clpXcompl
growth phase
exp
stat

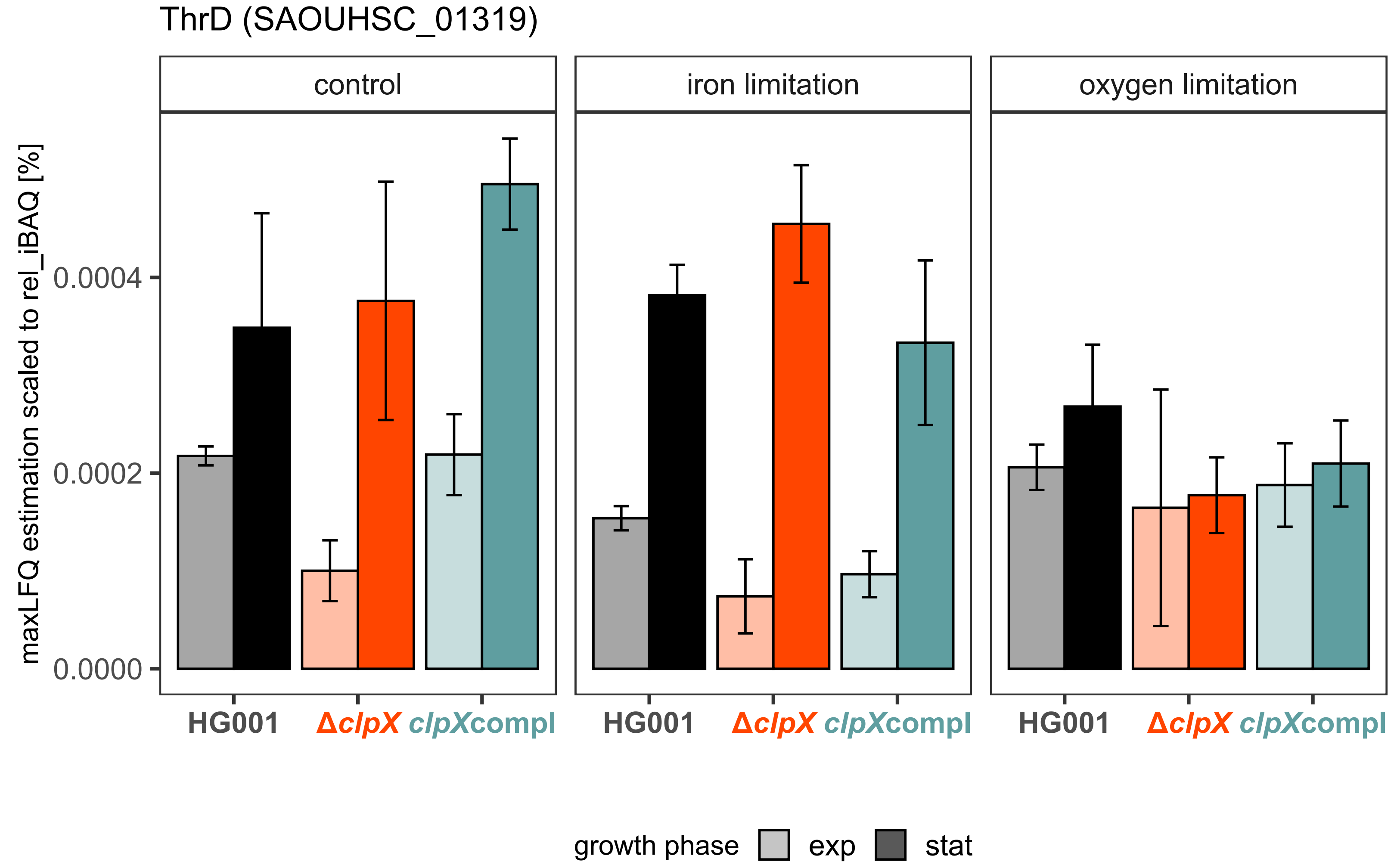

ThrD (SAOUHSC_01319)
control
iron limitation
oxygen limitation
maxLFQ estimation scaled to rel_iBAQ [%]
0.0004
0.0002
0.0000
HG001
ΔclpX
clpXcompl
growth phase
exp
stat

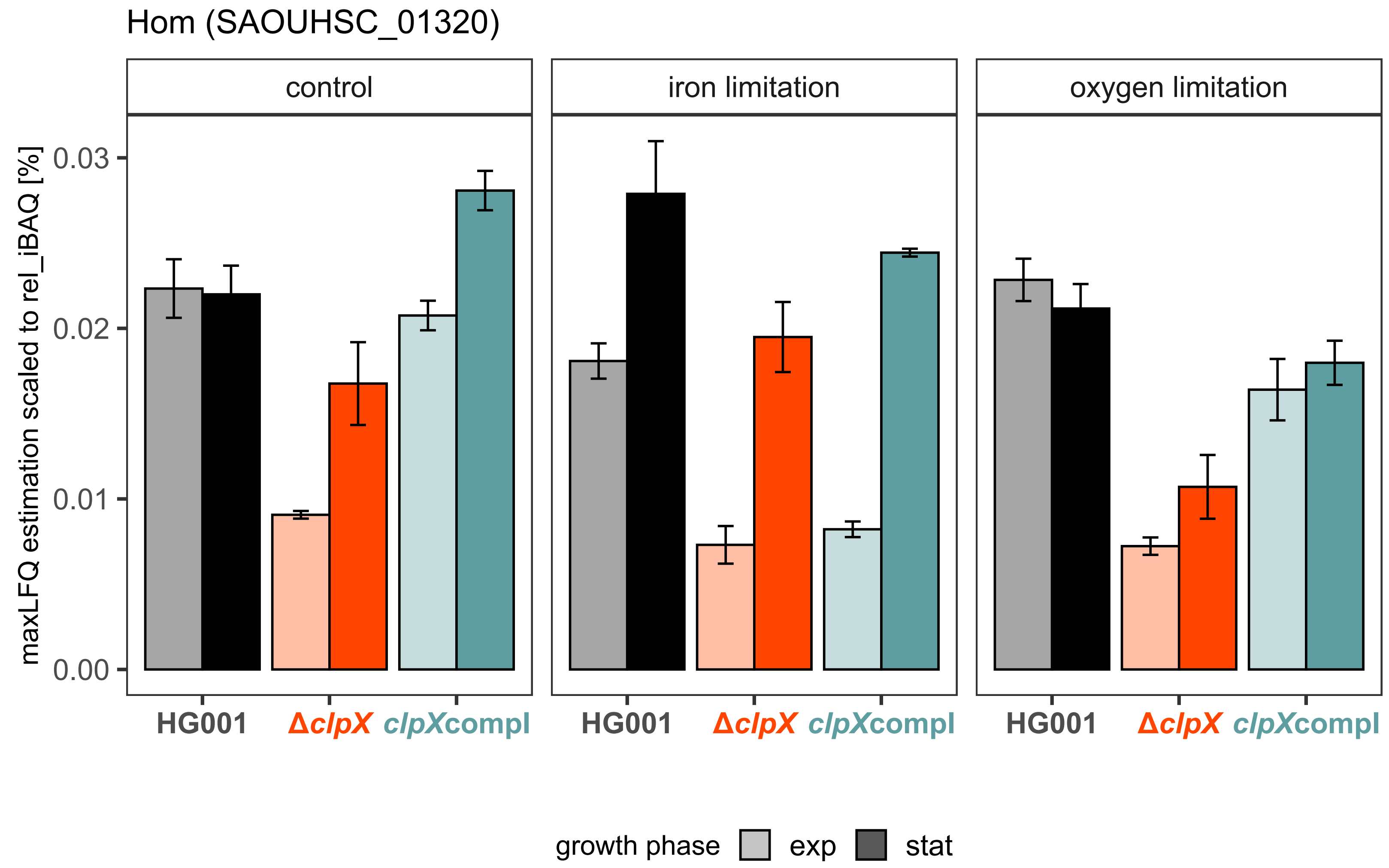
Hom (SAOUHSC_01320)
control
iron limitation
oxygen limitation
maxLFQ estimation scaled to rel_iBAQ [%]
0.03
0.02
0.01
0.00
HG001
ΔclpX
clpXcompl
growth phase
exp
stat

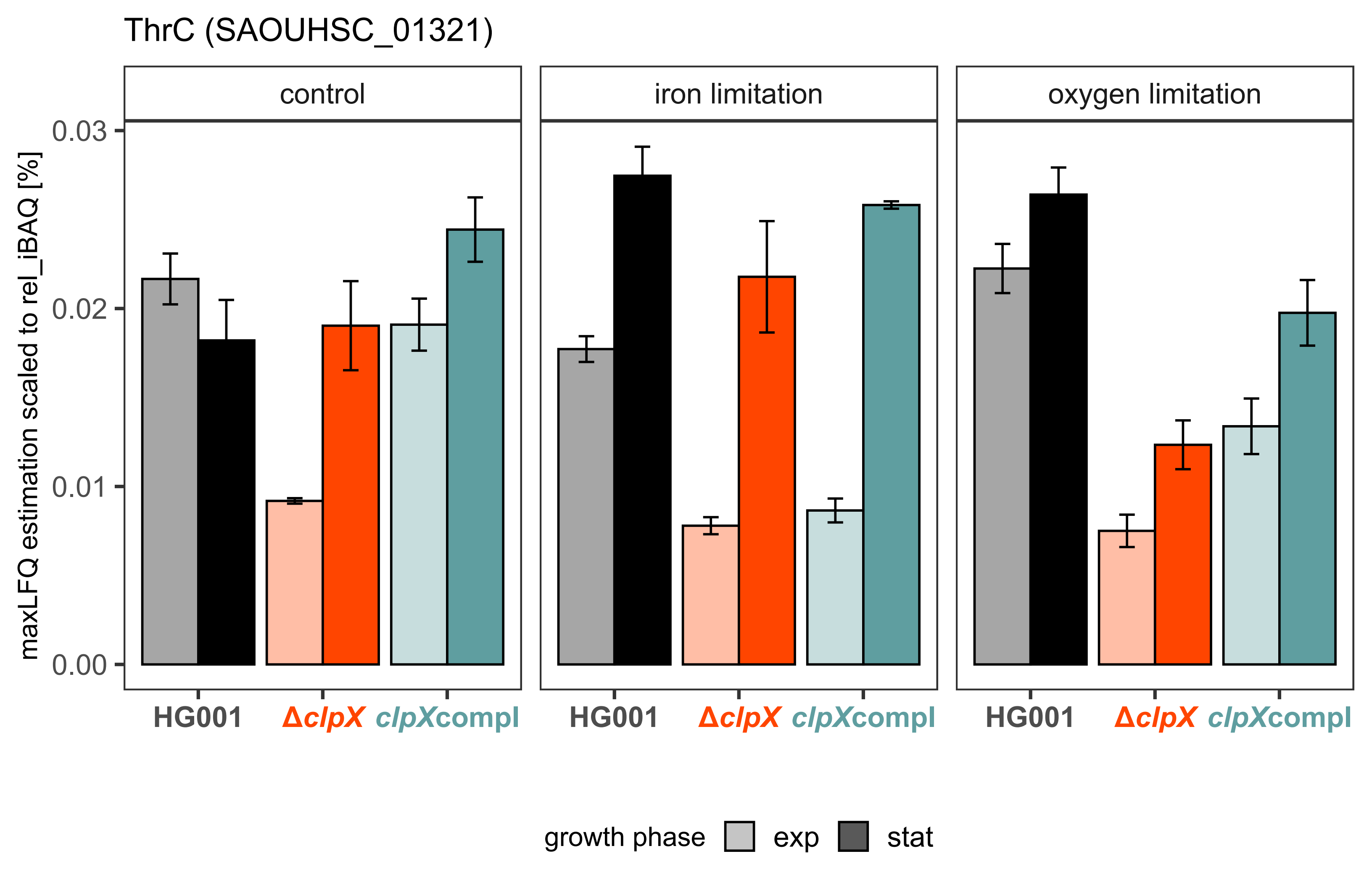
ThrC (SAOUHSC_01321)
control
iron limitation
oxygen limitation
maxLFQ estimation scaled to rel_iBAQ [%]
0.03
0.02
0.01
0.00
HG001
ΔclpX
clpXcompl
growth phase
exp
stat

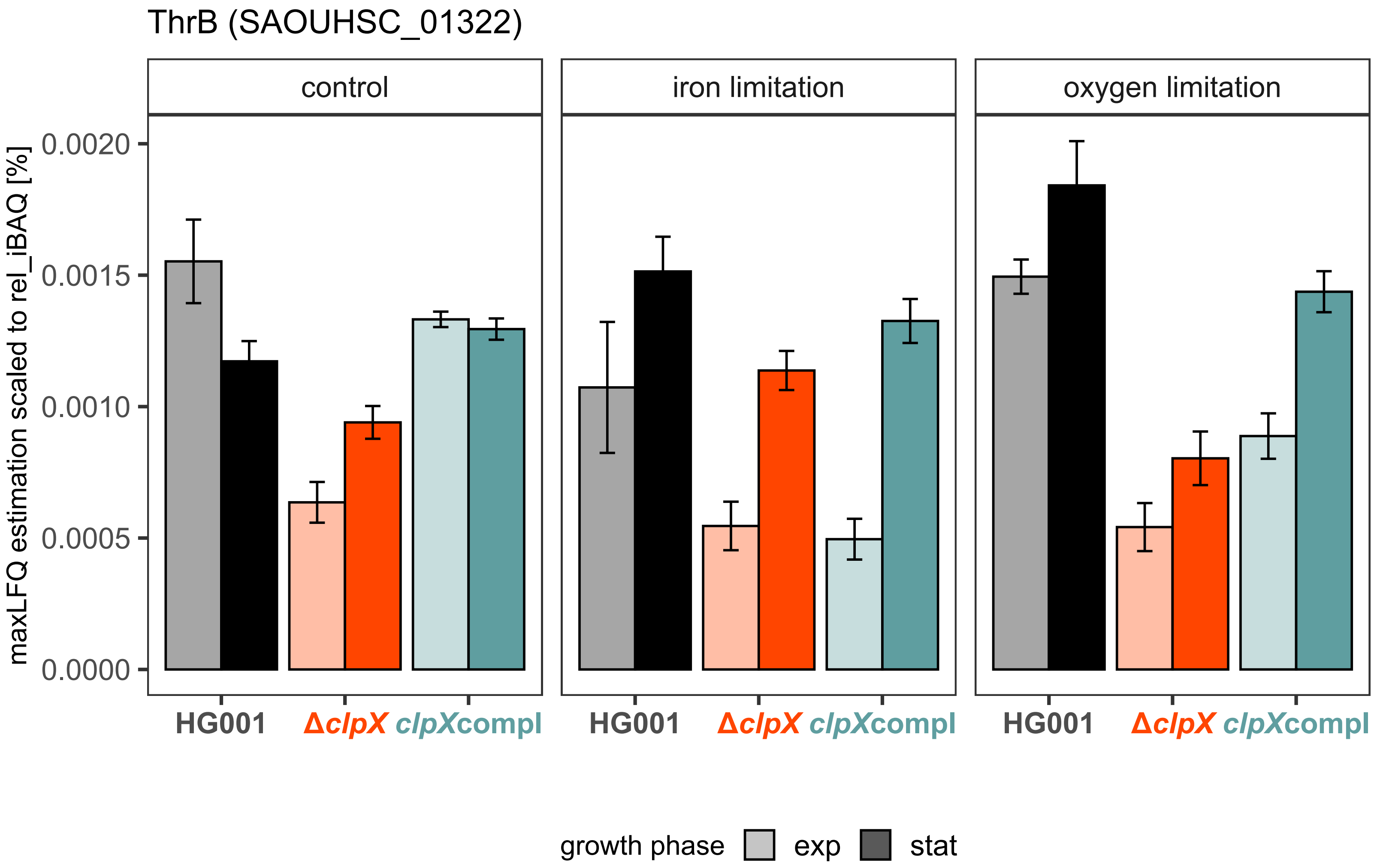

ThrB (SAOUHSC_01322)
control
iron limitation
oxygen limitation
maxLFQ estimation scaled to rel_iBAQ [%]
0.0020
0.0015
0.0010
0.0005
0.0000
HG001
ΔclpX
clpXcompl
growth phase
exp
stat

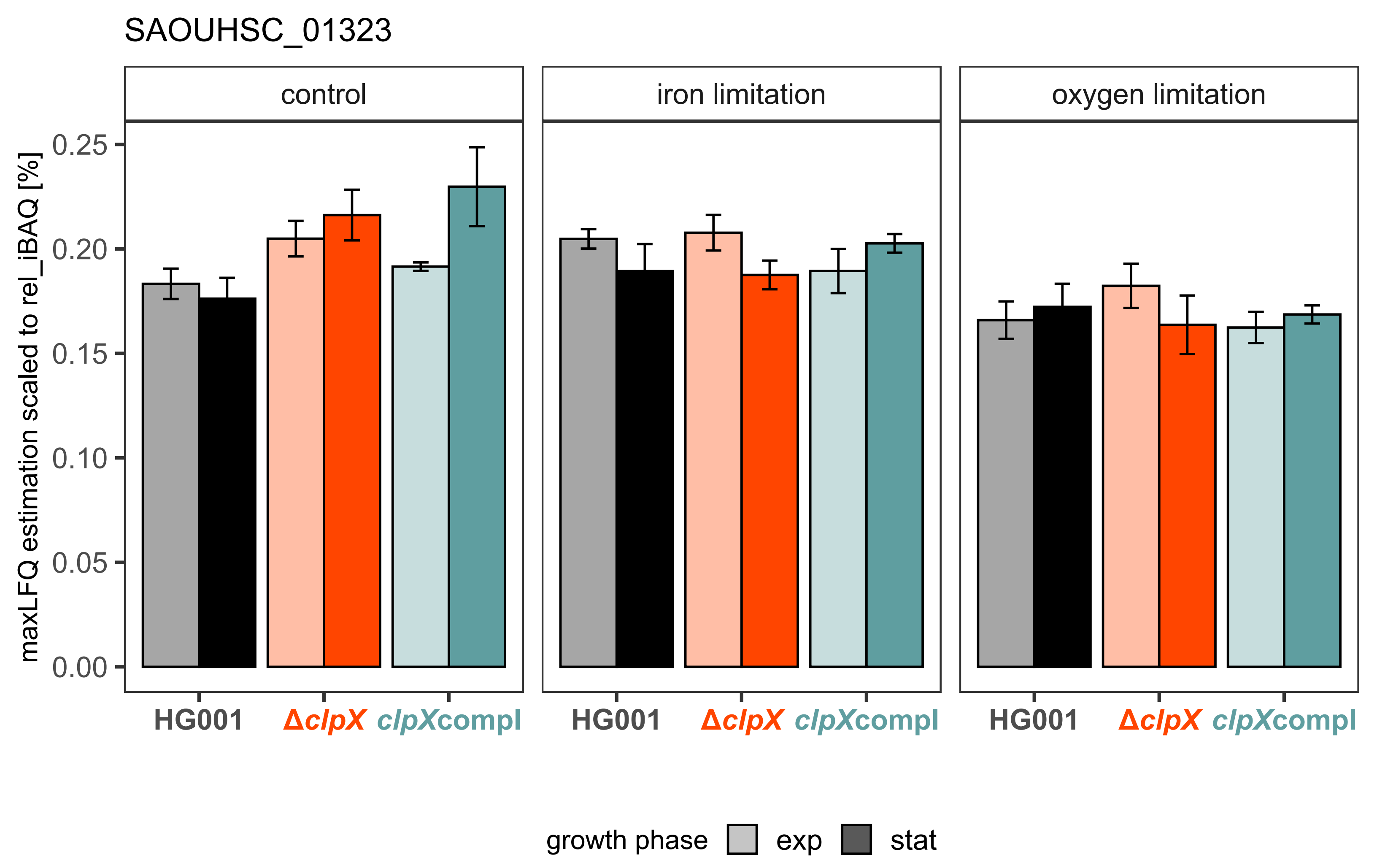
SAOUHSC_01323
control
iron limitation
oxygen limitation
maxLFQ estimation scaled to rel_iBAQ [%]
0.25
0.20
0.15
0.10
0.05
0.00
HG001
ΔclpX
clpXcompl
growth phase
exp
stat

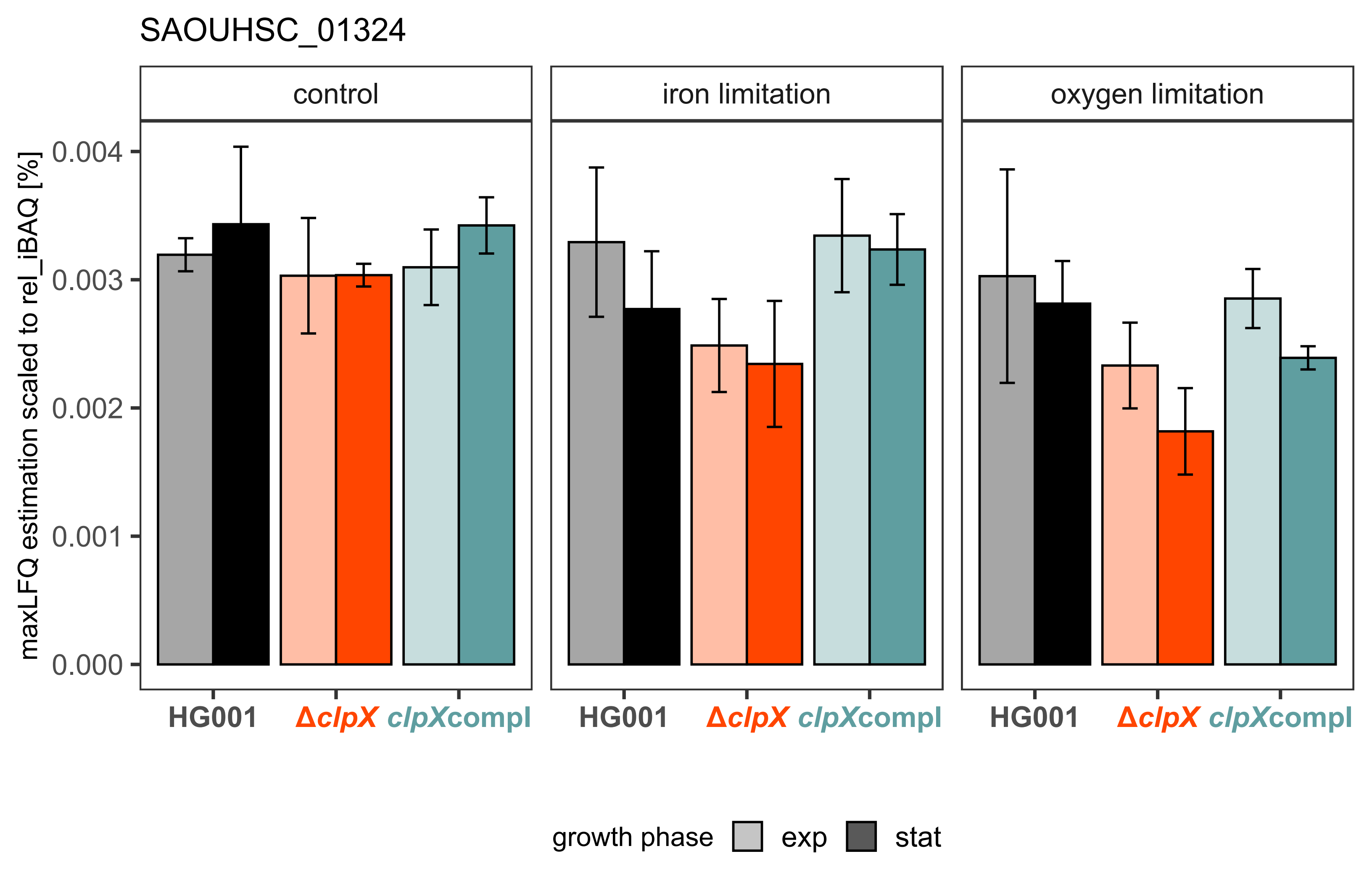

SAOUHSC_01324
control
iron limitation
oxygen limitation
maxLFQ estimation scaled to rel_iBAQ [%]
0.004
0.003
0.002
0.001
0.000
HG001
ΔclpX
clpXcompl
growth phase
exp
stat

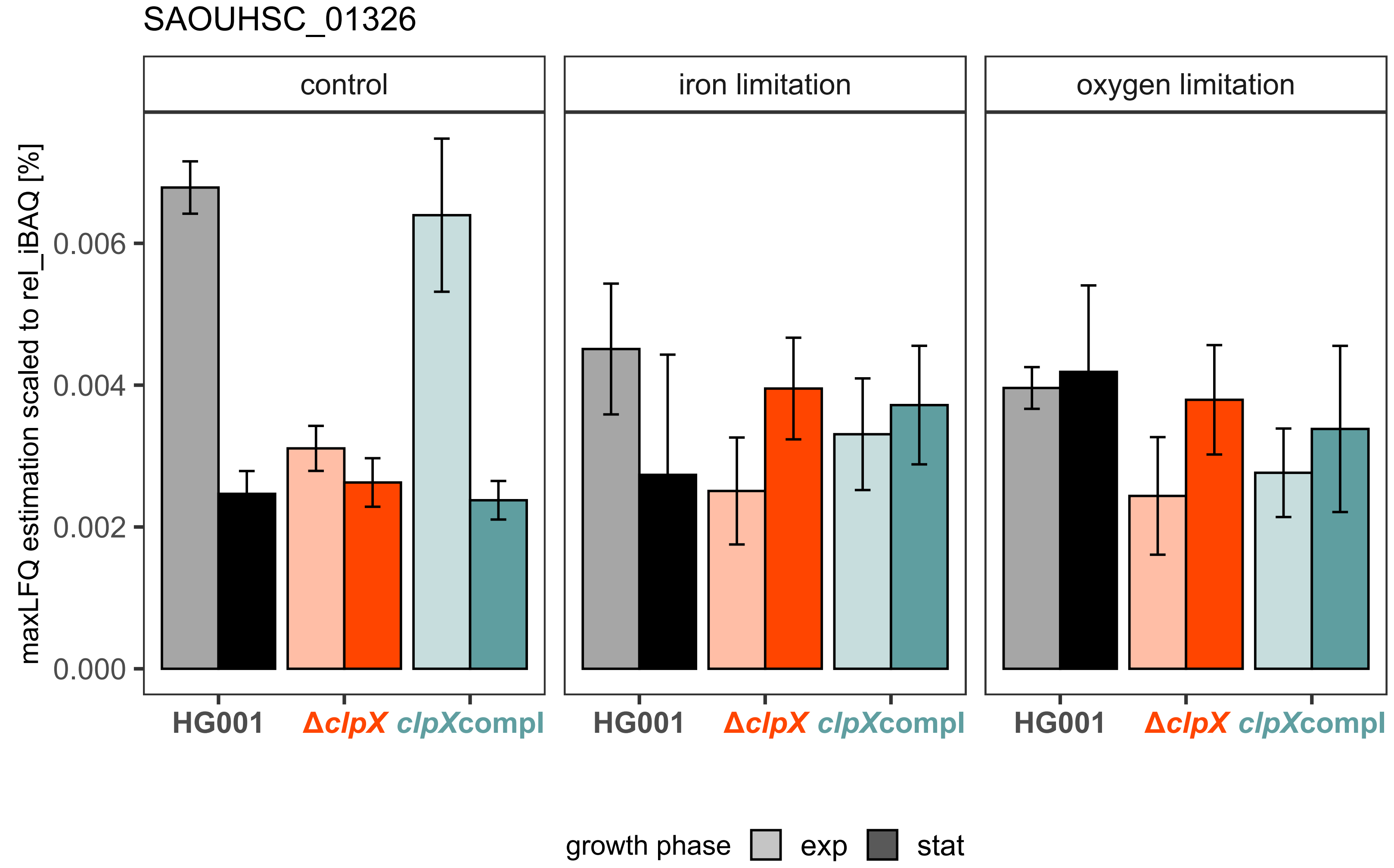

SAOUHSC_01326
control
iron limitation
oxygen limitation
maxLFQ estimation scaled to rel_iBAQ [%]
0.006
0.004
0.002
0.000
HG001
ΔclpX
clpXcompl
growth phase
exp
stat

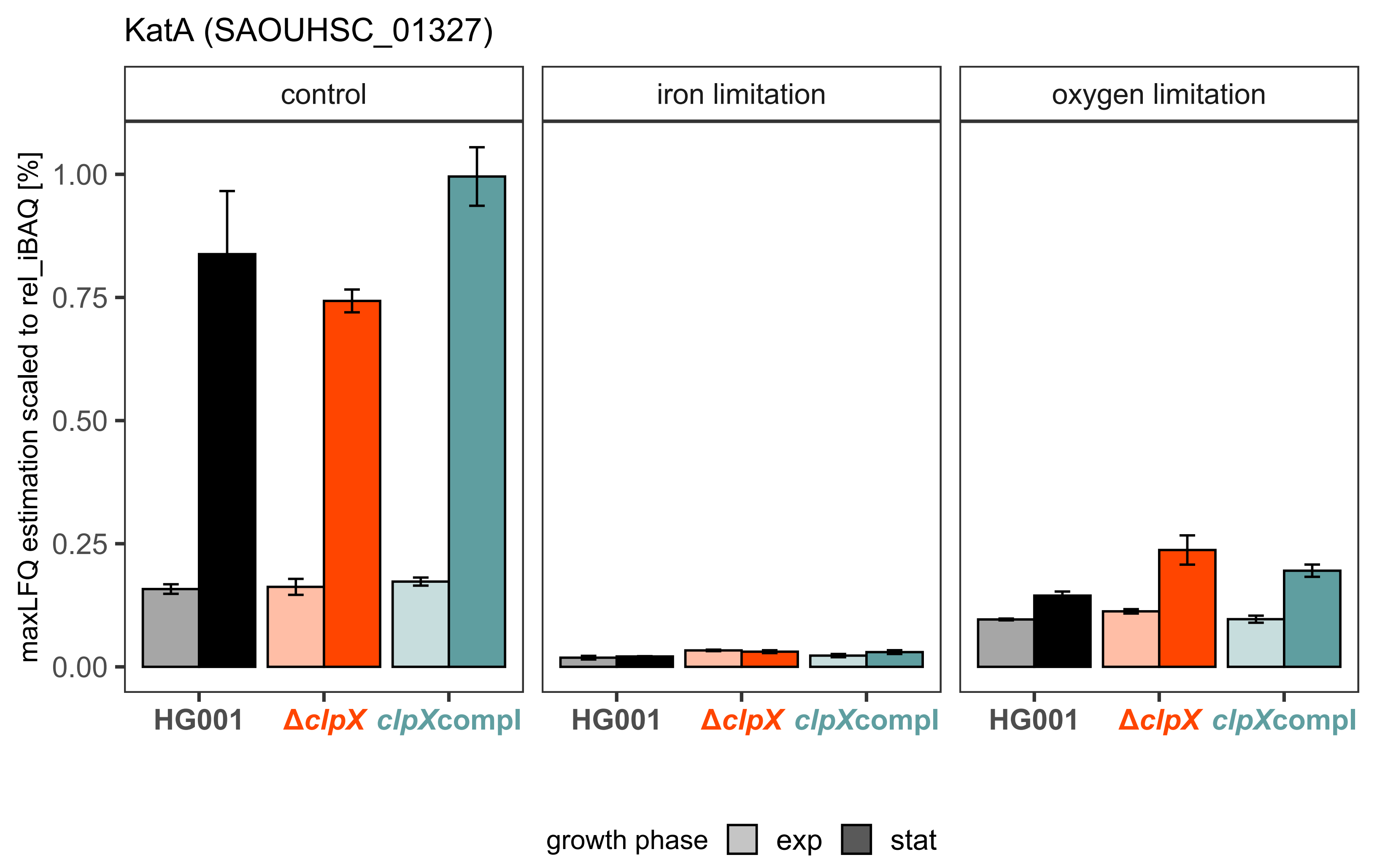
KatA (SAOUHSC_01327)
control
iron limitation
oxygen limitation
maxLFQ estimation scaled to rel_iBAQ [%]
1.00
0.75
0.50
0.25
0.00
HG001
ΔclpX
clpXcompl
growth phase
exp
stat

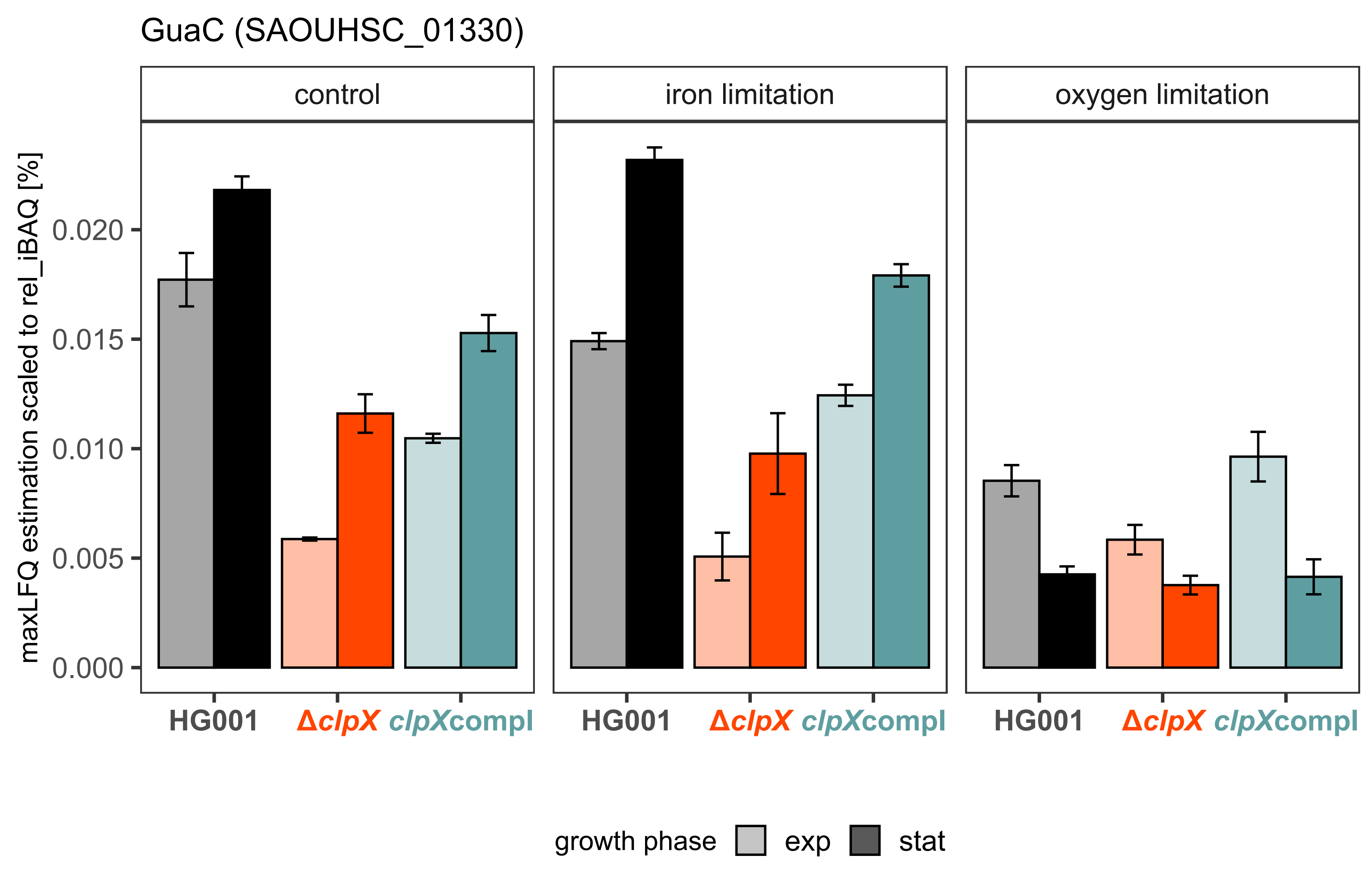
GuaC (SAOUHSC_01330)
control
iron limitation
oxygen limitation
maxLFQ estimation scaled to rel_iBAQ [%]
0.020
0.015
0.010
0.005
0.000
HG001
ΔclpX
clpXcompl
growth phase
exp
stat

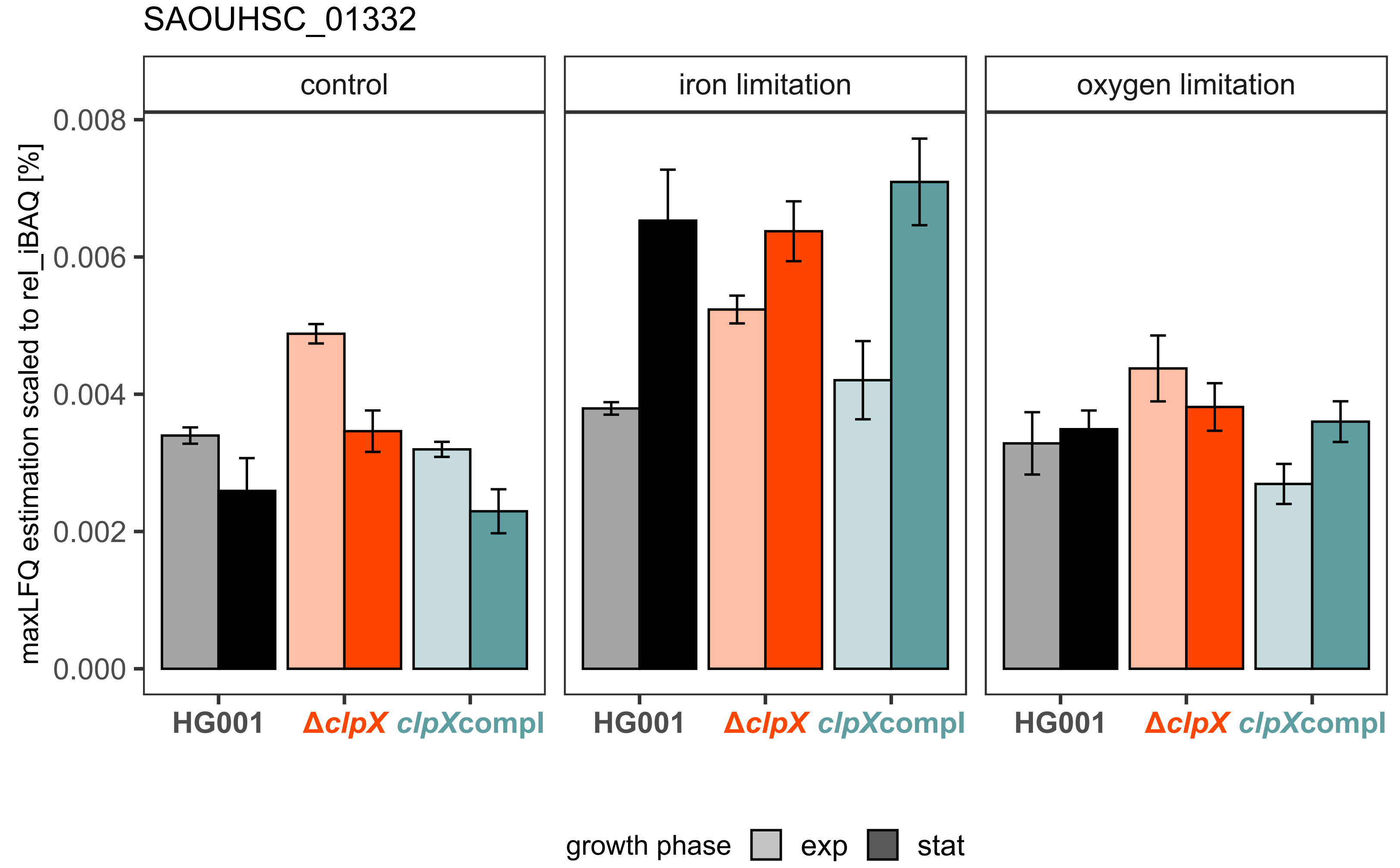
SAOUHSC_01332
control
iron limitation
oxygen limitation
maxLFQ estimation scaled to rel_iBAQ [%]
0.008
0.006
0.004
0.002
0.000
HG001
ΔclpX
clpXcompl
growth phase
exp
stat

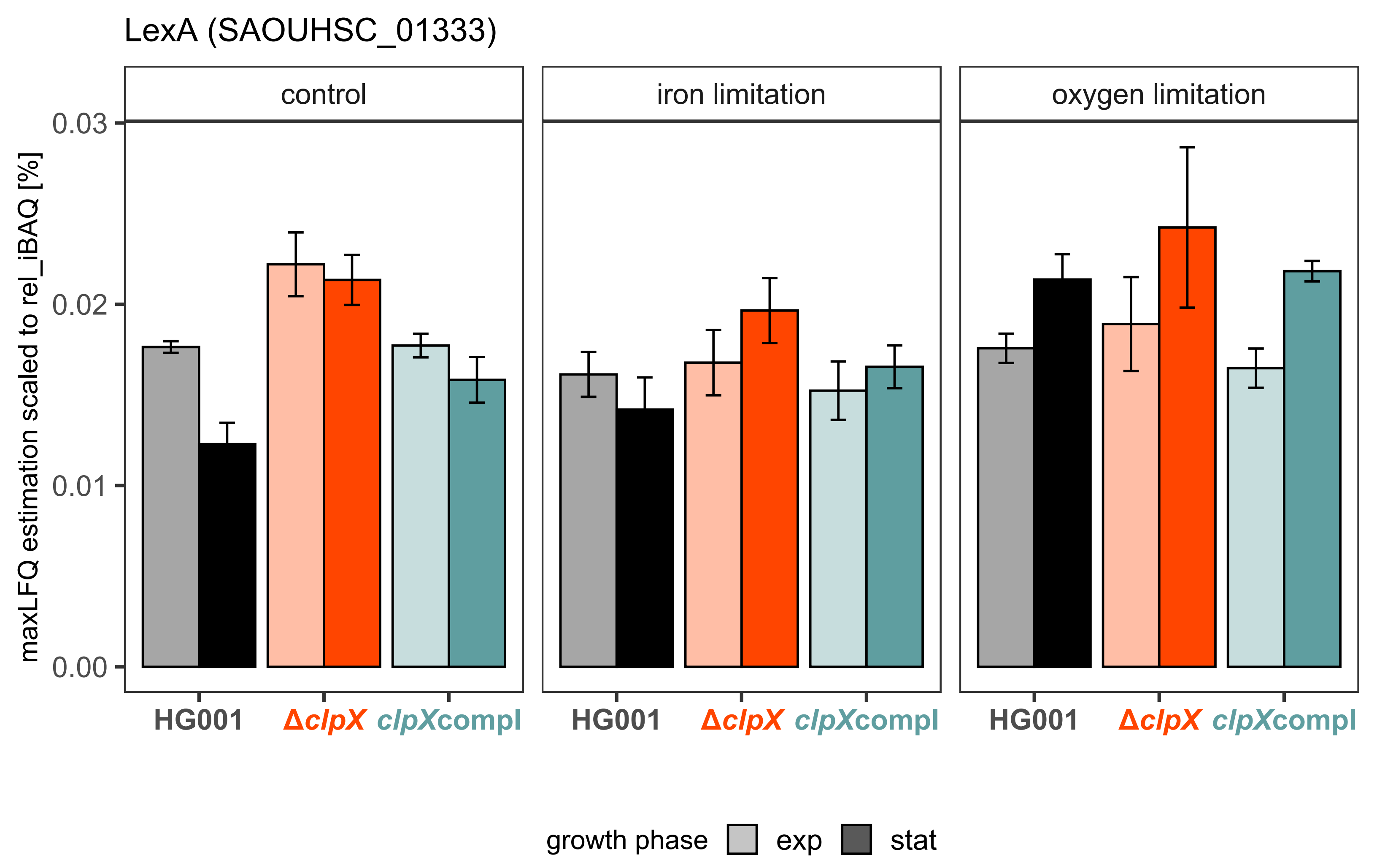
LexA (SAOUHSC_01333)
control
iron limitation
oxygen limitation
0.03
0.02
0.01
0.00
maxLFQ estimation scaled to rel_iBAQ [%]
HG001
ΔclpX
clpXcompl
growth phase
exp
stat

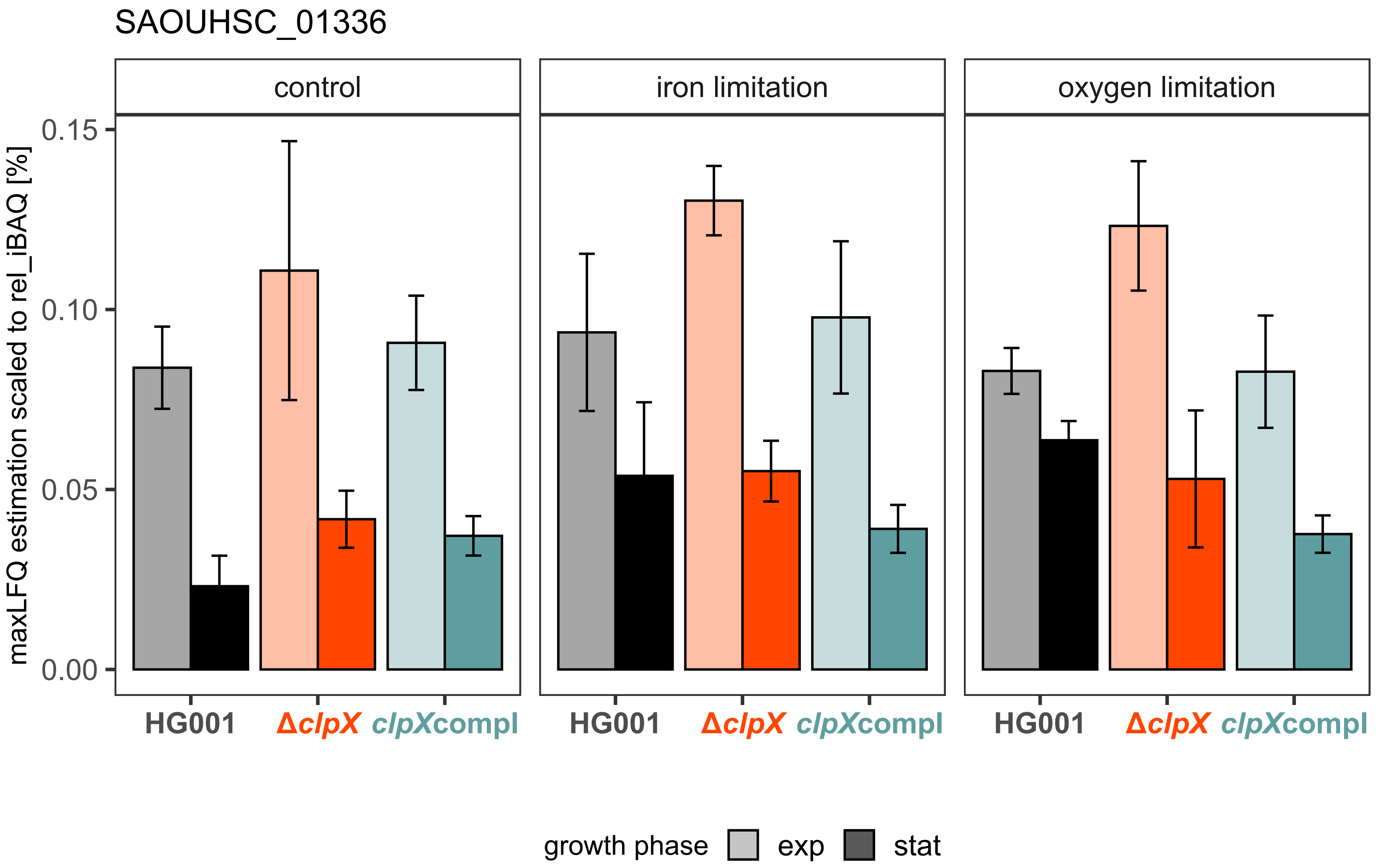

SAOUHSC_01336
control
iron limitation
oxygen limitation
maxLFQ estimation scaled to rel_iBAQ [%]
0.15
0.10
0.05
0.00
HG001
ΔclpX
clpXcompl
growth phase
exp
stat

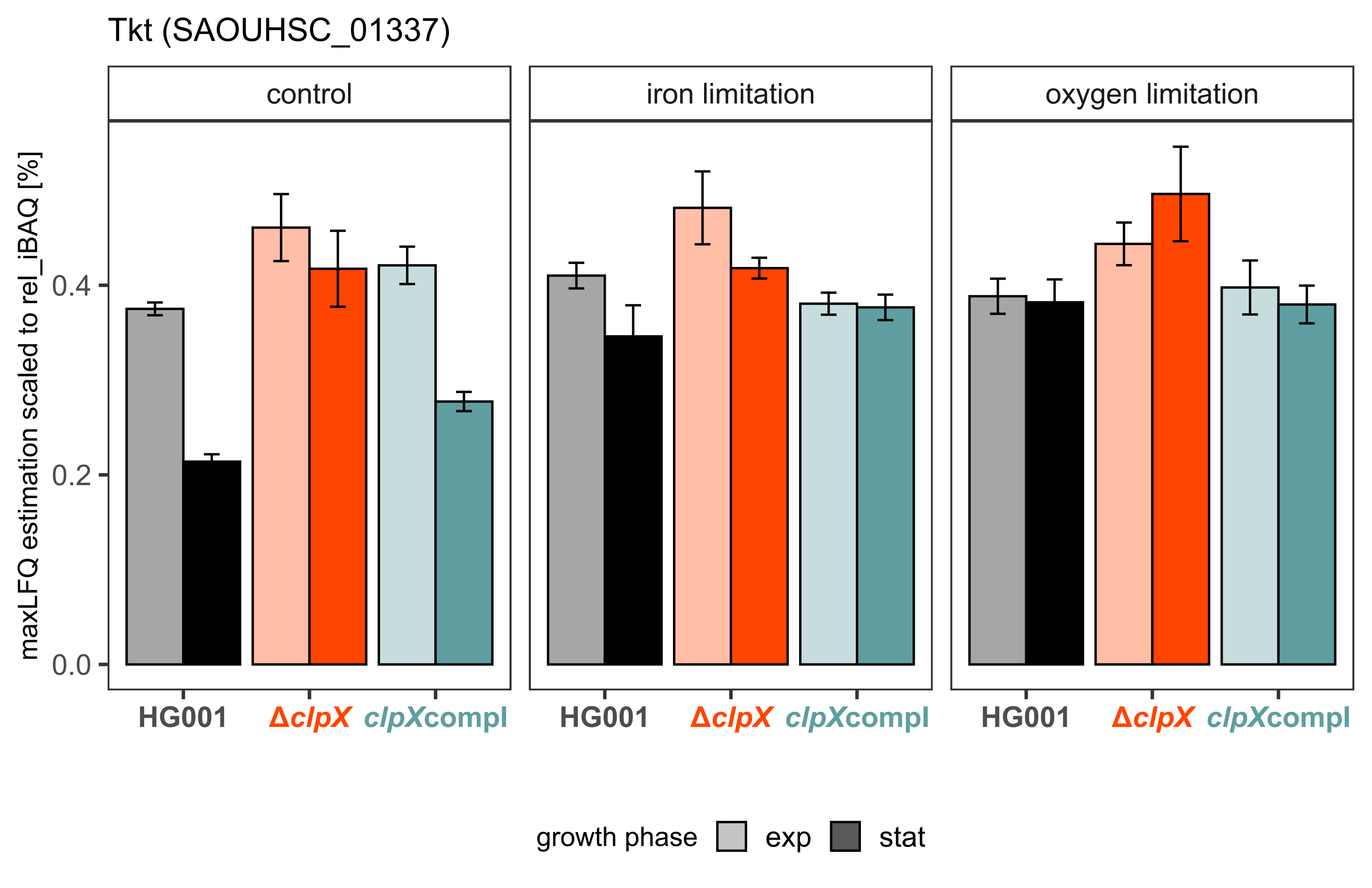
Tkt (SAOUHSC_01337)
control
iron limitation
oxygen limitation
maxLFQ estimation scaled to rel_iBAQ [%]
0.0
0.2
0.4
HG001
ΔclpX
clpXcompl
growth phase
exp
stat

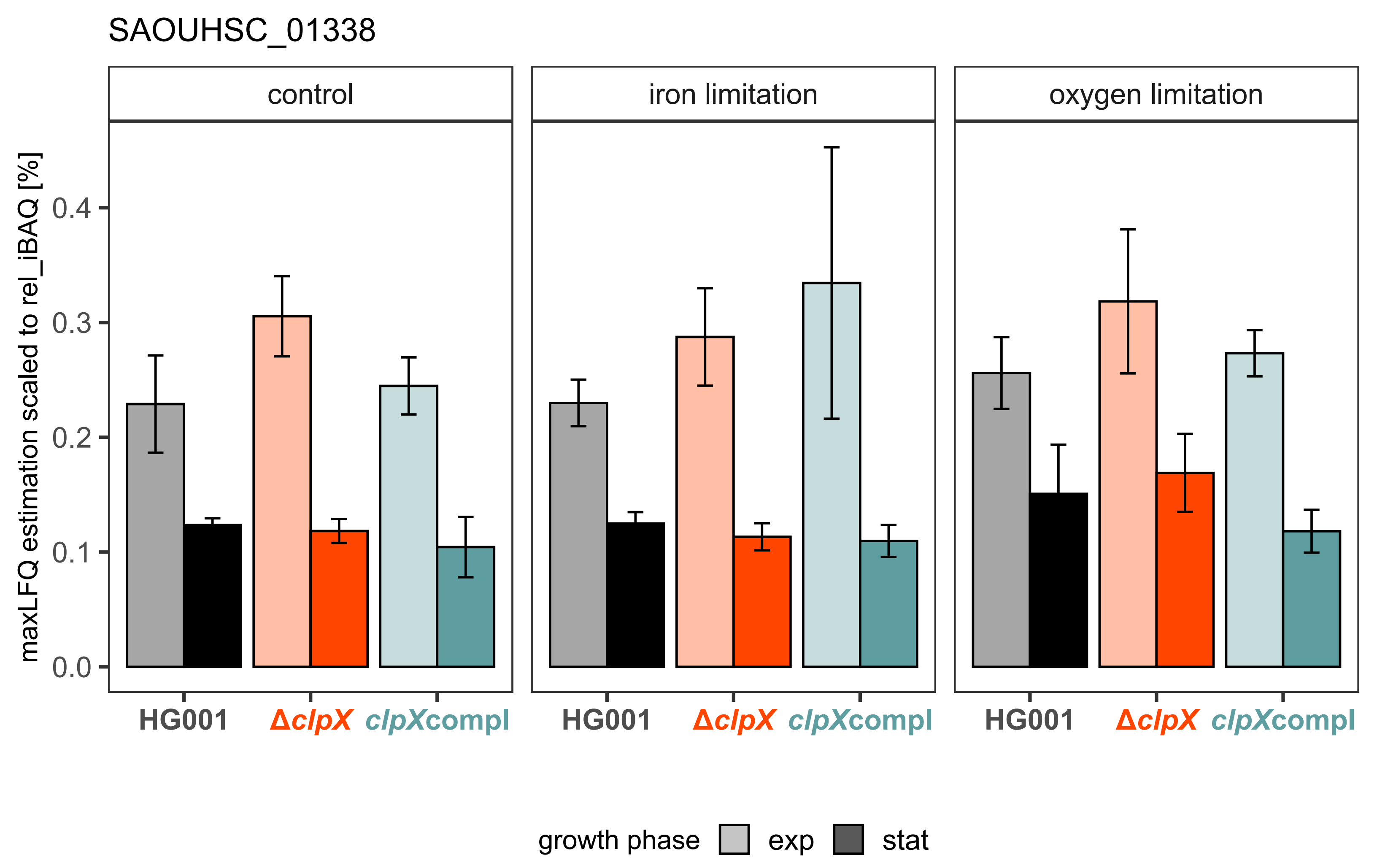
SAOUHSC_01338
control
iron limitation
oxygen limitation
maxLFQ estimation scaled to rel_iBAQ [%]
0.0
0.1
0.2
0.3
0.4
HG001
ΔclpX
clpXcompl
growth phase
exp
stat

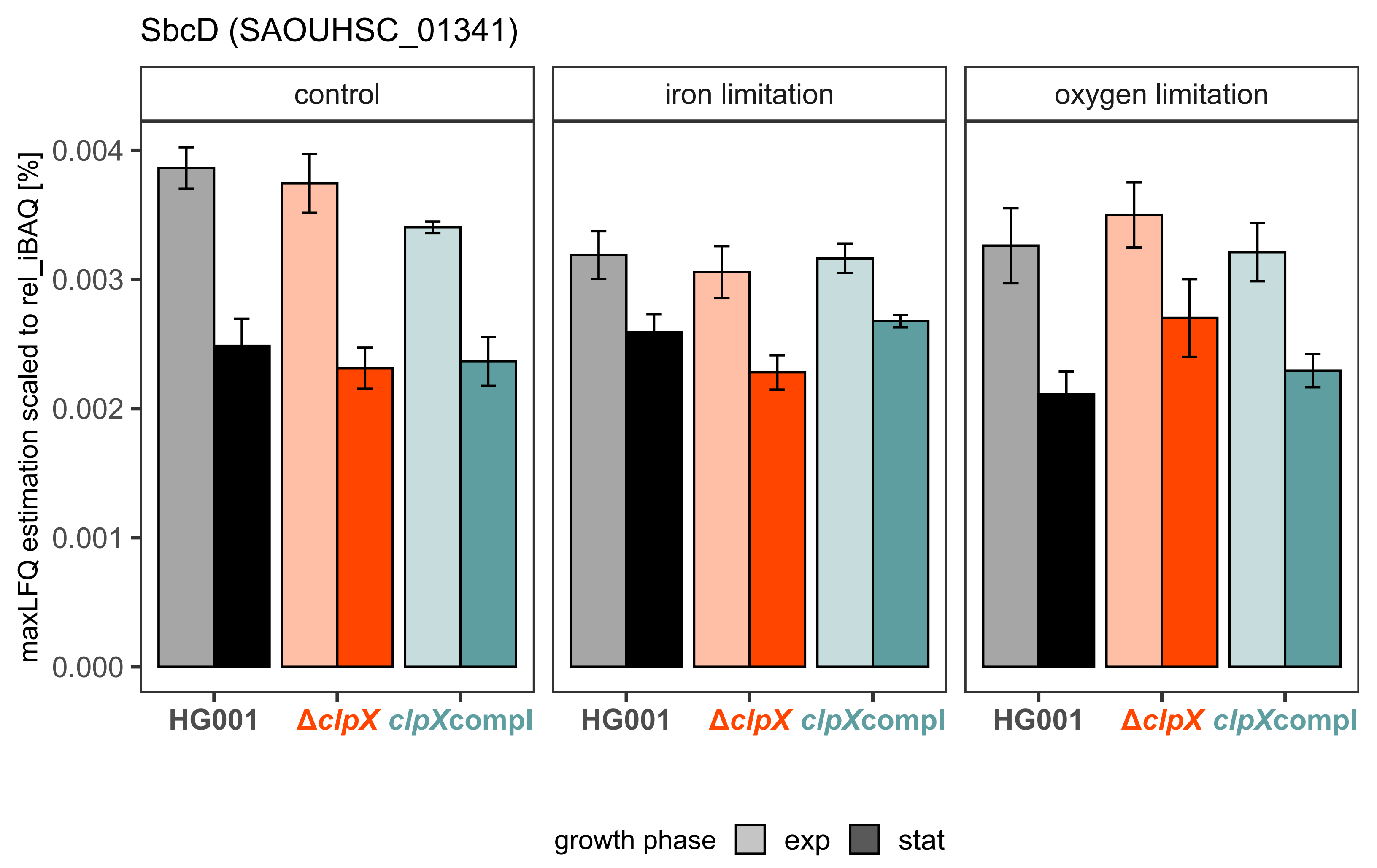
SbcD (SAOUHSC_01341)
control
iron limitation
oxygen limitation
maxLFQ estimation scaled to rel_iBAQ [%]
0.004
0.003
0.002
0.001
0.000
HG001
ΔclpX
clpXcompl
growth phase
exp
stat

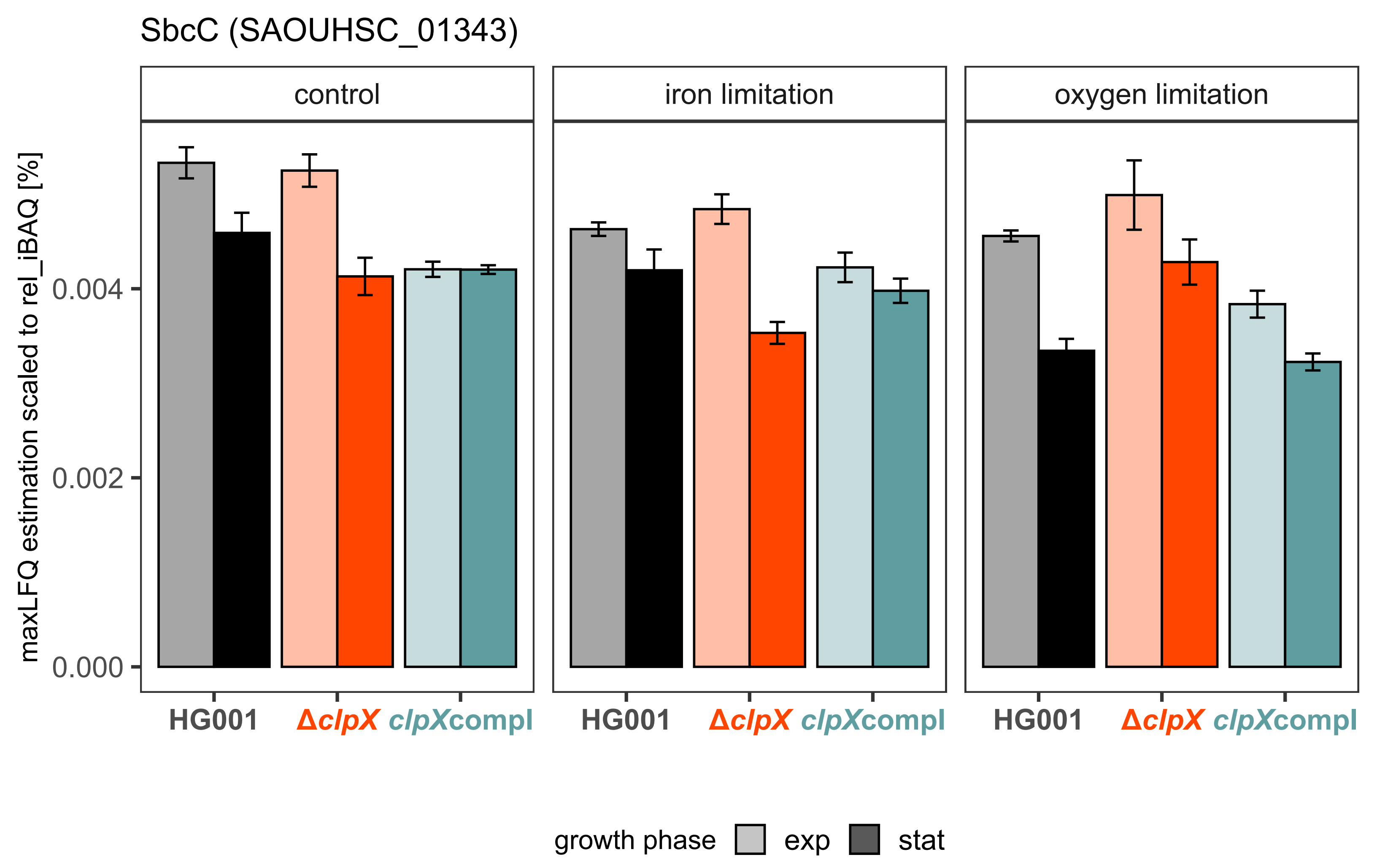

SbcC (SAOUHSC_01343)
control
iron limitation
oxygen limitation
maxLFQ estimation scaled to rel_iBAQ [%]
0.000
0.002
0.004
HG001
ΔclpX
clpXcompl
growth phase
exp
stat

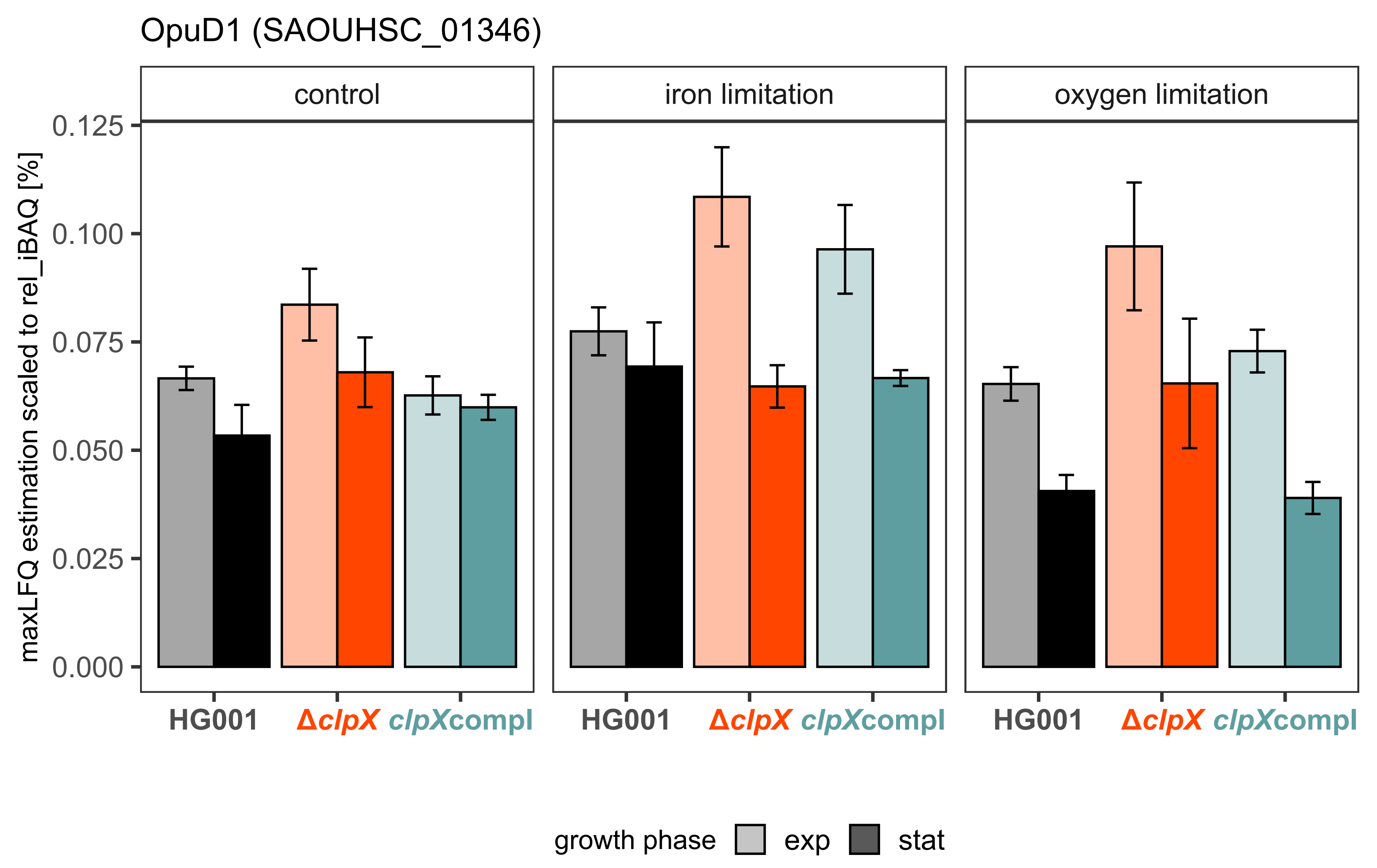
OpuD1 (SAOUHSC_01346)
control
iron limitation
oxygen limitation
maxLFQ estimation scaled to rel_iBAQ [%]
0.125
0.100
0.075
0.050
0.025
0.000
HG001
ΔclpX
clpXcompl
growth phase
exp
stat

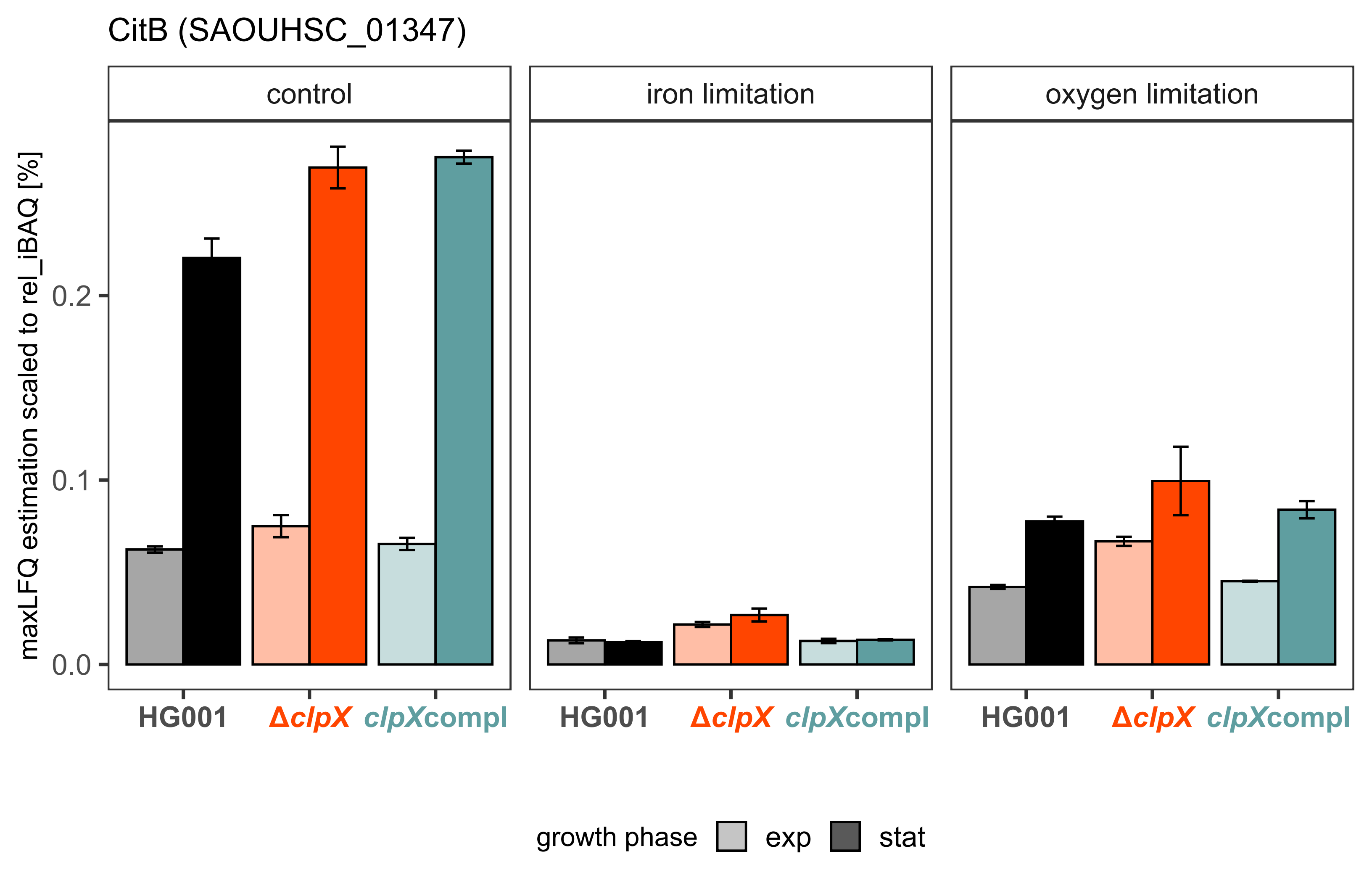
CitB (SAOUHSC_01347)
control
iron limitation
oxygen limitation
maxLFQ estimation scaled to rel_iBAQ [%]
0.0
0.1
0.2
HG001
ΔclpX
clpXcompl
growth phase
exp
stat

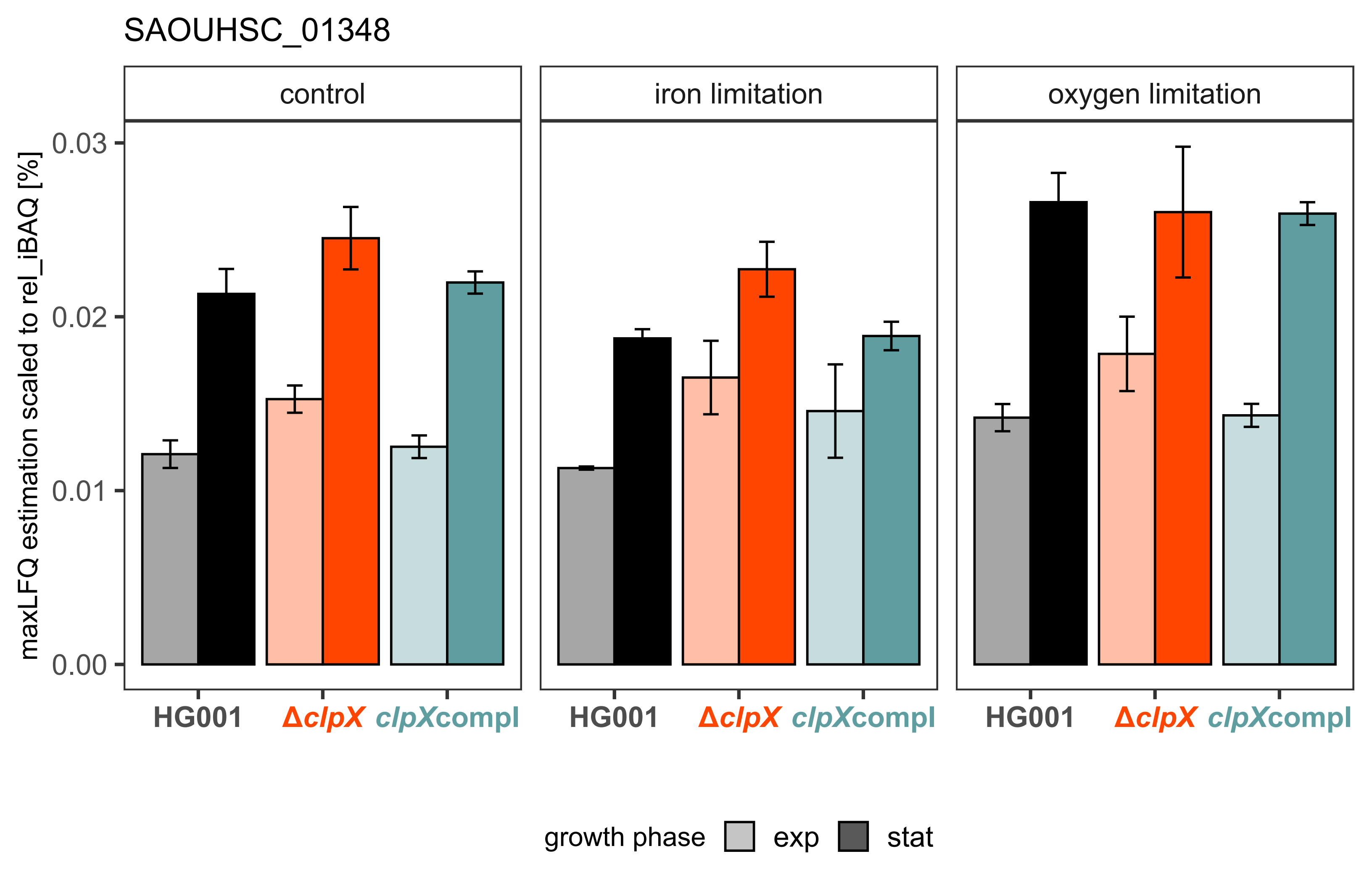
SAOUHSC_01348
control
iron limitation
oxygen limitation
maxLFQ estimation scaled to rel_iBAQ [%]
0.03
0.02
0.01
0.00
HG001
ΔclpX
clpXcompl
growth phase
exp
stat

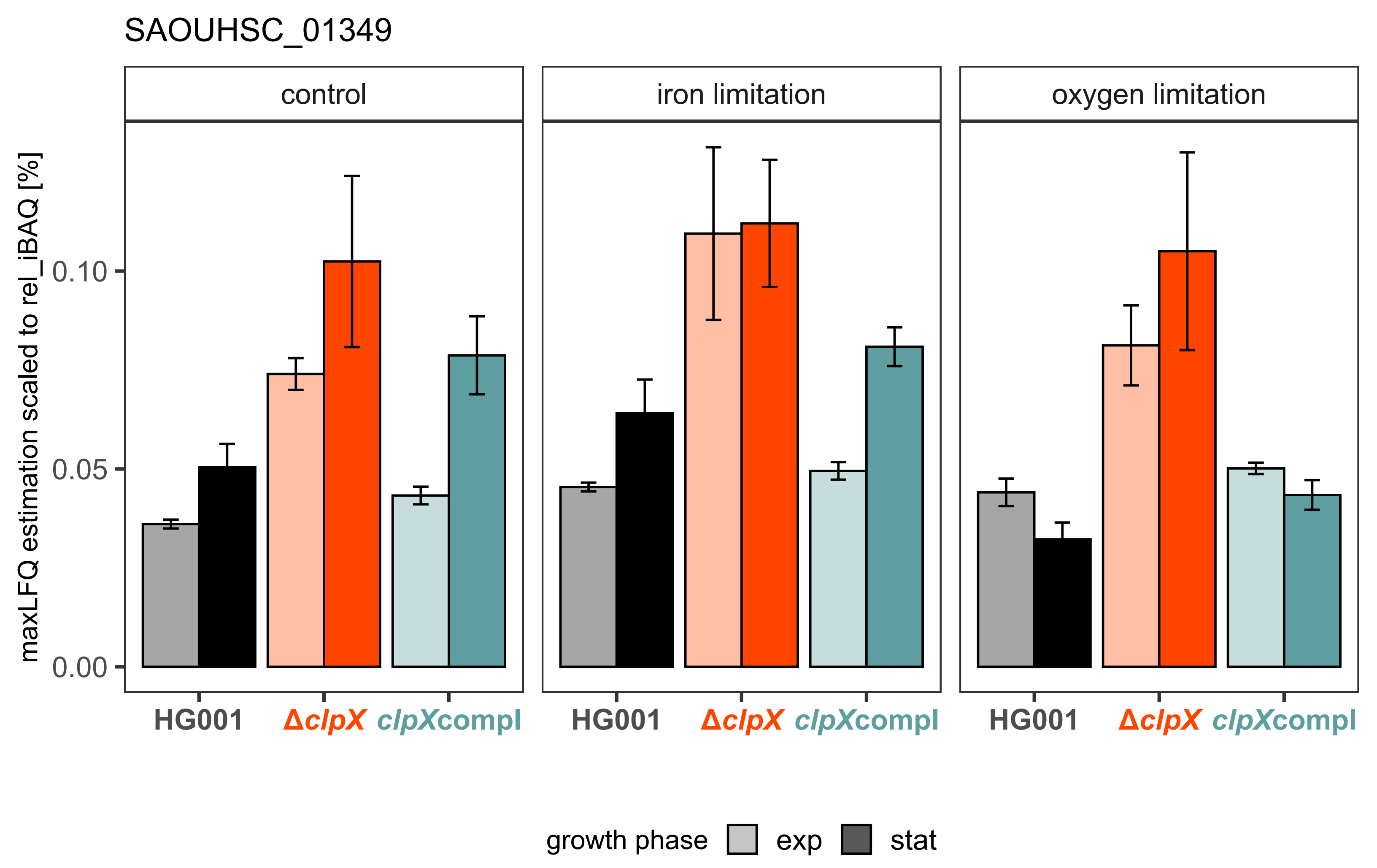
SAOUHSC_01349
control
iron limitation
oxygen limitation
maxLFQ estimation scaled to rel_iBAQ [%]
0.10
0.05
0.00
HG001
ΔclpX
clpXcompl
growth phase
exp
stat

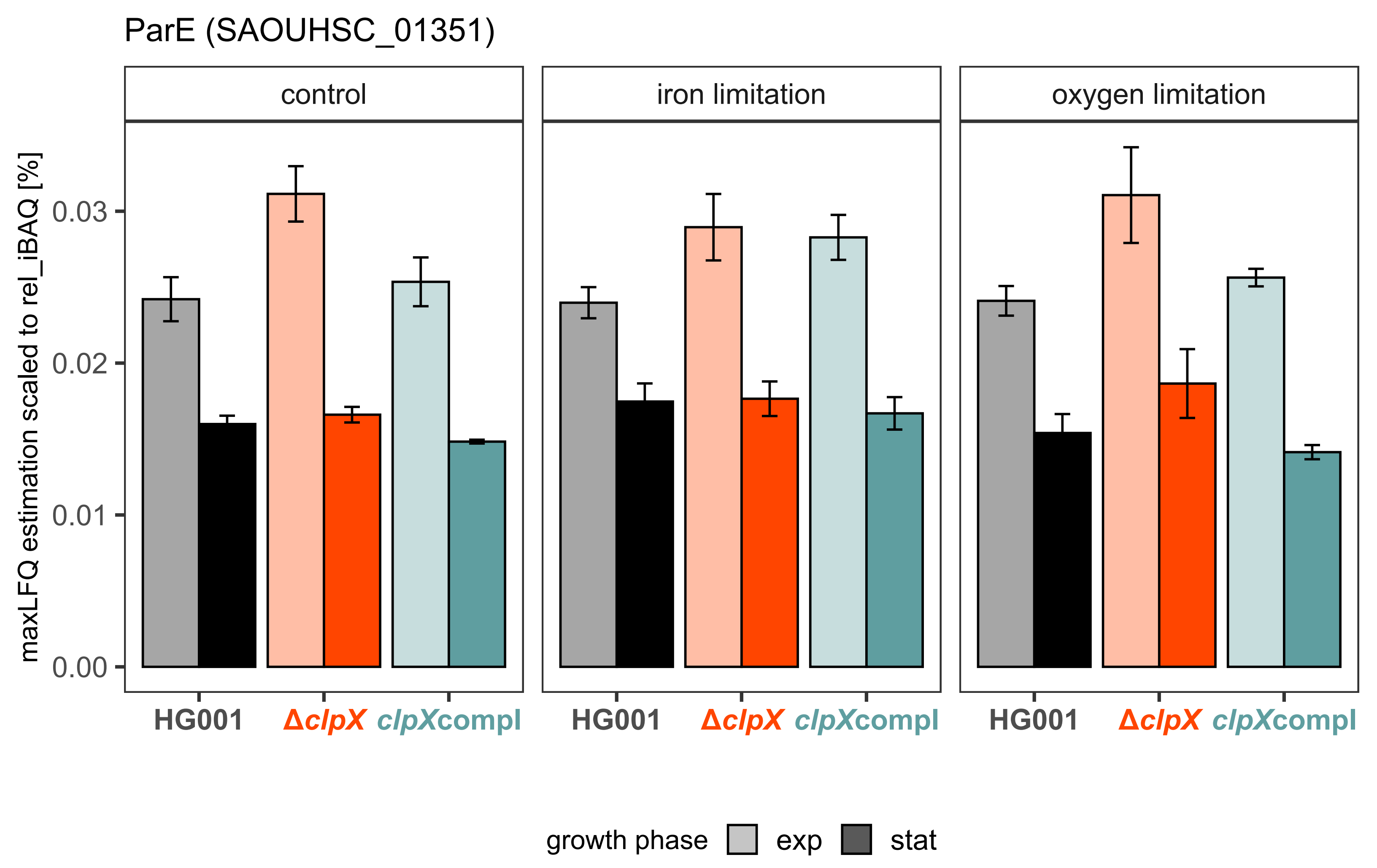

ParE (SAOUHSC_01351)
control
iron limitation
oxygen limitation
maxLFQ estimation scaled to rel_iBAQ [%]
0.03
0.02
0.01
0.00
HG001
ΔclpX
clpXcompl
growth phase
exp
stat

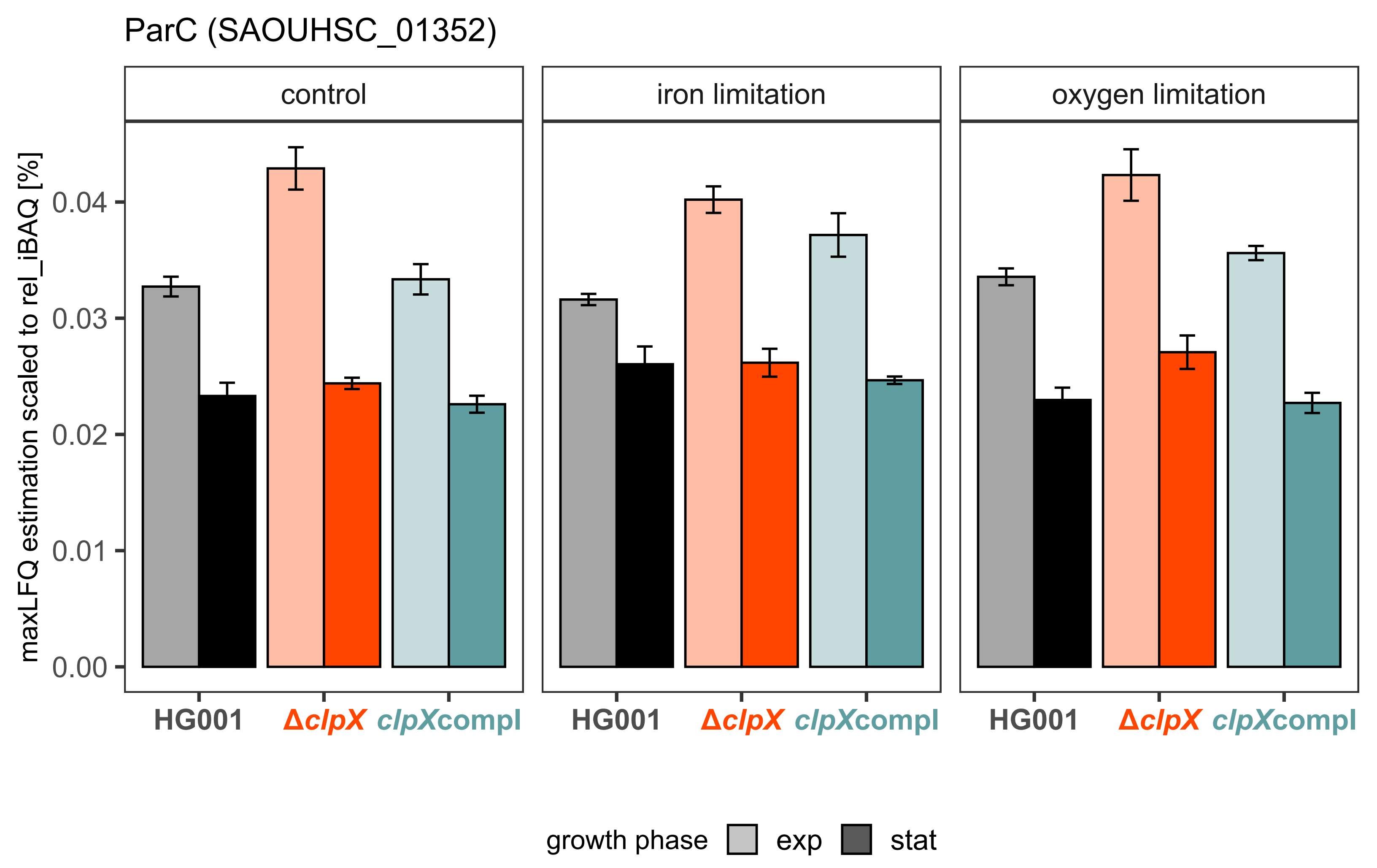
ParC (SAOUHSC_01352)
control
iron limitation
oxygen limitation
maxLFQ estimation scaled to rel_iBAQ [%]
0.00
0.01
0.02
0.03
0.04
HG001
ΔclpX
clpXcompl
growth phase
exp
stat

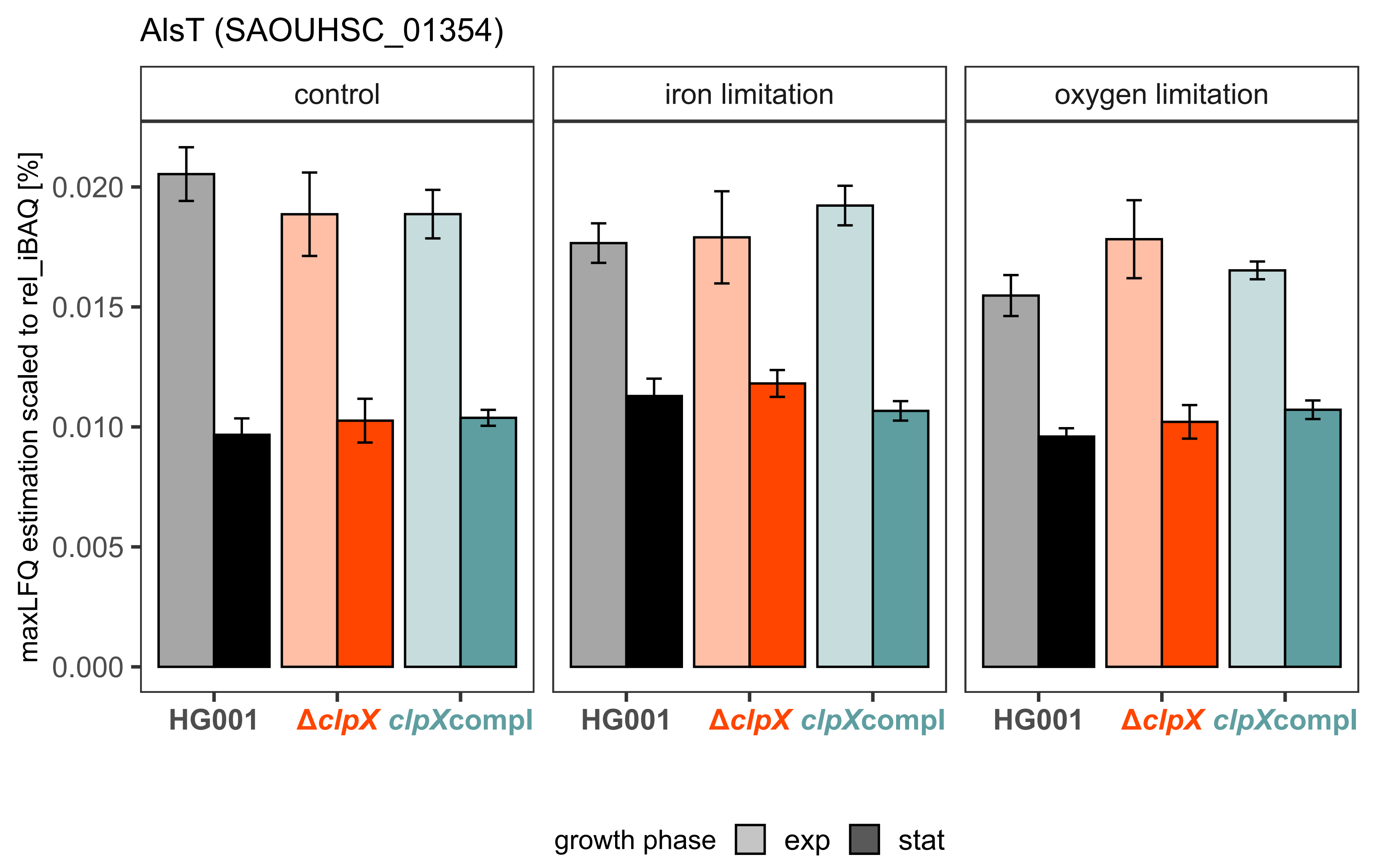
AlsT (SAOUHSC_01354)
control
iron limitation
oxygen limitation
maxLFQ estimation scaled to rel_iBAQ [%]
0.020
0.015
0.010
0.005
0.000
HG001
ΔclpX
clpXcompl
growth phase
exp
stat

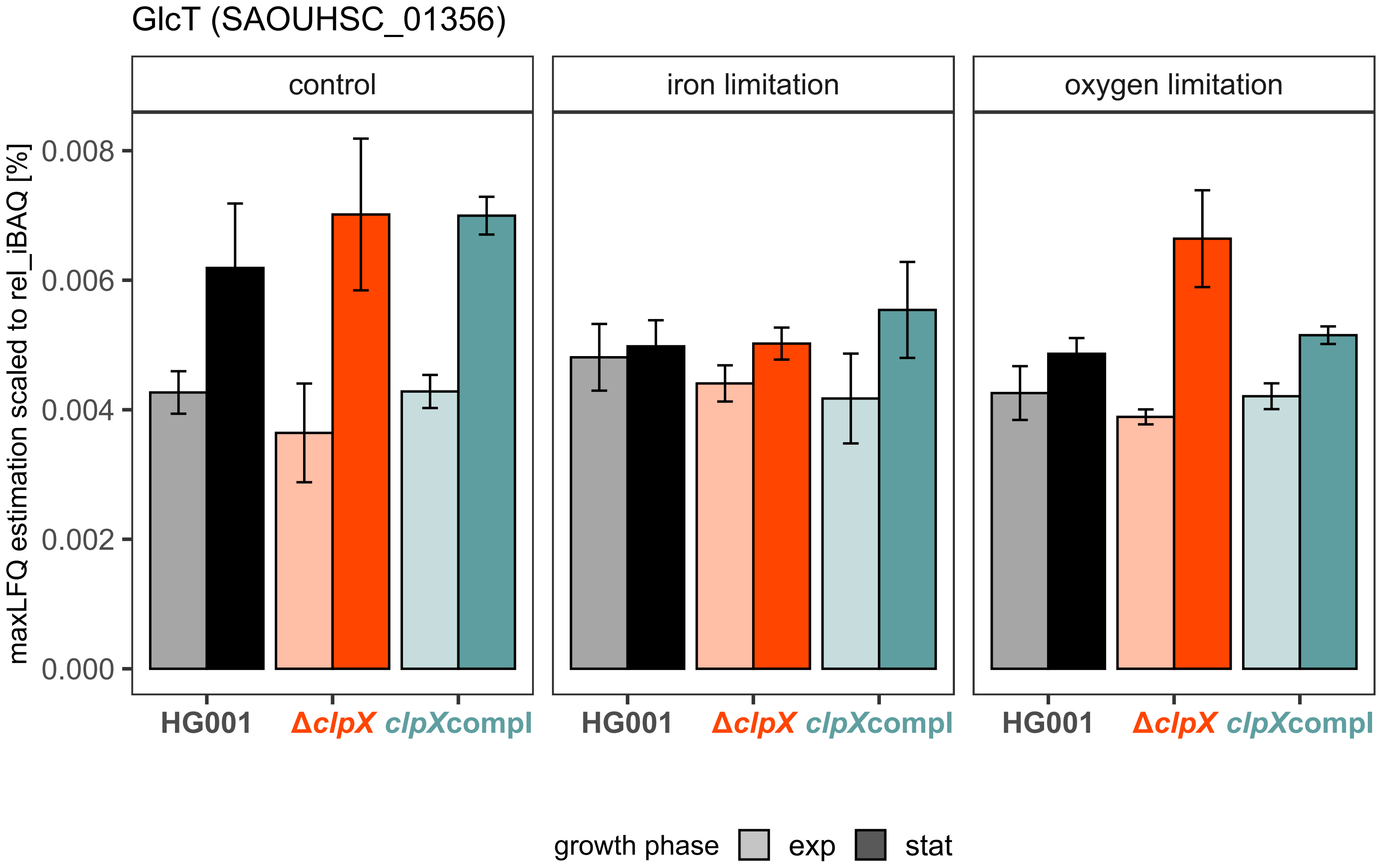
GlcT (SAOUHSC_01356)
control
iron limitation
oxygen limitation
maxLFQ estimation scaled to rel_iBAQ [%]
0.008
0.006
0.004
0.002
0.000
HG001
ΔclpX
clpXcompl
growth phase
exp
stat

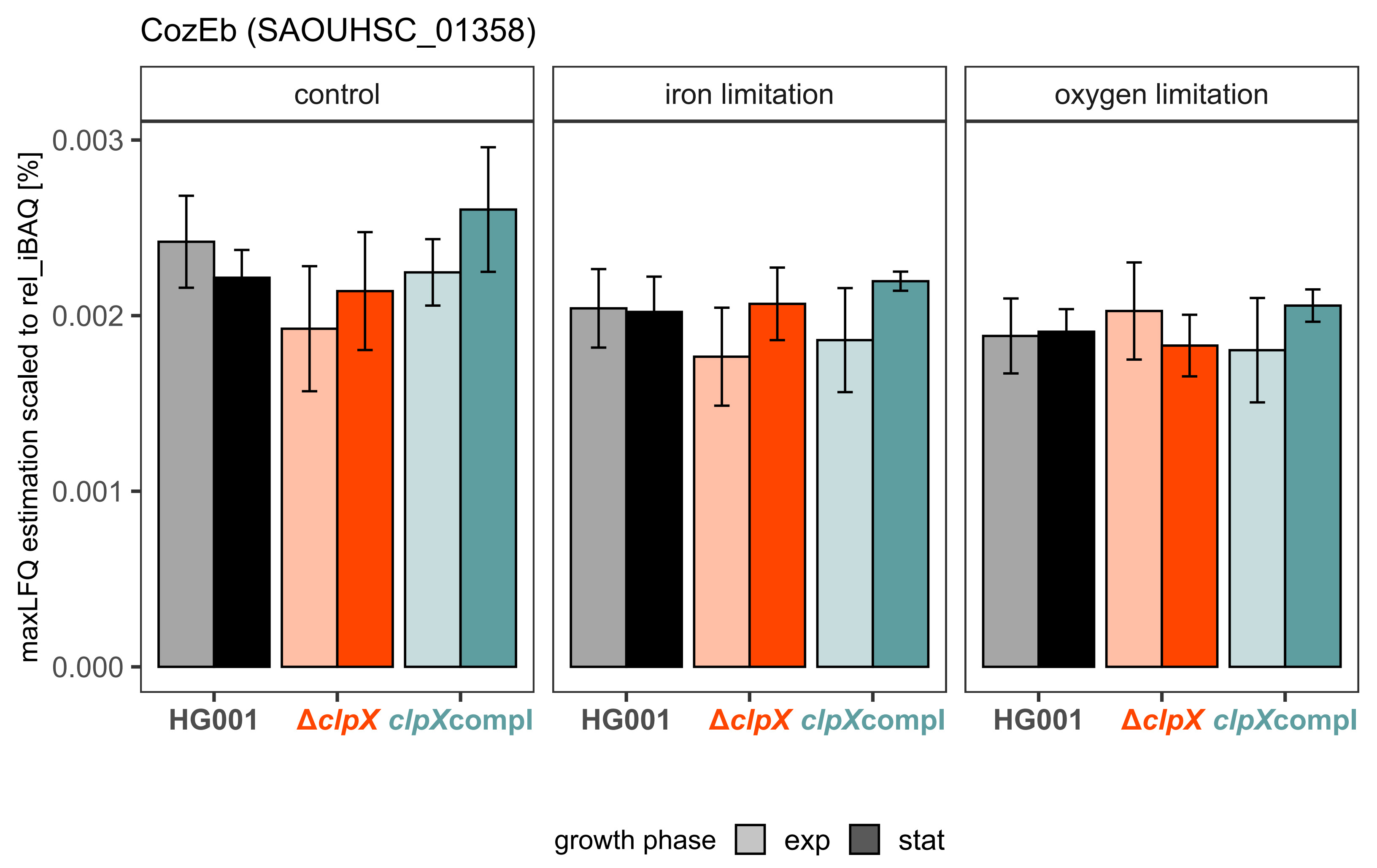

CozEb (SAOUHSC_01358)
control
iron limitation
oxygen limitation
maxLFQ estimation scaled to rel_iBAQ [%]
0.003
0.002
0.001
0.000
HG001
ΔclpX
clpXcompl
growth phase
exp
stat

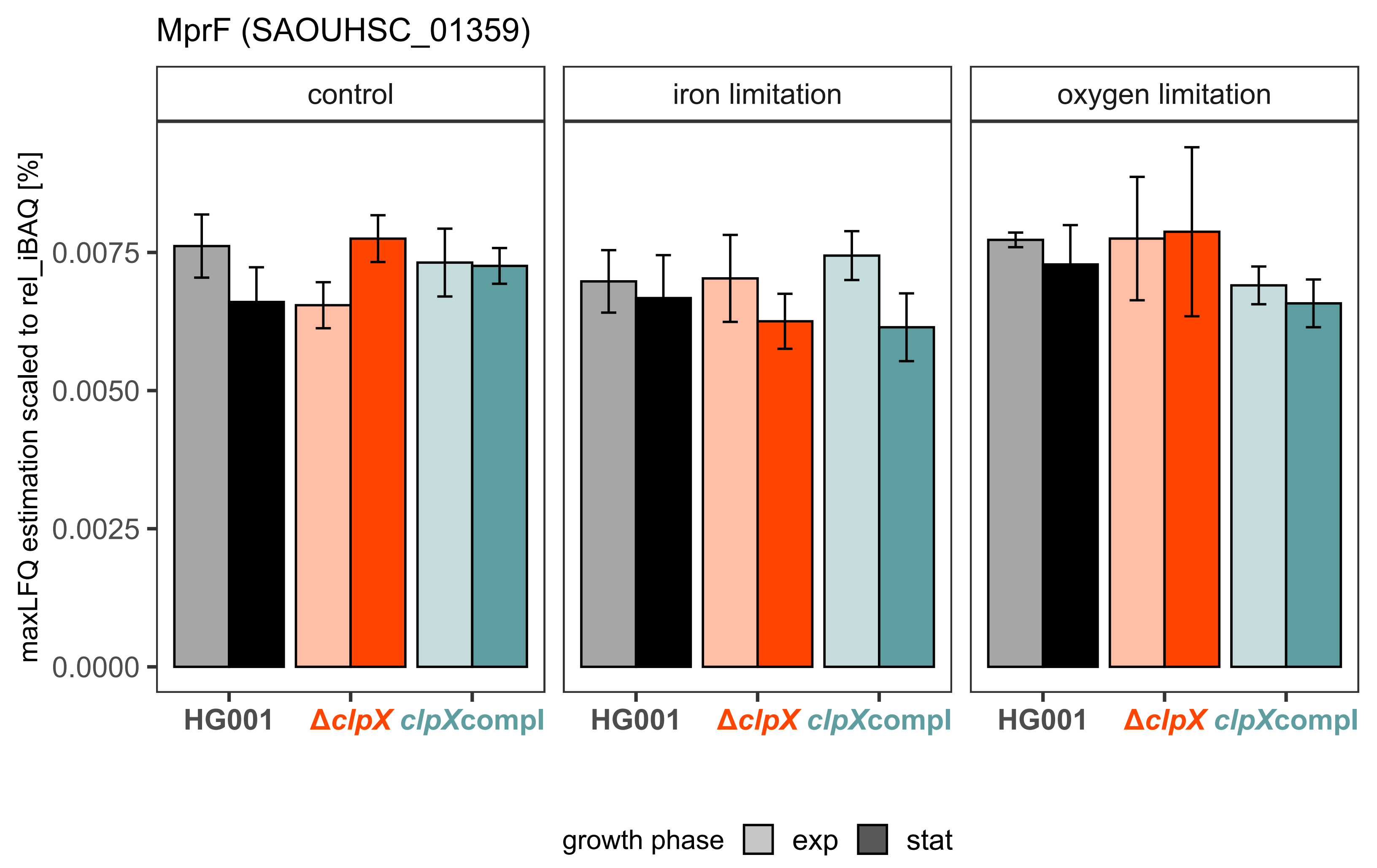

MprF (SAOUHSC_01359)
control
iron limitation
oxygen limitation
maxLFQ estimation scaled to rel_iBAQ [%]
0.0075
0.0050
0.0025
0.0000
HG001
ΔclpX
clpXcompl
growth phase
exp
stat

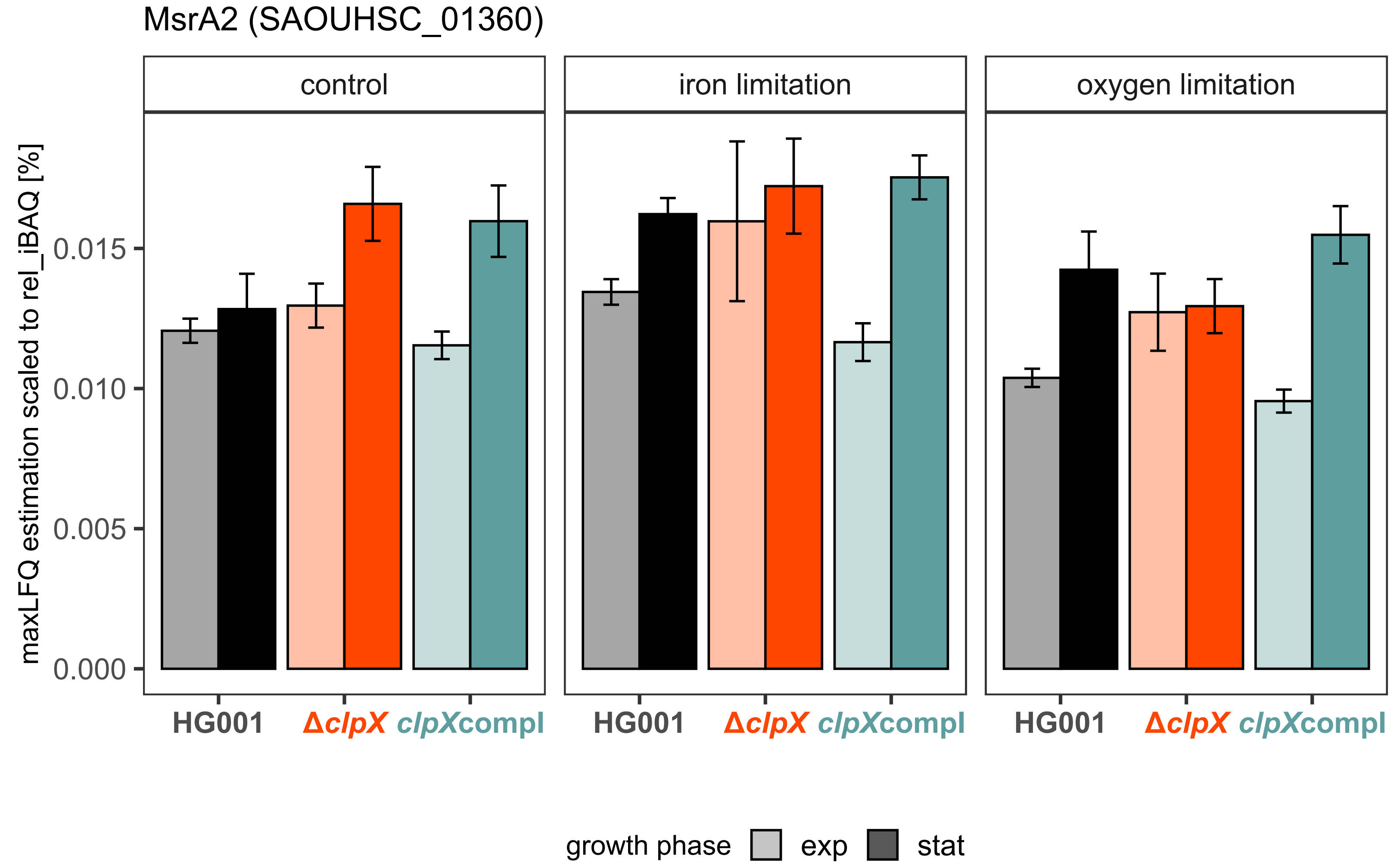

MsrA2 (SAOUHSC_01360)
control
iron limitation
oxygen limitation
maxLFQ estimation scaled to rel_iBAQ [%]
0.015
0.010
0.005
0.000
HG001
ΔclpX
clpXcompl
growth phase
exp
stat

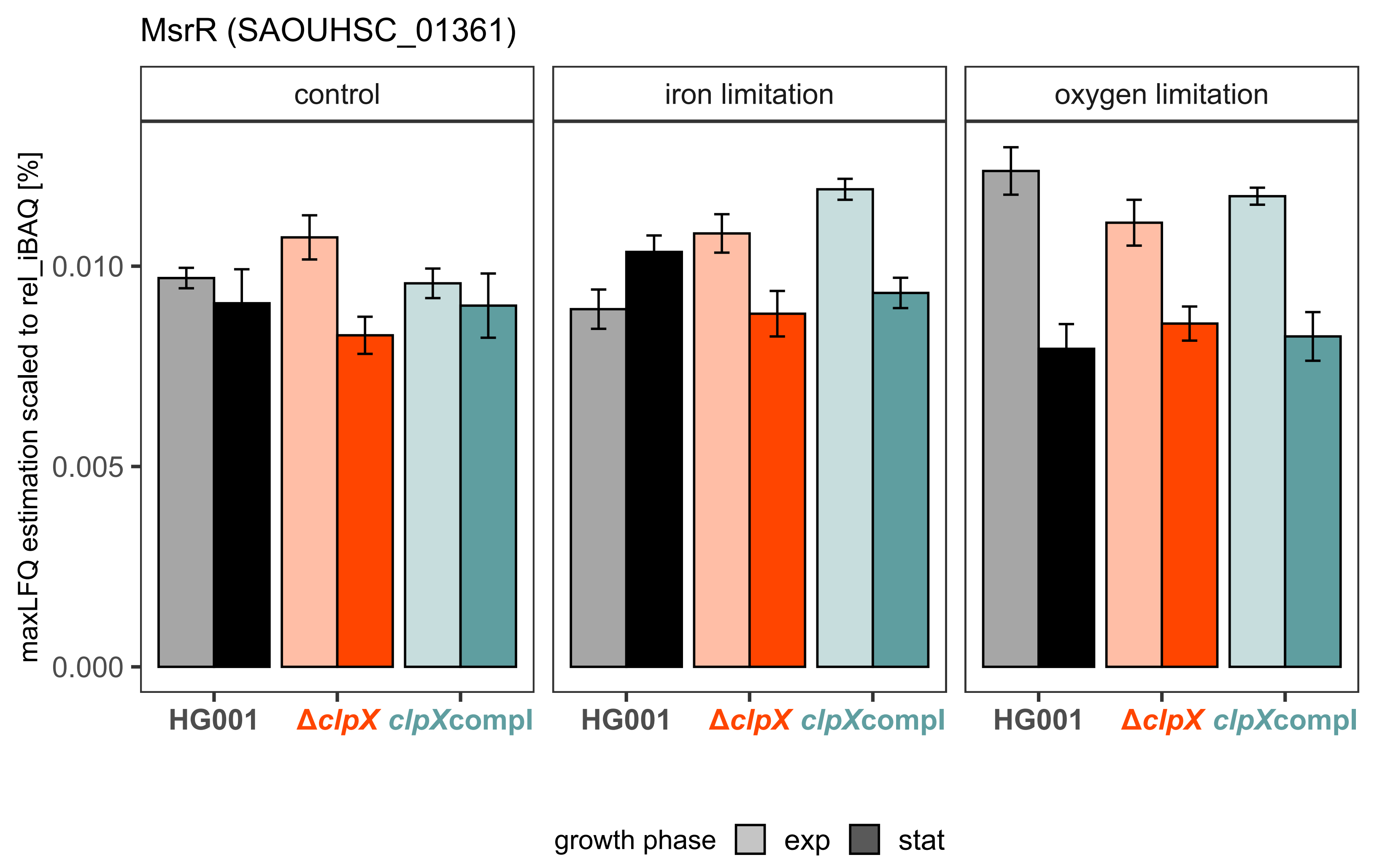

MsrR (SAOUHSC_01361)
control
iron limitation
oxygen limitation
maxLFQ estimation scaled to rel_iBAQ [%]
0.000
0.005
0.010
HG001
ΔclpX
clpXcompl
growth phase
exp
stat

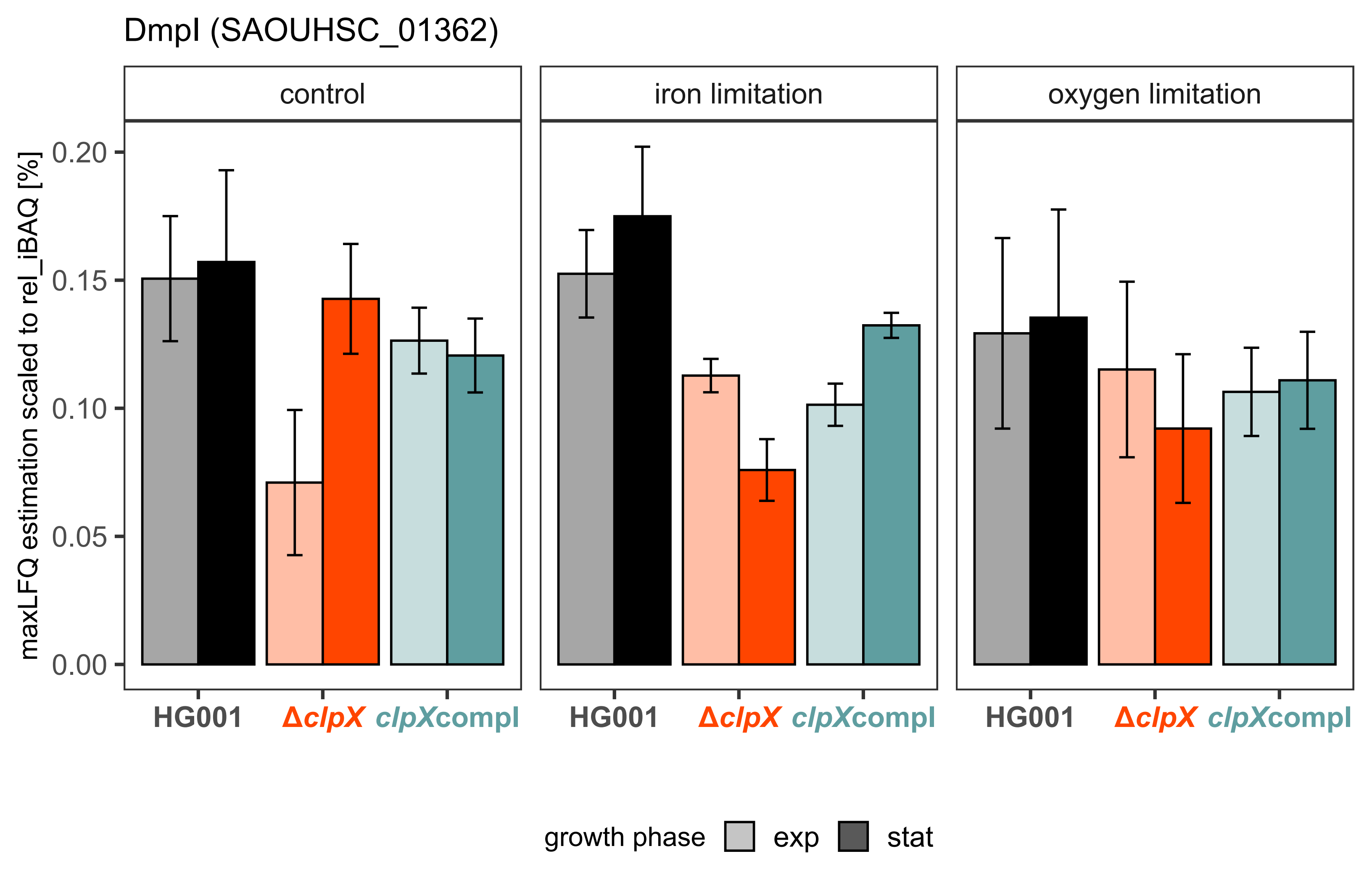

DmpI (SAOUHSC_01362)
control
iron limitation
oxygen limitation
maxLFQ estimation scaled to rel_iBAQ [%]
0.00
0.05
0.10
0.15
0.20
HG001
ΔclpX
clpXcompl
growth phase
exp
stat

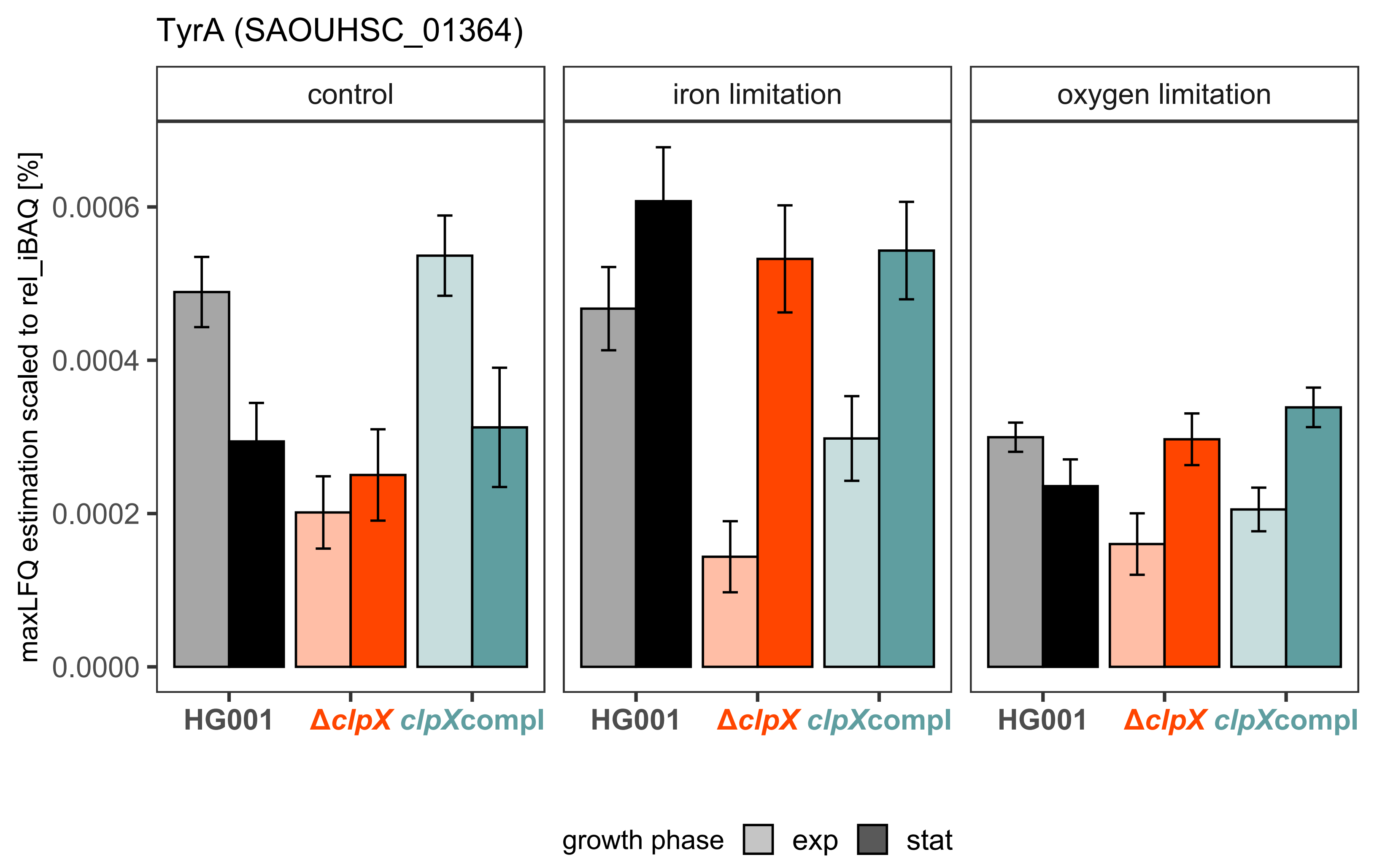
TyrA (SAOUHSC_01364)
control
iron limitation
oxygen limitation
maxLFQ estimation scaled to rel_iBAQ [%]
0.0006
0.0004
0.0002
0.0000
HG001
ΔclpX
clpXcompl
growth phase
exp
stat

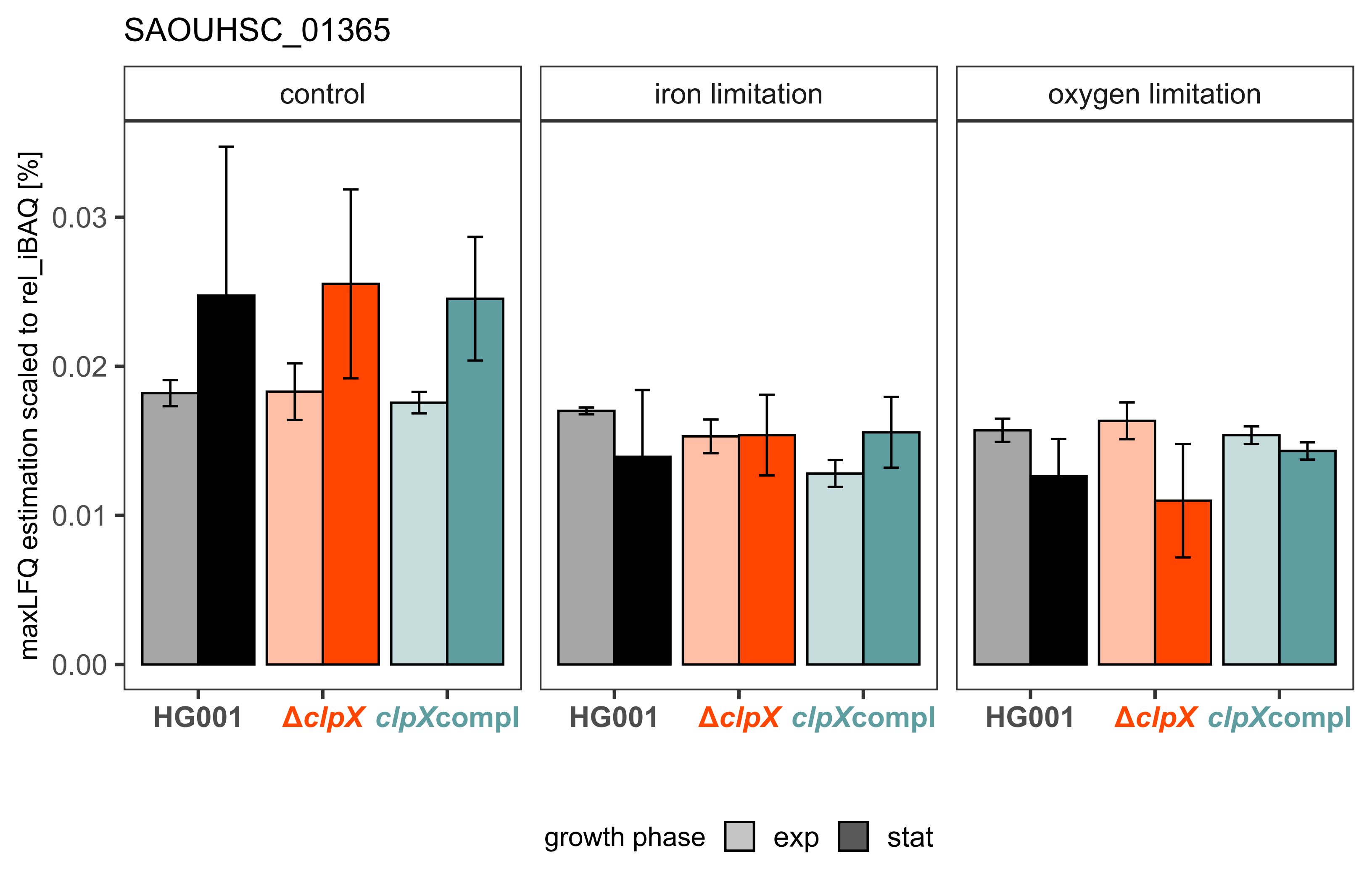
SAOUHSC_01365
control
iron limitation
oxygen limitation
maxLFQ estimation scaled to rel_iBAQ [%]
0.03
0.02
0.01
0.00
HG001
ΔclpX
clpXcompl
growth phase
exp
stat

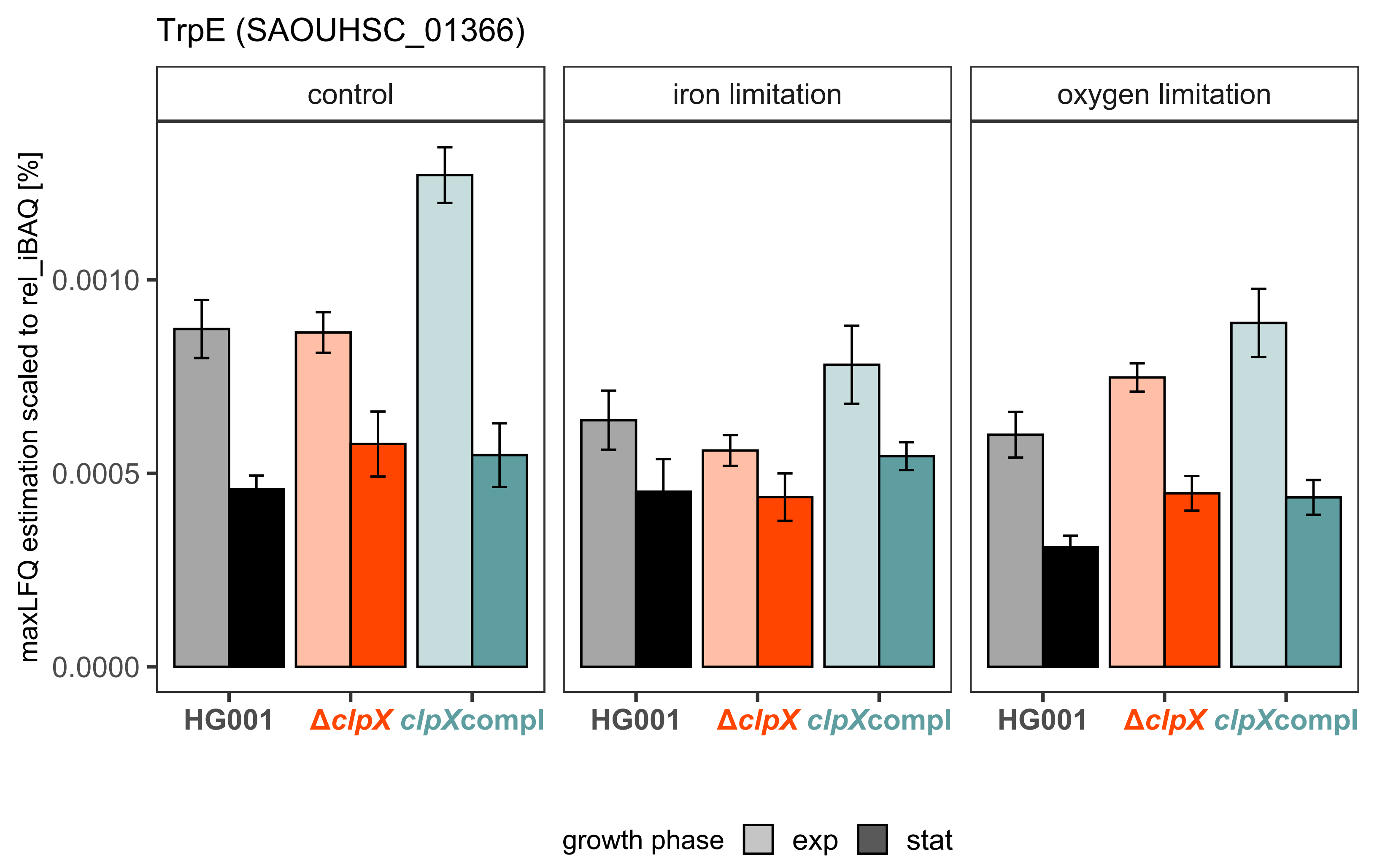

TrpE (SAOUHSC_01366)
control
iron limitation
oxygen limitation
maxLFQ estimation scaled to rel_iBAQ [%]
0.0010
0.0005
0.0000
HG001
ΔclpX
clpXcompl
growth phase
exp
stat

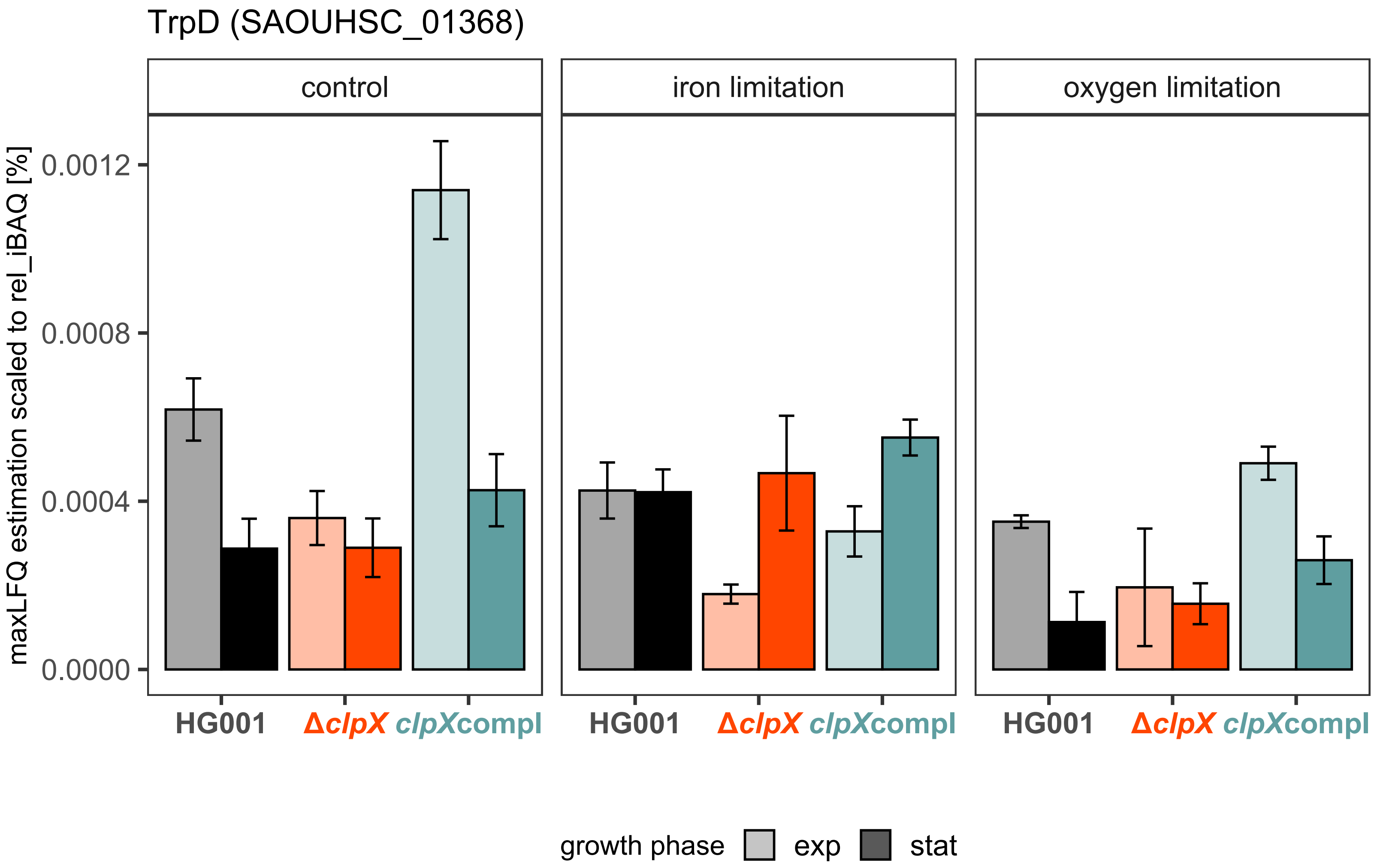

TrpD (SAOUHSC_01368)
control
iron limitation
oxygen limitation
maxLFQ estimation scaled to rel_iBAQ [%]
0.0012
0.0008
0.0004
0.0000
HG001
ΔclpX
clpXcompl
growth phase
exp
stat

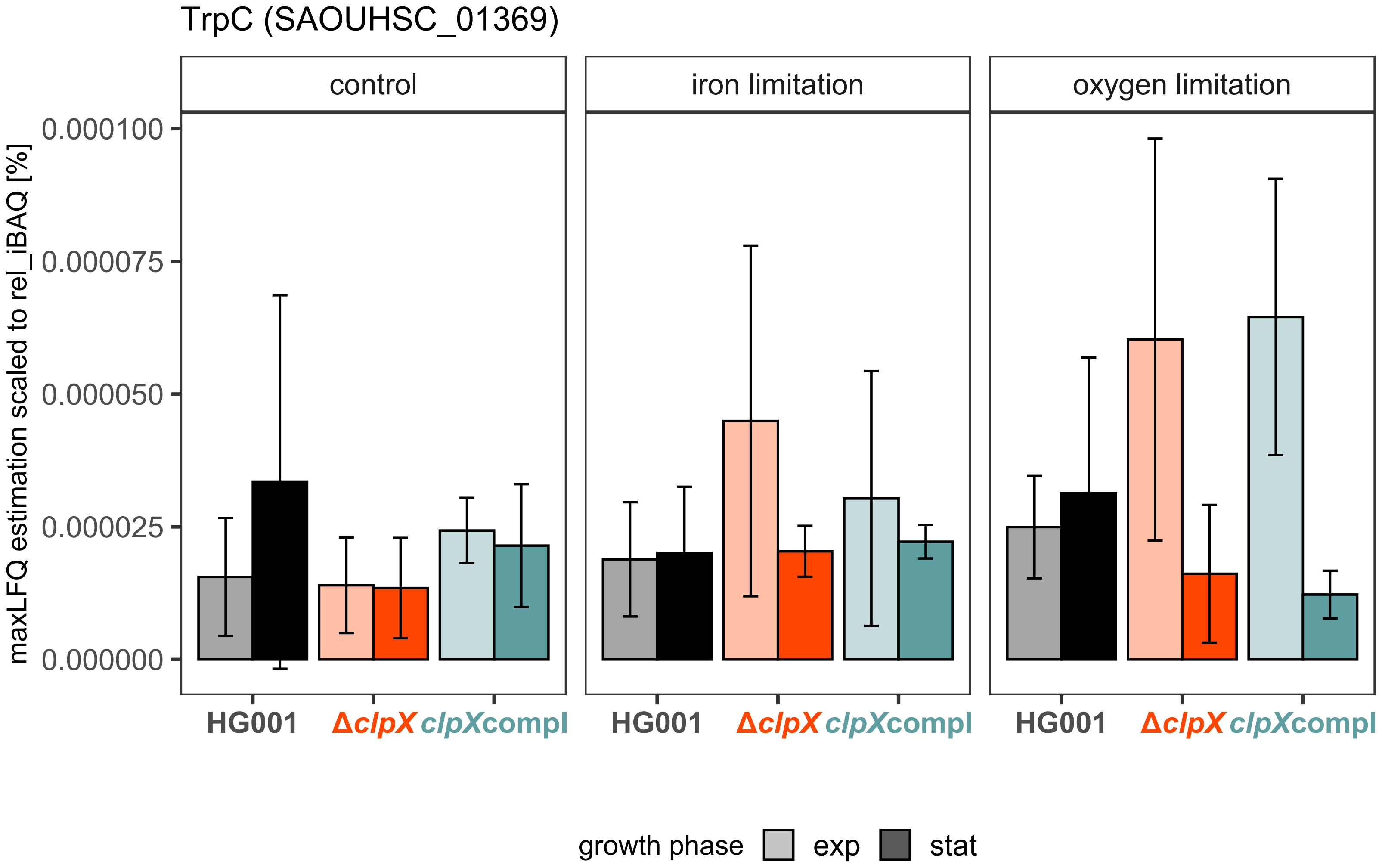

TrpC (SAOUHSC_01369)
control
iron limitation
oxygen limitation
0.000100
0.000075
0.000050
0.000025
0.000000
maxLFQ estimation scaled to rel_iBAQ [%]
HG001
ΔclpX
clpXcompl
HG001
ΔclpX
clpXcompl
HG001
ΔclpX
clpXcompl
growth phase
exp
stat

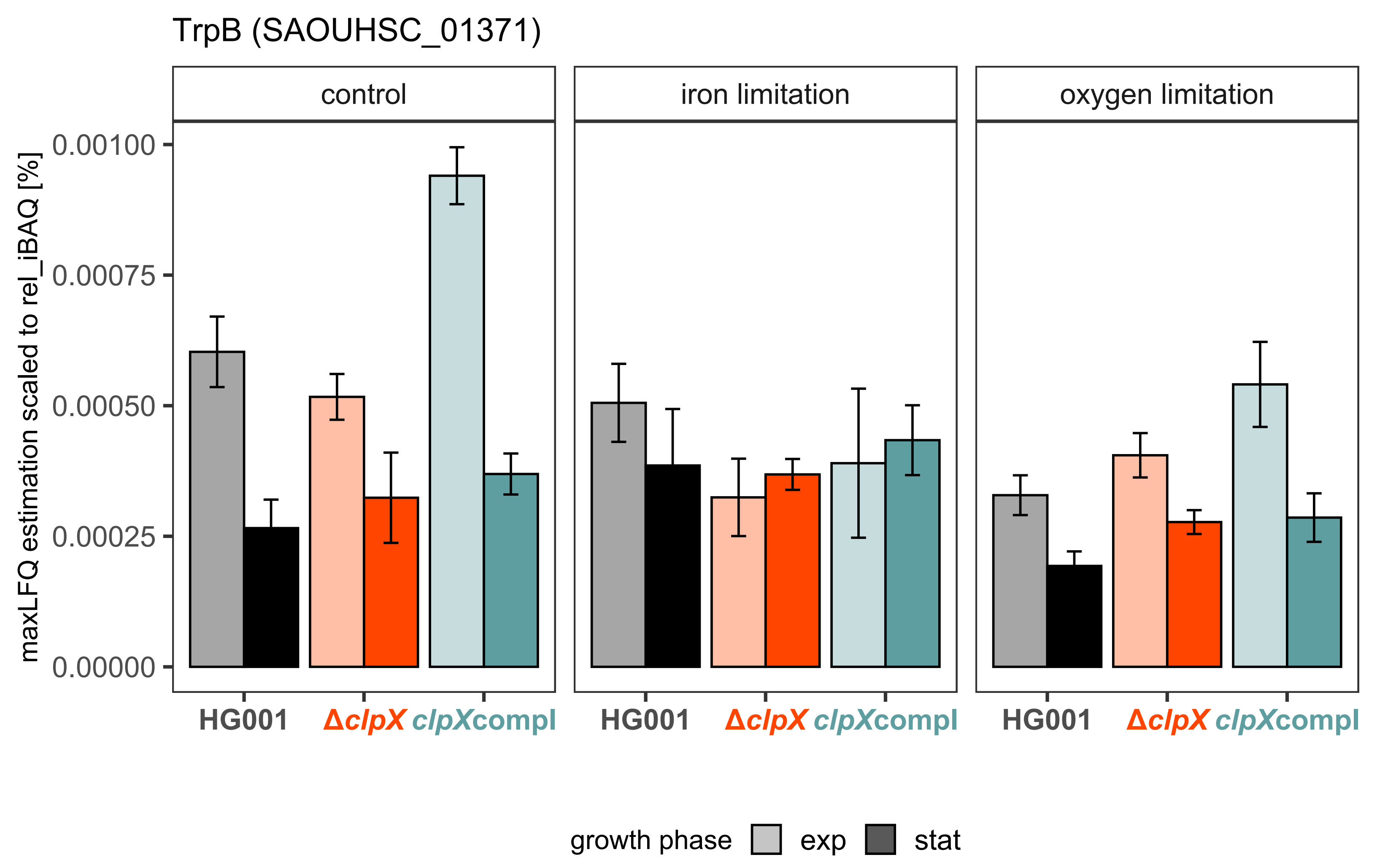
TrpB (SAOUHSC_01371)
control
iron limitation
oxygen limitation
maxLFQ estimation scaled to rel_iBAQ [%]
0.00100
0.00075
0.00050
0.00025
0.00000
HG001
ΔclpX
clpXcompl
growth phase
exp
stat

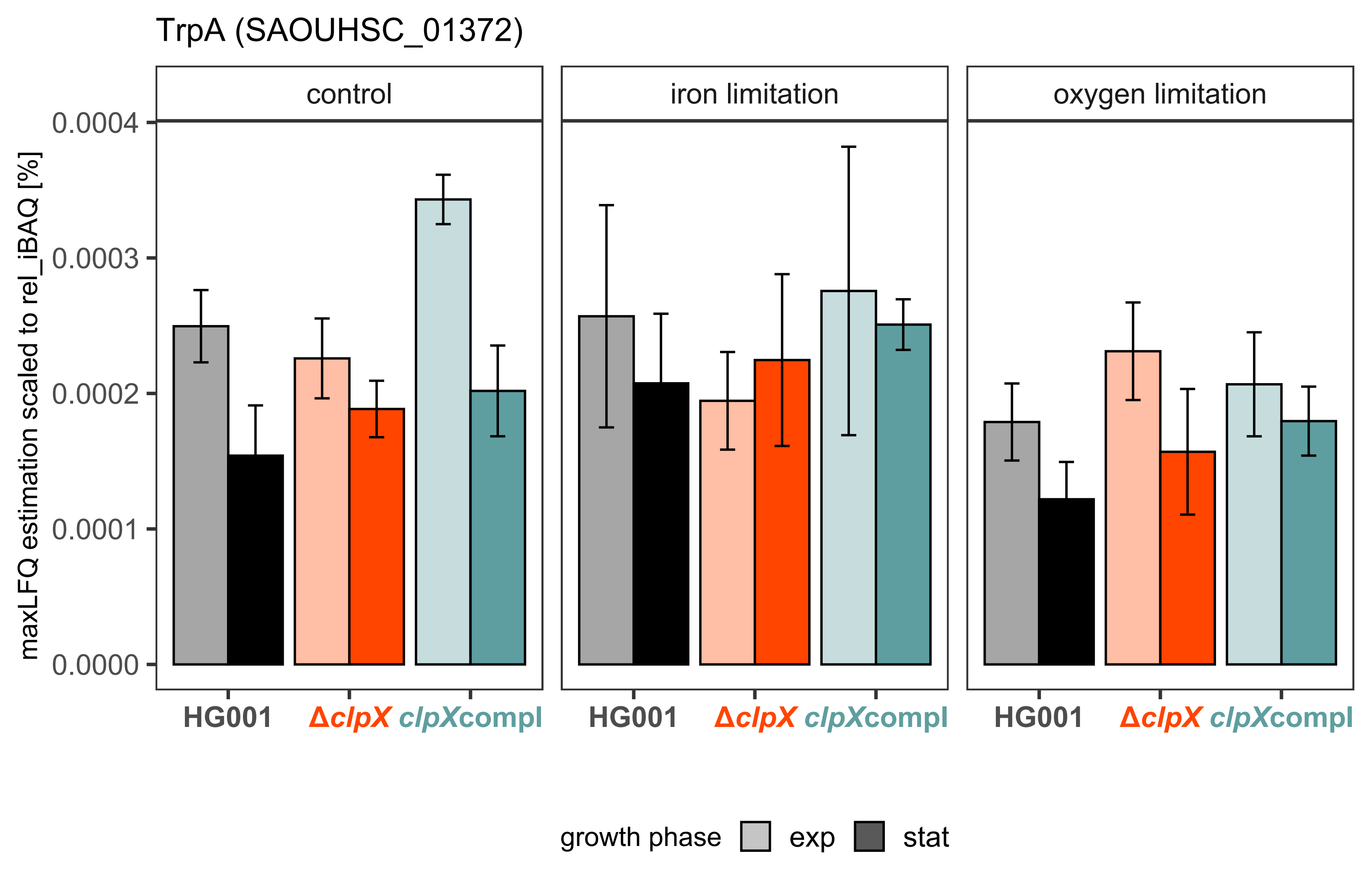
TrpA (SAOUHSC_01372)
control
iron limitation
oxygen limitation
maxLFQ estimation scaled to rel_iBAQ [%]
0.0004
0.0003
0.0002
0.0001
0.0000
HG001
ΔclpX
clpXcompl
growth phase
exp
stat

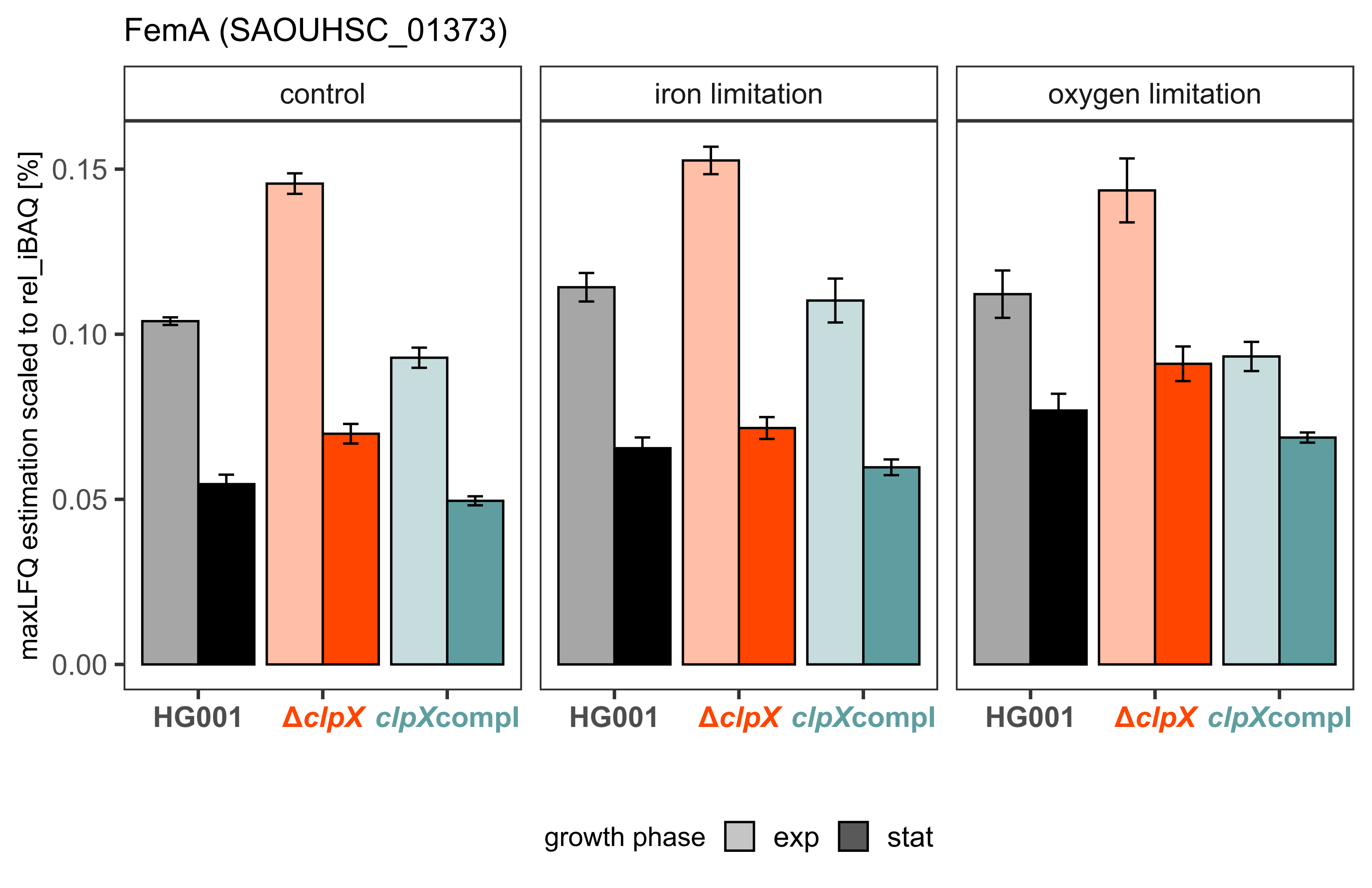
FemA (SAOUHSC_01373)
control
iron limitation
oxygen limitation
maxLFQ estimation scaled to rel_iBAQ [%]
0.15
0.10
0.05
0.00
HG001
ΔclpX
clpXcompl
growth phase
exp
stat

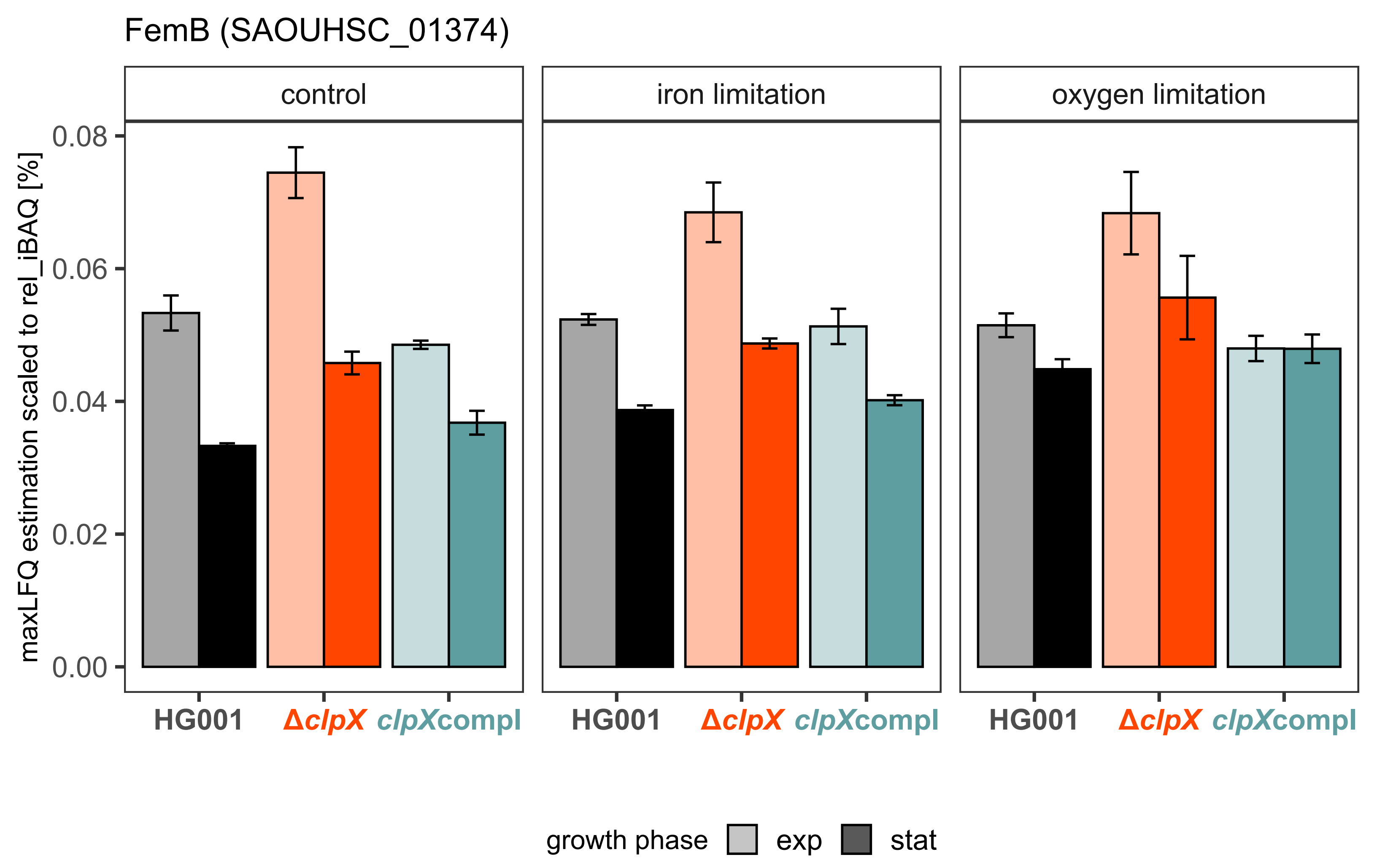

FemB (SAOUHSC_01374)
control
iron limitation
oxygen limitation
maxLFQ estimation scaled to rel_iBAQ [%]
0.08
0.06
0.04
0.02
0.00
HG001
ΔclpX
clpXcompl
growth phase
exp
stat

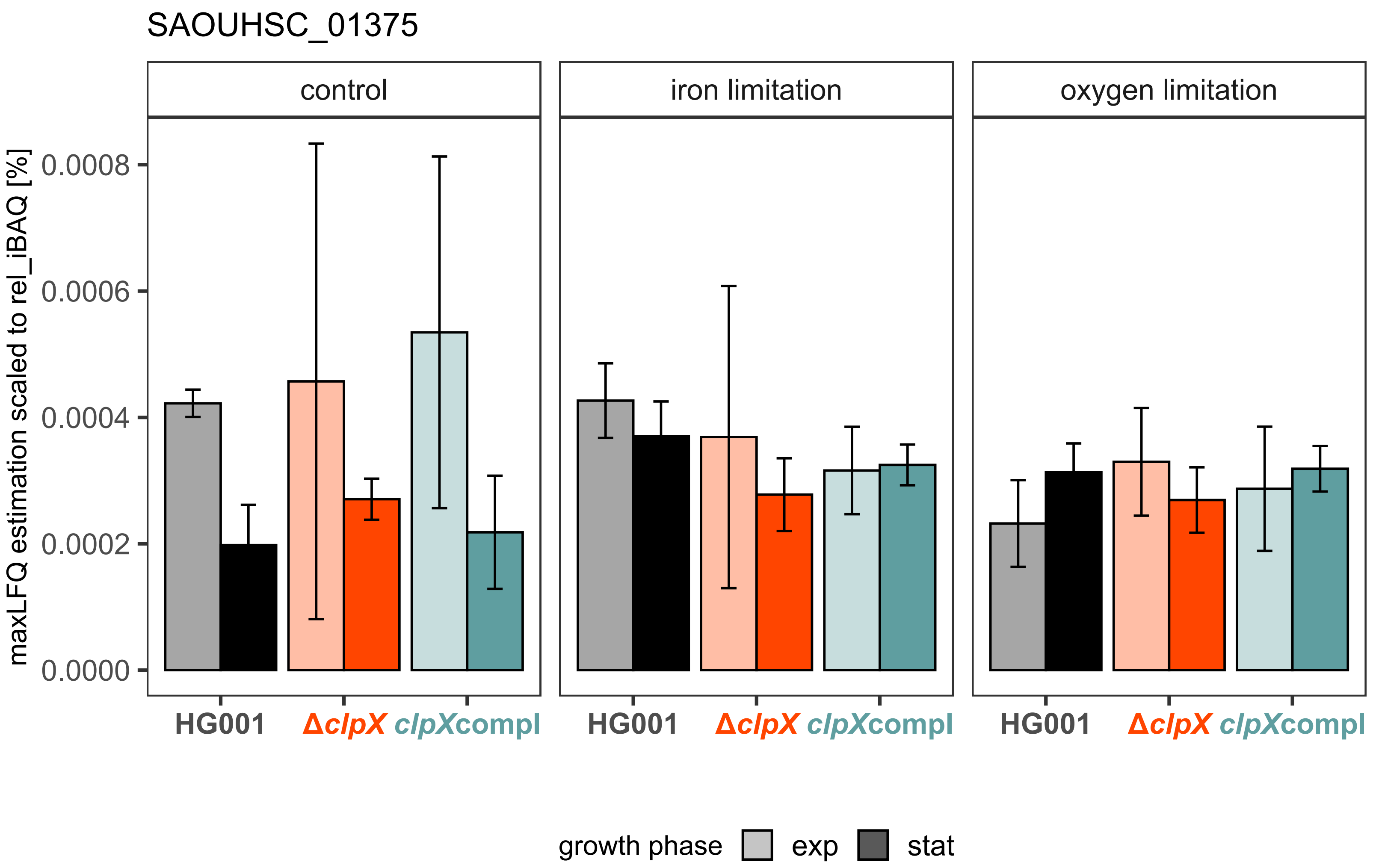
SAOUHSC_01375
control
iron limitation
oxygen limitation
maxLFQ estimation scaled to rel_iBAQ [%]
0.0008
0.0006
0.0004
0.0002
0.0000
HG001
ΔclpX
clpXcompl
growth phase
exp
stat

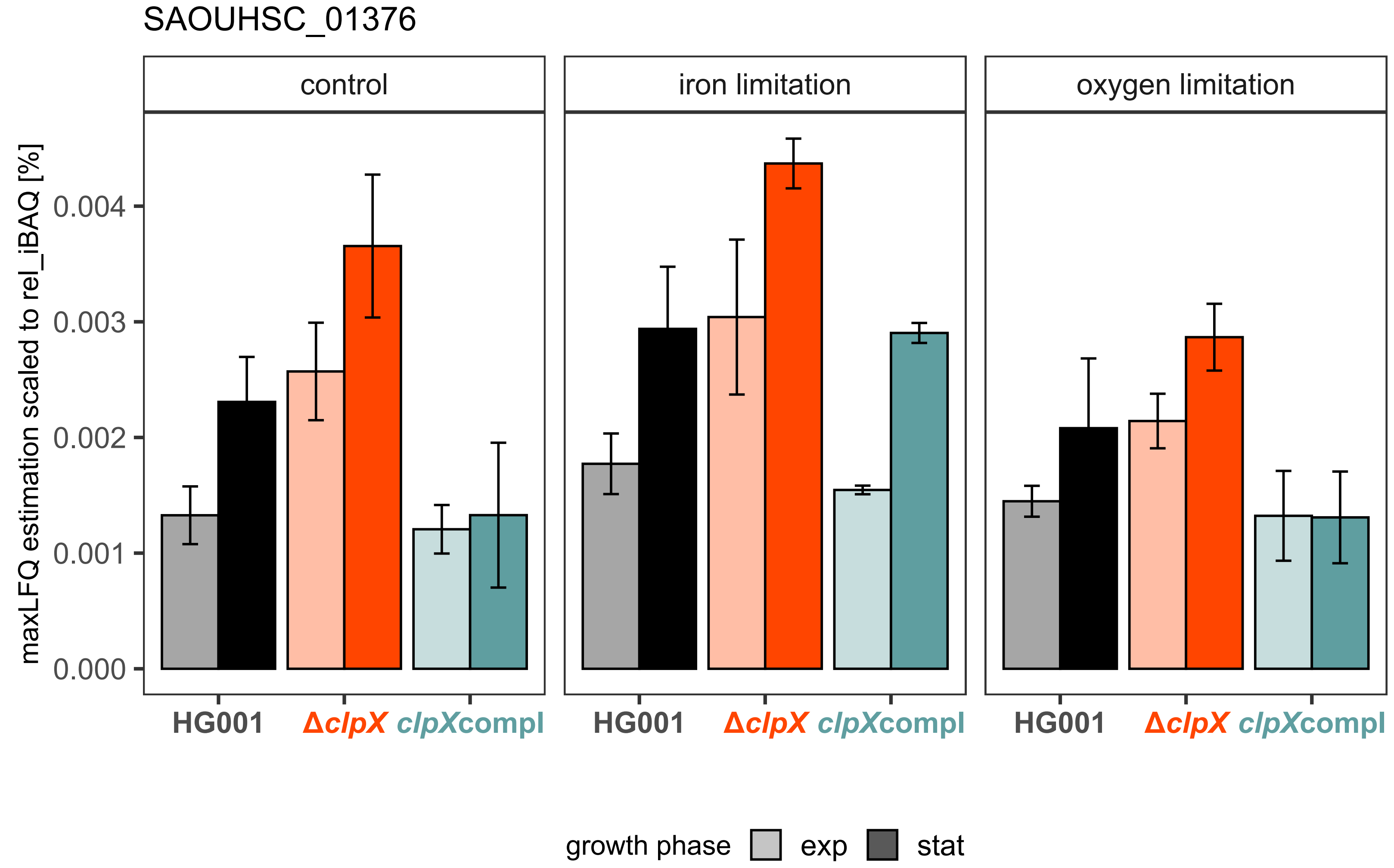
SAOUHSC_01376
control
iron limitation
oxygen limitation
maxLFQ estimation scaled to rel_iBAQ [%]
0.000
0.001
0.002
0.003
0.004
HG001
ΔclpX
clpXcompl
growth phase
exp
stat

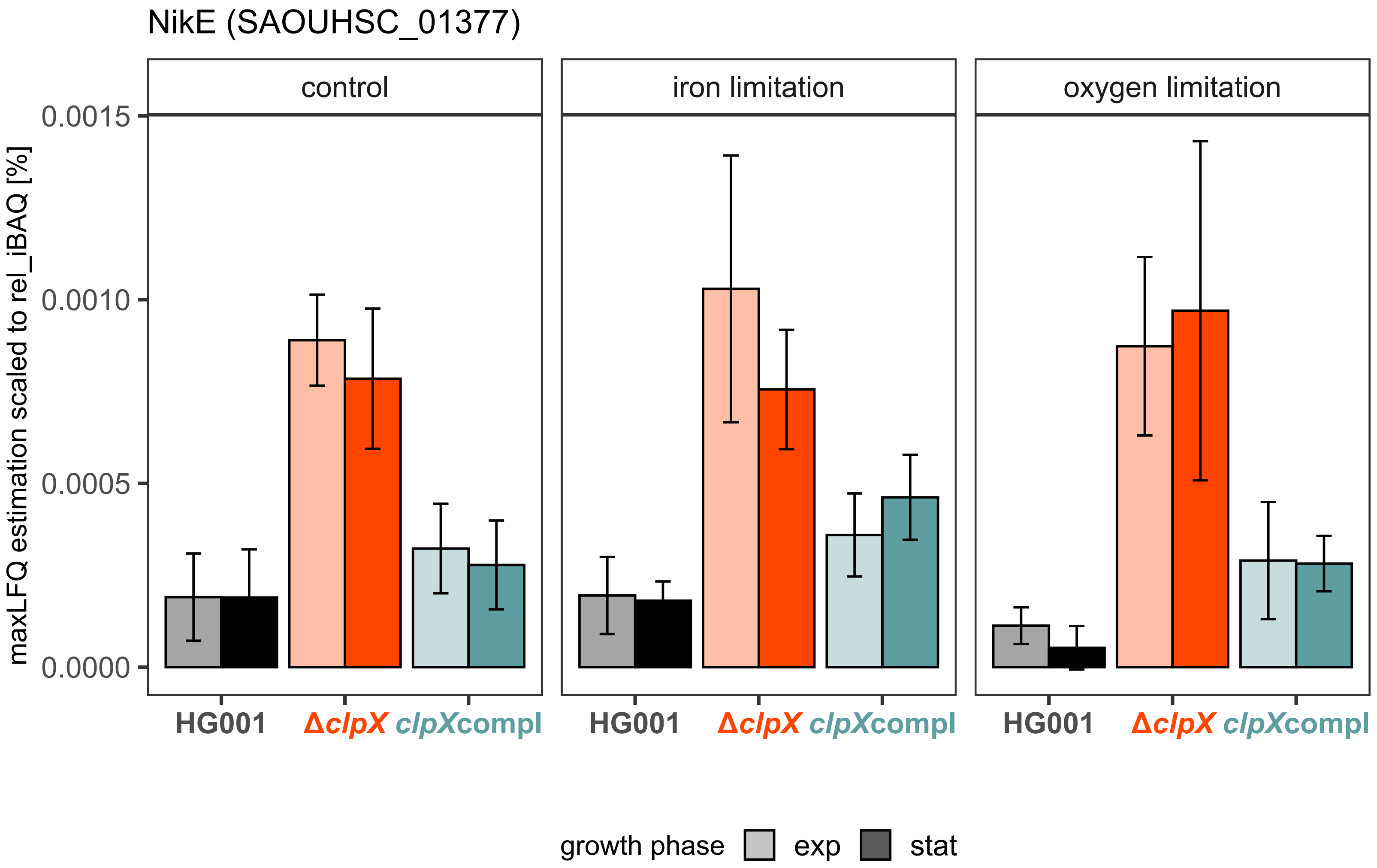

NikE (SAOUHSC_01377)
control
iron limitation
oxygen limitation
maxLFQ estimation scaled to rel_iBAQ [%]
0.0015
0.0010
0.0005
0.0000
HG001
ΔclpX
clpXcompl
growth phase
exp
stat

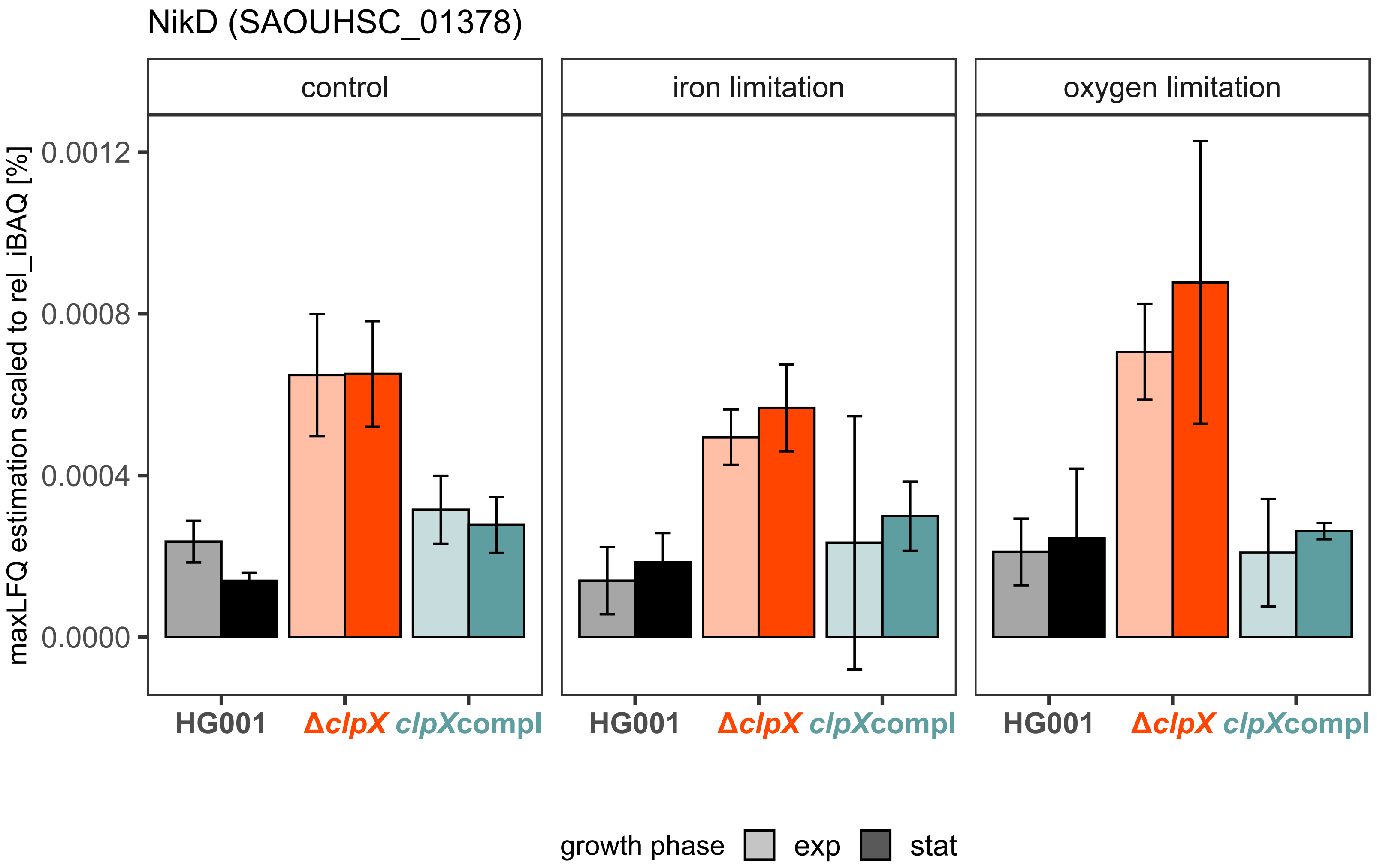

NikD (SAOUHSC_01378)
control
iron limitation
oxygen limitation
maxLFQ estimation scaled to rel_iBAQ [%]
0.0012
0.0008
0.0004
0.0000
HG001
ΔclpX
clpXcompl
growth phase
exp
stat

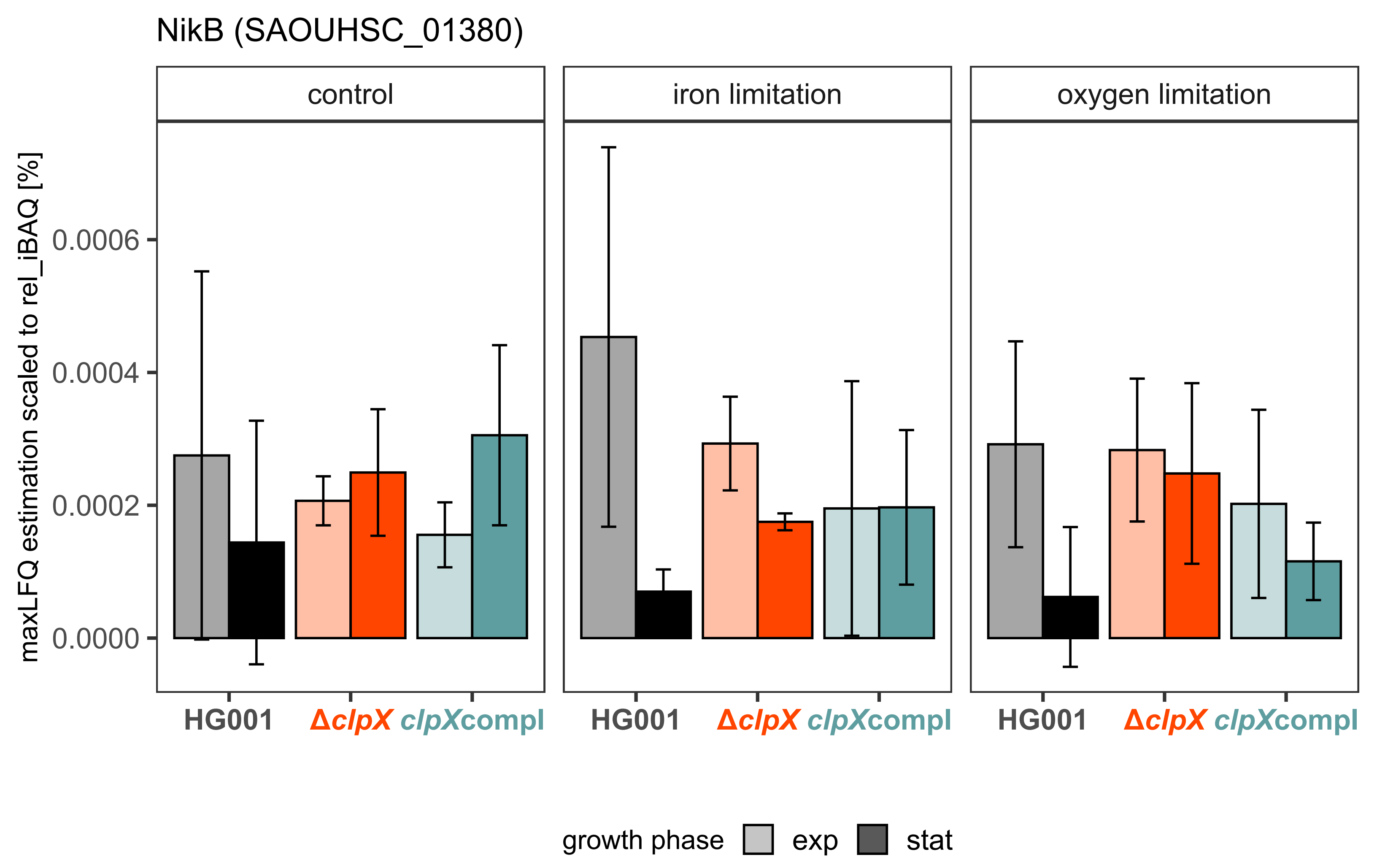
NikB (SAOUHSC_01380)
control
iron limitation
oxygen limitation
maxLFQ estimation scaled to rel_iBAQ [%]
0.0006
0.0004
0.0002
0.0000
HG001
ΔclpX
clpXcompl
growth phase
exp
stat

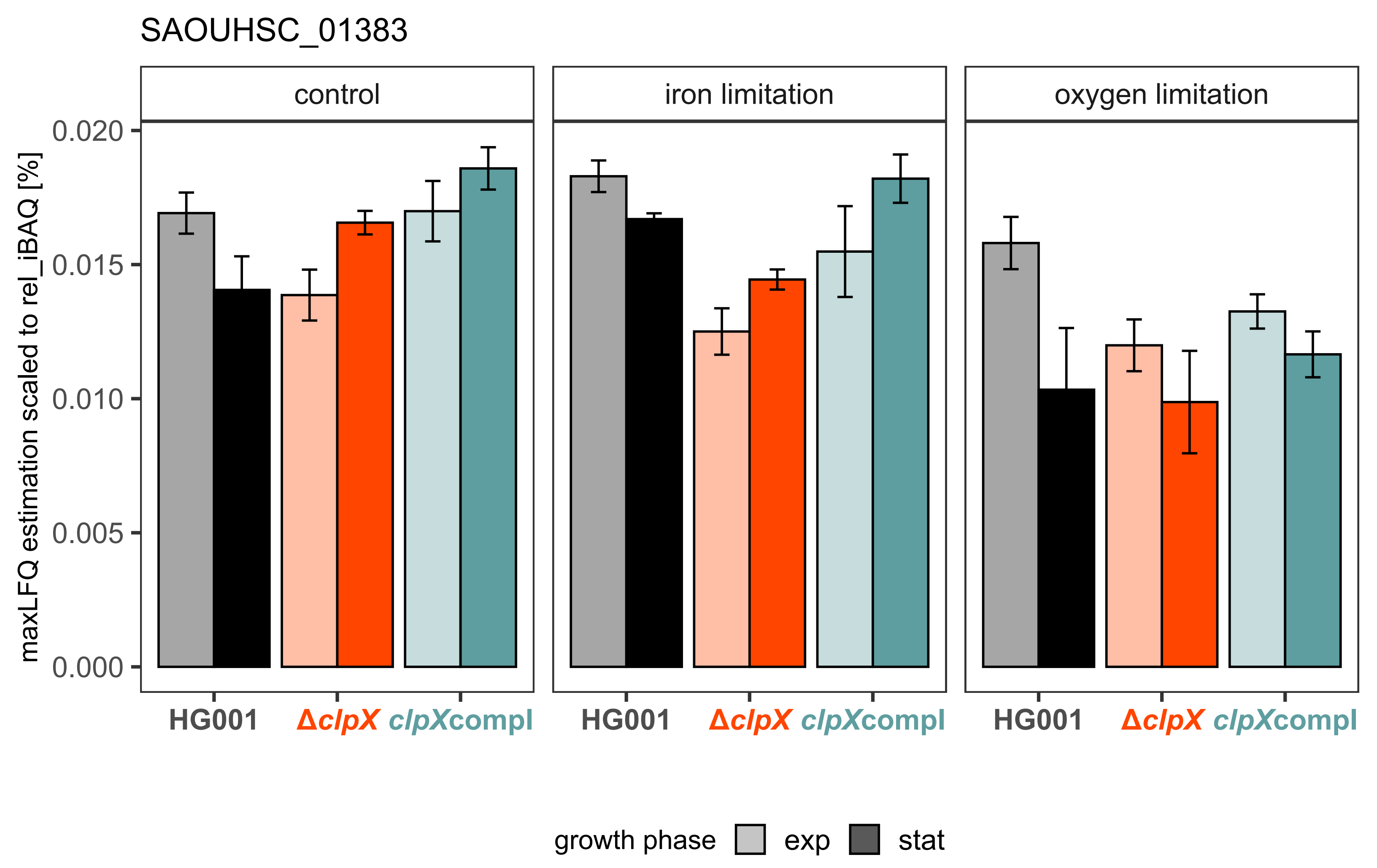
SAOUHSC_01383
control
iron limitation
oxygen limitation
maxLFQ estimation scaled to rel_iBAQ [%]
0.020
0.015
0.010
0.005
0.000
HG001
ΔclpX
clpXcompl
growth phase
exp
stat

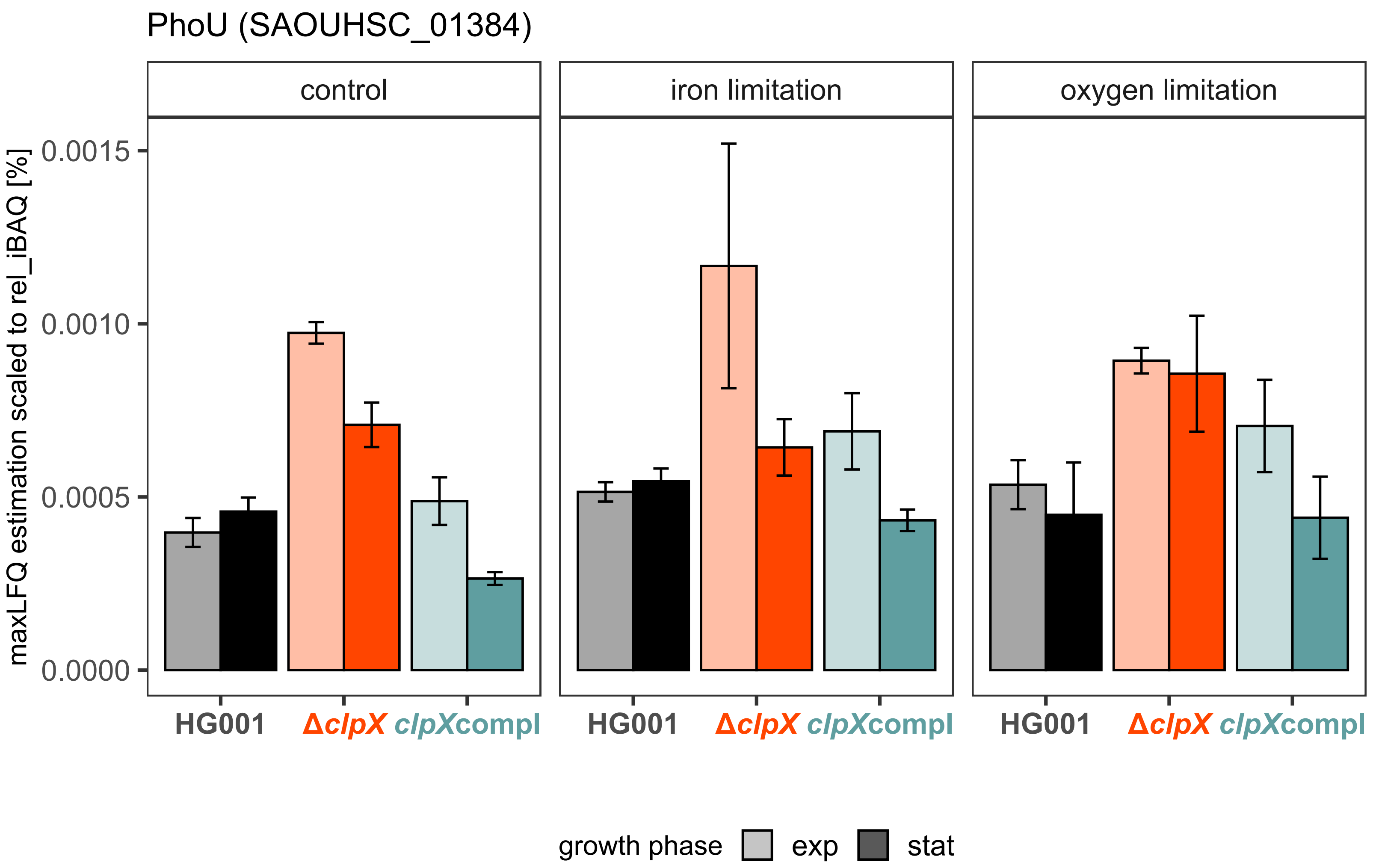
PhoU (SAOUHSC_01384)
control
iron limitation
oxygen limitation
maxLFQ estimation scaled to rel_iBAQ [%]
0.0015
0.0010
0.0005
0.0000
HG001
ΔclpX
clpXcompl
growth phase
exp
stat

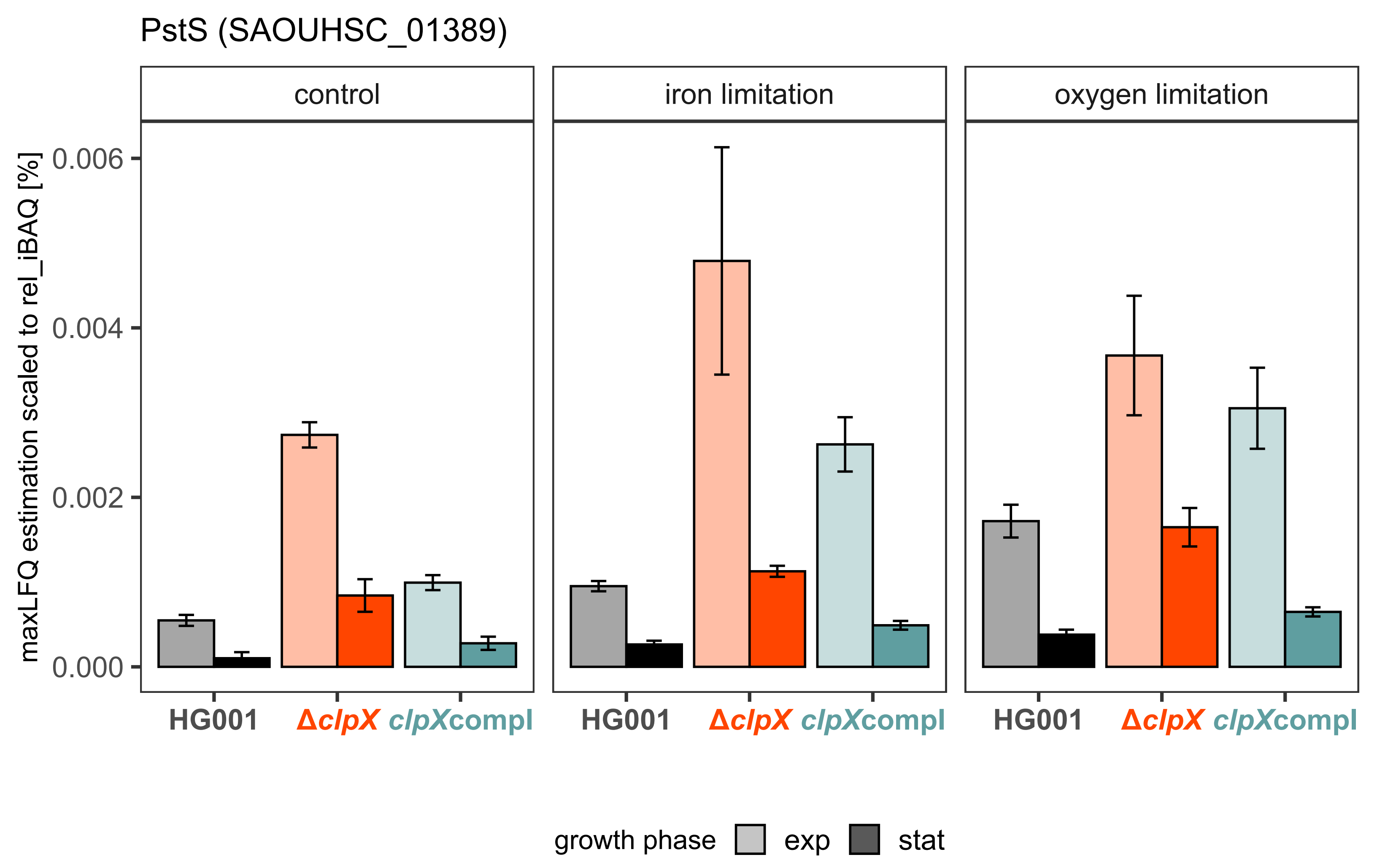
PstS (SAOUHSC_01389)
control
iron limitation
oxygen limitation
maxLFQ estimation scaled to rel_iBAQ [%]
0.006
0.004
0.002
0.000
HG001
ΔclpX
clpXcompl
growth phase
exp
stat

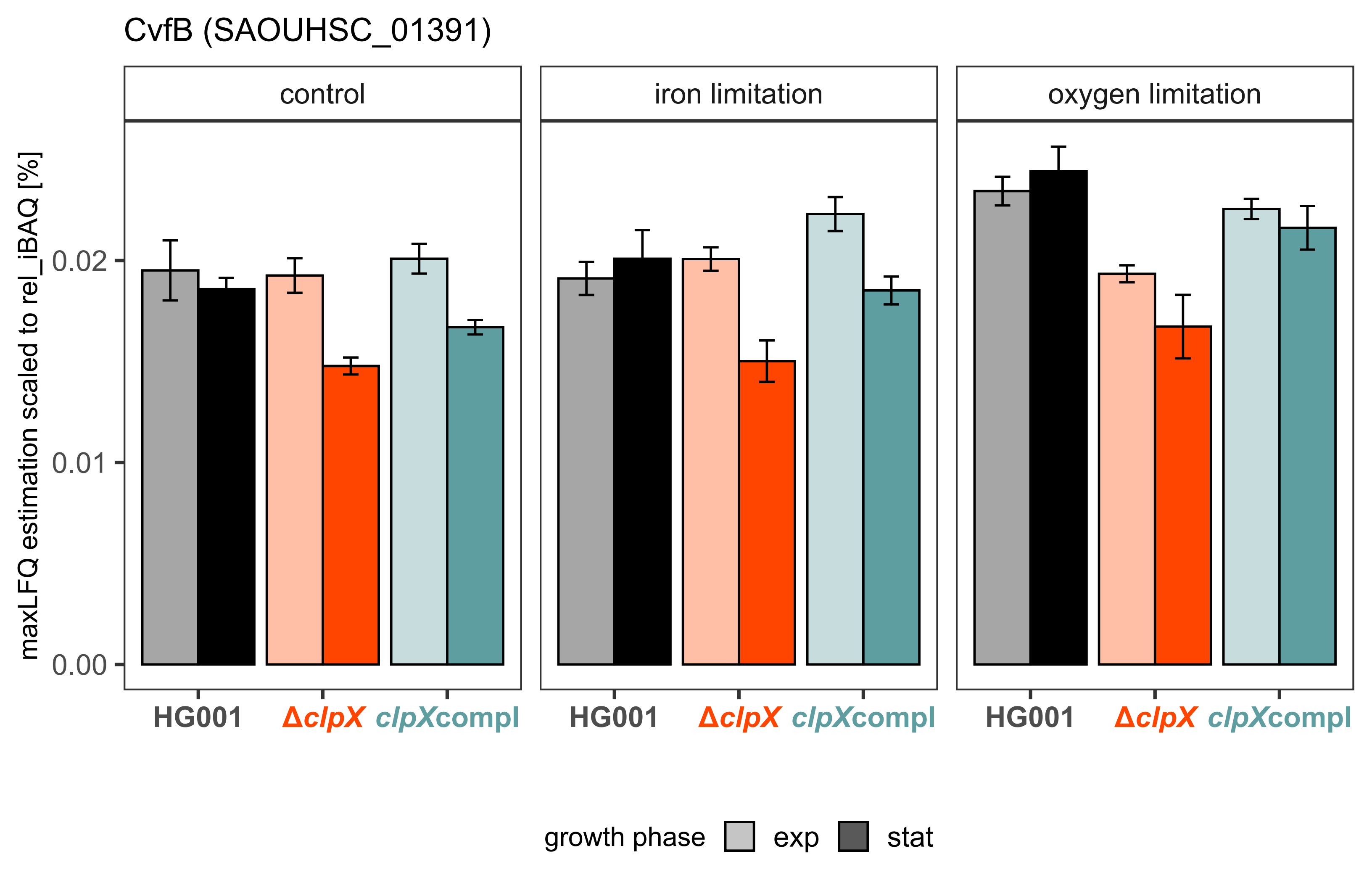
CvfB (SAOUHSC_01391)
control
iron limitation
oxygen limitation
maxLFQ estimation scaled to rel_iBAQ [%]
0.00
0.01
0.02
HG001
ΔclpX
clpXcompl
growth phase
exp
stat

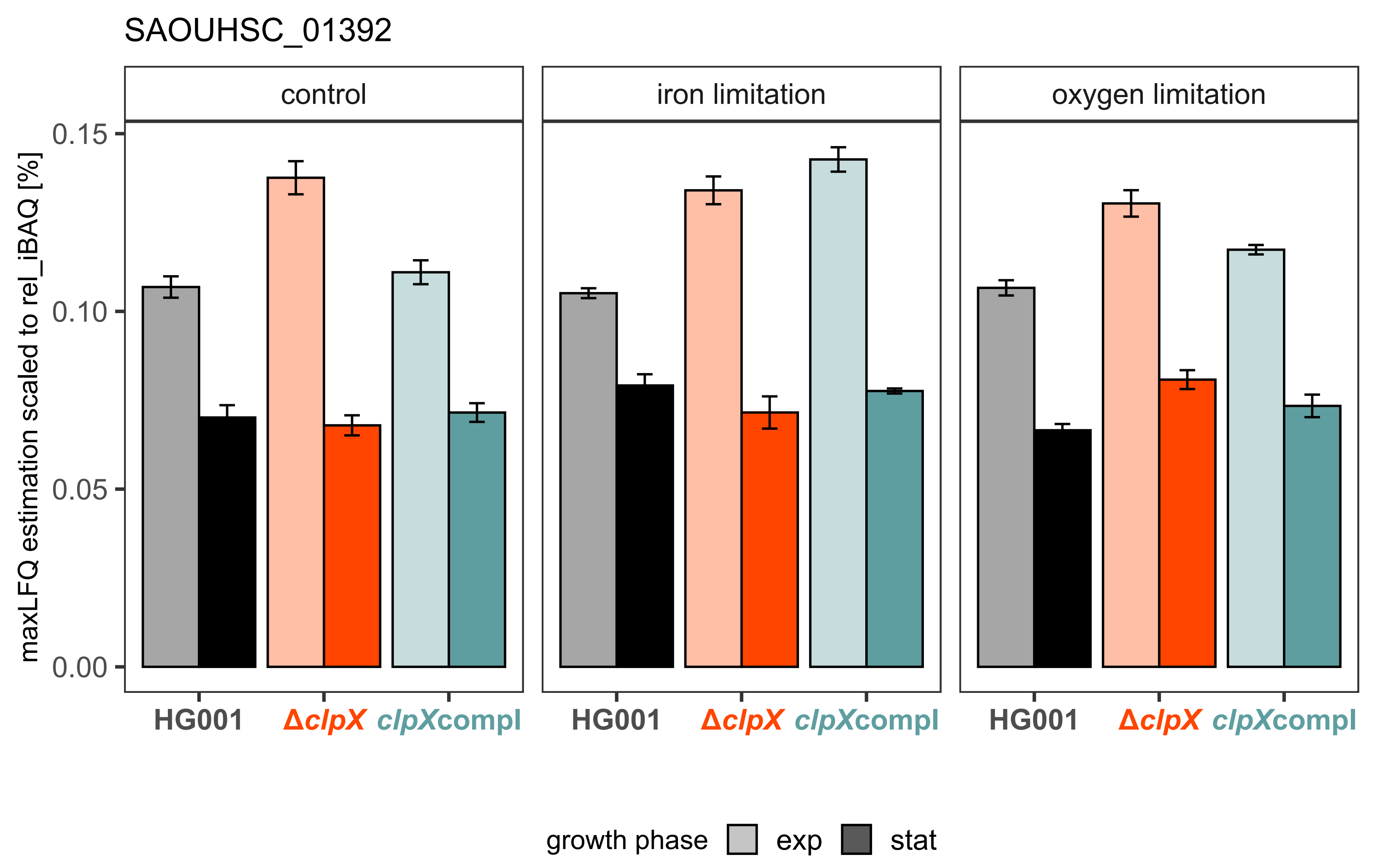
SAOUHSC_01392
control
iron limitation
oxygen limitation
maxLFQ estimation scaled to rel_iBAQ [%]
0.15
0.10
0.05
0.00
HG001
ΔclpX
clpXcompl
growth phase
exp
stat

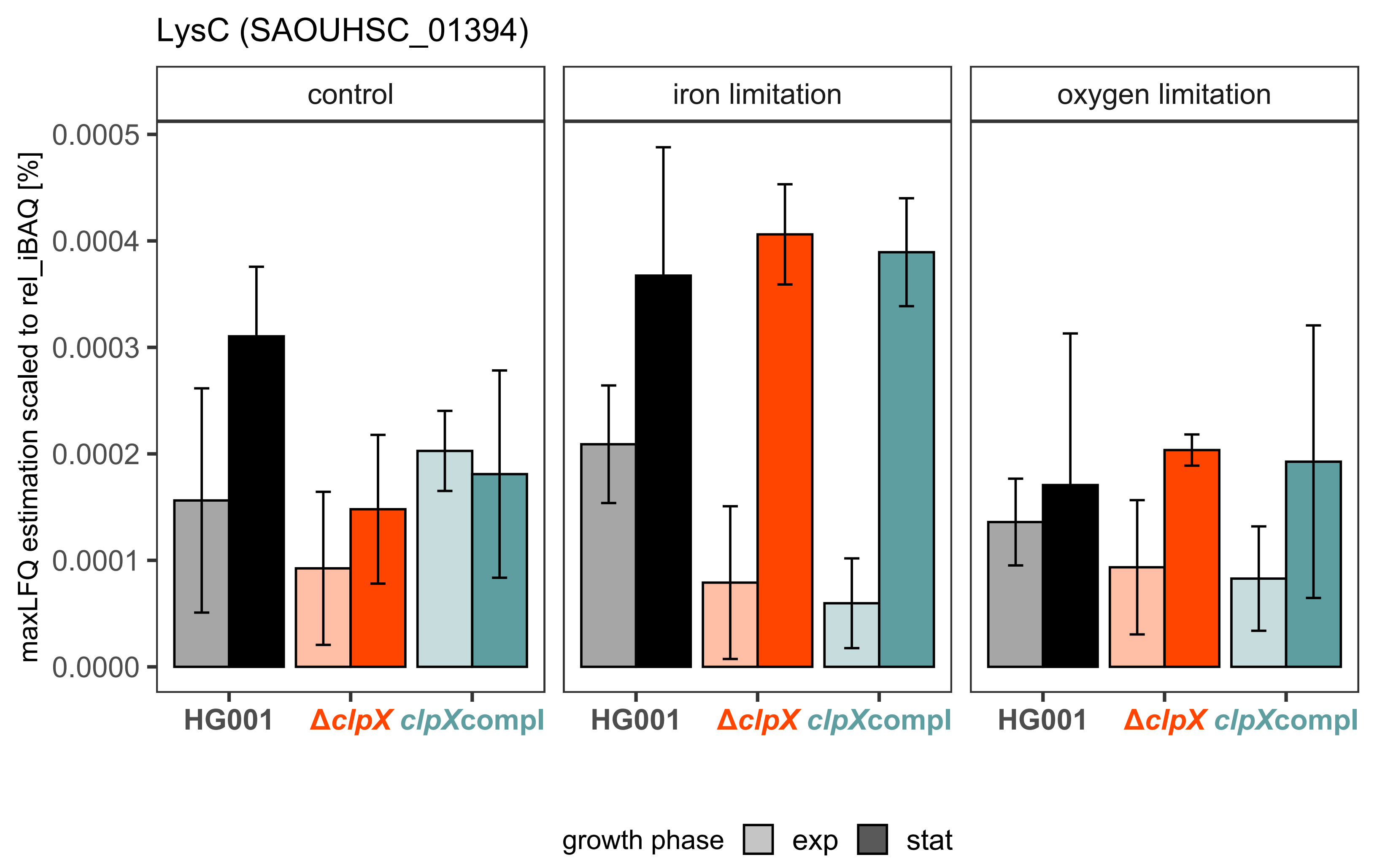
LysC (SAOUHSC_01394)
control
iron limitation
oxygen limitation
maxLFQ estimation scaled to rel_iBAQ [%]
0.0005
0.0004
0.0003
0.0002
0.0001
0.0000
HG001
ΔclpX
clpXcompl
growth phase
exp
stat

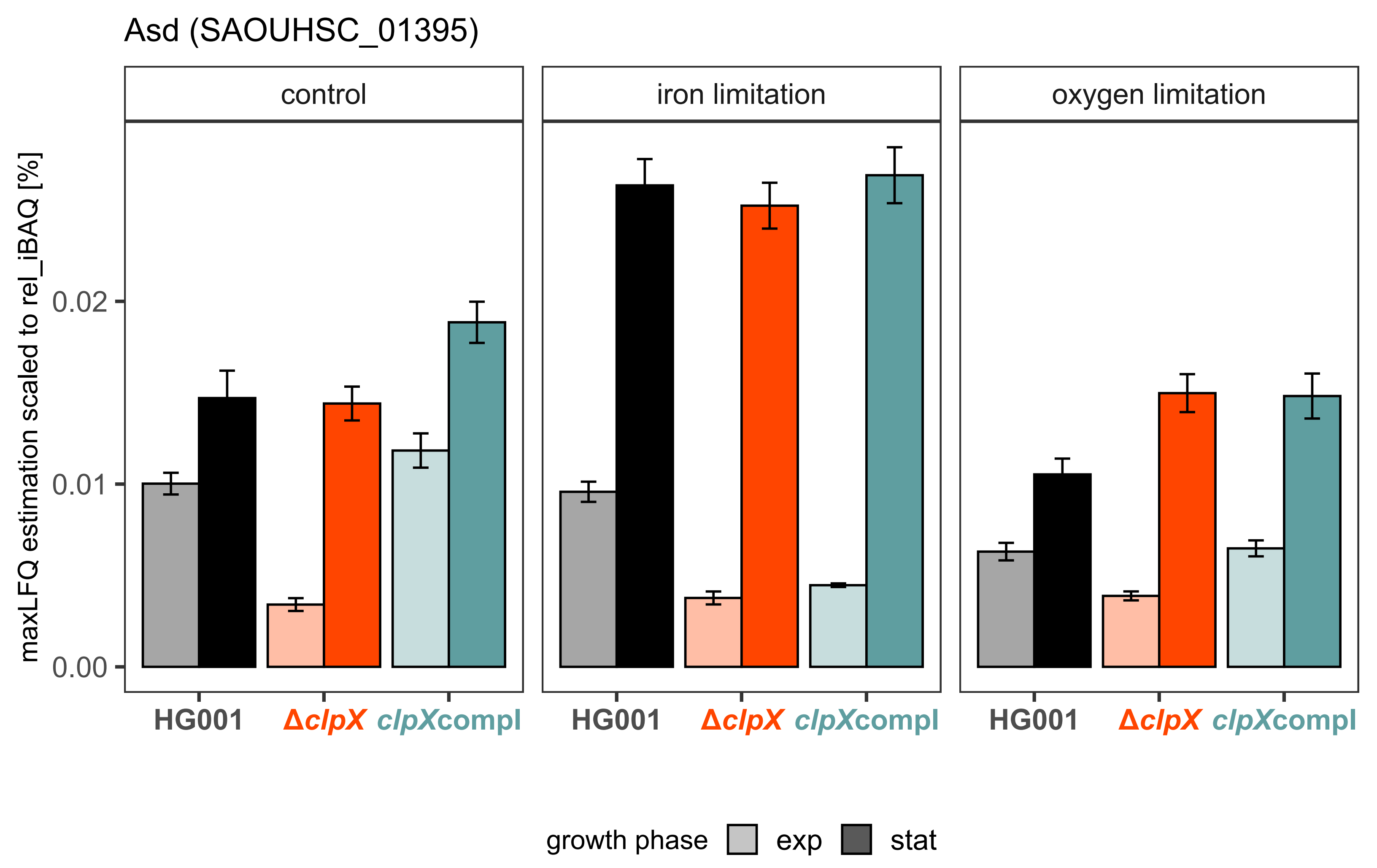
Asd (SAOUHSC_01395)
control
iron limitation
oxygen limitation
maxLFQ estimation scaled to rel_iBAQ [%]
0.00
0.01
0.02
HG001
ΔclpX
clpXcompl
growth phase
exp
stat

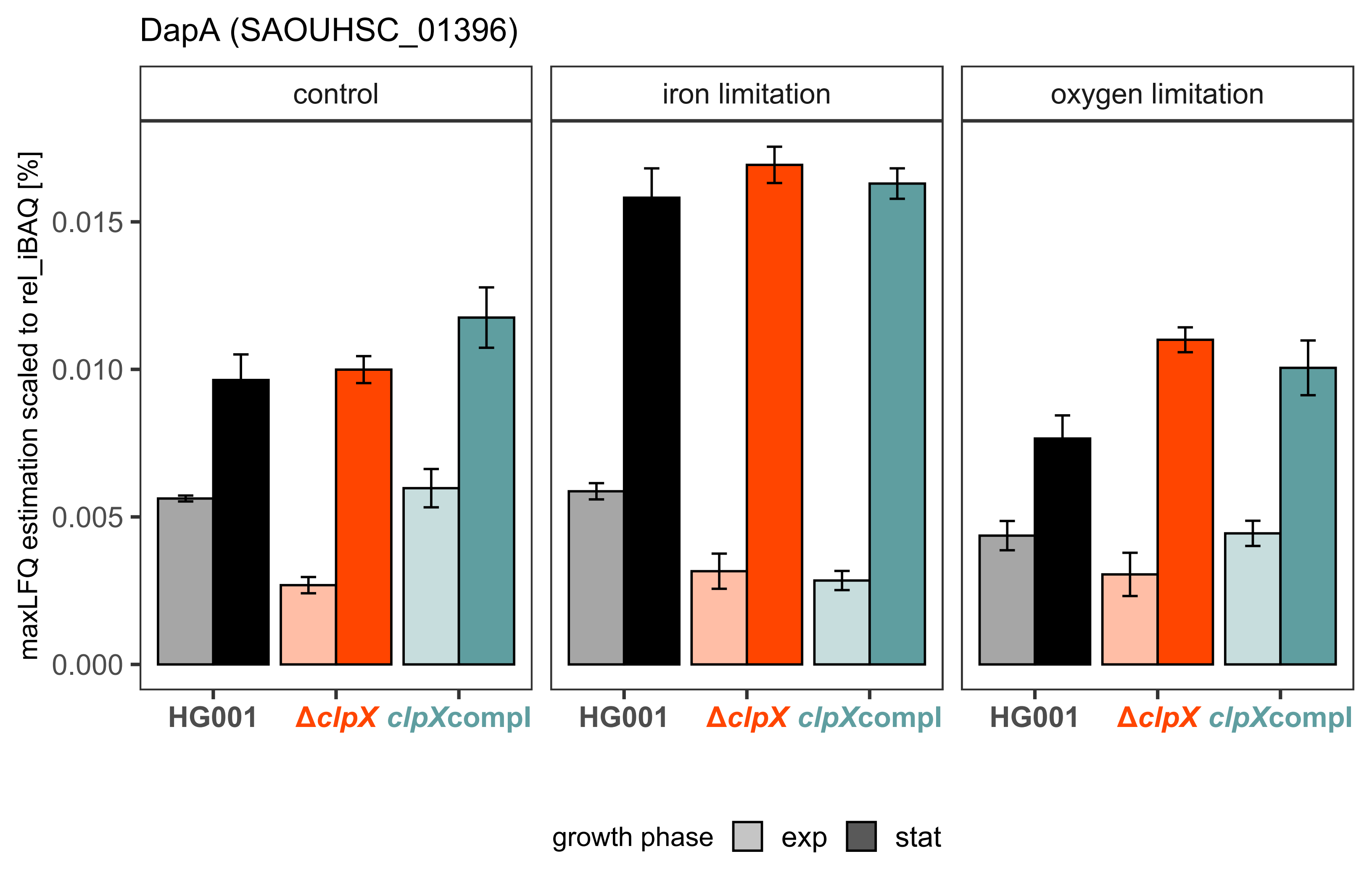

DapA (SAOUHSC_01396)
control
iron limitation
oxygen limitation
maxLFQ estimation scaled to rel_iBAQ [%]
0.015
0.010
0.005
0.000
HG001
ΔclpX
clpXcompl
growth phase
exp
stat

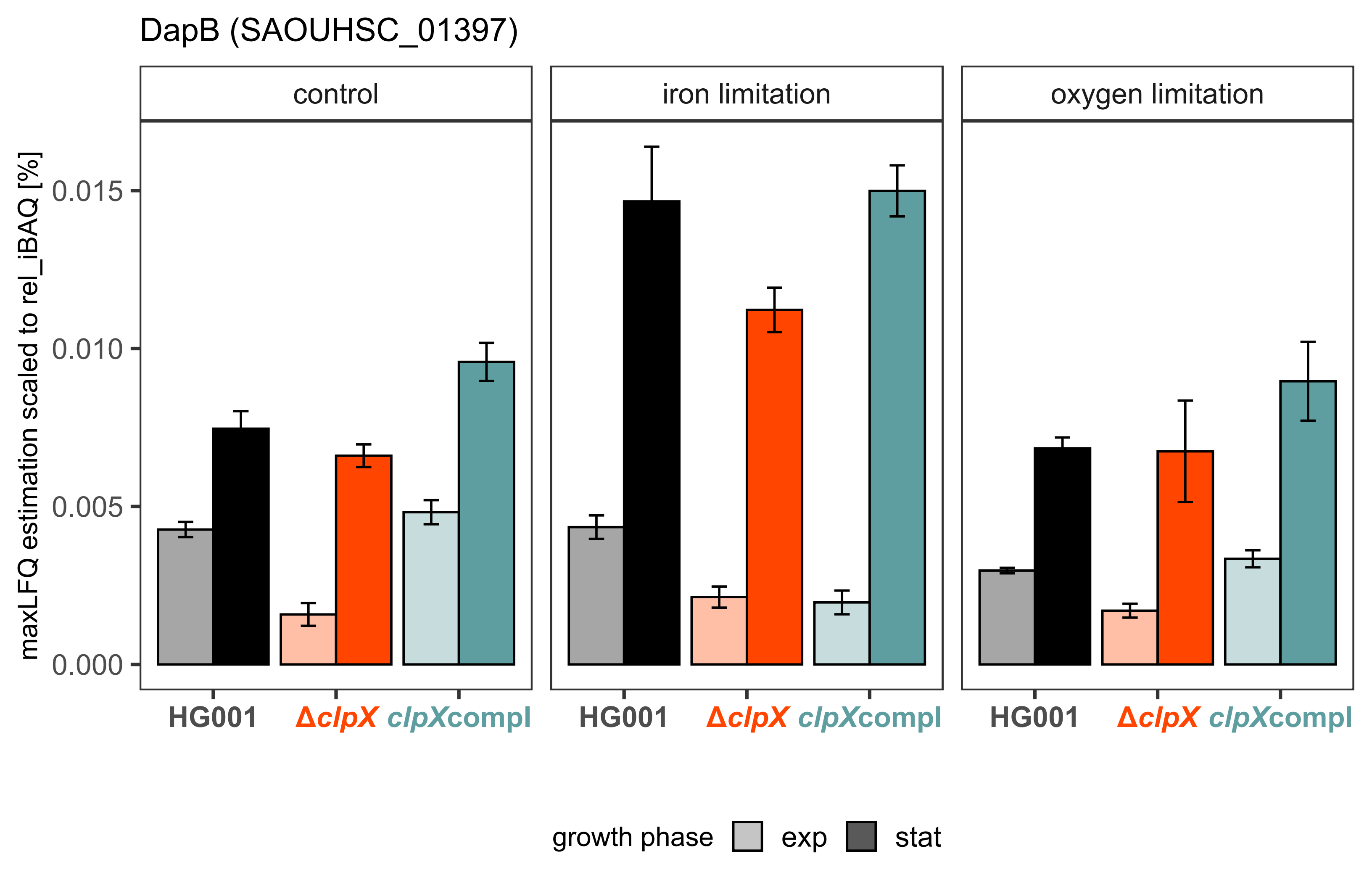
DapB (SAOUHSC_01397)
control
iron limitation
oxygen limitation
maxLFQ estimation scaled to rel_iBAQ [%]
0.015
0.010
0.005
0.000
HG001
ΔclpX
clpXcompl
growth phase
exp
stat

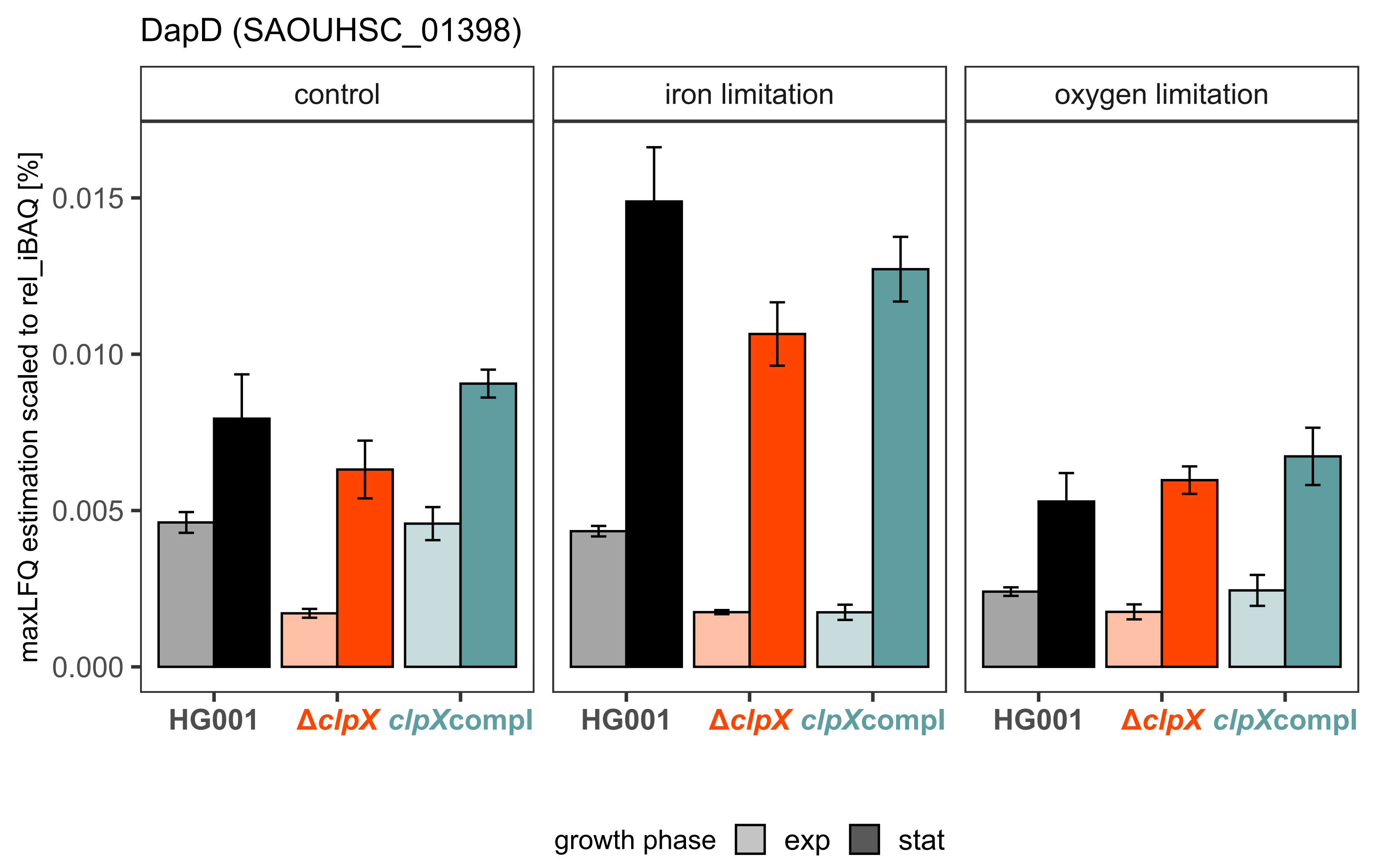
DapD (SAOUHSC_01398)
control
iron limitation
oxygen limitation
maxLFQ estimation scaled to rel_iBAQ [%]
0.015
0.010
0.005
0.000
HG001
ΔclpX
clpXcompl
growth phase
exp
stat

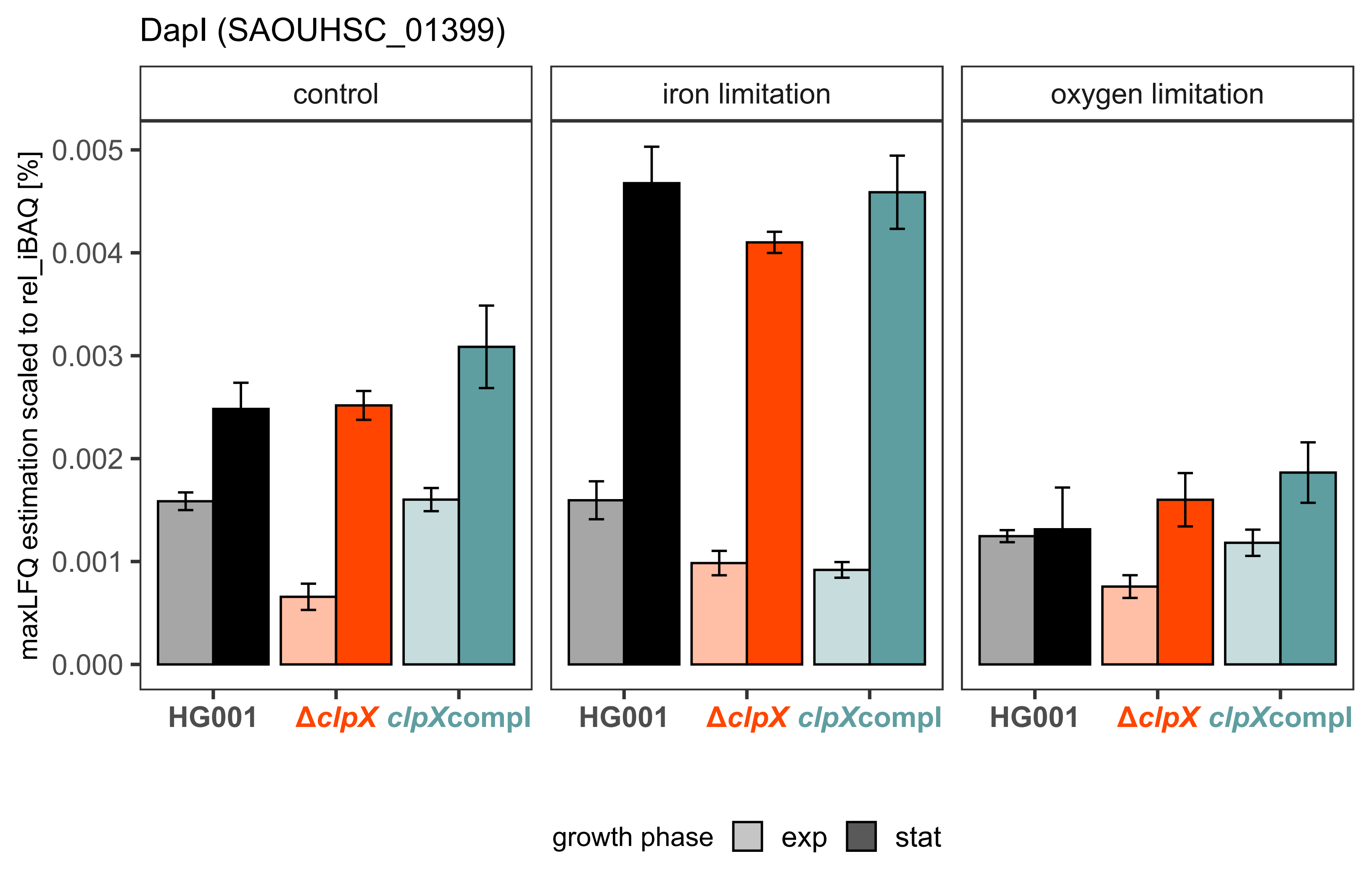

DapI (SAOUHSC_01399)
control
iron limitation
oxygen limitation
maxLFQ estimation scaled to rel_iBAQ [%]
0.005
0.004
0.003
0.002
0.001
0.000
HG001
ΔclpX
clpXcompl
growth phase
exp
stat

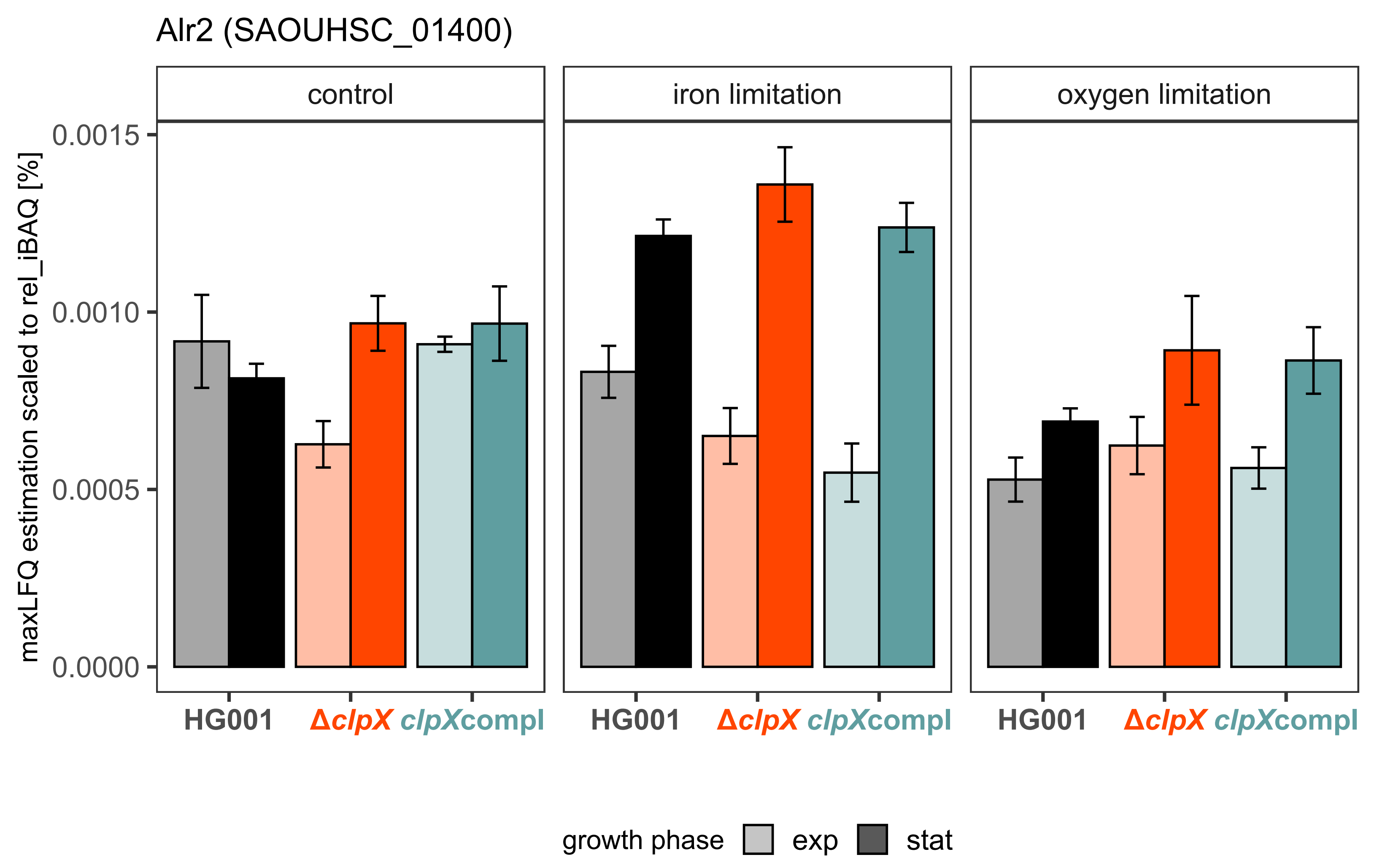

Alr2 (SAOUHSC_01400)
control
iron limitation
oxygen limitation
0.0015
0.0010
0.0005
0.0000
maxLFQ estimation scaled to rel_iBAQ [%]
HG001
ΔclpX
clpXcompl
growth phase
exp
stat

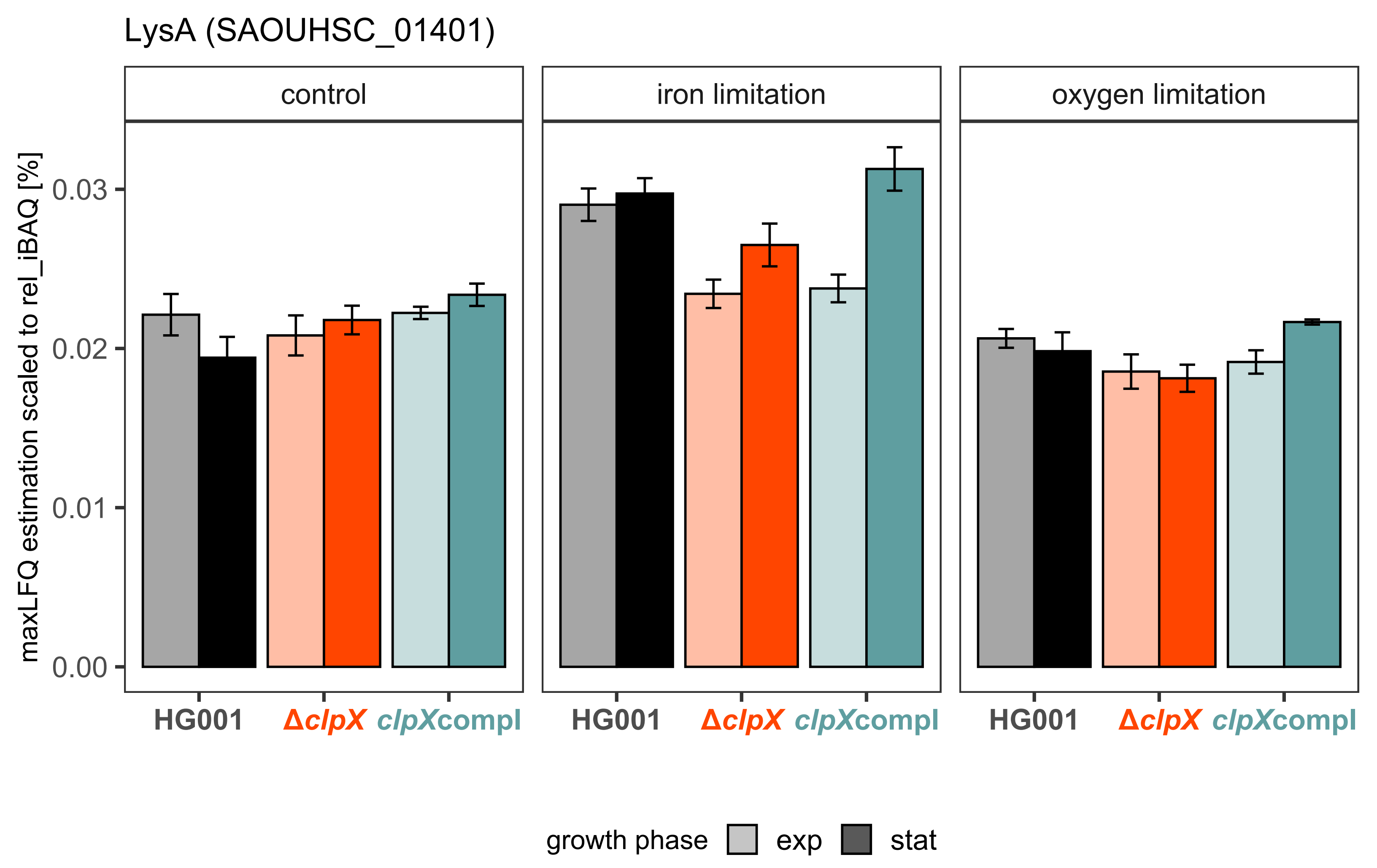
LysA (SAOUHSC_01401)
control
iron limitation
oxygen limitation
maxLFQ estimation scaled to rel_iBAQ [%]
0.03
0.02
0.01
0.00
HG001
ΔclpX
clpXcompl
HG001
ΔclpX
clpXcompl
HG001
ΔclpX
clpXcompl
growth phase
exp
stat

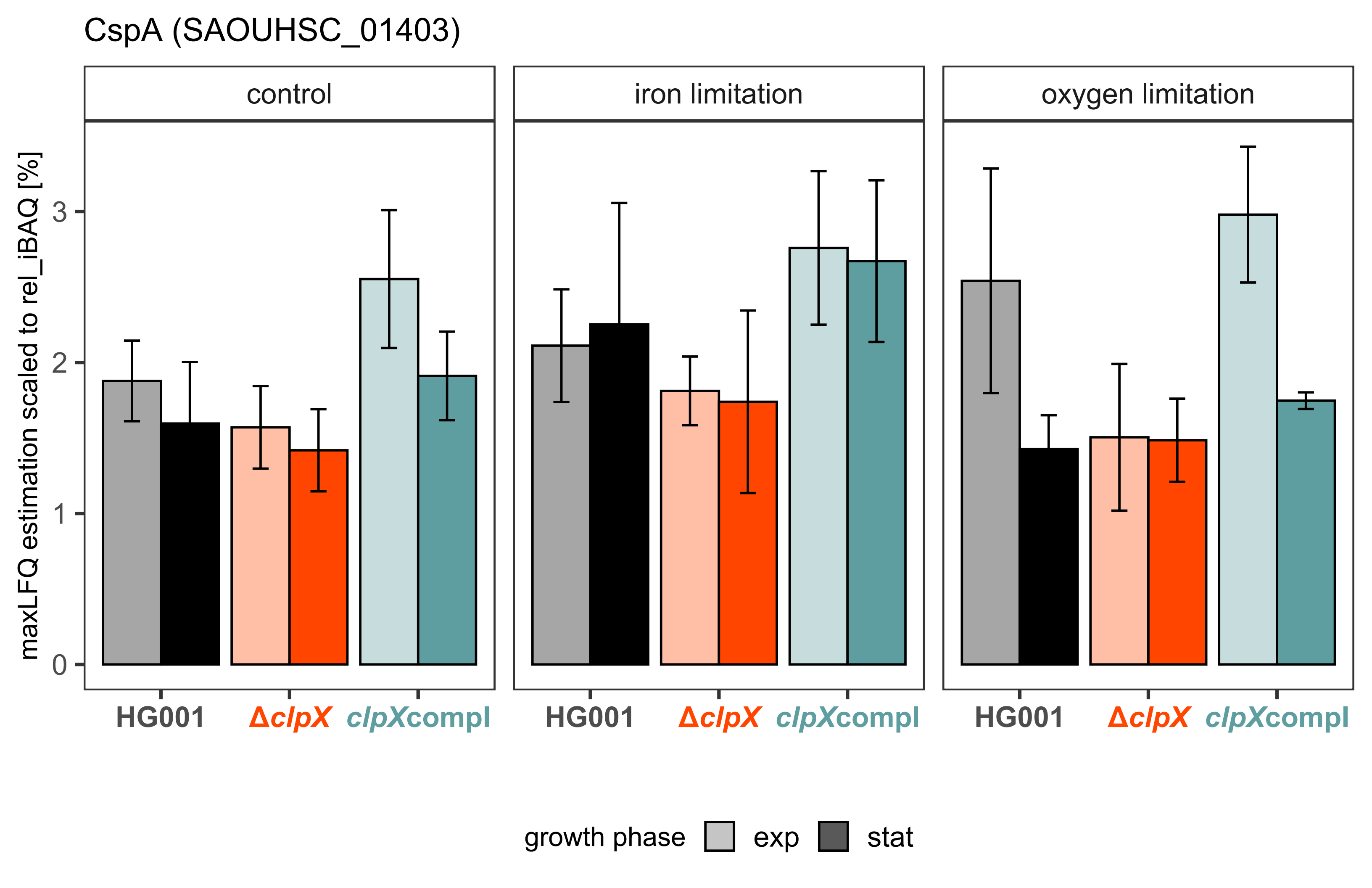
CspA (SAOUHSC_01403)
control
iron limitation
oxygen limitation
maxLFQ estimation scaled to rel_iBAQ [%]
0
1
2
3
HG001
ΔclpX
clpXcompl
growth phase
exp
stat

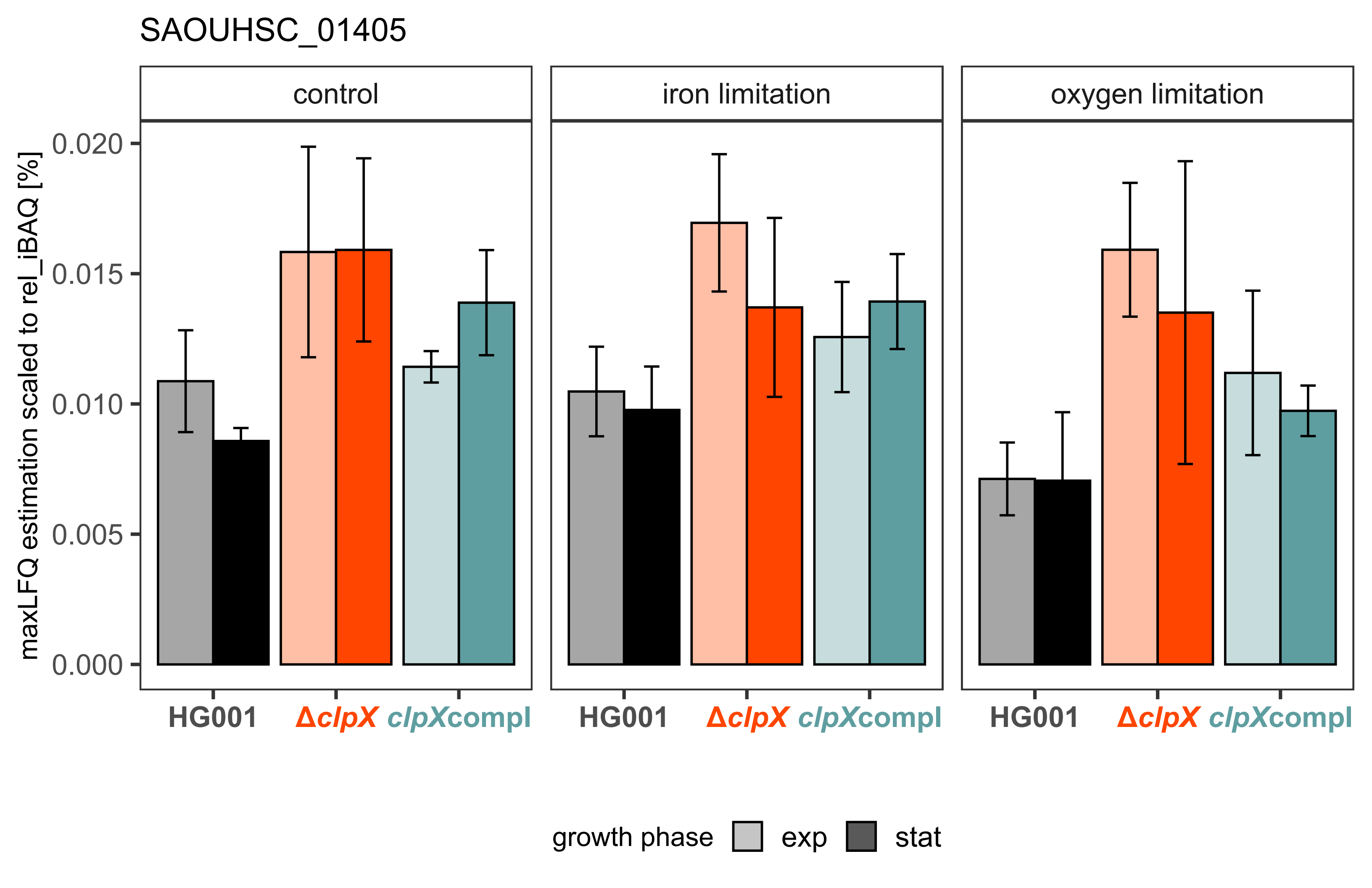
SAOUHSC_01405
control
iron limitation
oxygen limitation
maxLFQ estimation scaled to rel_iBAQ [%]
0.020
0.015
0.010
0.005
0.000
HG001
ΔclpX
clpXcompl
growth phase
exp
stat

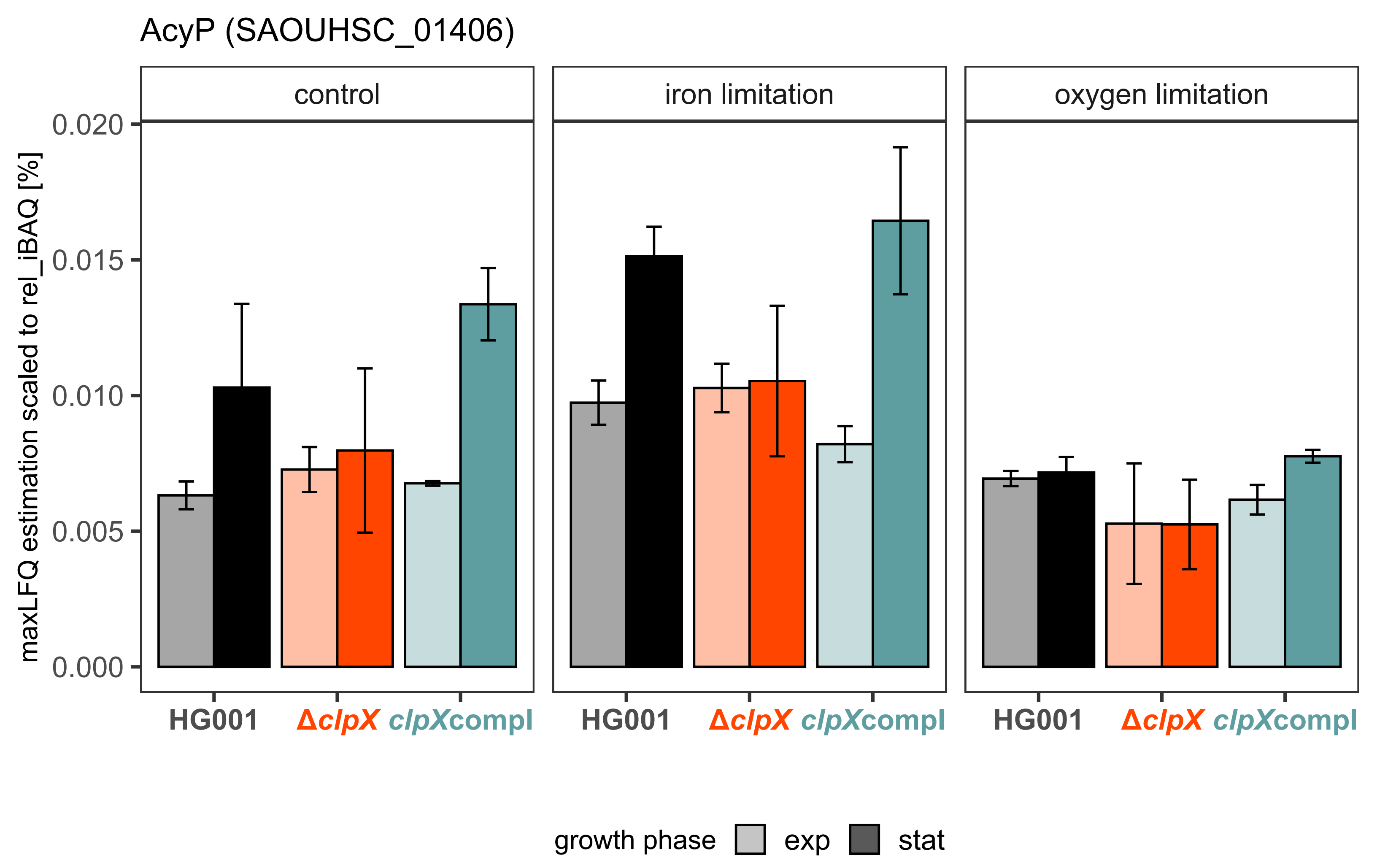

AcyP (SAOUHSC_01406)
control
iron limitation
oxygen limitation
maxLFQ estimation scaled to rel_iBAQ [%]
0.020
0.015
0.010
0.005
0.000
HG001
ΔclpX
clpXcompl
growth phase
exp
stat

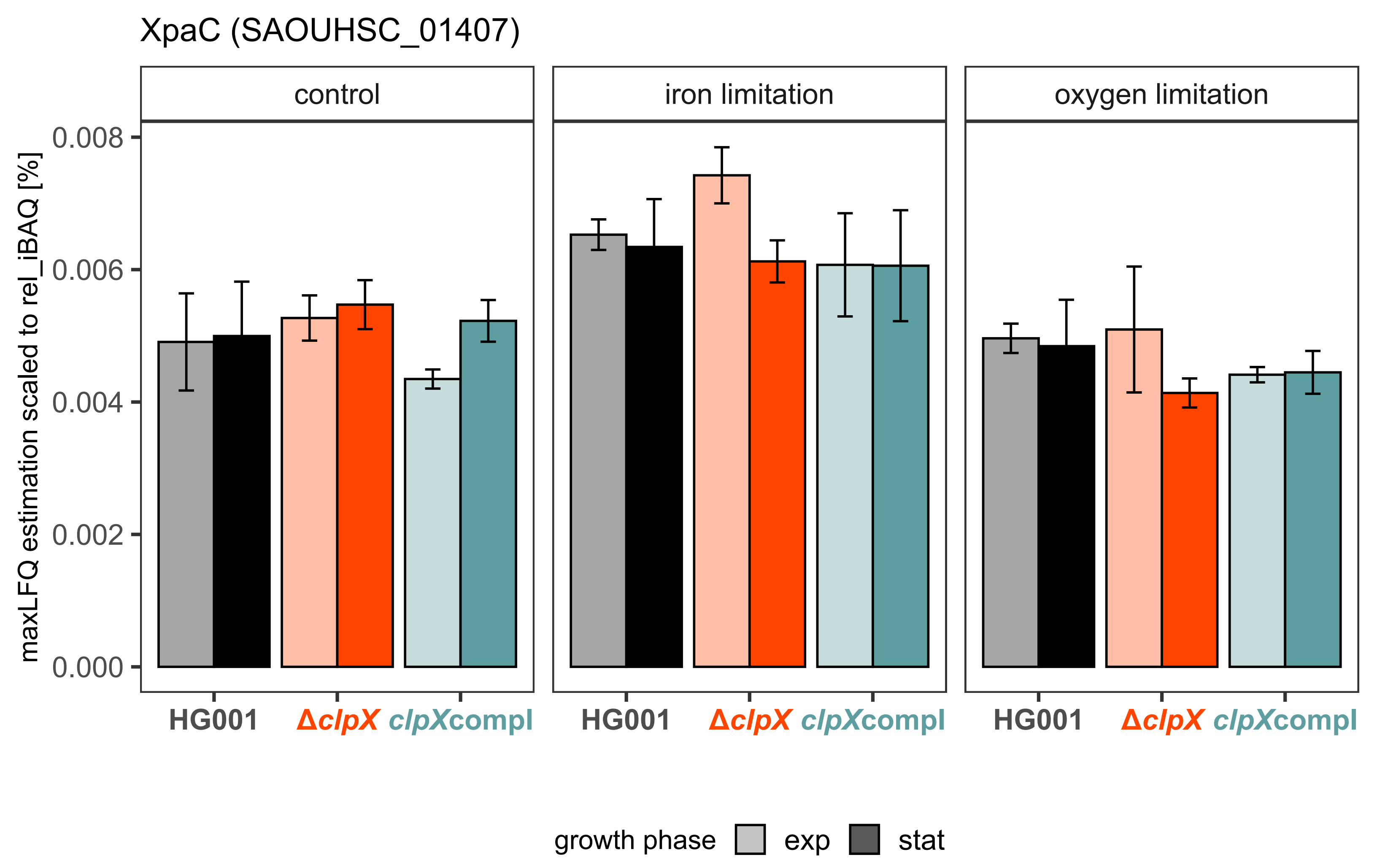

XpaC (SAOUHSC_01407)
control
iron limitation
oxygen limitation
maxLFQ estimation scaled to rel_iBAQ [%]
0.008
0.006
0.004
0.002
0.000
HG001
ΔclpX
clpXcompl
growth phase
exp
stat

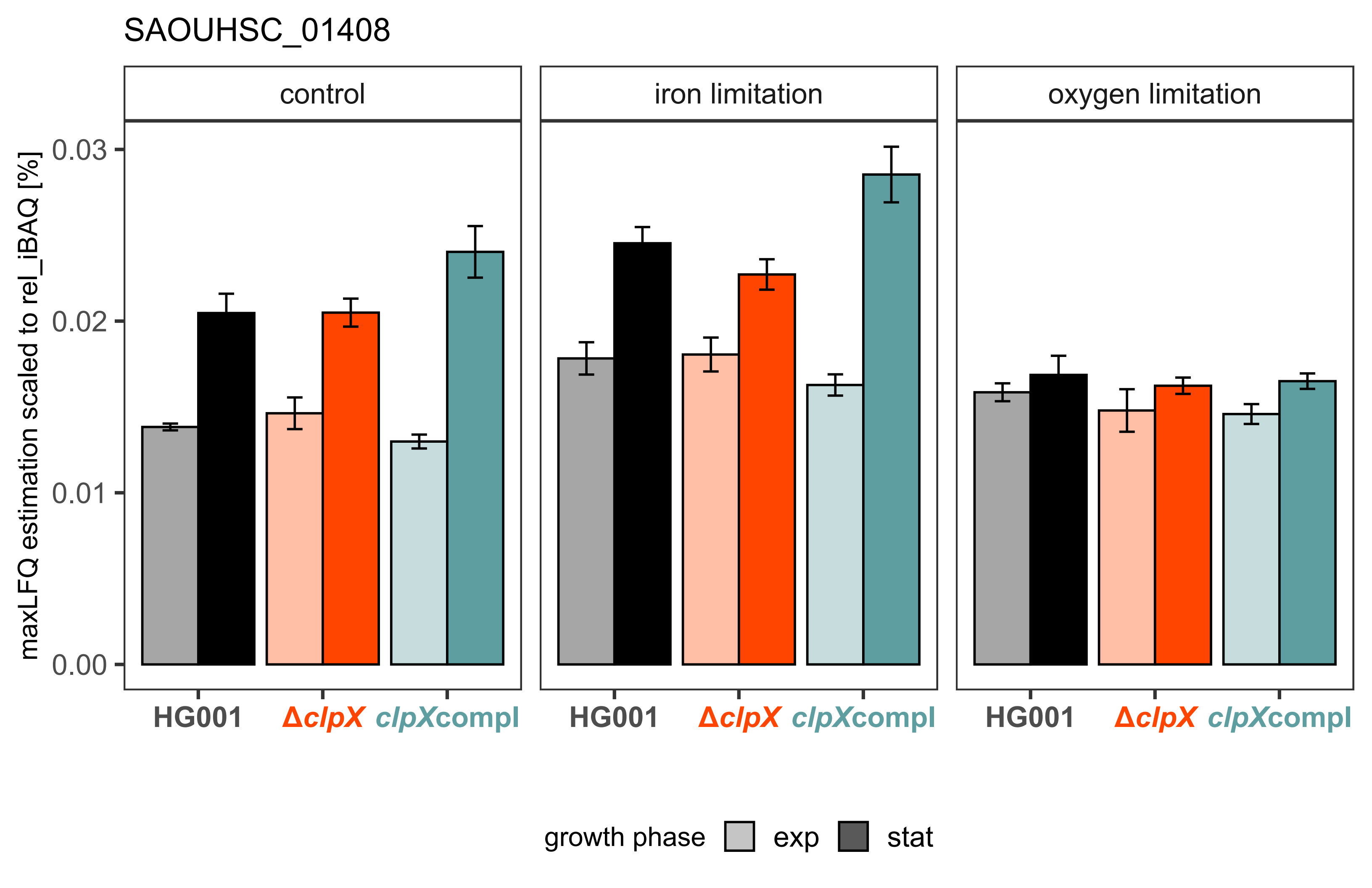

SAOUHSC_01408
control
iron limitation
oxygen limitation
maxLFQ estimation scaled to rel_iBAQ [%]
0.03
0.02
0.01
0.00
HG001
ΔclpX
clpXcompl
growth phase
exp
stat

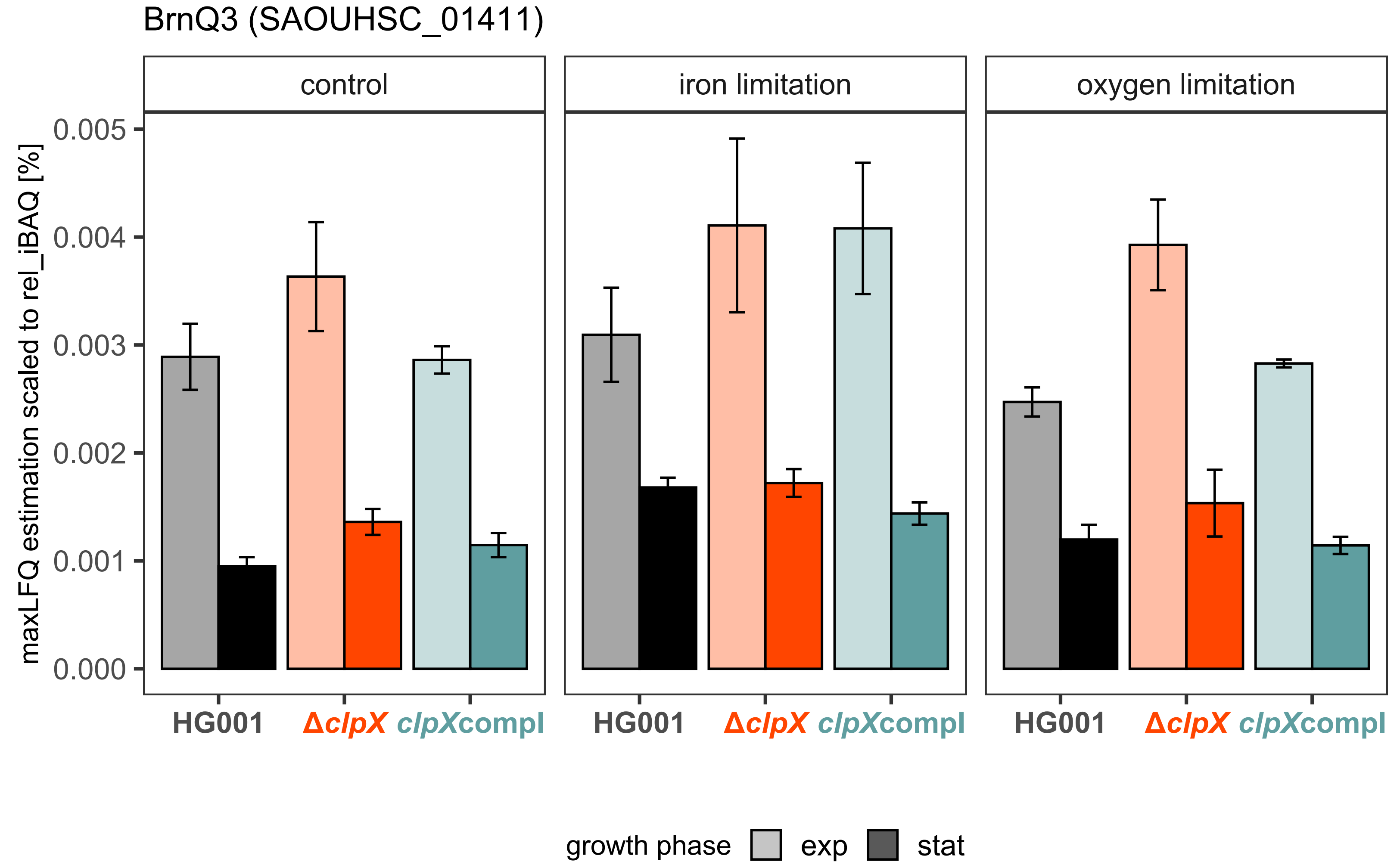
BrnQ3 (SAOUHSC_01411)
control
iron limitation
oxygen limitation
maxLFQ estimation scaled to rel_iBAQ [%]
0.005
0.004
0.003
0.002
0.001
0.000
HG001
ΔclpX
clpXcompl
growth phase
exp
stat

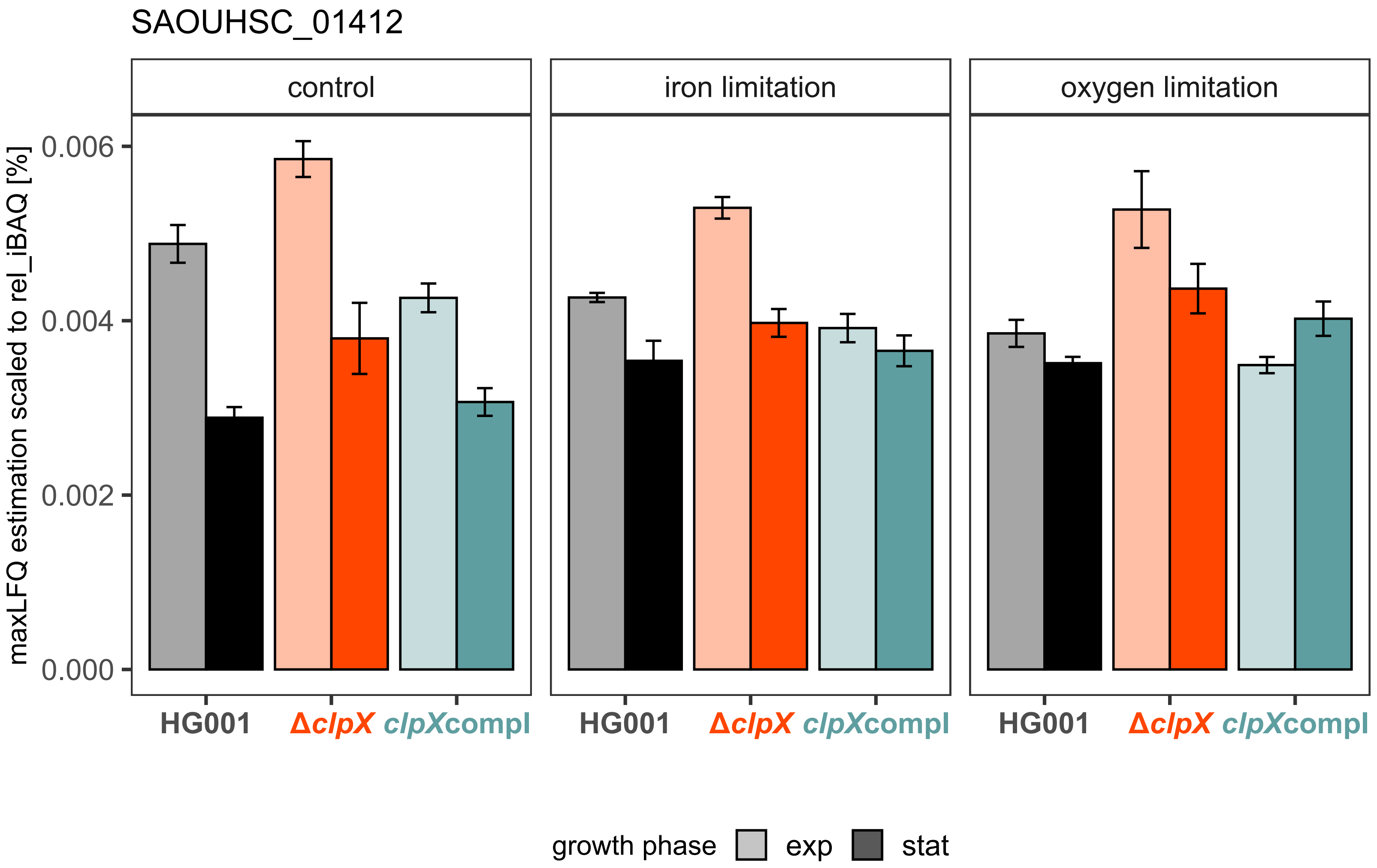

SAOUHSC_01412
control
iron limitation
oxygen limitation
maxLFQ estimation scaled to rel_iBAQ [%]
0.006
0.004
0.002
0.000
HG001
ΔclpX
clpXcompl
growth phase
exp
stat

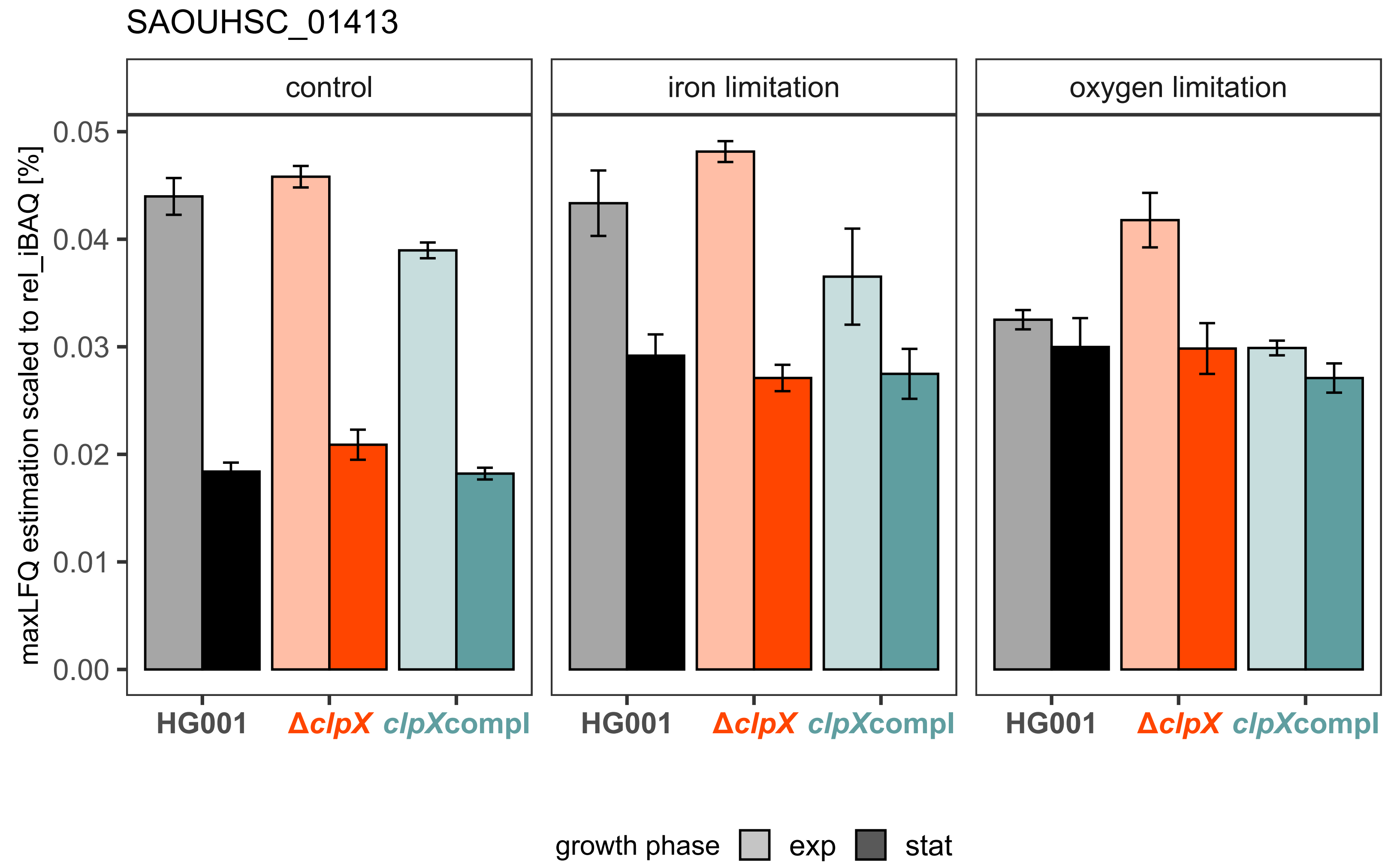

SAOUHSC_01413
control
iron limitation
oxygen limitation
maxLFQ estimation scaled to rel_iBAQ [%]
0.05
0.04
0.03
0.02
0.01
0.00
HG001
ΔclpX
clpXcompl
growth phase
exp
stat

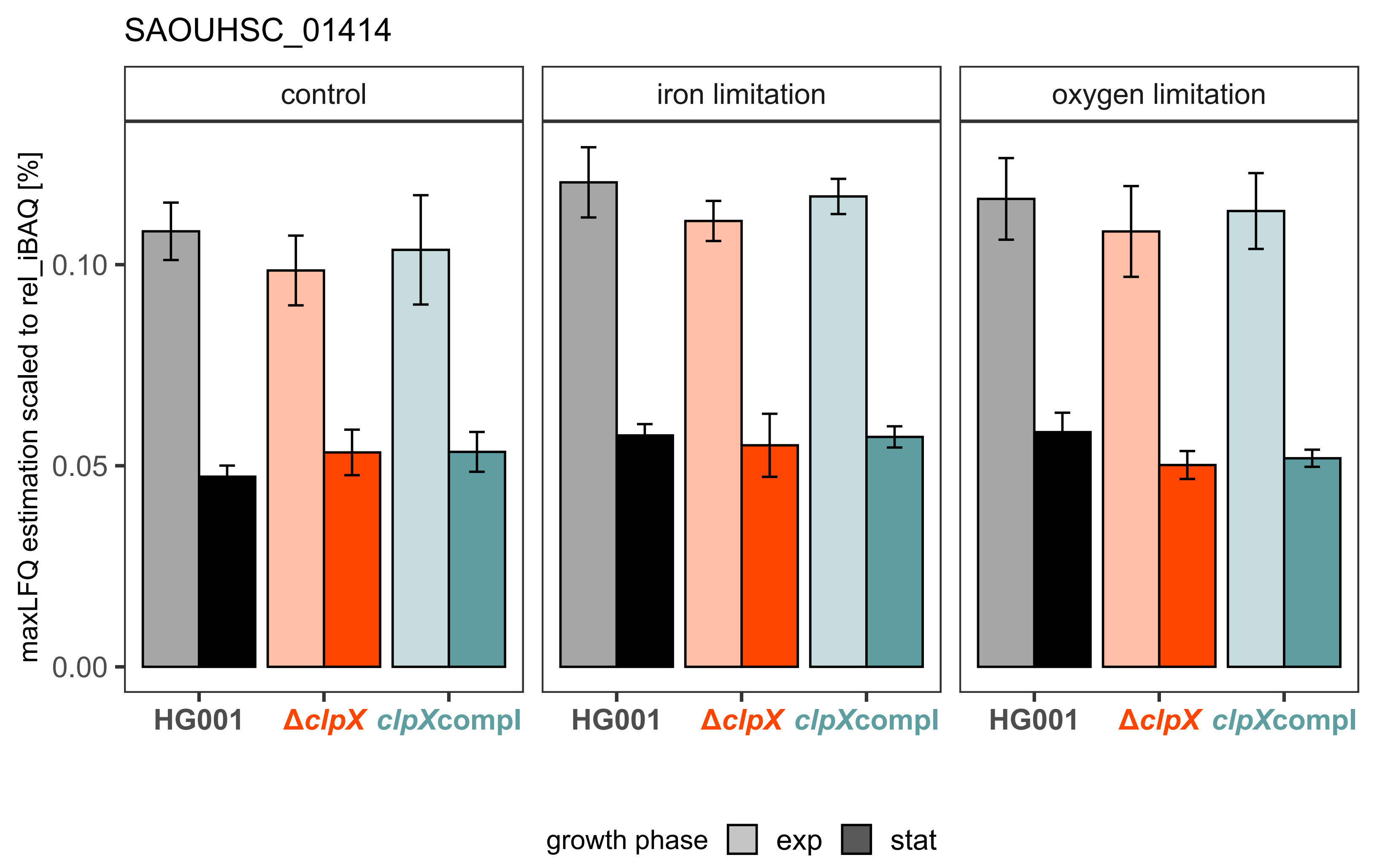
SAOUHSC_01414
control
iron limitation
oxygen limitation
maxLFQ estimation scaled to rel_iBAQ [%]
0.10
0.05
0.00
HG001
ΔclpX
clpXcompl
growth phase
exp
stat

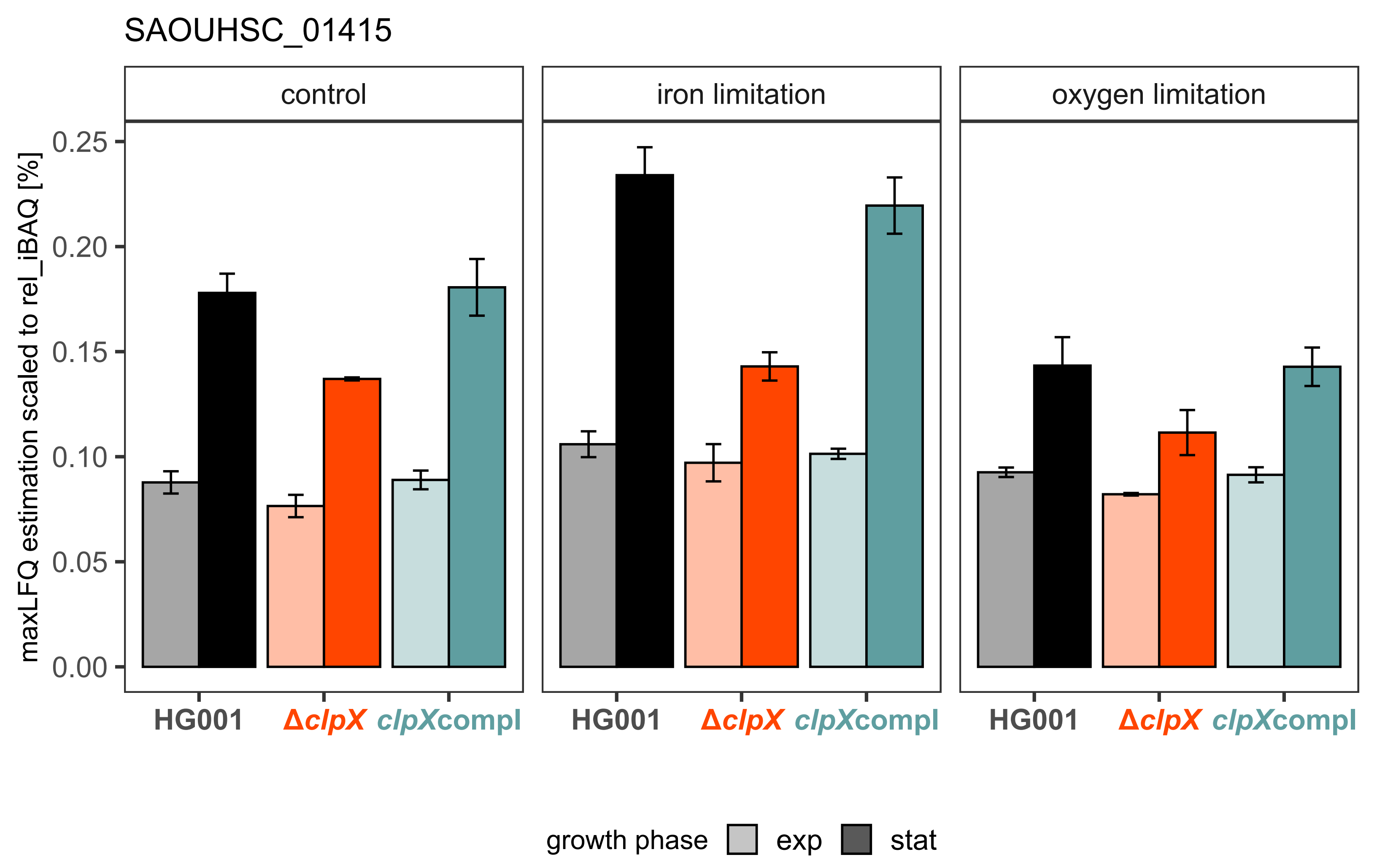
SAOUHSC_01415
control
iron limitation
oxygen limitation
maxLFQ estimation scaled to rel_iBAQ [%]
0.25
0.20
0.15
0.10
0.05
0.00
HG001
ΔclpX
clpXcompl
growth phase
exp
stat

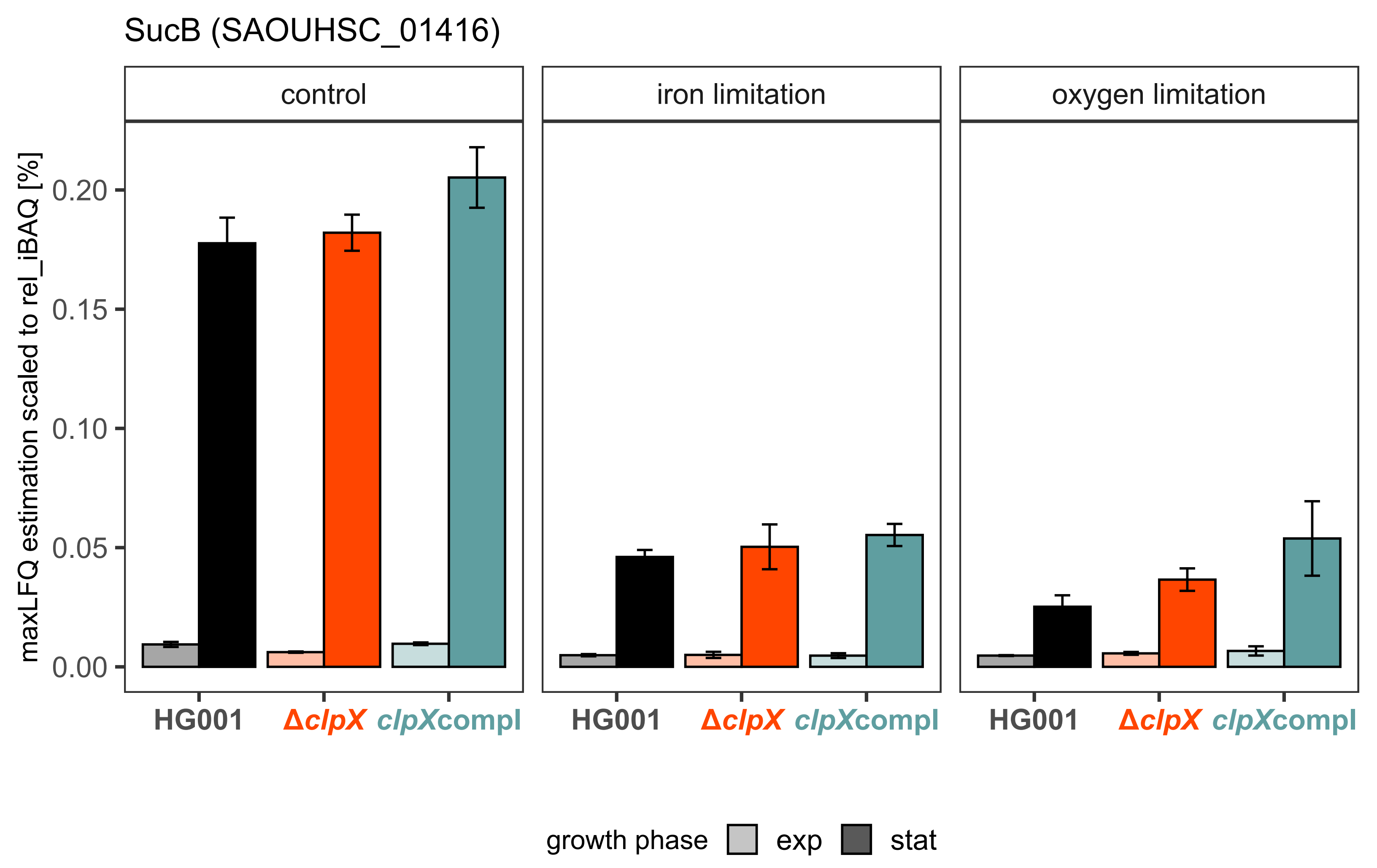

SucB (SAOUHSC_01416)
control
iron limitation
oxygen limitation
maxLFQ estimation scaled to rel_iBAQ [%]
0.20
0.15
0.10
0.05
0.00
HG001
ΔclpX
clpXcompl
growth phase
exp
stat

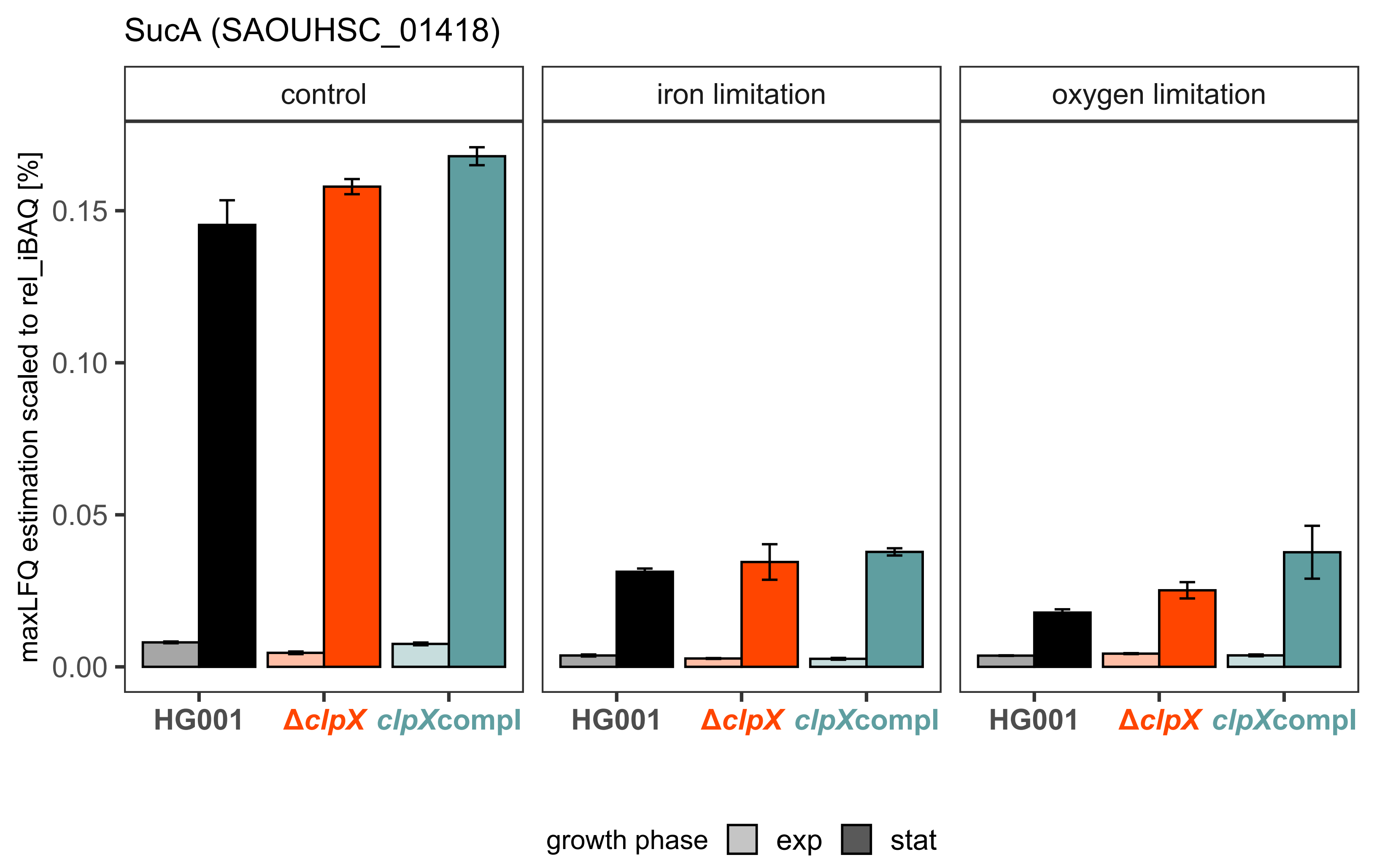

SucA (SAOUHSC_01418)
control
iron limitation
oxygen limitation
maxLFQ estimation scaled to rel_iBAQ [%]
0.00
0.05
0.10
0.15
HG001
ΔclpX
clpXcompl
growth phase
exp
stat

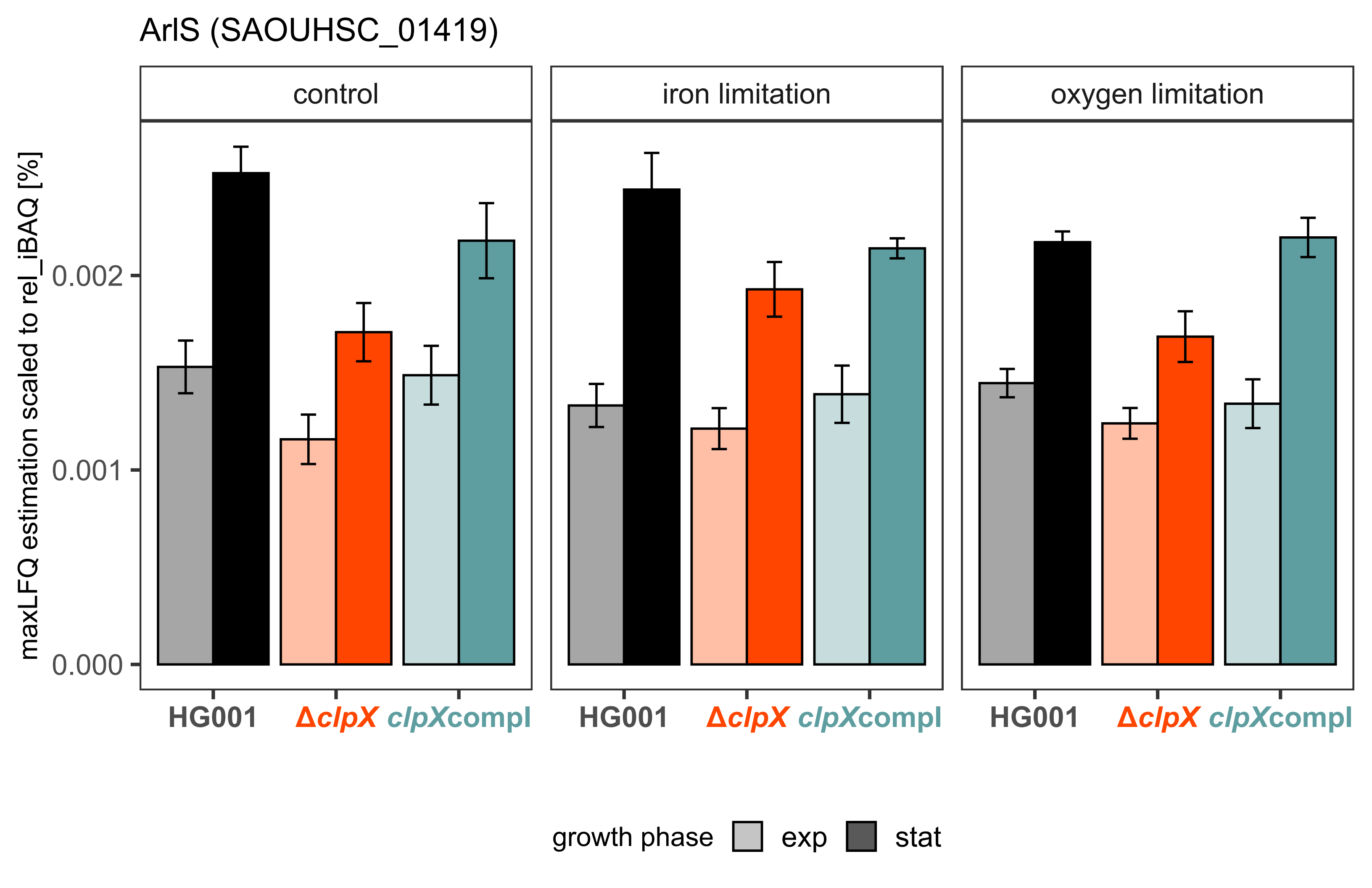

ArlS (SAOUHSC_01419)
control
iron limitation
oxygen limitation
maxLFQ estimation scaled to rel_iBAQ [%]
0.002
0.001
0.000
HG001
ΔclpX
clpXcompl
growth phase
exp
stat

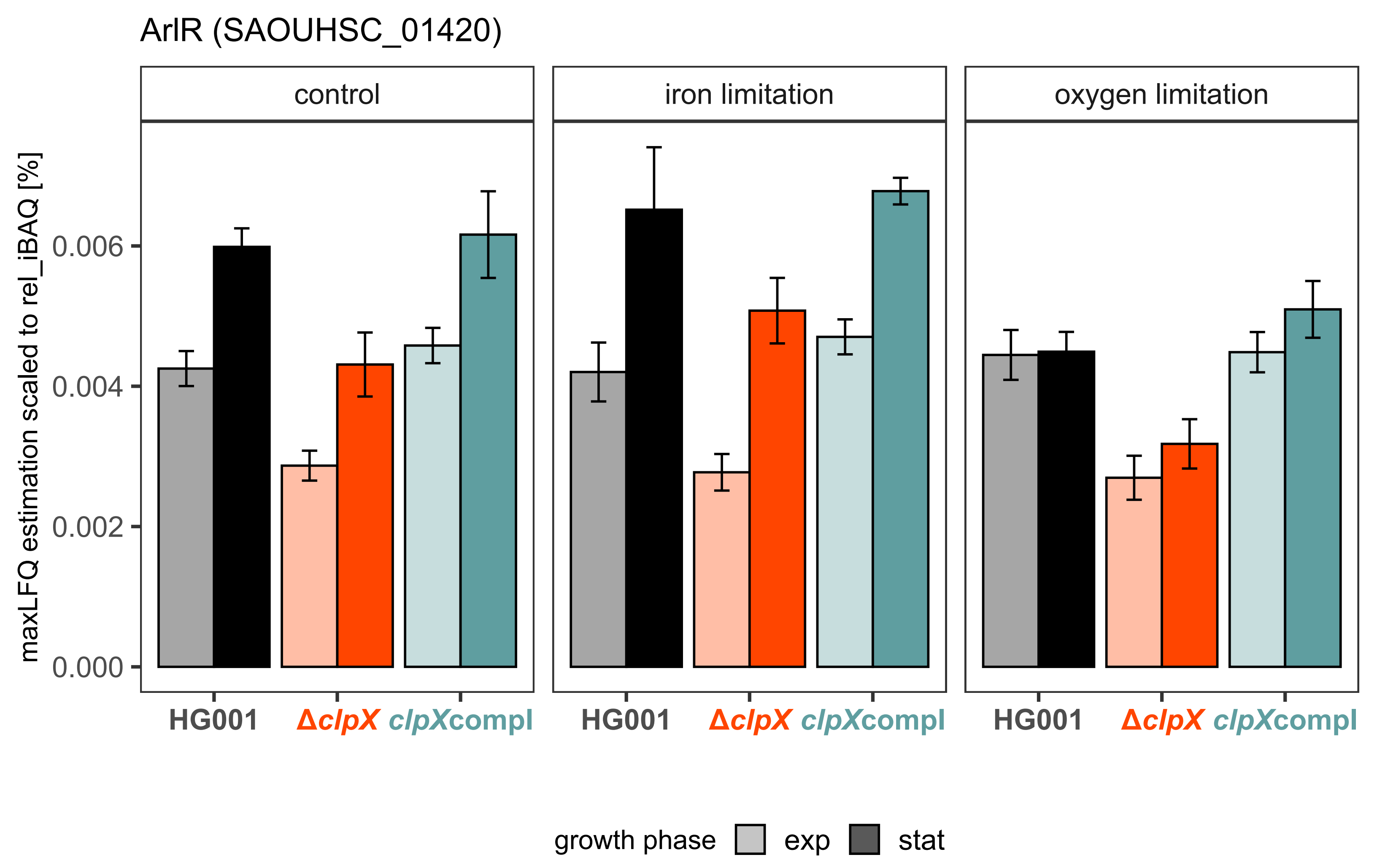

ArlR (SAOUHSC_01420)
control
iron limitation
oxygen limitation
maxLFQ estimation scaled to rel_iBAQ [%]
0.006
0.004
0.002
0.000
HG001
ΔclpX
clpXcompl
growth phase
exp
stat

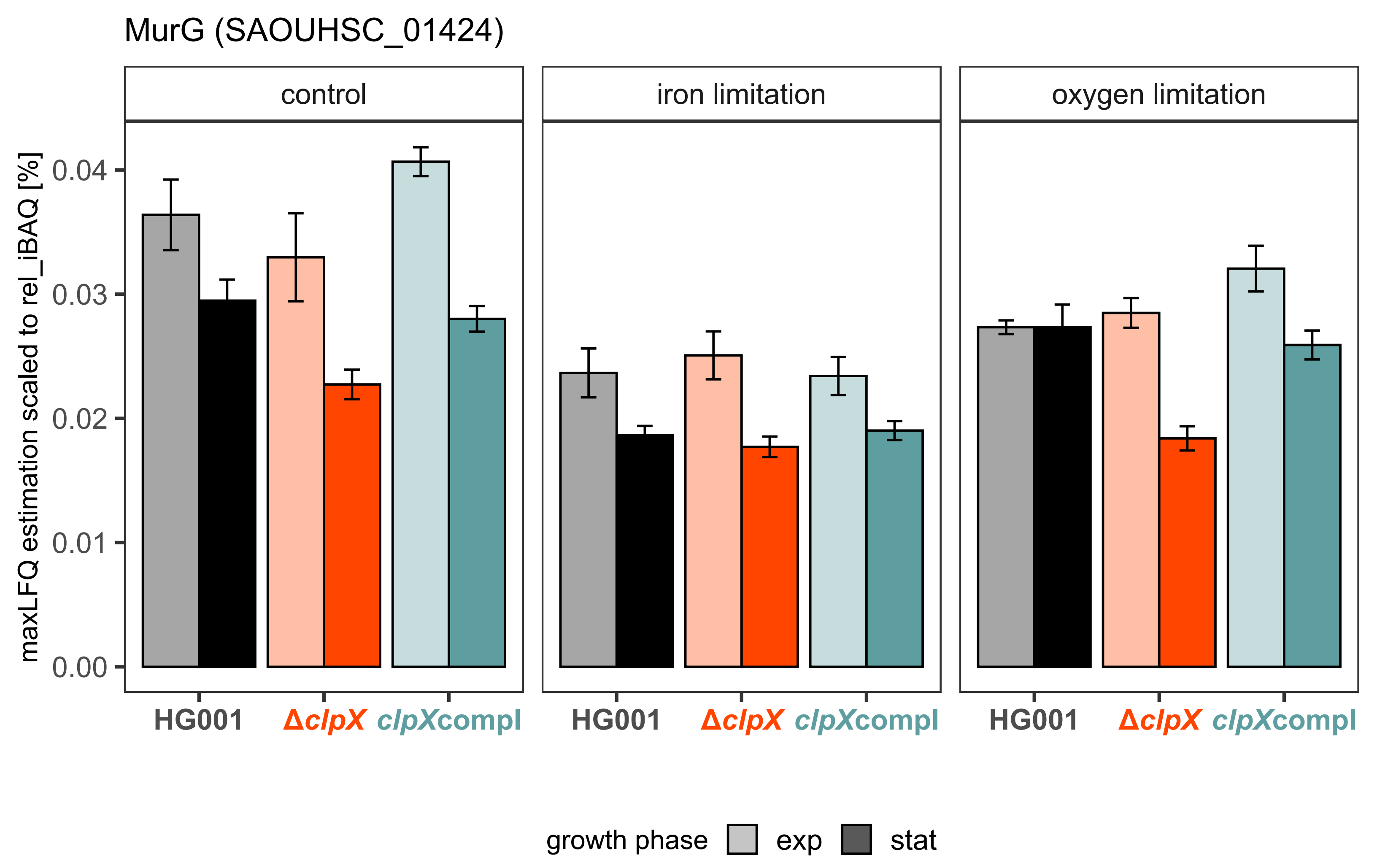

MurG (SAOUHSC_01424)
control
iron limitation
oxygen limitation
maxLFQ estimation scaled to rel_iBAQ [%]
0.04
0.03
0.02
0.01
0.00
HG001
ΔclpX
clpXcompl
growth phase
exp
stat

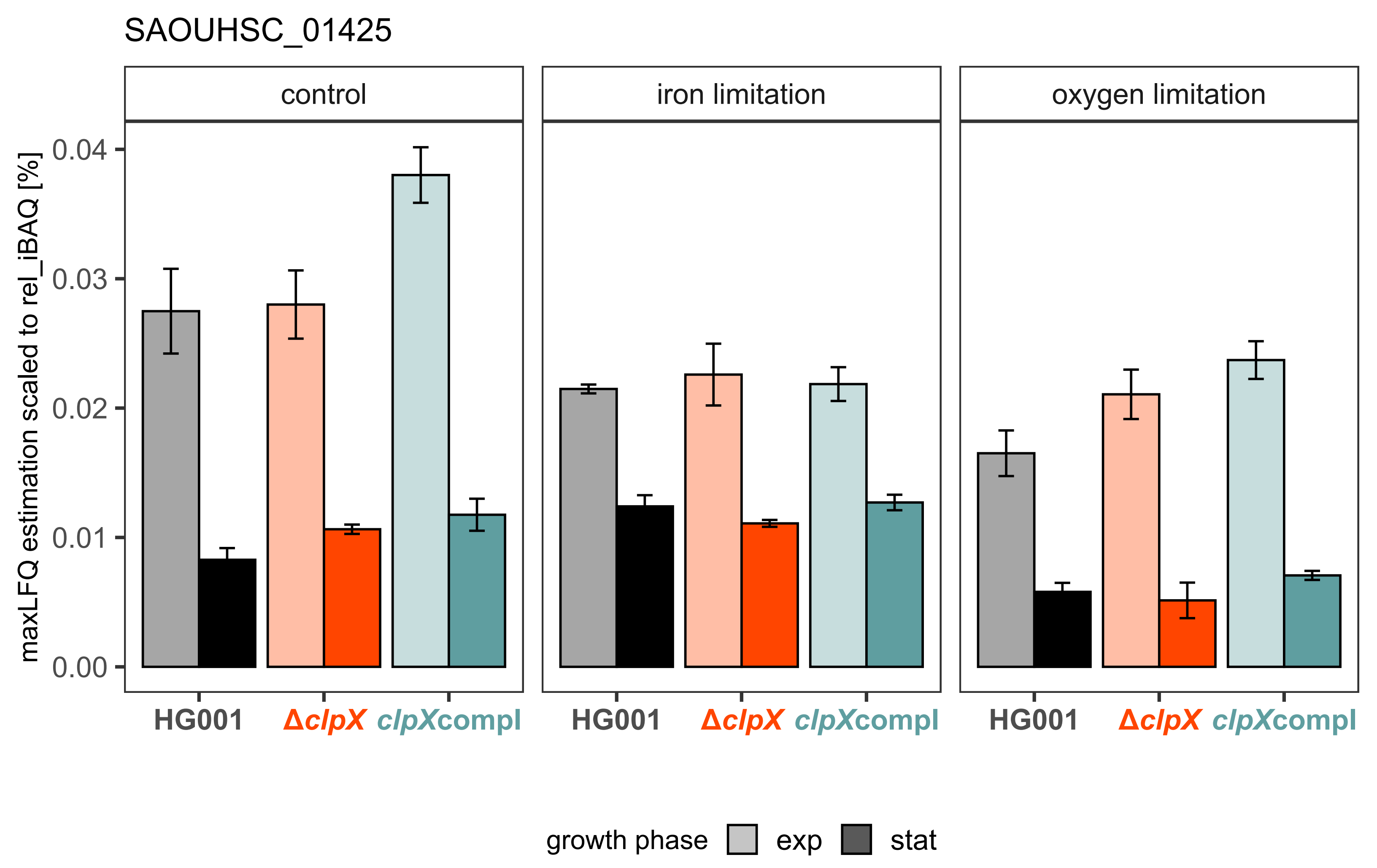
SAOUHSC_01425
control
iron limitation
oxygen limitation
maxLFQ estimation scaled to rel_iBAQ [%]
0.04
0.03
0.02
0.01
0.00
HG001
ΔclpX
clpXcompl
growth phase
exp
stat

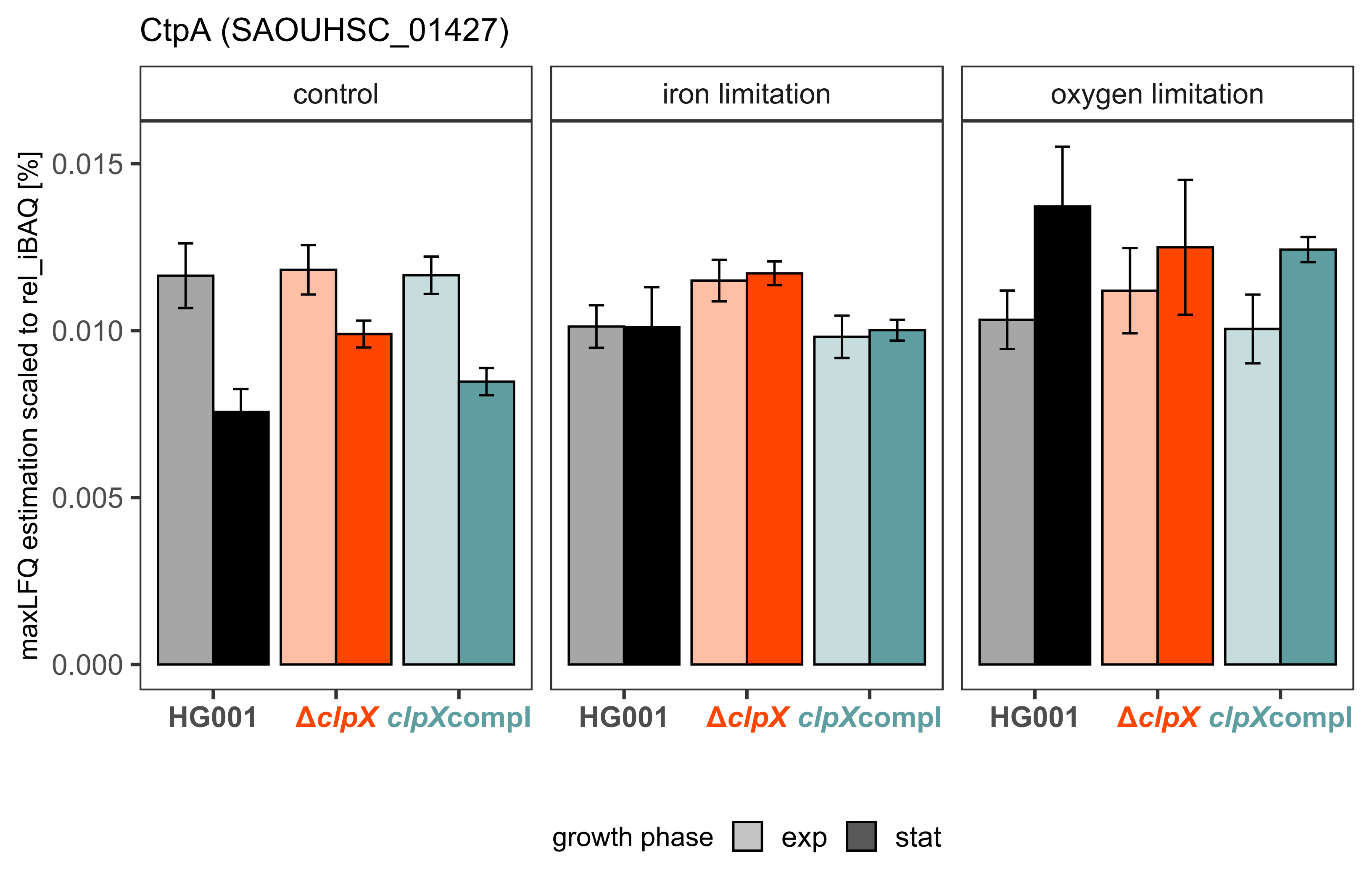

CtpA (SAOUHSC_01427)
control
iron limitation
oxygen limitation
maxLFQ estimation scaled to rel_iBAQ [%]
0.015
0.010
0.005
0.000
HG001
ΔclpX
clpXcompl
growth phase
exp
stat

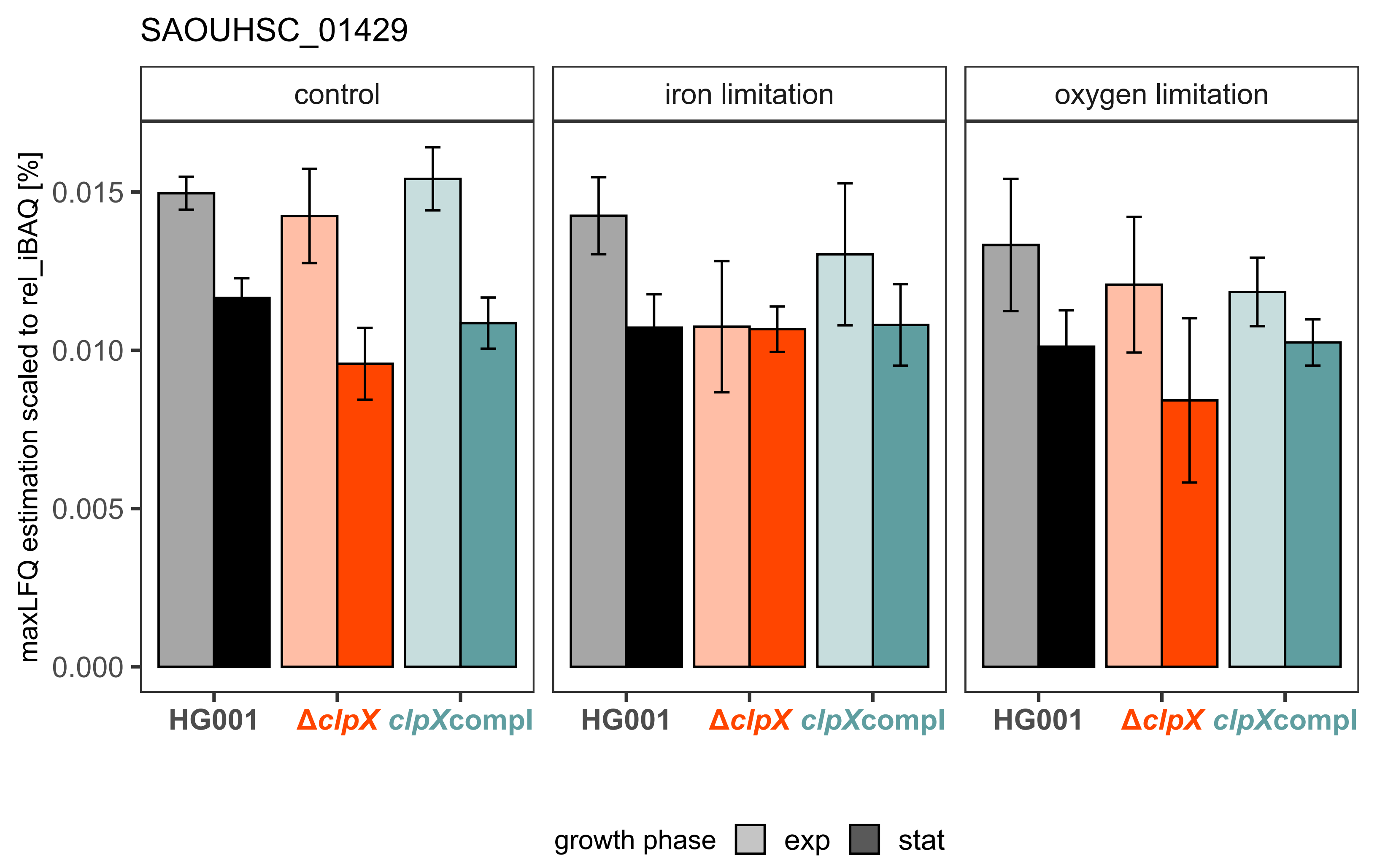
SAOUHSC_01429
control
iron limitation
oxygen limitation
maxLFQ estimation scaled to rel_iBAQ [%]
0.015
0.010
0.005
0.000
HG001
ΔclpX
clpXcompl
growth phase
exp
stat

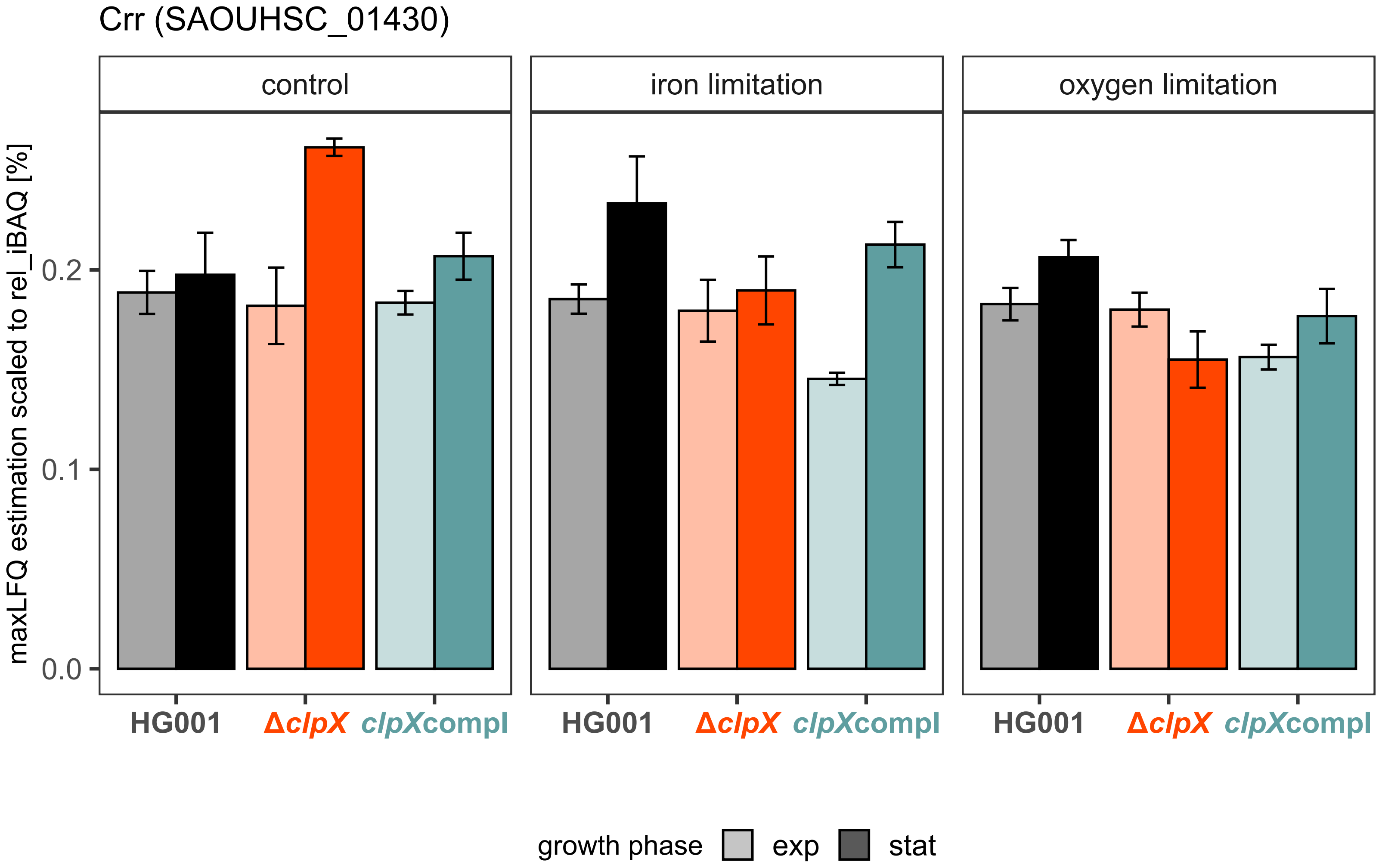
Crr (SAOUHSC_01430)
control
iron limitation
oxygen limitation
maxLFQ estimation scaled to rel_iBAQ [%]
0.0
0.1
0.2
HG001
ΔclpX
clpXcompl
growth phase
exp
stat

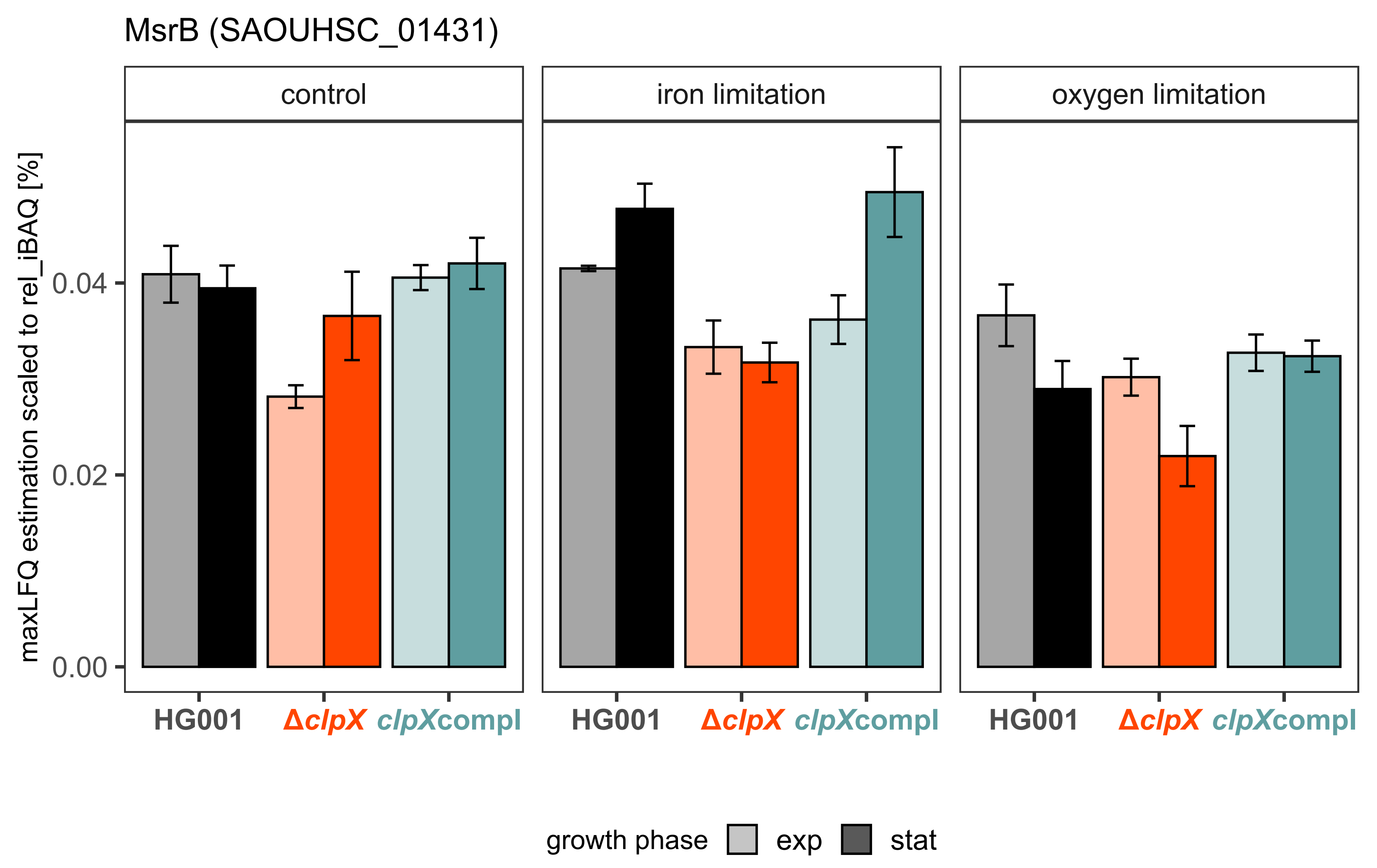
MsrB (SAOUHSC_01431)
control
iron limitation
oxygen limitation
maxLFQ estimation scaled to rel_iBAQ [%]
0.00
0.02
0.04
HG001
ΔclpX
clpXcompl
growth phase
exp
stat

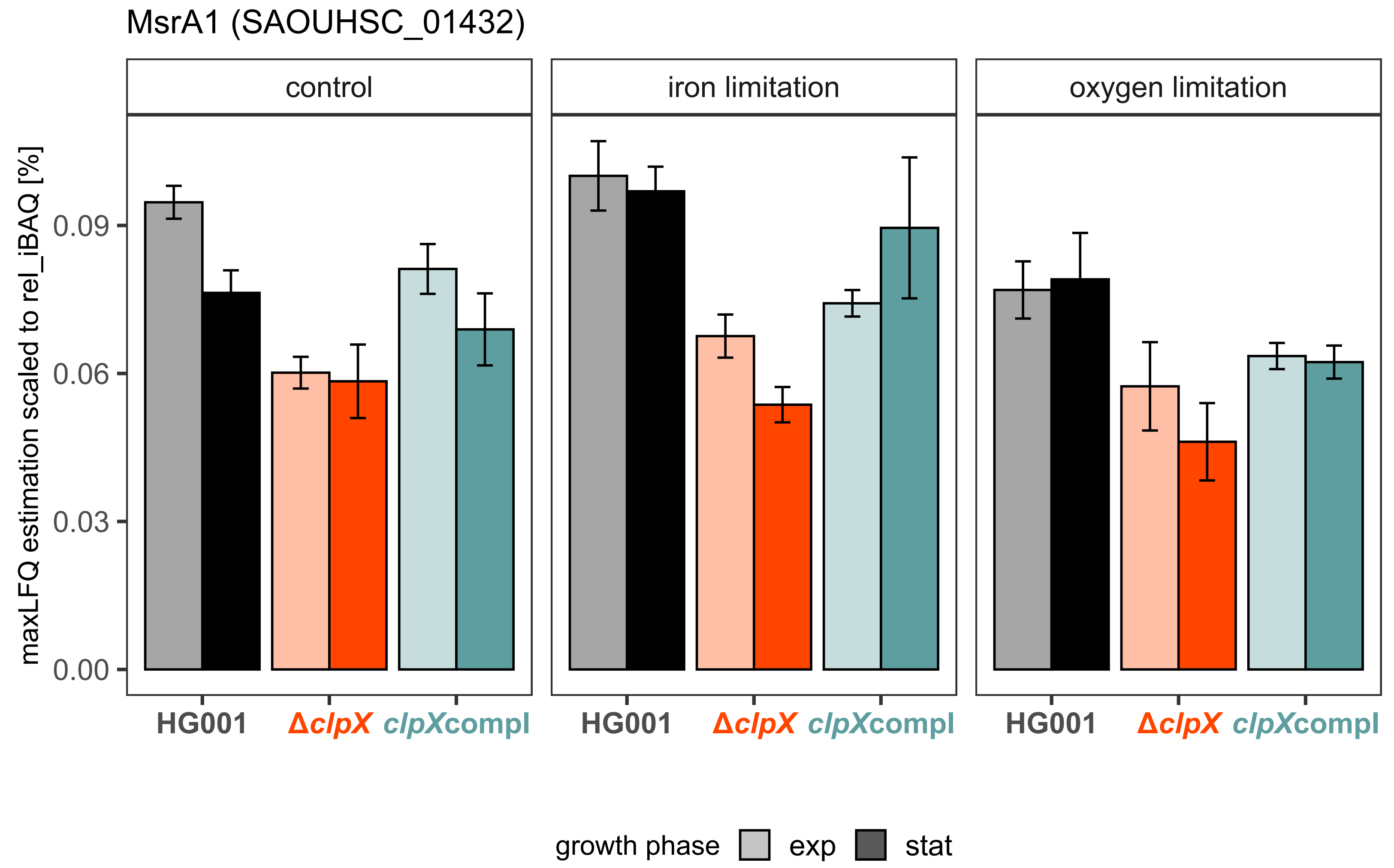

MsrA1 (SAOUHSC_01432)
control
iron limitation
oxygen limitation
maxLFQ estimation scaled to rel_iBAQ [%]
0.00
0.03
0.06
0.09
HG001
ΔclpX
clpXcompl
growth phase
exp
stat

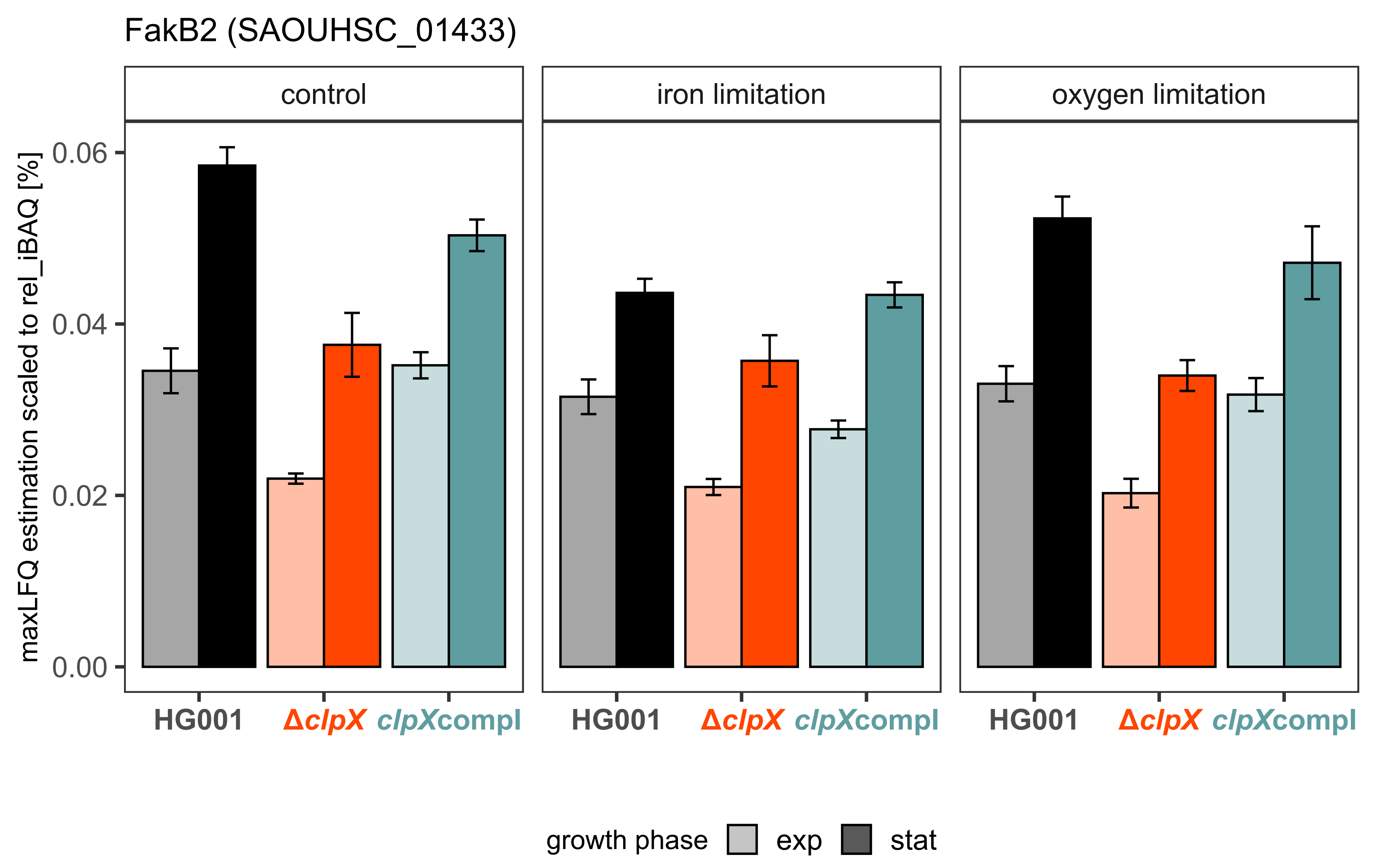
FakB2 (SAOUHSC_01433)
control
iron limitation
oxygen limitation
maxLFQ estimation scaled to rel_iBAQ [%]
0.06
0.04
0.02
0.00
HG001
ΔclpX
clpXcompl
growth phase
exp
stat

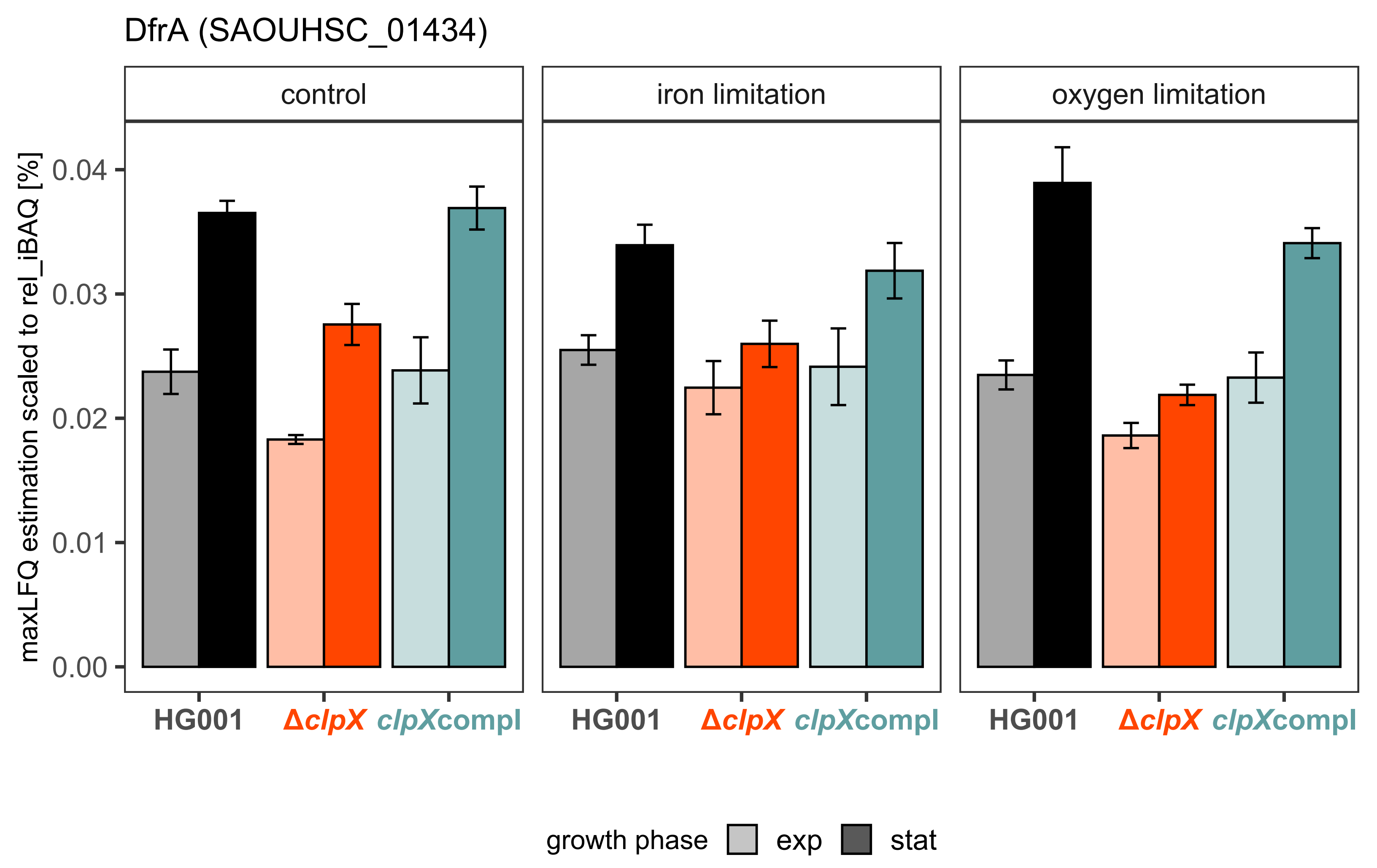
DfrA (SAOUHSC_01434)
control
iron limitation
oxygen limitation
maxLFQ estimation scaled to rel_iBAQ [%]
0.00
0.01
0.02
0.03
0.04
HG001
ΔclpX
clpXcompl
growth phase
exp
stat

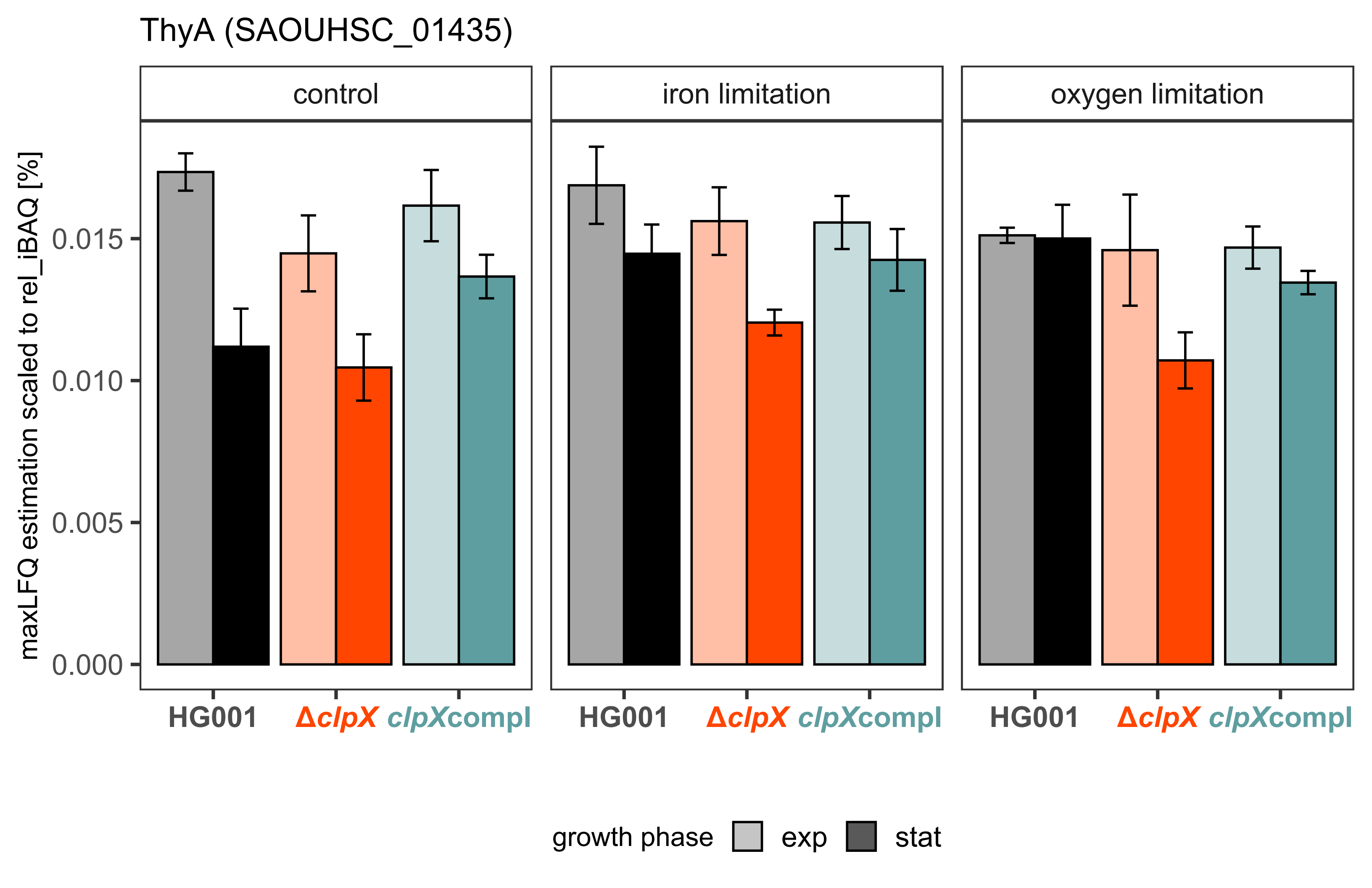

ThyA (SAOUHSC_01435)
control
iron limitation
oxygen limitation
maxLFQ estimation scaled to rel_iBAQ [%]
0.000
0.005
0.010
0.015
HG001
ΔclpX
clpXcompl
growth phase
exp
stat

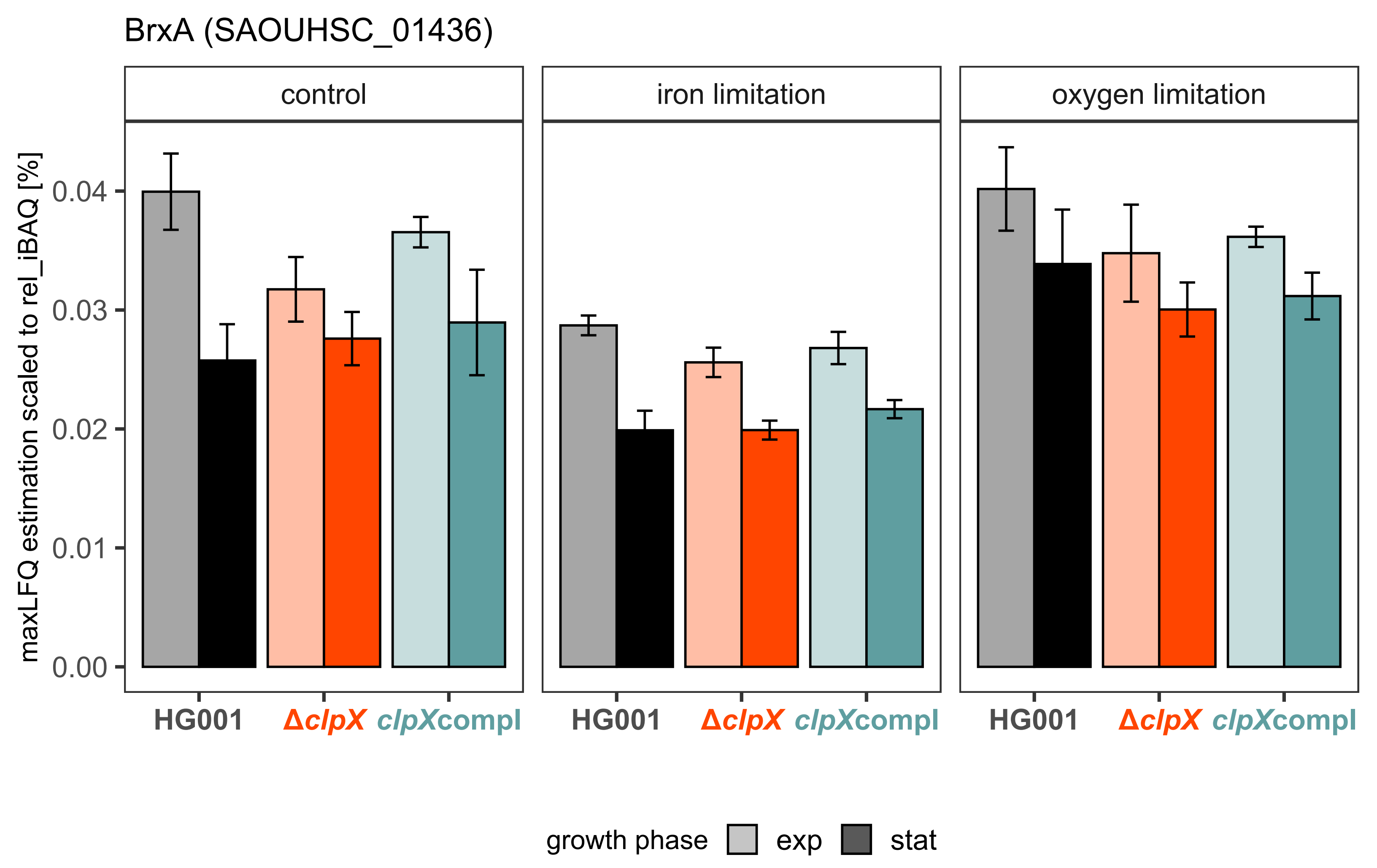

BrxA (SAOUHSC_01436)
control
iron limitation
oxygen limitation
maxLFQ estimation scaled to rel_iBAQ [%]
0.00
0.01
0.02
0.03
0.04
HG001
ΔclpX
clpXcompl
growth phase
exp
stat

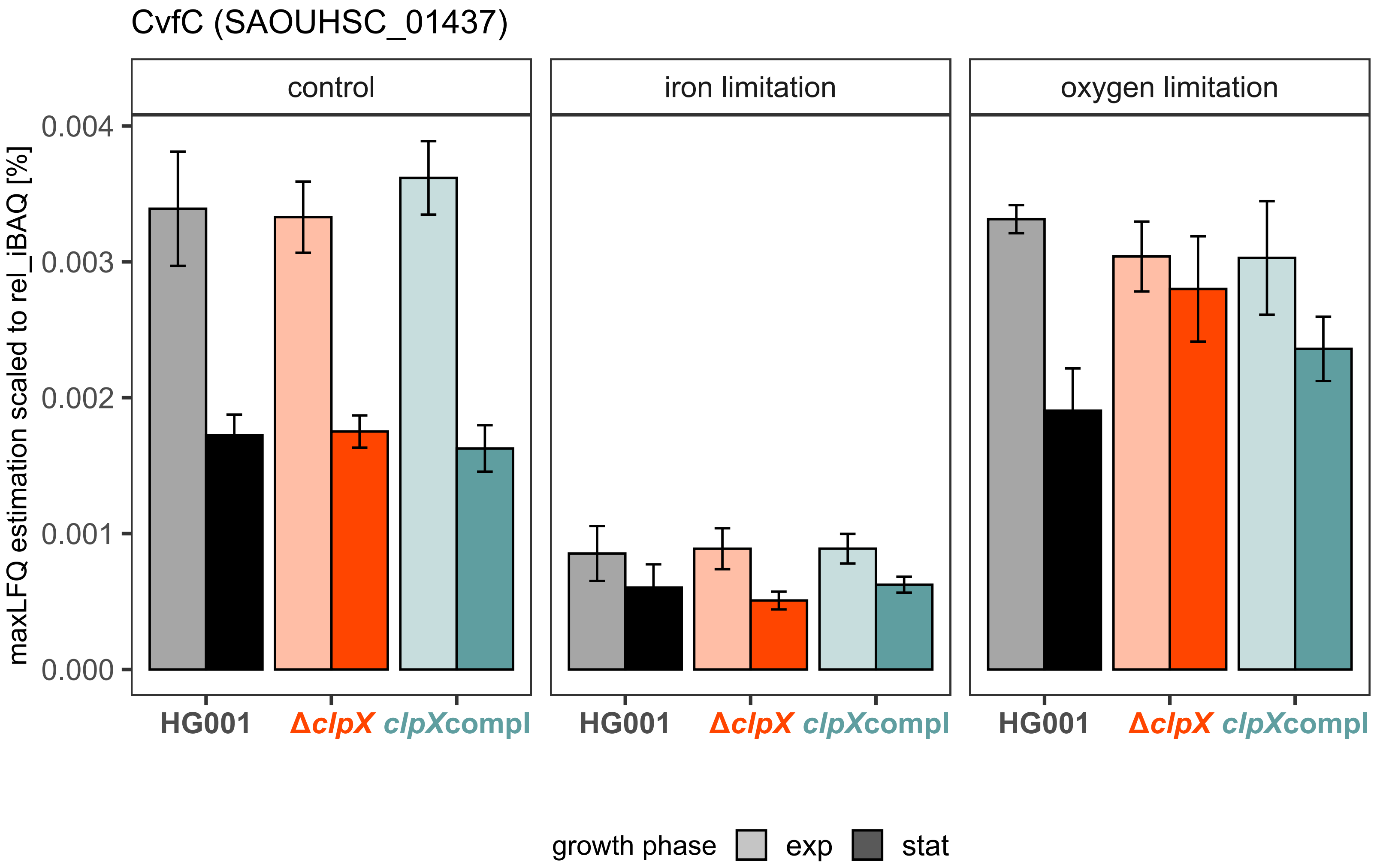
CvfC (SAOUHSC_01437)
control
iron limitation
oxygen limitation
maxLFQ estimation scaled to rel_iBAQ [%]
0.004
0.003
0.002
0.001
0.000
HG001
ΔclpX
clpXcompl
growth phase
exp
stat

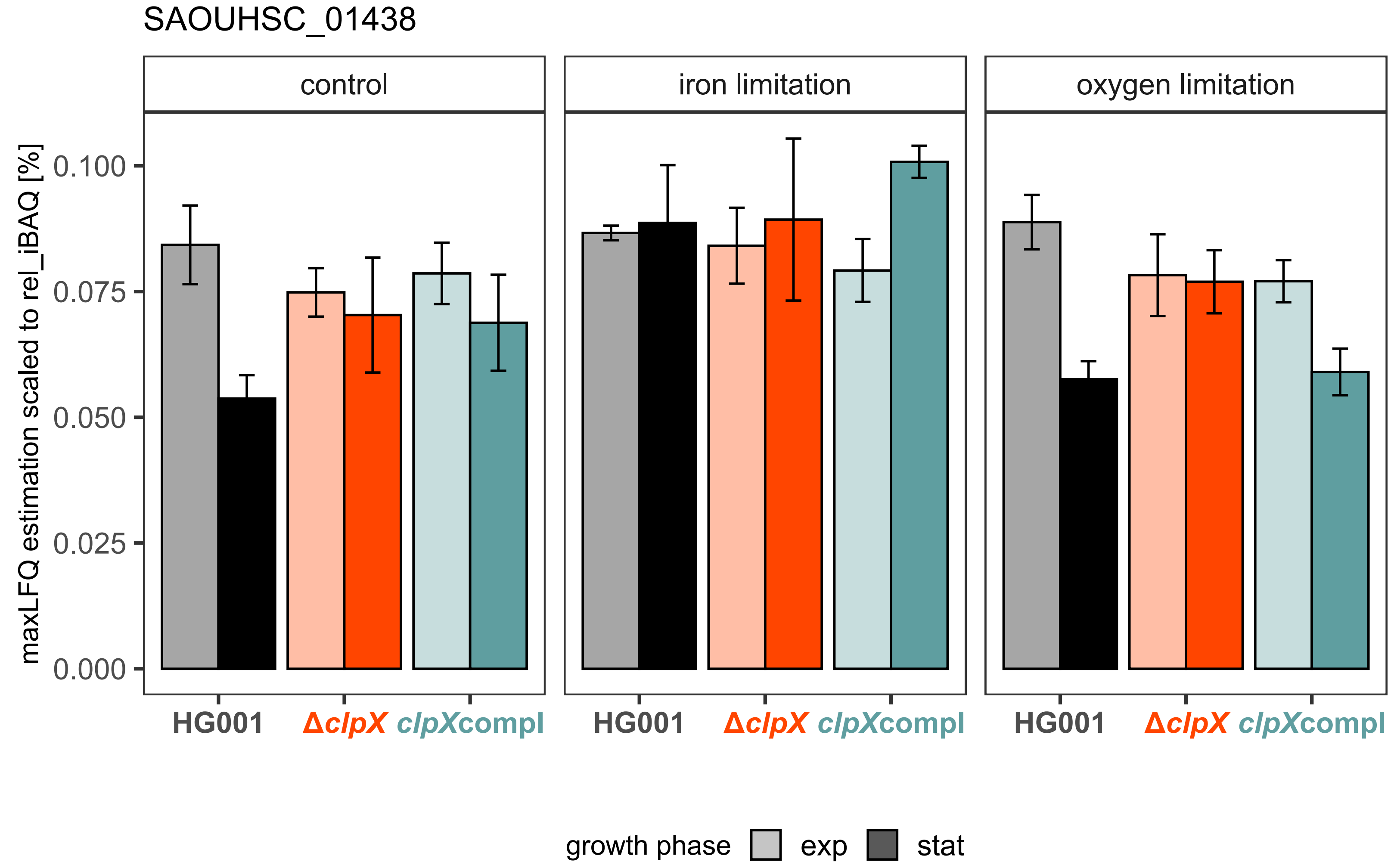
SAOUHSC_01438
control
iron limitation
oxygen limitation
maxLFQ estimation scaled to rel_iBAQ [%]
0.100
0.075
0.050
0.025
0.000
HG001
ΔclpX
clpXcompl
growth phase
exp
stat

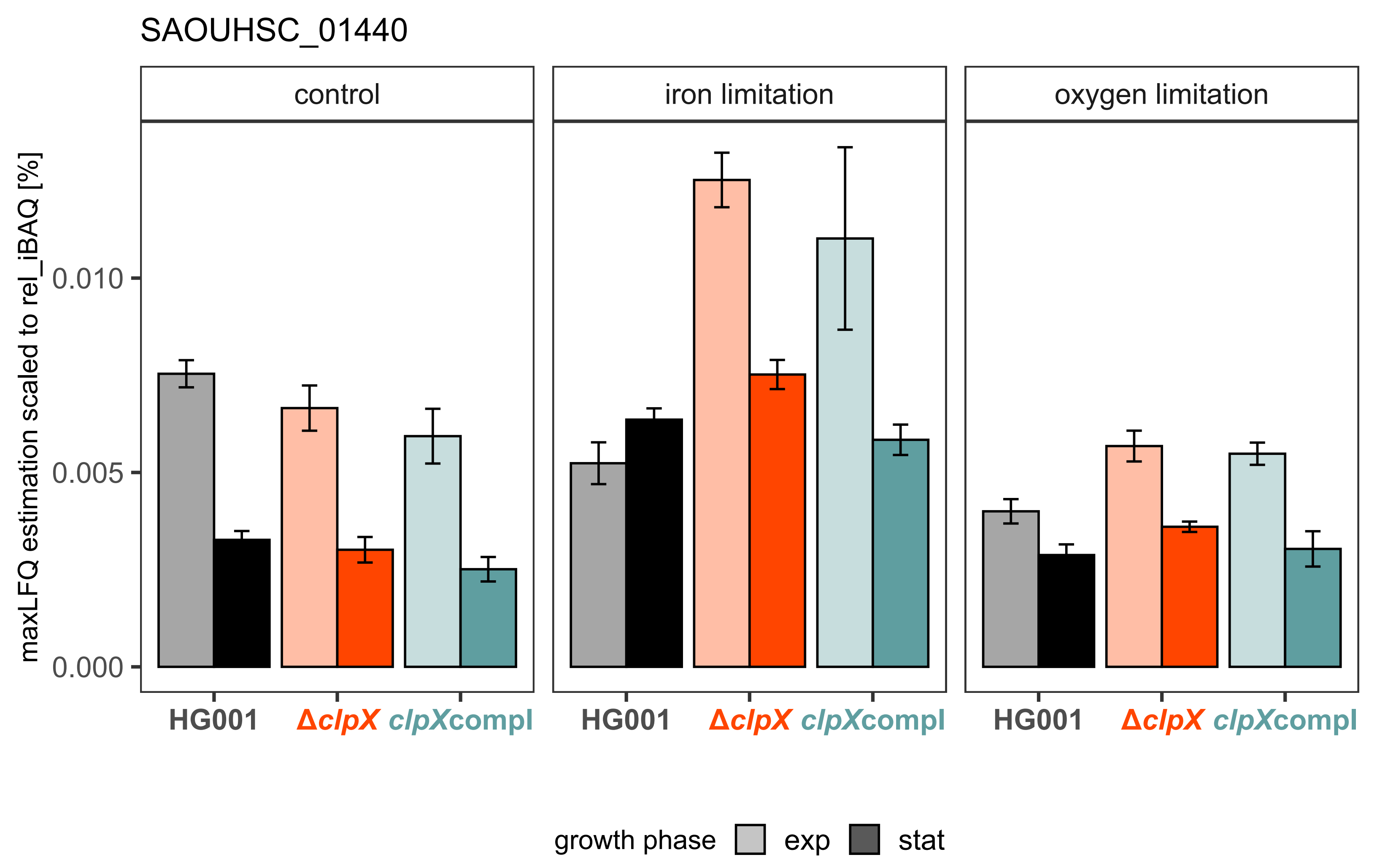

SAOUHSC_01440
control
iron limitation
oxygen limitation
maxLFQ estimation scaled to rel_iBAQ [%]
0.010
0.005
0.000
HG001
ΔclpX
clpXcompl
growth phase
exp
stat

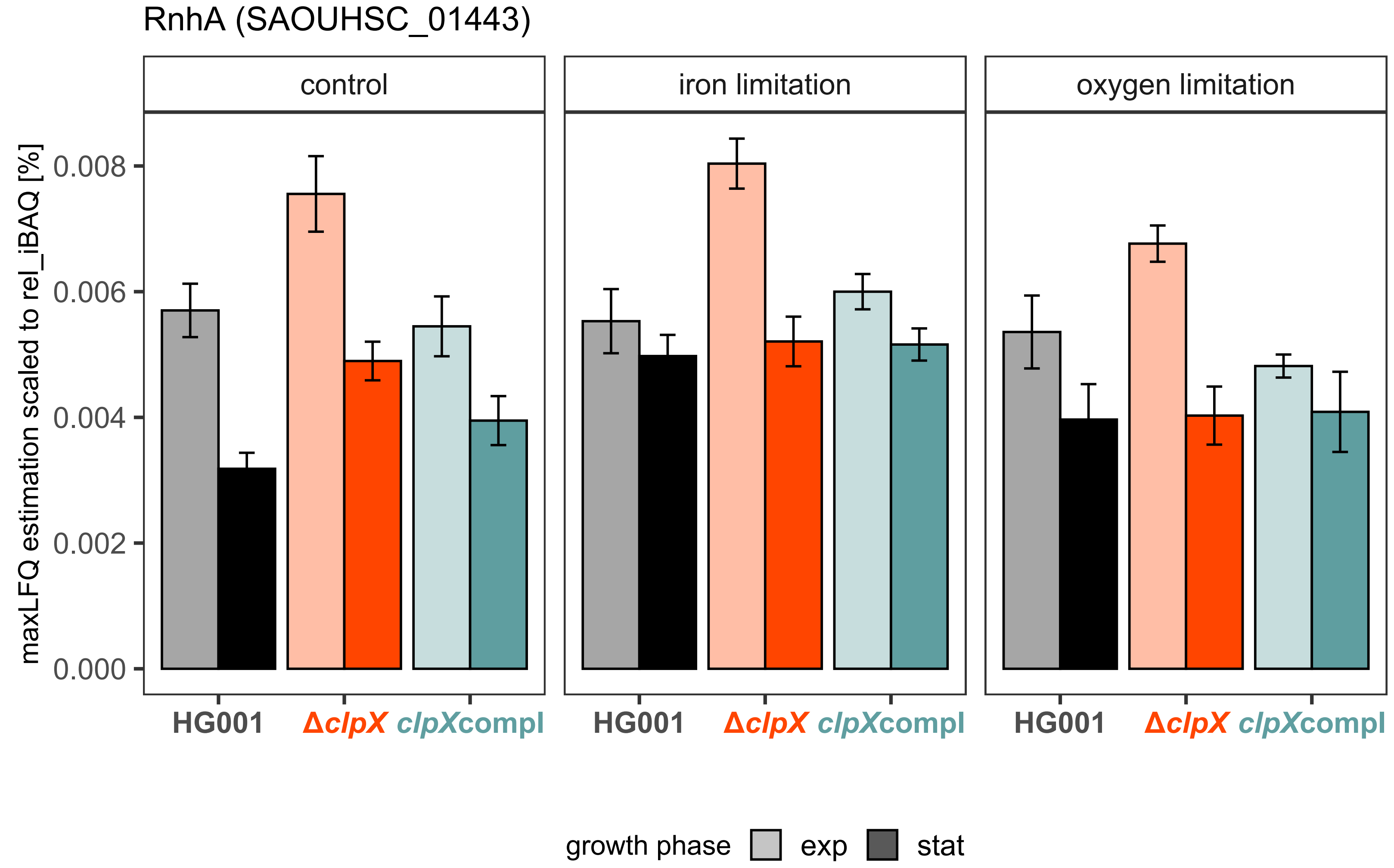
RnhA (SAOUHSC_01443)
control
iron limitation
oxygen limitation
maxLFQ estimation scaled to rel_iBAQ [%]
0.008
0.006
0.004
0.002
0.000
HG001
ΔclpX
clpXcompl
growth phase
exp
stat

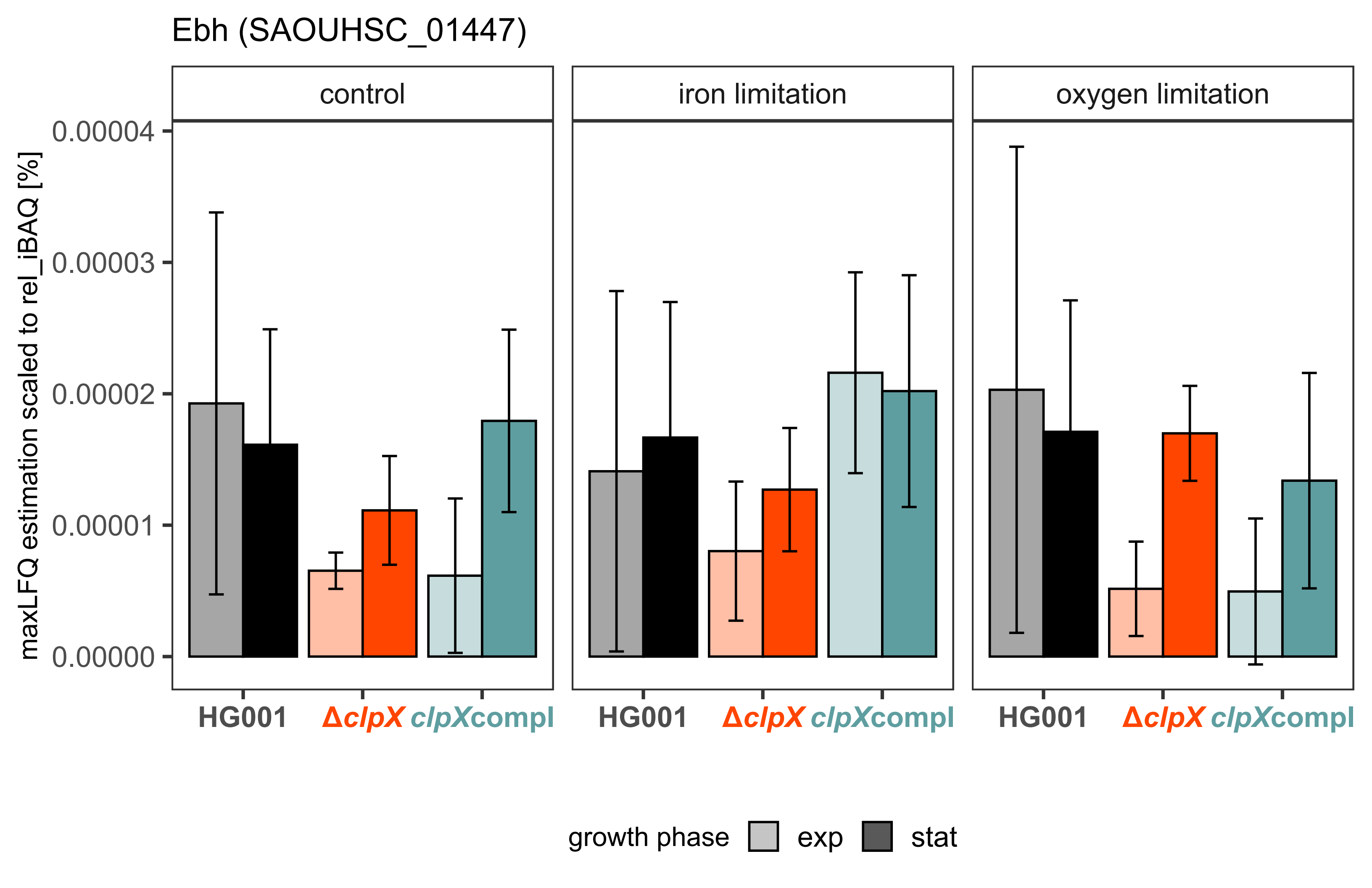
Ebh (SAOUHSC_01447)
control
iron limitation
oxygen limitation
maxLFQ estimation scaled to rel_iBAQ [%]
0.00004
0.00003
0.00002
0.00001
0.00000
HG001
ΔclpX
clpXcompl
growth phase
exp
stat

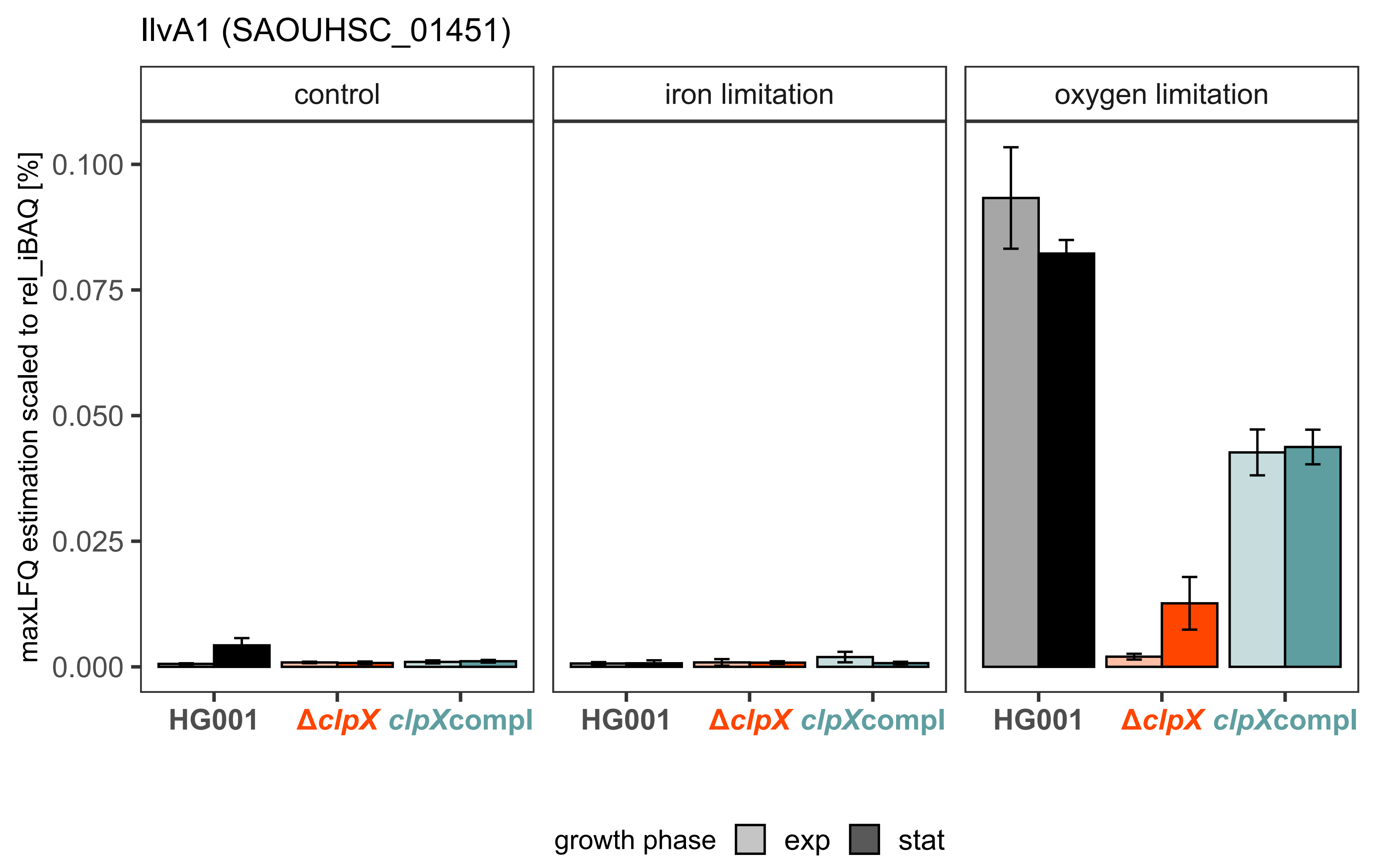
IlvA1 (SAOUHSC_01451)
control
iron limitation
oxygen limitation
maxLFQ estimation scaled to rel_iBAQ [%]
0.100
0.075
0.050
0.025
0.000
HG001
ΔclpX
clpXcompl
growth phase
exp
stat

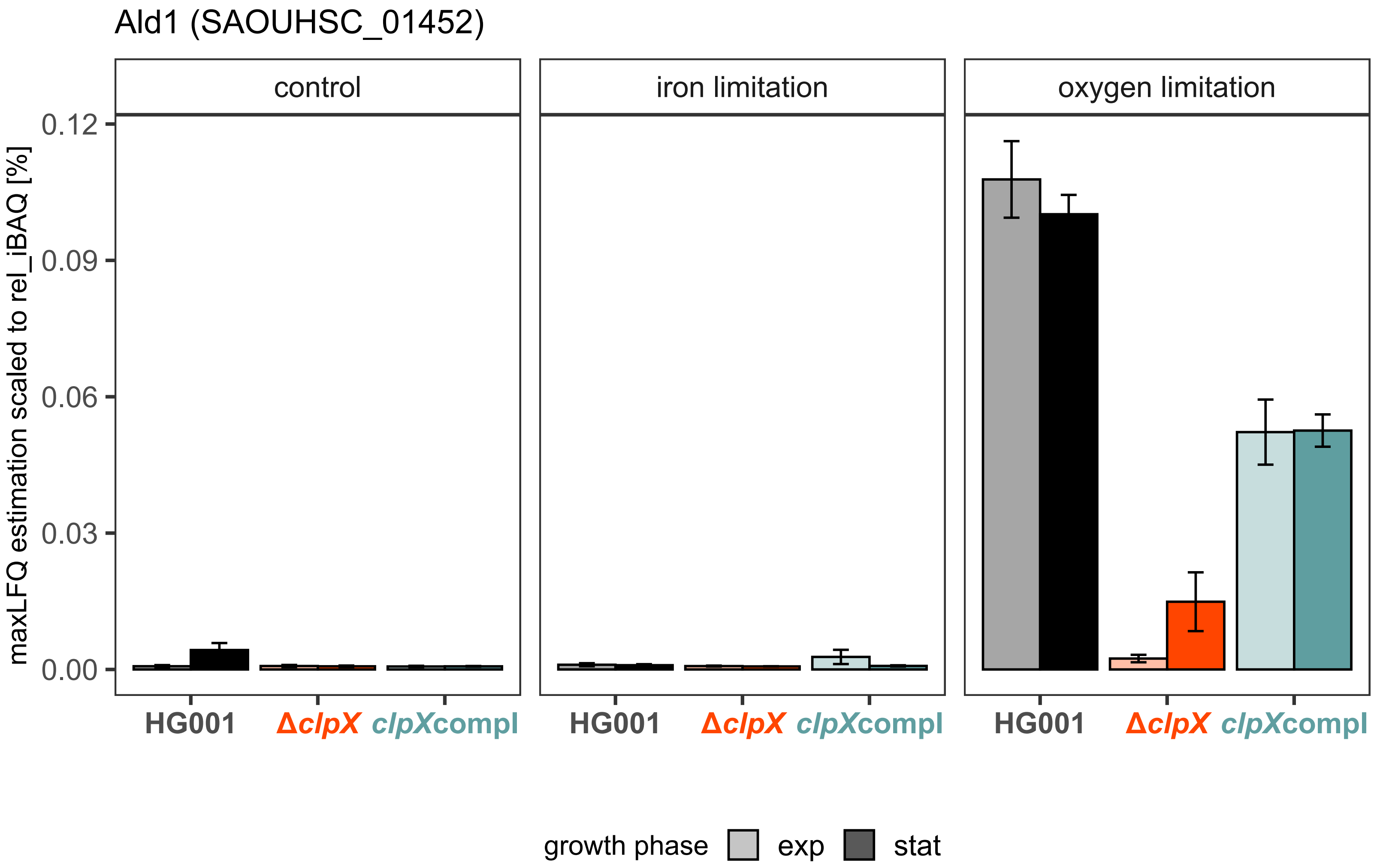
Ald1 (SAOUHSC_01452)
control
iron limitation
oxygen limitation
maxLFQ estimation scaled to rel_iBAQ [%]
0.12
0.09
0.06
0.03
0.00
HG001
ΔclpX
clpXcompl
growth phase
exp
stat

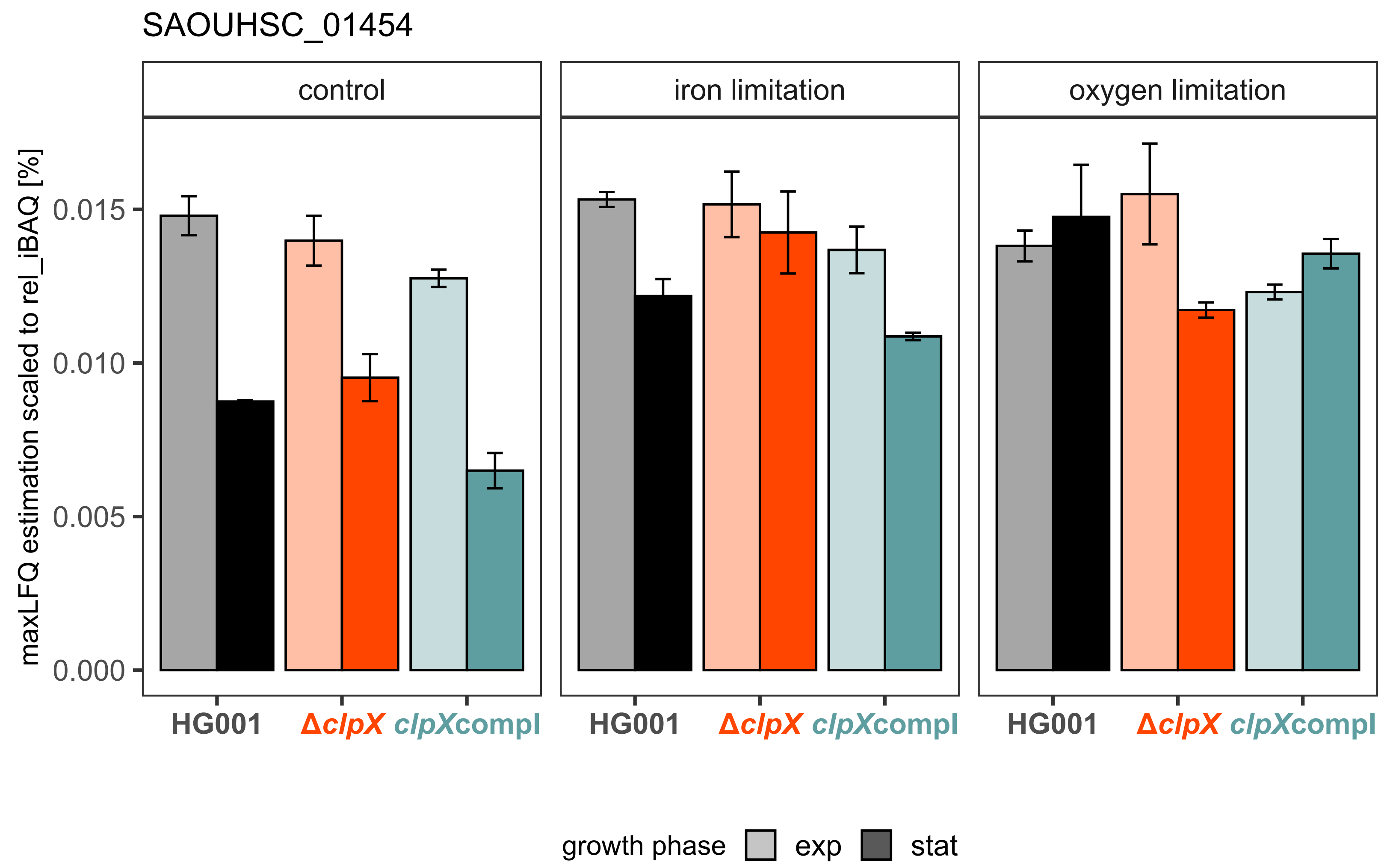

SAOUHSC_01454
control
iron limitation
oxygen limitation
maxLFQ estimation scaled to rel_iBAQ [%]
0.015
0.010
0.005
0.000
HG001
ΔclpX
clpXcompl
growth phase
exp
stat

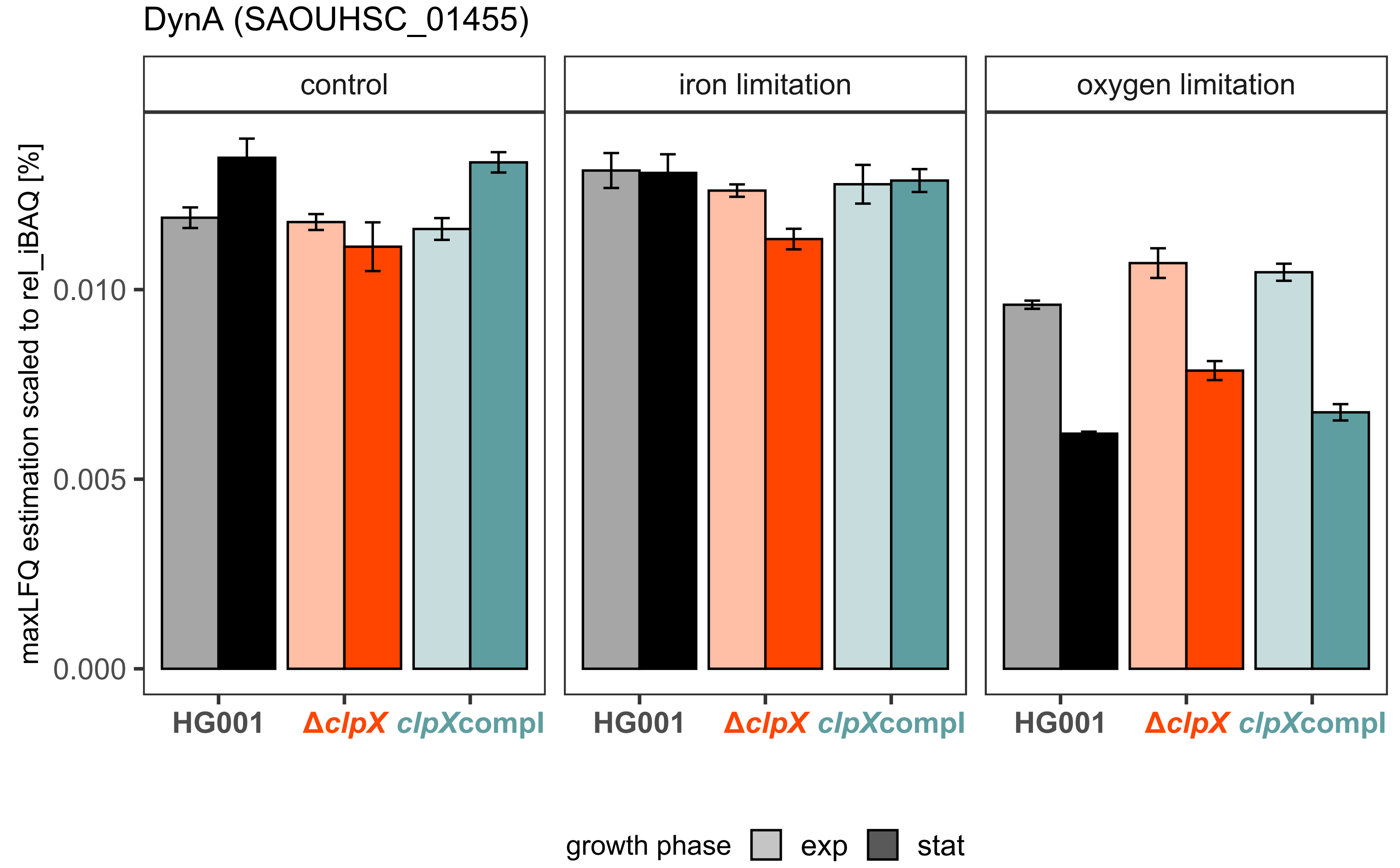
DynA (SAOUHSC_01455)
control
iron limitation
oxygen limitation
maxLFQ estimation scaled to rel_iBAQ [%]
0.010
0.005
0.000
HG001
ΔclpX
clpXcompl
growth phase
exp
stat

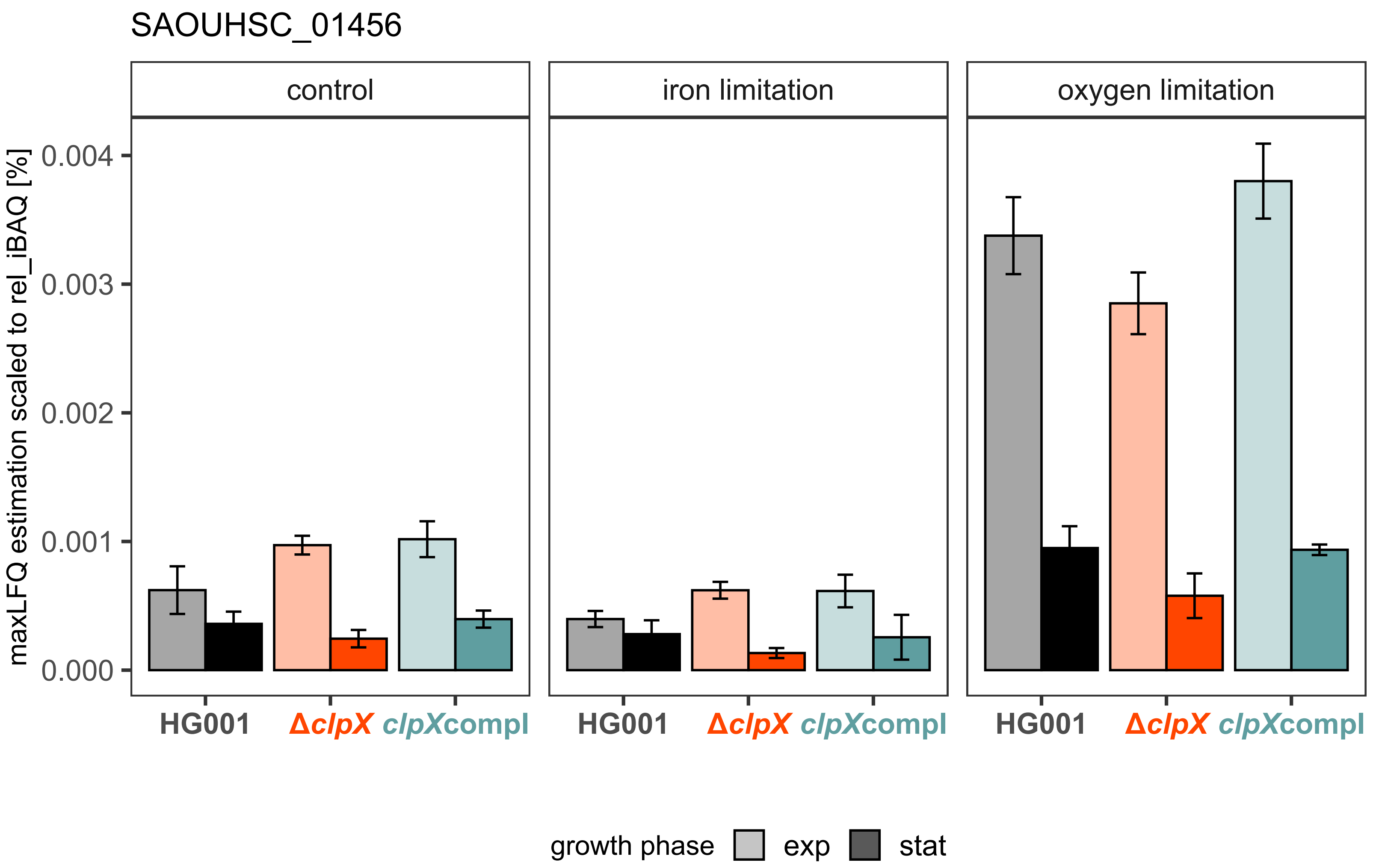
SAOUHSC_01456
control
iron limitation
oxygen limitation
maxLFQ estimation scaled to rel_iBAQ [%]
0.004
0.003
0.002
0.001
0.000
HG001
ΔclpX
clpXcompl
growth phase
exp
stat

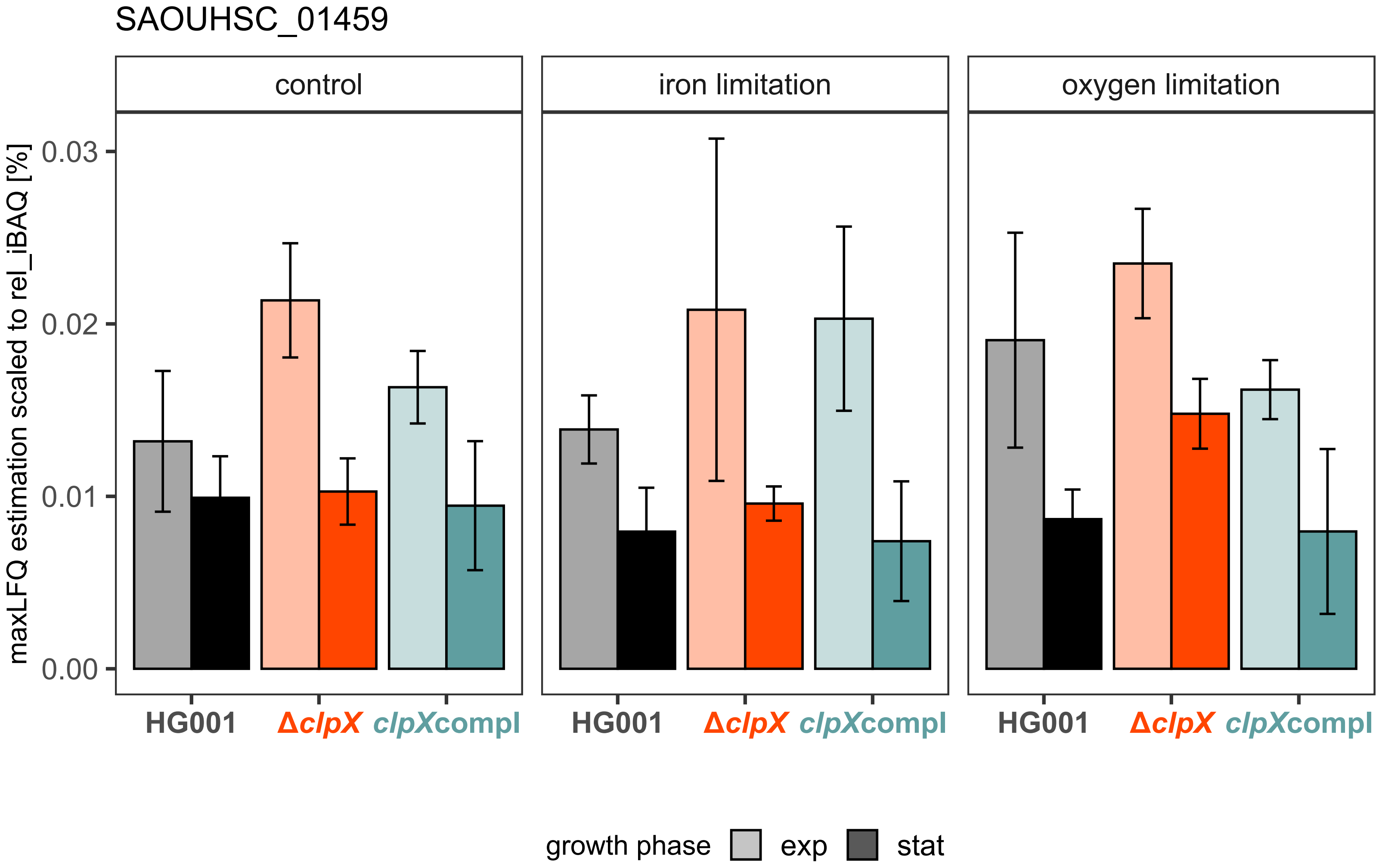
SAOUHSC_01459
control
iron limitation
oxygen limitation
maxLFQ estimation scaled to rel_iBAQ [%]
0.03
0.02
0.01
0.00
HG001
ΔclpX
clpXcompl
growth phase
exp
stat

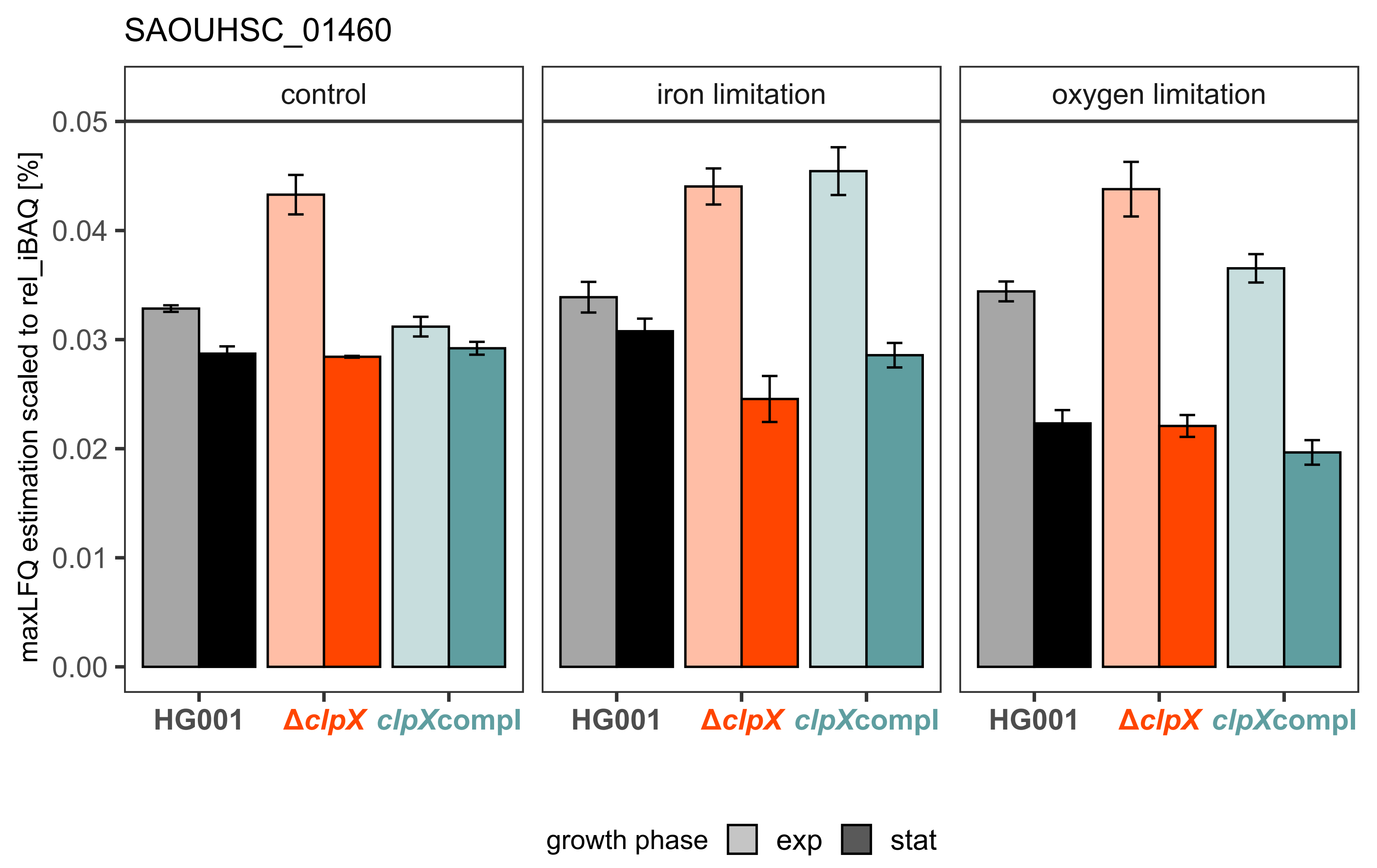

SAOUHSC_01460
control
iron limitation
oxygen limitation
0.05
0.04
0.03
0.02
0.01
0.00
maxLFQ estimation scaled to rel_iBAQ [%]
HG001
ΔclpX
clpXcompl
growth phase
exp
stat

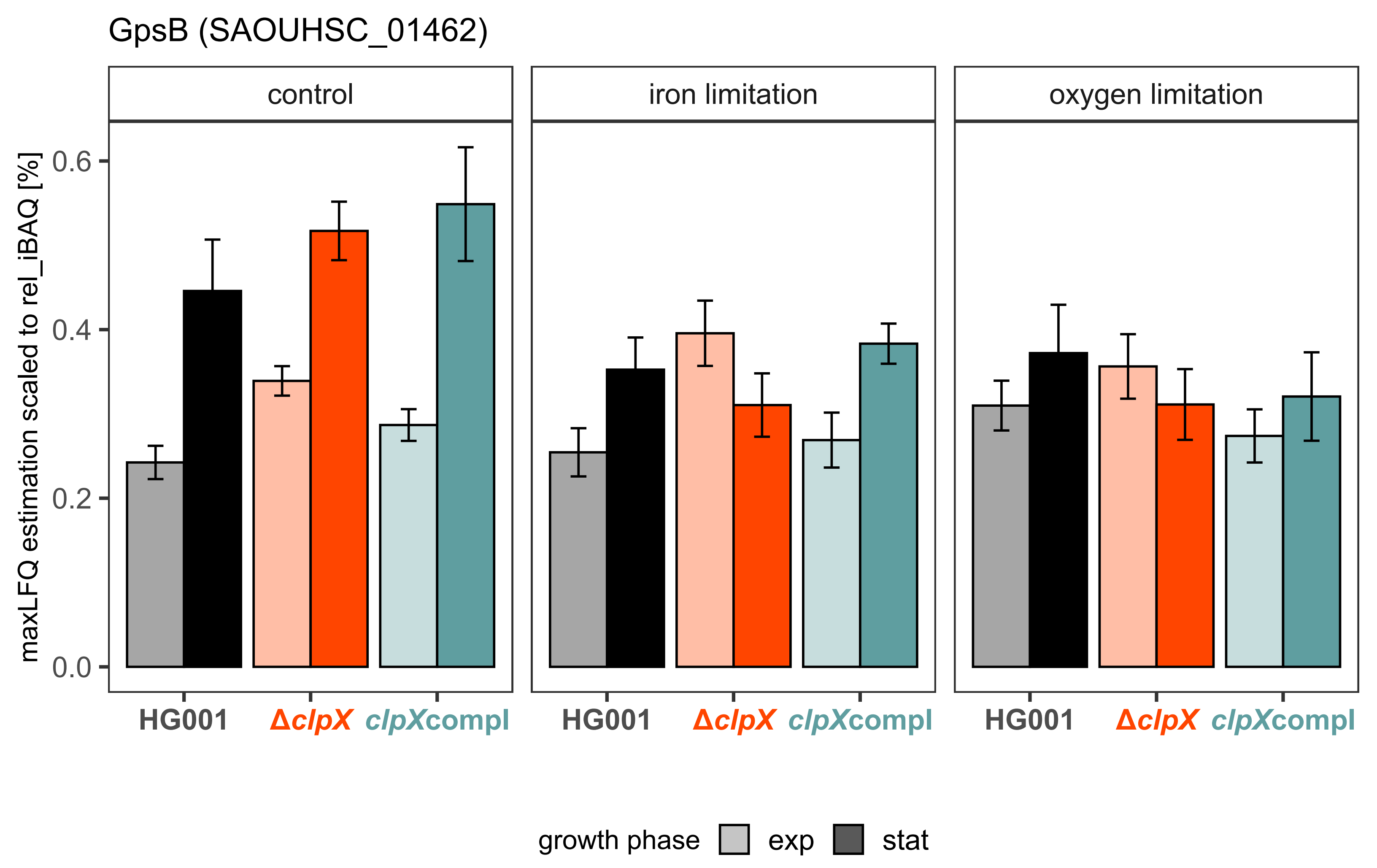

GpsB (SAOUHSC_01462)
control
iron limitation
oxygen limitation
maxLFQ estimation scaled to rel_iBAQ [%]
0.6
0.4
0.2
0.0
HG001
ΔclpX
clpXcompl
growth phase
exp
stat

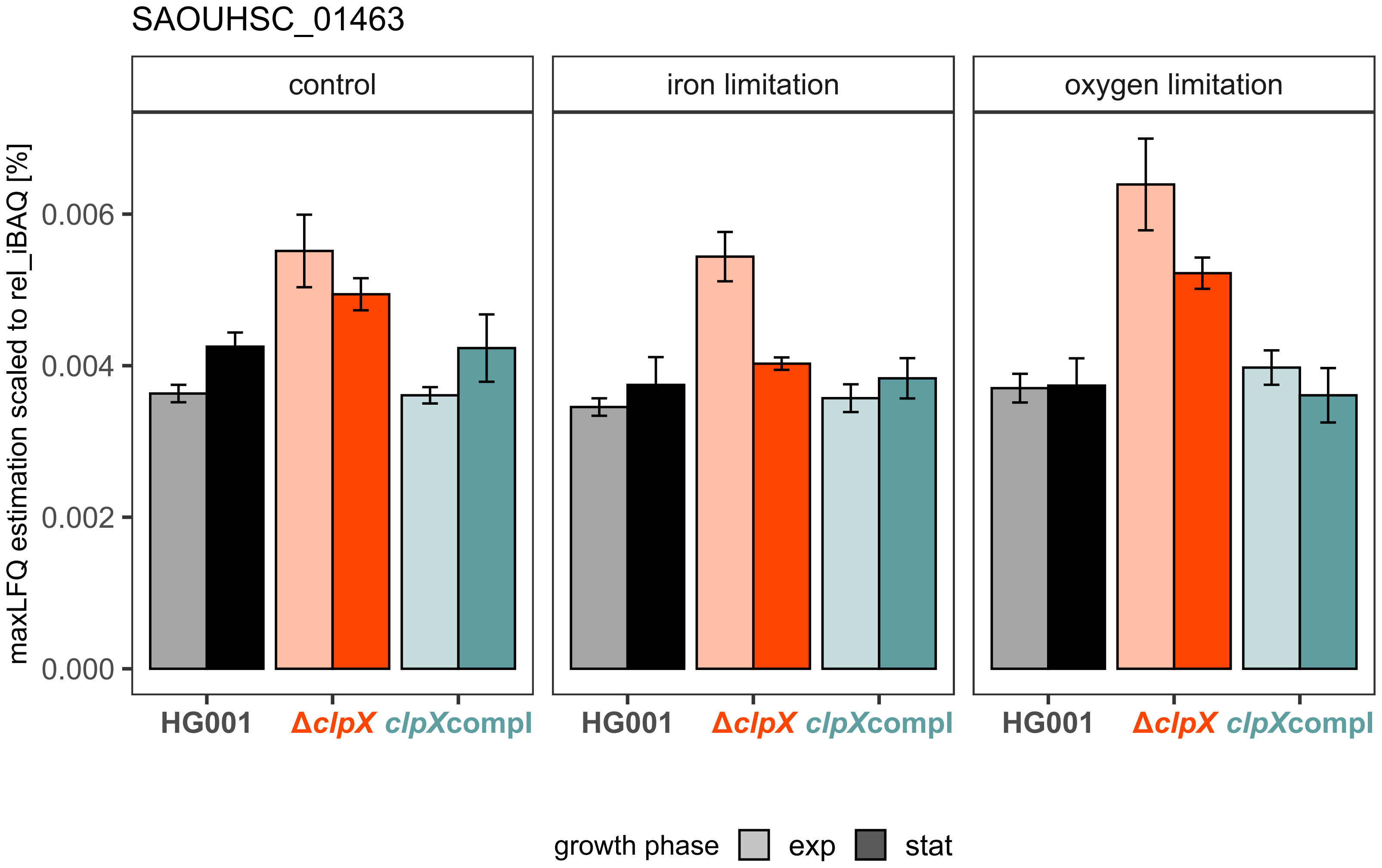
SAOUHSC_01463
control
iron limitation
oxygen limitation
maxLFQ estimation scaled to rel_iBAQ [%]
0.006
0.004
0.002
0.000
HG001
ΔclpX
clpXcompl
growth phase
exp
stat

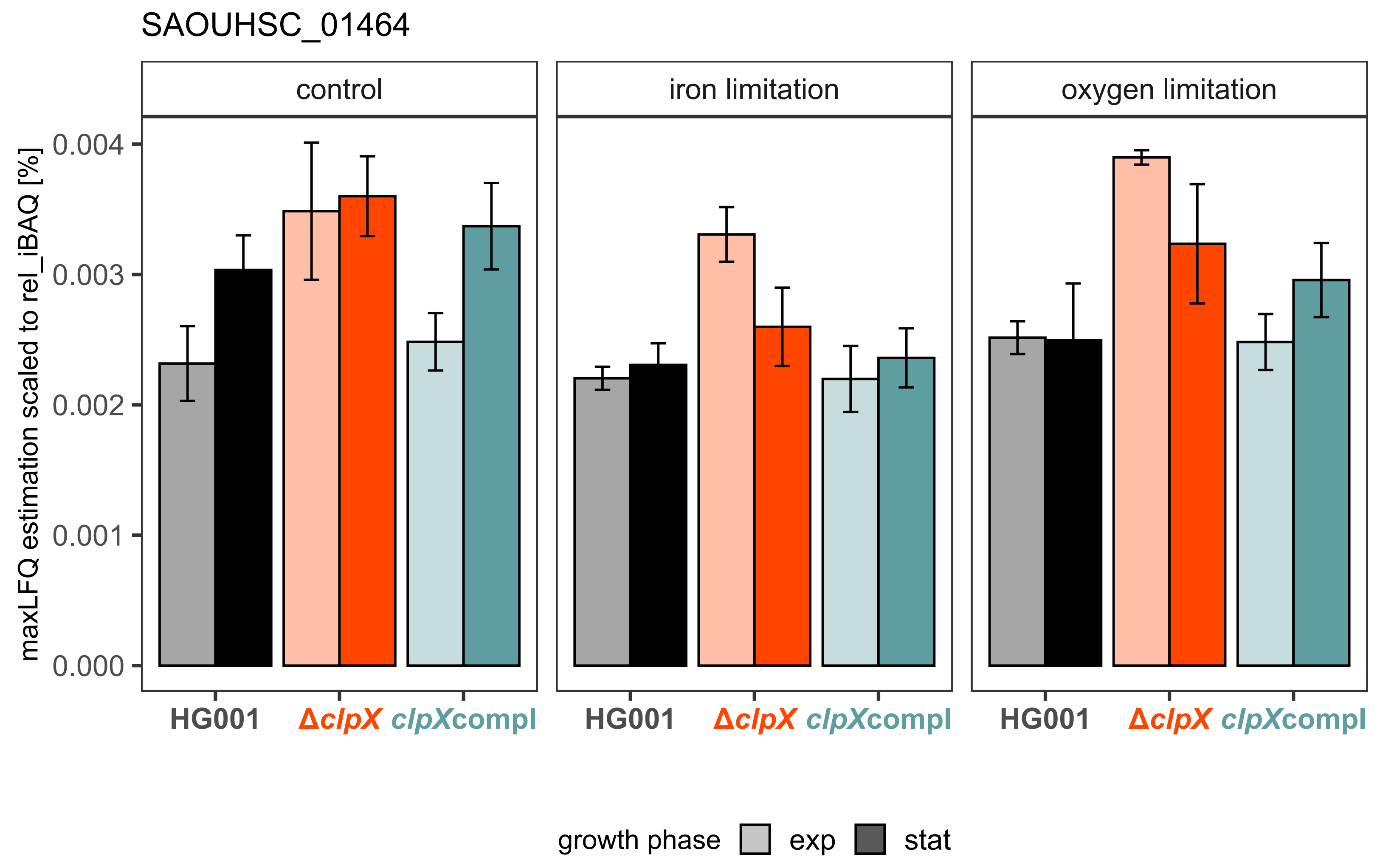
SAOUHSC_01464
control
iron limitation
oxygen limitation
maxLFQ estimation scaled to rel_iBAQ [%]
0.004
0.003
0.002
0.001
0.000
HG001
ΔclpX
clpXcompl
growth phase
exp
stat

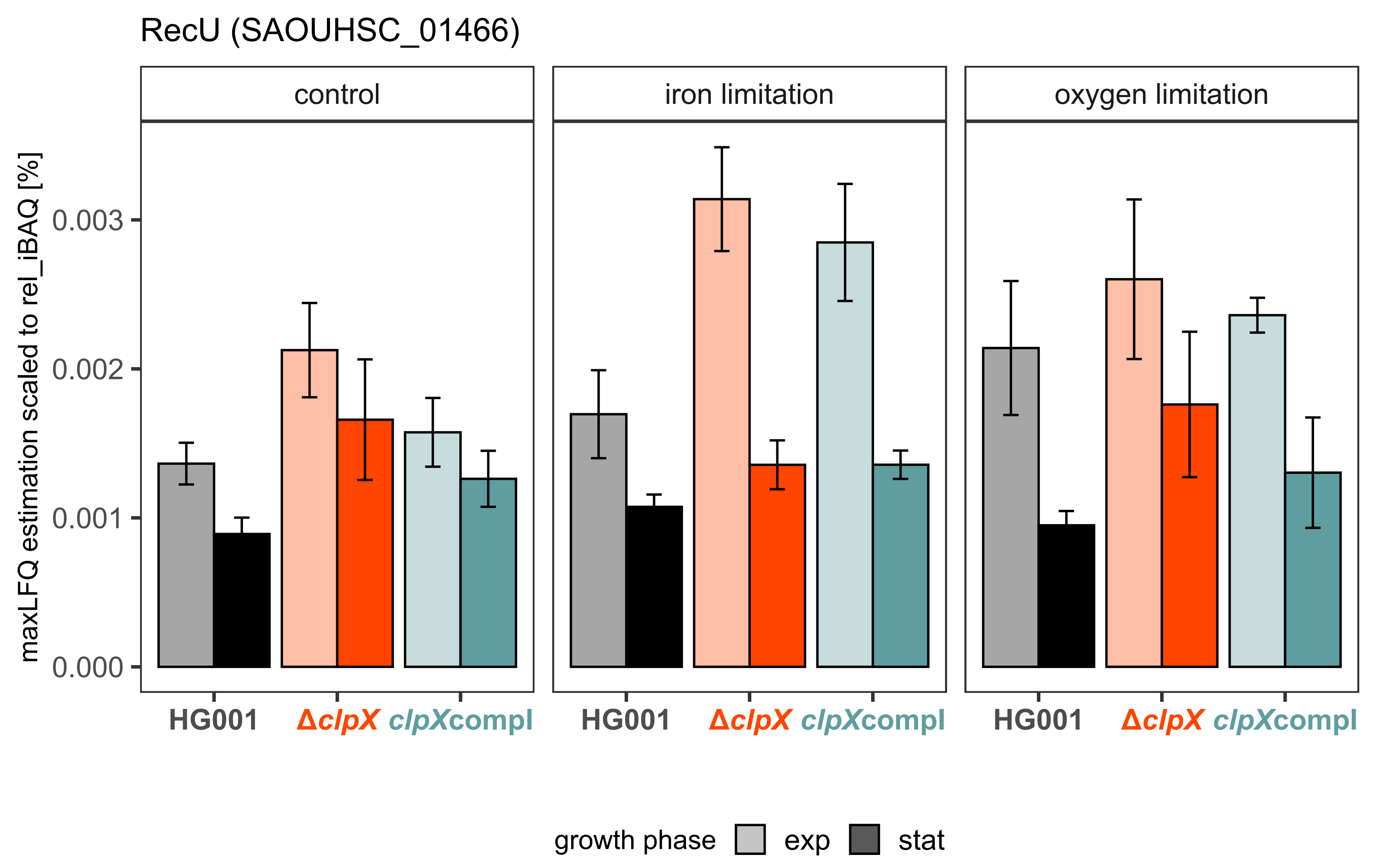

RecU (SAOUHSC_01466)
control
iron limitation
oxygen limitation
maxLFQ estimation scaled to rel_iBAQ [%]
0.003
0.002
0.001
0.000
HG001
ΔclpX
clpXcompl
growth phase
exp
stat

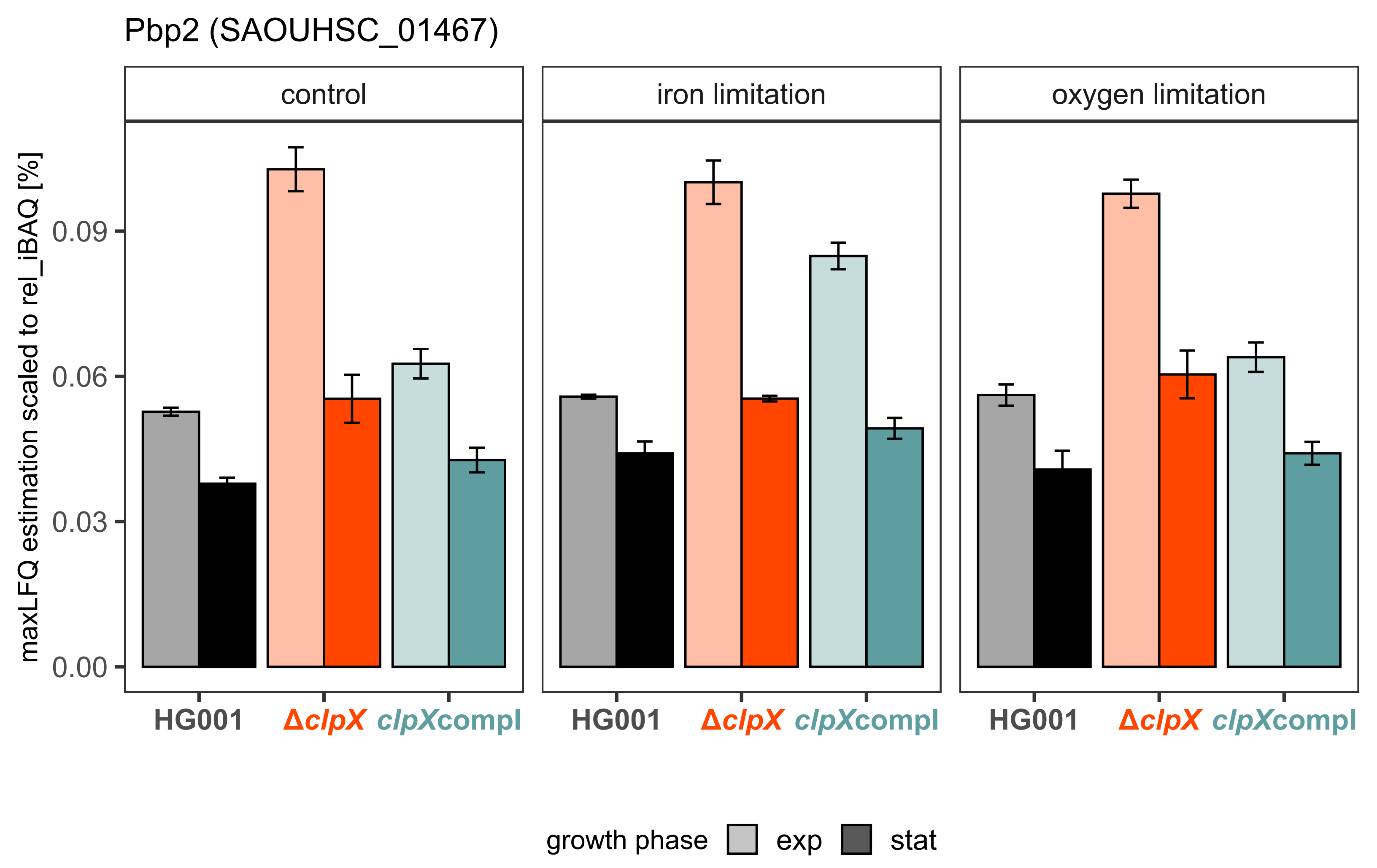
Pbp2 (SAOUHSC_01467)
control
iron limitation
oxygen limitation
maxLFQ estimation scaled to rel_iBAQ [%]
0.00
0.03
0.06
0.09
HG001
ΔclpX
clpXcompl
growth phase
exp
stat

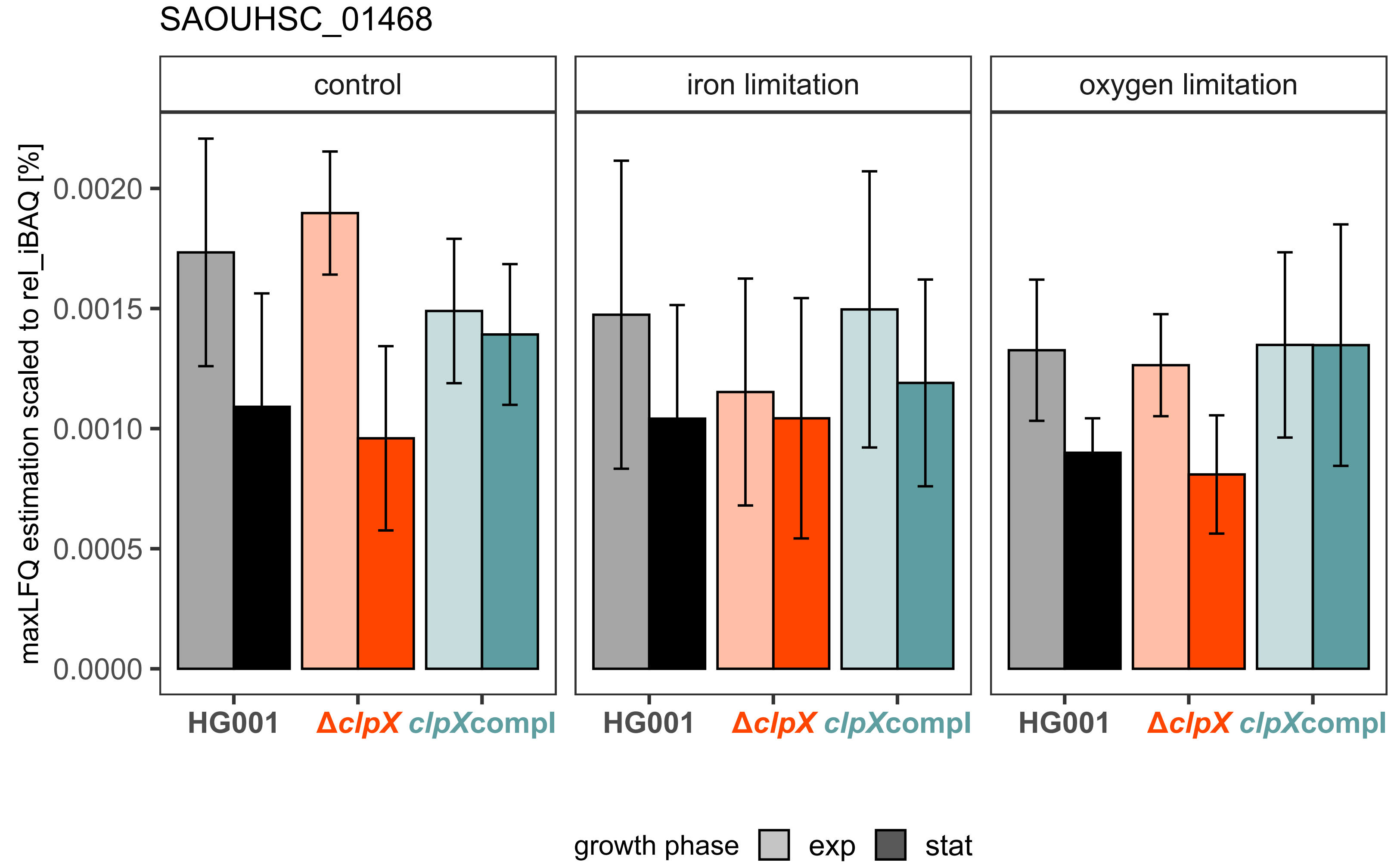
SAOUHSC_01468
control
iron limitation
oxygen limitation
maxLFQ estimation scaled to rel_iBAQ [%]
0.0020
0.0015
0.0010
0.0005
0.0000
HG001
ΔclpX
clpXcompl
growth phase
exp
stat

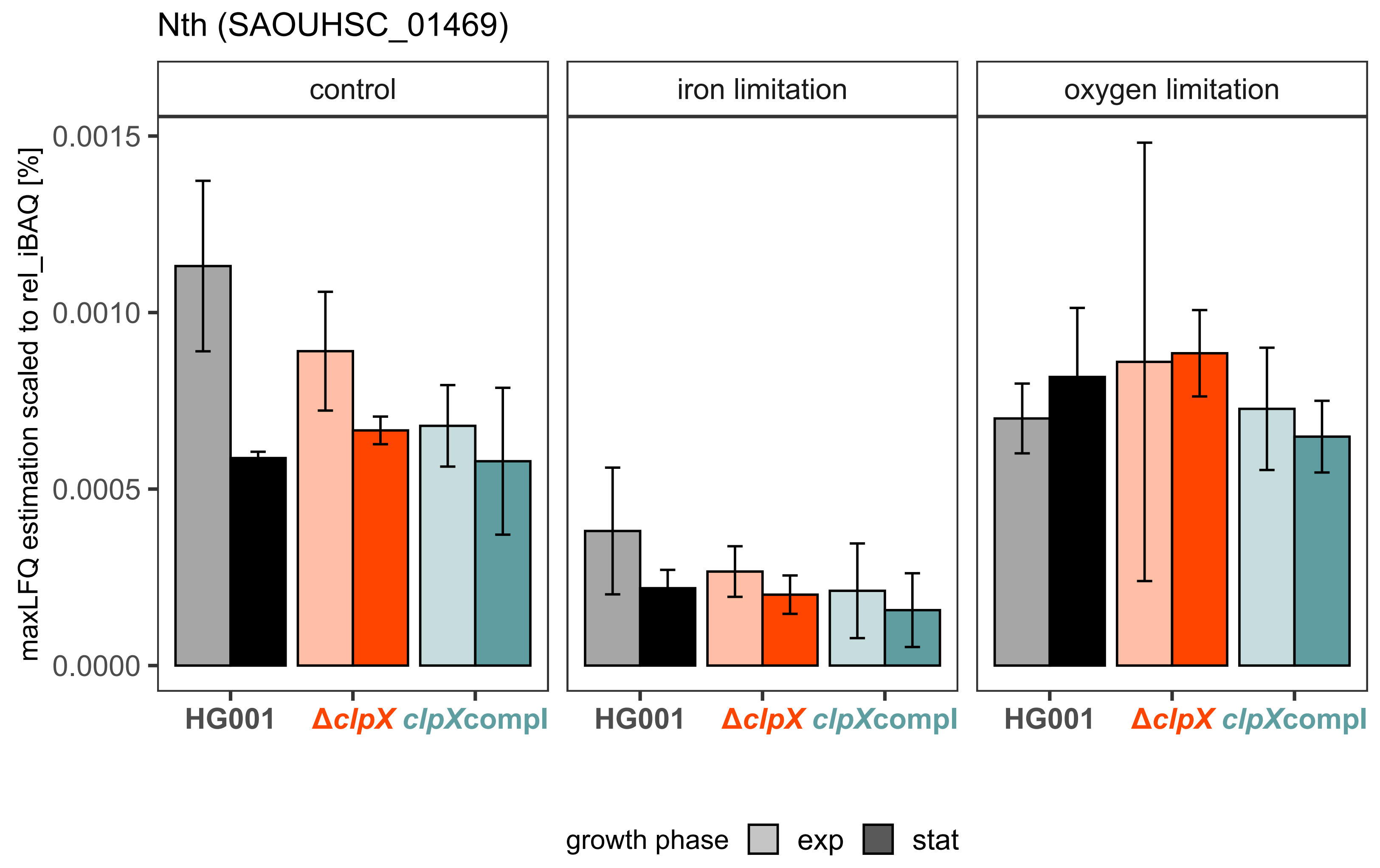

Nth (SAOUHSC_01469)
control
iron limitation
oxygen limitation
maxLFQ estimation scaled to rel_iBAQ [%]
0.0015
0.0010
0.0005
0.0000
HG001
ΔclpX
clpXcompl
growth phase
exp
stat

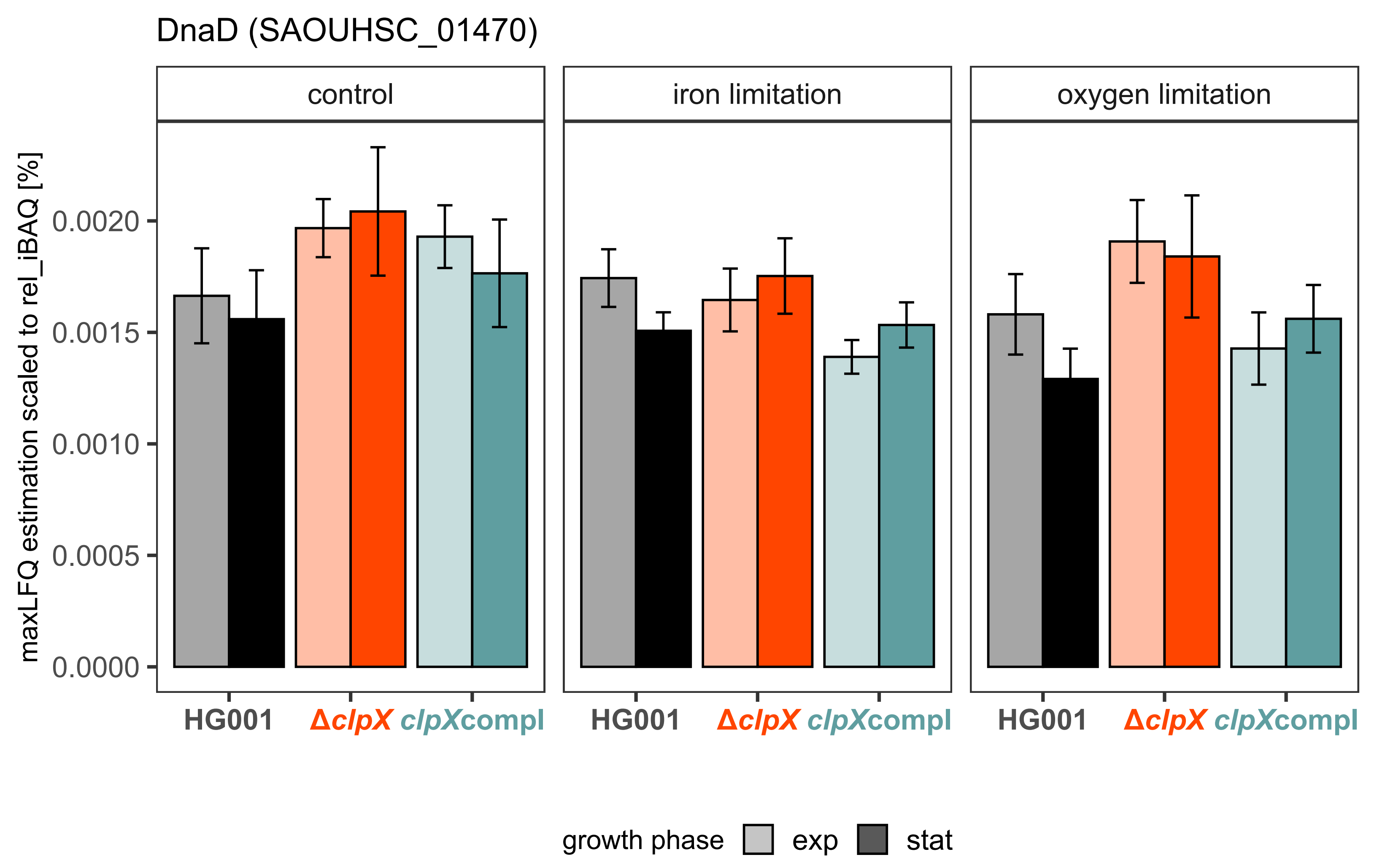
DnaD (SAOUHSC_01470)
control
iron limitation
oxygen limitation
maxLFQ estimation scaled to rel_iBAQ [%]
0.0020
0.0015
0.0010
0.0005
0.0000
HG001
ΔclpX
clpXcompl
growth phase
exp
stat

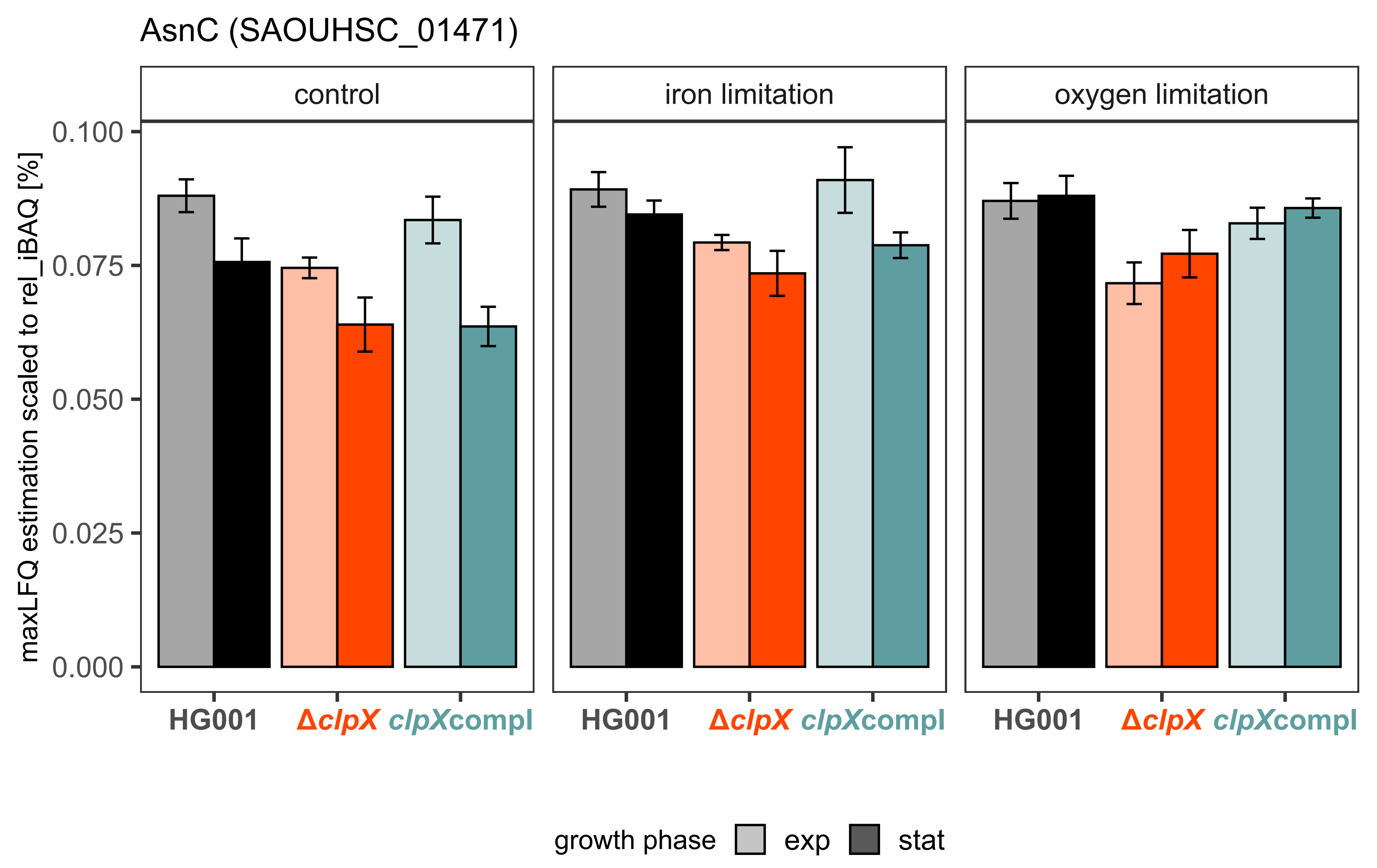

AsnC (SAOUHSC_01471)
control
iron limitation
oxygen limitation
maxLFQ estimation scaled to rel_iBAQ [%]
0.100
0.075
0.050
0.025
0.000
HG001
ΔclpX
clpXcompl
growth phase
exp
stat

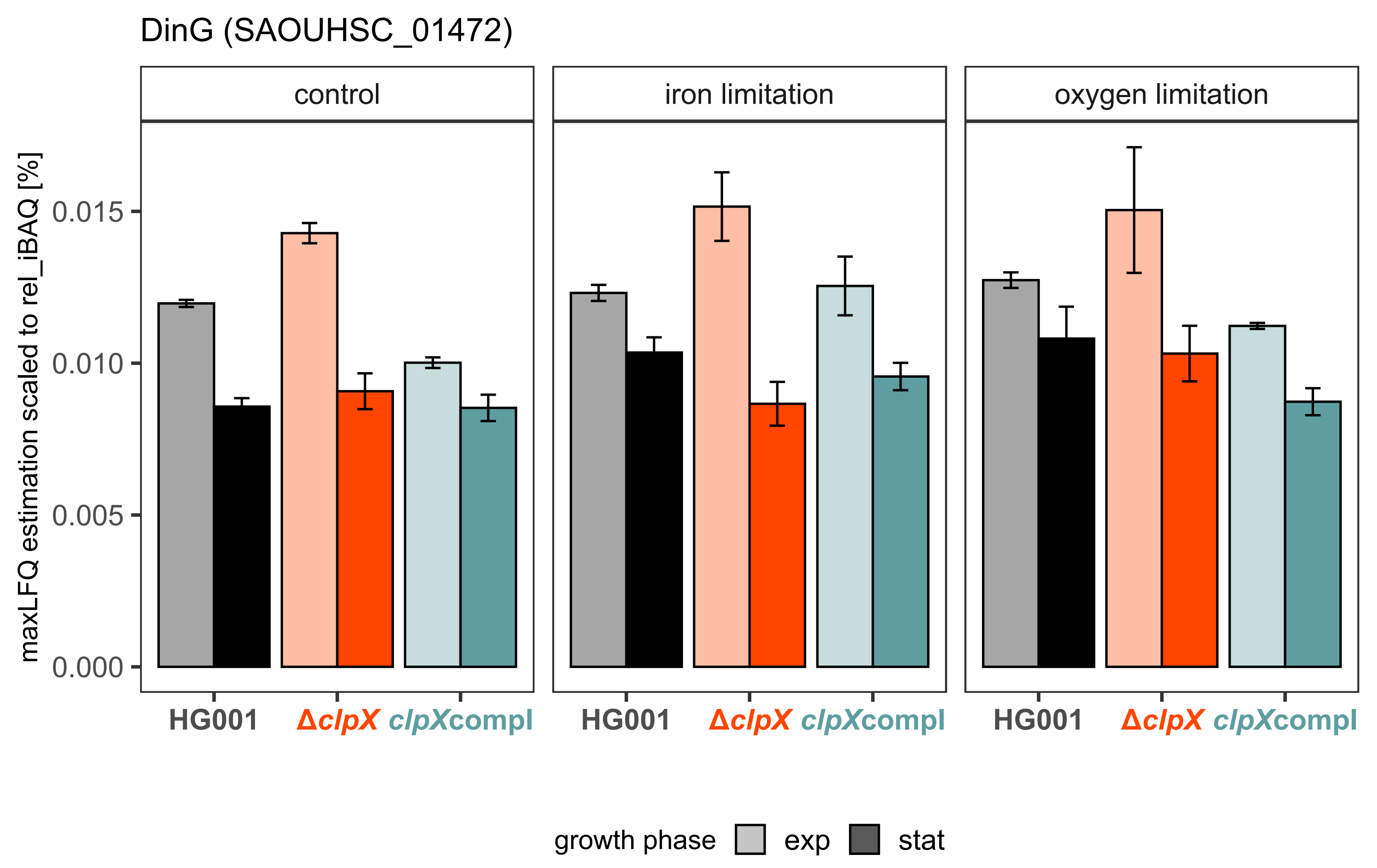

DinG (SAOUHSC_01472)
control
iron limitation
oxygen limitation
maxLFQ estimation scaled to rel_iBAQ [%]
0.015
0.010
0.005
0.000
HG001
ΔclpX
clpXcompl
growth phase
exp
stat

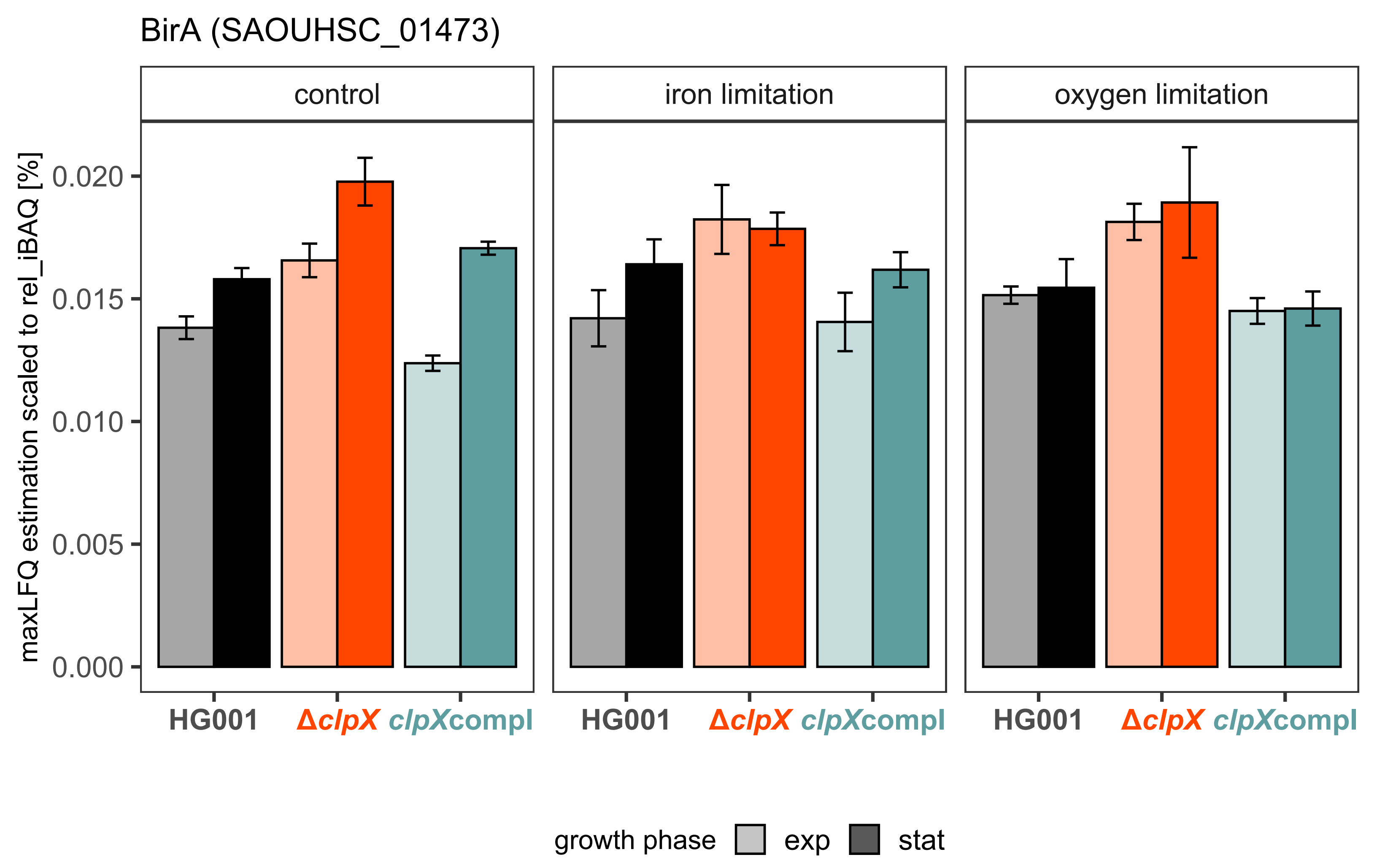
BirA (SAOUHSC_01473)
control
iron limitation
oxygen limitation
maxLFQ estimation scaled to rel_iBAQ [%]
0.000
0.005
0.010
0.015
0.020
HG001
ΔclpX
clpXcompl
growth phase
exp
stat

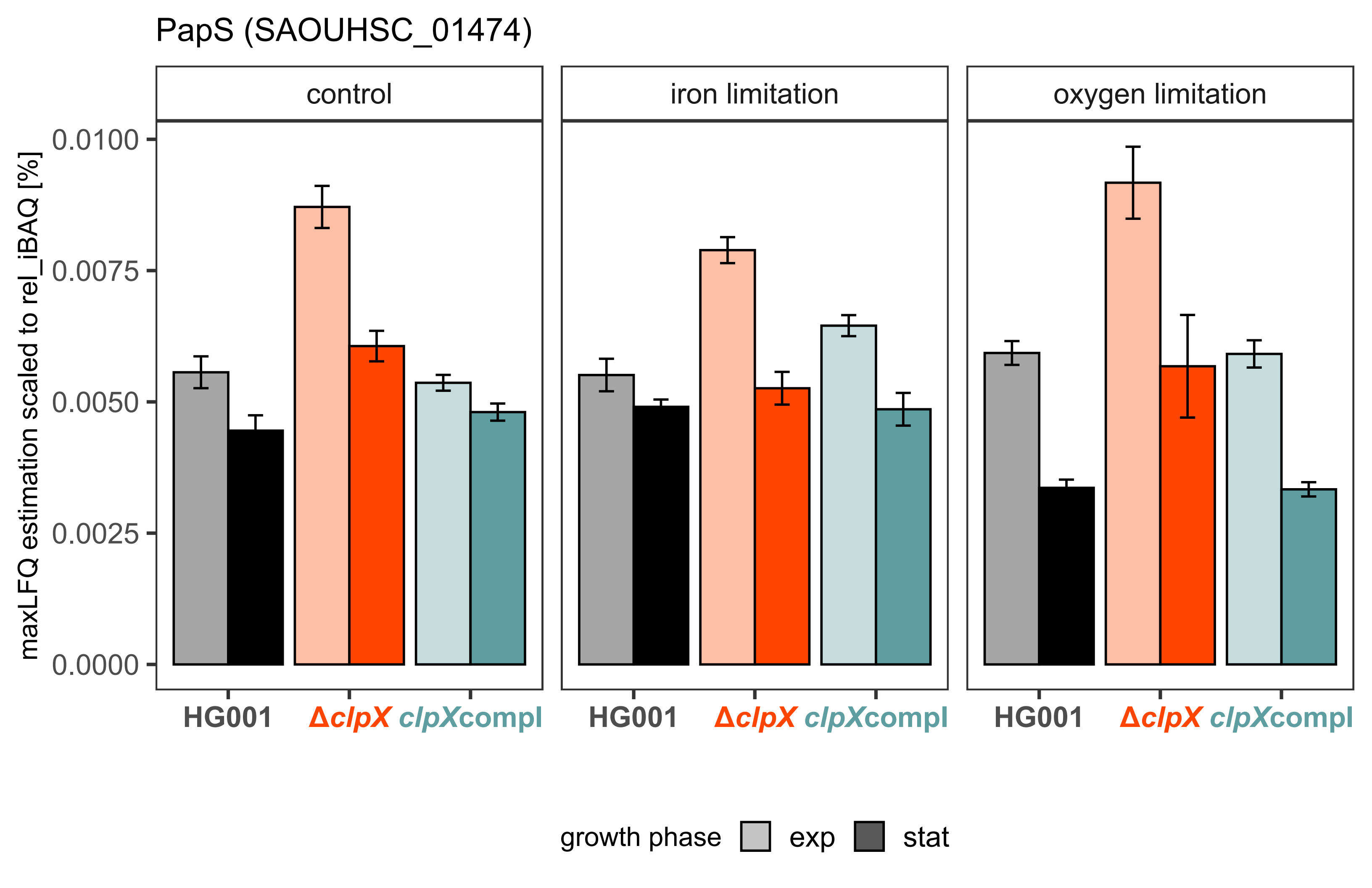
PapS (SAOUHSC_01474)
control
iron limitation
oxygen limitation
maxLFQ estimation scaled to rel_iBAQ [%]
0.0100
0.0075
0.0050
0.0025
0.0000
HG001
ΔclpX
clpXcompl
growth phase
exp
stat

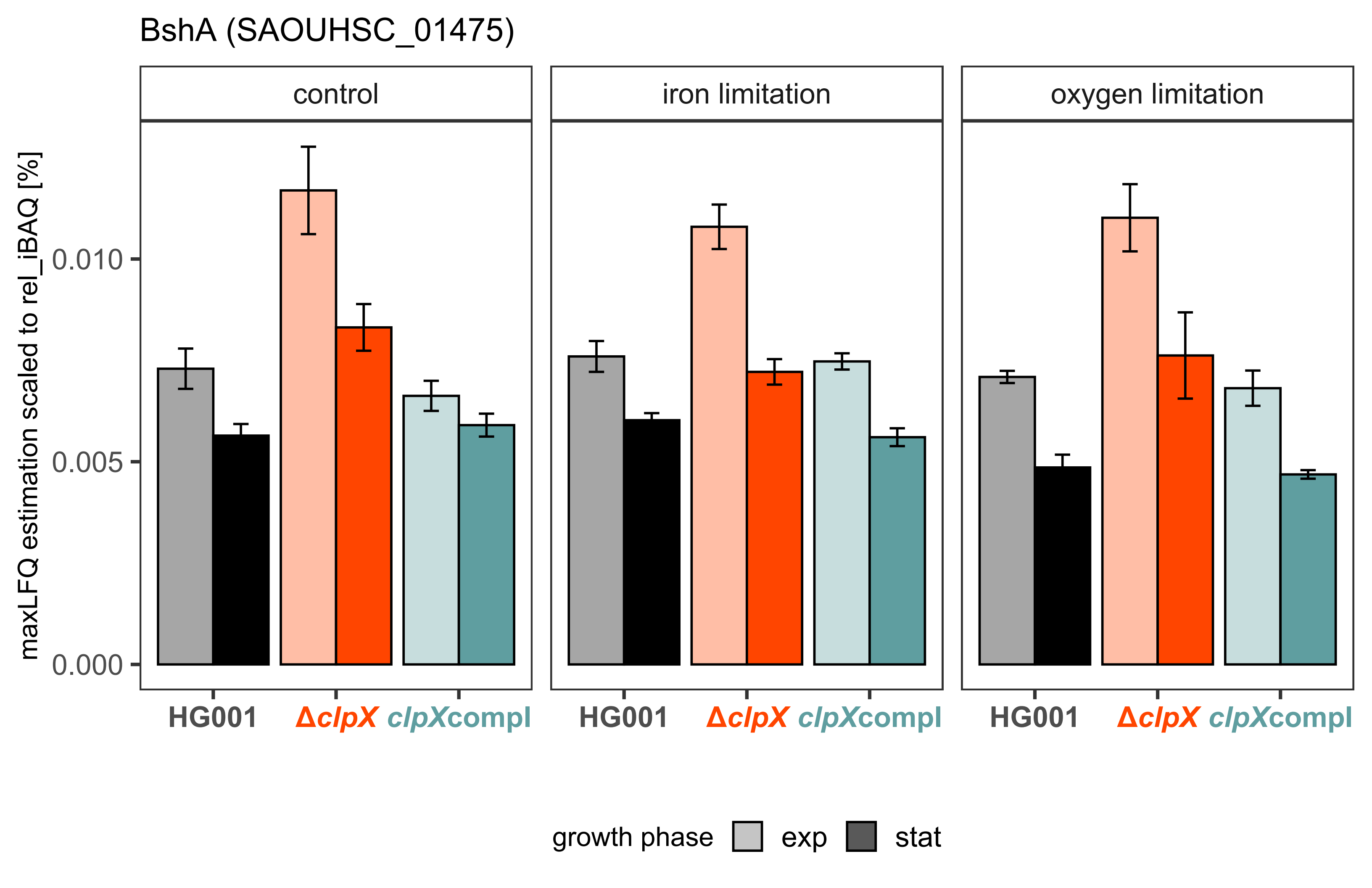

BshA (SAOUHSC_01475)
control
iron limitation
oxygen limitation
maxLFQ estimation scaled to rel_iBAQ [%]
0.000
0.005
0.010
HG001
ΔclpX
clpXcompl
growth phase
exp
stat

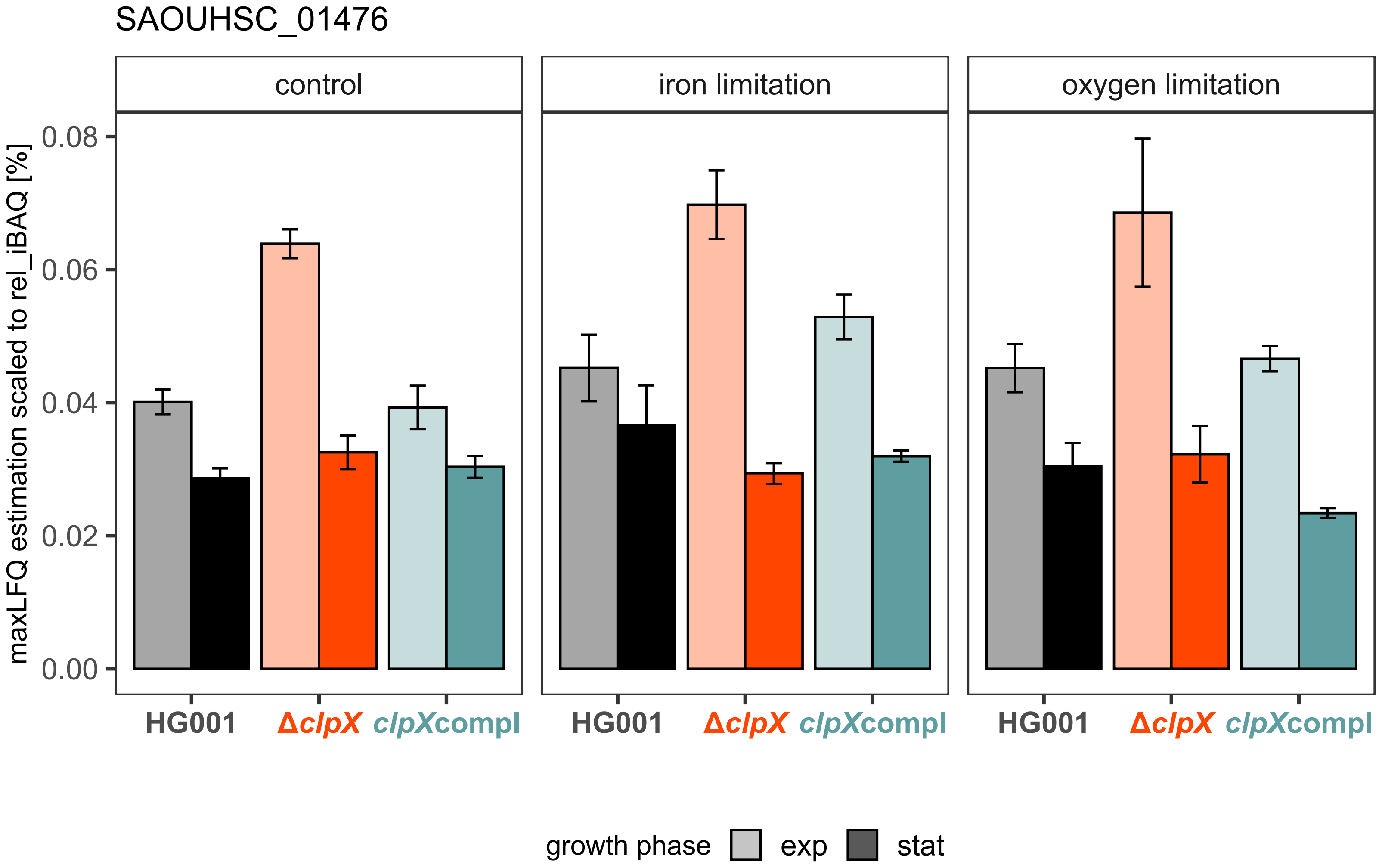

SAOUHSC_01476
control
iron limitation
oxygen limitation
maxLFQ estimation scaled to rel_iBAQ [%]
0.08
0.06
0.04
0.02
0.00
HG001
ΔclpX
clpXcompl
growth phase
exp
stat

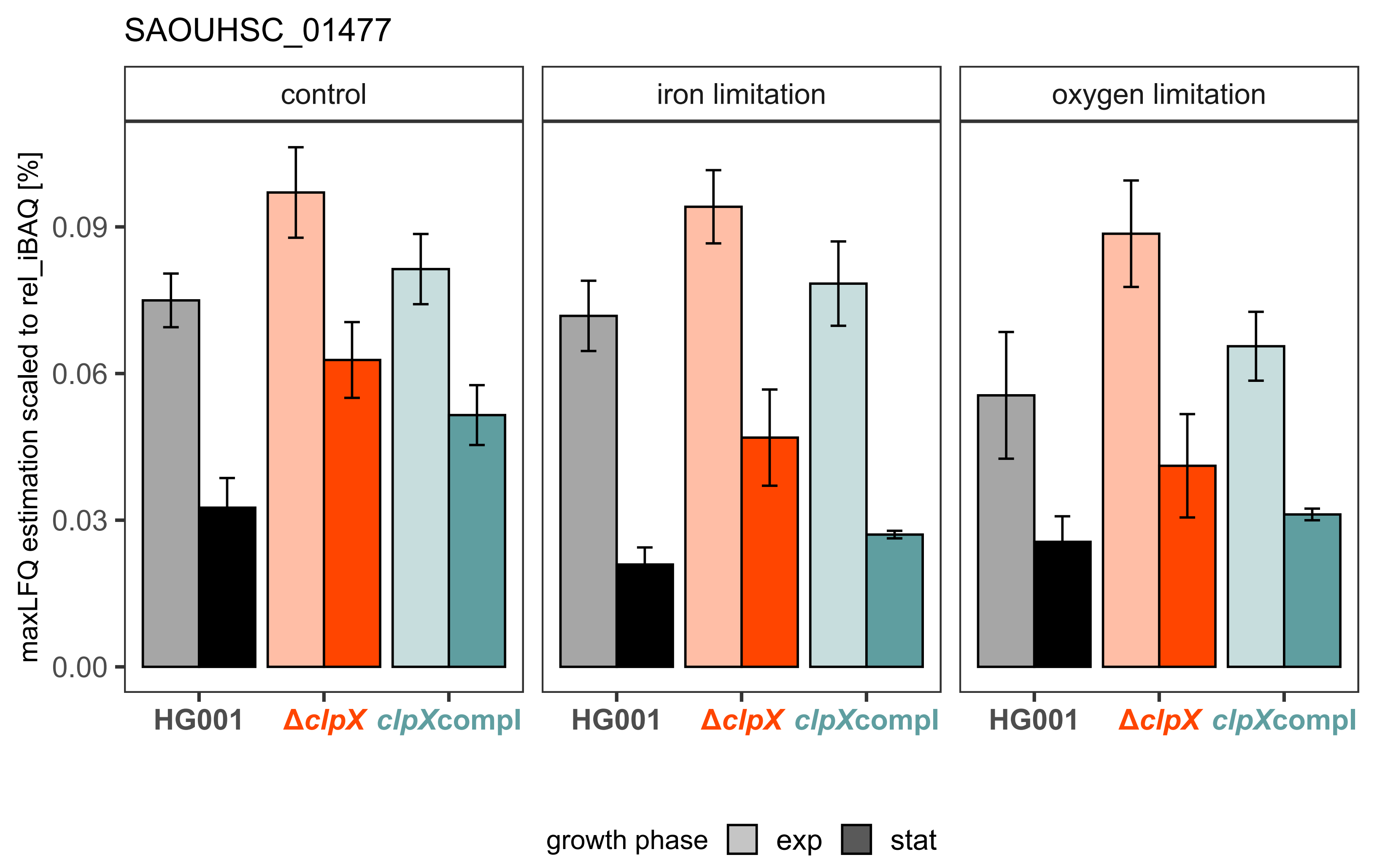

SAOUHSC_01477
control
iron limitation
oxygen limitation
maxLFQ estimation scaled to rel_iBAQ [%]
0.09
0.06
0.03
0.00
HG001
ΔclpX
clpXcompl
growth phase
exp
stat

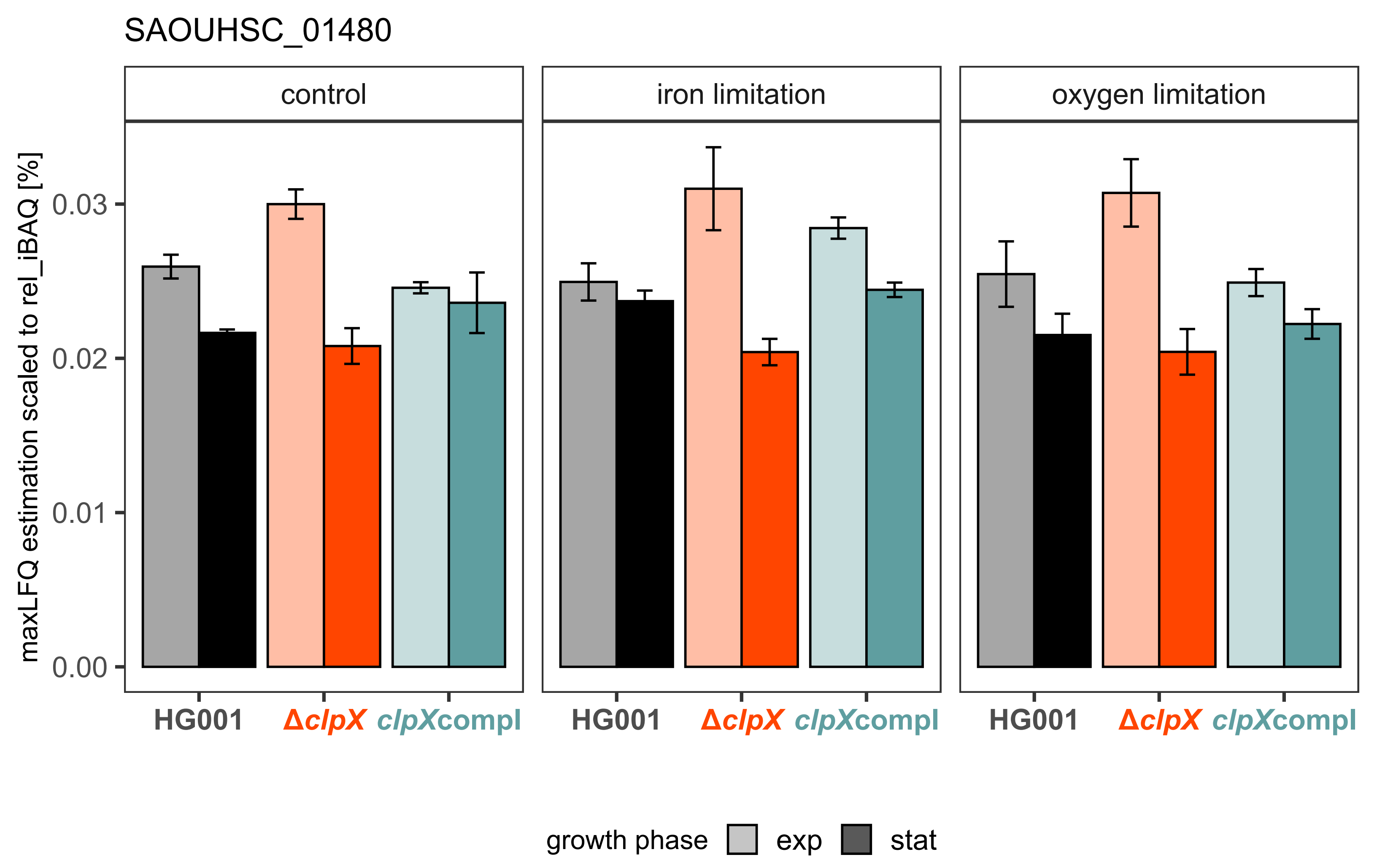

SAOUHSC_01480
control
iron limitation
oxygen limitation
maxLFQ estimation scaled to rel_iBAQ [%]
0.03
0.02
0.01
0.00
HG001
ΔclpX
clpXcompl
growth phase
exp
stat

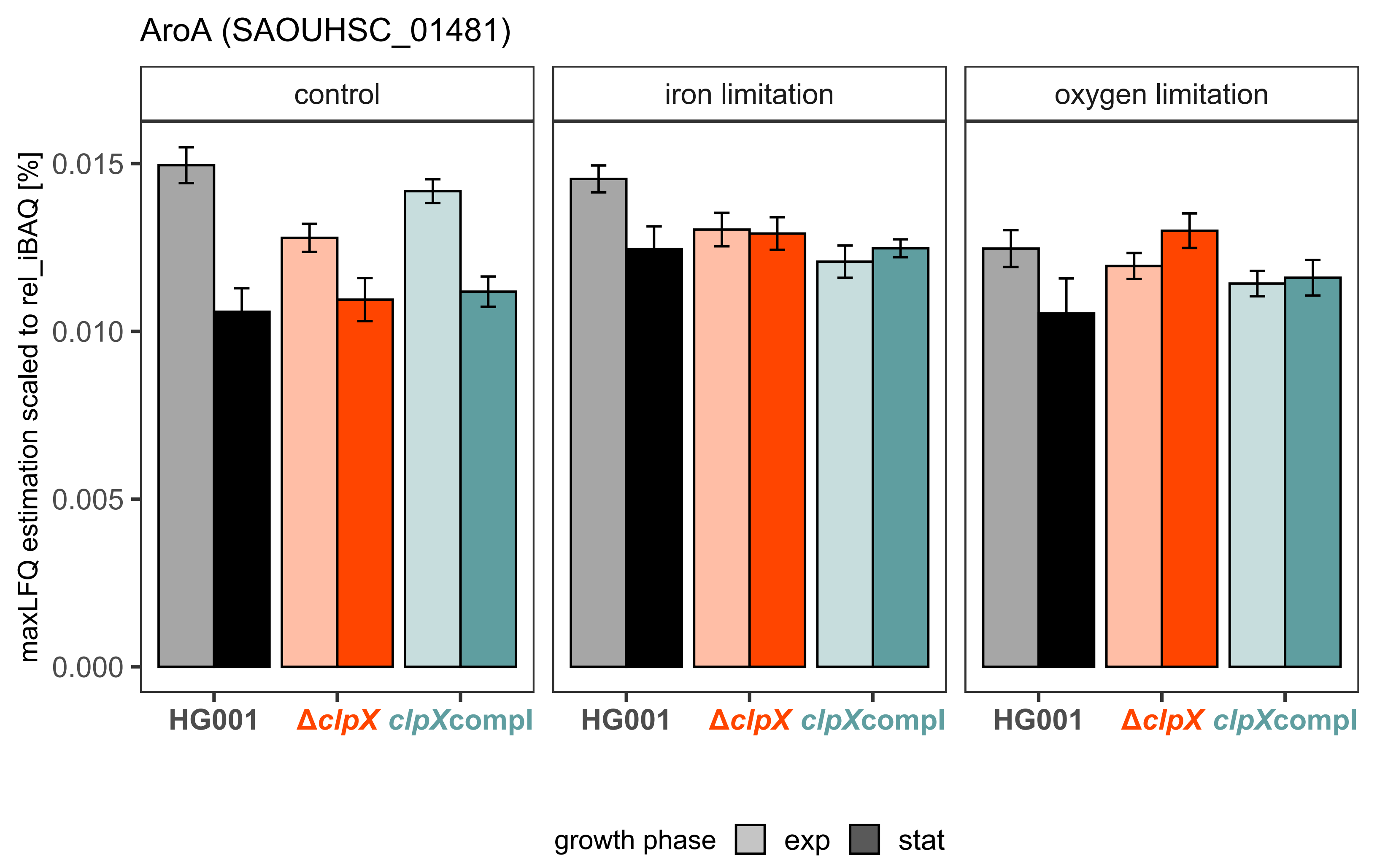
AroA (SAOUHSC_01481)
control
iron limitation
oxygen limitation
maxLFQ estimation scaled to rel_iBAQ [%]
0.015
0.010
0.005
0.000
HG001
ΔclpX
clpXcompl
growth phase
exp
stat

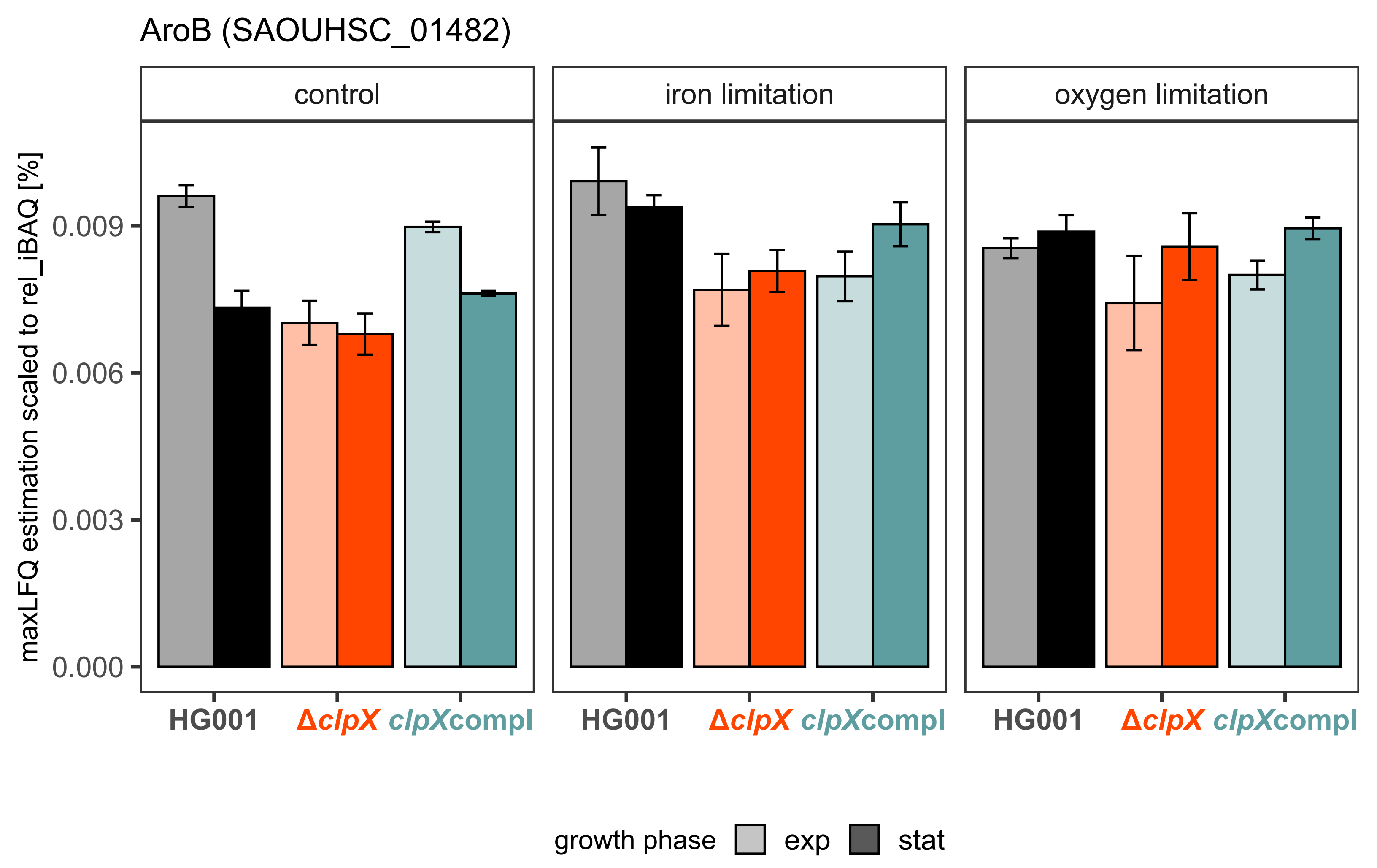
AroB (SAOUHSC_01482)
control
iron limitation
oxygen limitation
maxLFQ estimation scaled to rel_iBAQ [%]
0.009
0.006
0.003
0.000
HG001
ΔclpX
clpXcompl
growth phase
exp
stat

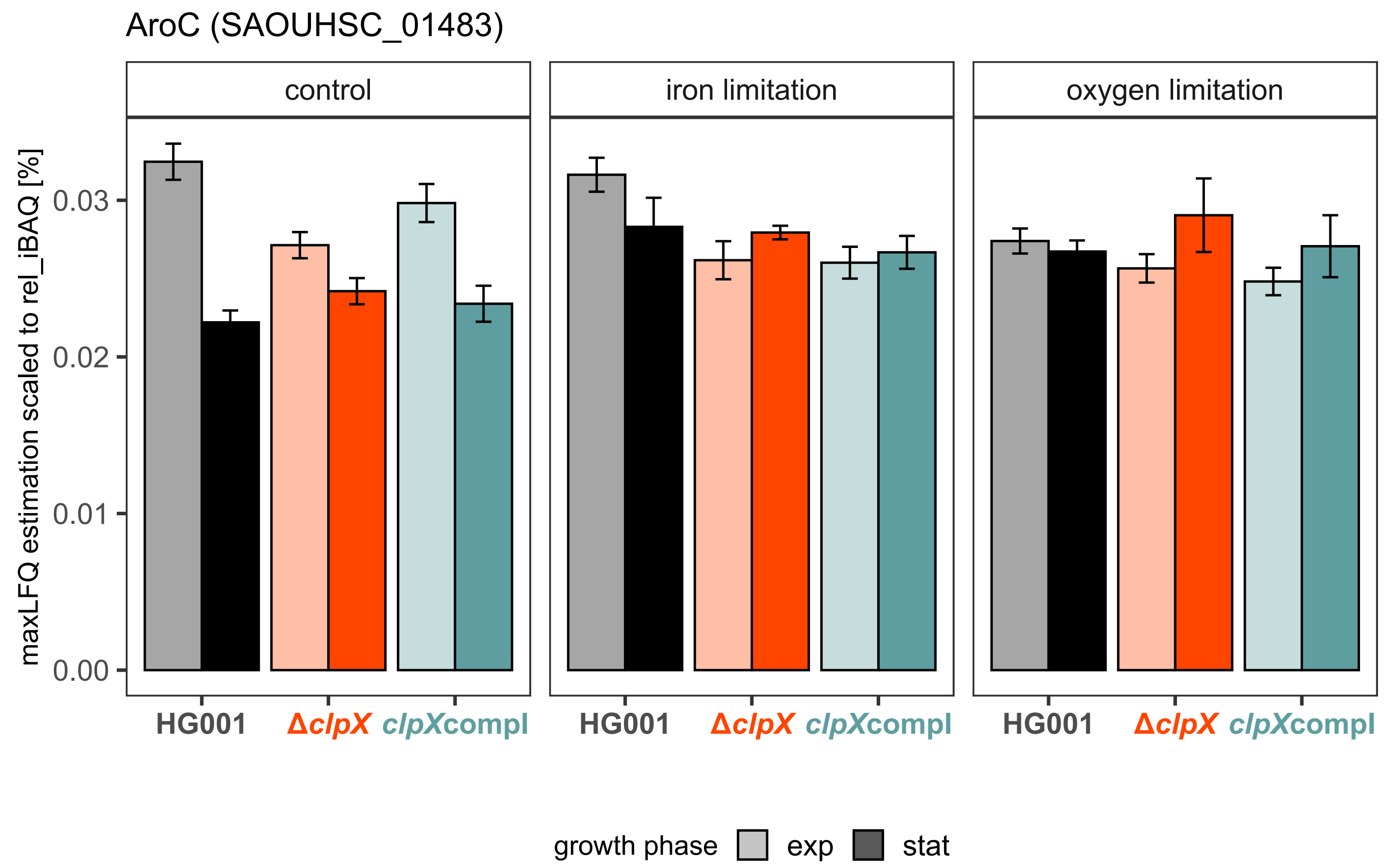

AroC (SAOUHSC_01483)
control
iron limitation
oxygen limitation
maxLFQ estimation scaled to rel_iBAQ [%]
0.03
0.02
0.01
0.00
HG001
ΔclpX
clpXcompl
growth phase
exp
stat

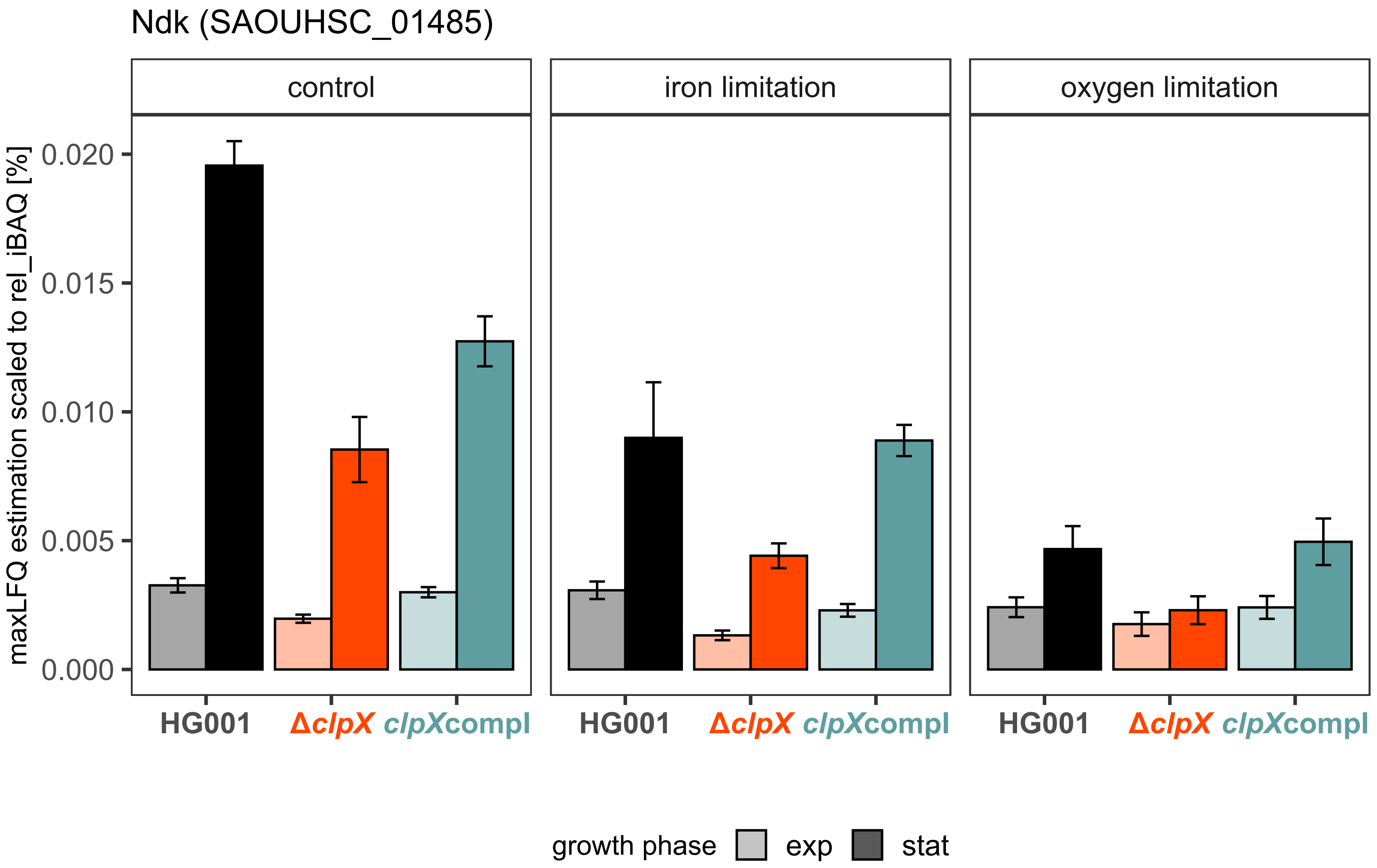

Ndk (SAOUHSC_01485)
control
iron limitation
oxygen limitation
maxLFQ estimation scaled to rel_iBAQ [%]
0.020
0.015
0.010
0.005
0.000
HG001
ΔclpX
clpXcompl
growth phase
exp
stat

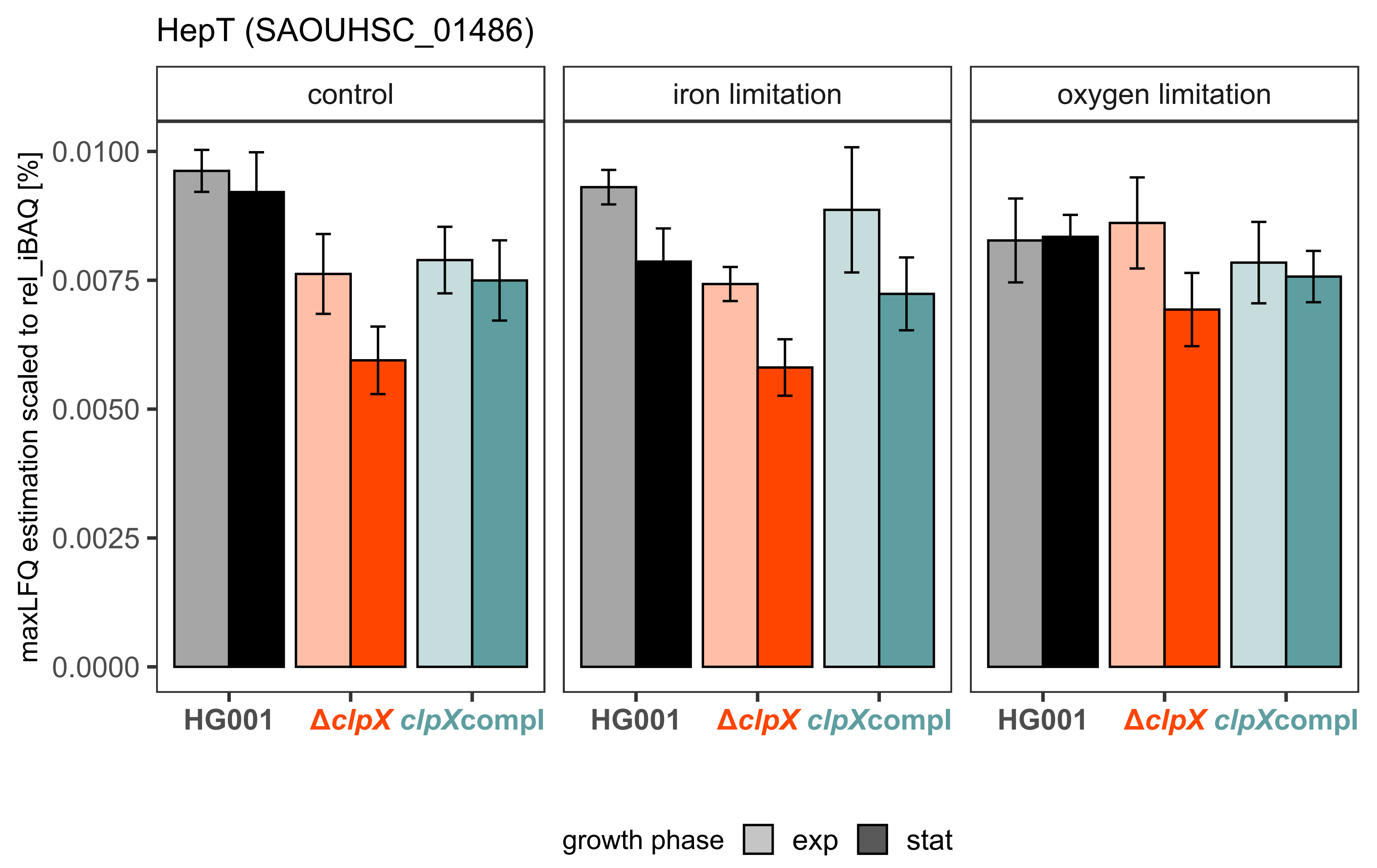
HepT (SAOUHSC_01486)
control
iron limitation
oxygen limitation
maxLFQ estimation scaled to rel_iBAQ [%]
0.0100
0.0075
0.0050
0.0025
0.0000
HG001
ΔclpX
clpXcompl
growth phase
exp
stat

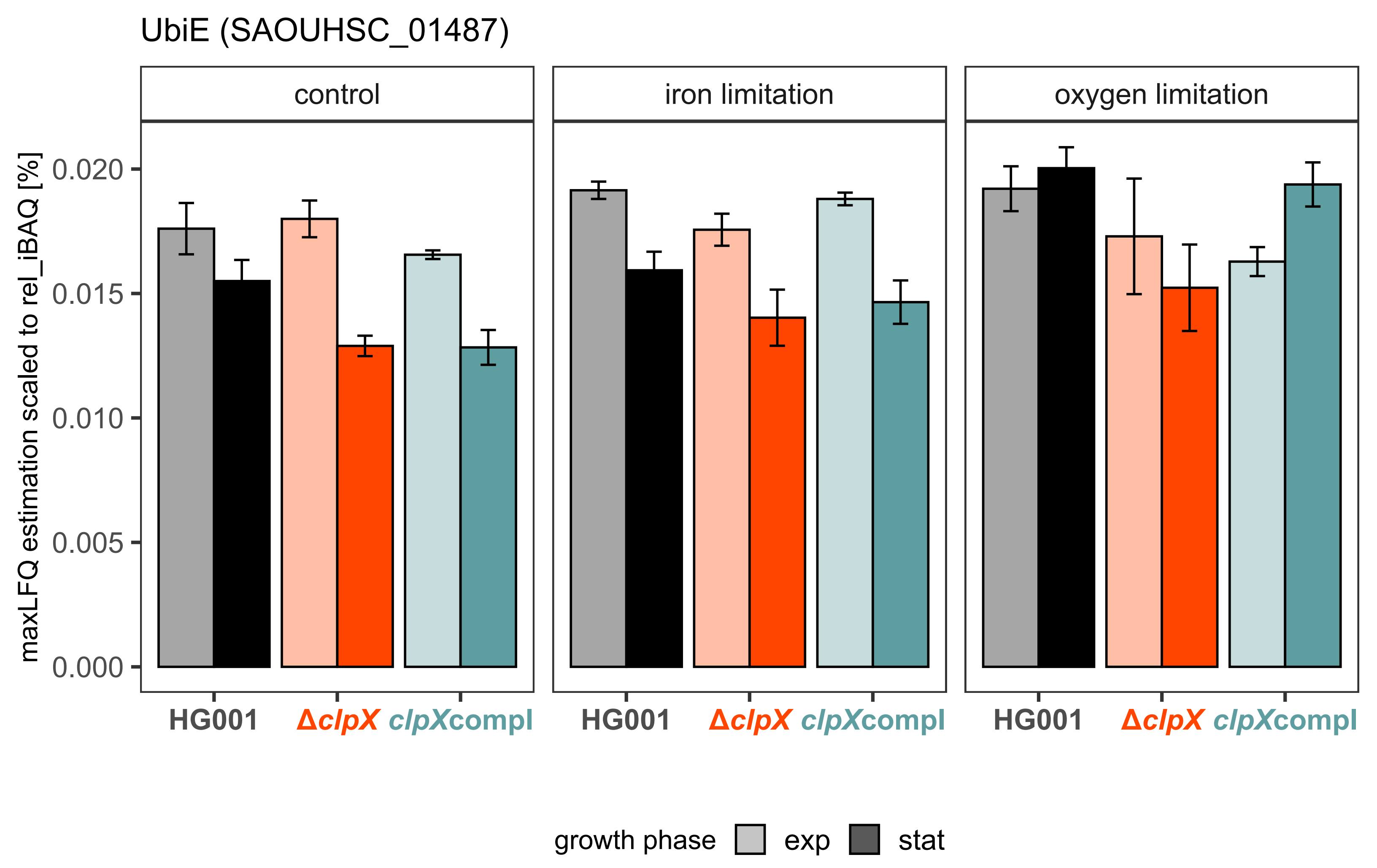
UbiE (SAOUHSC_01487)
control
iron limitation
oxygen limitation
maxLFQ estimation scaled to rel_iBAQ [%]
0.020
0.015
0.010
0.005
0.000
HG001
ΔclpX
clpXcompl
growth phase
exp
stat

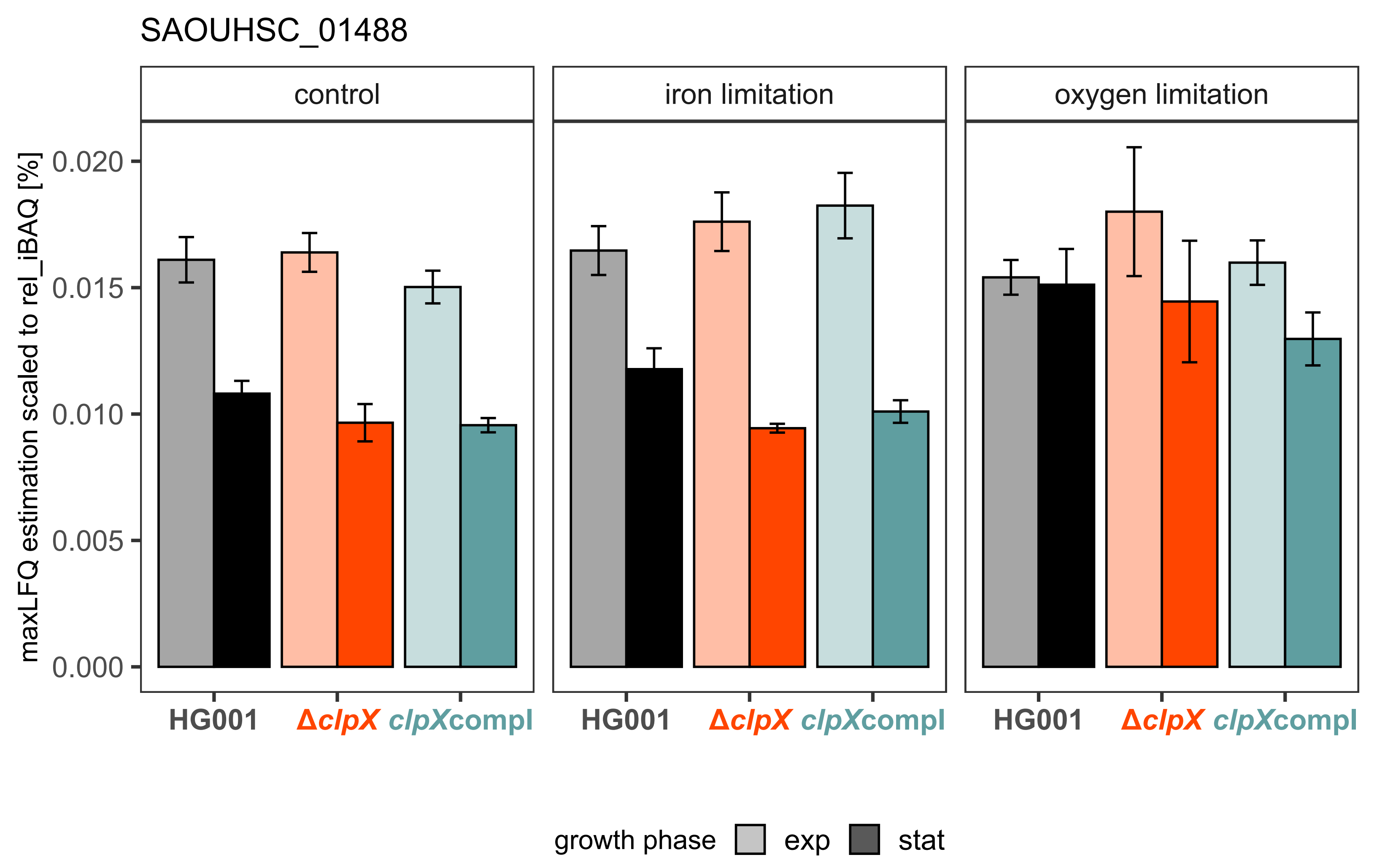
SAOUHSC_01488
control
iron limitation
oxygen limitation
maxLFQ estimation scaled to rel_iBAQ [%]
0.020
0.015
0.010
0.005
0.000
HG001
ΔclpX
clpXcompl
growth phase
exp
stat

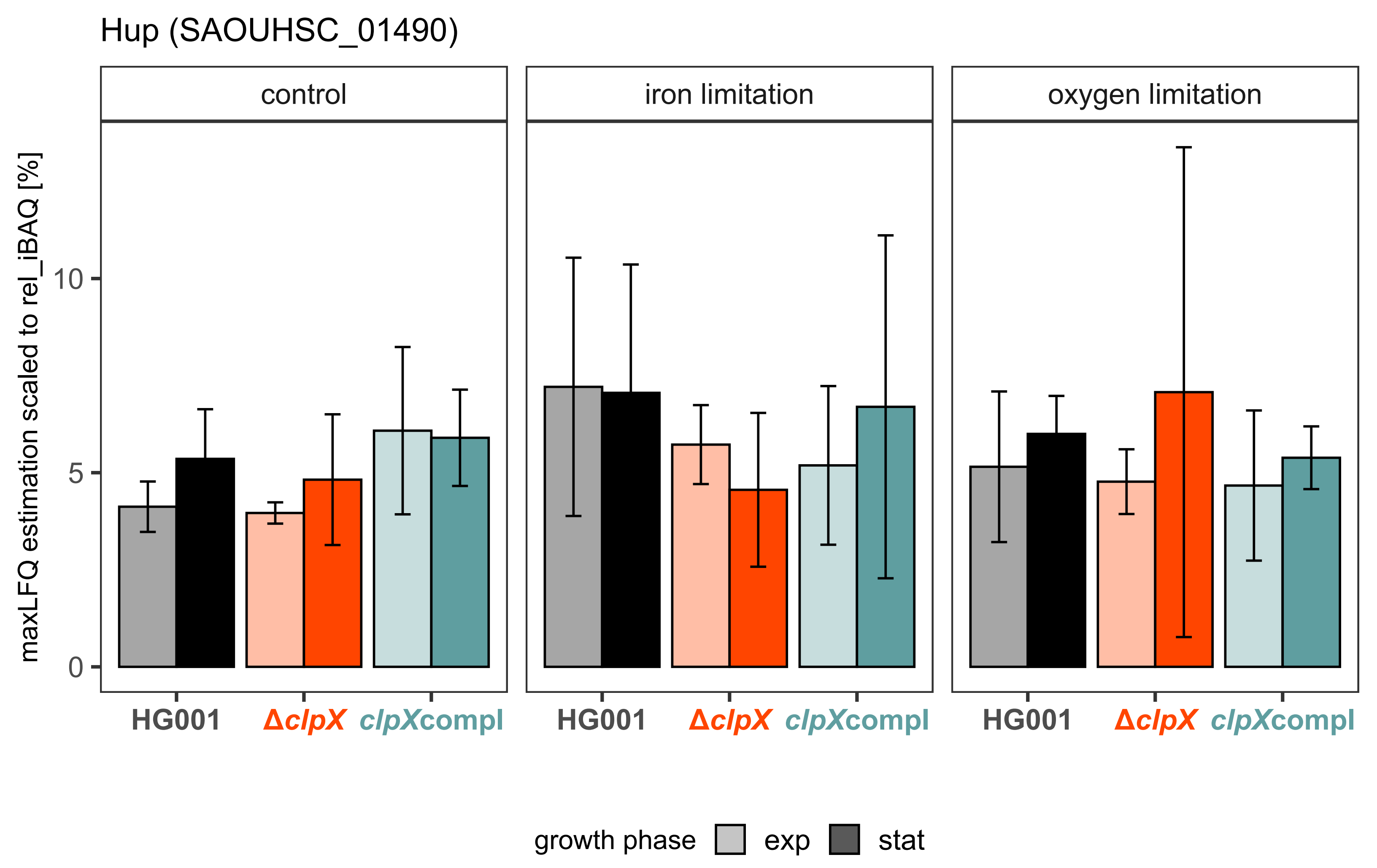

Hup (SAOUHSC_01490)
control
iron limitation
oxygen limitation
maxLFQ estimation scaled to rel_iBAQ [%]
0
5
10
HG001
ΔclpX
clpXcompl
growth phase
exp
stat

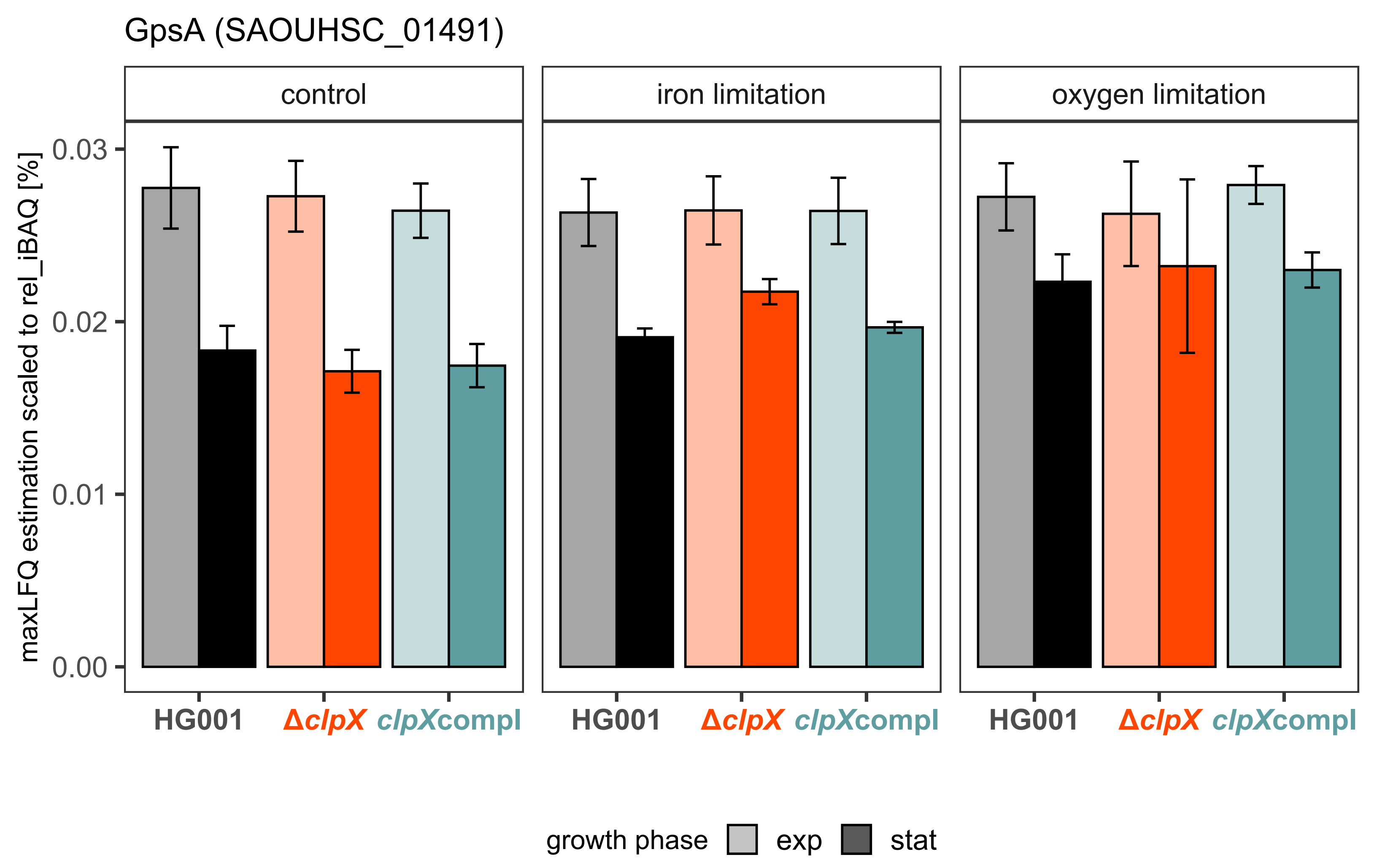

GpsA (SAOUHSC_01491)
control
iron limitation
oxygen limitation
maxLFQ estimation scaled to rel_iBAQ [%]
0.00
0.01
0.02
0.03
HG001
ΔclpX
clpXcompl
growth phase
exp
stat

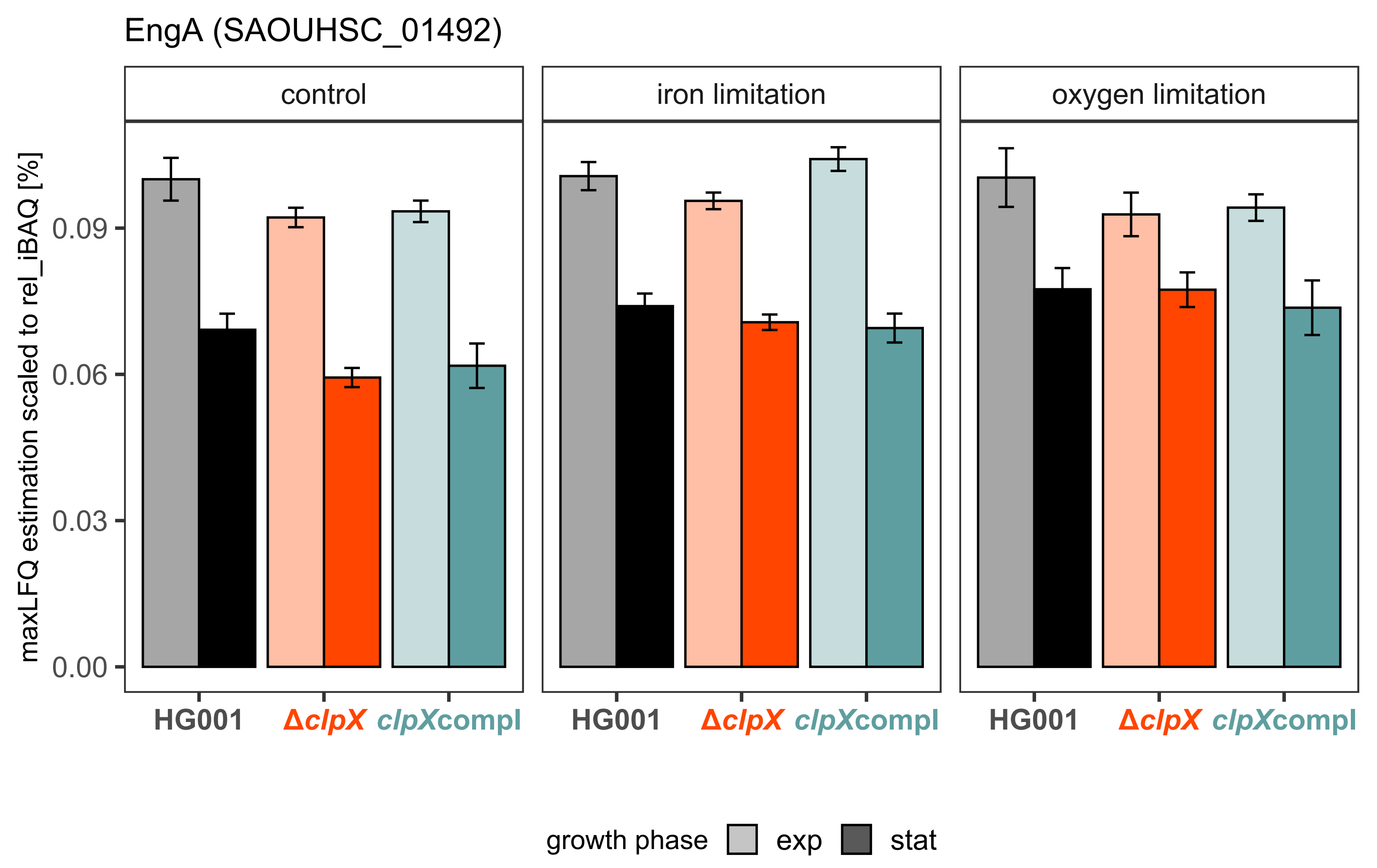
EngA (SAOUHSC_01492)
control
iron limitation
oxygen limitation
maxLFQ estimation scaled to rel_iBAQ [%]
0.00
0.03
0.06
0.09
HG001
ΔclpX
clpXcompl
growth phase
exp
stat

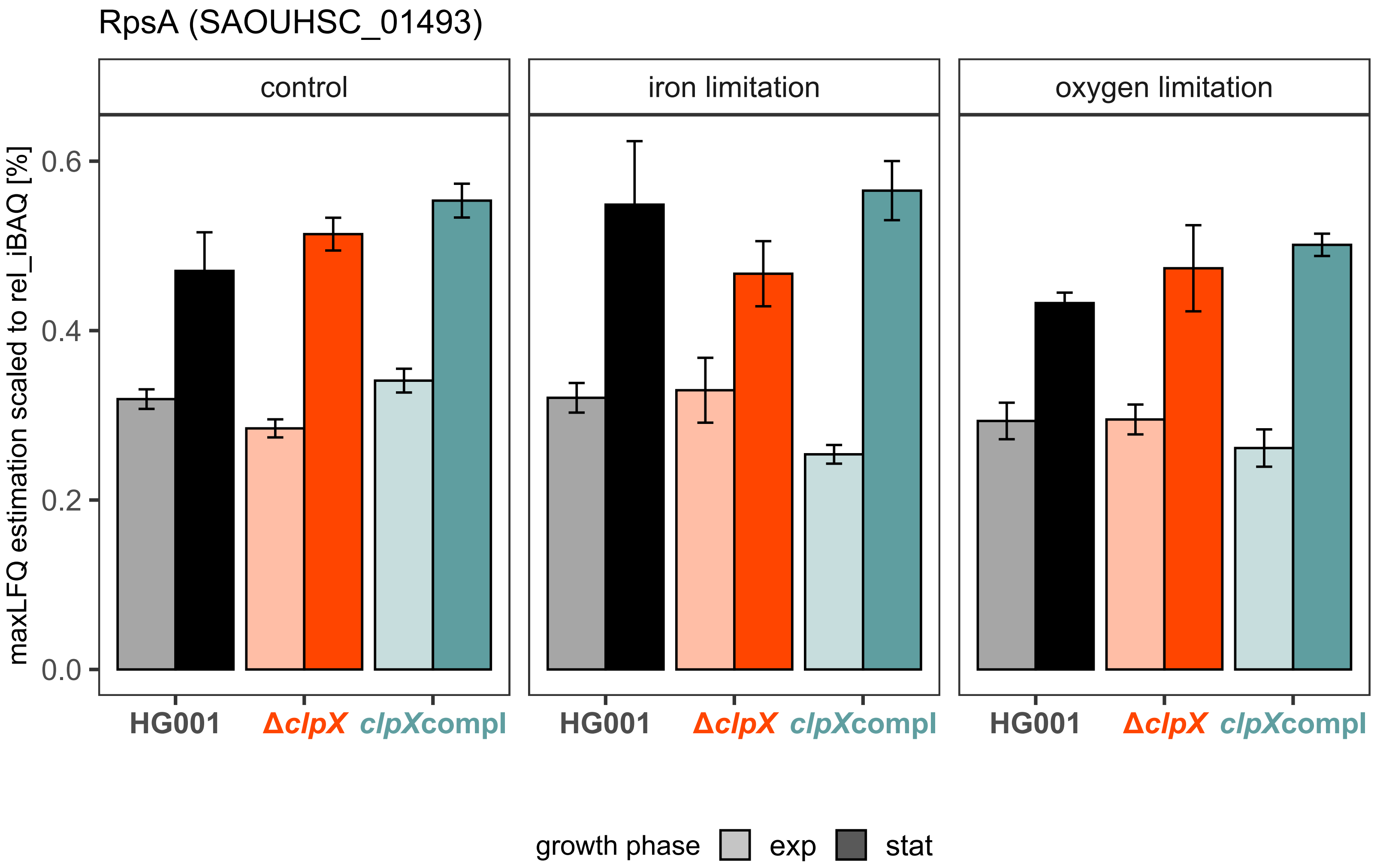
RpsA (SAOUHSC_01493)
control
iron limitation
oxygen limitation
maxLFQ estimation scaled to rel_iBAQ [%]
0.0
0.2
0.4
0.6
HG001
ΔclpX
clpXcompl
growth phase
exp
stat

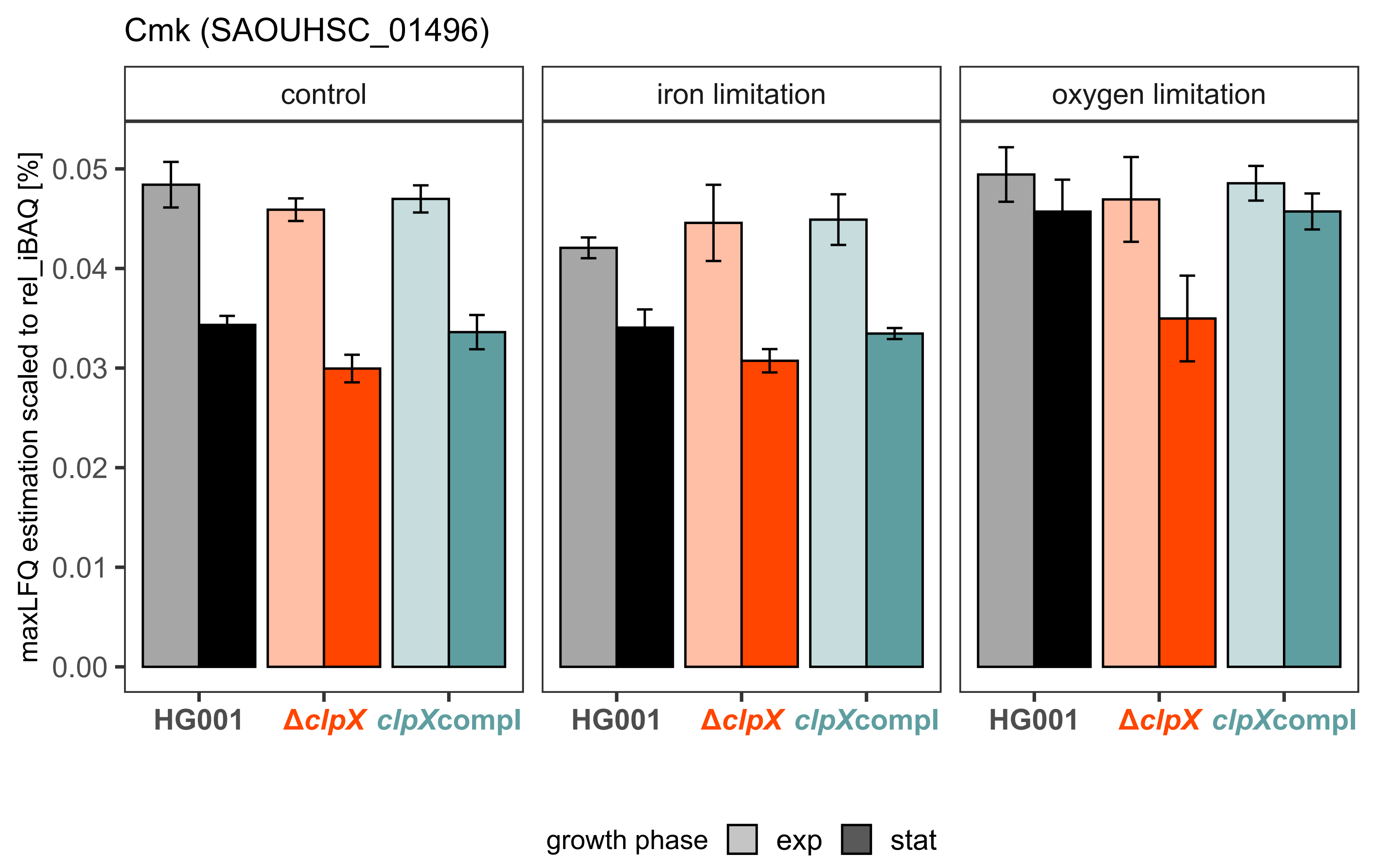

Cmk (SAOUHSC_01496)
control
iron limitation
oxygen limitation
maxLFQ estimation scaled to rel_iBAQ [%]
0.05
0.04
0.03
0.02
0.01
0.00
HG001
ΔclpX
clpXcompl
growth phase
exp
stat

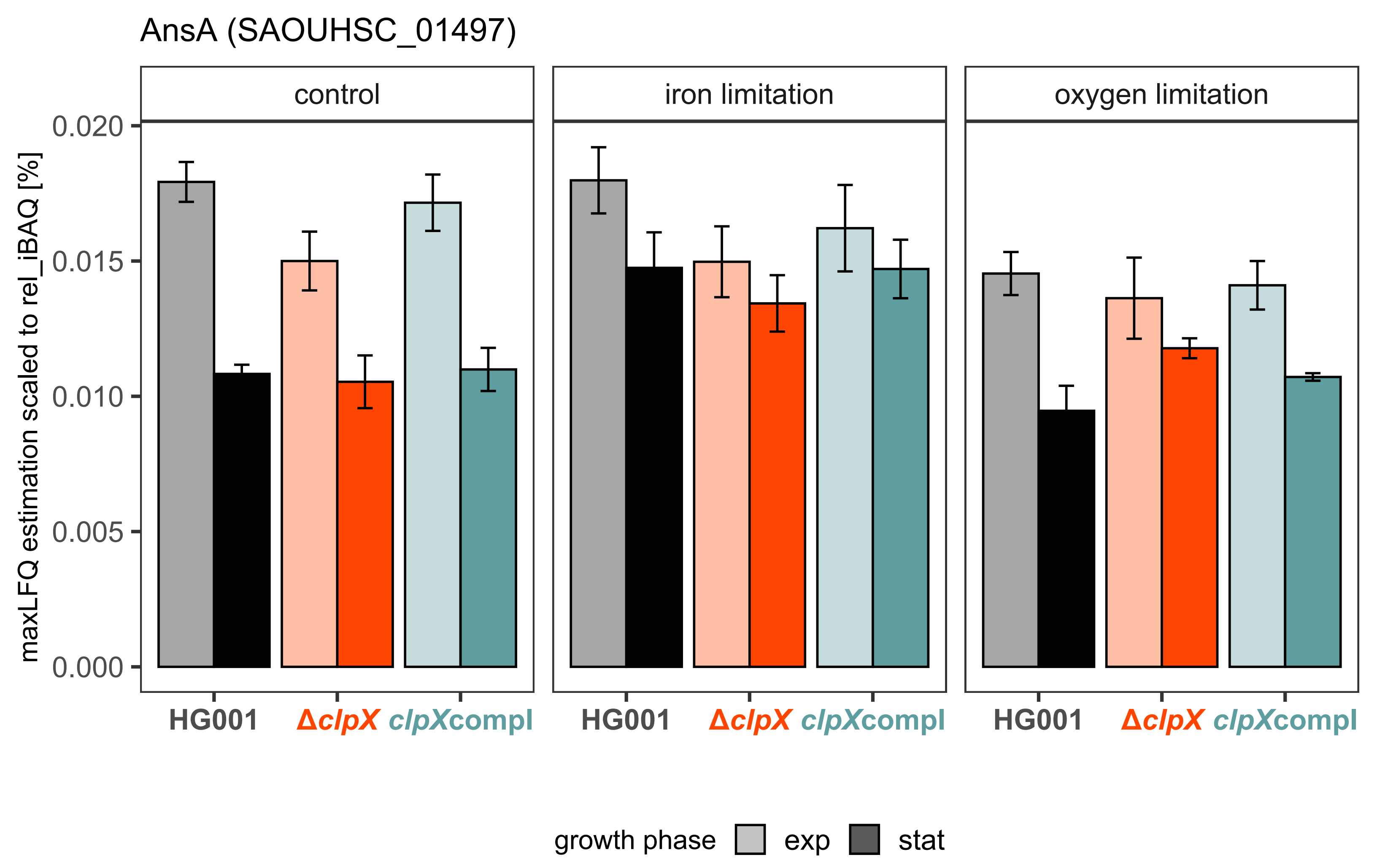
AnsA (SAOUHSC_01497)
control
iron limitation
oxygen limitation
maxLFQ estimation scaled to rel_iBAQ [%]
0.020
0.015
0.010
0.005
0.000
HG001
ΔclpX
clpXcompl
growth phase
exp
stat

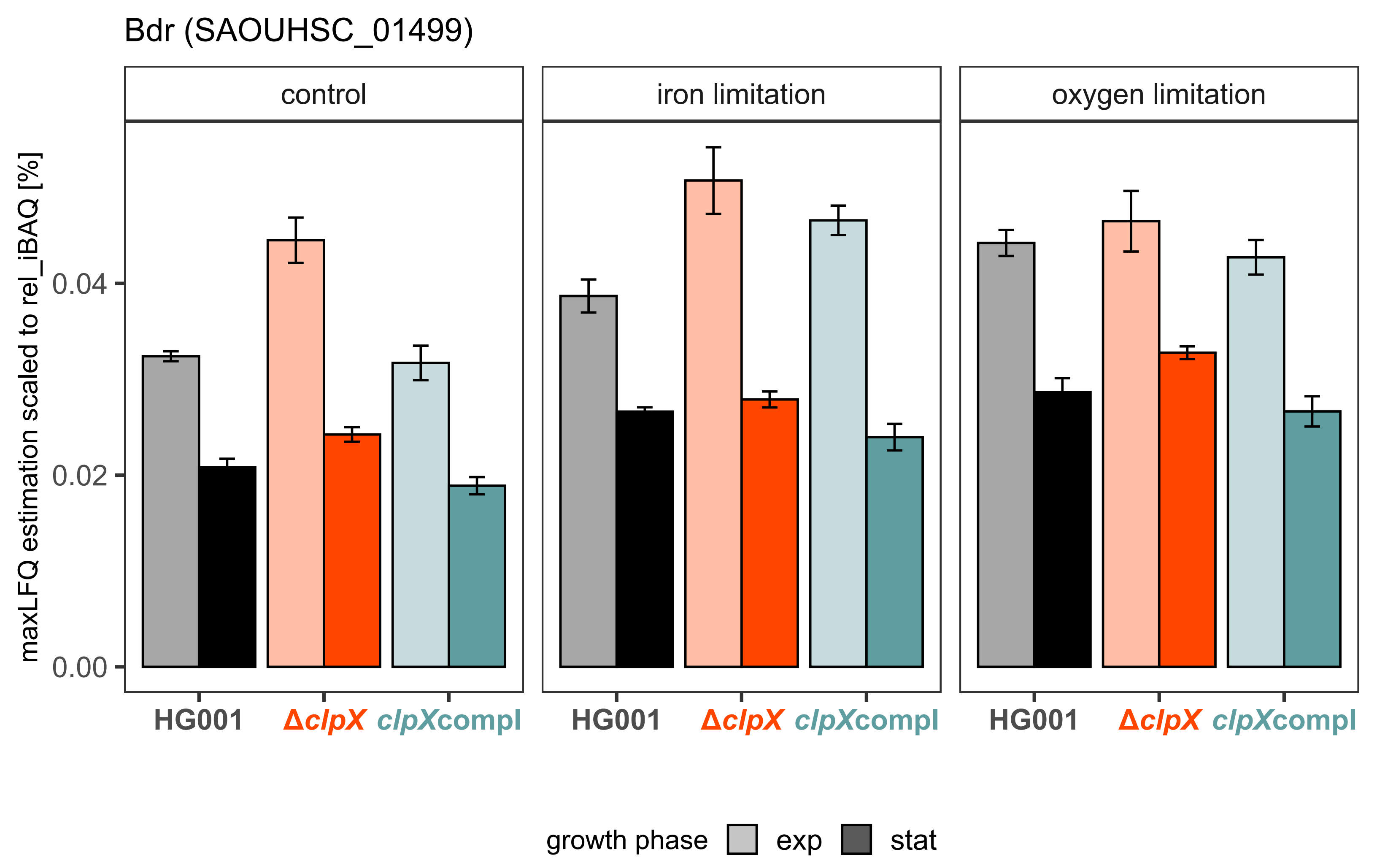

Bdr (SAOUHSC_01499)
control
iron limitation
oxygen limitation
maxLFQ estimation scaled to rel_iBAQ [%]
0.00
0.02
0.04
HG001
ΔclpX
clpXcompl
growth phase
exp
stat

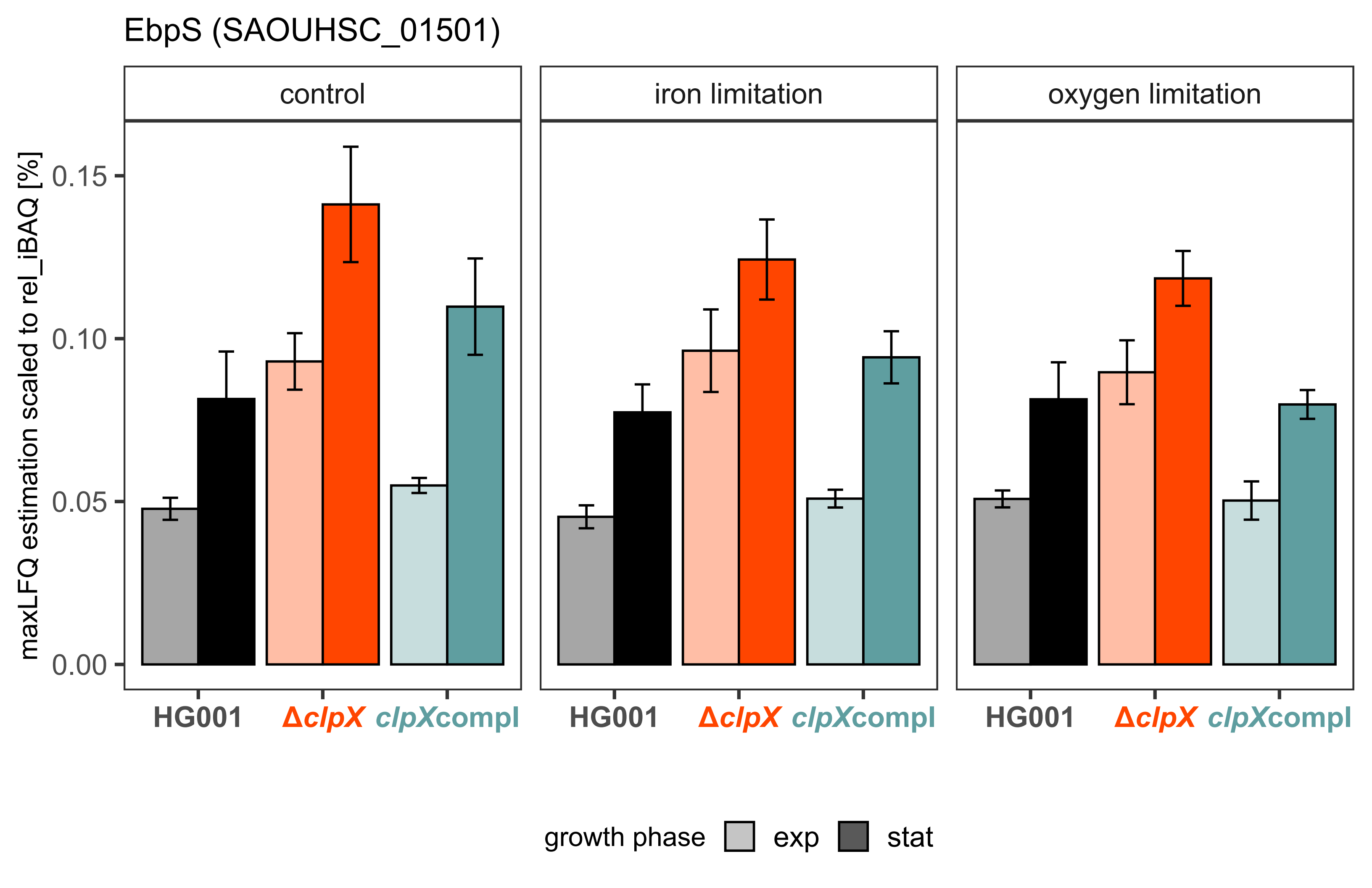
EbpS (SAOUHSC_01501)
control
iron limitation
oxygen limitation
maxLFQ estimation scaled to rel_iBAQ [%]
0.00
0.05
0.10
0.15
HG001
ΔclpX
clpXcompl
growth phase
exp
stat

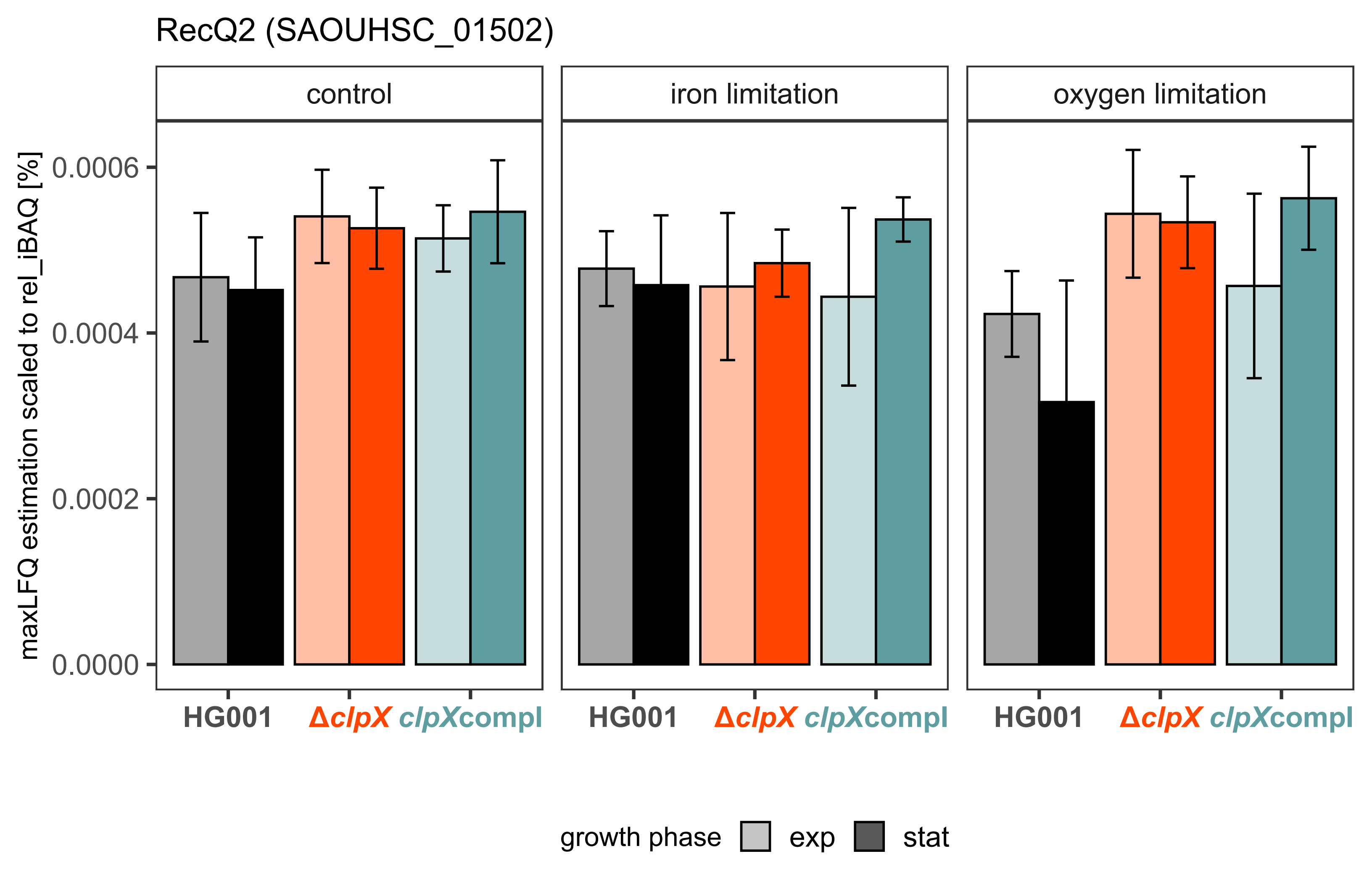
RecQ2 (SAOUHSC_01502)
control
iron limitation
oxygen limitation
maxLFQ estimation scaled to rel_iBAQ [%]
0.0006
0.0004
0.0002
0.0000
HG001
ΔclpX
clpXcompl
growth phase
exp
stat

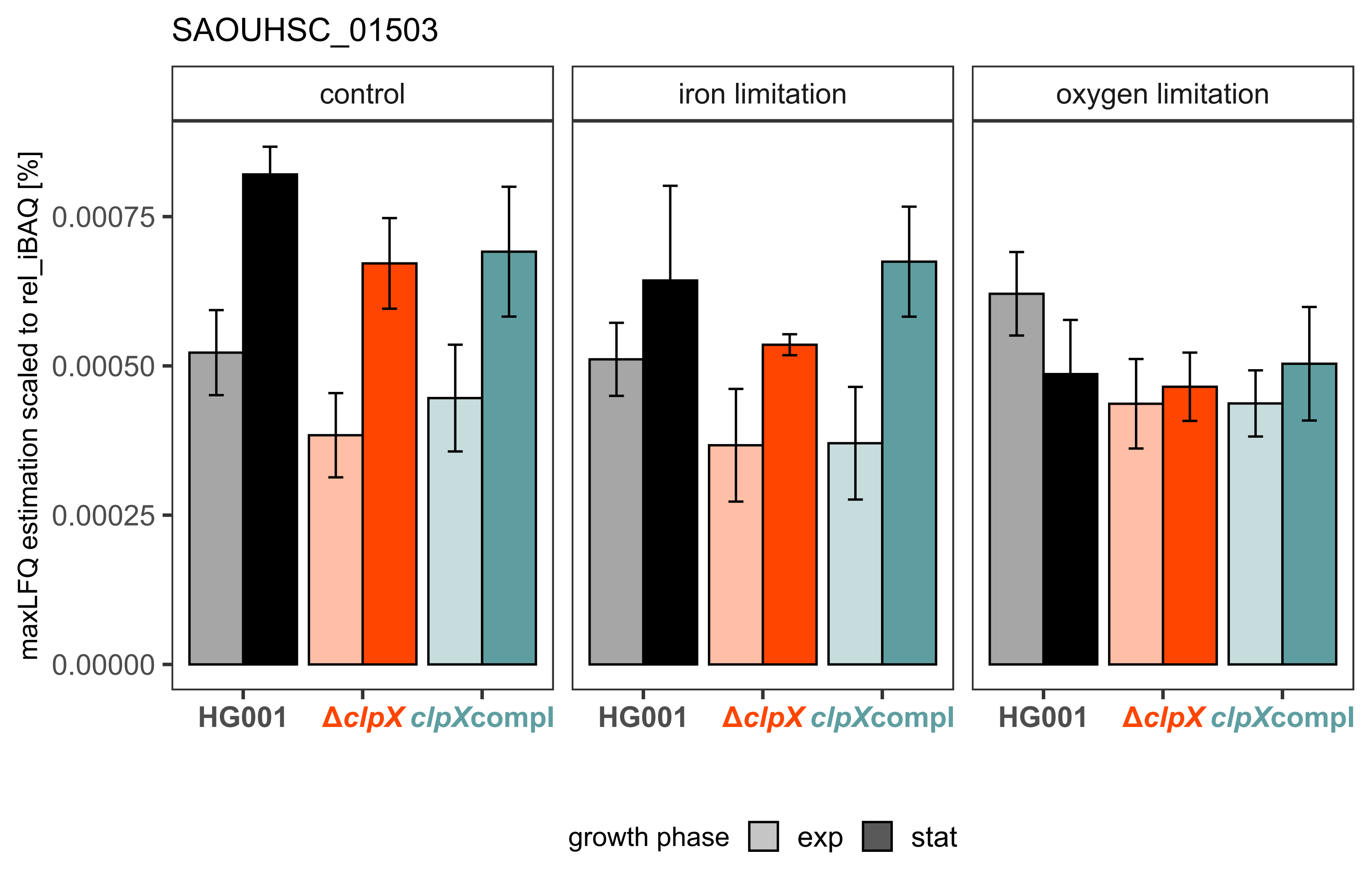

SAOUHSC_01503
control
iron limitation
oxygen limitation
maxLFQ estimation scaled to rel_iBAQ [%]
0.00075
0.00050
0.00025
0.00000
HG001
ΔclpX
clpXcompl
growth phase
exp
stat

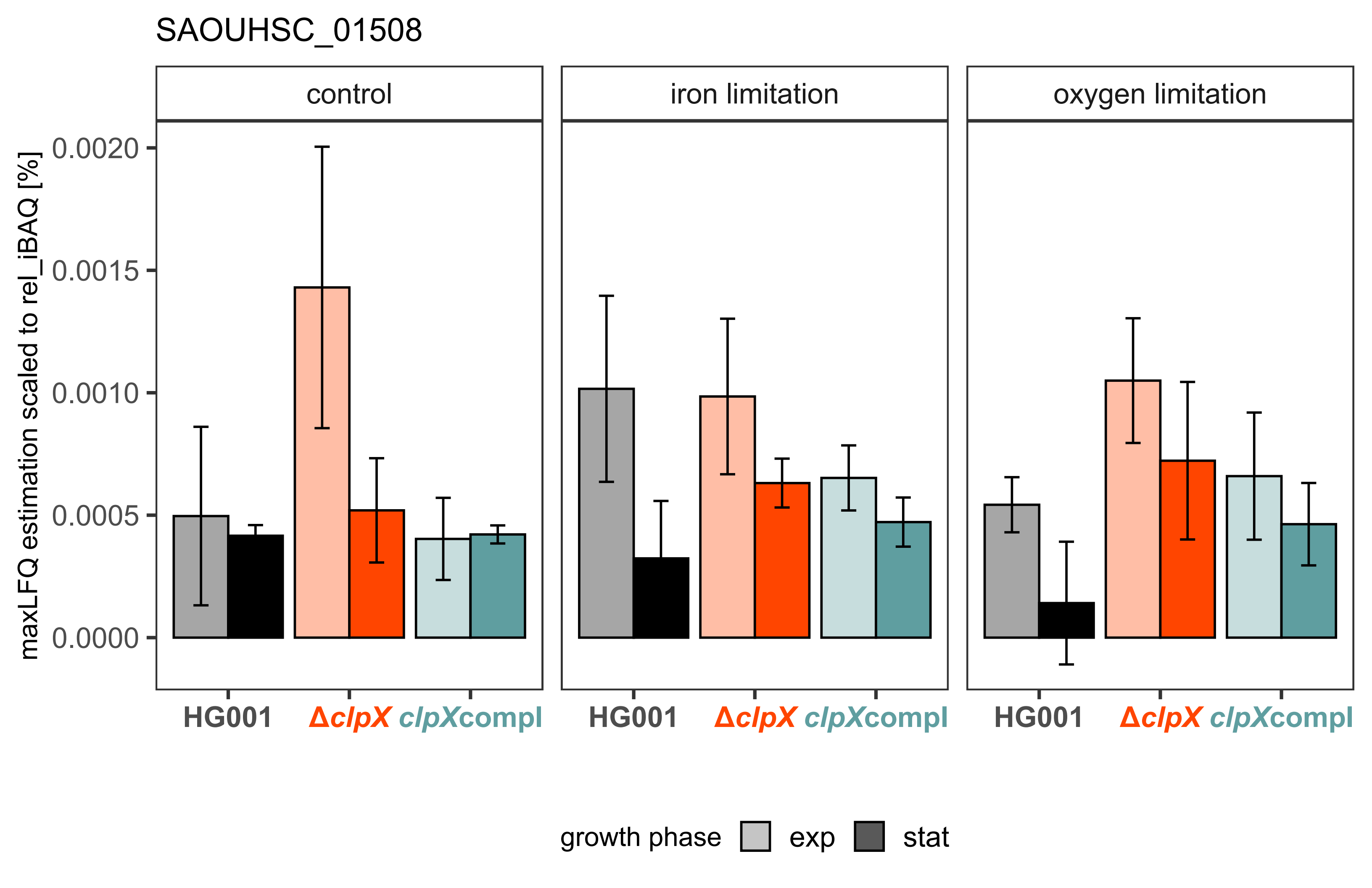

SAOUHSC_01508
control
iron limitation
oxygen limitation
maxLFQ estimation scaled to rel_iBAQ [%]
0.0020
0.0015
0.0010
0.0005
0.0000
HG001
ΔclpX
clpXcompl
growth phase
exp
stat

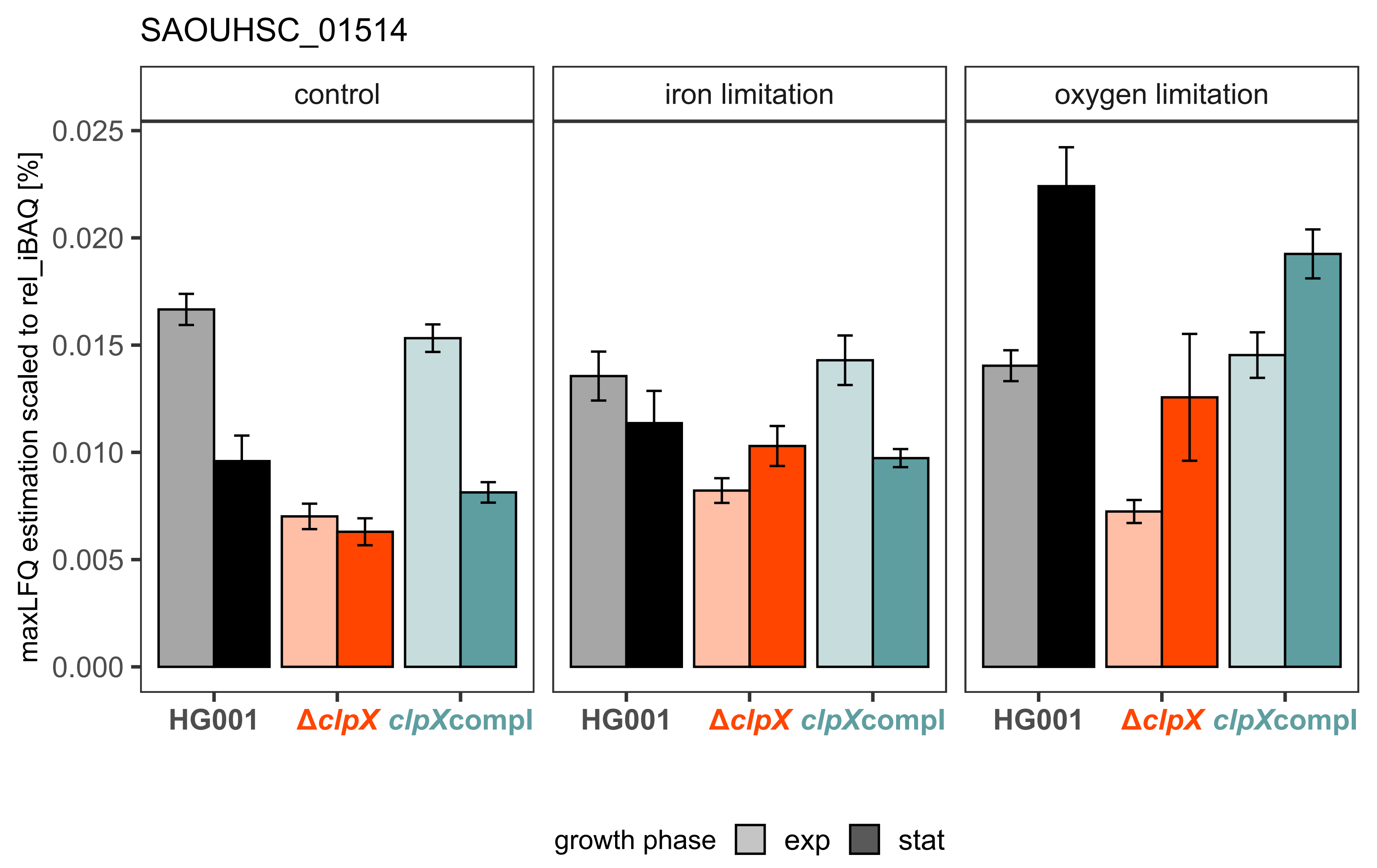
SAOUHSC_01514
control
iron limitation
oxygen limitation
maxLFQ estimation scaled to rel_iBAQ [%]
0.025
0.020
0.015
0.010
0.005
0.000
HG001
ΔclpX
clpXcompl
growth phase
exp
stat

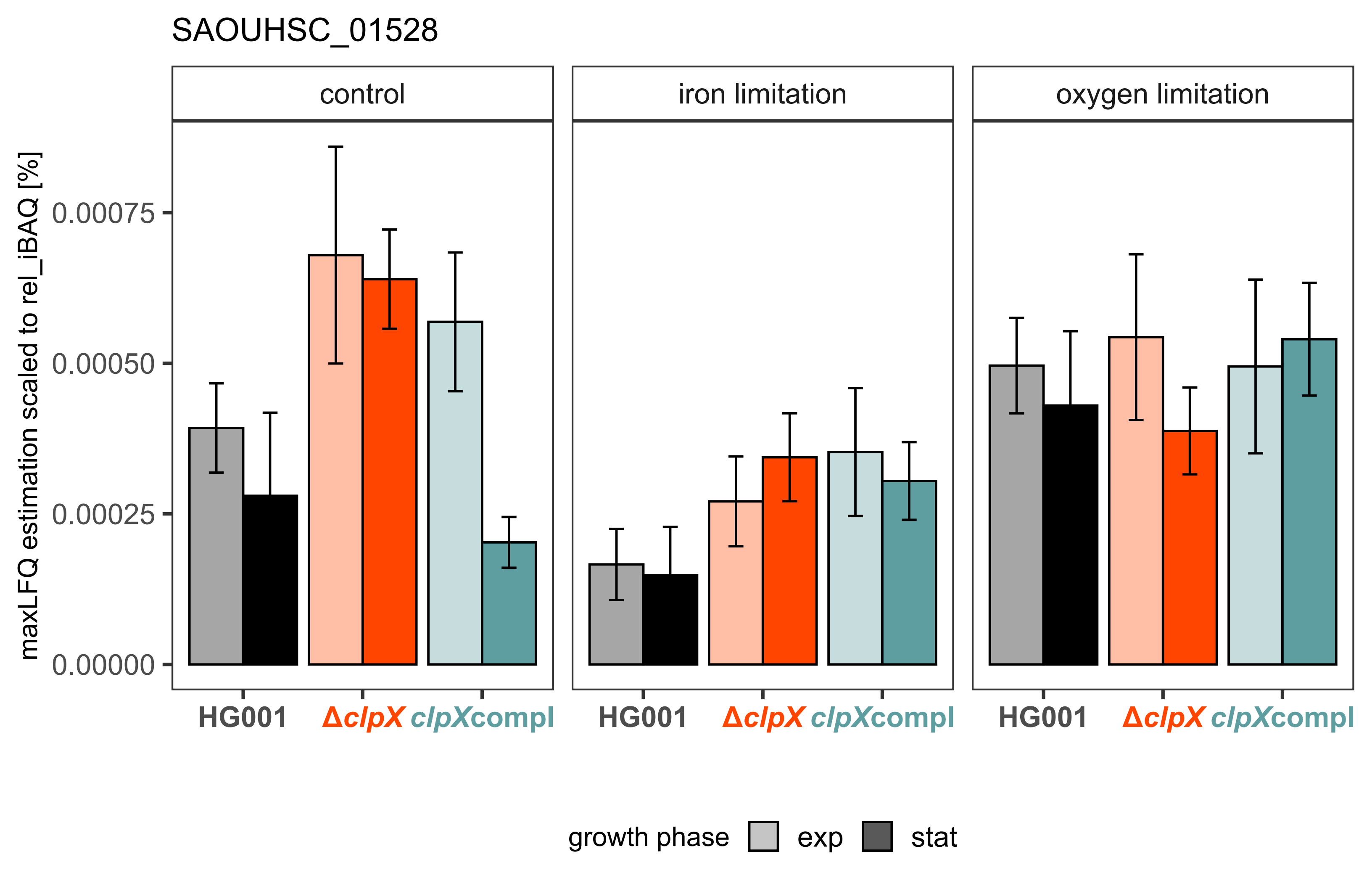

SAOUHSC_01528
control
iron limitation
oxygen limitation
maxLFQ estimation scaled to rel_iBAQ [%]
0.00075
0.00050
0.00025
0.00000
HG001
ΔclpX
clpXcompl
growth phase
exp
stat

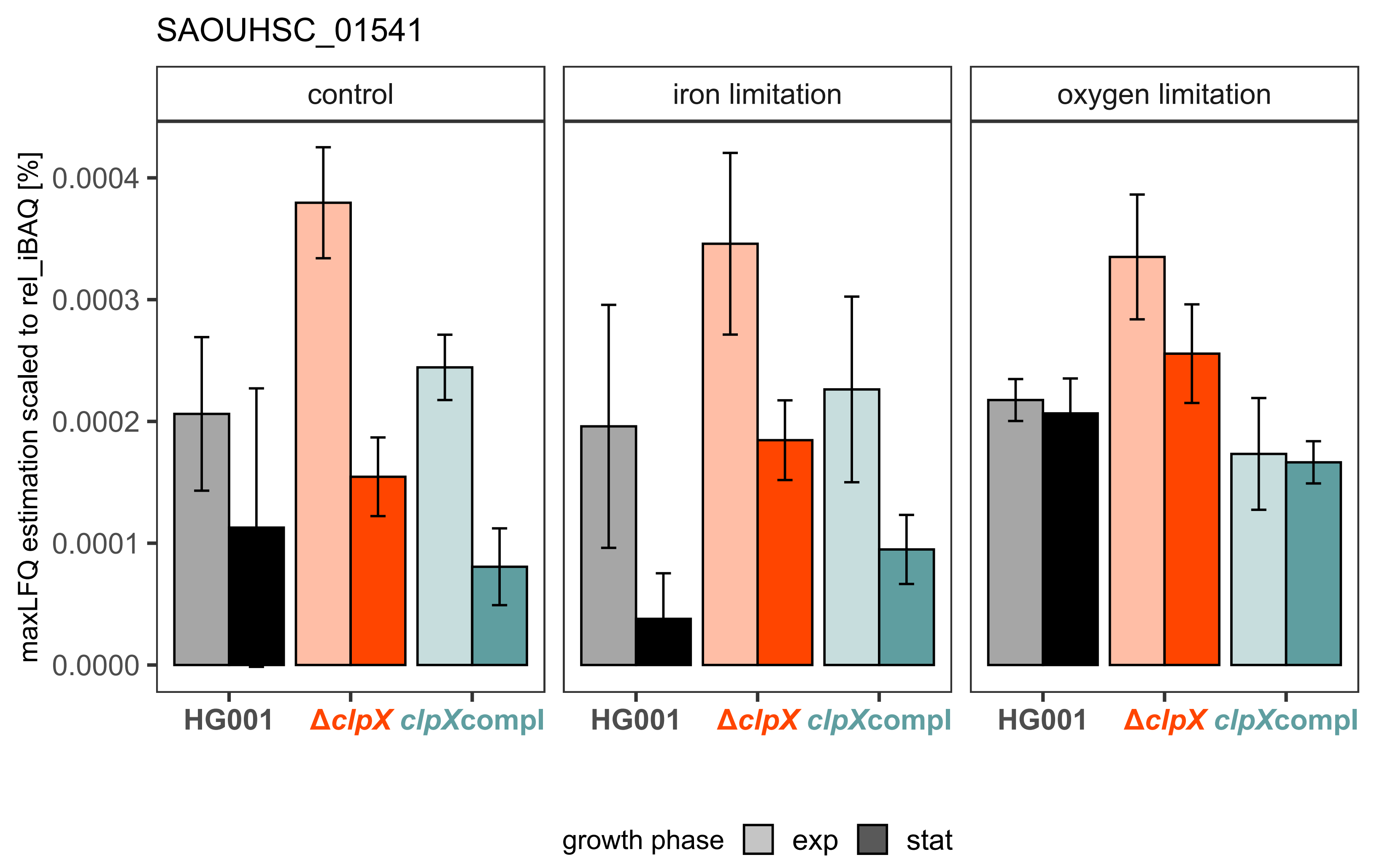
SAOUHSC_01541
control
iron limitation
oxygen limitation
maxLFQ estimation scaled to rel_iBAQ [%]
0.0004
0.0003
0.0002
0.0001
0.0000
HG001
ΔclpX
clpXcompl
growth phase
exp
stat

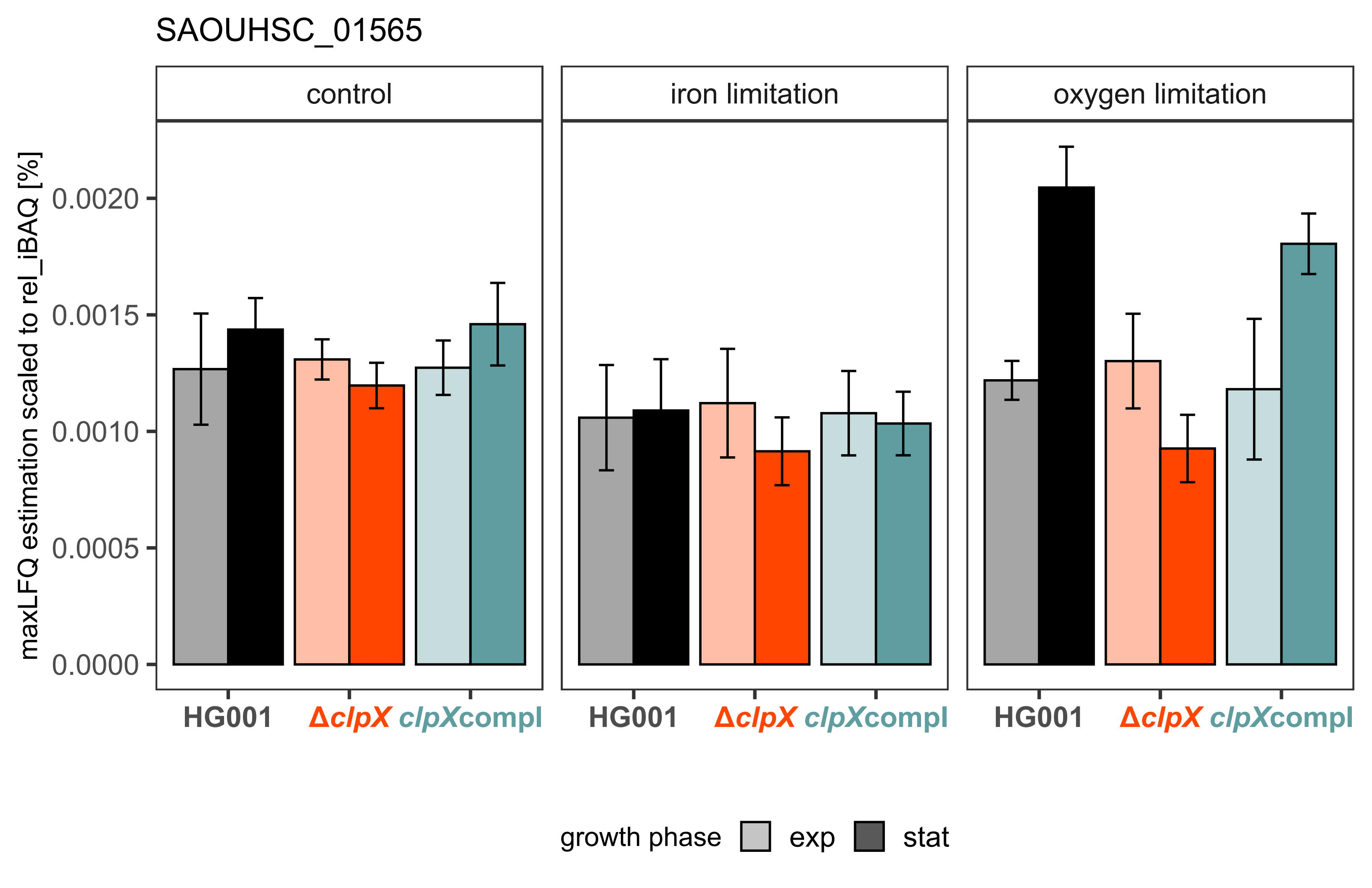
SAOUHSC_01565
control
iron limitation
oxygen limitation
maxLFQ estimation scaled to rel_iBAQ [%]
0.0020
0.0015
0.0010
0.0005
0.0000
HG001
ΔclpX
clpXcompl
growth phase
exp
stat

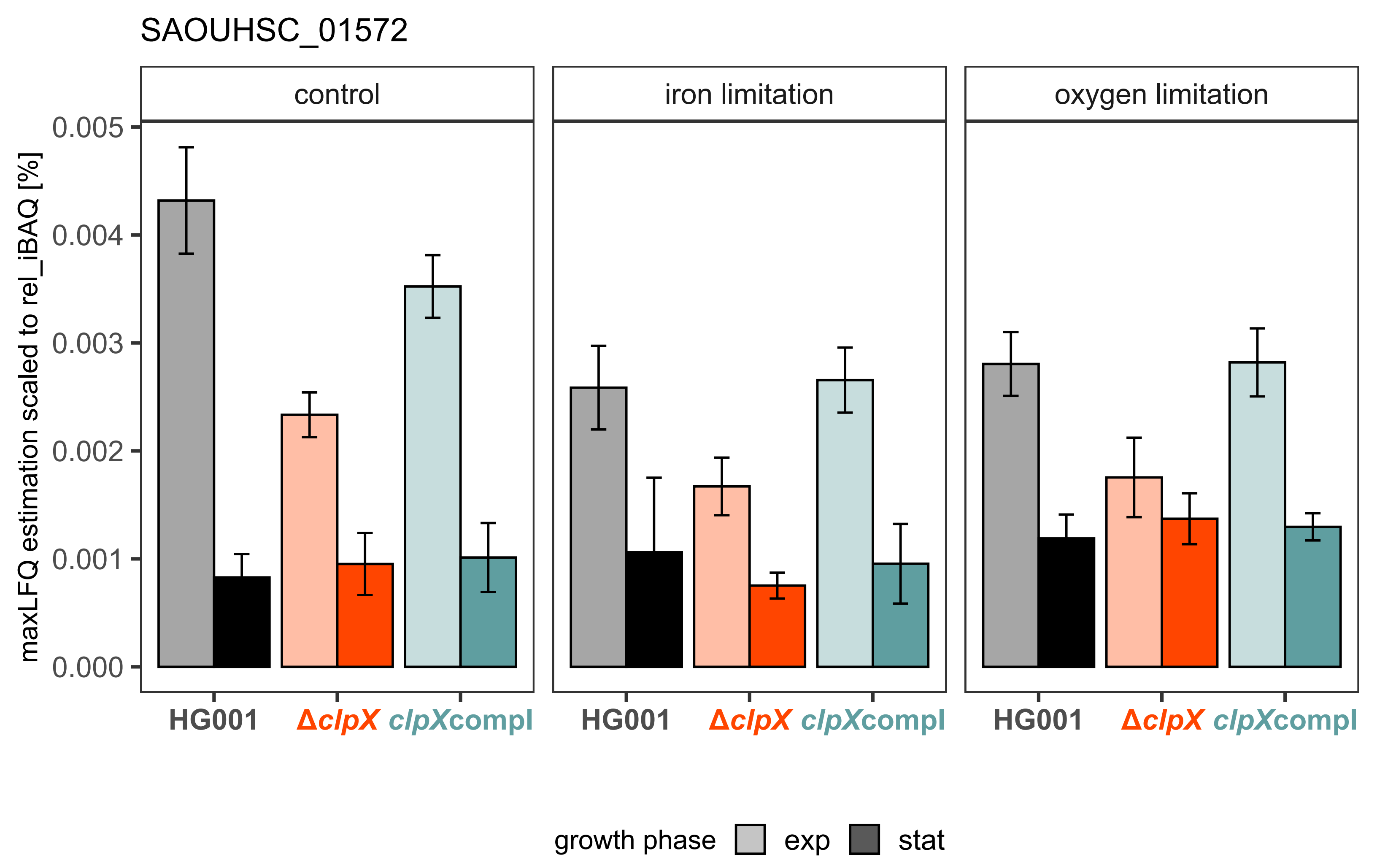
SAOUHSC_01572
control
iron limitation
oxygen limitation
maxLFQ estimation scaled to rel_iBAQ [%]
0.005
0.004
0.003
0.002
0.001
0.000
HG001
ΔclpX
clpXcompl
growth phase
exp
stat

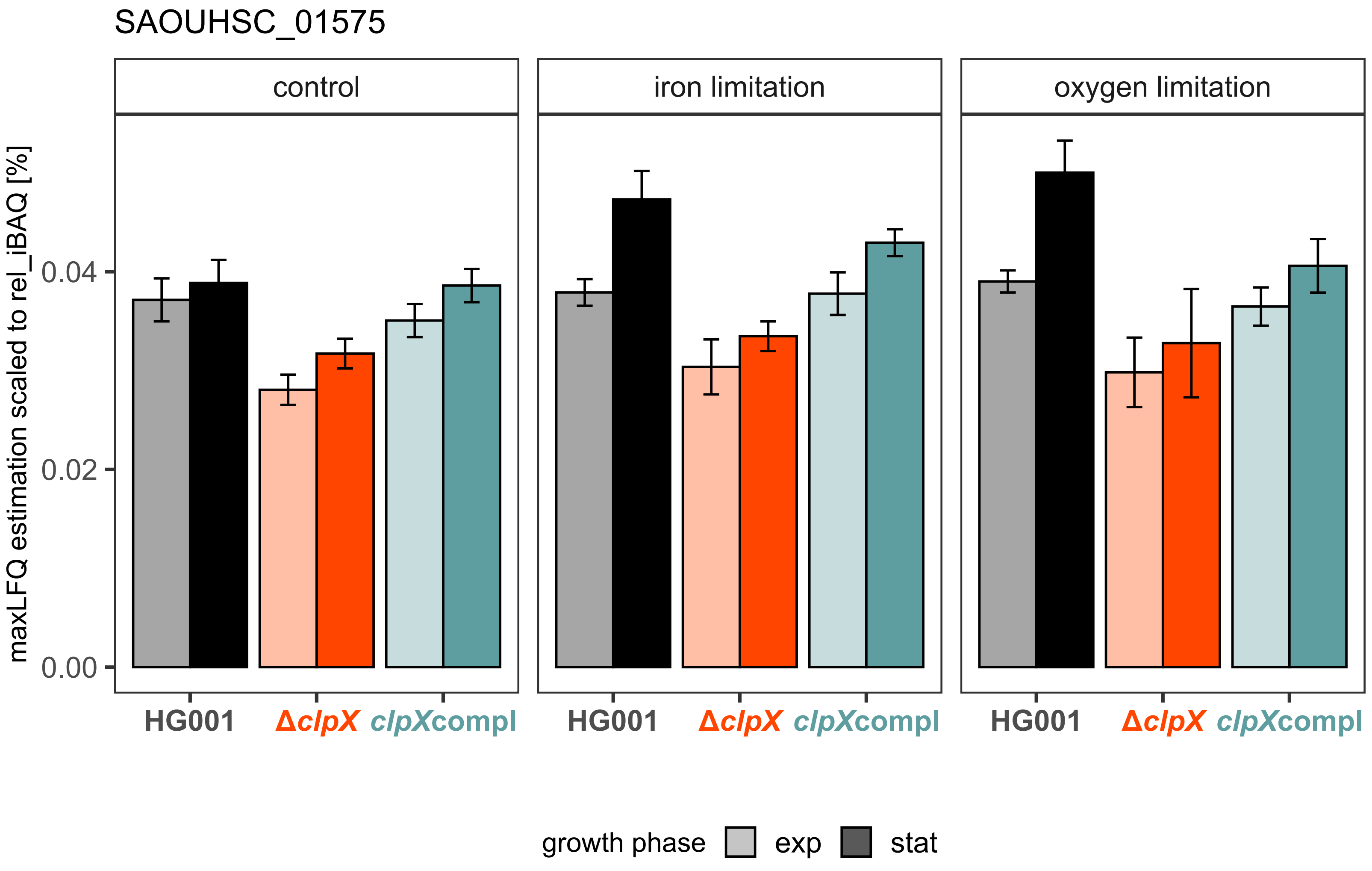
SAOUHSC_01575
control
iron limitation
oxygen limitation
maxLFQ estimation scaled to rel_iBAQ [%]
0.04
0.02
0.00
HG001
ΔclpX
clpXcompl
growth phase
exp
stat

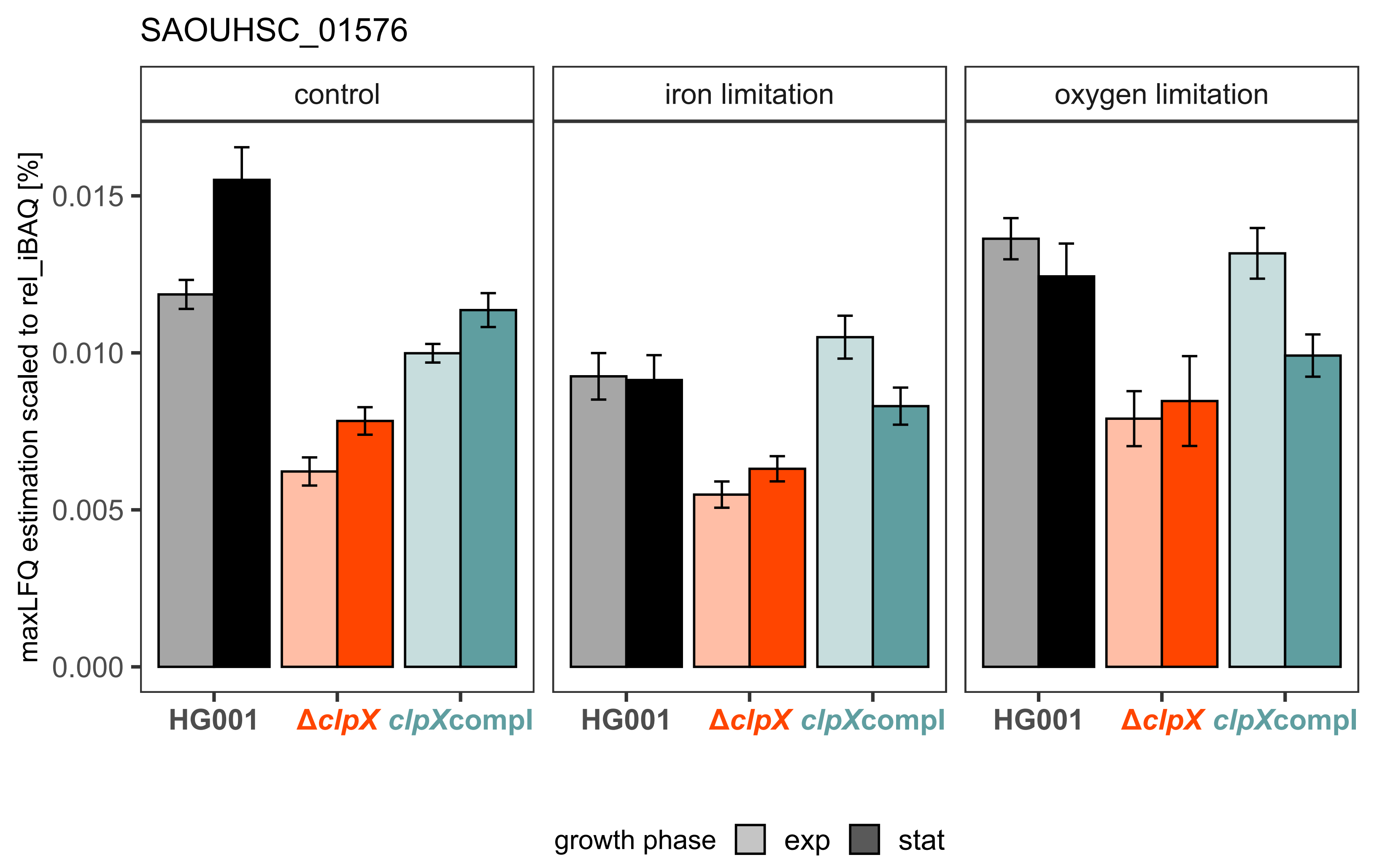

SAOUHSC_01576
control
iron limitation
oxygen limitation
maxLFQ estimation scaled to rel_iBAQ [%]
0.015
0.010
0.005
0.000
HG001
ΔclpX
clpXcompl
growth phase
exp
stat

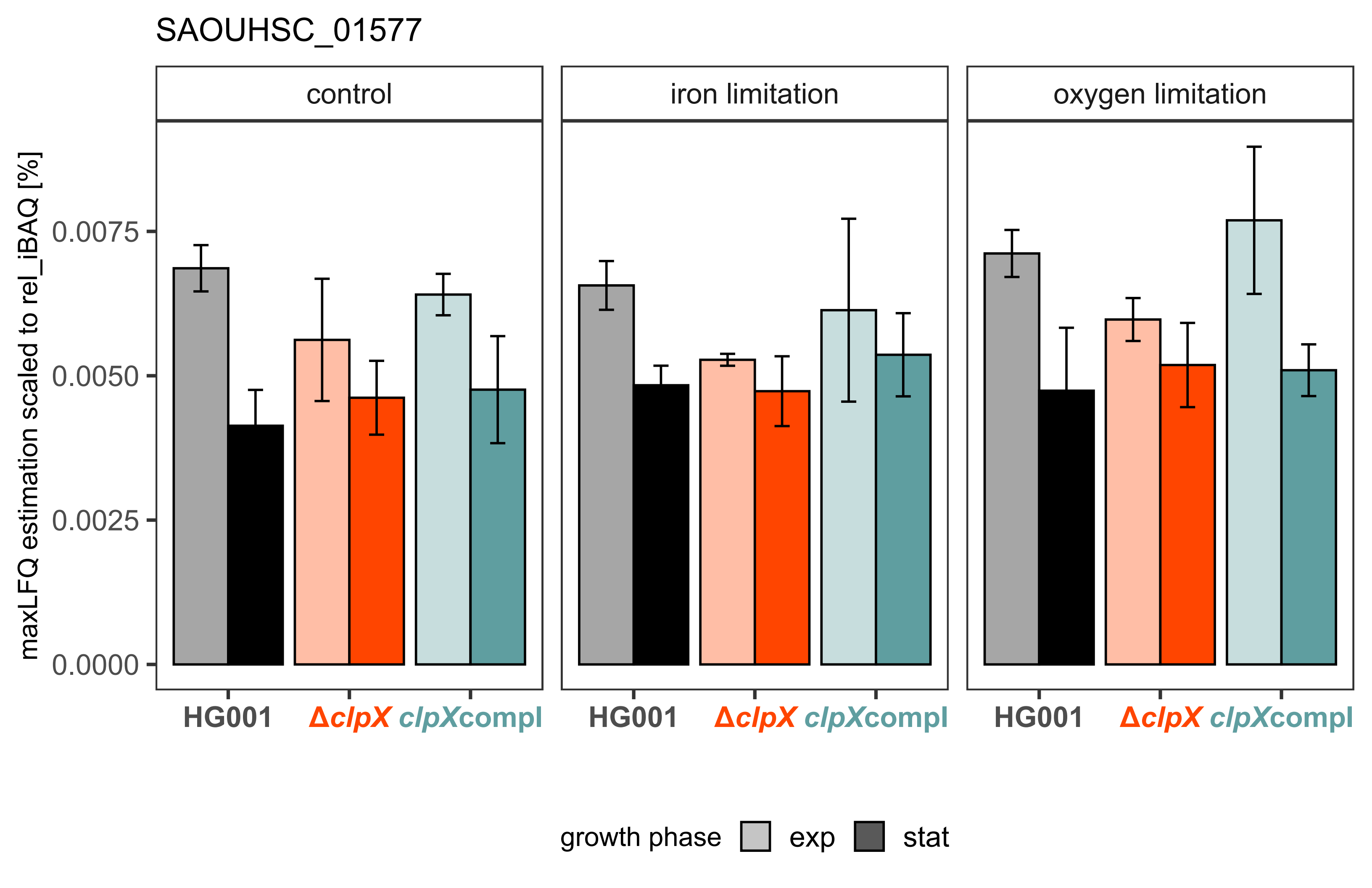
SAOUHSC_01577
control
iron limitation
oxygen limitation
maxLFQ estimation scaled to rel_iBAQ [%]
0.0075
0.0050
0.0025
0.0000
HG001
ΔclpX
clpXcompl
growth phase
exp
stat

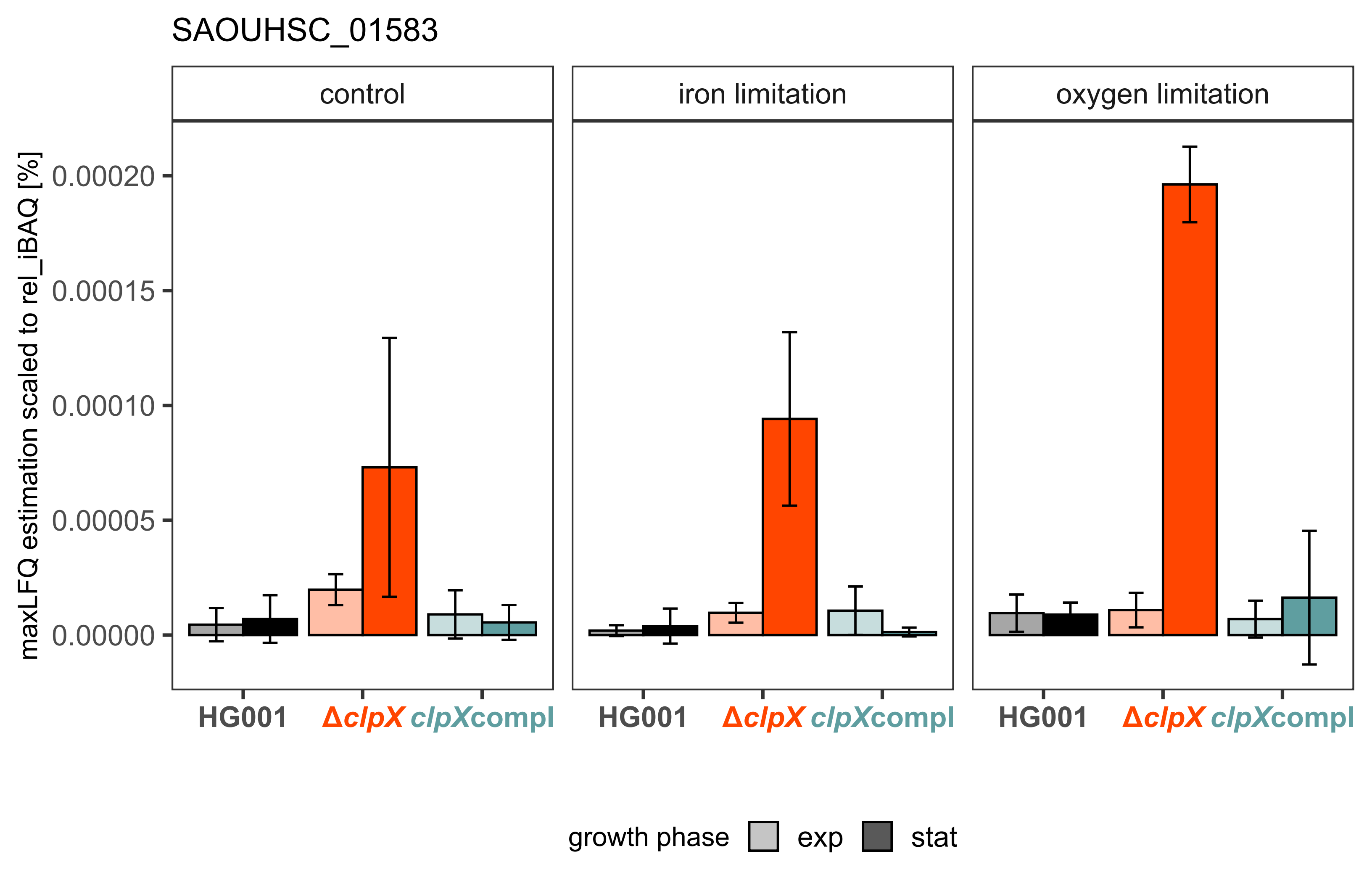

SAOUHSC_01583
control
iron limitation
oxygen limitation
maxLFQ estimation scaled to rel_iBAQ [%]
0.00020
0.00015
0.00010
0.00005
0.00000
HG001
ΔclpX
clpXcompl
growth phase
exp
stat

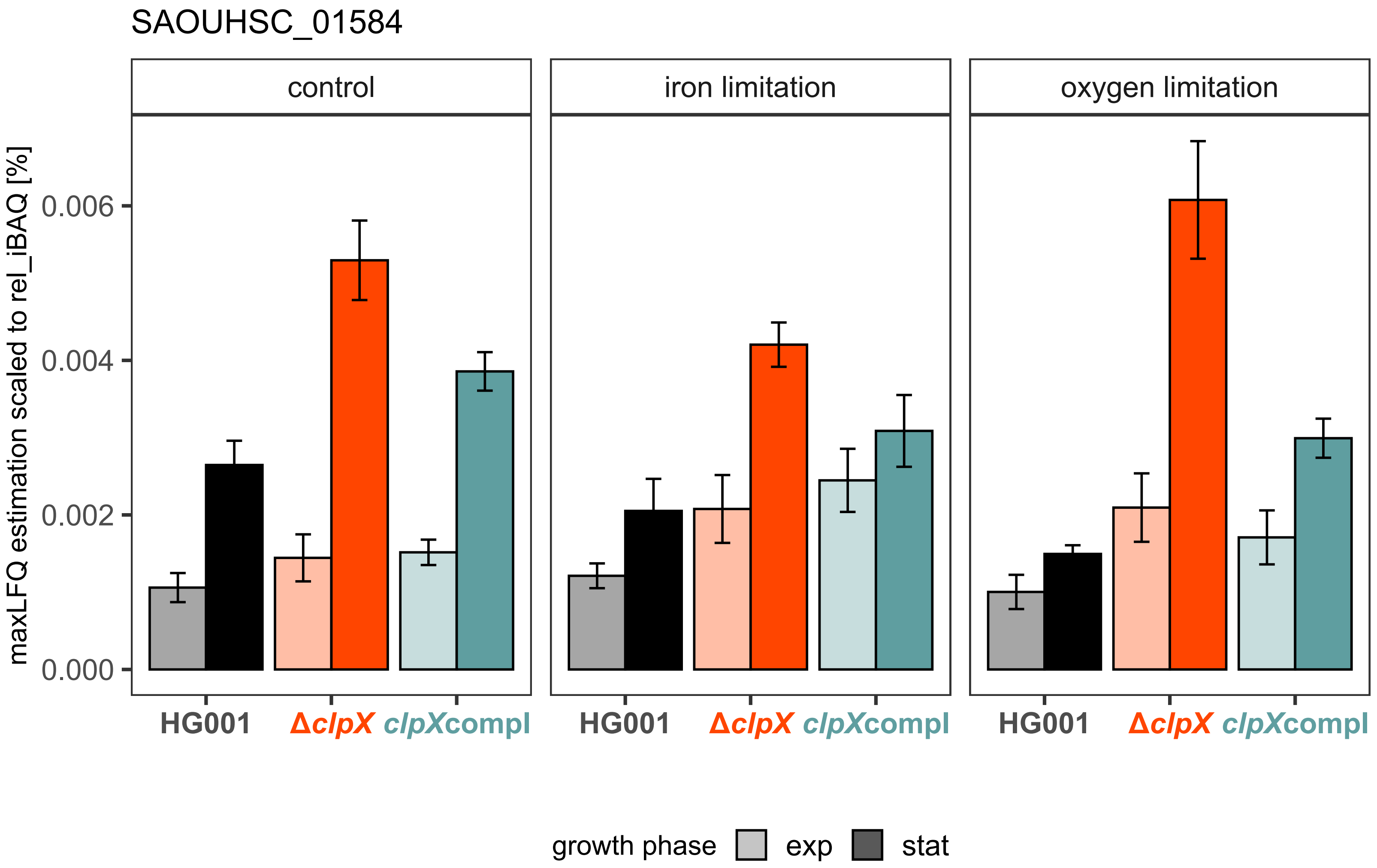
SAOUHSC_01584
control
iron limitation
oxygen limitation
maxLFQ estimation scaled to rel_iBAQ [%]
0.006
0.004
0.002
0.000
HG001
ΔclpX
clpXcompl
growth phase
exp
stat

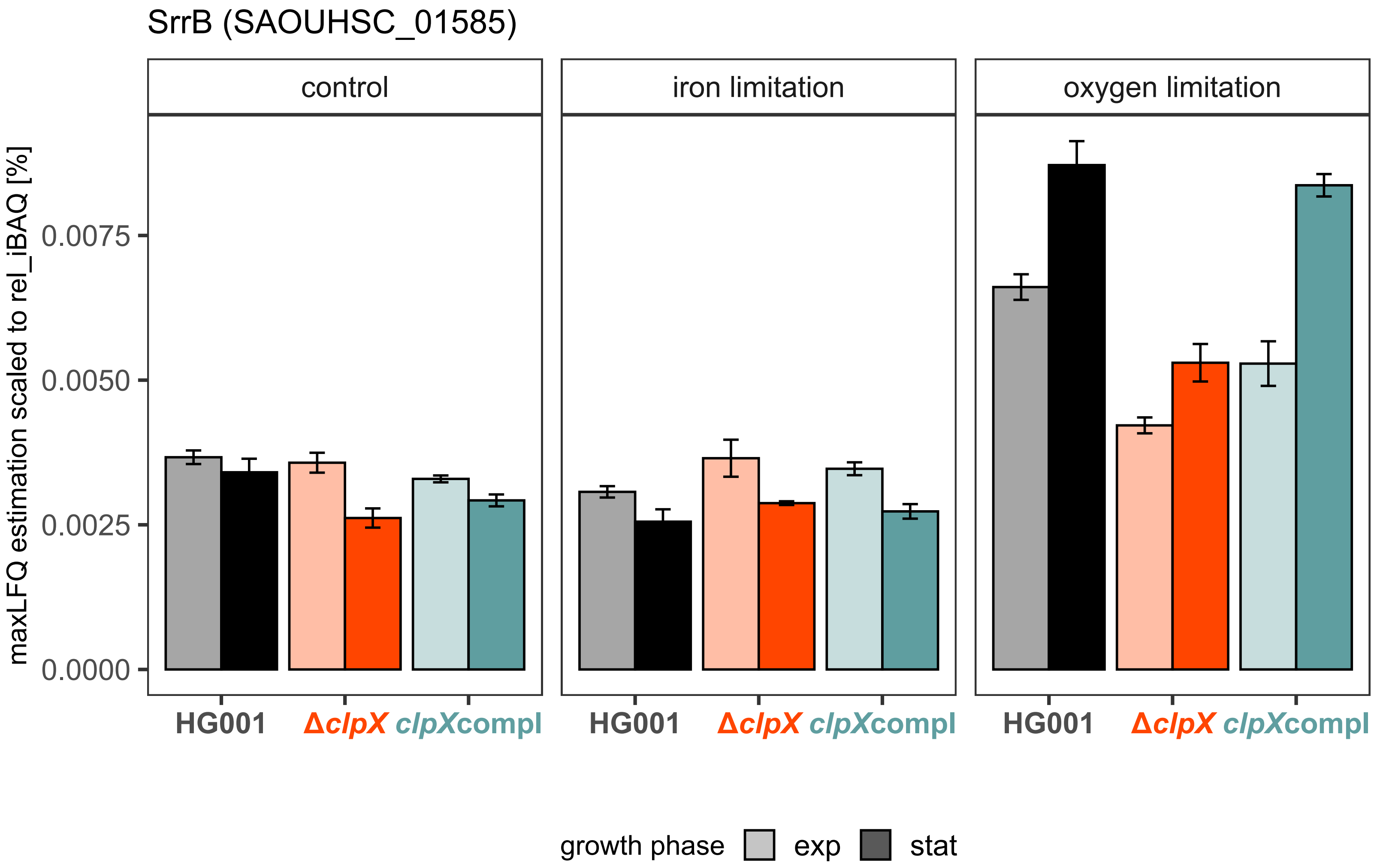
SrrB (SAOUHSC_01585)
control
iron limitation
oxygen limitation
maxLFQ estimation scaled to rel_iBAQ [%]
0.0075
0.0050
0.0025
0.0000
HG001
ΔclpX
clpXcompl
growth phase
exp
stat

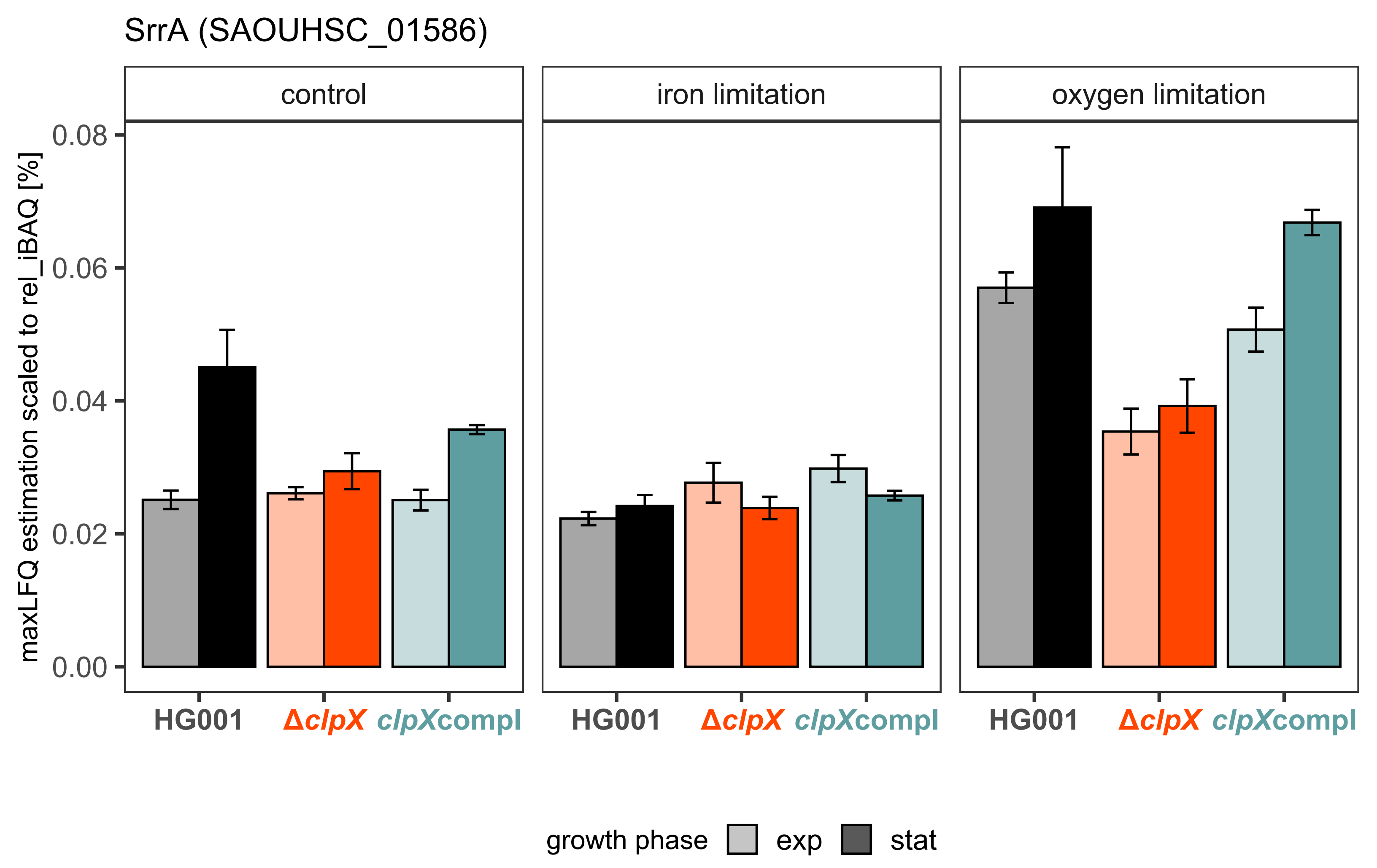
SrrA (SAOUHSC_01586)
control
iron limitation
oxygen limitation
maxLFQ estimation scaled to rel_iBAQ [%]
0.08
0.06
0.04
0.02
0.00
HG001
ΔclpX
clpXcompl
growth phase
exp
stat

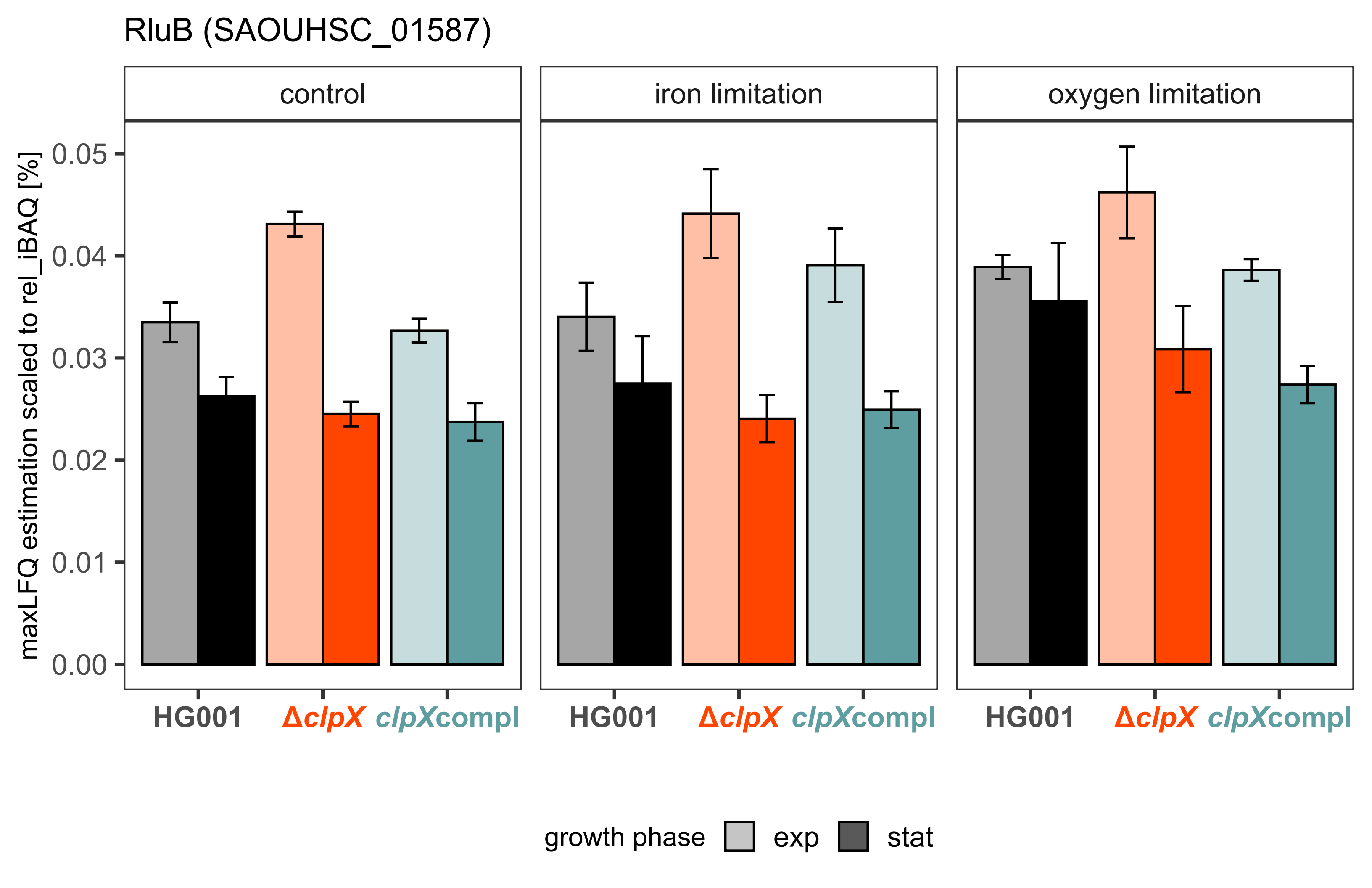
RluB (SAOUHSC_01587)
control
iron limitation
oxygen limitation
maxLFQ estimation scaled to rel_iBAQ [%]
0.05
0.04
0.03
0.02
0.01
0.00
HG001
ΔclpX
clpXcompl
growth phase
exp
stat

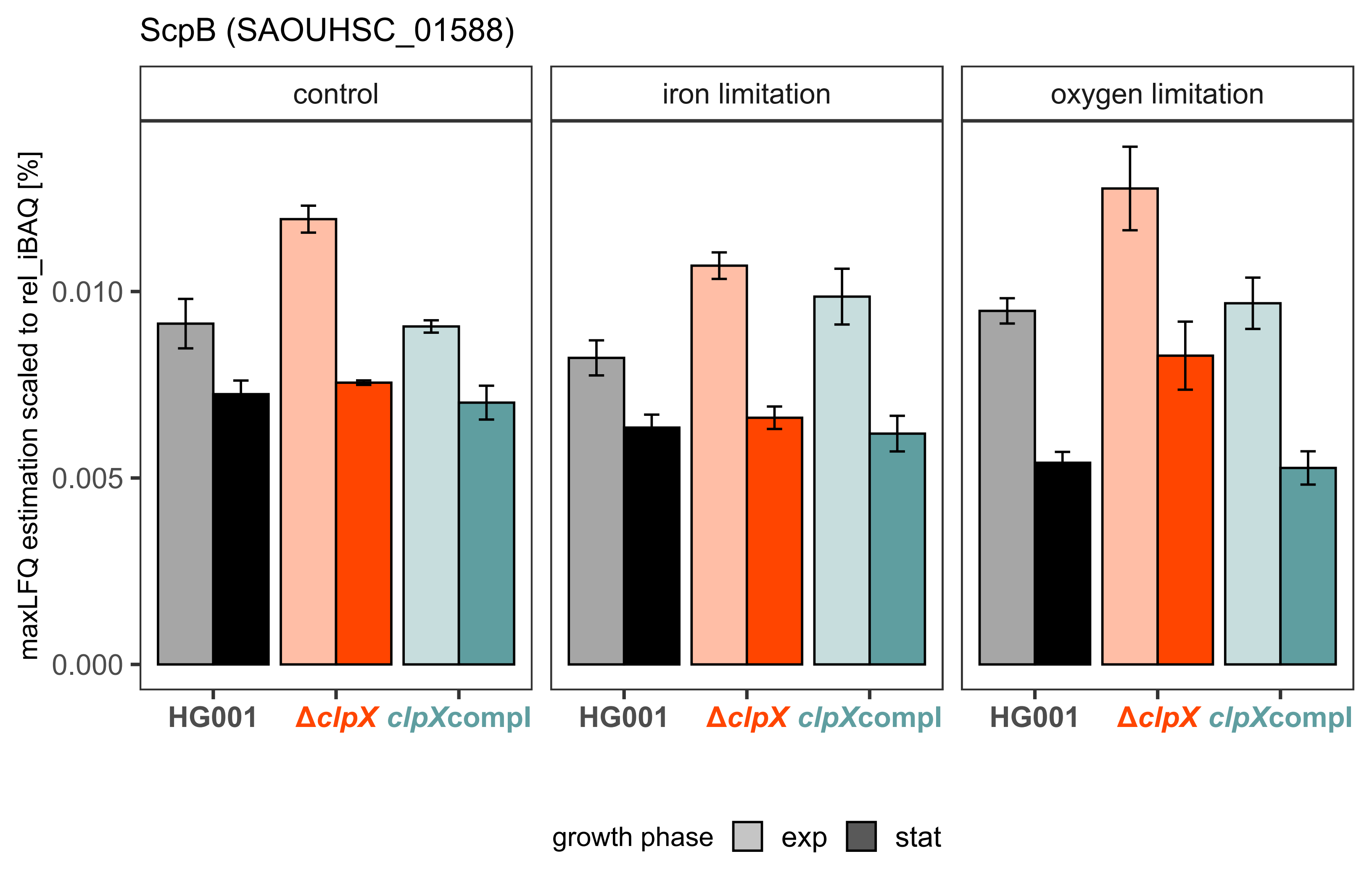

ScpB (SAOUHSC_01588)
control
iron limitation
oxygen limitation
maxLFQ estimation scaled to rel_iBAQ [%]
0.010
0.005
0.000
HG001
ΔclpX
clpXcompl
growth phase
exp
stat

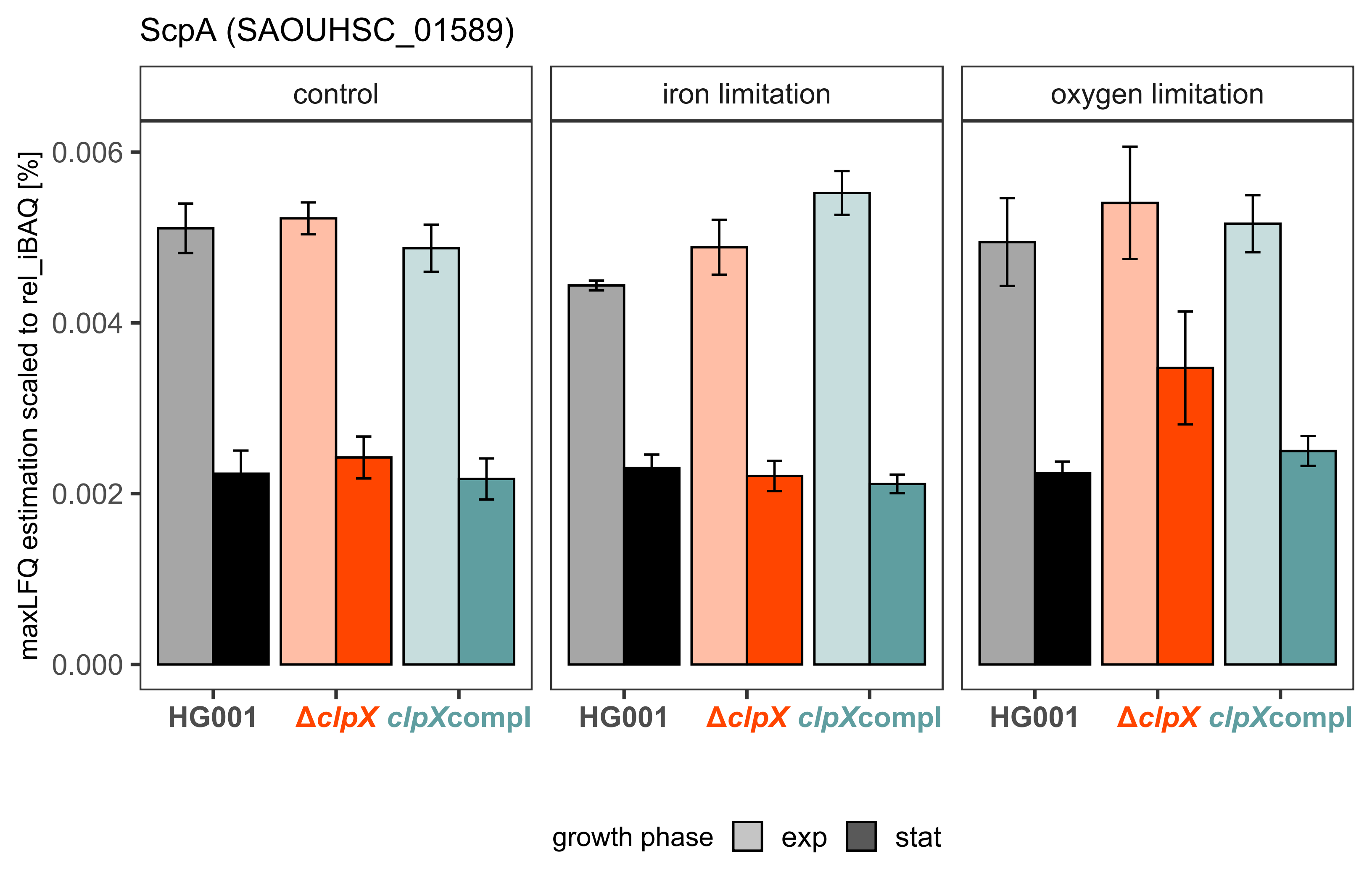

ScpA (SAOUHSC_01589)
control
iron limitation
oxygen limitation
maxLFQ estimation scaled to rel_iBAQ [%]
0.006
0.004
0.002
0.000
HG001
ΔclpX
clpXcompl
growth phase
exp
stat

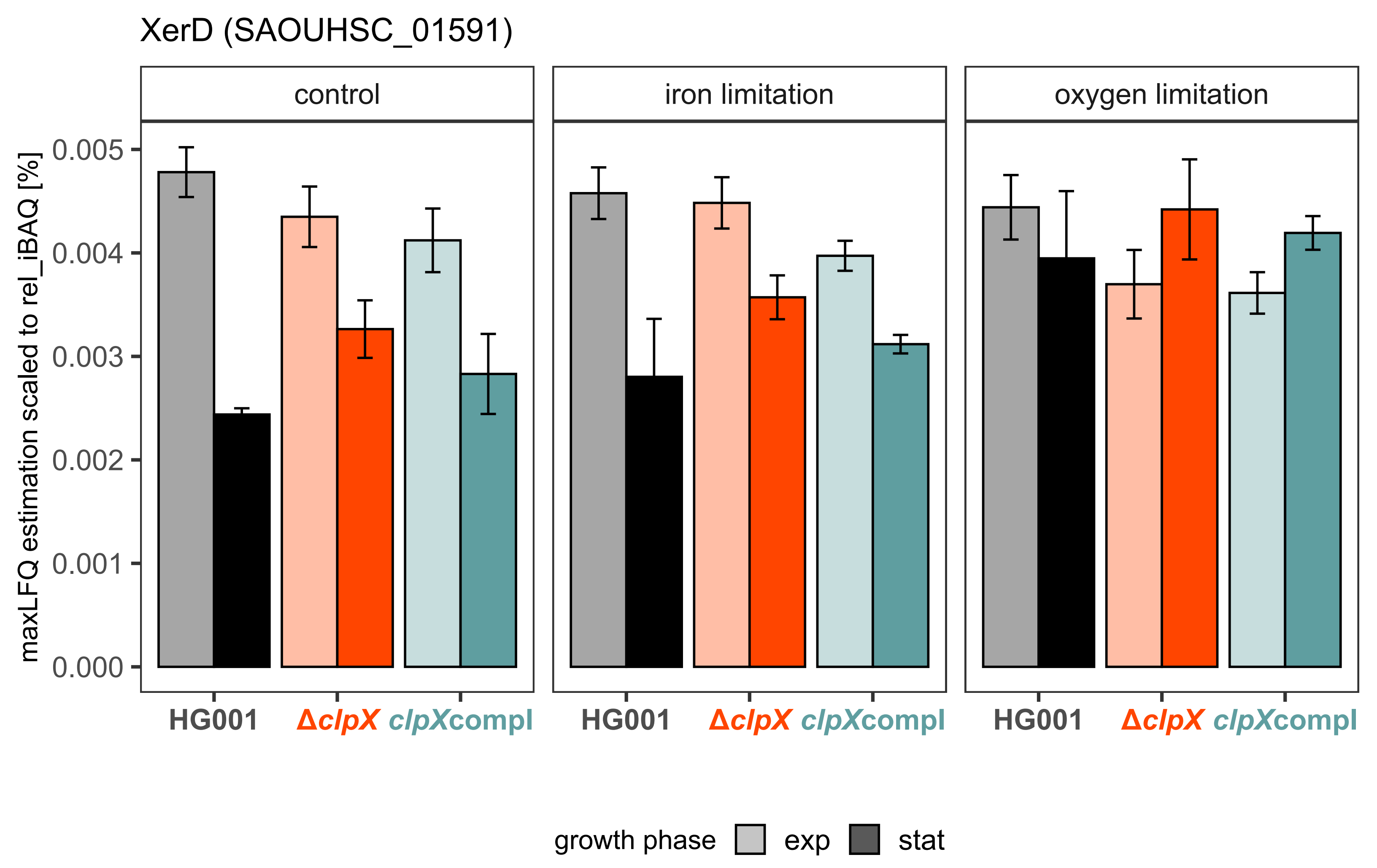
XerD (SAOUHSC_01591)
control
iron limitation
oxygen limitation
maxLFQ estimation scaled to rel_iBAQ [%]
0.005
0.004
0.003
0.002
0.001
0.000
HG001
ΔclpX
clpXcompl
growth phase
exp
stat

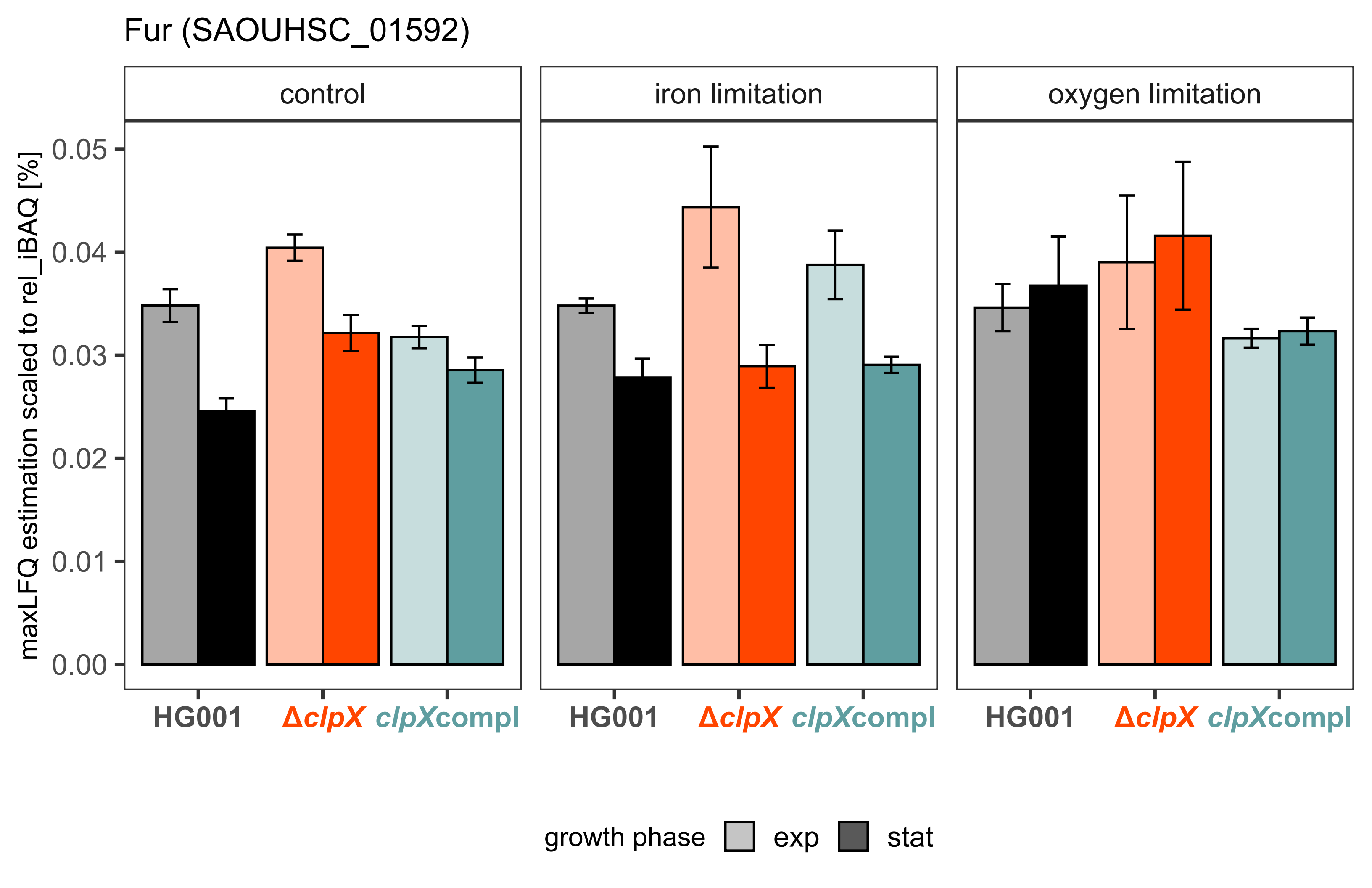

Fur (SAOUHSC_01592)
control
iron limitation
oxygen limitation
maxLFQ estimation scaled to rel_iBAQ [%]
0.05
0.04
0.03
0.02
0.01
0.00
HG001
ΔclpX
clpXcompl
growth phase
exp
stat

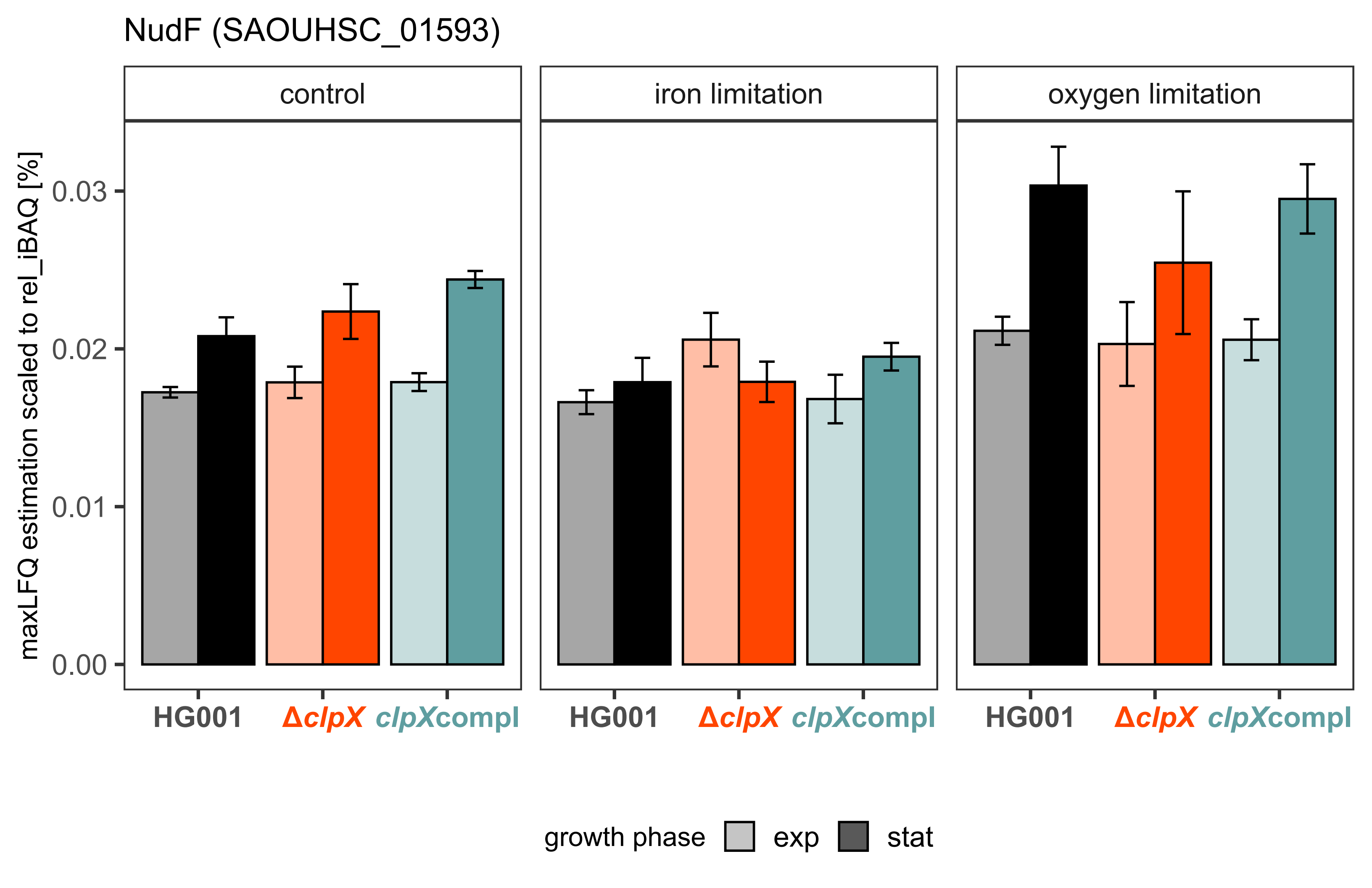

NudF (SAOUHSC_01593)
control
iron limitation
oxygen limitation
maxLFQ estimation scaled to rel_iBAQ [%]
0.00
0.01
0.02
0.03
HG001
ΔclpX
clpXcompl
growth phase
exp
stat

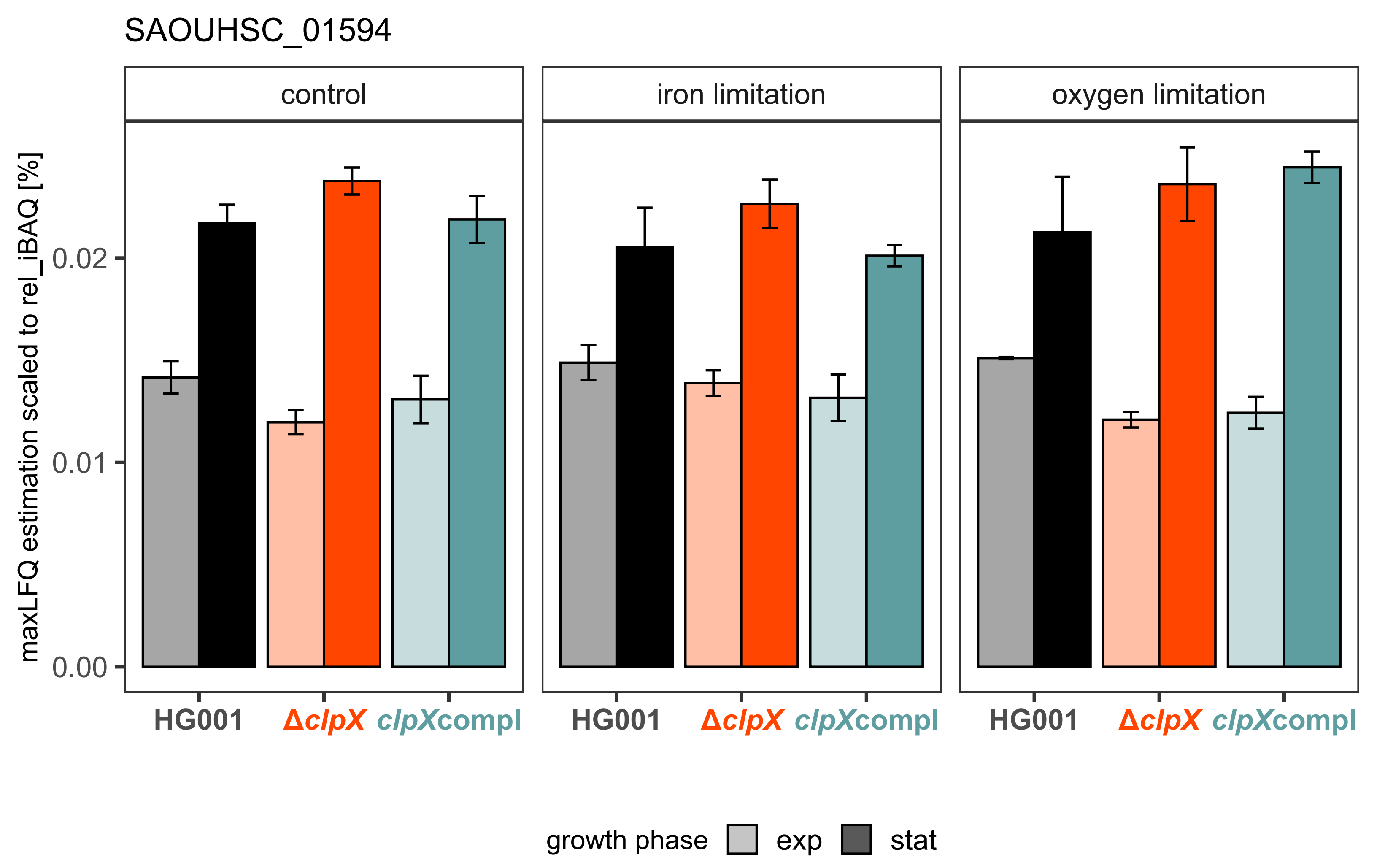

SAOUHSC_01594
control
iron limitation
oxygen limitation
maxLFQ estimation scaled to rel_iBAQ [%]
0.00
0.01
0.02
HG001
ΔclpX
clpXcompl
growth phase
exp
stat

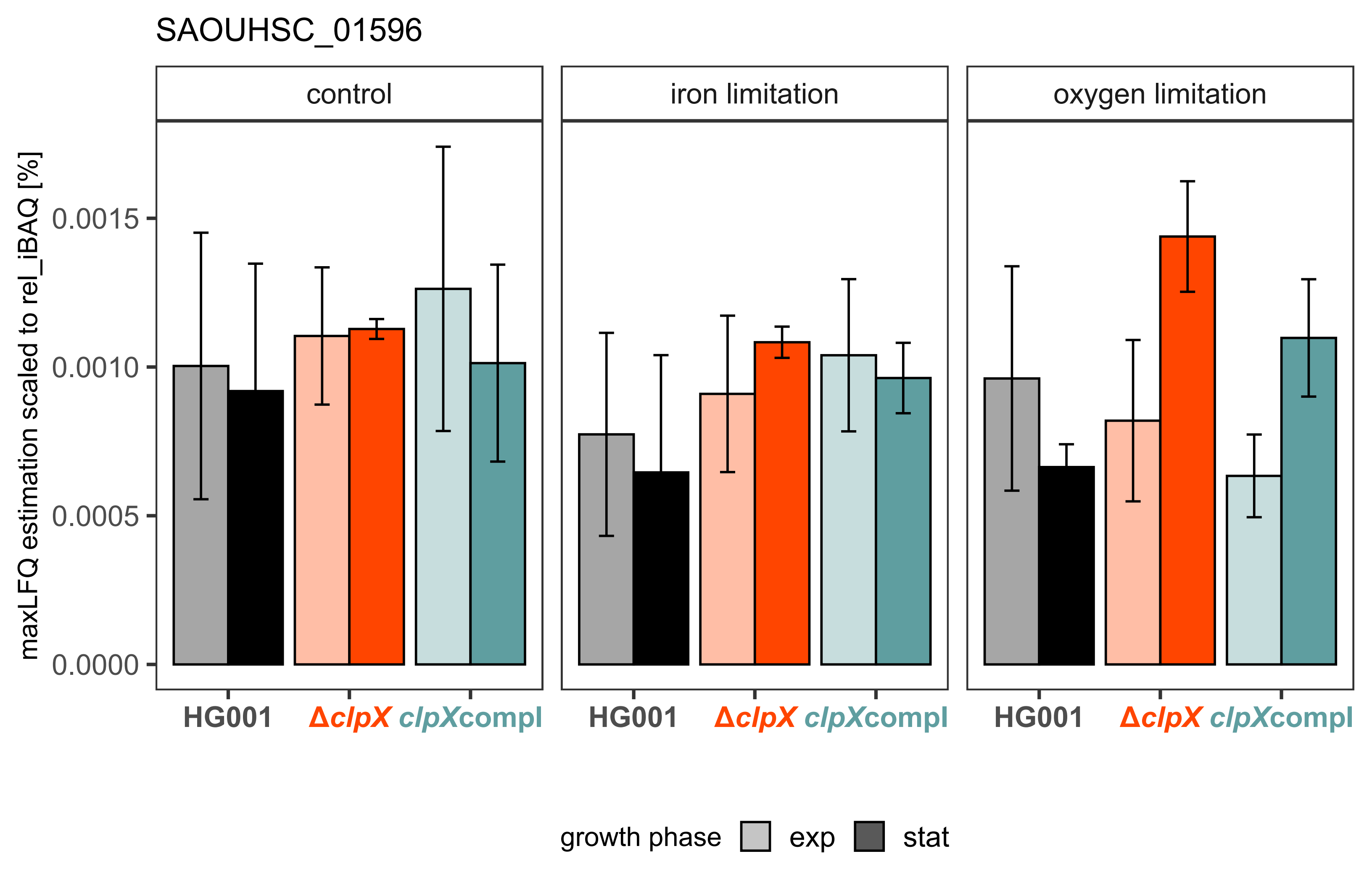

SAOUHSC_01596
control
iron limitation
oxygen limitation
maxLFQ estimation scaled to rel_iBAQ [%]
0.0015
0.0010
0.0005
0.0000
HG001
ΔclpX
clpXcompl
growth phase
exp
stat

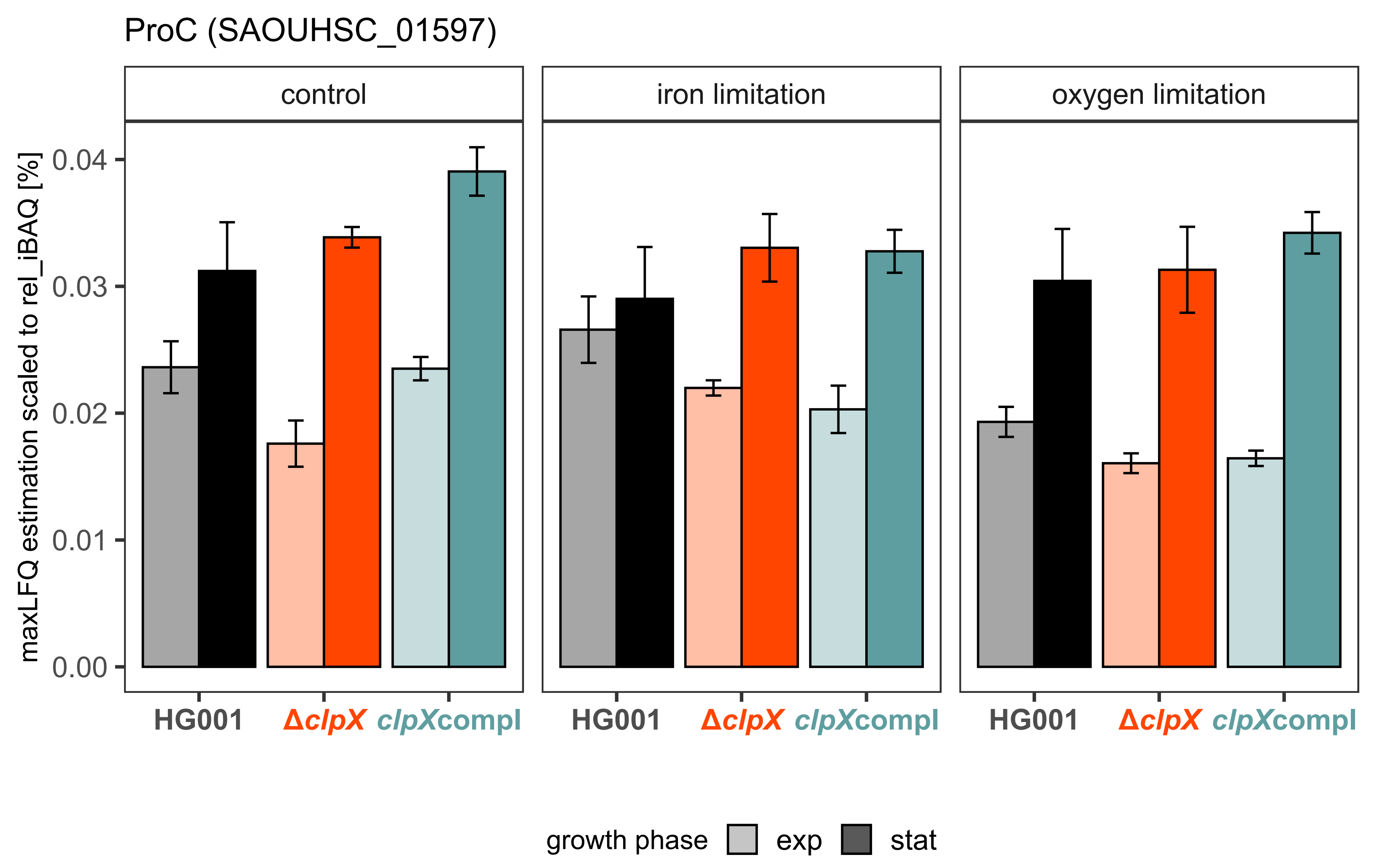
ProC (SAOUHSC_01597)
control
iron limitation
oxygen limitation
maxLFQ estimation scaled to rel_iBAQ [%]
0.04
0.03
0.02
0.01
0.00
HG001
ΔclpX
clpXcompl
growth phase
exp
stat

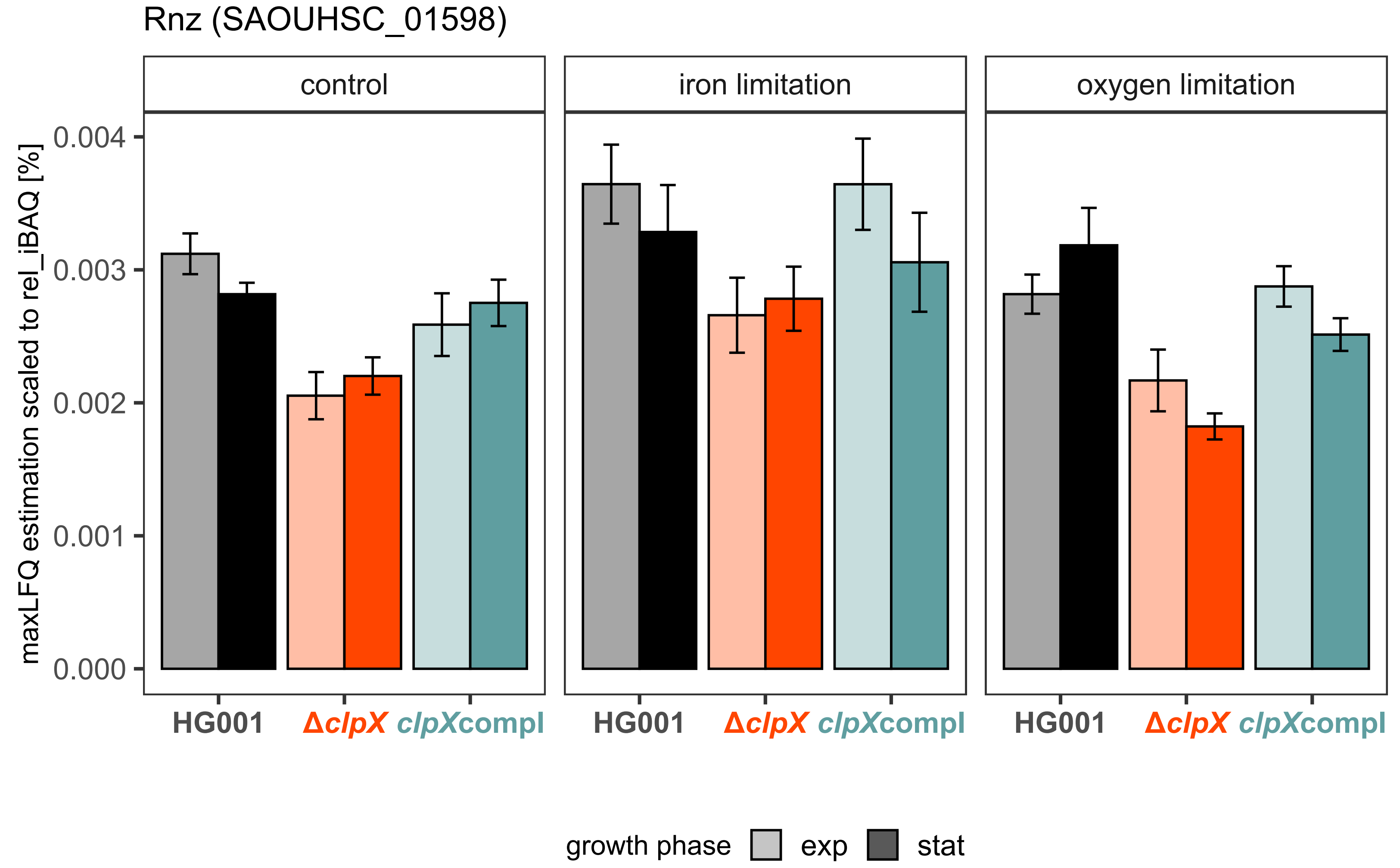
Rnz (SAOUHSC_01598)
control
iron limitation
oxygen limitation
maxLFQ estimation scaled to rel_iBAQ [%]
0.004
0.003
0.002
0.001
0.000
HG001
ΔclpX
clpXcompl
growth phase
exp
stat

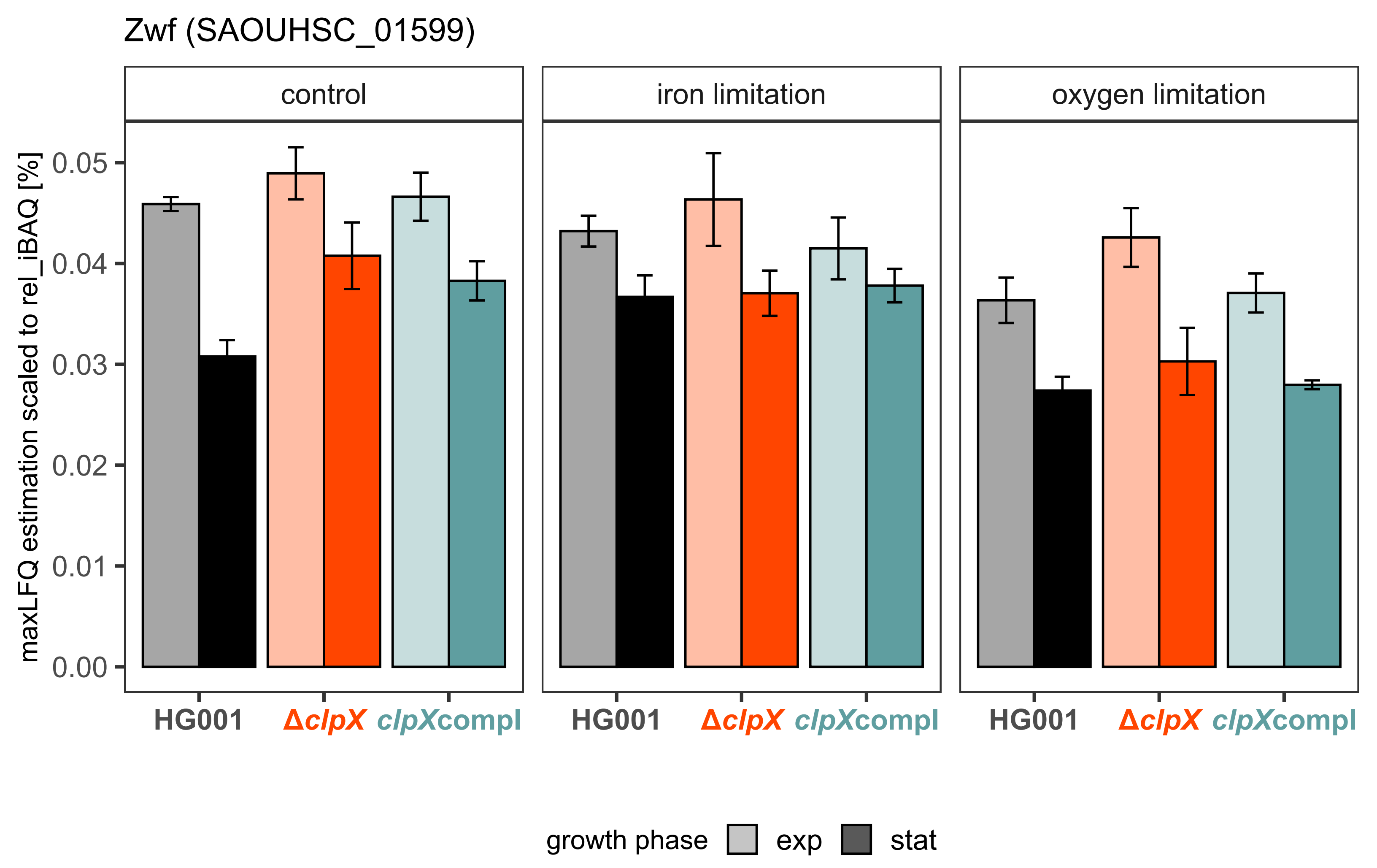
Zwf (SAOUHSC_01599)
control
iron limitation
oxygen limitation
maxLFQ estimation scaled to rel_iBAQ [%]
0.05
0.04
0.03
0.02
0.01
0.00
HG001
ΔclpX
clpXcompl
growth phase
exp
stat

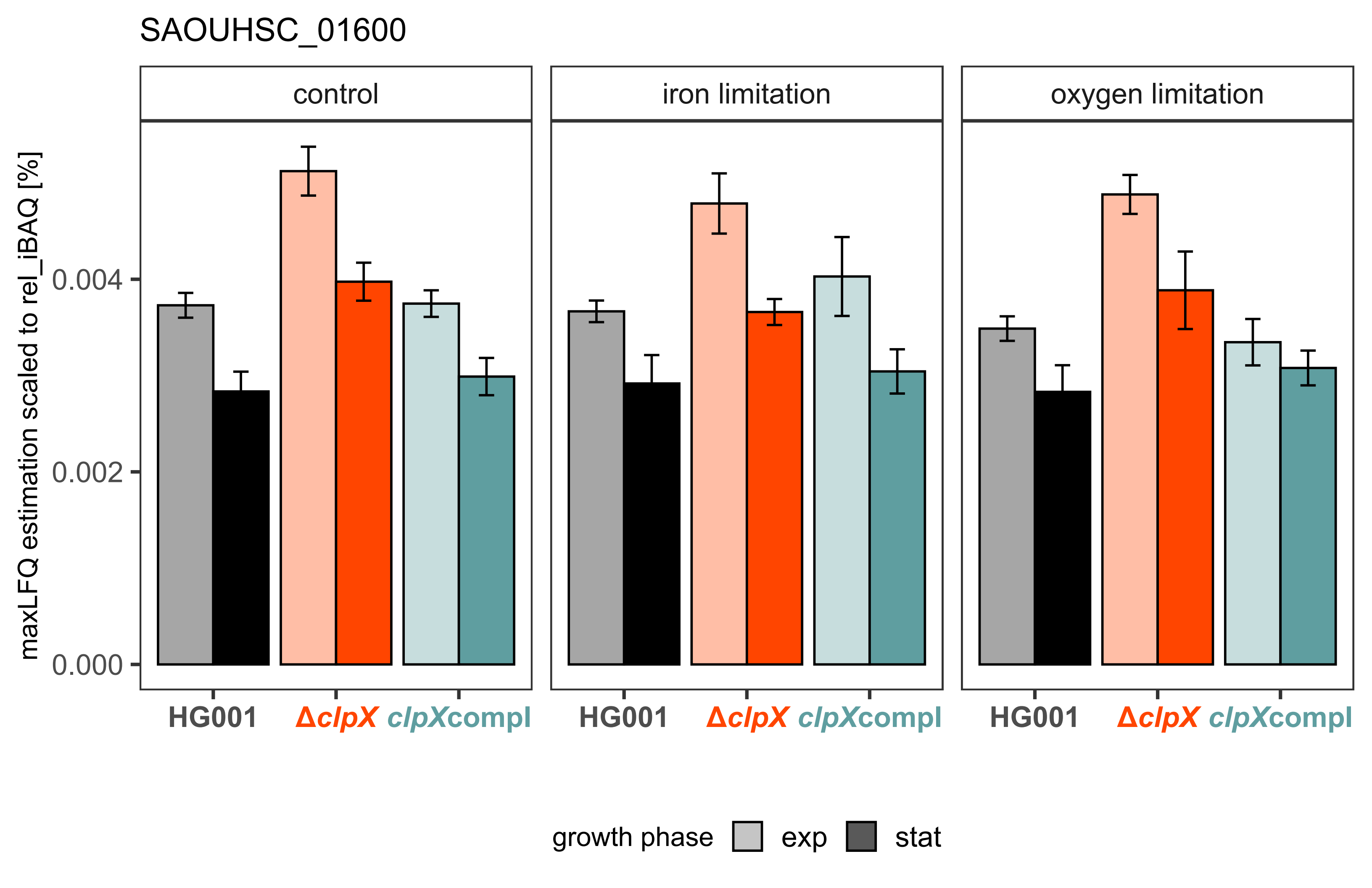
SAOUHSC_01600
control
iron limitation
oxygen limitation
maxLFQ estimation scaled to rel_iBAQ [%]
0.000
0.002
0.004
HG001
ΔclpX
clpXcompl
growth phase
exp
stat

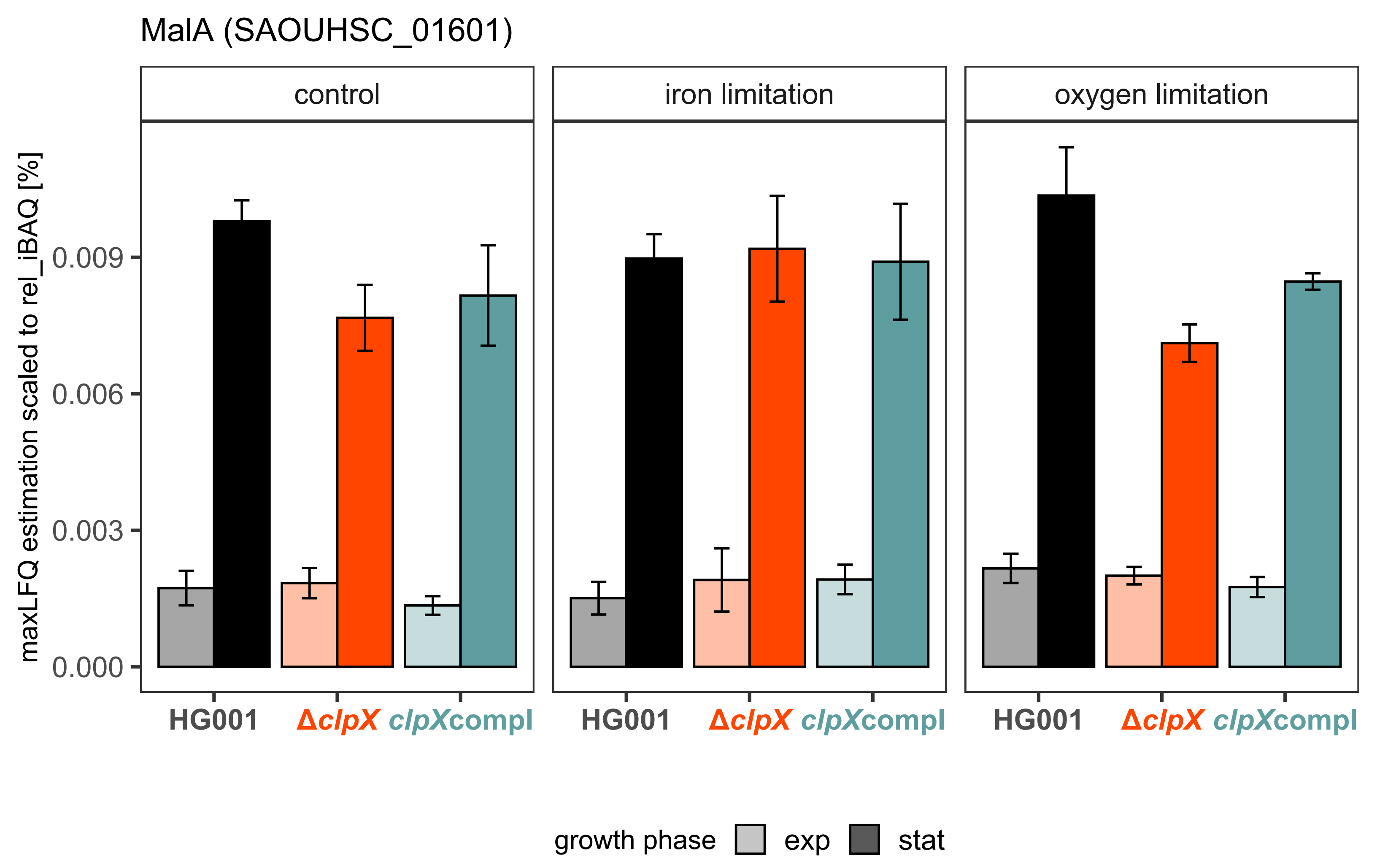
MalA (SAOUHSC_01601)
control
iron limitation
oxygen limitation
maxLFQ estimation scaled to rel_iBAQ [%]
0.009
0.006
0.003
0.000
HG001
ΔclpX
clpXcompl
growth phase
exp
stat

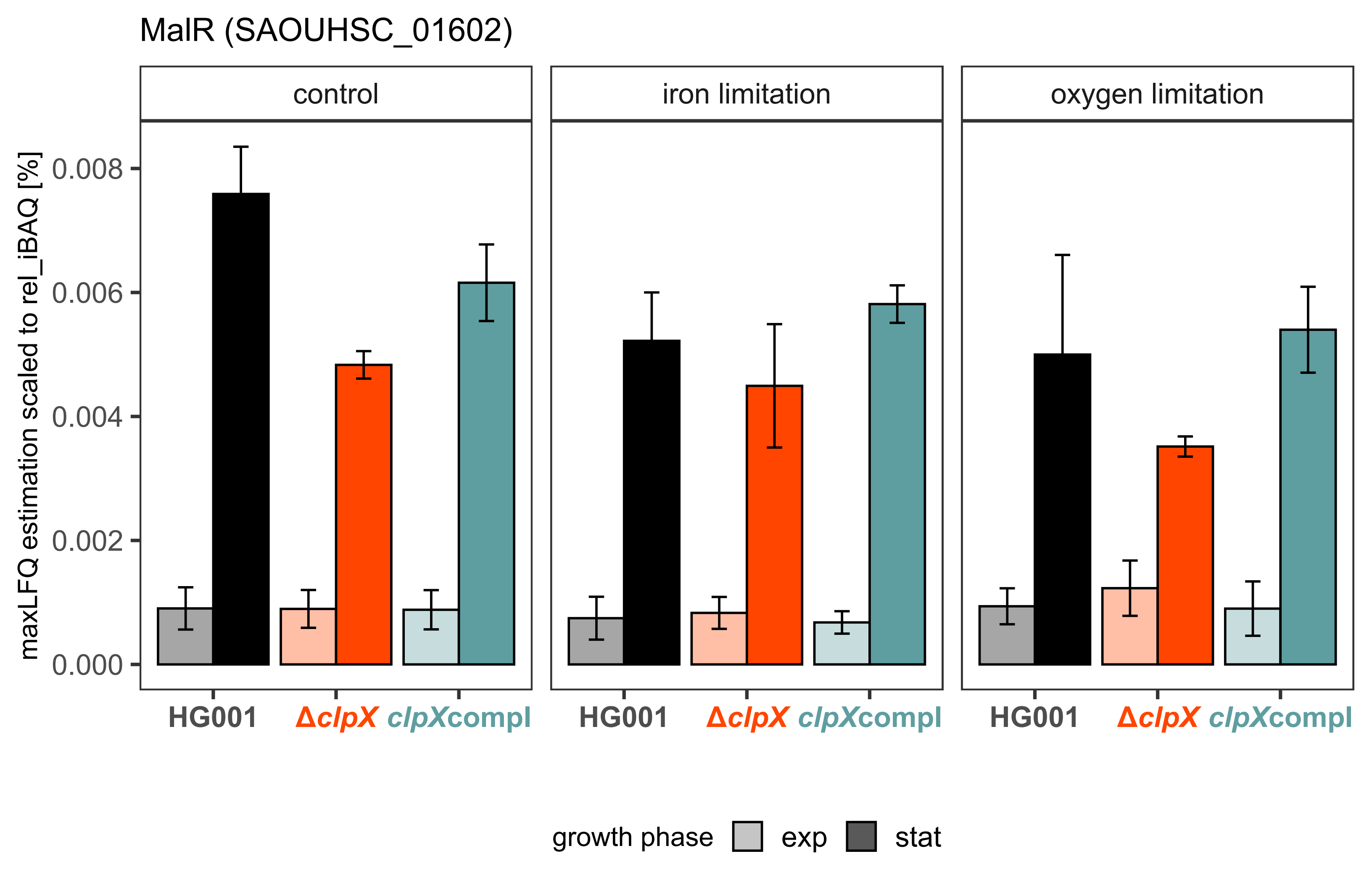

MalR (SAOUHSC_01602)
control
iron limitation
oxygen limitation
maxLFQ estimation scaled to rel_iBAQ [%]
0.000
0.002
0.004
0.006
0.008
HG001
ΔclpX
clpXcompl
growth phase
exp
stat

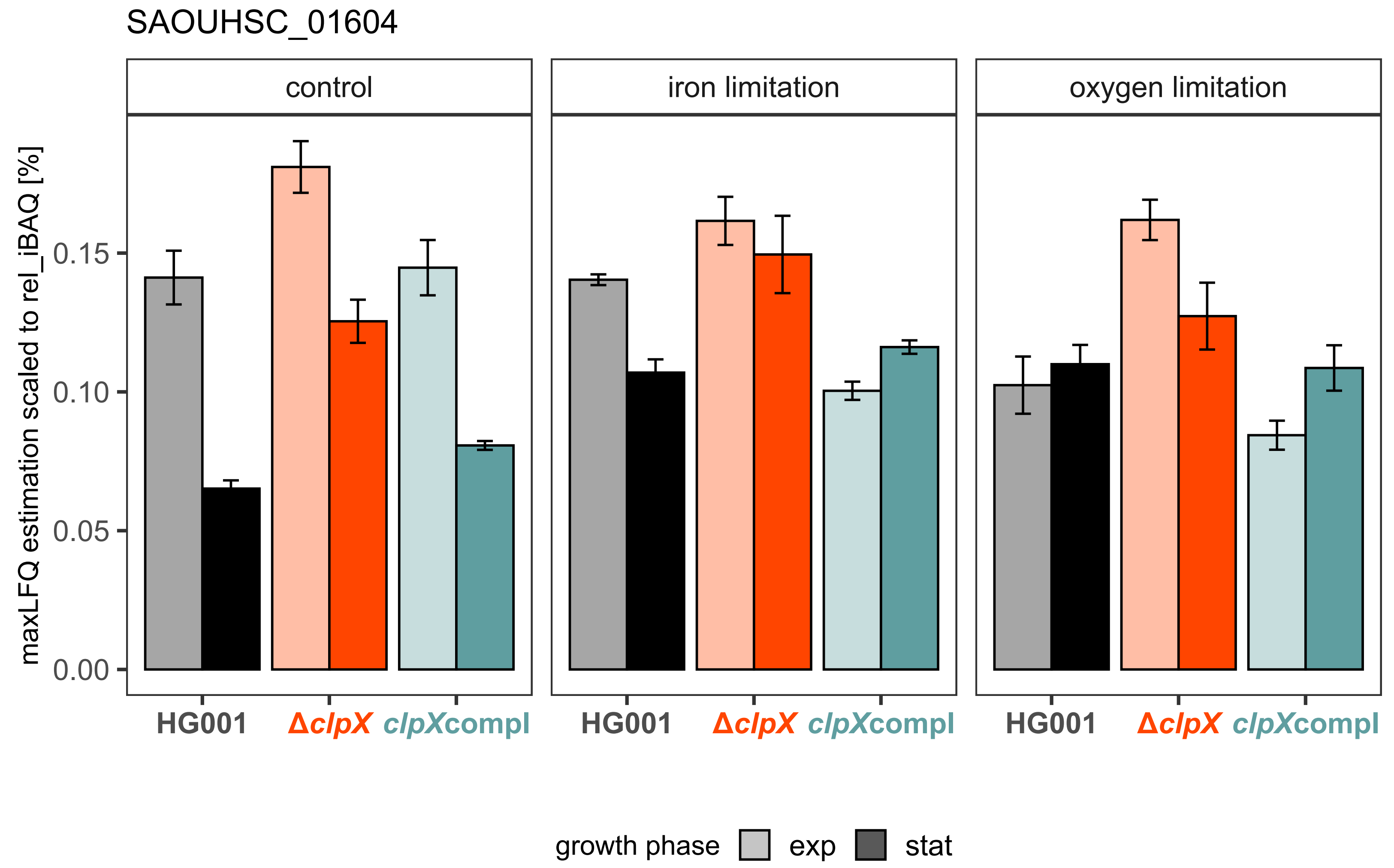
SAOUHSC_01604
control
iron limitation
oxygen limitation
maxLFQ estimation scaled to rel_iBAQ [%]
0.15
0.10
0.05
0.00
HG001
ΔclpX
clpXcompl
growth phase
exp
stat

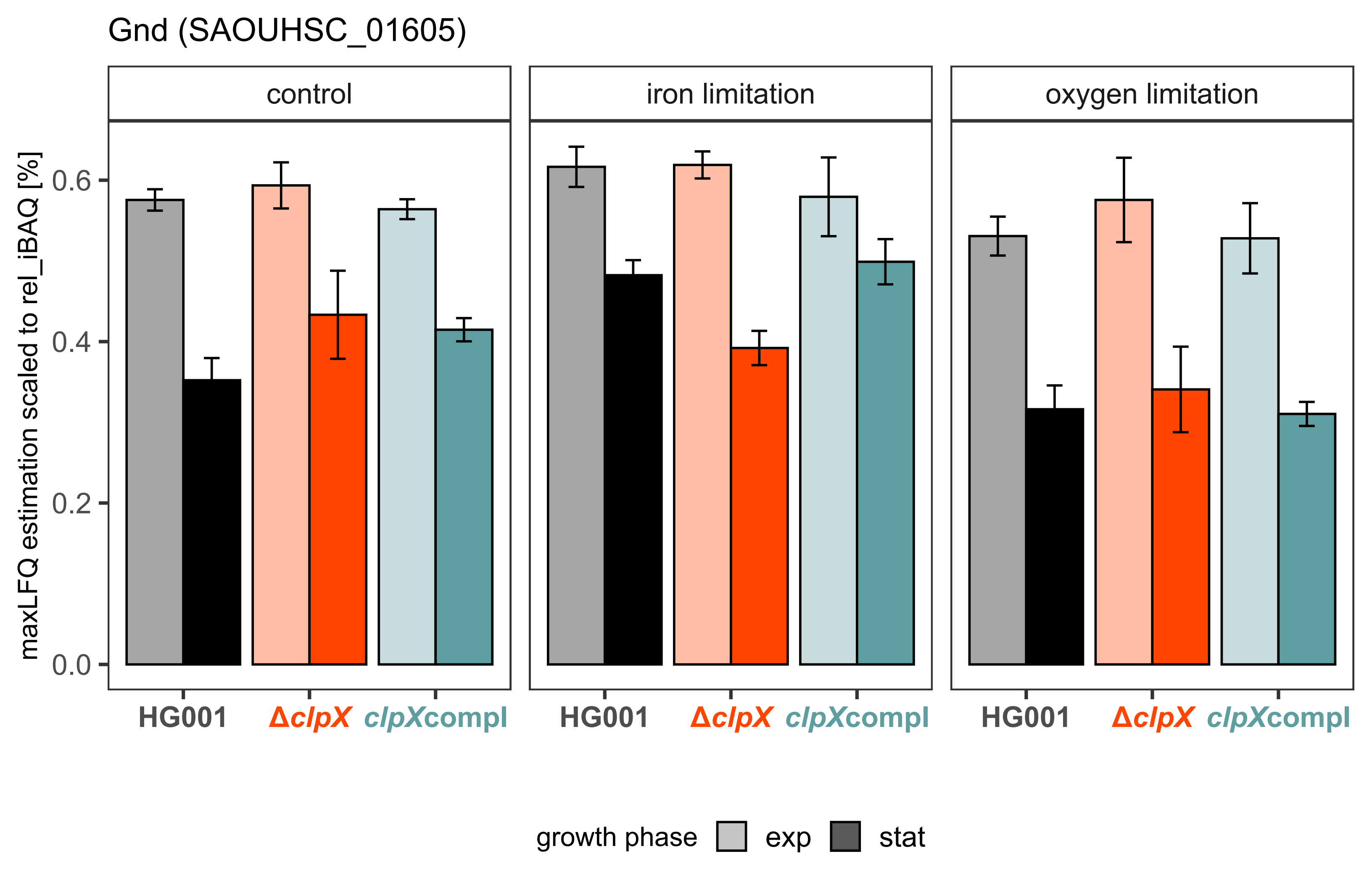
Gnd (SAOUHSC_01605)
control
iron limitation
oxygen limitation
maxLFQ estimation scaled to rel_iBAQ [%]
0.0
0.2
0.4
0.6
HG001
ΔclpX
clpXcompl
growth phase
exp
stat

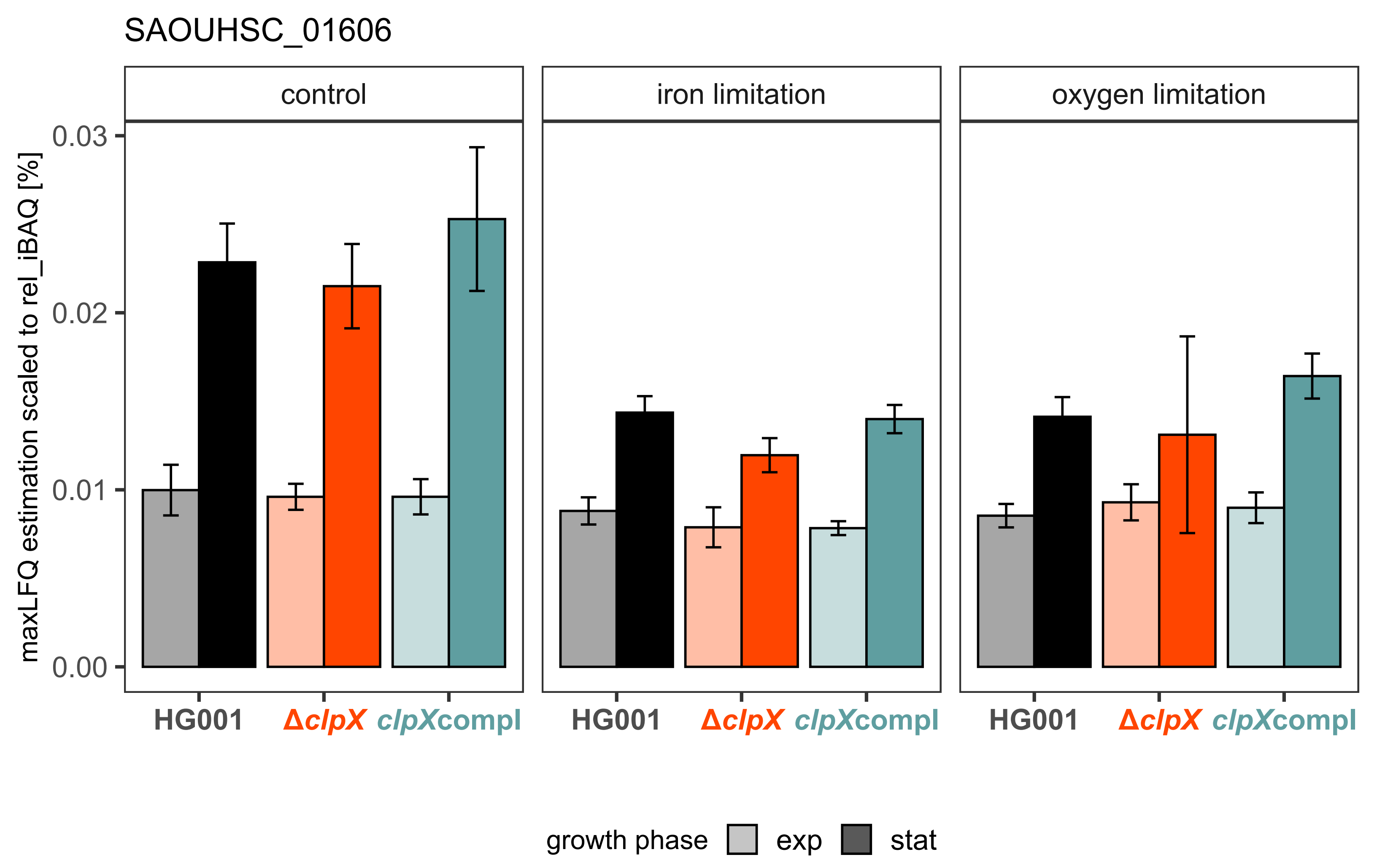
SAOUHSC_01606
control
iron limitation
oxygen limitation
maxLFQ estimation scaled to rel_iBAQ [%]
0.03
0.02
0.01
0.00
HG001
ΔclpX
clpXcompl
growth phase
exp
stat

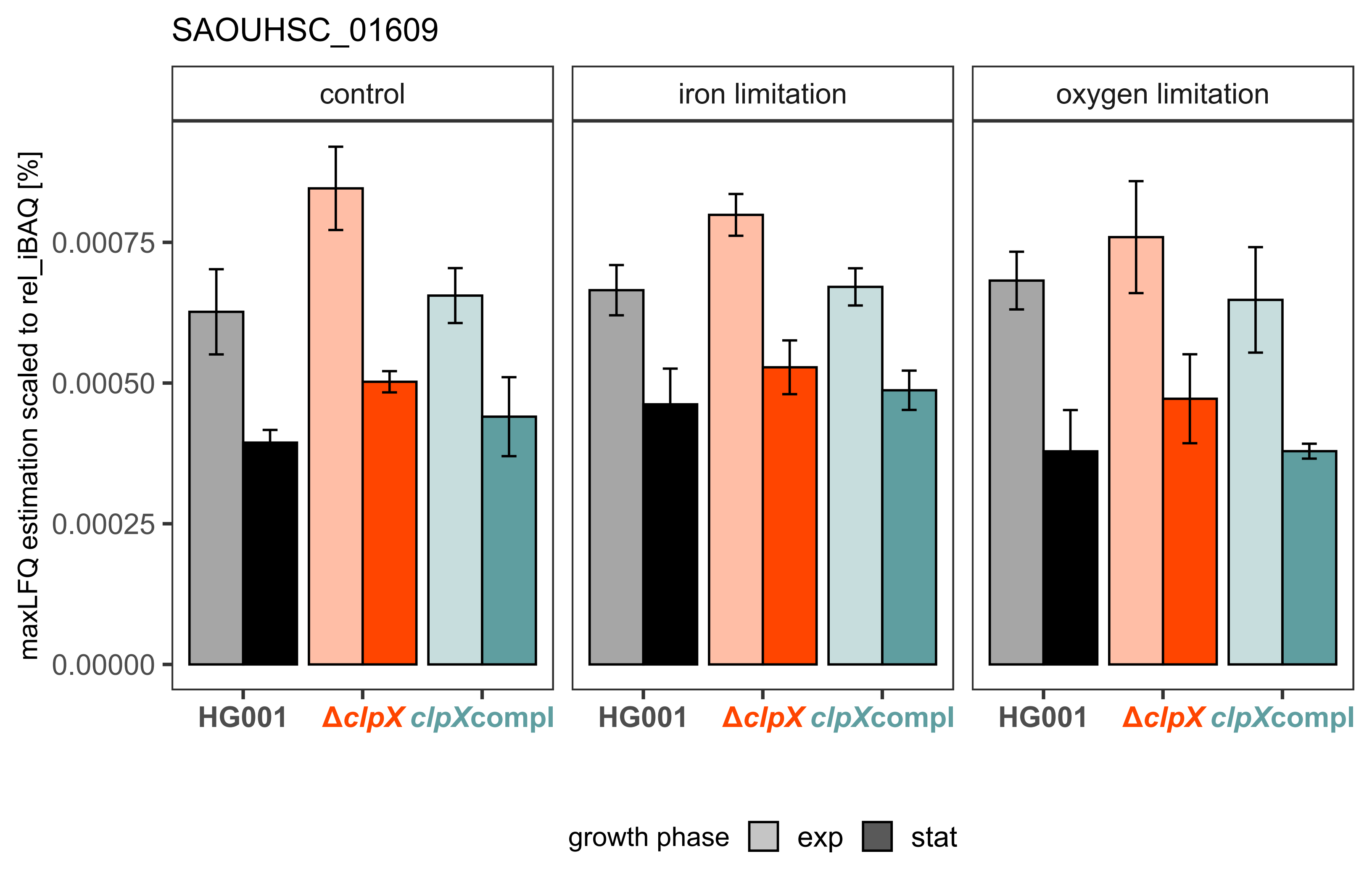
SAOUHSC_01609
control
iron limitation
oxygen limitation
maxLFQ estimation scaled to rel_iBAQ [%]
0.00075
0.00050
0.00025
0.00000
HG001
ΔclpX
clpXcompl
growth phase
exp
stat

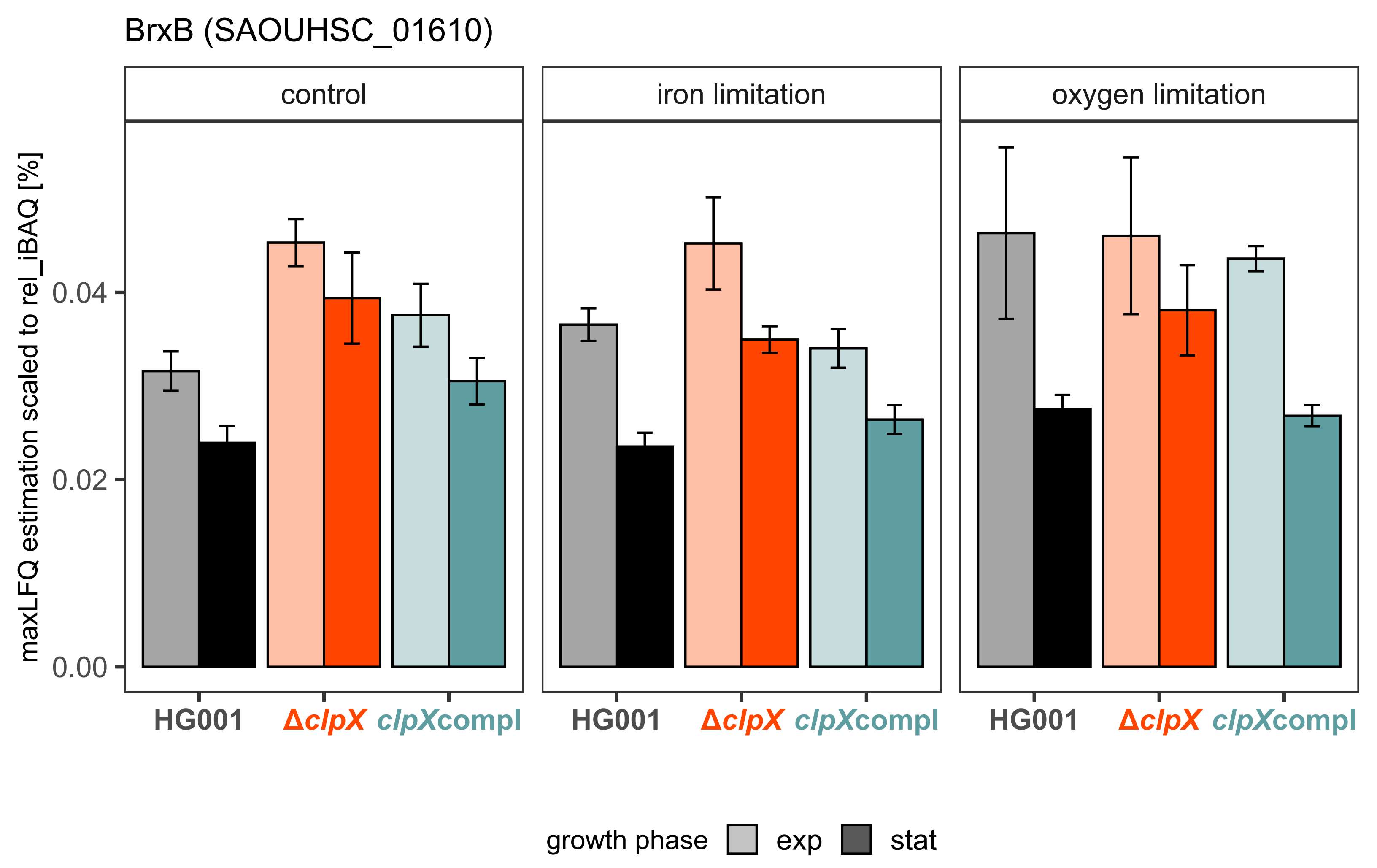
BrxB (SAOUHSC_01610)
control
iron limitation
oxygen limitation
maxLFQ estimation scaled to rel_iBAQ [%]
0.04
0.02
0.00
HG001
ΔclpX
clpXcompl
growth phase
exp
stat

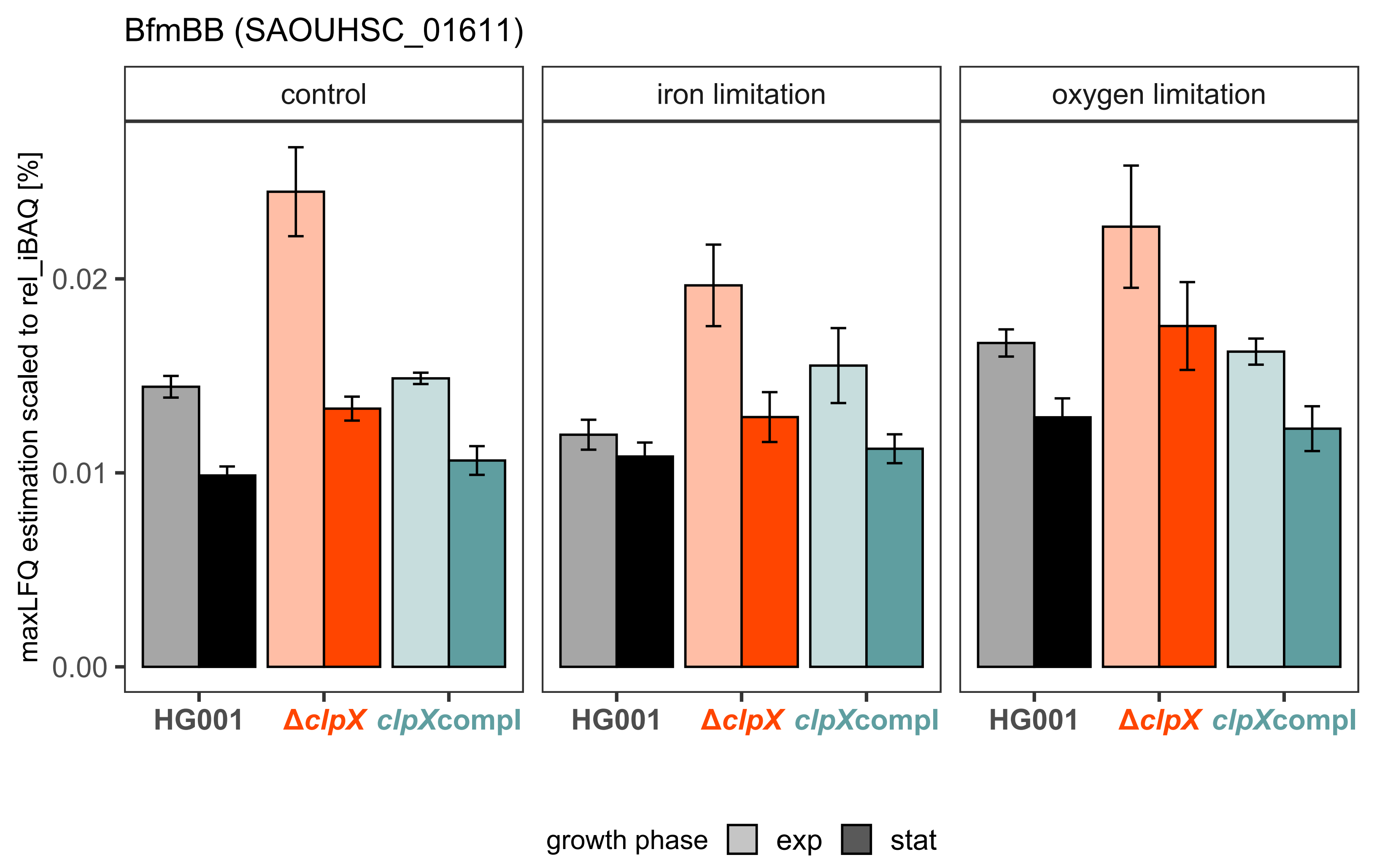
BfmBB (SAOUHSC_01611)
control
iron limitation
oxygen limitation
maxLFQ estimation scaled to rel_iBAQ [%]
0.00
0.01
0.02
HG001
ΔclpX
clpXcompl
growth phase
exp
stat

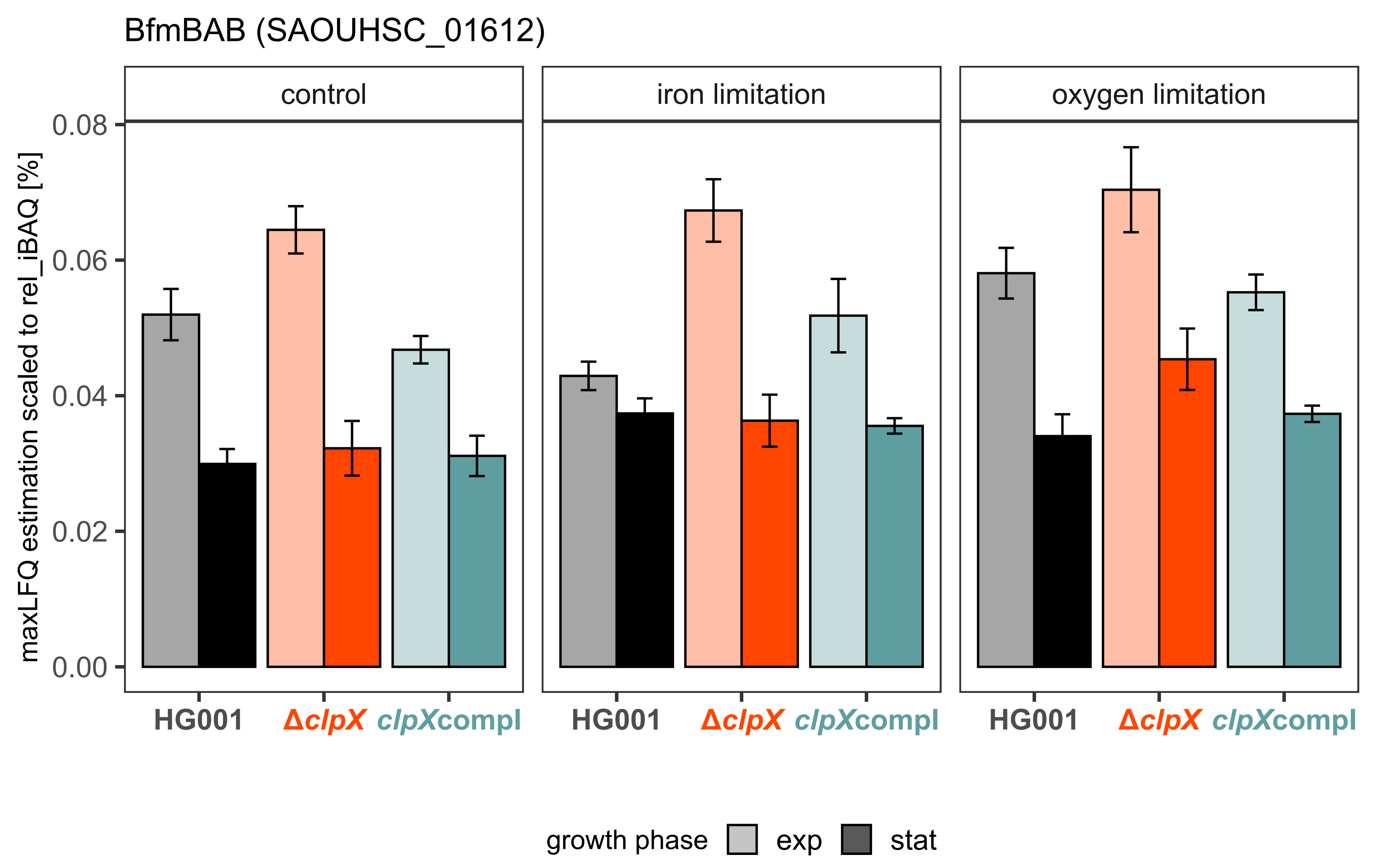
BfmBAB (SAOUHSC_01612)
control
iron limitation
oxygen limitation
maxLFQ estimation scaled to rel_iBAQ [%]
0.08
0.06
0.04
0.02
0.00
HG001
ΔclpX
clpXcompl
growth phase
exp
stat

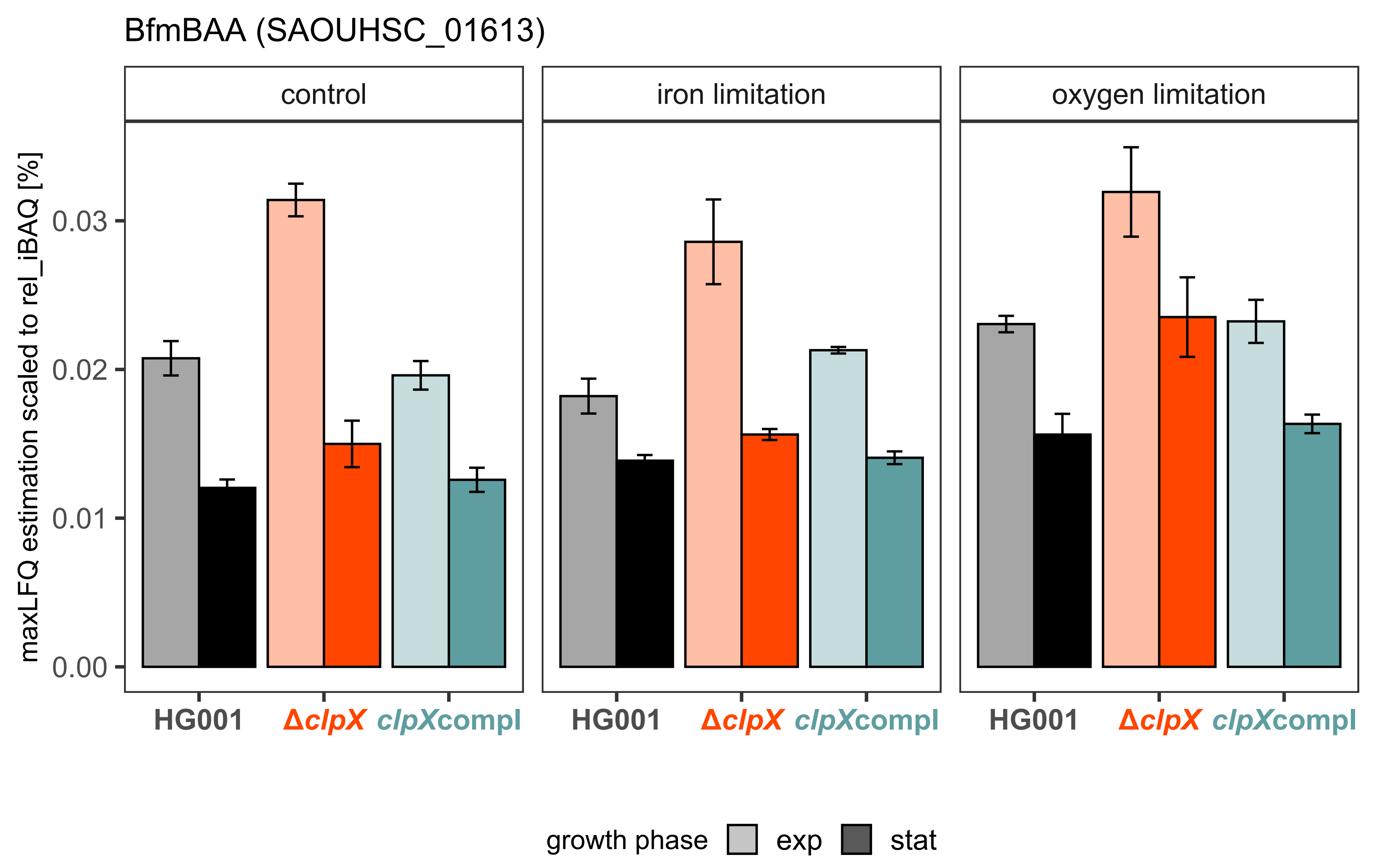

BfmBAA (SAOUHSC_01613)
control
iron limitation
oxygen limitation
maxLFQ estimation scaled to rel_iBAQ [%]
0.00
0.01
0.02
0.03
HG001
ΔclpX
clpXcompl
growth phase
exp
stat

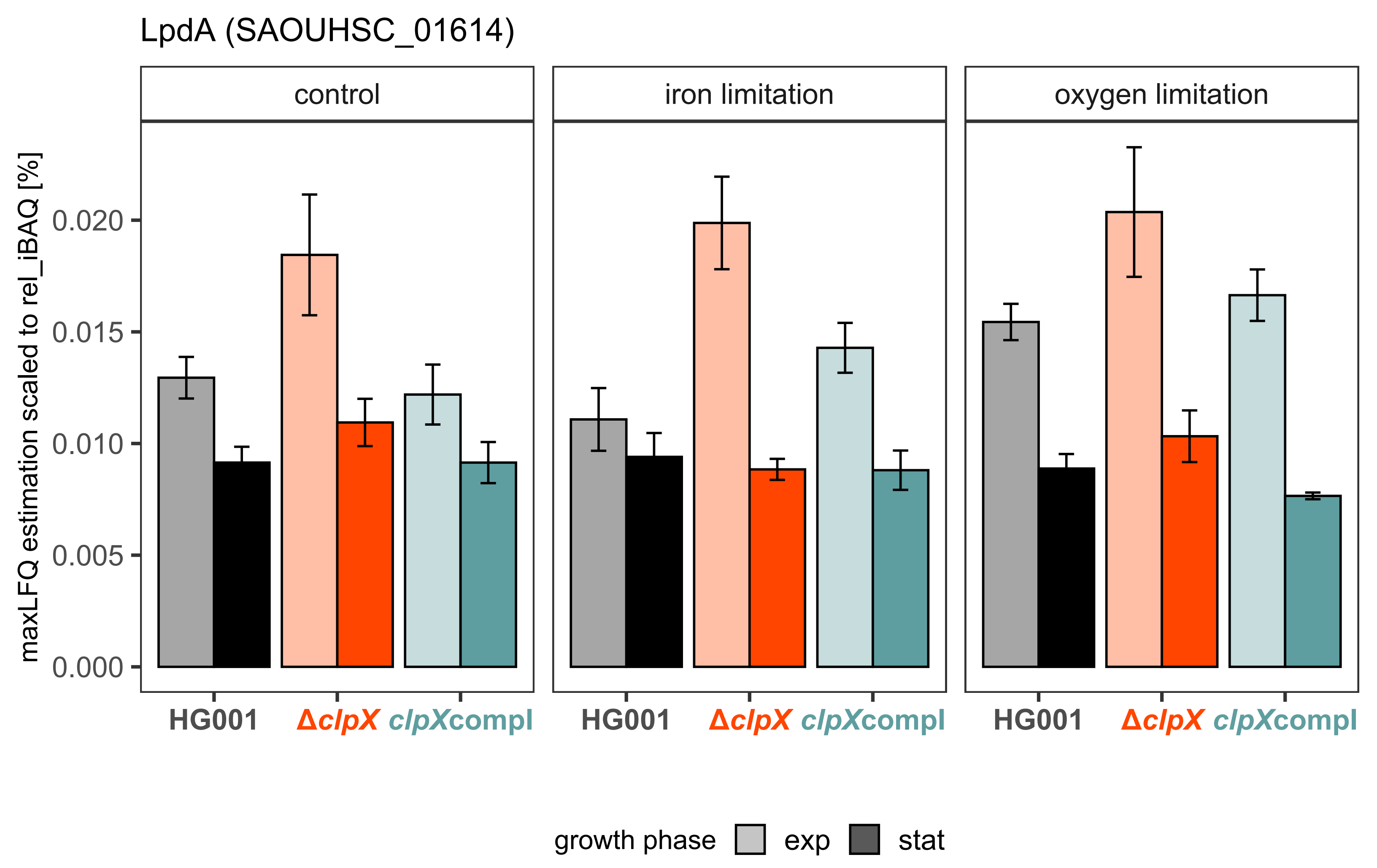

LpdA (SAOUHSC_01614)
control
iron limitation
oxygen limitation
maxLFQ estimation scaled to rel_iBAQ [%]
0.020
0.015
0.010
0.005
0.000
HG001
ΔclpX
clpXcompl
growth phase
exp
stat

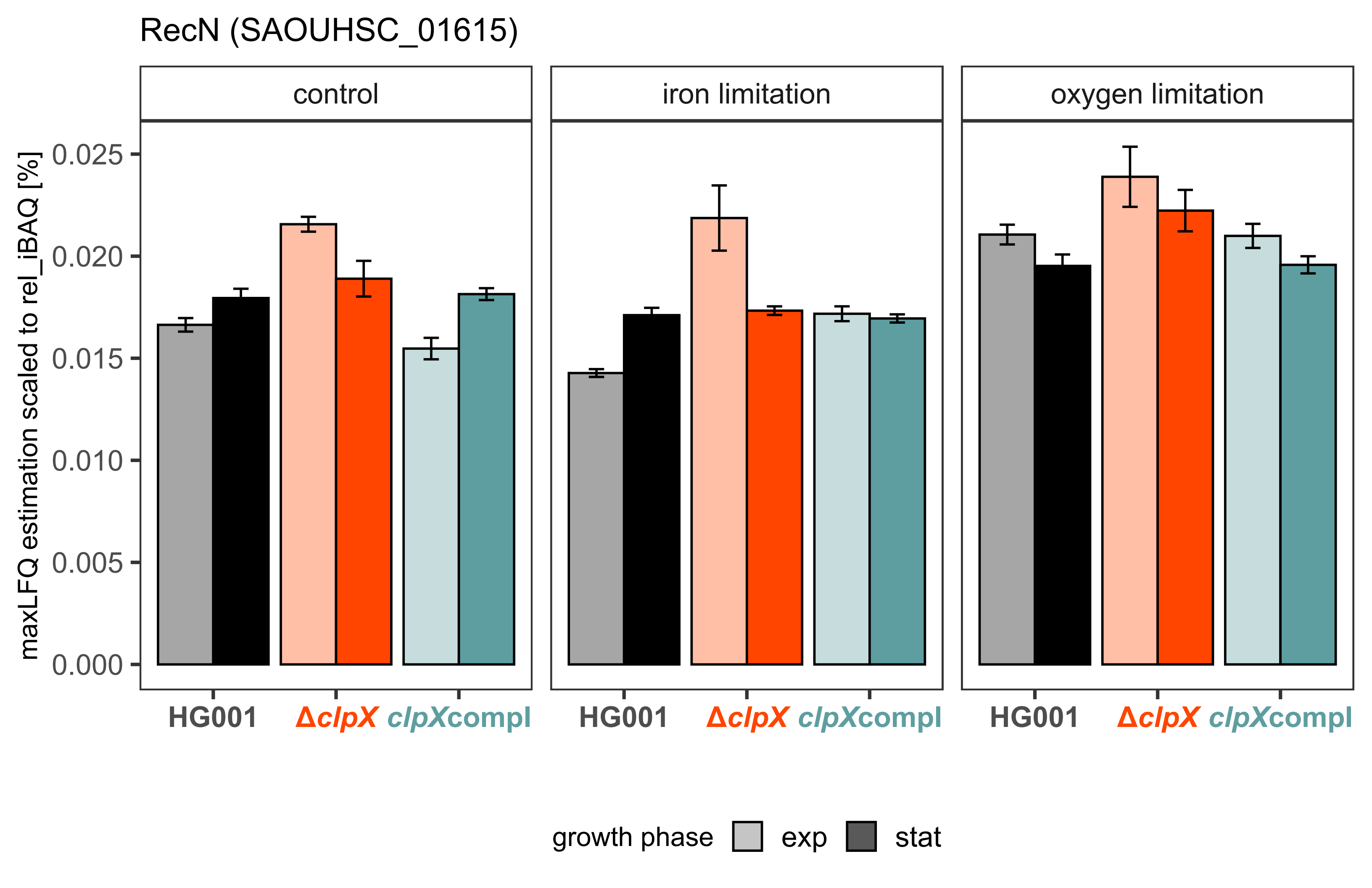

RecN (SAOUHSC_01615)
control
iron limitation
oxygen limitation
maxLFQ estimation scaled to rel_iBAQ [%]
0.025
0.020
0.015
0.010
0.005
0.000
HG001
ΔclpX
clpXcompl
growth phase
exp
stat

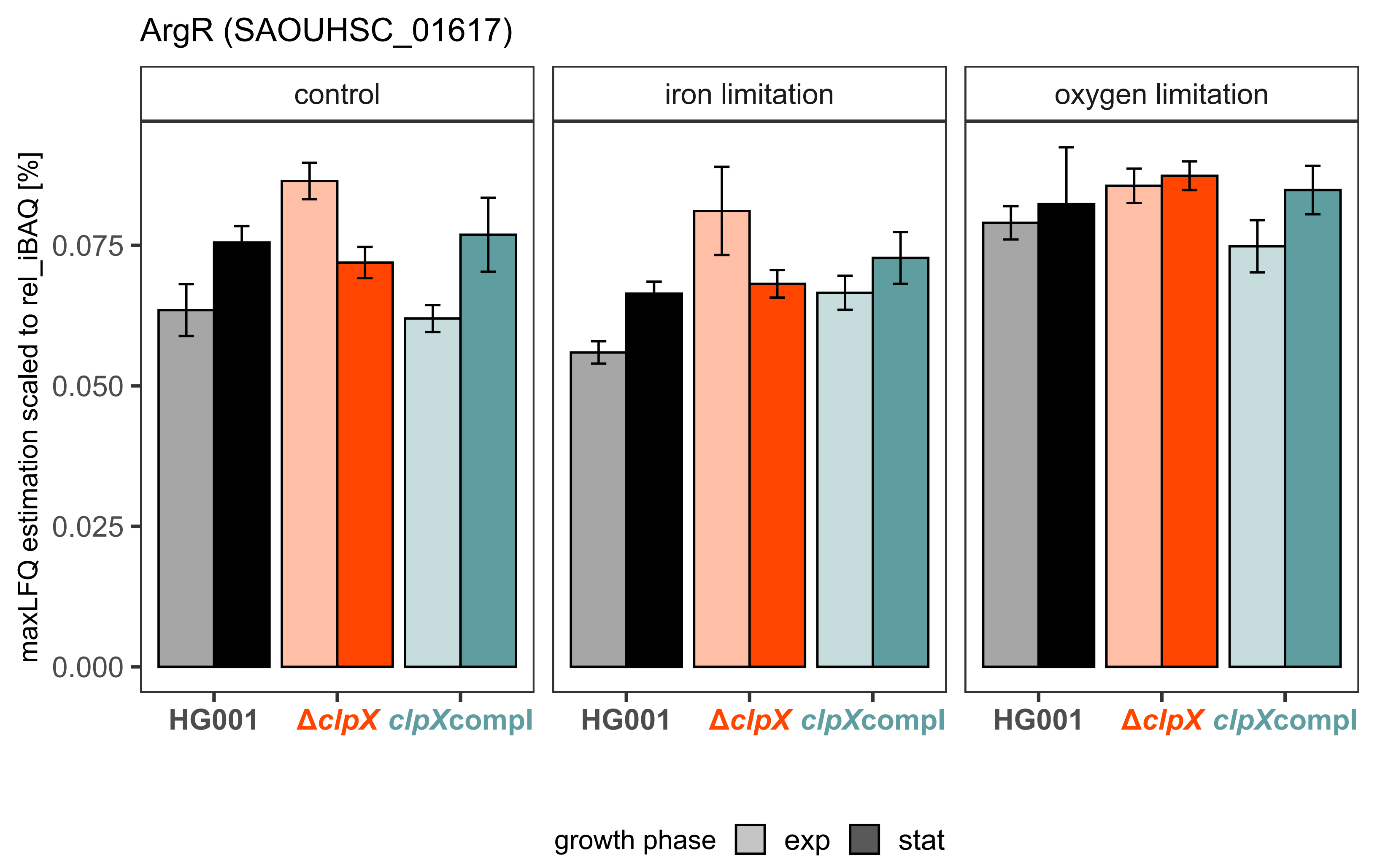
ArgR (SAOUHSC_01617)
control
iron limitation
oxygen limitation
maxLFQ estimation scaled to rel_iBAQ [%]
0.000
0.025
0.050
0.075
HG001
ΔclpX
clpXcompl
growth phase
exp
stat

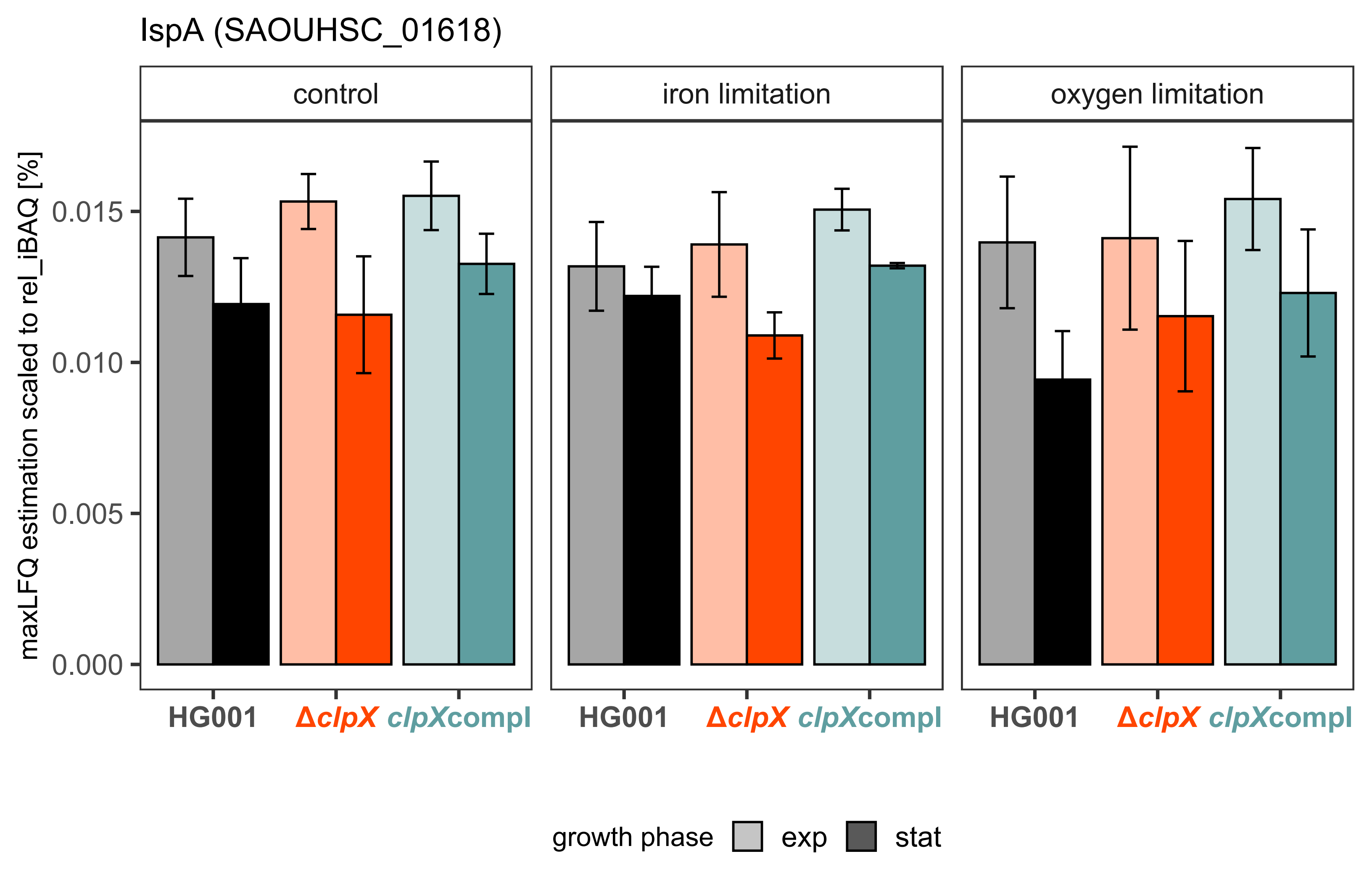

lspA (SAOUHSC_01618)
control
iron limitation
oxygen limitation
maxLFQ estimation scaled to rel_iBAQ [%]
0.015
0.010
0.005
0.000
HG001
ΔclpX
clpXcompl
growth phase
exp
stat

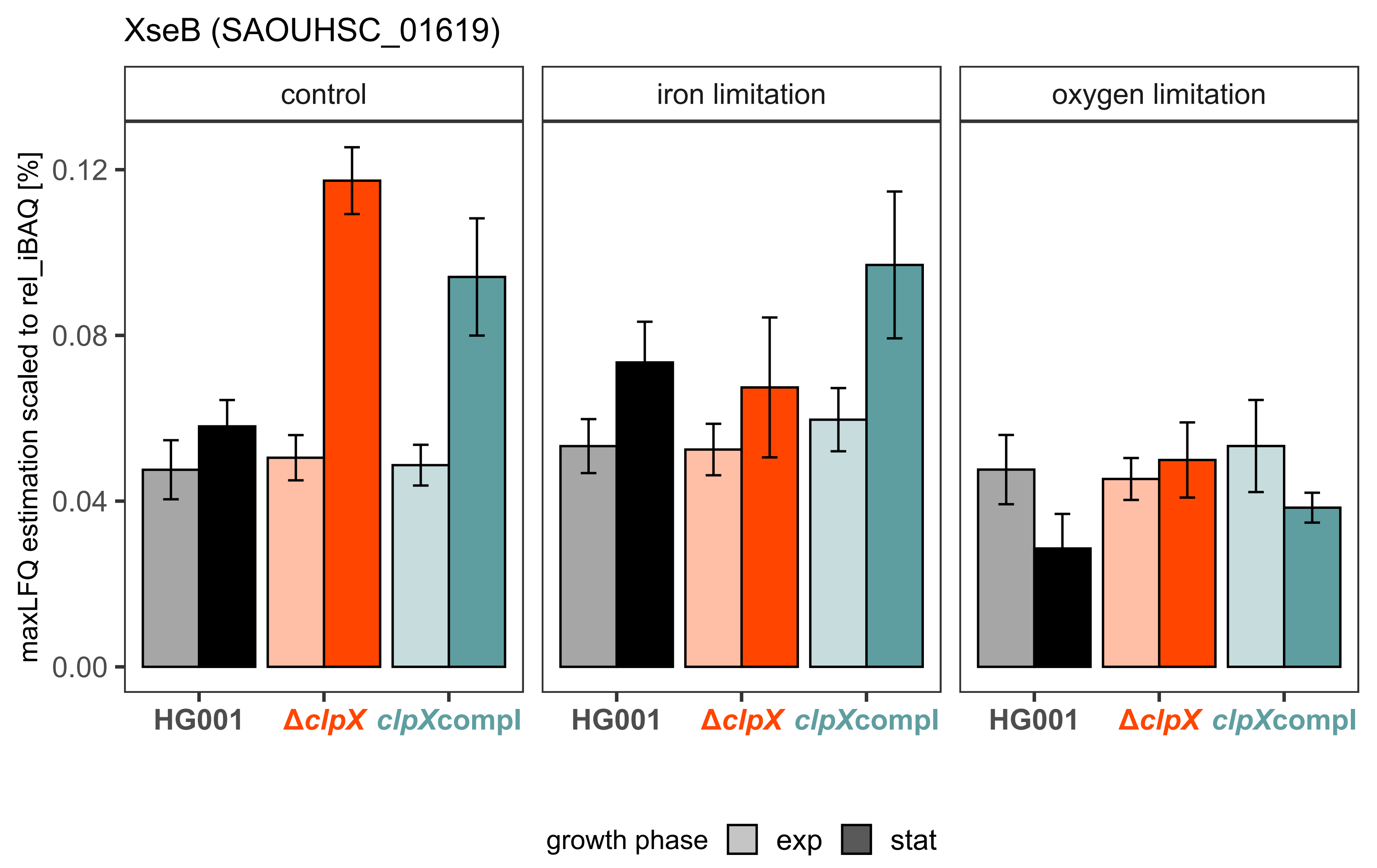
XseB (SAOUHSC_01619)
control
iron limitation
oxygen limitation
maxLFQ estimation scaled to rel_iBAQ [%]
0.12
0.08
0.04
0.00
HG001
ΔclpX
clpXcompl
growth phase
exp
stat

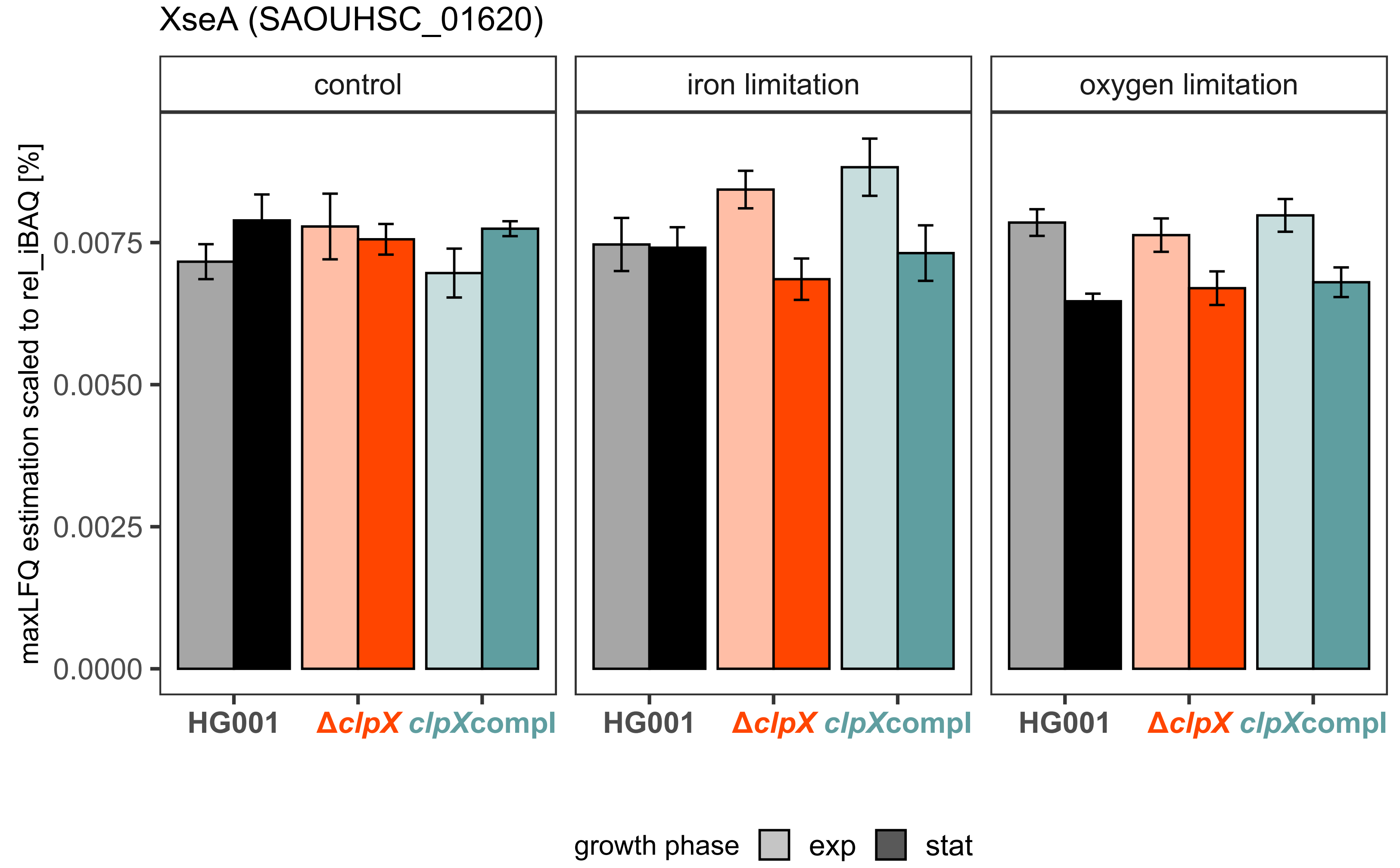
XseA (SAOUHSC_01620)
control
iron limitation
oxygen limitation
maxLFQ estimation scaled to rel_iBAQ [%]
0.0075
0.0050
0.0025
0.0000
HG001
ΔclpX
clpXcompl
growth phase
exp
stat

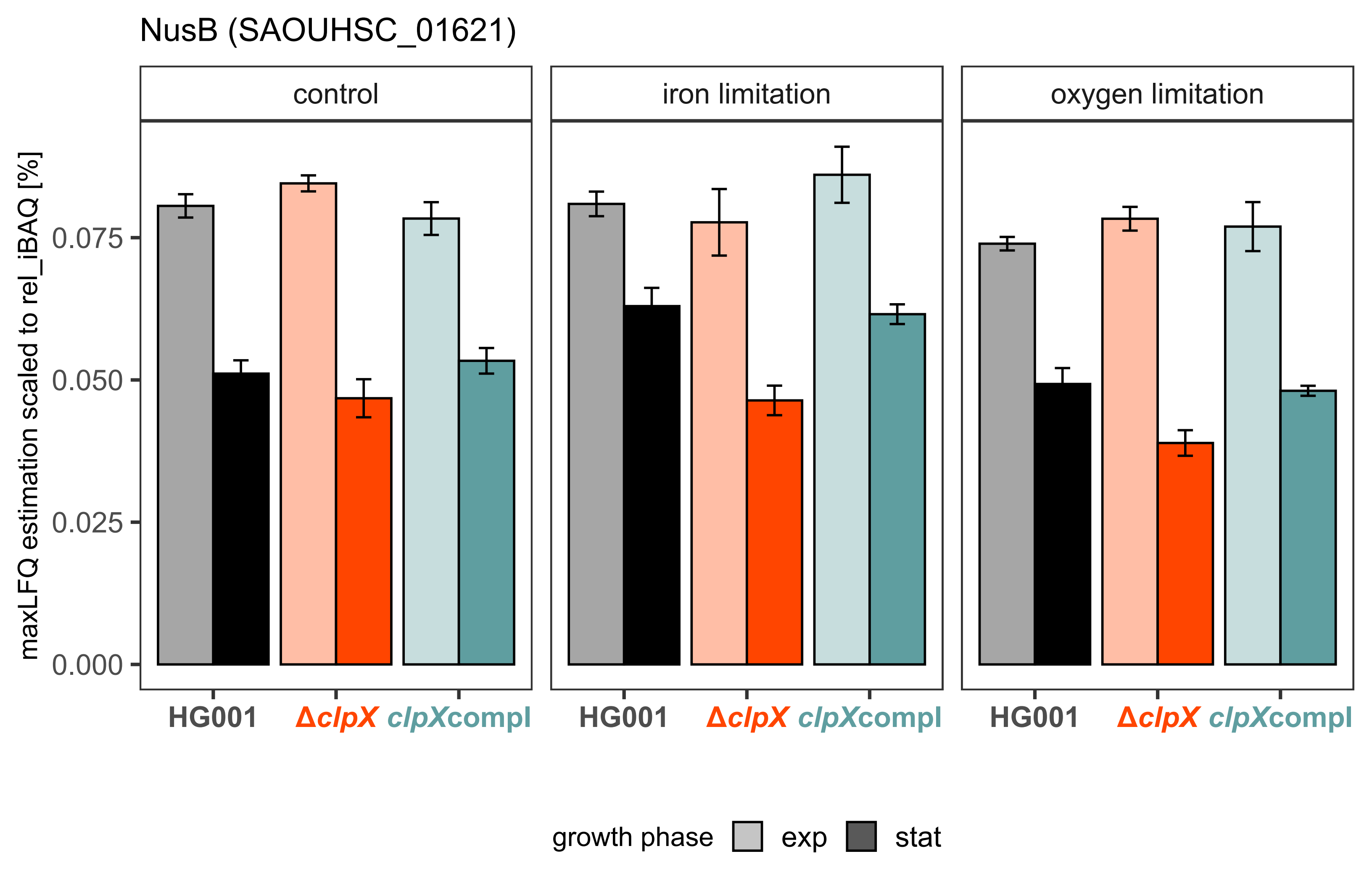
NusB (SAOUHSC_01621)
control
iron limitation
oxygen limitation
maxLFQ estimation scaled to rel_iBAQ [%]
0.075
0.050
0.025
0.000
HG001
ΔclpX
clpXcompl
growth phase
exp
stat

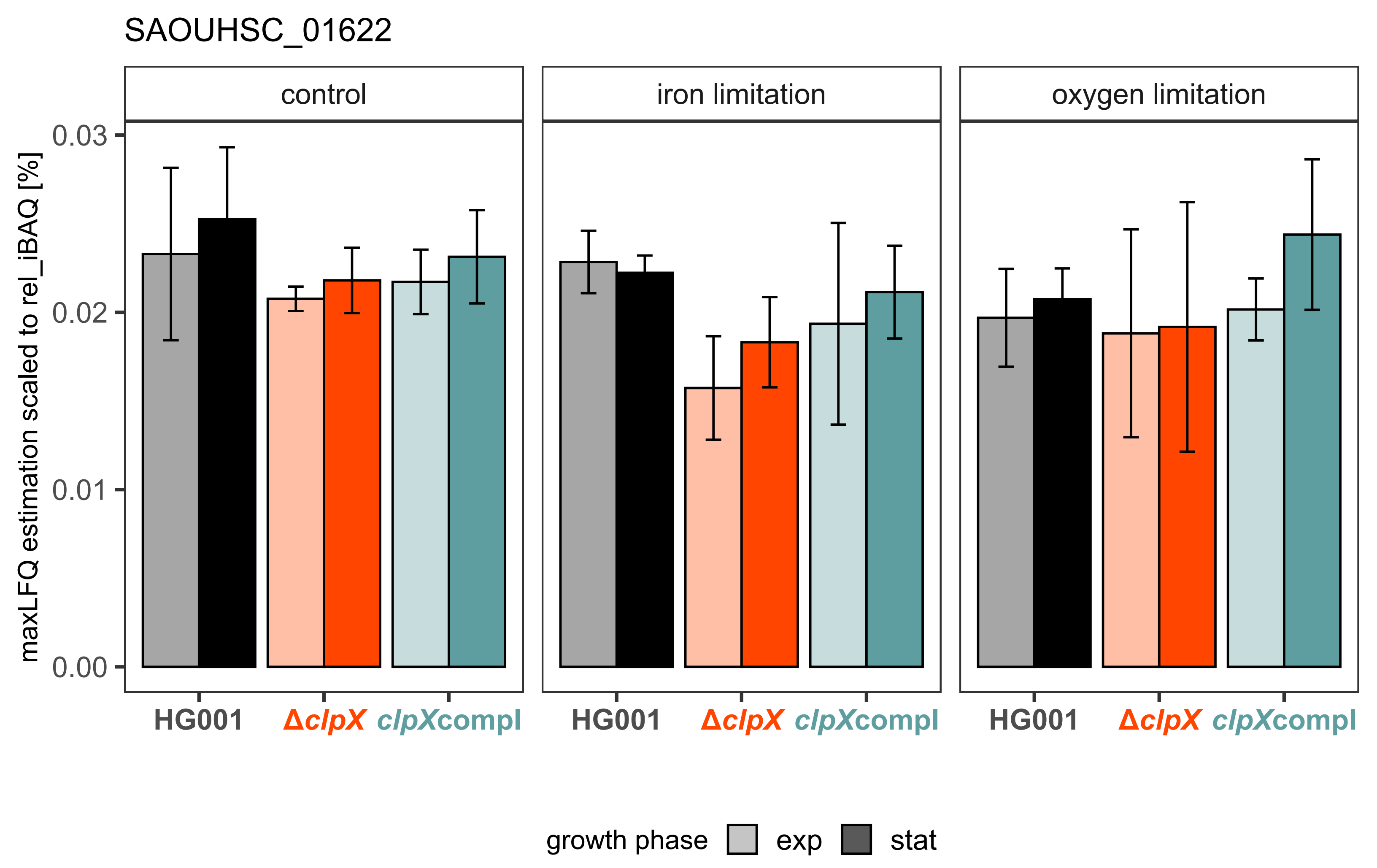
SAOUHSC_01622
control
iron limitation
oxygen limitation
maxLFQ estimation scaled to rel_iBAQ [%]
0.03
0.02
0.01
0.00
HG001
ΔclpX
clpXcompl
growth phase
exp
stat

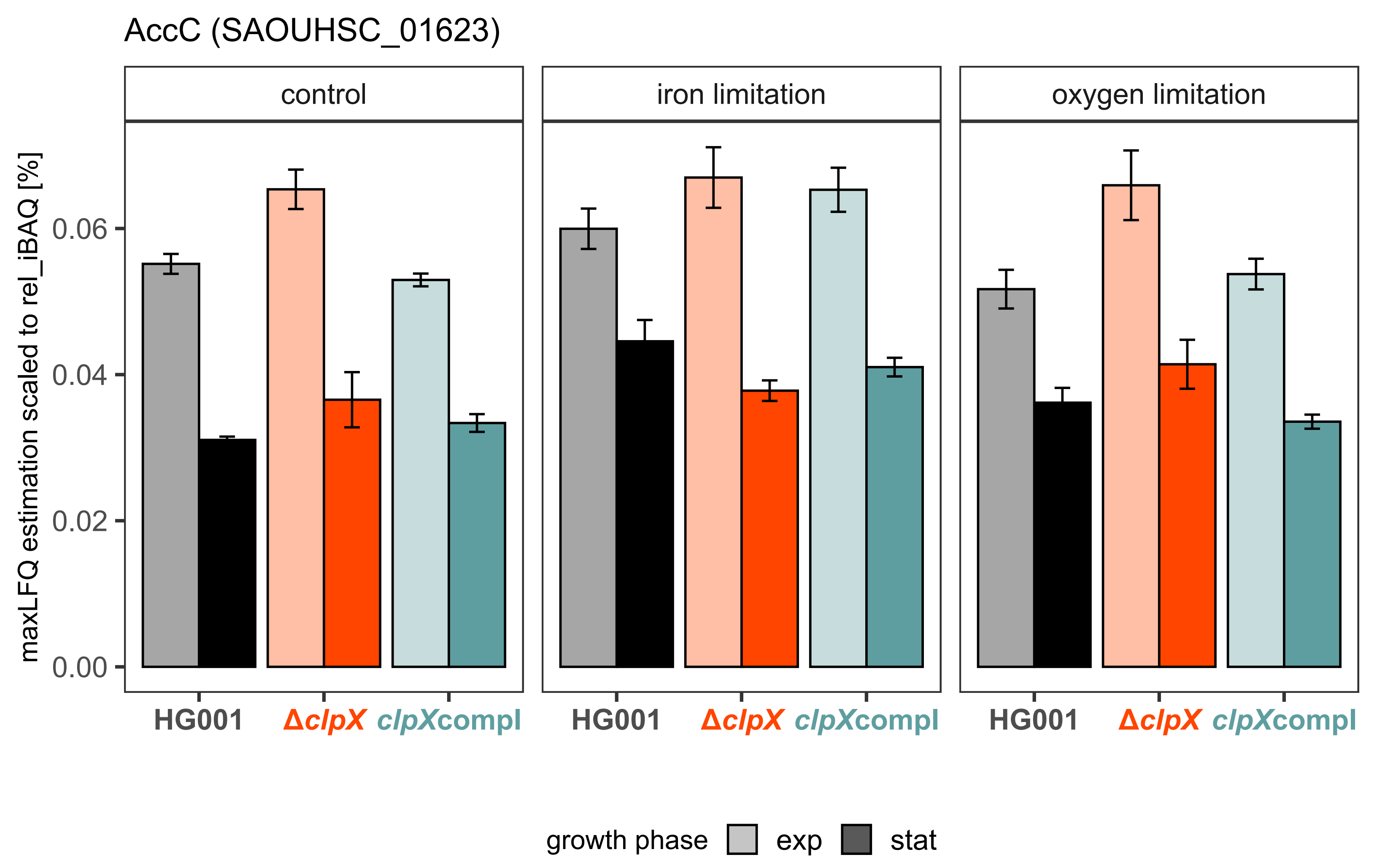
AccC (SAOUHSC_01623)
control
iron limitation
oxygen limitation
maxLFQ estimation scaled to rel_iBAQ [%]
0.06
0.04
0.02
0.00
HG001
ΔclpX
clpXcompl
growth phase
exp
stat

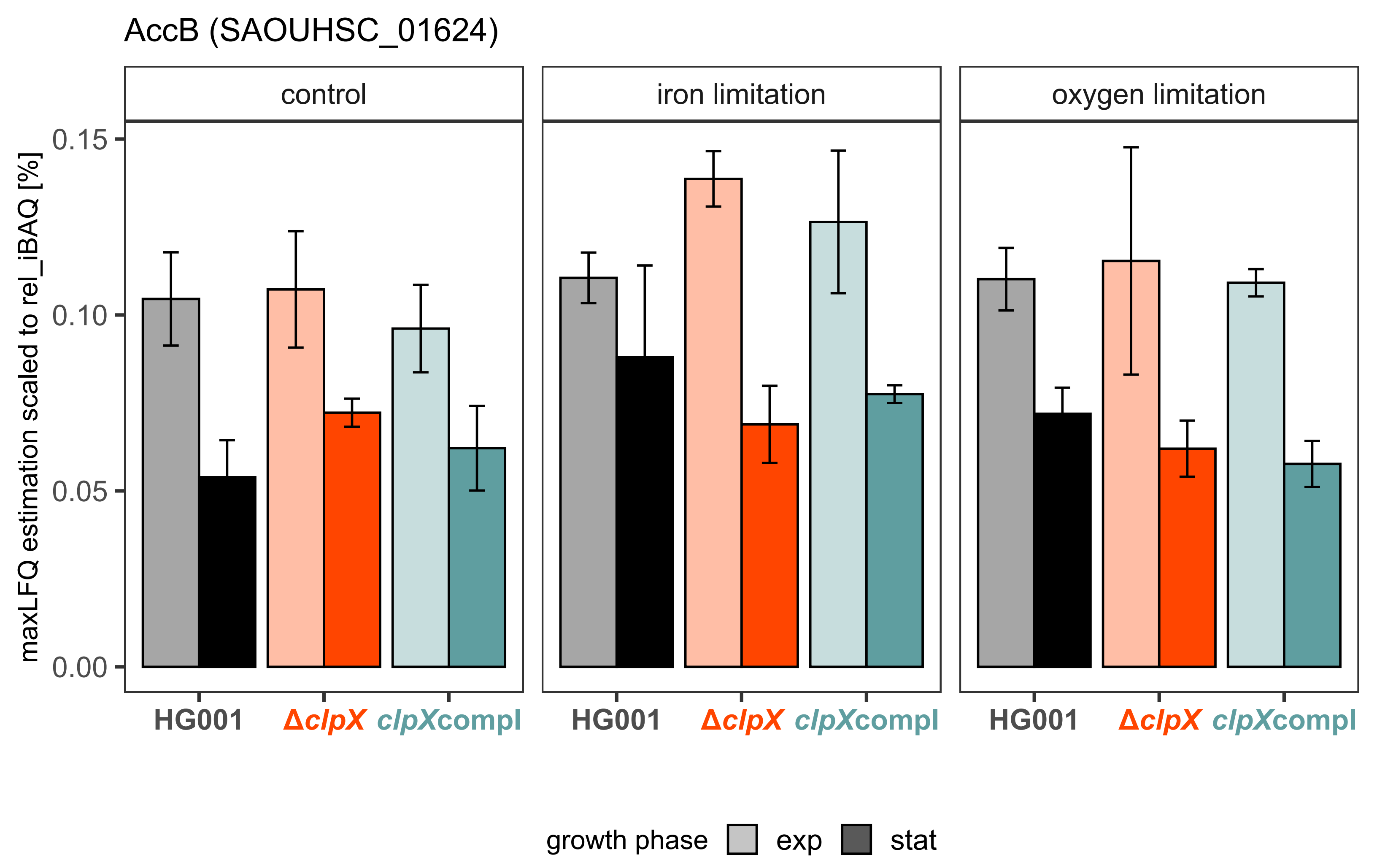
AccB (SAOUHSC_01624)
control
iron limitation
oxygen limitation
maxLFQ estimation scaled to rel_iBAQ [%]
0.15
0.10
0.05
0.00
HG001
ΔclpX
clpXcompl
growth phase
exp
stat

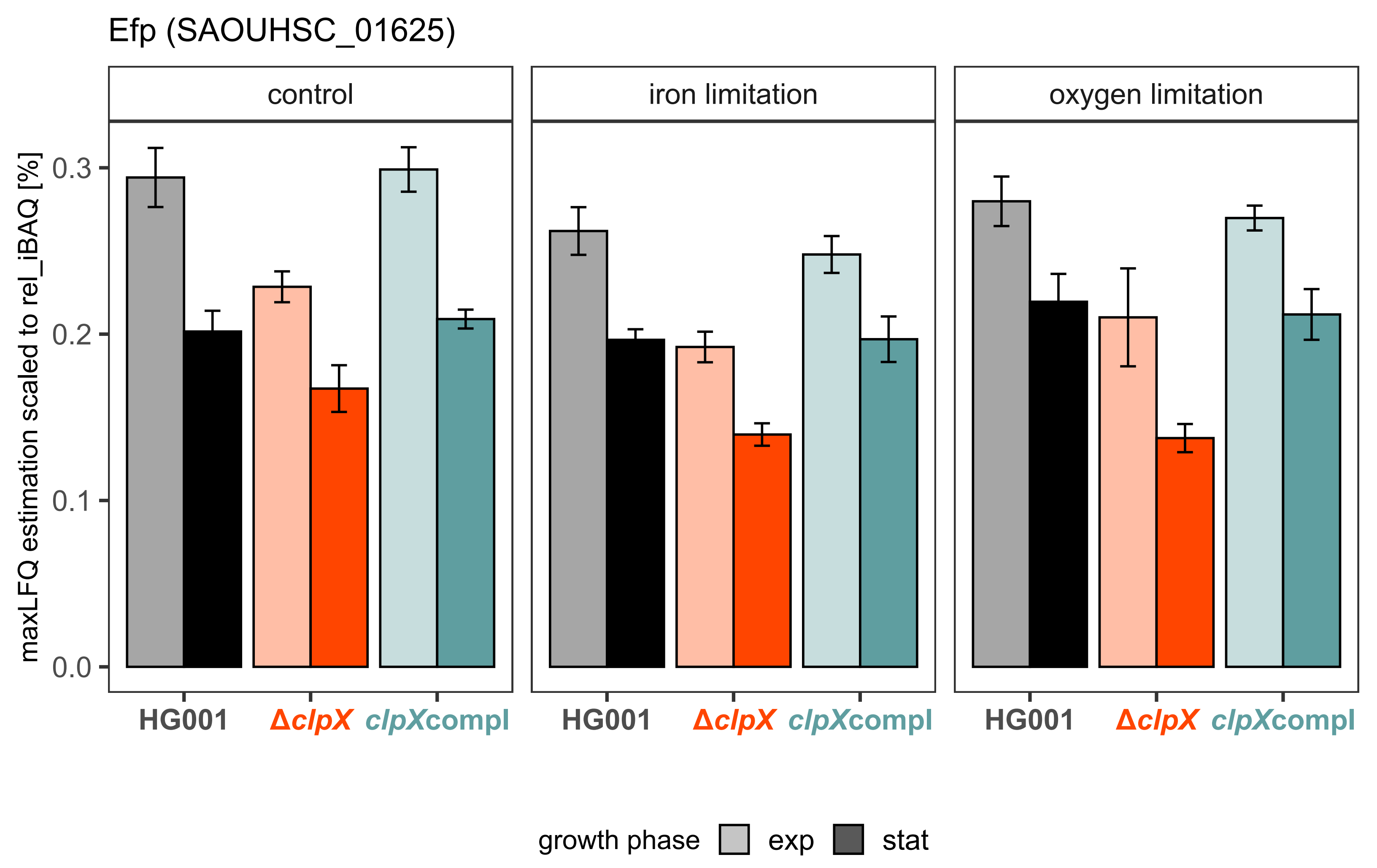

Efp (SAOUHSC_01625)
control
iron limitation
oxygen limitation
maxLFQ estimation scaled to rel_iBAQ [%]
0.0
0.1
0.2
0.3
HG001
ΔclpX
clpXcompl
growth phase
exp
stat

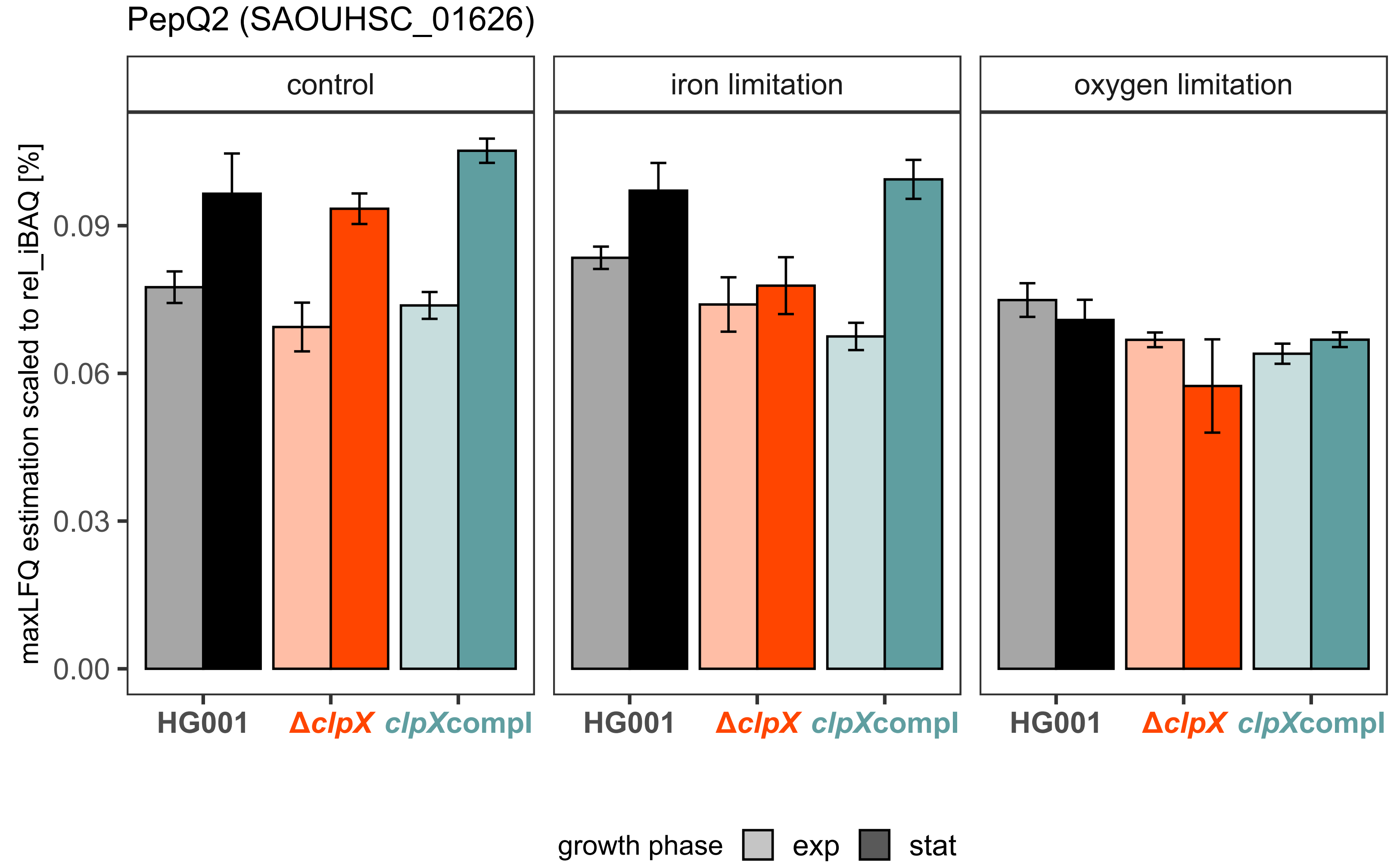
PepQ2 (SAOUHSC_01626)
control
iron limitation
oxygen limitation
maxLFQ estimation scaled to rel_iBAQ [%]
0.00
0.03
0.06
0.09
HG001
ΔclpX
clpXcompl
growth phase
exp
stat

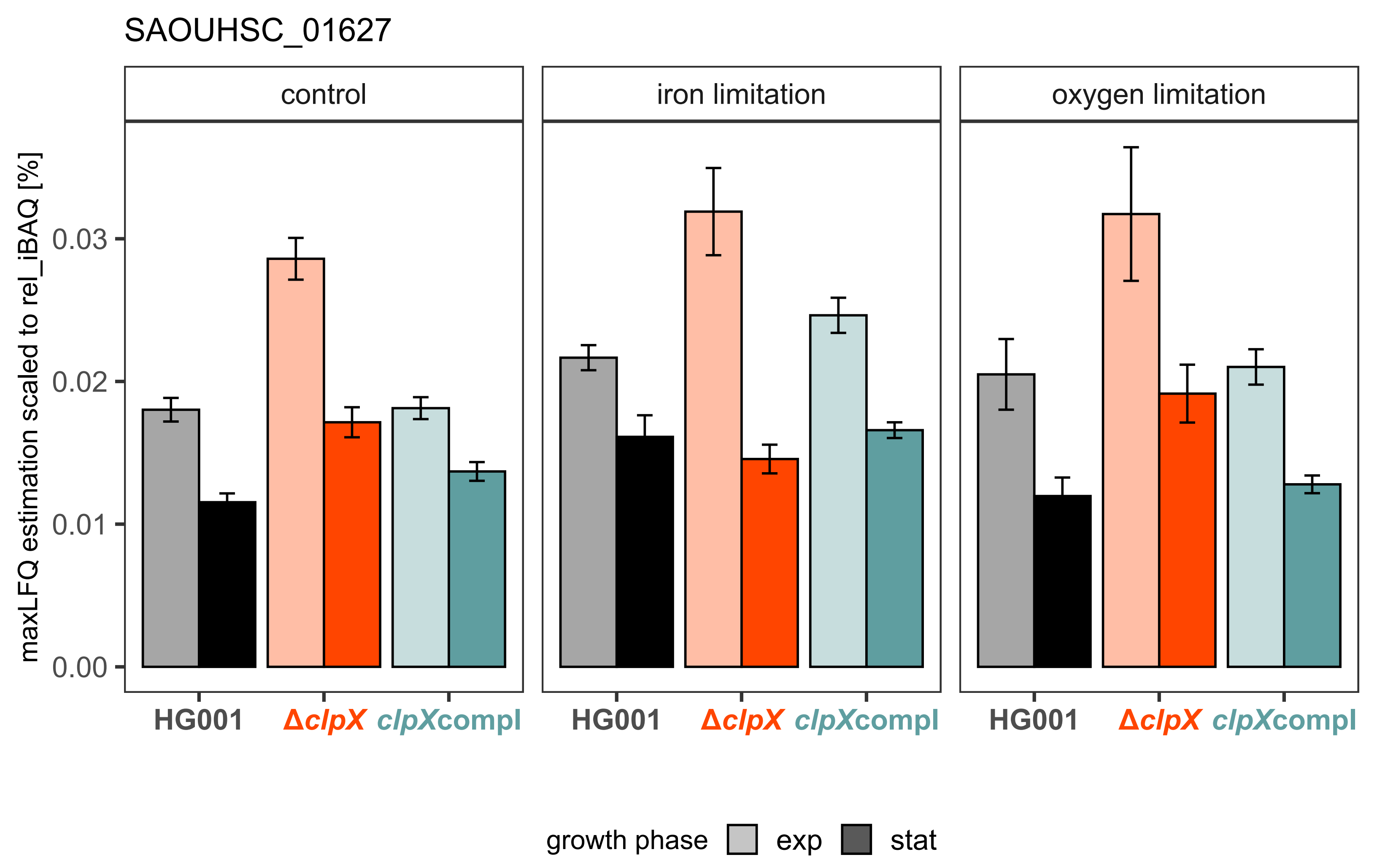

SAOUHSC_01627
control
iron limitation
oxygen limitation
maxLFQ estimation scaled to rel_iBAQ [%]
0.00
0.01
0.02
0.03
HG001
ΔclpX
clpXcompl
growth phase
exp
stat

LipM (SAOUHSC_01629)

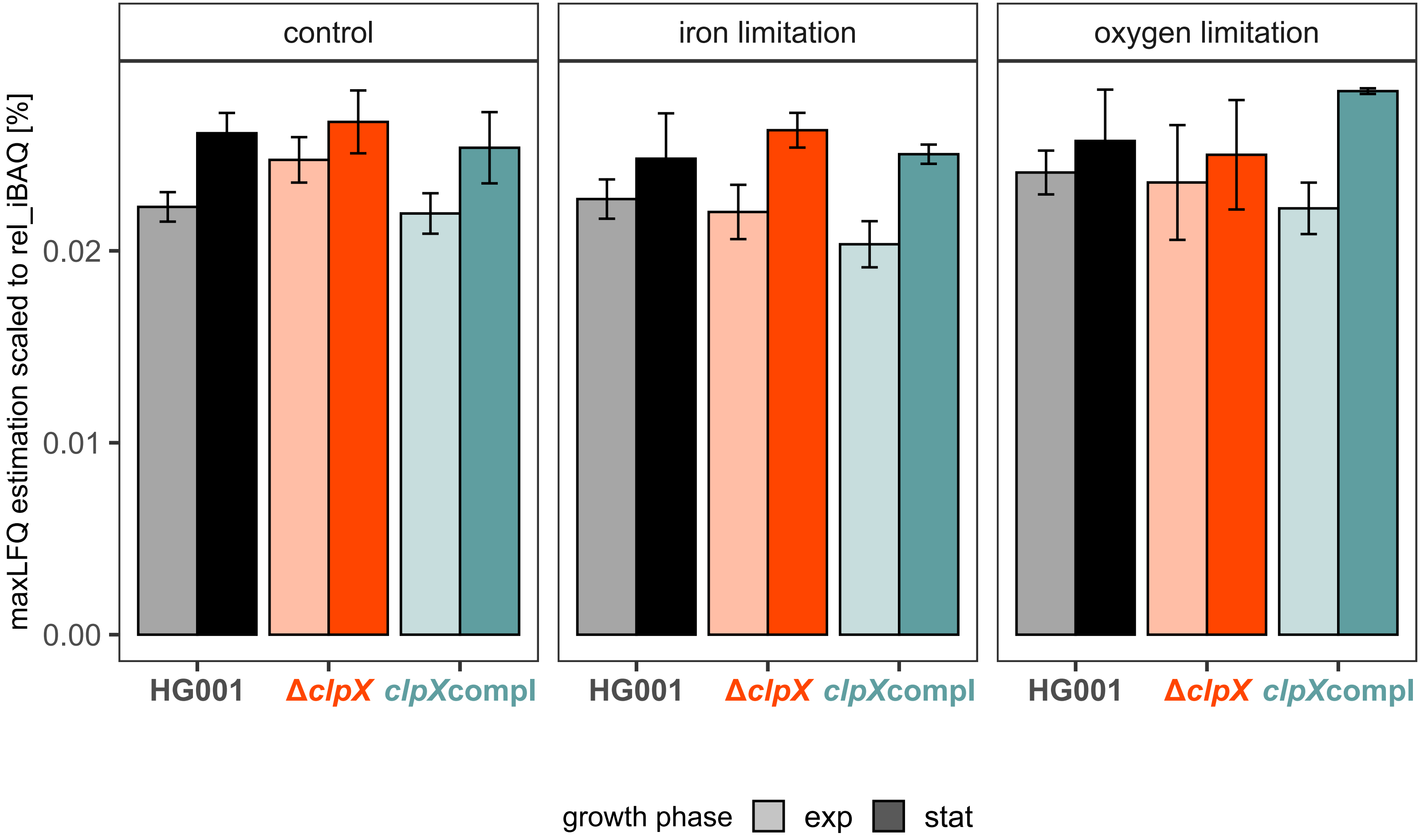

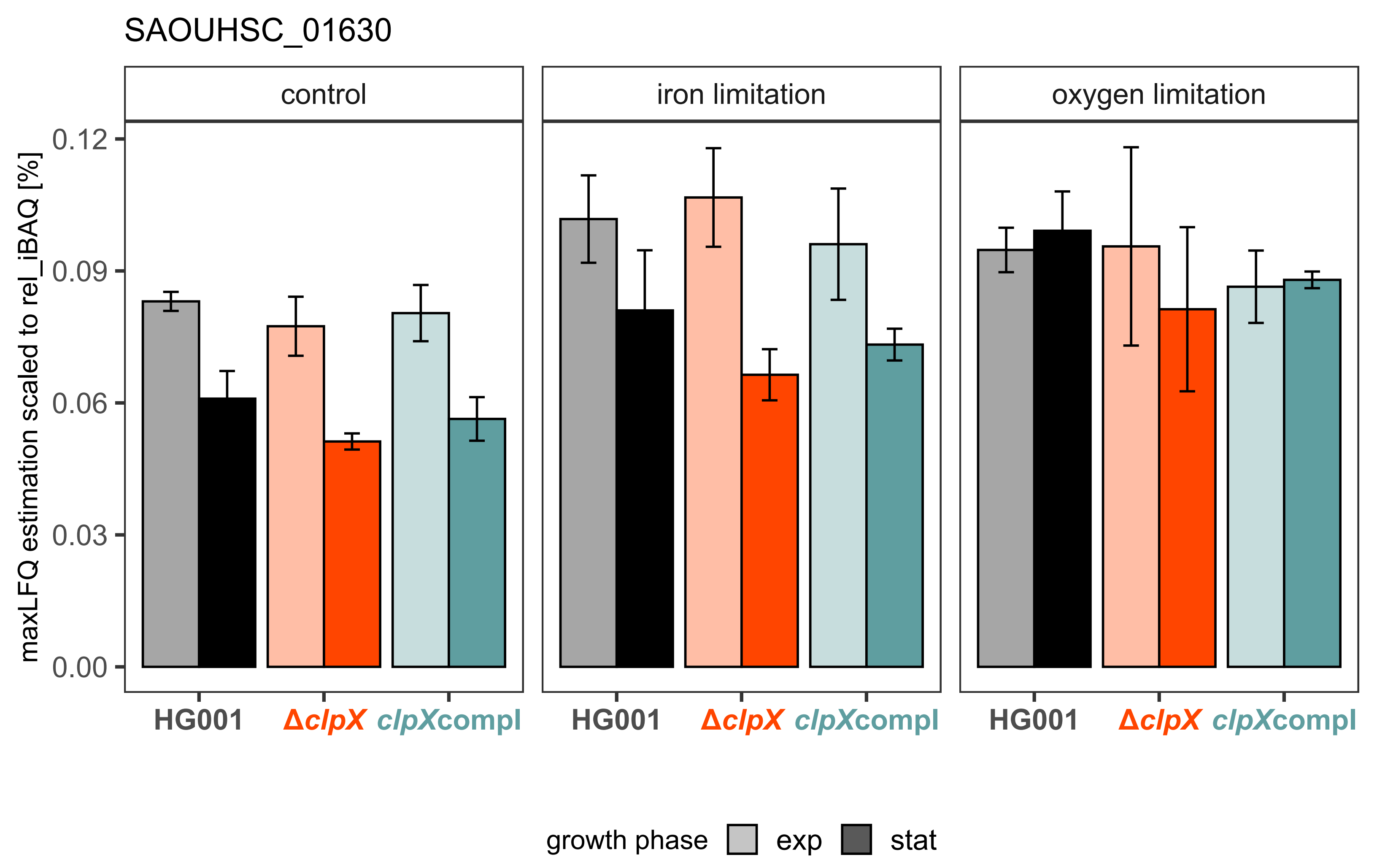

SAOUHSC_01630
control
iron limitation
oxygen limitation
maxLFQ estimation scaled to rel_iBAQ [%]
0.12
0.09
0.06
0.03
0.00
HG001
ΔclpX
clpXcompl
growth phase
exp
stat

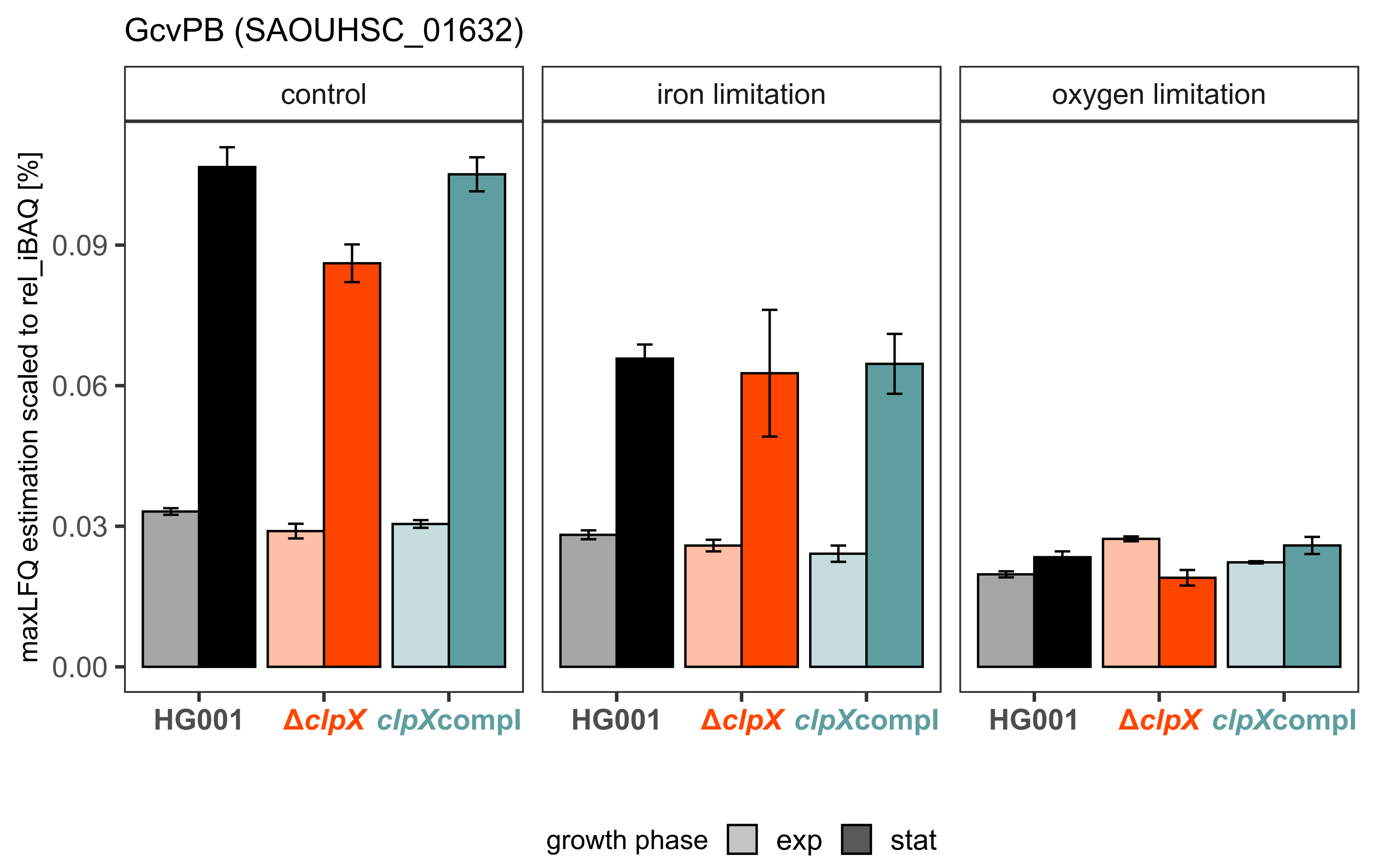
GcvPB (SAOUHSC_01632)
control
iron limitation
oxygen limitation
maxLFQ estimation scaled to rel_iBAQ [%]
0.09
0.06
0.03
0.00
HG001
ΔclpX
clpXcompl
growth phase
exp
stat

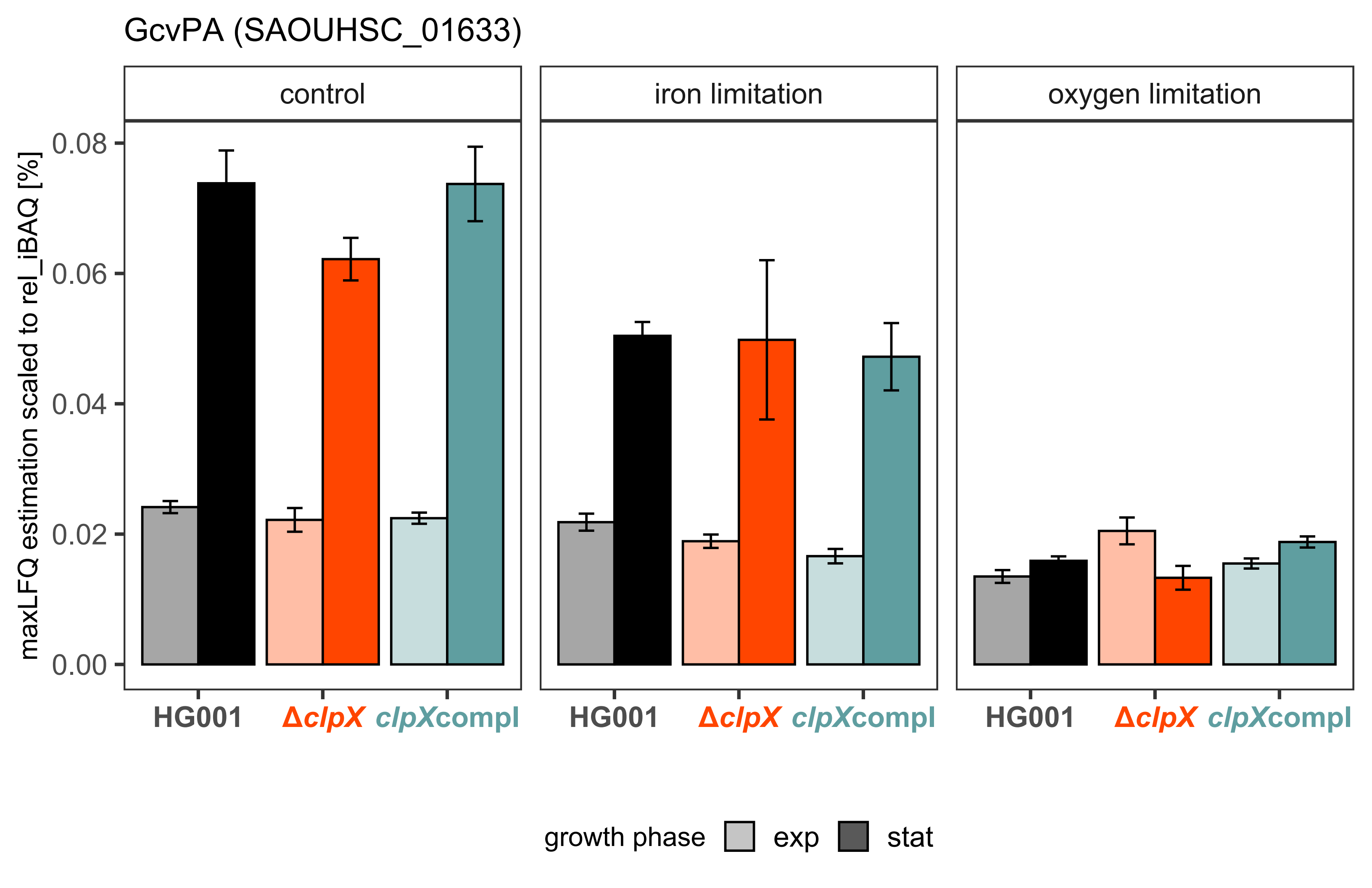
GcvPA (SAOUHSC_01633)
control
iron limitation
oxygen limitation
maxLFQ estimation scaled to rel_iBAQ [%]
0.08
0.06
0.04
0.02
0.00
HG001
ΔclpX
clpXcompl
growth phase
exp
stat

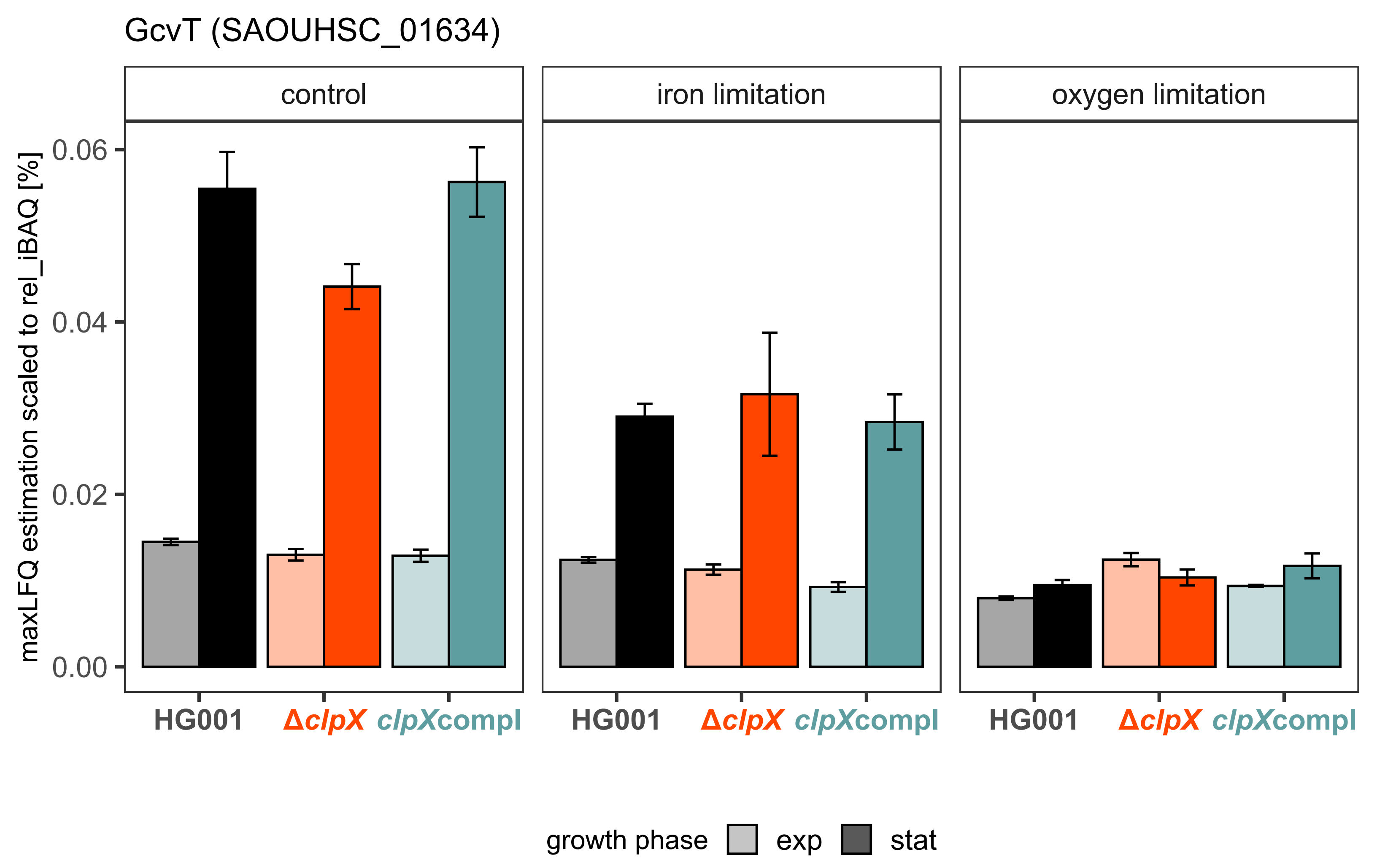

GcvT (SAOUHSC_01634)
control
iron limitation
oxygen limitation
maxLFQ estimation scaled to rel_iBAQ [%]
0.06
0.04
0.02
0.00
HG001
ΔclpX
clpXcompl
growth phase
exp
stat

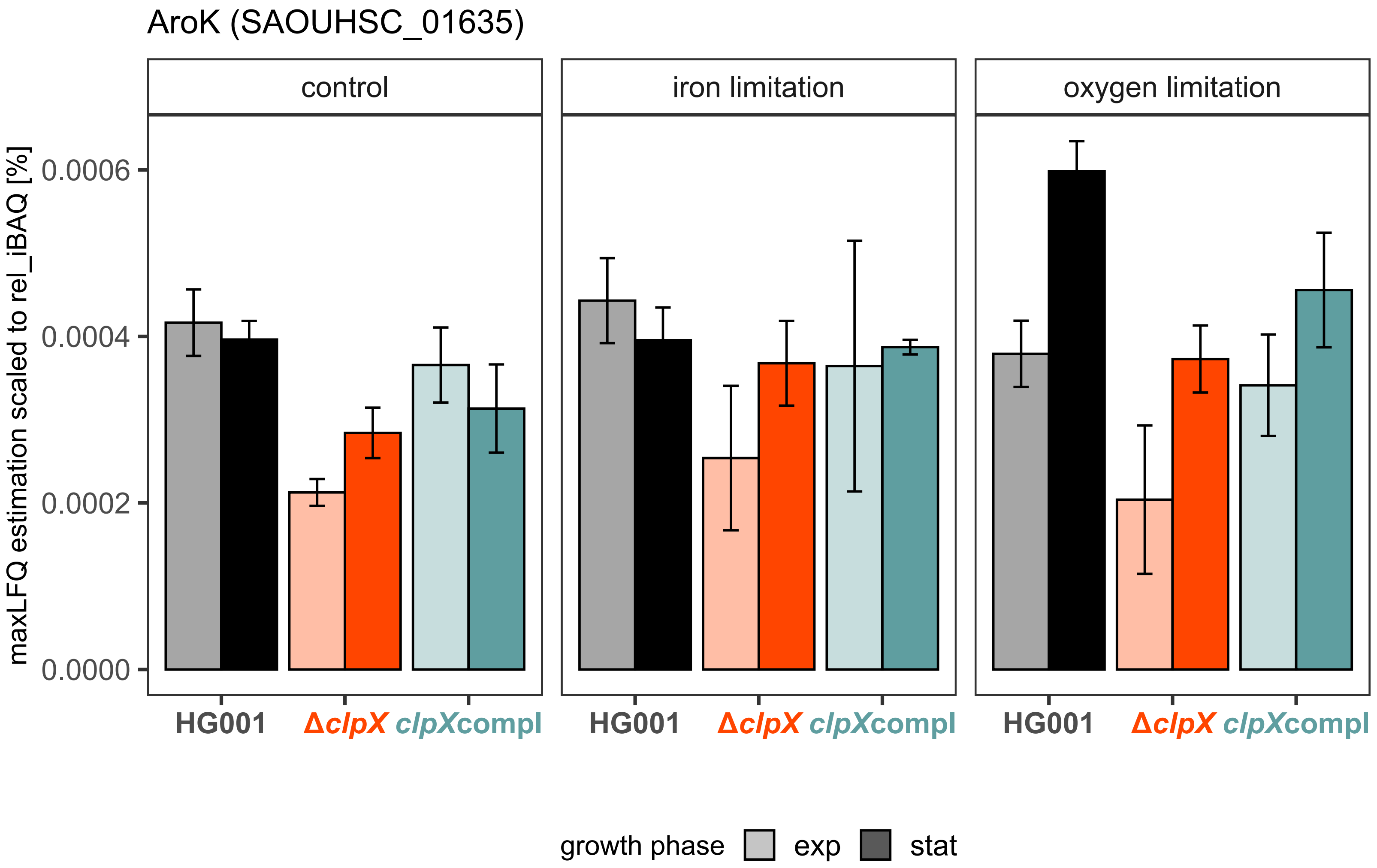
AroK (SAOUHSC_01635)
control
iron limitation
oxygen limitation
maxLFQ estimation scaled to rel_iBAQ [%]
0.0006
0.0004
0.0002
0.0000
HG001
ΔclpX
clpXcompl
growth phase
exp
stat

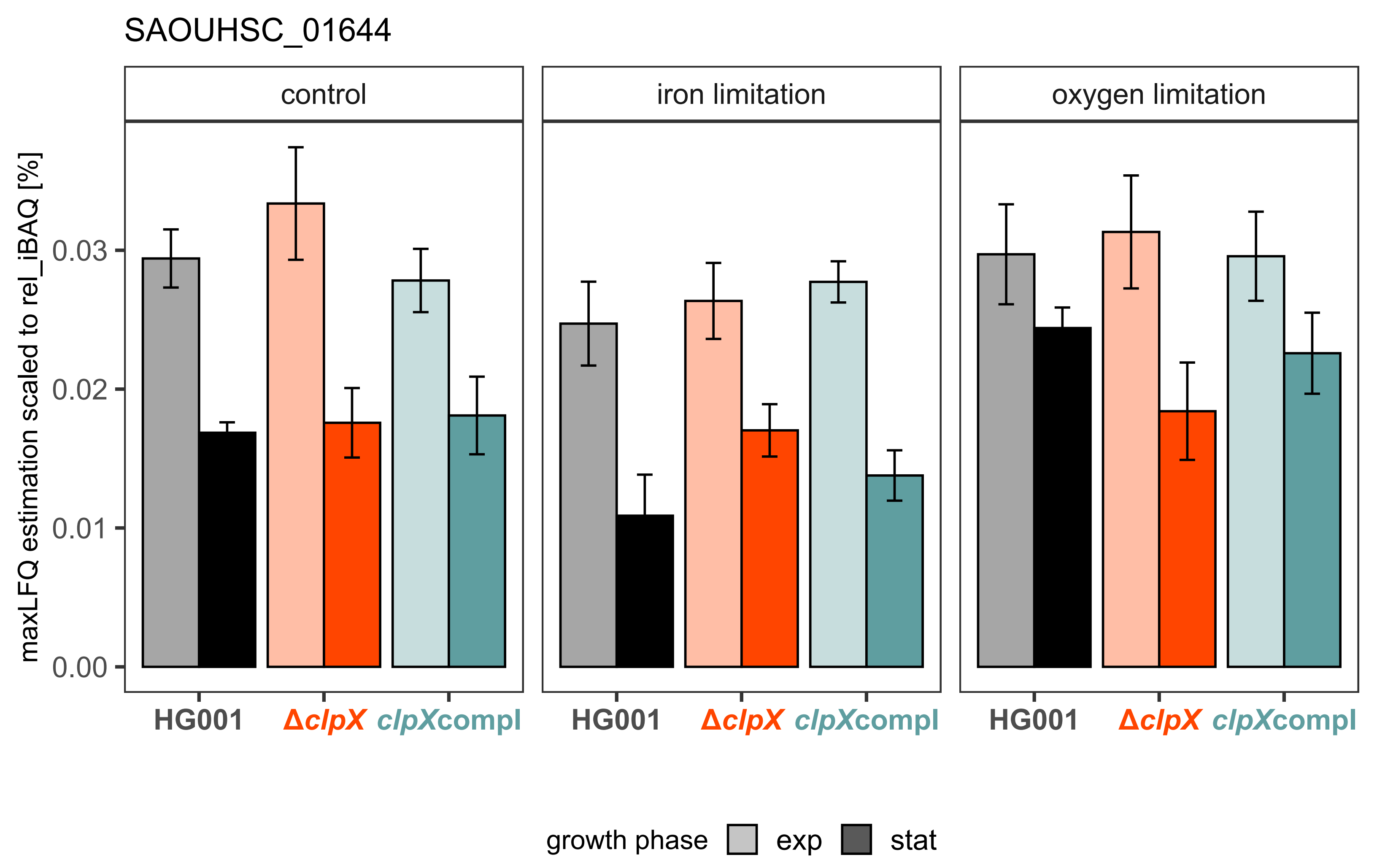
SAOUHSC_01644
control
iron limitation
oxygen limitation
maxLFQ estimation scaled to rel_iBAQ [%]
0.03
0.02
0.01
0.00
HG001
ΔclpX
clpXcompl
growth phase
exp
stat

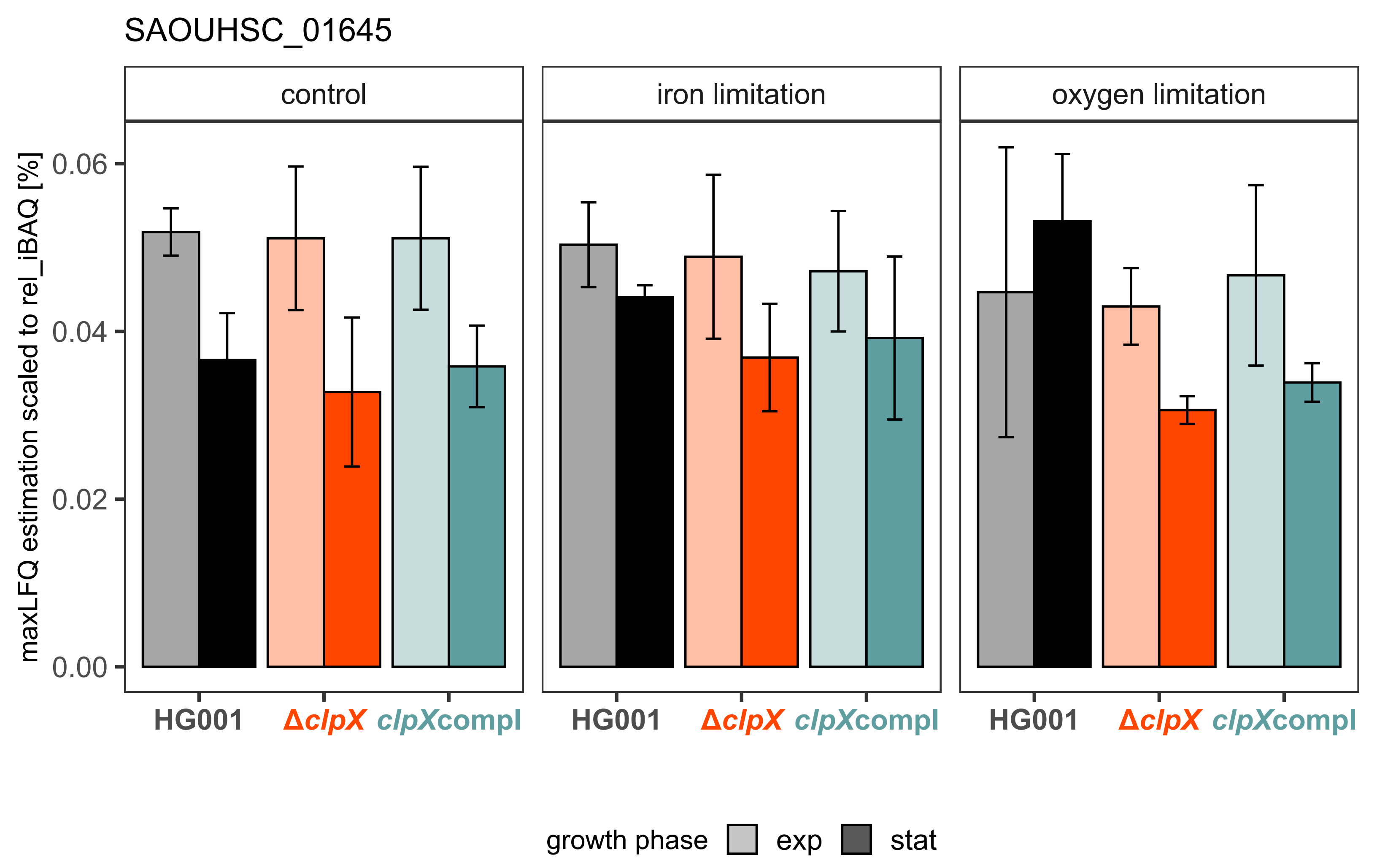
SAOUHSC_01645
control
iron limitation
oxygen limitation
maxLFQ estimation scaled to rel_iBAQ [%]
0.06
0.04
0.02
0.00
HG001
ΔclpX
clpXcompl
growth phase
exp
stat

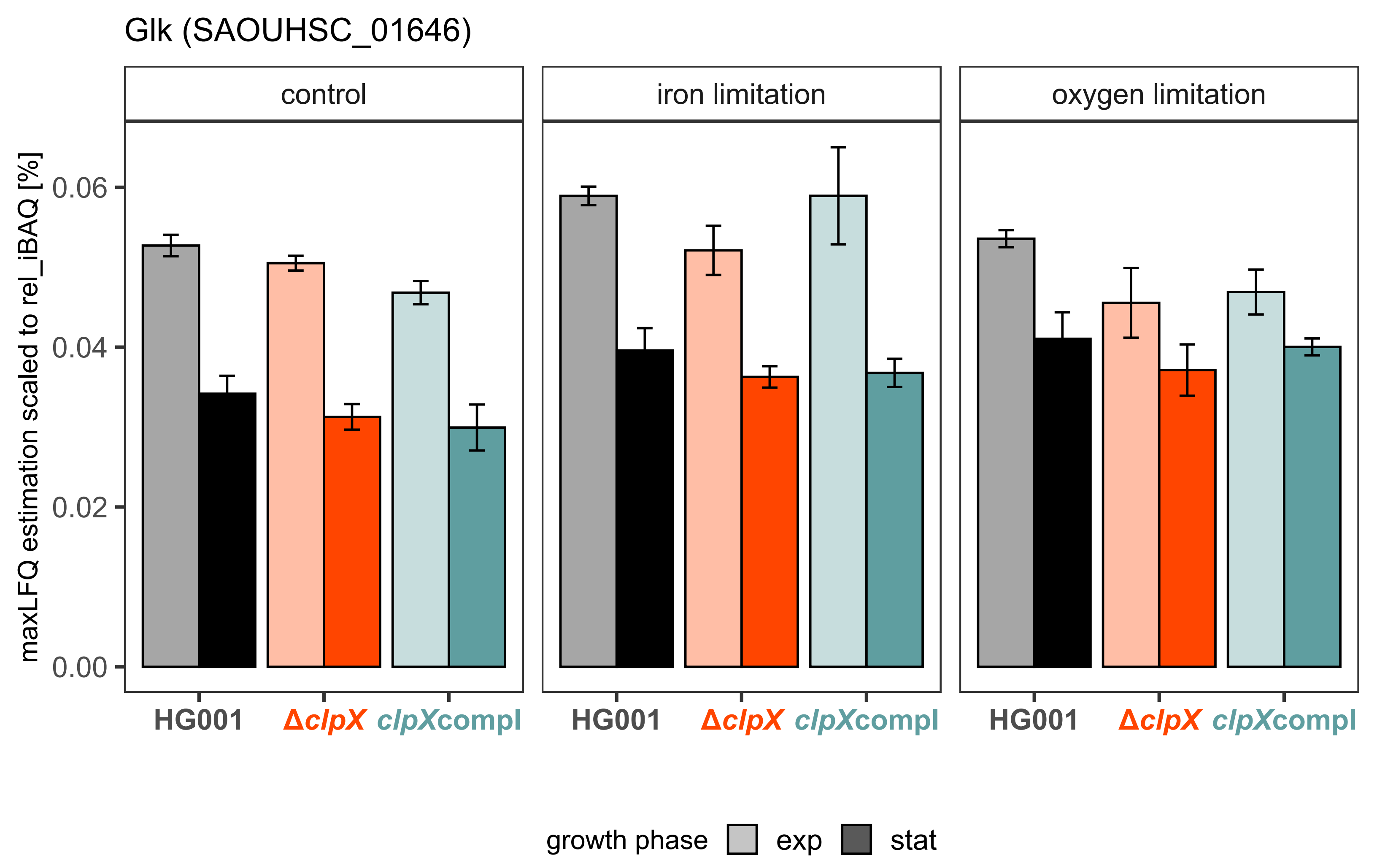
Glk (SAOUHSC_01646)
control
iron limitation
oxygen limitation
maxLFQ estimation scaled to rel_iBAQ [%]
0.06
0.04
0.02
0.00
HG001
ΔclpX
clpXcompl
growth phase
exp
stat

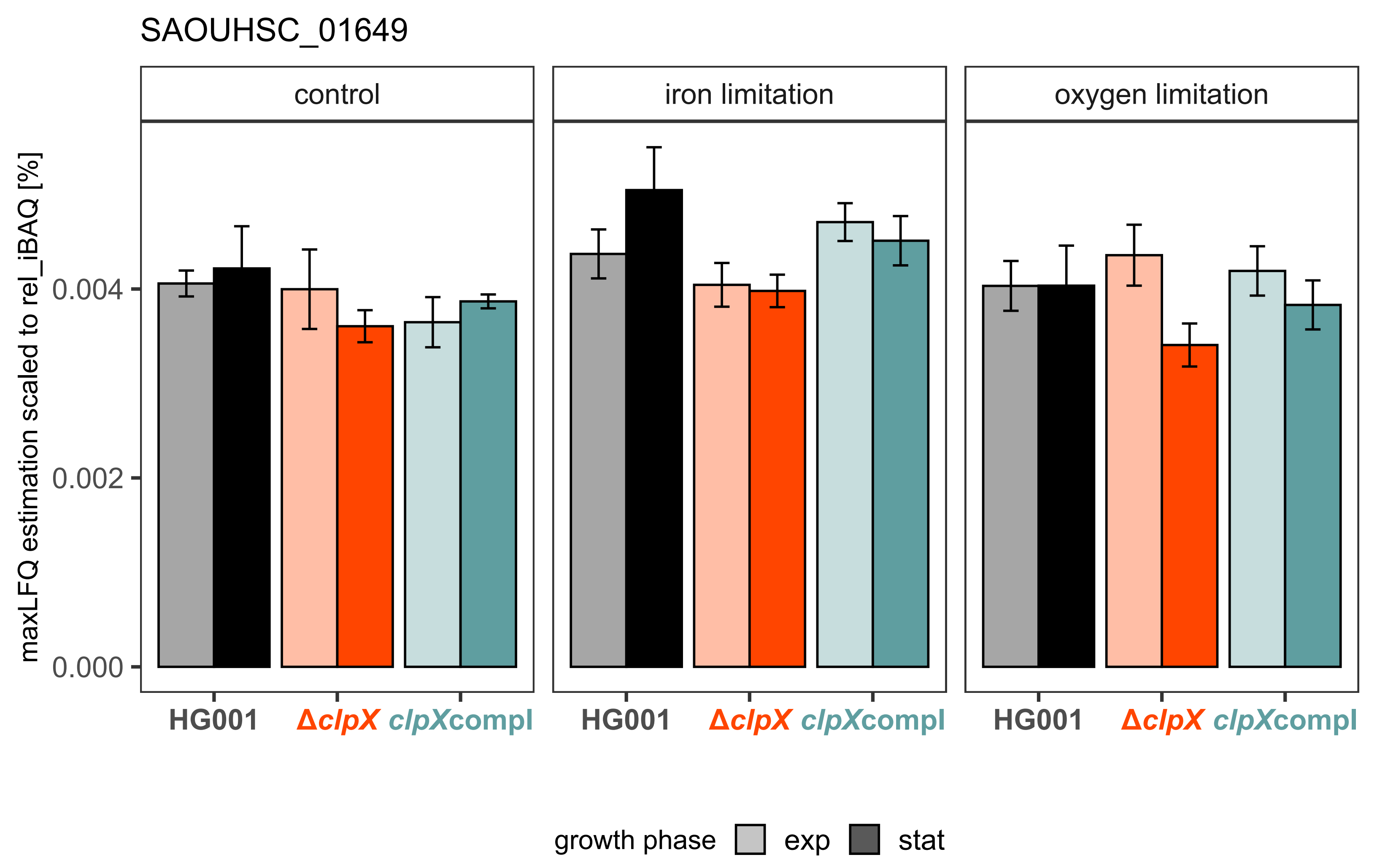

SAOUHSC_01649
control
iron limitation
oxygen limitation
maxLFQ estimation scaled to rel_iBAQ [%]
0.000
0.002
0.004
HG001
ΔclpX
clpXcompl
growth phase
exp
stat

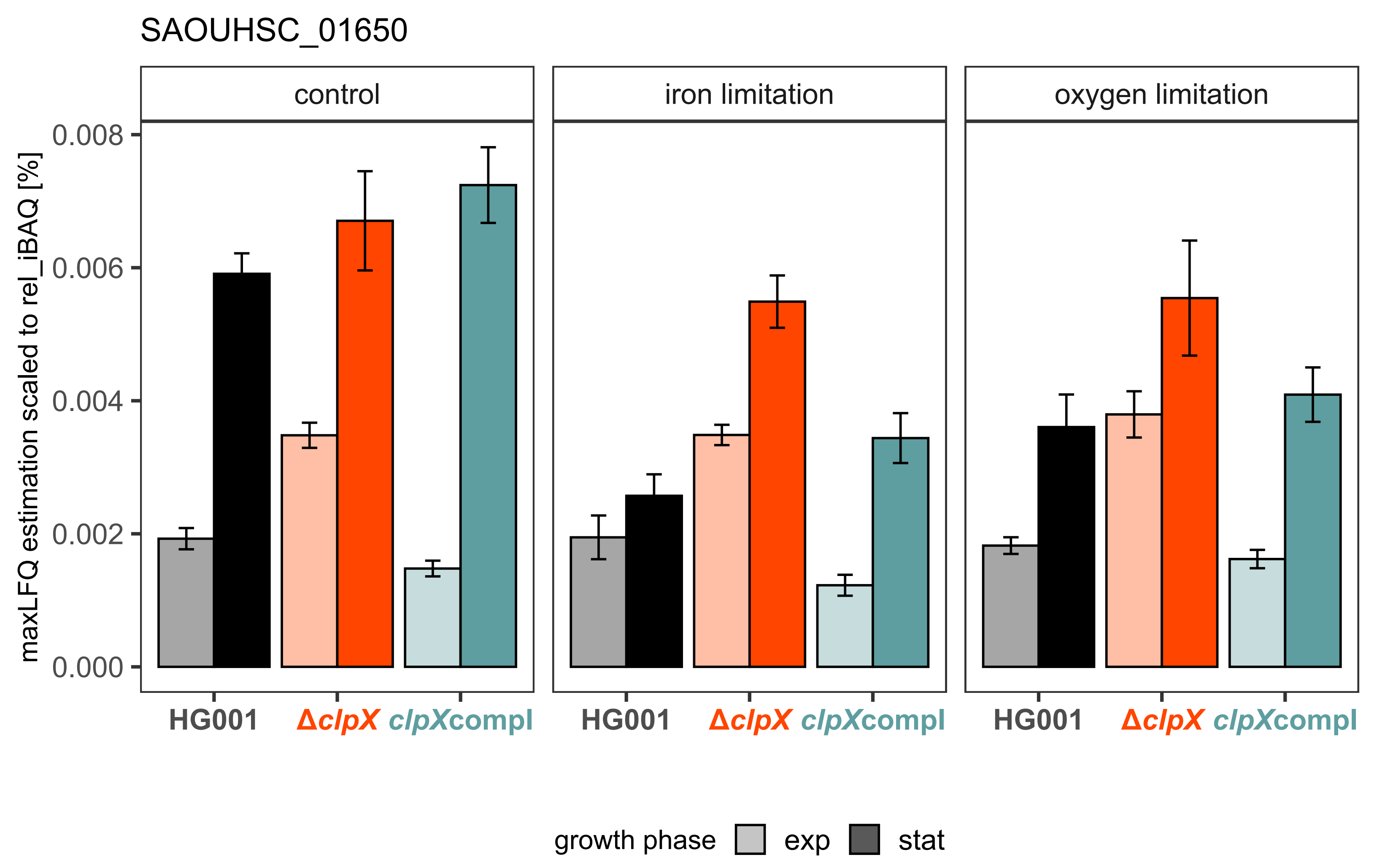
SAOUHSC_01650
control
iron limitation
oxygen limitation
maxLFQ estimation scaled to rel_iBAQ [%]
0.008
0.006
0.004
0.002
0.000
HG001
ΔclpX
clpXcompl
growth phase
exp
stat

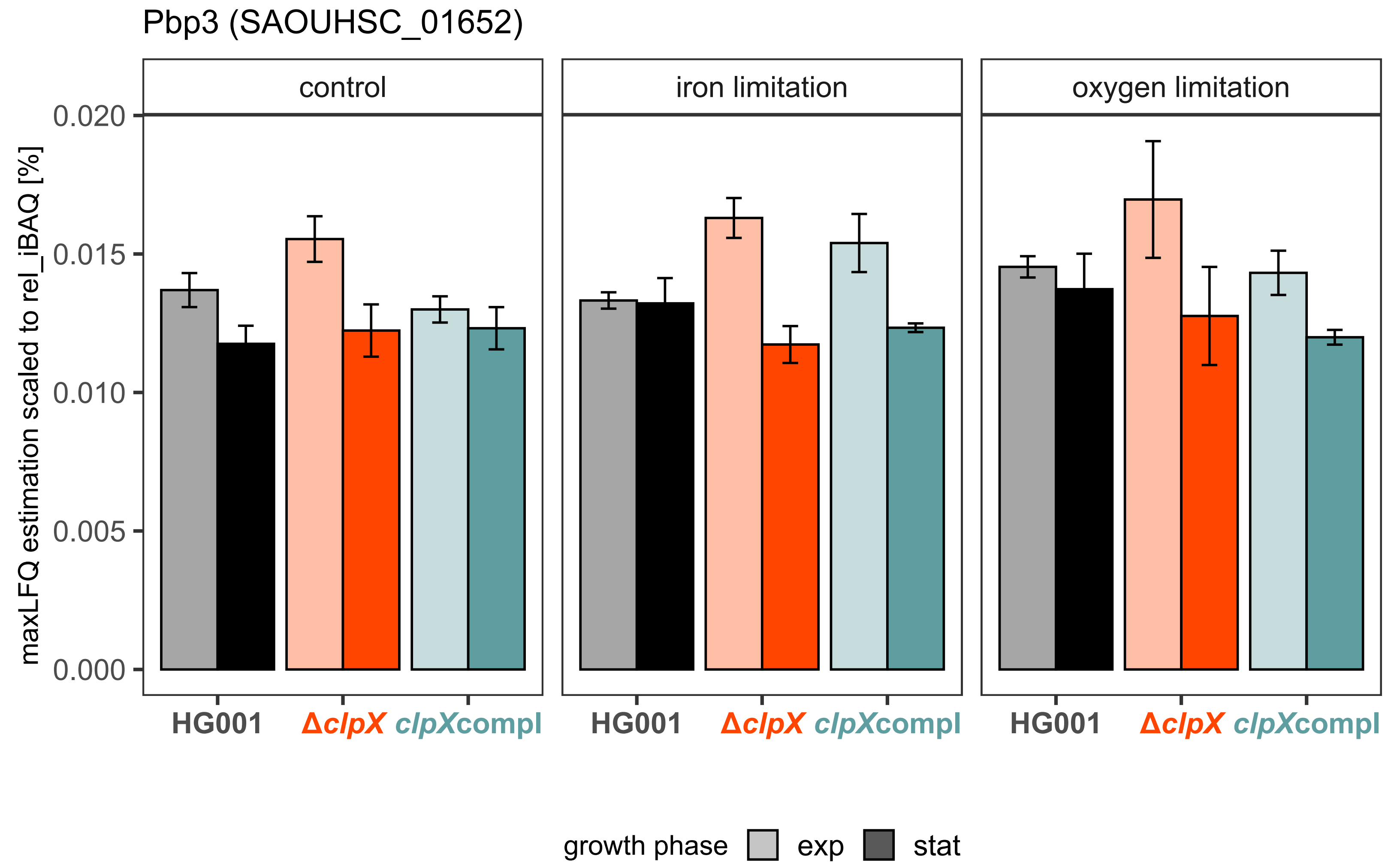
Pbp3 (SAOUHSC_01652)
control
iron limitation
oxygen limitation
maxLFQ estimation scaled to rel_iBAQ [%]
0.020
0.015
0.010
0.005
0.000
HG001
ΔclpX
clpXcompl
growth phase
exp
stat

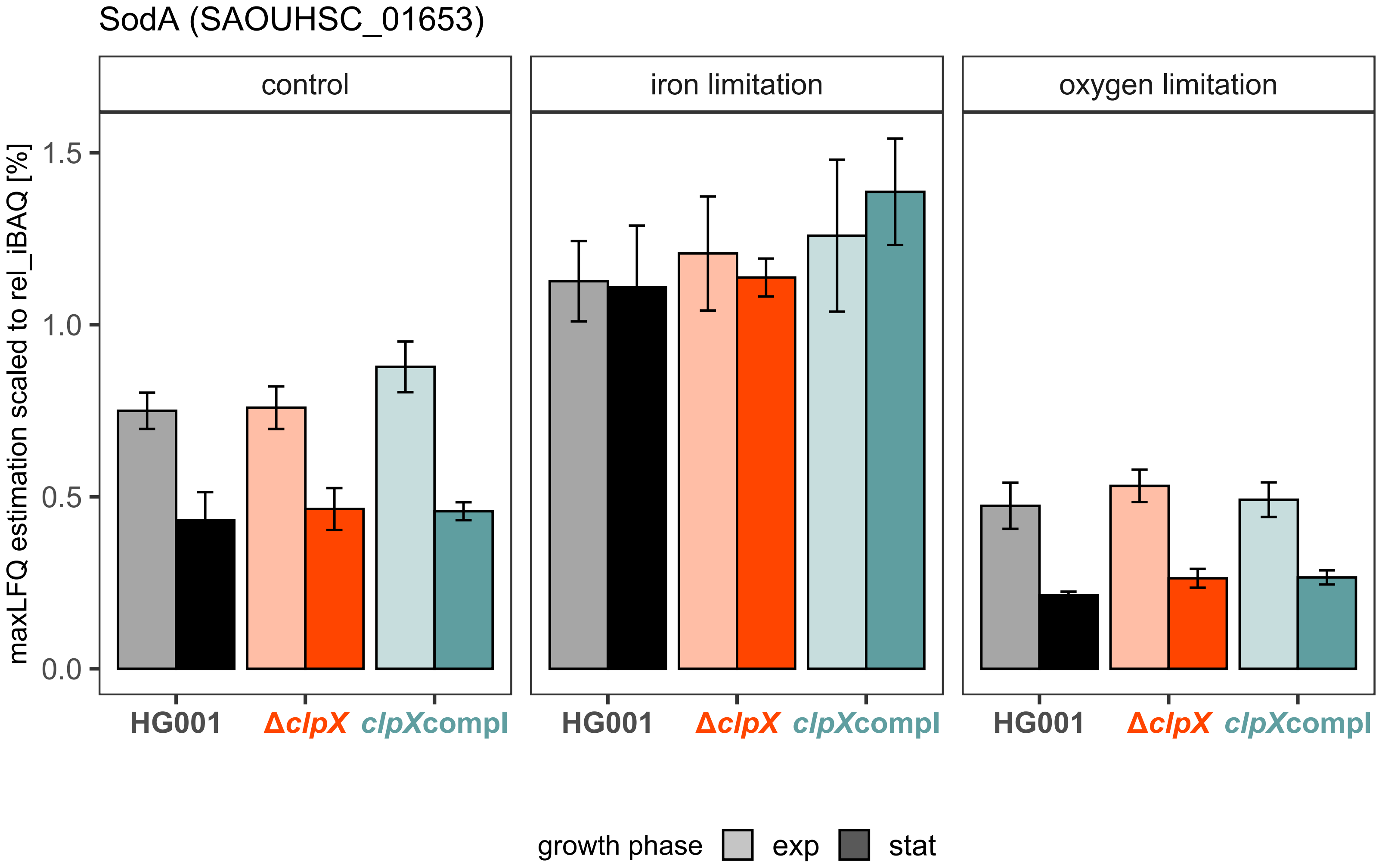
SodA (SAOUHSC_01653)
control
iron limitation
oxygen limitation
maxLFQ estimation scaled to rel_iBAQ [%]
0.0
0.5
1.0
1.5
HG001
ΔclpX
clpXcompl
growth phase
exp
stat

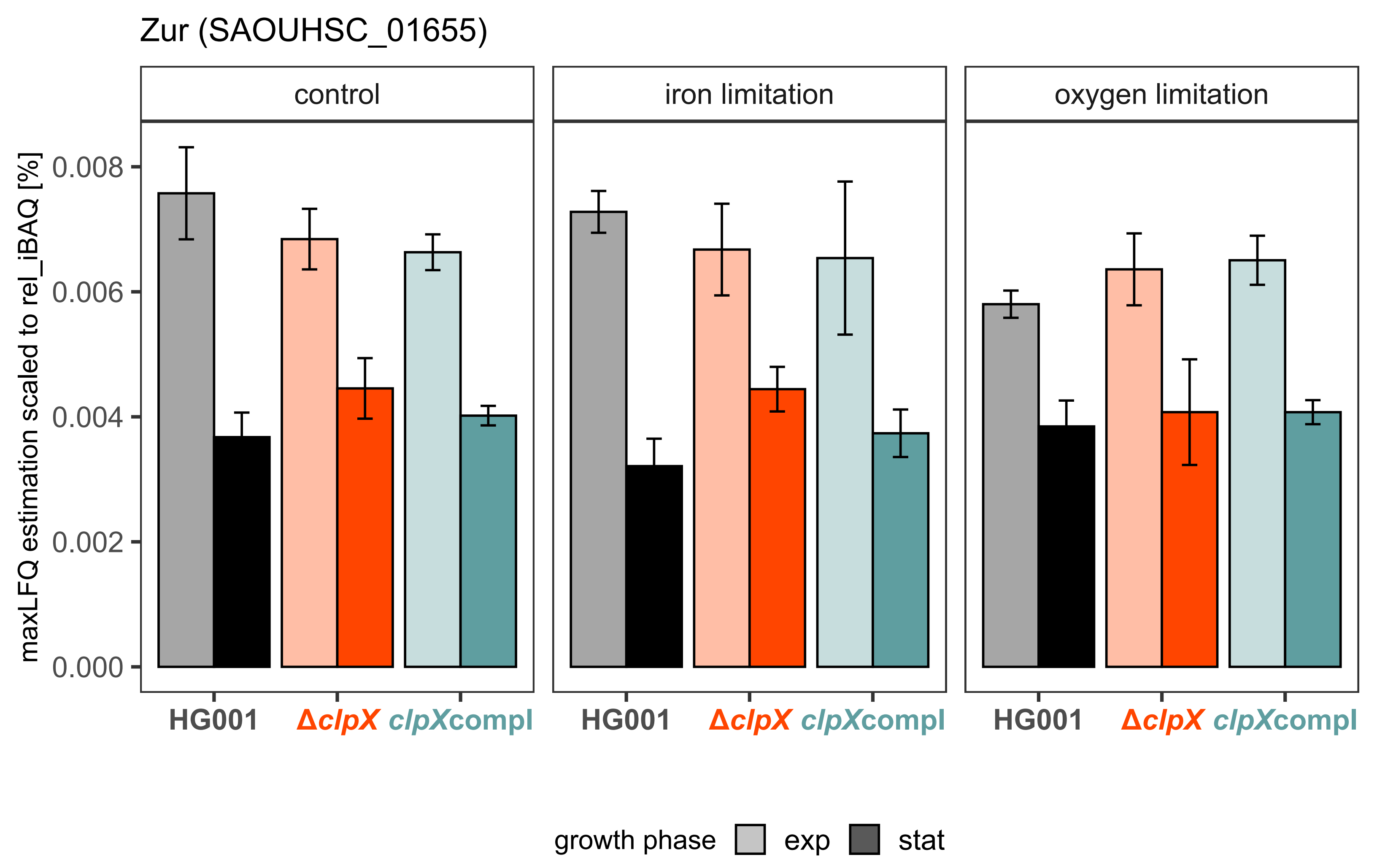

Zur (SAOUHSC_01655)
control
iron limitation
oxygen limitation
maxLFQ estimation scaled to rel_iBAQ [%]
0.000
0.002
0.004
0.006
0.008
HG001
ΔclpX
clpXcompl
growth phase
exp
stat

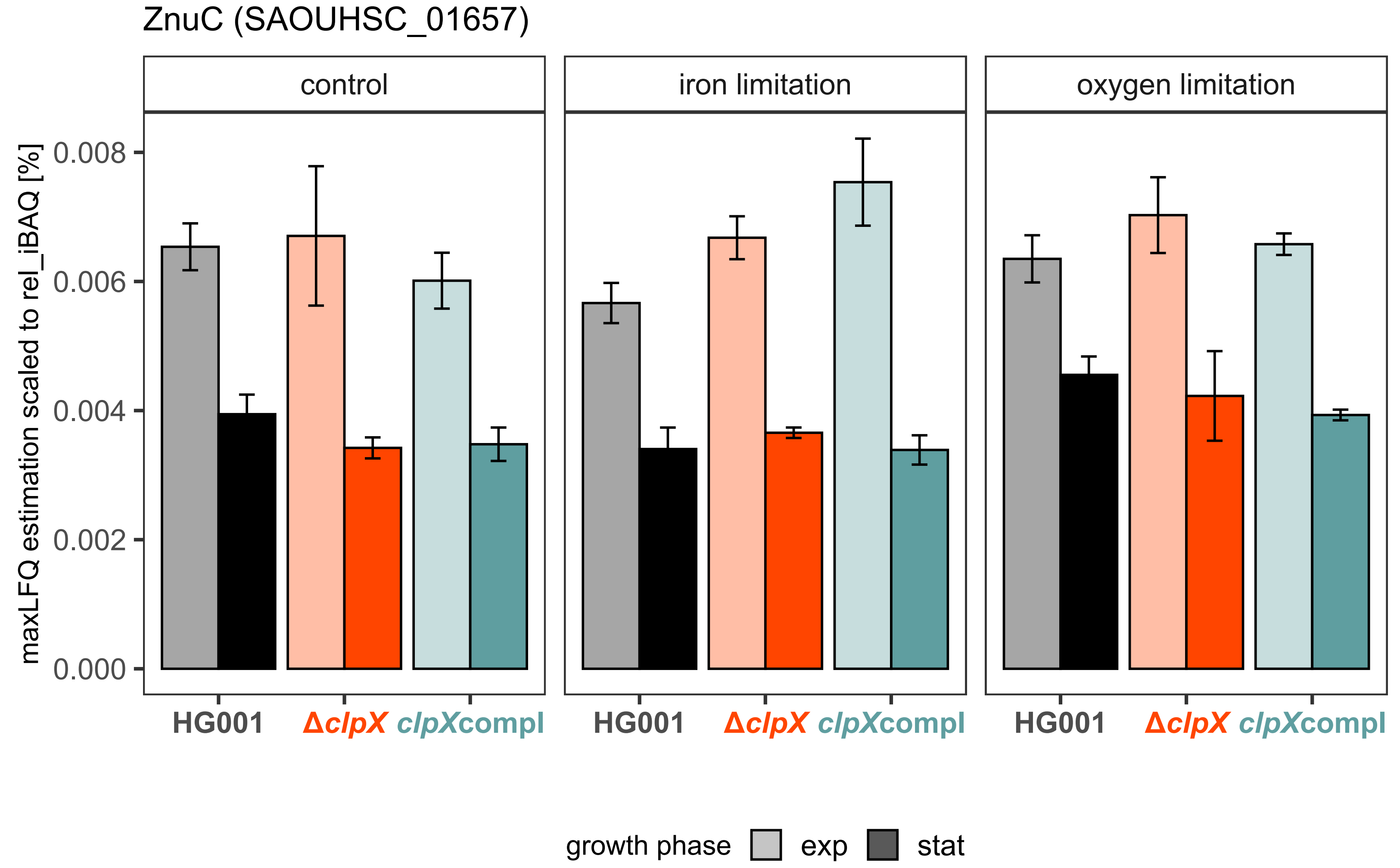

ZnuC (SAOUHSC_01657)
control
iron limitation
oxygen limitation
maxLFQ estimation scaled to rel_iBAQ [%]
0.008
0.006
0.004
0.002
0.000
HG001
ΔclpX
clpXcompl
growth phase
exp
stat

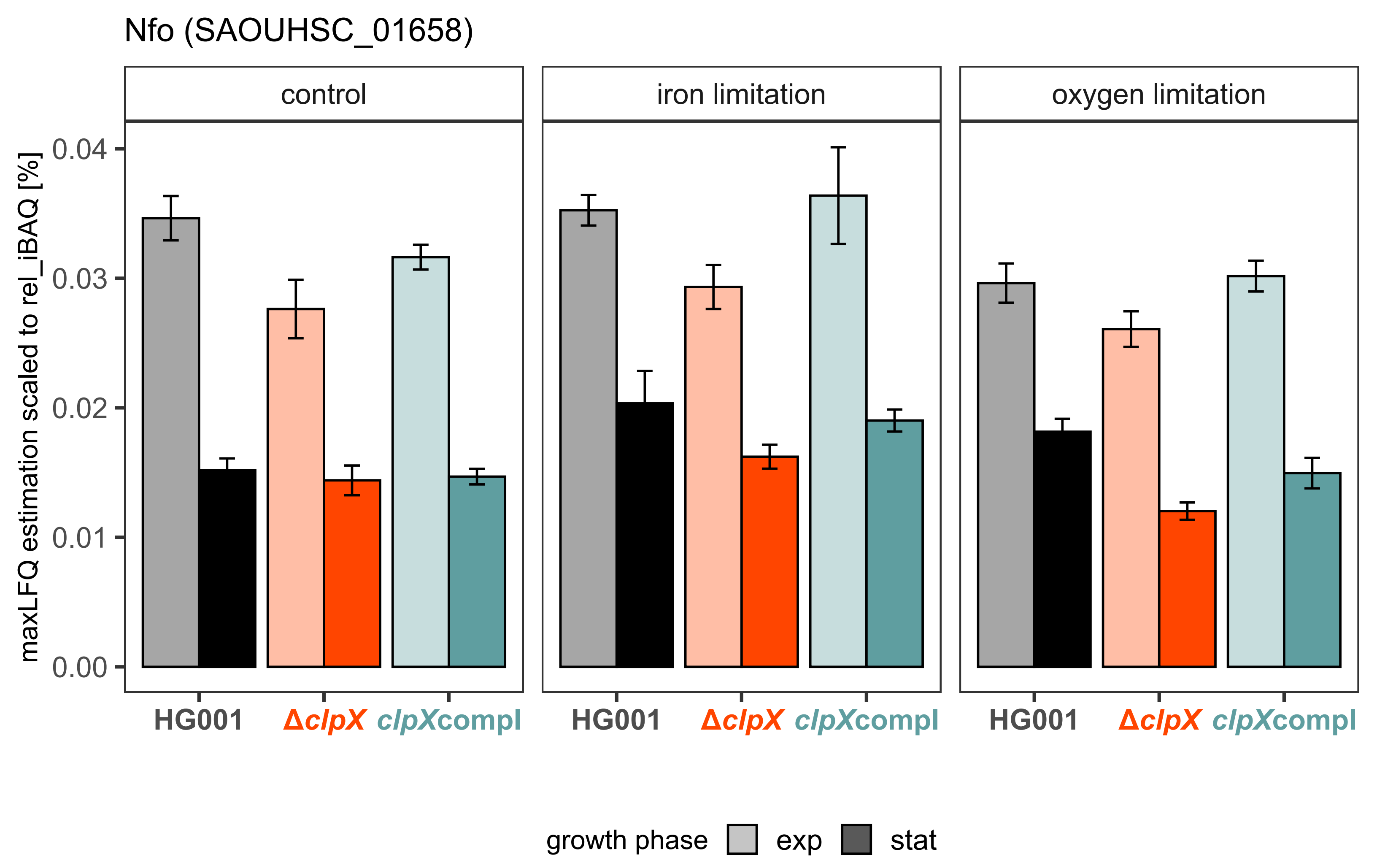
Nfo (SAOUHSC_01658)
control
iron limitation
oxygen limitation
maxLFQ estimation scaled to rel_iBAQ [%]
0.04
0.03
0.02
0.01
0.00
HG001
ΔclpX
clpXcompl
growth phase
exp
stat

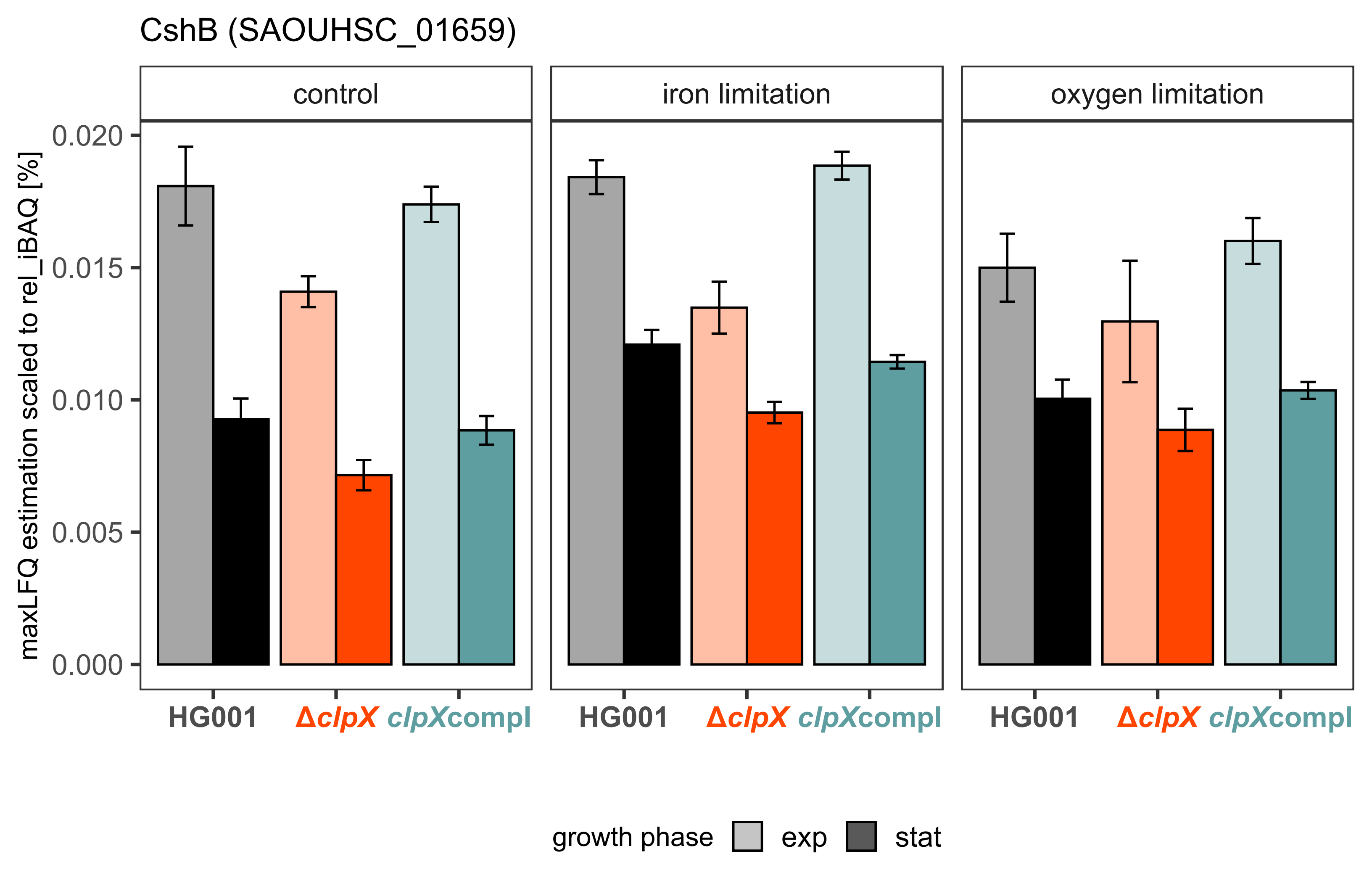
CshB (SAOUHSC_01659)
control
iron limitation
oxygen limitation
maxLFQ estimation scaled to rel_iBAQ [%]
0.020
0.015
0.010
0.005
0.000
HG001
ΔclpX
clpXcompl
growth phase
exp
stat

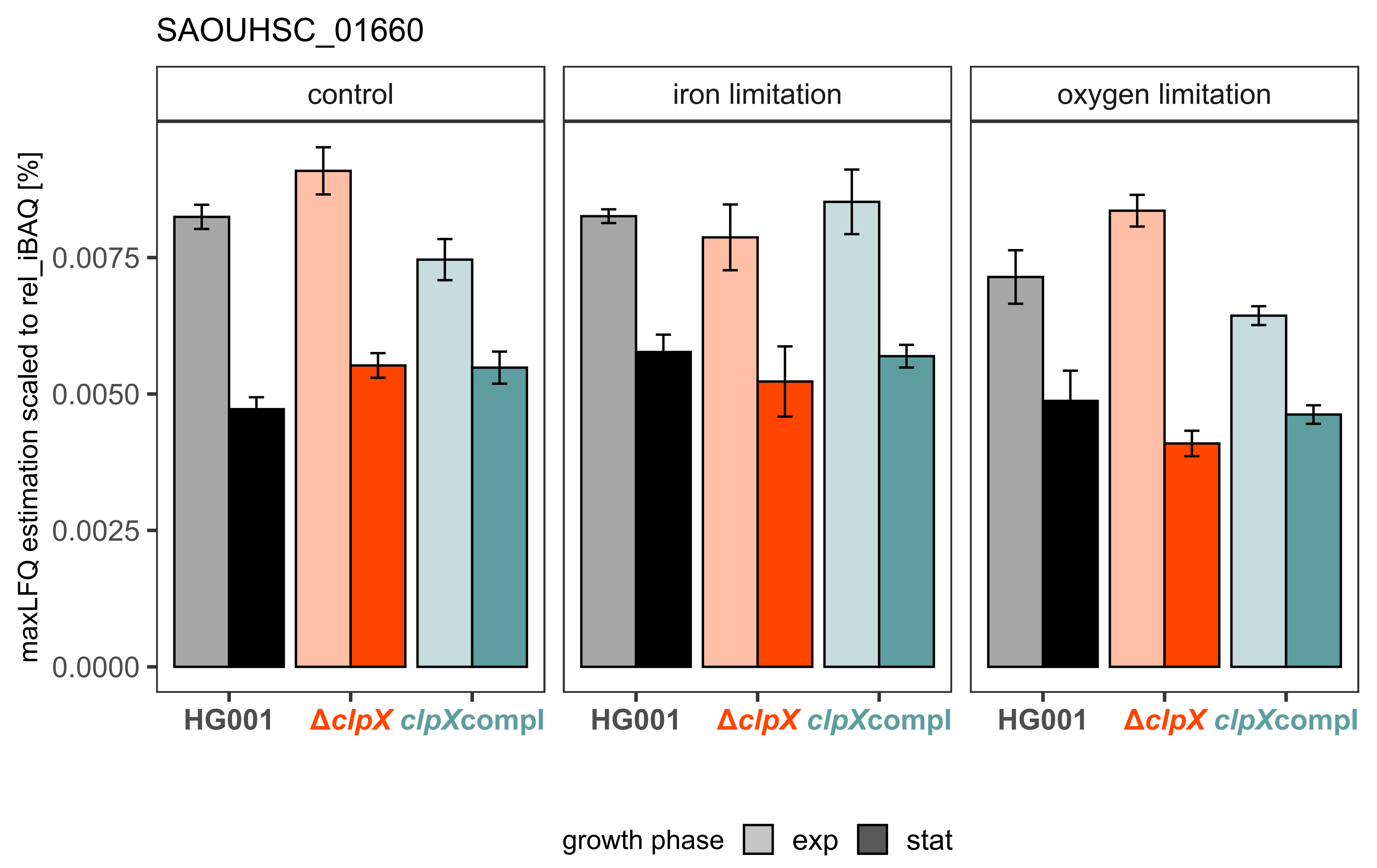
SAOUHSC_01660
control
iron limitation
oxygen limitation
maxLFQ estimation scaled to rel_iBAQ [%]
0.0075
0.0050
0.0025
0.0000
HG001
ΔclpX
clpXcompl
growth phase
exp
stat

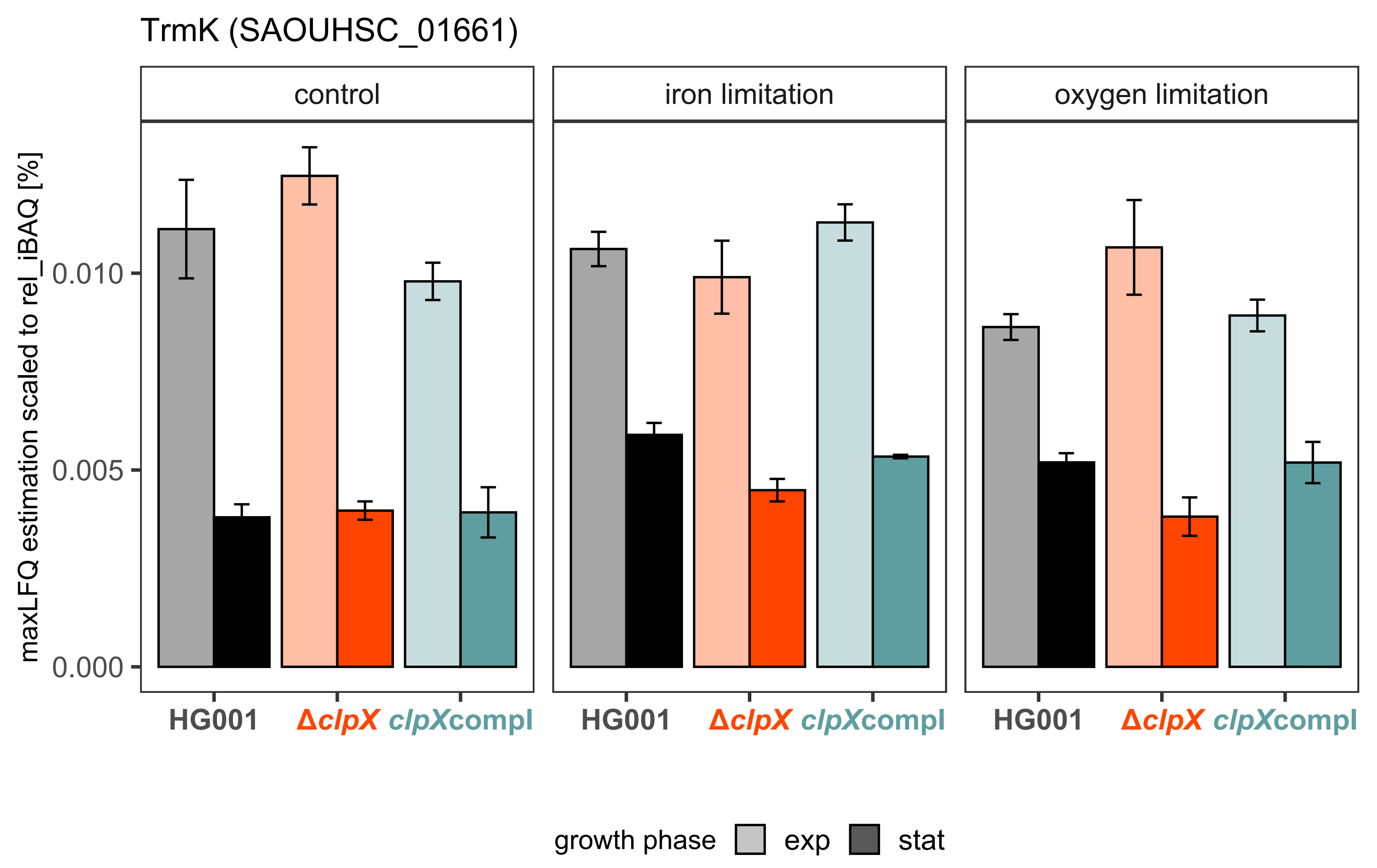

TrmK (SAOUHSC_01661)
control
iron limitation
oxygen limitation
maxLFQ estimation scaled to rel_iBAQ [%]
0.010
0.005
0.000
HG001
ΔclpX
clpXcompl
growth phase
exp
stat

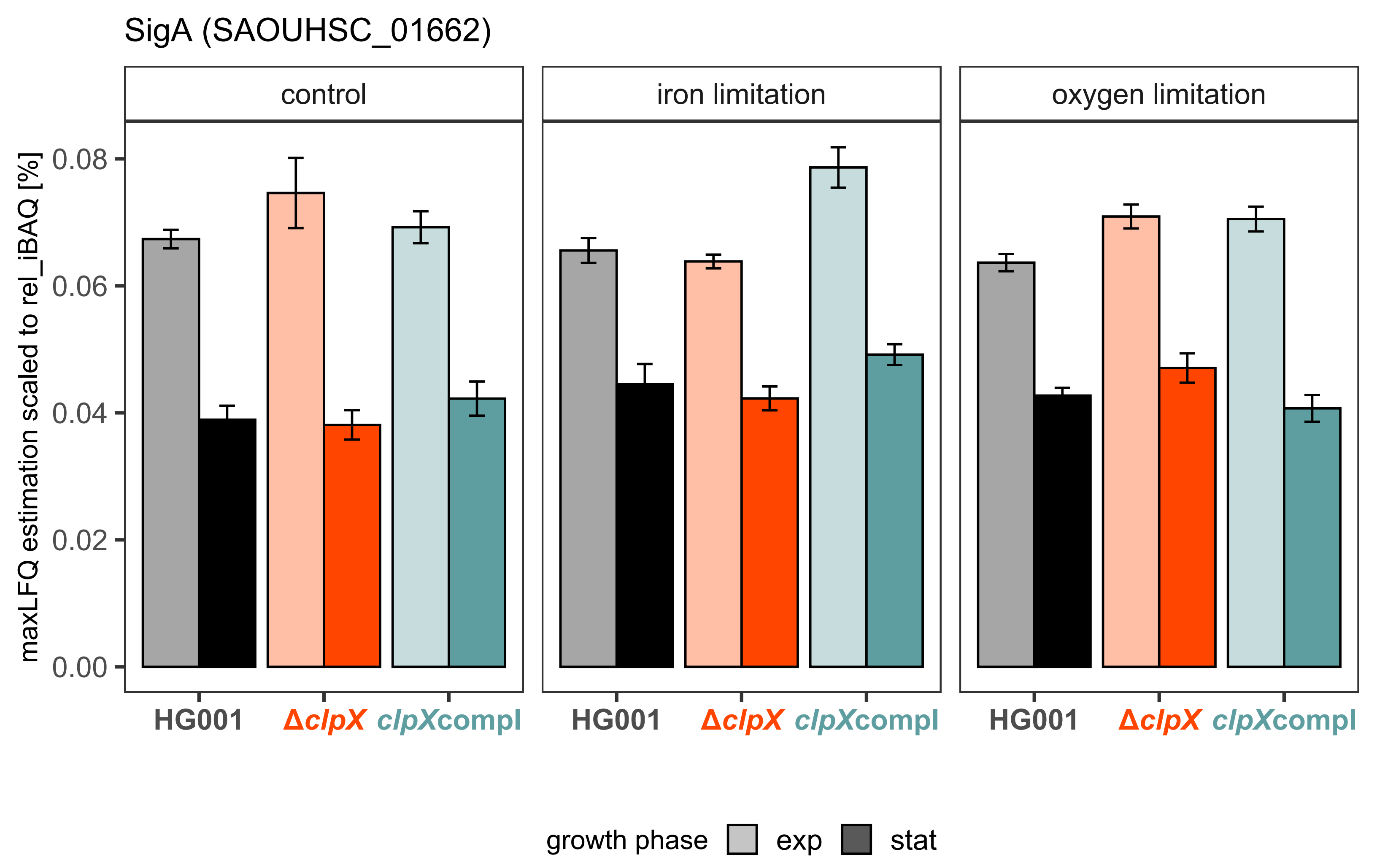
SigA (SAOUHSC_01662)
control
iron limitation
oxygen limitation
maxLFQ estimation scaled to rel_iBAQ [%]
0.00
0.02
0.04
0.06
0.08
HG001
ΔclpX
clpXcompl
growth phase
exp
stat

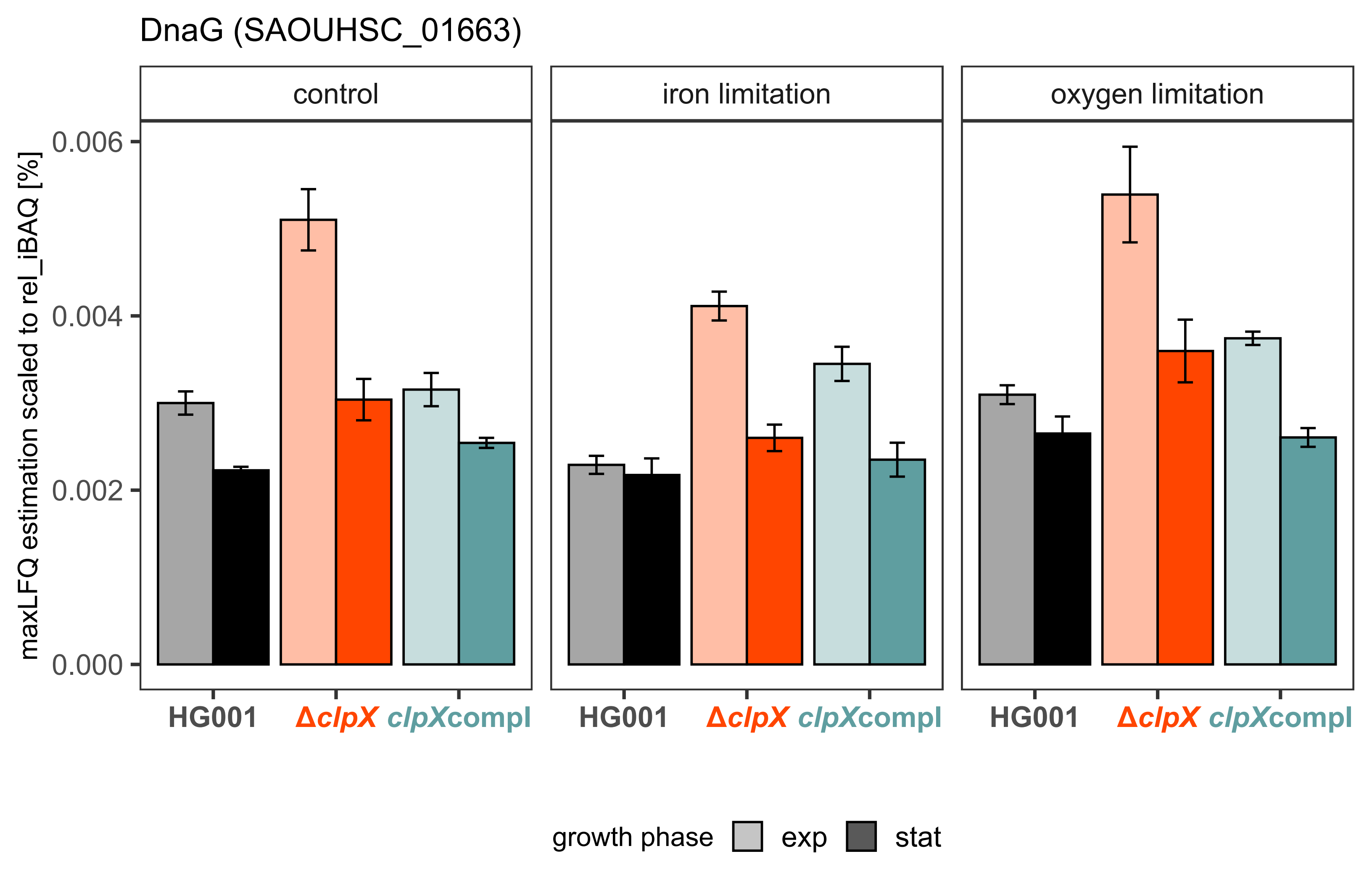
DnaG (SAOUHSC_01663)
control
iron limitation
oxygen limitation
maxLFQ estimation scaled to rel_iBAQ [%]
0.006
0.004
0.002
0.000
HG001
ΔclpX
clpXcompl
growth phase
exp
stat

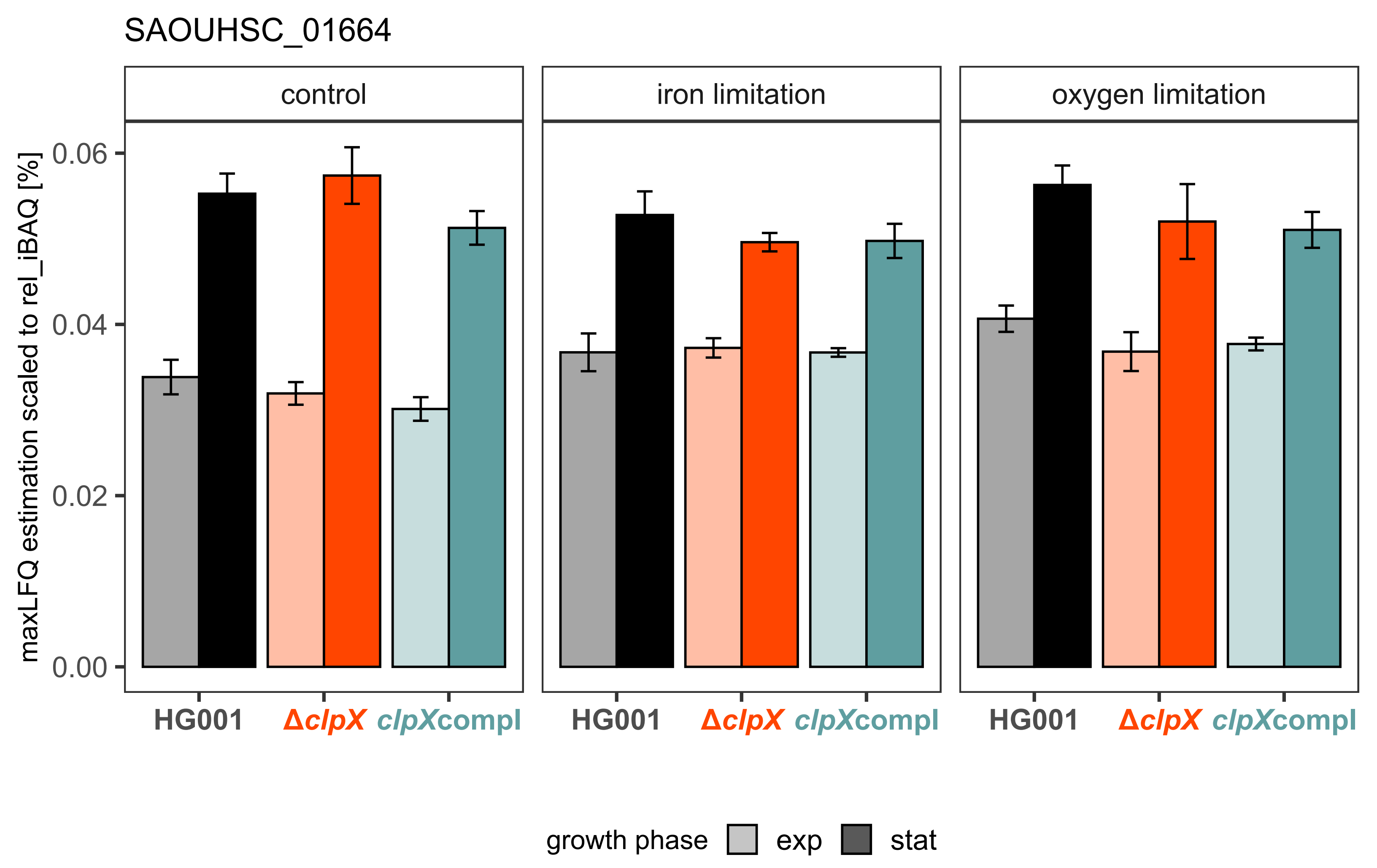
SAOUHSC_01664
control
iron limitation
oxygen limitation
maxLFQ estimation scaled to rel_iBAQ [%]
0.06
0.04
0.02
0.00
HG001
ΔclpX
clpXcompl
growth phase
exp
stat

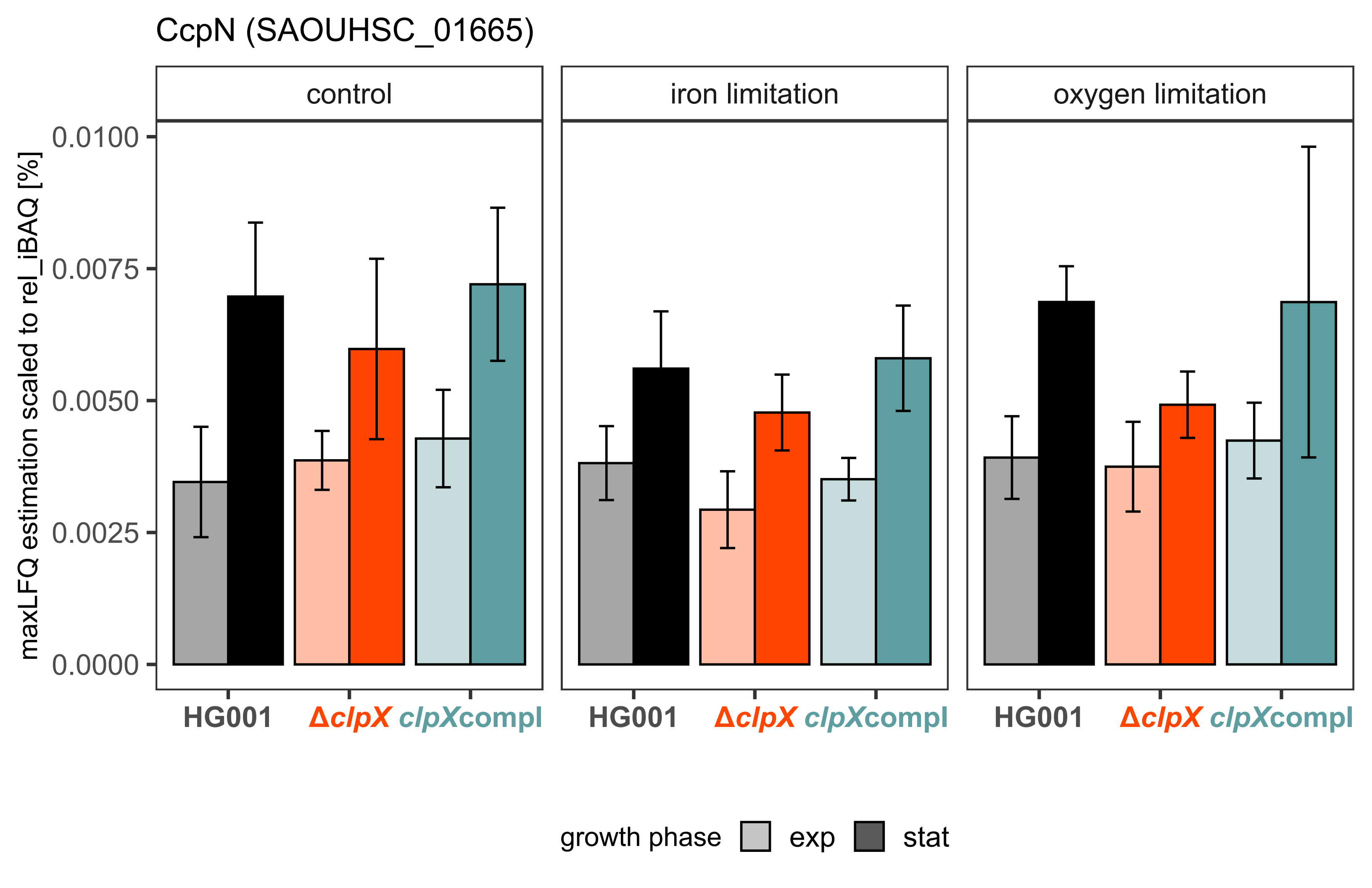

CcpN (SAOUHSC_01665)
control
iron limitation
oxygen limitation
maxLFQ estimation scaled to rel_iBAQ [%]
0.0100
0.0075
0.0050
0.0025
0.0000
HG001
ΔclpX
clpXcompl
growth phase
exp
stat

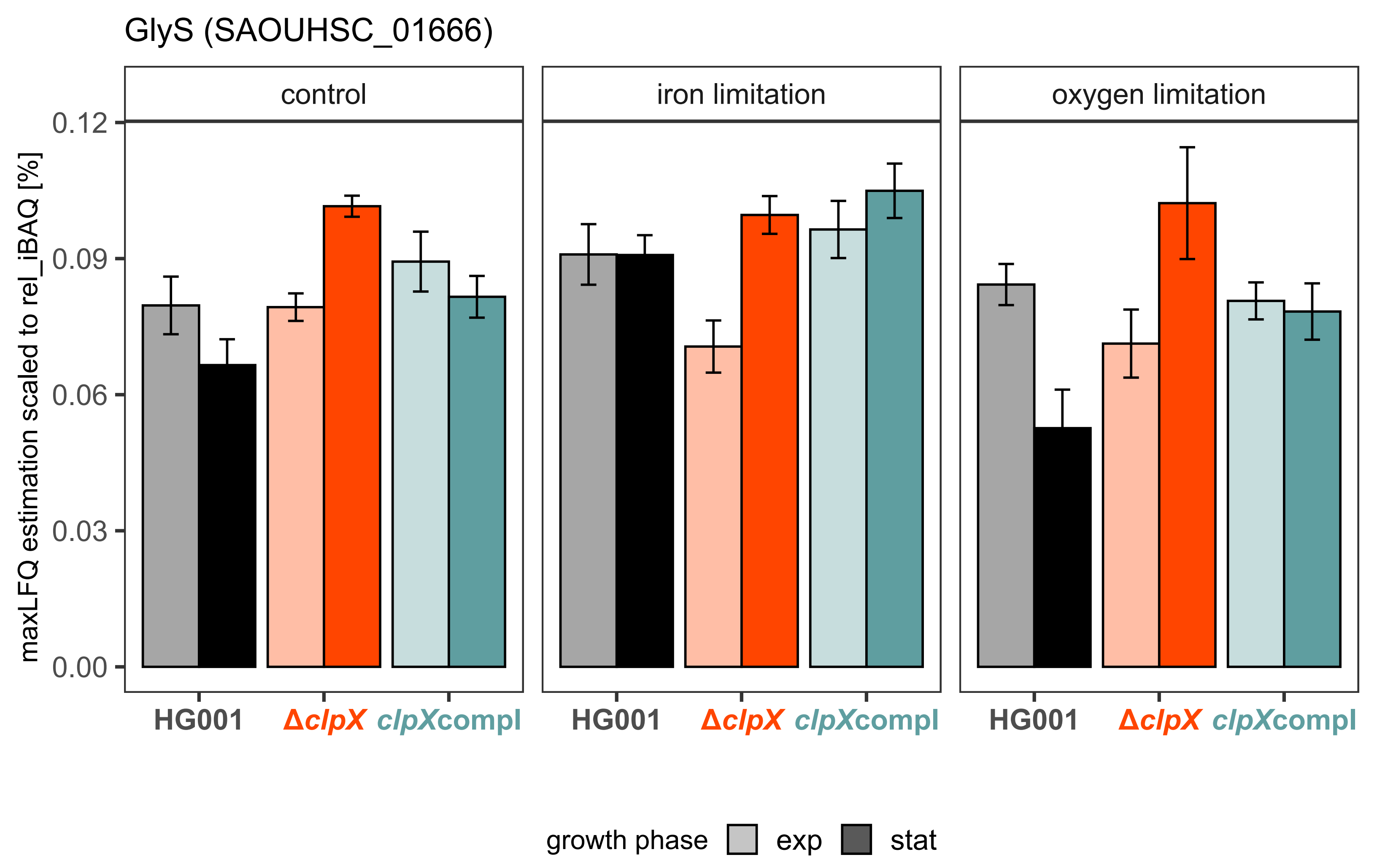
GlyS (SAOUHSC_01666)
control
iron limitation
oxygen limitation
maxLFQ estimation scaled to rel_iBAQ [%]
0.12
0.09
0.06
0.03
0.00
HG001
ΔclpX
clpXcompl
growth phase
exp
stat

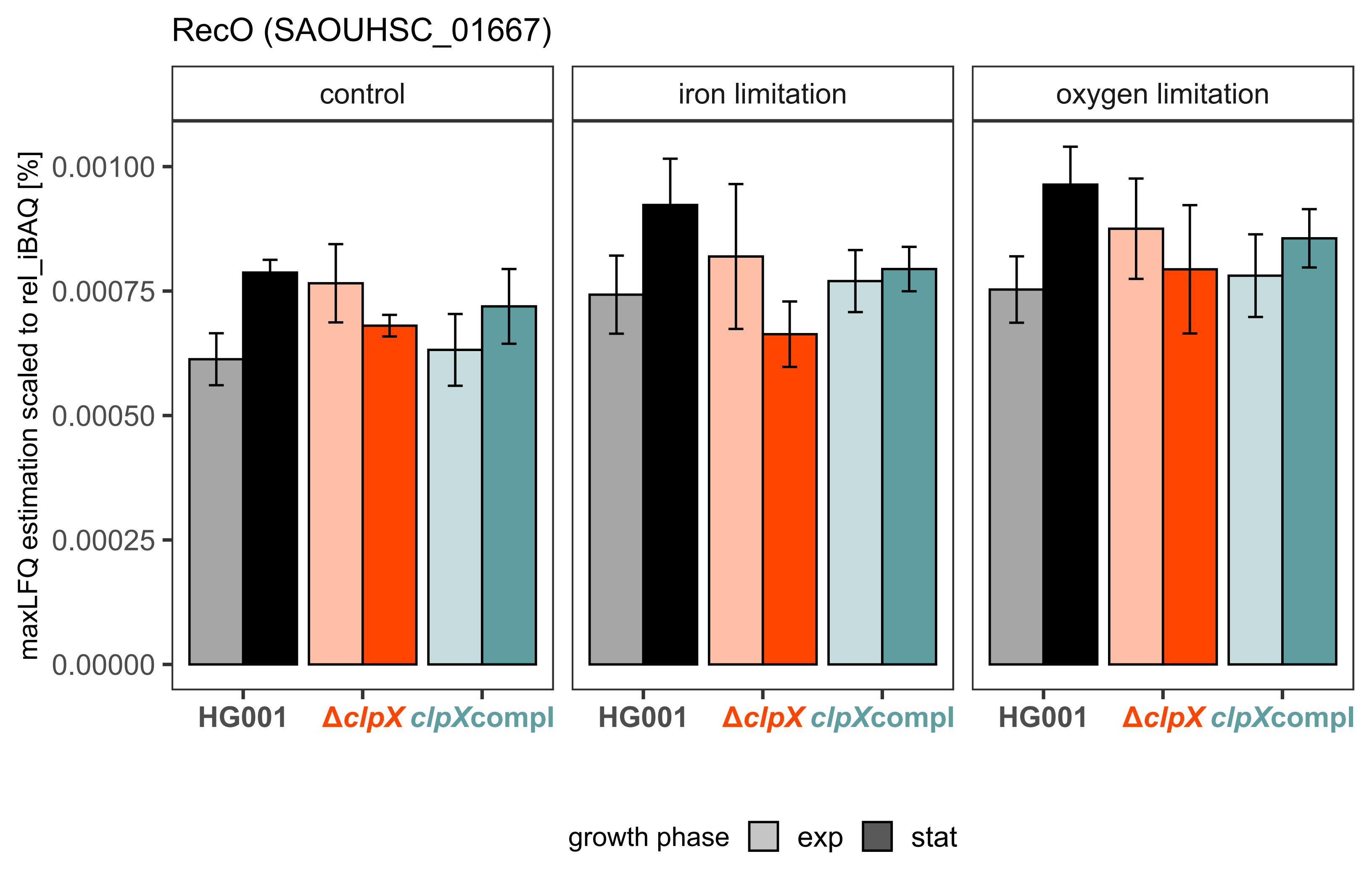

RecO (SAOUHSC_01667)
control
iron limitation
oxygen limitation
maxLFQ estimation scaled to rel_iBAQ [%]
0.00100
0.00075
0.00050
0.00025
0.00000
HG001
ΔclpX
clpXcompl
growth phase
exp
stat

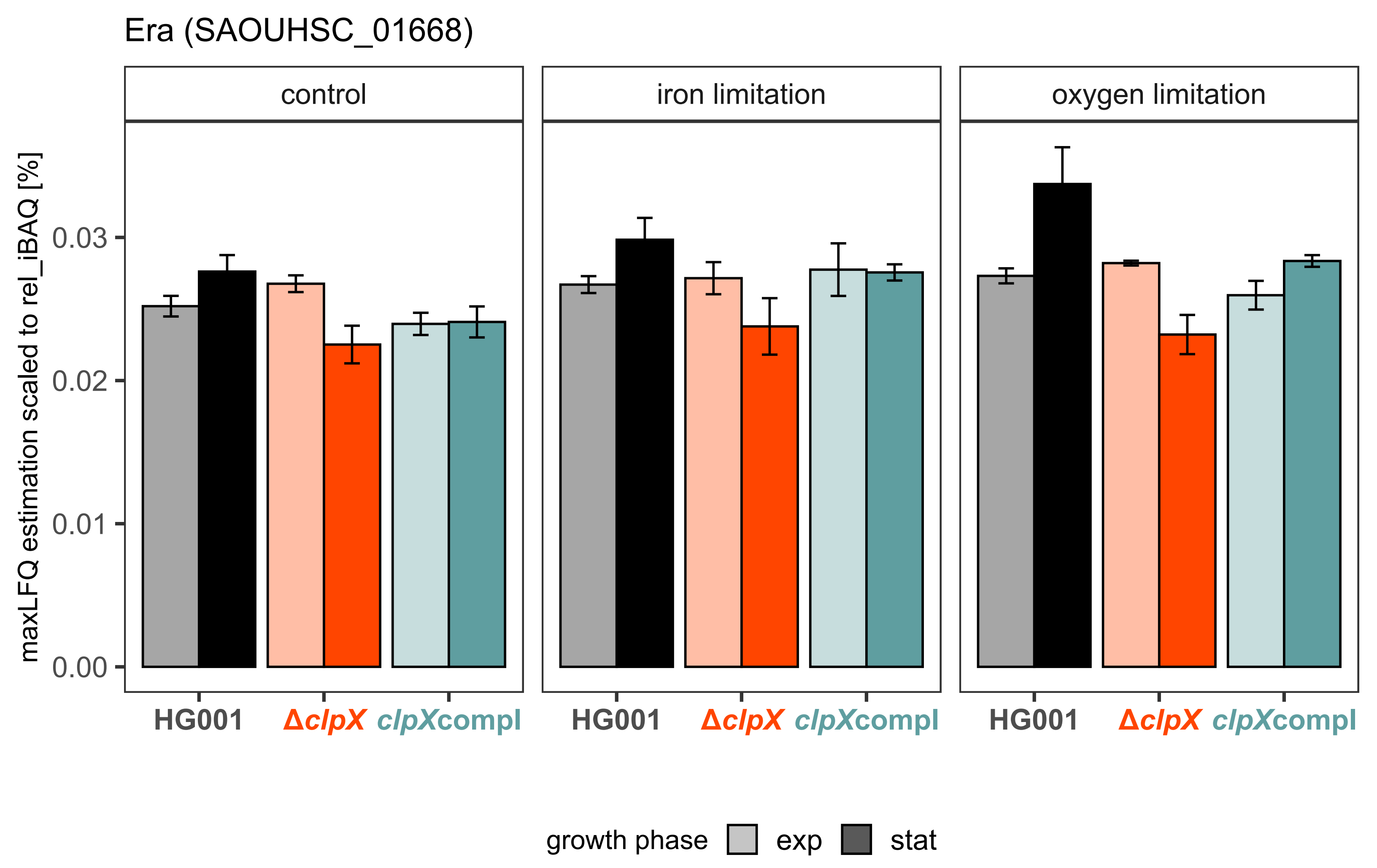

Era (SAOUHSC_01668)
control
iron limitation
oxygen limitation
maxLFQ estimation scaled to rel_iBAQ [%]
0.00
0.01
0.02
0.03
HG001
ΔclpX
clpXcompl
growth phase
exp
stat

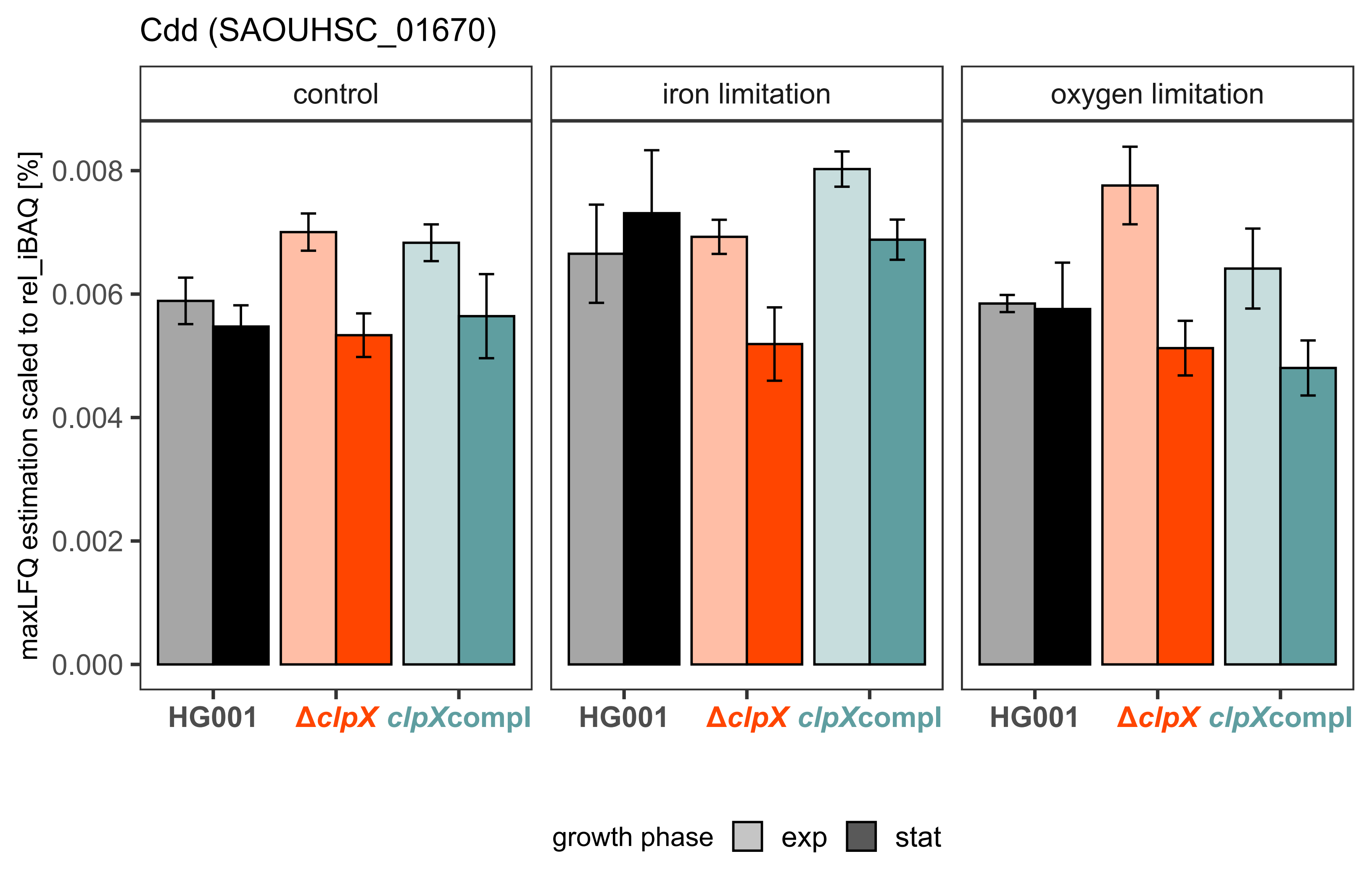

Cdd (SAOUHSC_01670)
control
iron limitation
oxygen limitation
maxLFQ estimation scaled to rel_iBAQ [%]
0.008
0.006
0.004
0.002
0.000
HG001
ΔclpX
clpXcompl
growth phase
exp
stat

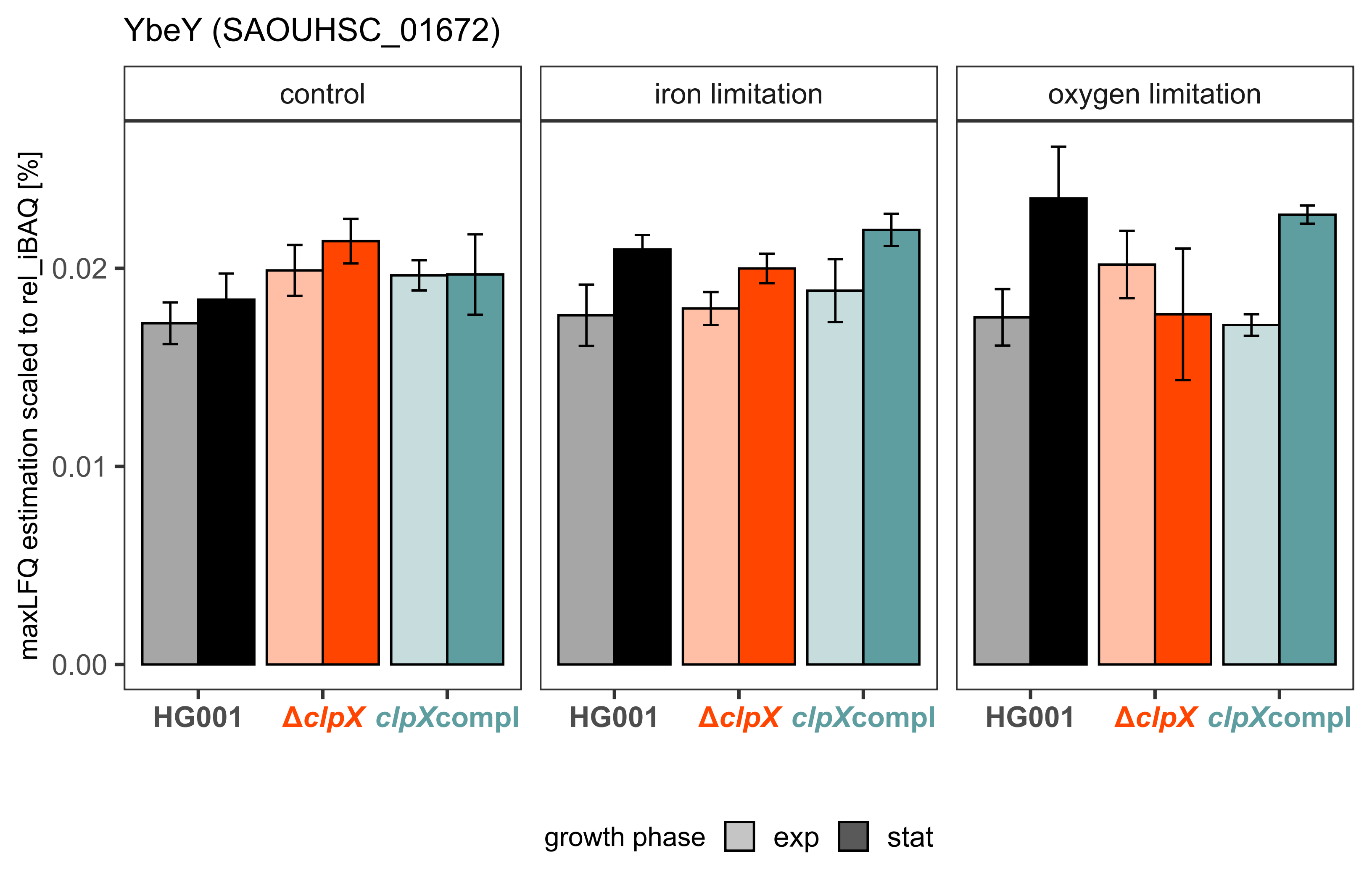

YbeY (SAOUHSC_01672)
control
iron limitation
oxygen limitation
maxLFQ estimation scaled to rel_iBAQ [%]
0.00
0.01
0.02
HG001
ΔclpX
clpXcompl
growth phase
exp
stat

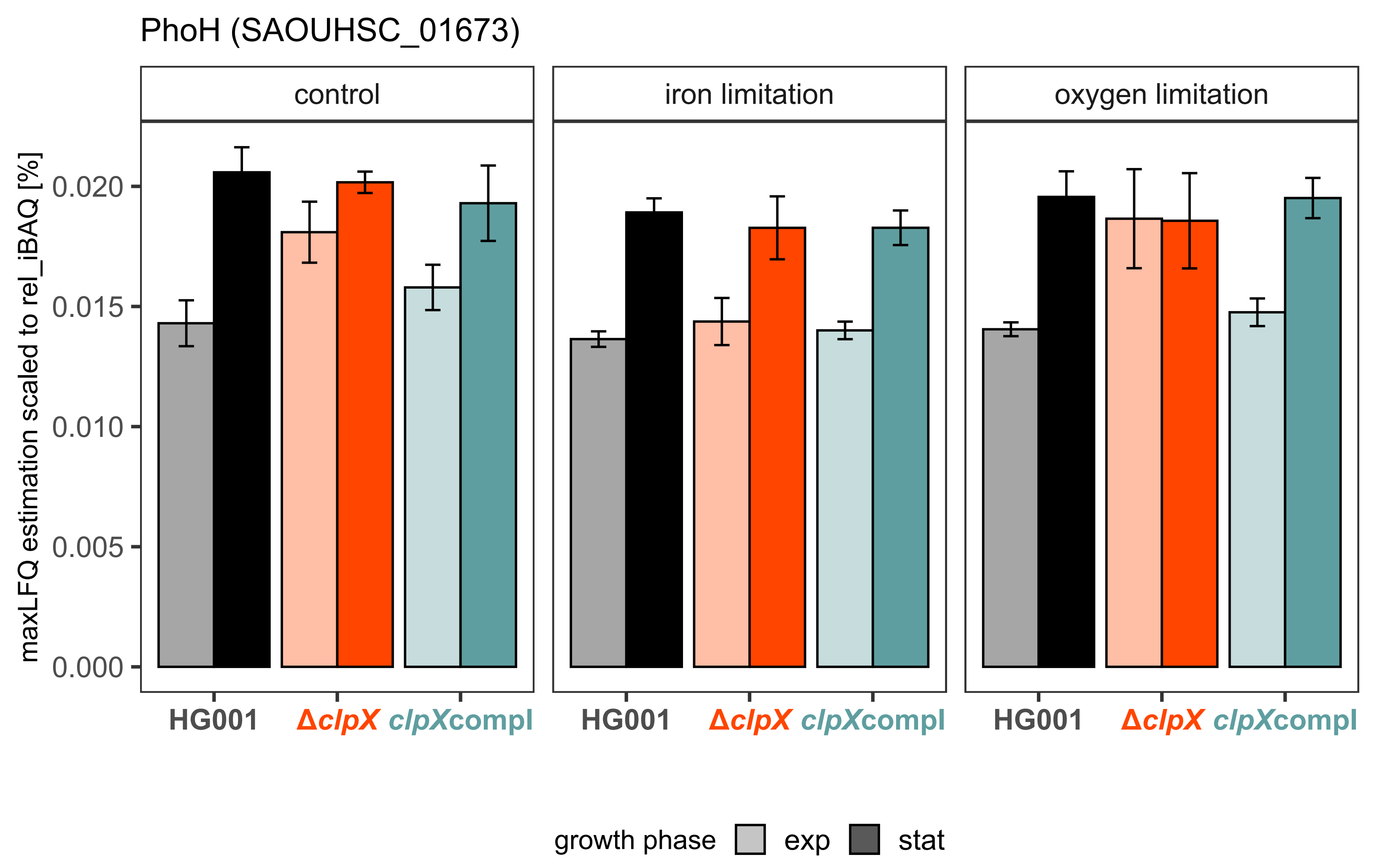

PhoH (SAOUHSC_01673)
control
iron limitation
oxygen limitation
maxLFQ estimation scaled to rel_iBAQ [%]
0.000
0.005
0.010
0.015
0.020
HG001
ΔclpX
clpXcompl
growth phase
exp
stat

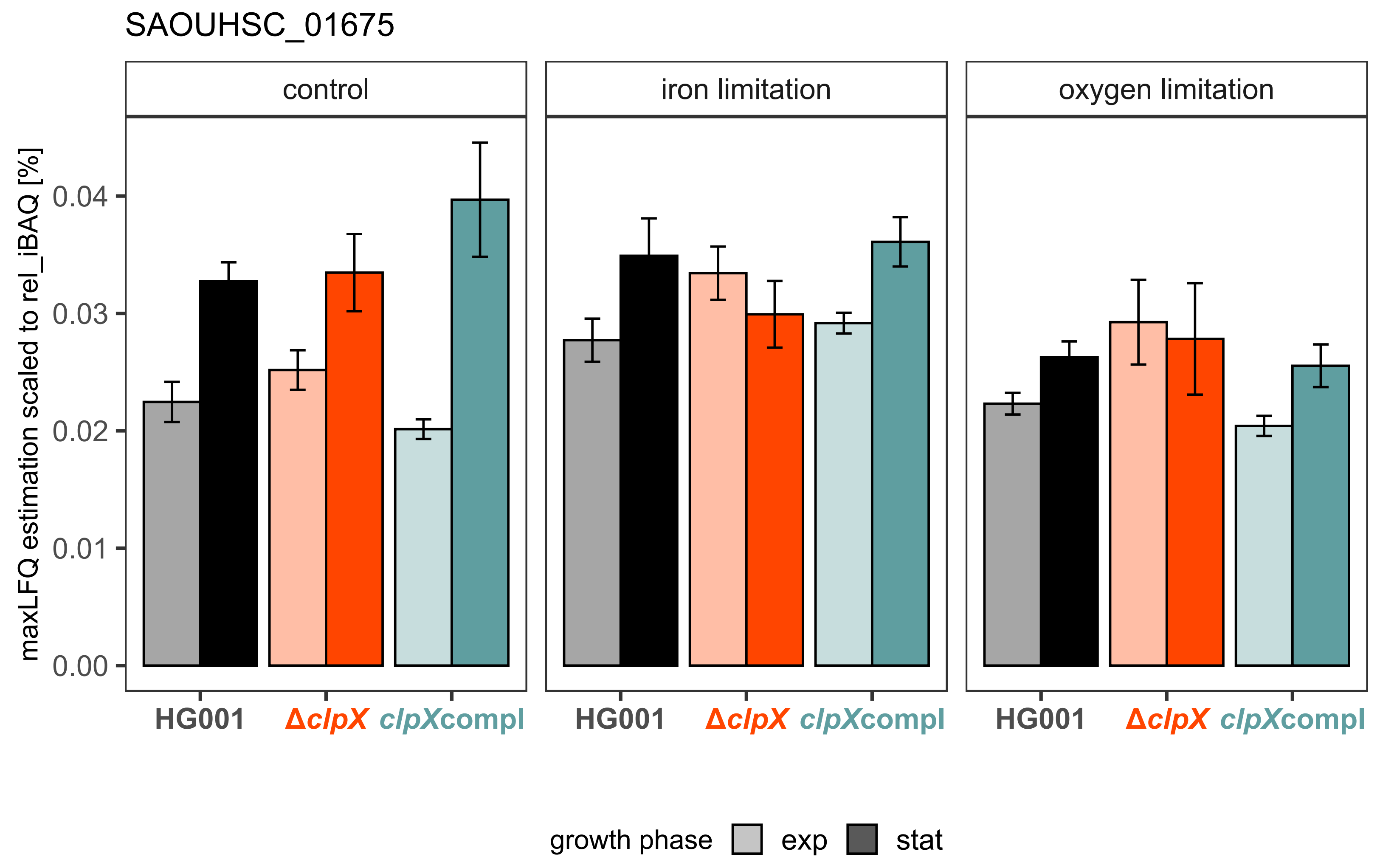
SAOUHSC_01675
control
iron limitation
oxygen limitation
maxLFQ estimation scaled to rel_iBAQ [%]
0.00
0.01
0.02
0.03
0.04
HG001
ΔclpX
clpXcompl
growth phase
exp
stat

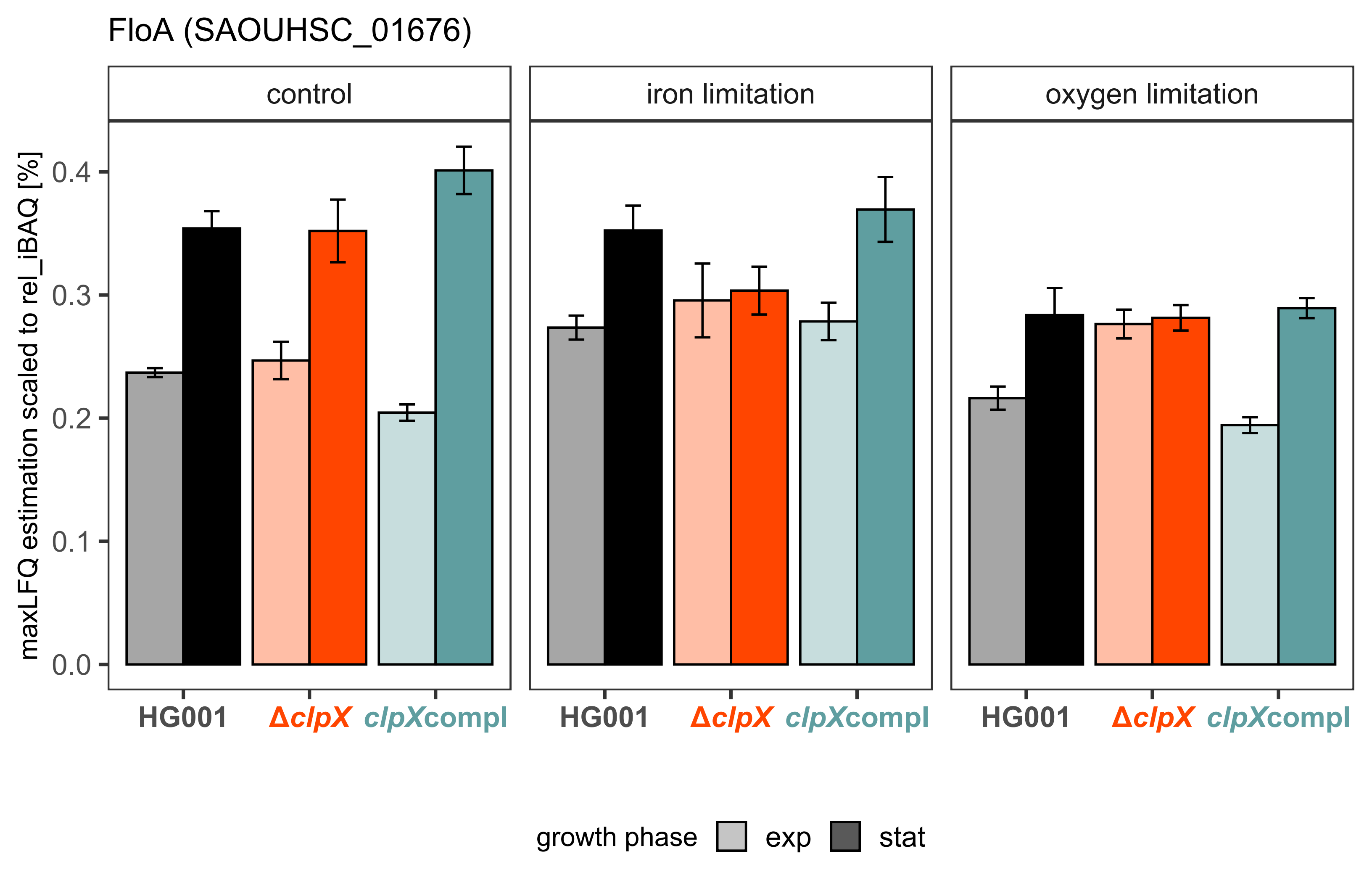

FloA (SAOUHSC_01676)
control
iron limitation
oxygen limitation
maxLFQ estimation scaled to rel_iBAQ [%]
0.0
0.1
0.2
0.3
0.4
HG001
ΔclpX
clpXcompl
growth phase
exp
stat

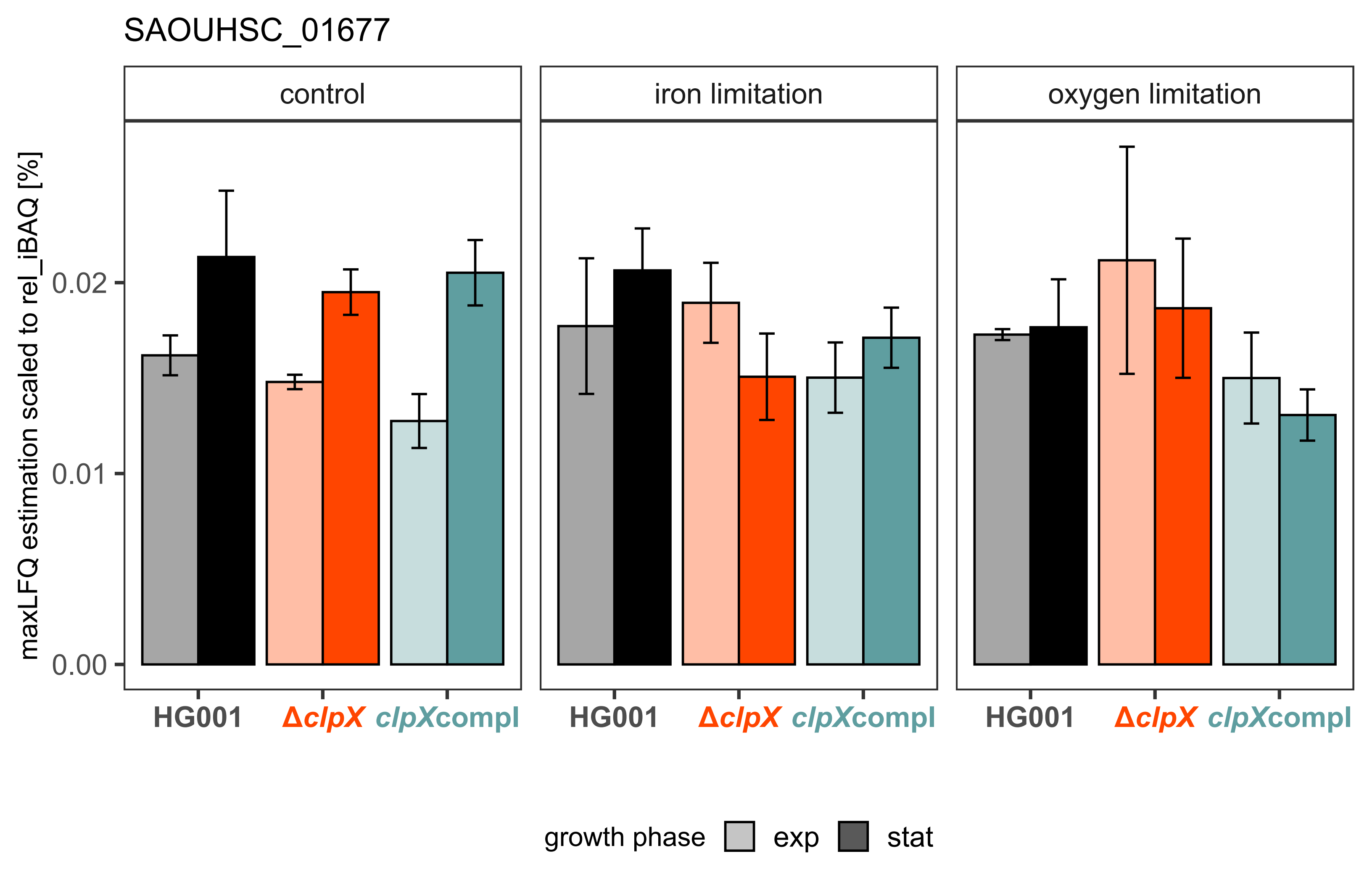
SAOUHSC_01677
control
iron limitation
oxygen limitation
maxLFQ estimation scaled to rel_iBAQ [%]
0.00
0.01
0.02
HG001
ΔclpX
clpXcompl
growth phase
exp
stat

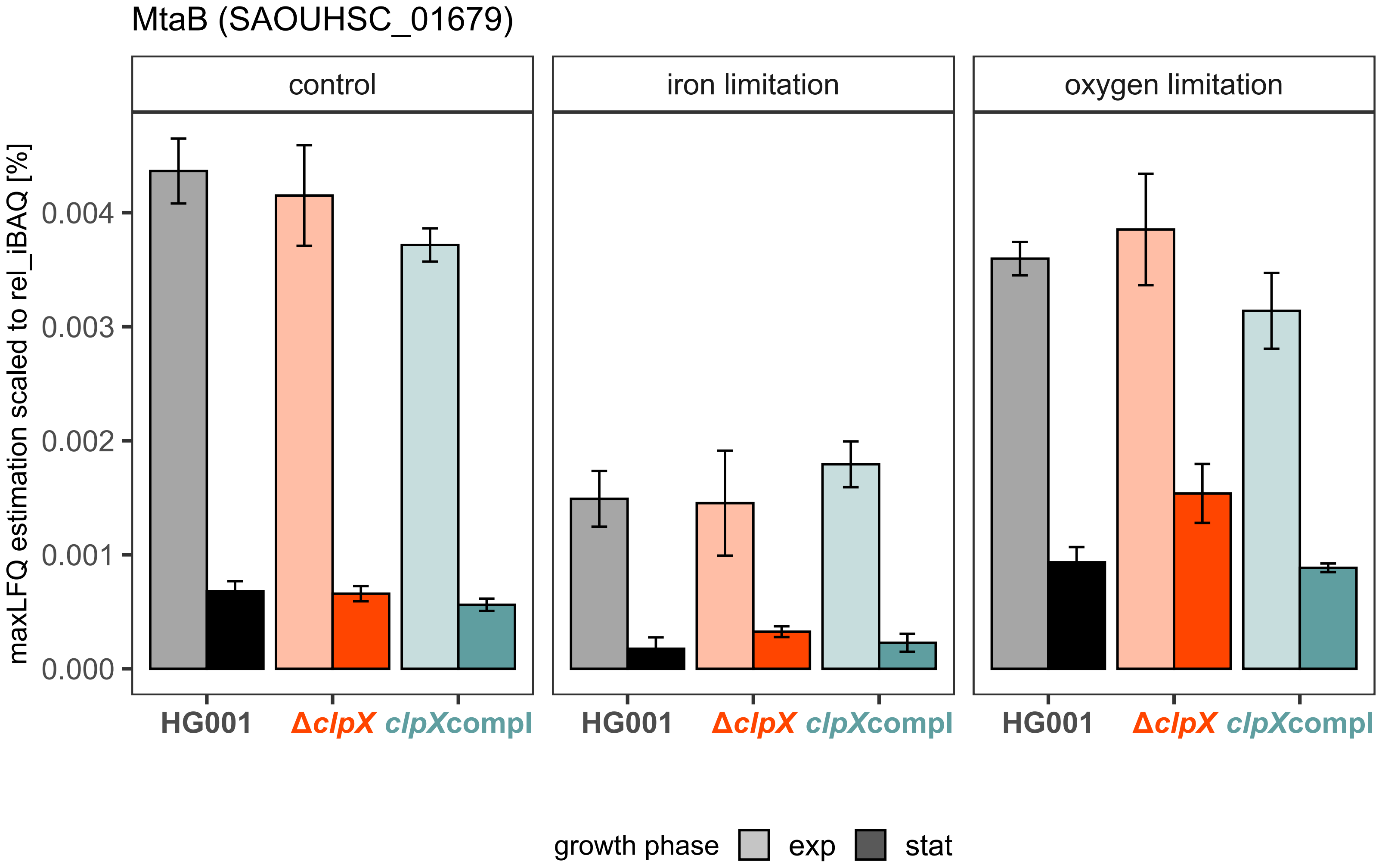
MtaB (SAOUHSC_01679)
control
iron limitation
oxygen limitation
maxLFQ estimation scaled to rel_iBAQ [%]
0.004
0.003
0.002
0.001
0.000
HG001
ΔclpX
clpXcompl
growth phase
exp
stat

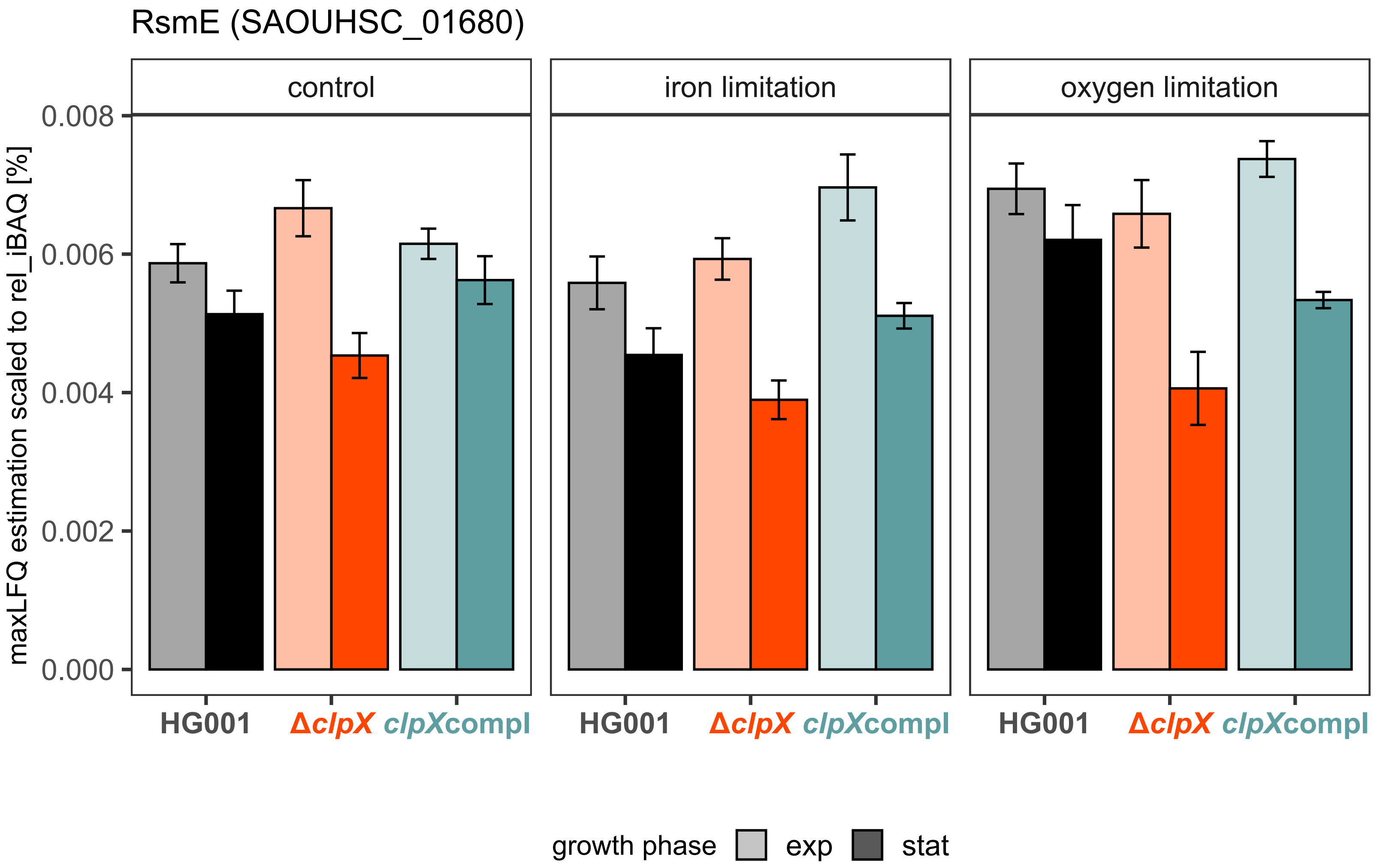

RsmE (SAOUHSC_01680)
control
iron limitation
oxygen limitation
maxLFQ estimation scaled to rel_iBAQ [%]
0.008
0.006
0.004
0.002
0.000
HG001
ΔclpX
clpXcompl
growth phase
exp
stat

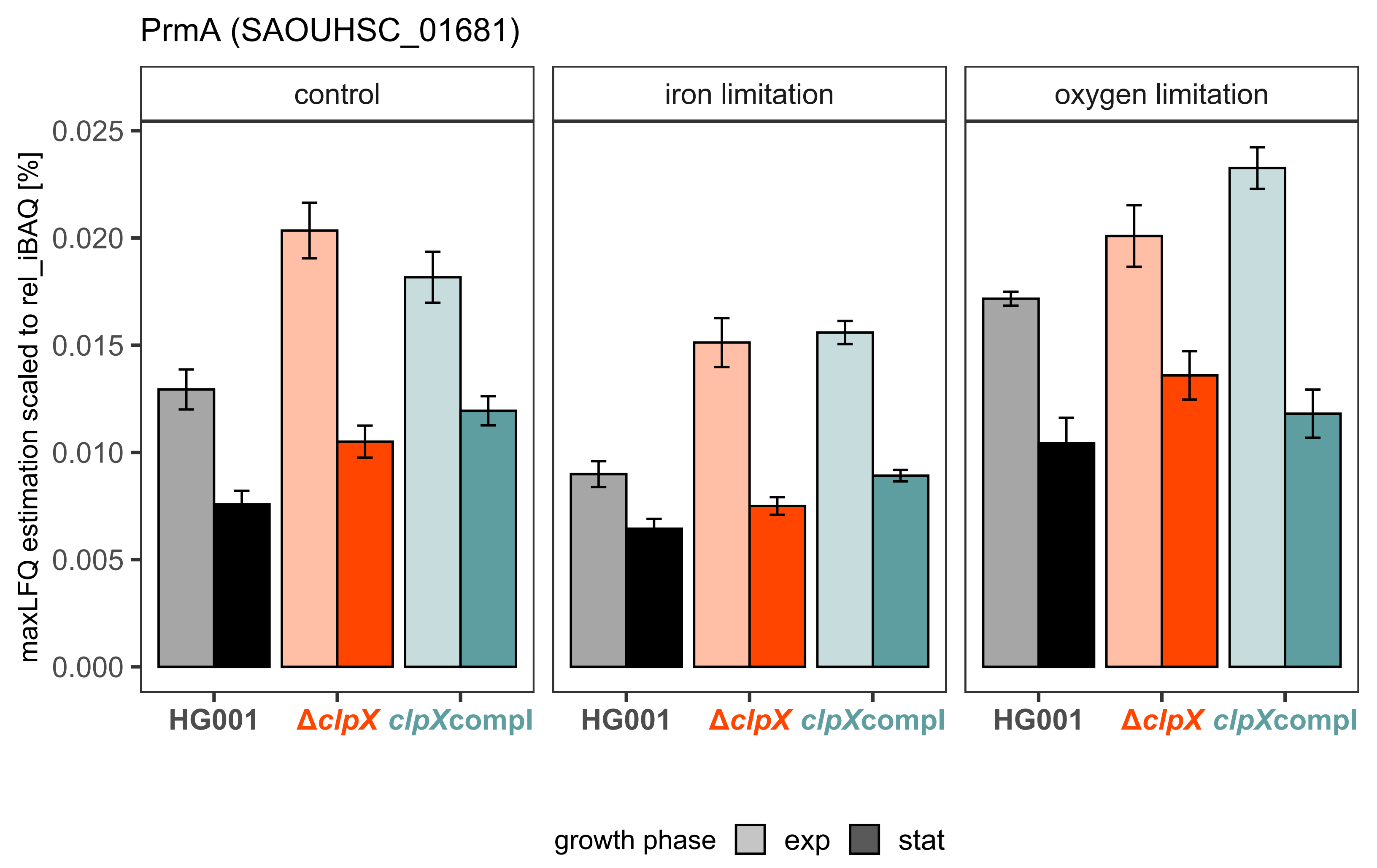
PrmA (SAOUHSC_01681)
control
iron limitation
oxygen limitation
maxLFQ estimation scaled to rel_iBAQ [%]
0.025
0.020
0.015
0.010
0.005
0.000
HG001
ΔclpX
clpXcompl
growth phase
exp
stat

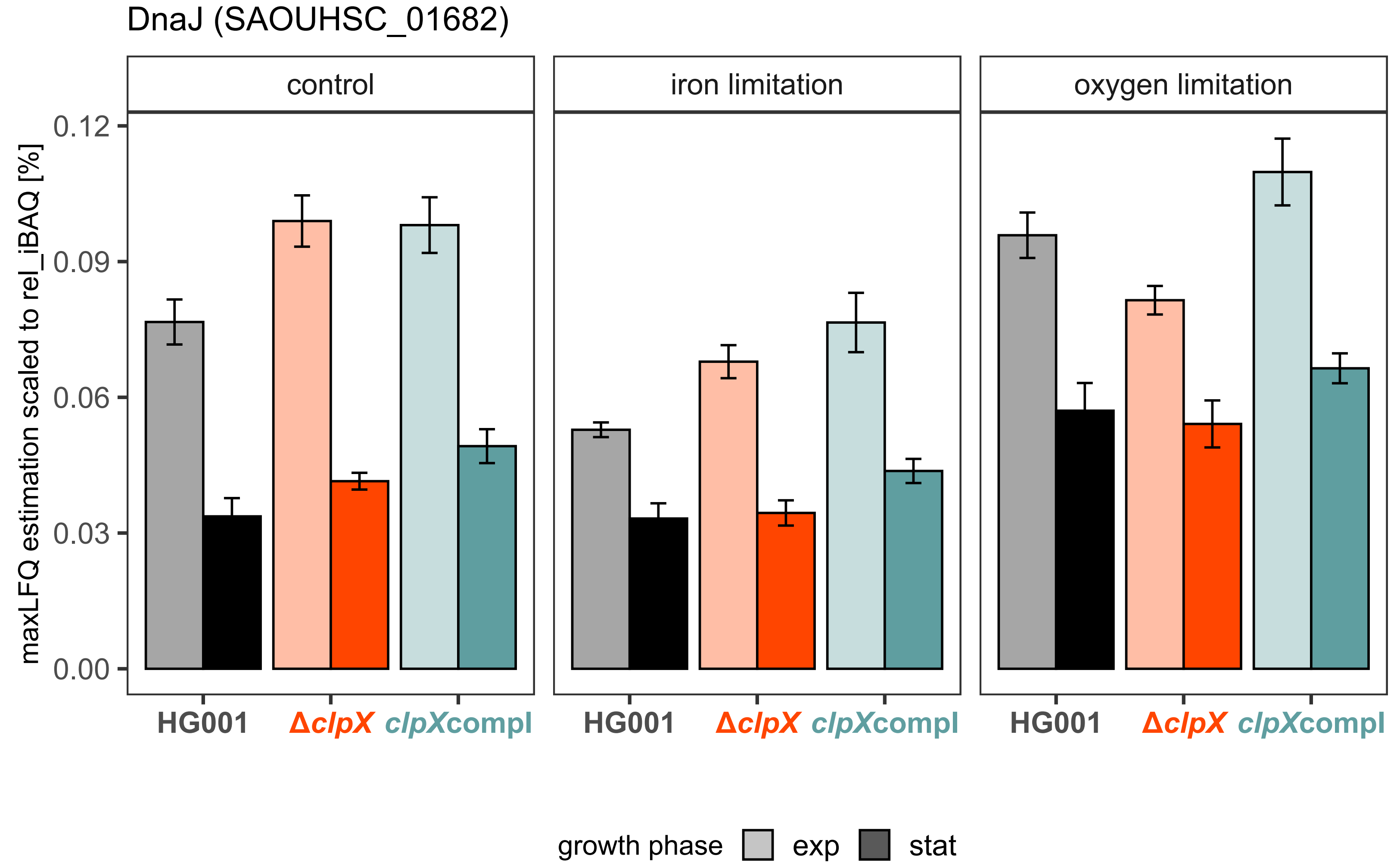

DnaJ (SAOUHSC_01682)
control
iron limitation
oxygen limitation
maxLFQ estimation scaled to rel_iBAQ [%]
0.12
0.09
0.06
0.03
0.00
HG001
ΔclpX
clpXcompl
growth phase
exp
stat

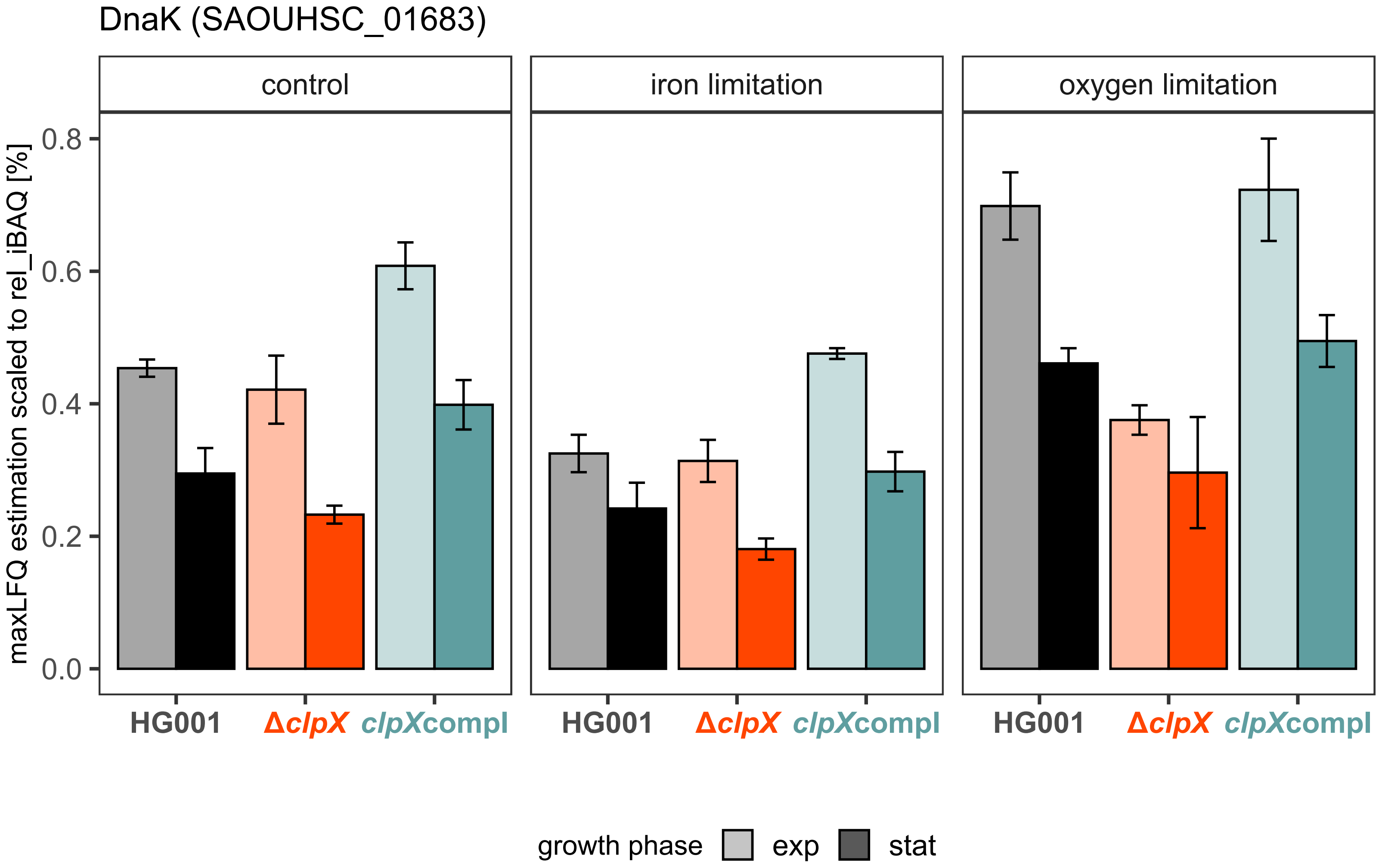
DnaK (SAOUHSC_01683)
control
iron limitation
oxygen limitation
maxLFQ estimation scaled to rel_iBAQ [%]
0.0
0.2
0.4
0.6
0.8
HG001
ΔclpX
clpXcompl
growth phase
exp
stat

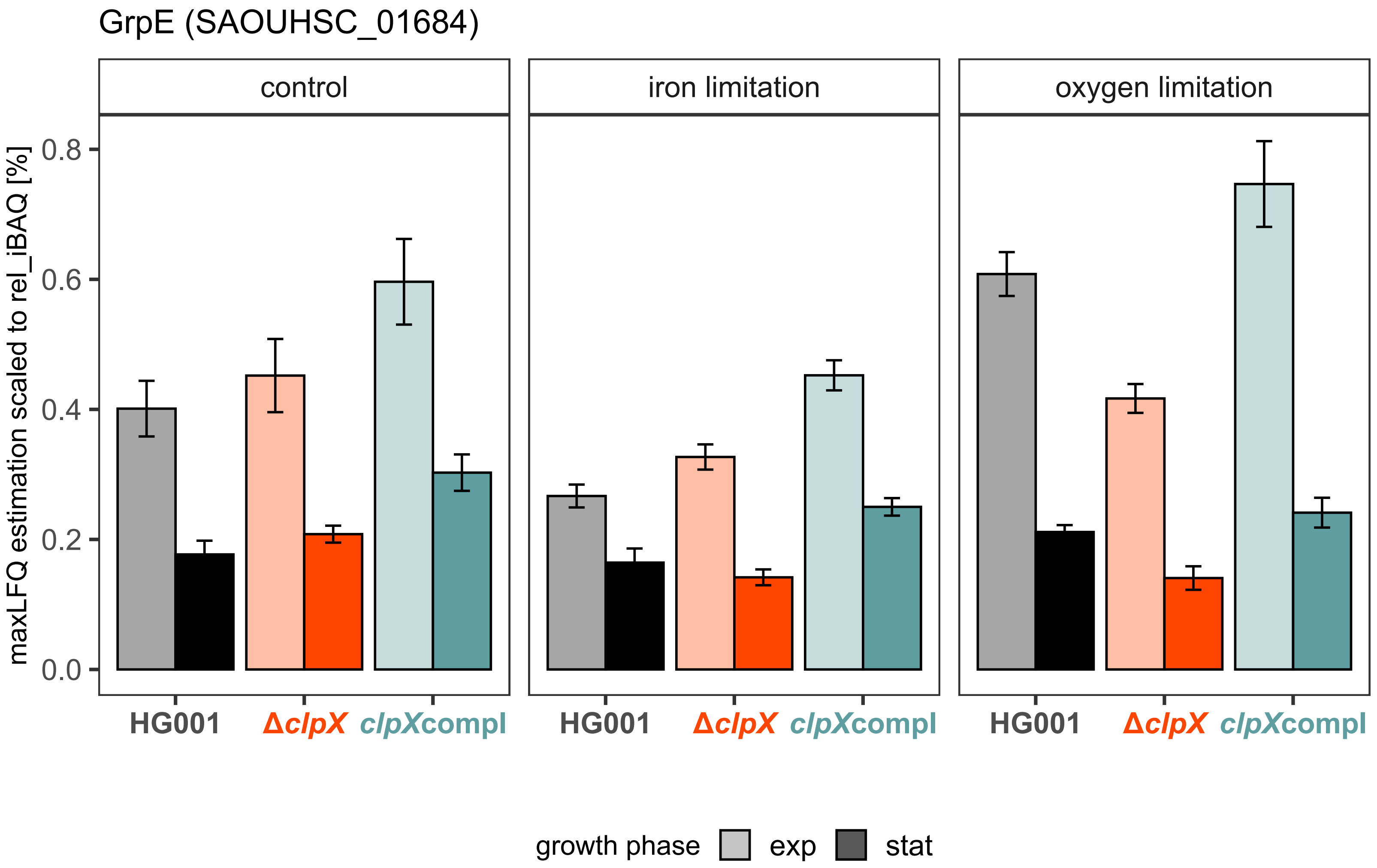
GrpE (SAOUHSC_01684)
control
iron limitation
oxygen limitation
maxLFQ estimation scaled to rel_iBAQ [%]
0.0
0.2
0.4
0.6
0.8
HG001
ΔclpX
clpXcompl
growth phase
exp
stat

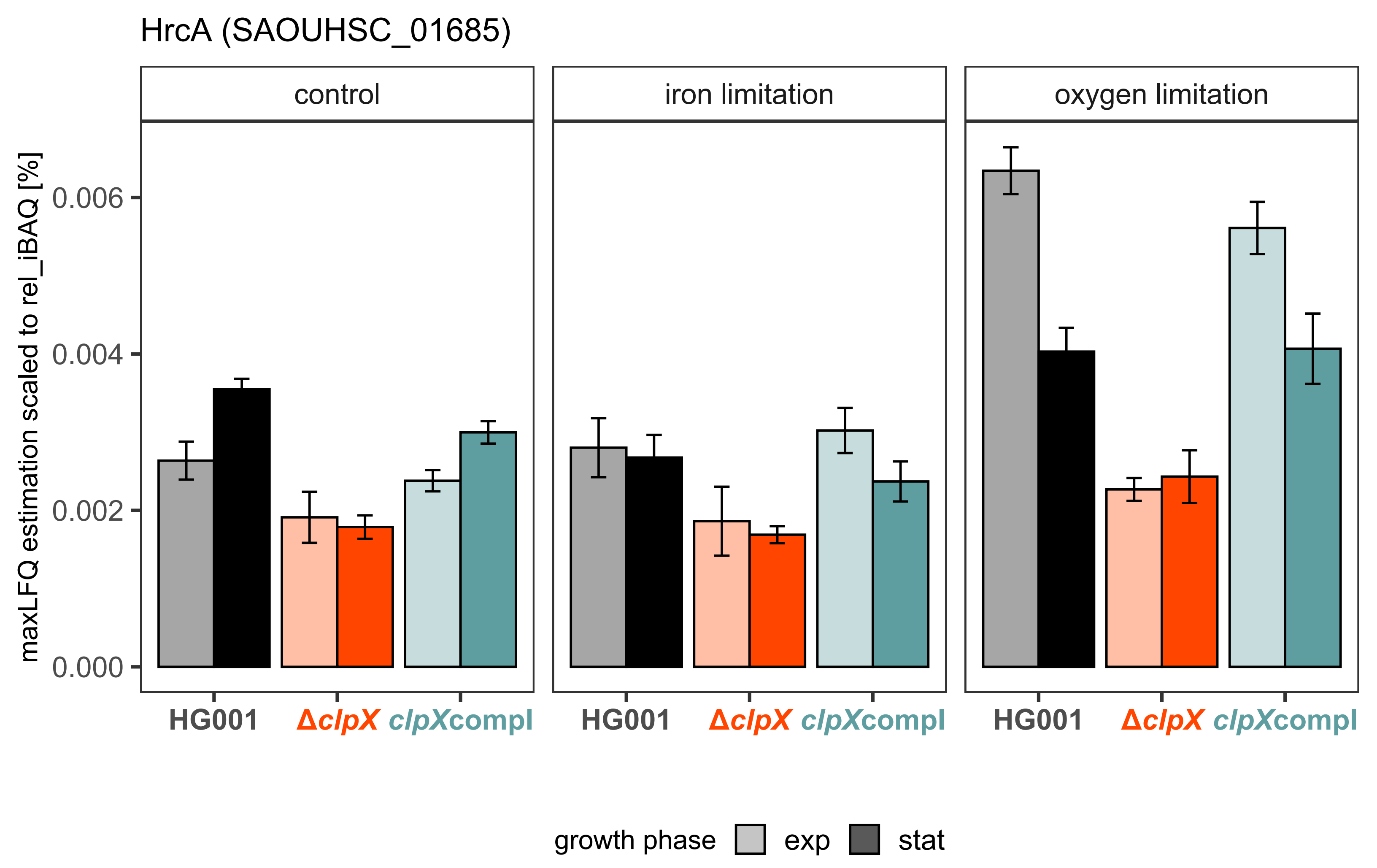

HrcA (SAOUHSC_01685)
control
iron limitation
oxygen limitation
maxLFQ estimation scaled to rel_iBAQ [%]
0.006
0.004
0.002
0.000
HG001
ΔclpX
clpXcompl
growth phase
exp
stat

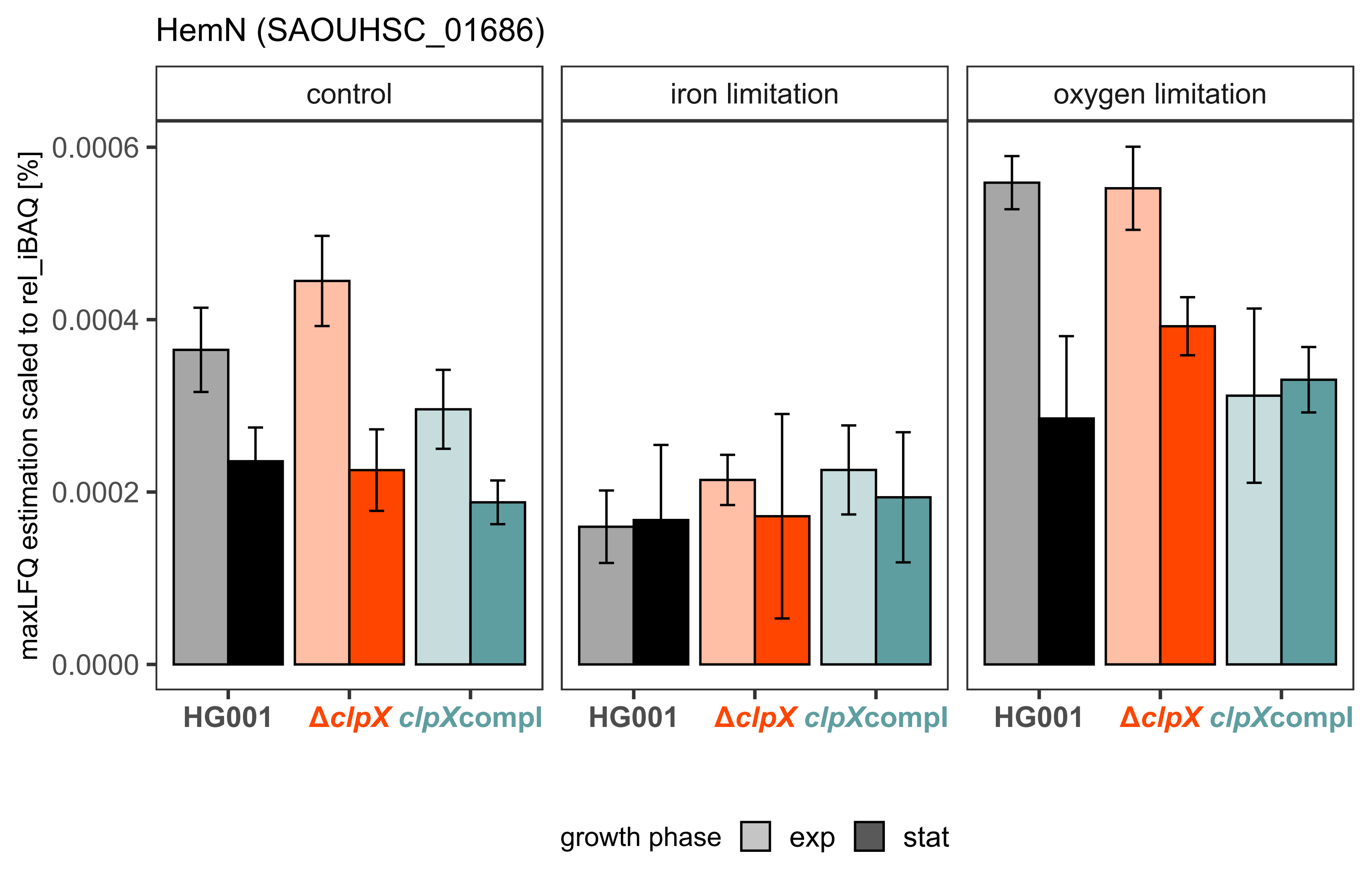

HemN (SAOUHSC_01686)
control
iron limitation
oxygen limitation
maxLFQ estimation scaled to rel_iBAQ [%]
0.0006
0.0004
0.0002
0.0000
HG001
ΔclpX
clpXcompl
growth phase
exp
stat

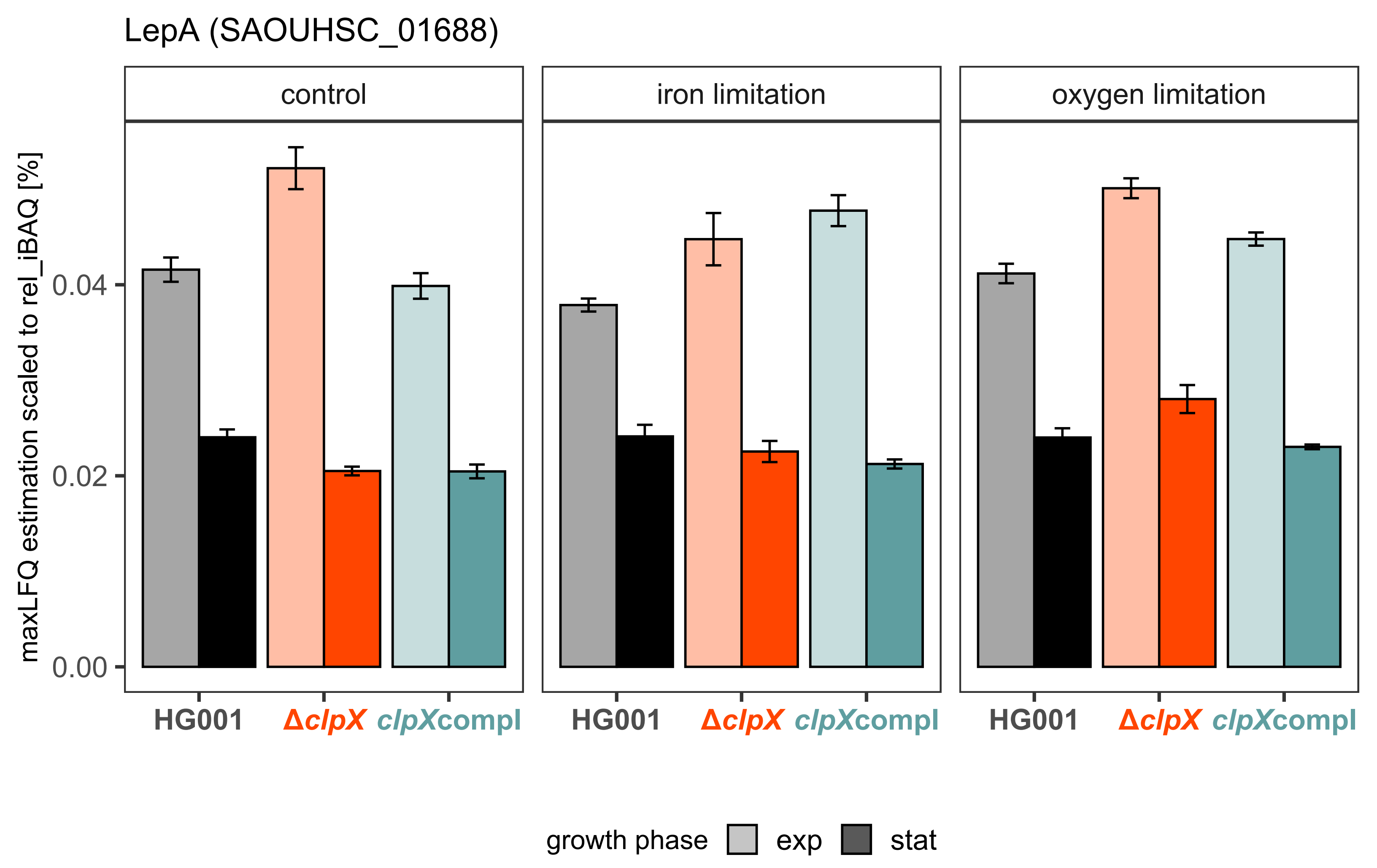
LepA (SAOUHSC_01688)
control
iron limitation
oxygen limitation
maxLFQ estimation scaled to rel_iBAQ [%]
0.04
0.02
0.00
HG001
ΔclpX
clpXcompl
growth phase
exp
stat

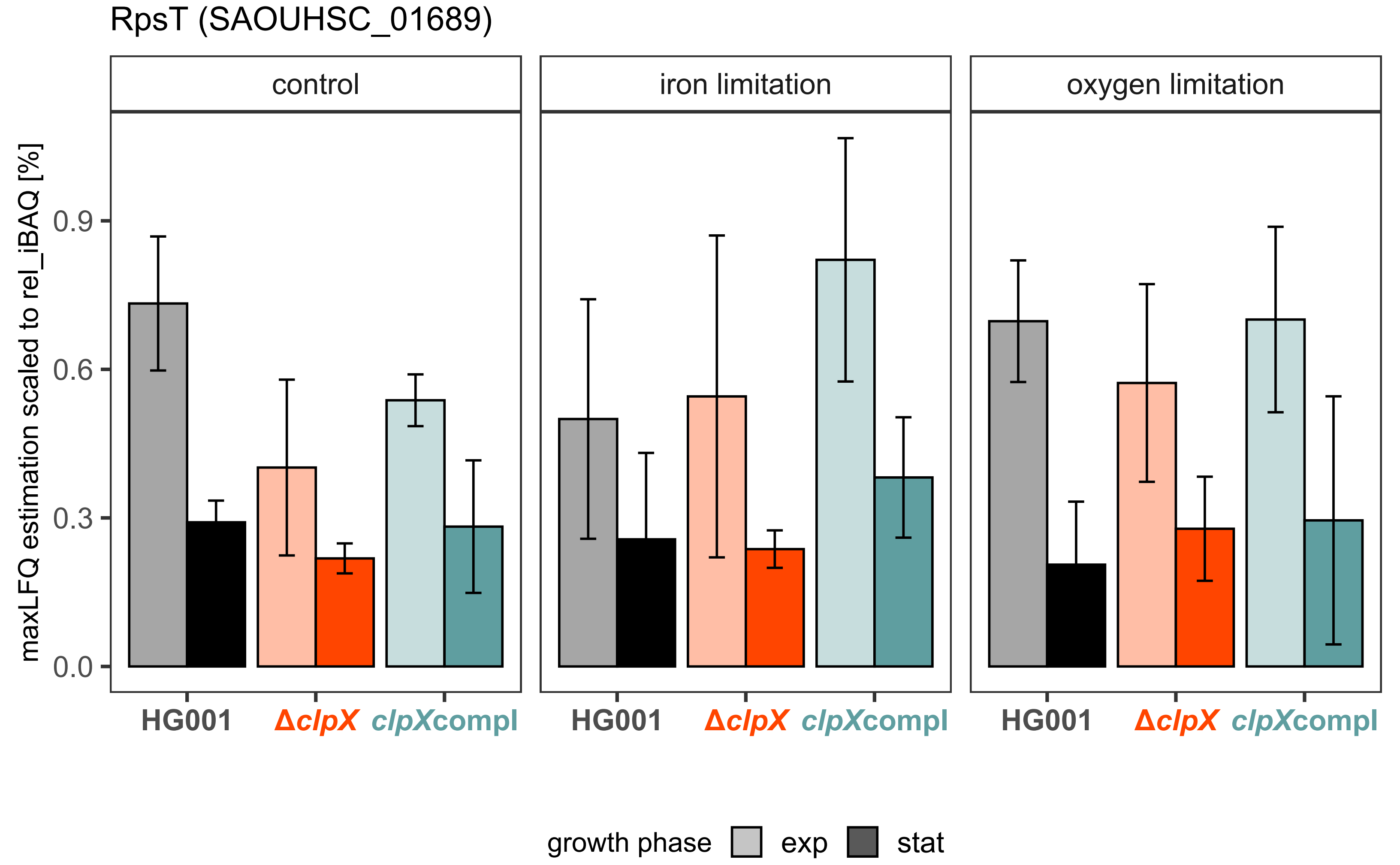

RpsT (SAOUHSC_01689)
control
iron limitation
oxygen limitation
maxLFQ estimation scaled to rel_iBAQ [%]
0.0
0.3
0.6
0.9
HG001
ΔclpX
clpXcompl
growth phase
exp
stat

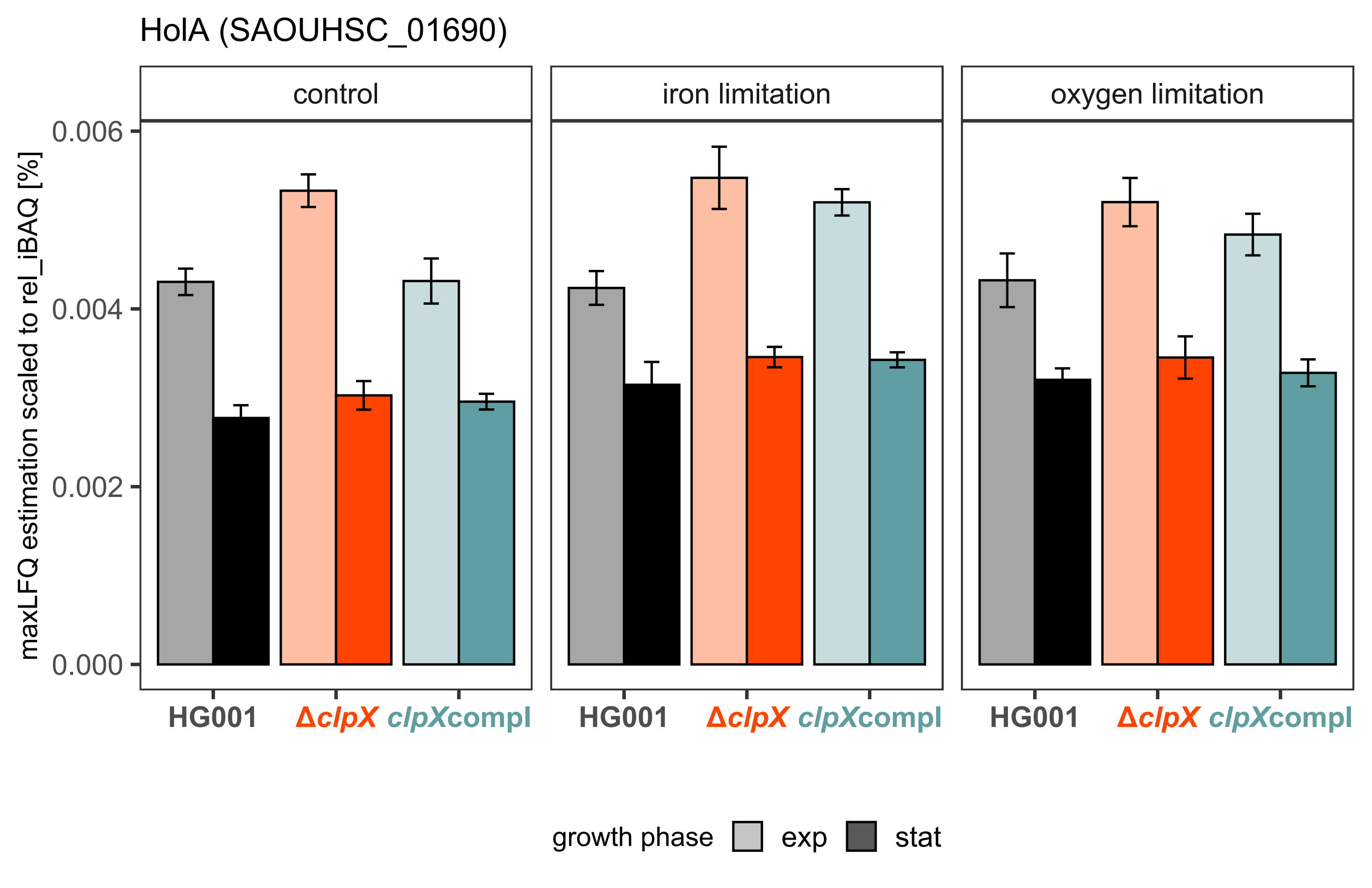

HolA (SAOUHSC_01690)
control
iron limitation
oxygen limitation
0.006
0.004
0.002
0.000
maxLFQ estimation scaled to rel_iBAQ [%]
HG001
ΔclpX
clpXcompl
growth phase
exp
stat

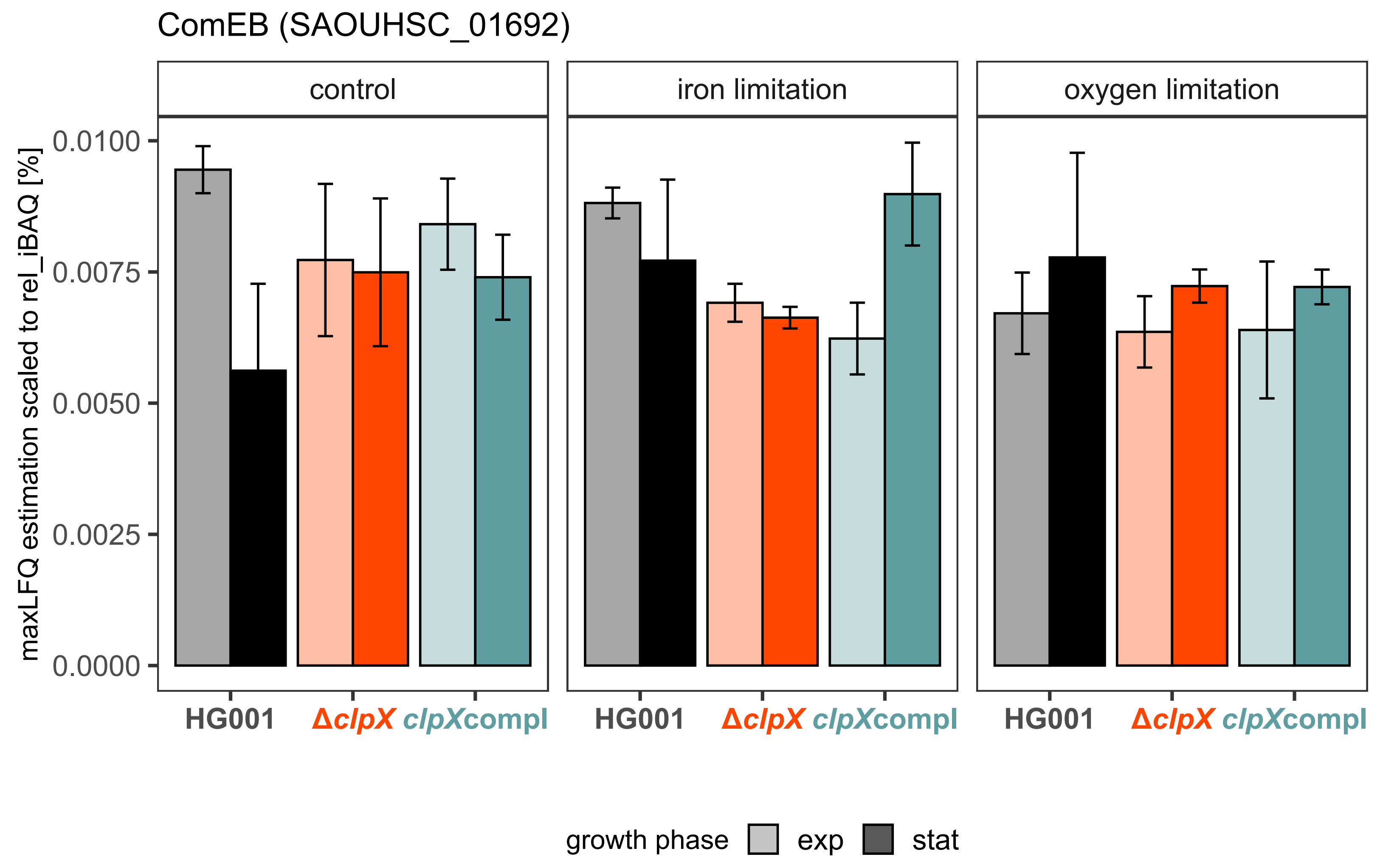
ComEB (SAOUHSC_01692)
control
iron limitation
oxygen limitation
maxLFQ estimation scaled to rel_iBAQ [%]
0.0100
0.0075
0.0050
0.0025
0.0000
HG001
ΔclpX
clpXcompl
growth phase
exp
stat

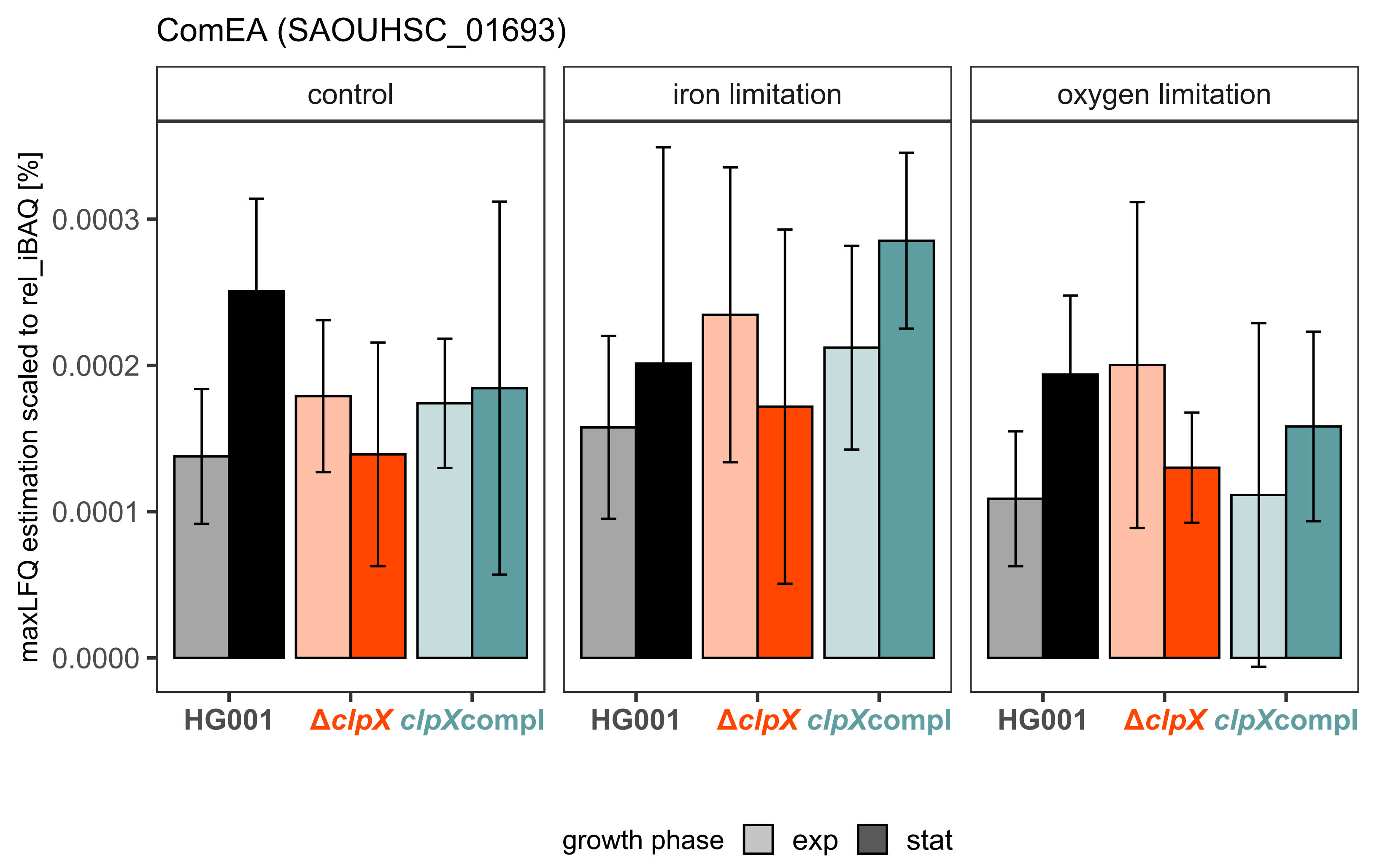

ComEA (SAOUHSC_01693)
control
iron limitation
oxygen limitation
maxLFQ estimation scaled to rel_iBAQ [%]
0.0003
0.0002
0.0001
0.0000
HG001
ΔclpX
clpXcompl
growth phase
exp
stat

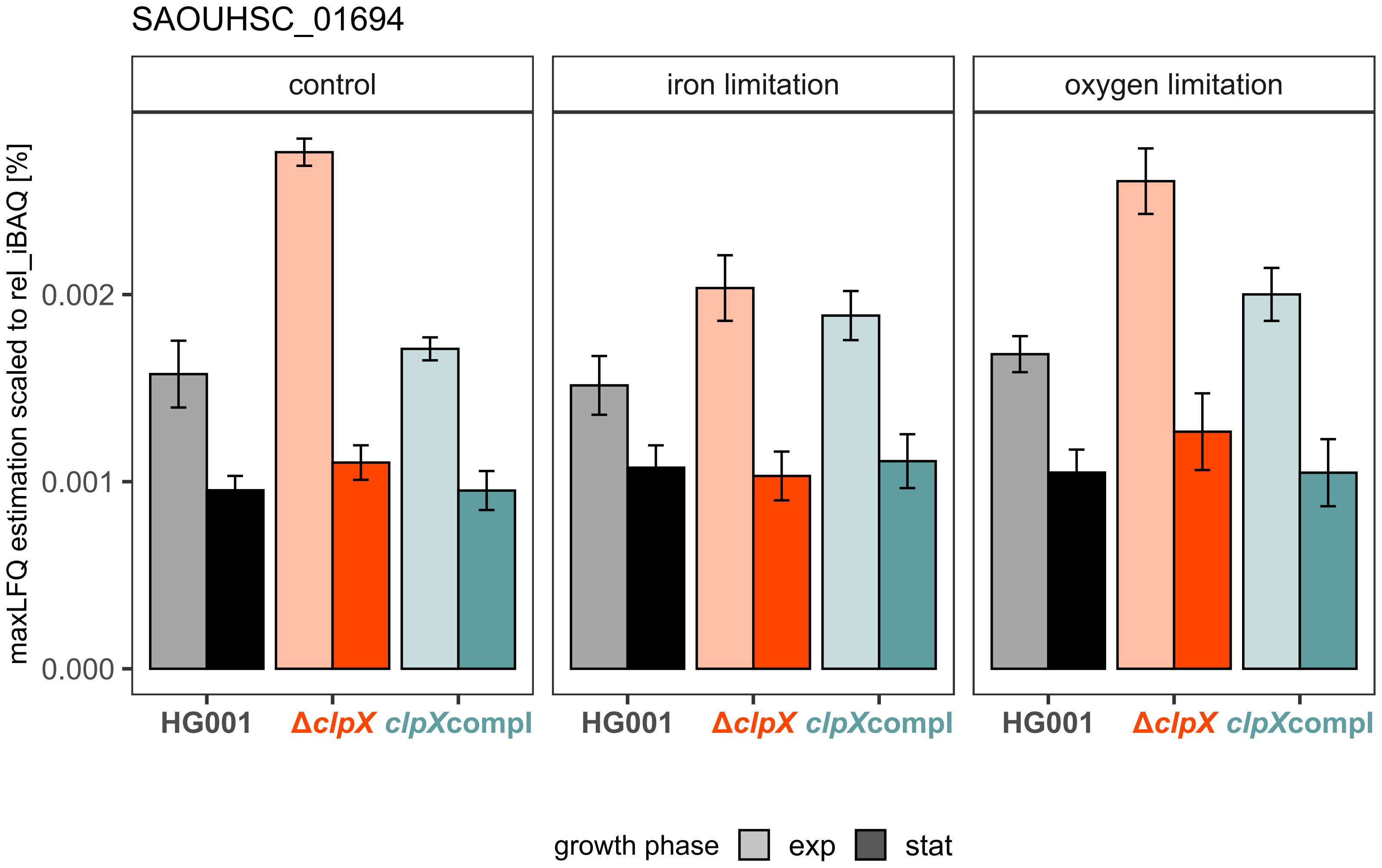

SAOUHSC_01694
control
iron limitation
oxygen limitation
maxLFQ estimation scaled to rel_iBAQ [%]
0.002
0.001
0.000
HG001
ΔclpX
clpXcompl
HG001
ΔclpX
clpXcompl
HG001
ΔclpX
clpXcompl
growth phase
exp
stat

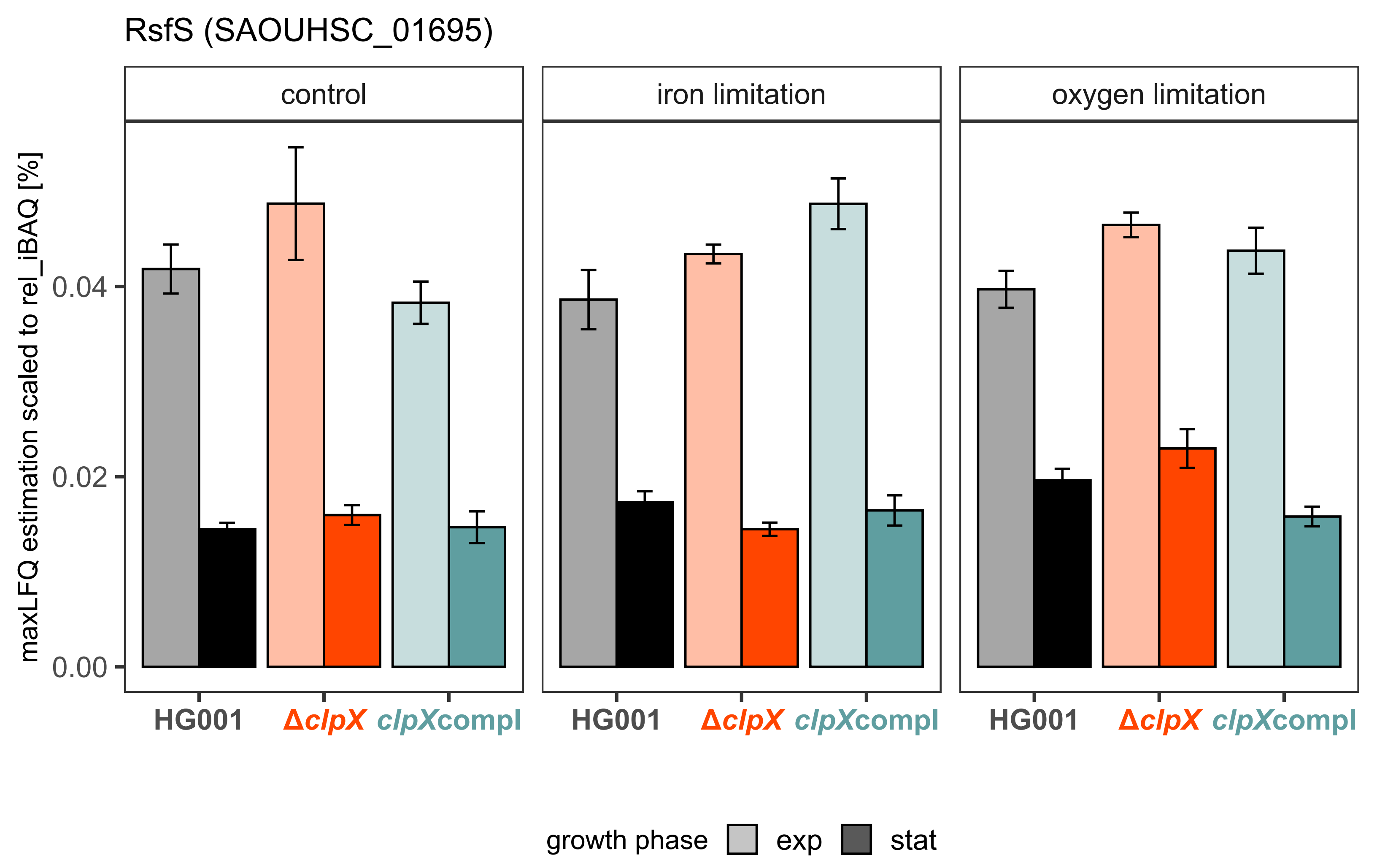
RsfS (SAOUHSC_01695)
control
iron limitation
oxygen limitation
maxLFQ estimation scaled to rel_iBAQ [%]
0.04
0.02
0.00
HG001
ΔclpX
clpXcompl
HG001
ΔclpX
clpXcompl
HG001
ΔclpX
clpXcompl
growth phase
exp
stat

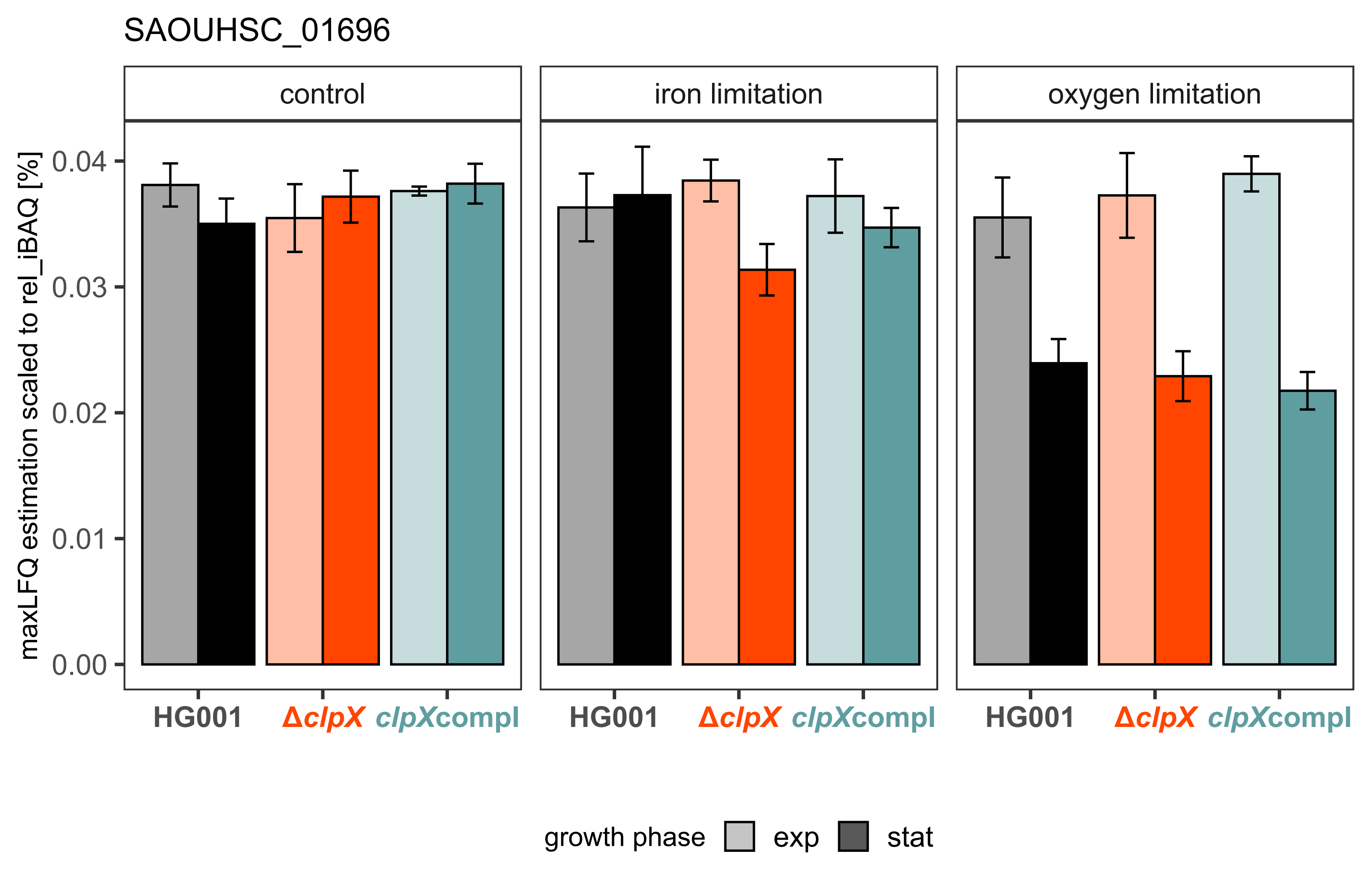
SAOUHSC_01696
control
iron limitation
oxygen limitation
maxLFQ estimation scaled to rel_iBAQ [%]
0.04
0.03
0.02
0.01
0.00
HG001
ΔclpX
clpXcompl
growth phase
exp
stat

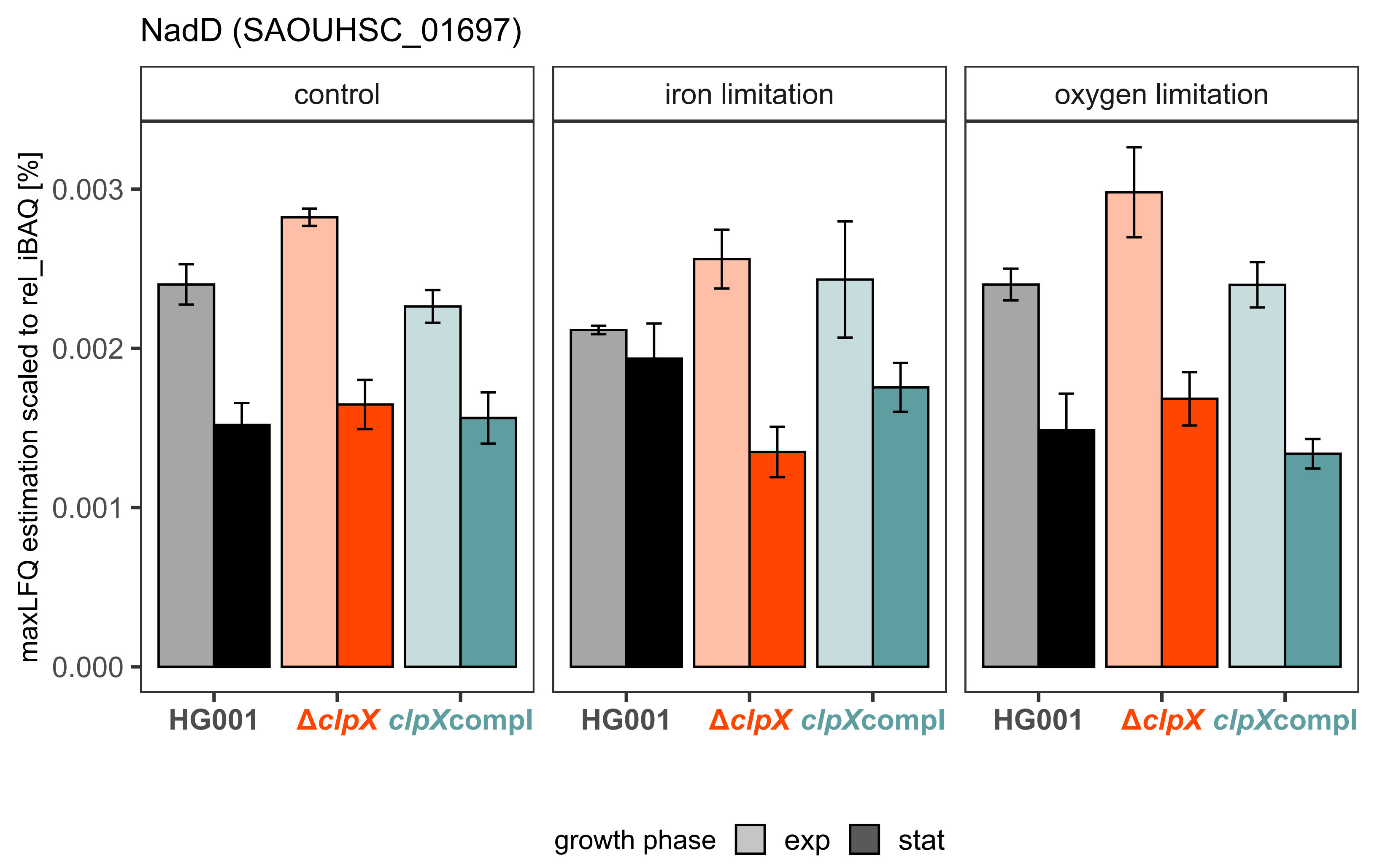

NadD (SAOUHSC_01697)
control
iron limitation
oxygen limitation
maxLFQ estimation scaled to rel_iBAQ [%]
0.003
0.002
0.001
0.000
HG001
ΔclpX
clpXcompl
growth phase
exp
stat

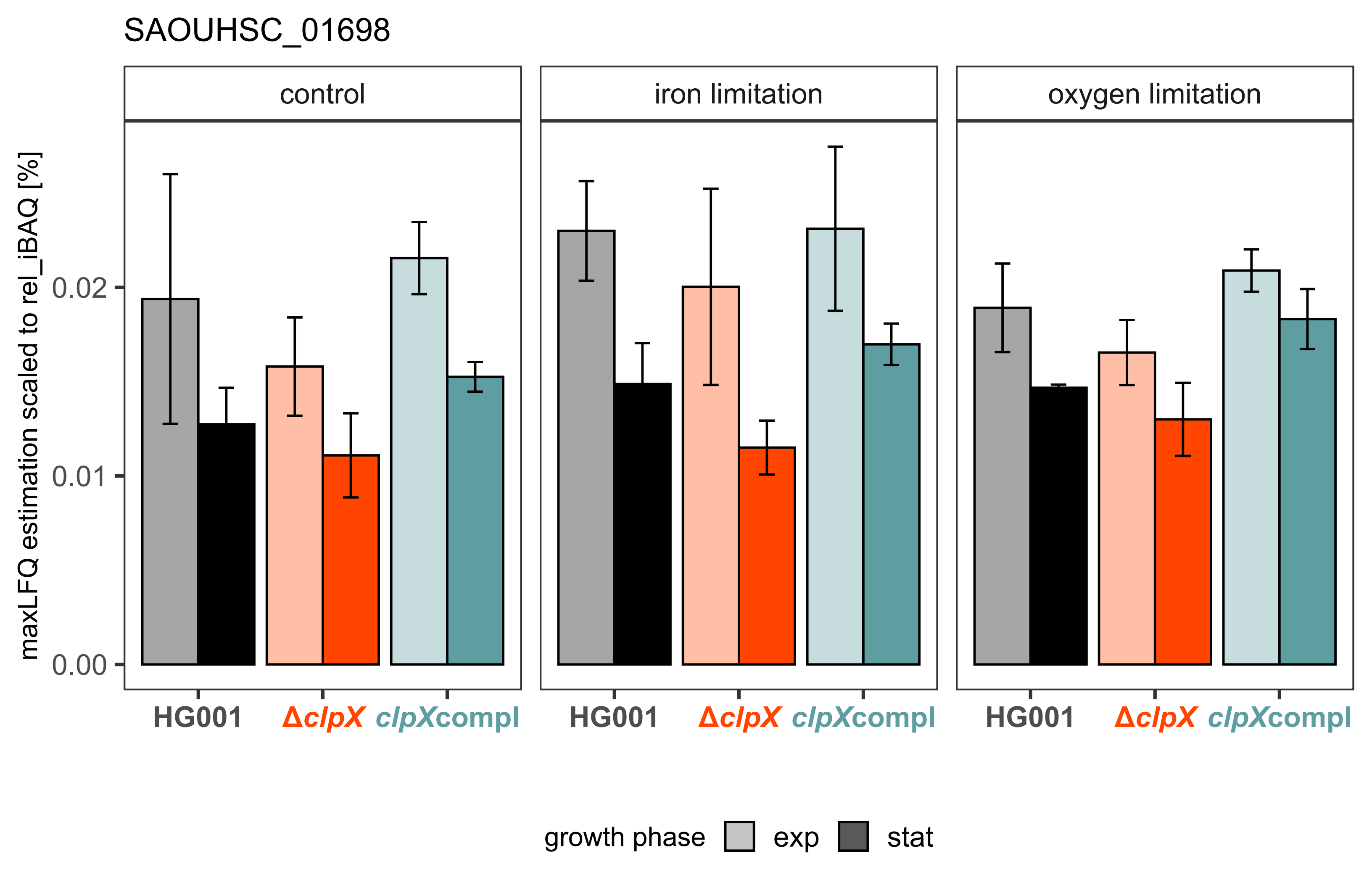

SAOUHSC_01698
control
iron limitation
oxygen limitation
maxLFQ estimation scaled to rel_iBAQ [%]
0.02
0.01
0.00
HG001
ΔclpX
clpXcompl
growth phase
exp
stat

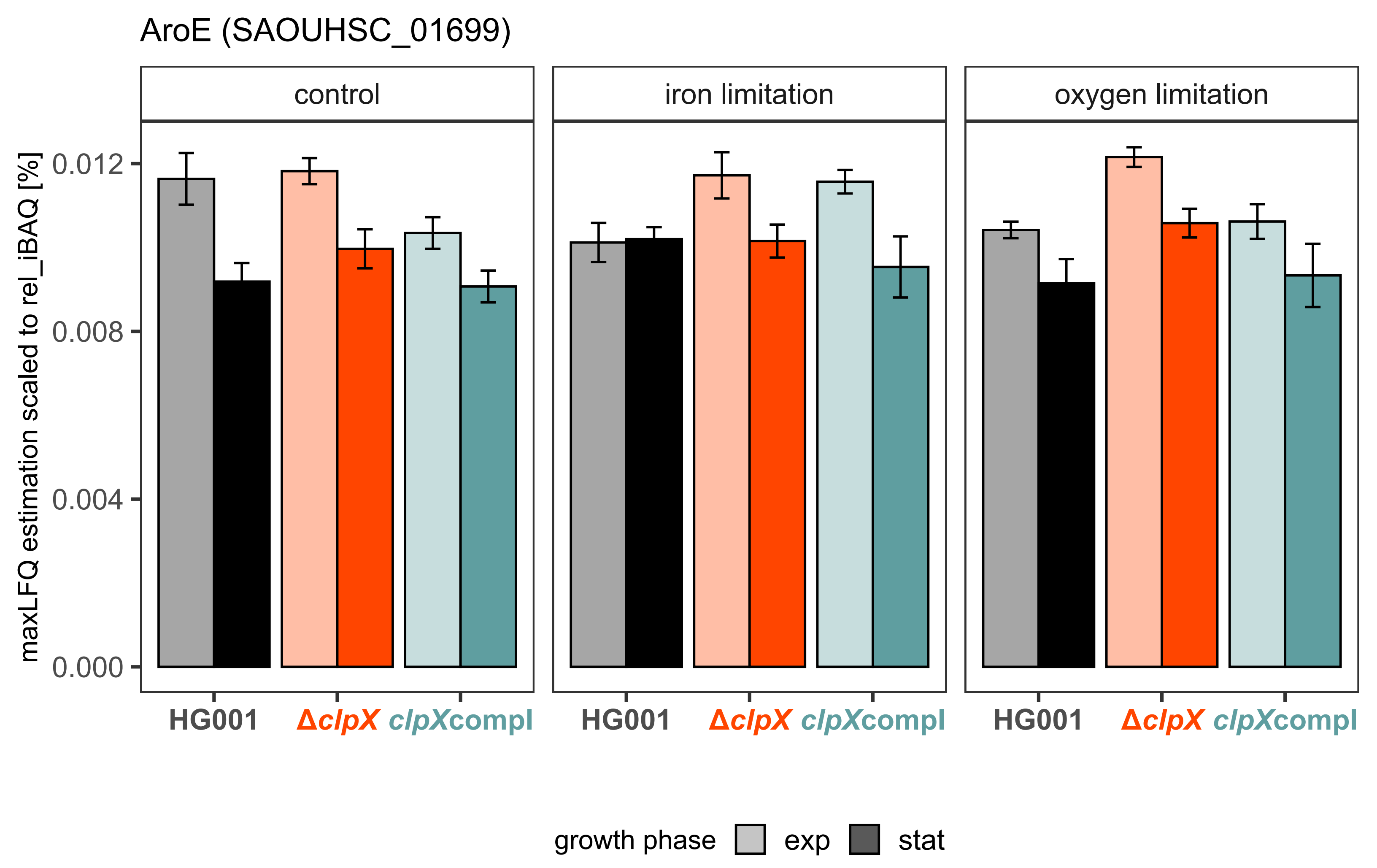
AroE (SAOUHSC_01699)
control
iron limitation
oxygen limitation
maxLFQ estimation scaled to rel_iBAQ [%]
0.012
0.008
0.004
0.000
HG001
ΔclpX
clpXcompl
growth phase
exp
stat

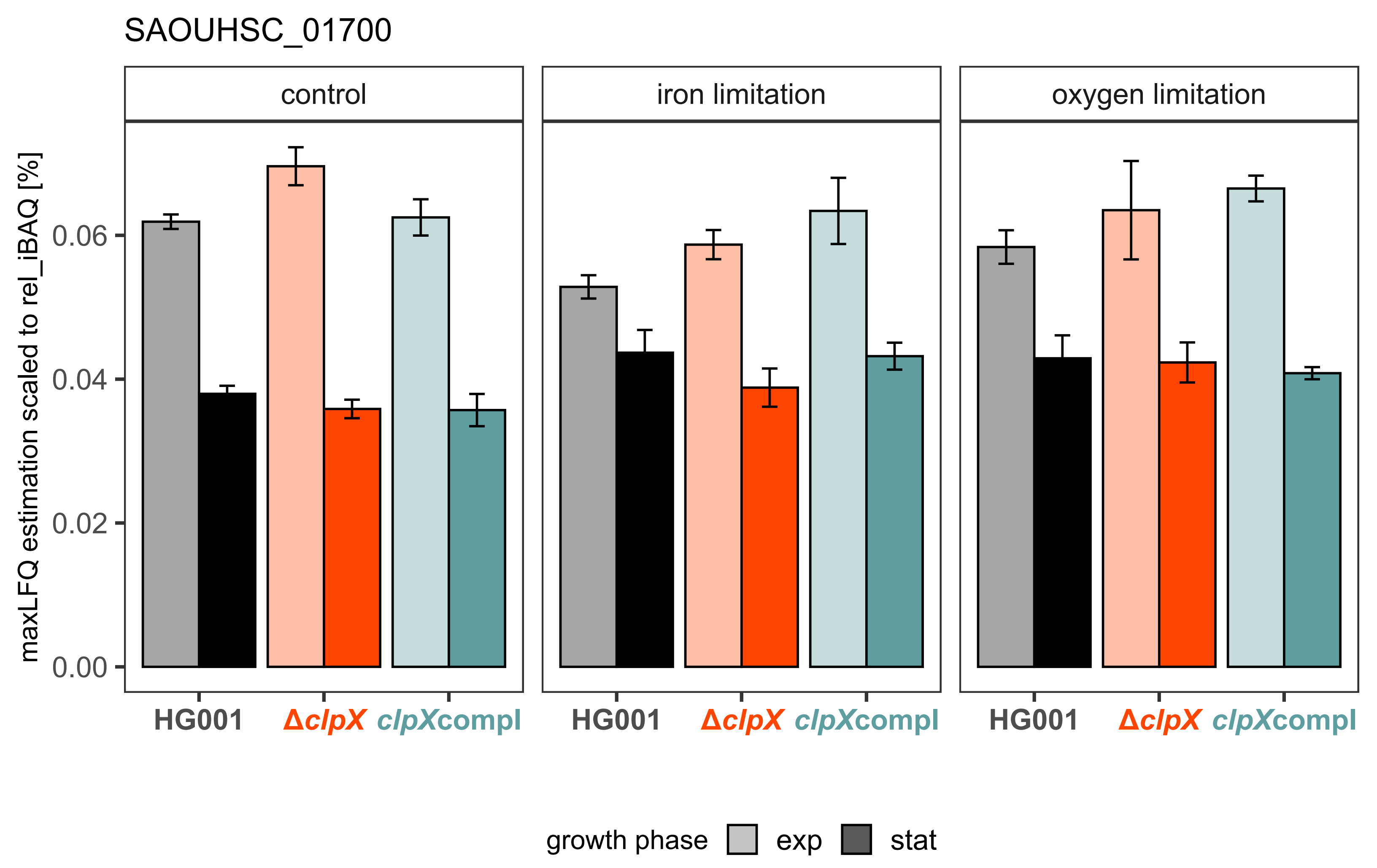

SAOUHSC_01700
control
iron limitation
oxygen limitation
maxLFQ estimation scaled to rel_iBAQ [%]
0.06
0.04
0.02
0.00
HG001
ΔclpX
clpXcompl
growth phase
exp
stat

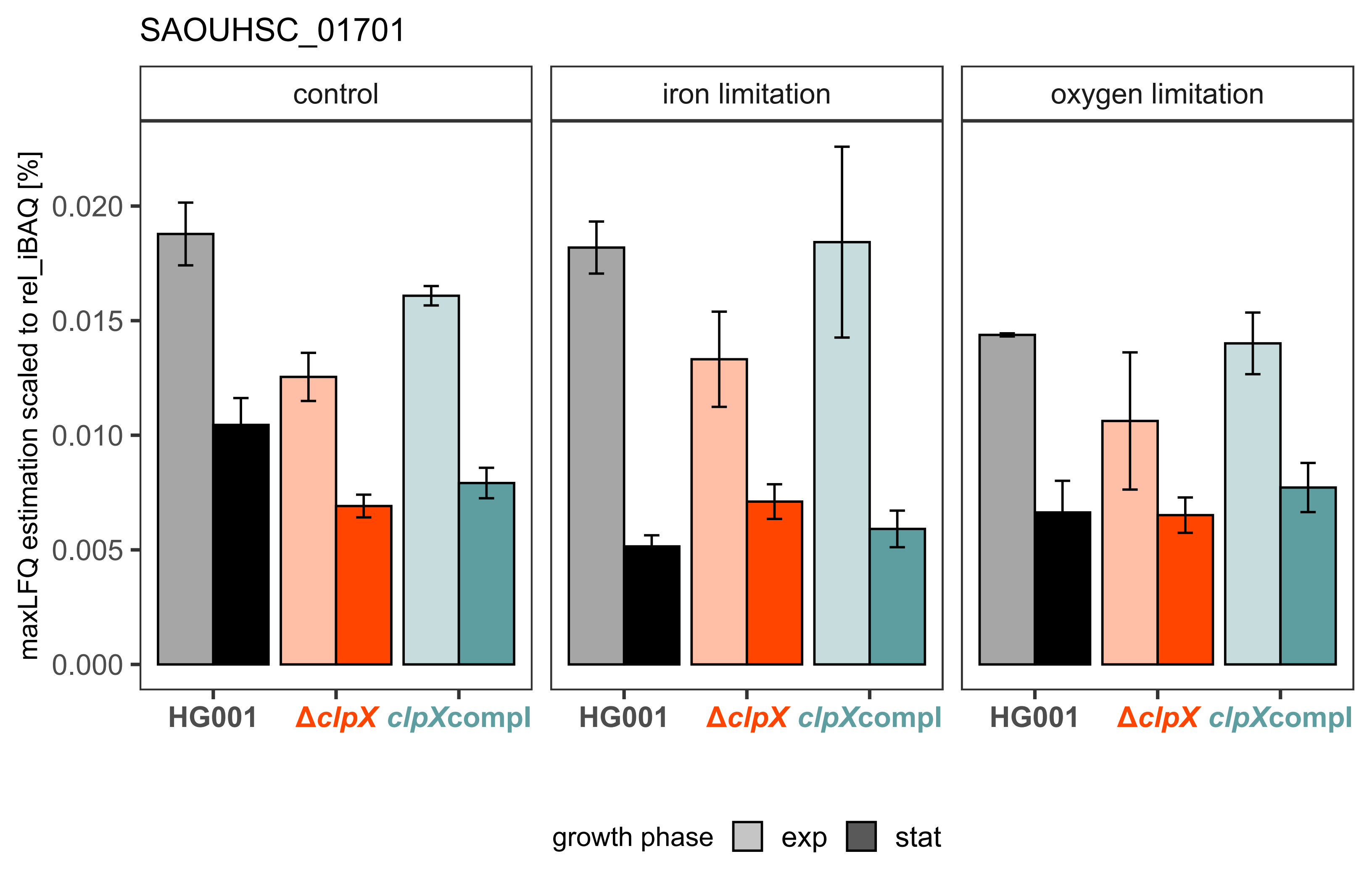
SAOUHSC_01701
control
iron limitation
oxygen limitation
maxLFQ estimation scaled to rel_iBAQ [%]
0.020
0.015
0.010
0.005
0.000
HG001
ΔclpX
clpXcompl
growth phase
exp
stat

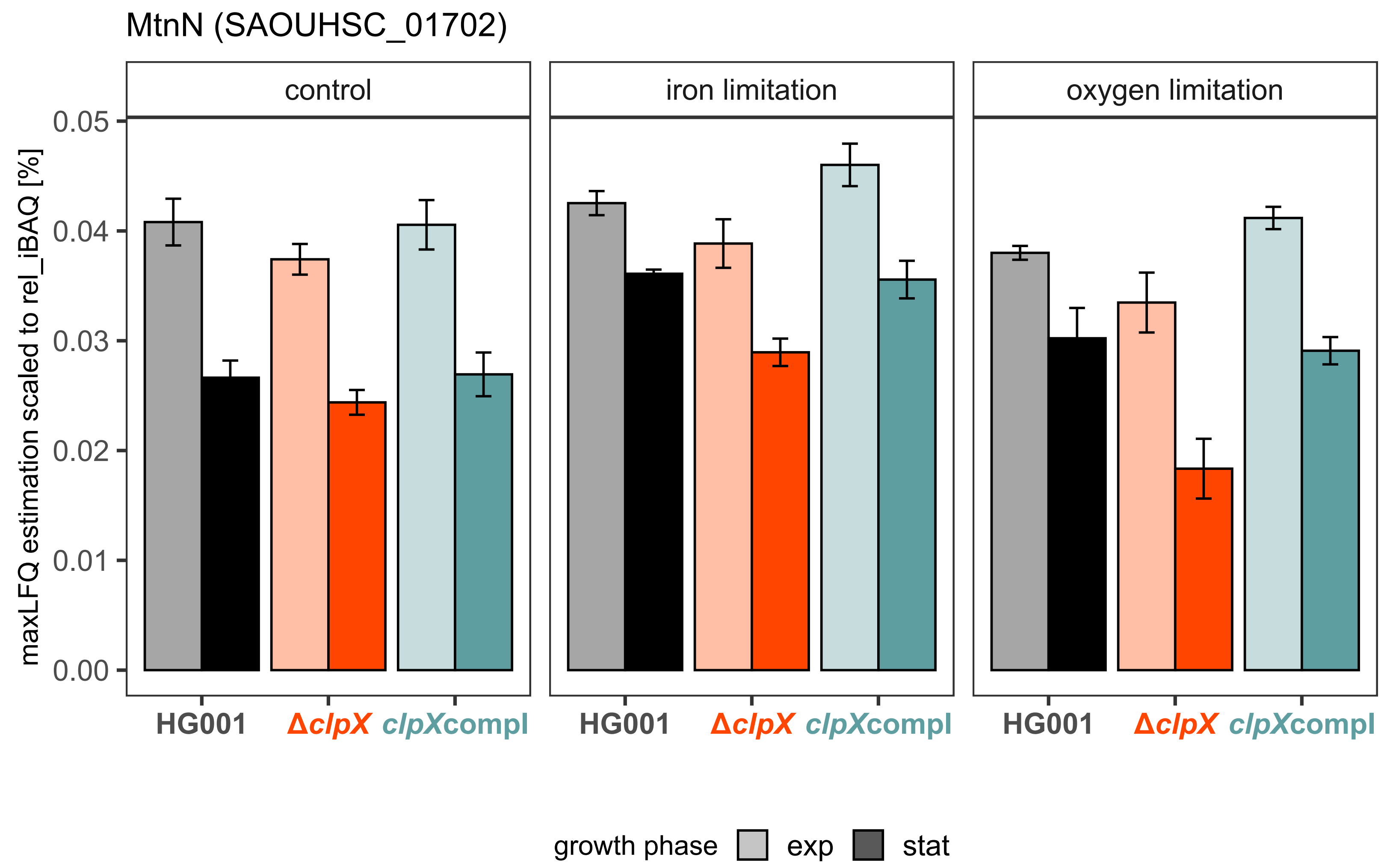

MtnN (SAOUHSC_01702)
control
iron limitation
oxygen limitation
maxLFQ estimation scaled to rel_iBAQ [%]
0.05
0.04
0.03
0.02
0.01
0.00
HG001
ΔclpX
clpXcompl
growth phase
exp
stat

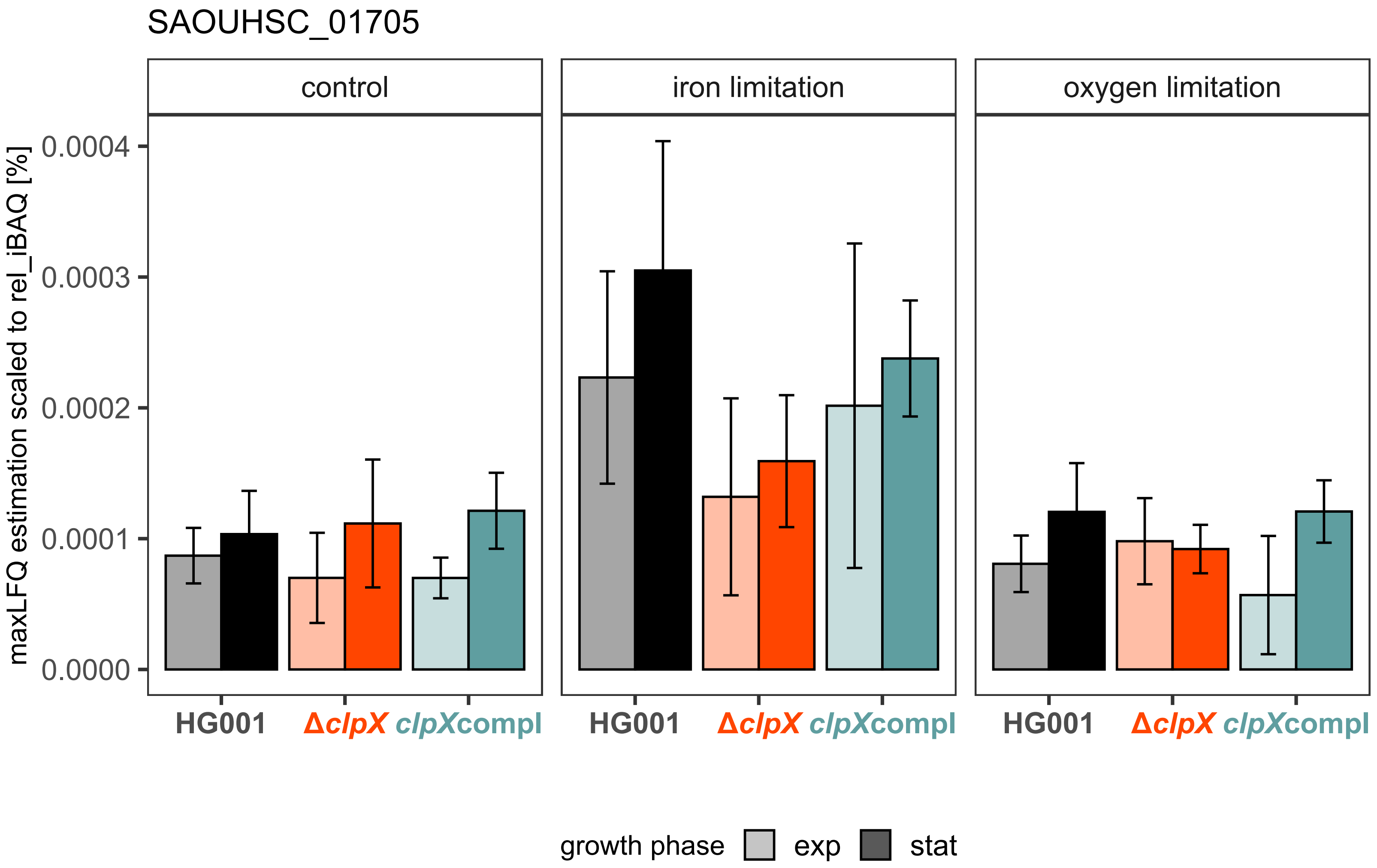
SAOUHSC_01705
control
iron limitation
oxygen limitation
maxLFQ estimation scaled to rel_iBAQ [%]
0.0004
0.0003
0.0002
0.0001
0.0000
HG001
ΔclpX
clpXcompl
growth phase
exp
stat

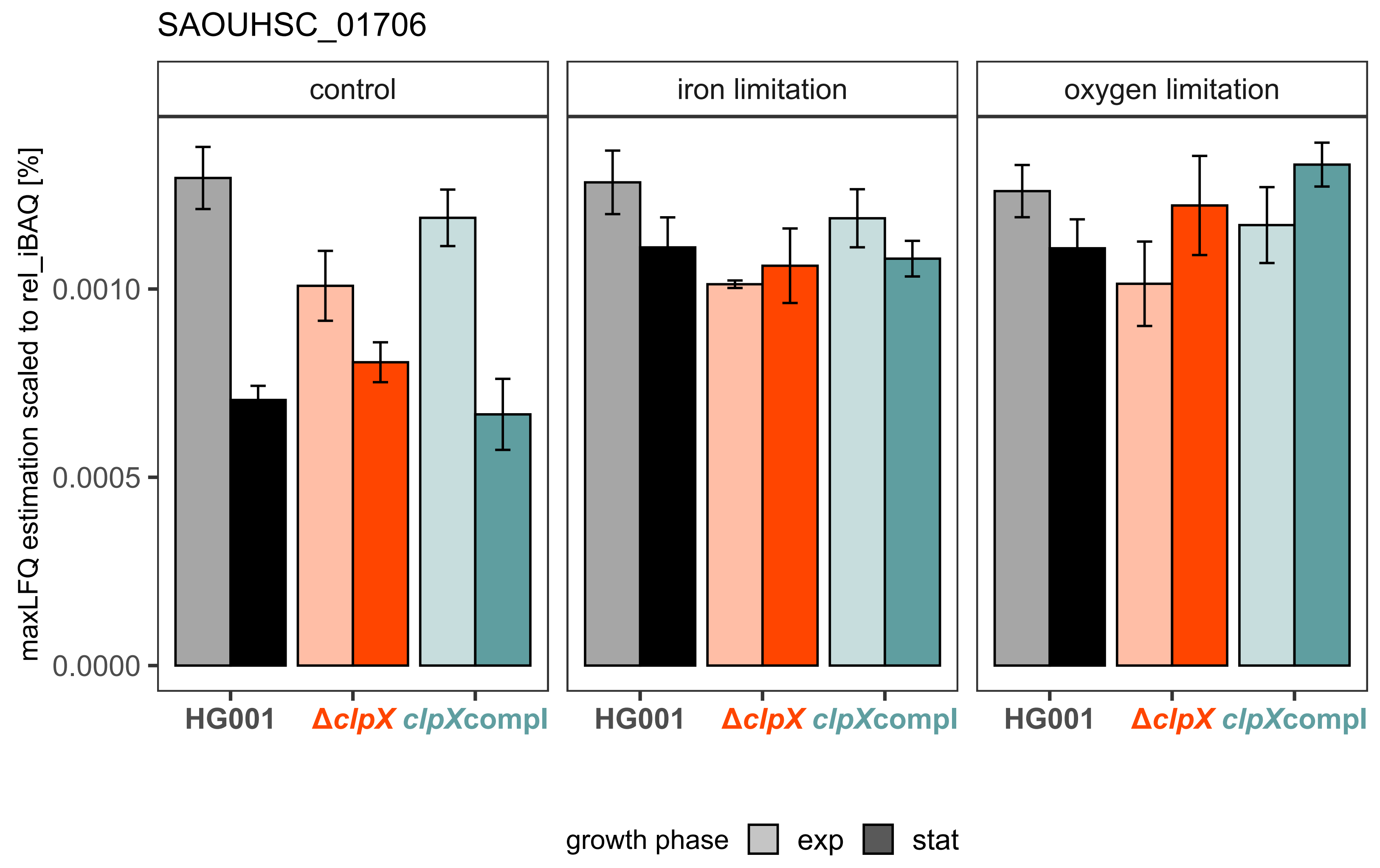

SAOUHSC_01706
control
iron limitation
oxygen limitation
maxLFQ estimation scaled to rel_iBAQ [%]
0.0010
0.0005
0.0000
HG001
ΔclpX
clpXcompl
growth phase
exp
stat

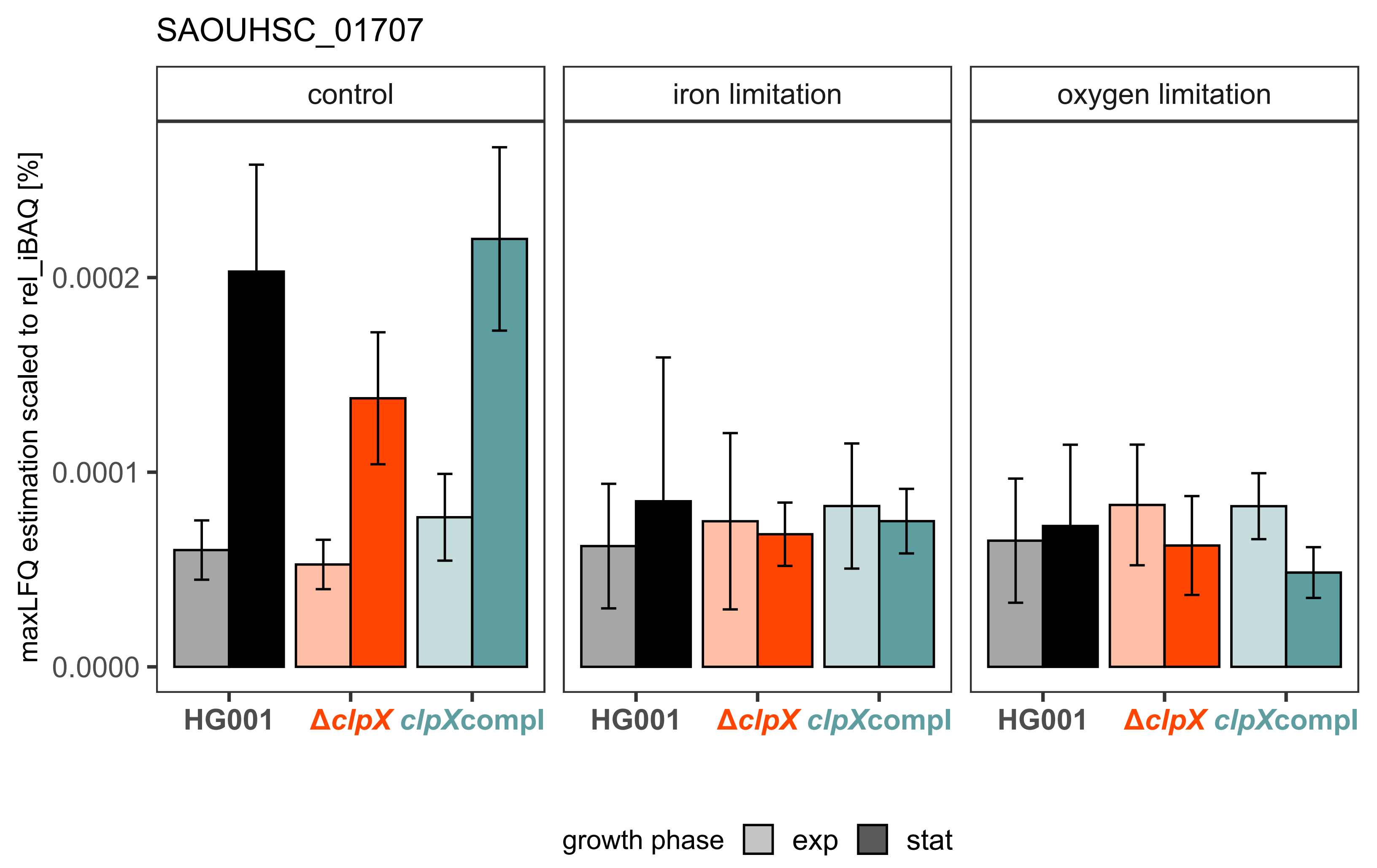
SAOUHSC_01707
control
iron limitation
oxygen limitation
maxLFQ estimation scaled to rel_iBAQ [%]
0.0002
0.0001
0.0000
HG001
ΔclpX
clpXcompl
growth phase
exp
stat

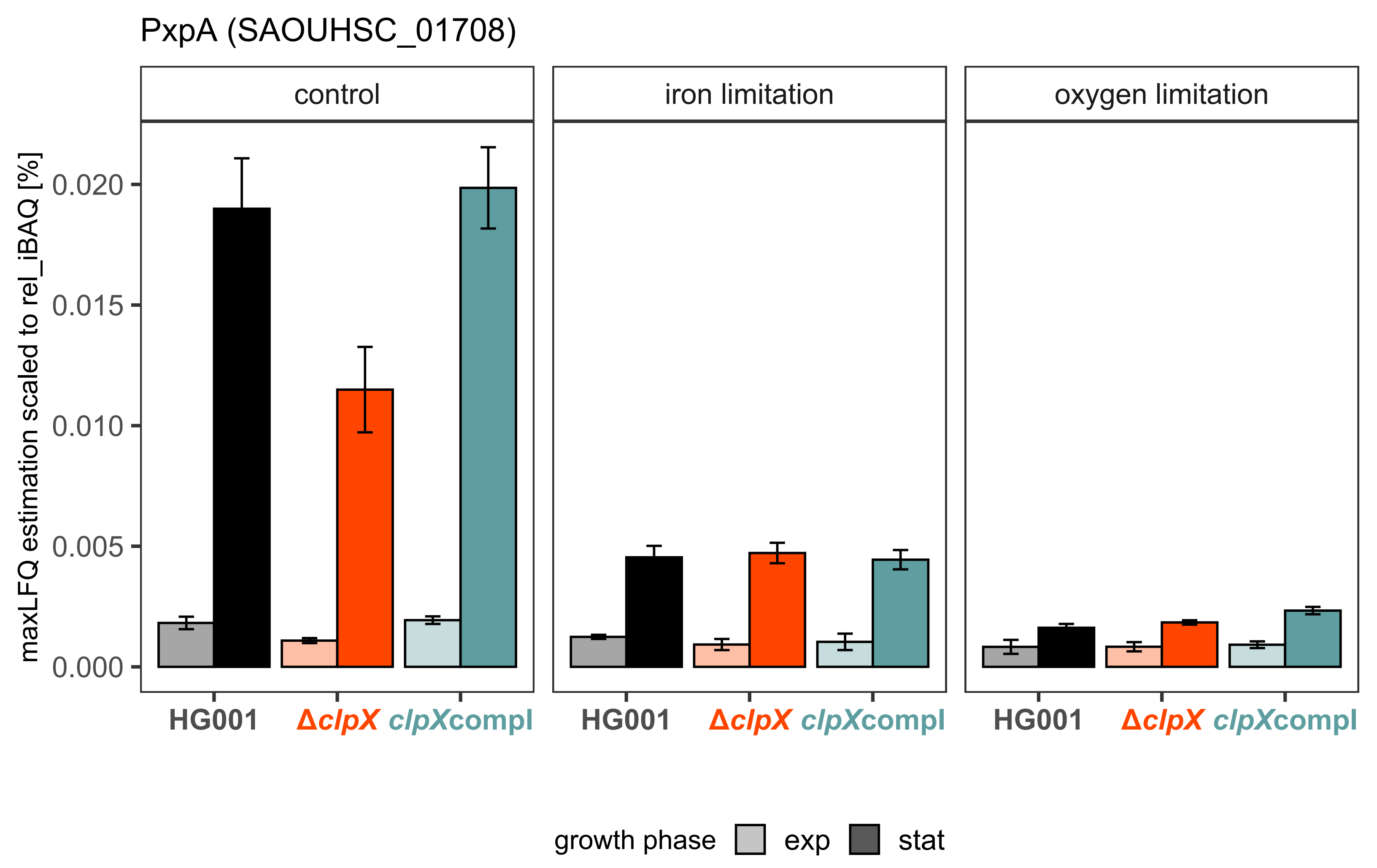
PxpA (SAOUHSC_01708)
control
iron limitation
oxygen limitation
maxLFQ estimation scaled to rel_iBAQ [%]
0.020
0.015
0.010
0.005
0.000
HG001
ΔclpX
clpXcompl
growth phase
exp
stat

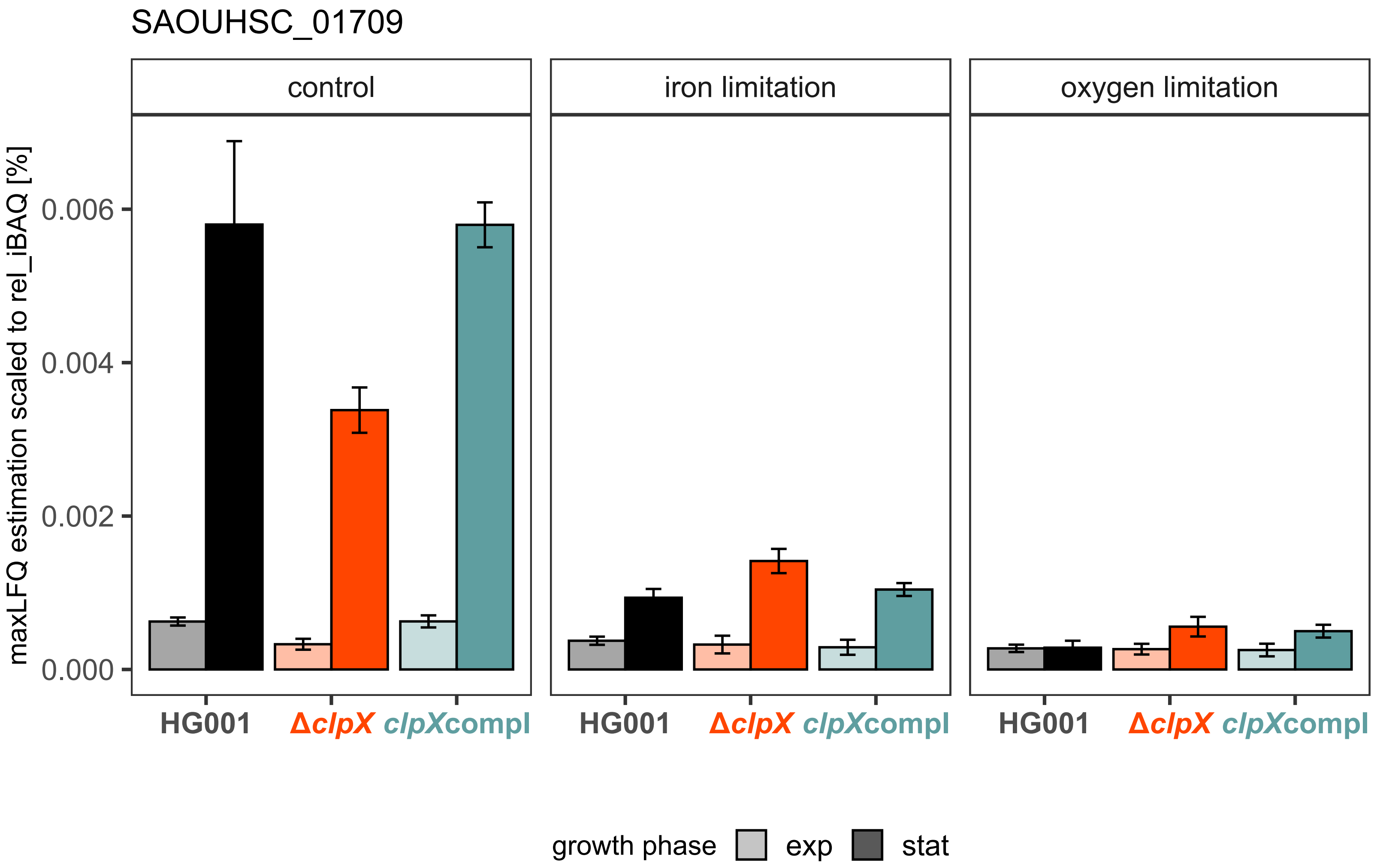

SAOUHSC_01709
control
iron limitation
oxygen limitation
maxLFQ estimation scaled to rel_iBAQ [%]
0.000
0.002
0.004
0.006
HG001
ΔclpX
clpXcompl
growth phase
exp
stat

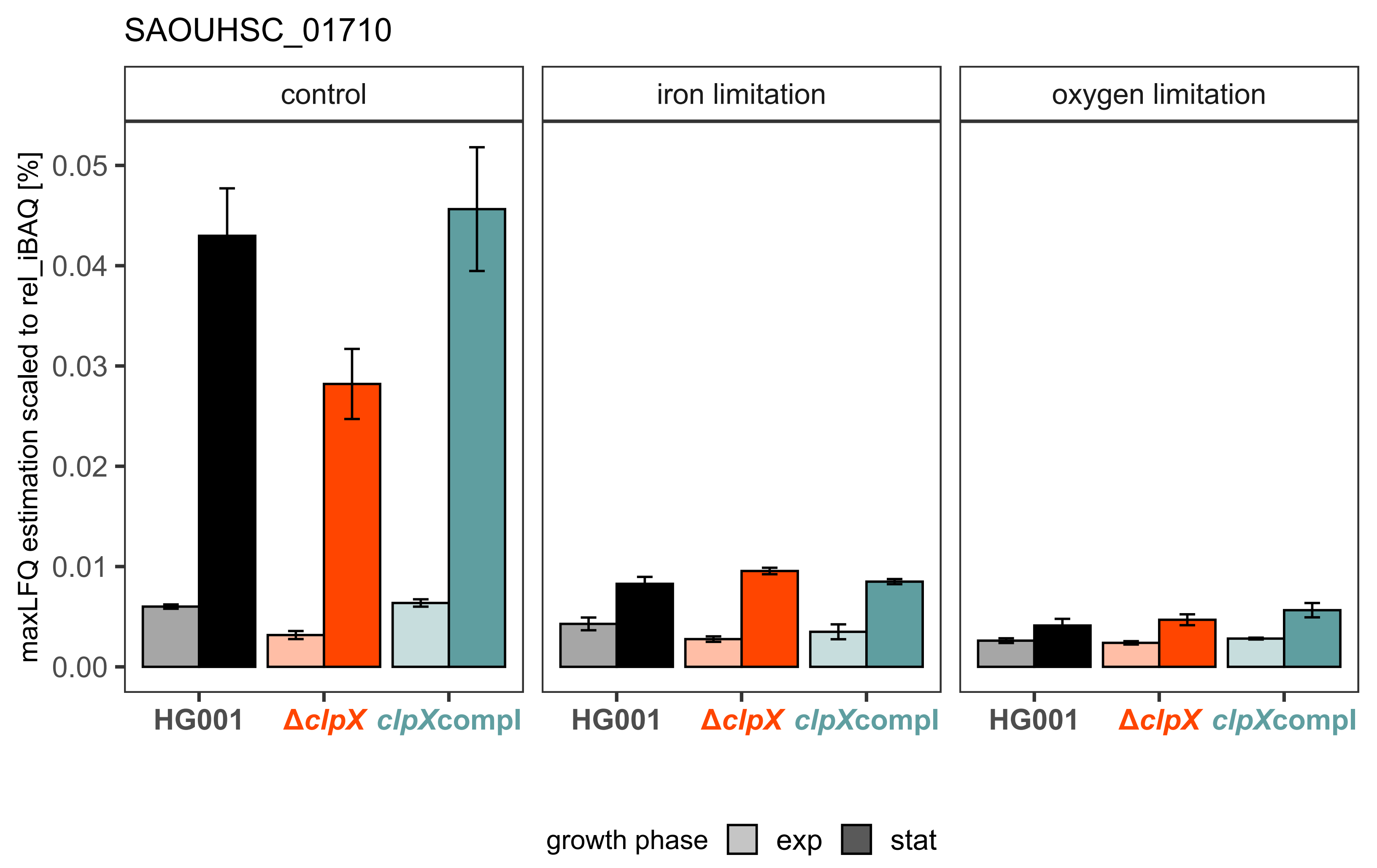
SAOUHSC_01710
control
iron limitation
oxygen limitation
maxLFQ estimation scaled to rel_iBAQ [%]
0.05
0.04
0.03
0.02
0.01
0.00
HG001
ΔclpX
clpXcompl
HG001
ΔclpX
clpXcompl
HG001
ΔclpX
clpXcompl
growth phase
exp
stat

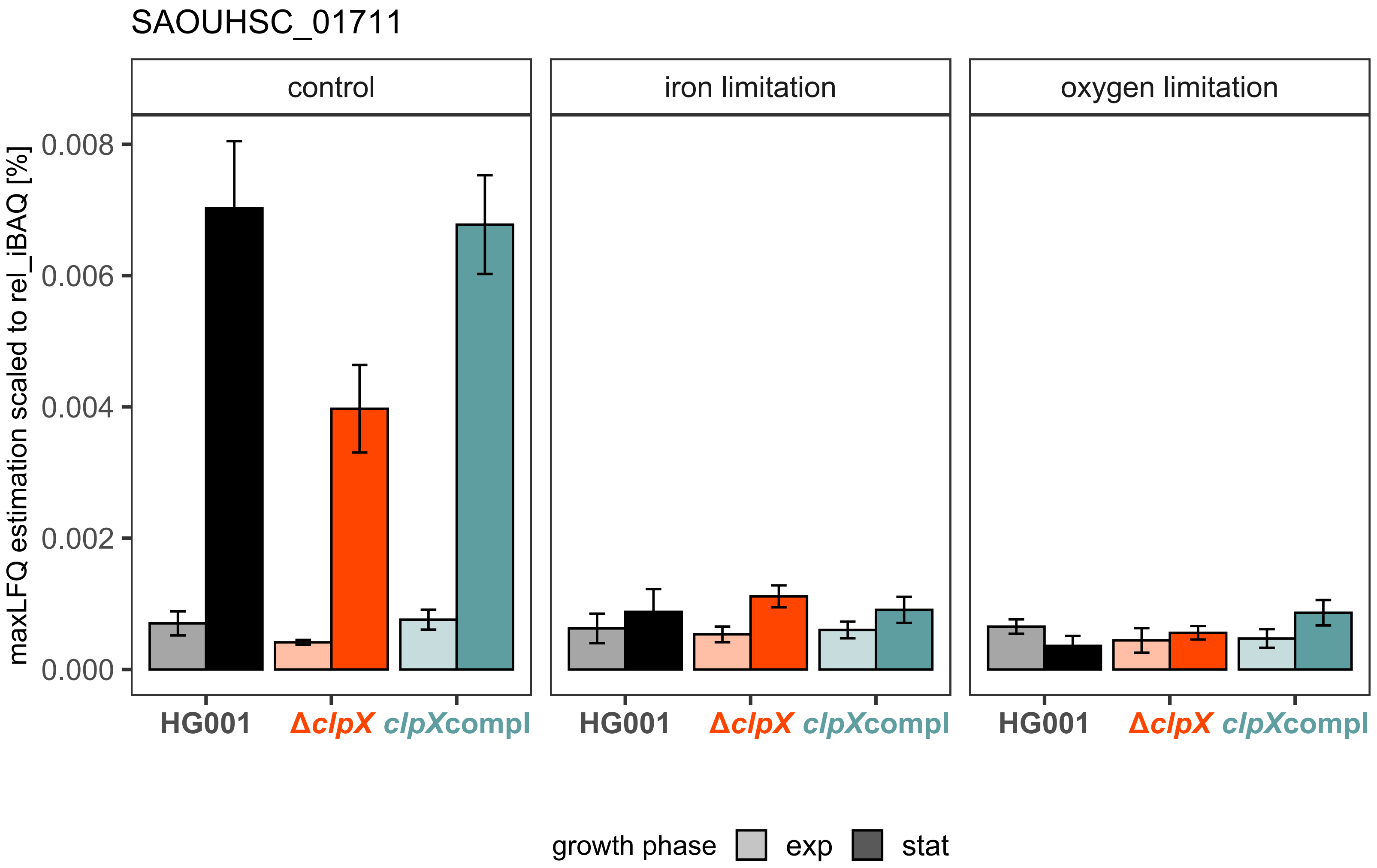
SAOUHSC_01711
control
iron limitation
oxygen limitation
maxLFQ estimation scaled to rel_iBAQ [%]
0.008
0.006
0.004
0.002
0.000
HG001
ΔclpX
clpXcompl
growth phase
exp
stat

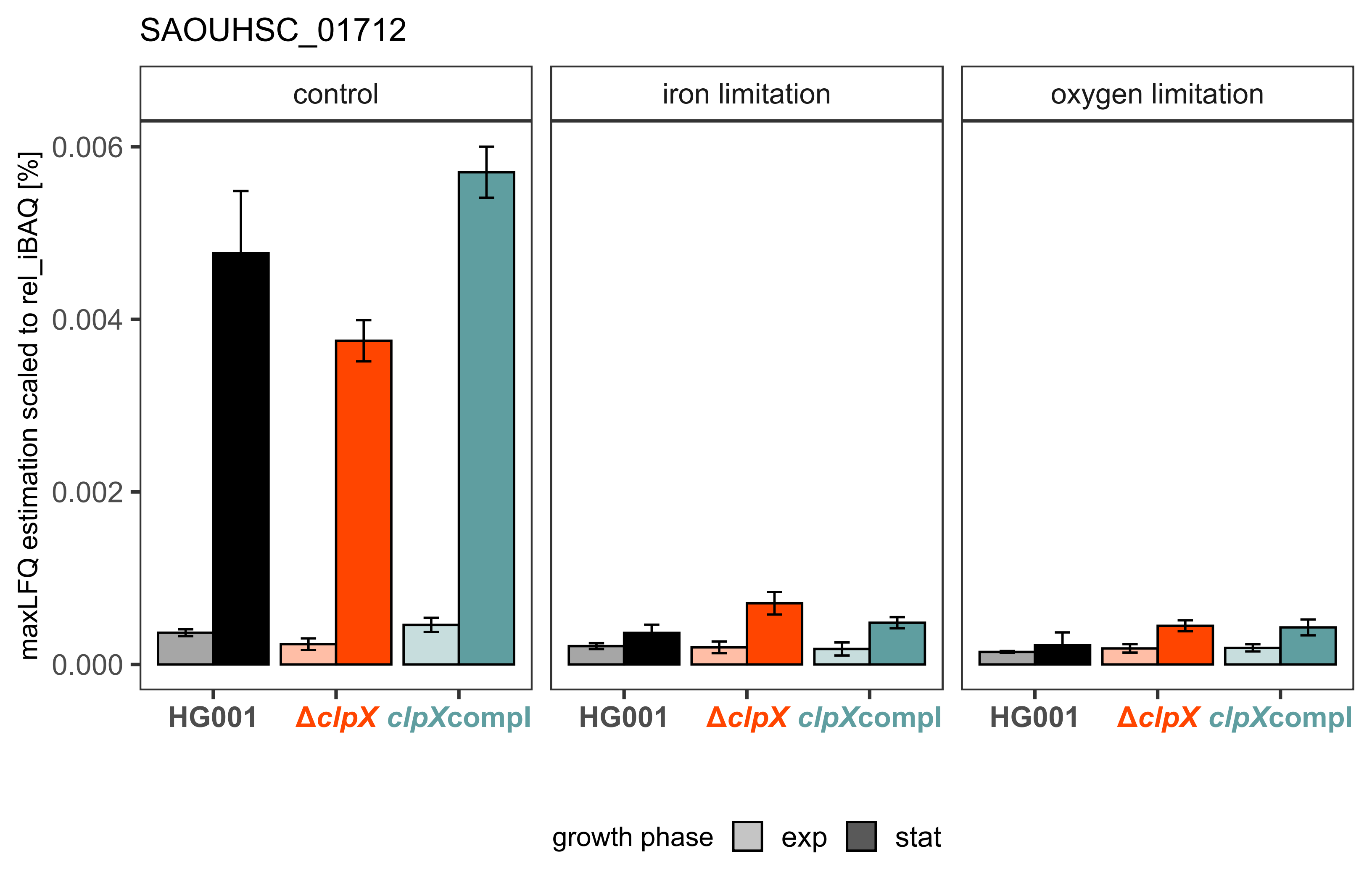

SAOUHSC_01712
control
iron limitation
oxygen limitation
maxLFQ estimation scaled to rel_iBAQ [%]
0.006
0.004
0.002
0.000
HG001
ΔclpX
clpXcompl
growth phase
exp
stat

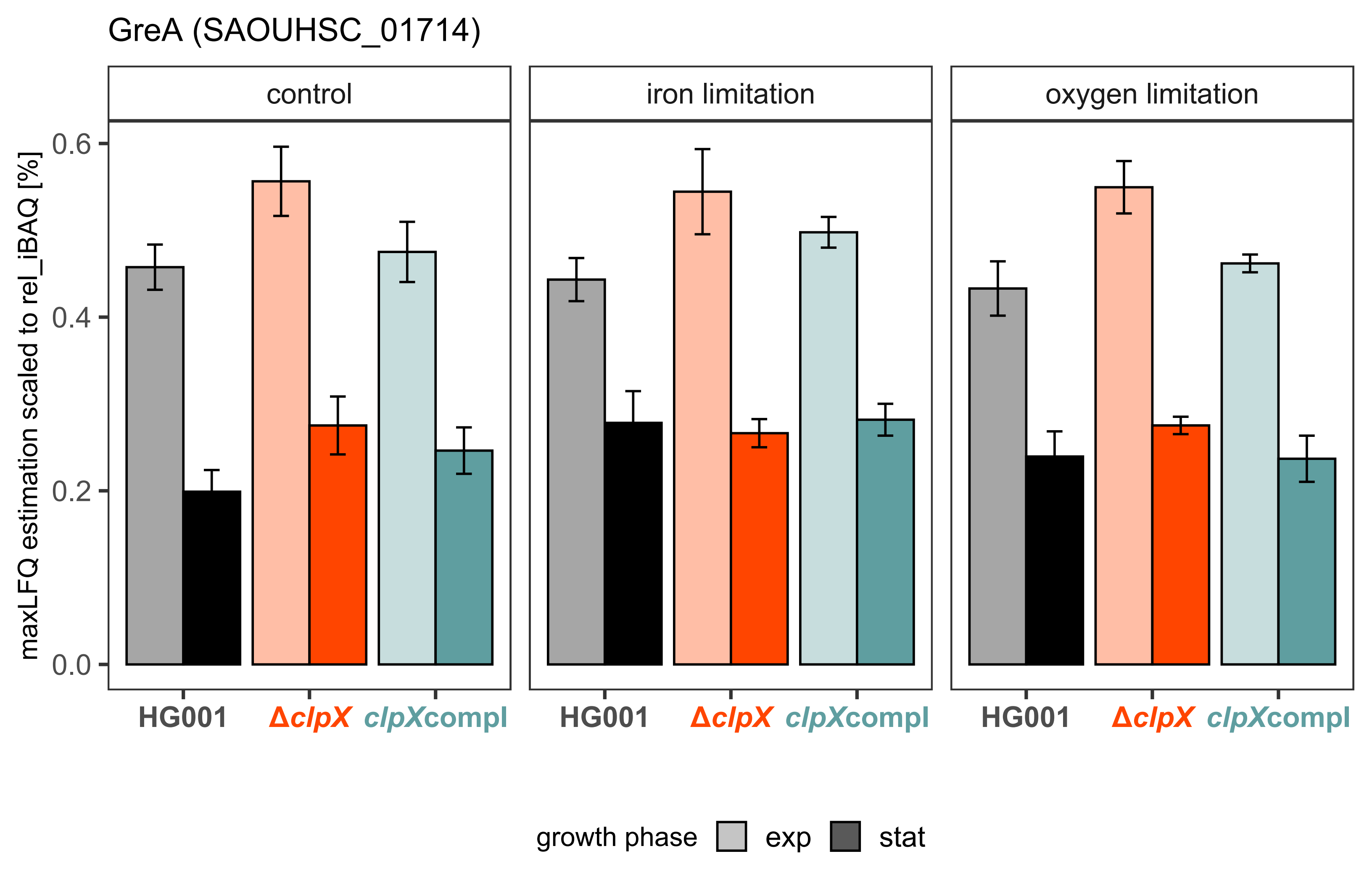
GreA (SAOUHSC_01714)
control
iron limitation
oxygen limitation
maxLFQ estimation scaled to rel_iBAQ [%]
0.0
0.2
0.4
0.6
HG001
ΔclpX
clpXcompl
growth phase
exp
stat

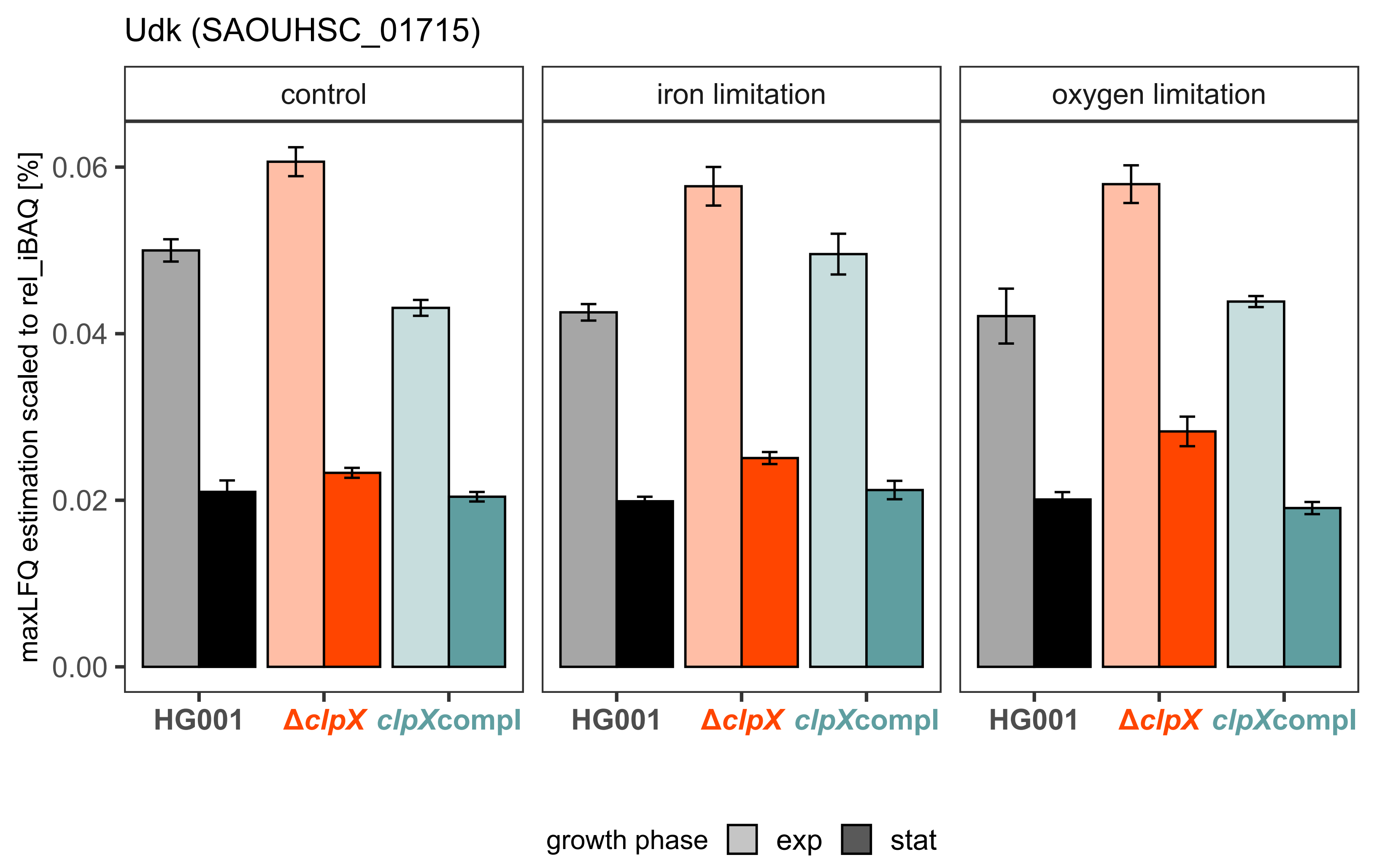
Udk (SAOUHSC_01715)
control
iron limitation
oxygen limitation
maxLFQ estimation scaled to rel_iBAQ [%]
0.06
0.04
0.02
0.00
HG001
ΔclpX
clpXcompl
growth phase
exp
stat

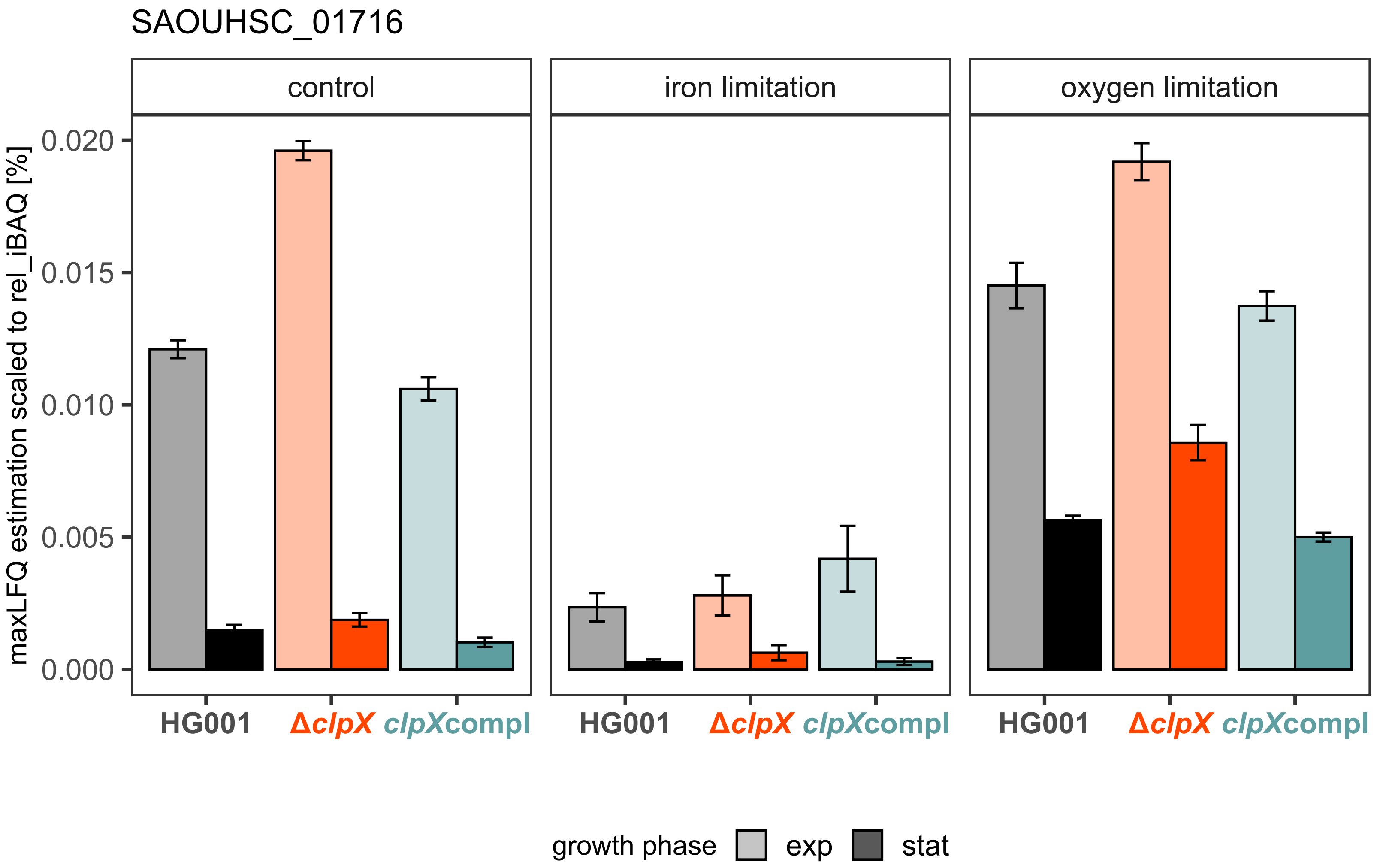
SAOUHSC_01716
control
iron limitation
oxygen limitation
maxLFQ estimation scaled to rel_iBAQ [%]
0.020
0.015
0.010
0.005
0.000
HG001
ΔclpX
clpXcompl
growth phase
exp
stat

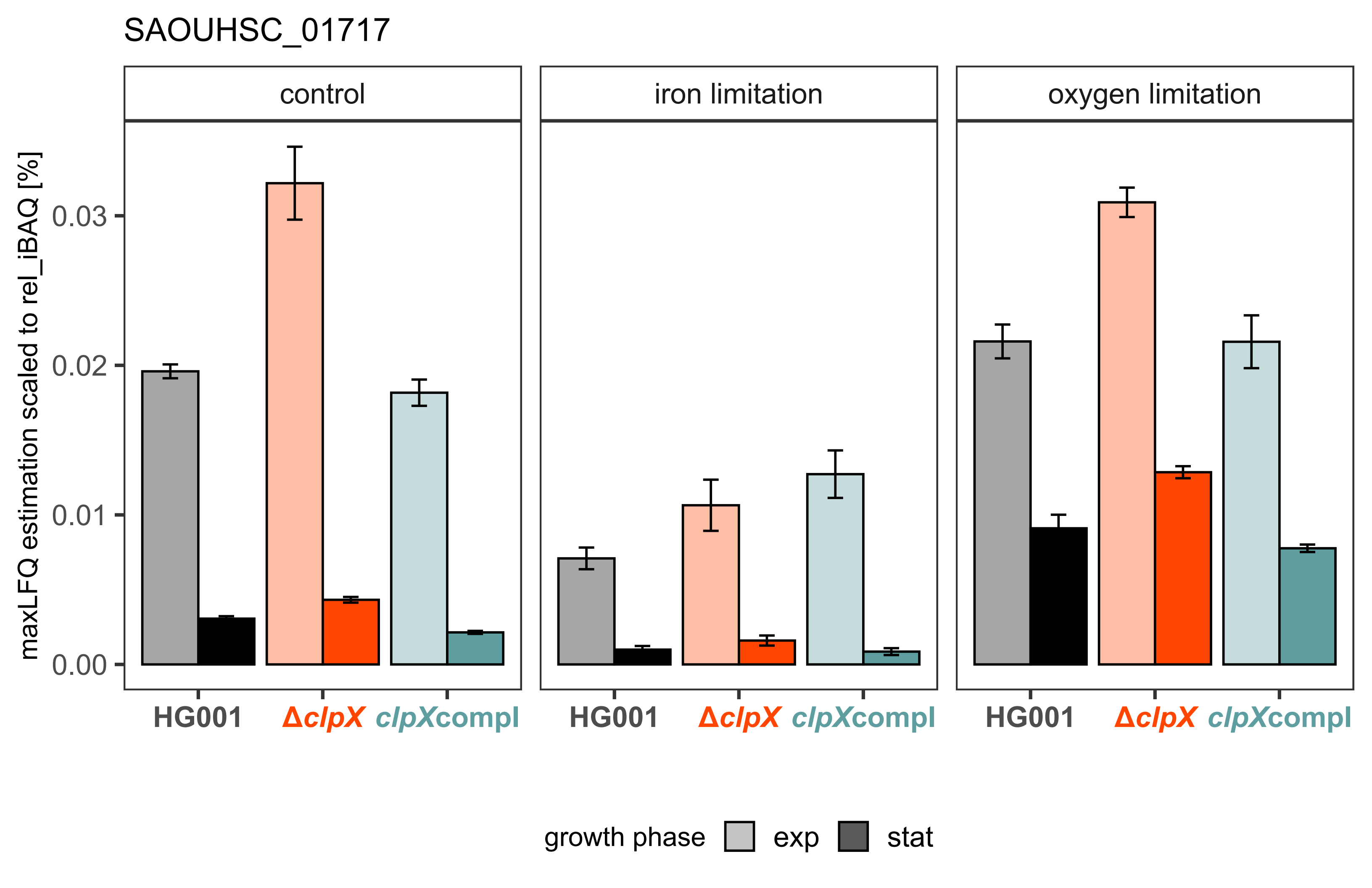
SAOUHSC_01717
control
iron limitation
oxygen limitation
maxLFQ estimation scaled to rel_iBAQ [%]
0.03
0.02
0.01
0.00
HG001
ΔclpX
clpXcompl
growth phase
exp
stat

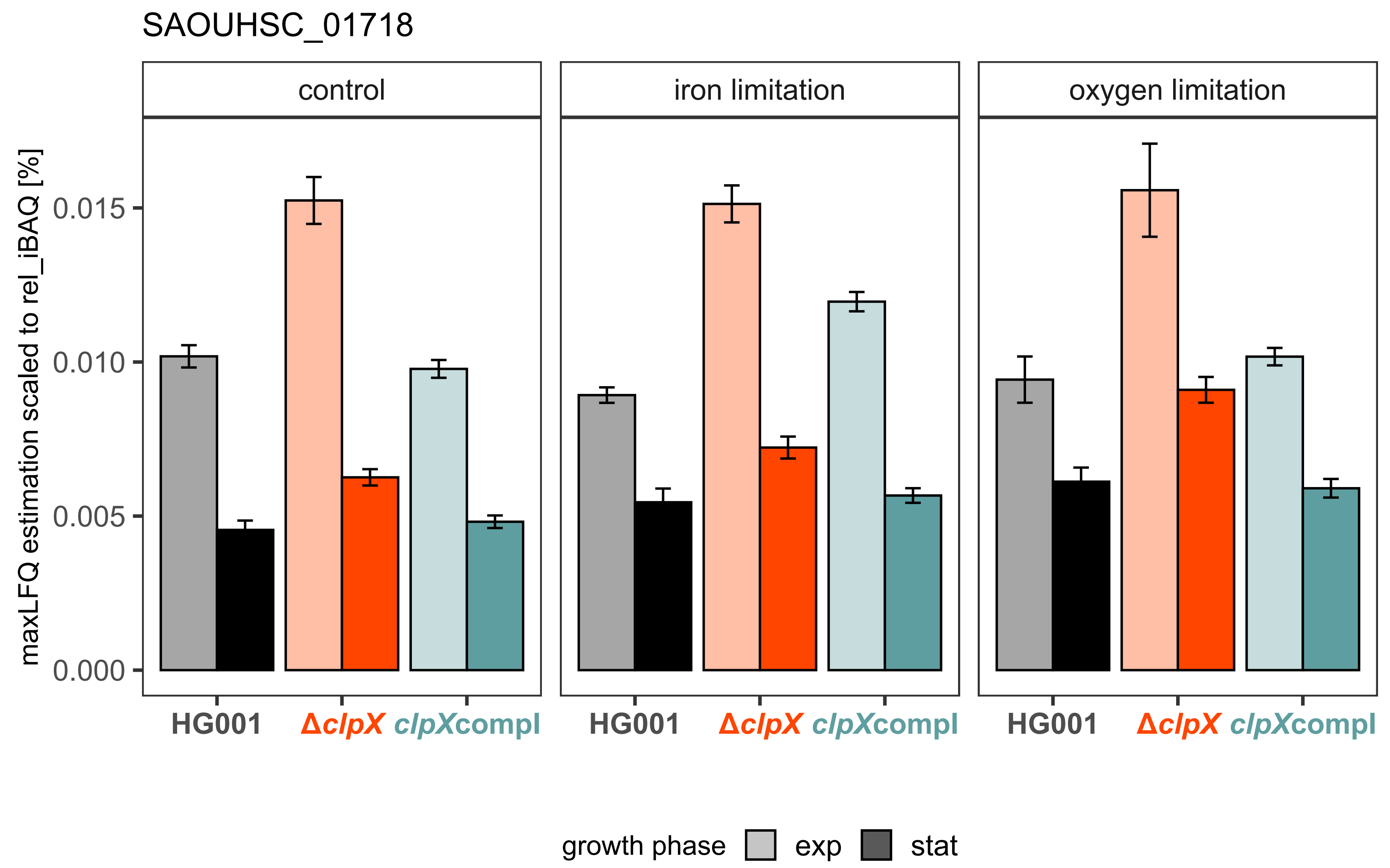
SAOUHSC_01718
control
iron limitation
oxygen limitation
maxLFQ estimation scaled to rel_iBAQ [%]
0.015
0.010
0.005
0.000
HG001
ΔclpX
clpXcompl
growth phase
exp
stat

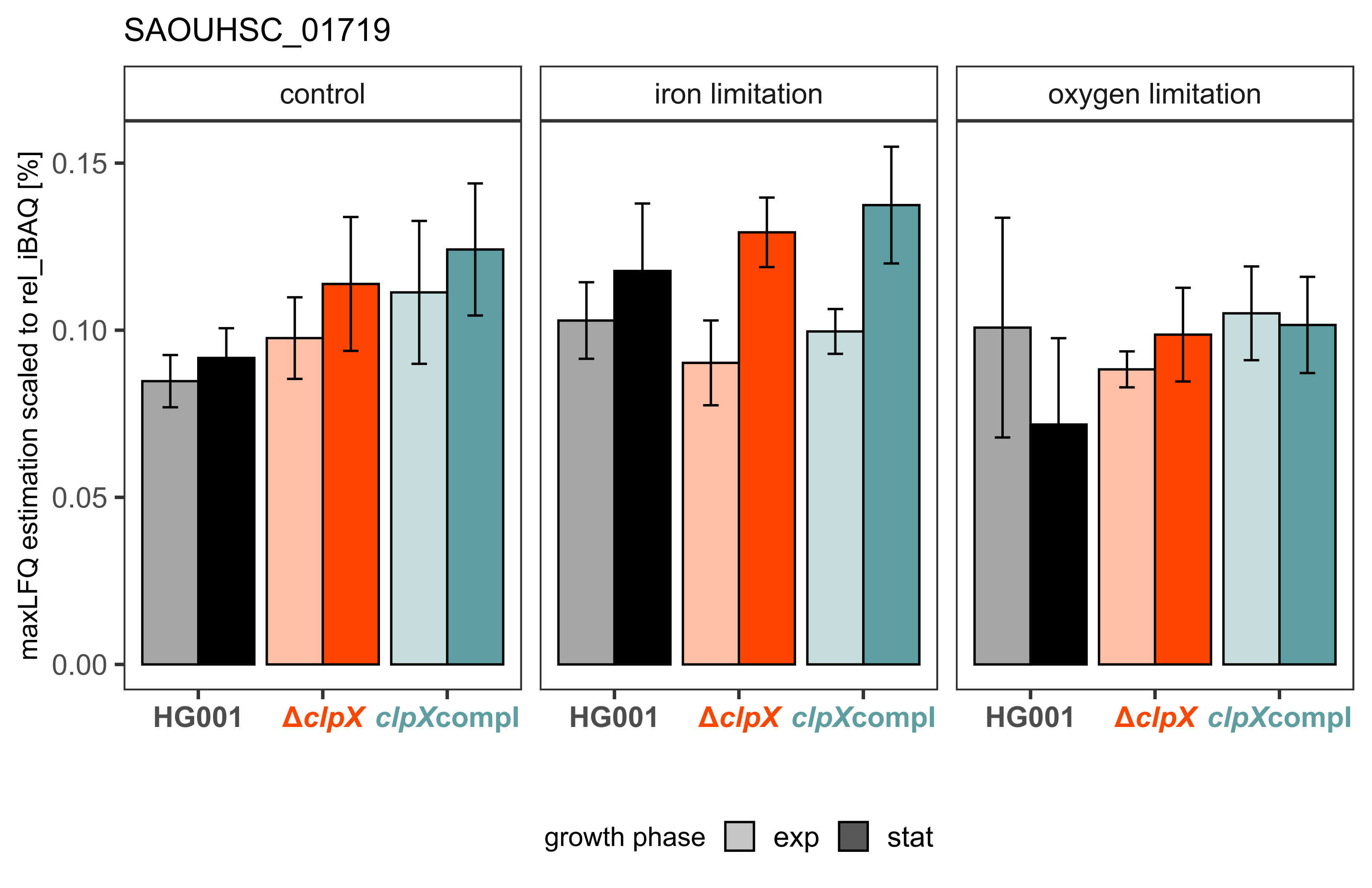

SAOUHSC_01719
control
iron limitation
oxygen limitation
maxLFQ estimation scaled to rel_iBAQ [%]
0.15
0.10
0.05
0.00
HG001
ΔclpX
clpXcompl
growth phase
exp
stat

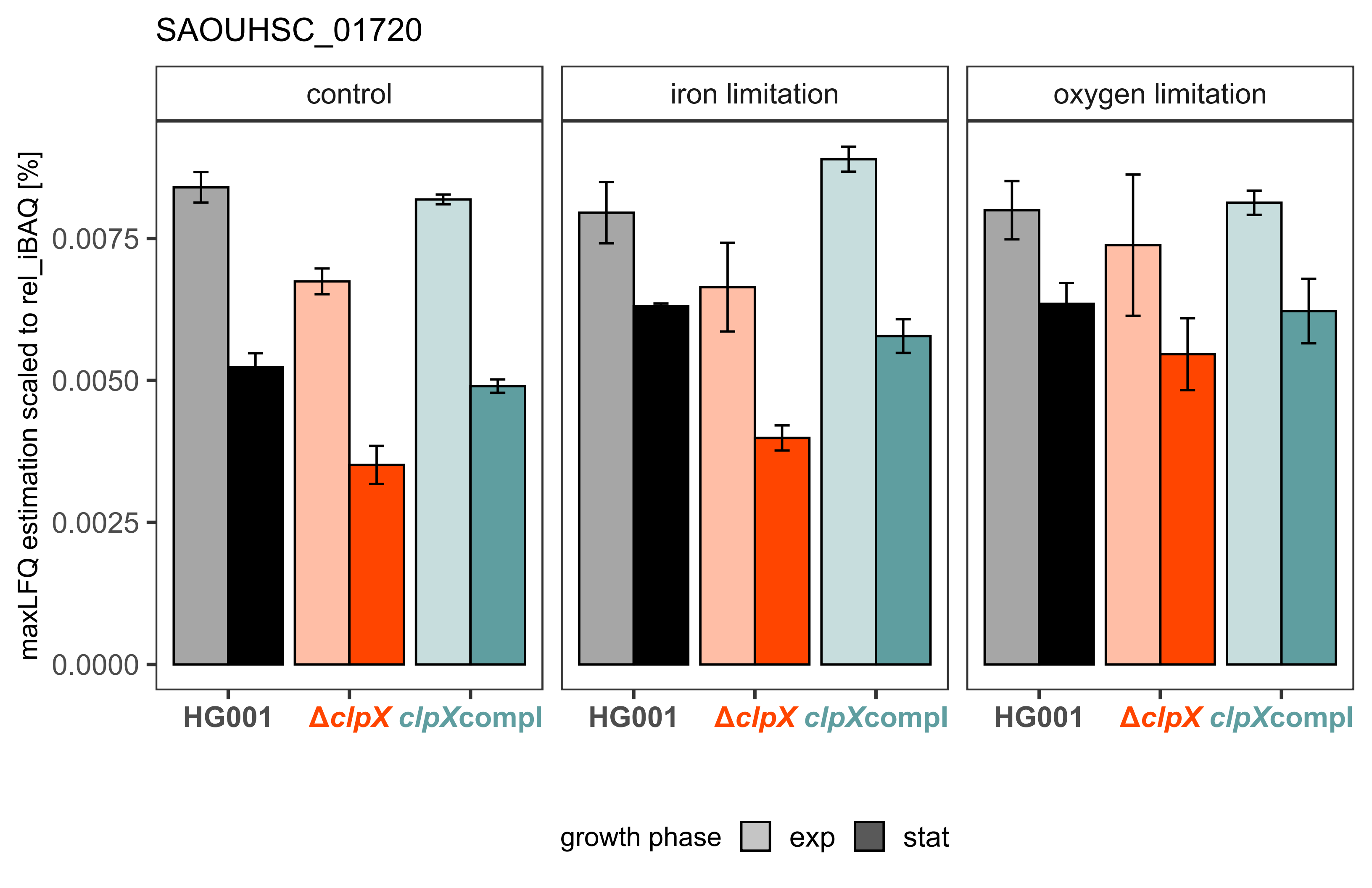

SAOUHSC_01720
control
iron limitation
oxygen limitation
maxLFQ estimation scaled to rel_iBAQ [%]
0.0075
0.0050
0.0025
0.0000
HG001
ΔclpX
clpXcompl
HG001
ΔclpX
clpXcompl
HG001
ΔclpX
clpXcompl
growth phase
exp
stat

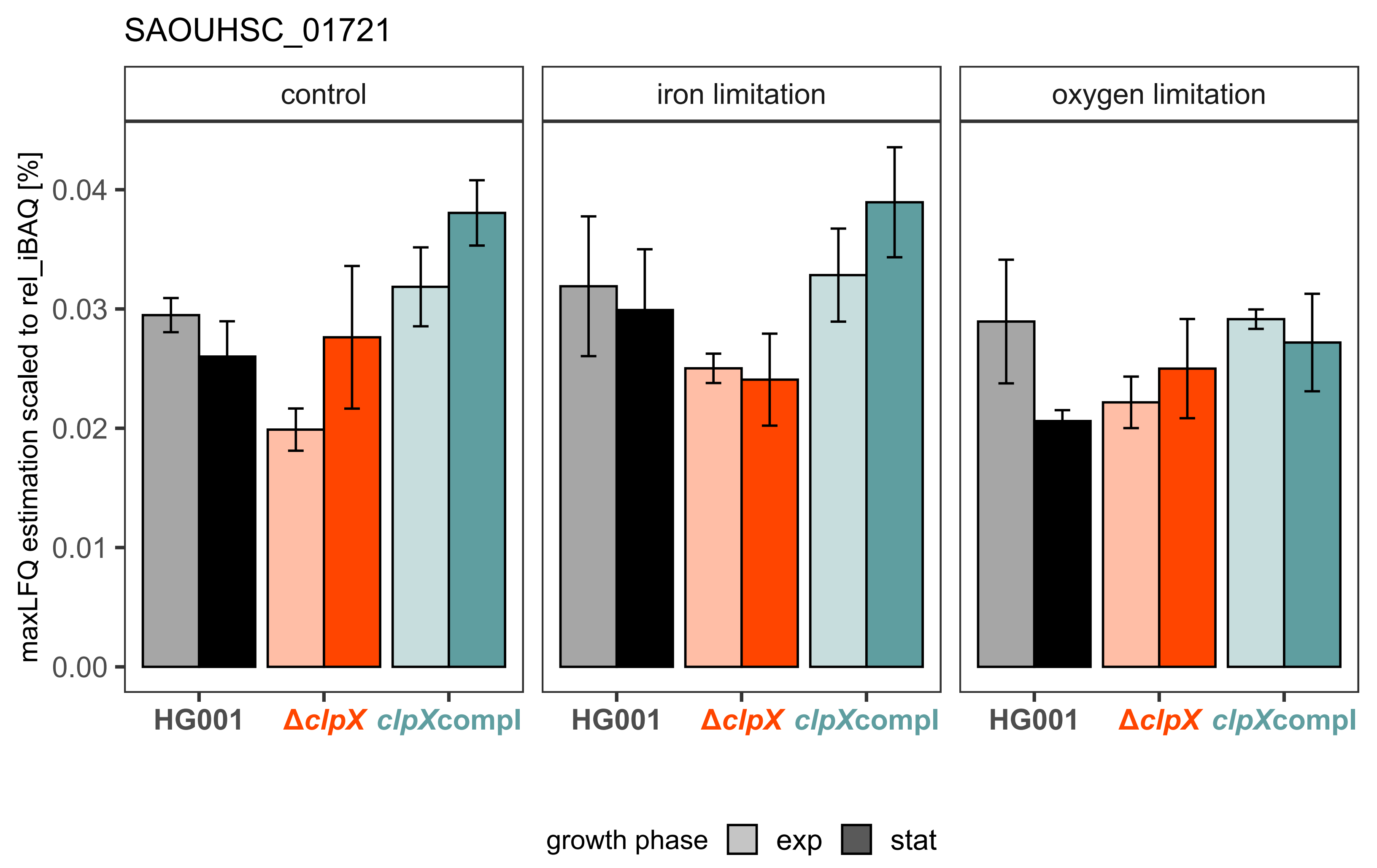
SAOUHSC_01721
control
iron limitation
oxygen limitation
maxLFQ estimation scaled to rel_iBAQ [%]
0.00
0.01
0.02
0.03
0.04
HG001
ΔclpX
clpXcompl
growth phase
exp
stat

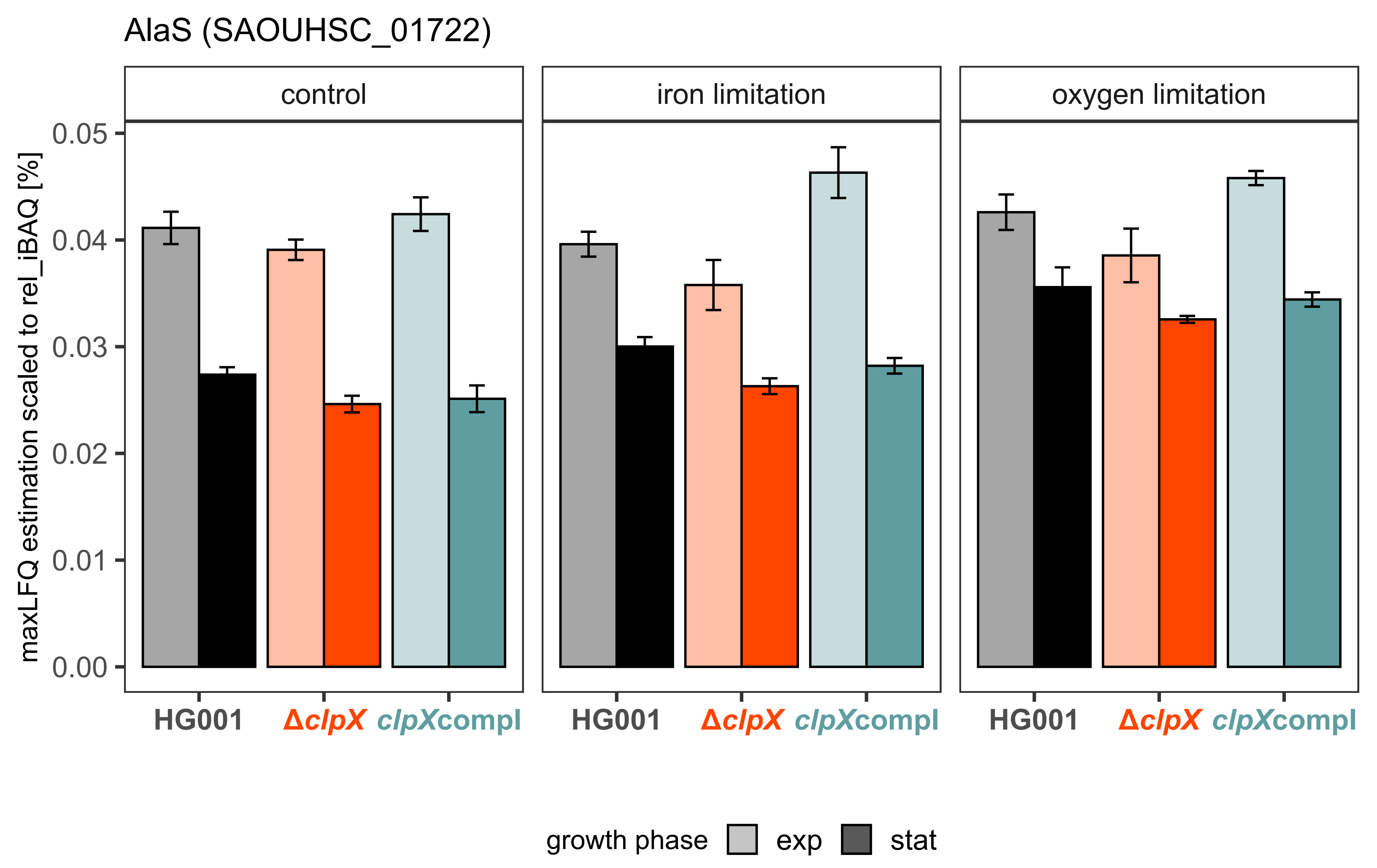

AlaS (SAOUHSC_01722)
control
iron limitation
oxygen limitation
maxLFQ estimation scaled to rel_iBAQ [%]
0.05
0.04
0.03
0.02
0.01
0.00
HG001
ΔclpX
clpXcompl
growth phase
exp
stat

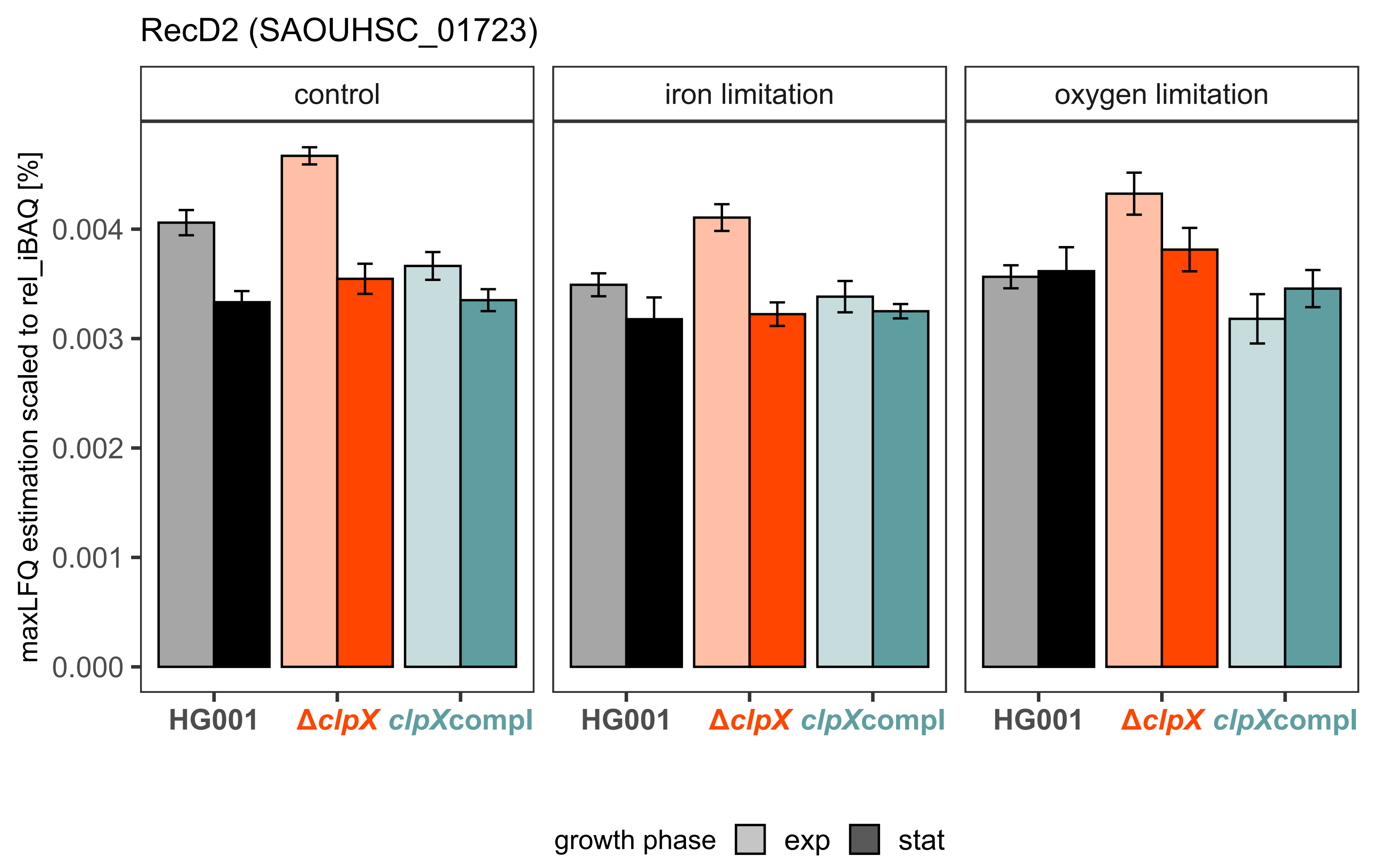
RecD2 (SAOUHSC_01723)
control
iron limitation
oxygen limitation
maxLFQ estimation scaled to rel_iBAQ [%]
0.004
0.003
0.002
0.001
0.000
HG001
ΔclpX
clpXcompl
growth phase
exp
stat

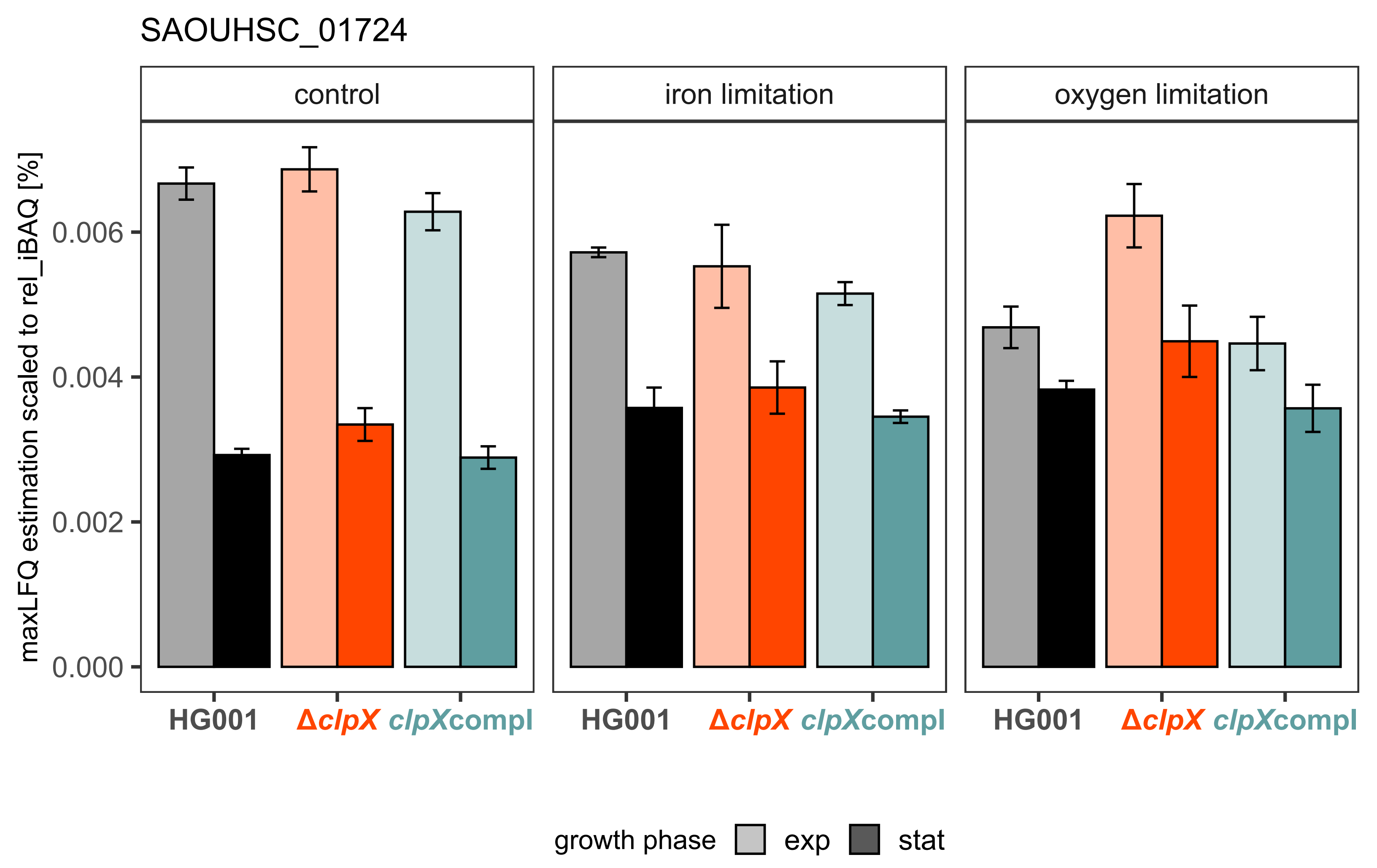

SAOUHSC_01724
control
iron limitation
oxygen limitation
maxLFQ estimation scaled to rel_iBAQ [%]
0.000
0.002
0.004
0.006
HG001
ΔclpX
clpXcompl
HG001
ΔclpX
clpXcompl
HG001
ΔclpX
clpXcompl
growth phase
exp
stat

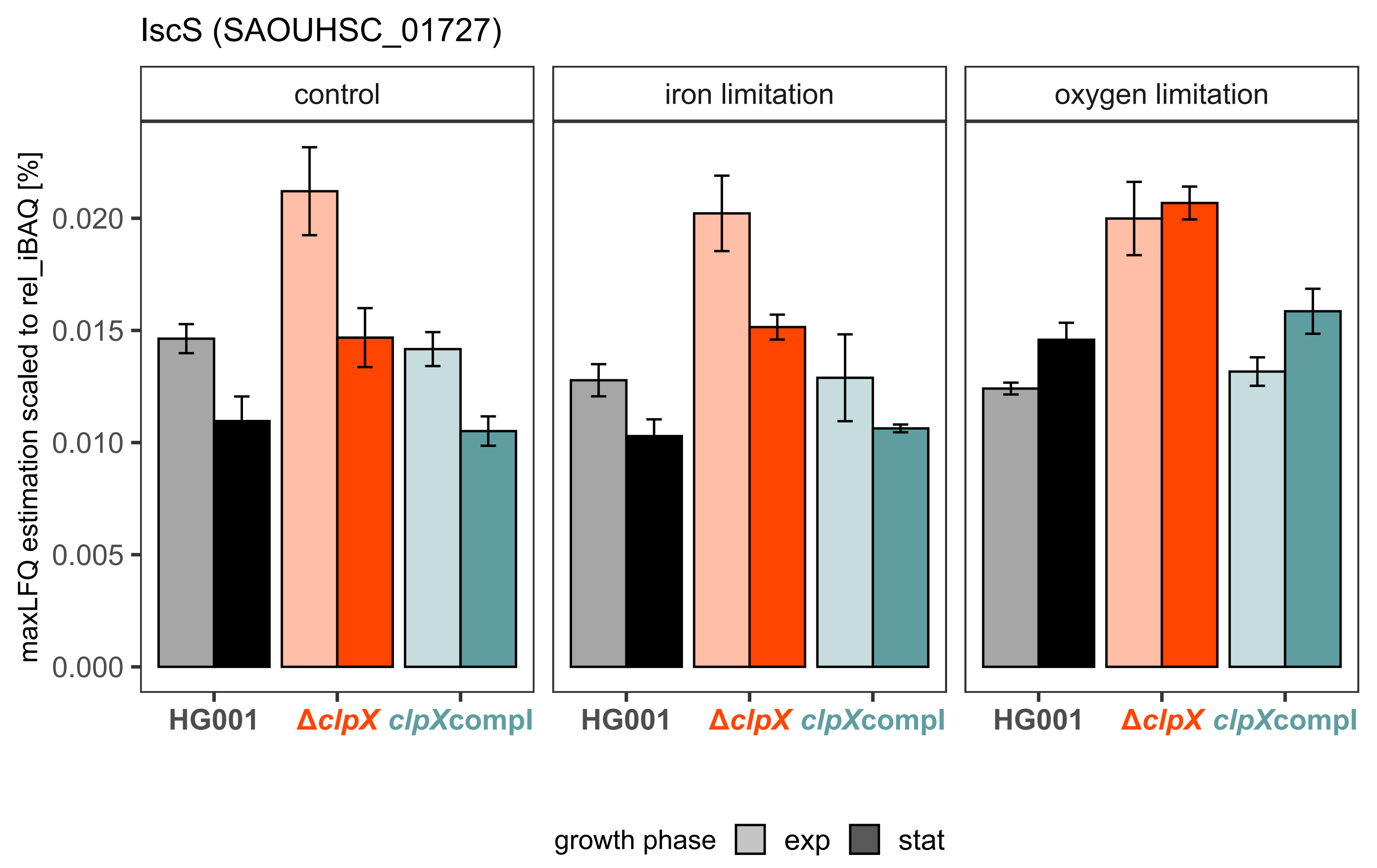
IscS (SAOUHSC_01727)
control
iron limitation
oxygen limitation
maxLFQ estimation scaled to rel_iBAQ [%]
0.020
0.015
0.010
0.005
0.000
HG001
ΔclpX
clpXcompl
growth phase
exp
stat

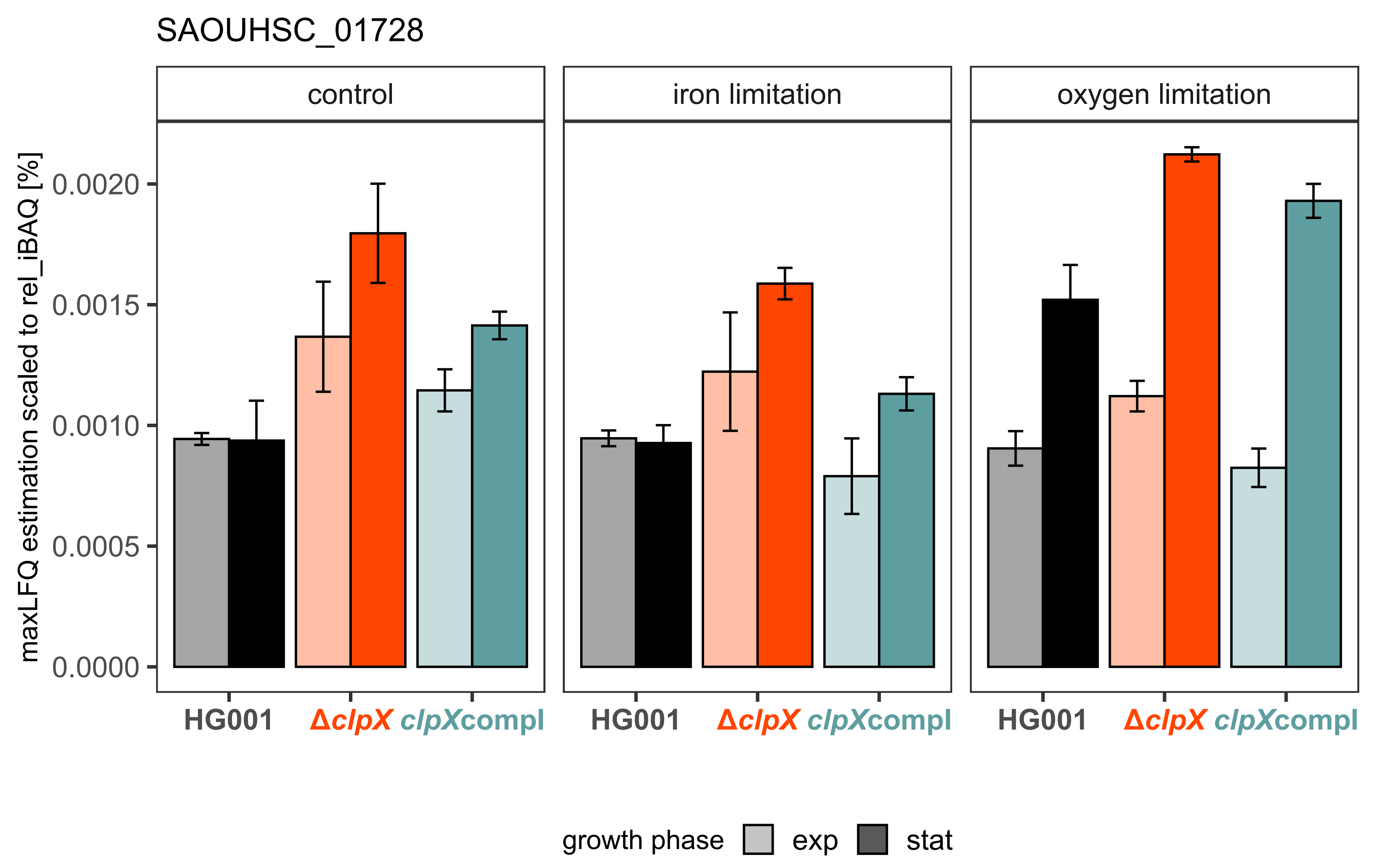

SAOUHSC_01728
control
iron limitation
oxygen limitation
maxLFQ estimation scaled to rel_iBAQ [%]
0.0020
0.0015
0.0010
0.0005
0.0000
HG001
ΔclpX
clpXcompl
growth phase
exp
stat

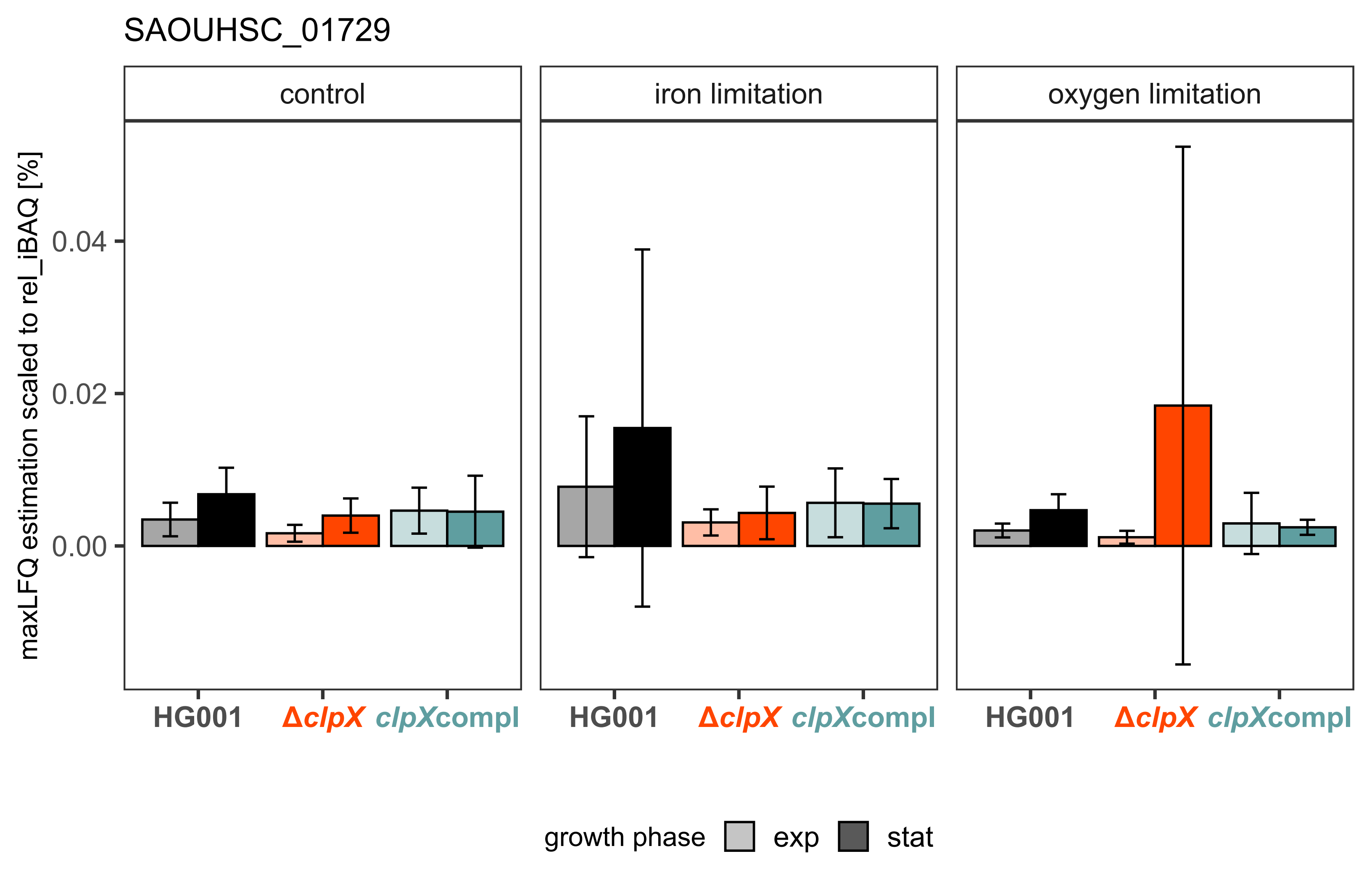
SAOUHSC_01729
control
iron limitation
oxygen limitation
maxLFQ estimation scaled to rel_iBAQ [%]
0.04
0.02
0.00
HG001
ΔclpX
clpXcompl
growth phase
exp
stat

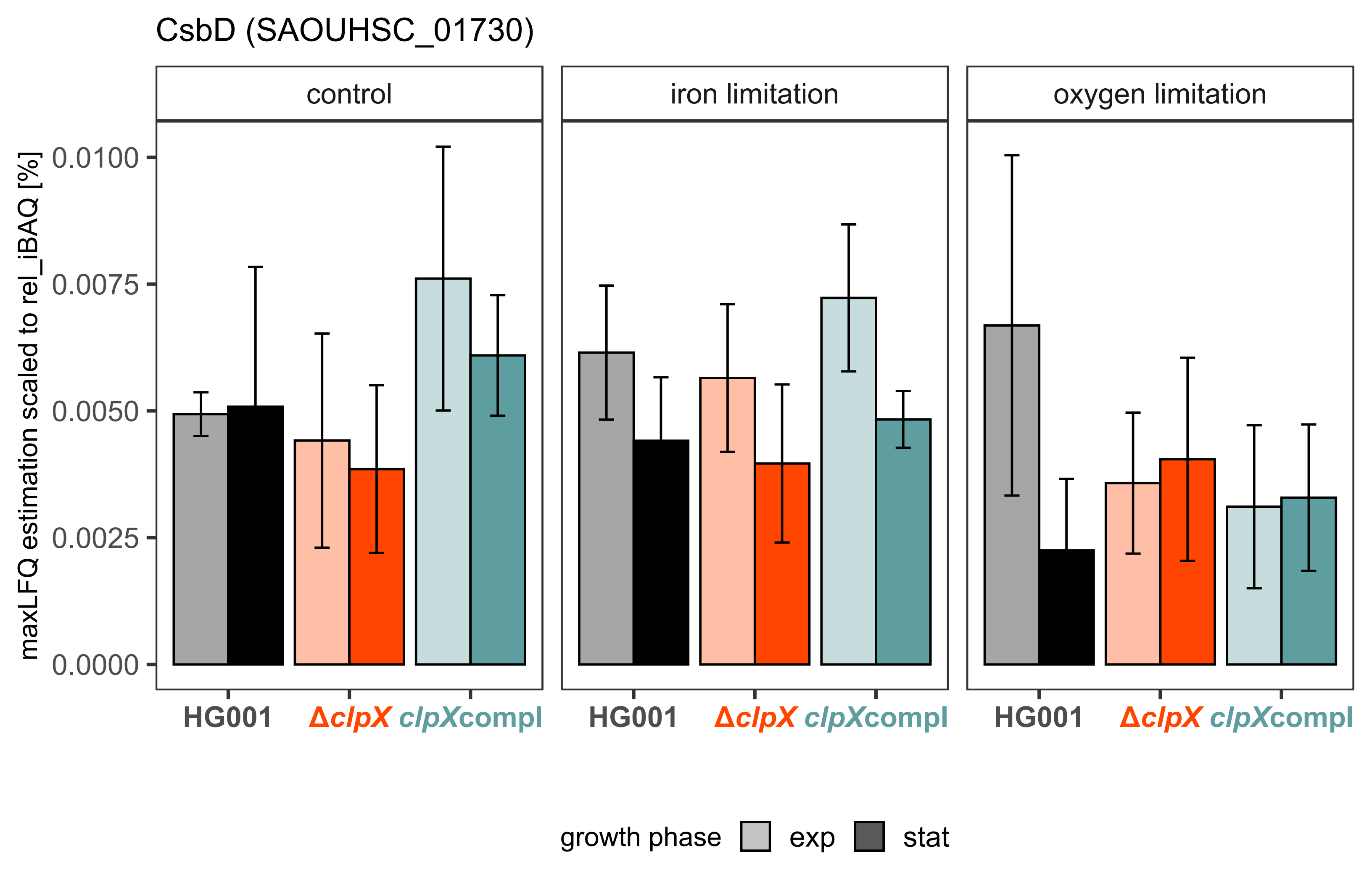

CsbD (SAOUHSC_01730)
control
iron limitation
oxygen limitation
maxLFQ estimation scaled to rel_iBAQ [%]
0.0100
0.0075
0.0050
0.0025
0.0000
HG001
ΔclpX
clpXcompl
growth phase
exp
stat

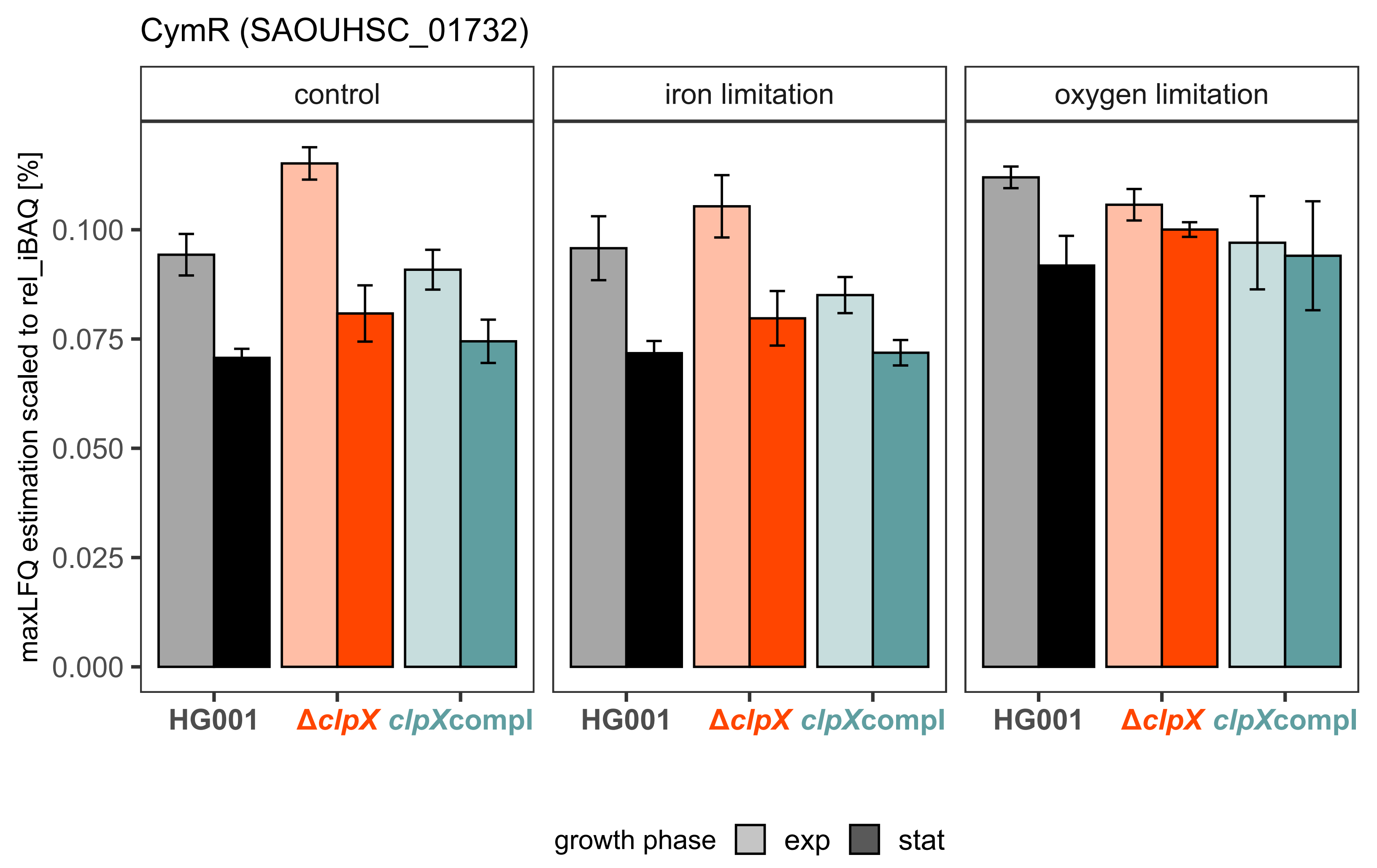

CymR (SAOUHSC_01732)
control
iron limitation
oxygen limitation
maxLFQ estimation scaled to rel_iBAQ [%]
0.000
0.025
0.050
0.075
0.100
HG001
ΔclpX
clpXcompl
growth phase
exp
stat

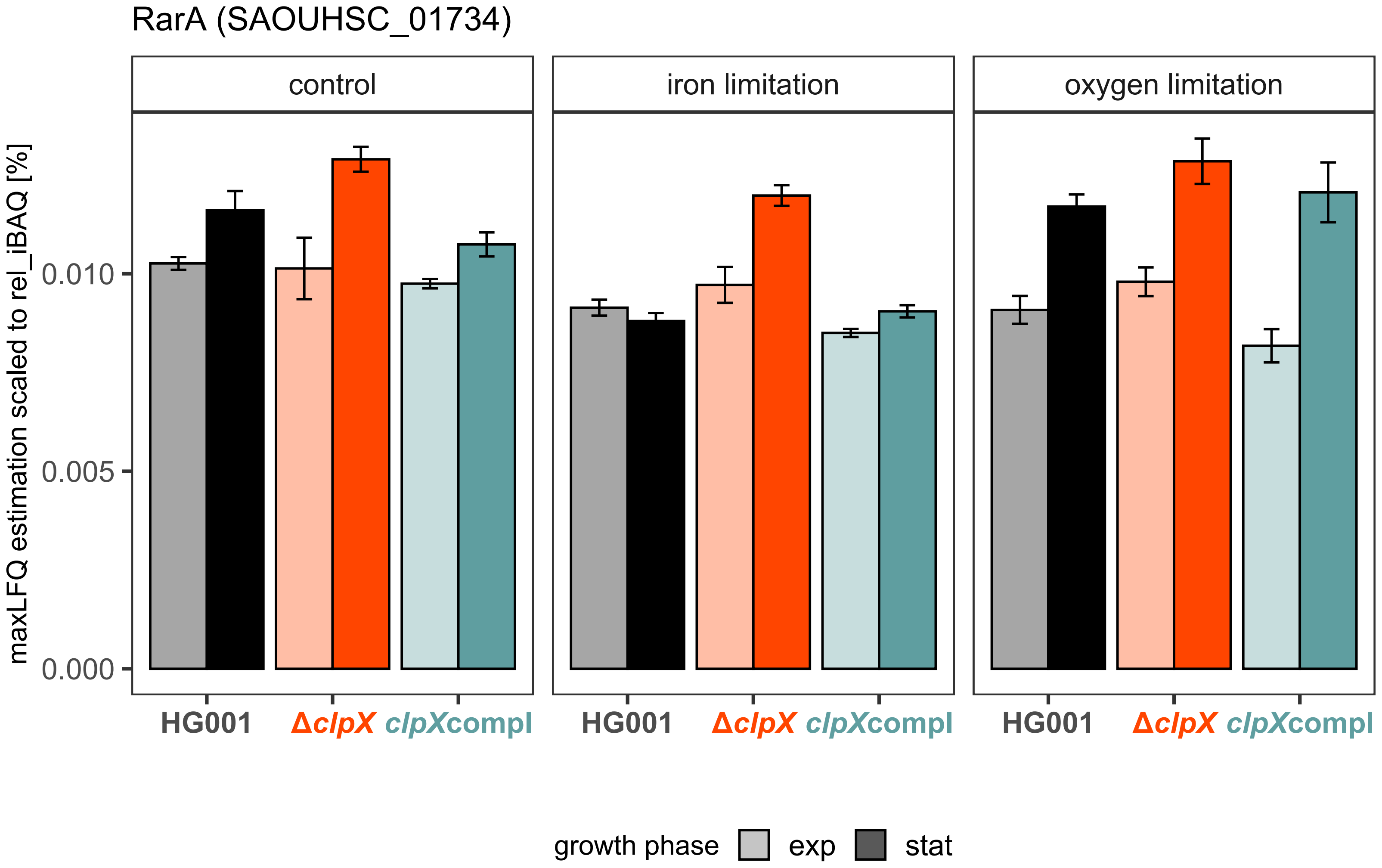
RarA (SAOUHSC_01734)
control
iron limitation
oxygen limitation
maxLFQ estimation scaled to rel_iBAQ [%]
0.010
0.005
0.000
HG001
ΔclpX
clpXcompl
growth phase
exp
stat

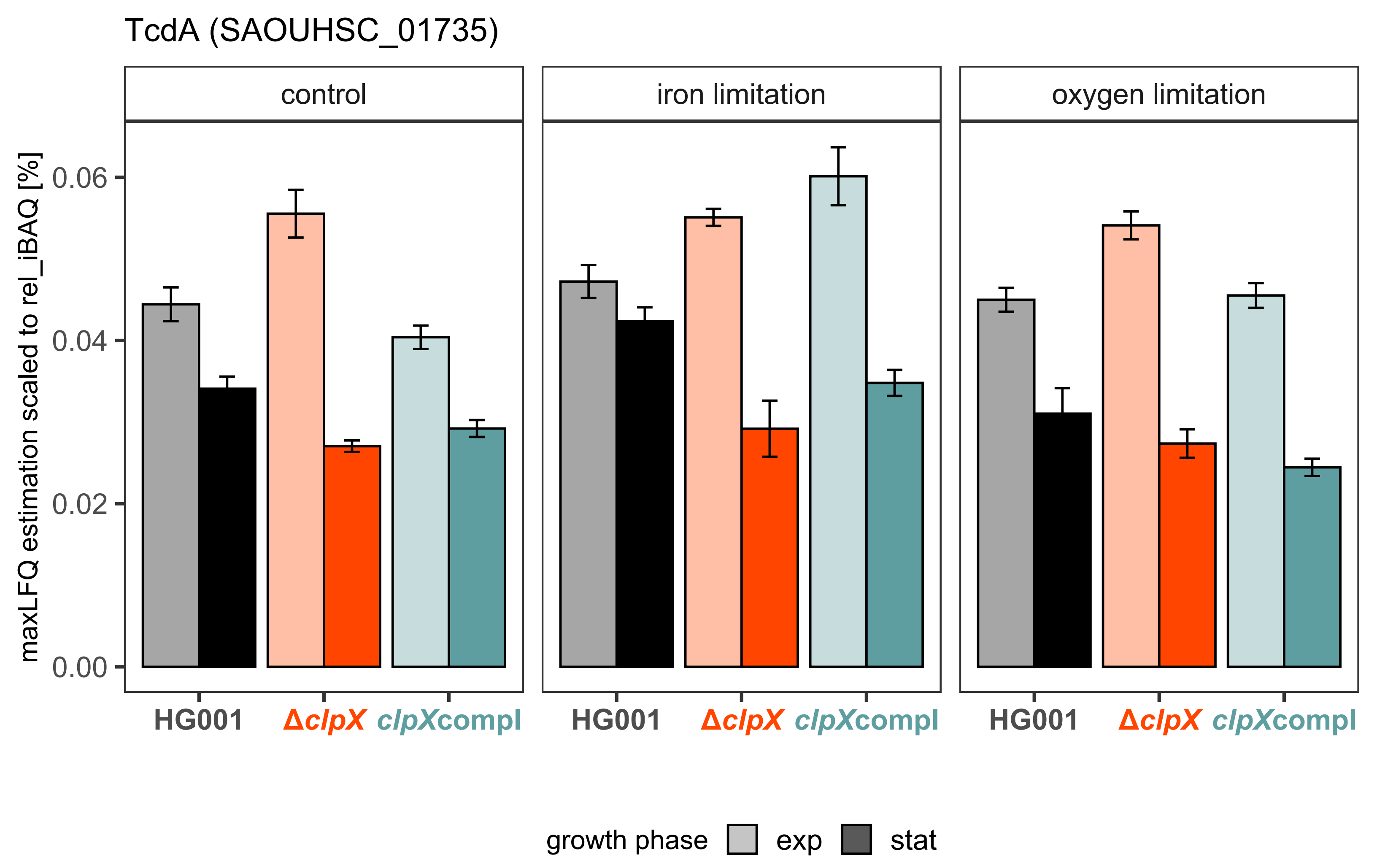
TcdA (SAOUHSC_01735)
control
iron limitation
oxygen limitation
maxLFQ estimation scaled to rel_iBAQ [%]
0.06
0.04
0.02
0.00
HG001
ΔclpX
clpXcompl
growth phase
exp
stat

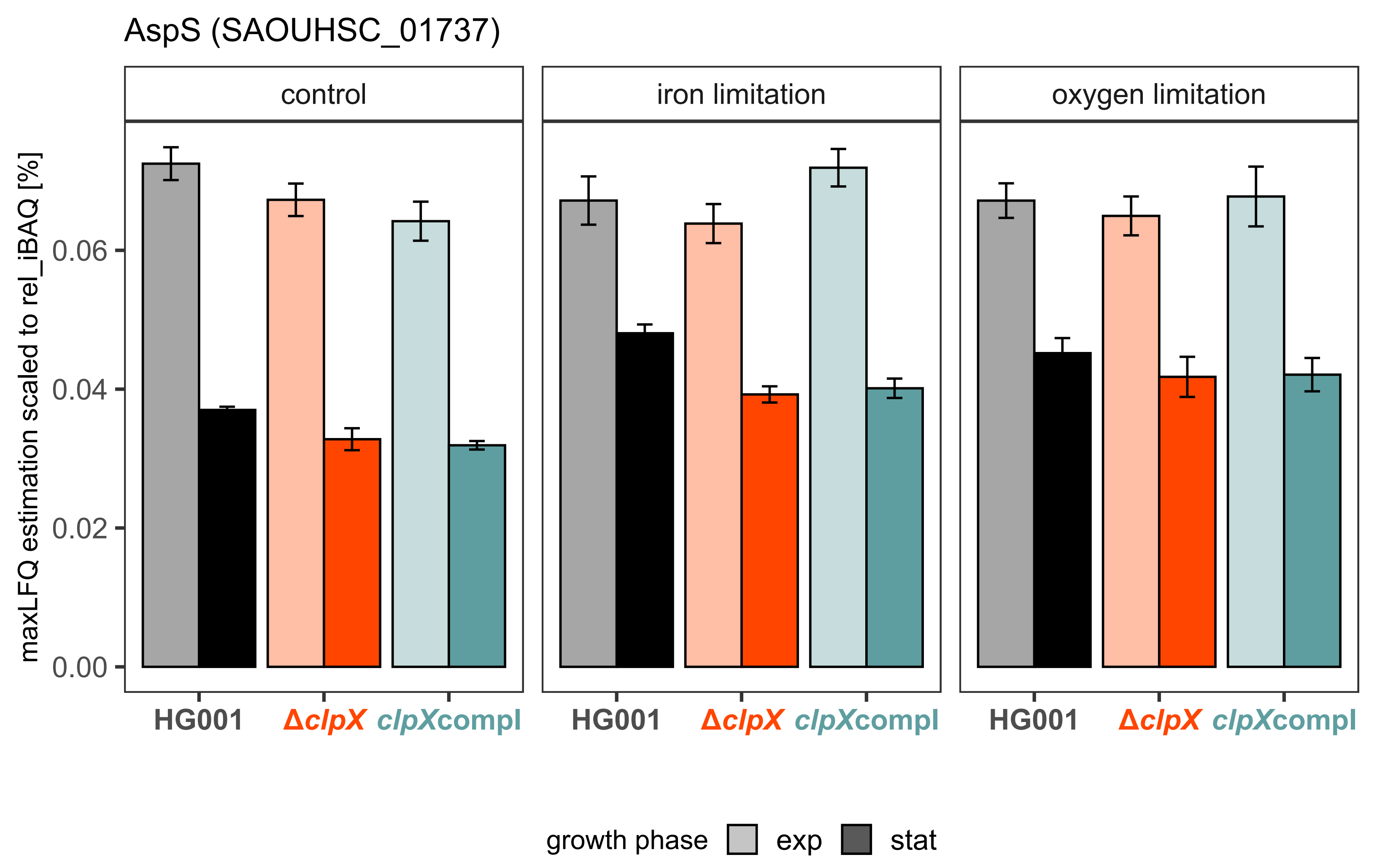
AspS (SAOUHSC_01737)
control
iron limitation
oxygen limitation
maxLFQ estimation scaled to rel_iBAQ [%]
0.00
0.02
0.04
0.06
HG001
ΔclpX
clpXcompl
growth phase
exp
stat

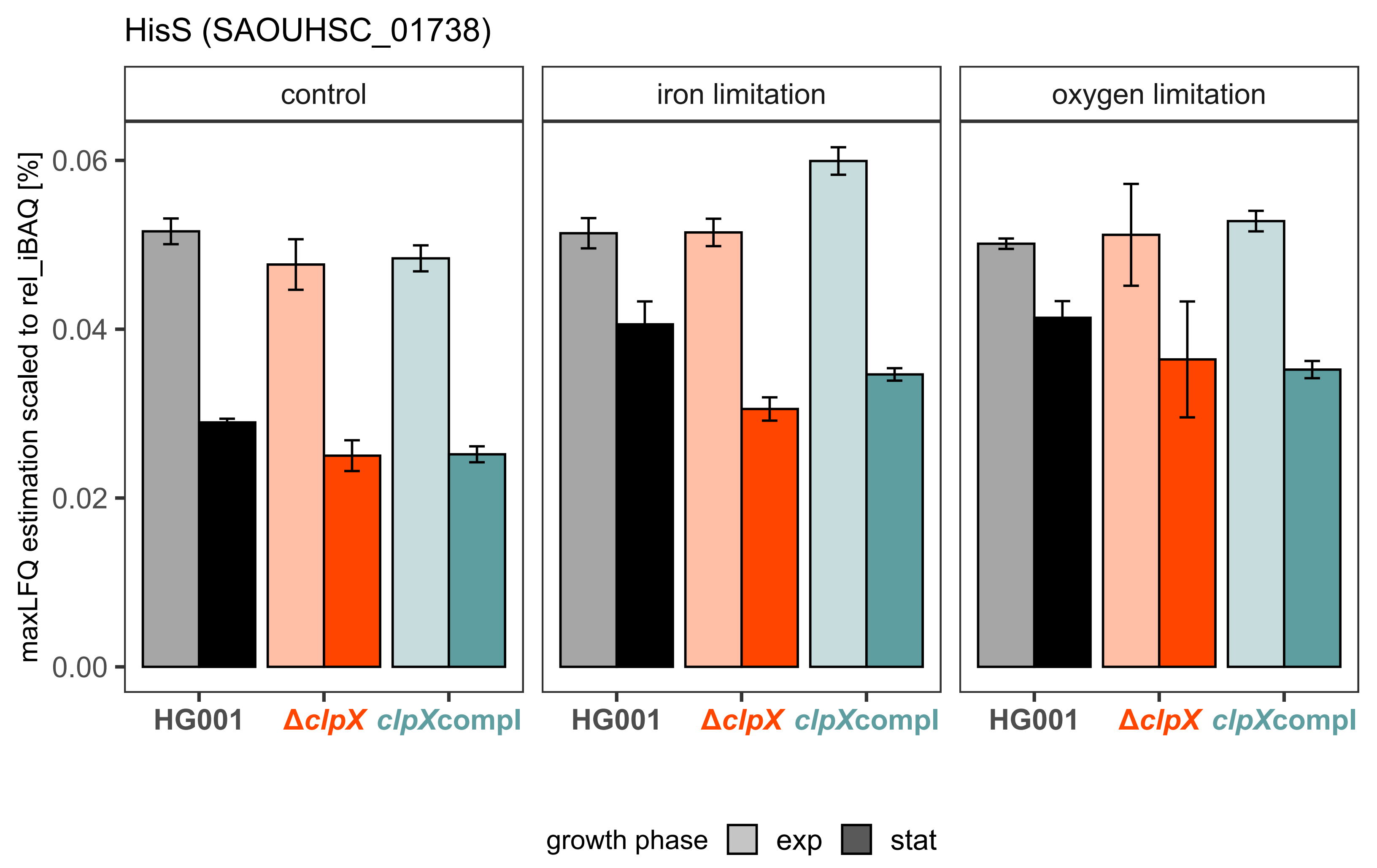
HisS (SAOUHSC_01738)
control
iron limitation
oxygen limitation
maxLFQ estimation scaled to rel_iBAQ [%]
0.06
0.04
0.02
0.00
HG001
ΔclpX
clpXcompl
growth phase
exp
stat

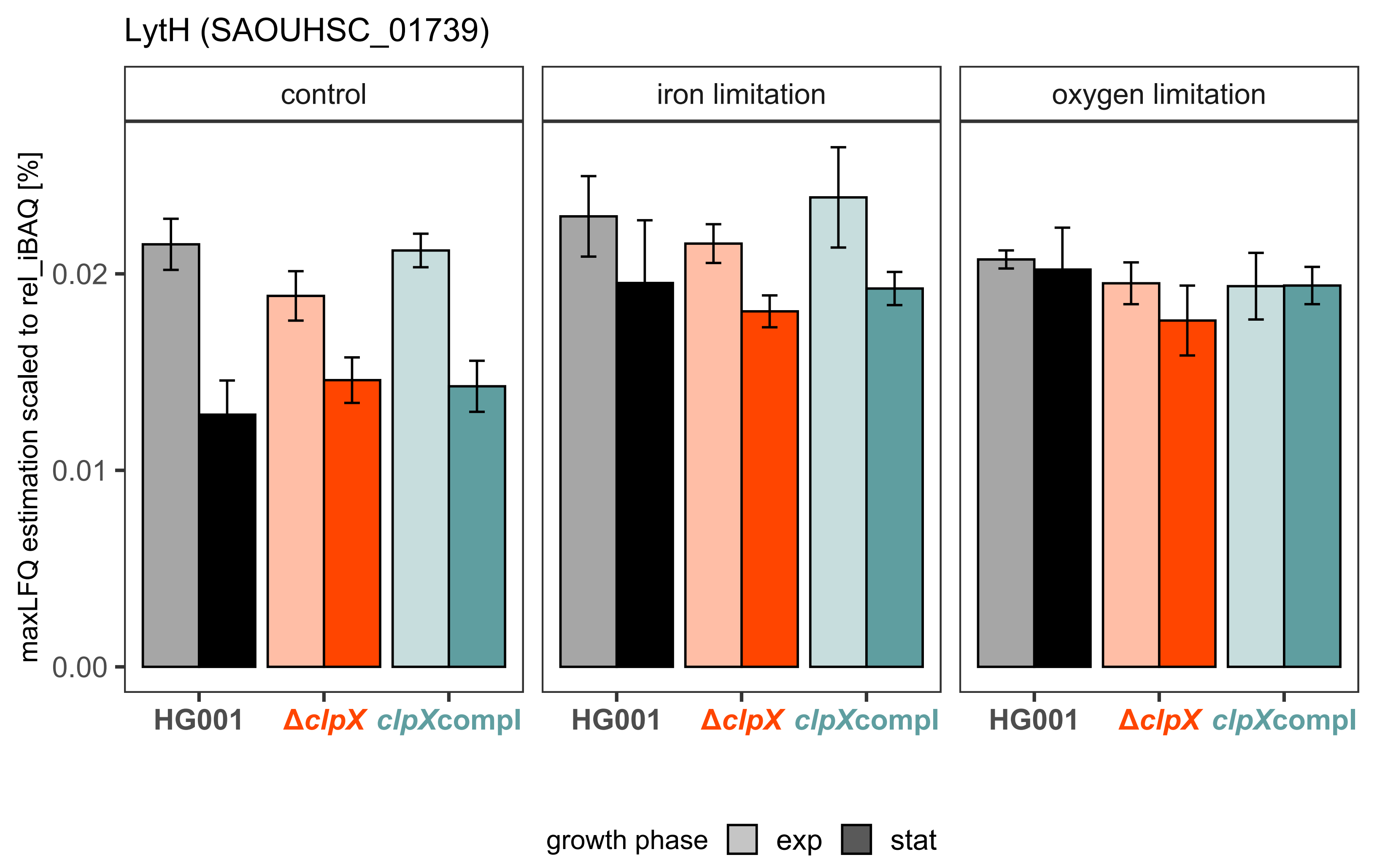

LytH (SAOUHSC_01739)
control
iron limitation
oxygen limitation
maxLFQ estimation scaled to rel_iBAQ [%]
0.00
0.01
0.02
HG001
ΔclpX
clpXcompl
growth phase
exp
stat

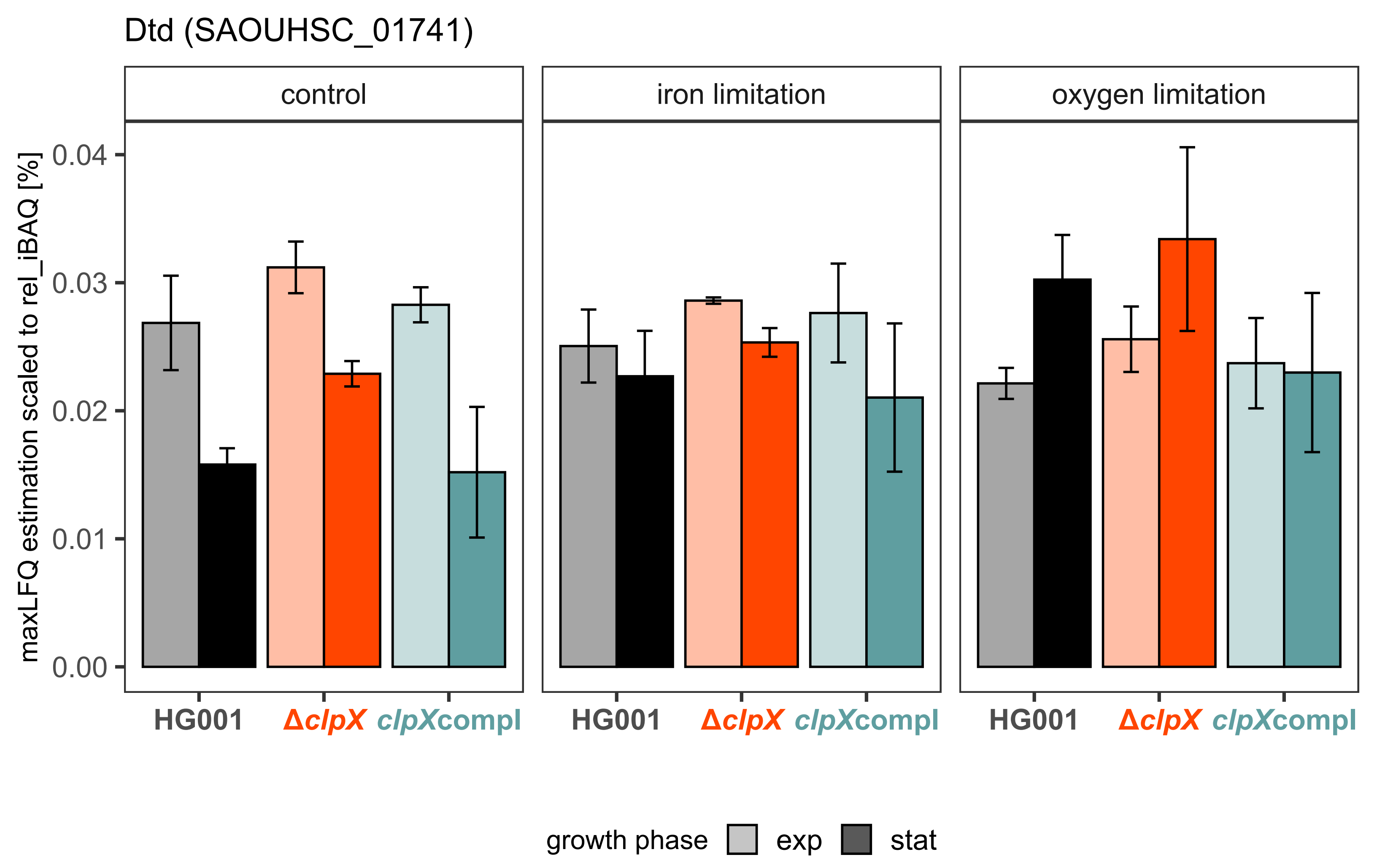
Dtd (SAOUHSC_01741)
control
iron limitation
oxygen limitation
maxLFQ estimation scaled to rel_iBAQ [%]
0.04
0.03
0.02
0.01
0.00
HG001
ΔclpX
clpXcompl
HG001
ΔclpX
clpXcompl
HG001
ΔclpX
clpXcompl
growth phase
exp
stat

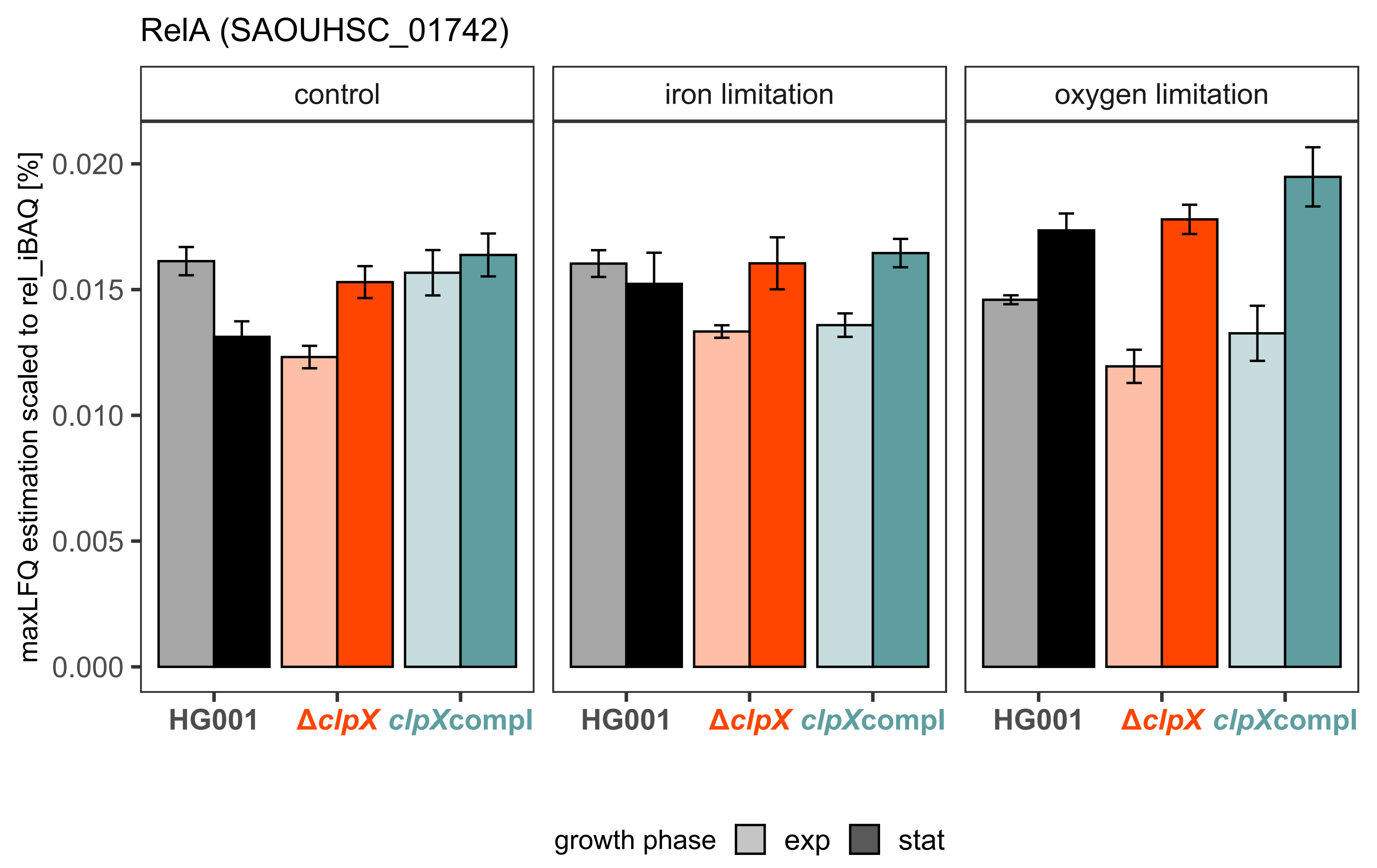

RelA (SAOUHSC_01742)
control
iron limitation
oxygen limitation
maxLFQ estimation scaled to rel_iBAQ [%]
0.020
0.015
0.010
0.005
0.000
HG001
ΔclpX
clpXcompl
growth phase
exp
stat

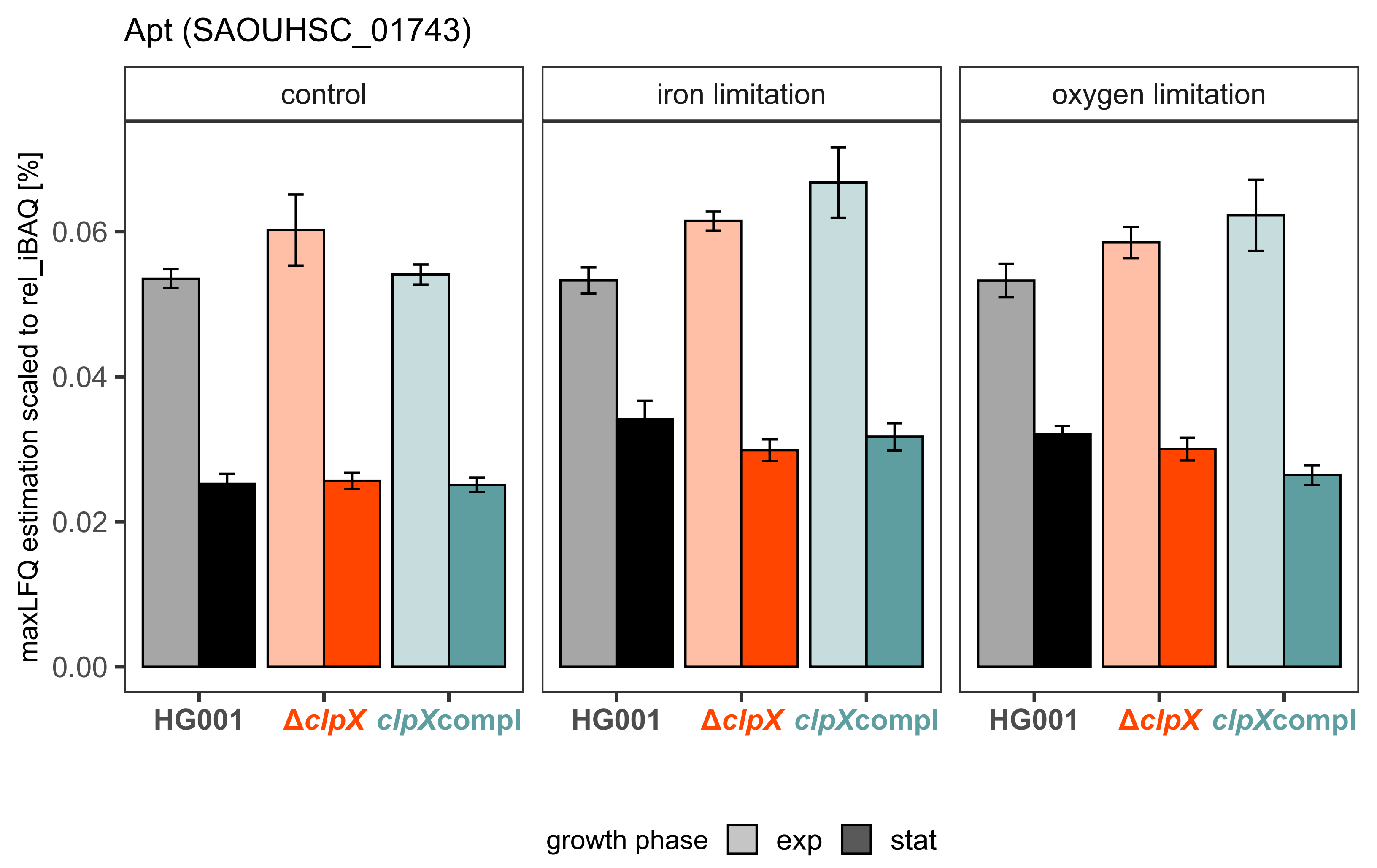

Apt (SAOUHSC_01743)
control
iron limitation
oxygen limitation
maxLFQ estimation scaled to rel_iBAQ [%]
0.06
0.04
0.02
0.00
HG001
ΔclpX
clpXcompl
growth phase
exp
stat

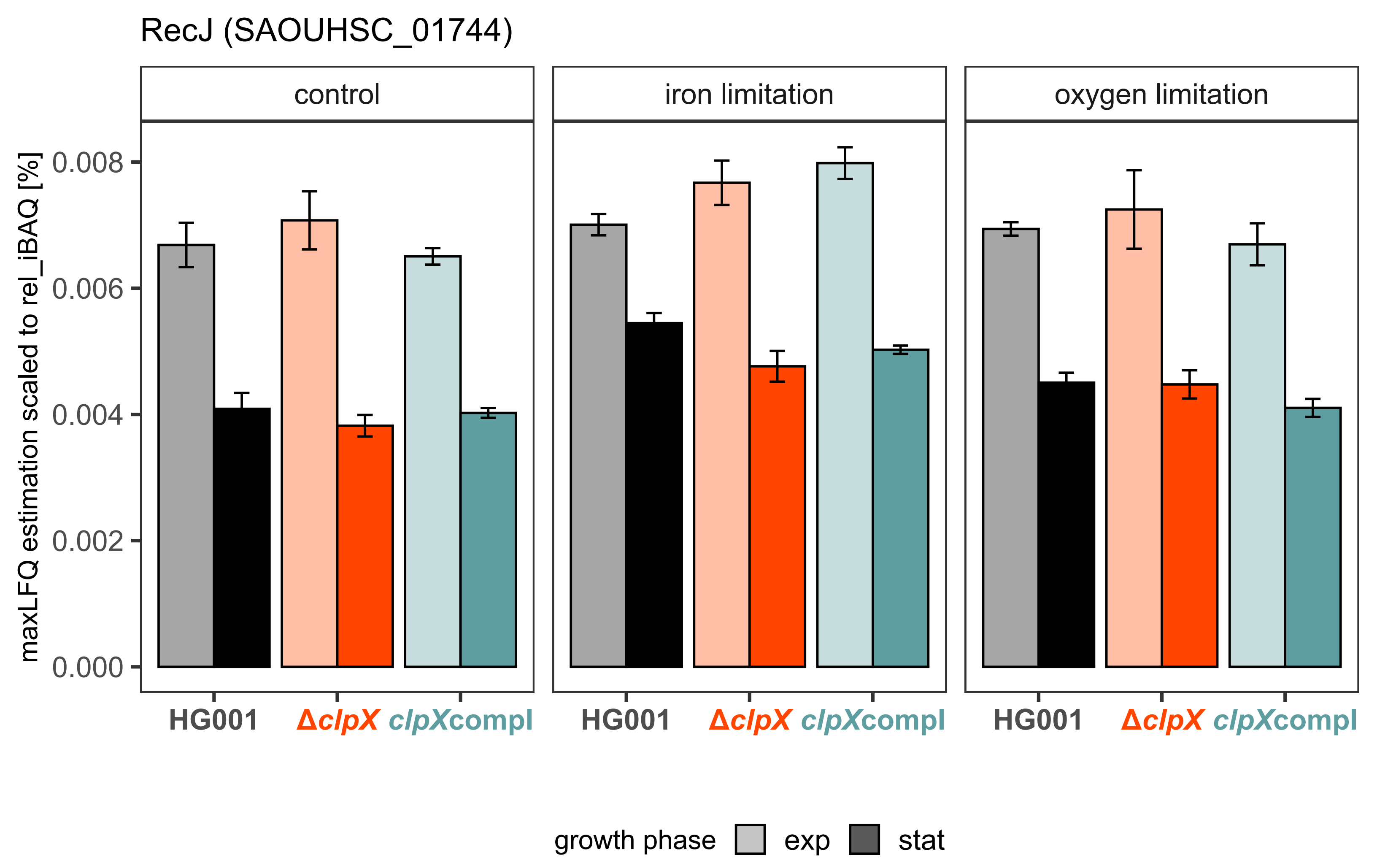

RecJ (SAOUHSC_01744)
control
iron limitation
oxygen limitation
maxLFQ estimation scaled to rel_iBAQ [%]
0.000
0.002
0.004
0.006
0.008
HG001
ΔclpX
clpXcompl
growth phase
exp
stat

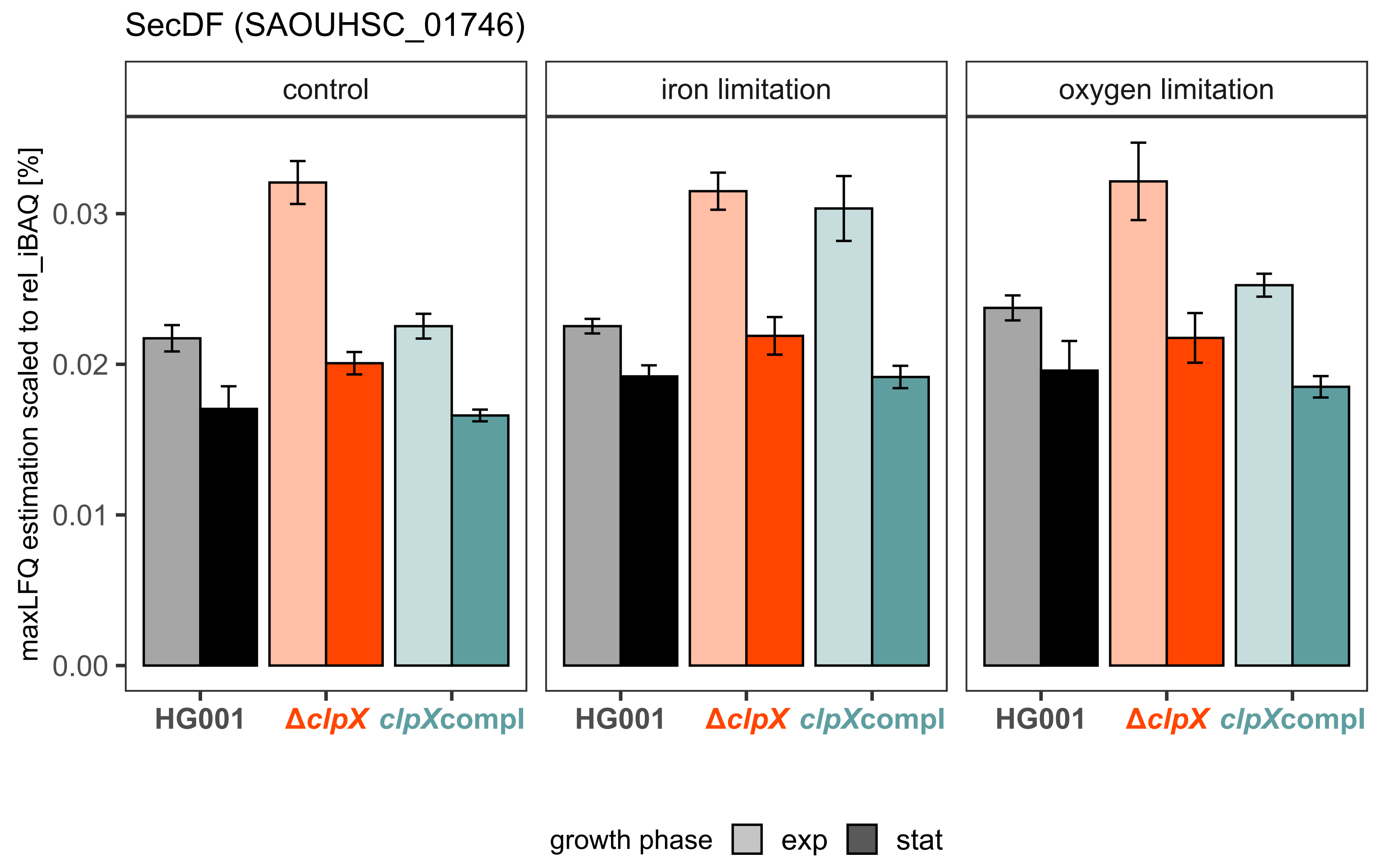

SecDF (SAOUHSC_01746)
control
iron limitation
oxygen limitation
maxLFQ estimation scaled to rel_iBAQ [%]
0.00
0.01
0.02
0.03
HG001
ΔclpX
clpXcompl
growth phase
exp
stat

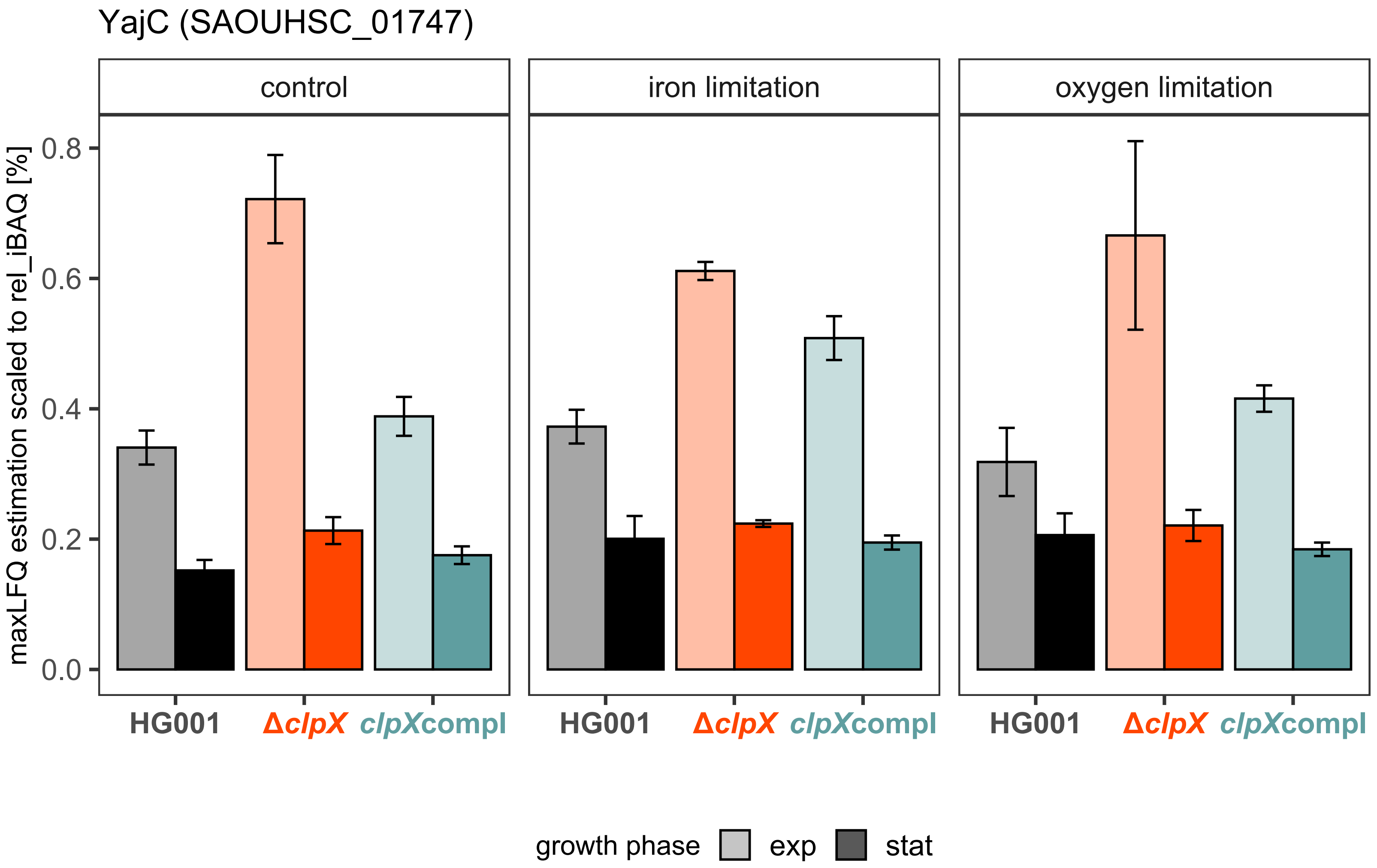

YajC (SAOUHSC_01747)
control
iron limitation
oxygen limitation
maxLFQ estimation scaled to rel_iBAQ [%]
0.0
0.2
0.4
0.6
0.8
HG001
ΔclpX
clpXcompl
growth phase
exp
stat

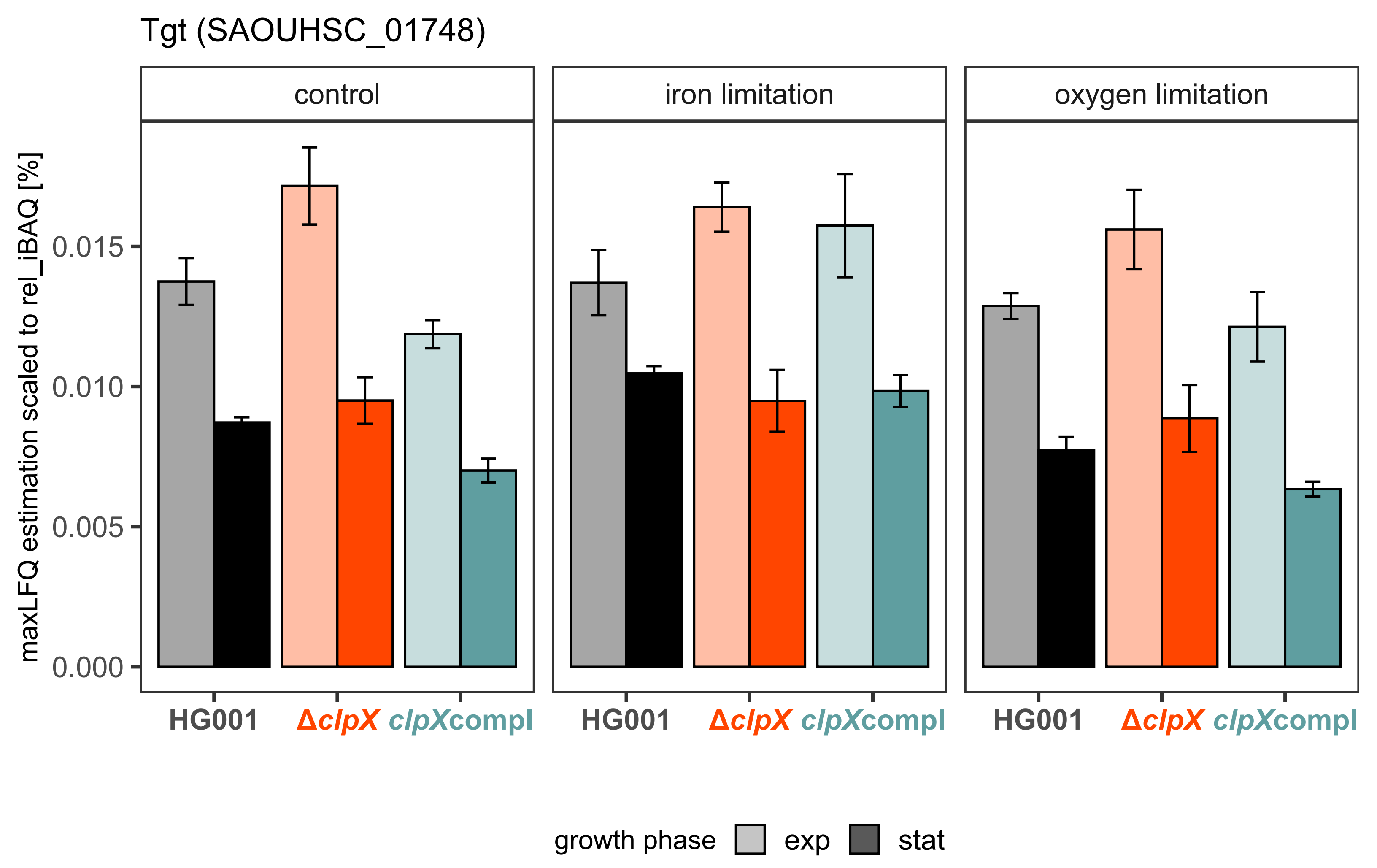

Tgt (SAOUHSC_01748)
control
iron limitation
oxygen limitation
maxLFQ estimation scaled to rel_iBAQ [%]
0.000
0.005
0.010
0.015
HG001
ΔclpX
clpXcompl
growth phase
exp
stat

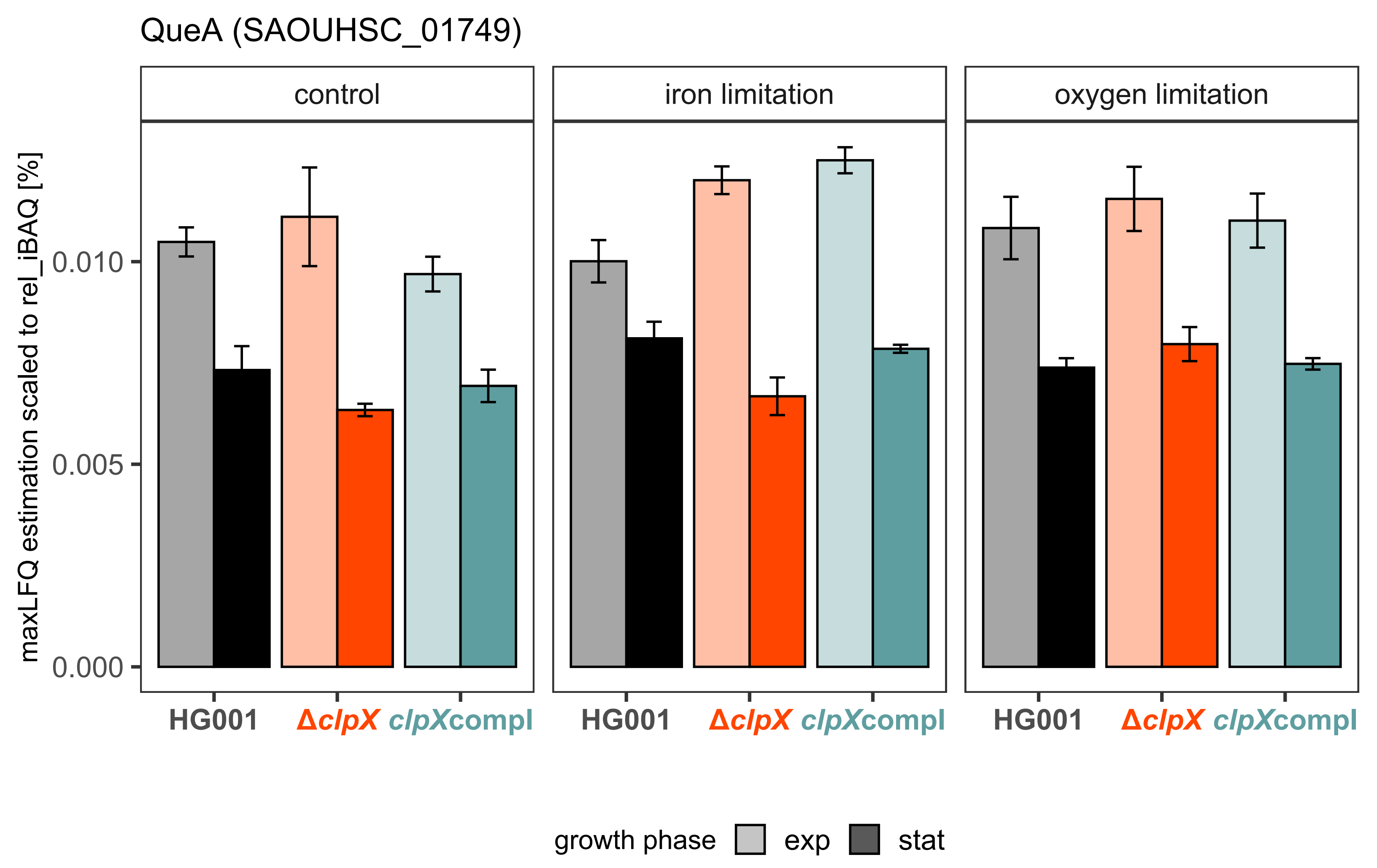

QueA (SAOUHSC_01749)
control
iron limitation
oxygen limitation
maxLFQ estimation scaled to rel_iBAQ [%]
0.010
0.005
0.000
HG001
ΔclpX
clpXcompl
growth phase
exp
stat

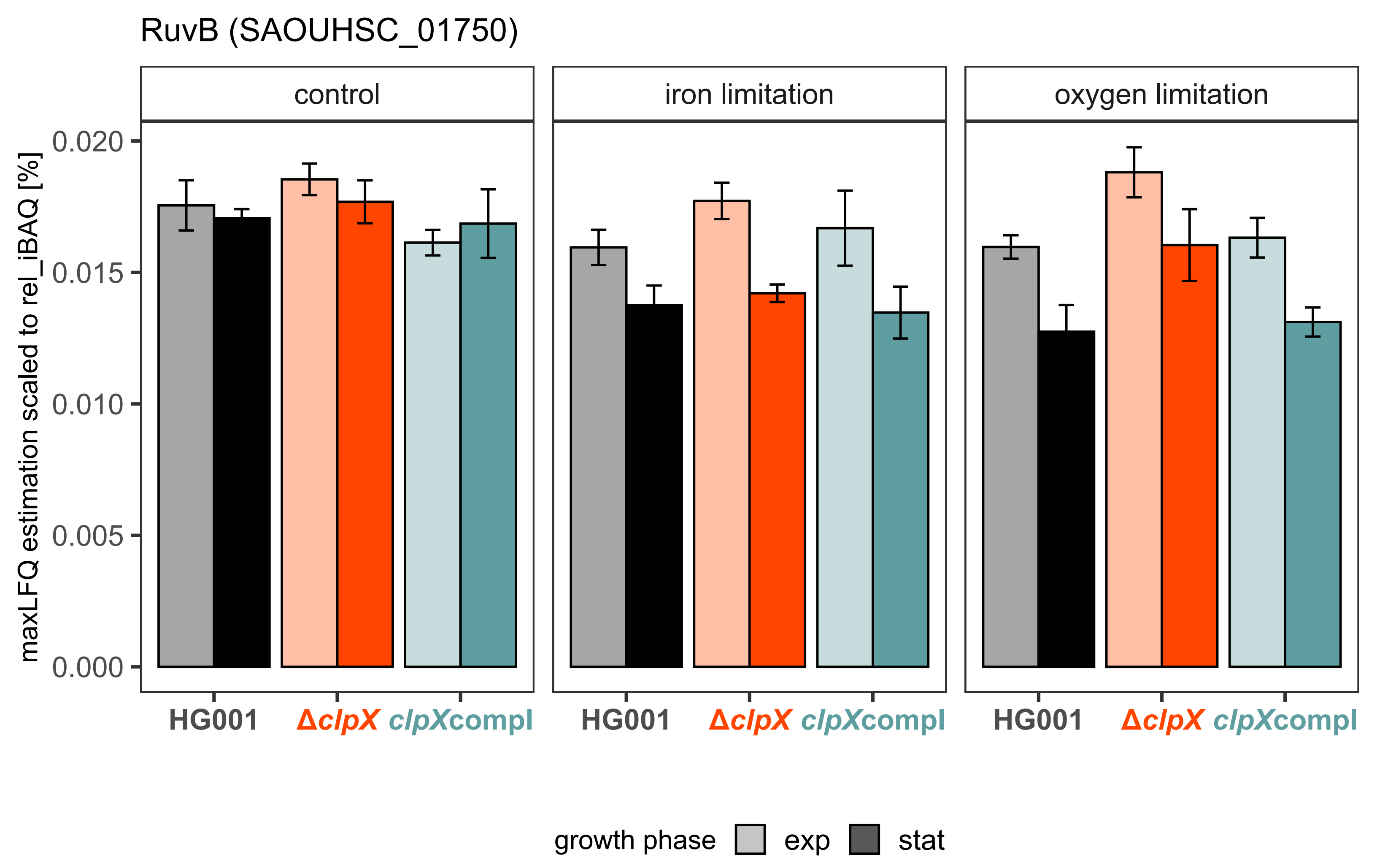
RuvB (SAOUHSC_01750)
control
iron limitation
oxygen limitation
maxLFQ estimation scaled to rel_iBAQ [%]
0.020
0.015
0.010
0.005
0.000
HG001
ΔclpX
clpXcompl
growth phase
exp
stat

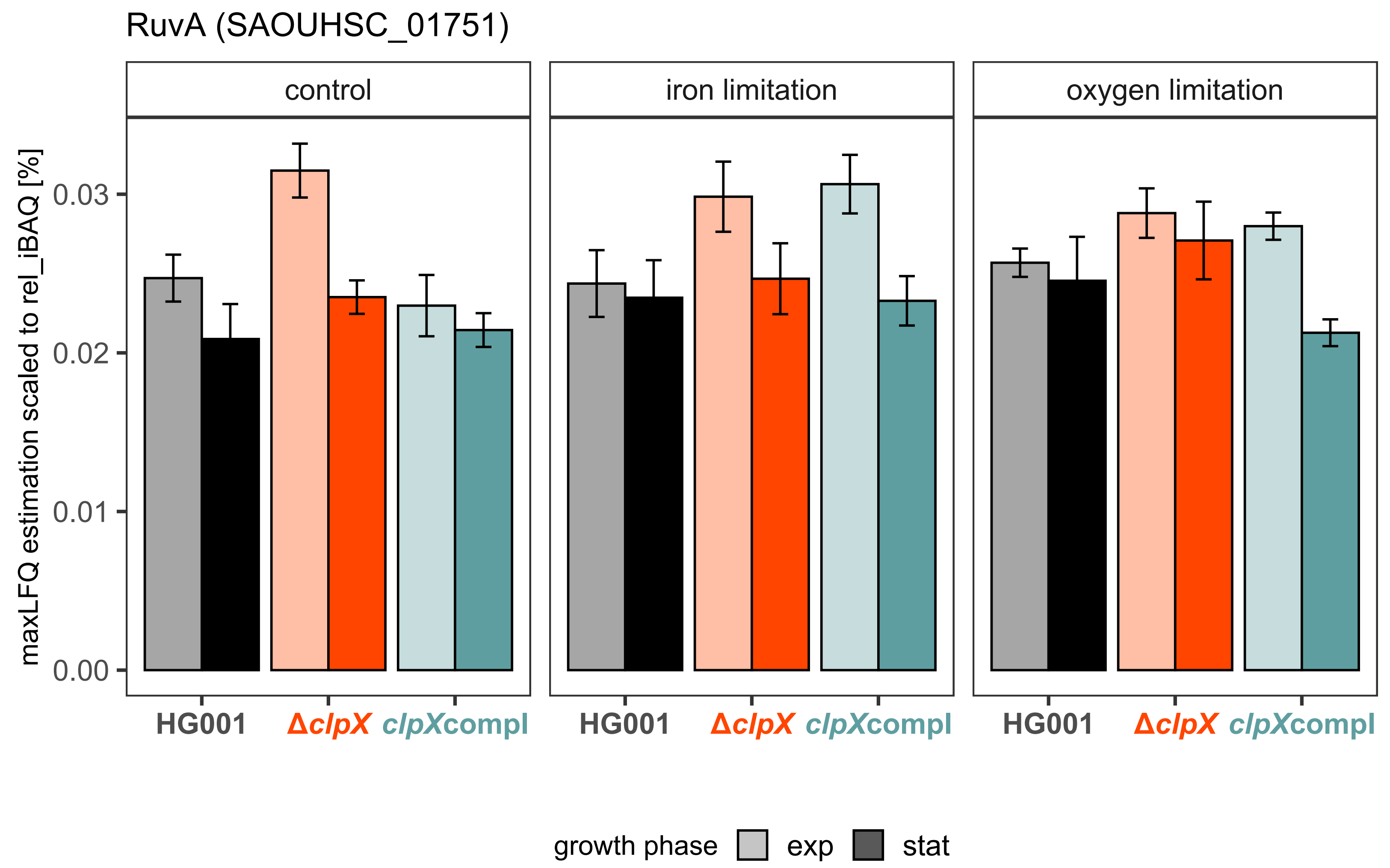

RuvA (SAOUHSC_01751)
control
iron limitation
oxygen limitation
maxLFQ estimation scaled to rel_iBAQ [%]
0.03
0.02
0.01
0.00
HG001
ΔclpX
clpXcompl
HG001
ΔclpX
clpXcompl
HG001
ΔclpX
clpXcompl
growth phase
exp
stat

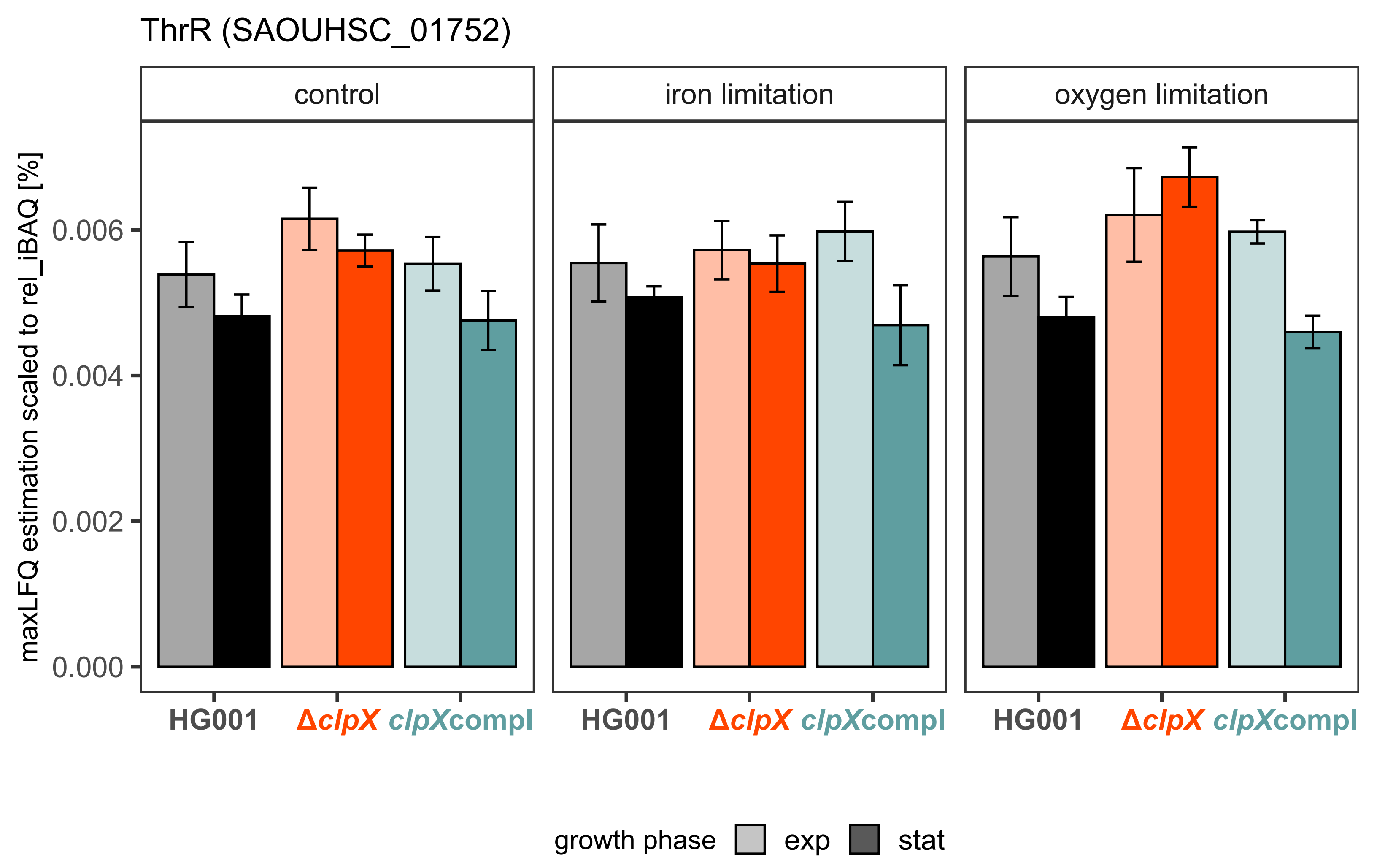
ThrR (SAOUHSC_01752)
control
iron limitation
oxygen limitation
maxLFQ estimation scaled to rel_iBAQ [%]
0.006
0.004
0.002
0.000
HG001
ΔclpX
clpXcompl
growth phase
exp
stat

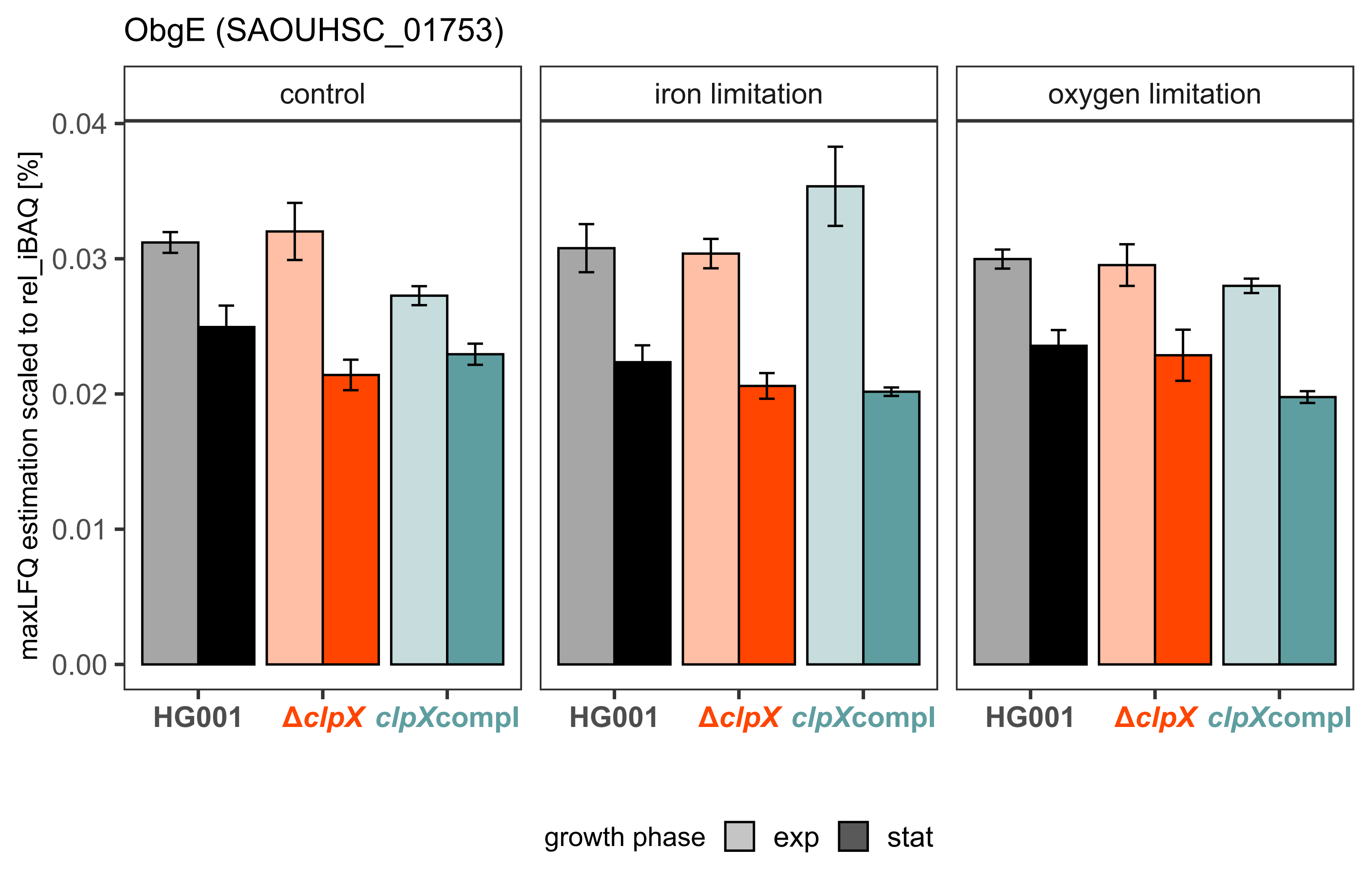

ObgE (SAOUHSC_01753)
control
iron limitation
oxygen limitation
maxLFQ estimation scaled to rel_iBAQ [%]
0.04
0.03
0.02
0.01
0.00
HG001
ΔclpX
clpXcompl
growth phase
exp
stat

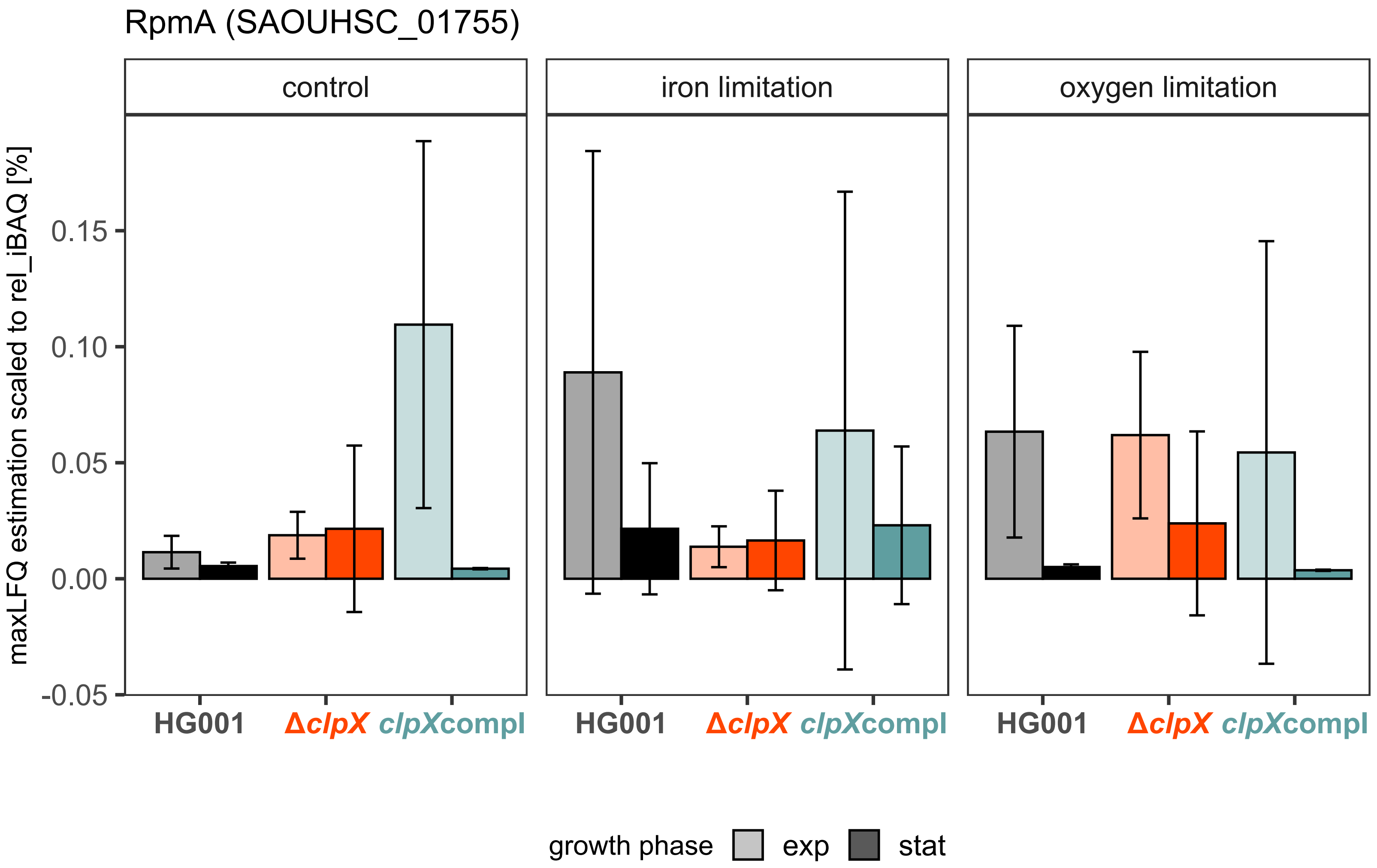

RpmA (SAOUHSC_01755)
control
iron limitation
oxygen limitation
maxLFQ estimation scaled to rel_iBAQ [%]
0.15
0.10
0.05
0.00
-0.05
HG001
ΔclpX
clpXcompl
growth phase
exp
stat

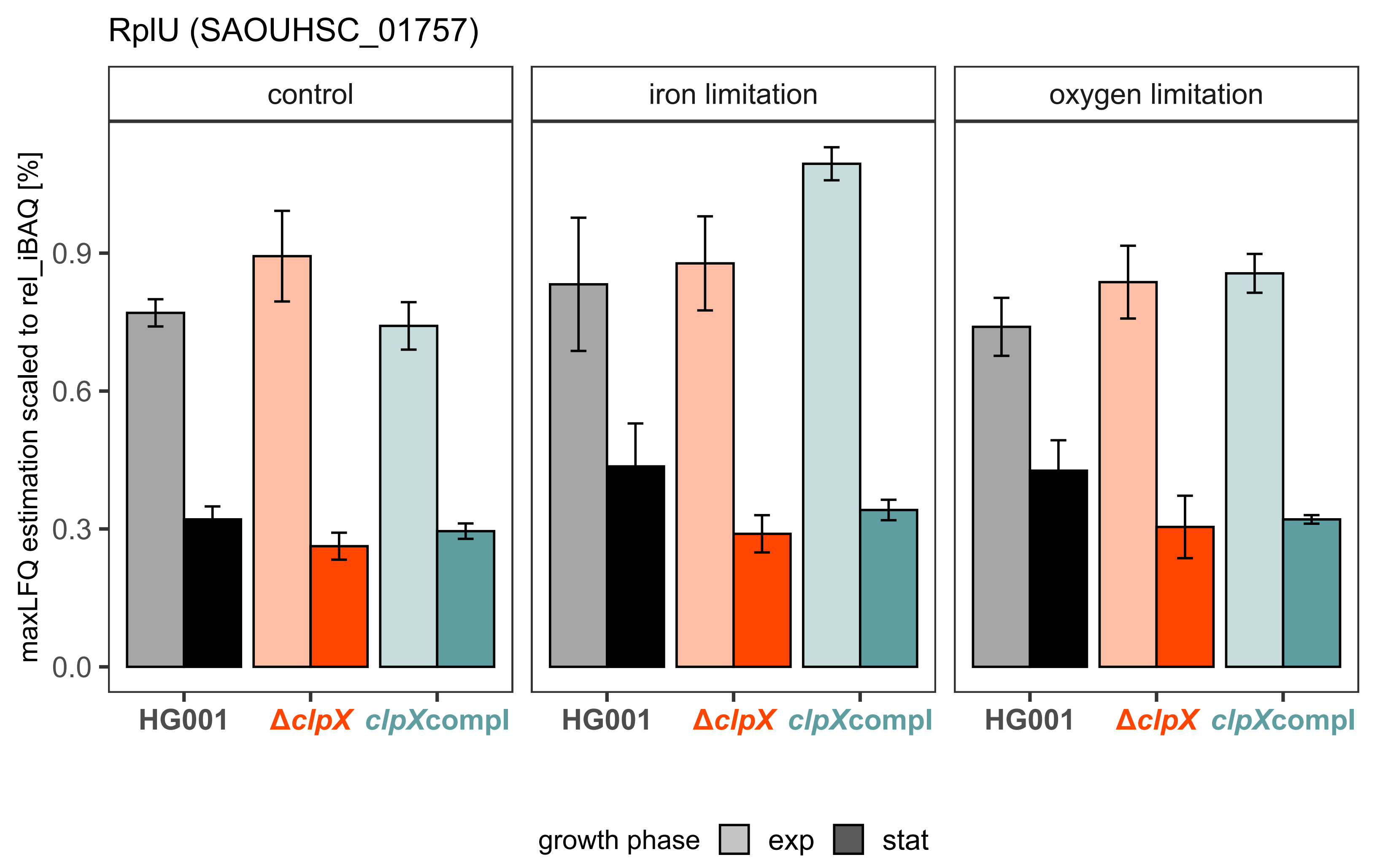
RplU (SAOUHSC_01757)
control
iron limitation
oxygen limitation
maxLFQ estimation scaled to rel_iBAQ [%]
0.0
0.3
0.6
0.9
HG001
ΔclpX
clpXcompl
growth phase
exp
stat

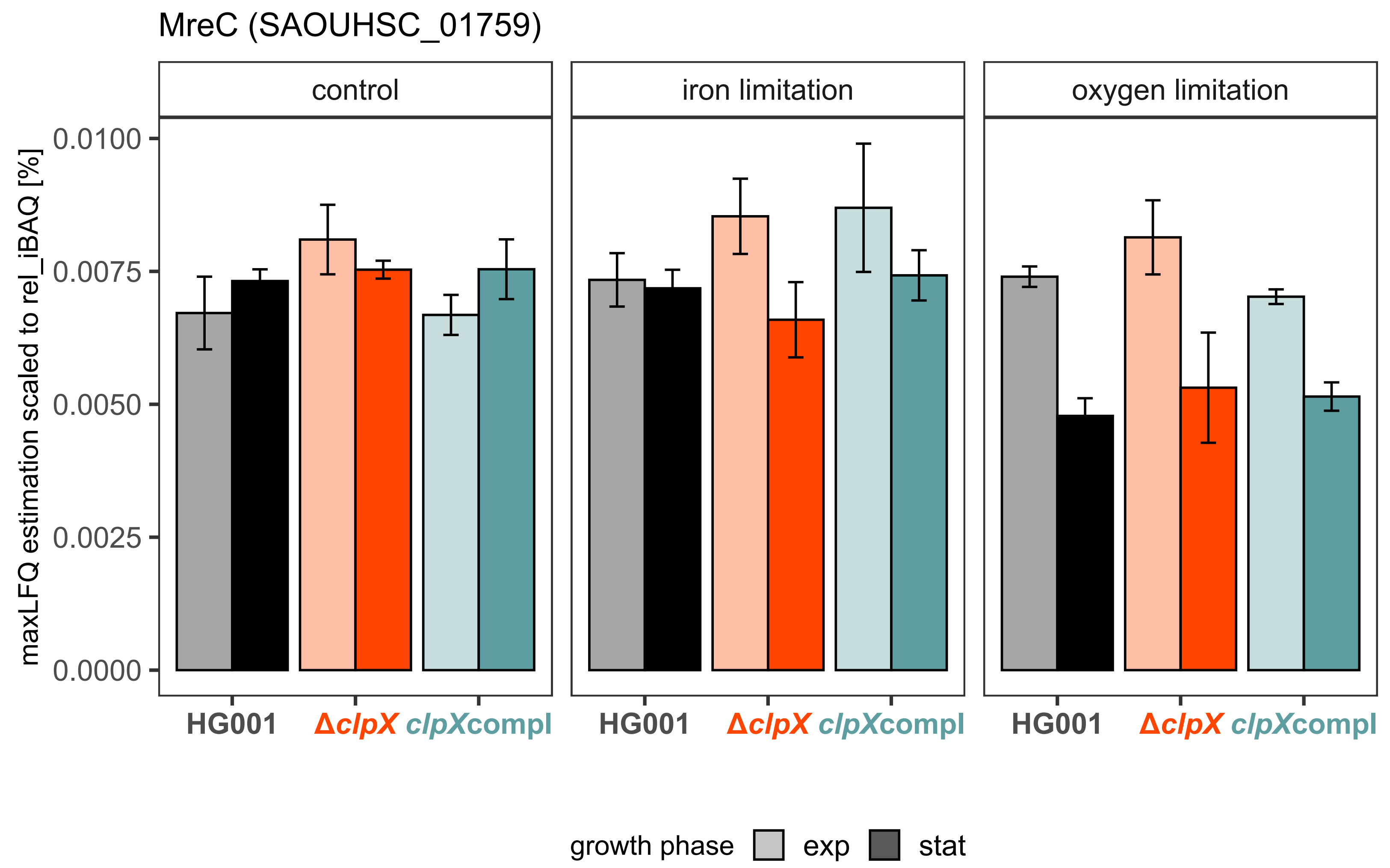
MreC (SAOUHSC_01759)
control
iron limitation
oxygen limitation
maxLFQ estimation scaled to rel_iBAQ [%]
0.0100
0.0075
0.0050
0.0025
0.0000
HG001
ΔclpX
clpXcompl
growth phase
exp
stat

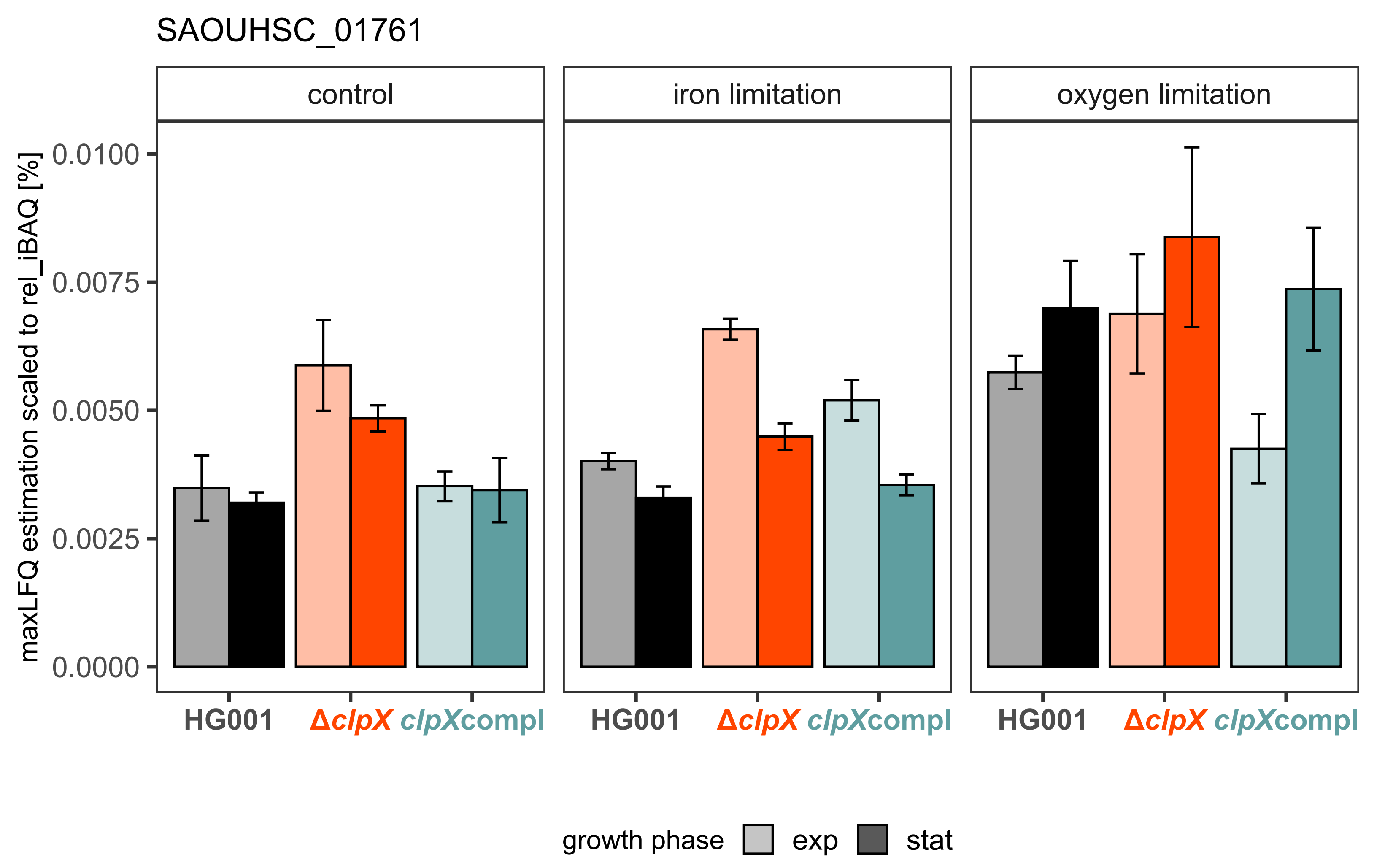
SAOUHSC_01761
control
iron limitation
oxygen limitation
maxLFQ estimation scaled to rel_iBAQ [%]
0.0100
0.0075
0.0050
0.0025
0.0000
HG001
ΔclpX
clpXcompl
growth phase
exp
stat

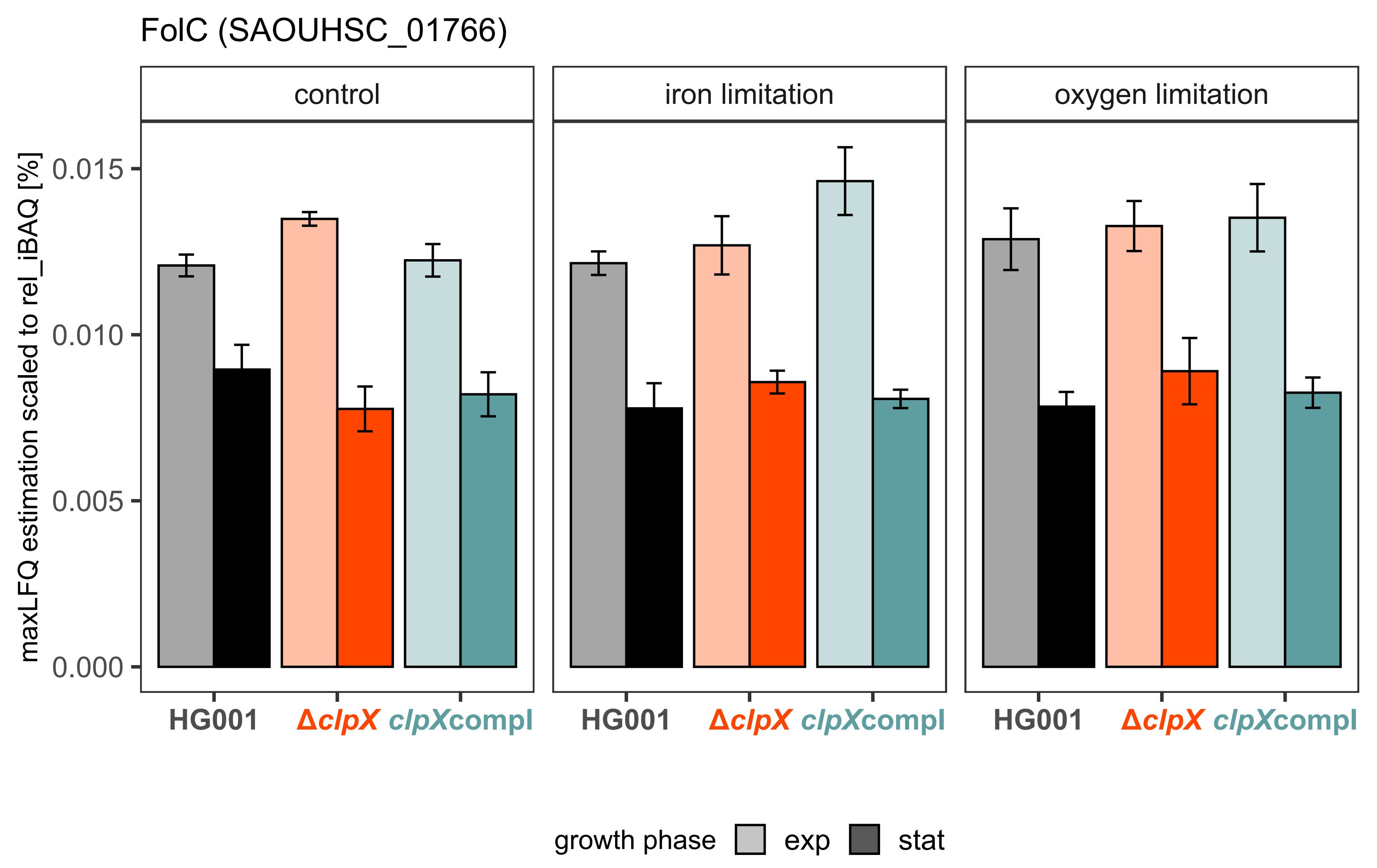

FolC (SAOUHSC_01766)
control
iron limitation
oxygen limitation
maxLFQ estimation scaled to rel_iBAQ [%]
0.015
0.010
0.005
0.000
HG001
ΔclpX
clpXcompl
growth phase
exp
stat

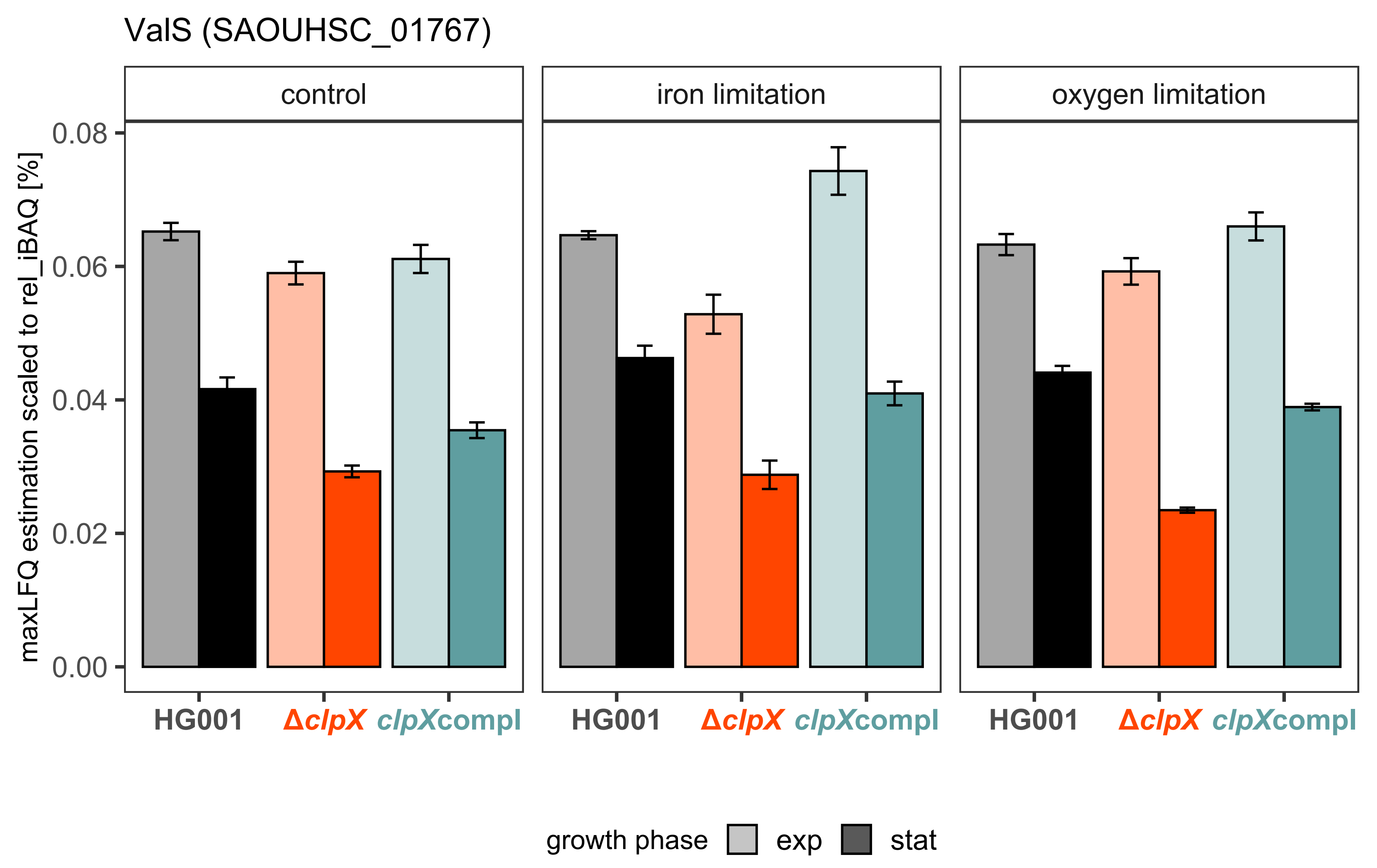
ValS (SAOUHSC_01767)
control
iron limitation
oxygen limitation
maxLFQ estimation scaled to rel_iBAQ [%]
0.08
0.06
0.04
0.02
0.00
HG001
ΔclpX
clpXcompl
growth phase
exp
stat

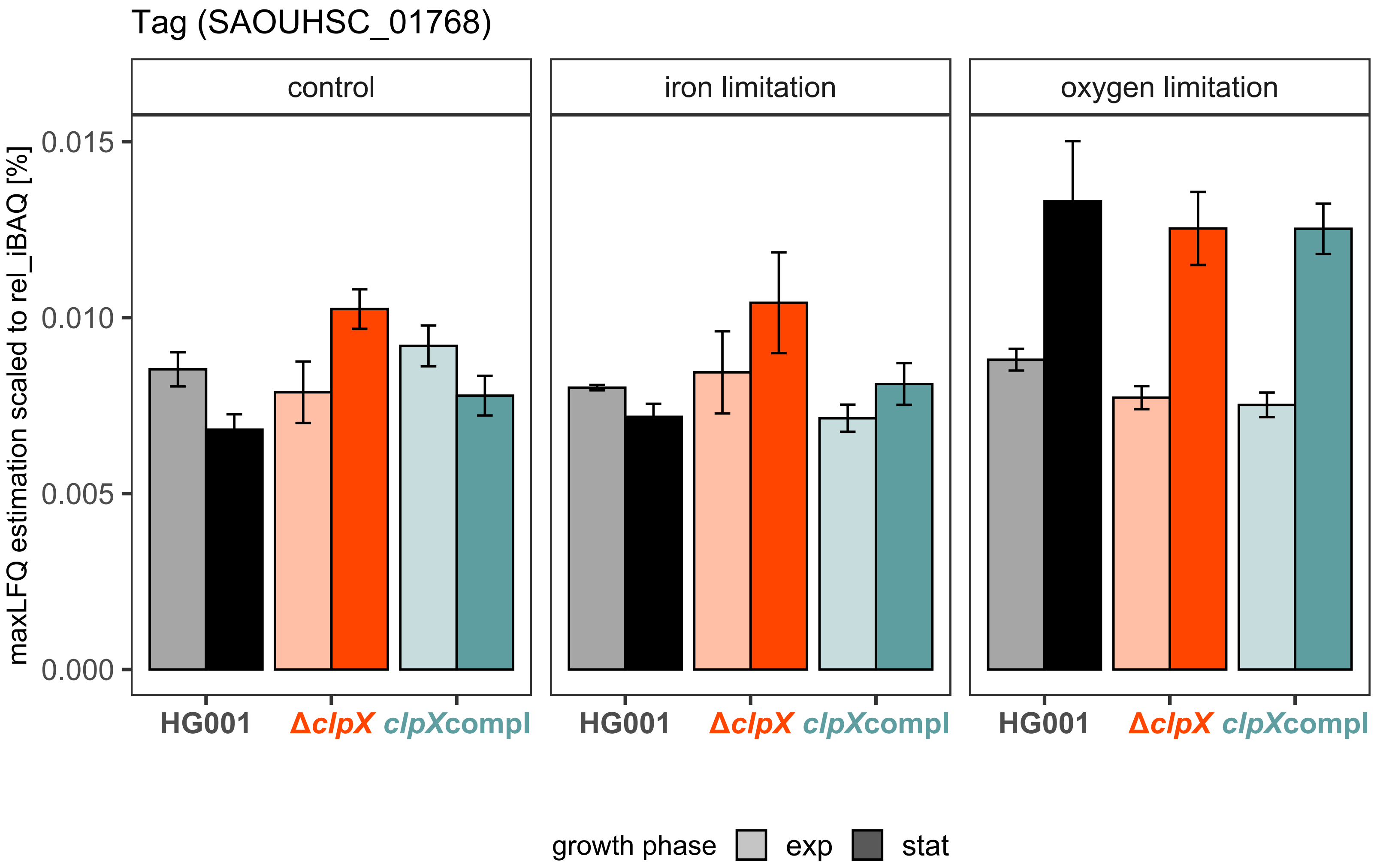
Tag (SAOUHSC_01768)
control
iron limitation
oxygen limitation
maxLFQ estimation scaled to rel_iBAQ [%]
0.015
0.010
0.005
0.000
HG001
ΔclpX
clpXcompl
growth phase
exp
stat

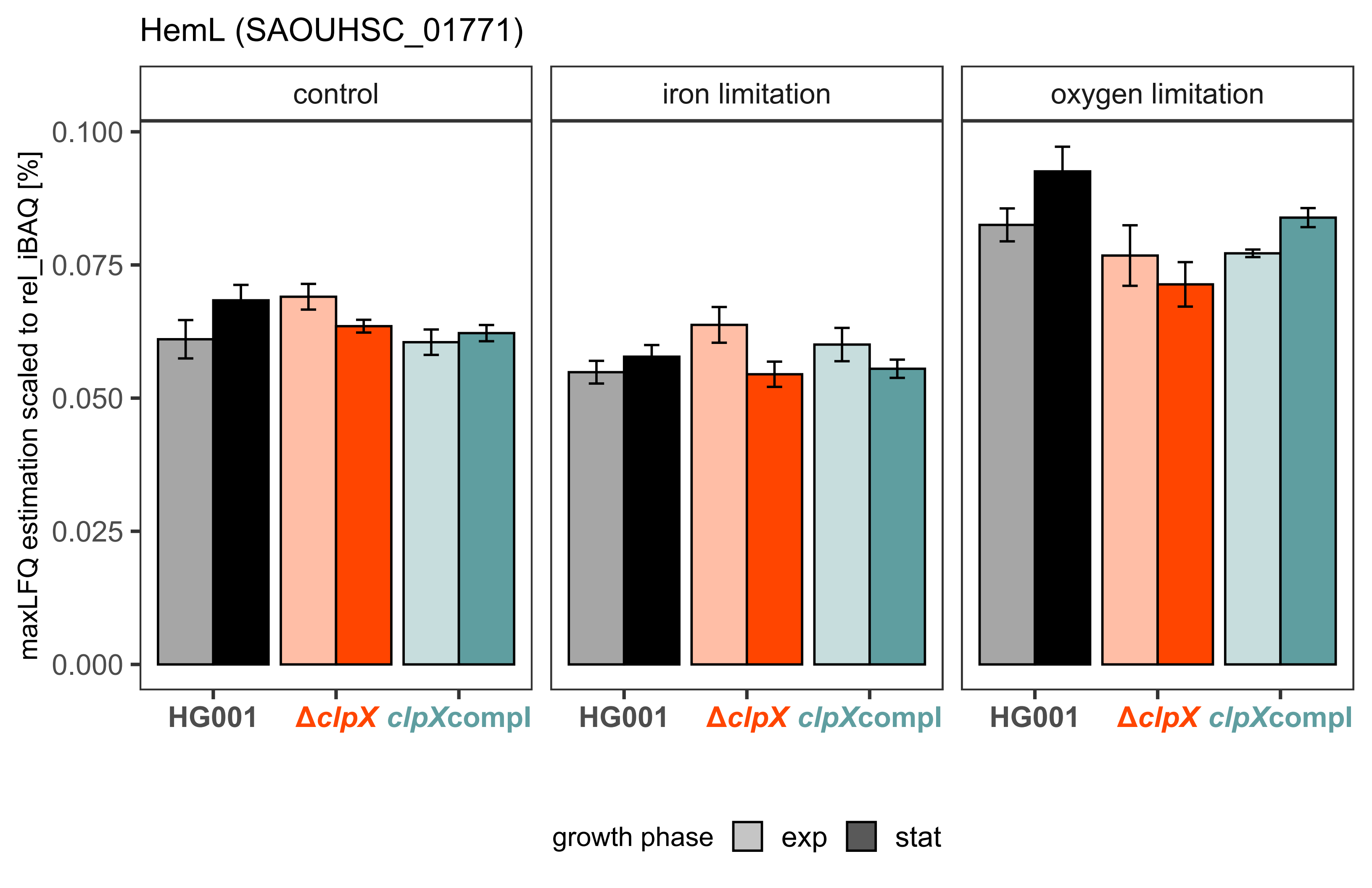

HemL (SAOUHSC_01771)
control
iron limitation
oxygen limitation
maxLFQ estimation scaled to rel_iBAQ [%]
0.100
0.075
0.050
0.025
0.000
HG001
ΔclpX
clpXcompl
growth phase
exp
stat

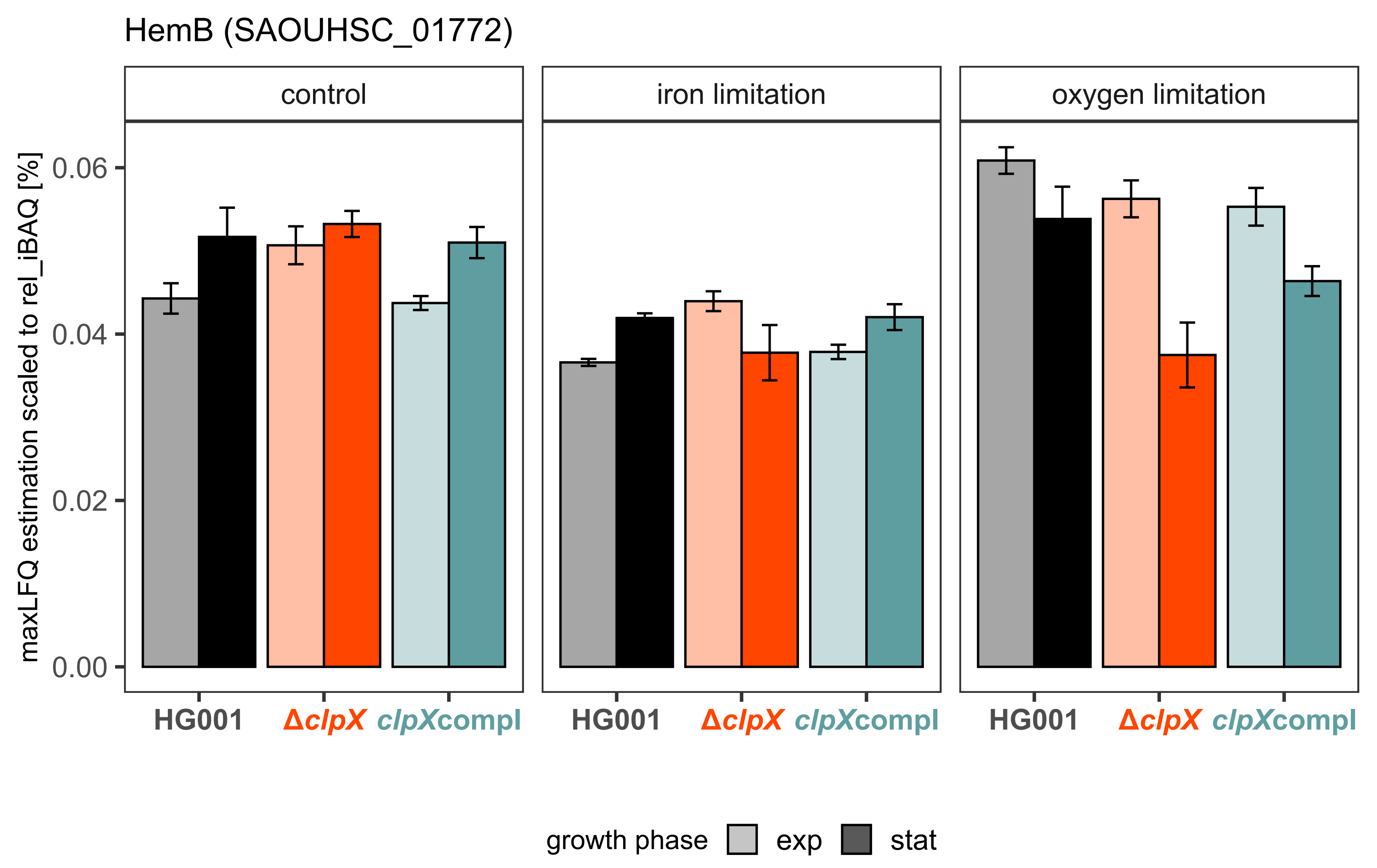
HemB (SAOUHSC_01772)
control
iron limitation
oxygen limitation
maxLFQ estimation scaled to rel_iBAQ [%]
0.06
0.04
0.02
0.00
HG001
ΔclpX
clpXcompl
growth phase
exp
stat

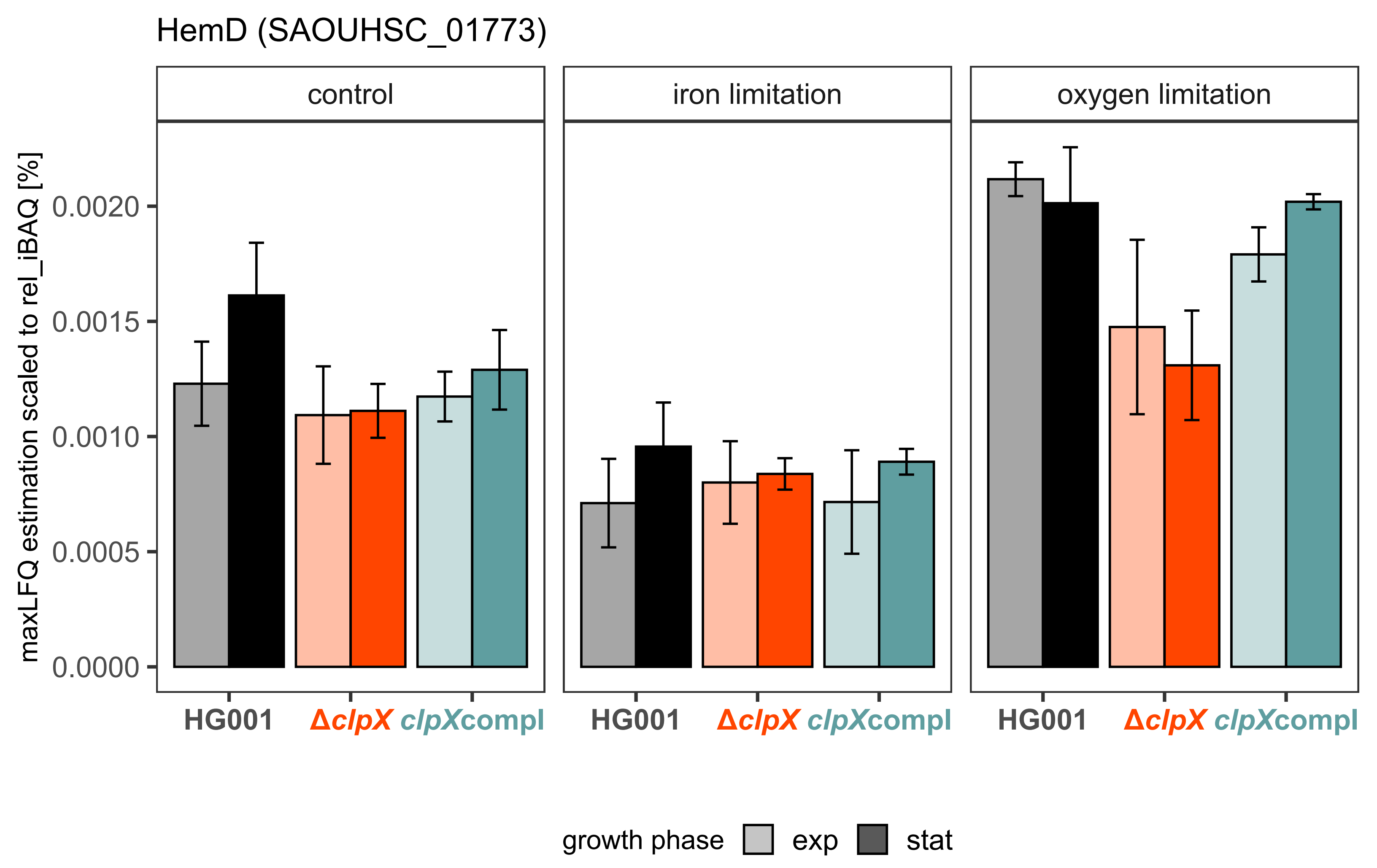
HemD (SAOUHSC_01773)
control
iron limitation
oxygen limitation
maxLFQ estimation scaled to rel_iBAQ [%]
0.0020
0.0015
0.0010
0.0005
0.0000
HG001
ΔclpX
clpXcompl
growth phase
exp
stat

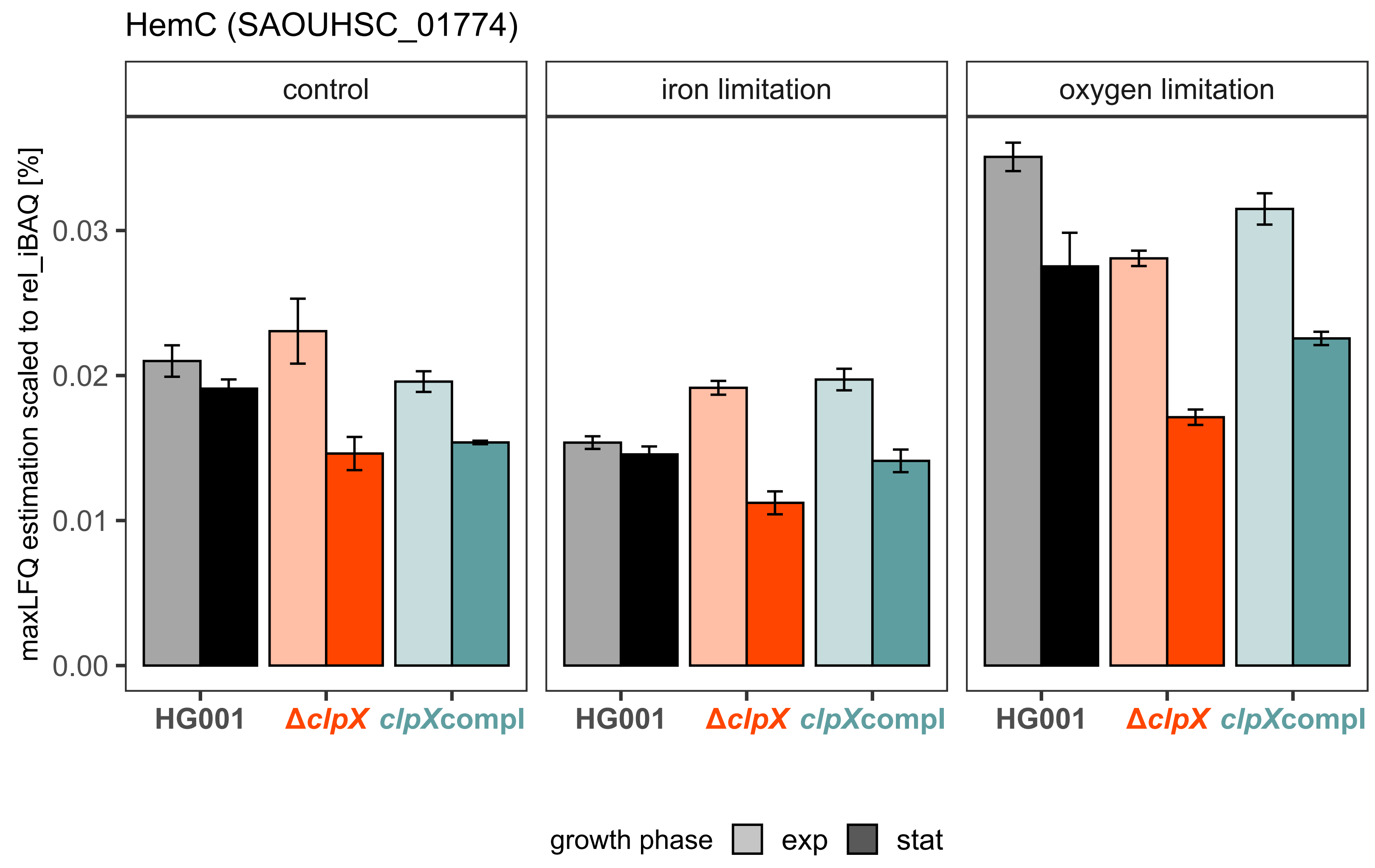

HemC (SAOUHSC_01774)
control
iron limitation
oxygen limitation
maxLFQ estimation scaled to rel_iBAQ [%]
0.03
0.02
0.01
0.00
HG001
ΔclpX
clpXcompl
growth phase
exp
stat

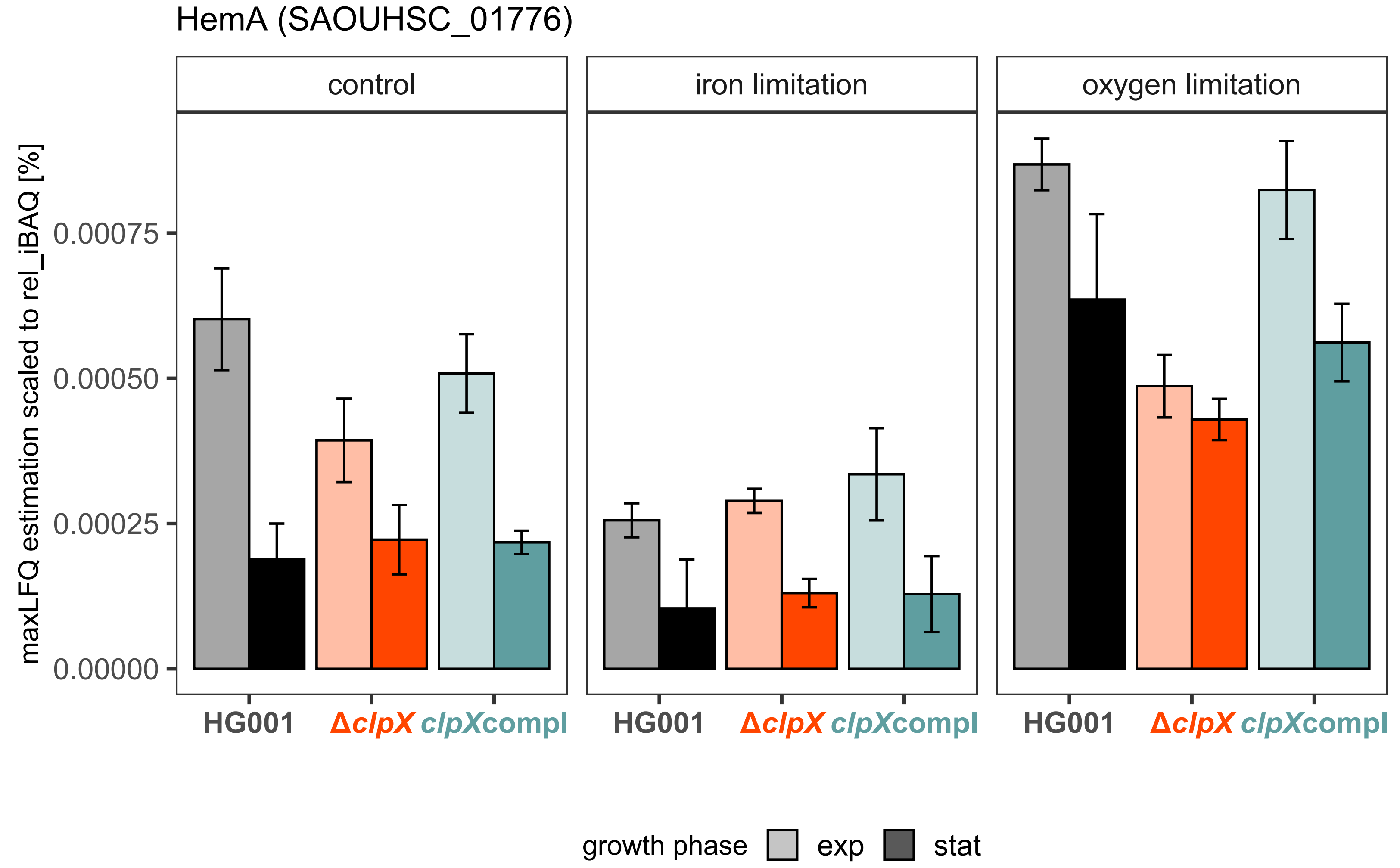
HemA (SAOUHSC_01776)
control
iron limitation
oxygen limitation
maxLFQ estimation scaled to rel_iBAQ [%]
0.00075
0.00050
0.00025
0.00000
HG001
ΔclpX
clpXcompl
growth phase
exp
stat

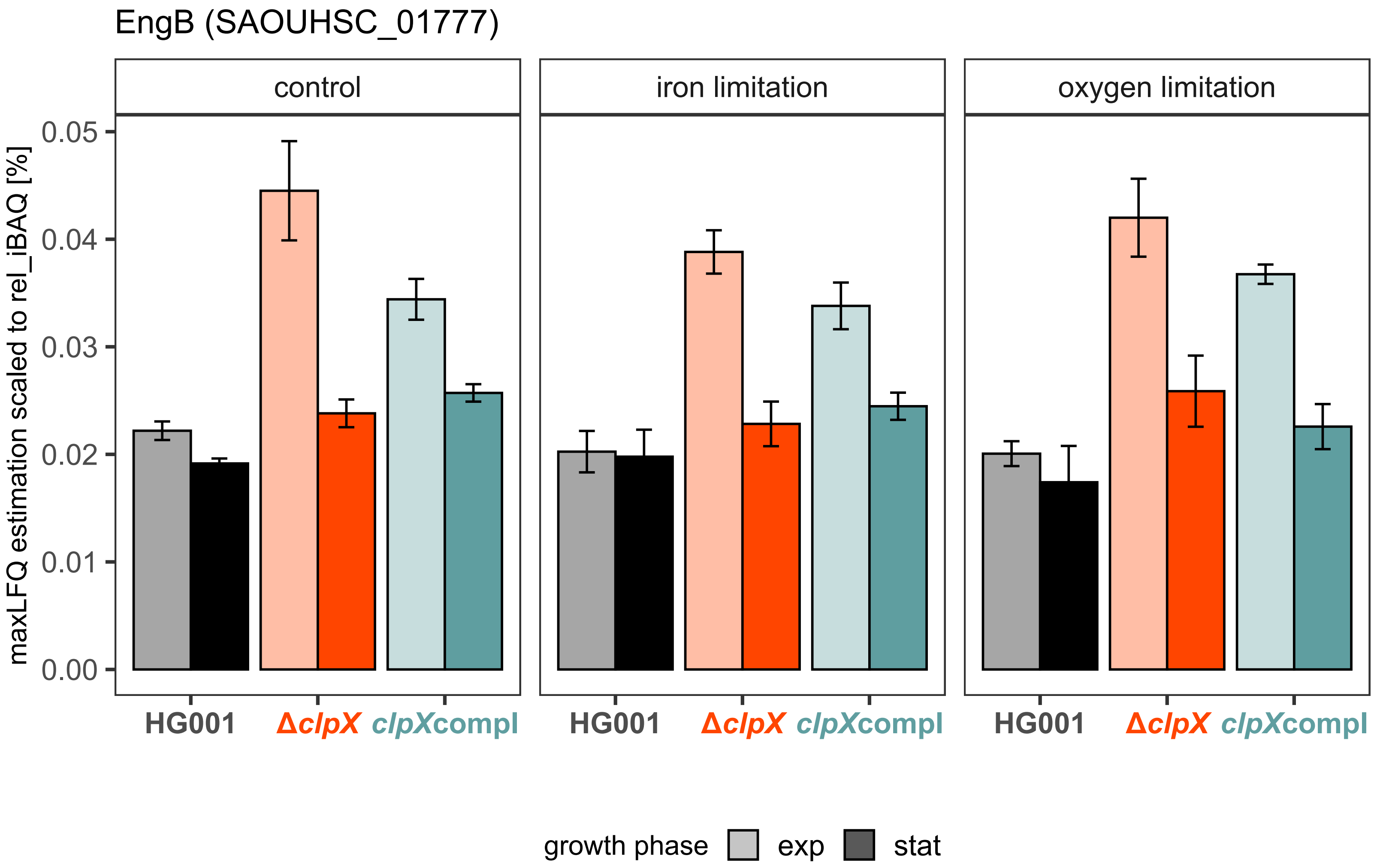

EngB (SAOUHSC_01777)
control
iron limitation
oxygen limitation
maxLFQ estimation scaled to rel_iBAQ [%]
0.05
0.04
0.03
0.02
0.01
0.00
HG001
ΔclpX
clpXcompl
growth phase
exp
stat

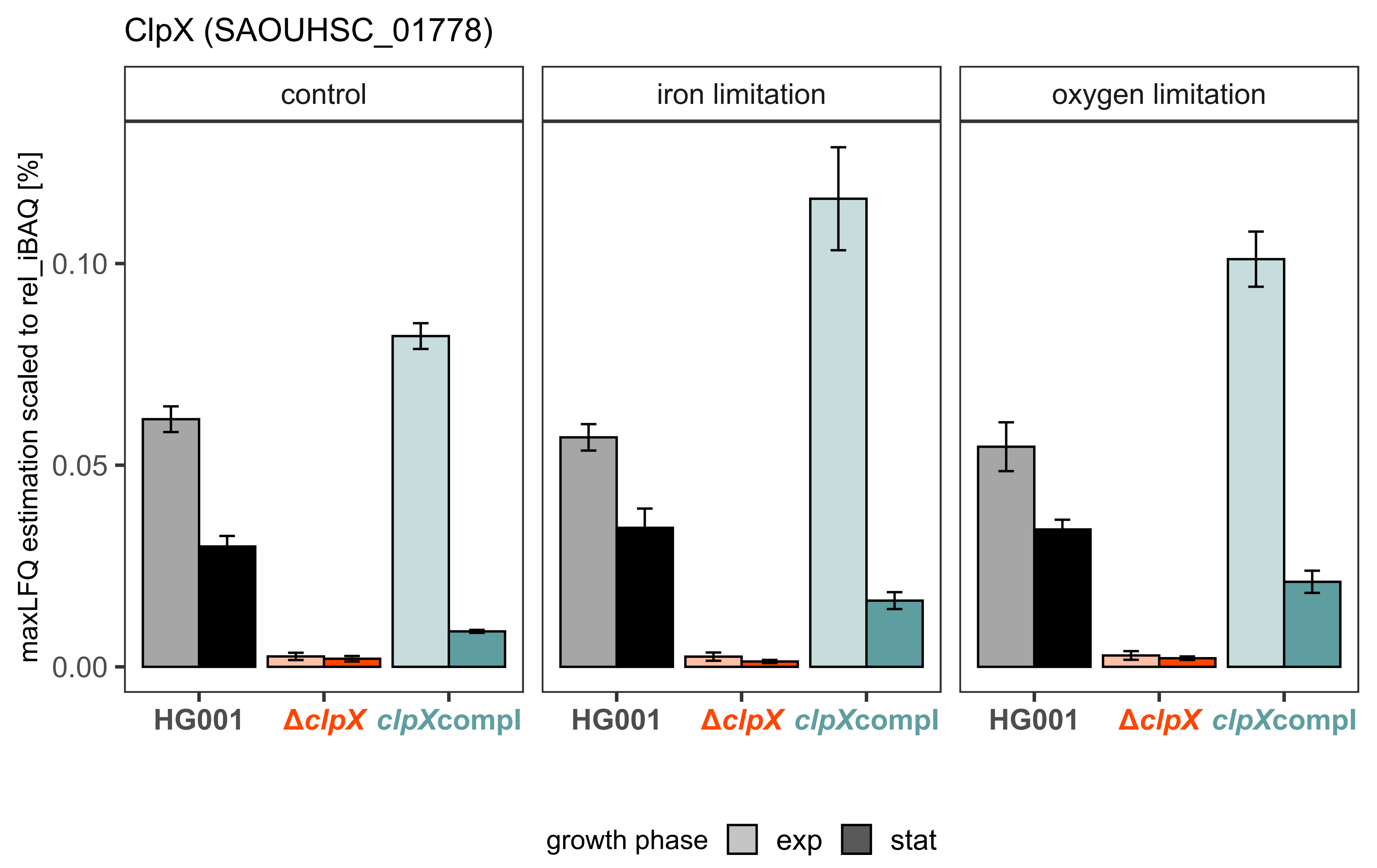
ClpX (SAOUHSC_01778)
control
iron limitation
oxygen limitation
maxLFQ estimation scaled to rel_iBAQ [%]
0.10
0.05
0.00
HG001
ΔclpX
clpXcompl
growth phase
exp
stat

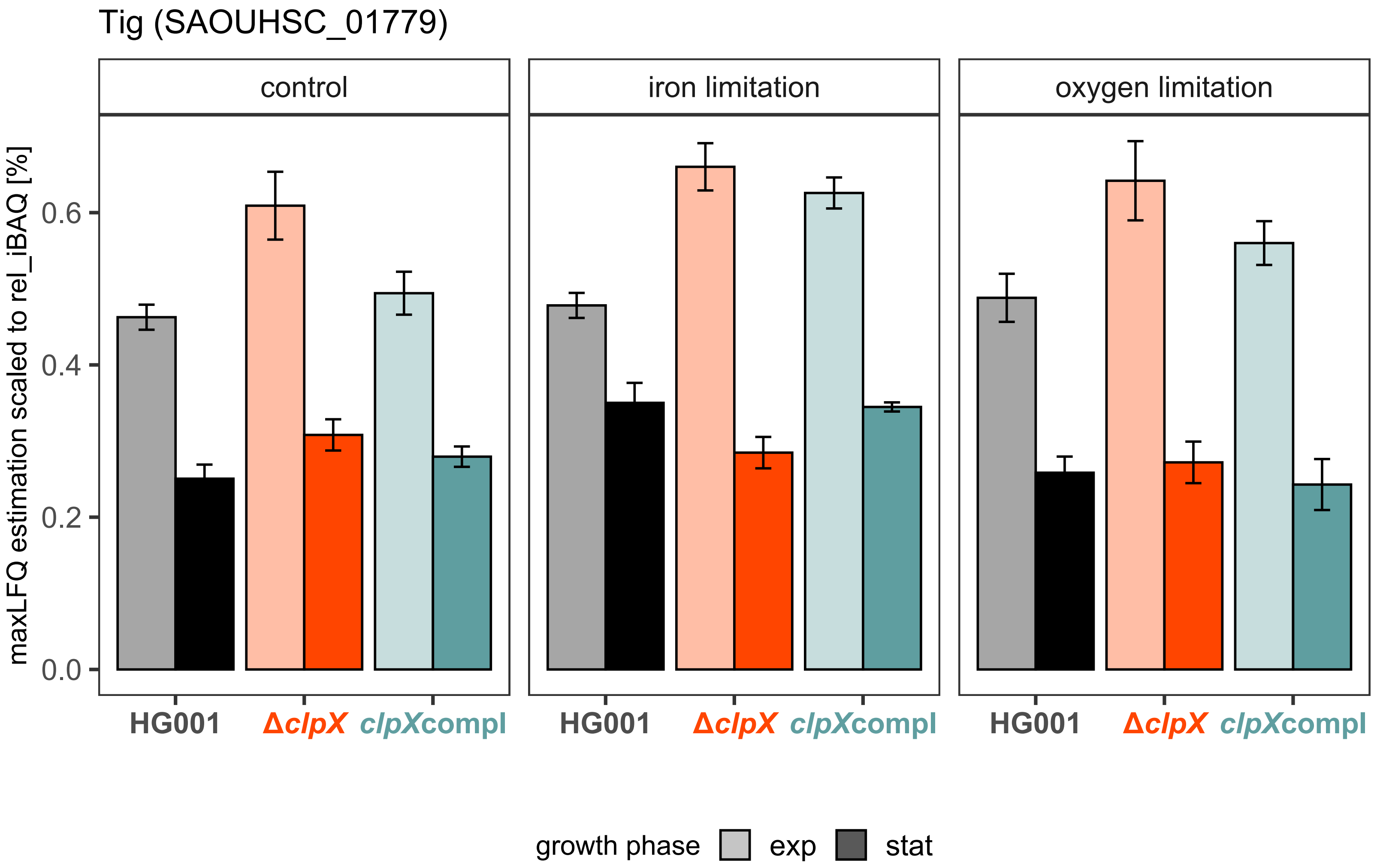

Tig (SAOUHSC_01779)
control
iron limitation
oxygen limitation
maxLFQ estimation scaled to rel_iBAQ [%]
0.0
0.2
0.4
0.6
HG001
ΔclpX
clpXcompl
growth phase
exp
stat

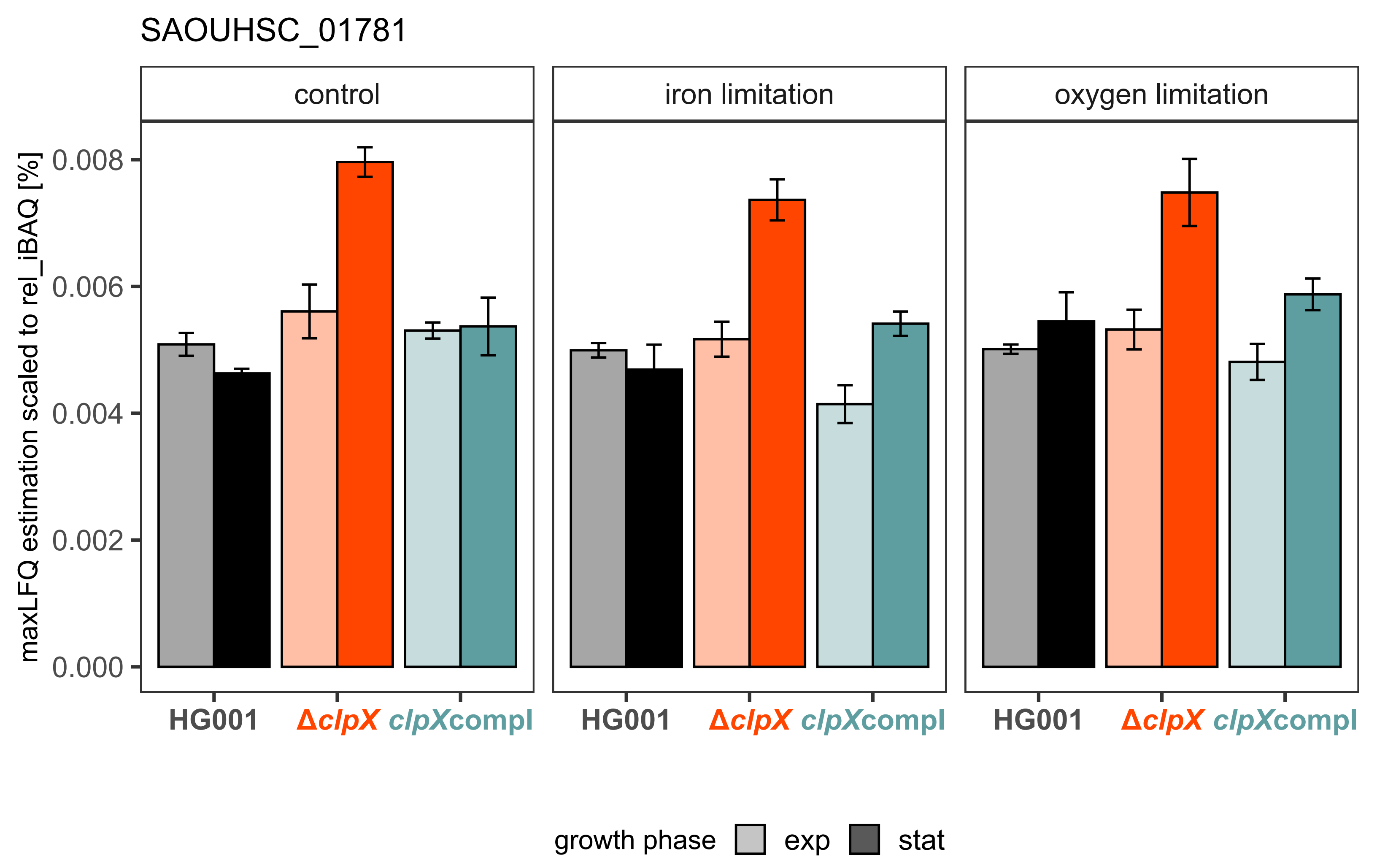
SAOUHSC_01781
control
iron limitation
oxygen limitation
maxLFQ estimation scaled to rel_iBAQ [%]
0.008
0.006
0.004
0.002
0.000
HG001
ΔclpX
clpXcompl
growth phase
exp
stat

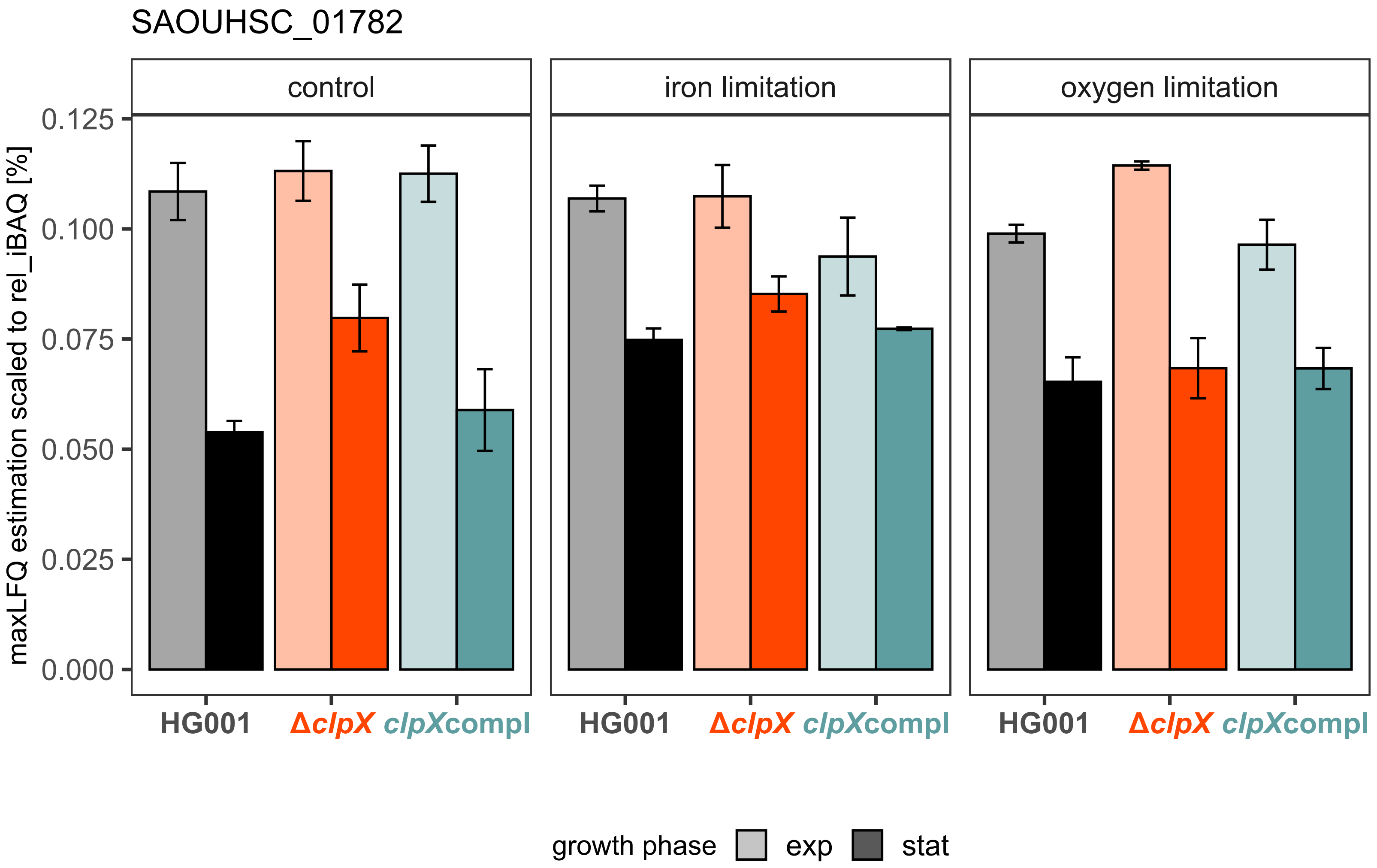
SAOUHSC_01782
control
iron limitation
oxygen limitation
maxLFQ estimation scaled to rel_iBAQ [%]
0.125
0.100
0.075
0.050
0.025
0.000
HG001
ΔclpX
clpXcompl
growth phase
exp
stat

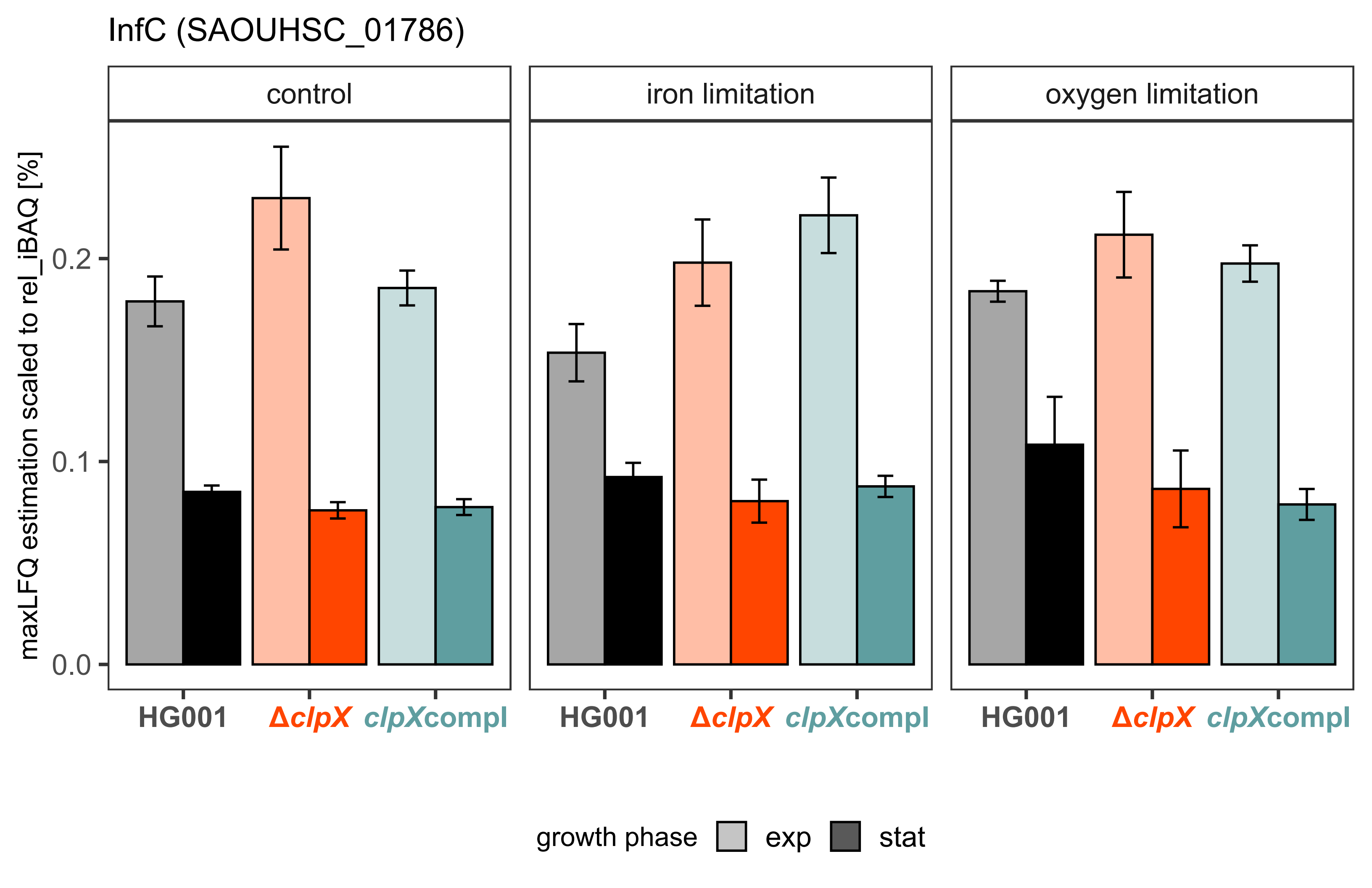
InfC (SAOUHSC_01786)
control
iron limitation
oxygen limitation
maxLFQ estimation scaled to rel_iBAQ [%]
0.0
0.1
0.2
HG001
ΔclpX
clpXcompl
growth phase
exp
stat

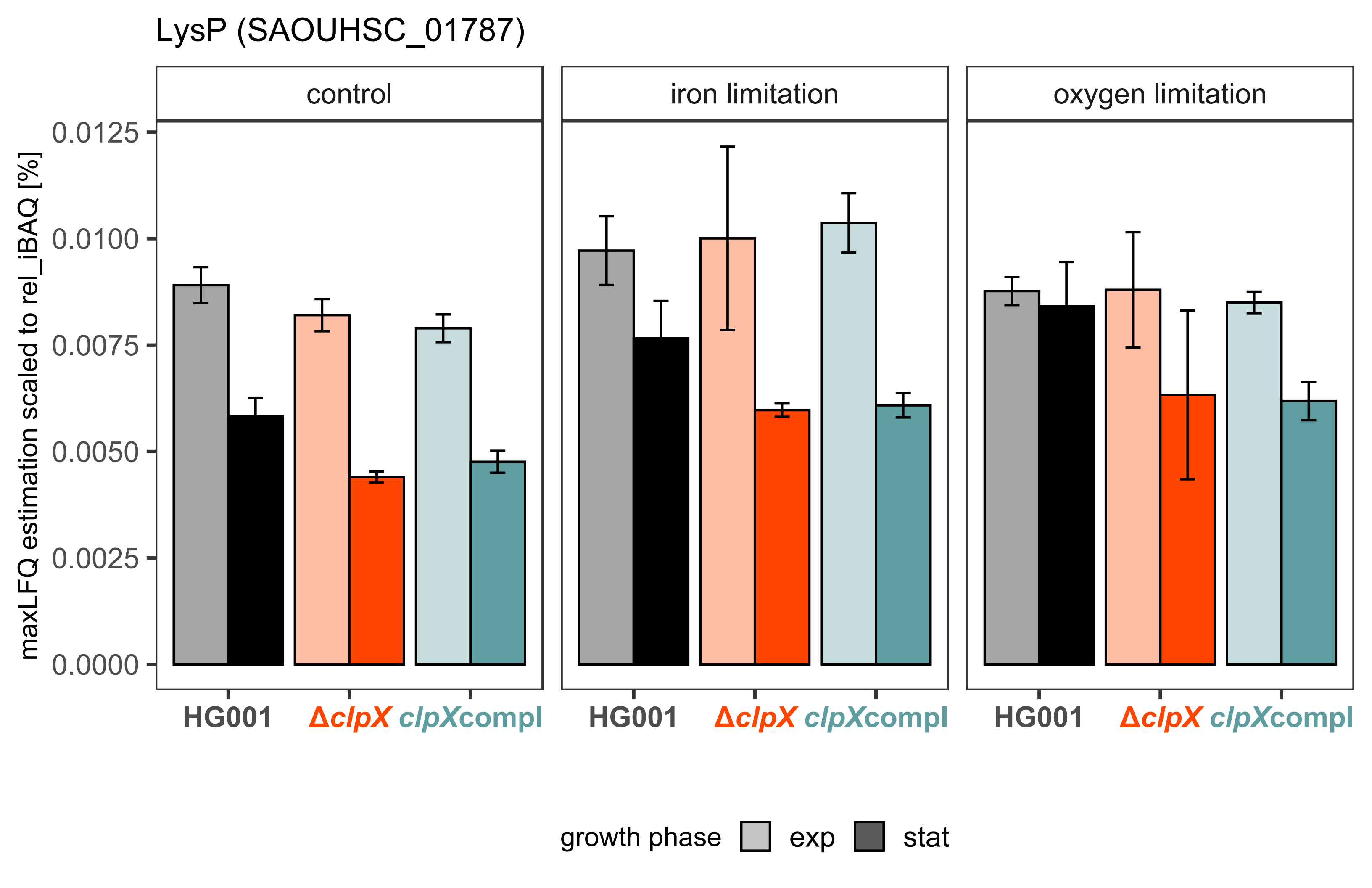
LysP (SAOUHSC_01787)
control
iron limitation
oxygen limitation
maxLFQ estimation scaled to rel_iBAQ [%]
0.0125
0.0100
0.0075
0.0050
0.0025
0.0000
HG001
ΔclpX
clpXcompl
growth phase
exp
stat

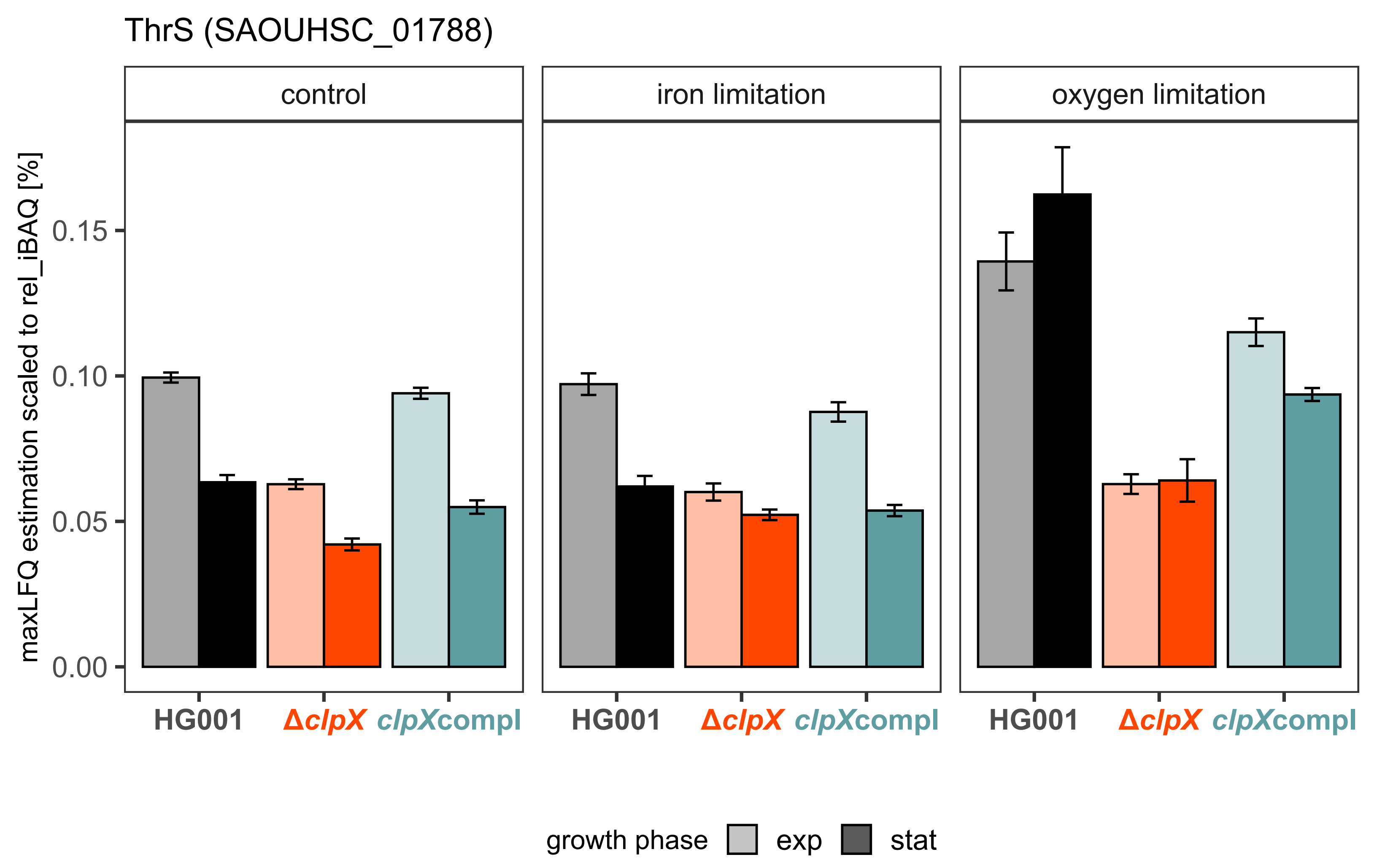
ThrS (SAOUHSC_01788)
control
iron limitation
oxygen limitation
maxLFQ estimation scaled to rel_iBAQ [%]
0.15
0.10
0.05
0.00
HG001
ΔclpX
clpXcompl
growth phase
exp
stat

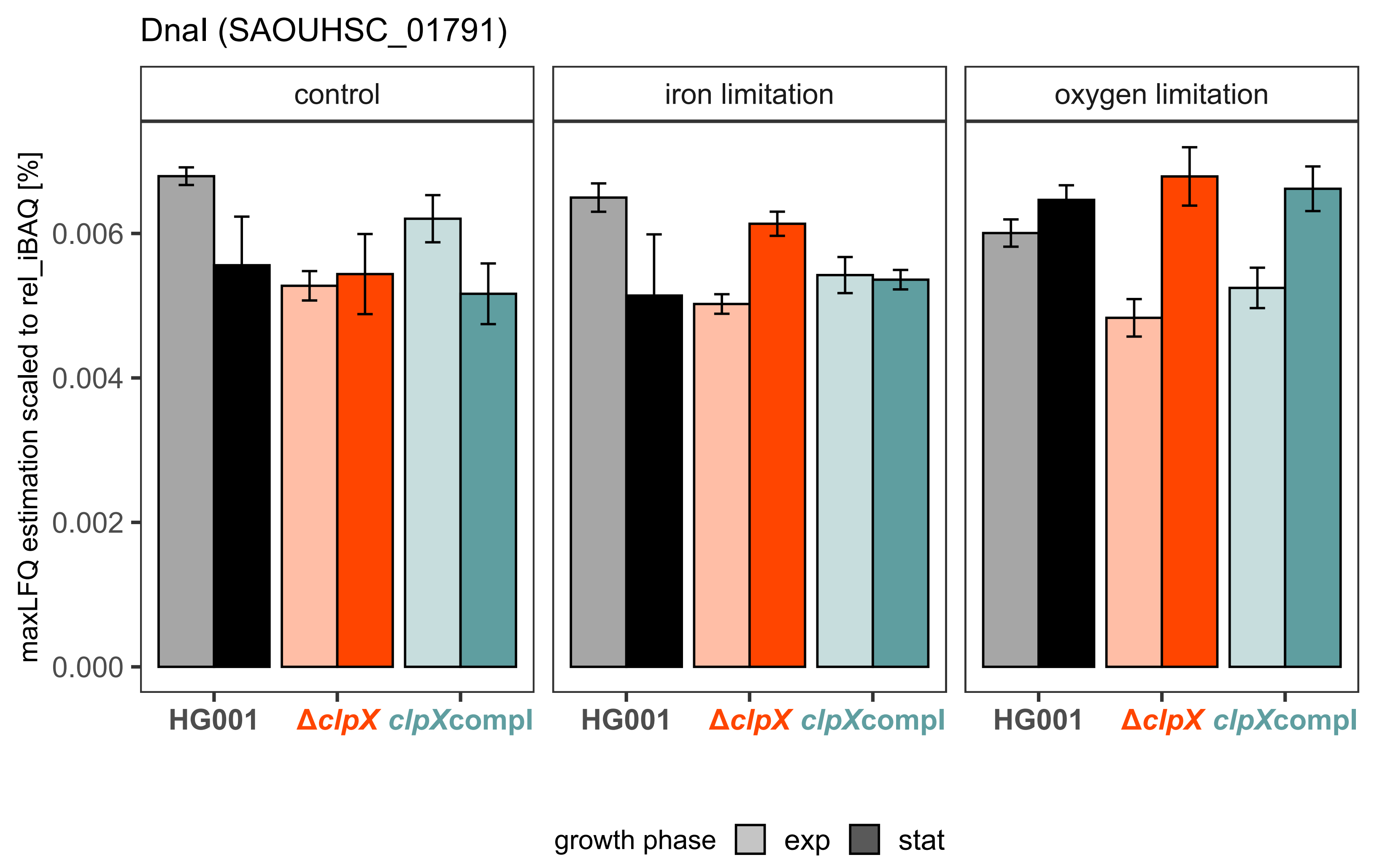
DnaI (SAOUHSC_01791)
control
iron limitation
oxygen limitation
maxLFQ estimation scaled to rel_iBAQ [%]
0.006
0.004
0.002
0.000
HG001
ΔclpX
clpXcompl
growth phase
exp
stat

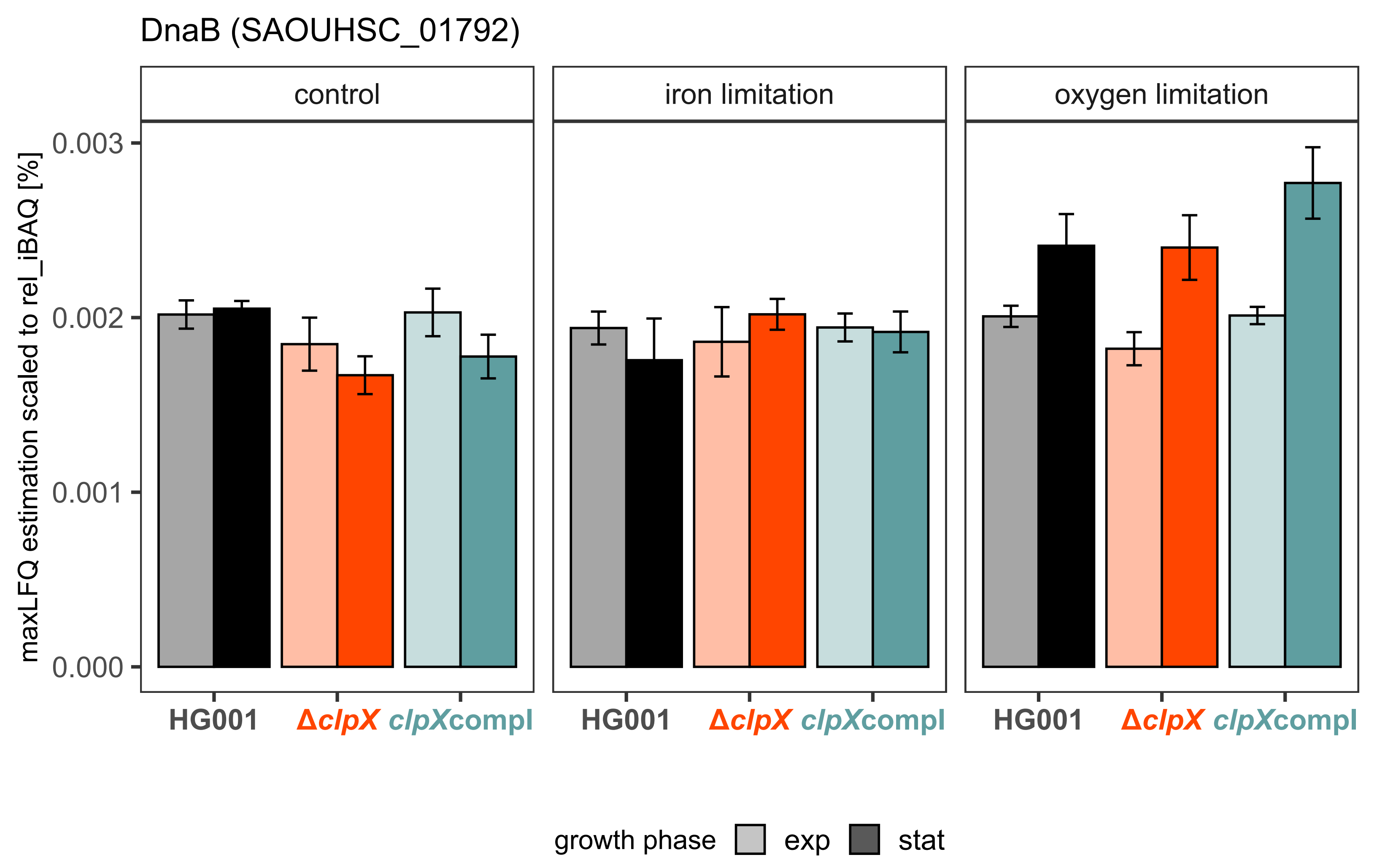
DnaB (SAOUHSC_01792)
control
iron limitation
oxygen limitation
maxLFQ estimation scaled to rel_iBAQ [%]
0.003
0.002
0.001
0.000
HG001
ΔclpX
clpXcompl
growth phase
exp
stat

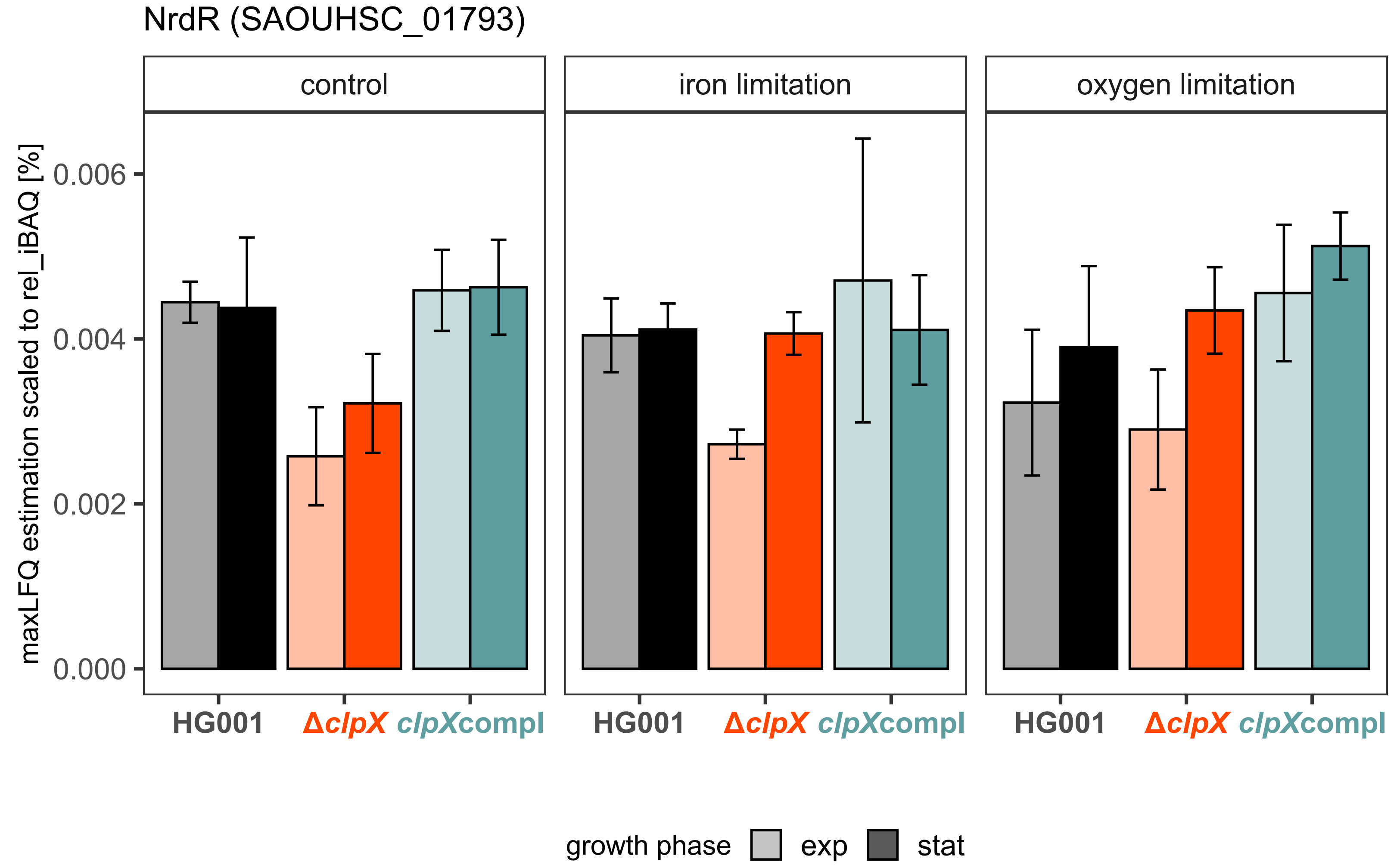

NrdR (SAOUHSC_01793)
control
iron limitation
oxygen limitation
maxLFQ estimation scaled to rel_iBAQ [%]
0.006
0.004
0.002
0.000
HG001
ΔclpX
clpXcompl
growth phase
exp
stat

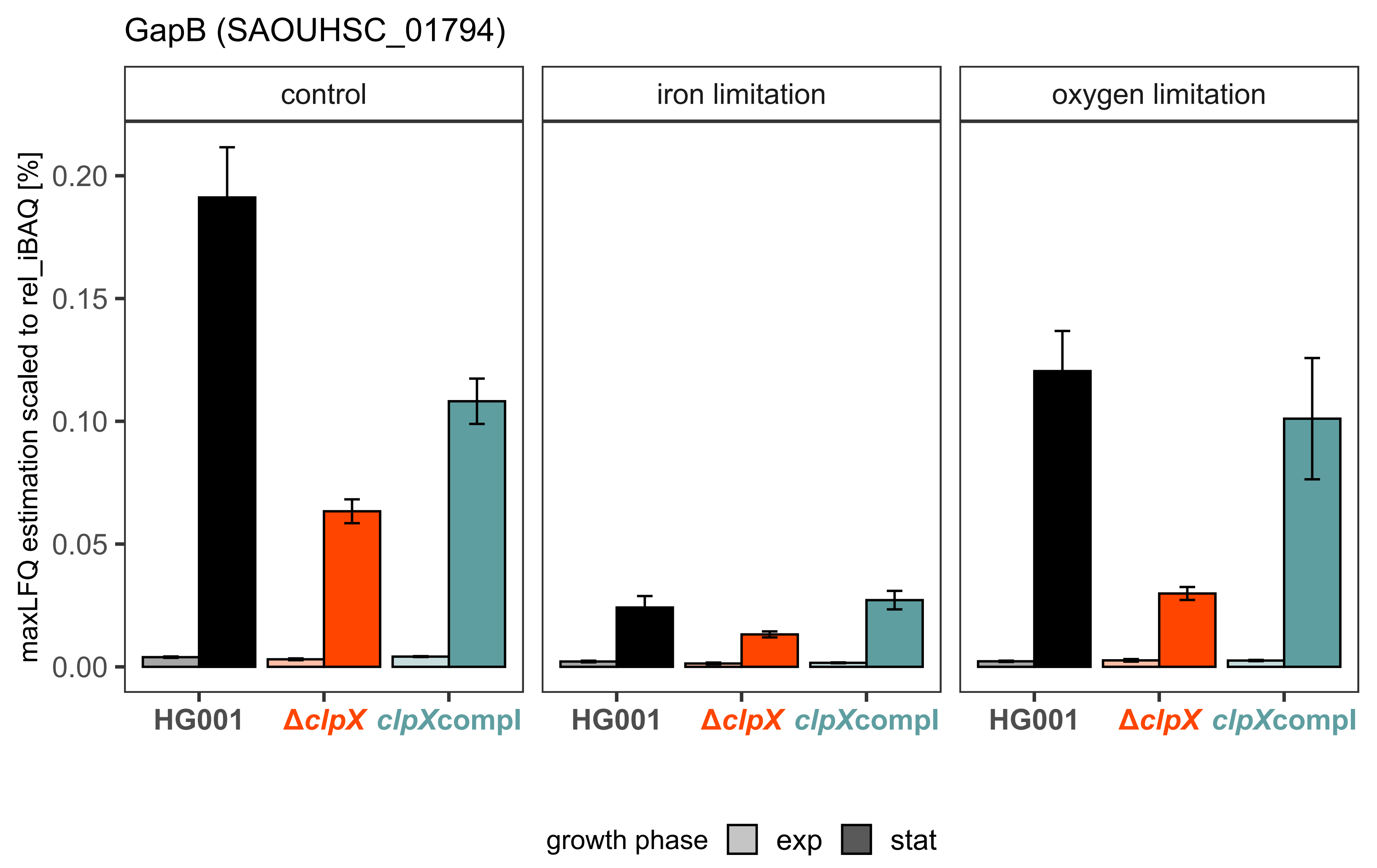

GapB (SAOUHSC_01794)
control
iron limitation
oxygen limitation
maxLFQ estimation scaled to rel_iBAQ [%]
0.00
0.05
0.10
0.15
0.20
HG001
ΔclpX
clpXcompl
growth phase
exp
stat

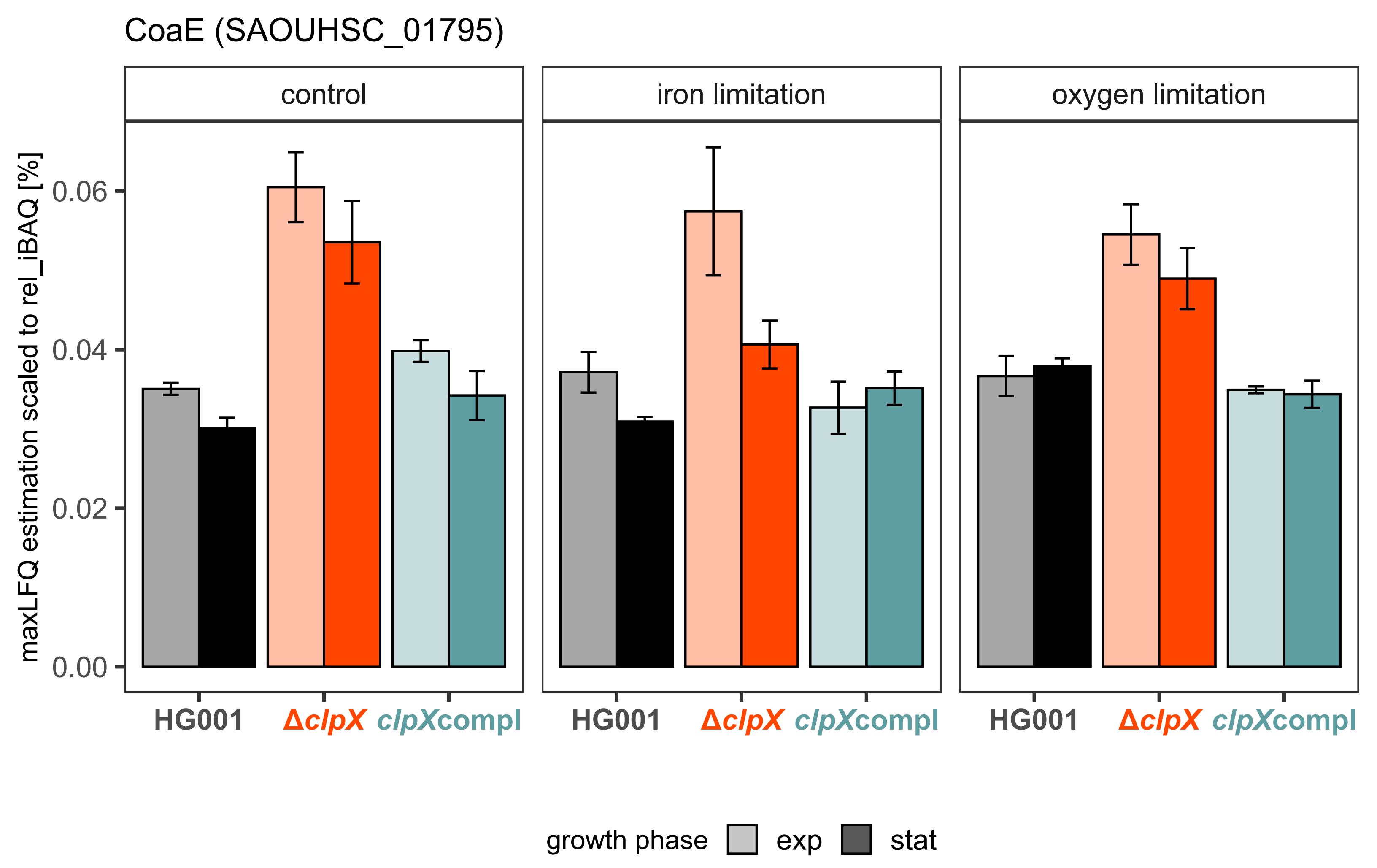
CoaE (SAOUHSC_01795)
control
iron limitation
oxygen limitation
maxLFQ estimation scaled to rel_iBAQ [%]
0.06
0.04
0.02
0.00
HG001
ΔclpX
clpXcompl
growth phase
exp
stat

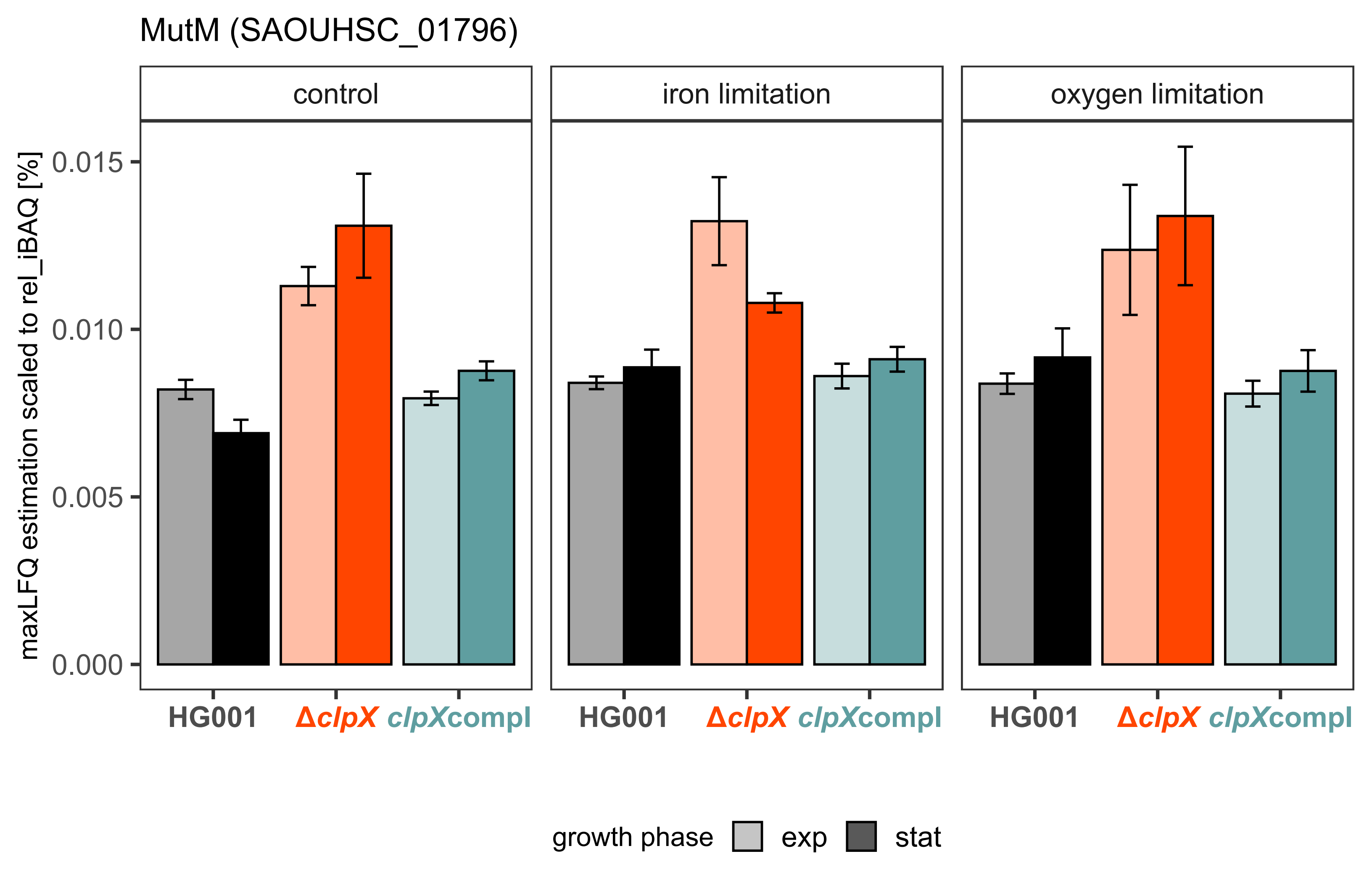

MutM (SAOUHSC_01796)
control
iron limitation
oxygen limitation
maxLFQ estimation scaled to rel_iBAQ [%]
0.015
0.010
0.005
0.000
HG001
ΔclpX
clpXcompl
growth phase
exp
stat

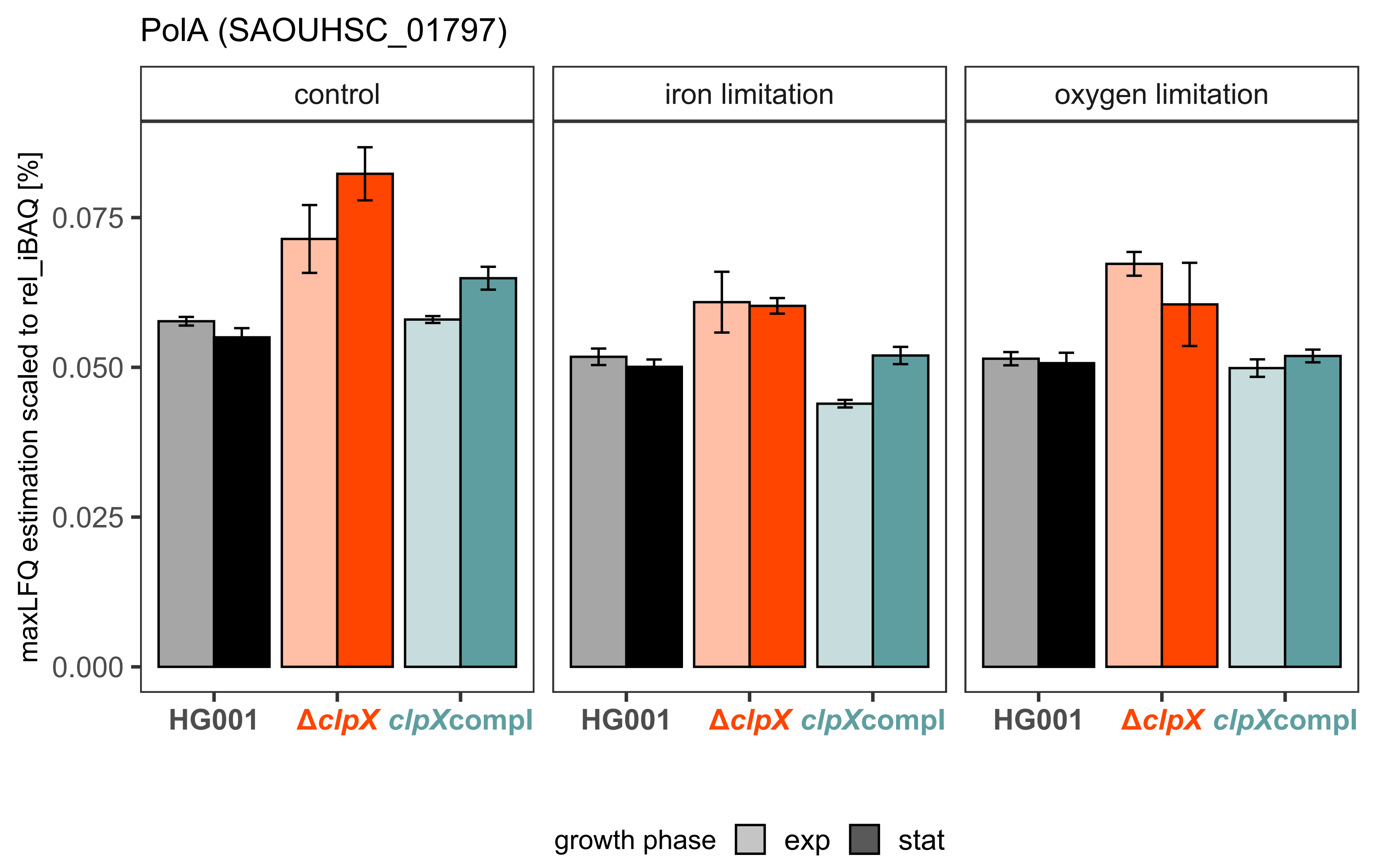
PolA (SAOUHSC_01797)
control
iron limitation
oxygen limitation
maxLFQ estimation scaled to rel_iBAQ [%]
0.000
0.025
0.050
0.075
HG001
ΔclpX
clpXcompl
growth phase
exp
stat

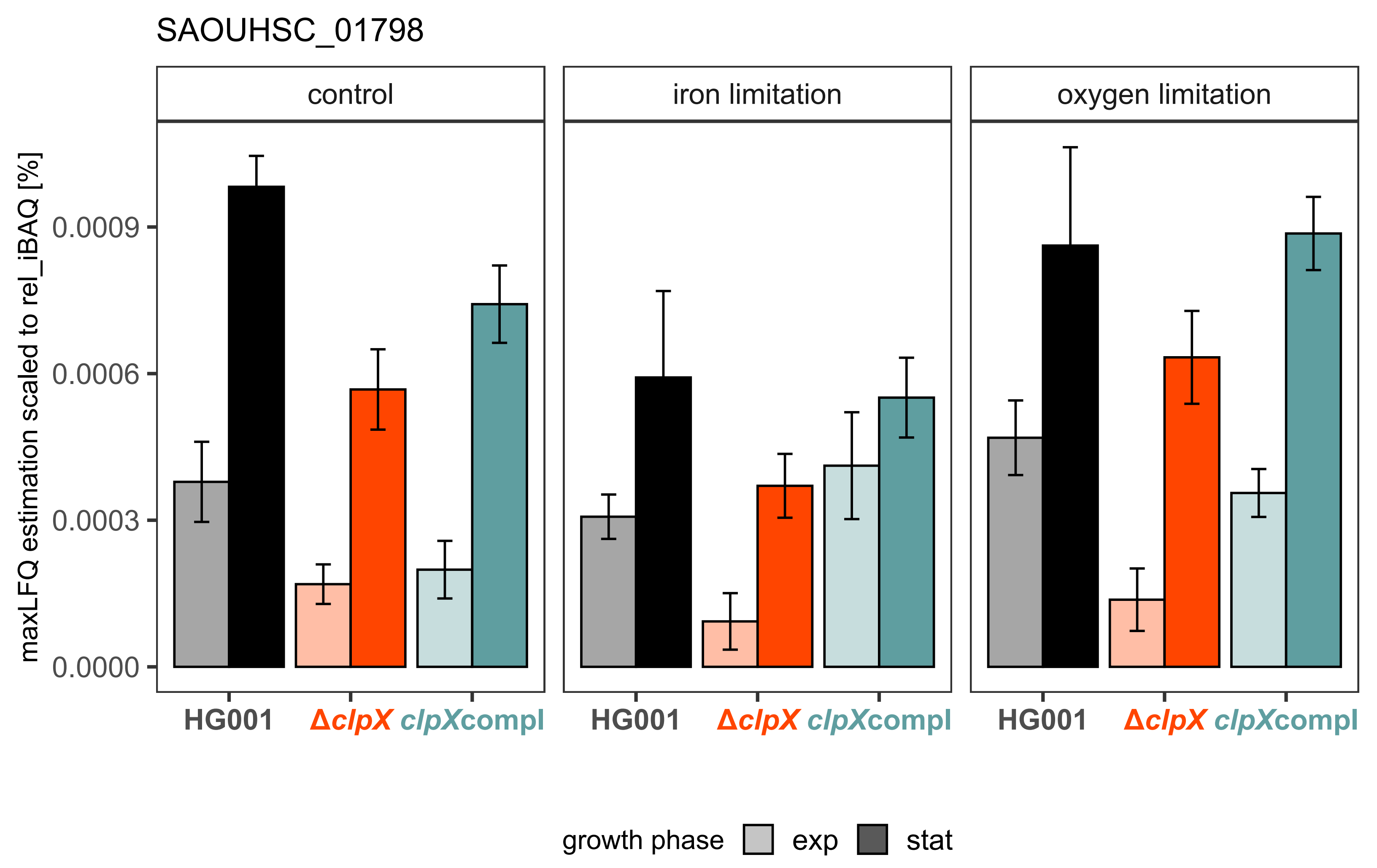

SAOUHSC_01798
control
iron limitation
oxygen limitation
maxLFQ estimation scaled to rel_iBAQ [%]
0.0009
0.0006
0.0003
0.0000
HG001
ΔclpX
clpXcompl
growth phase
exp
stat

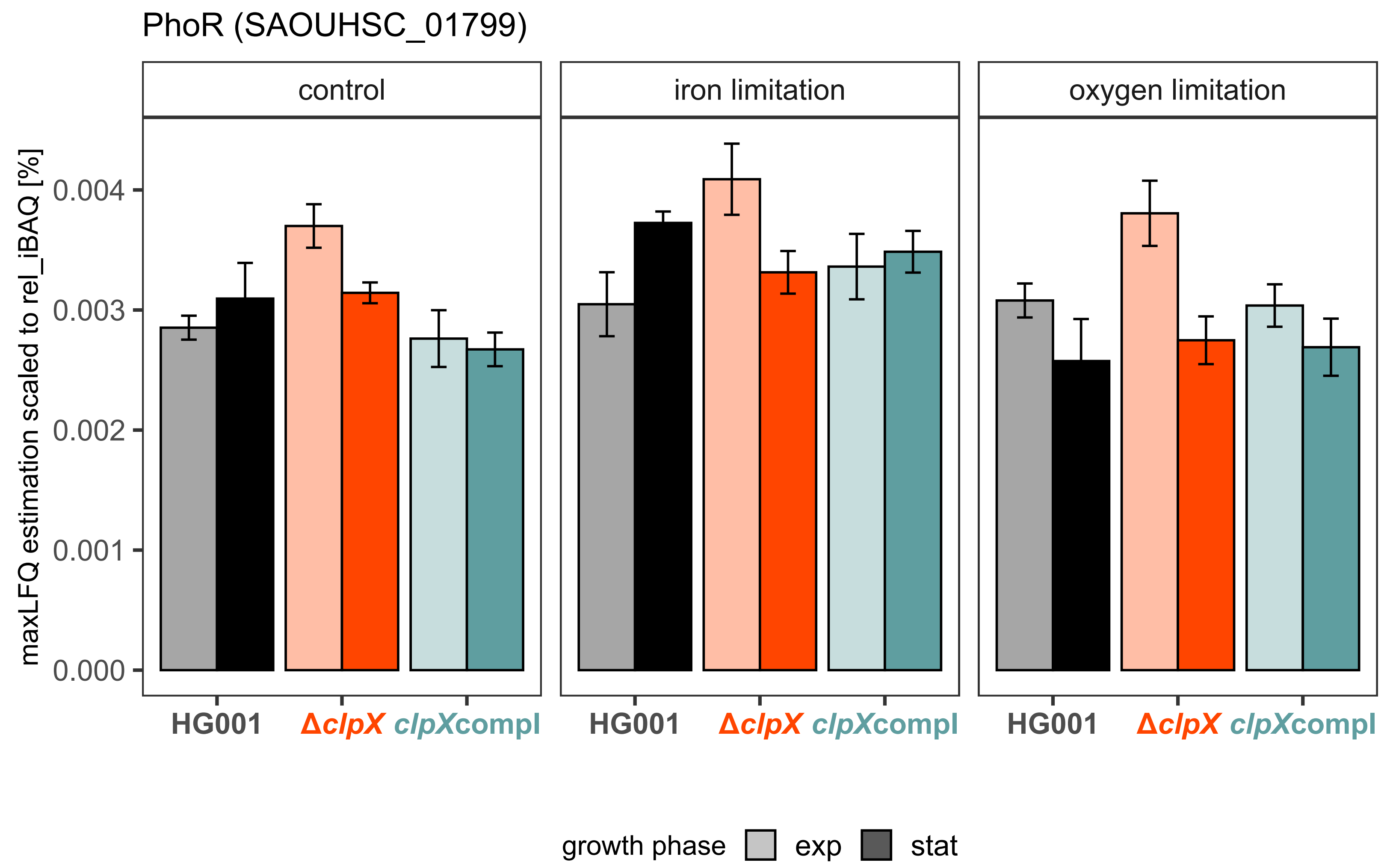
PhoR (SAOUHSC_01799)
control
iron limitation
oxygen limitation
maxLFQ estimation scaled to rel_iBAQ [%]
0.004
0.003
0.002
0.001
0.000
HG001
ΔclpX
clpXcompl
growth phase
exp
stat

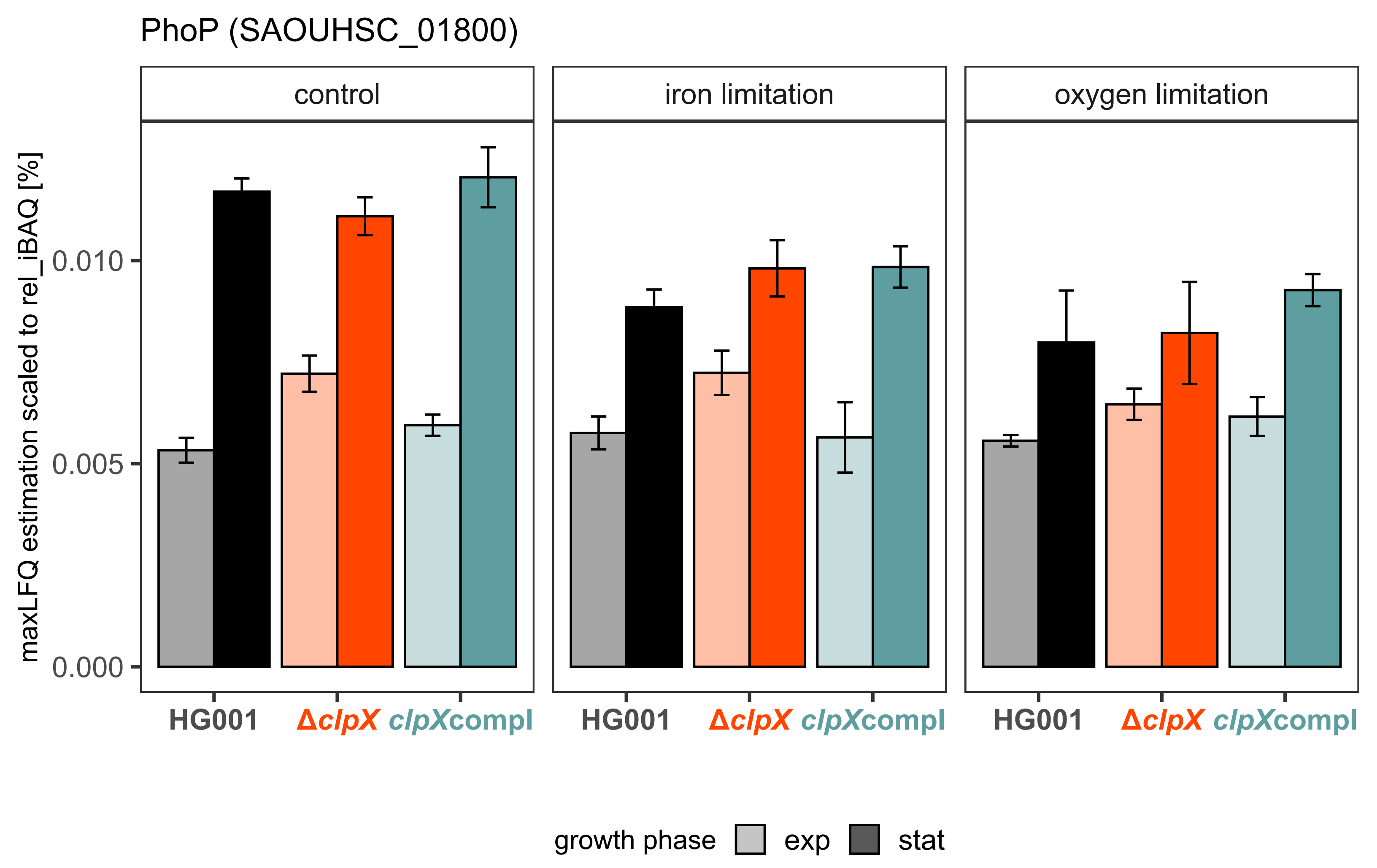

PhoP (SAOUHSC_01800)
control
iron limitation
oxygen limitation
maxLFQ estimation scaled to rel_iBAQ [%]
0.010
0.005
0.000
HG001
ΔclpX
clpXcompl
growth phase
exp
stat

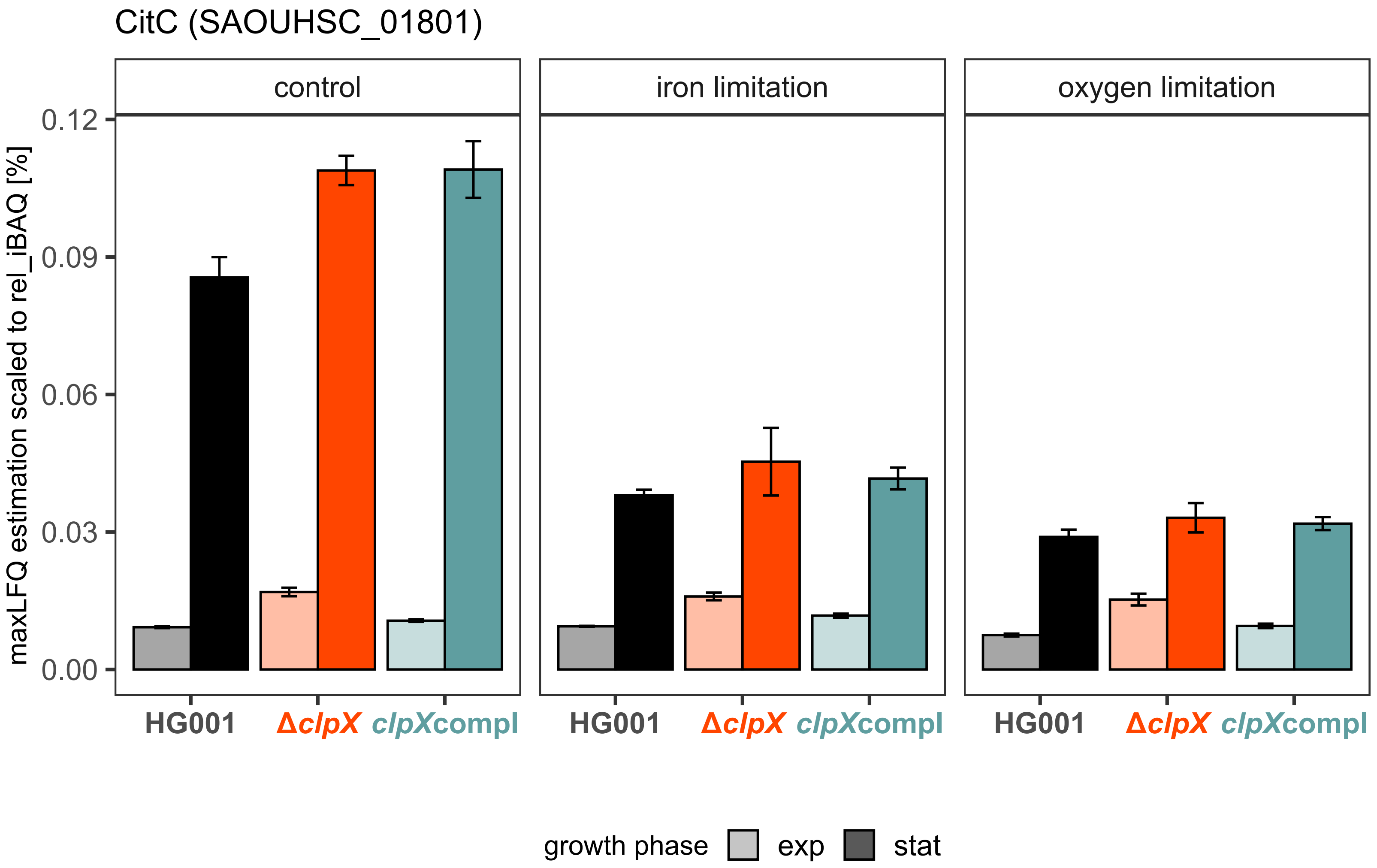

CitC (SAOUHSC_01801)
control
iron limitation
oxygen limitation
maxLFQ estimation scaled to rel_iBAQ [%]
0.12
0.09
0.06
0.03
0.00
HG001
ΔclpX
clpXcompl
growth phase
exp
stat

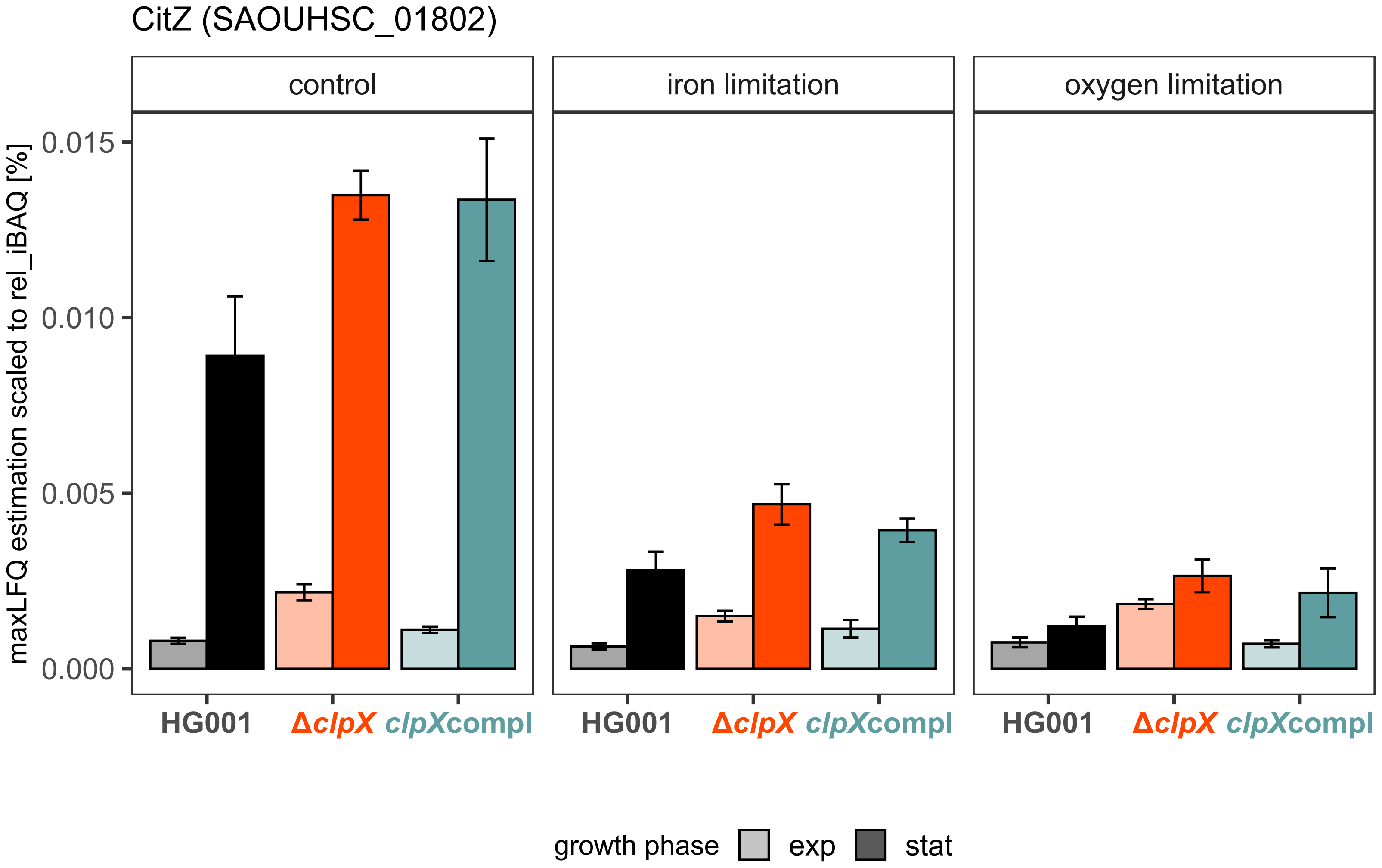
CitZ (SAOUHSC_01802)
control
iron limitation
oxygen limitation
maxLFQ estimation scaled to rel_iBAQ [%]
0.015
0.010
0.005
0.000
HG001
ΔclpX
clpXcompl
growth phase
exp
stat

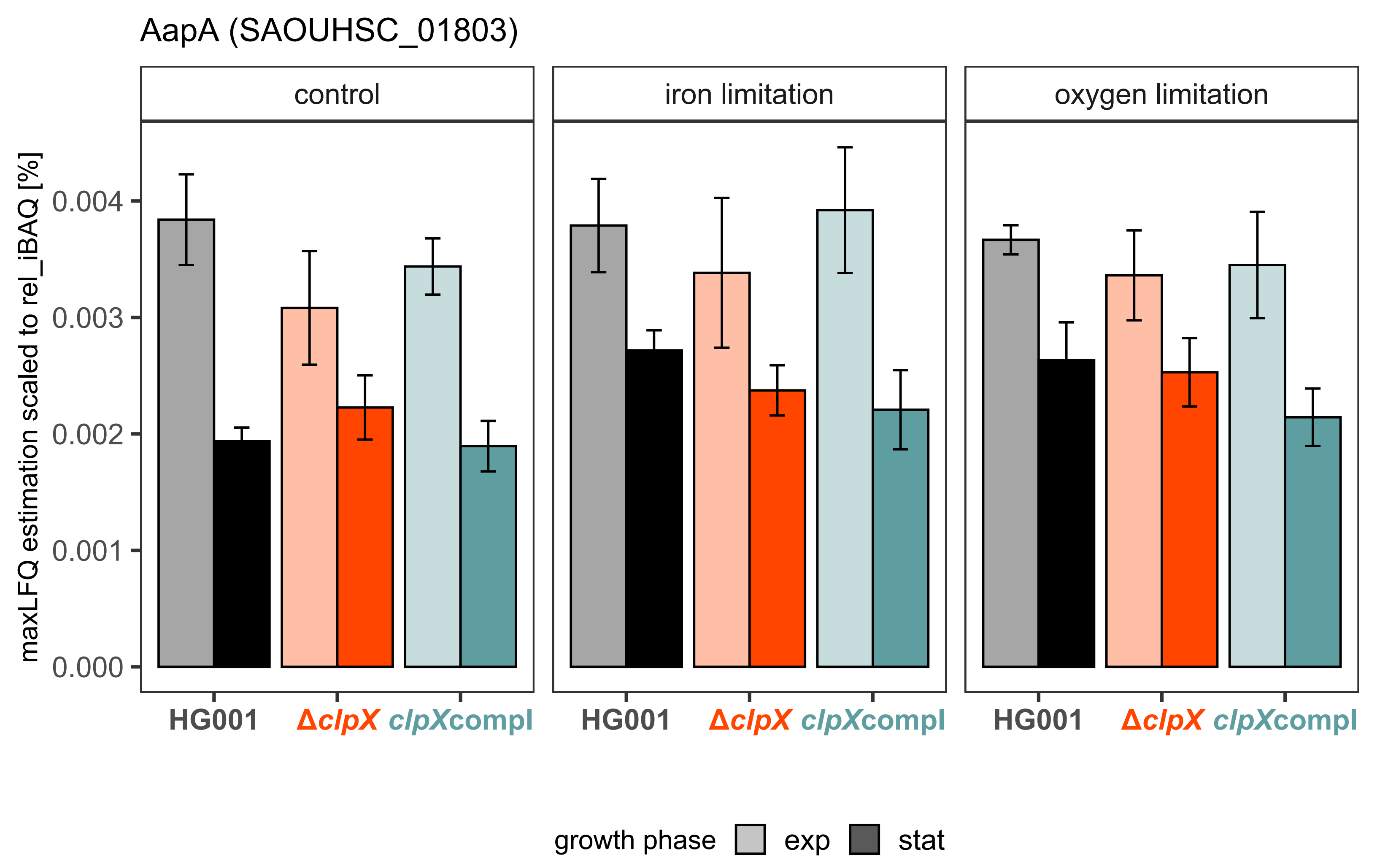
AapA (SAOUHSC_01803)
control
iron limitation
oxygen limitation
maxLFQ estimation scaled to rel_iBAQ [%]
0.004
0.003
0.002
0.001
0.000
HG001
ΔclpX
clpXcompl
growth phase
exp
stat

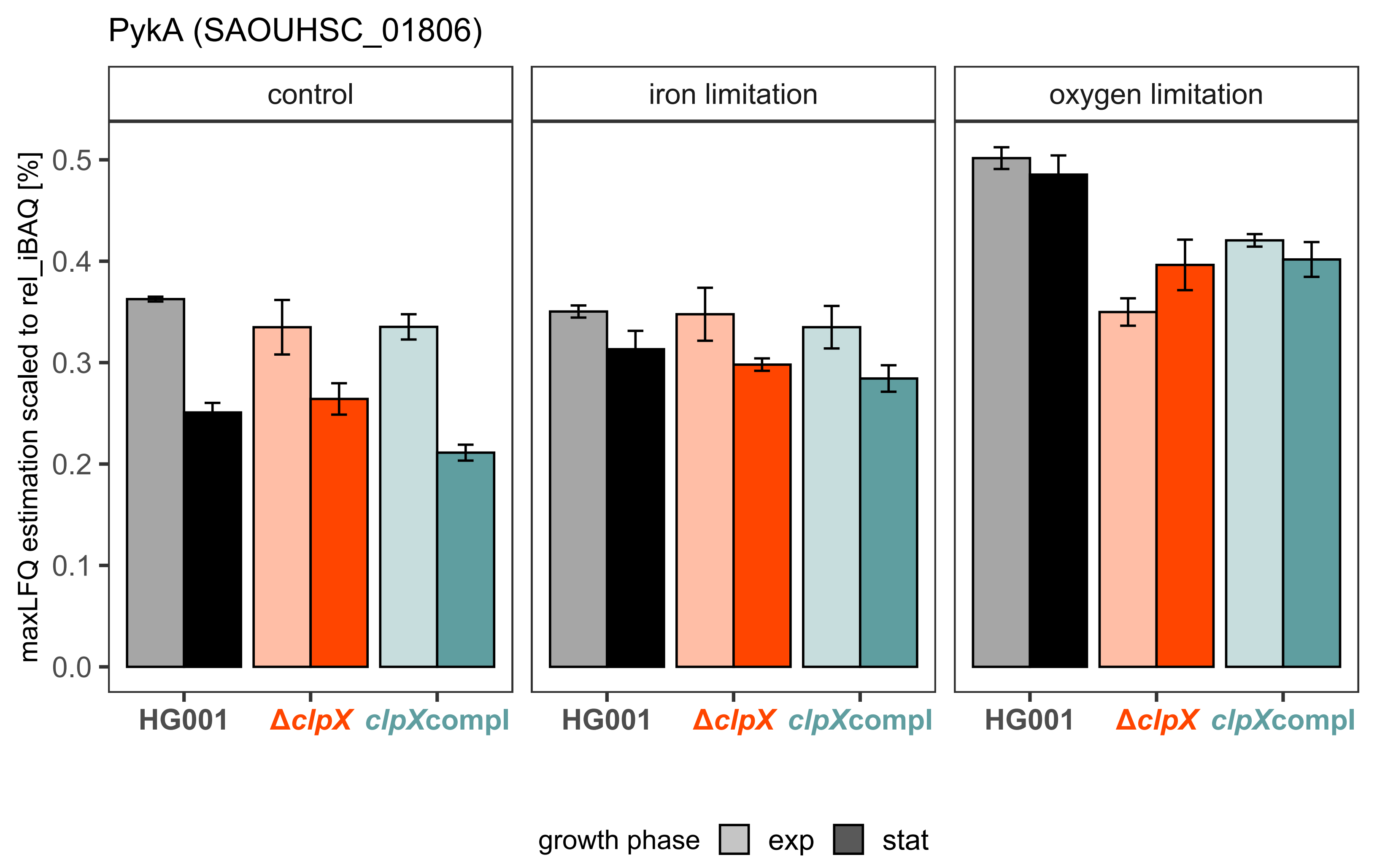

PykA (SAOUHSC_01806)
control
iron limitation
oxygen limitation
maxLFQ estimation scaled to rel_iBAQ [%]
0.5
0.4
0.3
0.2
0.1
0.0
HG001
ΔclpX
clpXcompl
growth phase
exp
stat

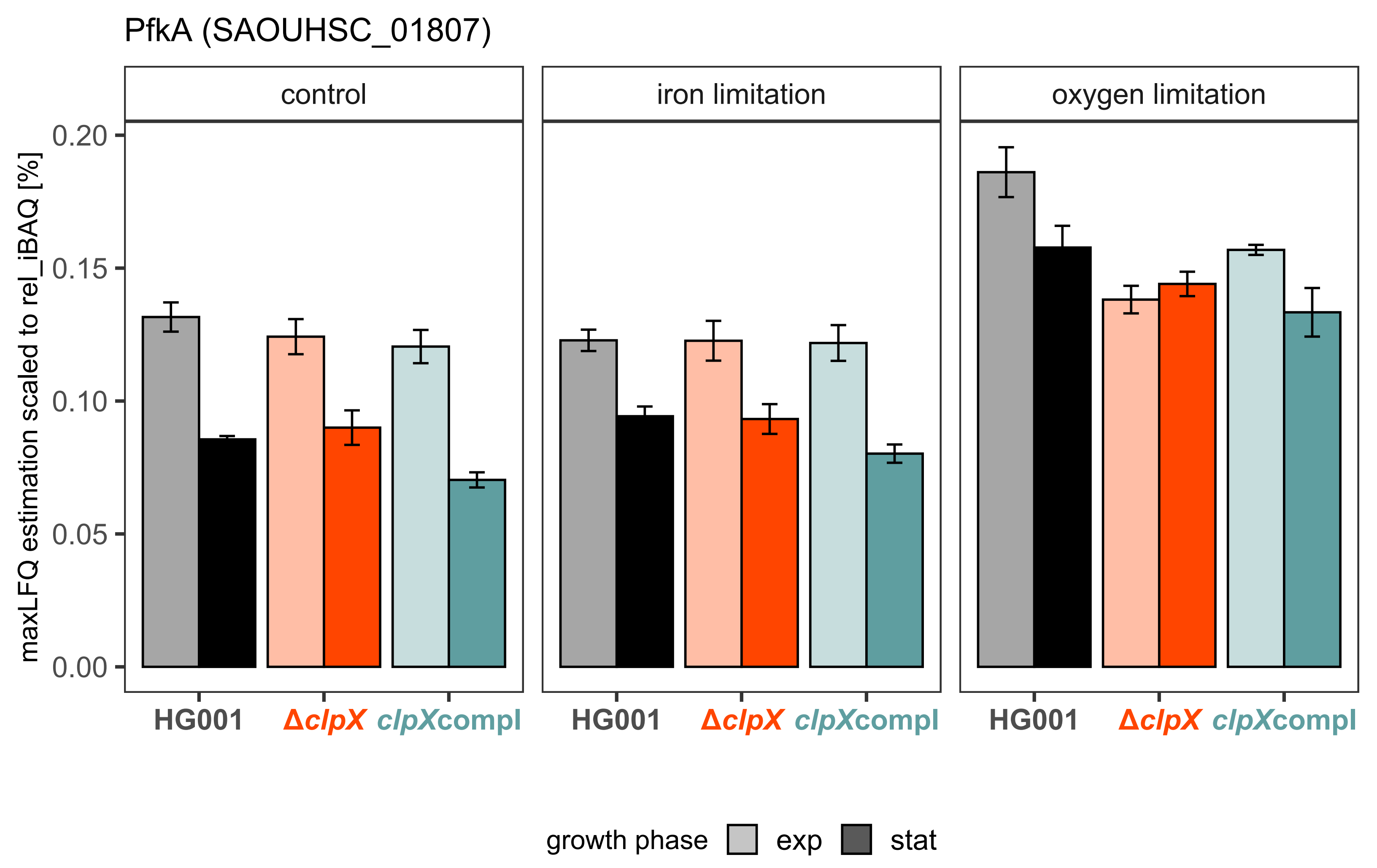

PfkA (SAOUHSC_01807)
control
iron limitation
oxygen limitation
maxLFQ estimation scaled to rel_iBAQ [%]
0.20
0.15
0.10
0.05
0.00
HG001
ΔclpX
clpXcompl
growth phase
exp
stat

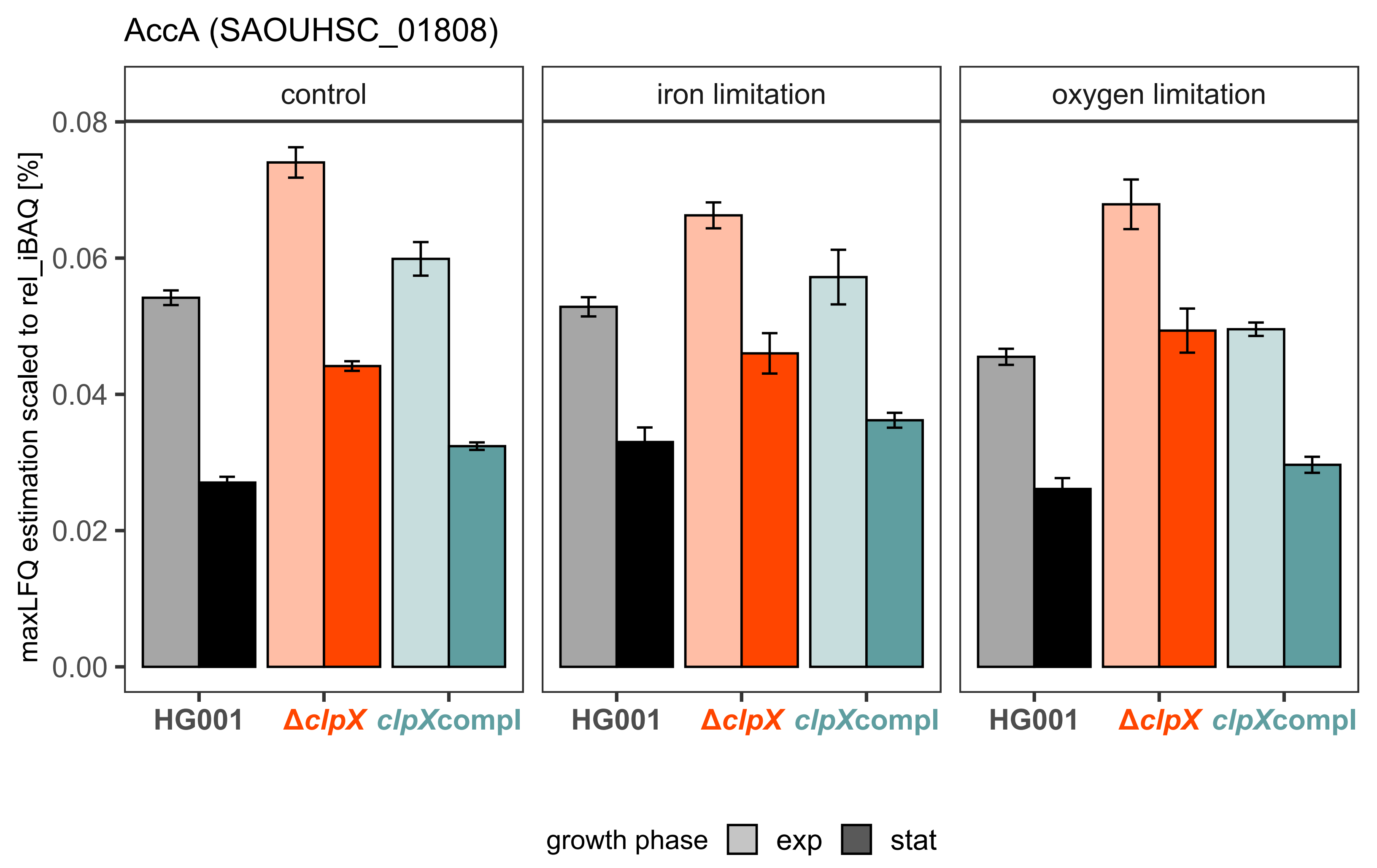

AccA (SAOUHSC_01808)
control
iron limitation
oxygen limitation
0.08
0.06
0.04
0.02
0.00
maxLFQ estimation scaled to rel_iBAQ [%]
HG001
ΔclpX
clpXcompl
growth phase
exp
stat

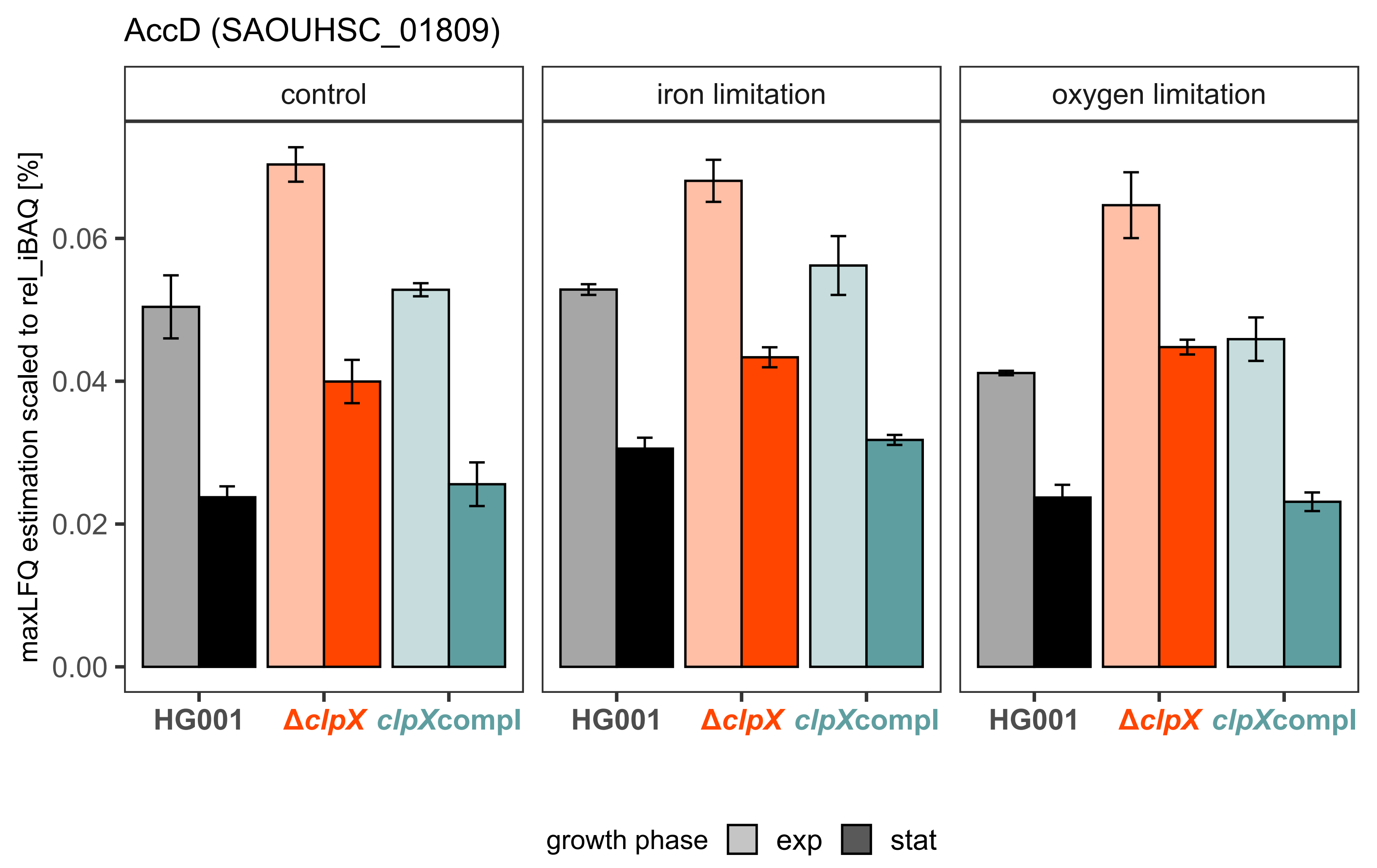

AccD (SAOUHSC_01809)
control
iron limitation
oxygen limitation
maxLFQ estimation scaled to rel_iBAQ [%]
0.06
0.04
0.02
0.00
HG001
ΔclpX
clpXcompl
growth phase
exp
stat

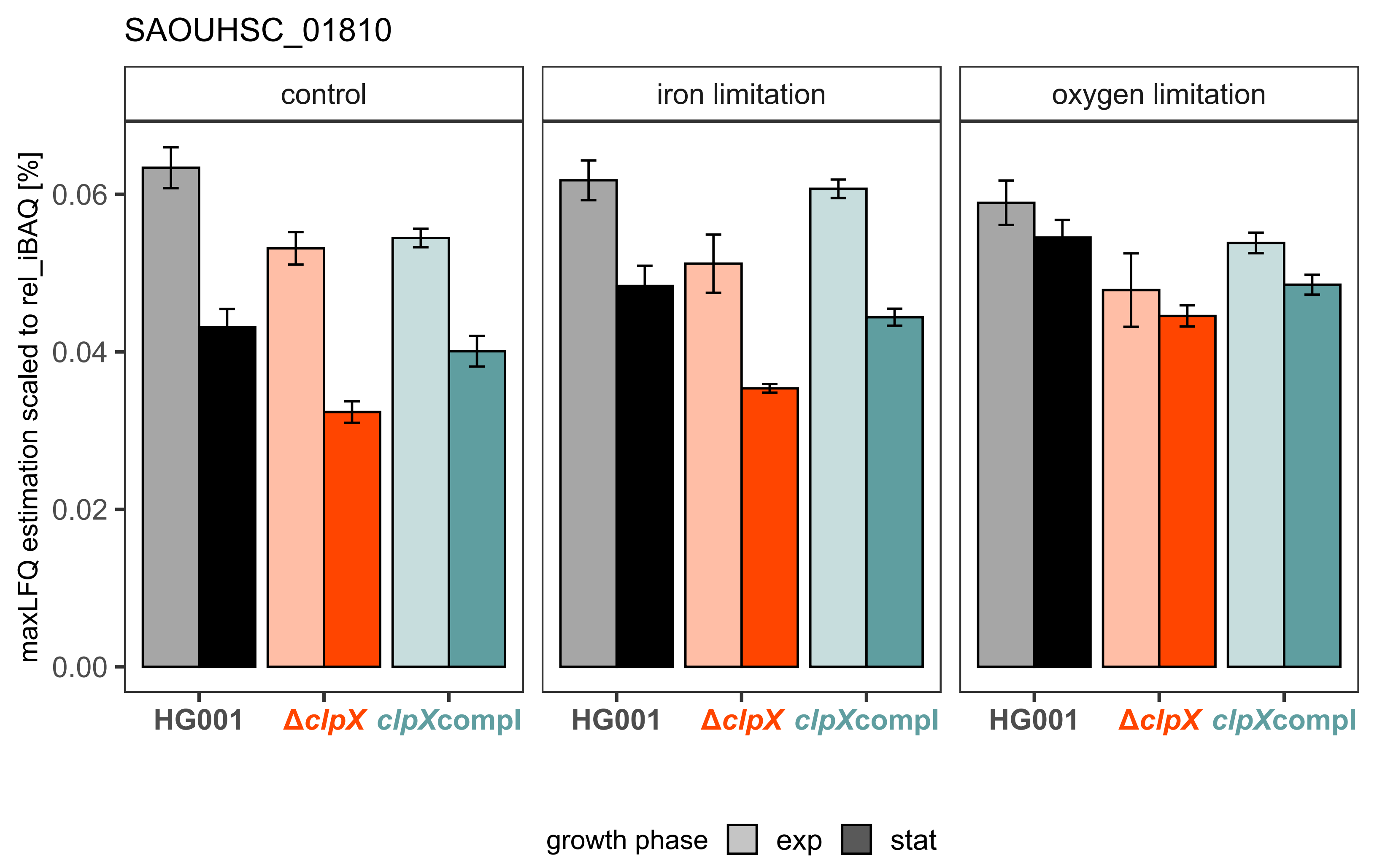
SAOUHSC_01810
control
iron limitation
oxygen limitation
maxLFQ estimation scaled to rel_iBAQ [%]
0.06
0.04
0.02
0.00
HG001
ΔclpX
clpXcompl
growth phase
exp
stat

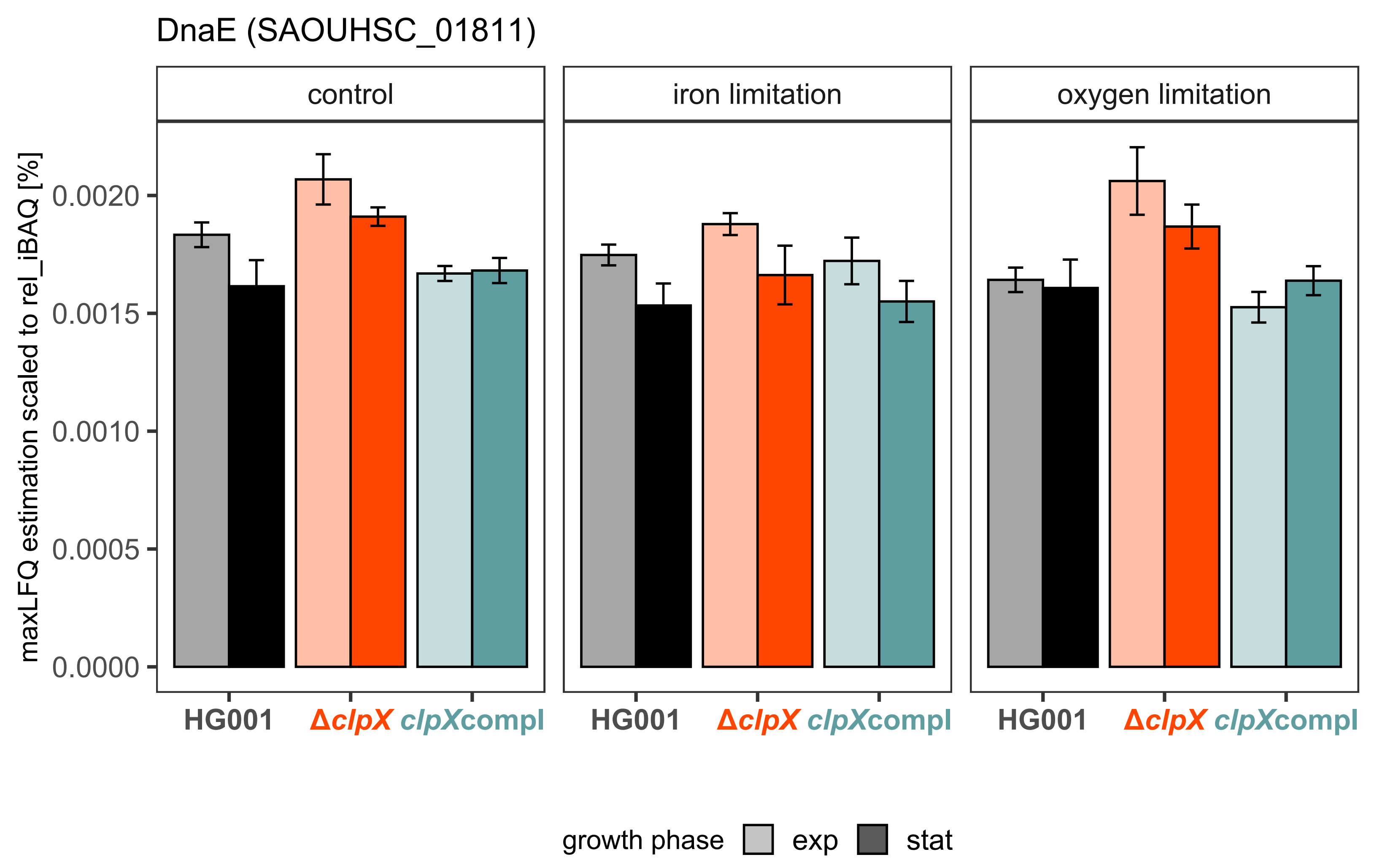
DnaE (SAOUHSC_01811)
control
iron limitation
oxygen limitation
maxLFQ estimation scaled to rel_iBAQ [%]
0.0020
0.0015
0.0010
0.0005
0.0000
HG001
ΔclpX
clpXcompl
growth phase
exp
stat

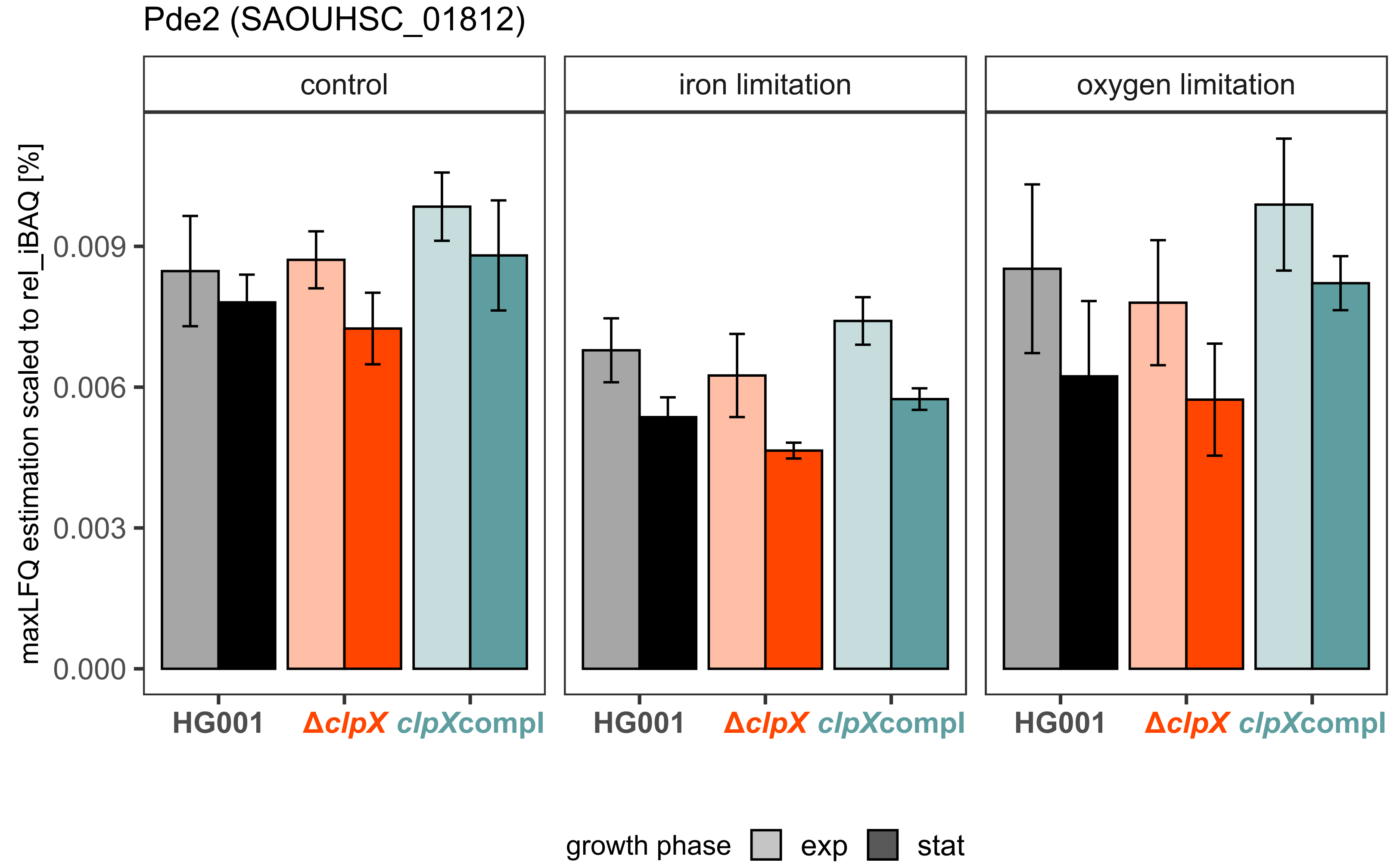

Pde2 (SAOUHSC_01812)
control
iron limitation
oxygen limitation
maxLFQ estimation scaled to rel_iBAQ [%]
0.009
0.006
0.003
0.000
HG001
ΔclpX
clpXcompl
growth phase
exp
stat

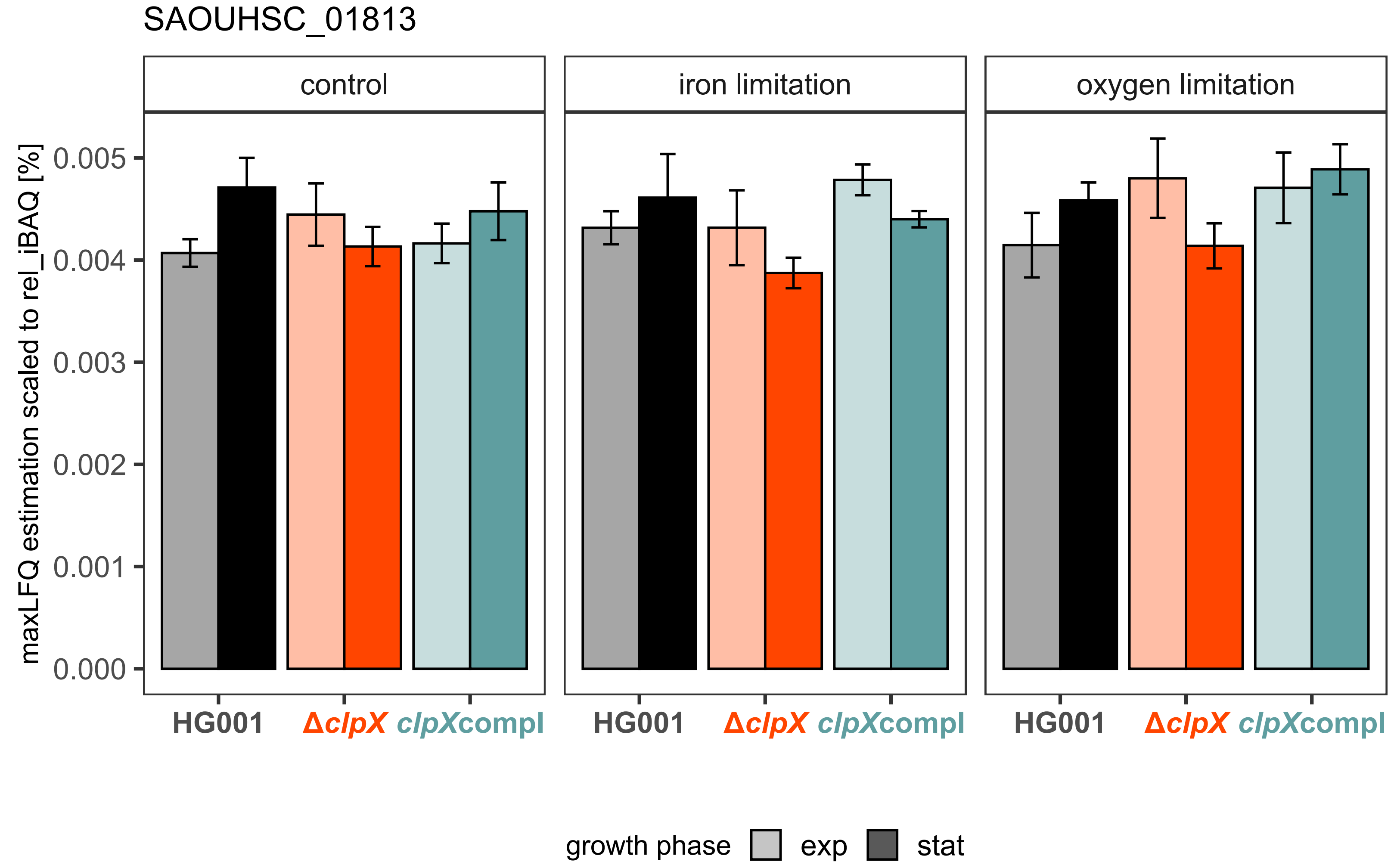

SAOUHSC_01813
control
iron limitation
oxygen limitation
maxLFQ estimation scaled to rel_iBAQ [%]
0.005
0.004
0.003
0.002
0.001
0.000
HG001
ΔclpX
clpXcompl
growth phase
exp
stat

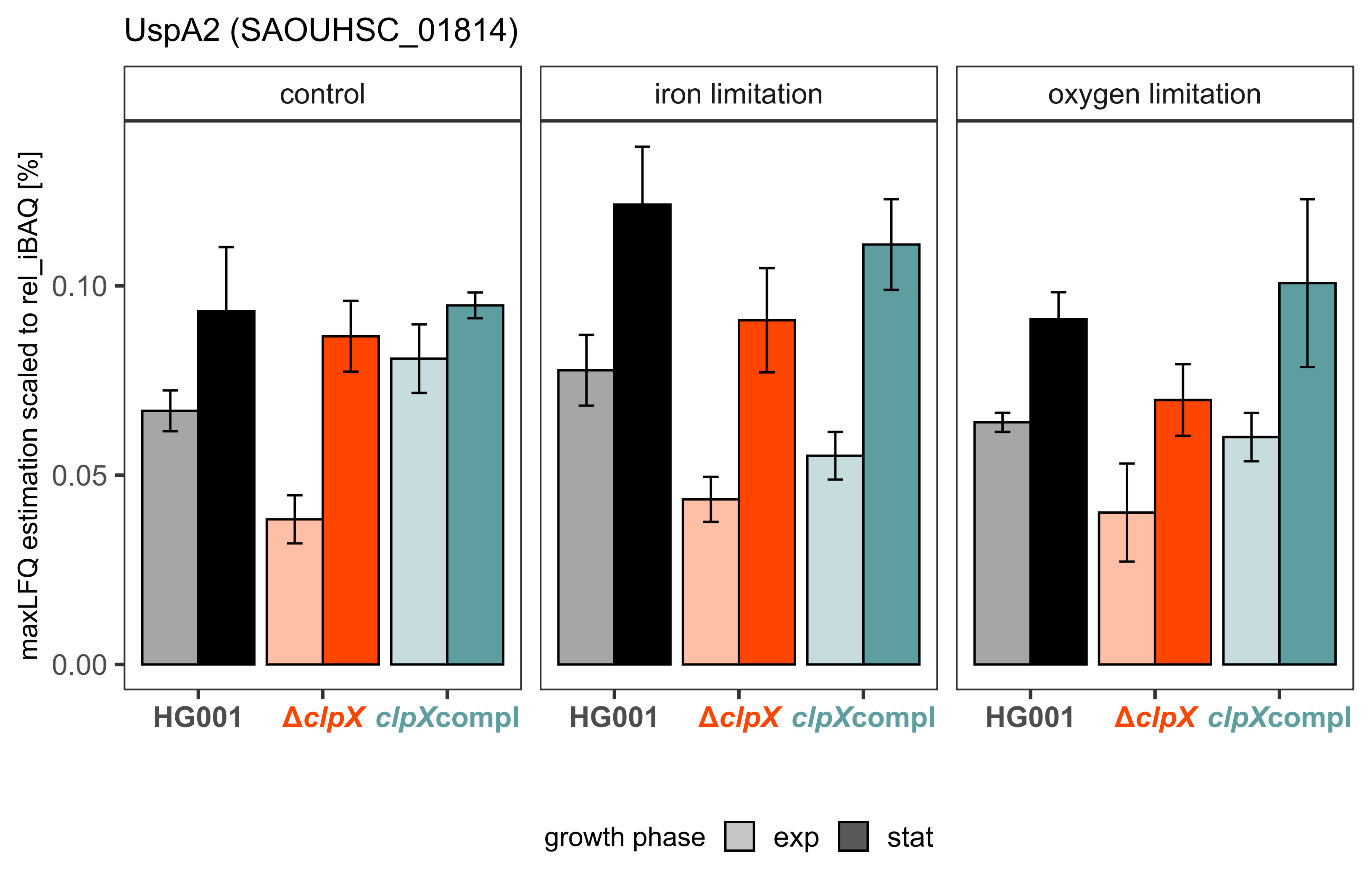

UspA2 (SAOUHSC_01814)
control
iron limitation
oxygen limitation
maxLFQ estimation scaled to rel_iBAQ [%]
0.10
0.05
0.00
HG001
ΔclpX
clpXcompl
growth phase
exp
stat

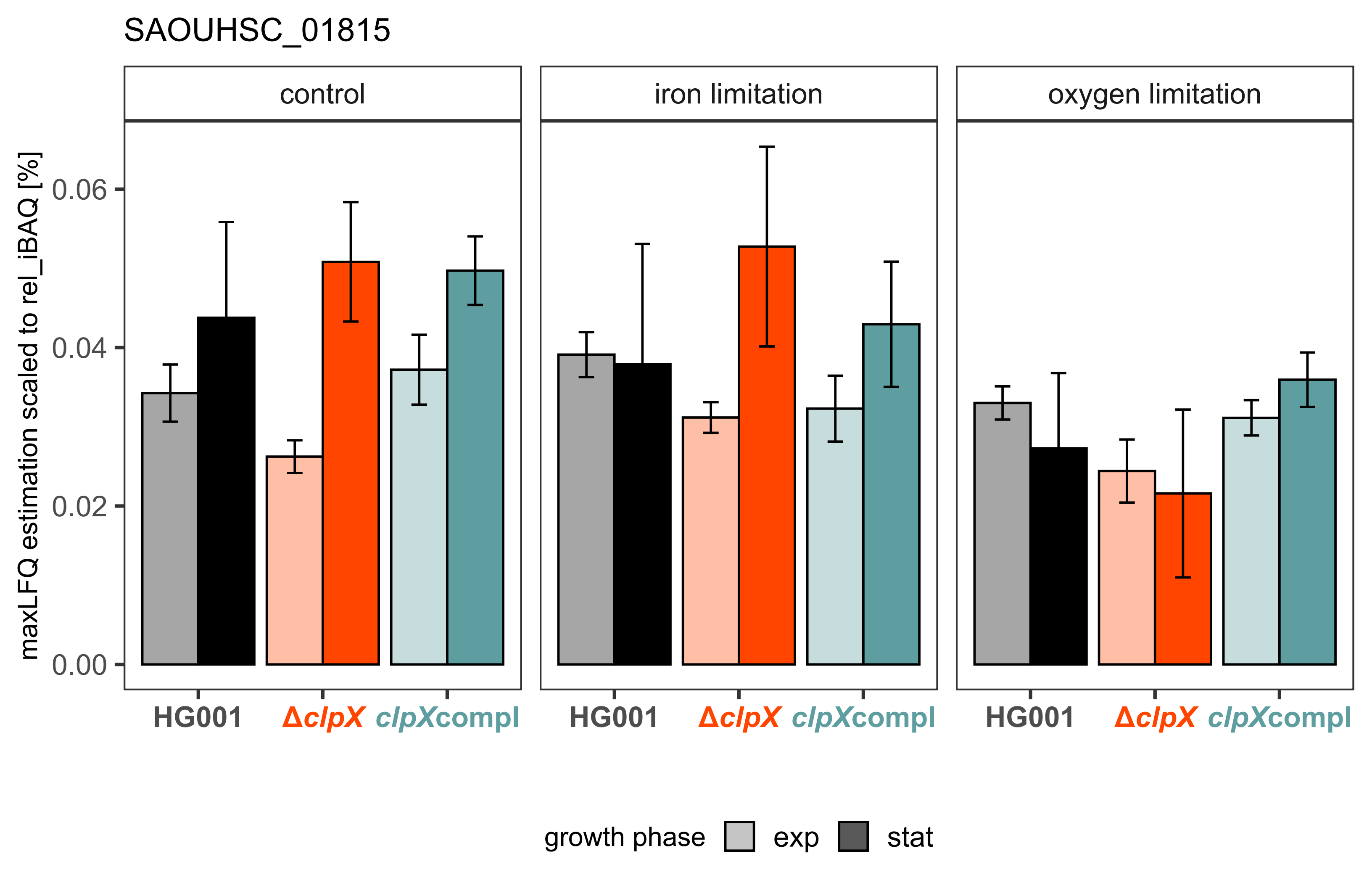

SAOUHSC_01815
control
iron limitation
oxygen limitation
maxLFQ estimation scaled to rel_iBAQ [%]
0.06
0.04
0.02
0.00
HG001
ΔclpX
clpXcompl
growth phase
exp
stat

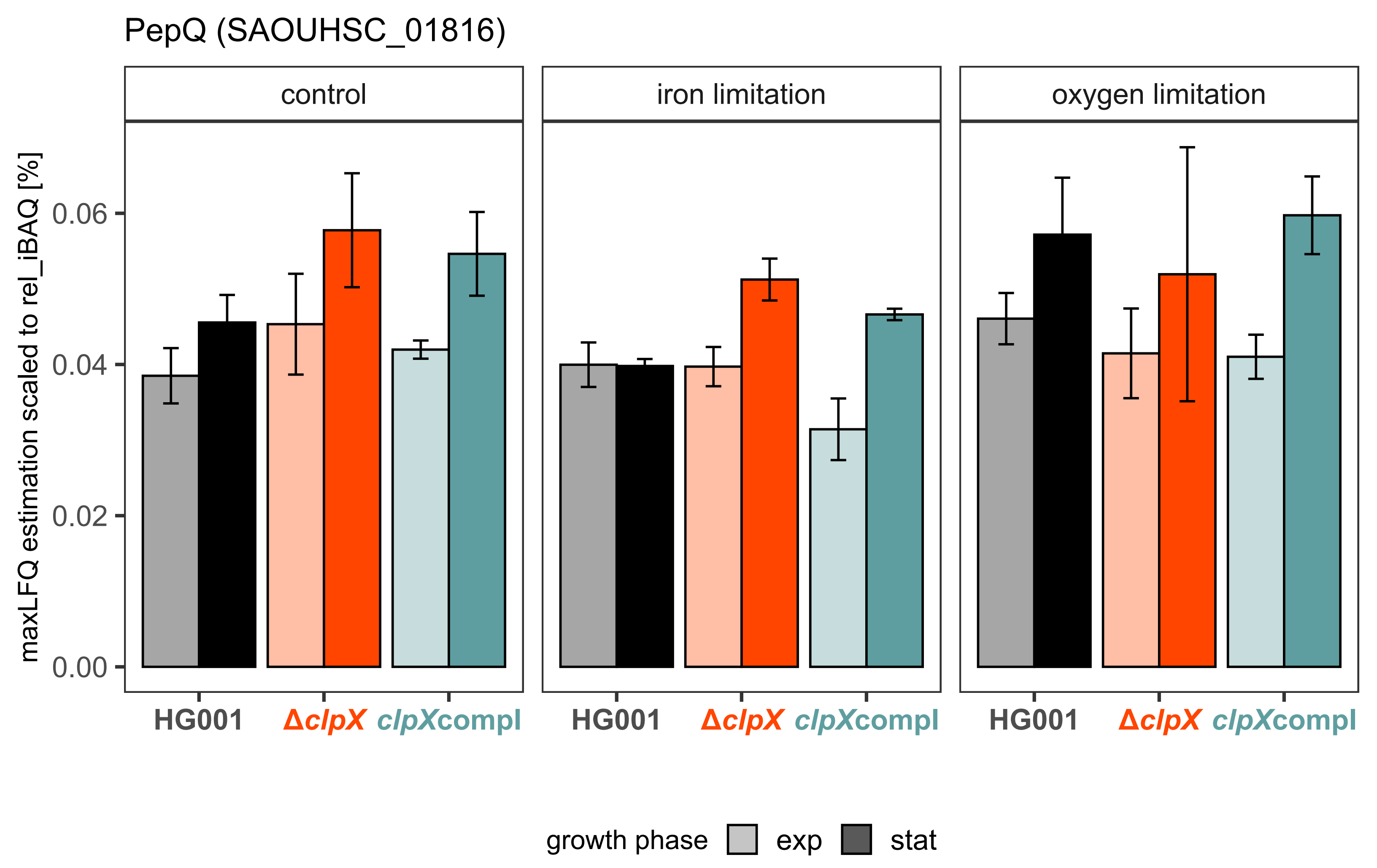

PepQ (SAOUHSC_01816)
control
iron limitation
oxygen limitation
maxLFQ estimation scaled to rel_iBAQ [%]
0.06
0.04
0.02
0.00
HG001
ΔclpX
clpXcompl
growth phase
exp
stat

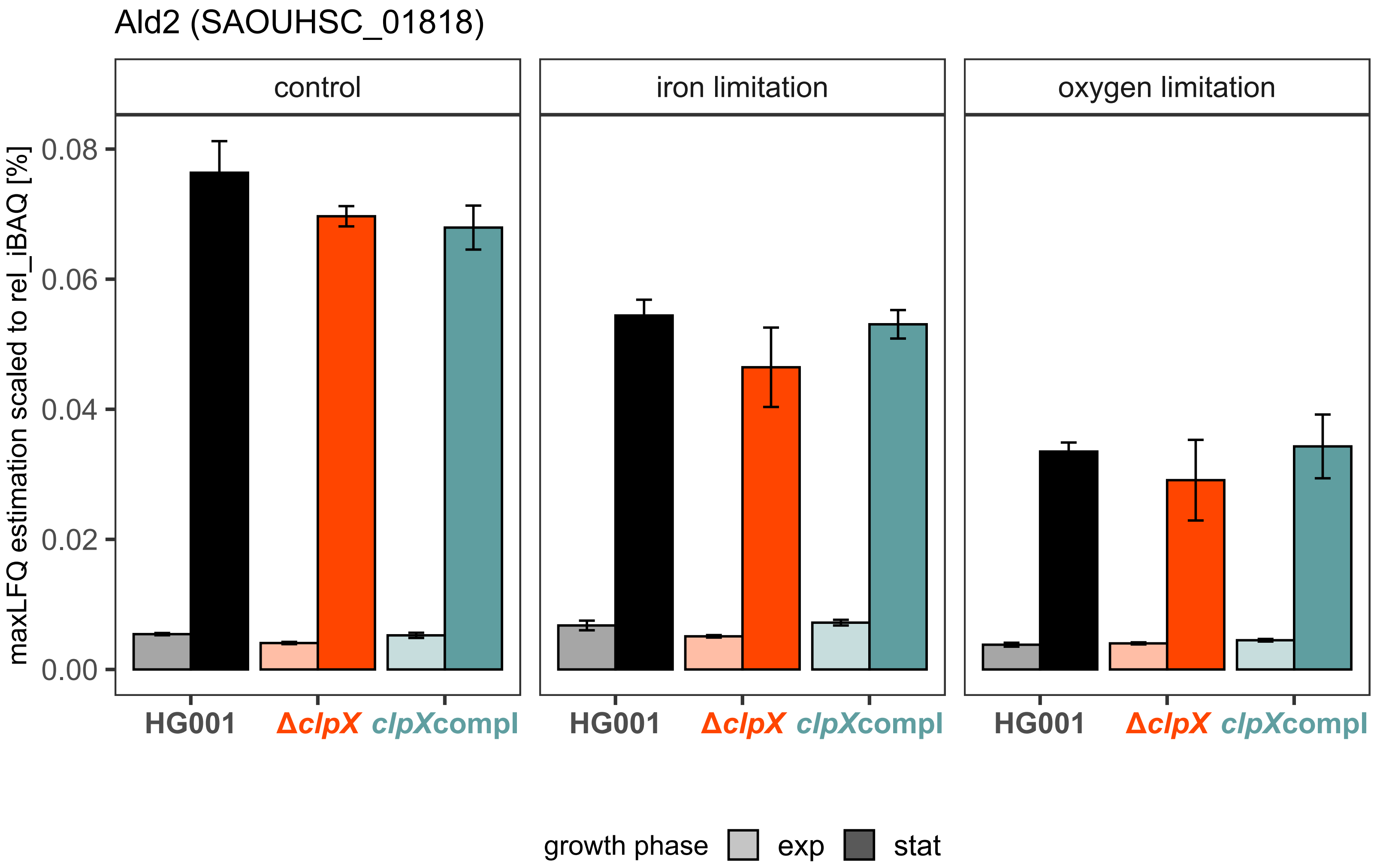

Ald2 (SAOUHSC_01818)
control
iron limitation
oxygen limitation
maxLFQ estimation scaled to rel_iBAQ [%]
0.08
0.06
0.04
0.02
0.00
HG001
ΔclpX
clpXcompl
growth phase
exp
stat

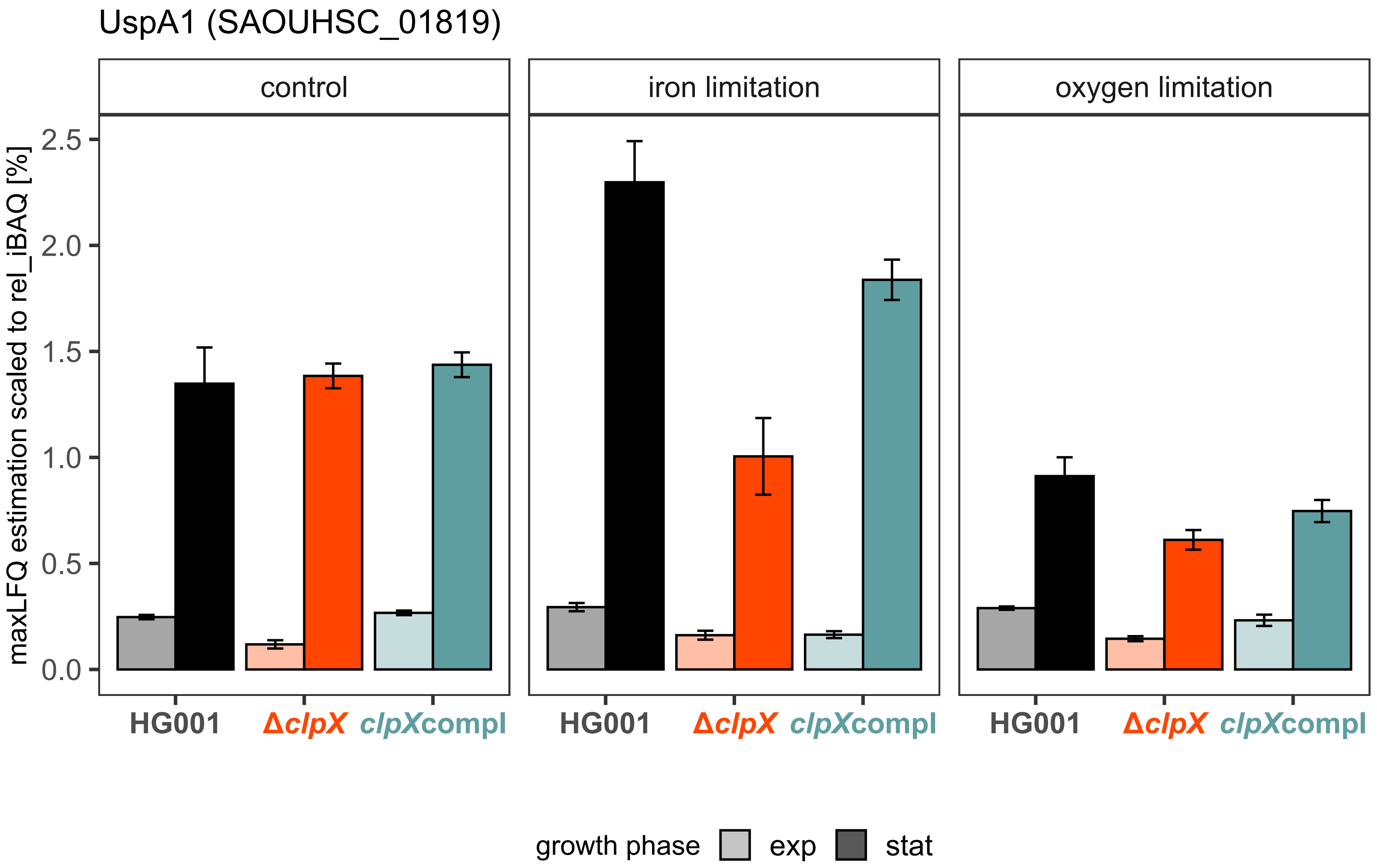

UspA1 (SAOUHSC_01819)
control
iron limitation
oxygen limitation
maxLFQ estimation scaled to rel_iBAQ [%]
0.0
0.5
1.0
1.5
2.0
2.5
HG001
ΔclpX
clpXcompl
growth phase
exp
stat

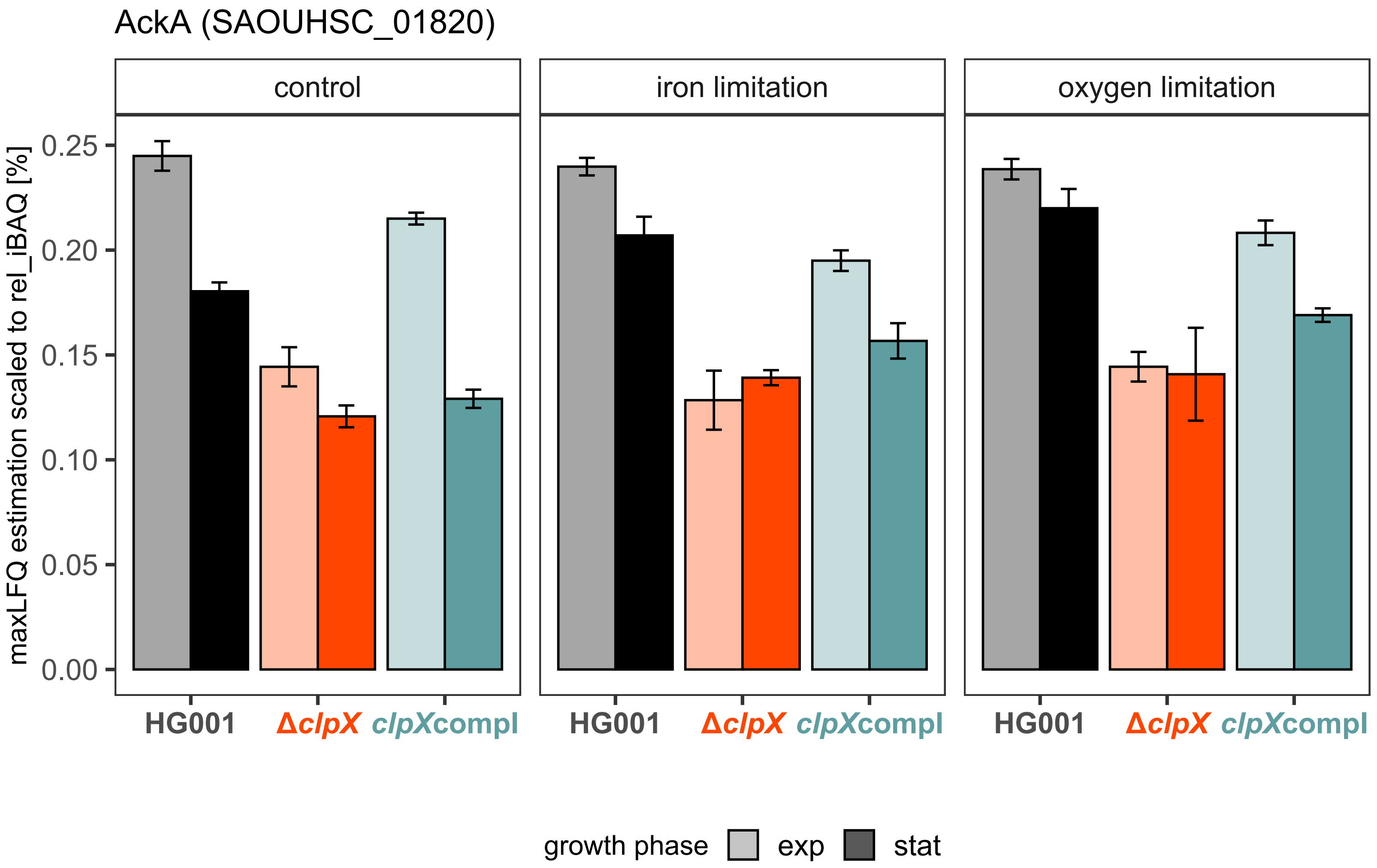
AckA (SAOUHSC_01820)
control
iron limitation
oxygen limitation
maxLFQ estimation scaled to rel_iBAQ [%]
0.25
0.20
0.15
0.10
0.05
0.00
HG001
ΔclpX
clpXcompl
growth phase
exp
stat

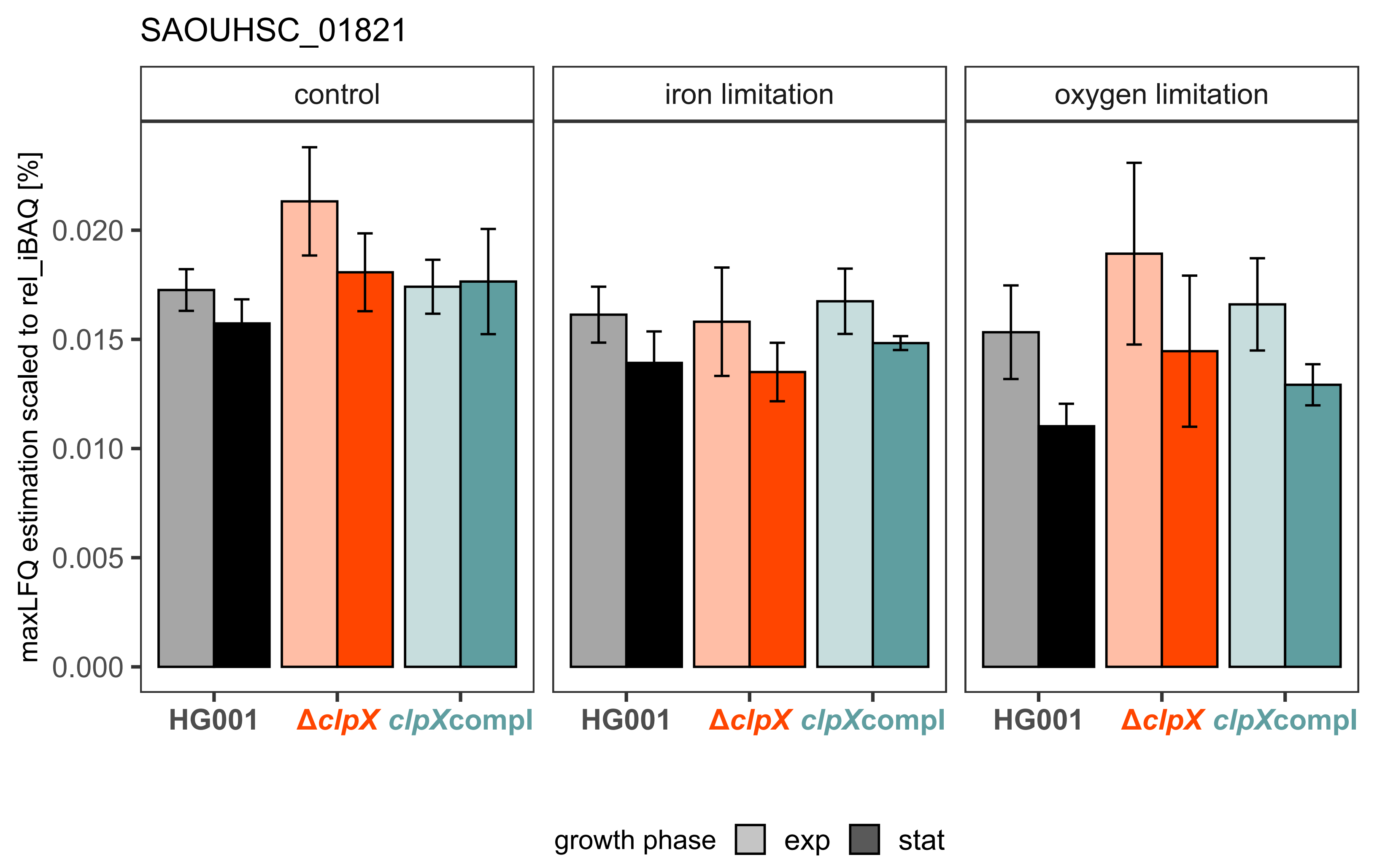
SAOUHSC_01821
control
iron limitation
oxygen limitation
maxLFQ estimation scaled to rel_iBAQ [%]
0.020
0.015
0.010
0.005
0.000
HG001
ΔclpX
clpXcompl
growth phase
exp
stat

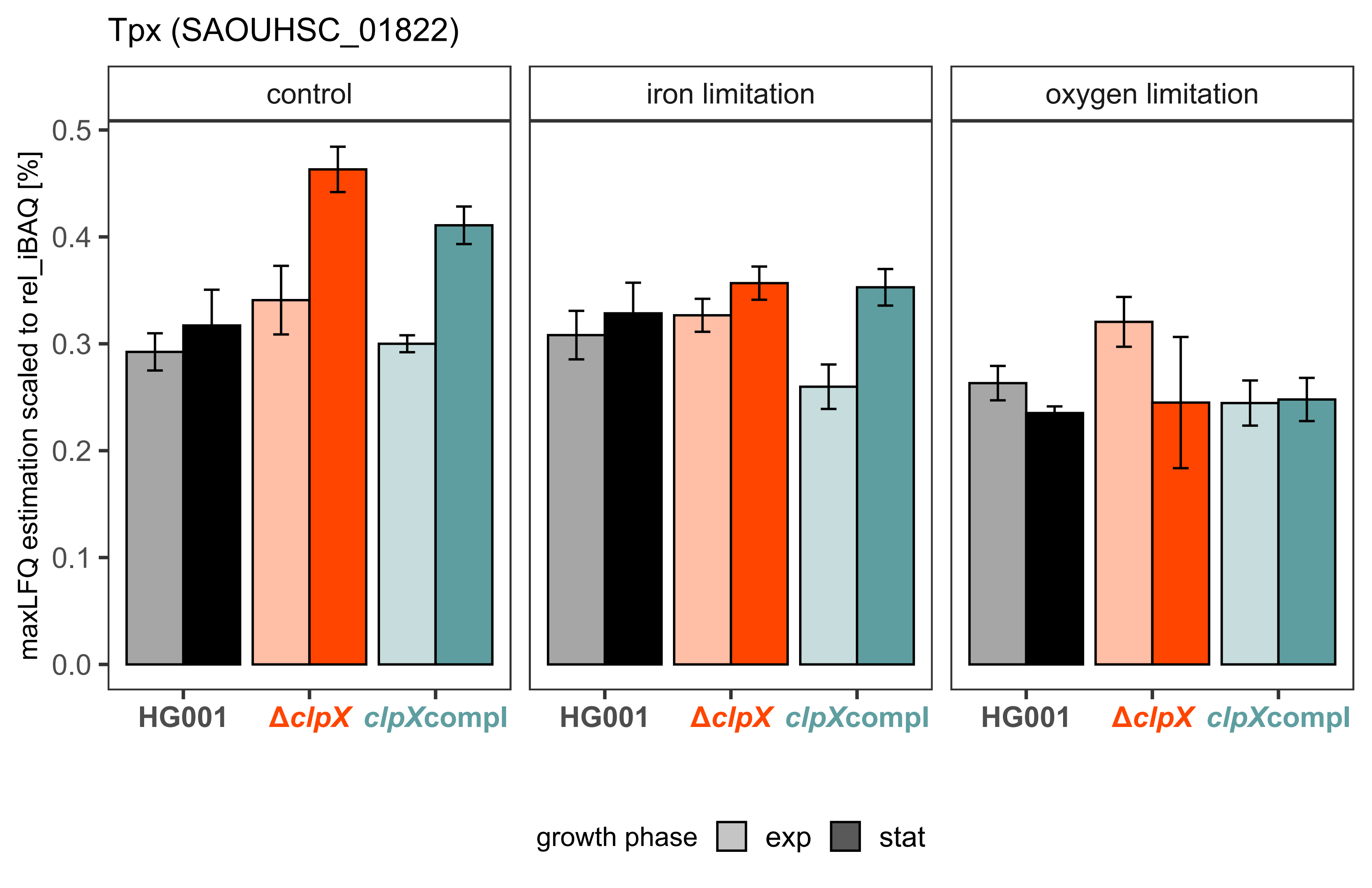

Tpx (SAOUHSC_01822)
control
iron limitation
oxygen limitation
maxLFQ estimation scaled to rel_iBAQ [%]
0.5
0.4
0.3
0.2
0.1
0.0
HG001
ΔclpX
clpXcompl
growth phase
exp
stat

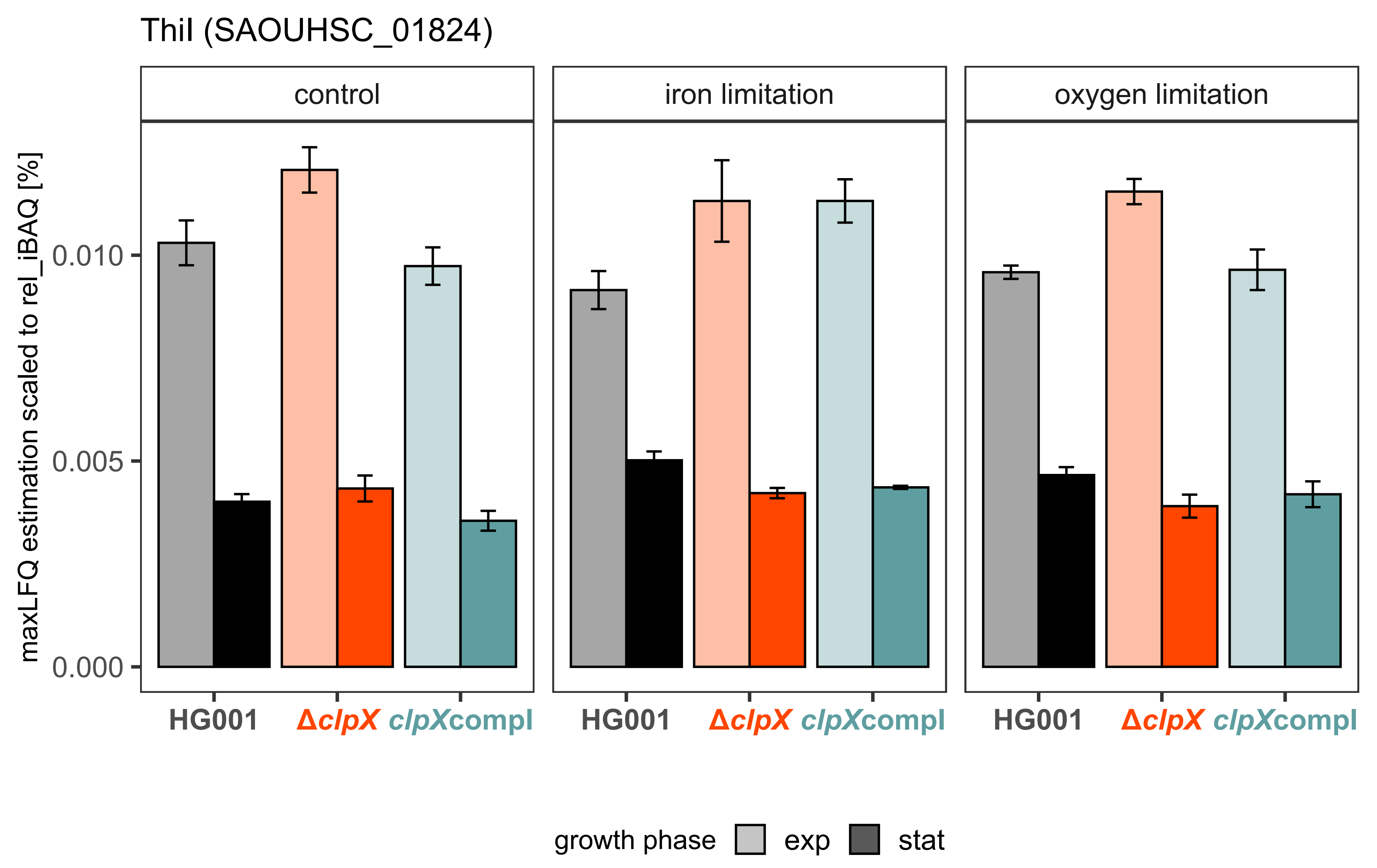

ThiI (SAOUHSC_01824)
control
iron limitation
oxygen limitation
maxLFQ estimation scaled to rel_iBAQ [%]
0.010
0.005
0.000
HG001
ΔclpX
clpXcompl
growth phase
exp
stat

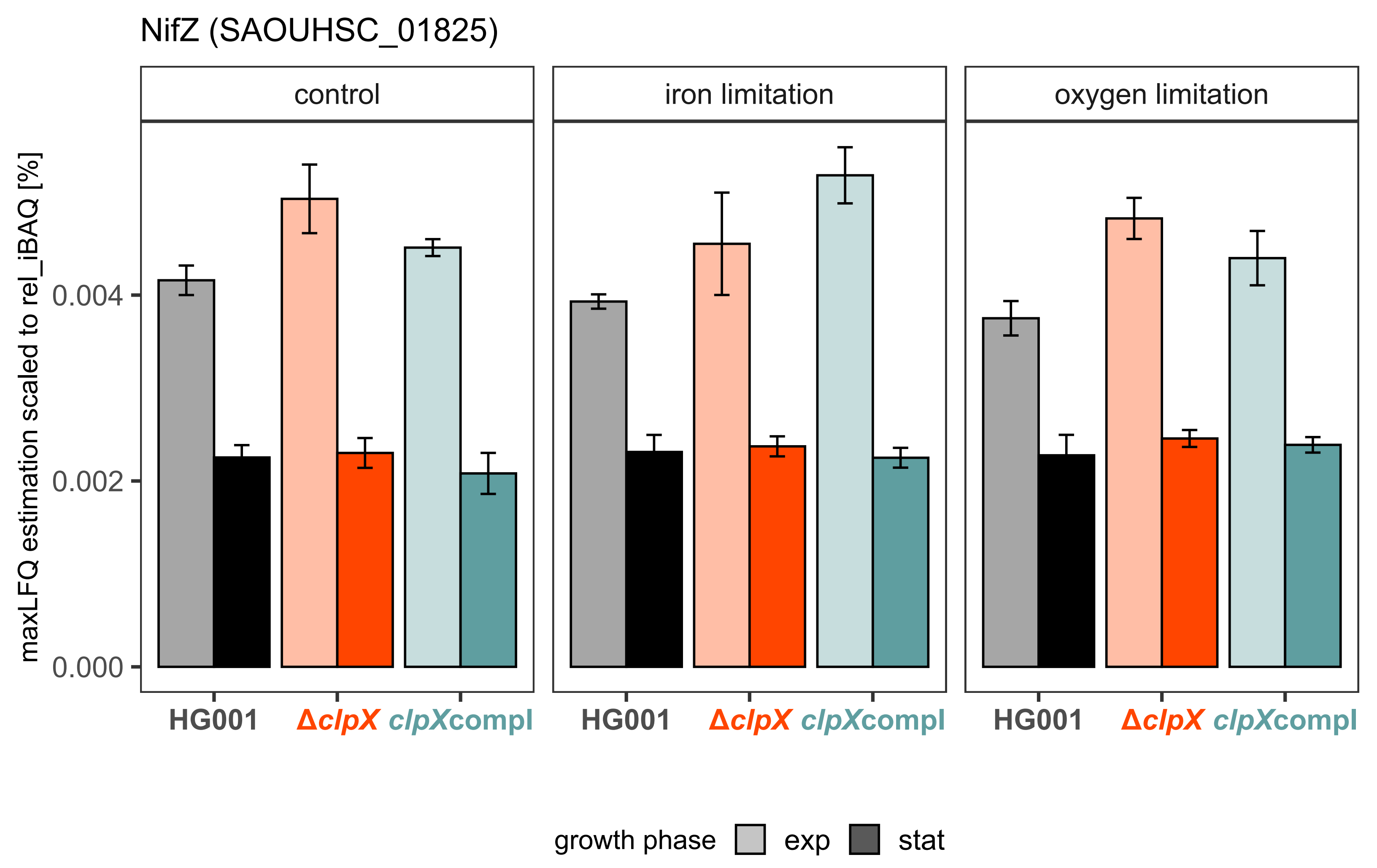
NifZ (SAOUHSC_01825)
control
iron limitation
oxygen limitation
maxLFQ estimation scaled to rel_iBAQ [%]
0.004
0.002
0.000
HG001
ΔclpX
clpXcompl
growth phase
exp
stat

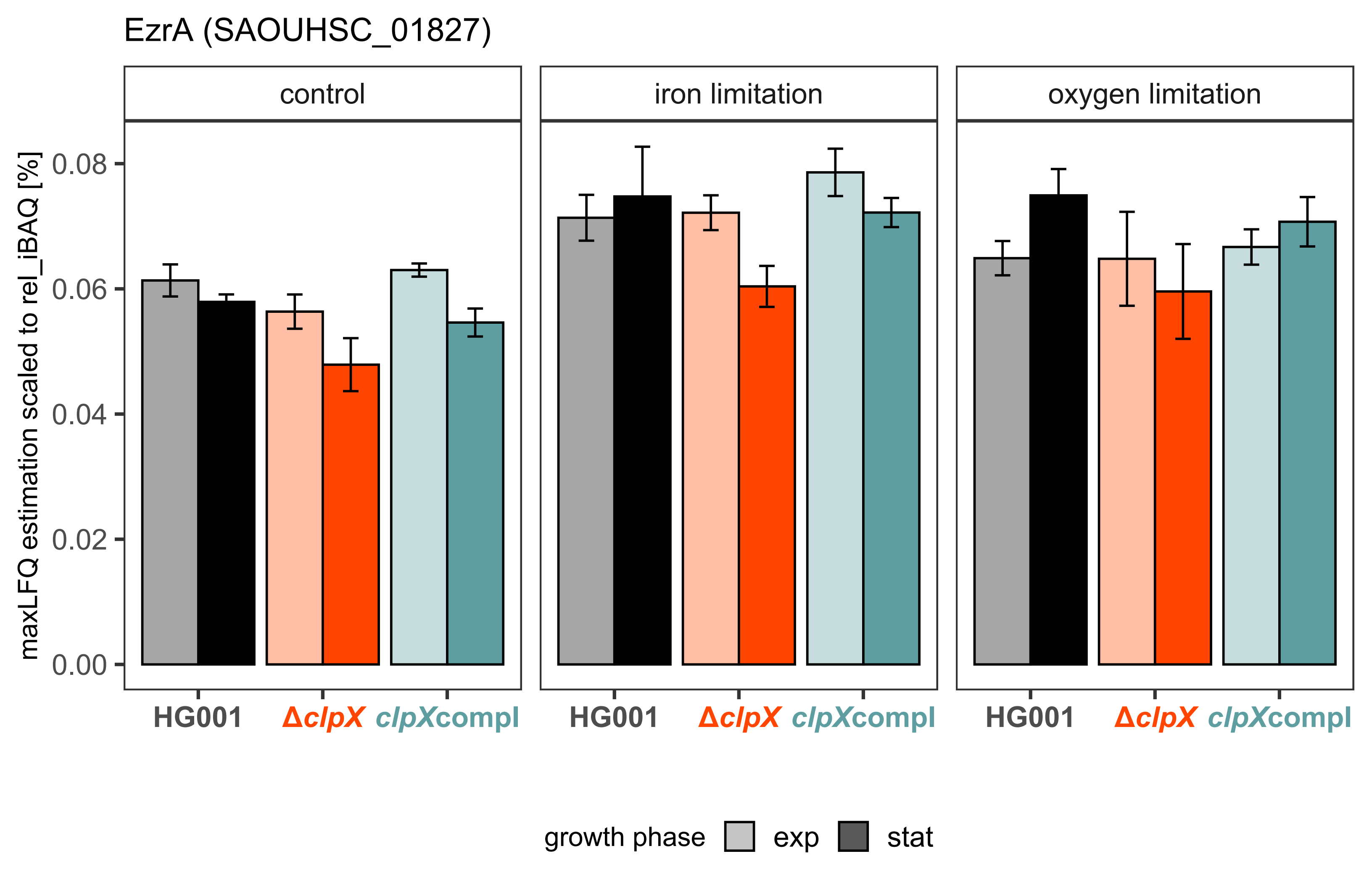

EzrA (SAOUHSC_01827)
control
iron limitation
oxygen limitation
maxLFQ estimation scaled to rel_iBAQ [%]
0.08
0.06
0.04
0.02
0.00
HG001
ΔclpX
clpXcompl
growth phase
exp
stat

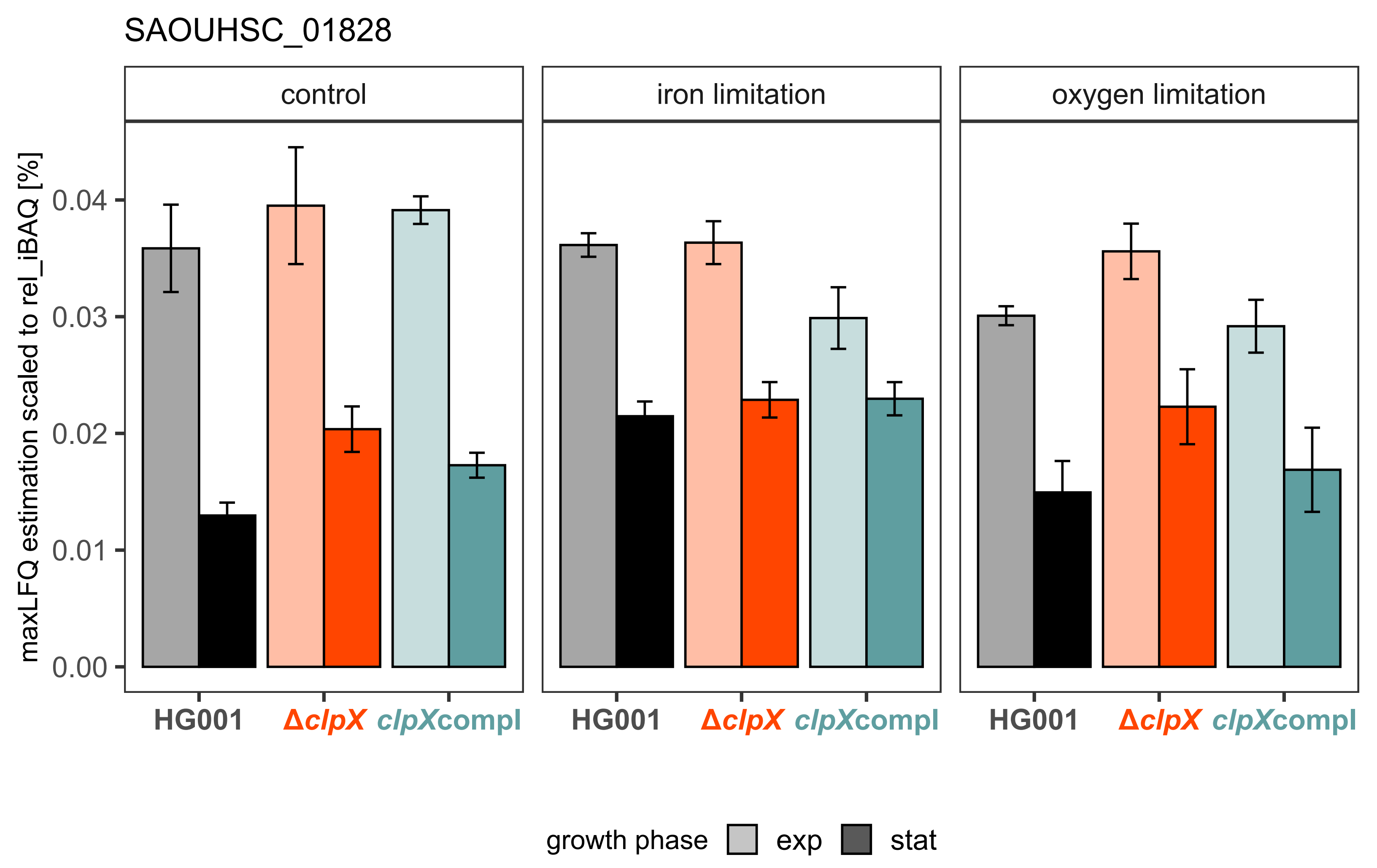
SAOUHSC_01828
control
iron limitation
oxygen limitation
maxLFQ estimation scaled to rel_iBAQ [%]
0.00
0.01
0.02
0.03
0.04
HG001
ΔclpX
clpXcompl
growth phase
exp
stat

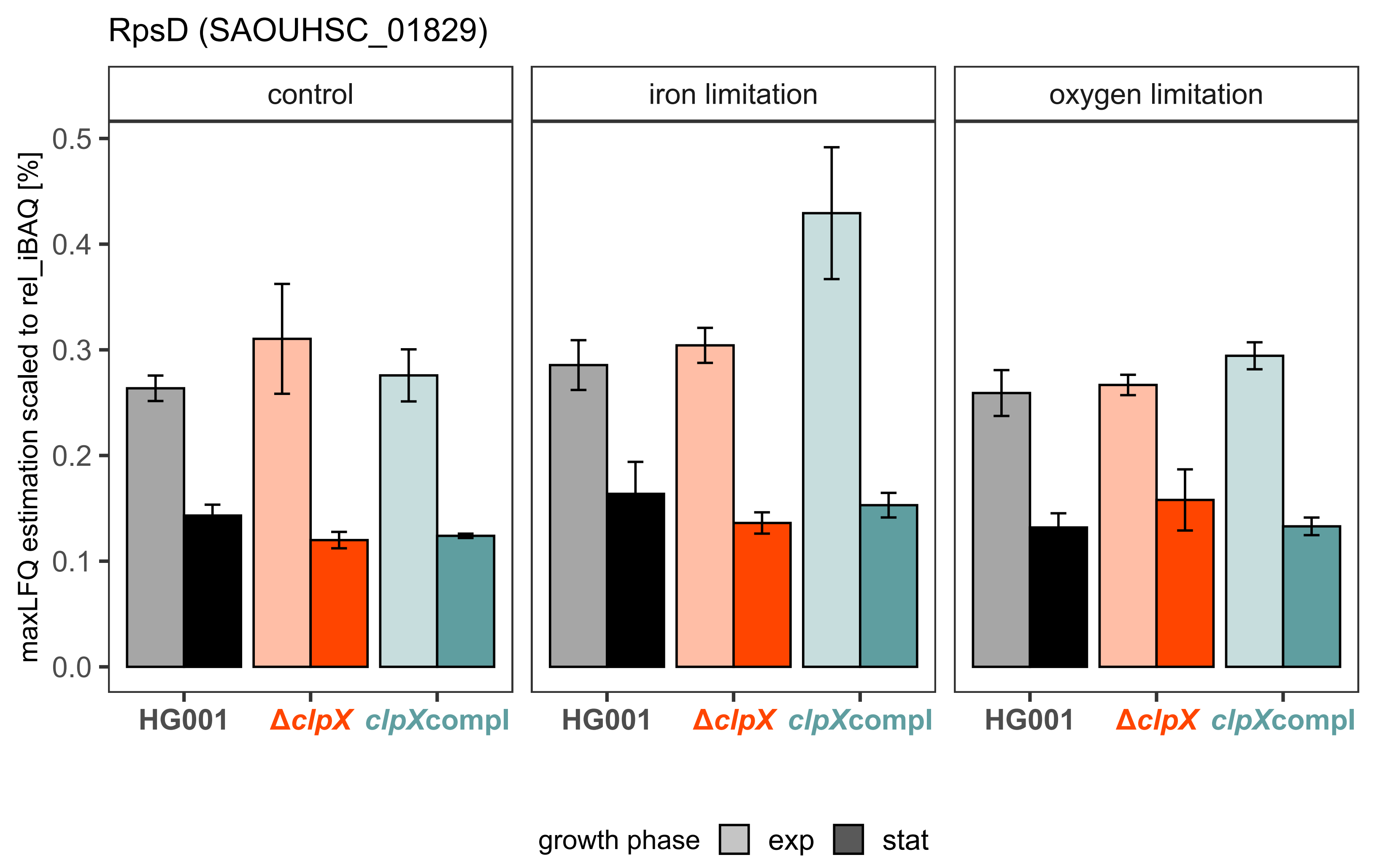
RpsD (SAOUHSC_01829)
control
iron limitation
oxygen limitation
maxLFQ estimation scaled to rel_iBAQ [%]
0.0
0.1
0.2
0.3
0.4
0.5
HG001
ΔclpX
clpXcompl
growth phase
exp
stat

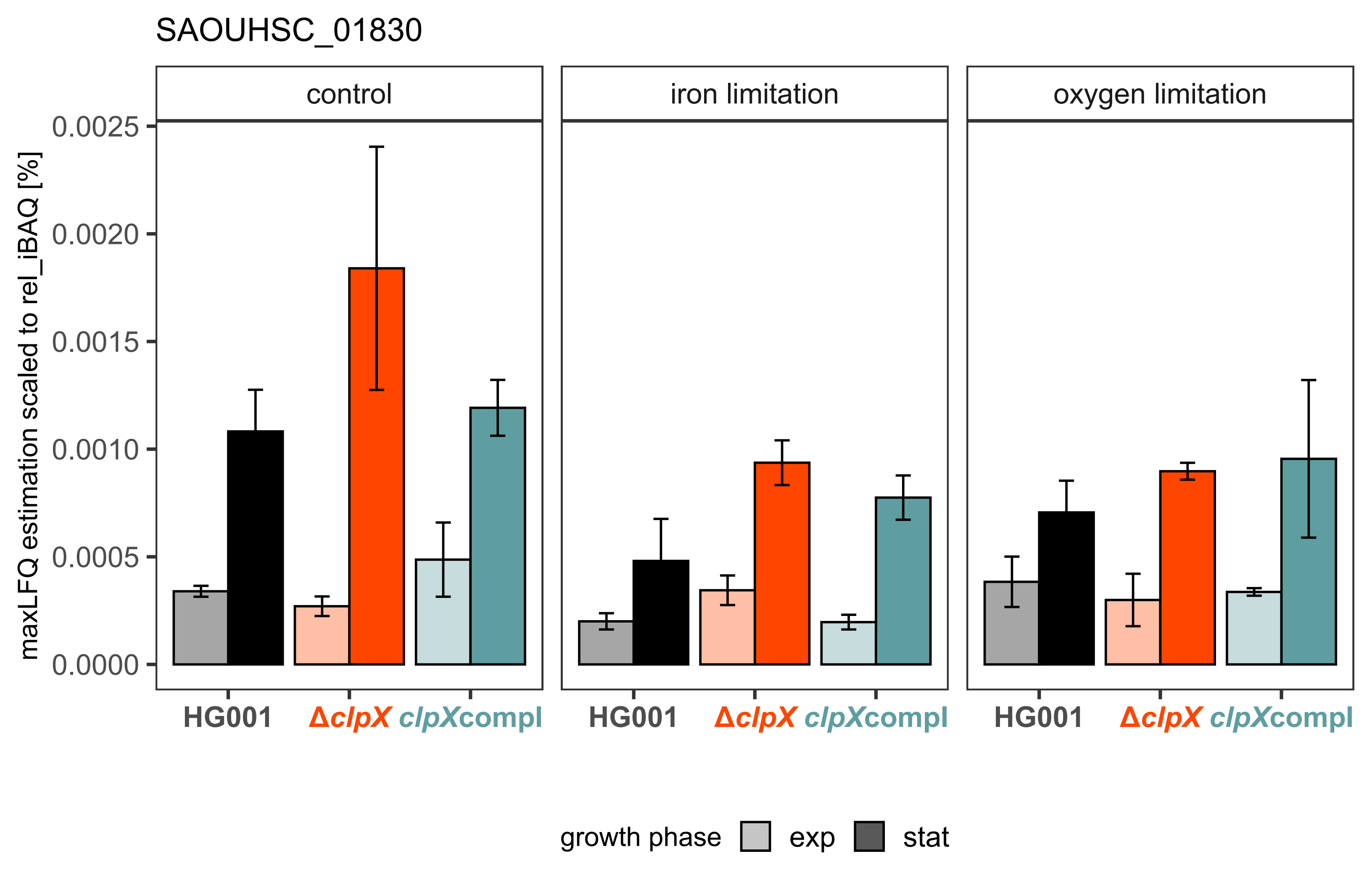

SAOUHSC_01830
control
iron limitation
oxygen limitation
maxLFQ estimation scaled to rel_iBAQ [%]
0.0025
0.0020
0.0015
0.0010
0.0005
0.0000
HG001
ΔclpX
clpXcompl
growth phase
exp
stat

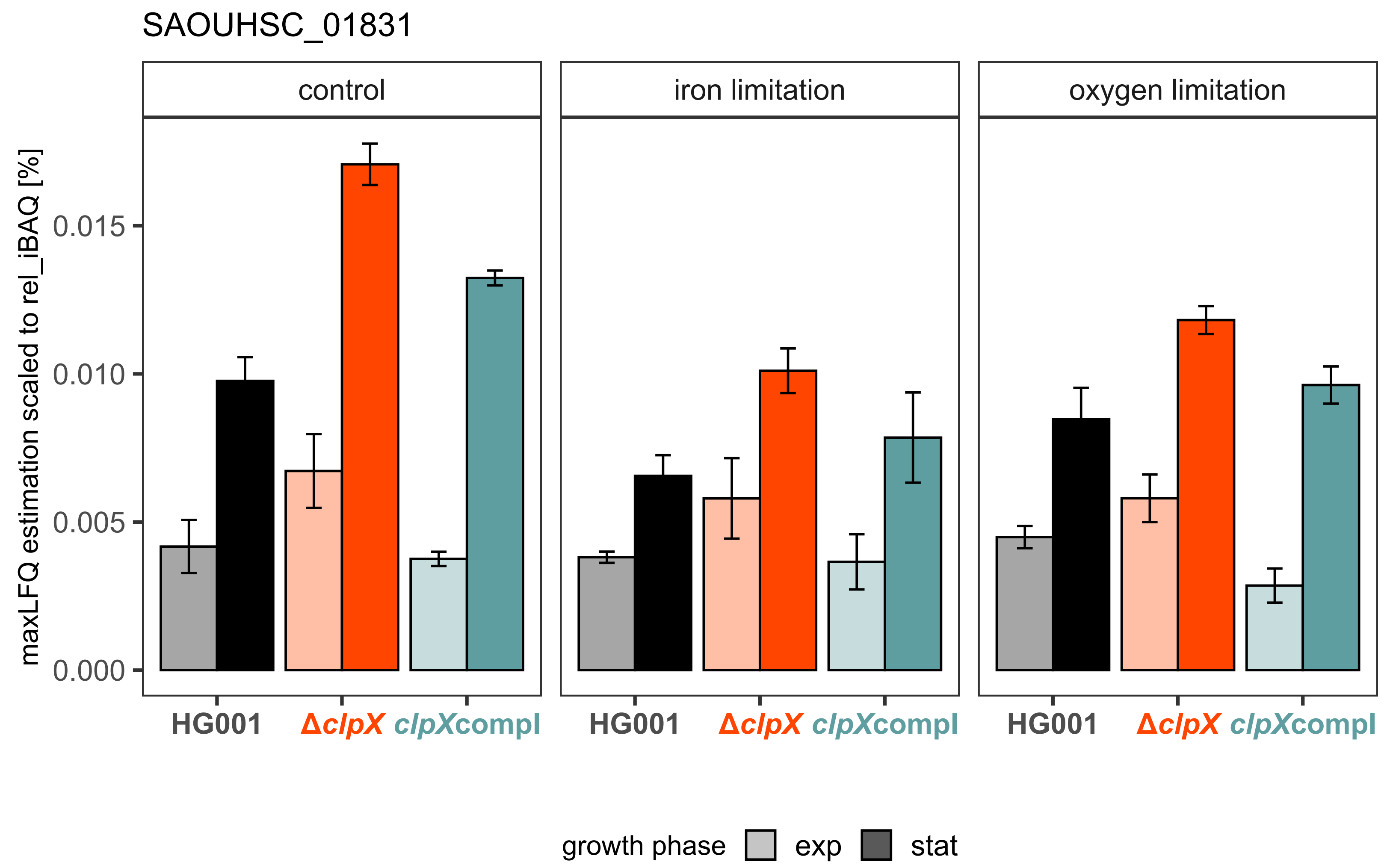
SAOUHSC_01831
control
iron limitation
oxygen limitation
maxLFQ estimation scaled to rel_iBAQ [%]
0.000
0.005
0.010
0.015
HG001
ΔclpX
clpXcompl
growth phase
exp
stat

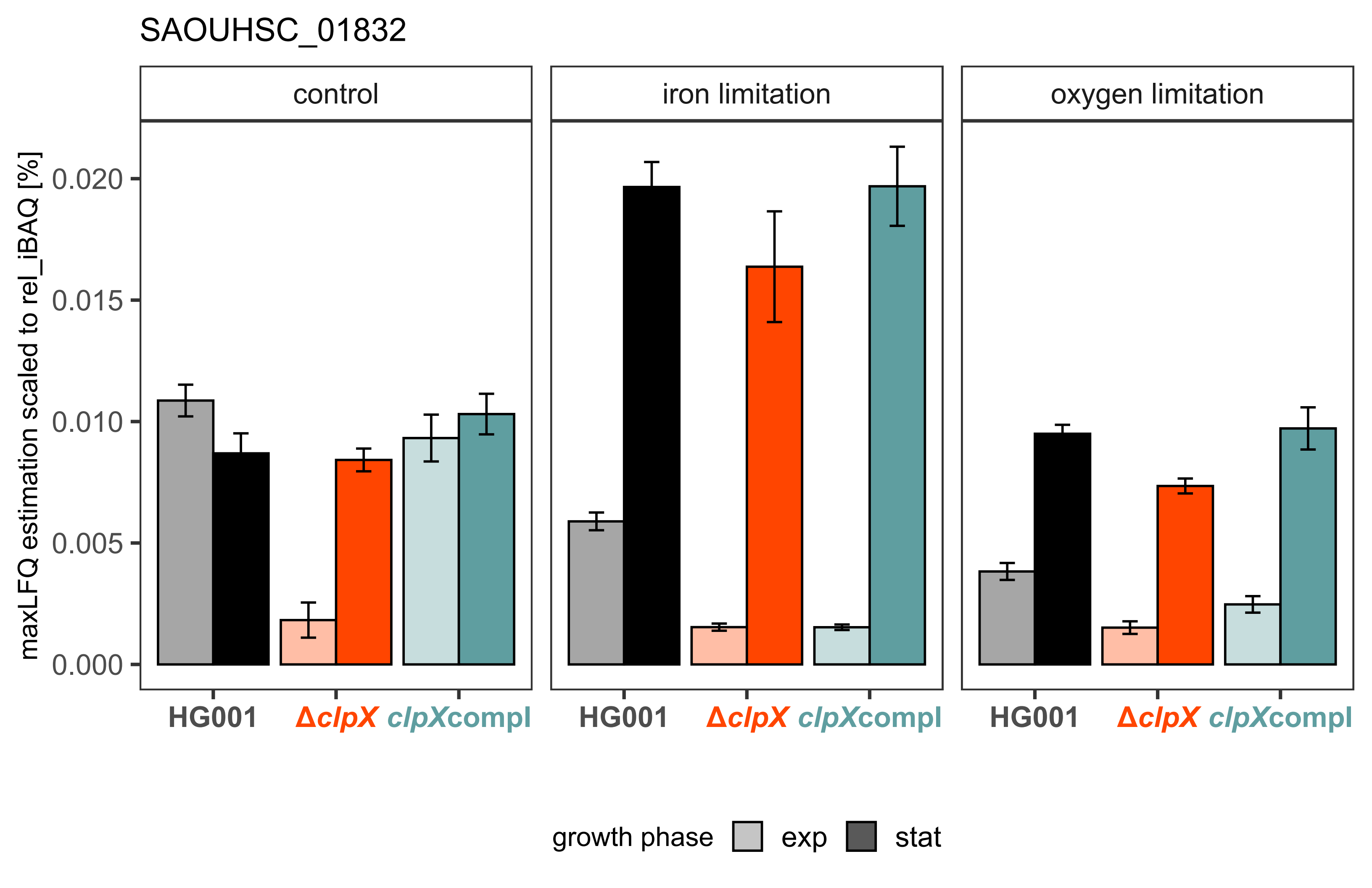
SAOUHSC_01832
control
iron limitation
oxygen limitation
maxLFQ estimation scaled to rel_iBAQ [%]
0.020
0.015
0.010
0.005
0.000
HG001
ΔclpX
clpXcompl
growth phase
exp
stat

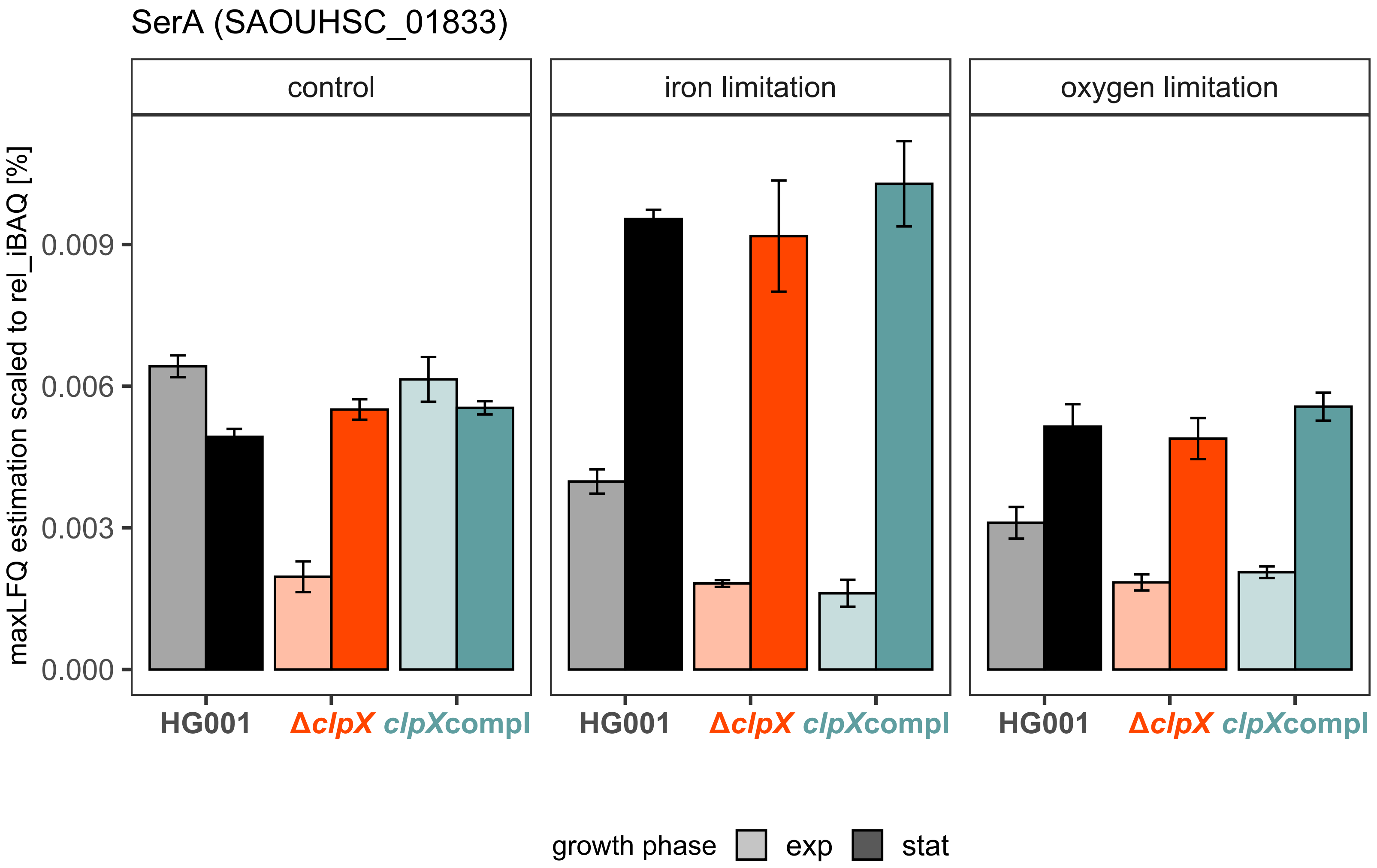

SerA (SAOUHSC_01833)
control
iron limitation
oxygen limitation
maxLFQ estimation scaled to rel_iBAQ [%]
0.009
0.006
0.003
0.000
HG001
ΔclpX
clpXcompl
growth phase
exp
stat

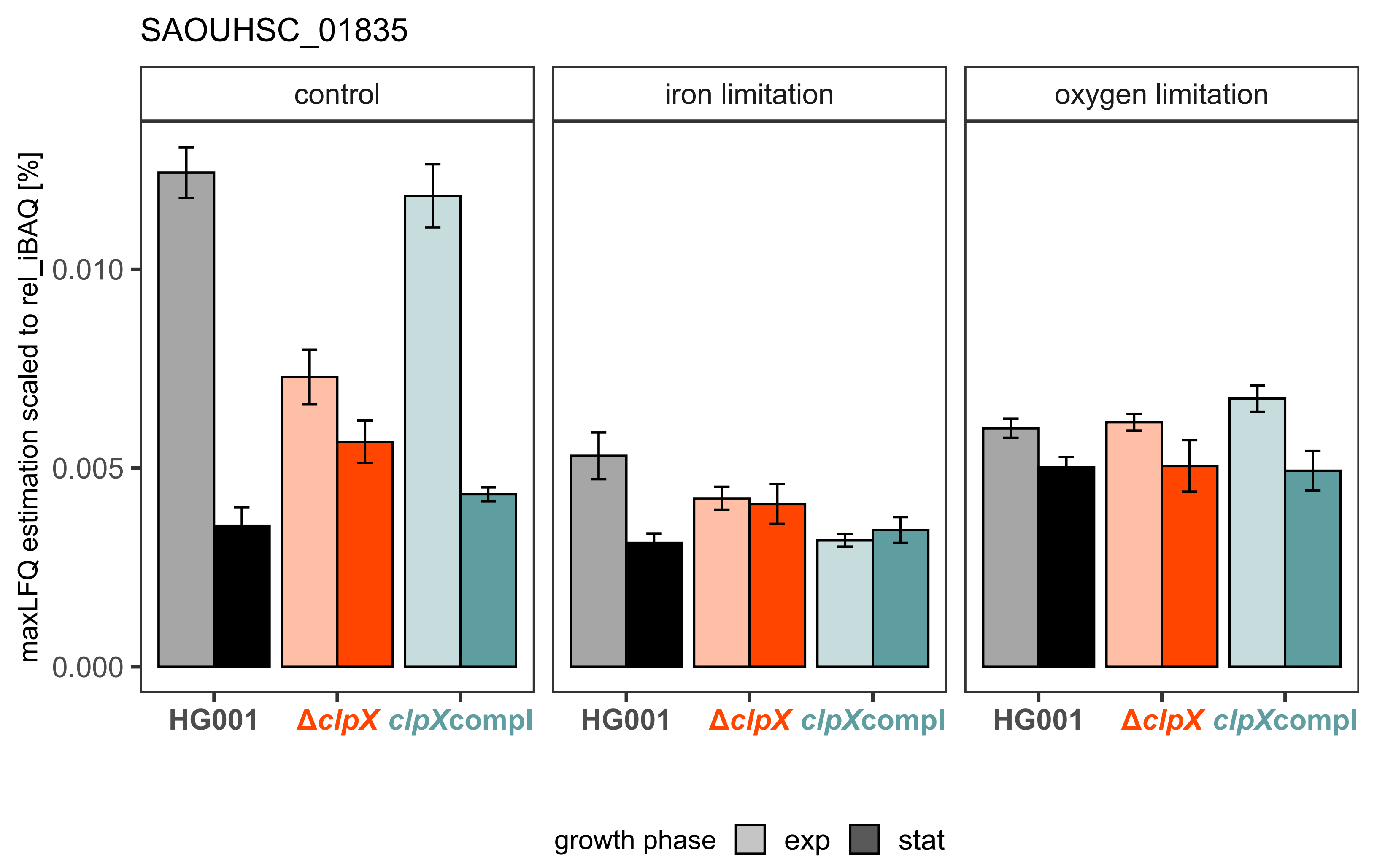

SAOUHSC_01835
control
iron limitation
oxygen limitation
maxLFQ estimation scaled to rel_iBAQ [%]
0.010
0.005
0.000
HG001
ΔclpX
clpXcompl
growth phase
exp
stat

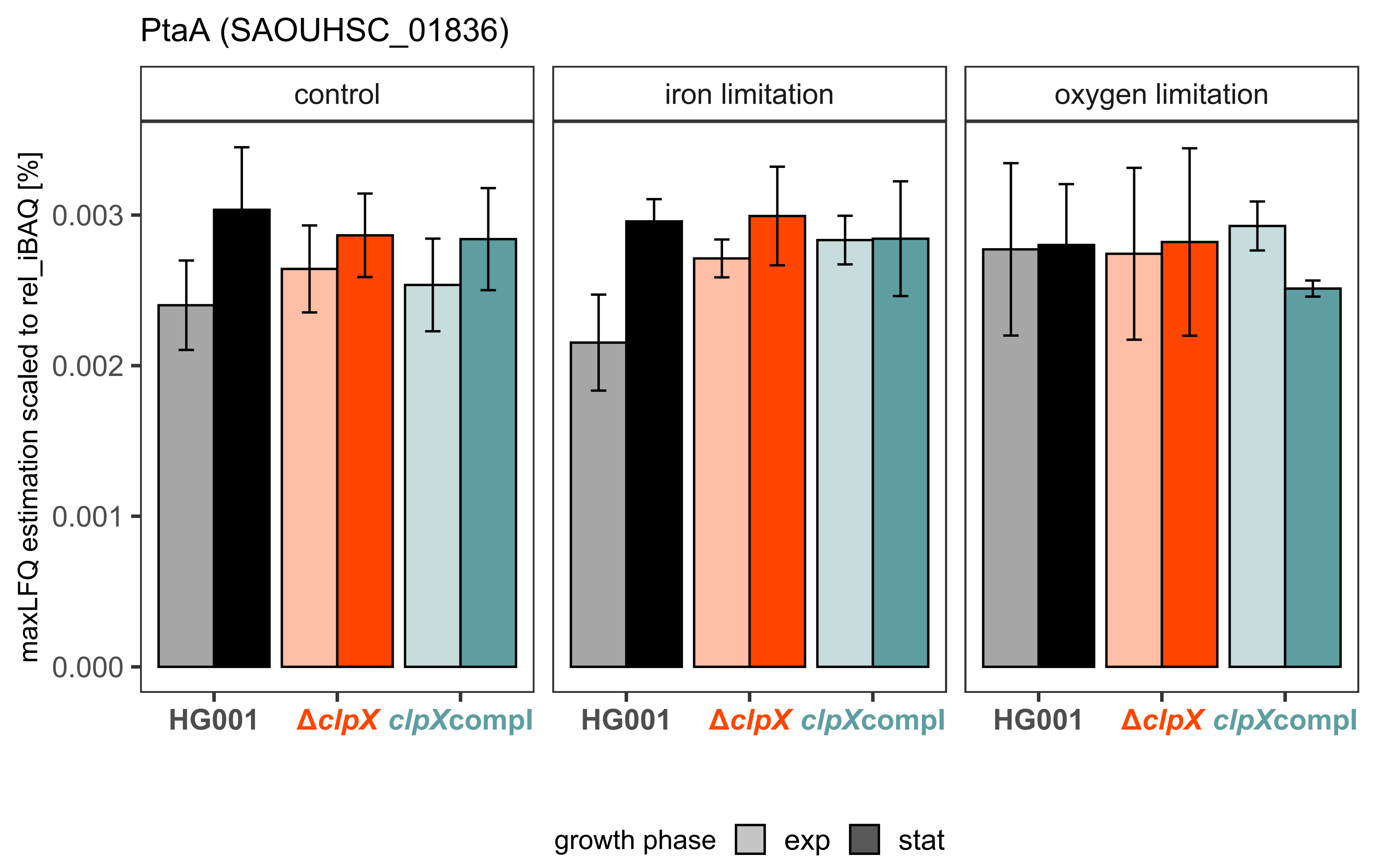
PtaA (SAOUHSC_01836)
control
iron limitation
oxygen limitation
maxLFQ estimation scaled to rel_iBAQ [%]
0.003
0.002
0.001
0.000
HG001
ΔclpX
clpXcompl
growth phase
exp
stat

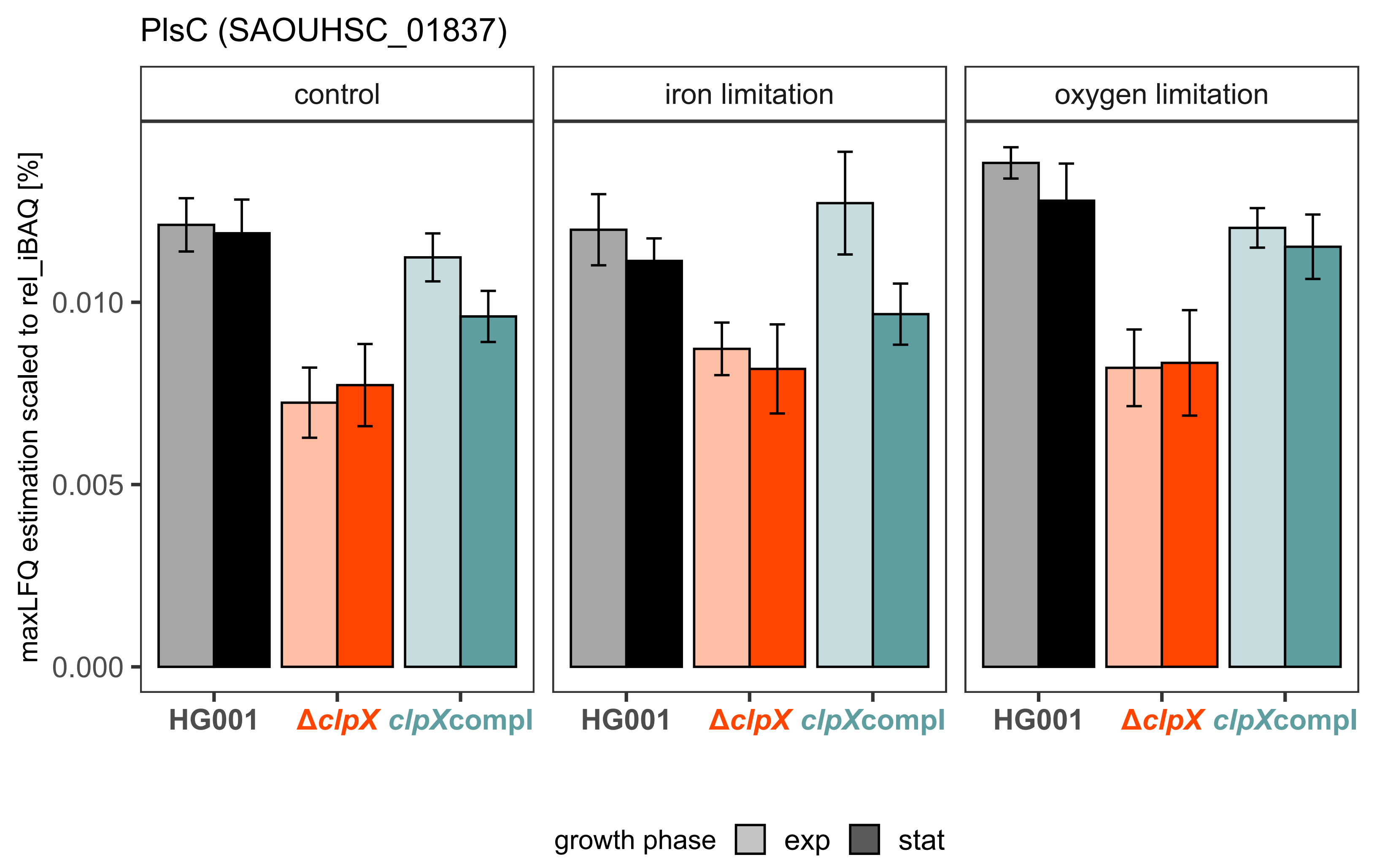
PlsC (SAOUHSC_01837)
control
iron limitation
oxygen limitation
maxLFQ estimation scaled to rel_iBAQ [%]
0.010
0.005
0.000
HG001
ΔclpX
clpXcompl
growth phase
exp
stat

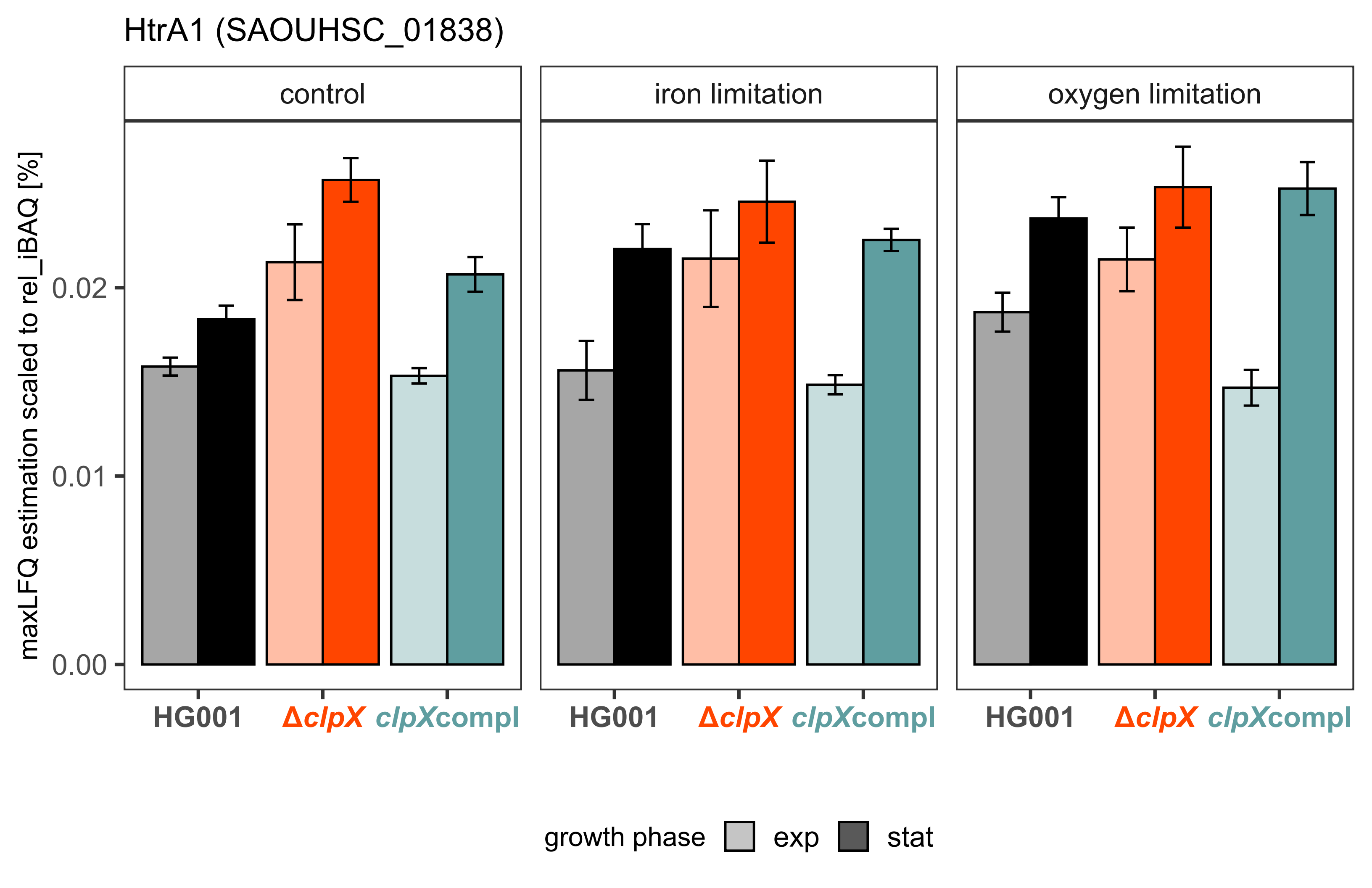

HtrA1 (SAOUHSC_01838)
control
iron limitation
oxygen limitation
maxLFQ estimation scaled to rel_iBAQ [%]
0.00
0.01
0.02
HG001
ΔclpX
clpXcompl
growth phase
exp
stat

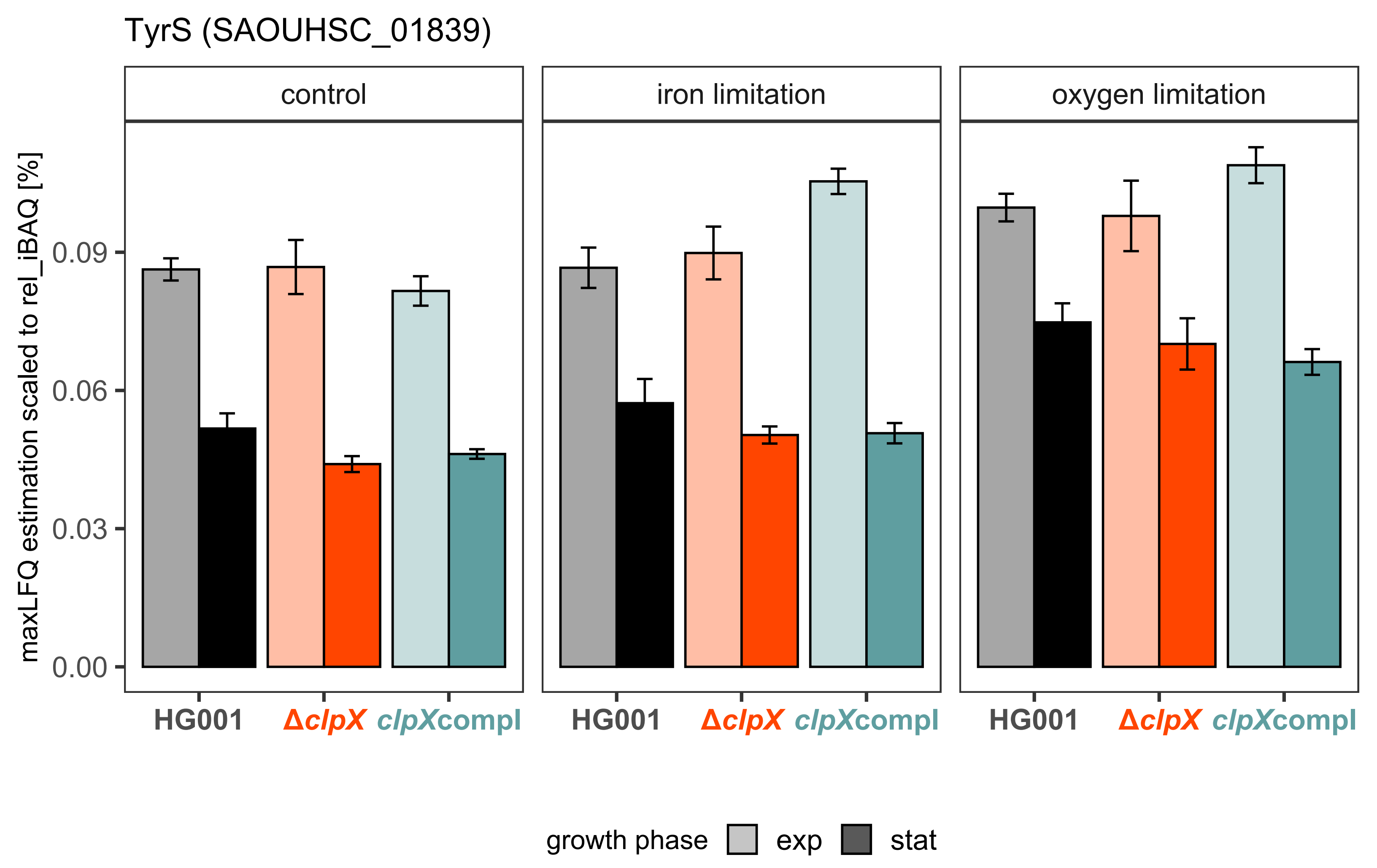
TyrS (SAOUHSC_01839)
control
iron limitation
oxygen limitation
maxLFQ estimation scaled to rel_iBAQ [%]
0.00
0.03
0.06
0.09
HG001
ΔclpX
clpXcompl
growth phase
exp
stat

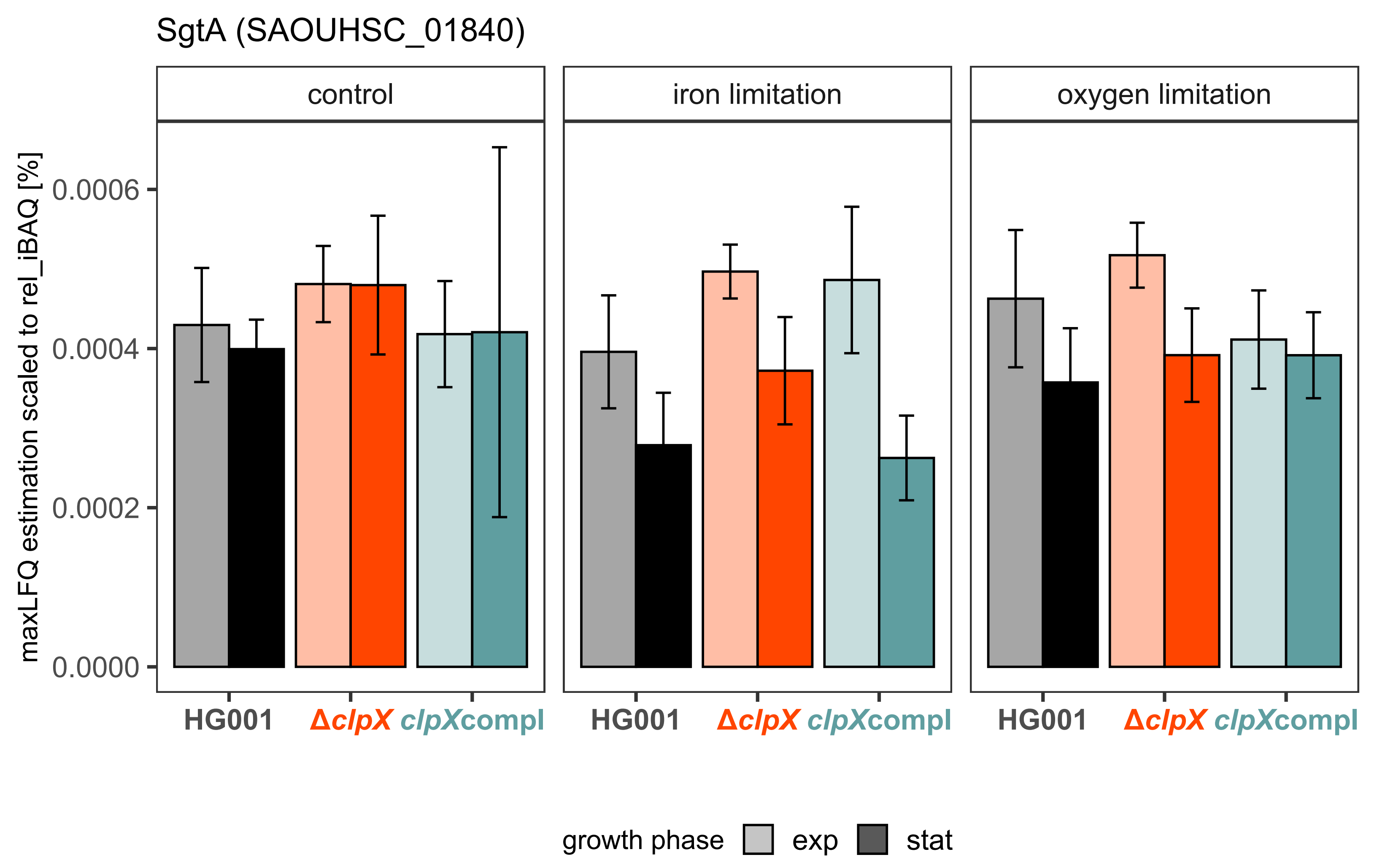
SgtA (SAOUHSC_01840)
control
iron limitation
oxygen limitation
maxLFQ estimation scaled to rel_iBAQ [%]
0.0006
0.0004
0.0002
0.0000
HG001
ΔclpX
clpXcompl
growth phase
exp
stat

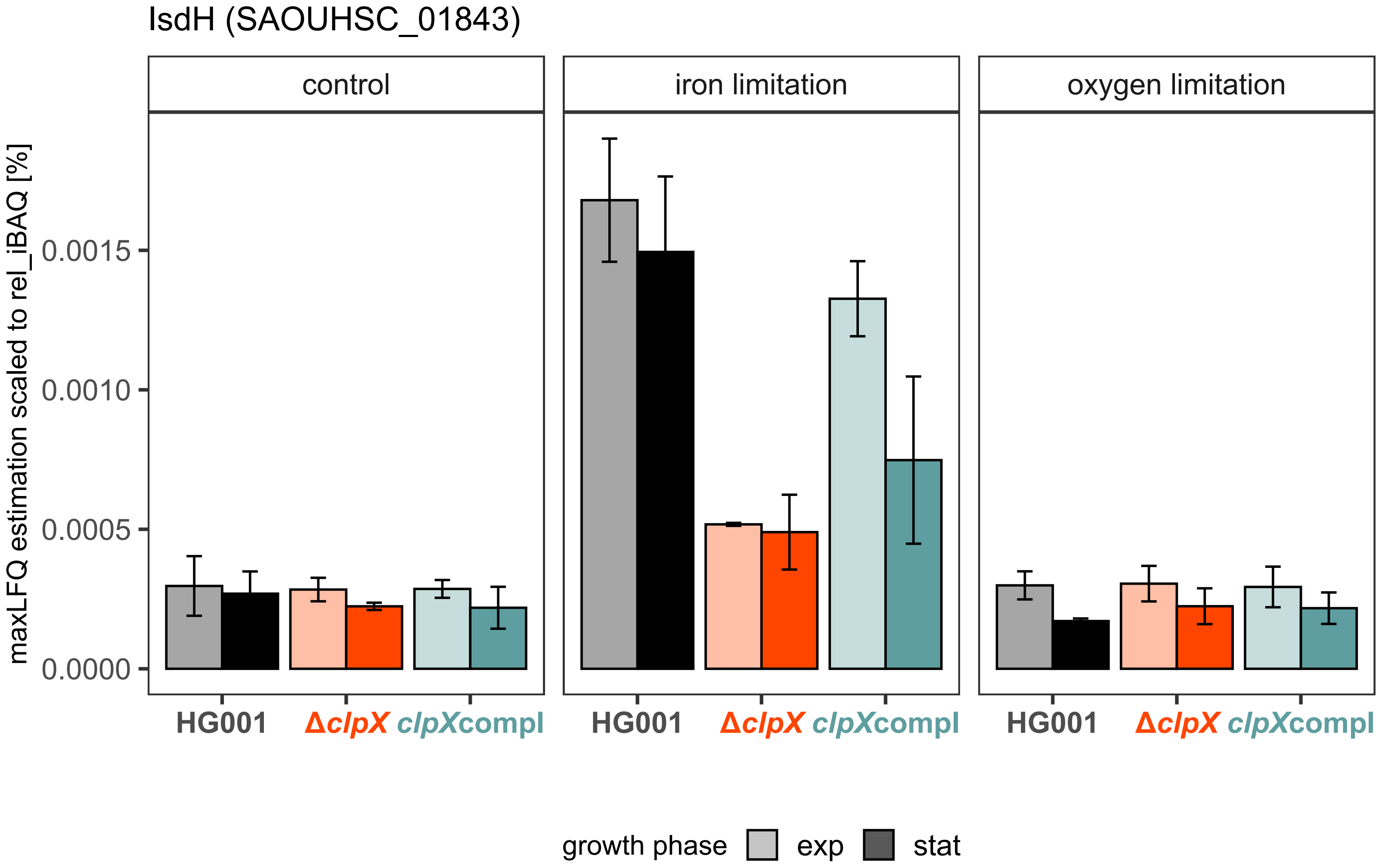

IsdH (SAOUHSC_01843)
control
iron limitation
oxygen limitation
maxLFQ estimation scaled to rel_iBAQ [%]
0.0015
0.0010
0.0005
0.0000
HG001
ΔclpX
clpXcompl
growth phase
exp
stat

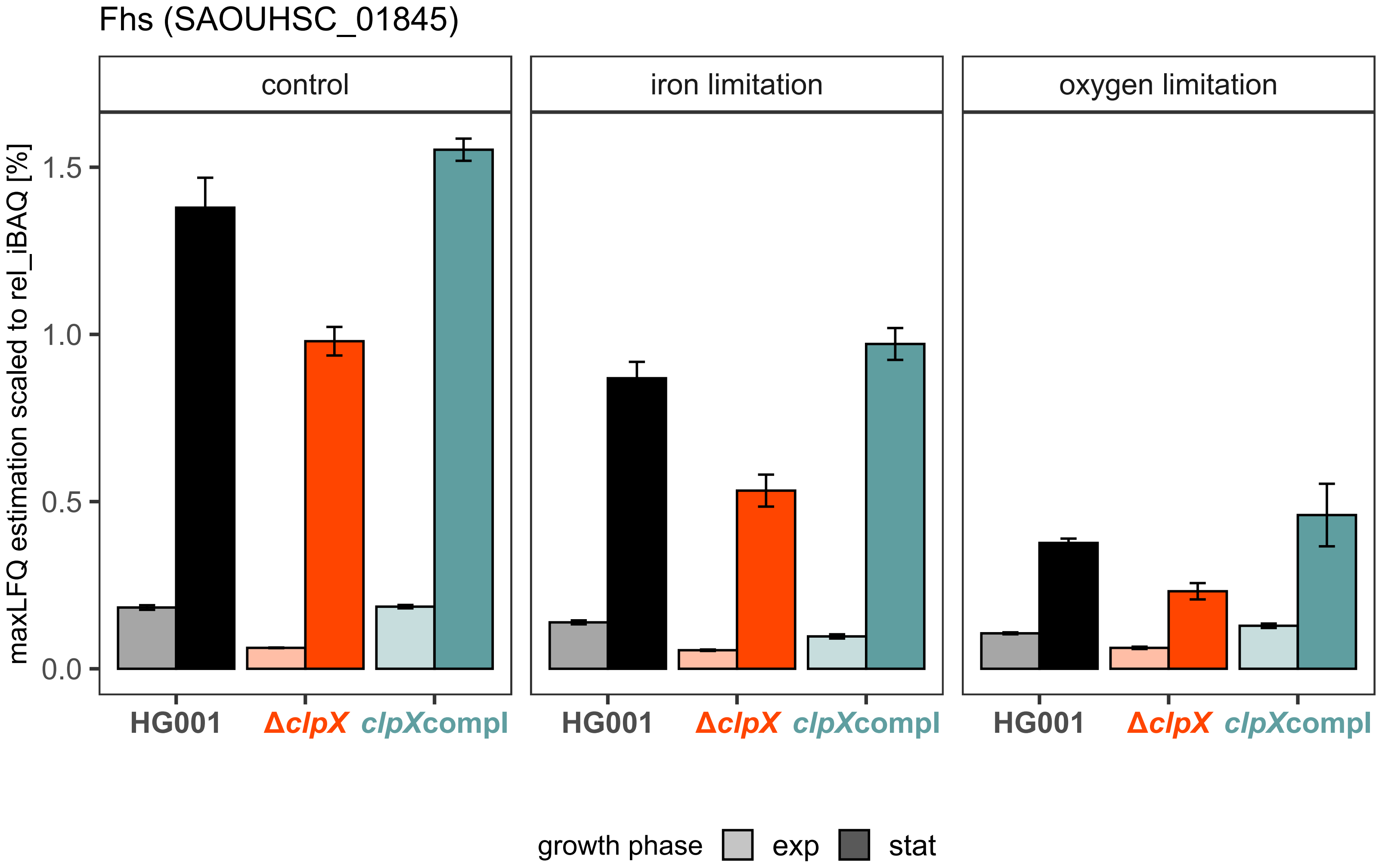
Fhs (SAOUHSC_01845)
control
iron limitation
oxygen limitation
maxLFQ estimation scaled to rel_iBAQ [%]
0.0
0.5
1.0
1.5
HG001
ΔclpX
clpXcompl
growth phase
exp
stat

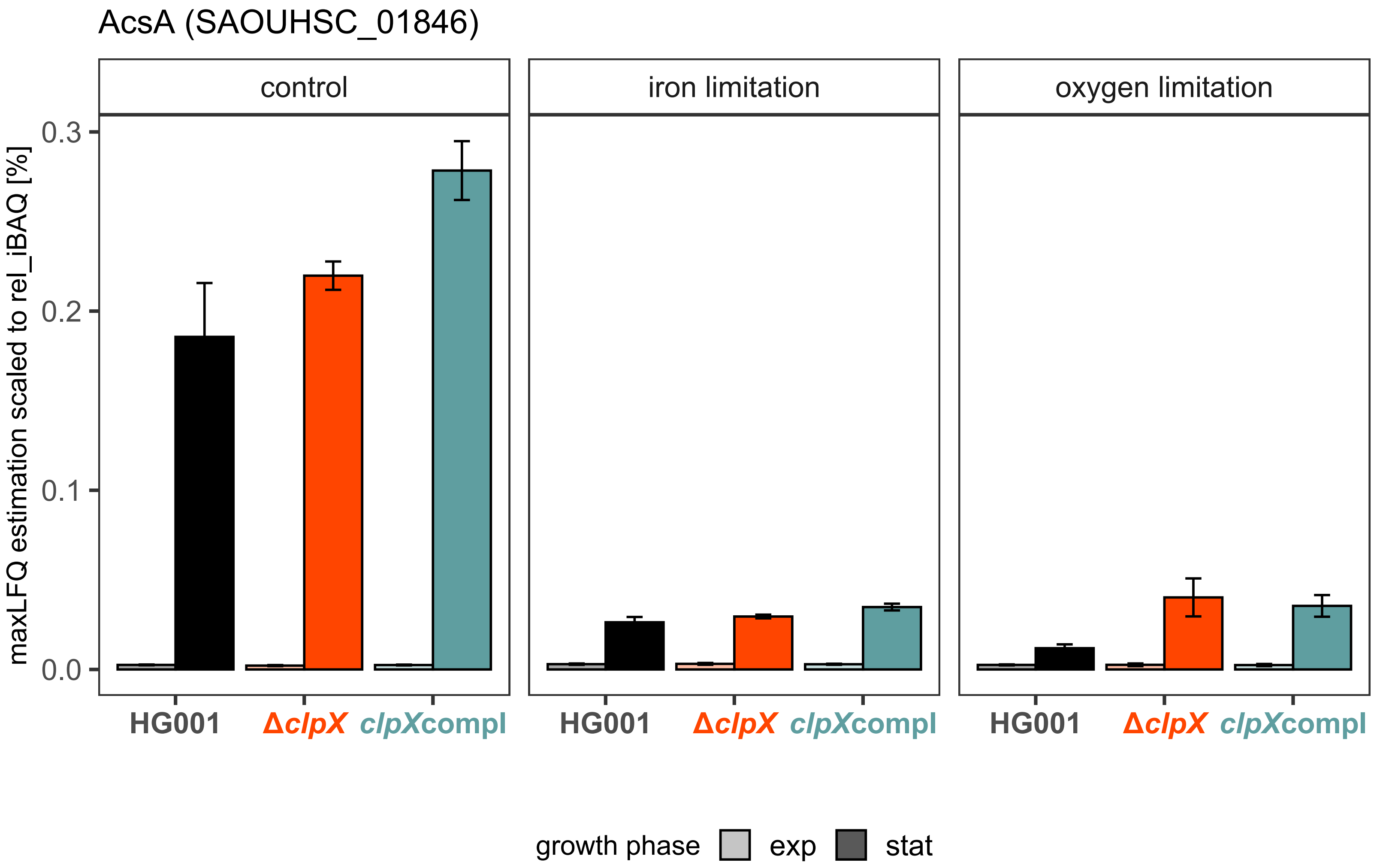
AcsA (SAOUHSC_01846)
control
iron limitation
oxygen limitation
maxLFQ estimation scaled to rel_iBAQ [%]
0.3
0.2
0.1
0.0
HG001
ΔclpX
clpXcompl
growth phase
exp
stat

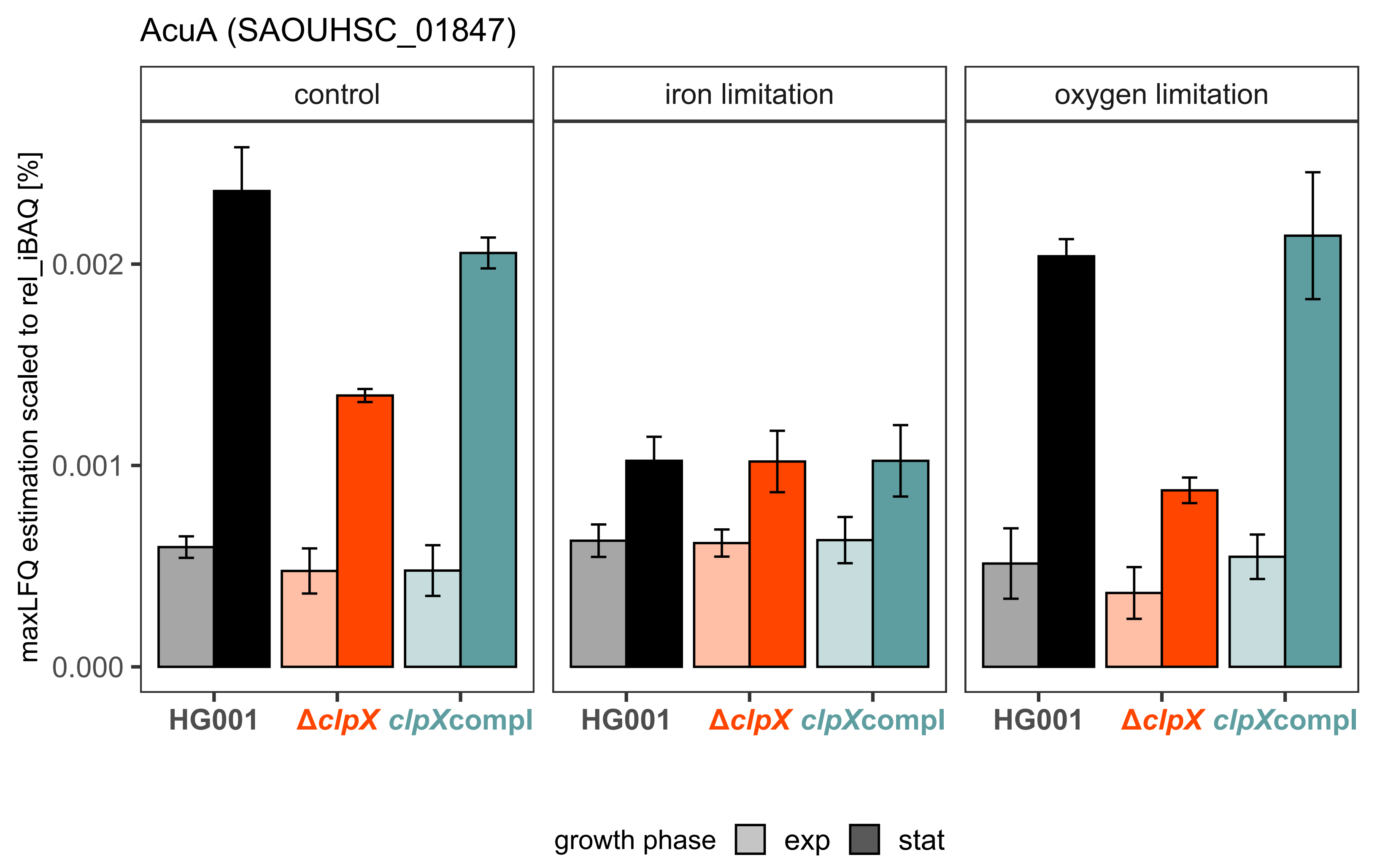
AcuA (SAOUHSC_01847)
control
iron limitation
oxygen limitation
maxLFQ estimation scaled to rel_iBAQ [%]
0.000
0.001
0.002
HG001
ΔclpX
clpXcompl
growth phase
exp
stat

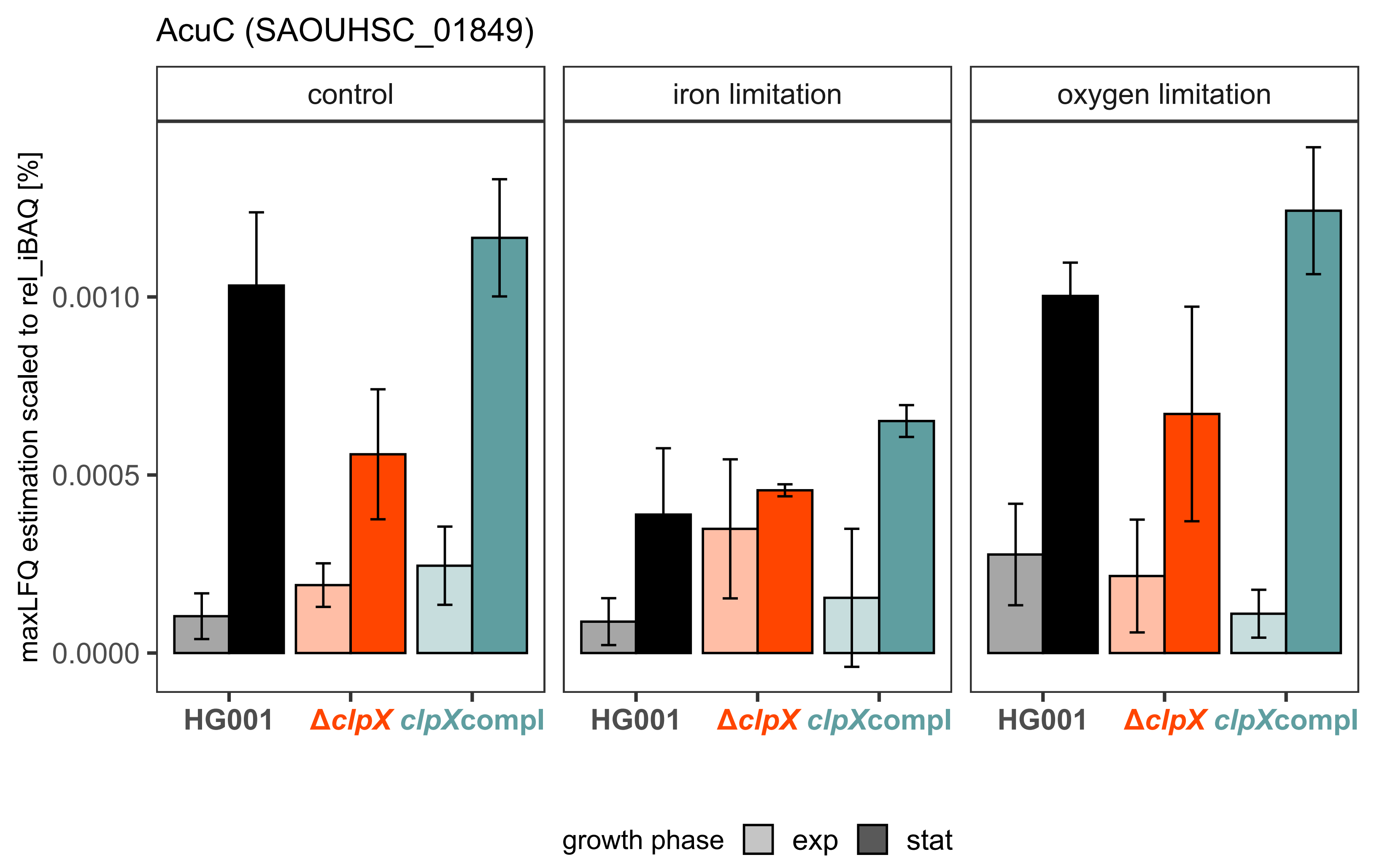

AcuC (SAOUHSC_01849)
control
iron limitation
oxygen limitation
maxLFQ estimation scaled to rel_iBAQ [%]
0.0010
0.0005
0.0000
HG001
ΔclpX
clpXcompl
growth phase
exp
stat

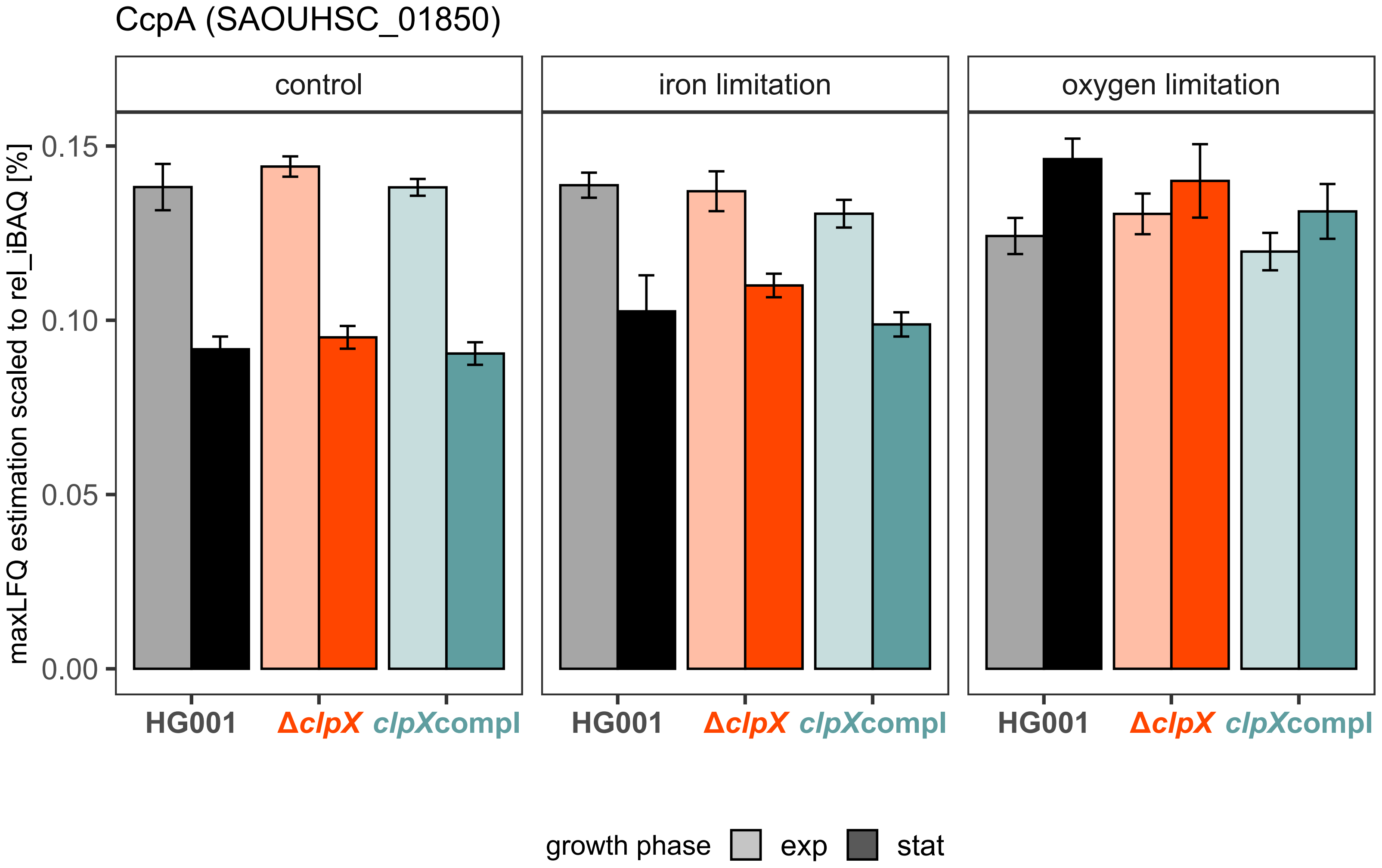

CcpA (SAOUHSC_01850)
control
iron limitation
oxygen limitation
maxLFQ estimation scaled to rel_iBAQ [%]
0.15
0.10
0.05
0.00
HG001
ΔclpX
clpXcompl
growth phase
exp
stat

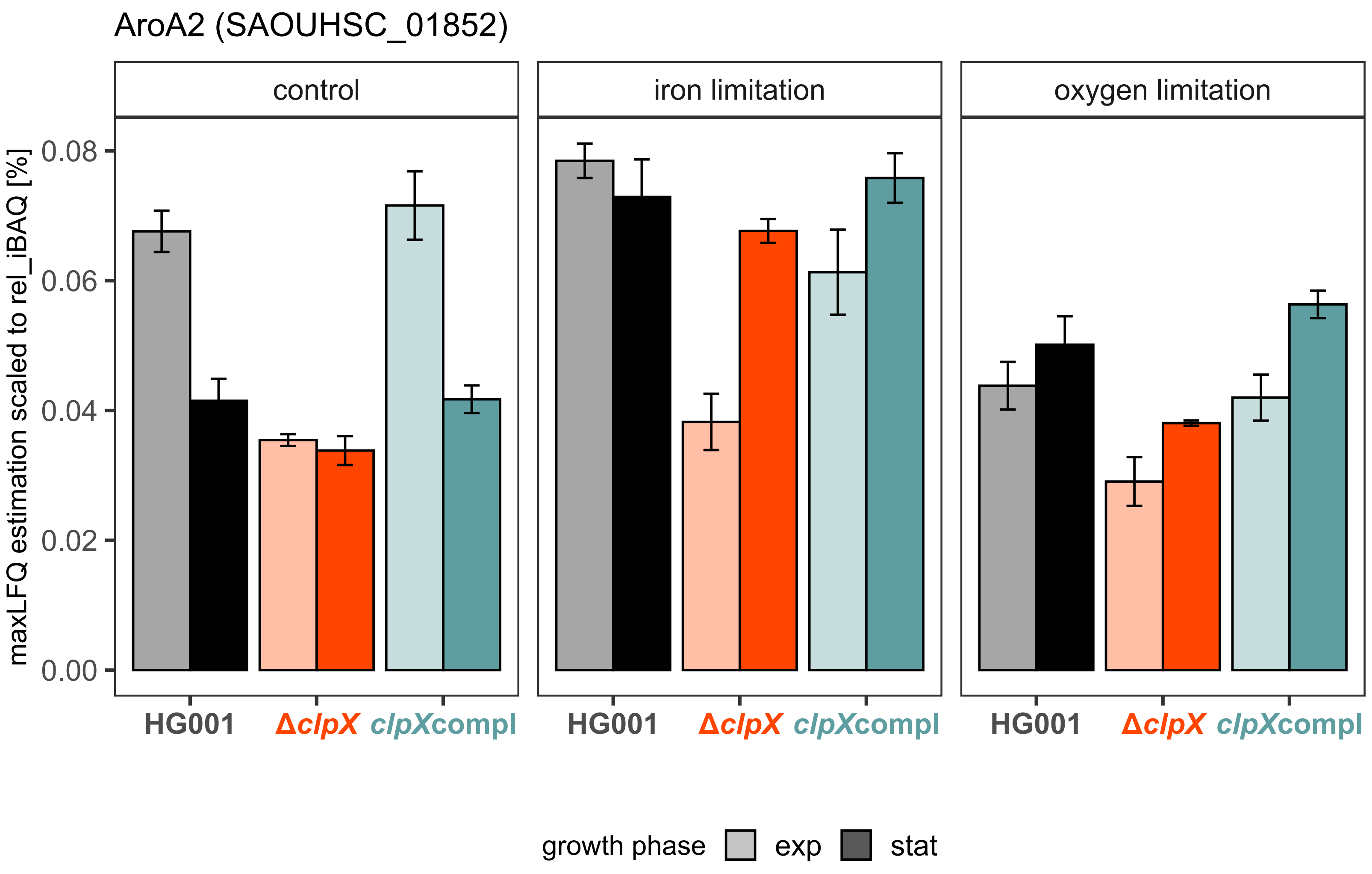

AroA2 (SAOUHSC_01852)
control
iron limitation
oxygen limitation
maxLFQ estimation scaled to rel_iBAQ [%]
0.08
0.06
0.04
0.02
0.00
HG001
ΔclpX
clpXcompl
growth phase
exp
stat

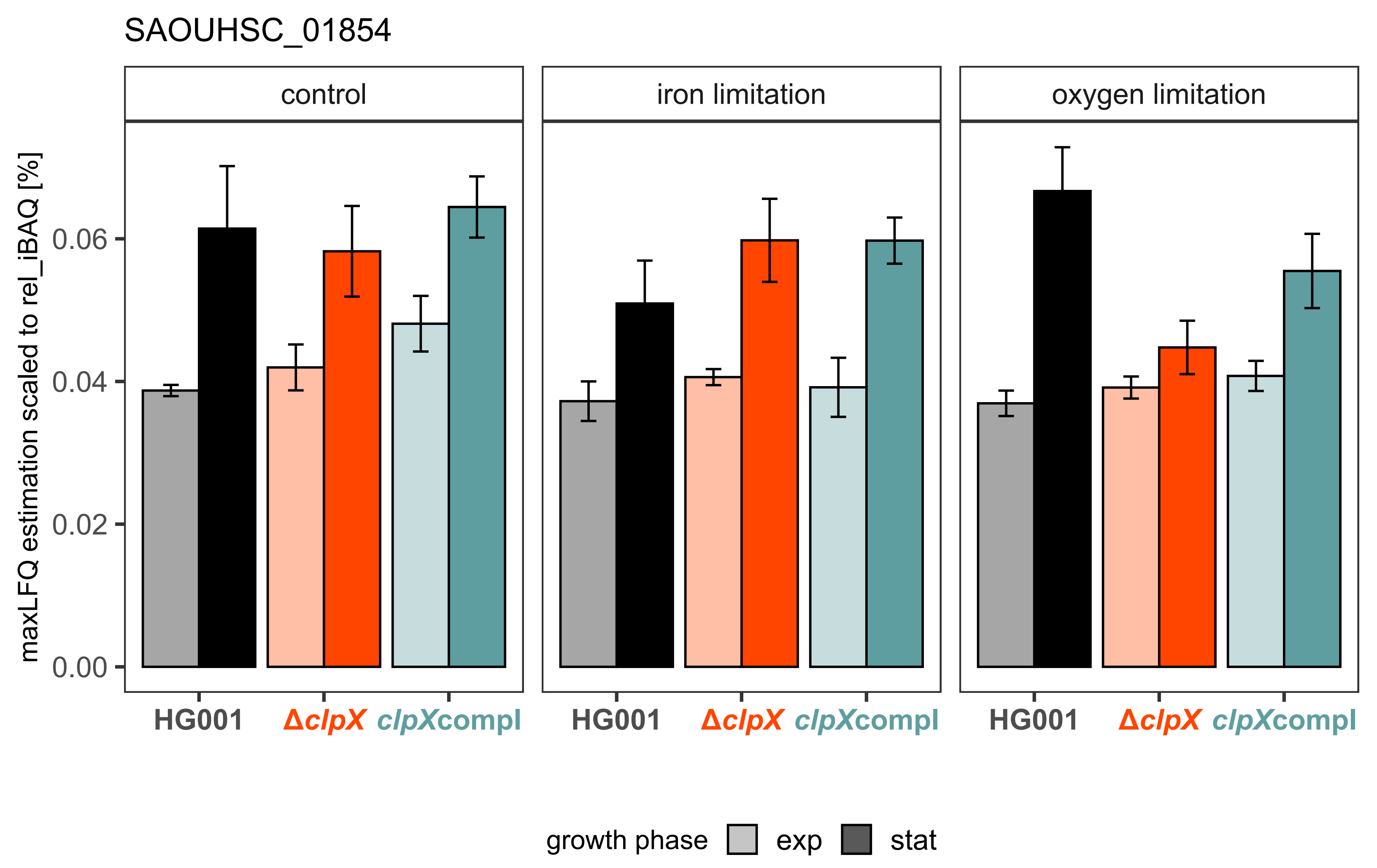

SAOUHSC_01854
control
iron limitation
oxygen limitation
maxLFQ estimation scaled to rel_iBAQ [%]
0.00
0.02
0.04
0.06
HG001
ΔclpX
clpXcompl
growth phase
exp
stat

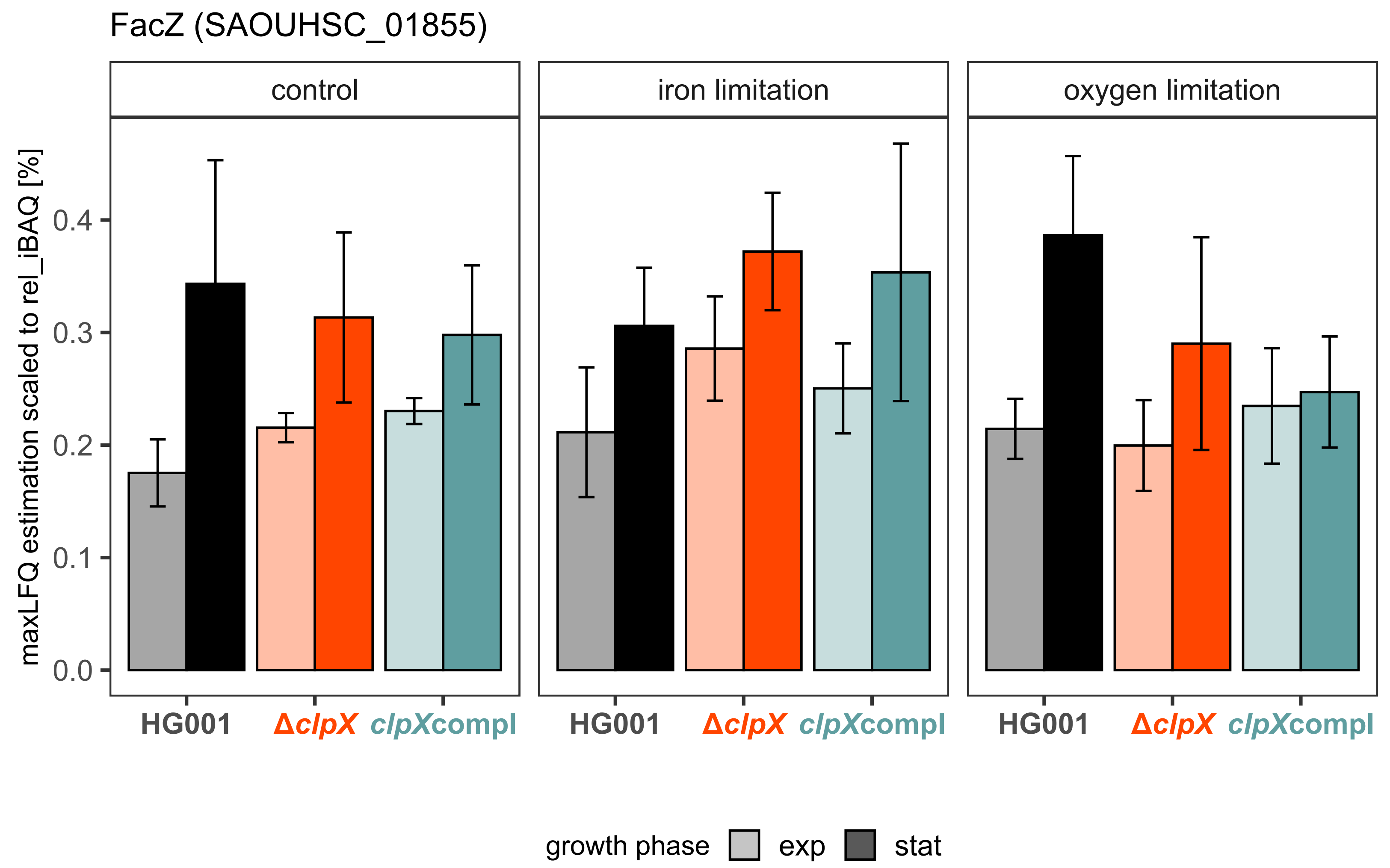
FacZ (SAOUHSC_01855)
control
iron limitation
oxygen limitation
maxLFQ estimation scaled to rel_iBAQ [%]
0.0
0.1
0.2
0.3
0.4
HG001
ΔclpX
clpXcompl
growth phase
exp
stat

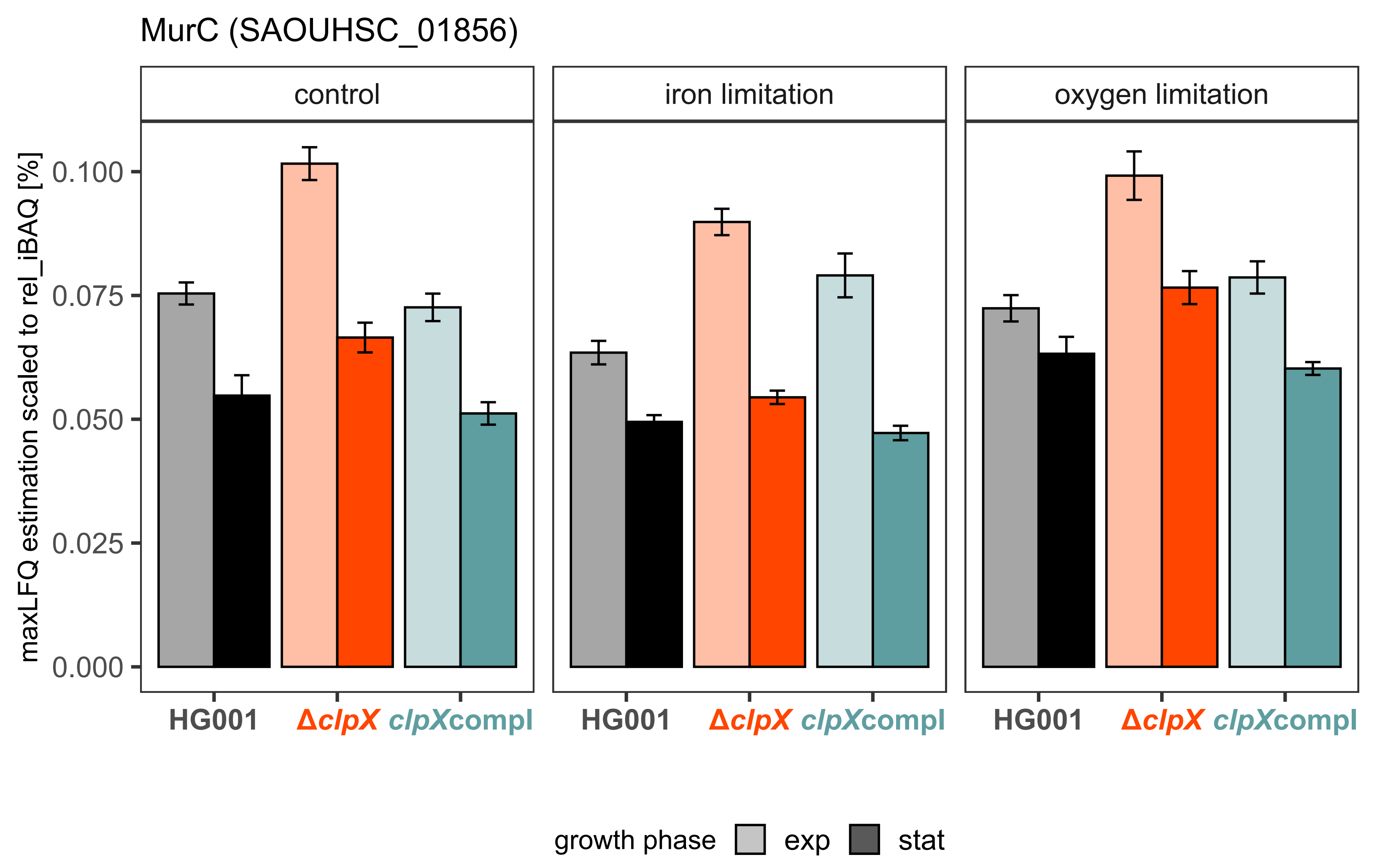

MurC (SAOUHSC_01856)
control
iron limitation
oxygen limitation
maxLFQ estimation scaled to rel_iBAQ [%]
0.100
0.075
0.050
0.025
0.000
HG001
ΔclpX
clpXcompl
growth phase
exp
stat

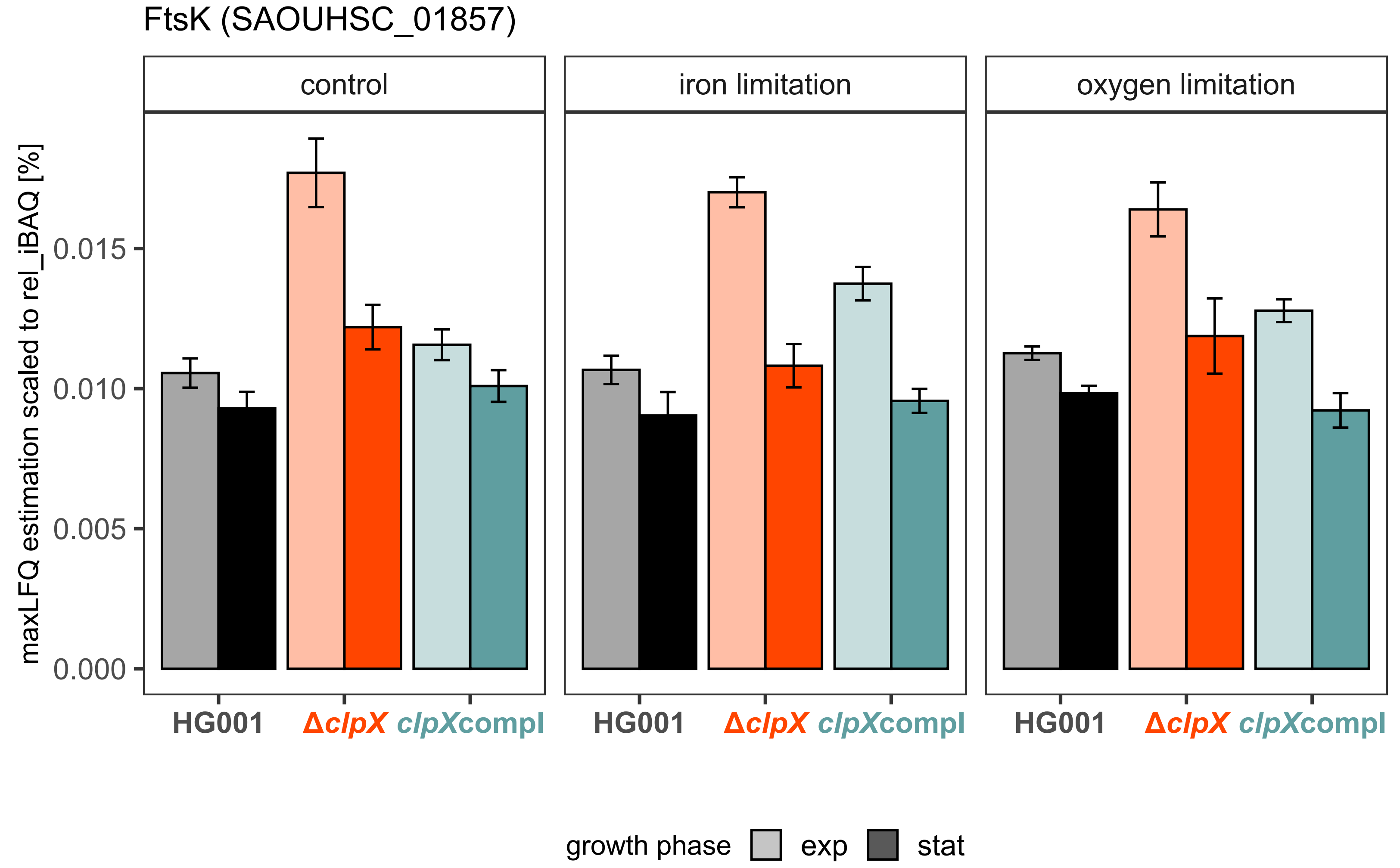

FtsK (SAOUHSC_01857)
control
iron limitation
oxygen limitation
maxLFQ estimation scaled to rel_iBAQ [%]
0.015
0.010
0.005
0.000
HG001
ΔclpX
clpXcompl
growth phase
exp
stat

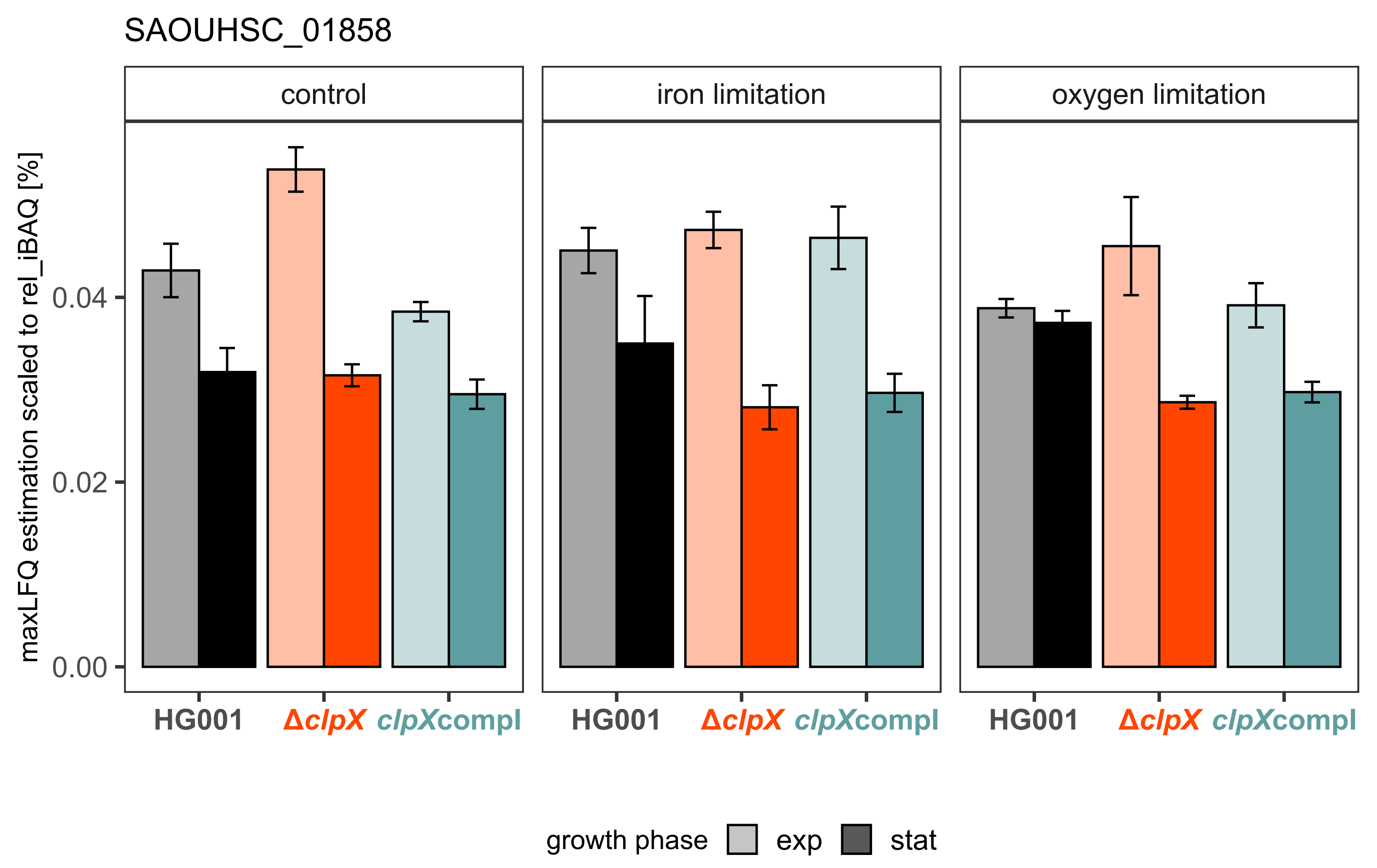
SAOUHSC_01858
control
iron limitation
oxygen limitation
maxLFQ estimation scaled to rel_iBAQ [%]
0.04
0.02
0.00
HG001
ΔclpX
clpXcompl
growth phase
exp
stat

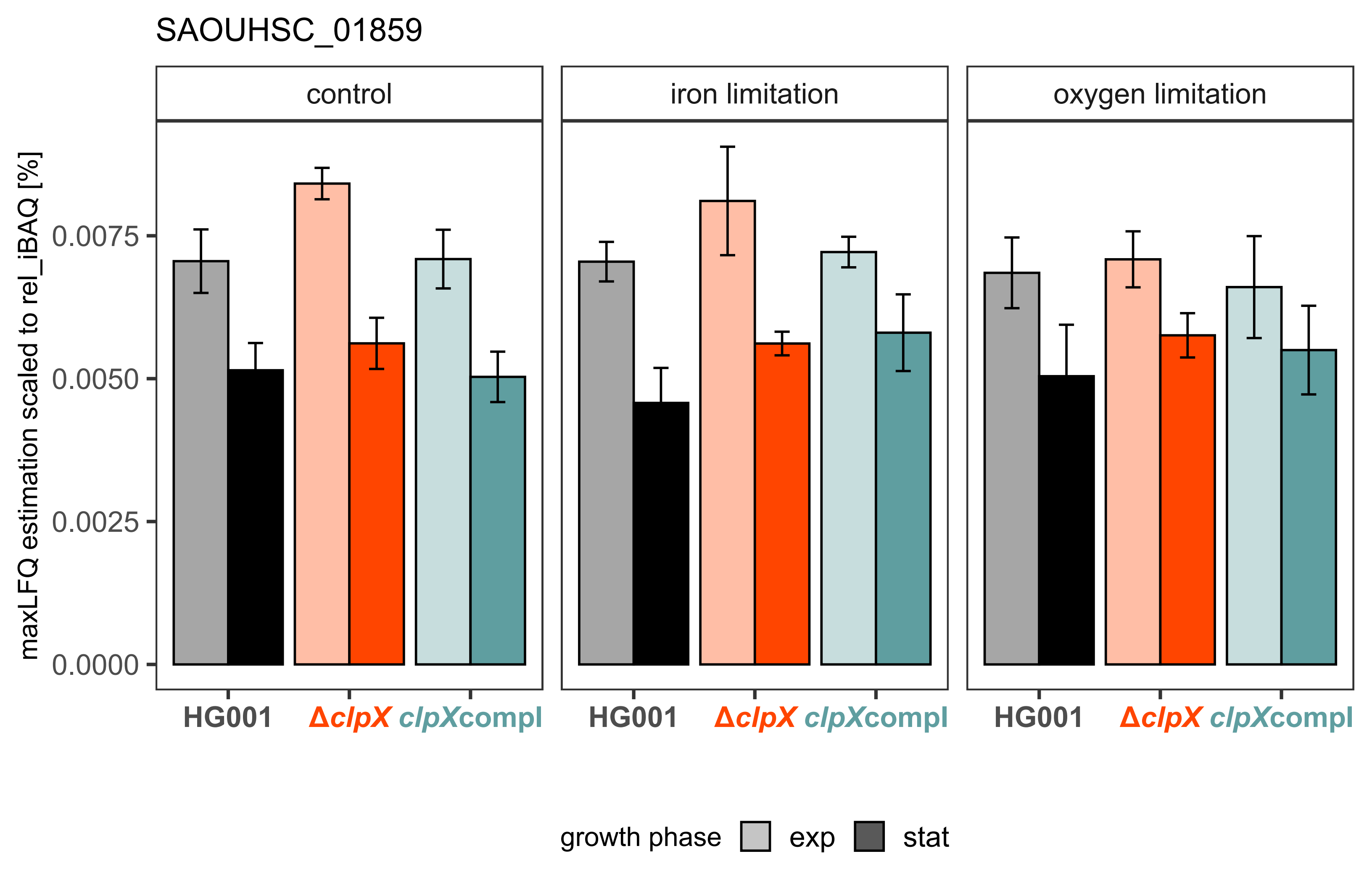

SAOUHSC_01859
control
iron limitation
oxygen limitation
maxLFQ estimation scaled to rel_iBAQ [%]
0.0075
0.0050
0.0025
0.0000
HG001
ΔclpX
clpXcompl
growth phase
exp
stat

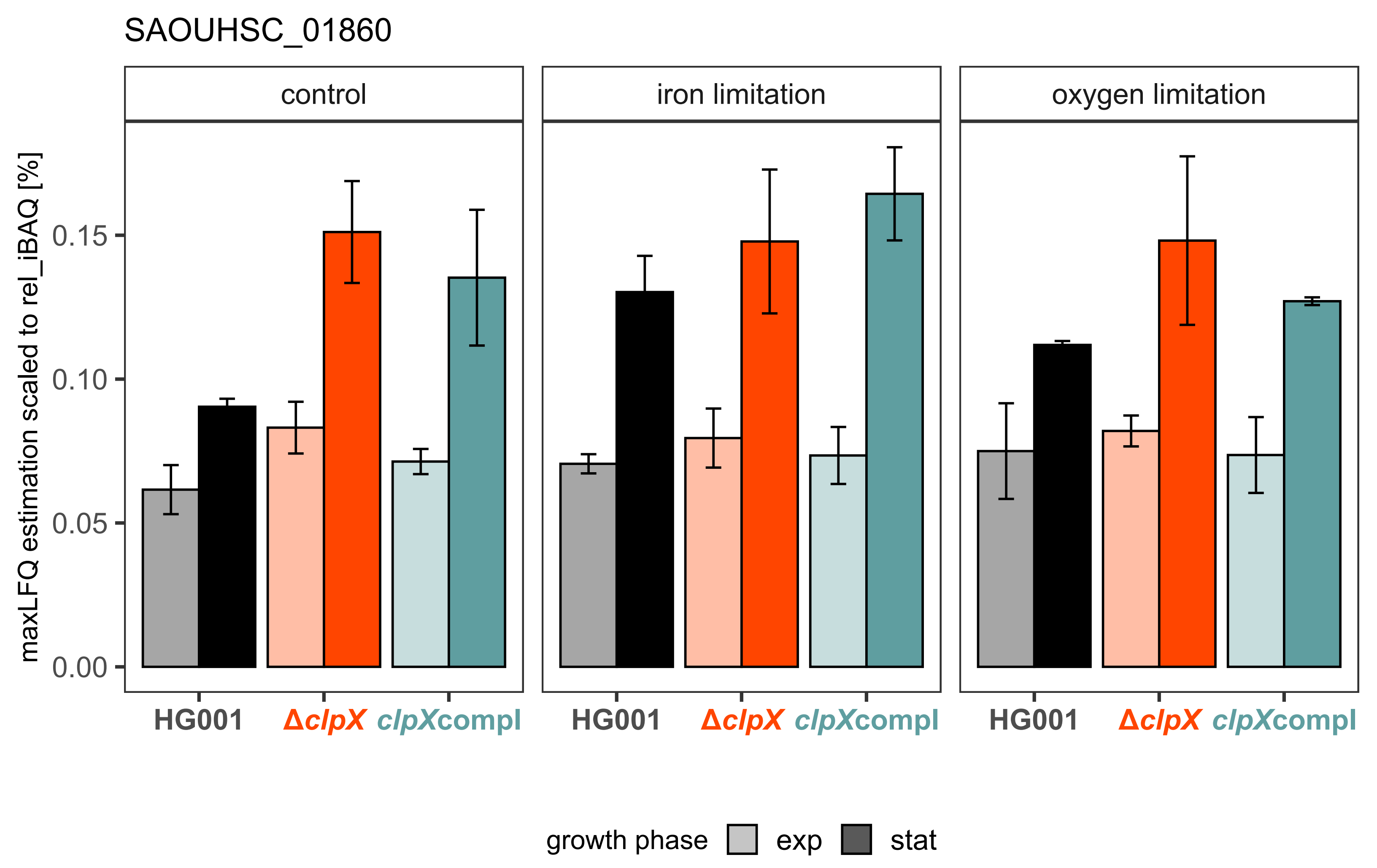
SAOUHSC_01860
control
iron limitation
oxygen limitation
maxLFQ estimation scaled to rel_iBAQ [%]
0.00
0.05
0.10
0.15
HG001
ΔclpX
clpXcompl
growth phase
exp
stat

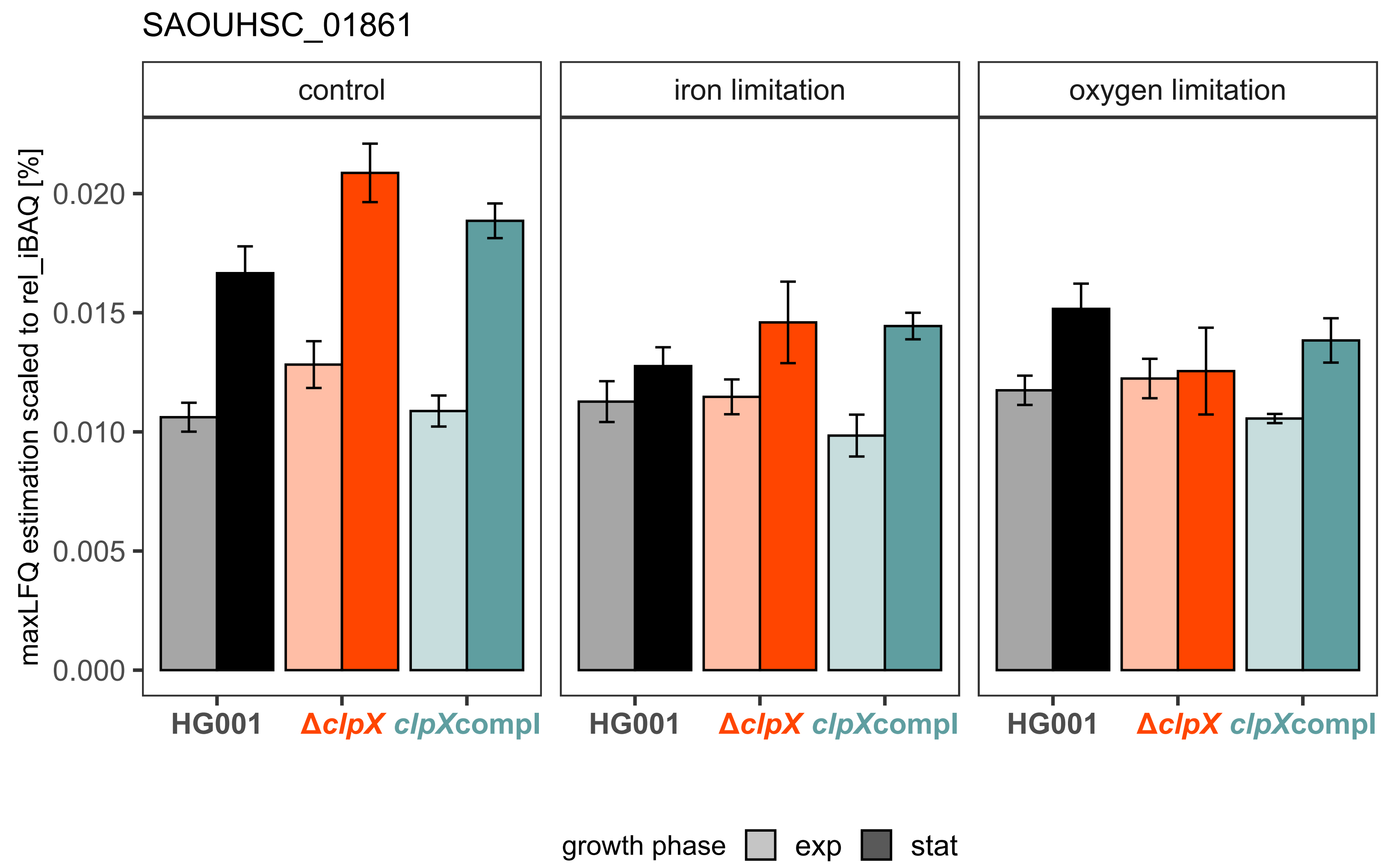
SAOUHSC_01861
control
iron limitation
oxygen limitation
maxLFQ estimation scaled to rel_iBAQ [%]
0.020
0.015
0.010
0.005
0.000
HG001
ΔclpX
clpXcompl
growth phase
exp
stat

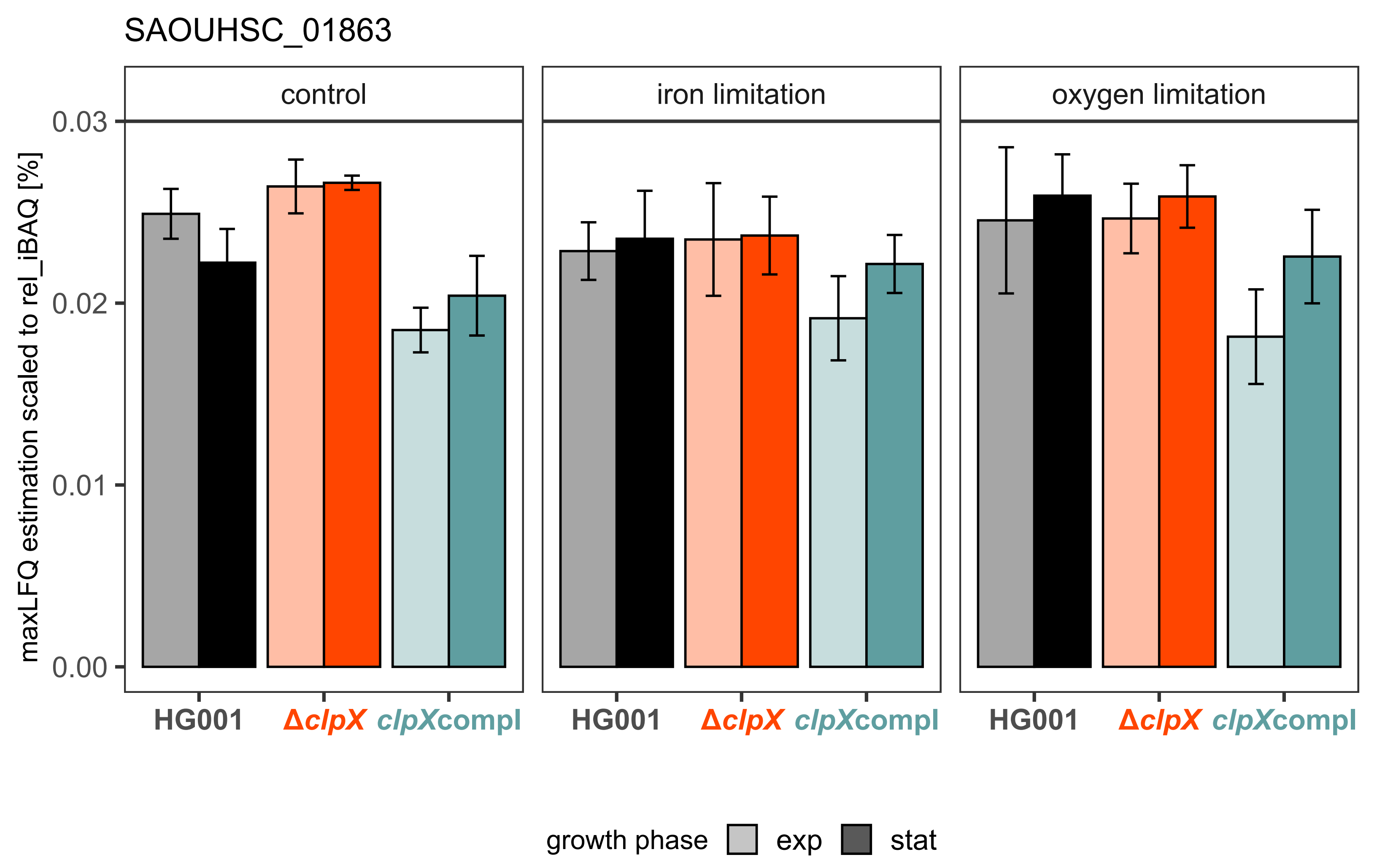
SAOUHSC_01863
control
iron limitation
oxygen limitation
maxLFQ estimation scaled to rel_iBAQ [%]
0.03
0.02
0.01
0.00
HG001
ΔclpX
clpXcompl
growth phase
exp
stat

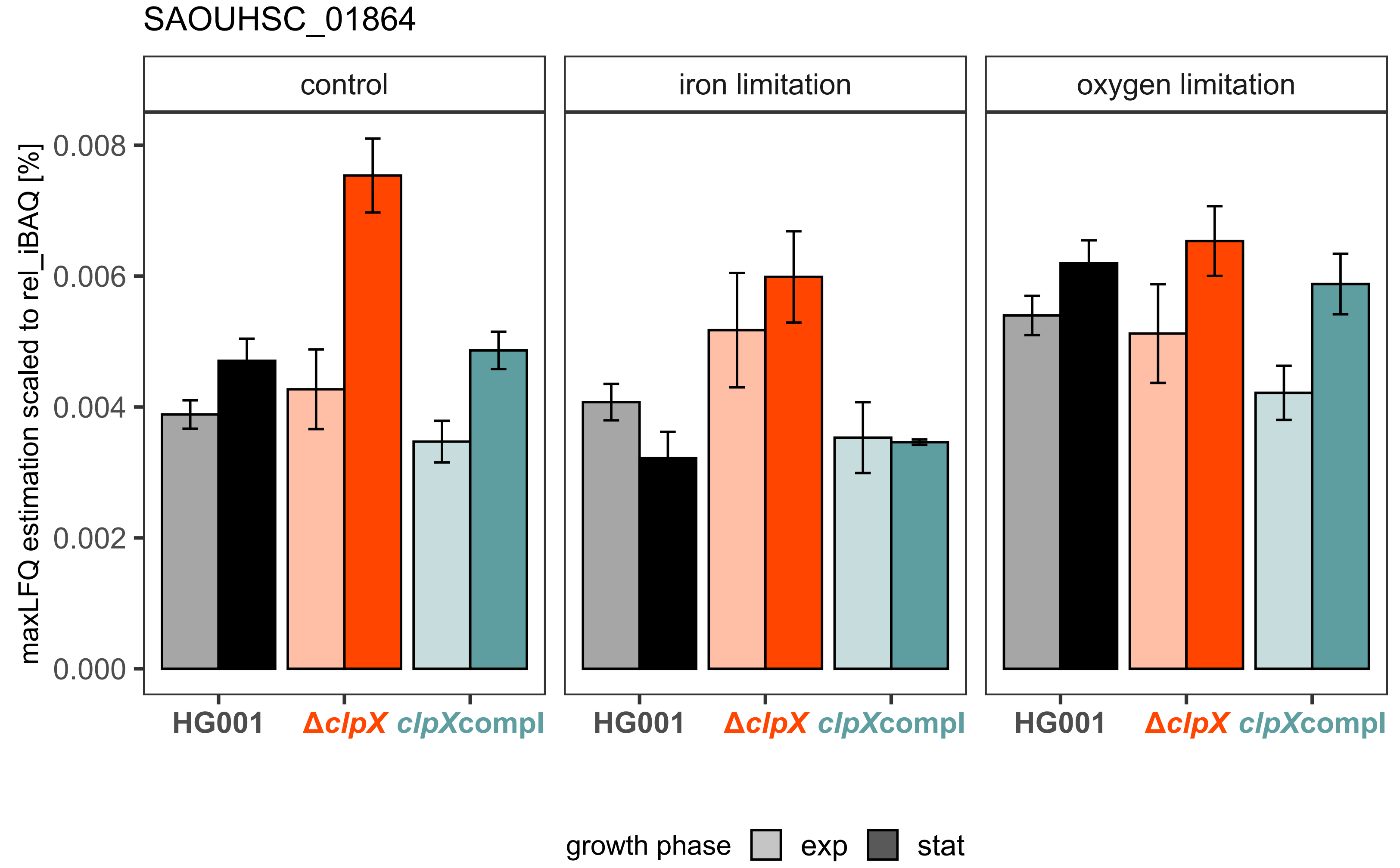
SAOUHSC_01864
control
iron limitation
oxygen limitation
maxLFQ estimation scaled to rel_iBAQ [%]
0.008
0.006
0.004
0.002
0.000
HG001
ΔclpX
clpXcompl
growth phase
exp
stat

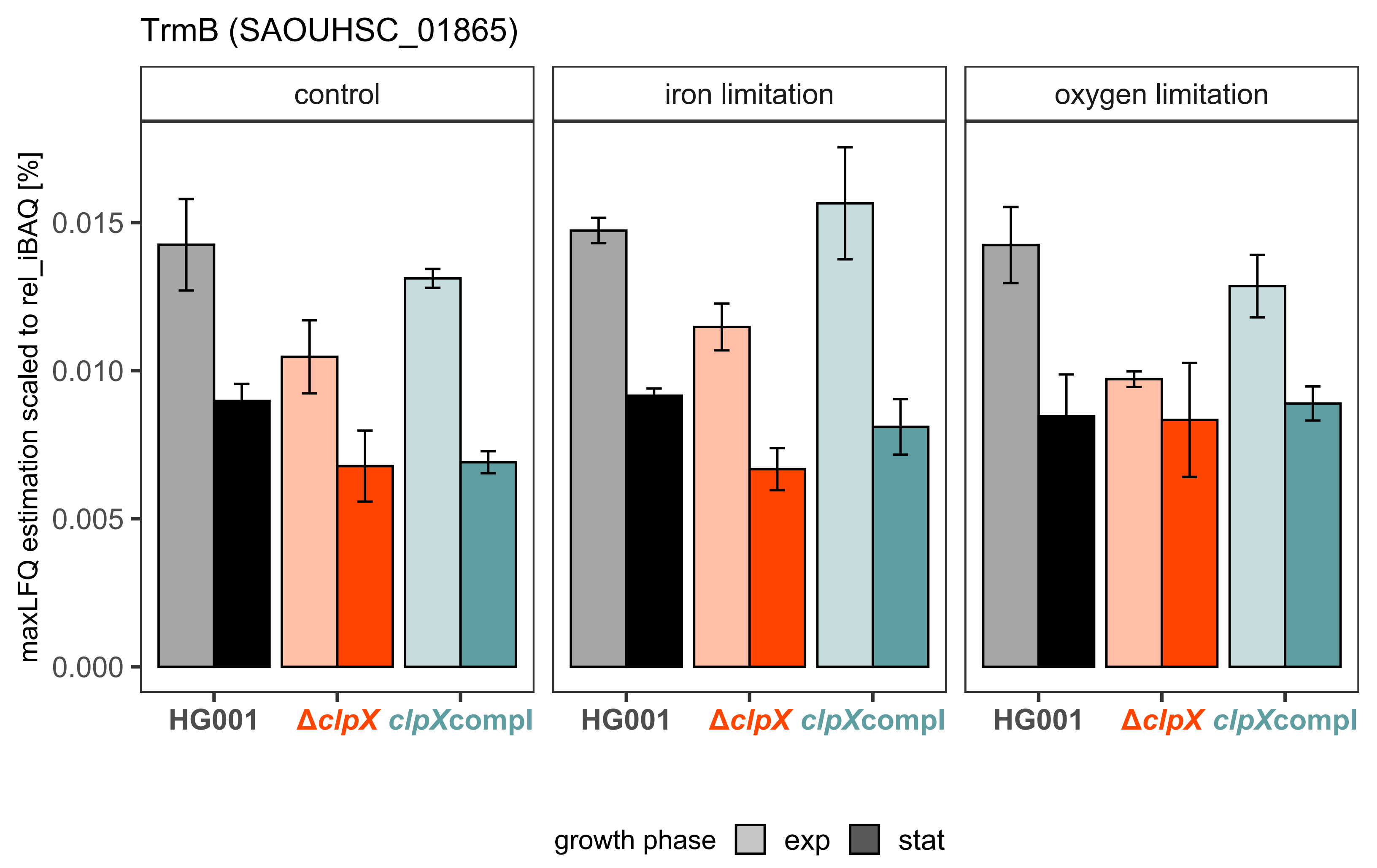
TrmB (SAOUHSC_01865)
control
iron limitation
oxygen limitation
maxLFQ estimation scaled to rel_iBAQ [%]
0.015
0.010
0.005
0.000
HG001
ΔclpX
clpXcompl
growth phase
exp
stat

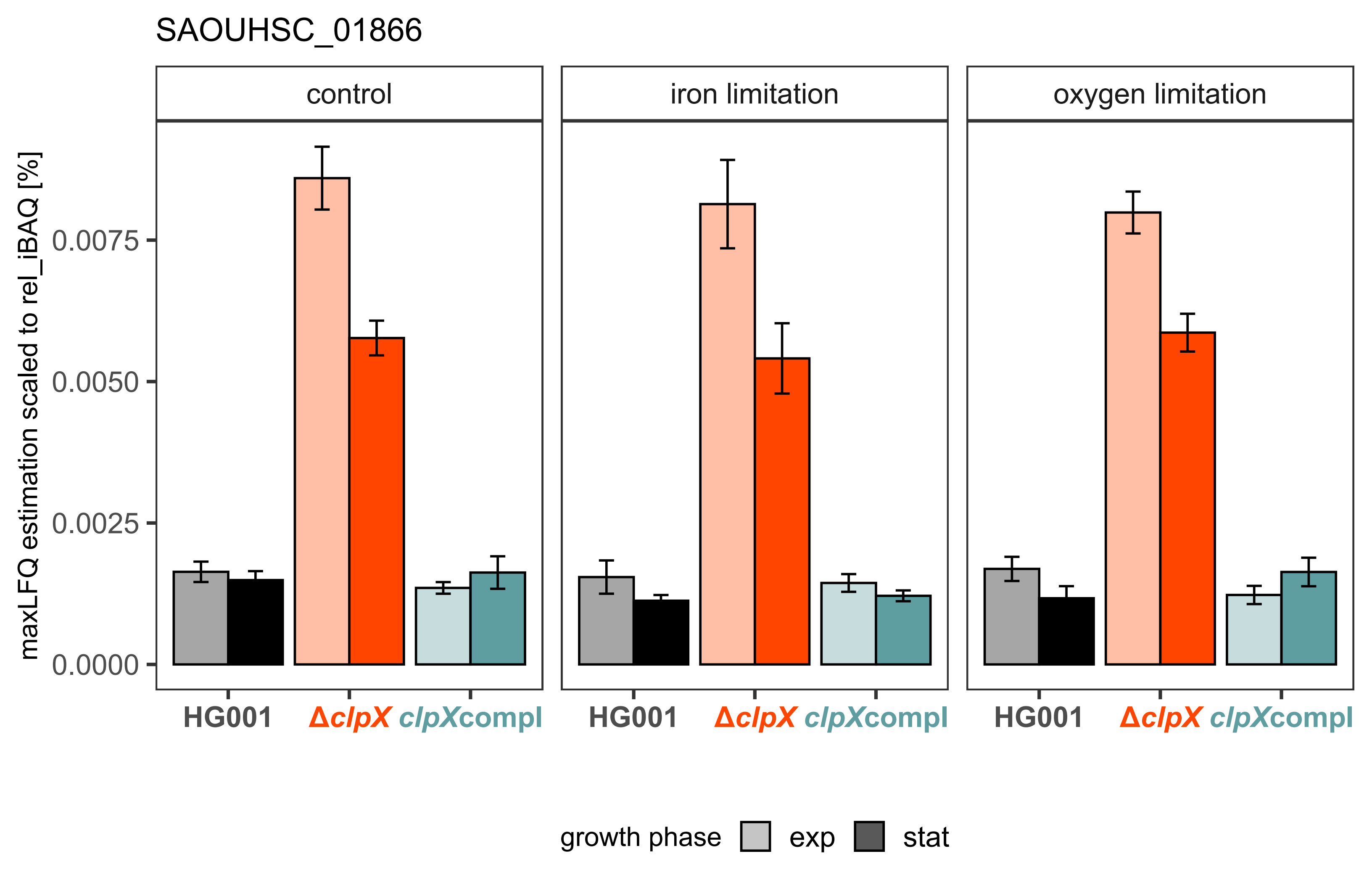

SAOUHSC_01866
control
iron limitation
oxygen limitation
maxLFQ estimation scaled to rel_iBAQ [%]
0.0075
0.0050
0.0025
0.0000
HG001
ΔclpX
clpXcompl
growth phase
exp
stat

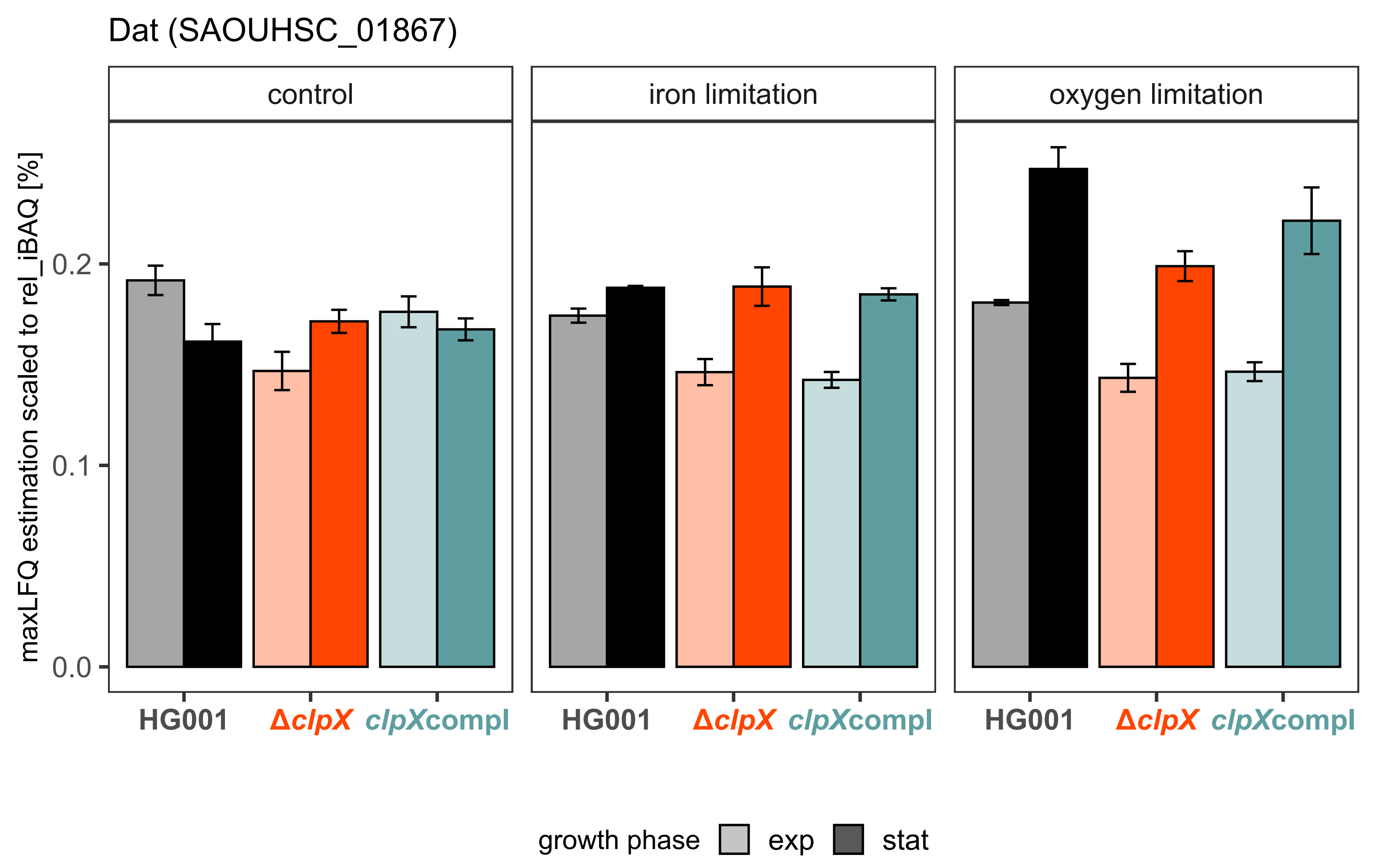

Dat (SAOUHSC_01867)
control
iron limitation
oxygen limitation
maxLFQ estimation scaled to rel_iBAQ [%]
0.0
0.1
0.2
HG001
ΔclpX
clpXcompl
growth phase
exp
stat

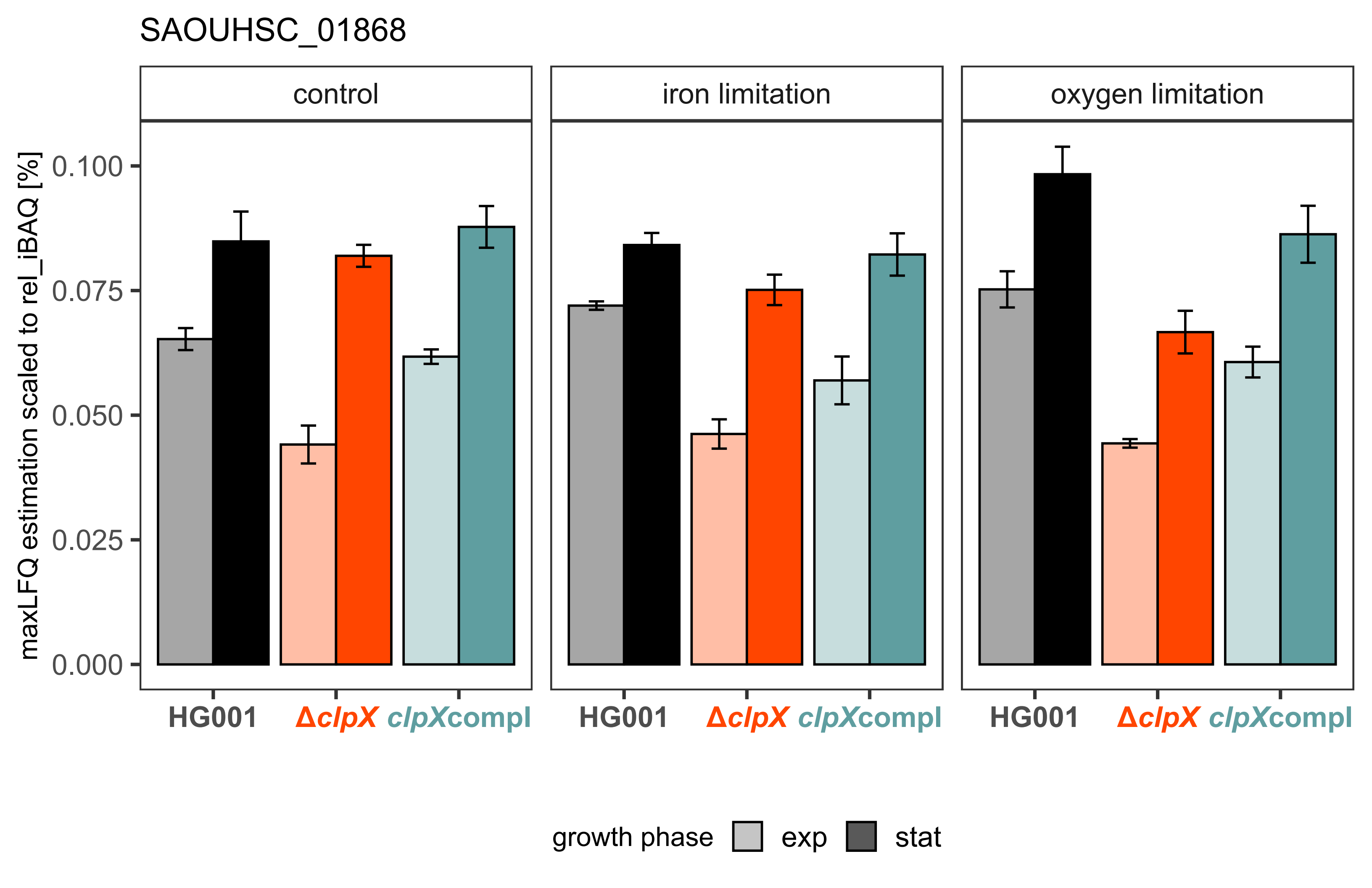
SAOUHSC_01868
control
iron limitation
oxygen limitation
maxLFQ estimation scaled to rel_iBAQ [%]
0.100
0.075
0.050
0.025
0.000
HG001
ΔclpX
clpXcompl
growth phase
exp
stat

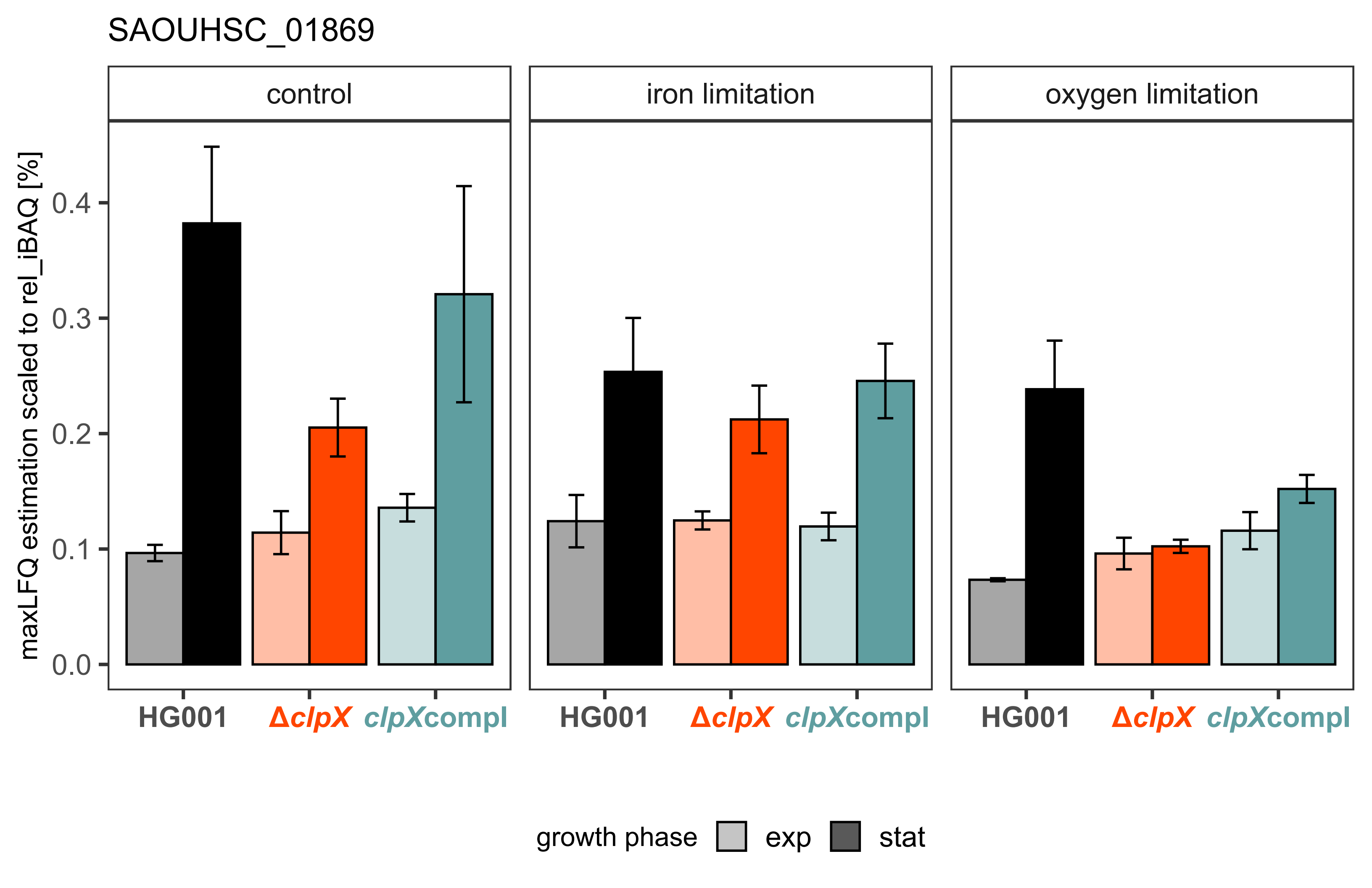

SAOUHSC_01869
control
iron limitation
oxygen limitation
maxLFQ estimation scaled to rel_iBAQ [%]
0.0
0.1
0.2
0.3
0.4
HG001
ΔclpX
clpXcompl
growth phase
exp
stat

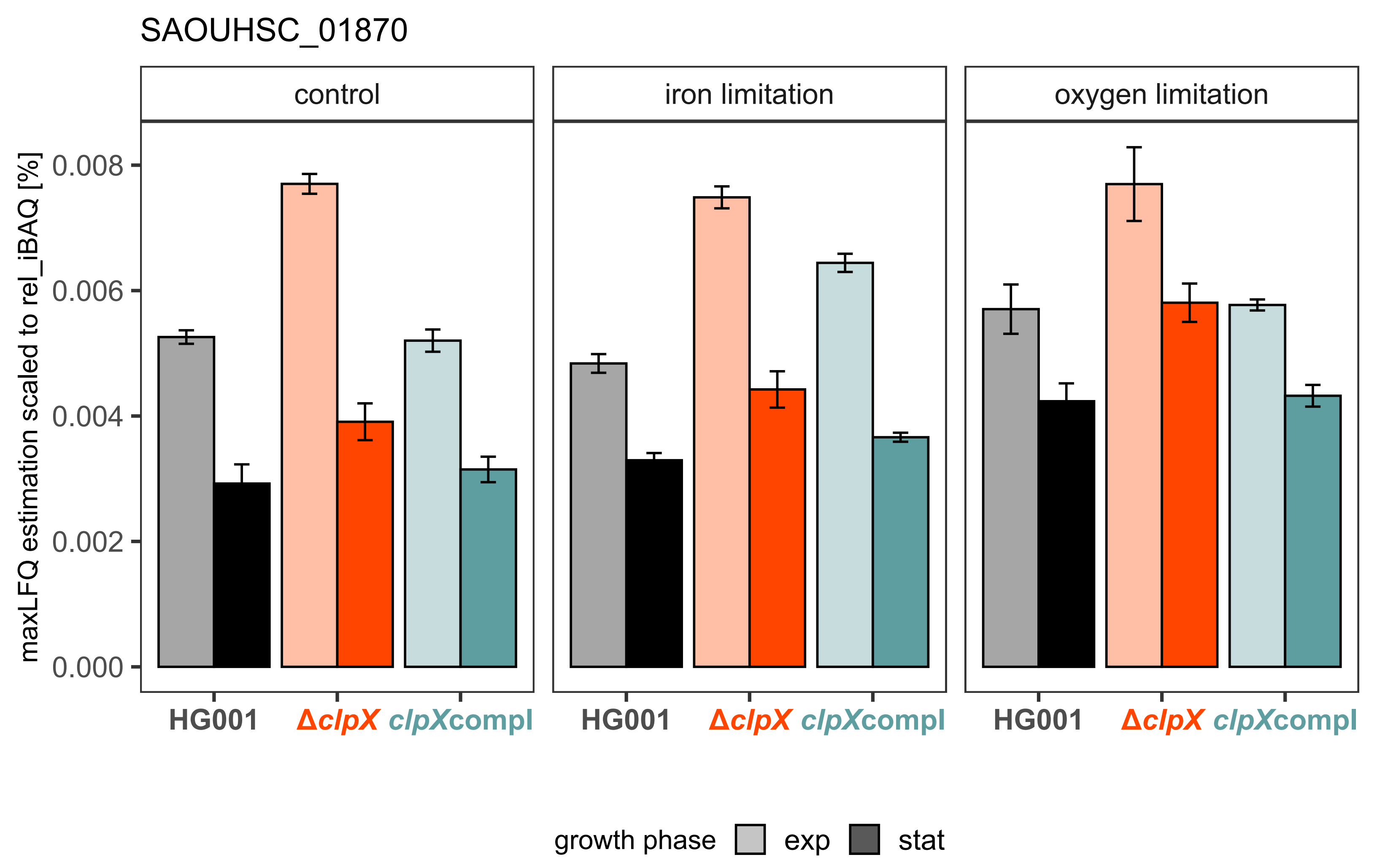

SAOUHSC_01870
control
iron limitation
oxygen limitation
maxLFQ estimation scaled to rel_iBAQ [%]
0.008
0.006
0.004
0.002
0.000
HG001
ΔclpX
clpXcompl
growth phase
exp
stat

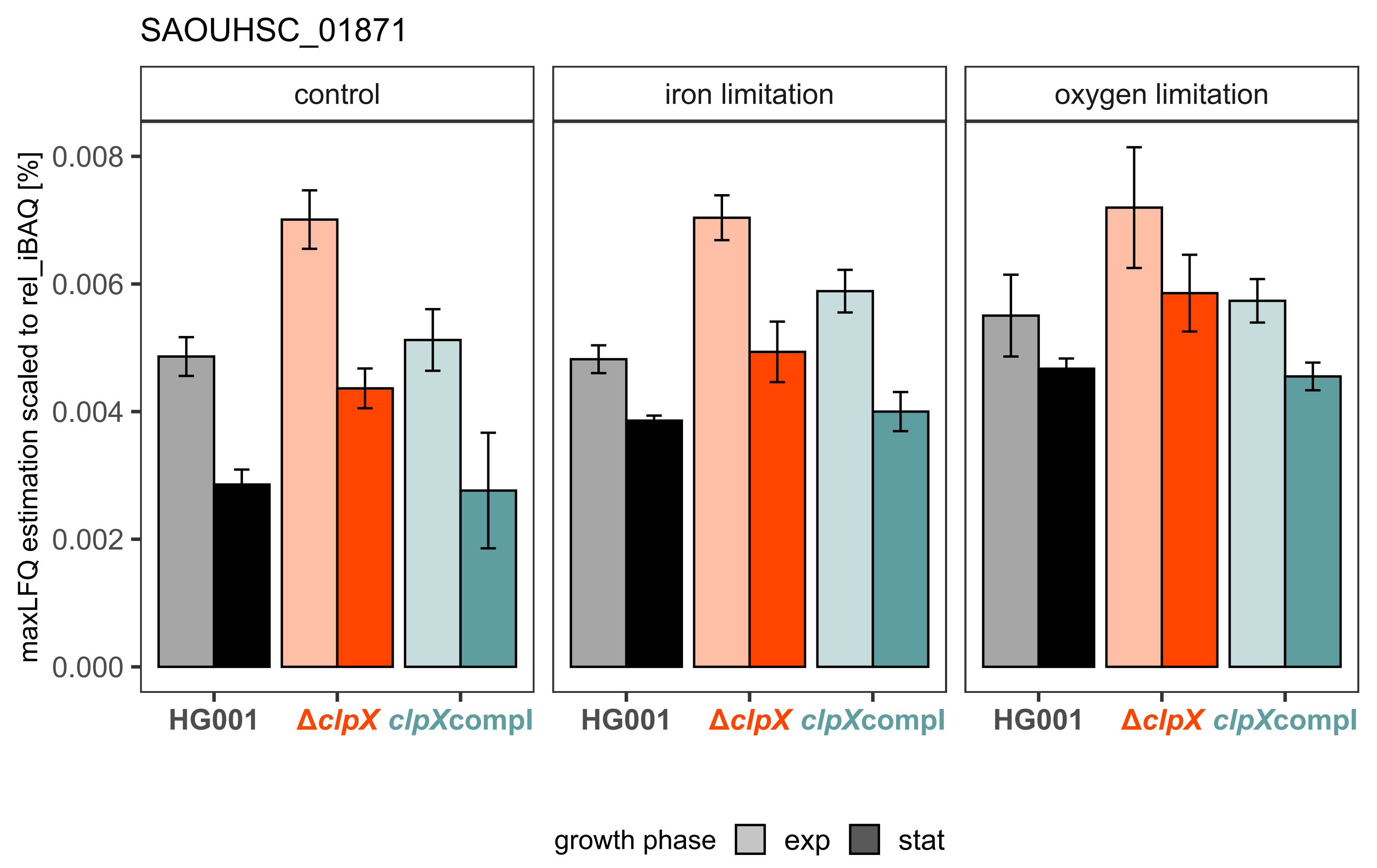
SAOUHSC_01871
control
iron limitation
oxygen limitation
maxLFQ estimation scaled to rel_iBAQ [%]
0.008
0.006
0.004
0.002
0.000
HG001
ΔclpX
clpXcompl
growth phase
exp
stat

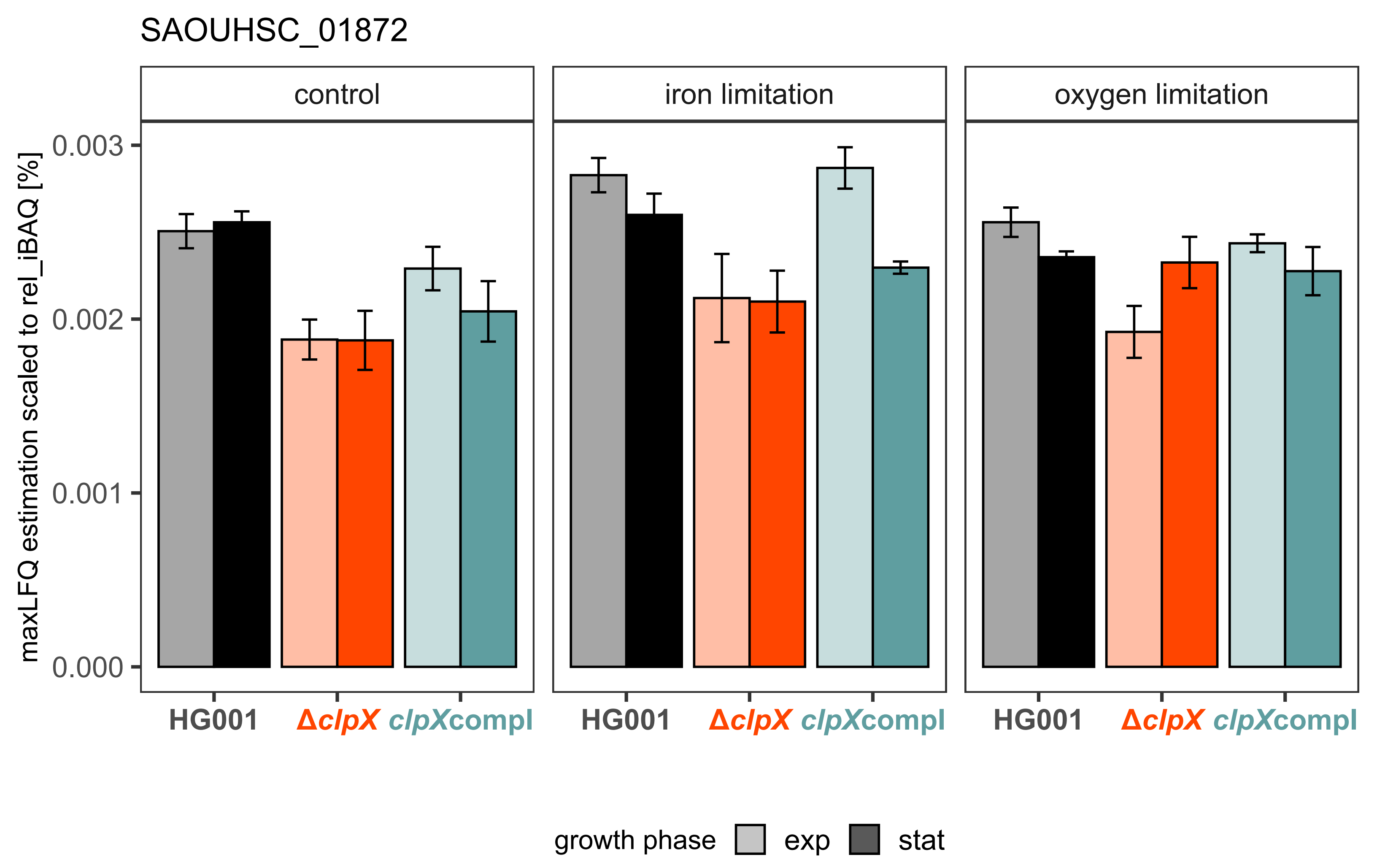
SAOUHSC_01872
control
iron limitation
oxygen limitation
maxLFQ estimation scaled to rel_iBAQ [%]
0.003
0.002
0.001
0.000
HG001
ΔclpX
clpXcompl
growth phase
exp
stat

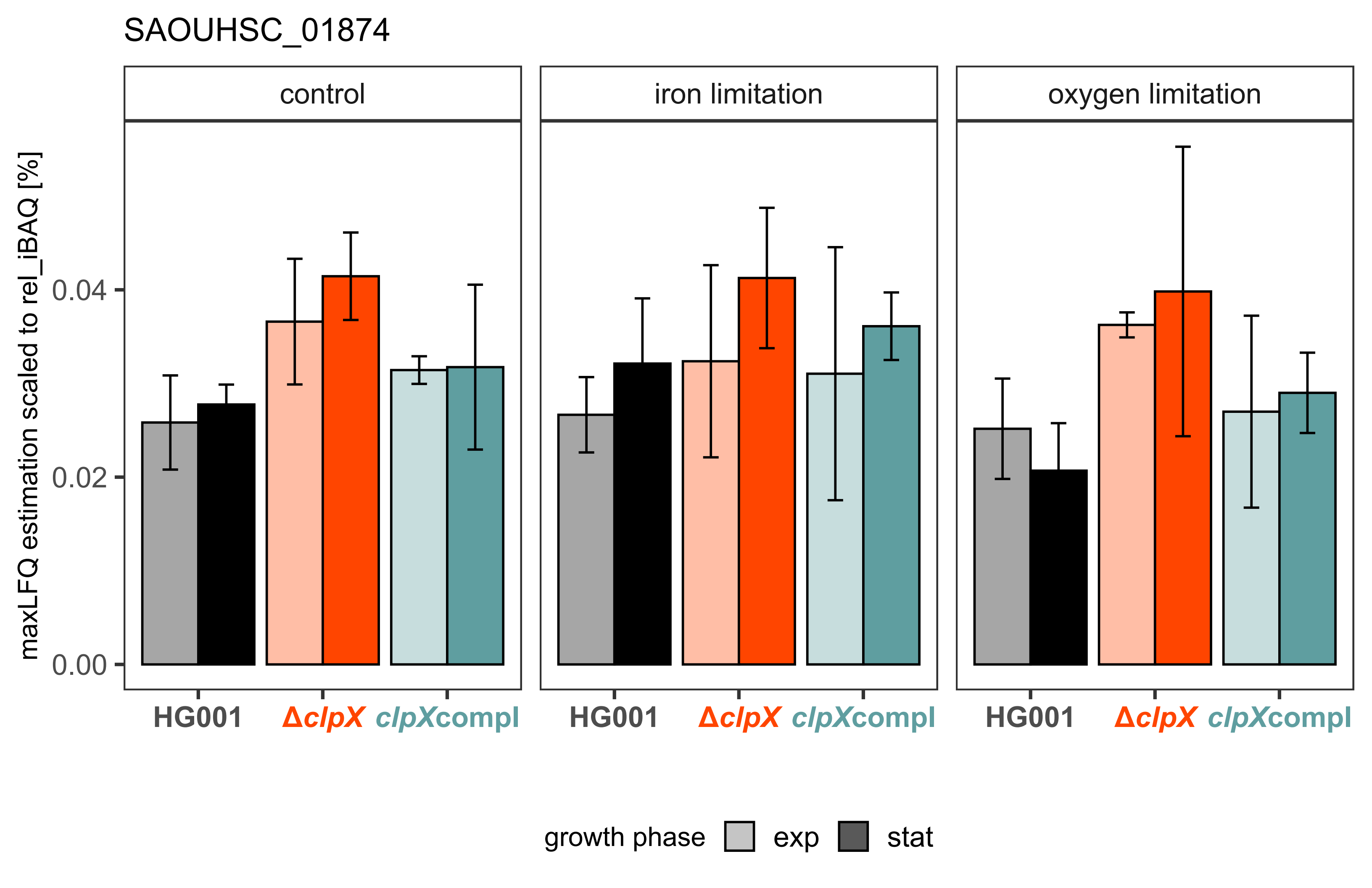
SAOUHSC_01874
control
iron limitation
oxygen limitation
maxLFQ estimation scaled to rel_iBAQ [%]
0.04
0.02
0.00
HG001
ΔclpX
clpXcompl
growth phase
exp
stat

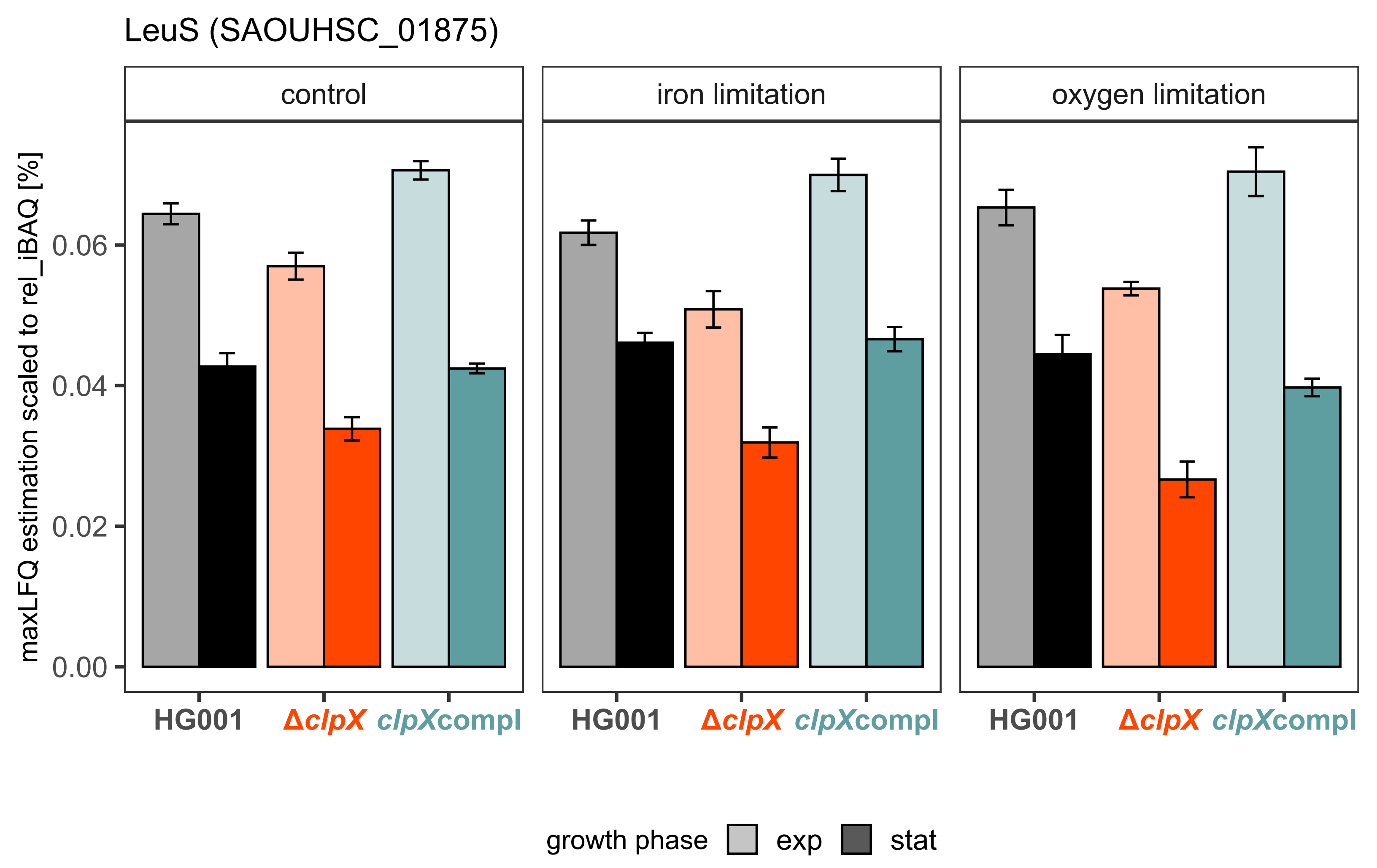
LeuS (SAOUHSC_01875)
control
iron limitation
oxygen limitation
maxLFQ estimation scaled to rel_iBAQ [%]
0.06
0.04
0.02
0.00
HG001
ΔclpX
clpXcompl
growth phase
exp
stat

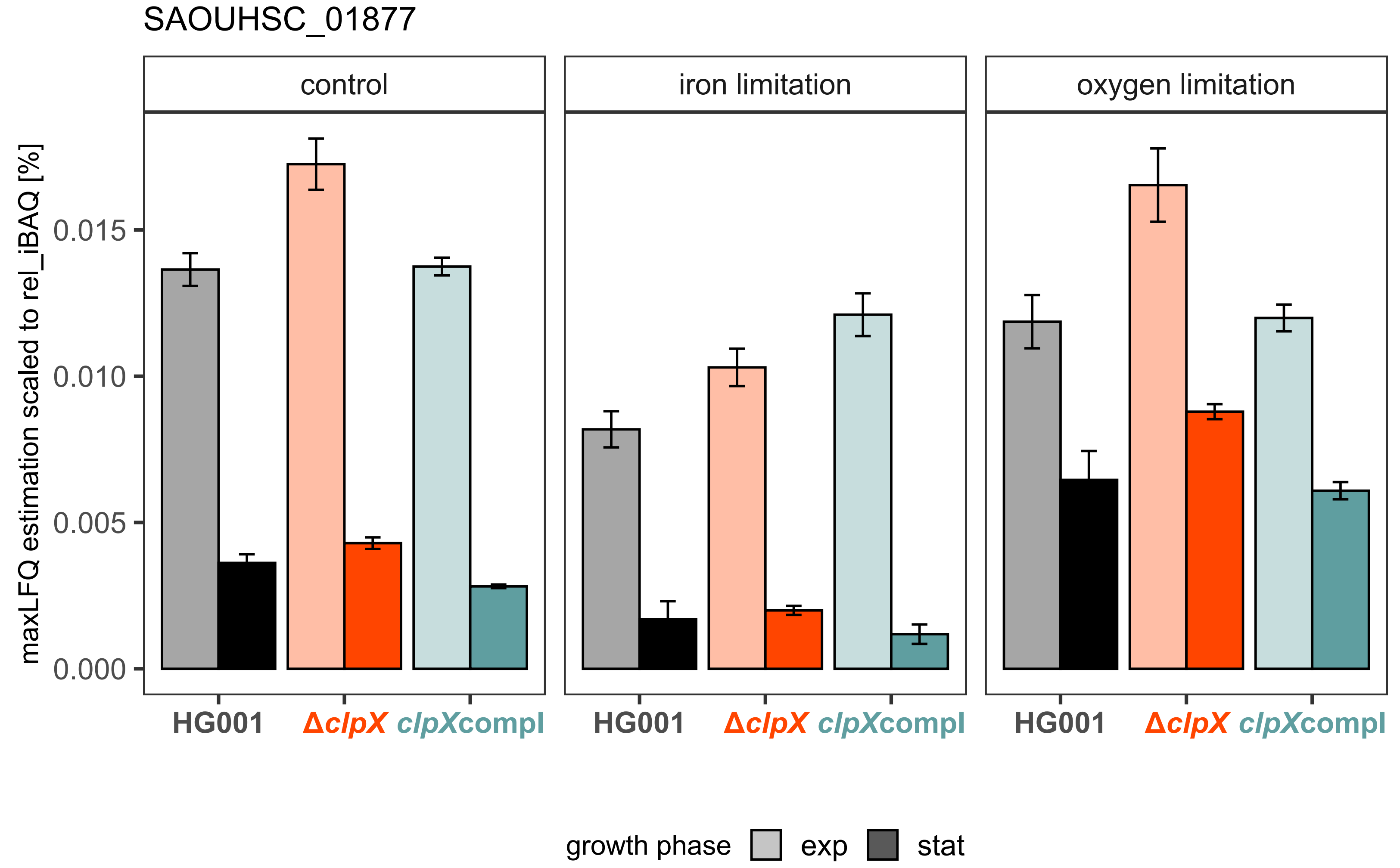
SAOUHSC_01877
control
iron limitation
oxygen limitation
maxLFQ estimation scaled to rel_iBAQ [%]
0.015
0.010
0.005
0.000
HG001
ΔclpX
clpXcompl
growth phase
exp
stat

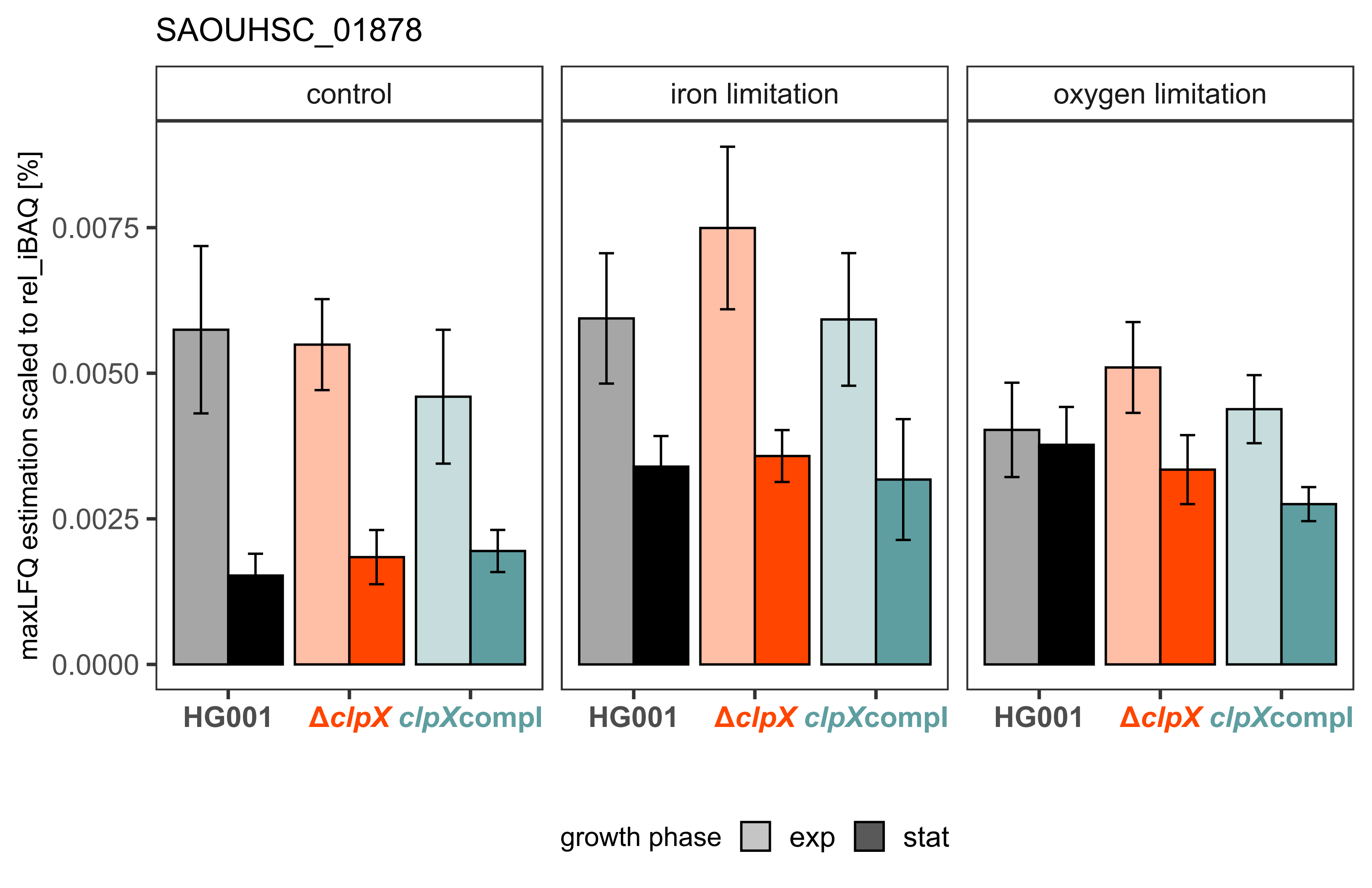

SAOUHSC_01878
control
iron limitation
oxygen limitation
maxLFQ estimation scaled to rel_iBAQ [%]
0.0075
0.0050
0.0025
0.0000
HG001
ΔclpX
clpXcompl
growth phase
exp
stat

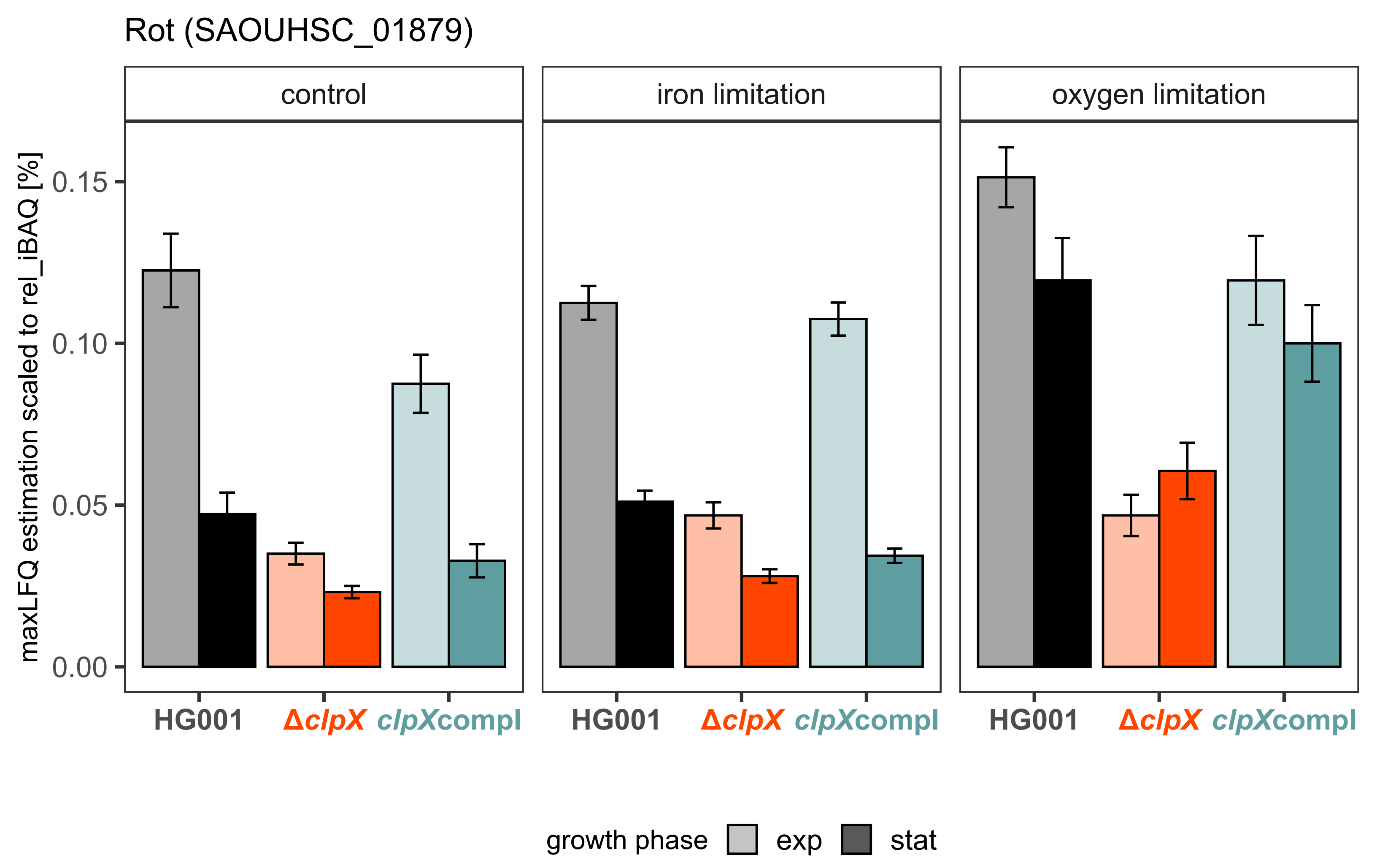

Rot (SAOUHSC_01879)
control
iron limitation
oxygen limitation
maxLFQ estimation scaled to rel_iBAQ [%]
0.15
0.10
0.05
0.00
HG001
ΔclpX
clpXcompl
growth phase
exp
stat

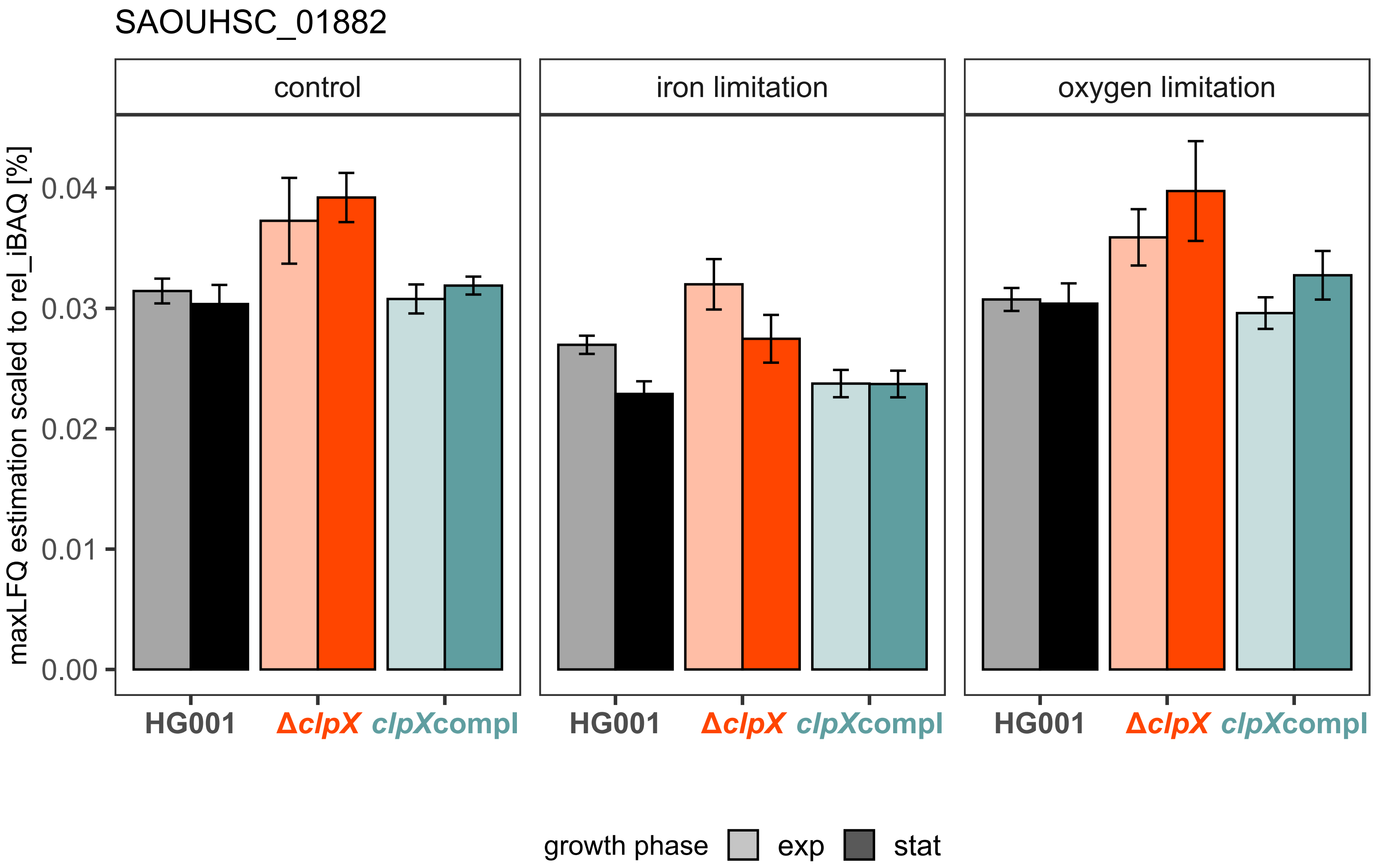

SAOUHSC_01882
control
iron limitation
oxygen limitation
maxLFQ estimation scaled to rel_iBAQ [%]
0.04
0.03
0.02
0.01
0.00
HG001
ΔclpX
clpXcompl
growth phase
exp
stat

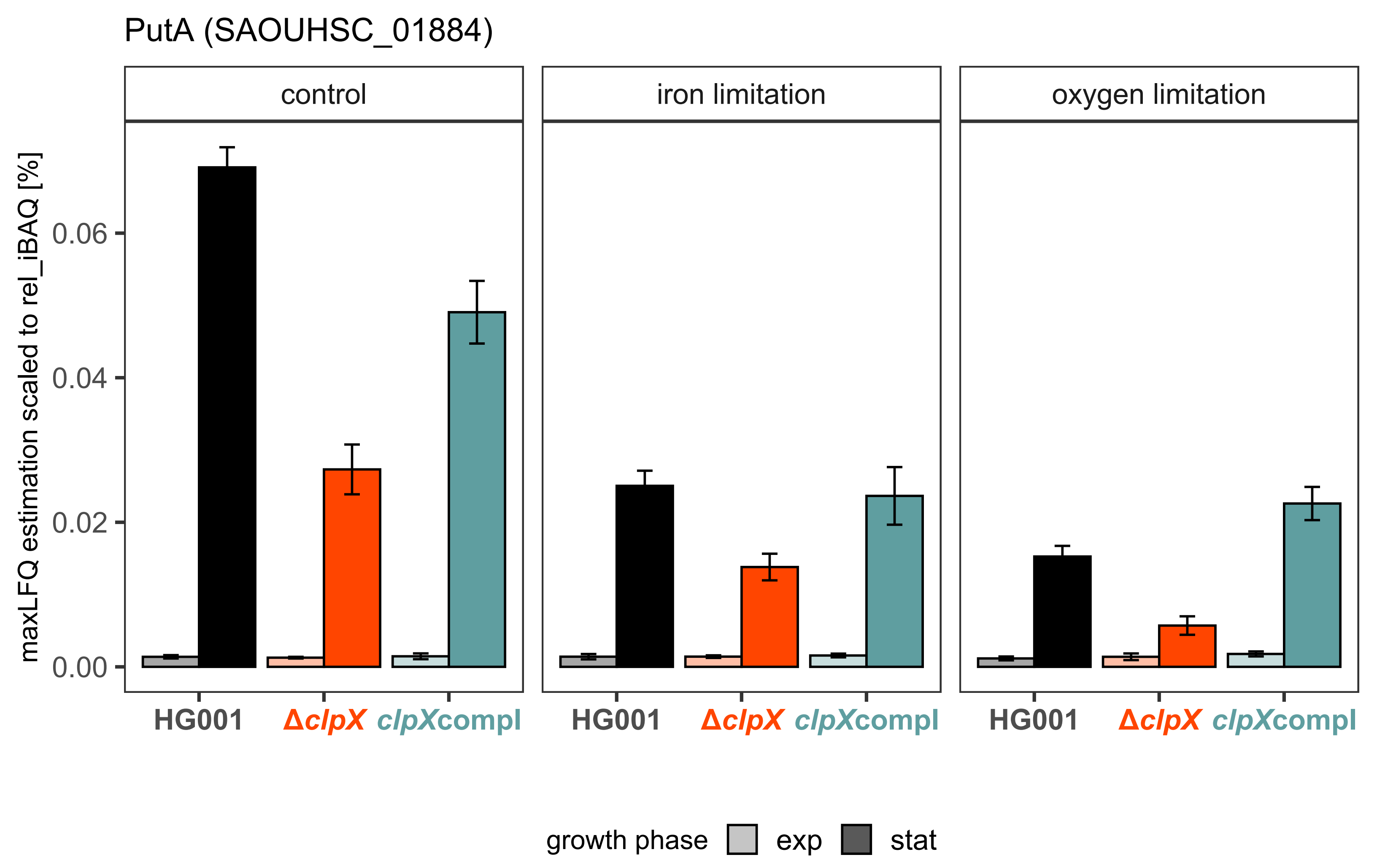
PutA (SAOUHSC_01884)
control
iron limitation
oxygen limitation
maxLFQ estimation scaled to rel_iBAQ [%]
0.00
0.02
0.04
0.06
HG001
ΔclpX
clpXcompl
growth phase
exp
stat

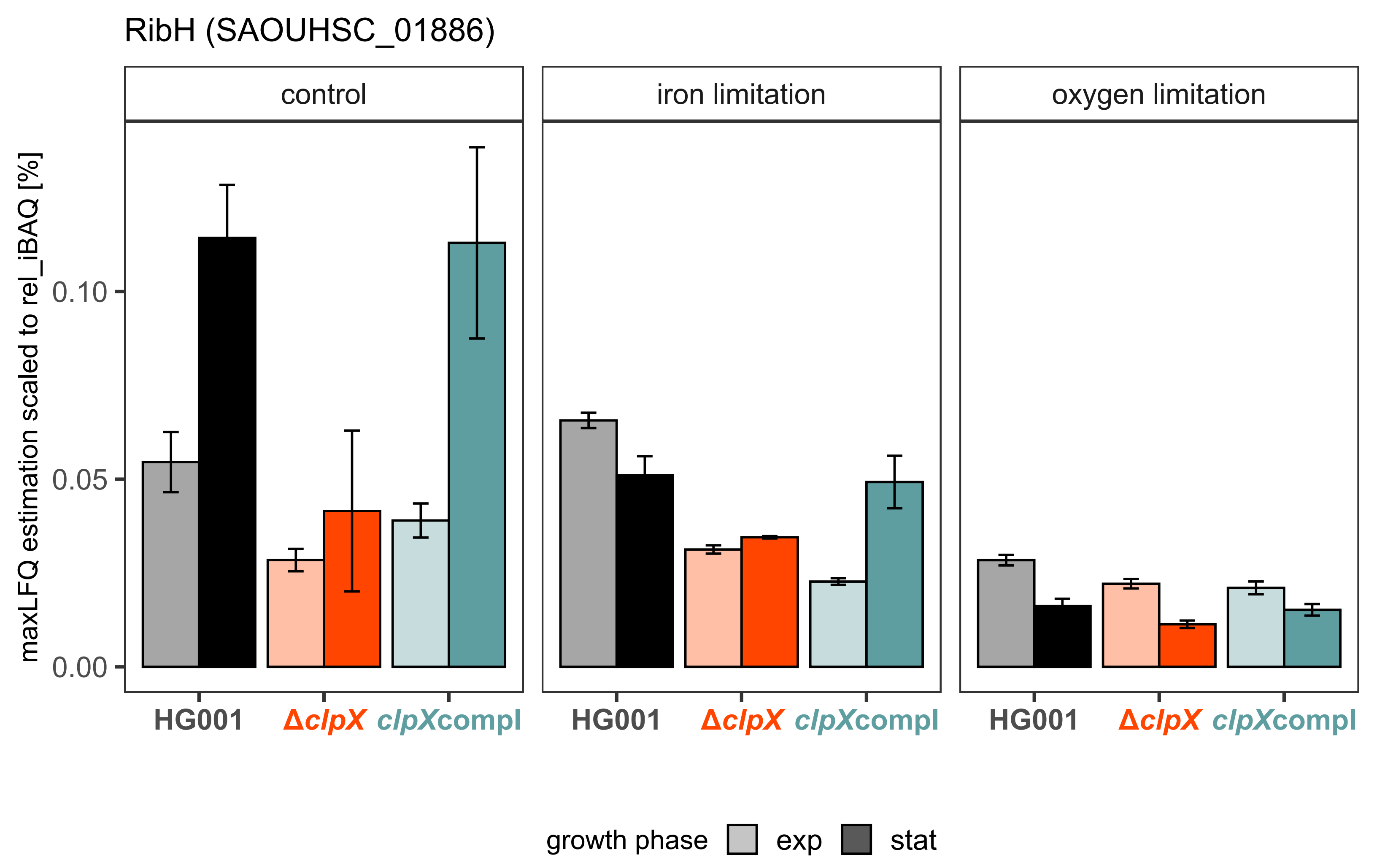

RibH (SAOUHSC_01886)
control
iron limitation
oxygen limitation
maxLFQ estimation scaled to rel_iBAQ [%]
0.10
0.05
0.00
HG001
ΔclpX
clpXcompl
growth phase
exp
stat

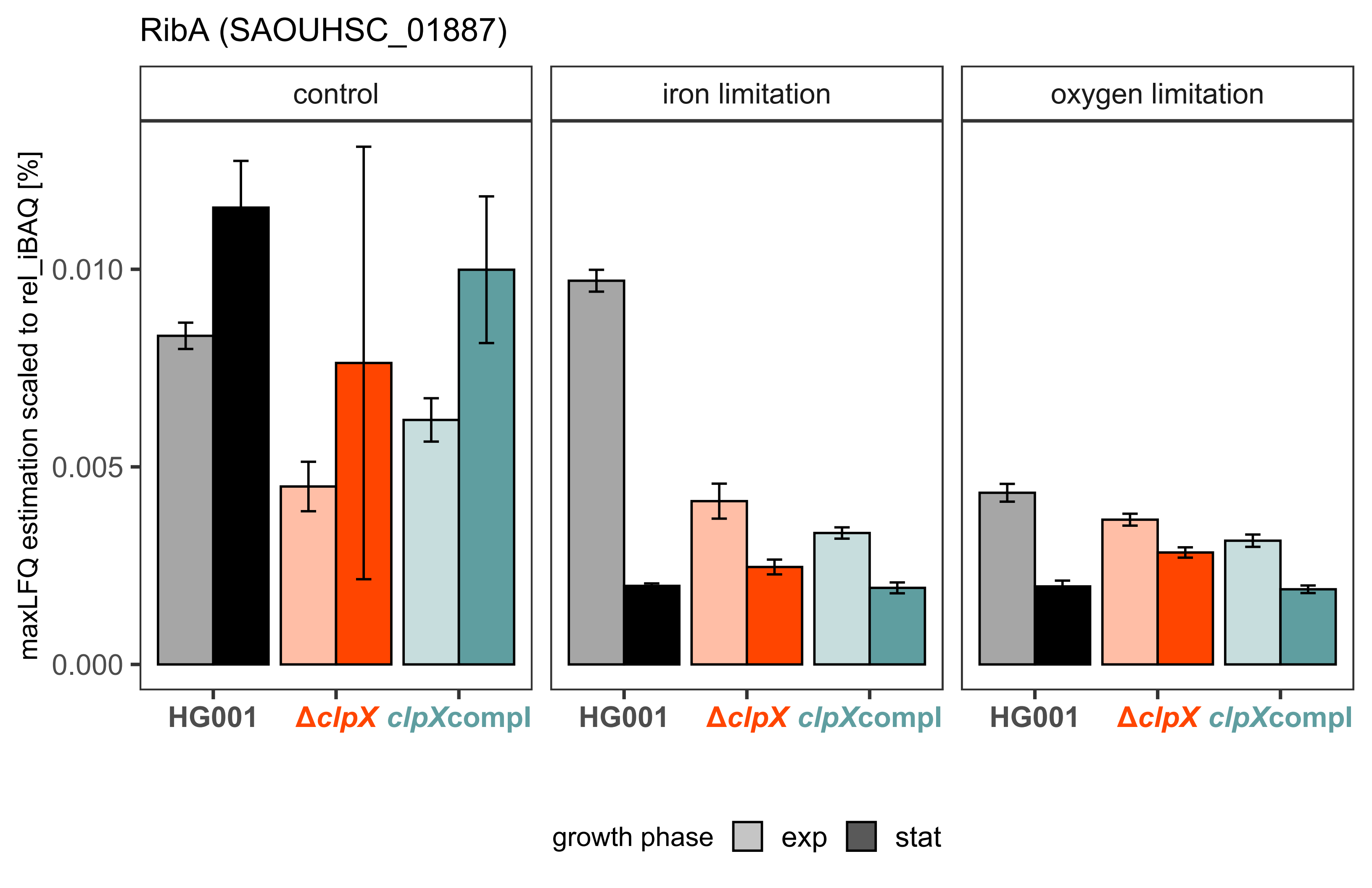
RibA (SAOUHSC_01887)
control
iron limitation
oxygen limitation
maxLFQ estimation scaled to rel_iBAQ [%]
0.000
0.005
0.010
HG001
ΔclpX
clpXcompl
growth phase
exp
stat

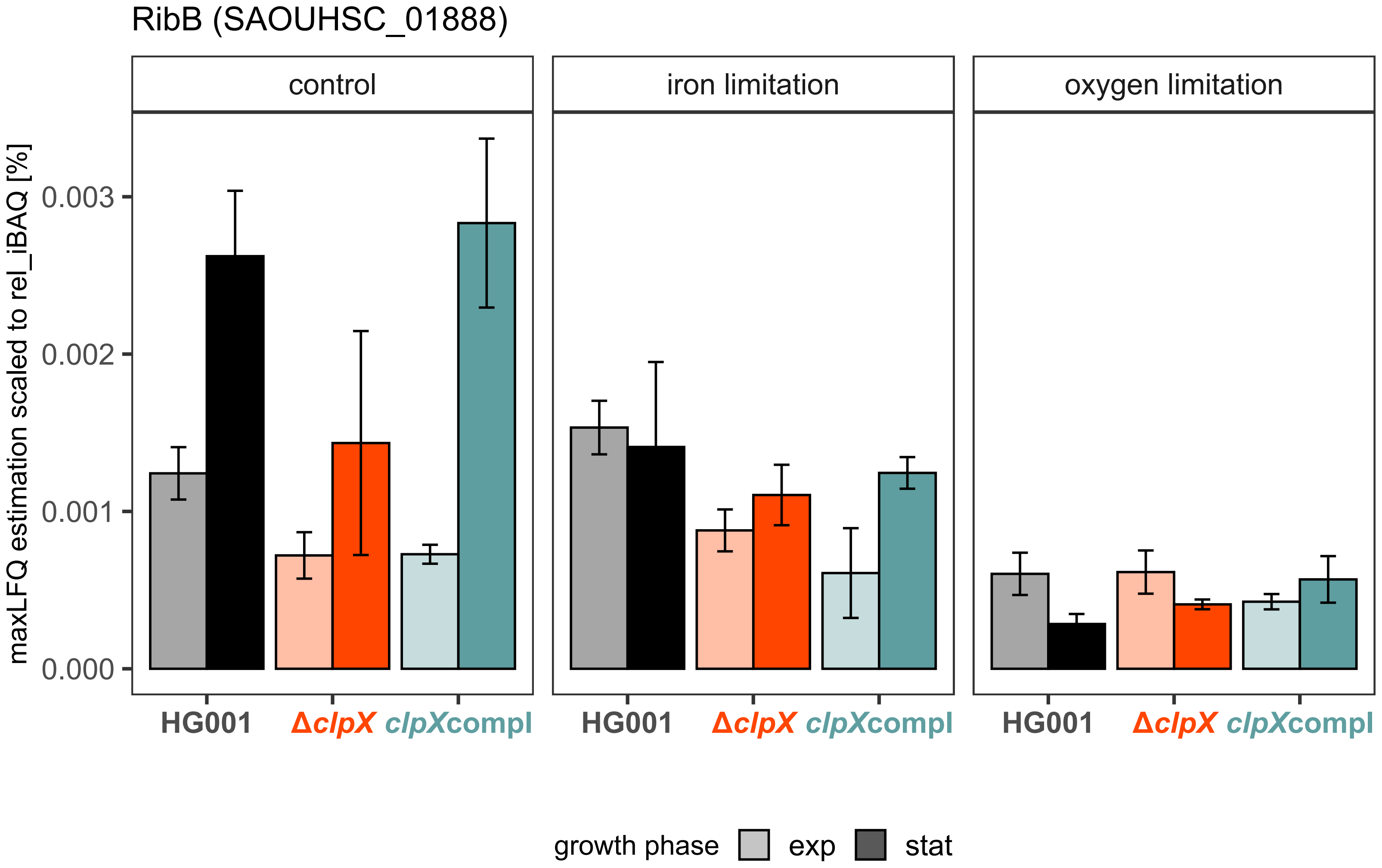

RibB (SAOUHSC_01888)
control
iron limitation
oxygen limitation
maxLFQ estimation scaled to rel_iBAQ [%]
0.000
0.001
0.002
0.003
HG001
ΔclpX
clpXcompl
growth phase
exp
stat

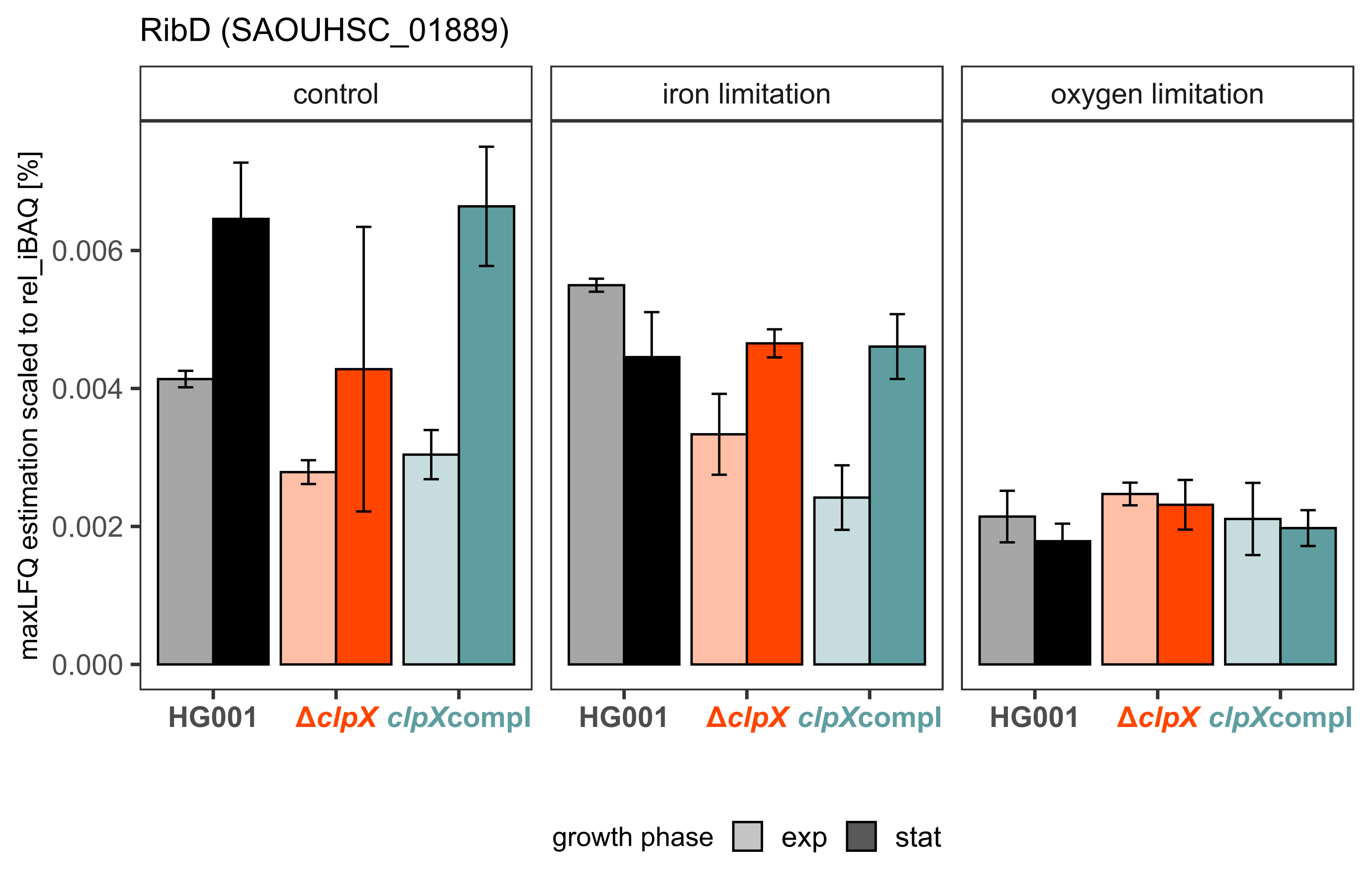

RibD (SAOUHSC_01889)
control
iron limitation
oxygen limitation
maxLFQ estimation scaled to rel_iBAQ [%]
0.000
0.002
0.004
0.006
HG001
ΔclpX
clpXcompl
growth phase
exp
stat

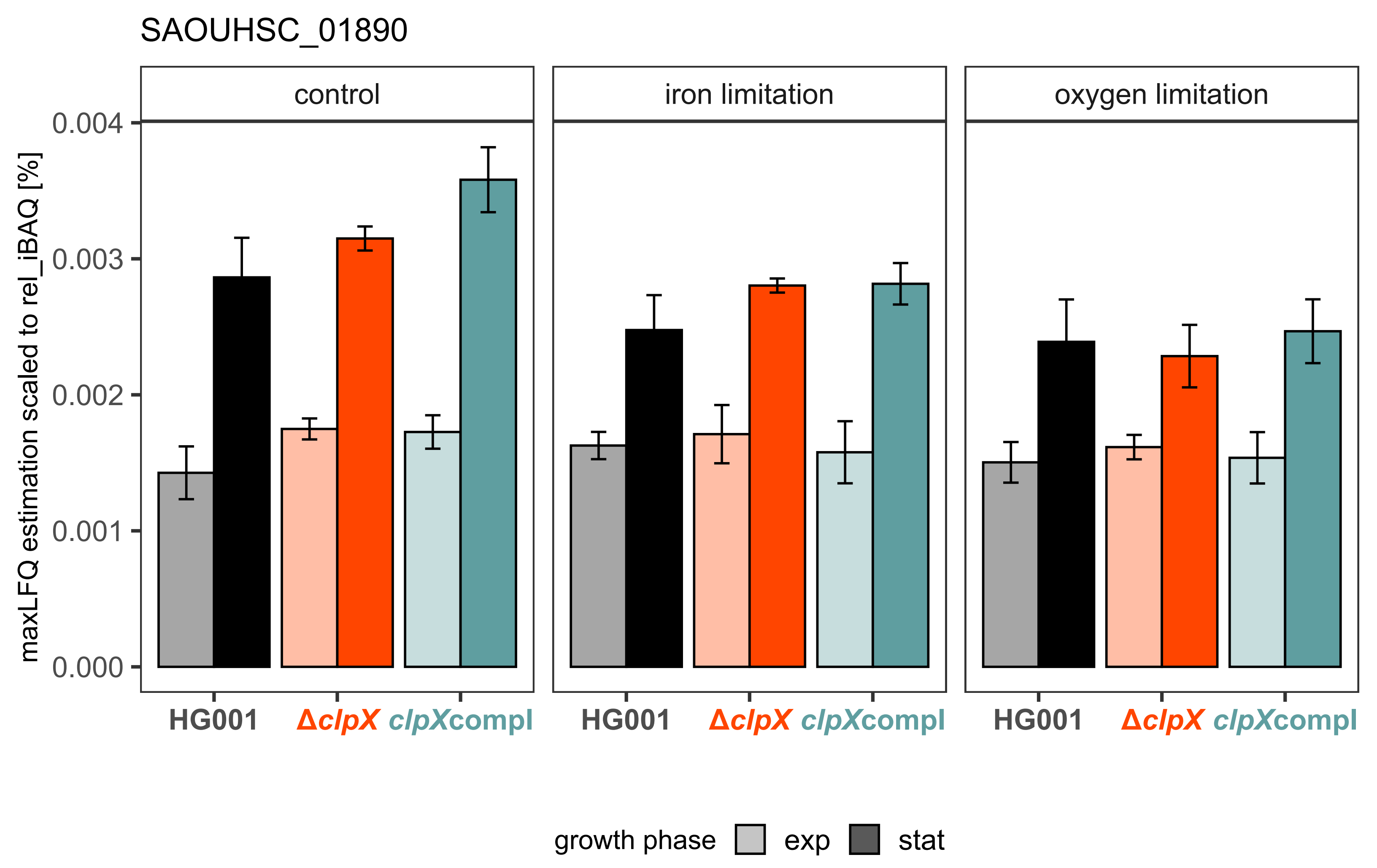
SAOUHSC_01890
control
iron limitation
oxygen limitation
maxLFQ estimation scaled to rel_iBAQ [%]
0.004
0.003
0.002
0.001
0.000
HG001
ΔclpX
clpXcompl
growth phase
exp
stat

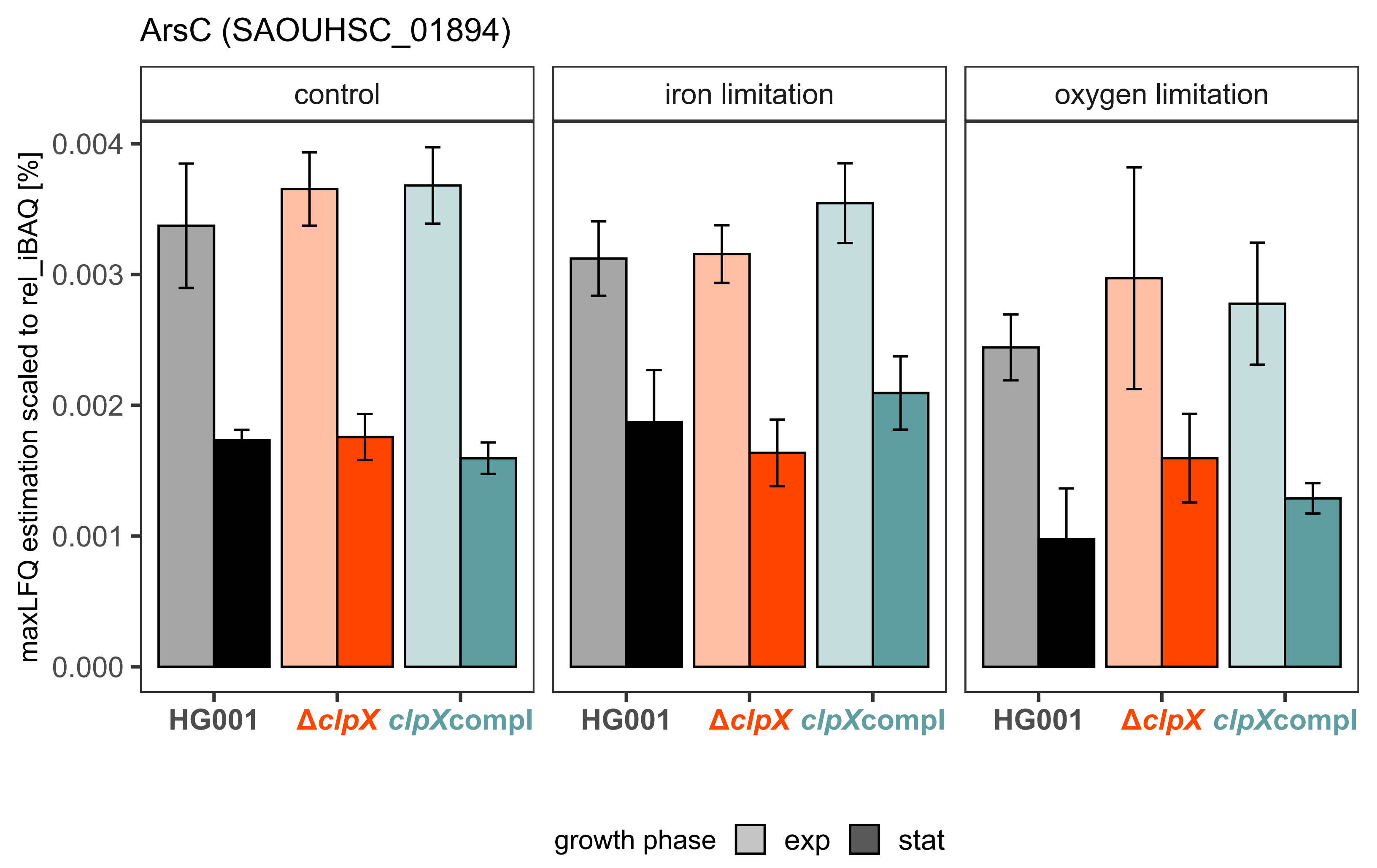
ArsC (SAOUHSC_01894)
control
iron limitation
oxygen limitation
maxLFQ estimation scaled to rel_iBAQ [%]
0.004
0.003
0.002
0.001
0.000
HG001
ΔclpX
clpXcompl
growth phase
exp
stat

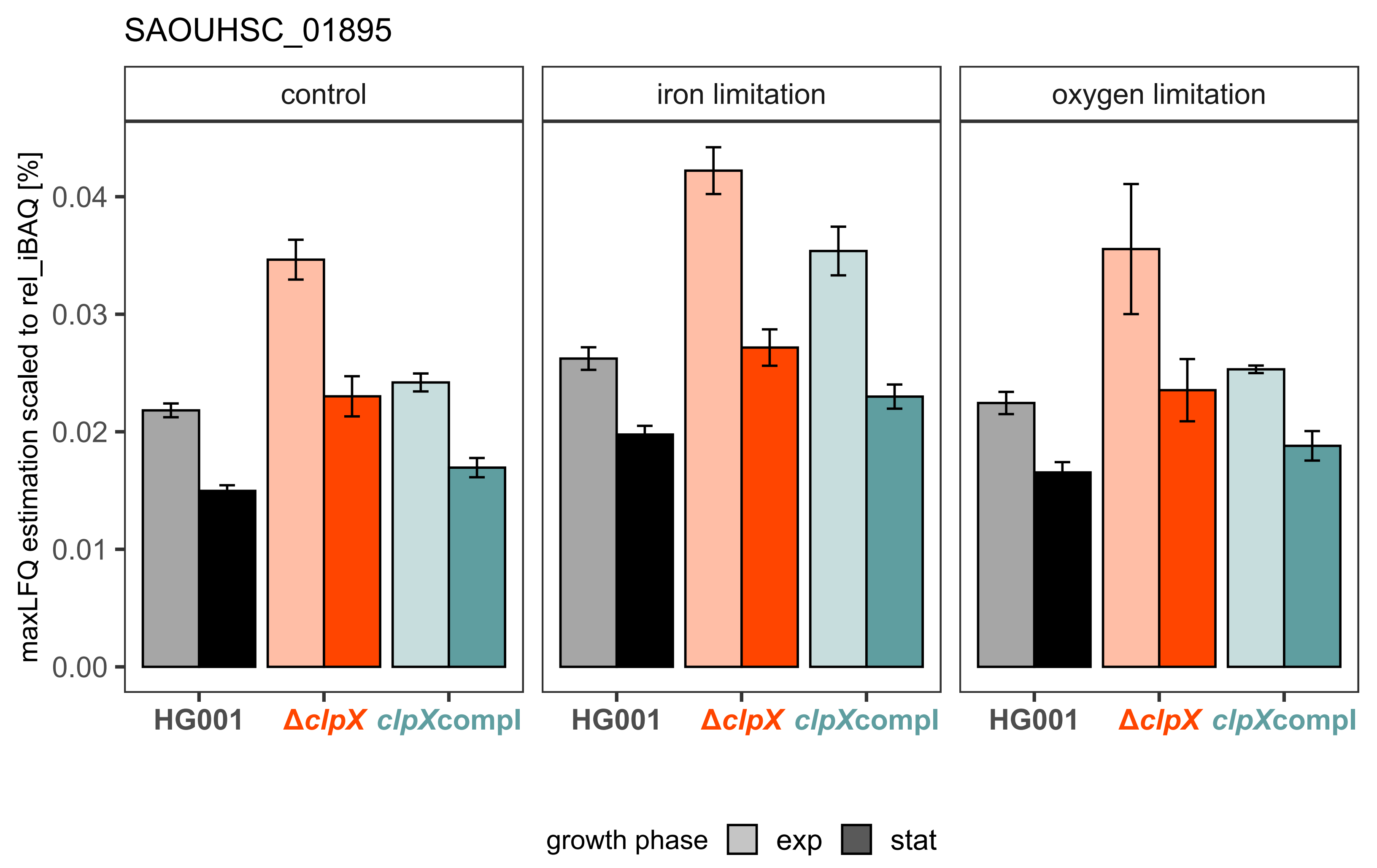
SAOUHSC_01895
control
iron limitation
oxygen limitation
maxLFQ estimation scaled to rel_iBAQ [%]
0.04
0.03
0.02
0.01
0.00
HG001
ΔclpX
clpXcompl
growth phase
exp
stat

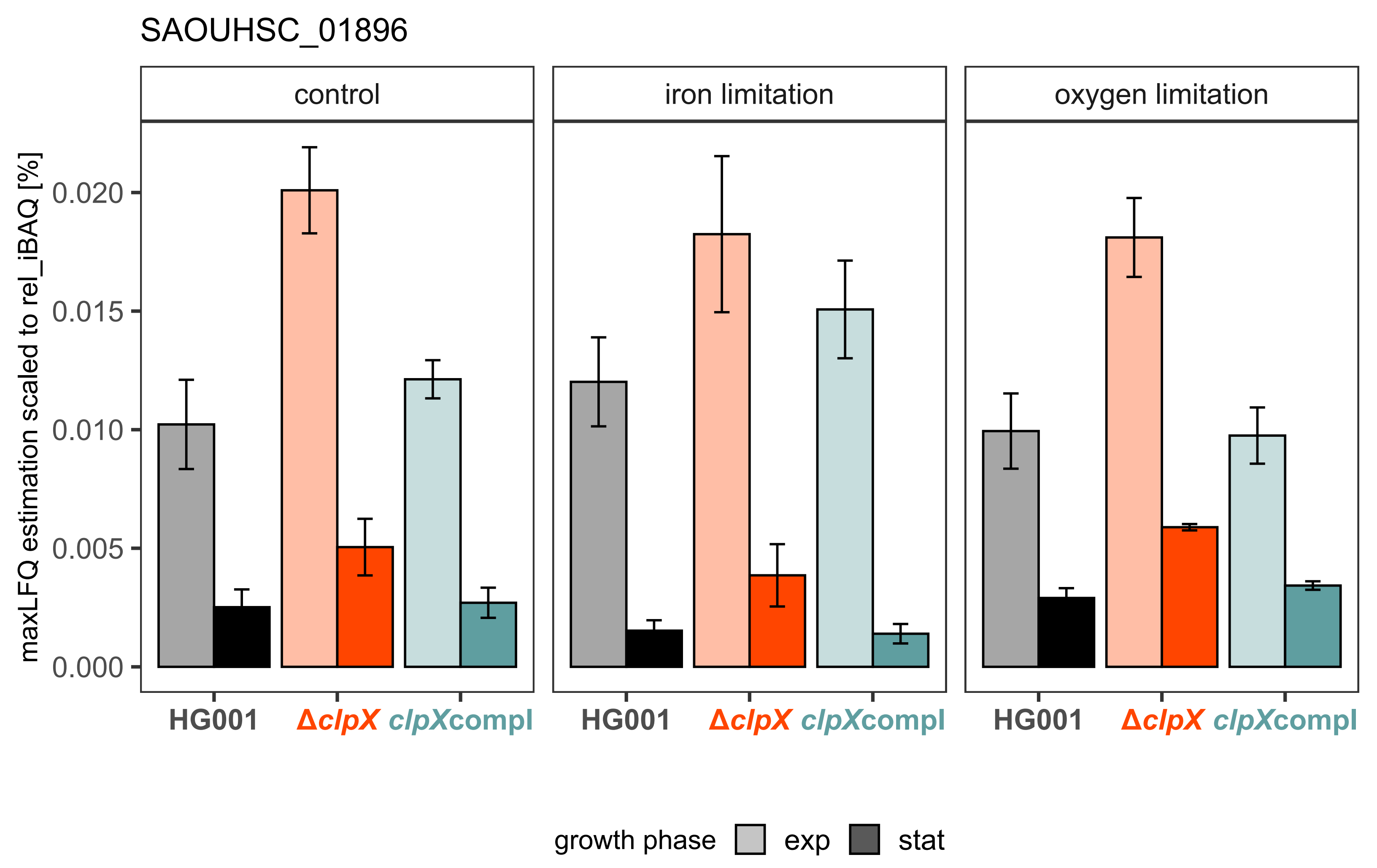

SAOUHSC_01896
control
iron limitation
oxygen limitation
maxLFQ estimation scaled to rel_iBAQ [%]
0.020
0.015
0.010
0.005
0.000
HG001
ΔclpX
clpXcompl
growth phase
exp
stat

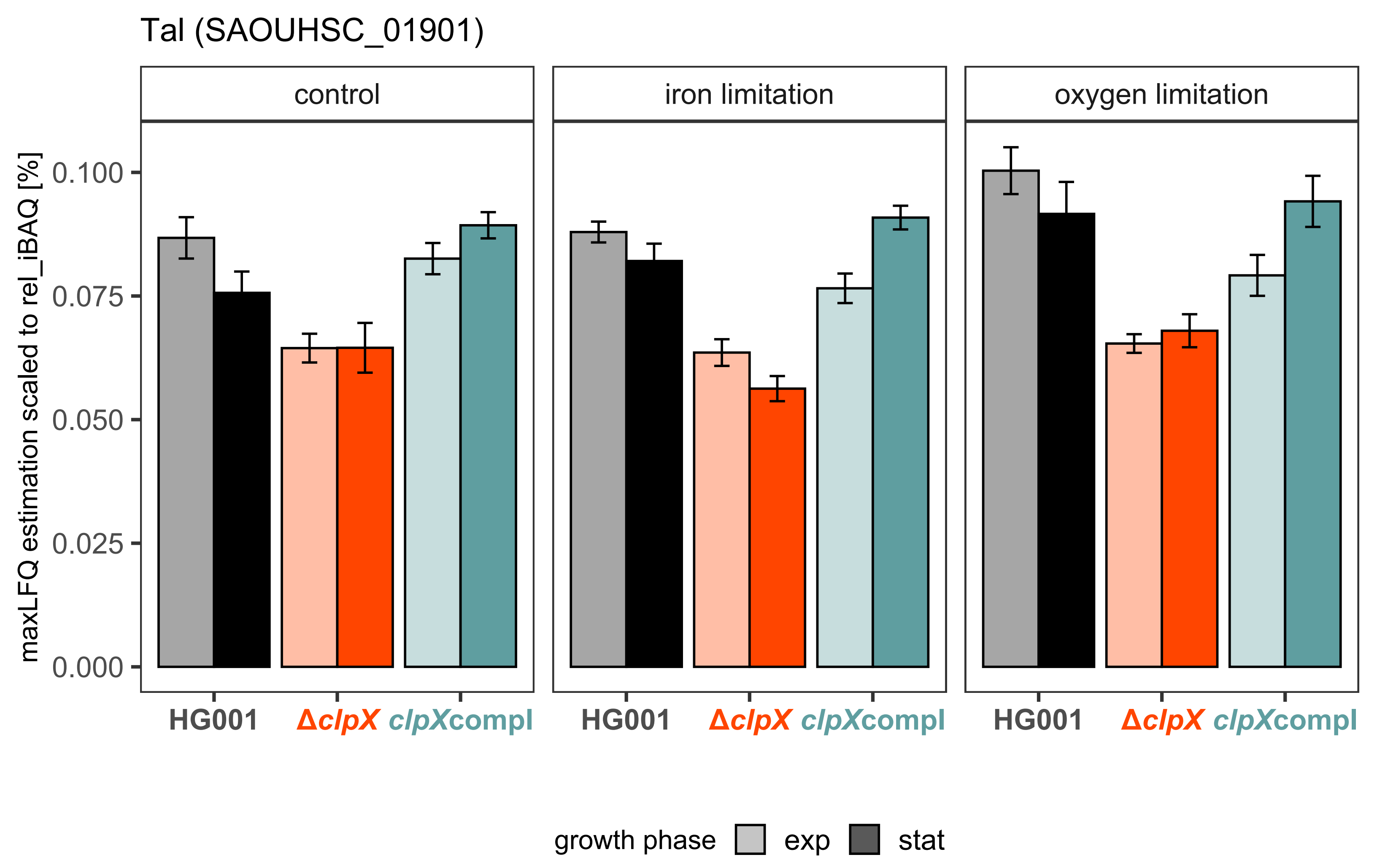
Tal (SAOUHSC_01901)
control
iron limitation
oxygen limitation
maxLFQ estimation scaled to rel_iBAQ [%]
0.100
0.075
0.050
0.025
0.000
HG001
ΔclpX
clpXcompl
growth phase
exp
stat

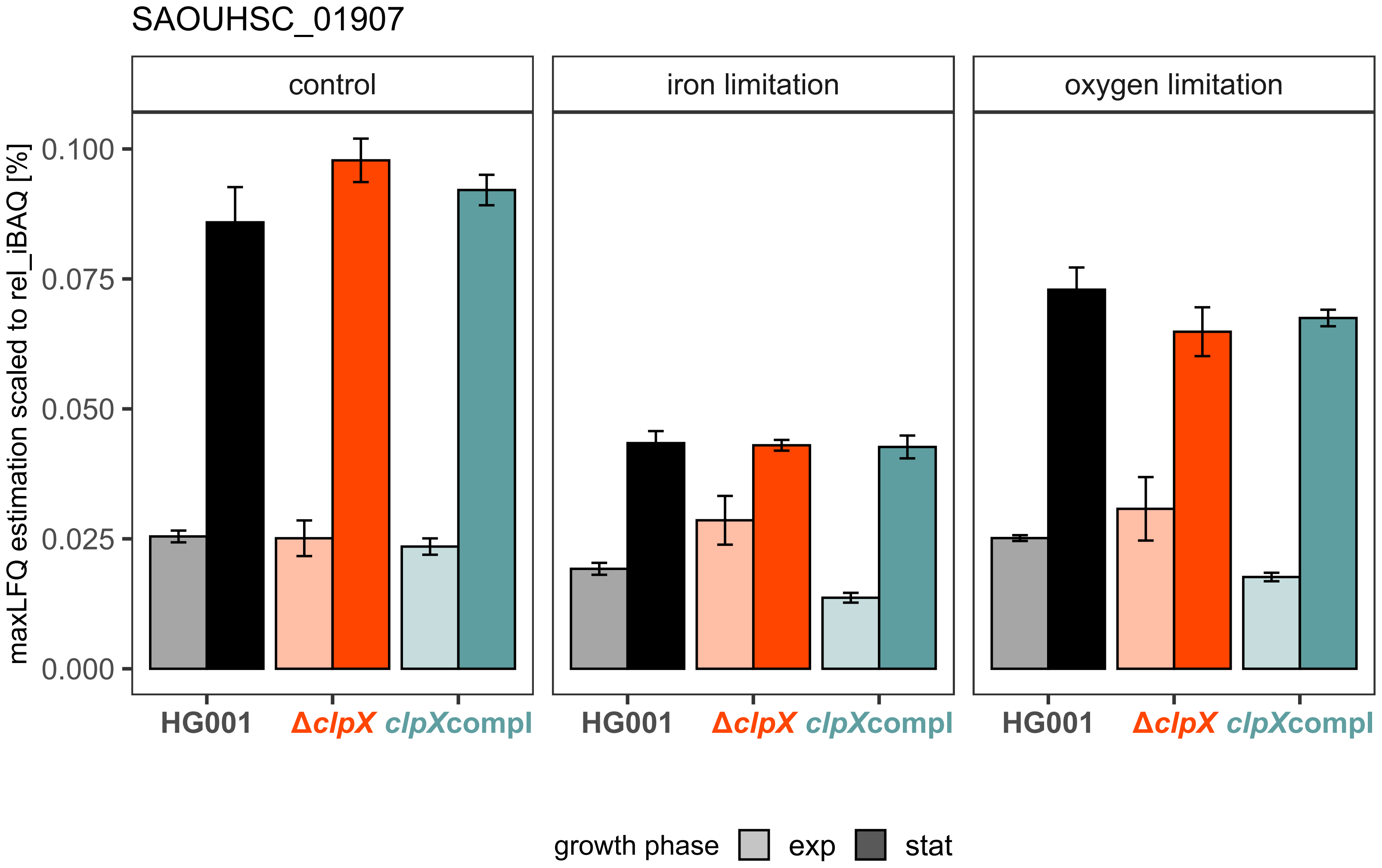
SAOUHSC_01907
control
iron limitation
oxygen limitation
maxLFQ estimation scaled to rel_iBAQ [%]
0.100
0.075
0.050
0.025
0.000
HG001
ΔclpX
clpXcompl
growth phase
exp
stat

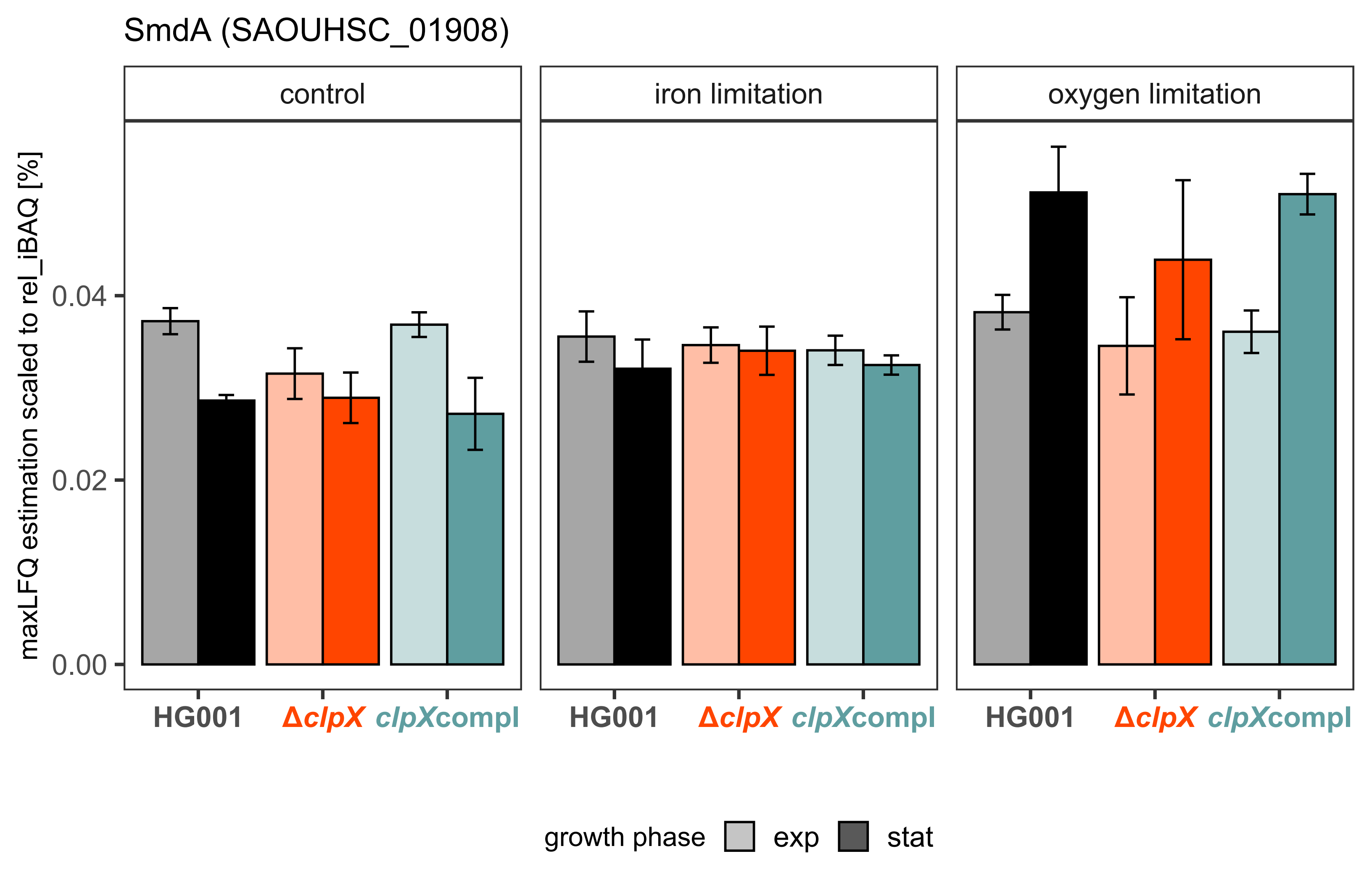

SmdA (SAOUHSC_01908)
control
iron limitation
oxygen limitation
maxLFQ estimation scaled to rel_iBAQ [%]
0.04
0.02
0.00
HG001
ΔclpX
clpXcompl
growth phase
exp
stat

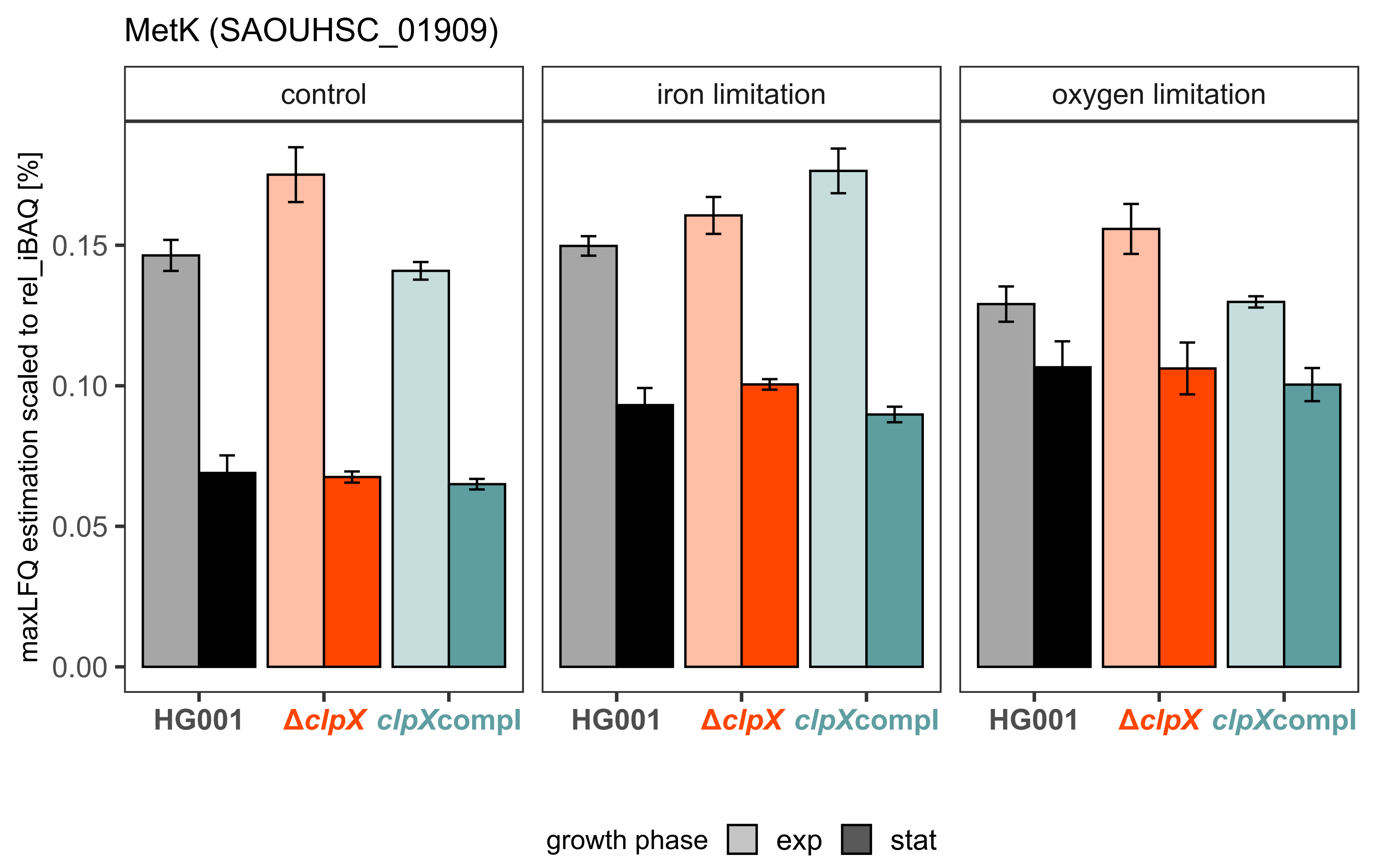

MetK (SAOUHSC_01909)
control
iron limitation
oxygen limitation
maxLFQ estimation scaled to rel_iBAQ [%]
0.15
0.10
0.05
0.00
HG001
ΔclpX
clpXcompl
growth phase
exp
stat

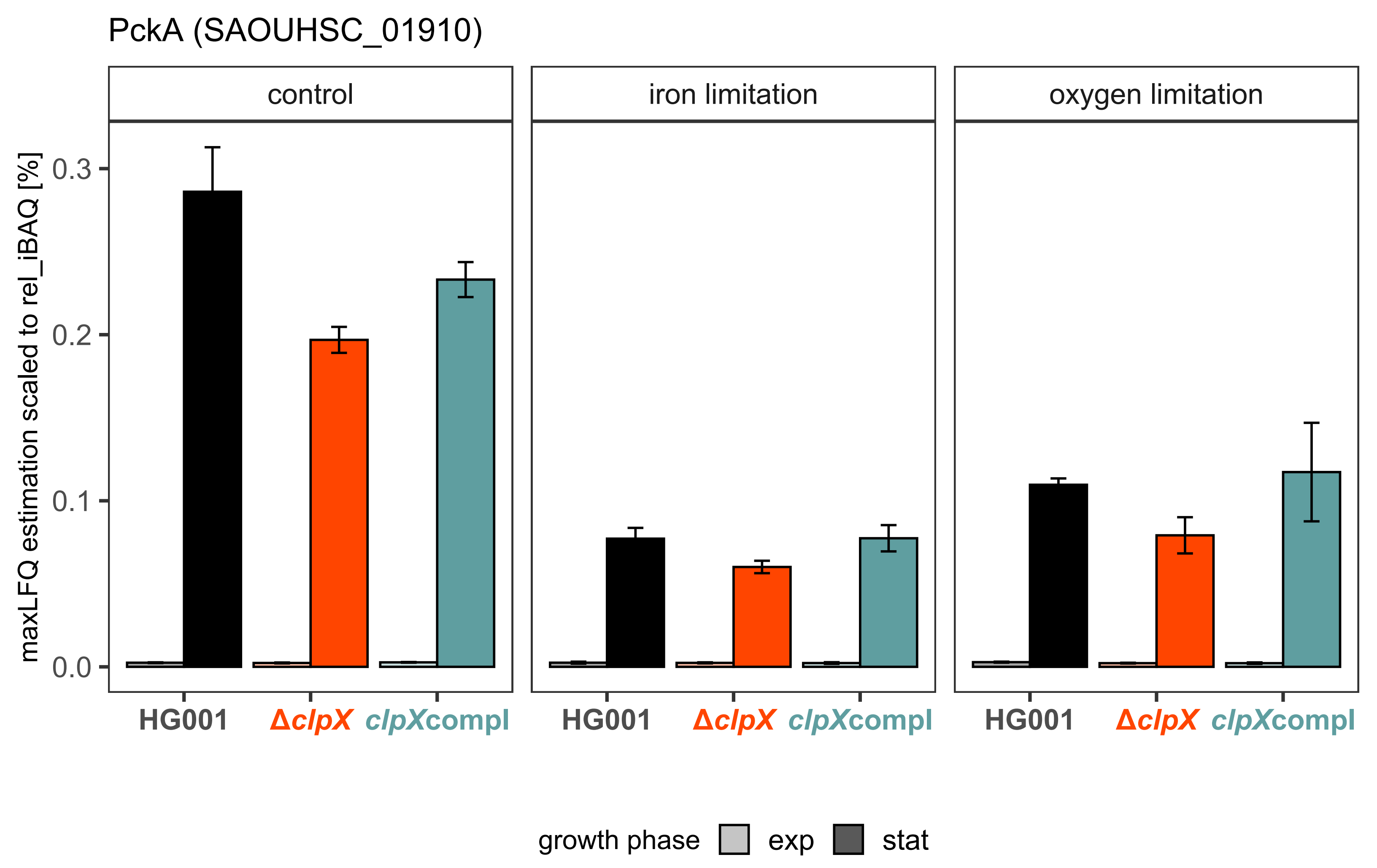
PckA (SAOUHSC_01910)
control
iron limitation
oxygen limitation
maxLFQ estimation scaled to rel_iBAQ [%]
0.3
0.2
0.1
0.0
HG001
ΔclpX
clpXcompl
growth phase
exp
stat

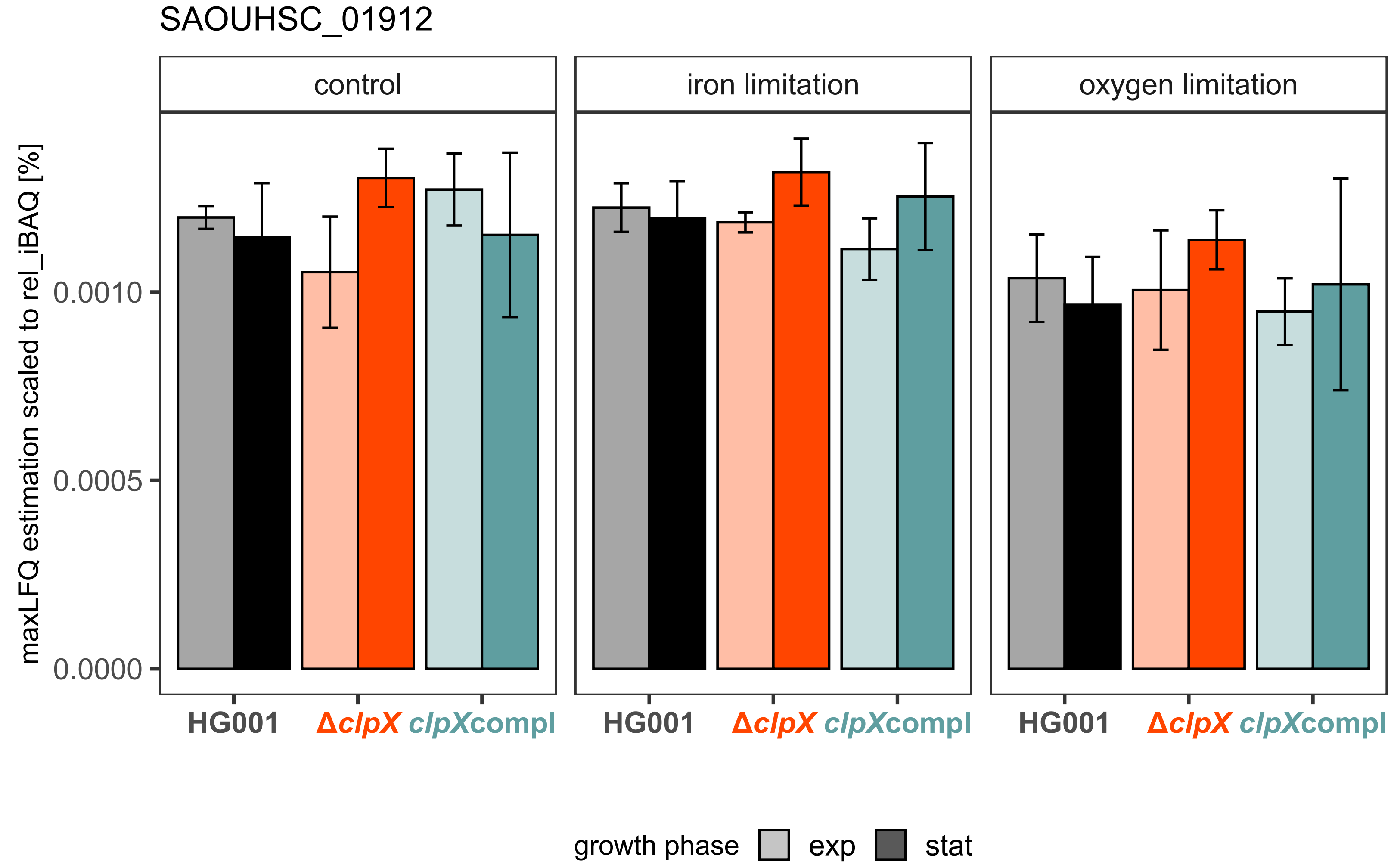
SAOUHSC_01912
control
iron limitation
oxygen limitation
maxLFQ estimation scaled to rel_iBAQ [%]
0.0010
0.0005
0.0000
HG001
ΔclpX
clpXcompl
growth phase
exp
stat

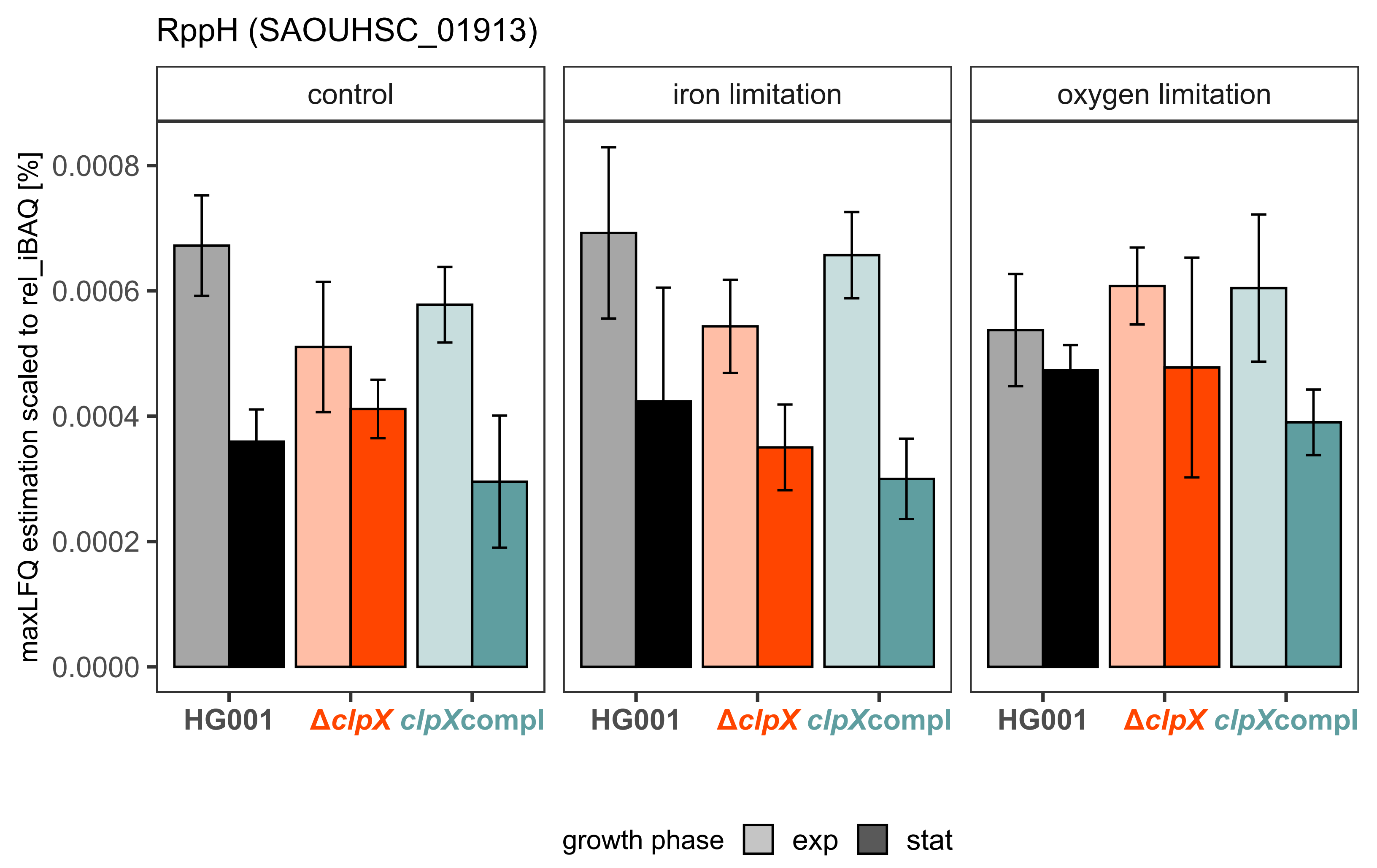
RppH (SAOUHSC_01913)
control
iron limitation
oxygen limitation
maxLFQ estimation scaled to rel_iBAQ [%]
0.0008
0.0006
0.0004
0.0002
0.0000
HG001
ΔclpX
clpXcompl
growth phase
exp
stat

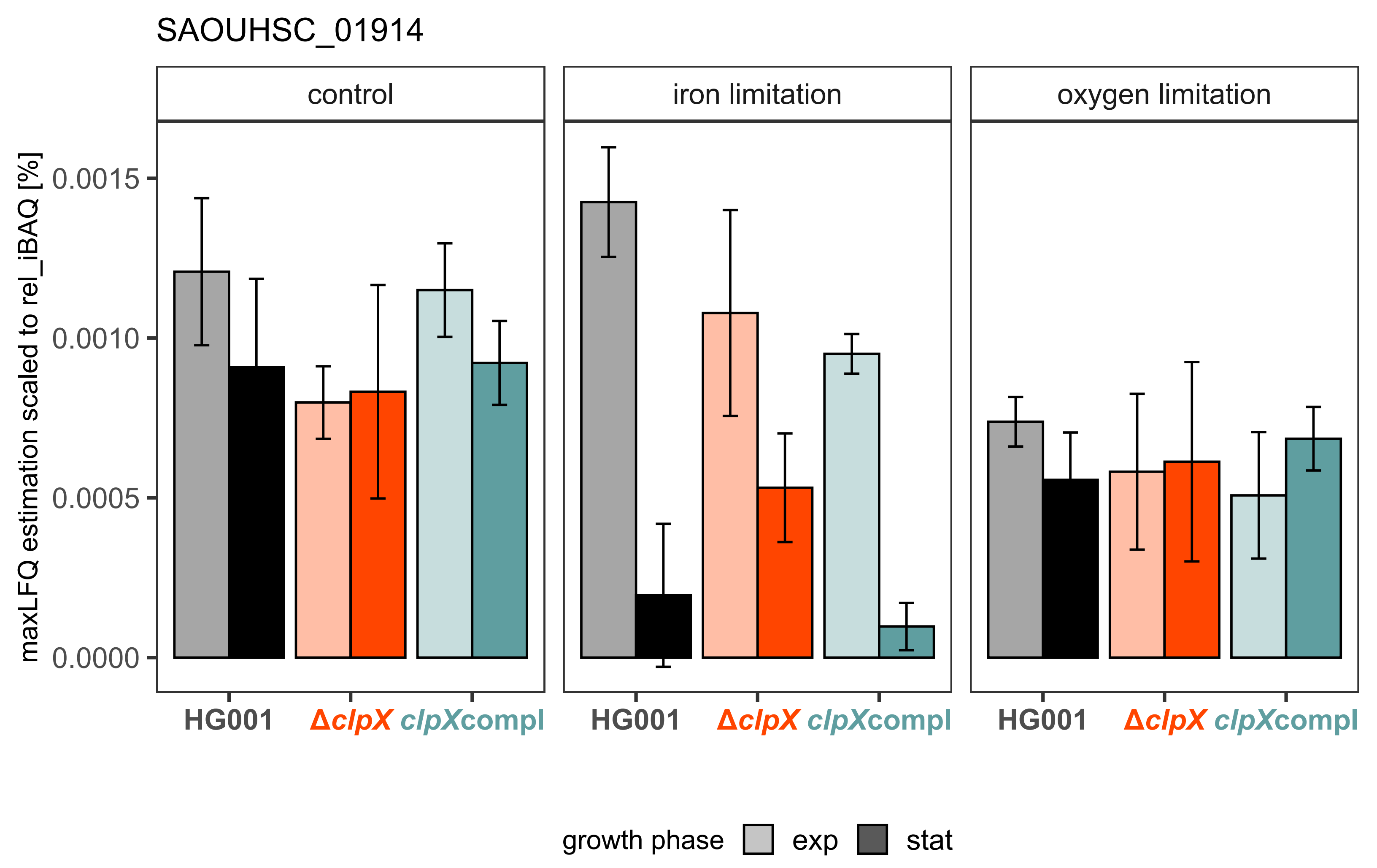
SAOUHSC_01914
control
iron limitation
oxygen limitation
maxLFQ estimation scaled to rel_iBAQ [%]
0.0015
0.0010
0.0005
0.0000
HG001
ΔclpX
clpXcompl
growth phase
exp
stat

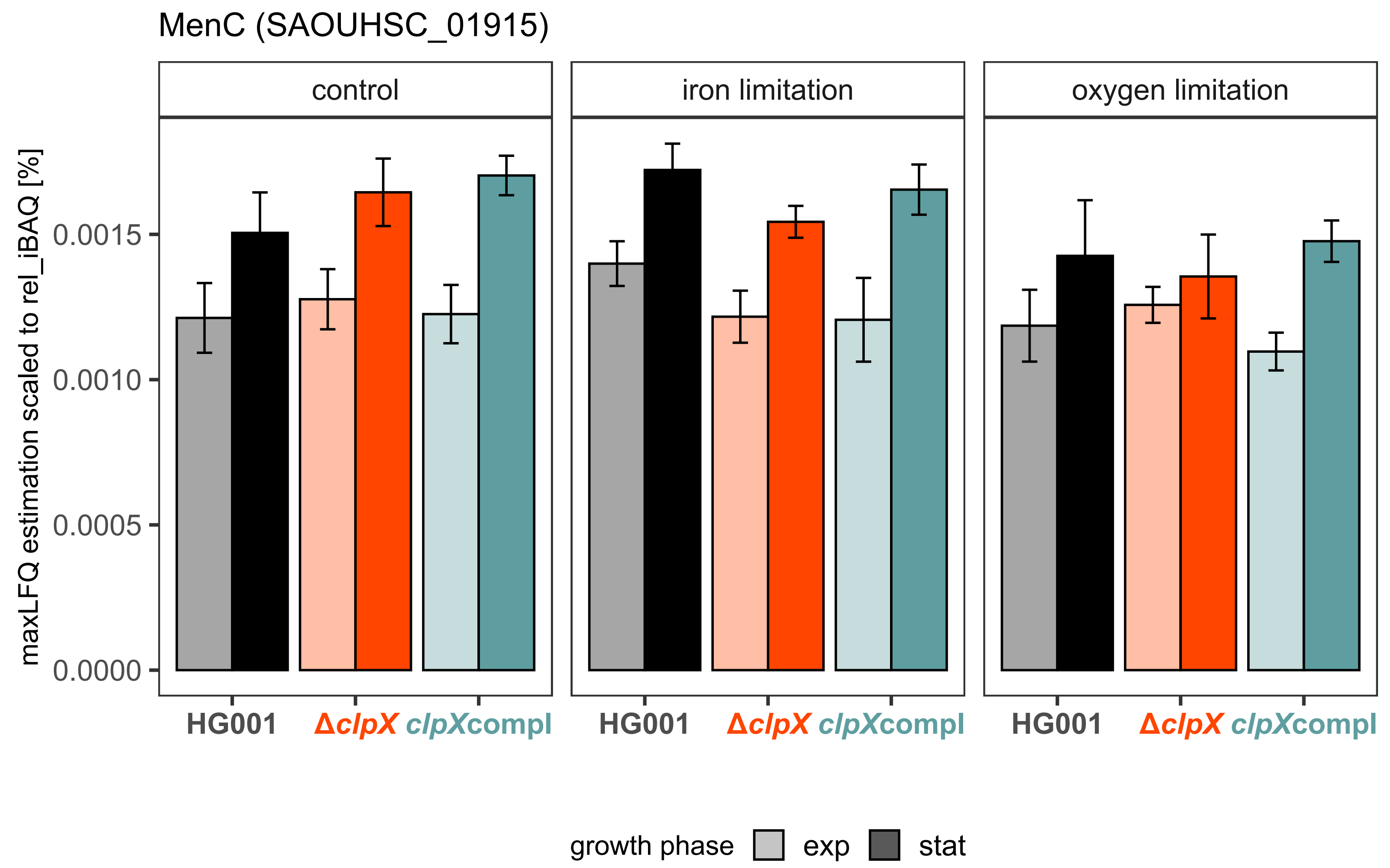

MenC (SAOUHSC_01915)
control
iron limitation
oxygen limitation
maxLFQ estimation scaled to rel_iBAQ [%]
0.0015
0.0010
0.0005
0.0000
HG001
ΔclpX
clpXcompl
growth phase
exp
stat

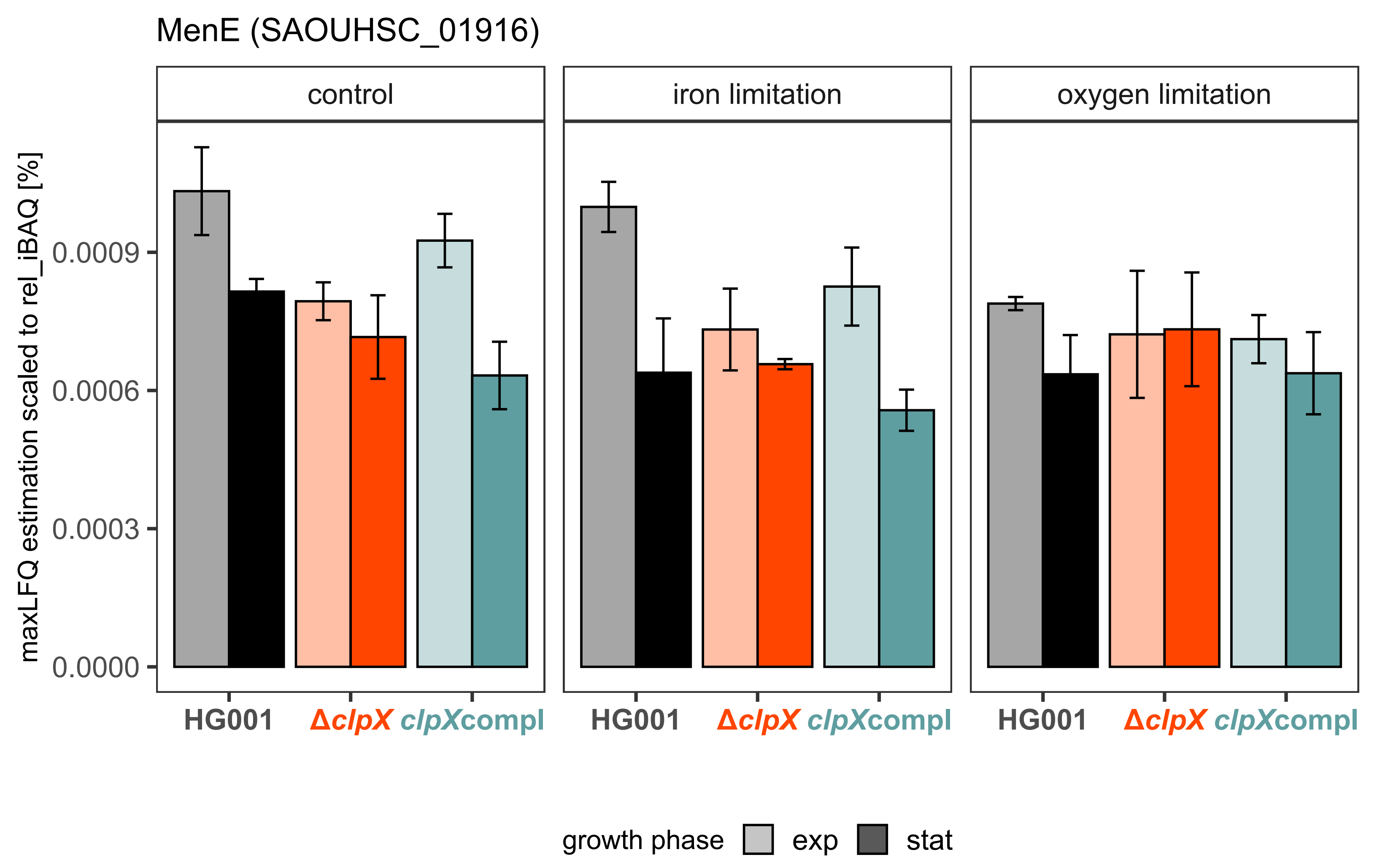

MenE (SAOUHSC_01916)
control
iron limitation
oxygen limitation
maxLFQ estimation scaled to rel_iBAQ [%]
0.0009
0.0006
0.0003
0.0000
HG001
ΔclpX
clpXcompl
growth phase
exp
stat

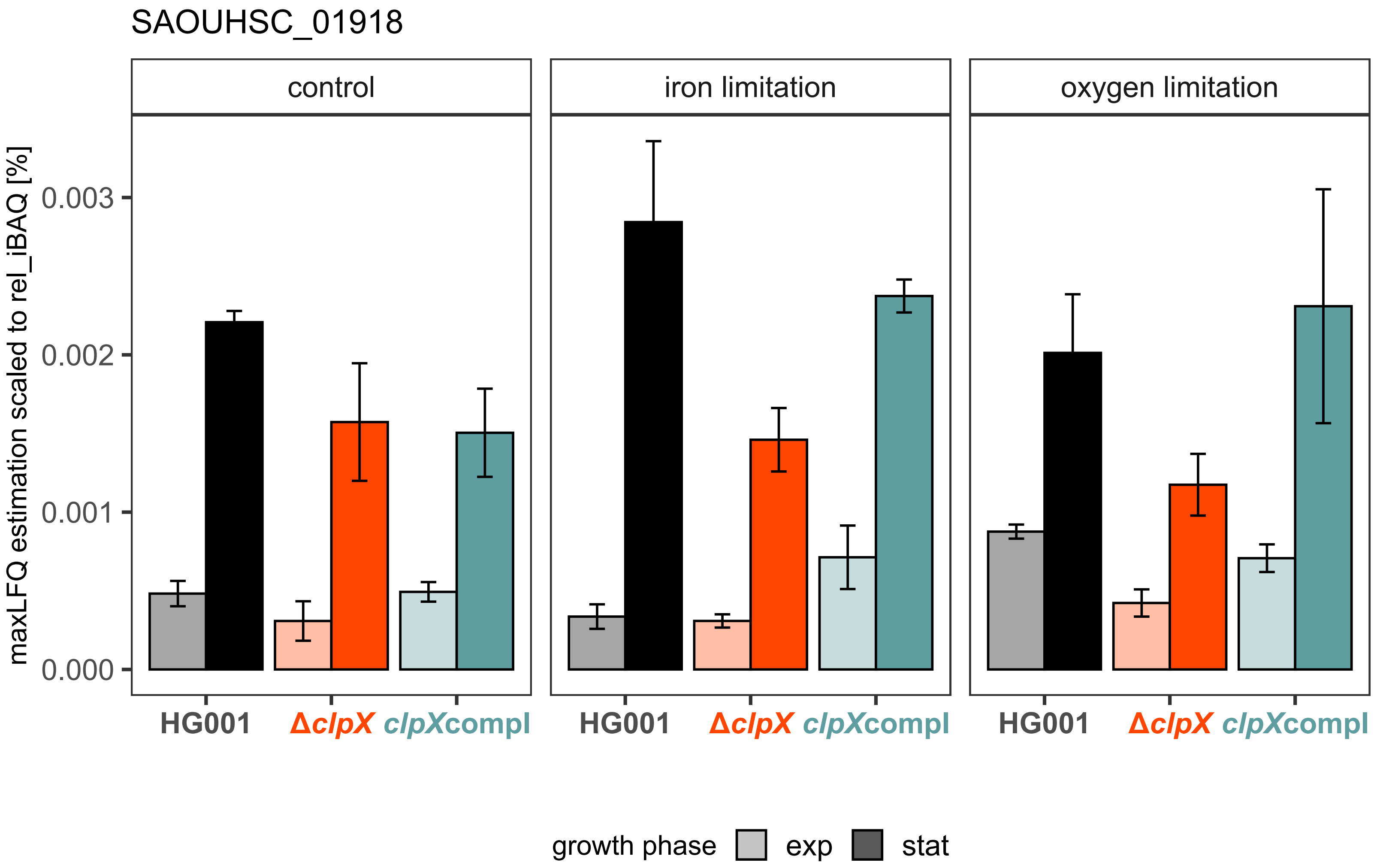

SAOUHSC_01918
control
iron limitation
oxygen limitation
maxLFQ estimation scaled to rel_iBAQ [%]
0.000
0.001
0.002
0.003
HG001
ΔclpX
clpXcompl
growth phase
exp
stat

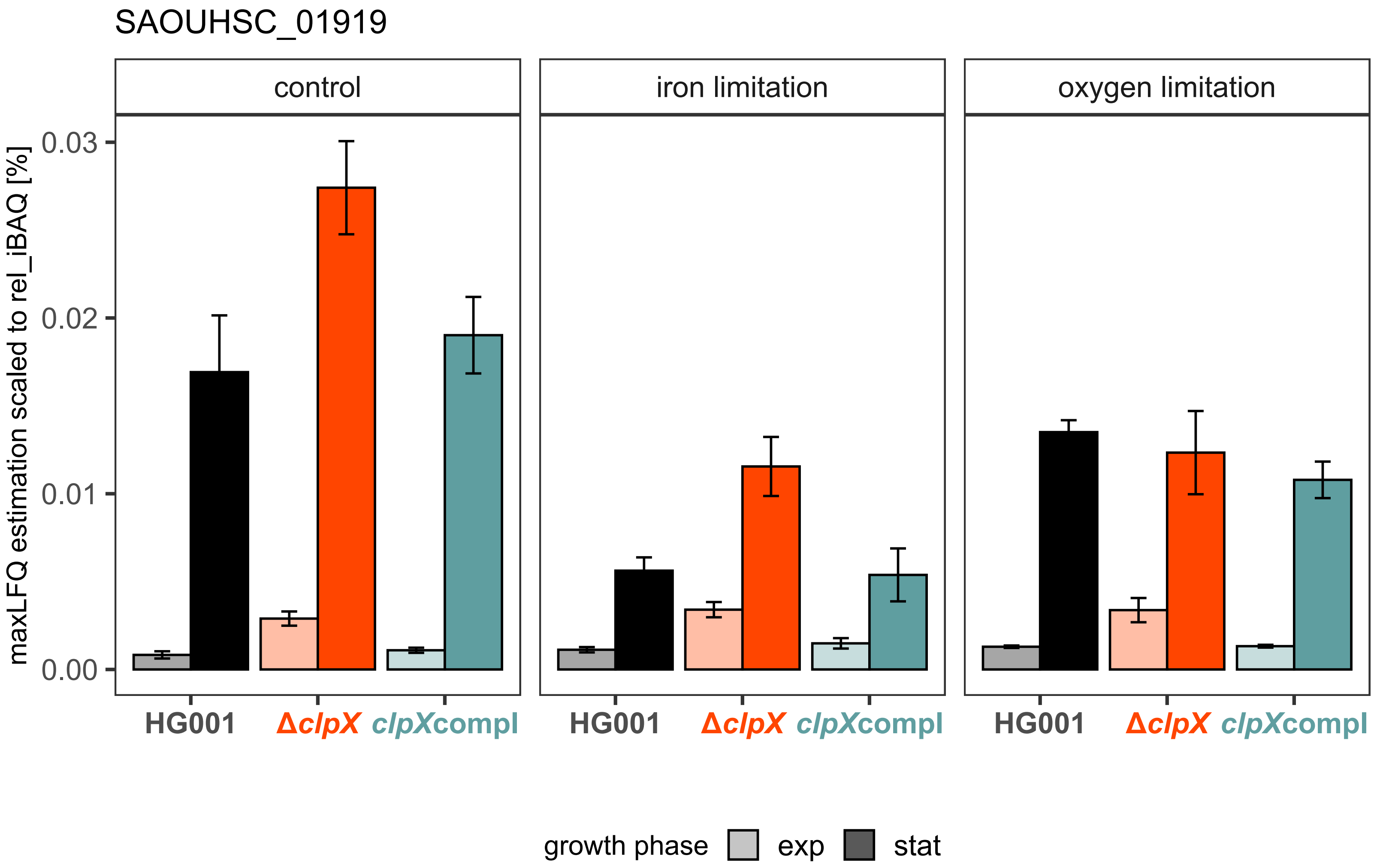

SAOUHSC_01919
control
iron limitation
oxygen limitation
maxLFQ estimation scaled to rel_iBAQ [%]
0.03
0.02
0.01
0.00
HG001
ΔclpX
clpXcompl
growth phase
exp
stat

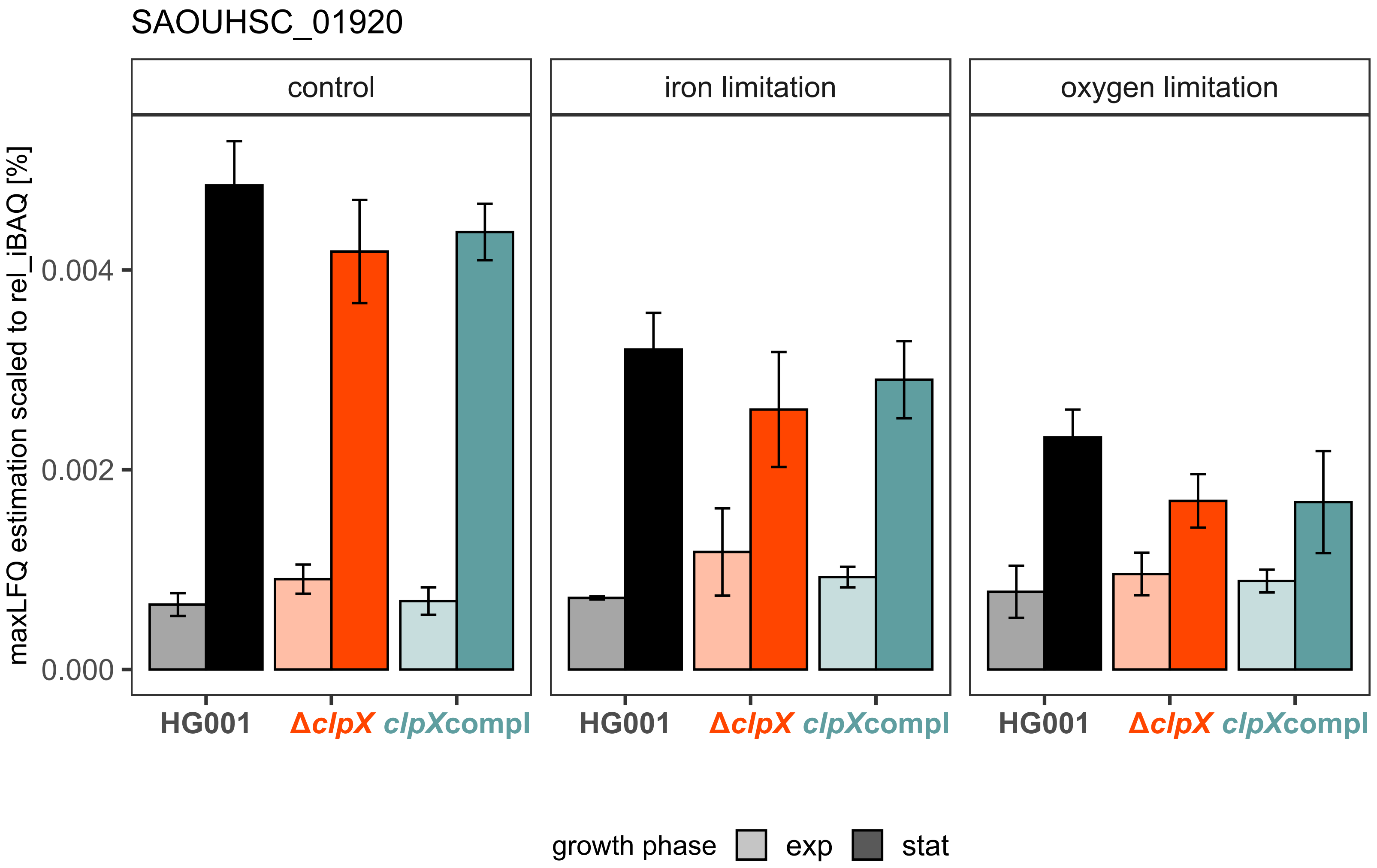

SAOUHSC_01920
control
iron limitation
oxygen limitation
maxLFQ estimation scaled to rel_iBAQ [%]
0.004
0.002
0.000
HG001
ΔclpX
clpXcompl
growth phase
exp
stat

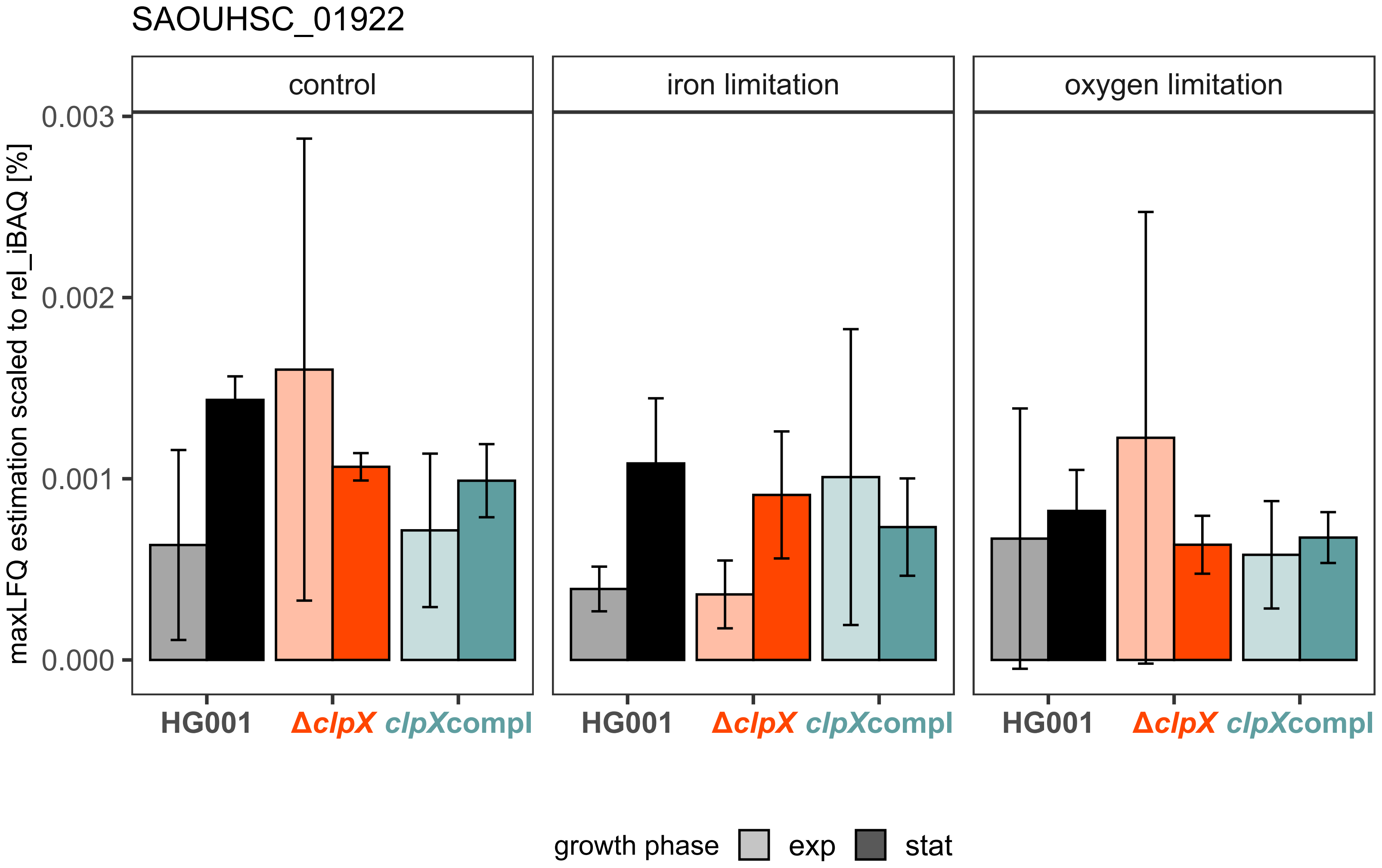
SAOUHSC_01922
control
iron limitation
oxygen limitation
maxLFQ estimation scaled to rel_iBAQ [%]
0.003
0.002
0.001
0.000
HG001
ΔclpX
clpXcompl
growth phase
exp
stat

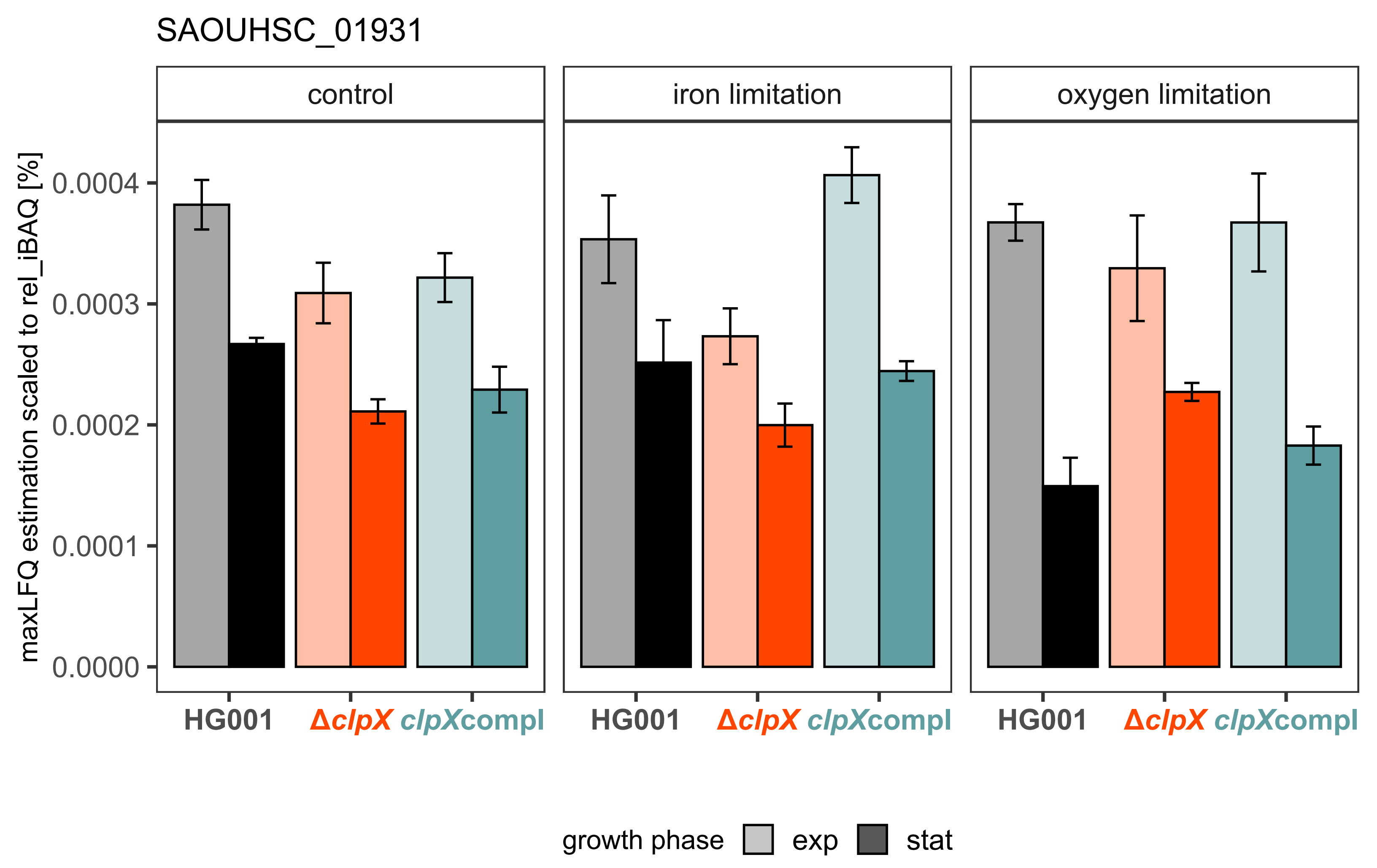

SAOUHSC_01931
control
iron limitation
oxygen limitation
maxLFQ estimation scaled to rel_iBAQ [%]
0.0004
0.0003
0.0002
0.0001
0.0000
HG001
ΔclpX
clpXcompl
growth phase
exp
stat

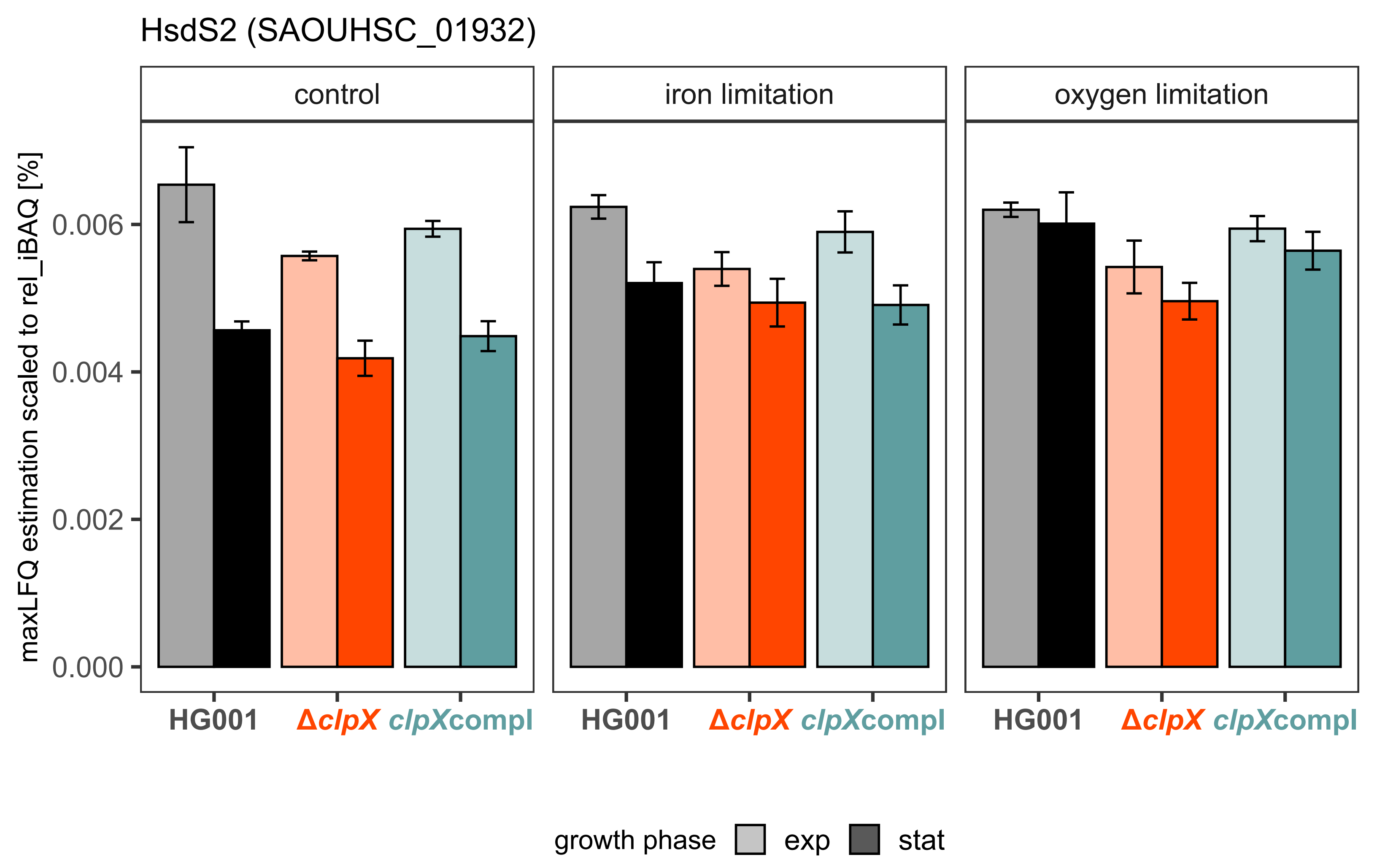

HsdS2 (SAOUHSC_01932)
control
iron limitation
oxygen limitation
maxLFQ estimation scaled to rel_iBAQ [%]
0.006
0.004
0.002
0.000
HG001
ΔclpX
clpXcompl
growth phase
exp
stat

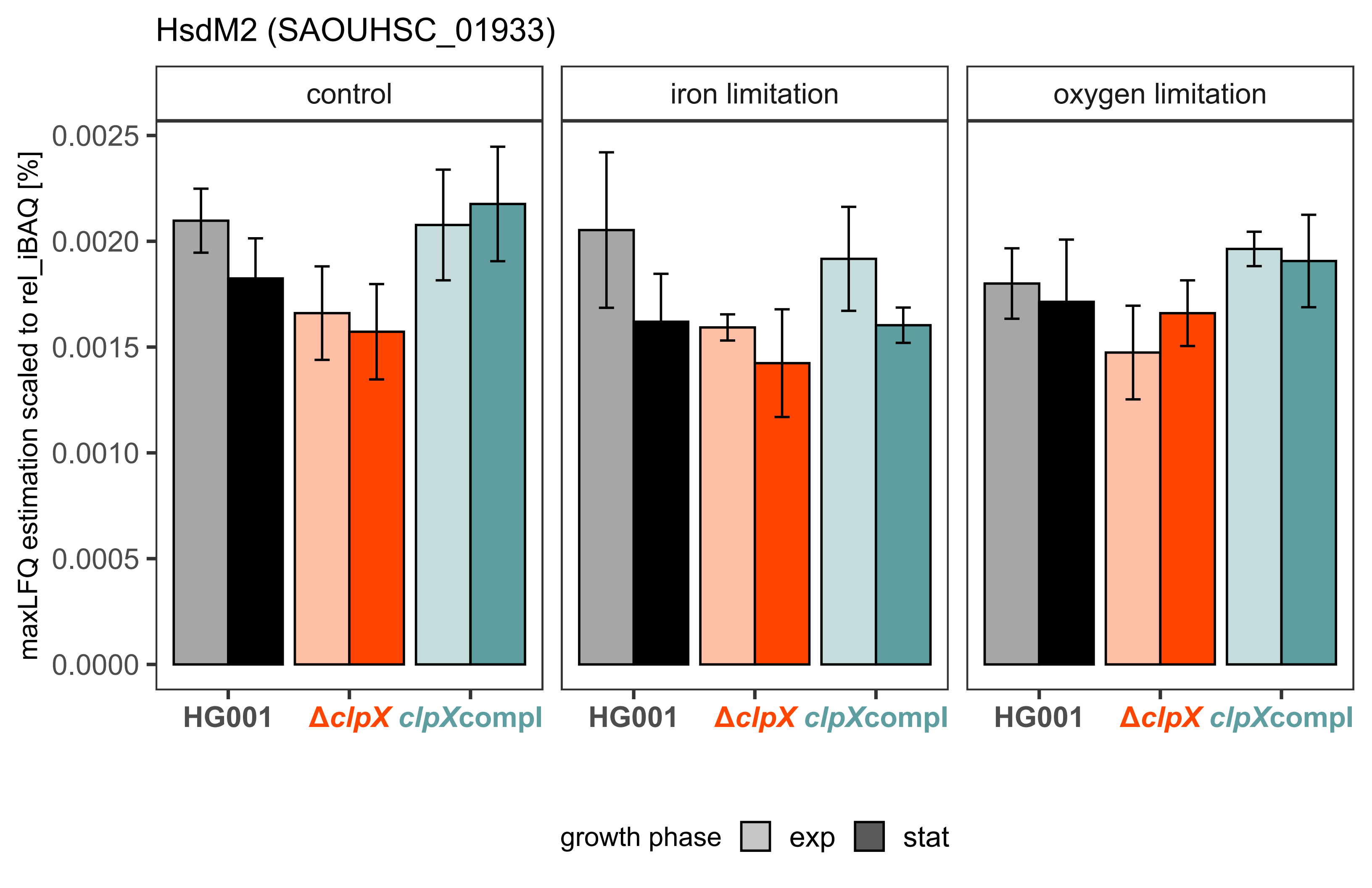

HsdM2 (SAOUHSC_01933)
control
iron limitation
oxygen limitation
maxLFQ estimation scaled to rel_iBAQ [%]
0.0025
0.0020
0.0015
0.0010
0.0005
0.0000
HG001
ΔclpX
clpXcompl
growth phase
exp
stat

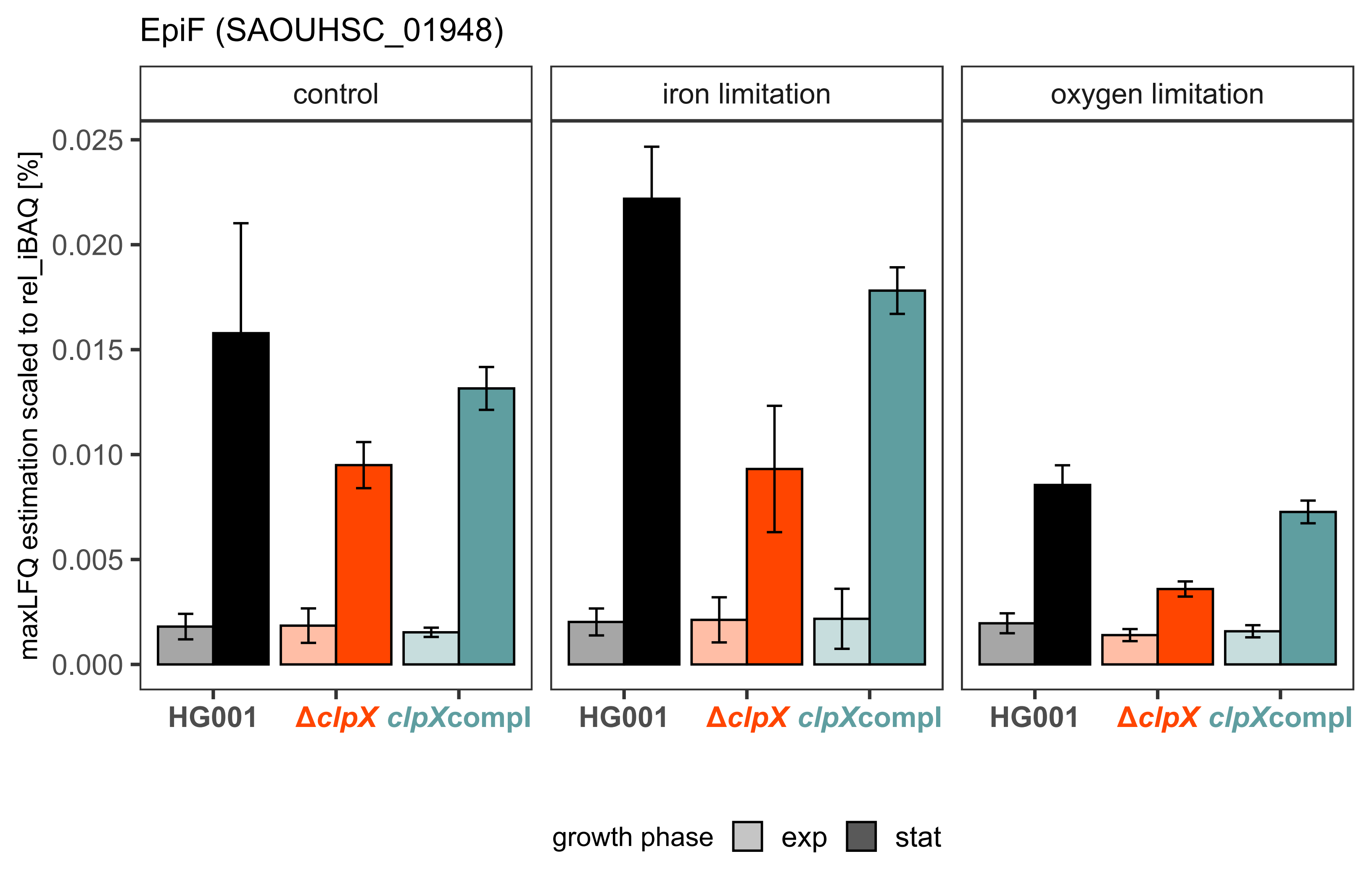

EpiF (SAOUHSC_01948)
control
iron limitation
oxygen limitation
maxLFQ estimation scaled to rel_iBAQ [%]
0.025
0.020
0.015
0.010
0.005
0.000
HG001
ΔclpX
clpXcompl
growth phase
exp
stat

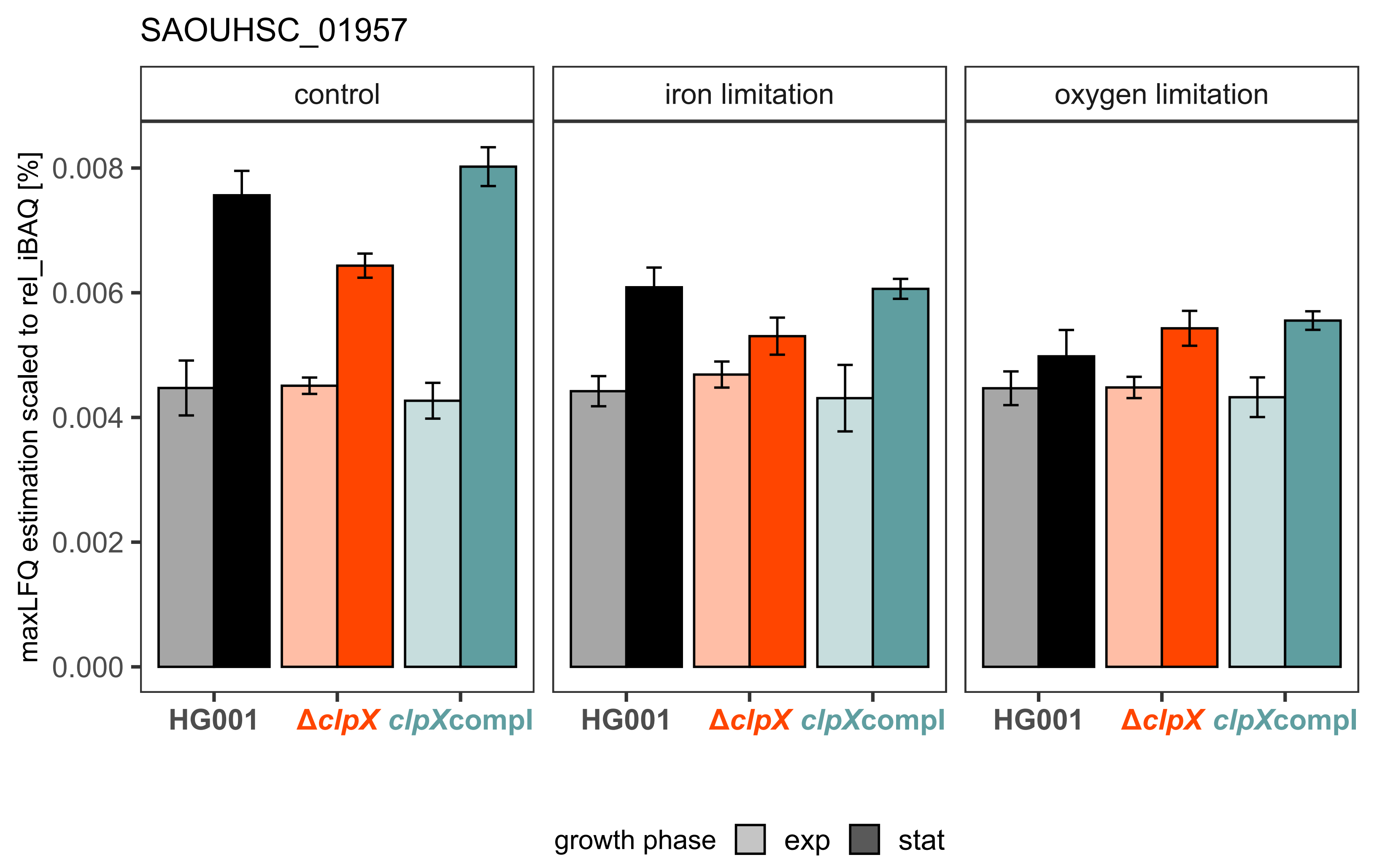
SAOUHSC_01957
control
iron limitation
oxygen limitation
maxLFQ estimation scaled to rel_iBAQ [%]
0.008
0.006
0.004
0.002
0.000
HG001
ΔclpX
clpXcompl
growth phase
exp
stat

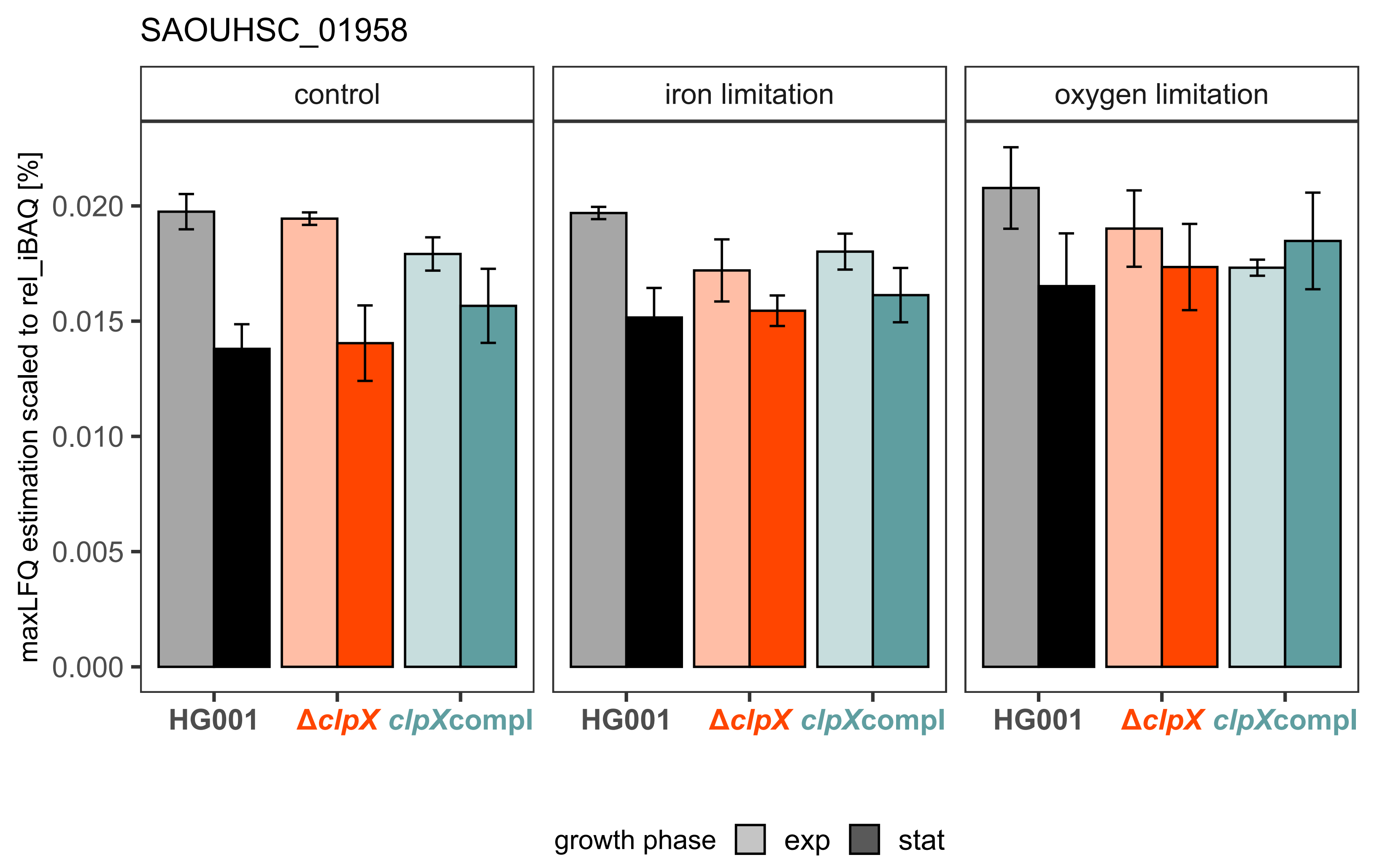

SAOUHSC_01958
control
iron limitation
oxygen limitation
maxLFQ estimation scaled to rel_iBAQ [%]
0.020
0.015
0.010
0.005
0.000
HG001
ΔclpX
clpXcompl
growth phase
exp
stat

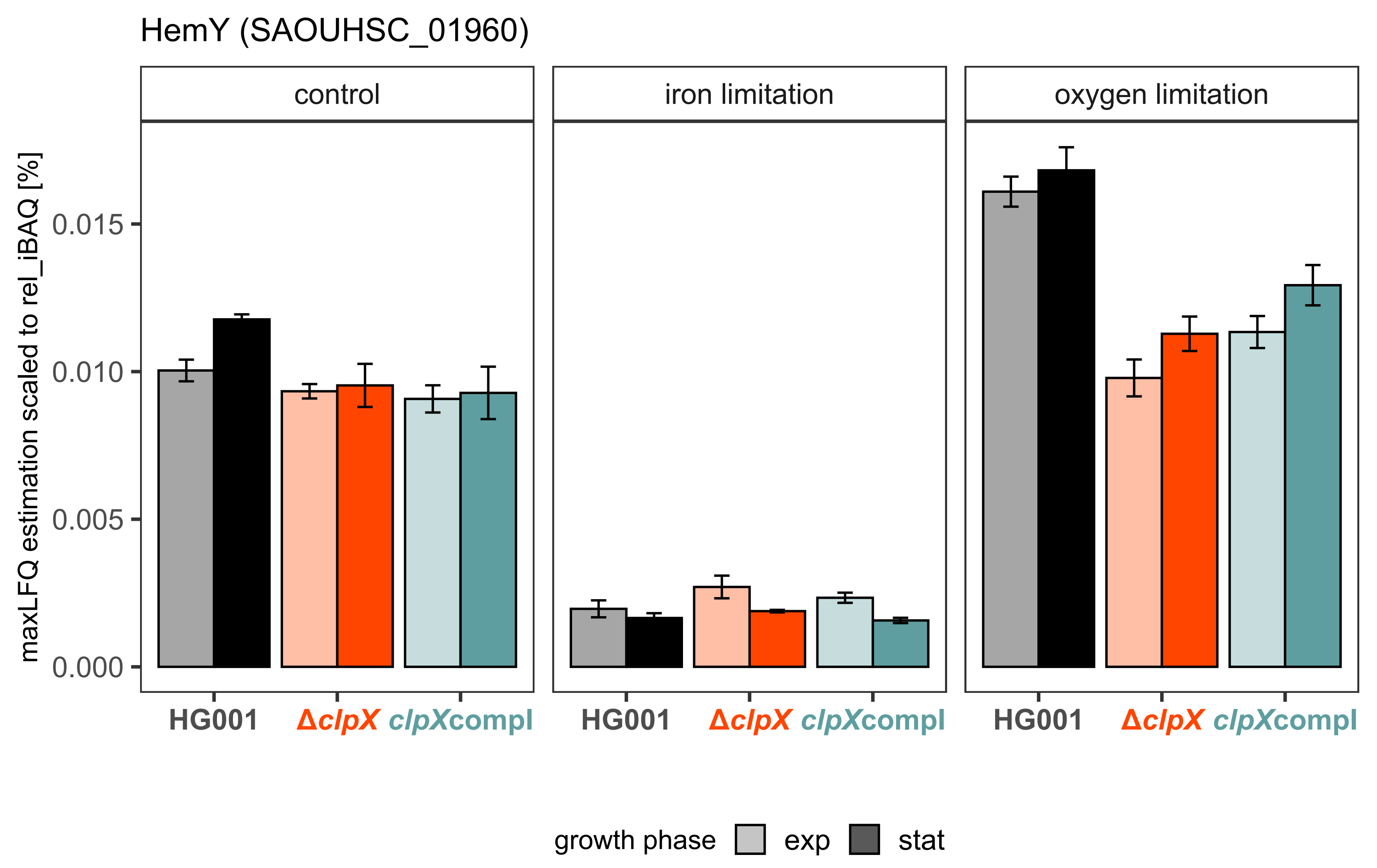

HemY (SAOUHSC_01960)
control
iron limitation
oxygen limitation
maxLFQ estimation scaled to rel_iBAQ [%]
0.015
0.010
0.005
0.000
HG001
ΔclpX
clpXcompl
growth phase
exp
stat

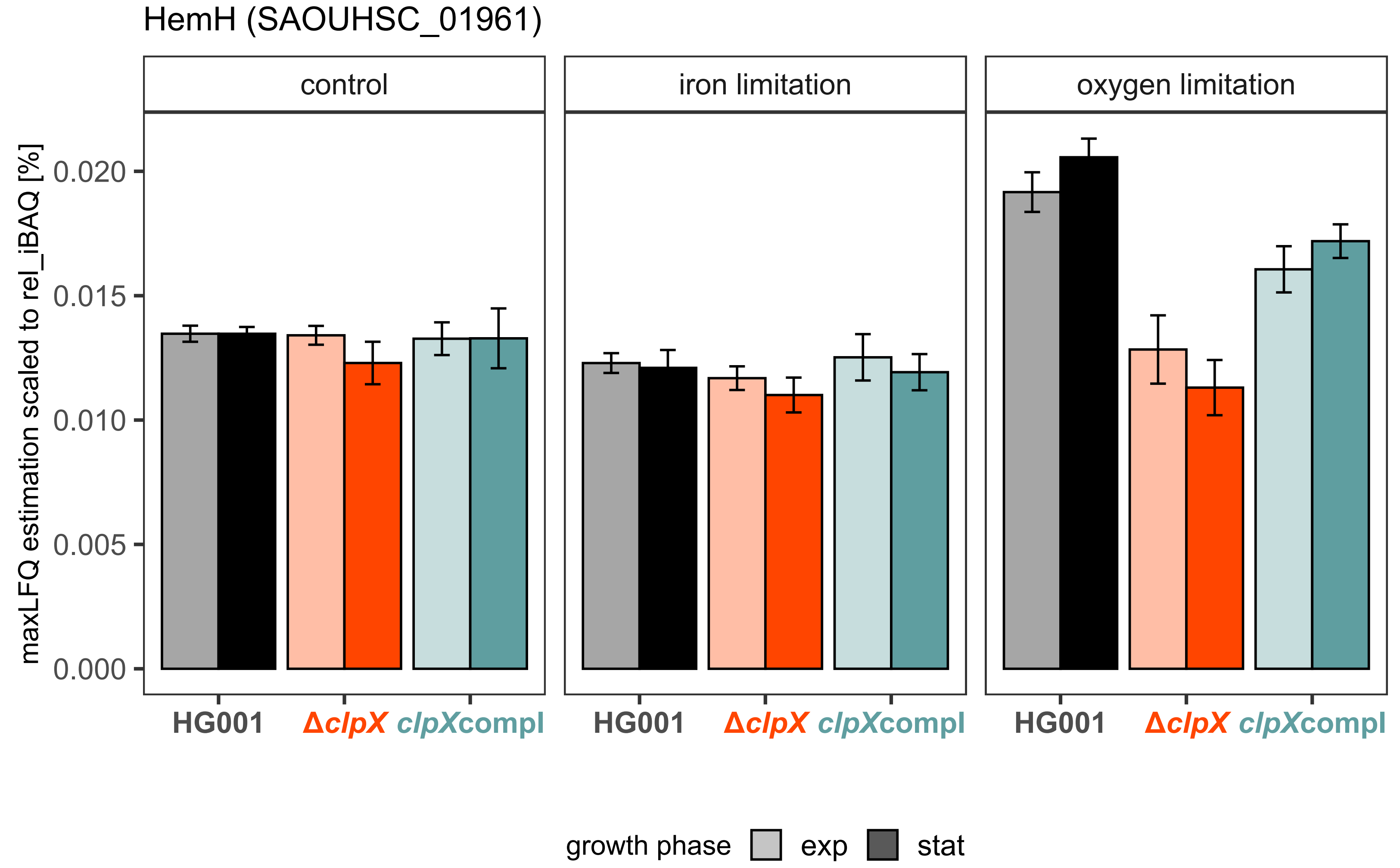
HemH (SAOUHSC_01961)
control
iron limitation
oxygen limitation
maxLFQ estimation scaled to rel_iBAQ [%]
0.020
0.015
0.010
0.005
0.000
HG001
ΔclpX
clpXcompl
growth phase
exp
stat

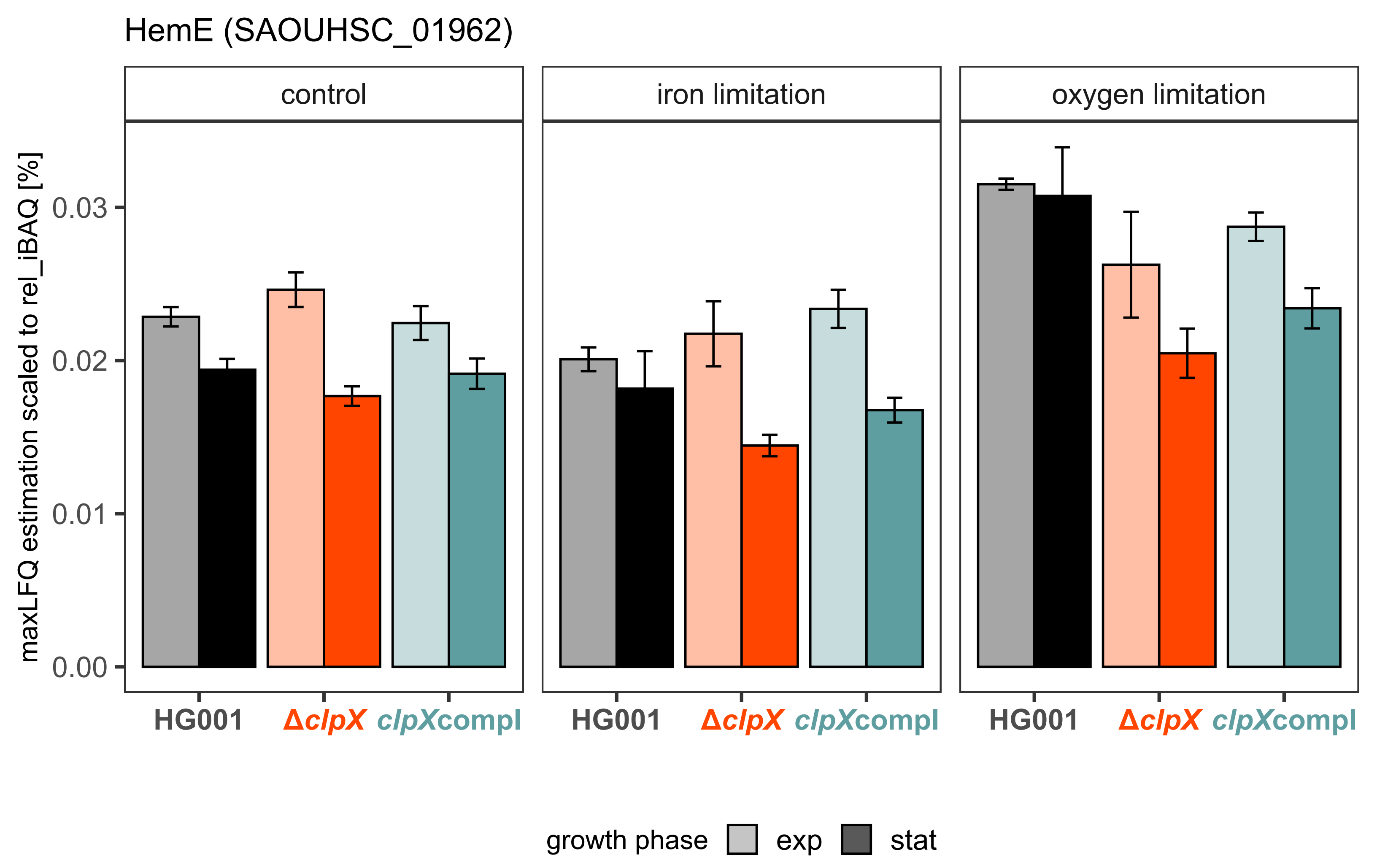
HemE (SAOUHSC_01962)
control
iron limitation
oxygen limitation
maxLFQ estimation scaled to rel_iBAQ [%]
0.03
0.02
0.01
0.00
HG001
ΔclpX
clpXcompl
growth phase
exp
stat

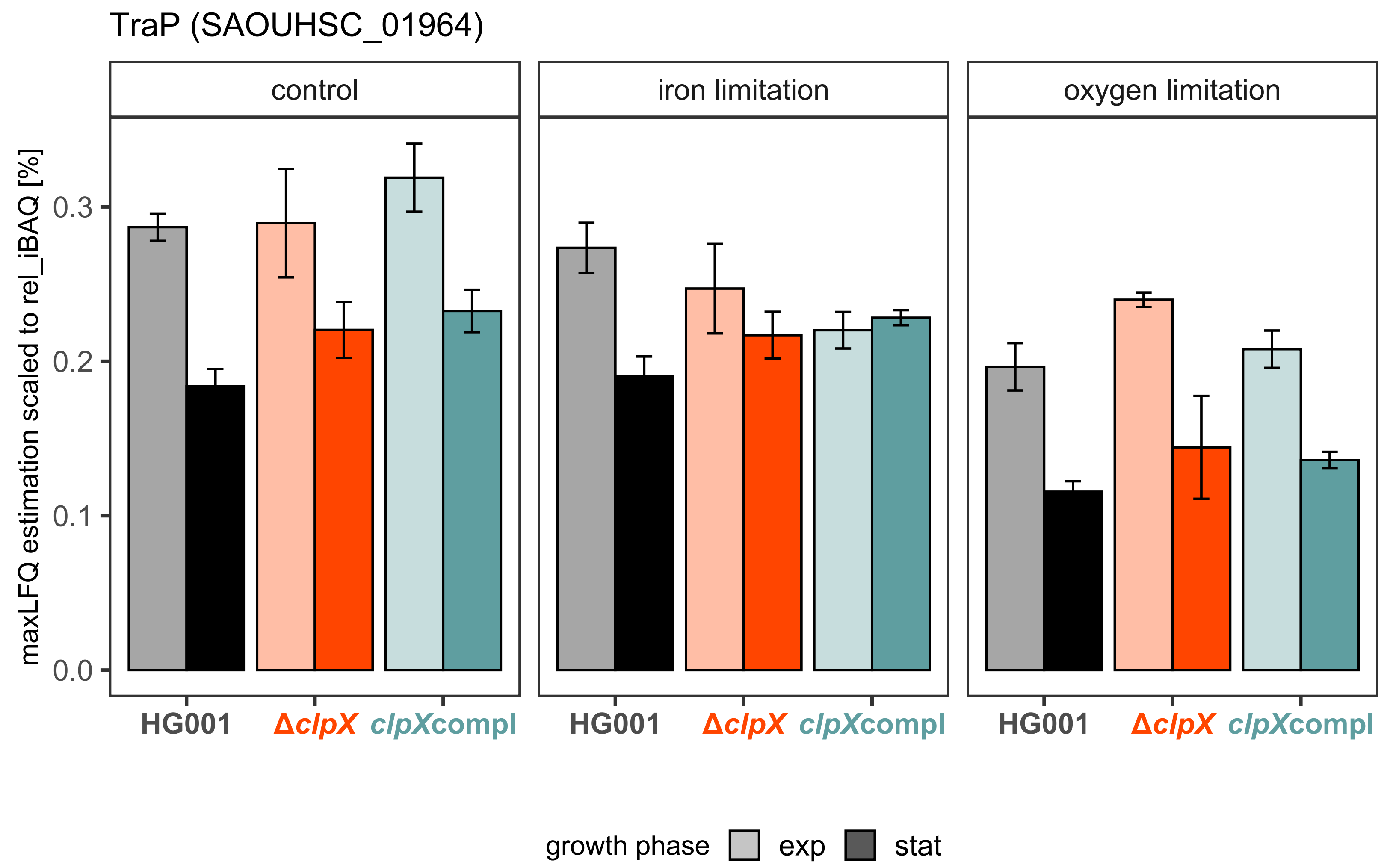

TraP (SAOUHSC_01964)
control
iron limitation
oxygen limitation
maxLFQ estimation scaled to rel_iBAQ [%]
0.0
0.1
0.2
0.3
HG001
ΔclpX
clpXcompl
growth phase
exp
stat

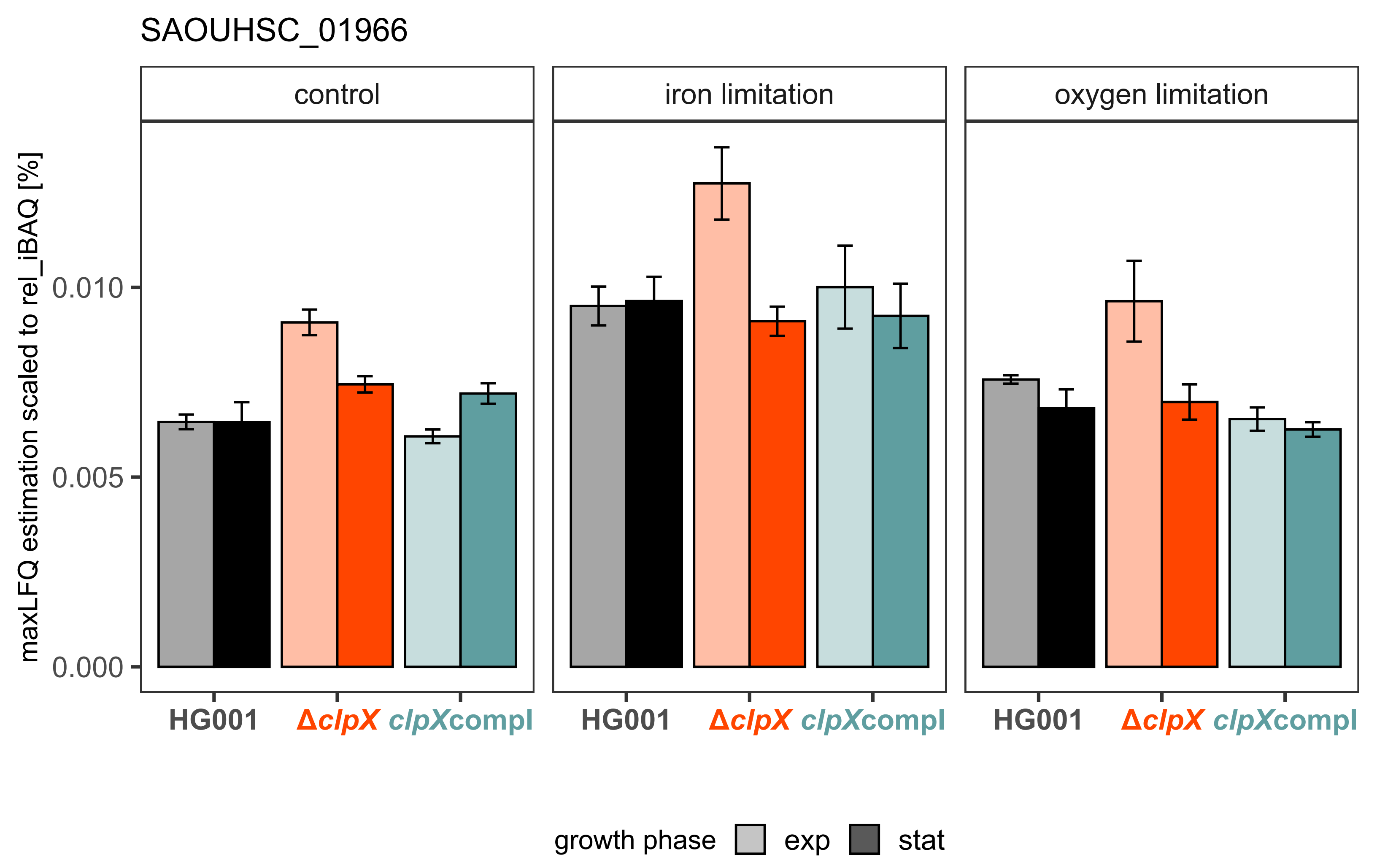

SAOUHSC_01966
control
iron limitation
oxygen limitation
maxLFQ estimation scaled to rel_iBAQ [%]
0.010
0.005
0.000
HG001
ΔclpX
clpXcompl
growth phase
exp
stat

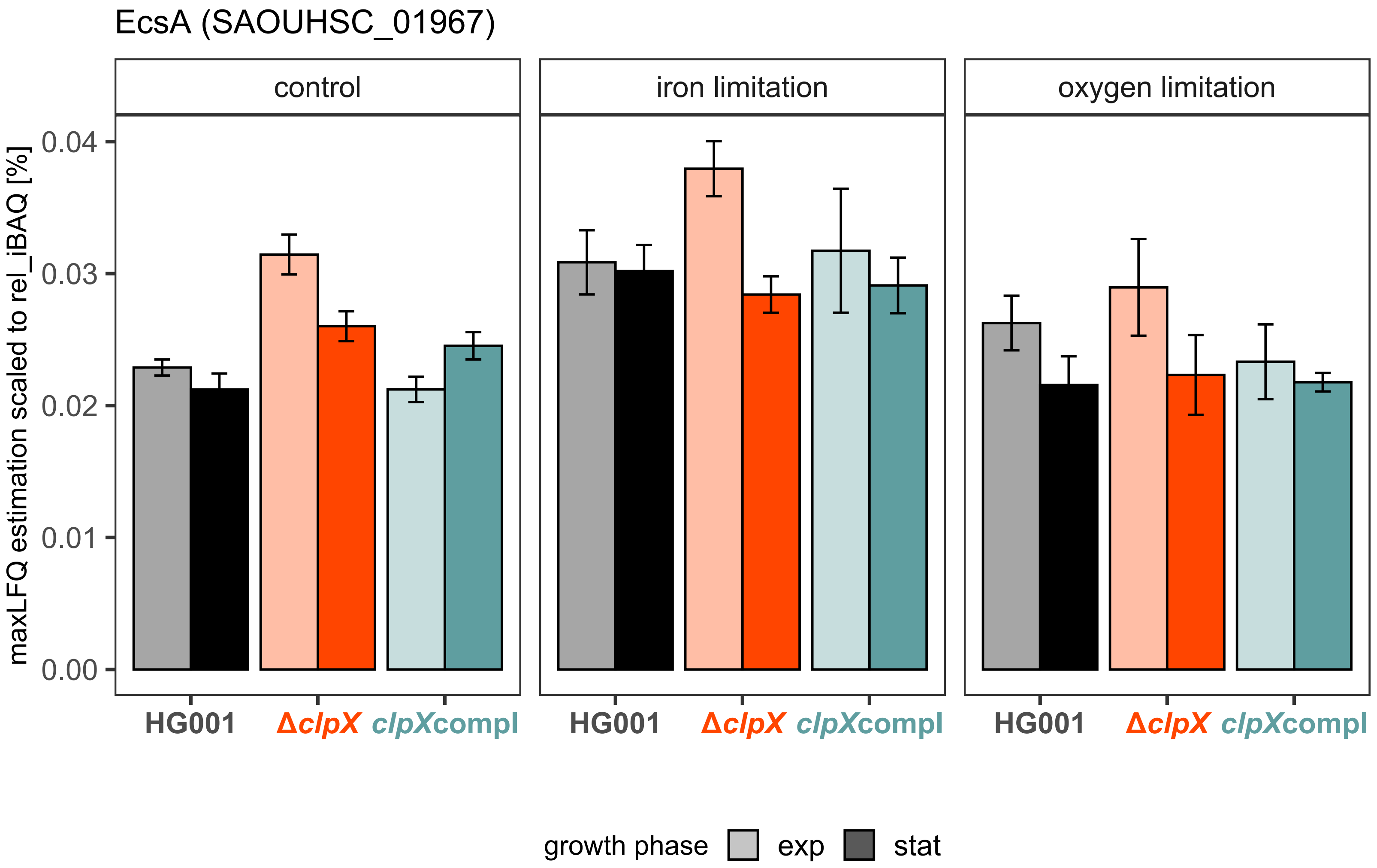

EcsA (SAOUHSC_01967)
control
iron limitation
oxygen limitation
maxLFQ estimation scaled to rel_iBAQ [%]
0.04
0.03
0.02
0.01
0.00
HG001
ΔclpX
clpXcompl
growth phase
exp
stat

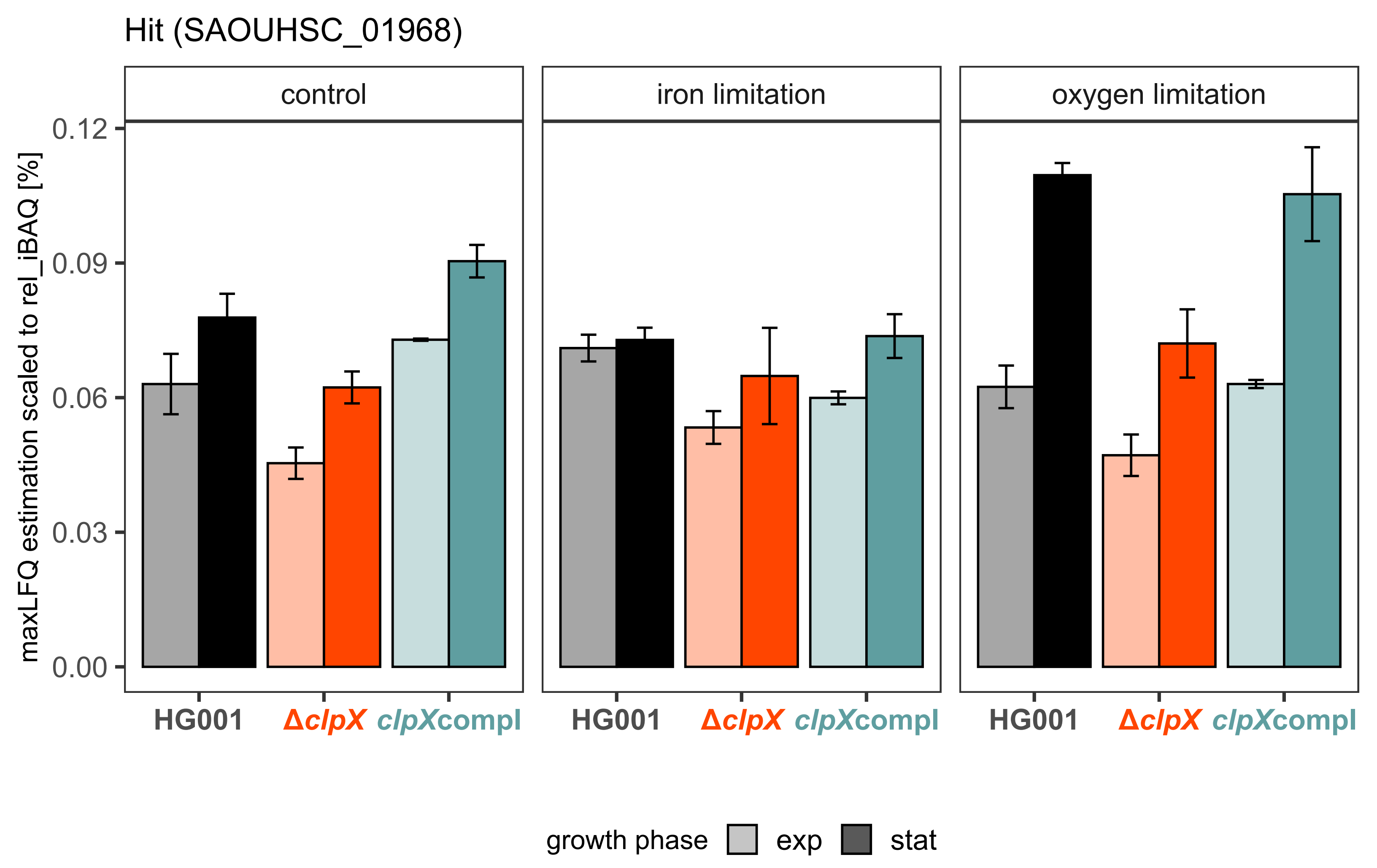

Hit (SAOUHSC_01968)
control
iron limitation
oxygen limitation
maxLFQ estimation scaled to rel_iBAQ [%]
0.12
0.09
0.06
0.03
0.00
HG001
ΔclpX
clpXcompl
growth phase
exp
stat

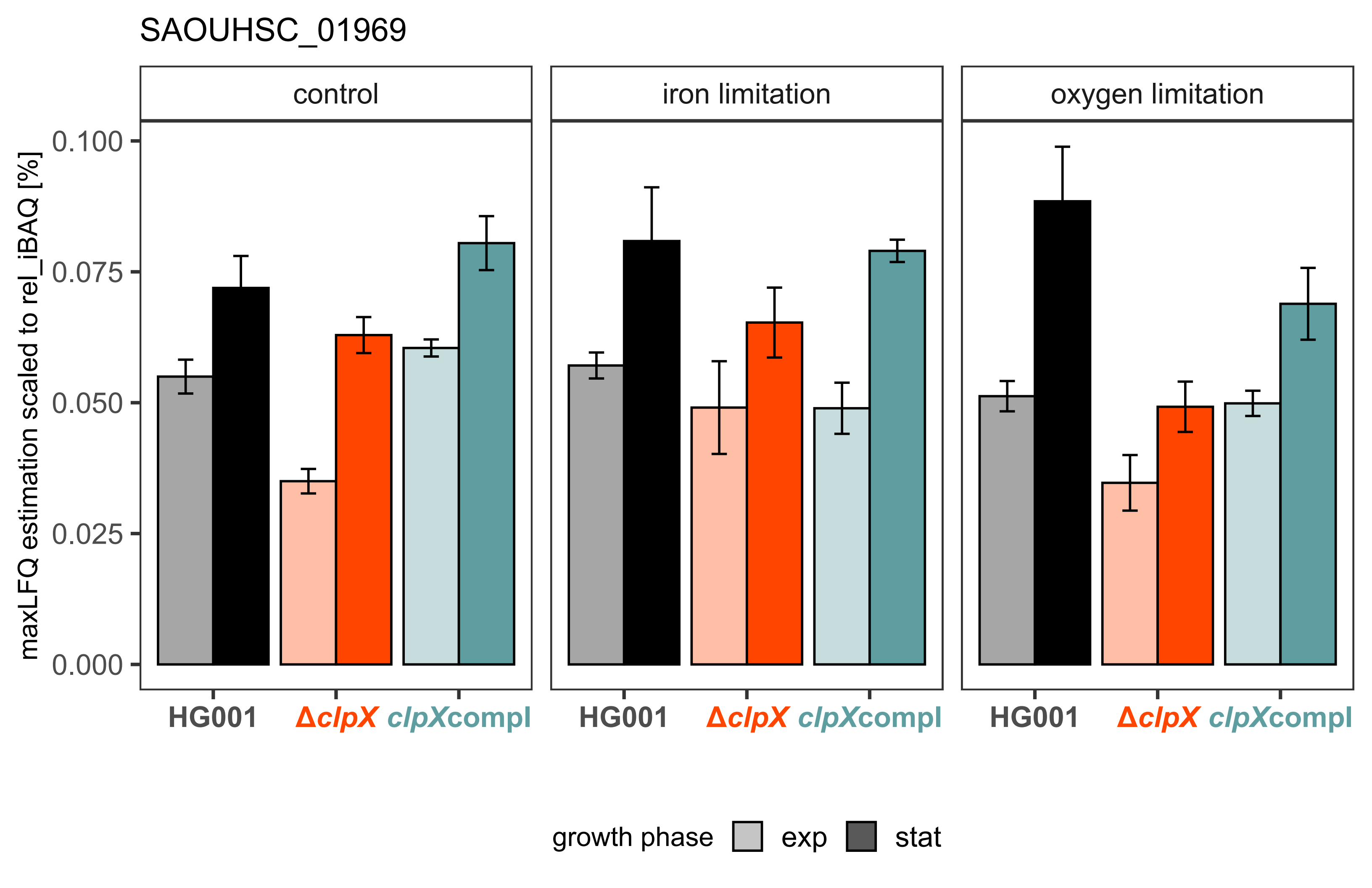
SAOUHSC_01969
control
iron limitation
oxygen limitation
maxLFQ estimation scaled to rel_iBAQ [%]
0.100
0.075
0.050
0.025
0.000
HG001
ΔclpX
clpXcompl
growth phase
exp
stat

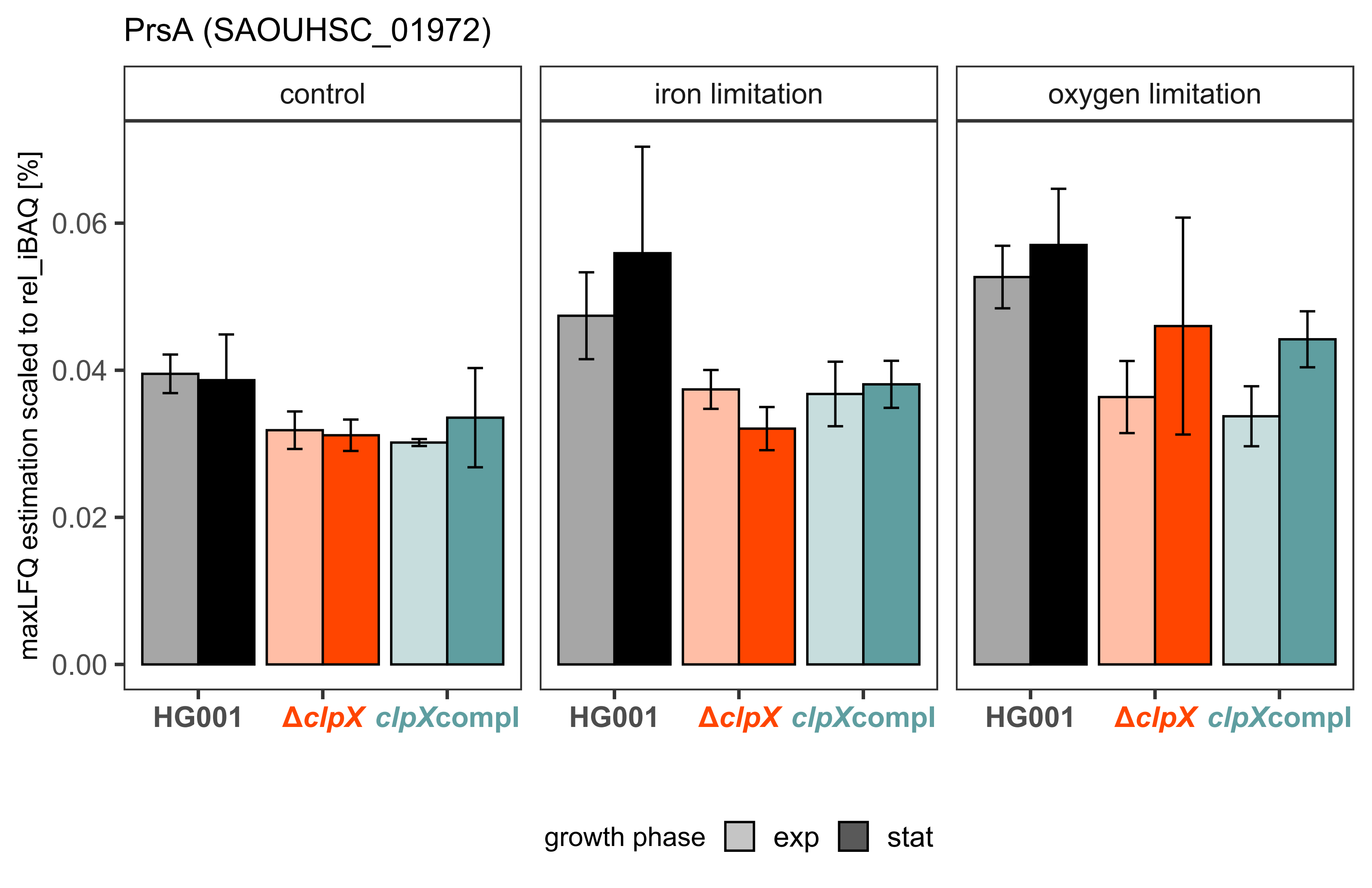
PrsA (SAOUHSC_01972)
control
iron limitation
oxygen limitation
maxLFQ estimation scaled to rel_iBAQ [%]
0.00
0.02
0.04
0.06
HG001
ΔclpX
clpXcompl
growth phase
exp
stat

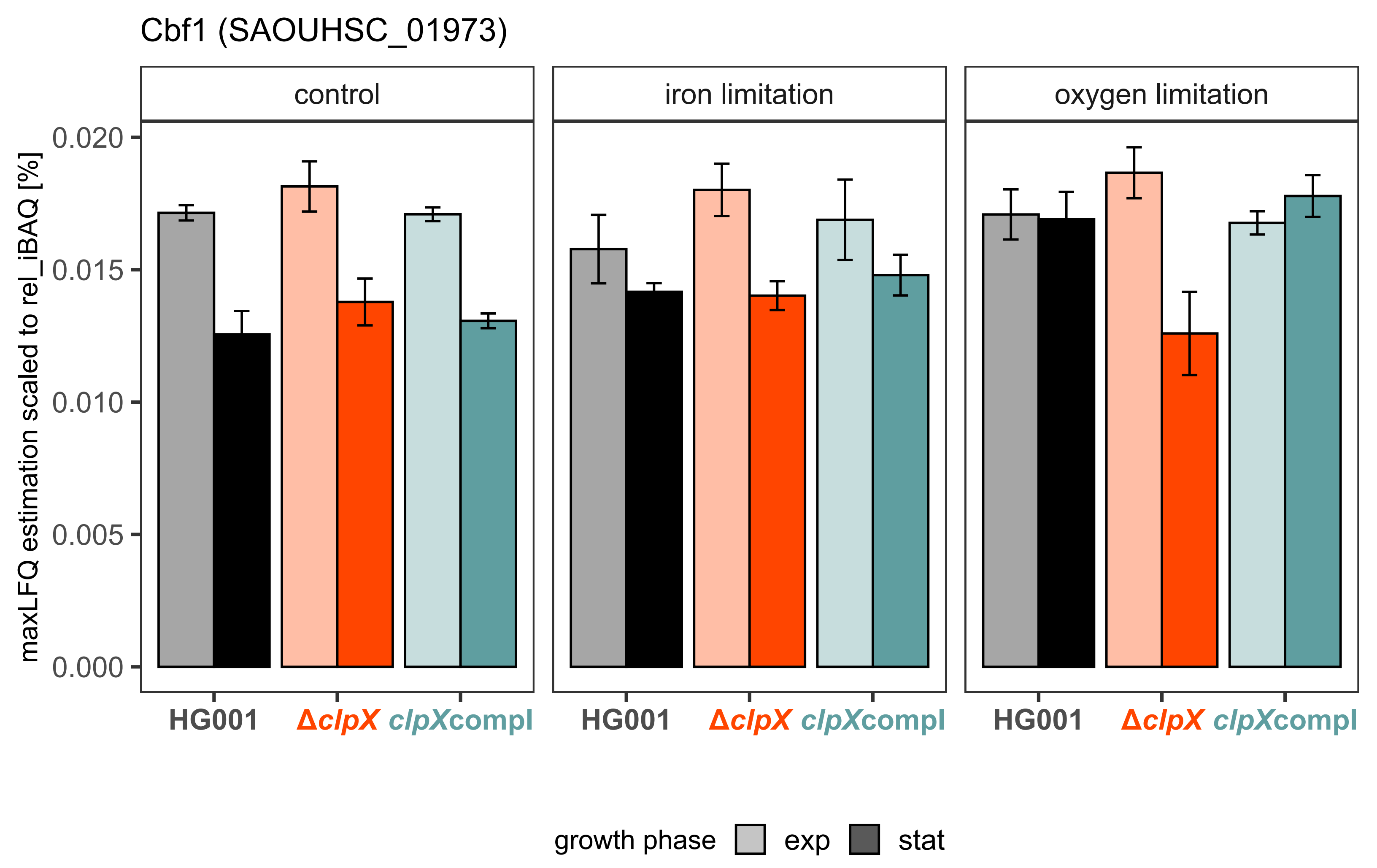
Cbf1 (SAOUHSC_01973)
control
iron limitation
oxygen limitation
maxLFQ estimation scaled to rel_iBAQ [%]
0.020
0.015
0.010
0.005
0.000
HG001
ΔclpX
clpXcompl
growth phase
exp
stat

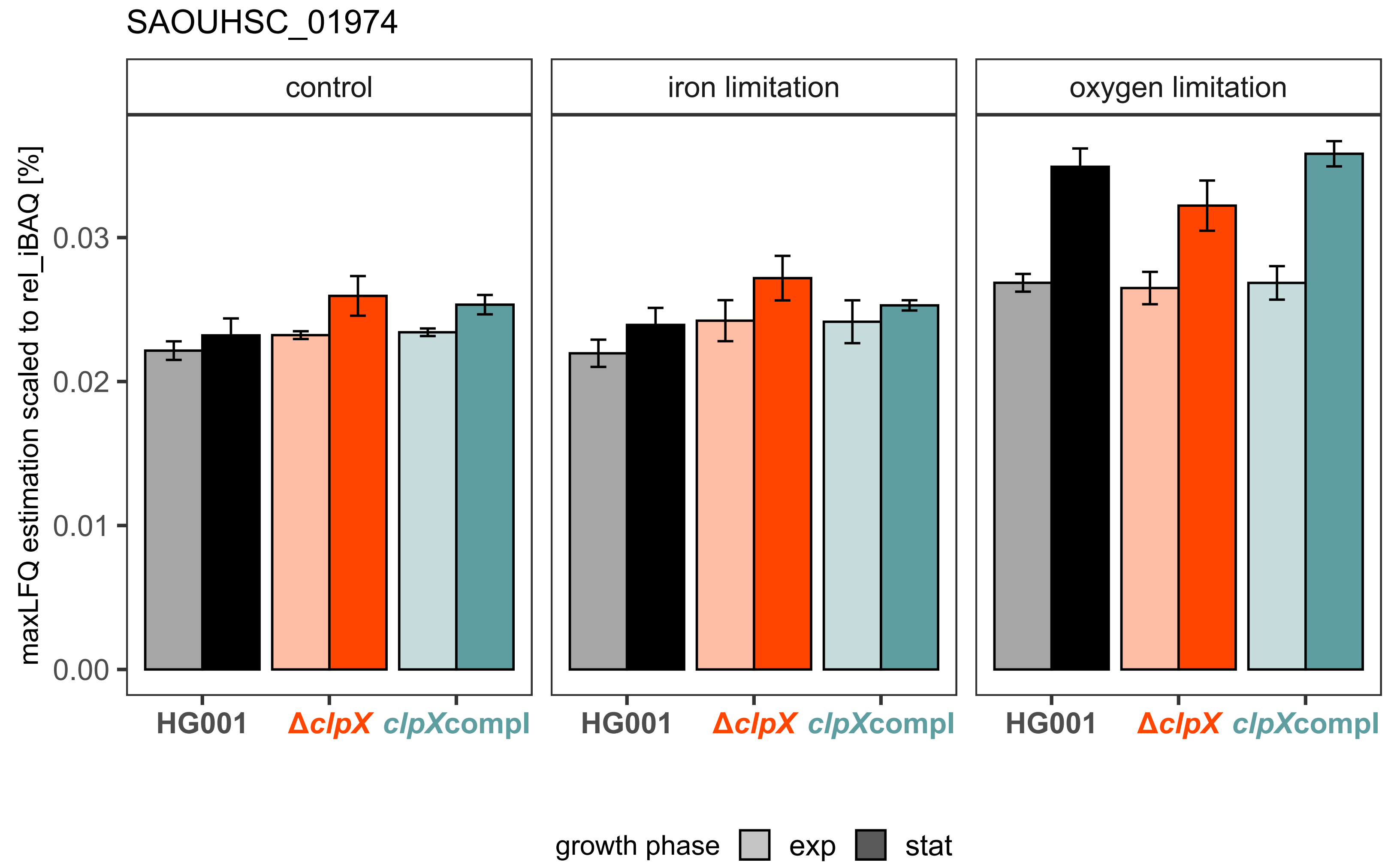

SAOUHSC_01974
control
iron limitation
oxygen limitation
maxLFQ estimation scaled to rel_iBAQ [%]
0.00
0.01
0.02
0.03
HG001
ΔclpX
clpXcompl
growth phase
exp
stat

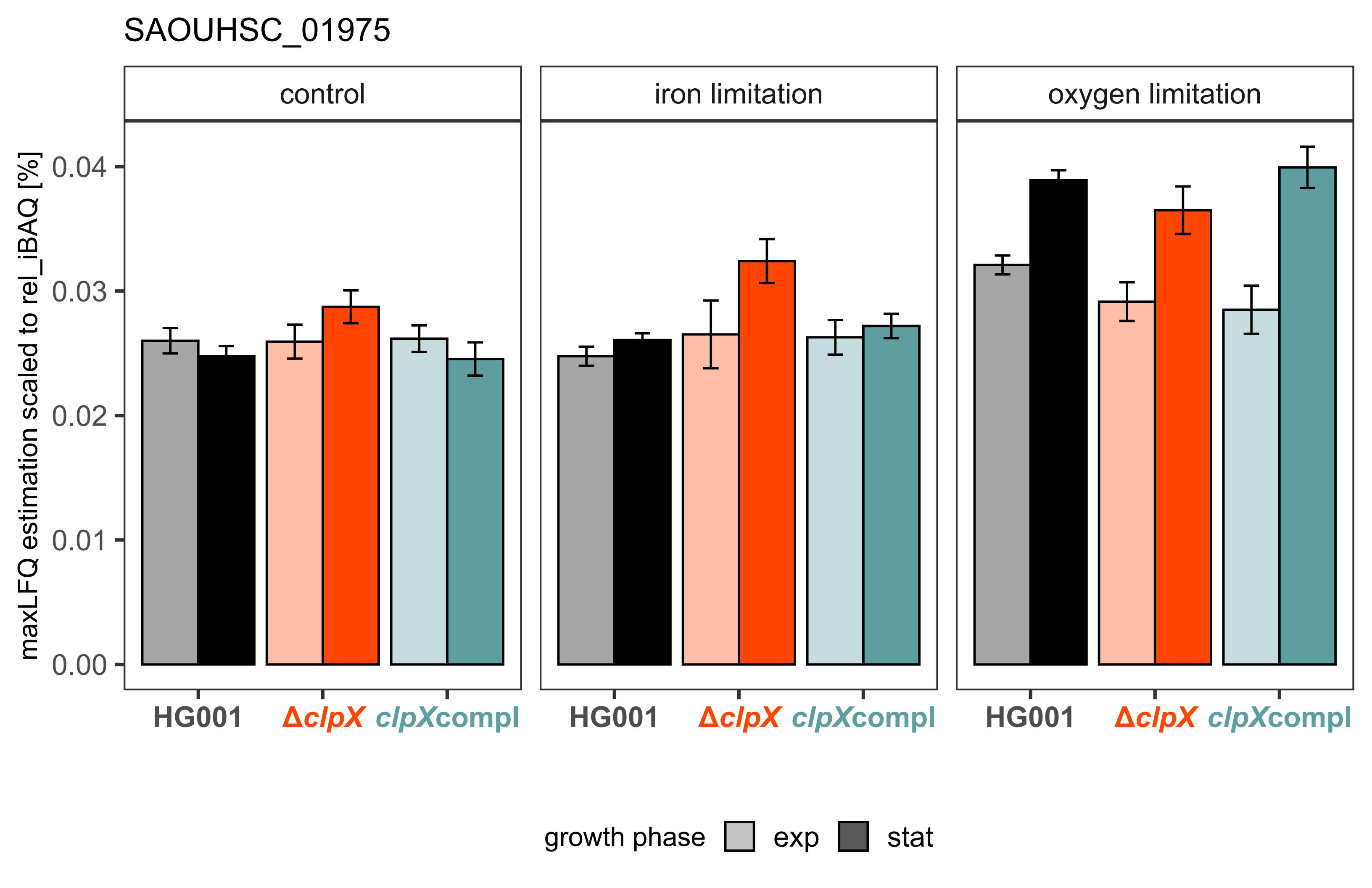

SAOUHSC_01975
control
iron limitation
oxygen limitation
maxLFQ estimation scaled to rel_iBAQ [%]
0.04
0.03
0.02
0.01
0.00
HG001
ΔclpX
clpXcompl
growth phase
exp
stat

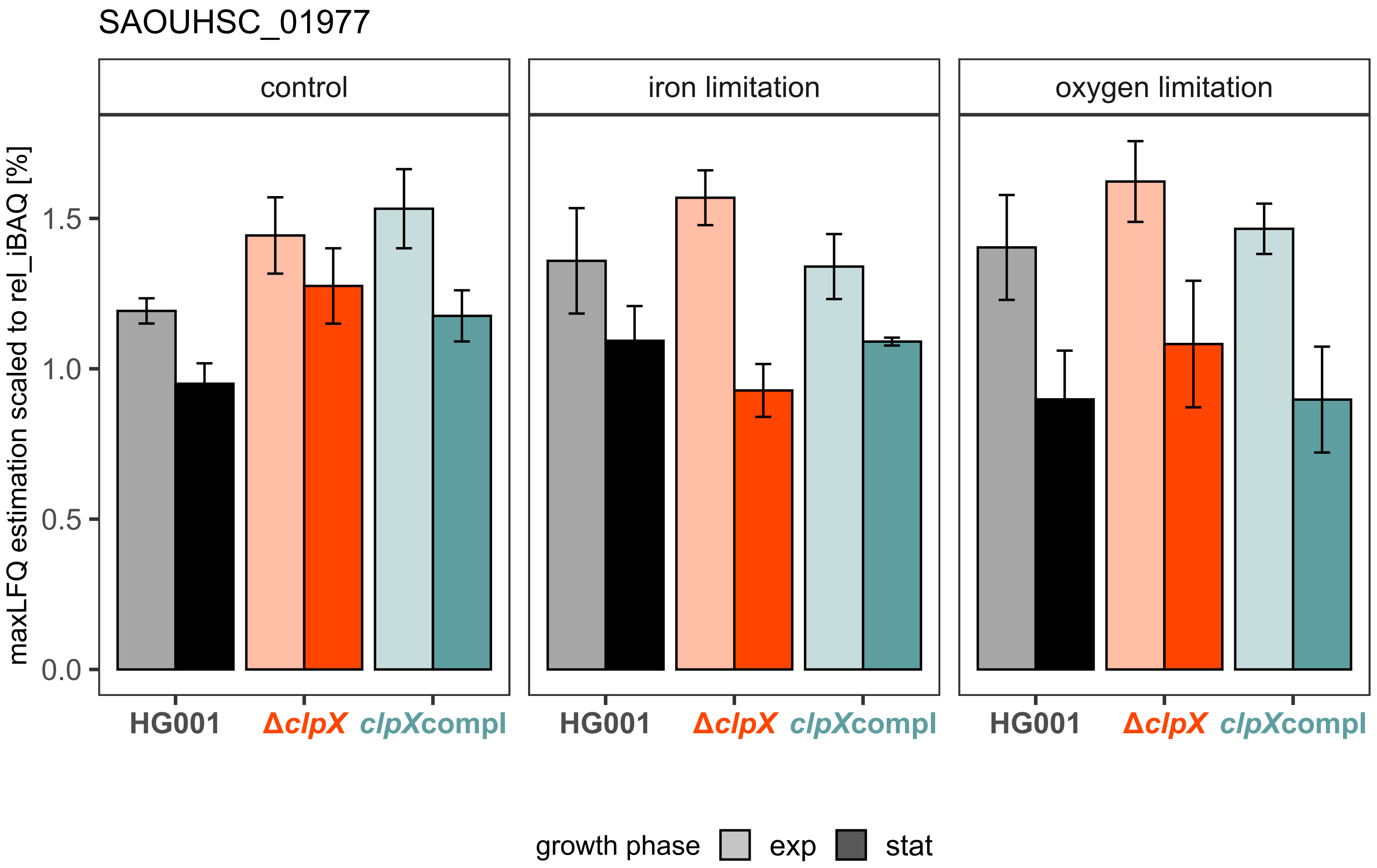

SAOUHSC_01977
control
iron limitation
oxygen limitation
maxLFQ estimation scaled to rel_iBAQ [%]
0.0
0.5
1.0
1.5
HG001
ΔclpX
clpXcompl
growth phase
exp
stat

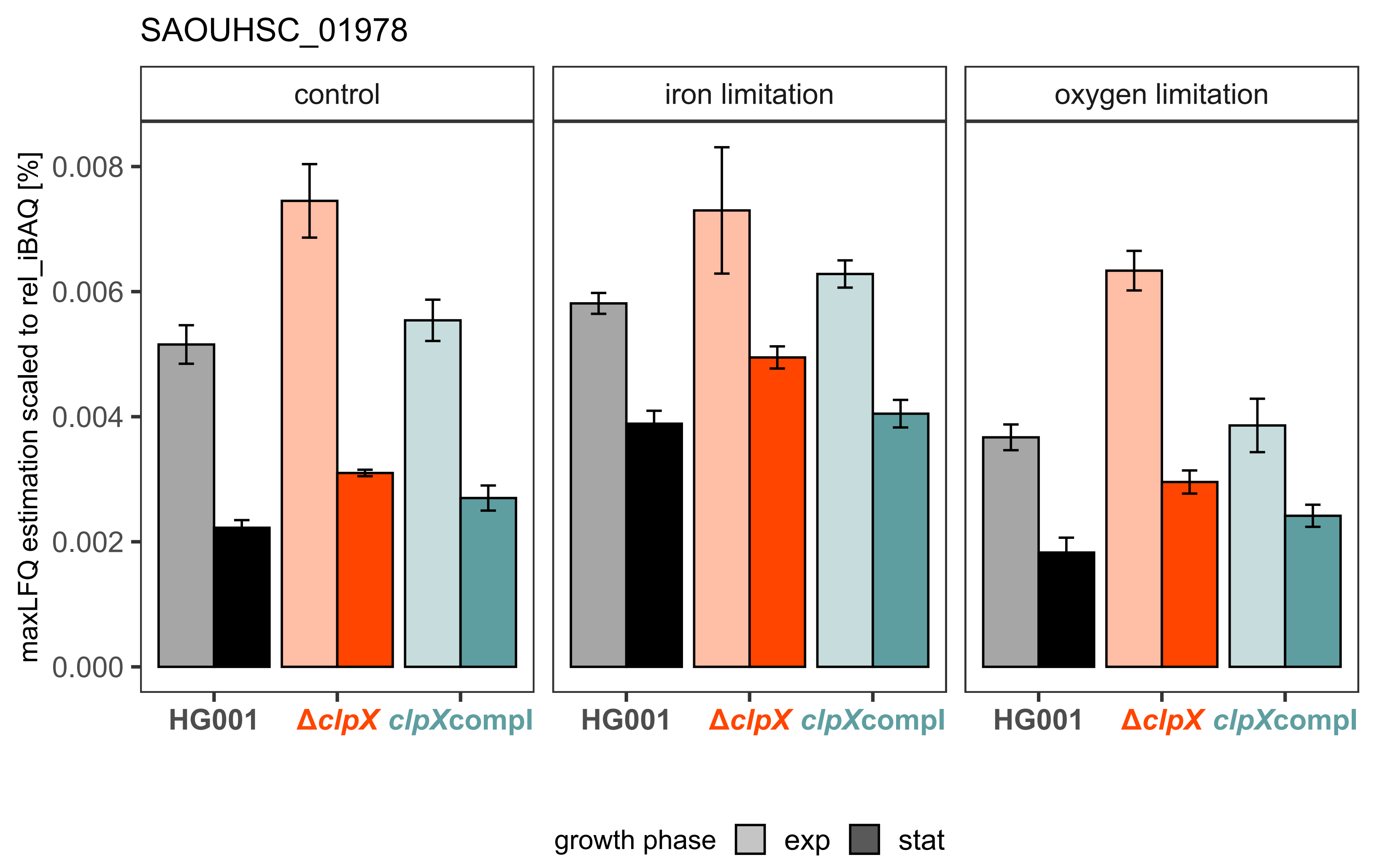
SAOUHSC_01978
control
iron limitation
oxygen limitation
maxLFQ estimation scaled to rel_iBAQ [%]
0.008
0.006
0.004
0.002
0.000
HG001
ΔclpX
clpXcompl
growth phase
exp
stat

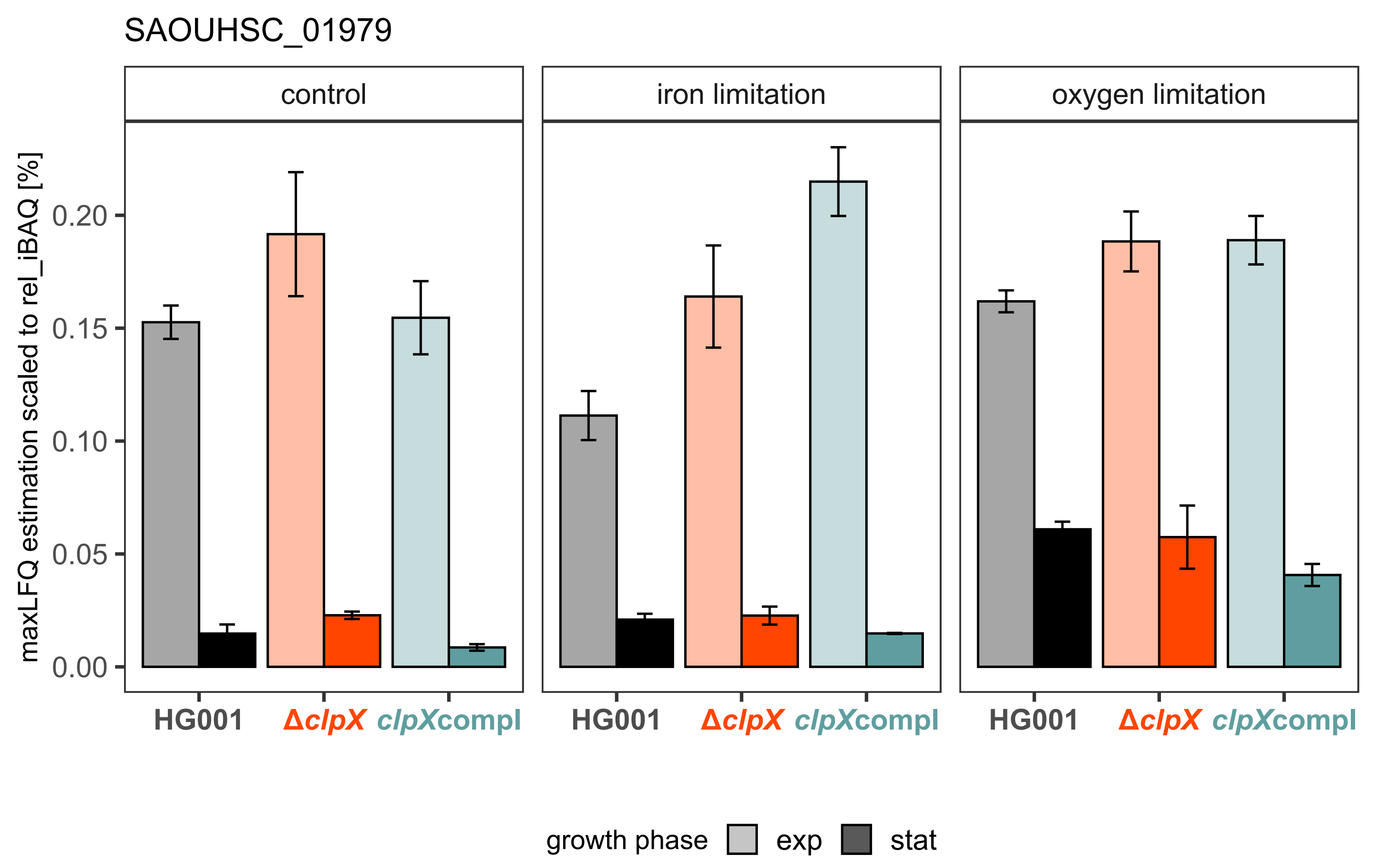
SAOUHSC_01979
control
iron limitation
oxygen limitation
maxLFQ estimation scaled to rel_iBAQ [%]
0.00
0.05
0.10
0.15
0.20
HG001
ΔclpX
clpXcompl
growth phase
exp
stat

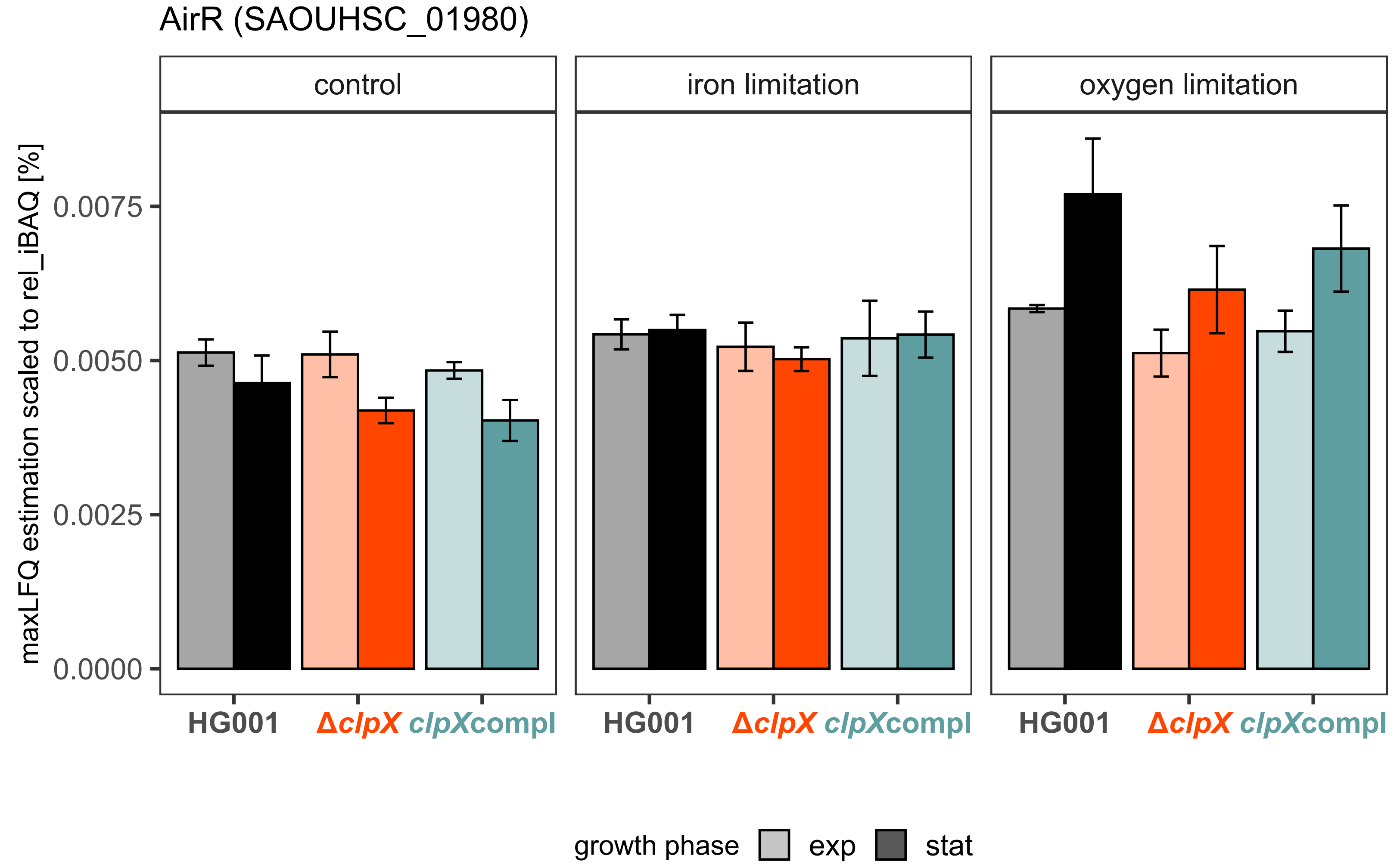

AirR (SAOUHSC_01980)
control
iron limitation
oxygen limitation
maxLFQ estimation scaled to rel_iBAQ [%]
0.0075
0.0050
0.0025
0.0000
HG001
ΔclpX
clpXcompl
growth phase
exp
stat

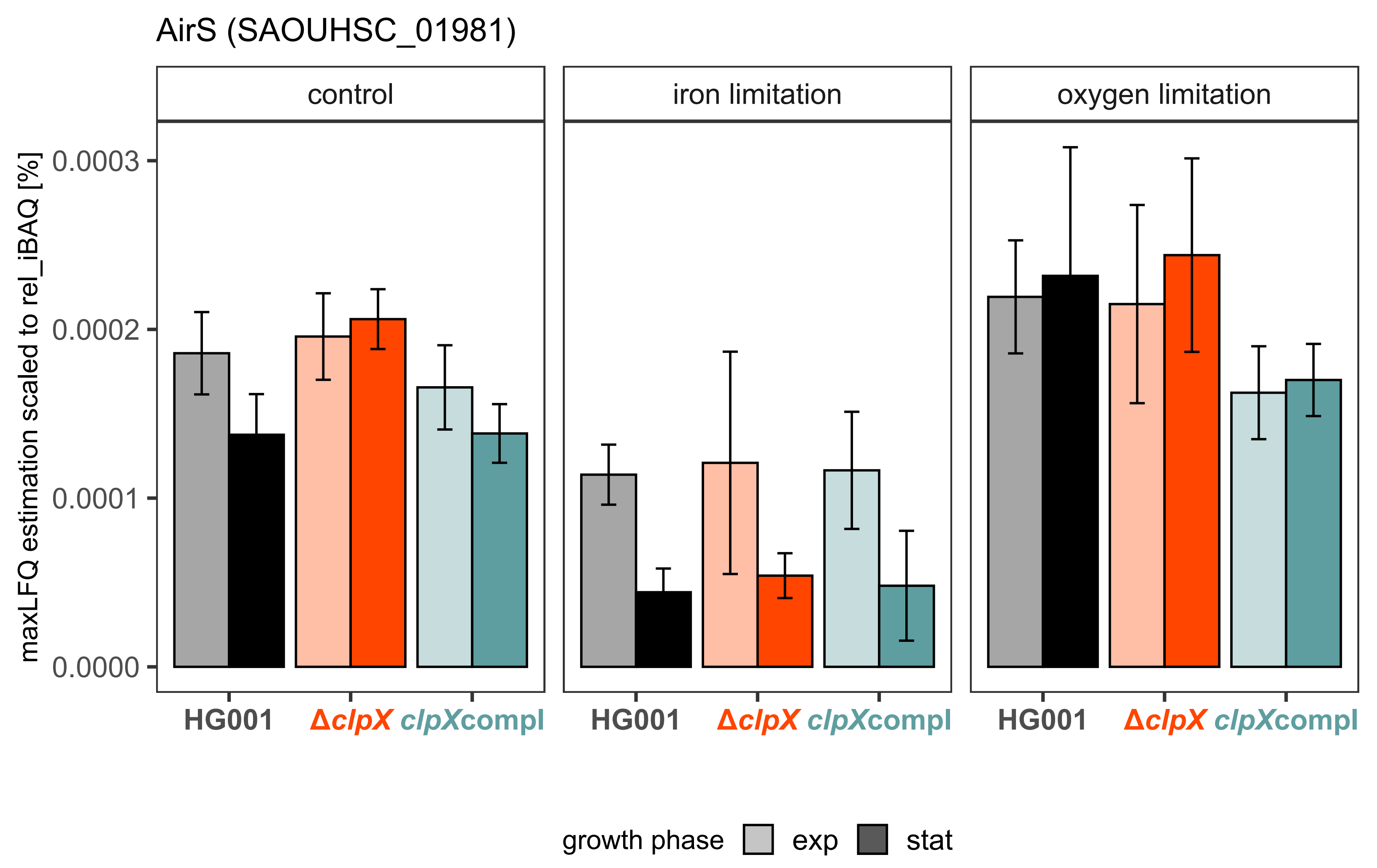

AirS (SAOUHSC_01981)
control
iron limitation
oxygen limitation
maxLFQ estimation scaled to rel_iBAQ [%]
0.0003
0.0002
0.0001
0.0000
HG001
ΔclpX
clpXcompl
growth phase
exp
stat

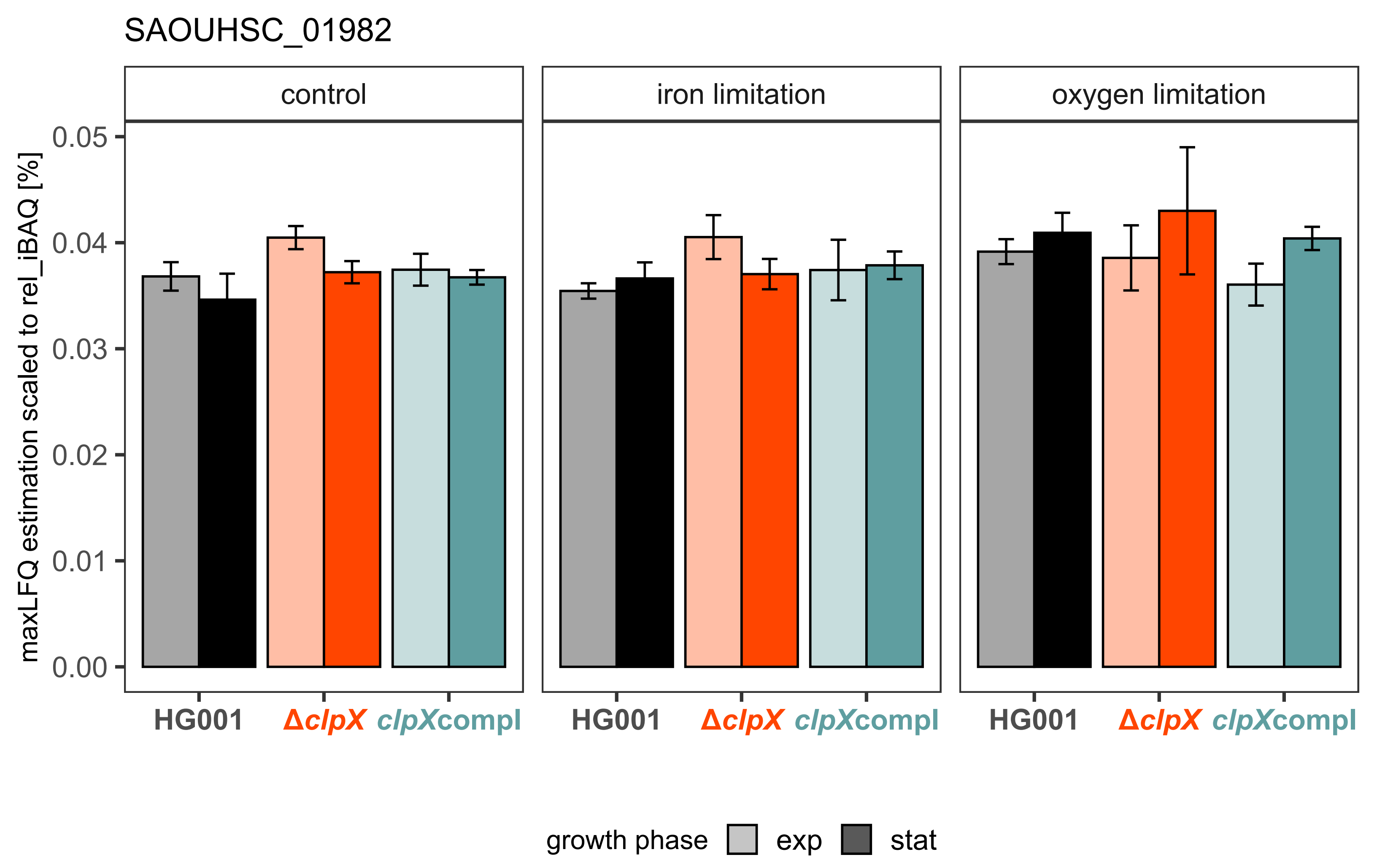

SAOUHSC_01982
control
iron limitation
oxygen limitation
maxLFQ estimation scaled to rel_iBAQ [%]
0.05
0.04
0.03
0.02
0.01
0.00
HG001
ΔclpX
clpXcompl
growth phase
exp
stat

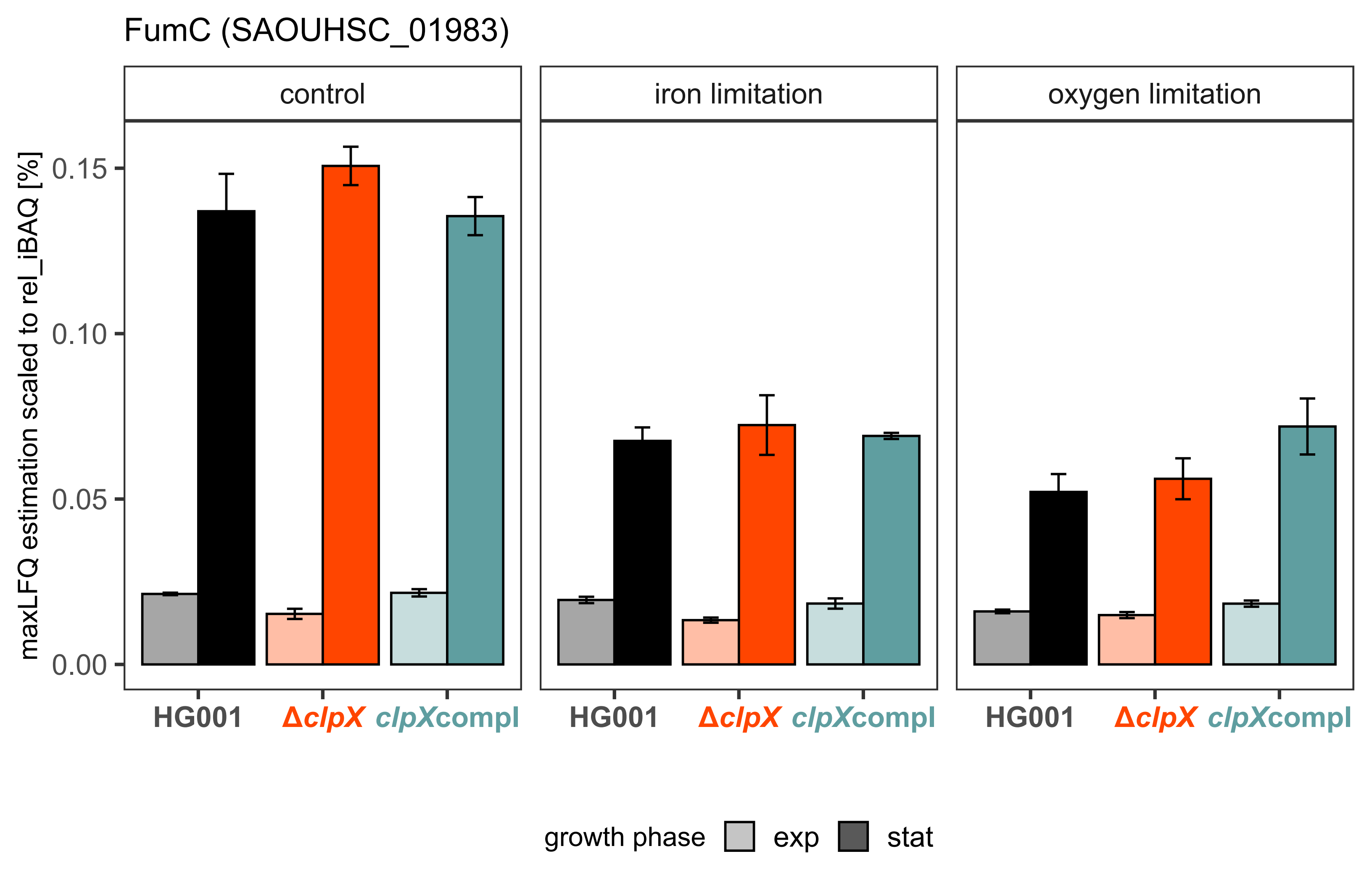
FumC (SAOUHSC_01983)
control
iron limitation
oxygen limitation
maxLFQ estimation scaled to rel_iBAQ [%]
0.15
0.10
0.05
0.00
HG001
ΔclpX
clpXcompl
growth phase
exp
stat

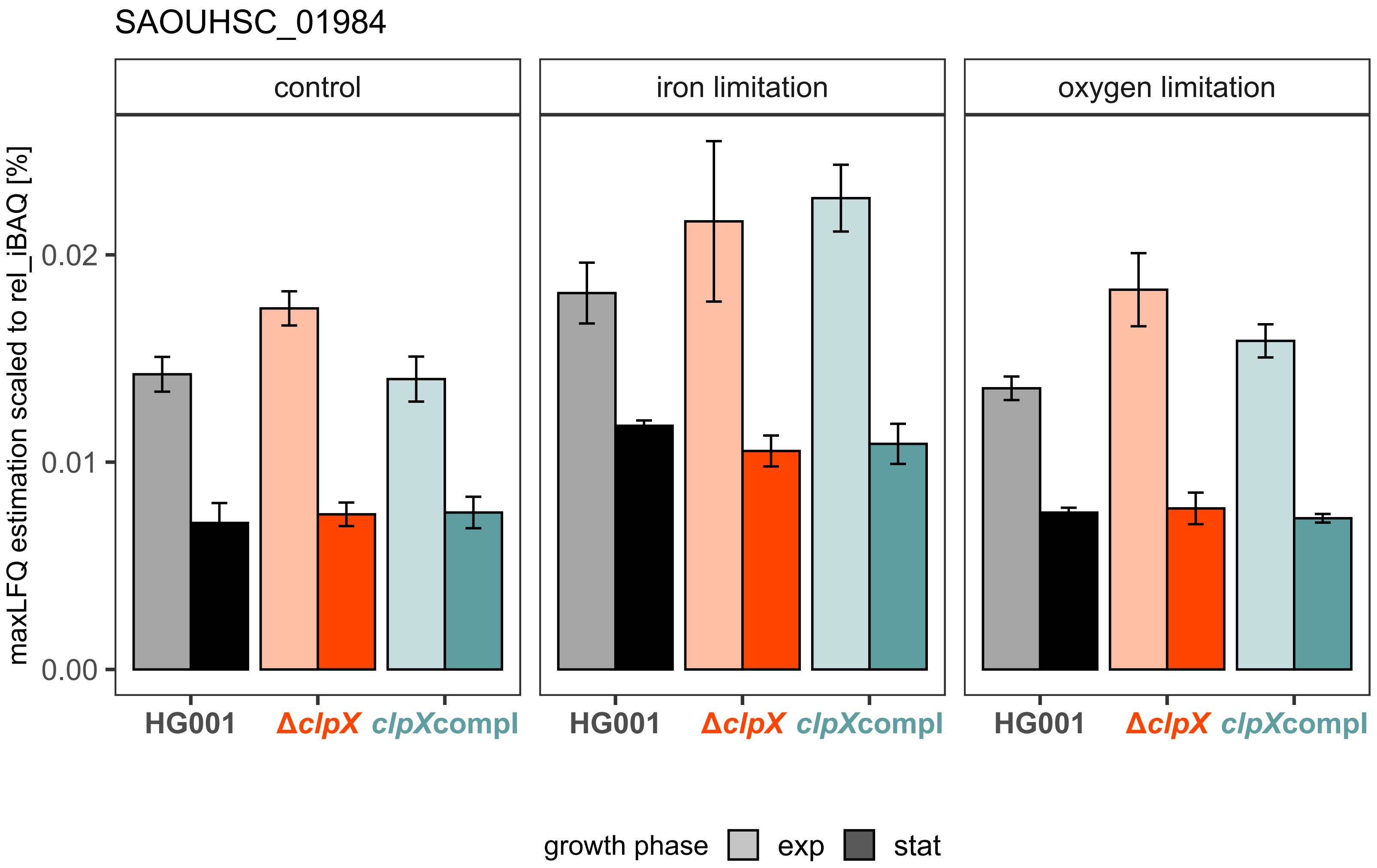
SAOUHSC_01984
control
iron limitation
oxygen limitation
maxLFQ estimation scaled to rel_iBAQ [%]
0.02
0.01
0.00
HG001
ΔclpX
clpXcompl
growth phase
exp
stat

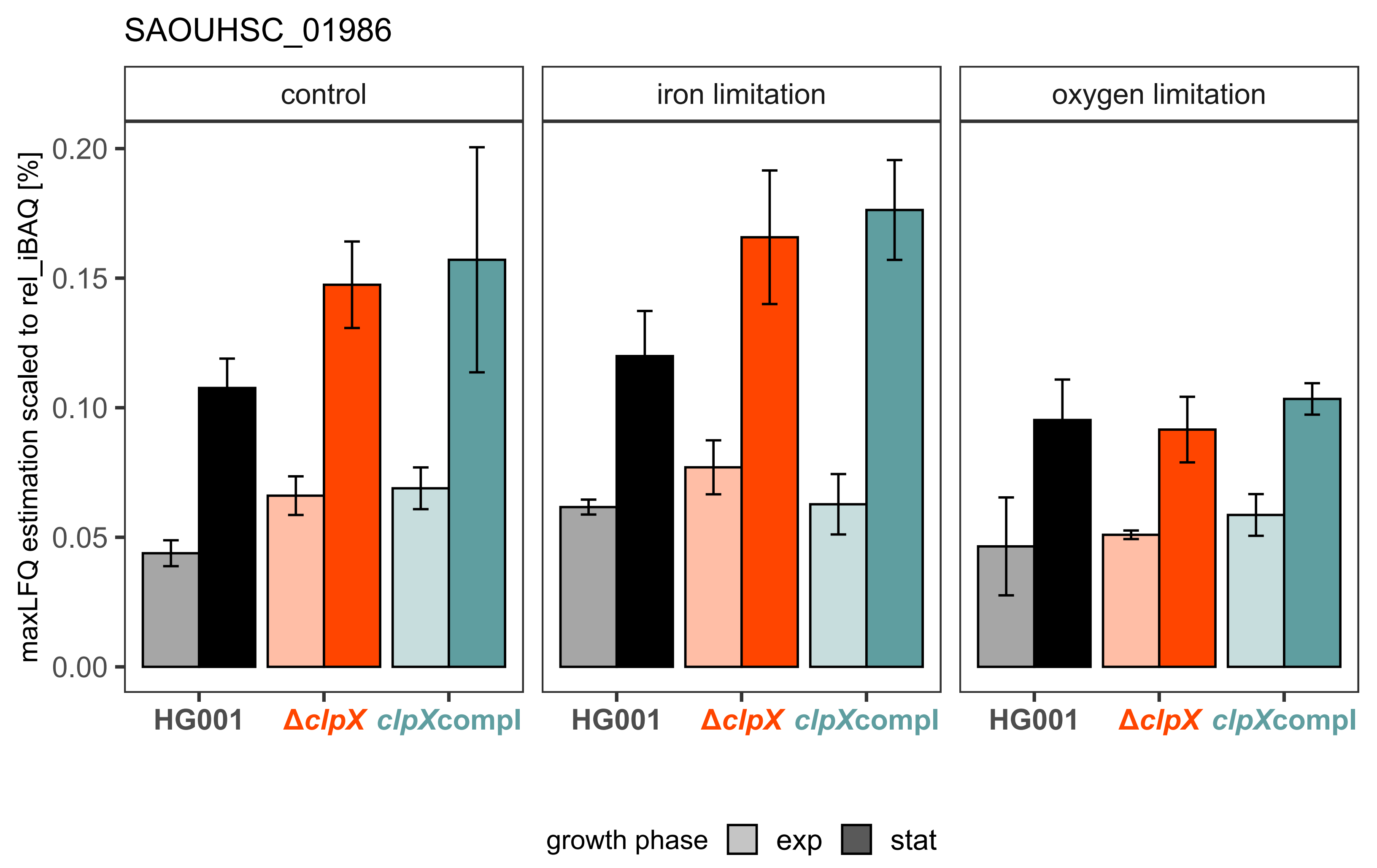
SAOUHSC_01986
control
iron limitation
oxygen limitation
maxLFQ estimation scaled to rel_iBAQ [%]
0.20
0.15
0.10
0.05
0.00
HG001
ΔclpX
clpXcompl
growth phase
exp
stat

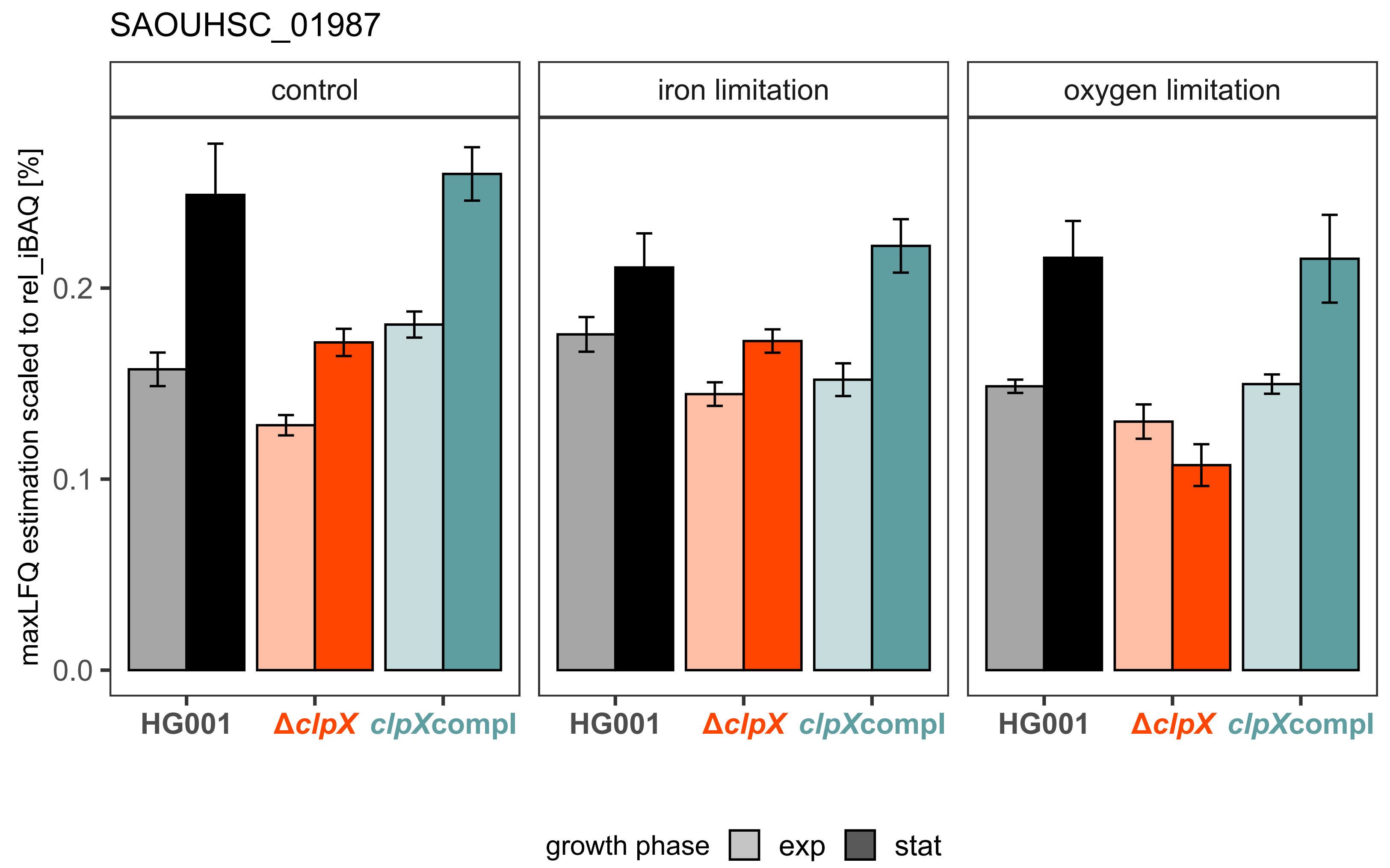
SAOUHSC_01987
control
iron limitation
oxygen limitation
maxLFQ estimation scaled to rel_iBAQ [%]
0.0
0.1
0.2
HG001
ΔclpX
clpXcompl
growth phase
exp
stat

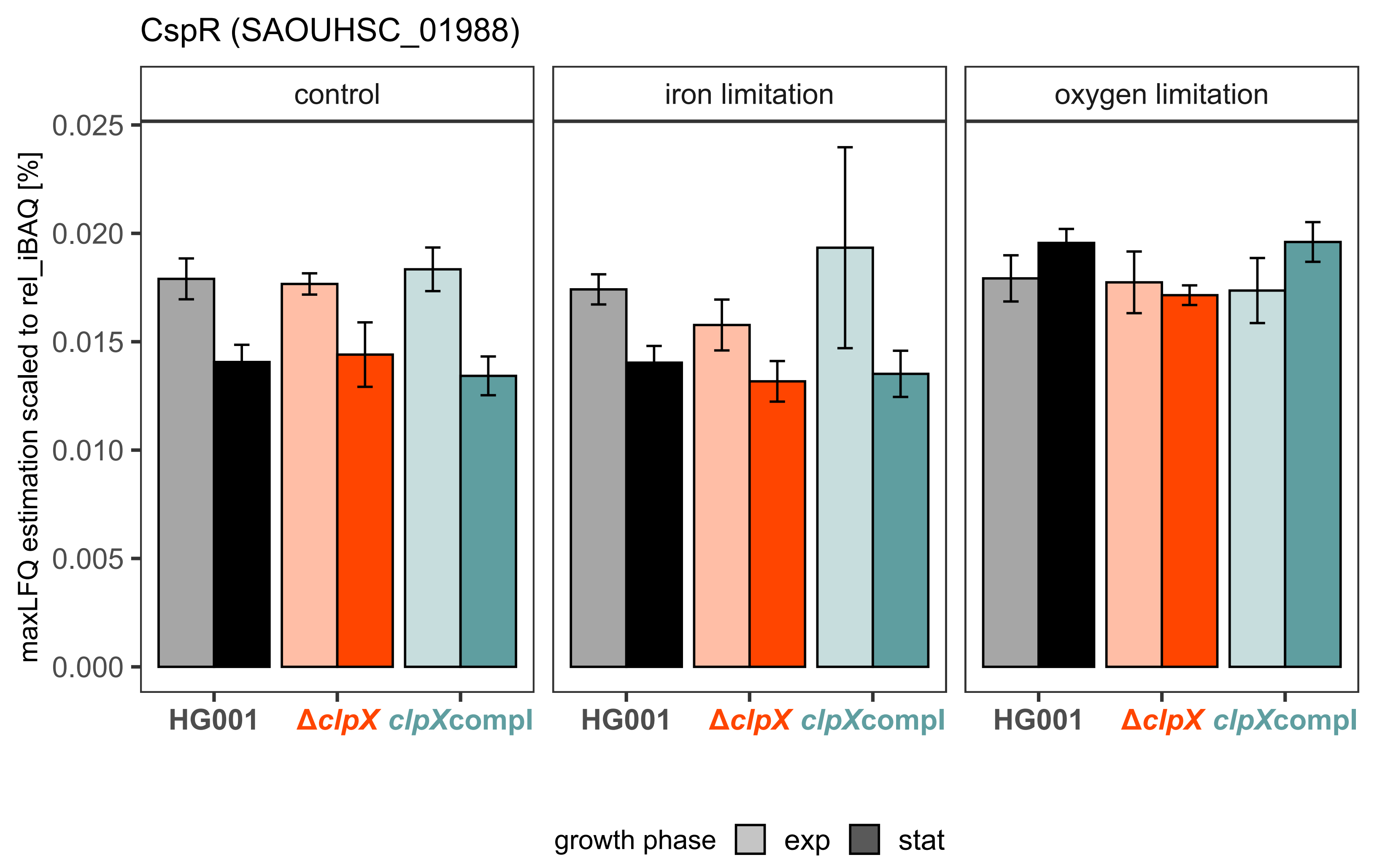
CspR (SAOUHSC_01988)
control
iron limitation
oxygen limitation
maxLFQ estimation scaled to rel_iBAQ [%]
0.025
0.020
0.015
0.010
0.005
0.000
HG001
ΔclpX
clpXcompl
growth phase
exp
stat

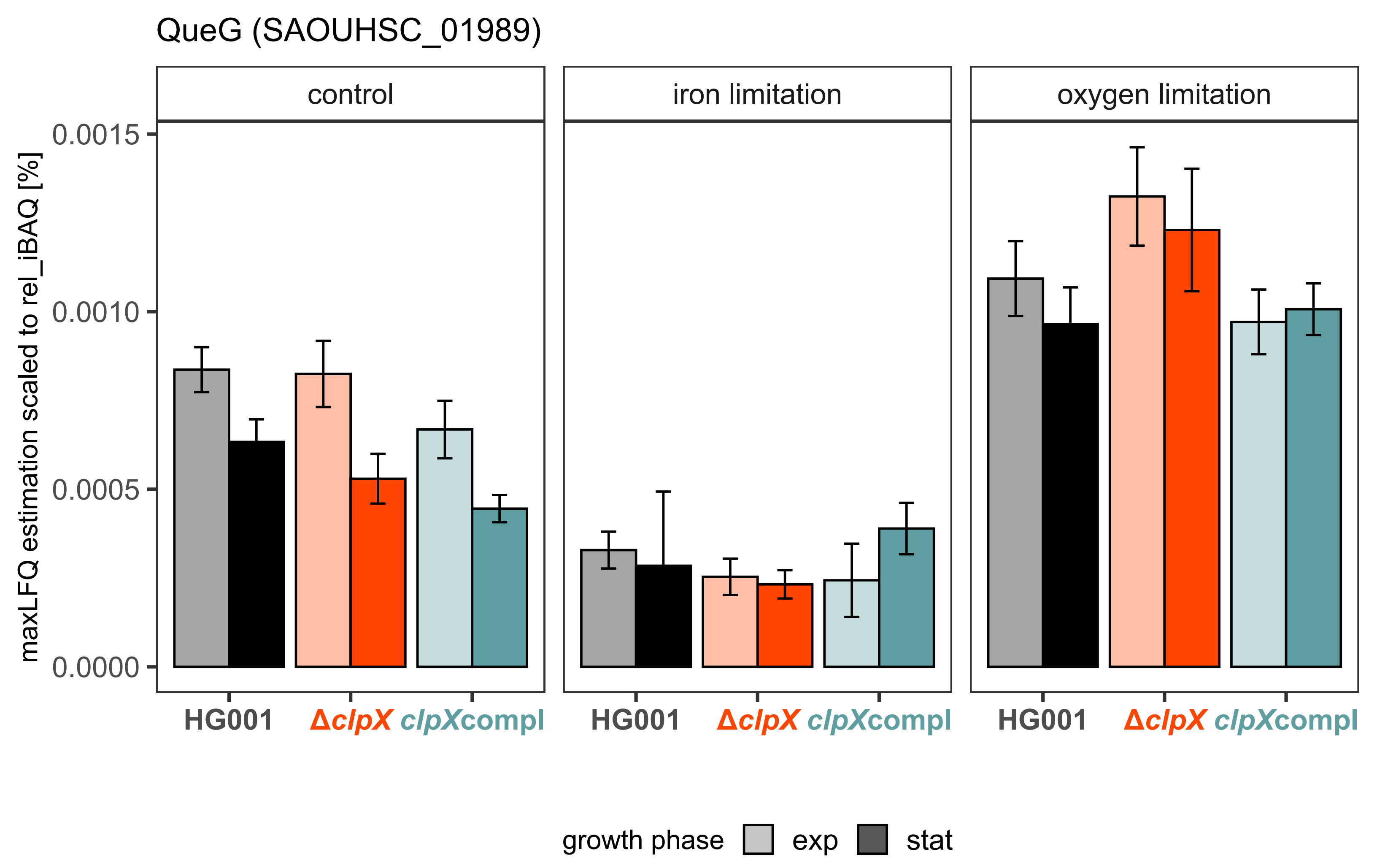

QueG (SAOUHSC_01989)
control
iron limitation
oxygen limitation
maxLFQ estimation scaled to rel_iBAQ [%]
0.0015
0.0010
0.0005
0.0000
HG001
ΔclpX
clpXcompl
growth phase
exp
stat

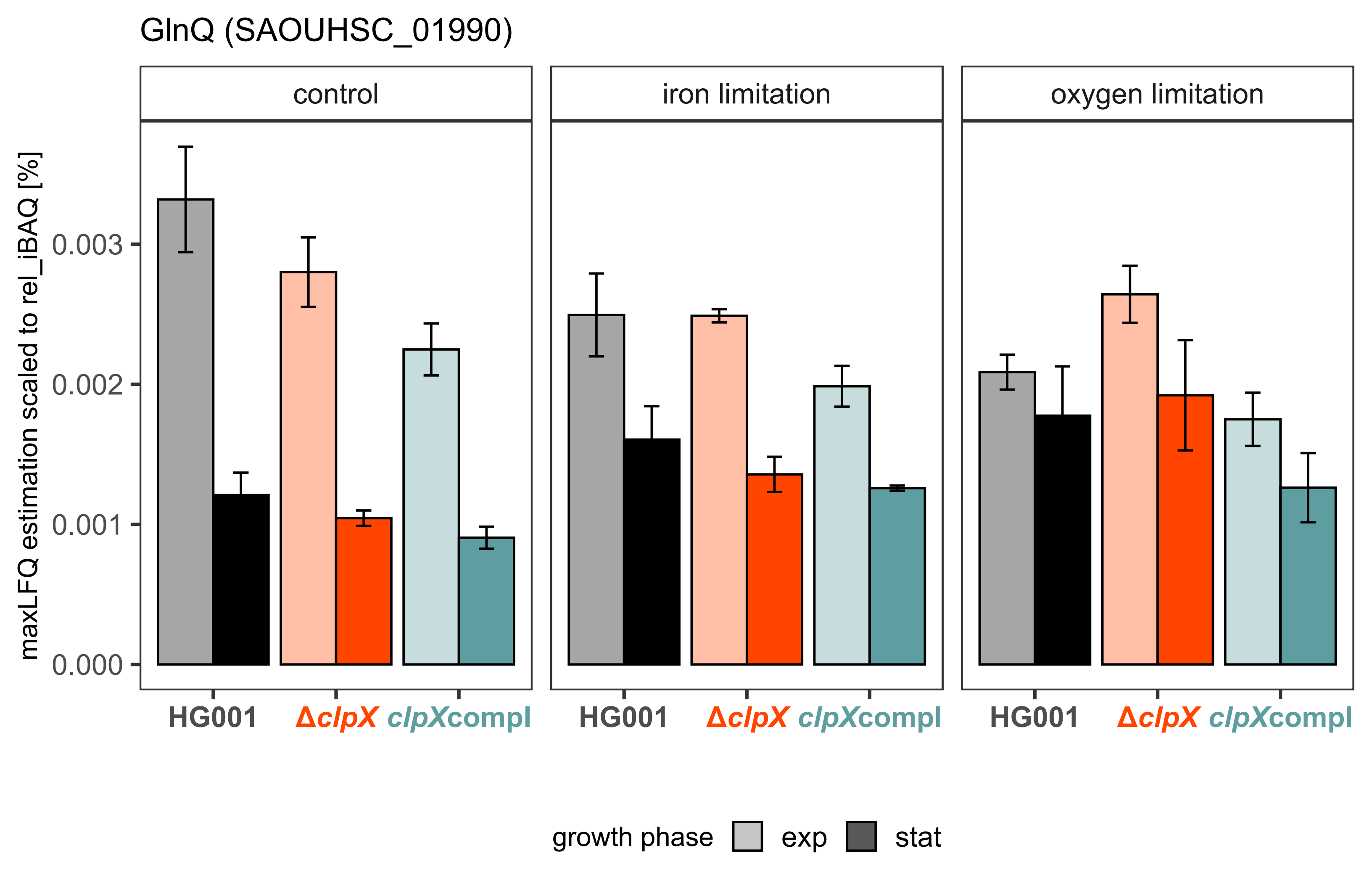

GlnQ (SAOUHSC_01990)
control
iron limitation
oxygen limitation
maxLFQ estimation scaled to rel_iBAQ [%]
0.003
0.002
0.001
0.000
HG001
ΔclpX
clpXcompl
growth phase
exp
stat

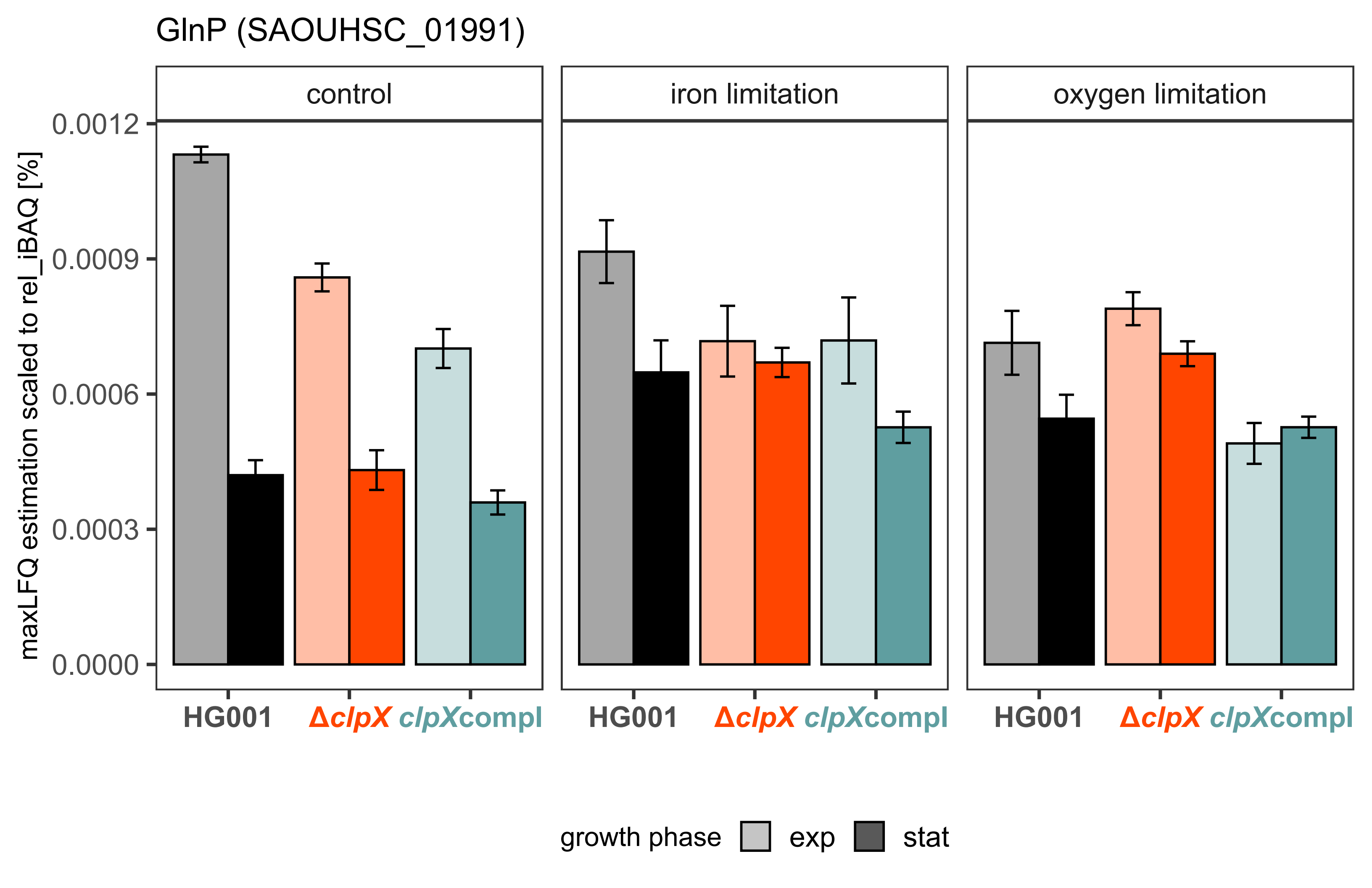

GlnP (SAOUHSC_01991)
control
iron limitation
oxygen limitation
maxLFQ estimation scaled to rel_iBAQ [%]
0.0012
0.0009
0.0006
0.0003
0.0000
HG001
ΔclpX
clpXcompl
growth phase
exp
stat

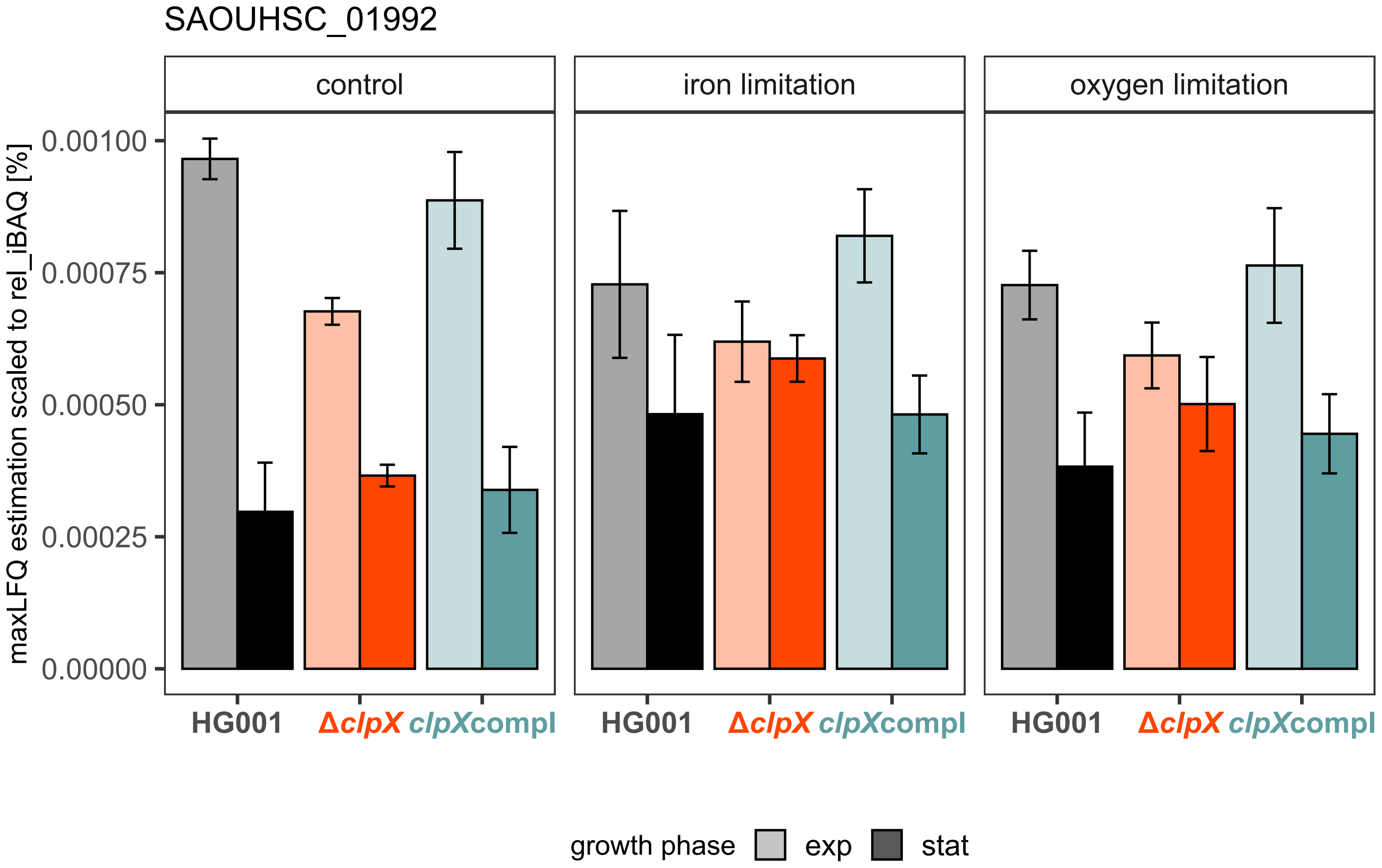
SAOUHSC_01992
control
iron limitation
oxygen limitation
maxLFQ estimation scaled to rel_iBAQ [%]
0.00100
0.00075
0.00050
0.00025
0.00000
HG001
ΔclpX
clpXcompl
growth phase
exp
stat

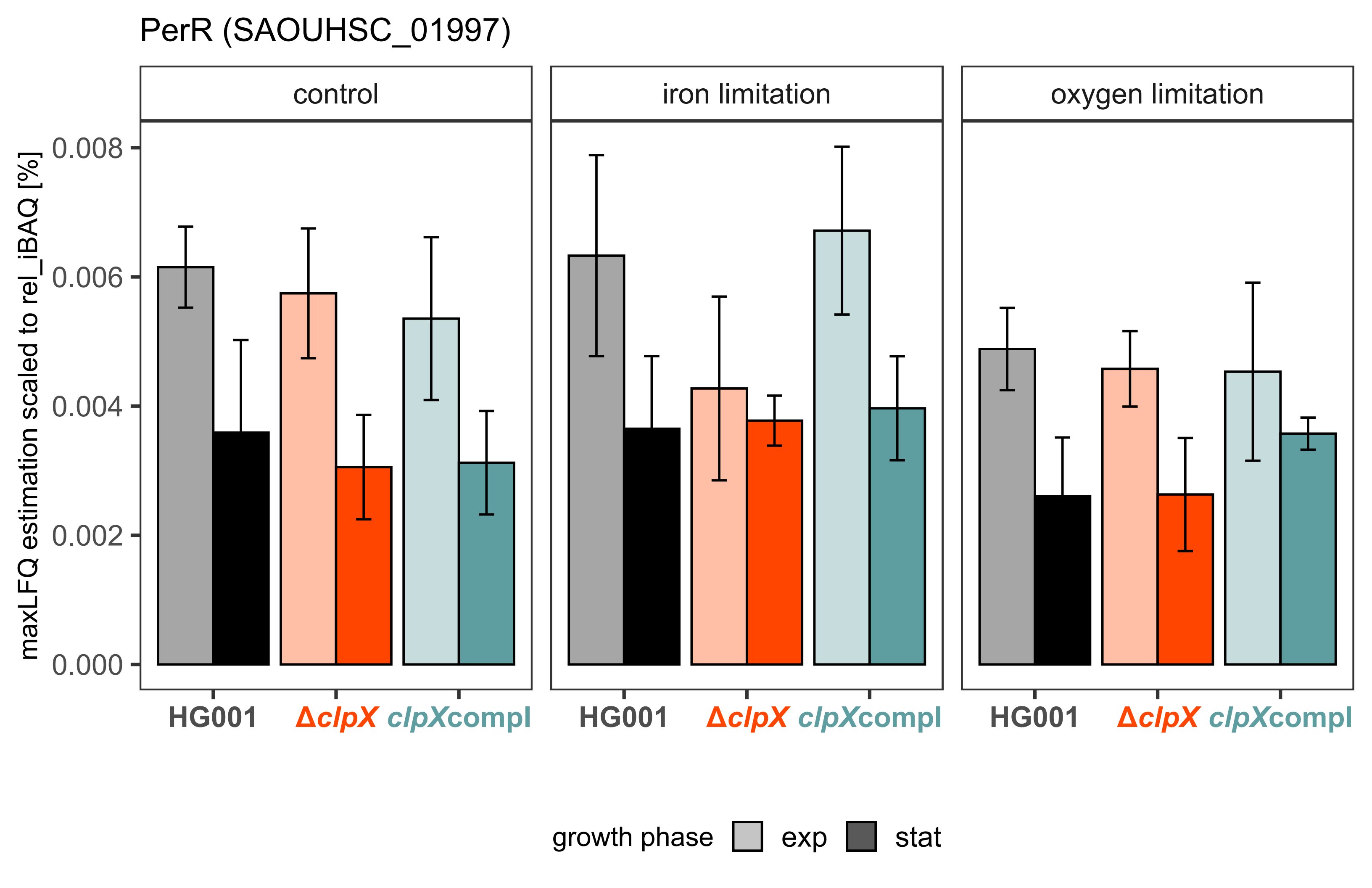
PerR (SAOUHSC_01997)
control
iron limitation
oxygen limitation
maxLFQ estimation scaled to rel_iBAQ [%]
0.008
0.006
0.004
0.002
0.000
HG001
ΔclpX
clpXcompl
growth phase
exp
stat

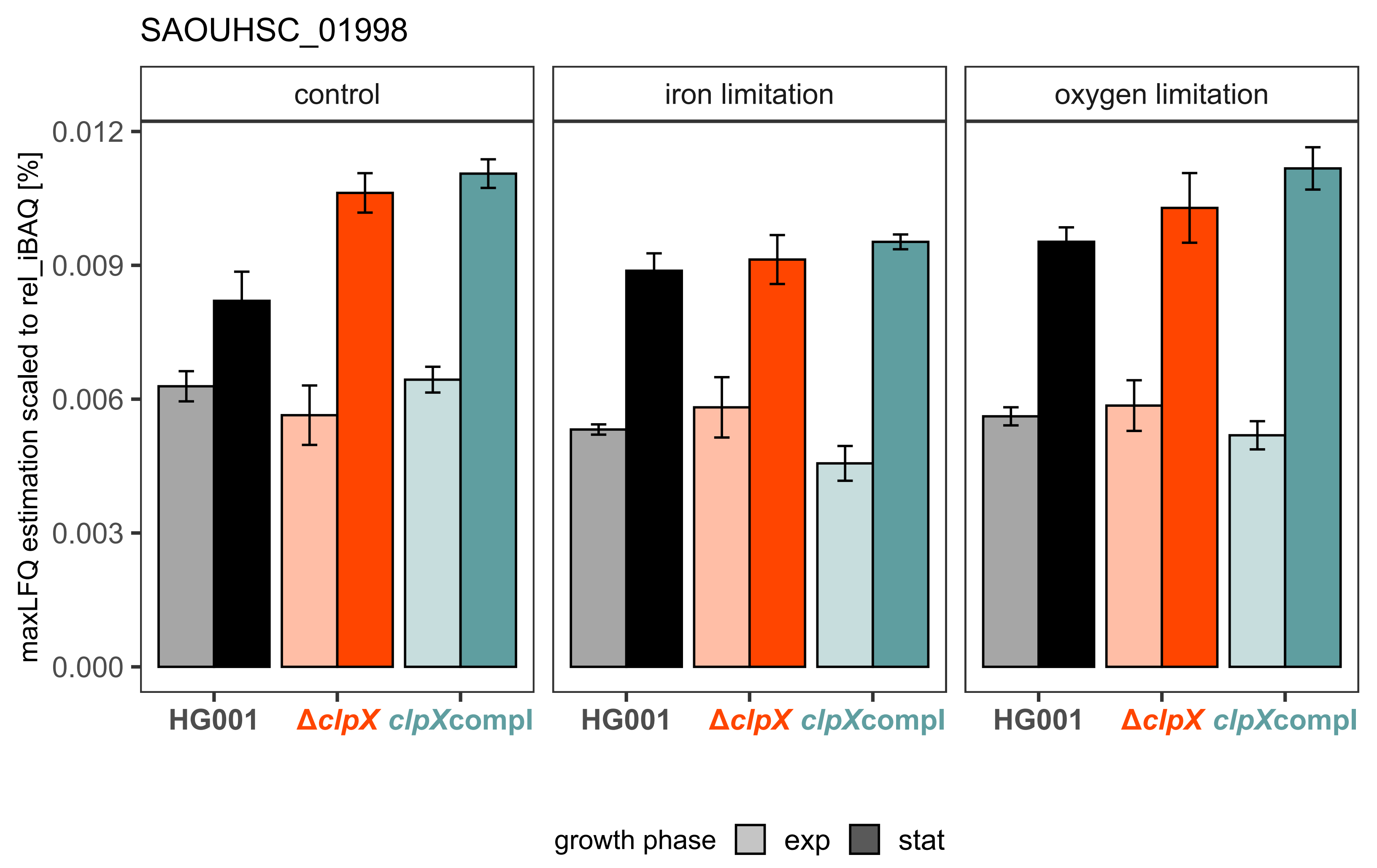

SAOUHSC_01998
control
iron limitation
oxygen limitation
maxLFQ estimation scaled to rel_iBAQ [%]
0.012
0.009
0.006
0.003
0.000
HG001
ΔclpX
clpXcompl
growth phase
exp
stat

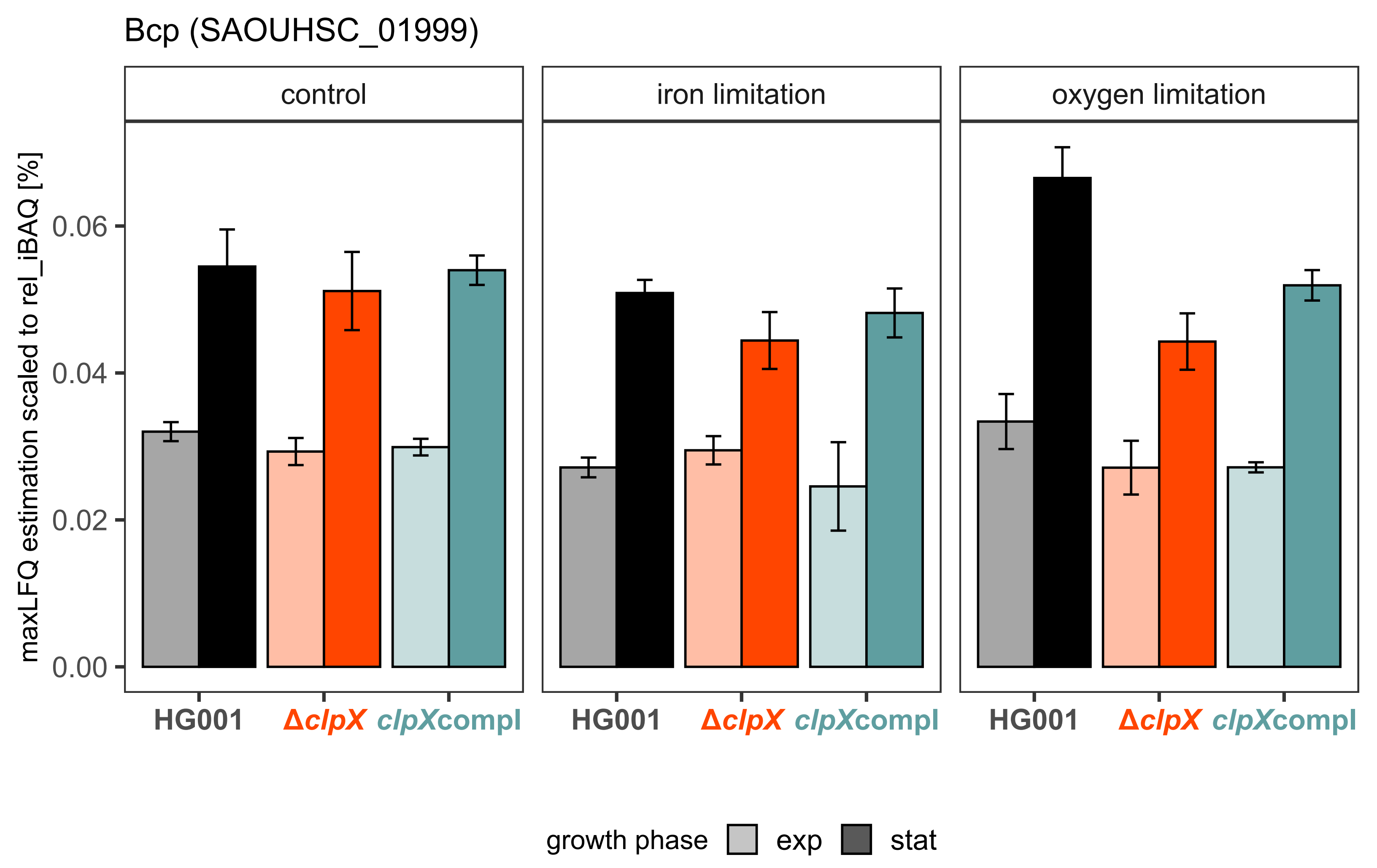
Bcp (SAOUHSC_01999)
control
iron limitation
oxygen limitation
maxLFQ estimation scaled to rel_iBAQ [%]
0.00
0.02
0.04
0.06
HG001
ΔclpX
clpXcompl
growth phase
exp
stat

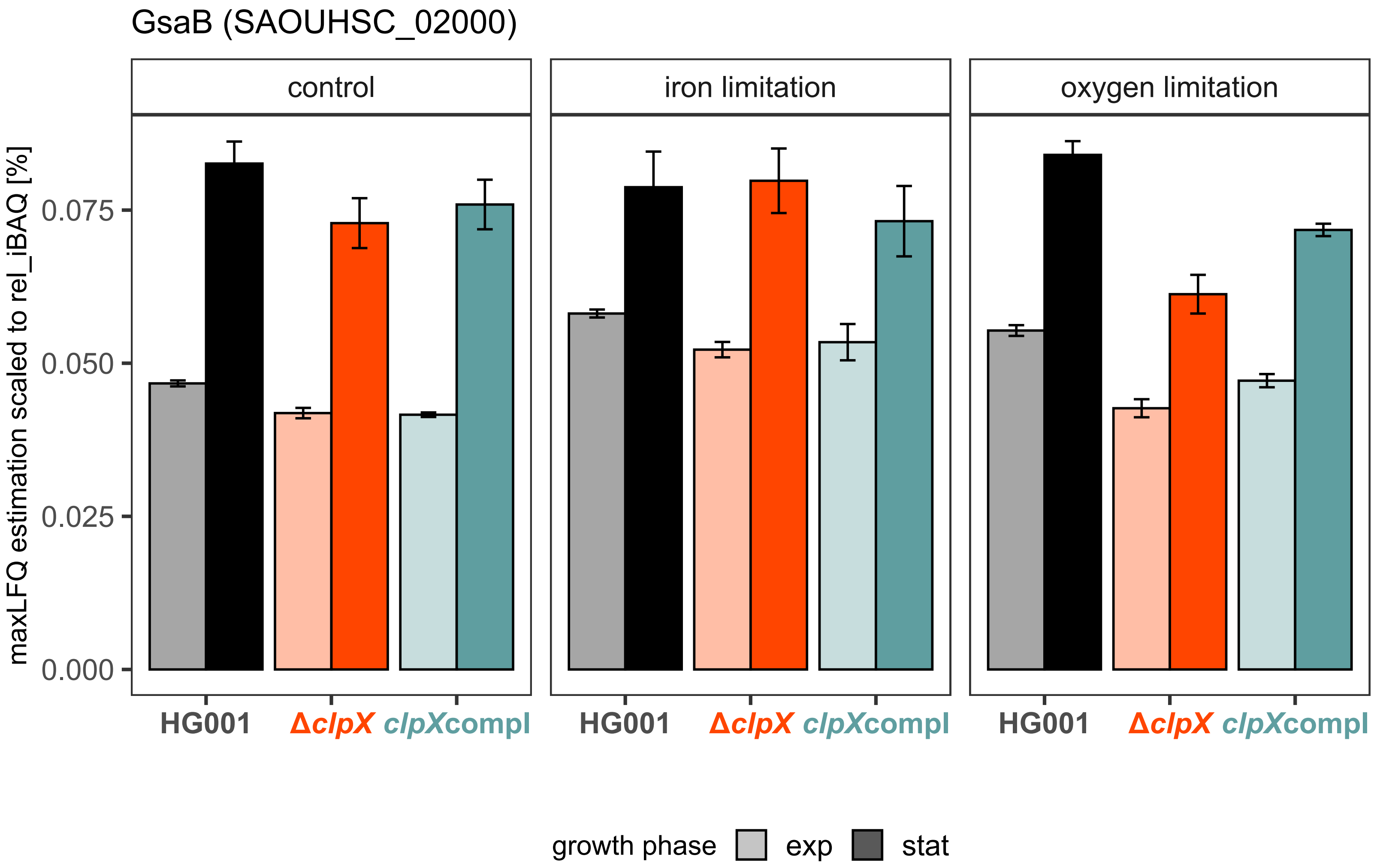

GsaB (SAOUHSC_02000)
control
iron limitation
oxygen limitation
maxLFQ estimation scaled to rel_iBAQ [%]
0.000
0.025
0.050
0.075
HG001
ΔclpX
clpXcompl
growth phase
exp
stat

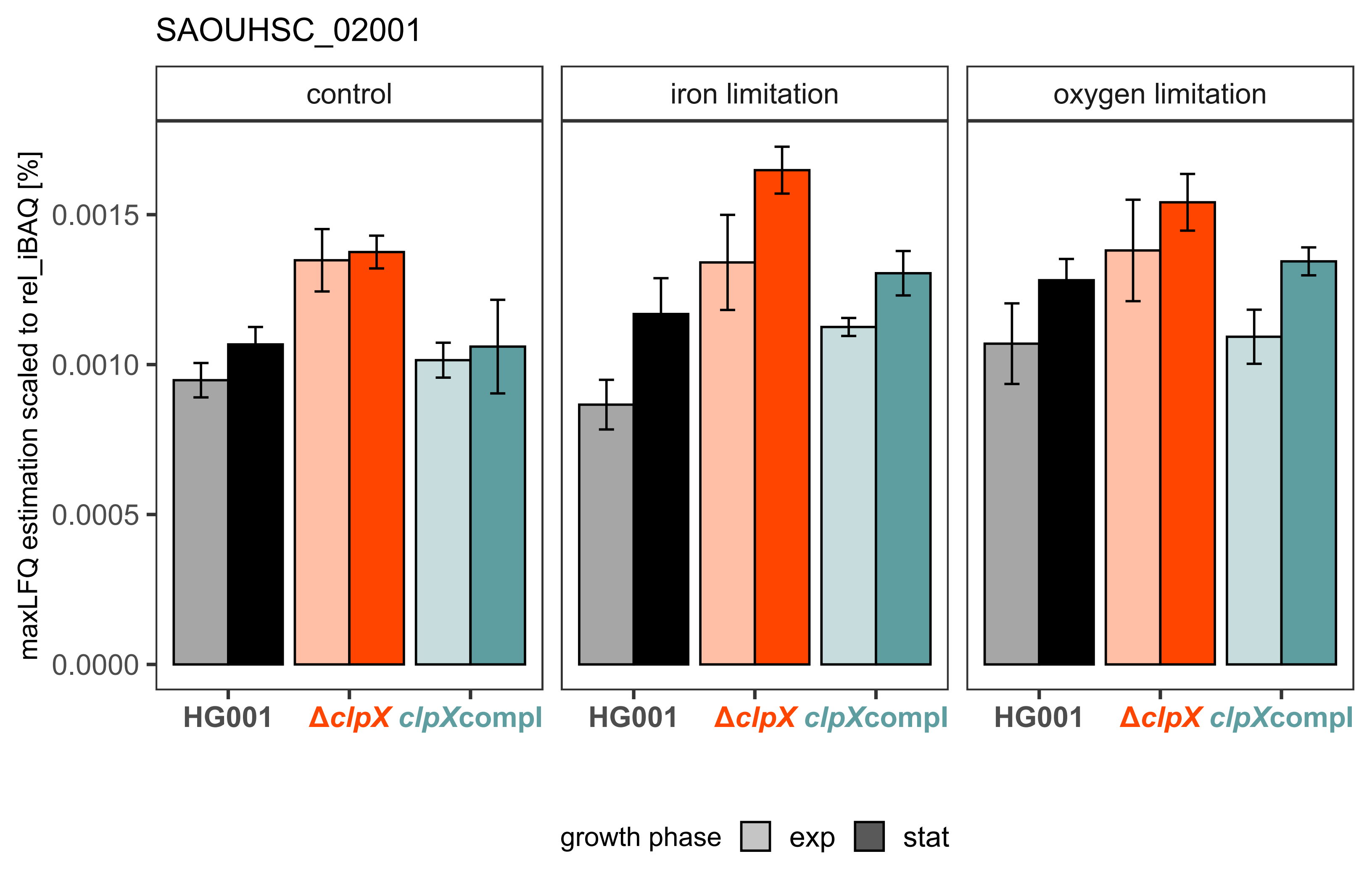

SAOUHSC_02001
control
iron limitation
oxygen limitation
maxLFQ estimation scaled to rel_iBAQ [%]
0.0015
0.0010
0.0005
0.0000
HG001
ΔclpX
clpXcompl
growth phase
exp
stat

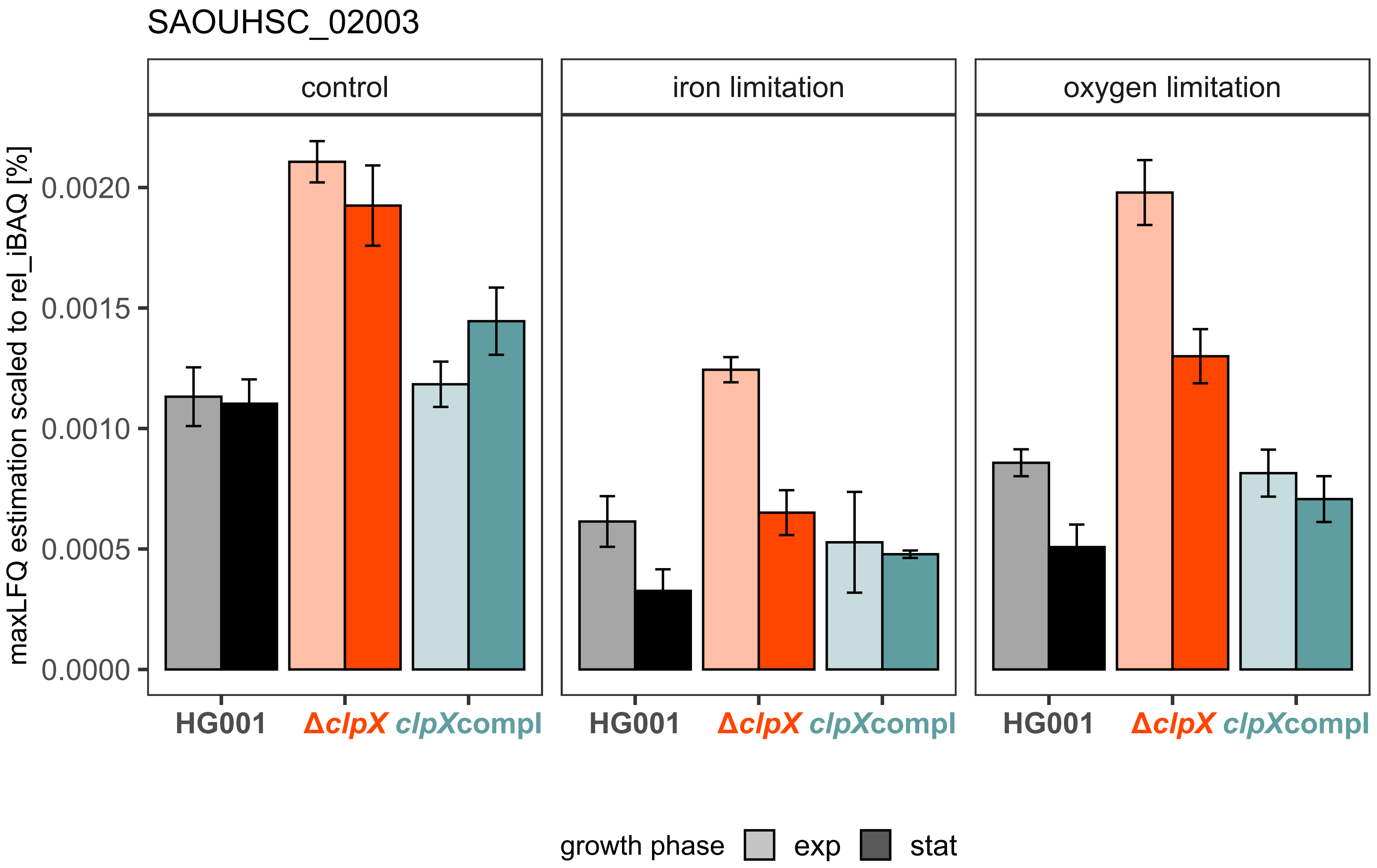
SAOUHSC_02003
control
iron limitation
oxygen limitation
maxLFQ estimation scaled to rel_iBAQ [%]
0.0020
0.0015
0.0010
0.0005
0.0000
HG001
ΔclpX
clpXcompl
growth phase
exp
stat

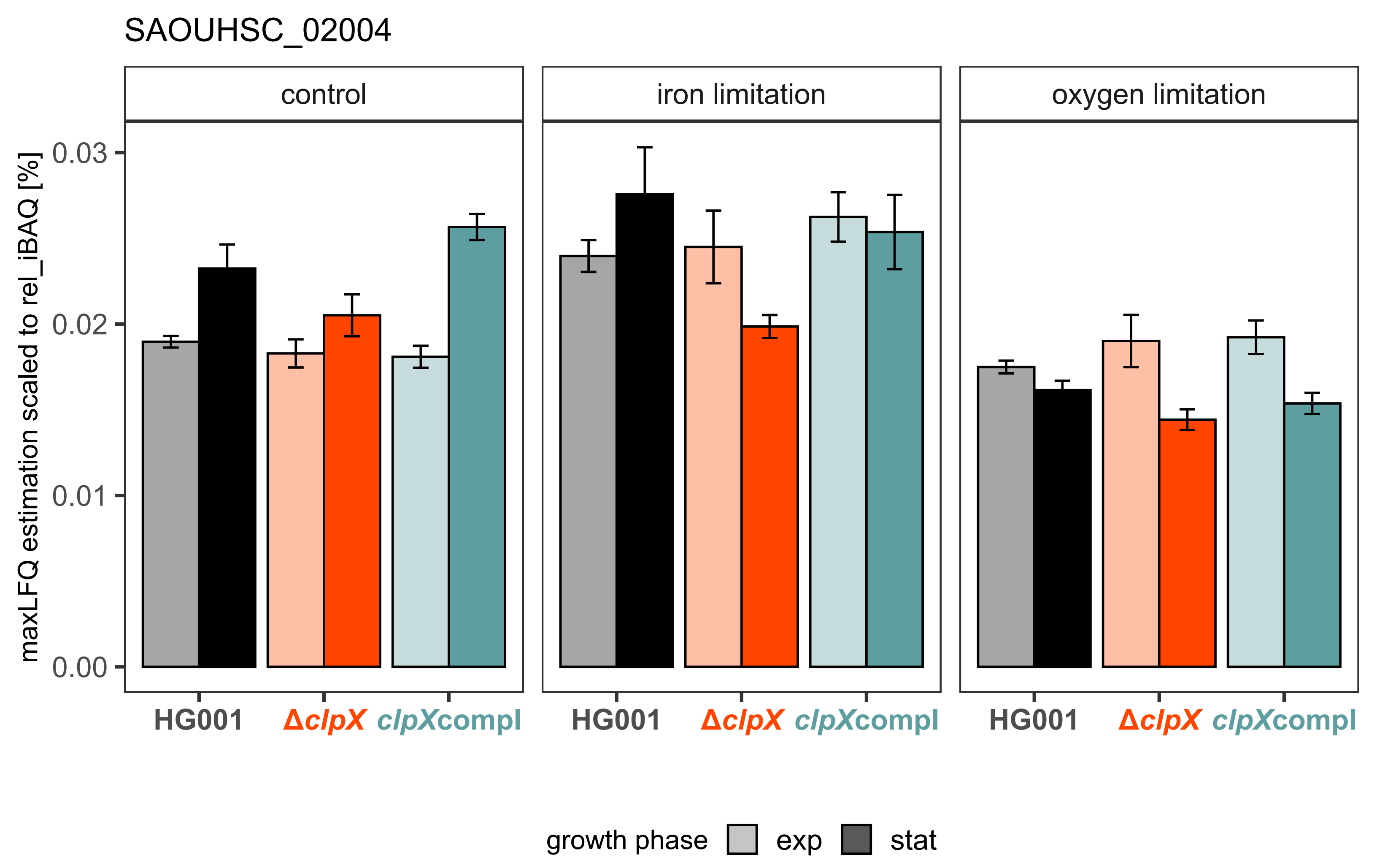

SAOUHSC_02004
control
iron limitation
oxygen limitation
maxLFQ estimation scaled to rel_iBAQ [%]
0.03
0.02
0.01
0.00
HG001
ΔclpX
clpXcompl
growth phase
exp
stat

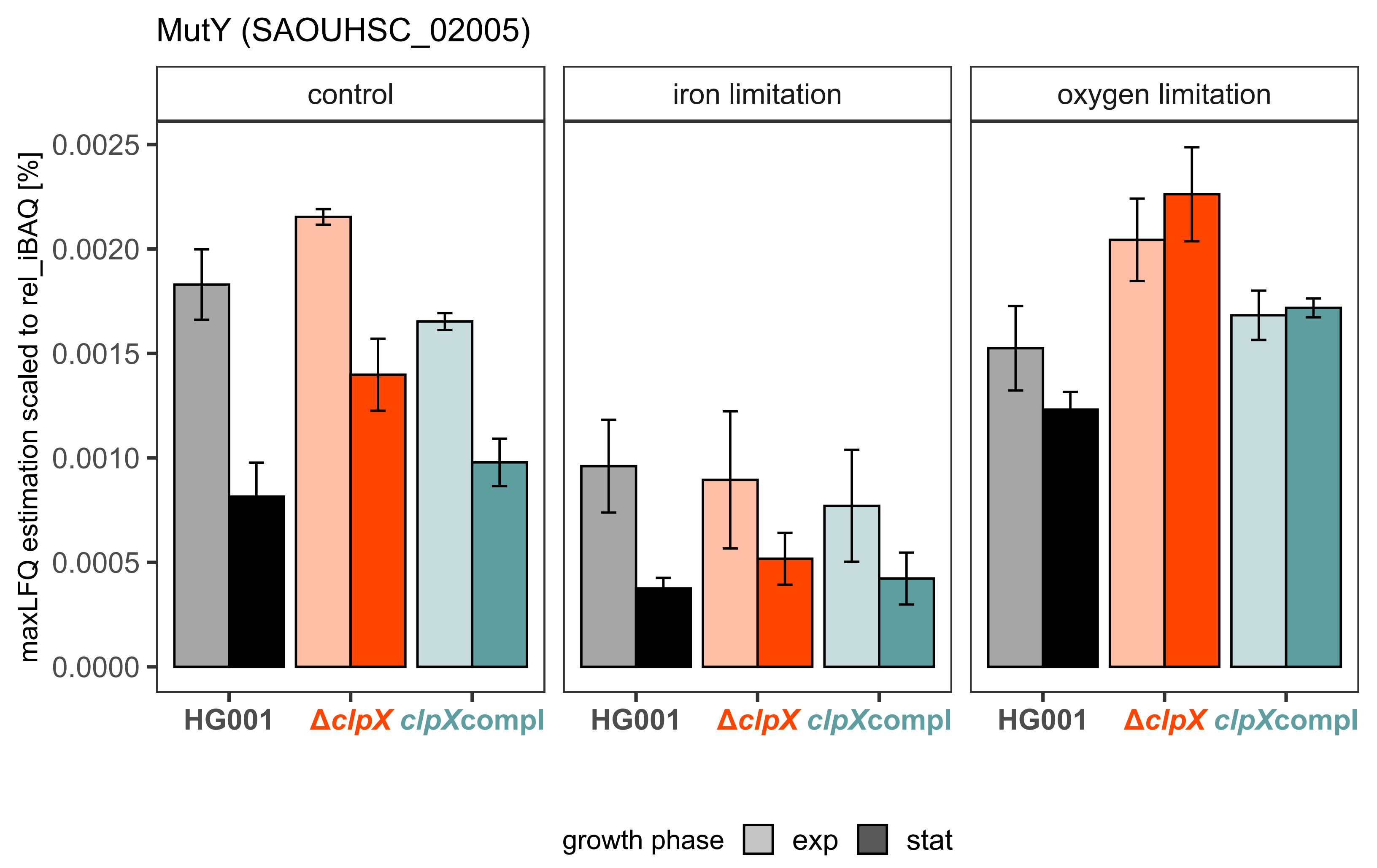
MutY (SAOUHSC_02005)
control
iron limitation
oxygen limitation
maxLFQ estimation scaled to rel_iBAQ [%]
0.0025
0.0020
0.0015
0.0010
0.0005
0.0000
HG001
ΔclpX
clpXcompl
growth phase
exp
stat

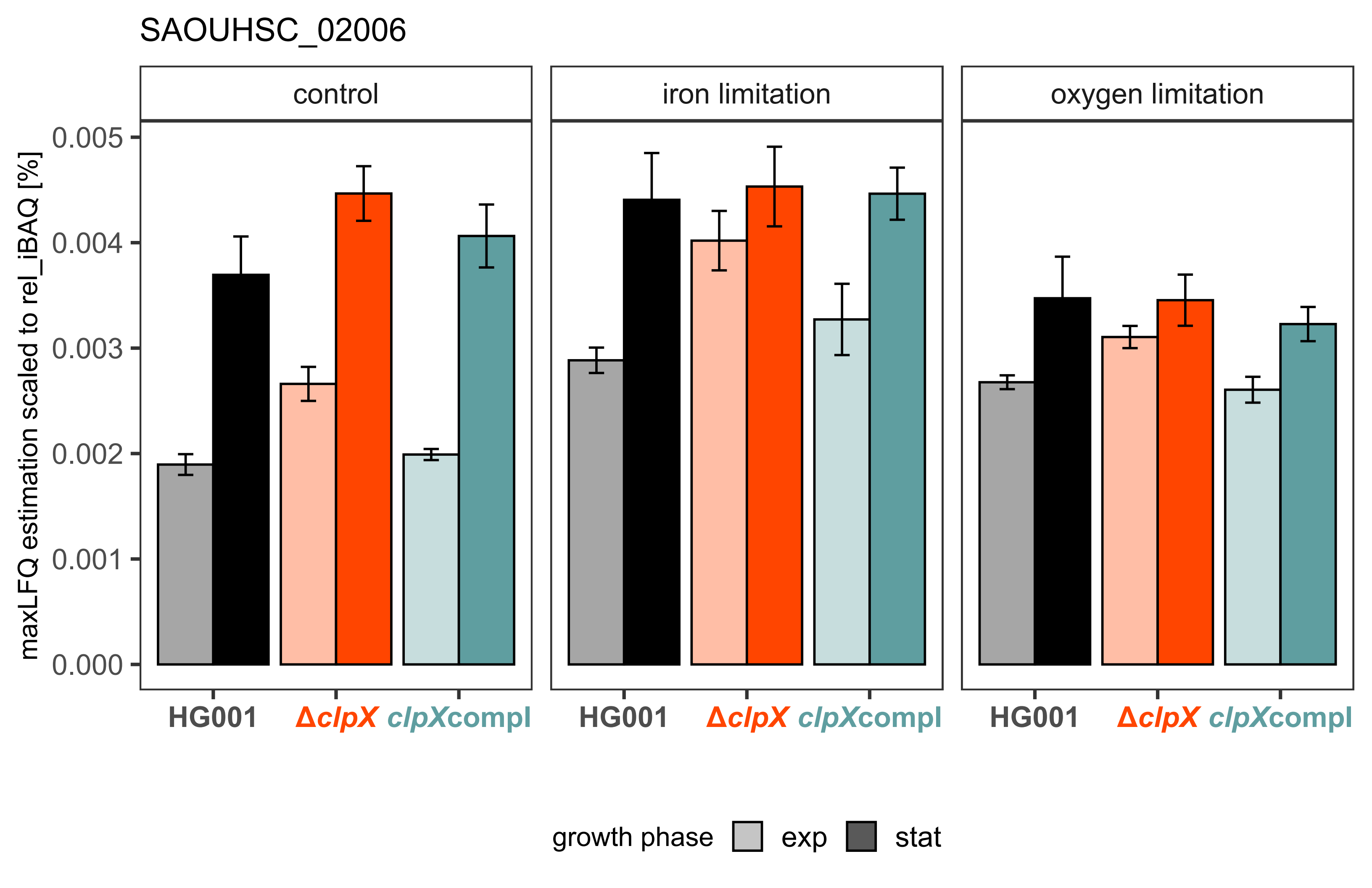

SAOUHSC_02006
control
iron limitation
oxygen limitation
maxLFQ estimation scaled to rel_iBAQ [%]
0.005
0.004
0.003
0.002
0.001
0.000
HG001
ΔclpX
clpXcompl
growth phase
exp
stat

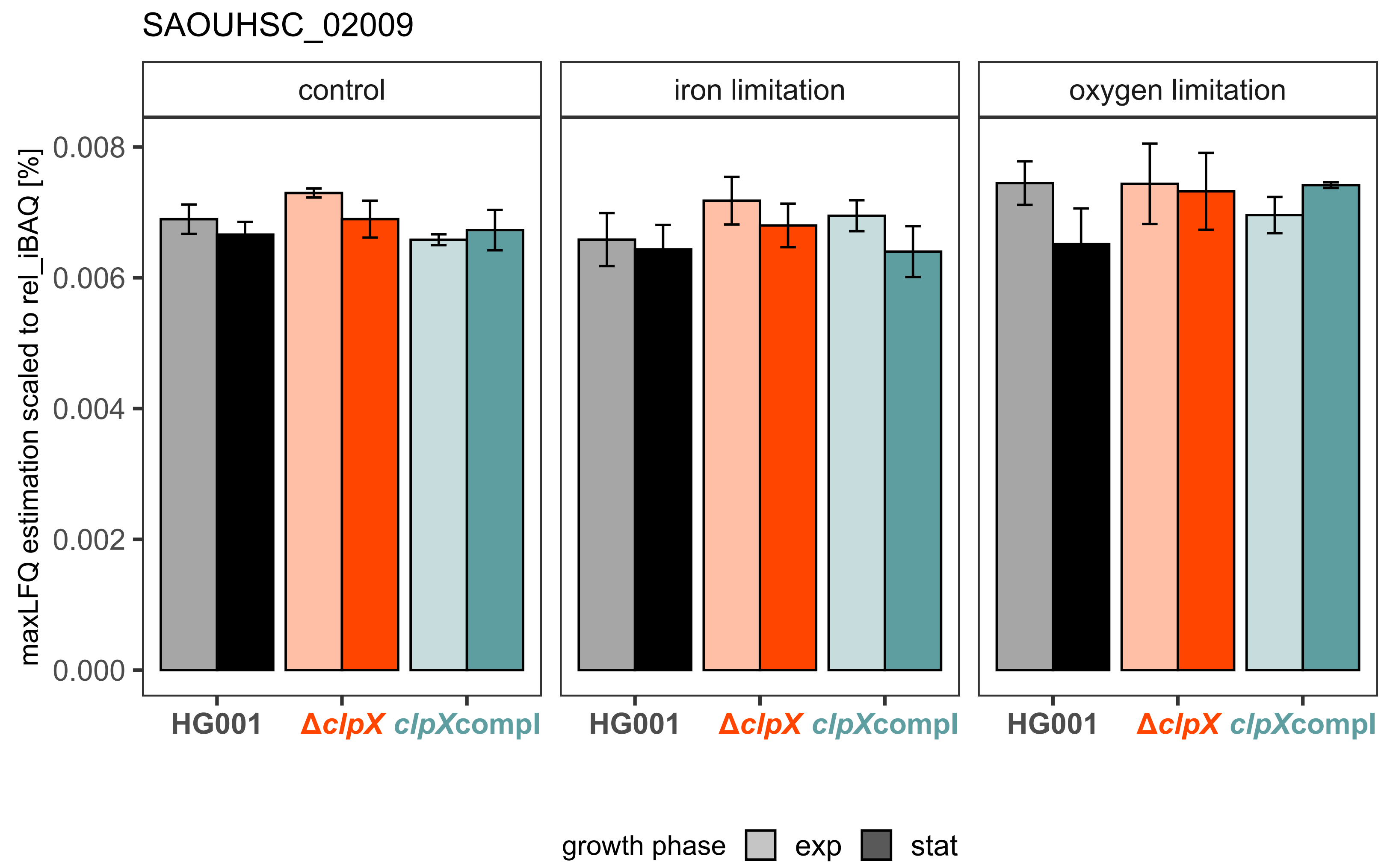
SAOUHSC_02009
control
iron limitation
oxygen limitation
maxLFQ estimation scaled to rel_iBAQ [%]
0.008
0.006
0.004
0.002
0.000
HG001
ΔclpX
clpXcompl
growth phase
exp
stat

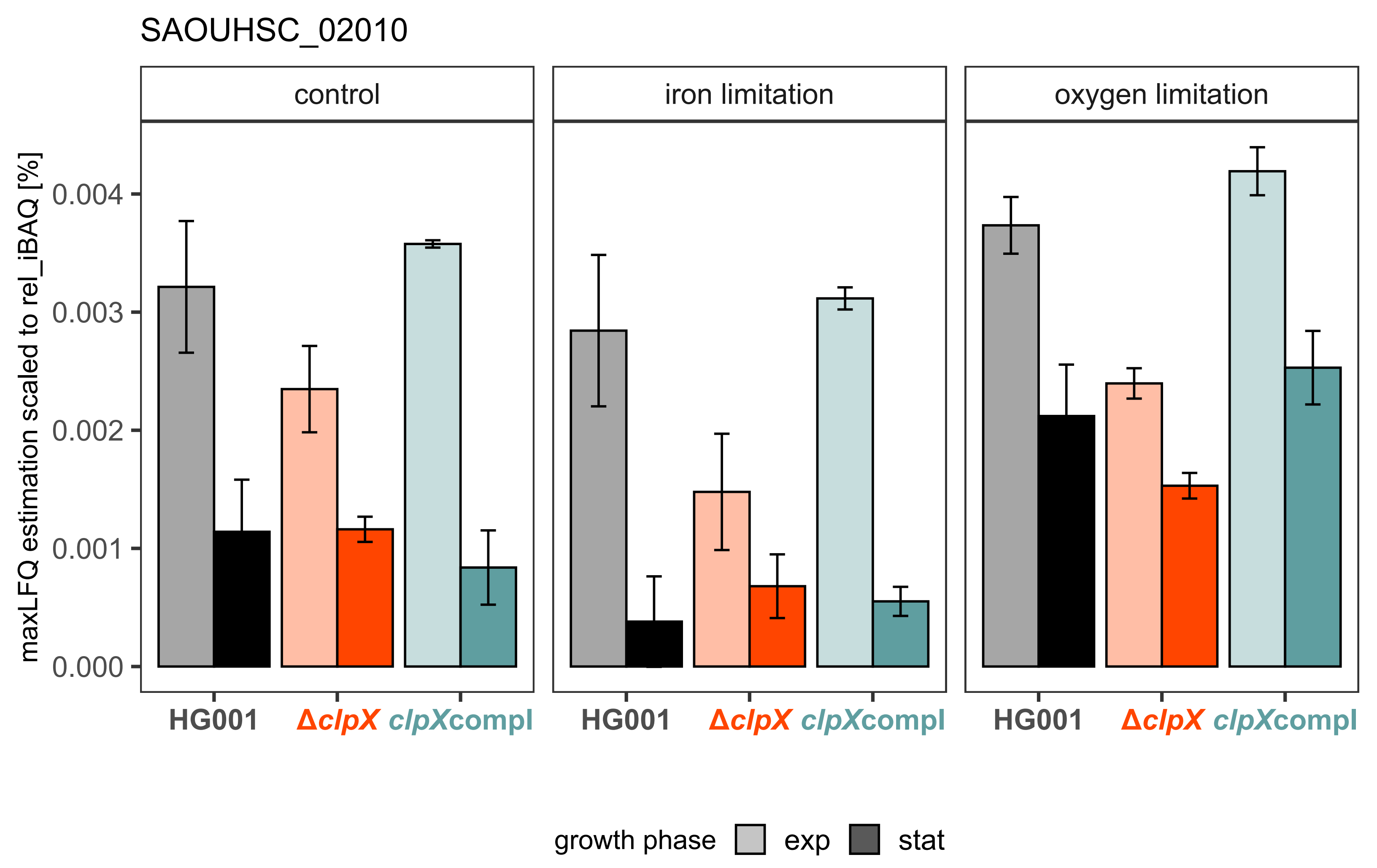
SAOUHSC_02010
control
iron limitation
oxygen limitation
maxLFQ estimation scaled to rel_iBAQ [%]
0.004
0.003
0.002
0.001
0.000
HG001
ΔclpX
clpXcompl
growth phase
exp
stat

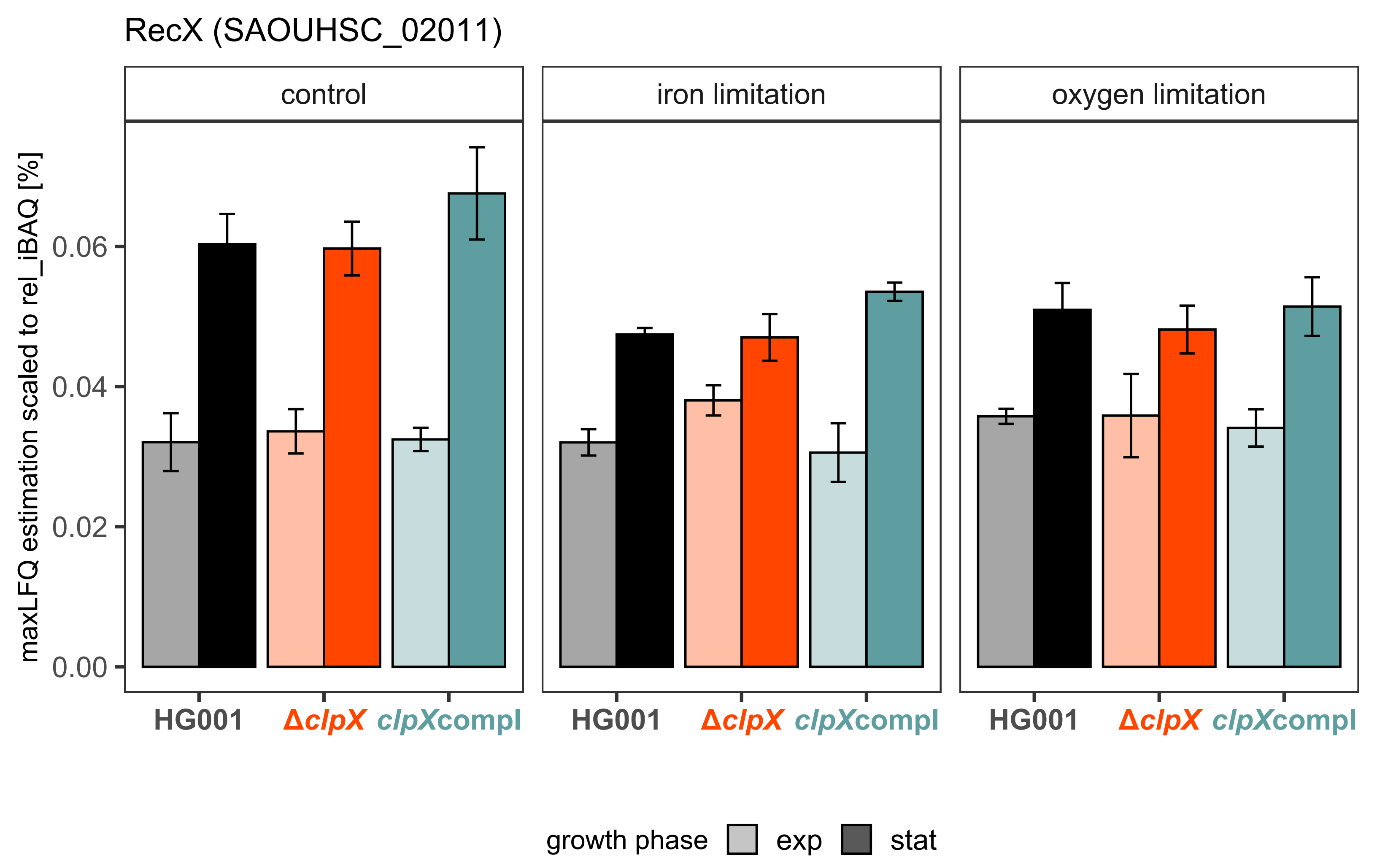
RecX (SAOUHSC_02011)
control
iron limitation
oxygen limitation
maxLFQ estimation scaled to rel_iBAQ [%]
0.00
0.02
0.04
0.06
HG001
ΔclpX
clpXcompl
HG001
ΔclpX
clpXcompl
HG001
ΔclpX
clpXcompl
growth phase
exp
stat

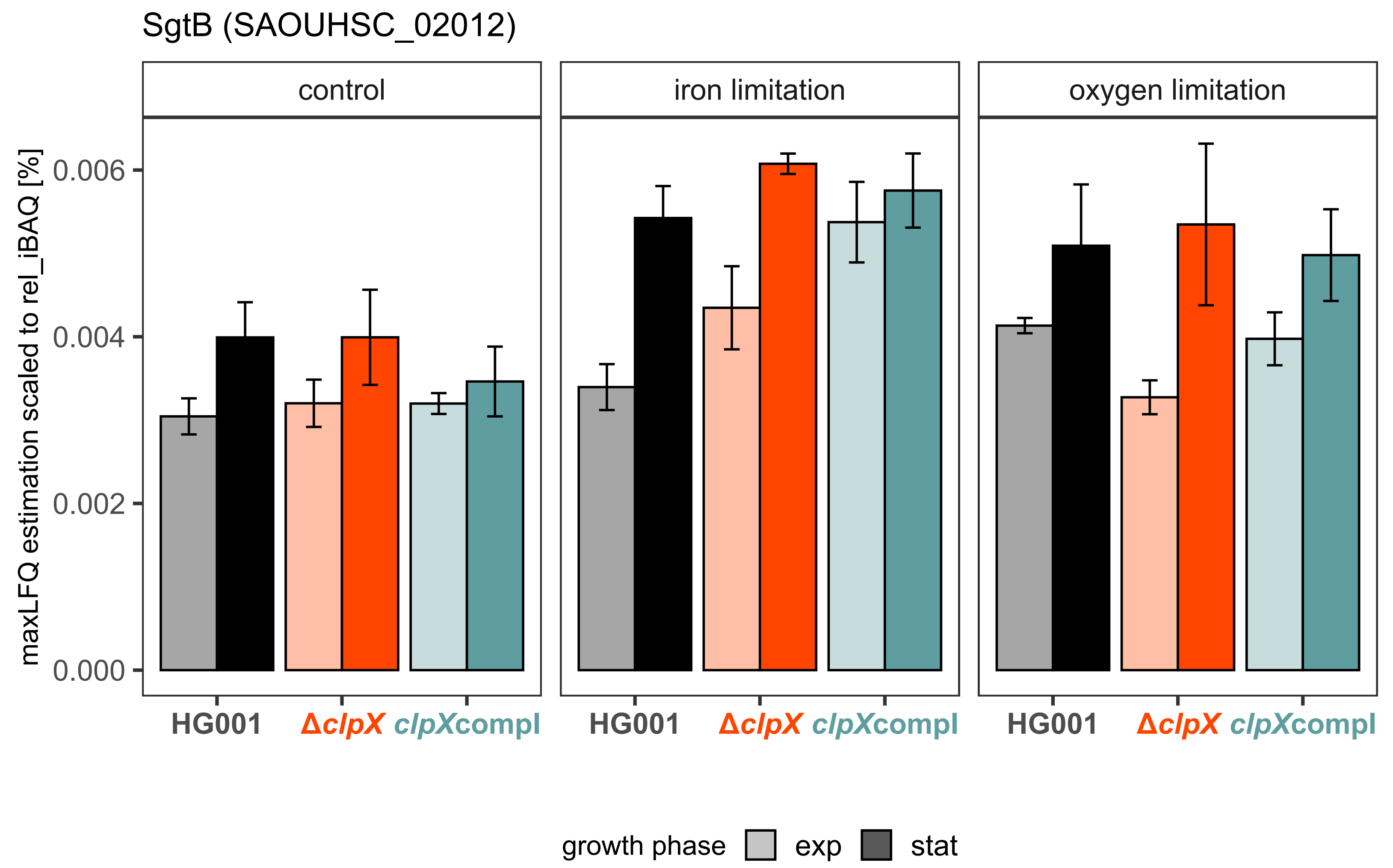
SgtB (SAOUHSC_02012)
control
iron limitation
oxygen limitation
maxLFQ estimation scaled to rel_iBAQ [%]
0.006
0.004
0.002
0.000
HG001
ΔclpX
clpXcompl
growth phase
exp
stat

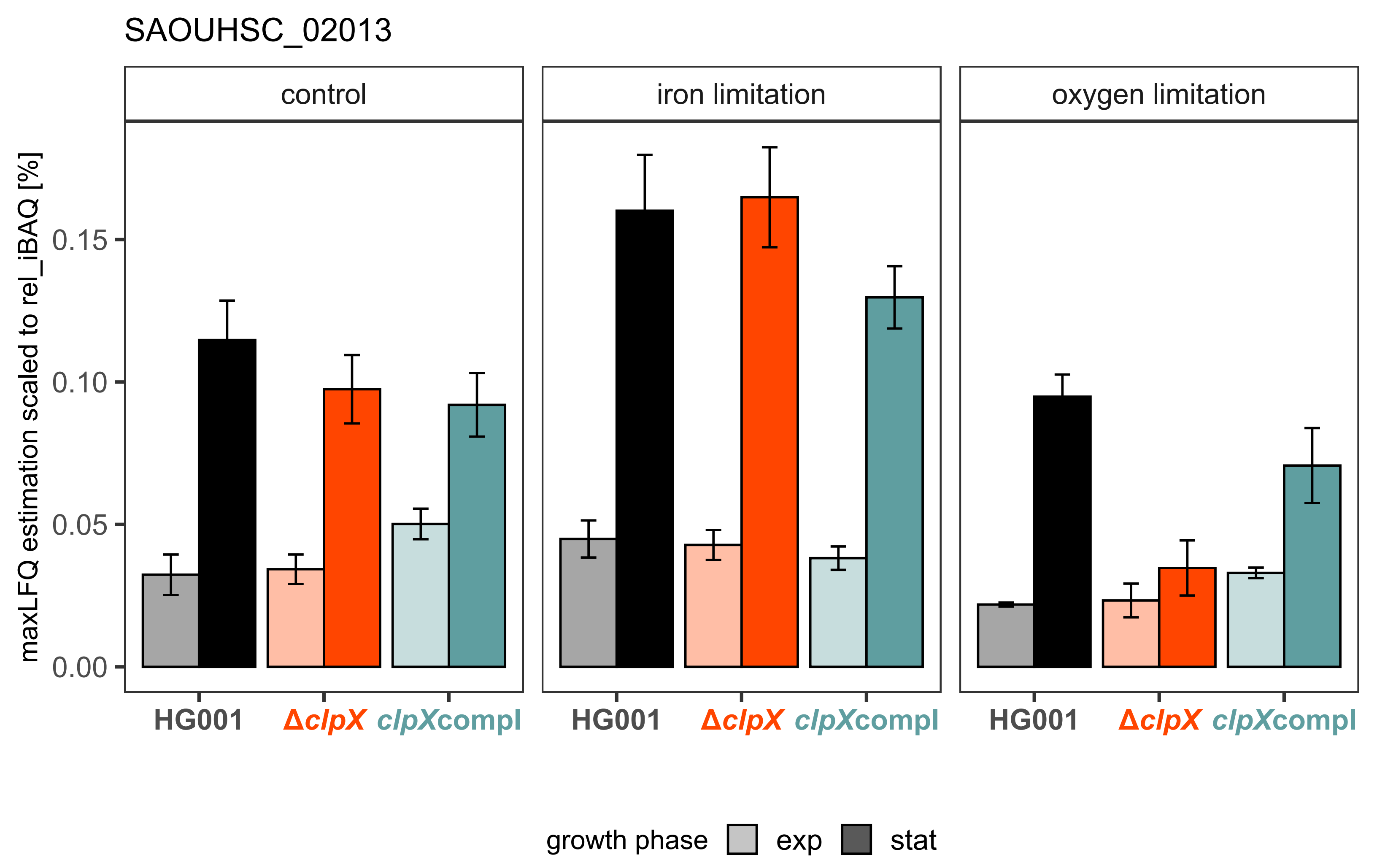
SAOUHSC_02013
control
iron limitation
oxygen limitation
maxLFQ estimation scaled to rel_iBAQ [%]
0.00
0.05
0.10
0.15
HG001
ΔclpX
clpXcompl
growth phase
exp
stat

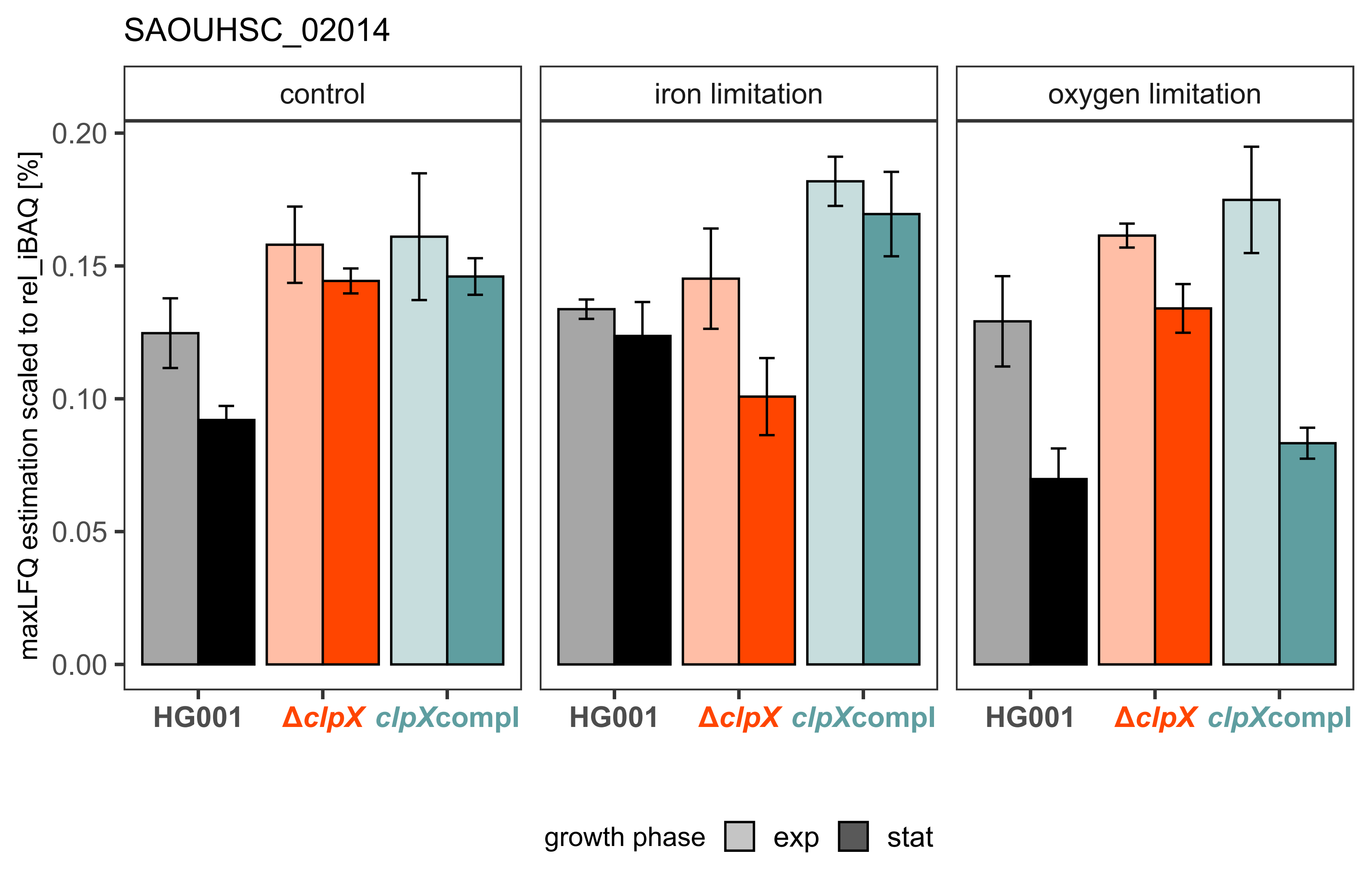

SAOUHSC_02014
control
iron limitation
oxygen limitation
maxLFQ estimation scaled to rel_iBAQ [%]
0.00
0.05
0.10
0.15
0.20
HG001
ΔclpX
clpXcompl
growth phase
exp
stat

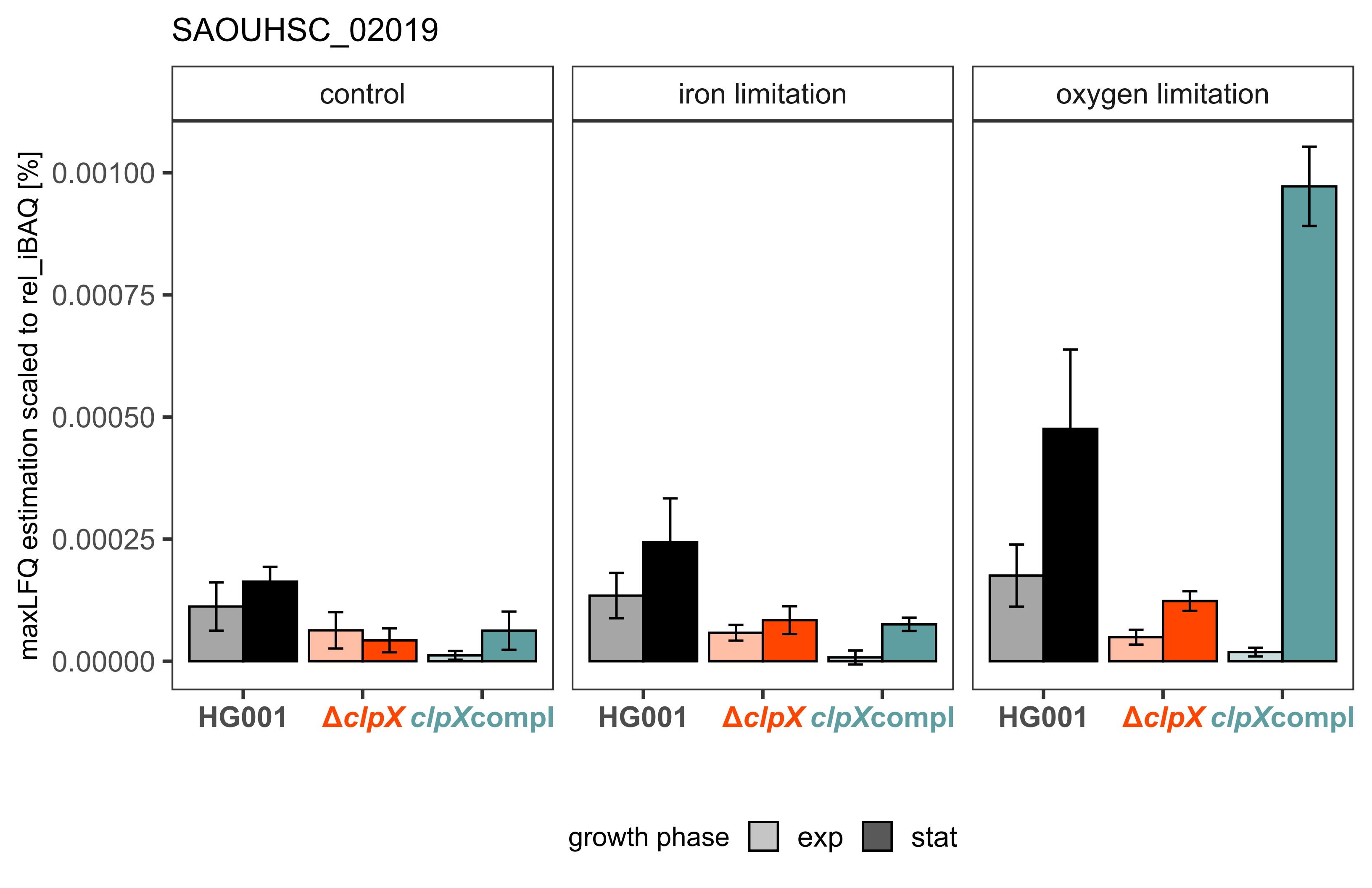

SAOUHSC_02019
control
iron limitation
oxygen limitation
maxLFQ estimation scaled to rel_iBAQ [%]
0.00100
0.00075
0.00050
0.00025
0.00000
HG001
ΔclpX
clpXcompl
growth phase
exp
stat

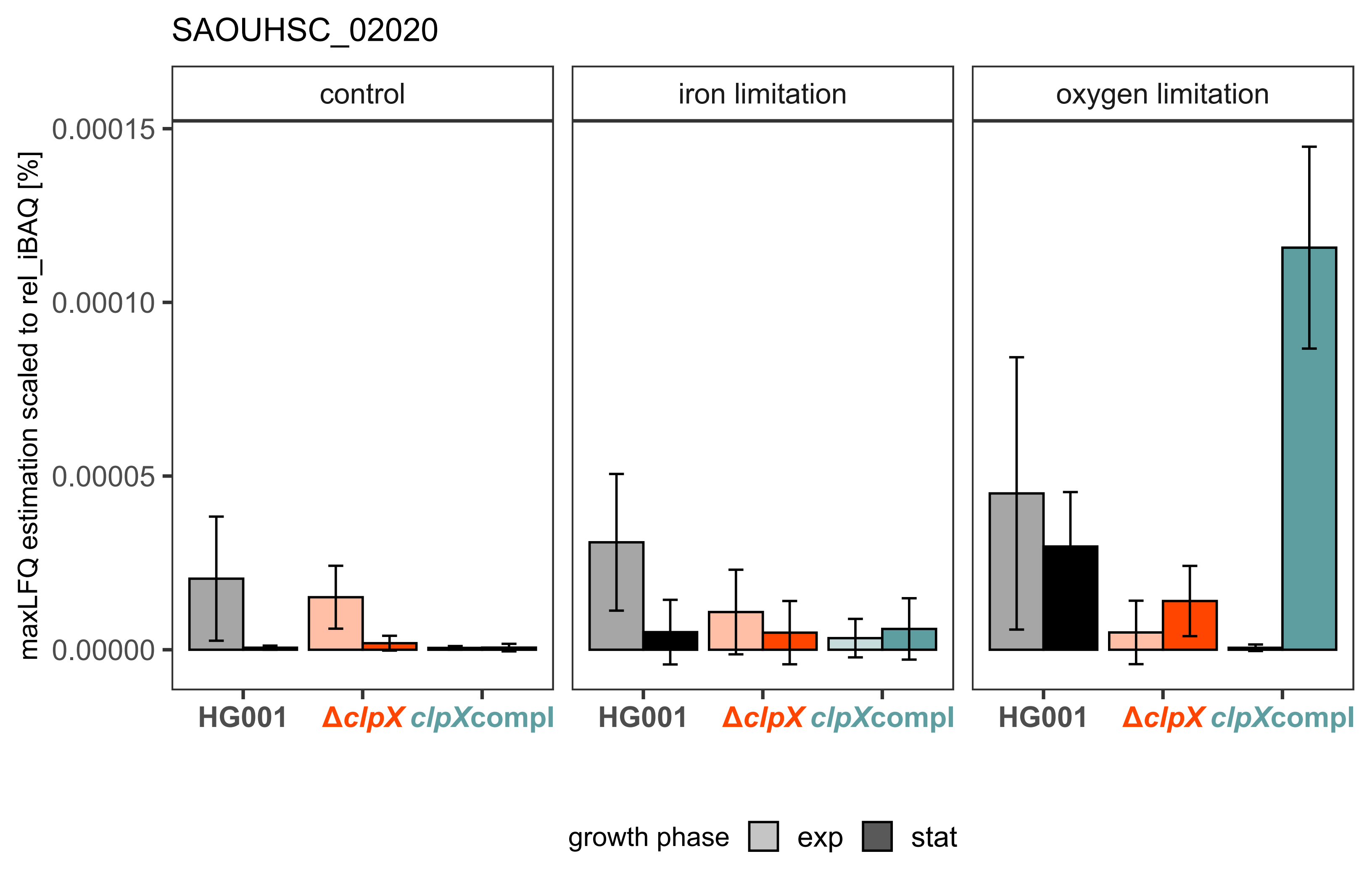

SAOUHSC_02020
control
iron limitation
oxygen limitation
maxLFQ estimation scaled to rel_iBAQ [%]
0.00015
0.00010
0.00005
0.00000
HG001
ΔclpX
clpXcompl
growth phase
exp
stat

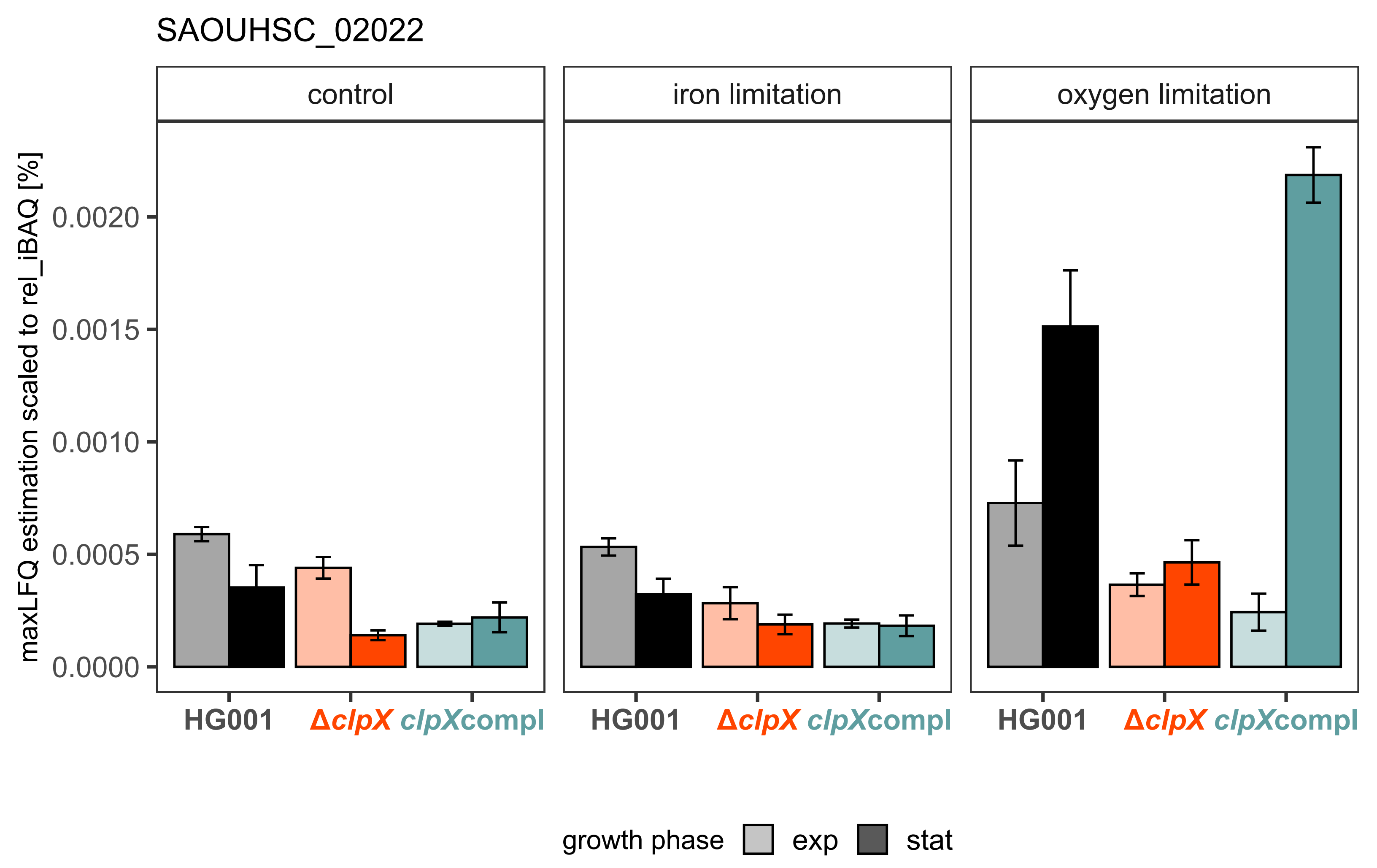
SAOUHSC_02022
control
iron limitation
oxygen limitation
maxLFQ estimation scaled to rel_iBAQ [%]
0.0020
0.0015
0.0010
0.0005
0.0000
HG001
ΔclpX
clpXcompl
growth phase
exp
stat

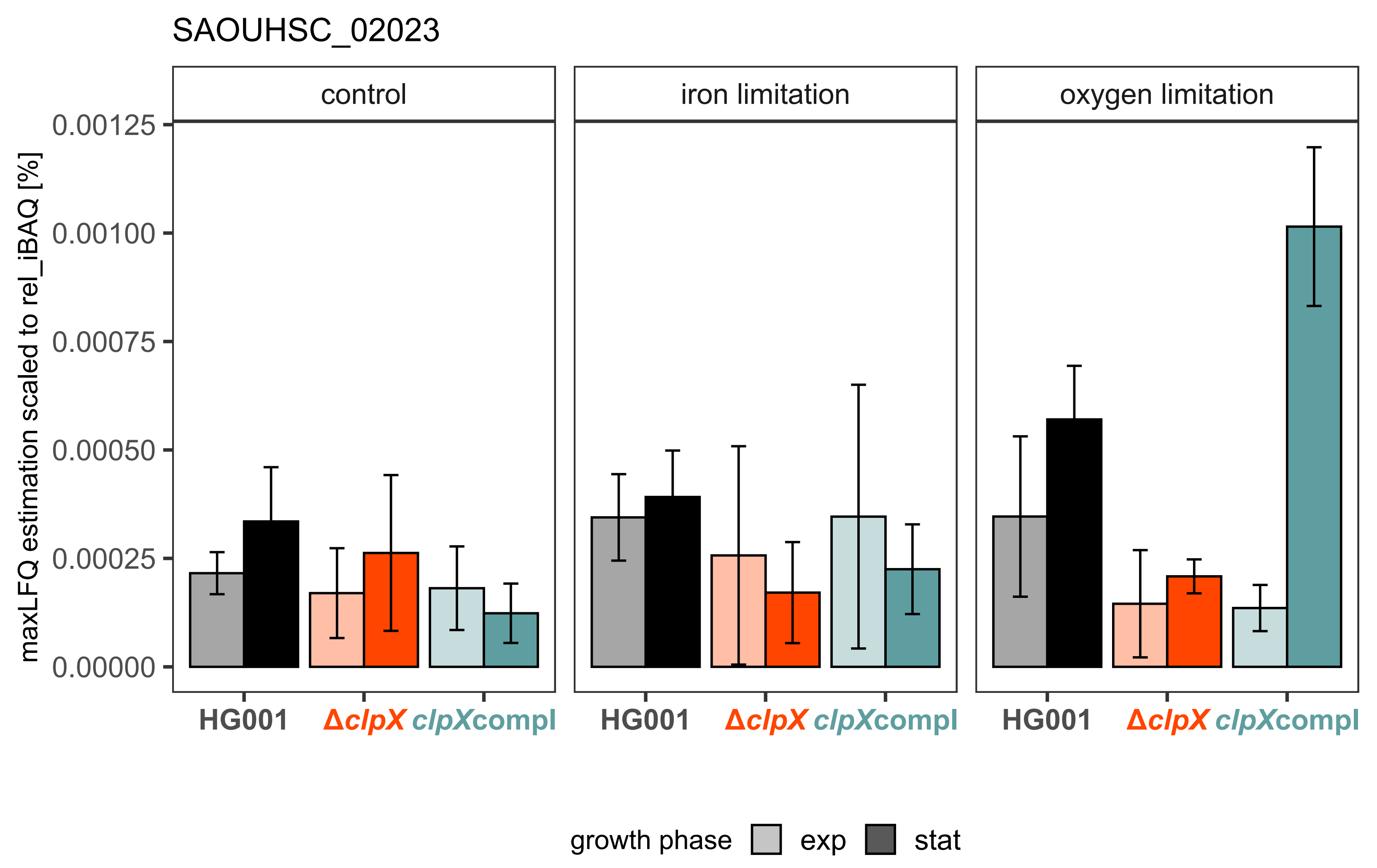
SAOUHSC_02023
control
iron limitation
oxygen limitation
maxLFQ estimation scaled to rel_iBAQ [%]
0.00125
0.00100
0.00075
0.00050
0.00025
0.00000
HG001
ΔclpX
clpXcompl
growth phase
exp
stat

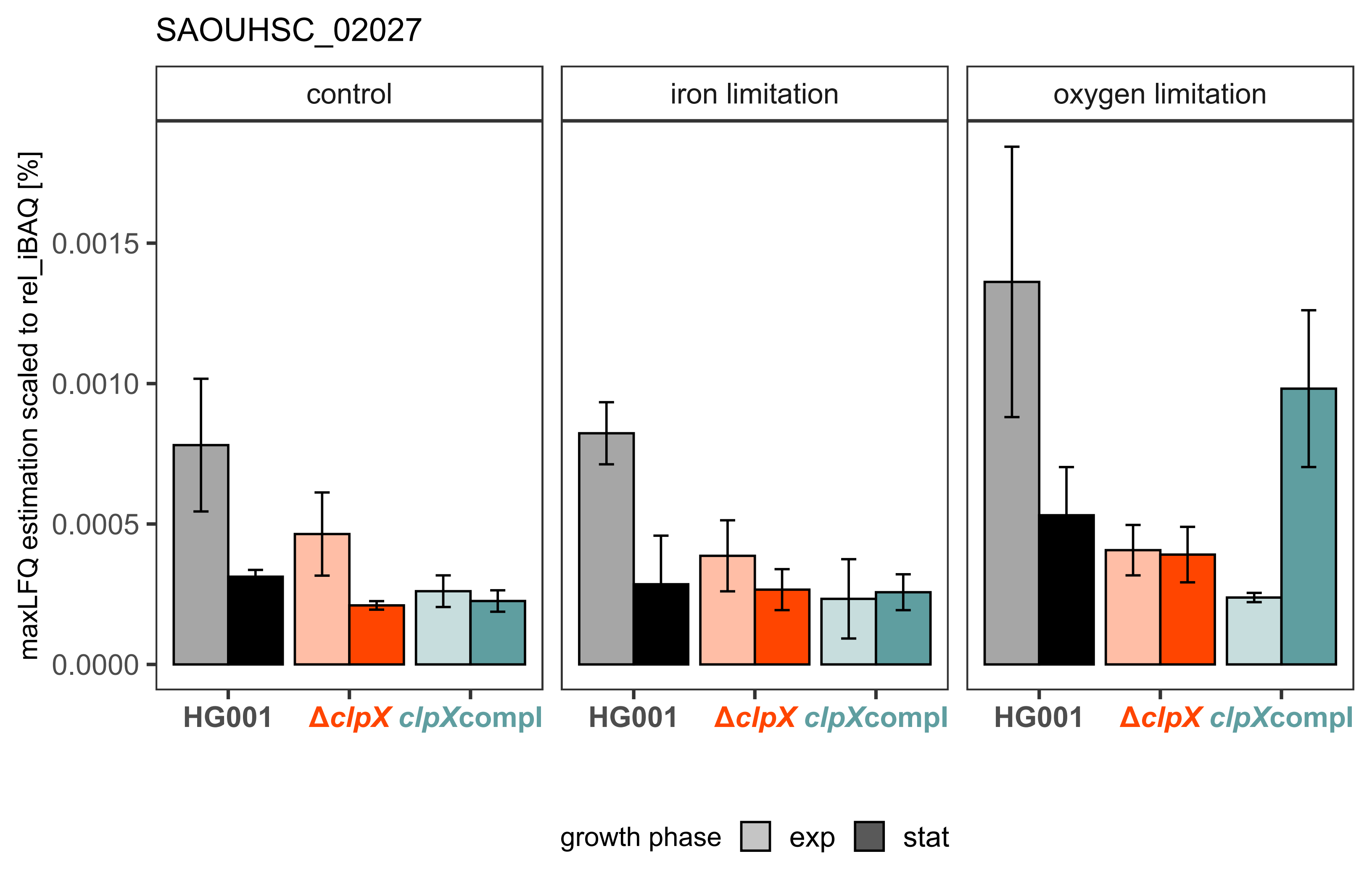
SAOUHSC_02027
control
iron limitation
oxygen limitation
maxLFQ estimation scaled to rel_iBAQ [%]
0.0015
0.0010
0.0005
0.0000
HG001
ΔclpX
clpXcompl
growth phase
exp
stat

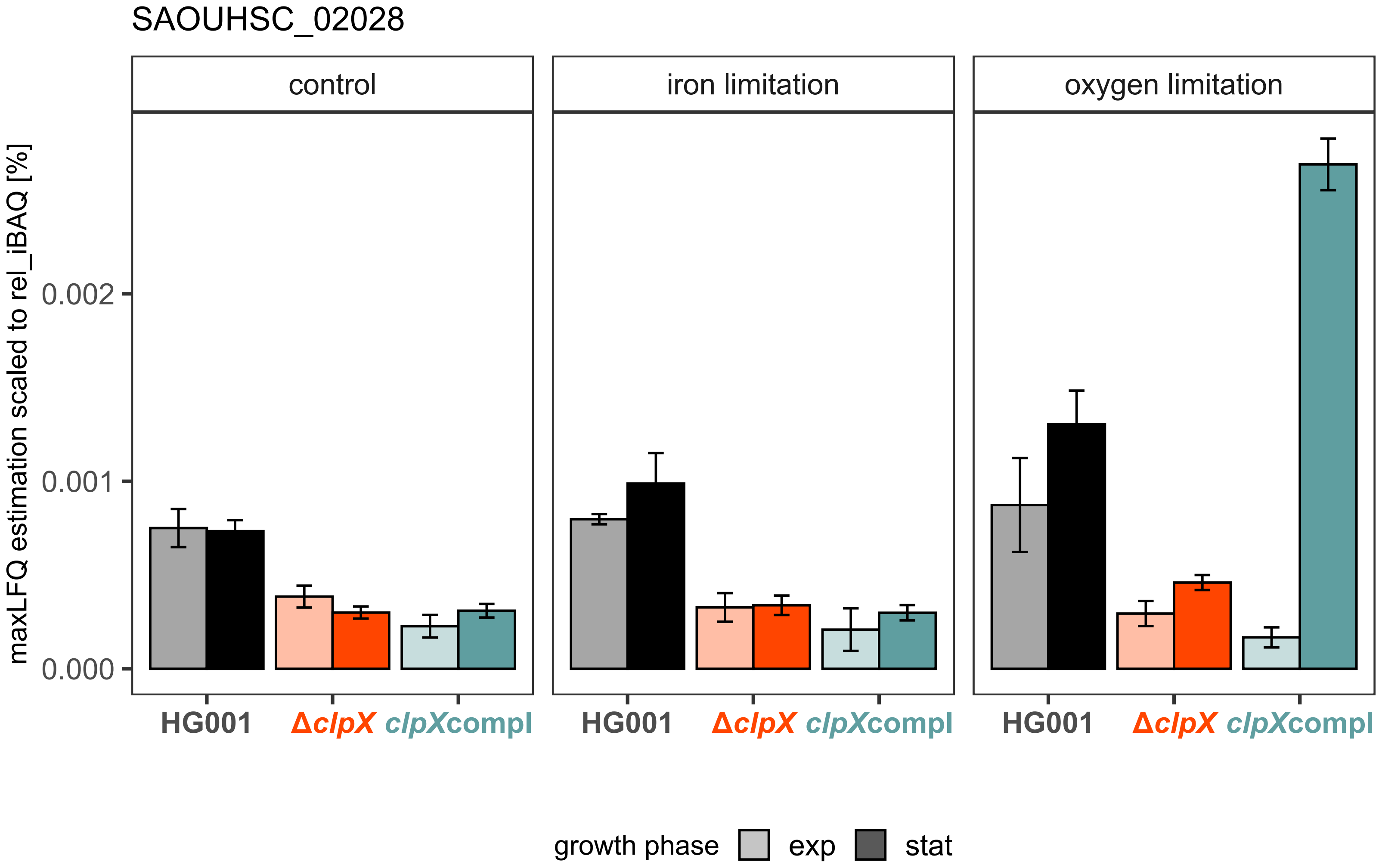
SAOUHSC_02028
control
iron limitation
oxygen limitation
maxLFQ estimation scaled to rel_iBAQ [%]
0.000
0.001
0.002
HG001
ΔclpX
clpXcompl
growth phase
exp
stat

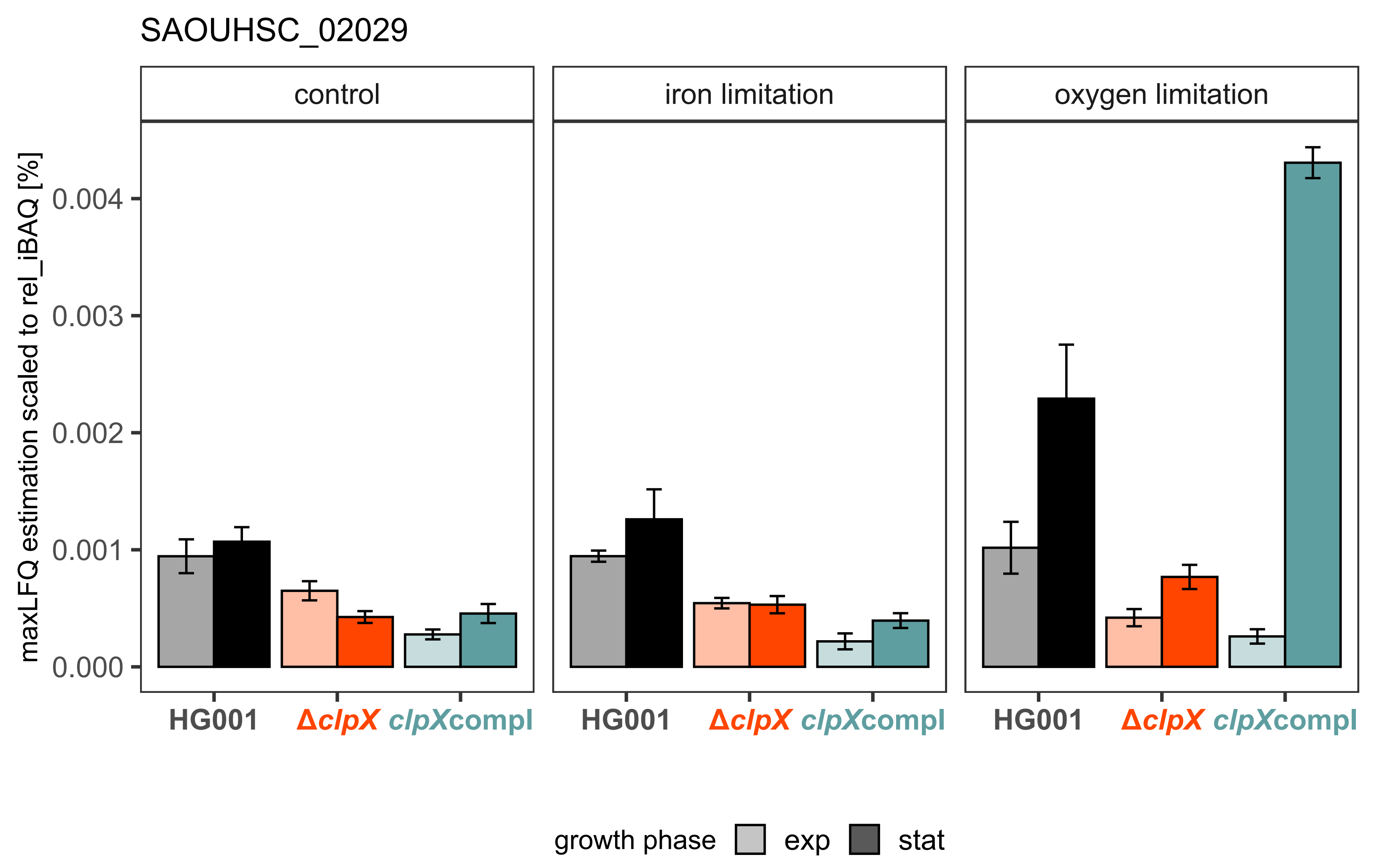

SAOUHSC_02029
control
iron limitation
oxygen limitation
maxLFQ estimation scaled to rel_iBAQ [%]
0.000
0.001
0.002
0.003
0.004
HG001
ΔclpX
clpXcompl
growth phase
exp
stat

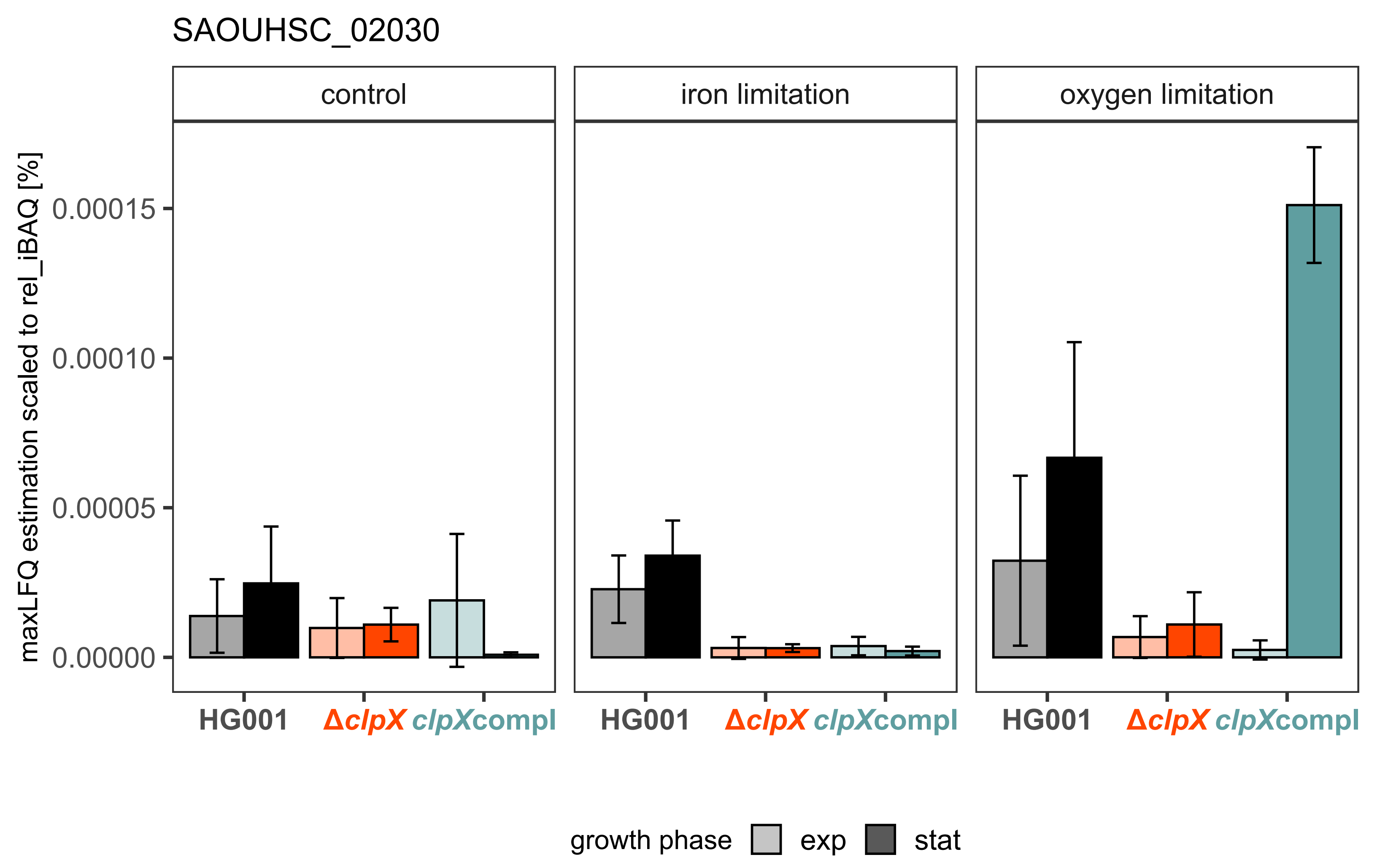

SAOUHSC_02030
control
iron limitation
oxygen limitation
maxLFQ estimation scaled to rel_iBAQ [%]
0.00015
0.00010
0.00005
0.00000
HG001
ΔclpX
clpXcompl
growth phase
exp
stat

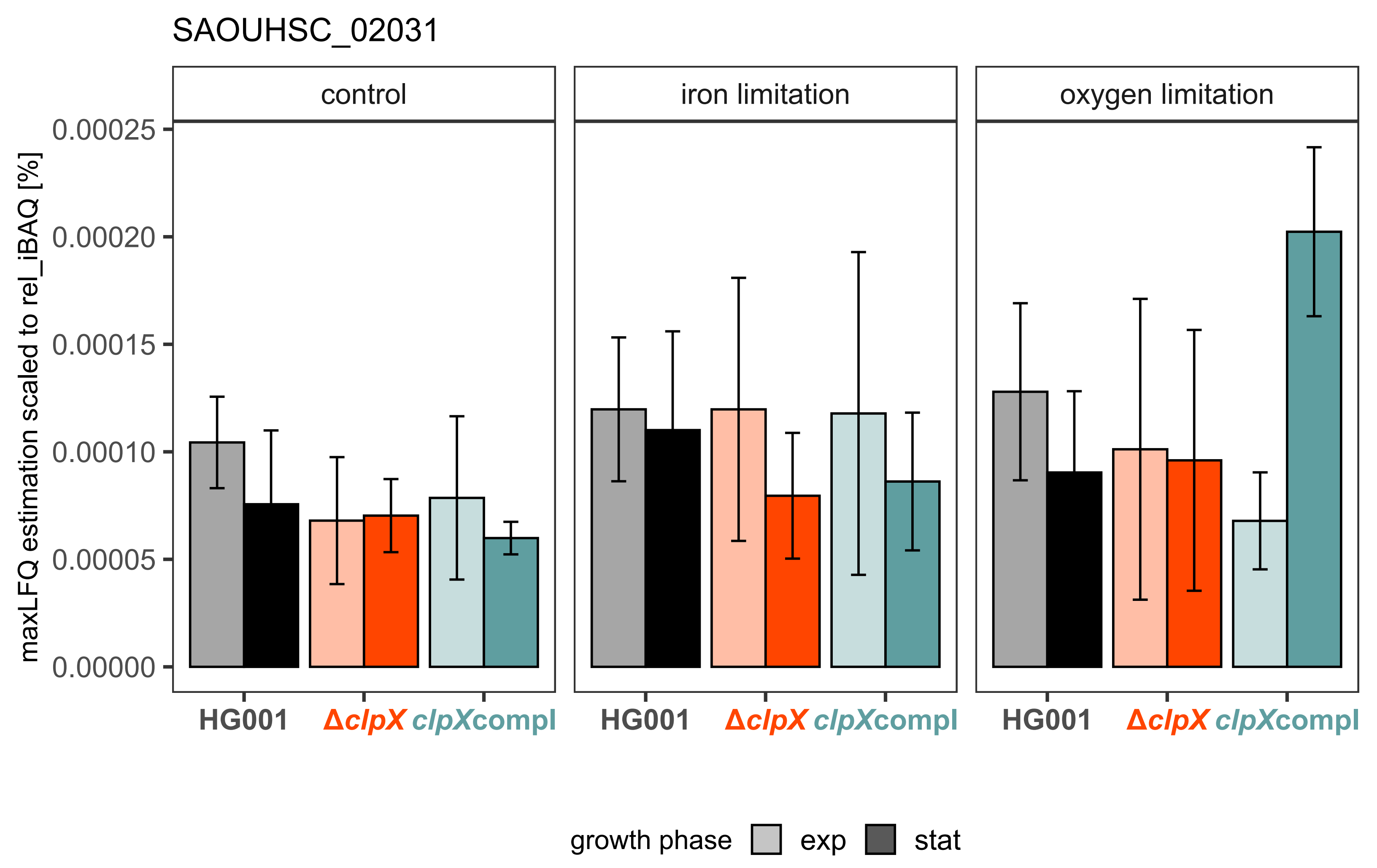

SAOUHSC_02031
control
iron limitation
oxygen limitation
0.00025
0.00020
0.00015
0.00010
0.00005
0.00000
maxLFQ estimation scaled to rel_iBAQ [%]
HG001
ΔclpX
clpXcompl
growth phase
exp
stat

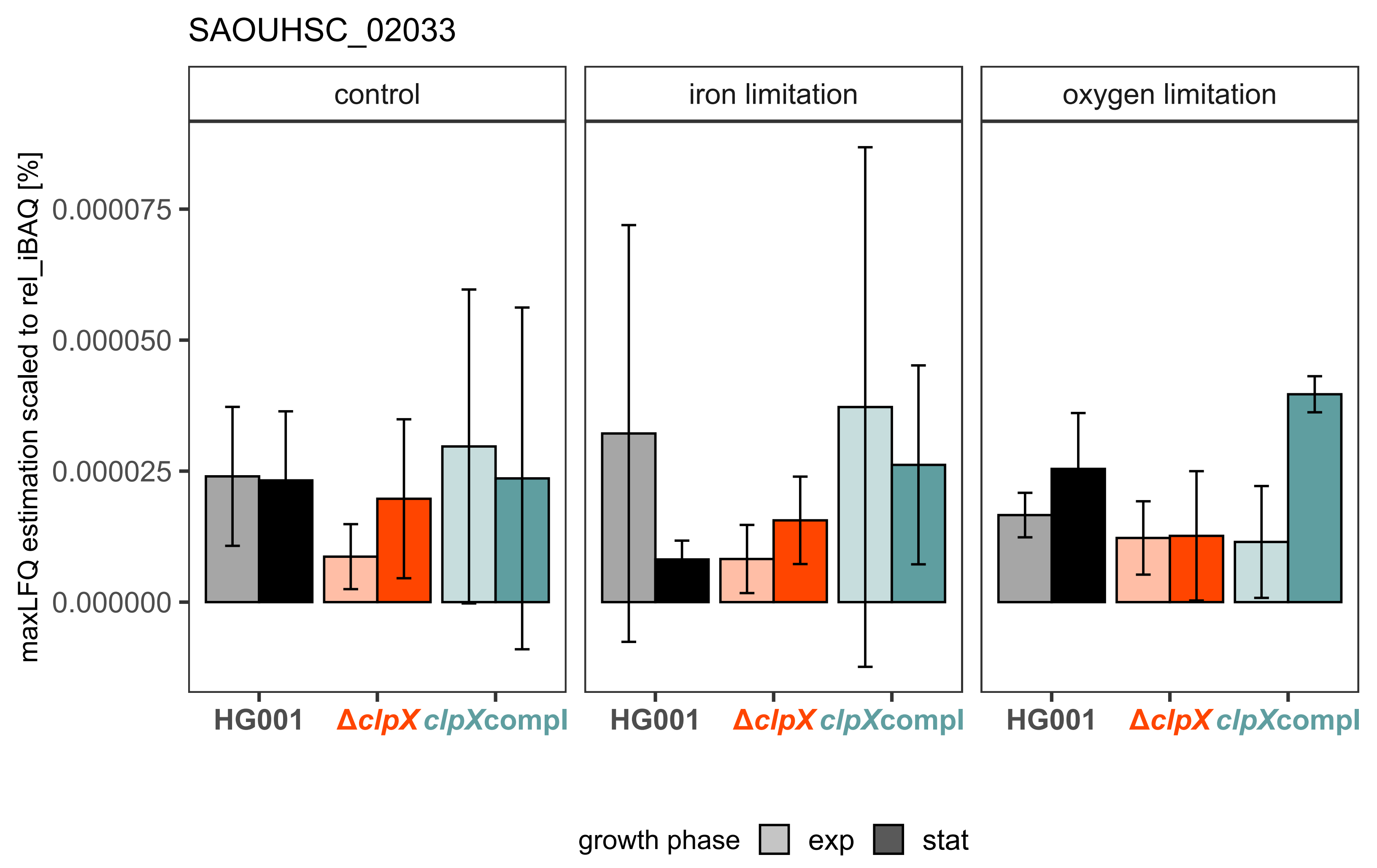

SAOUHSC_02033
control
iron limitation
oxygen limitation
maxLFQ estimation scaled to rel_iBAQ [%]
0.000075
0.000050
0.000025
0.000000
HG001
ΔclpX
clpXcompl
growth phase
exp
stat

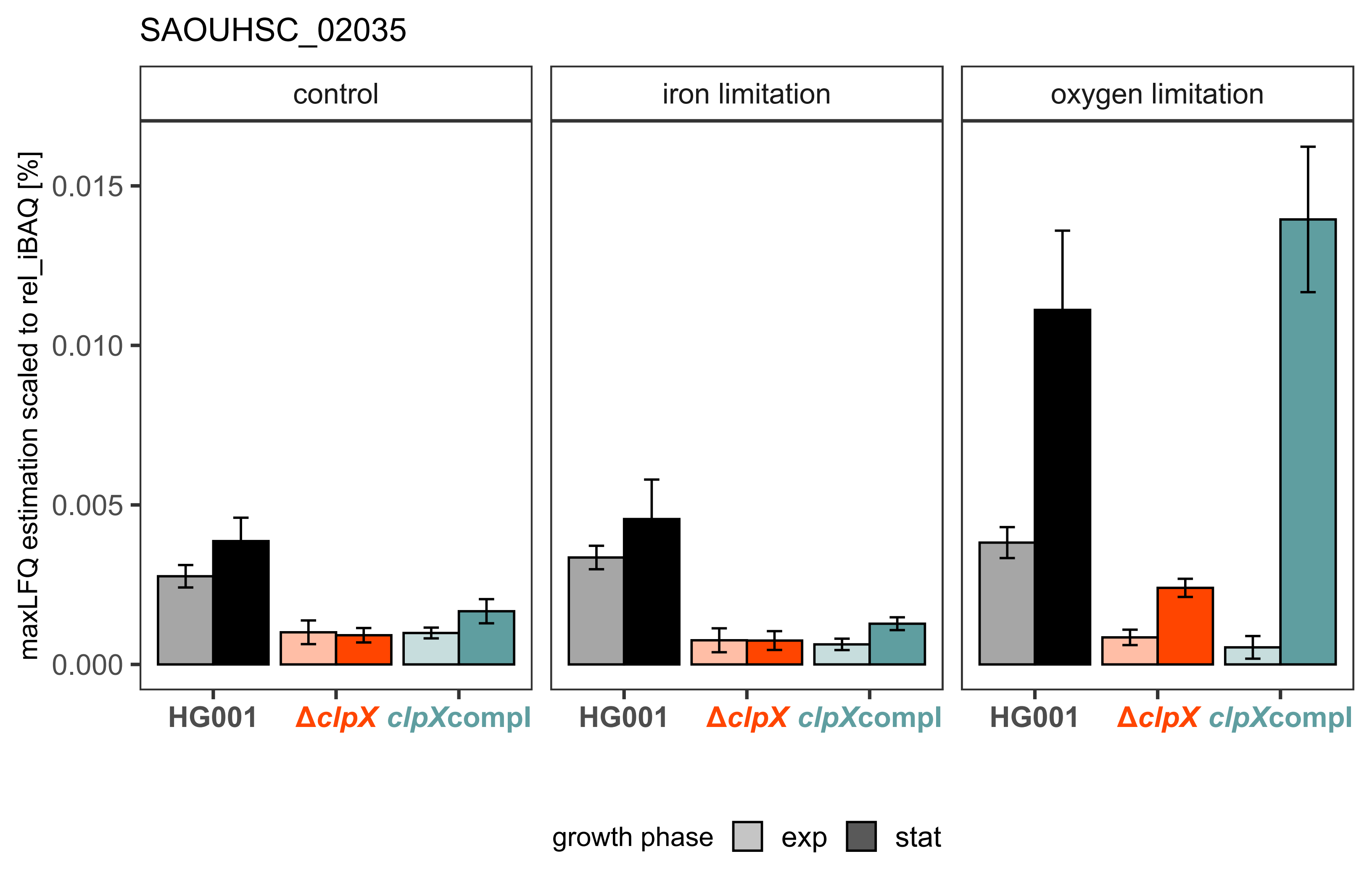
SAOUHSC_02035
control
iron limitation
oxygen limitation
maxLFQ estimation scaled to rel_iBAQ [%]
0.015
0.010
0.005
0.000
HG001
ΔclpX
clpXcompl
growth phase
exp
stat

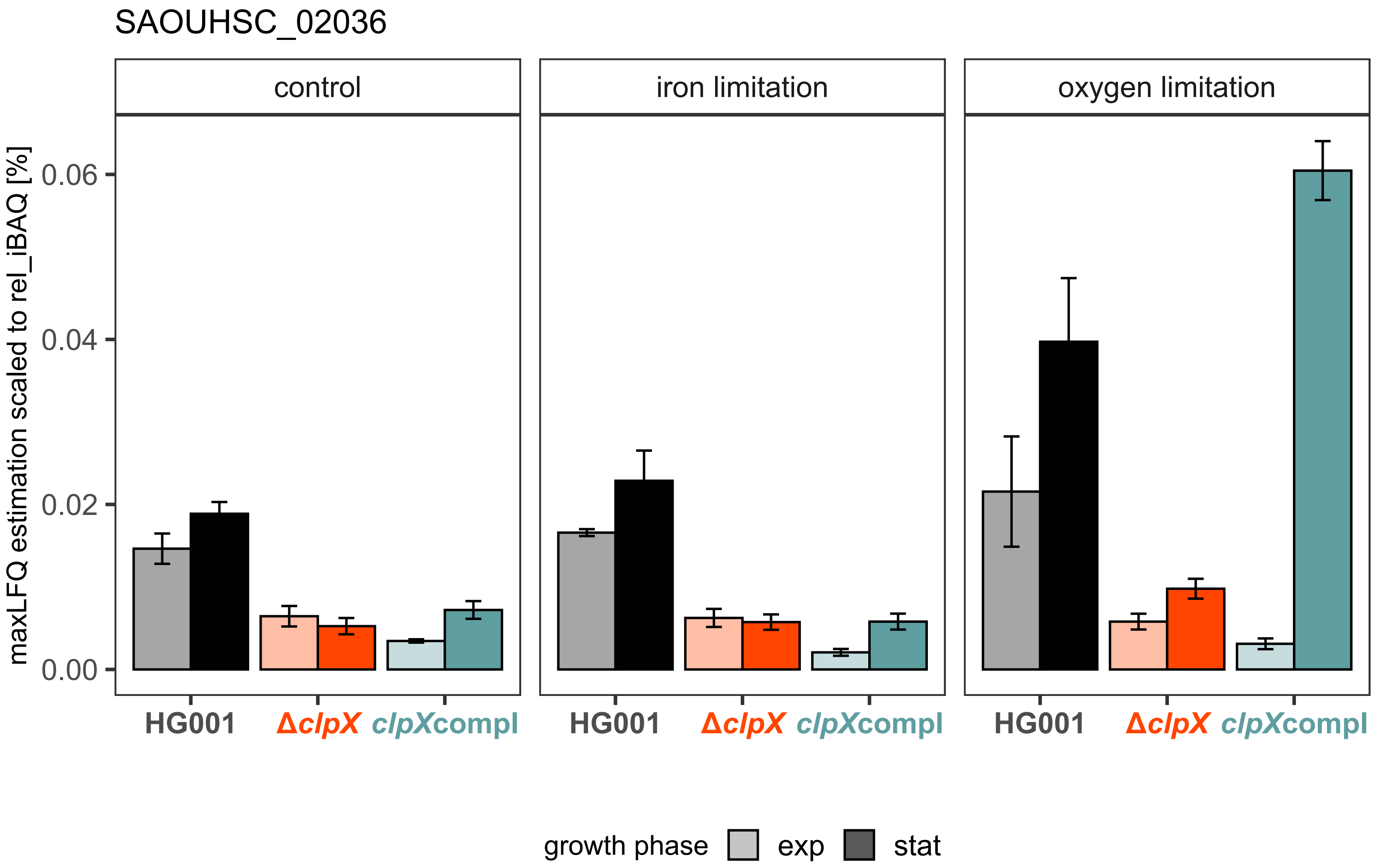
SAOUHSC_02036
control
iron limitation
oxygen limitation
maxLFQ estimation scaled to rel_iBAQ [%]
0.06
0.04
0.02
0.00
HG001
ΔclpX
clpXcompl
growth phase
exp
stat

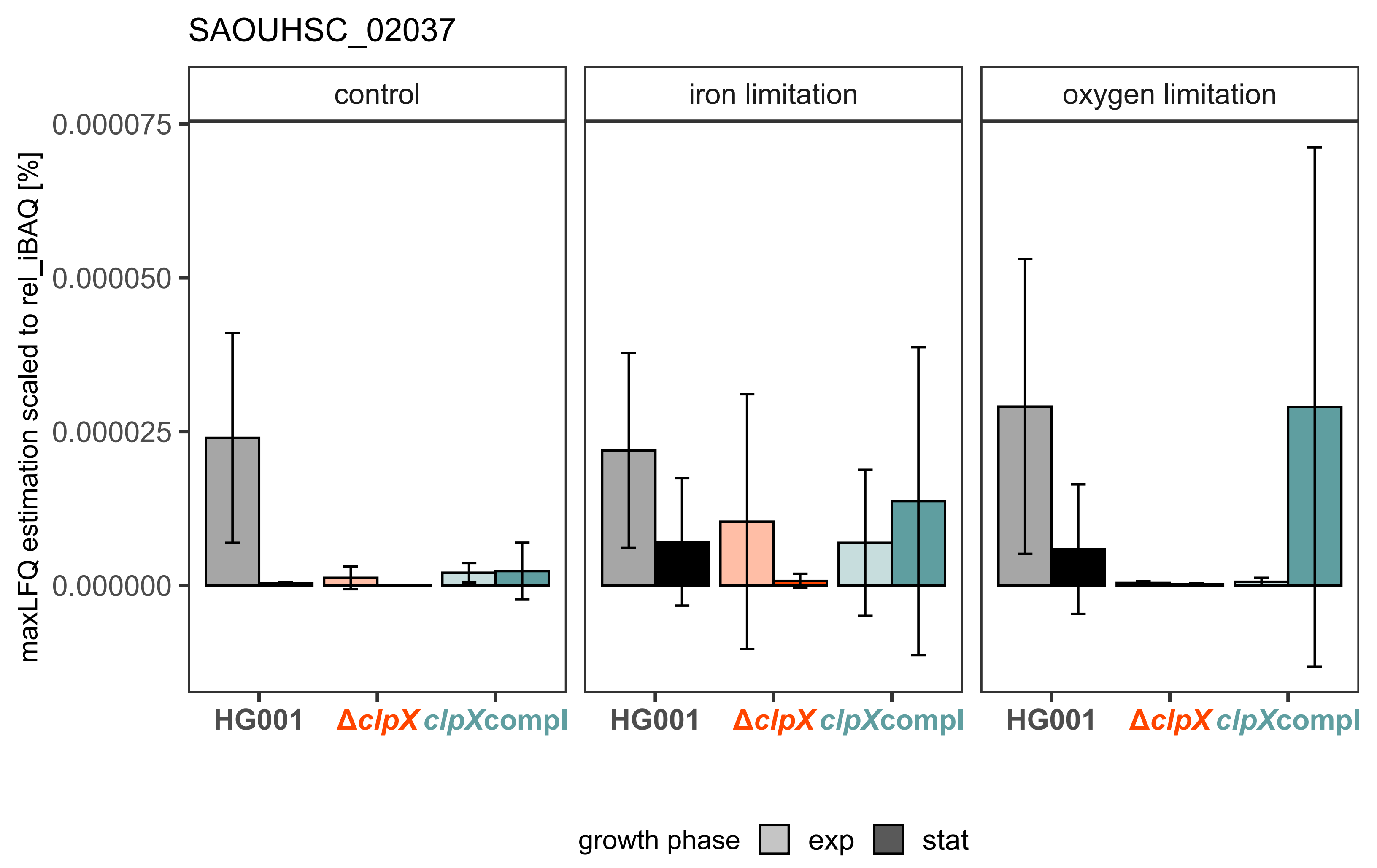
SAOUHSC_02037
control
iron limitation
oxygen limitation
0.000075
0.000050
0.000025
0.000000
maxLFQ estimation scaled to rel_iBAQ [%]
HG001
ΔclpX
clpXcompl
growth phase
exp
stat

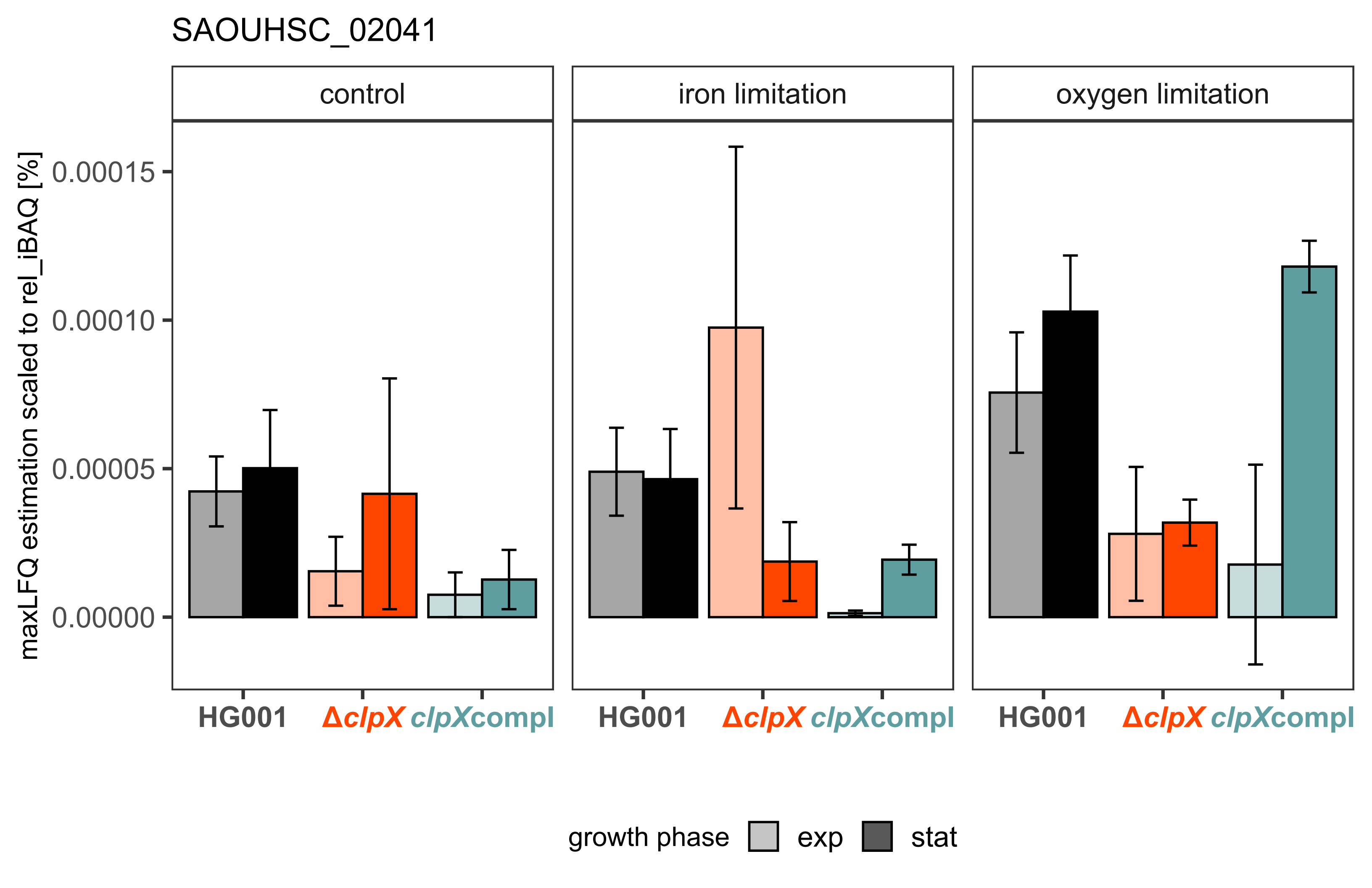
SAOUHSC_02041
control
iron limitation
oxygen limitation
maxLFQ estimation scaled to rel_iBAQ [%]
0.00015
0.00010
0.00005
0.00000
HG001
ΔclpX
clpXcompl
growth phase
exp
stat

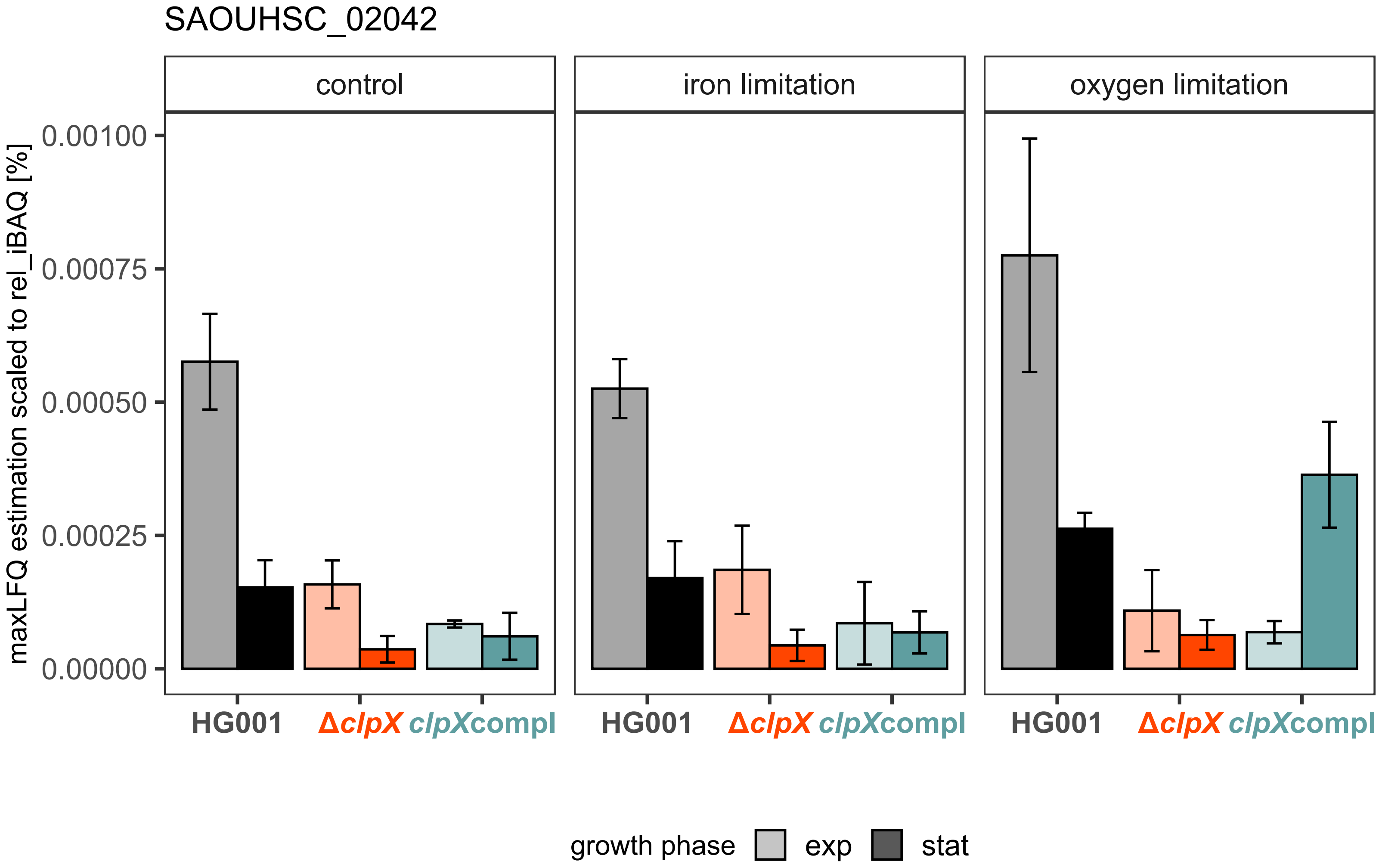

SAOUHSC_02042
control
iron limitation
oxygen limitation
maxLFQ estimation scaled to rel_iBAQ [%]
0.00100
0.00075
0.00050
0.00025
0.00000
HG001
ΔclpX
clpXcompl
growth phase
exp
stat

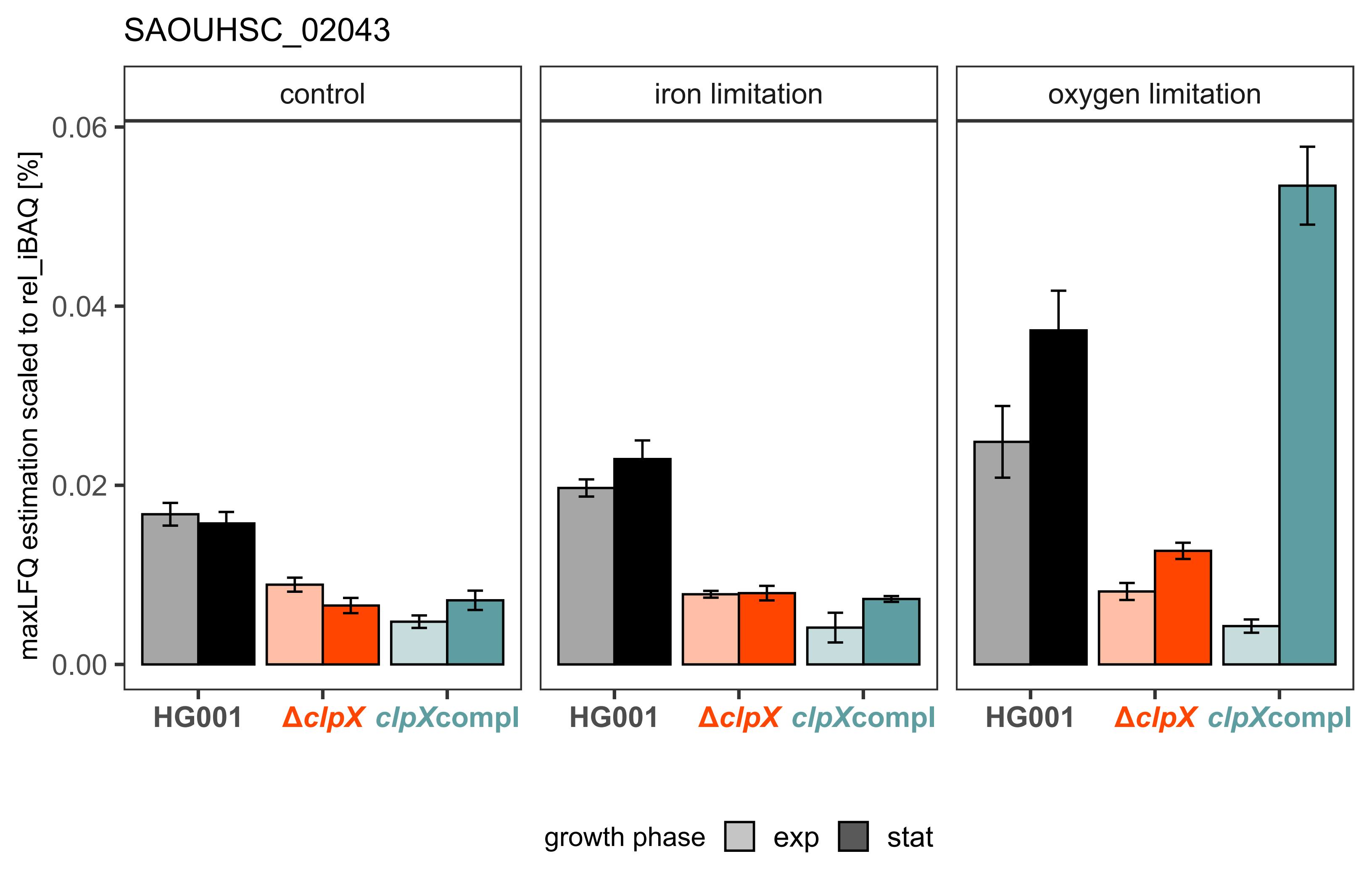
SAOUHSC_02043
control
iron limitation
oxygen limitation
maxLFQ estimation scaled to rel_iBAQ [%]
0.06
0.04
0.02
0.00
HG001
ΔclpX
clpXcompl
growth phase
exp
stat

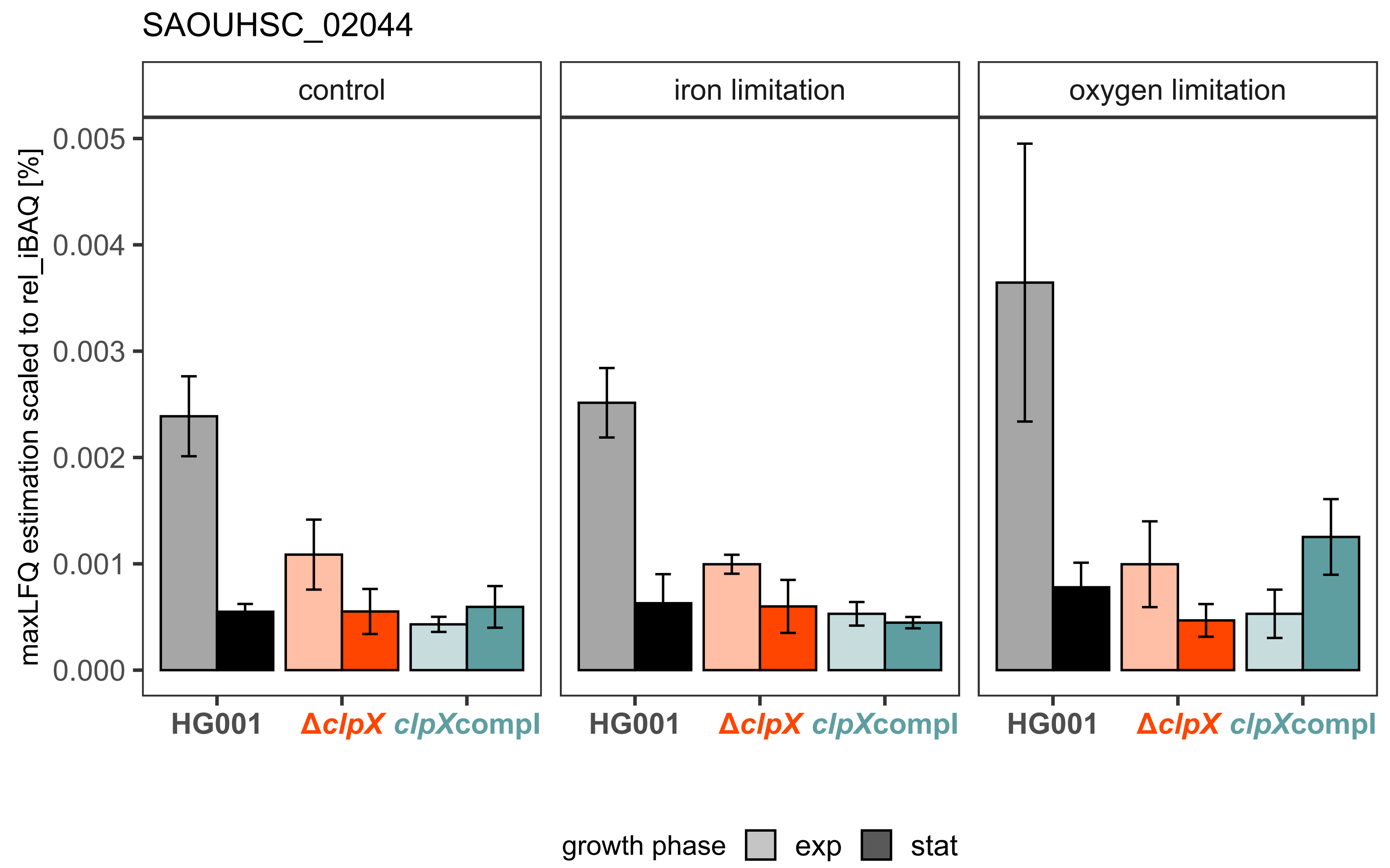
SAOUHSC_02044
control
iron limitation
oxygen limitation
maxLFQ estimation scaled to rel_iBAQ [%]
0.005
0.004
0.003
0.002
0.001
0.000
HG001
ΔclpX
clpXcompl
growth phase
exp
stat

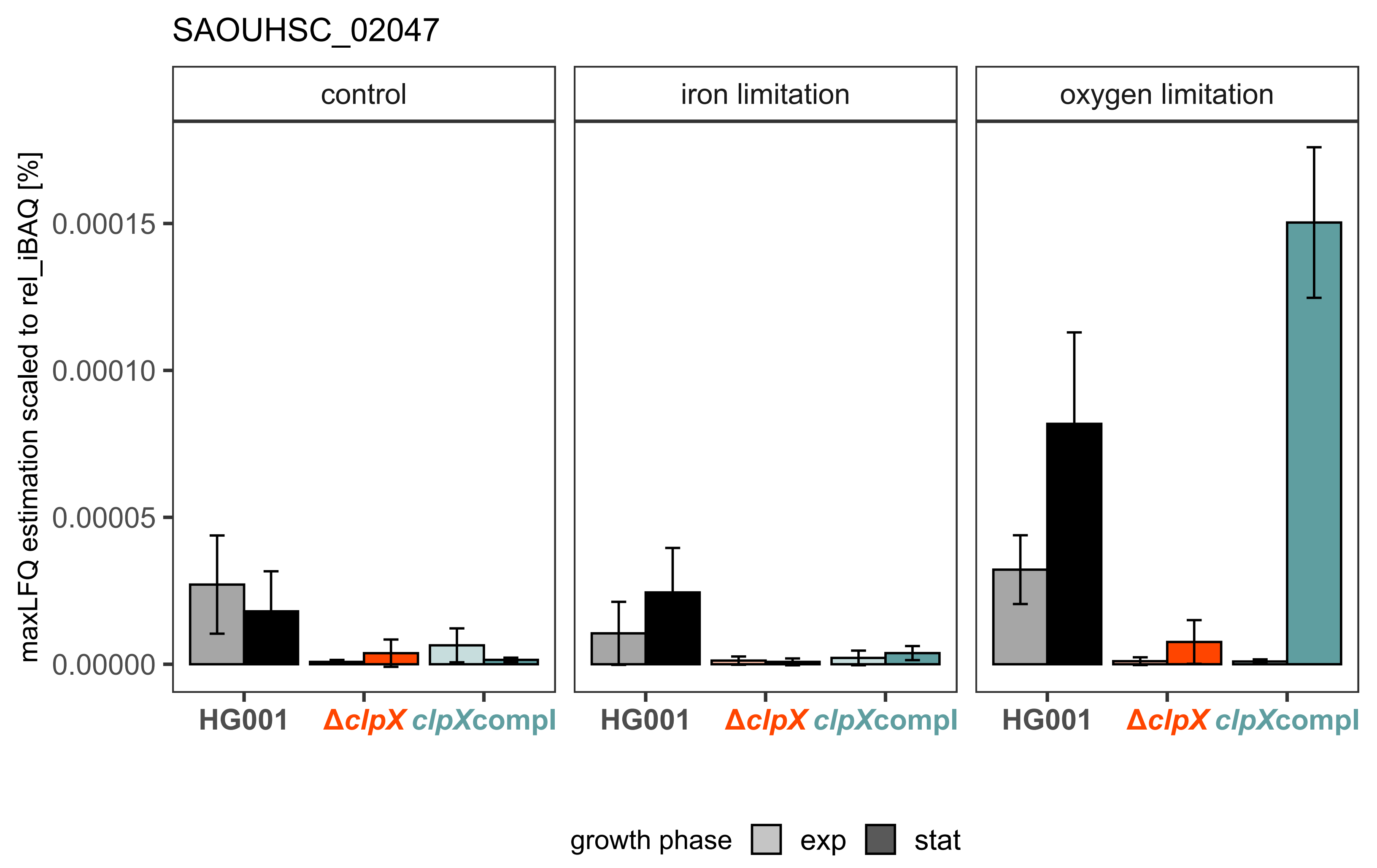
SAOUHSC_02047
control
iron limitation
oxygen limitation
maxLFQ estimation scaled to rel_iBAQ [%]
0.00015
0.00010
0.00005
0.00000
HG001
ΔclpX
clpXcompl
growth phase
exp
stat

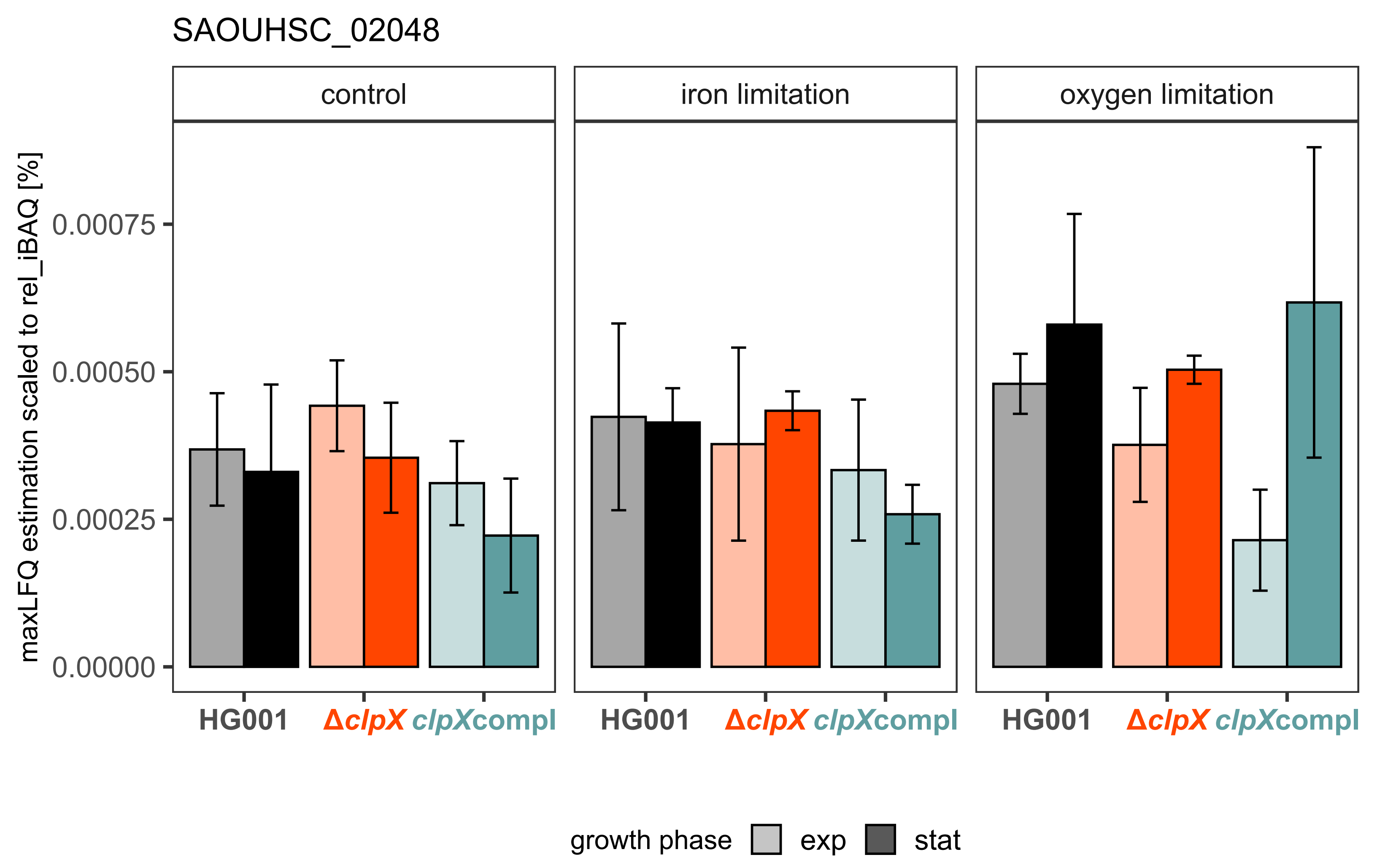

SAOUHSC_02048
control
iron limitation
oxygen limitation
maxLFQ estimation scaled to rel_iBAQ [%]
0.00075
0.00050
0.00025
0.00000
HG001
ΔclpX
clpXcompl
growth phase
exp
stat

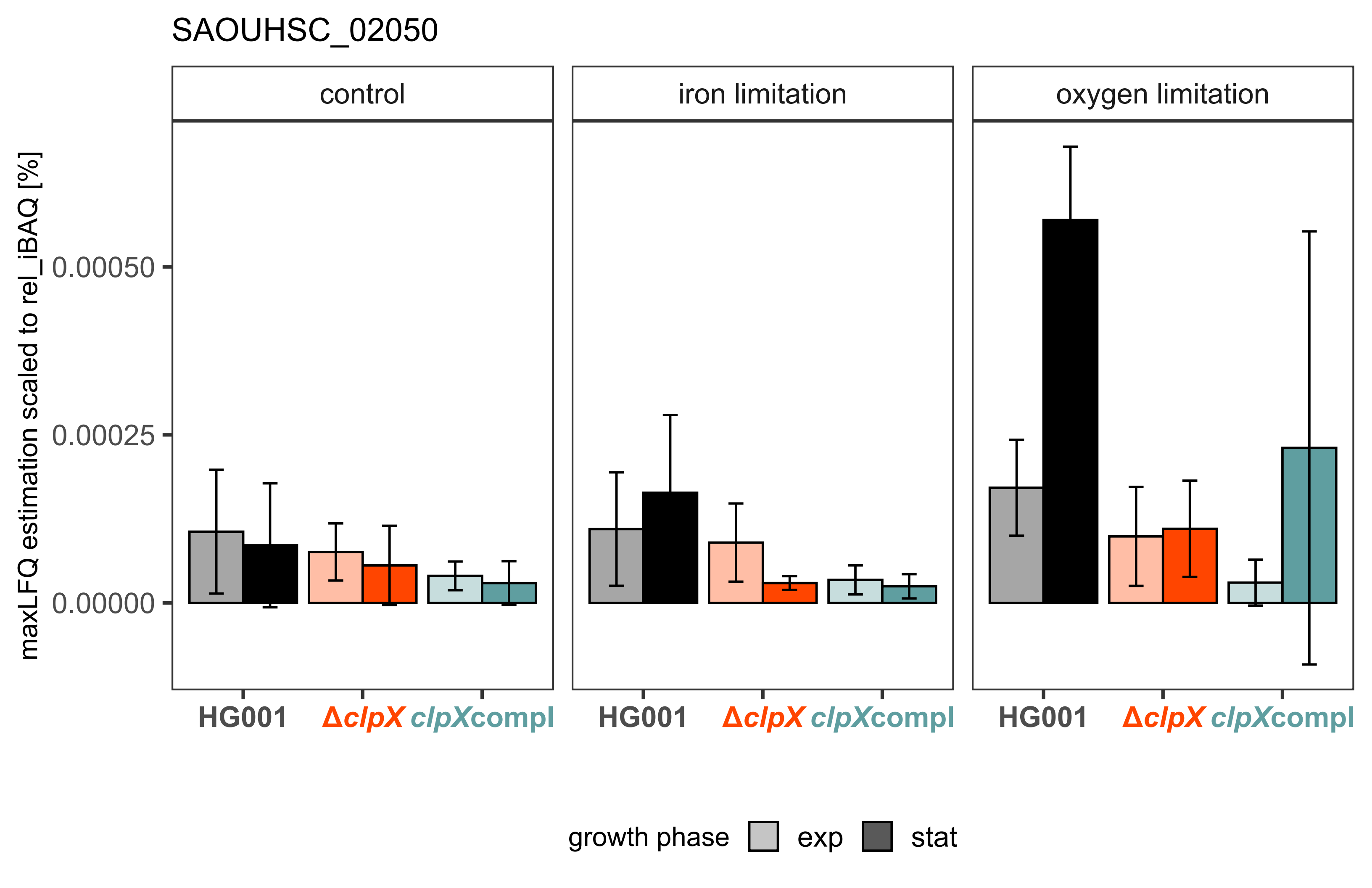

SAOUHSC_02050
control
iron limitation
oxygen limitation
maxLFQ estimation scaled to rel_iBAQ [%]
0.00050
0.00025
0.00000
HG001
ΔclpX
clpXcompl
growth phase
exp
stat

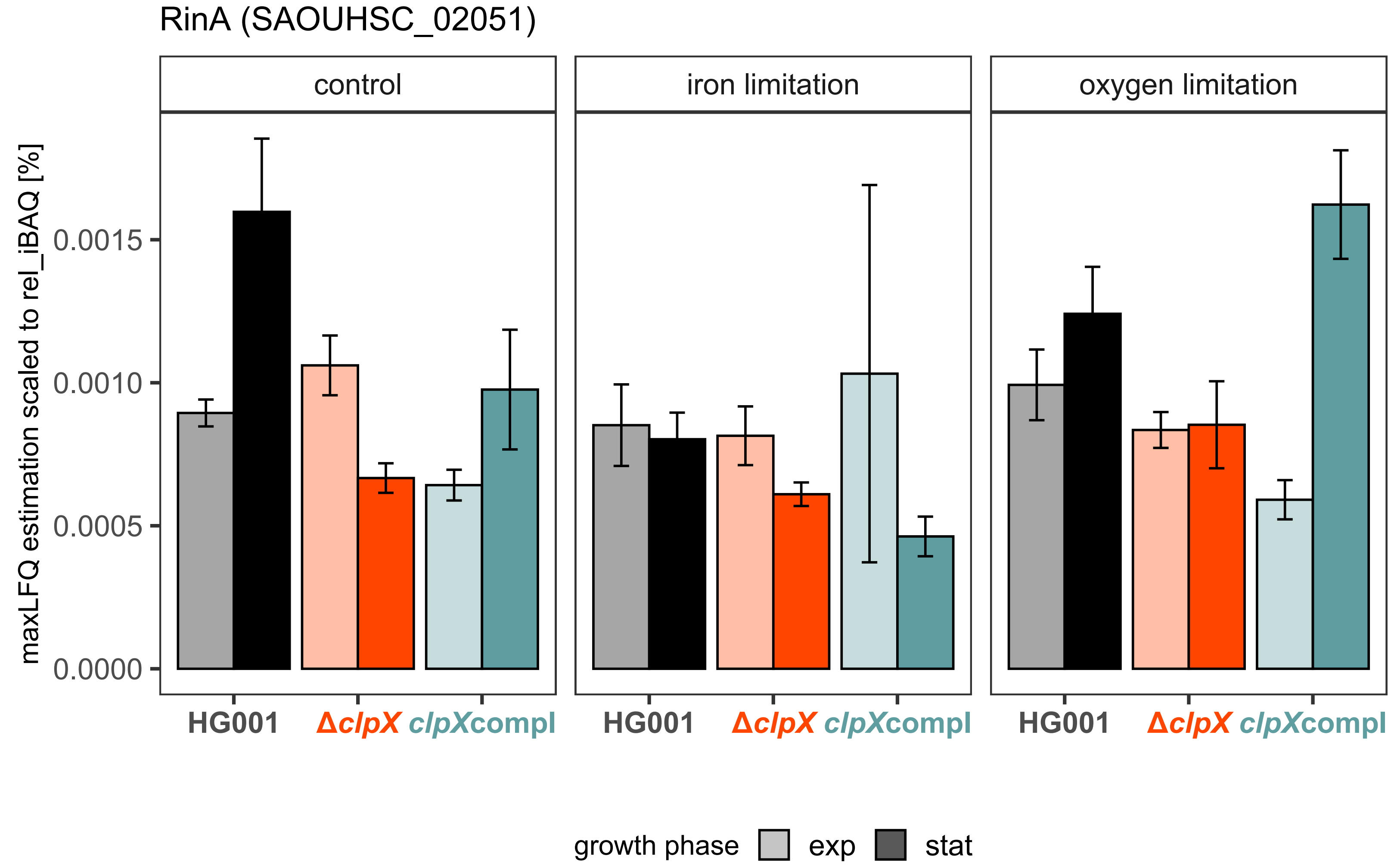
RinA (SAOUHSC_02051)
control
iron limitation
oxygen limitation
maxLFQ estimation scaled to rel_iBAQ [%]
0.0015
0.0010
0.0005
0.0000
HG001
ΔclpX
clpXcompl
growth phase
exp
stat

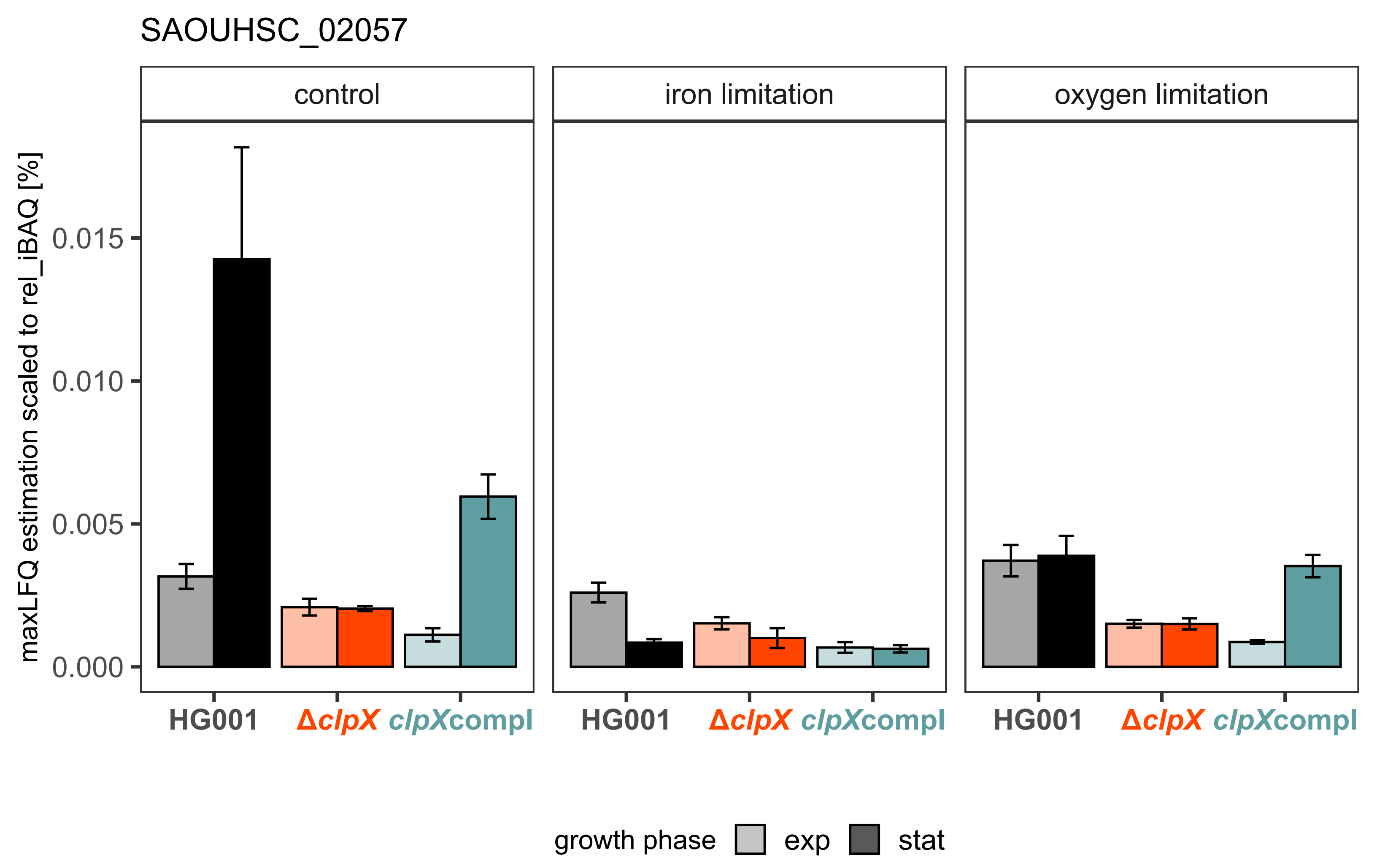
SAOUHSC_02057
control
iron limitation
oxygen limitation
maxLFQ estimation scaled to rel_iBAQ [%]
0.015
0.010
0.005
0.000
HG001
ΔclpX
clpXcompl
growth phase
exp
stat

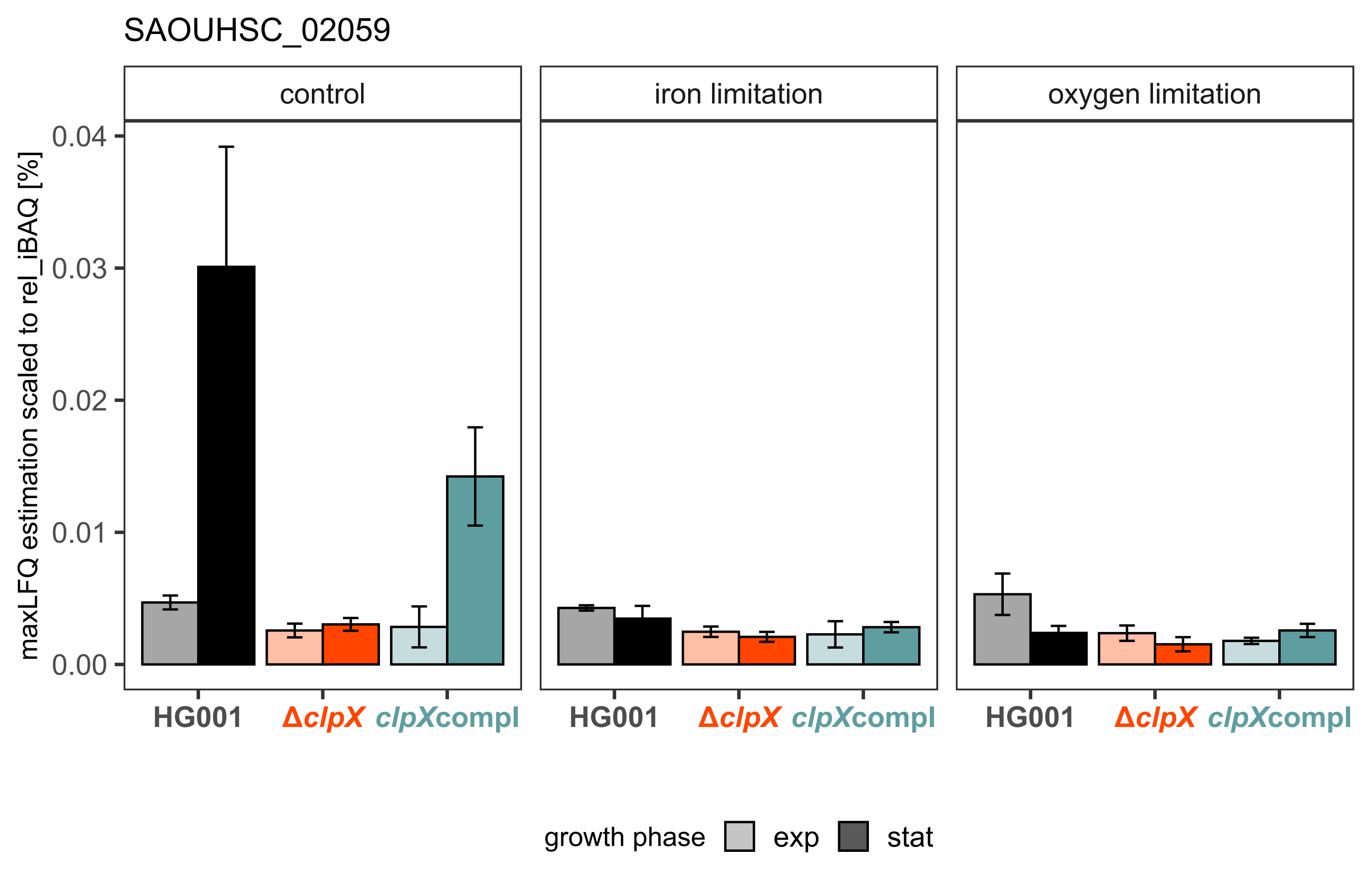

SAOUHSC_02059
control
iron limitation
oxygen limitation
maxLFQ estimation scaled to rel_iBAQ [%]
0.04
0.03
0.02
0.01
0.00
HG001
ΔclpX
clpXcompl
growth phase
exp
stat

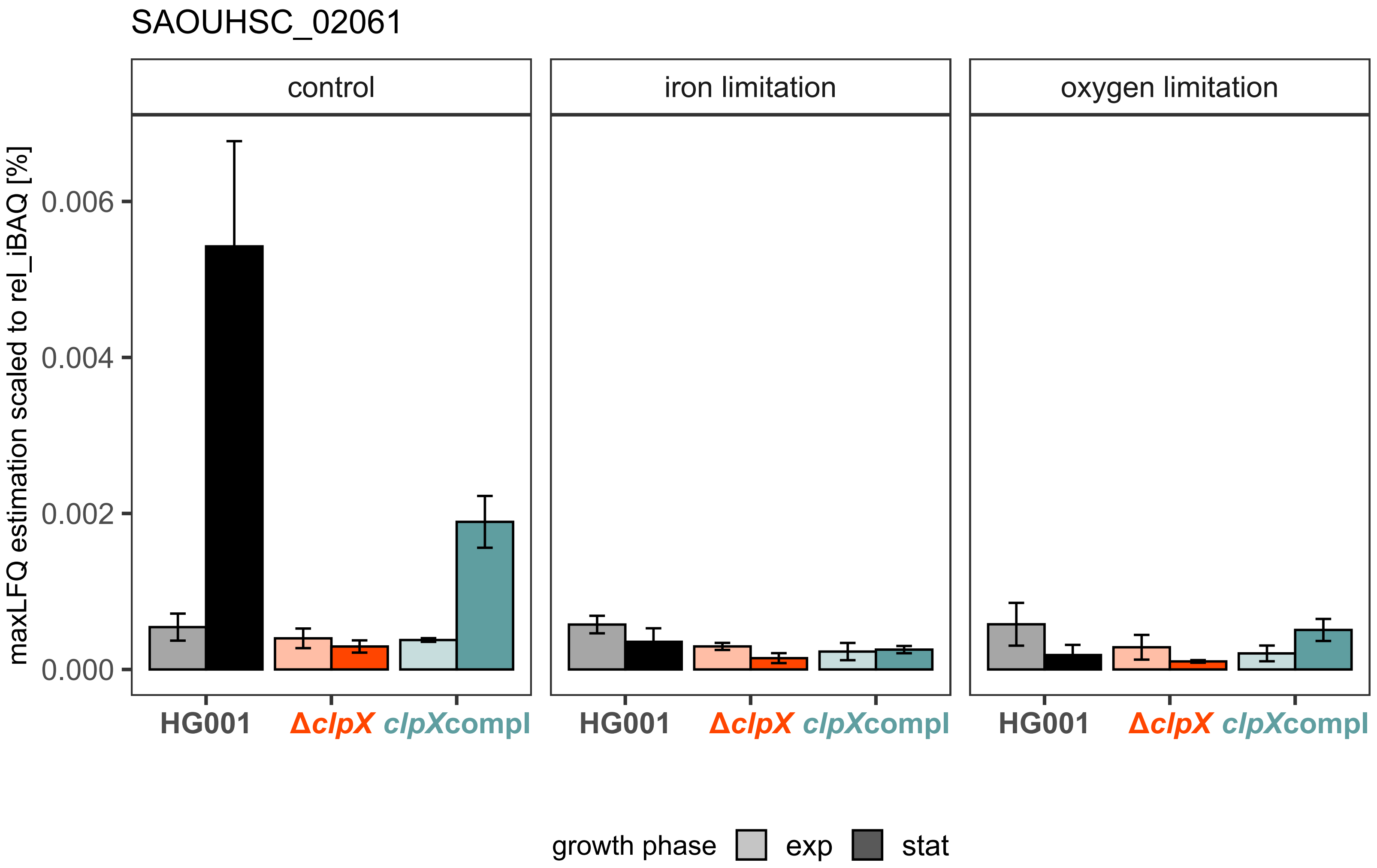
SAOUHSC_02061
control
iron limitation
oxygen limitation
maxLFQ estimation scaled to rel_iBAQ [%]
0.000
0.002
0.004
0.006
HG001
ΔclpX
clpXcompl
growth phase
exp
stat

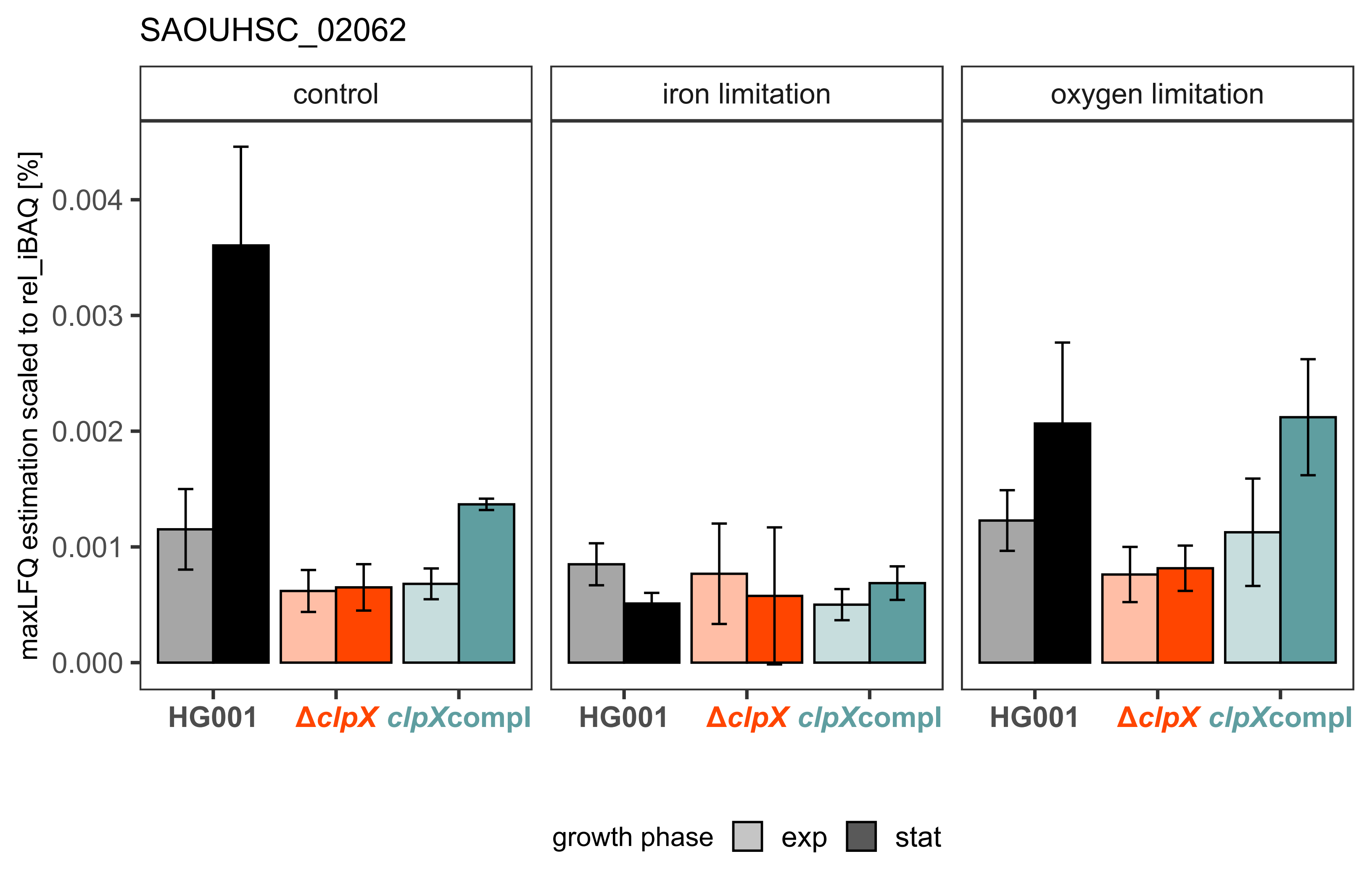
SAOUHSC_02062
control
iron limitation
oxygen limitation
maxLFQ estimation scaled to rel_iBAQ [%]
0.004
0.003
0.002
0.001
0.000
HG001
ΔclpX
clpXcompl
growth phase
exp
stat

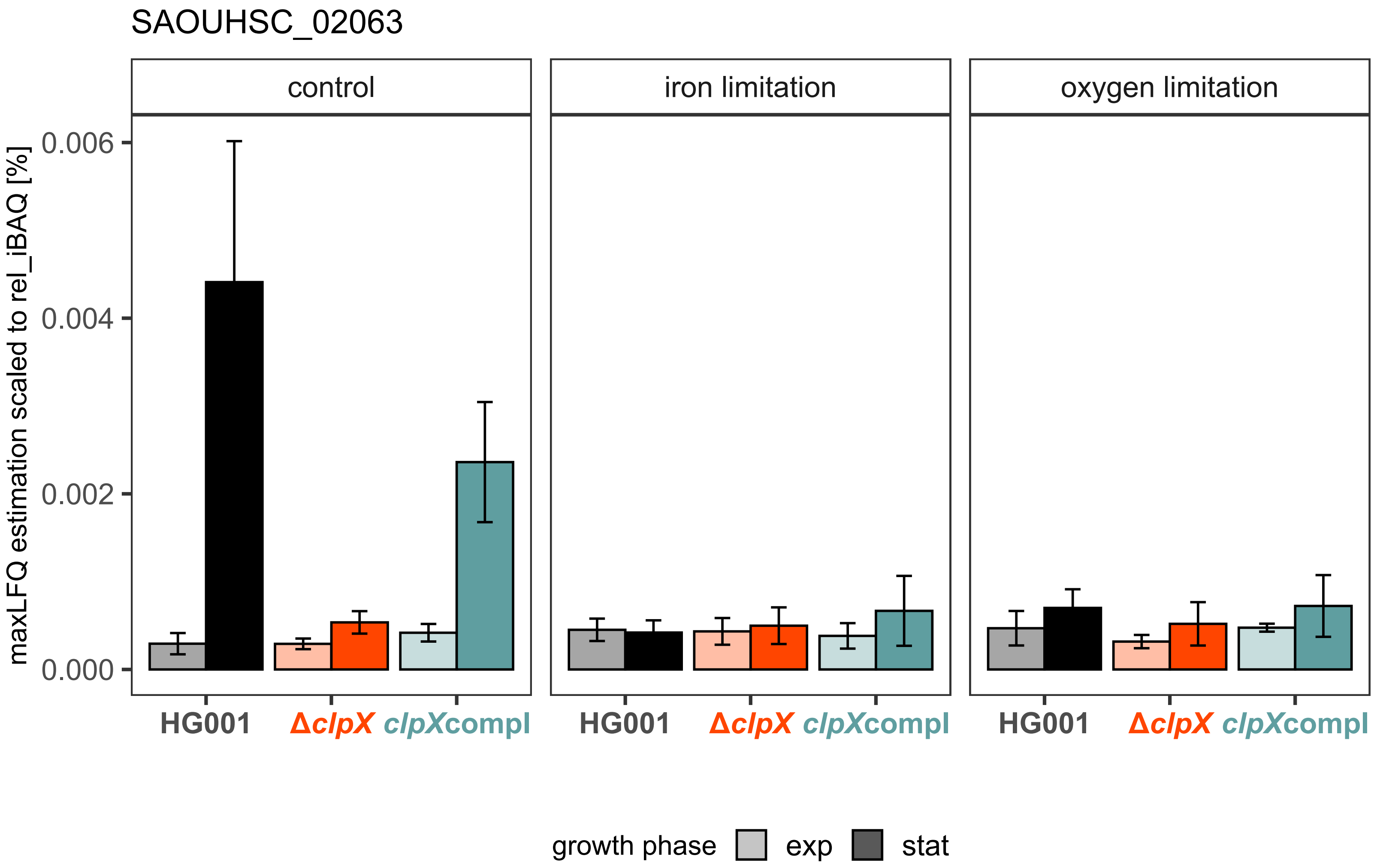

SAOUHSC_02063
control
iron limitation
oxygen limitation
maxLFQ estimation scaled to rel_iBAQ [%]
0.006
0.004
0.002
0.000
HG001
ΔclpX
clpXcompl
growth phase
exp
stat

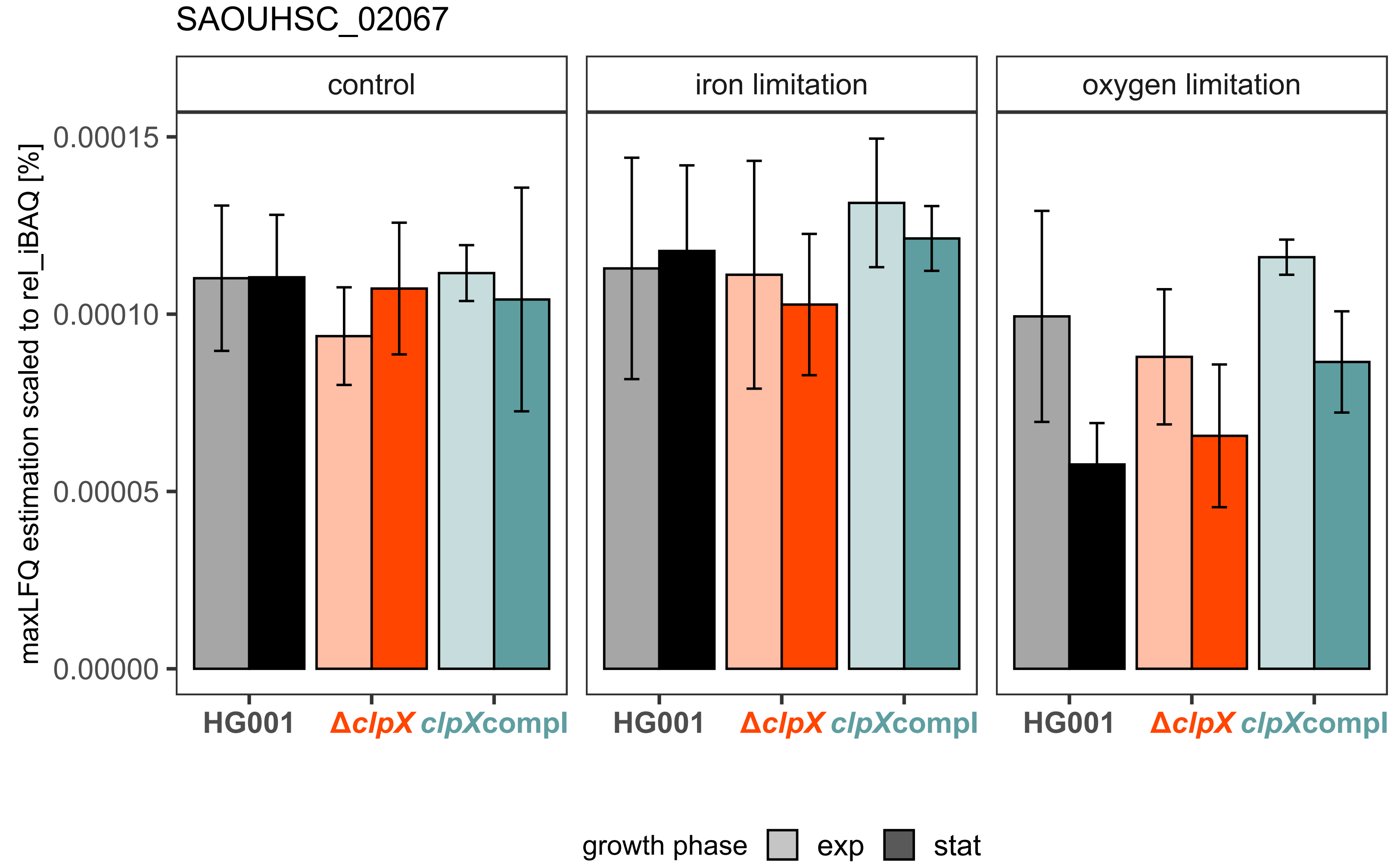

SAOUHSC_02067
control
iron limitation
oxygen limitation
maxLFQ estimation scaled to rel_iBAQ [%]
0.00015
0.00010
0.00005
0.00000
HG001
ΔclpX
clpXcompl
growth phase
exp
stat

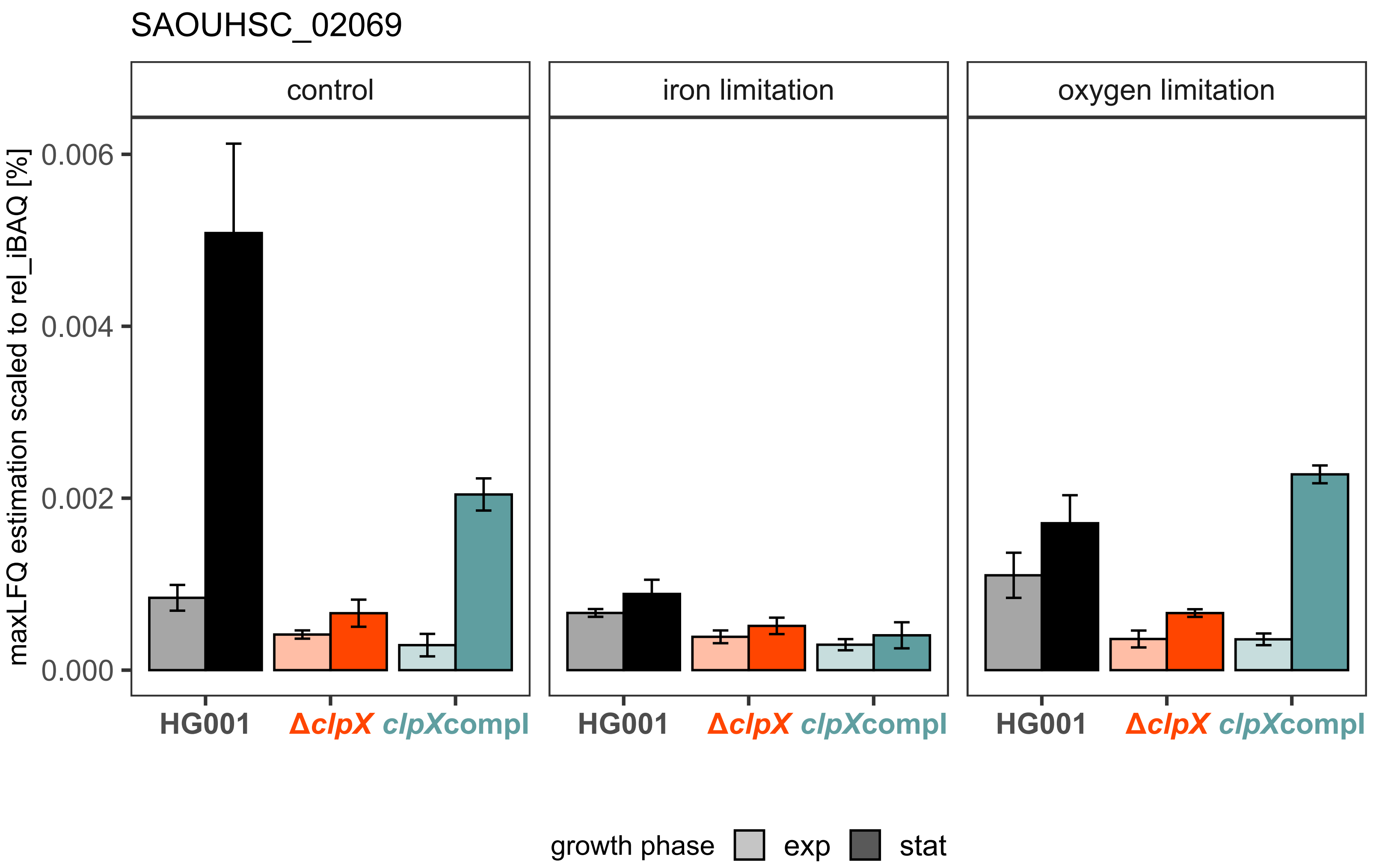

SAOUHSC_02069
control
iron limitation
oxygen limitation
maxLFQ estimation scaled to rel_iBAQ [%]
0.006
0.004
0.002
0.000
HG001
ΔclpX
clpXcompl
growth phase
exp
stat

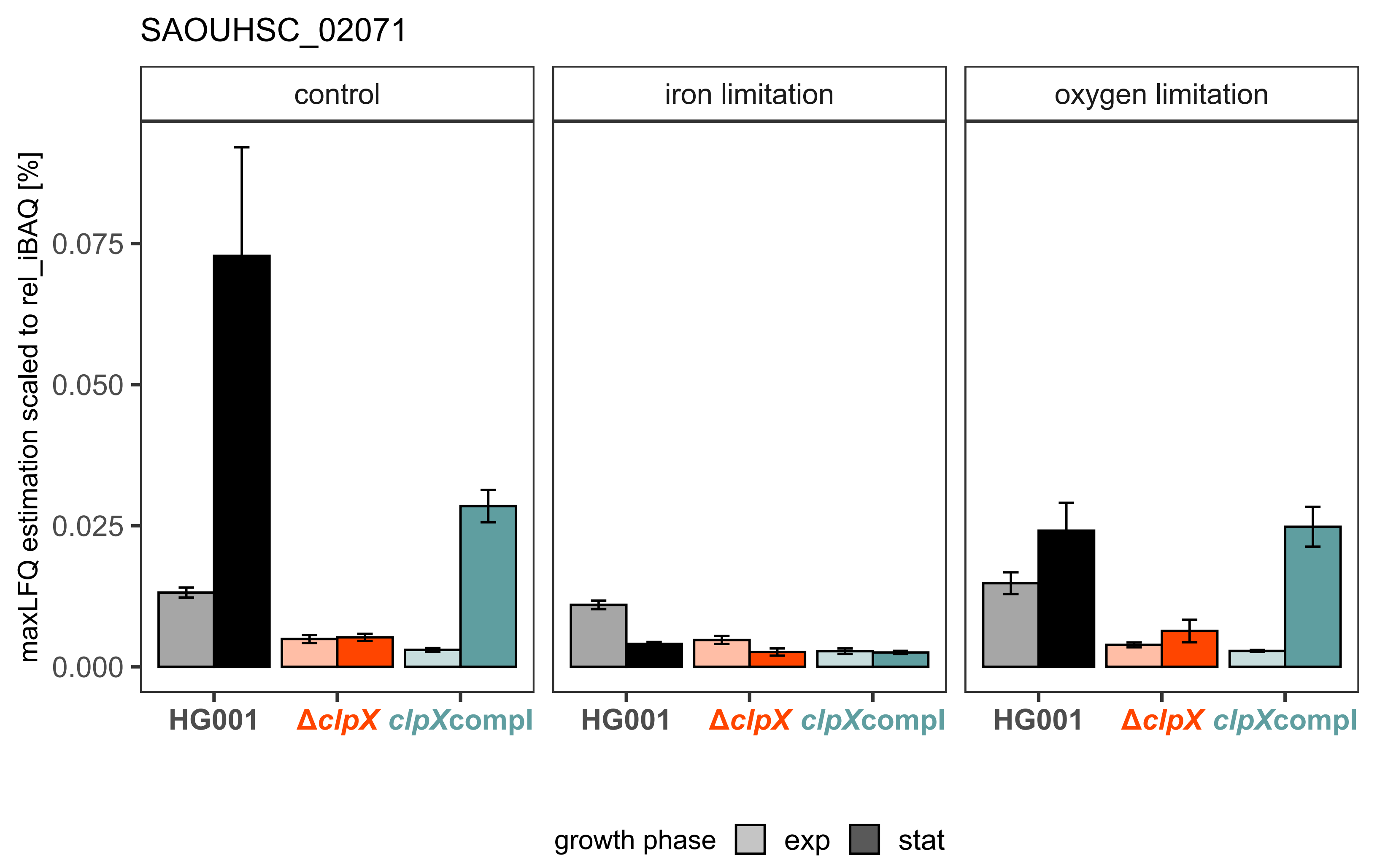
SAOUHSC_02071
control
iron limitation
oxygen limitation
maxLFQ estimation scaled to rel_iBAQ [%]
0.075
0.050
0.025
0.000
HG001
ΔclpX
clpXcompl
growth phase
exp
stat

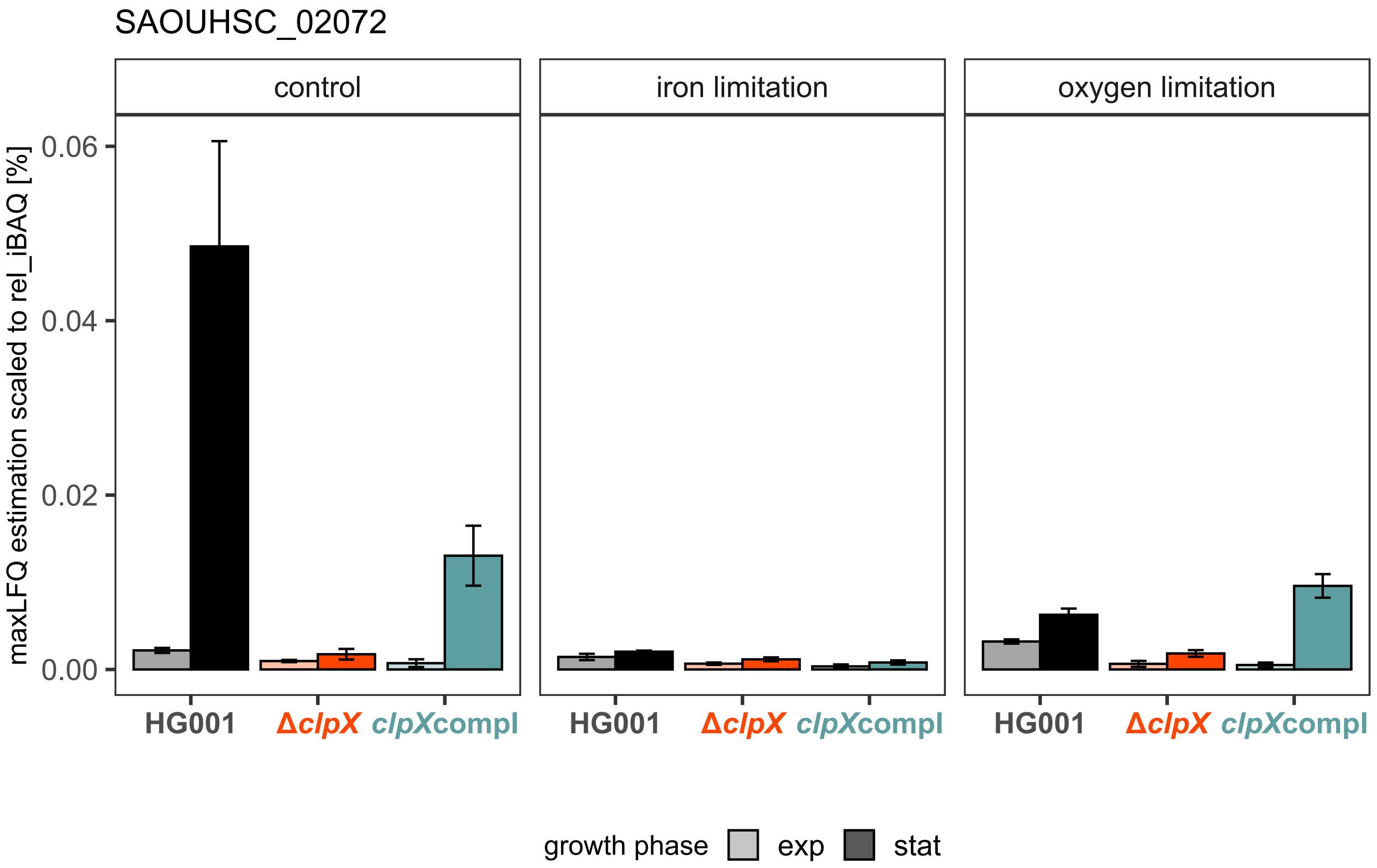
SAOUHSC_02072
control
iron limitation
oxygen limitation
maxLFQ estimation scaled to rel_iBAQ [%]
0.06
0.04
0.02
0.00
HG001
ΔclpX
clpXcompl
growth phase
exp
stat

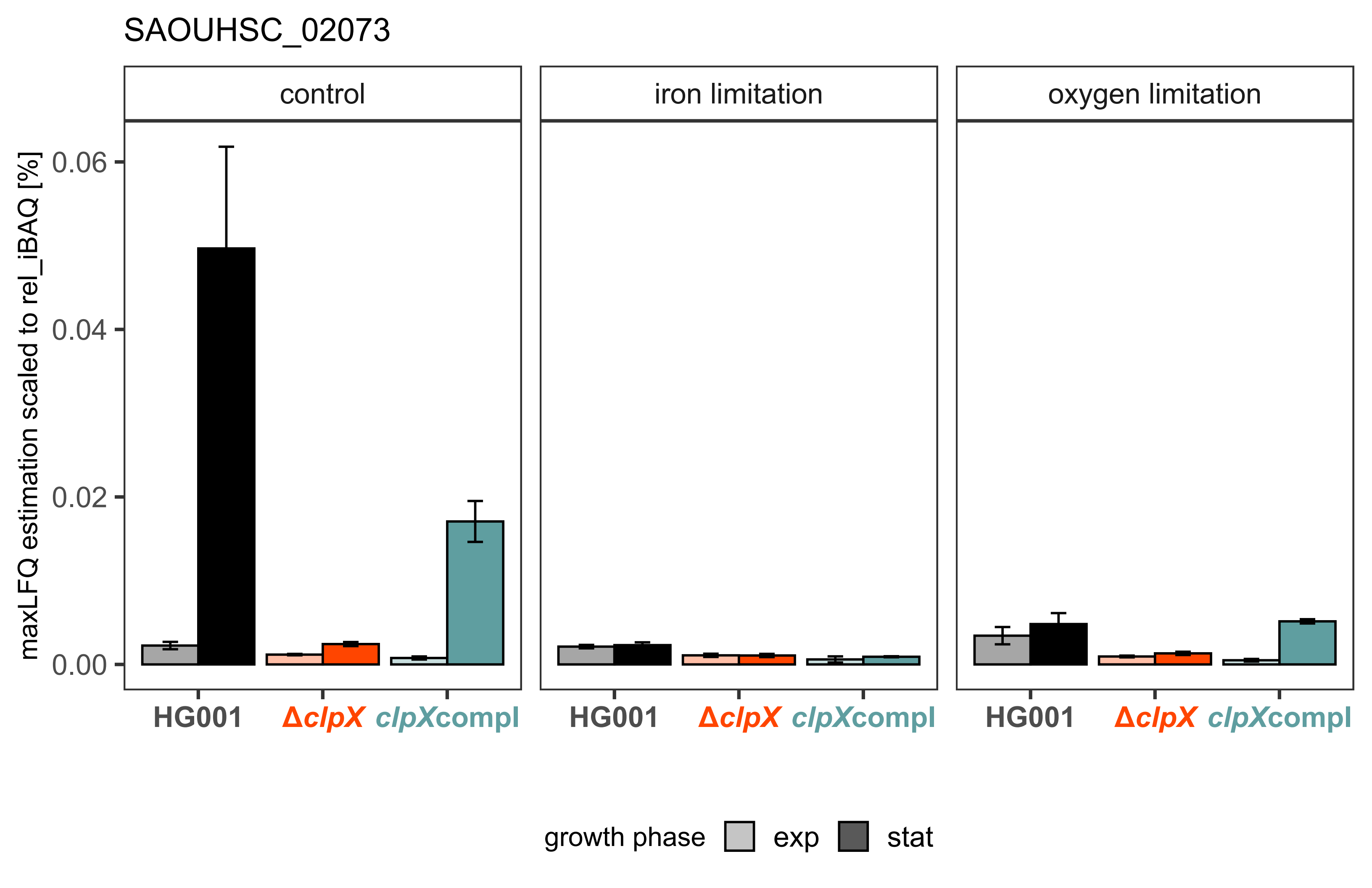
SAOUHSC_02073
control
iron limitation
oxygen limitation
maxLFQ estimation scaled to rel_iBAQ [%]
0.06
0.04
0.02
0.00
HG001
ΔclpX
clpXcompl
growth phase
exp
stat

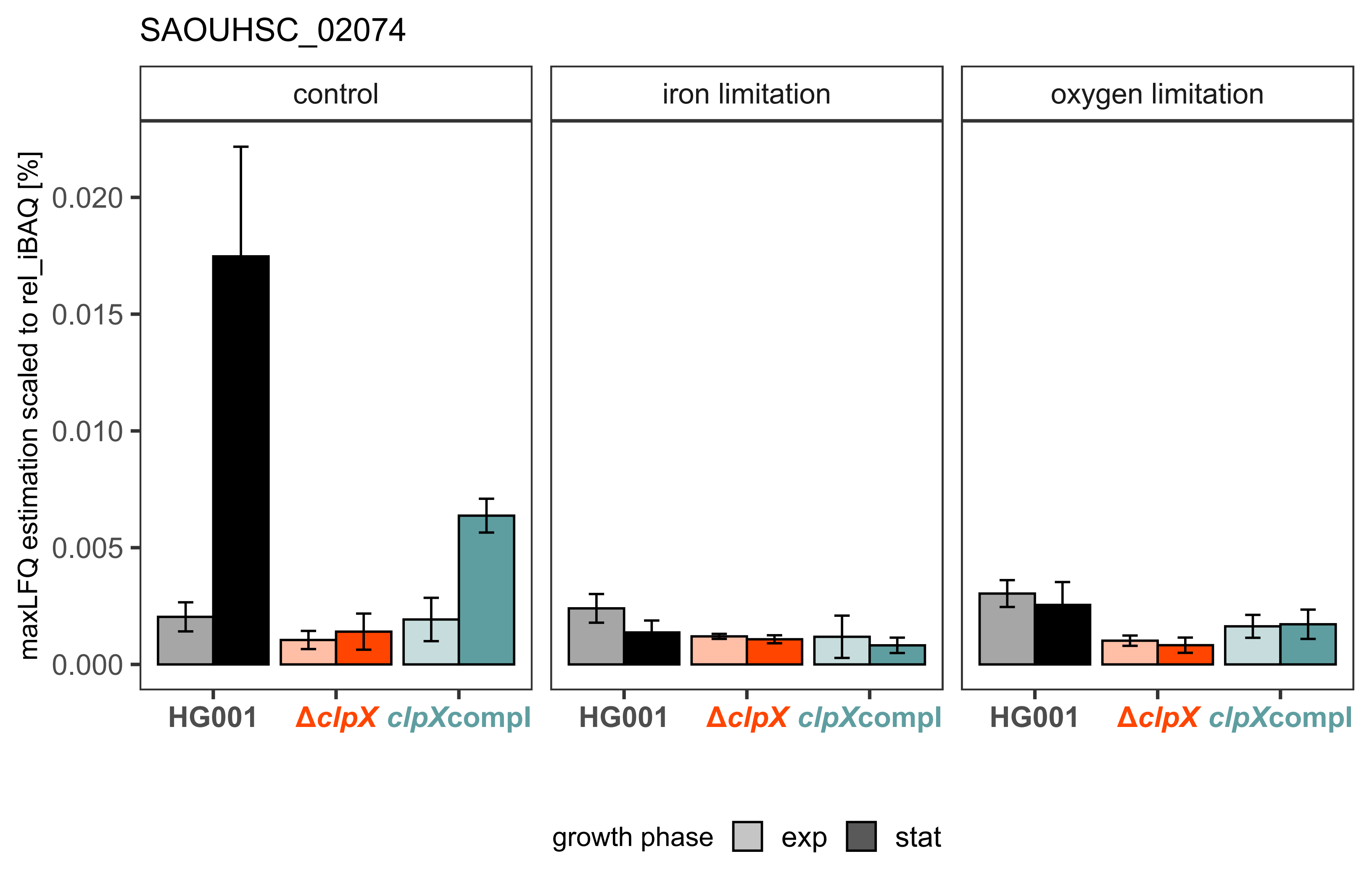

SAOUHSC_02074
control
iron limitation
oxygen limitation
maxLFQ estimation scaled to rel_iBAQ [%]
0.020
0.015
0.010
0.005
0.000
HG001
ΔclpX
clpXcompl
growth phase
exp
stat

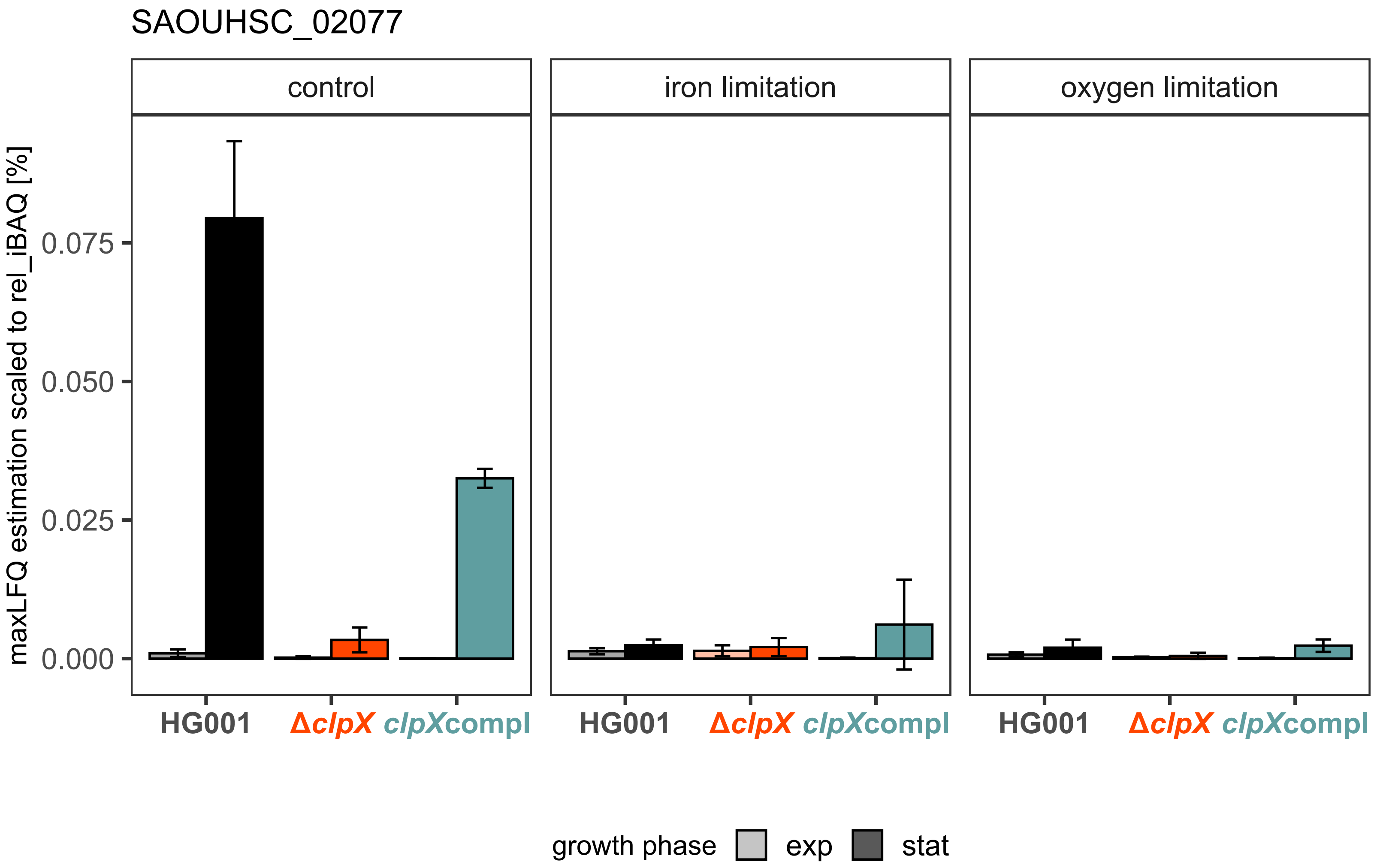

SAOUHSC_02077
control
iron limitation
oxygen limitation
maxLFQ estimation scaled to rel_iBAQ [%]
0.075
0.050
0.025
0.000
HG001
ΔclpX
clpXcompl
growth phase
exp
stat

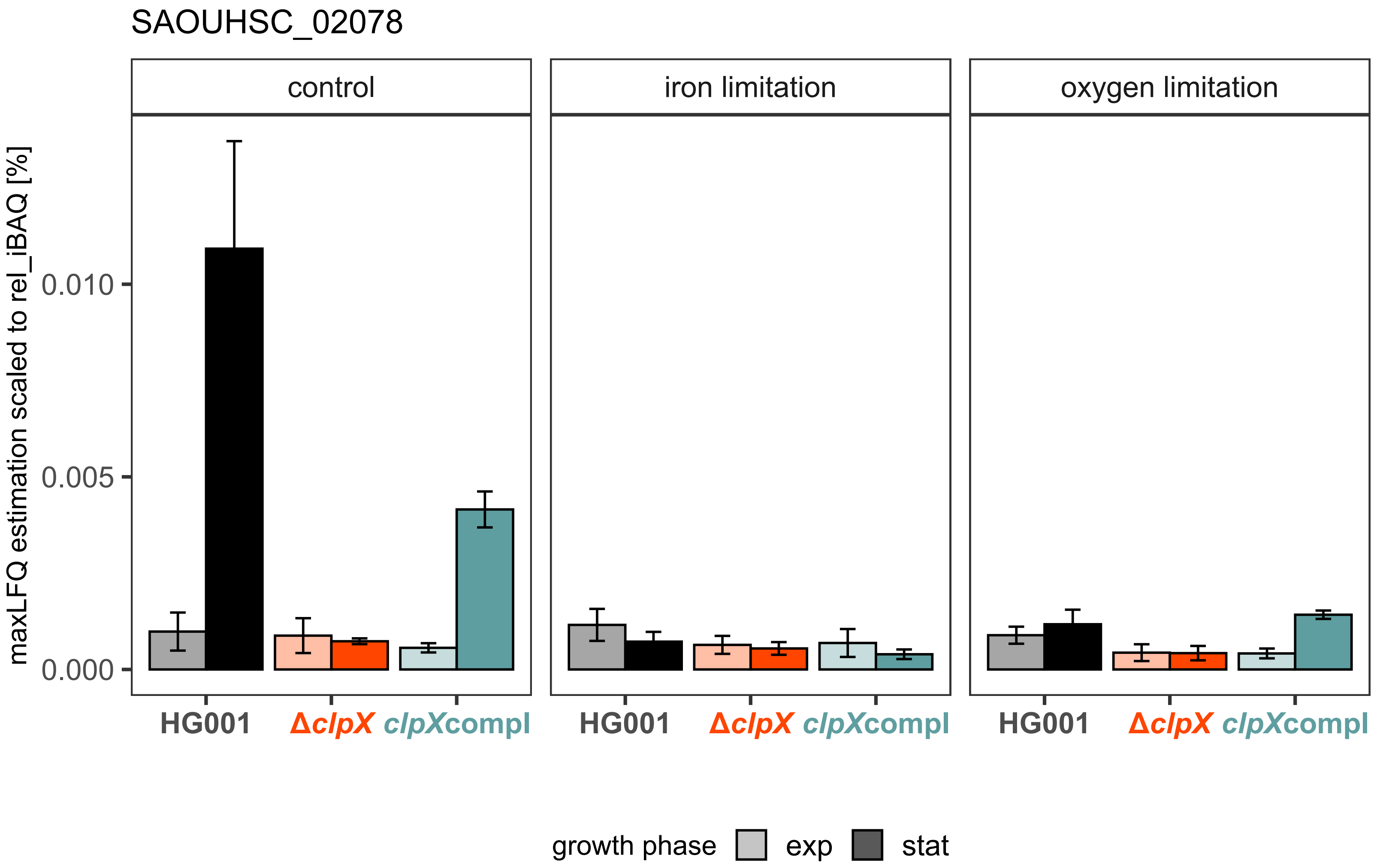
SAOUHSC_02078
control
iron limitation
oxygen limitation
maxLFQ estimation scaled to rel_iBAQ [%]
0.010
0.005
0.000
HG001
ΔclpX
clpXcompl
growth phase
exp
stat

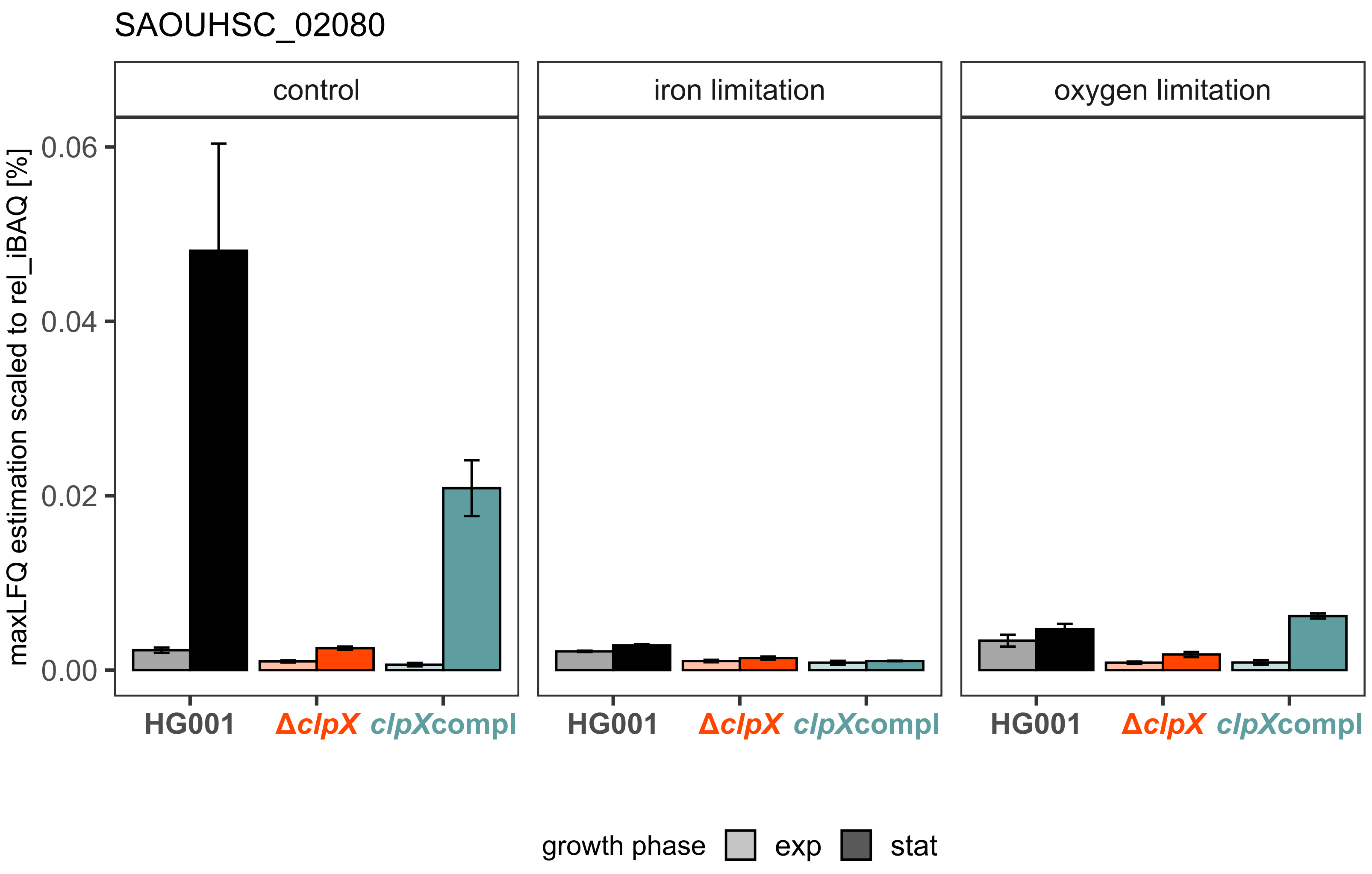
SAOUHSC_02080
control
iron limitation
oxygen limitation
maxLFQ estimation scaled to rel_iBAQ [%]
0.06
0.04
0.02
0.00
HG001
ΔclpX
clpXcompl
growth phase
exp
stat

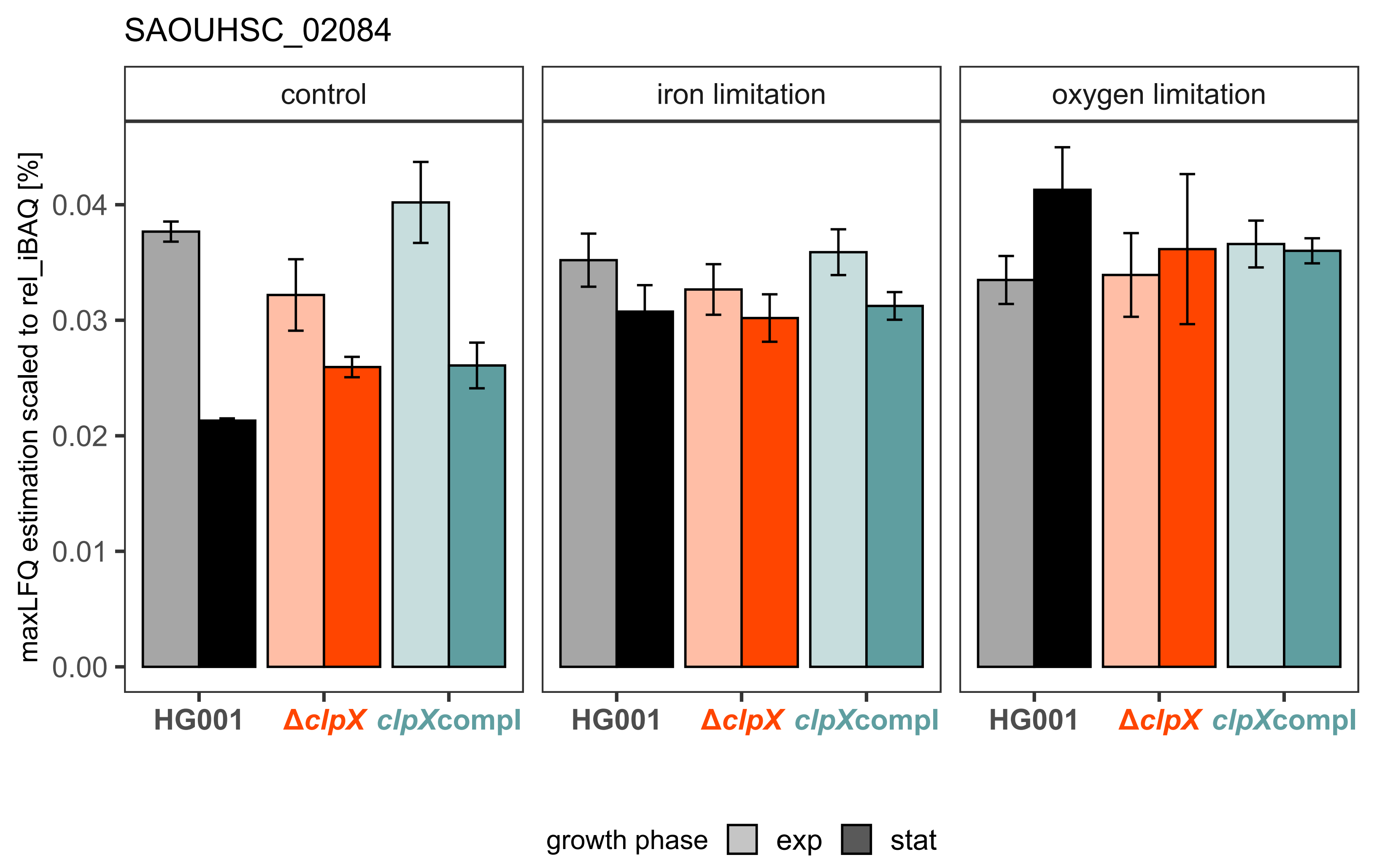
SAOUHSC_02084
control
iron limitation
oxygen limitation
maxLFQ estimation scaled to rel_iBAQ [%]
0.04
0.03
0.02
0.01
0.00
HG001
ΔclpX
clpXcompl
growth phase
exp
stat

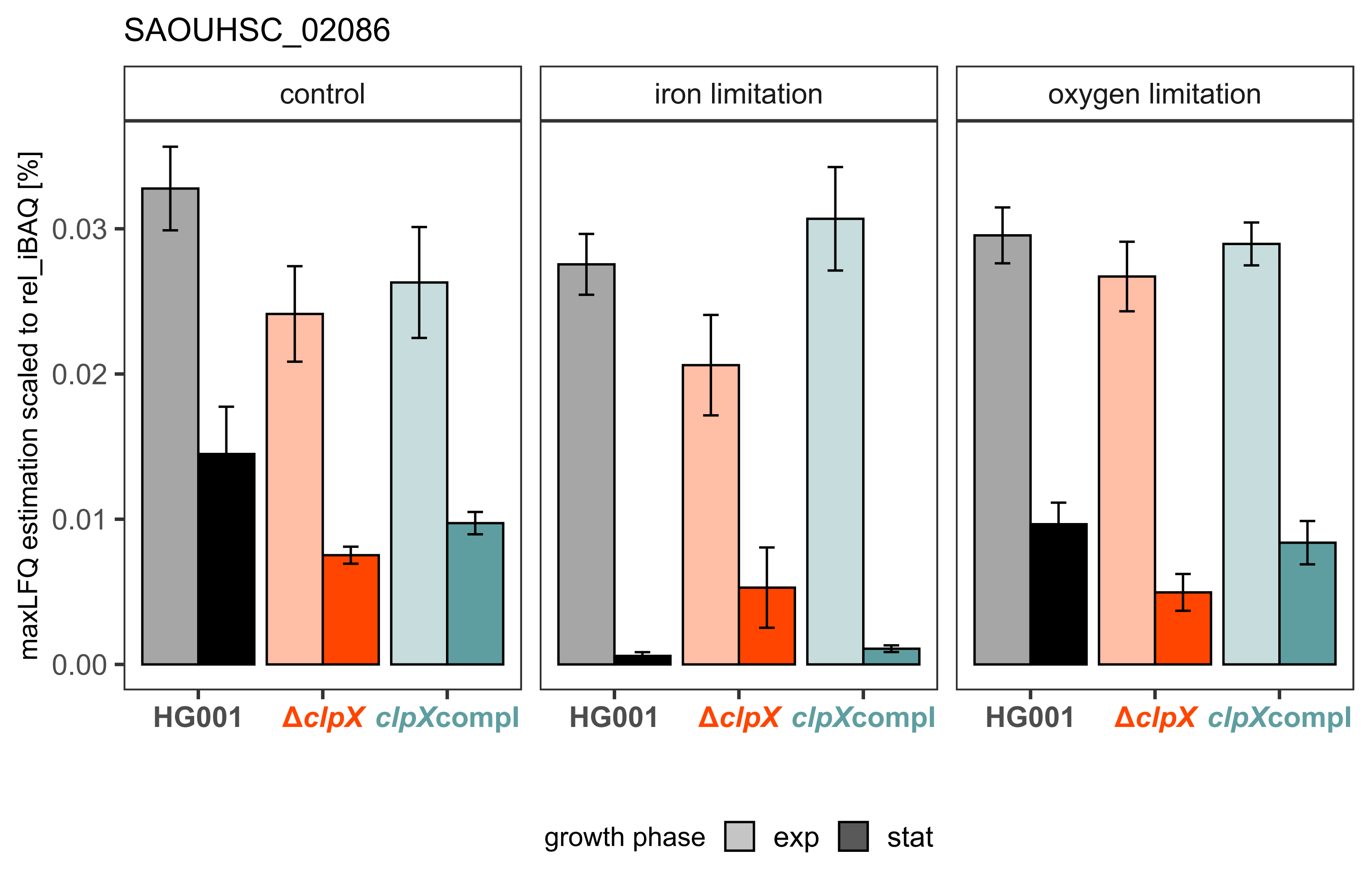

SAOUHSC_02086
control
iron limitation
oxygen limitation
maxLFQ estimation scaled to rel_iBAQ [%]
0.00
0.01
0.02
0.03
HG001
ΔclpX
clpXcompl
growth phase
exp
stat

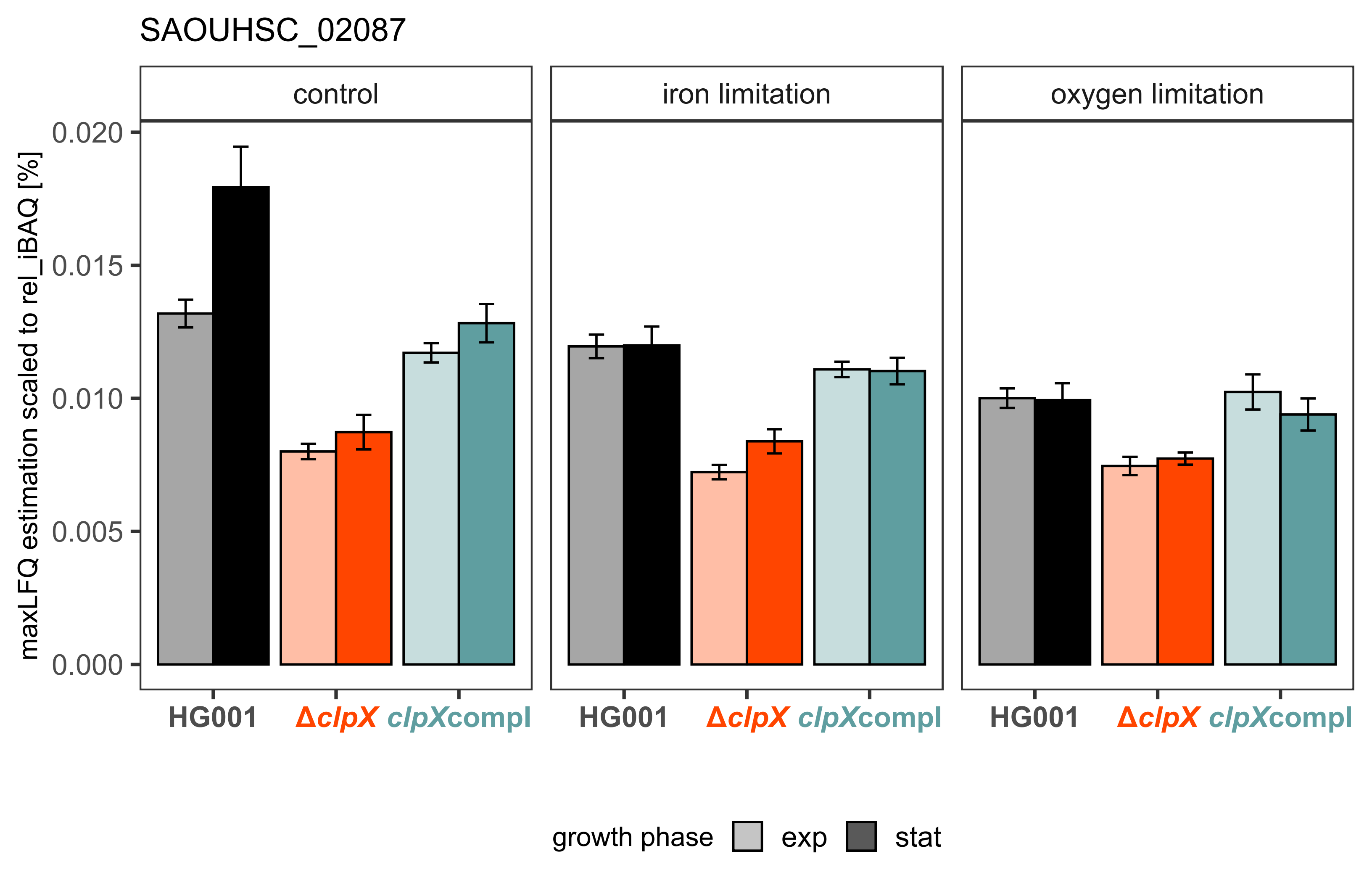

SAOUHSC_02087
control
iron limitation
oxygen limitation
maxLFQ estimation scaled to rel_iBAQ [%]
0.020
0.015
0.010
0.005
0.000
HG001
ΔclpX
clpXcompl
growth phase
exp
stat

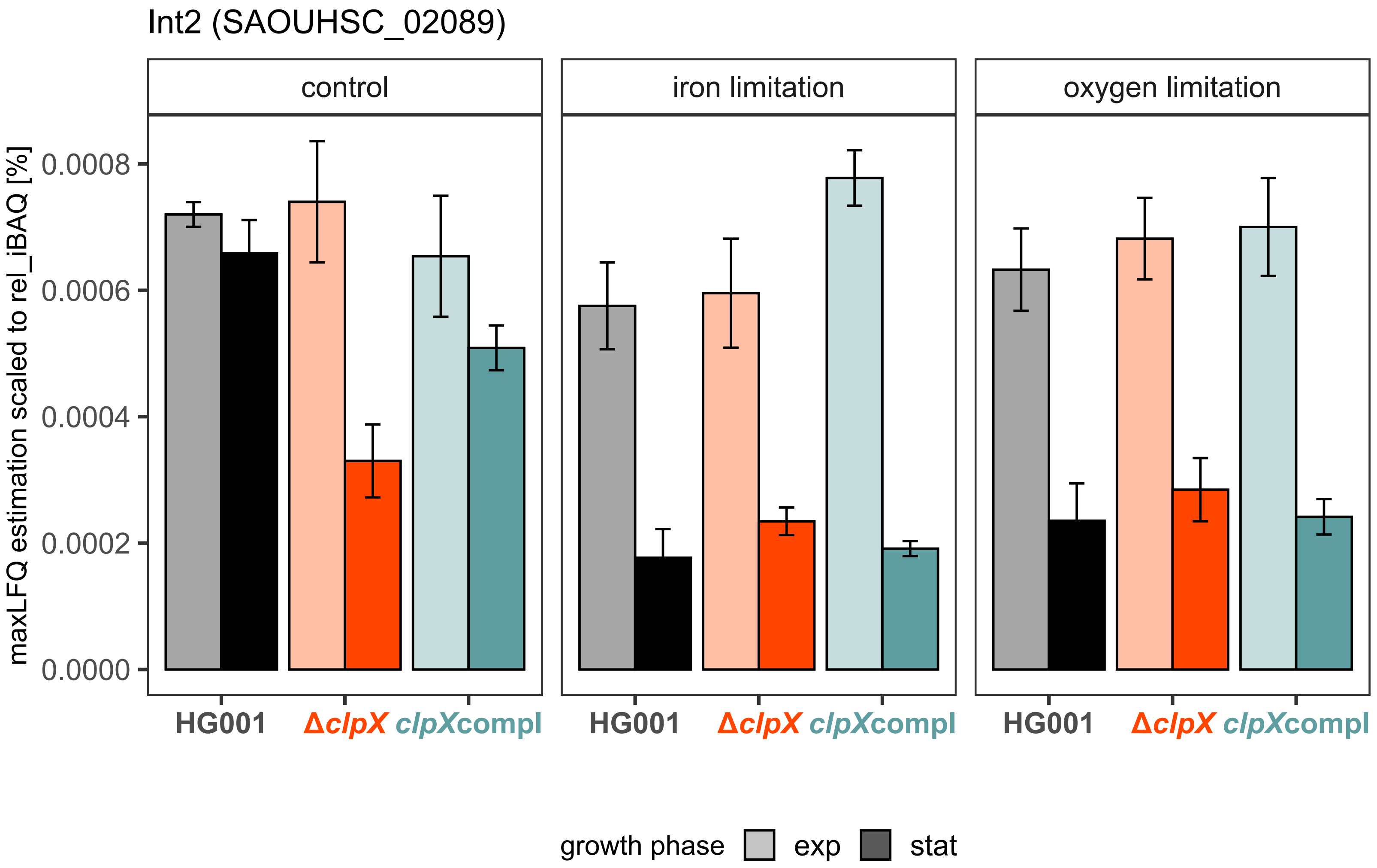

Int2 (SAOUHSC_02089)
control
iron limitation
oxygen limitation
maxLFQ estimation scaled to rel_iBAQ [%]
0.0008
0.0006
0.0004
0.0002
0.0000
HG001
ΔclpX
clpXcompl
growth phase
exp
stat

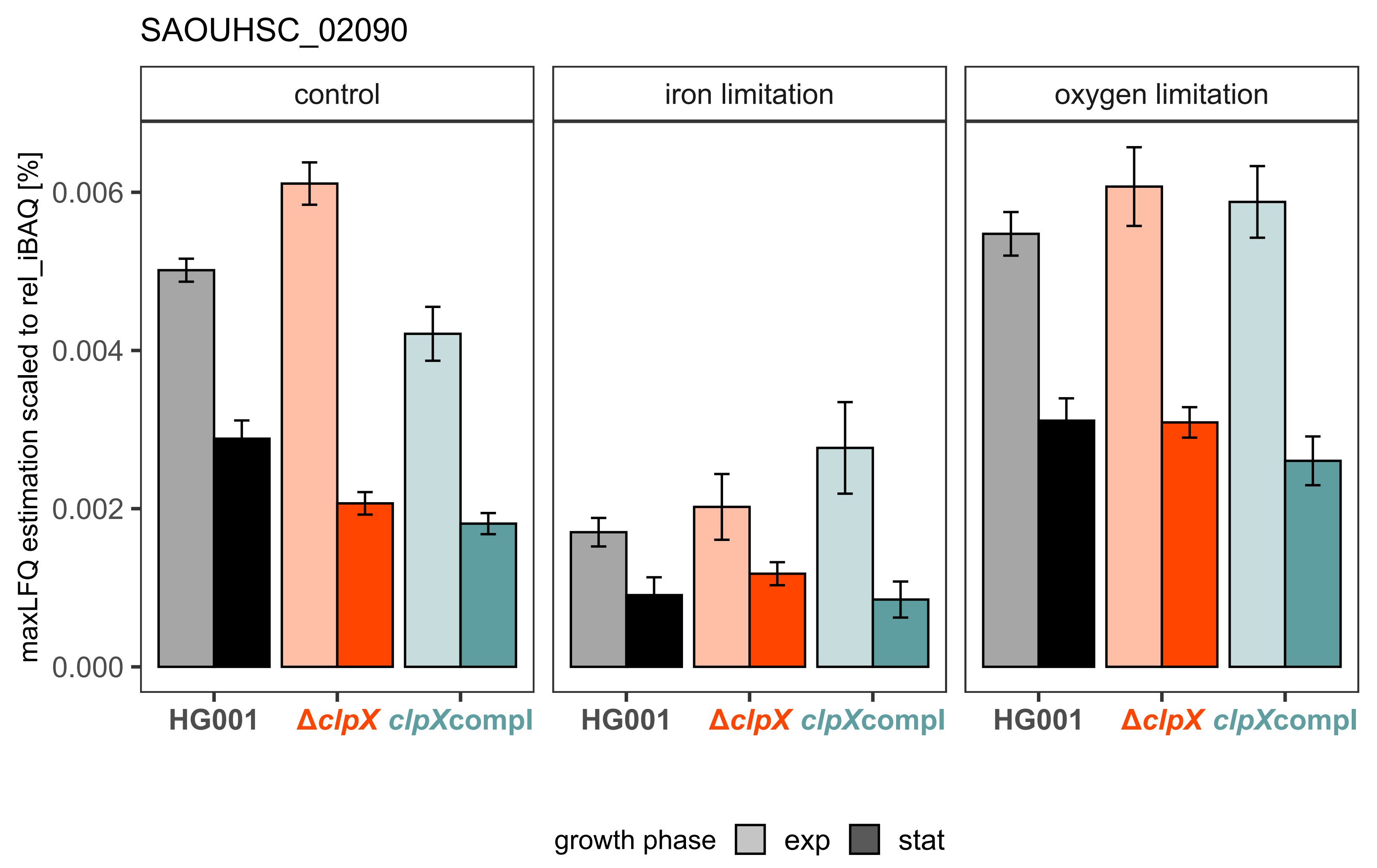

SAOUHSC_02090
control
iron limitation
oxygen limitation
maxLFQ estimation scaled to rel_iBAQ [%]
0.006
0.004
0.002
0.000
HG001
ΔclpX
clpXcompl
growth phase
exp
stat

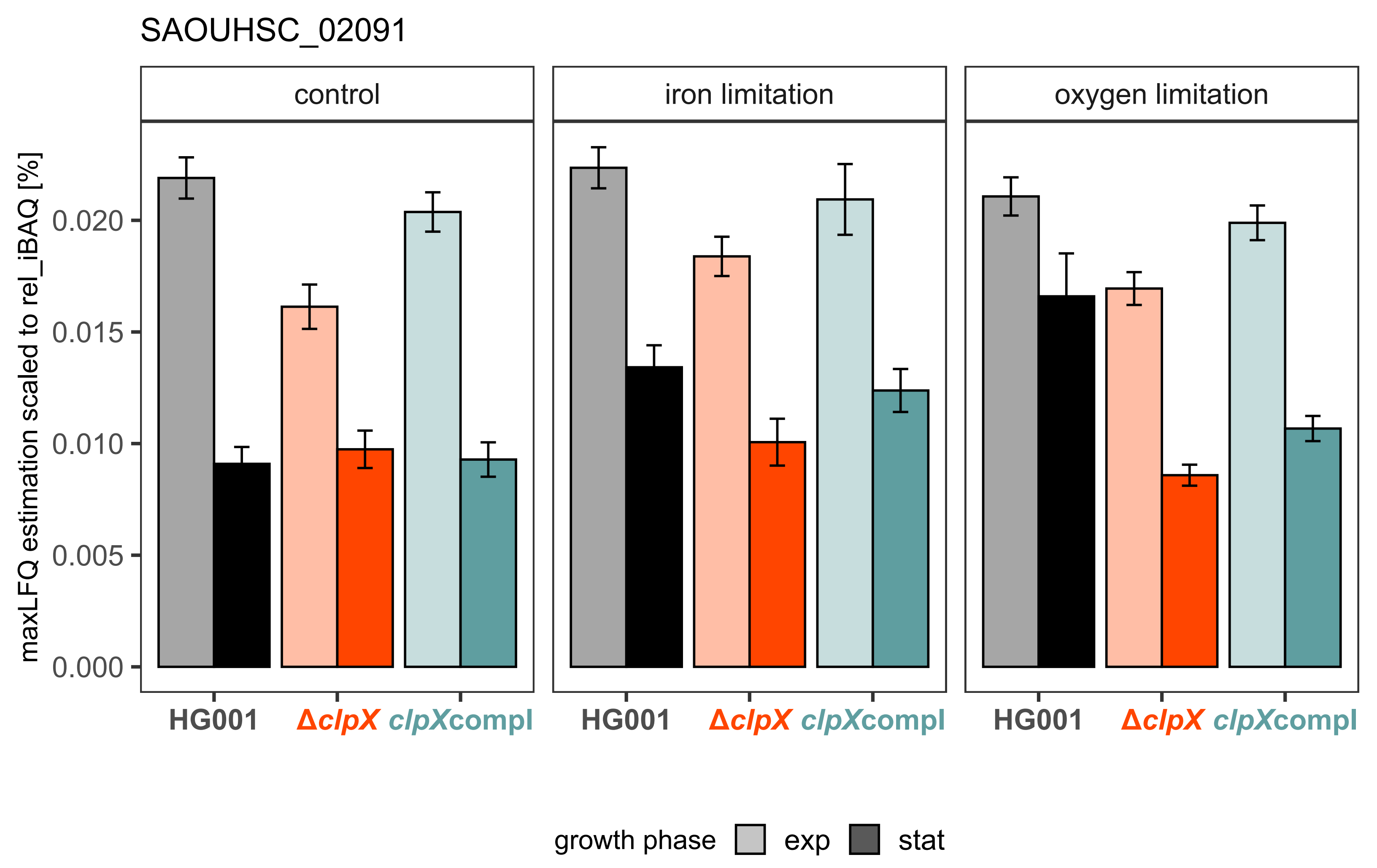

SAOUHSC_02091
control
iron limitation
oxygen limitation
maxLFQ estimation scaled to rel_iBAQ [%]
0.000
0.005
0.010
0.015
0.020
HG001
ΔclpX
clpXcompl
growth phase
exp
stat

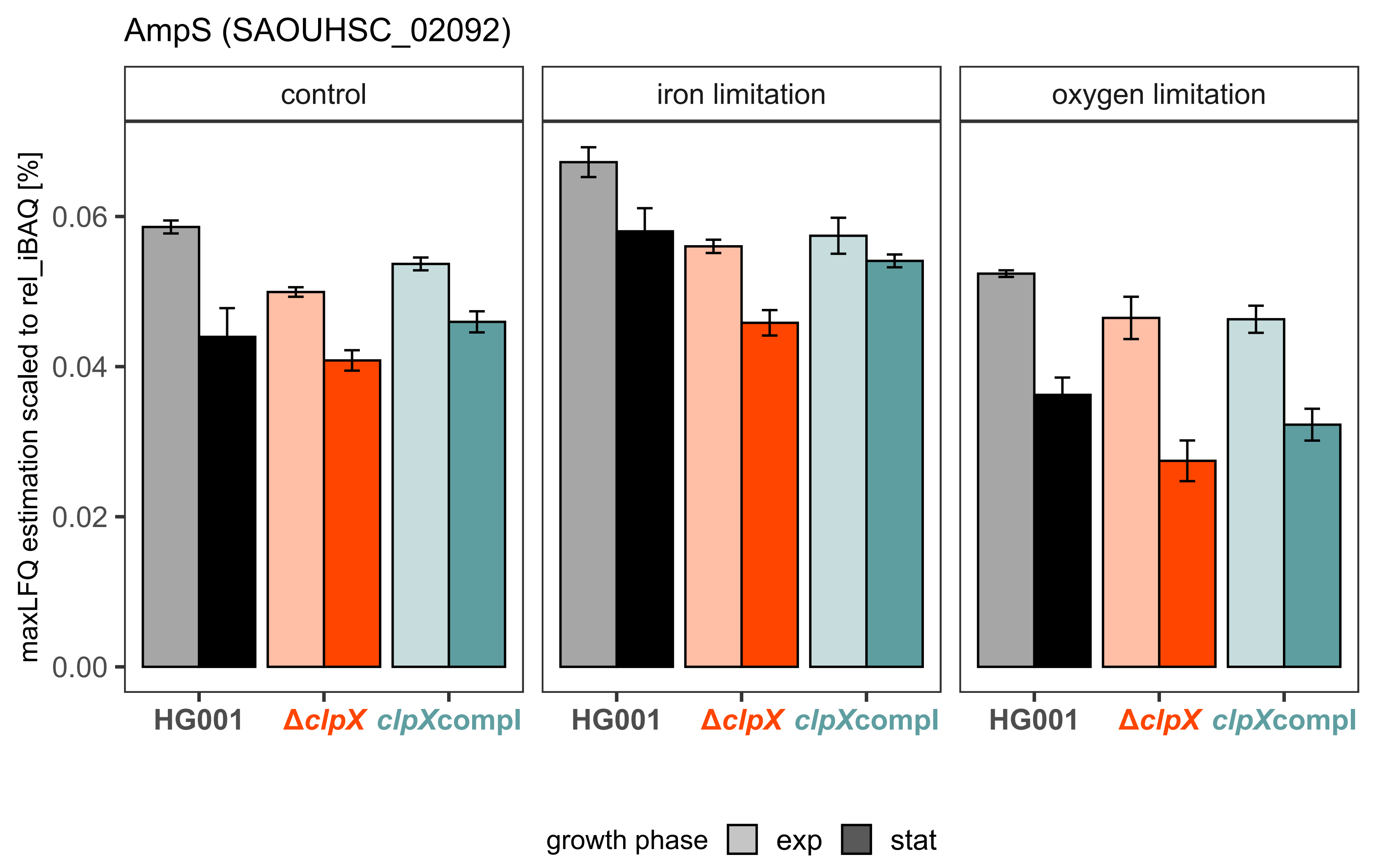

AmpS (SAOUHSC_02092)
control
iron limitation
oxygen limitation
maxLFQ estimation scaled to rel_iBAQ [%]
0.00
0.02
0.04
0.06
HG001
ΔclpX
clpXcompl
growth phase
exp
stat

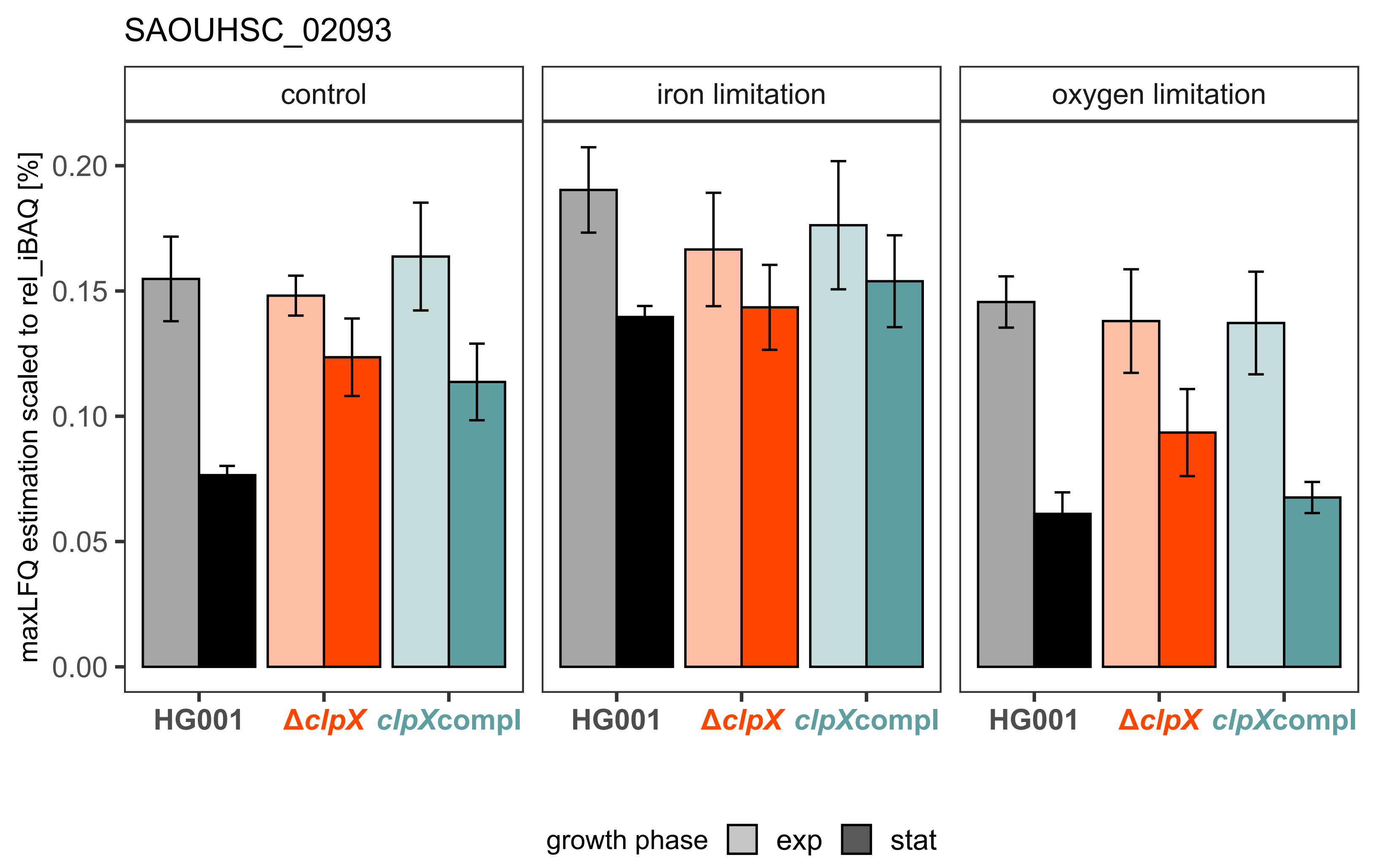

SAOUHSC_02093
control
iron limitation
oxygen limitation
maxLFQ estimation scaled to rel_iBAQ [%]
0.00
0.05
0.10
0.15
0.20
HG001
ΔclpX
clpXcompl
growth phase
exp
stat

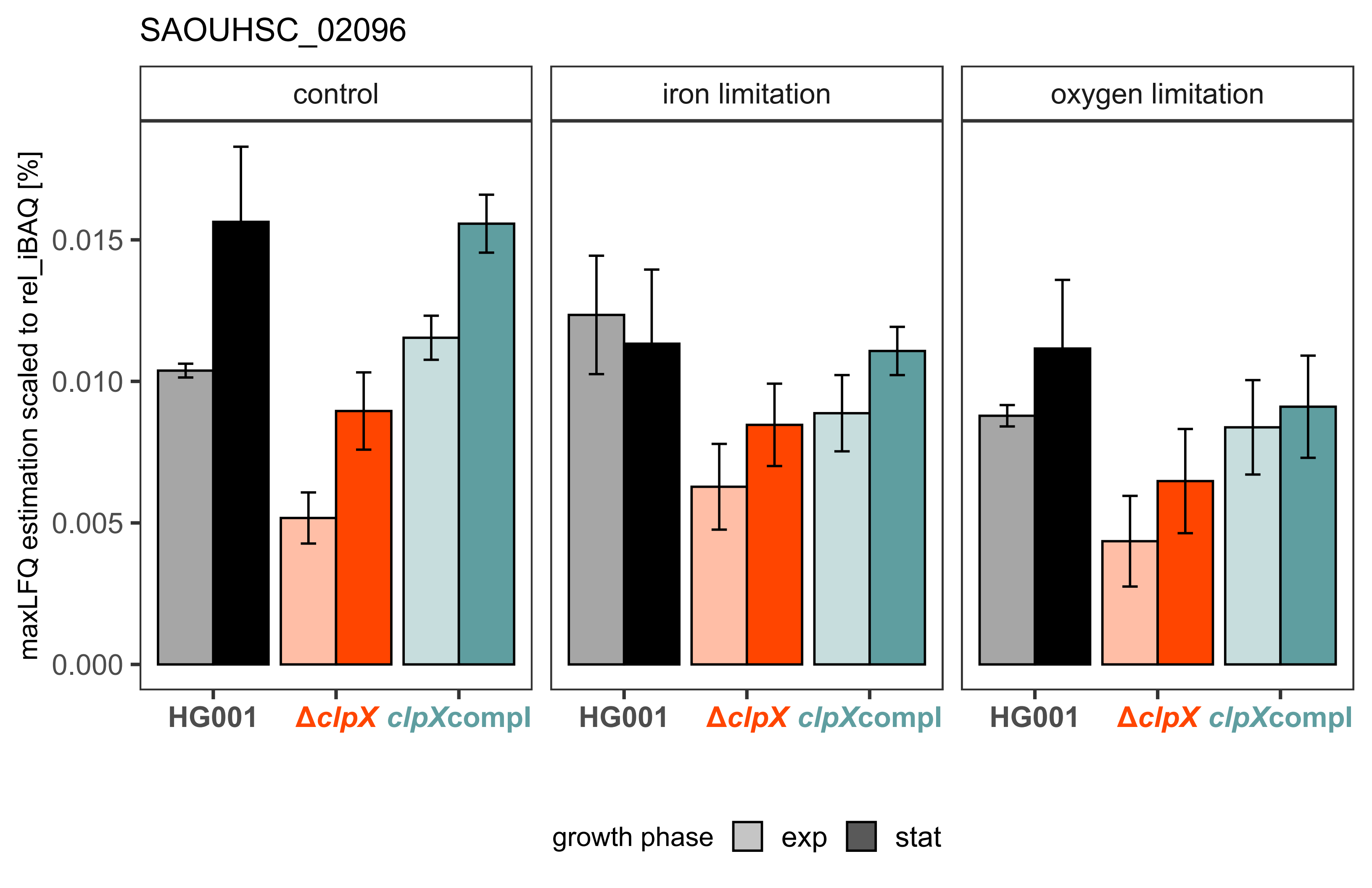
SAOUHSC_02096
control
iron limitation
oxygen limitation
maxLFQ estimation scaled to rel_iBAQ [%]
0.015
0.010
0.005
0.000
HG001
ΔclpX
clpXcompl
growth phase
exp
stat

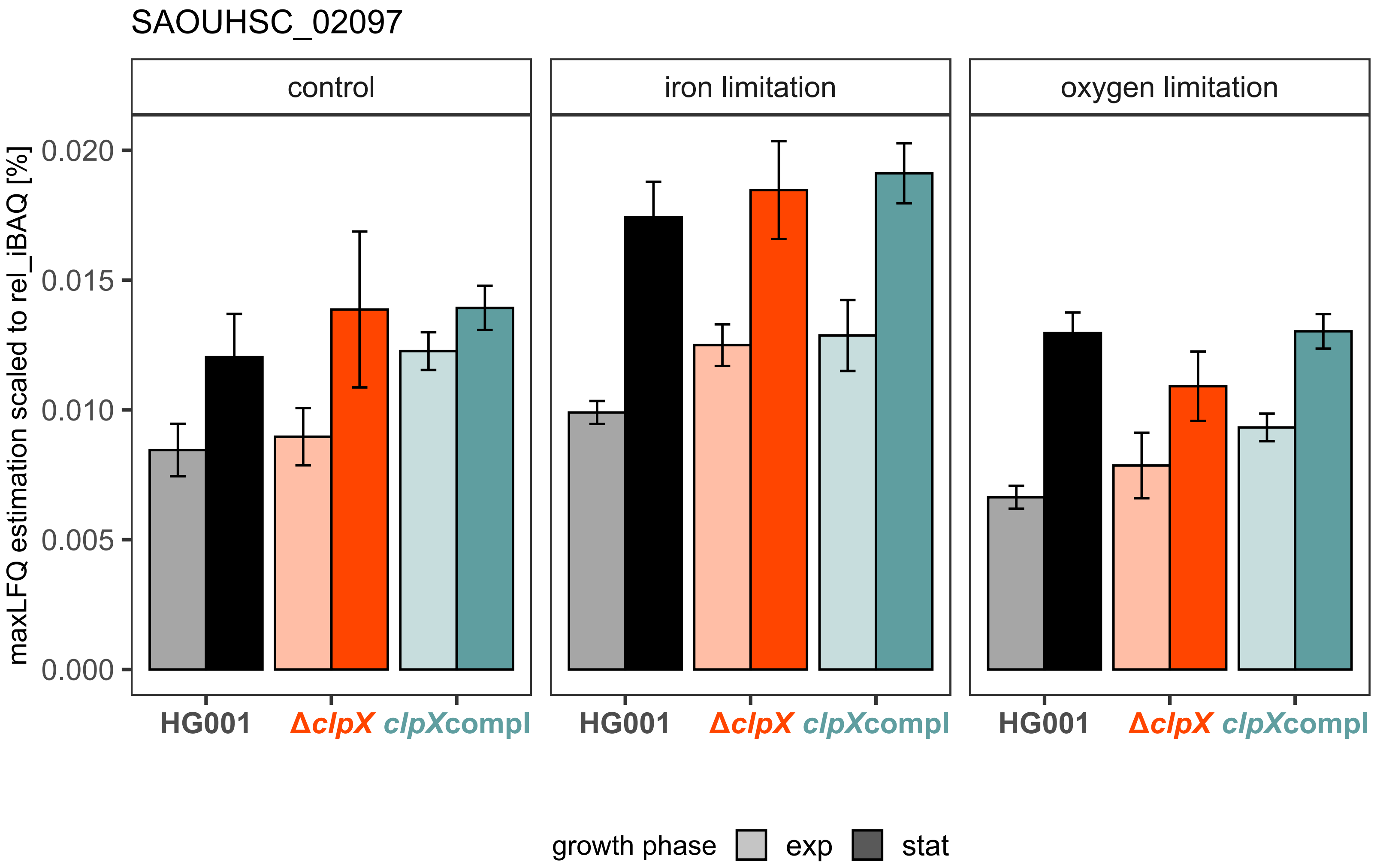

SAOUHSC_02097
control
iron limitation
oxygen limitation
maxLFQ estimation scaled to rel_iBAQ [%]
0.020
0.015
0.010
0.005
0.000
HG001
ΔclpX
clpXcompl
growth phase
exp
stat

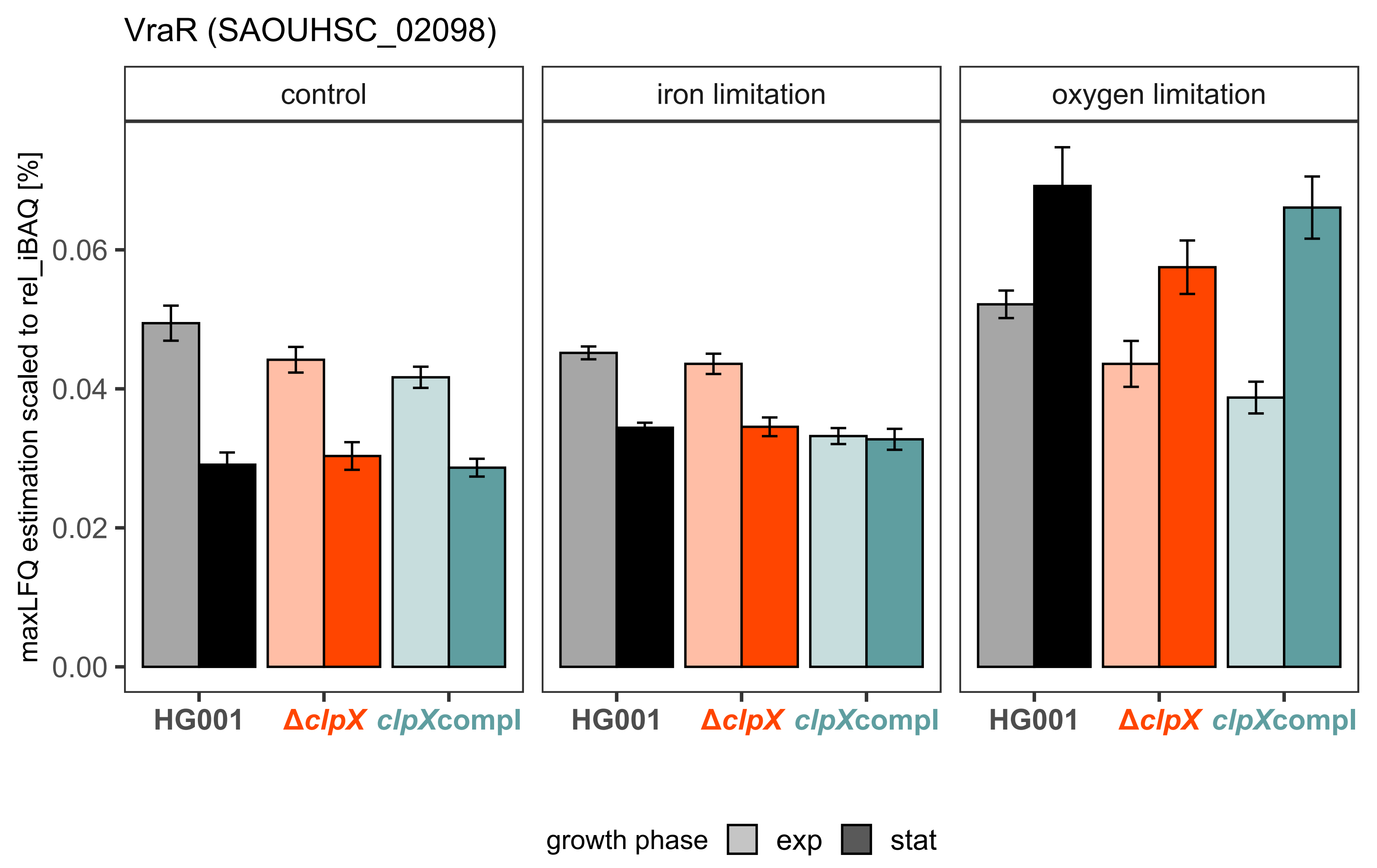

VraR (SAOUHSC_02098)
control
iron limitation
oxygen limitation
maxLFQ estimation scaled to rel_iBAQ [%]
0.06
0.04
0.02
0.00
HG001
ΔclpX
clpXcompl
growth phase
exp
stat

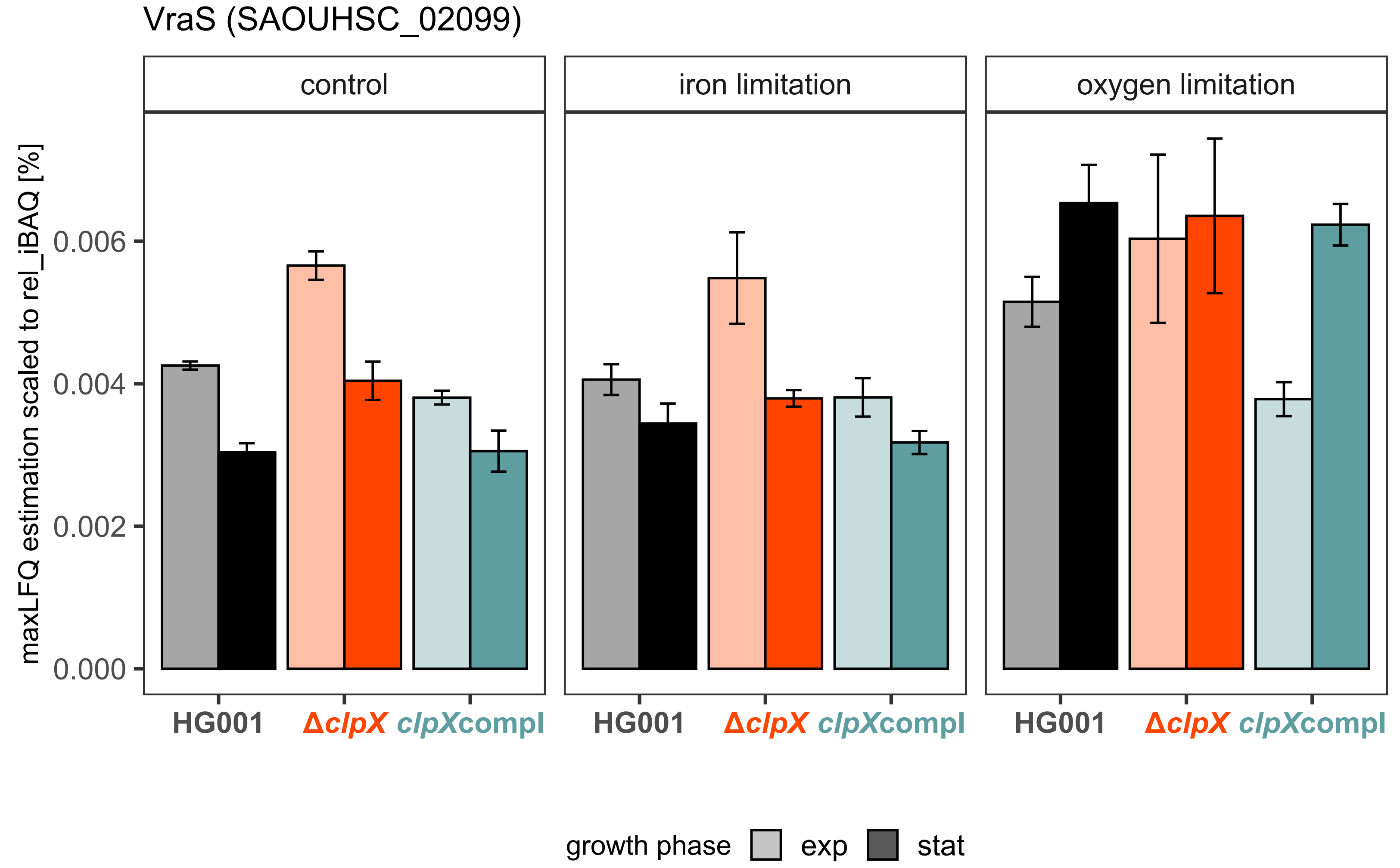
VraS (SAOUHSC_02099)
control
iron limitation
oxygen limitation
maxLFQ estimation scaled to rel_iBAQ [%]
0.006
0.004
0.002
0.000
HG001
ΔclpX
clpXcompl
growth phase
exp
stat

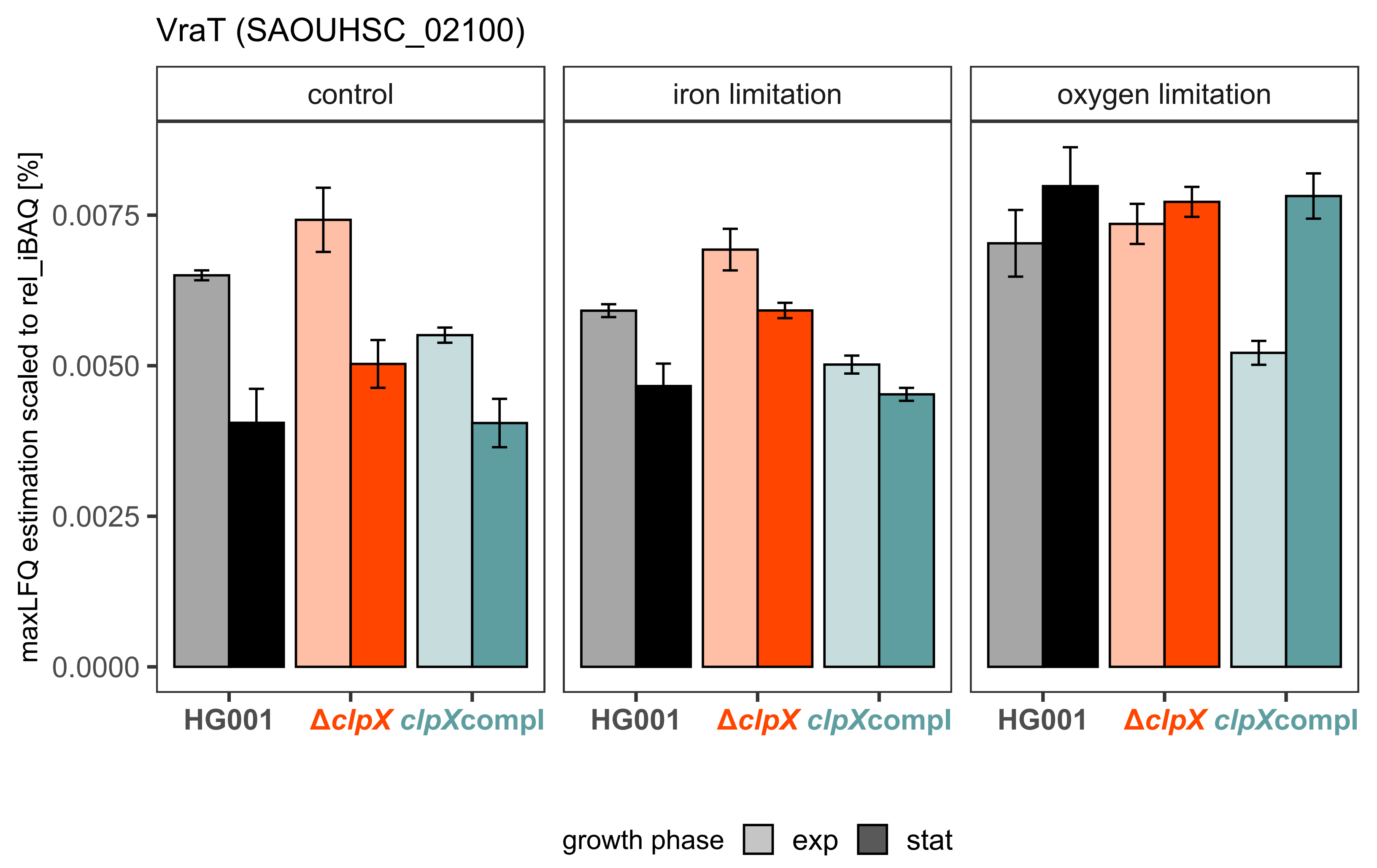

VraT (SAOUHSC_02100)
control
iron limitation
oxygen limitation
maxLFQ estimation scaled to rel_iBAQ [%]
0.0075
0.0050
0.0025
0.0000
HG001
ΔclpX
clpXcompl
growth phase
exp
stat

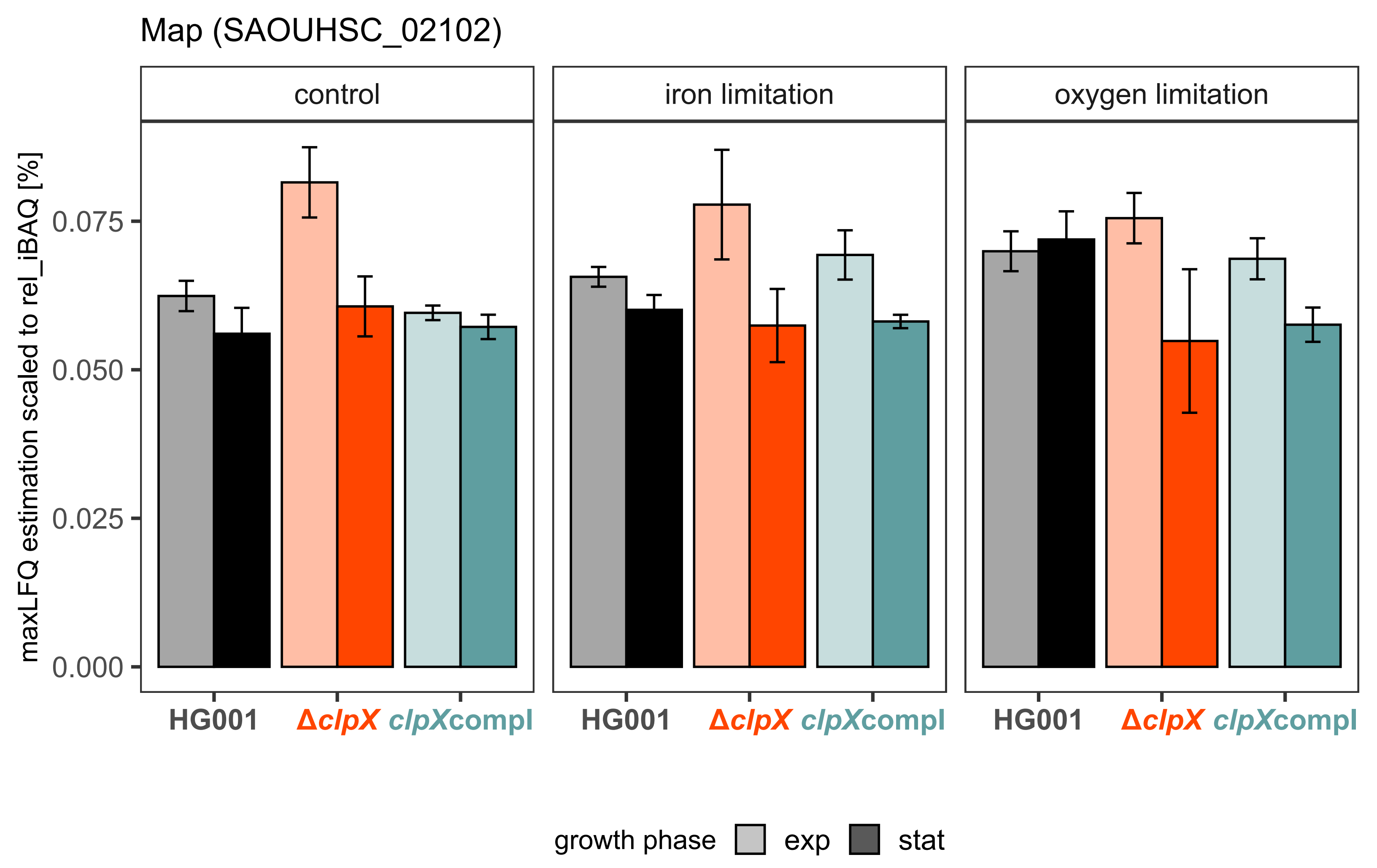
Map (SAOUHSC_02102)
control
iron limitation
oxygen limitation
maxLFQ estimation scaled to rel_iBAQ [%]
0.075
0.050
0.025
0.000
HG001
ΔclpX
clpXcompl
growth phase
exp
stat

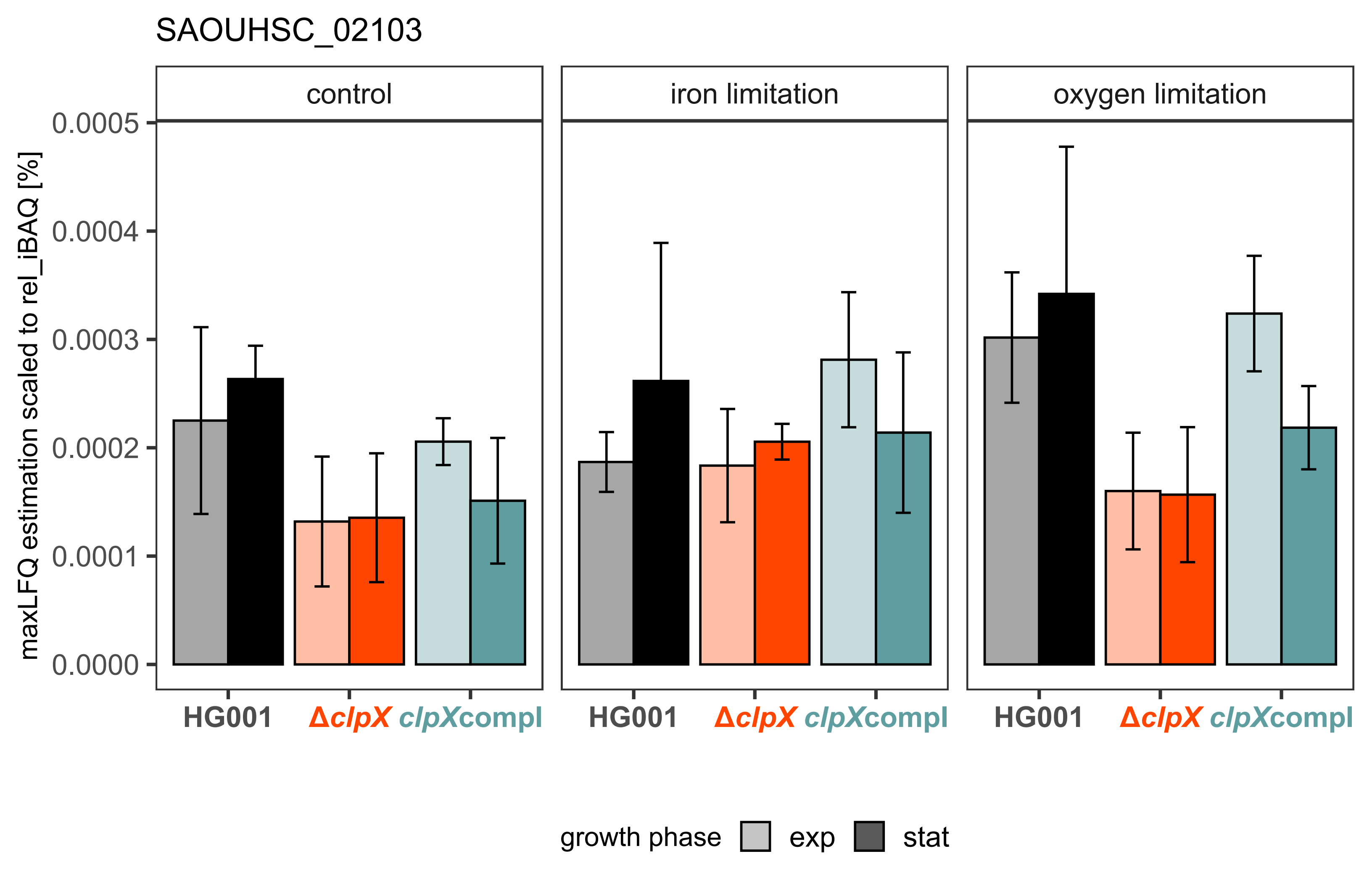
SAOUHSC_02103
control
iron limitation
oxygen limitation
maxLFQ estimation scaled to rel_iBAQ [%]
0.0005
0.0004
0.0003
0.0002
0.0001
0.0000
HG001
ΔclpX
clpXcompl
growth phase
exp
stat

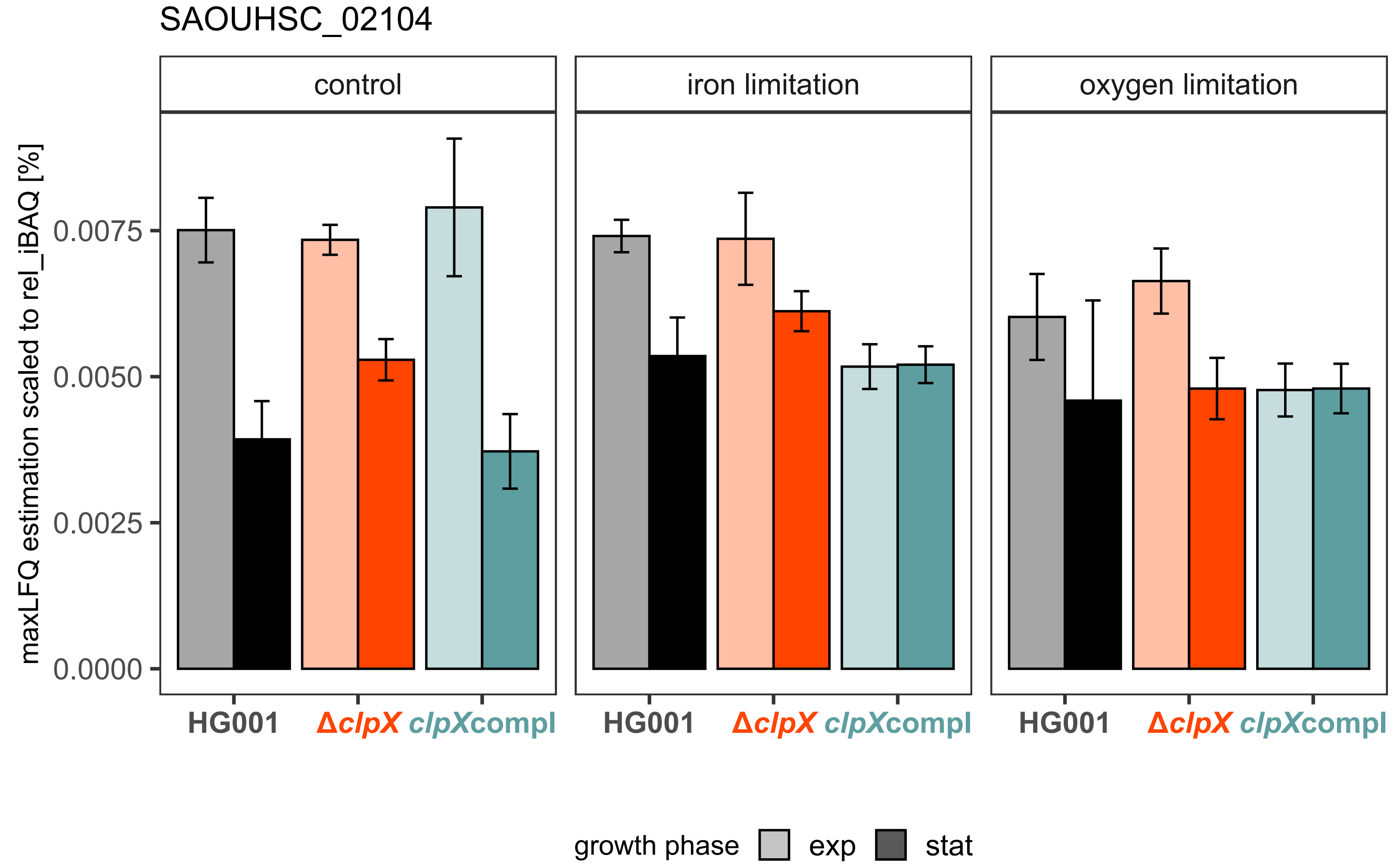
SAOUHSC_02104
control
iron limitation
oxygen limitation
maxLFQ estimation scaled to rel_iBAQ [%]
0.0075
0.0050
0.0025
0.0000
HG001
ΔclpX
clpXcompl
growth phase
exp
stat

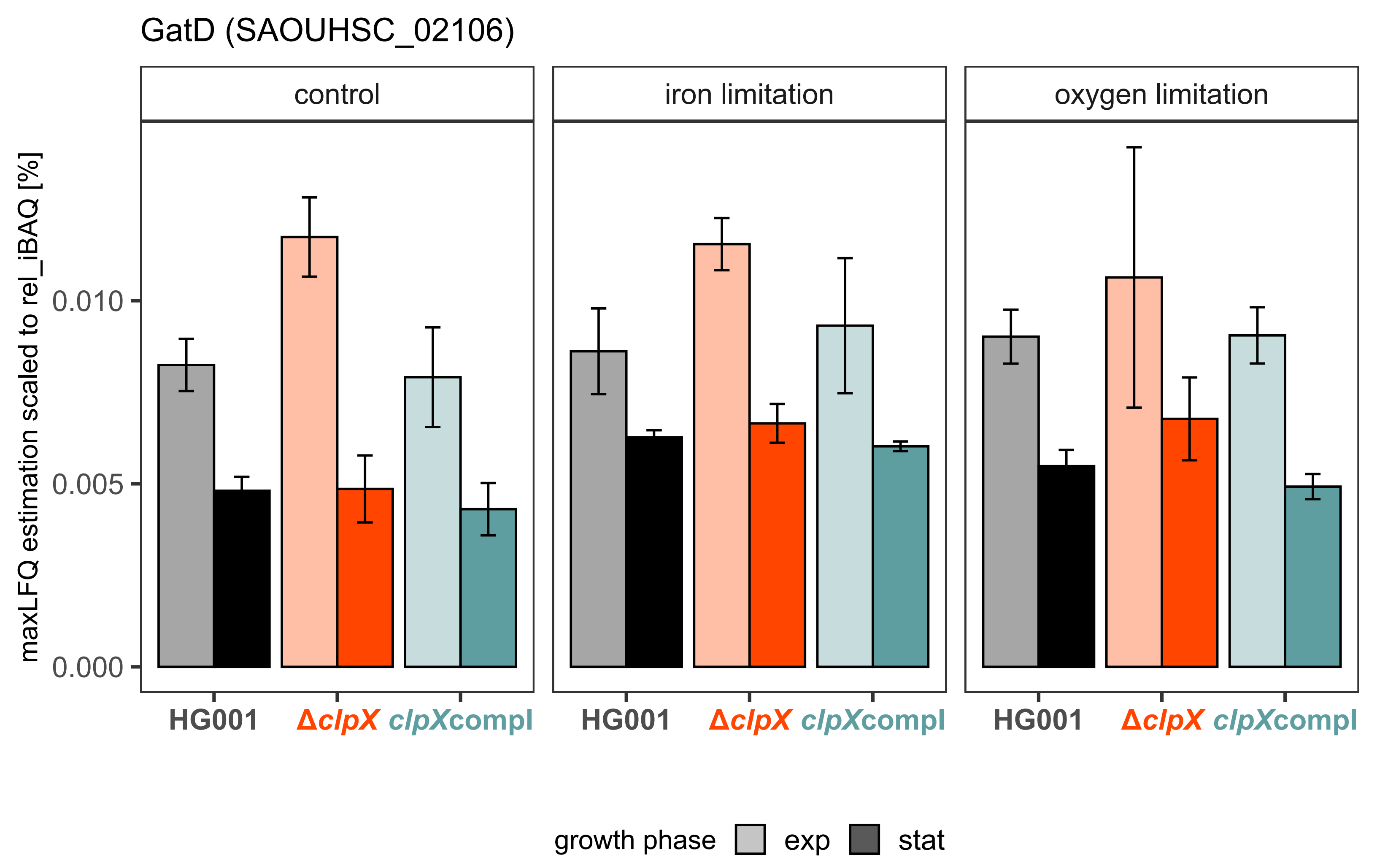

GatD (SAOUHSC_02106)
control
iron limitation
oxygen limitation
maxLFQ estimation scaled to rel_iBAQ [%]
0.010
0.005
0.000
HG001
ΔclpX
clpXcompl
growth phase
exp
stat

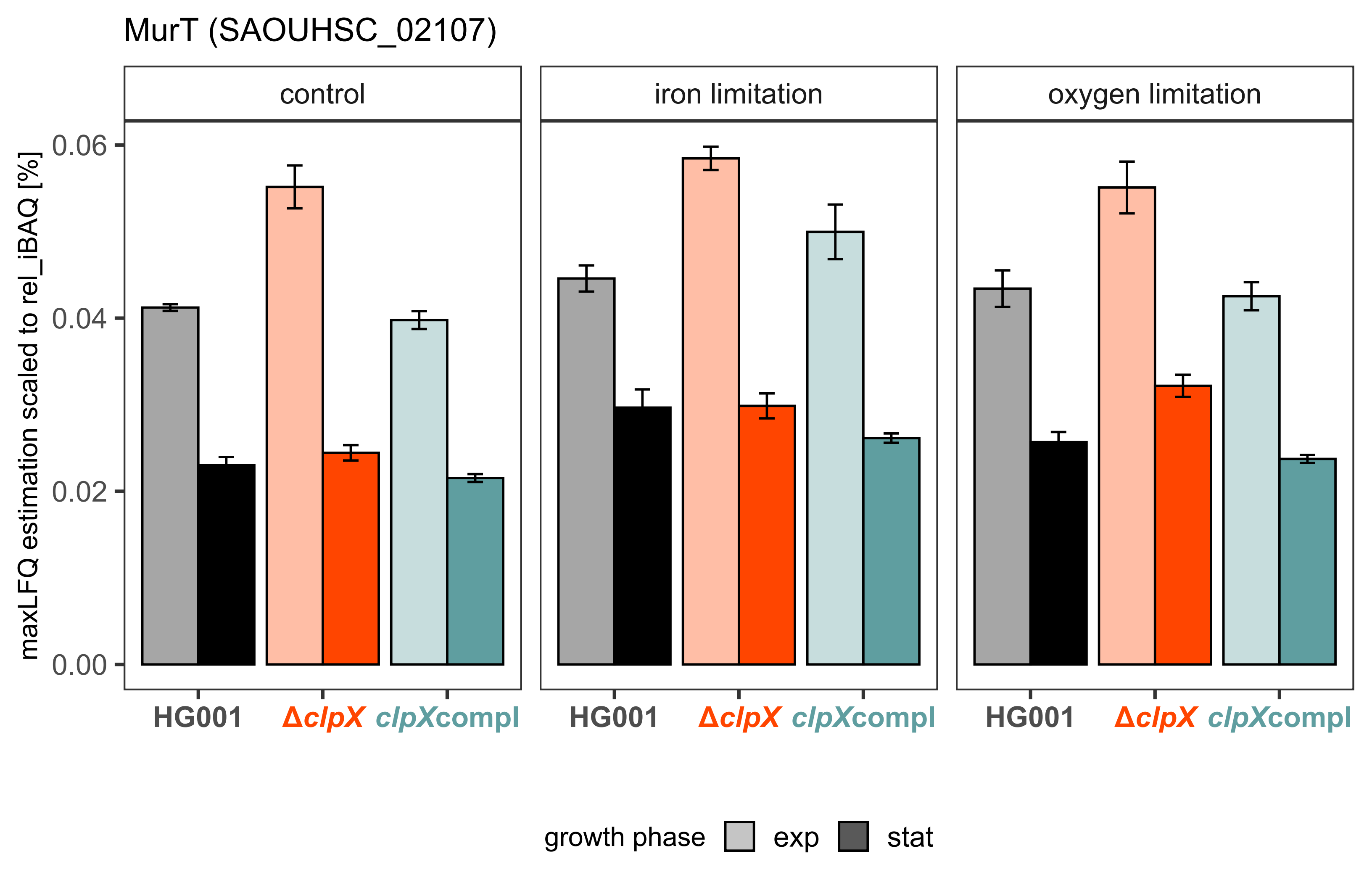

MurT (SAOUHSC_02107)
control
iron limitation
oxygen limitation
maxLFQ estimation scaled to rel_iBAQ [%]
0.06
0.04
0.02
0.00
HG001
ΔclpX
clpXcompl
growth phase
exp
stat

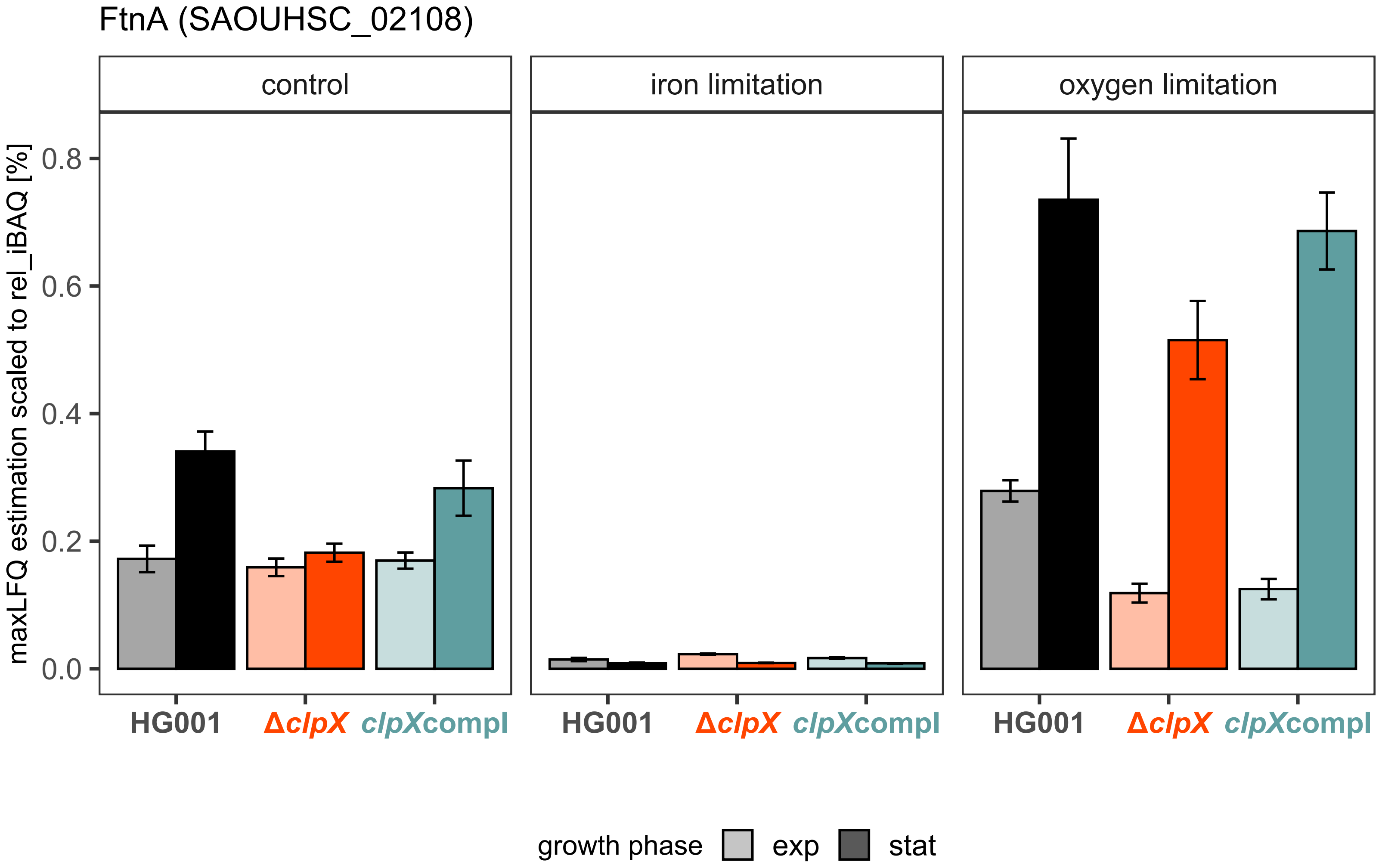
FtnA (SAOUHSC_02108)
control
iron limitation
oxygen limitation
maxLFQ estimation scaled to rel_iBAQ [%]
0.0
0.2
0.4
0.6
0.8
HG001
ΔclpX
clpXcompl
growth phase
exp
stat

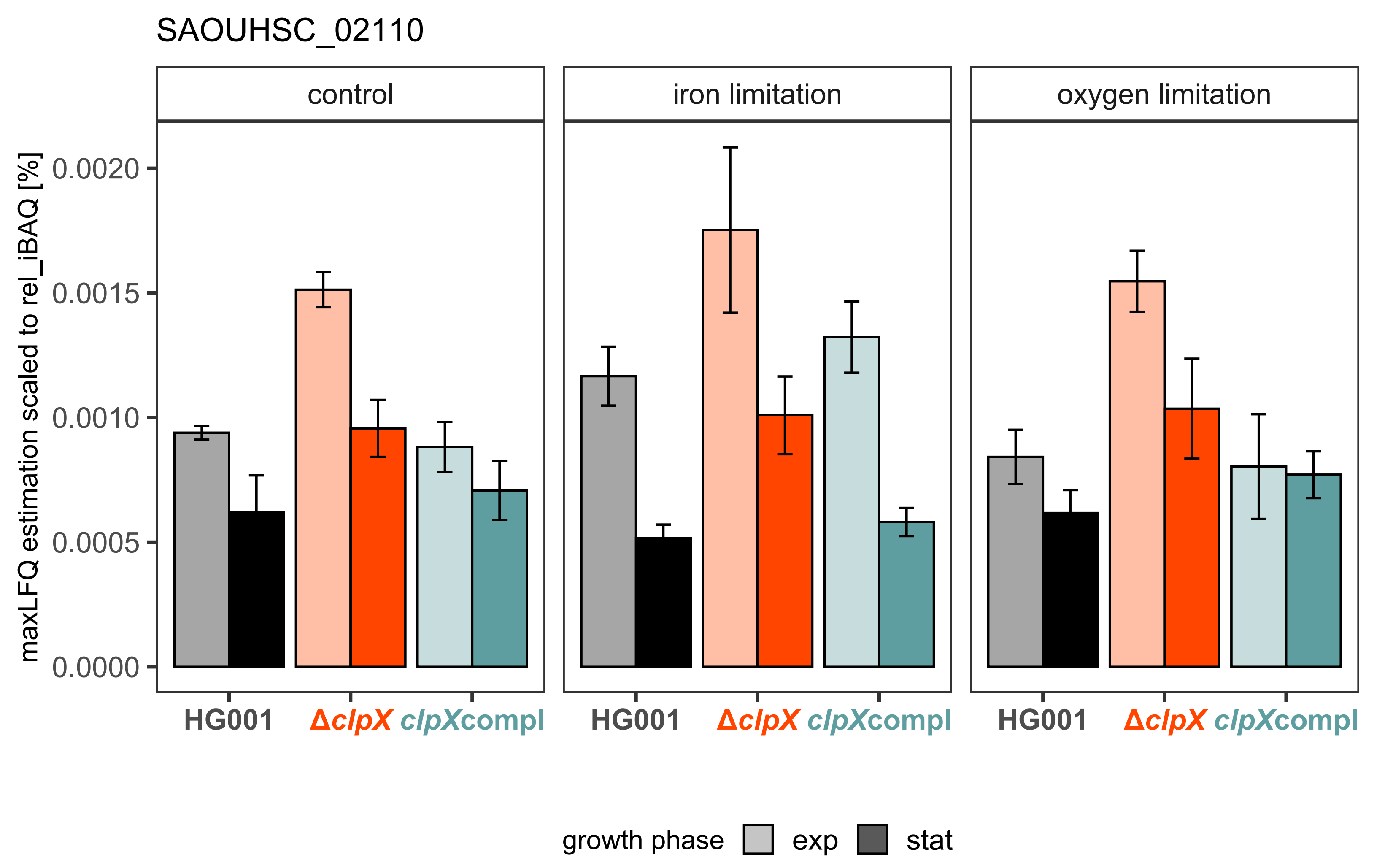

SAOUHSC_02110
control
iron limitation
oxygen limitation
maxLFQ estimation scaled to rel_iBAQ [%]
0.0020
0.0015
0.0010
0.0005
0.0000
HG001
ΔclpX
clpXcompl
growth phase
exp
stat

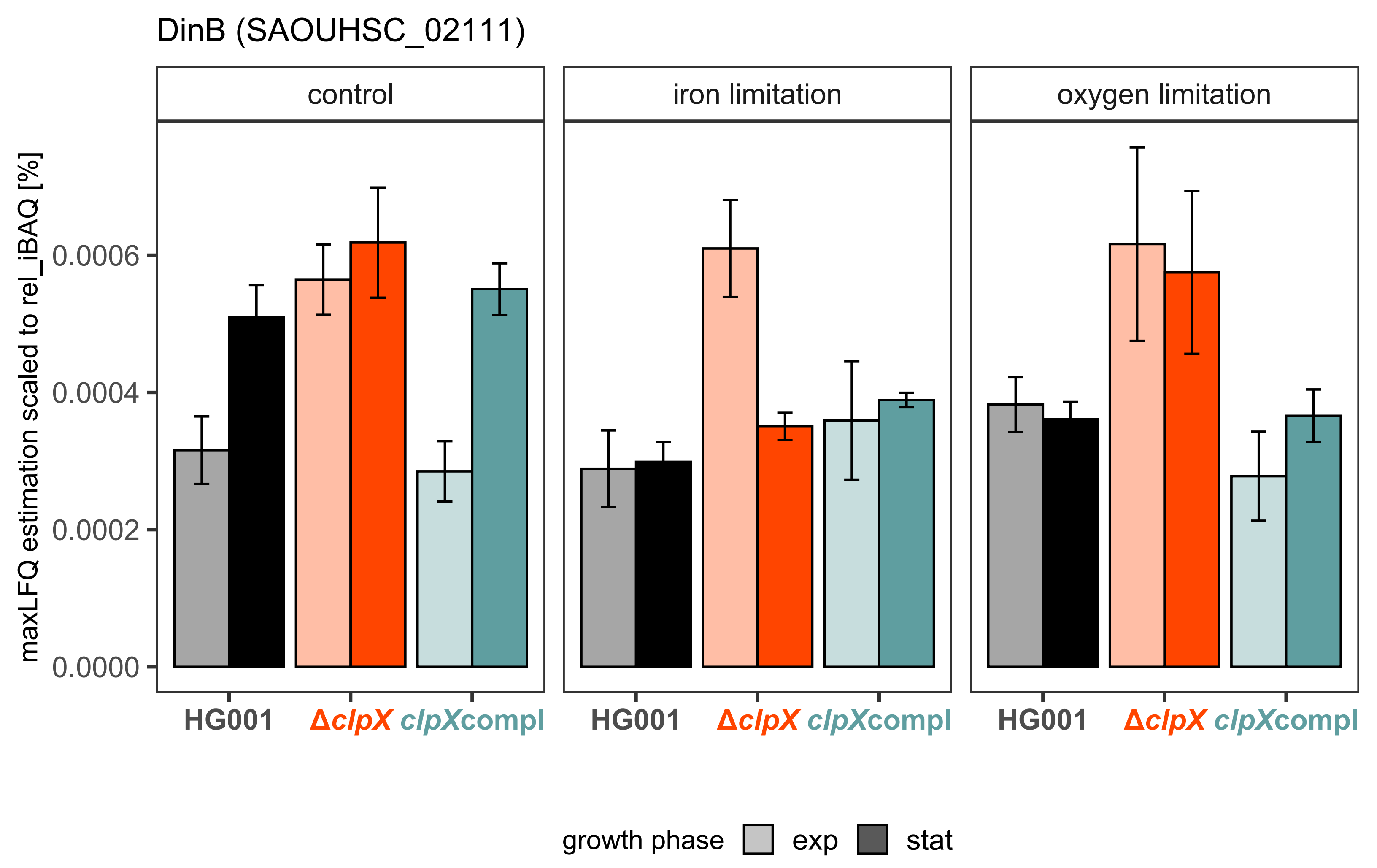
DinB (SAOUHSC_02111)
control
iron limitation
oxygen limitation
maxLFQ estimation scaled to rel_iBAQ [%]
0.0000
0.0002
0.0004
0.0006
HG001
ΔclpX
clpXcompl
growth phase
exp
stat

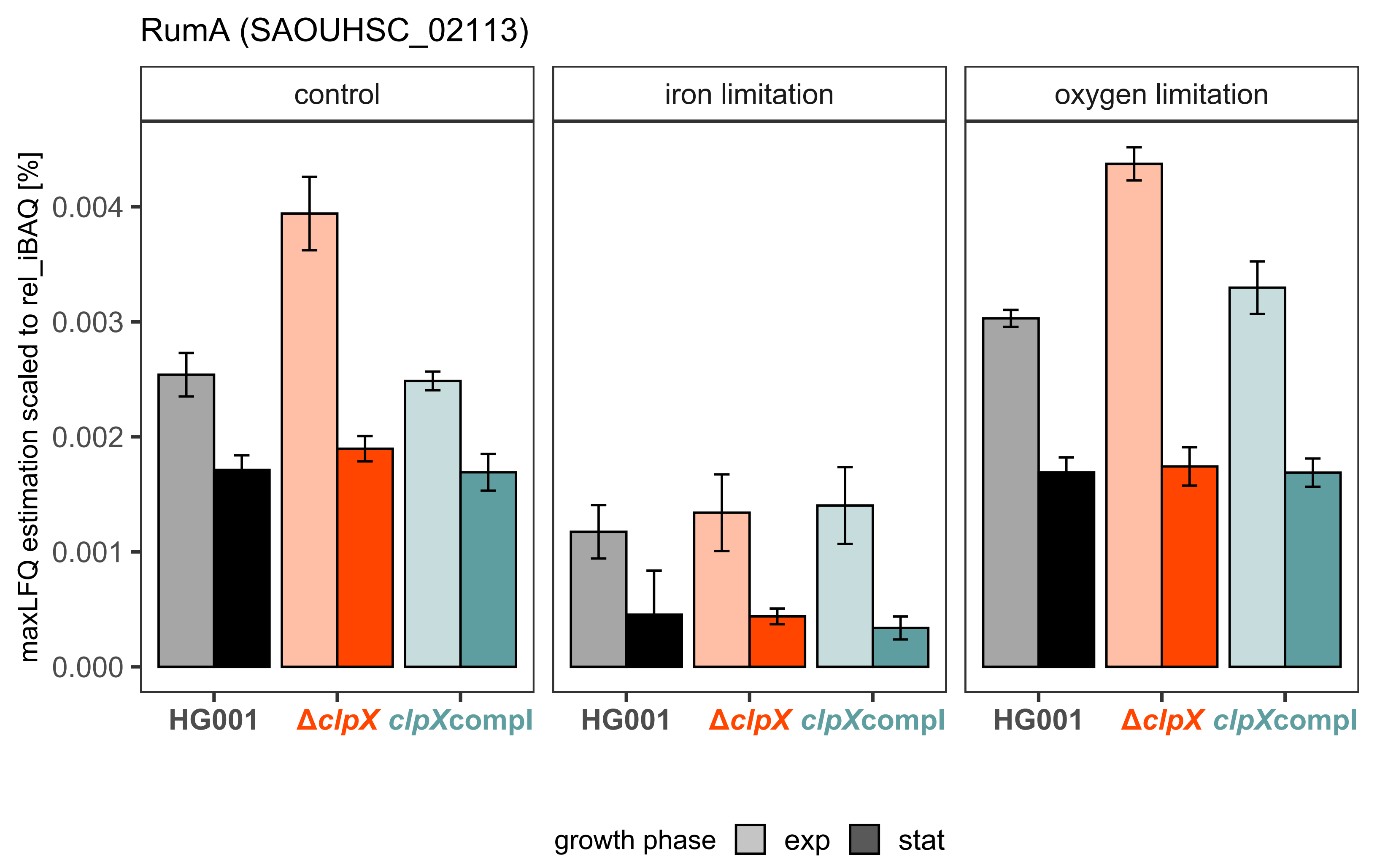
RumA (SAOUHSC_02113)
control
iron limitation
oxygen limitation
maxLFQ estimation scaled to rel_iBAQ [%]
0.004
0.003
0.002
0.001
0.000
HG001
ΔclpX
clpXcompl
growth phase
exp
stat

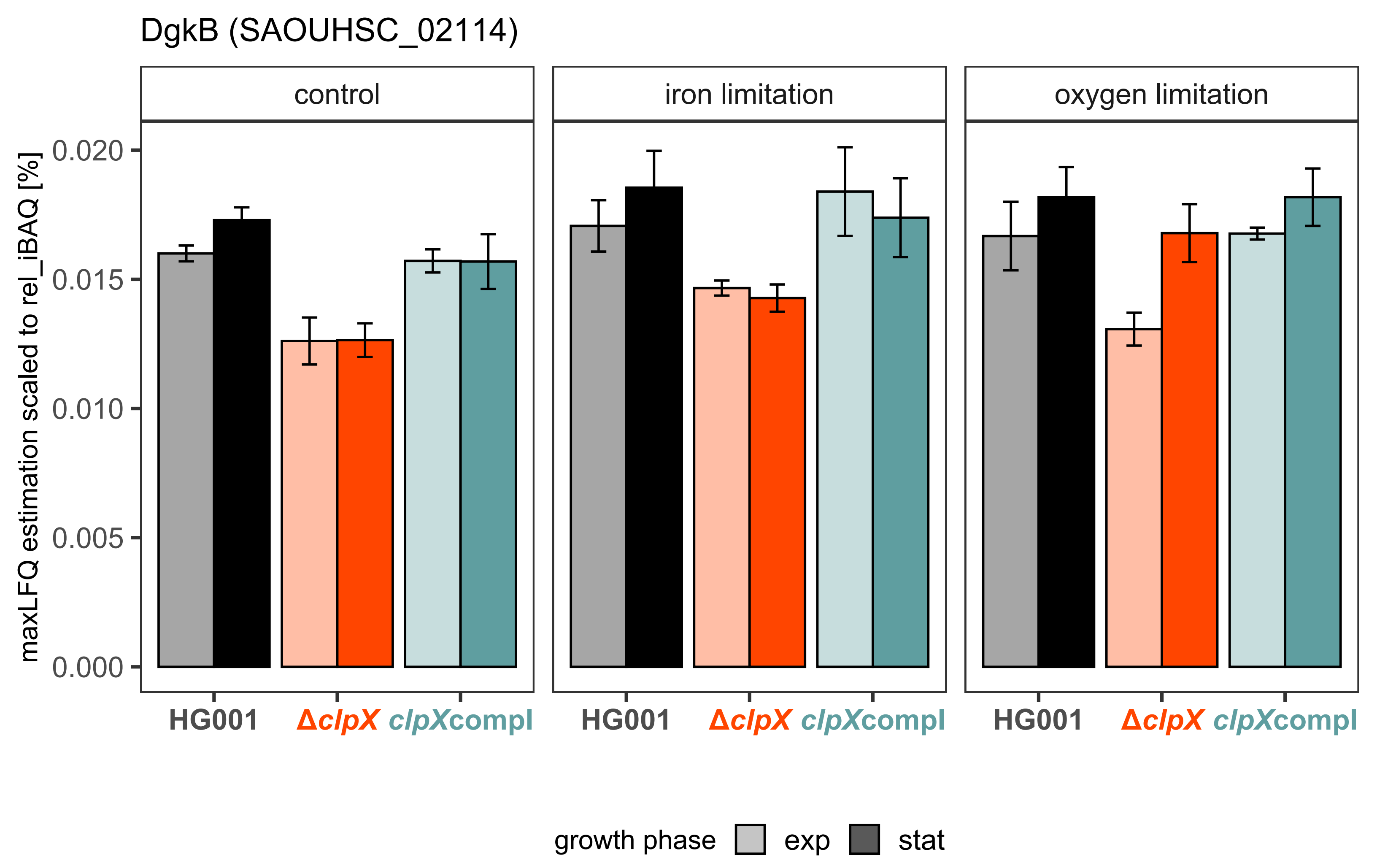
DgkB (SAOUHSC_02114)
control
iron limitation
oxygen limitation
maxLFQ estimation scaled to rel_iBAQ [%]
0.020
0.015
0.010
0.005
0.000
HG001
ΔclpX
clpXcompl
growth phase
exp
stat

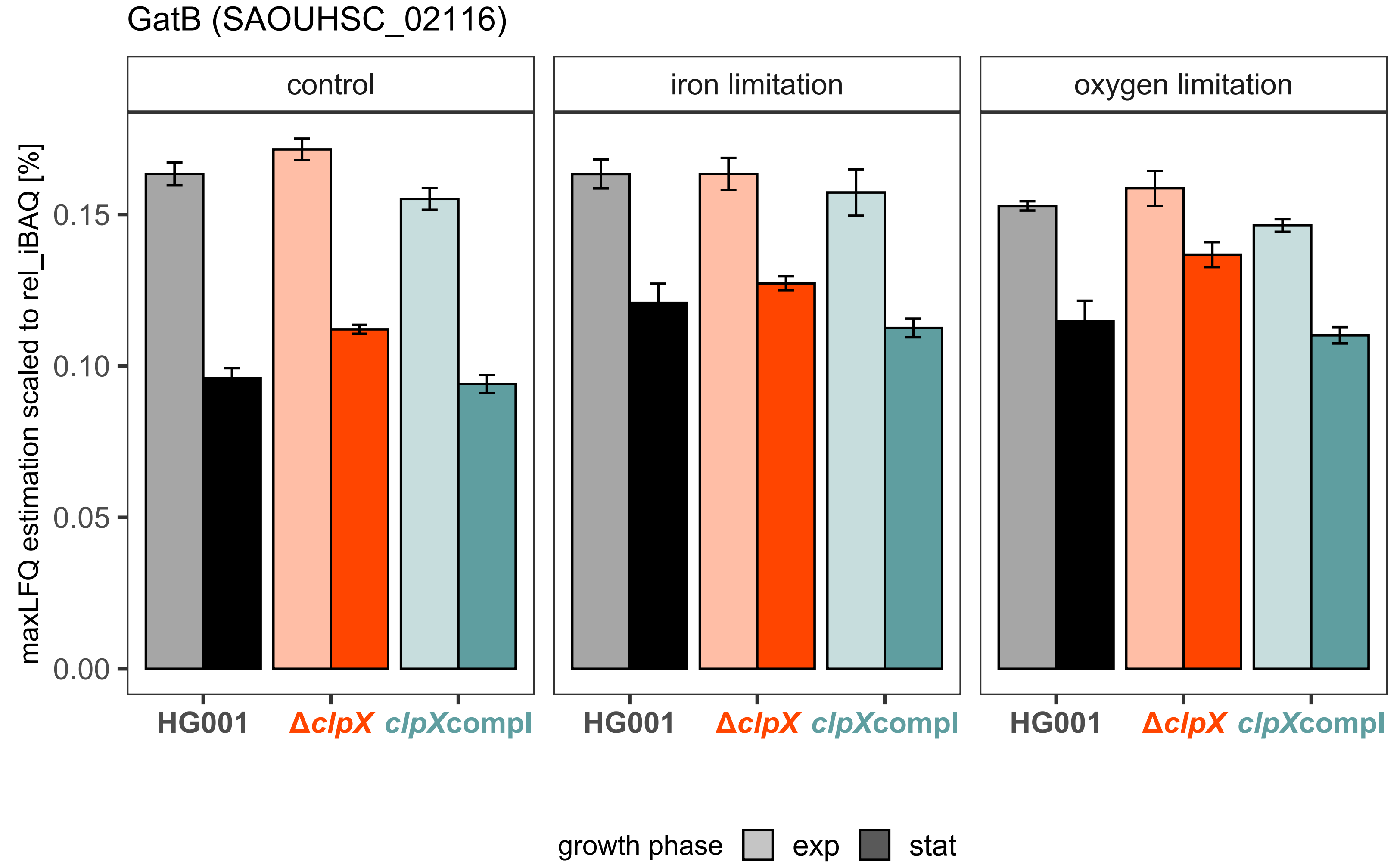
GatB (SAOUHSC_02116)
control
iron limitation
oxygen limitation
maxLFQ estimation scaled to rel_iBAQ [%]
0.15
0.10
0.05
0.00
HG001
ΔclpX
clpXcompl
growth phase
exp
stat

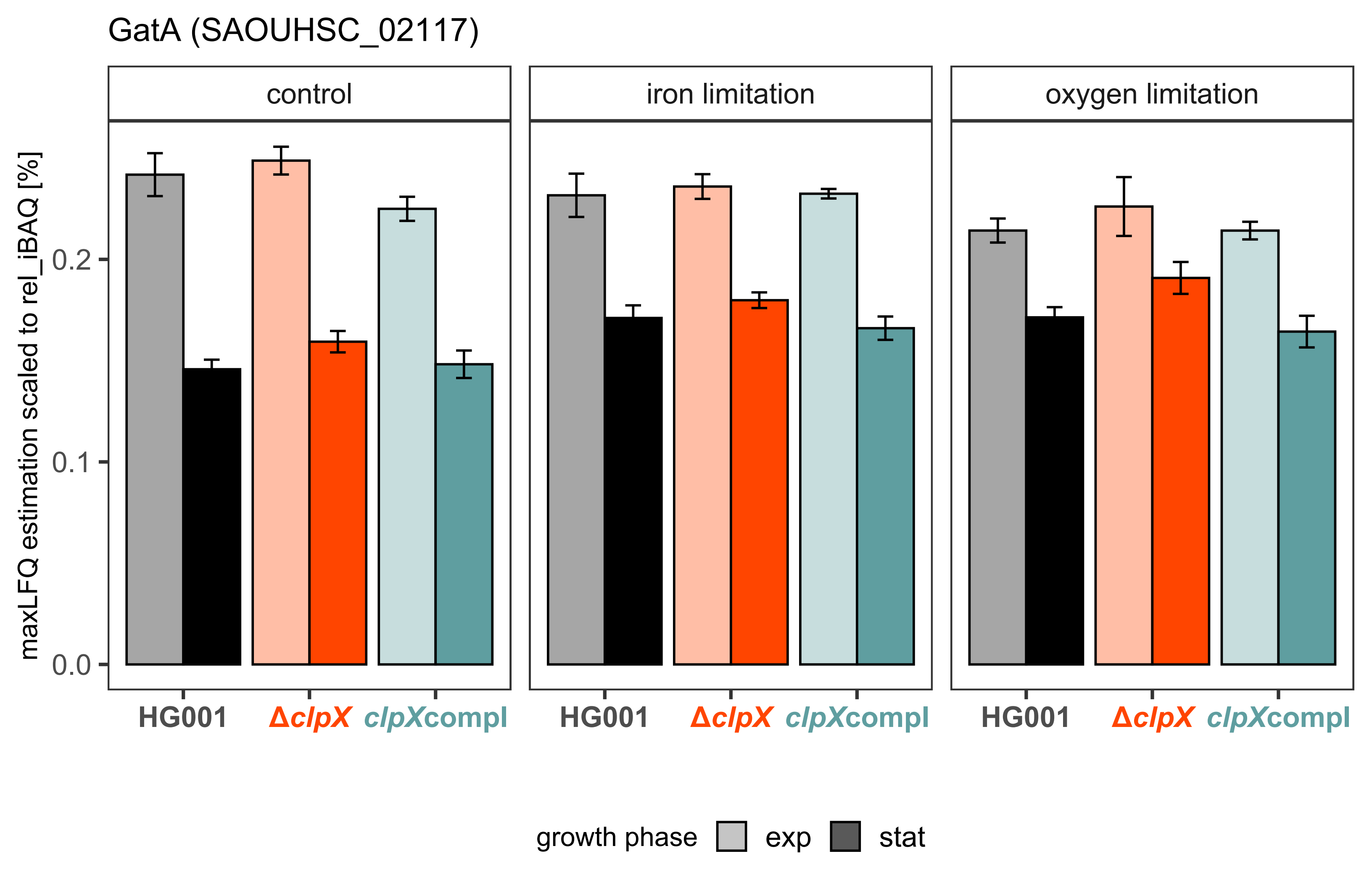
GatA (SAOUHSC_02117)
control
iron limitation
oxygen limitation
maxLFQ estimation scaled to rel_iBAQ [%]
0.2
0.1
0.0
HG001
ΔclpX
clpXcompl
growth phase
exp
stat

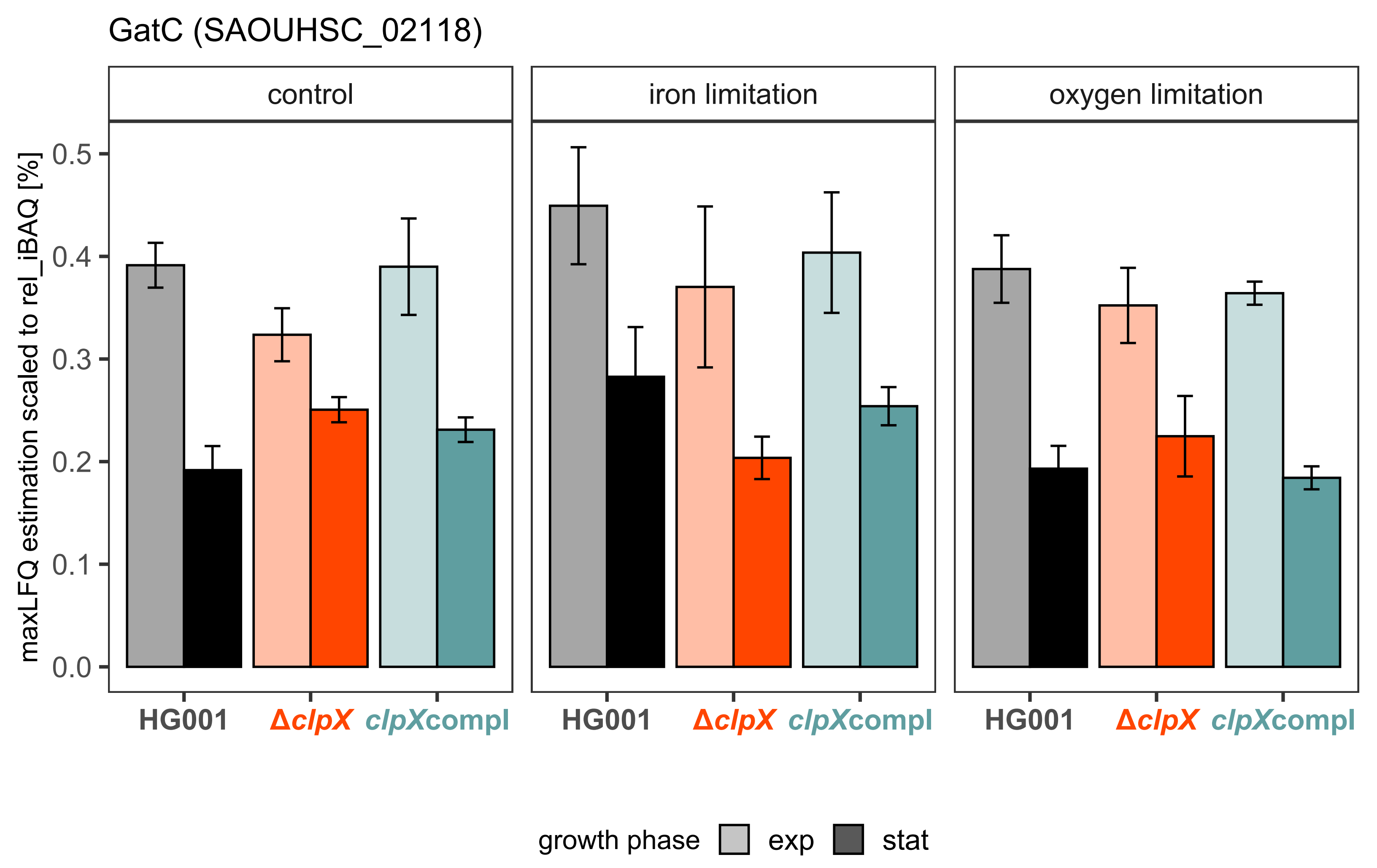
GatC (SAOUHSC_02118)
control
iron limitation
oxygen limitation
maxLFQ estimation scaled to rel_iBAQ [%]
0.0
0.1
0.2
0.3
0.4
0.5
HG001
ΔclpX
clpXcompl
growth phase
exp
stat

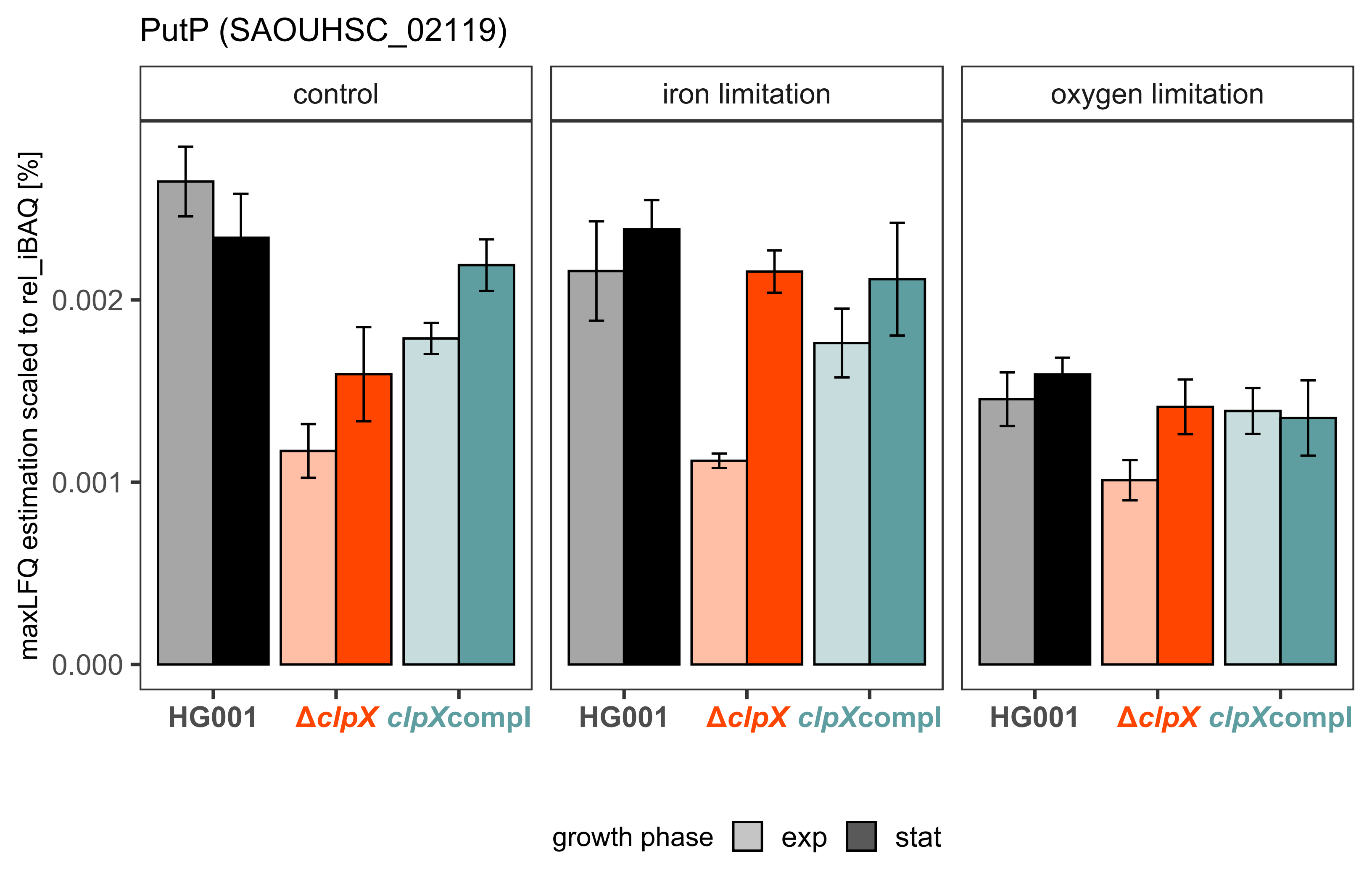

PutP (SAOUHSC_02119)
control
iron limitation
oxygen limitation
maxLFQ estimation scaled to rel_iBAQ [%]
0.002
0.001
0.000
HG001
ΔclpX
clpXcompl
growth phase
exp
stat

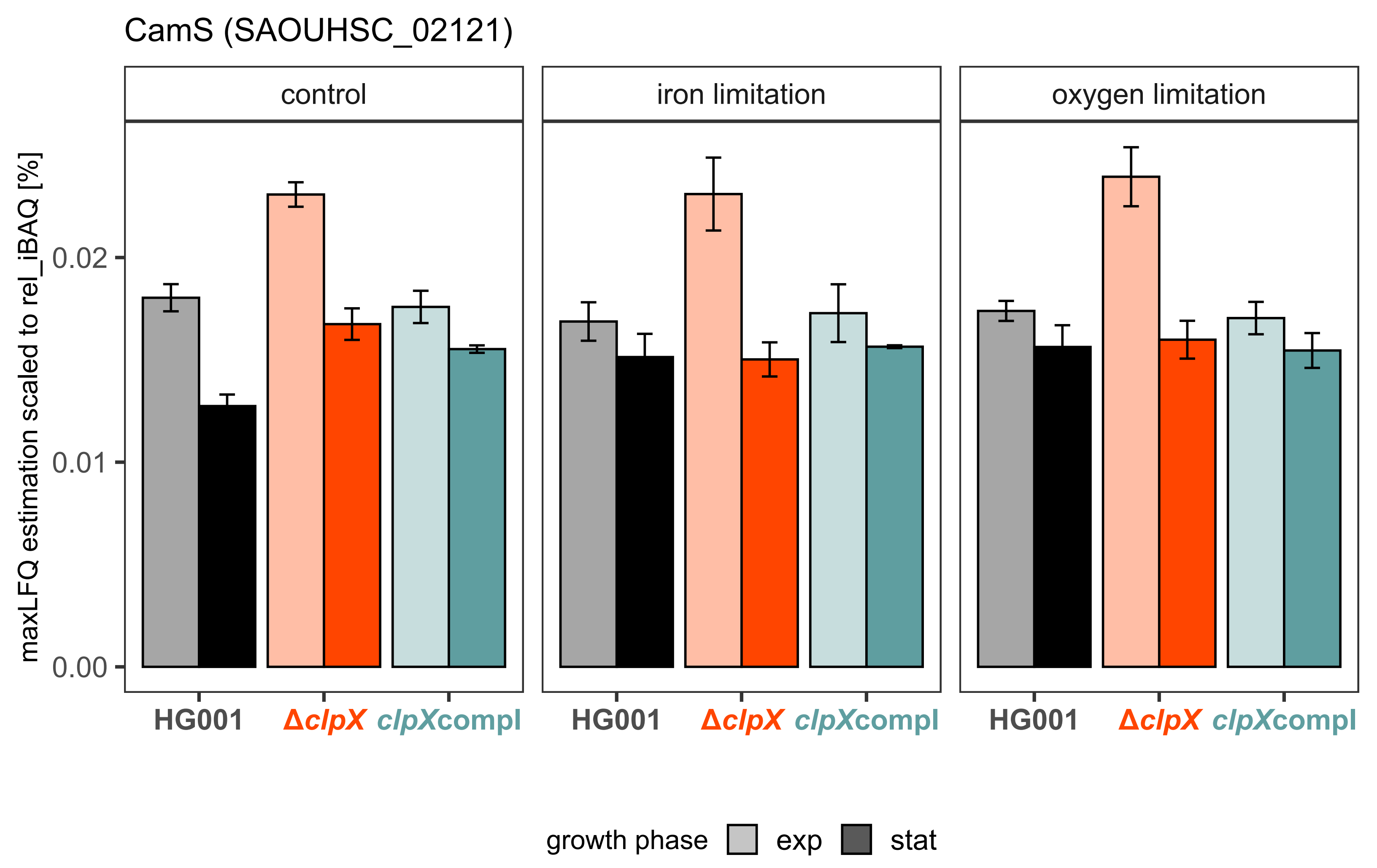
CamS (SAOUHSC_02121)
control
iron limitation
oxygen limitation
maxLFQ estimation scaled to rel_iBAQ [%]
0.00
0.01
0.02
HG001
ΔclpX
clpXcompl
growth phase
exp
stat

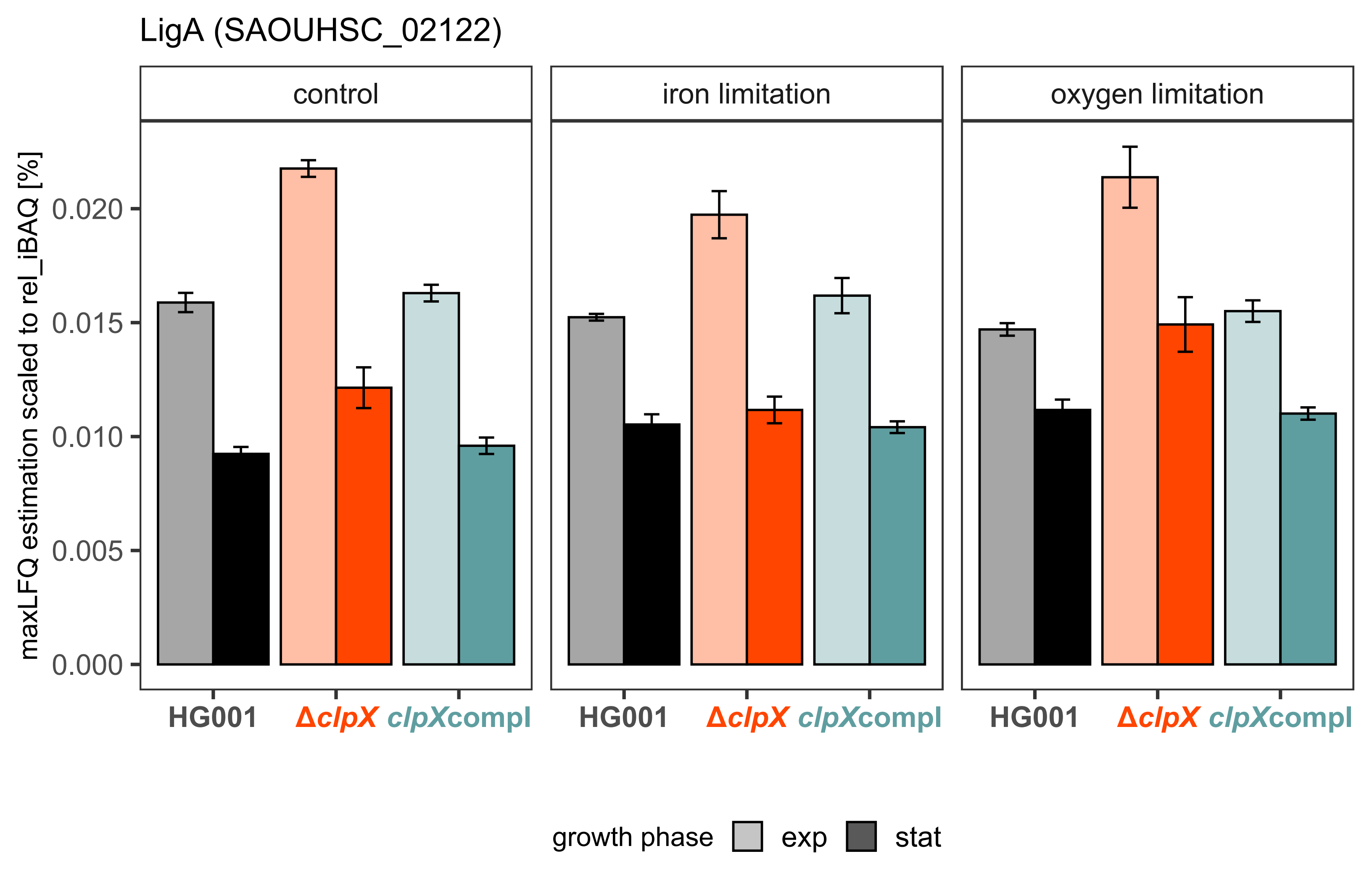

LigA (SAOUHSC_02122)
control
iron limitation
oxygen limitation
maxLFQ estimation scaled to rel_iBAQ [%]
0.000
0.005
0.010
0.015
0.020
HG001
ΔclpX
clpXcompl
growth phase
exp
stat

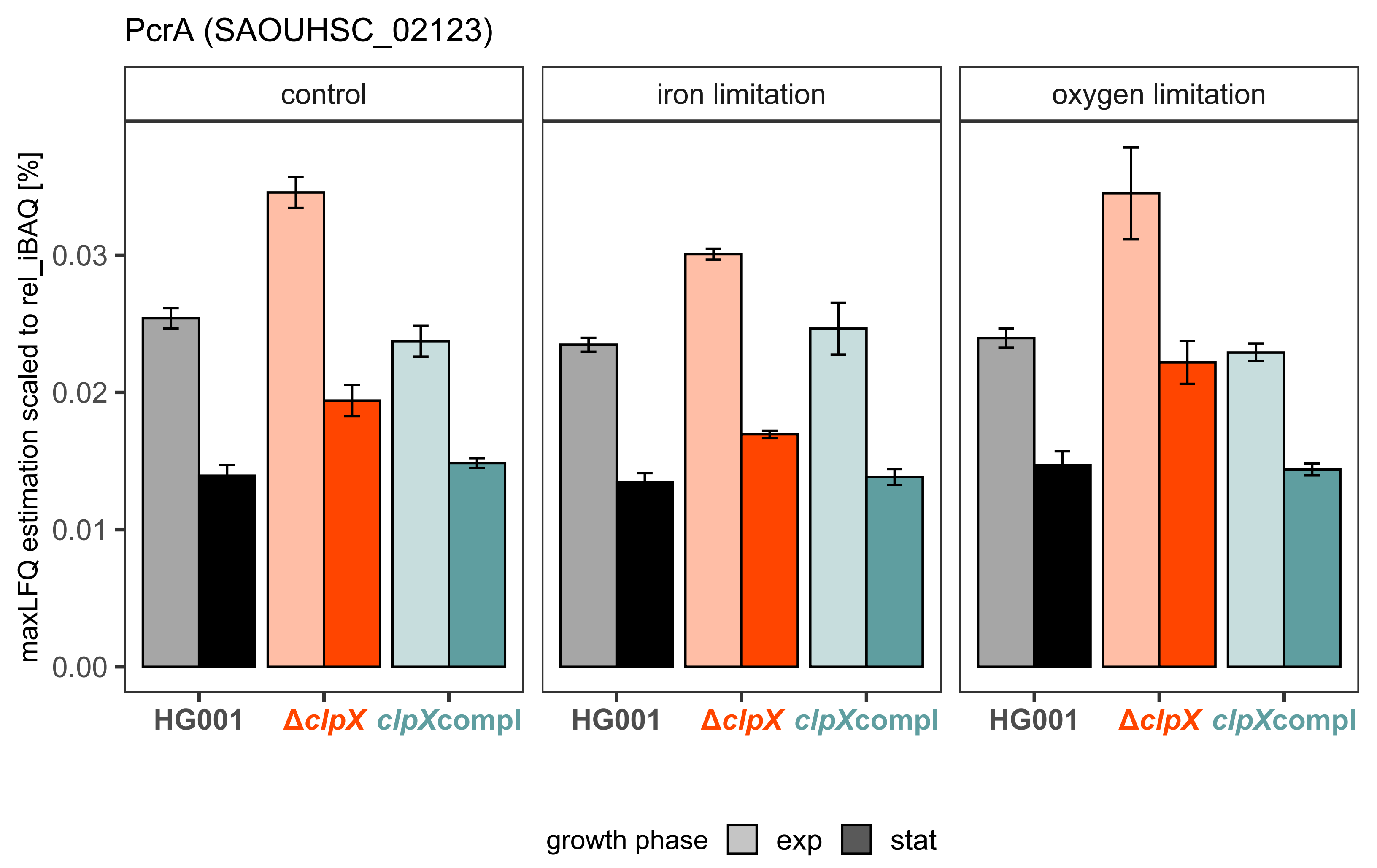
PcrA (SAOUHSC_02123)
control
iron limitation
oxygen limitation
maxLFQ estimation scaled to rel_iBAQ [%]
0.03
0.02
0.01
0.00
HG001
ΔclpX
clpXcompl
growth phase
exp
stat

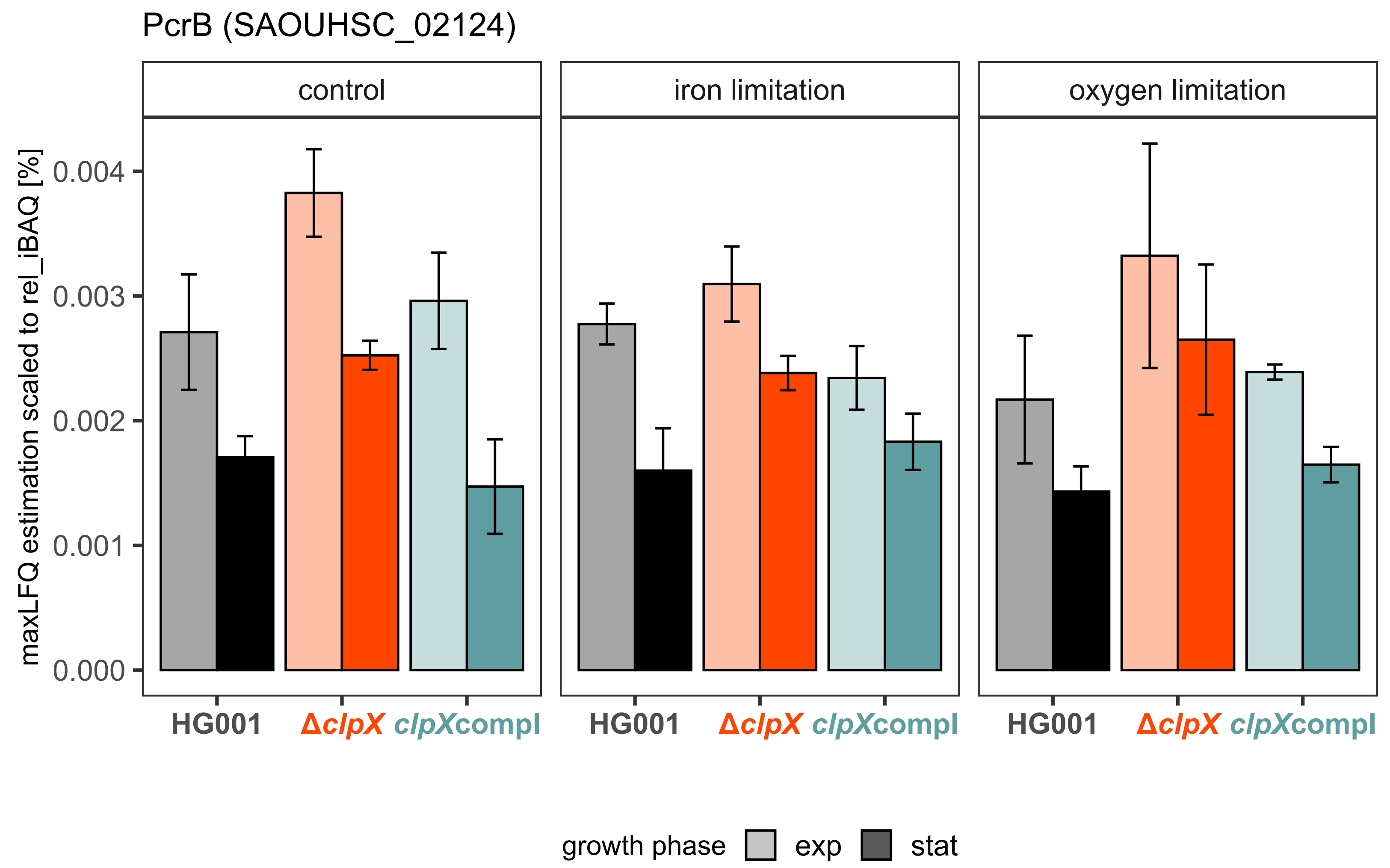

PcrB (SAOUHSC_02124)
control
iron limitation
oxygen limitation
maxLFQ estimation scaled to rel_iBAQ [%]
0.004
0.003
0.002
0.001
0.000
HG001
ΔclpX
clpXcompl
growth phase
exp
stat

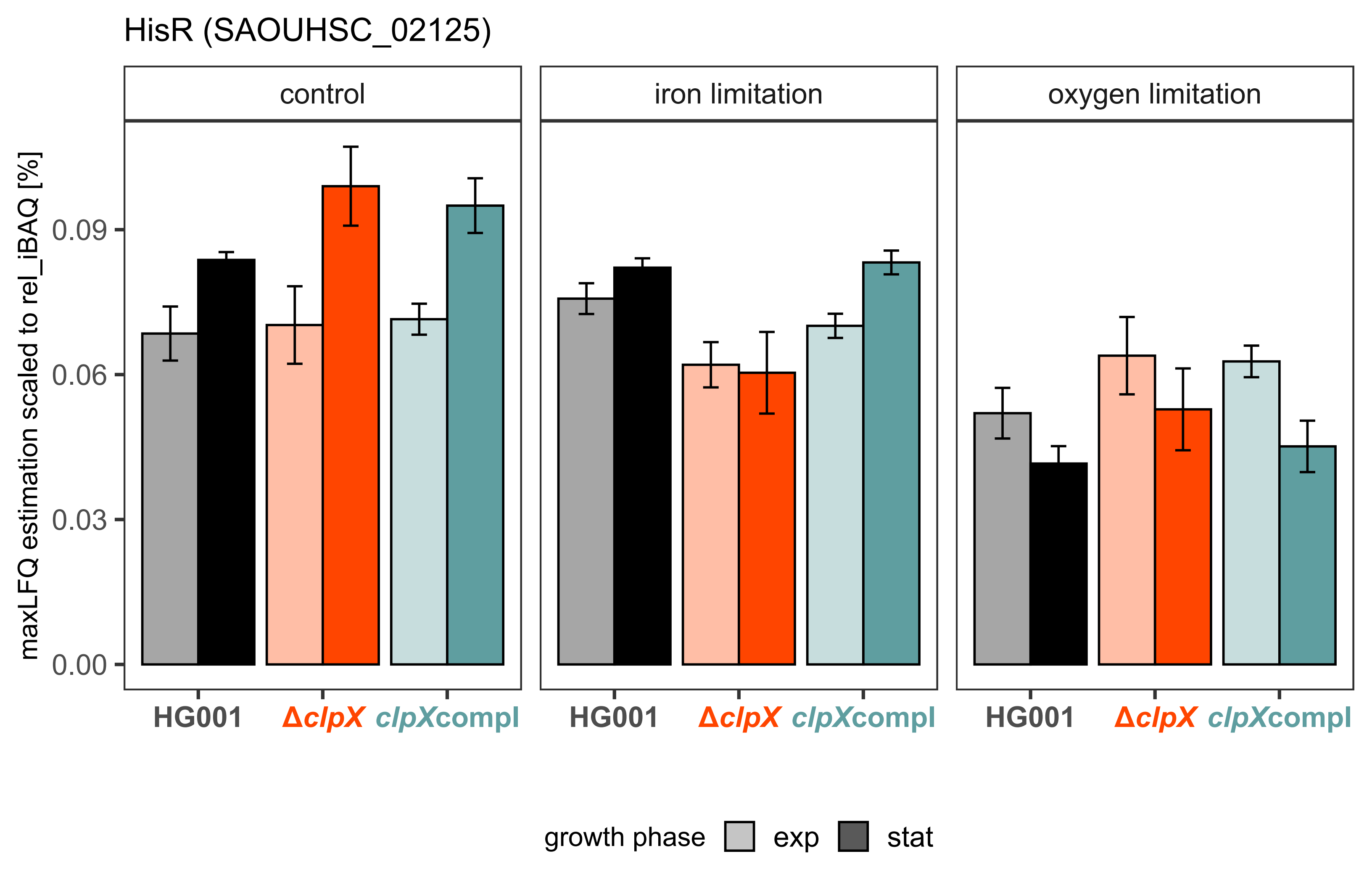

HisR (SAOUHSC_02125)
control
iron limitation
oxygen limitation
maxLFQ estimation scaled to rel_iBAQ [%]
0.09
0.06
0.03
0.00
HG001
ΔclpX
clpXcompl
growth phase
exp
stat

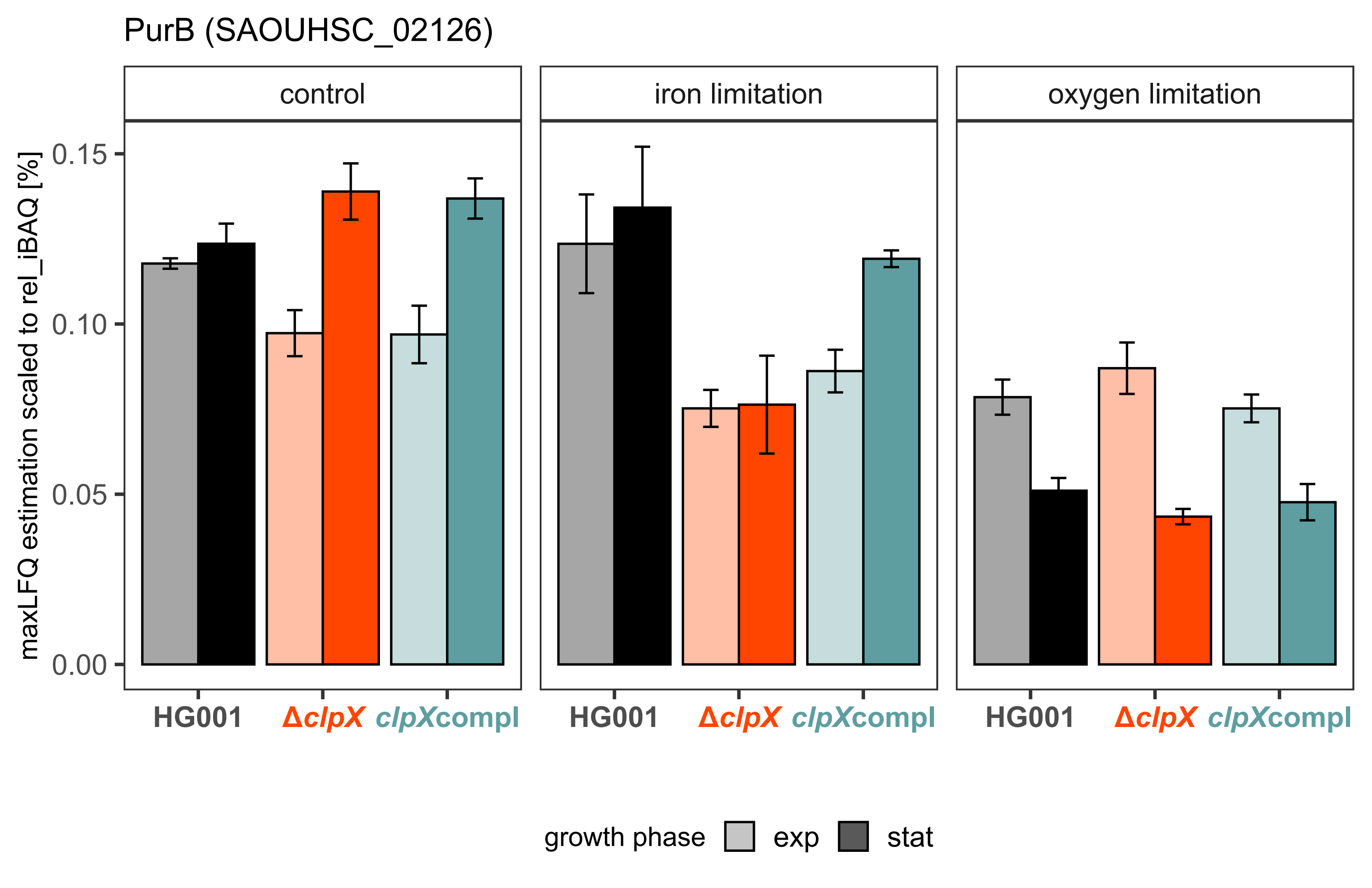

PurB (SAOUHSC_02126)
control
iron limitation
oxygen limitation
maxLFQ estimation scaled to rel_iBAQ [%]
0.15
0.10
0.05
0.00
HG001
ΔclpX
clpXcompl
growth phase
exp
stat

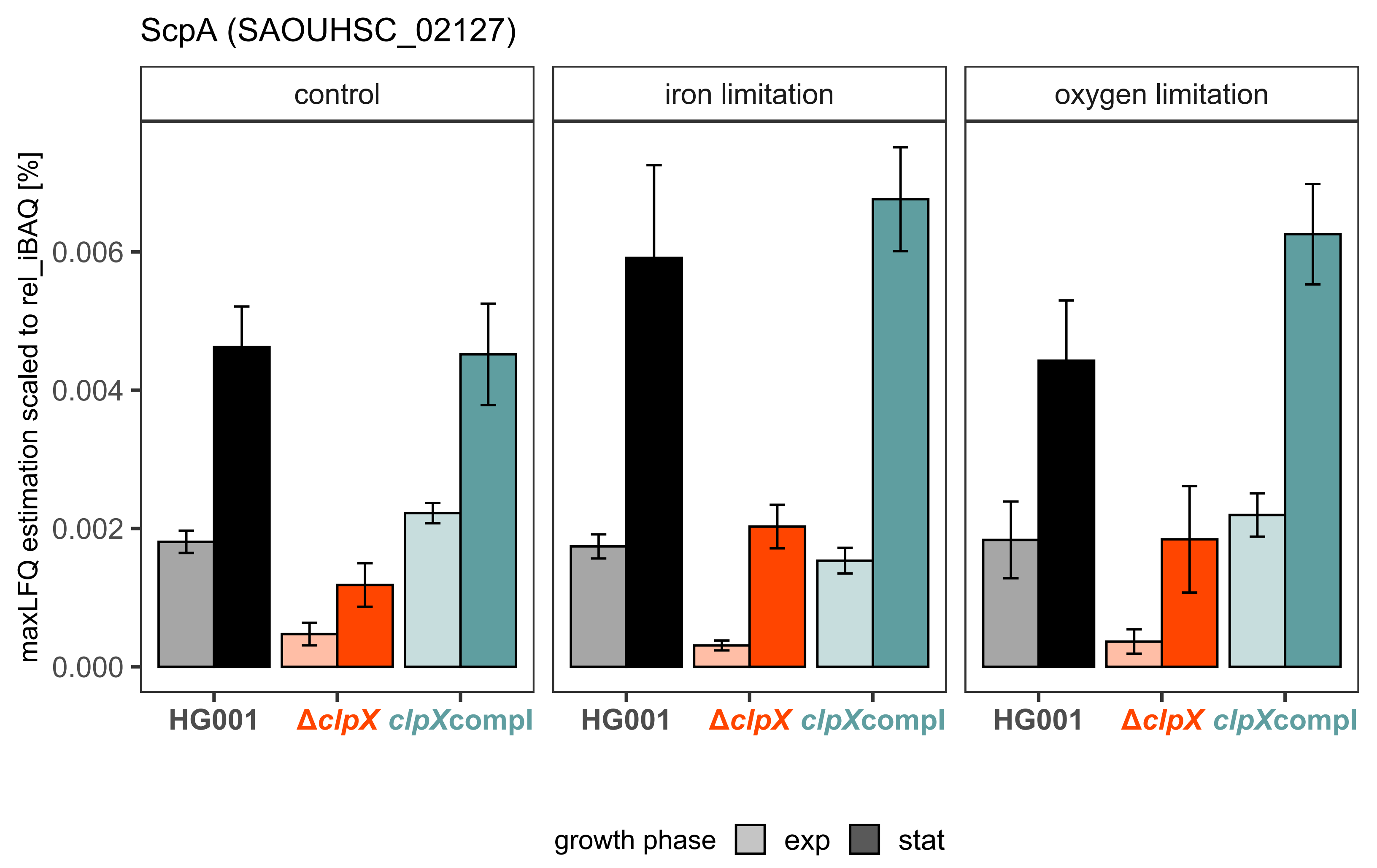
ScpA (SAOUHSC_02127)
control
iron limitation
oxygen limitation
maxLFQ estimation scaled to rel_iBAQ [%]
0.006
0.004
0.002
0.000
HG001
ΔclpX
clpXcompl
growth phase
exp
stat

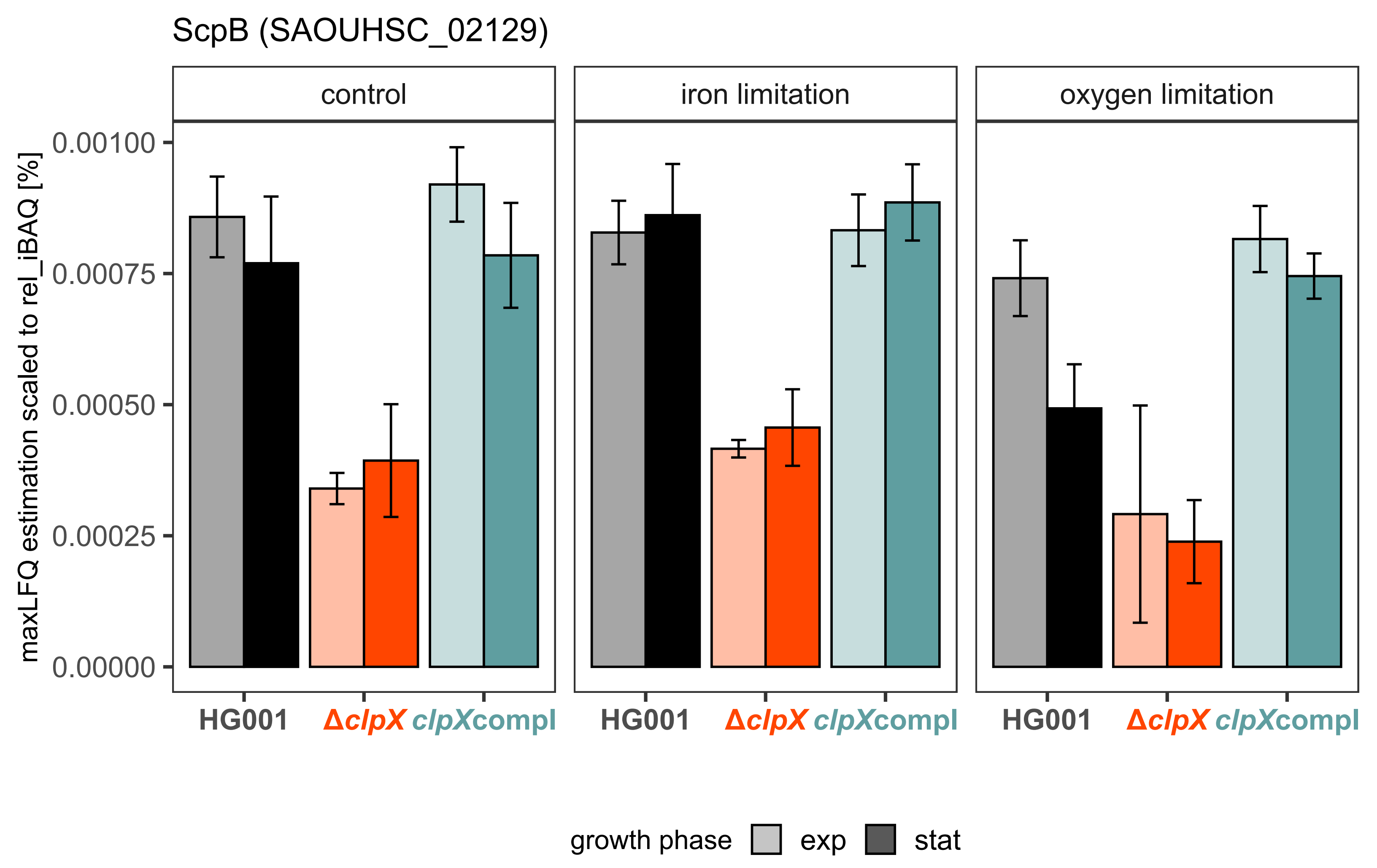

ScpB (SAOUHSC_02129)
control
iron limitation
oxygen limitation
maxLFQ estimation scaled to rel_iBAQ [%]
0.00100
0.00075
0.00050
0.00025
0.00000
HG001
ΔclpX
clpXcompl
growth phase
exp
stat

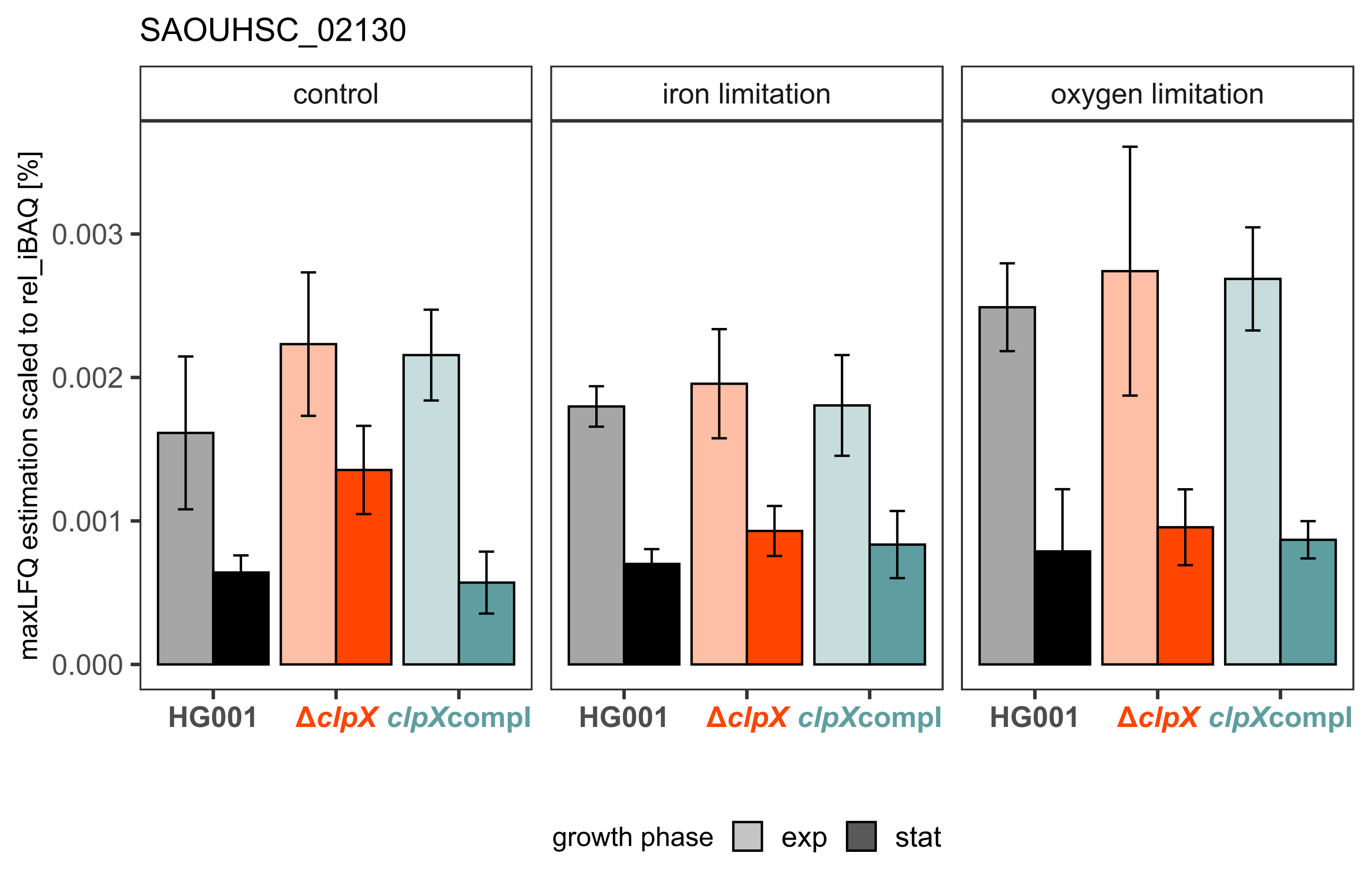

SAOUHSC_02130
control
iron limitation
oxygen limitation
maxLFQ estimation scaled to rel_iBAQ [%]
0.003
0.002
0.001
0.000
HG001
ΔclpX
clpXcompl
growth phase
exp
stat

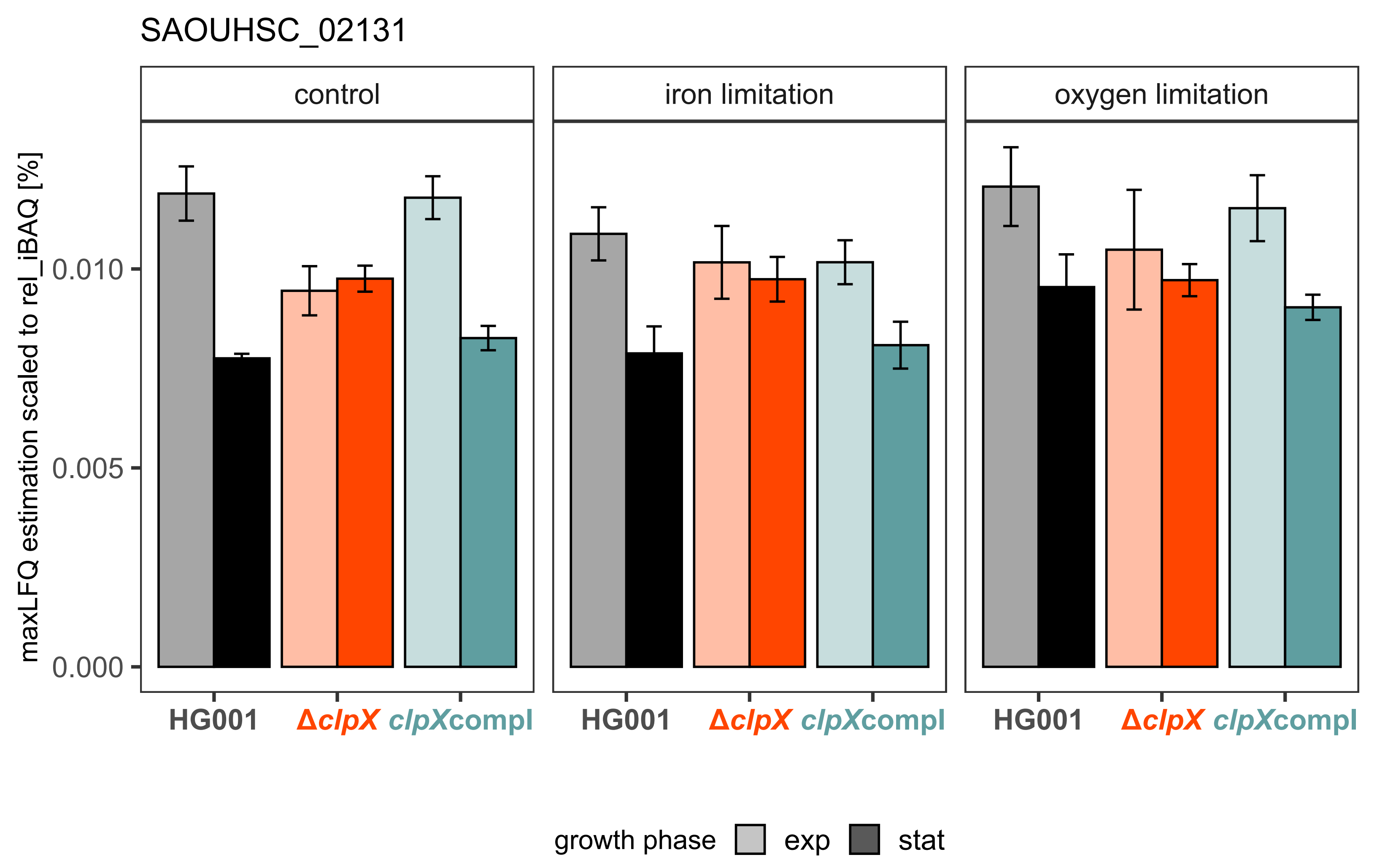
SAOUHSC_02131
control
iron limitation
oxygen limitation
maxLFQ estimation scaled to rel_iBAQ [%]
0.010
0.005
0.000
HG001
ΔclpX
clpXcompl
growth phase
exp
stat

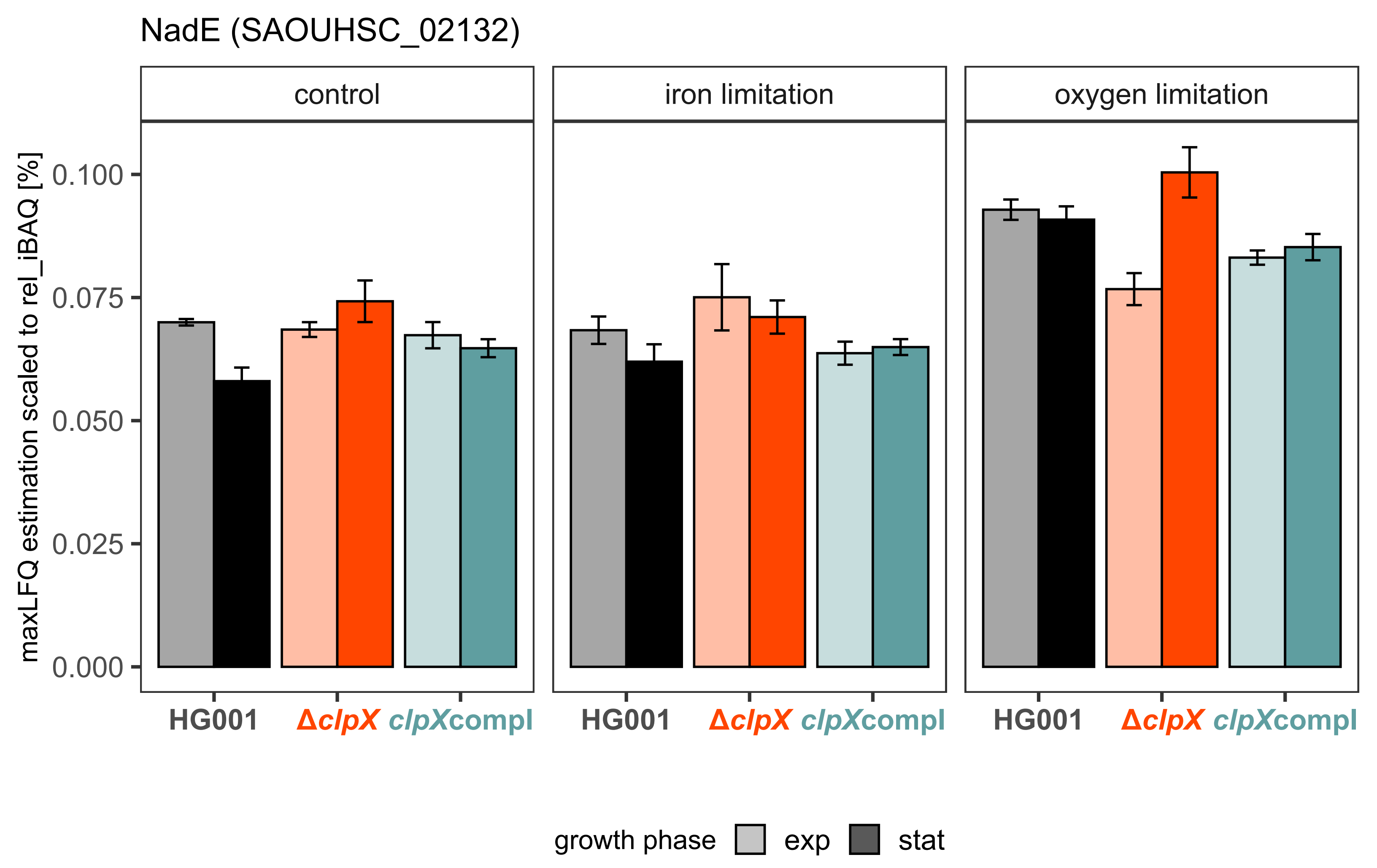

NadE (SAOUHSC_02132)
control
iron limitation
oxygen limitation
maxLFQ estimation scaled to rel_iBAQ [%]
0.100
0.075
0.050
0.025
0.000
HG001
ΔclpX
clpXcompl
growth phase
exp
stat

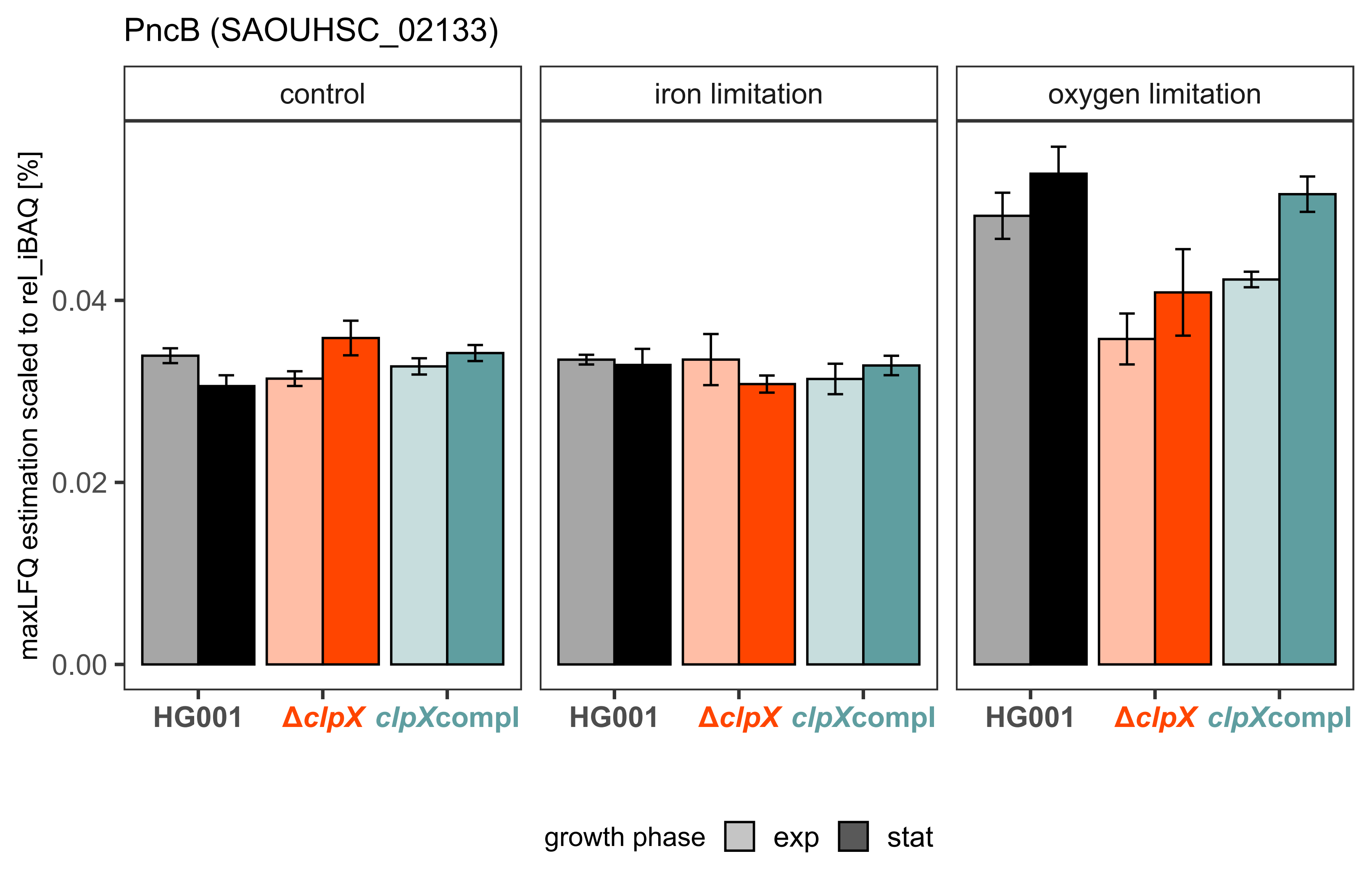

PncB (SAOUHSC_02133)
control
iron limitation
oxygen limitation
maxLFQ estimation scaled to rel_iBAQ [%]
0.04
0.02
0.00
HG001
ΔclpX
clpXcompl
growth phase
exp
stat

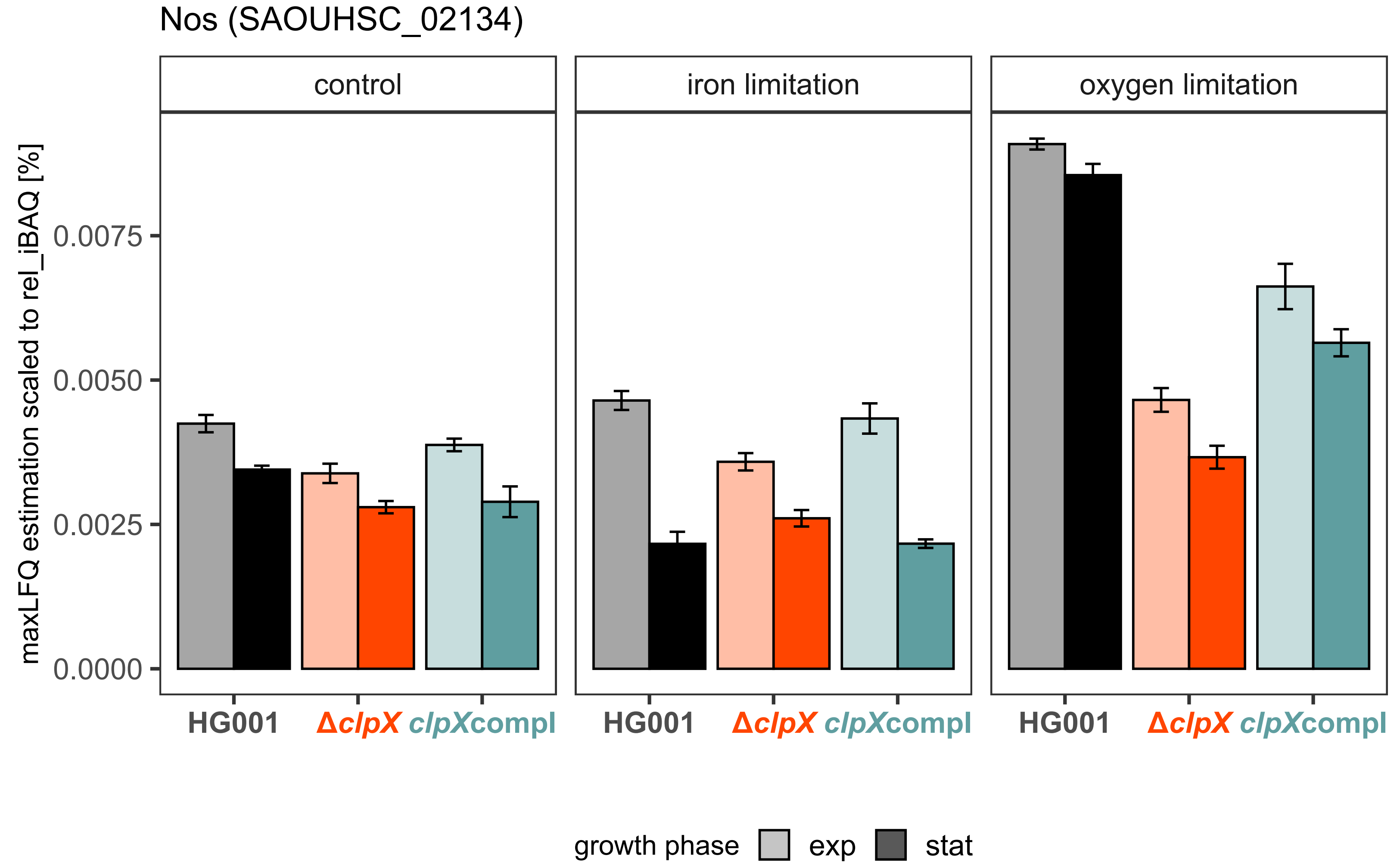
Nos (SAOUHSC_02134)
control
iron limitation
oxygen limitation
maxLFQ estimation scaled to rel_iBAQ [%]
0.0075
0.0050
0.0025
0.0000
HG001
ΔclpX
clpXcompl
growth phase
exp
stat

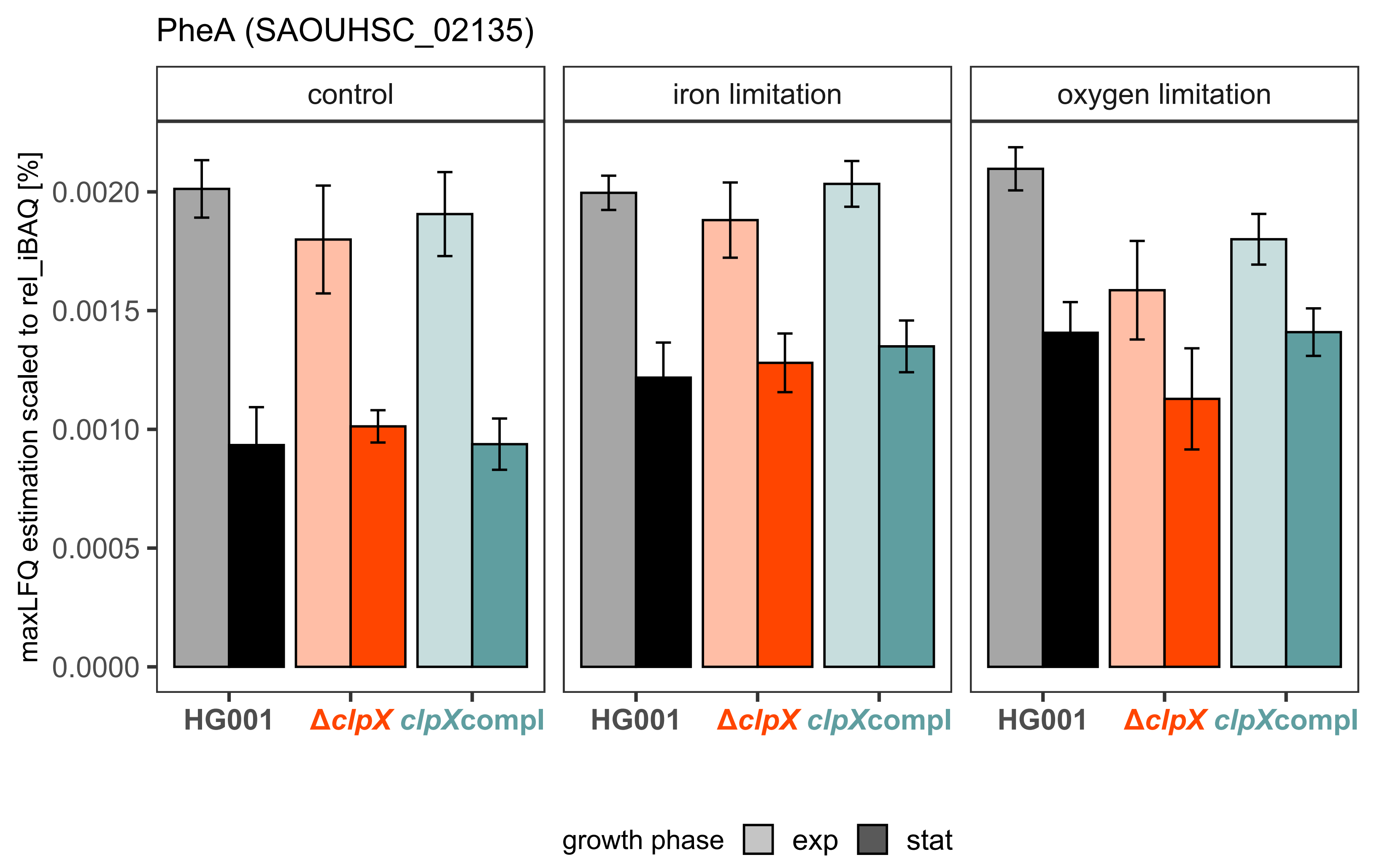
PheA (SAOUHSC_02135)
control
iron limitation
oxygen limitation
maxLFQ estimation scaled to rel_iBAQ [%]
0.0020
0.0015
0.0010
0.0005
0.0000
HG001
ΔclpX
clpXcompl
growth phase
exp
stat

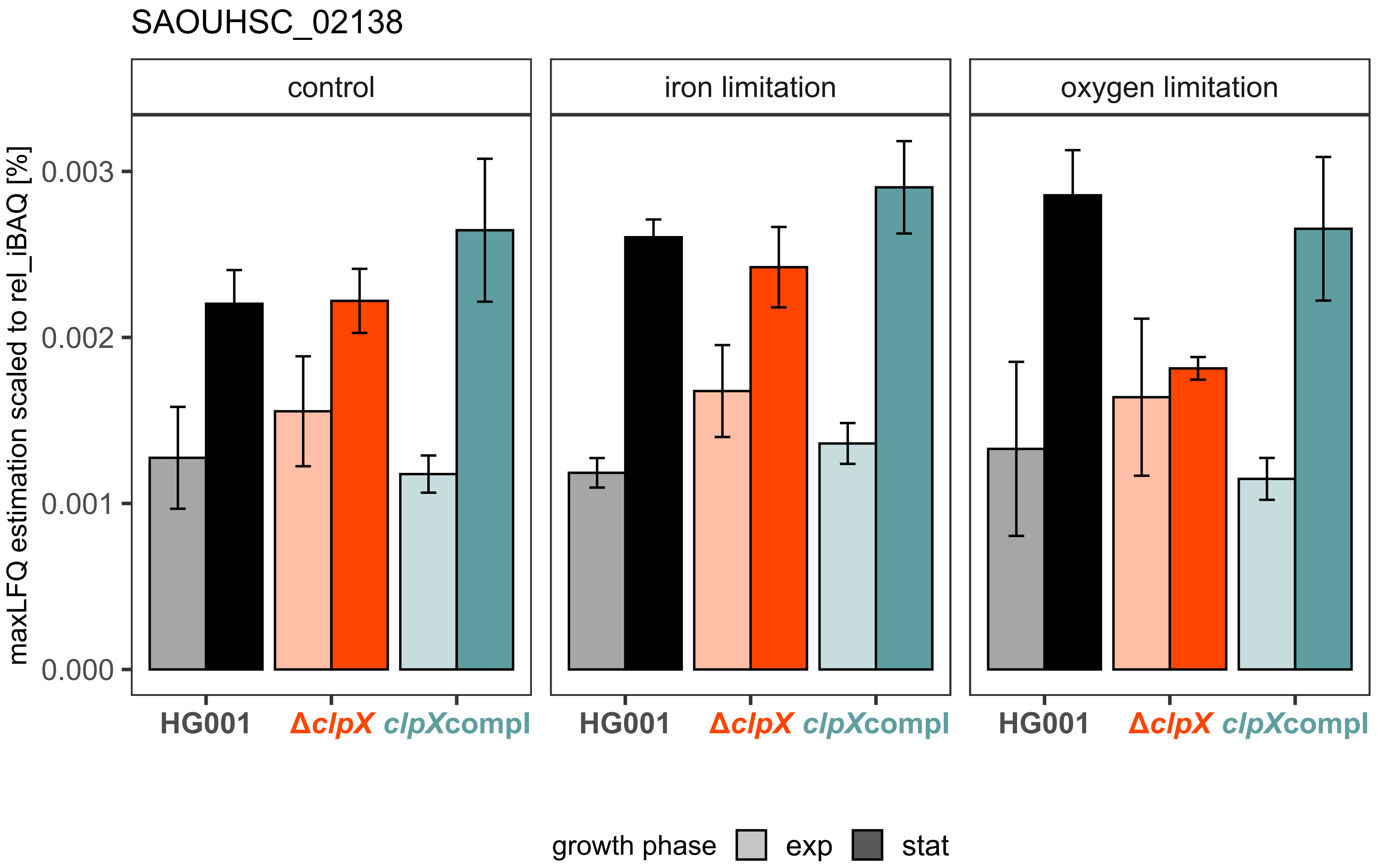
SAOUHSC_02138
control
iron limitation
oxygen limitation
maxLFQ estimation scaled to rel_iBAQ [%]
0.003
0.002
0.001
0.000
HG001
ΔclpX
clpXcompl
growth phase
exp
stat

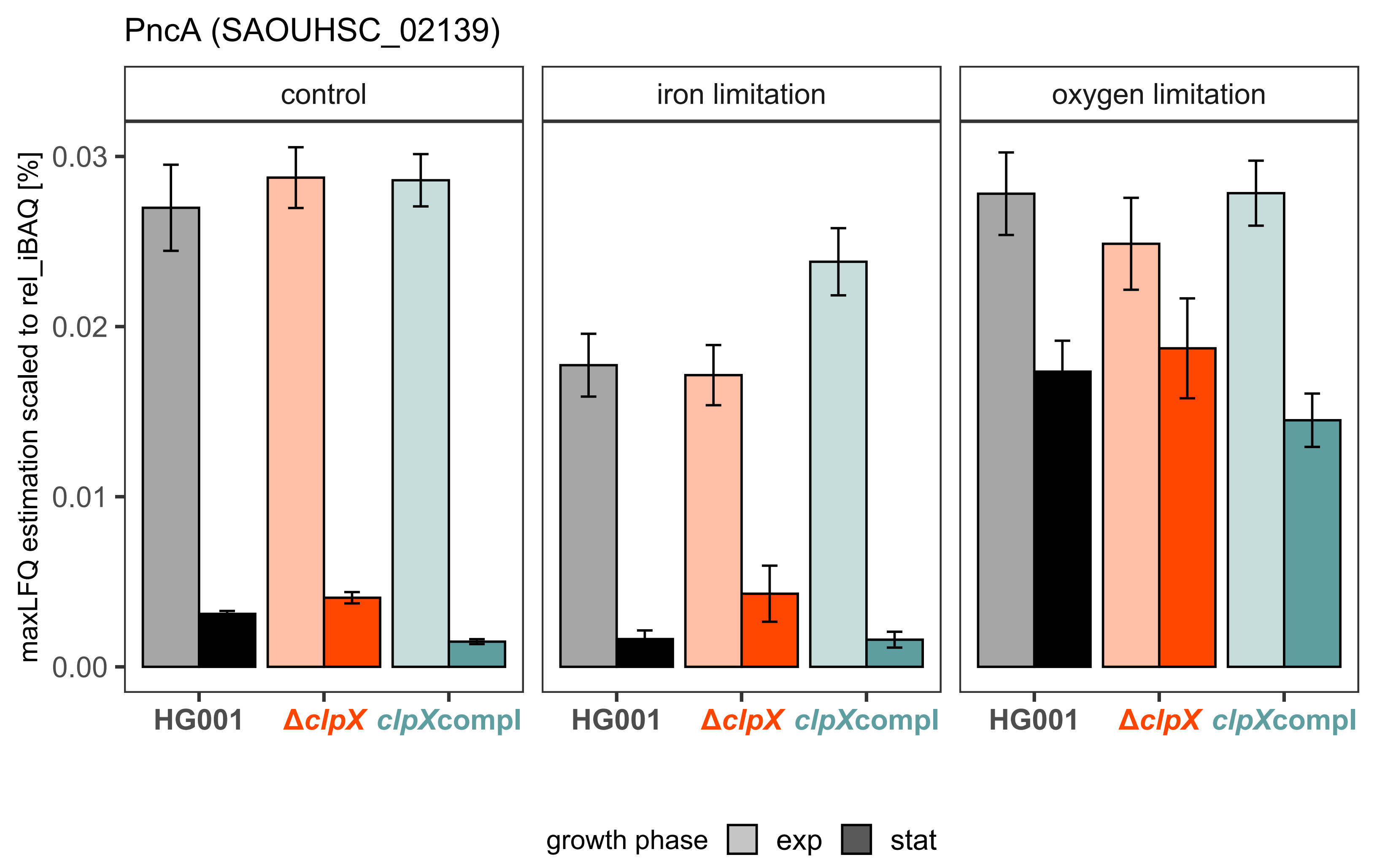

PncA (SAOUHSC_02139)
control
iron limitation
oxygen limitation
maxLFQ estimation scaled to rel_iBAQ [%]
0.03
0.02
0.01
0.00
HG001
ΔclpX
clpXcompl
growth phase
exp
stat

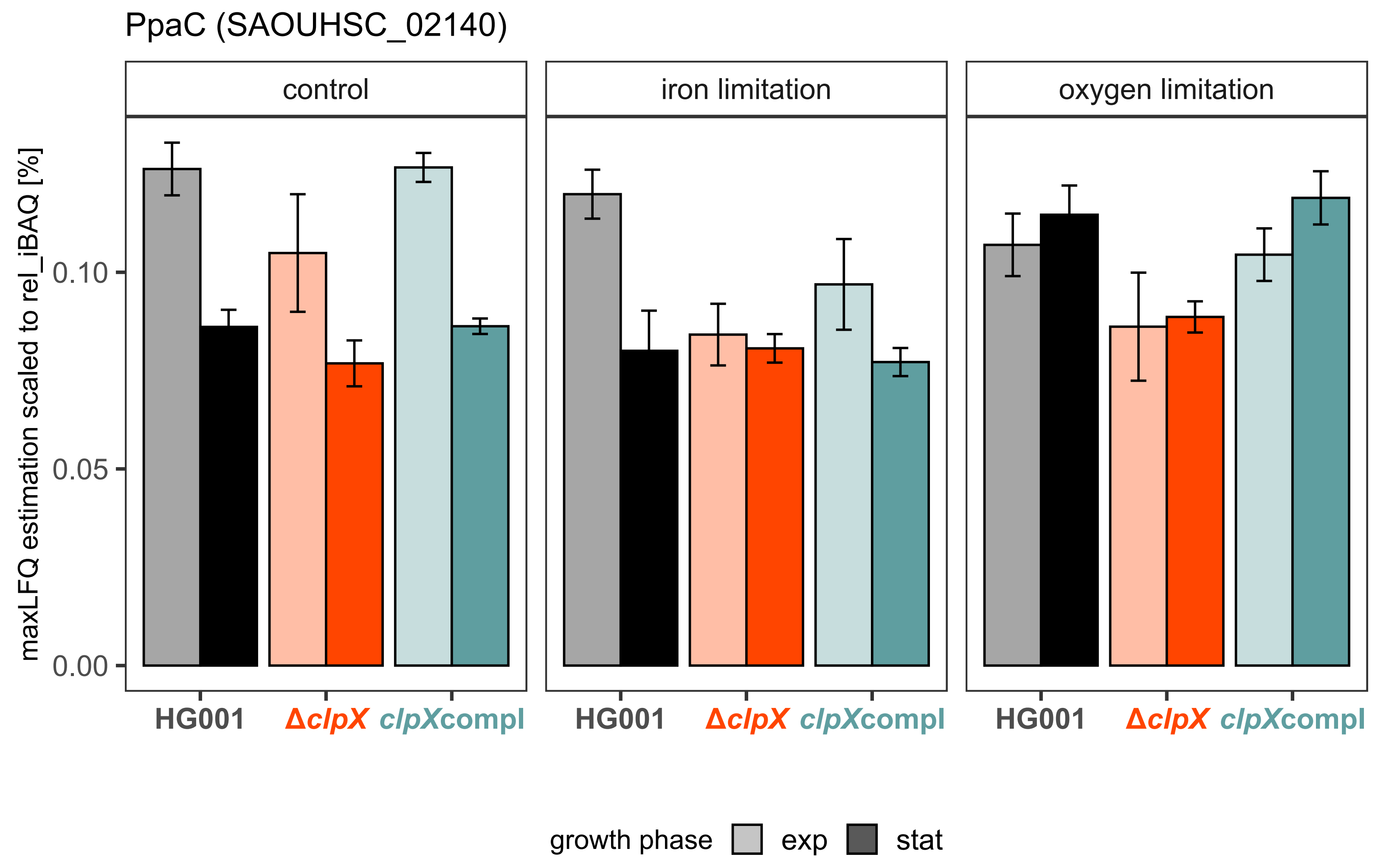
PpaC (SAOUHSC_02140)
control
iron limitation
oxygen limitation
maxLFQ estimation scaled to rel_iBAQ [%]
0.10
0.05
0.00
HG001
ΔclpX
clpXcompl
growth phase
exp
stat

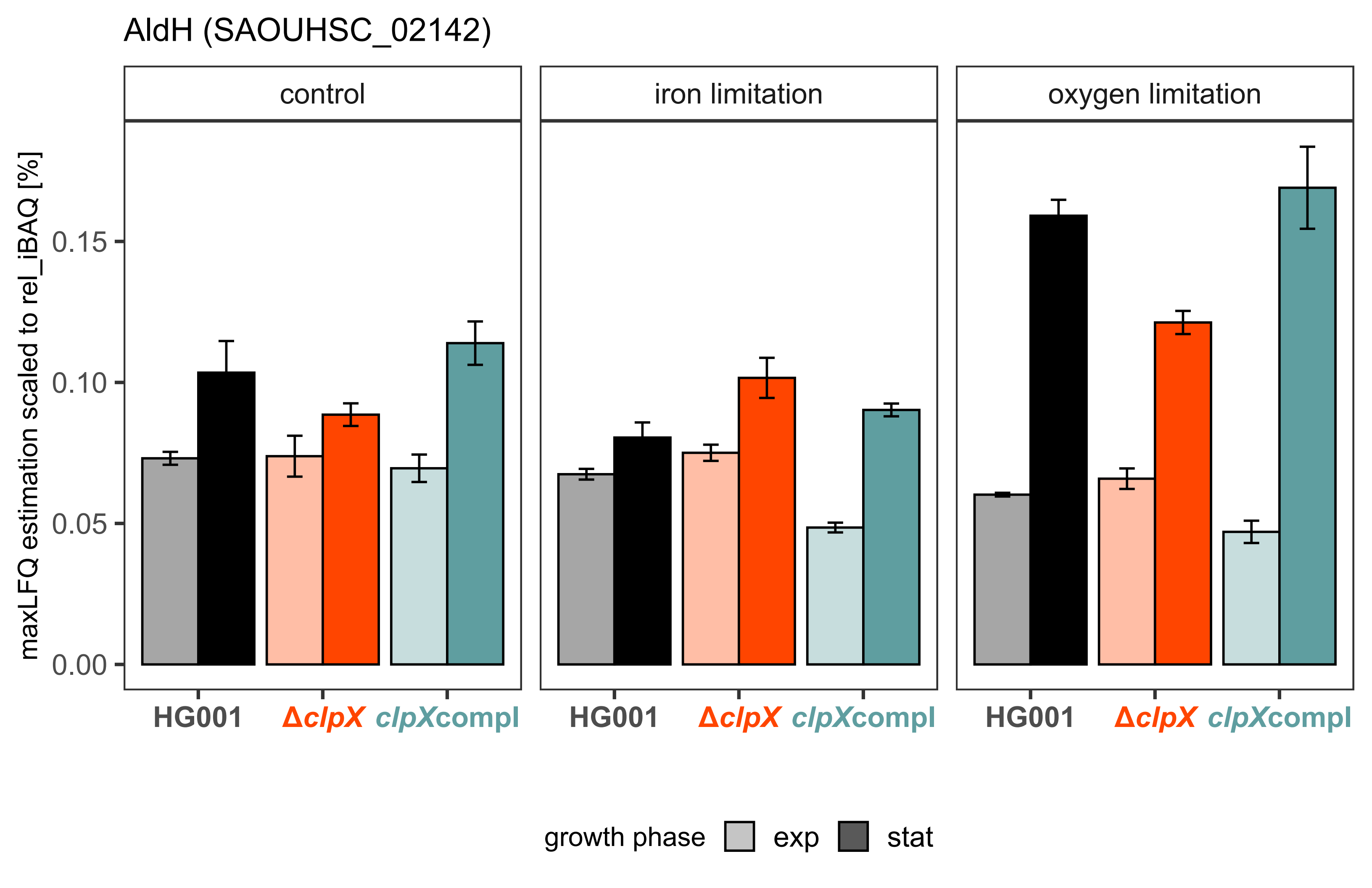

AldH (SAOUHSC_02142)
control
iron limitation
oxygen limitation
maxLFQ estimation scaled to rel_iBAQ [%]
0.00
0.05
0.10
0.15
HG001
ΔclpX
clpXcompl
growth phase
exp
stat

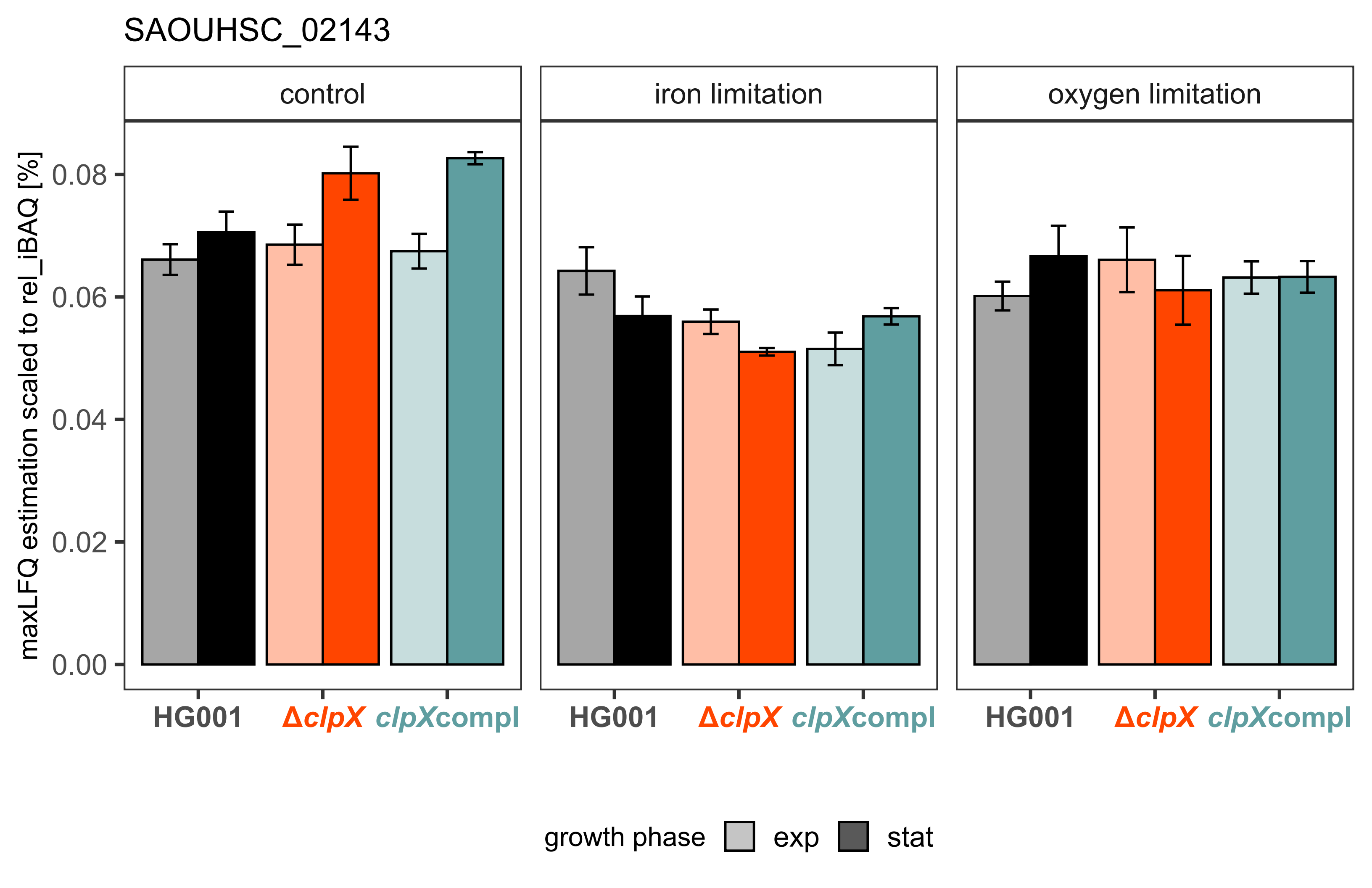

SAOUHSC_02143
control
iron limitation
oxygen limitation
maxLFQ estimation scaled to rel_iBAQ [%]
0.00
0.02
0.04
0.06
0.08
HG001
ΔclpX
clpXcompl
growth phase
exp
stat

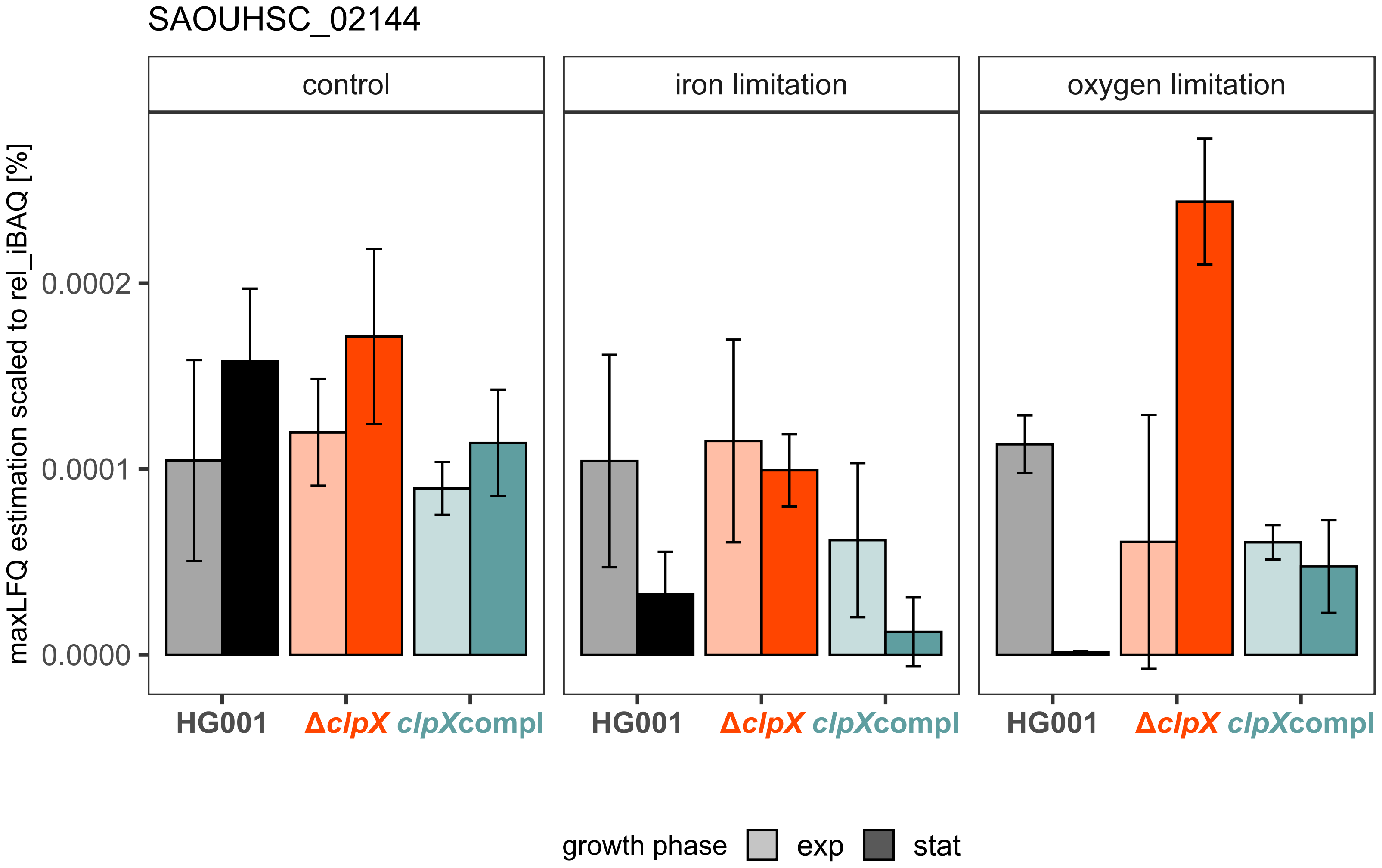

SAOUHSC_02144
control
iron limitation
oxygen limitation
maxLFQ estimation scaled to rel_iBAQ [%]
0.0002
0.0001
0.0000
HG001
ΔclpX
clpXcompl
growth phase
exp
stat

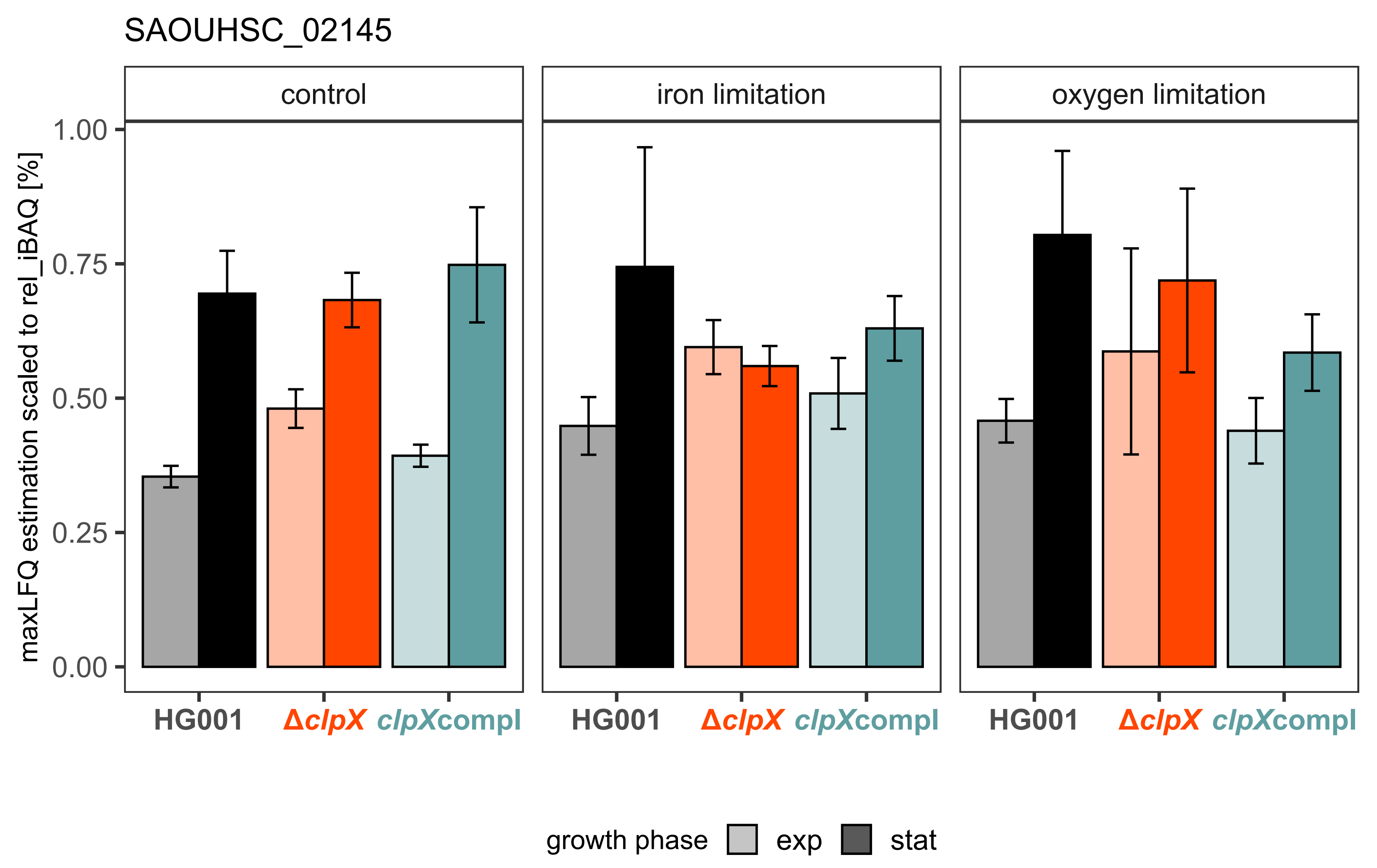
SAOUHSC_02145
control
iron limitation
oxygen limitation
maxLFQ estimation scaled to rel_iBAQ [%]
1.00
0.75
0.50
0.25
0.00
HG001
ΔclpX
clpXcompl
growth phase
exp
stat

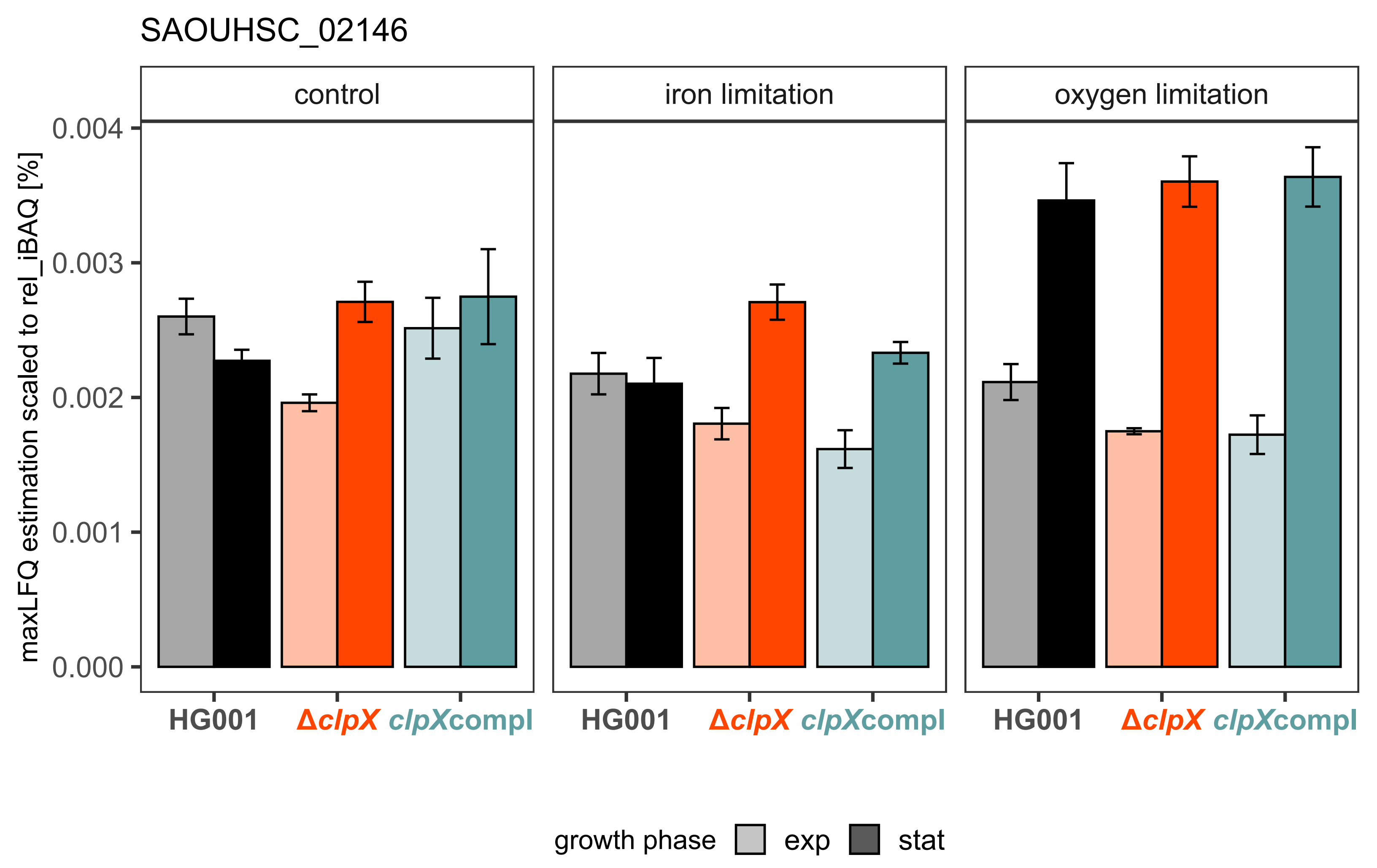
SAOUHSC_02146
control
iron limitation
oxygen limitation
maxLFQ estimation scaled to rel_iBAQ [%]
0.004
0.003
0.002
0.001
0.000
HG001
ΔclpX
clpXcompl
growth phase
exp
stat

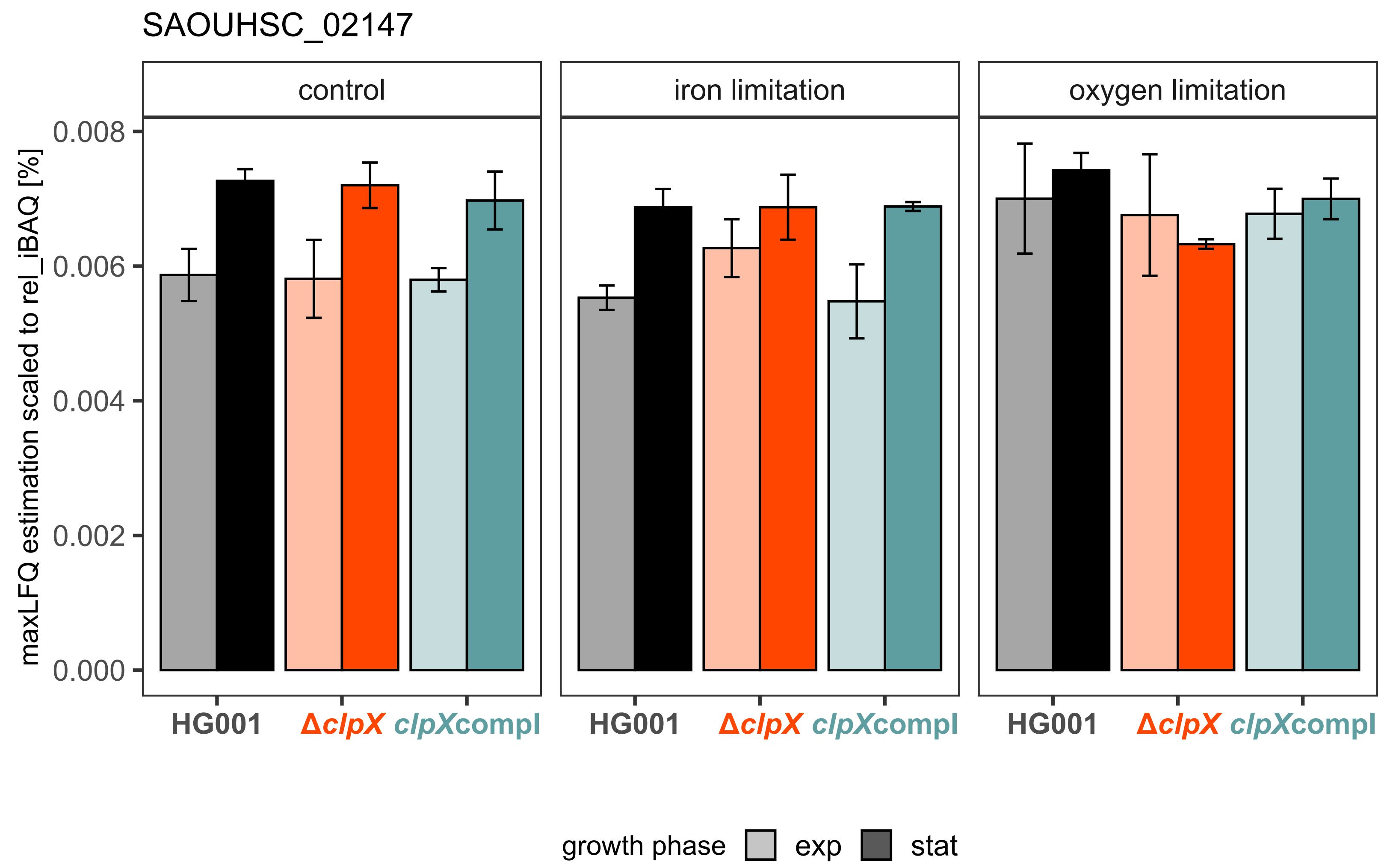
SAOUHSC_02147
control
iron limitation
oxygen limitation
maxLFQ estimation scaled to rel_iBAQ [%]
0.008
0.006
0.004
0.002
0.000
HG001
ΔclpX
clpXcompl
growth phase
exp
stat

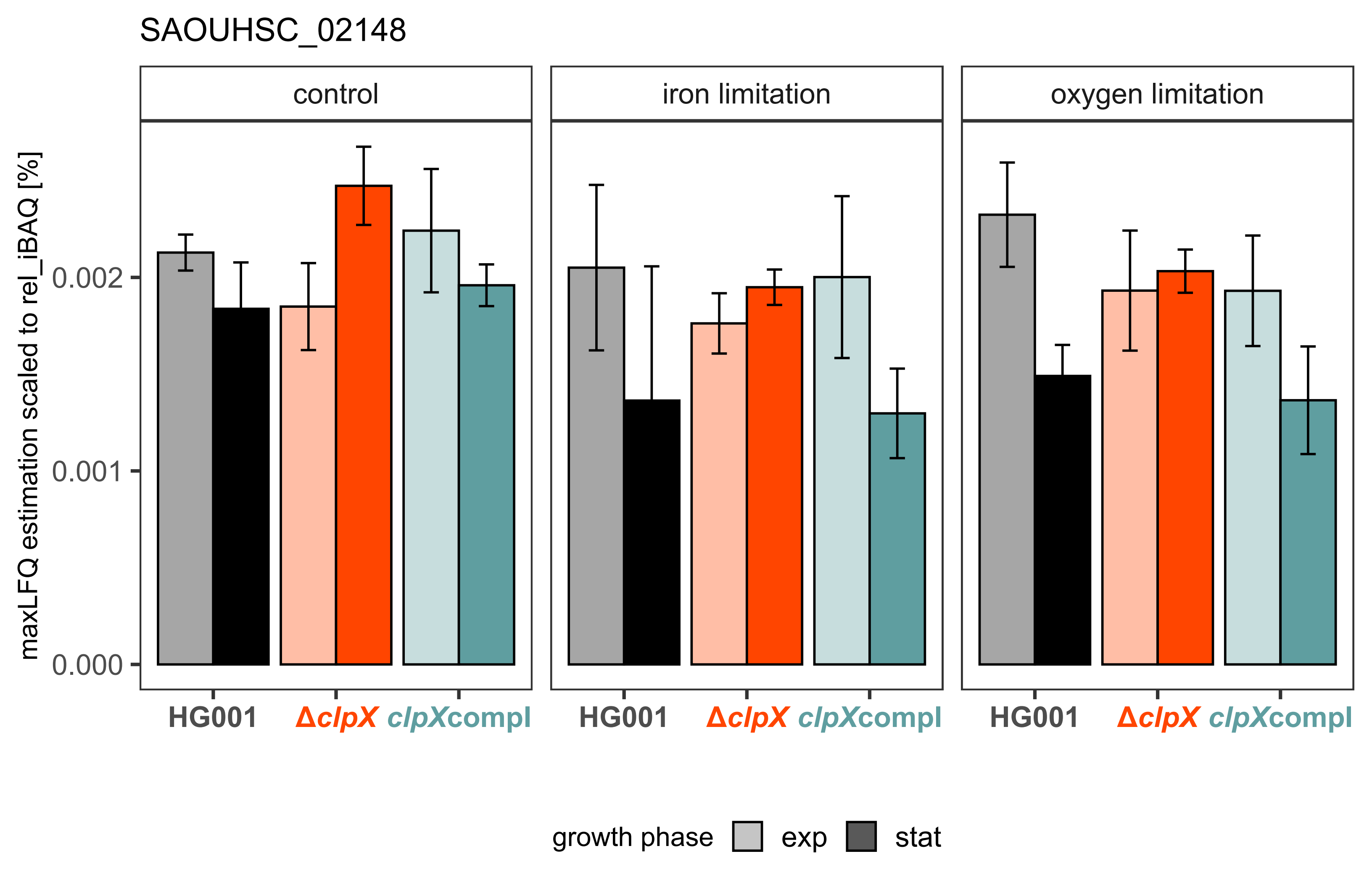

SAOUHSC_02148
control
iron limitation
oxygen limitation
maxLFQ estimation scaled to rel_iBAQ [%]
0.000
0.001
0.002
HG001
ΔclpX
clpXcompl
growth phase
exp
stat

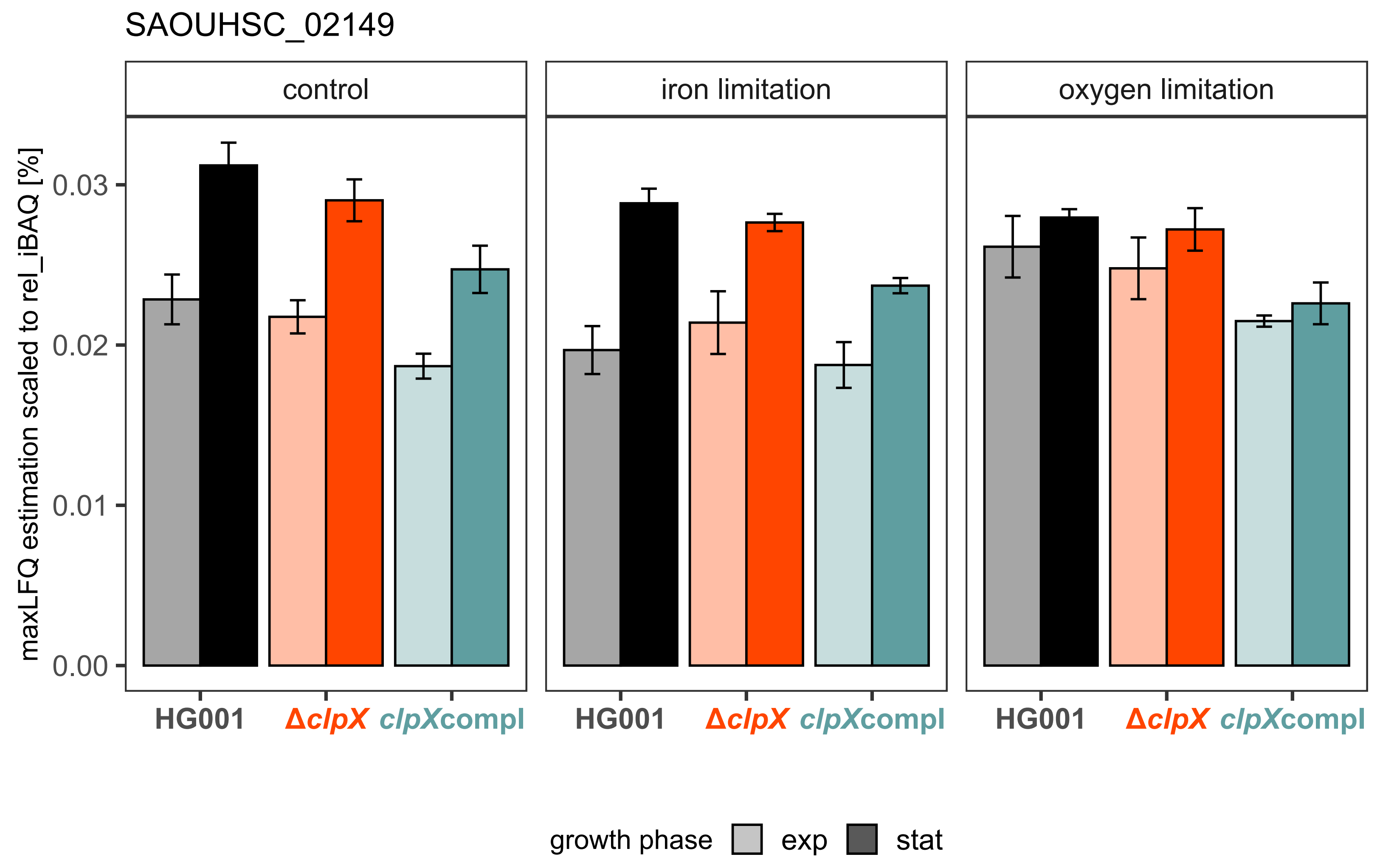

SAOUHSC_02149
control
iron limitation
oxygen limitation
maxLFQ estimation scaled to rel_iBAQ [%]
0.03
0.02
0.01
0.00
HG001
ΔclpX
clpXcompl
growth phase
exp
stat

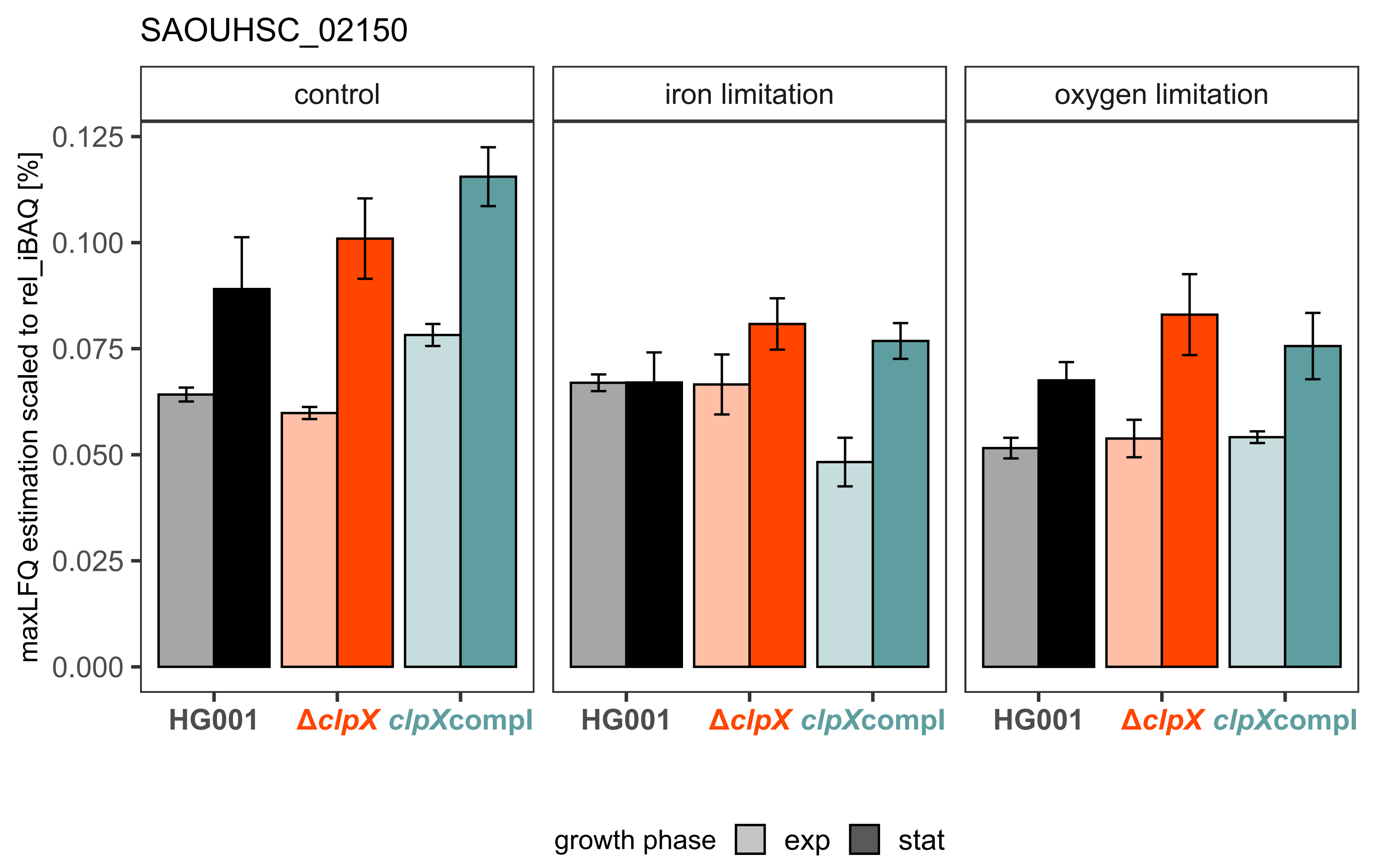

SAOUHSC_02150
control
iron limitation
oxygen limitation
maxLFQ estimation scaled to rel_iBAQ [%]
0.125
0.100
0.075
0.050
0.025
0.000
HG001
ΔclpX
clpXcompl
growth phase
exp
stat

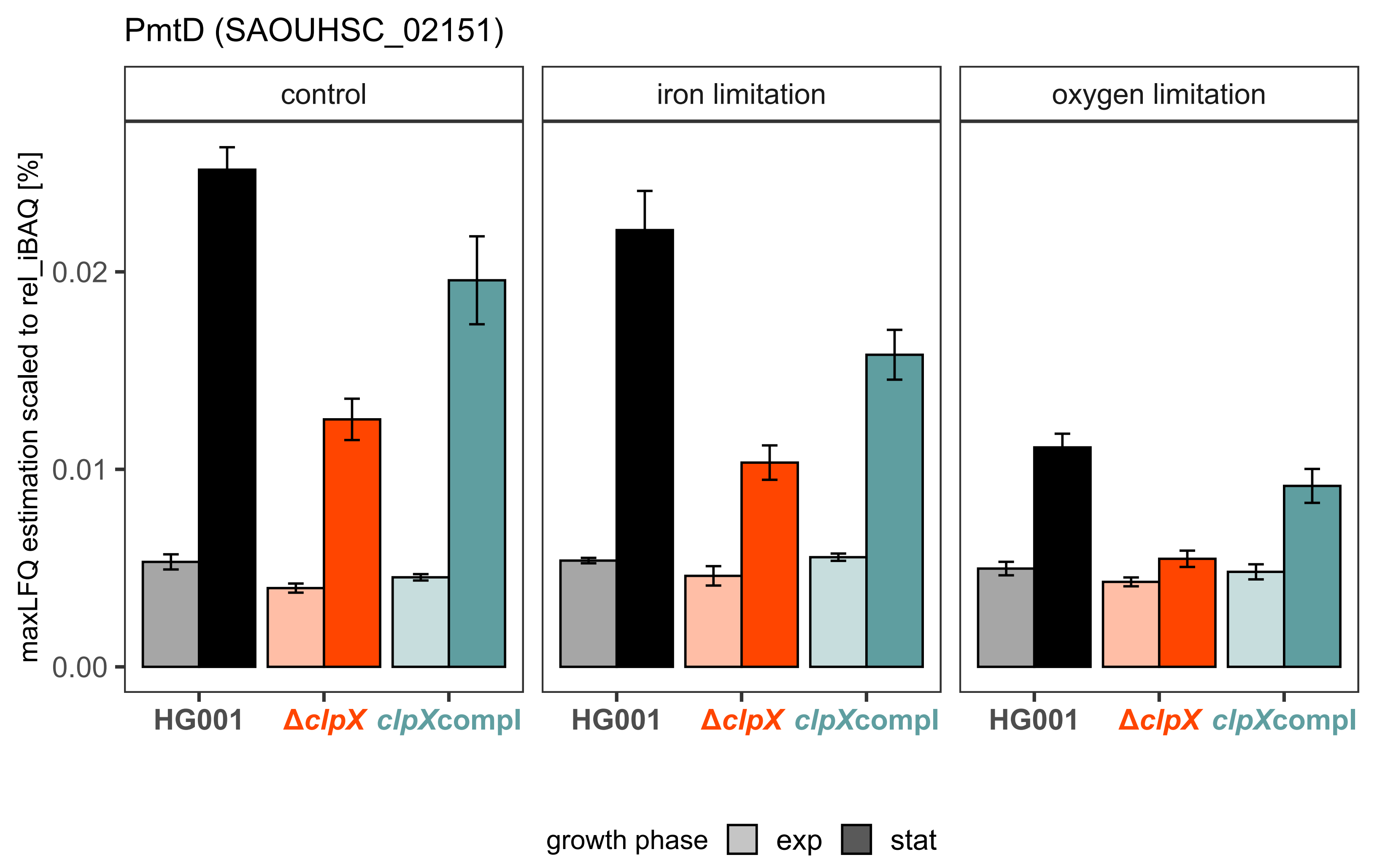
PmtD (SAOUHSC_02151)
control
iron limitation
oxygen limitation
maxLFQ estimation scaled to rel_iBAQ [%]
0.00
0.01
0.02
HG001
ΔclpX
clpXcompl
growth phase
exp
stat

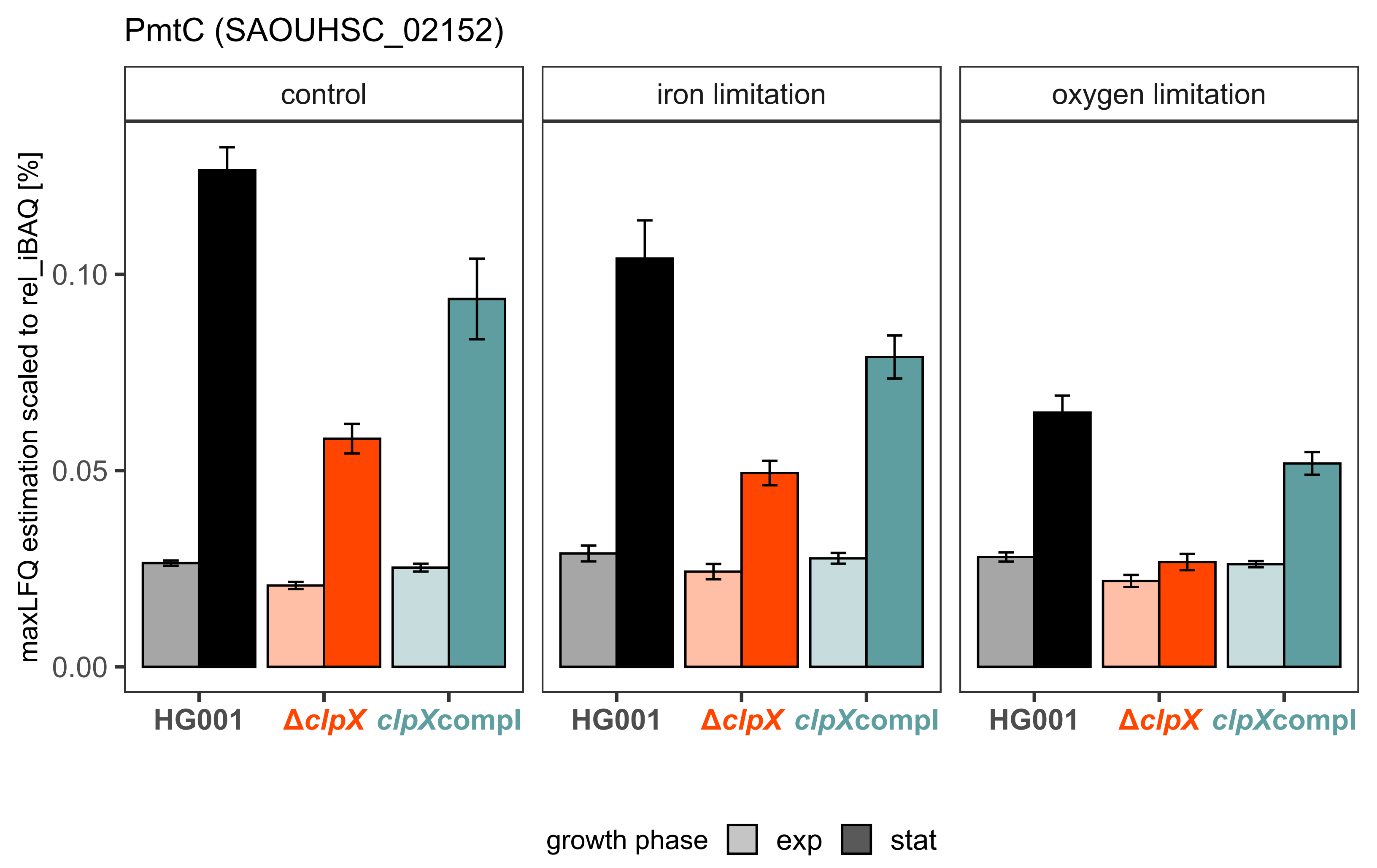
PmtC (SAOUHSC_02152)
control
iron limitation
oxygen limitation
maxLFQ estimation scaled to rel_iBAQ [%]
0.10
0.05
0.00
HG001
ΔclpX
clpXcompl
growth phase
exp
stat

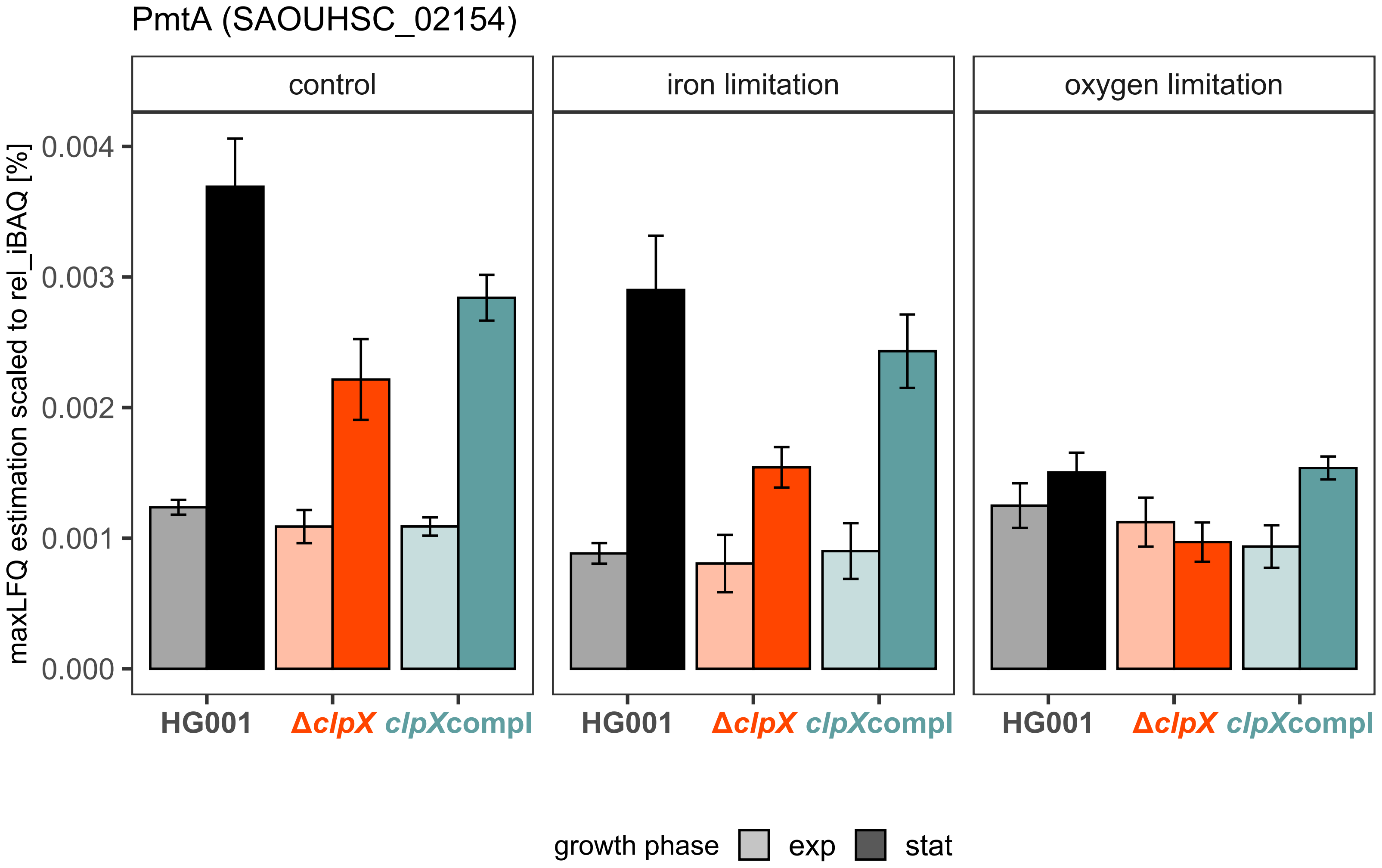
PmtA (SAOUHSC_02154)
control
iron limitation
oxygen limitation
maxLFQ estimation scaled to rel_iBAQ [%]
0.004
0.003
0.002
0.001
0.000
HG001
ΔclpX
clpXcompl
growth phase
exp
stat

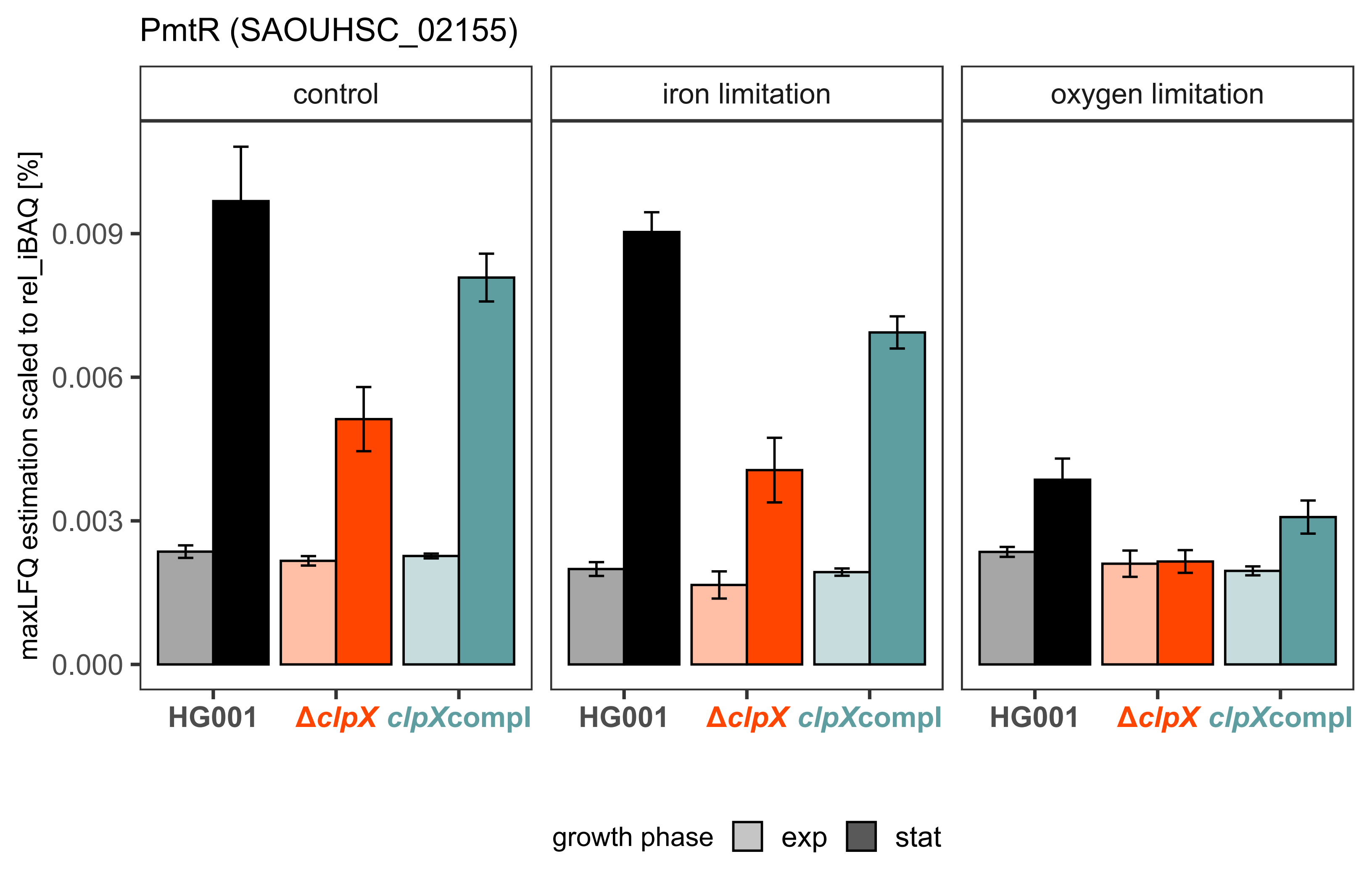
PmtR (SAOUHSC_02155)
control
iron limitation
oxygen limitation
maxLFQ estimation scaled to rel_iBAQ [%]
0.009
0.006
0.003
0.000
HG001
ΔclpX
clpXcompl
growth phase
exp
stat

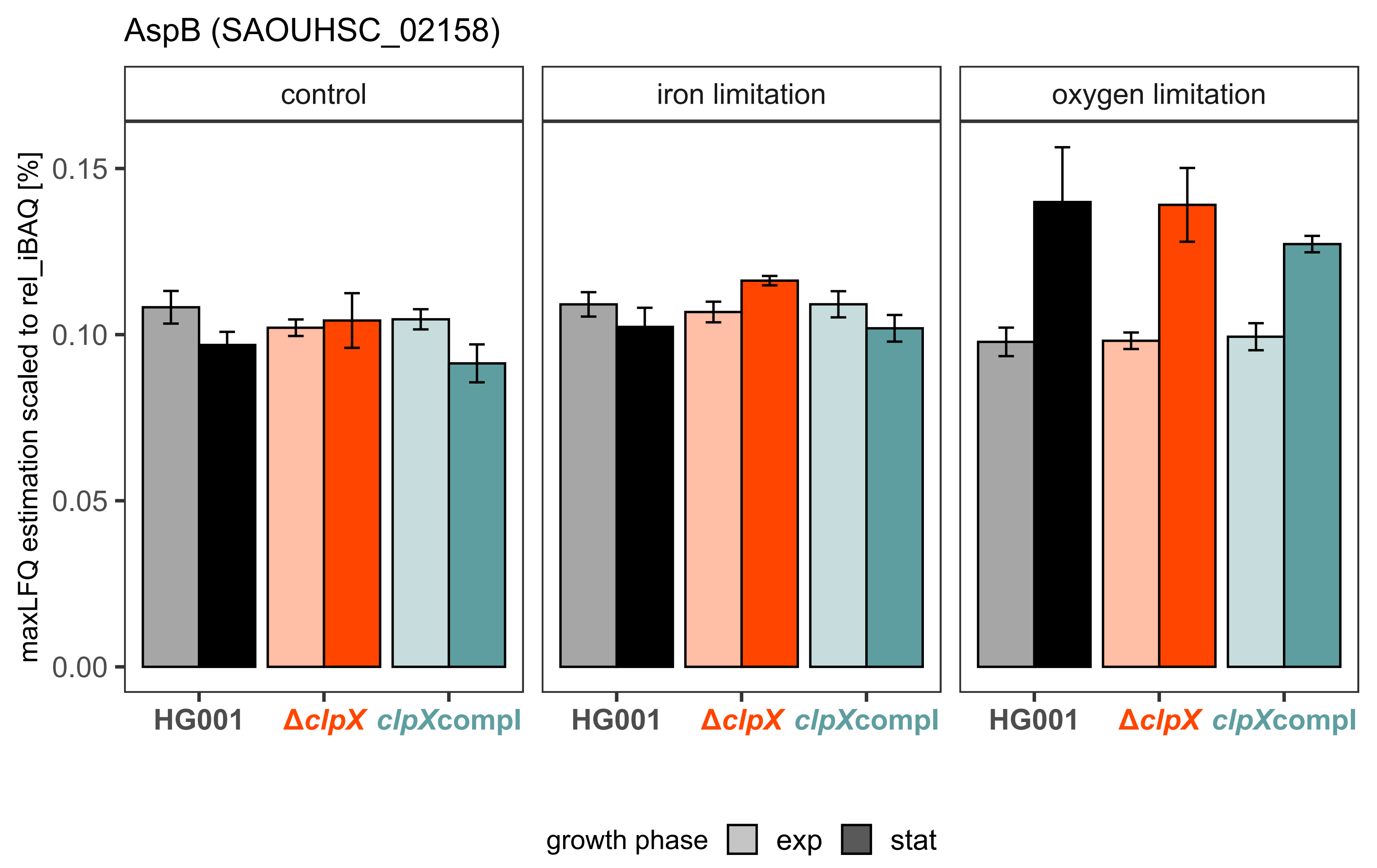

AspB (SAOUHSC_02158)
control
iron limitation
oxygen limitation
maxLFQ estimation scaled to rel_iBAQ [%]
0.15
0.10
0.05
0.00
HG001
ΔclpX
clpXcompl
growth phase
exp
stat

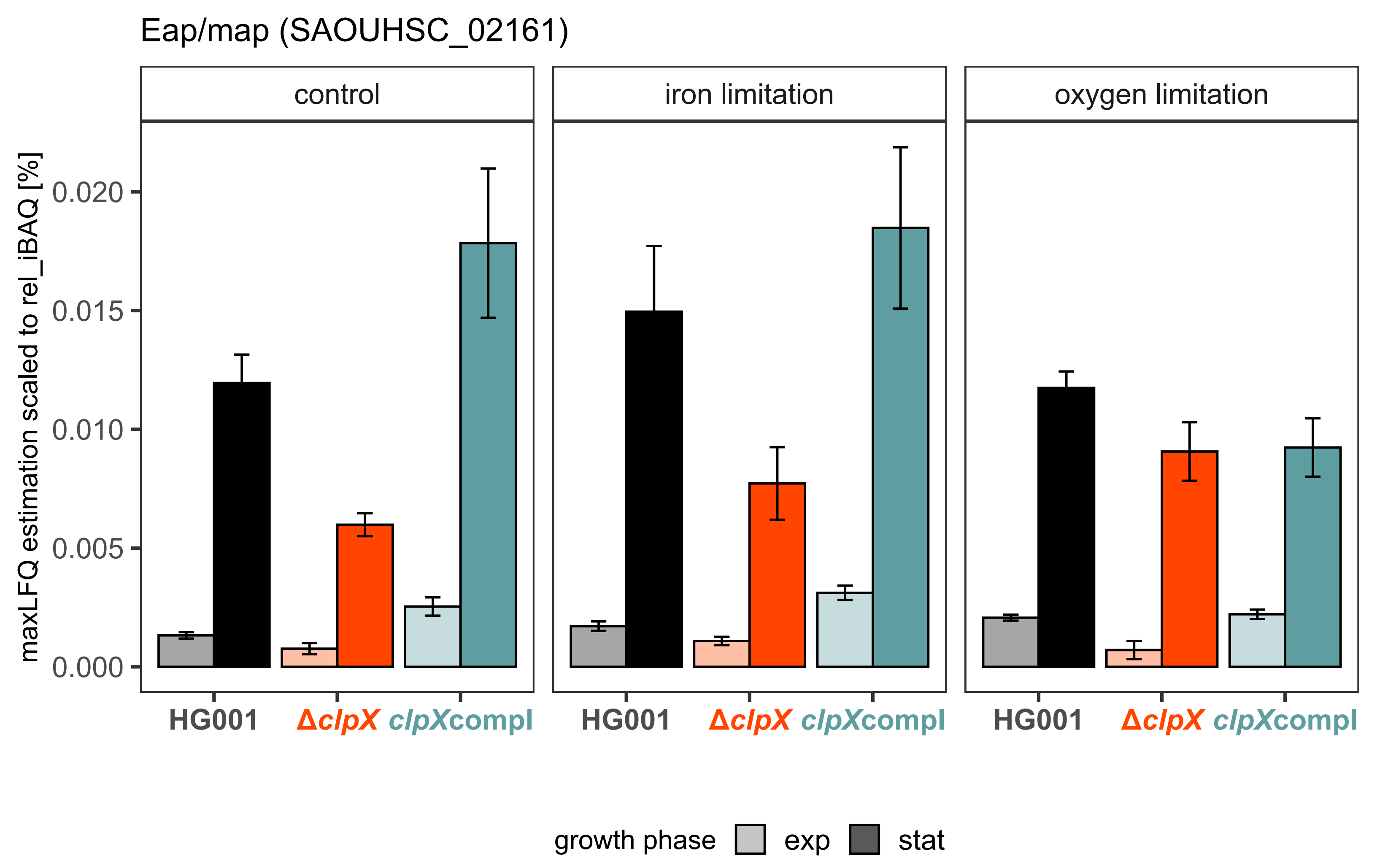

Eap/map (SAOUHSC_02161)
control
iron limitation
oxygen limitation
maxLFQ estimation scaled to rel_iBAQ [%]
0.020
0.015
0.010
0.005
0.000
HG001
ΔclpX
clpXcompl
growth phase
exp
stat

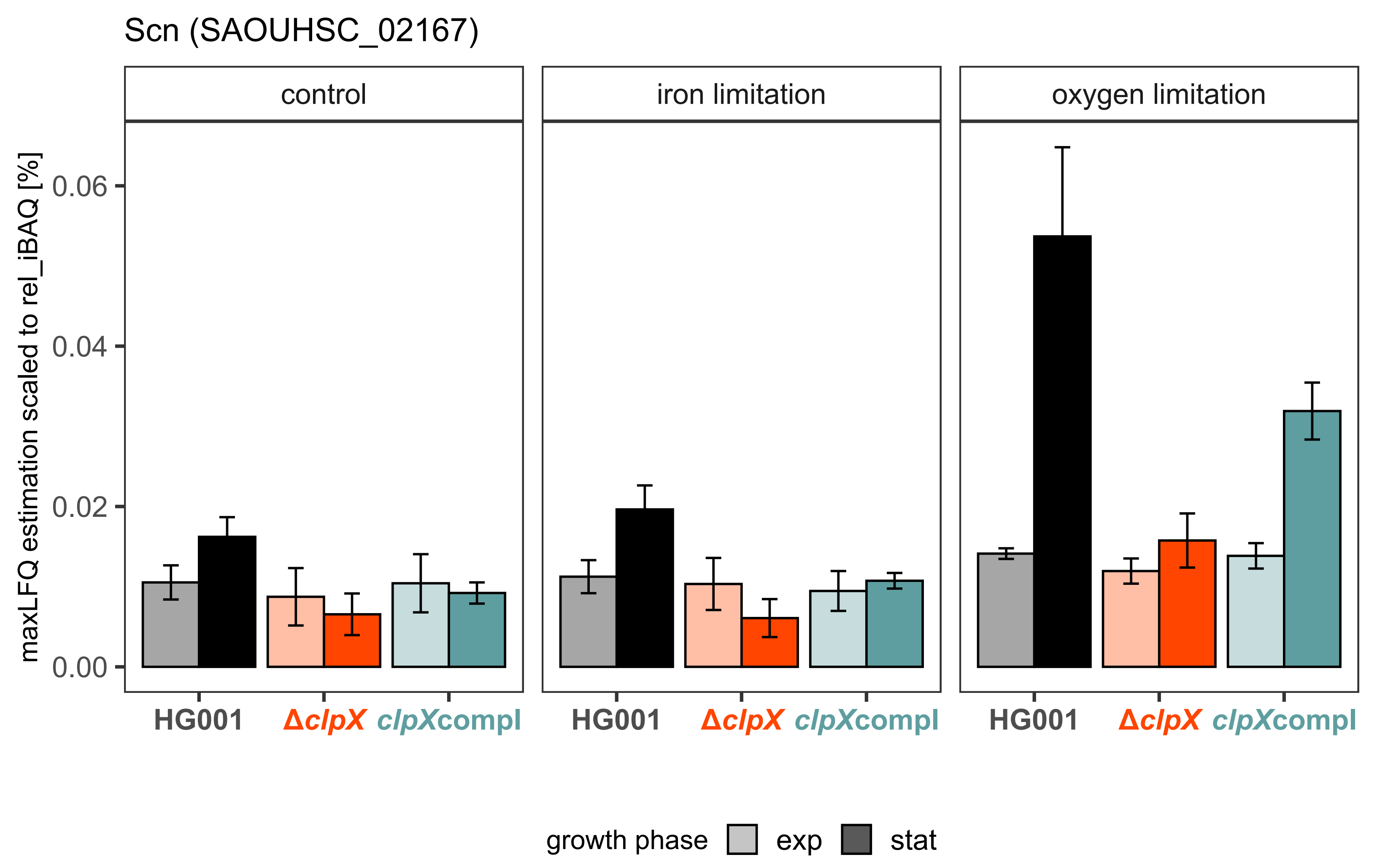

Scn (SAOUHSC_02167)
control
iron limitation
oxygen limitation
maxLFQ estimation scaled to rel_iBAQ [%]
0.06
0.04
0.02
0.00
HG001
ΔclpX
clpXcompl
HG001
ΔclpX
clpXcompl
HG001
ΔclpX
clpXcompl
growth phase
exp
stat

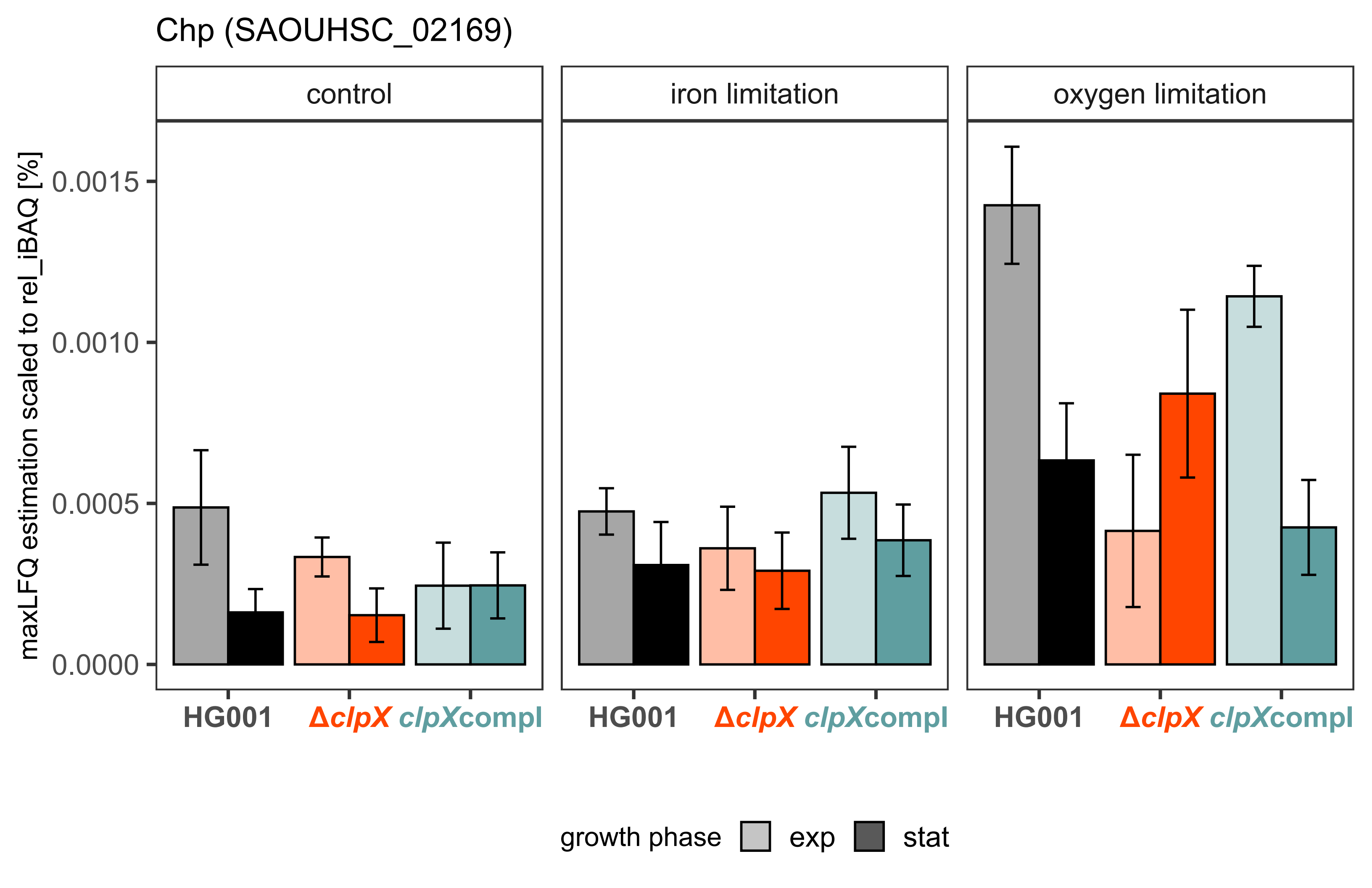

Chp (SAOUHSC_02169)
control
iron limitation
oxygen limitation
maxLFQ estimation scaled to rel_iBAQ [%]
0.0015
0.0010
0.0005
0.0000
HG001
ΔclpX
clpXcompl
growth phase
exp
stat

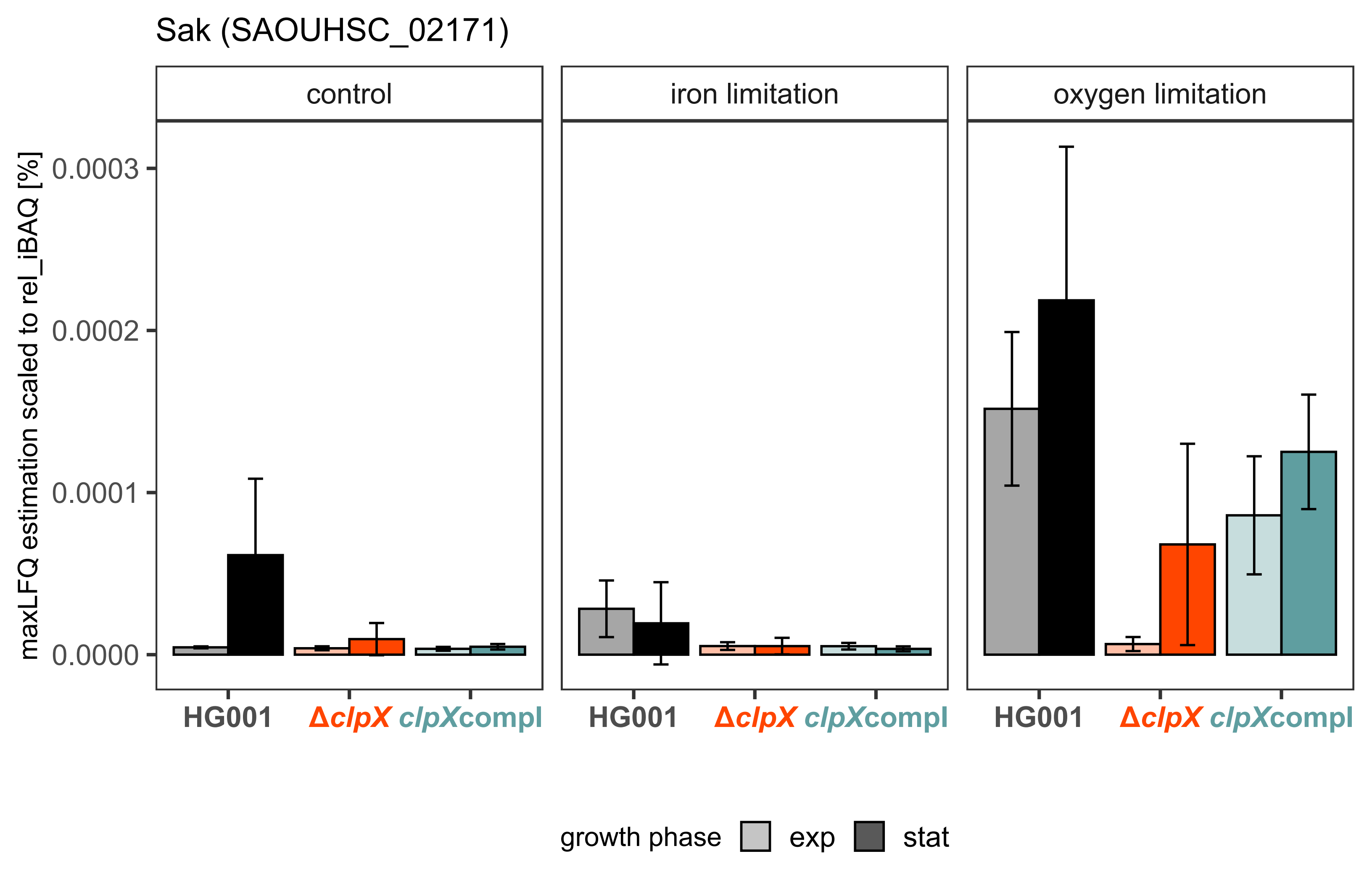

Sak (SAOUHSC_02171)
control
iron limitation
oxygen limitation
maxLFQ estimation scaled to rel_iBAQ [%]
0.0003
0.0002
0.0001
0.0000
HG001
ΔclpX
clpXcompl
growth phase
exp
stat

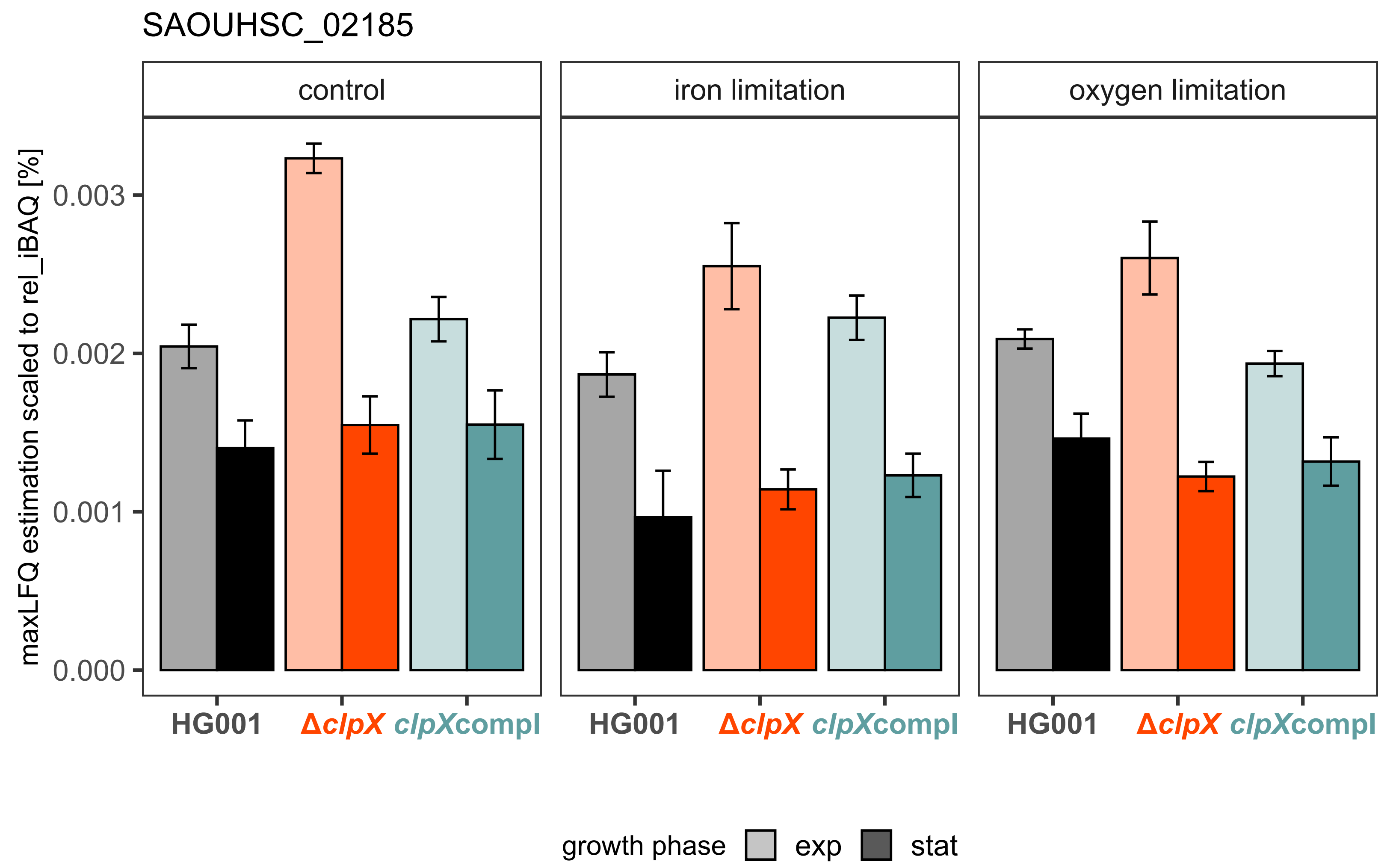
SAOUHSC_02185
control
iron limitation
oxygen limitation
maxLFQ estimation scaled to rel_iBAQ [%]
0.003
0.002
0.001
0.000
HG001
ΔclpX
clpXcompl
growth phase
exp
stat

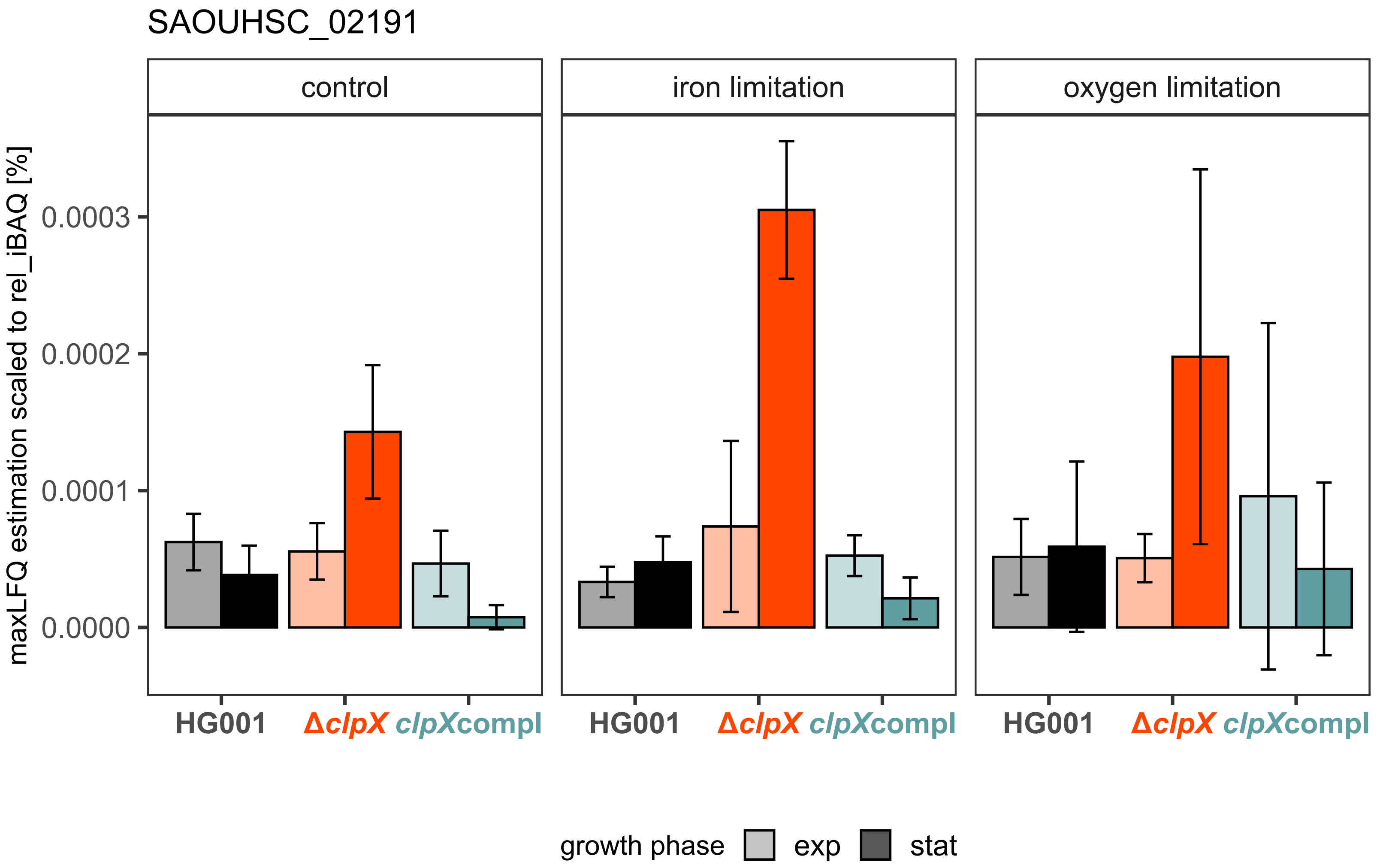

SAOUHSC_02191
control
iron limitation
oxygen limitation
maxLFQ estimation scaled to rel_iBAQ [%]
0.0003
0.0002
0.0001
0.0000
HG001
ΔclpX
clpXcompl
growth phase
exp
stat

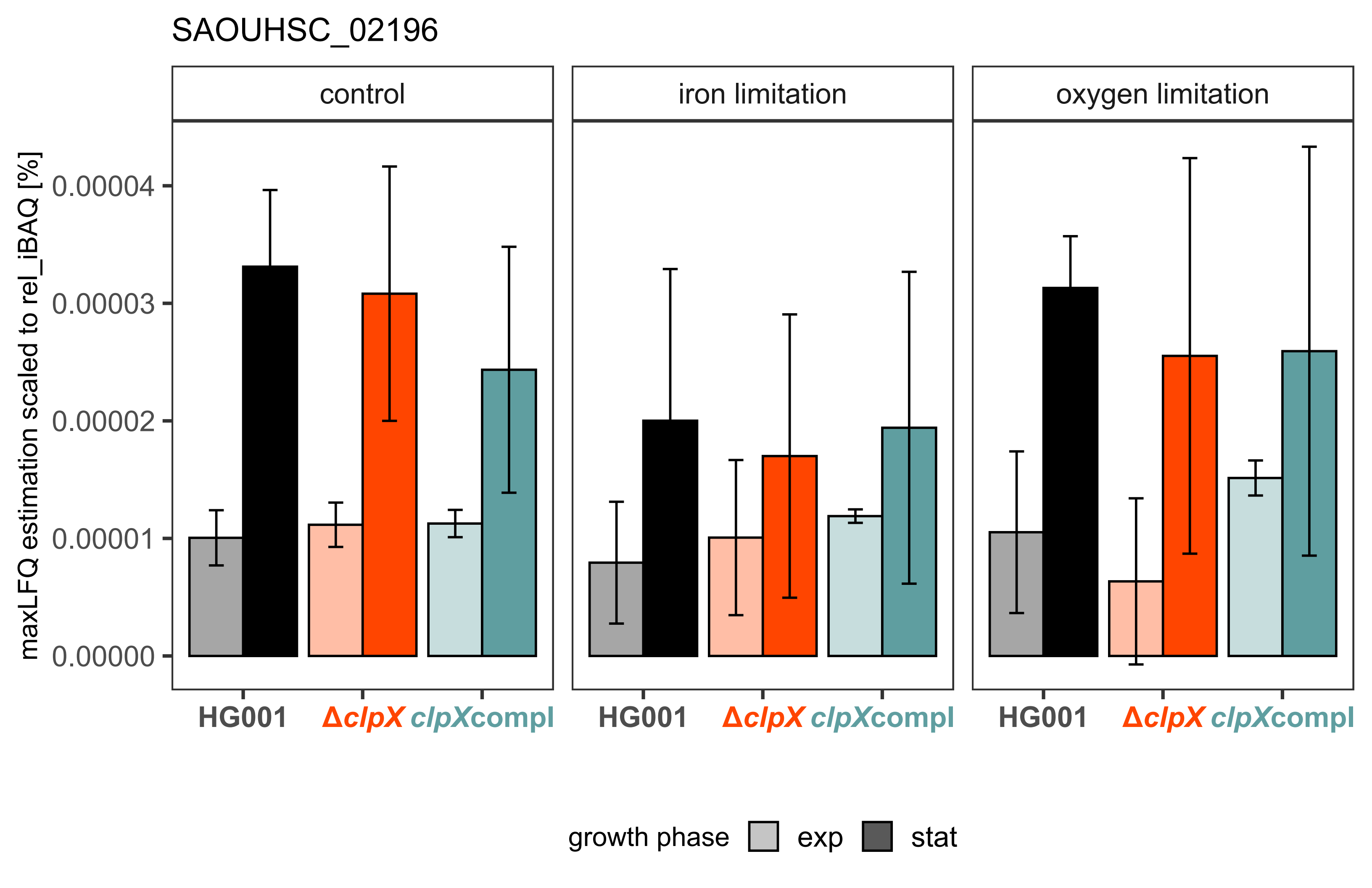

SAOUHSC_02196
control
iron limitation
oxygen limitation
maxLFQ estimation scaled to rel_iBAQ [%]
0.00004
0.00003
0.00002
0.00001
0.00000
HG001
ΔclpX
clpXcompl
growth phase
exp
stat

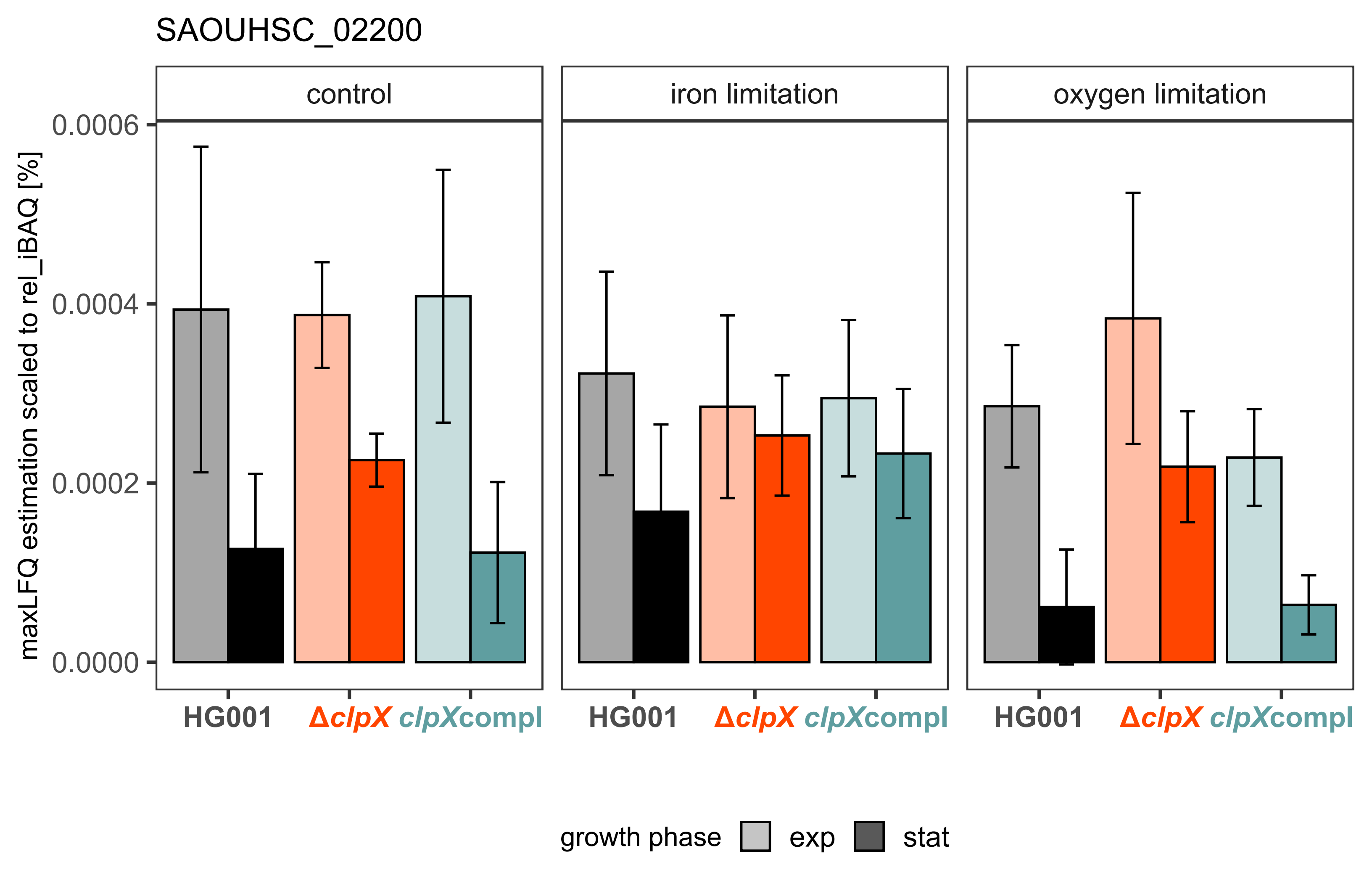
SAOUHSC_02200
control
iron limitation
oxygen limitation
maxLFQ estimation scaled to rel_iBAQ [%]
0.0006
0.0004
0.0002
0.0000
HG001
ΔclpX
clpXcompl
growth phase
exp
stat

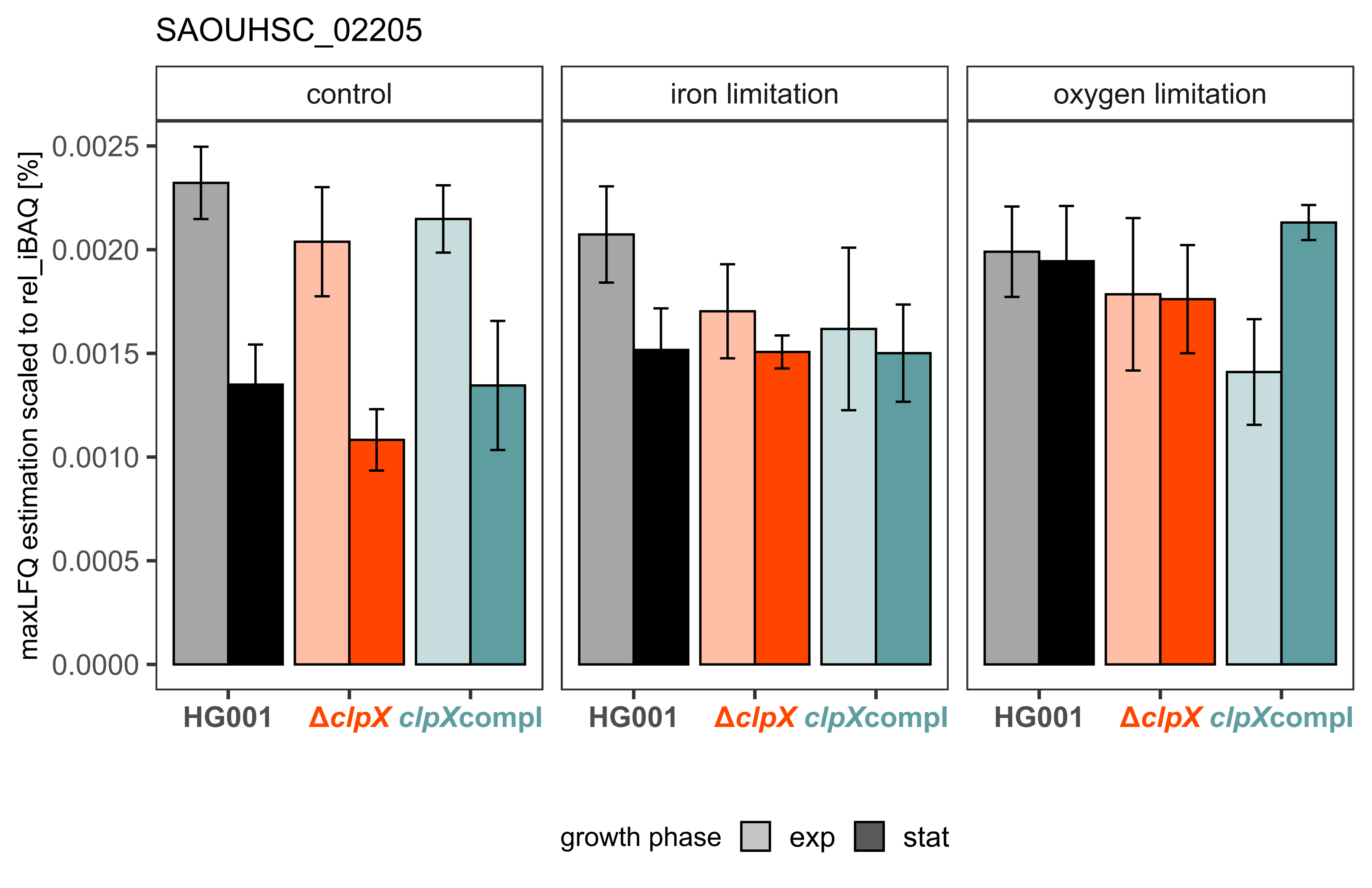

SAOUHSC_02205
control
iron limitation
oxygen limitation
maxLFQ estimation scaled to rel_iBAQ [%]
0.0025
0.0020
0.0015
0.0010
0.0005
0.0000
HG001
ΔclpX
clpXcompl
growth phase
exp
stat

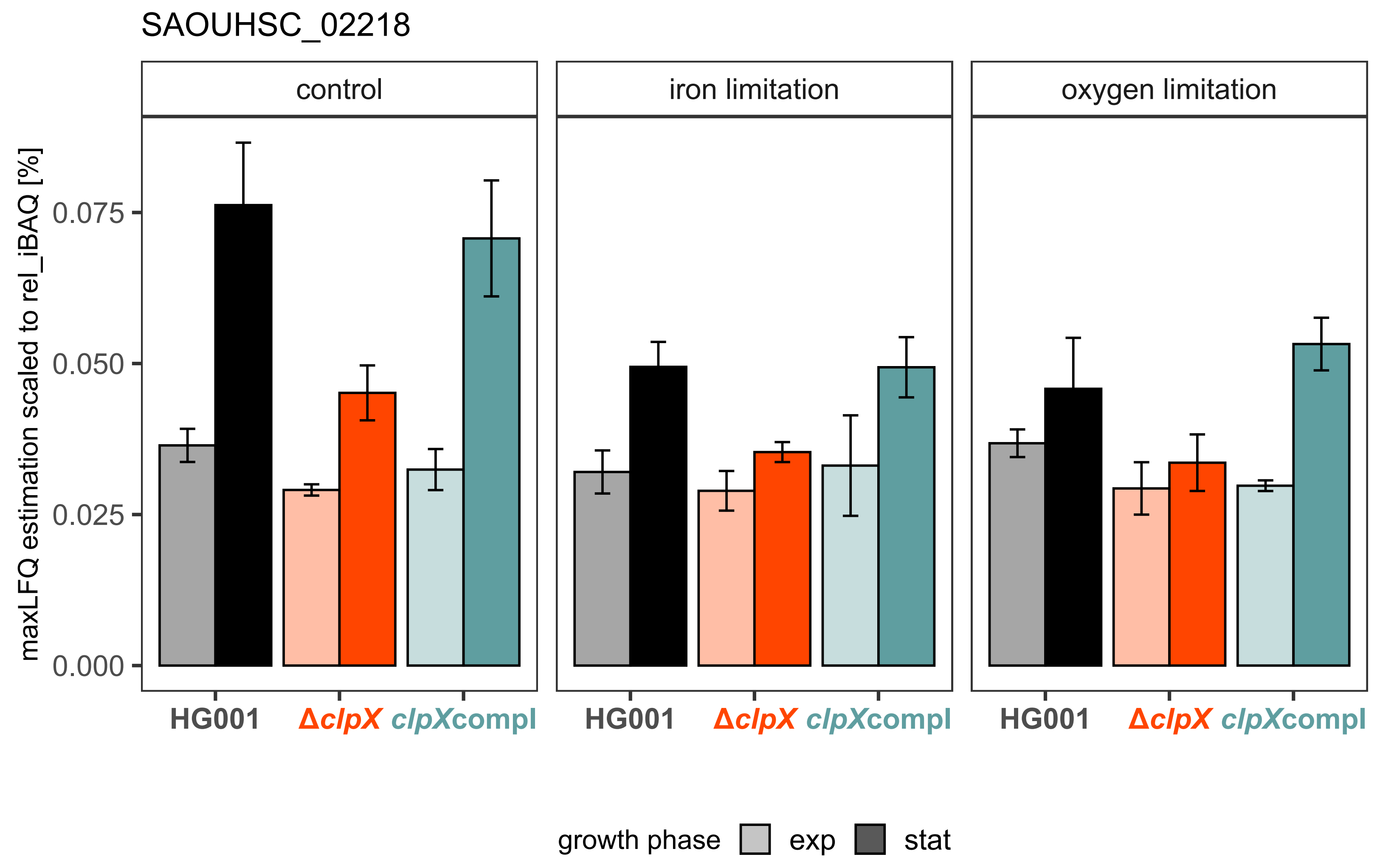

SAOUHSC_02218
control
iron limitation
oxygen limitation
maxLFQ estimation scaled to rel_iBAQ [%]
0.075
0.050
0.025
0.000
HG001
ΔclpX
clpXcompl
growth phase
exp
stat

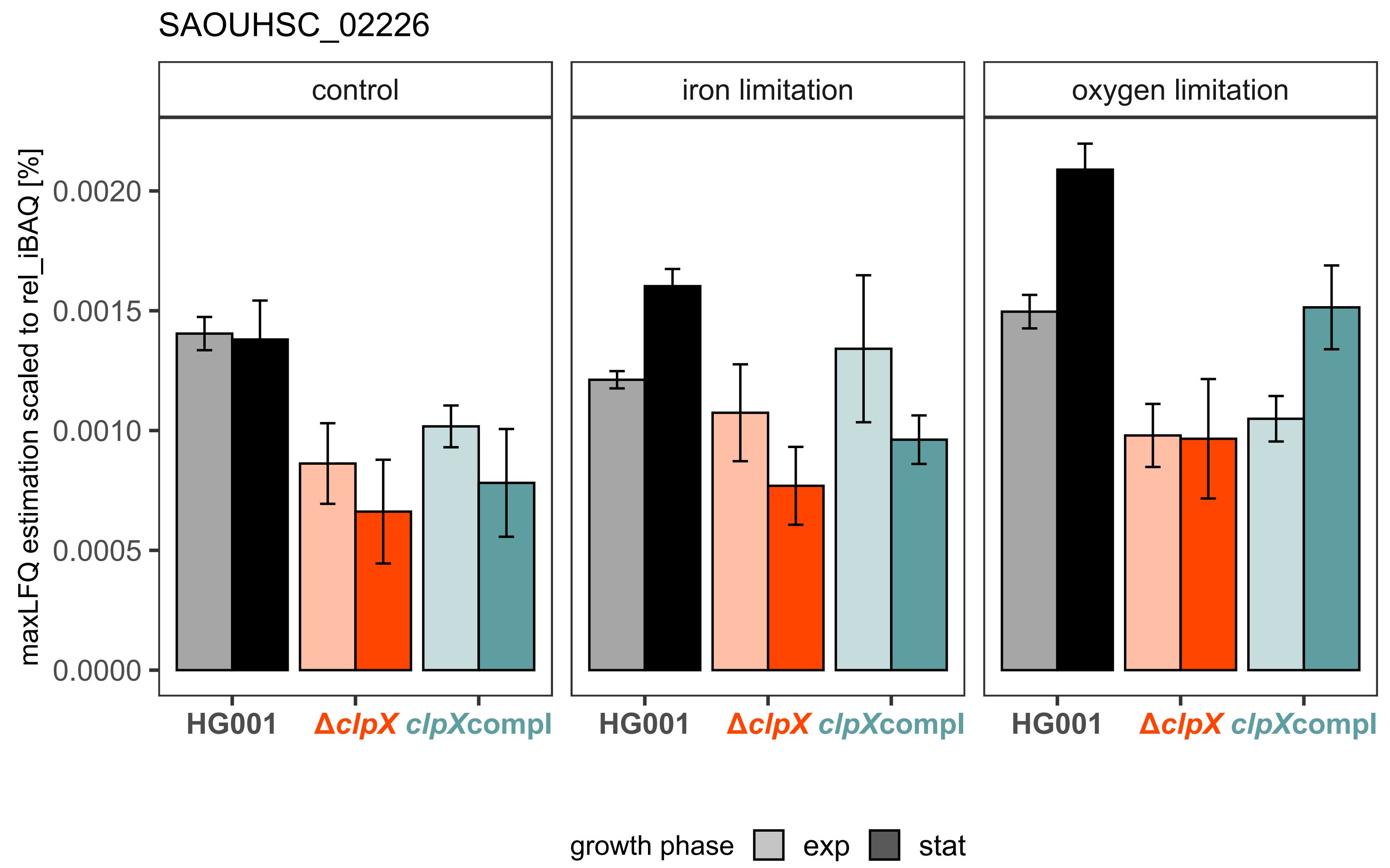
SAOUHSC_02226
control
iron limitation
oxygen limitation
maxLFQ estimation scaled to rel_iBAQ [%]
0.0020
0.0015
0.0010
0.0005
0.0000
HG001
ΔclpX
clpXcompl
growth phase
exp
stat

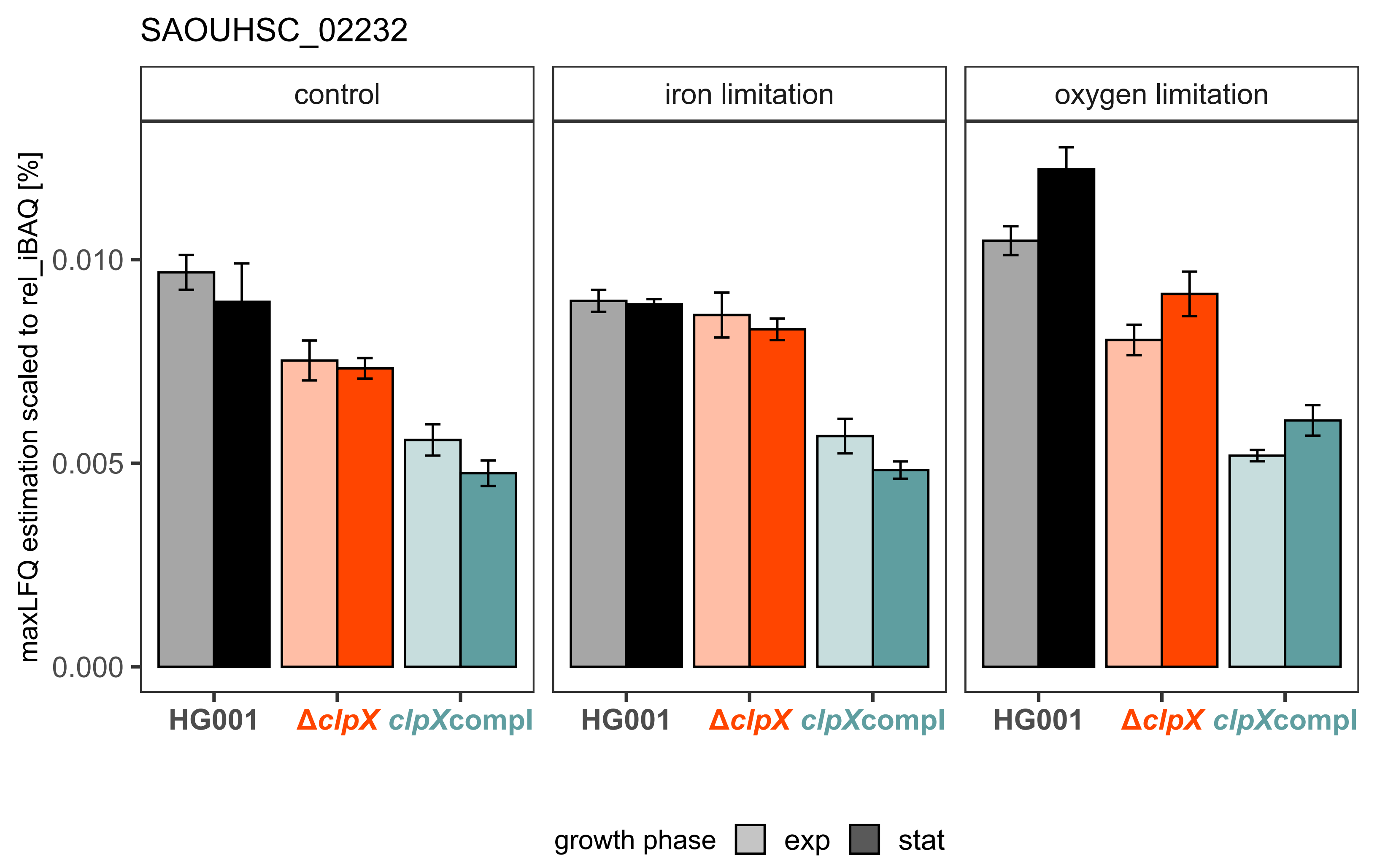

SAOUHSC_02232
control
iron limitation
oxygen limitation
maxLFQ estimation scaled to rel_iBAQ [%]
0.010
0.005
0.000
HG001
ΔclpX
clpXcompl
growth phase
exp
stat

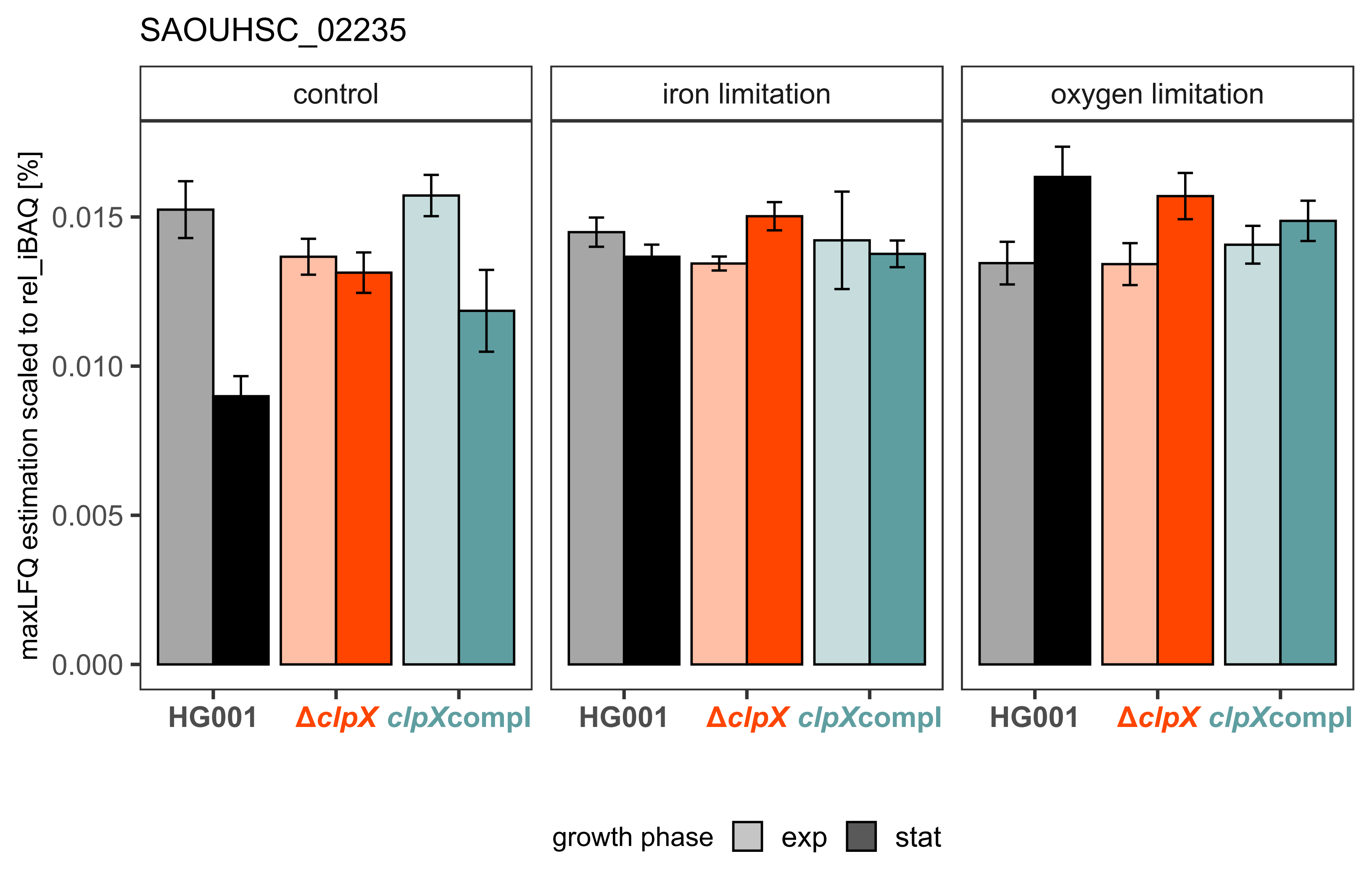
SAOUHSC_02235
control
iron limitation
oxygen limitation
maxLFQ estimation scaled to rel_iBAQ [%]
0.015
0.010
0.005
0.000
HG001
ΔclpX
clpXcompl
growth phase
exp
stat

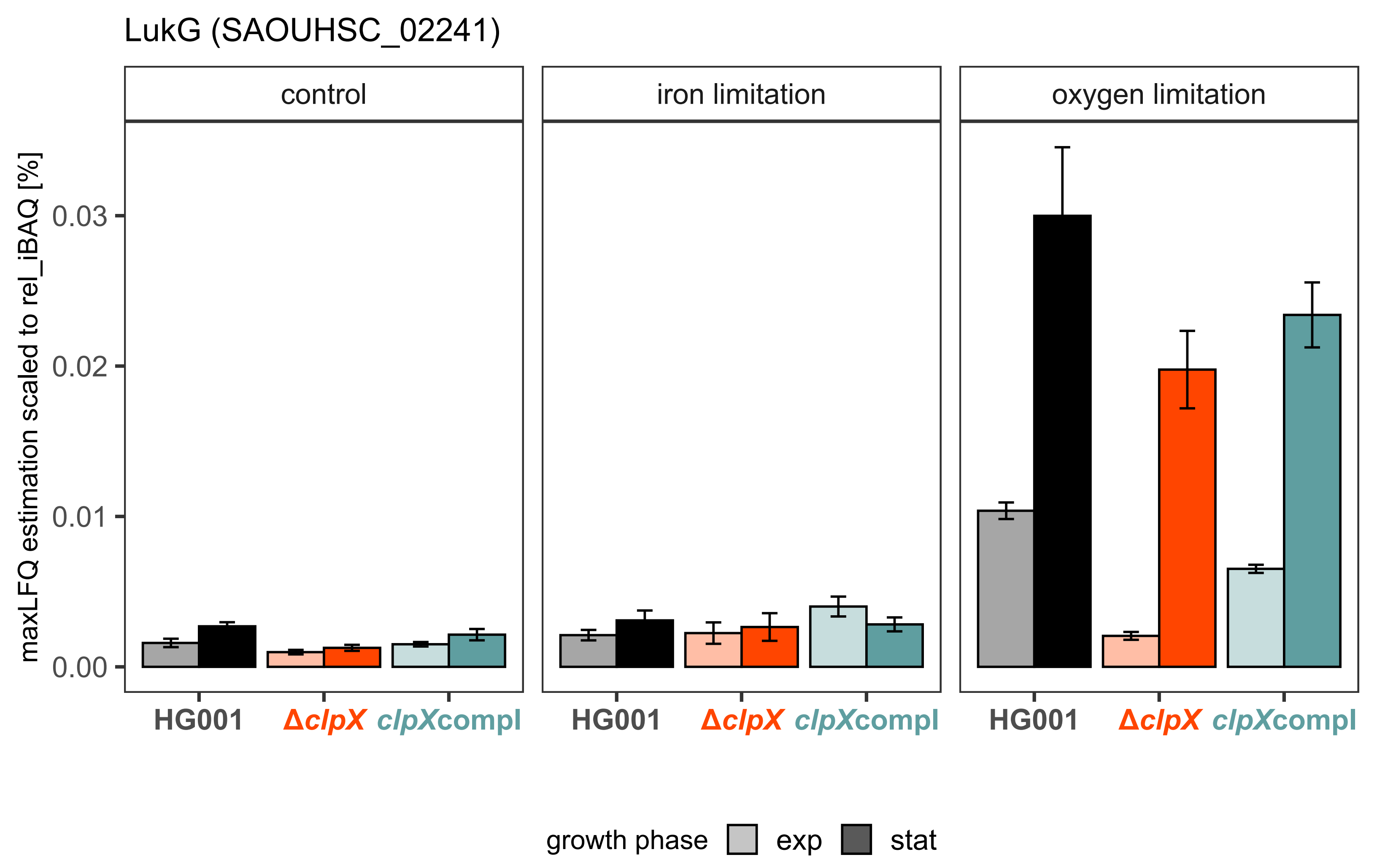

LukG (SAOUHSC_02241)
control
iron limitation
oxygen limitation
maxLFQ estimation scaled to rel_iBAQ [%]
0.03
0.02
0.01
0.00
HG001
ΔclpX
clpXcompl
growth phase
exp
stat

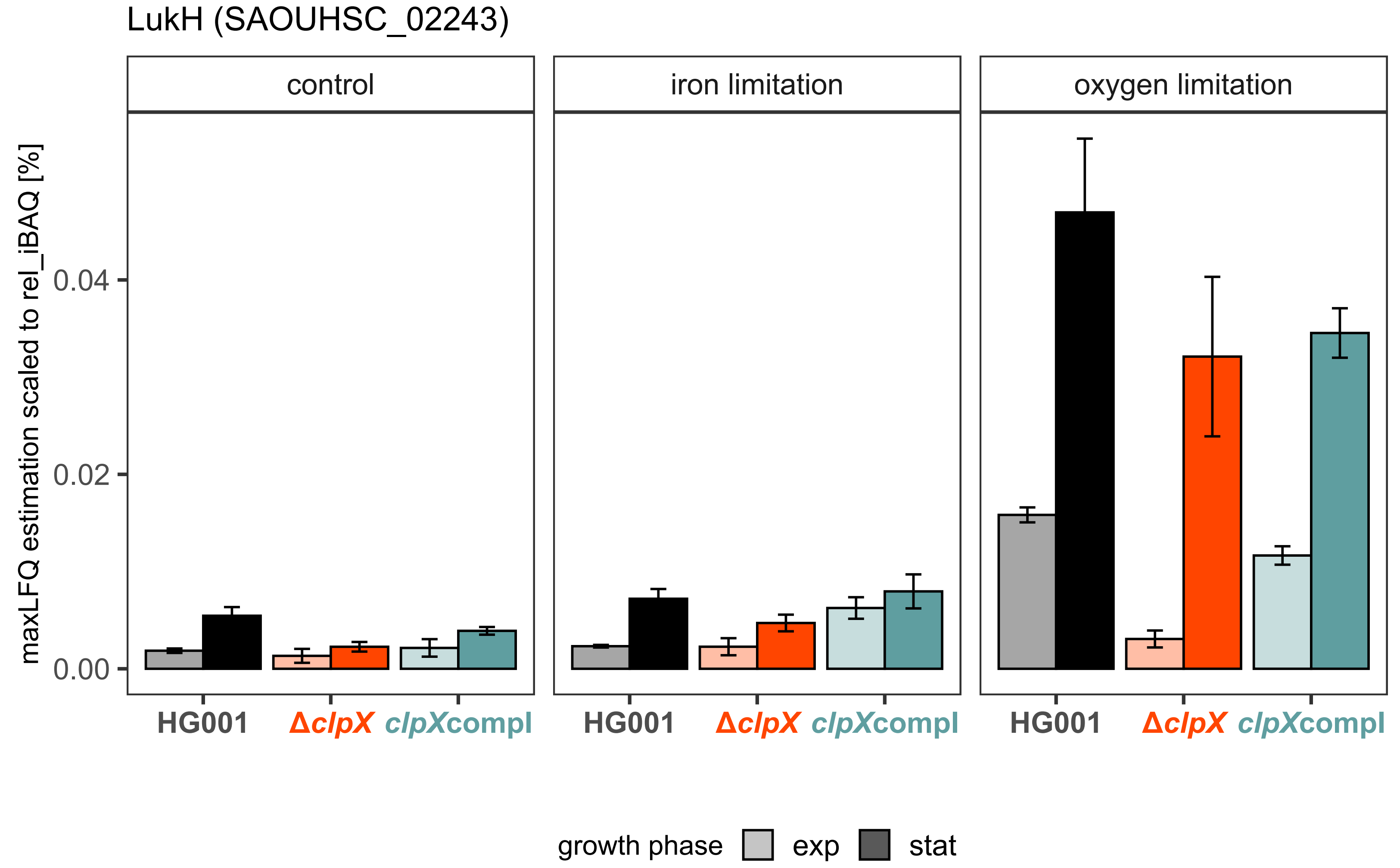
LukH (SAOUHSC_02243)
control
iron limitation
oxygen limitation
maxLFQ estimation scaled to rel_iBAQ [%]
0.04
0.02
0.00
HG001
ΔclpX
clpXcompl
growth phase
exp
stat

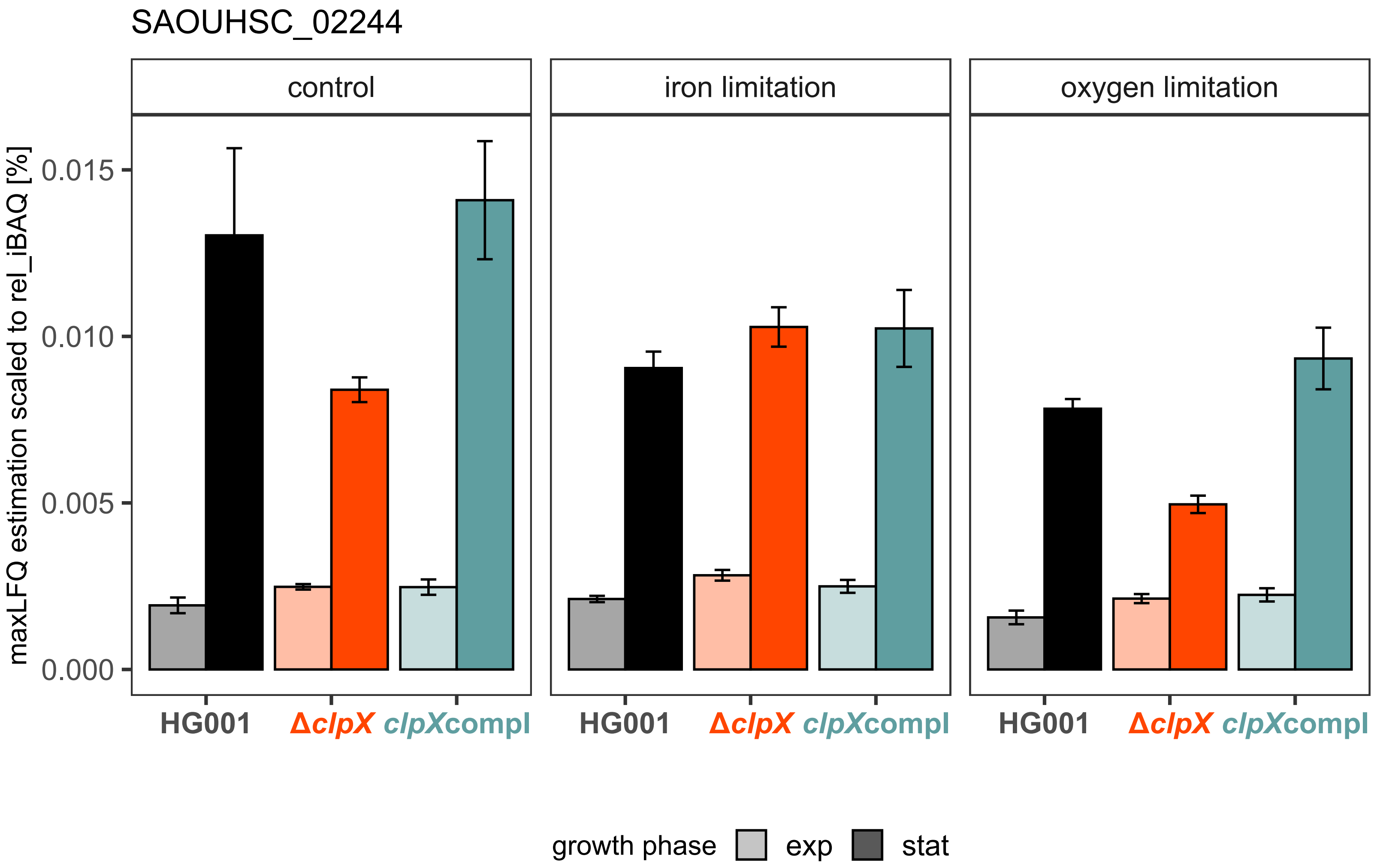
SAOUHSC_02244
control
iron limitation
oxygen limitation
maxLFQ estimation scaled to rel_iBAQ [%]
0.015
0.010
0.005
0.000
HG001
ΔclpX
clpXcompl
growth phase
exp
stat

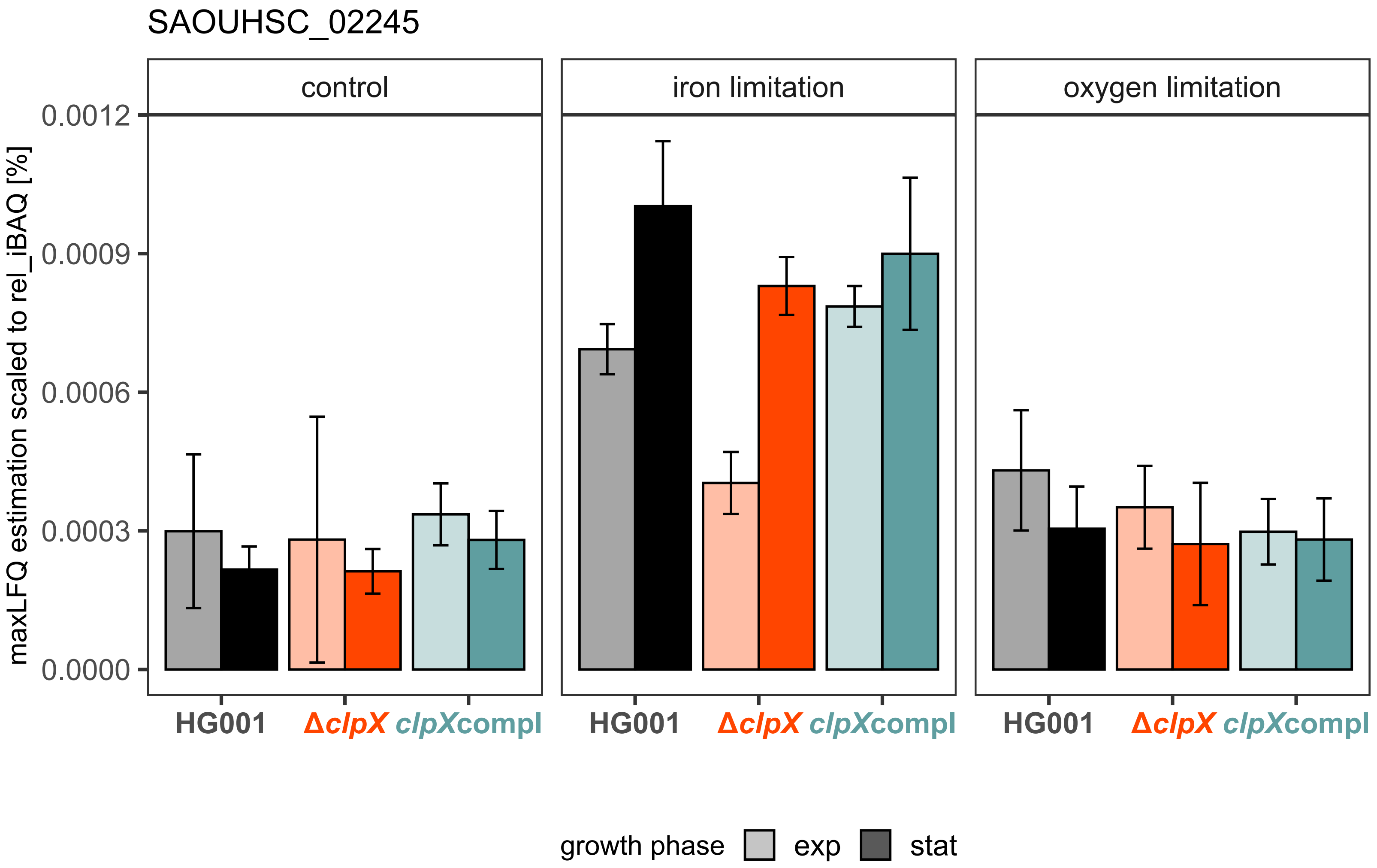
SAOUHSC_02245
control
iron limitation
oxygen limitation
maxLFQ estimation scaled to rel_iBAQ [%]
0.0012
0.0009
0.0006
0.0003
0.0000
HG001
ΔclpX
clpXcompl
growth phase
exp
stat

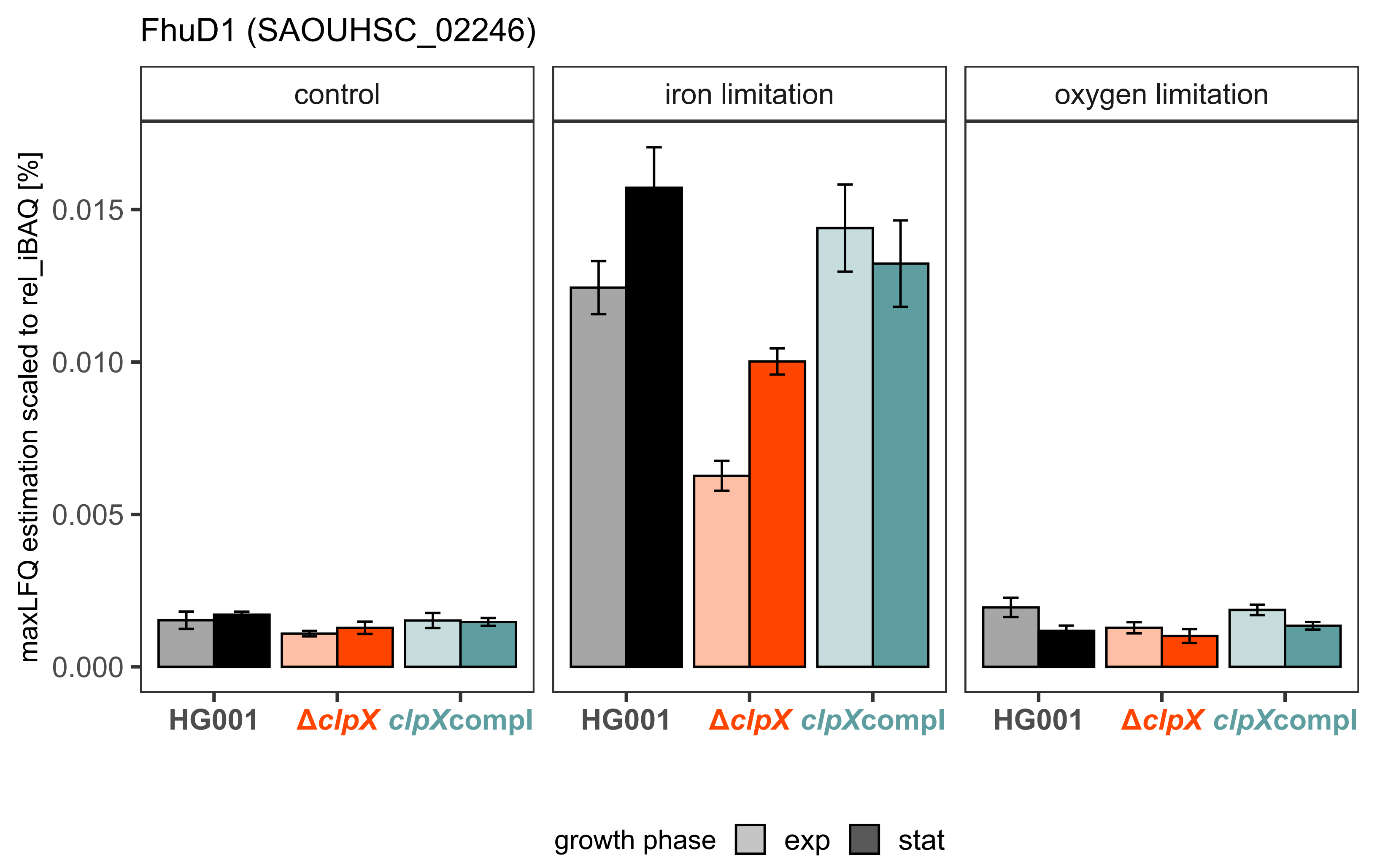

FhuD1 (SAOUHSC_02246)
control
iron limitation
oxygen limitation
maxLFQ estimation scaled to rel_iBAQ [%]
0.015
0.010
0.005
0.000
HG001
ΔclpX
clpXcompl
growth phase
exp
stat

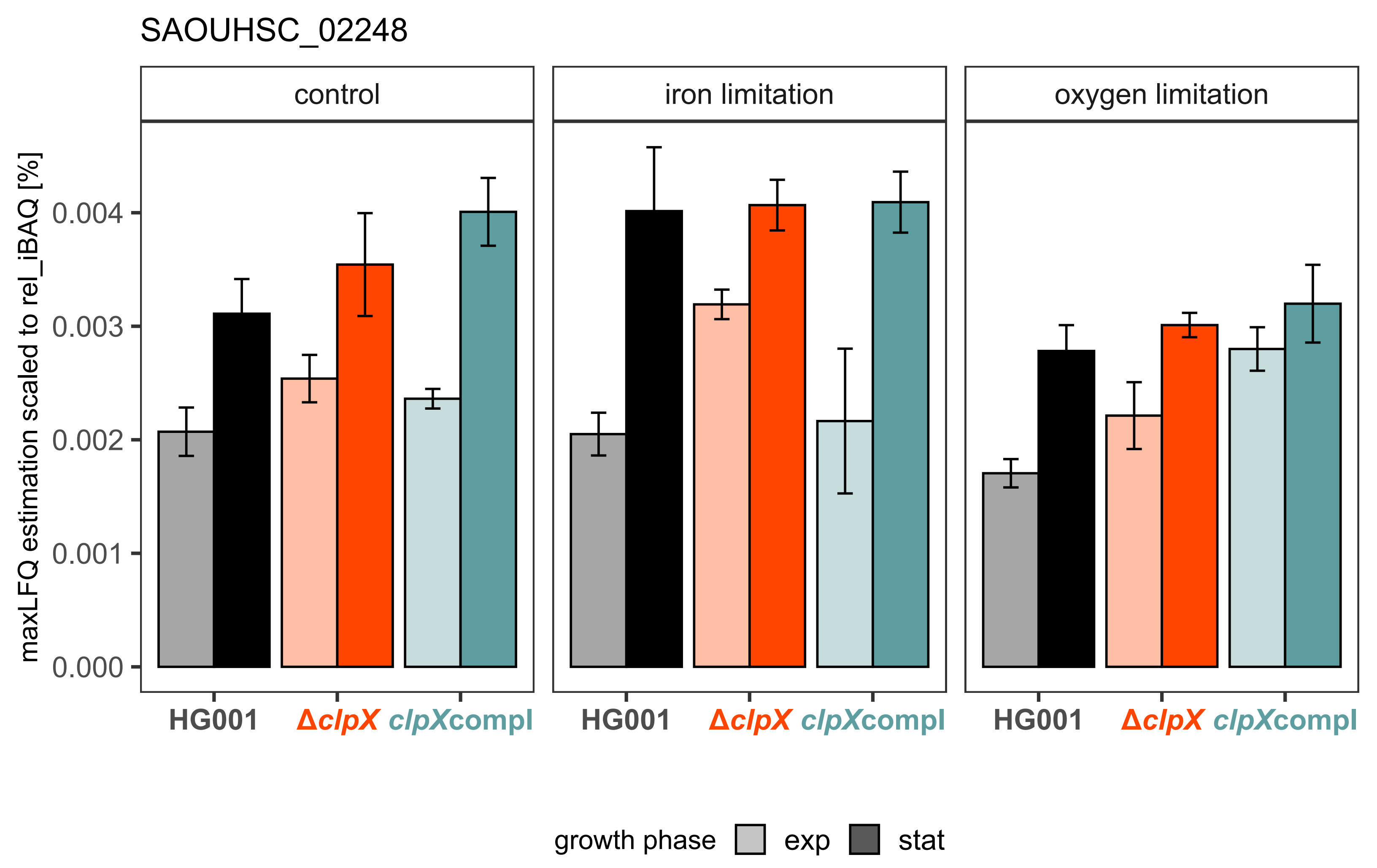
SAOUHSC_02248
control
iron limitation
oxygen limitation
maxLFQ estimation scaled to rel_iBAQ [%]
0.004
0.003
0.002
0.001
0.000
HG001
ΔclpX
clpXcompl
growth phase
exp
stat

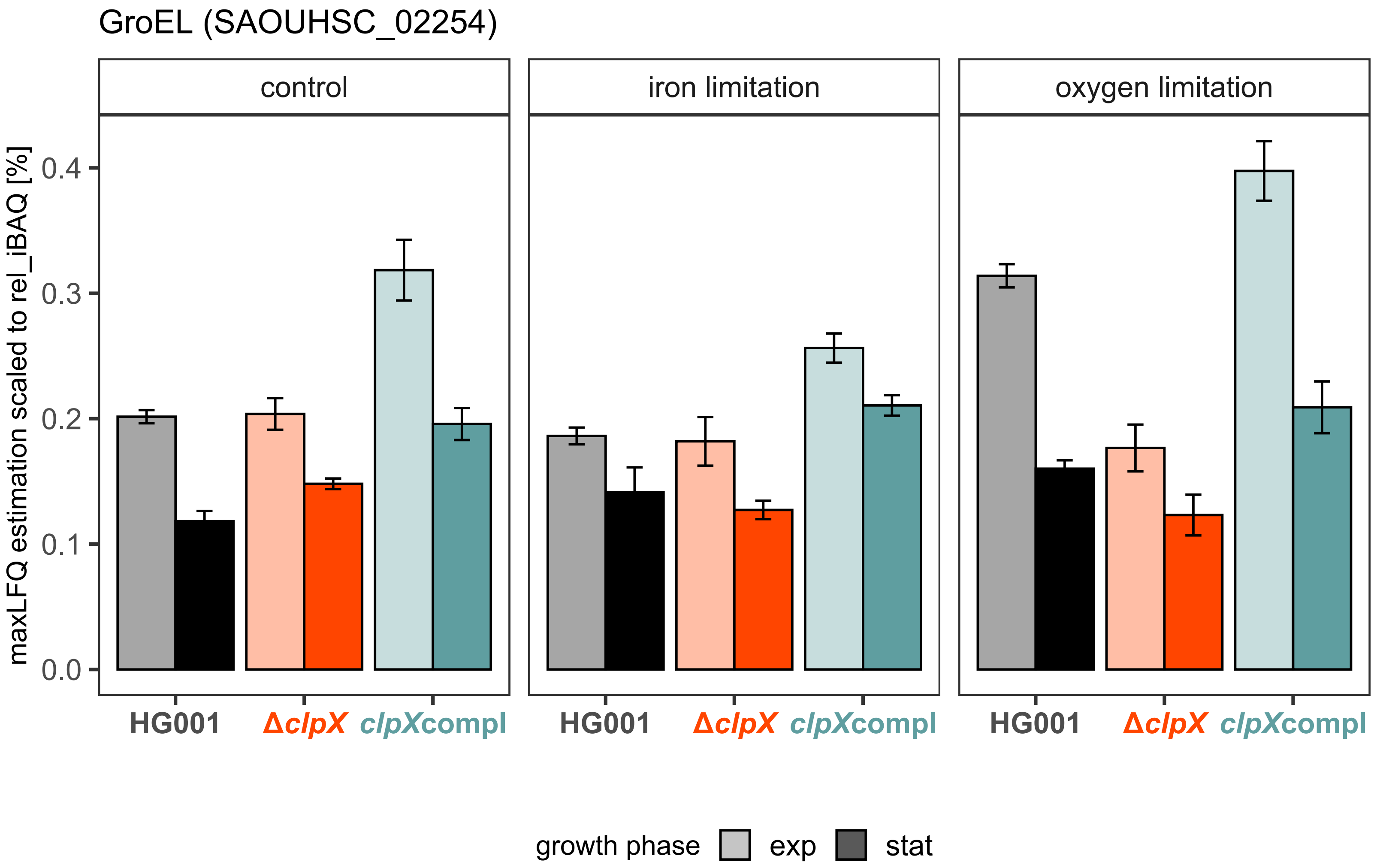
GroEL (SAOUHSC_02254)
control
iron limitation
oxygen limitation
maxLFQ estimation scaled to rel_iBAQ [%]
0.0
0.1
0.2
0.3
0.4
HG001
ΔclpX
clpXcompl
growth phase
exp
stat

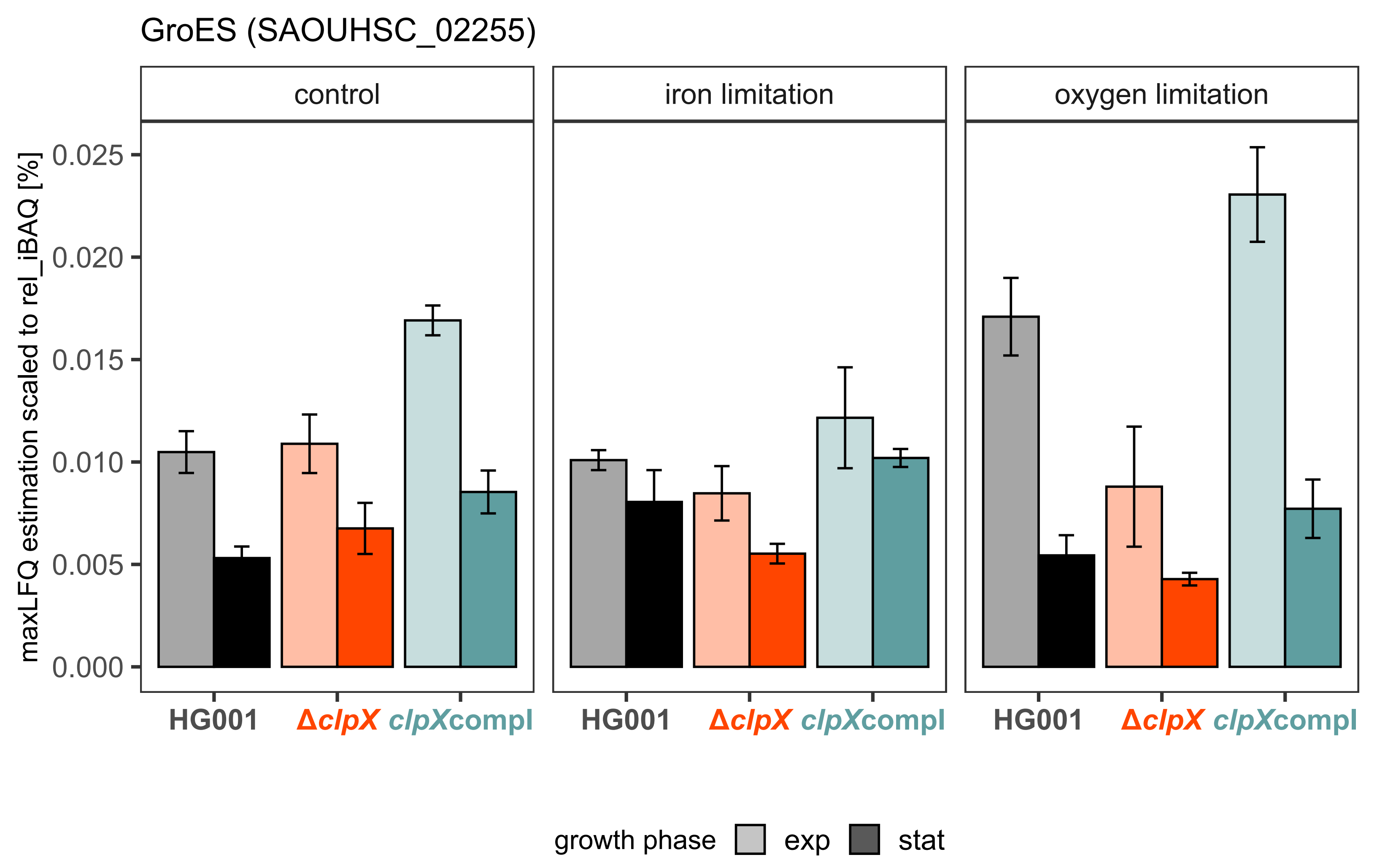
GroES (SAOUHSC_02255)
control
iron limitation
oxygen limitation
maxLFQ estimation scaled to rel_iBAQ [%]
0.025
0.020
0.015
0.010
0.005
0.000
HG001
ΔclpX
clpXcompl
growth phase
exp
stat

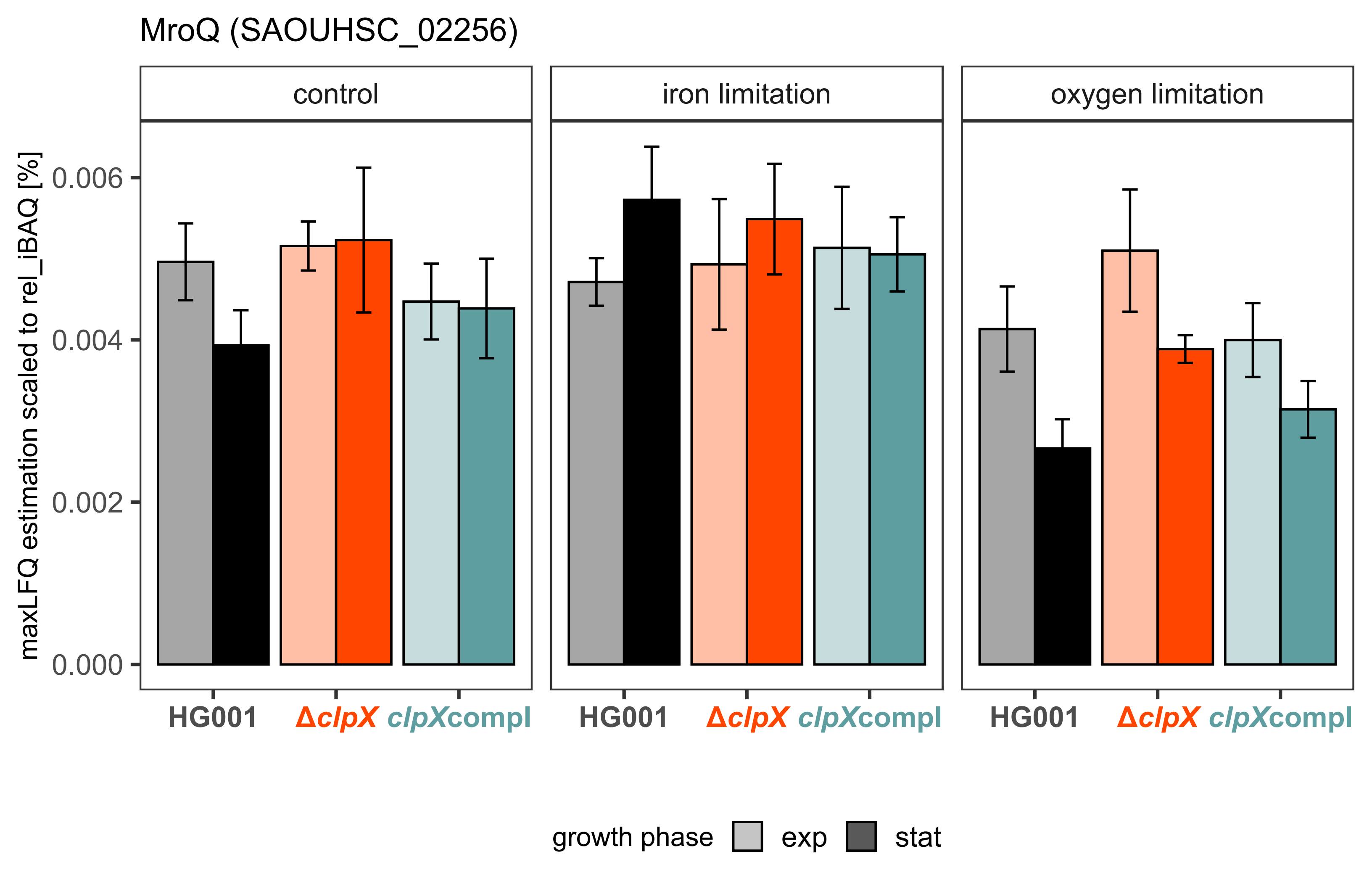

MroQ (SAOUHSC_02256)
control
iron limitation
oxygen limitation
maxLFQ estimation scaled to rel_iBAQ [%]
0.006
0.004
0.002
0.000
HG001
ΔclpX
clpXcompl
growth phase
exp
stat

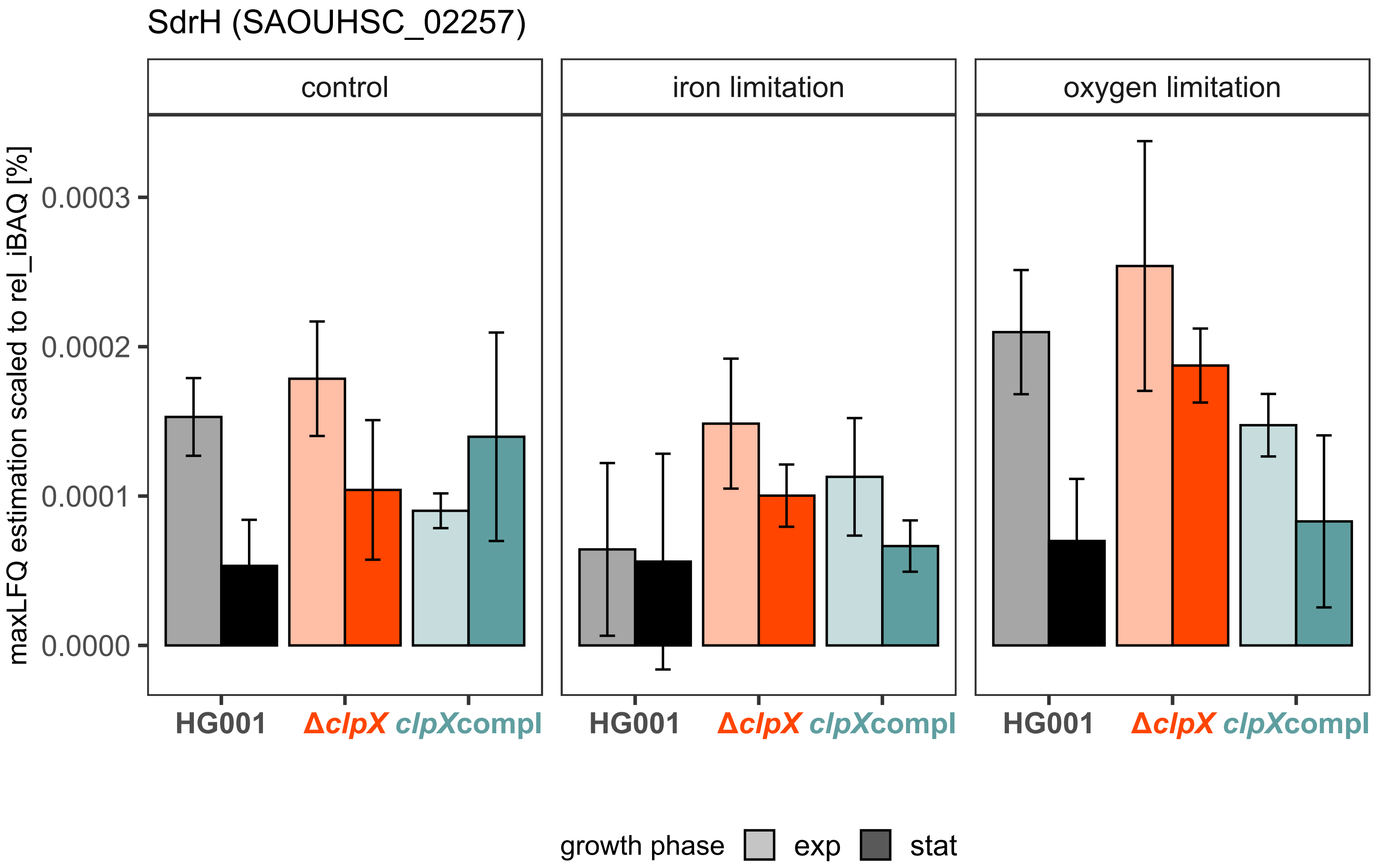
SdrH (SAOUHSC_02257)
control
iron limitation
oxygen limitation
maxLFQ estimation scaled to rel_iBAQ [%]
0.0003
0.0002
0.0001
0.0000
HG001
ΔclpX
clpXcompl
growth phase
exp
stat

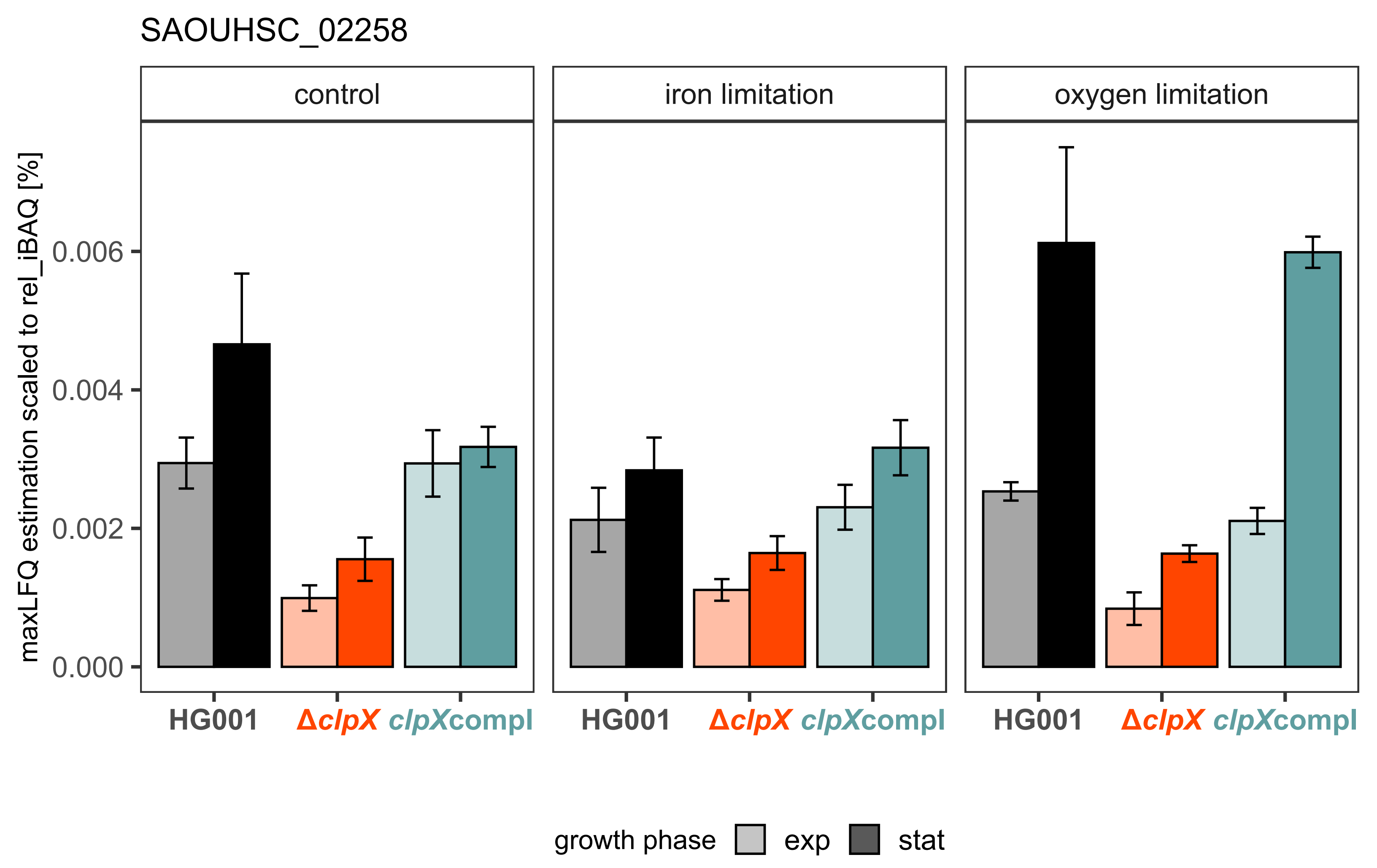

SAOUHSC_02258
control
iron limitation
oxygen limitation
maxLFQ estimation scaled to rel_iBAQ [%]
0.000
0.002
0.004
0.006
HG001
ΔclpX
clpXcompl
growth phase
exp
stat

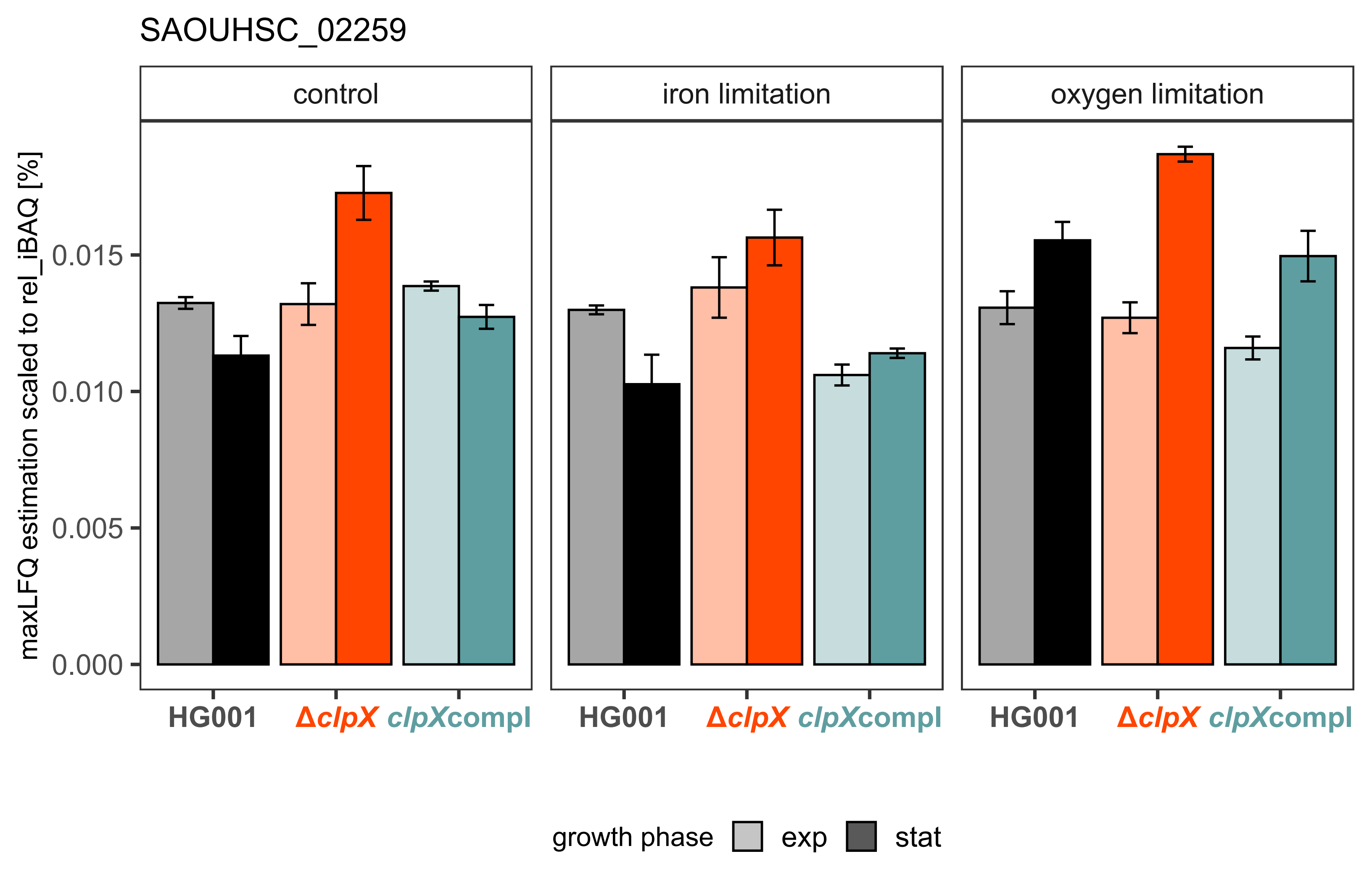
SAOUHSC_02259
control
iron limitation
oxygen limitation
maxLFQ estimation scaled to rel_iBAQ [%]
0.015
0.010
0.005
0.000
HG001
ΔclpX
clpXcompl
growth phase
exp
stat

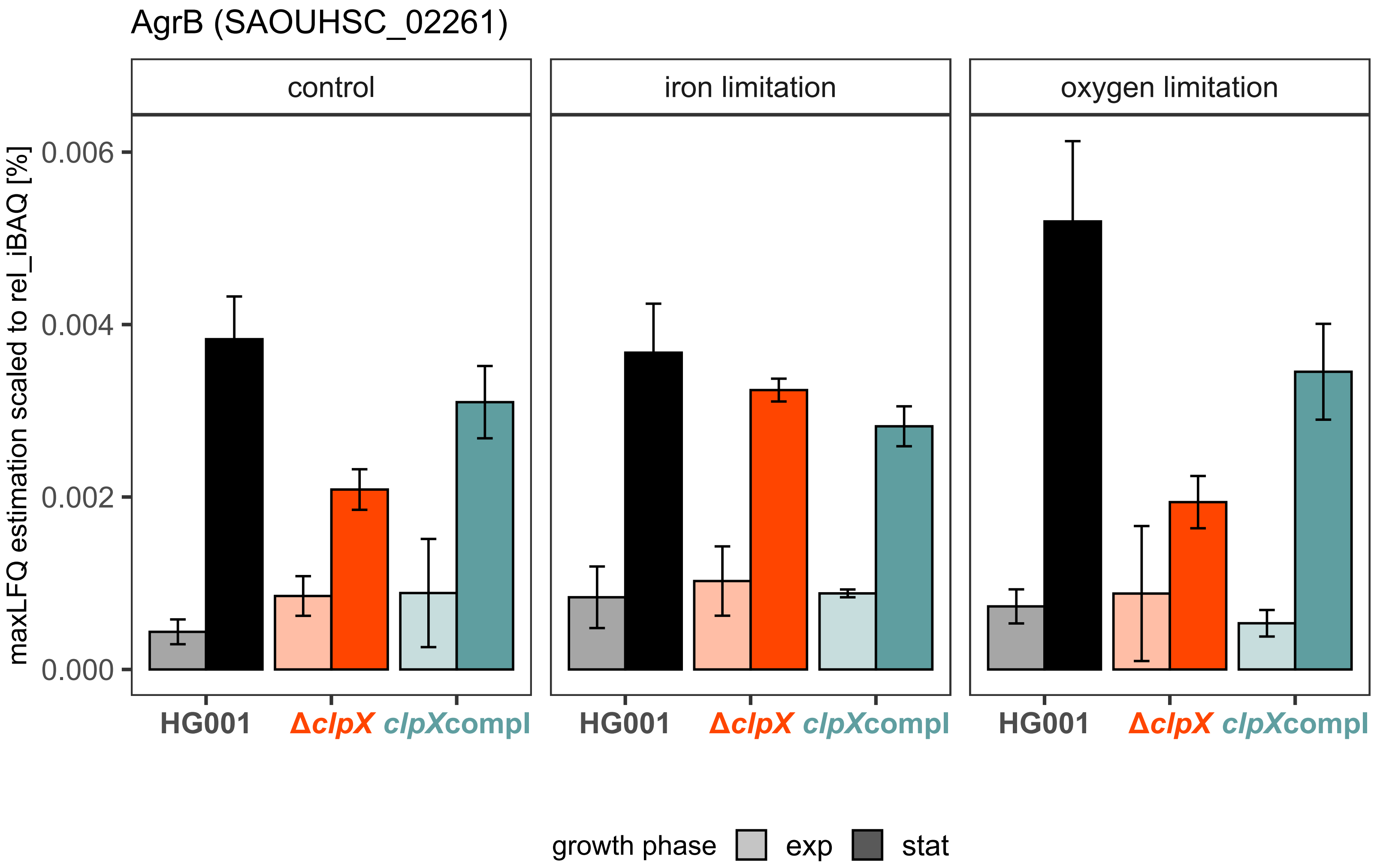
AgrB (SAOUHSC_02261)
control
iron limitation
oxygen limitation
maxLFQ estimation scaled to rel_iBAQ [%]
0.006
0.004
0.002
0.000
HG001
ΔclpX
clpXcompl
growth phase
exp
stat

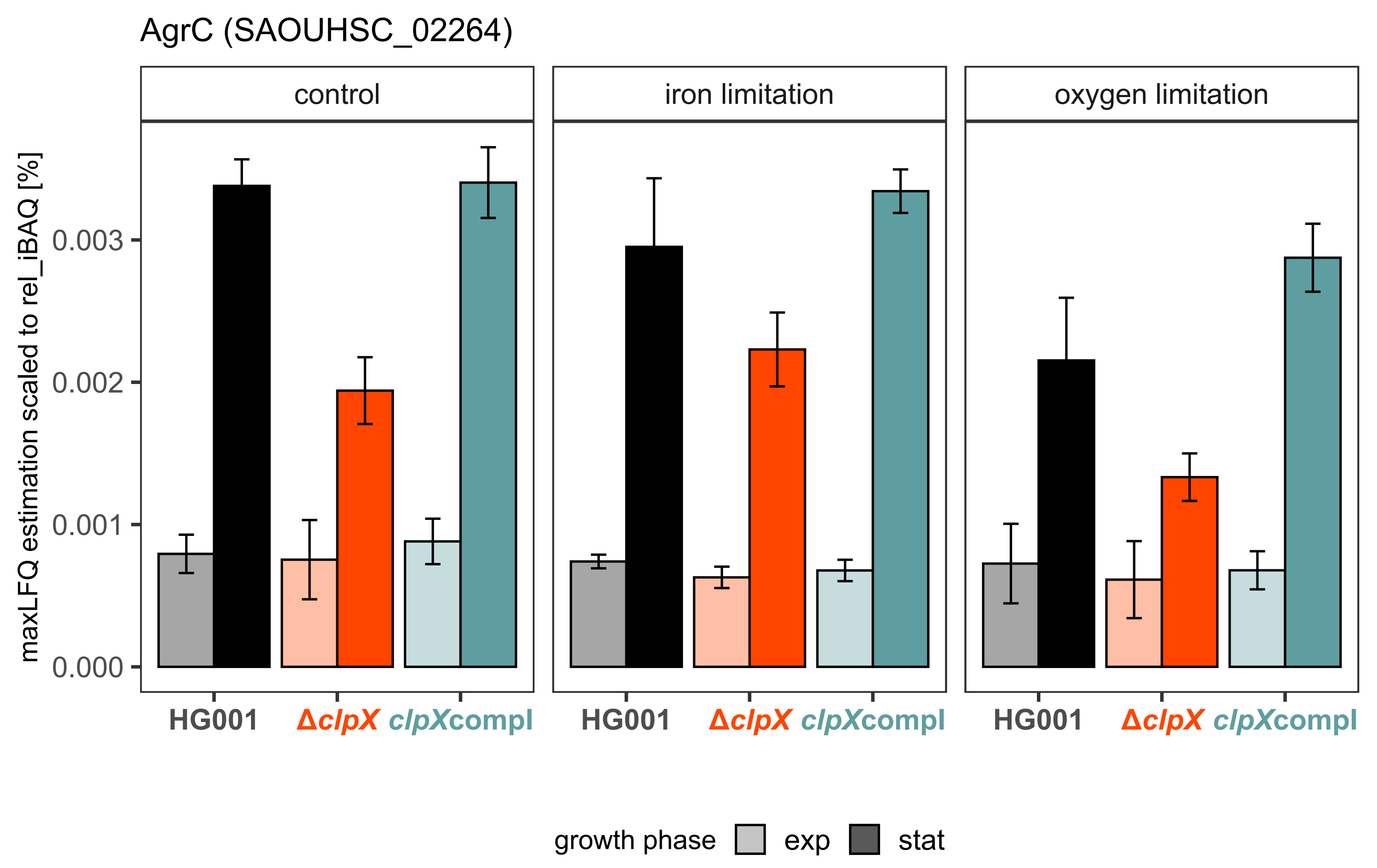

AgrC (SAOUHSC_02264)
control
iron limitation
oxygen limitation
maxLFQ estimation scaled to rel_iBAQ [%]
0.000
0.001
0.002
0.003
HG001
ΔclpX
clpXcompl
growth phase
exp
stat

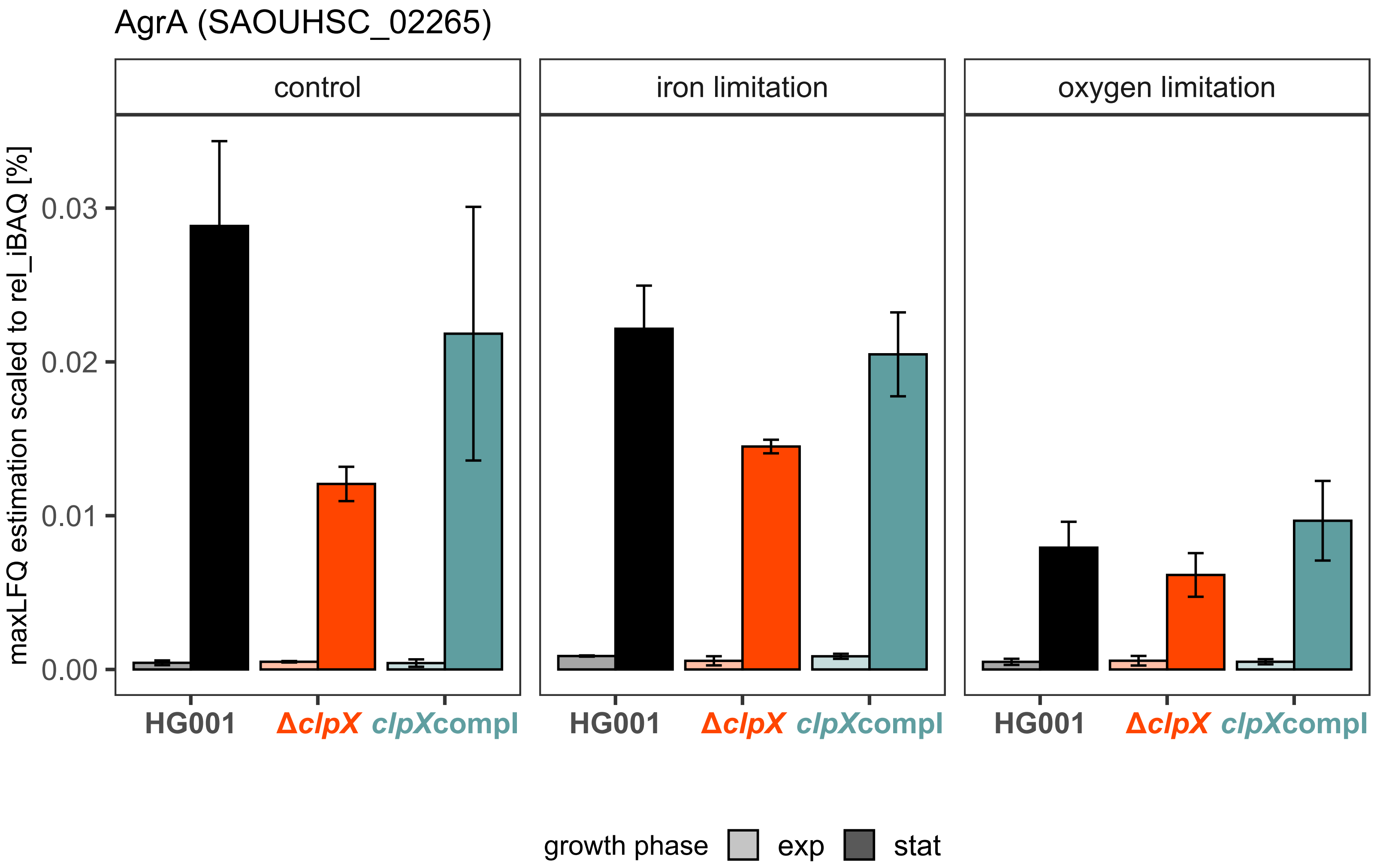

AgrA (SAOUHSC_02265)
control
iron limitation
oxygen limitation
maxLFQ estimation scaled to rel_iBAQ [%]
0.03
0.02
0.01
0.00
HG001
ΔclpX
clpXcompl
growth phase
exp
stat

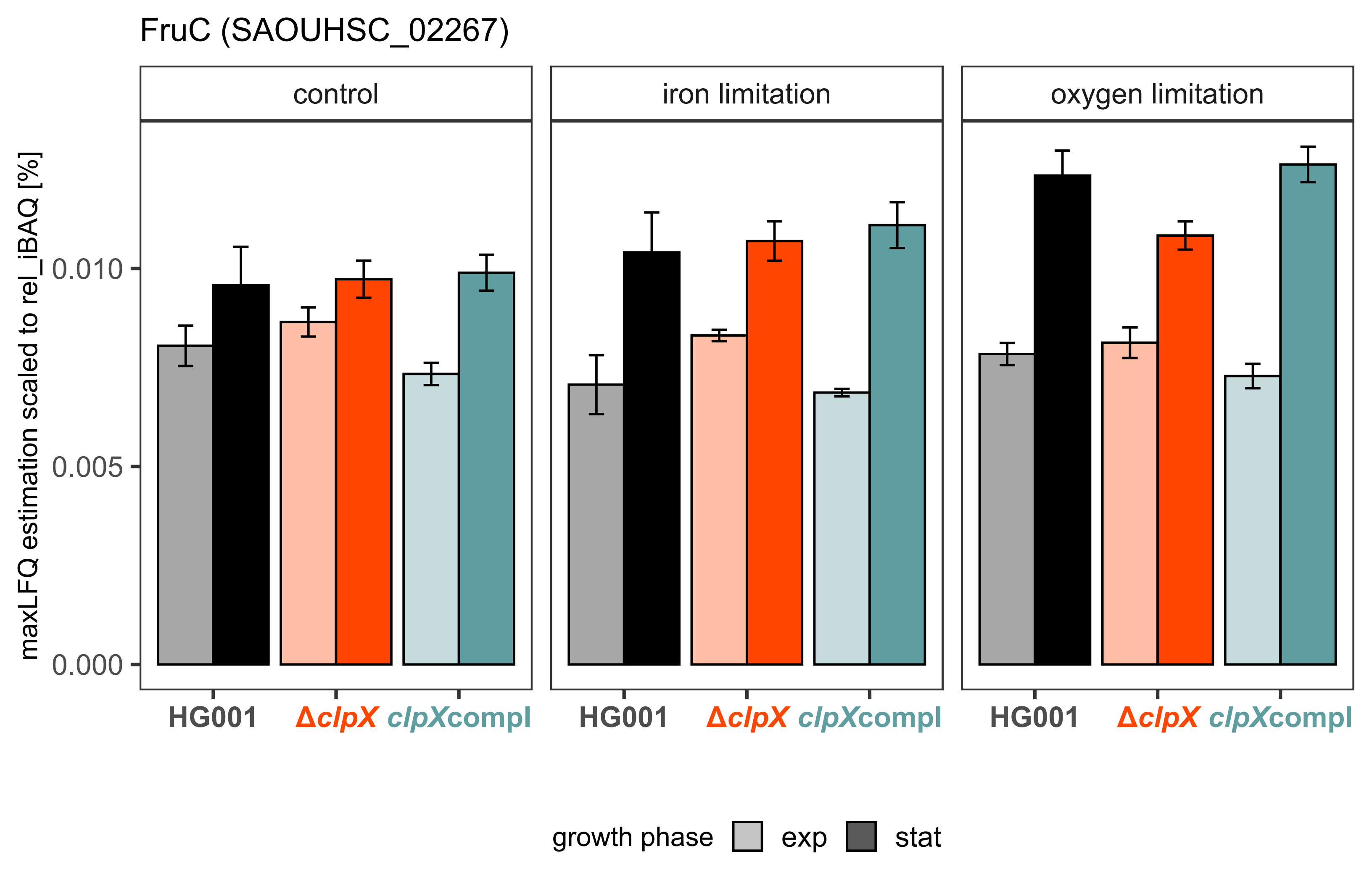
FruC (SAOUHSC_02267)
control
iron limitation
oxygen limitation
maxLFQ estimation scaled to rel_iBAQ [%]
0.010
0.005
0.000
HG001
ΔclpX
clpXcompl
growth phase
exp
stat

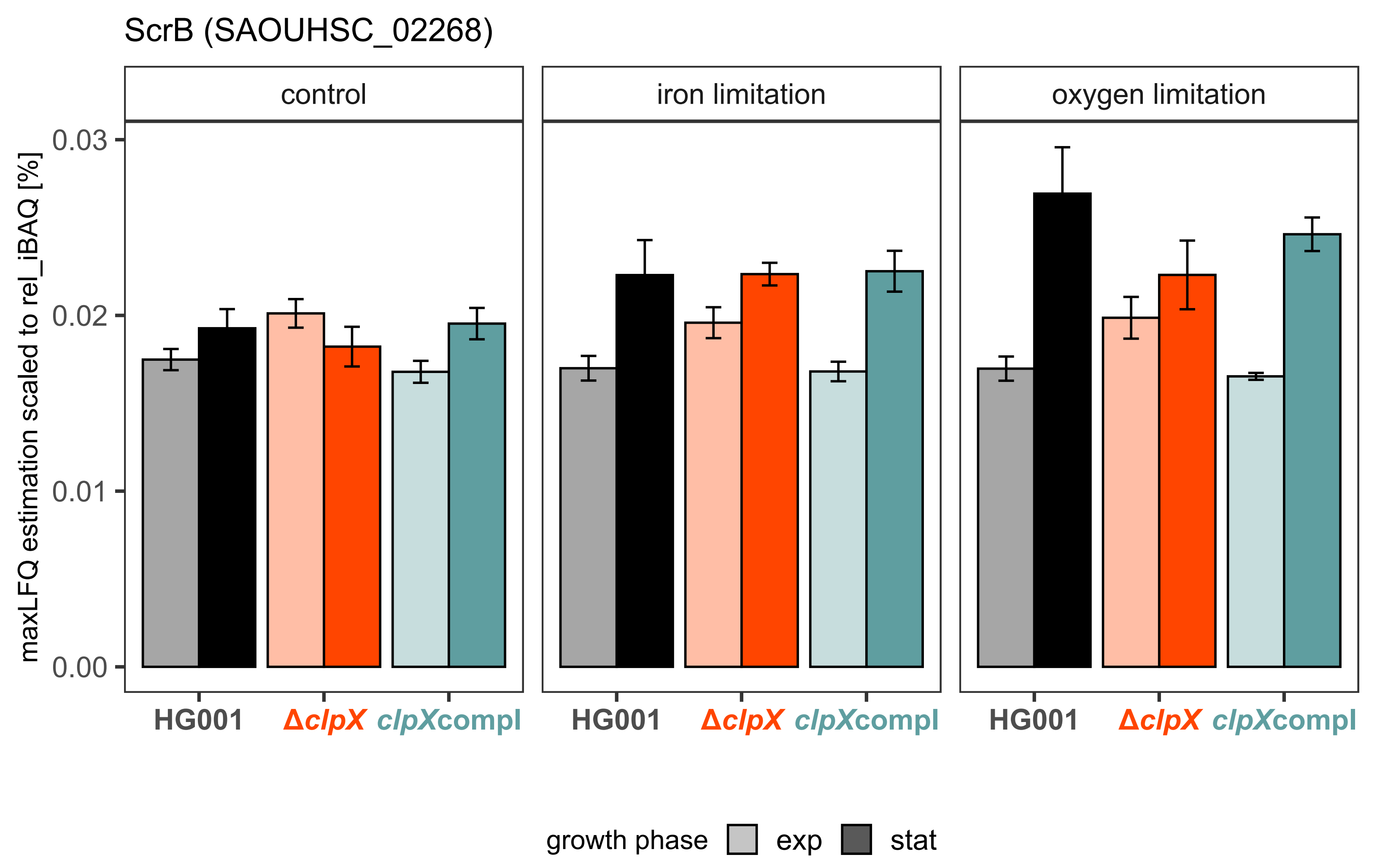

ScrB (SAOUHSC_02268)
control
iron limitation
oxygen limitation
maxLFQ estimation scaled to rel_iBAQ [%]
0.03
0.02
0.01
0.00
HG001
ΔclpX
clpXcompl
growth phase
exp
stat

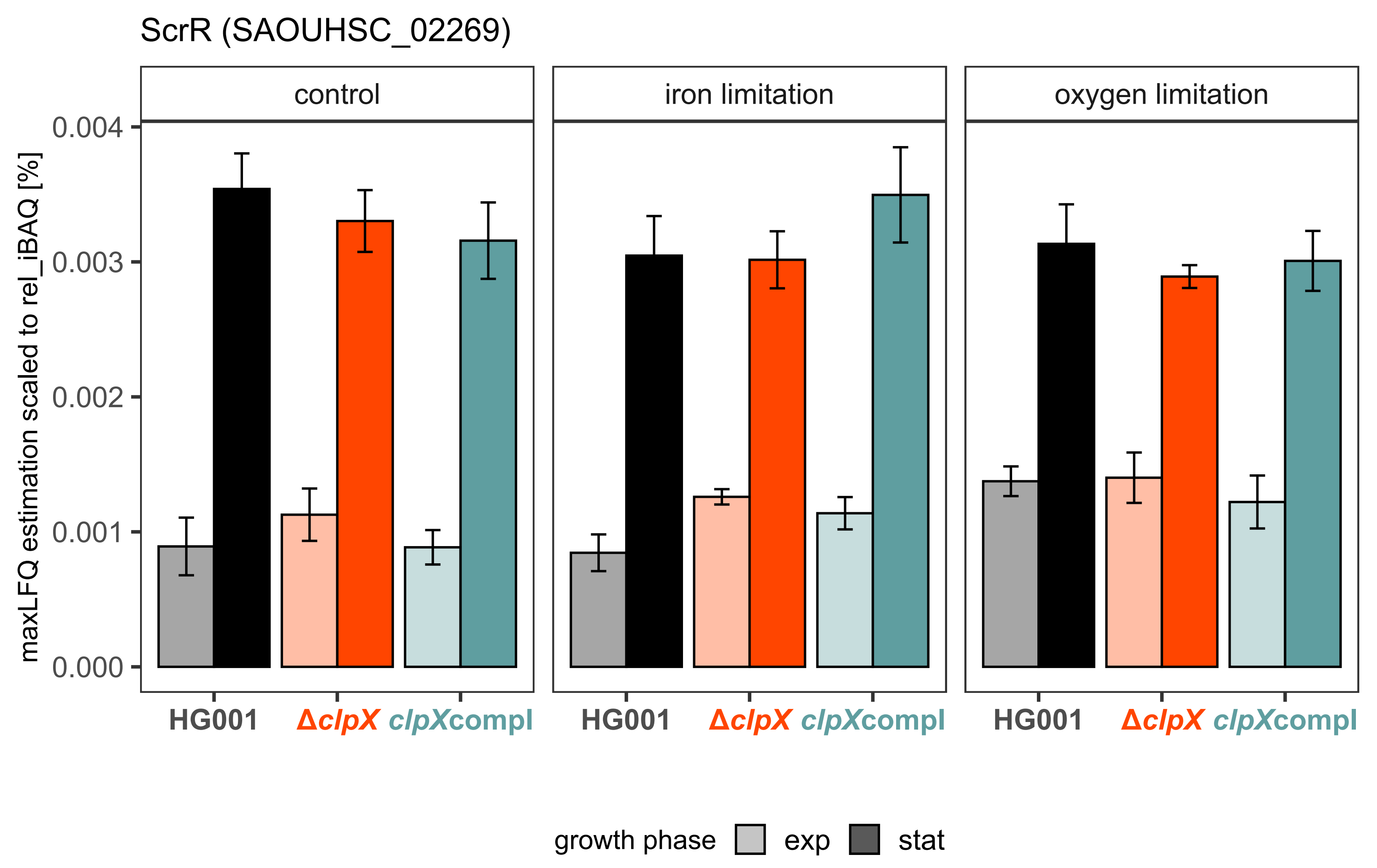
ScrR (SAOUHSC_02269)
control
iron limitation
oxygen limitation
maxLFQ estimation scaled to rel_iBAQ [%]
0.004
0.003
0.002
0.001
0.000
HG001
ΔclpX
clpXcompl
growth phase
exp
stat

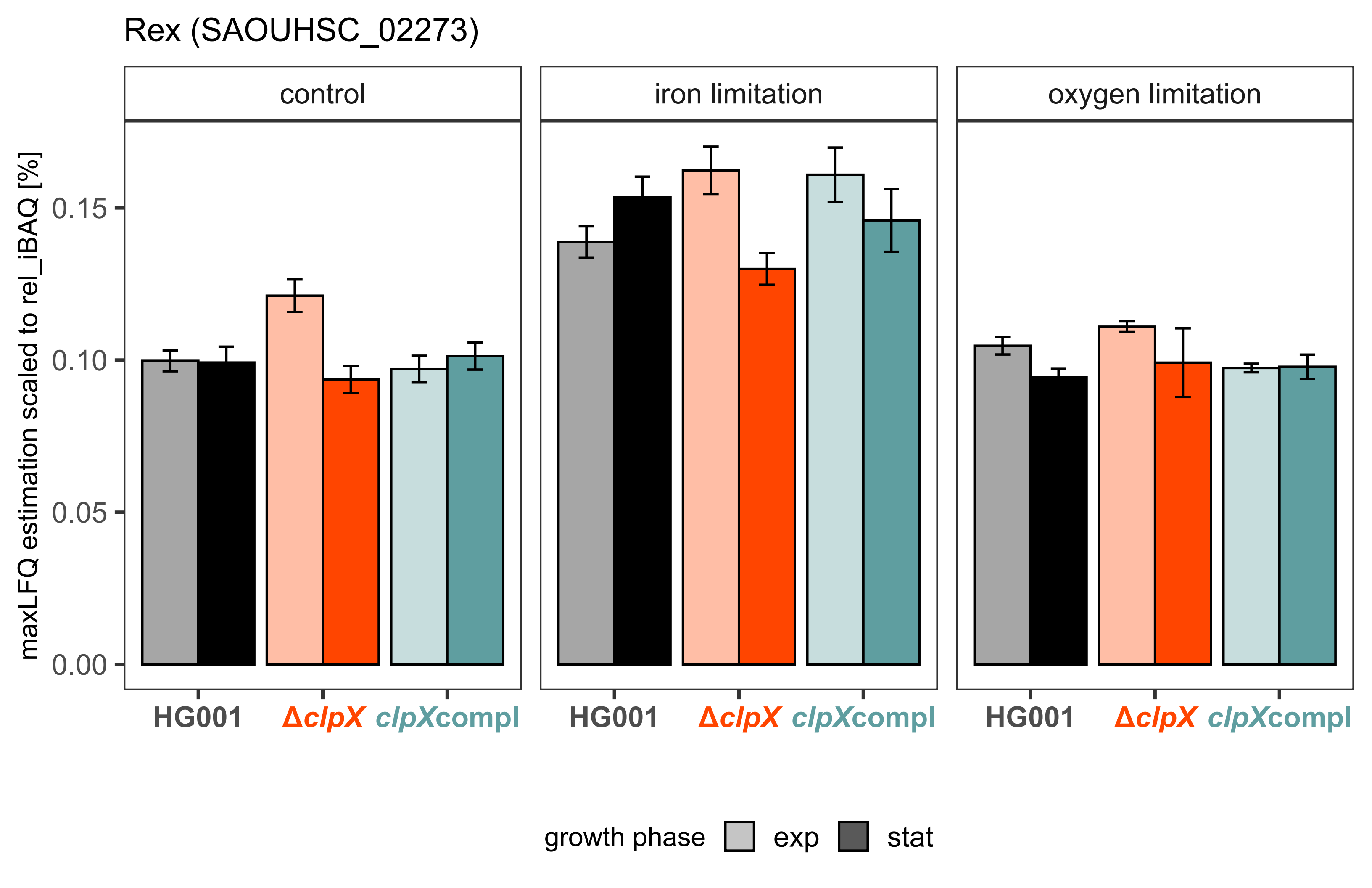

Rex (SAOUHSC_02273)
control
iron limitation
oxygen limitation
maxLFQ estimation scaled to rel_iBAQ [%]
0.15
0.10
0.05
0.00
HG001
ΔclpX
clpXcompl
growth phase
exp
stat

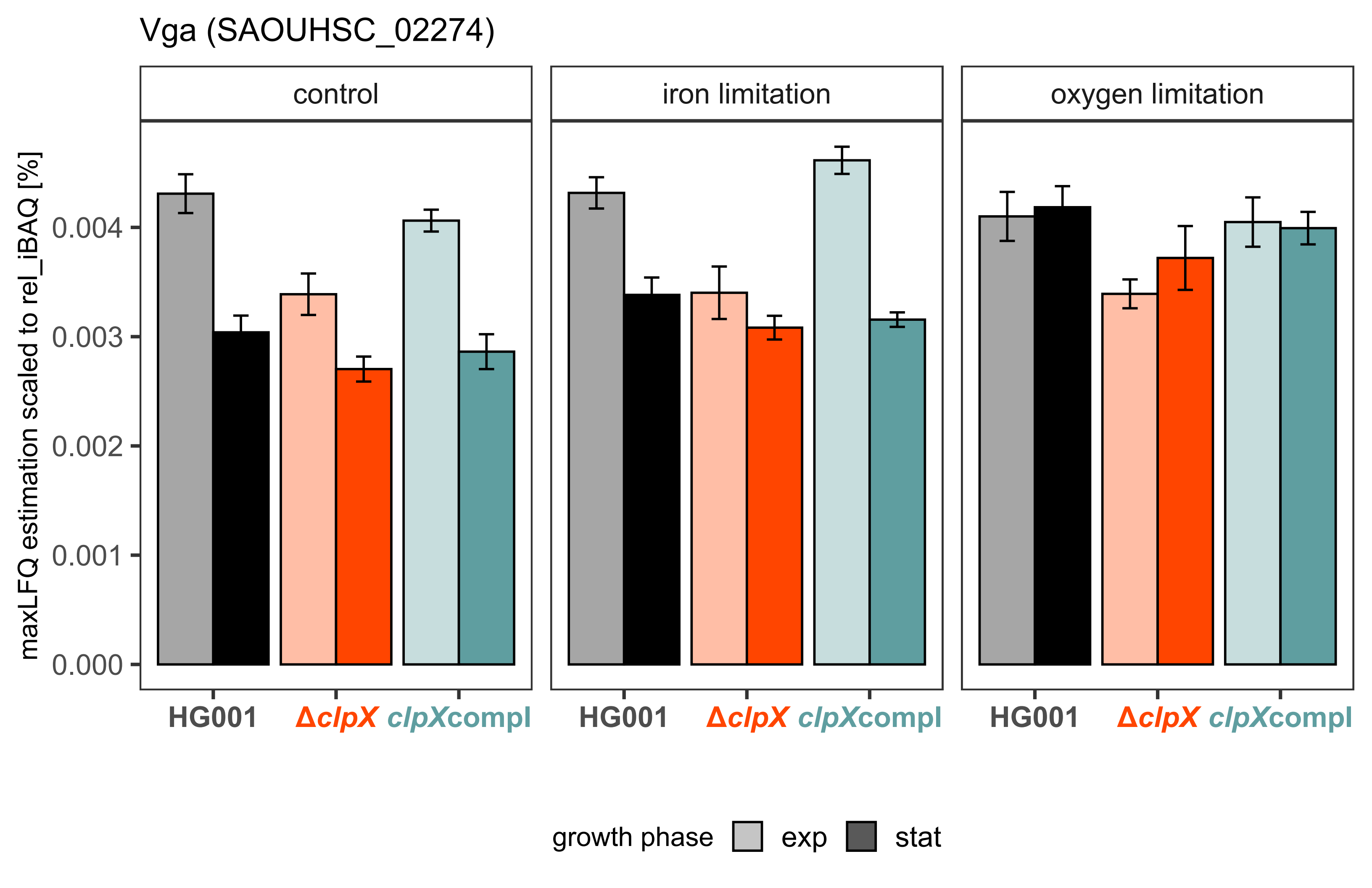

Vga (SAOUHSC_02274)
control
iron limitation
oxygen limitation
maxLFQ estimation scaled to rel_iBAQ [%]
0.004
0.003
0.002
0.001
0.000
HG001
ΔclpX
clpXcompl
growth phase
exp
stat

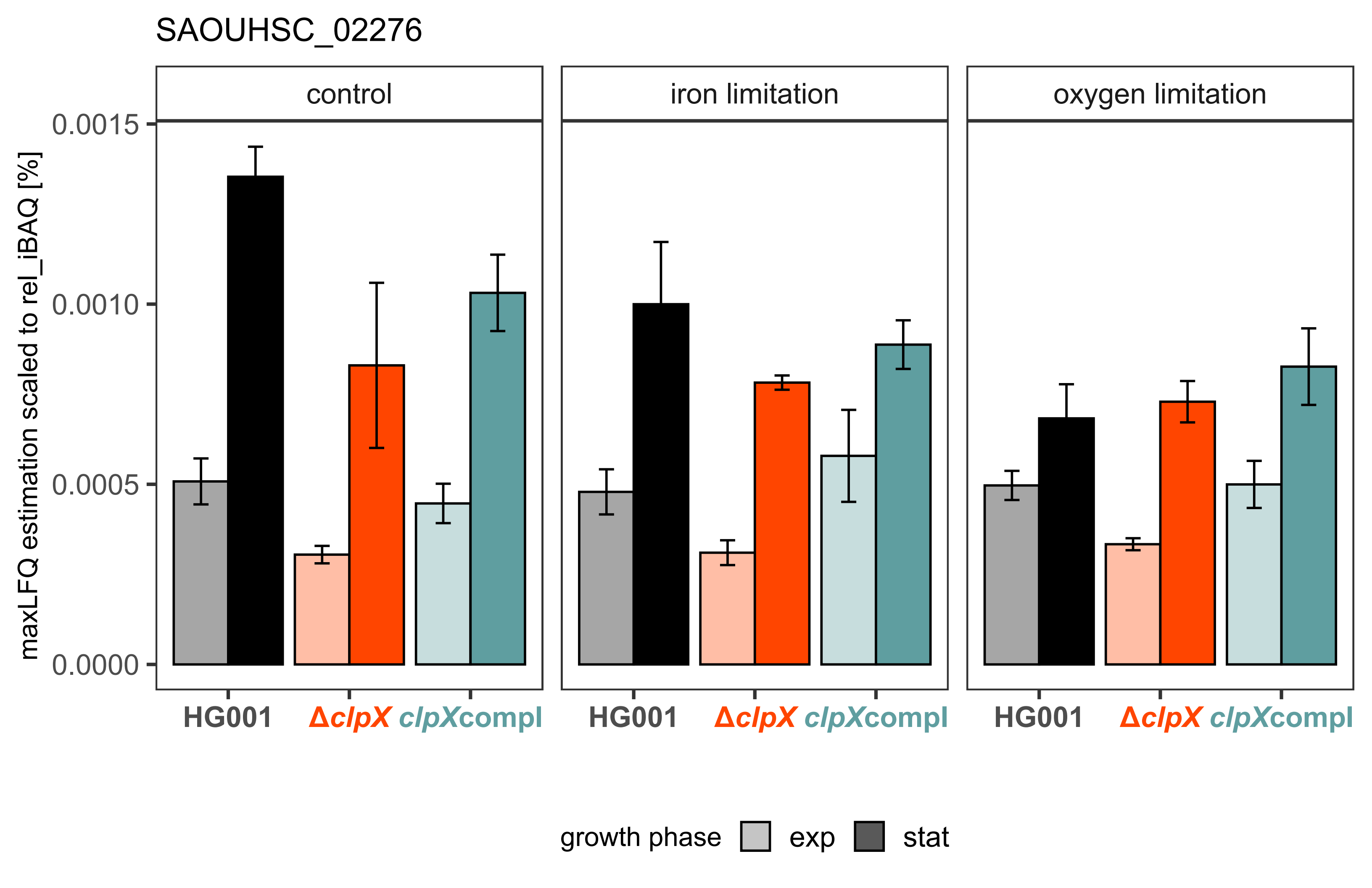

SAOUHSC_02276
control
iron limitation
oxygen limitation
maxLFQ estimation scaled to rel_iBAQ [%]
0.0015
0.0010
0.0005
0.0000
HG001
ΔclpX
clpXcompl
growth phase
exp
stat

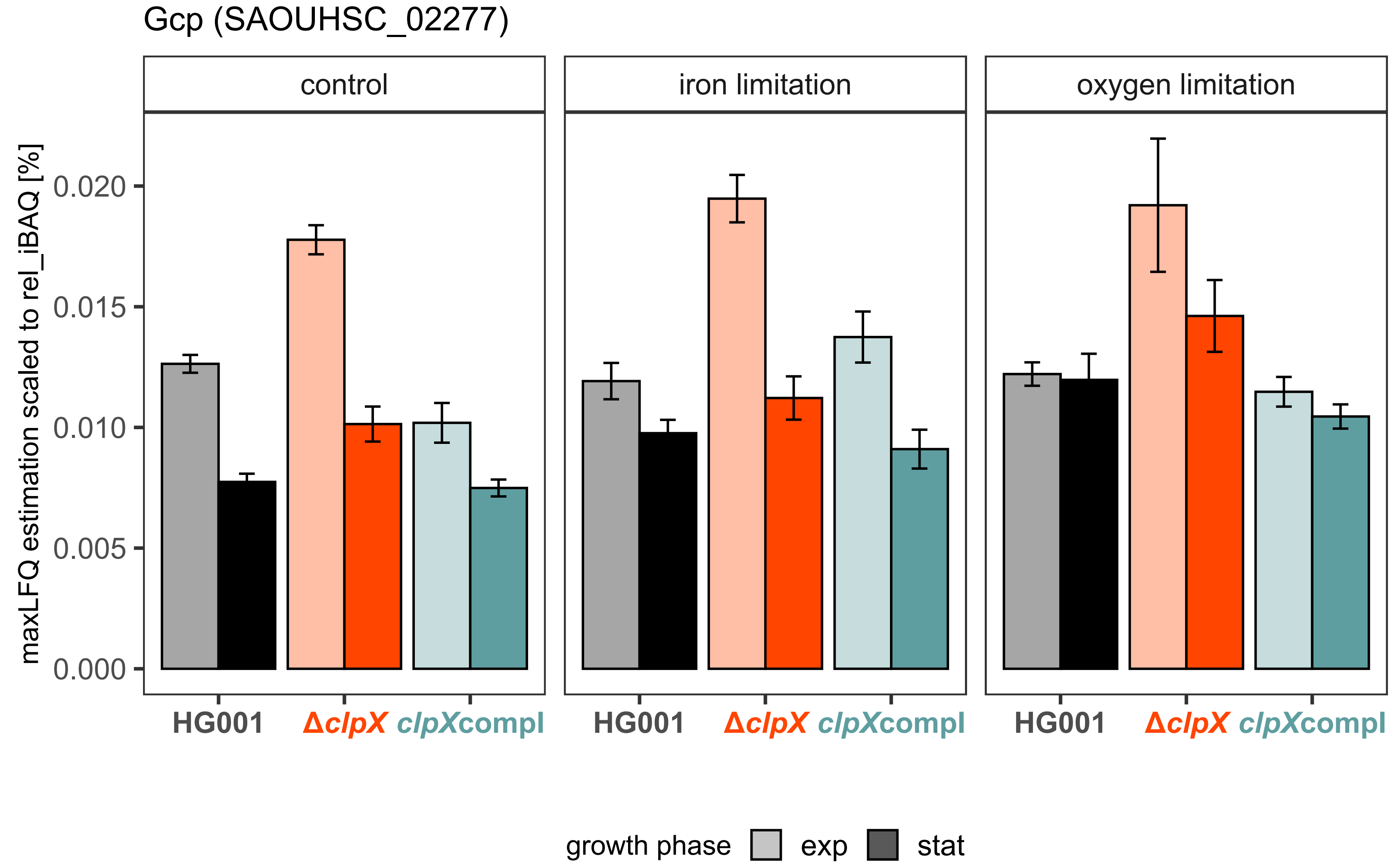

Gcp (SAOUHSC_02277)
control
iron limitation
oxygen limitation
maxLFQ estimation scaled to rel_iBAQ [%]
0.020
0.015
0.010
0.005
0.000
HG001
ΔclpX
clpXcompl
growth phase
exp
stat

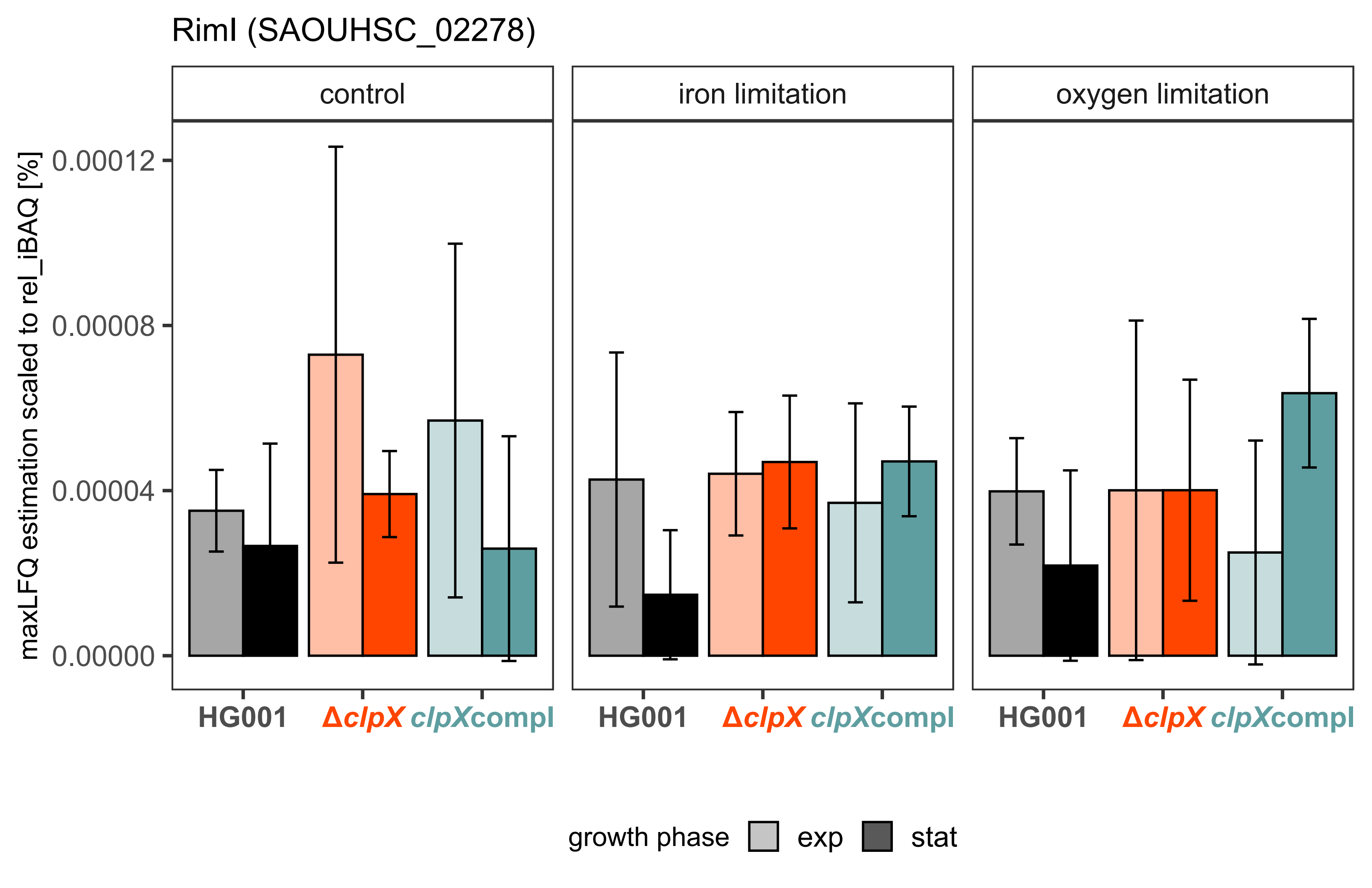

RimI (SAOUHSC_02278)
control
iron limitation
oxygen limitation
maxLFQ estimation scaled to rel_iBAQ [%]
0.00012
0.00008
0.00004
0.00000
HG001
ΔclpX
clpXcompl
growth phase
exp
stat

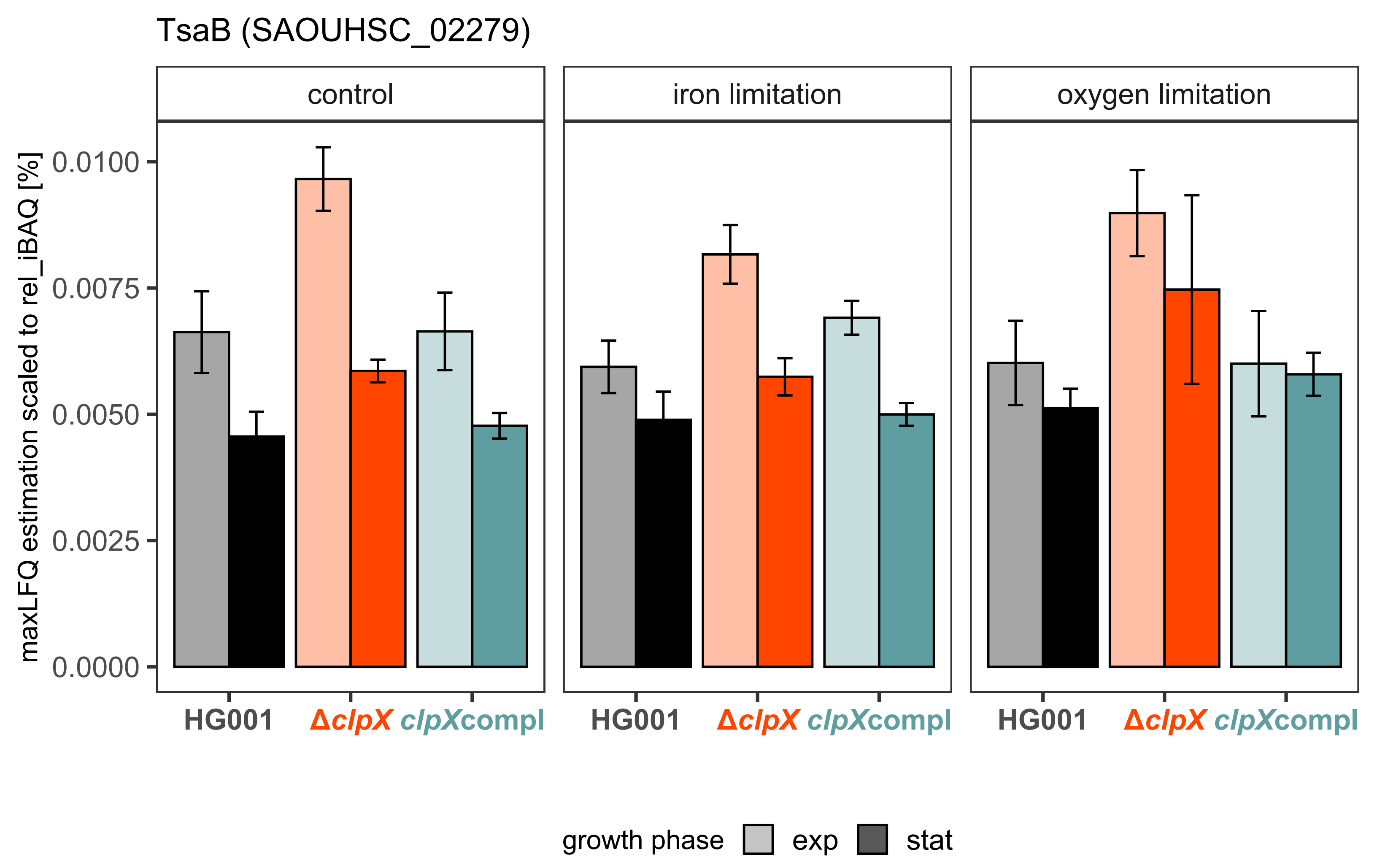

TsaB (SAOUHSC_02279)
control
iron limitation
oxygen limitation
maxLFQ estimation scaled to rel_iBAQ [%]
0.0100
0.0075
0.0050
0.0025
0.0000
HG001
ΔclpX
clpXcompl
growth phase
exp
stat

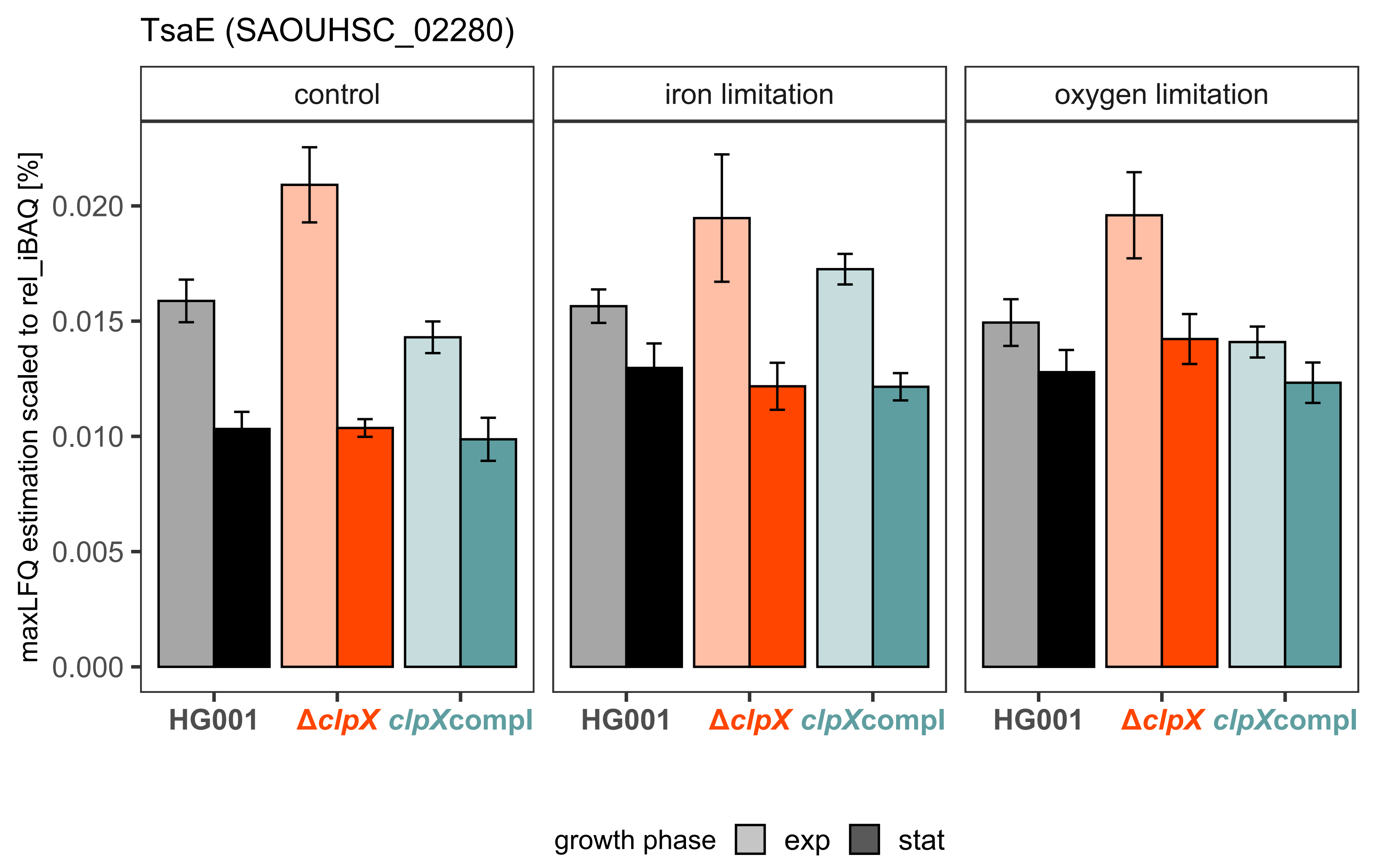

TsaE (SAOUHSC_02280)
control
iron limitation
oxygen limitation
maxLFQ estimation scaled to rel_iBAQ [%]
0.000
0.005
0.010
0.015
0.020
HG001
ΔclpX
clpXcompl
growth phase
exp
stat

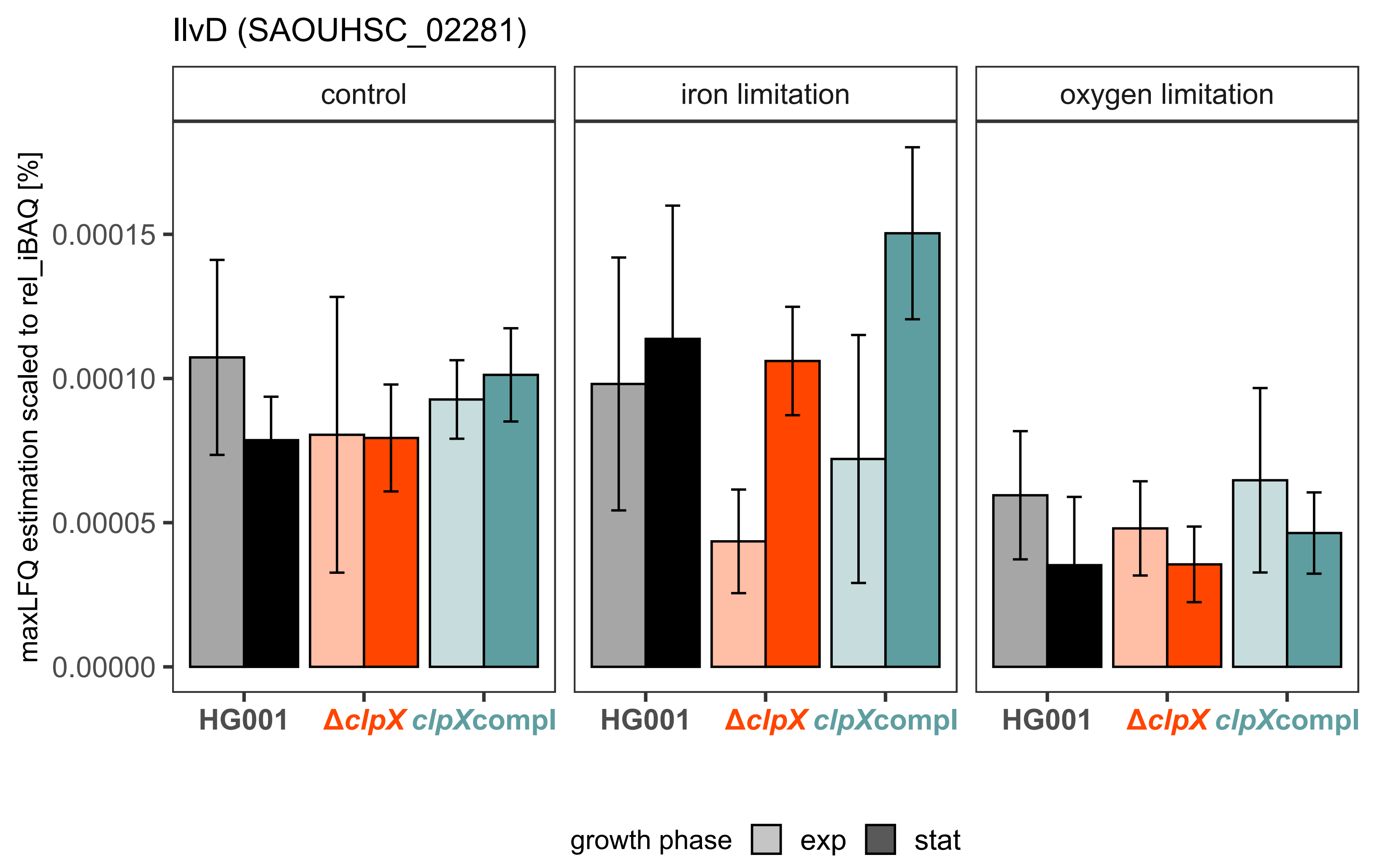
IlvD (SAOUHSC_02281)
control
iron limitation
oxygen limitation
maxLFQ estimation scaled to rel_iBAQ [%]
0.00015
0.00010
0.00005
0.00000
HG001
ΔclpX
clpXcompl
growth phase
exp
stat

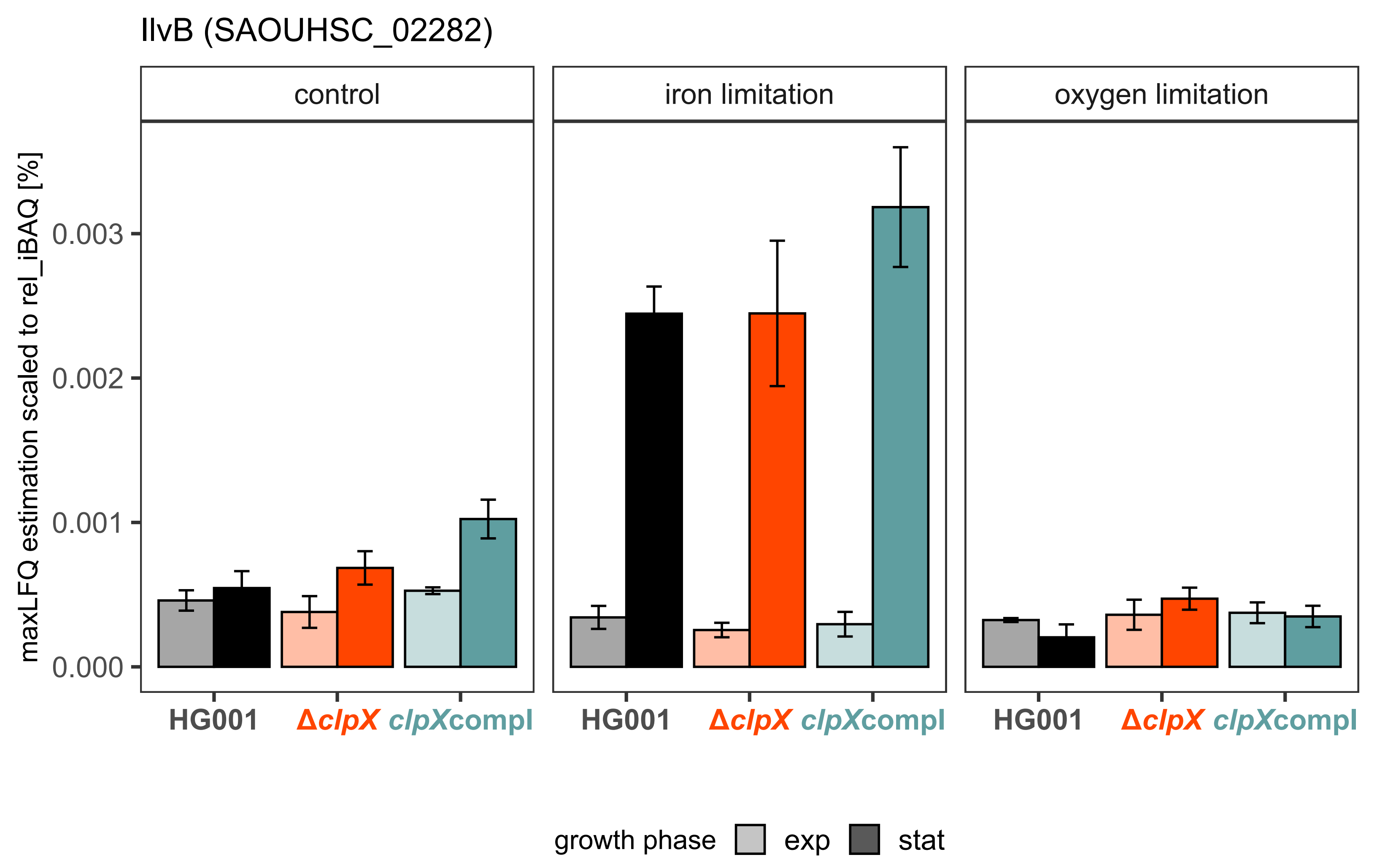
IlvB (SAOUHSC_02282)
control
iron limitation
oxygen limitation
maxLFQ estimation scaled to rel_iBAQ [%]
0.003
0.002
0.001
0.000
HG001
ΔclpX
clpXcompl
growth phase
exp
stat

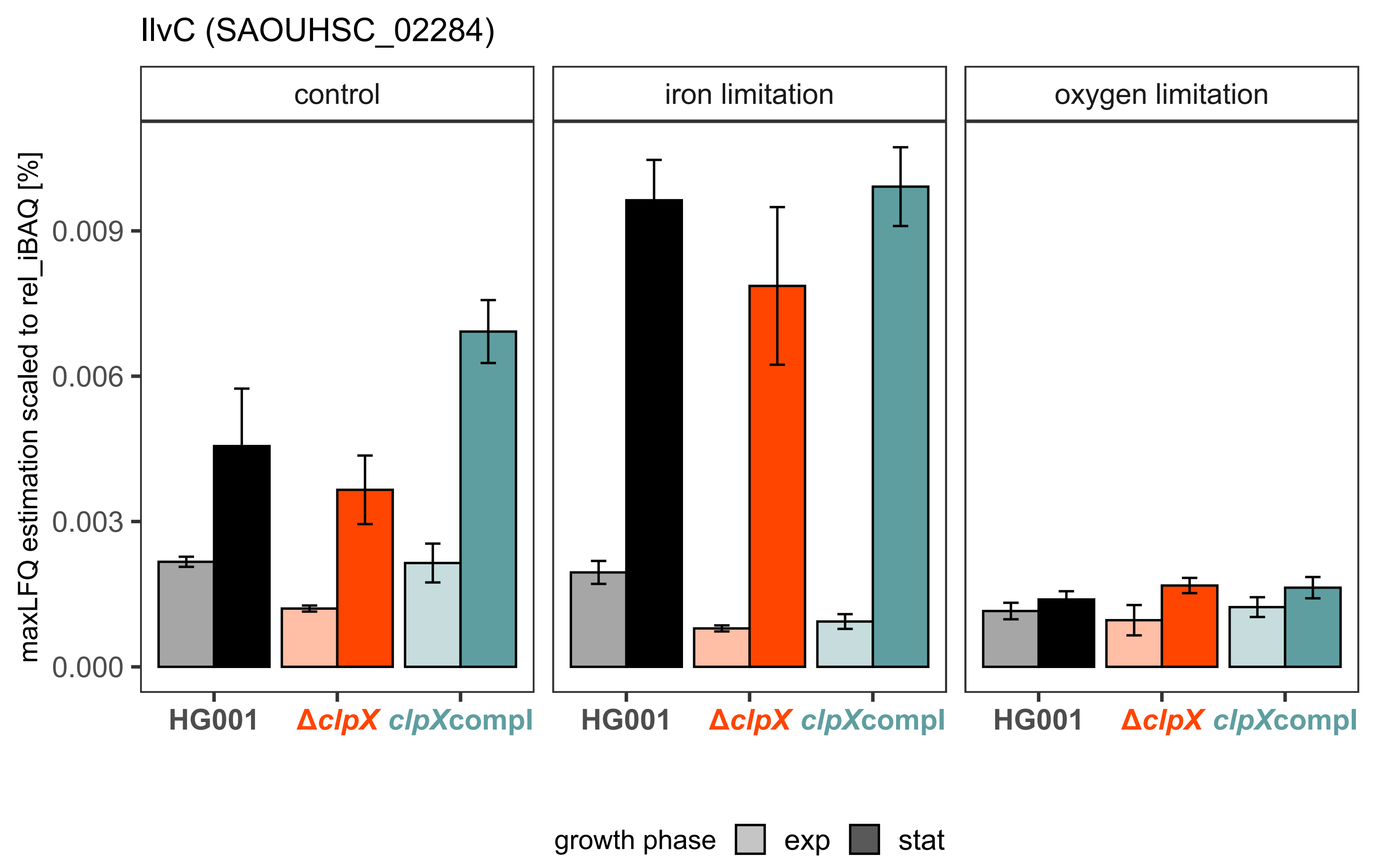
IlvC (SAOUHSC_02284)
control
iron limitation
oxygen limitation
maxLFQ estimation scaled to rel_iBAQ [%]
0.009
0.006
0.003
0.000
HG001
ΔclpX
clpXcompl
growth phase
exp
stat

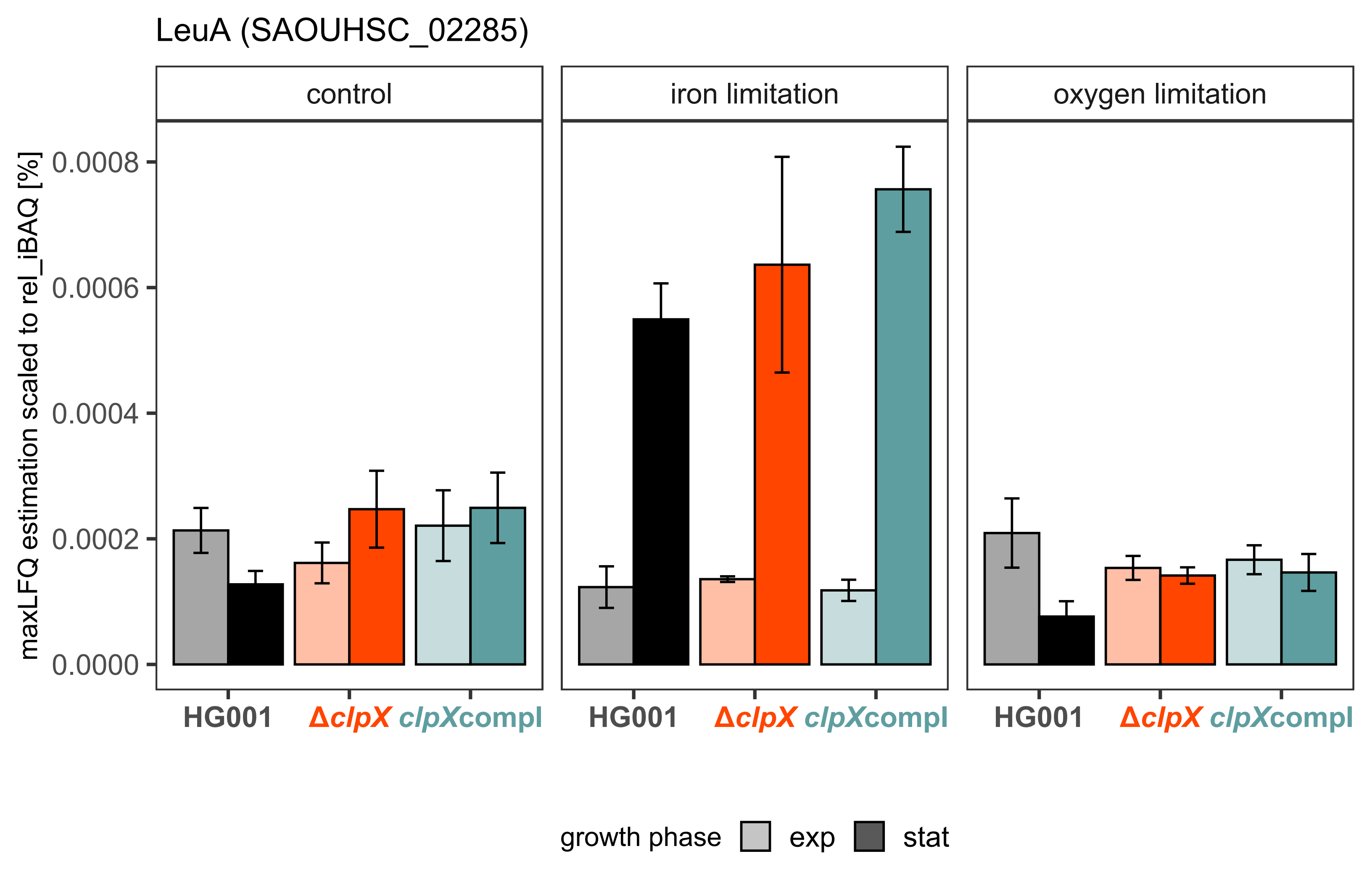

LeuA (SAOUHSC_02285)
control
iron limitation
oxygen limitation
maxLFQ estimation scaled to rel_iBAQ [%]
0.0008
0.0006
0.0004
0.0002
0.0000
HG001
ΔclpX
clpXcompl
growth phase
exp
stat

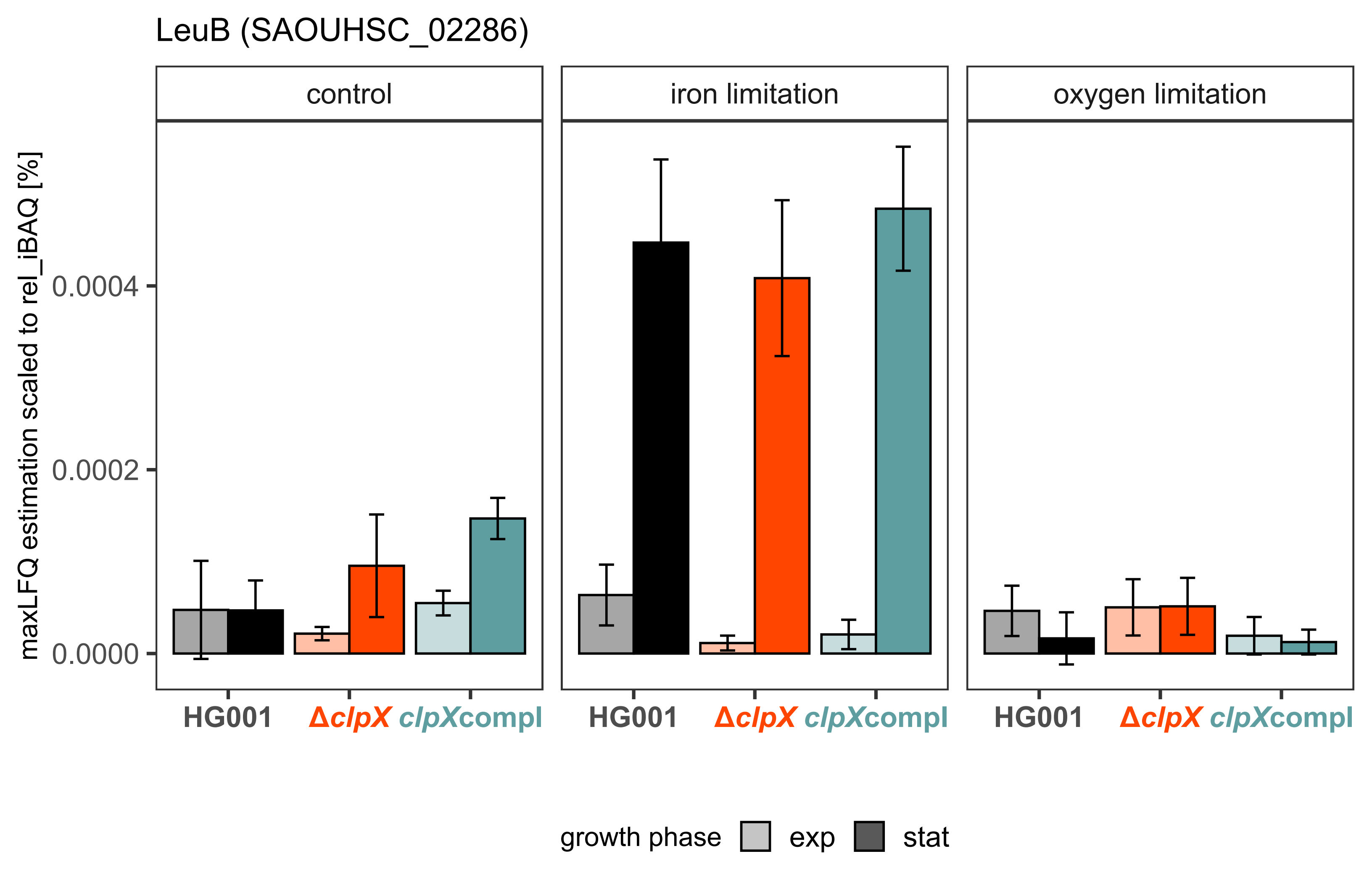
LeuB (SAOUHSC_02286)
control
iron limitation
oxygen limitation
maxLFQ estimation scaled to rel_iBAQ [%]
0.0004
0.0002
0.0000
HG001
ΔclpX
clpXcompl
growth phase
exp
stat

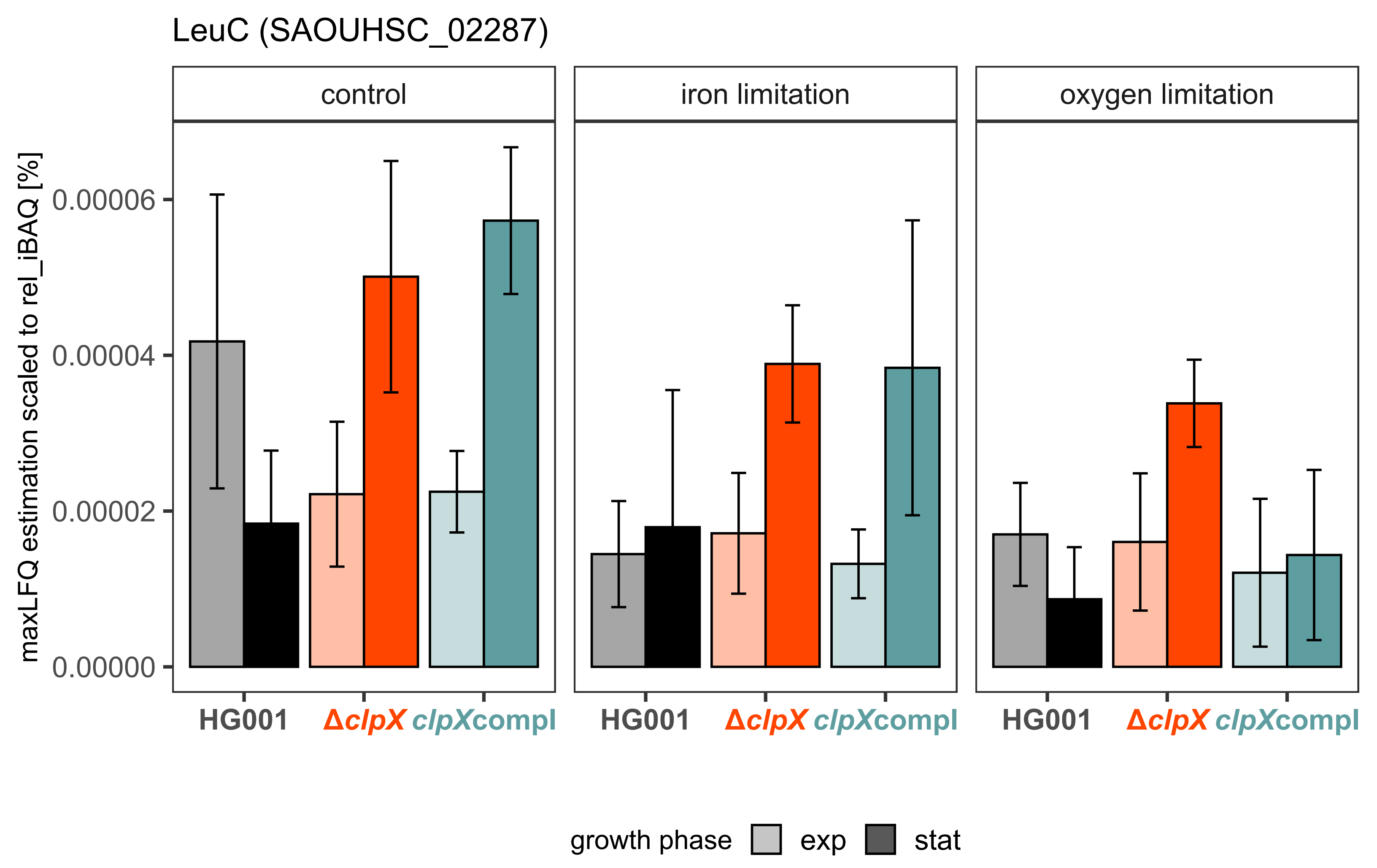
LeuC (SAOUHSC_02287)
control
iron limitation
oxygen limitation
maxLFQ estimation scaled to rel_iBAQ [%]
0.00006
0.00004
0.00002
0.00000
HG001
ΔclpX
clpXcompl
growth phase
exp
stat

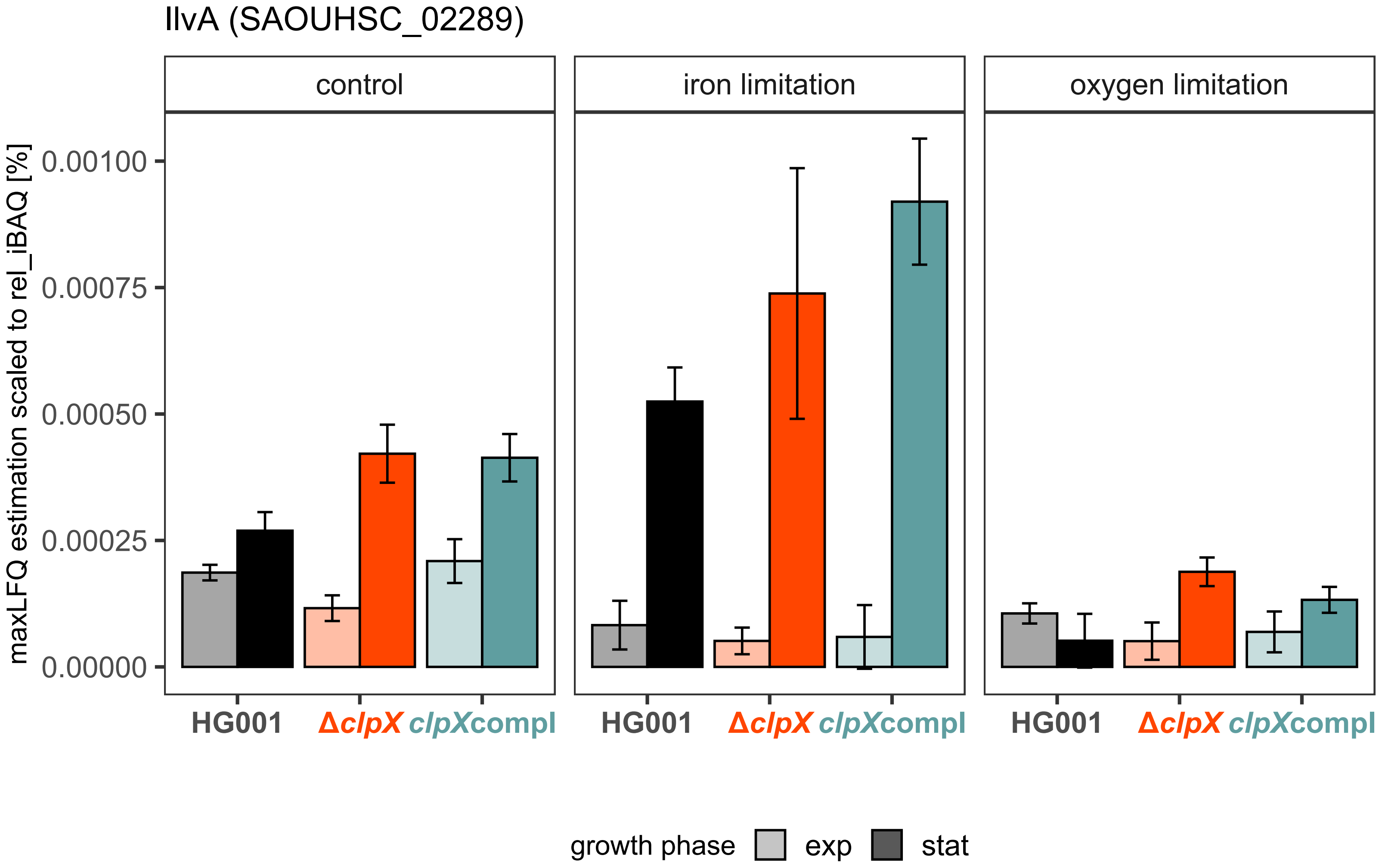

IlvA (SAOUHSC_02289)
control
iron limitation
oxygen limitation
maxLFQ estimation scaled to rel_iBAQ [%]
0.00100
0.00075
0.00050
0.00025
0.00000
HG001
ΔclpX
clpXcompl
growth phase
exp
stat

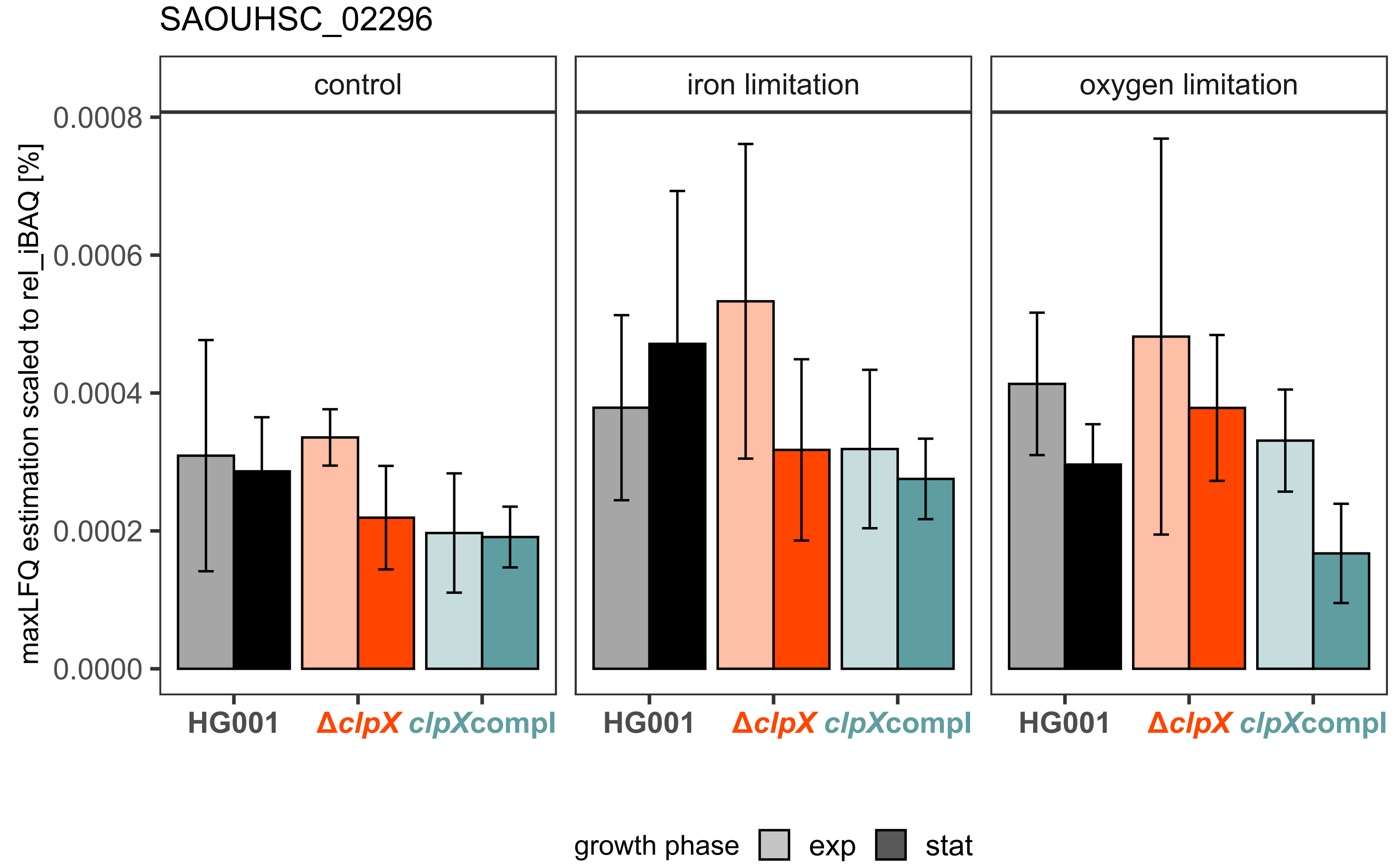
SAOUHSC_02296
control
iron limitation
oxygen limitation
maxLFQ estimation scaled to rel_iBAQ [%]
0.0008
0.0006
0.0004
0.0002
0.0000
HG001
ΔclpX
clpXcompl
growth phase
exp
stat

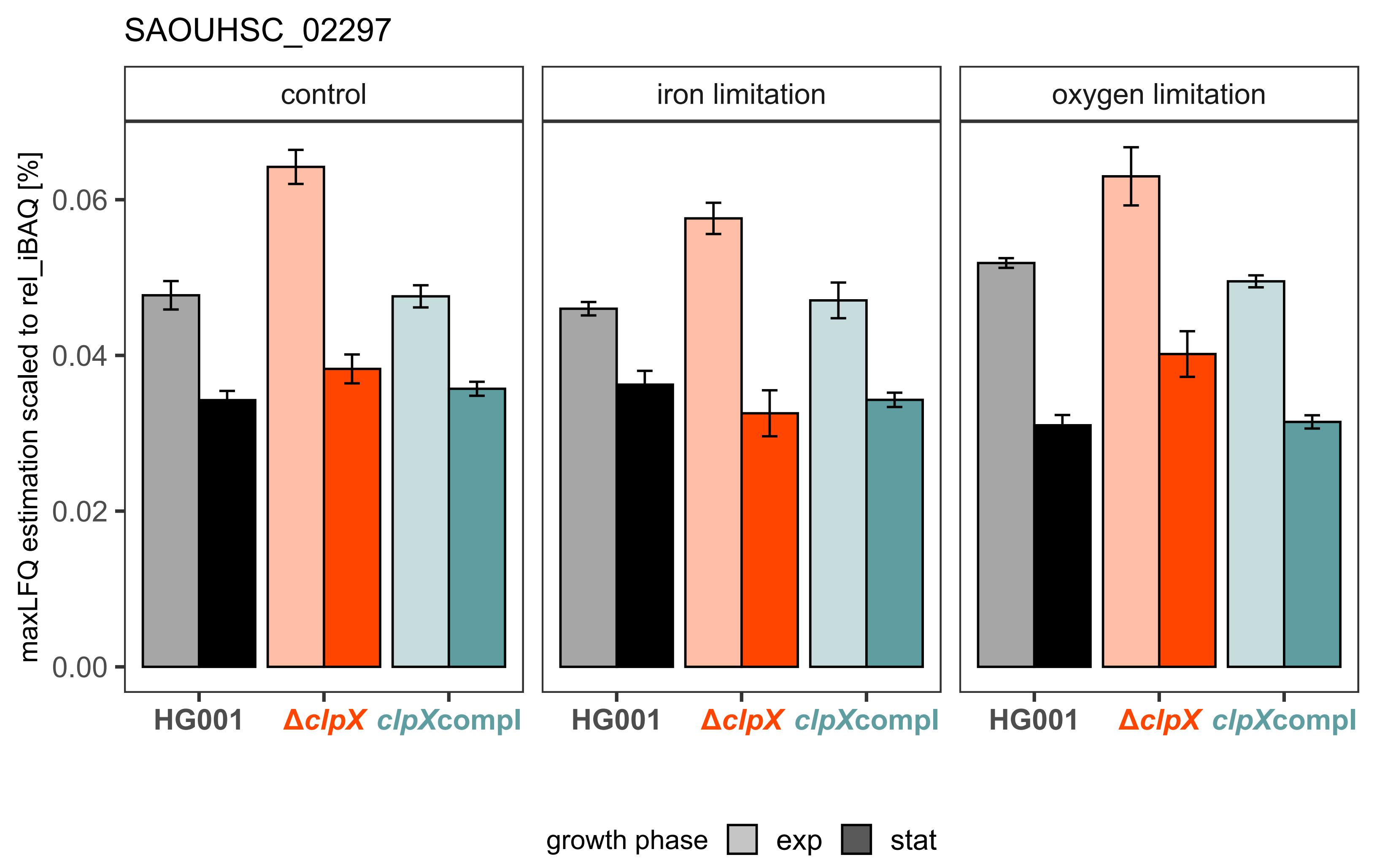
SAOUHSC_02297
control
iron limitation
oxygen limitation
maxLFQ estimation scaled to rel_iBAQ [%]
0.00
0.02
0.04
0.06
HG001
ΔclpX
clpXcompl
HG001
ΔclpX
clpXcompl
HG001
ΔclpX
clpXcompl
growth phase
exp
stat

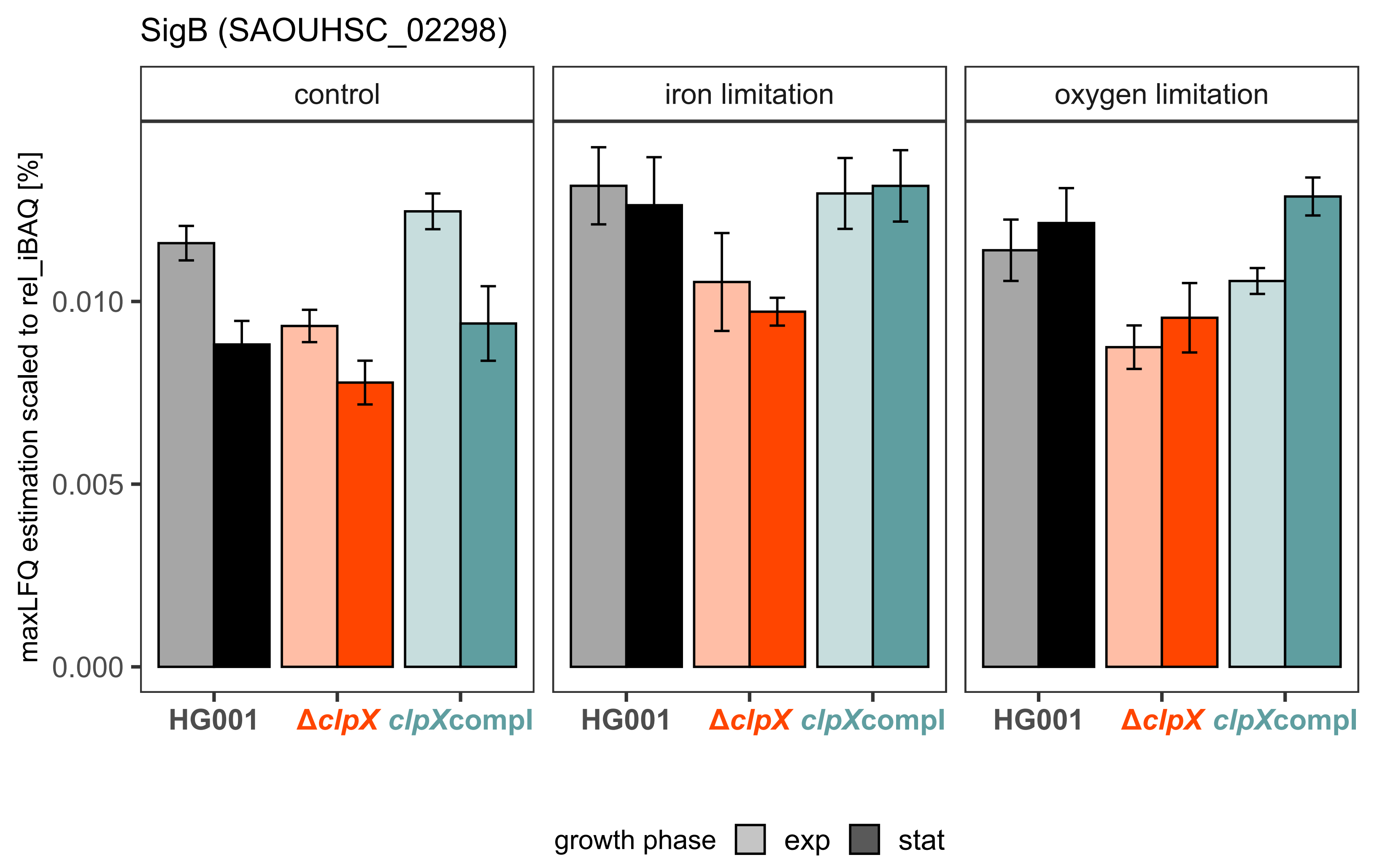

SigB (SAOUHSC_02298)
control
iron limitation
oxygen limitation
maxLFQ estimation scaled to rel_iBAQ [%]
0.010
0.005
0.000
HG001
ΔclpX
clpXcompl
growth phase
exp
stat

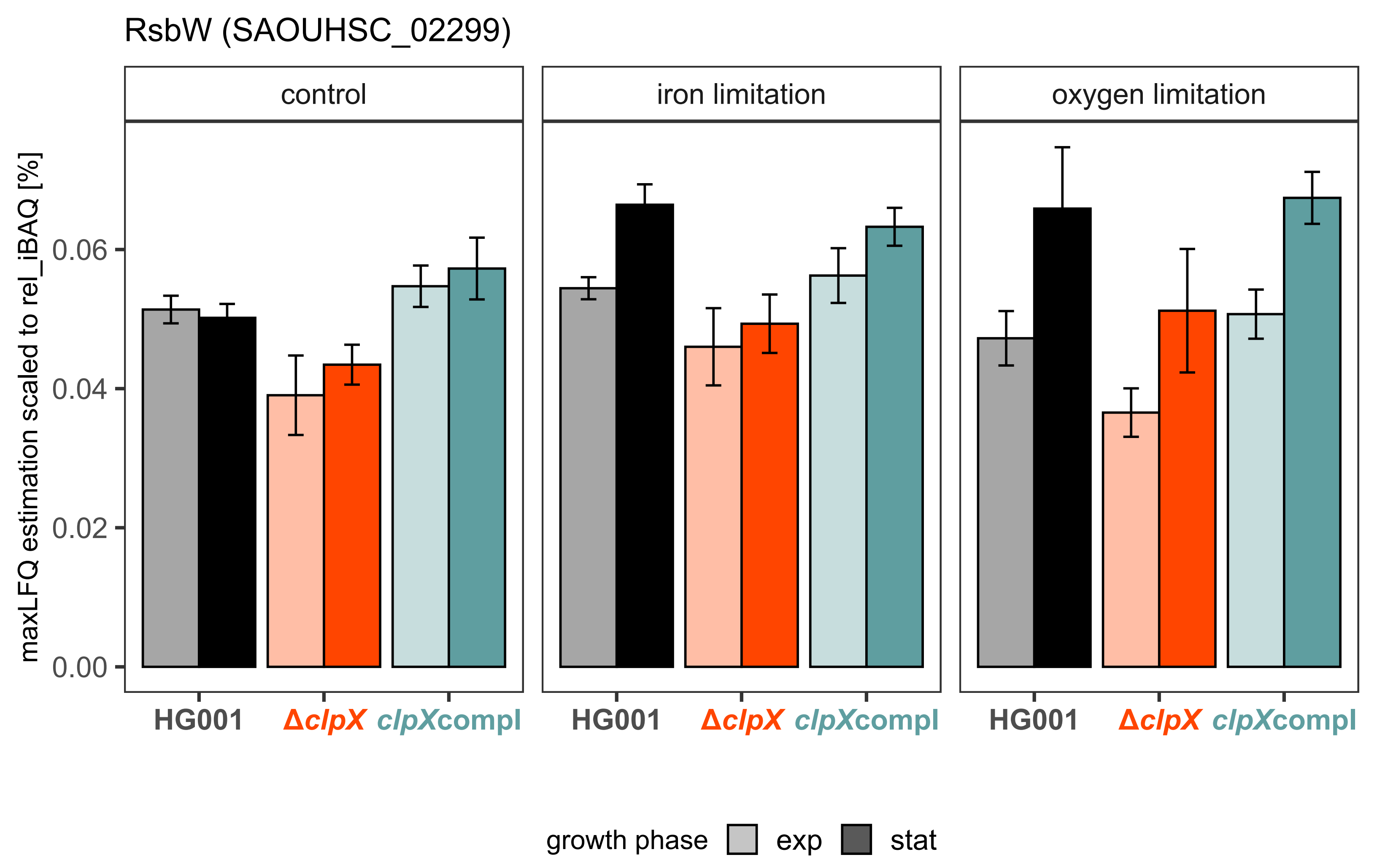
RsbW (SAOUHSC_02299)
control
iron limitation
oxygen limitation
maxLFQ estimation scaled to rel_iBAQ [%]
0.00
0.02
0.04
0.06
HG001
ΔclpX
clpXcompl
growth phase
exp
stat

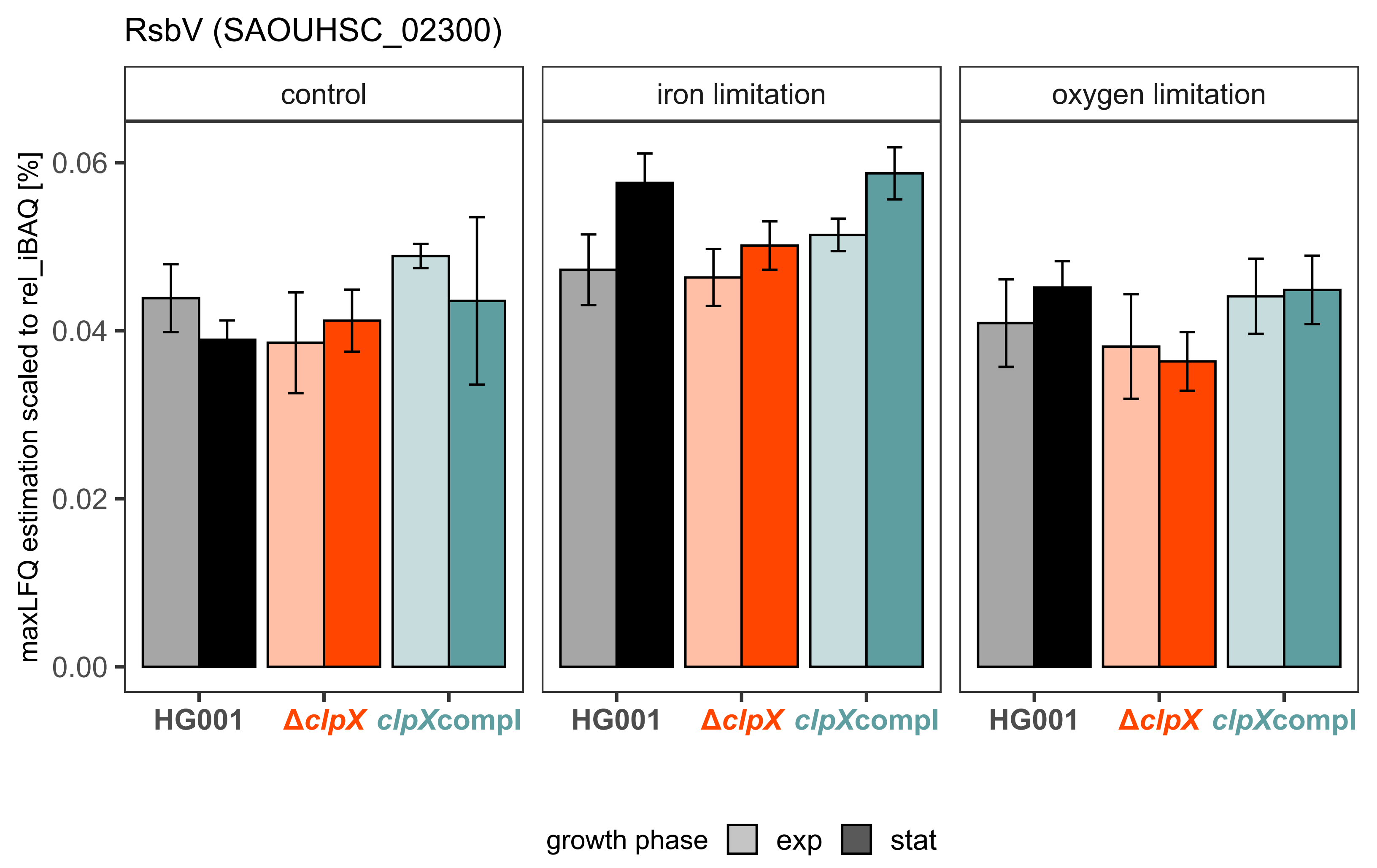

RsbV (SAOUHSC_02300)
control
iron limitation
oxygen limitation
maxLFQ estimation scaled to rel_iBAQ [%]
0.06
0.04
0.02
0.00
HG001
ΔclpX
clpXcompl
growth phase
exp
stat

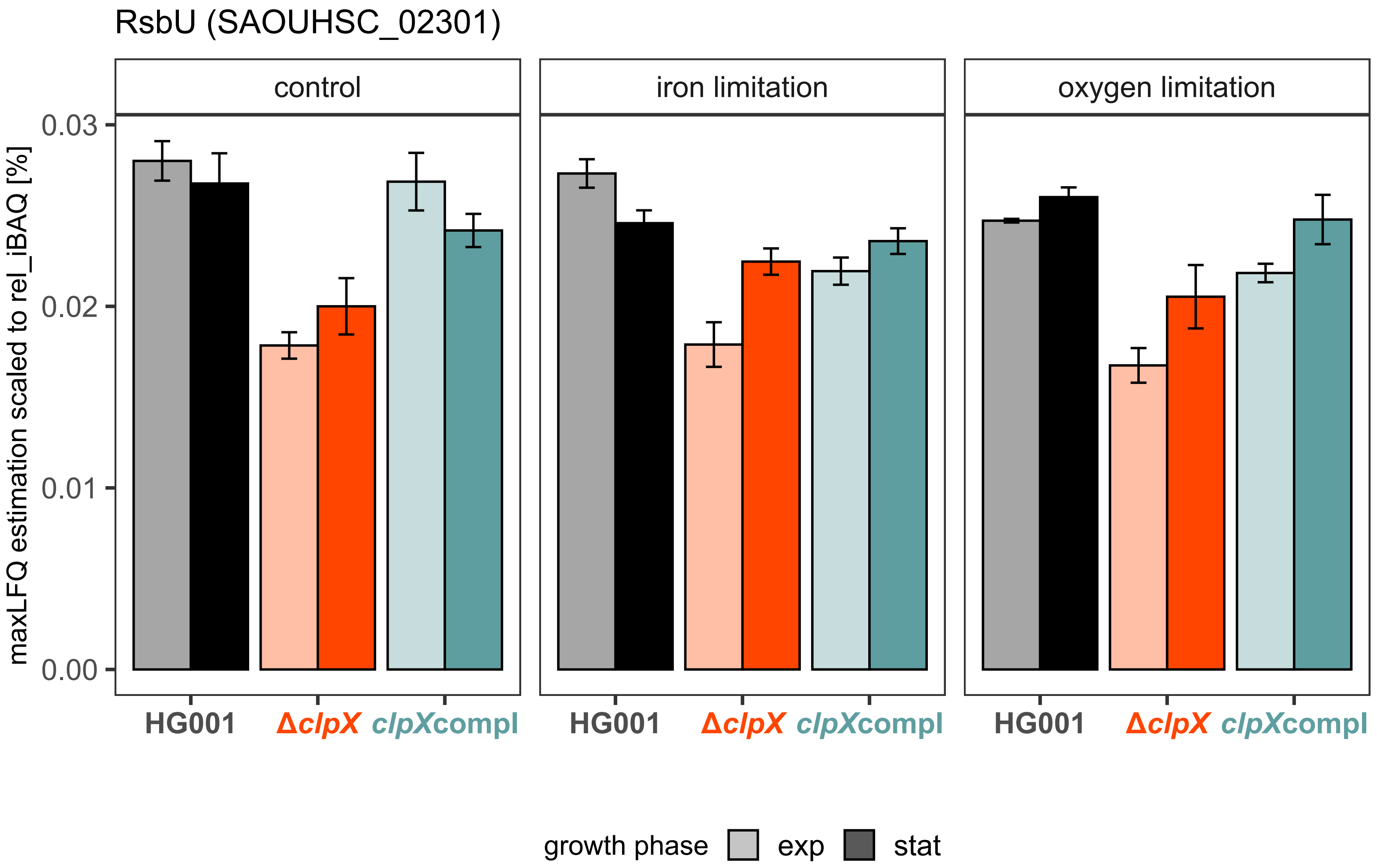
RsbU (SAOUHSC_02301)
control
iron limitation
oxygen limitation
maxLFQ estimation scaled to rel_iBAQ [%]
0.03
0.02
0.01
0.00
HG001
ΔclpX
clpXcompl
growth phase
exp
stat

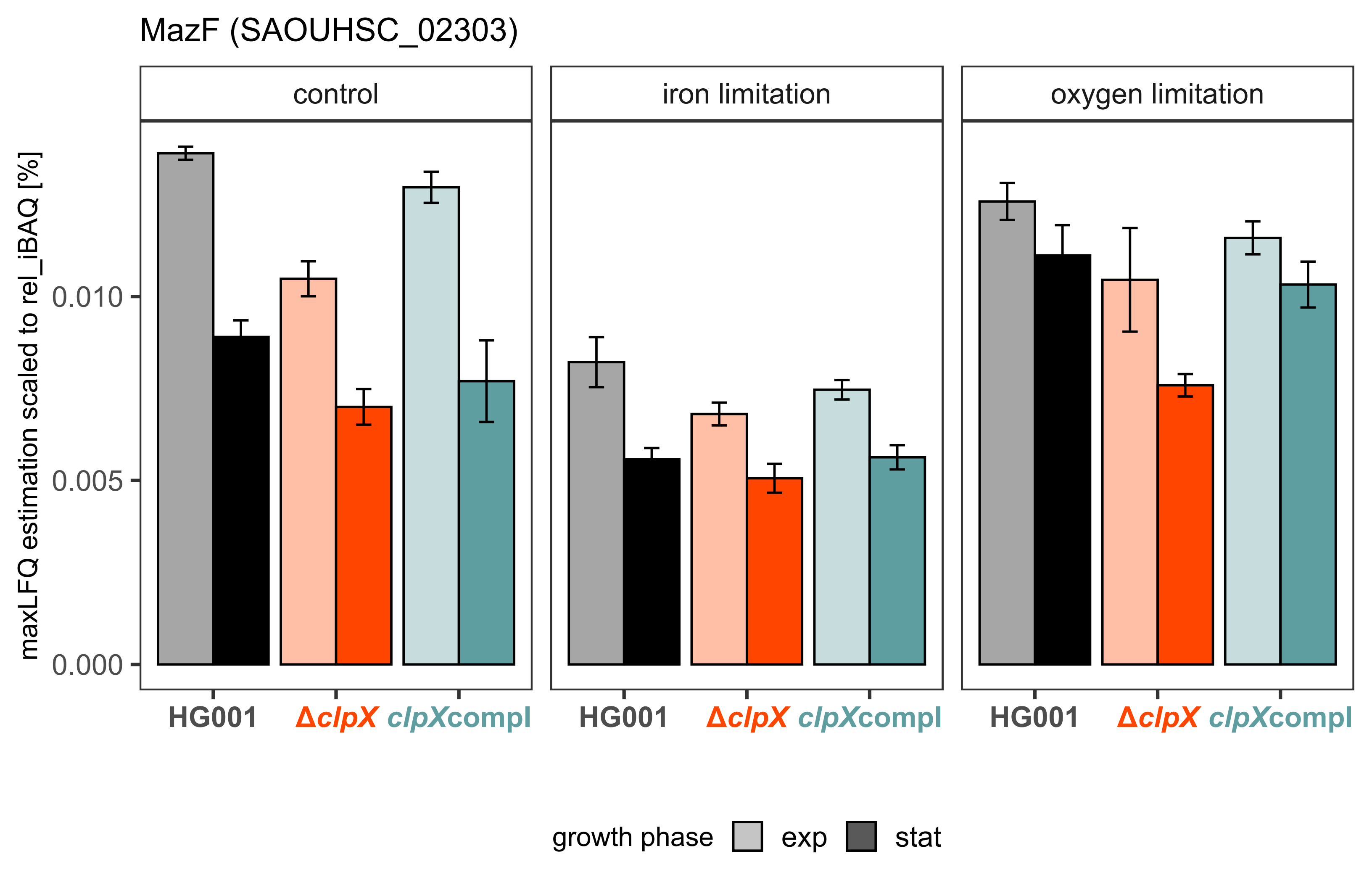
MazF (SAOUHSC_02303)
control
iron limitation
oxygen limitation
maxLFQ estimation scaled to rel_iBAQ [%]
0.010
0.005
0.000
HG001
ΔclpX
clpXcompl
growth phase
exp
stat

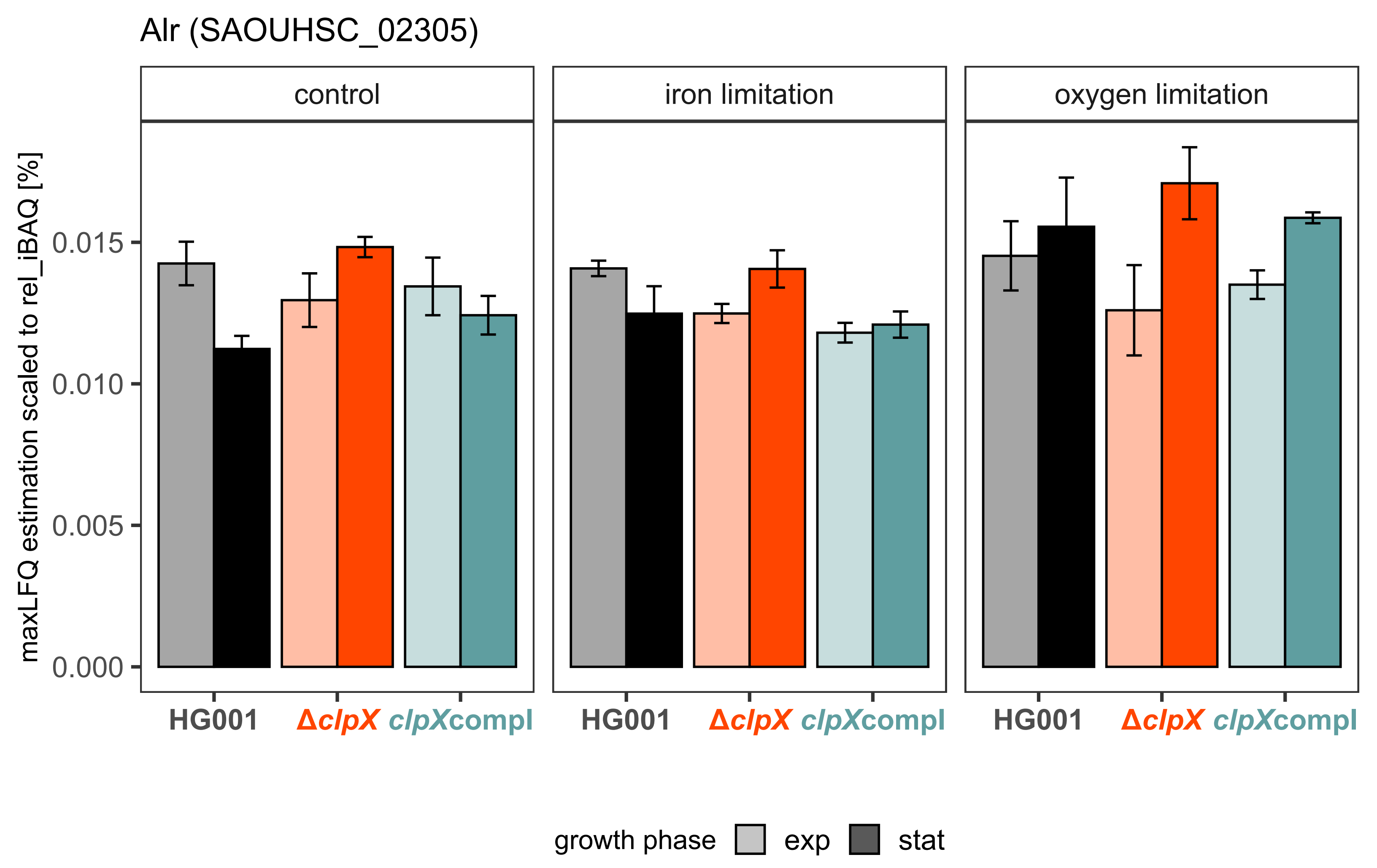

Alr (SAOUHSC_02305)
control
iron limitation
oxygen limitation
maxLFQ estimation scaled to rel_iBAQ [%]
0.015
0.010
0.005
0.000
HG001
ΔclpX
clpXcompl
growth phase
exp
stat

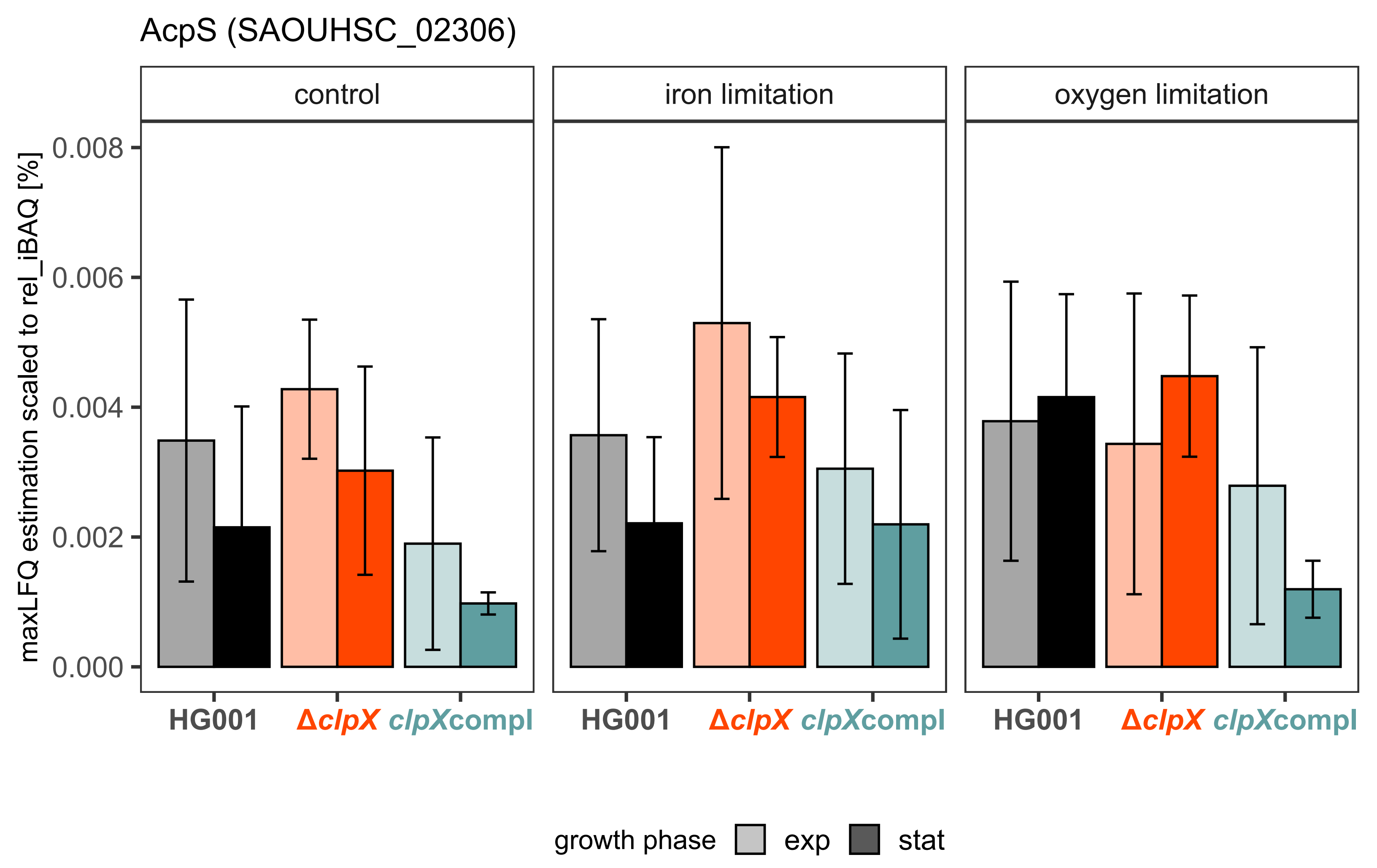

AcpS (SAOUHSC_02306)
control
iron limitation
oxygen limitation
maxLFQ estimation scaled to rel_iBAQ [%]
0.008
0.006
0.004
0.002
0.000
HG001
ΔclpX
clpXcompl
growth phase
exp
stat

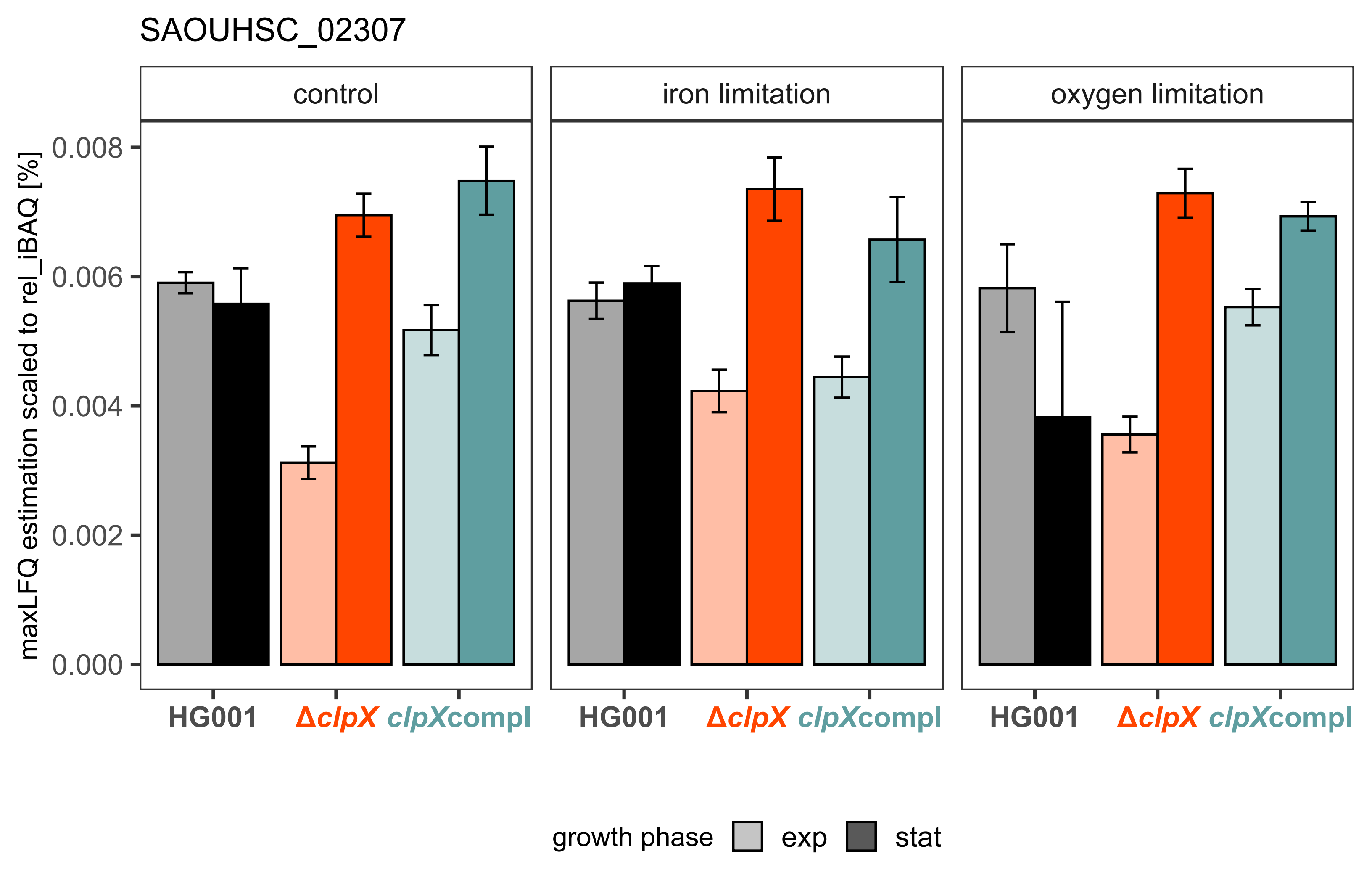

SAOUHSC_02307
control
iron limitation
oxygen limitation
maxLFQ estimation scaled to rel_iBAQ [%]
0.000
0.002
0.004
0.006
0.008
HG001
ΔclpX
clpXcompl
growth phase
exp
stat

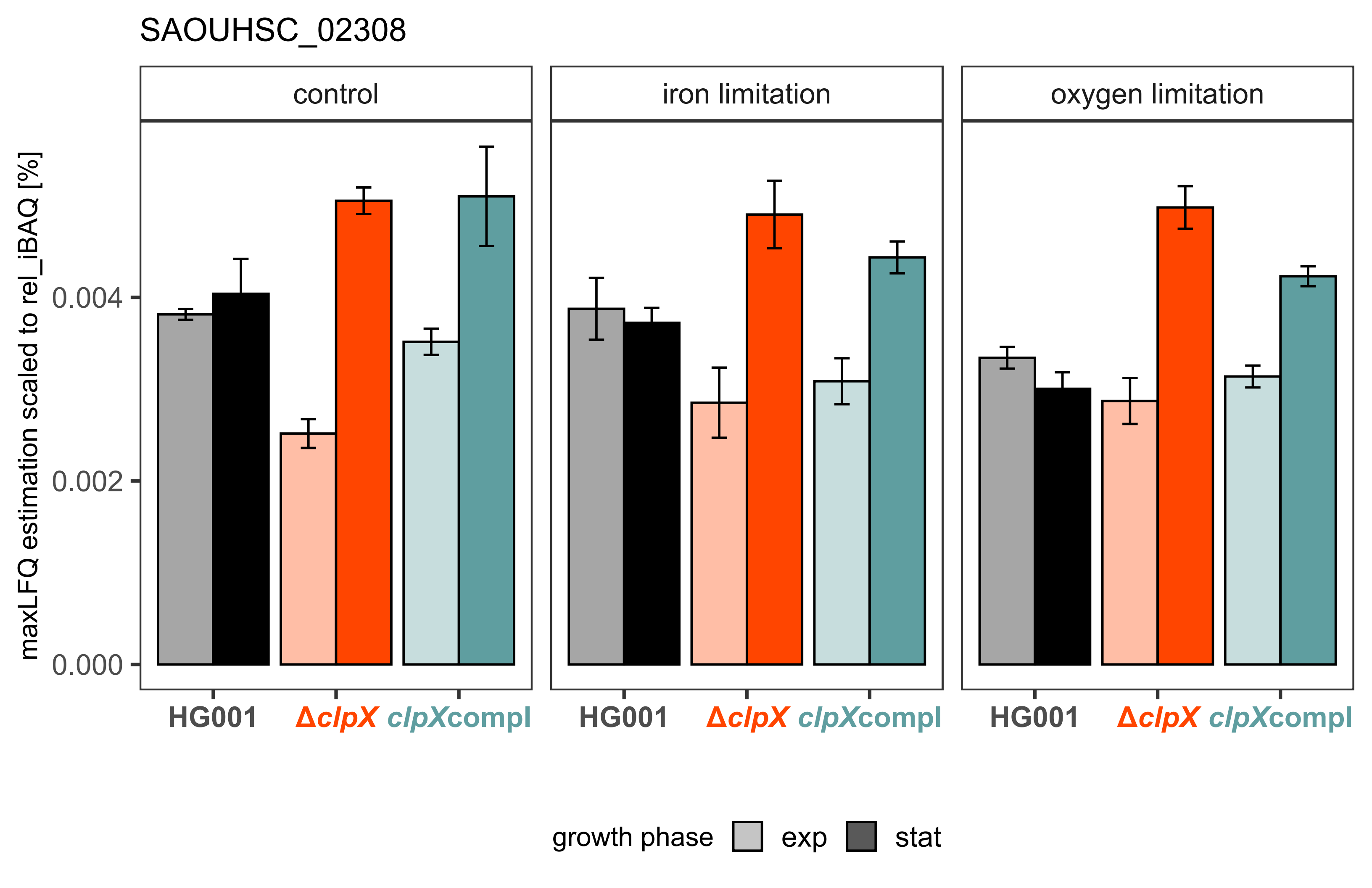
SAOUHSC_02308
control
iron limitation
oxygen limitation
maxLFQ estimation scaled to rel_iBAQ [%]
0.000
0.002
0.004
HG001
ΔclpX
clpXcompl
growth phase
exp
stat

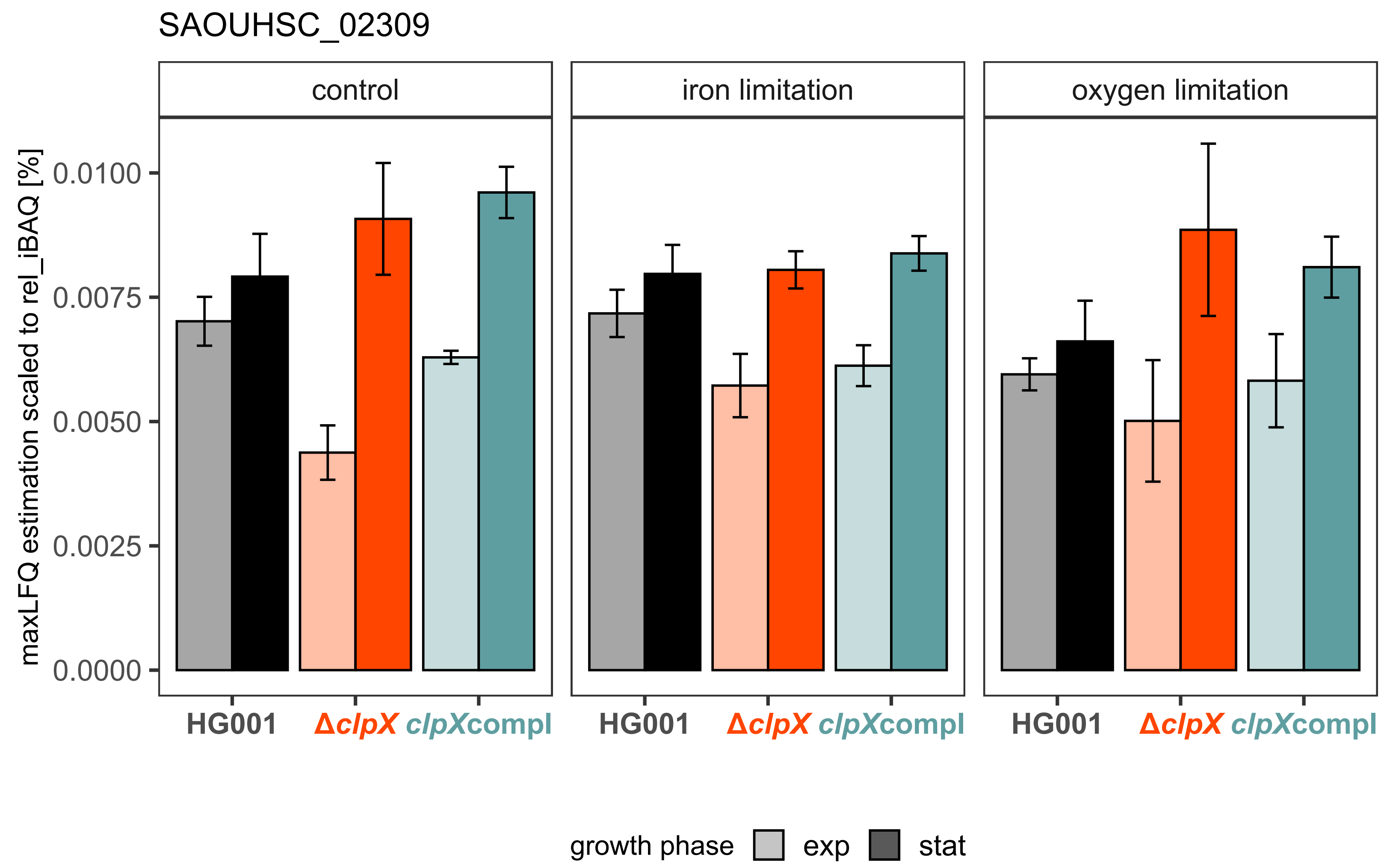

SAOUHSC_02309
control
iron limitation
oxygen limitation
maxLFQ estimation scaled to rel_iBAQ [%]
0.0100
0.0075
0.0050
0.0025
0.0000
HG001
ΔclpX
clpXcompl
growth phase
exp
stat

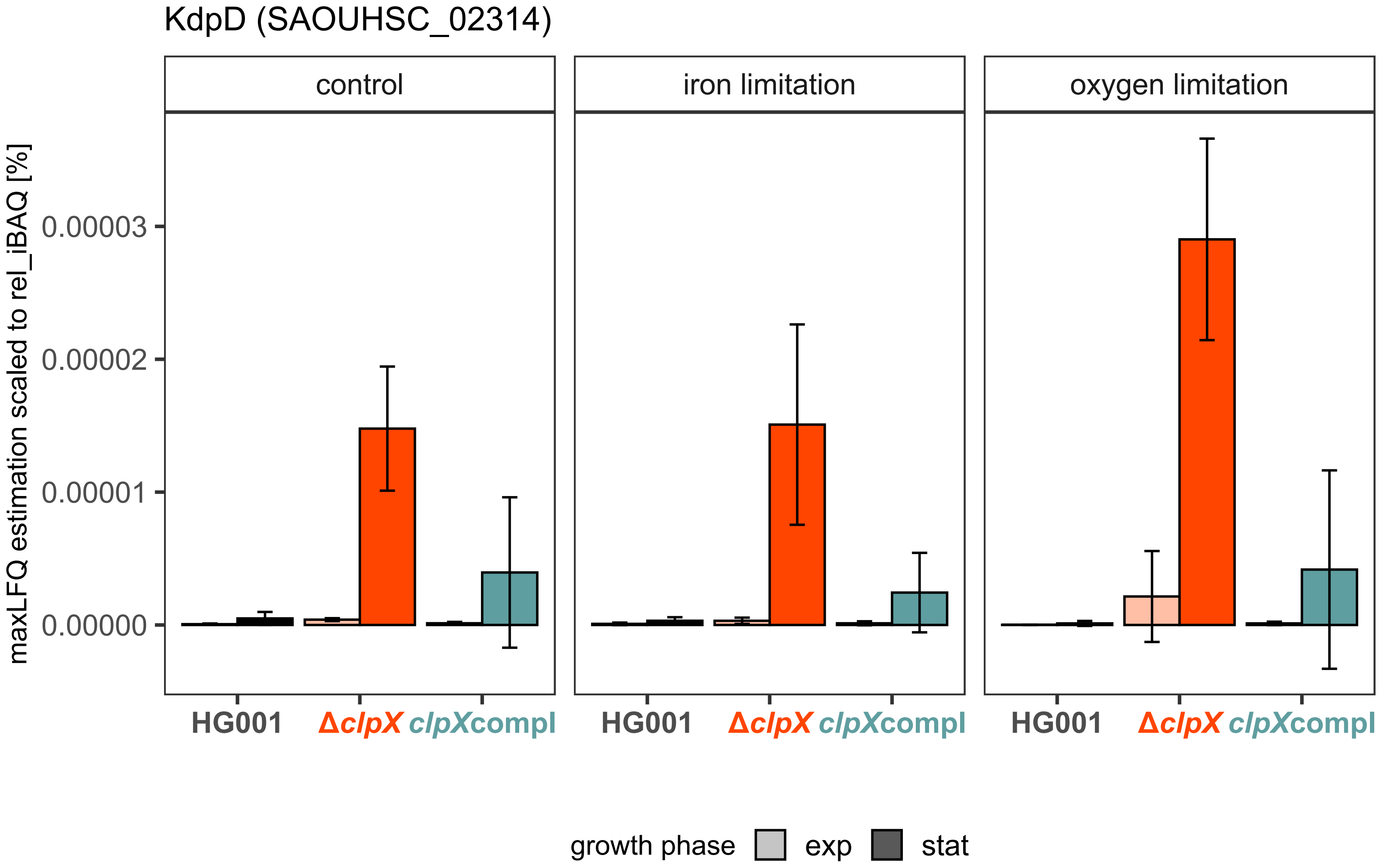
KdpD (SAOUHSC_02314)
control
iron limitation
oxygen limitation
maxLFQ estimation scaled to rel_iBAQ [%]
0.00003
0.00002
0.00001
0.00000
HG001
ΔclpX
clpXcompl
growth phase
exp
stat

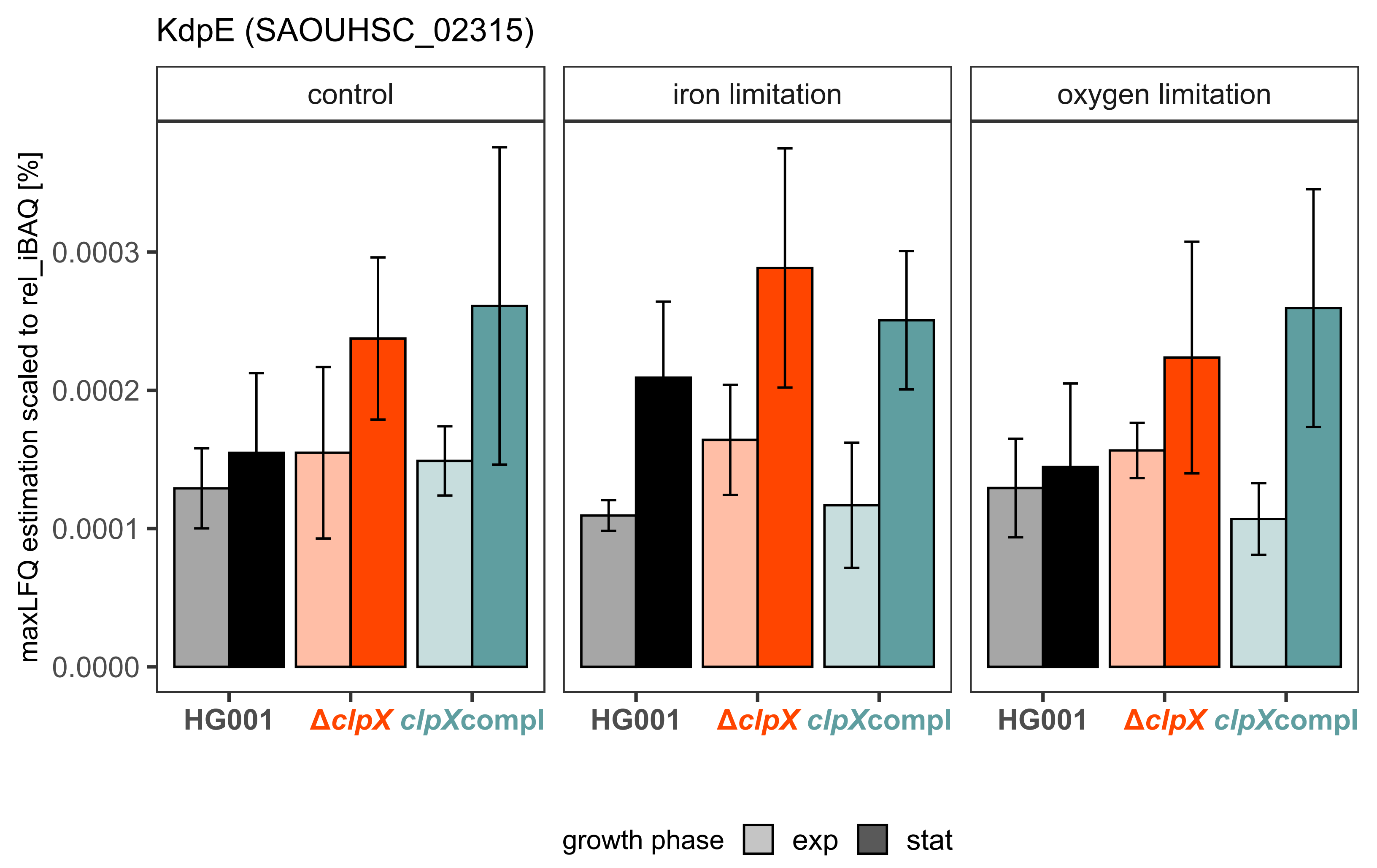

KdpE (SAOUHSC_02315)
control
iron limitation
oxygen limitation
maxLFQ estimation scaled to rel_iBAQ [%]
0.0003
0.0002
0.0001
0.0000
HG001
ΔclpX
clpXcompl
growth phase
exp
stat

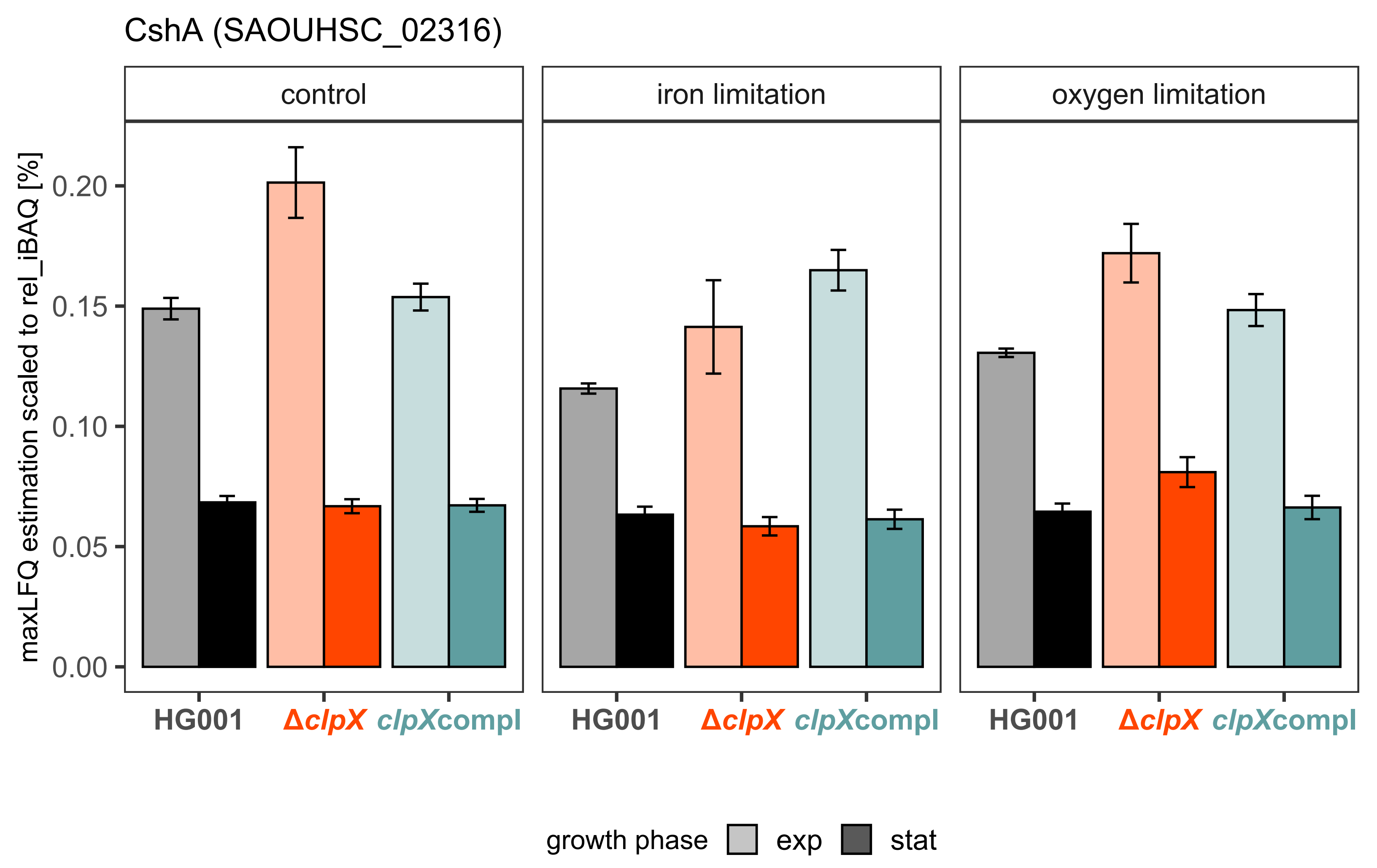

CshA (SAOUHSC_02316)
control
iron limitation
oxygen limitation
maxLFQ estimation scaled to rel_iBAQ [%]
0.00
0.05
0.10
0.15
0.20
HG001
ΔclpX
clpXcompl
growth phase
exp
stat

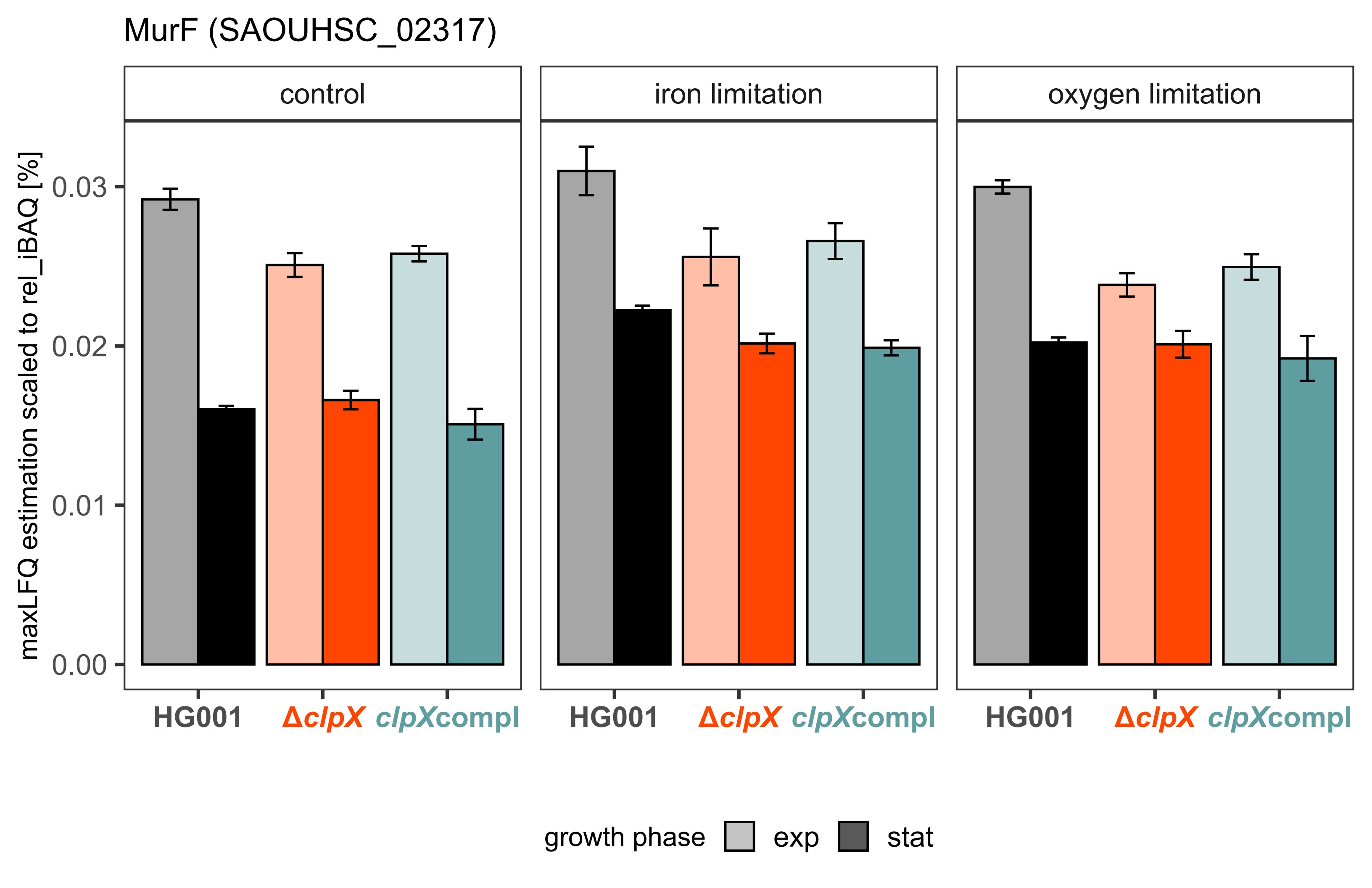
MurF (SAOUHSC_02317)
control
iron limitation
oxygen limitation
maxLFQ estimation scaled to rel_iBAQ [%]
0.03
0.02
0.01
0.00
HG001
ΔclpX
clpXcompl
growth phase
exp
stat

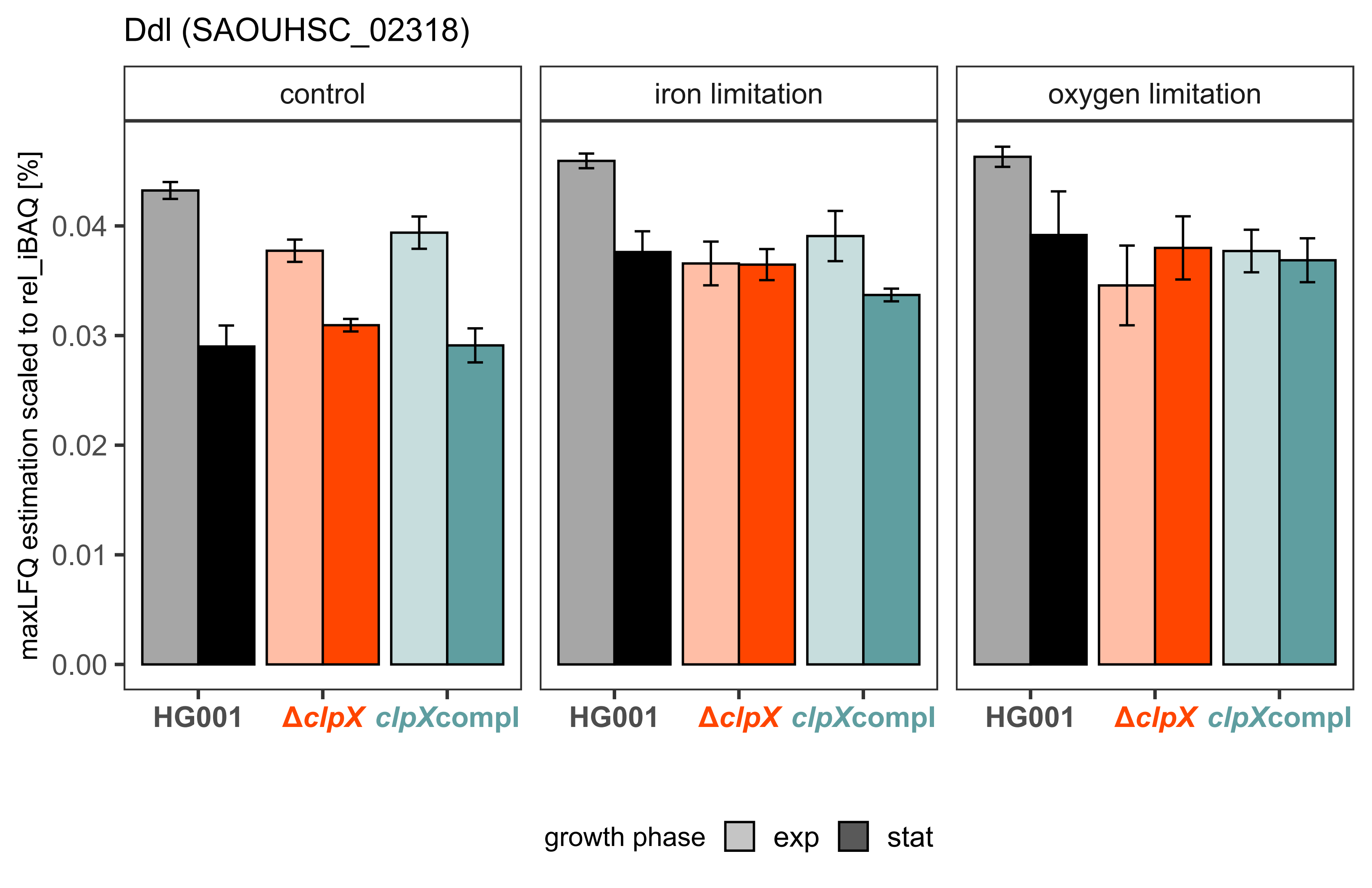

Ddl (SAOUHSC_02318)
control
iron limitation
oxygen limitation
maxLFQ estimation scaled to rel_iBAQ [%]
0.00
0.01
0.02
0.03
0.04
HG001
ΔclpX
clpXcompl
growth phase
exp
stat

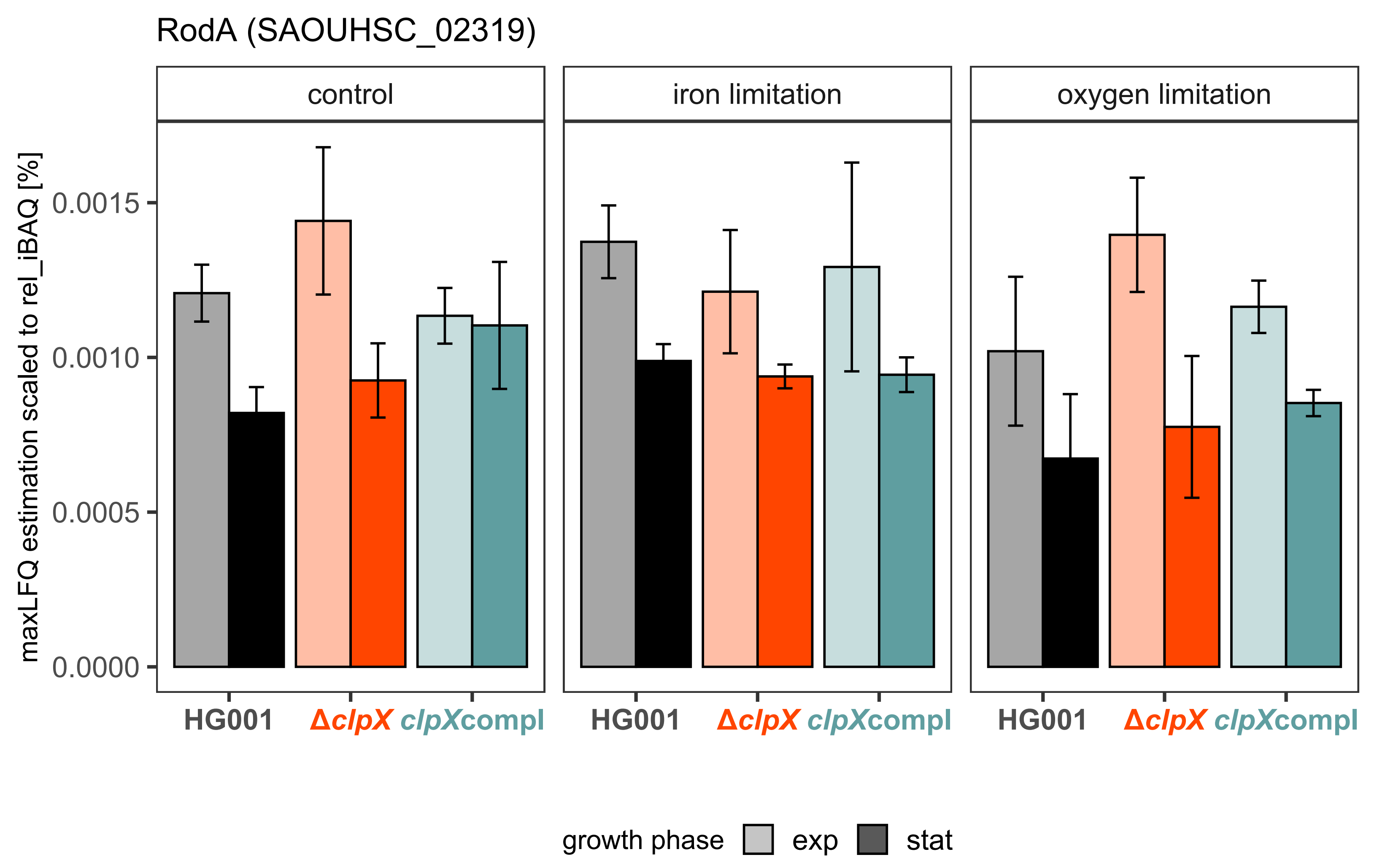
RodA (SAOUHSC_02319)
control
iron limitation
oxygen limitation
maxLFQ estimation scaled to rel_iBAQ [%]
0.0015
0.0010
0.0005
0.0000
HG001
ΔclpX
clpXcompl
growth phase
exp
stat

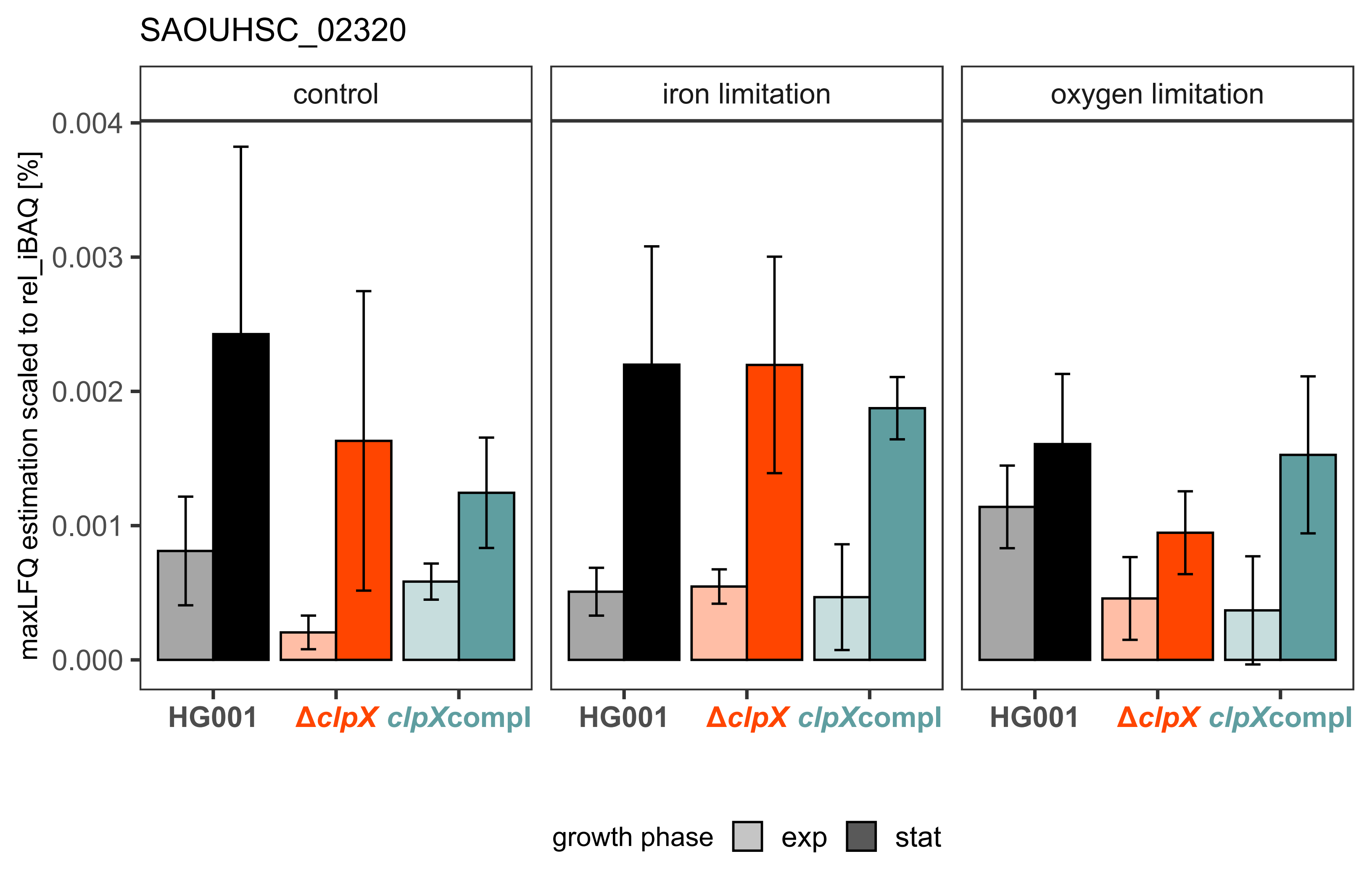
SAOUHSC_02320
control
iron limitation
oxygen limitation
maxLFQ estimation scaled to rel_iBAQ [%]
0.004
0.003
0.002
0.001
0.000
HG001
ΔclpX
clpXcompl
growth phase
exp
stat

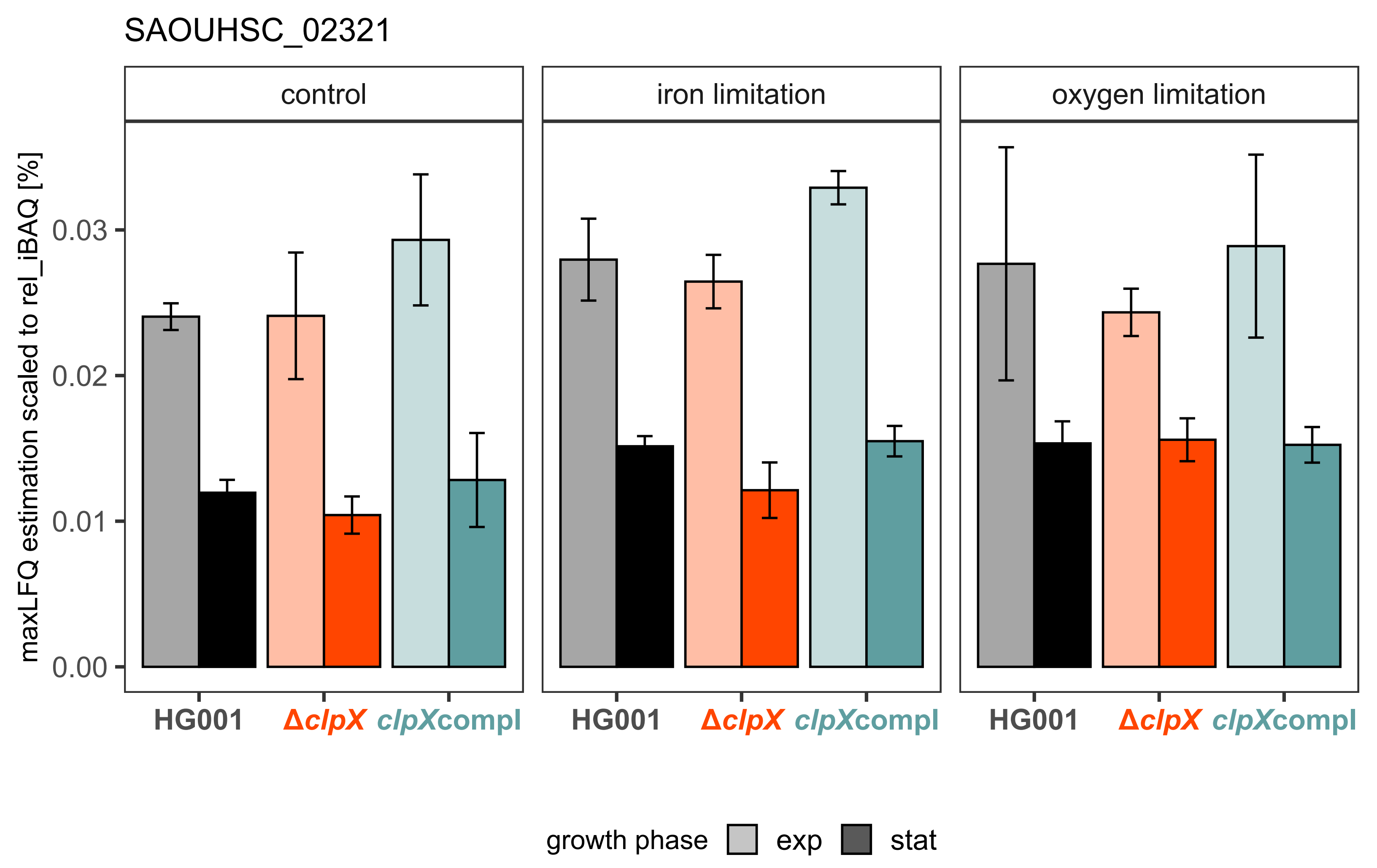
SAOUHSC_02321
control
iron limitation
oxygen limitation
maxLFQ estimation scaled to rel_iBAQ [%]
0.03
0.02
0.01
0.00
HG001
ΔclpX
clpXcompl
growth phase
exp
stat

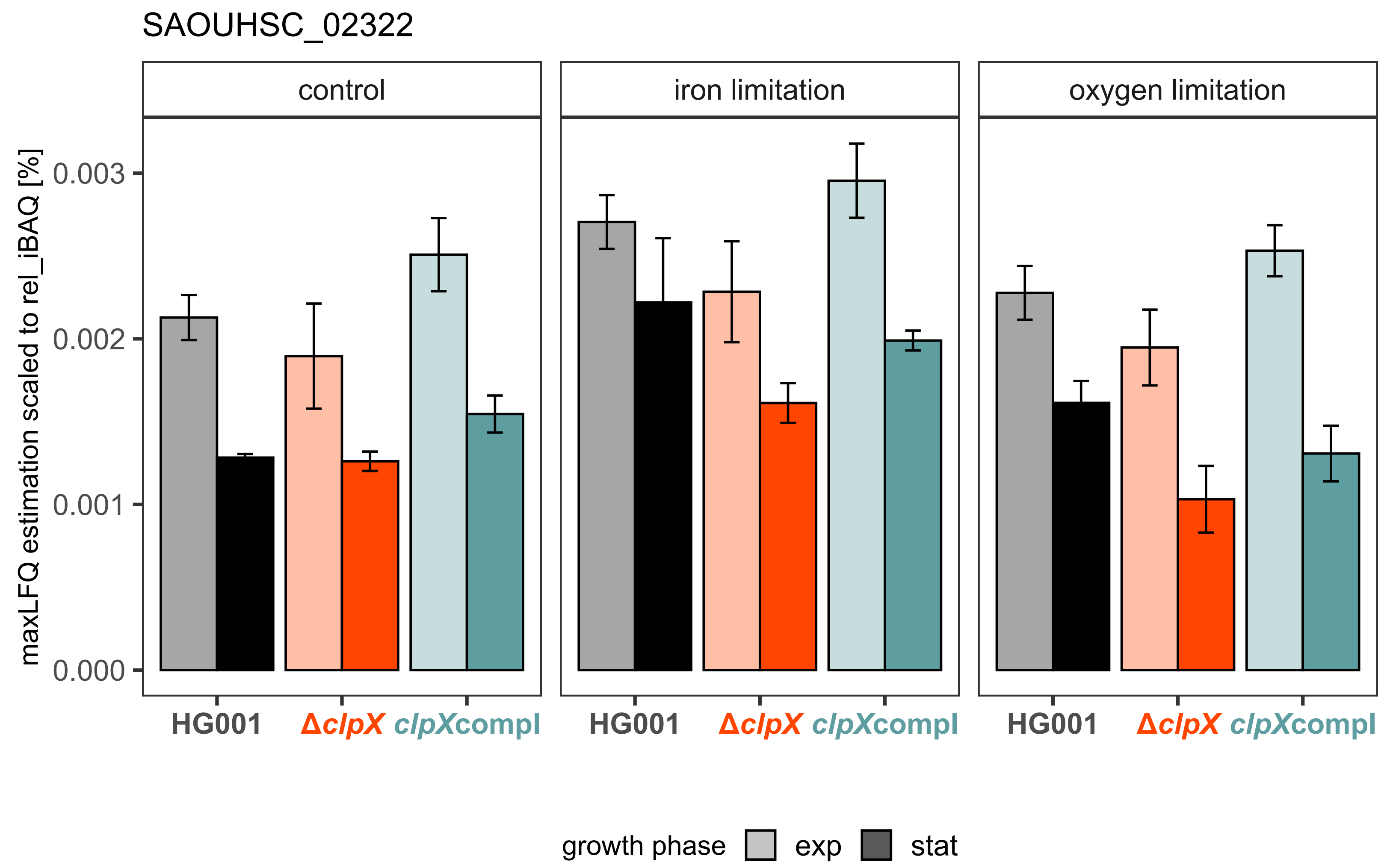
SAOUHSC_02322
control
iron limitation
oxygen limitation
maxLFQ estimation scaled to rel_iBAQ [%]
0.003
0.002
0.001
0.000
HG001
ΔclpX
clpXcompl
growth phase
exp
stat

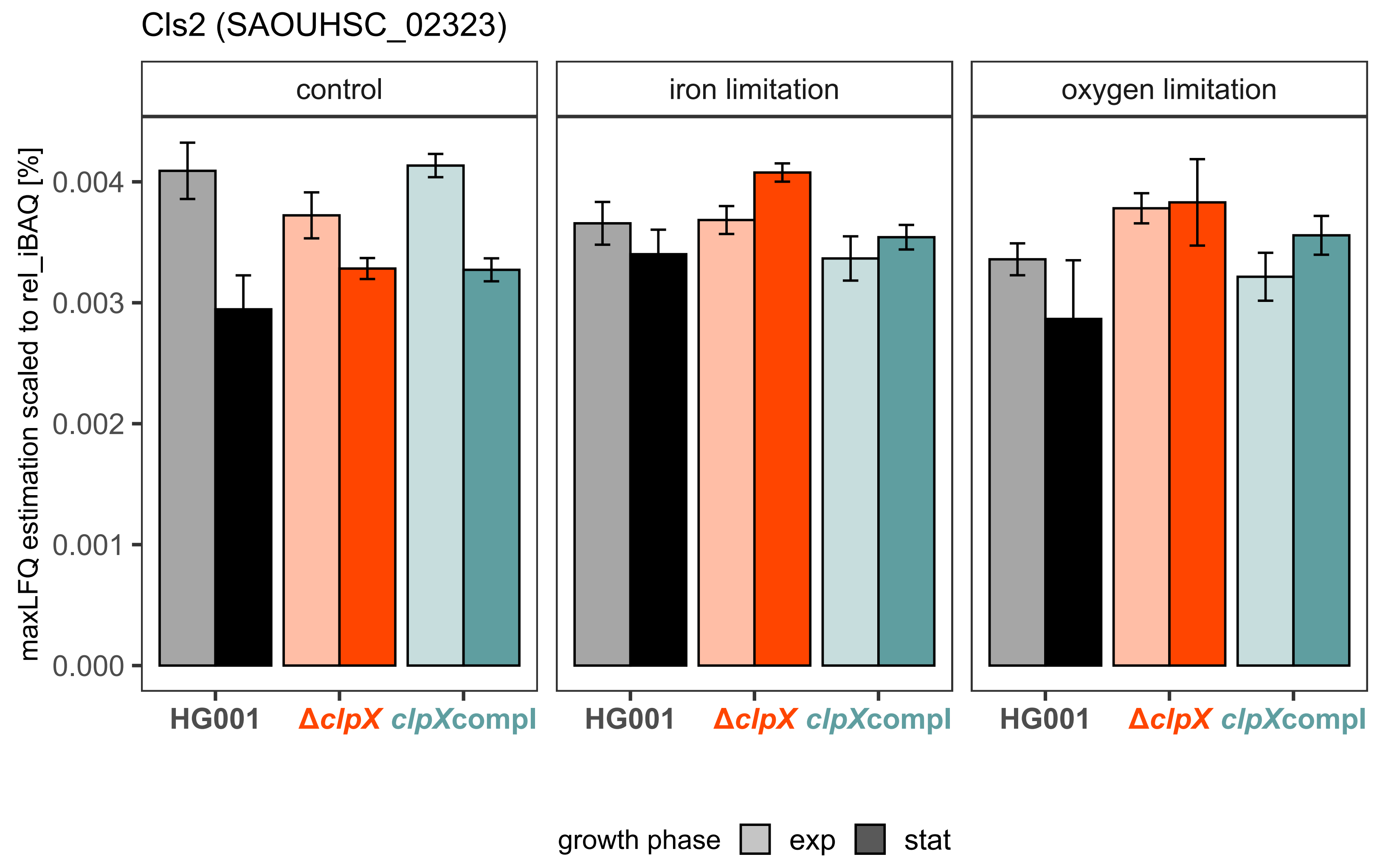

Cls2 (SAOUHSC_02323)
control
iron limitation
oxygen limitation
maxLFQ estimation scaled to rel_iBAQ [%]
0.004
0.003
0.002
0.001
0.000
HG001
ΔclpX
clpXcompl
growth phase
exp
stat

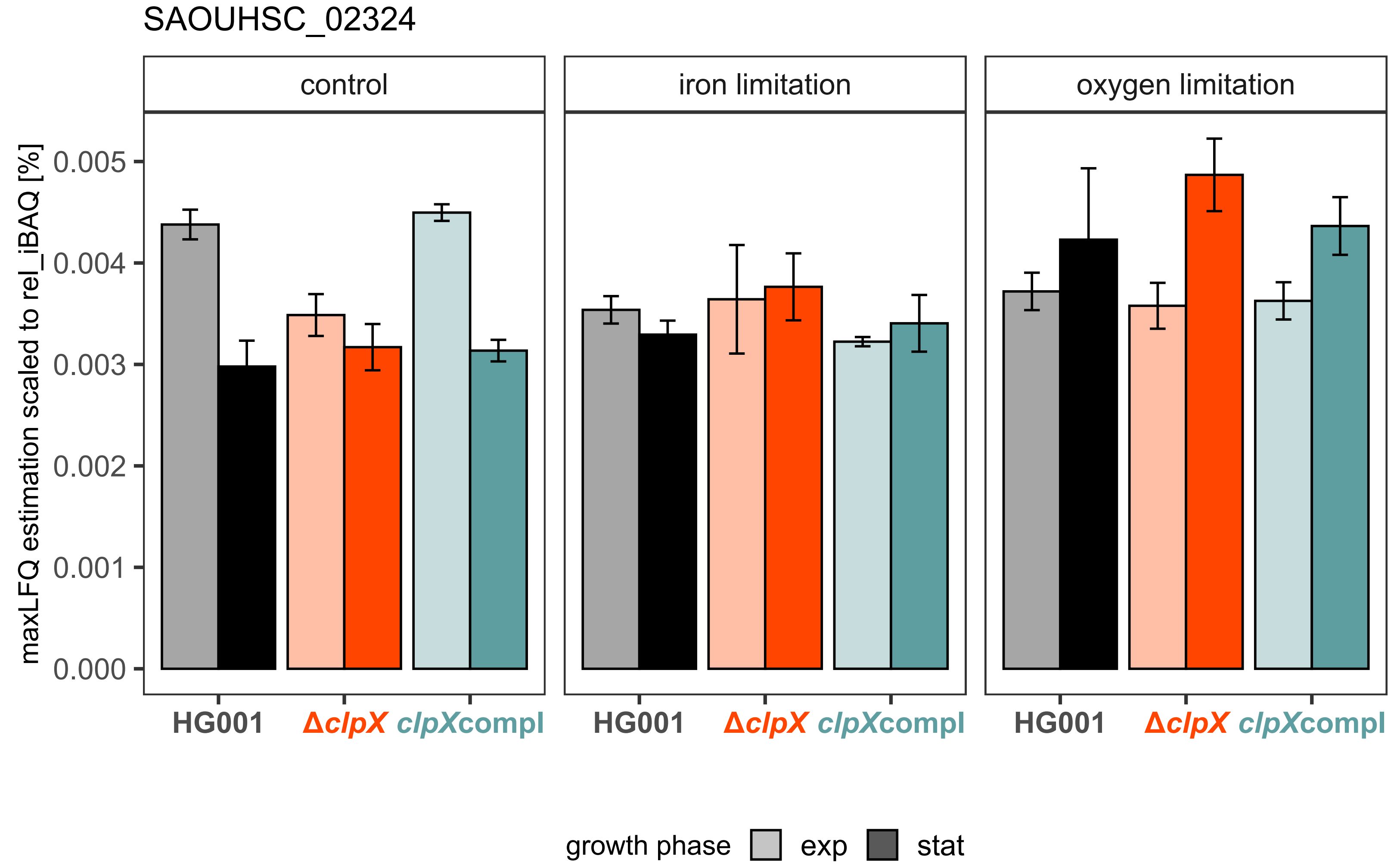

SAOUHSC_02324
control
iron limitation
oxygen limitation
maxLFQ estimation scaled to rel_iBAQ [%]
0.005
0.004
0.003
0.002
0.001
0.000
HG001
ΔclpX
clpXcompl
growth phase
exp
stat

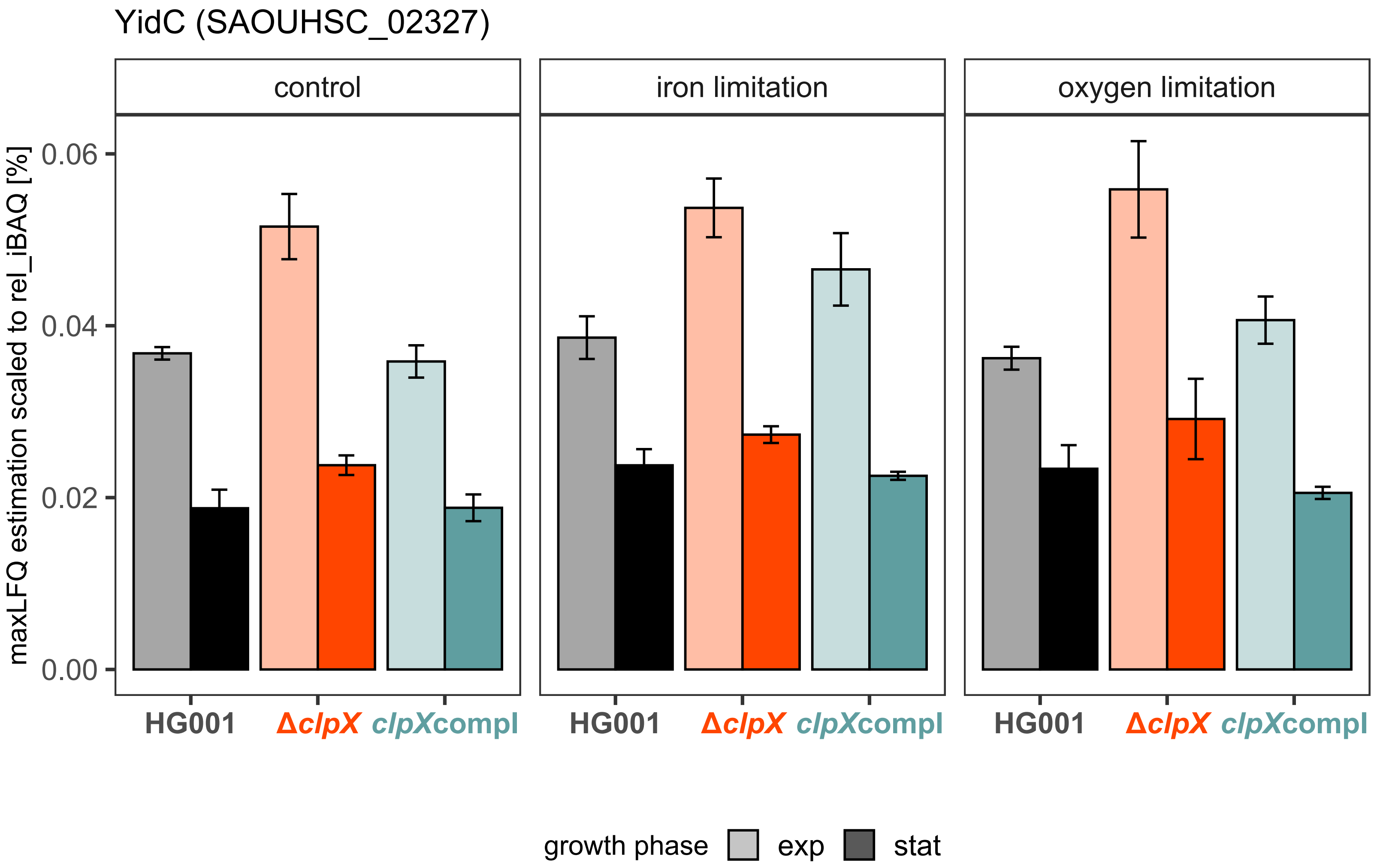

YidC (SAOUHSC_02327)
control
iron limitation
oxygen limitation
maxLFQ estimation scaled to rel_iBAQ [%]
0.06
0.04
0.02
0.00
HG001
ΔclpX
clpXcompl
growth phase
exp
stat

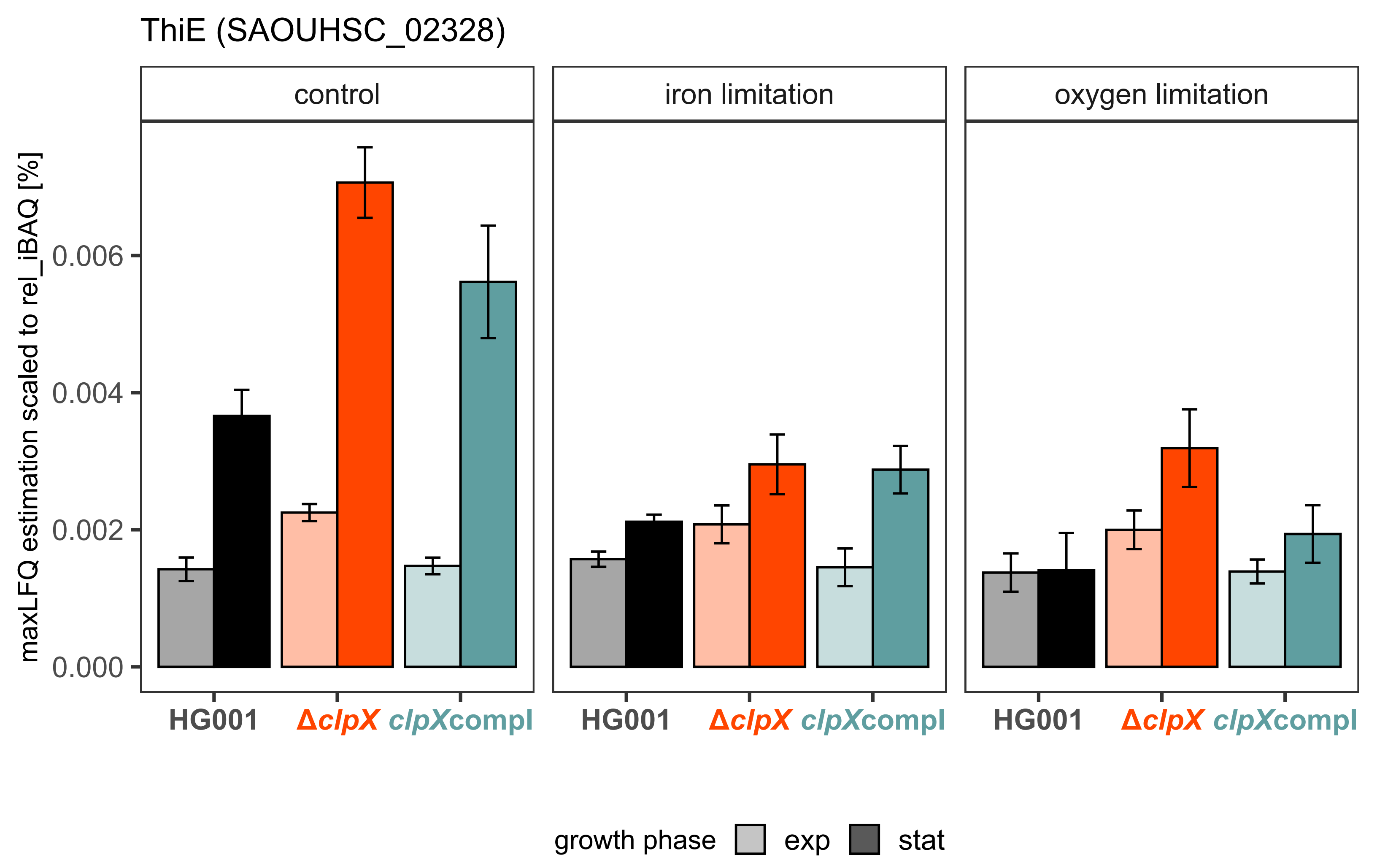
ThiE (SAOUHSC_02328)
control
iron limitation
oxygen limitation
maxLFQ estimation scaled to rel_iBAQ [%]
0.006
0.004
0.002
0.000
HG001
ΔclpX
clpXcompl
growth phase
exp
stat

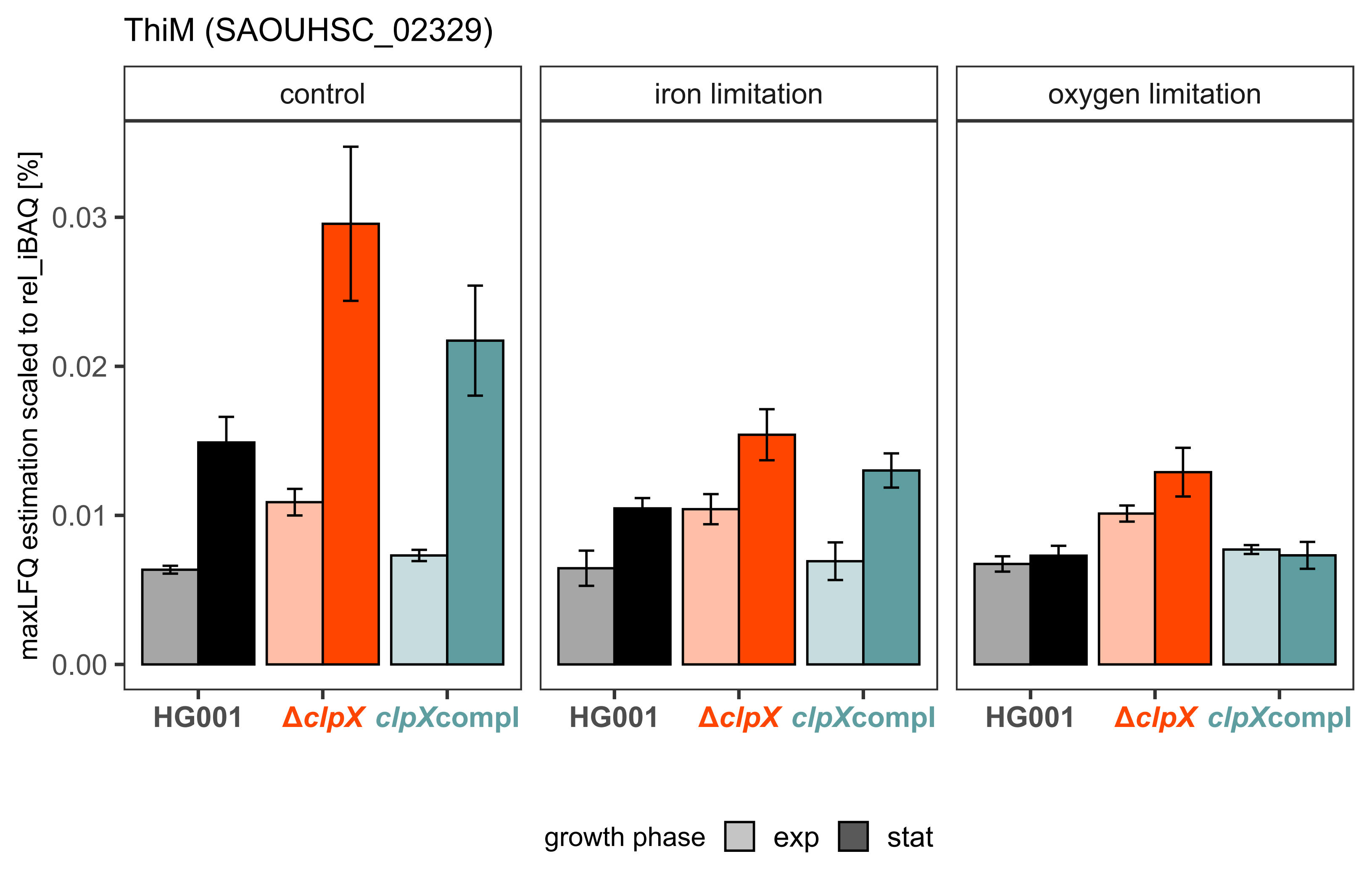

ThiM (SAOUHSC_02329)
control
iron limitation
oxygen limitation
maxLFQ estimation scaled to rel_iBAQ [%]
0.00
0.01
0.02
0.03
HG001
ΔclpX
clpXcompl
growth phase
exp
stat

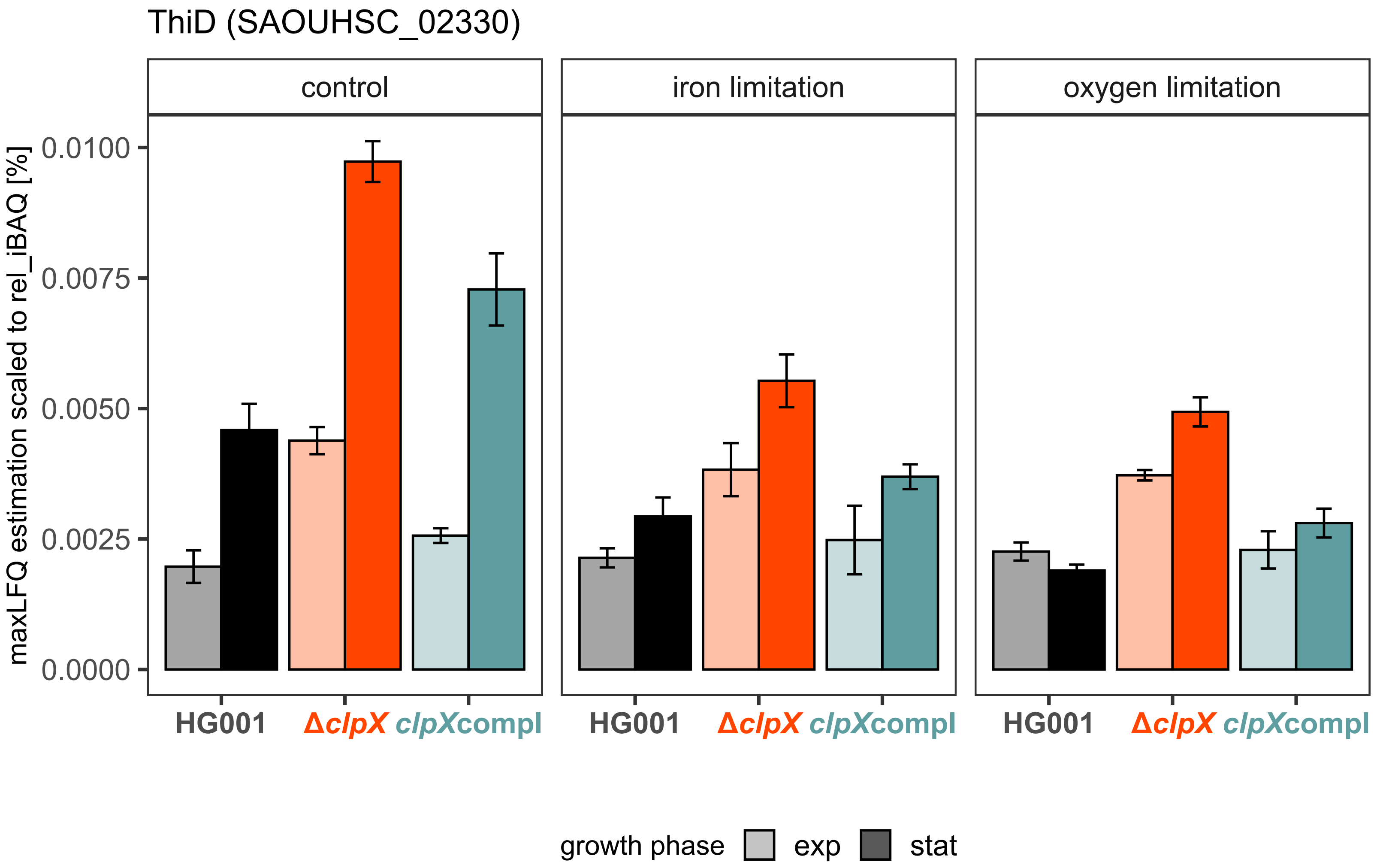
ThiD (SAOUHSC_02330)
control
iron limitation
oxygen limitation
maxLFQ estimation scaled to rel_iBAQ [%]
0.0100
0.0075
0.0050
0.0025
0.0000
HG001
ΔclpX
clpXcompl
growth phase
exp
stat

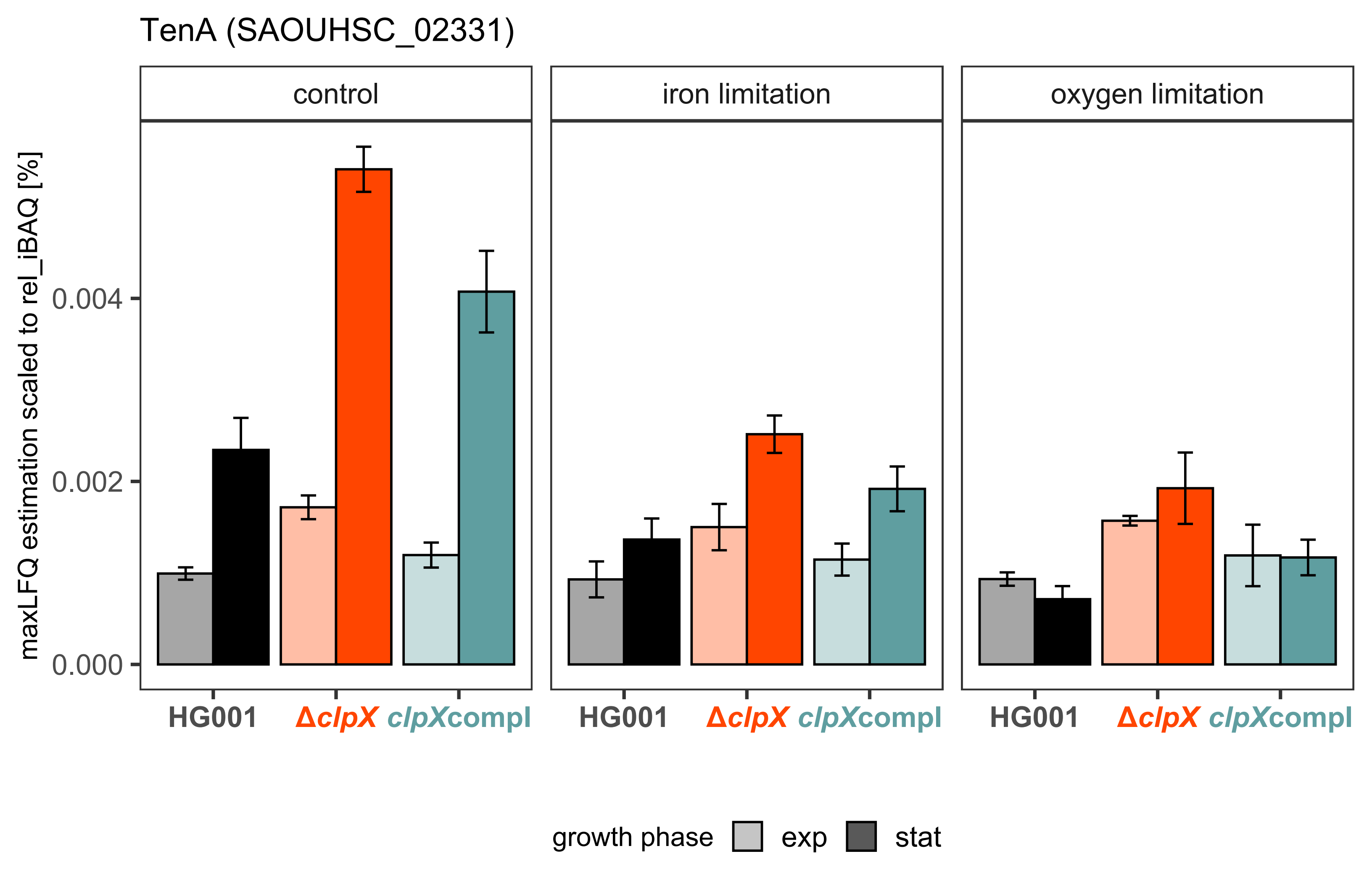

TenA (SAOUHSC_02331)
control
iron limitation
oxygen limitation
maxLFQ estimation scaled to rel_iBAQ [%]
0.000
0.002
0.004
HG001
ΔclpX
clpXcompl
growth phase
exp
stat

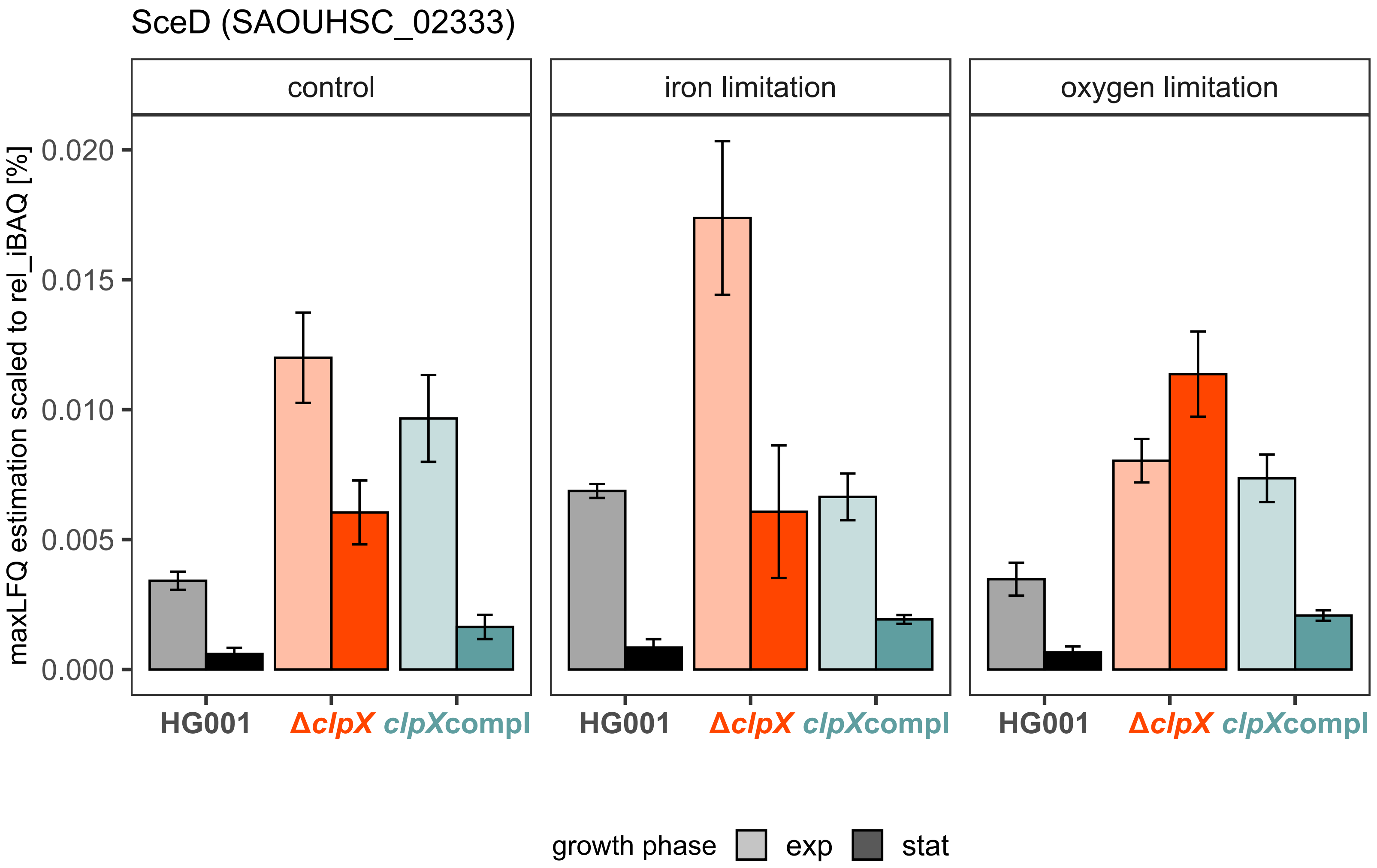

SceD (SAOUHSC_02333)
control
iron limitation
oxygen limitation
maxLFQ estimation scaled to rel_iBAQ [%]
0.020
0.015
0.010
0.005
0.000
HG001
ΔclpX
clpXcompl
growth phase
exp
stat

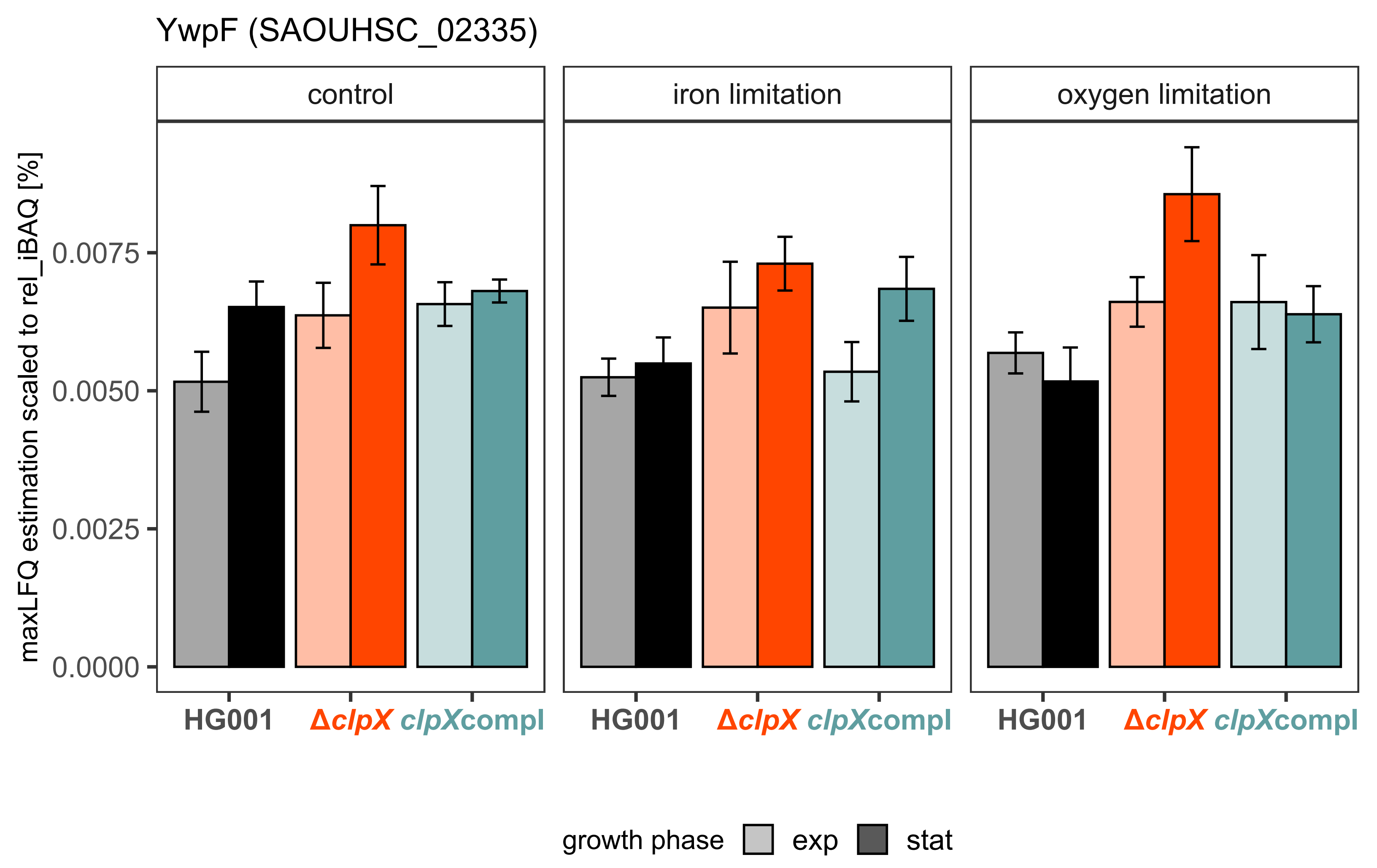
YwpF (SAOUHSC_02335)
control
iron limitation
oxygen limitation
maxLFQ estimation scaled to rel_iBAQ [%]
0.0075
0.0050
0.0025
0.0000
HG001
ΔclpX
clpXcompl
growth phase
exp
stat

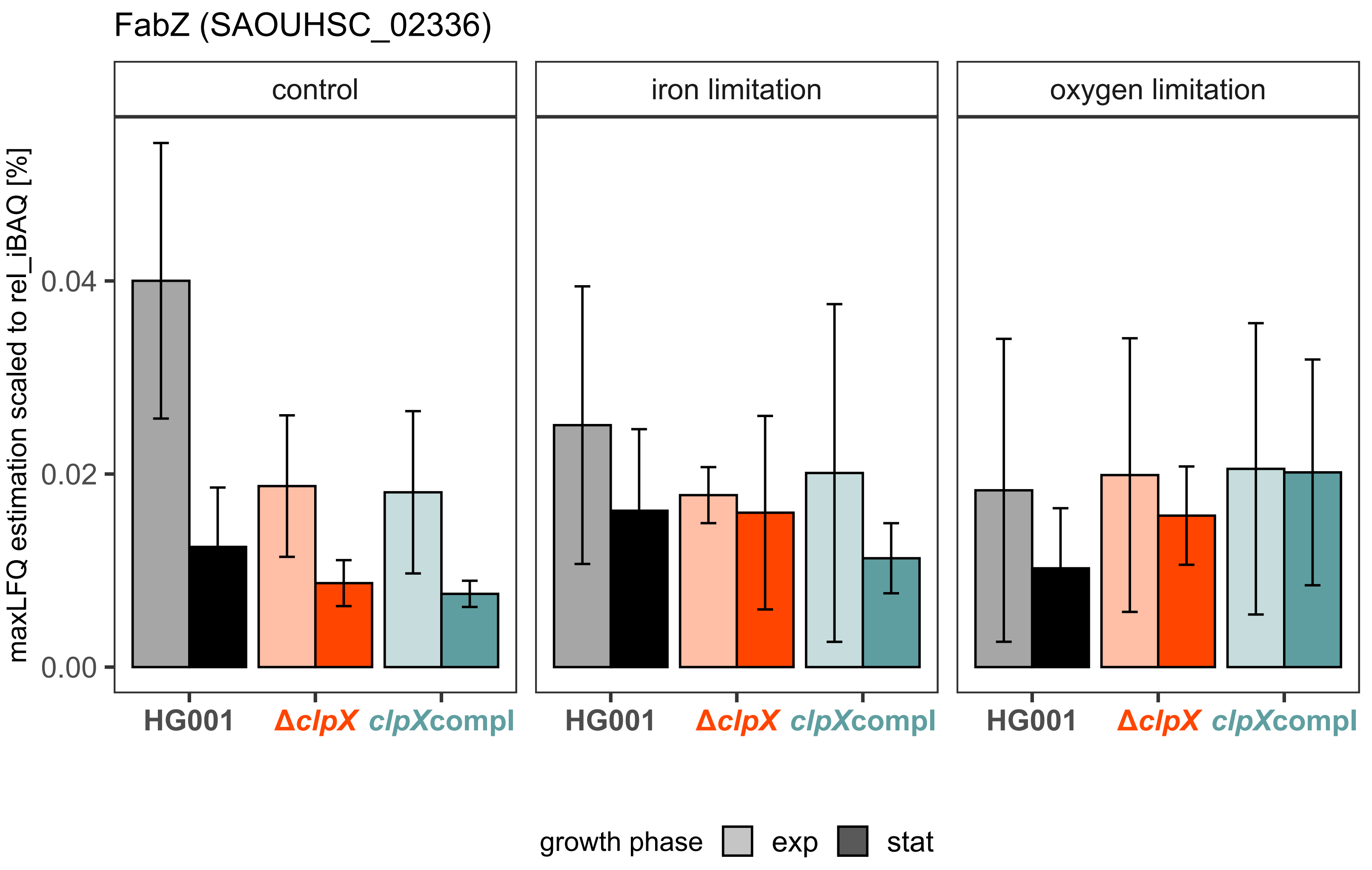
FabZ (SAOUHSC_02336)
control
iron limitation
oxygen limitation
maxLFQ estimation scaled to rel_iBAQ [%]
0.04
0.02
0.00
HG001
ΔclpX
clpXcompl
growth phase
exp
stat

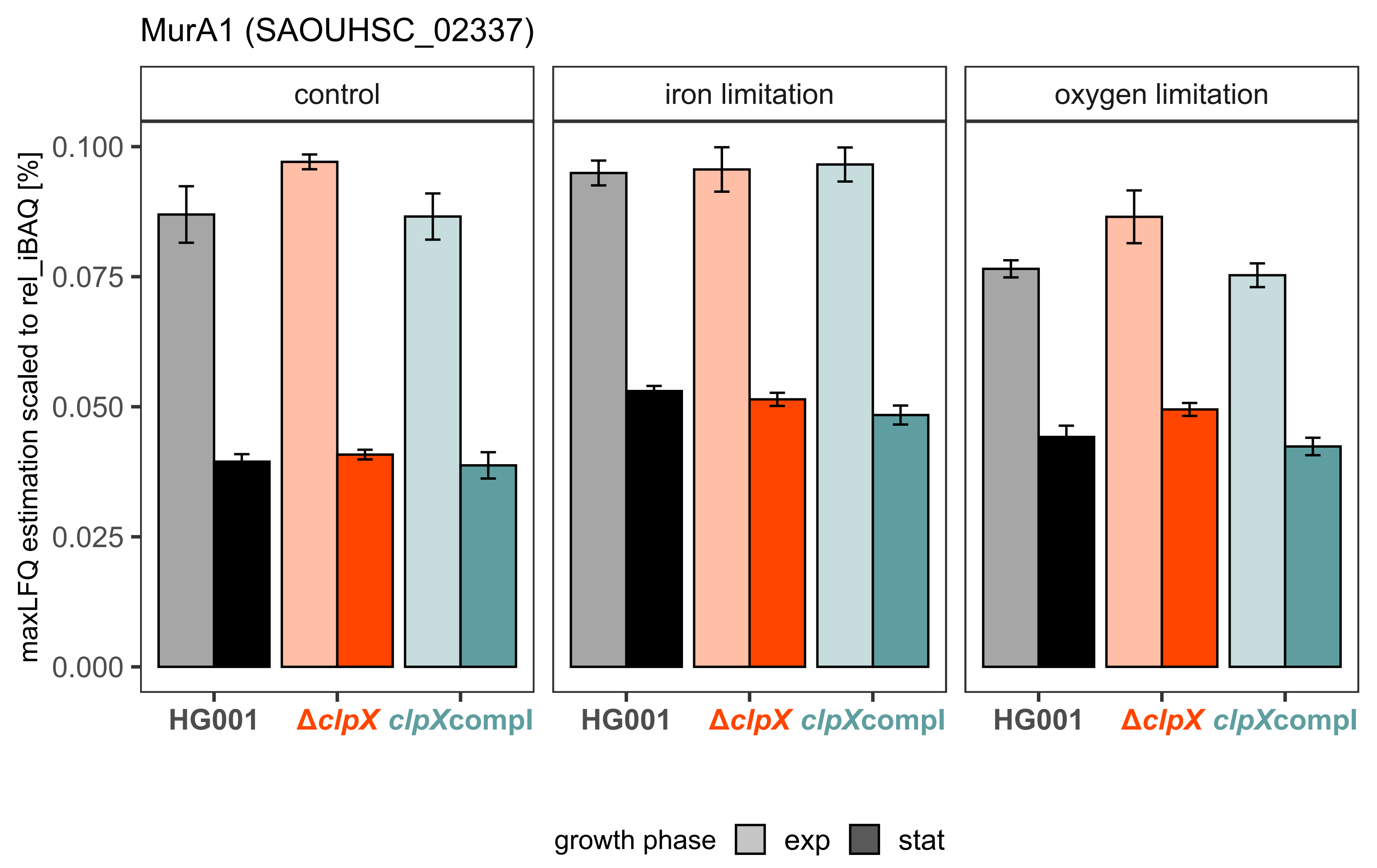

MurA1 (SAOUHSC_02337)
control
iron limitation
oxygen limitation
maxLFQ estimation scaled to rel_iBAQ [%]
0.100
0.075
0.050
0.025
0.000
HG001
ΔclpX
clpXcompl
growth phase
exp
stat

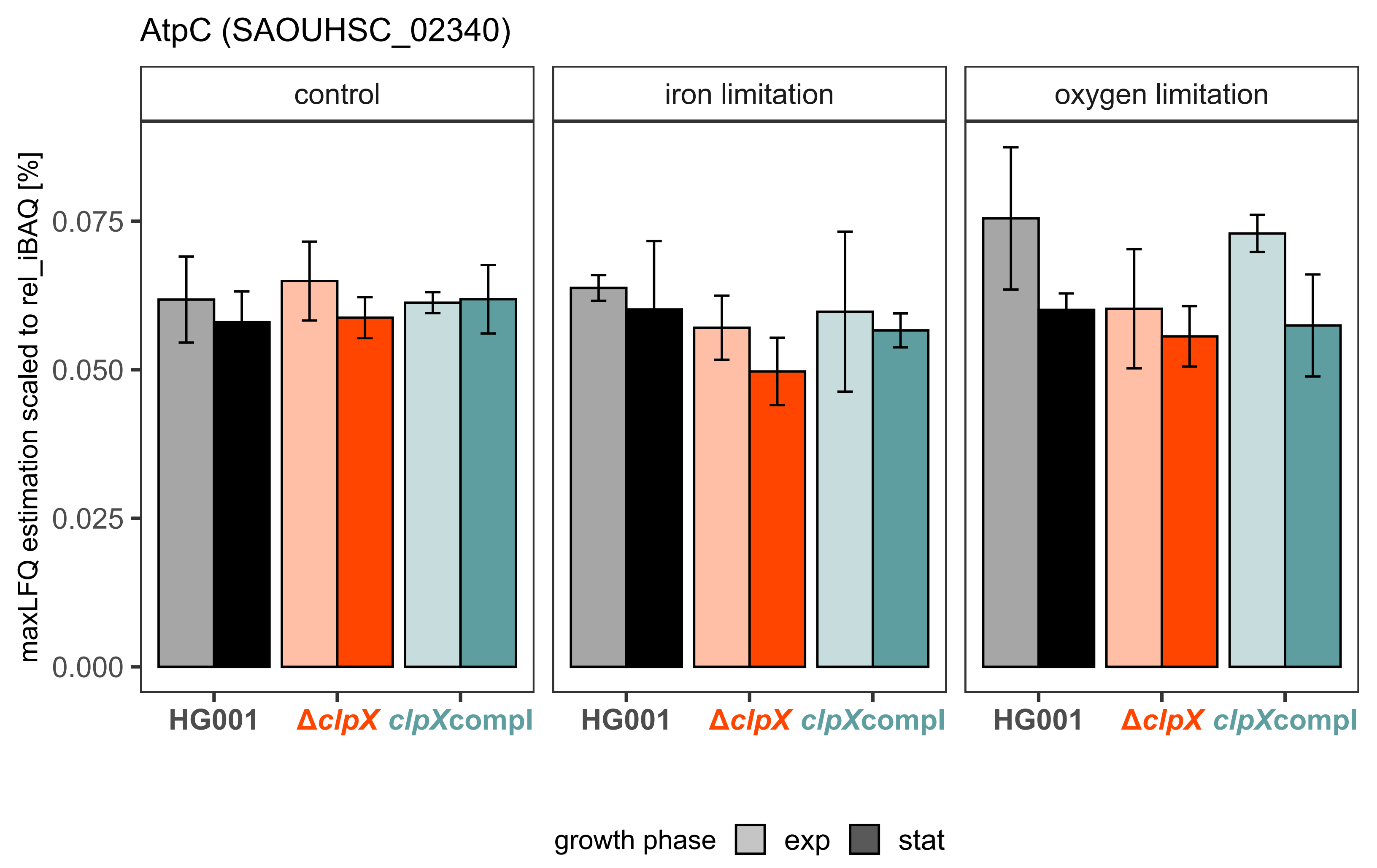
AtpC (SAOUHSC_02340)
control
iron limitation
oxygen limitation
maxLFQ estimation scaled to rel_iBAQ [%]
0.075
0.050
0.025
0.000
HG001
ΔclpX
clpXcompl
growth phase
exp
stat

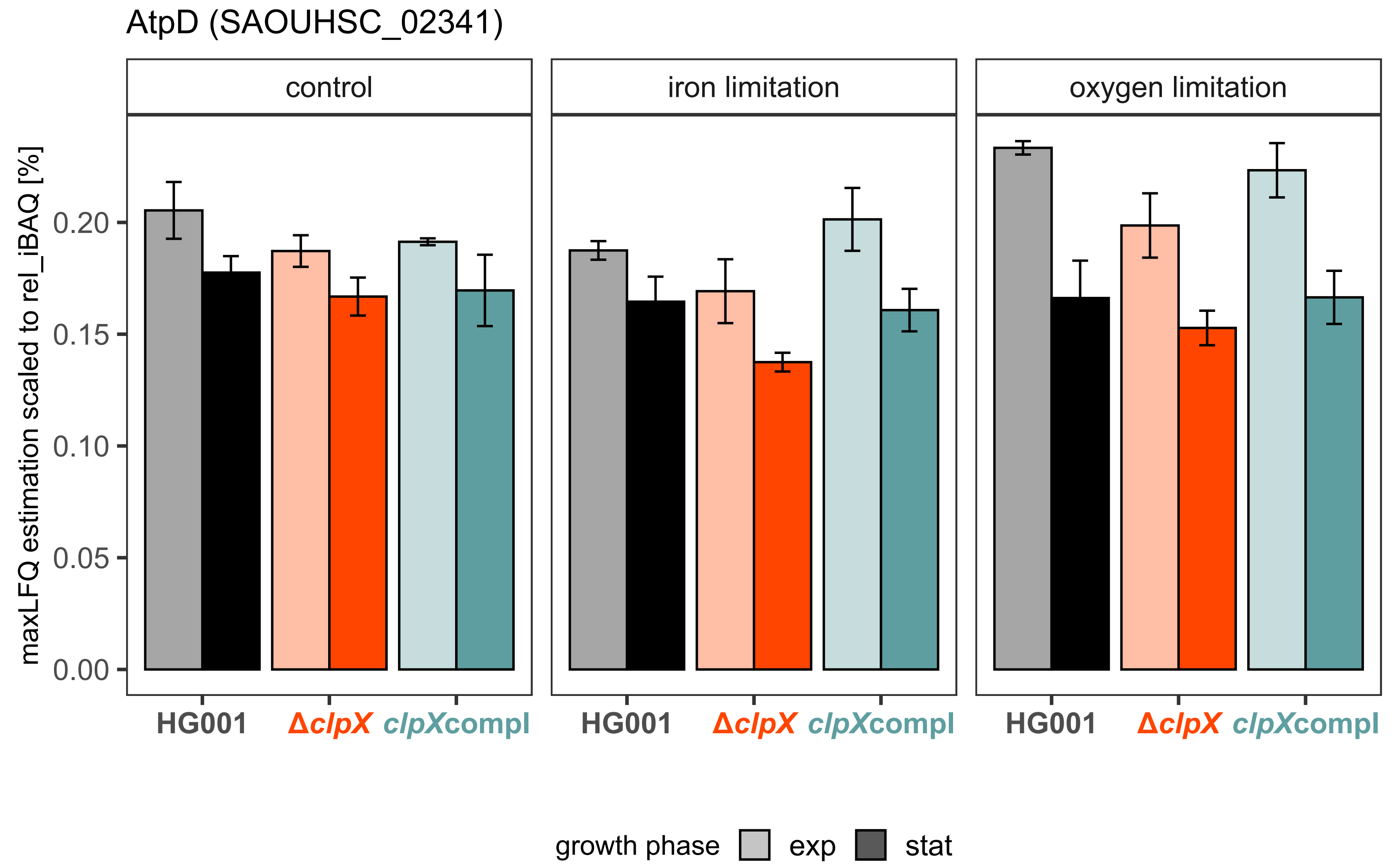

AtpD (SAOUHSC_02341)
control
iron limitation
oxygen limitation
maxLFQ estimation scaled to rel_iBAQ [%]
0.20
0.15
0.10
0.05
0.00
HG001
ΔclpX
clpXcompl
growth phase
exp
stat

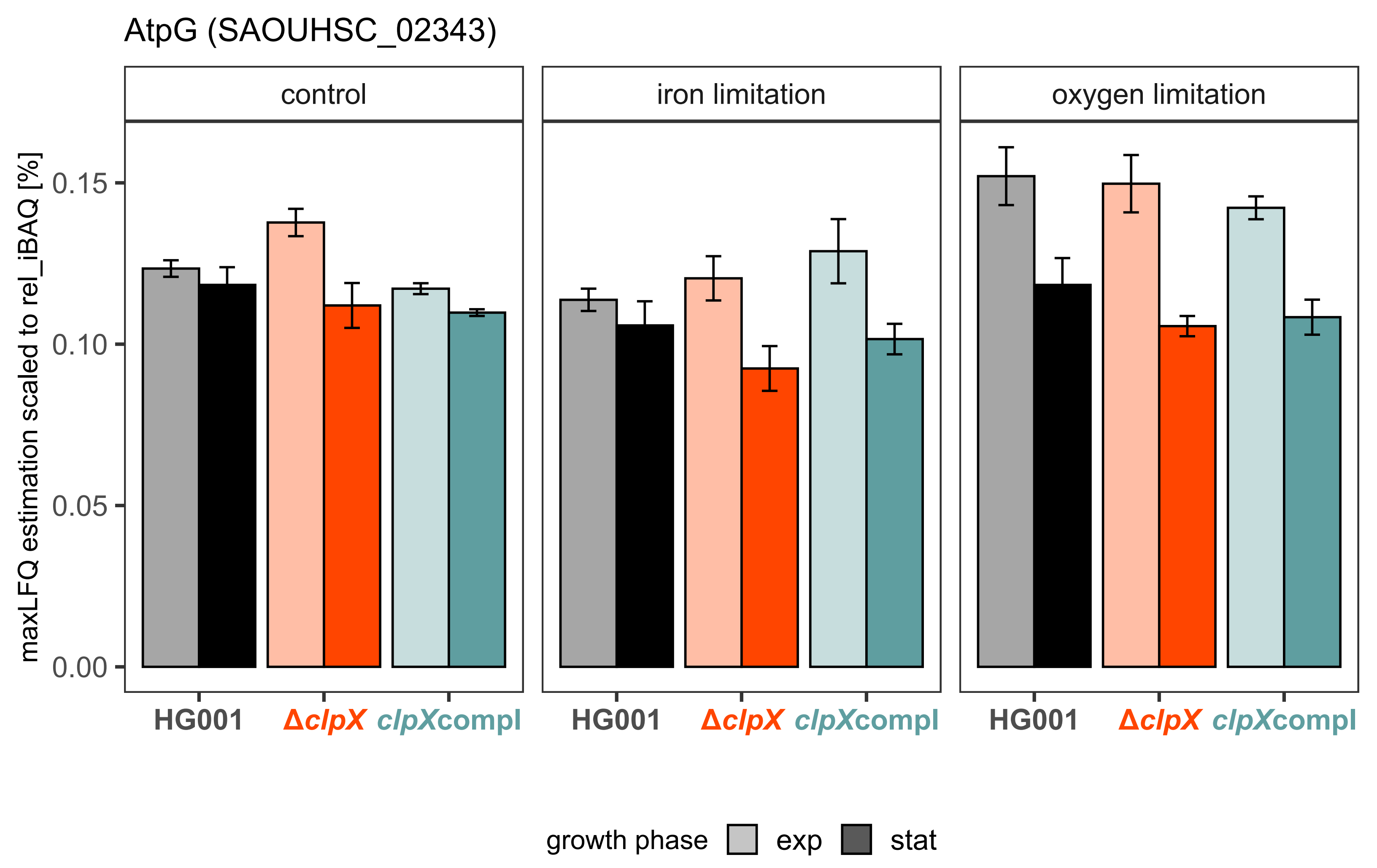

AtpG (SAOUHSC_02343)
control
iron limitation
oxygen limitation
maxLFQ estimation scaled to rel_iBAQ [%]
0.15
0.10
0.05
0.00
HG001
ΔclpX
clpXcompl
growth phase
exp
stat

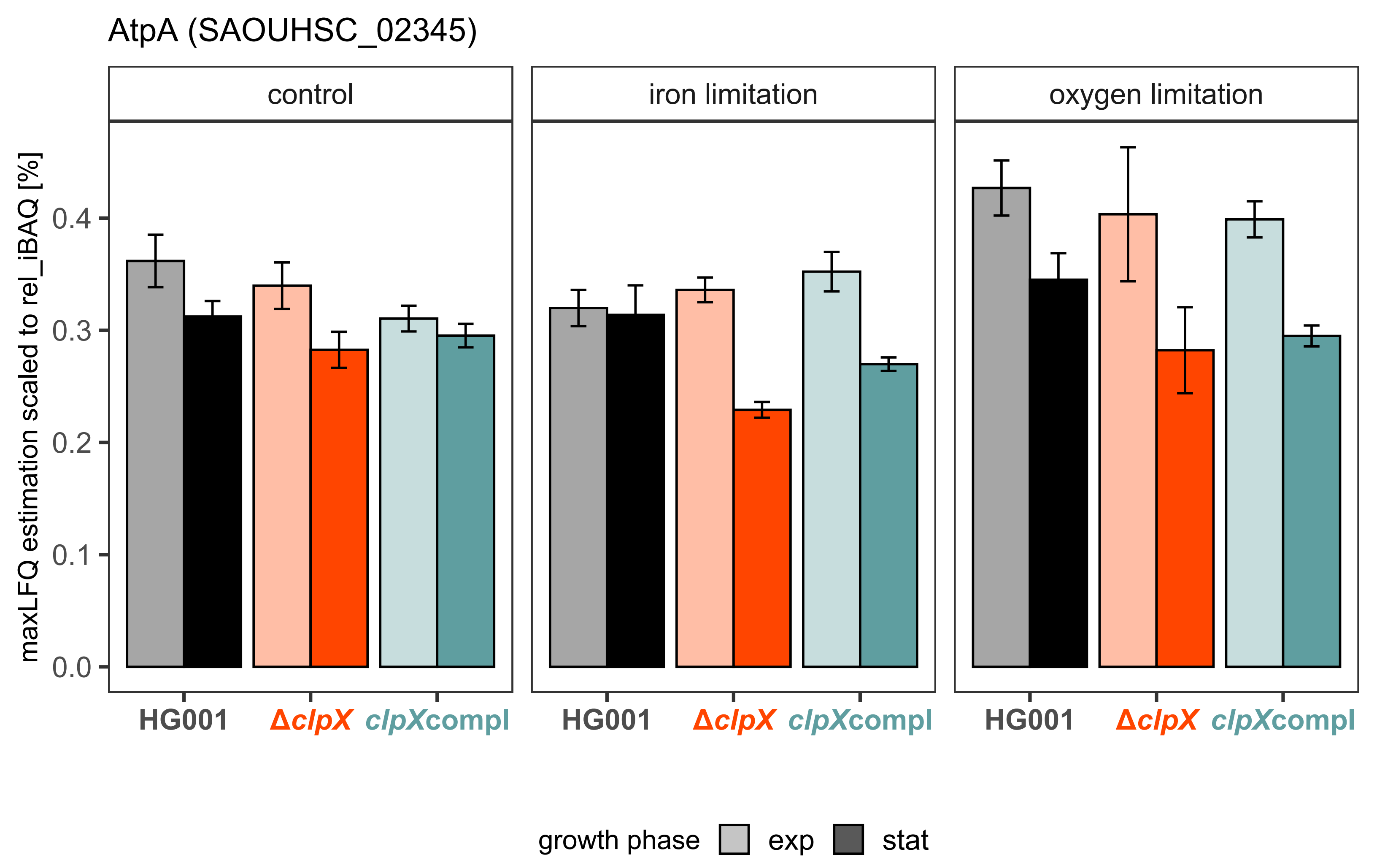

AtpA (SAOUHSC_02345)
control
iron limitation
oxygen limitation
maxLFQ estimation scaled to rel_iBAQ [%]
0.0
0.1
0.2
0.3
0.4
HG001
ΔclpX
clpXcompl
growth phase
exp
stat

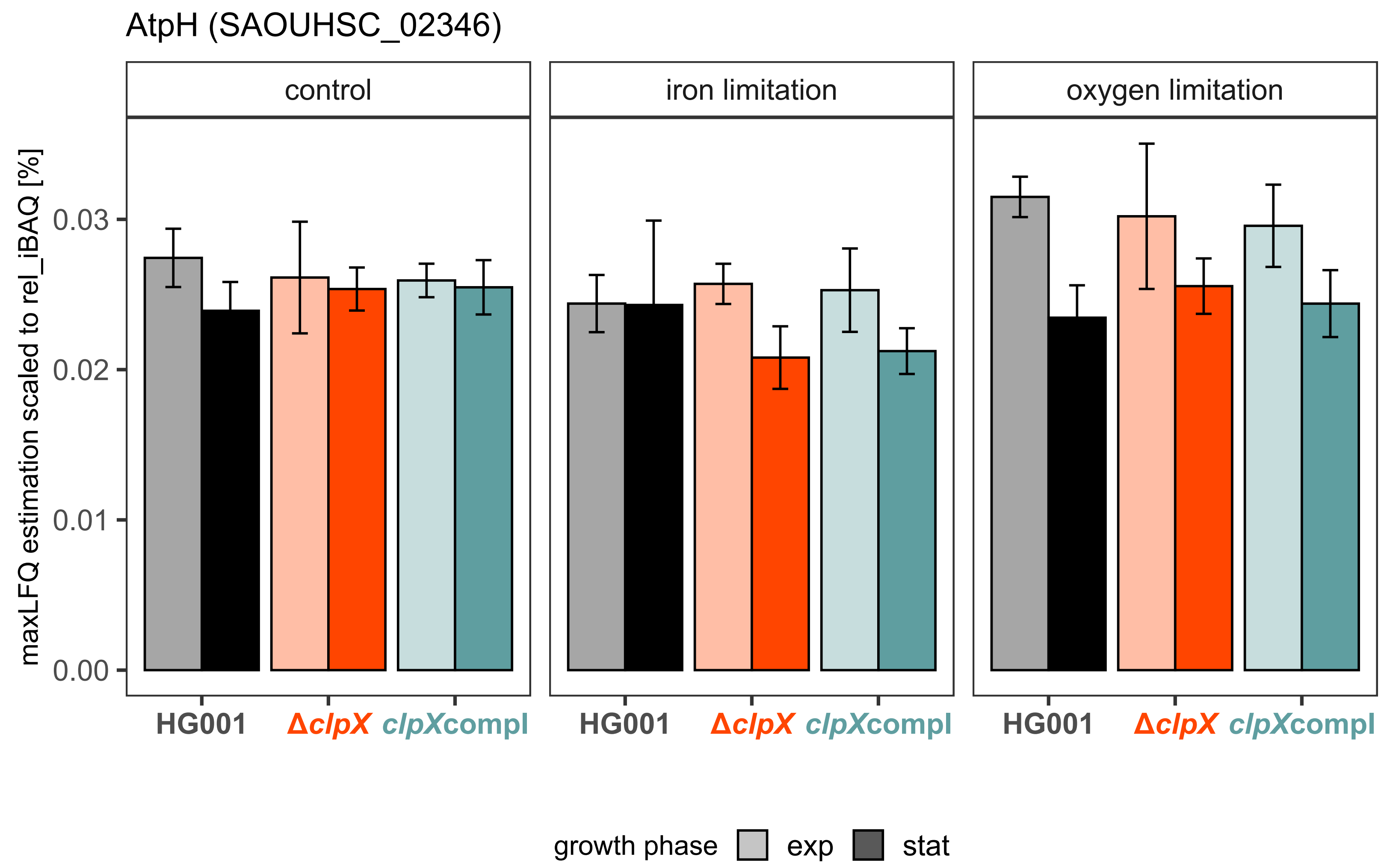

AtpH (SAOUHSC_02346)
control
iron limitation
oxygen limitation
maxLFQ estimation scaled to rel_iBAQ [%]
0.00
0.01
0.02
0.03
HG001
ΔclpX
clpXcompl
growth phase
exp
stat

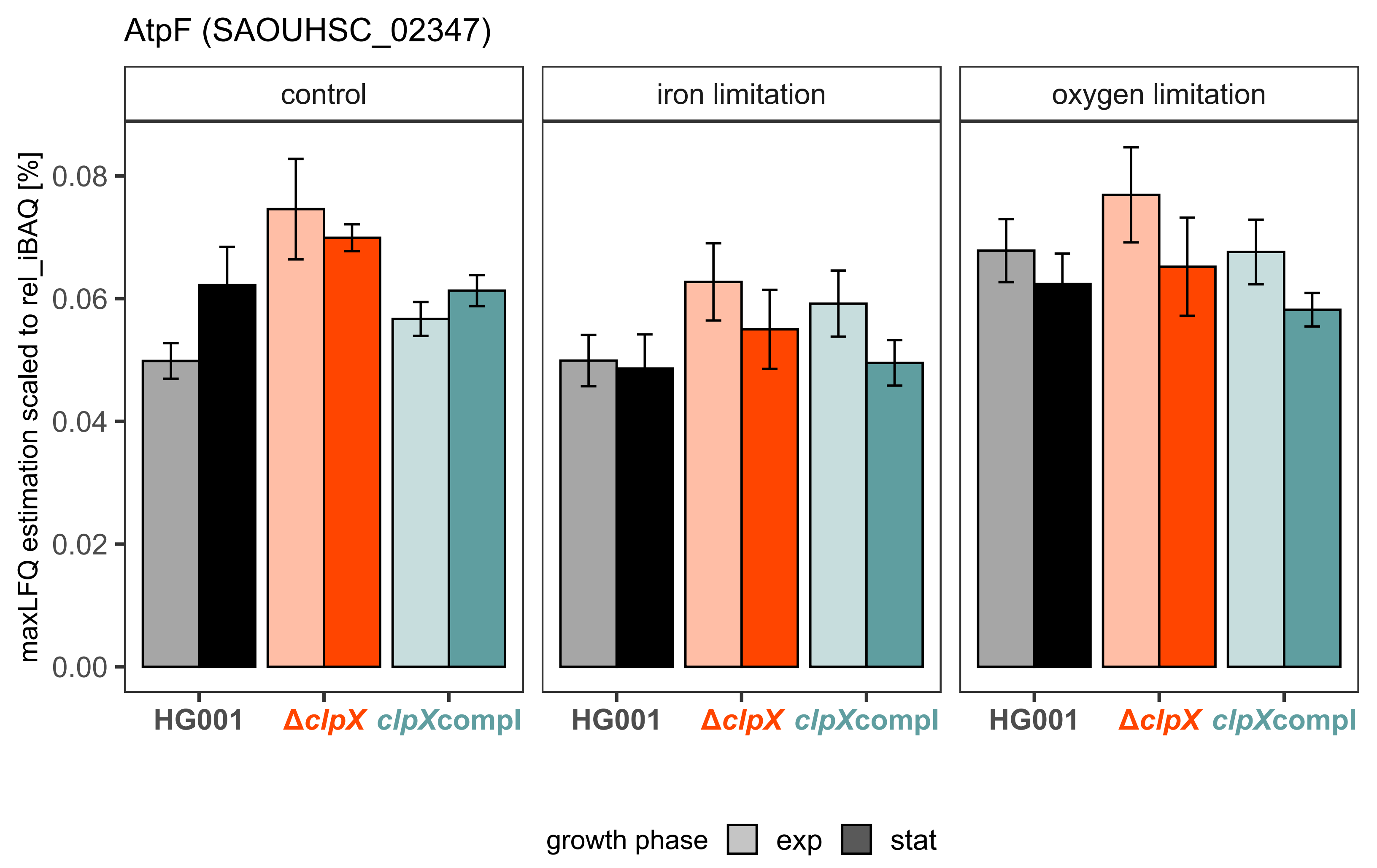
AtpF (SAOUHSC_02347)
control
iron limitation
oxygen limitation
maxLFQ estimation scaled to rel_iBAQ [%]
0.00
0.02
0.04
0.06
0.08
HG001
ΔclpX
clpXcompl
growth phase
exp
stat

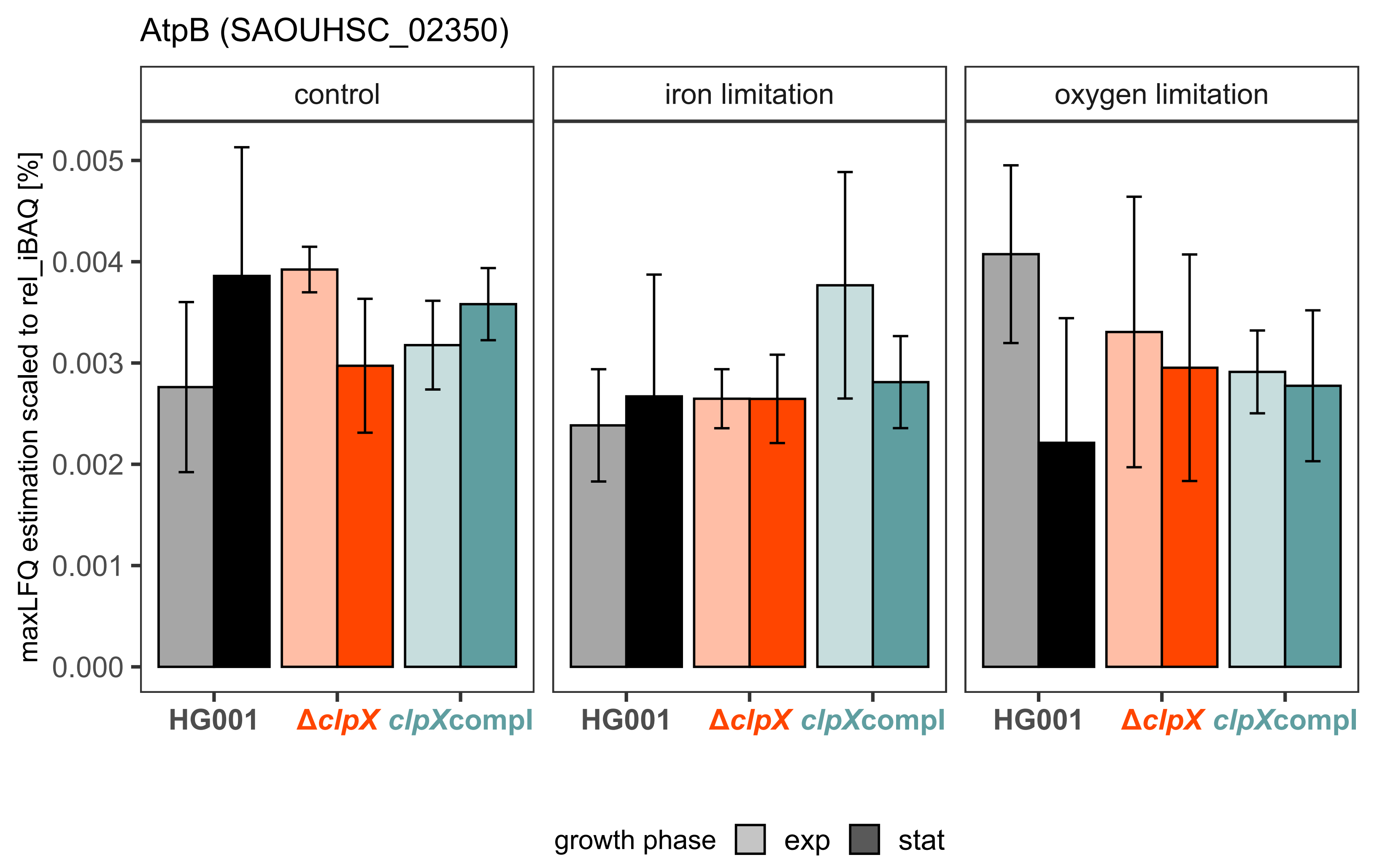
AtpB (SAOUHSC_02350)
control
iron limitation
oxygen limitation
maxLFQ estimation scaled to rel_iBAQ [%]
0.005
0.004
0.003
0.002
0.001
0.000
HG001
ΔclpX
clpXcompl
growth phase
exp
stat

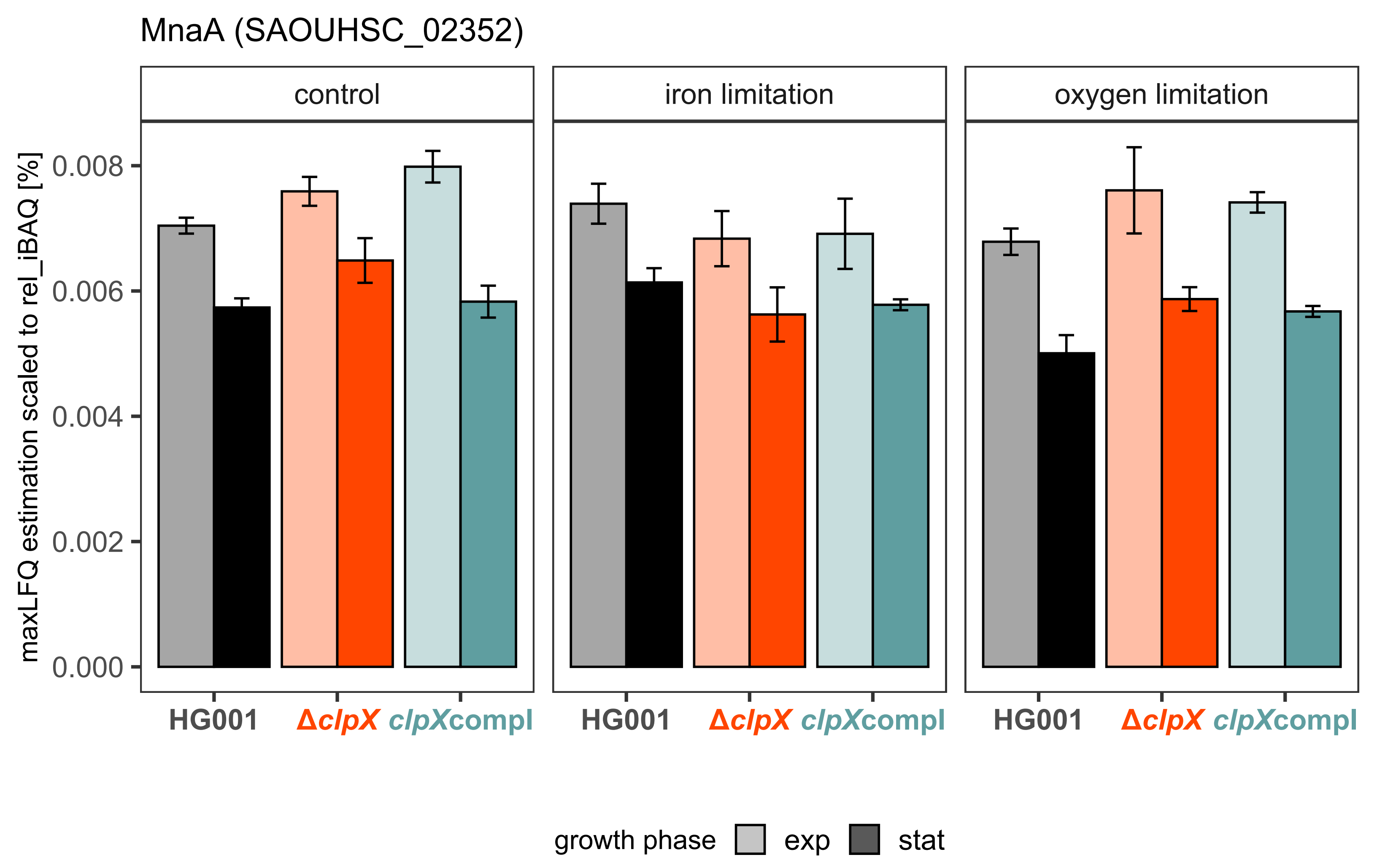

MnaA (SAOUHSC_02352)
control
iron limitation
oxygen limitation
maxLFQ estimation scaled to rel_iBAQ [%]
0.008
0.006
0.004
0.002
0.000
HG001
ΔclpX
clpXcompl
growth phase
exp
stat

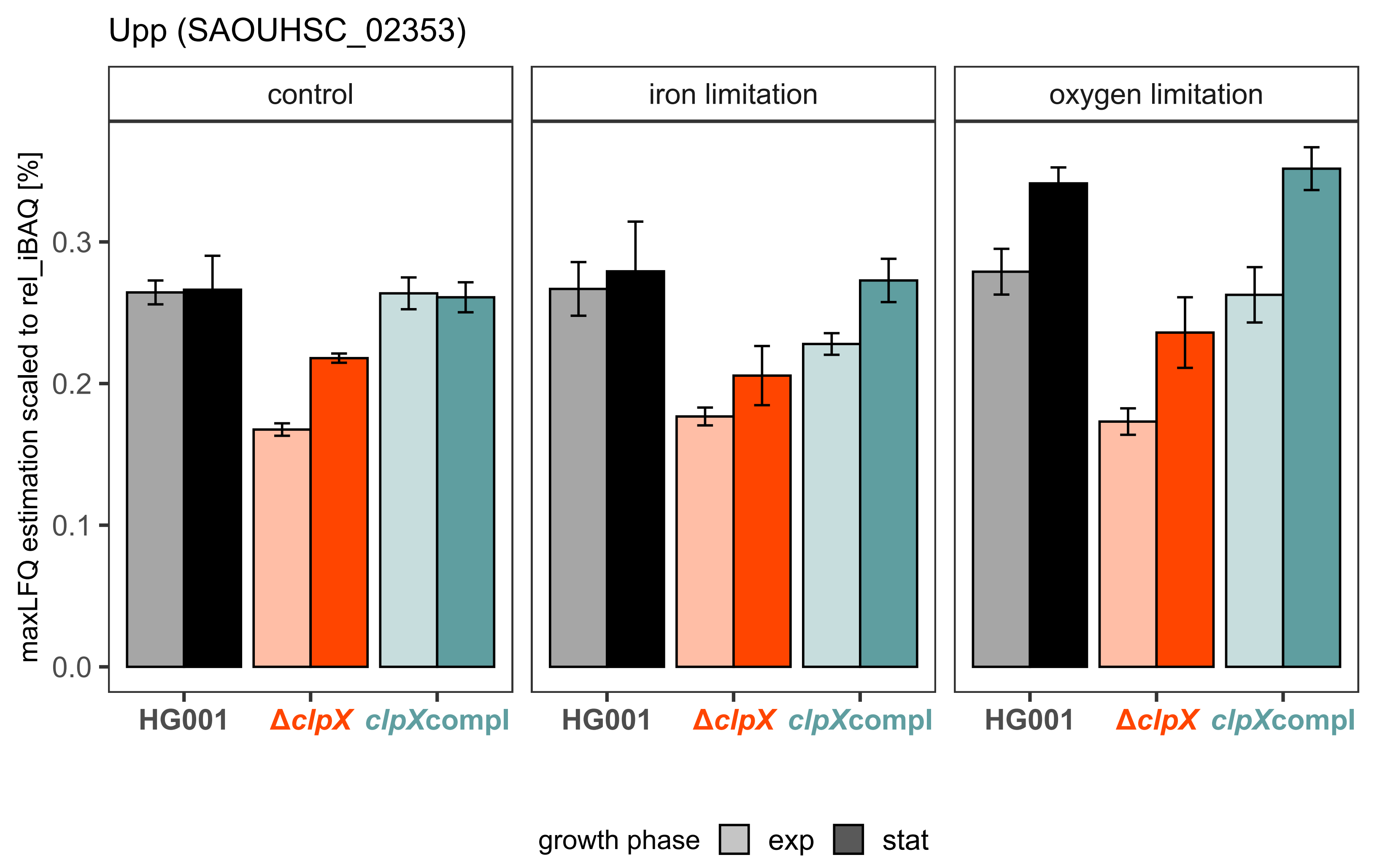

Upp (SAOUHSC_02353)
control
iron limitation
oxygen limitation
maxLFQ estimation scaled to rel_iBAQ [%]
0.0
0.1
0.2
0.3
HG001
ΔclpX
clpXcompl
growth phase
exp
stat

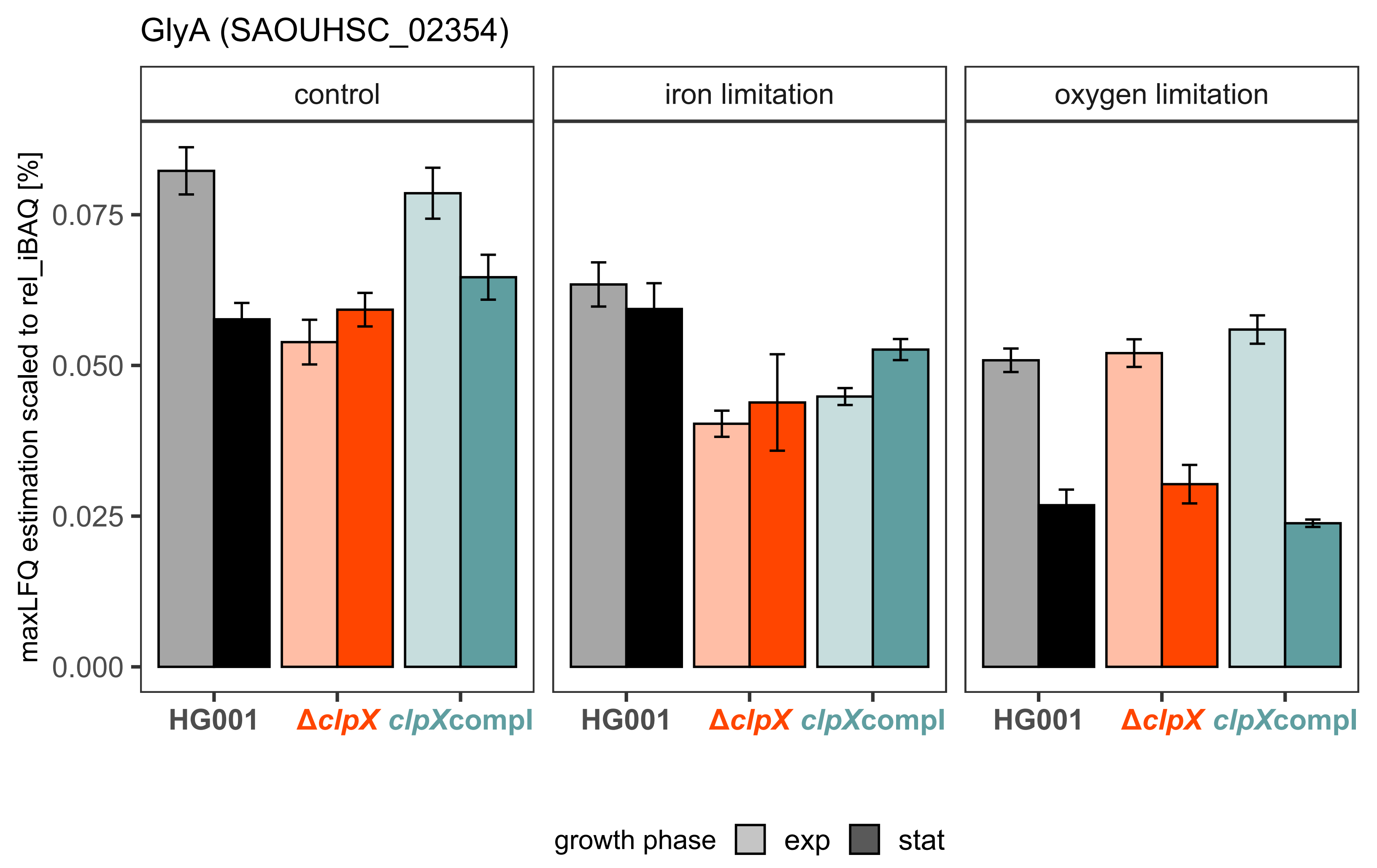

GlyA (SAOUHSC_02354)
control
iron limitation
oxygen limitation
maxLFQ estimation scaled to rel_iBAQ [%]
0.075
0.050
0.025
0.000
HG001
ΔclpX
clpXcompl
growth phase
exp
stat

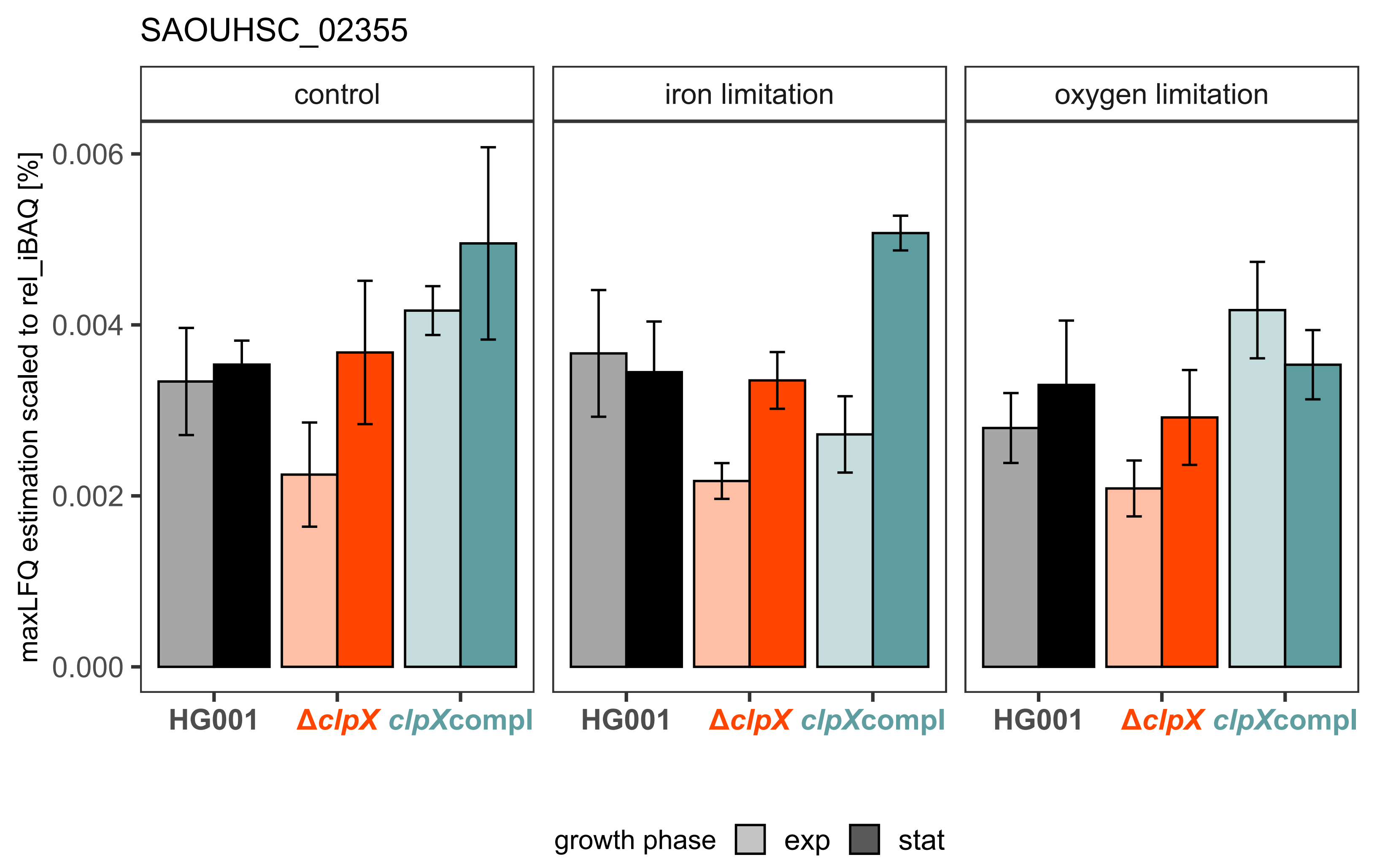

SAOUHSC_02355
control
iron limitation
oxygen limitation
maxLFQ estimation scaled to rel_iBAQ [%]
0.006
0.004
0.002
0.000
HG001
ΔclpX
clpXcompl
growth phase
exp
stat

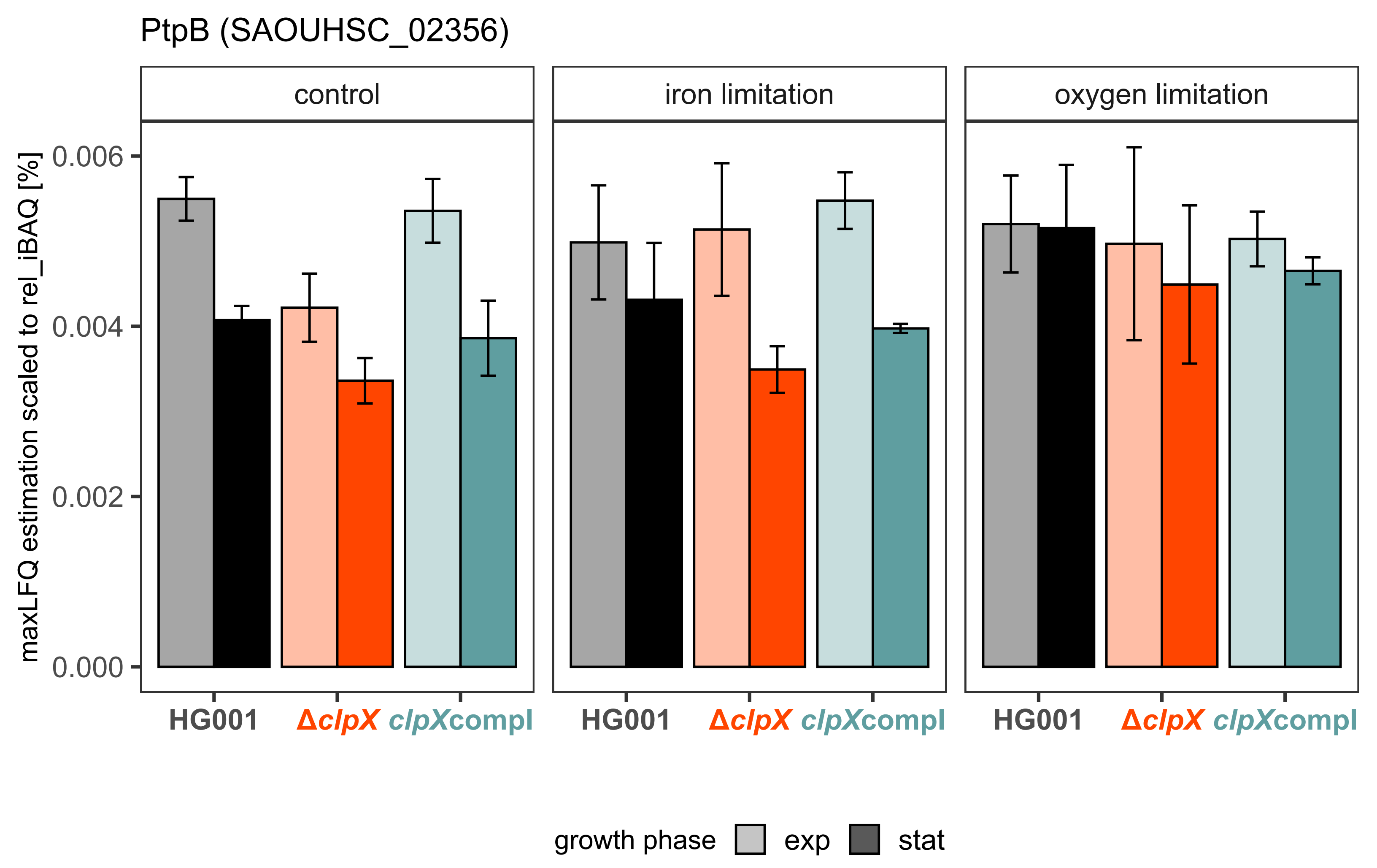

PtpB (SAOUHSC_02356)
control
iron limitation
oxygen limitation
maxLFQ estimation scaled to rel_iBAQ [%]
0.006
0.004
0.002
0.000
HG001
ΔclpX
clpXcompl
growth phase
exp
stat

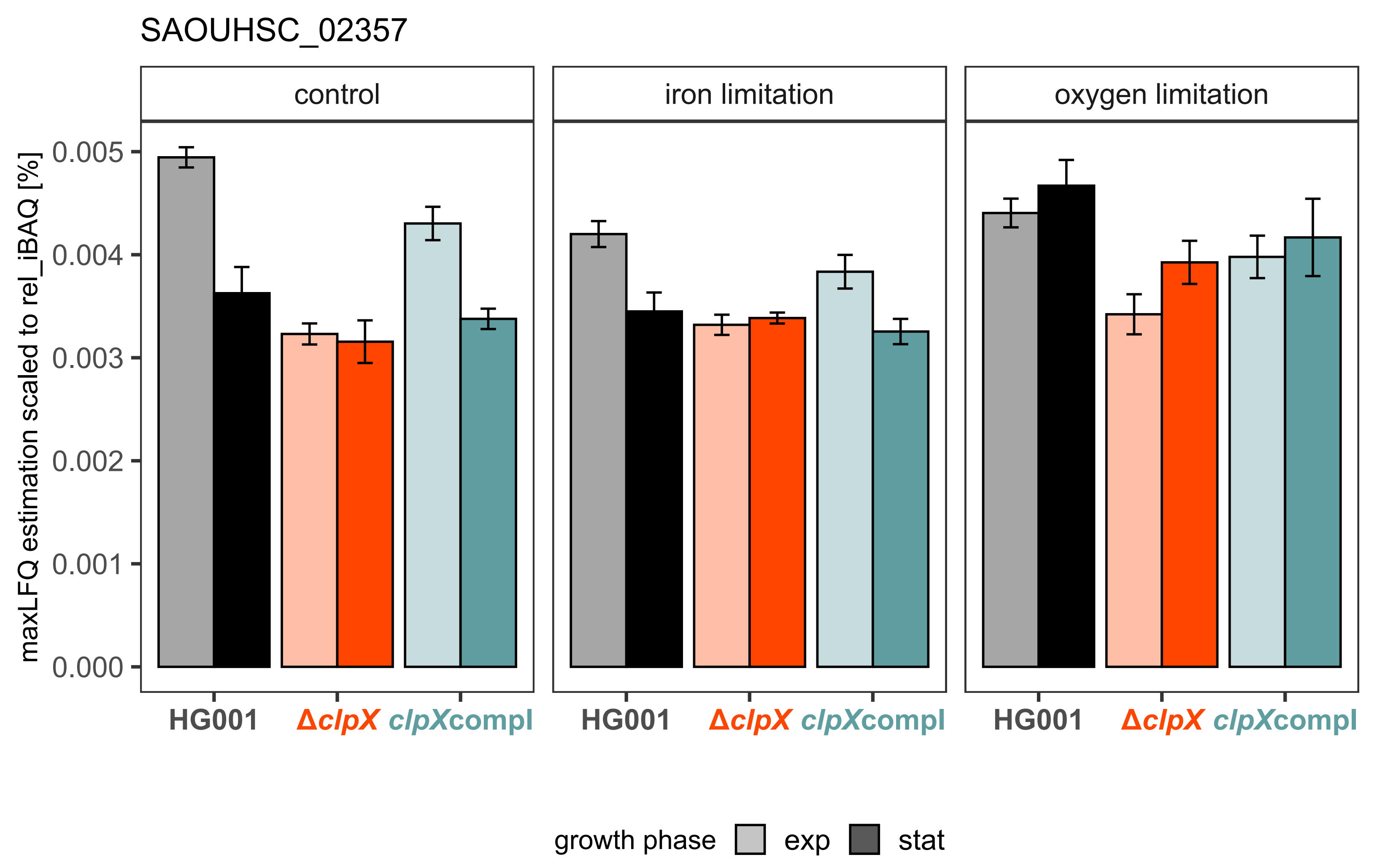
SAOUHSC_02357
control
iron limitation
oxygen limitation
maxLFQ estimation scaled to rel_iBAQ [%]
0.005
0.004
0.003
0.002
0.001
0.000
HG001
ΔclpX
clpXcompl
growth phase
exp
stat

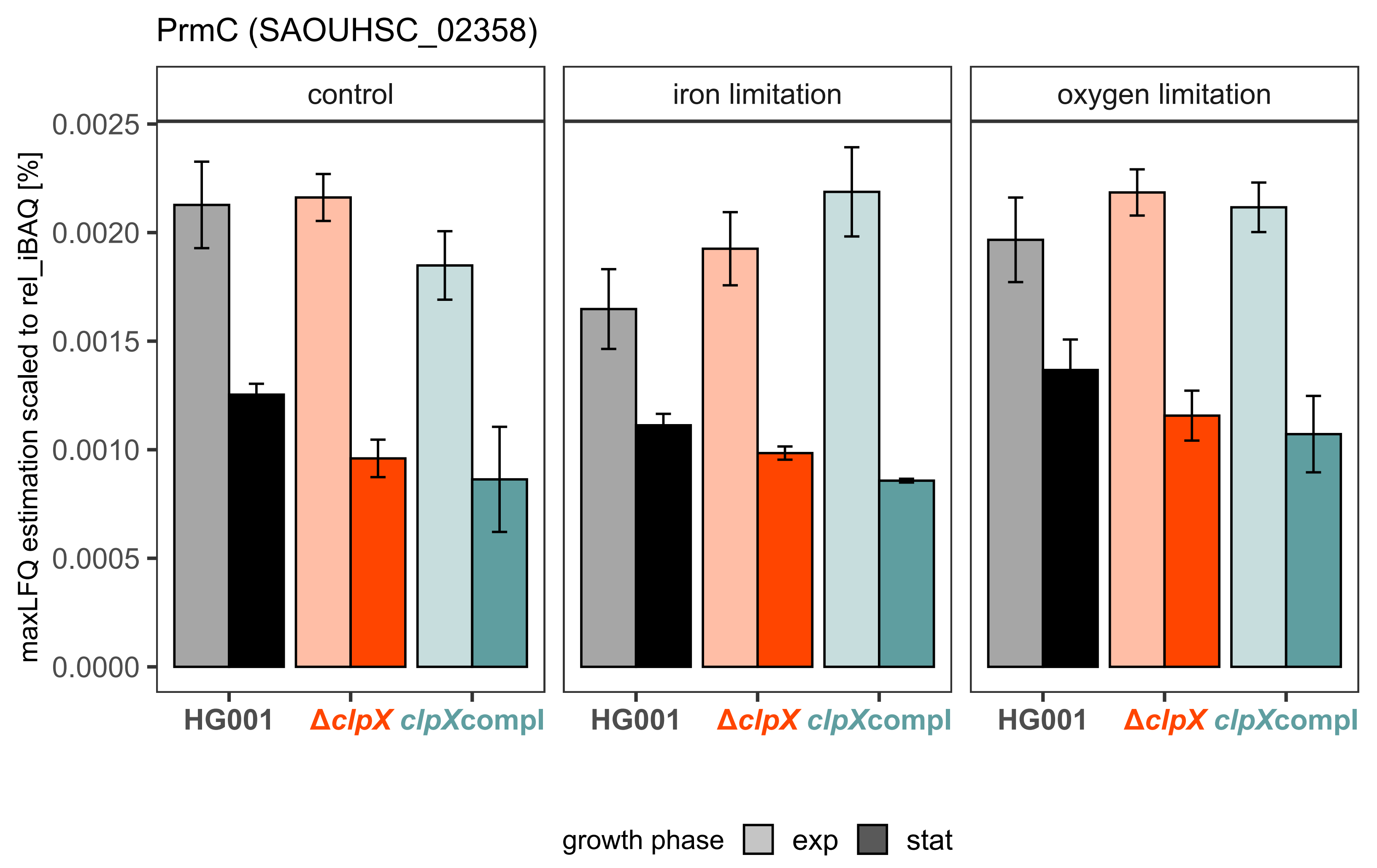
PrmC (SAOUHSC_02358)
control
iron limitation
oxygen limitation
maxLFQ estimation scaled to rel_iBAQ [%]
0.0025
0.0020
0.0015
0.0010
0.0005
0.0000
HG001
ΔclpX
clpXcompl
growth phase
exp
stat

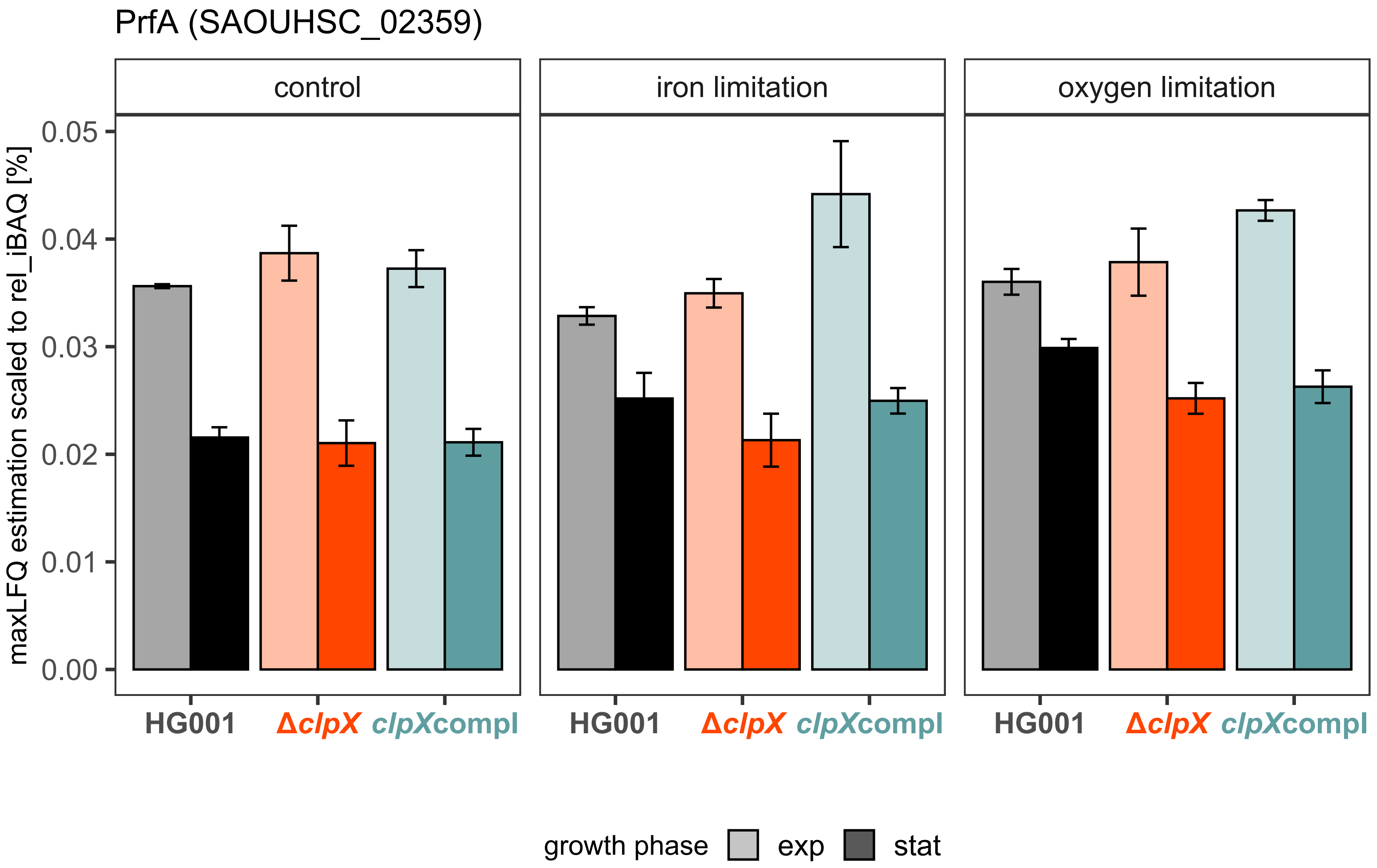
PrfA (SAOUHSC_02359)
control
iron limitation
oxygen limitation
maxLFQ estimation scaled to rel_iBAQ [%]
0.05
0.04
0.03
0.02
0.01
0.00
HG001
ΔclpX
clpXcompl
growth phase
exp
stat

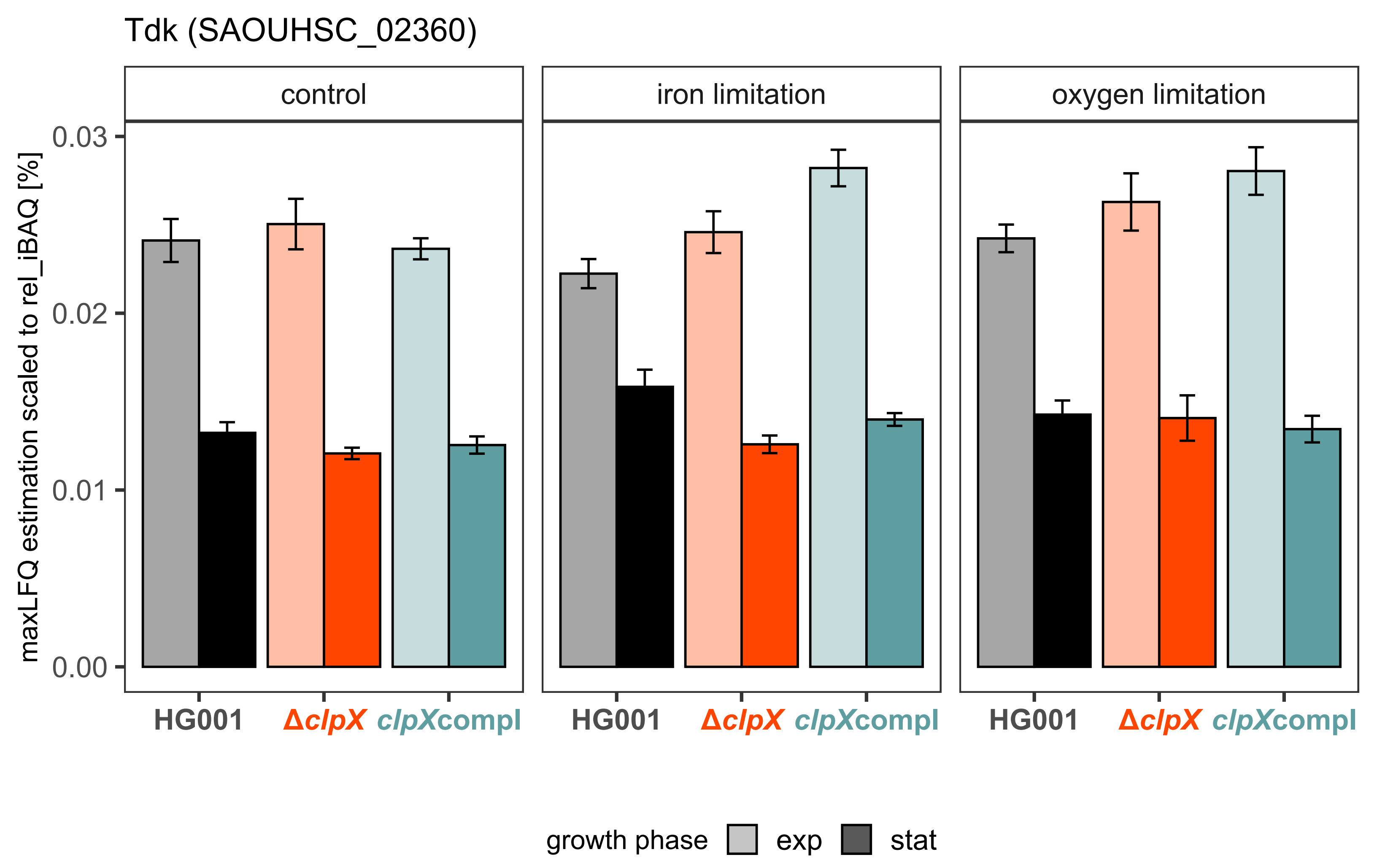

Tdk (SAOUHSC_02360)
control
iron limitation
oxygen limitation
maxLFQ estimation scaled to rel_iBAQ [%]
0.03
0.02
0.01
0.00
HG001
ΔclpX
clpXcompl
growth phase
exp
stat

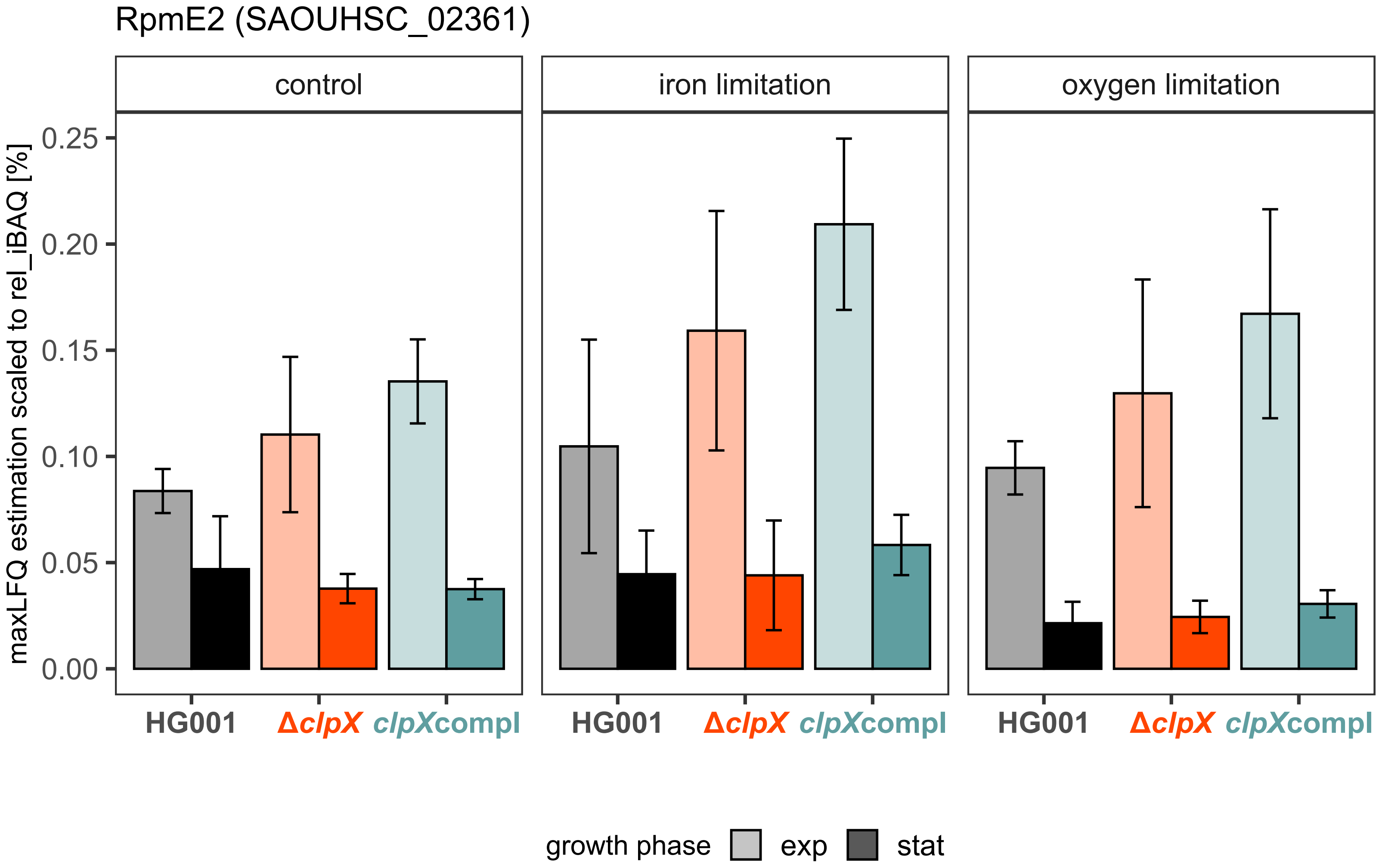
RpmE2 (SAOUHSC_02361)
control
iron limitation
oxygen limitation
maxLFQ estimation scaled to rel_iBAQ [%]
0.25
0.20
0.15
0.10
0.05
0.00
HG001
ΔclpX
clpXcompl
growth phase
exp
stat

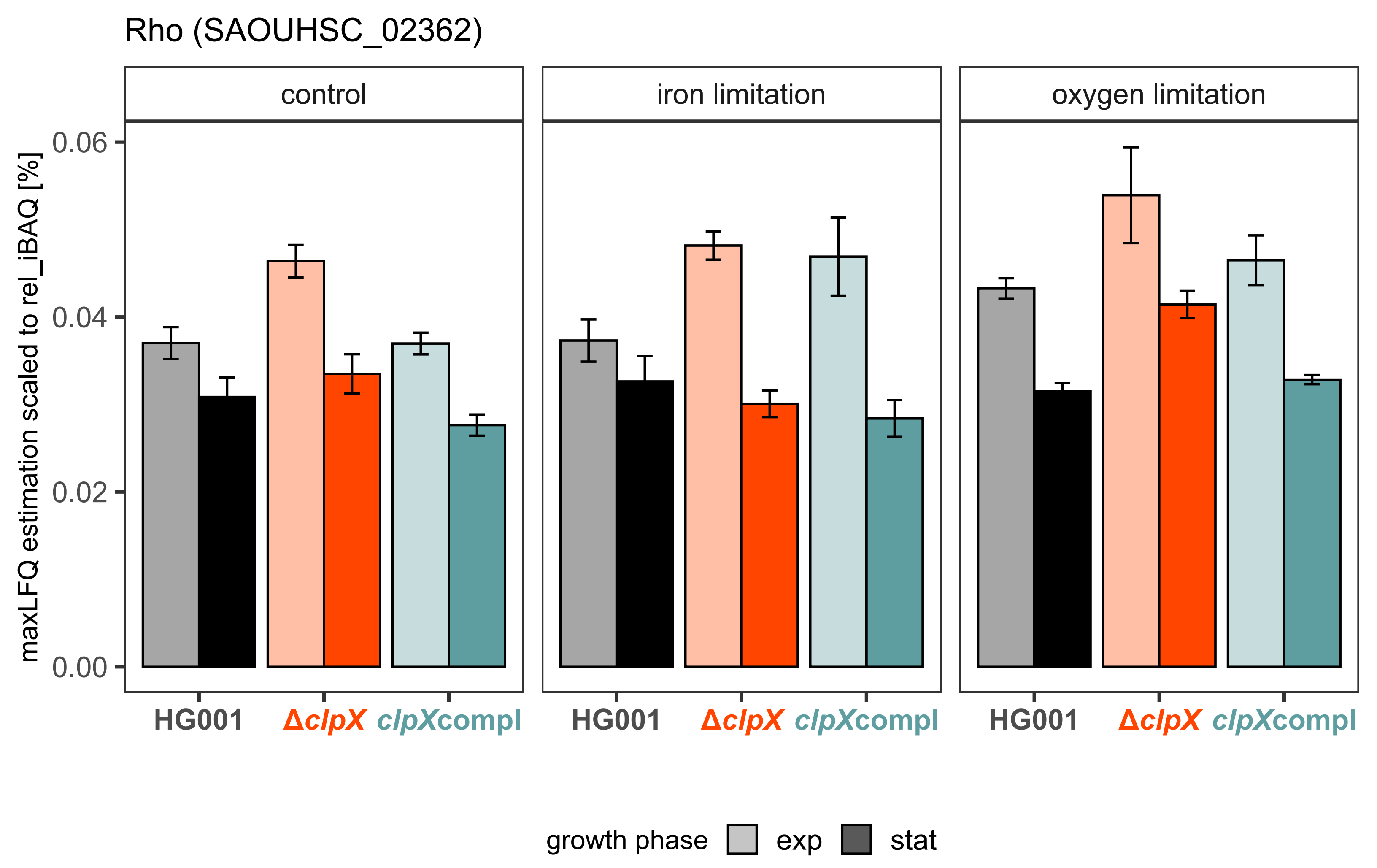

Rho (SAOUHSC_02362)
control
iron limitation
oxygen limitation
maxLFQ estimation scaled to rel_iBAQ [%]
0.06
0.04
0.02
0.00
HG001
ΔclpX
clpXcompl
growth phase
exp
stat

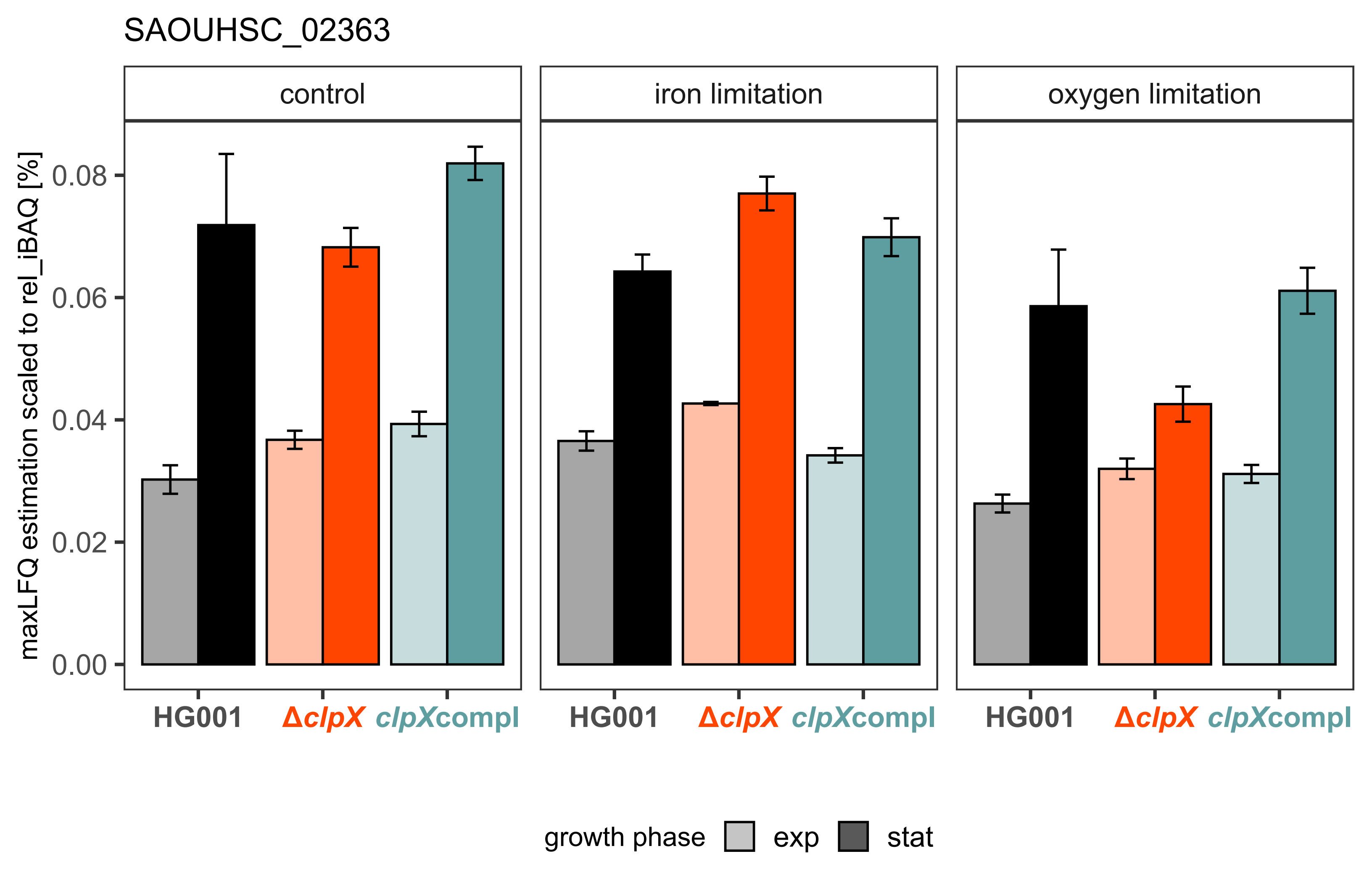

SAOUHSC_02363
control
iron limitation
oxygen limitation
maxLFQ estimation scaled to rel_iBAQ [%]
0.08
0.06
0.04
0.02
0.00
HG001
ΔclpX
clpXcompl
growth phase
exp
stat

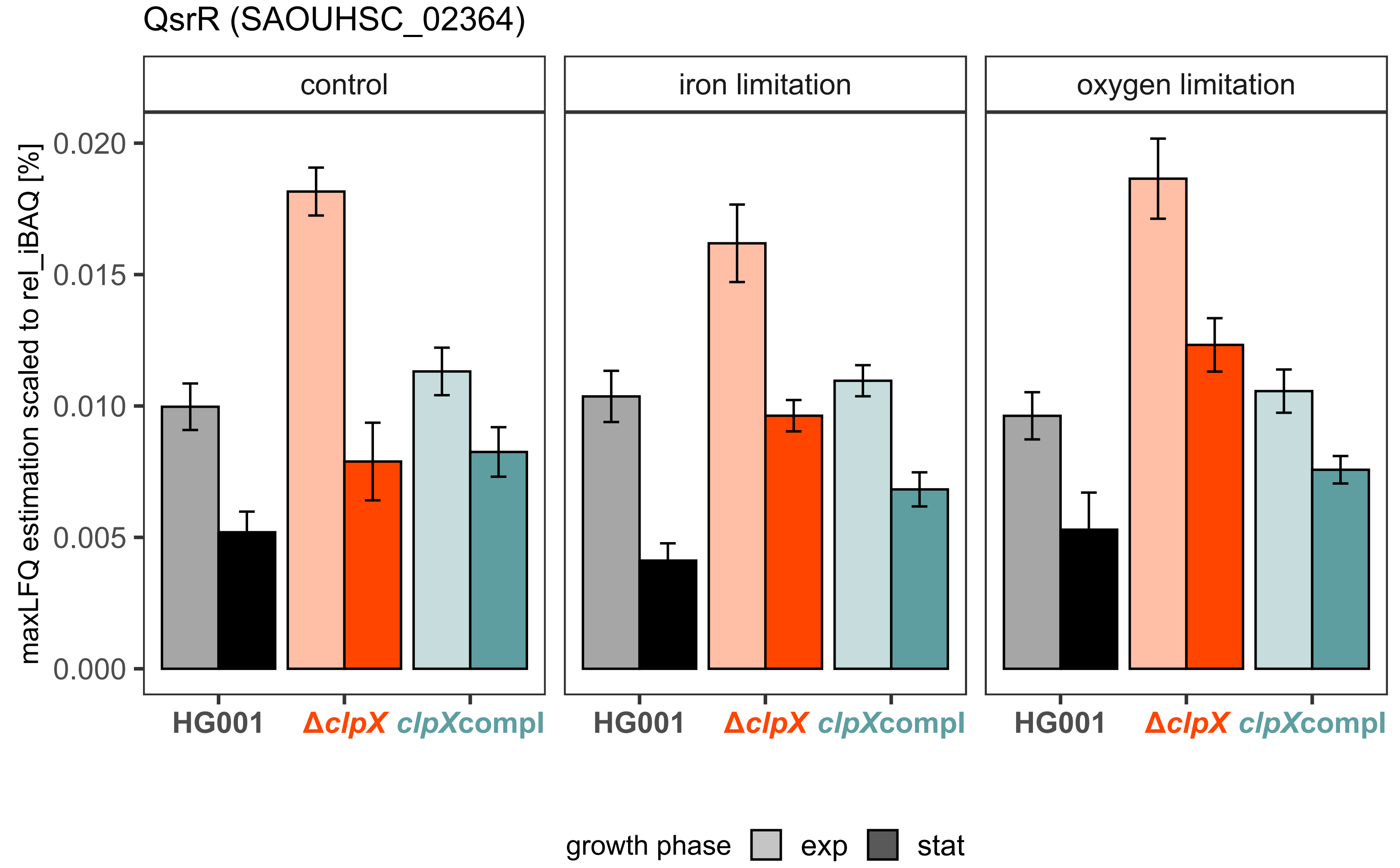

QsrR (SAOUHSC_02364)
control
iron limitation
oxygen limitation
maxLFQ estimation scaled to rel_iBAQ [%]
0.020
0.015
0.010
0.005
0.000
HG001
ΔclpX
clpXcompl
growth phase
exp
stat

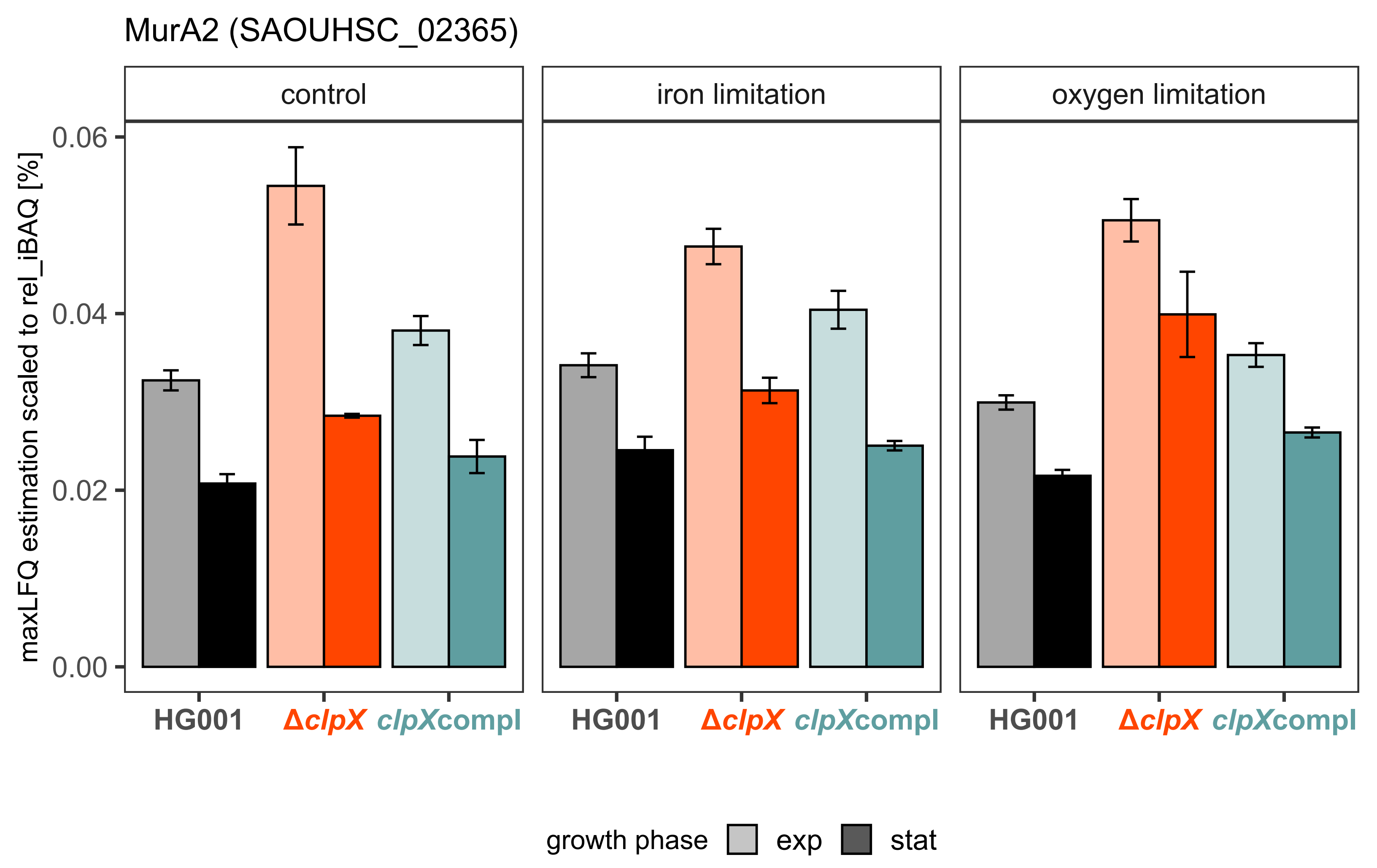
MurA2 (SAOUHSC_02365)
control
iron limitation
oxygen limitation
maxLFQ estimation scaled to rel_iBAQ [%]
0.06
0.04
0.02
0.00
HG001
ΔclpX
clpXcompl
growth phase
exp
stat

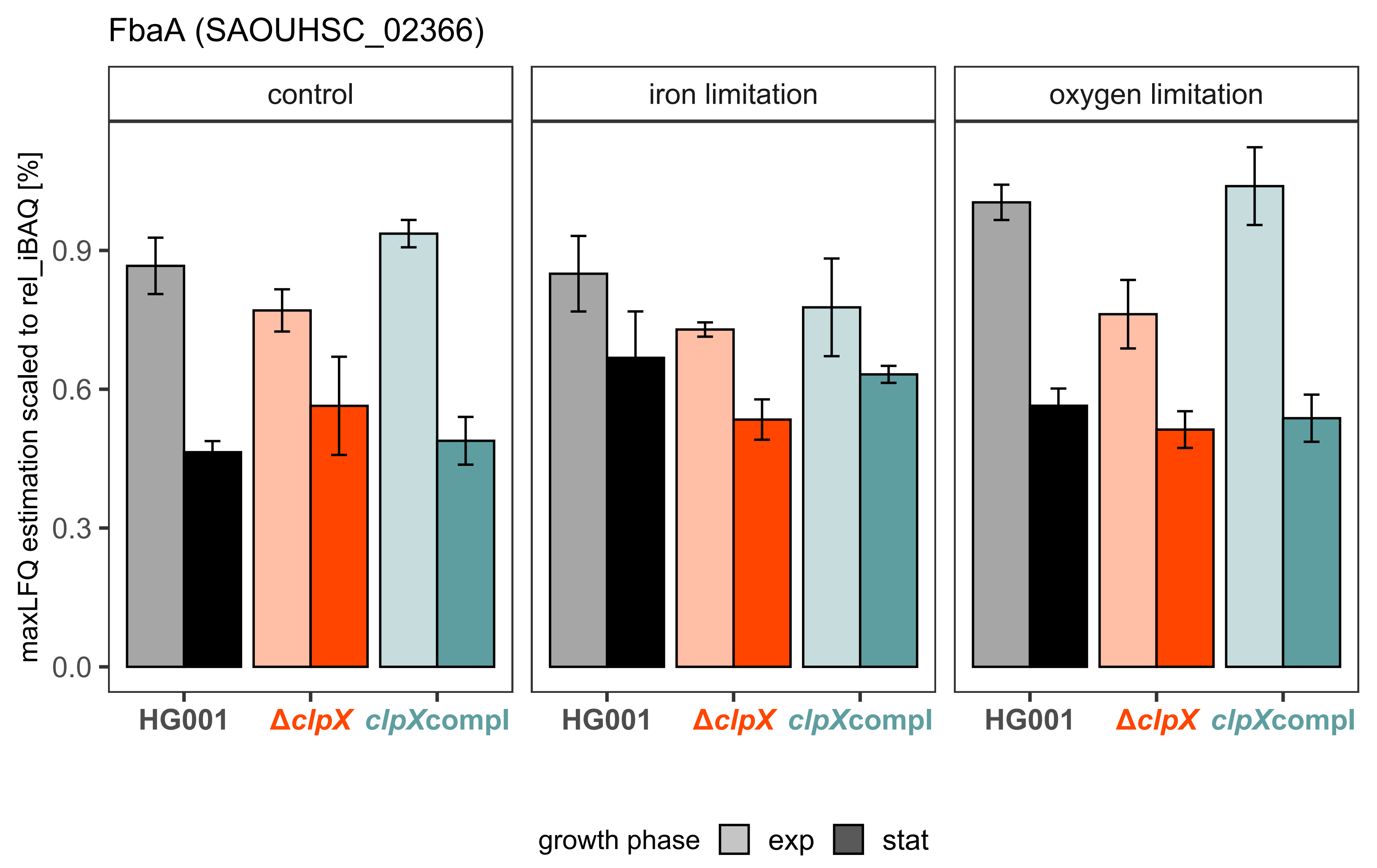

FbaA (SAOUHSC_02366)
control
iron limitation
oxygen limitation
maxLFQ estimation scaled to rel_iBAQ [%]
0.0
0.3
0.6
0.9
HG001
ΔclpX
clpXcompl
growth phase
exp
stat

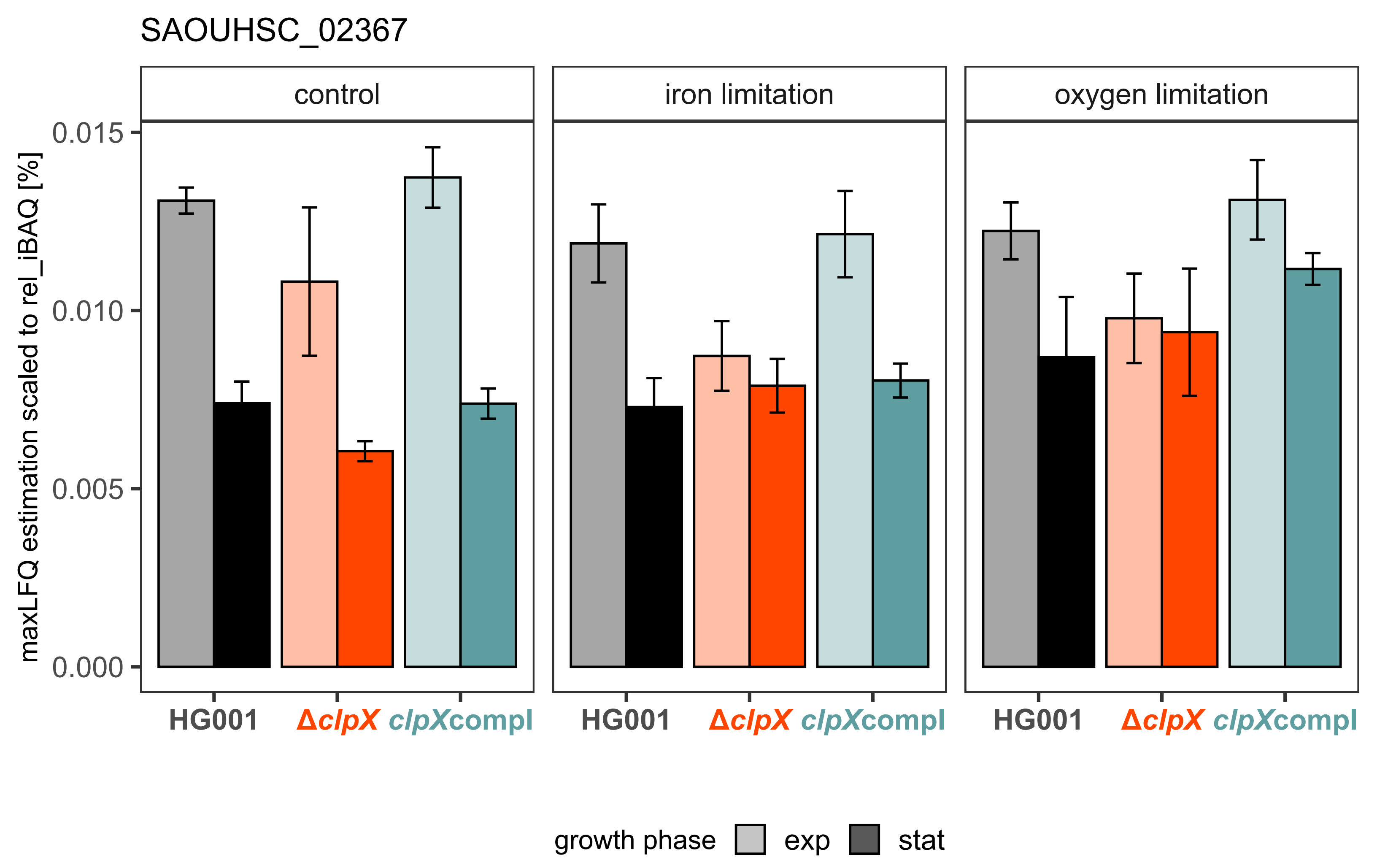

SAOUHSC_02367
control
iron limitation
oxygen limitation
maxLFQ estimation scaled to rel_iBAQ [%]
0.015
0.010
0.005
0.000
HG001
ΔclpX
clpXcompl
growth phase
exp
stat

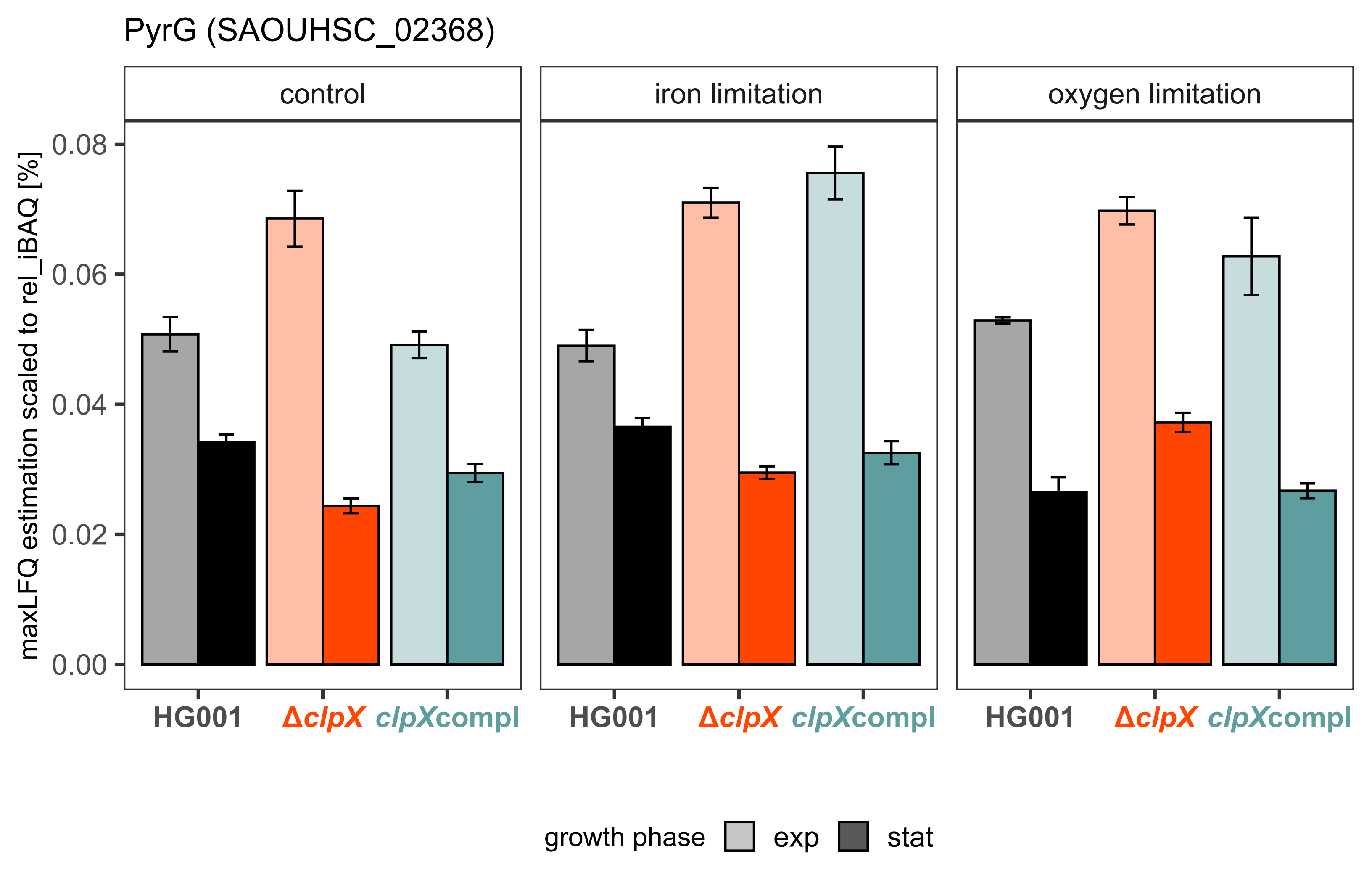
PyrG (SAOUHSC_02368)
control
iron limitation
oxygen limitation
maxLFQ estimation scaled to rel_iBAQ [%]
0.08
0.06
0.04
0.02
0.00
HG001
ΔclpX
clpXcompl
growth phase
exp
stat

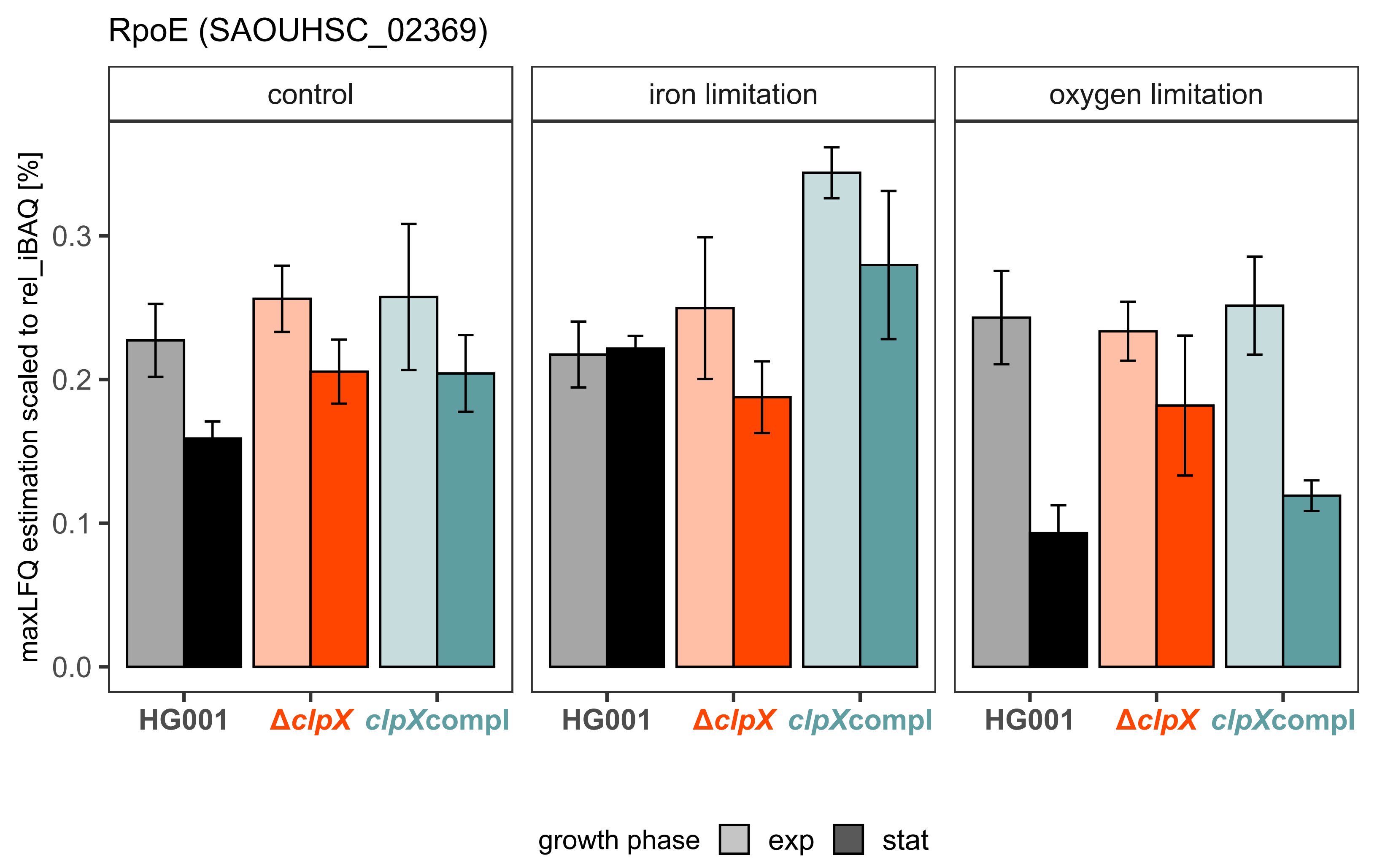

RpoE (SAOUHSC_02369)
control
iron limitation
oxygen limitation
maxLFQ estimation scaled to rel_iBAQ [%]
0.0
0.1
0.2
0.3
HG001
ΔclpX
clpXcompl
growth phase
exp
stat

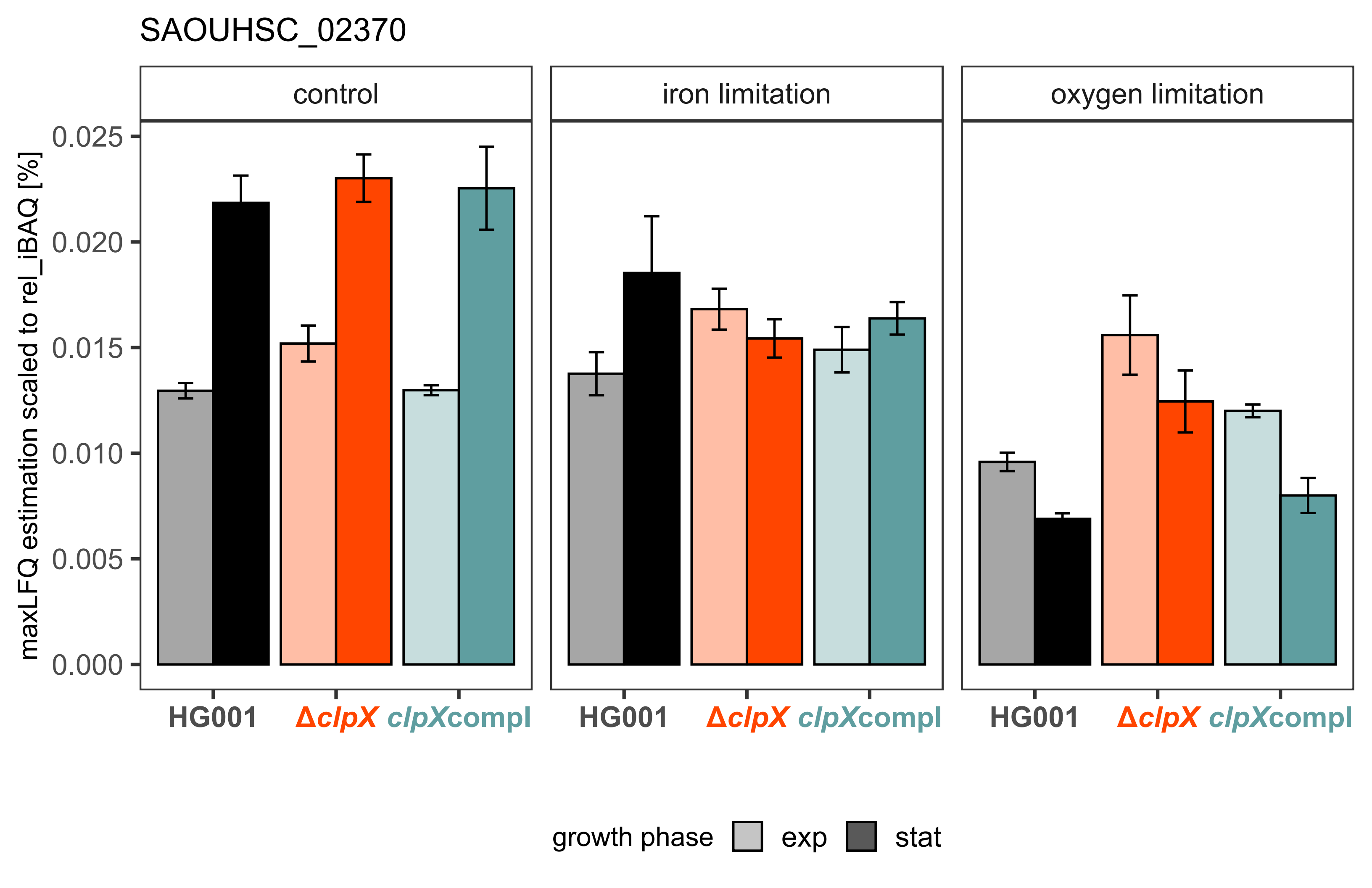
SAOUHSC_02370
control
iron limitation
oxygen limitation
maxLFQ estimation scaled to rel_iBAQ [%]
0.025
0.020
0.015
0.010
0.005
0.000
HG001
ΔclpX
clpXcompl
growth phase
exp
stat

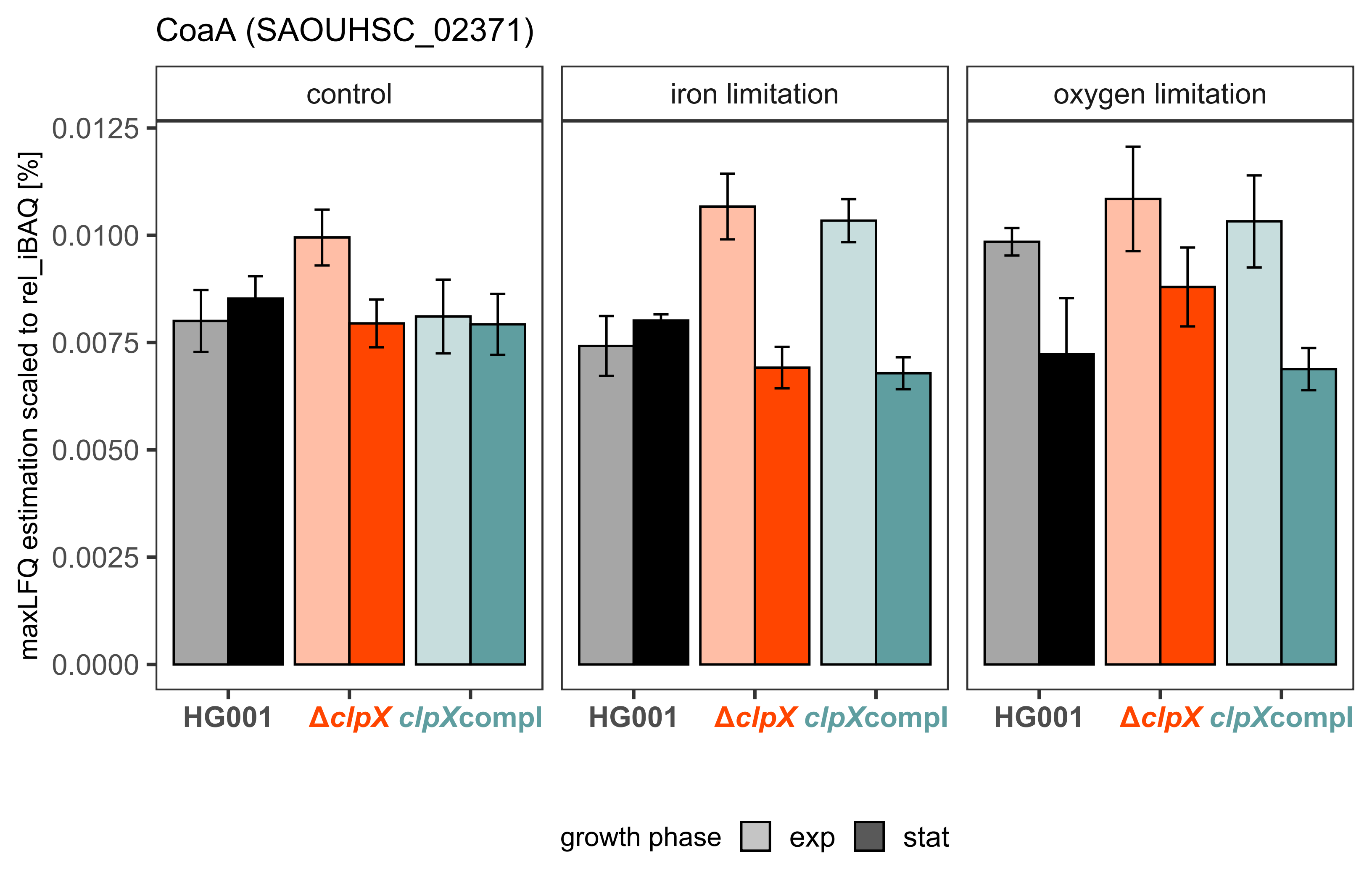

CoaA (SAOUHSC_02371)
control
iron limitation
oxygen limitation
maxLFQ estimation scaled to rel_iBAQ [%]
0.0125
0.0100
0.0075
0.0050
0.0025
0.0000
HG001
ΔclpX
clpXcompl
growth phase
exp
stat

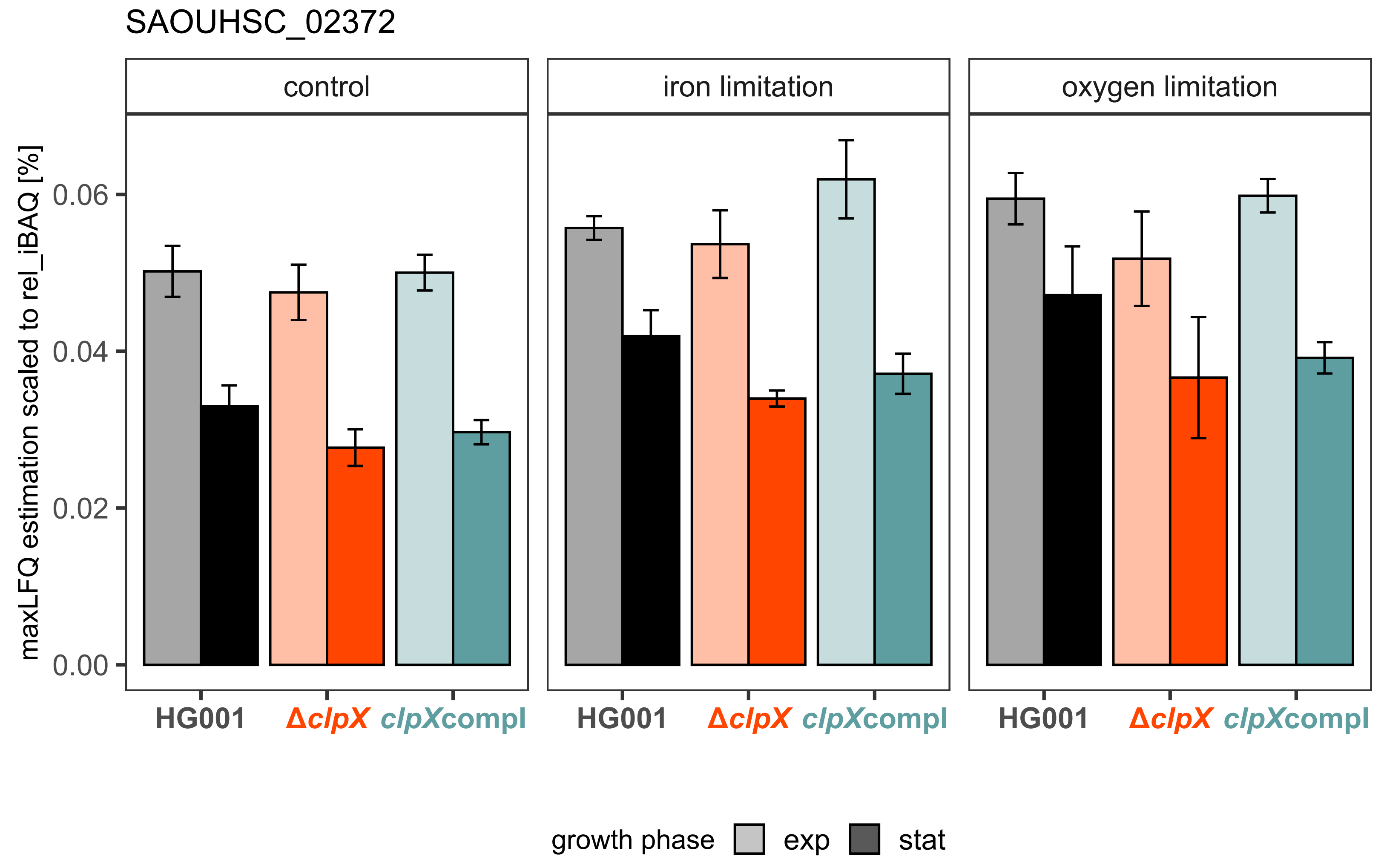
SAOUHSC_02372
control
iron limitation
oxygen limitation
maxLFQ estimation scaled to rel_iBAQ [%]
0.06
0.04
0.02
0.00
HG001
ΔclpX
clpXcompl
growth phase
exp
stat

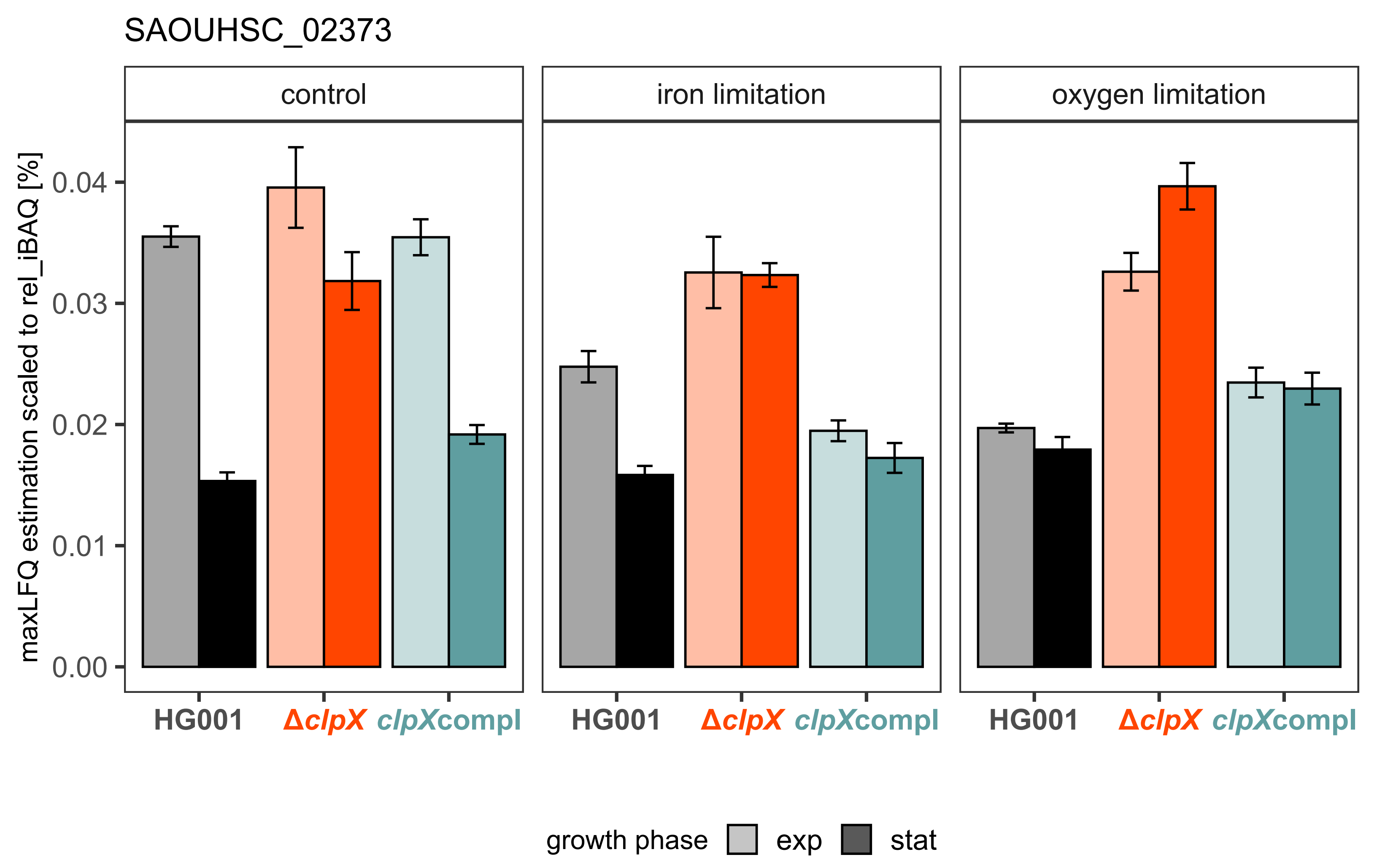
SAOUHSC_02373
control
iron limitation
oxygen limitation
maxLFQ estimation scaled to rel_iBAQ [%]
0.00
0.01
0.02
0.03
0.04
HG001
ΔclpX
clpXcompl
growth phase
exp
stat

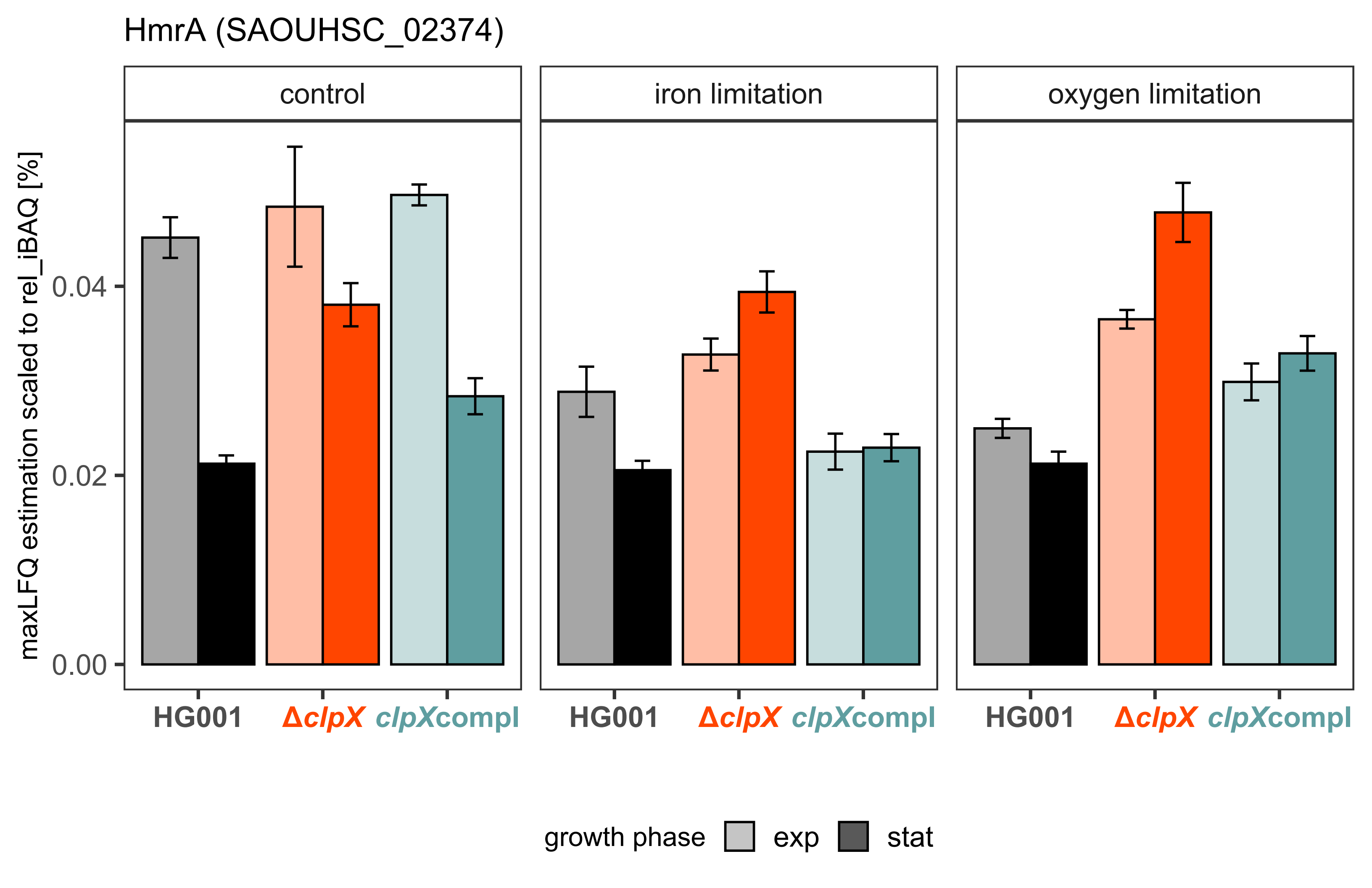

HmrA (SAOUHSC_02374)
control
iron limitation
oxygen limitation
maxLFQ estimation scaled to rel_iBAQ [%]
0.00
0.02
0.04
HG001
ΔclpX
clpXcompl
growth phase
exp
stat

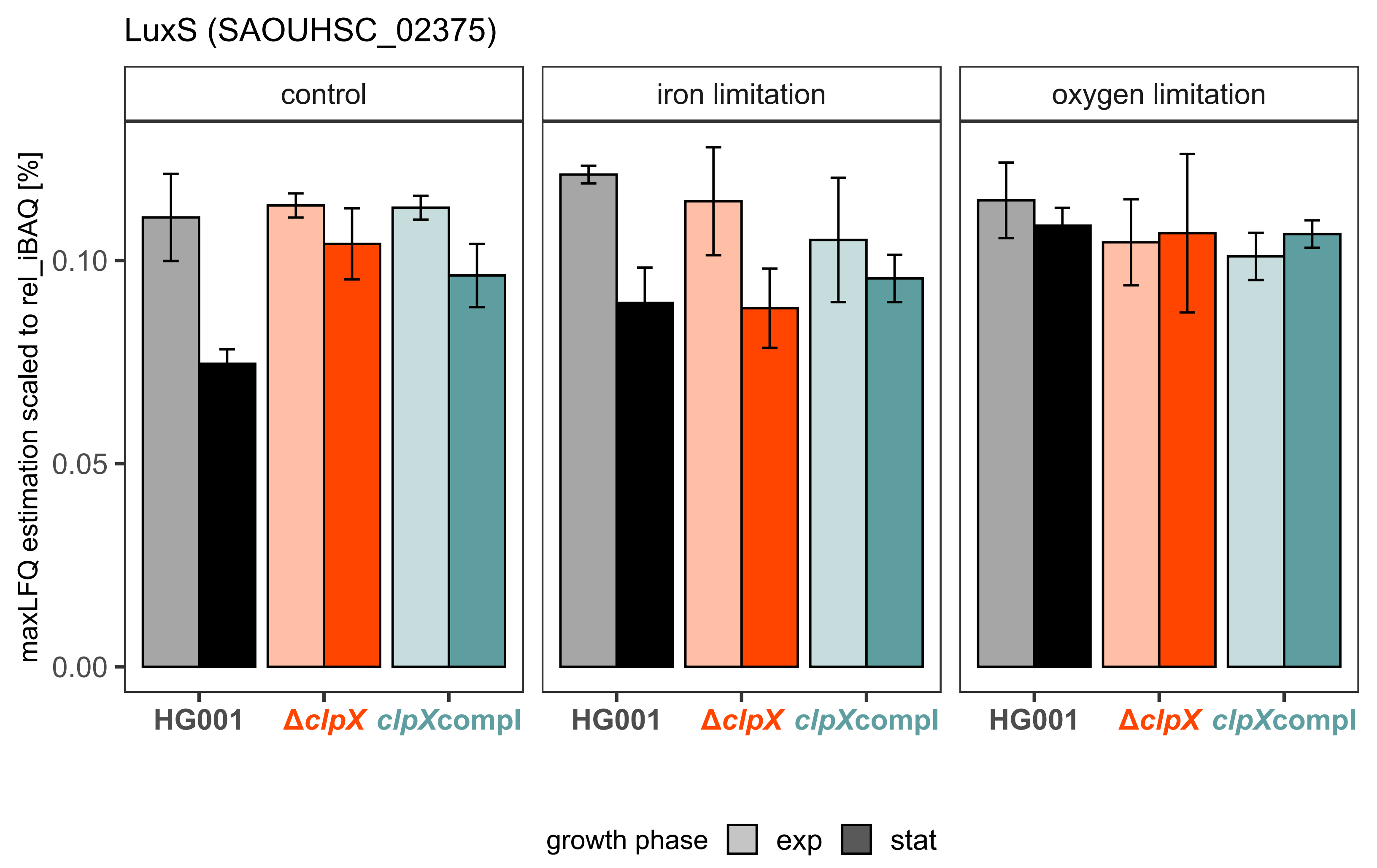
LuxS (SAOUHSC_02375)
control
iron limitation
oxygen limitation
maxLFQ estimation scaled to rel_iBAQ [%]
0.10
0.05
0.00
HG001
ΔclpX
clpXcompl
growth phase
exp
stat

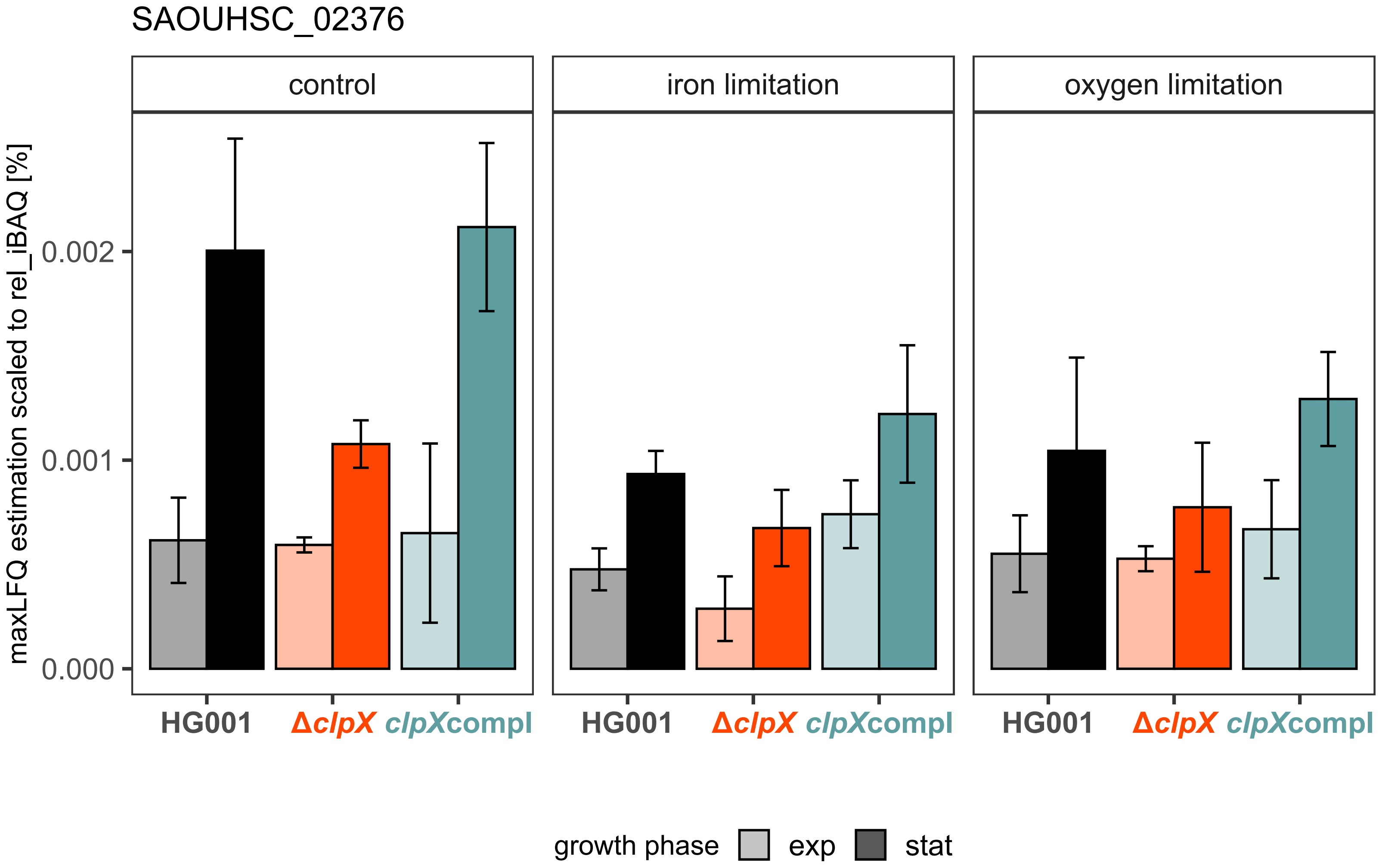

SAOUHSC_02376
control
iron limitation
oxygen limitation
maxLFQ estimation scaled to rel_iBAQ [%]
0.002
0.001
0.000
HG001
ΔclpX
clpXcompl
growth phase
exp
stat

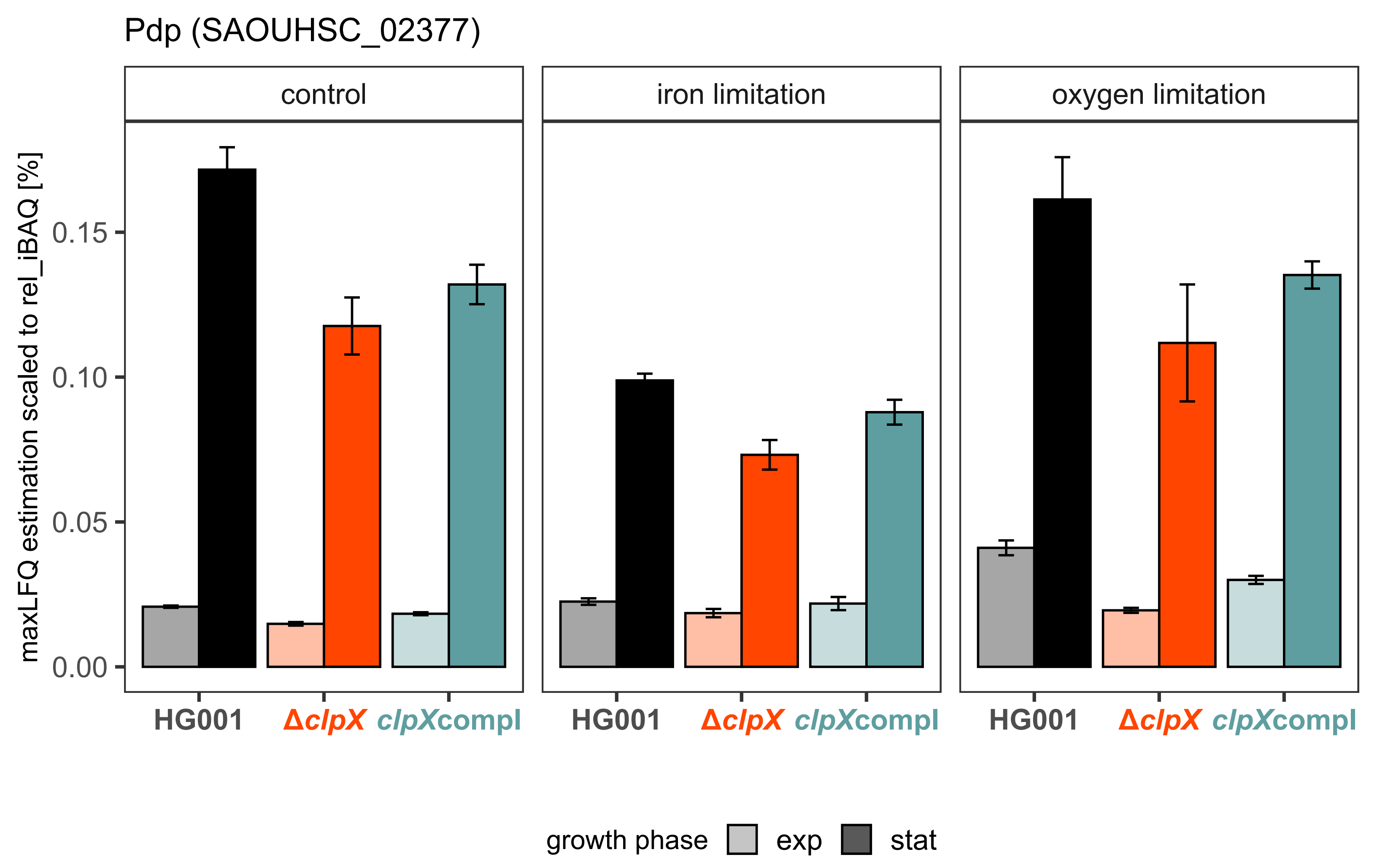
Pdp (SAOUHSC_02377)
control
iron limitation
oxygen limitation
maxLFQ estimation scaled to rel_iBAQ [%]
0.15
0.10
0.05
0.00
HG001
ΔclpX
clpXcompl
growth phase
exp
stat

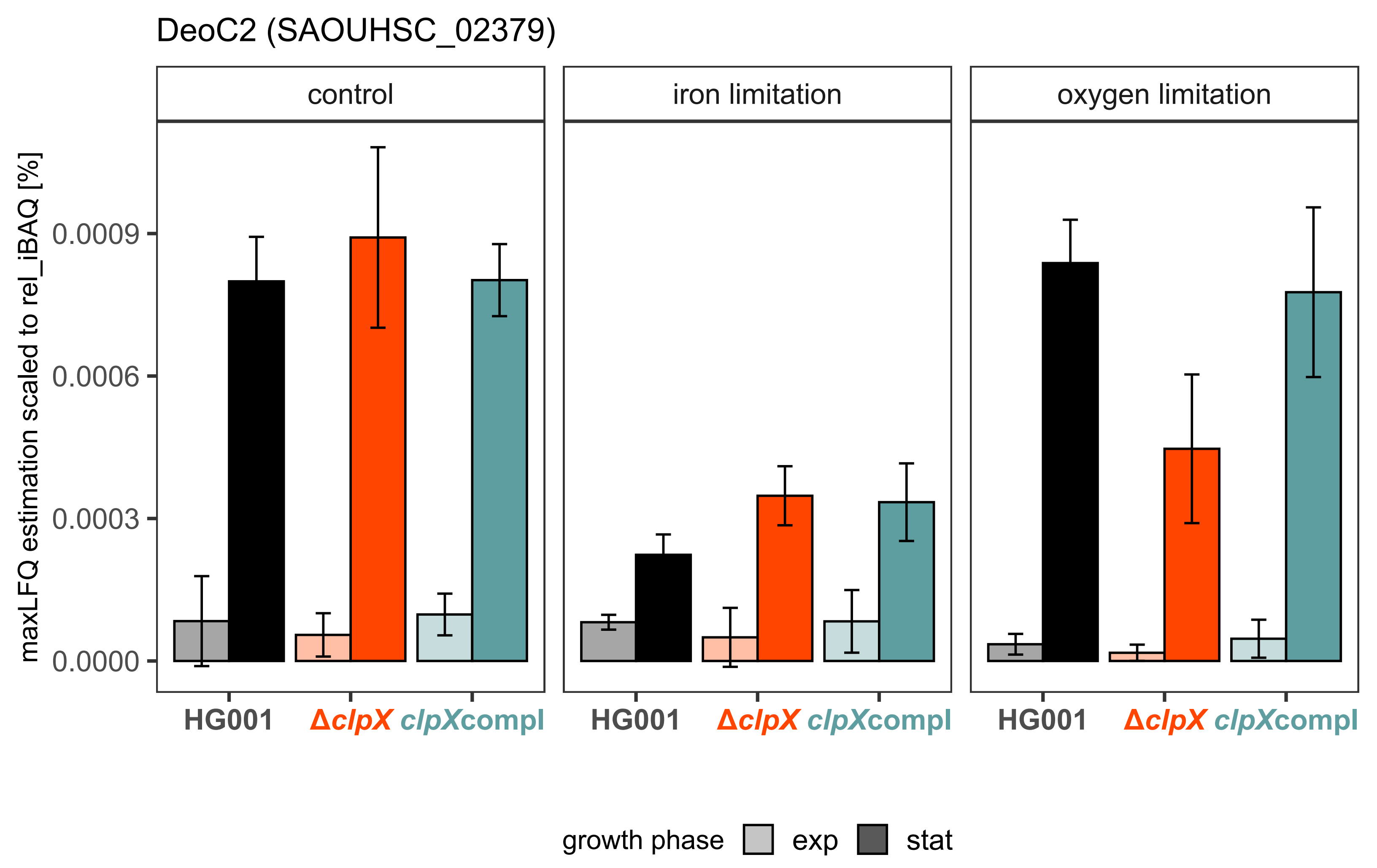

DeoC2 (SAOUHSC_02379)
control
iron limitation
oxygen limitation
maxLFQ estimation scaled to rel_iBAQ [%]
0.0009
0.0006
0.0003
0.0000
HG001
ΔclpX
clpXcompl
growth phase
exp
stat

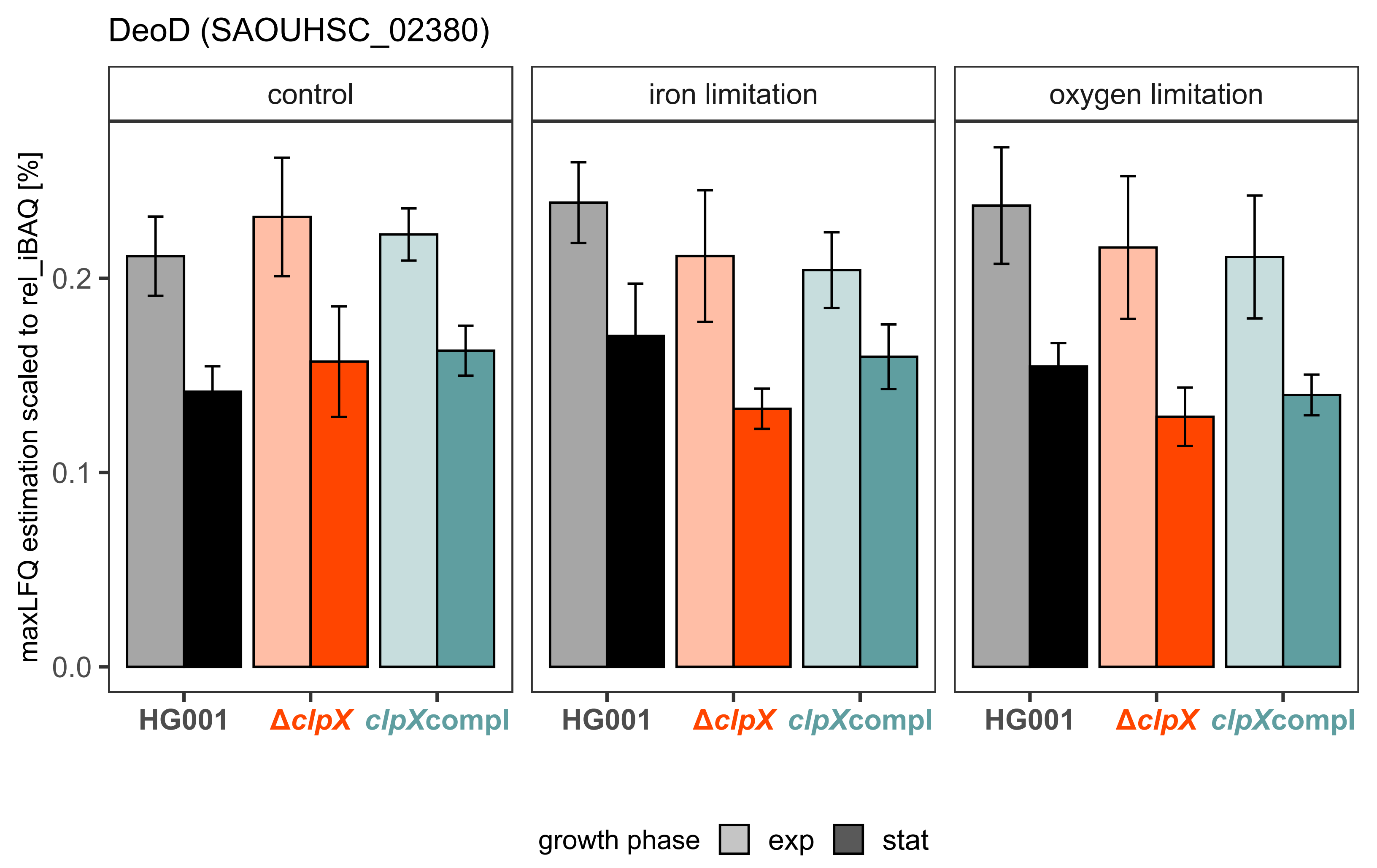

DeoD (SAOUHSC_02380)
control
iron limitation
oxygen limitation
maxLFQ estimation scaled to rel_iBAQ [%]
0.0
0.1
0.2
HG001
ΔclpX
clpXcompl
growth phase
exp
stat

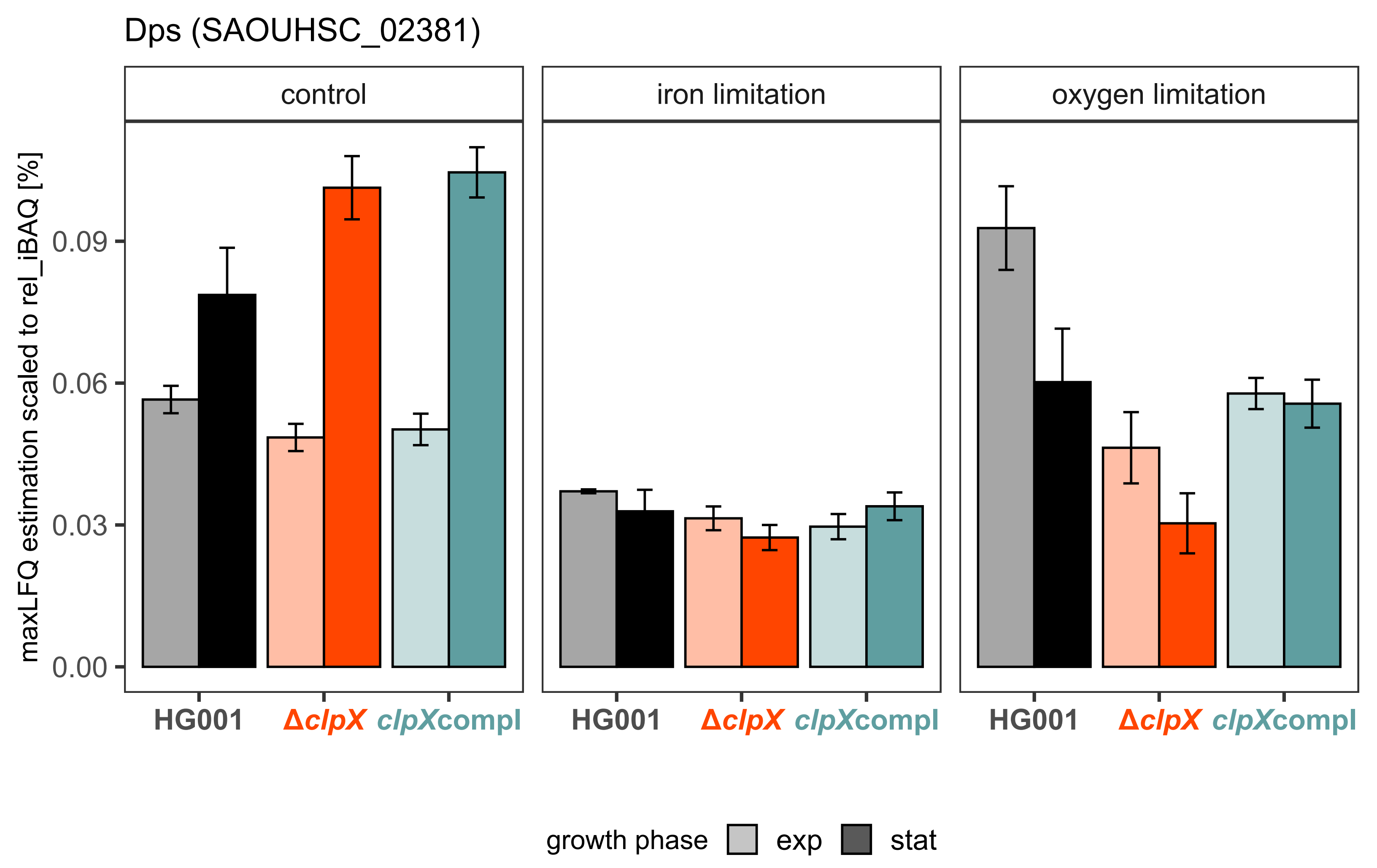
Dps (SAOUHSC_02381)
control
iron limitation
oxygen limitation
maxLFQ estimation scaled to rel_iBAQ [%]
0.09
0.06
0.03
0.00
HG001
ΔclpX
clpXcompl
growth phase
exp
stat

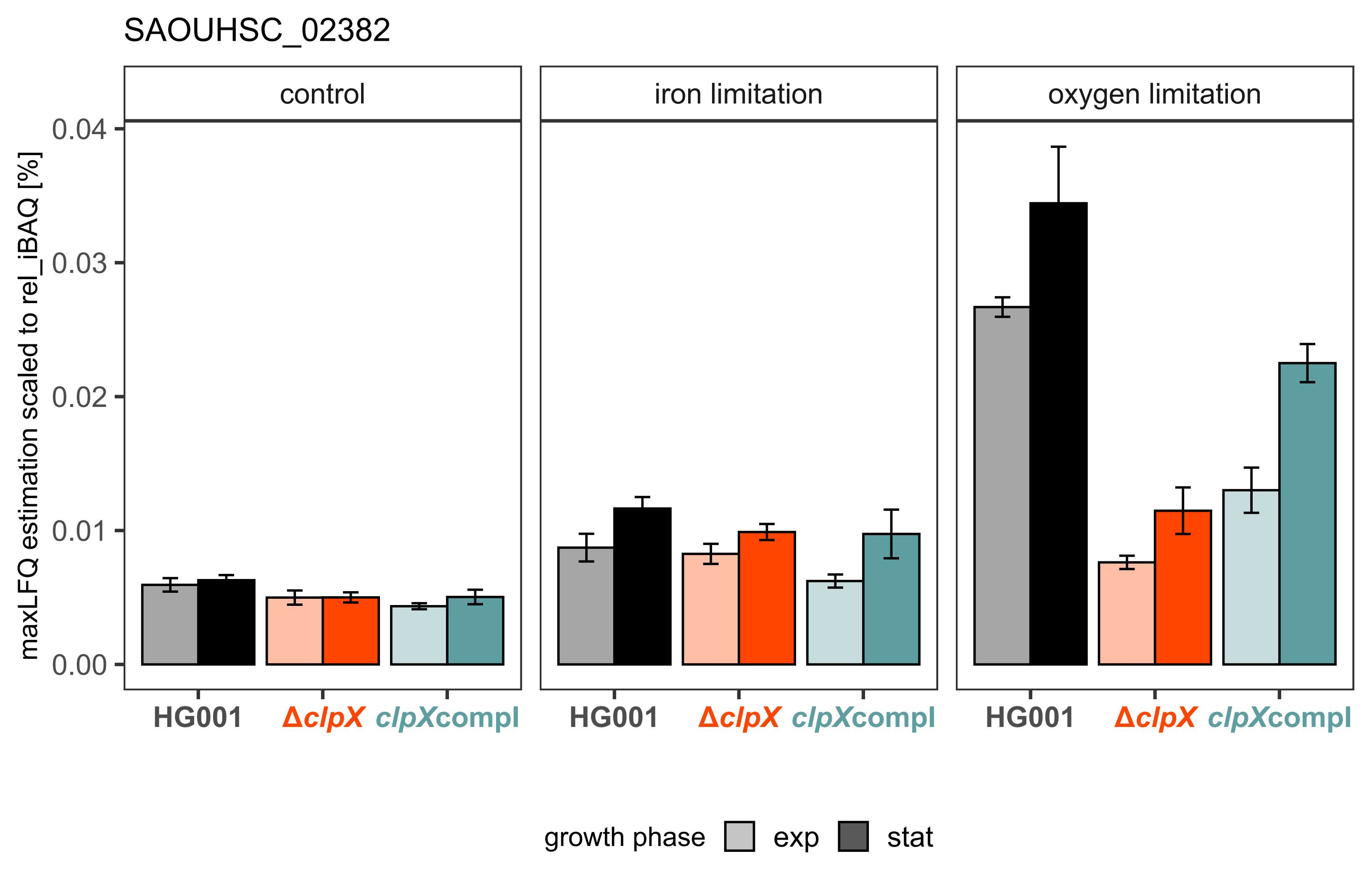

SAOUHSC_02382
control
iron limitation
oxygen limitation
0.04
0.03
0.02
0.01
0.00
maxLFQ estimation scaled to rel_iBAQ [%]
HG001
ΔclpX
clpXcompl
growth phase
exp
stat

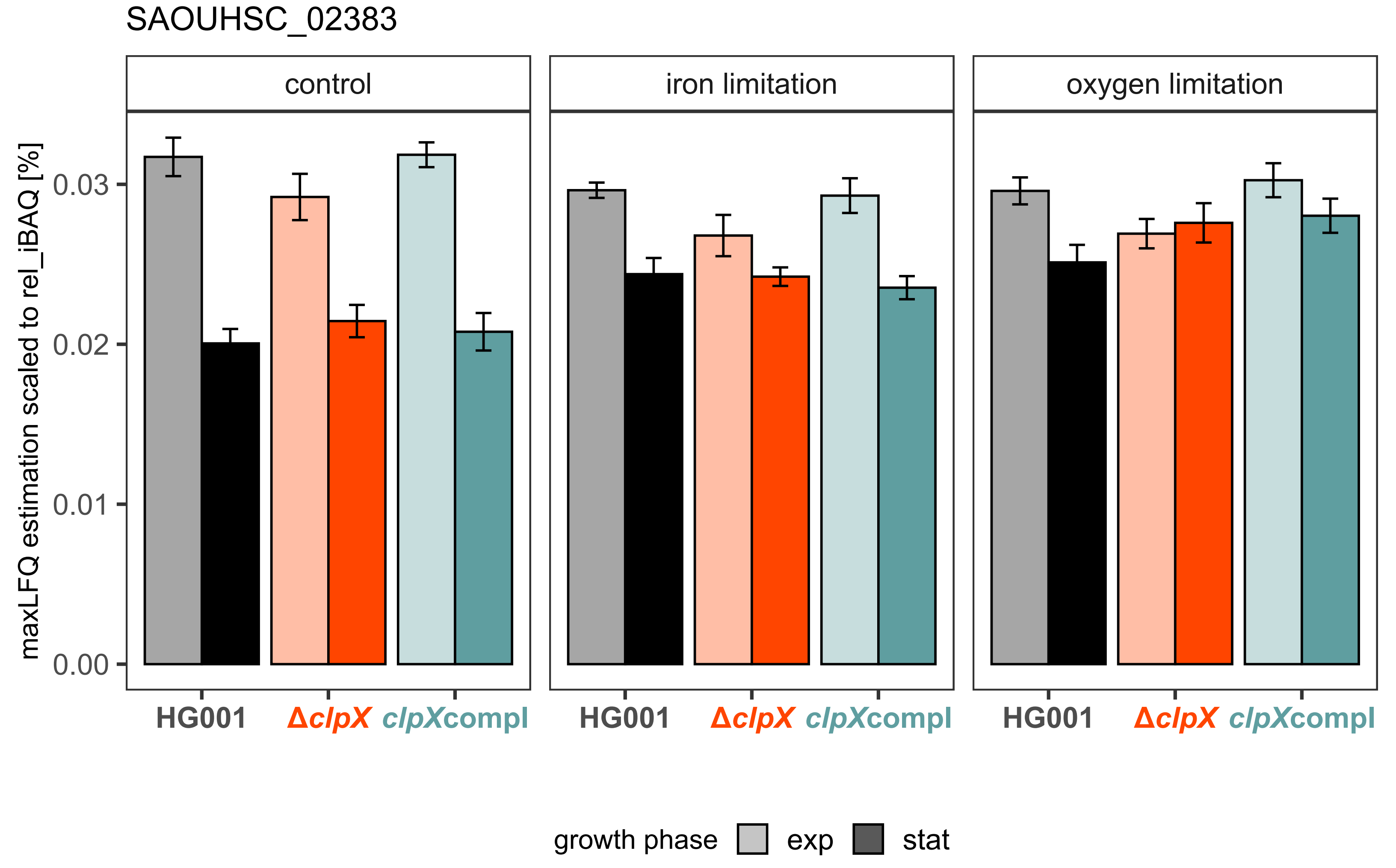

SAOUHSC_02383
control
iron limitation
oxygen limitation
maxLFQ estimation scaled to rel_iBAQ [%]
0.03
0.02
0.01
0.00
HG001
ΔclpX
clpXcompl
growth phase
exp
stat

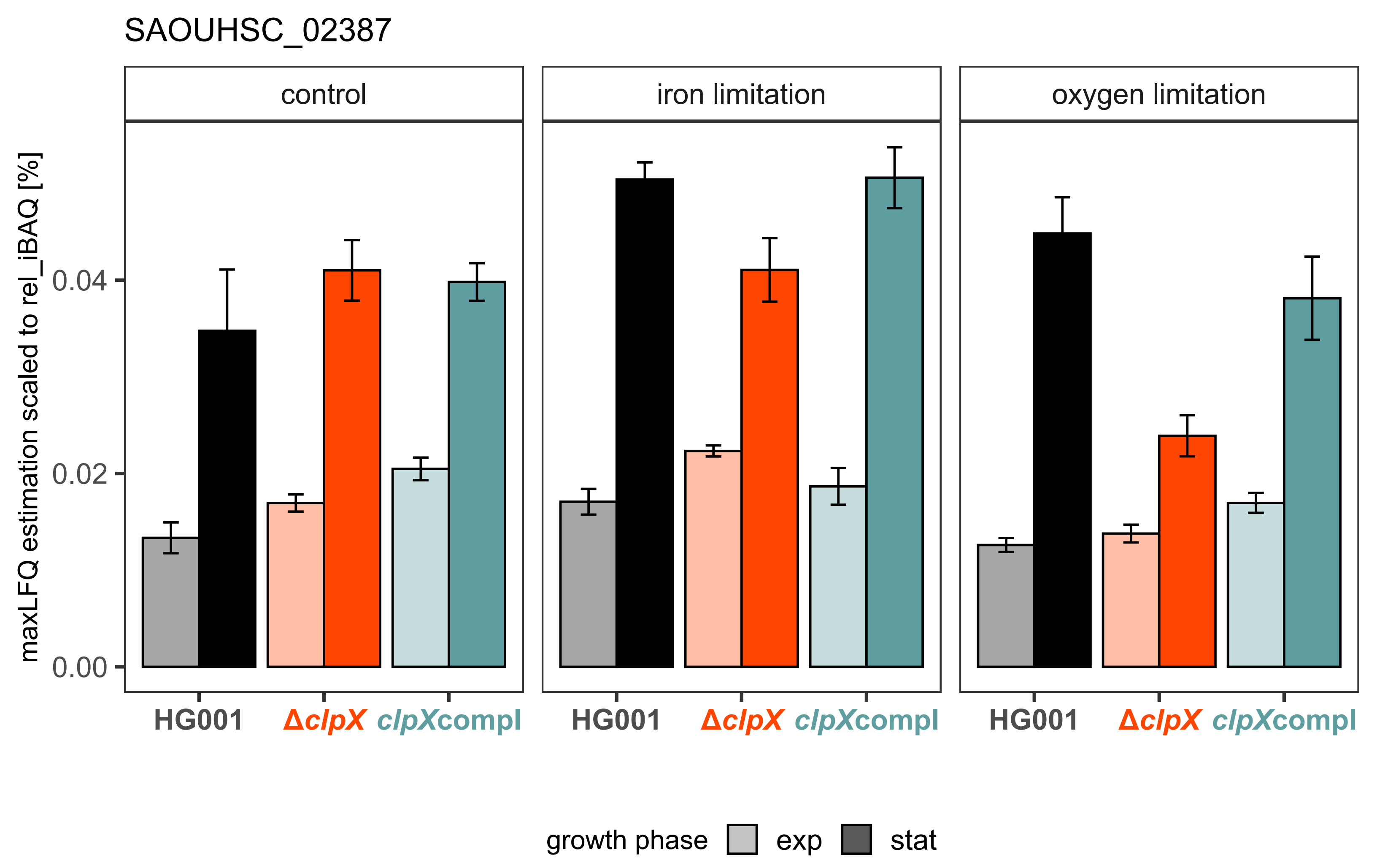
SAOUHSC_02387
control
iron limitation
oxygen limitation
maxLFQ estimation scaled to rel_iBAQ [%]
0.00
0.02
0.04
HG001
ΔclpX
clpXcompl
growth phase
exp
stat

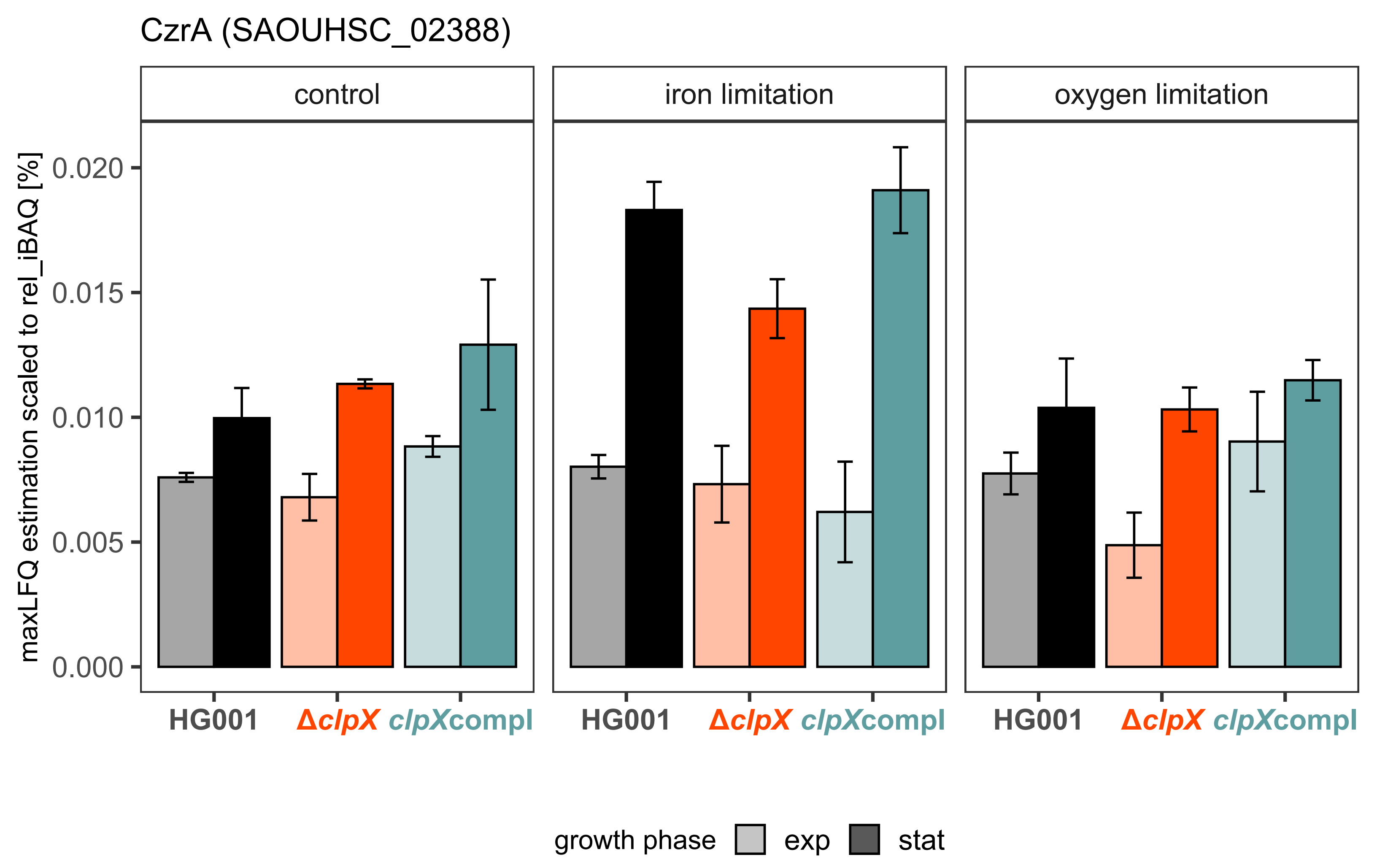

CzrA (SAOUHSC_02388)
control
iron limitation
oxygen limitation
maxLFQ estimation scaled to rel_iBAQ [%]
0.020
0.015
0.010
0.005
0.000
HG001
ΔclpX
clpXcompl
growth phase
exp
stat

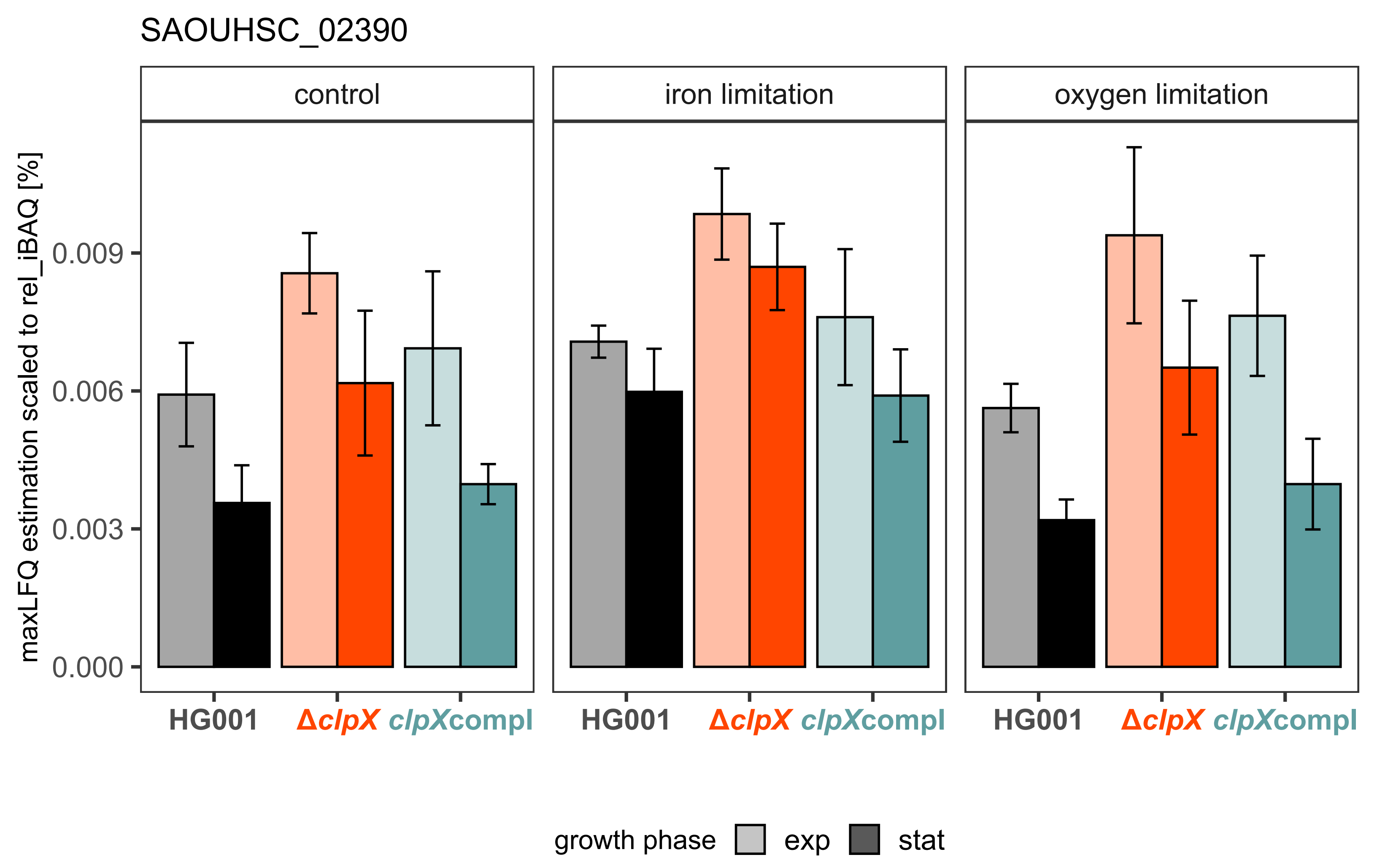

SAOUHSC_02390
control
iron limitation
oxygen limitation
maxLFQ estimation scaled to rel_iBAQ [%]
0.009
0.006
0.003
0.000
HG001
ΔclpX
clpXcompl
growth phase
exp
stat

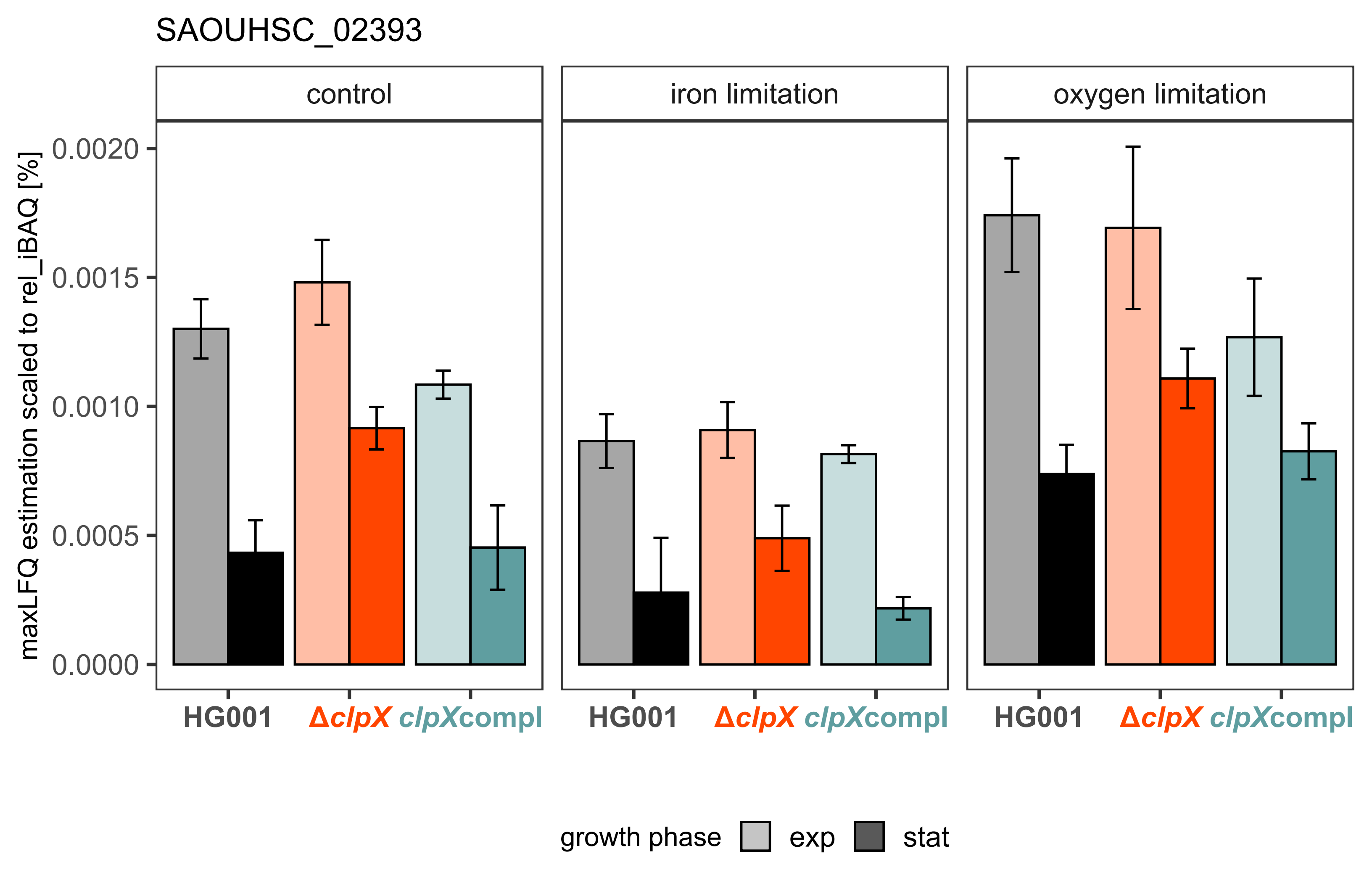
SAOUHSC_02393
control
iron limitation
oxygen limitation
maxLFQ estimation scaled to rel_iBAQ [%]
0.0020
0.0015
0.0010
0.0005
0.0000
HG001
ΔclpX
clpXcompl
growth phase
exp
stat

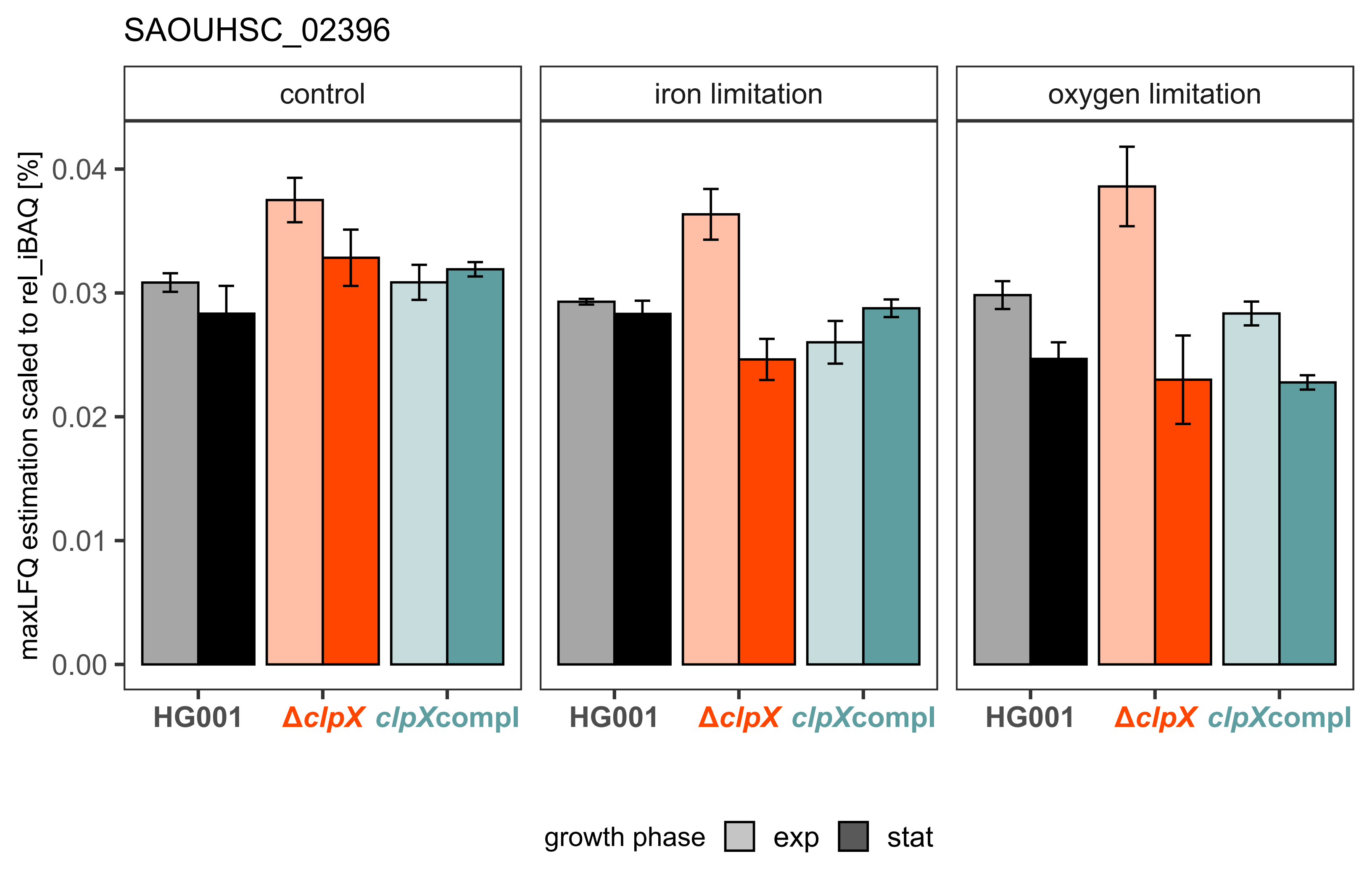
SAOUHSC_02396
control
iron limitation
oxygen limitation
maxLFQ estimation scaled to rel_iBAQ [%]
0.00
0.01
0.02
0.03
0.04
HG001
ΔclpX
clpXcompl
growth phase
exp
stat

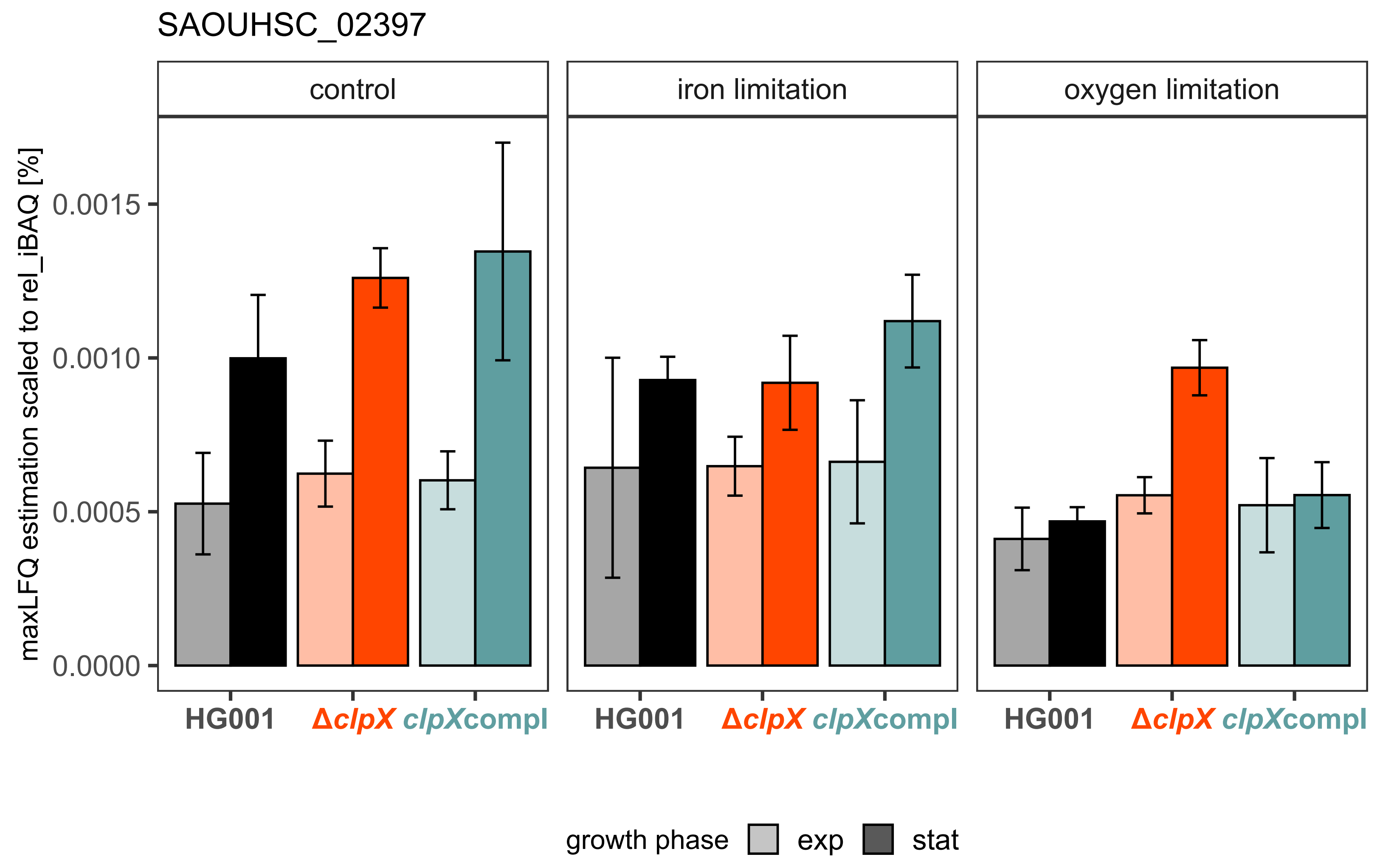
SAOUHSC_02397
control
iron limitation
oxygen limitation
maxLFQ estimation scaled to rel_iBAQ [%]
0.0015
0.0010
0.0005
0.0000
HG001
ΔclpX
clpXcompl
growth phase
exp
stat

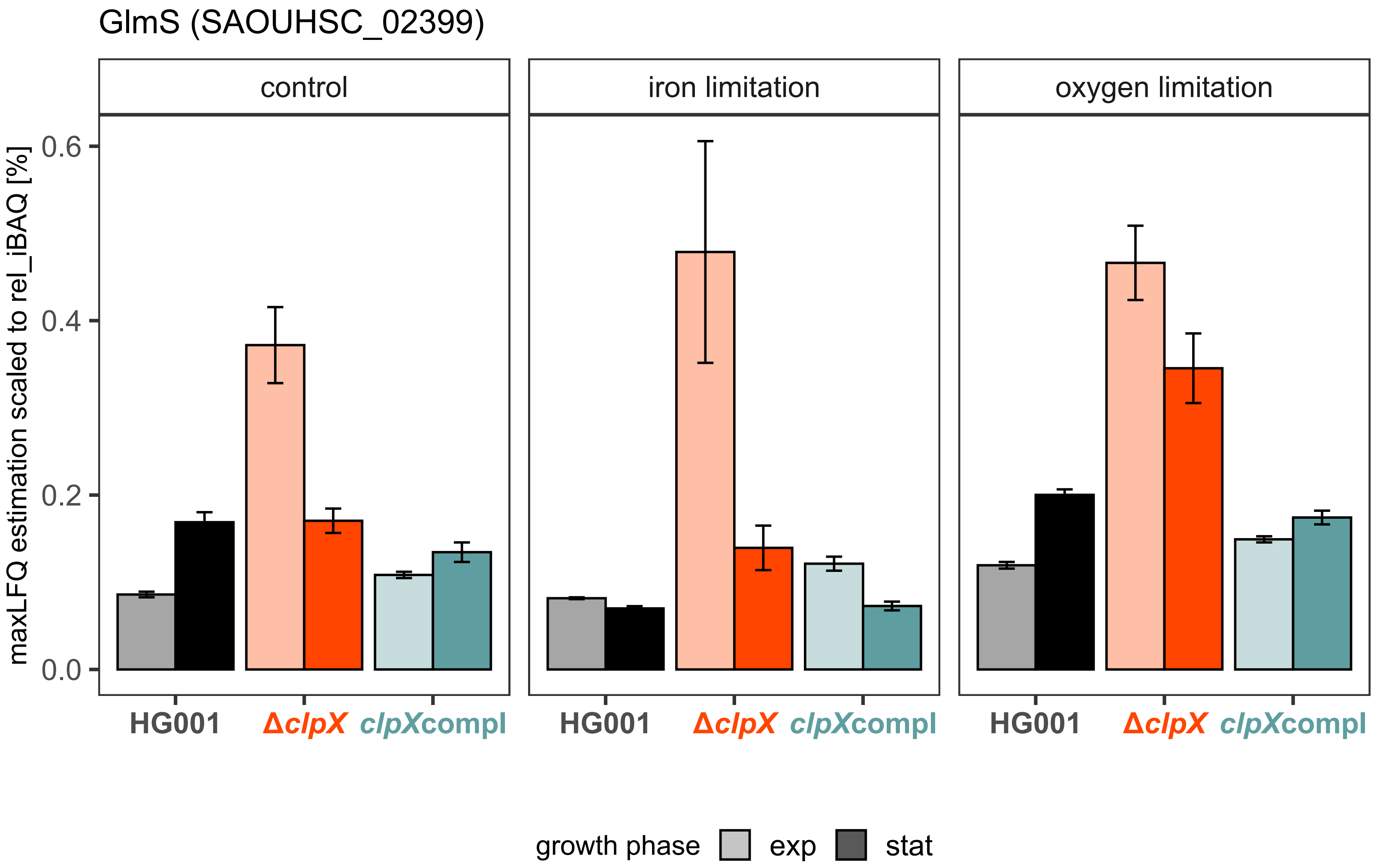

GlmS (SAOUHSC_02399)
control
iron limitation
oxygen limitation
maxLFQ estimation scaled to rel_iBAQ [%]
0.6
0.4
0.2
0.0
HG001
ΔclpX
clpXcompl
growth phase
exp
stat

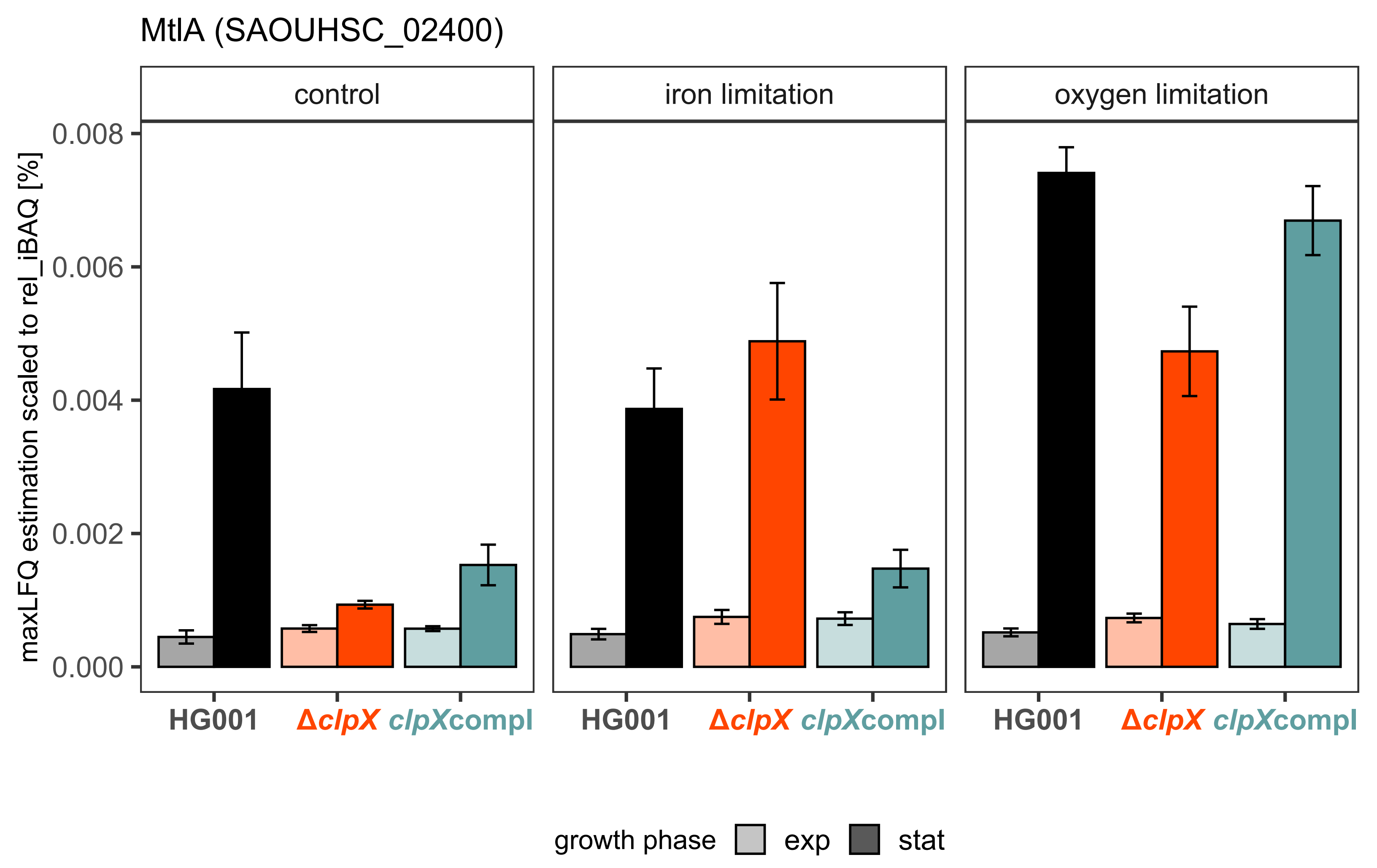
MtlA (SAOUHSC_02400)
control
iron limitation
oxygen limitation
maxLFQ estimation scaled to rel_iBAQ [%]
0.008
0.006
0.004
0.002
0.000
HG001
ΔclpX
clpXcompl
growth phase
exp
stat

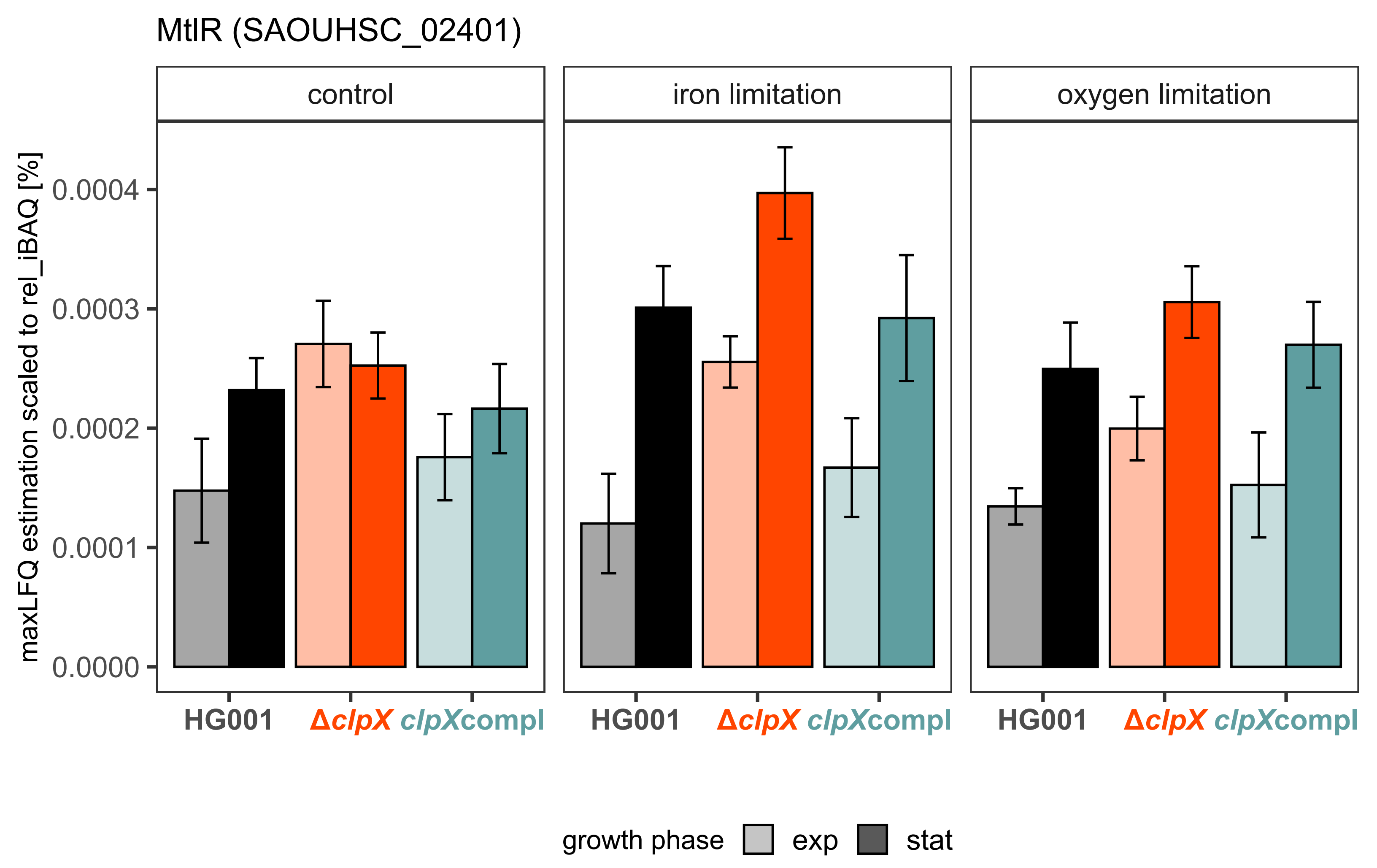
MtlR (SAOUHSC_02401)
control
iron limitation
oxygen limitation
maxLFQ estimation scaled to rel_iBAQ [%]
0.0004
0.0003
0.0002
0.0001
0.0000
HG001
ΔclpX
clpXcompl
growth phase
exp
stat

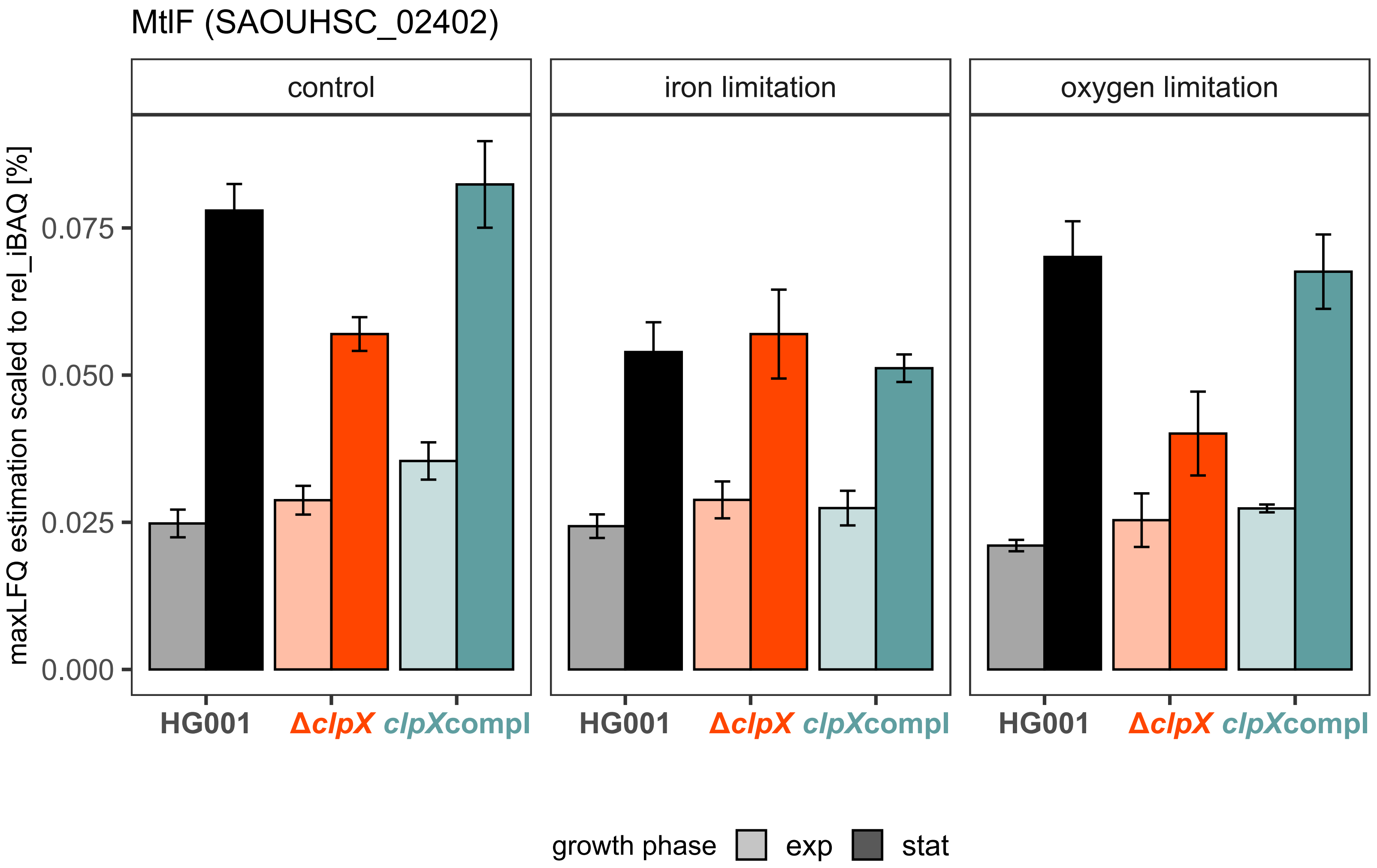

MtlF (SAOUHSC_02402)
control
iron limitation
oxygen limitation
maxLFQ estimation scaled to rel_iBAQ [%]
0.075
0.050
0.025
0.000
HG001
ΔclpX
clpXcompl
HG001
ΔclpX
clpXcompl
HG001
ΔclpX
clpXcompl
growth phase
exp
stat

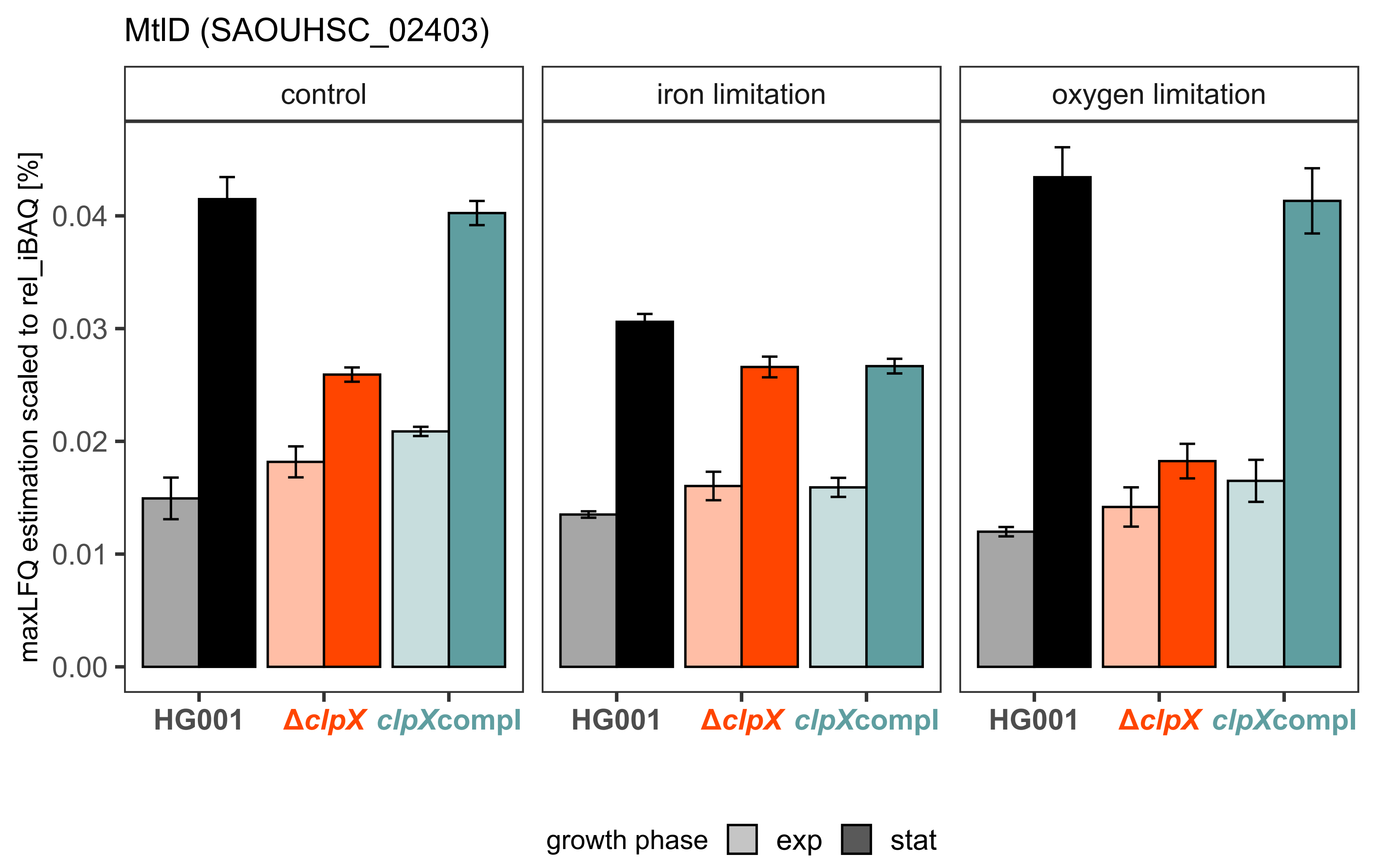
MtlD (SAOUHSC_02403)
control
iron limitation
oxygen limitation
maxLFQ estimation scaled to rel_iBAQ [%]
0.00
0.01
0.02
0.03
0.04
HG001
ΔclpX
clpXcompl
growth phase
exp
stat

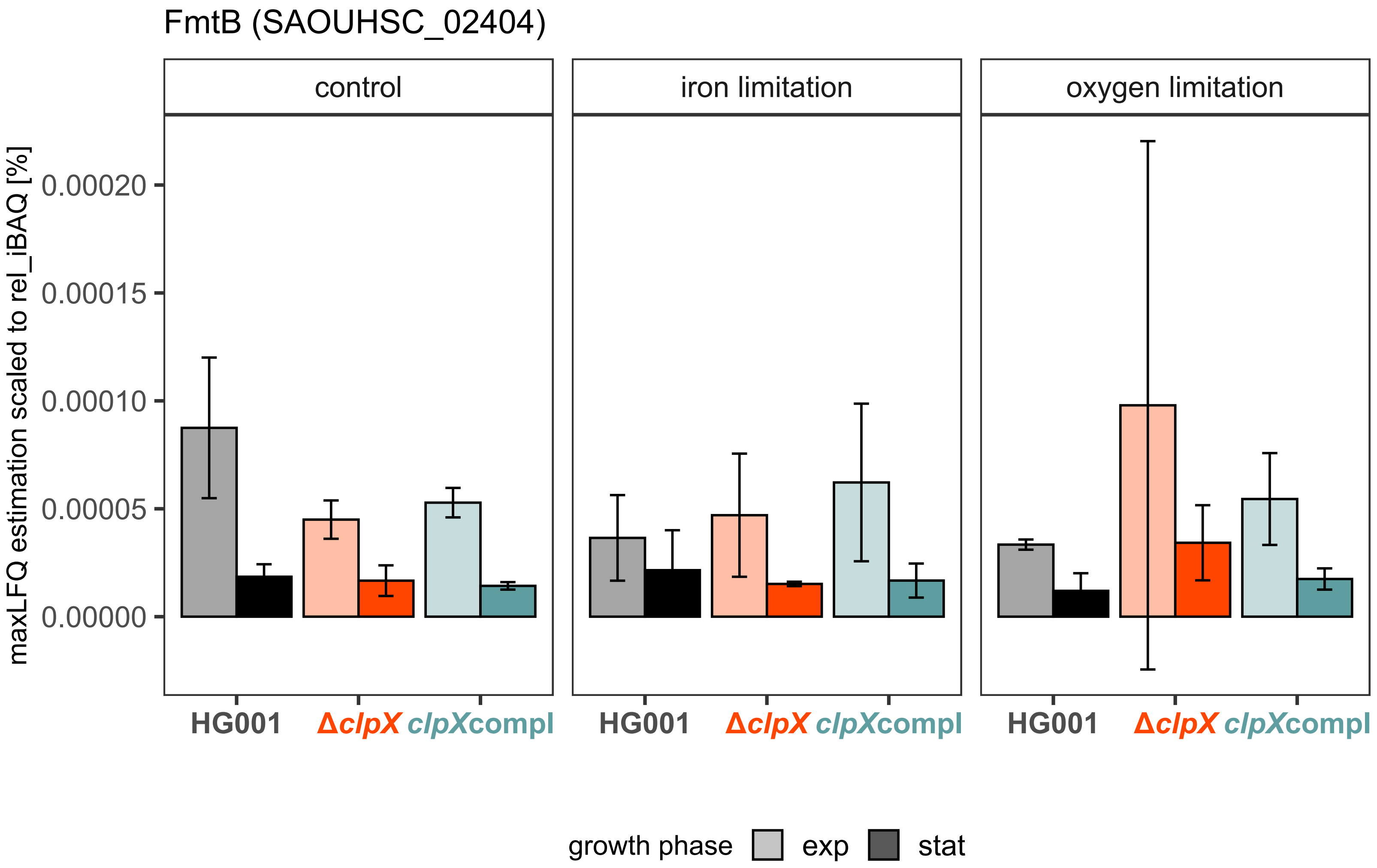
FmtB (SAOUHSC_02404)
control
iron limitation
oxygen limitation
maxLFQ estimation scaled to rel_iBAQ [%]
0.00020
0.00015
0.00010
0.00005
0.00000
HG001
ΔclpX
clpXcompl
growth phase
exp
stat

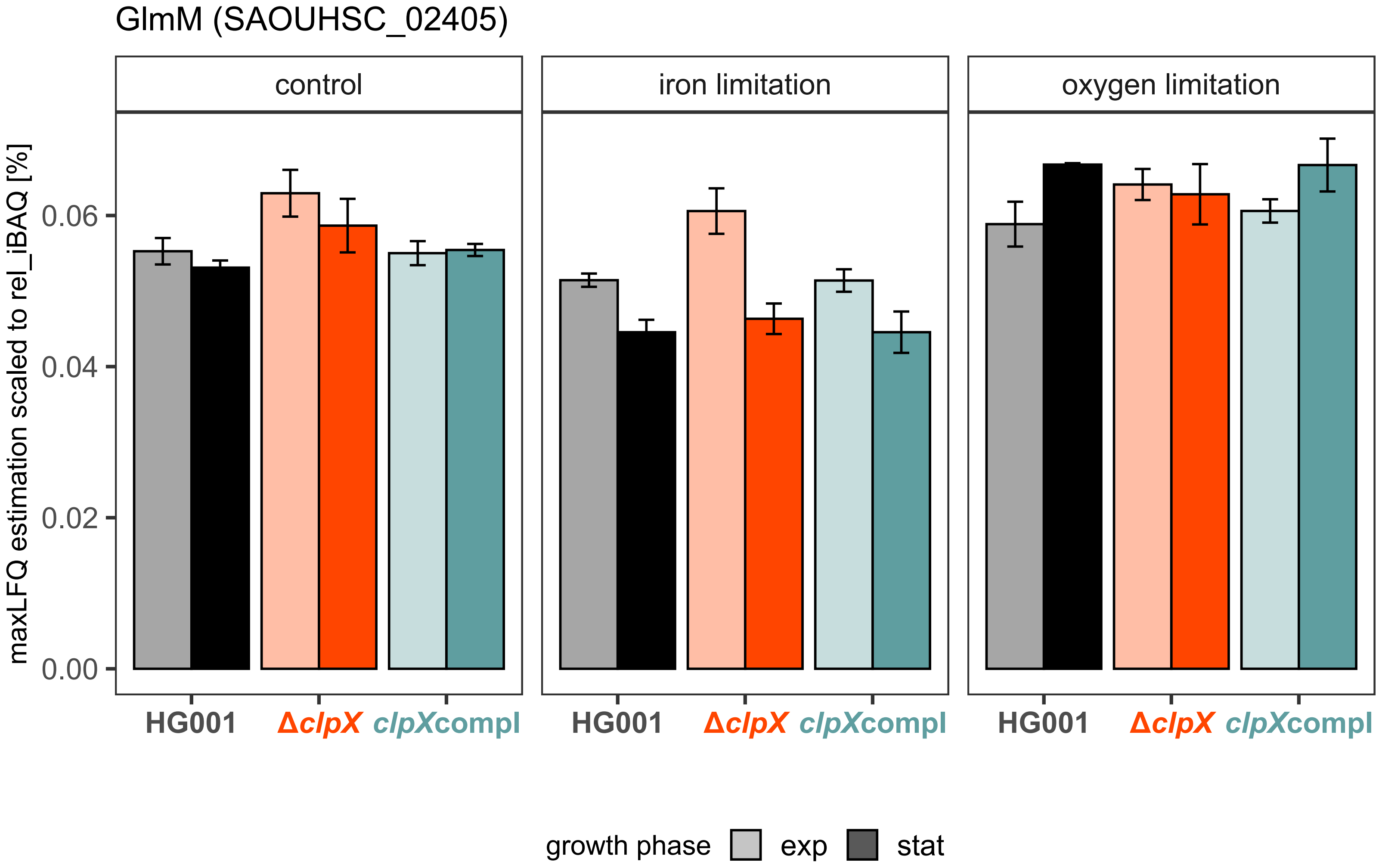

GlmM (SAOUHSC_02405)
control
iron limitation
oxygen limitation
maxLFQ estimation scaled to rel_iBAQ [%]
0.00
0.02
0.04
0.06
HG001
ΔclpX
clpXcompl
growth phase
exp
stat

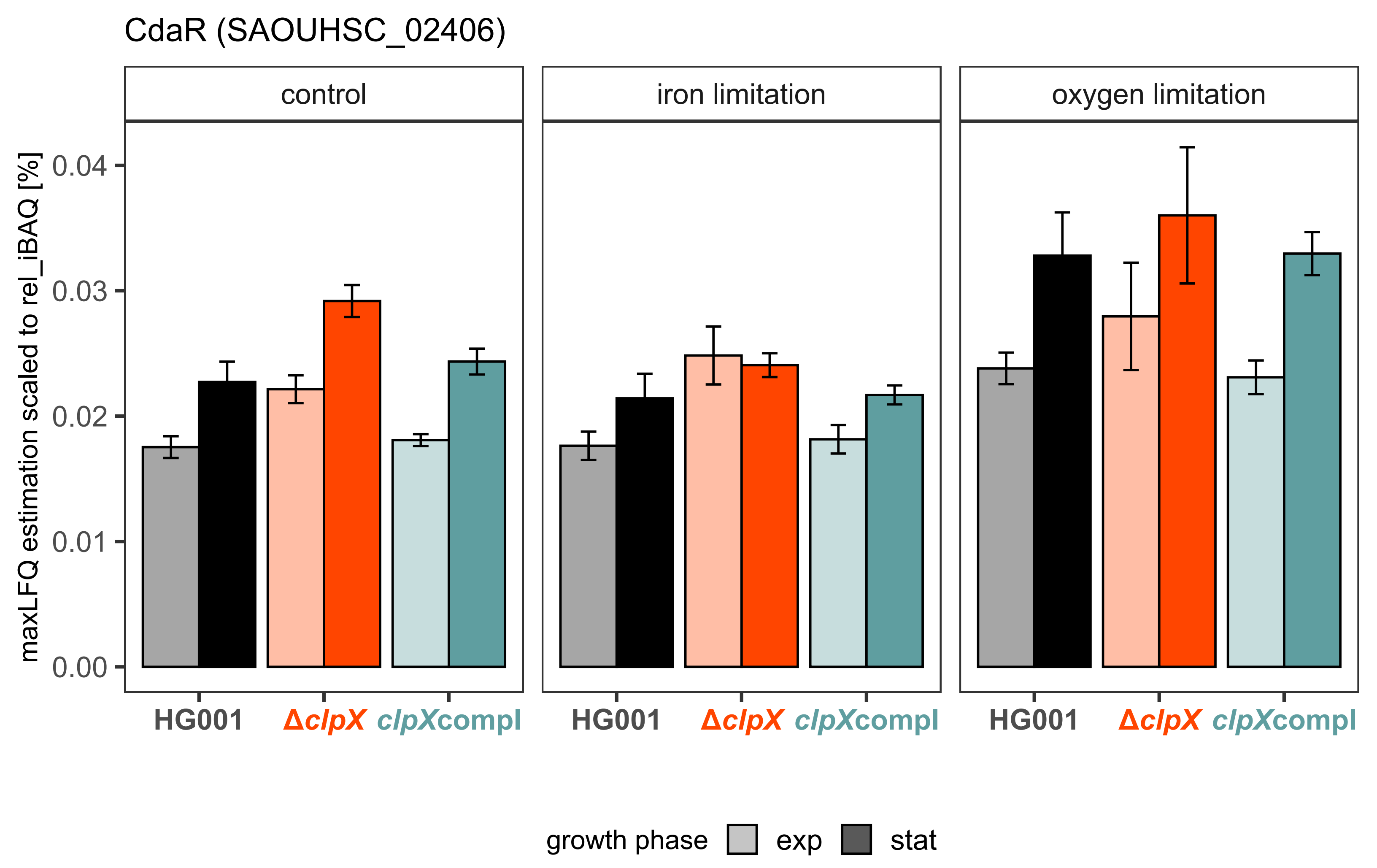
CdaR (SAOUHSC_02406)
control
iron limitation
oxygen limitation
maxLFQ estimation scaled to rel_iBAQ [%]
0.04
0.03
0.02
0.01
0.00
HG001
ΔclpX
clpXcompl
growth phase
exp
stat

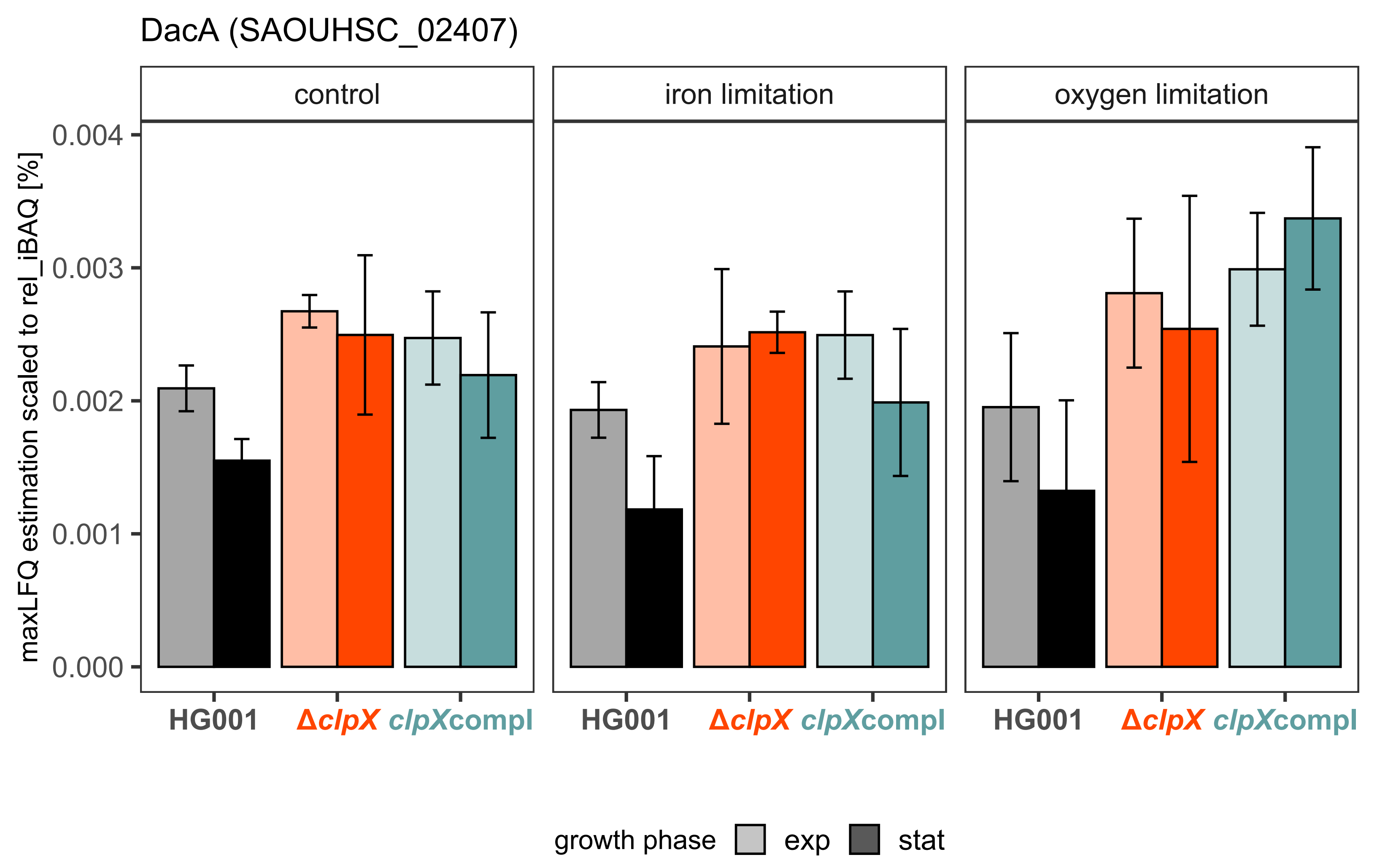
DacA (SAOUHSC_02407)
control
iron limitation
oxygen limitation
maxLFQ estimation scaled to rel_iBAQ [%]
0.004
0.003
0.002
0.001
0.000
HG001
ΔclpX
clpXcompl
growth phase
exp
stat

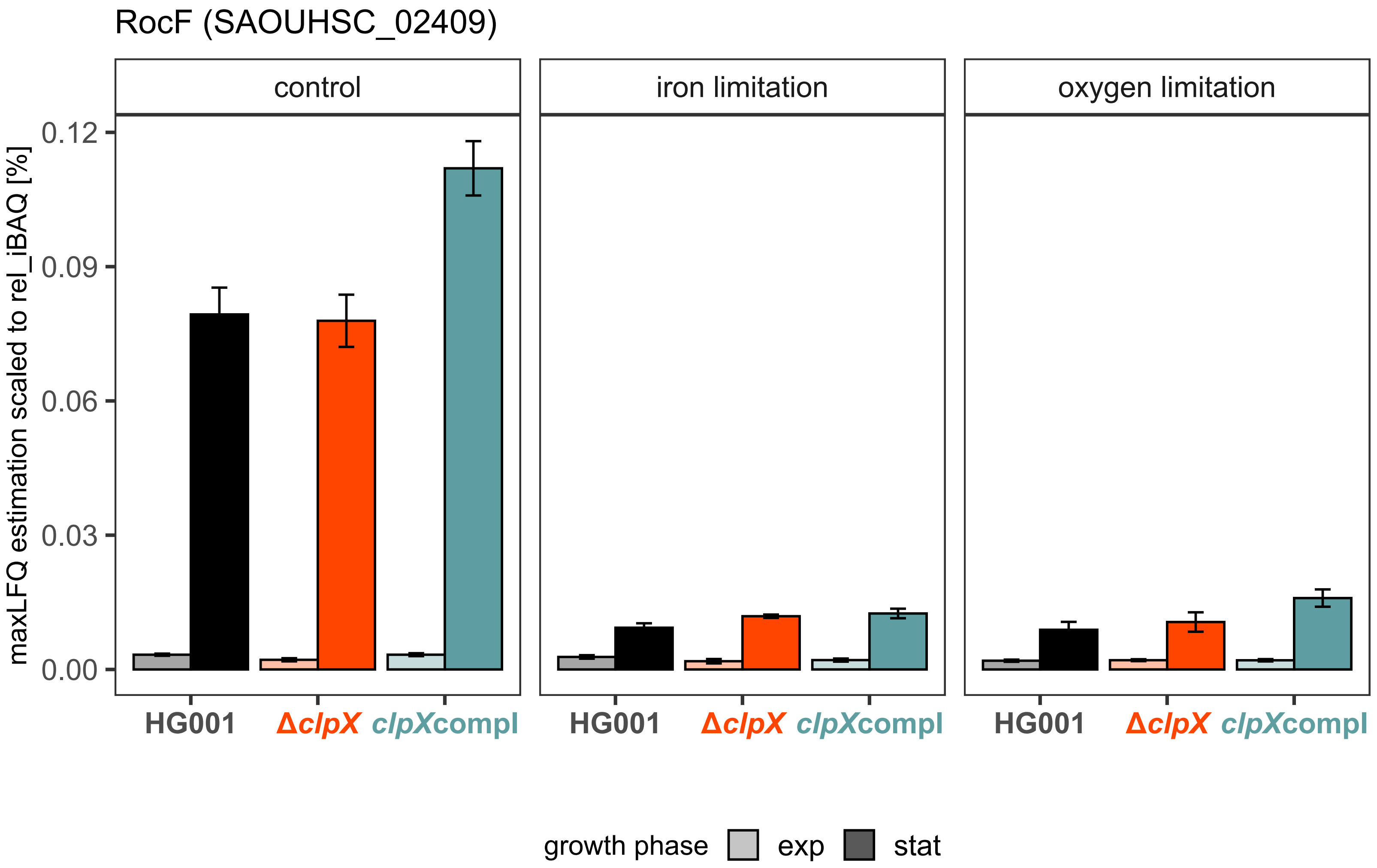

RocF (SAOUHSC_02409)
control
iron limitation
oxygen limitation
maxLFQ estimation scaled to rel_iBAQ [%]
0.12
0.09
0.06
0.03
0.00
HG001
ΔclpX
clpXcompl
growth phase
exp
stat

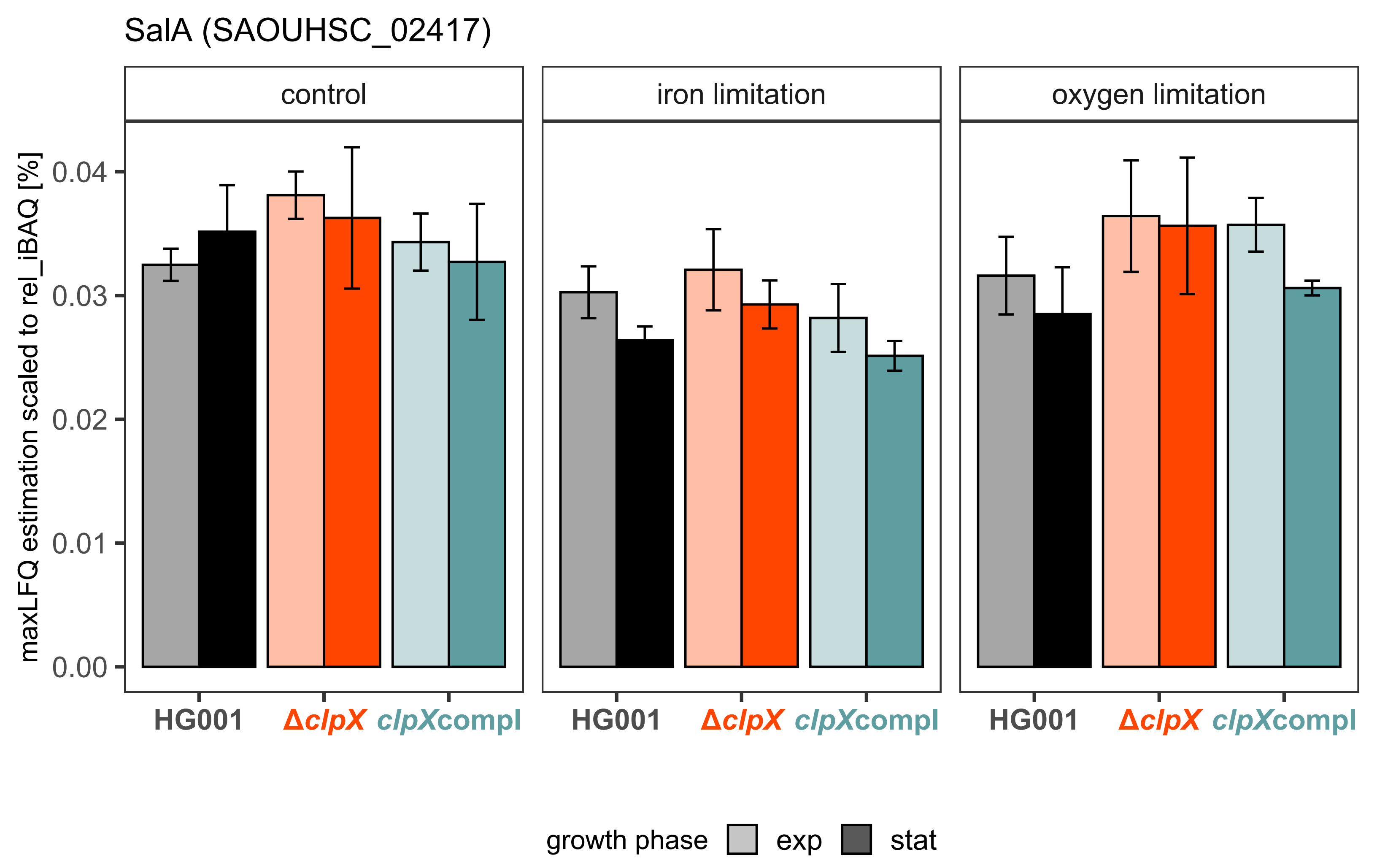
SalA (SAOUHSC_02417)
control
iron limitation
oxygen limitation
maxLFQ estimation scaled to rel_iBAQ [%]
0.00
0.01
0.02
0.03
0.04
HG001
ΔclpX
clpXcompl
growth phase
exp
stat

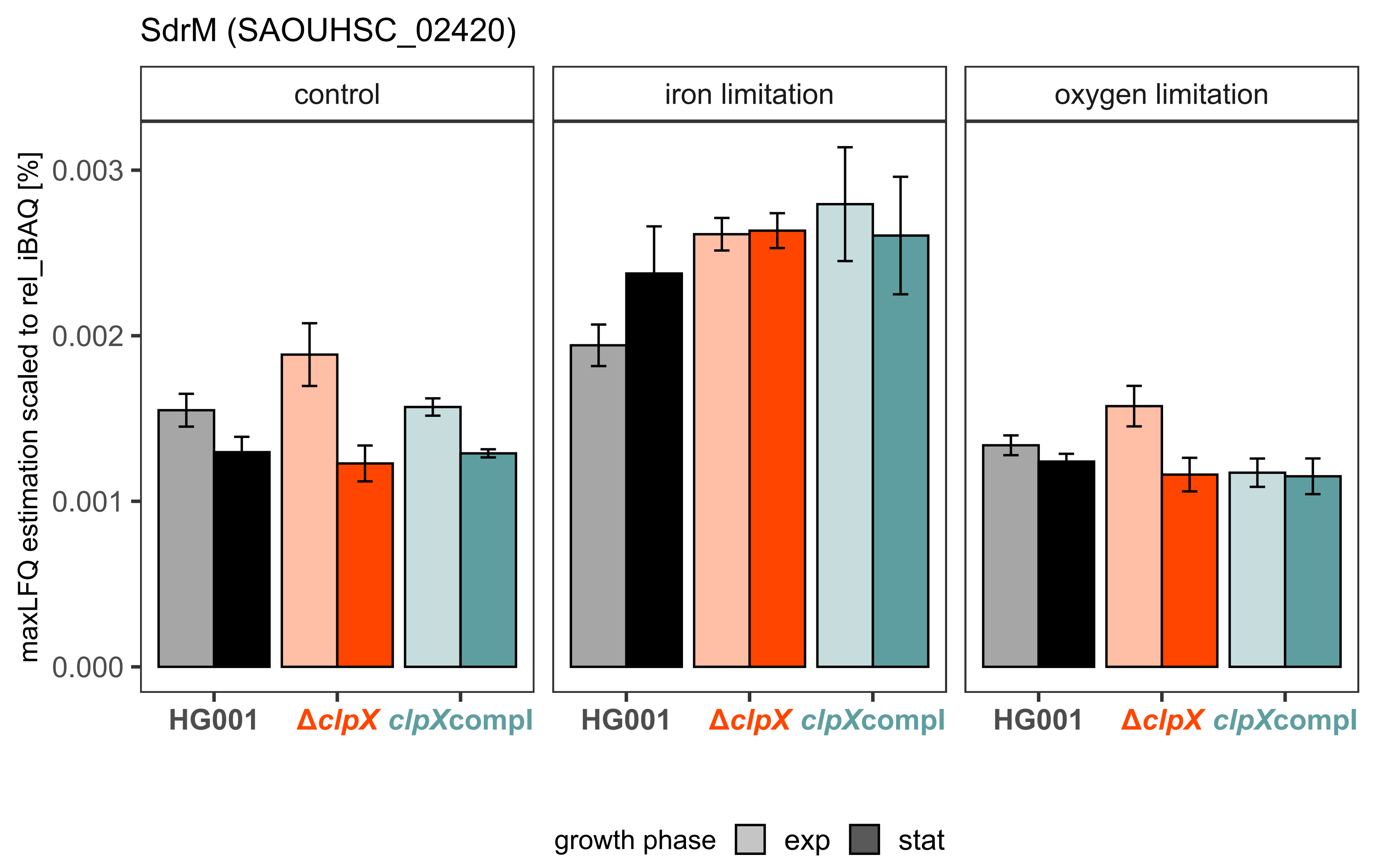

SdrM (SAOUHSC_02420)
control
iron limitation
oxygen limitation
maxLFQ estimation scaled to rel_iBAQ [%]
0.003
0.002
0.001
0.000
HG001
ΔclpX
clpXcompl
growth phase
exp
stat

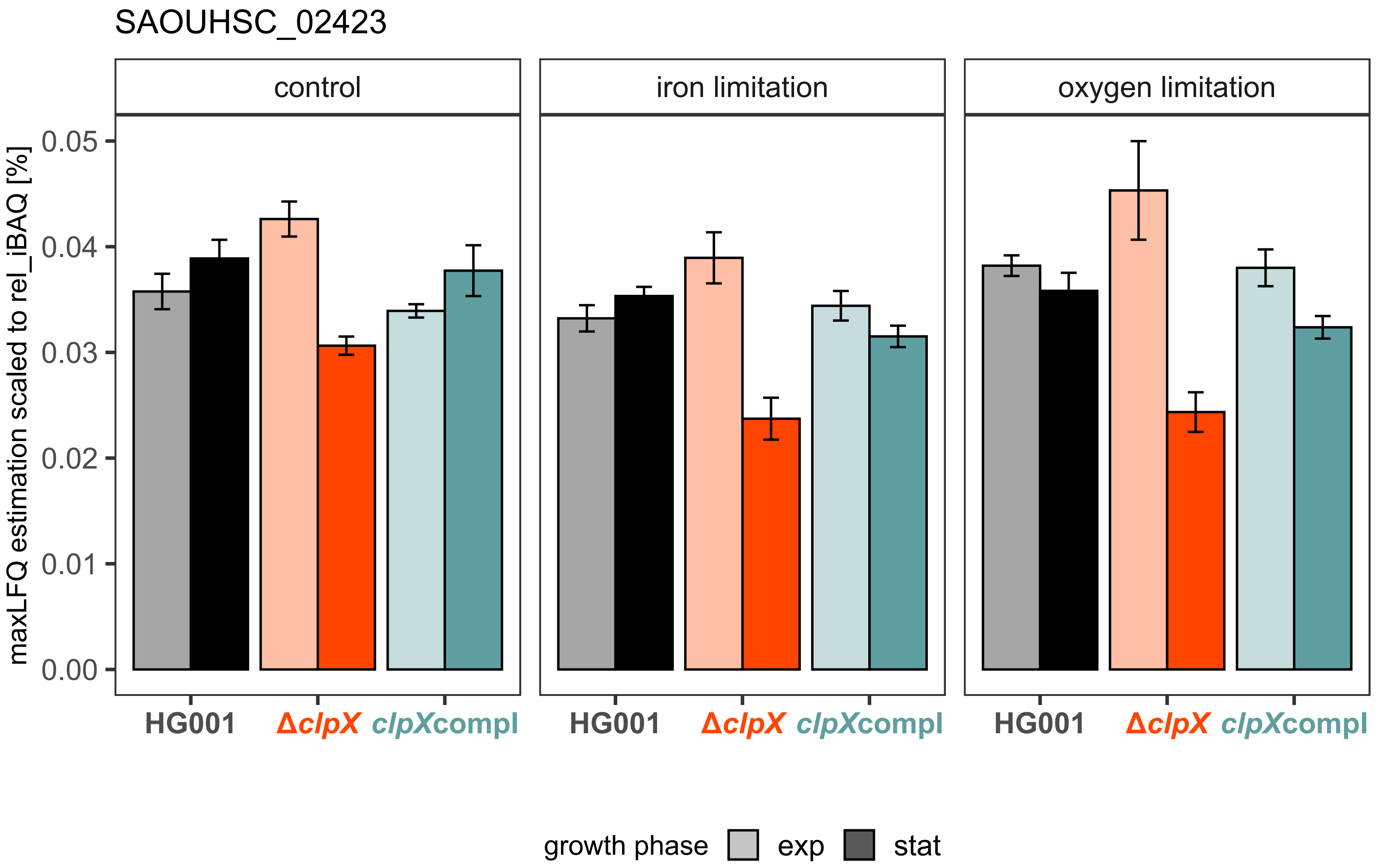

SAOUHSC_02423
control
iron limitation
oxygen limitation
maxLFQ estimation scaled to rel_iBAQ [%]
0.05
0.04
0.03
0.02
0.01
0.00
HG001
ΔclpX
clpXcompl
growth phase
exp
stat

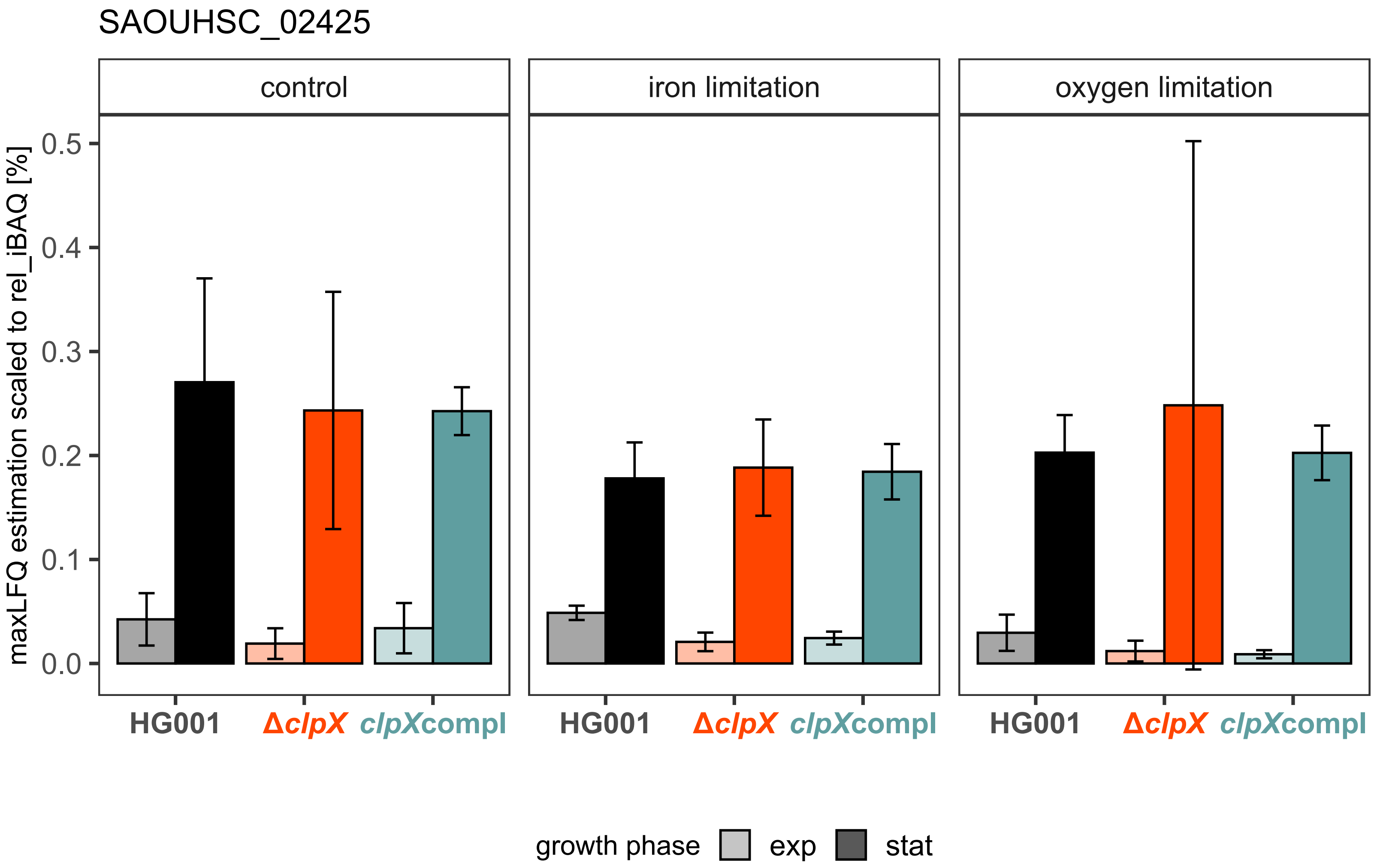

SAOUHSC_02425
control
iron limitation
oxygen limitation
maxLFQ estimation scaled to rel_iBAQ [%]
0.0
0.1
0.2
0.3
0.4
0.5
HG001
ΔclpX
clpXcompl
growth phase
exp
stat

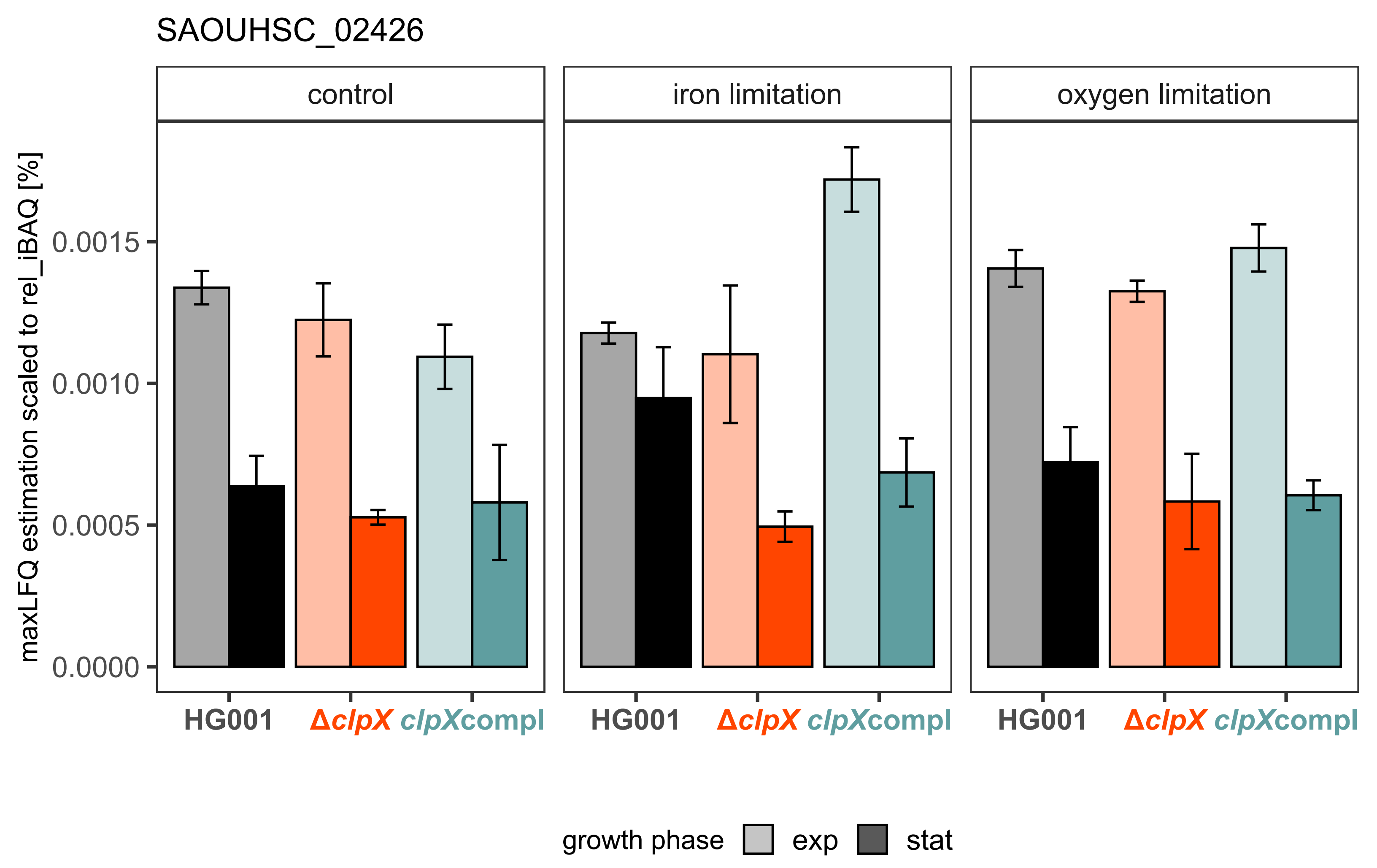
SAOUHSC_02426
control
iron limitation
oxygen limitation
maxLFQ estimation scaled to rel_iBAQ [%]
0.0015
0.0010
0.0005
0.0000
HG001
ΔclpX
clpXcompl
growth phase
exp
stat

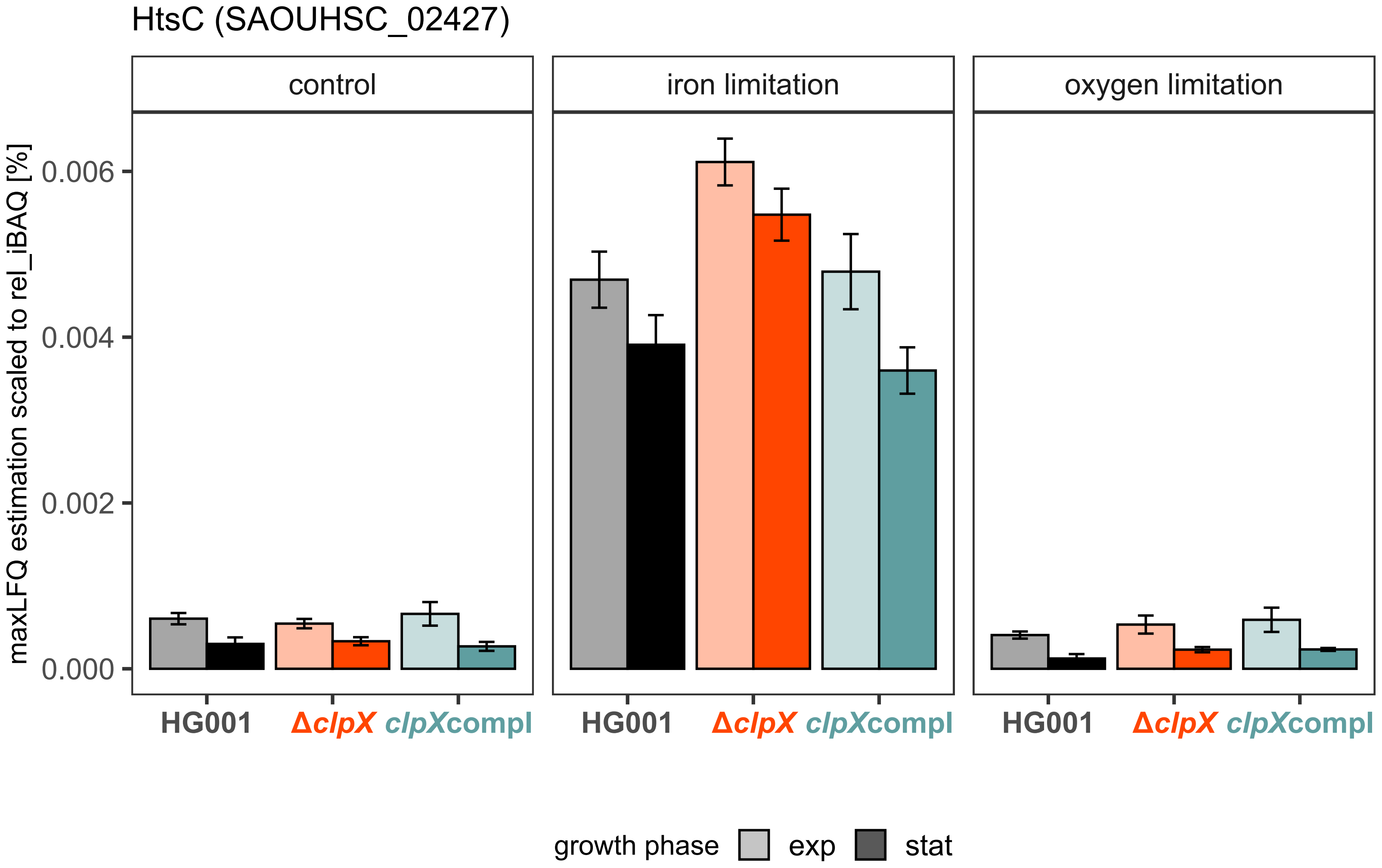

HtsC (SAOUHSC_02427)
control
iron limitation
oxygen limitation
maxLFQ estimation scaled to rel_iBAQ [%]
0.006
0.004
0.002
0.000
HG001
ΔclpX
clpXcompl
growth phase
exp
stat

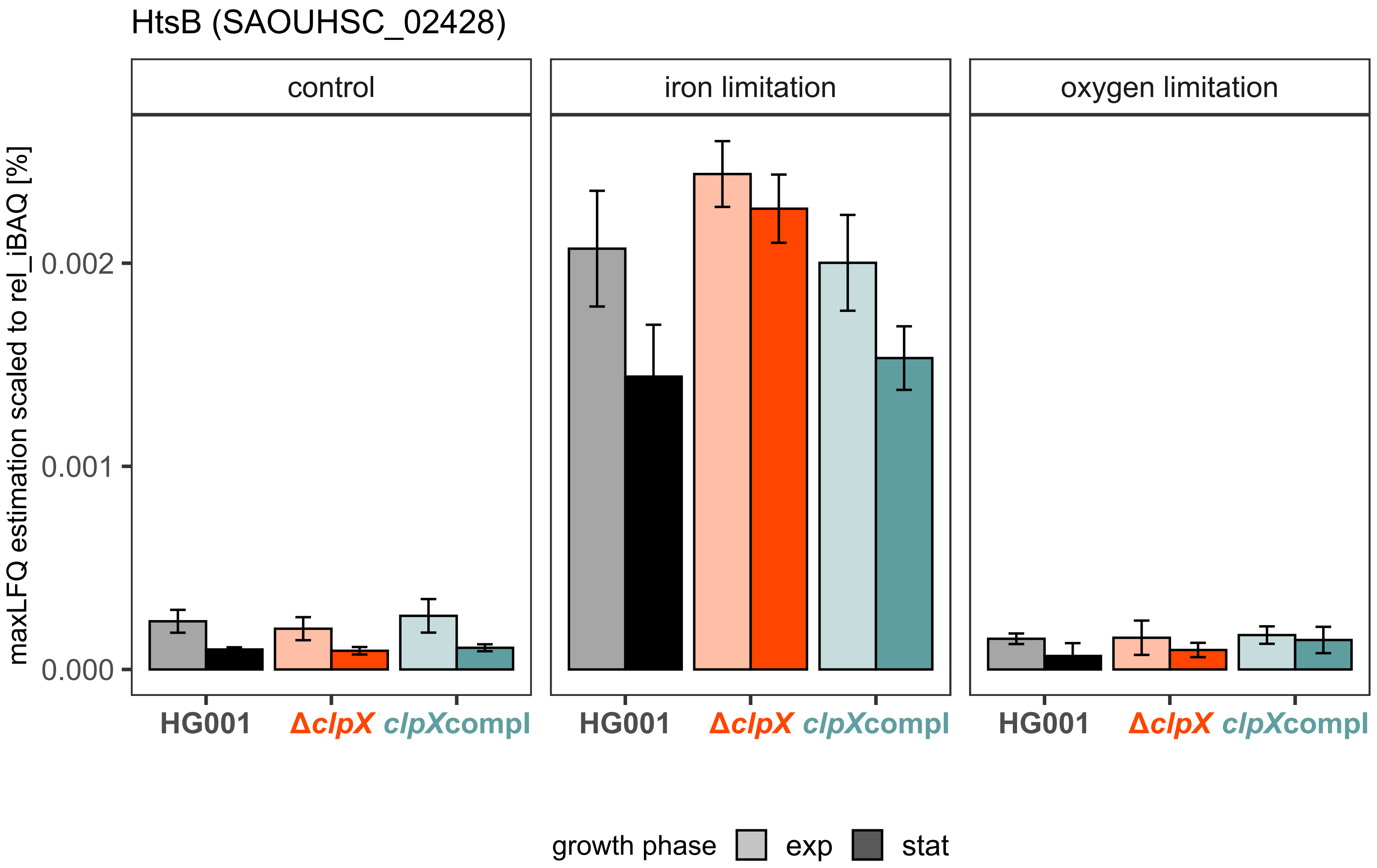
HtsB (SAOUHSC_02428)
control
iron limitation
oxygen limitation
maxLFQ estimation scaled to rel_iBAQ [%]
0.002
0.001
0.000
HG001
ΔclpX
clpXcompl
growth phase
exp
stat

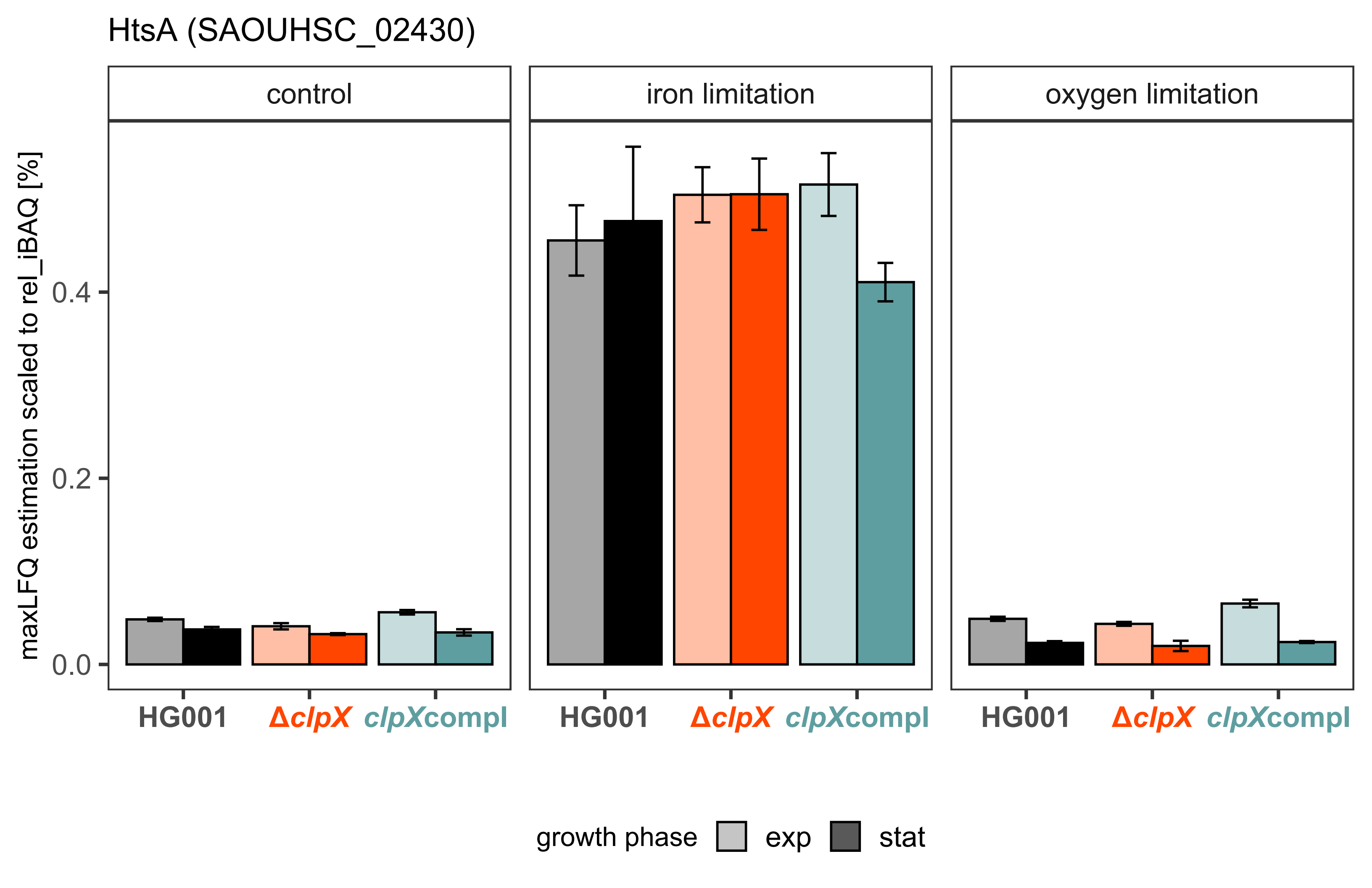

HtsA (SAOUHSC_02430)
control
iron limitation
oxygen limitation
maxLFQ estimation scaled to rel_iBAQ [%]
0.0
0.2
0.4
HG001
ΔclpX
clpXcompl
growth phase
exp
stat

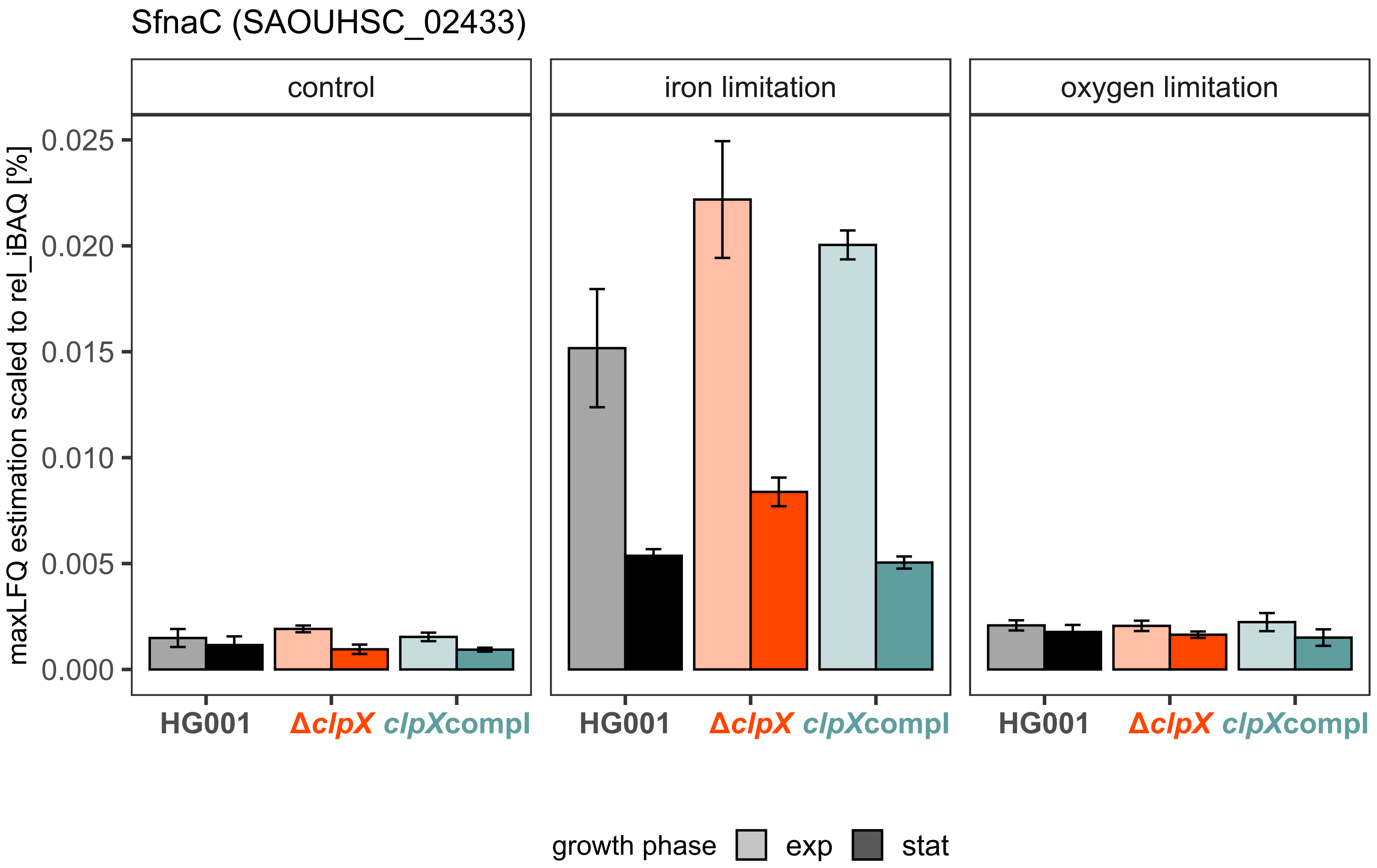

SfnaC (SAOUHSC_02433)
control
iron limitation
oxygen limitation
maxLFQ estimation scaled to rel_iBAQ [%]
0.025
0.020
0.015
0.010
0.005
0.000
HG001
ΔclpX
clpXcompl
growth phase
exp
stat

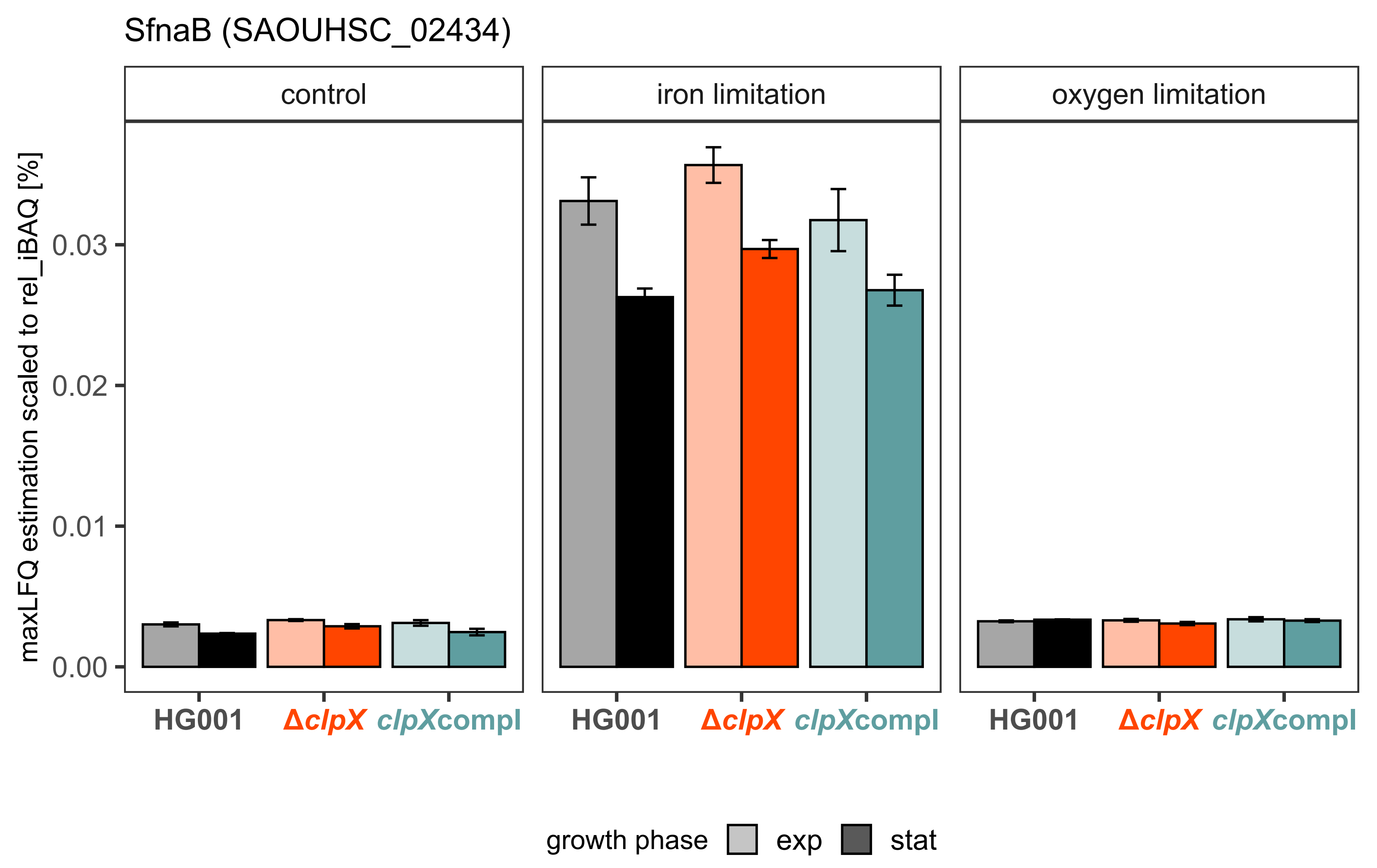
SfnaB (SAOUHSC_02434)
control
iron limitation
oxygen limitation
maxLFQ estimation scaled to rel_iBAQ [%]
0.03
0.02
0.01
0.00
HG001
ΔclpX
clpXcompl
growth phase
exp
stat

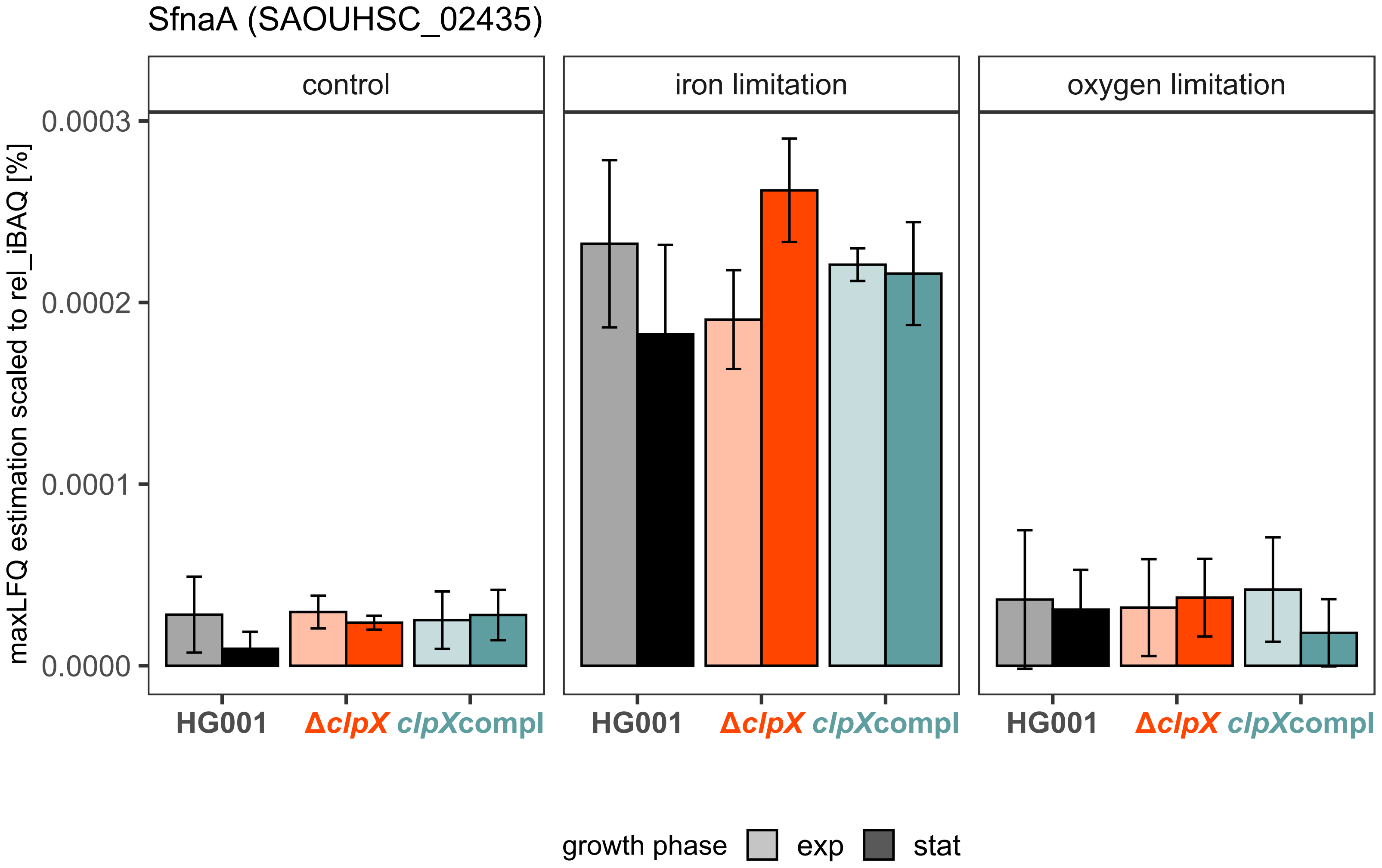

SfnaA (SAOUHSC_02435)
control
iron limitation
oxygen limitation
maxLFQ estimation scaled to rel_iBAQ [%]
0.0003
0.0002
0.0001
0.0000
HG001
ΔclpX
clpXcompl
growth phase
exp
stat

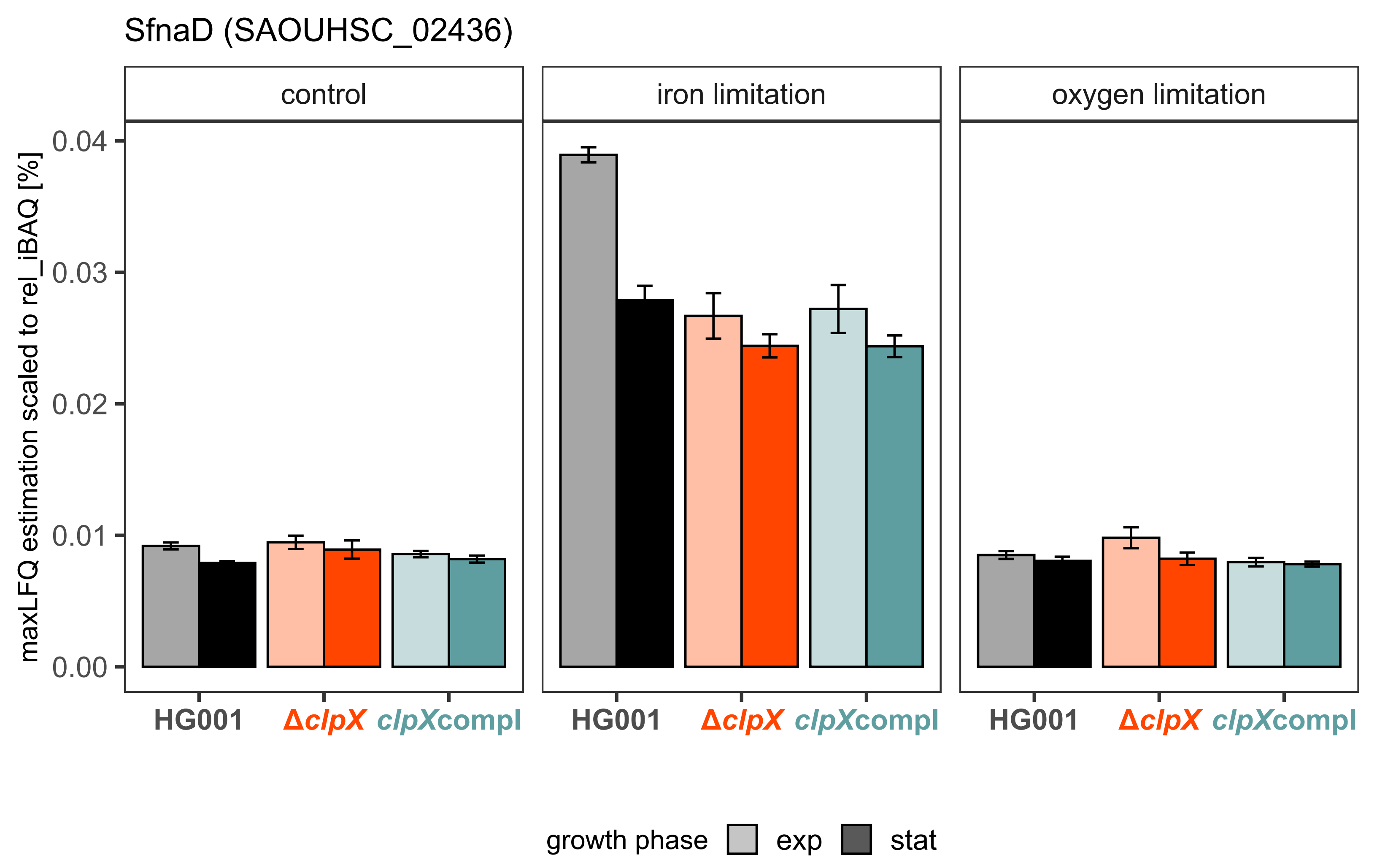

SfnaD (SAOUHSC_02436)
control
iron limitation
oxygen limitation
maxLFQ estimation scaled to rel_iBAQ [%]
0.04
0.03
0.02
0.01
0.00
HG001
ΔclpX
clpXcompl
growth phase
exp
stat

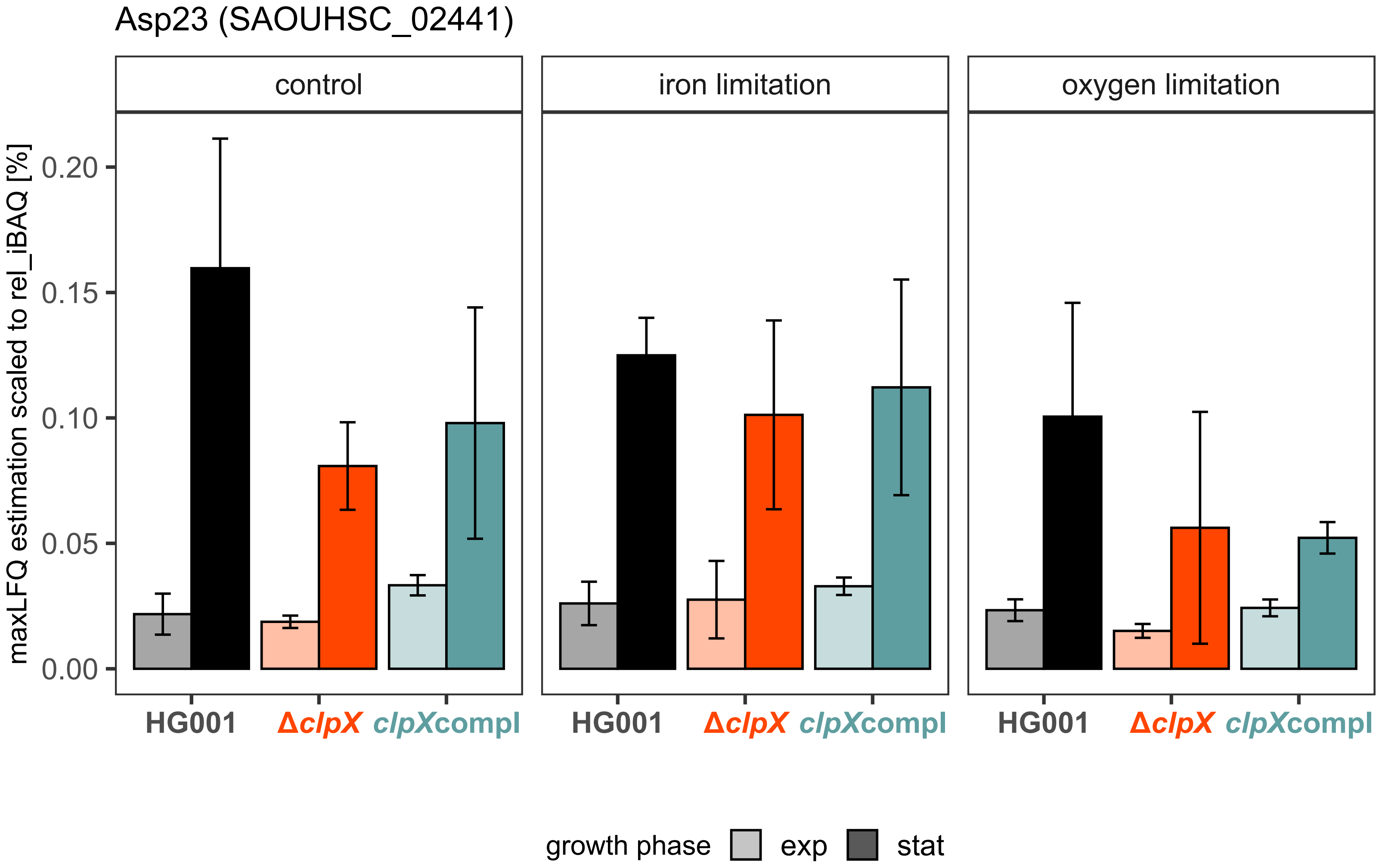
Asp23 (SAOUHSC_02441)
control
iron limitation
oxygen limitation
maxLFQ estimation scaled to rel_iBAQ [%]
0.00
0.05
0.10
0.15
0.20
HG001
ΔclpX
clpXcompl
growth phase
exp
stat

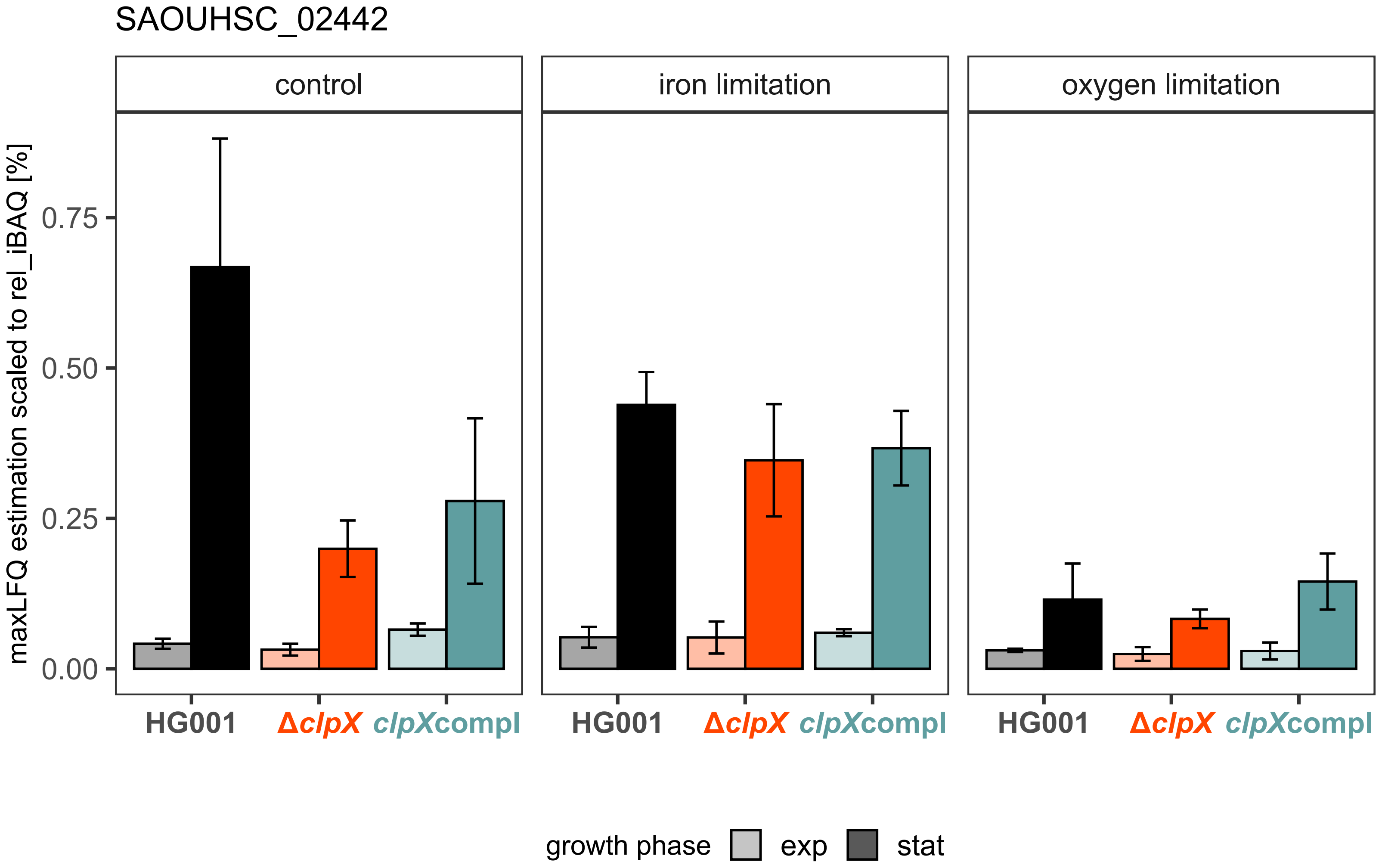

SAOUHSC_02442
control
iron limitation
oxygen limitation
maxLFQ estimation scaled to rel_iBAQ [%]
0.75
0.50
0.25
0.00
HG001
ΔclpX
clpXcompl
growth phase
exp
stat

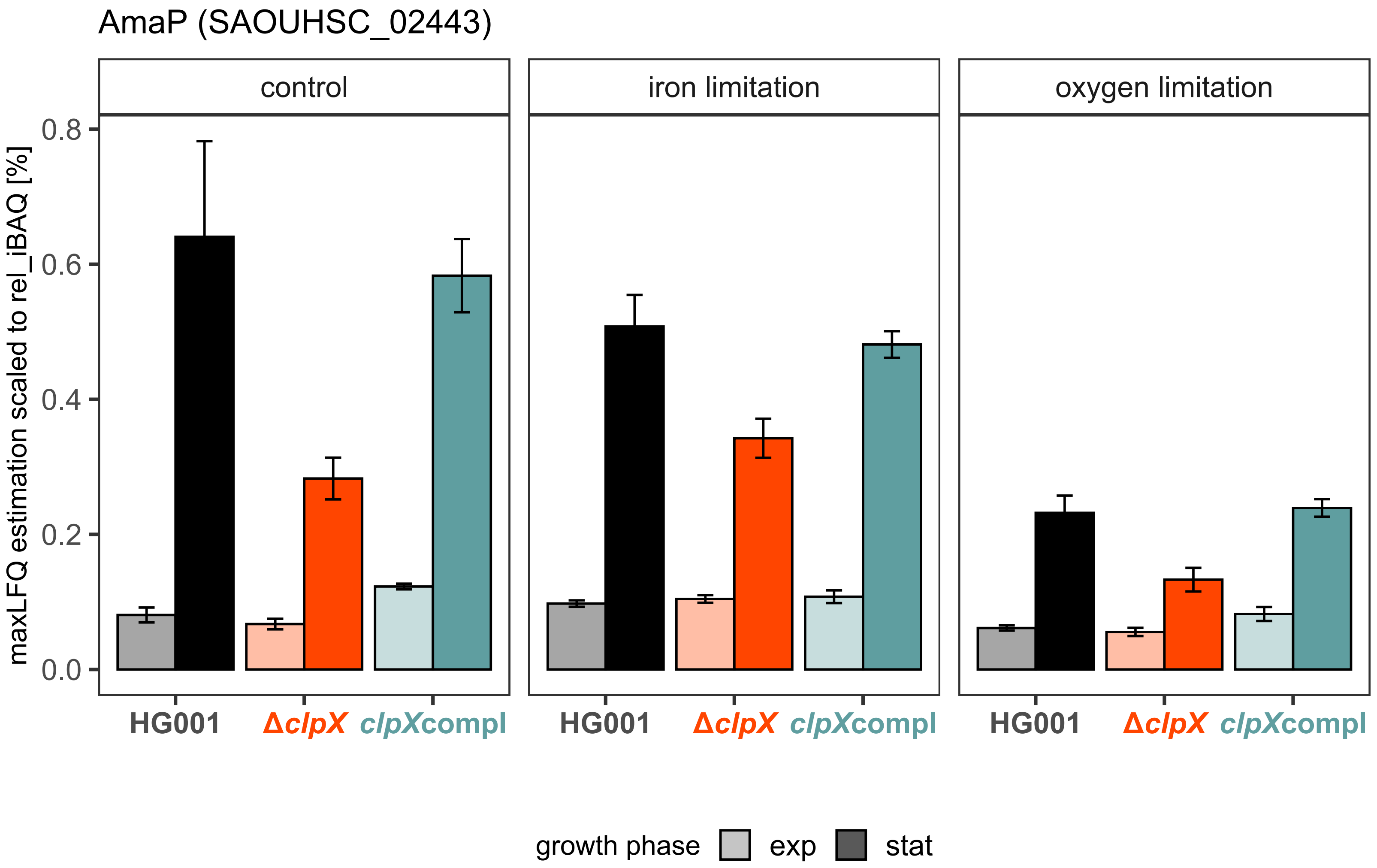

AmaP (SAOUHSC_02443)
control
iron limitation
oxygen limitation
maxLFQ estimation scaled to rel_iBAQ [%]
0.0
0.2
0.4
0.6
0.8
HG001
ΔclpX
clpXcompl
growth phase
exp
stat

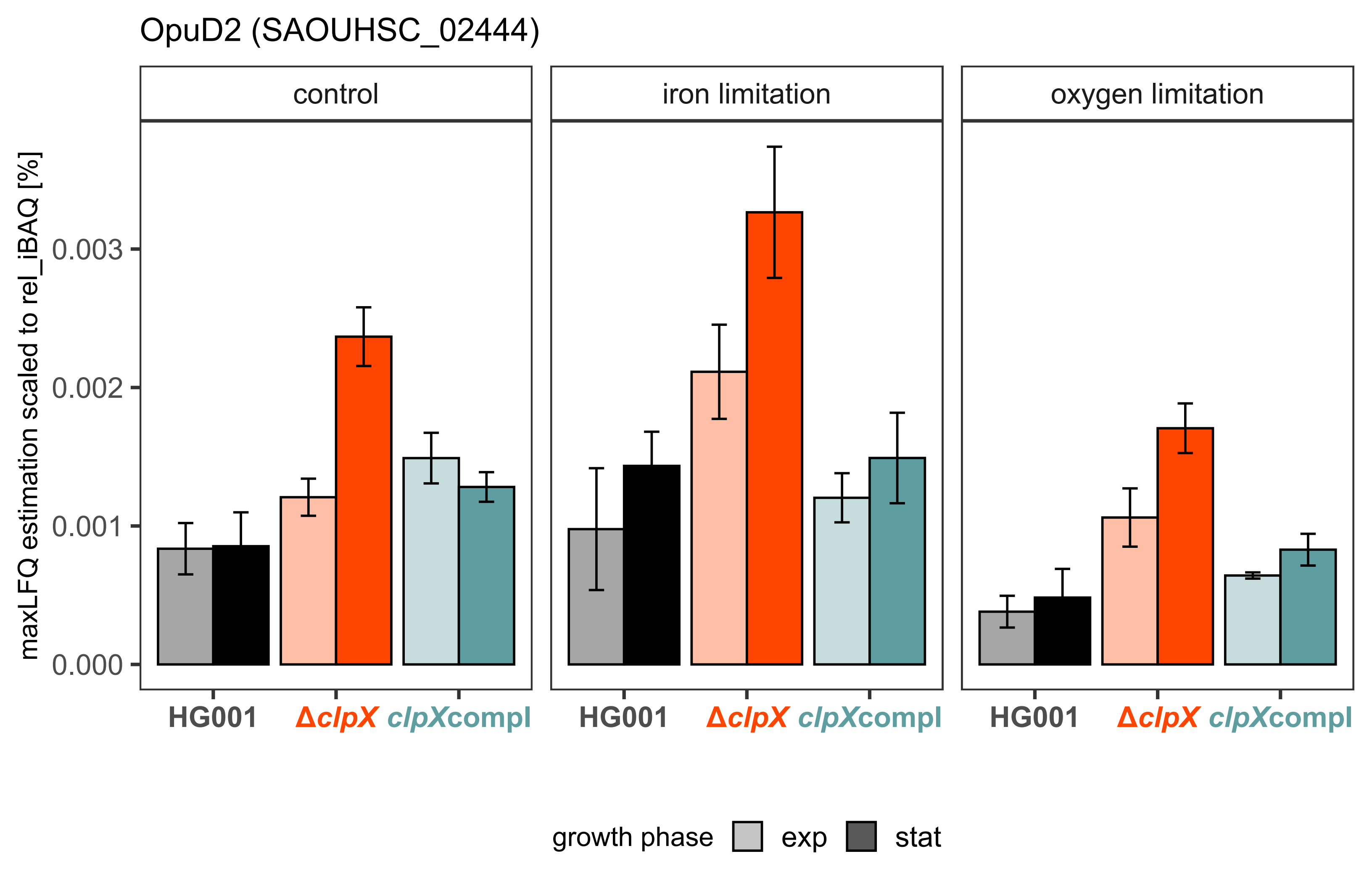

OpuD2 (SAOUHSC_02444)
control
iron limitation
oxygen limitation
maxLFQ estimation scaled to rel_iBAQ [%]
0.000
0.001
0.002
0.003
HG001
ΔclpX
clpXcompl
growth phase
exp
stat

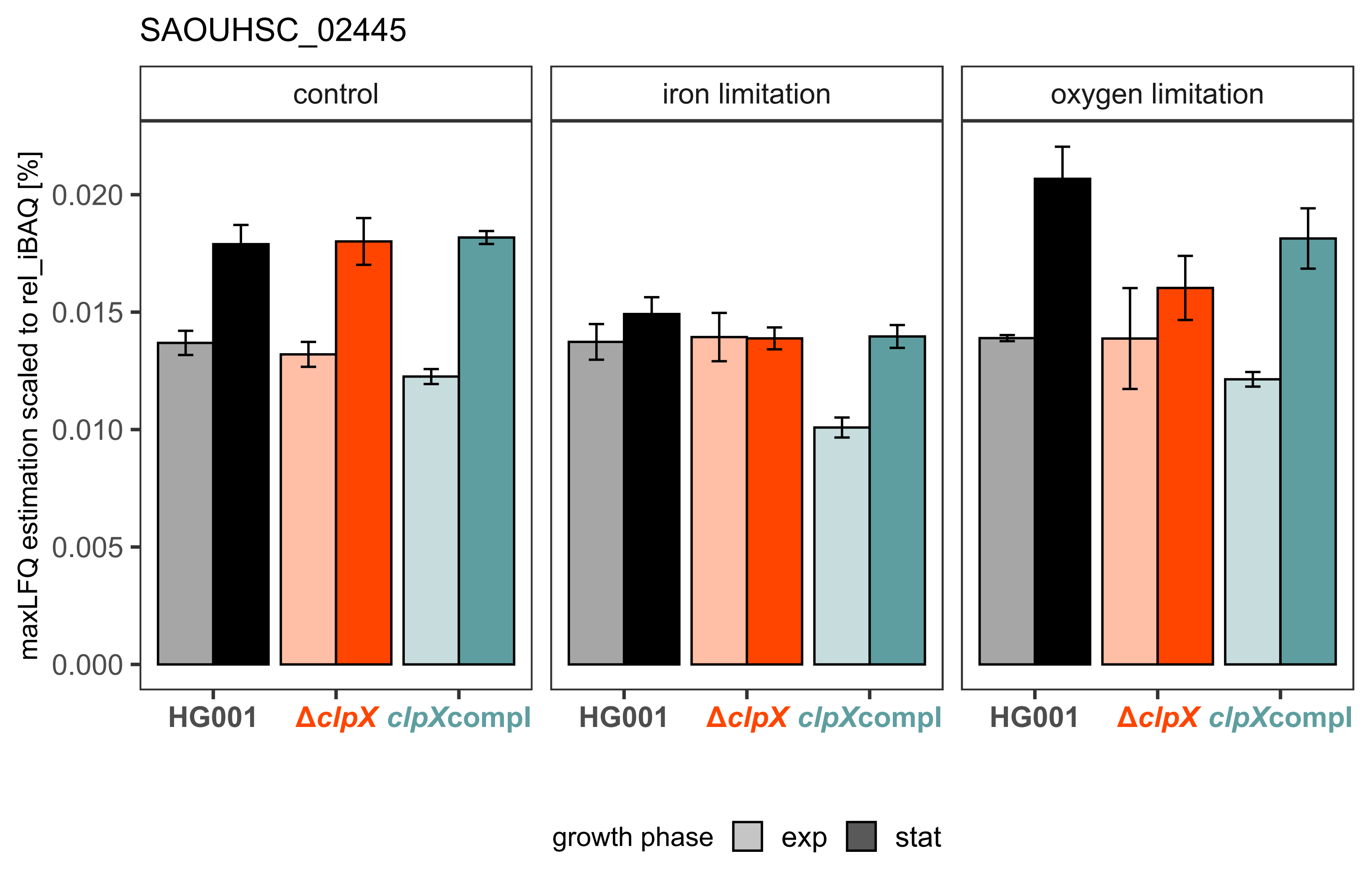

SAOUHSC_02445
control
iron limitation
oxygen limitation
maxLFQ estimation scaled to rel_iBAQ [%]
0.020
0.015
0.010
0.005
0.000
HG001
ΔclpX
clpXcompl
growth phase
exp
stat

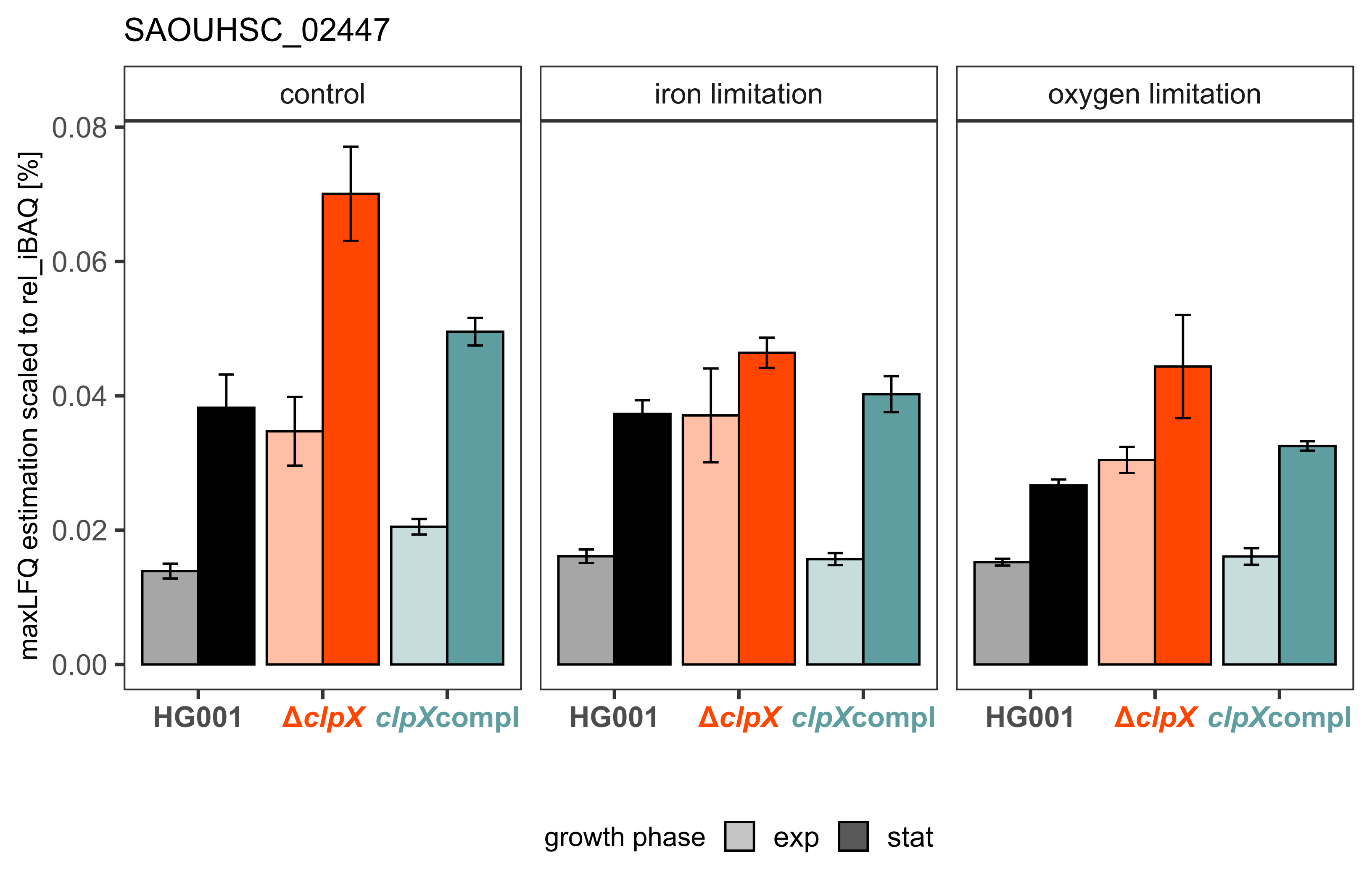

SAOUHSC_02447
control
iron limitation
oxygen limitation
maxLFQ estimation scaled to rel_iBAQ [%]
0.08
0.06
0.04
0.02
0.00
HG001
ΔclpX
clpXcompl
growth phase
exp
stat

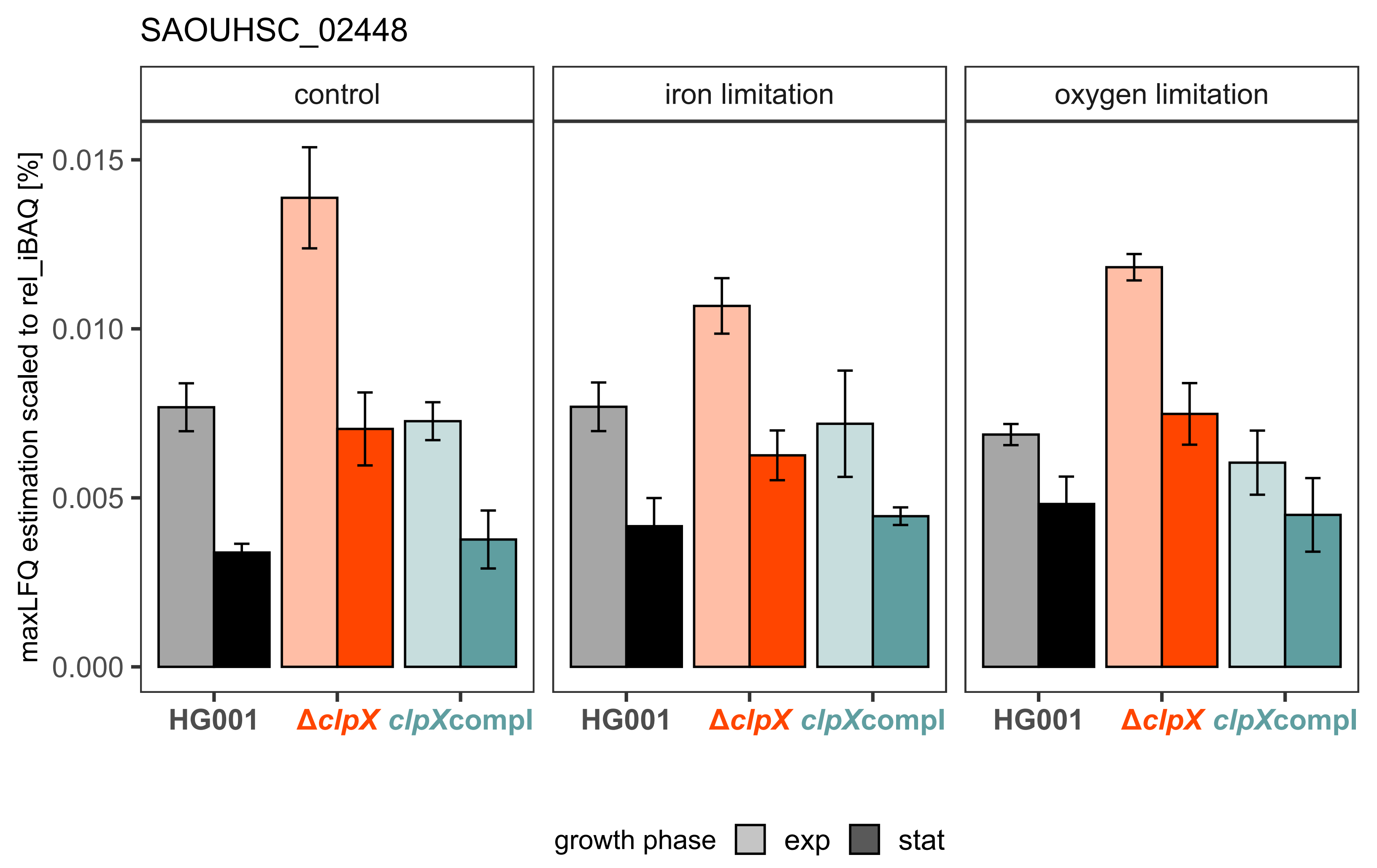

SAOUHSC_02448
control
iron limitation
oxygen limitation
maxLFQ estimation scaled to rel_iBAQ [%]
0.015
0.010
0.005
0.000
HG001
ΔclpX
clpXcompl
growth phase
exp
stat

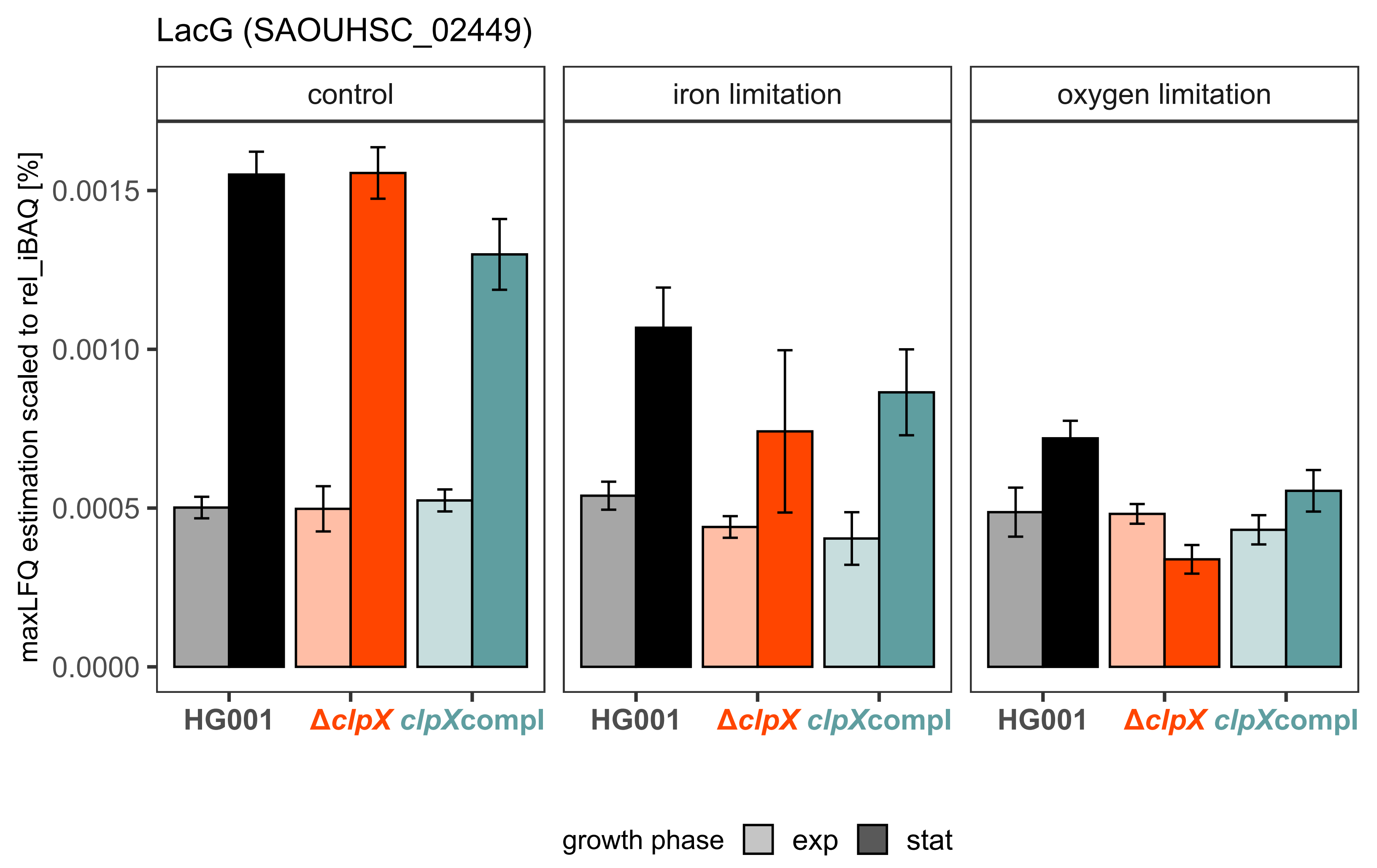

LacG (SAOUHSC_02449)
control
iron limitation
oxygen limitation
maxLFQ estimation scaled to rel_iBAQ [%]
0.0015
0.0010
0.0005
0.0000
HG001
ΔclpX
clpXcompl
growth phase
exp
stat

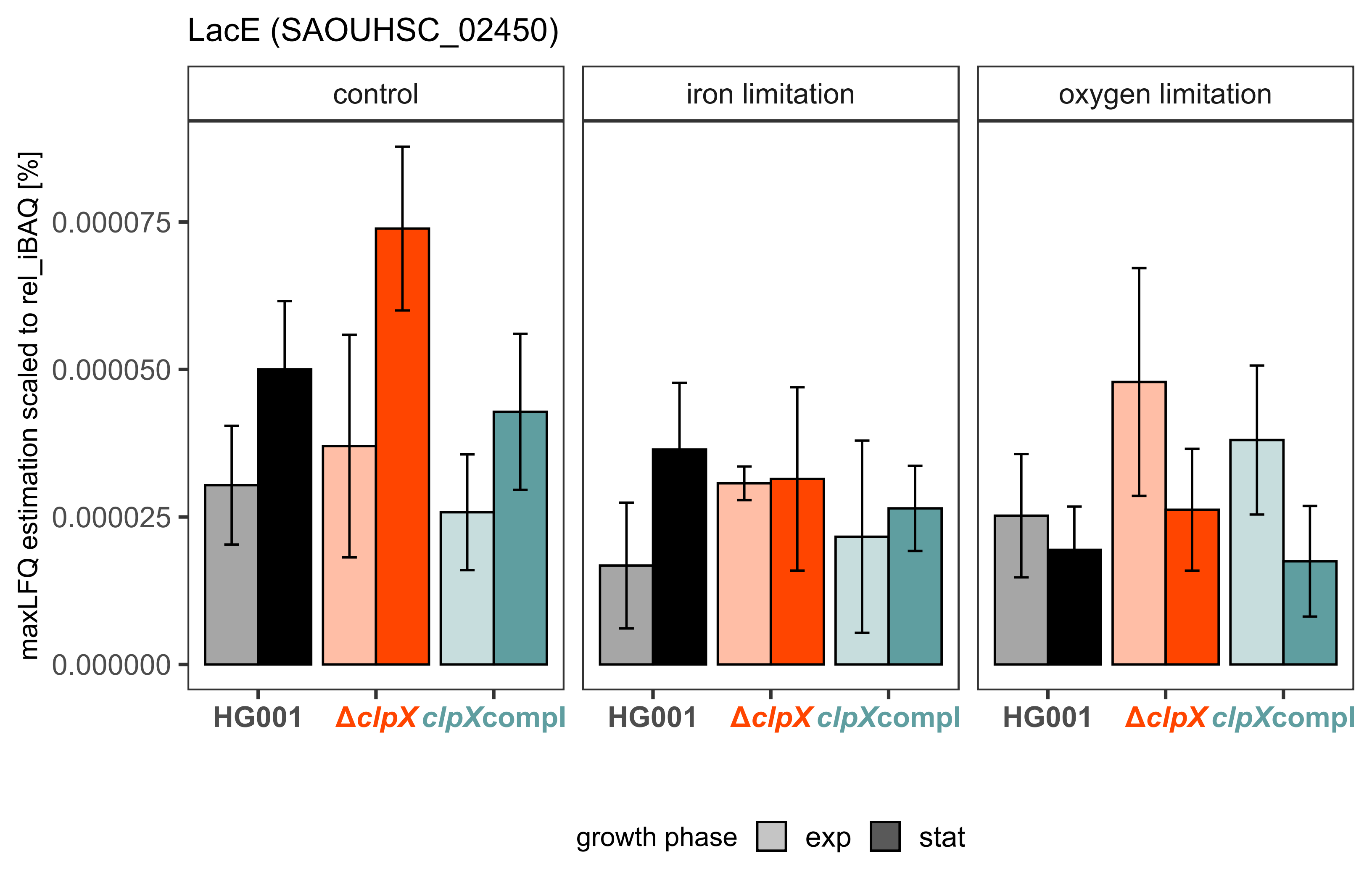

LacE (SAOUHSC_02450)
control
iron limitation
oxygen limitation
maxLFQ estimation scaled to rel_iBAQ [%]
0.000075
0.000050
0.000025
0.000000
HG001
ΔclpX
clpXcompl
growth phase
exp
stat

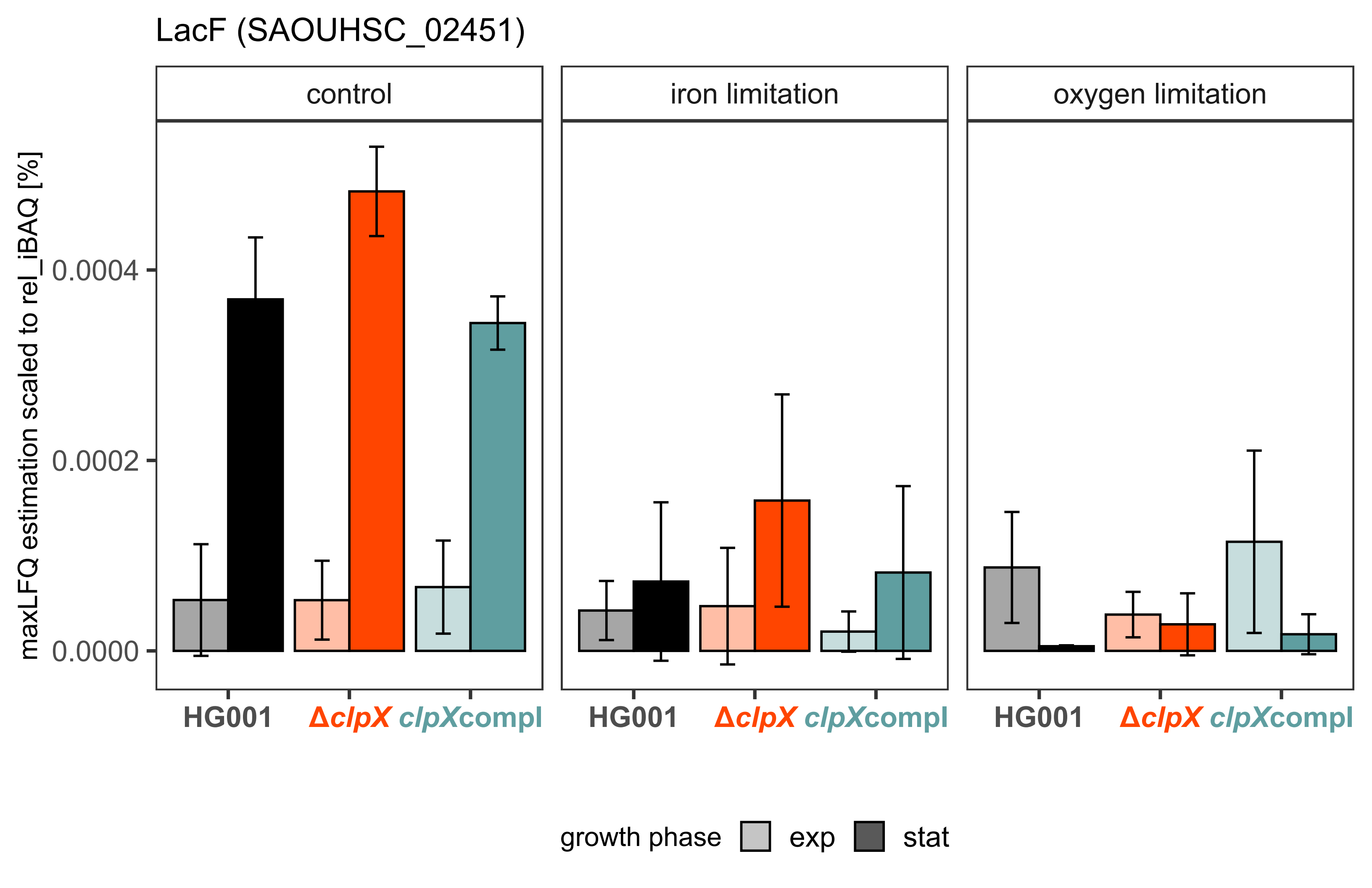

LacF (SAOUHSC_02451)
control
iron limitation
oxygen limitation
maxLFQ estimation scaled to rel_iBAQ [%]
0.0004
0.0002
0.0000
HG001
ΔclpX
clpXcompl
growth phase
exp
stat

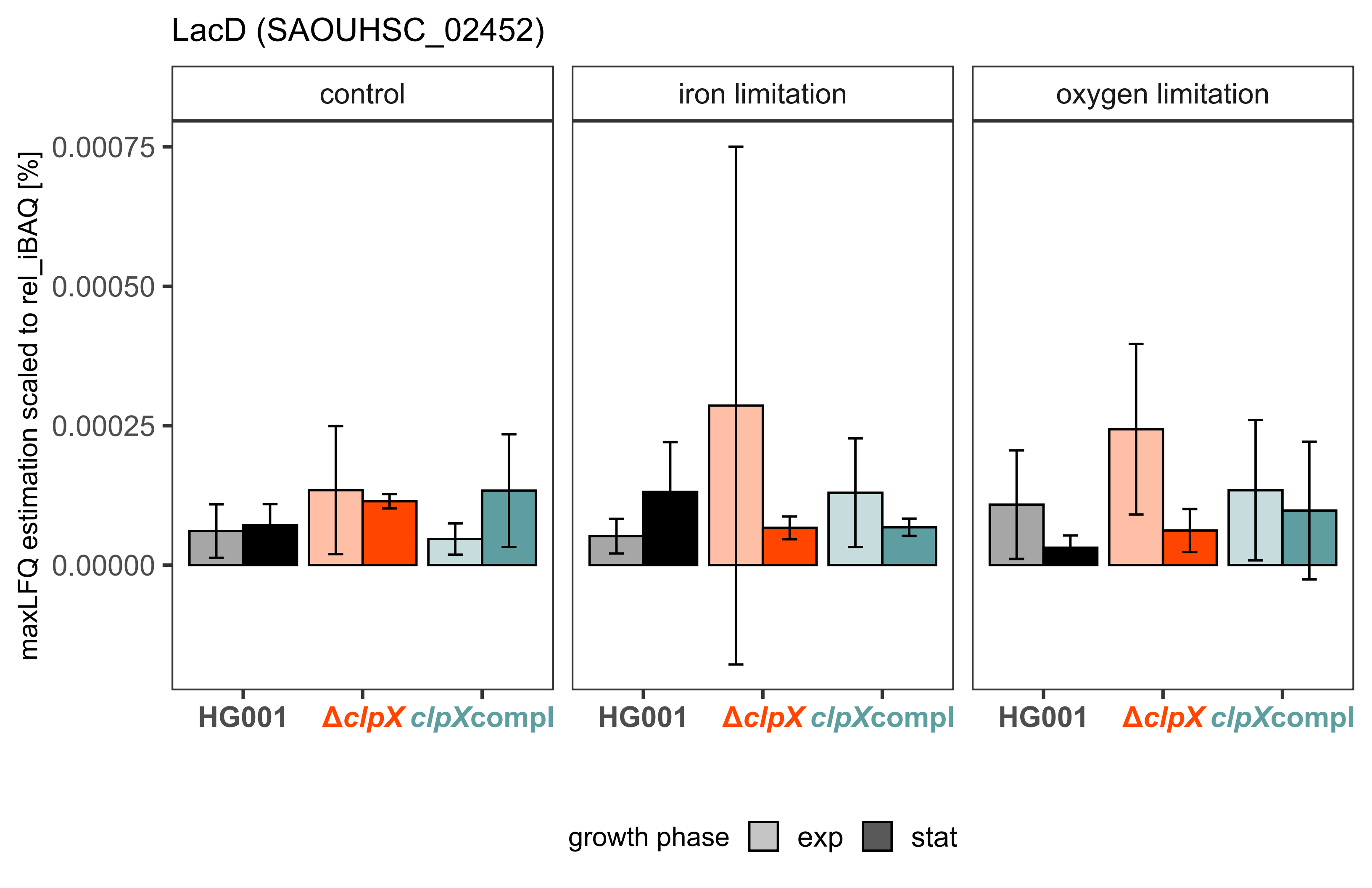

LacD (SAOUHSC_02452)
control
iron limitation
oxygen limitation
maxLFQ estimation scaled to rel_iBAQ [%]
0.00075
0.00050
0.00025
0.00000
HG001
ΔclpX
clpXcompl
growth phase
exp
stat

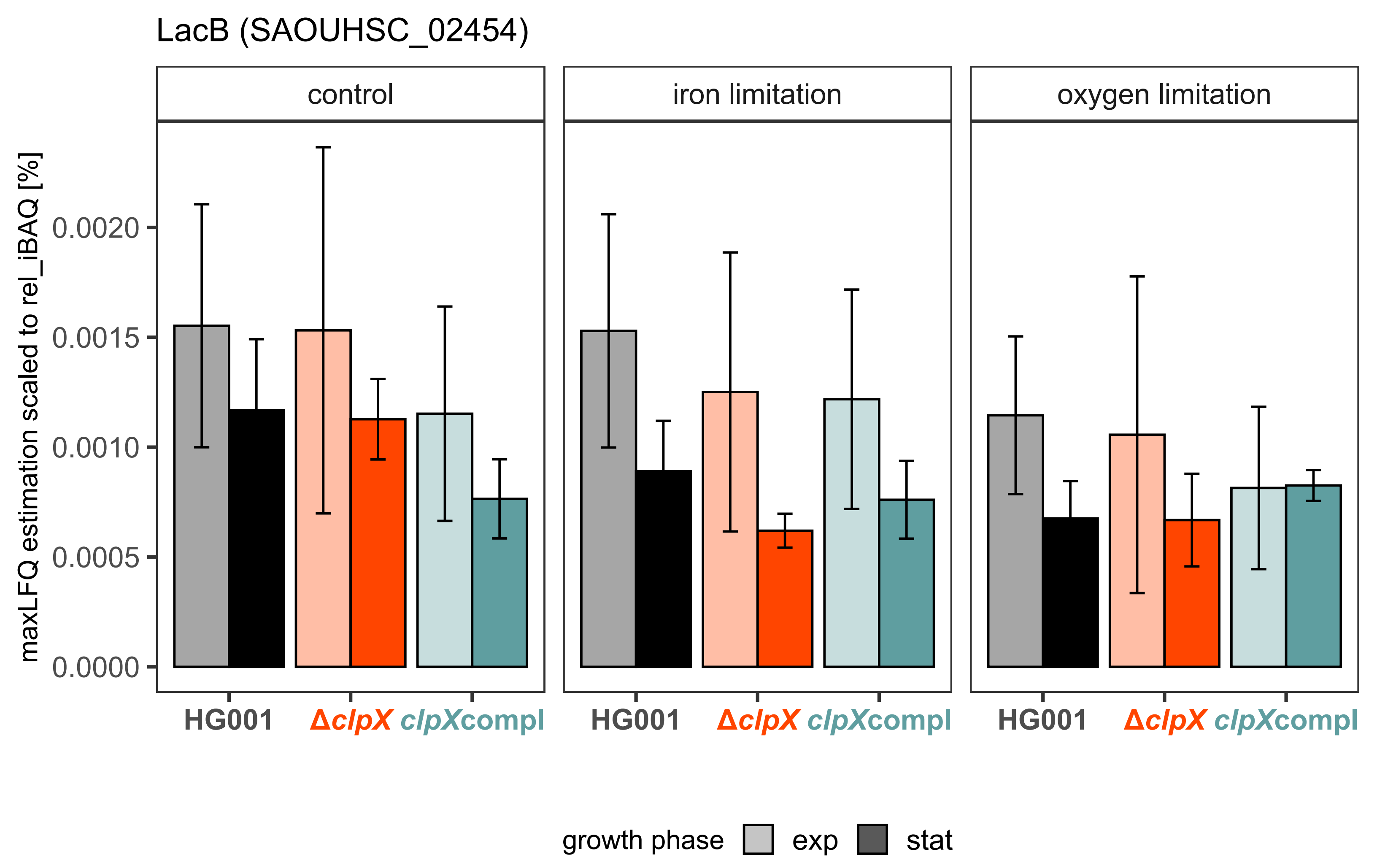

LacB (SAOUHSC_02454)
control
iron limitation
oxygen limitation
maxLFQ estimation scaled to rel_iBAQ [%]
0.0020
0.0015
0.0010
0.0005
0.0000
HG001
ΔclpX
clpXcompl
growth phase
exp
stat

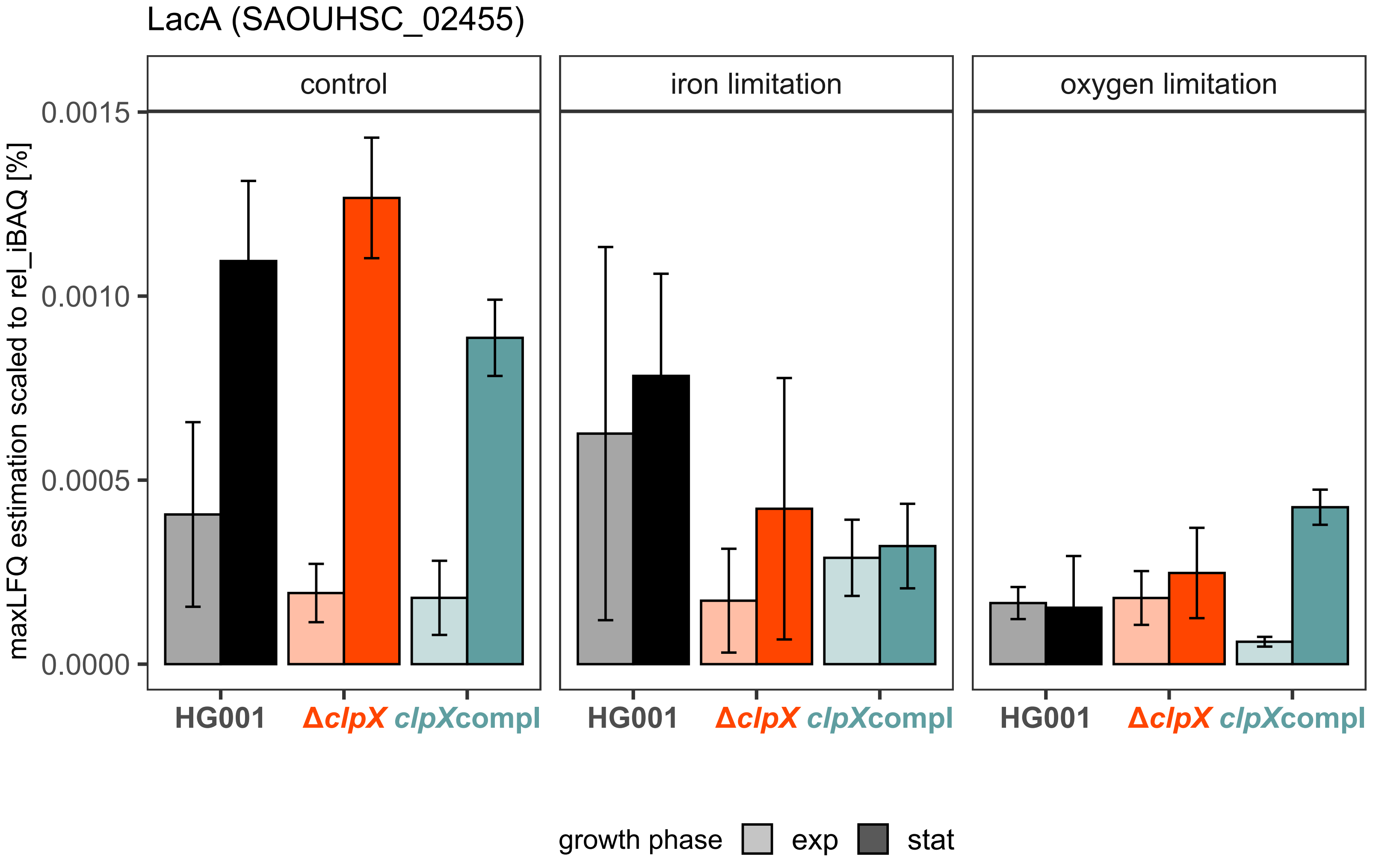

LacA (SAOUHSC_02455)
control
iron limitation
oxygen limitation
0.0015
0.0010
0.0005
0.0000
maxLFQ estimation scaled to rel_iBAQ [%]
HG001
ΔclpX
clpXcompl
growth phase
exp
stat

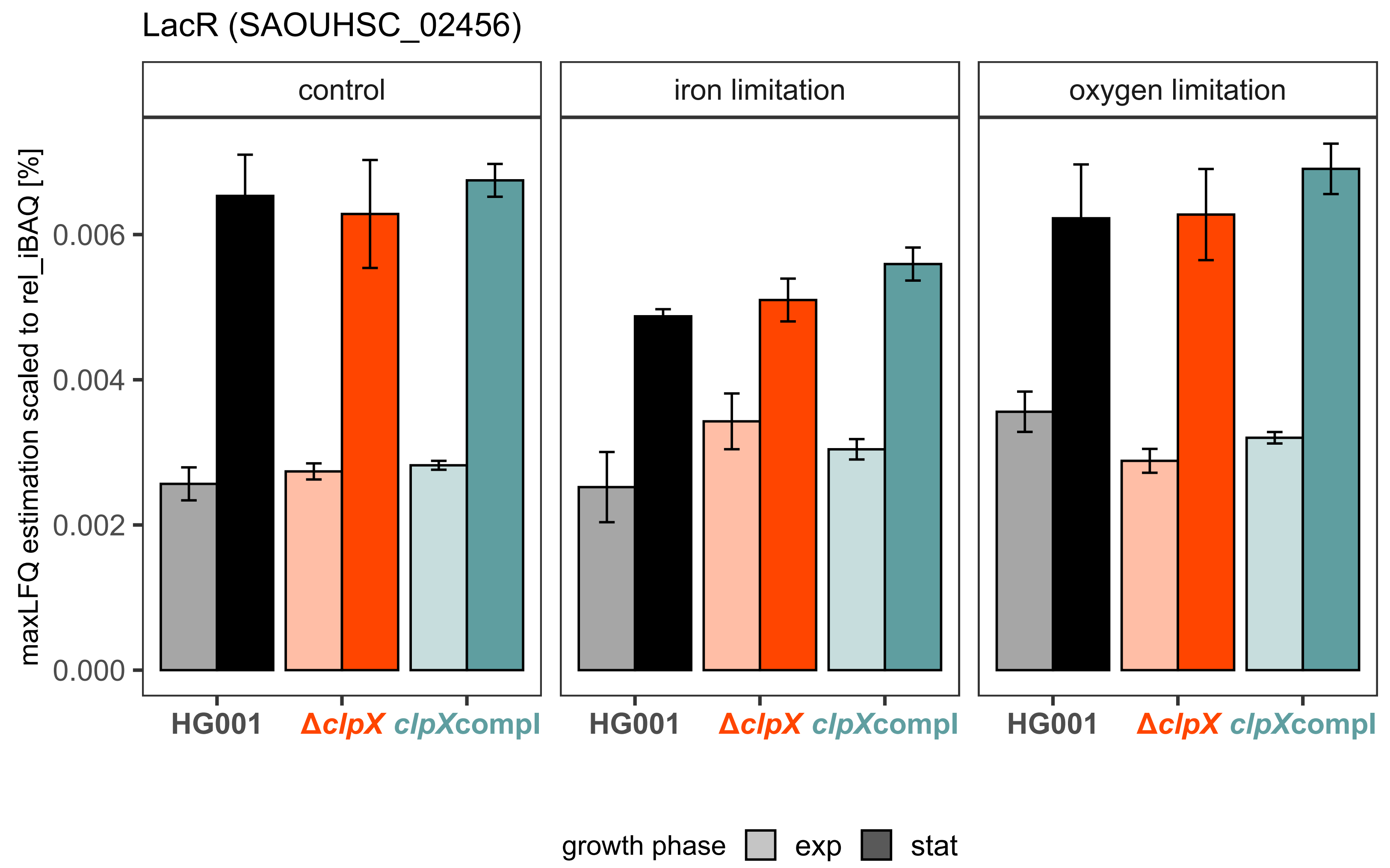
LacR (SAOUHSC_02456)
control
iron limitation
oxygen limitation
maxLFQ estimation scaled to rel_iBAQ [%]
0.000
0.002
0.004
0.006
HG001
ΔclpX
clpXcompl
growth phase
exp
stat

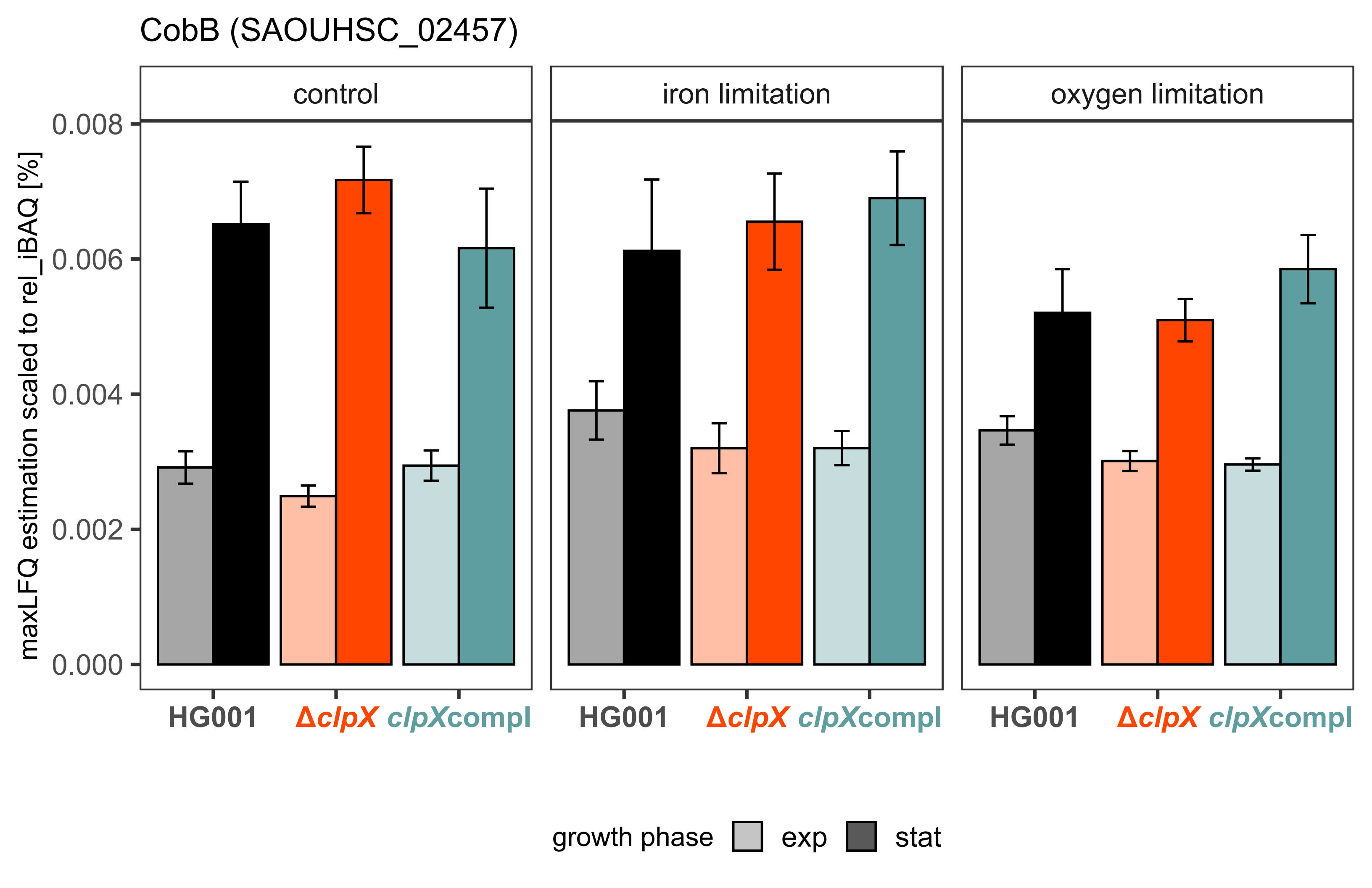
CobB (SAOUHSC_02457)
control
iron limitation
oxygen limitation
maxLFQ estimation scaled to rel_iBAQ [%]
0.008
0.006
0.004
0.002
0.000
HG001
ΔclpX
clpXcompl
growth phase
exp
stat

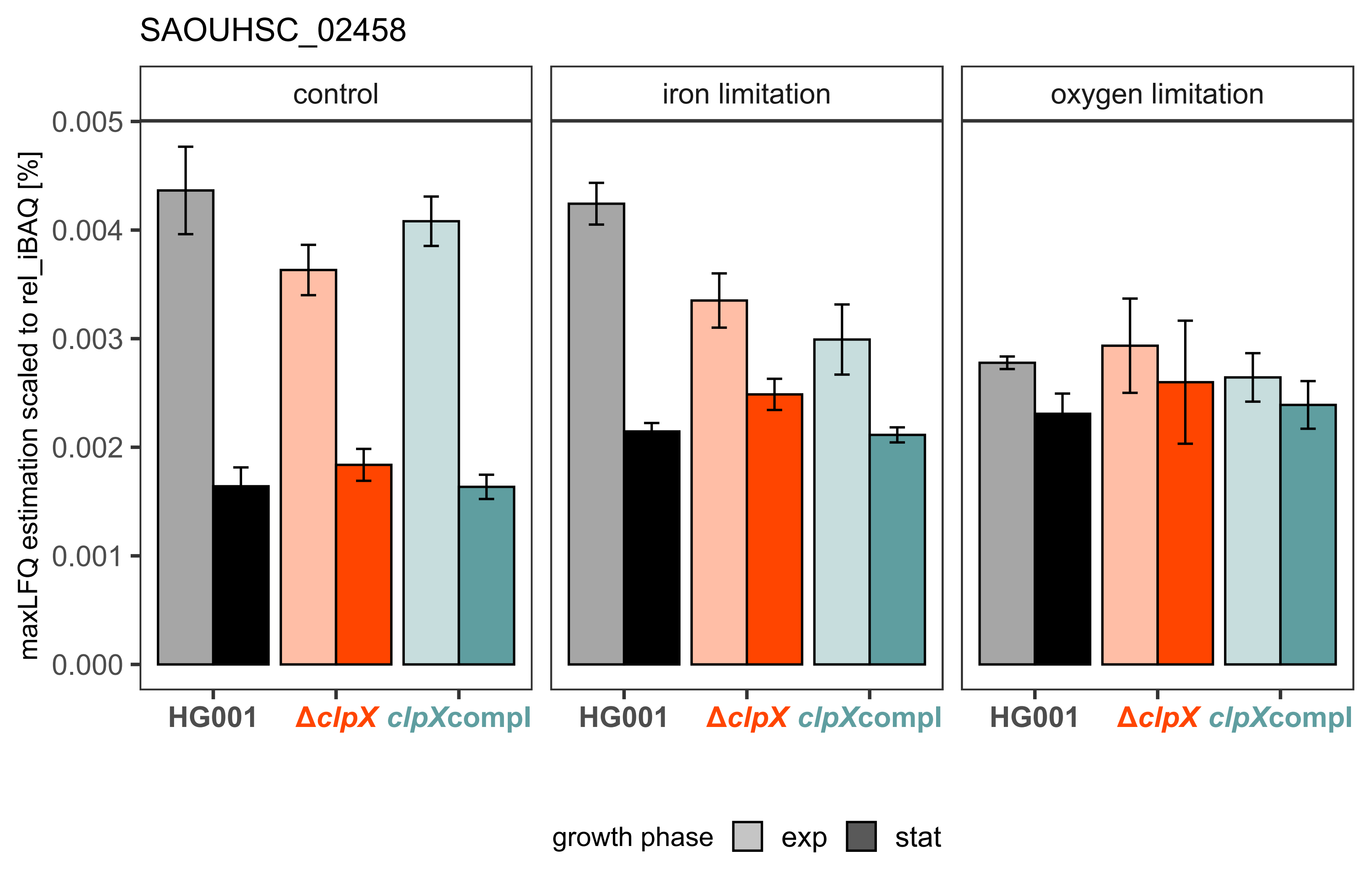
SAOUHSC_02458
control
iron limitation
oxygen limitation
maxLFQ estimation scaled to rel_iBAQ [%]
0.005
0.004
0.003
0.002
0.001
0.000
HG001
ΔclpX
clpXcompl
growth phase
exp
stat

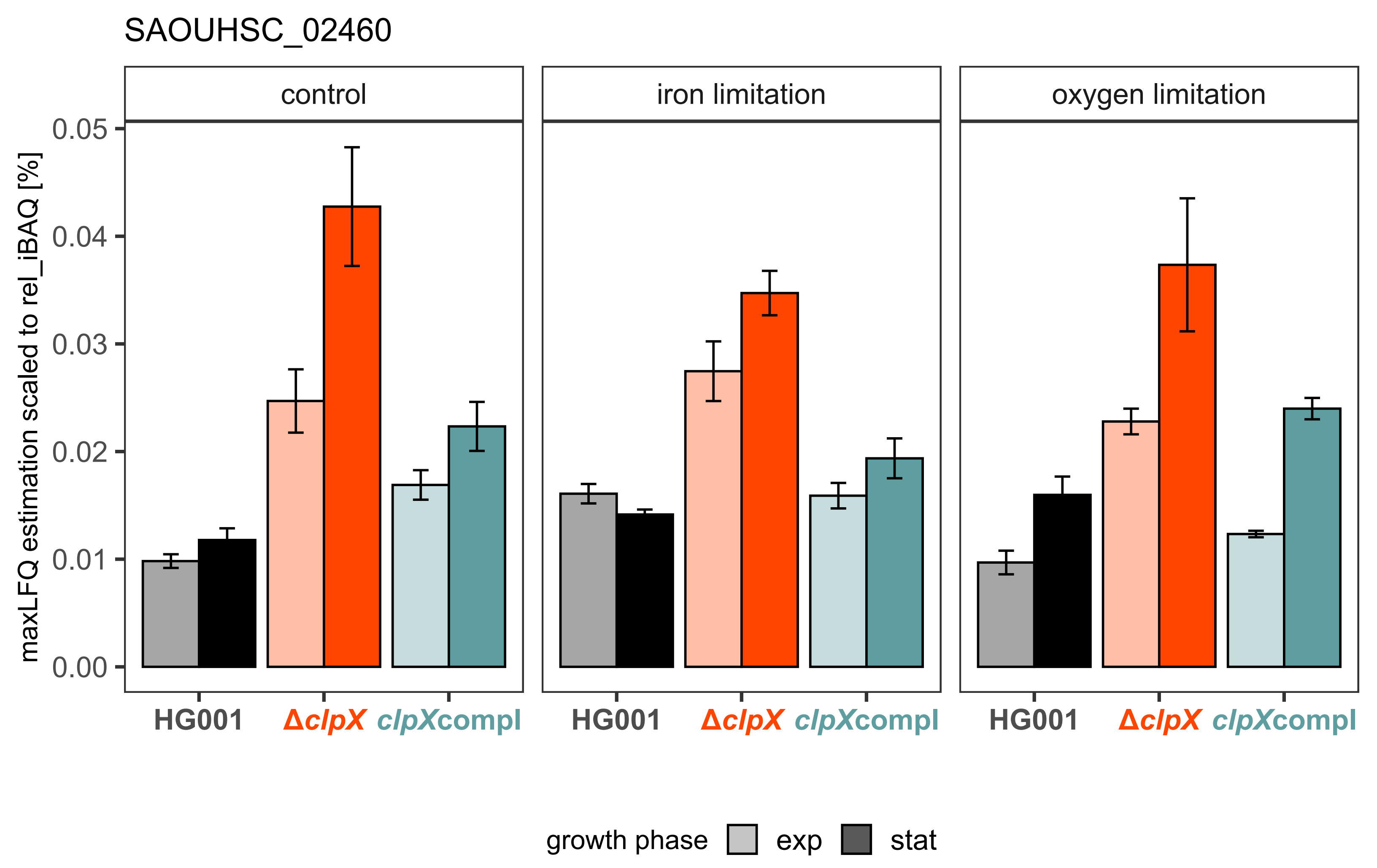

SAOUHSC_02460
control
iron limitation
oxygen limitation
maxLFQ estimation scaled to rel_iBAQ [%]
0.05
0.04
0.03
0.02
0.01
0.00
HG001
ΔclpX
clpXcompl
growth phase
exp
stat

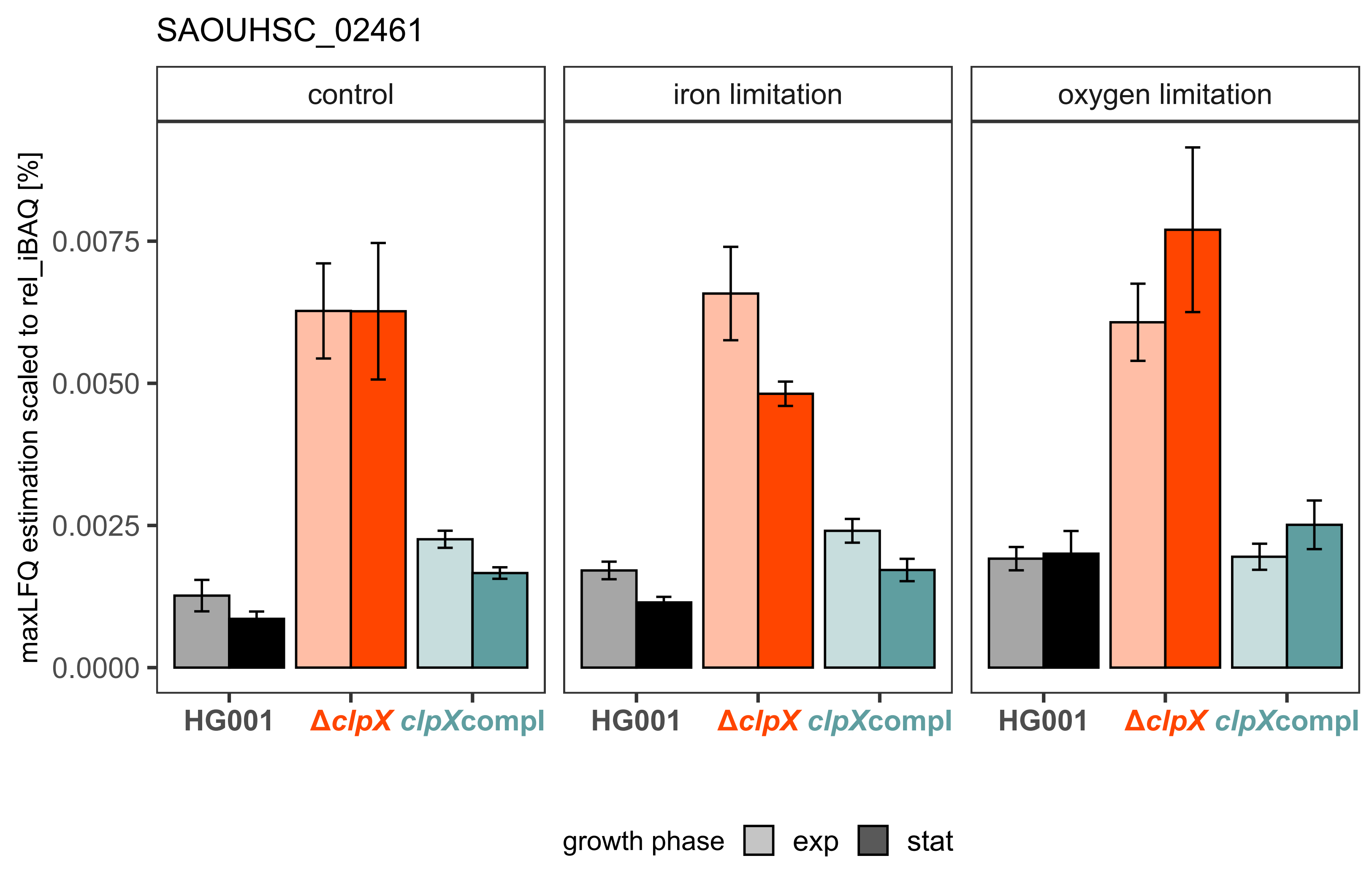
SAOUHSC_02461
control
iron limitation
oxygen limitation
maxLFQ estimation scaled to rel_iBAQ [%]
0.0075
0.0050
0.0025
0.0000
HG001
ΔclpX
clpXcompl
growth phase
exp
stat

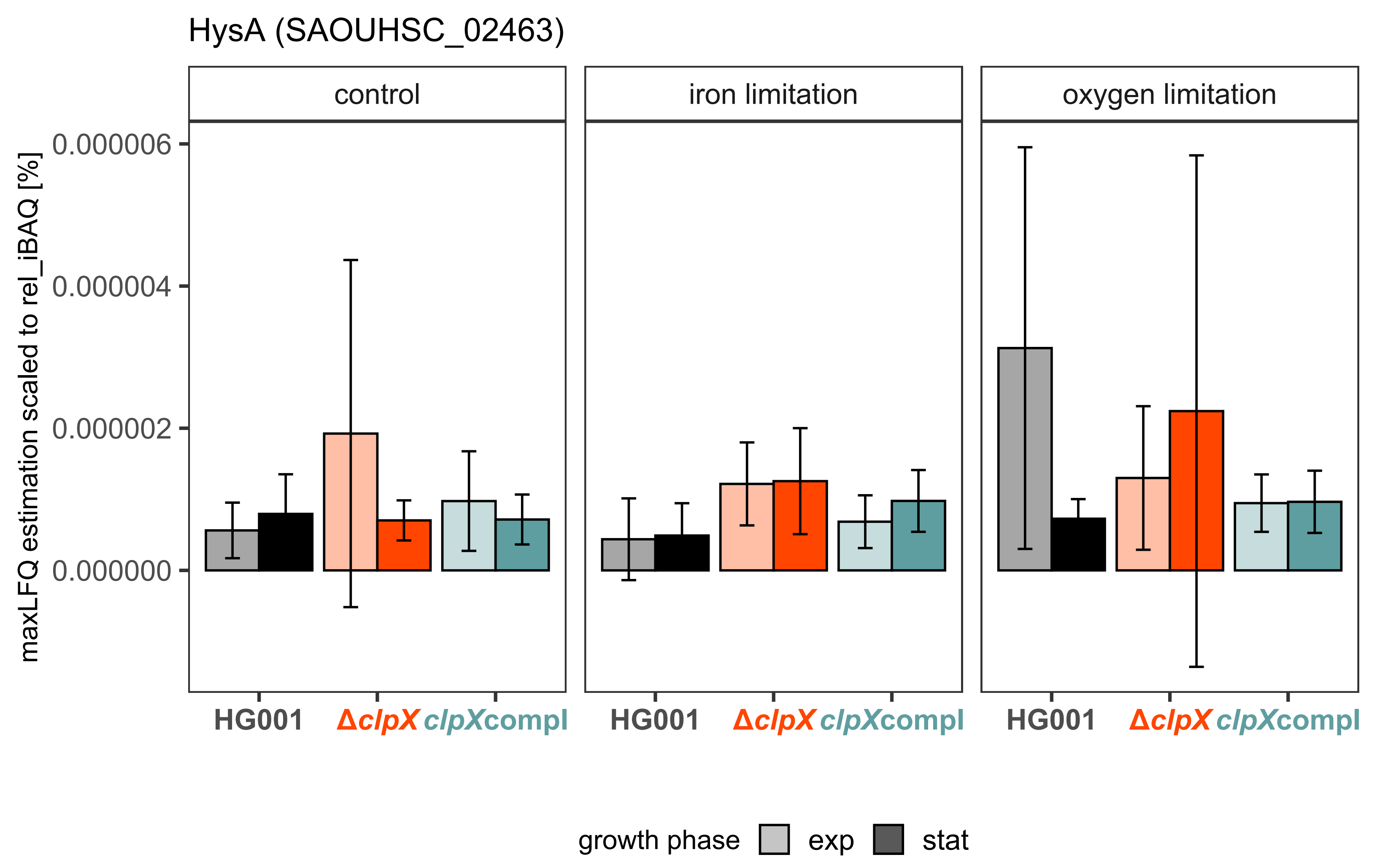
HysA (SAOUHSC_02463)
control
iron limitation
oxygen limitation
maxLFQ estimation scaled to rel_iBAQ [%]
0.000006
0.000004
0.000002
0.000000
HG001
ΔclpX
clpXcompl
growth phase
exp
stat

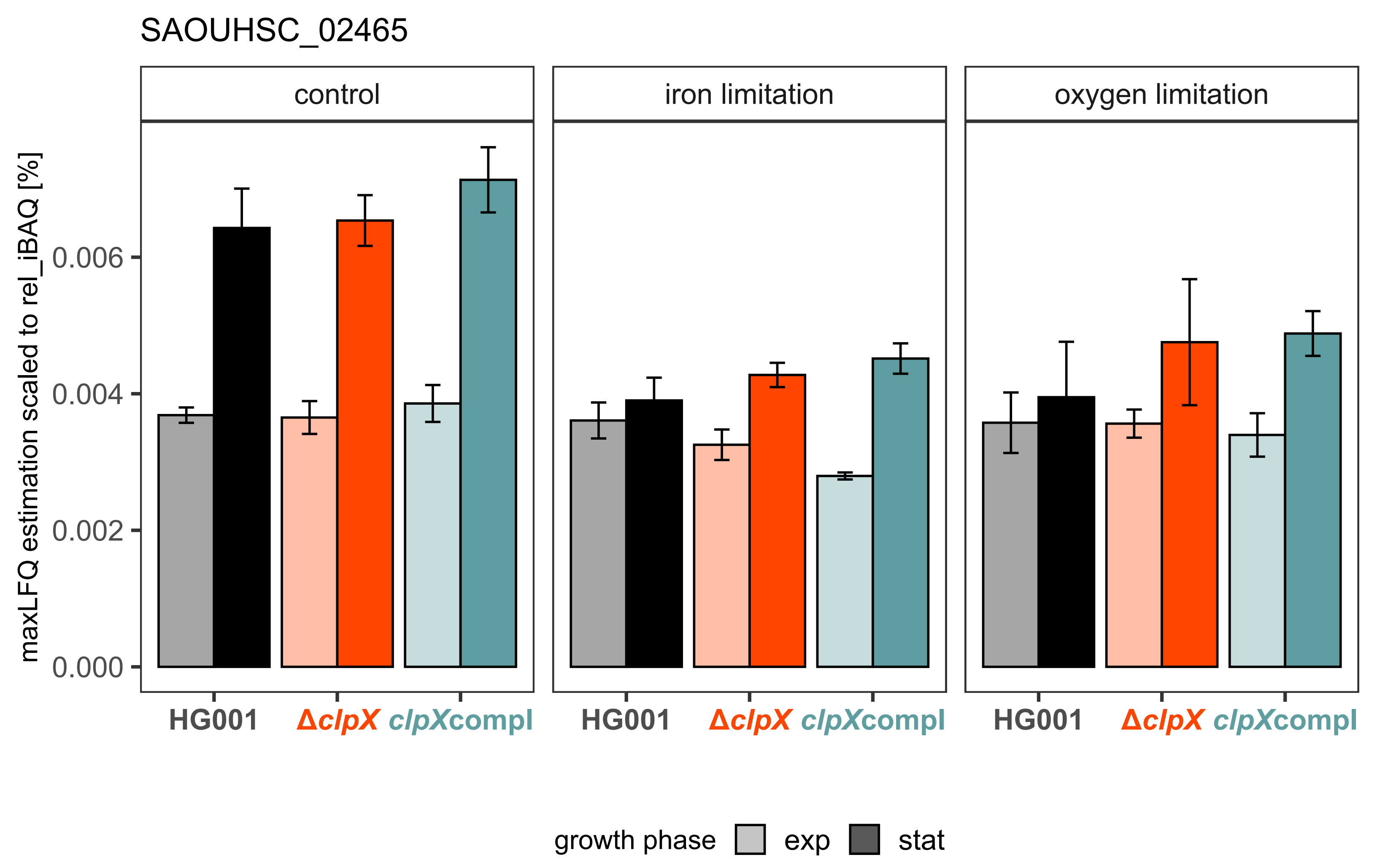

SAOUHSC_02465
control
iron limitation
oxygen limitation
maxLFQ estimation scaled to rel_iBAQ [%]
0.006
0.004
0.002
0.000
HG001
ΔclpX
clpXcompl
growth phase
exp
stat

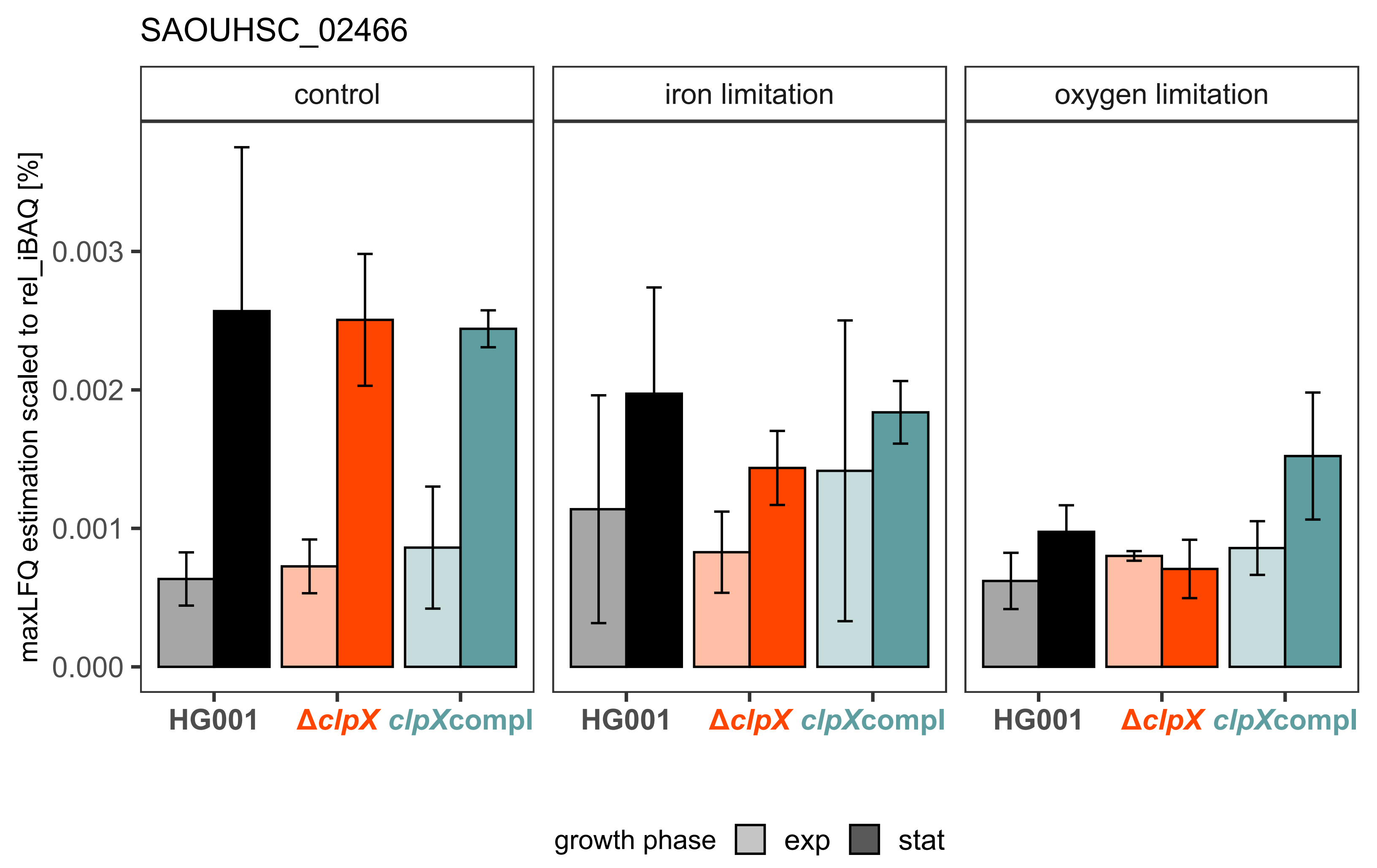

SAOUHSC_02466
control
iron limitation
oxygen limitation
maxLFQ estimation scaled to rel_iBAQ [%]
0.003
0.002
0.001
0.000
HG001
ΔclpX
clpXcompl
growth phase
exp
stat

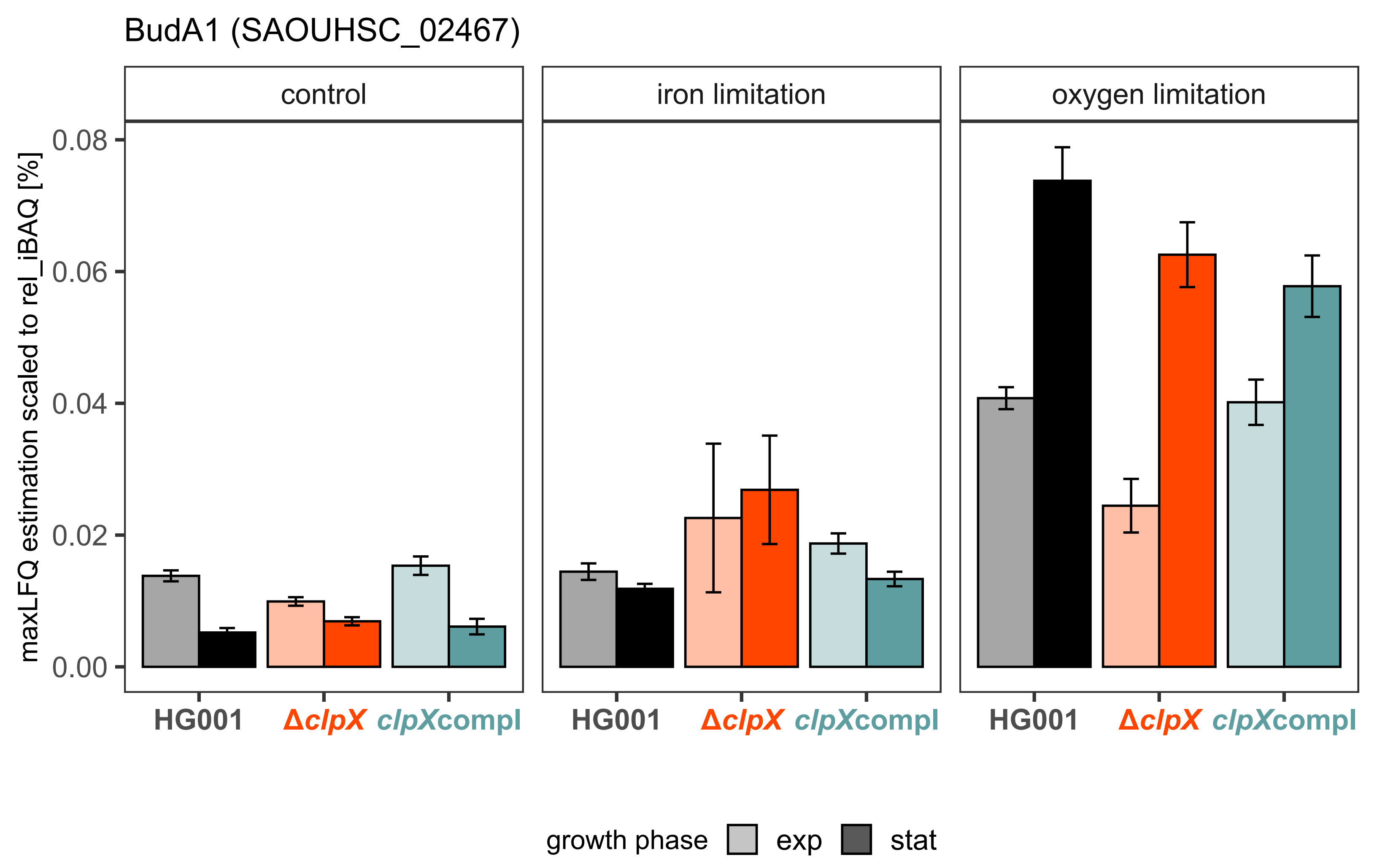

BudA1 (SAOUHSC_02467)
control
iron limitation
oxygen limitation
maxLFQ estimation scaled to rel_iBAQ [%]
0.00
0.02
0.04
0.06
0.08
HG001
ΔclpX
clpXcompl
growth phase
exp
stat

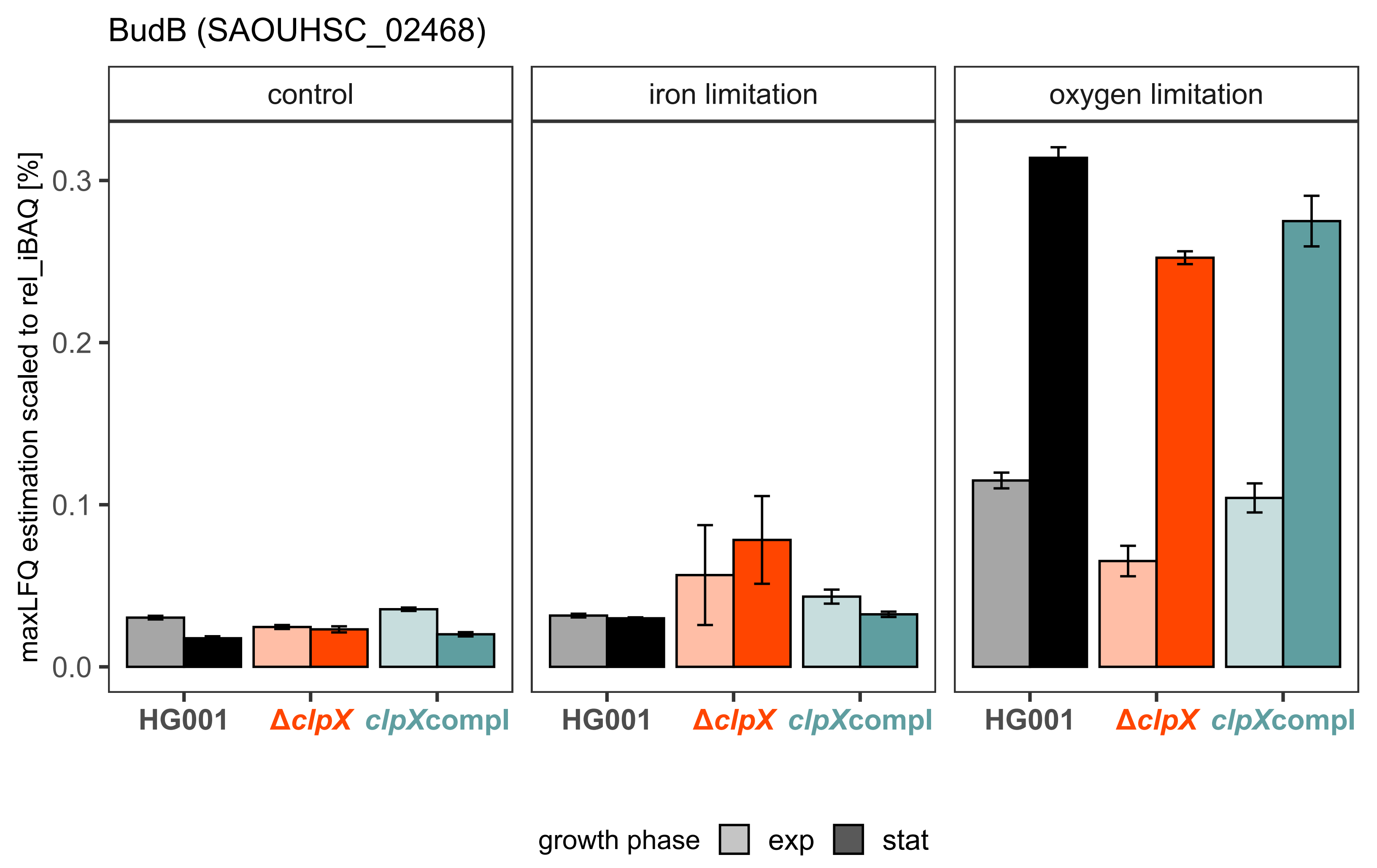
BudB (SAOUHSC_02468)
control
iron limitation
oxygen limitation
maxLFQ estimation scaled to rel_iBAQ [%]
0.0
0.1
0.2
0.3
HG001
ΔclpX
clpXcompl
growth phase
exp
stat

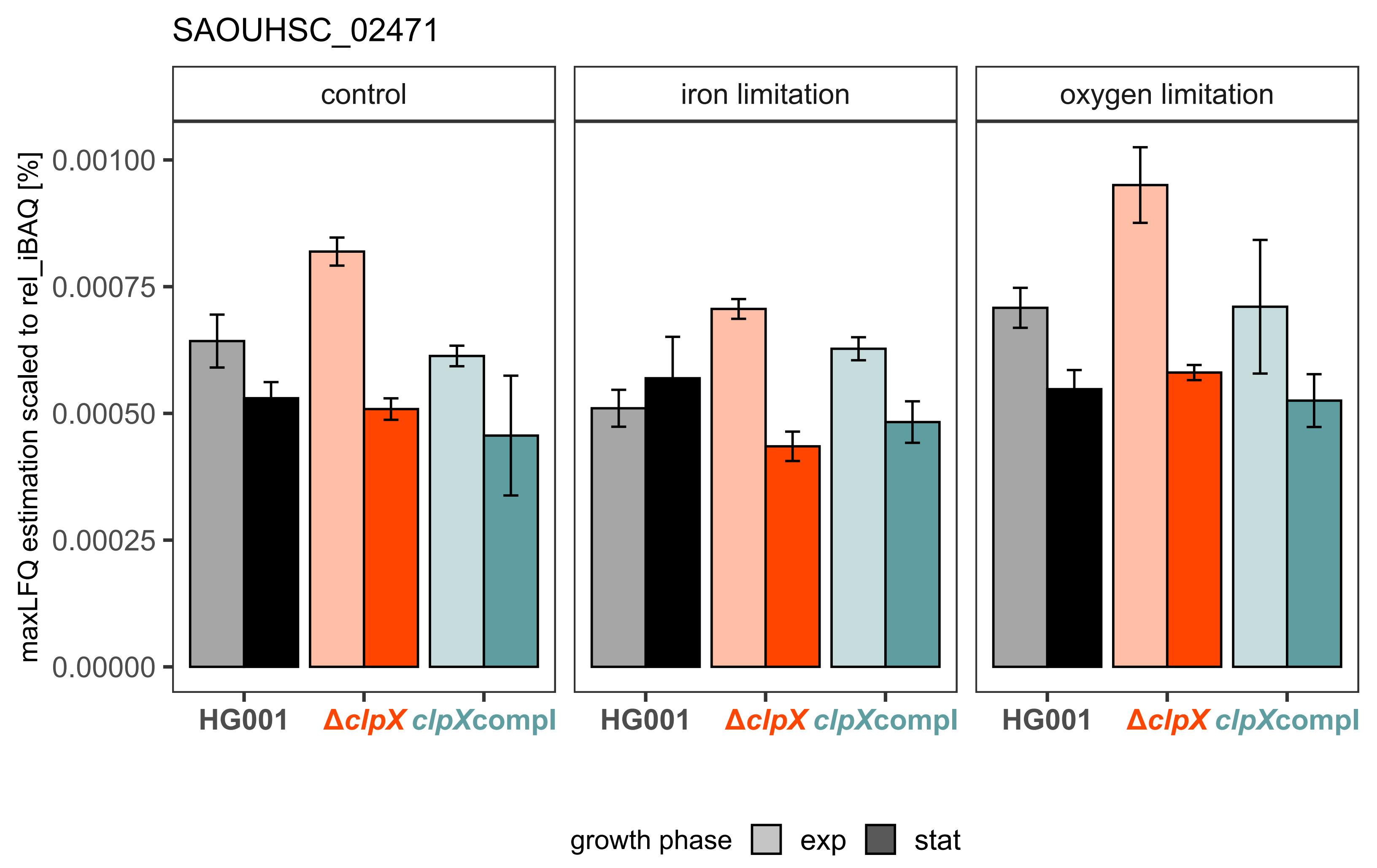

SAOUHSC_02471
control
iron limitation
oxygen limitation
maxLFQ estimation scaled to rel_iBAQ [%]
0.00100
0.00075
0.00050
0.00025
0.00000
HG001
ΔclpX
clpXcompl
growth phase
exp
stat

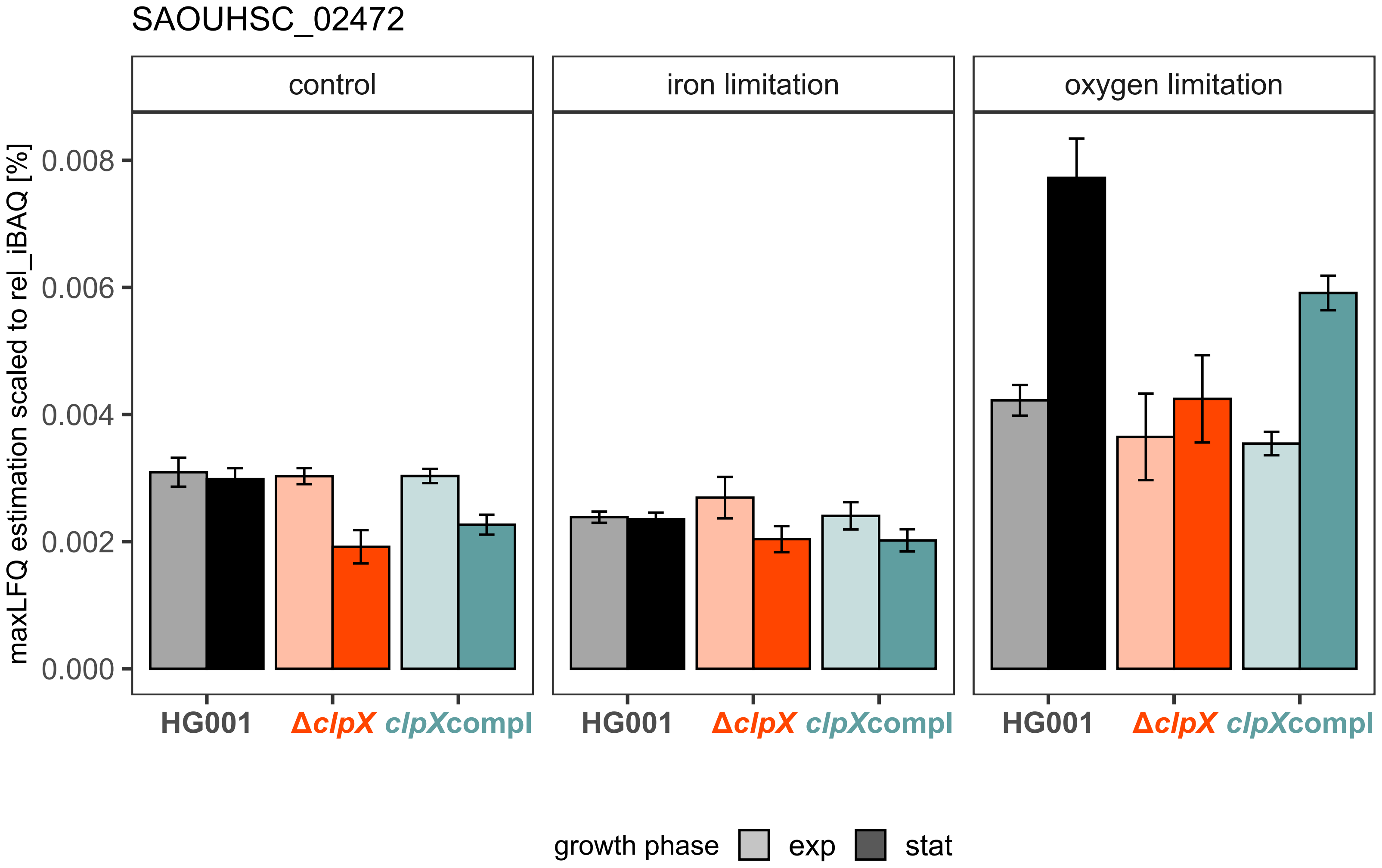
SAOUHSC_02472
control
iron limitation
oxygen limitation
maxLFQ estimation scaled to rel_iBAQ [%]
0.008
0.006
0.004
0.002
0.000
HG001
ΔclpX
clpXcompl
growth phase
exp
stat

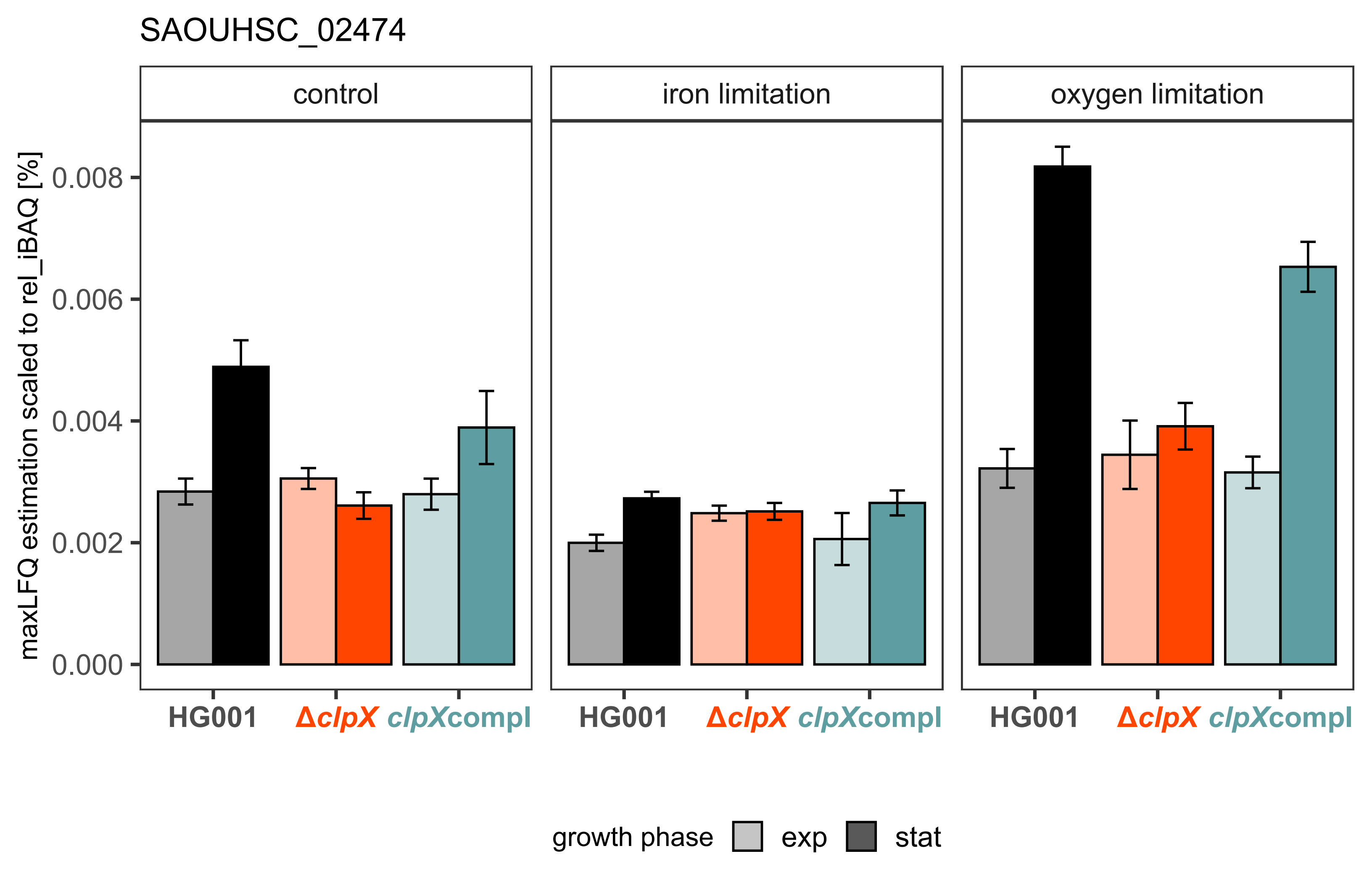

SAOUHSC_02474
control
iron limitation
oxygen limitation
maxLFQ estimation scaled to rel_iBAQ [%]
0.008
0.006
0.004
0.002
0.000
HG001
ΔclpX
clpXcompl
growth phase
exp
stat

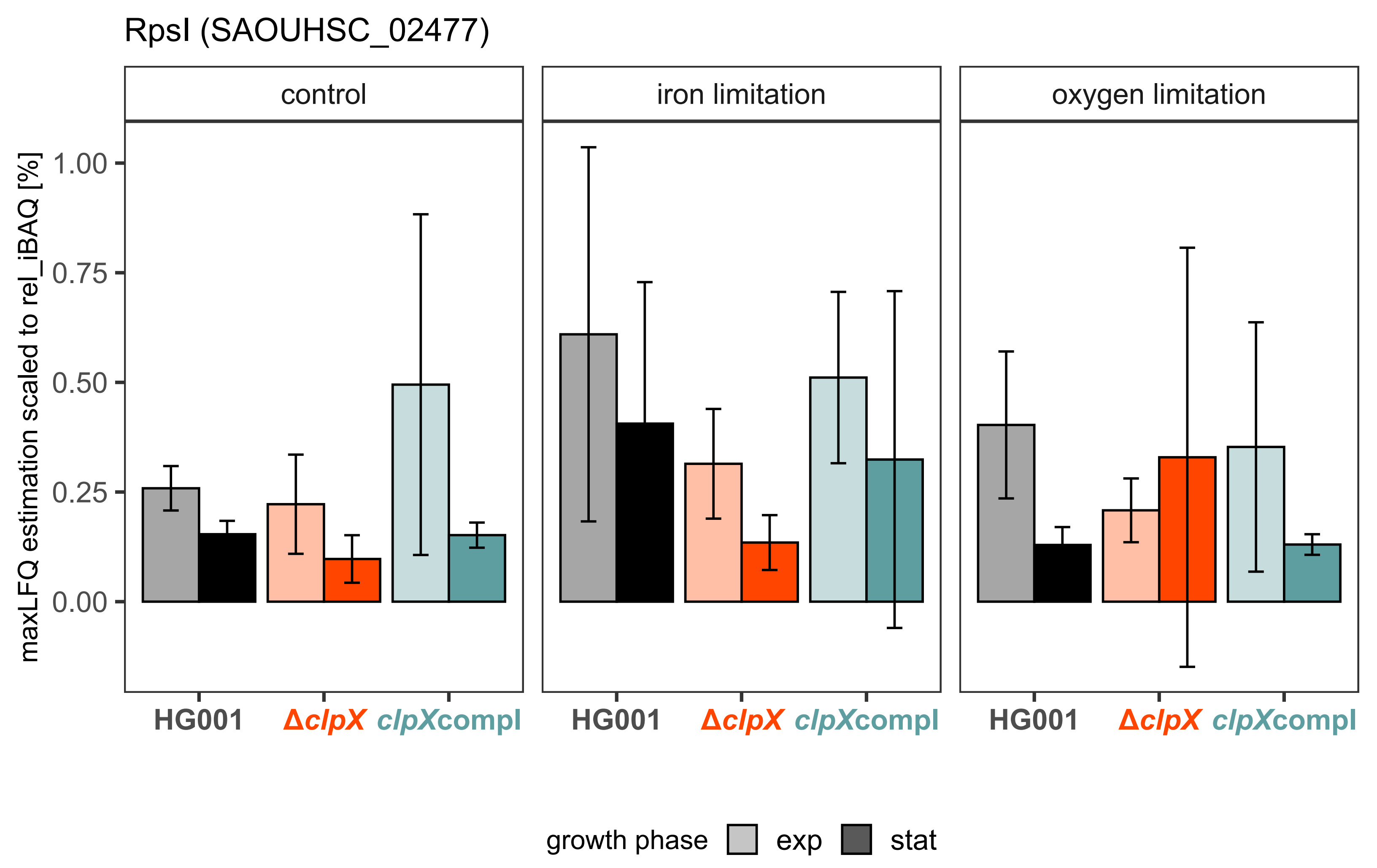

RpsI (SAOUHSC_02477)
control
iron limitation
oxygen limitation
maxLFQ estimation scaled to rel_iBAQ [%]
1.00
0.75
0.50
0.25
0.00
HG001
ΔclpX
clpXcompl
growth phase
exp
stat

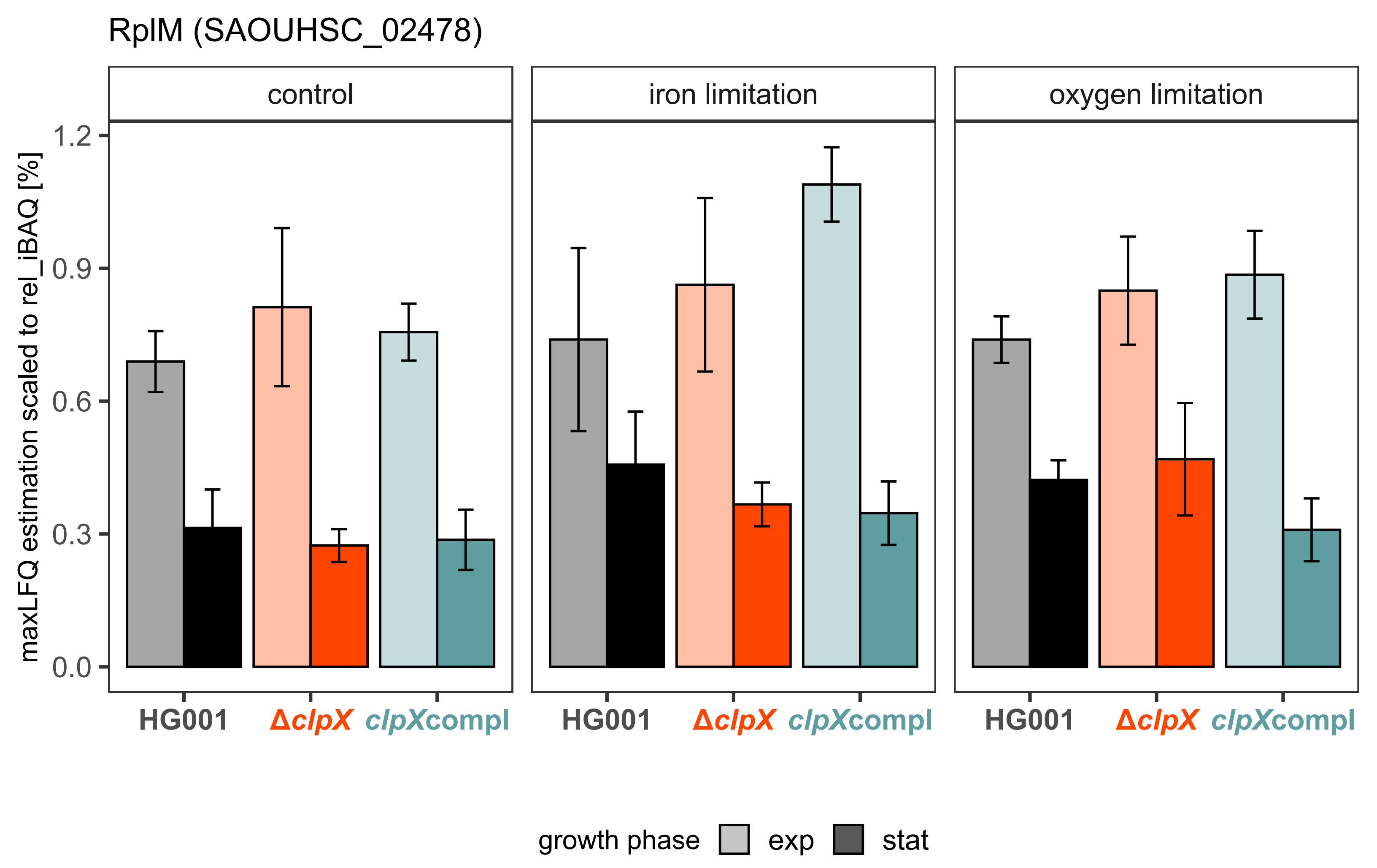
RplM (SAOUHSC_02478)
control
iron limitation
oxygen limitation
maxLFQ estimation scaled to rel_iBAQ [%]
1.2
0.9
0.6
0.3
0.0
HG001
ΔclpX
clpXcompl
growth phase
exp
stat

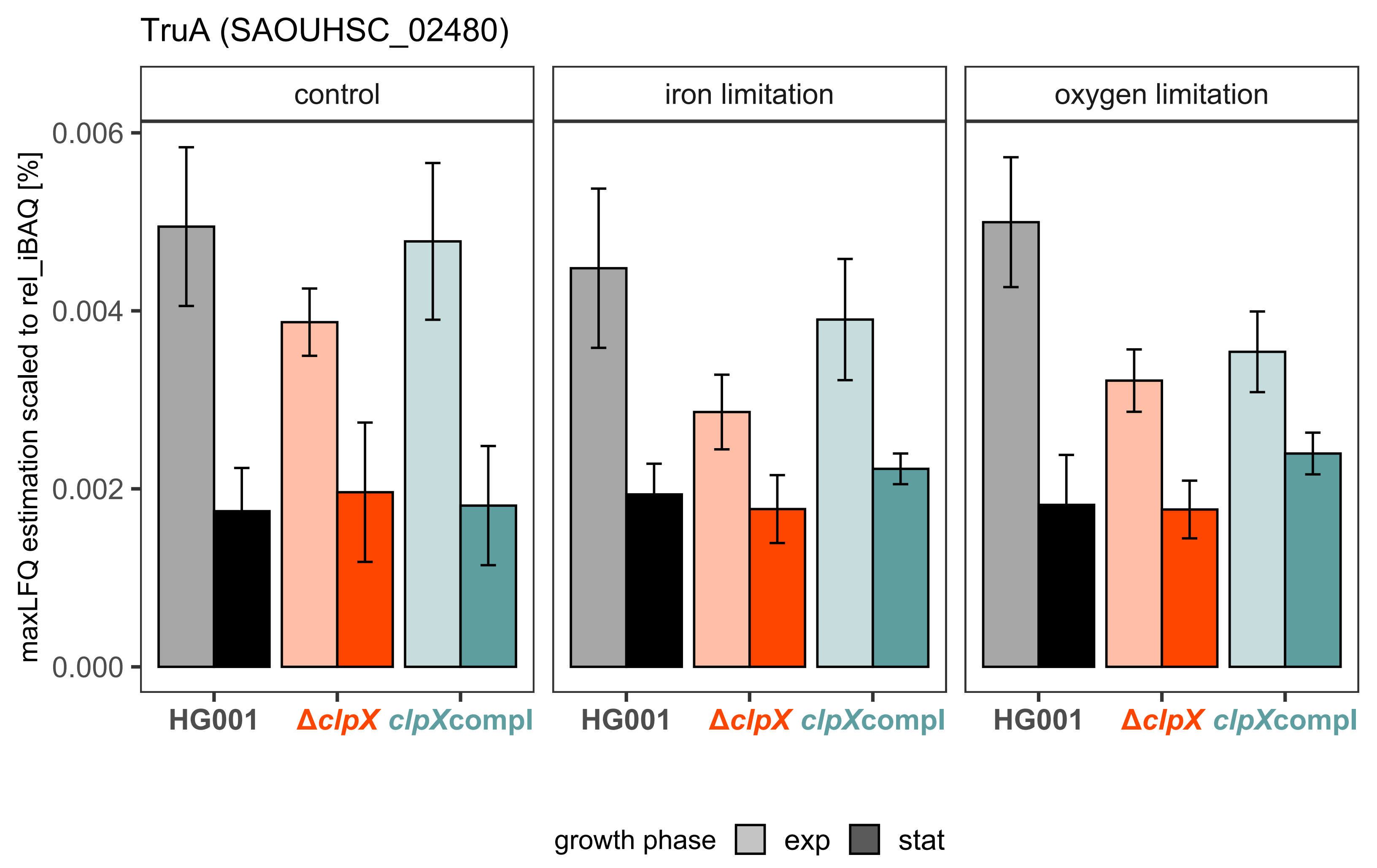
TruA (SAOUHSC_02480)
control
iron limitation
oxygen limitation
0.006
0.004
0.002
0.000
maxLFQ estimation scaled to rel_iBAQ [%]
HG001
ΔclpX
clpXcompl
growth phase
exp
stat

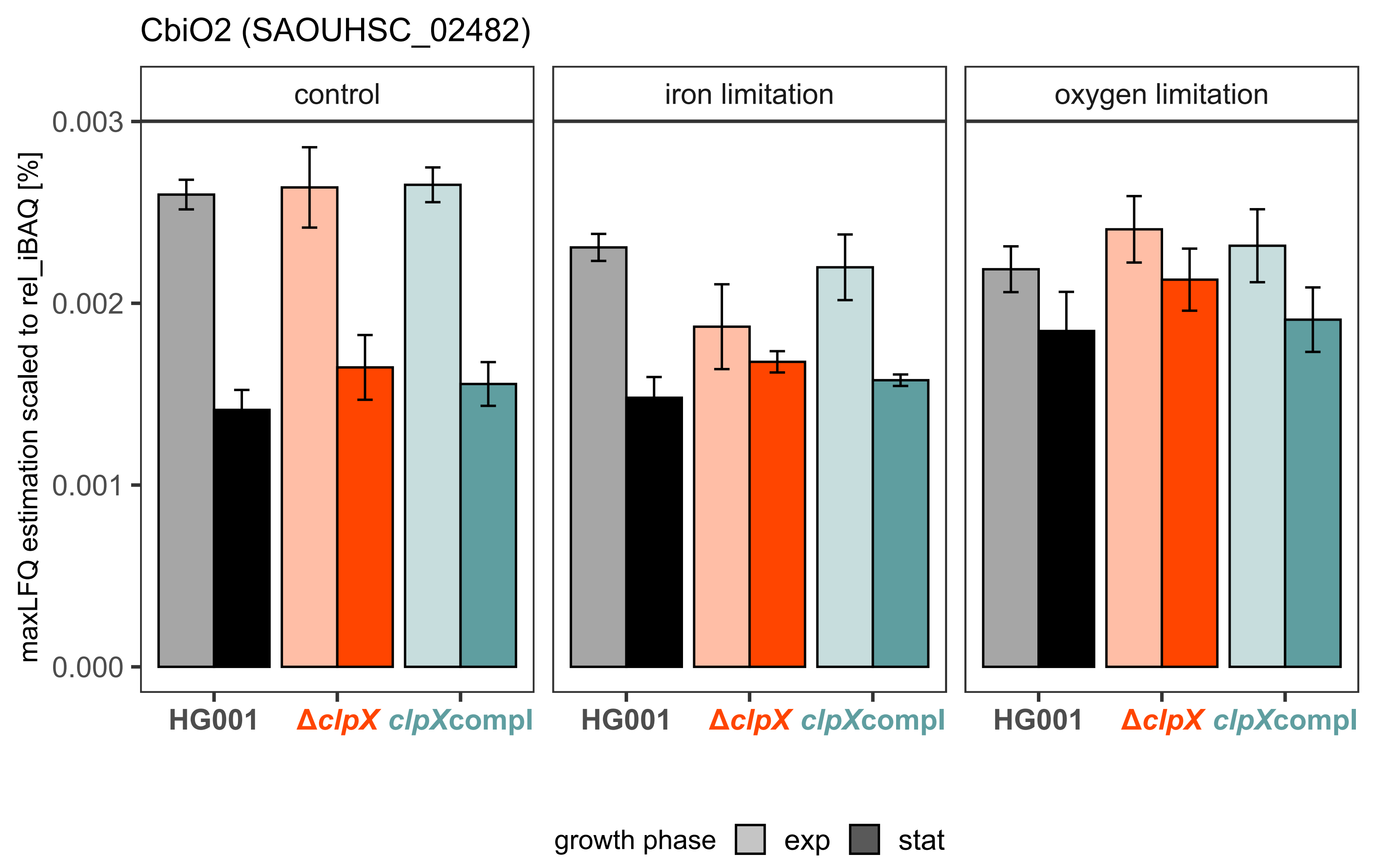

CbiO2 (SAOUHSC_02482)
control
iron limitation
oxygen limitation
0.003
0.002
0.001
0.000
maxLFQ estimation scaled to rel_iBAQ [%]
HG001
ΔclpX
clpXcompl
growth phase
exp
stat

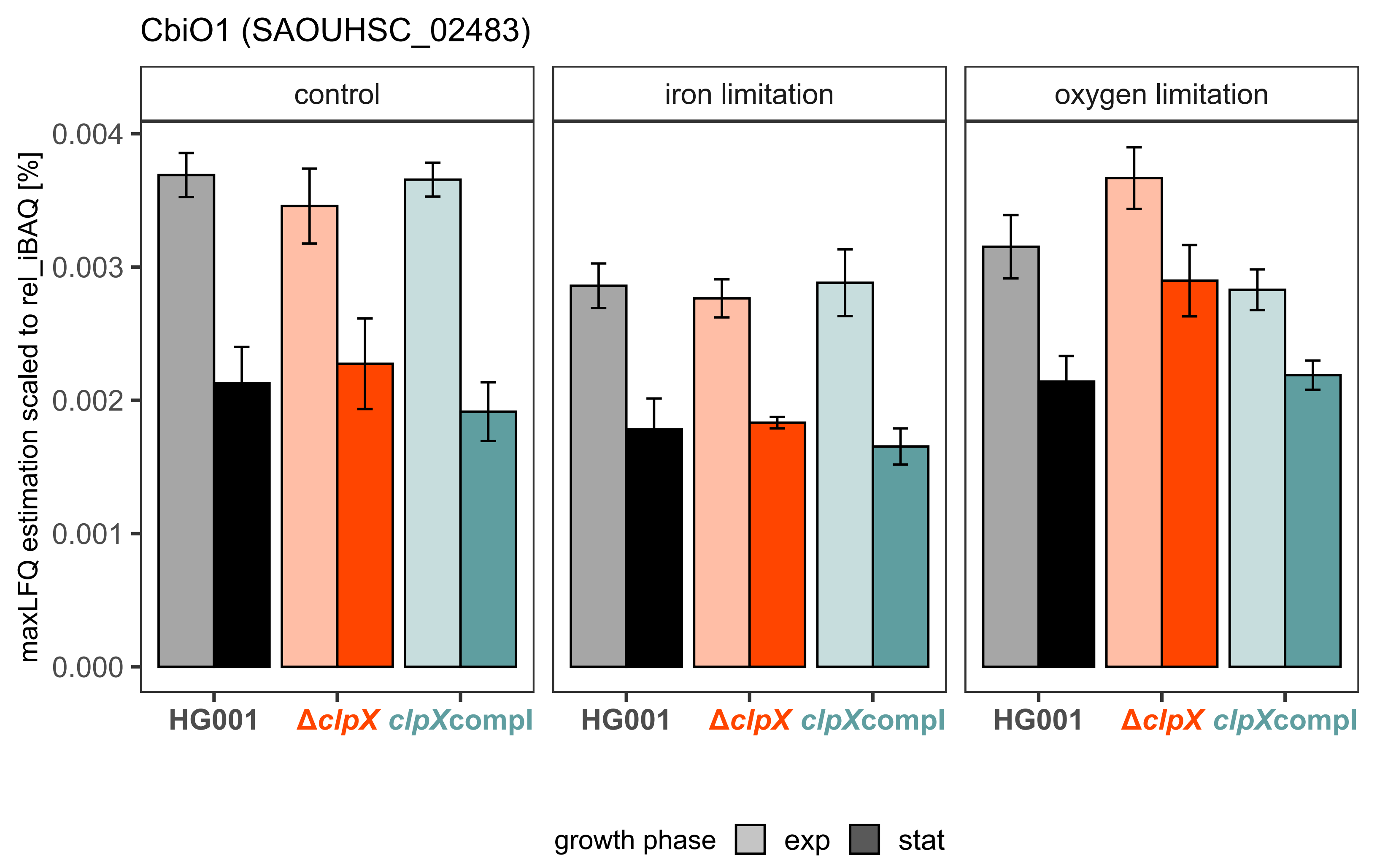

CbiO1 (SAOUHSC_02483)
control
iron limitation
oxygen limitation
maxLFQ estimation scaled to rel_iBAQ [%]
0.004
0.003
0.002
0.001
0.000
HG001
ΔclpX
clpXcompl
growth phase
exp
stat

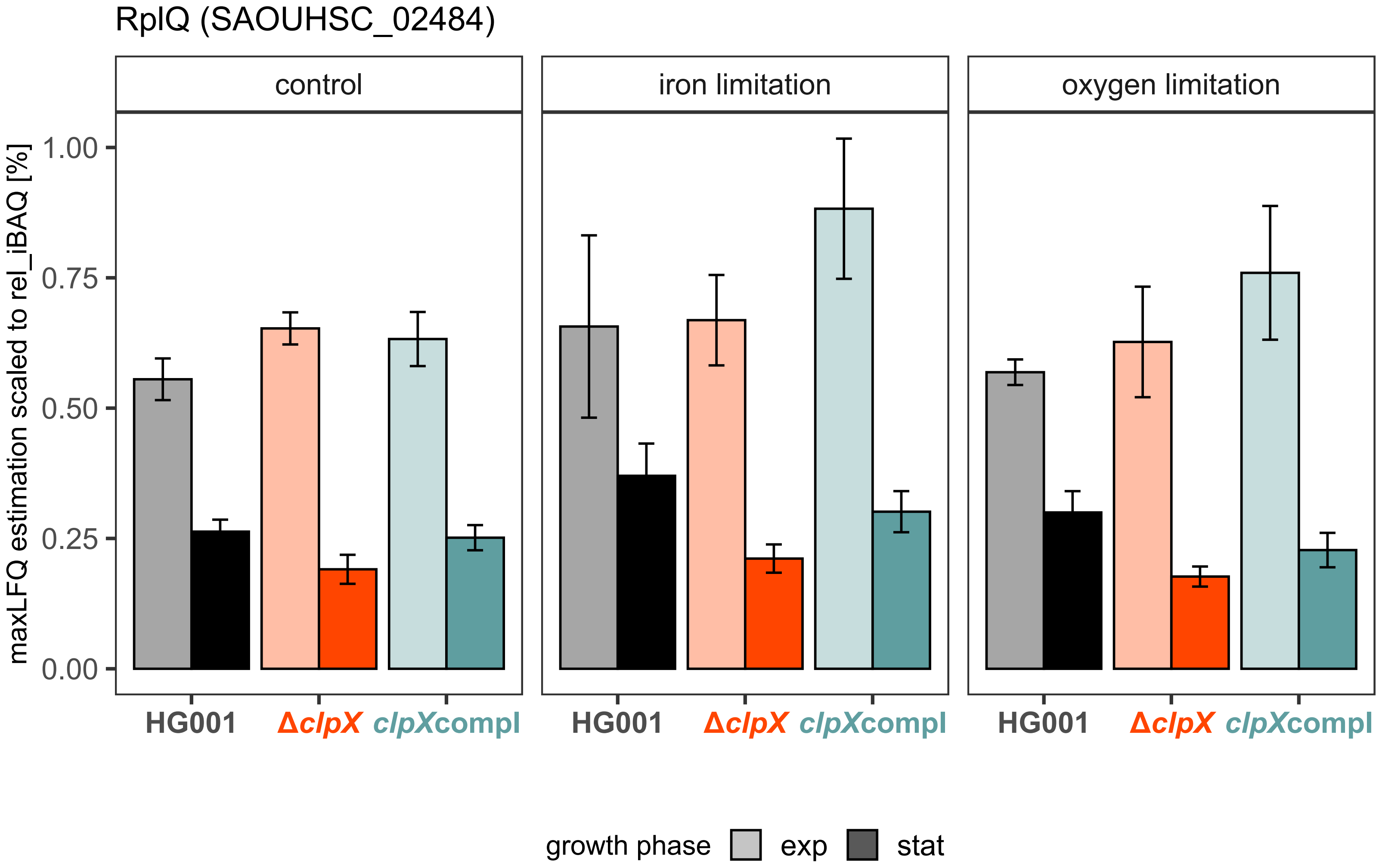
RplQ (SAOUHSC_02484)
control
iron limitation
oxygen limitation
maxLFQ estimation scaled to rel_iBAQ [%]
1.00
0.75
0.50
0.25
0.00
HG001
ΔclpX
clpXcompl
growth phase
exp
stat

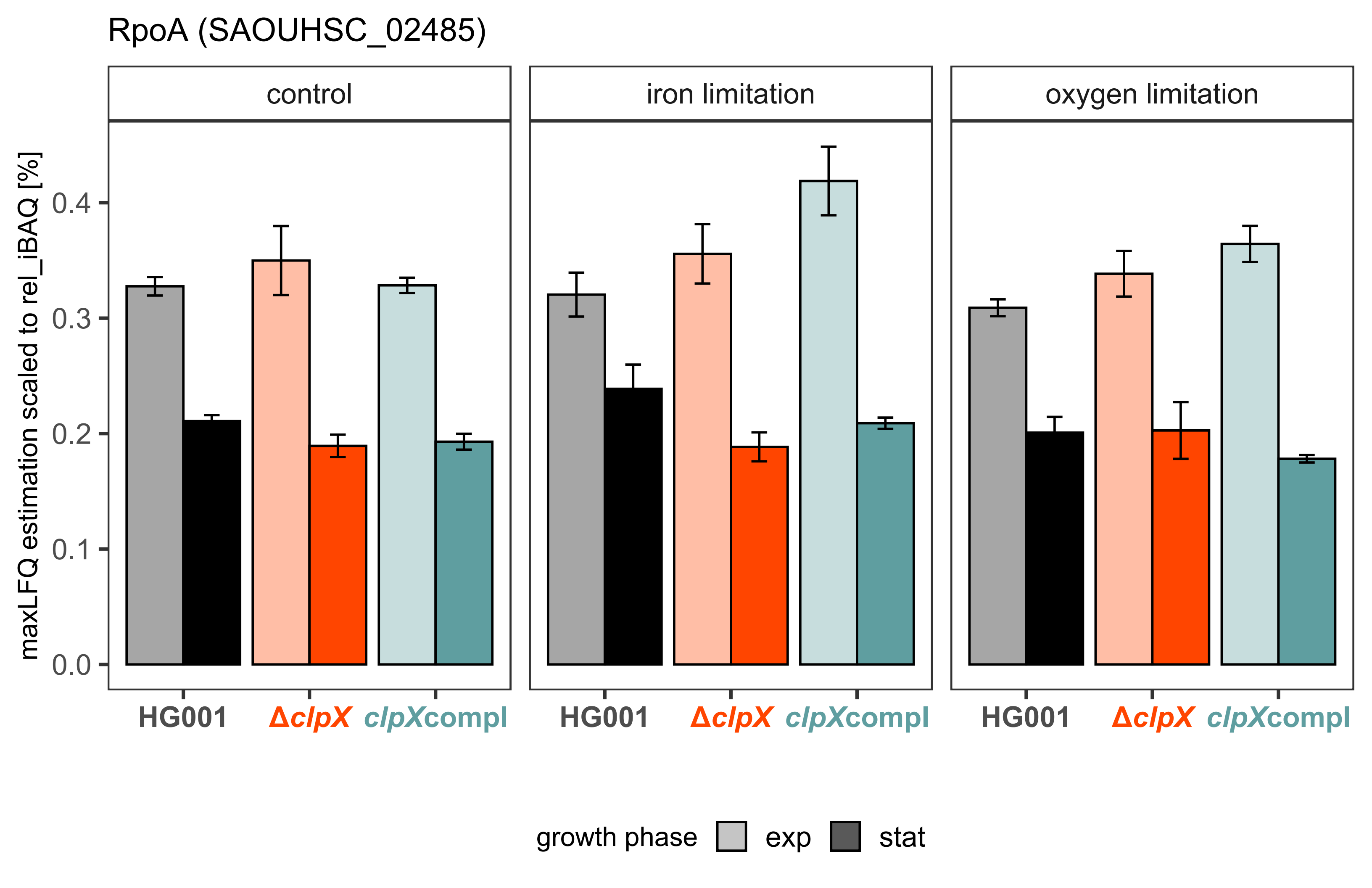

RpoA (SAOUHSC_02485)
control
iron limitation
oxygen limitation
maxLFQ estimation scaled to rel_iBAQ [%]
0.0
0.1
0.2
0.3
0.4
HG001
ΔclpX
clpXcompl
growth phase
exp
stat

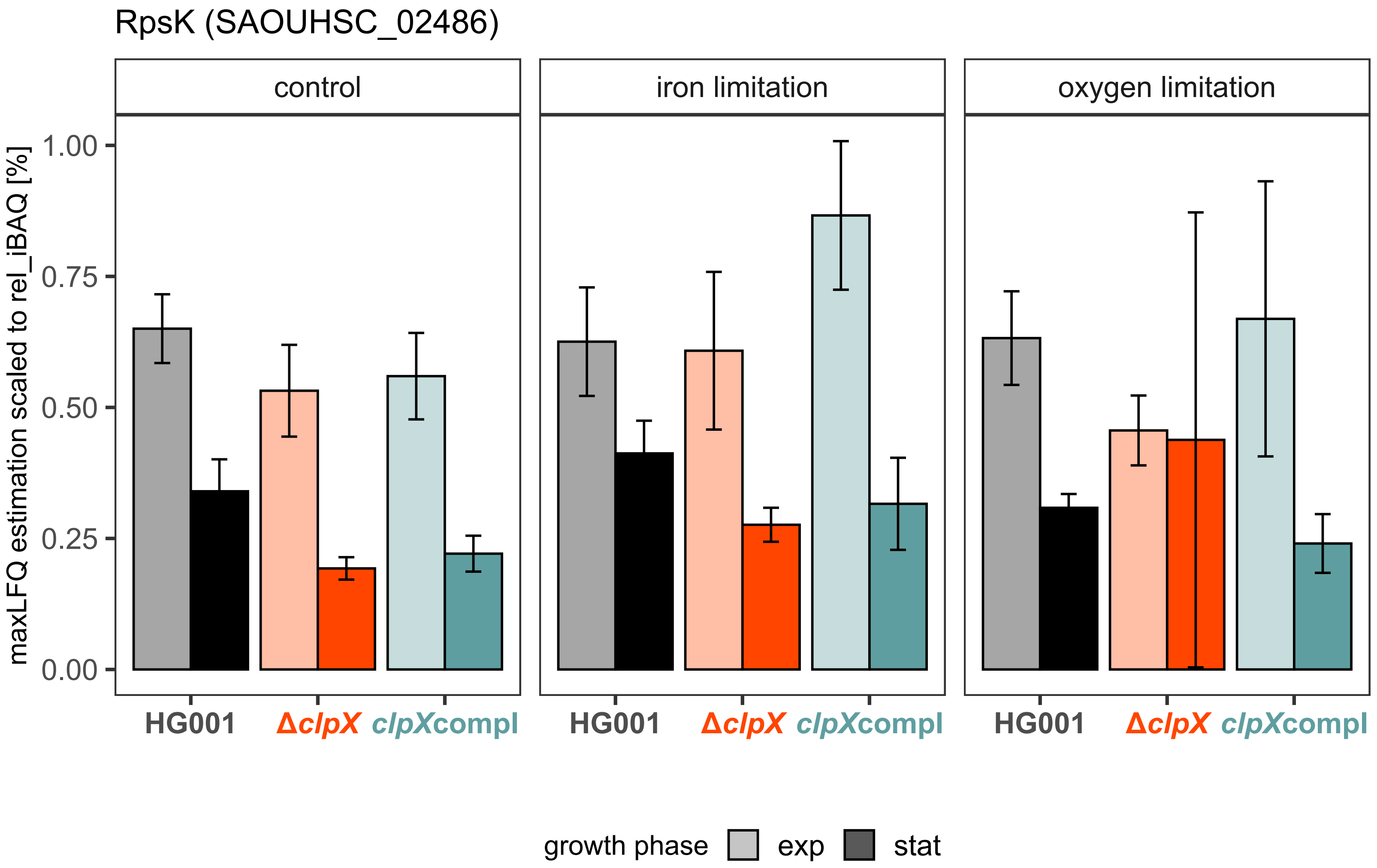
RpsK (SAOUHSC_02486)
control
iron limitation
oxygen limitation
maxLFQ estimation scaled to rel_iBAQ [%]
1.00
0.75
0.50
0.25
0.00
HG001
ΔclpX
clpXcompl
growth phase
exp
stat

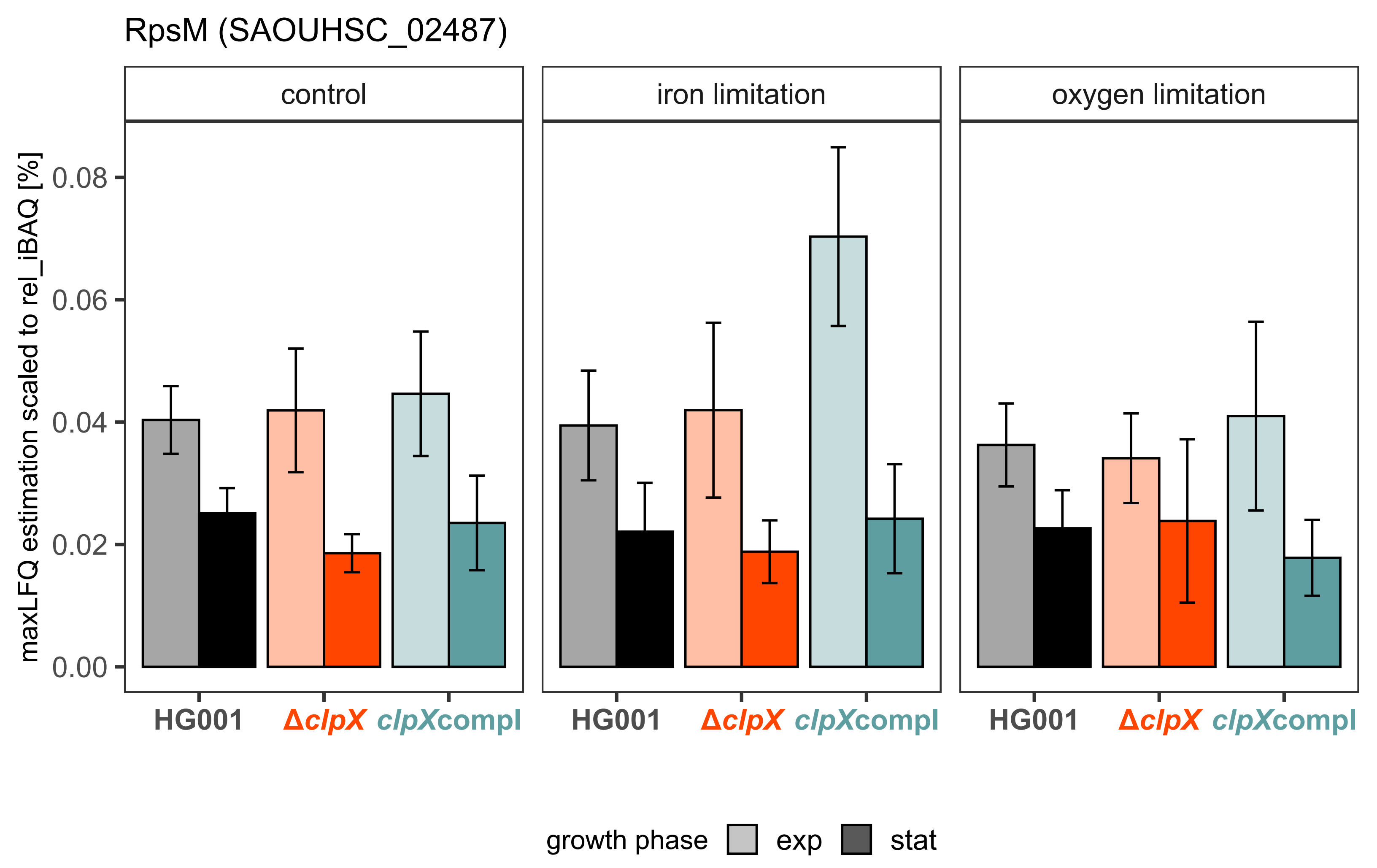

RpsM (SAOUHSC_02487)
control
iron limitation
oxygen limitation
maxLFQ estimation scaled to rel_iBAQ [%]
0.00
0.02
0.04
0.06
0.08
HG001
ΔclpX
clpXcompl
growth phase
exp
stat

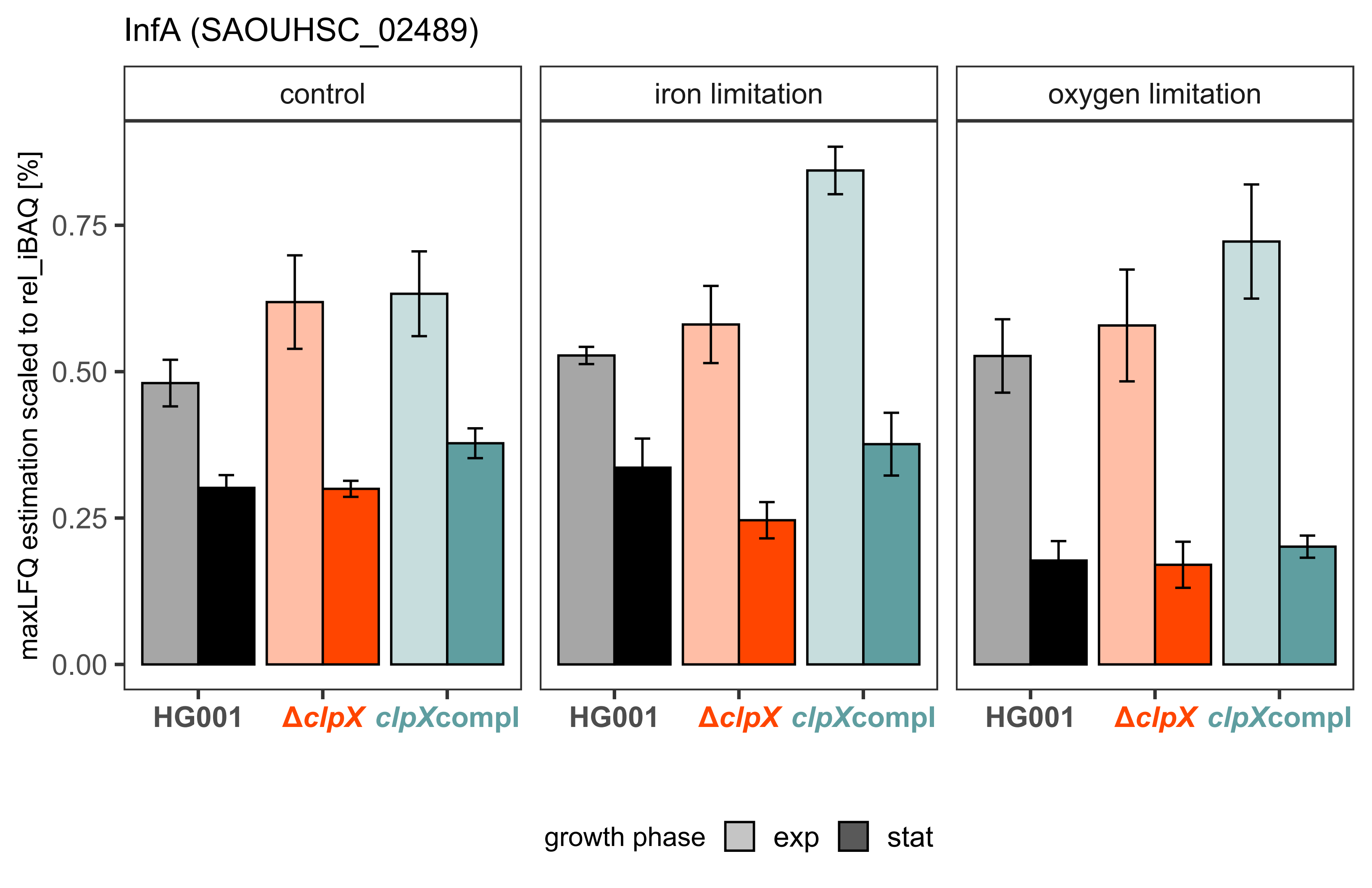

InfA (SAOUHSC_02489)
control
iron limitation
oxygen limitation
maxLFQ estimation scaled to rel_iBAQ [%]
0.00
0.25
0.50
0.75
HG001
ΔclpX
clpXcompl
growth phase
exp
stat

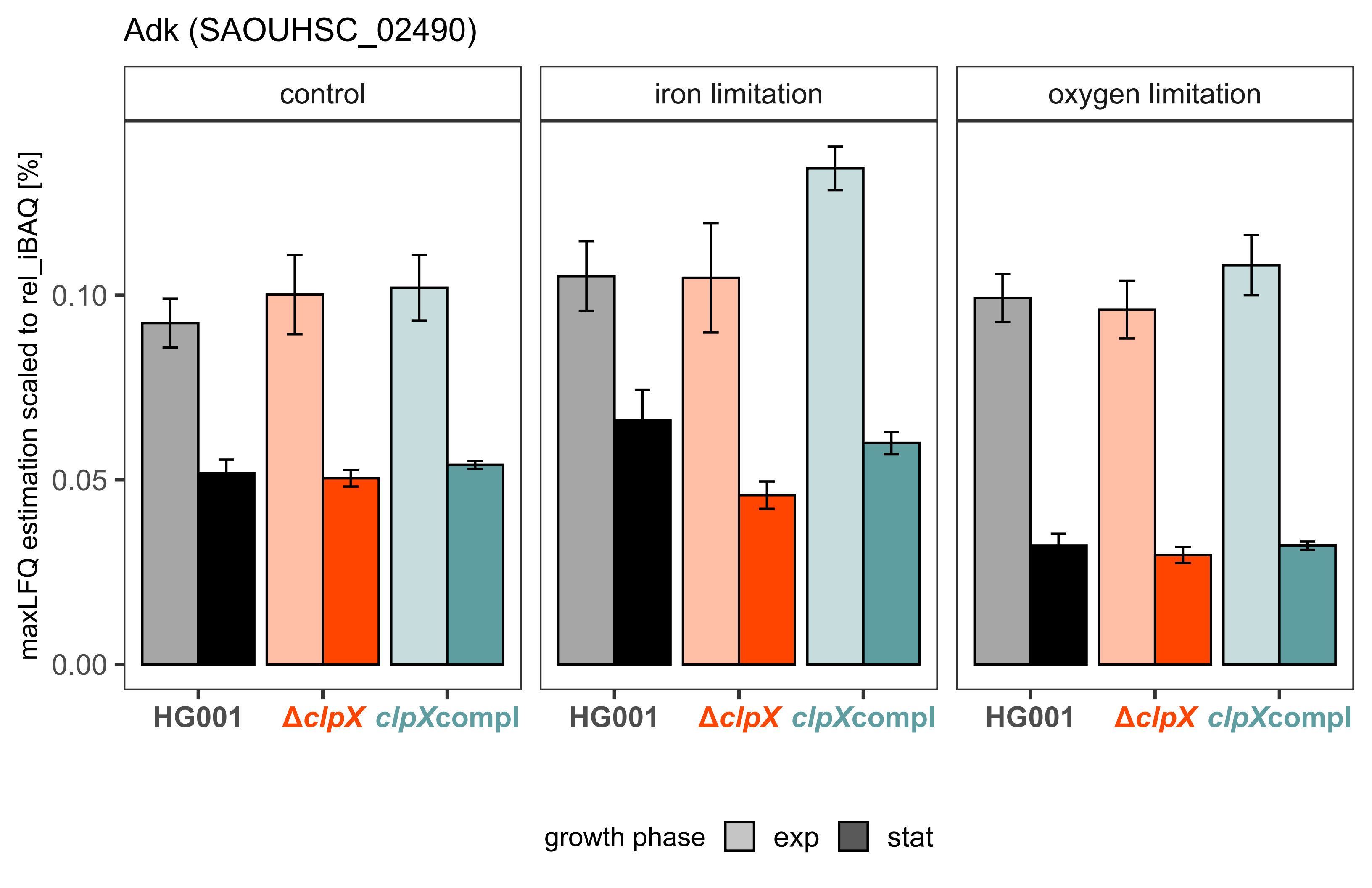

Adk (SAOUHSC_02490)
control
iron limitation
oxygen limitation
maxLFQ estimation scaled to rel_iBAQ [%]
0.10
0.05
0.00
HG001
ΔclpX
clpXcompl
growth phase
exp
stat

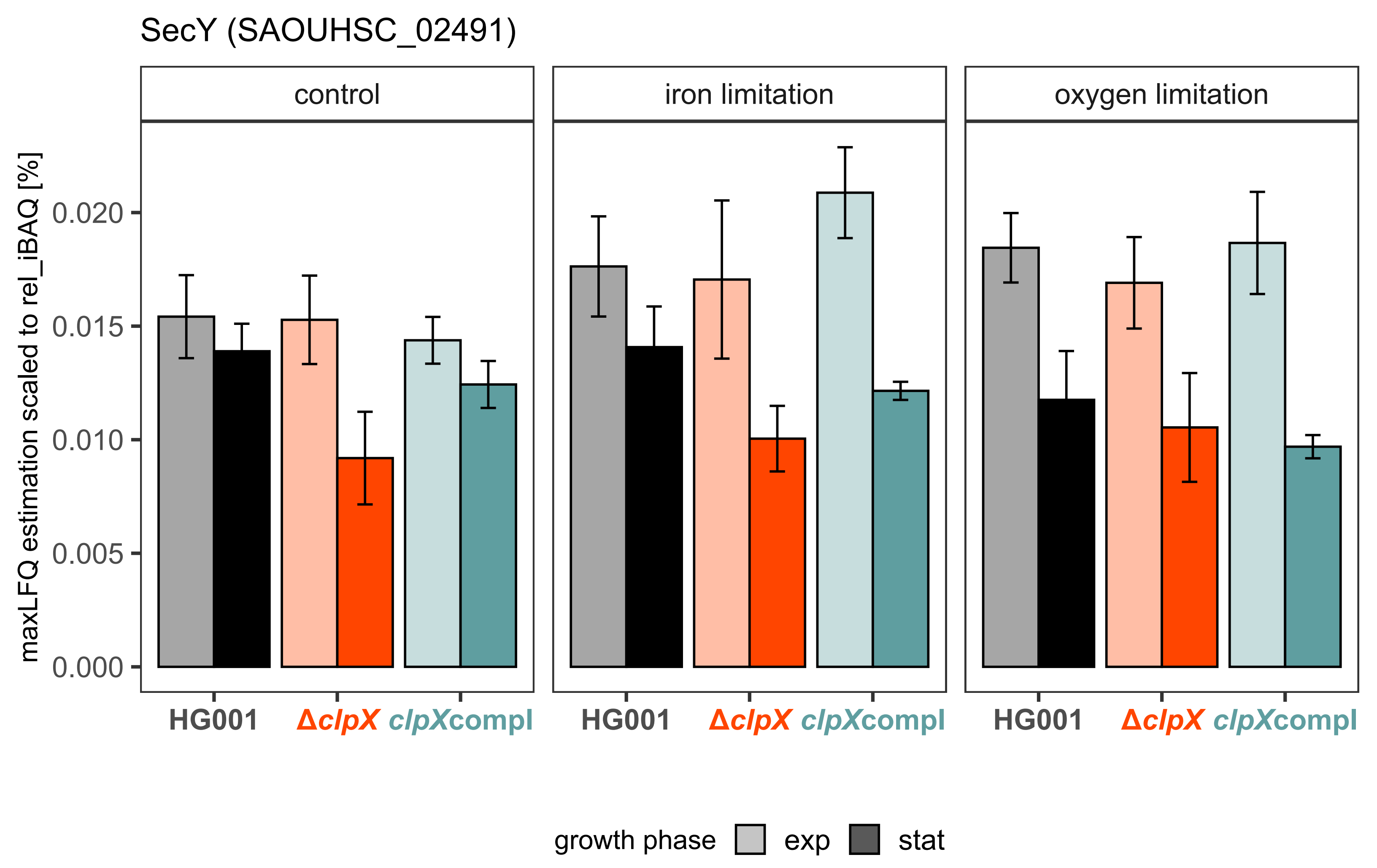

SecY (SAOUHSC_02491)
control
iron limitation
oxygen limitation
maxLFQ estimation scaled to rel_iBAQ [%]
0.020
0.015
0.010
0.005
0.000
HG001
ΔclpX
clpXcompl
growth phase
exp
stat

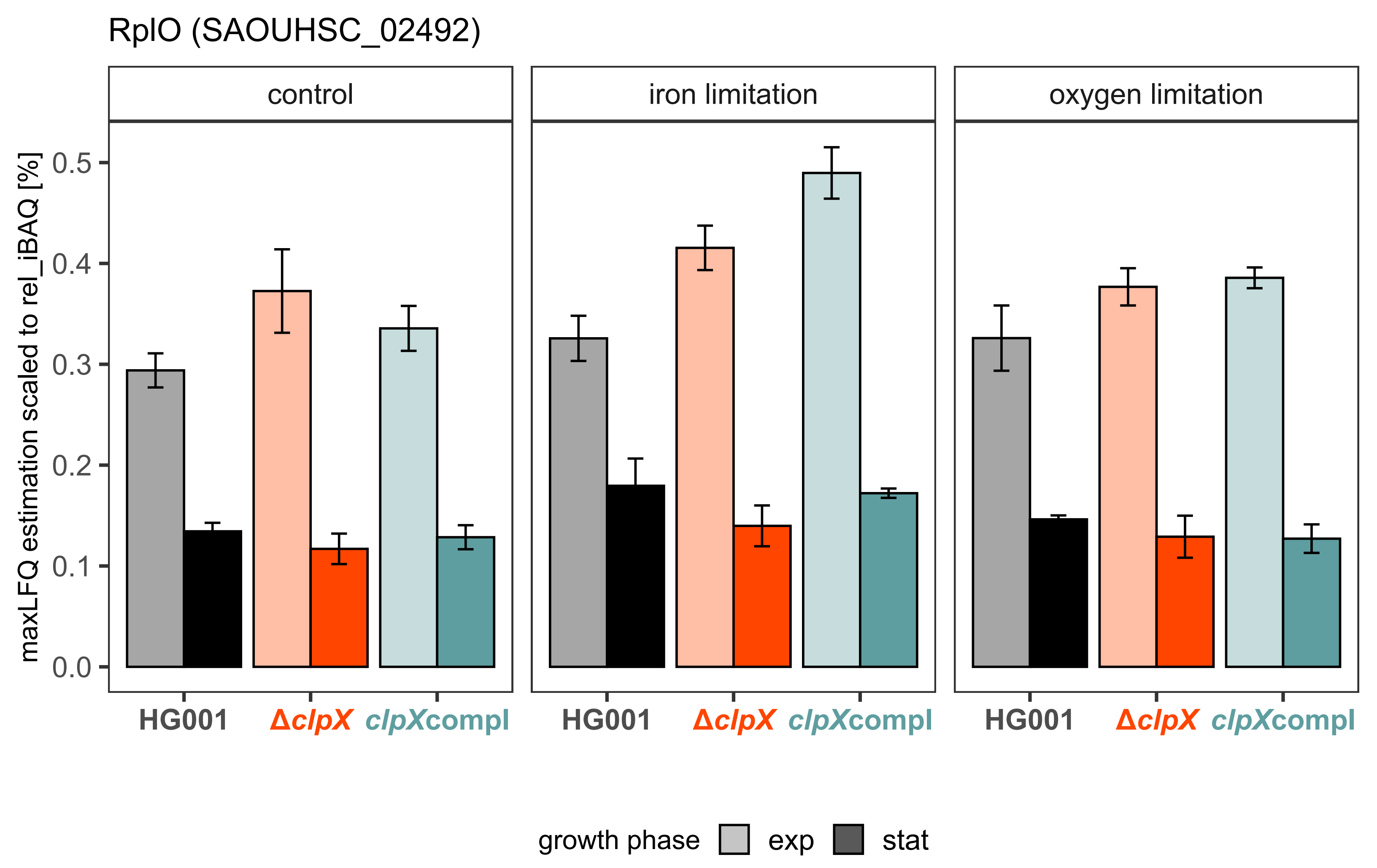
RplO (SAOUHSC_02492)
control
iron limitation
oxygen limitation
maxLFQ estimation scaled to rel_iBAQ [%]
0.0
0.1
0.2
0.3
0.4
0.5
HG001
ΔclpX
clpXcompl
growth phase
exp
stat

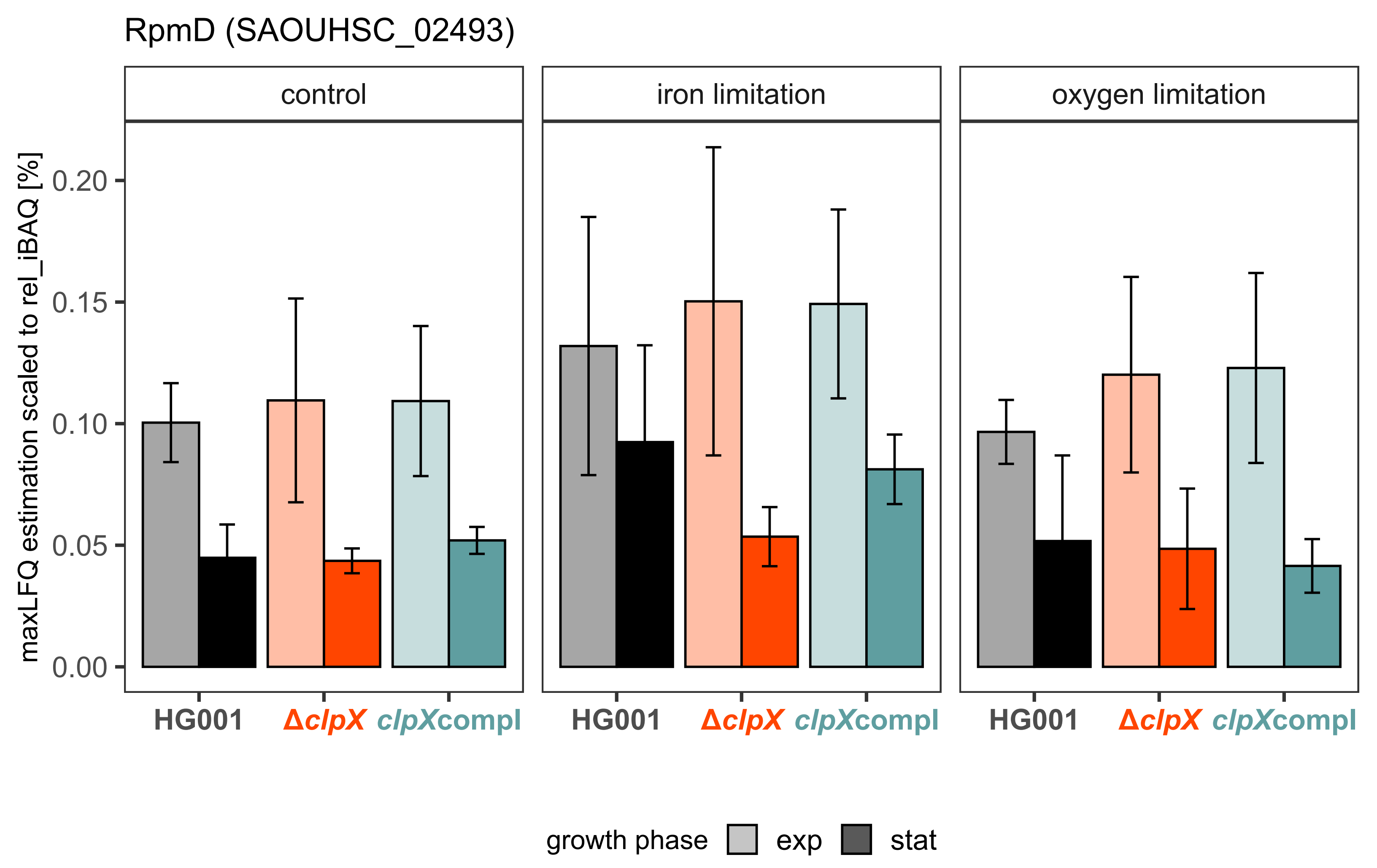

RpmD (SAOUHSC_02493)
control
iron limitation
oxygen limitation
maxLFQ estimation scaled to rel_iBAQ [%]
0.20
0.15
0.10
0.05
0.00
HG001
ΔclpX
clpXcompl
growth phase
exp
stat

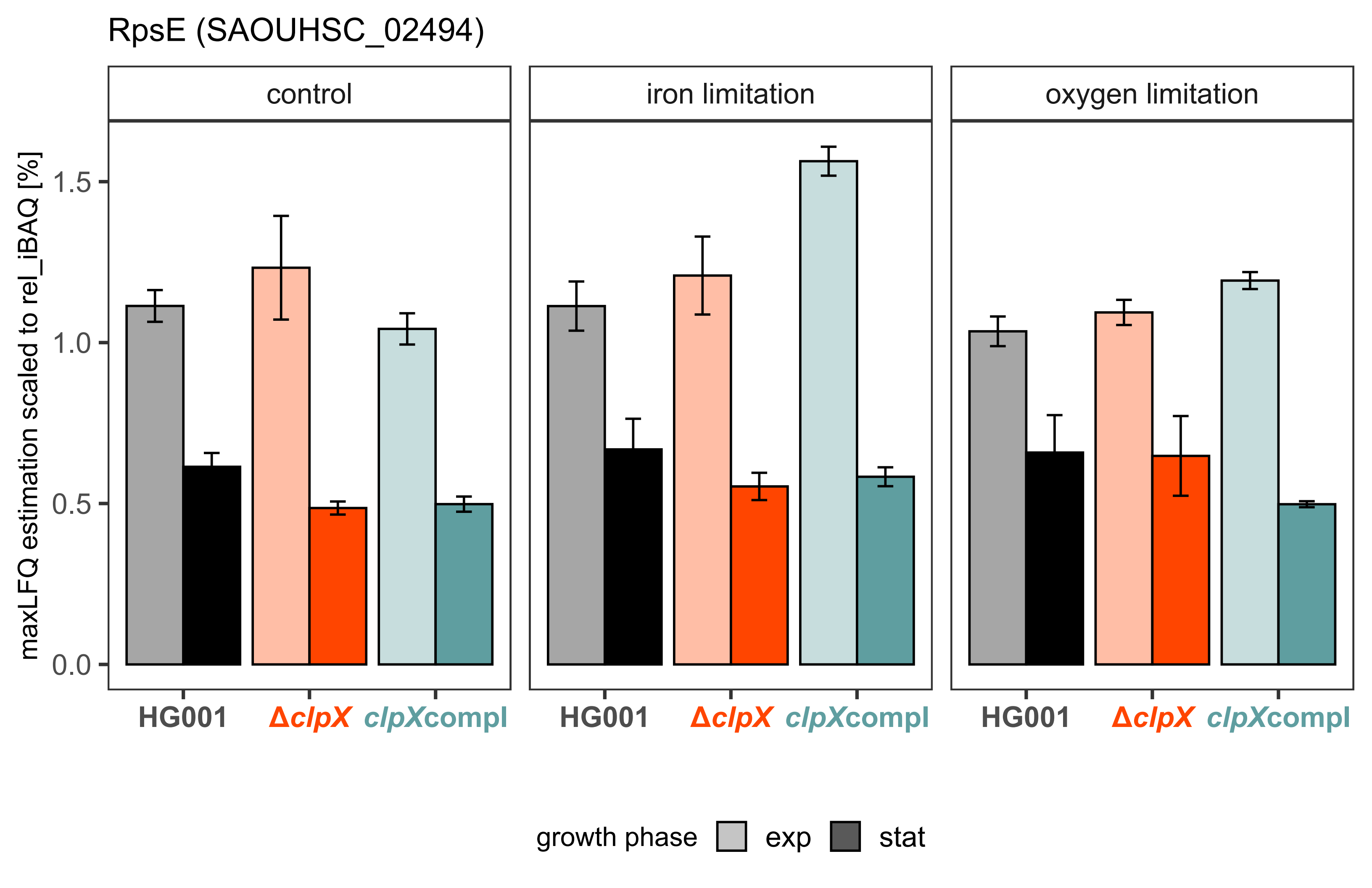

RpsE (SAOUHSC_02494)
control
iron limitation
oxygen limitation
maxLFQ estimation scaled to rel_iBAQ [%]
0.0
0.5
1.0
1.5
HG001
ΔclpX
clpXcompl
growth phase
exp
stat

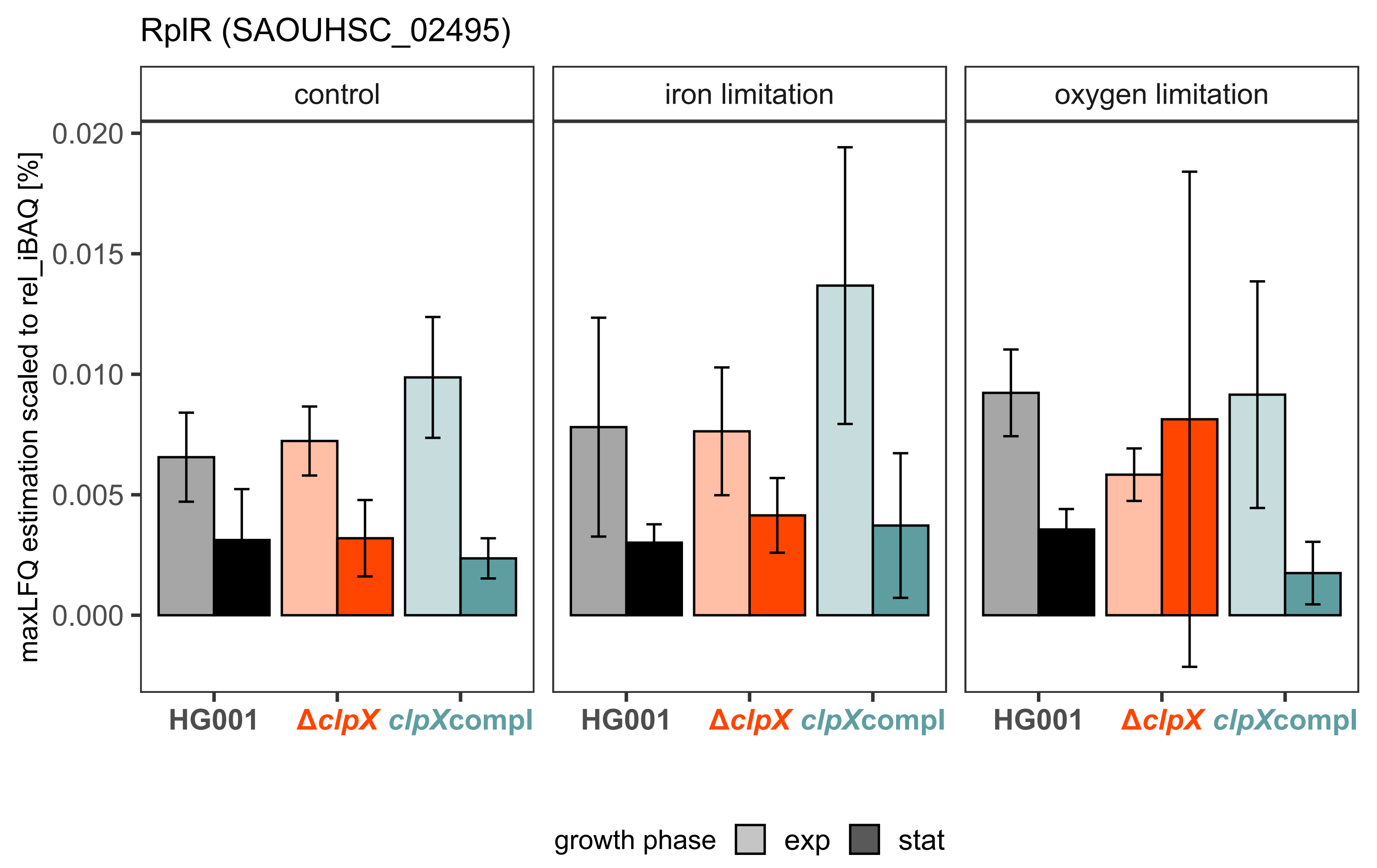

RplR (SAOUHSC_02495)
control
iron limitation
oxygen limitation
maxLFQ estimation scaled to rel_iBAQ [%]
0.020
0.015
0.010
0.005
0.000
HG001
ΔclpX
clpXcompl
growth phase
exp
stat

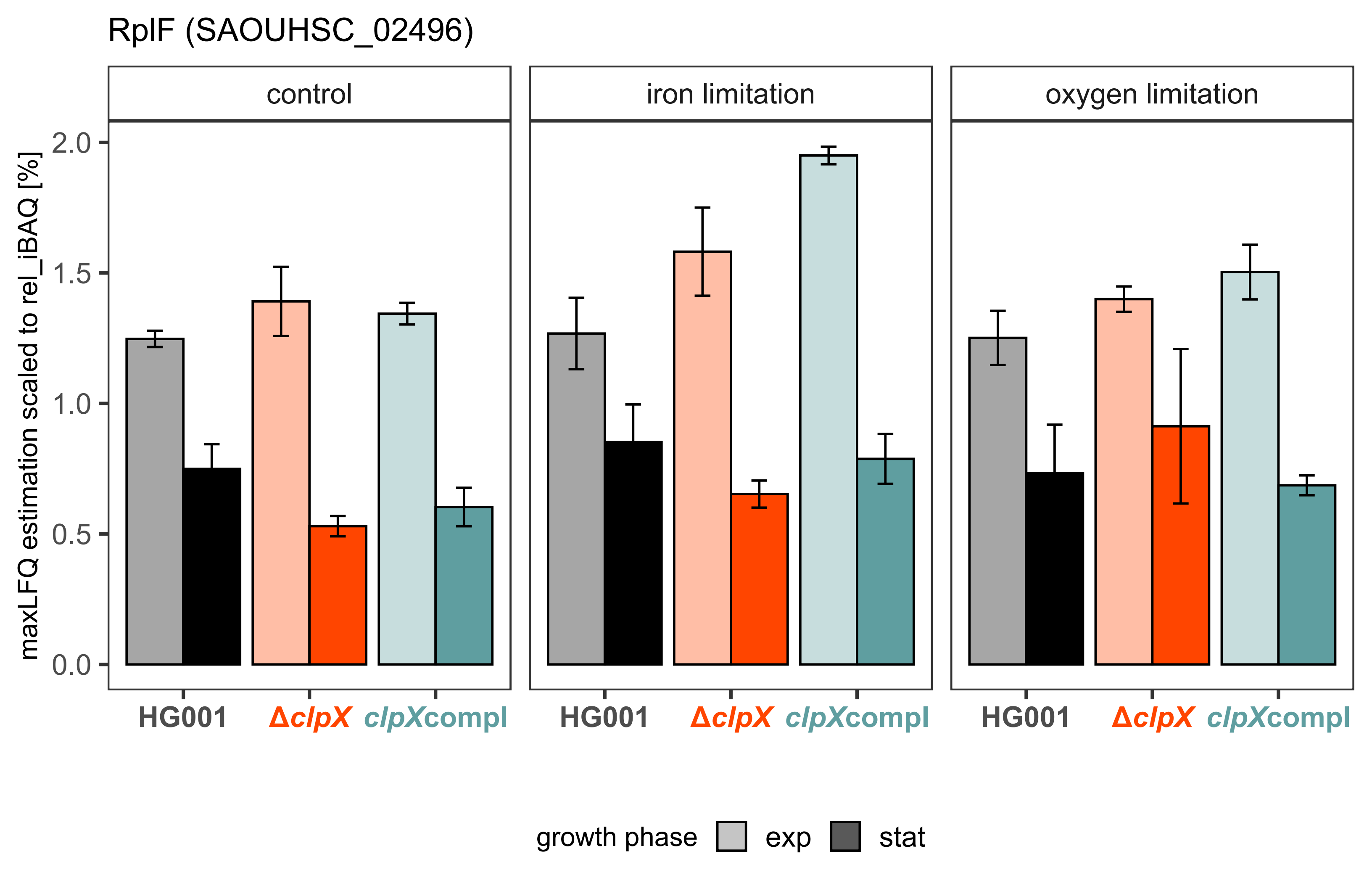
RplF (SAOUHSC_02496)
control
iron limitation
oxygen limitation
maxLFQ estimation scaled to rel_iBAQ [%]
2.0
1.5
1.0
0.5
0.0
HG001
ΔclpX
clpXcompl
growth phase
exp
stat

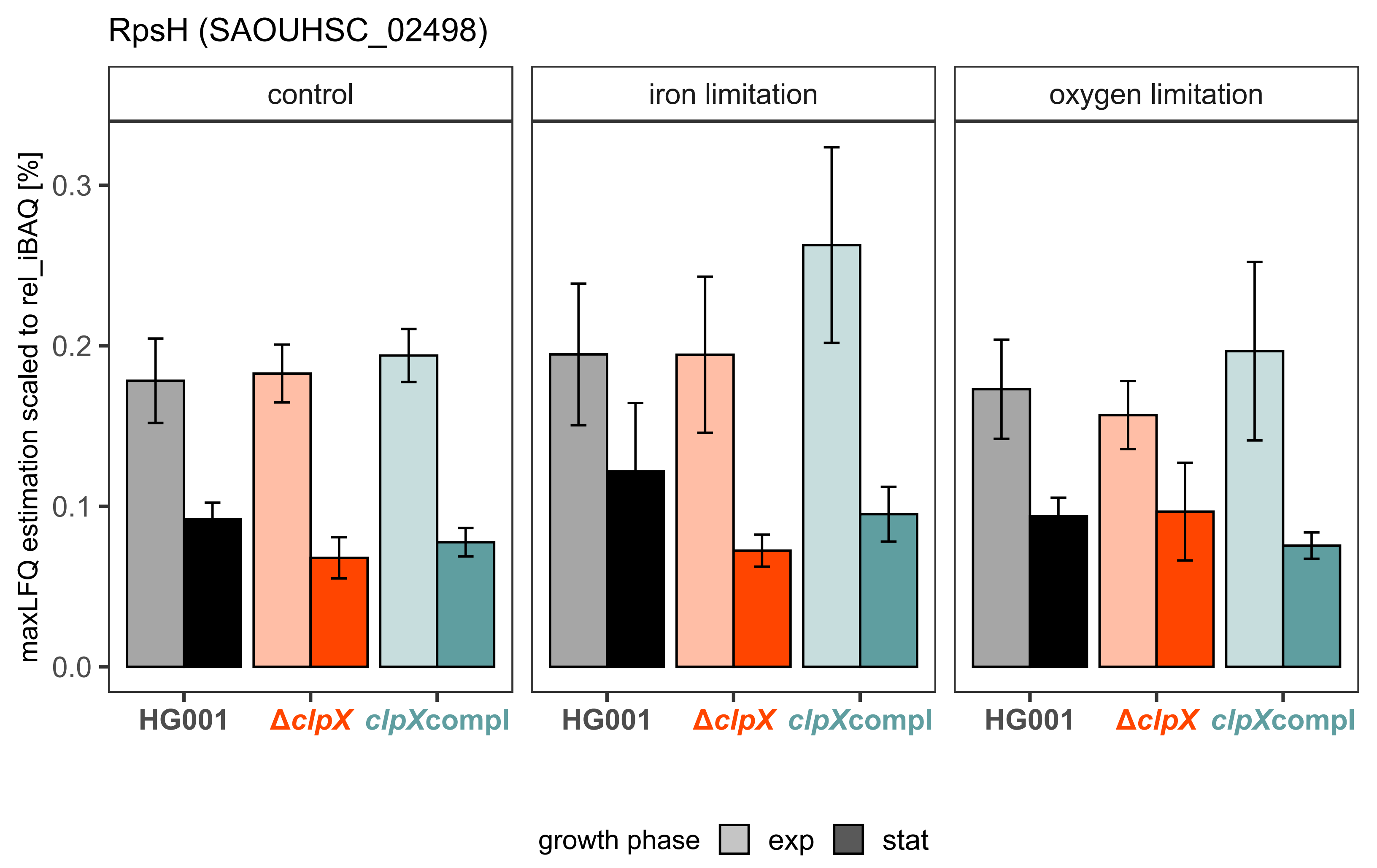

RpsH (SAOUHSC_02498)
control
iron limitation
oxygen limitation
maxLFQ estimation scaled to rel_iBAQ [%]
0.0
0.1
0.2
0.3
HG001
ΔclpX
clpXcompl
growth phase
exp
stat

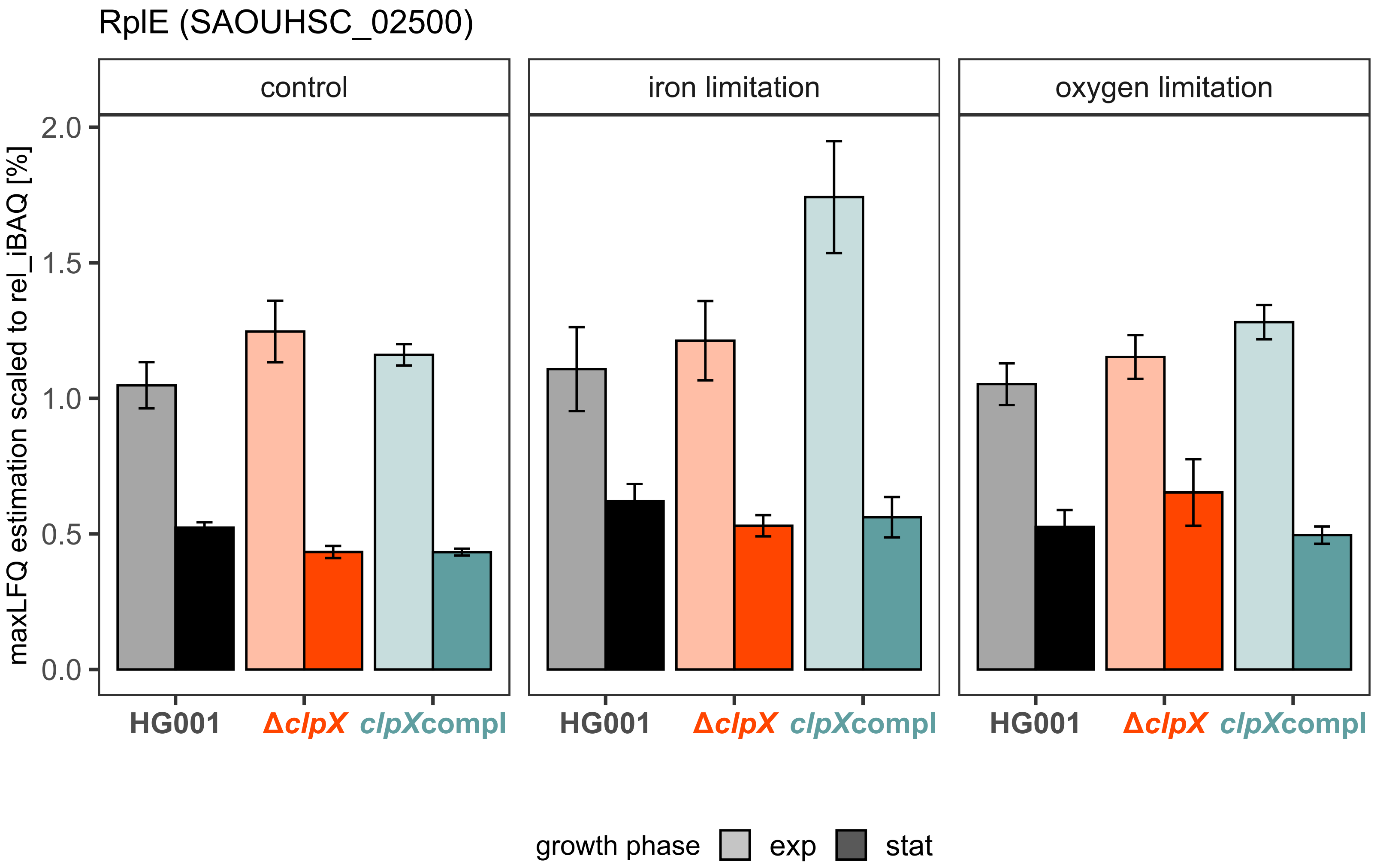
RplE (SAOUHSC_02500)
control
iron limitation
oxygen limitation
maxLFQ estimation scaled to rel_iBAQ [%]
2.0
1.5
1.0
0.5
0.0
HG001
ΔclpX
clpXcompl
growth phase
exp
stat

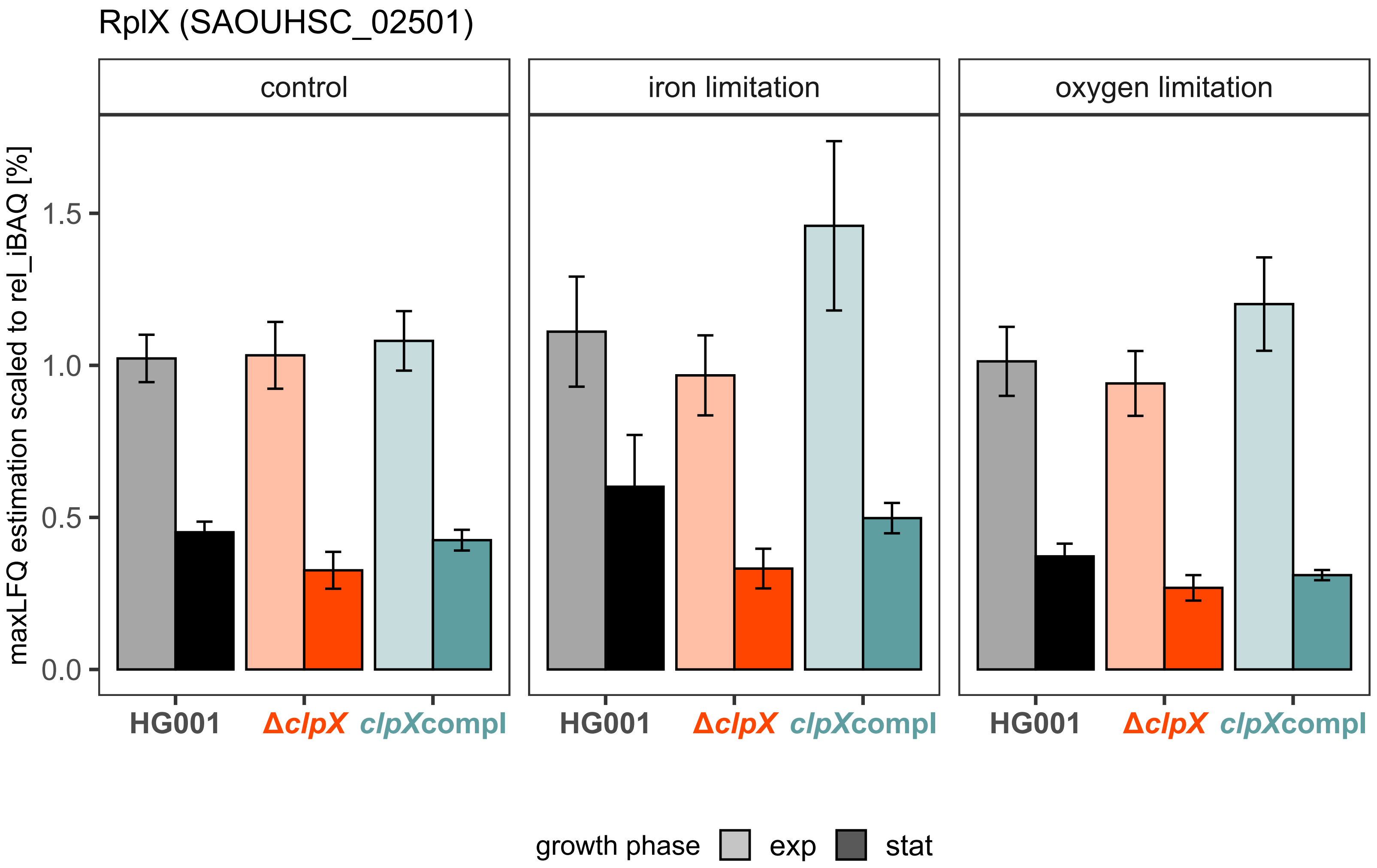

RplX (SAOUHSC_02501)
control
iron limitation
oxygen limitation
maxLFQ estimation scaled to rel_iBAQ [%]
1.5
1.0
0.5
0.0
HG001
ΔclpX
clpXcompl
HG001
ΔclpX
clpXcompl
HG001
ΔclpX
clpXcompl
growth phase
exp
stat

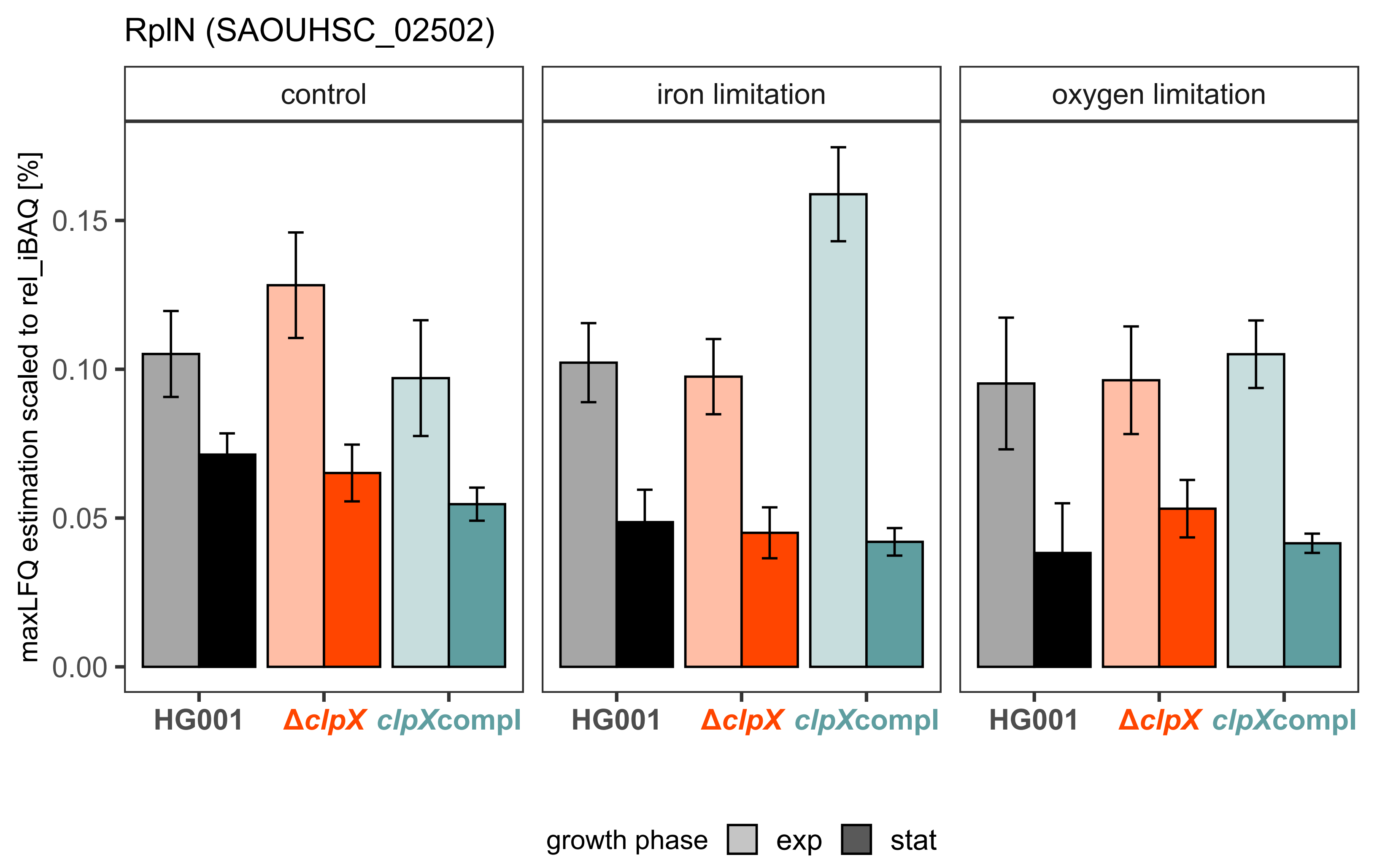

RplN (SAOUHSC_02502)
control
iron limitation
oxygen limitation
maxLFQ estimation scaled to rel_iBAQ [%]
0.00
0.05
0.10
0.15
HG001
ΔclpX
clpXcompl
growth phase
exp
stat

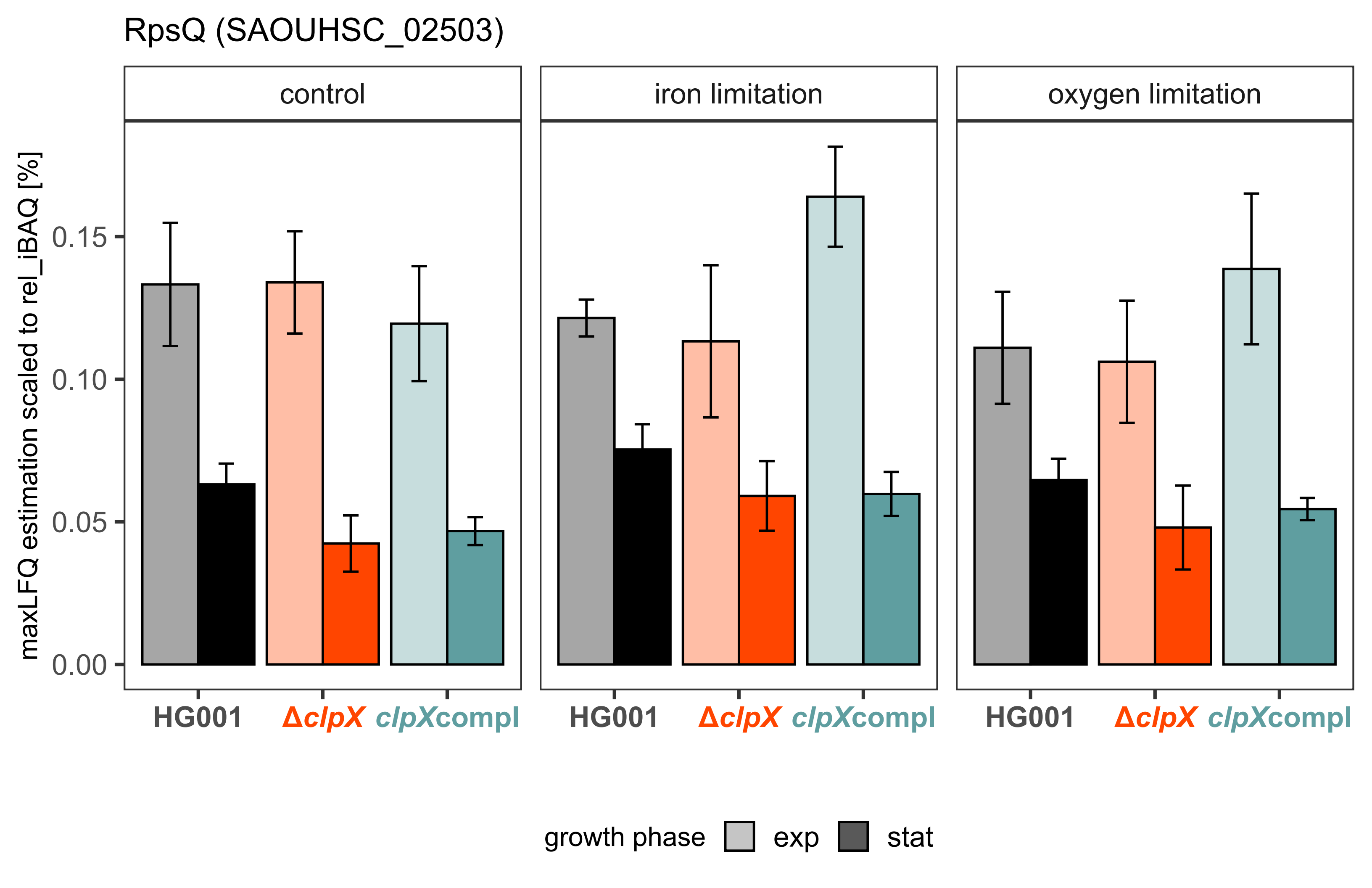

RpsQ (SAOUHSC_02503)
control
iron limitation
oxygen limitation
maxLFQ estimation scaled to rel_iBAQ [%]
0.15
0.10
0.05
0.00
HG001
ΔclpX
clpXcompl
growth phase
exp
stat

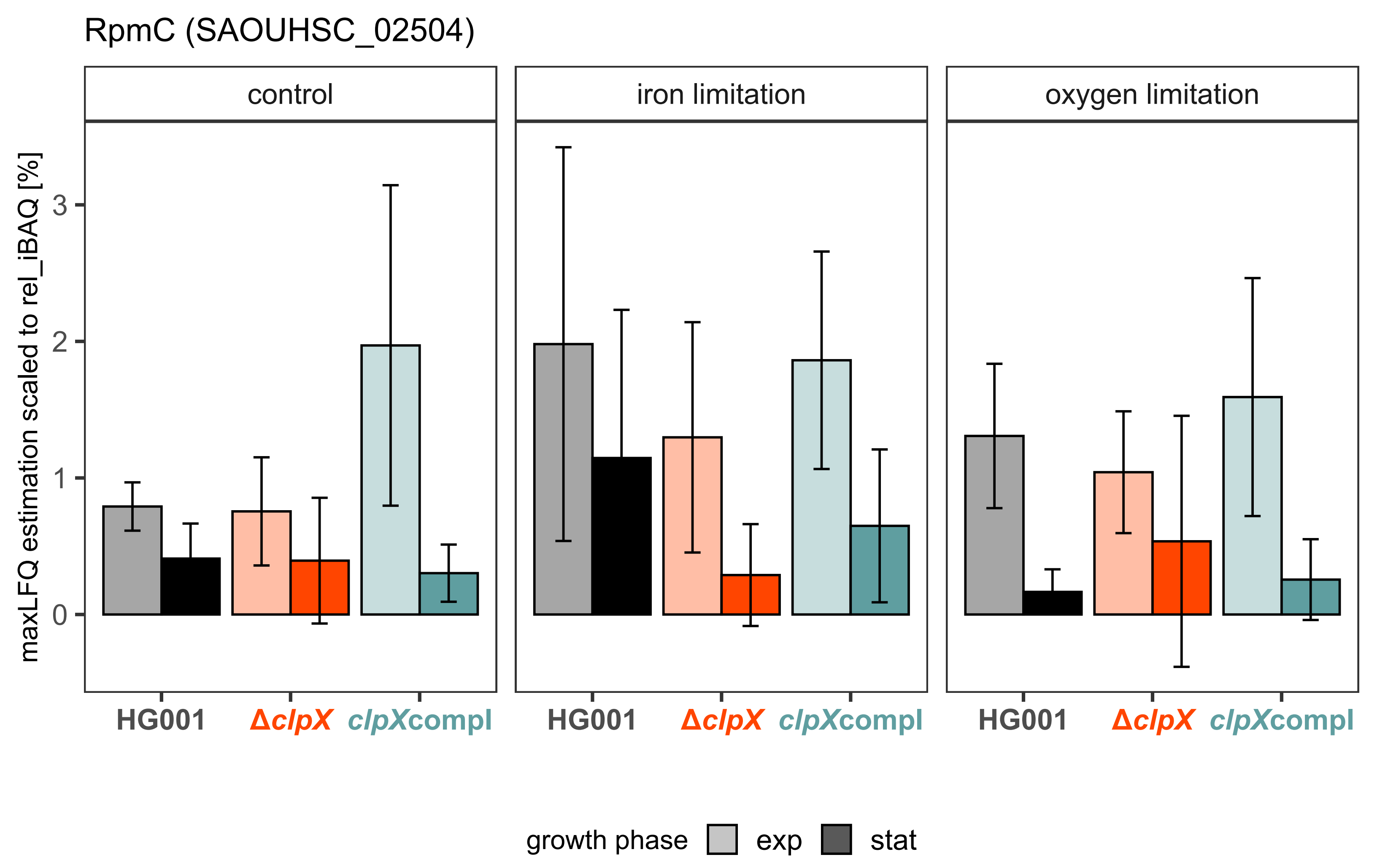

RpmC (SAOUHSC_02504)
control
iron limitation
oxygen limitation
maxLFQ estimation scaled to rel_iBAQ [%]
0
1
2
3
HG001
ΔclpX
clpXcompl
growth phase
exp
stat

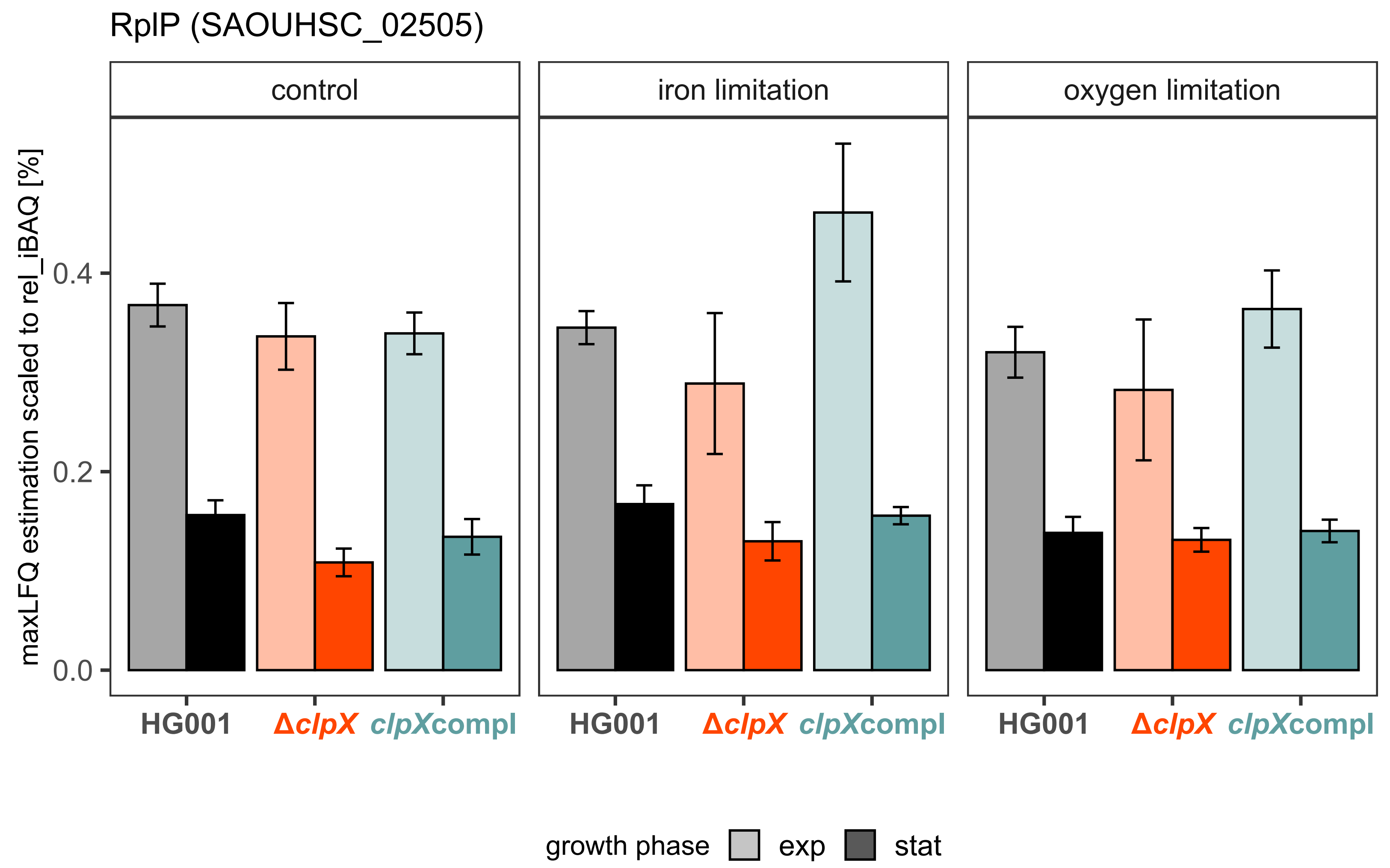

RplP (SAOUHSC_02505)
control
iron limitation
oxygen limitation
maxLFQ estimation scaled to rel_iBAQ [%]
0.4
0.2
0.0
HG001
ΔclpX
clpXcompl
growth phase
exp
stat

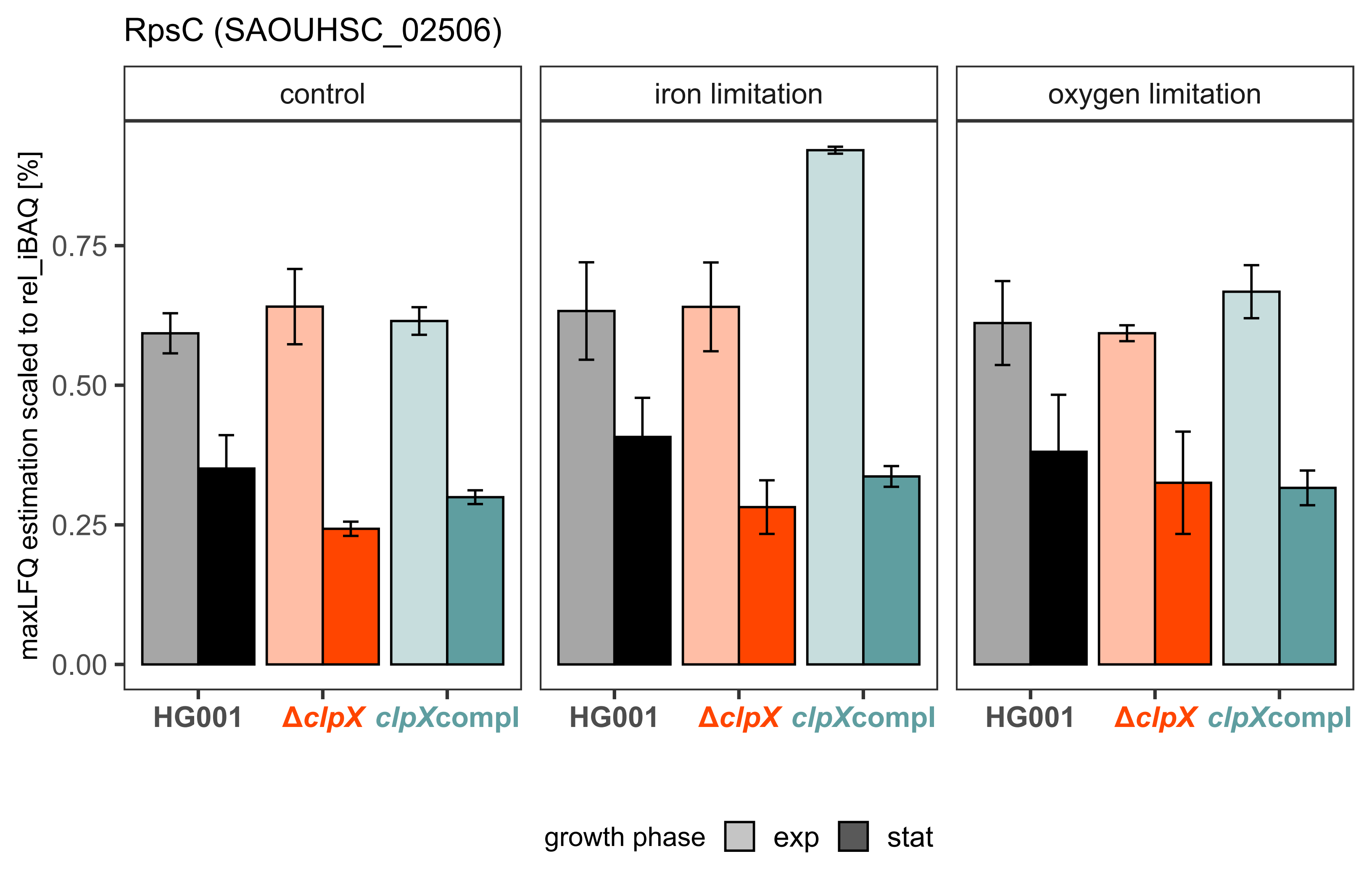

RpsC (SAOUHSC_02506)
control
iron limitation
oxygen limitation
maxLFQ estimation scaled to rel_iBAQ [%]
0.75
0.50
0.25
0.00
HG001
ΔclpX
clpXcompl
growth phase
exp
stat

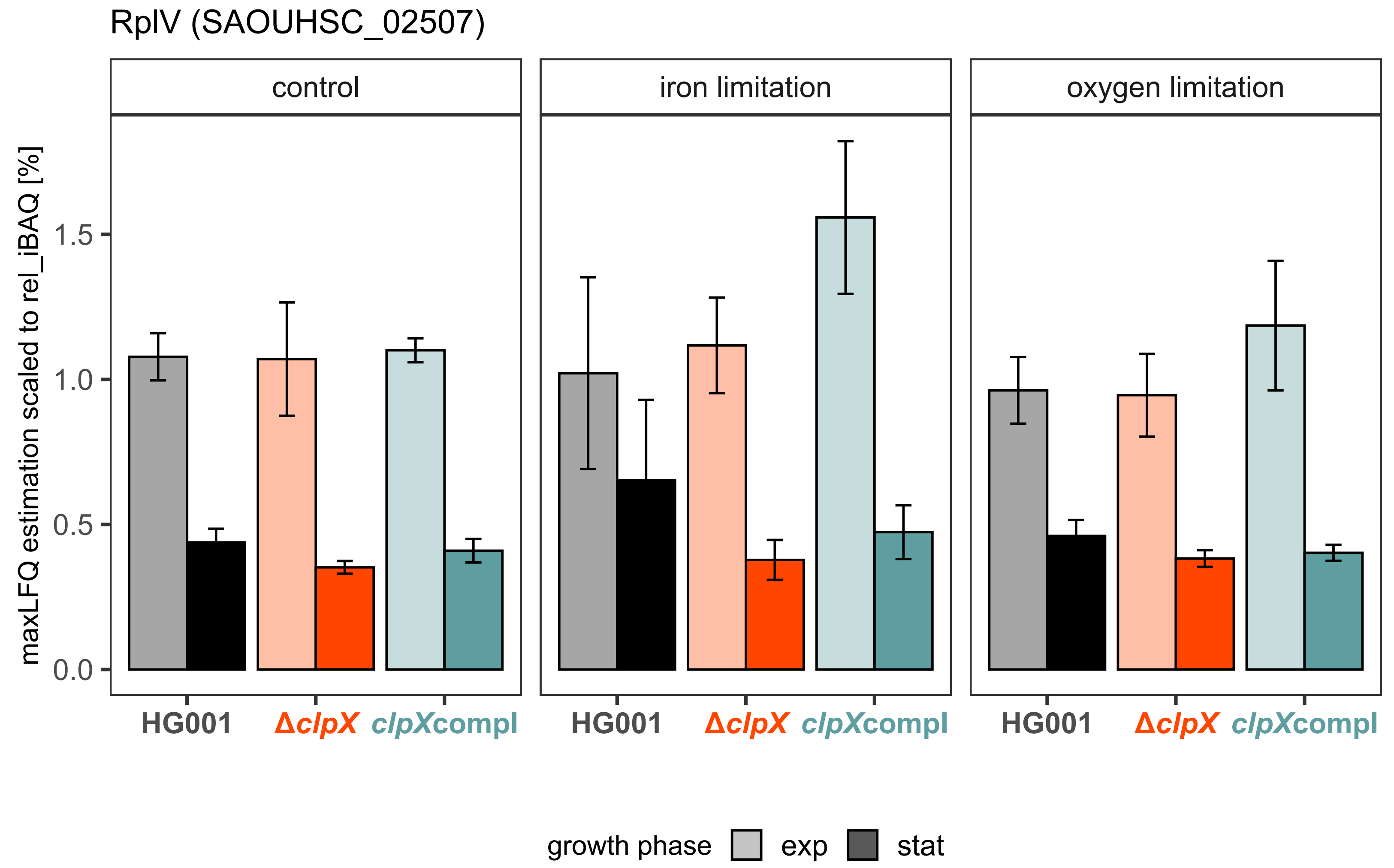
RplV (SAOUHSC_02507)
control
iron limitation
oxygen limitation
maxLFQ estimation scaled to rel_iBAQ [%]
0.0
0.5
1.0
1.5
HG001
ΔclpX
clpXcompl
growth phase
exp
stat

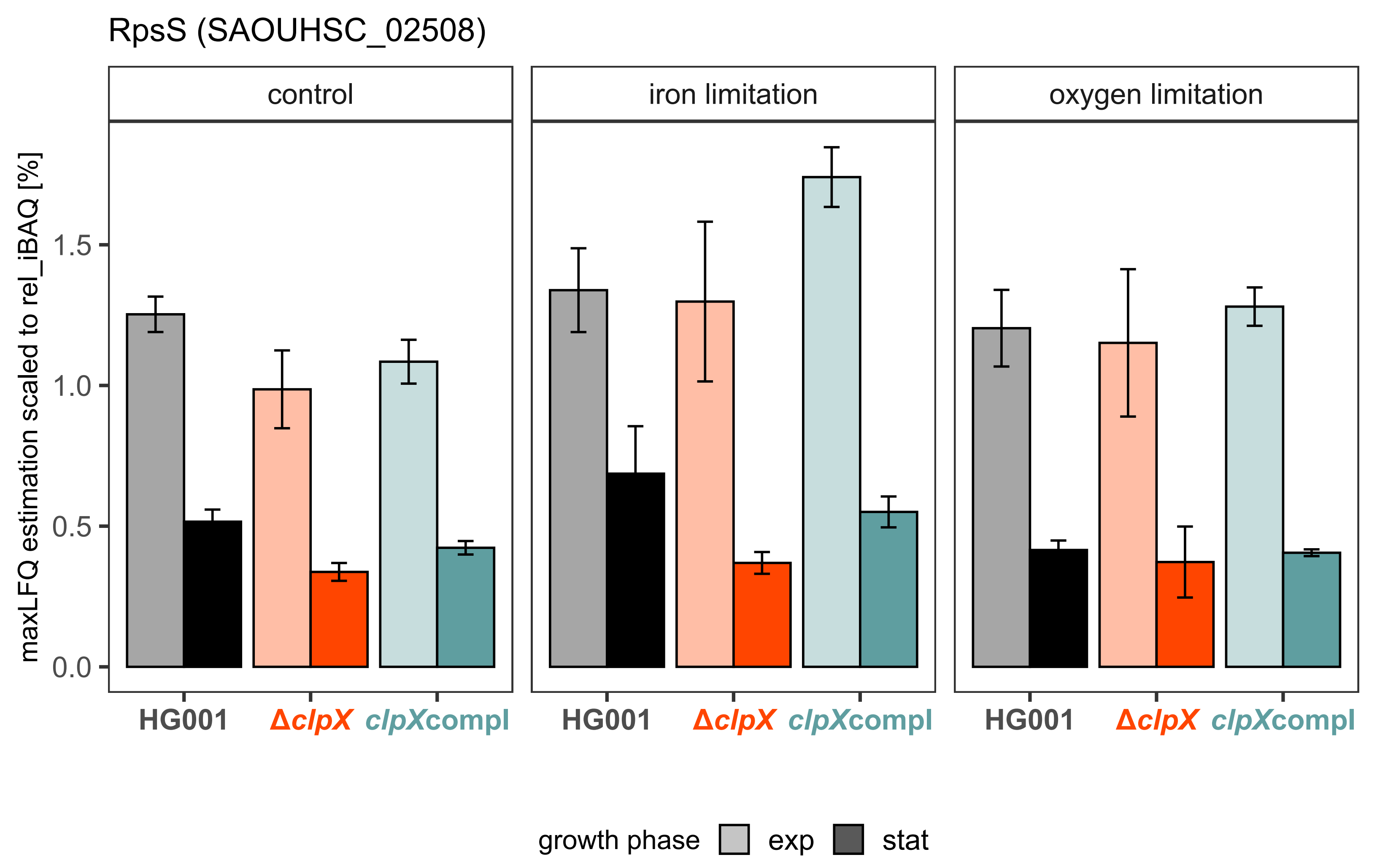
RpsS (SAOUHSC_02508)
control
iron limitation
oxygen limitation
maxLFQ estimation scaled to rel_iBAQ [%]
0.0
0.5
1.0
1.5
HG001
ΔclpX
clpXcompl
growth phase
exp
stat

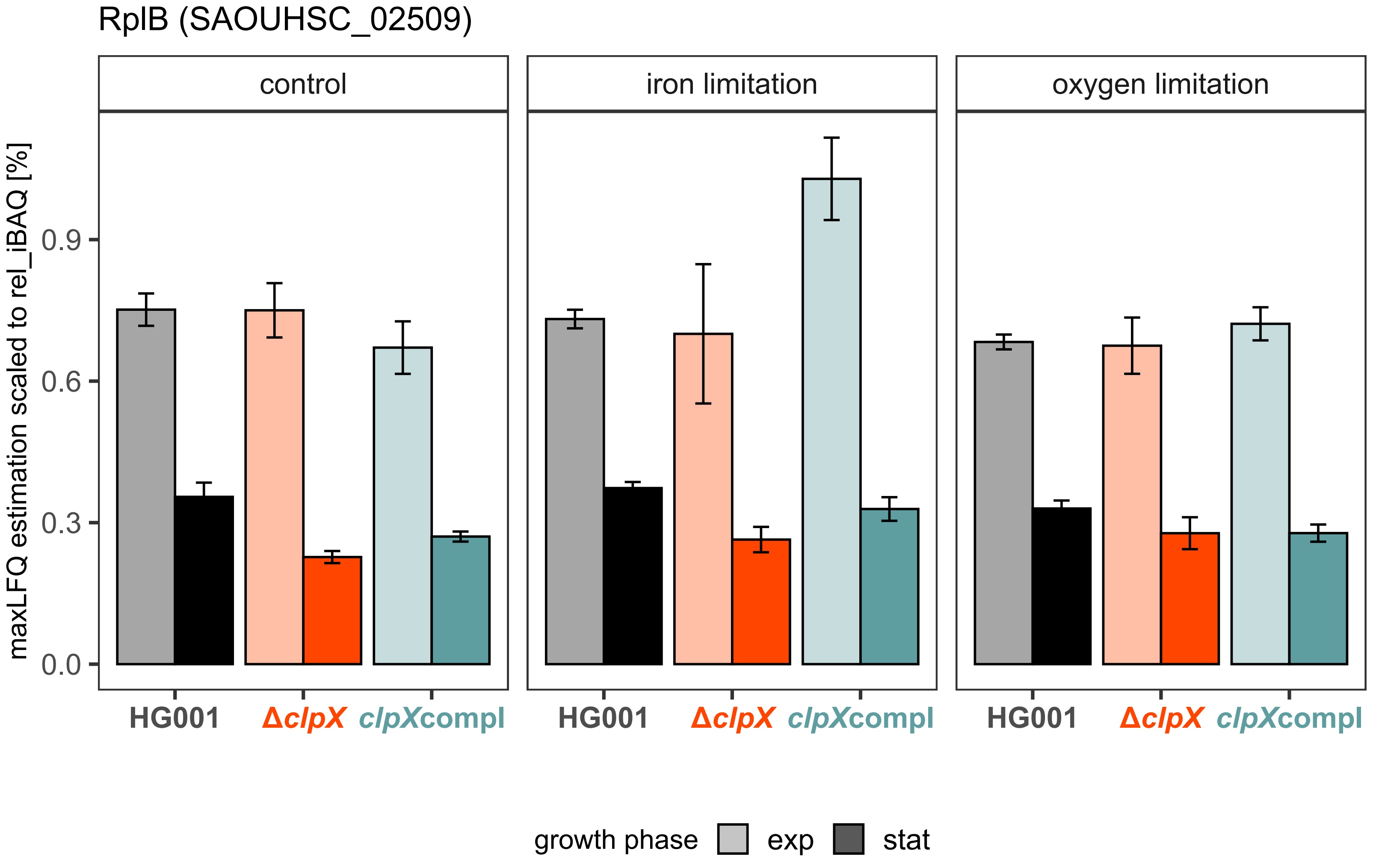

RplB (SAOUHSC_02509)
control
iron limitation
oxygen limitation
maxLFQ estimation scaled to rel_iBAQ [%]
0.0
0.3
0.6
0.9
HG001
ΔclpX
clpXcompl
growth phase
exp
stat

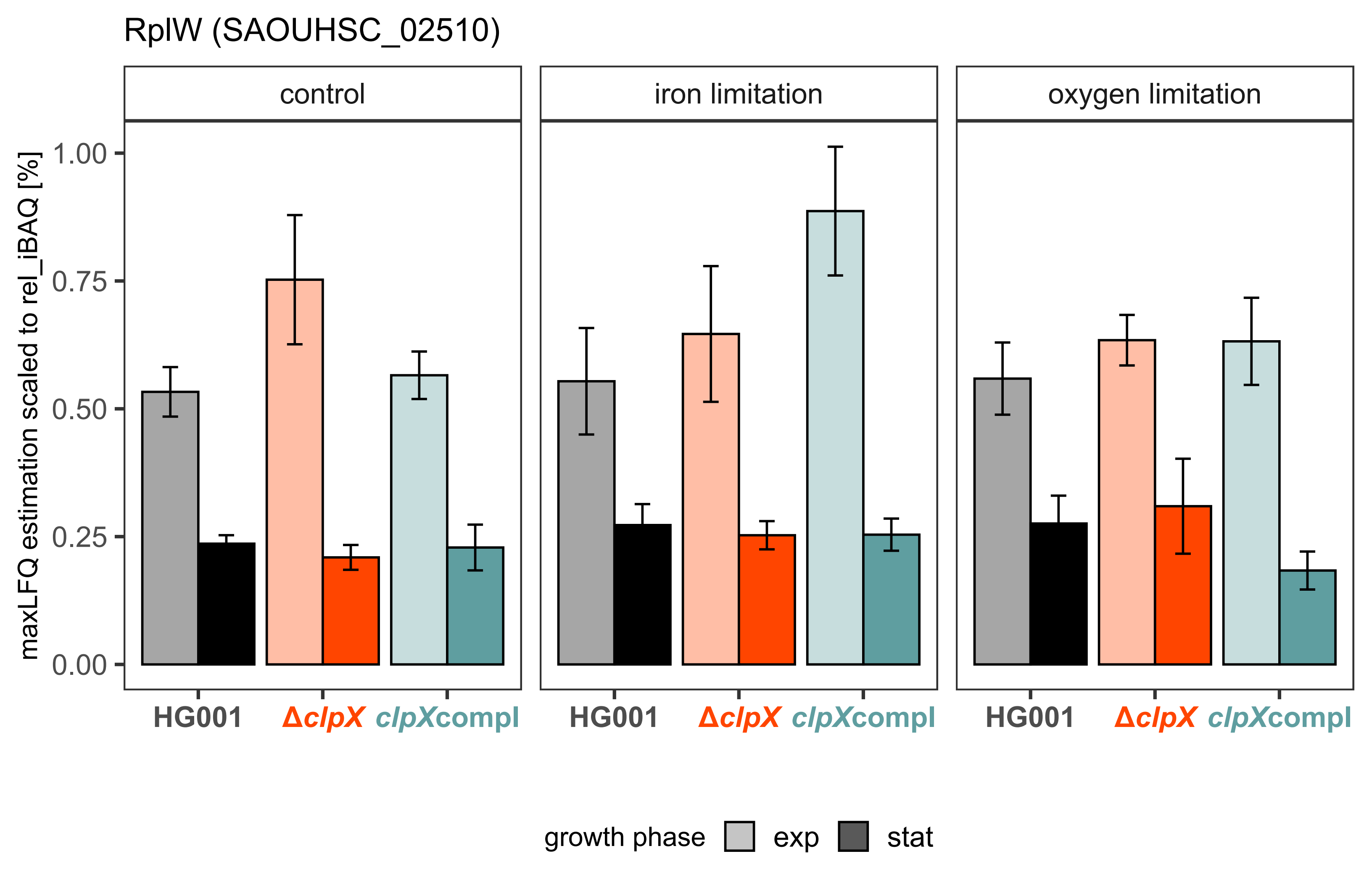

RplW (SAOUHSC_02510)
control
iron limitation
oxygen limitation
maxLFQ estimation scaled to rel_iBAQ [%]
1.00
0.75
0.50
0.25
0.00
HG001
ΔclpX
clpXcompl
growth phase
exp
stat

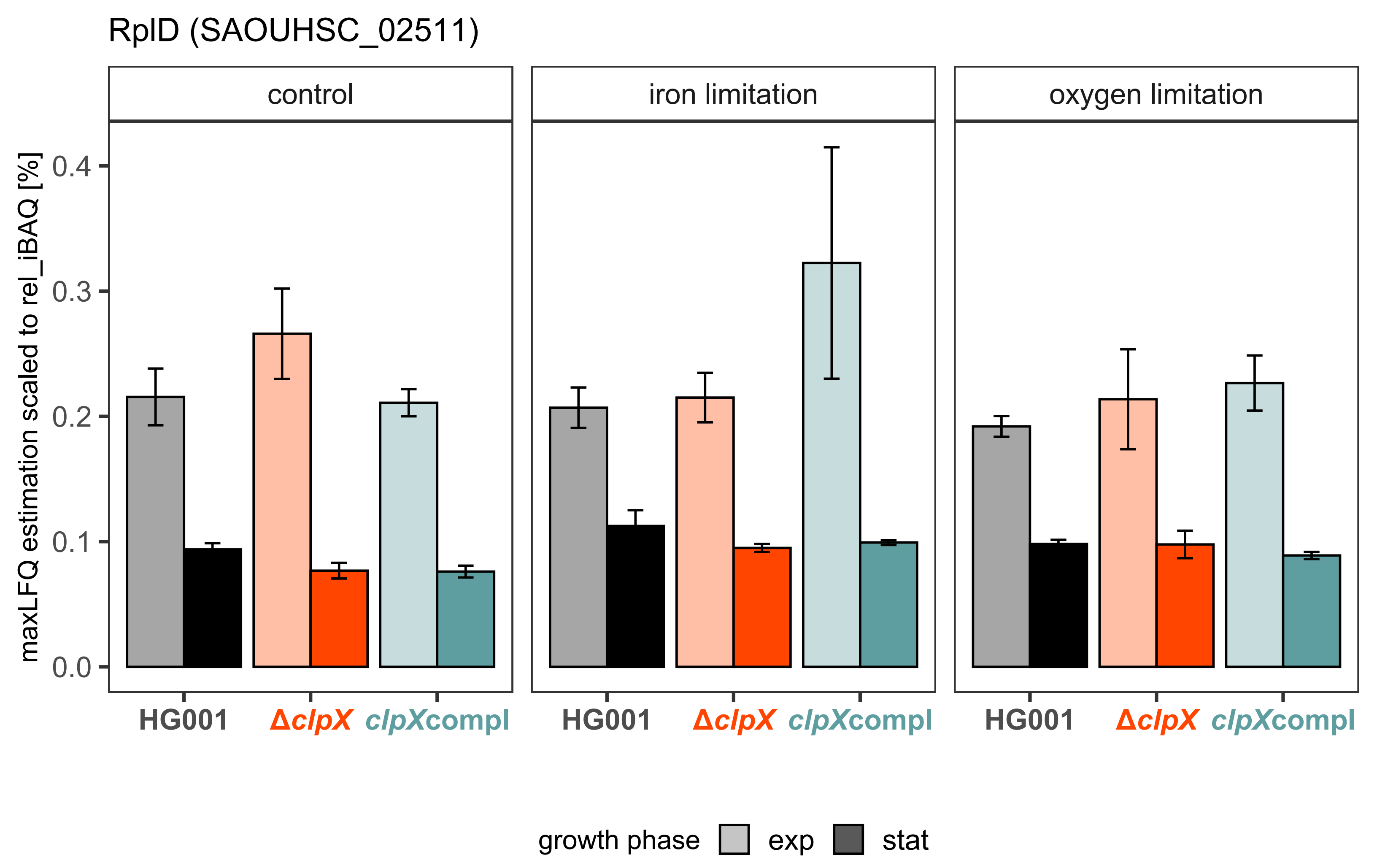

RplD (SAOUHSC_02511)
control
iron limitation
oxygen limitation
maxLFQ estimation scaled to rel_iBAQ [%]
0.0
0.1
0.2
0.3
0.4
HG001
ΔclpX
clpXcompl
growth phase
exp
stat

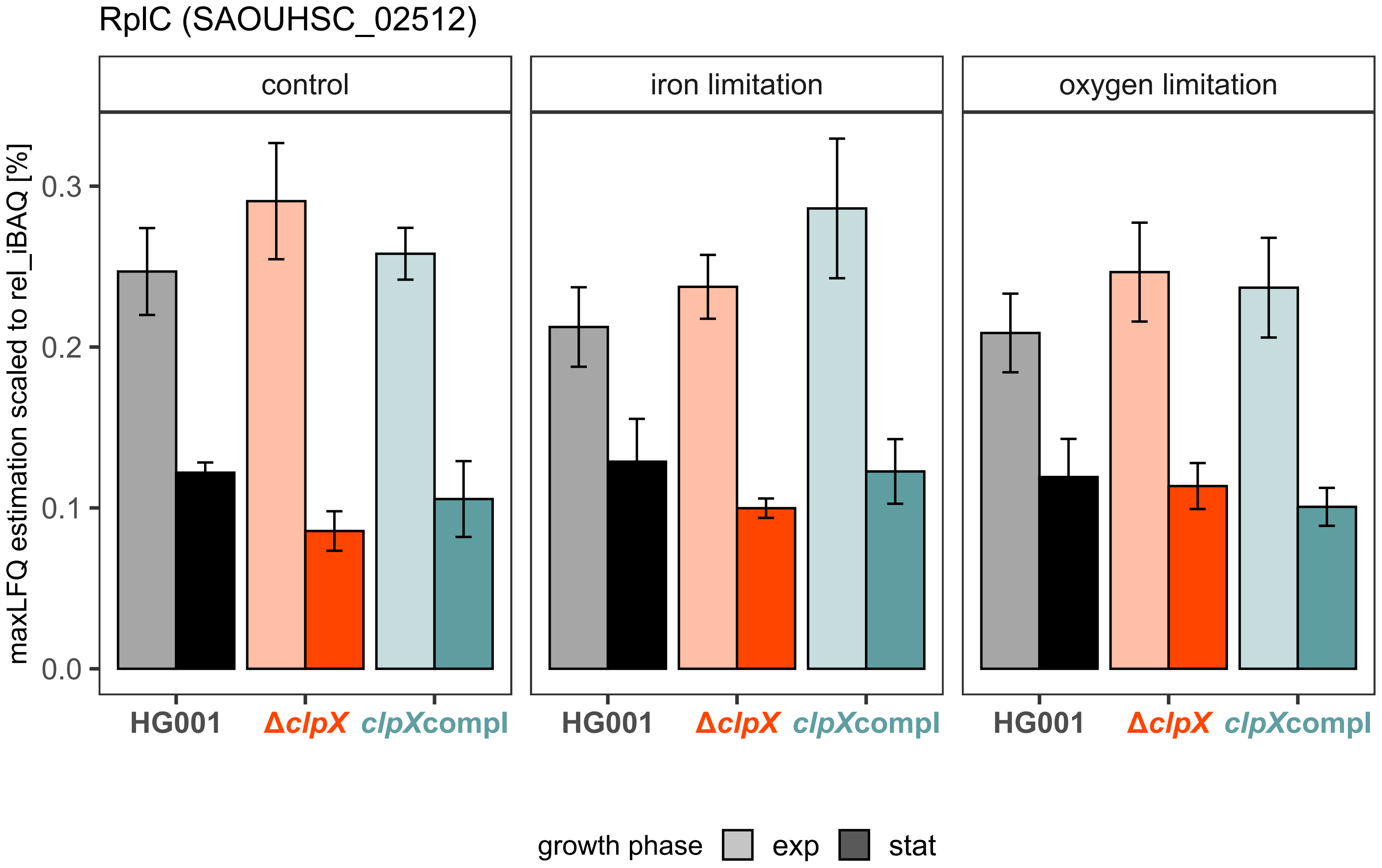

RplC (SAOUHSC_02512)
control
iron limitation
oxygen limitation
maxLFQ estimation scaled to rel_iBAQ [%]
0.0
0.1
0.2
0.3
HG001
ΔclpX
clpXcompl
growth phase
exp
stat

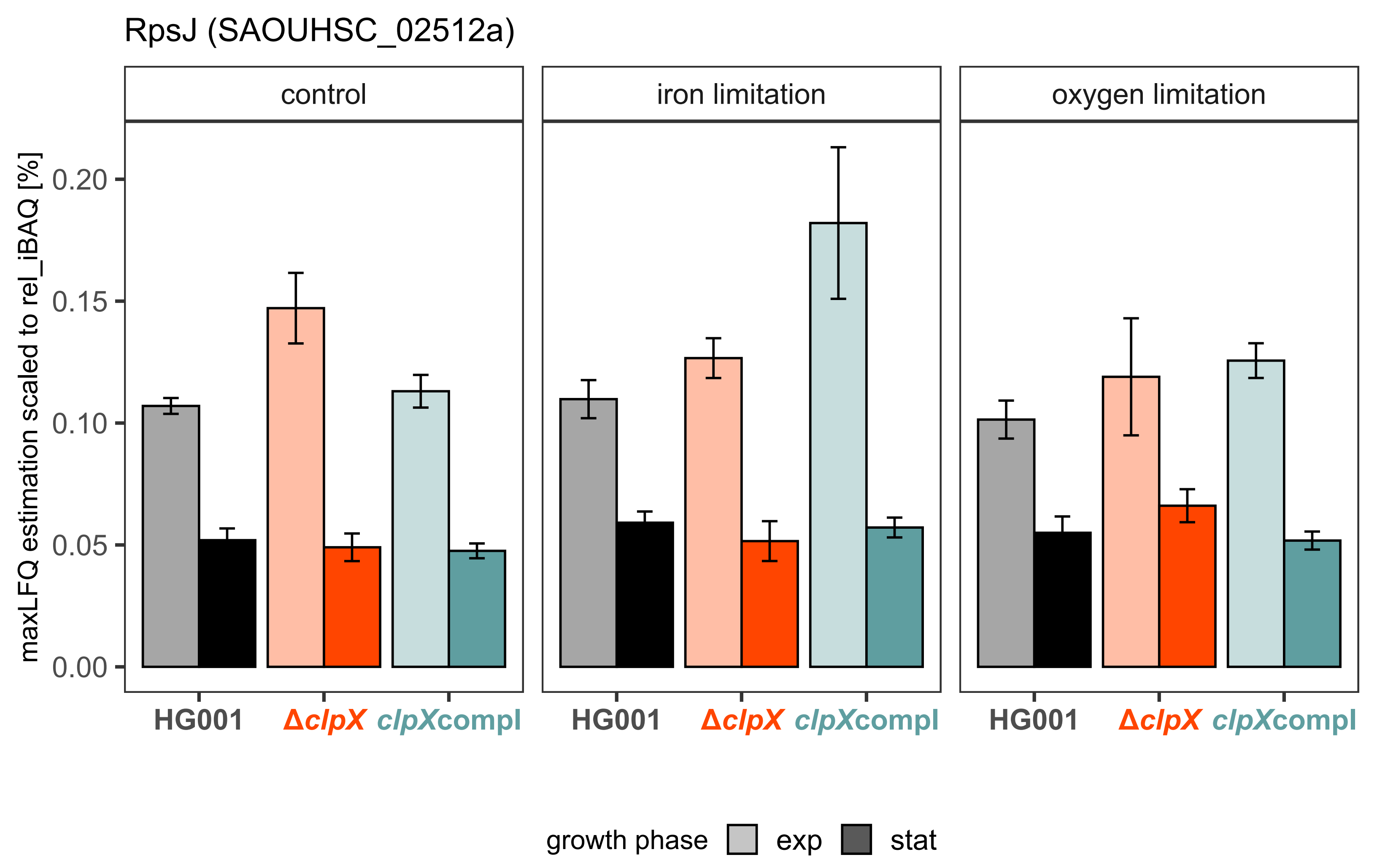
RpsJ (SAOUHSC_02512a)
control
iron limitation
oxygen limitation
maxLFQ estimation scaled to rel_iBAQ [%]
0.00
0.05
0.10
0.15
0.20
HG001
ΔclpX
clpXcompl
growth phase
exp
stat

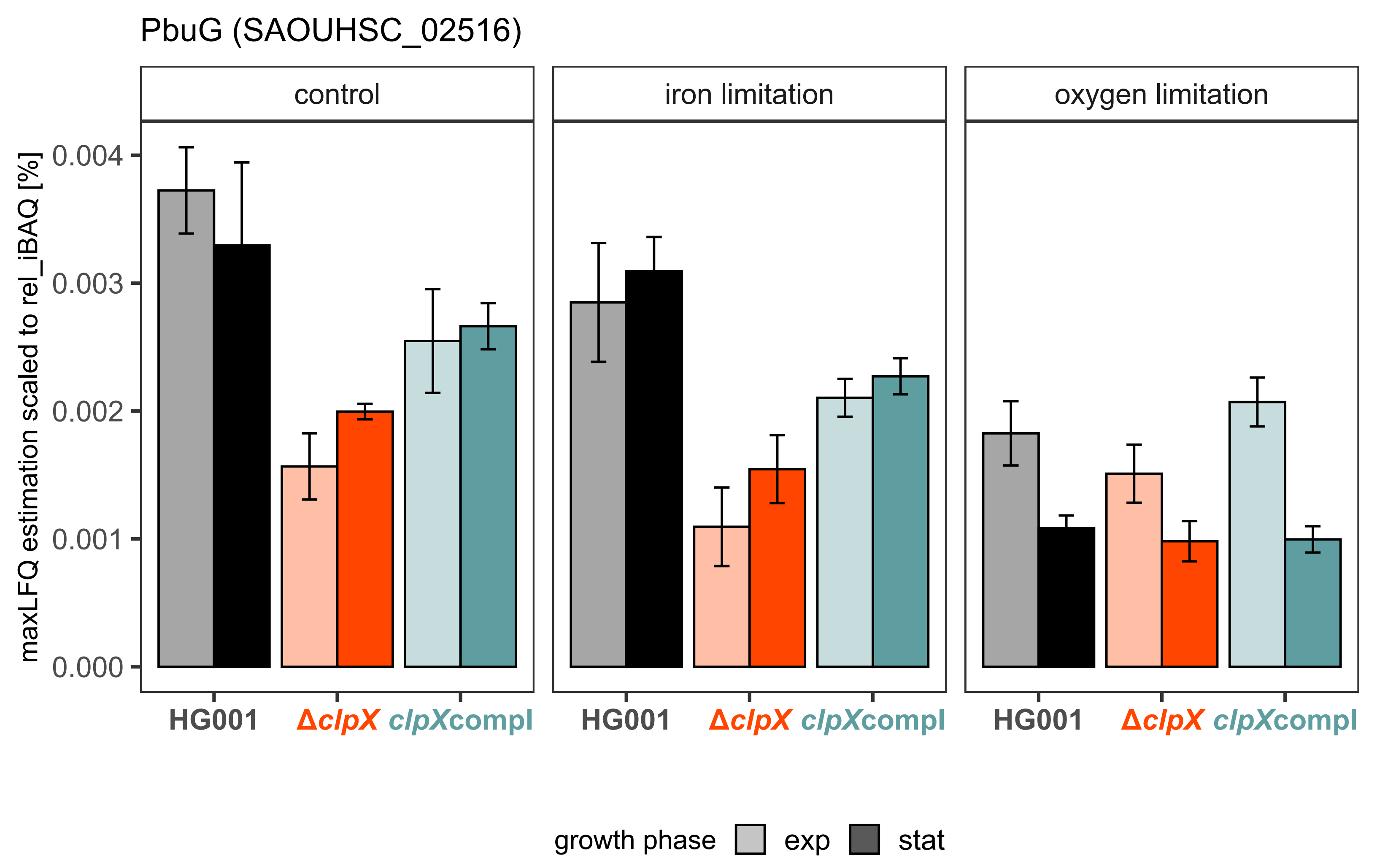
PbuG (SAOUHSC_02516)
control
iron limitation
oxygen limitation
maxLFQ estimation scaled to rel_iBAQ [%]
0.004
0.003
0.002
0.001
0.000
HG001
ΔclpX
clpXcompl
growth phase
exp
stat

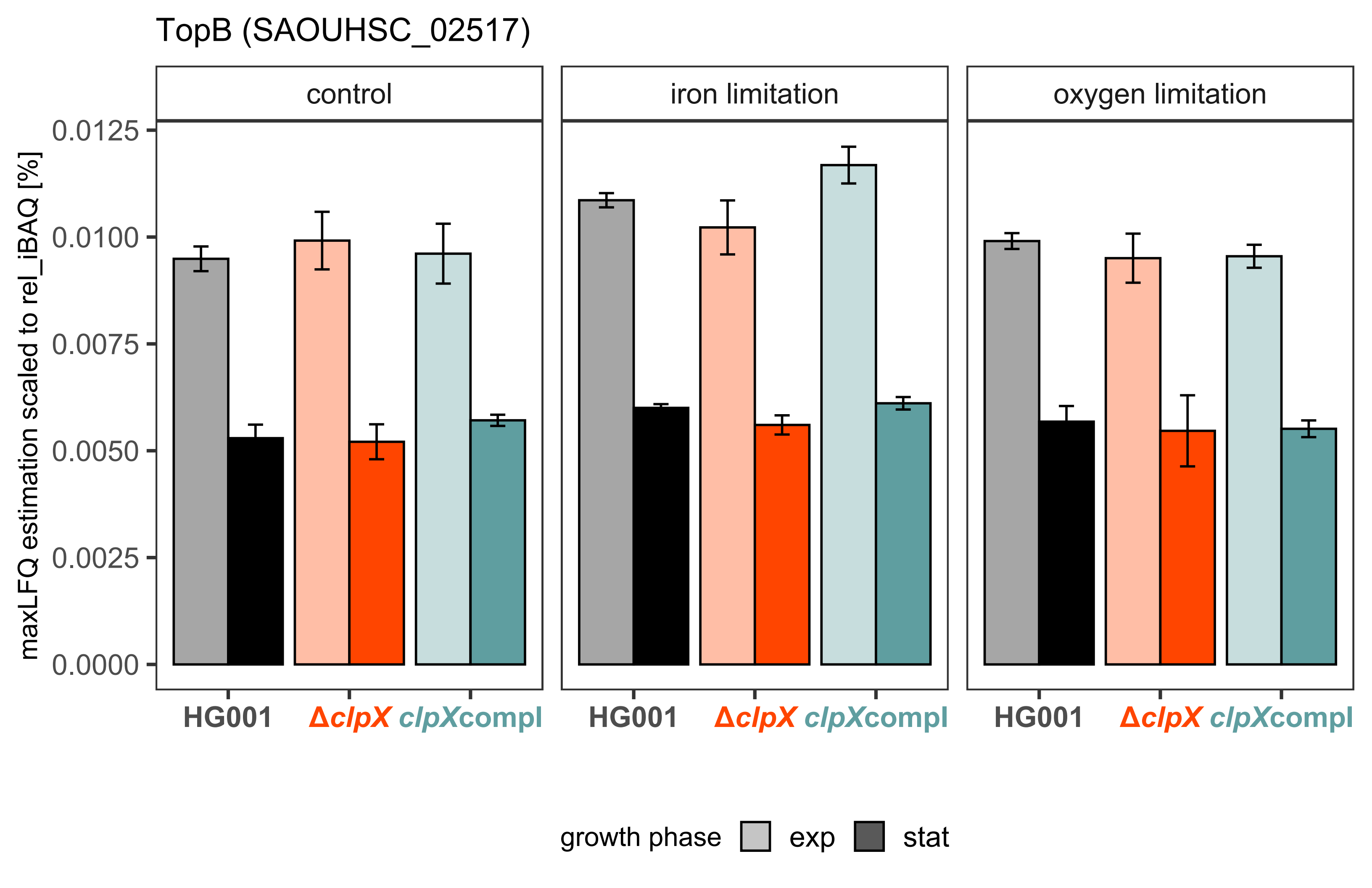

TopB (SAOUHSC_02517)
control
iron limitation
oxygen limitation
maxLFQ estimation scaled to rel_iBAQ [%]
0.0125
0.0100
0.0075
0.0050
0.0025
0.0000
HG001
ΔclpX
clpXcompl
growth phase
exp
stat

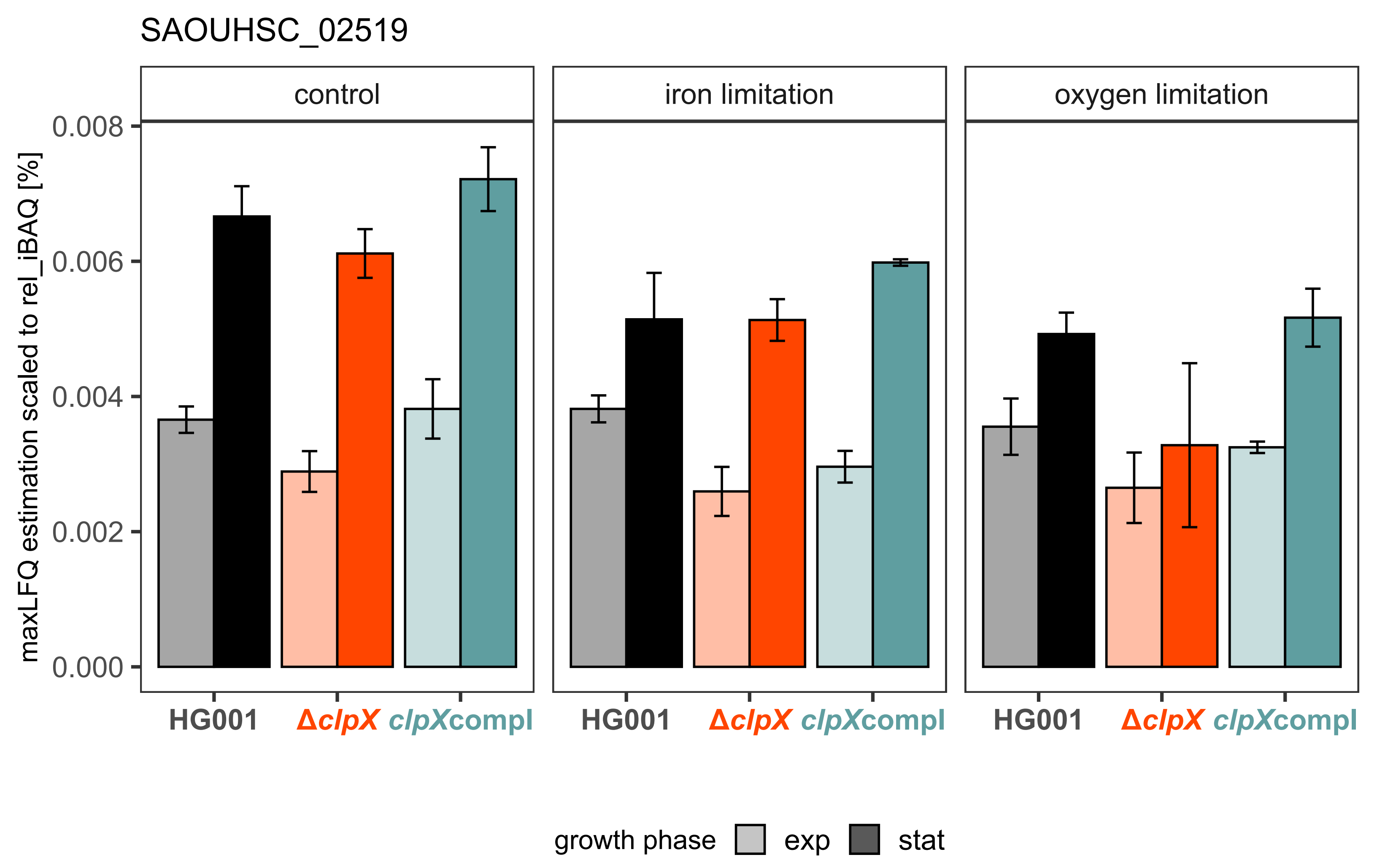

SAOUHSC_02519
control
iron limitation
oxygen limitation
maxLFQ estimation scaled to rel_iBAQ [%]
0.008
0.006
0.004
0.002
0.000
HG001
ΔclpX
clpXcompl
growth phase
exp
stat

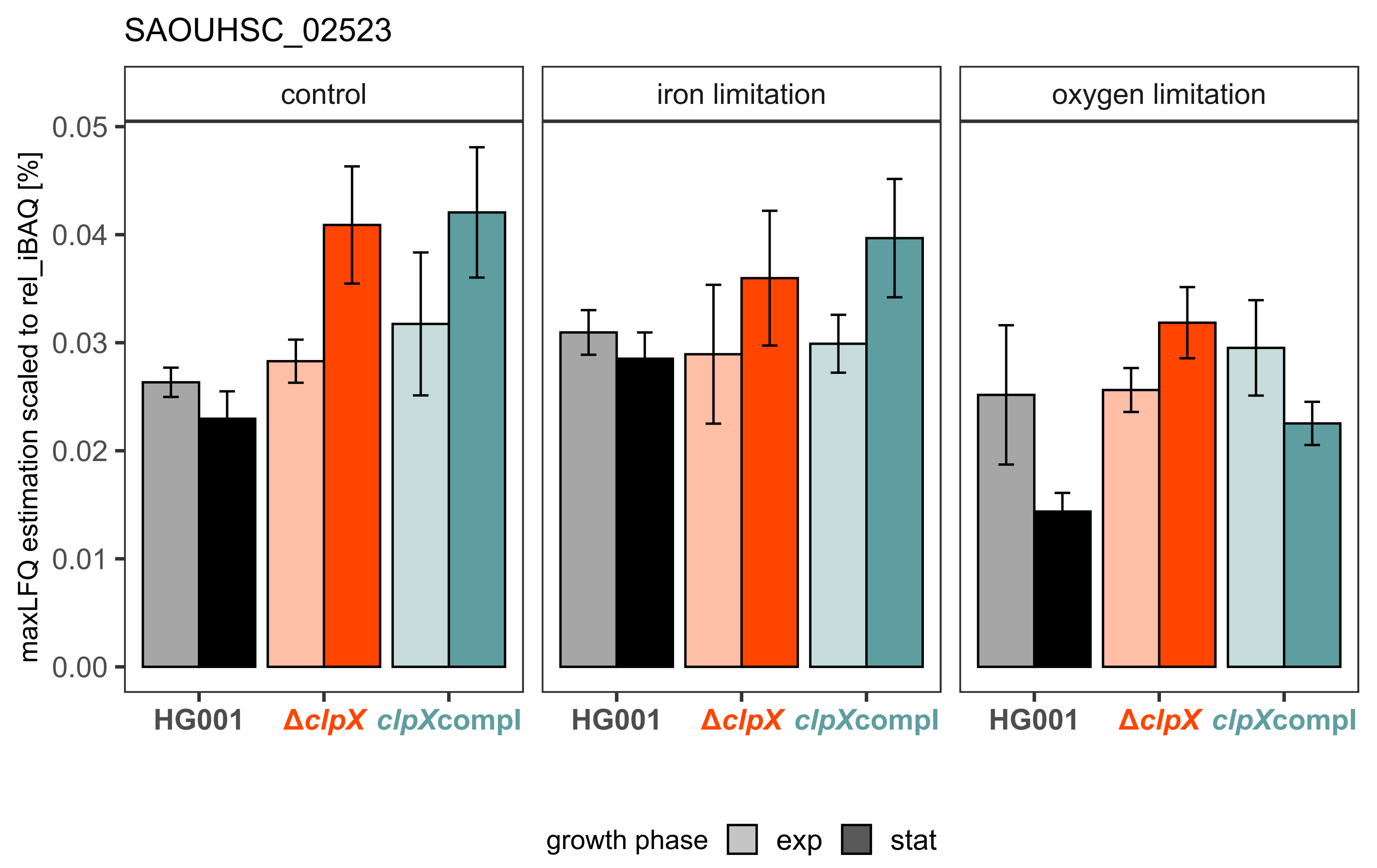
SAOUHSC_02523
control
iron limitation
oxygen limitation
maxLFQ estimation scaled to rel_iBAQ [%]
0.05
0.04
0.03
0.02
0.01
0.00
HG001
ΔclpX
clpXcompl
HG001
ΔclpX
clpXcompl
HG001
ΔclpX
clpXcompl
growth phase
exp
stat

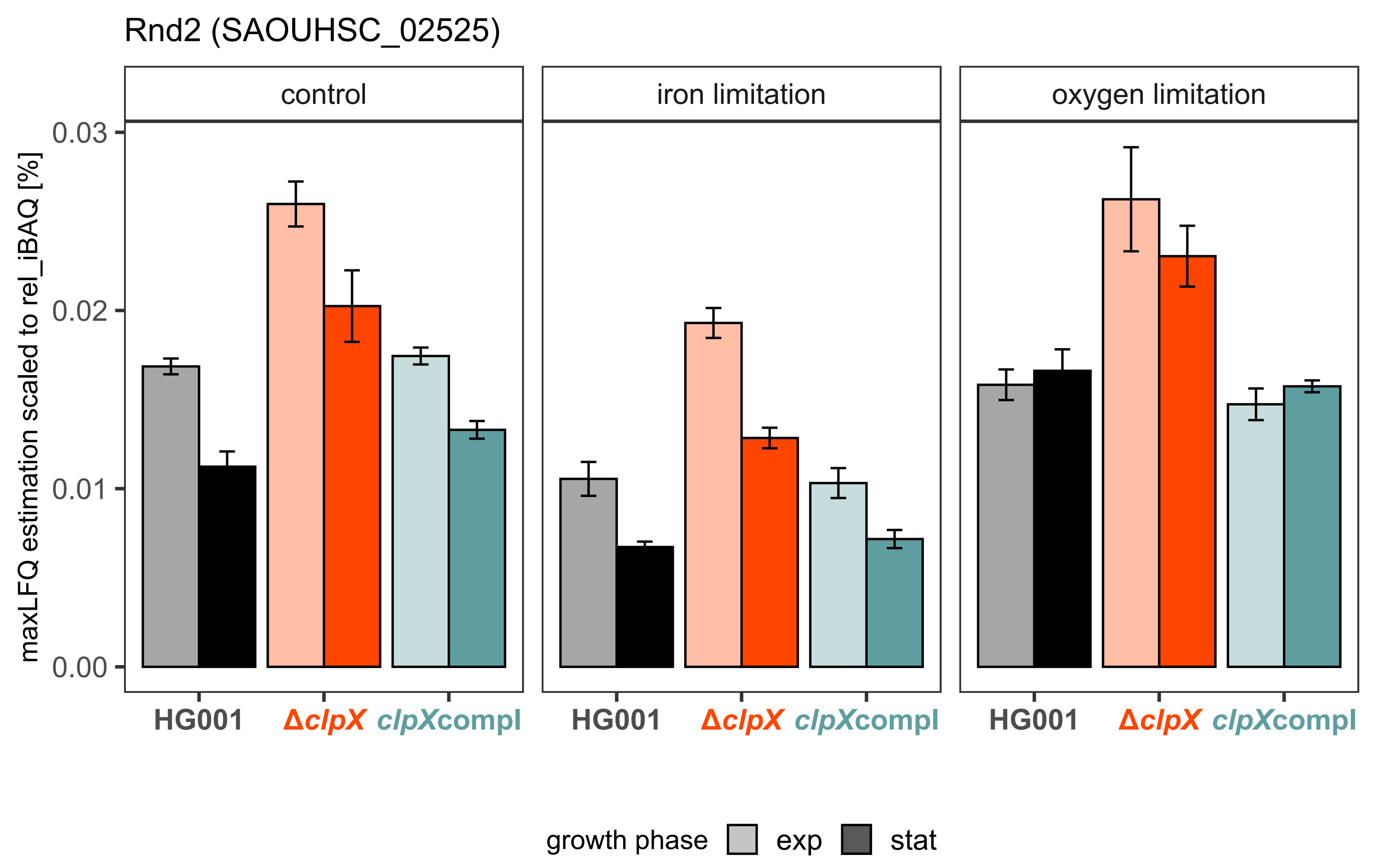

Rnd2 (SAOUHSC_02525)
control
iron limitation
oxygen limitation
maxLFQ estimation scaled to rel_iBAQ [%]
0.03
0.02
0.01
0.00
HG001
ΔclpX
clpXcompl
growth phase
exp
stat

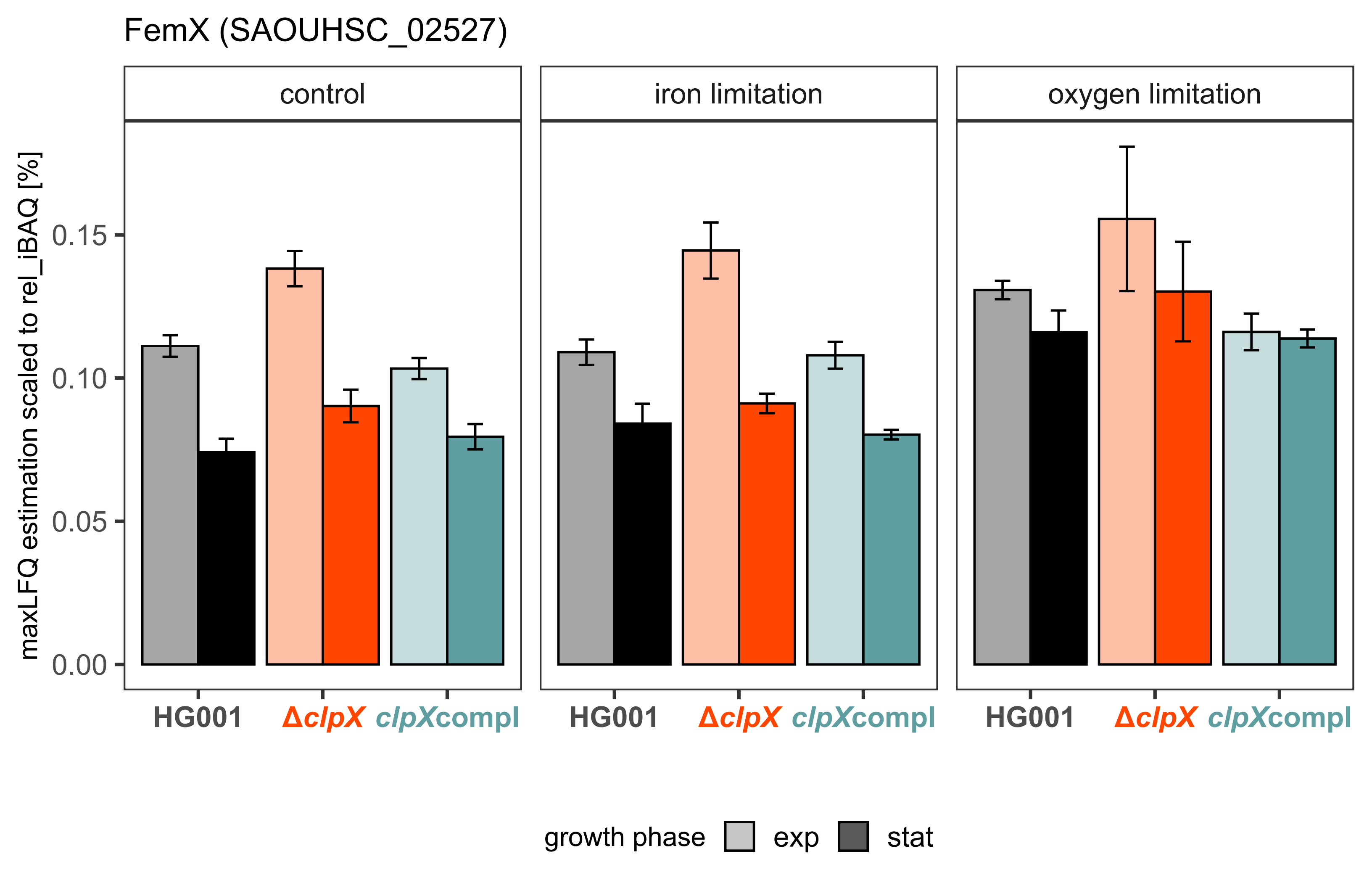
FemX (SAOUHSC_02527)
control
iron limitation
oxygen limitation
maxLFQ estimation scaled to rel_iBAQ [%]
0.15
0.10
0.05
0.00
HG001
ΔclpX
clpXcompl
growth phase
exp
stat

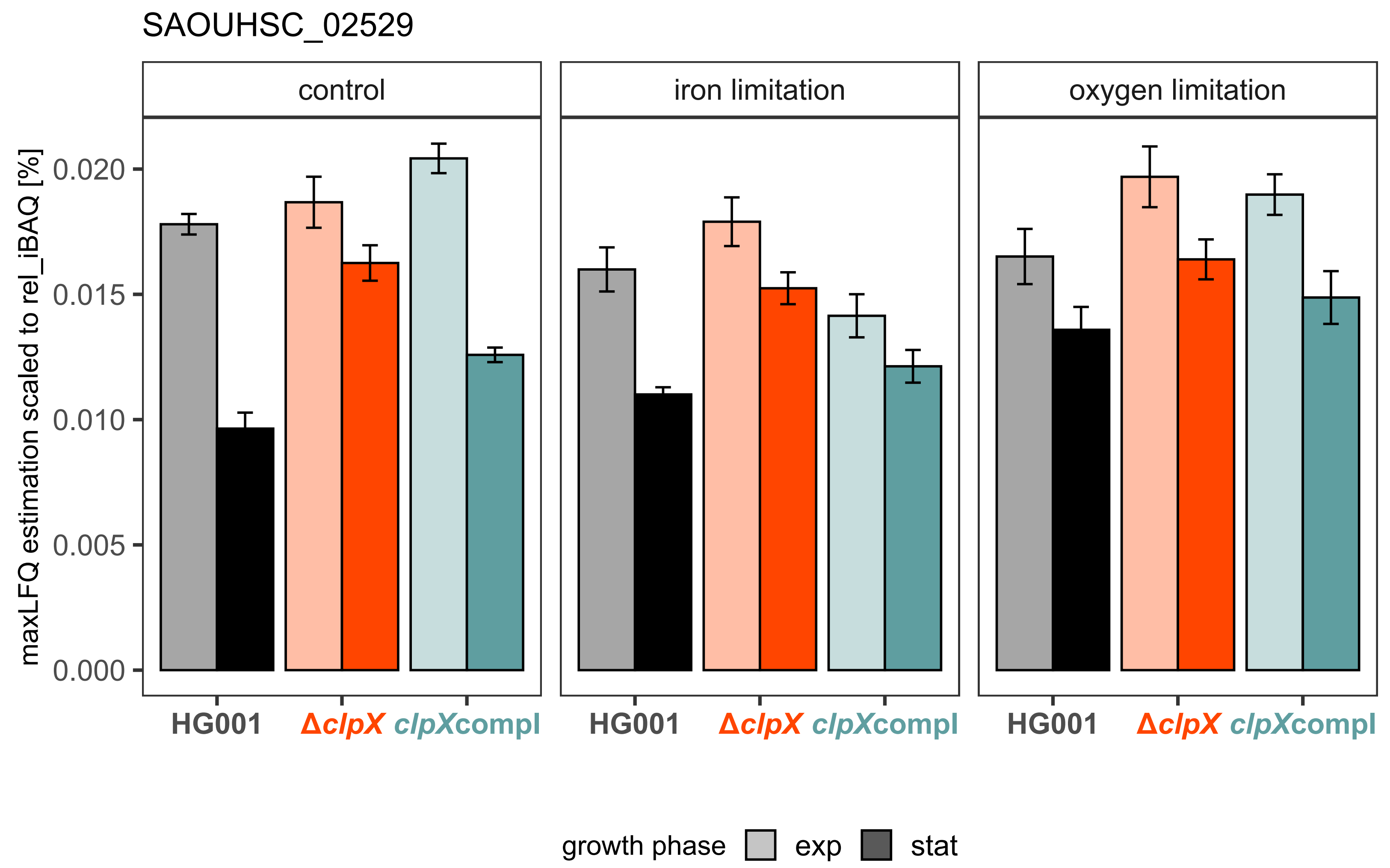
SAOUHSC_02529
control
iron limitation
oxygen limitation
maxLFQ estimation scaled to rel_iBAQ [%]
0.020
0.015
0.010
0.005
0.000
HG001
ΔclpX
clpXcompl
growth phase
exp
stat

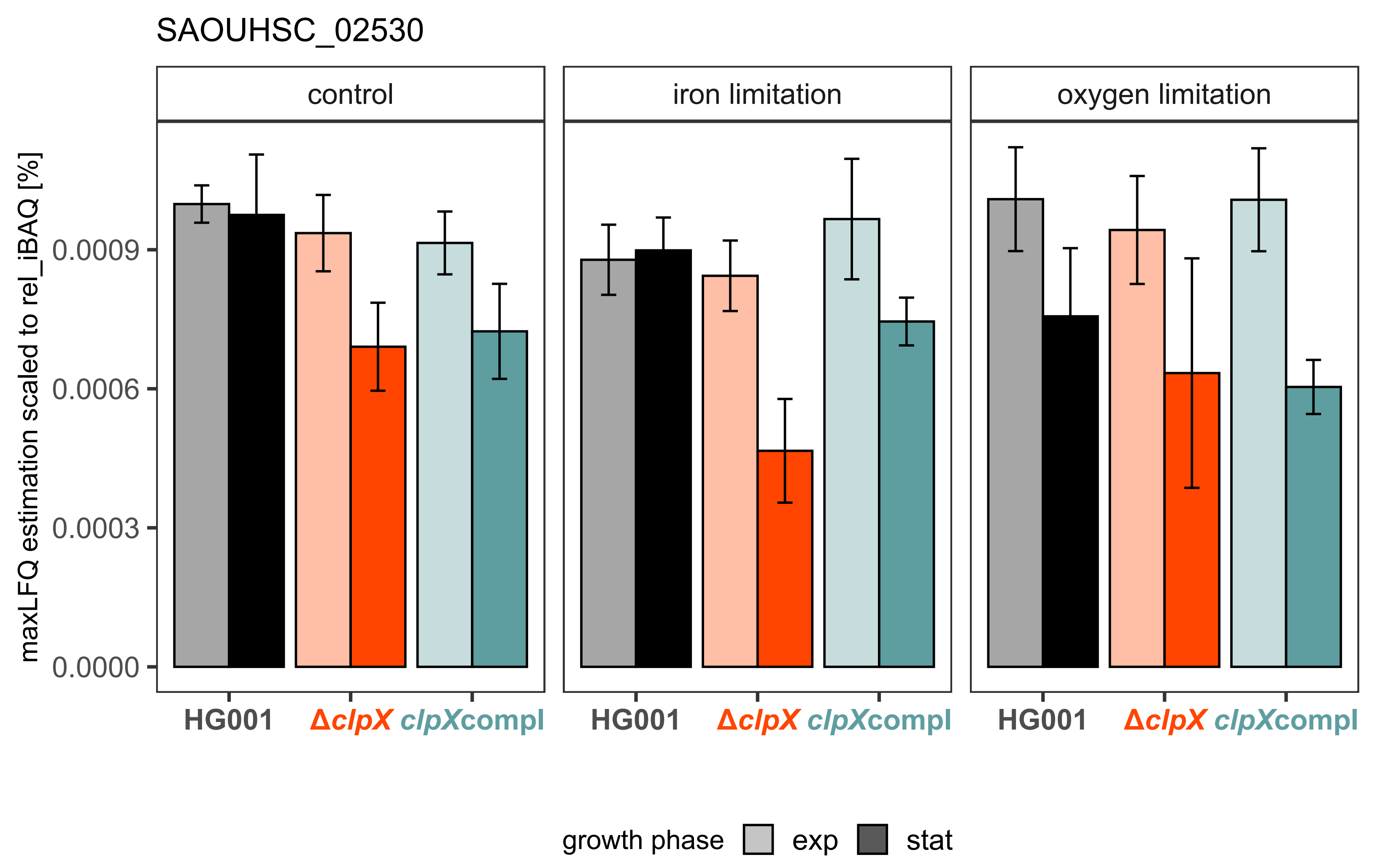

SAOUHSC_02530
control
iron limitation
oxygen limitation
maxLFQ estimation scaled to rel_iBAQ [%]
0.0000
0.0003
0.0006
0.0009
HG001
ΔclpX
clpXcompl
growth phase
exp
stat

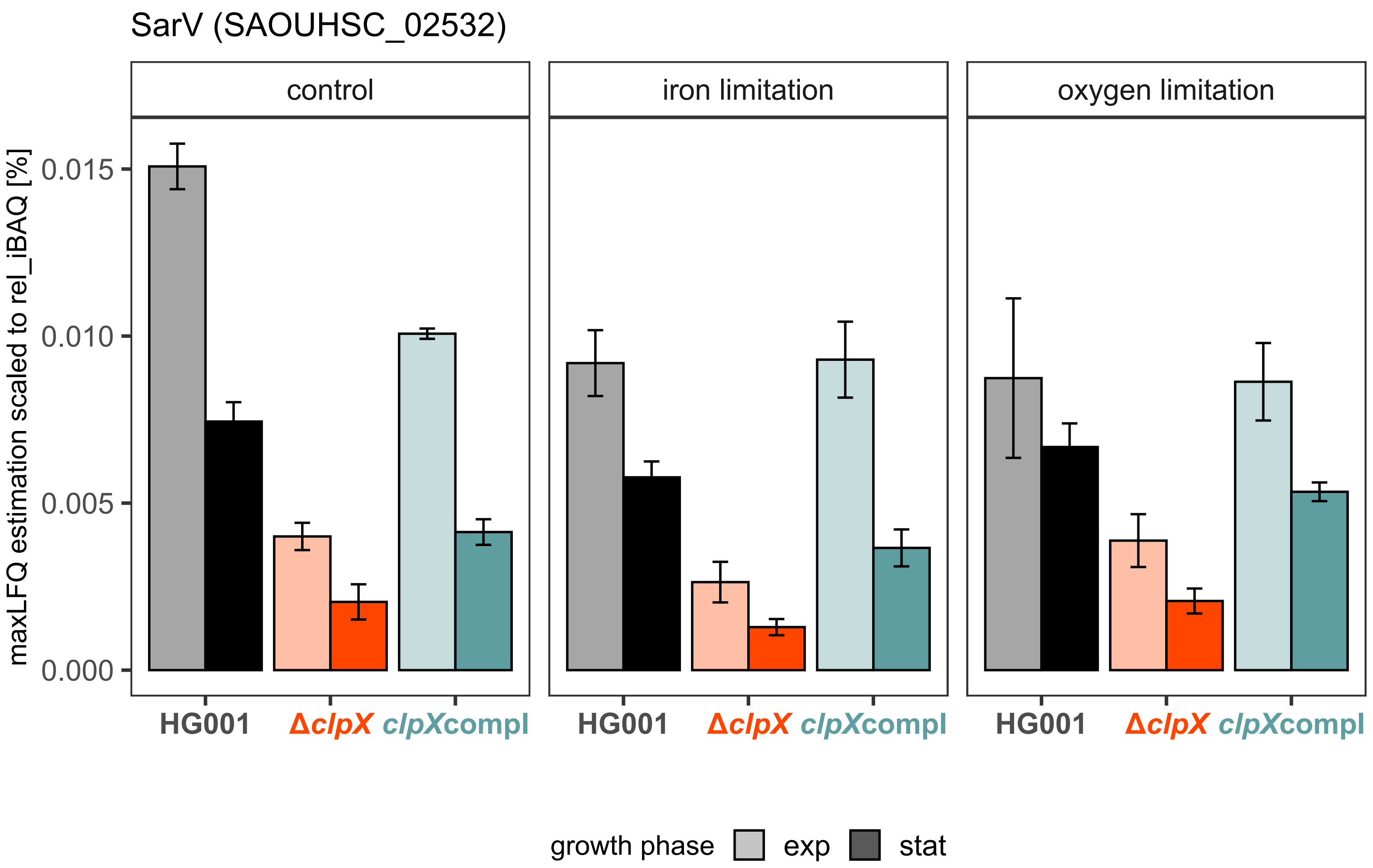
SarV (SAOUHSC_02532)
control
iron limitation
oxygen limitation
maxLFQ estimation scaled to rel_iBAQ [%]
0.015
0.010
0.005
0.000
HG001
ΔclpX
clpXcompl
growth phase
exp
stat

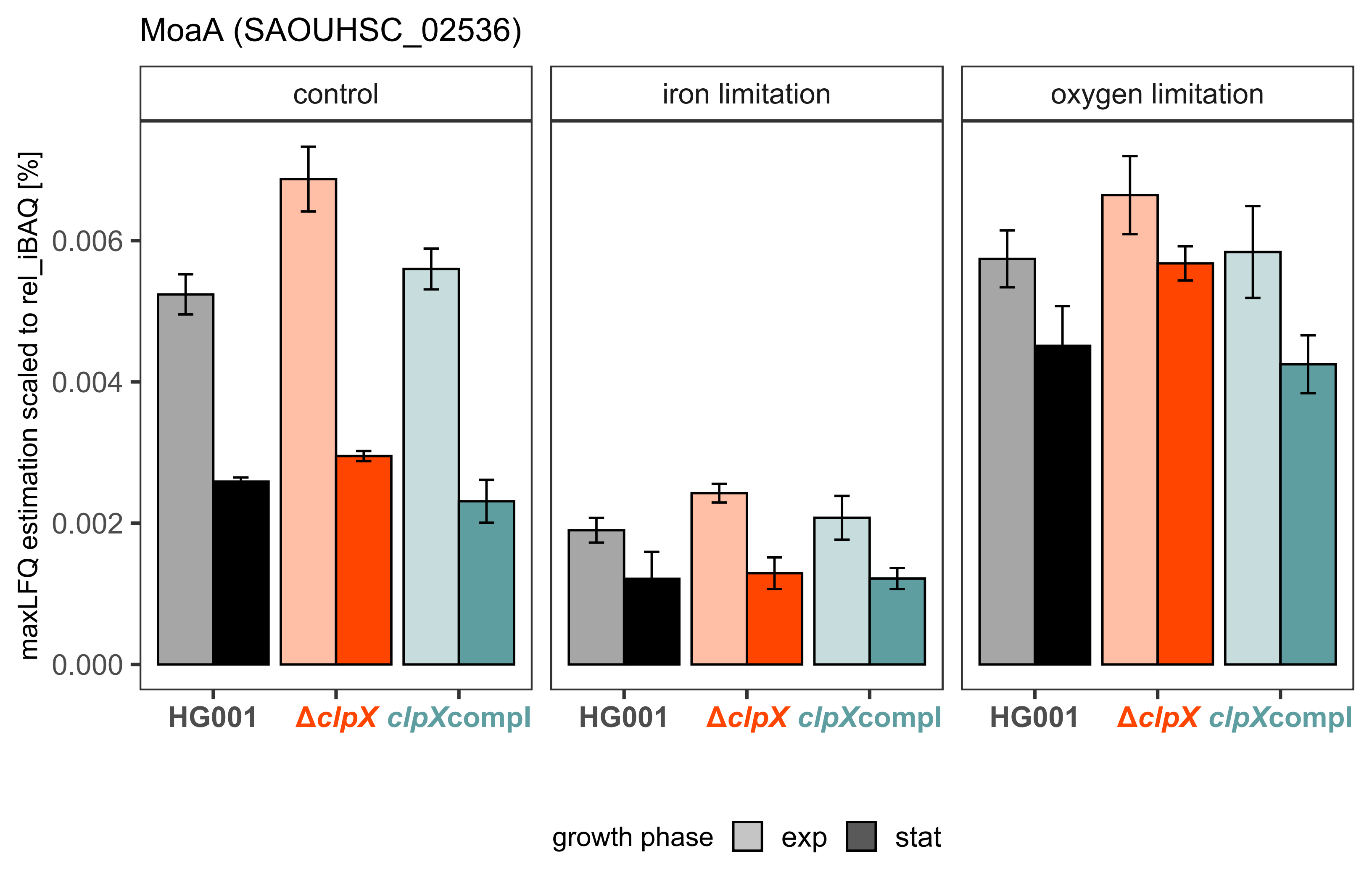

MoaA (SAOUHSC_02536)
control
iron limitation
oxygen limitation
maxLFQ estimation scaled to rel_iBAQ [%]
0.000
0.002
0.004
0.006
HG001
ΔclpX
clpXcompl
growth phase
exp
stat

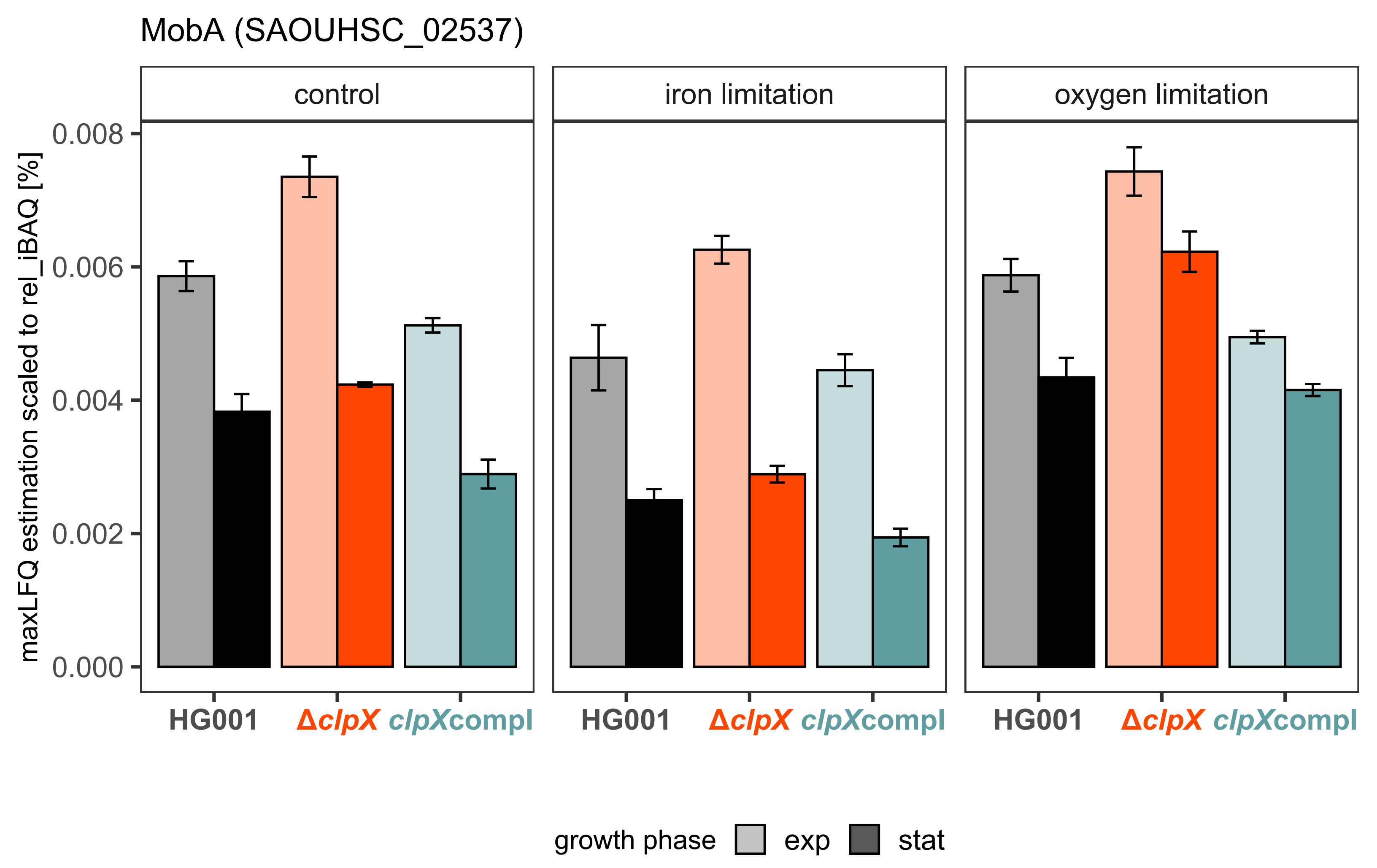

MobA (SAOUHSC_02537)
control
iron limitation
oxygen limitation
maxLFQ estimation scaled to rel_iBAQ [%]
0.008
0.006
0.004
0.002
0.000
HG001
ΔclpX
clpXcompl
growth phase
exp
stat

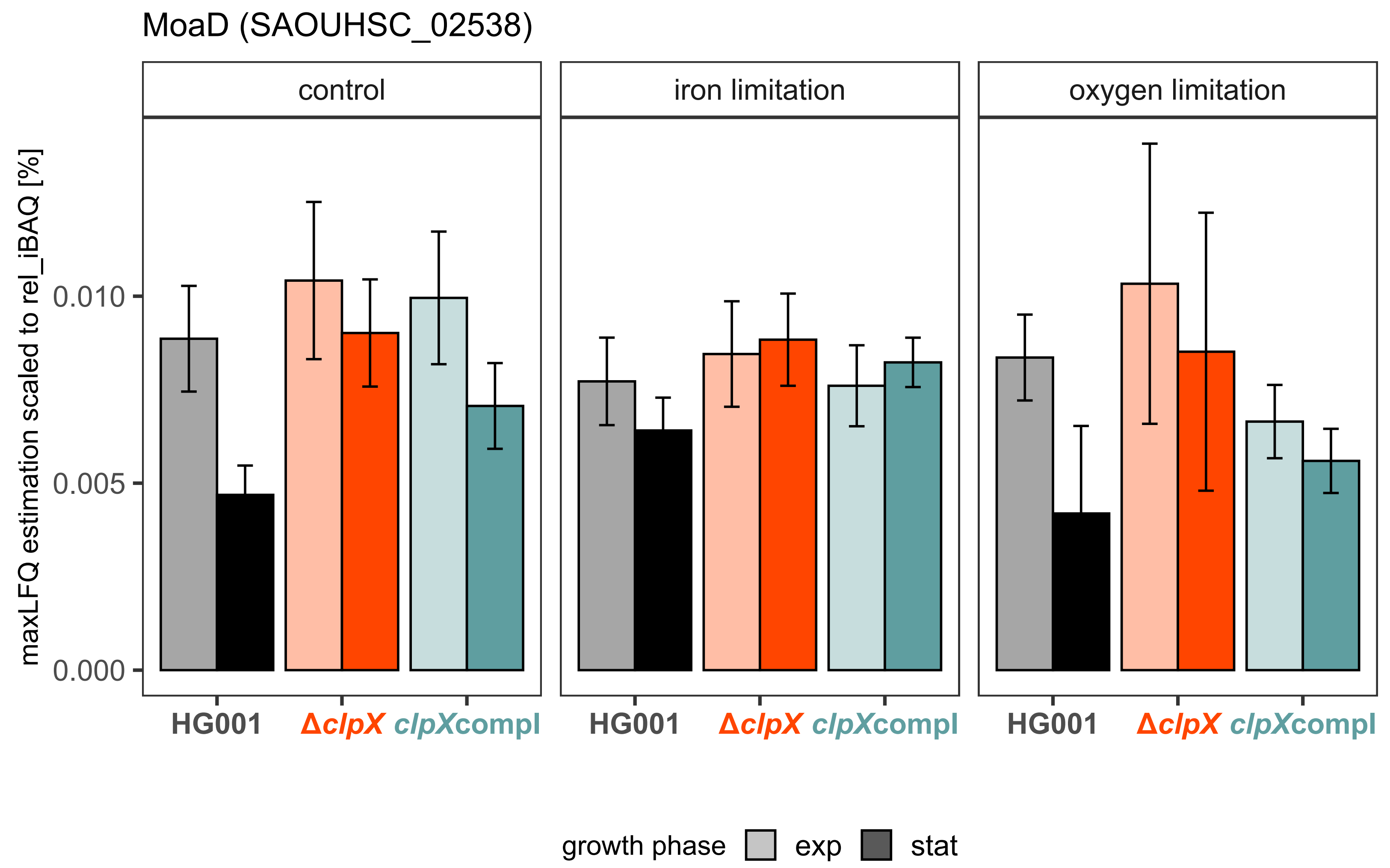
MoaD (SAOUHSC_02538)
control
iron limitation
oxygen limitation
maxLFQ estimation scaled to rel_iBAQ [%]
0.010
0.005
0.000
HG001
ΔclpX
clpXcompl
growth phase
exp
stat

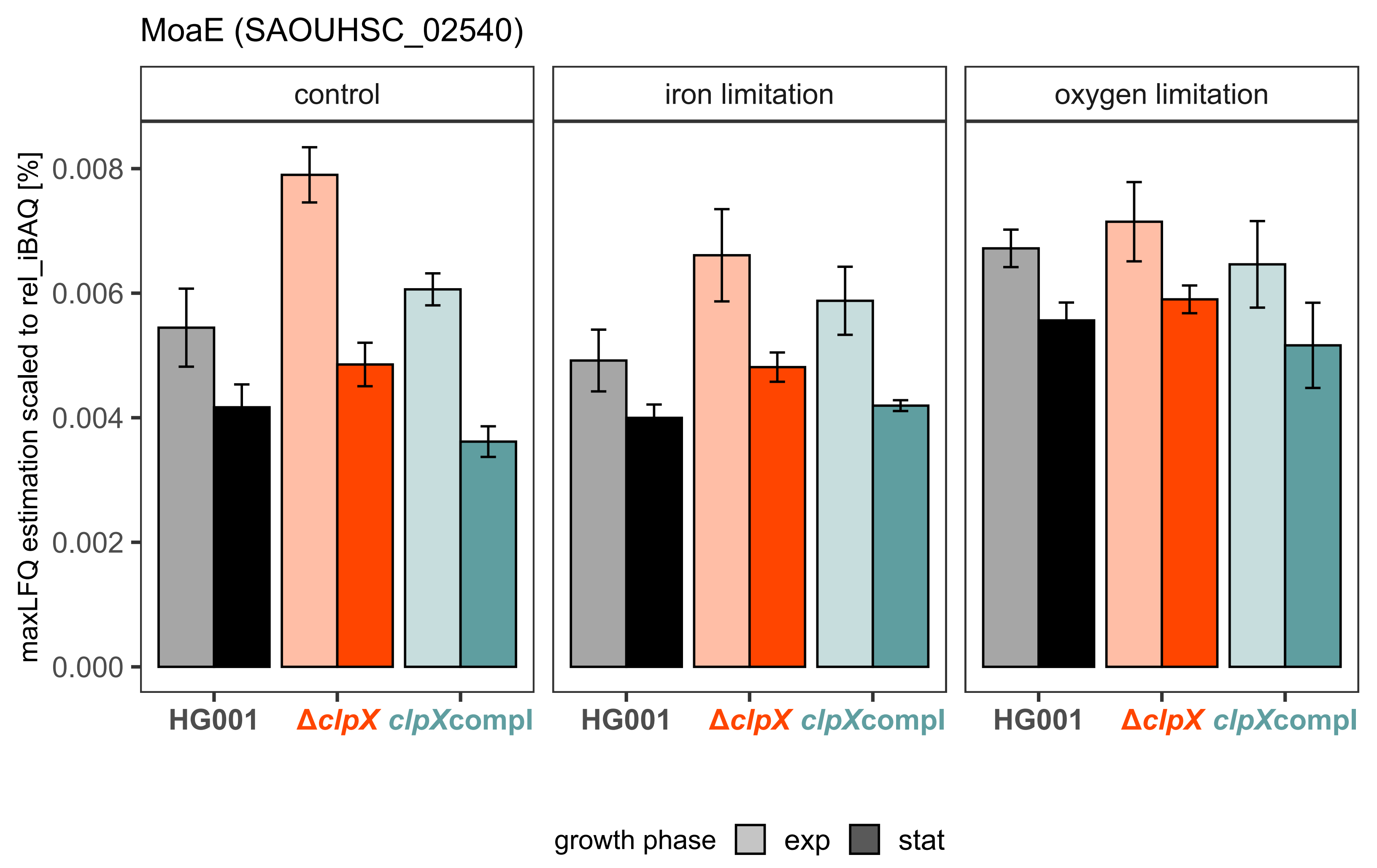

MoaE (SAOUHSC_02540)
control
iron limitation
oxygen limitation
maxLFQ estimation scaled to rel_iBAQ [%]
0.008
0.006
0.004
0.002
0.000
HG001
ΔclpX
clpXcompl
growth phase
exp
stat

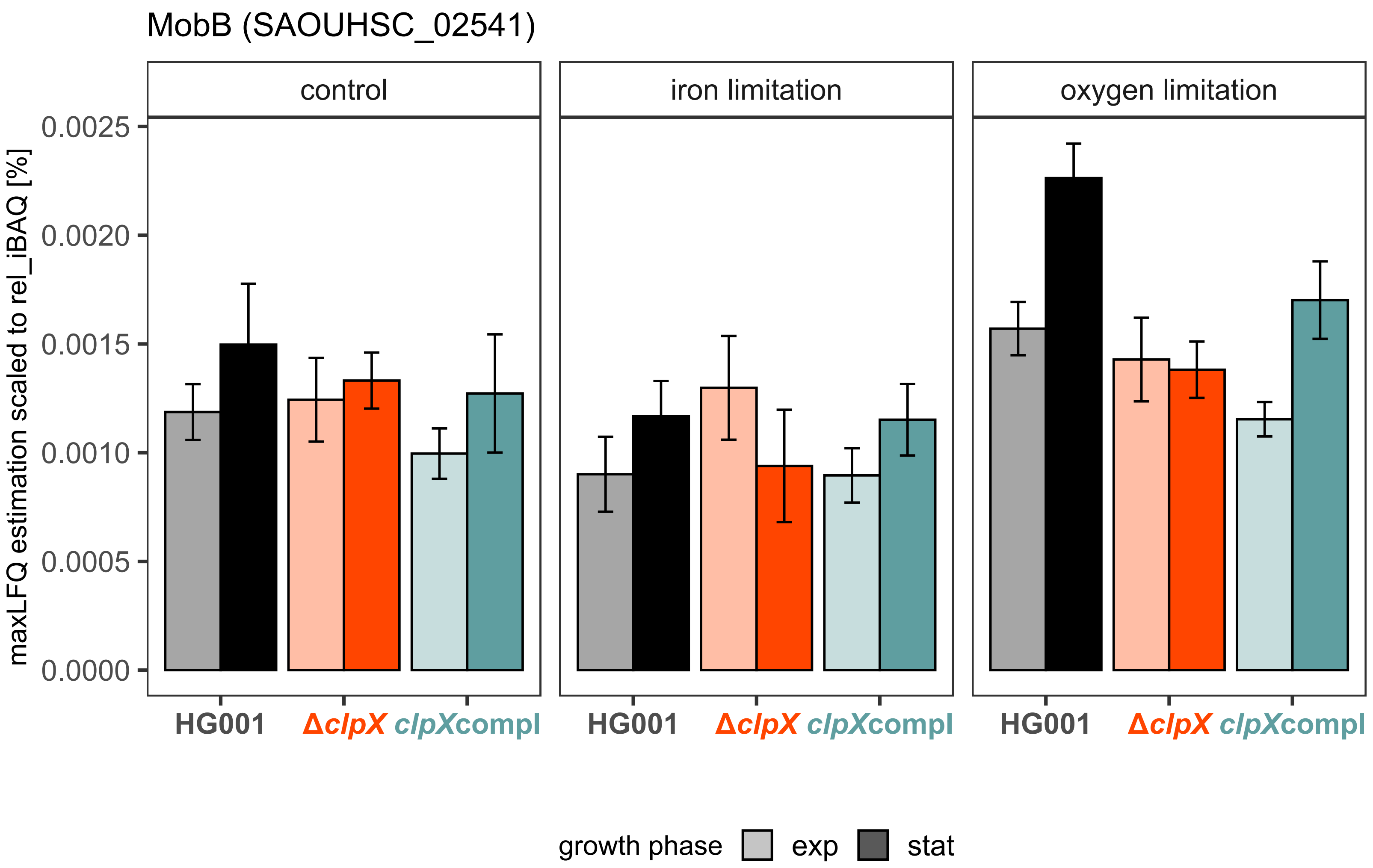

MobB (SAOUHSC_02541)
control
iron limitation
oxygen limitation
maxLFQ estimation scaled to rel_iBAQ [%]
0.0025
0.0020
0.0015
0.0010
0.0005
0.0000
HG001
ΔclpX
clpXcompl
growth phase
exp
stat

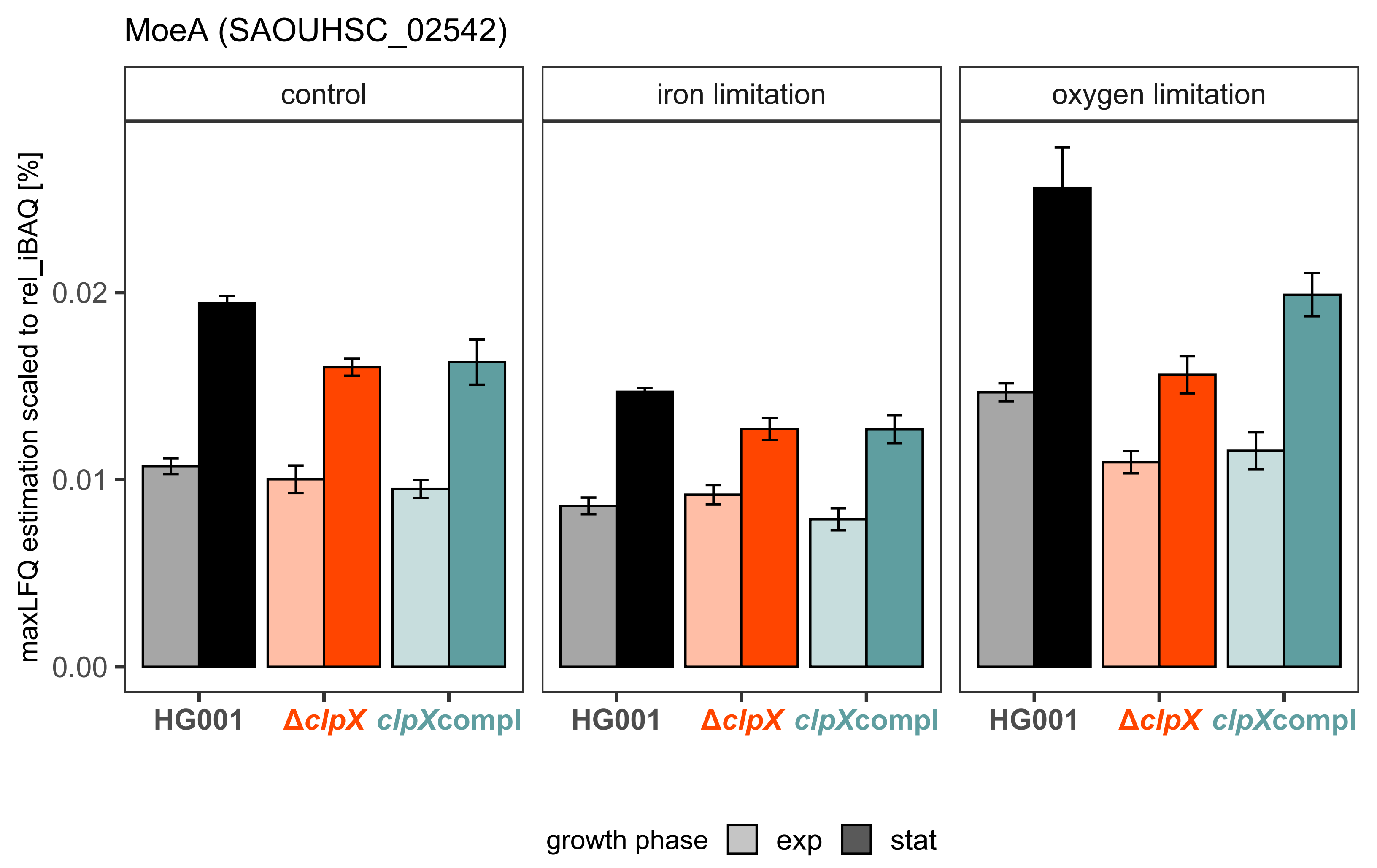
MoeA (SAOUHSC_02542)
control
iron limitation
oxygen limitation
maxLFQ estimation scaled to rel_iBAQ [%]
0.00
0.01
0.02
HG001
ΔclpX
clpXcompl
growth phase
exp
stat

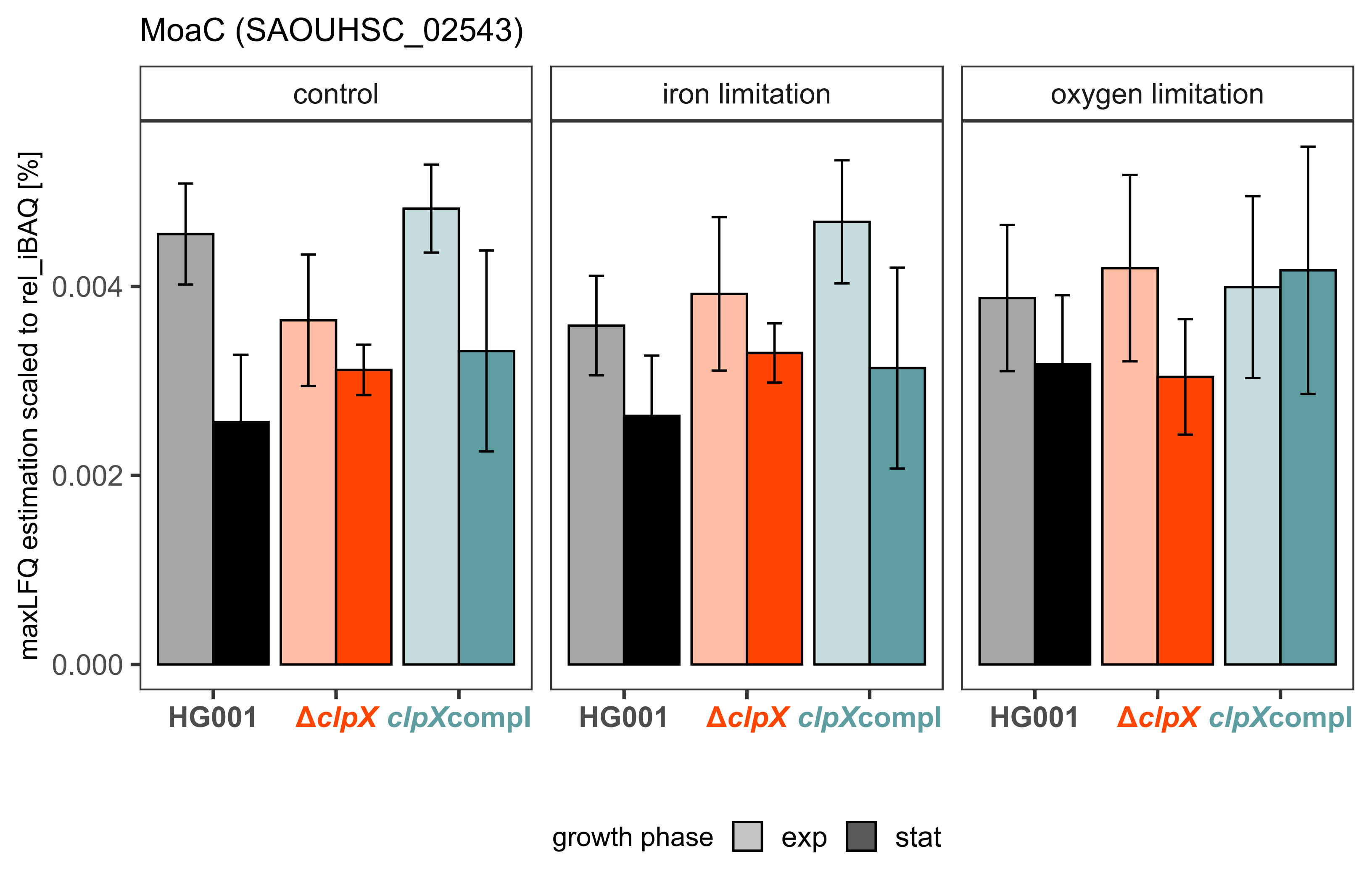

MoaC (SAOUHSC_02543)
control
iron limitation
oxygen limitation
maxLFQ estimation scaled to rel_iBAQ [%]
0.000
0.002
0.004
HG001
ΔclpX
clpXcompl
growth phase
exp
stat

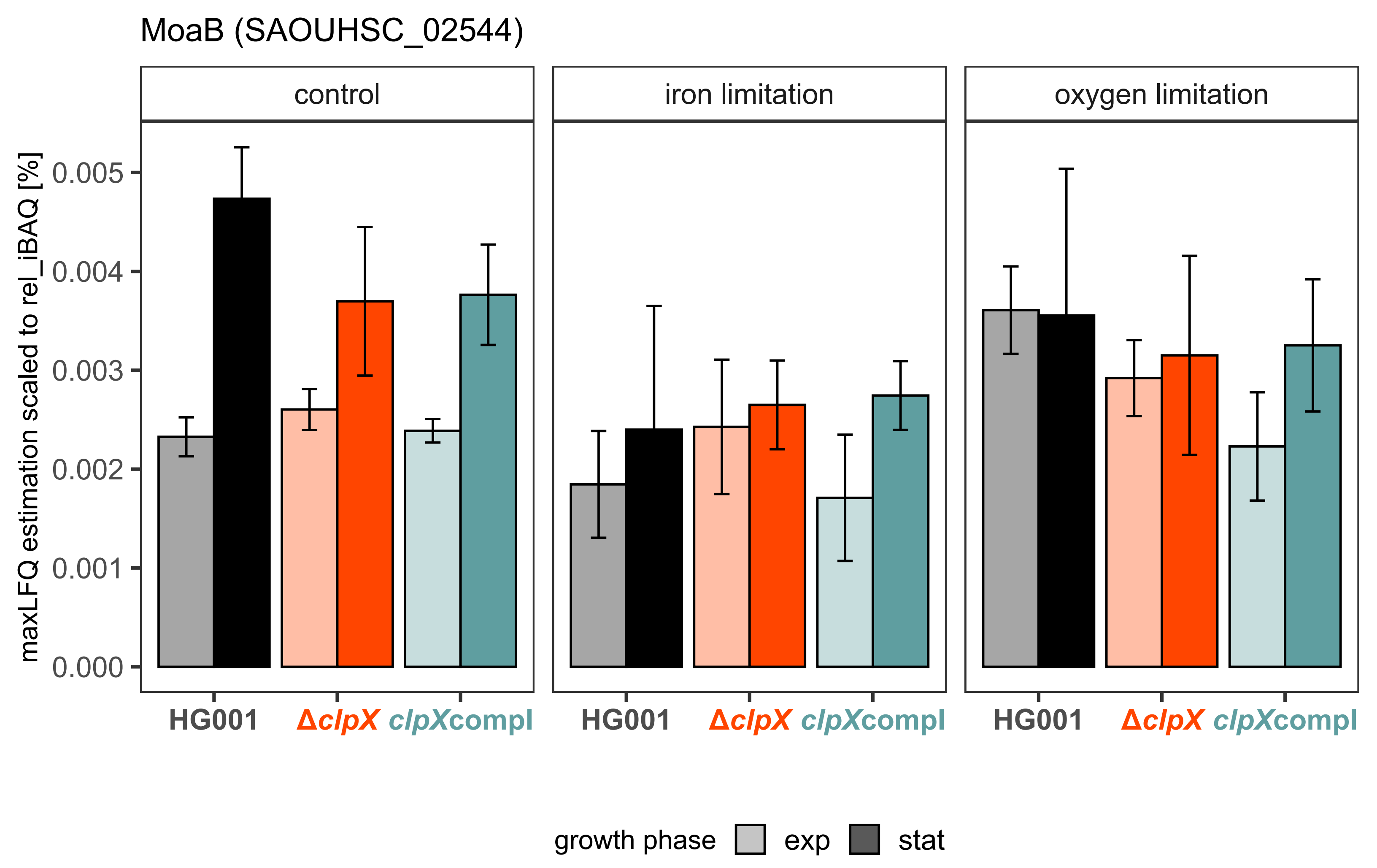

MoaB (SAOUHSC_02544)
control
iron limitation
oxygen limitation
maxLFQ estimation scaled to rel_iBAQ [%]
0.005
0.004
0.003
0.002
0.001
0.000
HG001
ΔclpX
clpXcompl
growth phase
exp
stat

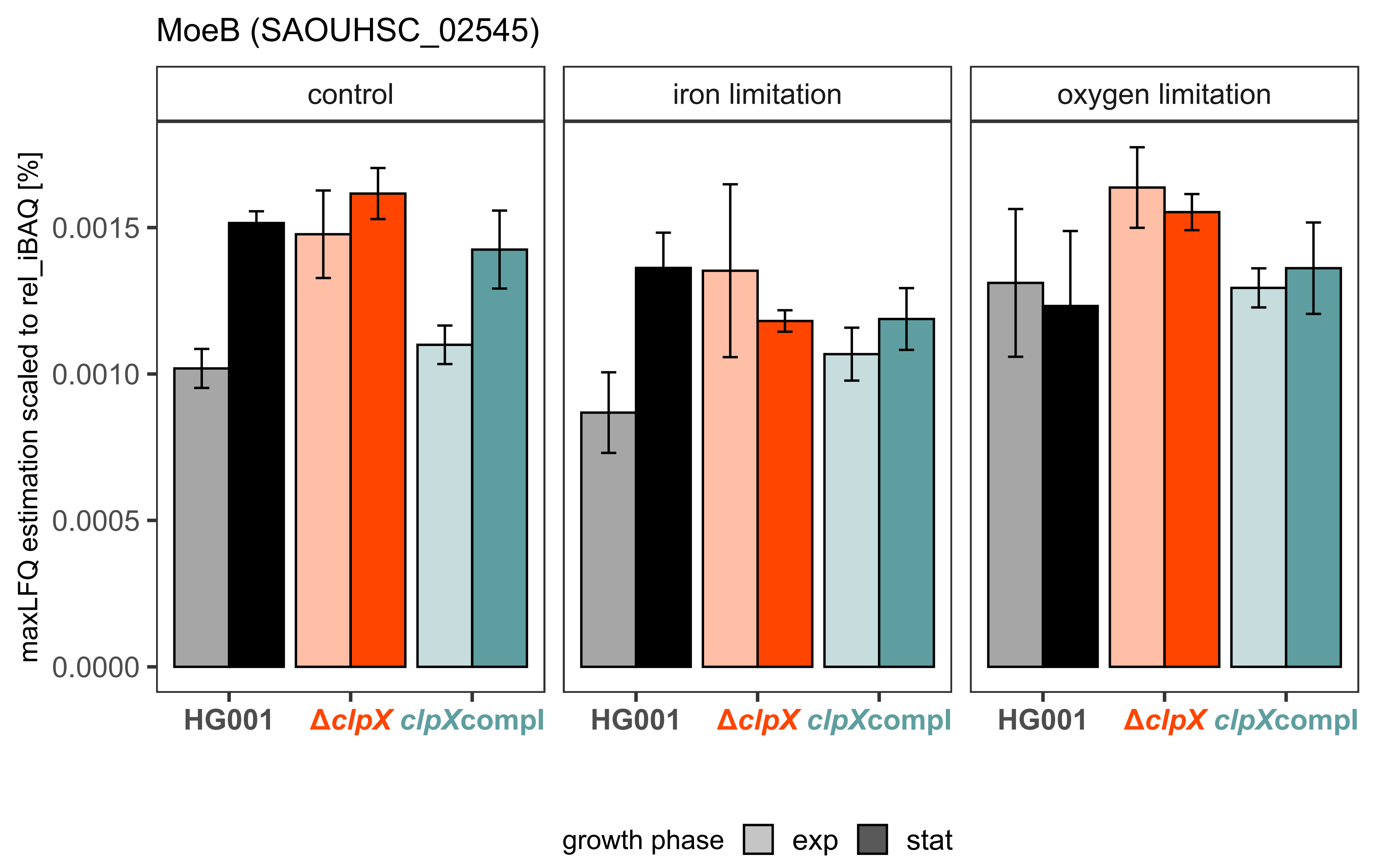
MoeB (SAOUHSC_02545)
control
iron limitation
oxygen limitation
maxLFQ estimation scaled to rel_iBAQ [%]
0.0015
0.0010
0.0005
0.0000
HG001
ΔclpX
clpXcompl
growth phase
exp
stat

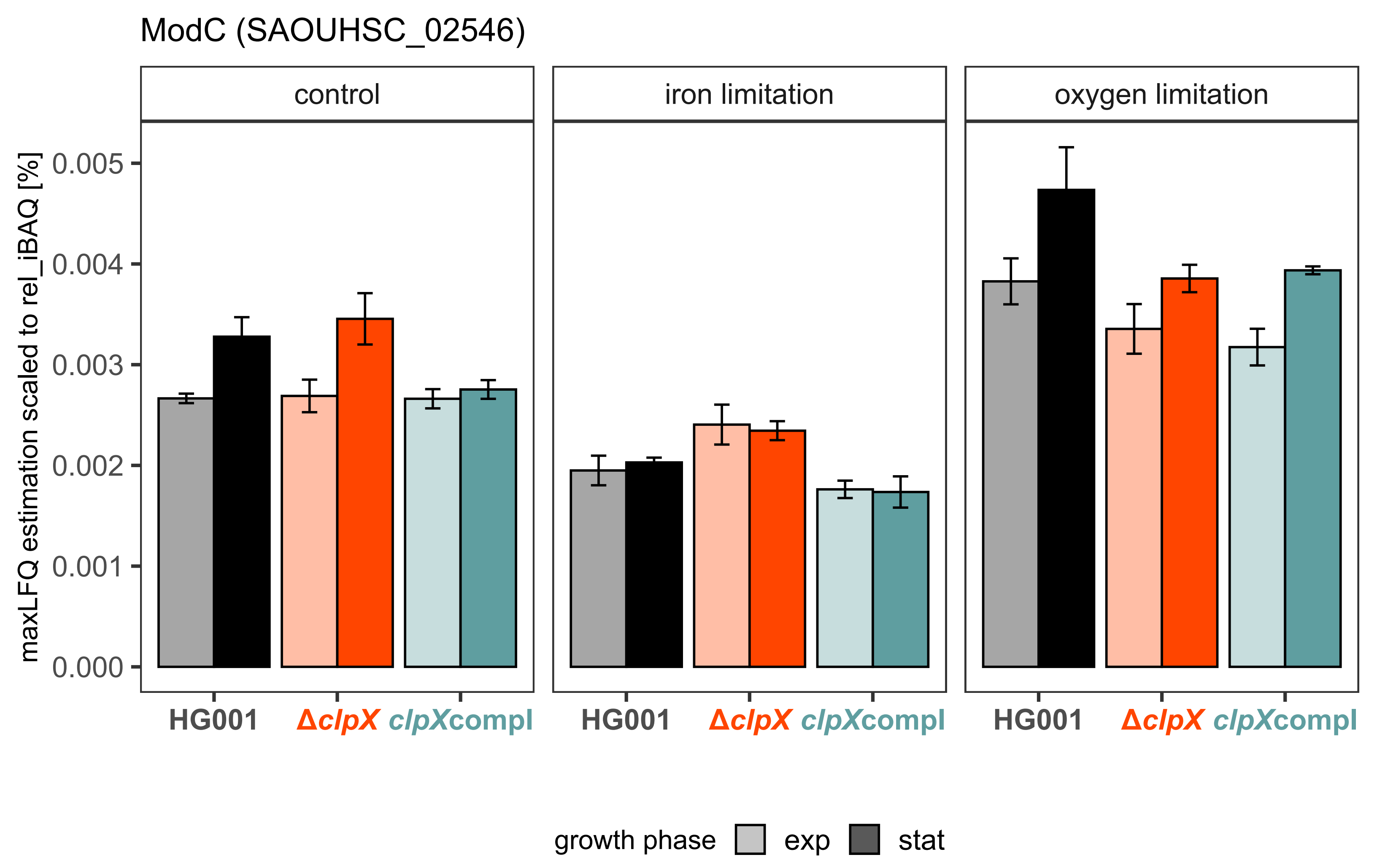
ModC (SAOUHSC_02546)
control
iron limitation
oxygen limitation
maxLFQ estimation scaled to rel_iBAQ [%]
0.005
0.004
0.003
0.002
0.001
0.000
HG001
ΔclpX
clpXcompl
growth phase
exp
stat

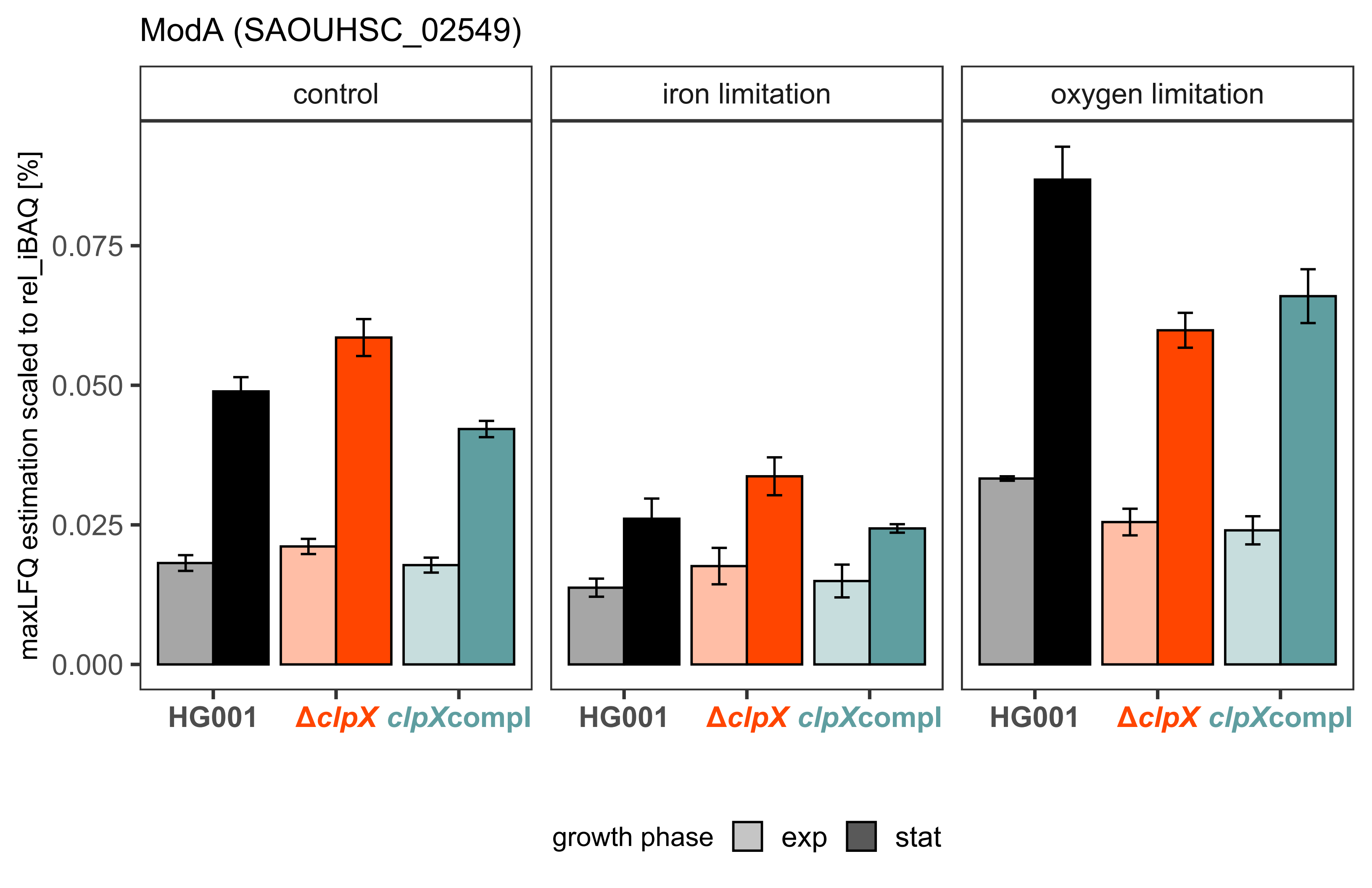

ModA (SAOUHSC_02549)
control
iron limitation
oxygen limitation
maxLFQ estimation scaled to rel_iBAQ [%]
0.075
0.050
0.025
0.000
HG001
ΔclpX
clpXcompl
growth phase
exp
stat

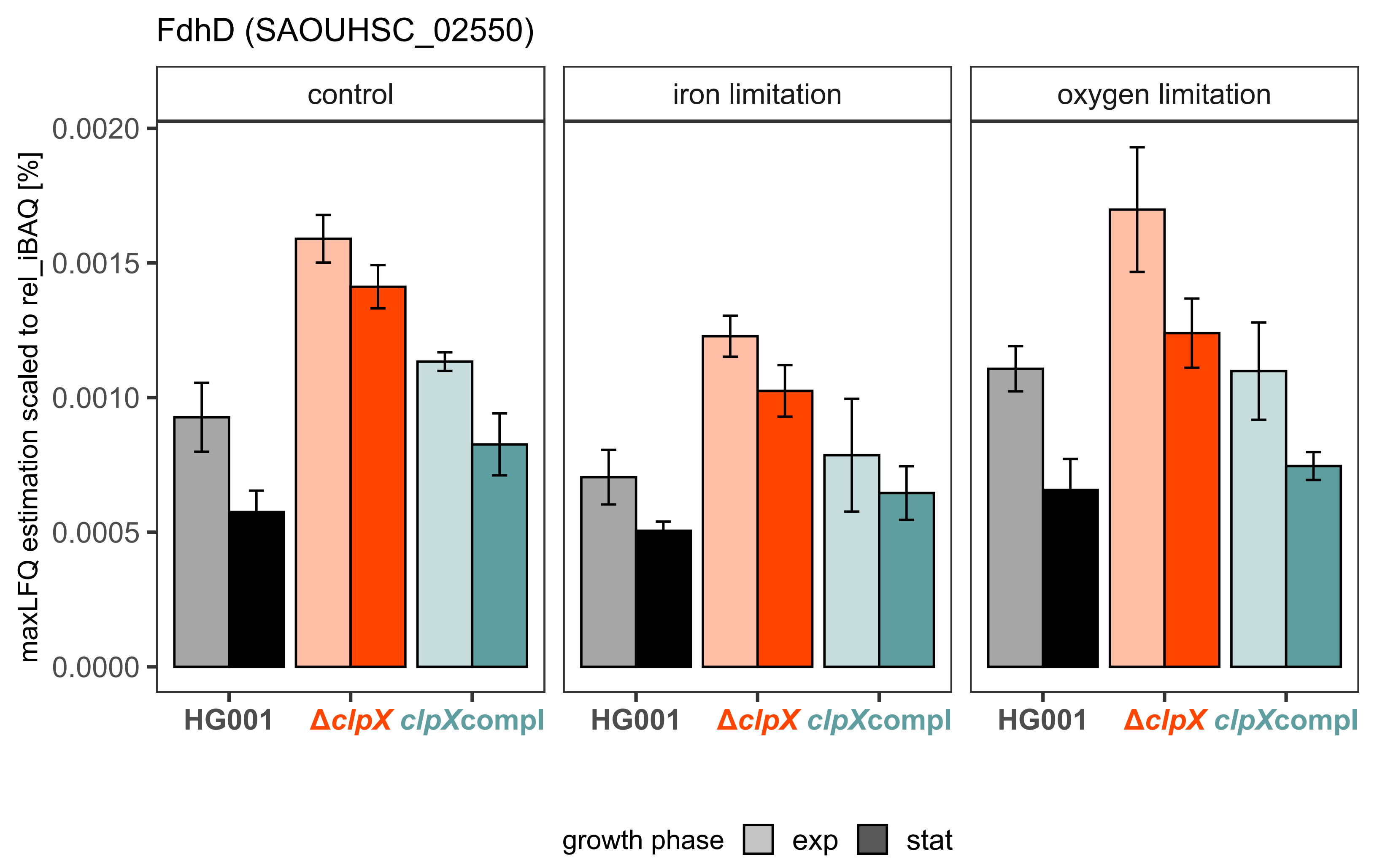

FdhD (SAOUHSC_02550)
control
iron limitation
oxygen limitation
0.0020
0.0015
0.0010
0.0005
0.0000
maxLFQ estimation scaled to rel_iBAQ [%]
HG001
ΔclpX
clpXcompl
growth phase
exp
stat

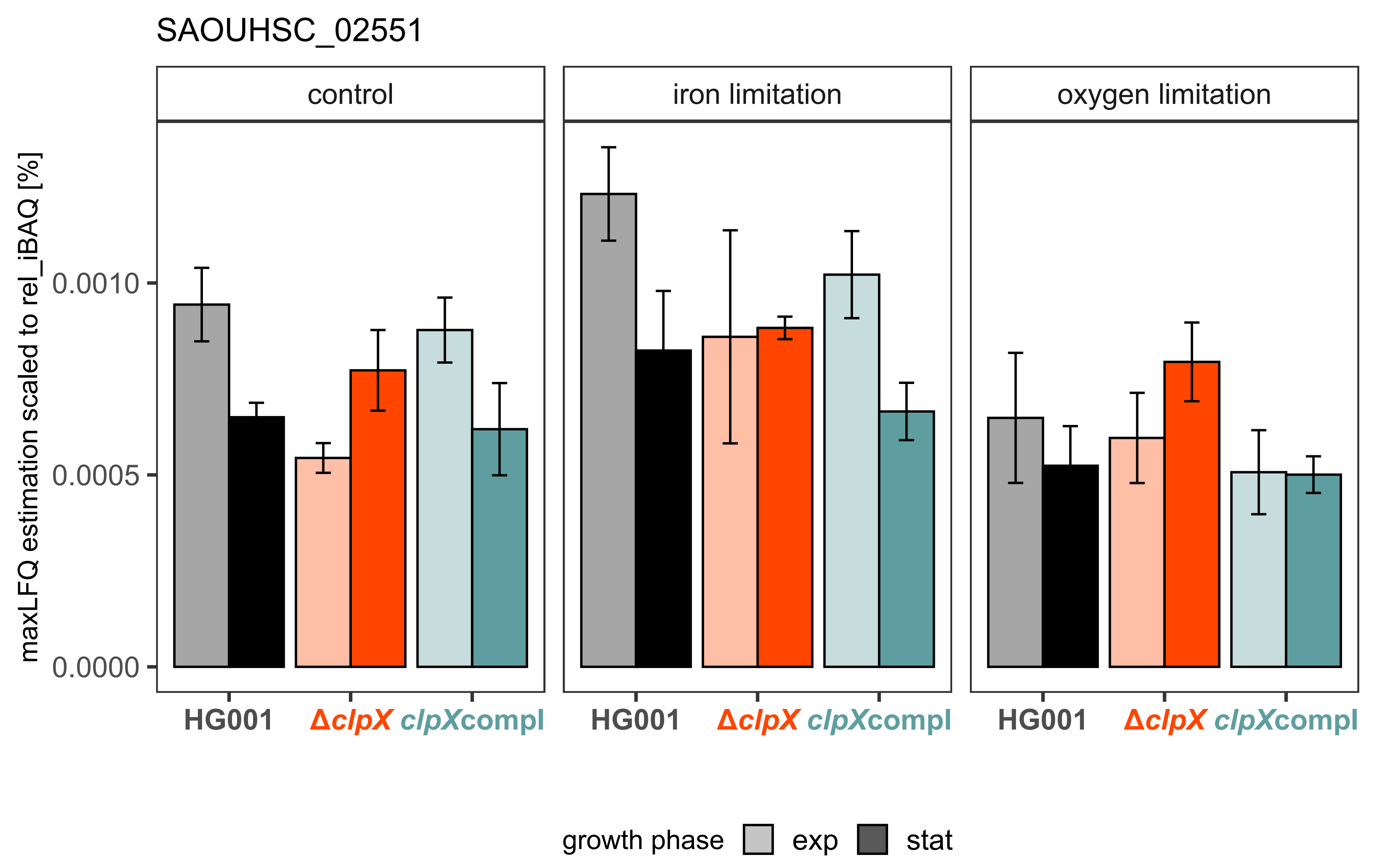

SAOUHSC_02551
control
iron limitation
oxygen limitation
maxLFQ estimation scaled to rel_iBAQ [%]
0.0010
0.0005
0.0000
HG001
ΔclpX
clpXcompl
growth phase
exp
stat

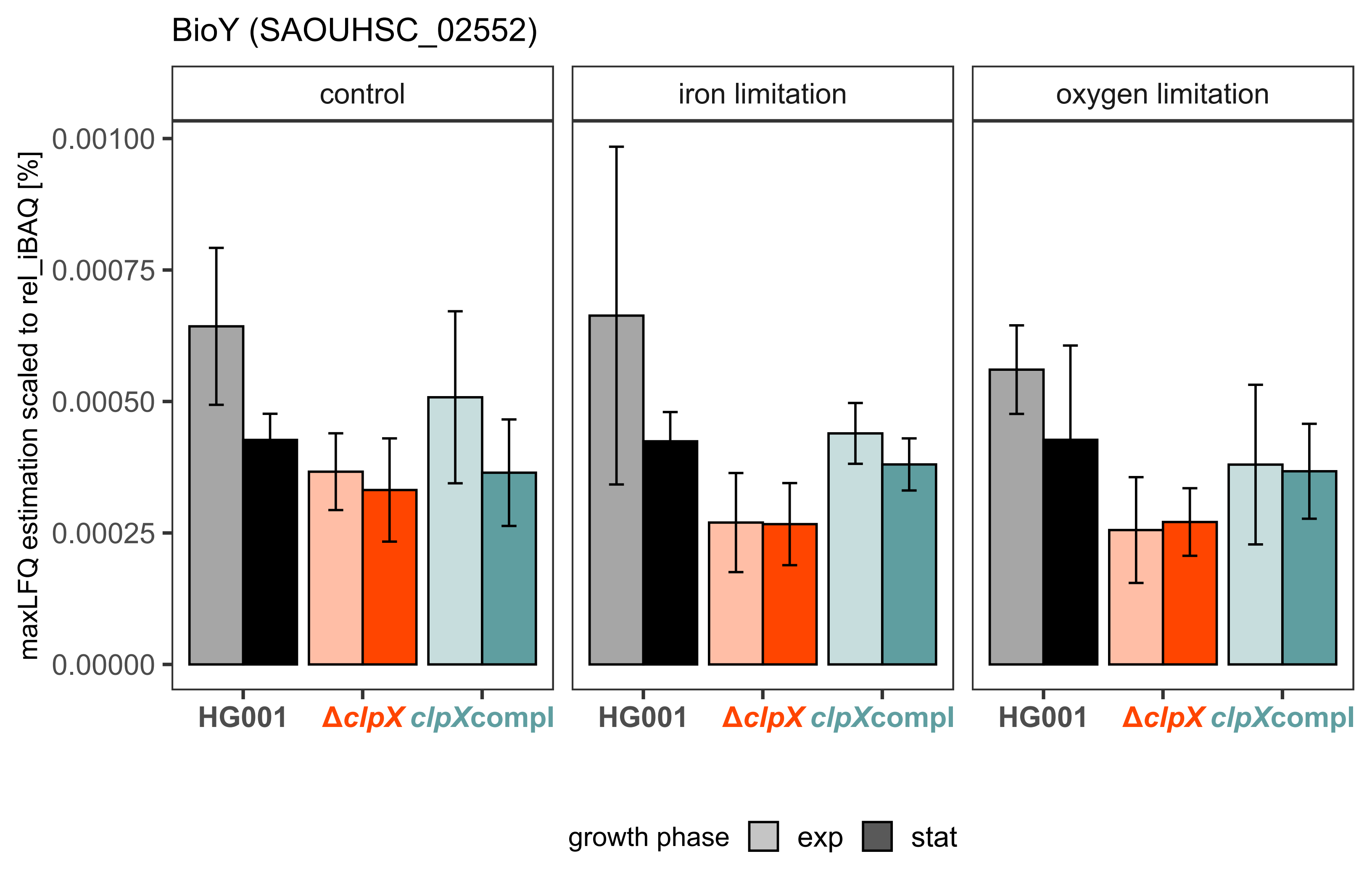
BioY (SAOUHSC_02552)
control
iron limitation
oxygen limitation
maxLFQ estimation scaled to rel_iBAQ [%]
0.00100
0.00075
0.00050
0.00025
0.00000
HG001
ΔclpX
clpXcompl
growth phase
exp
stat

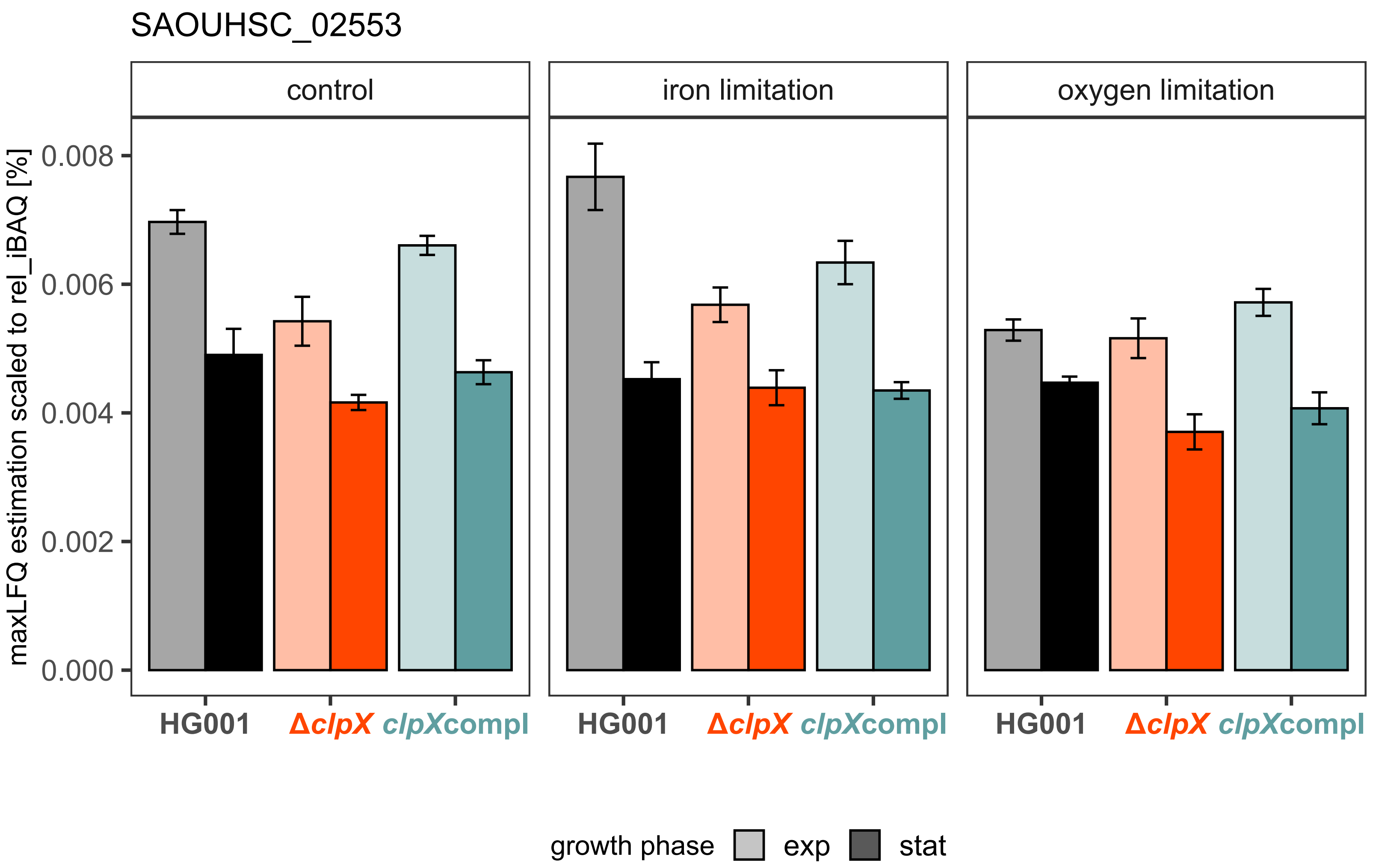

SAOUHSC_02553
control
iron limitation
oxygen limitation
maxLFQ estimation scaled to rel_iBAQ [%]
0.008
0.006
0.004
0.002
0.000
HG001
ΔclpX
clpXcompl
growth phase
exp
stat

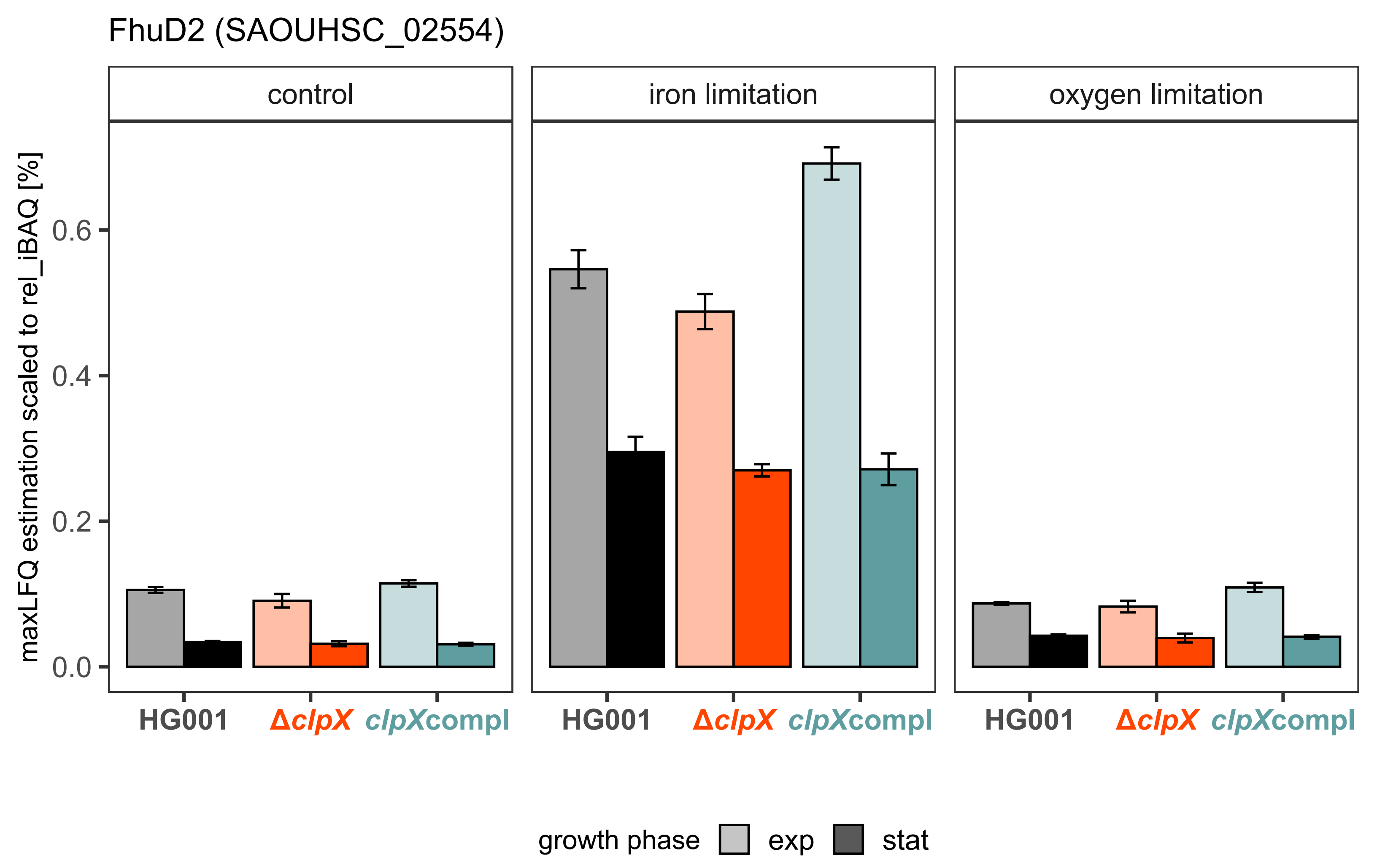

FhuD2 (SAOUHSC_02554)
control
iron limitation
oxygen limitation
maxLFQ estimation scaled to rel_iBAQ [%]
0.0
0.2
0.4
0.6
HG001
ΔclpX
clpXcompl
growth phase
exp
stat

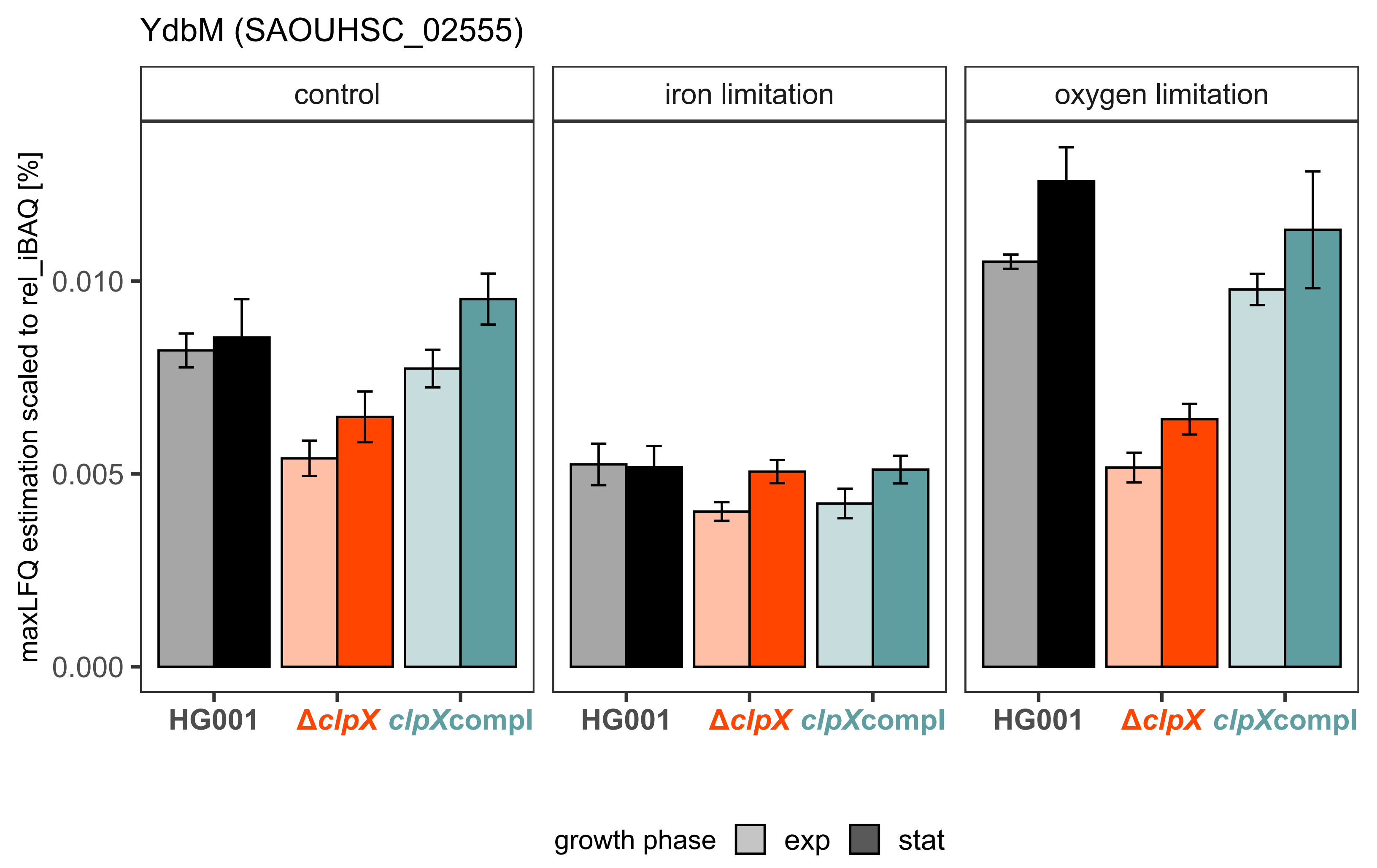
YdbM (SAOUHSC_02555)
control
iron limitation
oxygen limitation
maxLFQ estimation scaled to rel_iBAQ [%]
0.010
0.005
0.000
HG001
ΔclpX
clpXcompl
growth phase
exp
stat

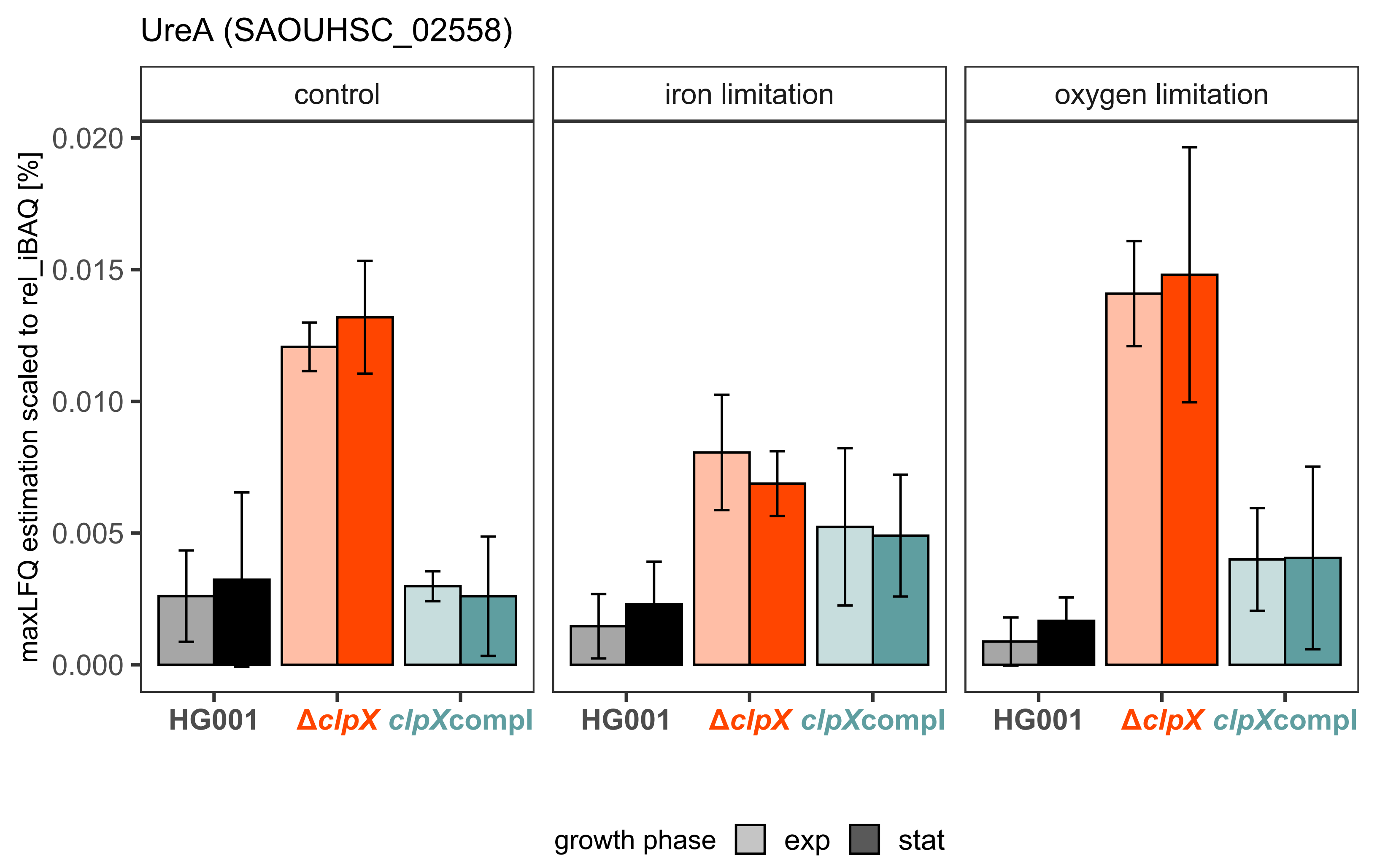

UreA (SAOUHSC_02558)
control
iron limitation
oxygen limitation
maxLFQ estimation scaled to rel_iBAQ [%]
0.020
0.015
0.010
0.005
0.000
HG001
ΔclpX
clpXcompl
growth phase
exp
stat

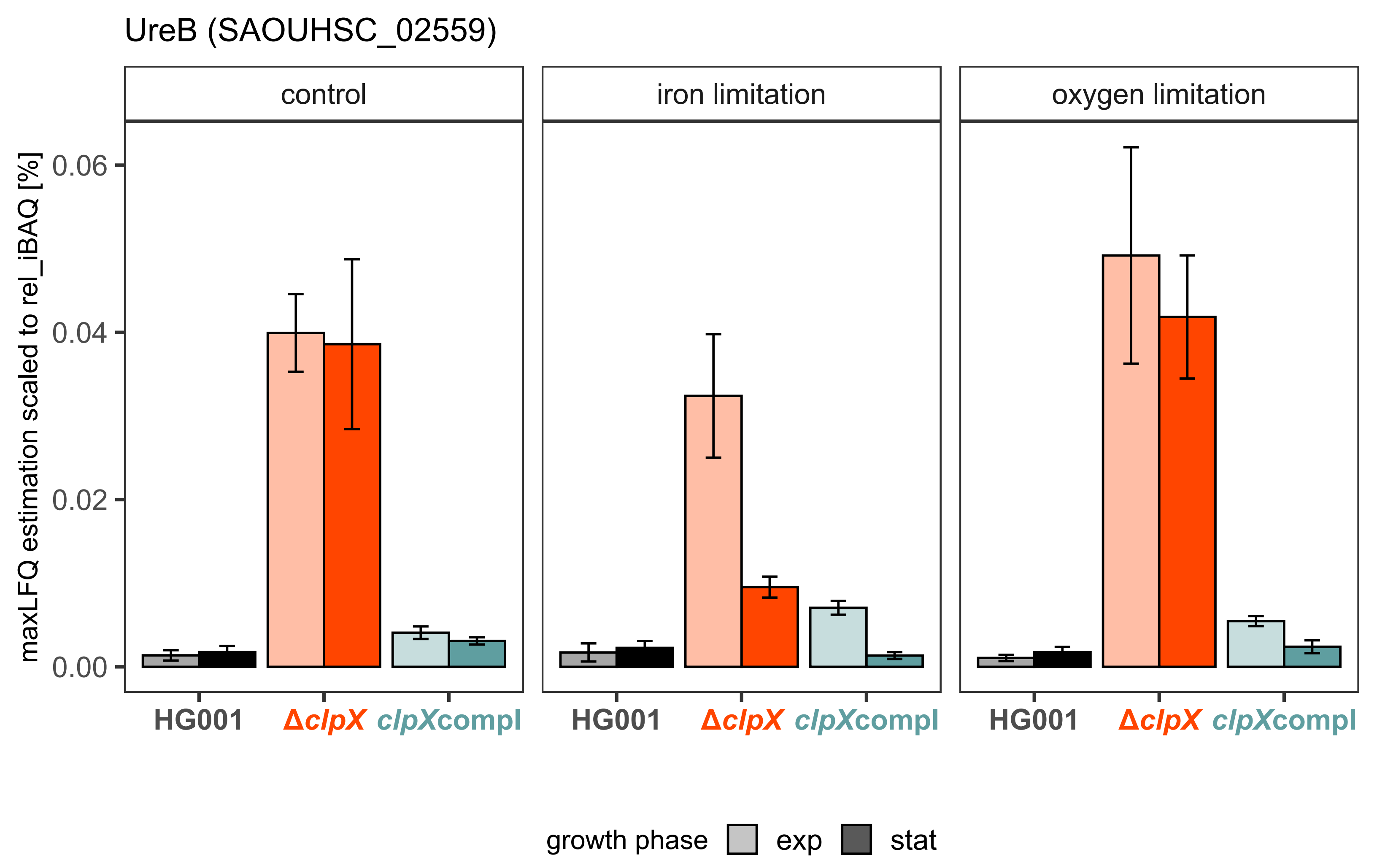

UreB (SAOUHSC_02559)
control
iron limitation
oxygen limitation
maxLFQ estimation scaled to rel_iBAQ [%]
0.06
0.04
0.02
0.00
HG001
ΔclpX
clpXcompl
growth phase
exp
stat

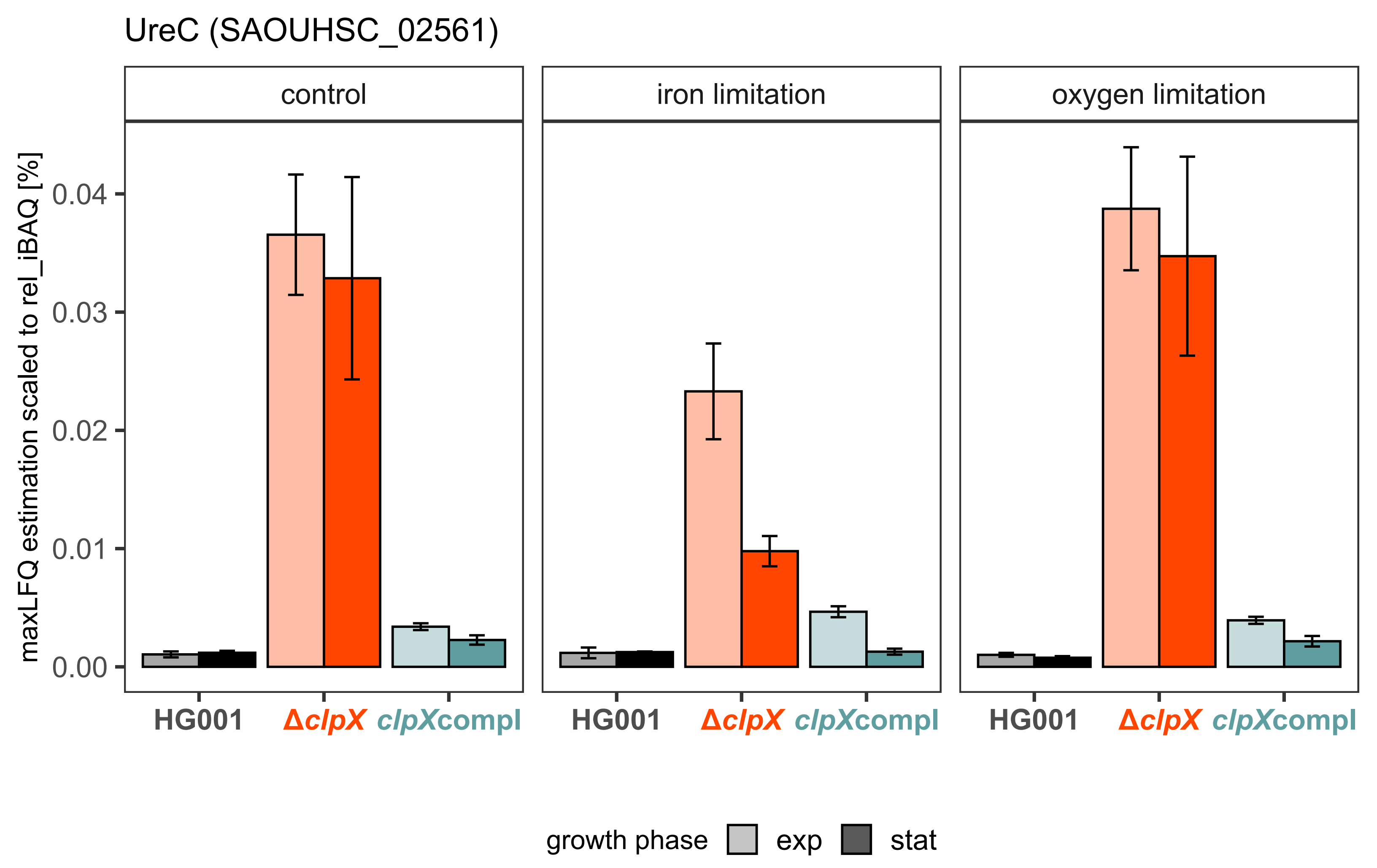
UreC (SAOUHSC_02561)
control
iron limitation
oxygen limitation
maxLFQ estimation scaled to rel_iBAQ [%]
0.04
0.03
0.02
0.01
0.00
HG001
ΔclpX
clpXcompl
growth phase
exp
stat

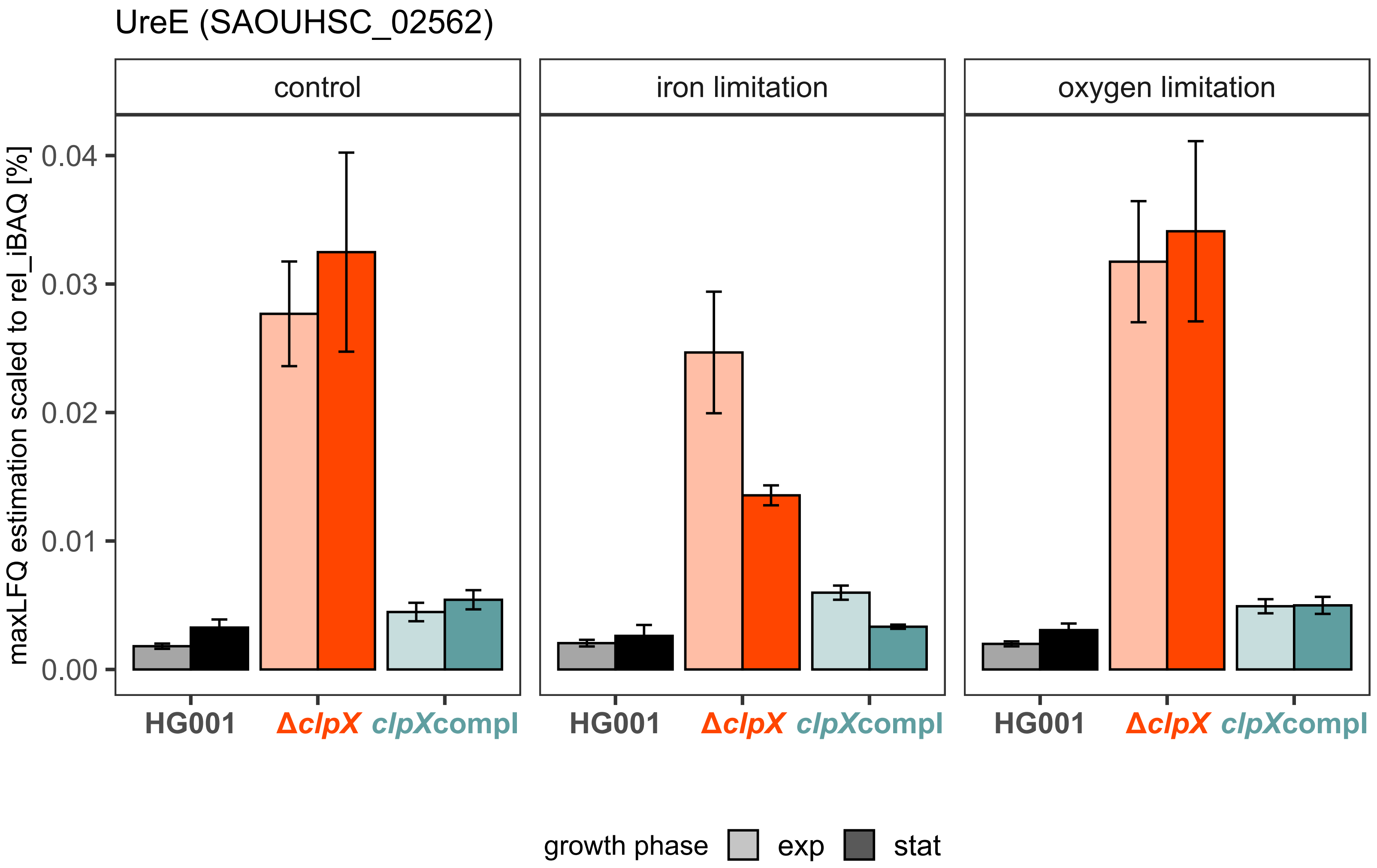
UreE (SAOUHSC_02562)
control
iron limitation
oxygen limitation
maxLFQ estimation scaled to rel_iBAQ [%]
0.00
0.01
0.02
0.03
0.04
HG001
ΔclpX
clpXcompl
growth phase
exp
stat

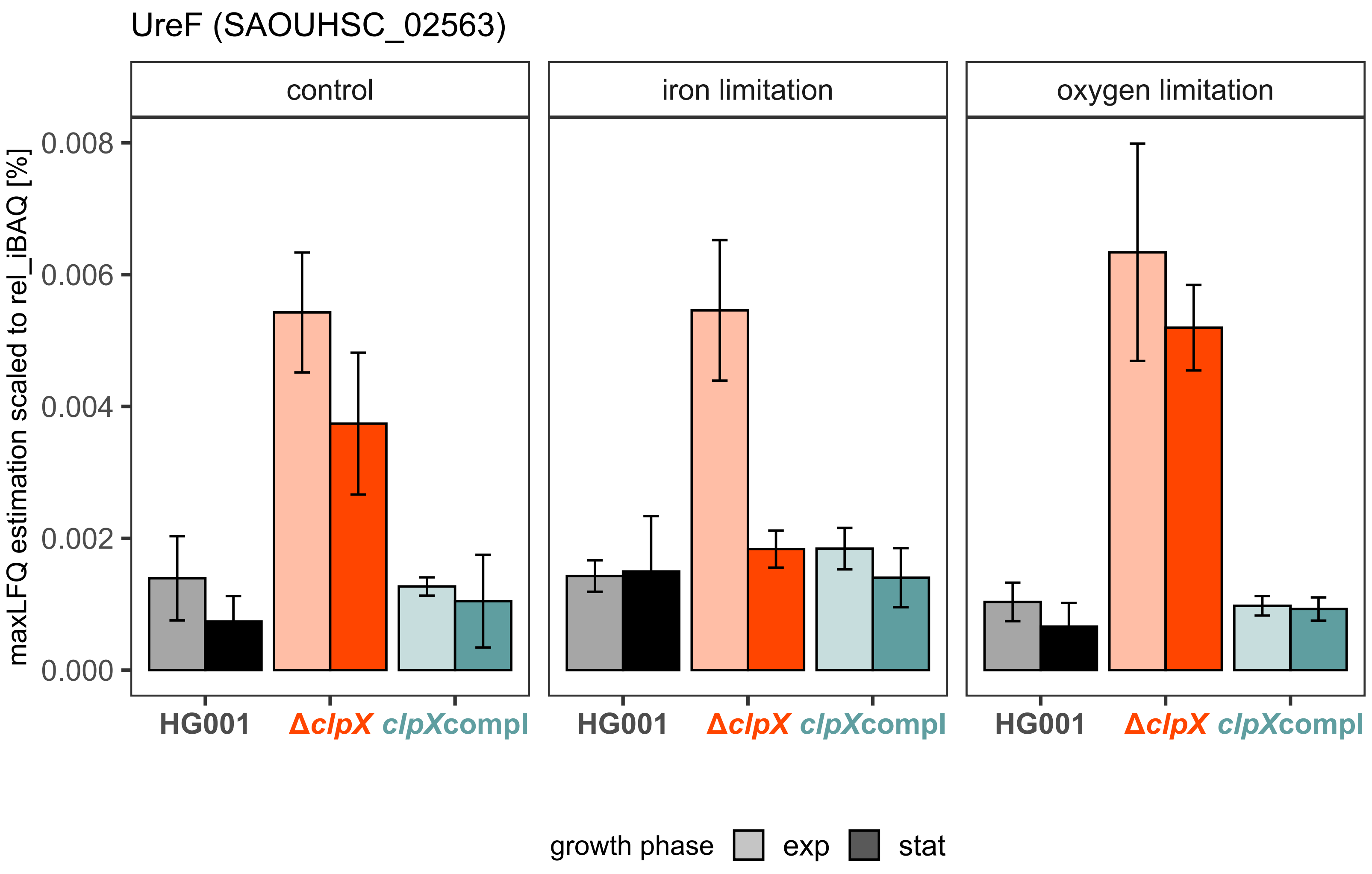
UreF (SAOUHSC_02563)
control
iron limitation
oxygen limitation
maxLFQ estimation scaled to rel_iBAQ [%]
0.008
0.006
0.004
0.002
0.000
HG001
ΔclpX
clpXcompl
growth phase
exp
stat

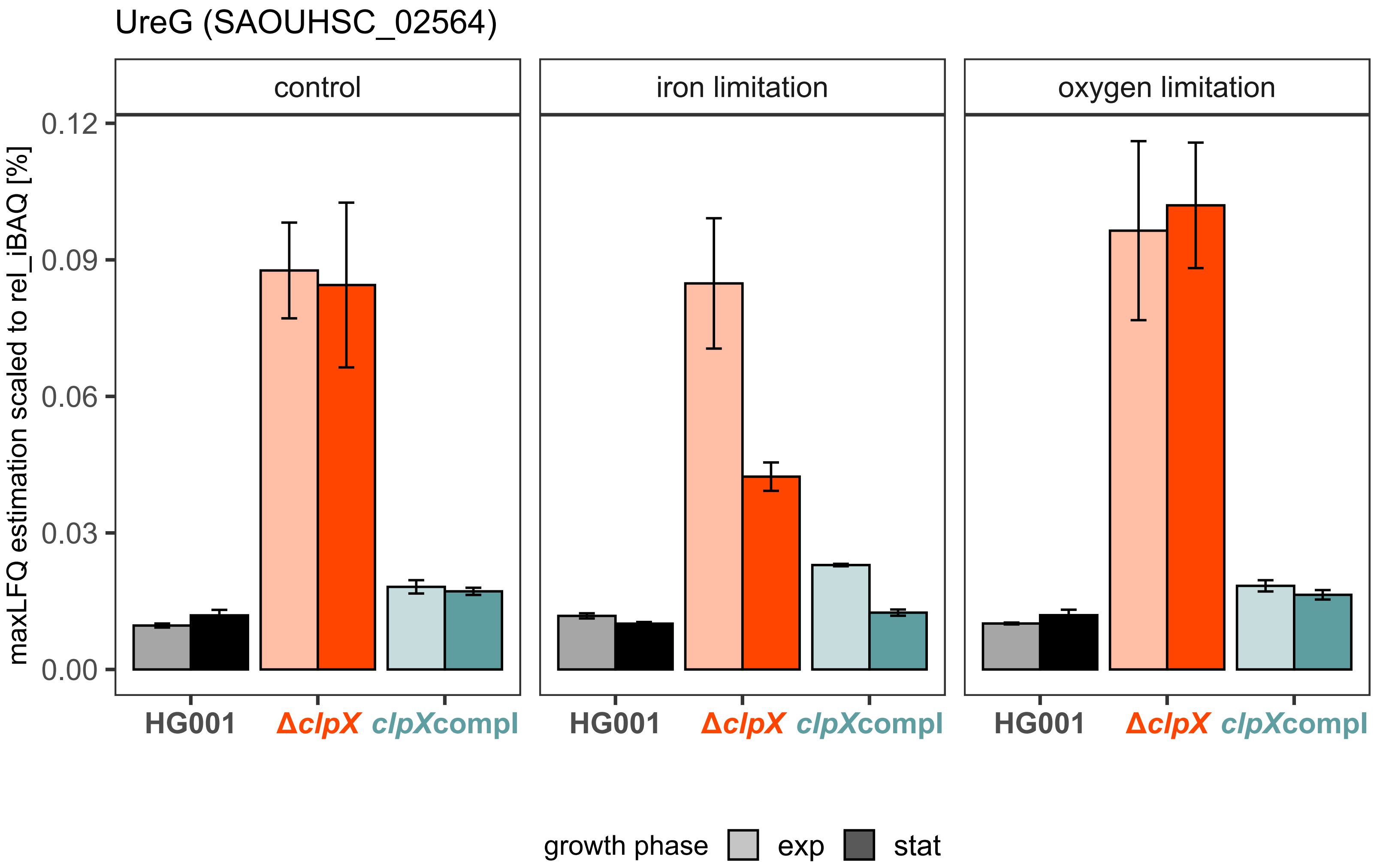
UreG (SAOUHSC_02564)
control
iron limitation
oxygen limitation
maxLFQ estimation scaled to rel_iBAQ [%]
0.12
0.09
0.06
0.03
0.00
HG001
ΔclpX
clpXcompl
growth phase
exp
stat

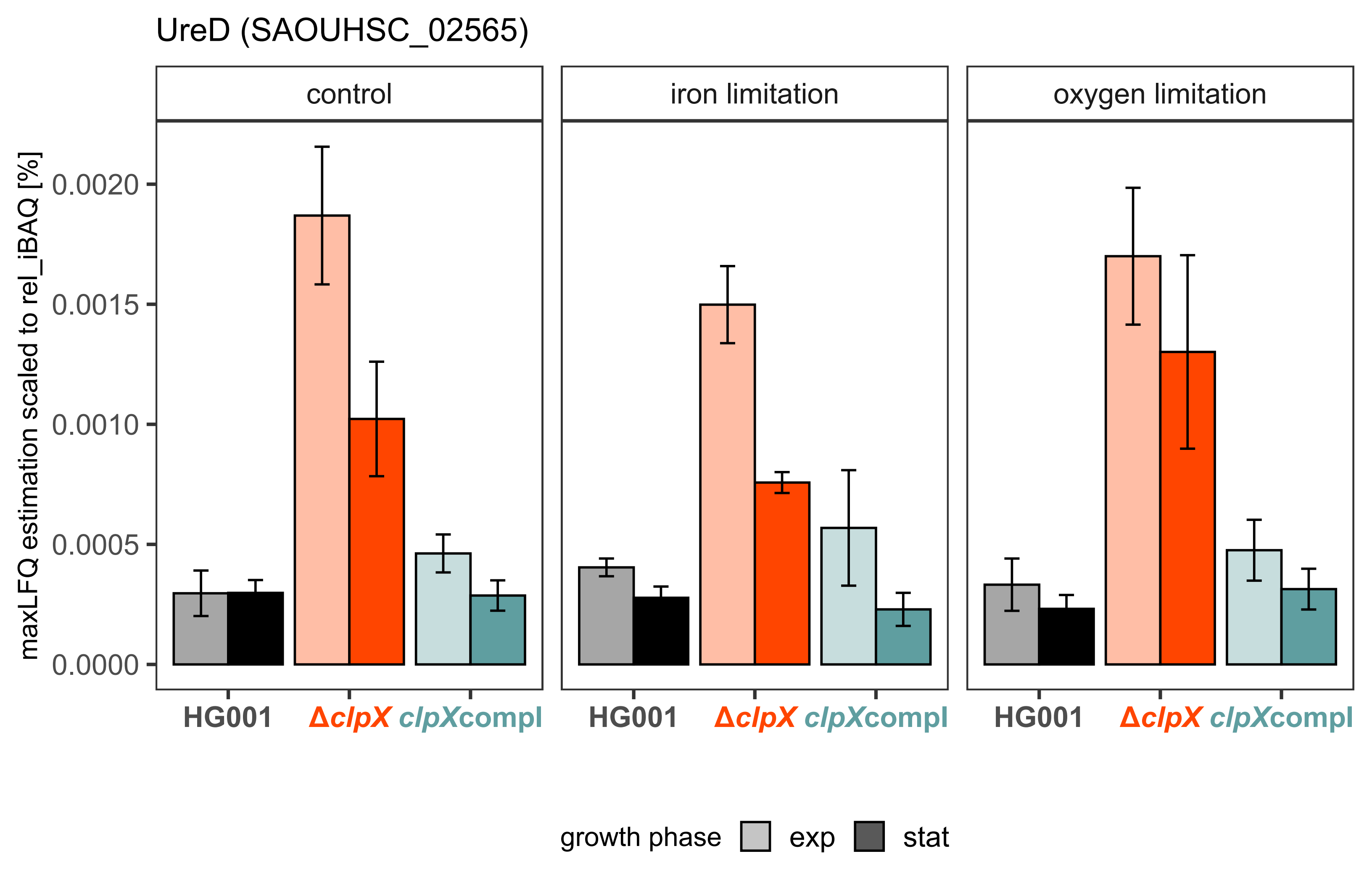

UreD (SAOUHSC_02565)
control
iron limitation
oxygen limitation
maxLFQ estimation scaled to rel_iBAQ [%]
0.0020
0.0015
0.0010
0.0005
0.0000
HG001
ΔclpX
clpXcompl
growth phase
exp
stat

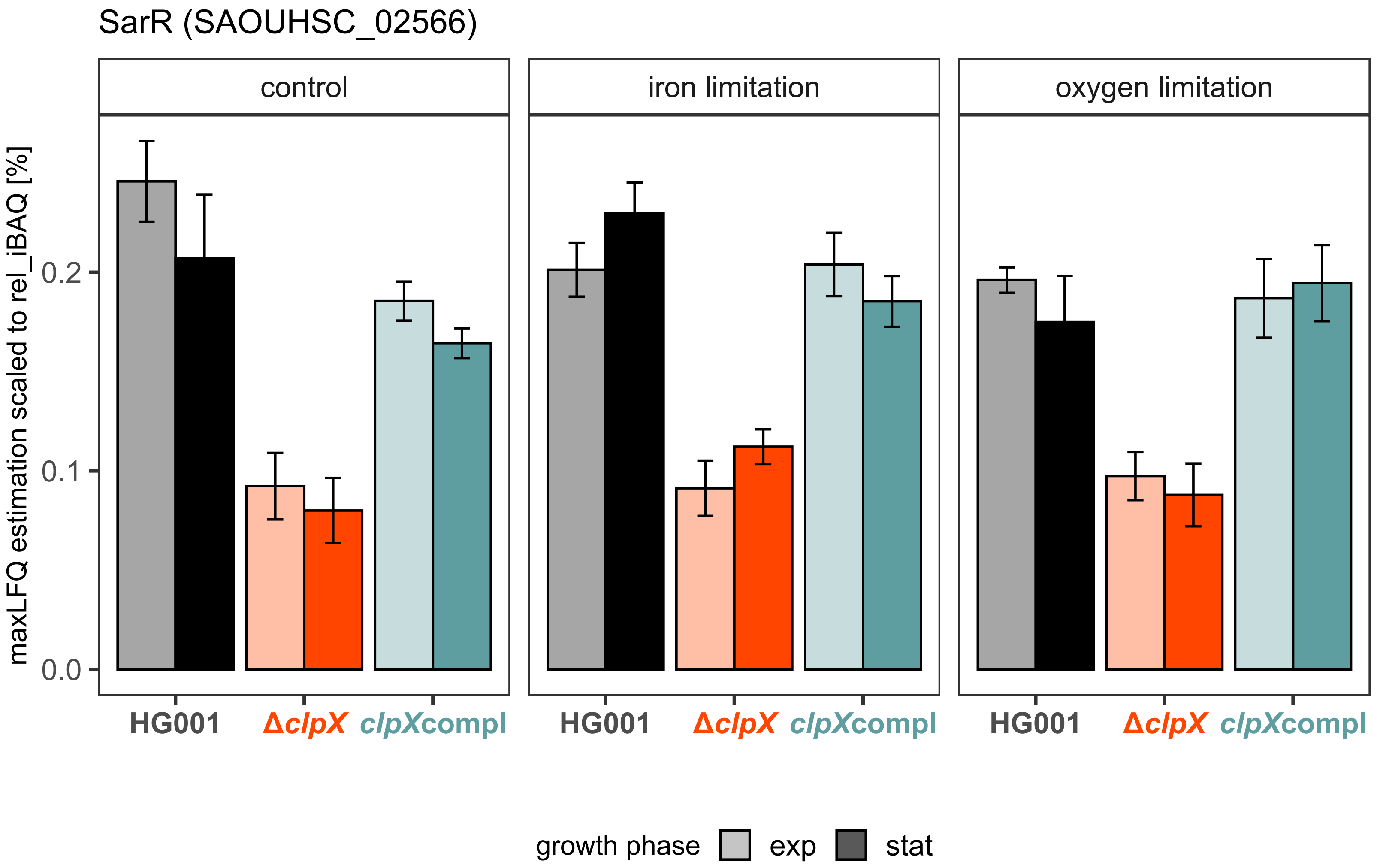

SarR (SAOUHSC_02566)
control
iron limitation
oxygen limitation
maxLFQ estimation scaled to rel_iBAQ [%]
0.0
0.1
0.2
HG001
ΔclpX
clpXcompl
growth phase
exp
stat

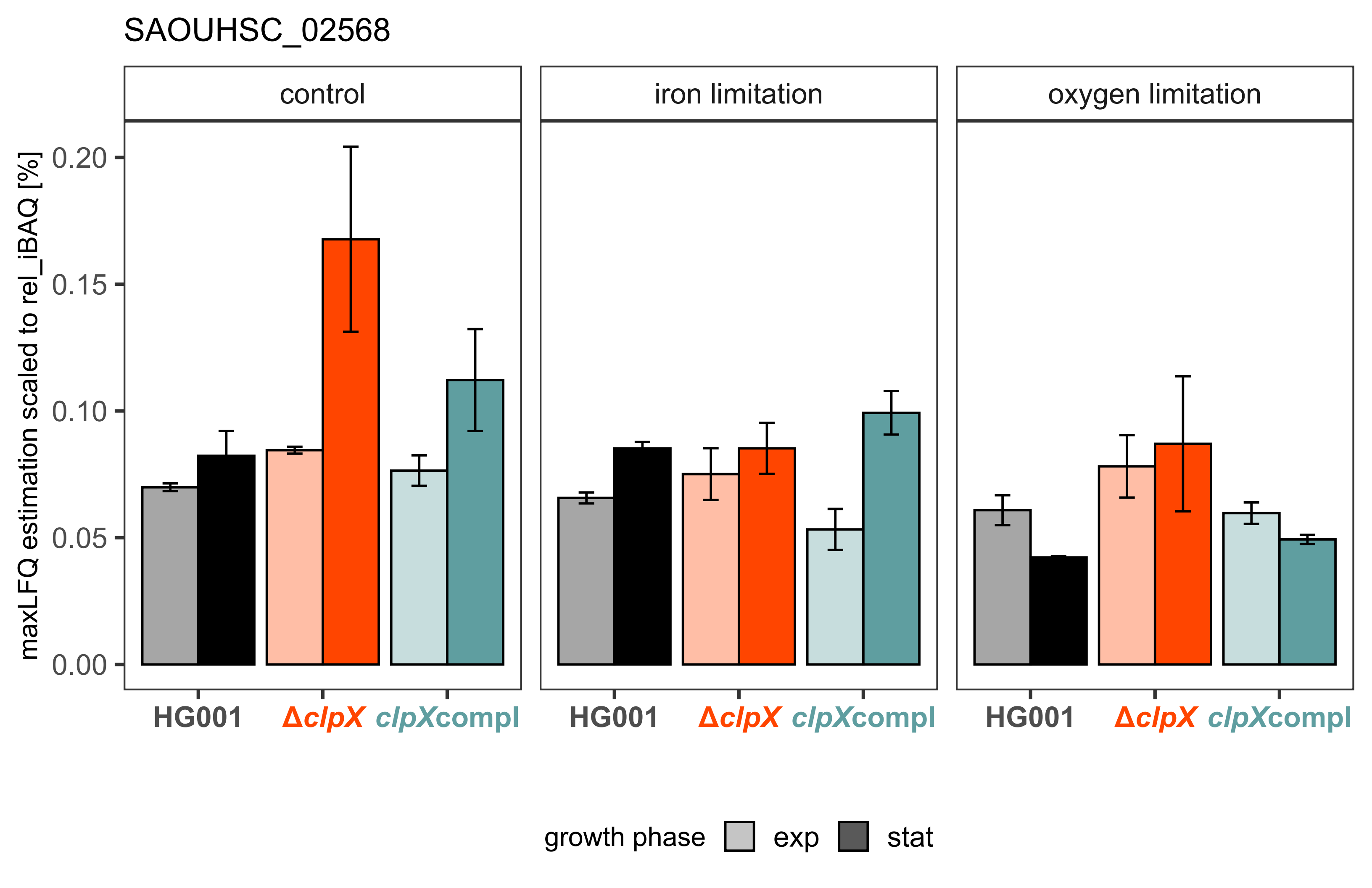

SAOUHSC_02568
control
iron limitation
oxygen limitation
maxLFQ estimation scaled to rel_iBAQ [%]
0.00
0.05
0.10
0.15
0.20
HG001
ΔclpX
clpXcompl
growth phase
exp
stat

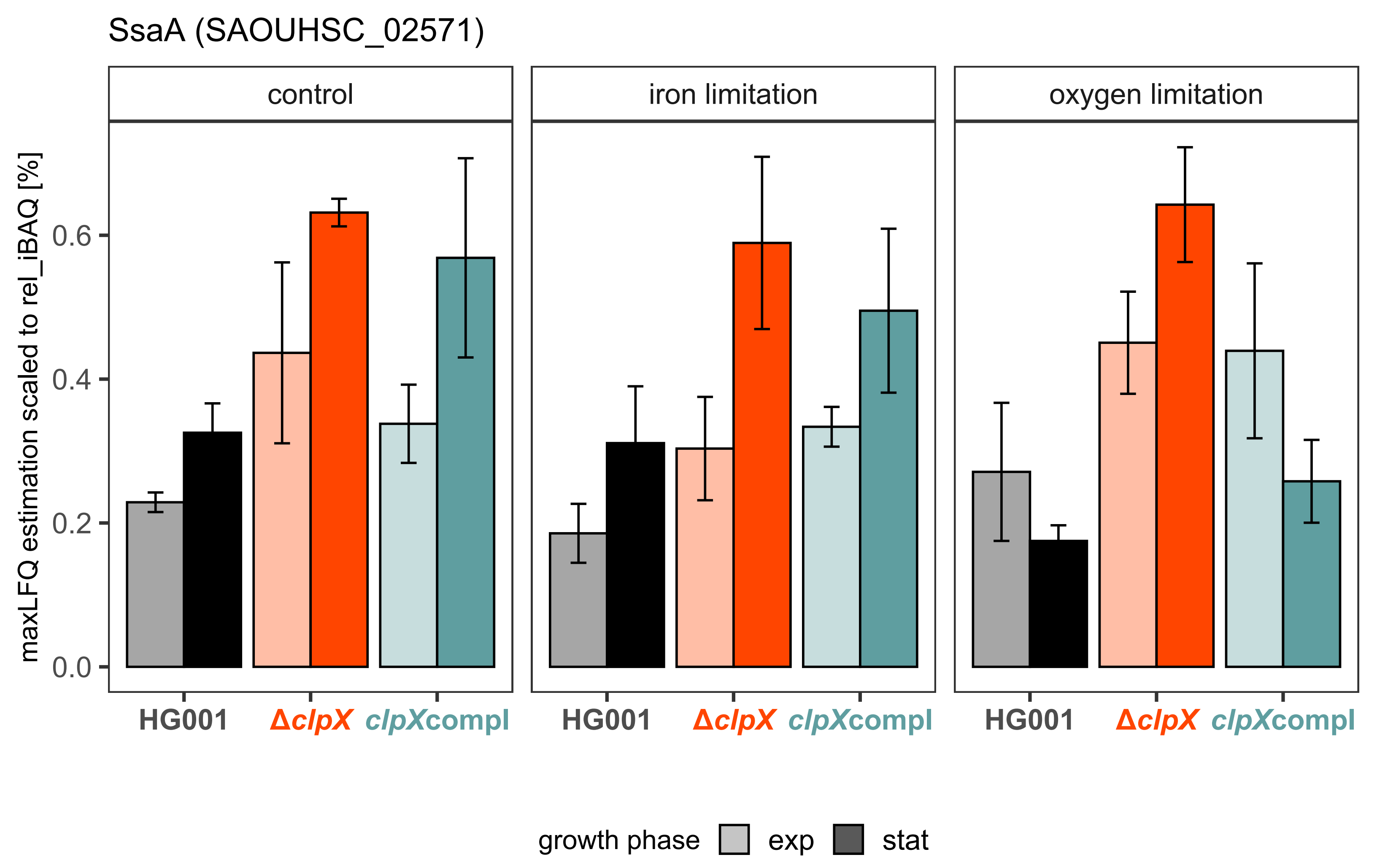

SsaA (SAOUHSC_02571)
control
iron limitation
oxygen limitation
maxLFQ estimation scaled to rel_iBAQ [%]
0.0
0.2
0.4
0.6
HG001
ΔclpX
clpXcompl
growth phase
exp
stat

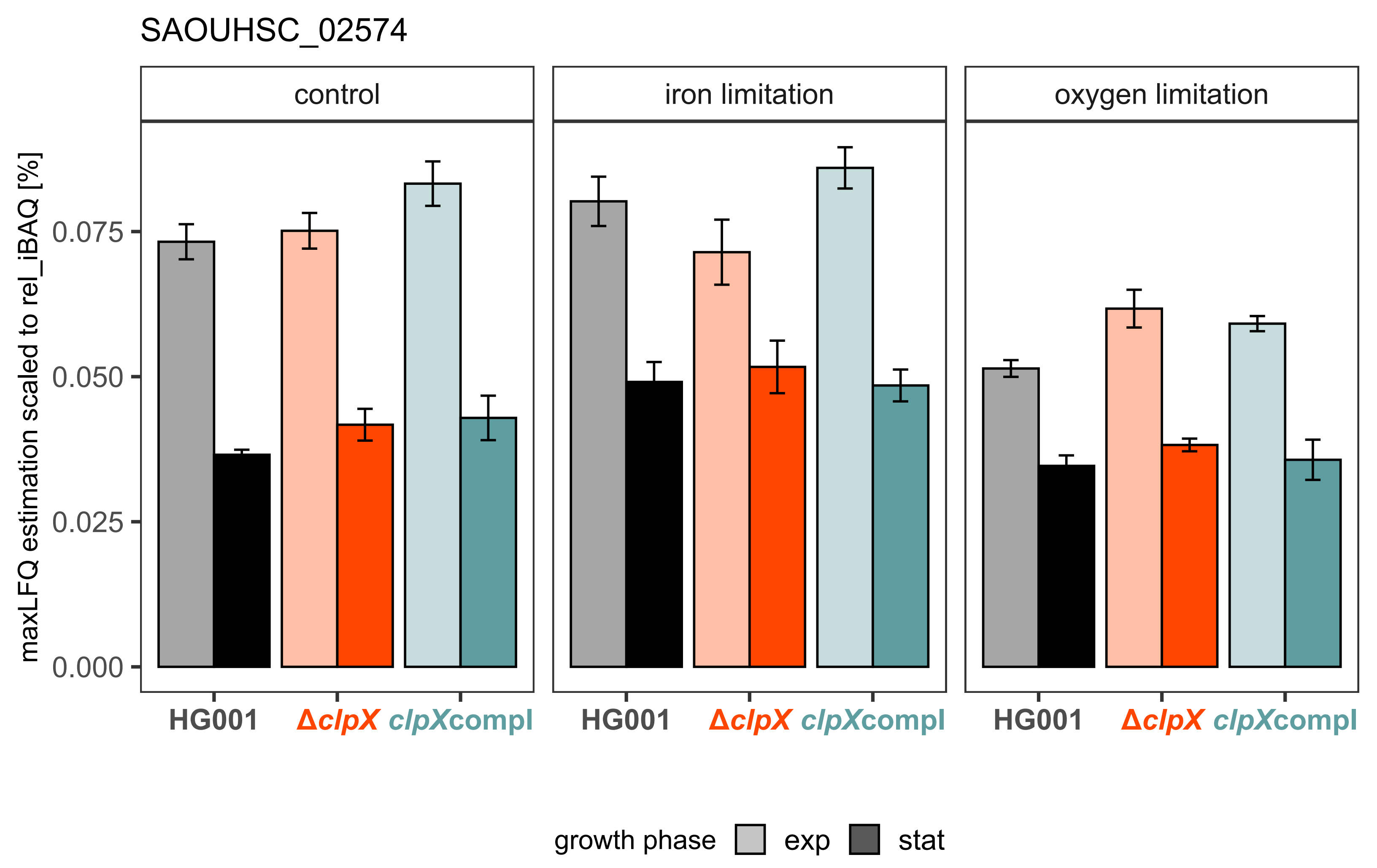
SAOUHSC_02574
control
iron limitation
oxygen limitation
maxLFQ estimation scaled to rel_iBAQ [%]
0.075
0.050
0.025
0.000
HG001
ΔclpX
clpXcompl
growth phase
exp
stat

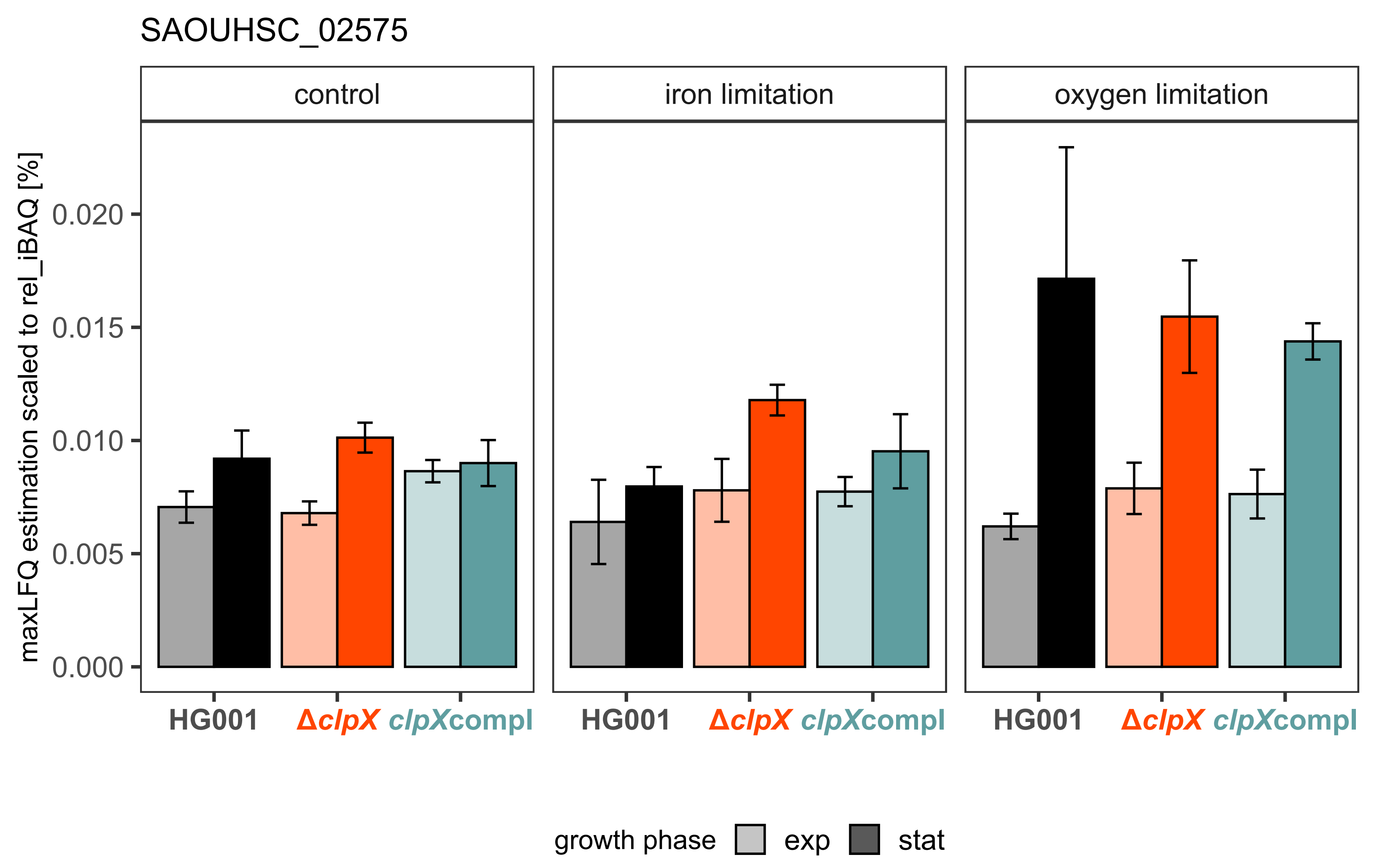

SAOUHSC_02575
control
iron limitation
oxygen limitation
maxLFQ estimation scaled to rel_iBAQ [%]
0.020
0.015
0.010
0.005
0.000
HG001
ΔclpX
clpXcompl
growth phase
exp
stat

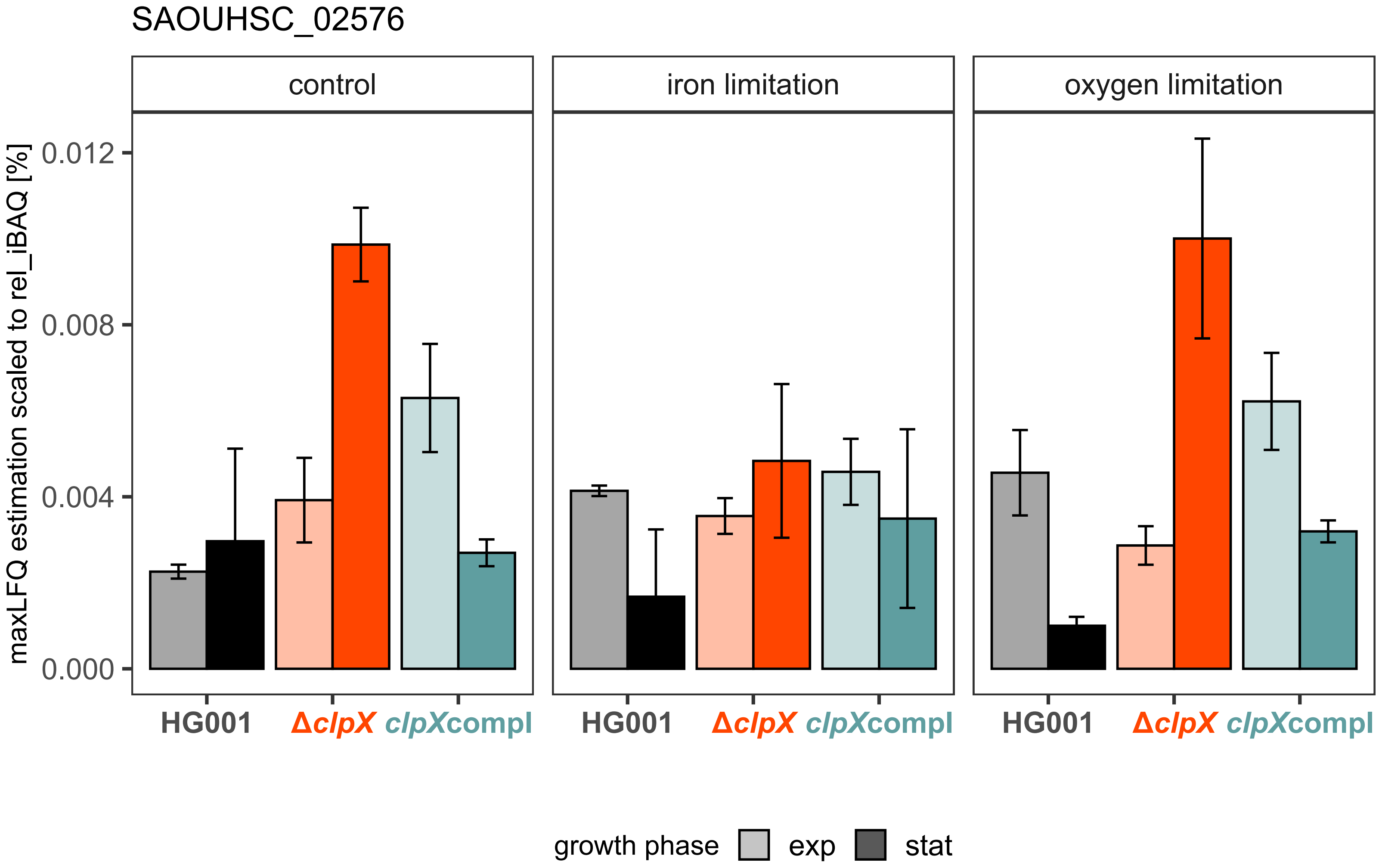

SAOUHSC_02576
control
iron limitation
oxygen limitation
maxLFQ estimation scaled to rel_iBAQ [%]
0.012
0.008
0.004
0.000
HG001
ΔclpX
clpXcompl
HG001
ΔclpX
clpXcompl
HG001
ΔclpX
clpXcompl
growth phase
exp
stat

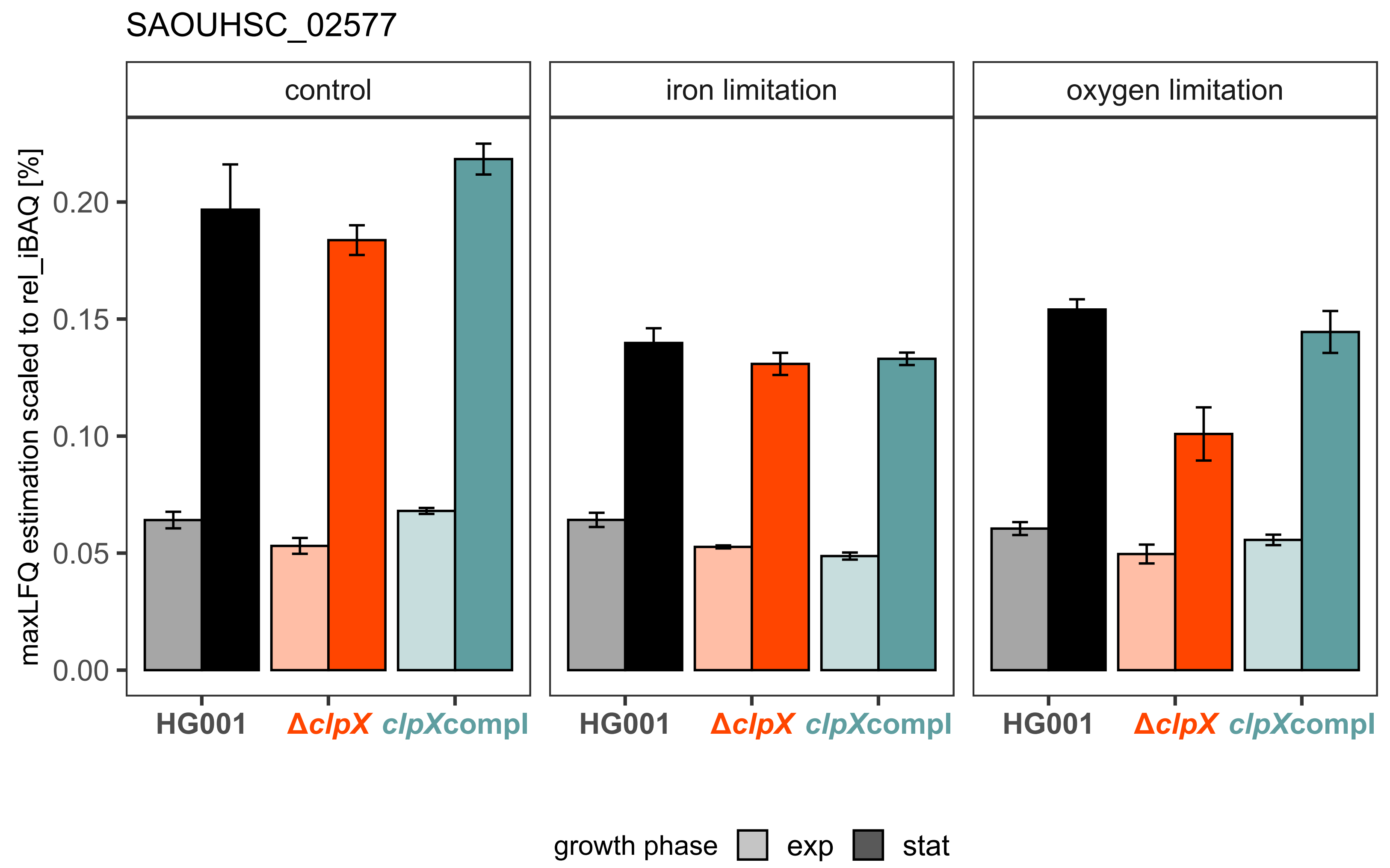
SAOUHSC_02577
control
iron limitation
oxygen limitation
maxLFQ estimation scaled to rel_iBAQ [%]
0.20
0.15
0.10
0.05
0.00
HG001
ΔclpX
clpXcompl
growth phase
exp
stat

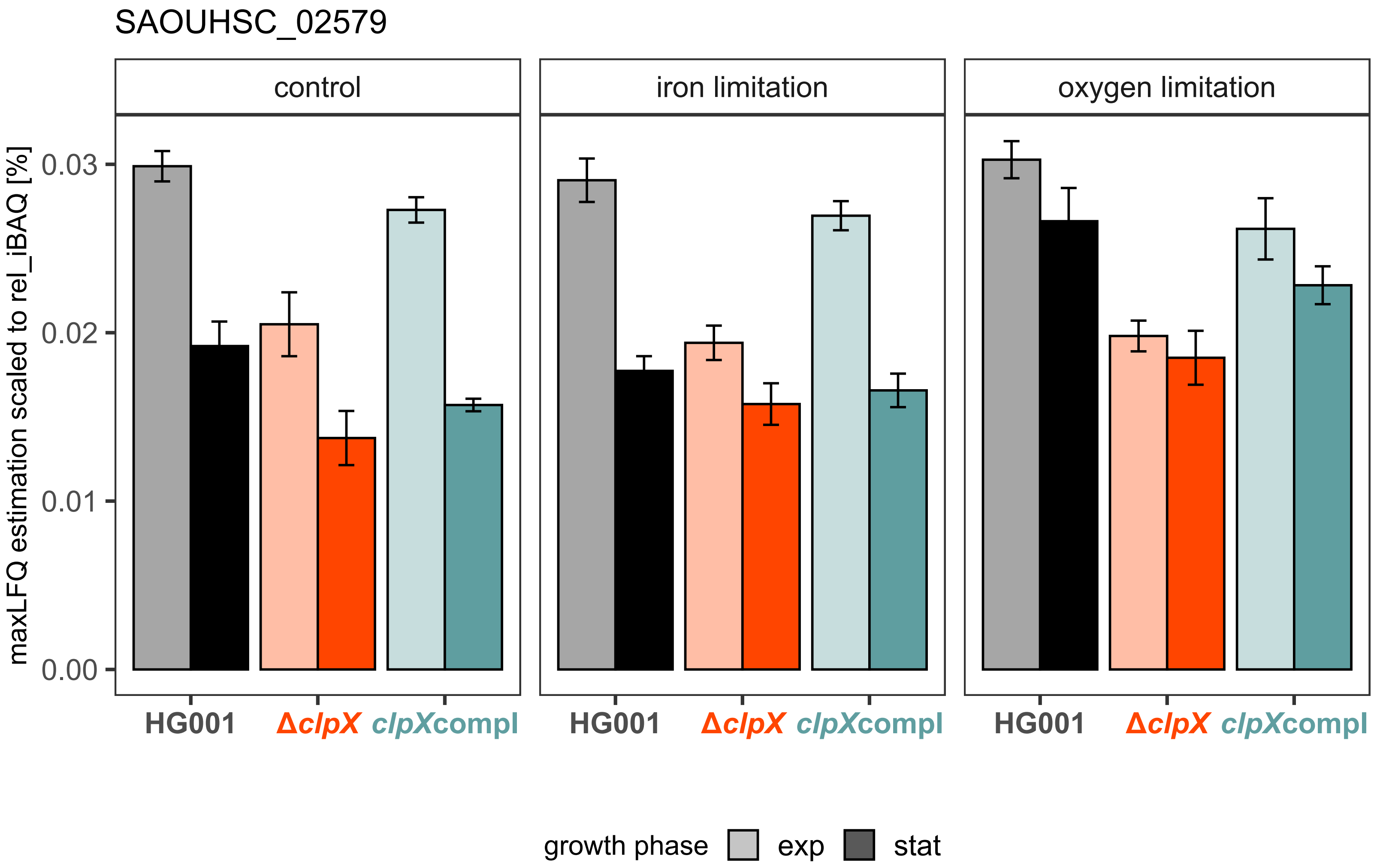

SAOUHSC_02579
control
iron limitation
oxygen limitation
maxLFQ estimation scaled to rel_iBAQ [%]
0.03
0.02
0.01
0.00
HG001
ΔclpX
clpXcompl
growth phase
exp
stat

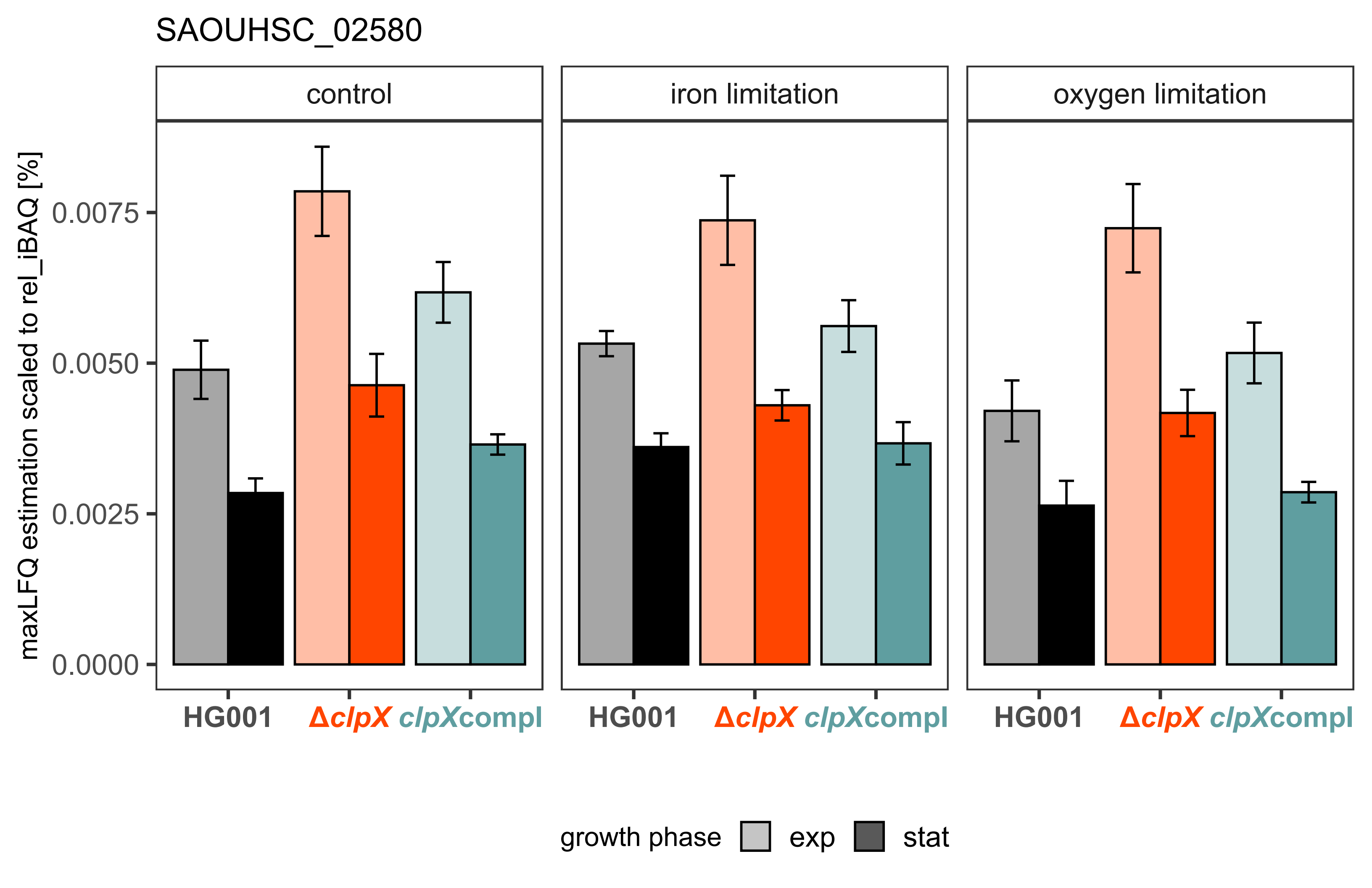
SAOUHSC_02580
control
iron limitation
oxygen limitation
maxLFQ estimation scaled to rel_iBAQ [%]
0.0075
0.0050
0.0025
0.0000
HG001
ΔclpX
clpXcompl
growth phase
exp
stat

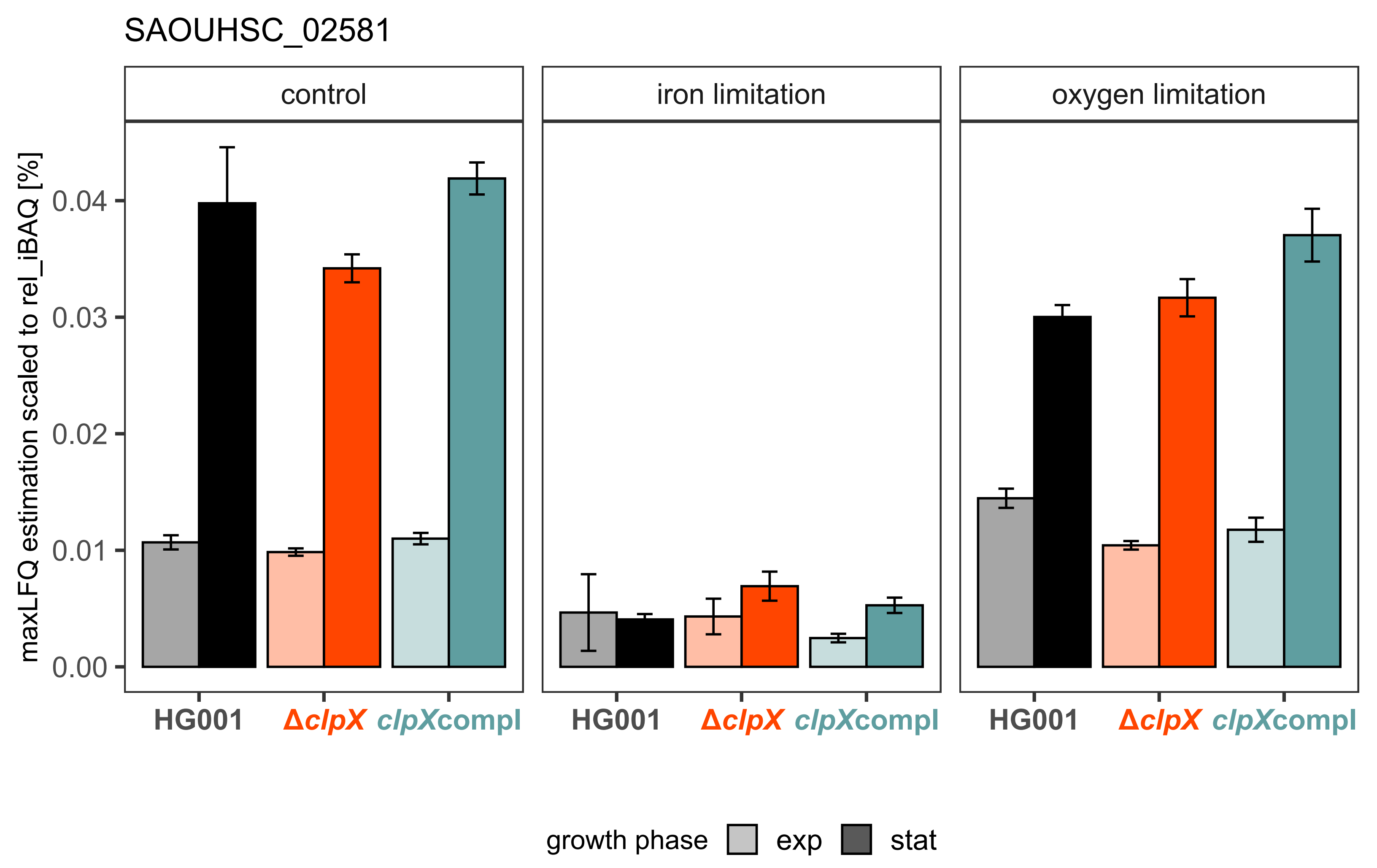
SAOUHSC_02581
control
iron limitation
oxygen limitation
maxLFQ estimation scaled to rel_iBAQ [%]
0.00
0.01
0.02
0.03
0.04
HG001
ΔclpX
clpXcompl
growth phase
exp
stat

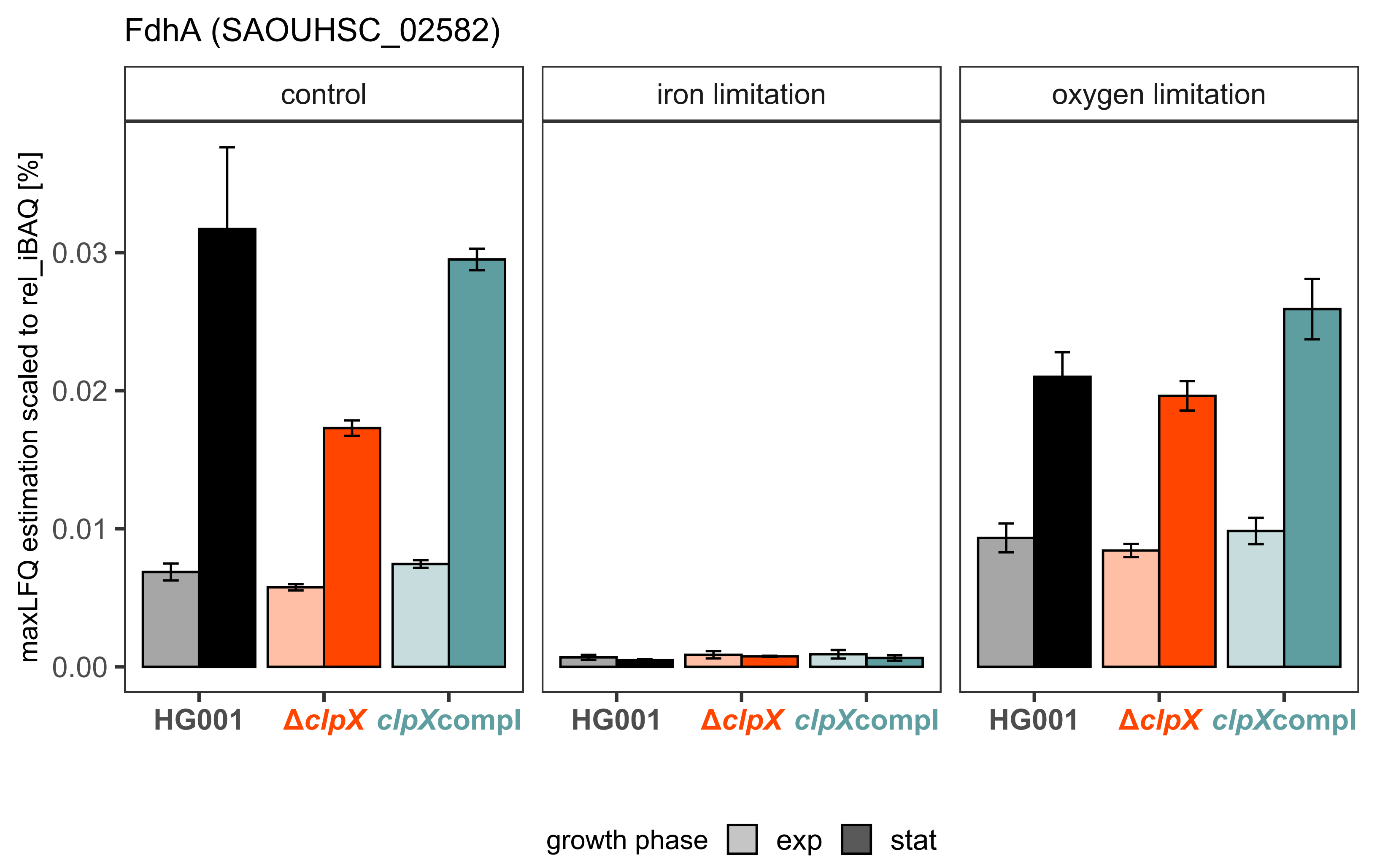

FdhA (SAOUHSC_02582)
control
iron limitation
oxygen limitation
maxLFQ estimation scaled to rel_iBAQ [%]
0.03
0.02
0.01
0.00
HG001
ΔclpX
clpXcompl
growth phase
exp
stat

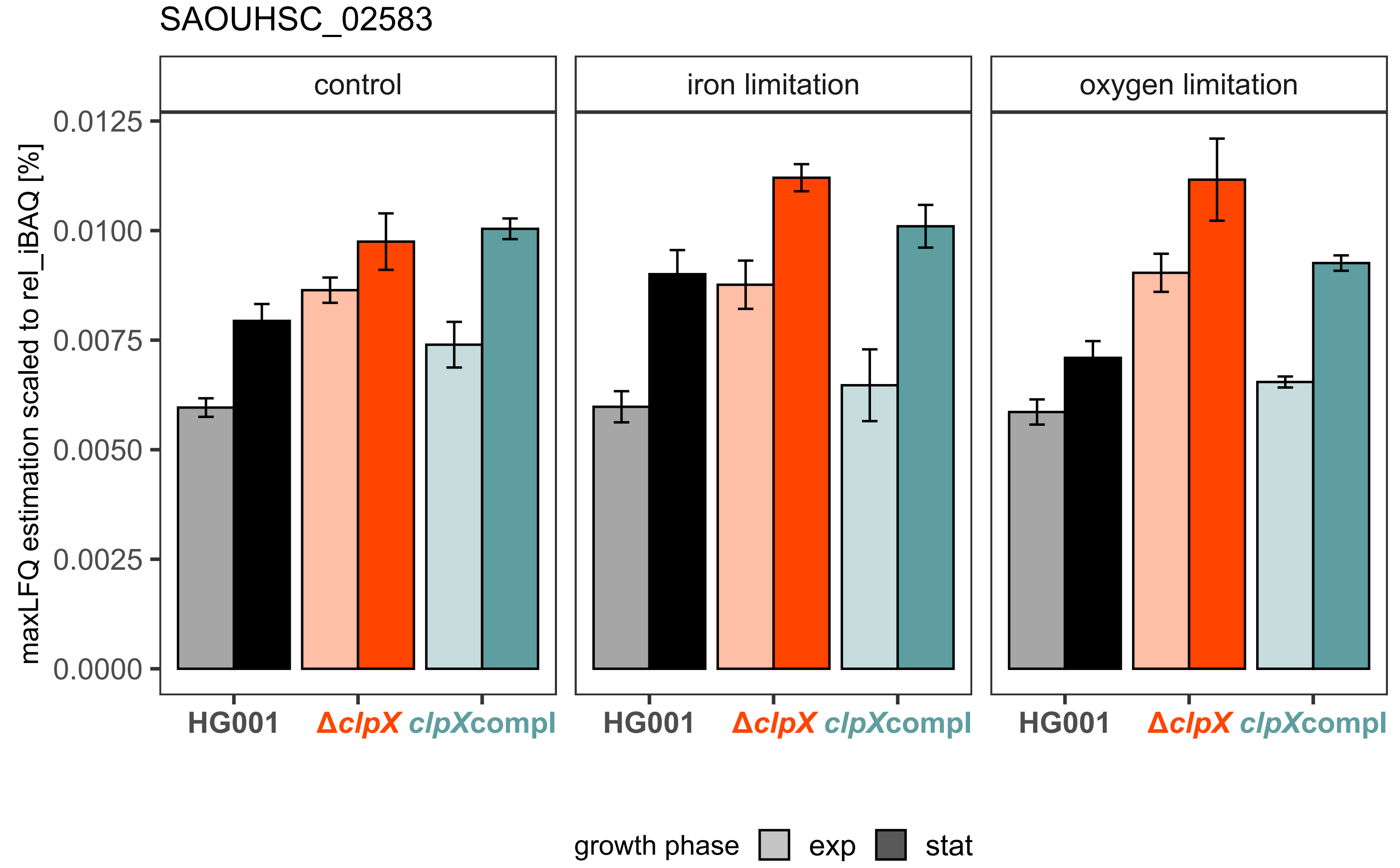
SAOUHSC_02583
control
iron limitation
oxygen limitation
maxLFQ estimation scaled to rel_iBAQ [%]
0.0125
0.0100
0.0075
0.0050
0.0025
0.0000
HG001
ΔclpX
clpXcompl
growth phase
exp
stat

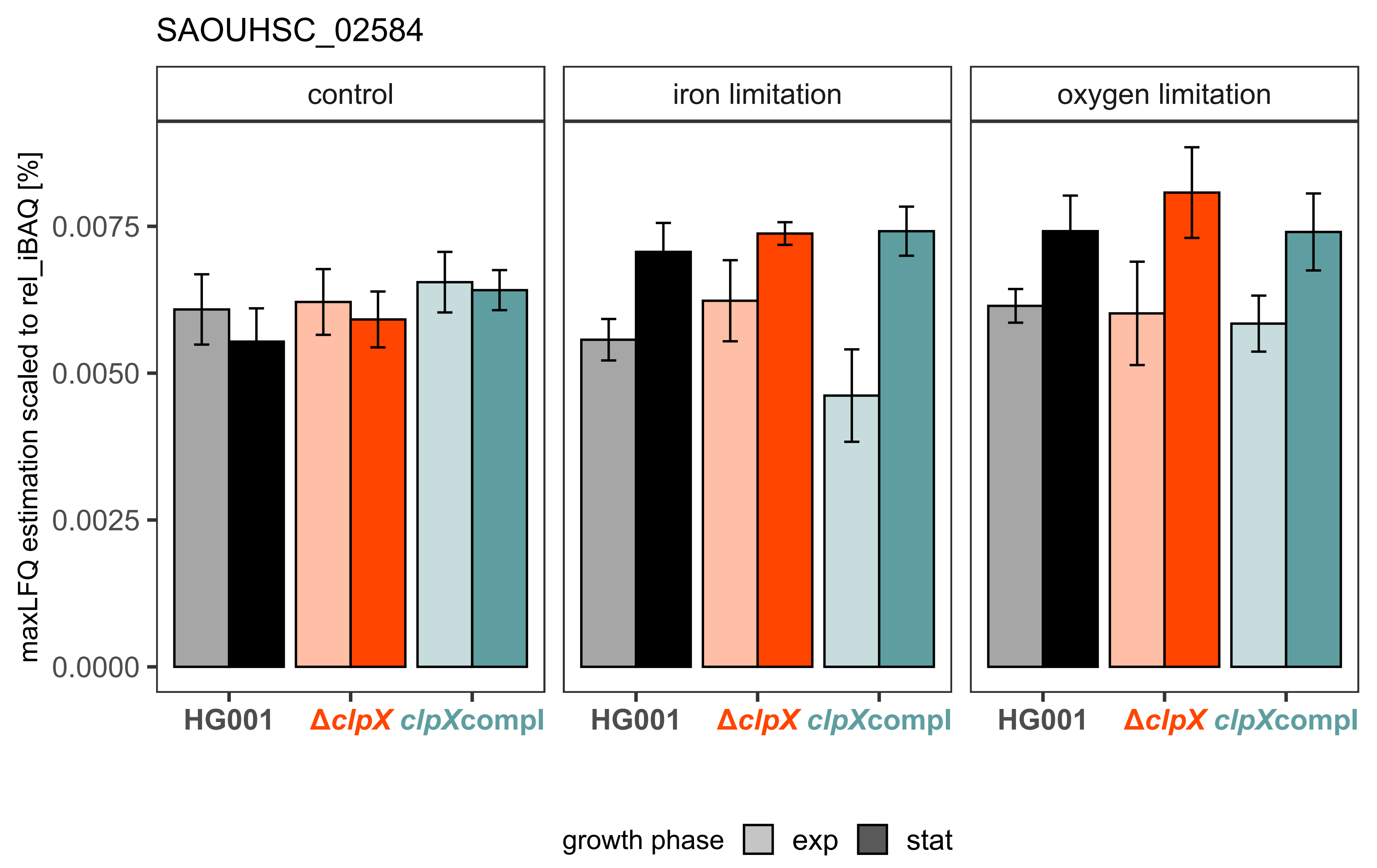

SAOUHSC_02584
control
iron limitation
oxygen limitation
maxLFQ estimation scaled to rel_iBAQ [%]
0.0075
0.0050
0.0025
0.0000
HG001
ΔclpX
clpXcompl
growth phase
exp
stat

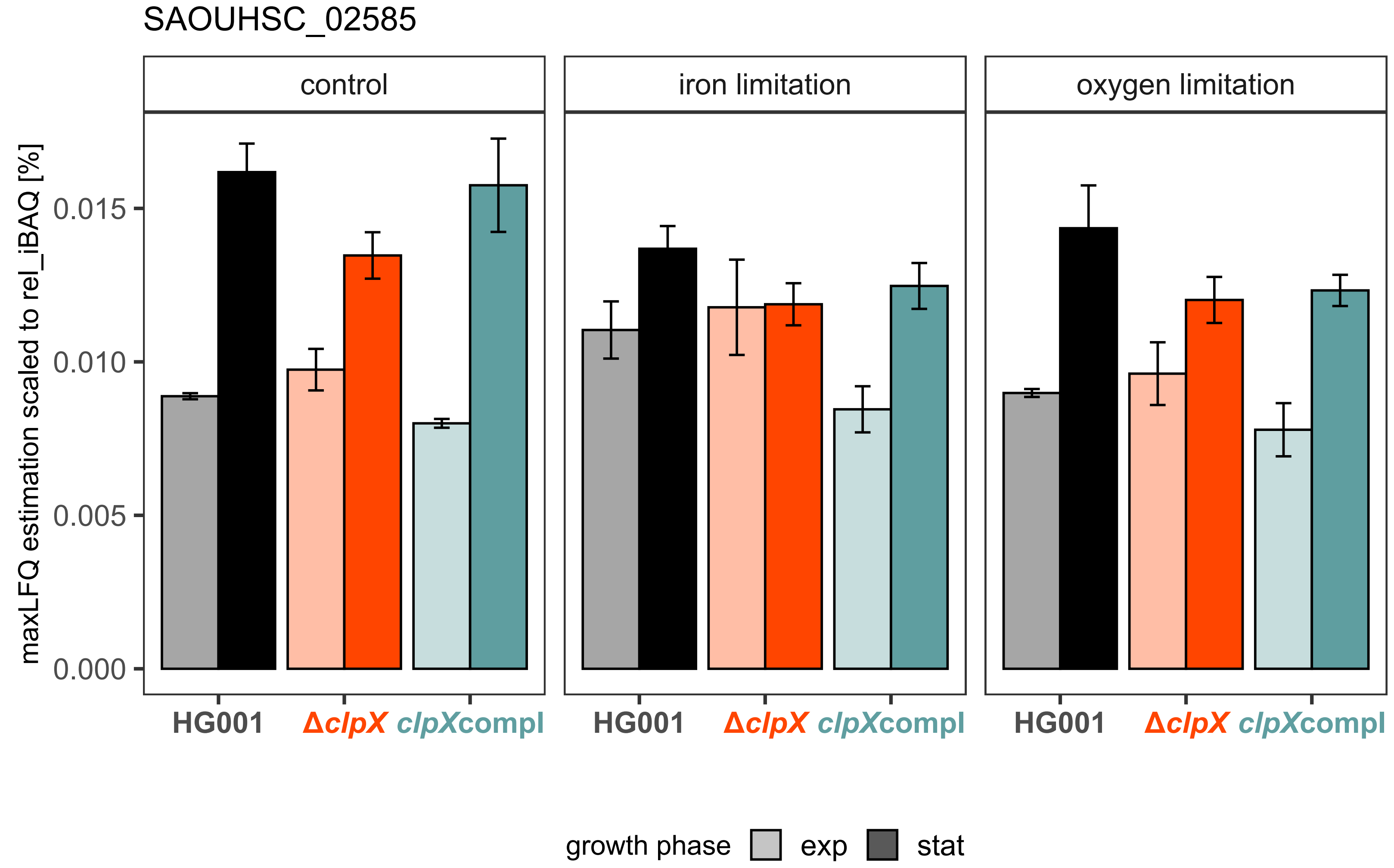

SAOUHSC_02585
control
iron limitation
oxygen limitation
maxLFQ estimation scaled to rel_iBAQ [%]
0.015
0.010
0.005
0.000
HG001
ΔclpX
clpXcompl
growth phase
exp
stat

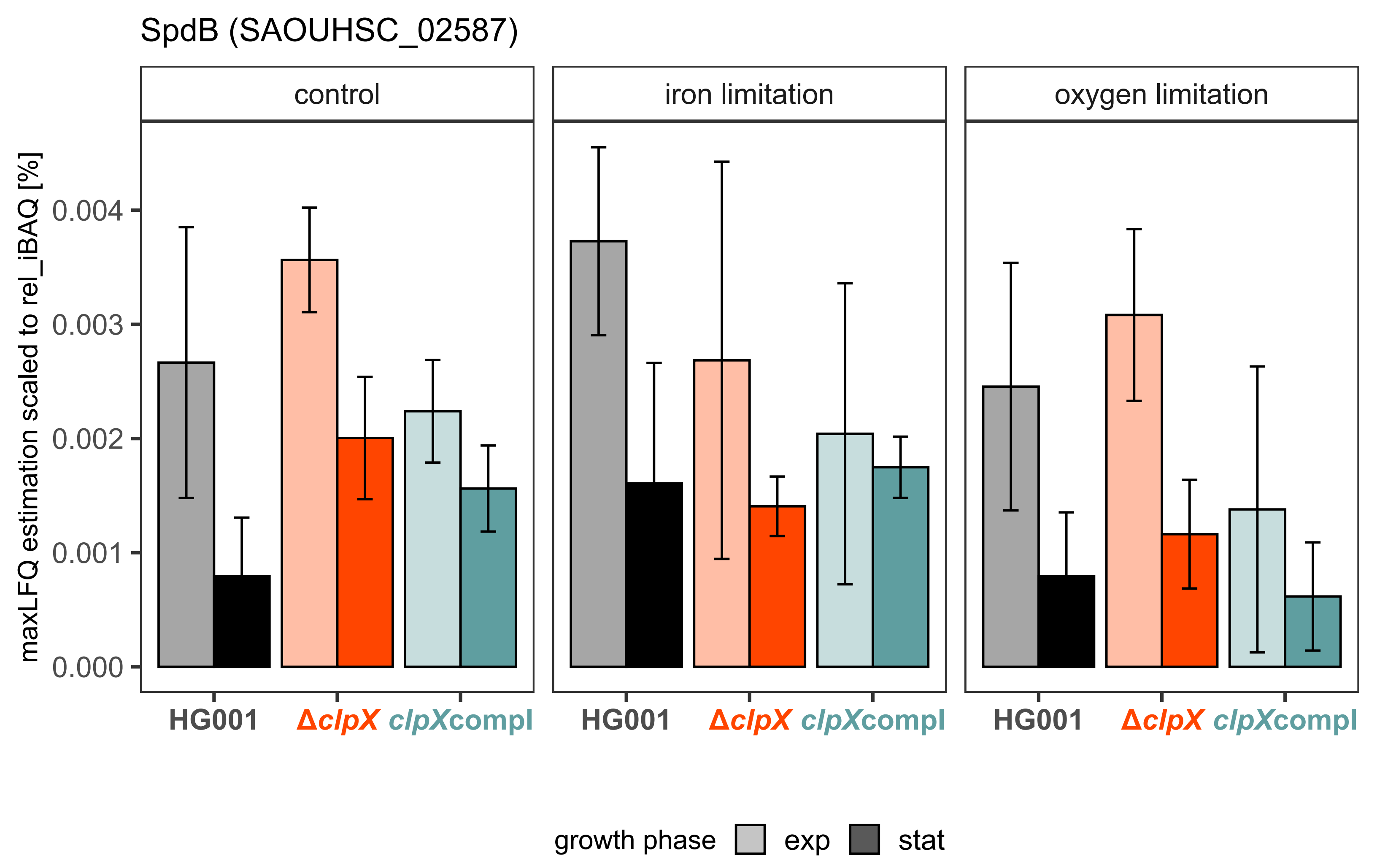
SpdB (SAOUHSC_02587)
control
iron limitation
oxygen limitation
maxLFQ estimation scaled to rel_iBAQ [%]
0.004
0.003
0.002
0.001
0.000
HG001
ΔclpX
clpXcompl
growth phase
exp
stat

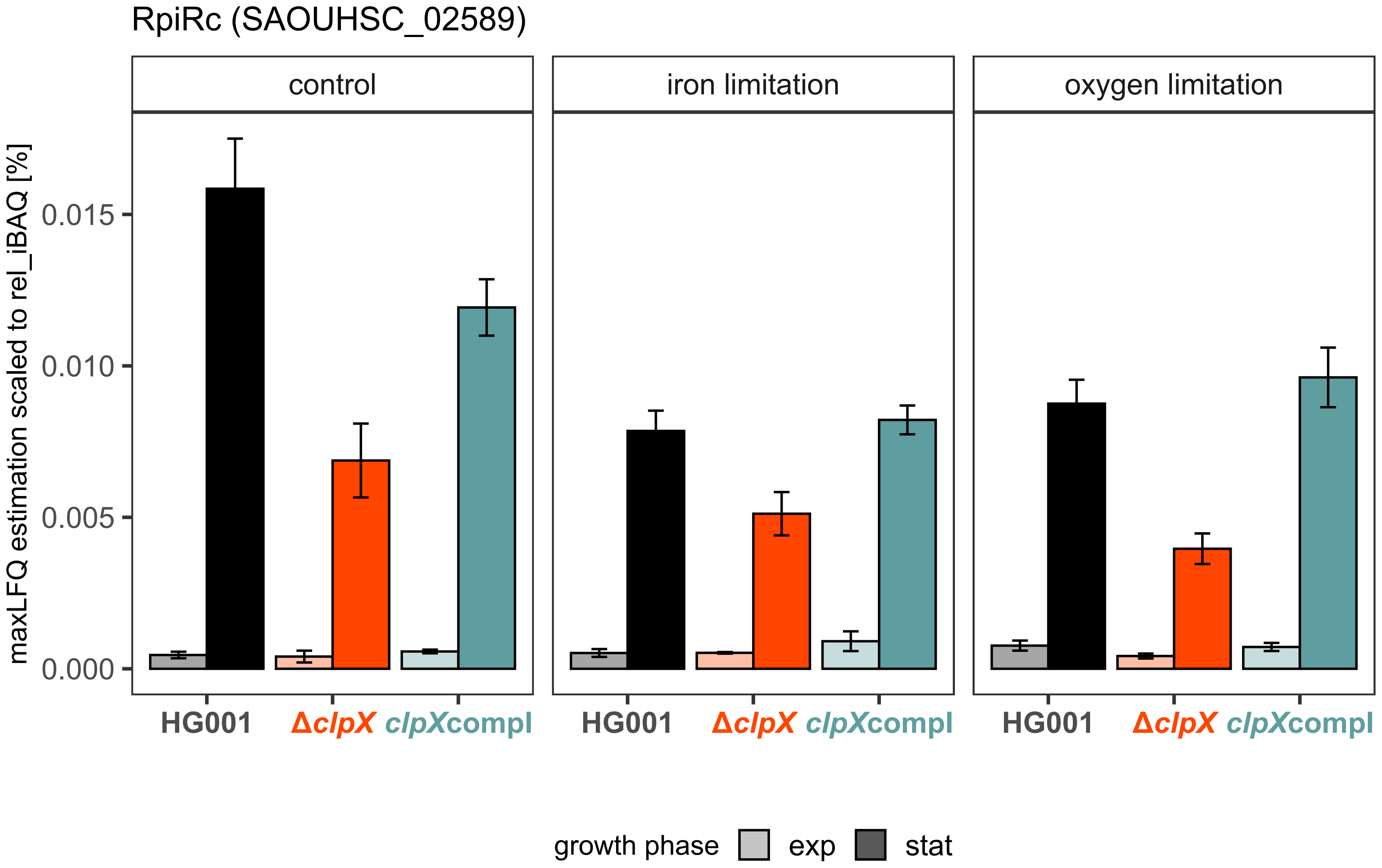
RpiRc (SAOUHSC_02589)
control
iron limitation
oxygen limitation
maxLFQ estimation scaled to rel_iBAQ [%]
0.000
0.005
0.010
0.015
HG001
ΔclpX
clpXcompl
growth phase
exp
stat

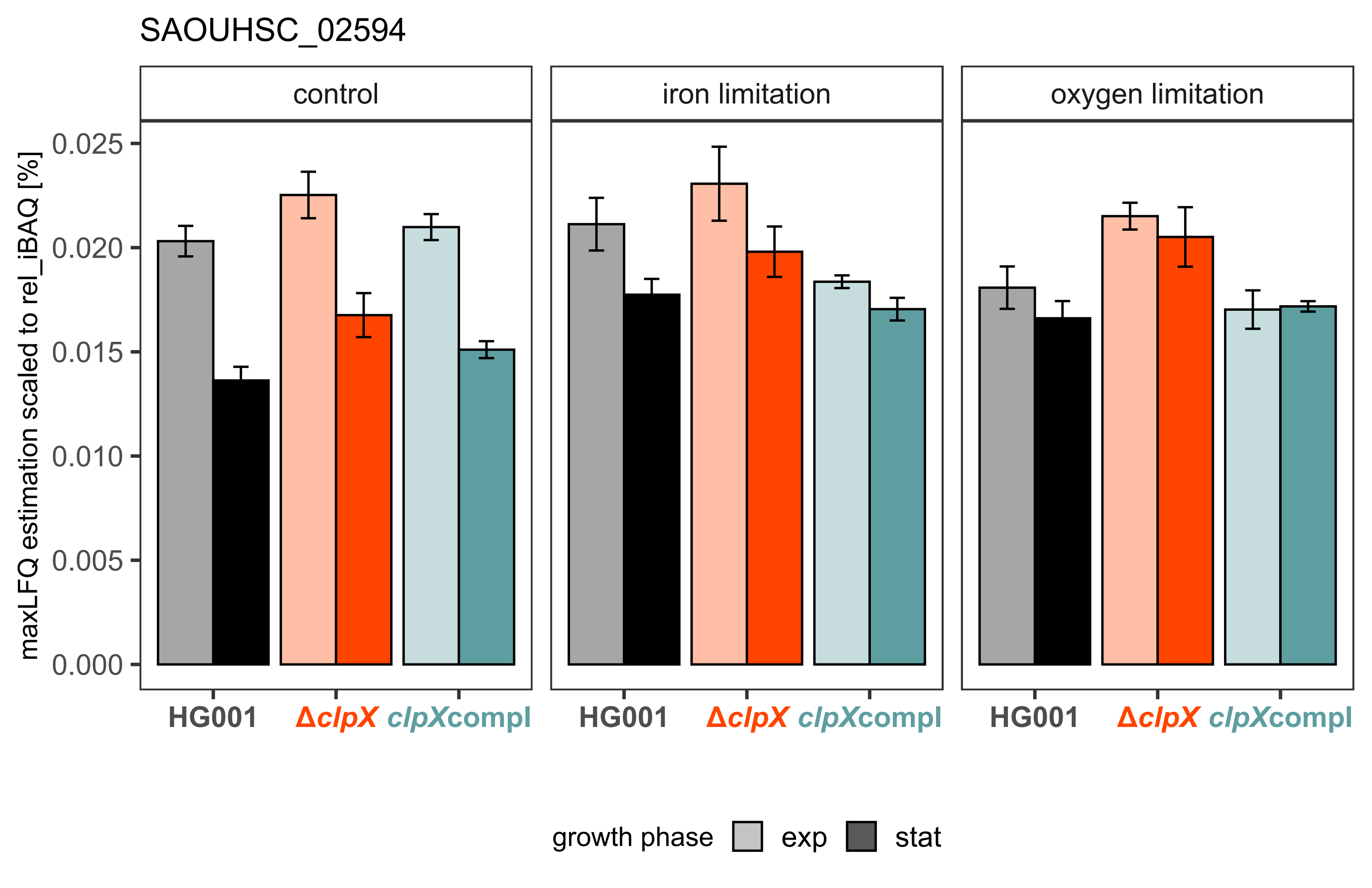

SAOUHSC_02594
control
iron limitation
oxygen limitation
maxLFQ estimation scaled to rel_iBAQ [%]
0.025
0.020
0.015
0.010
0.005
0.000
HG001
ΔclpX
clpXcompl
growth phase
exp
stat

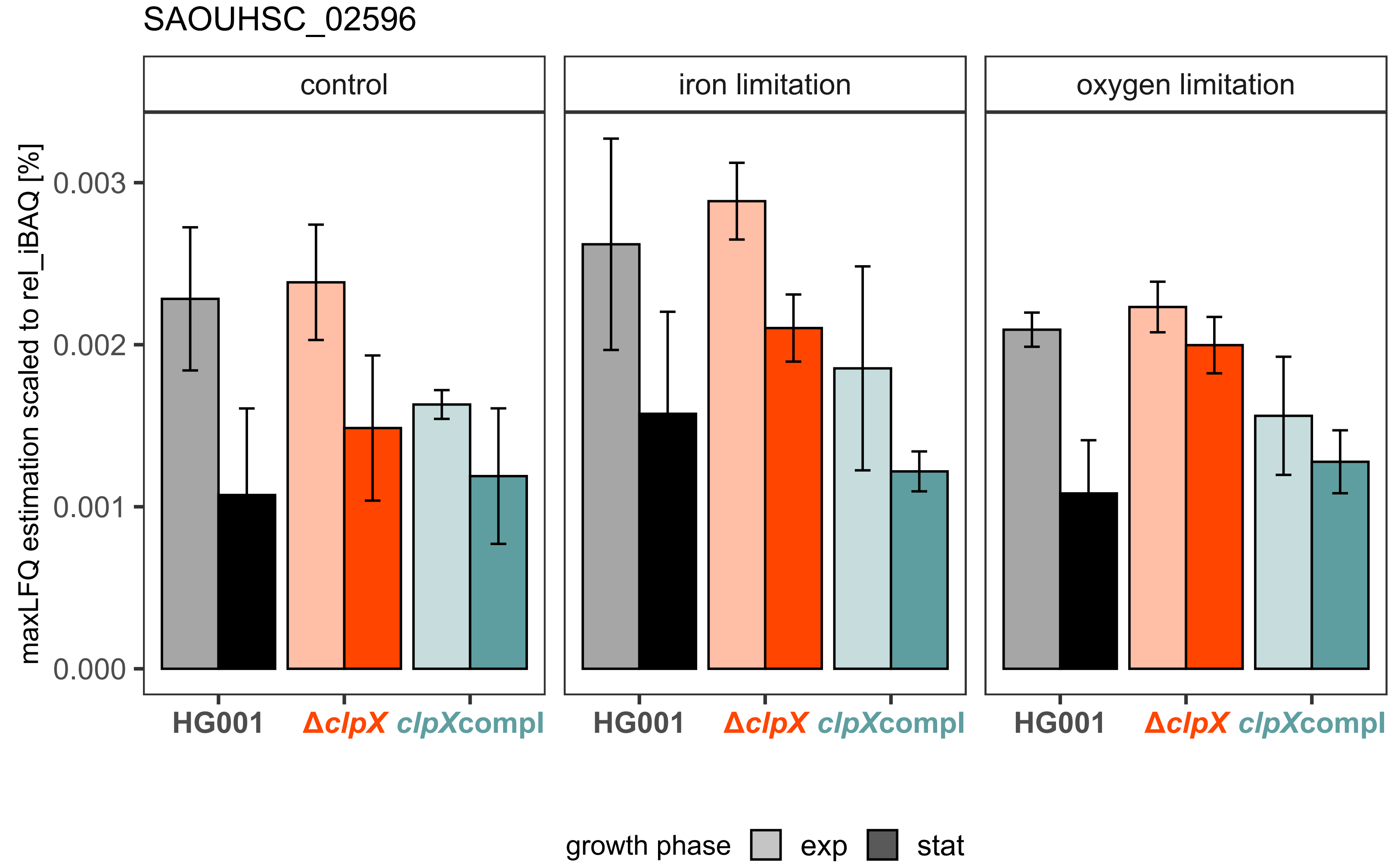

SAOUHSC_02596
control
iron limitation
oxygen limitation
maxLFQ estimation scaled to rel_iBAQ [%]
0.003
0.002
0.001
0.000
HG001
ΔclpX
clpXcompl
growth phase
exp
stat

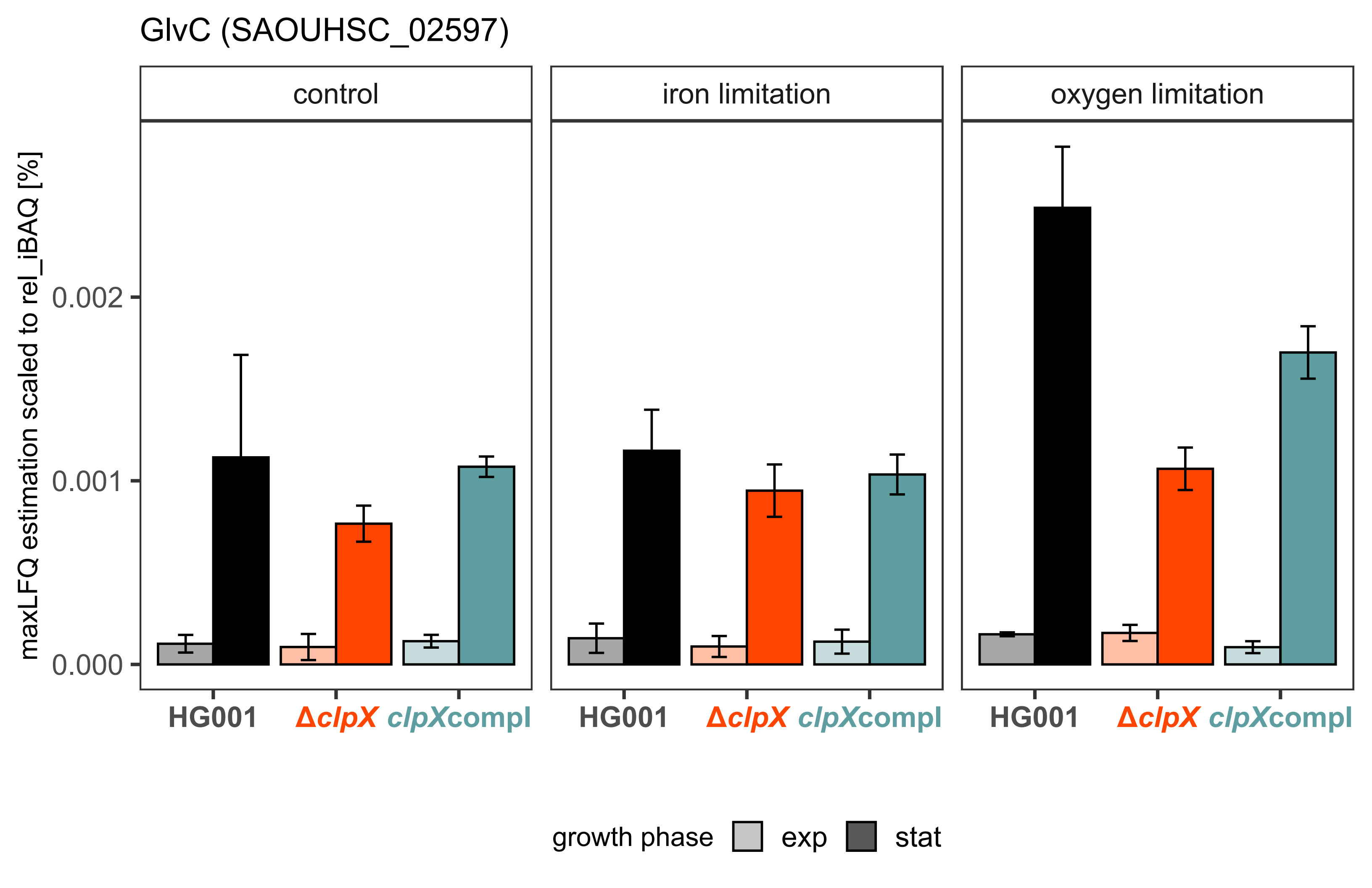

GlvC (SAOUHSC_02597)
control
iron limitation
oxygen limitation
maxLFQ estimation scaled to rel_iBAQ [%]
0.000
0.001
0.002
HG001
ΔclpX
clpXcompl
growth phase
exp
stat

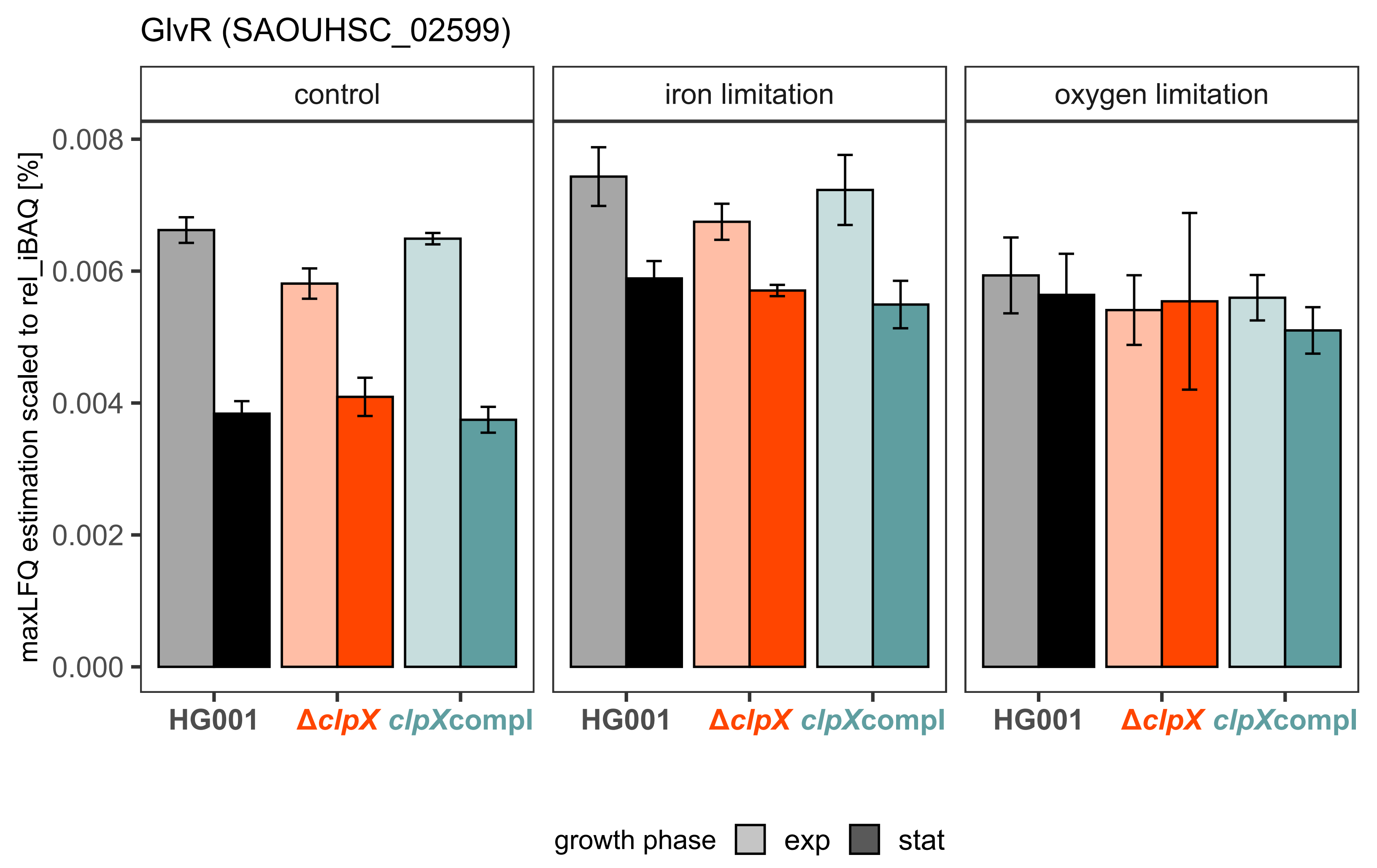

GlvR (SAOUHSC_02599)
control
iron limitation
oxygen limitation
maxLFQ estimation scaled to rel_iBAQ [%]
0.008
0.006
0.004
0.002
0.000
HG001
ΔclpX
clpXcompl
growth phase
exp
stat

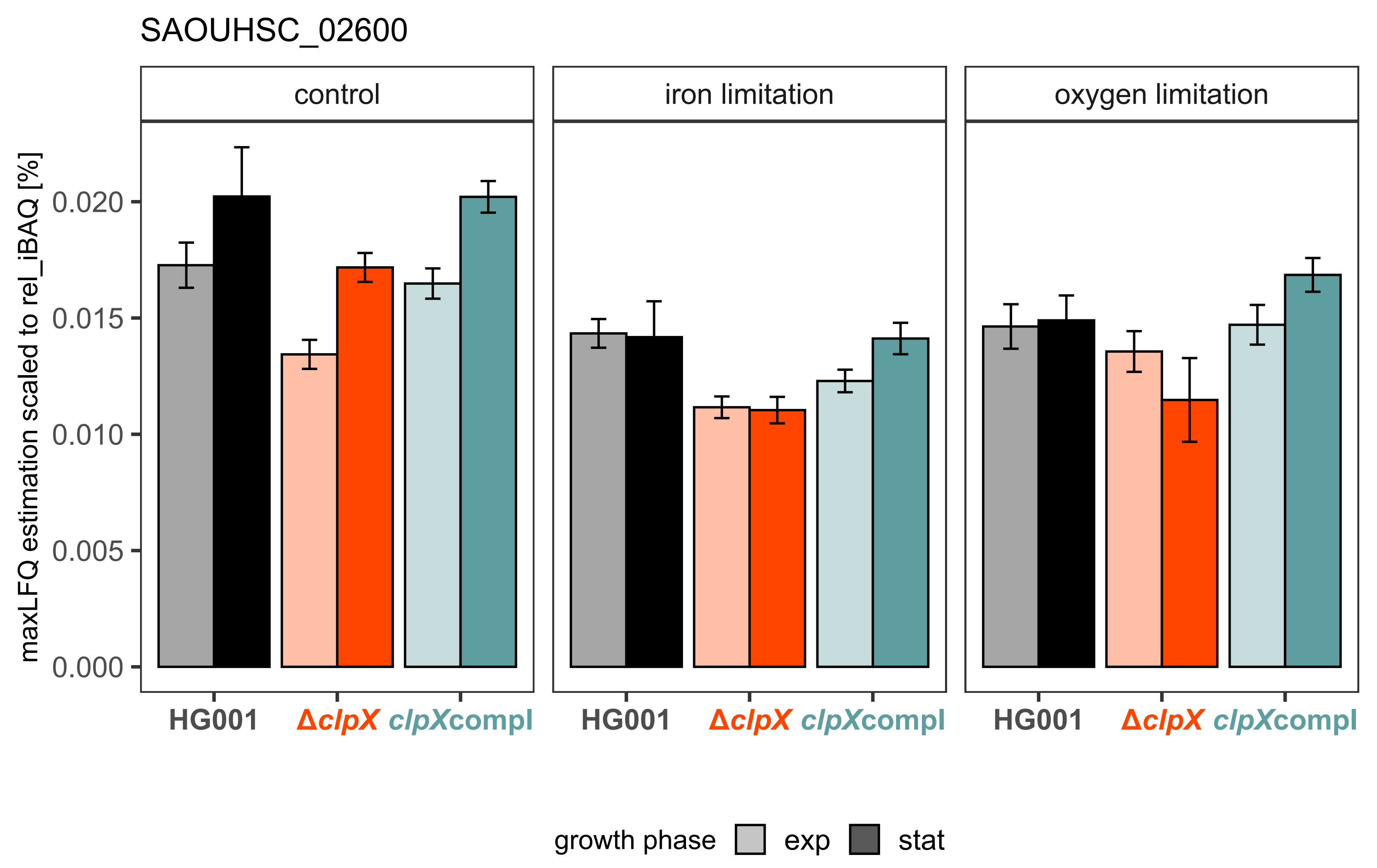
SAOUHSC_02600
control
iron limitation
oxygen limitation
maxLFQ estimation scaled to rel_iBAQ [%]
0.000
0.005
0.010
0.015
0.020
HG001
ΔclpX
clpXcompl
growth phase
exp
stat

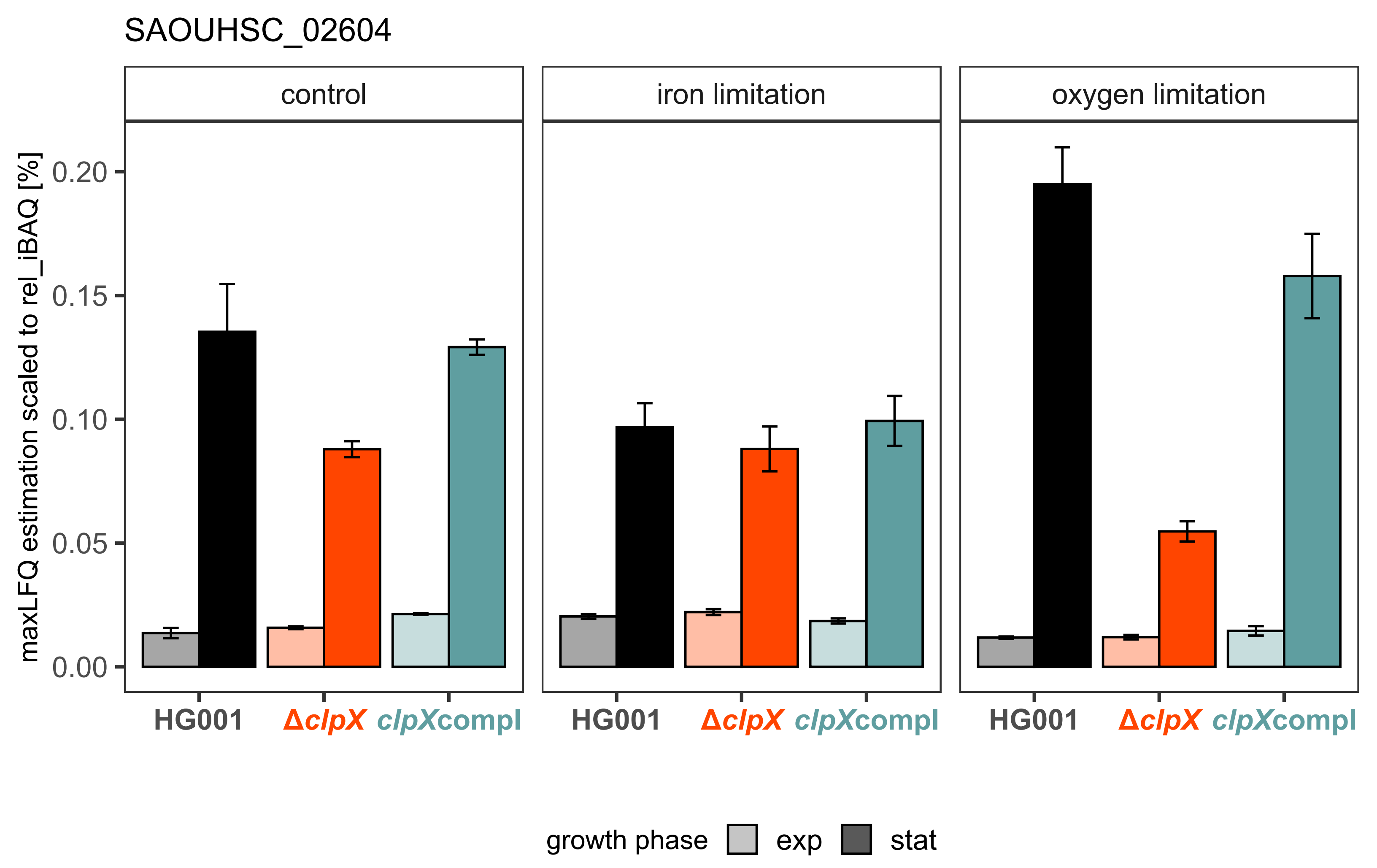

SAOUHSC_02604
control
iron limitation
oxygen limitation
maxLFQ estimation scaled to rel_iBAQ [%]
0.20
0.15
0.10
0.05
0.00
HG001
ΔclpX
clpXcompl
growth phase
exp
stat

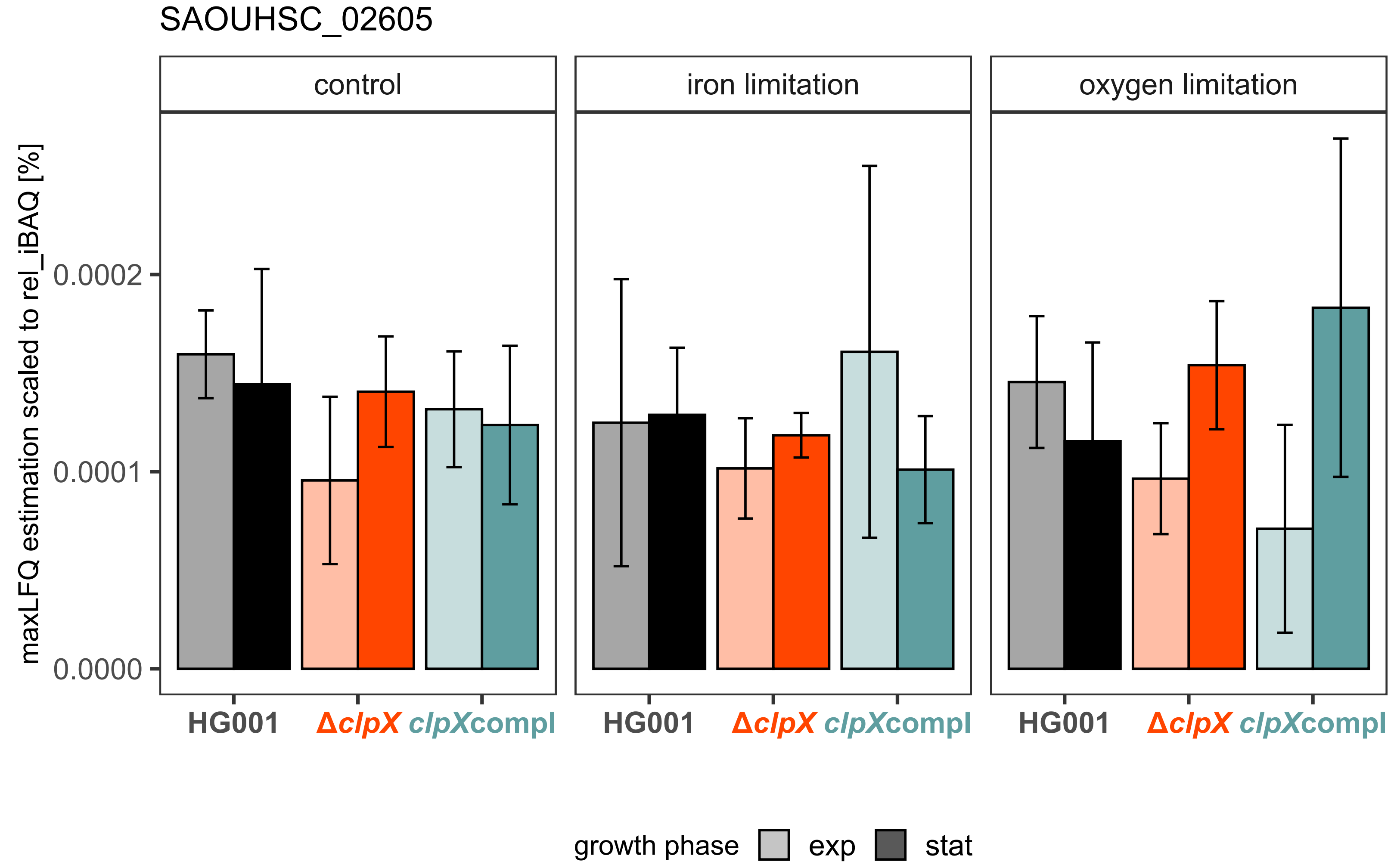

SAOUHSC_02605
control
iron limitation
oxygen limitation
maxLFQ estimation scaled to rel_iBAQ [%]
0.0002
0.0001
0.0000
HG001
ΔclpX
clpXcompl
growth phase
exp
stat

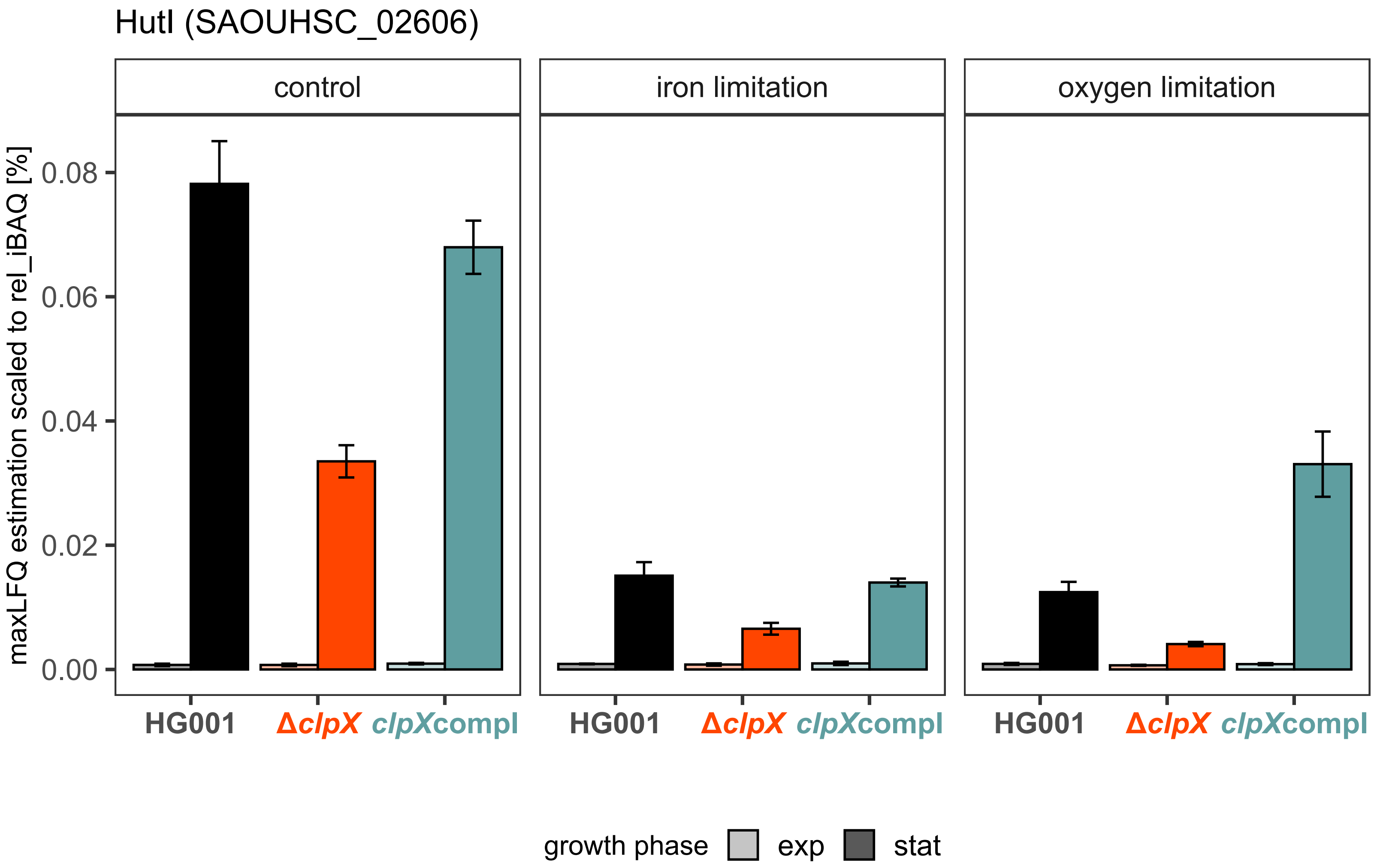

HutI (SAOUHSC_02606)
control
iron limitation
oxygen limitation
maxLFQ estimation scaled to rel_iBAQ [%]
0.00
0.02
0.04
0.06
0.08
HG001
ΔclpX
clpXcompl
growth phase
exp
stat

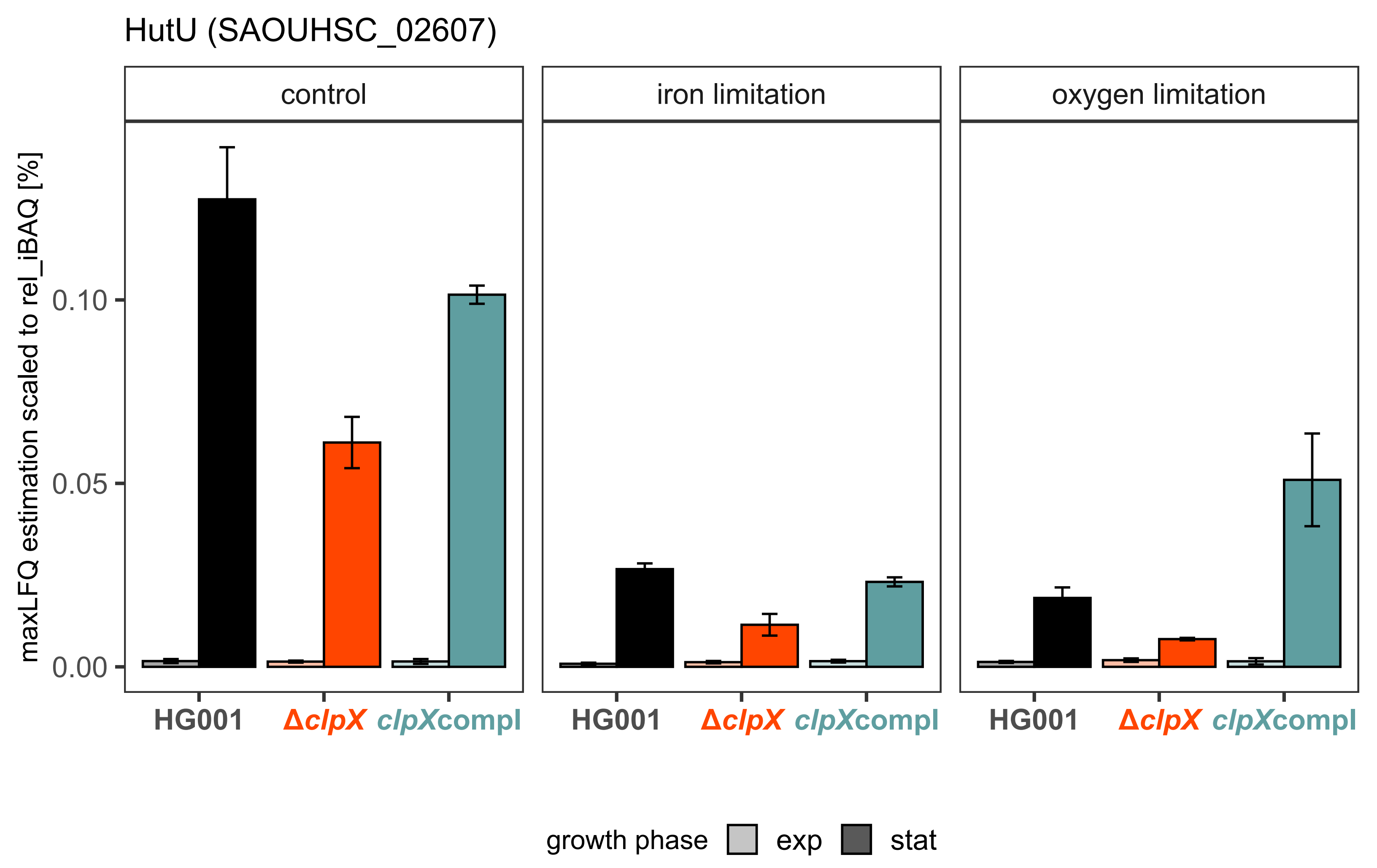
HutU (SAOUHSC_02607)
control
iron limitation
oxygen limitation
maxLFQ estimation scaled to rel_iBAQ [%]
0.10
0.05
0.00
HG001
ΔclpX
clpXcompl
growth phase
exp
stat

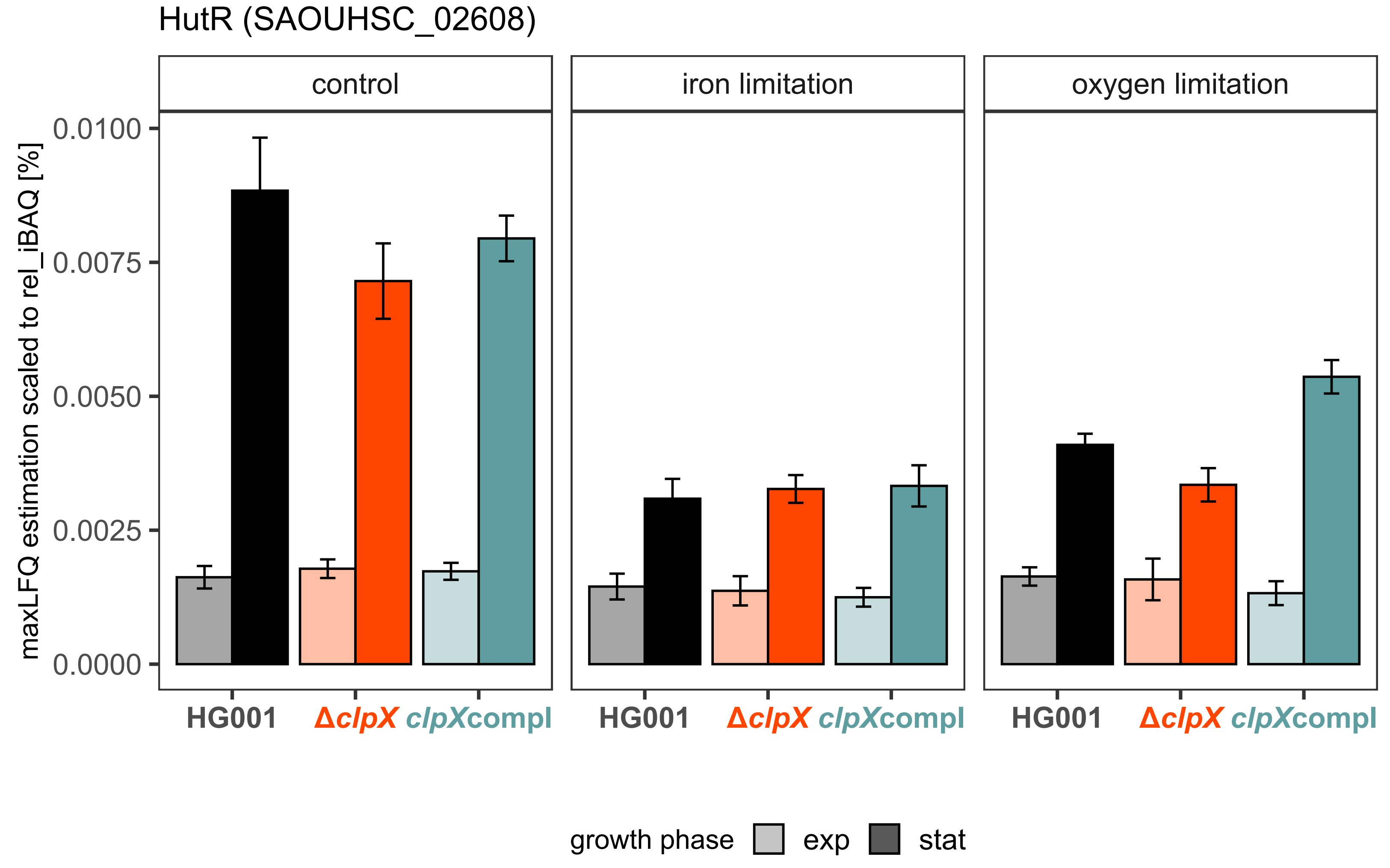
HutR (SAOUHSC_02608)
control
iron limitation
oxygen limitation
maxLFQ estimation scaled to rel_iBAQ [%]
0.0100
0.0075
0.0050
0.0025
0.0000
HG001
ΔclpX
clpXcompl
growth phase
exp
stat

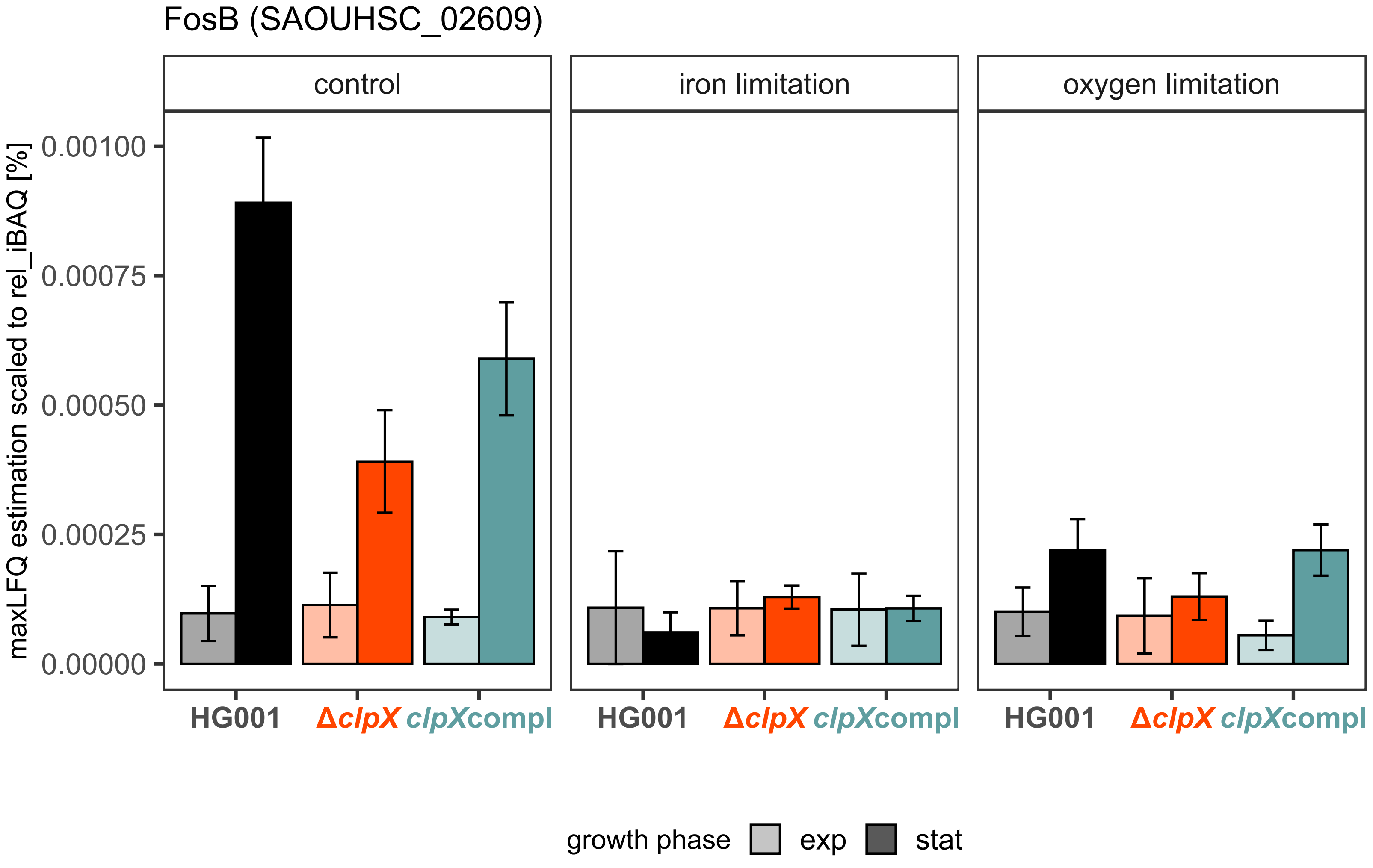
FosB (SAOUHSC_02609)
control
iron limitation
oxygen limitation
maxLFQ estimation scaled to rel_iBAQ [%]
0.00100
0.00075
0.00050
0.00025
0.00000
HG001
ΔclpX
clpXcompl
growth phase
exp
stat

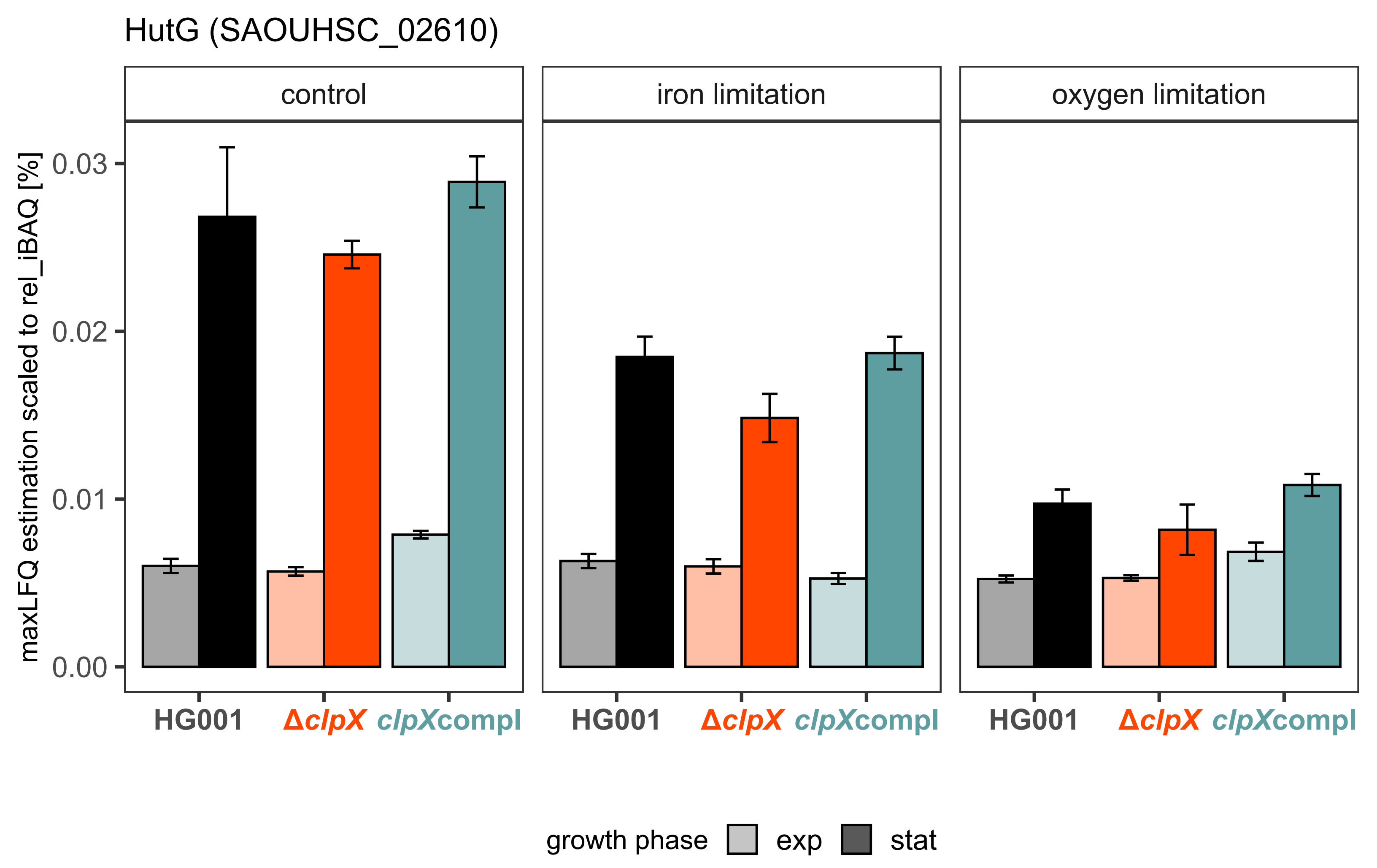
HutG (SAOUHSC_02610)
control
iron limitation
oxygen limitation
maxLFQ estimation scaled to rel_iBAQ [%]
0.00
0.01
0.02
0.03
HG001
ΔclpX
clpXcompl
growth phase
exp
stat

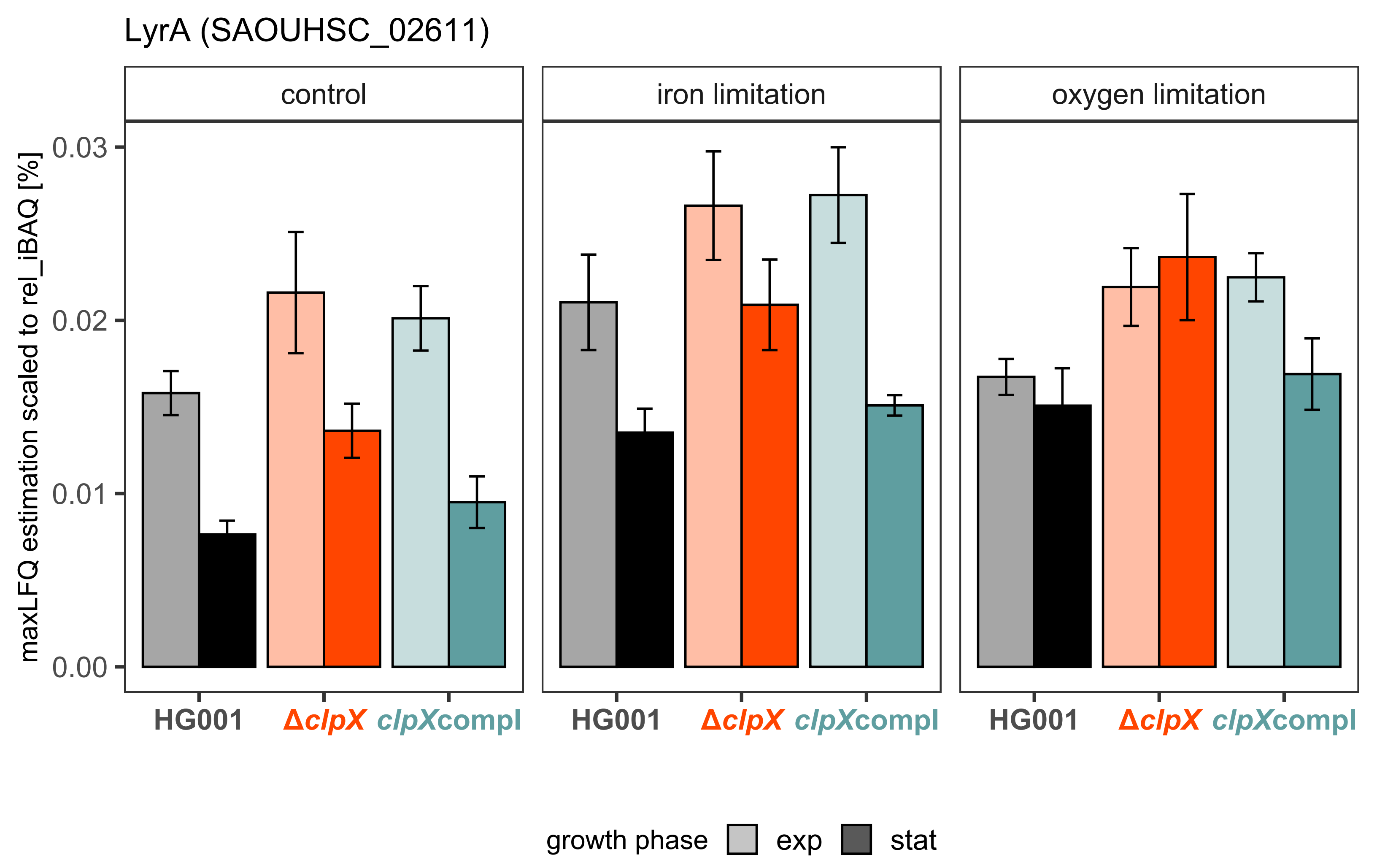

LyrA (SAOUHSC_02611)
control
iron limitation
oxygen limitation
maxLFQ estimation scaled to rel_iBAQ [%]
0.00
0.01
0.02
0.03
HG001
ΔclpX
clpXcompl
growth phase
exp
stat

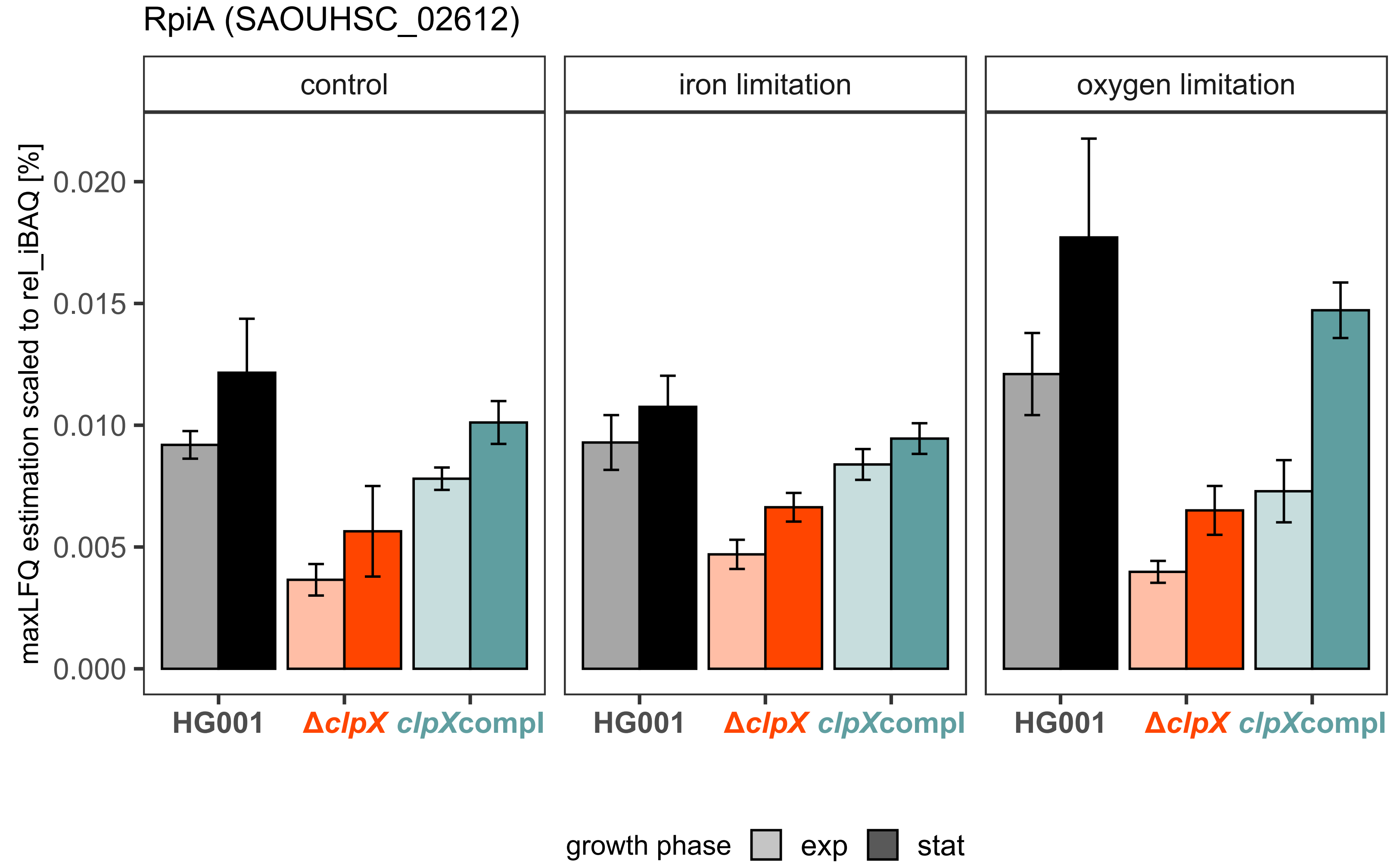
RpiA (SAOUHSC_02612)
control
iron limitation
oxygen limitation
maxLFQ estimation scaled to rel_iBAQ [%]
0.020
0.015
0.010
0.005
0.000
HG001
ΔclpX
clpXcompl
growth phase
exp
stat

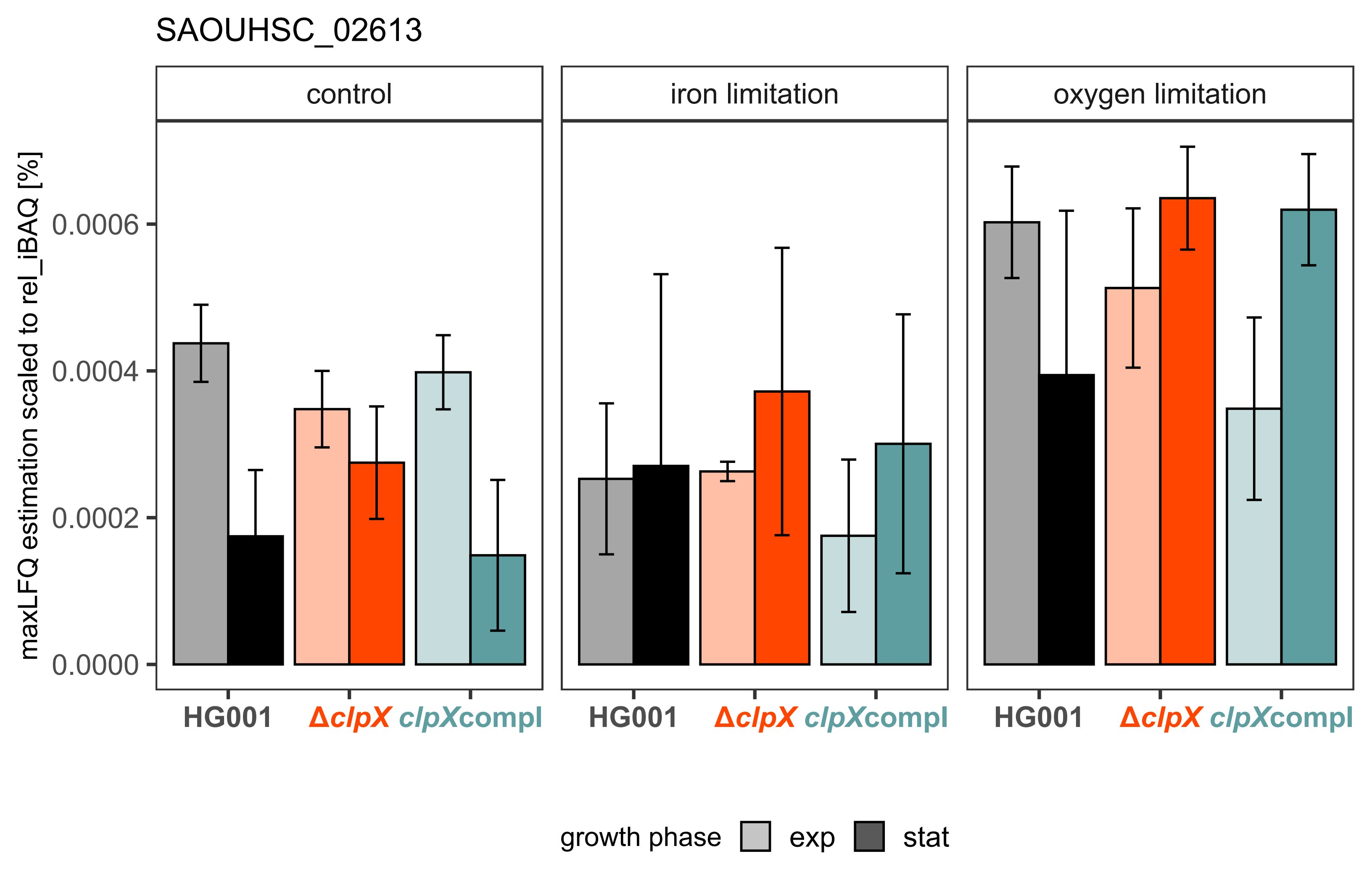
SAOUHSC_02613
control
iron limitation
oxygen limitation
maxLFQ estimation scaled to rel_iBAQ [%]
0.0006
0.0004
0.0002
0.0000
HG001
ΔclpX
clpXcompl
growth phase
exp
stat

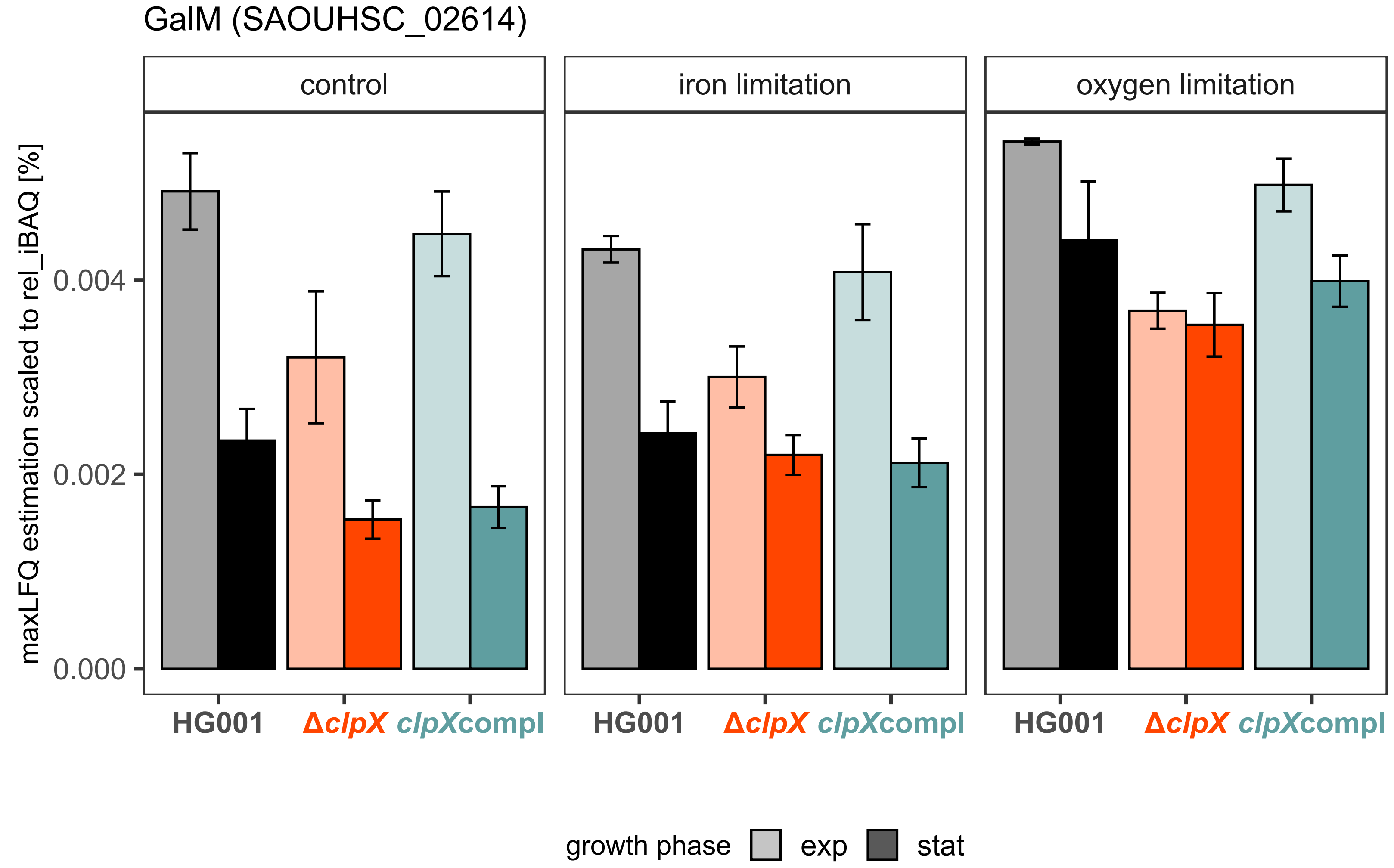

GalM (SAOUHSC_02614)
control
iron limitation
oxygen limitation
maxLFQ estimation scaled to rel_iBAQ [%]
0.004
0.002
0.000
HG001
ΔclpX
clpXcompl
growth phase
exp
stat

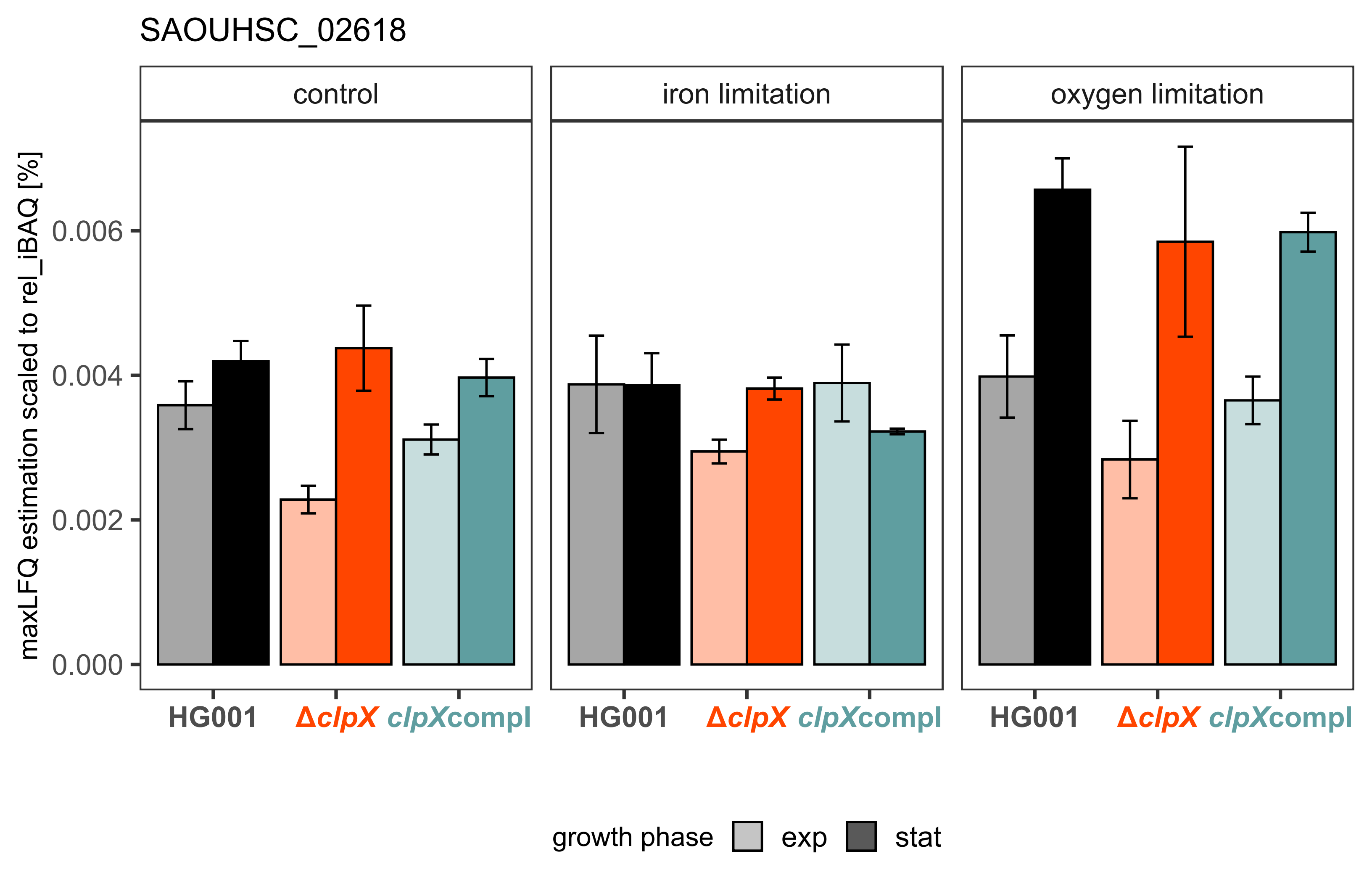
SAOUHSC_02618
control
iron limitation
oxygen limitation
maxLFQ estimation scaled to rel_iBAQ [%]
0.000
0.002
0.004
0.006
HG001
ΔclpX
clpXcompl
growth phase
exp
stat

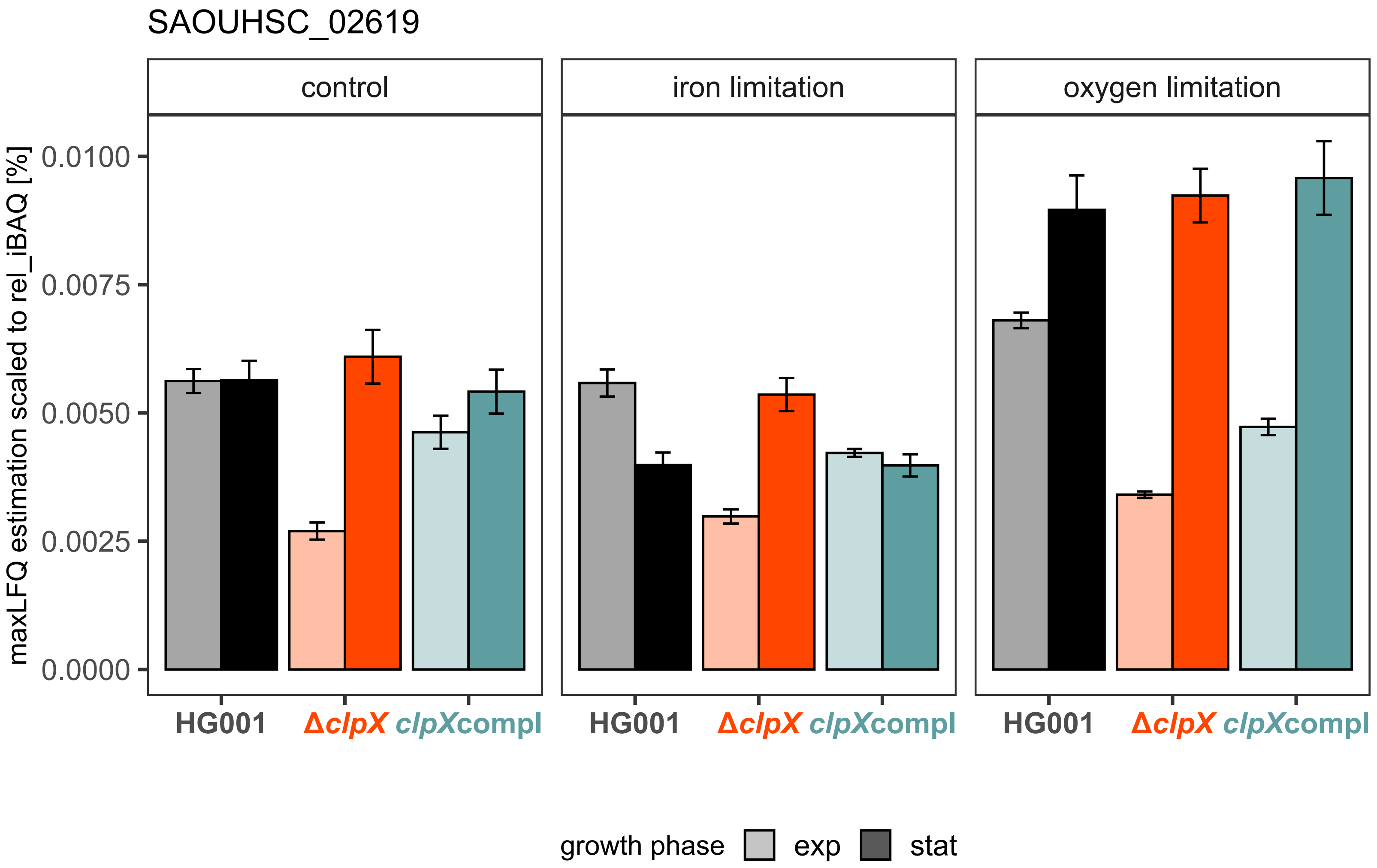
SAOUHSC_02619
control
iron limitation
oxygen limitation
maxLFQ estimation scaled to rel_iBAQ [%]
0.0100
0.0075
0.0050
0.0025
0.0000
HG001
ΔclpX
clpXcompl
growth phase
exp
stat

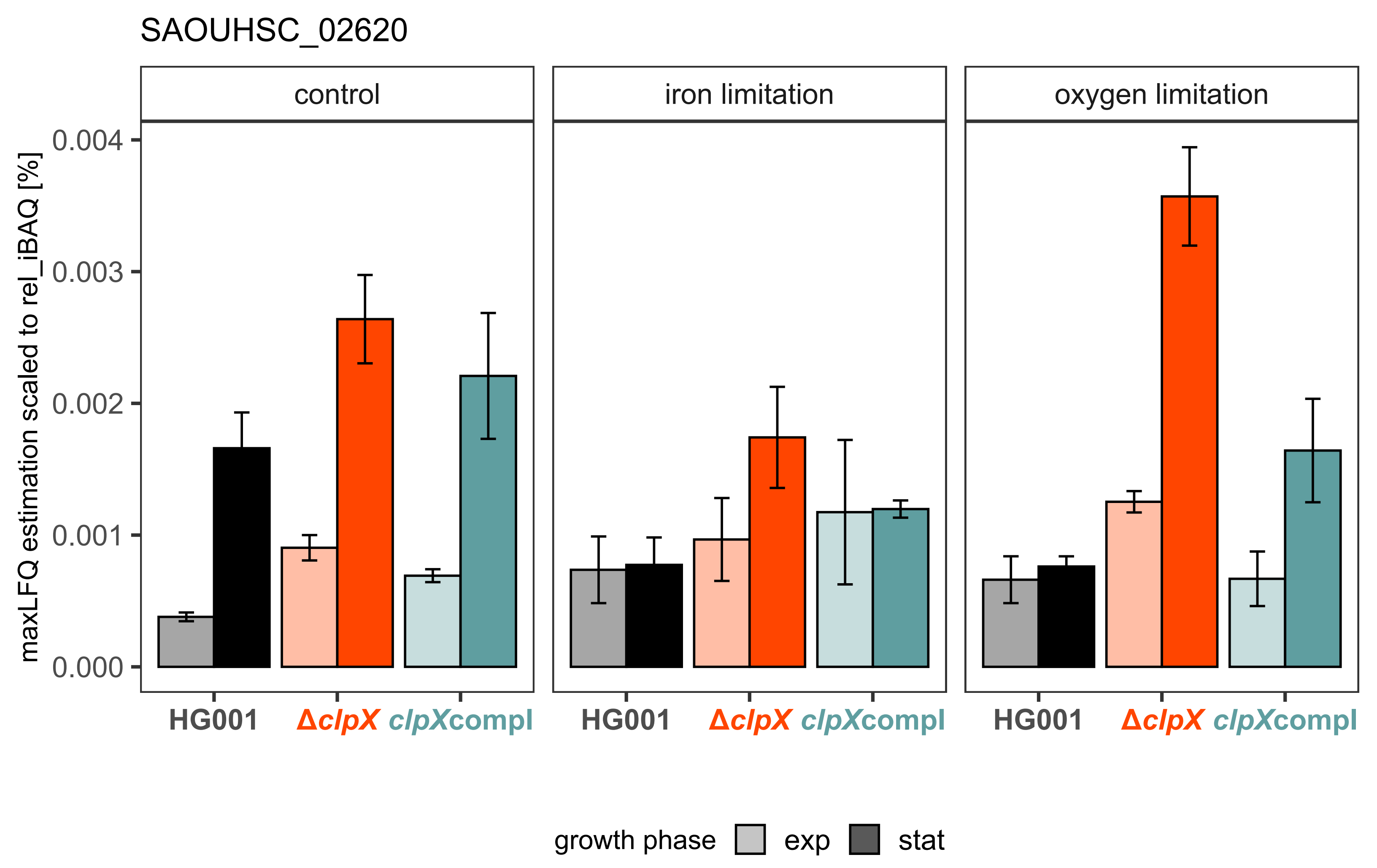
SAOUHSC_02620
control
iron limitation
oxygen limitation
maxLFQ estimation scaled to rel_iBAQ [%]
0.004
0.003
0.002
0.001
0.000
HG001
ΔclpX
clpXcompl
growth phase
exp
stat

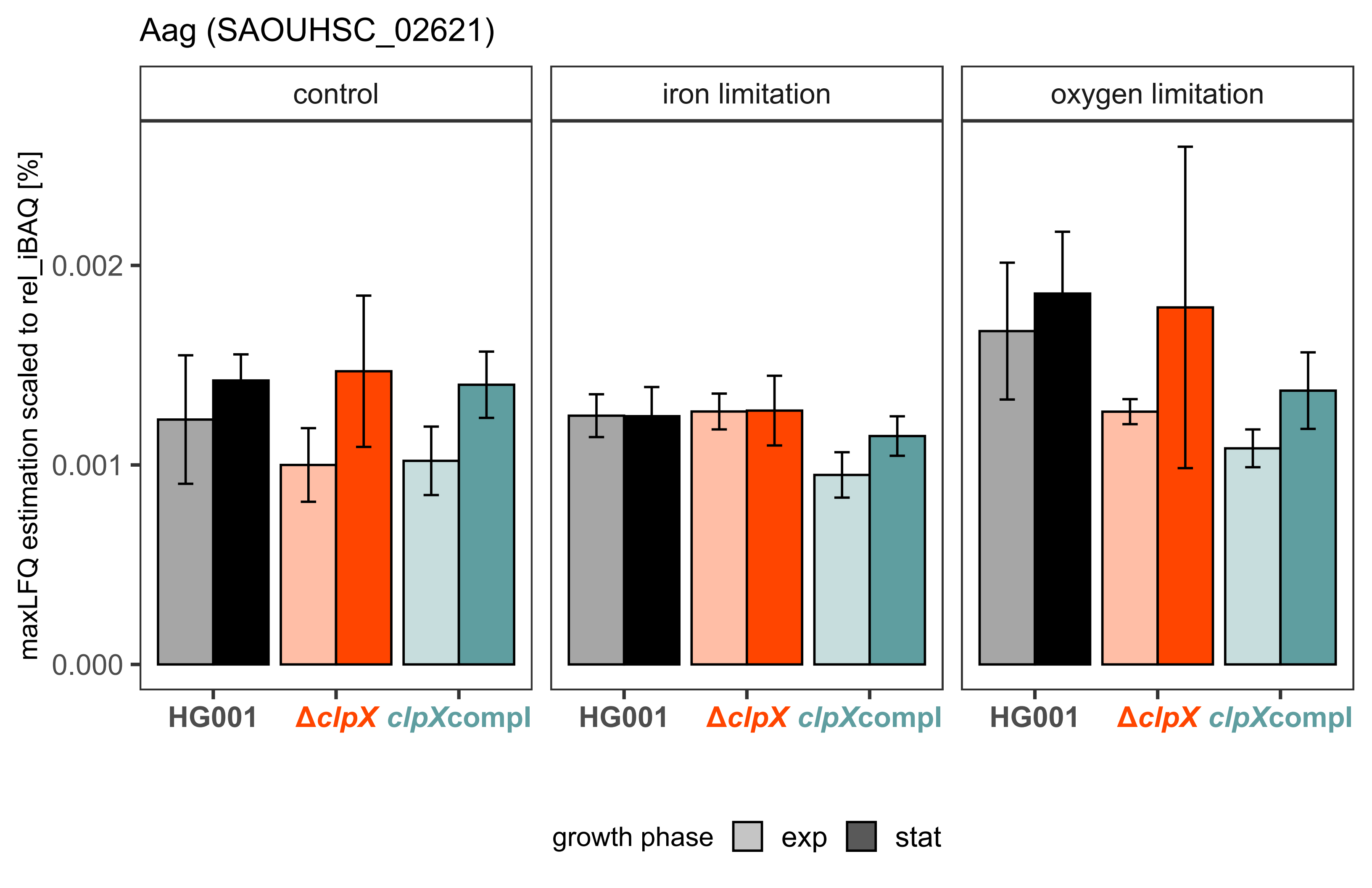

Aag (SAOUHSC_02621)
control
iron limitation
oxygen limitation
maxLFQ estimation scaled to rel_iBAQ [%]
0.000
0.001
0.002
HG001
ΔclpX
clpXcompl
growth phase
exp
stat

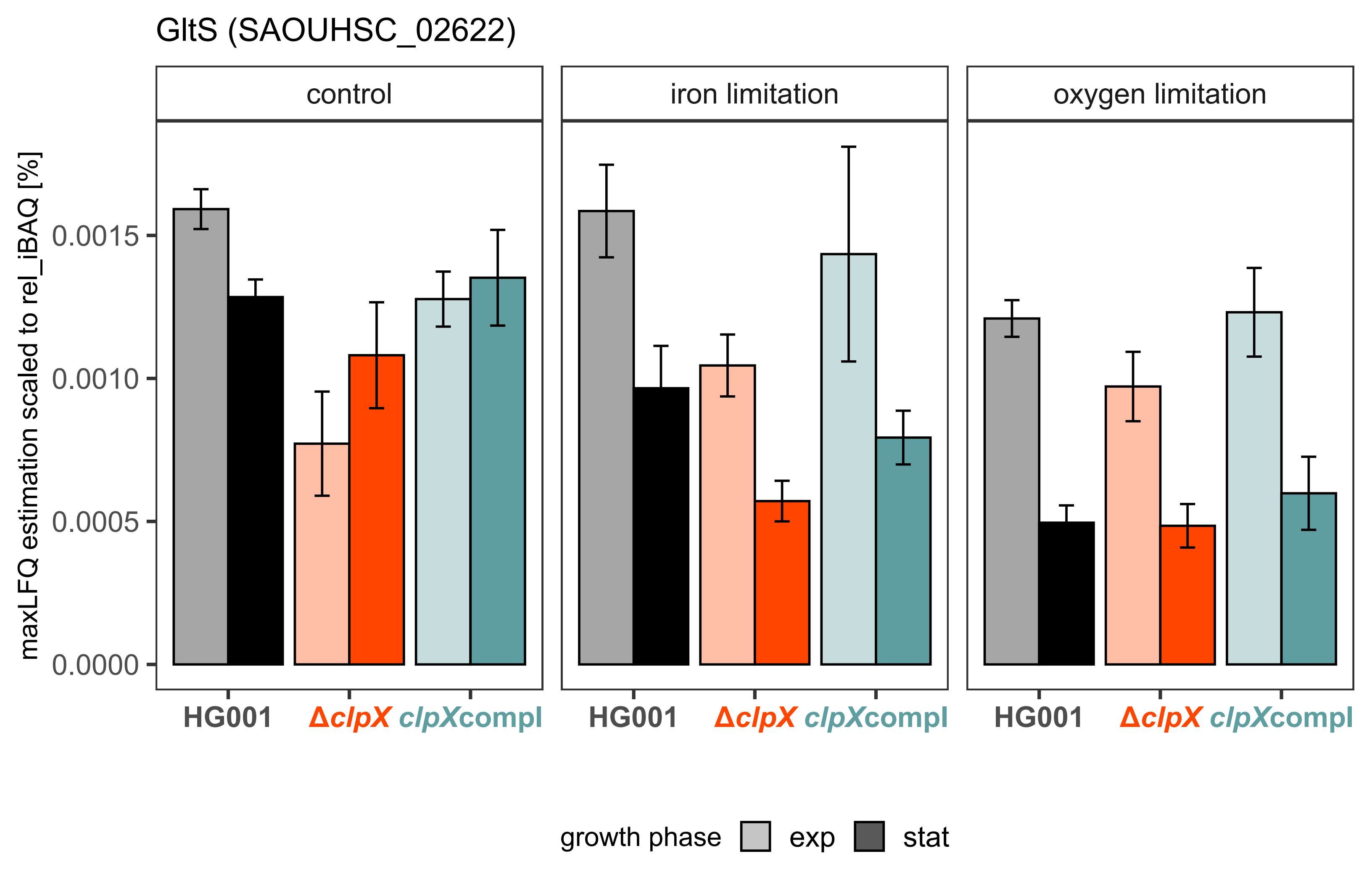

GltS (SAOUHSC_02622)
control
iron limitation
oxygen limitation
maxLFQ estimation scaled to rel_iBAQ [%]
0.0015
0.0010
0.0005
0.0000
HG001
ΔclpX
clpXcompl
growth phase
exp
stat

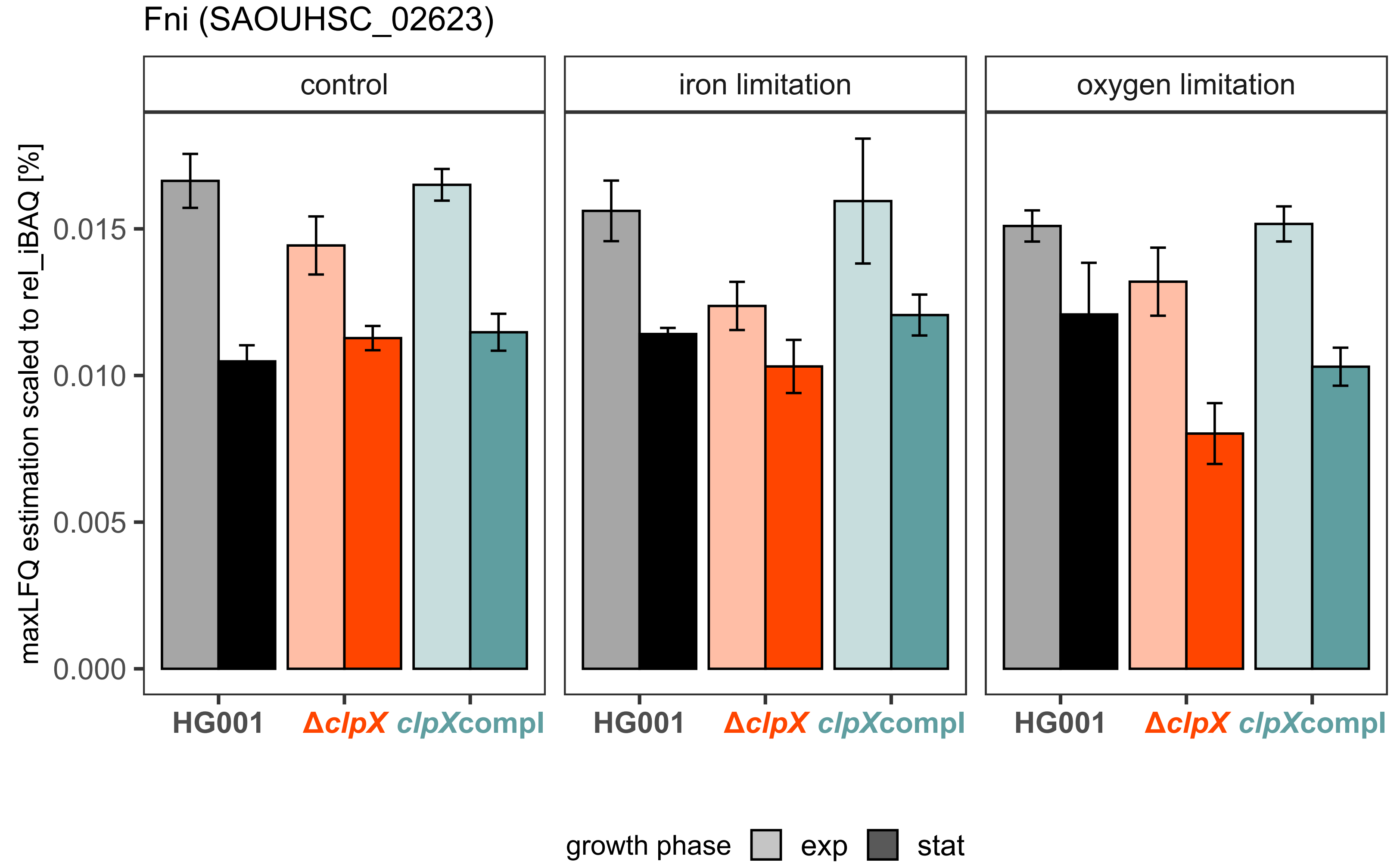
Fni (SAOUHSC_02623)
control
iron limitation
oxygen limitation
maxLFQ estimation scaled to rel_iBAQ [%]
0.015
0.010
0.005
0.000
HG001
ΔclpX
clpXcompl
growth phase
exp
stat

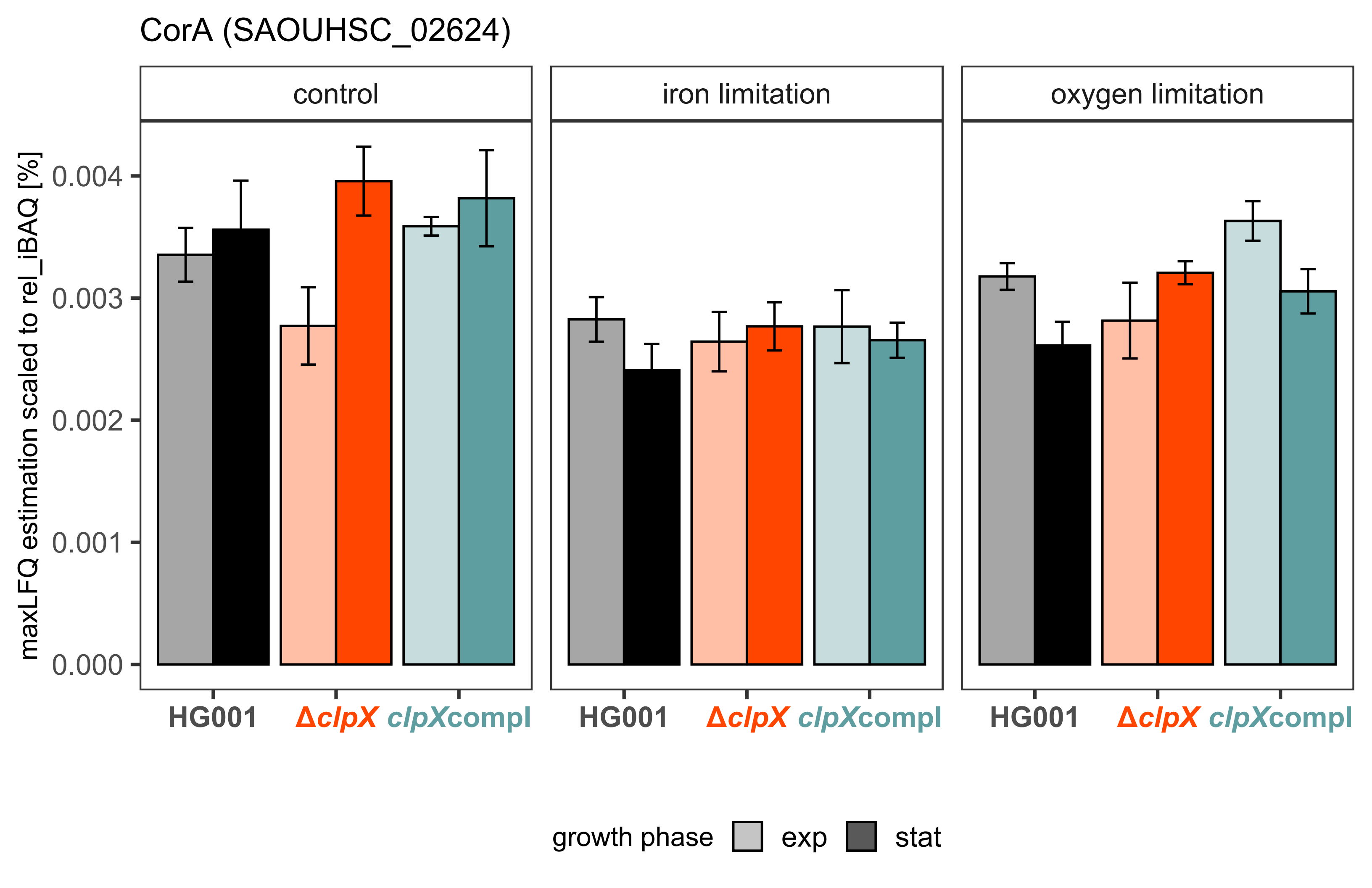

CorA (SAOUHSC_02624)
control
iron limitation
oxygen limitation
maxLFQ estimation scaled to rel_iBAQ [%]
0.004
0.003
0.002
0.001
0.000
HG001
ΔclpX
clpXcompl
growth phase
exp
stat

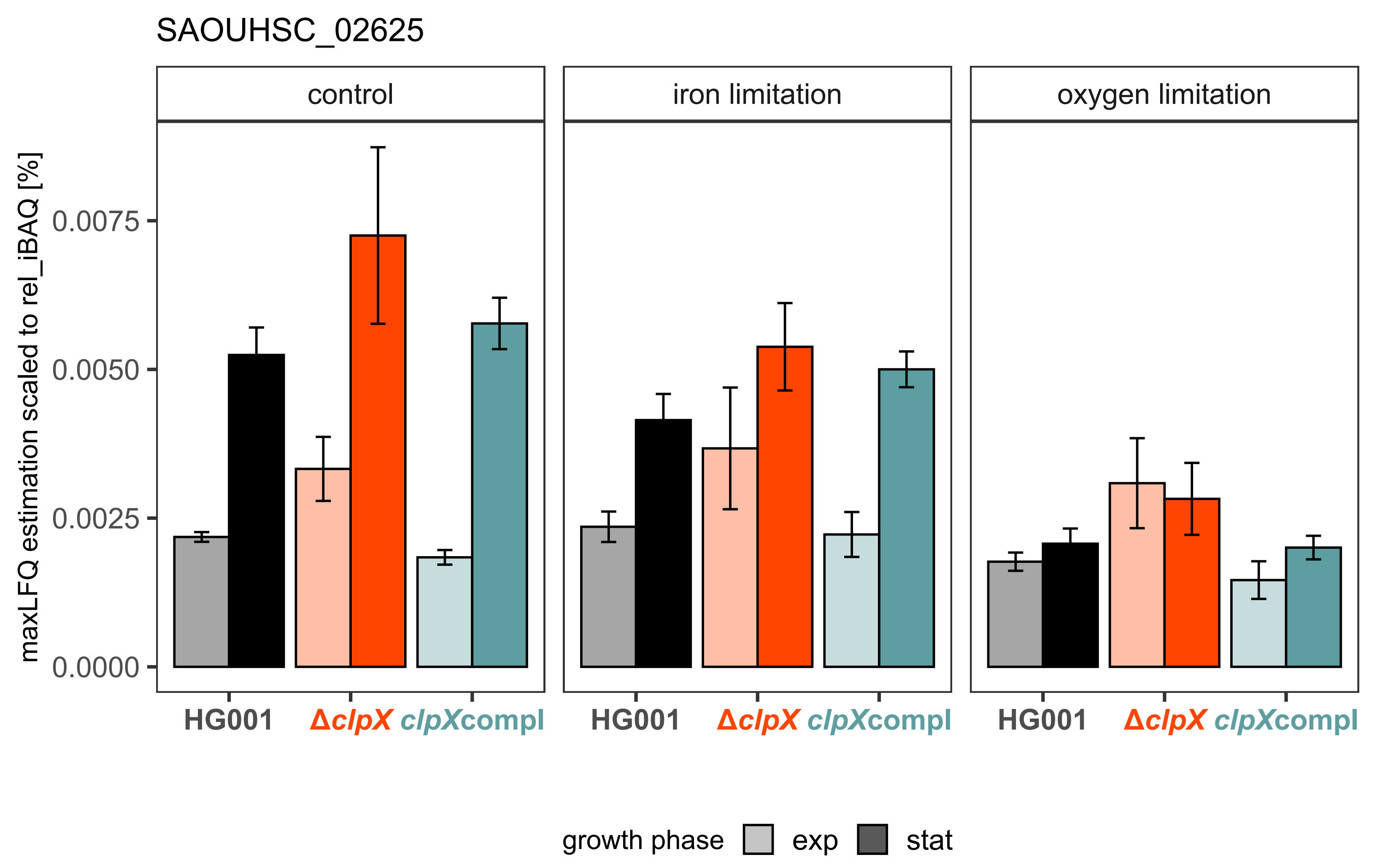
SAOUHSC_02625
control
iron limitation
oxygen limitation
maxLFQ estimation scaled to rel_iBAQ [%]
0.0075
0.0050
0.0025
0.0000
HG001
ΔclpX
clpXcompl
growth phase
exp
stat

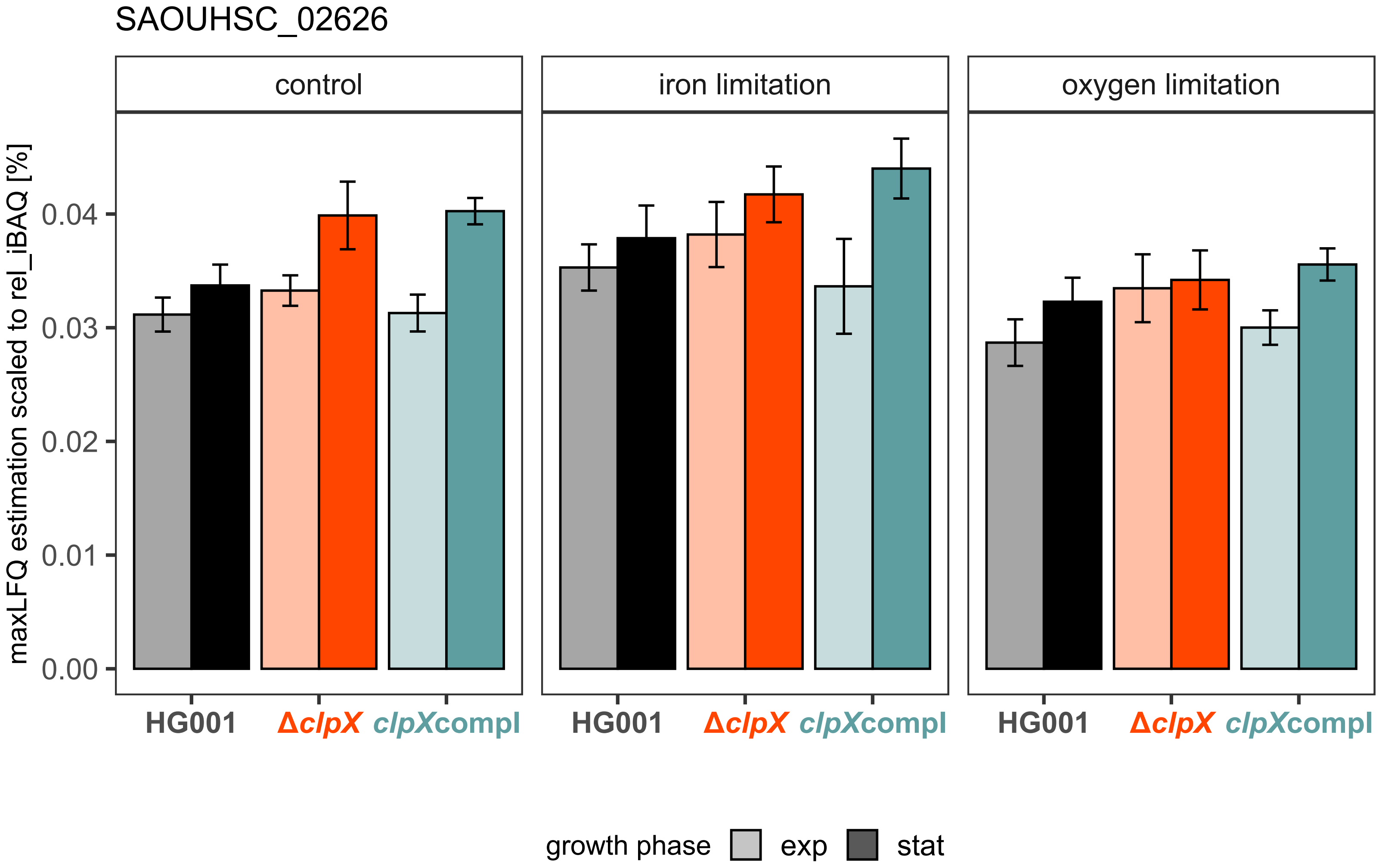
SAOUHSC_02626
control
iron limitation
oxygen limitation
maxLFQ estimation scaled to rel_iBAQ [%]
0.00
0.01
0.02
0.03
0.04
HG001
ΔclpX
clpXcompl
growth phase
exp
stat

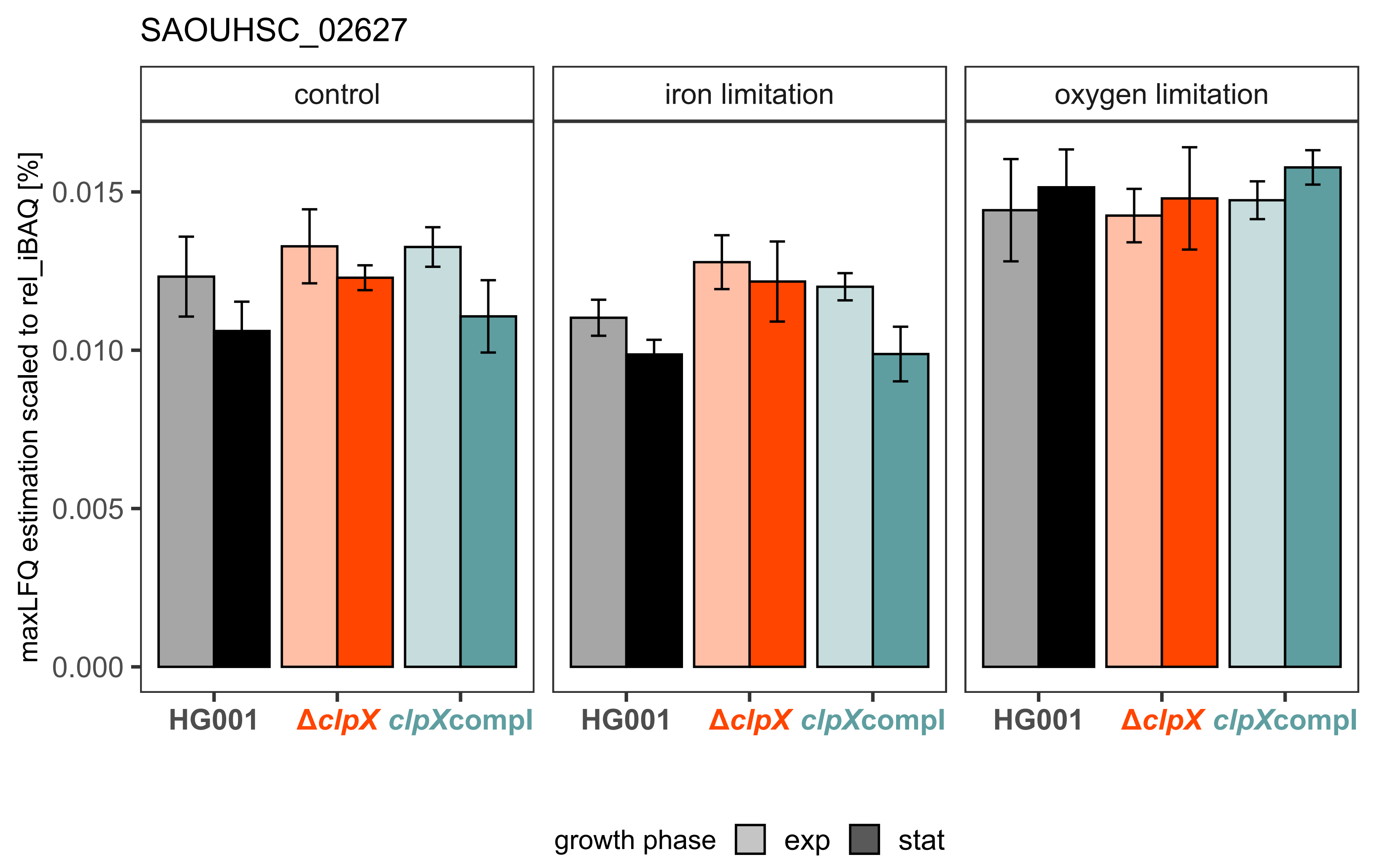

SAOUHSC_02627
control
iron limitation
oxygen limitation
maxLFQ estimation scaled to rel_iBAQ [%]
0.015
0.010
0.005
0.000
HG001
ΔclpX
clpXcompl
growth phase
exp
stat

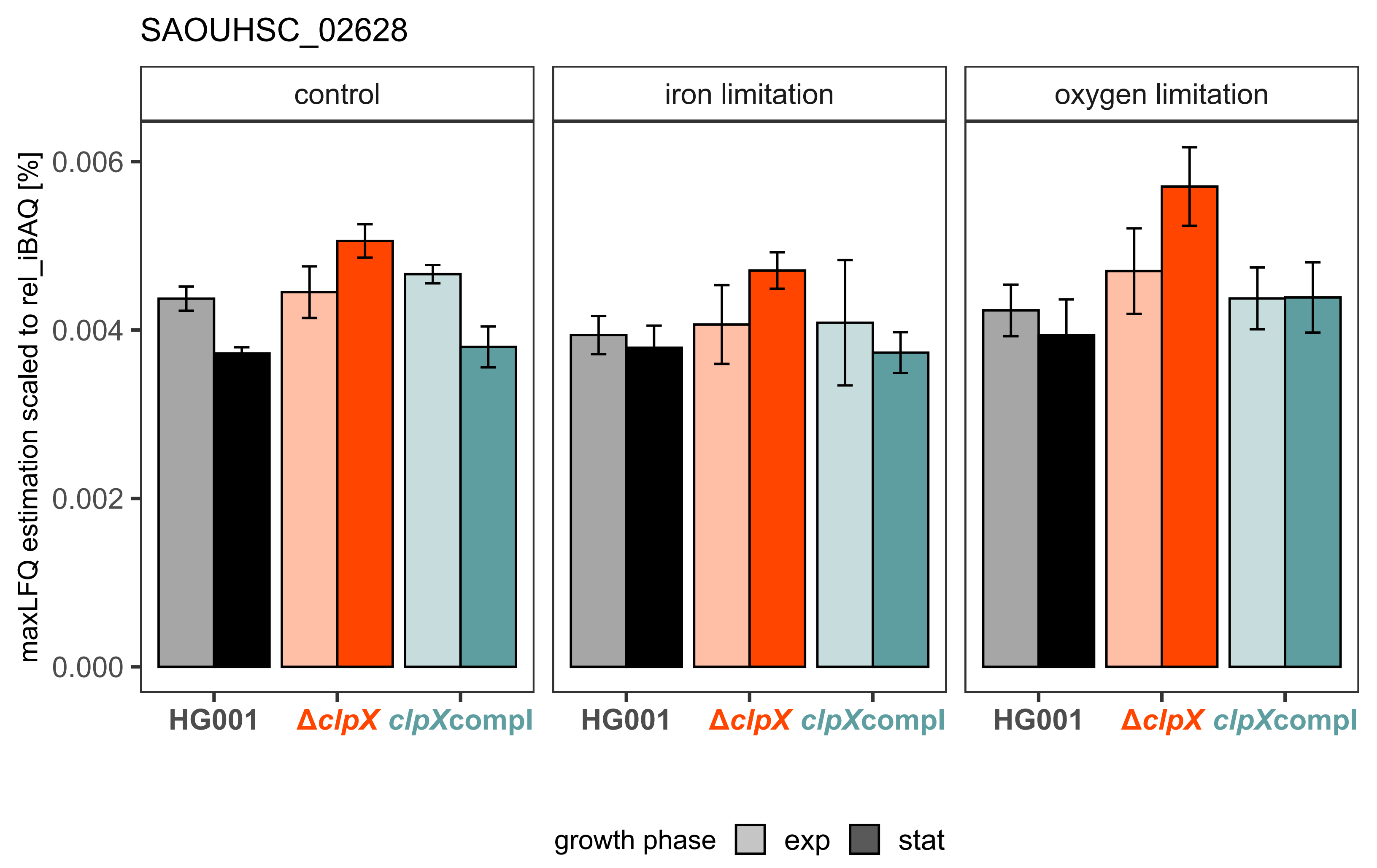
SAOUHSC_02628
control
iron limitation
oxygen limitation
maxLFQ estimation scaled to rel_iBAQ [%]
0.006
0.004
0.002
0.000
HG001
ΔclpX
clpXcompl
growth phase
exp
stat

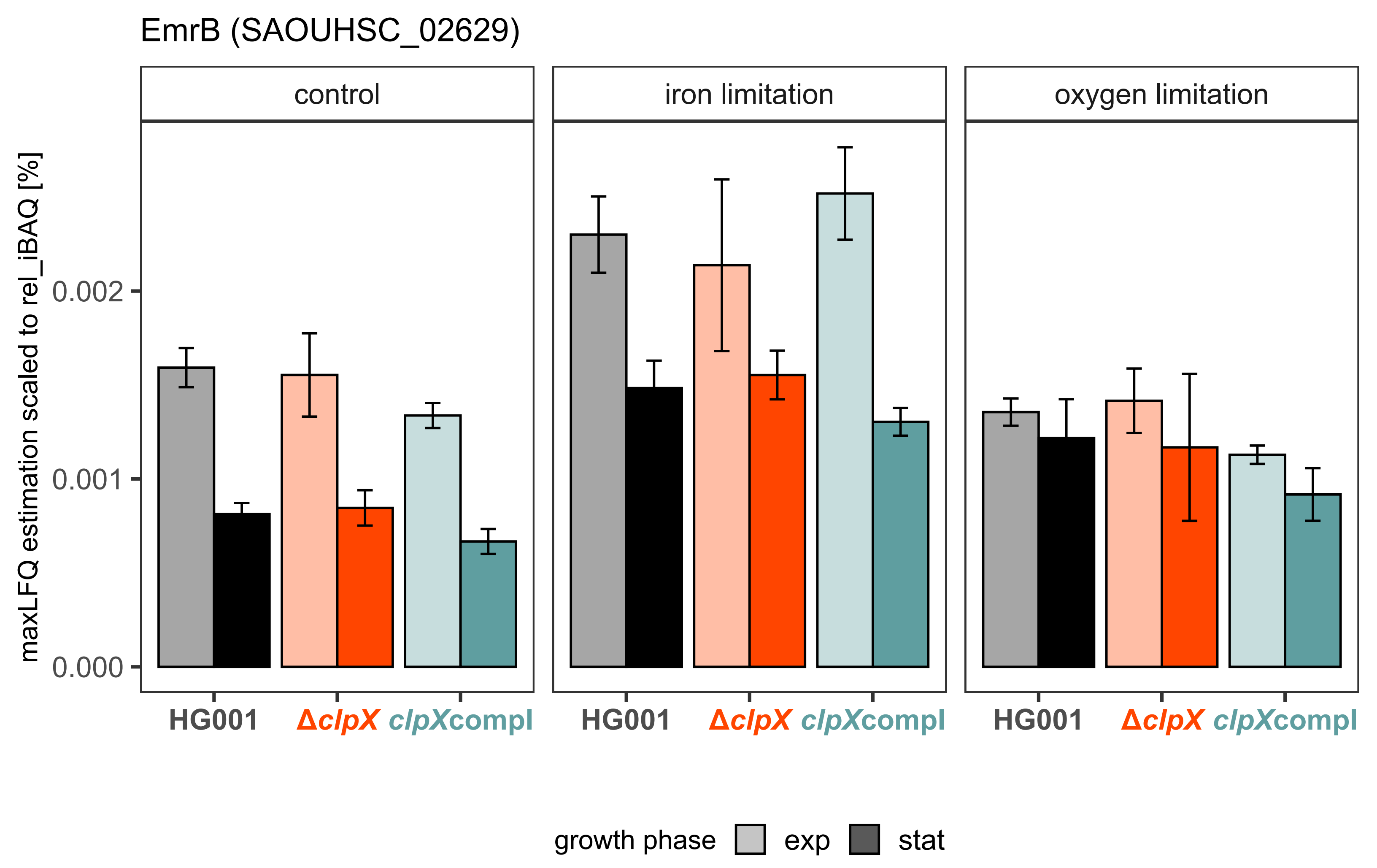
EmrB (SAOUHSC_02629)
control
iron limitation
oxygen limitation
maxLFQ estimation scaled to rel_iBAQ [%]
0.002
0.001
0.000
HG001
ΔclpX
clpXcompl
growth phase
exp
stat

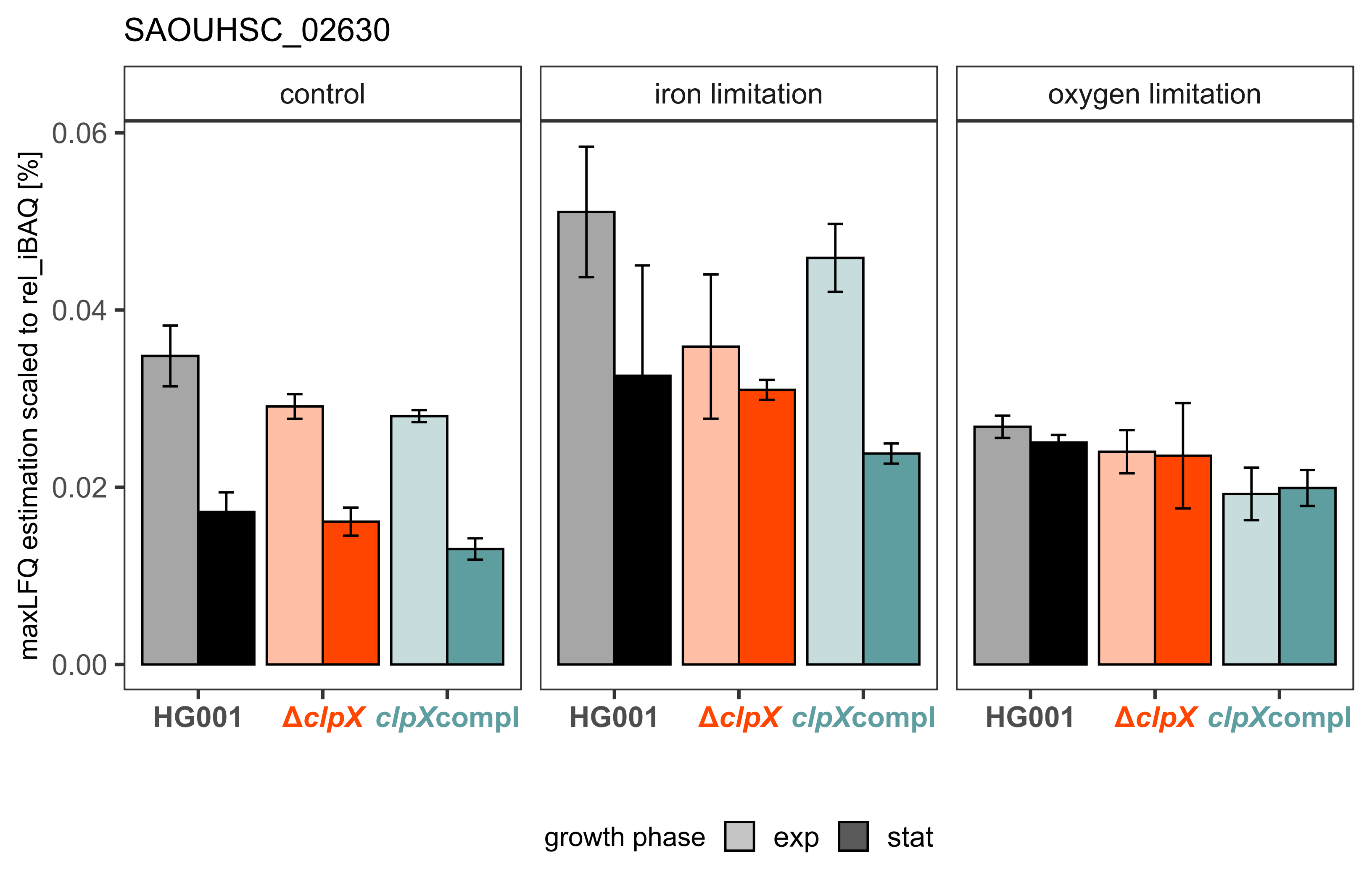

SAOUHSC_02630
control
iron limitation
oxygen limitation
maxLFQ estimation scaled to rel_iBAQ [%]
0.06
0.04
0.02
0.00
HG001
ΔclpX
clpXcompl
growth phase
exp
stat

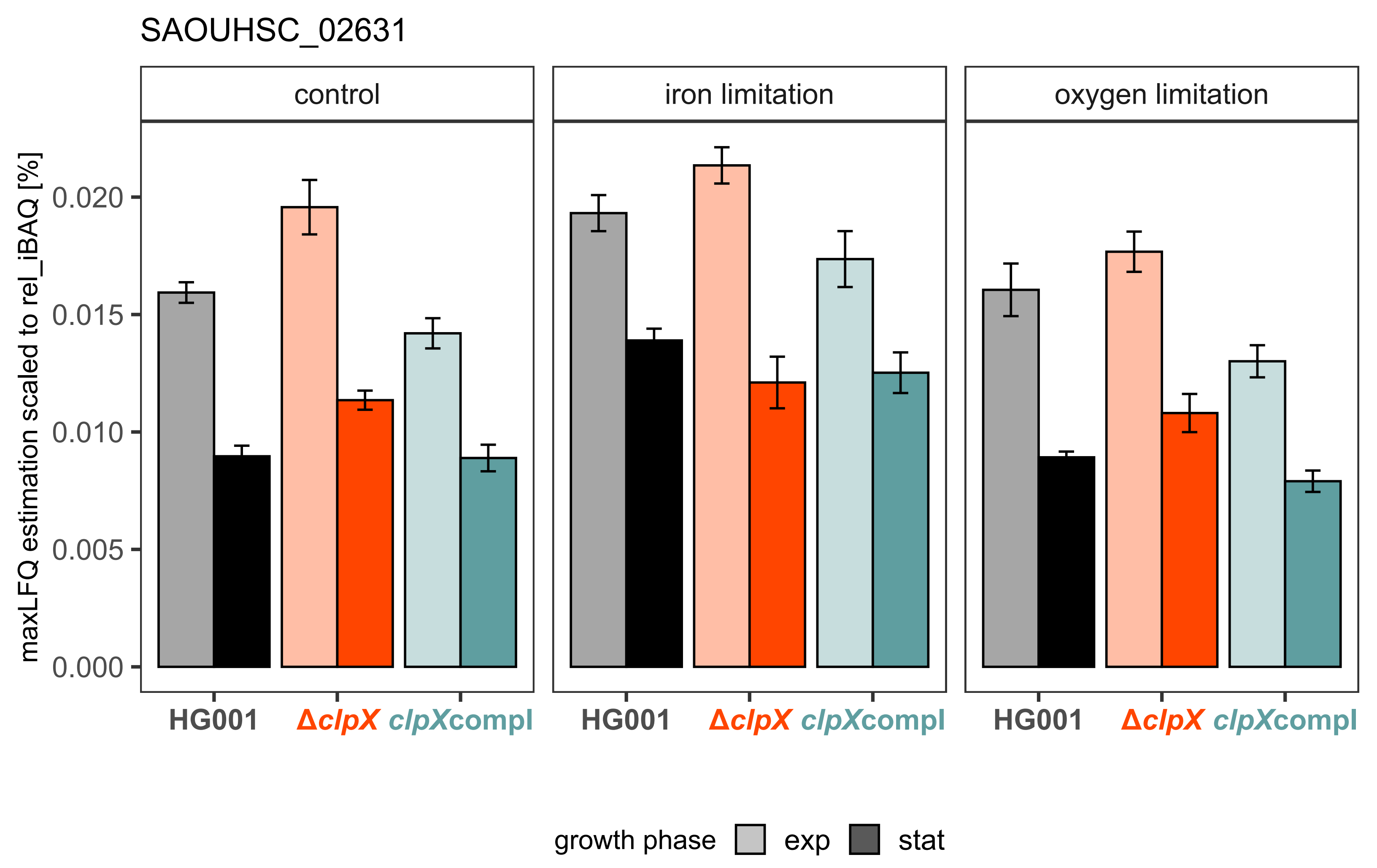
SAOUHSC_02631
control
iron limitation
oxygen limitation
maxLFQ estimation scaled to rel_iBAQ [%]
0.020
0.015
0.010
0.005
0.000
HG001
ΔclpX
clpXcompl
growth phase
exp
stat

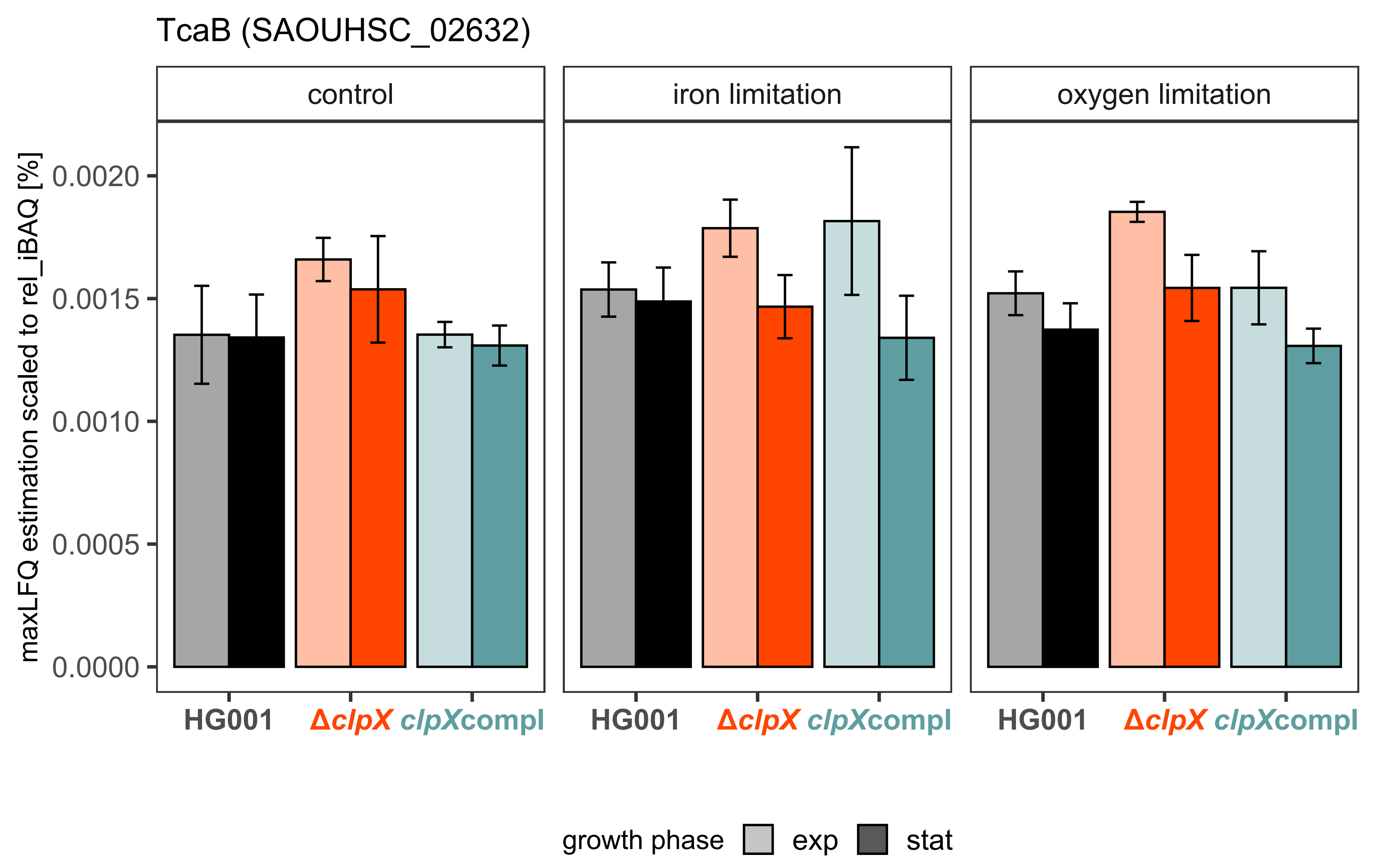
TcaB (SAOUHSC_02632)
control
iron limitation
oxygen limitation
maxLFQ estimation scaled to rel_iBAQ [%]
0.0020
0.0015
0.0010
0.0005
0.0000
HG001
ΔclpX
clpXcompl
growth phase
exp
stat

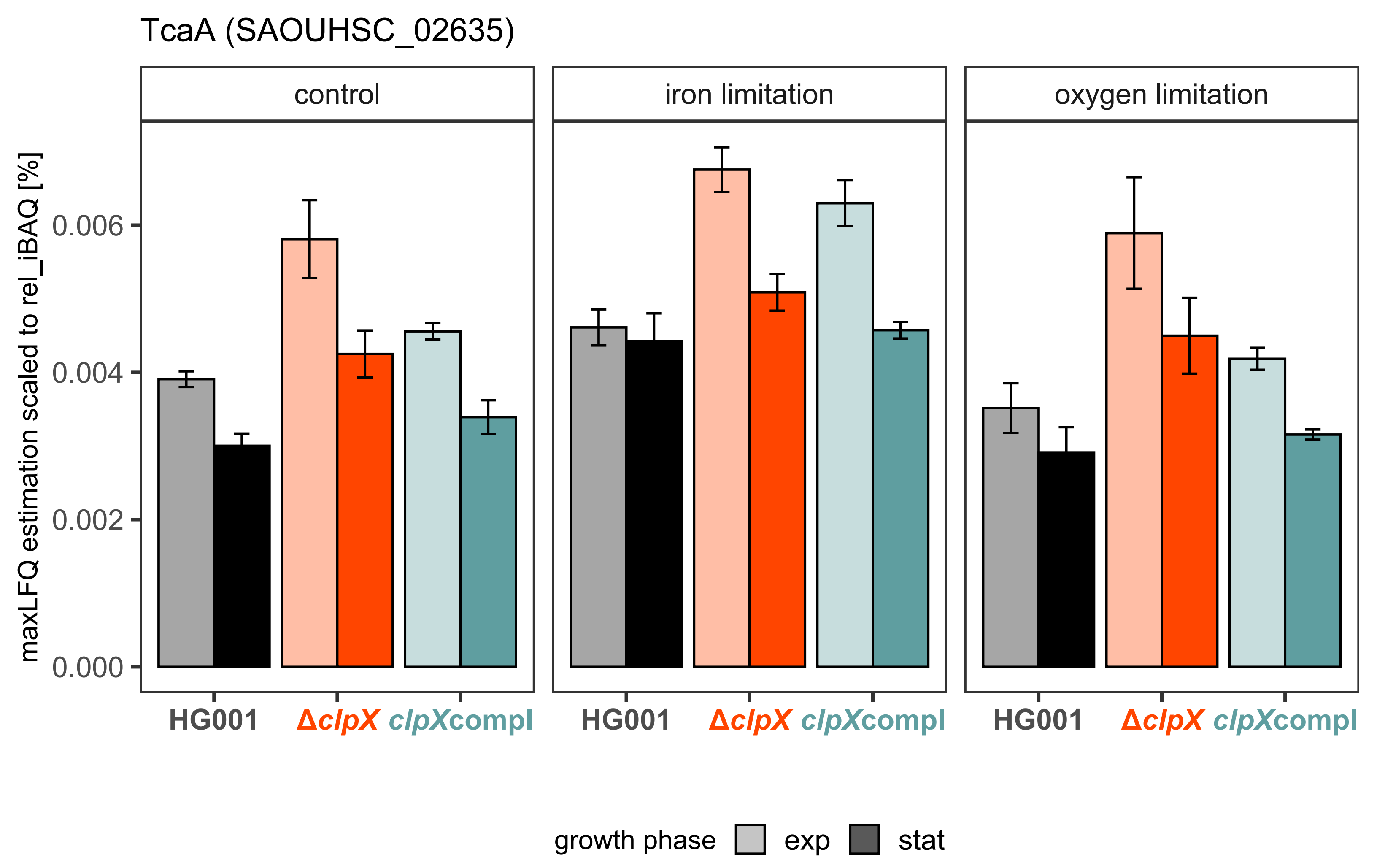
TcaA (SAOUHSC_02635)
control
iron limitation
oxygen limitation
maxLFQ estimation scaled to rel_iBAQ [%]
0.000
0.002
0.004
0.006
HG001
ΔclpX
clpXcompl
growth phase
exp
stat

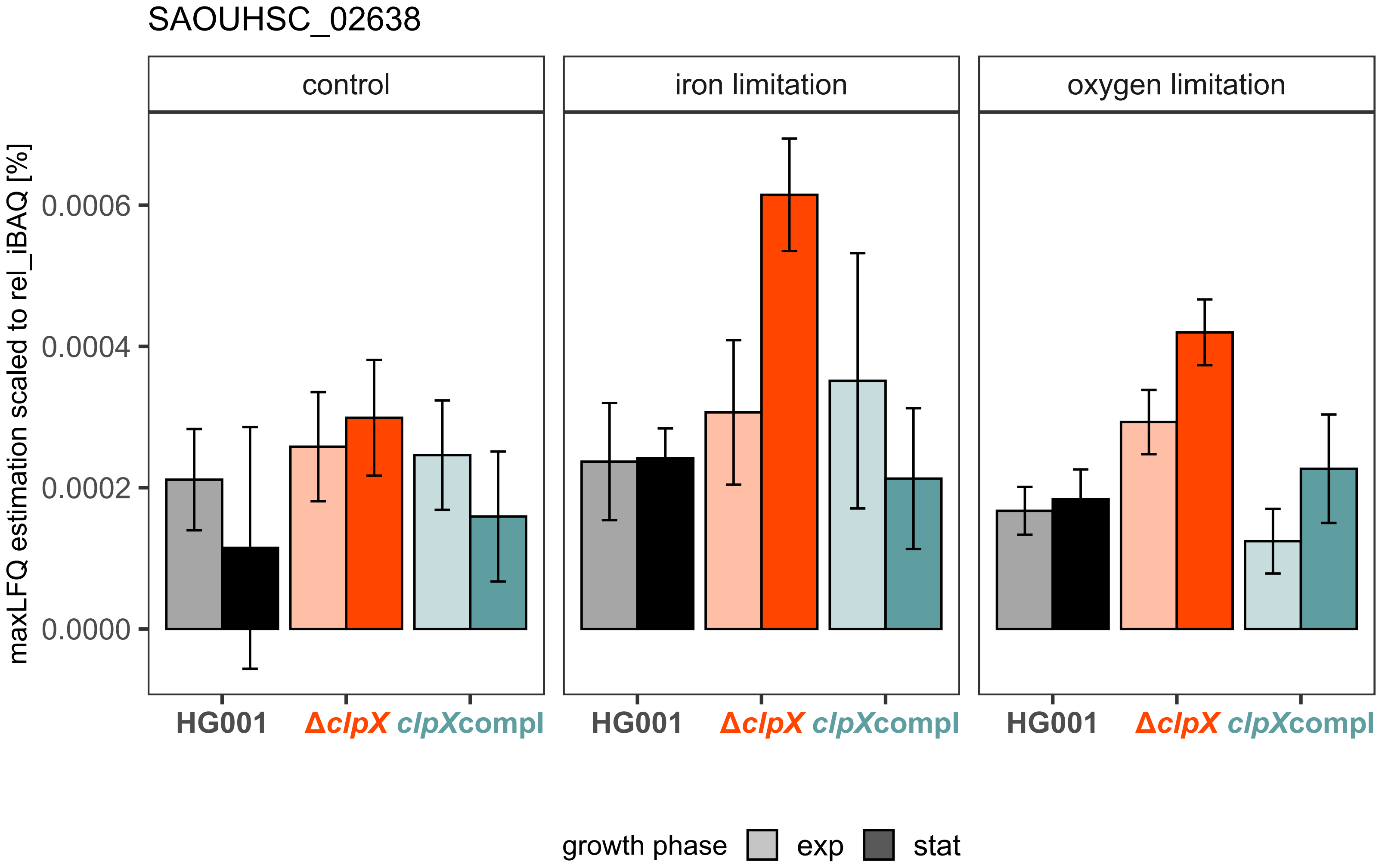
SAOUHSC_02638
control
iron limitation
oxygen limitation
maxLFQ estimation scaled to rel_iBAQ [%]
0.0006
0.0004
0.0002
0.0000
HG001
ΔclpX
clpXcompl
growth phase
exp
stat

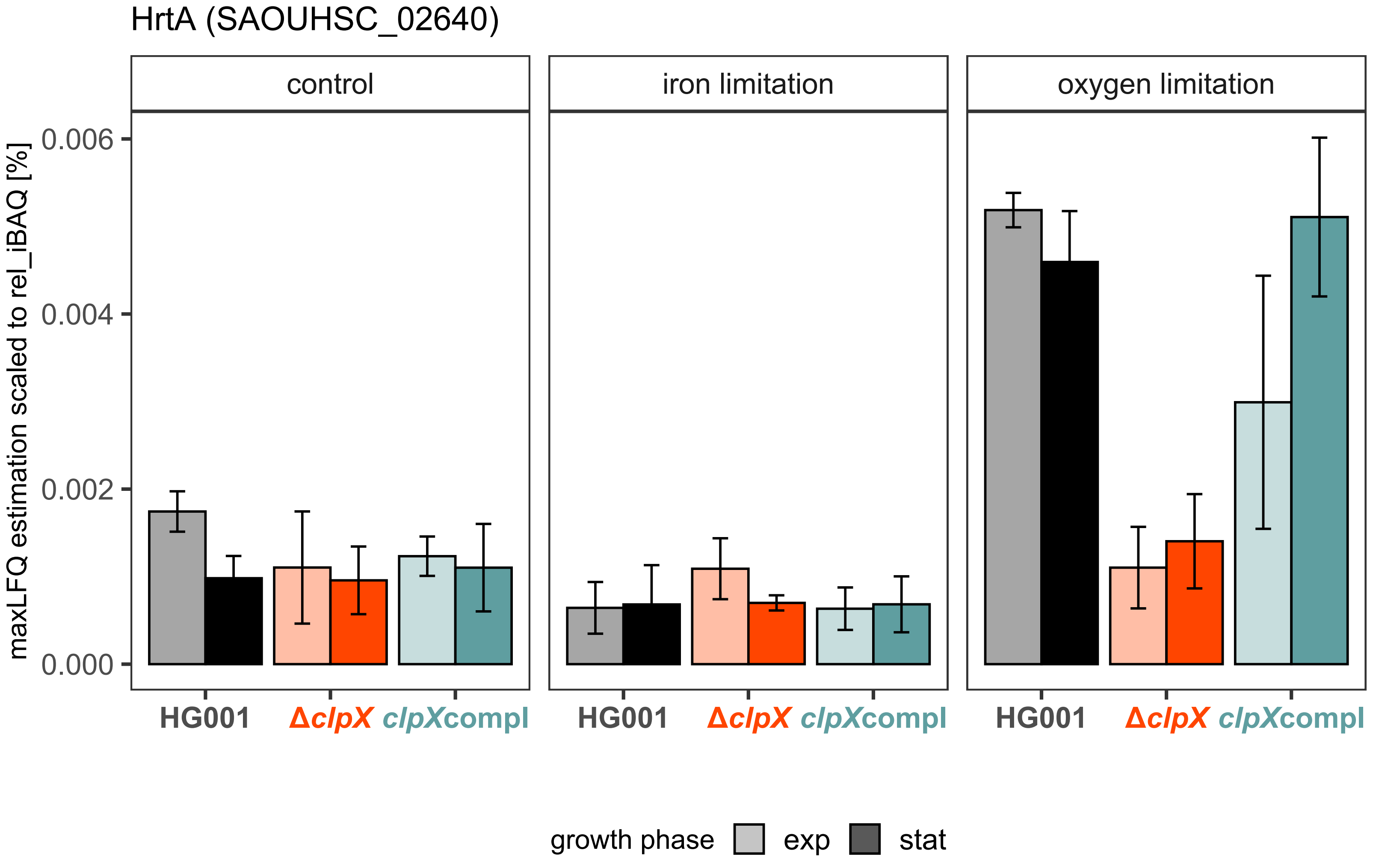

HrtA (SAOUHSC_02640)
control
iron limitation
oxygen limitation
maxLFQ estimation scaled to rel_iBAQ [%]
0.006
0.004
0.002
0.000
HG001
ΔclpX
clpXcompl
growth phase
exp
stat

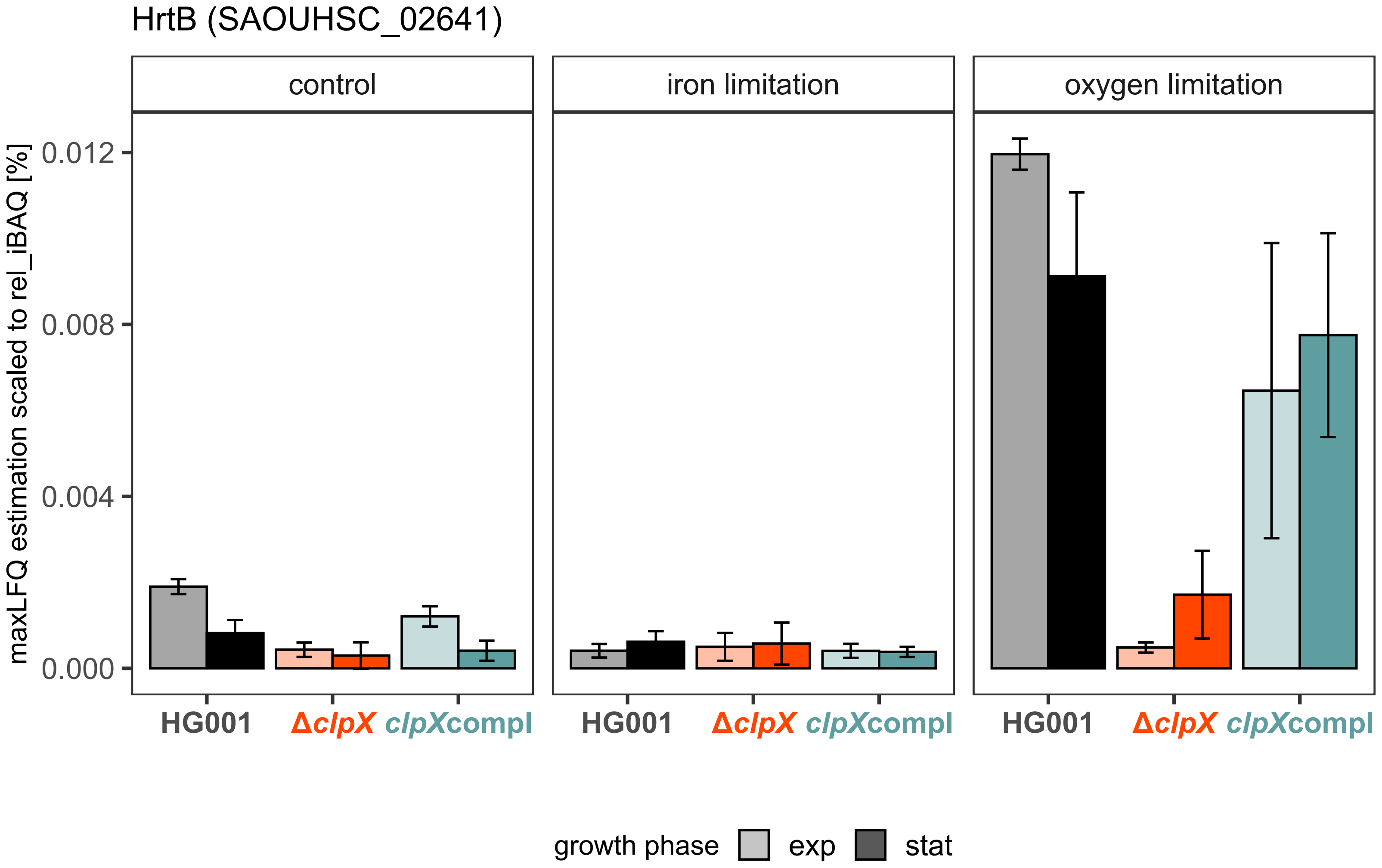
HrtB (SAOUHSC_02641)
control
iron limitation
oxygen limitation
maxLFQ estimation scaled to rel_iBAQ [%]
0.012
0.008
0.004
0.000
HG001
ΔclpX
clpXcompl
growth phase
exp
stat

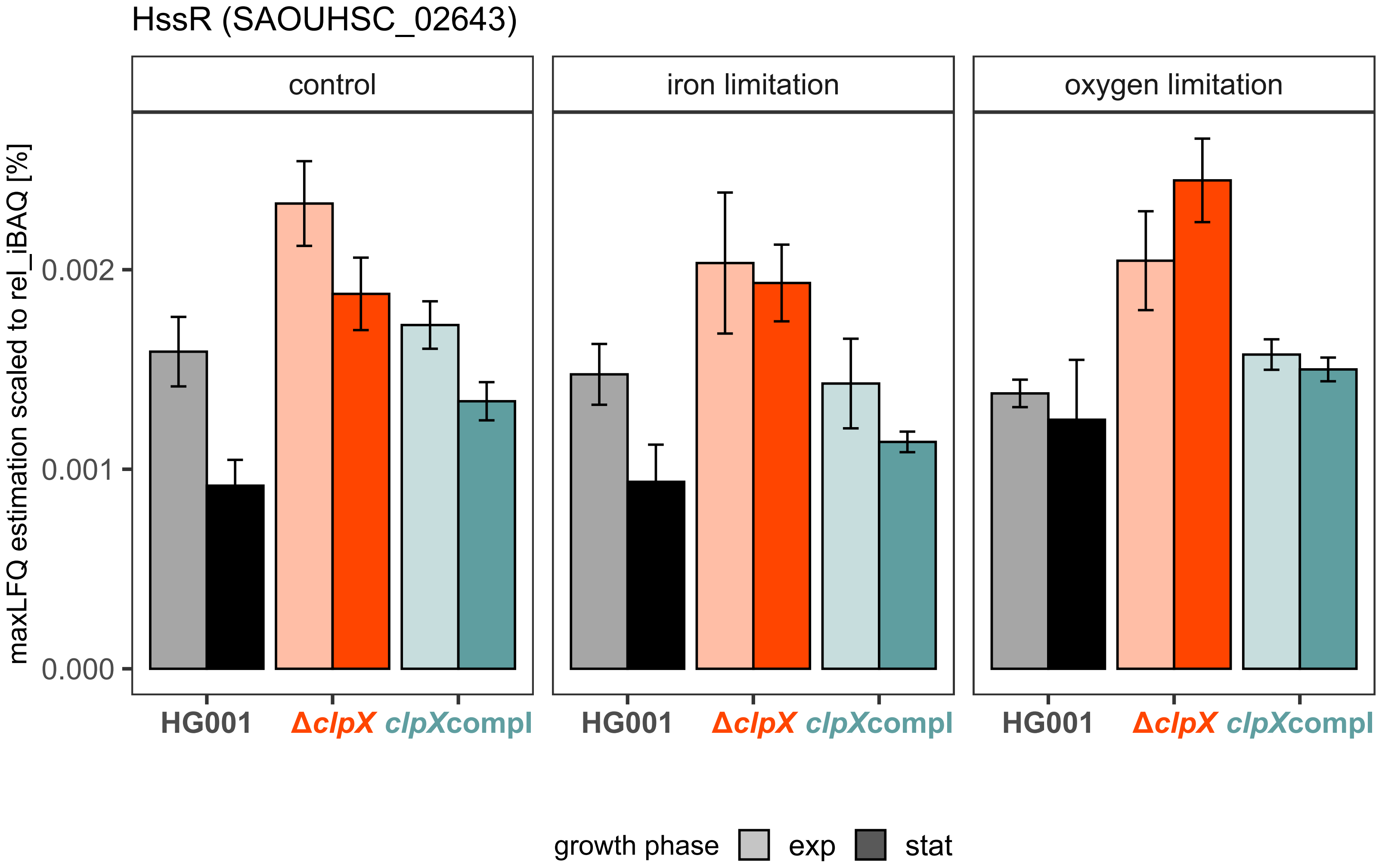
HssR (SAOUHSC_02643)
control
iron limitation
oxygen limitation
maxLFQ estimation scaled to rel_iBAQ [%]
0.002
0.001
0.000
HG001
ΔclpX
clpXcompl
growth phase
exp
stat

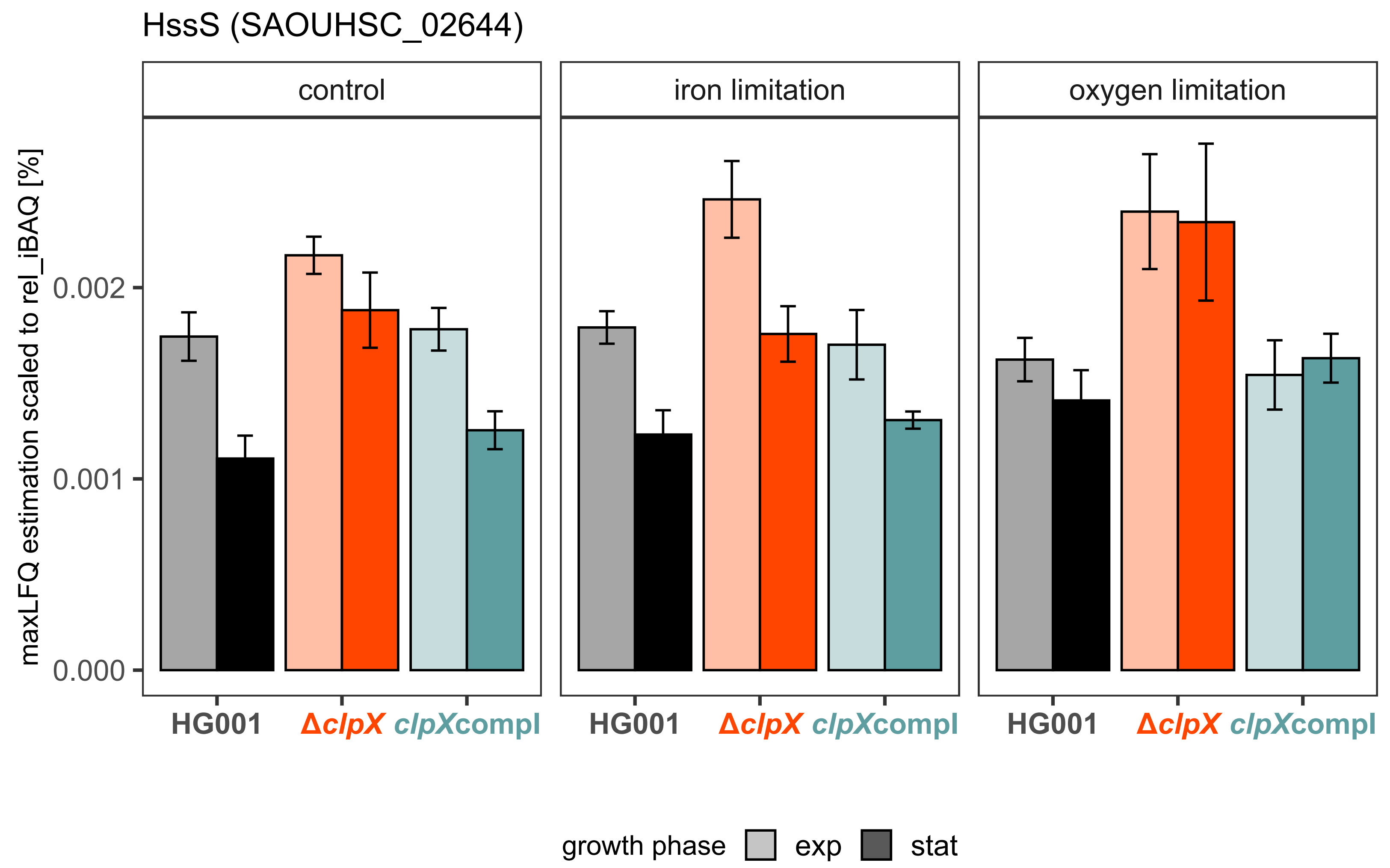

HssS (SAOUHSC_02644)
control
iron limitation
oxygen limitation
maxLFQ estimation scaled to rel_iBAQ [%]
0.002
0.001
0.000
HG001
ΔclpX
clpXcompl
growth phase
exp
stat

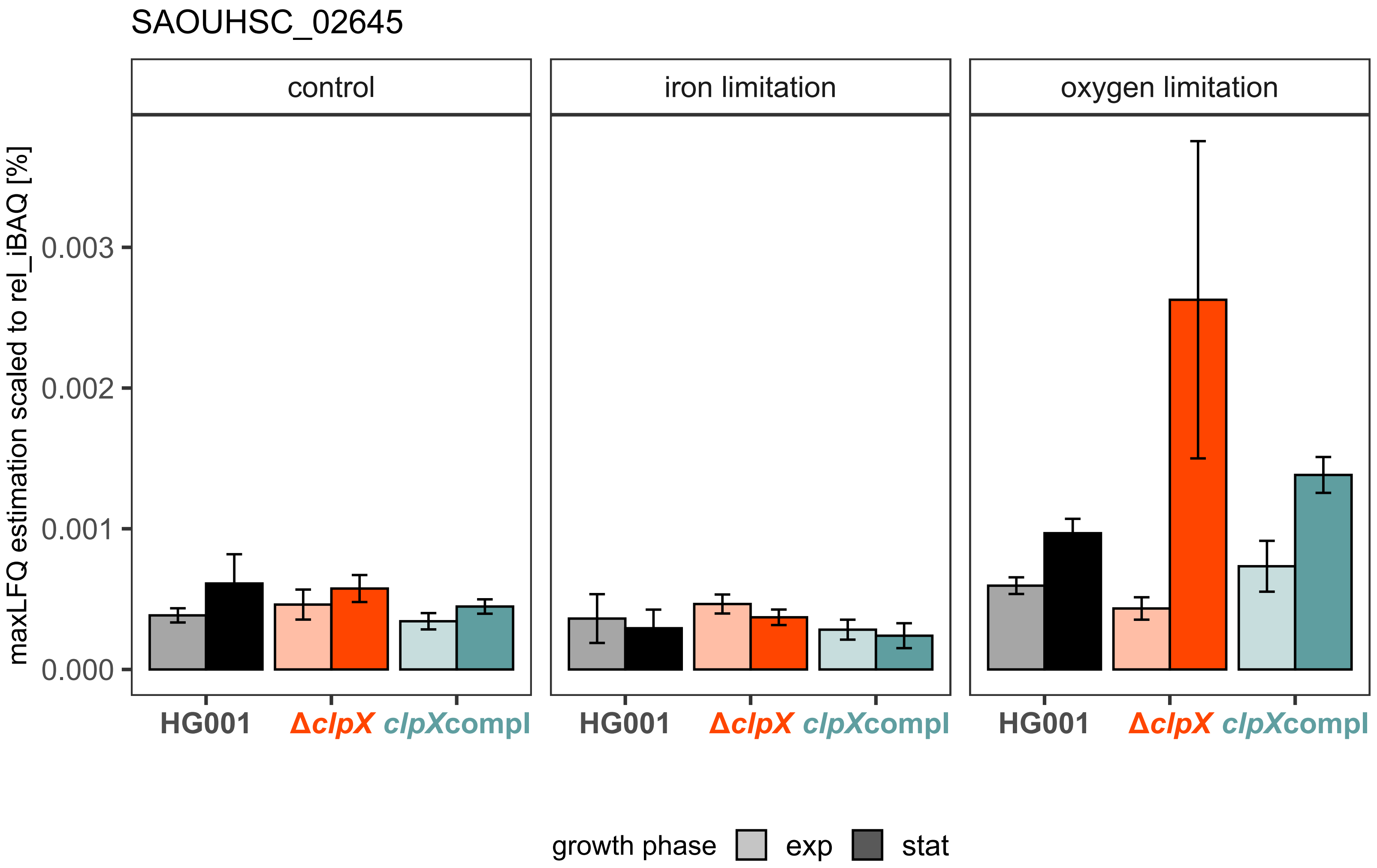
SAOUHSC_02645
control
iron limitation
oxygen limitation
maxLFQ estimation scaled to rel_iBAQ [%]
0.003
0.002
0.001
0.000
HG001
ΔclpX
clpXcompl
growth phase
exp
stat

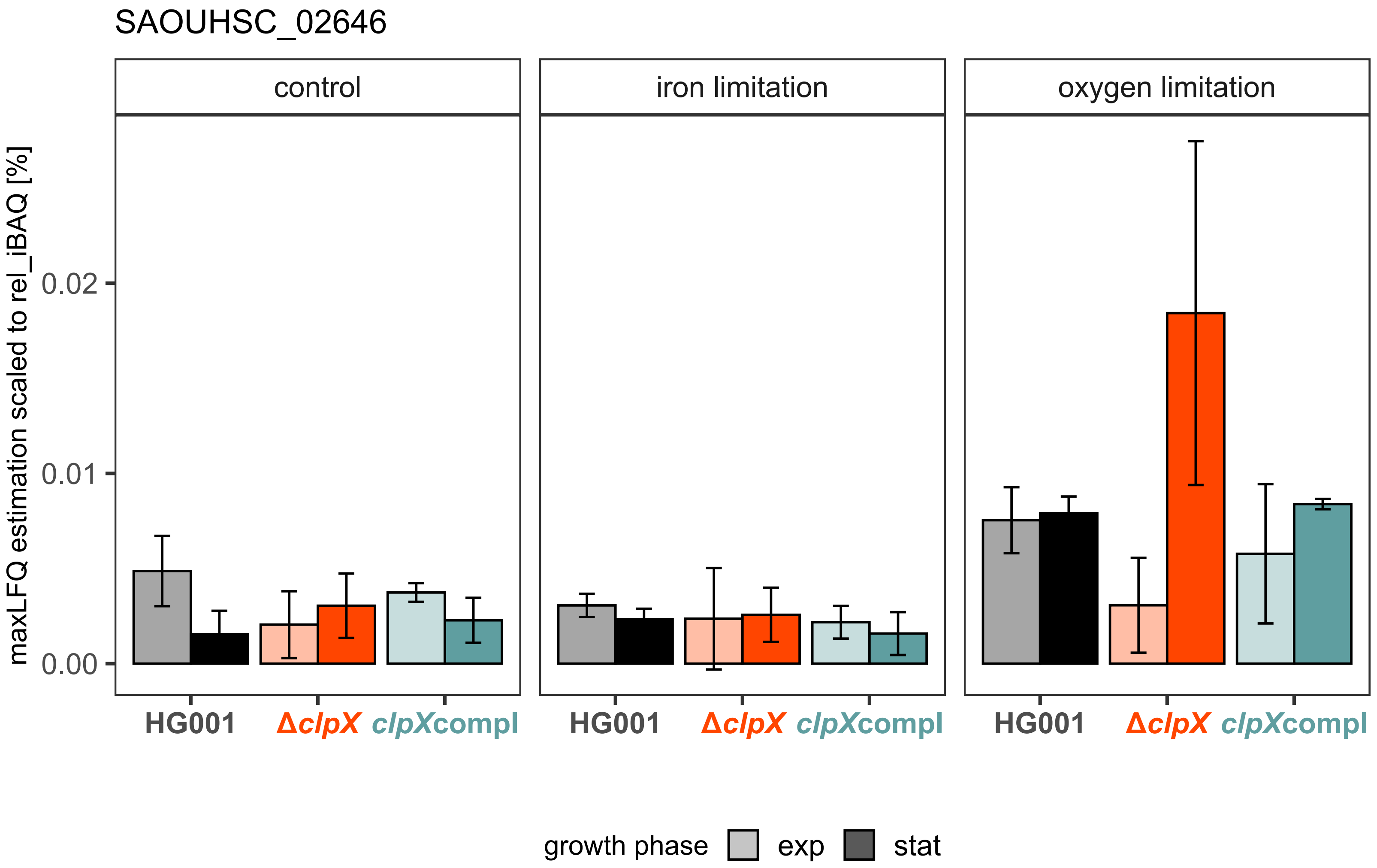
SAOUHSC_02646
control
iron limitation
oxygen limitation
maxLFQ estimation scaled to rel_iBAQ [%]
0.00
0.01
0.02
HG001
ΔclpX
clpXcompl
growth phase
exp
stat

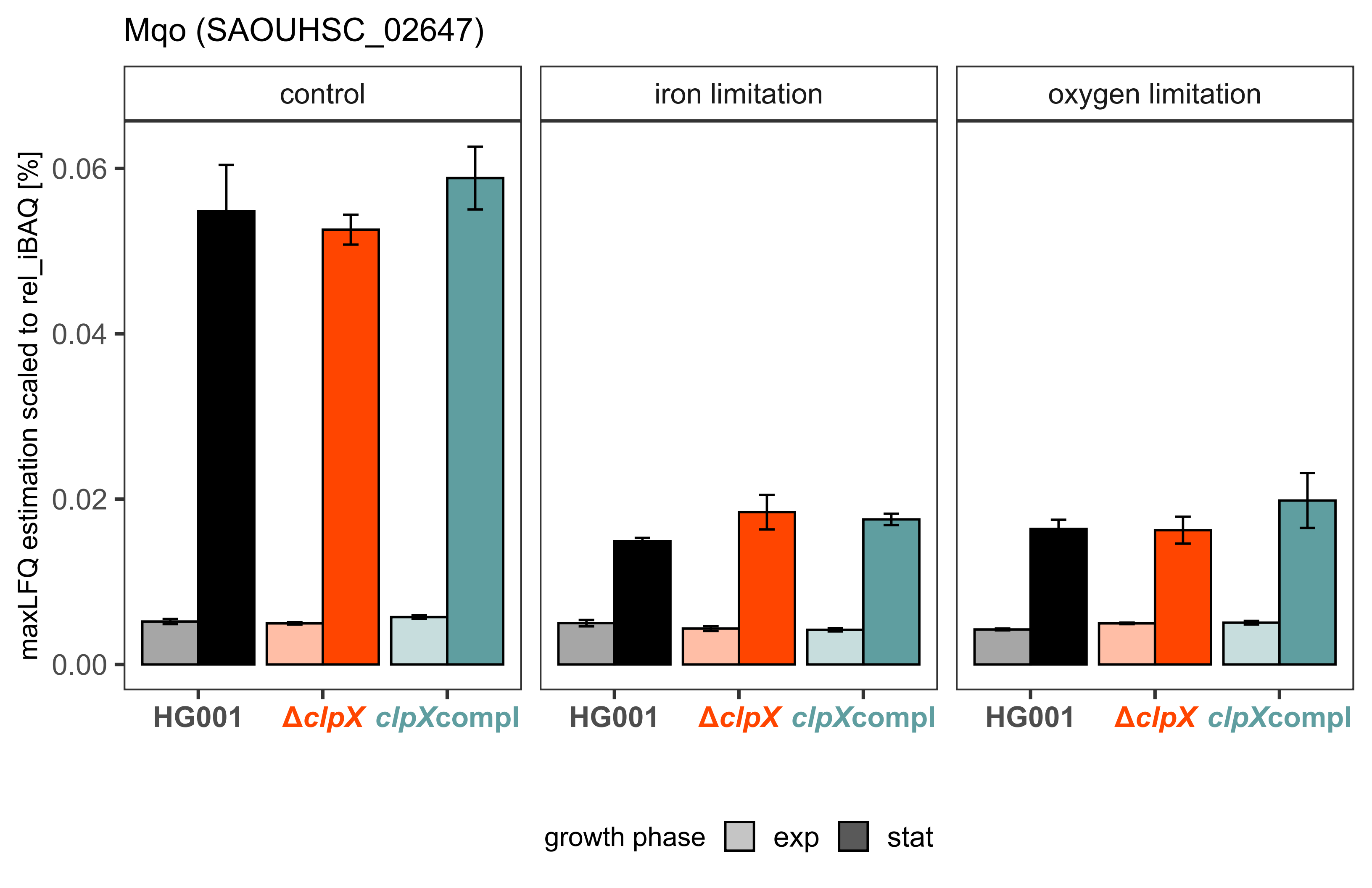

Mqo (SAOUHSC_02647)
control
iron limitation
oxygen limitation
maxLFQ estimation scaled to rel_iBAQ [%]
0.06
0.04
0.02
0.00
HG001
ΔclpX
clpXcompl
growth phase
exp
stat

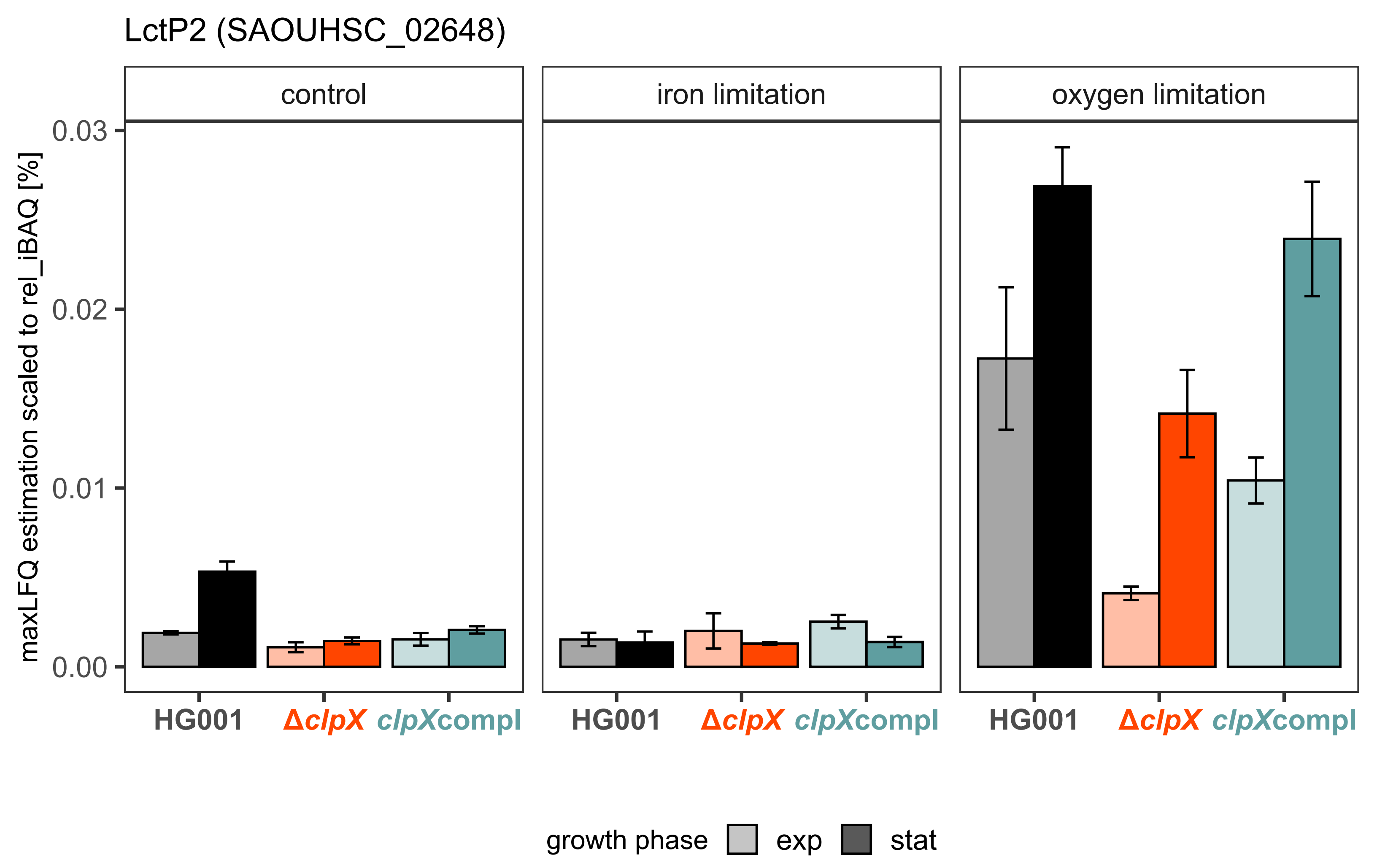

LctP2 (SAOUHSC_02648)
control
iron limitation
oxygen limitation
maxLFQ estimation scaled to rel_iBAQ [%]
0.03
0.02
0.01
0.00
HG001
ΔclpX
clpXcompl
growth phase
exp
stat

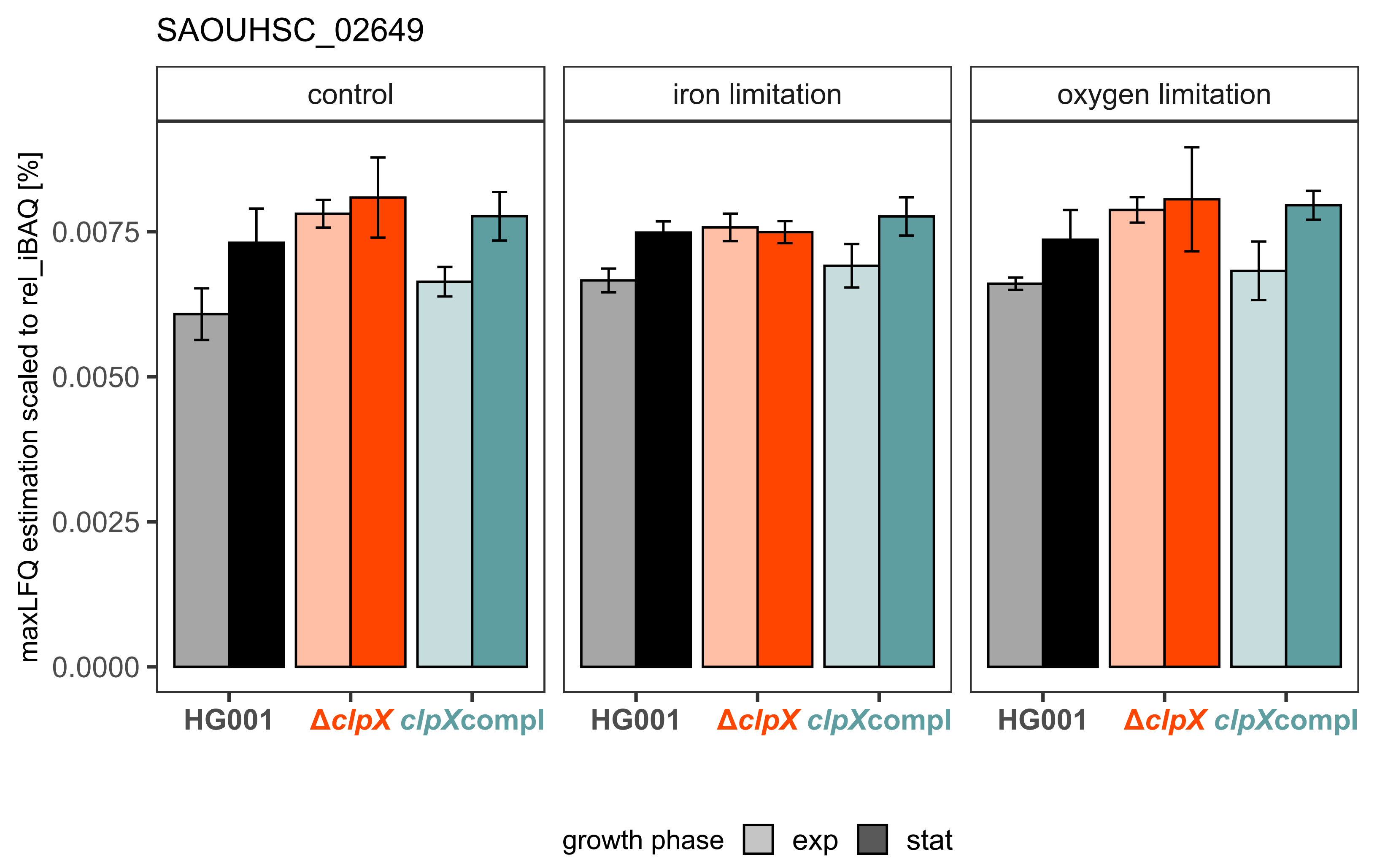
SAOUHSC_02649
control
iron limitation
oxygen limitation
maxLFQ estimation scaled to rel_iBAQ [%]
0.0075
0.0050
0.0025
0.0000
HG001
ΔclpX
clpXcompl
growth phase
exp
stat

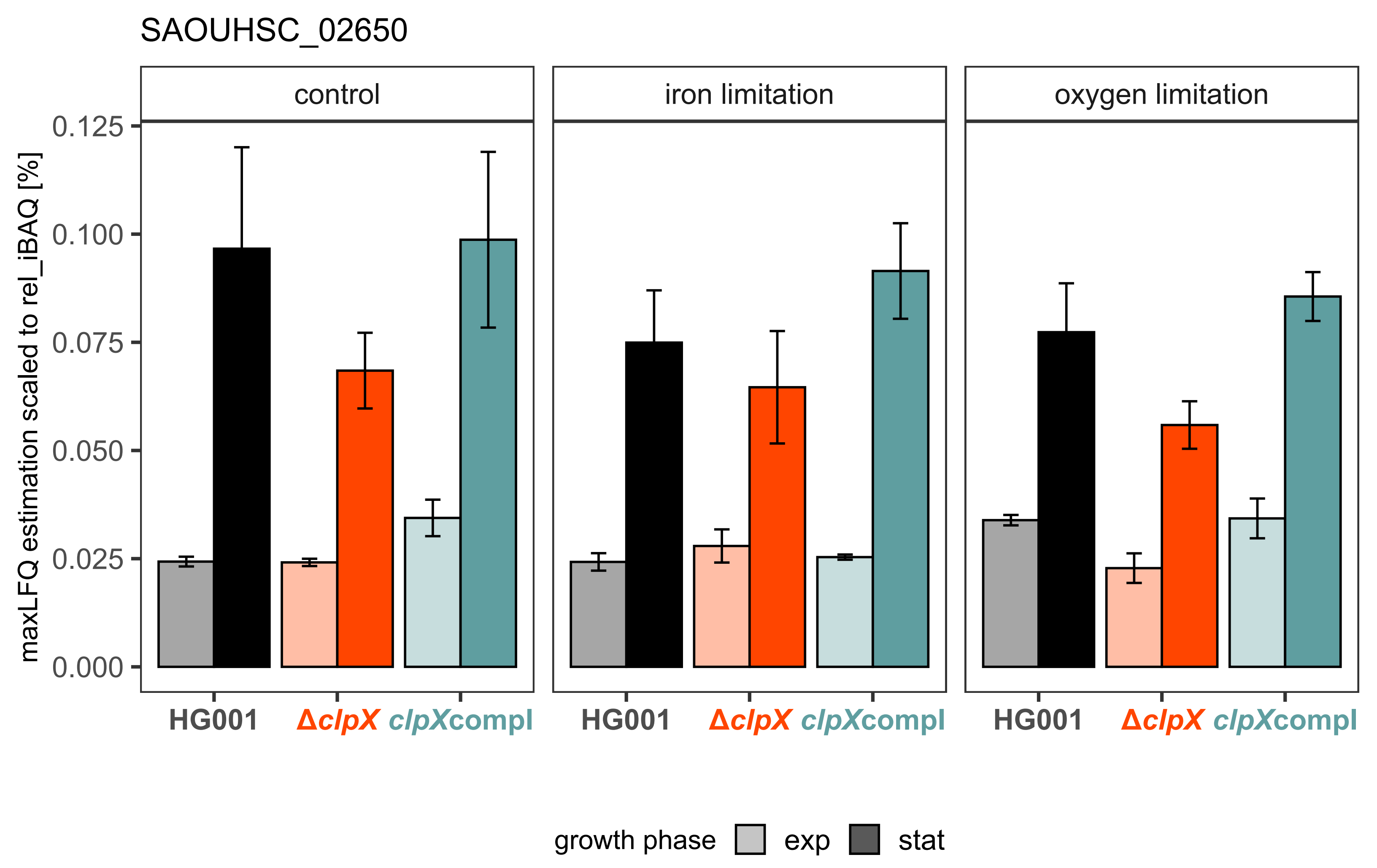
SAOUHSC_02650
control
iron limitation
oxygen limitation
maxLFQ estimation scaled to rel_iBAQ [%]
0.125
0.100
0.075
0.050
0.025
0.000
HG001
ΔclpX
clpXcompl
growth phase
exp
stat

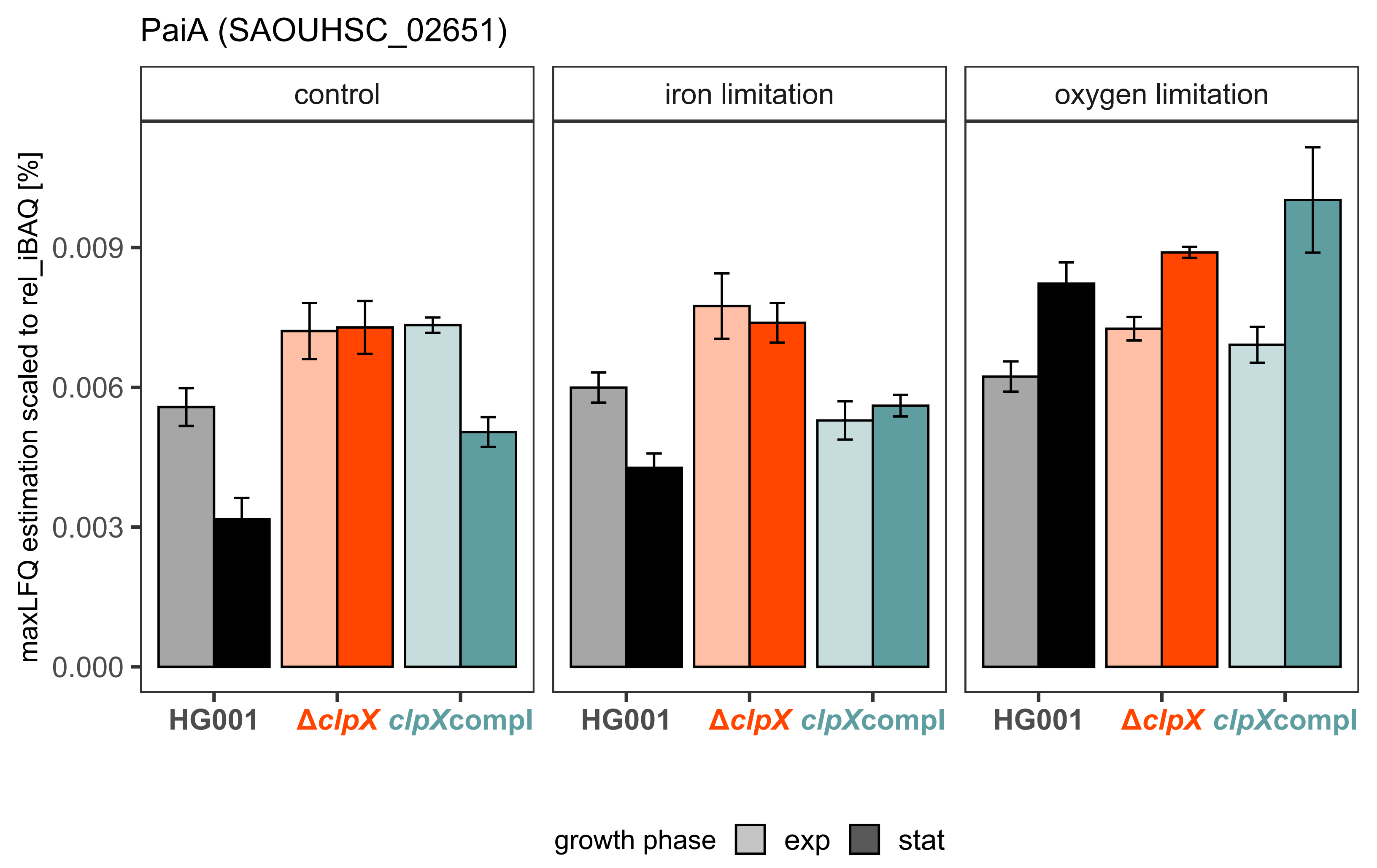
PaiA (SAOUHSC_02651)
control
iron limitation
oxygen limitation
maxLFQ estimation scaled to rel_iBAQ [%]
0.009
0.006
0.003
0.000
HG001
ΔclpX
clpXcompl
growth phase
exp
stat

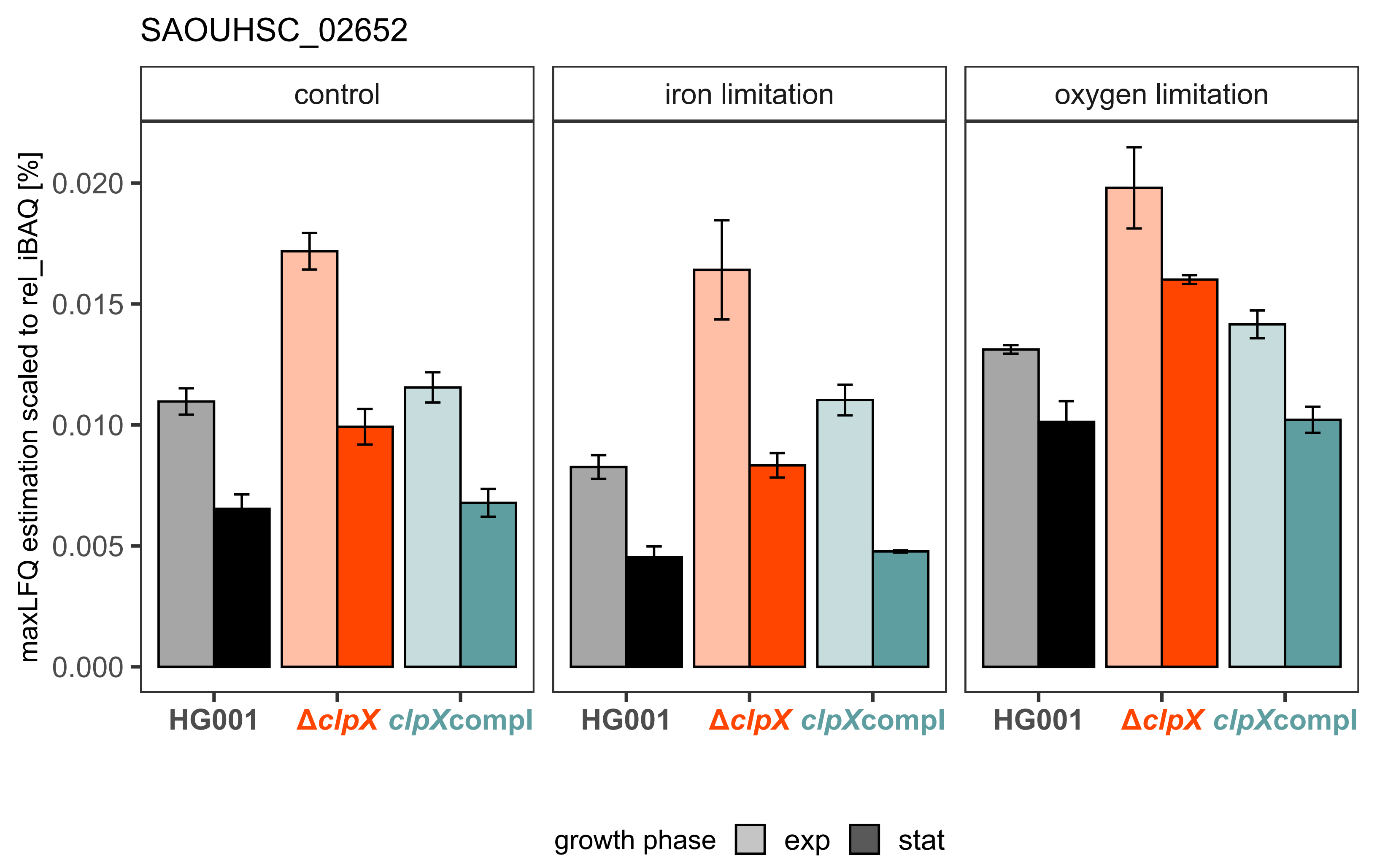

SAOUHSC_02652
control
iron limitation
oxygen limitation
maxLFQ estimation scaled to rel_iBAQ [%]
0.000
0.005
0.010
0.015
0.020
HG001
ΔclpX
clpXcompl
growth phase
exp
stat

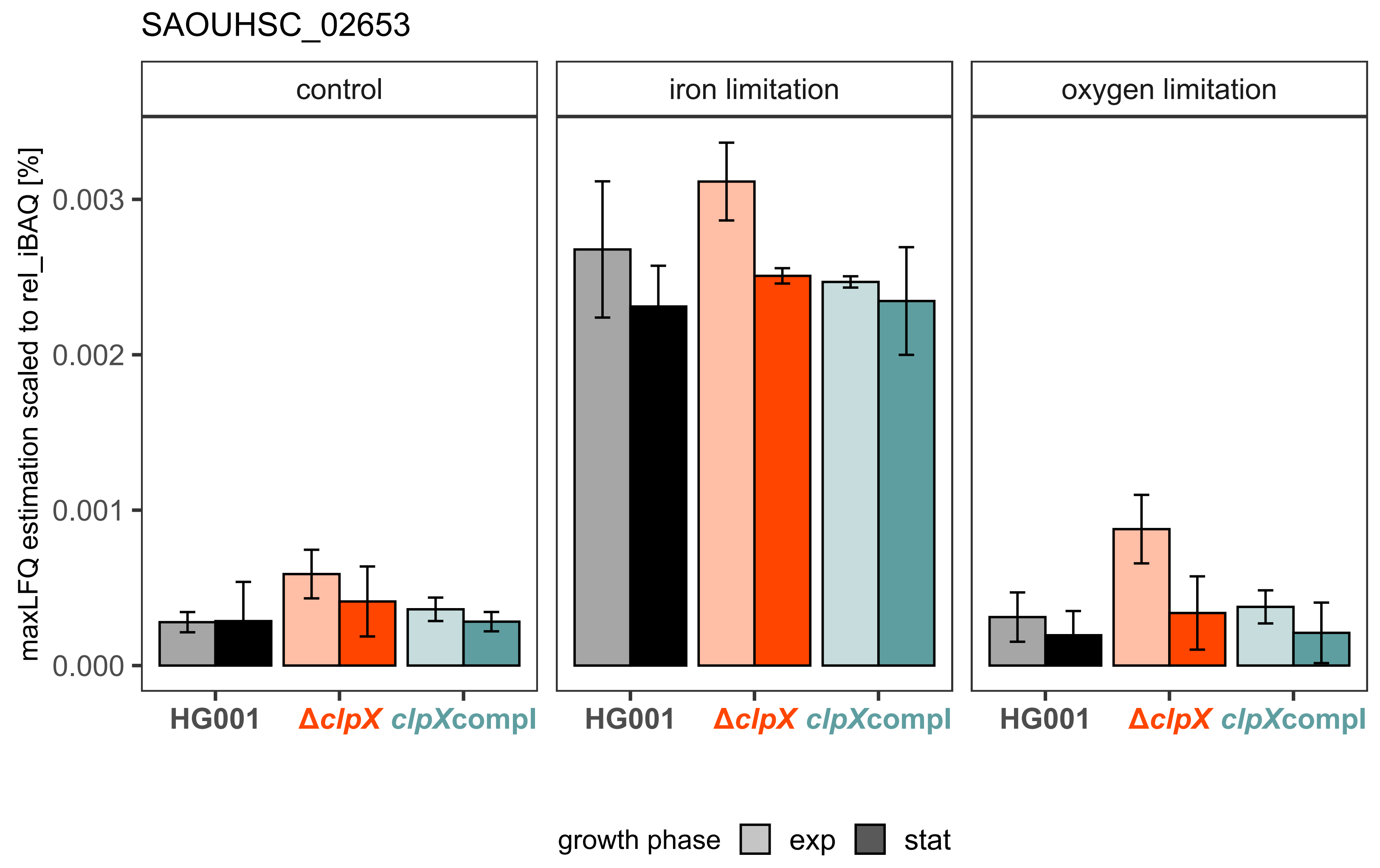
SAOUHSC_02653
control
iron limitation
oxygen limitation
maxLFQ estimation scaled to rel_iBAQ [%]
0.003
0.002
0.001
0.000
HG001
ΔclpX
clpXcompl
growth phase
exp
stat

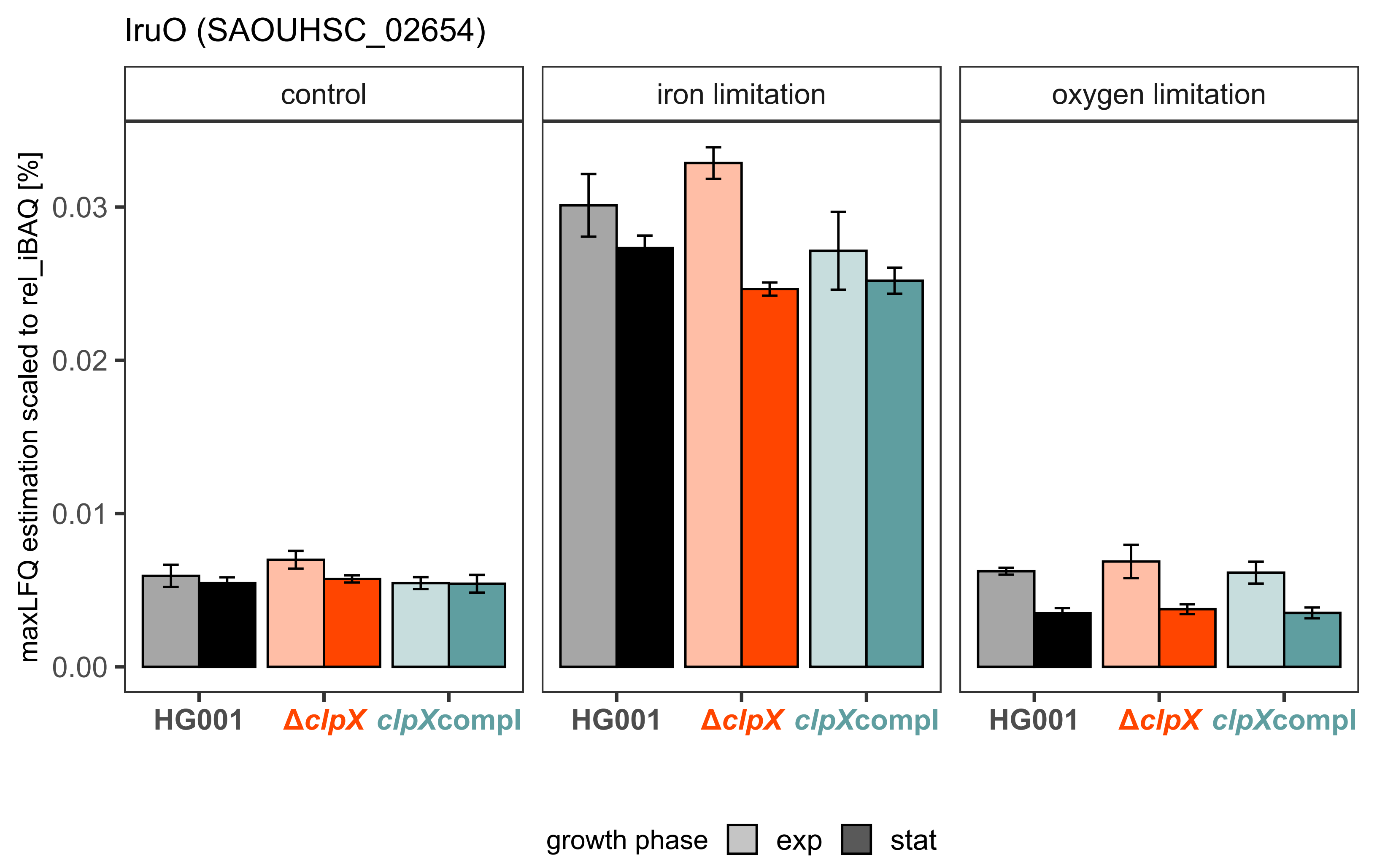

IruO (SAOUHSC_02654)
control
iron limitation
oxygen limitation
maxLFQ estimation scaled to rel_iBAQ [%]
0.00
0.01
0.02
0.03
HG001
ΔclpX
clpXcompl
growth phase
exp
stat

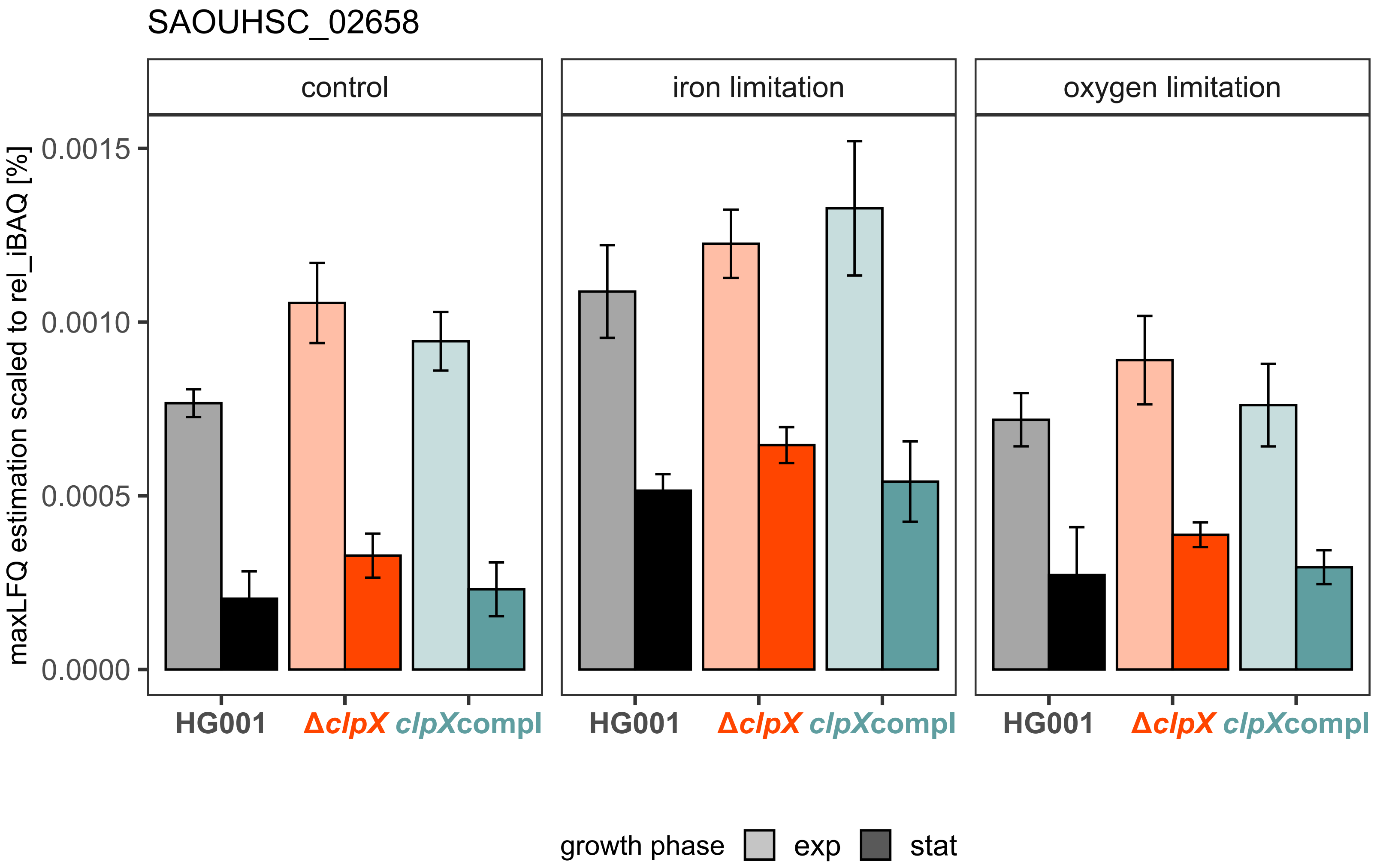
SAOUHSC_02658
control
iron limitation
oxygen limitation
maxLFQ estimation scaled to rel_iBAQ [%]
0.0015
0.0010
0.0005
0.0000
HG001
ΔclpX
clpXcompl
growth phase
exp
stat

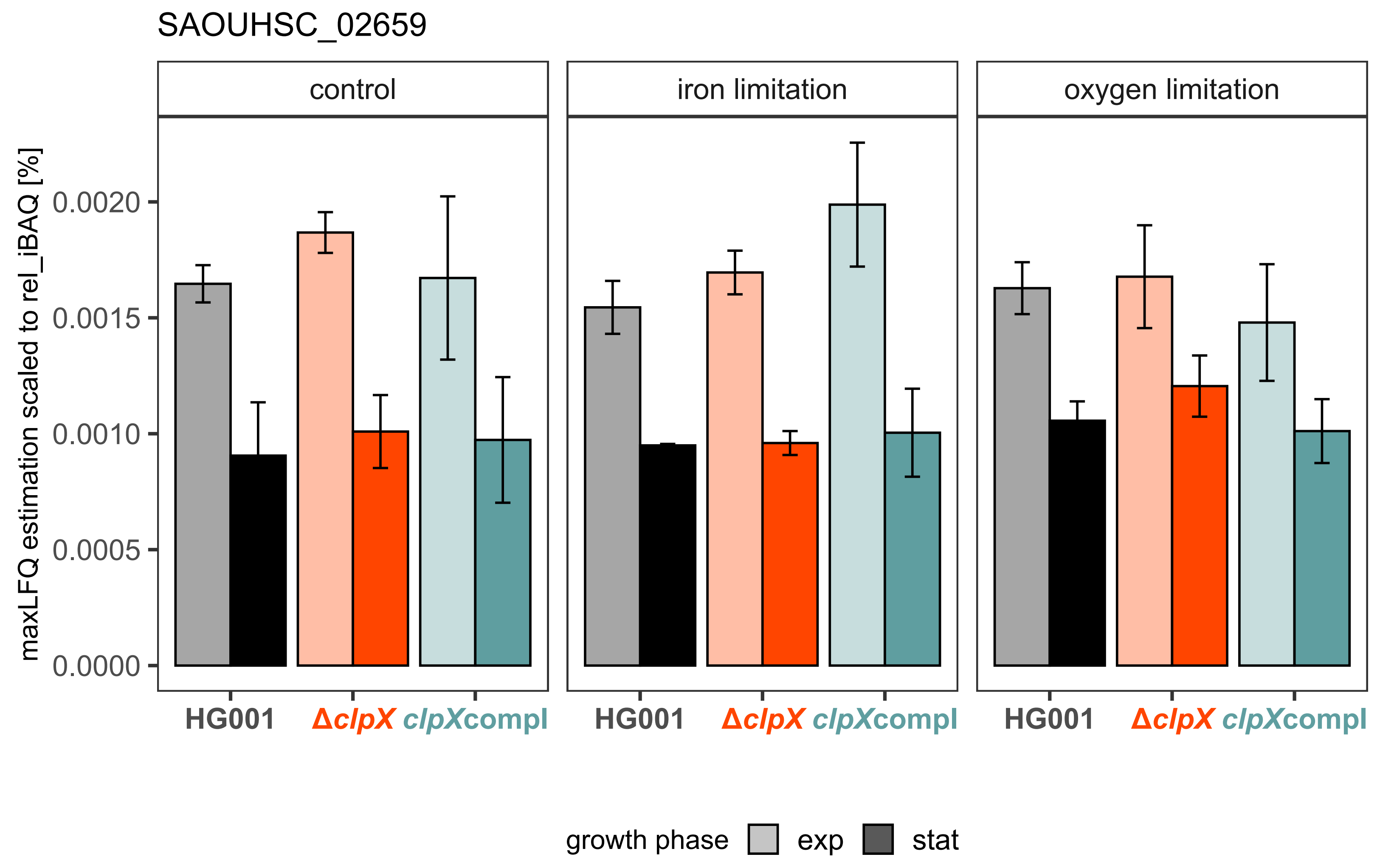
SAOUHSC_02659
control
iron limitation
oxygen limitation
maxLFQ estimation scaled to rel_iBAQ [%]
0.0020
0.0015
0.0010
0.0005
0.0000
HG001
ΔclpX
clpXcompl
growth phase
exp
stat

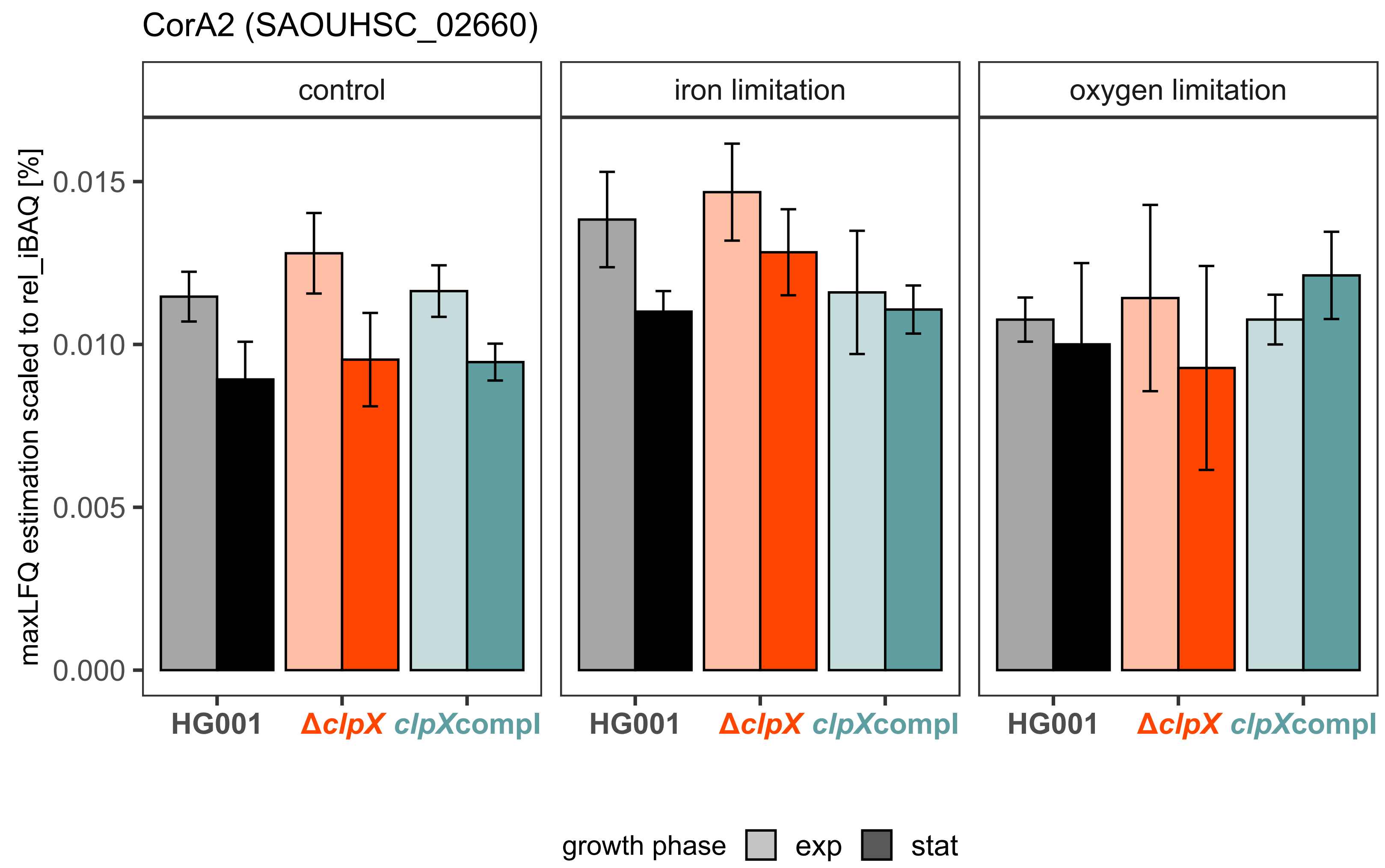

CorA2 (SAOUHSC_02660)
control
iron limitation
oxygen limitation
maxLFQ estimation scaled to rel_iBAQ [%]
0.015
0.010
0.005
0.000
HG001
ΔclpX
clpXcompl
growth phase
exp
stat

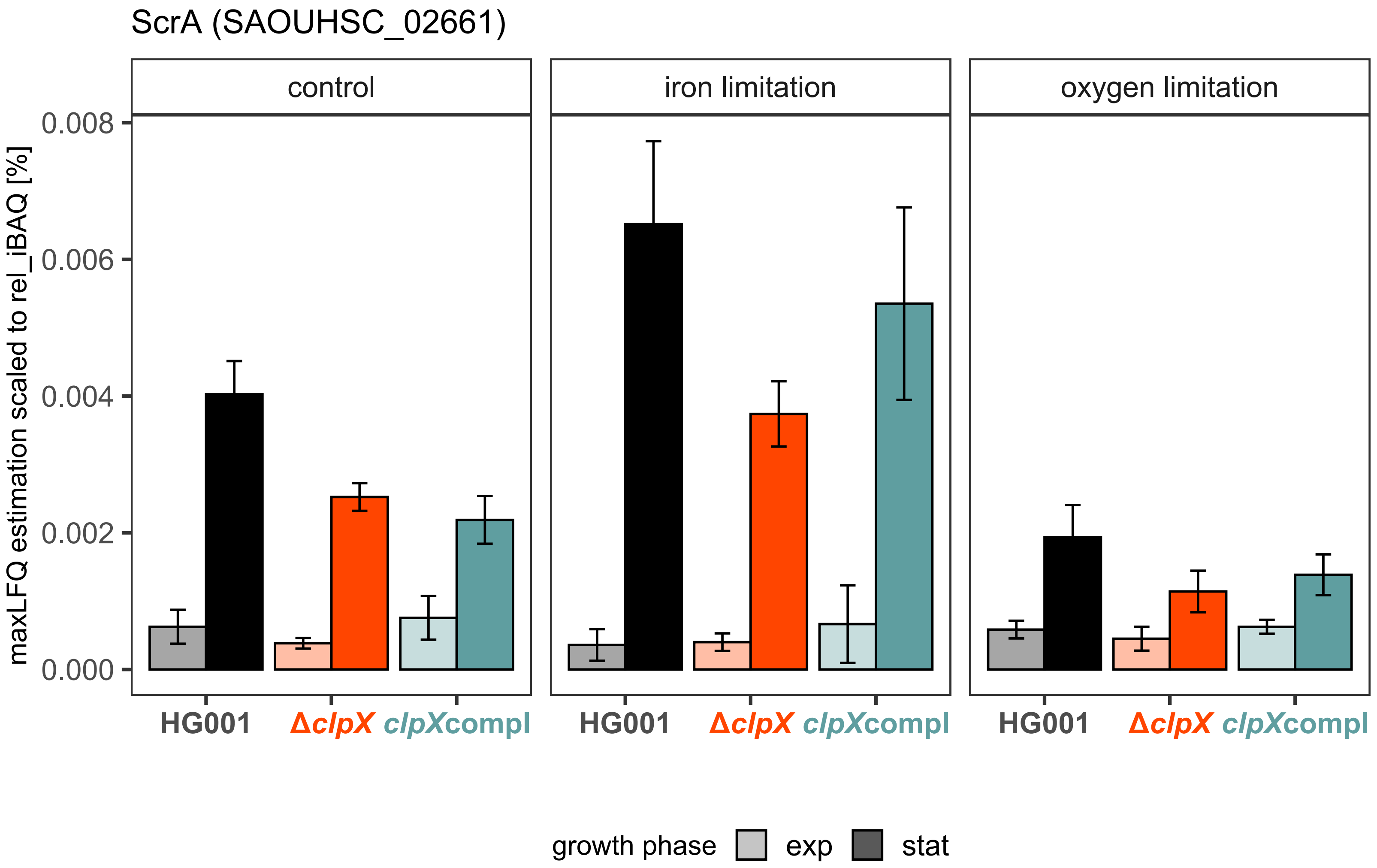
ScrA (SAOUHSC_02661)
control
iron limitation
oxygen limitation
maxLFQ estimation scaled to rel_iBAQ [%]
0.008
0.006
0.004
0.002
0.000
HG001
ΔclpX
clpXcompl
growth phase
exp
stat

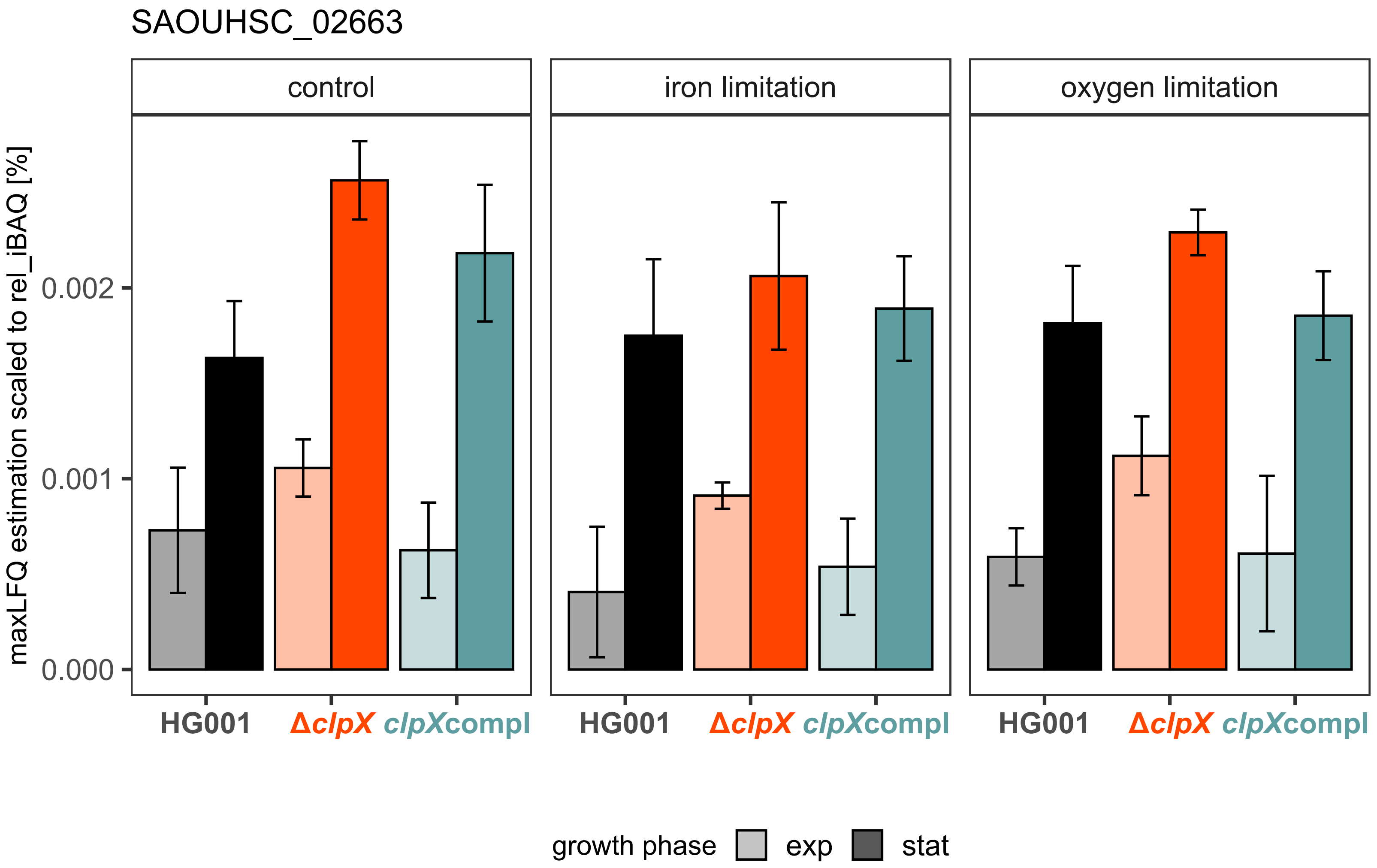
SAOUHSC_02663
control
iron limitation
oxygen limitation
maxLFQ estimation scaled to rel_iBAQ [%]
0.002
0.001
0.000
HG001
ΔclpX
clpXcompl
growth phase
exp
stat

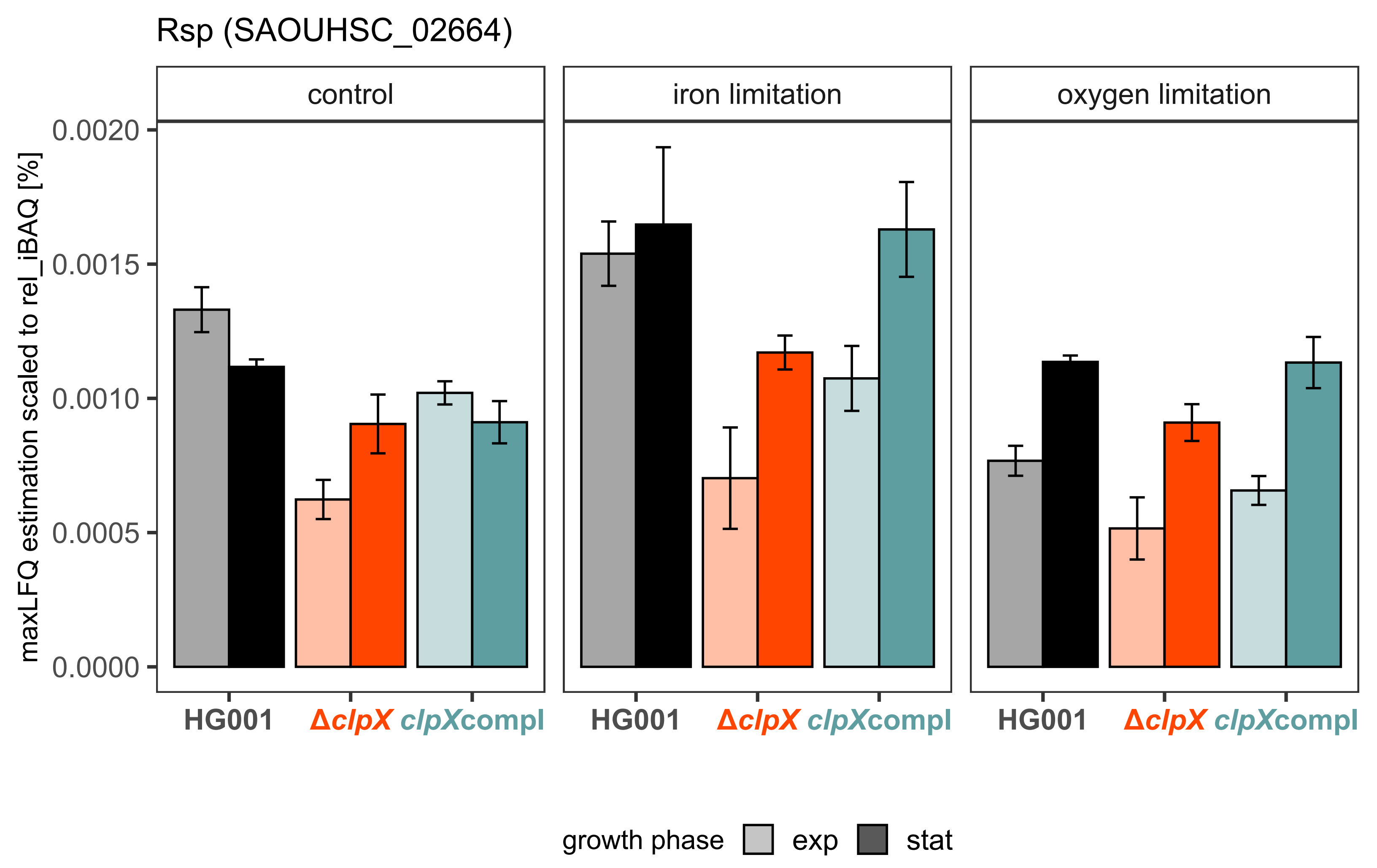

Rsp (SAOUHSC_02664)
control
iron limitation
oxygen limitation
maxLFQ estimation scaled to rel_iBAQ [%]
0.0020
0.0015
0.0010
0.0005
0.0000
HG001
ΔclpX
clpXcompl
growth phase
exp
stat

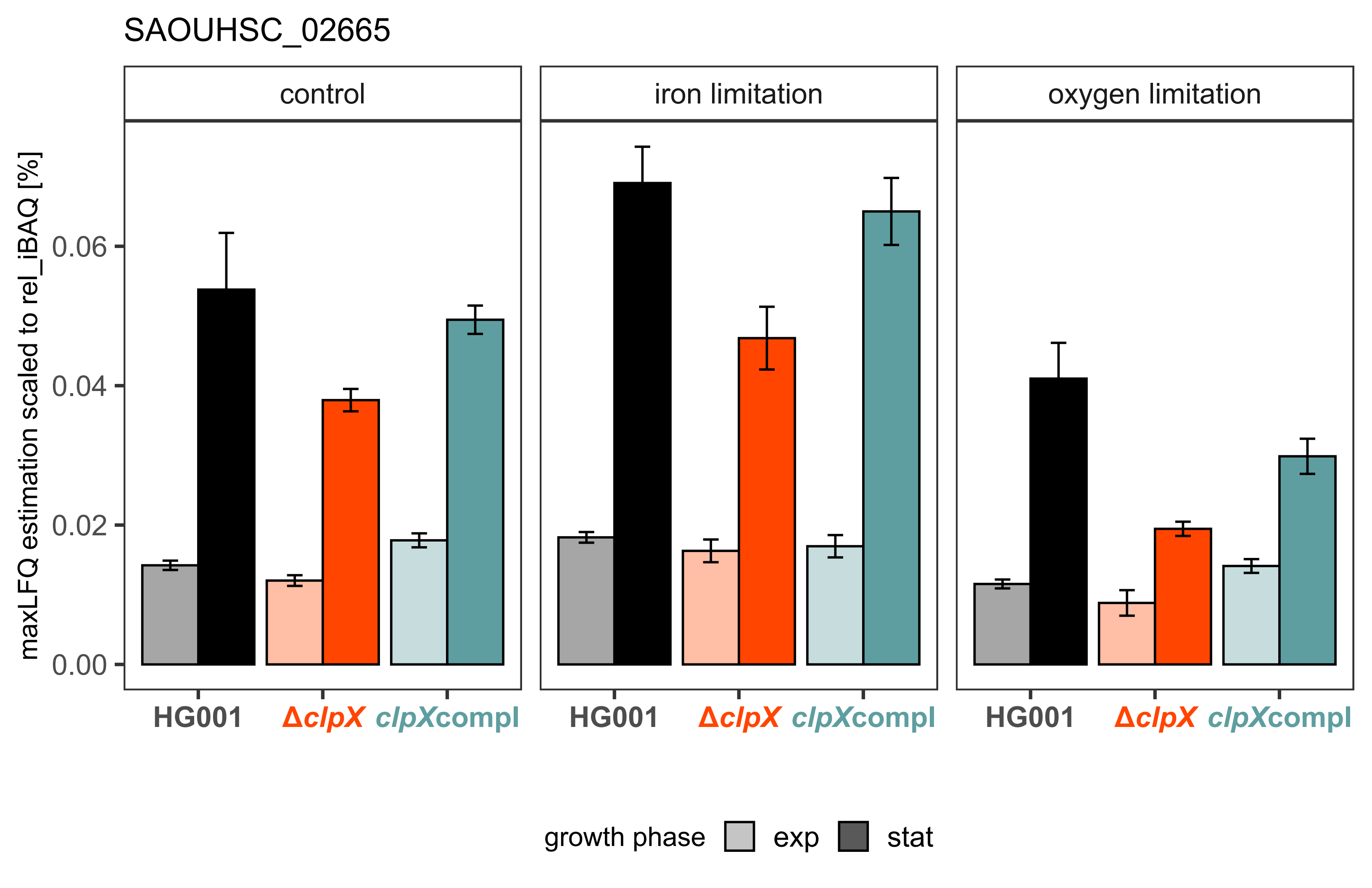

SAOUHSC_02665
control
iron limitation
oxygen limitation
maxLFQ estimation scaled to rel_iBAQ [%]
0.06
0.04
0.02
0.00
HG001
ΔclpX
clpXcompl
growth phase
exp
stat

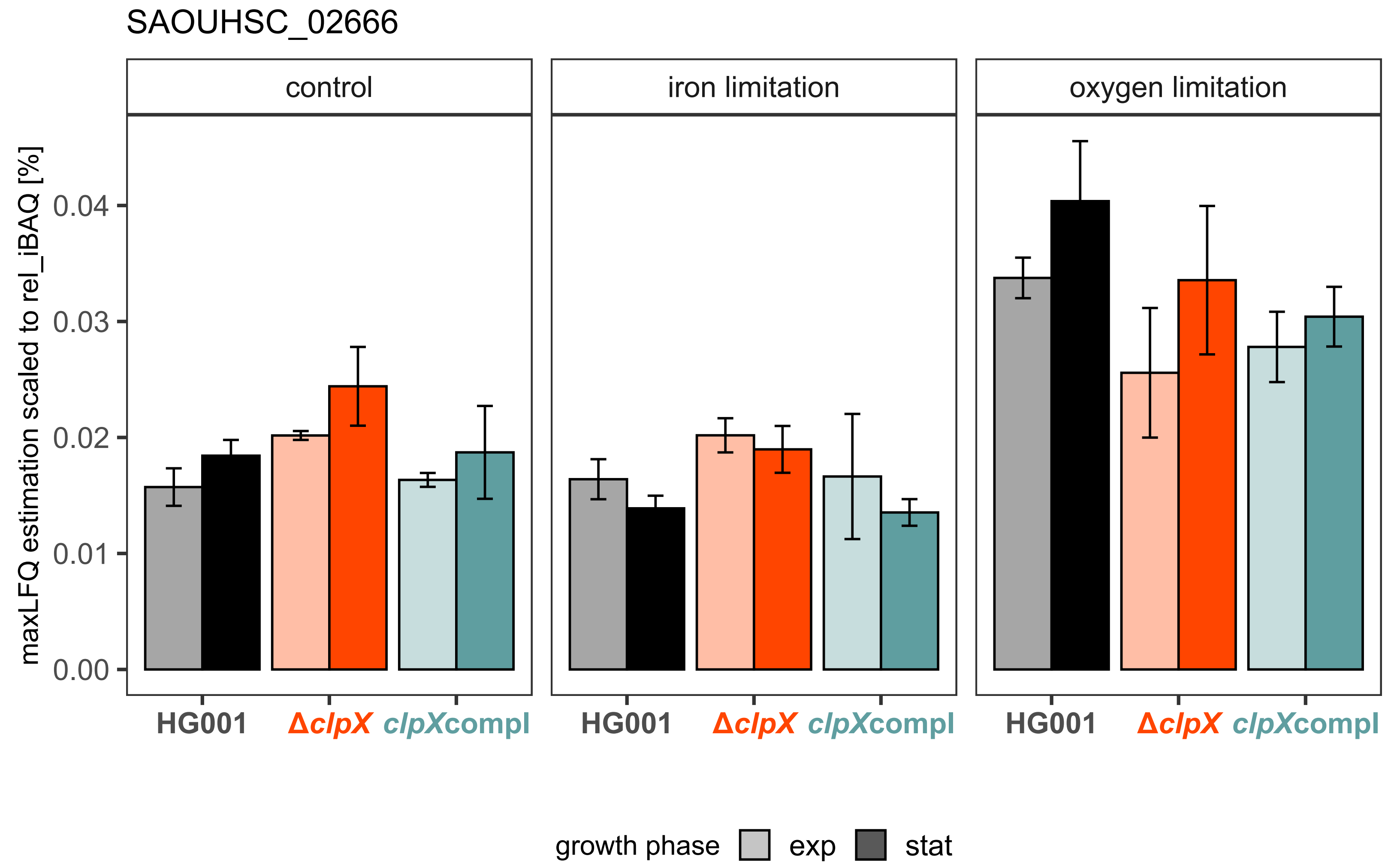

SAOUHSC_02666
control
iron limitation
oxygen limitation
maxLFQ estimation scaled to rel_iBAQ [%]
0.00
0.01
0.02
0.03
0.04
HG001
ΔclpX
clpXcompl
growth phase
exp
stat

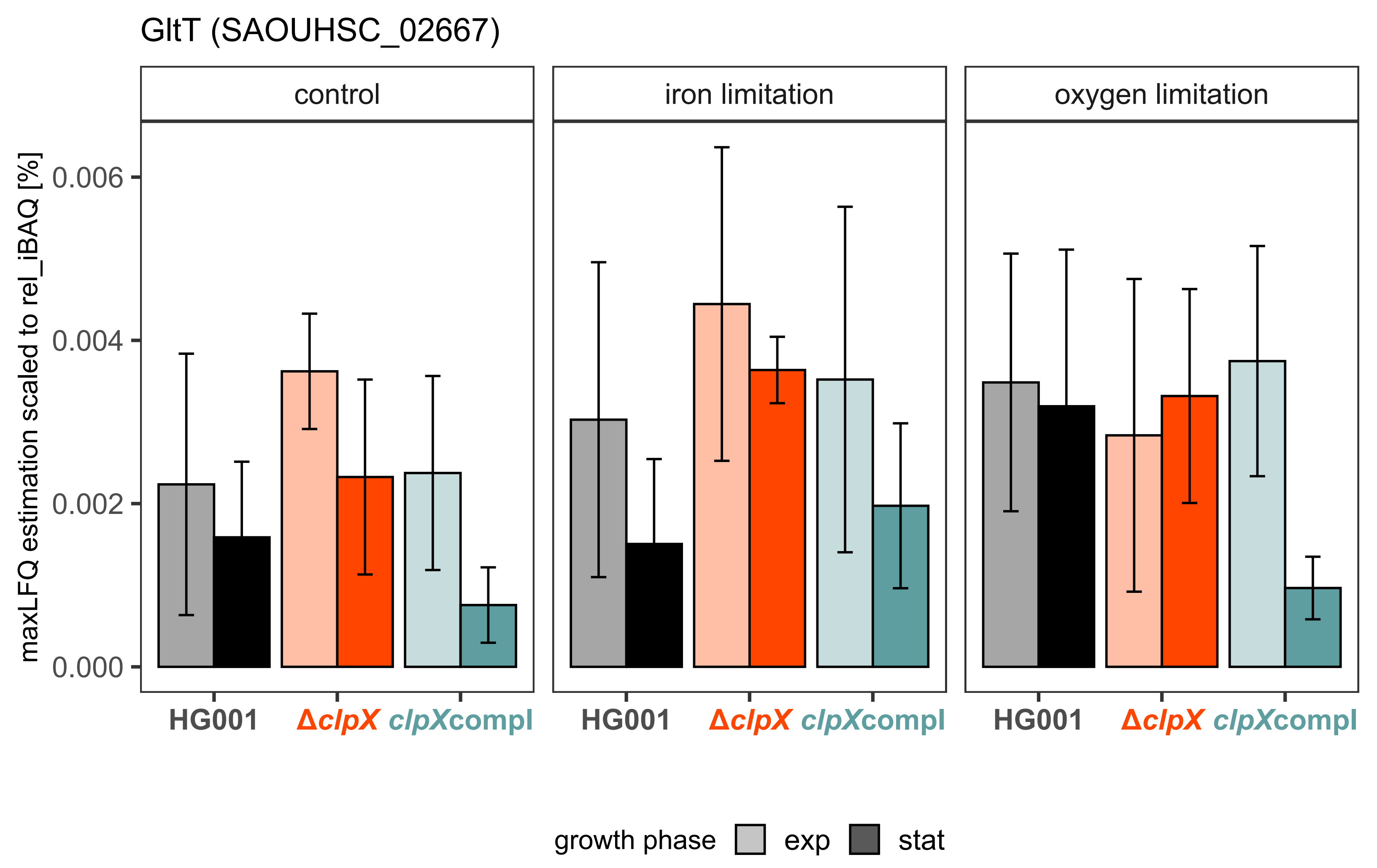

GltT (SAOUHSC_02667)
control
iron limitation
oxygen limitation
maxLFQ estimation scaled to rel_iBAQ [%]
0.000
0.002
0.004
0.006
HG001
ΔclpX
clpXcompl
growth phase
exp
stat

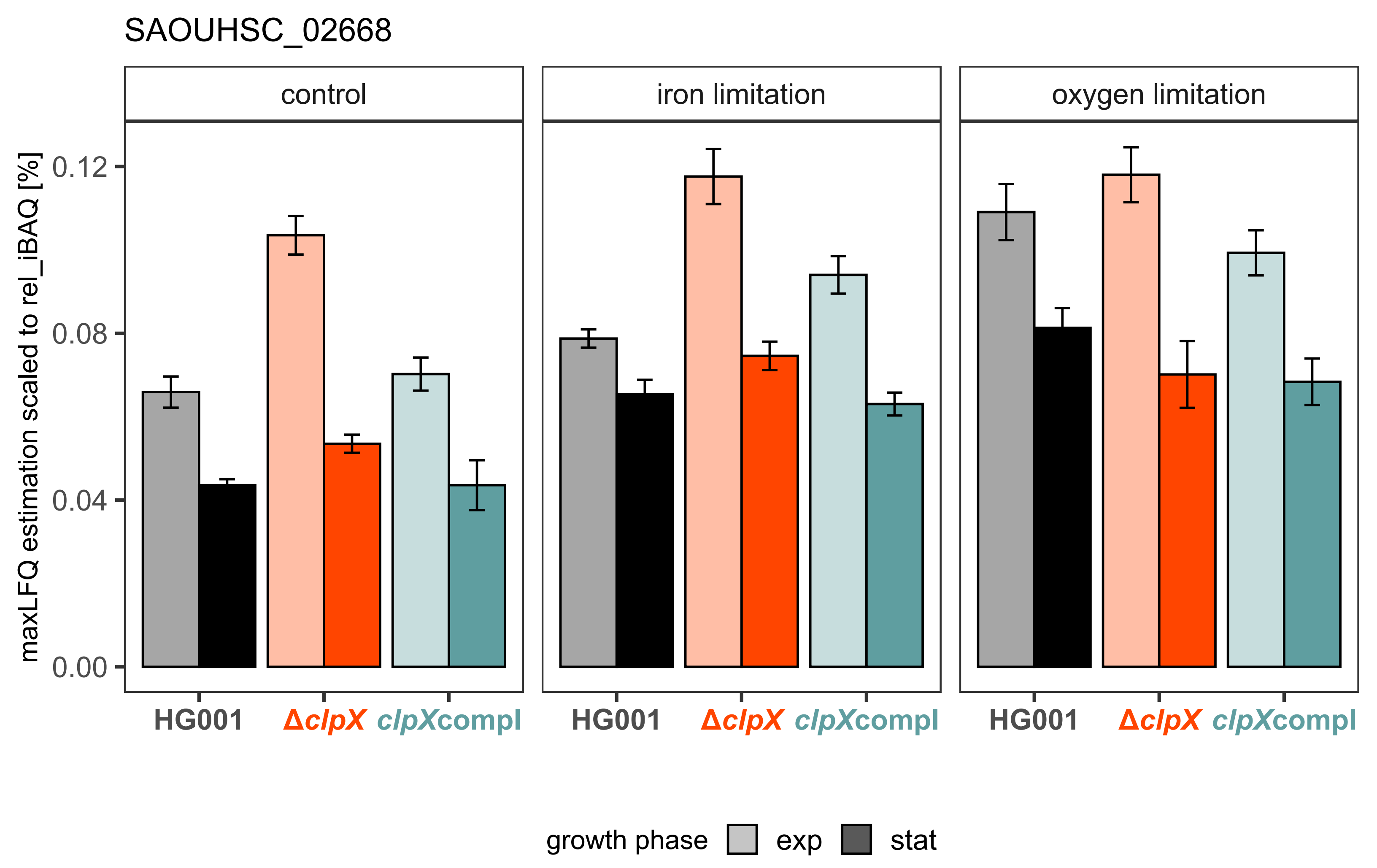
SAOUHSC_02668
control
iron limitation
oxygen limitation
maxLFQ estimation scaled to rel_iBAQ [%]
0.12
0.08
0.04
0.00
HG001
ΔclpX
clpXcompl
growth phase
exp
stat

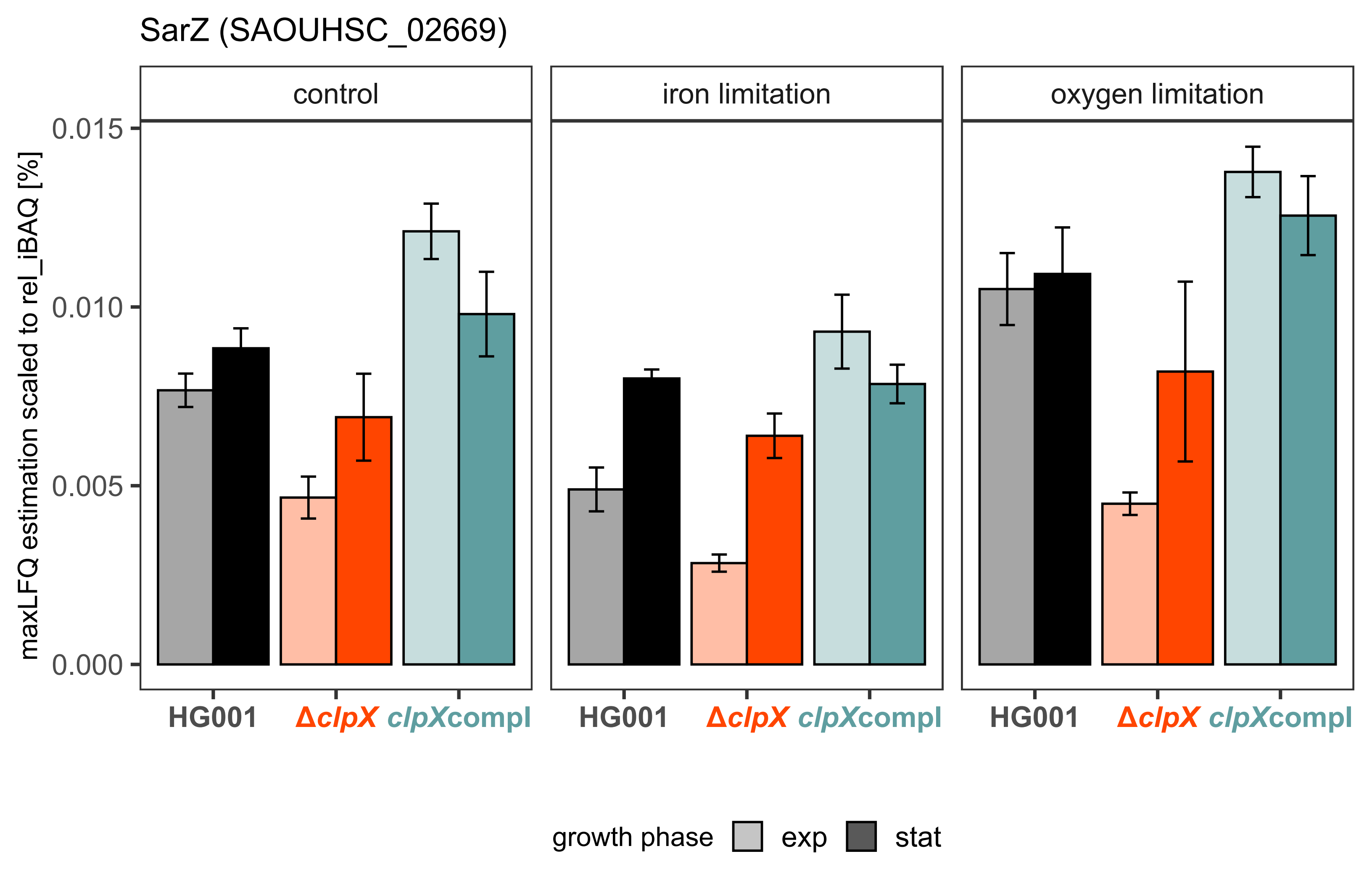

SarZ (SAOUHSC_02669)
control
iron limitation
oxygen limitation
maxLFQ estimation scaled to rel_iBAQ [%]
0.015
0.010
0.005
0.000
HG001
ΔclpX
clpXcompl
growth phase
exp
stat

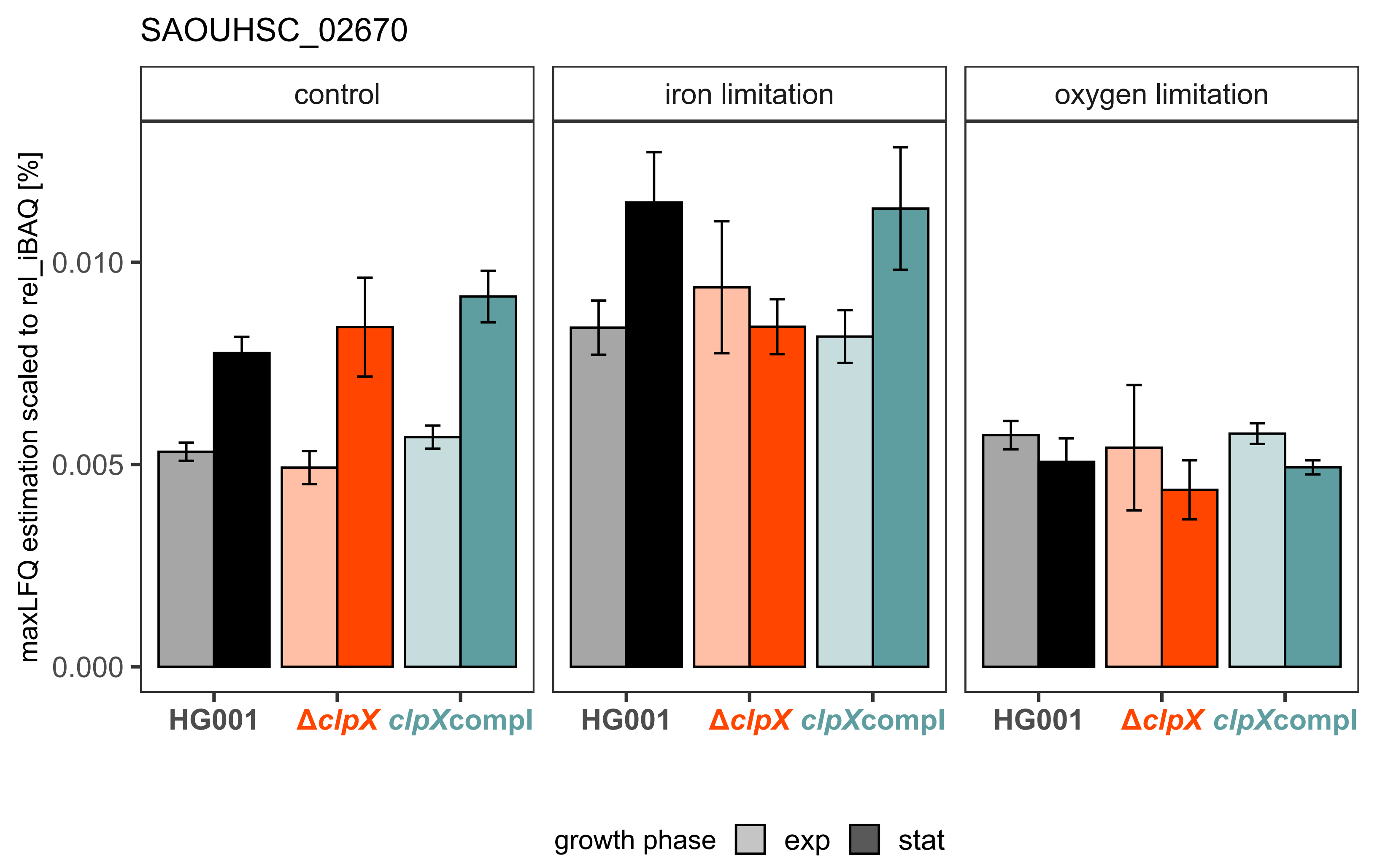
SAOUHSC_02670
control
iron limitation
oxygen limitation
maxLFQ estimation scaled to rel_iBAQ [%]
0.010
0.005
0.000
HG001
ΔclpX
clpXcompl
growth phase
exp
stat

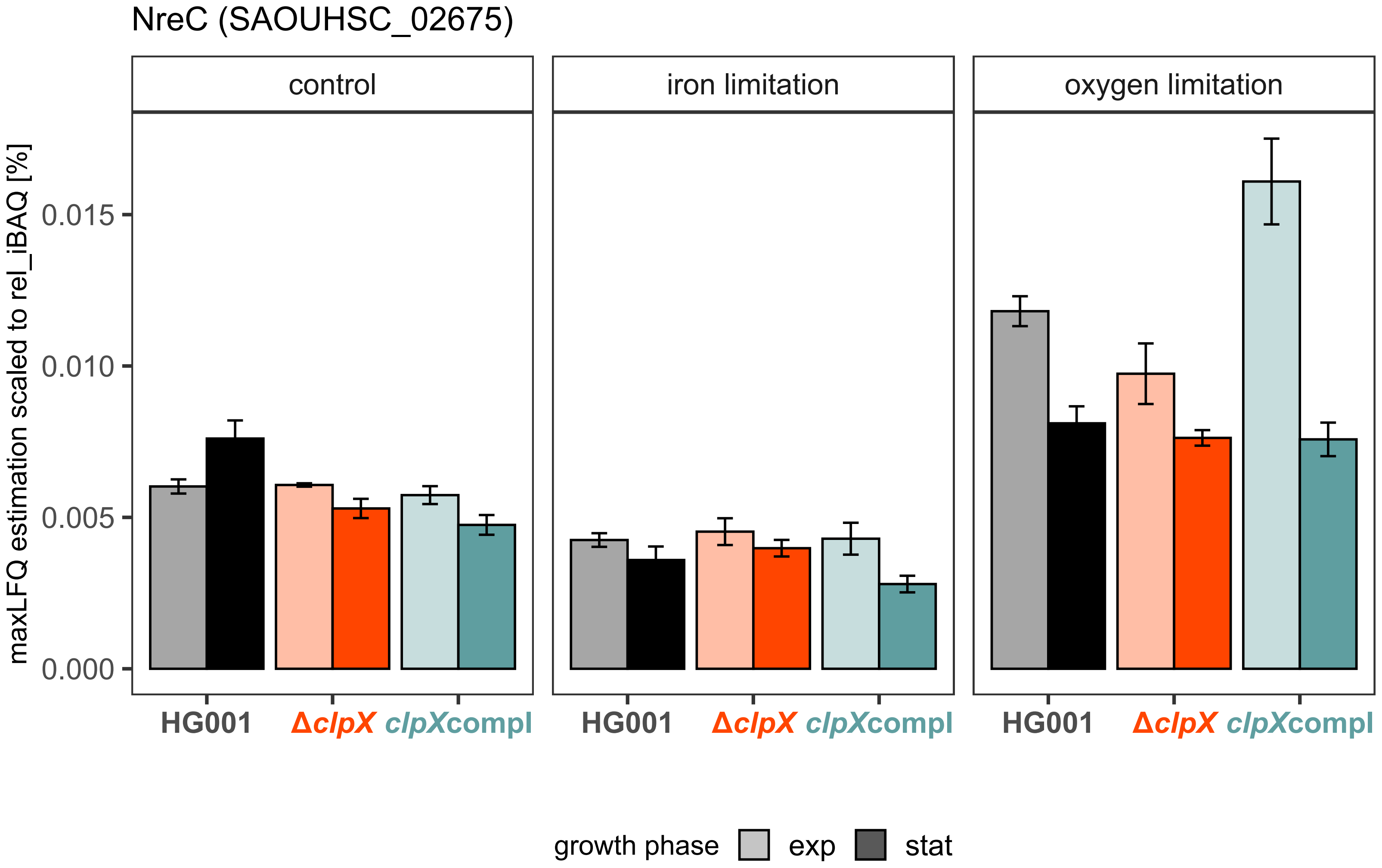

NreC (SAOUHSC_02675)
control
iron limitation
oxygen limitation
maxLFQ estimation scaled to rel_iBAQ [%]
0.015
0.010
0.005
0.000
HG001
ΔclpX
clpXcompl
growth phase
exp
stat

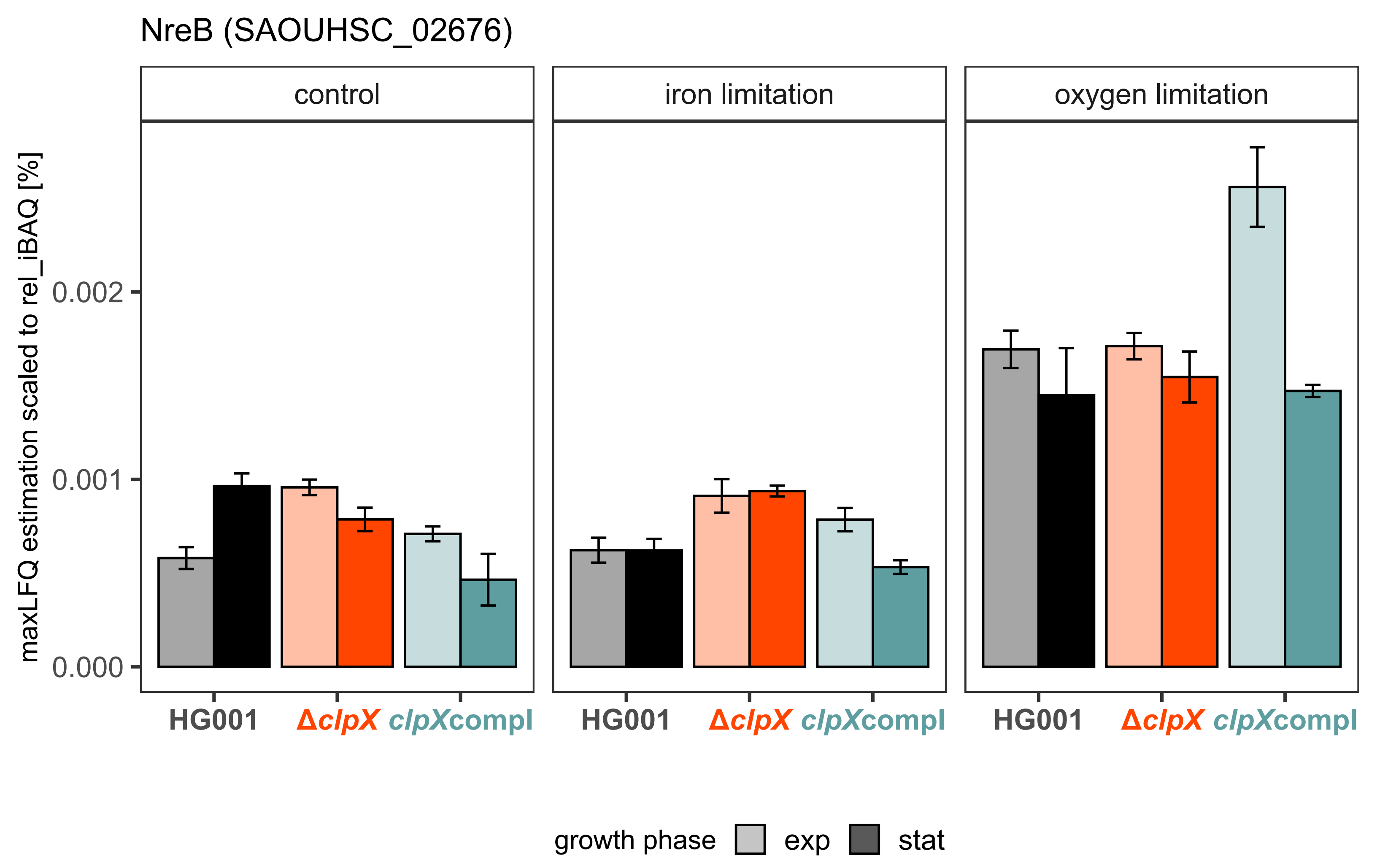

NreB (SAOUHSC_02676)
control
iron limitation
oxygen limitation
maxLFQ estimation scaled to rel_iBAQ [%]
0.000
0.001
0.002
HG001
ΔclpX
clpXcompl
growth phase
exp
stat

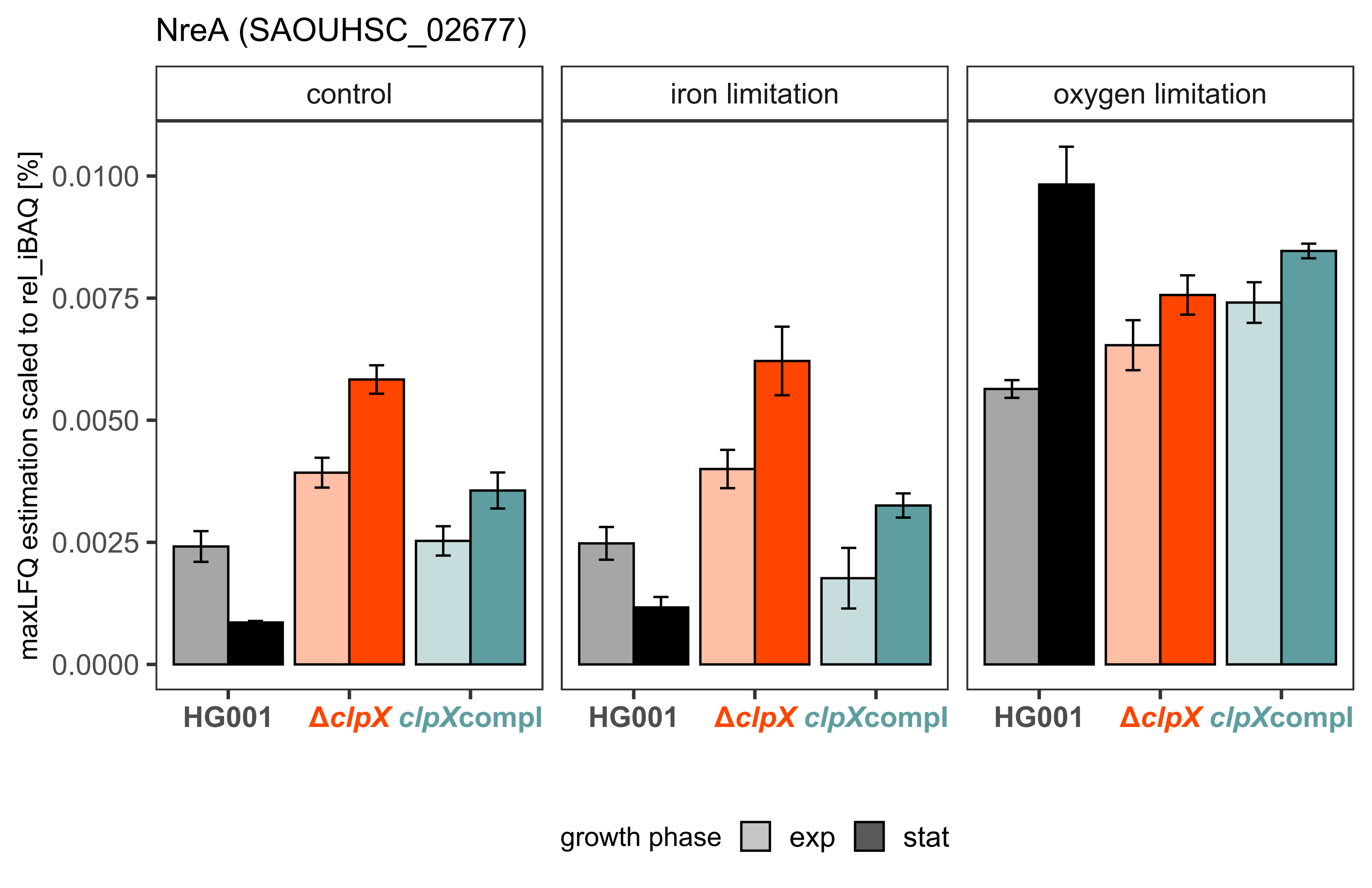

NreA (SAOUHSC_02677)
control
iron limitation
oxygen limitation
maxLFQ estimation scaled to rel_iBAQ [%]
0.0100
0.0075
0.0050
0.0025
0.0000
HG001
ΔclpX
clpXcompl
growth phase
exp
stat

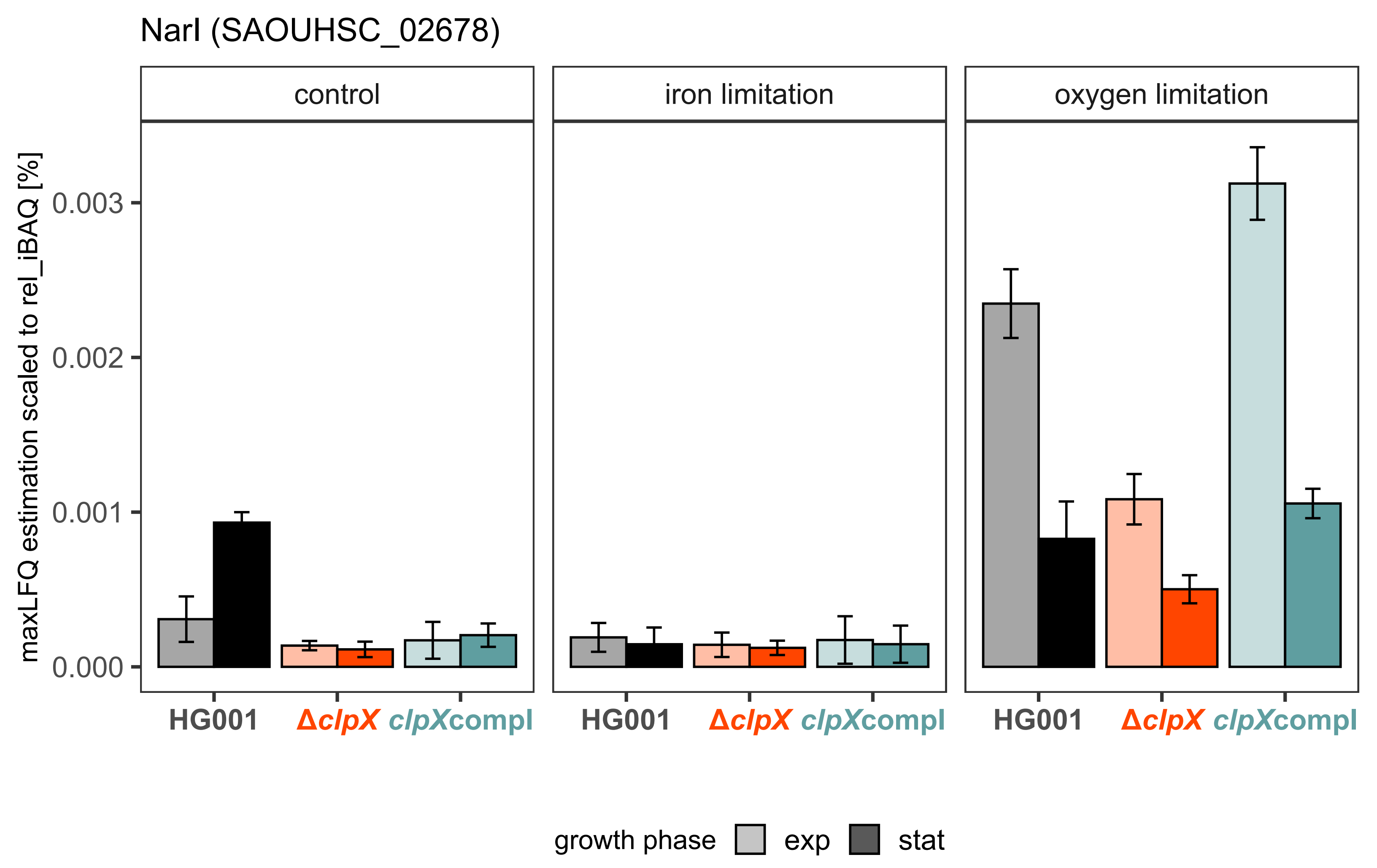
NarI (SAOUHSC_02678)
control
iron limitation
oxygen limitation
maxLFQ estimation scaled to rel_iBAQ [%]
0.003
0.002
0.001
0.000
HG001
ΔclpX
clpXcompl
growth phase
exp
stat

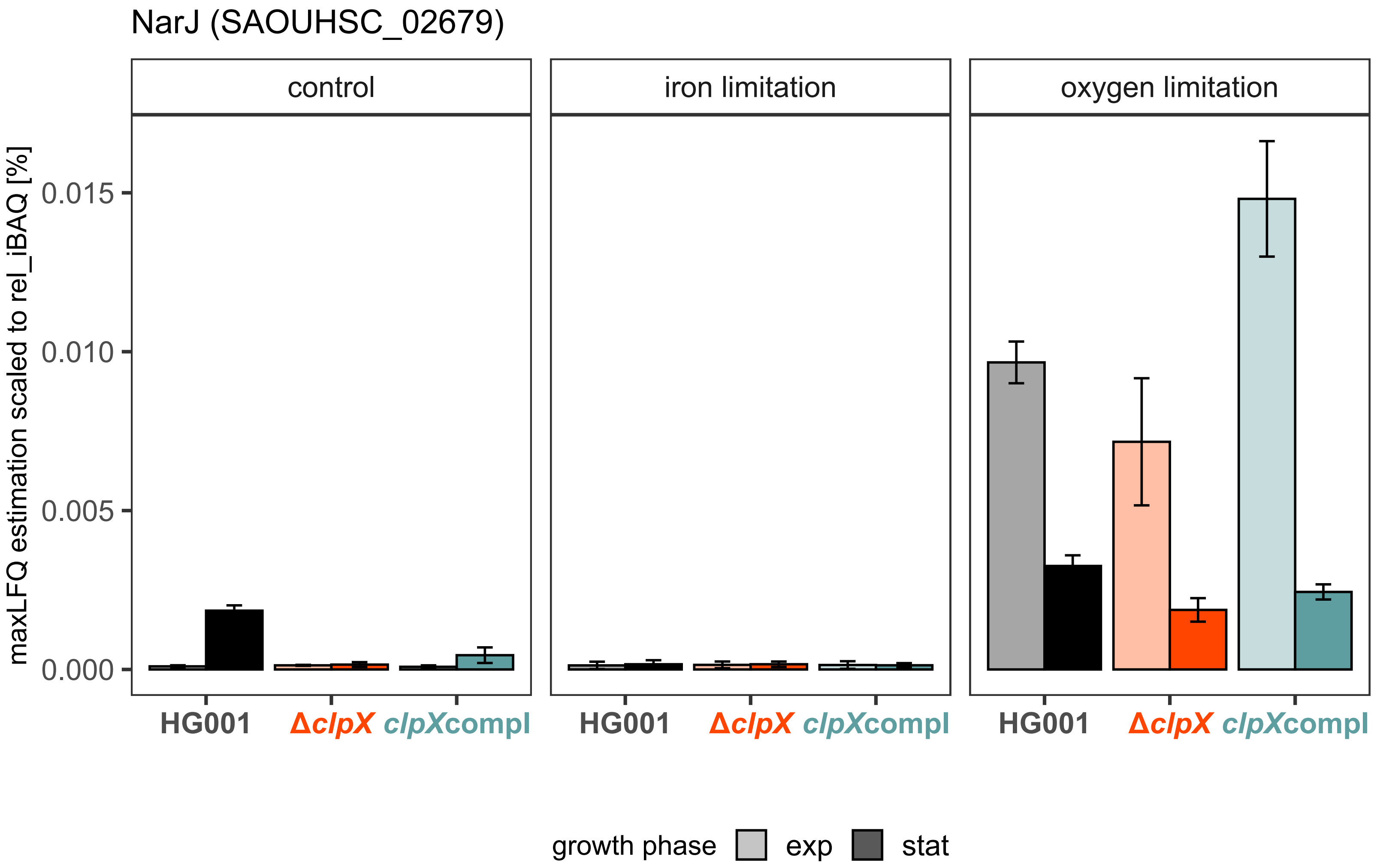
NarJ (SAOUHSC_02679)
control
iron limitation
oxygen limitation
maxLFQ estimation scaled to rel_iBAQ [%]
0.015
0.010
0.005
0.000
HG001
ΔclpX
clpXcompl
growth phase
exp
stat

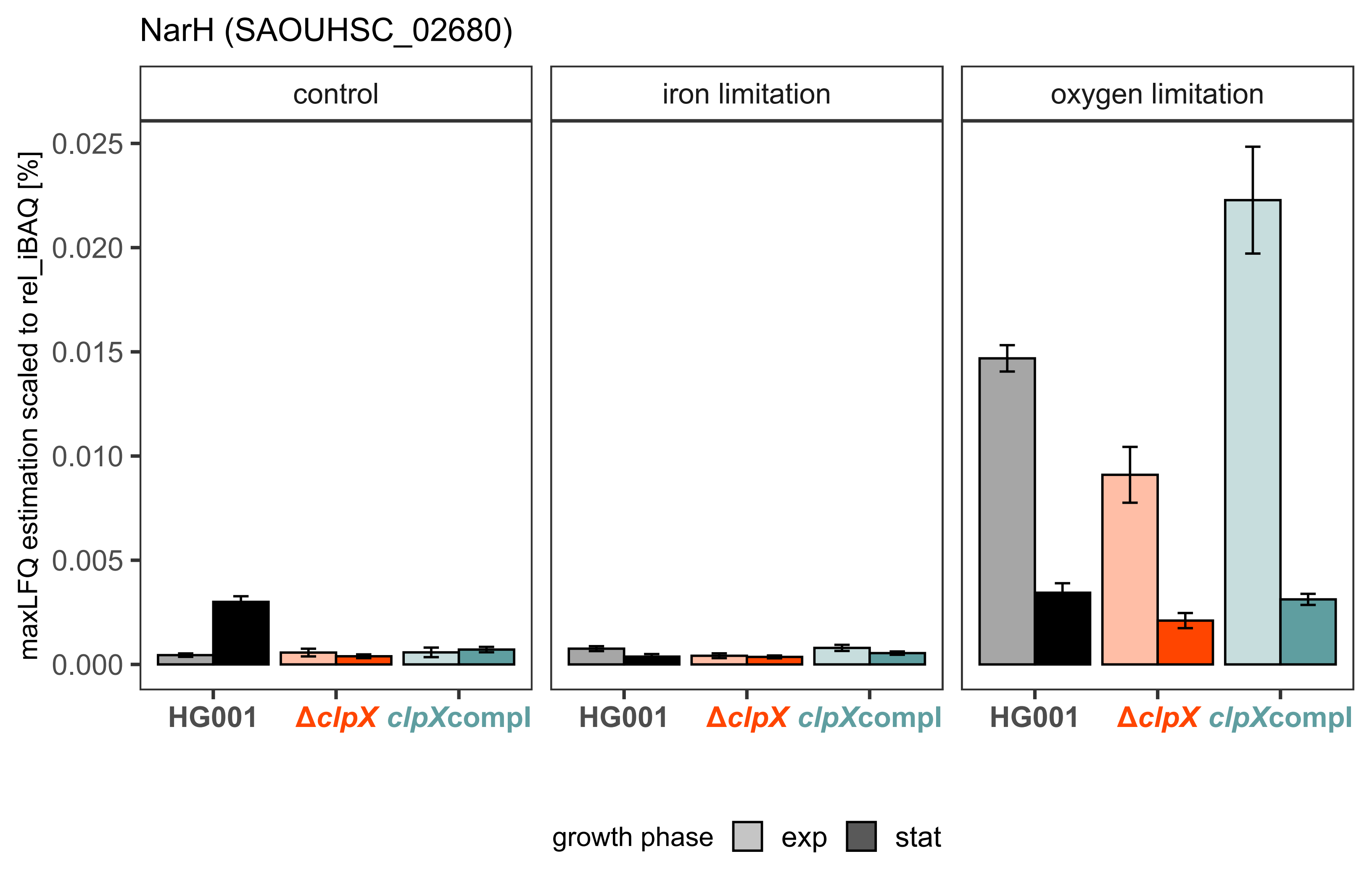

NarH (SAOUHSC_02680)
control
iron limitation
oxygen limitation
maxLFQ estimation scaled to rel_iBAQ [%]
0.025
0.020
0.015
0.010
0.005
0.000
HG001
ΔclpX
clpXcompl
growth phase
exp
stat

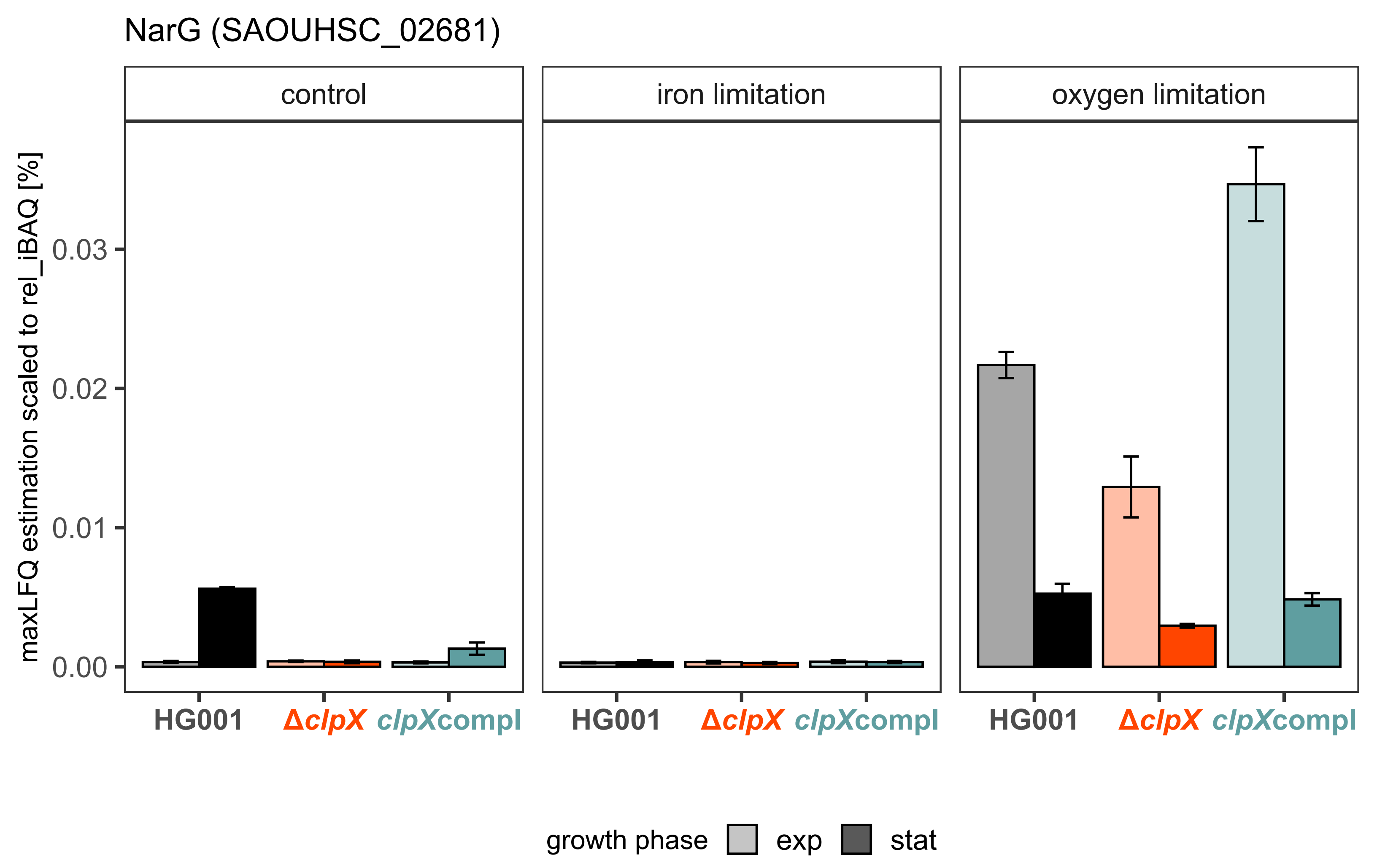

NarG (SAOUHSC_02681)
control
iron limitation
oxygen limitation
maxLFQ estimation scaled to rel_iBAQ [%]
0.00
0.01
0.02
0.03
HG001
ΔclpX
clpXcompl
growth phase
exp
stat

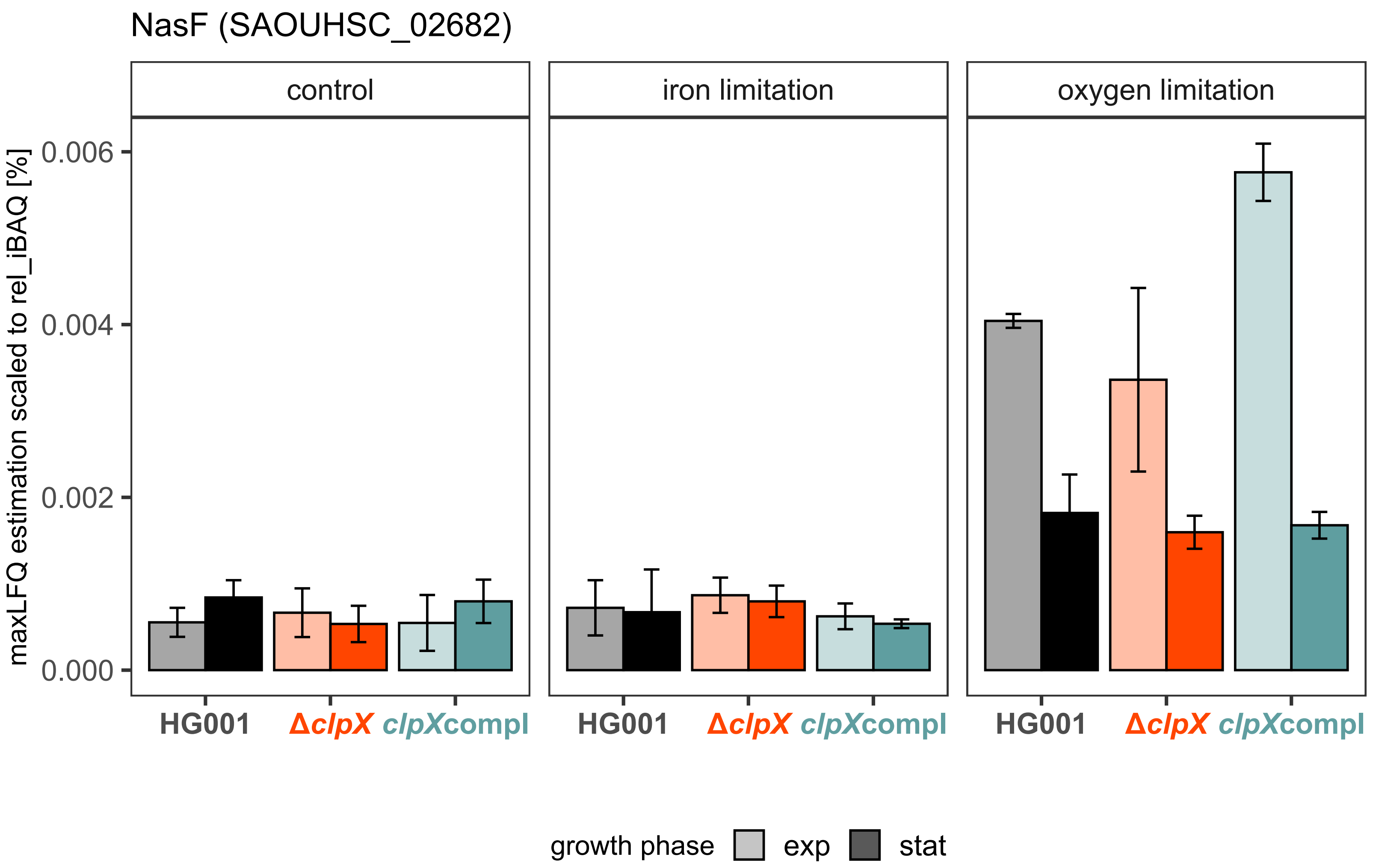
NasF (SAOUHSC_02682)
control
iron limitation
oxygen limitation
maxLFQ estimation scaled to rel_iBAQ [%]
0.006
0.004
0.002
0.000
HG001
ΔclpX
clpXcompl
growth phase
exp
stat

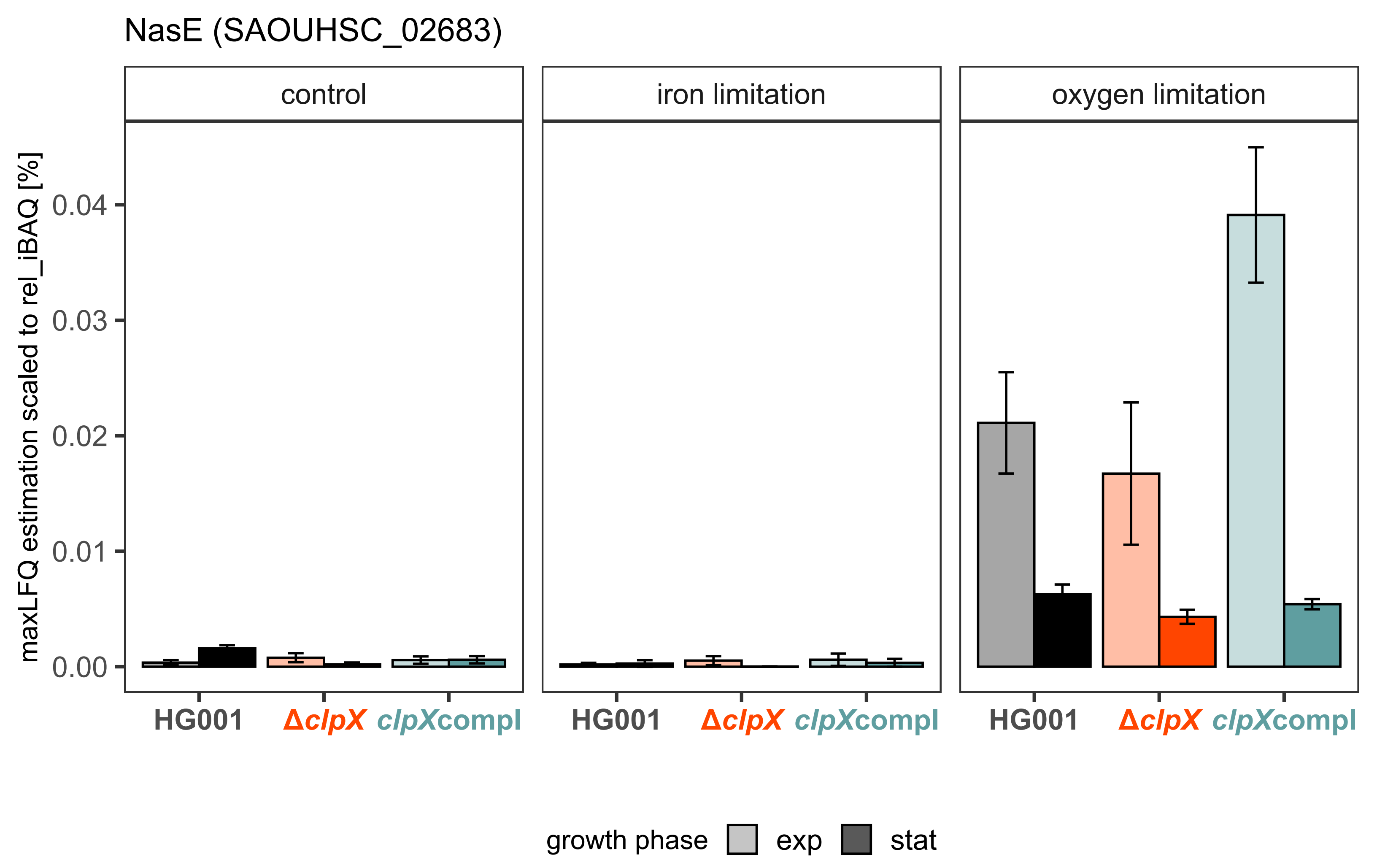

NasE (SAOUHSC_02683)
control
iron limitation
oxygen limitation
maxLFQ estimation scaled to rel_iBAQ [%]
0.04
0.03
0.02
0.01
0.00
HG001
ΔclpX
clpXcompl
growth phase
exp
stat

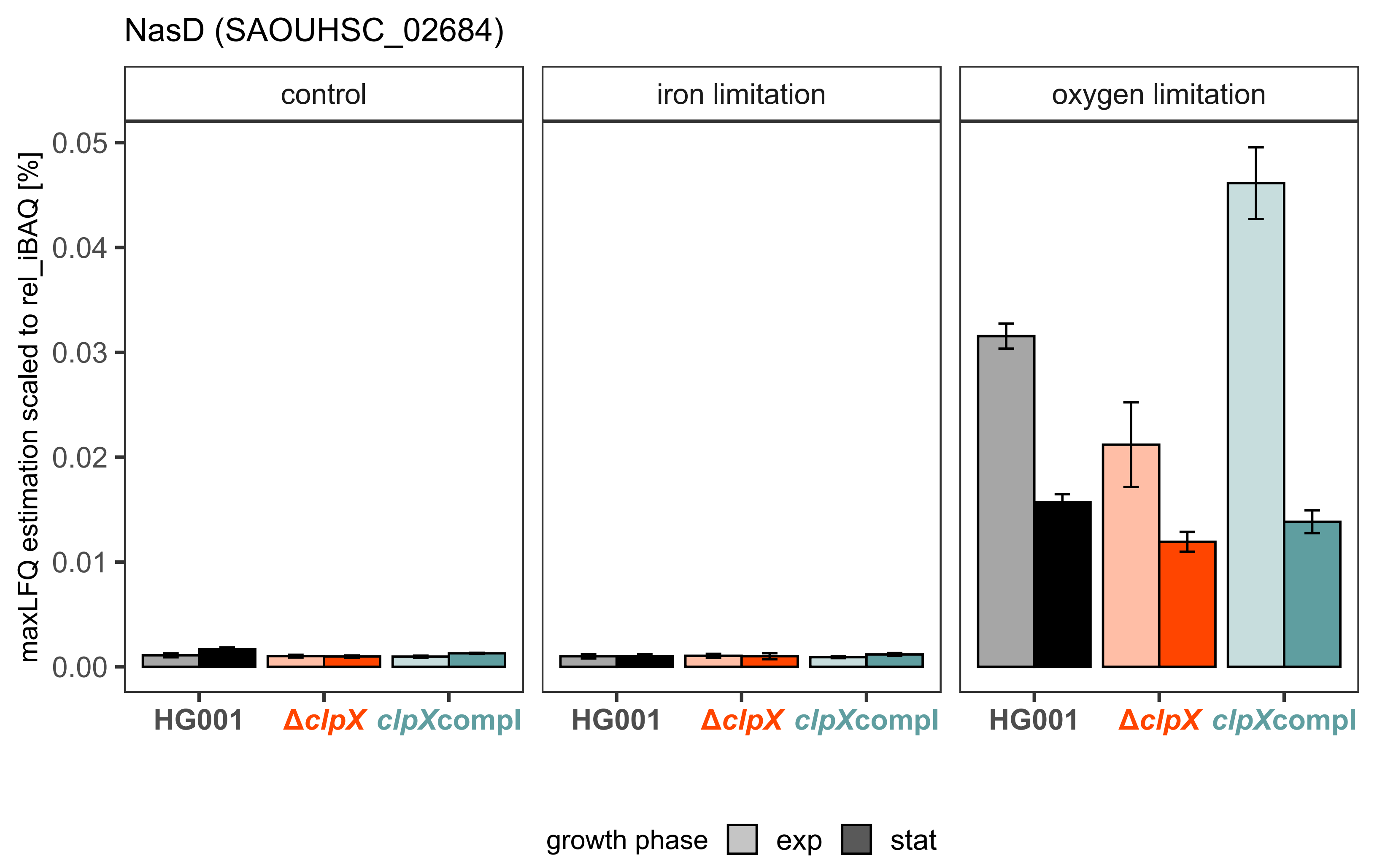
NasD (SAOUHSC_02684)
control
iron limitation
oxygen limitation
maxLFQ estimation scaled to rel_iBAQ [%]
0.05
0.04
0.03
0.02
0.01
0.00
HG001
ΔclpX
clpXcompl
growth phase
exp
stat

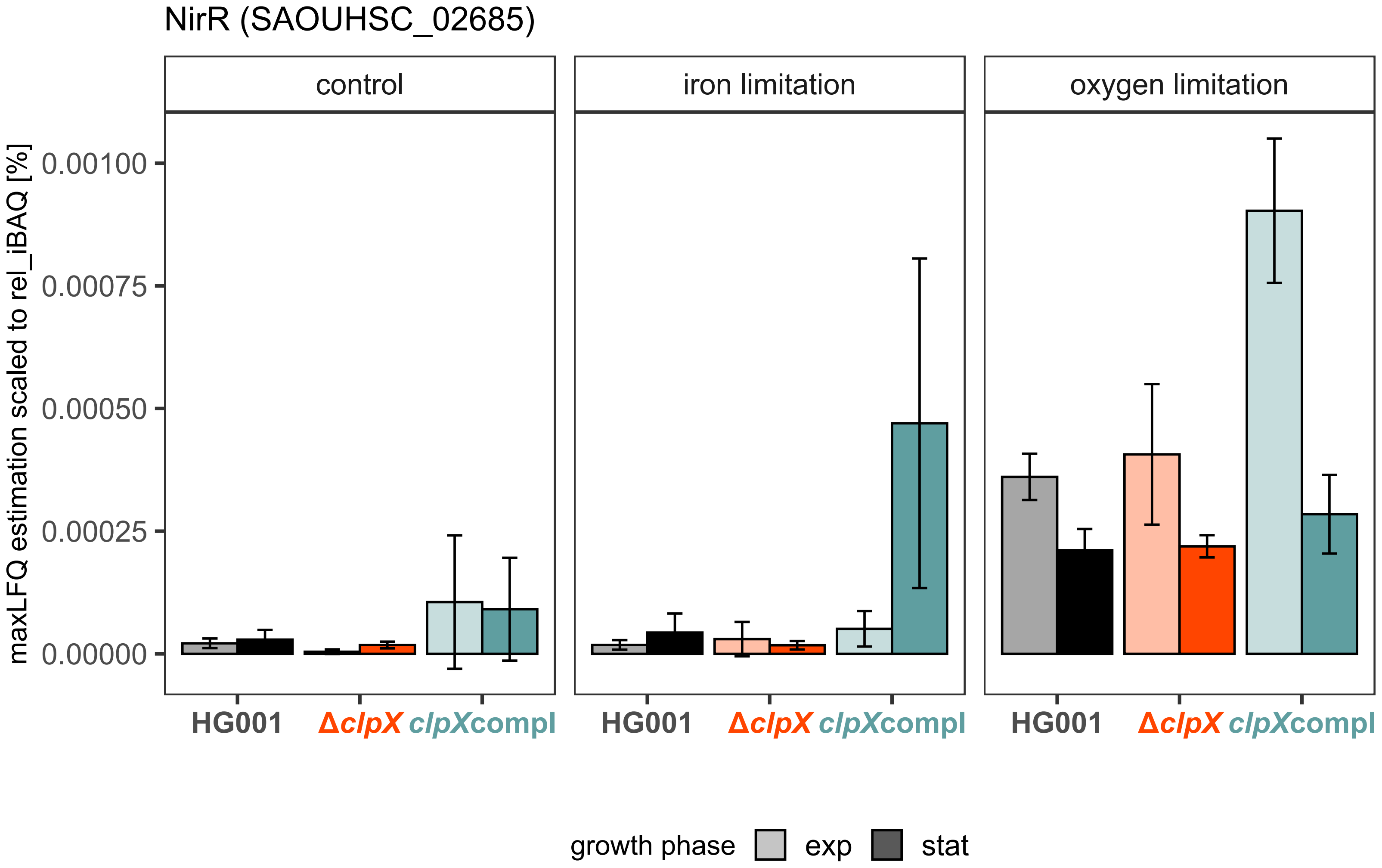
NirR (SAOUHSC_02685)
control
iron limitation
oxygen limitation
maxLFQ estimation scaled to rel_iBAQ [%]
0.00100
0.00075
0.00050
0.00025
0.00000
HG001
ΔclpX
clpXcompl
growth phase
exp
stat

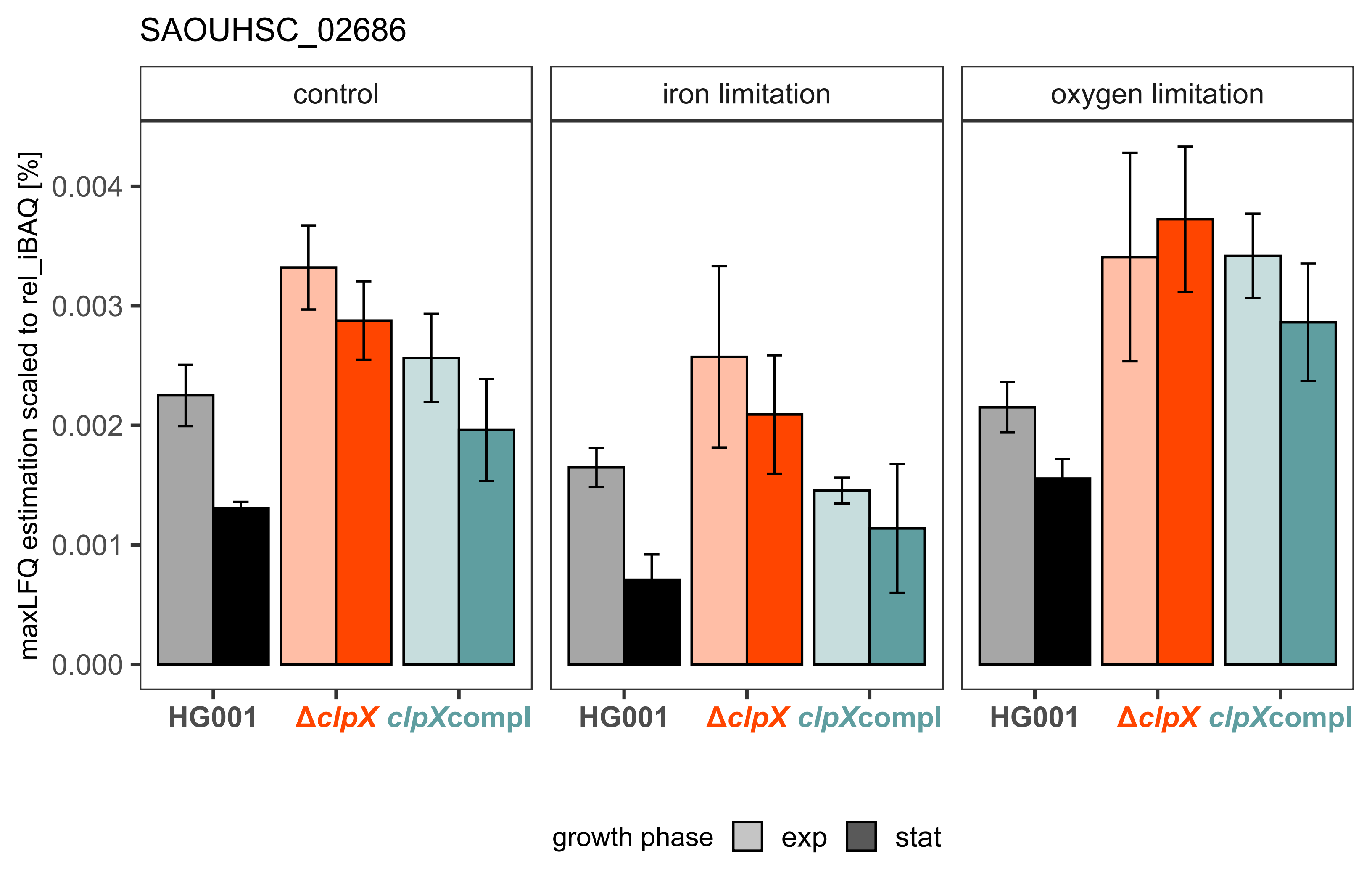

SAOUHSC_02686
control
iron limitation
oxygen limitation
maxLFQ estimation scaled to rel_iBAQ [%]
0.004
0.003
0.002
0.001
0.000
HG001
ΔclpX
clpXcompl
growth phase
exp
stat

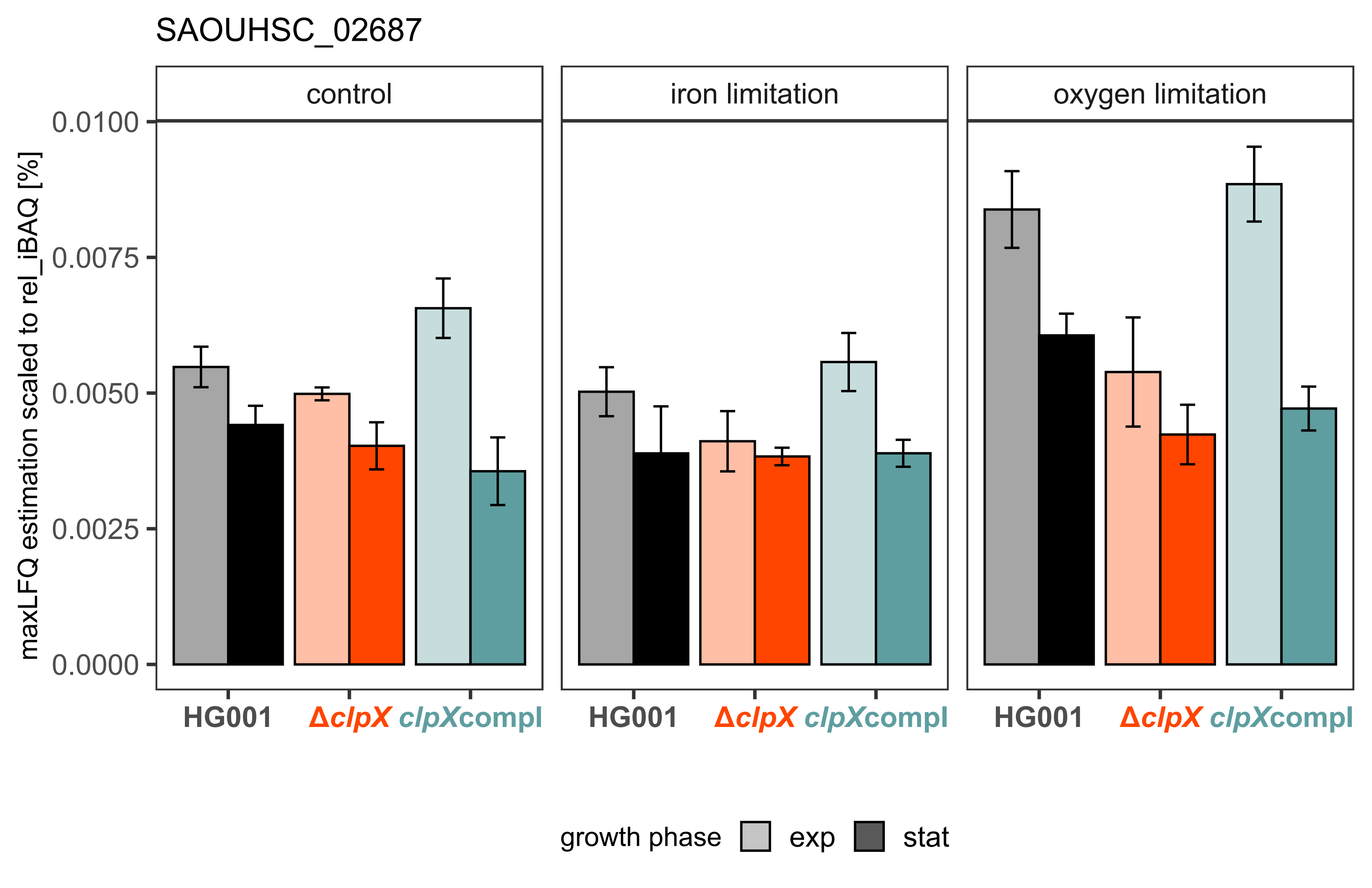

SAOUHSC_02687
control
iron limitation
oxygen limitation
maxLFQ estimation scaled to rel_iBAQ [%]
0.0100
0.0075
0.0050
0.0025
0.0000
HG001
ΔclpX
clpXcompl
growth phase
exp
stat

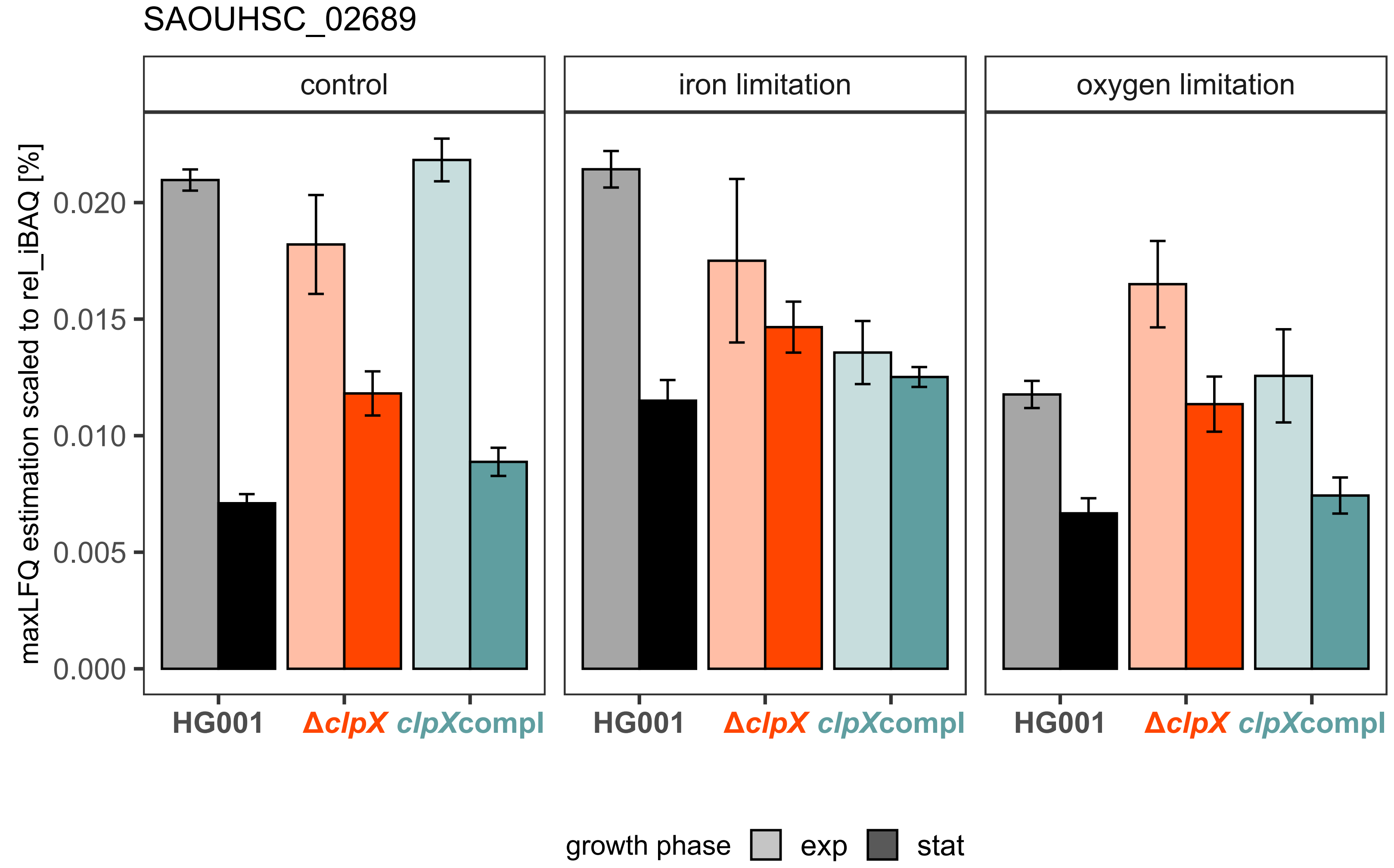
SAOUHSC_02689
control
iron limitation
oxygen limitation
maxLFQ estimation scaled to rel_iBAQ [%]
0.020
0.015
0.010
0.005
0.000
HG001
ΔclpX
clpXcompl
growth phase
exp
stat

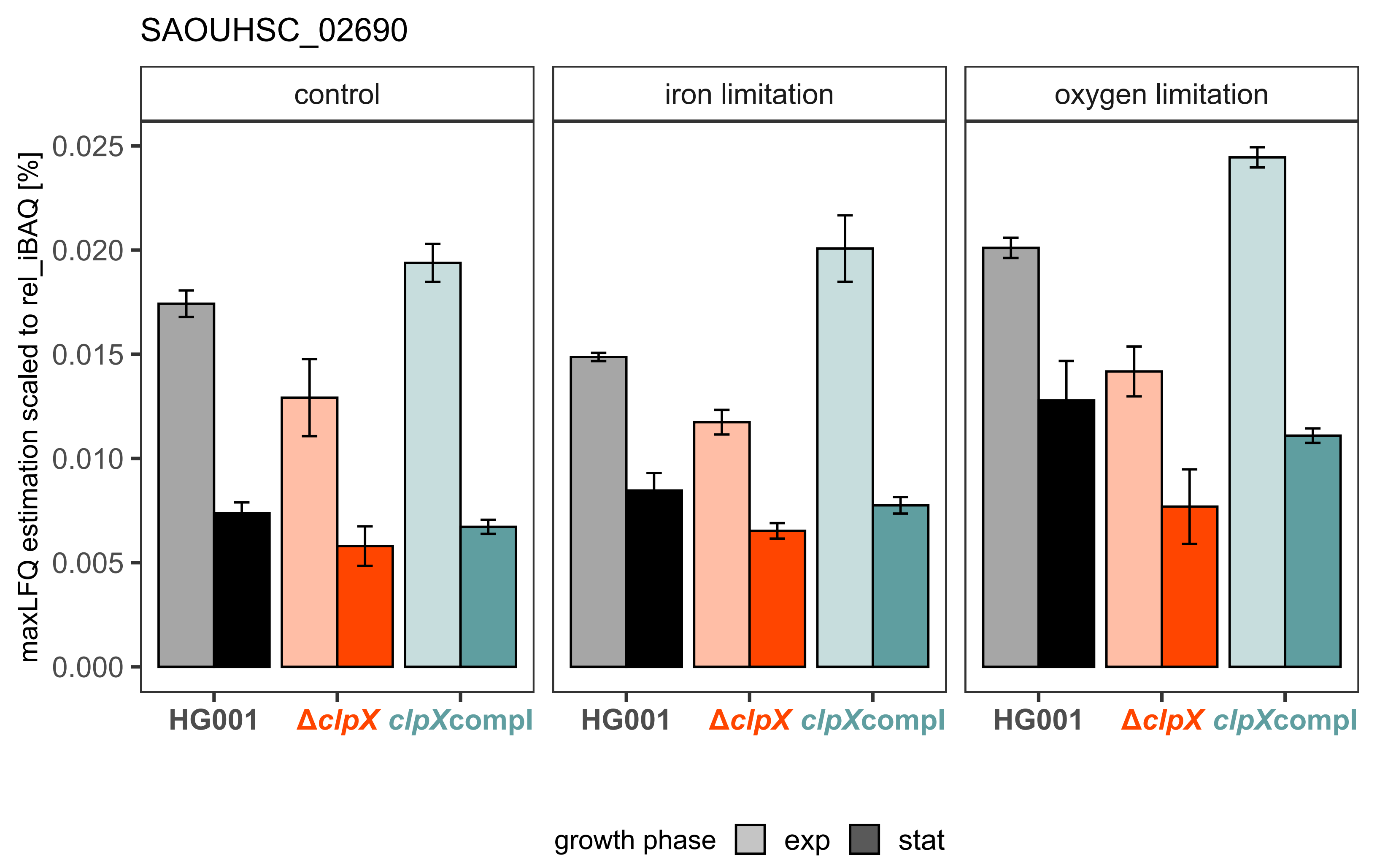
SAOUHSC_02690
control
iron limitation
oxygen limitation
maxLFQ estimation scaled to rel_iBAQ [%]
0.025
0.020
0.015
0.010
0.005
0.000
HG001
ΔclpX
clpXcompl
growth phase
exp
stat

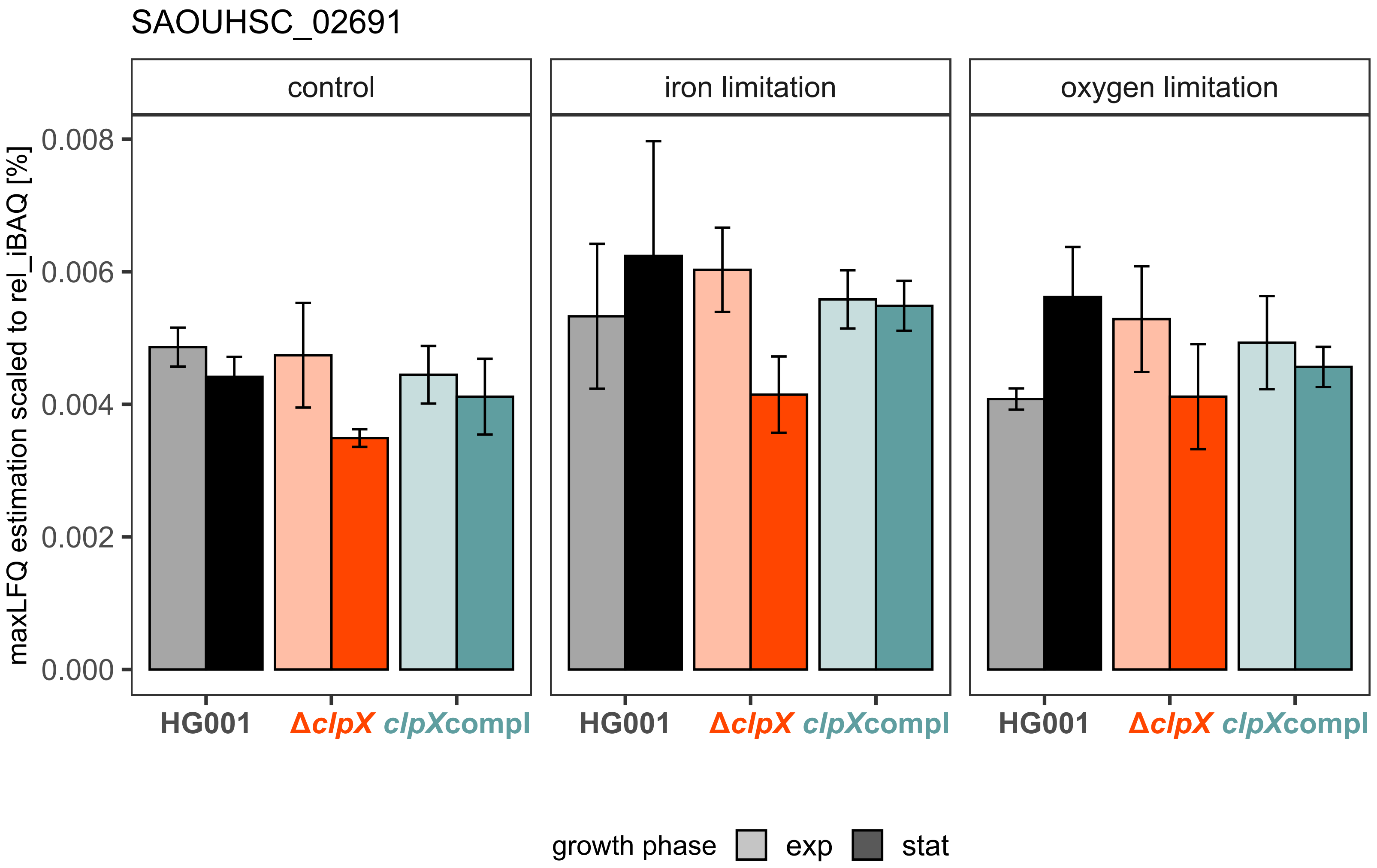
SAOUHSC_02691
control
iron limitation
oxygen limitation
maxLFQ estimation scaled to rel_iBAQ [%]
0.008
0.006
0.004
0.002
0.000
HG001
ΔclpX
clpXcompl
growth phase
exp
stat

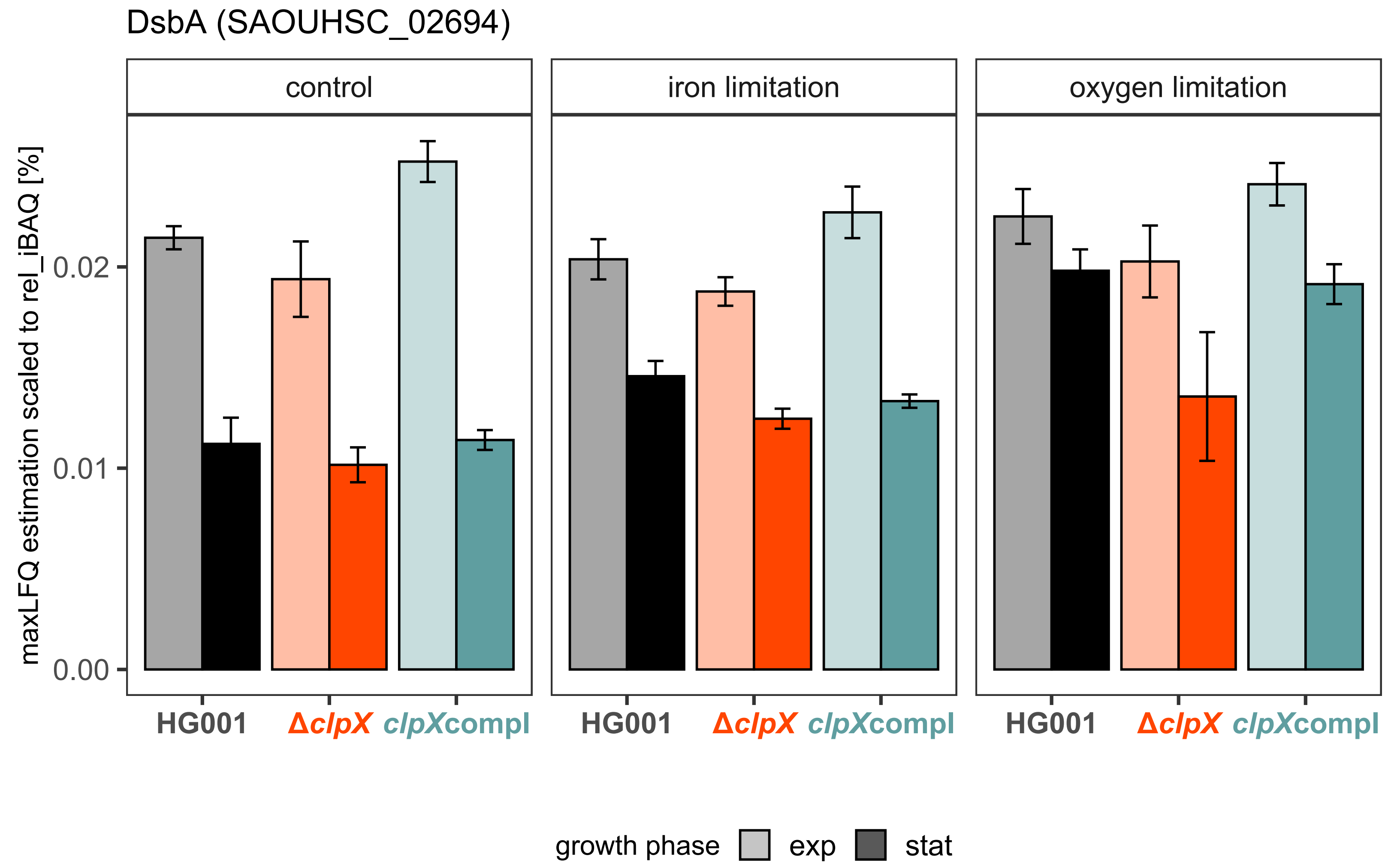
DsbA (SAOUHSC_02694)
control
iron limitation
oxygen limitation
maxLFQ estimation scaled to rel_iBAQ [%]
0.00
0.01
0.02
HG001
ΔclpX
clpXcompl
growth phase
exp
stat

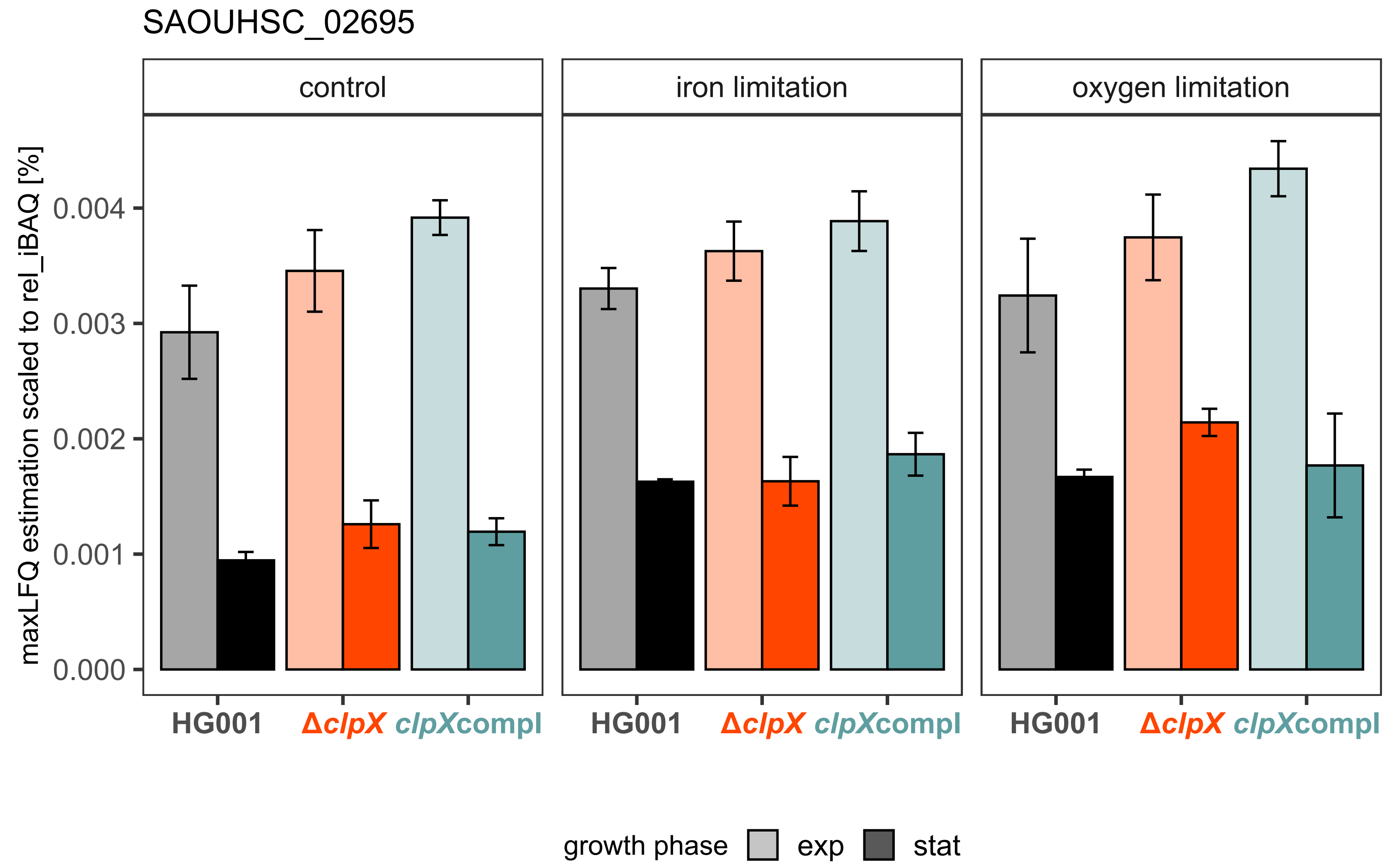
SAOUHSC_02695
control
iron limitation
oxygen limitation
maxLFQ estimation scaled to rel_iBAQ [%]
0.004
0.003
0.002
0.001
0.000
HG001
ΔclpX
clpXcompl
growth phase
exp
stat

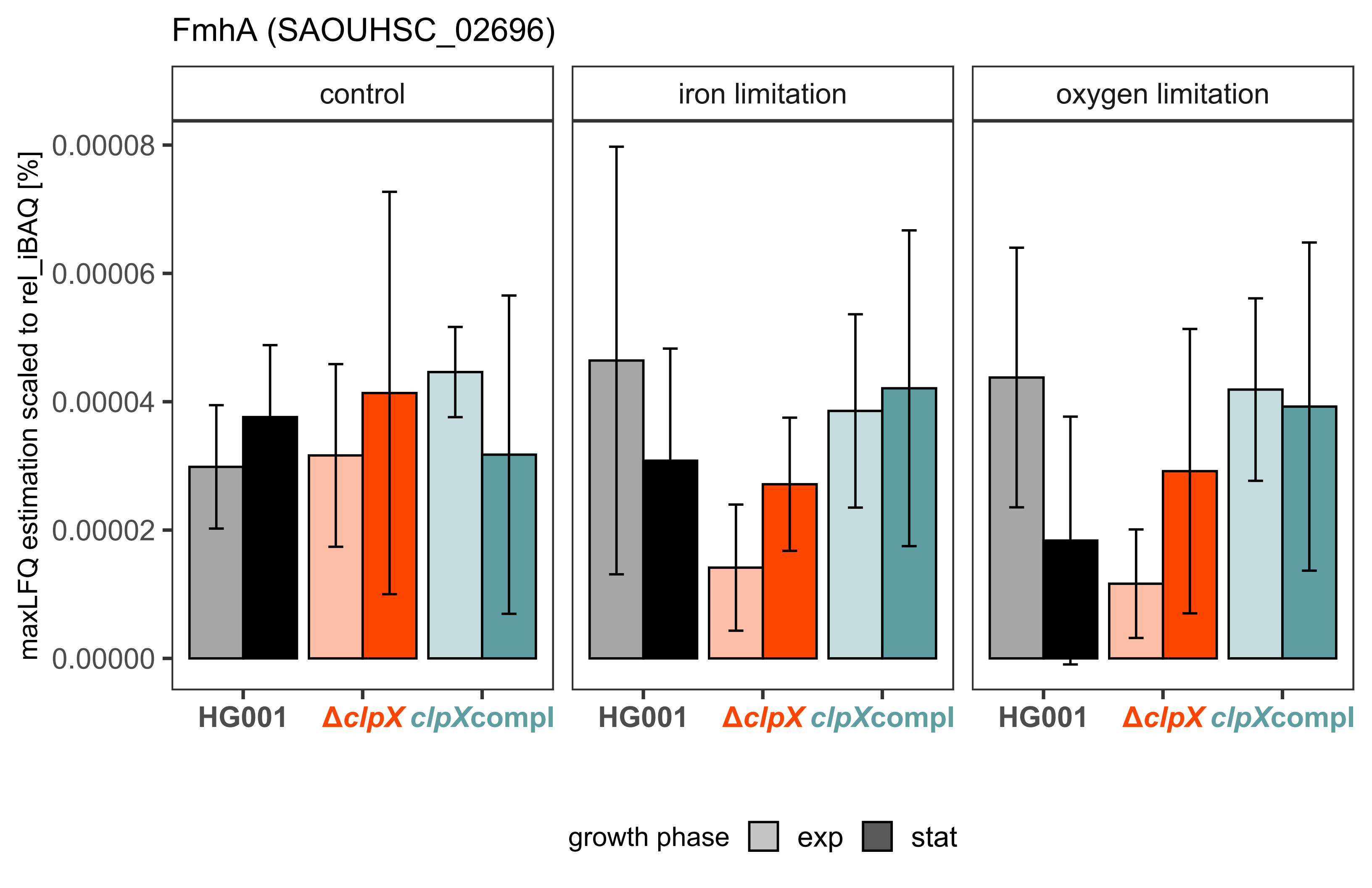
FmhA (SAOUHSC_02696)
control
iron limitation
oxygen limitation
maxLFQ estimation scaled to rel_iBAQ [%]
0.00008
0.00006
0.00004
0.00002
0.00000
HG001
ΔclpX
clpXcompl
growth phase
exp
stat

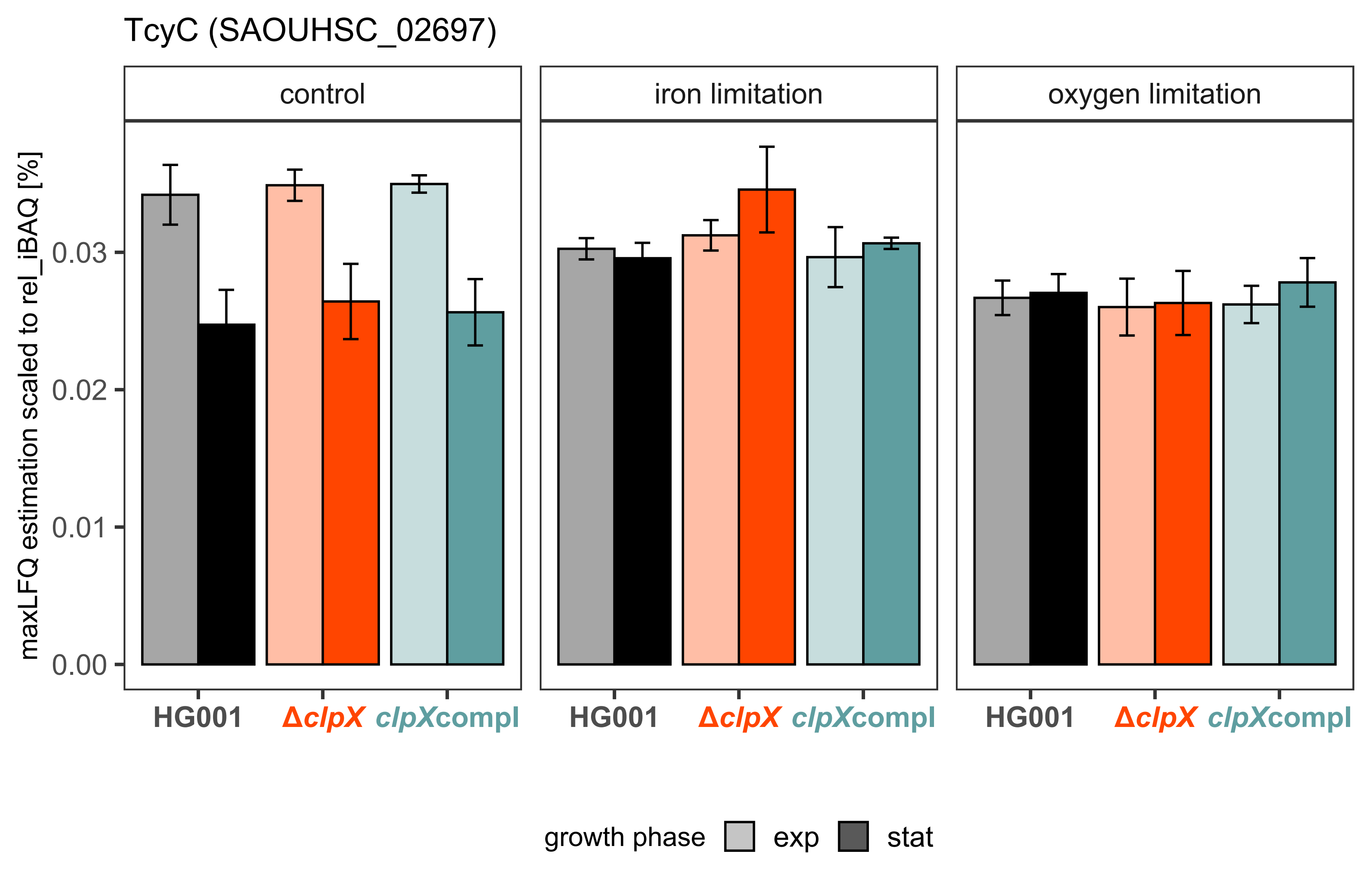

TcyC (SAOUHSC_02697)
control
iron limitation
oxygen limitation
maxLFQ estimation scaled to rel_iBAQ [%]
0.00
0.01
0.02
0.03
HG001
ΔclpX
clpXcompl
growth phase
exp
stat

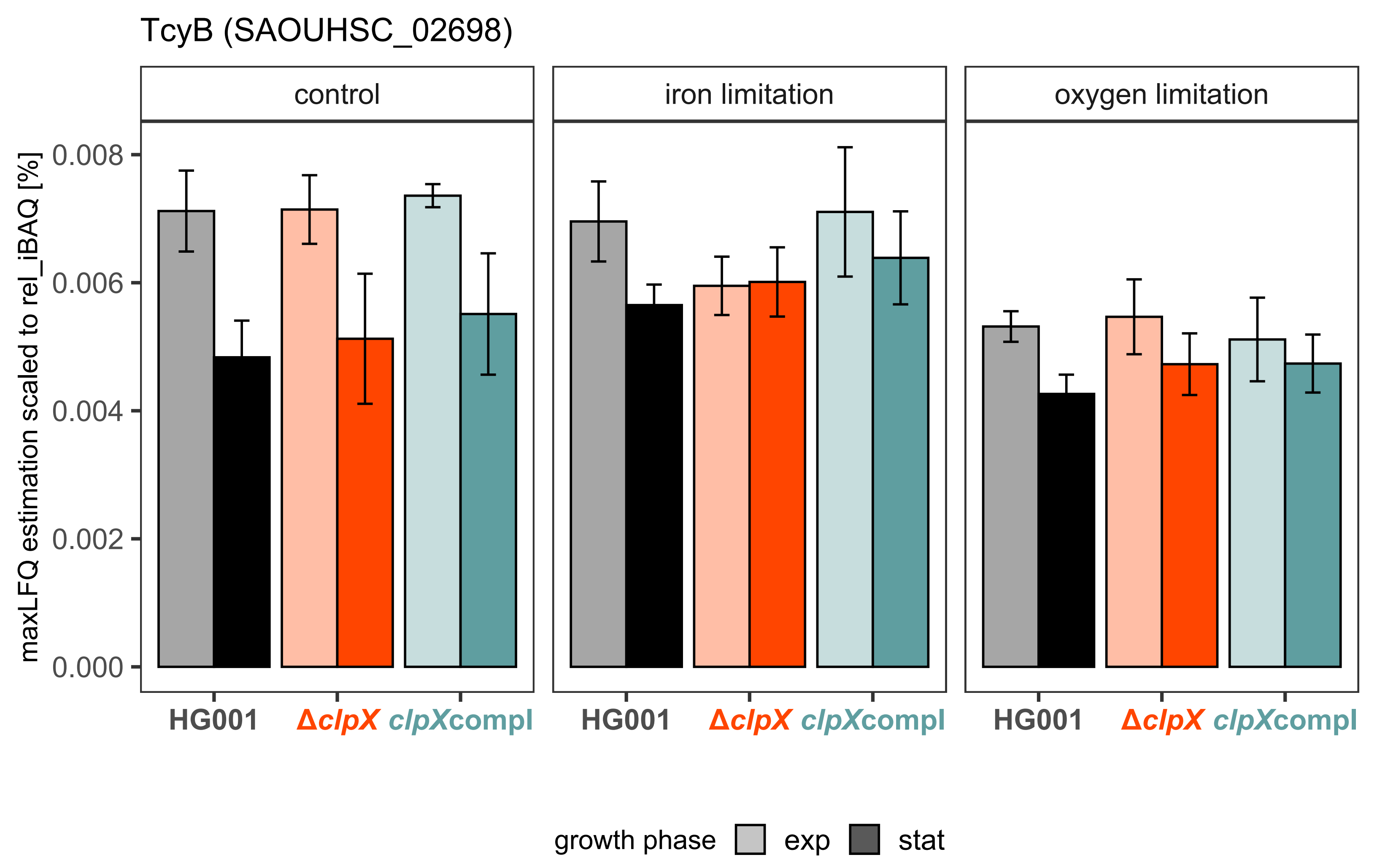

TcyB (SAOUHSC_02698)
control
iron limitation
oxygen limitation
maxLFQ estimation scaled to rel_iBAQ [%]
0.008
0.006
0.004
0.002
0.000
HG001
ΔclpX
clpXcompl
growth phase
exp
stat

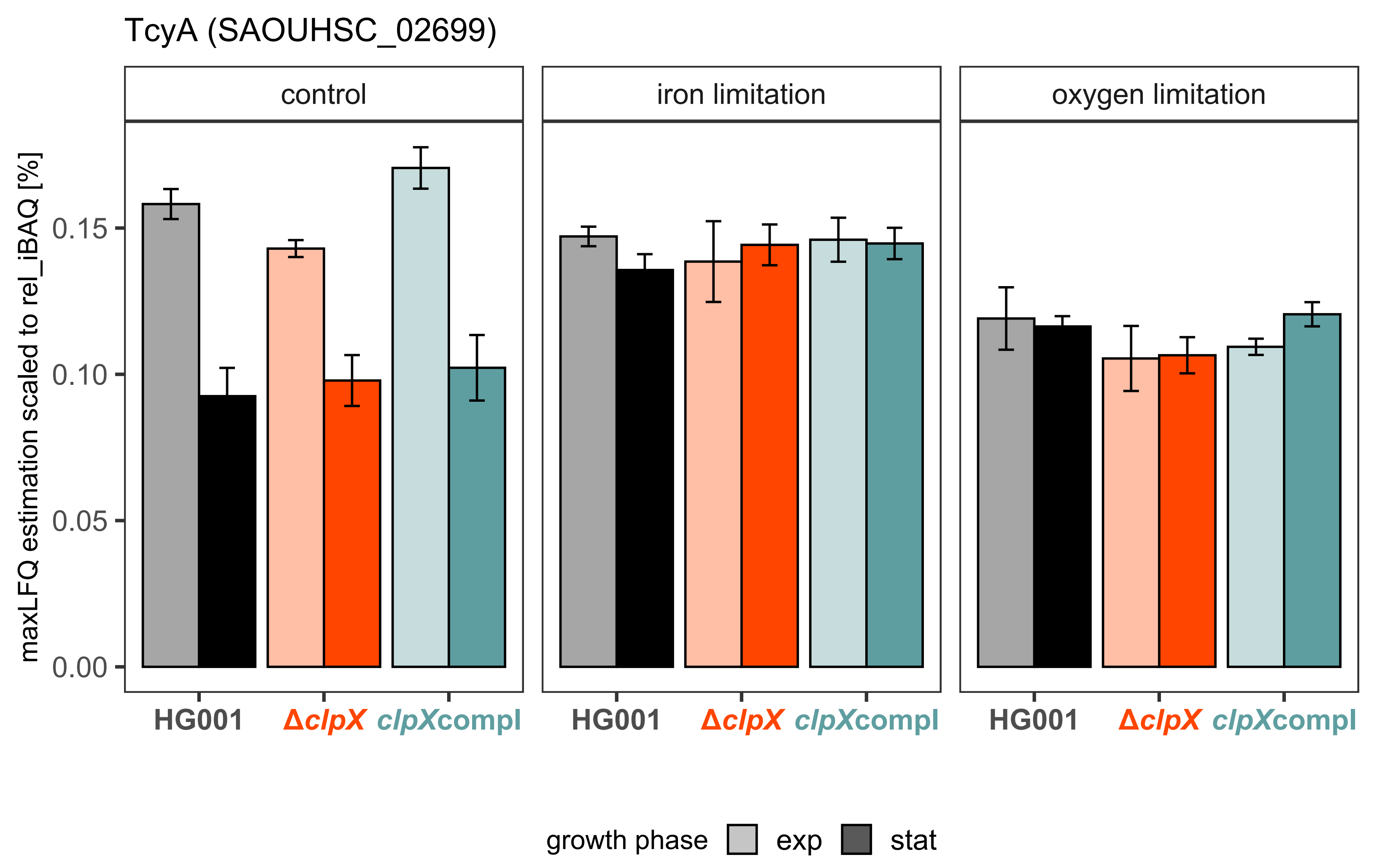
TcyA (SAOUHSC_02699)
control
iron limitation
oxygen limitation
maxLFQ estimation scaled to rel_iBAQ [%]
0.15
0.10
0.05
0.00
HG001
ΔclpX
clpXcompl
growth phase
exp
stat

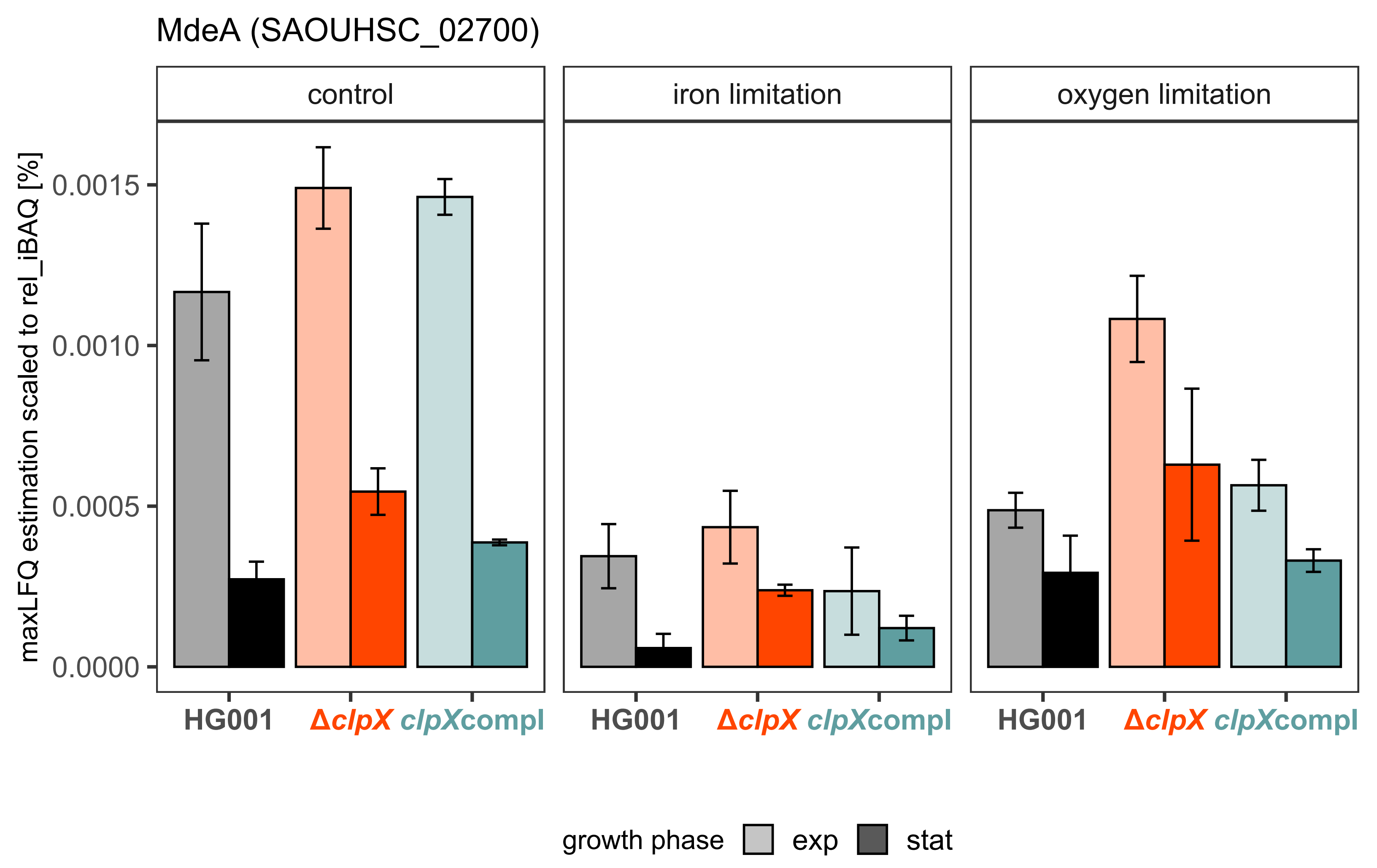

MdeA (SAOUHSC_02700)
control
iron limitation
oxygen limitation
maxLFQ estimation scaled to rel_iBAQ [%]
0.0015
0.0010
0.0005
0.0000
HG001
ΔclpX
clpXcompl
growth phase
exp
stat

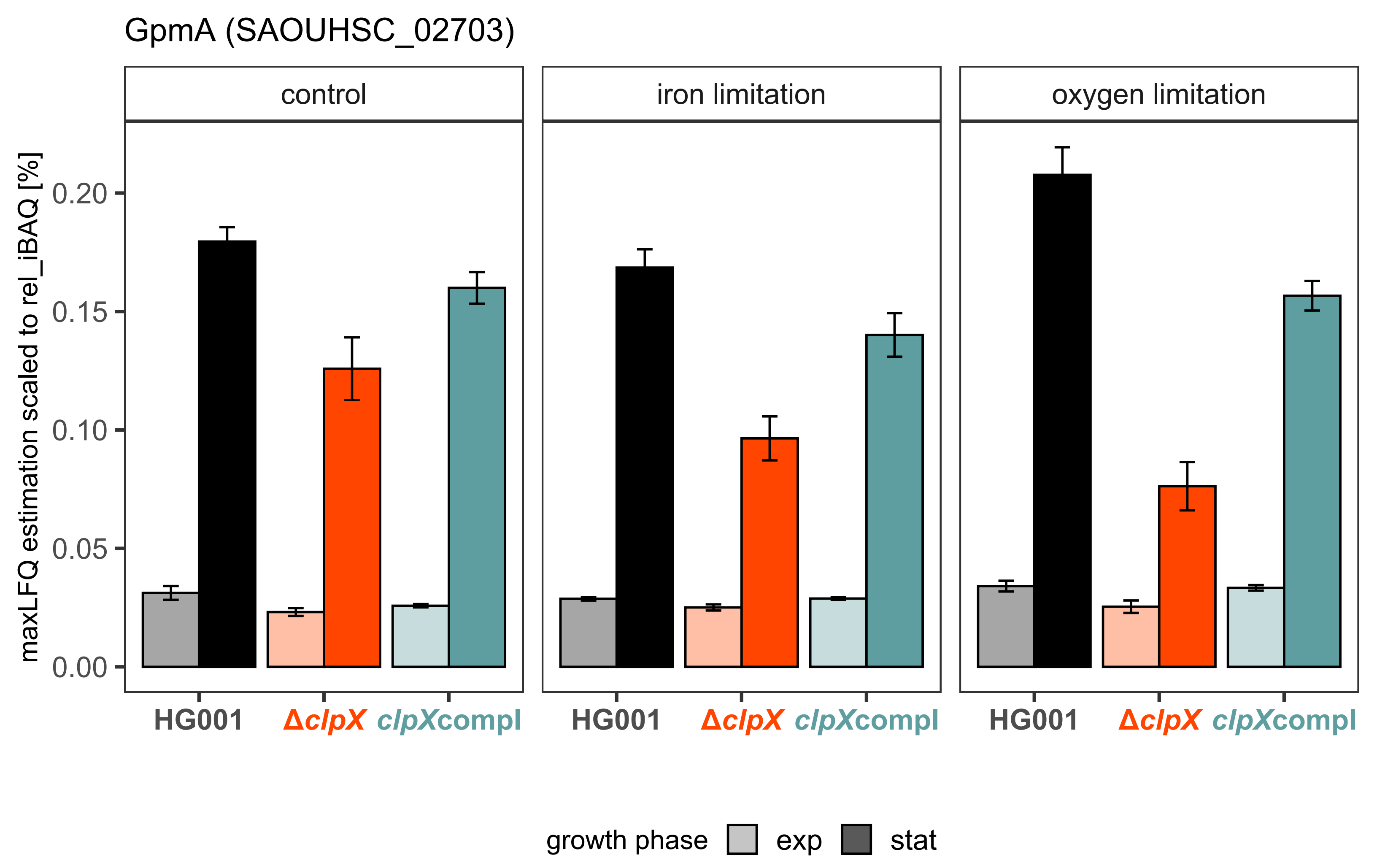

GpmA (SAOUHSC_02703)
control
iron limitation
oxygen limitation
maxLFQ estimation scaled to rel_iBAQ [%]
0.20
0.15
0.10
0.05
0.00
HG001
ΔclpX
clpXcompl
growth phase
exp
stat

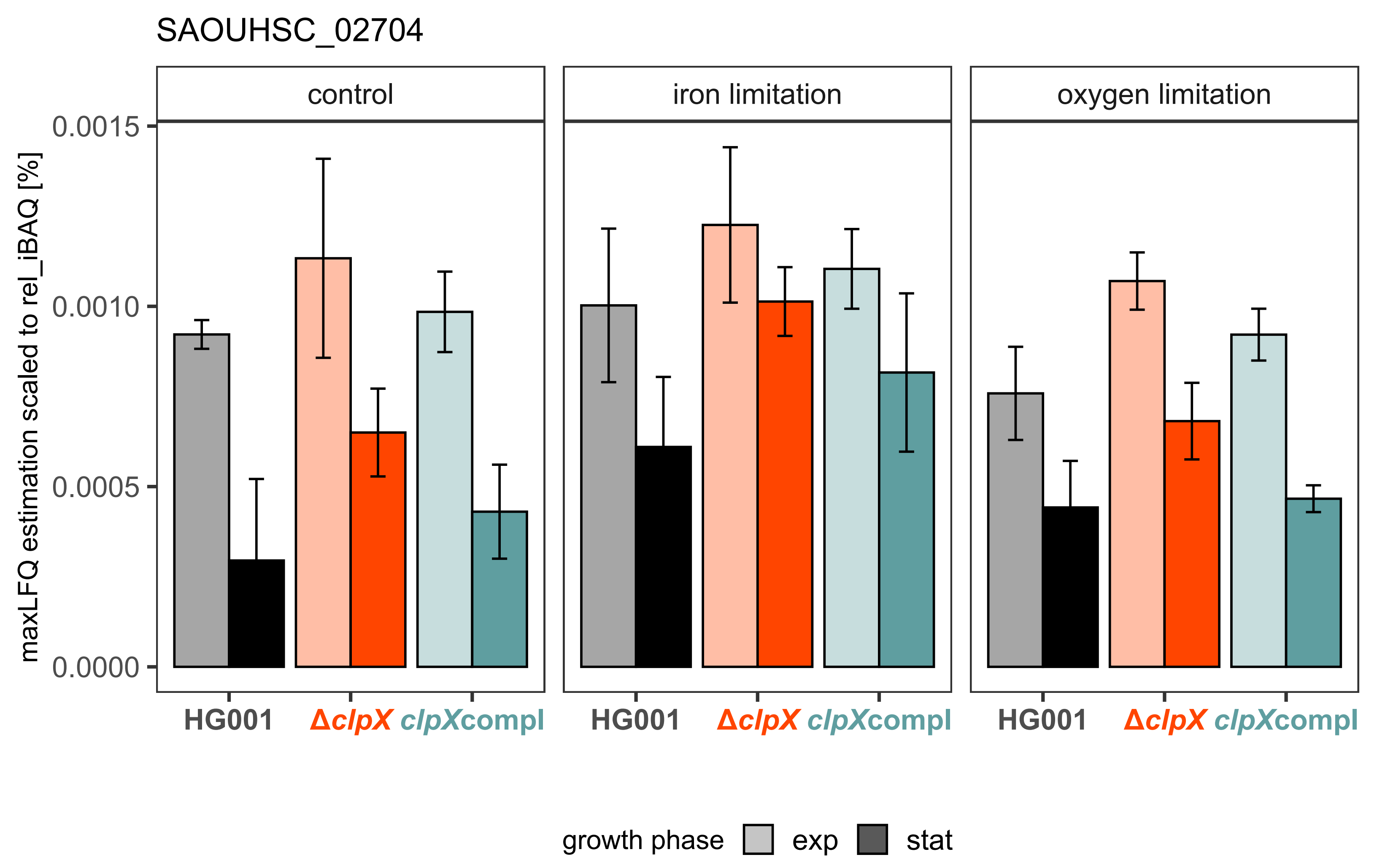
SAOUHSC_02704
control
iron limitation
oxygen limitation
maxLFQ estimation scaled to rel_iBAQ [%]
0.0015
0.0010
0.0005
0.0000
HG001
ΔclpX
clpXcompl
growth phase
exp
stat

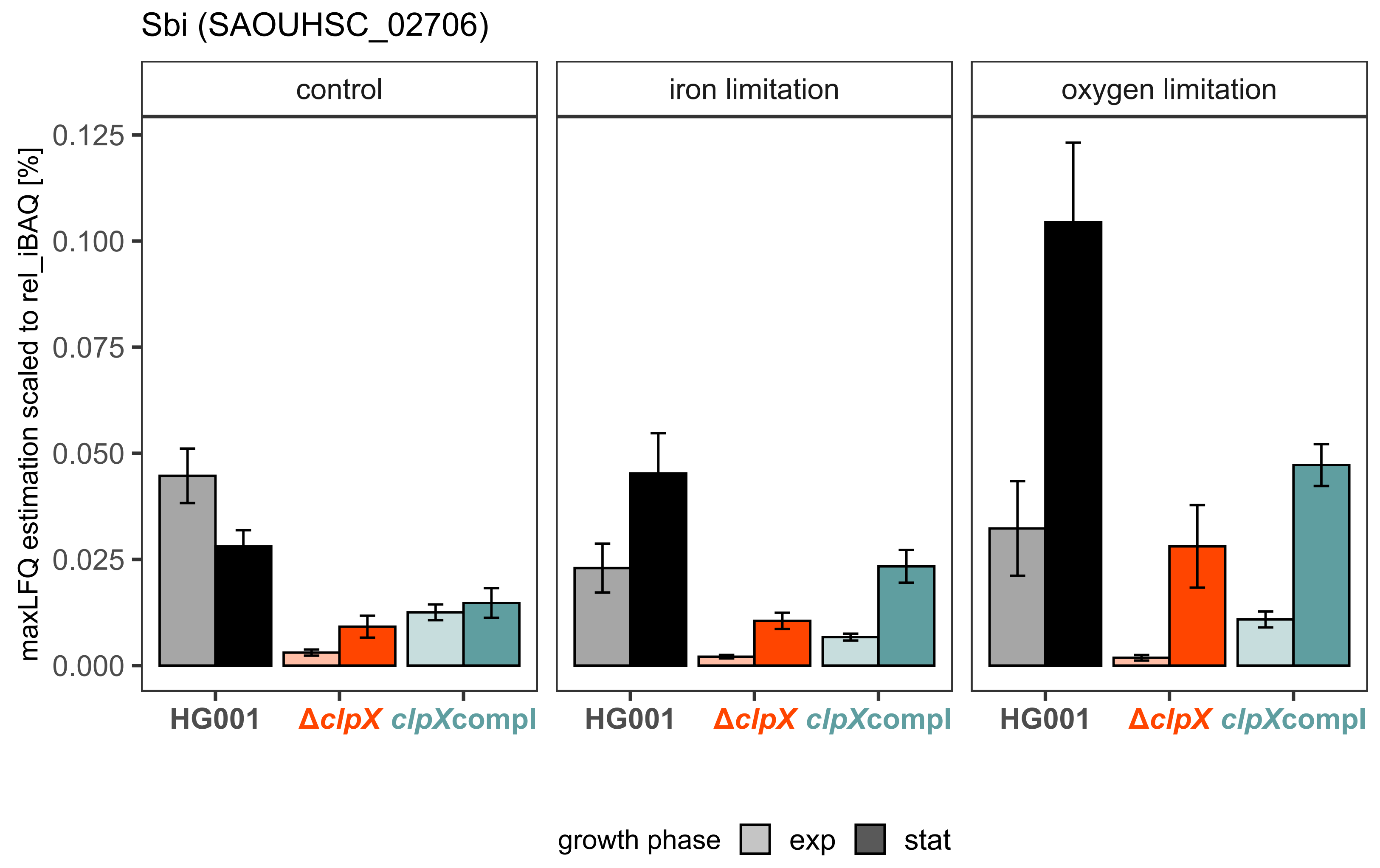

Sbi (SAOUHSC_02706)
control
iron limitation
oxygen limitation
maxLFQ estimation scaled to rel_iBAQ [%]
0.125
0.100
0.075
0.050
0.025
0.000
HG001
ΔclpX
clpXcompl
growth phase
exp
stat

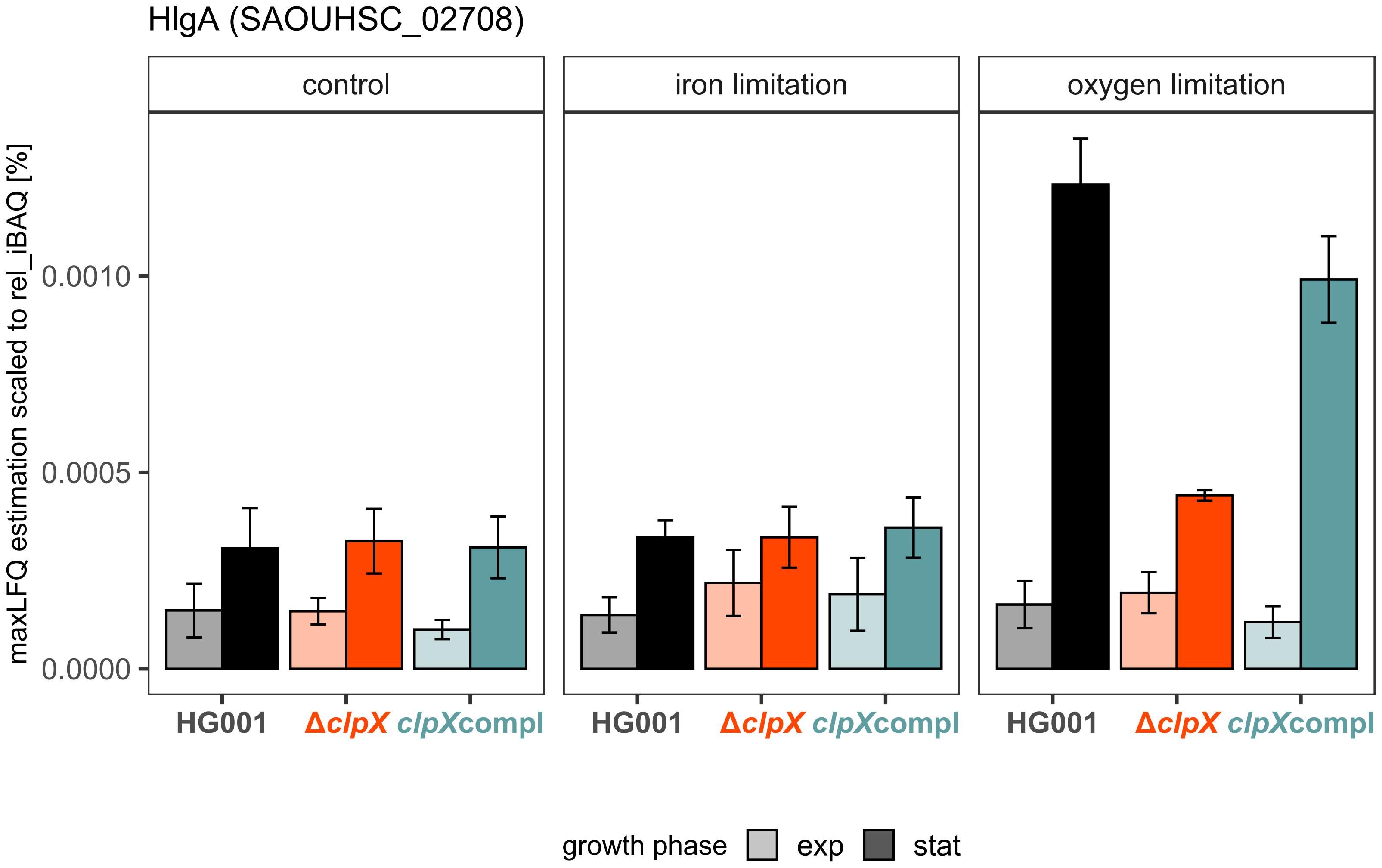

HlgA (SAOUHSC_02708)
control
iron limitation
oxygen limitation
maxLFQ estimation scaled to rel_iBAQ [%]
0.0010
0.0005
0.0000
HG001
ΔclpX
clpXcompl
growth phase
exp
stat

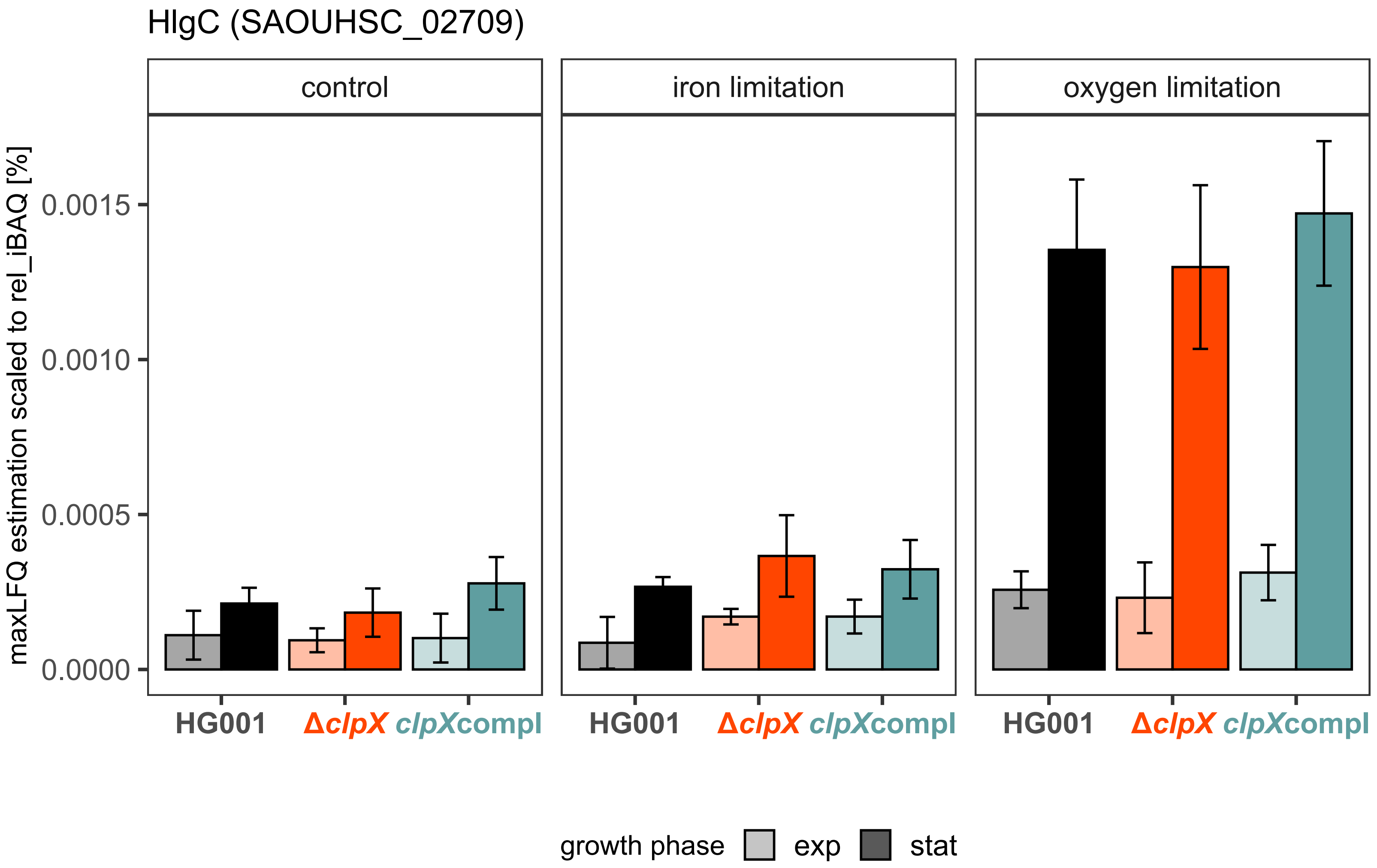

HlgC (SAOUHSC_02709)
control
iron limitation
oxygen limitation
maxLFQ estimation scaled to rel_iBAQ [%]
0.0015
0.0010
0.0005
0.0000
HG001
ΔclpX
clpXcompl
growth phase
exp
stat

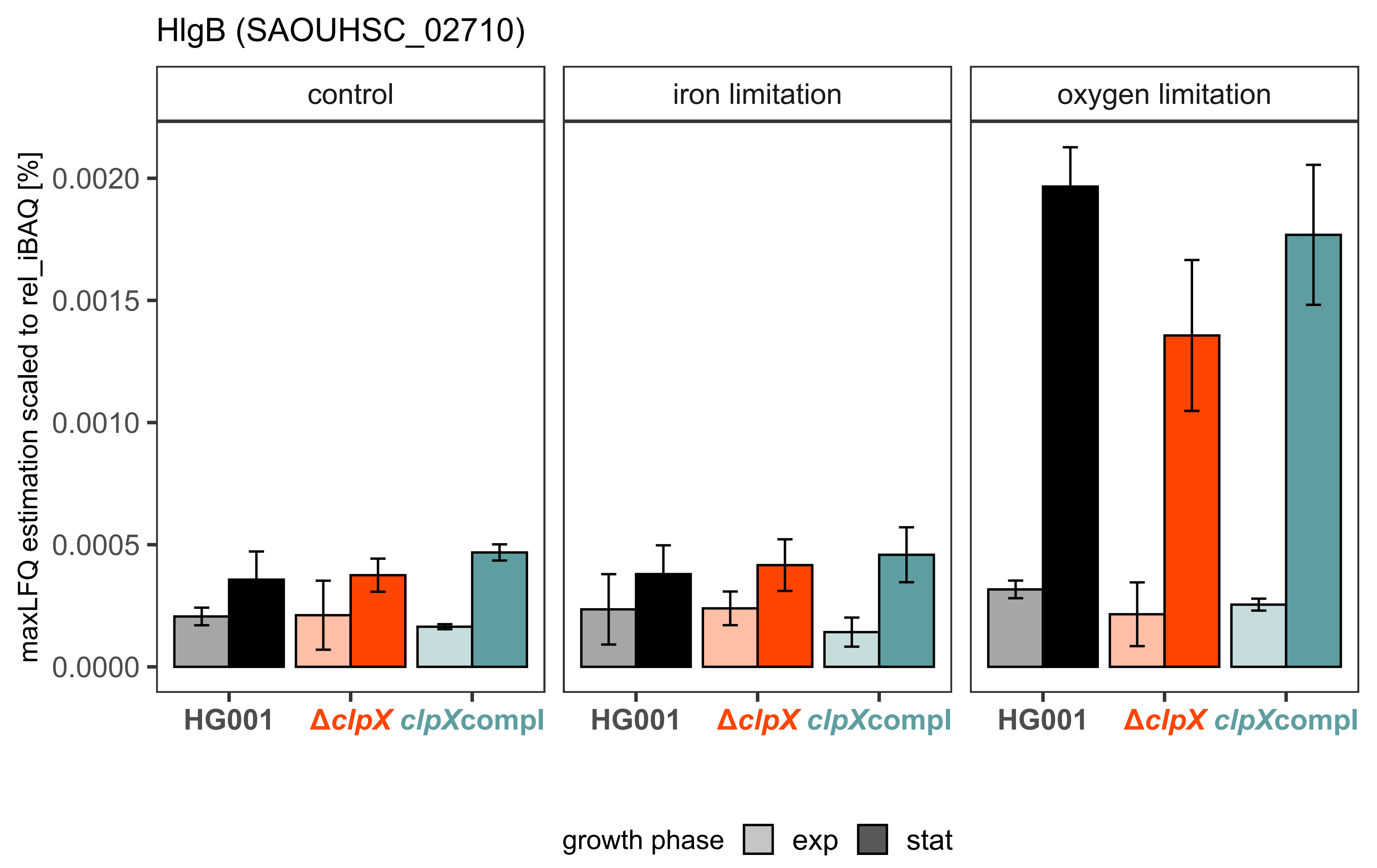

HlgB (SAOUHSC_02710)
control
iron limitation
oxygen limitation
maxLFQ estimation scaled to rel_iBAQ [%]
0.0020
0.0015
0.0010
0.0005
0.0000
HG001
ΔclpX
clpXcompl
growth phase
exp
stat

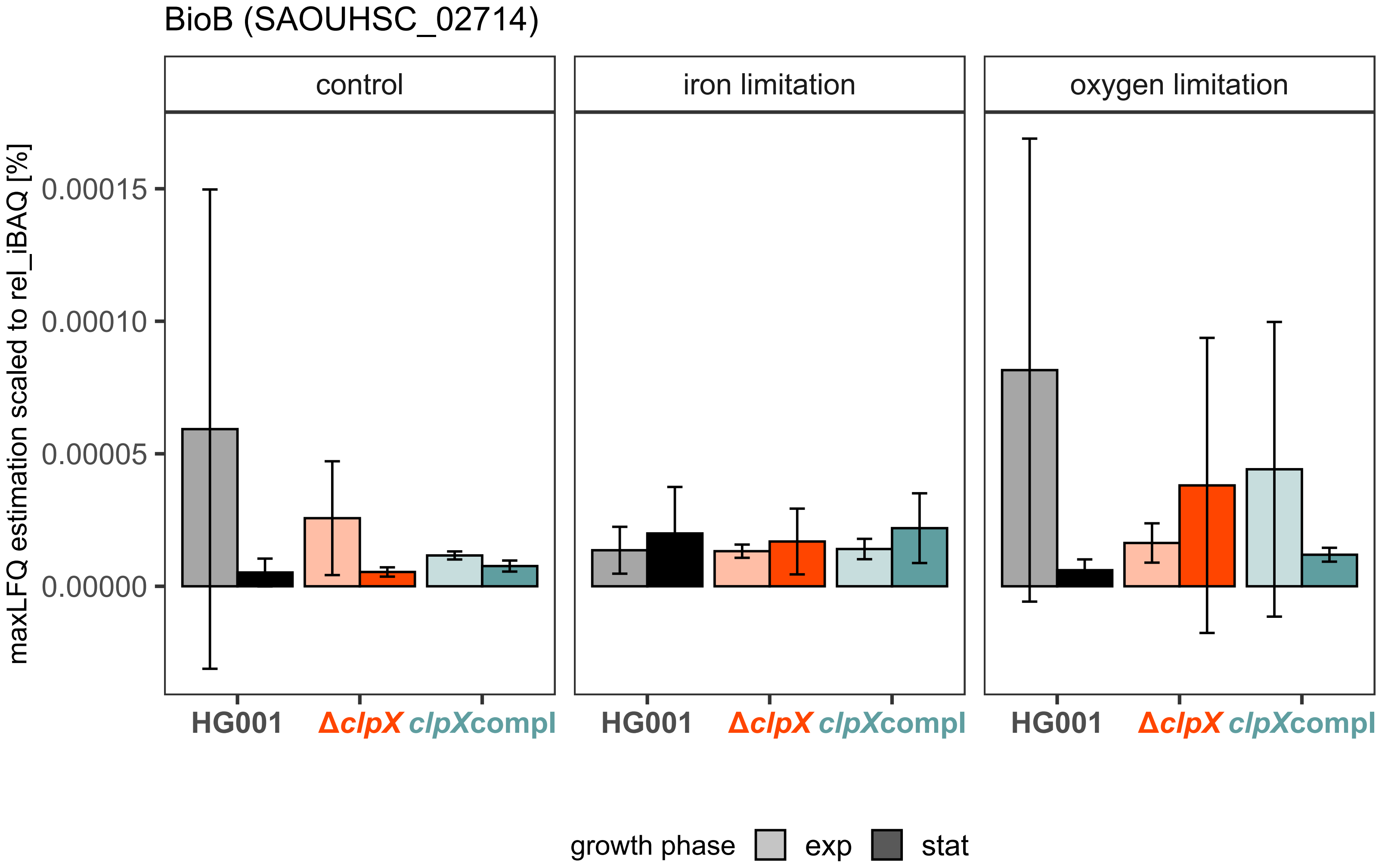

BioB (SAOUHSC_02714)
control
iron limitation
oxygen limitation
maxLFQ estimation scaled to rel_iBAQ [%]
0.00015
0.00010
0.00005
0.00000
HG001
ΔclpX
clpXcompl
growth phase
exp
stat

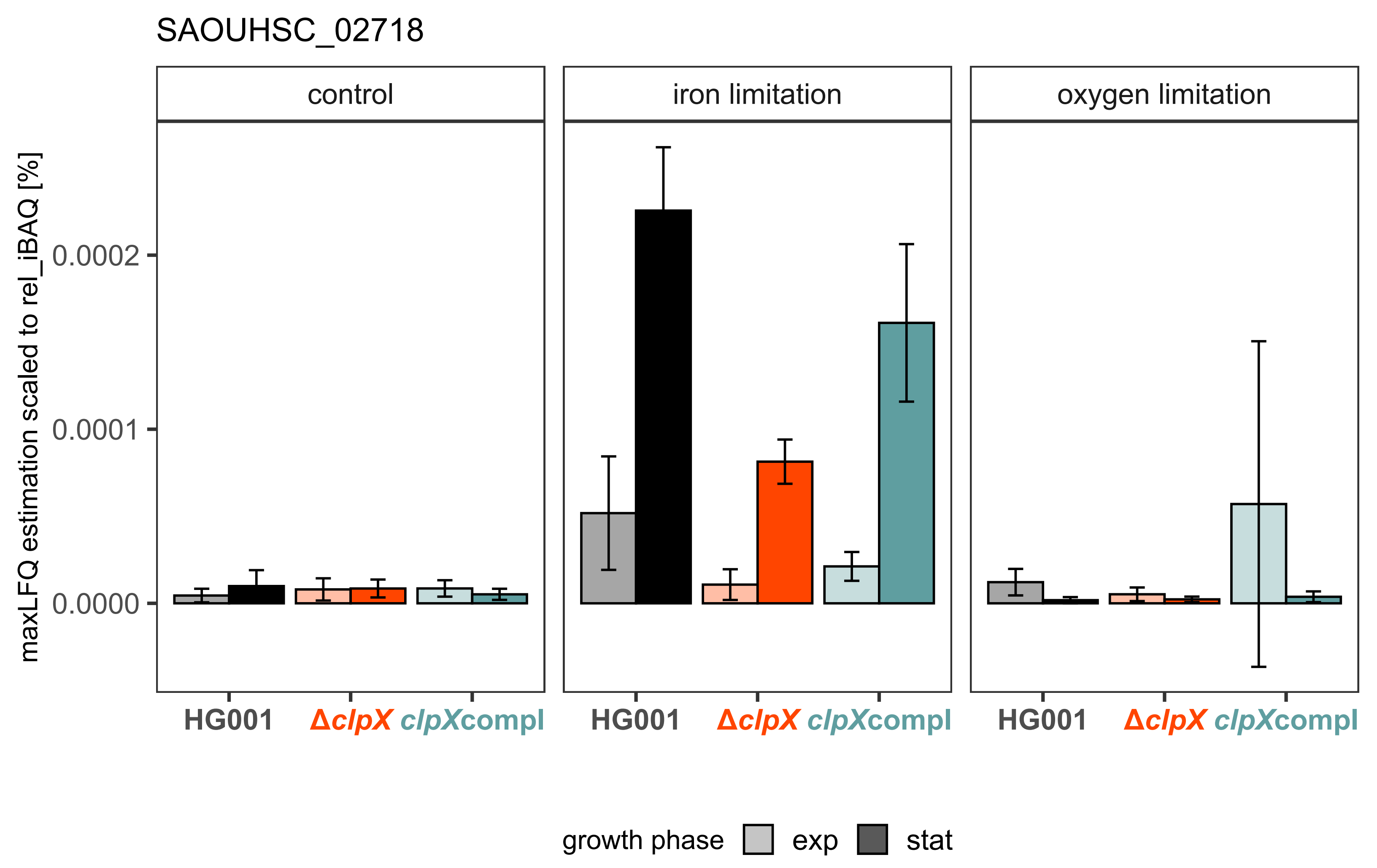

SAOUHSC_02718
control
iron limitation
oxygen limitation
maxLFQ estimation scaled to rel_iBAQ [%]
0.0002
0.0001
0.0000
HG001
ΔclpX
clpXcompl
growth phase
exp
stat

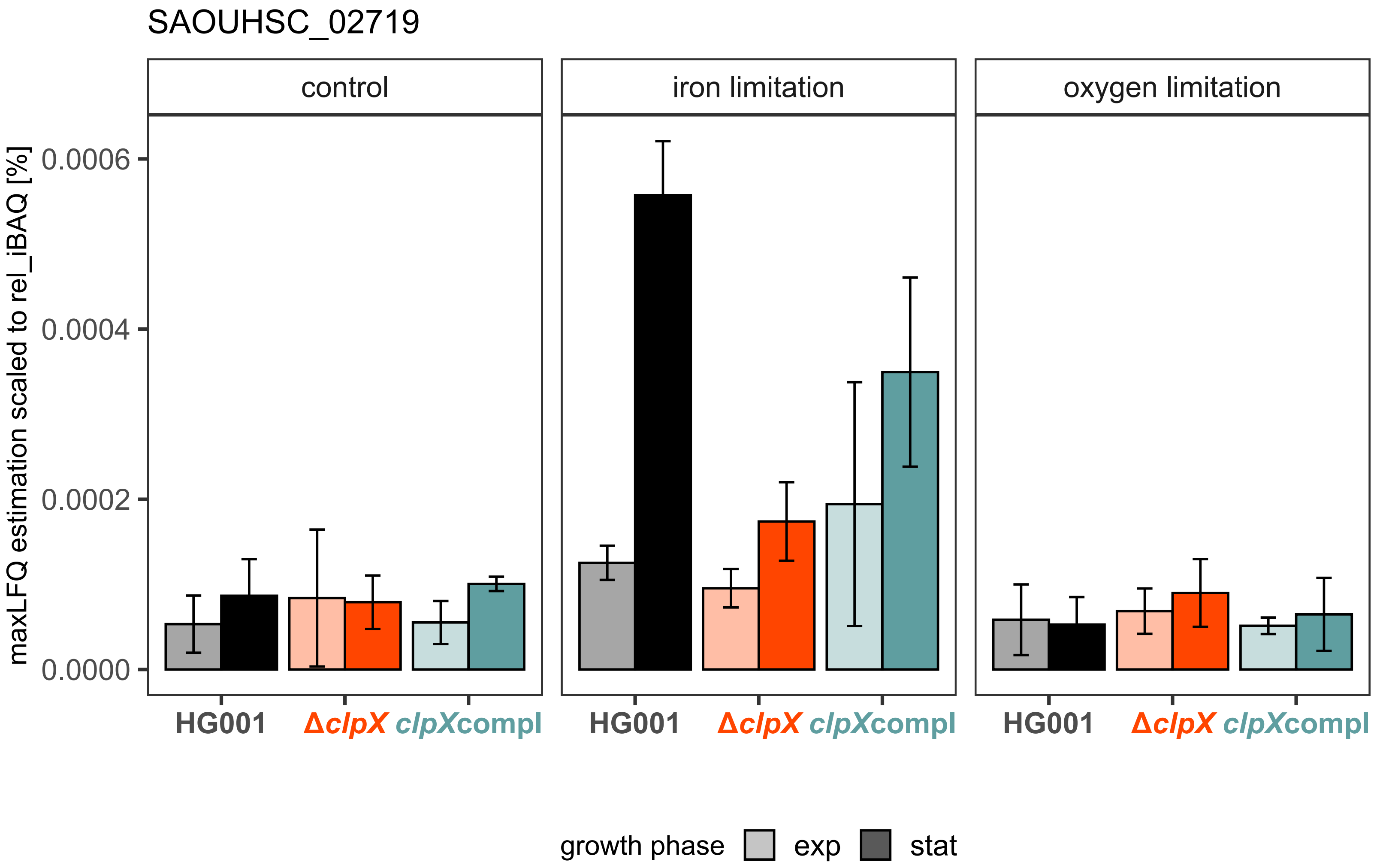
SAOUHSC_02719
control
iron limitation
oxygen limitation
maxLFQ estimation scaled to rel_iBAQ [%]
0.0006
0.0004
0.0002
0.0000
HG001
ΔclpX
clpXcompl
growth phase
exp
stat

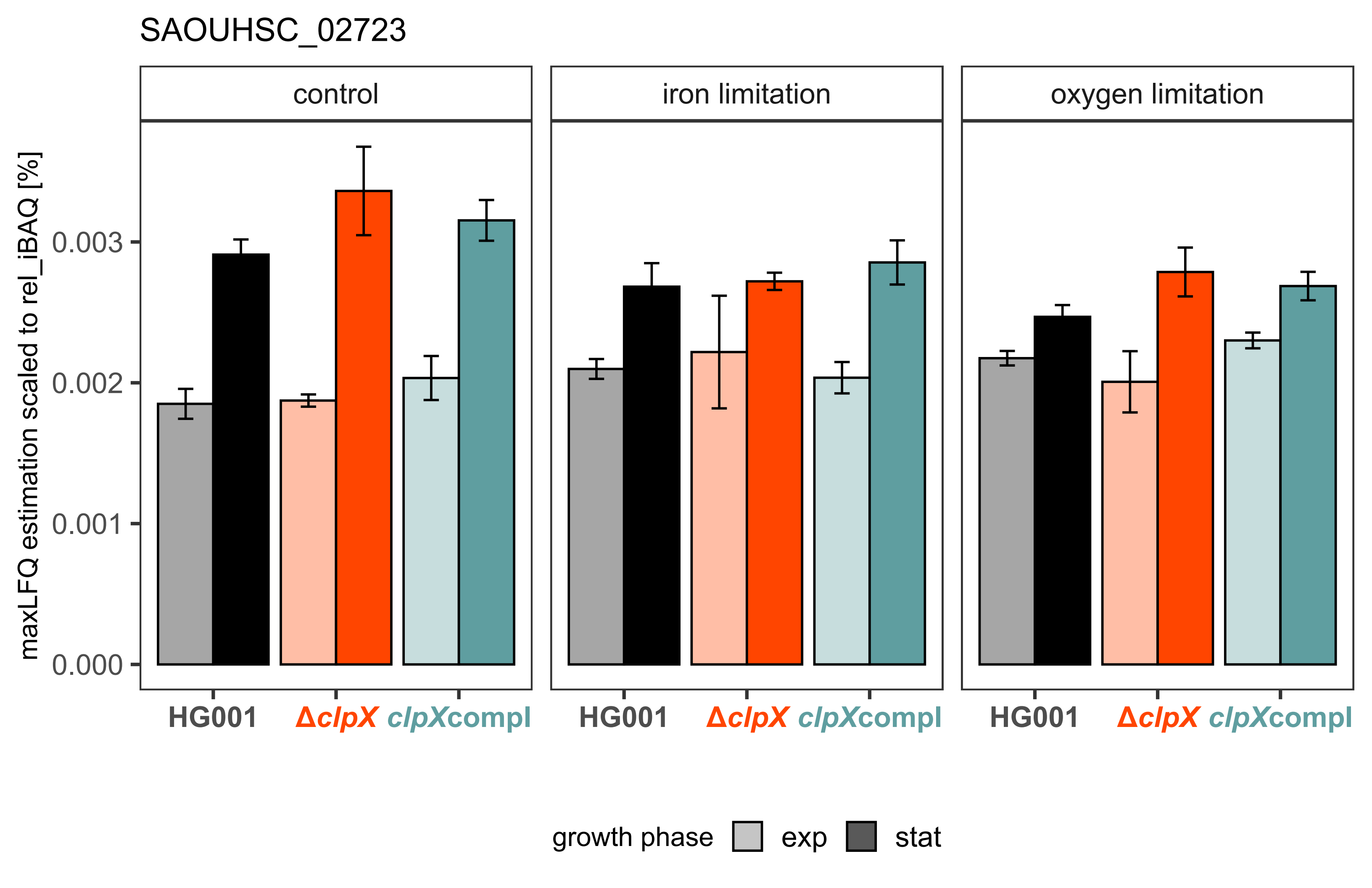
SAOUHSC_02723
control
iron limitation
oxygen limitation
maxLFQ estimation scaled to rel_iBAQ [%]
0.003
0.002
0.001
0.000
HG001
ΔclpX
clpXcompl
growth phase
exp
stat

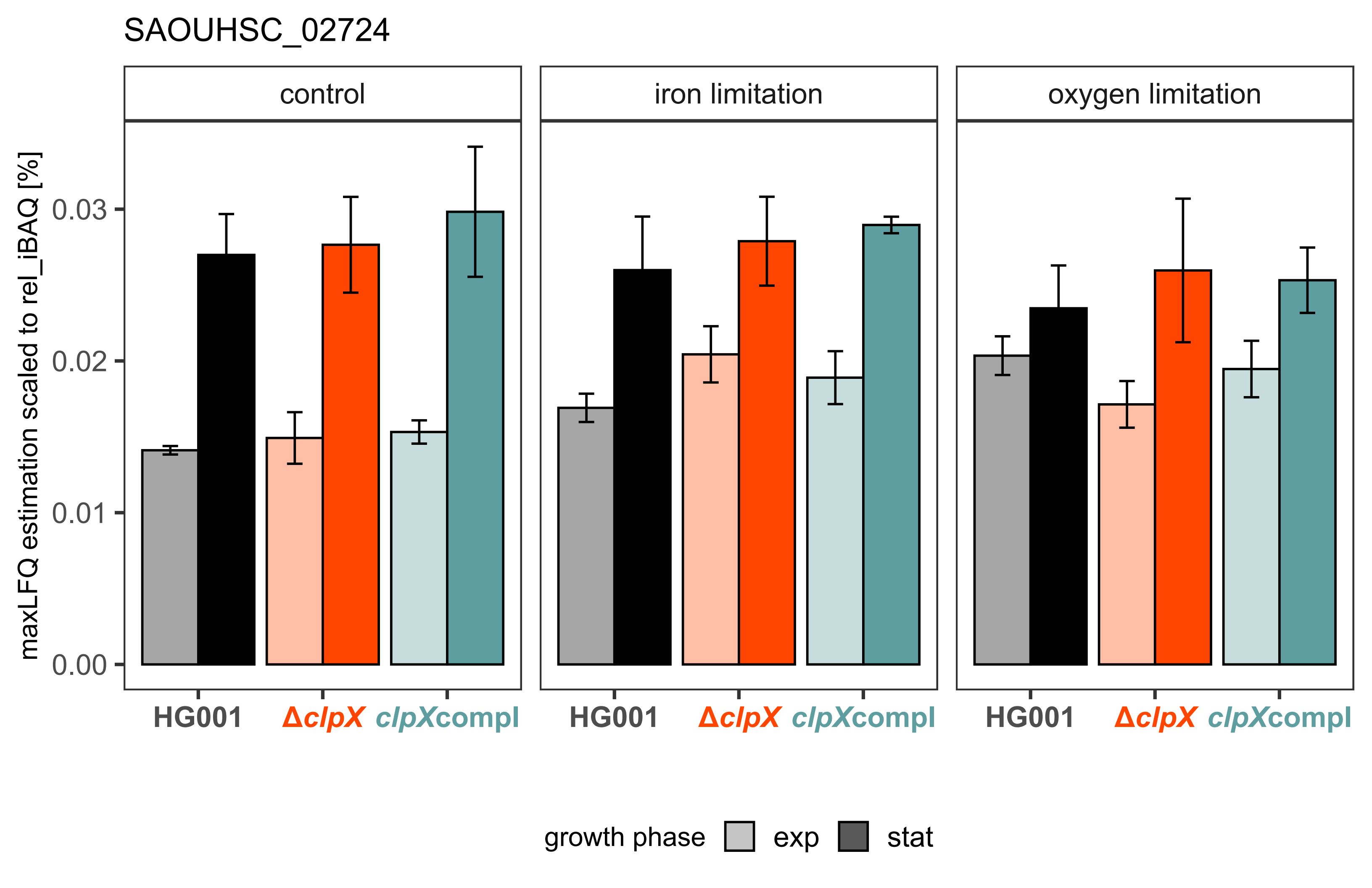
SAOUHSC_02724
control
iron limitation
oxygen limitation
maxLFQ estimation scaled to rel_iBAQ [%]
0.00
0.01
0.02
0.03
HG001
ΔclpX
clpXcompl
growth phase
exp
stat

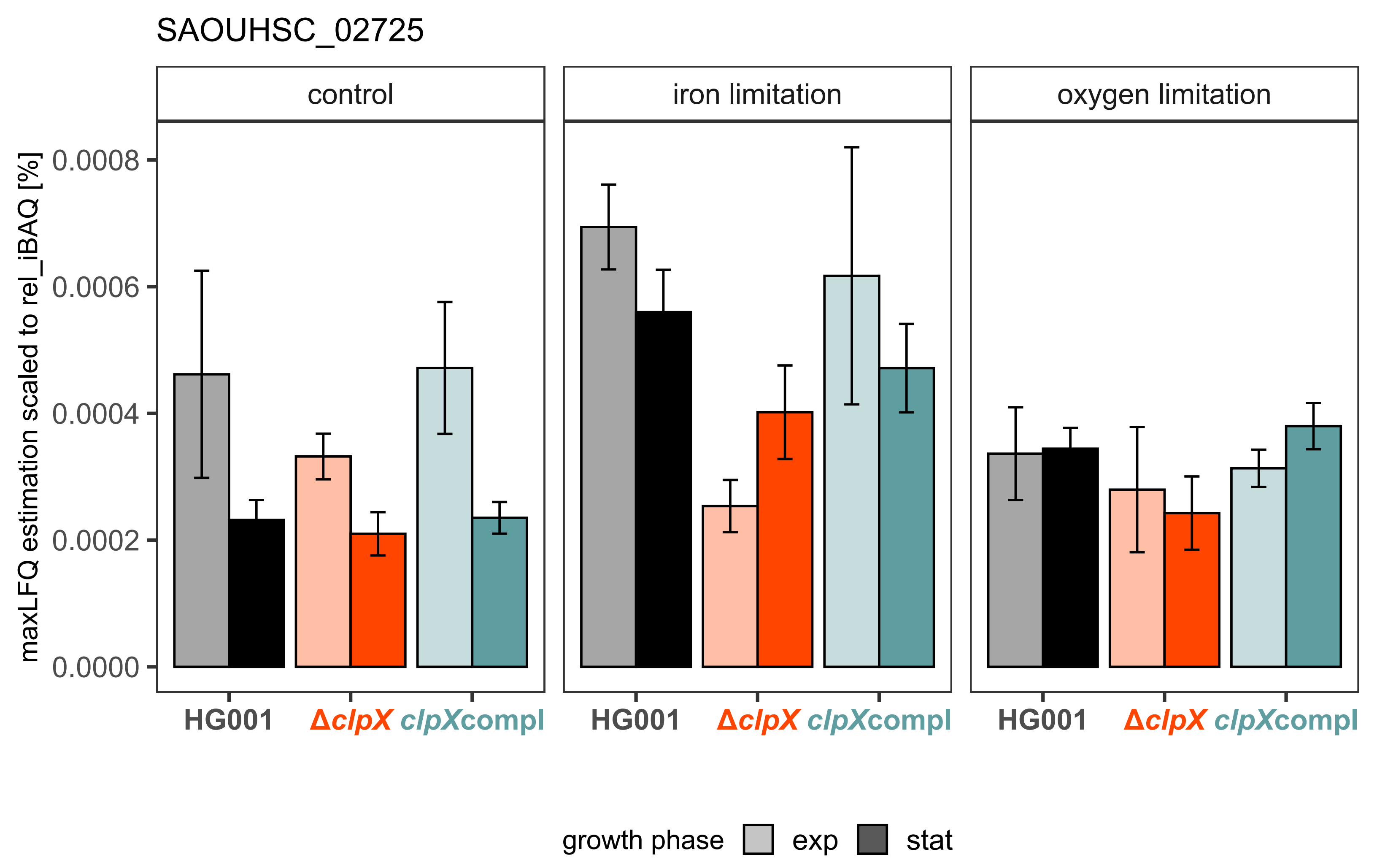

SAOUHSC_02725
control
iron limitation
oxygen limitation
maxLFQ estimation scaled to rel_iBAQ [%]
0.0008
0.0006
0.0004
0.0002
0.0000
HG001
ΔclpX
clpXcompl
growth phase
exp
stat

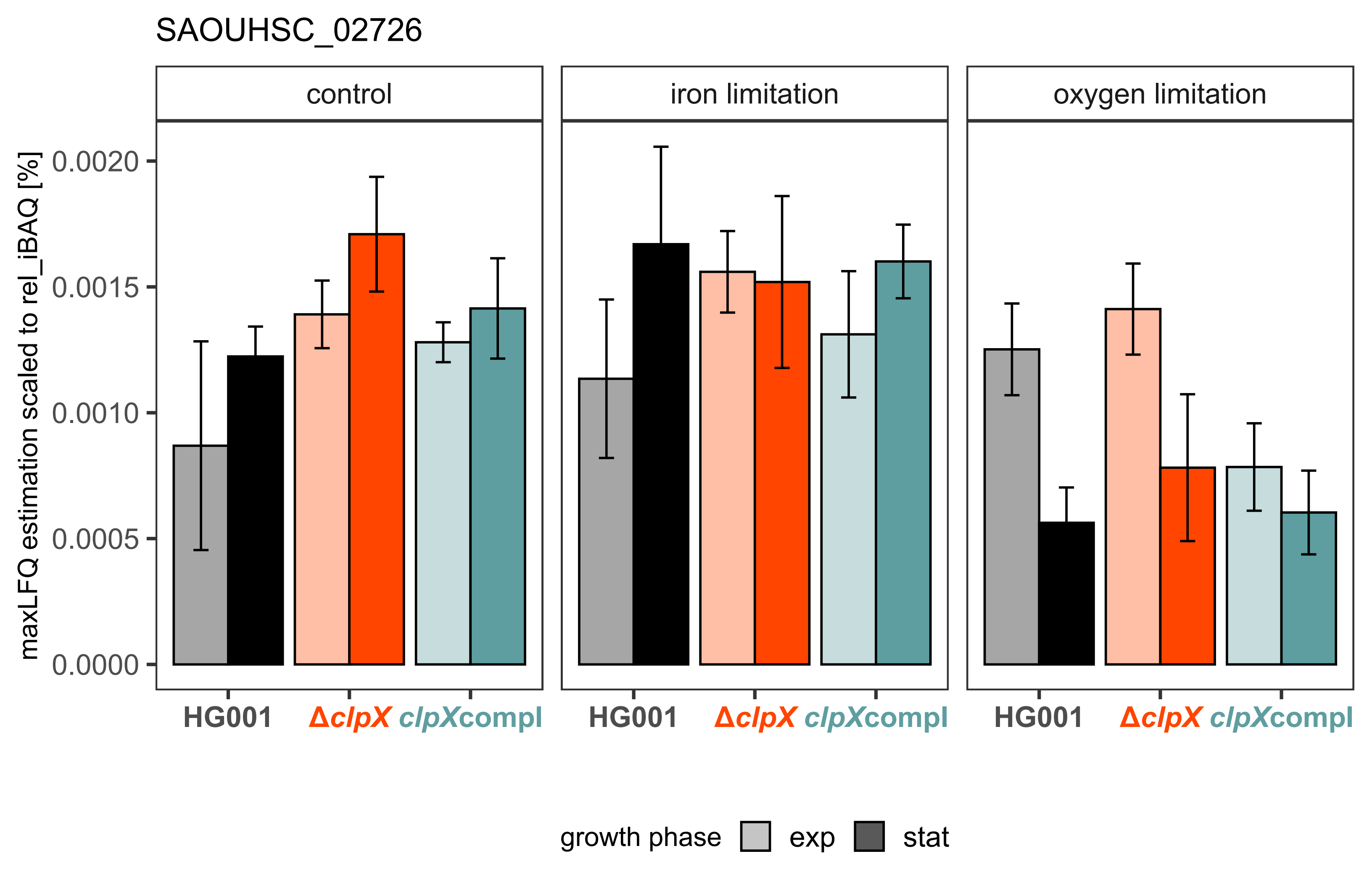
SAOUHSC_02726
control
iron limitation
oxygen limitation
maxLFQ estimation scaled to rel_iBAQ [%]
0.0020
0.0015
0.0010
0.0005
0.0000
HG001
ΔclpX
clpXcompl
growth phase
exp
stat

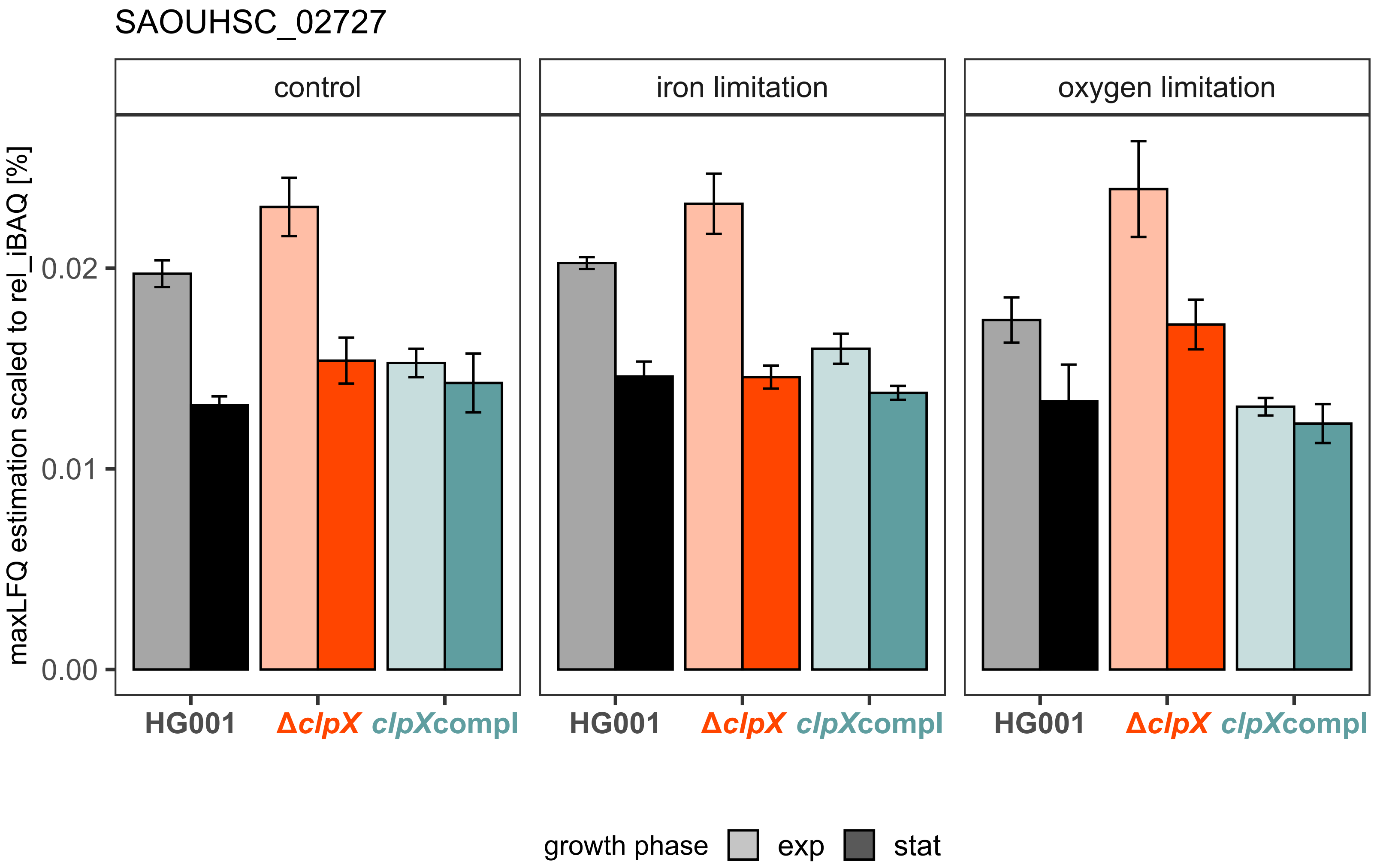
SAOUHSC_02727
control
iron limitation
oxygen limitation
maxLFQ estimation scaled to rel_iBAQ [%]
0.02
0.01
0.00
HG001
ΔclpX
clpXcompl
growth phase
exp
stat

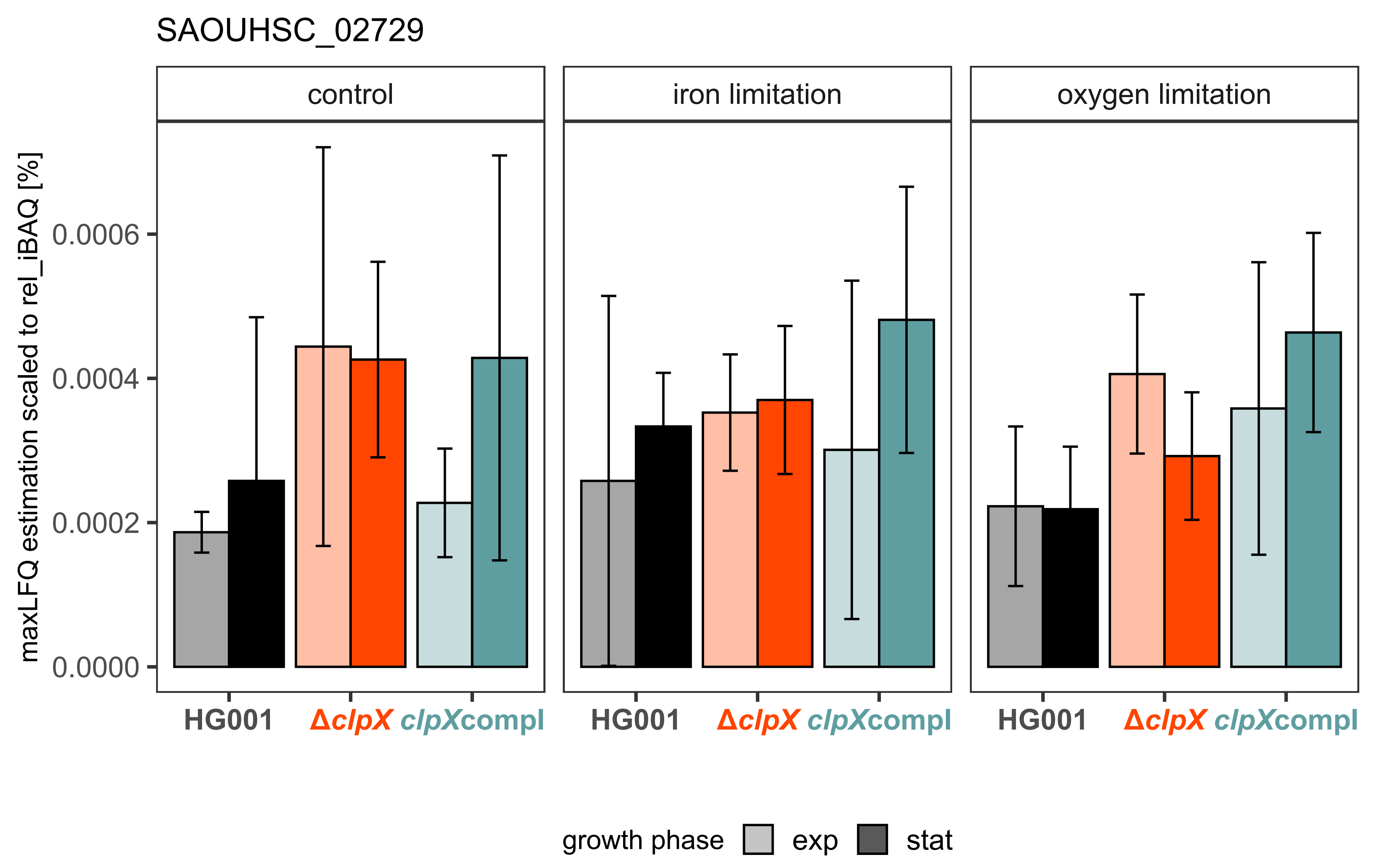

SAOUHSC_02729
control
iron limitation
oxygen limitation
maxLFQ estimation scaled to rel_iBAQ [%]
0.0006
0.0004
0.0002
0.0000
HG001
ΔclpX
clpXcompl
growth phase
exp
stat

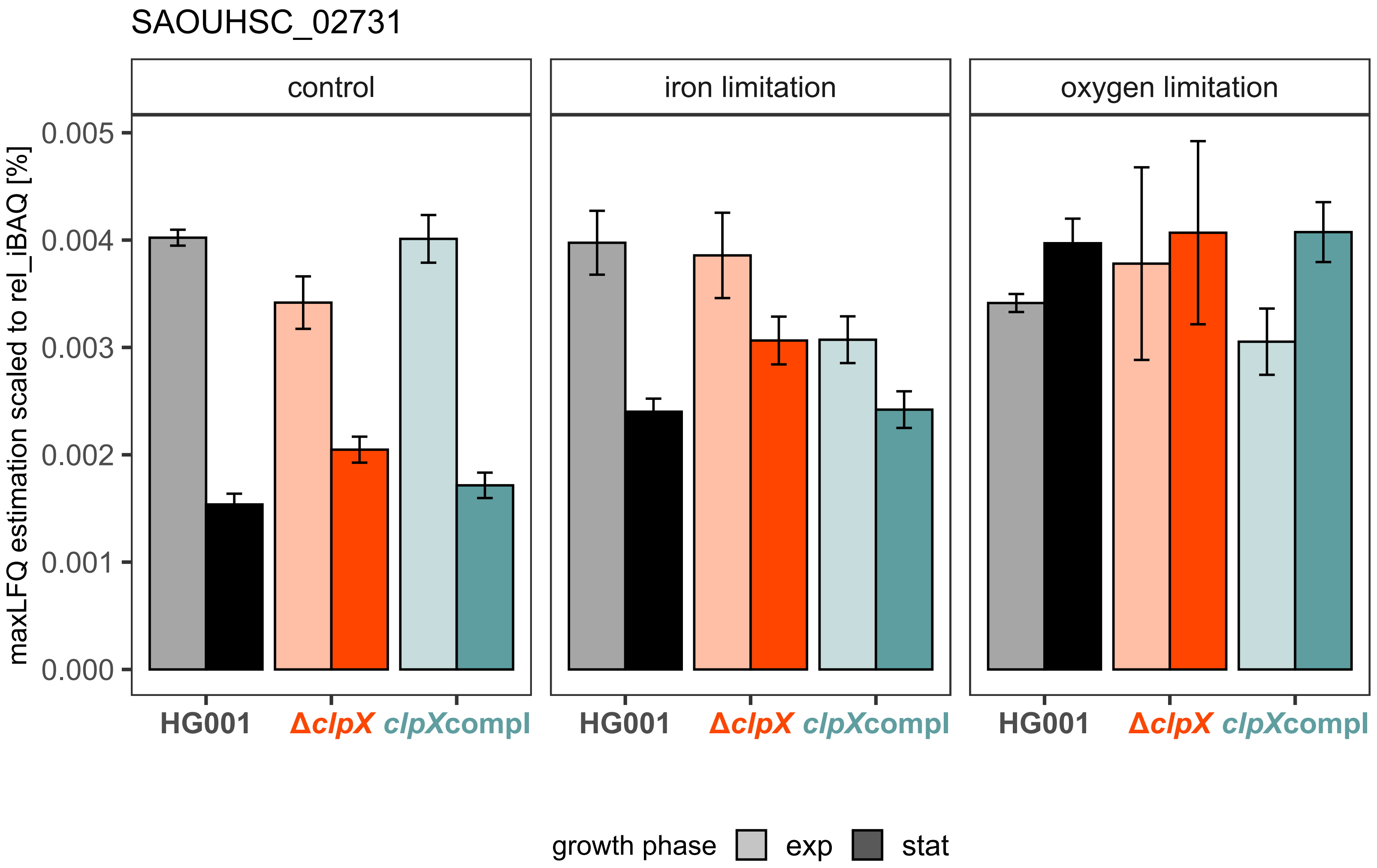
SAOUHSC_02731
control
iron limitation
oxygen limitation
maxLFQ estimation scaled to rel_iBAQ [%]
0.005
0.004
0.003
0.002
0.001
0.000
HG001
ΔclpX
clpXcompl
growth phase
exp
stat

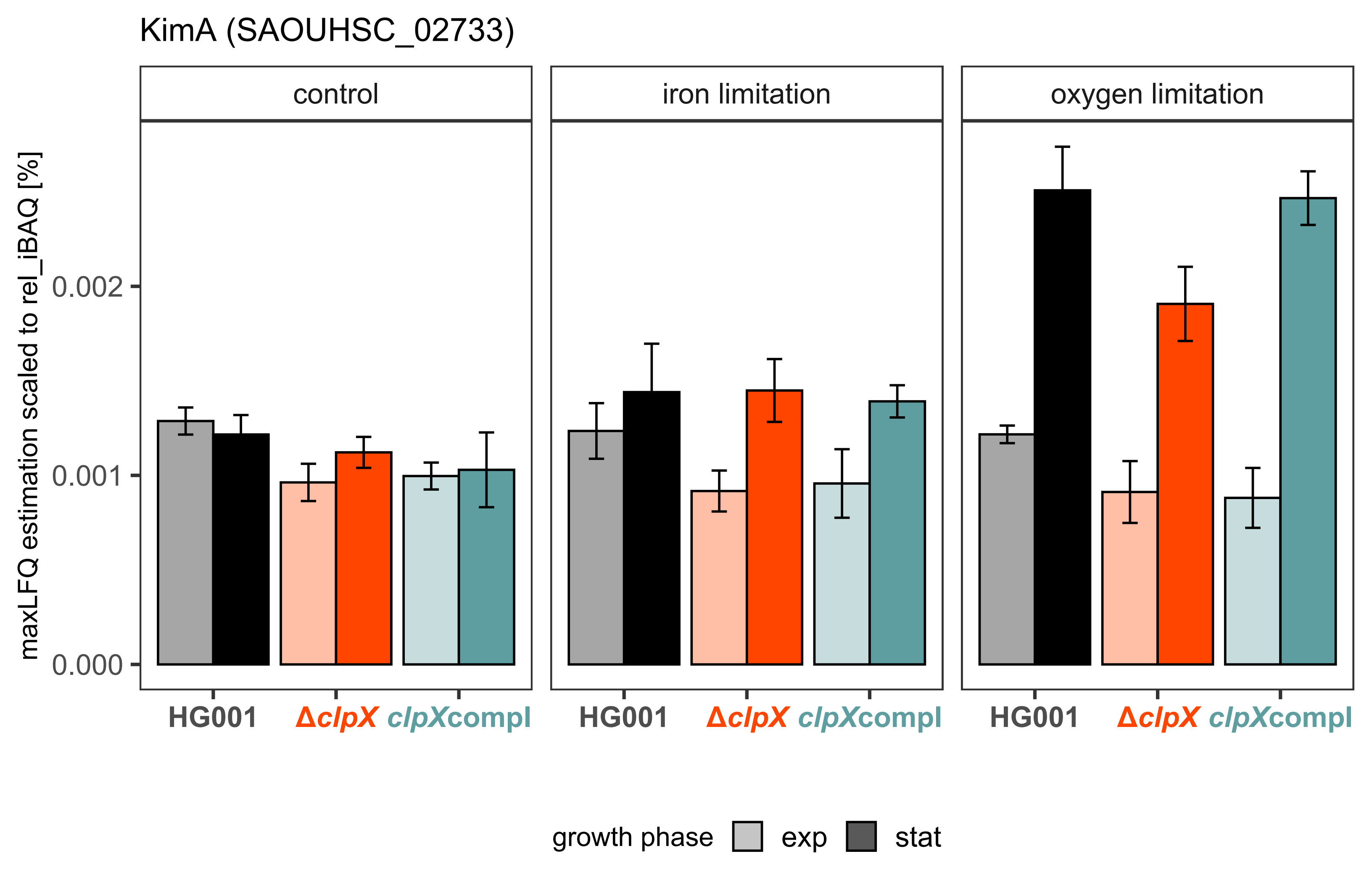
KimA (SAOUHSC_02733)
control
iron limitation
oxygen limitation
maxLFQ estimation scaled to rel_iBAQ [%]
0.002
0.001
0.000
HG001
ΔclpX
clpXcompl
growth phase
exp
stat

Flp (SAOUHSC_02736)

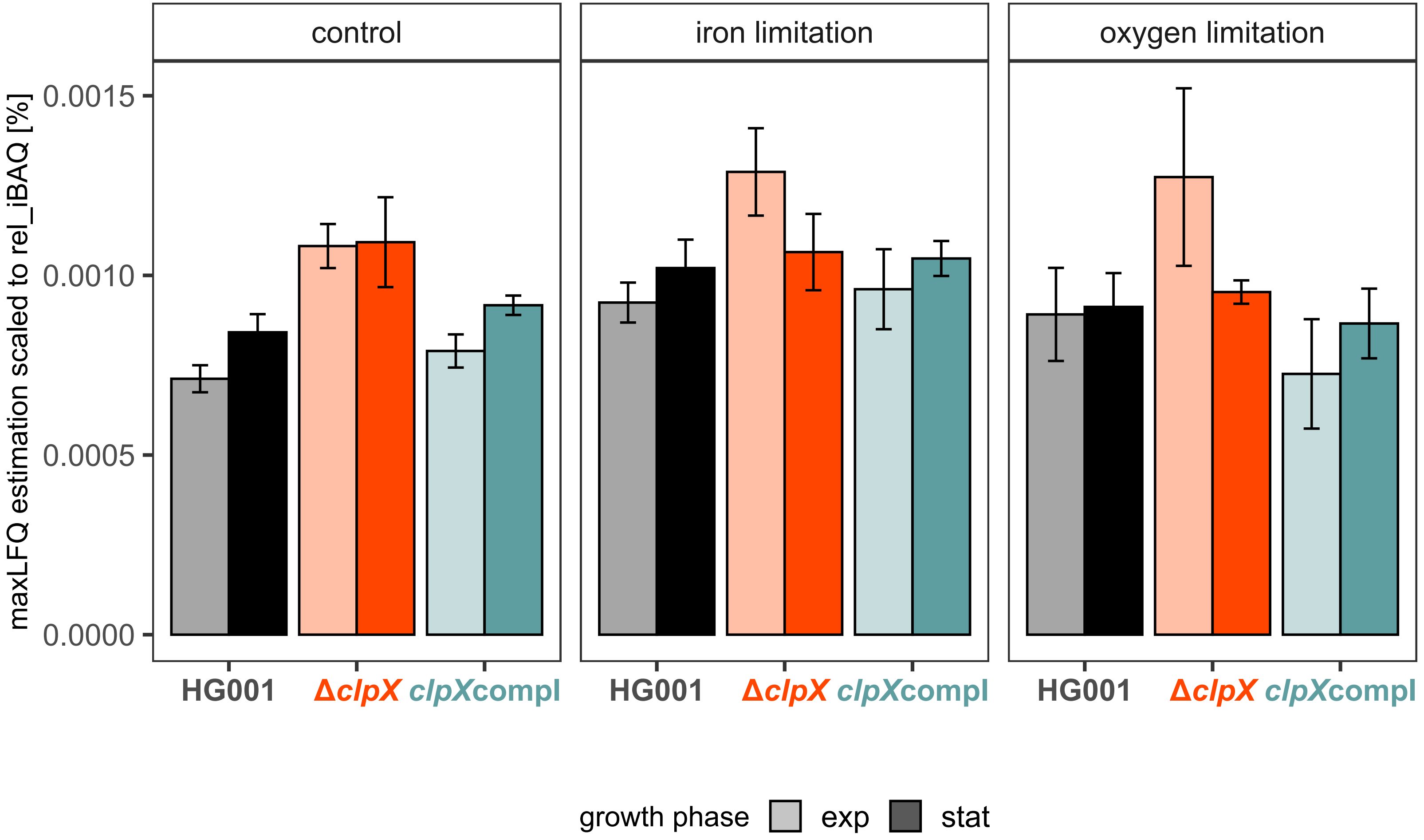

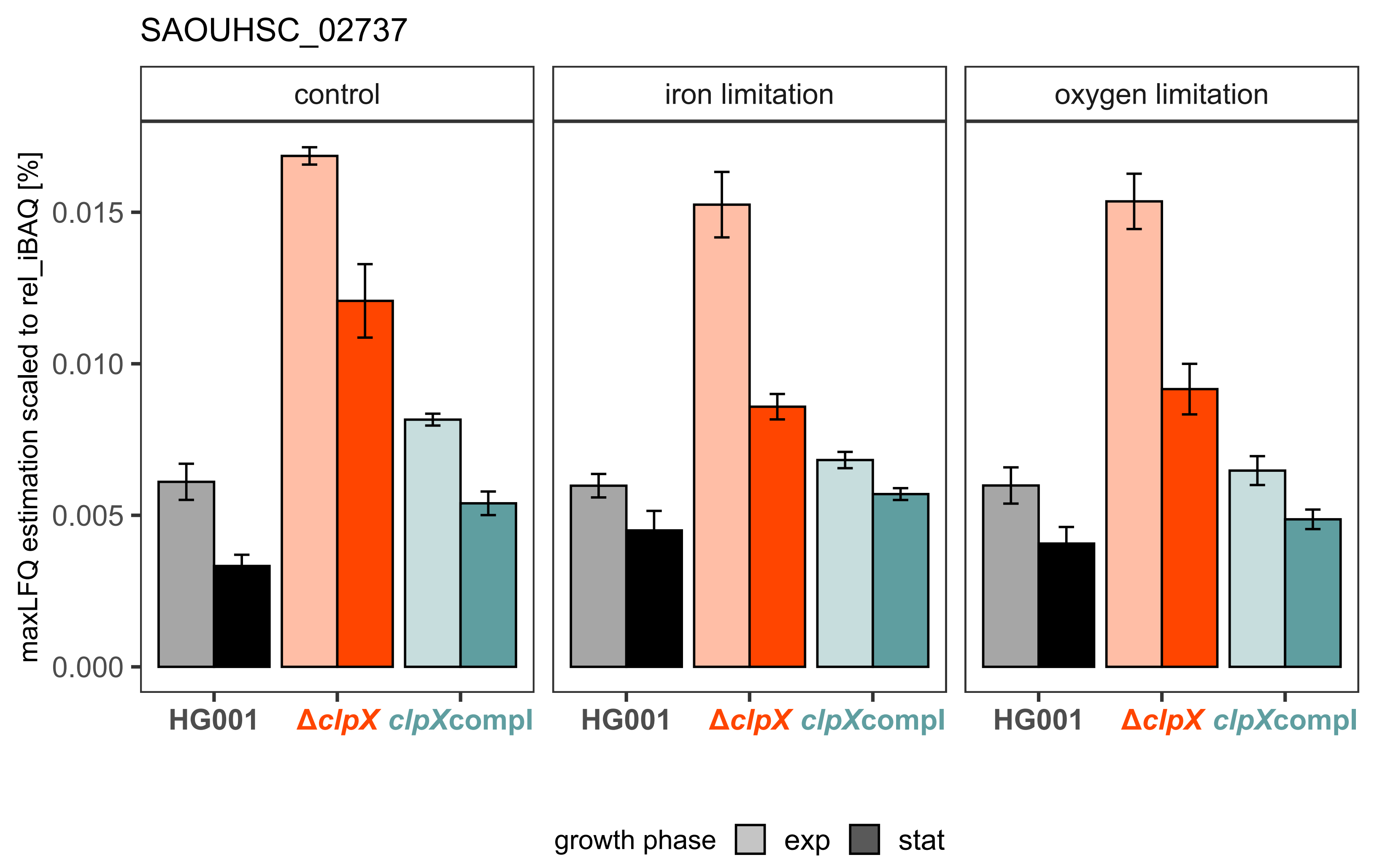
SAOUHSC_02737
control
iron limitation
oxygen limitation
maxLFQ estimation scaled to rel_iBAQ [%]
0.015
0.010
0.005
0.000
HG001
ΔclpX
clpXcompl
growth phase
exp
stat

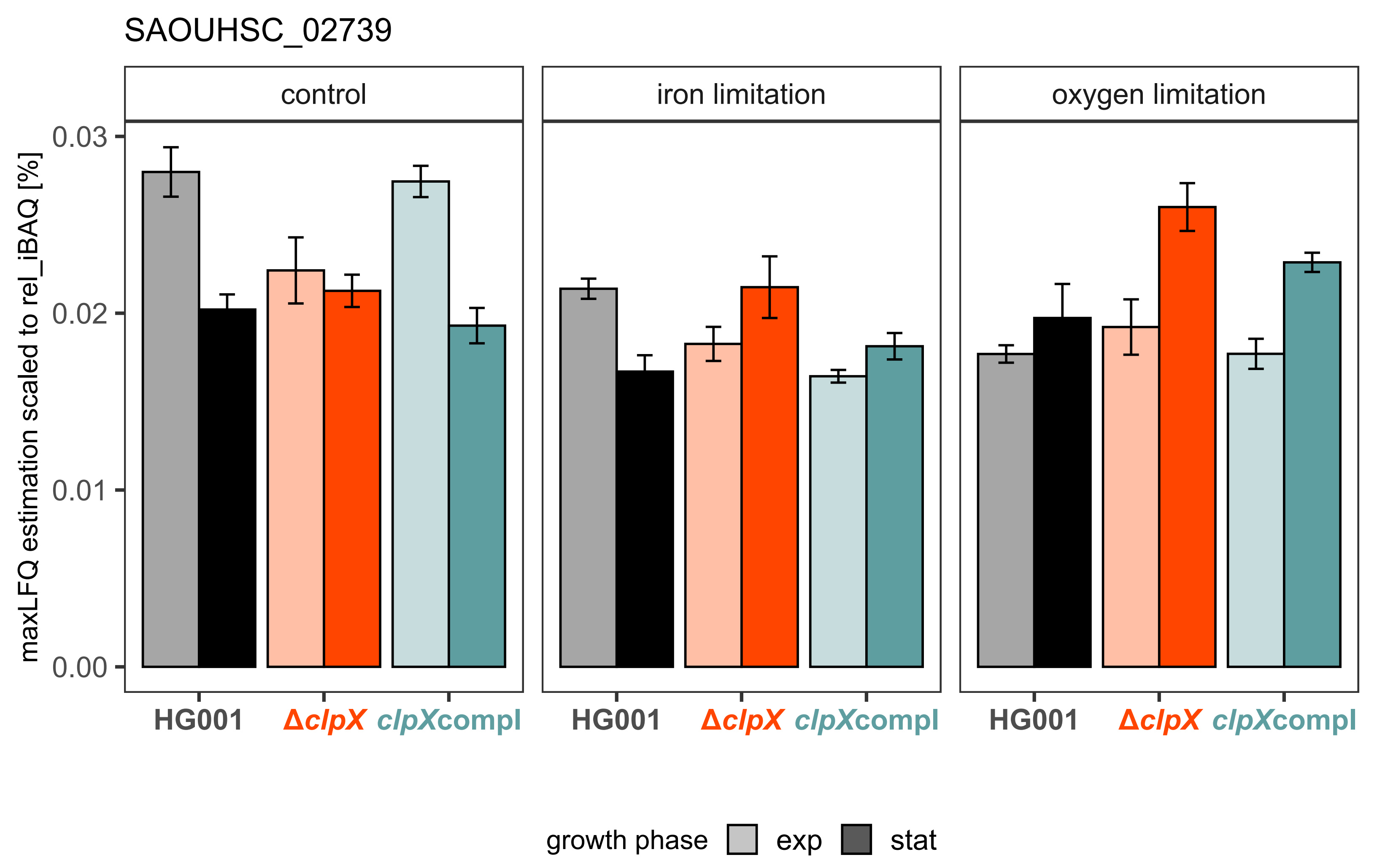

SAOUHSC_02739
control
iron limitation
oxygen limitation
maxLFQ estimation scaled to rel_iBAQ [%]
0.03
0.02
0.01
0.00
HG001
ΔclpX
clpXcompl
growth phase
exp
stat

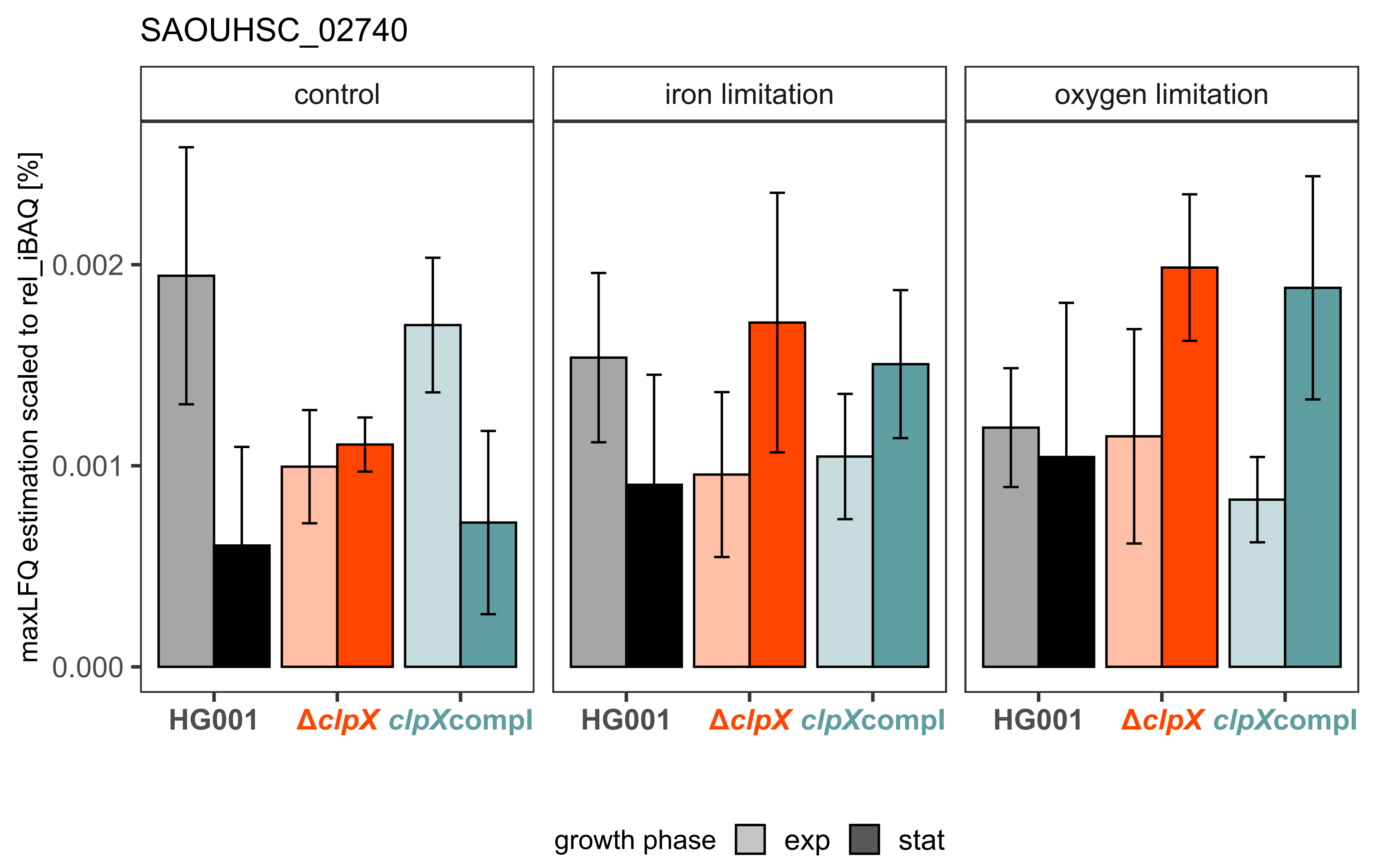

SAOUHSC_02740
control
iron limitation
oxygen limitation
maxLFQ estimation scaled to rel_iBAQ [%]
0.002
0.001
0.000
HG001
ΔclpX
clpXcompl
growth phase
exp
stat

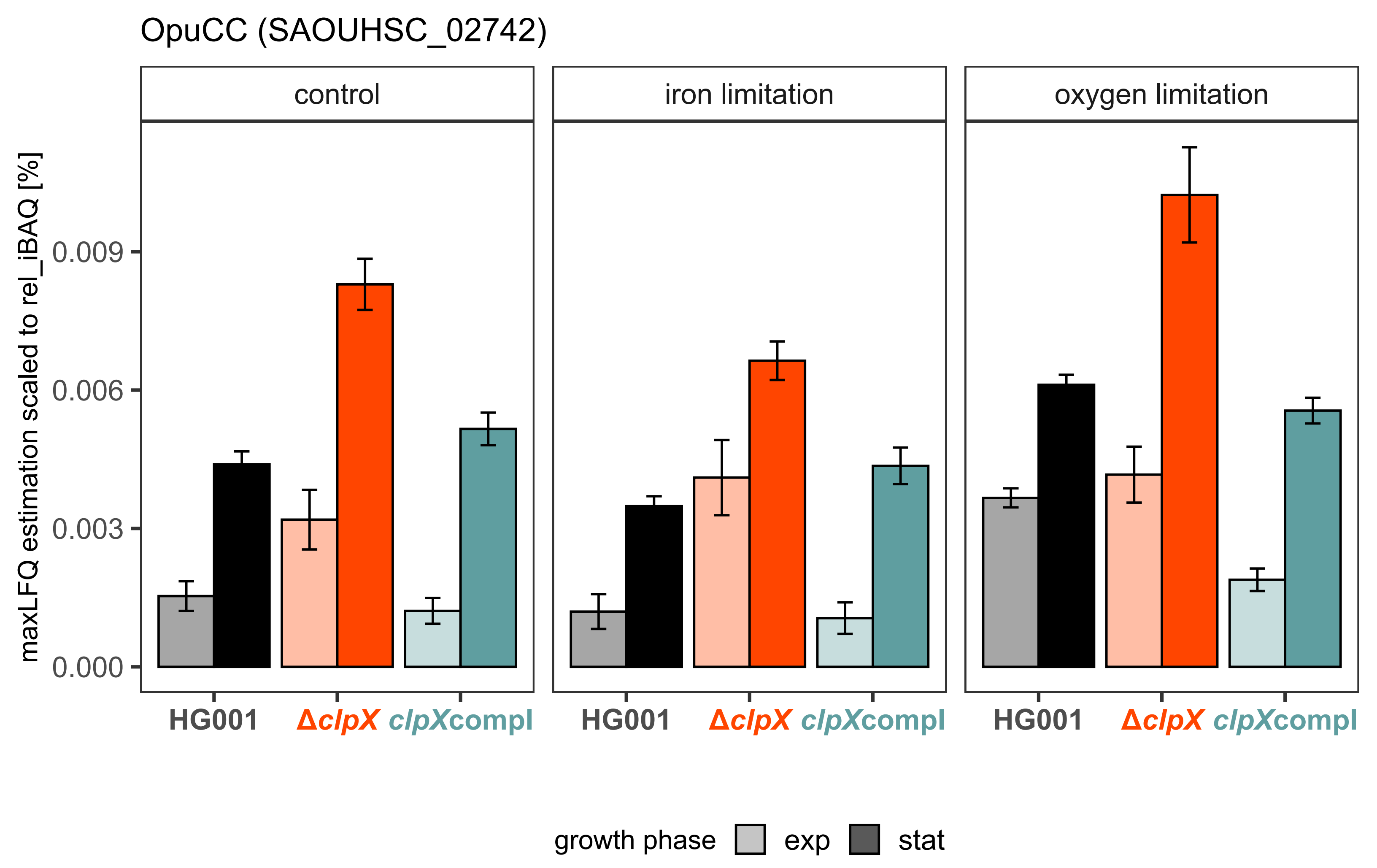

OpuCC (SAOUHSC_02742)
control
iron limitation
oxygen limitation
maxLFQ estimation scaled to rel_iBAQ [%]
0.009
0.006
0.003
0.000
HG001
ΔclpX
clpXcompl
growth phase
exp
stat

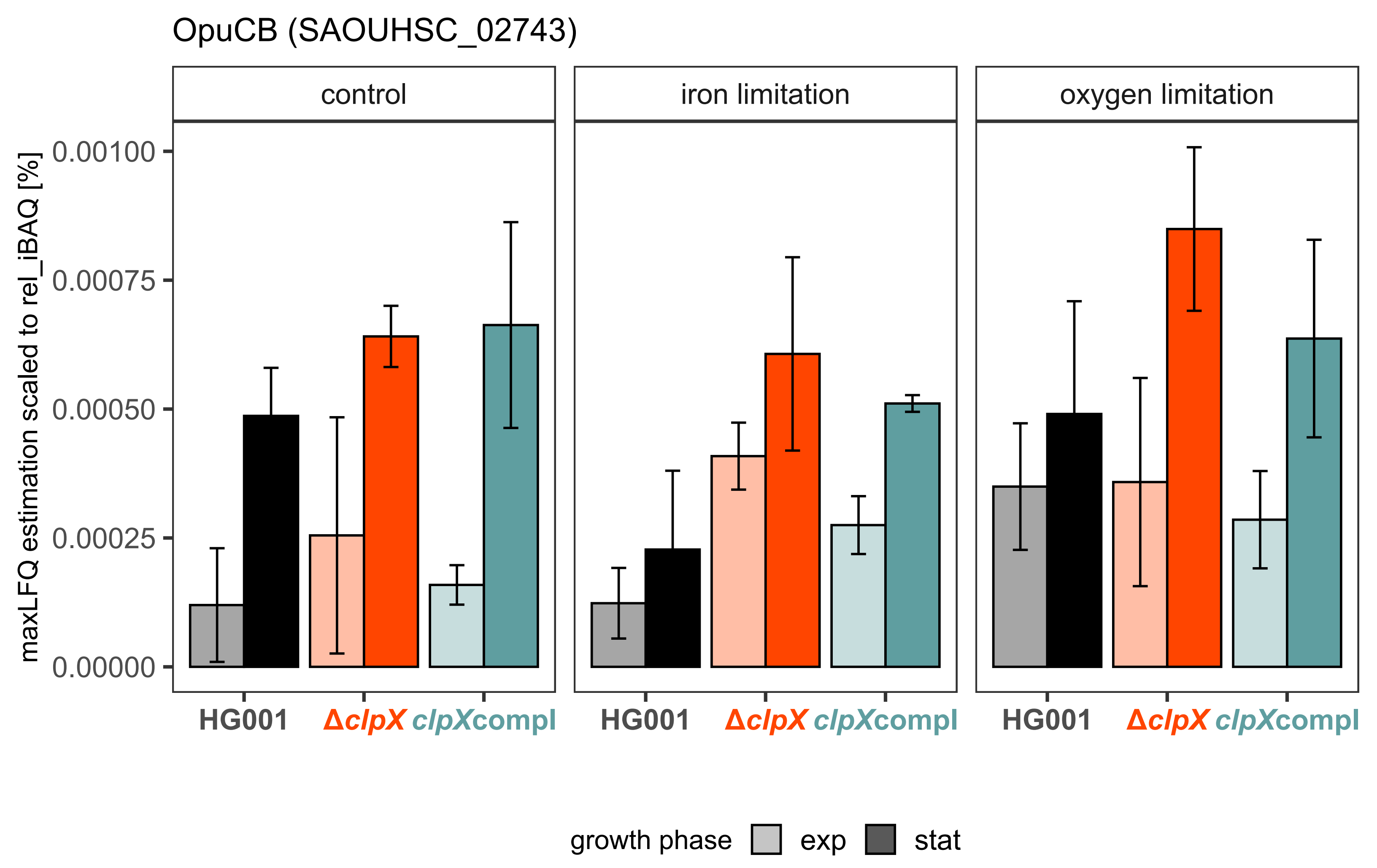

OpuCB (SAOUHSC_02743)
control
iron limitation
oxygen limitation
maxLFQ estimation scaled to rel_iBAQ [%]
0.00100
0.00075
0.00050
0.00025
0.00000
HG001
ΔclpX
clpXcompl
growth phase
exp
stat

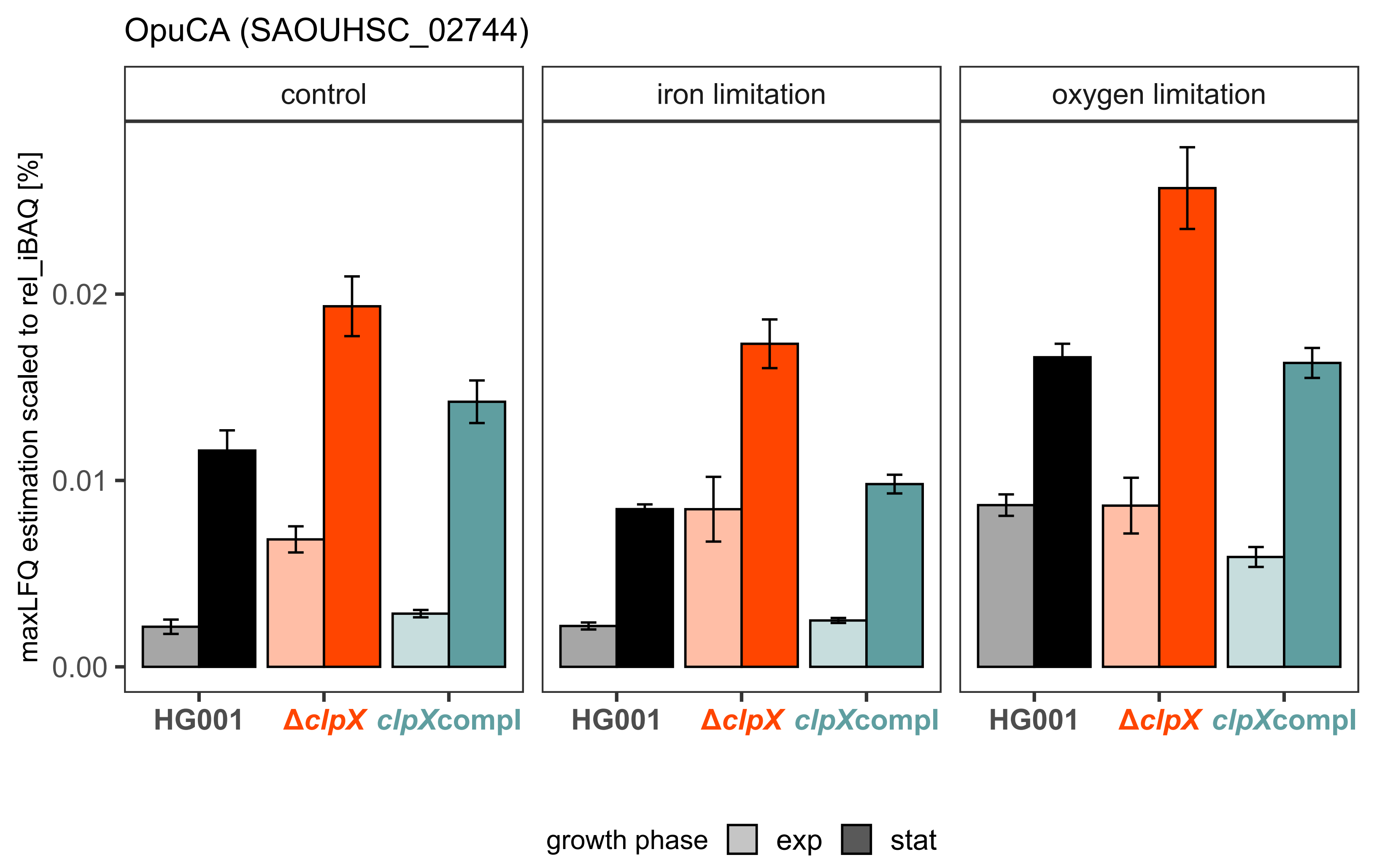

OpuCA (SAOUHSC_02744)
control
iron limitation
oxygen limitation
maxLFQ estimation scaled to rel_iBAQ [%]
0.00
0.01
0.02
HG001
ΔclpX
clpXcompl
growth phase
exp
stat

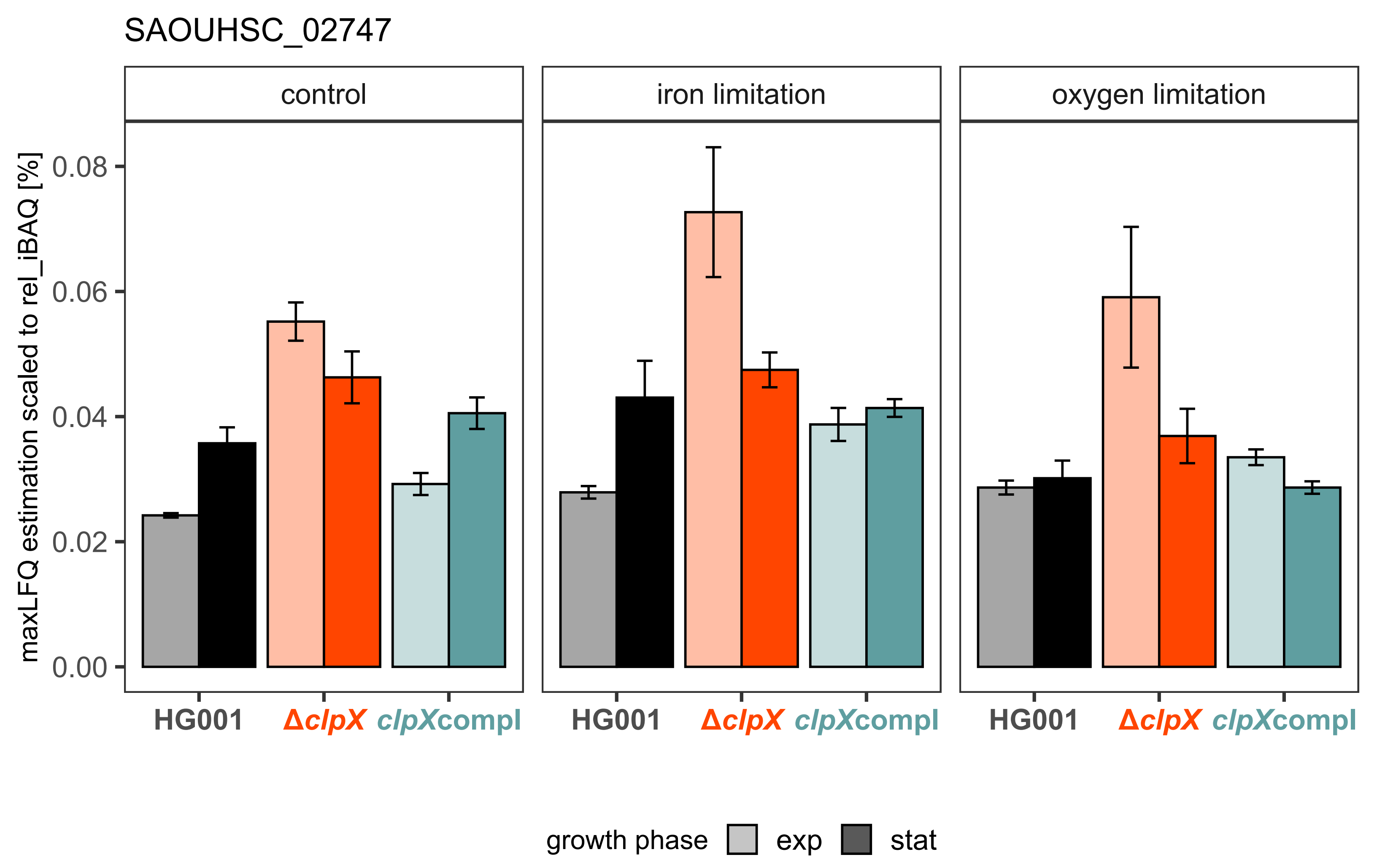

SAOUHSC_02747
control
iron limitation
oxygen limitation
maxLFQ estimation scaled to rel_iBAQ [%]
0.00
0.02
0.04
0.06
0.08
HG001
ΔclpX
clpXcompl
growth phase
exp
stat

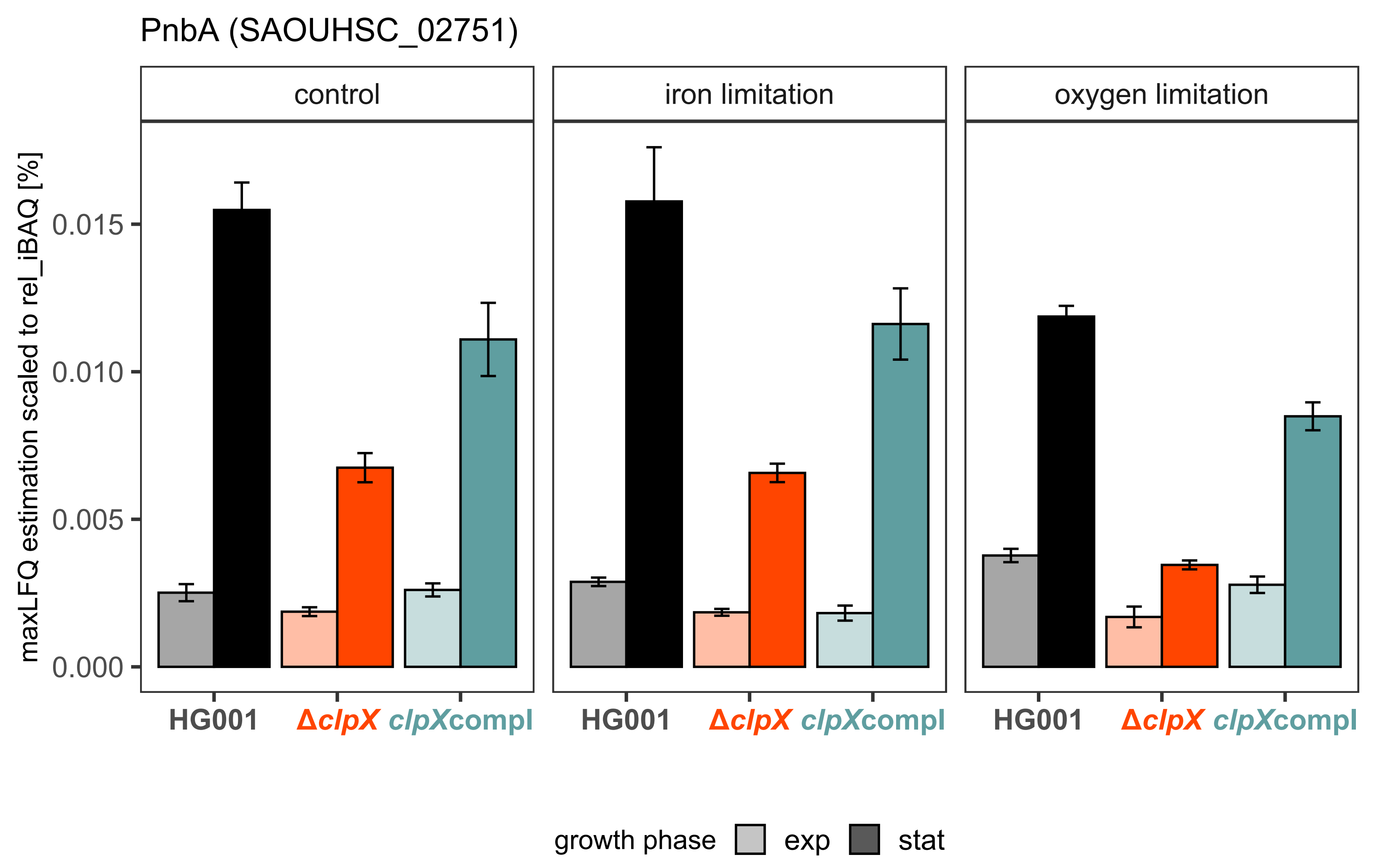

PnbA (SAOUHSC_02751)
control
iron limitation
oxygen limitation
maxLFQ estimation scaled to rel_iBAQ [%]
0.000
0.005
0.010
0.015
HG001
ΔclpX
clpXcompl
growth phase
exp
stat

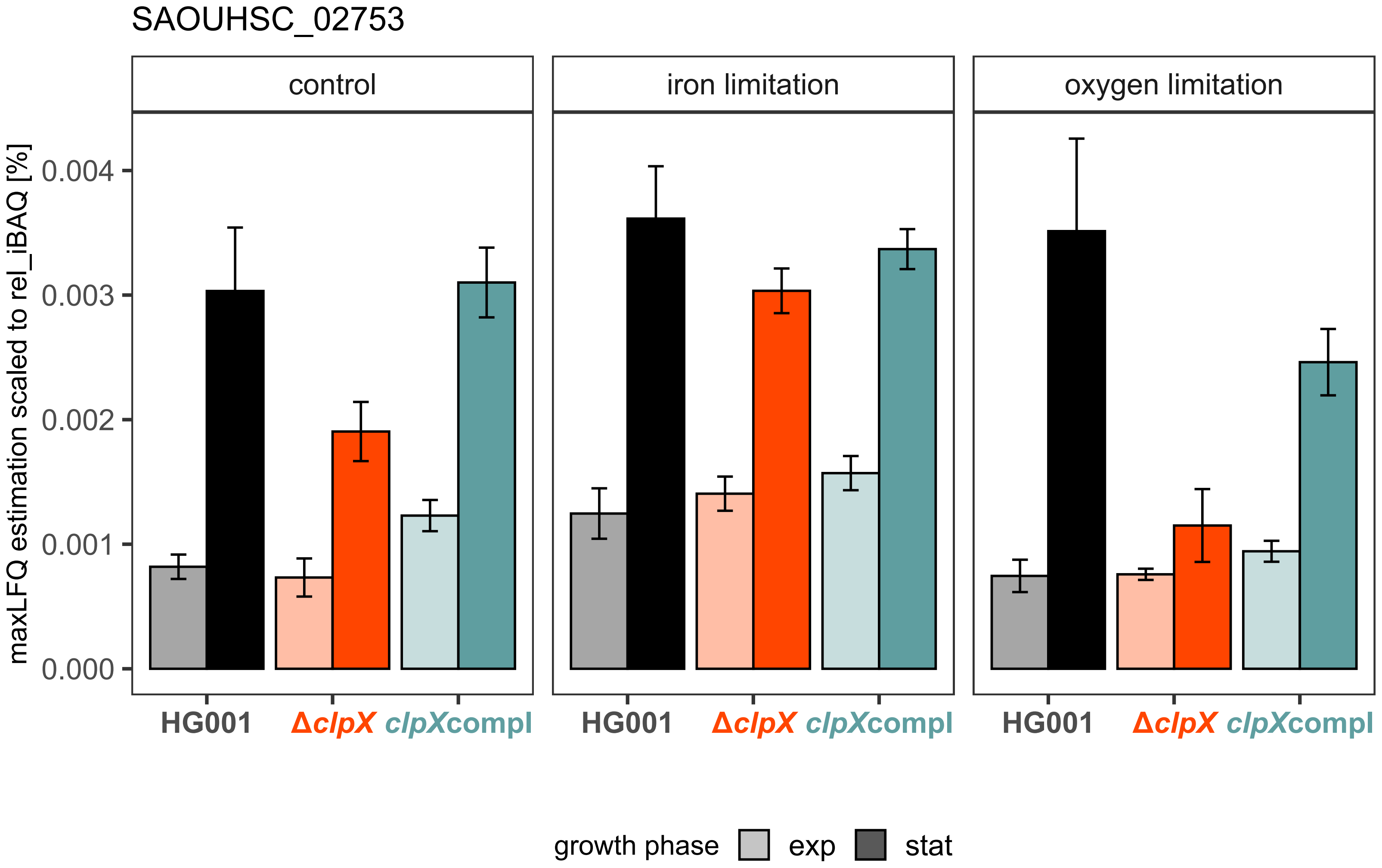
SAOUHSC_02753
control
iron limitation
oxygen limitation
maxLFQ estimation scaled to rel_iBAQ [%]
0.004
0.003
0.002
0.001
0.000
HG001
ΔclpX
clpXcompl
growth phase
exp
stat

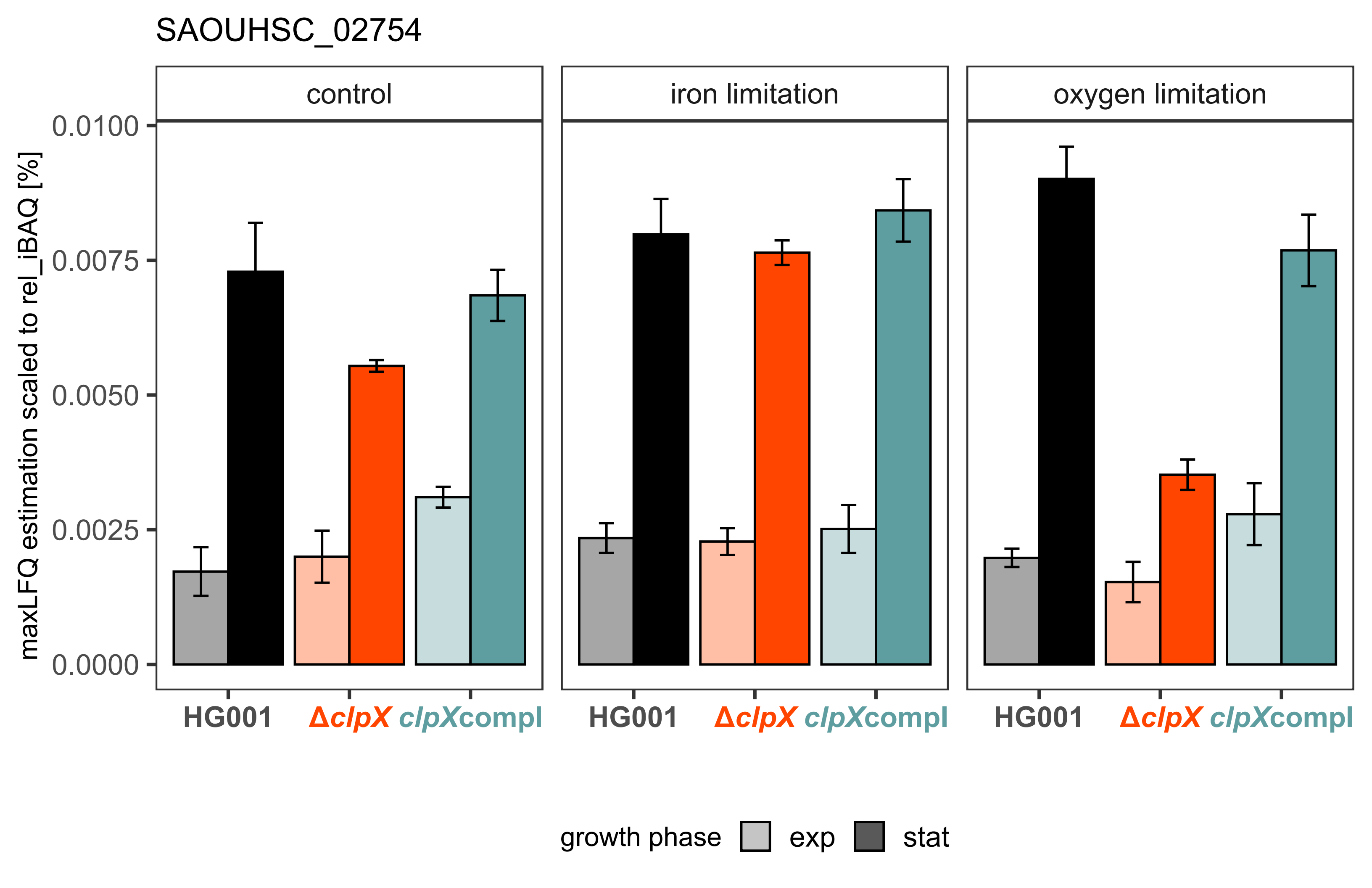

SAOUHSC_02754
control
iron limitation
oxygen limitation
maxLFQ estimation scaled to rel_iBAQ [%]
0.0100
0.0075
0.0050
0.0025
0.0000
HG001
ΔclpX
clpXcompl
growth phase
exp
stat

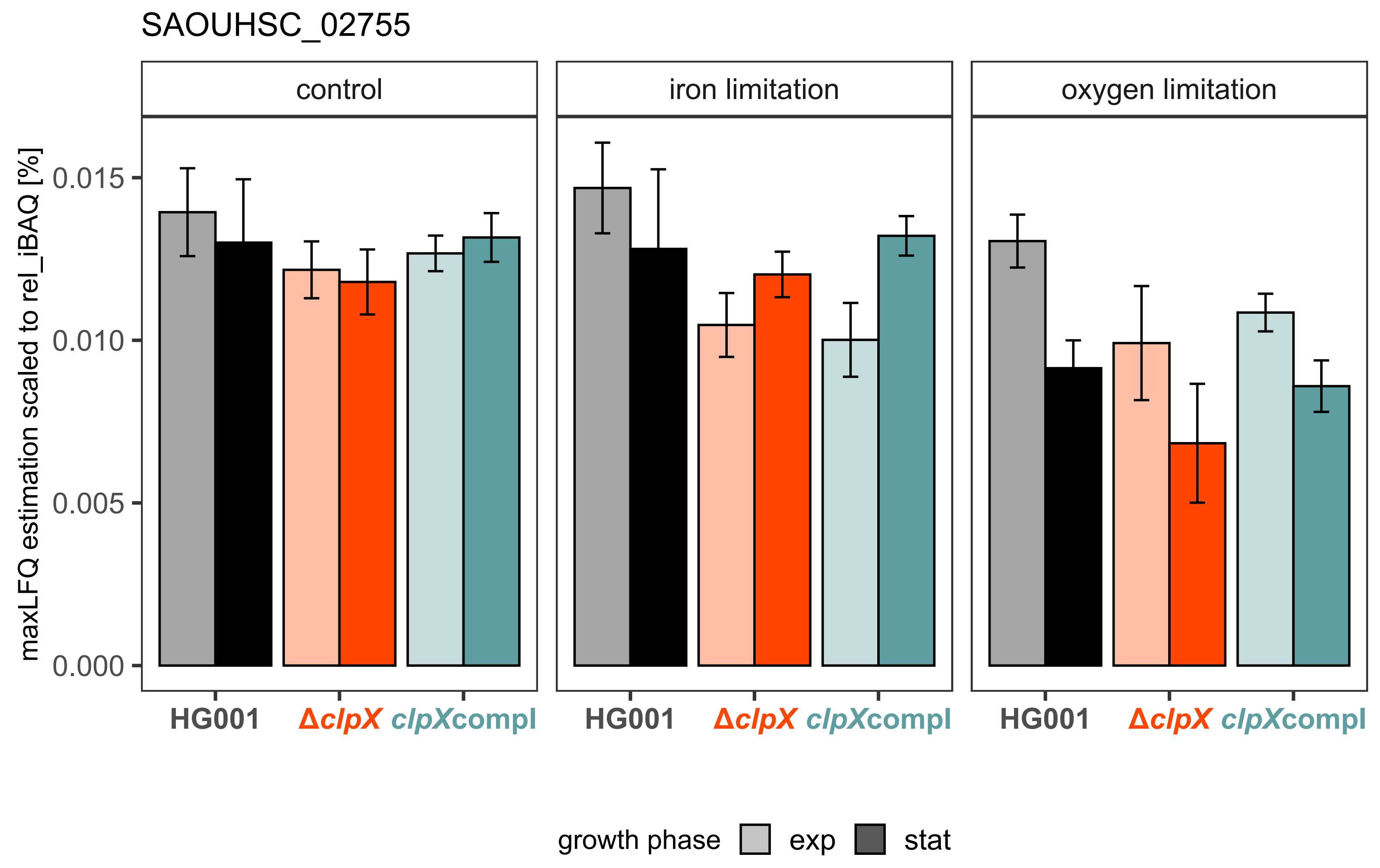

SAOUHSC_02755
control
iron limitation
oxygen limitation
maxLFQ estimation scaled to rel_iBAQ [%]
0.015
0.010
0.005
0.000
HG001
ΔclpX
clpXcompl
growth phase
exp
stat

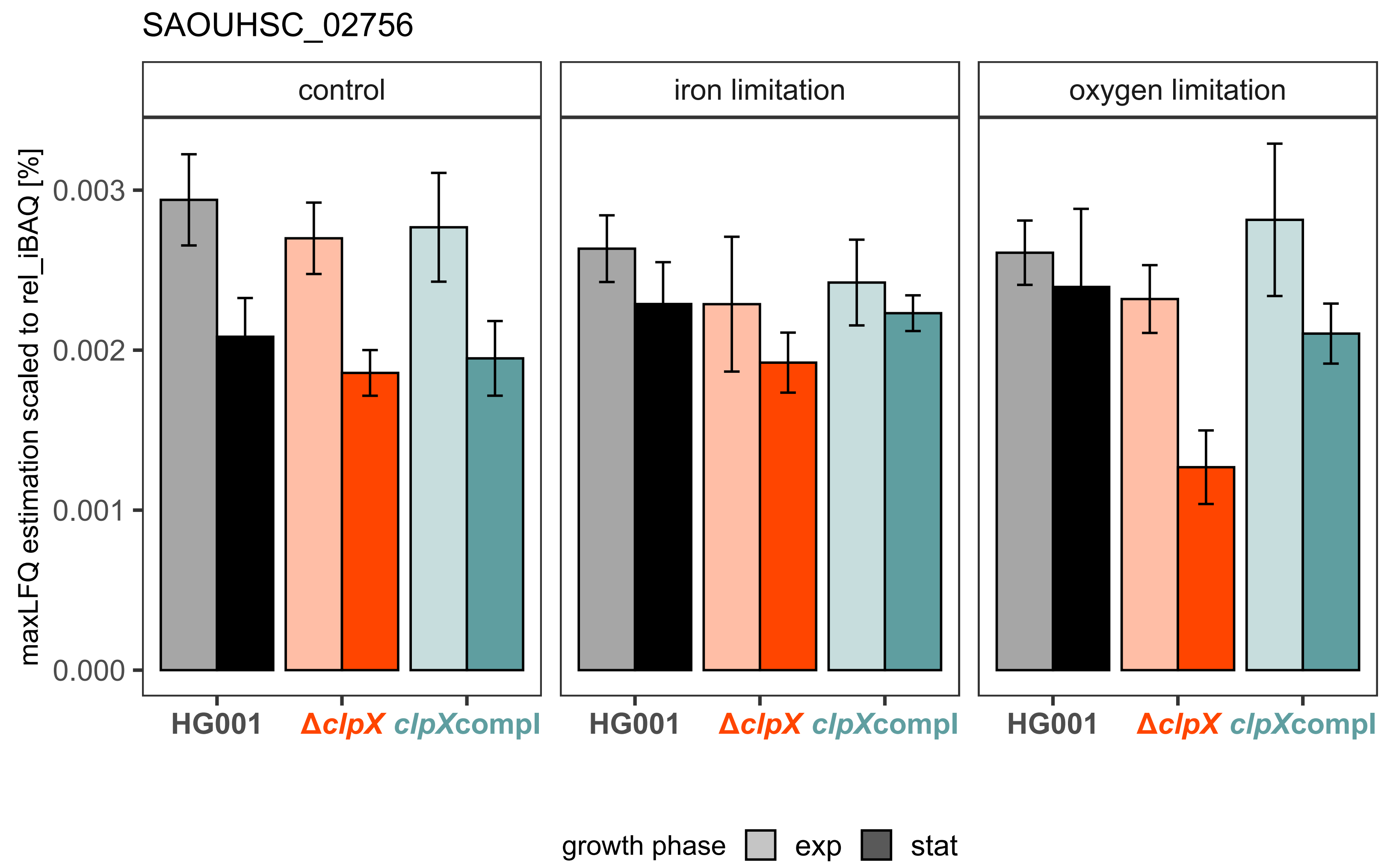

SAOUHSC_02756
control
iron limitation
oxygen limitation
maxLFQ estimation scaled to rel_iBAQ [%]
0.003
0.002
0.001
0.000
HG001
ΔclpX
clpXcompl
growth phase
exp
stat

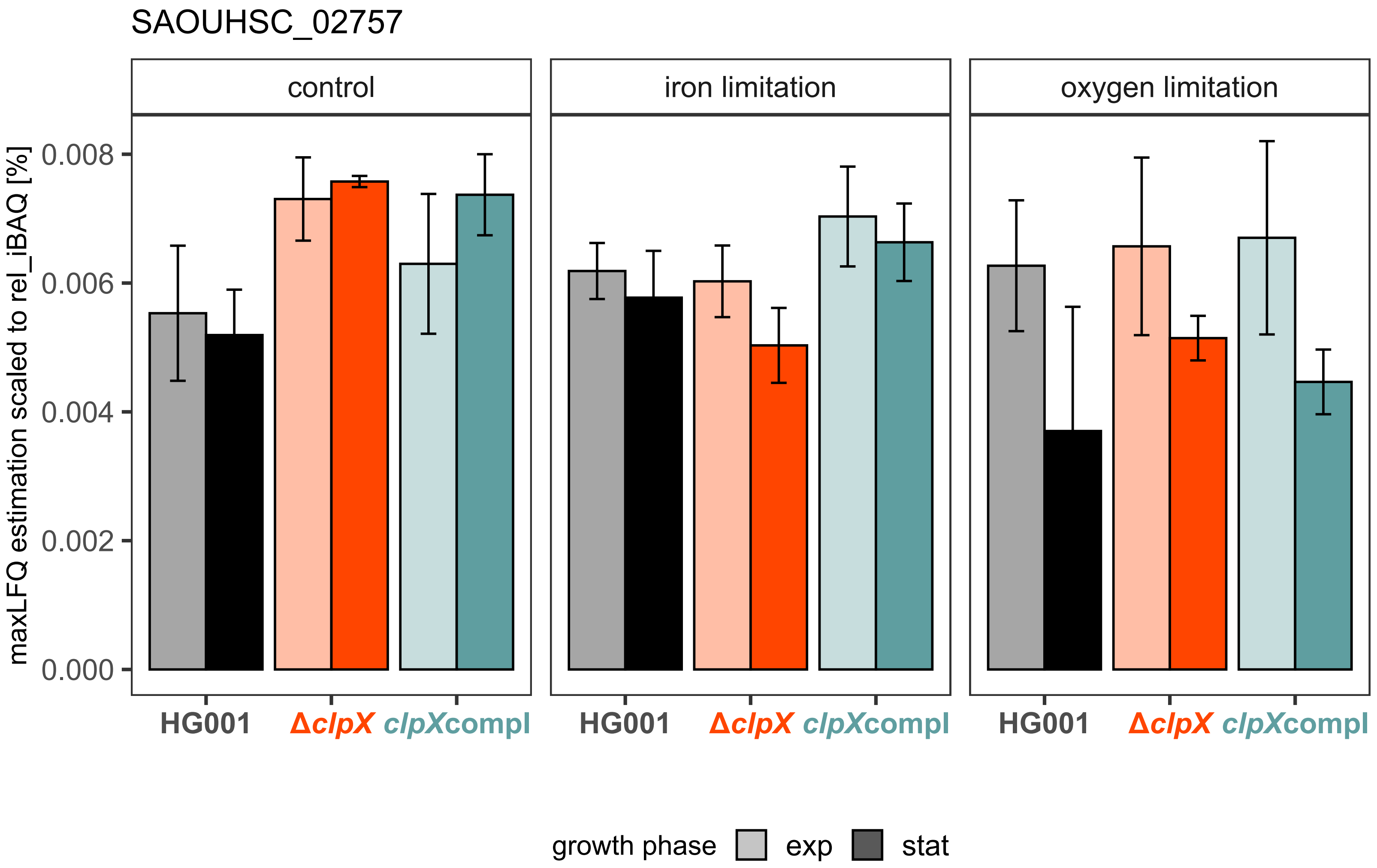
SAOUHSC_02757
control
iron limitation
oxygen limitation
maxLFQ estimation scaled to rel_iBAQ [%]
0.008
0.006
0.004
0.002
0.000
HG001
ΔclpX
clpXcompl
growth phase
exp
stat

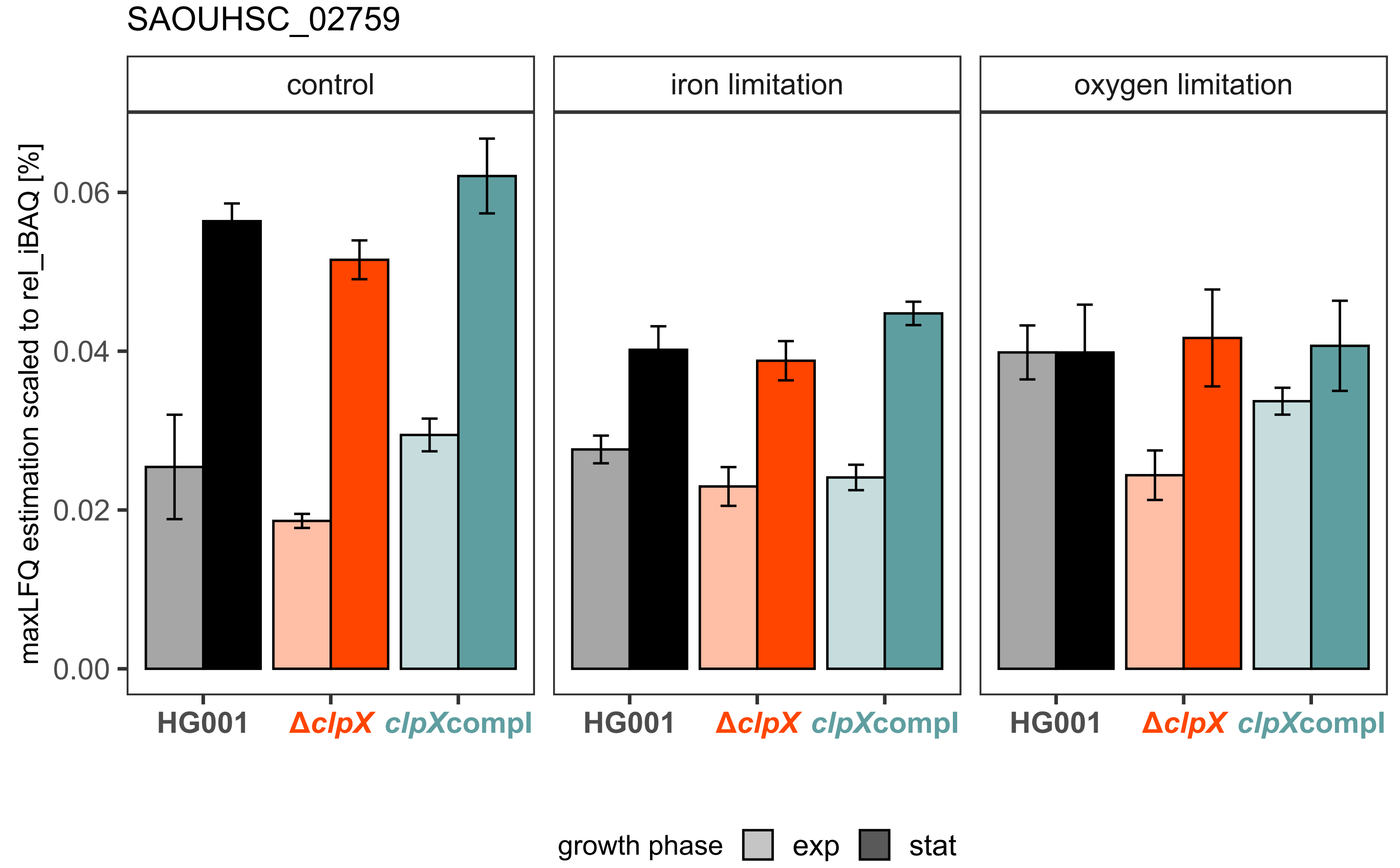

SAOUHSC_02759
control
iron limitation
oxygen limitation
maxLFQ estimation scaled to rel_iBAQ [%]
0.06
0.04
0.02
0.00
HG001
ΔclpX
clpXcompl
HG001
ΔclpX
clpXcompl
HG001
ΔclpX
clpXcompl
growth phase
exp
stat

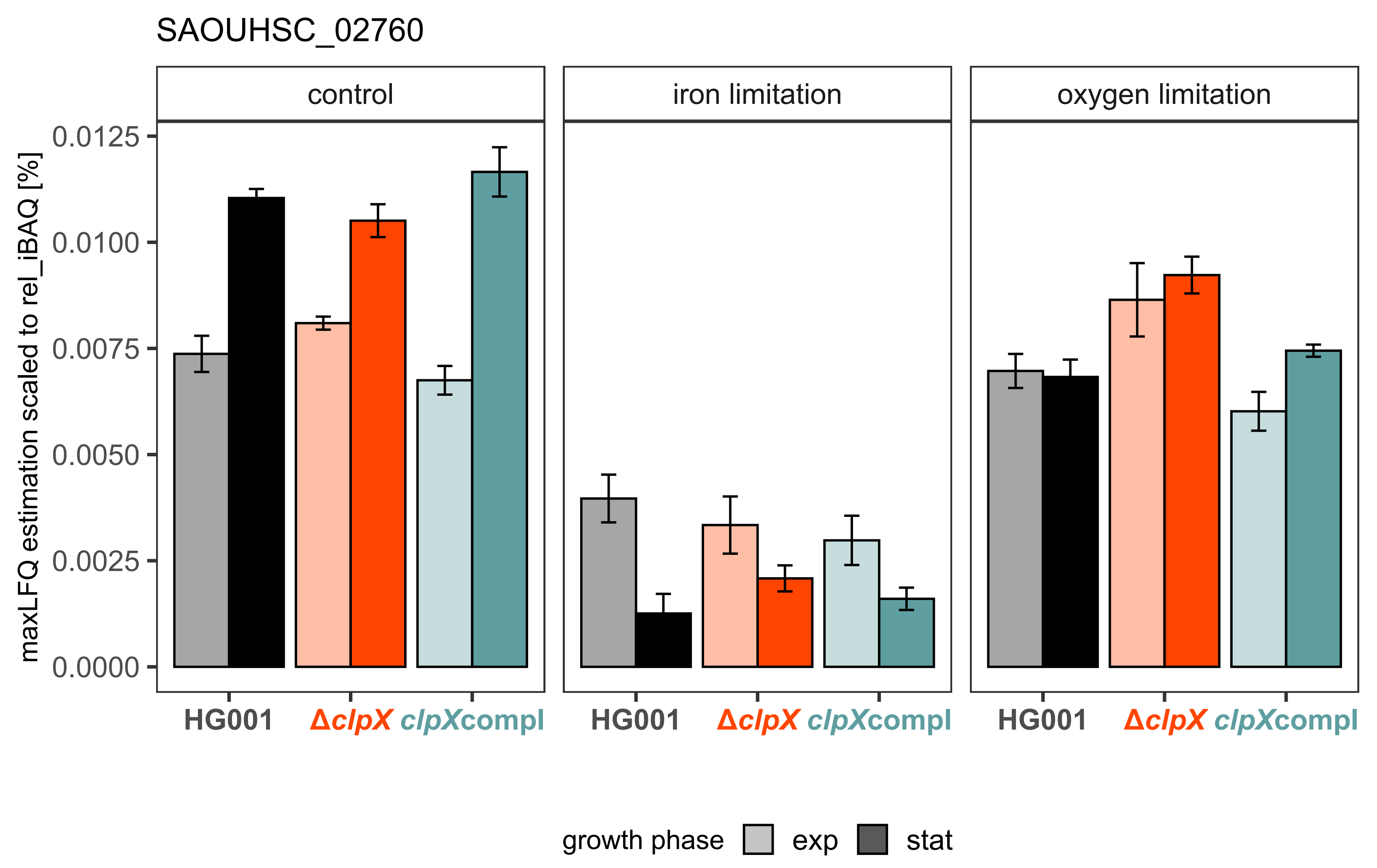

SAOUHSC_02760
control
iron limitation
oxygen limitation
maxLFQ estimation scaled to rel_iBAQ [%]
0.0125
0.0100
0.0075
0.0050
0.0025
0.0000
HG001
ΔclpX
clpXcompl
growth phase
exp
stat

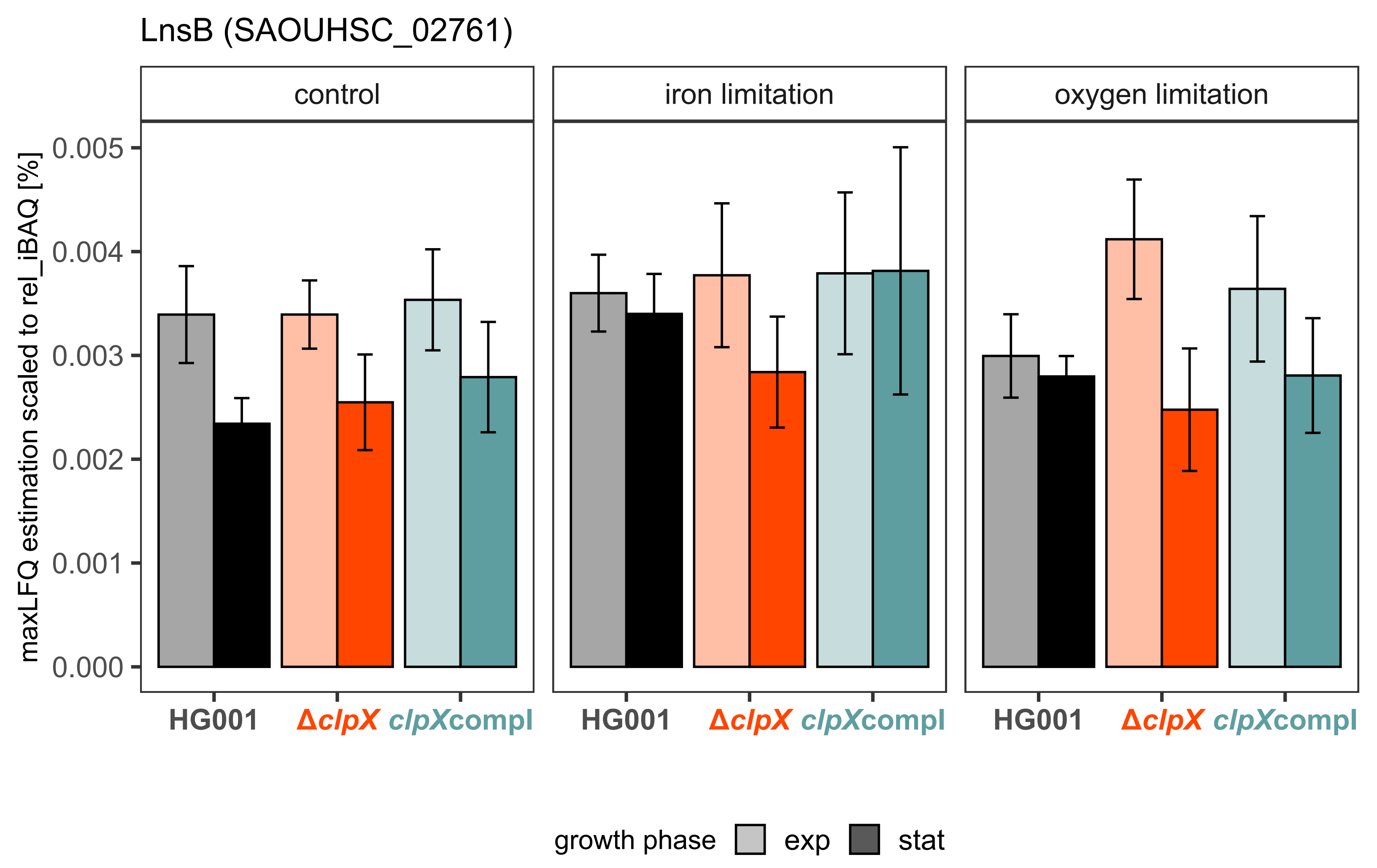

LnsB (SAOUHSC_02761)
control
iron limitation
oxygen limitation
maxLFQ estimation scaled to rel_iBAQ [%]
0.005
0.004
0.003
0.002
0.001
0.000
HG001
ΔclpX
clpXcompl
growth phase
exp
stat

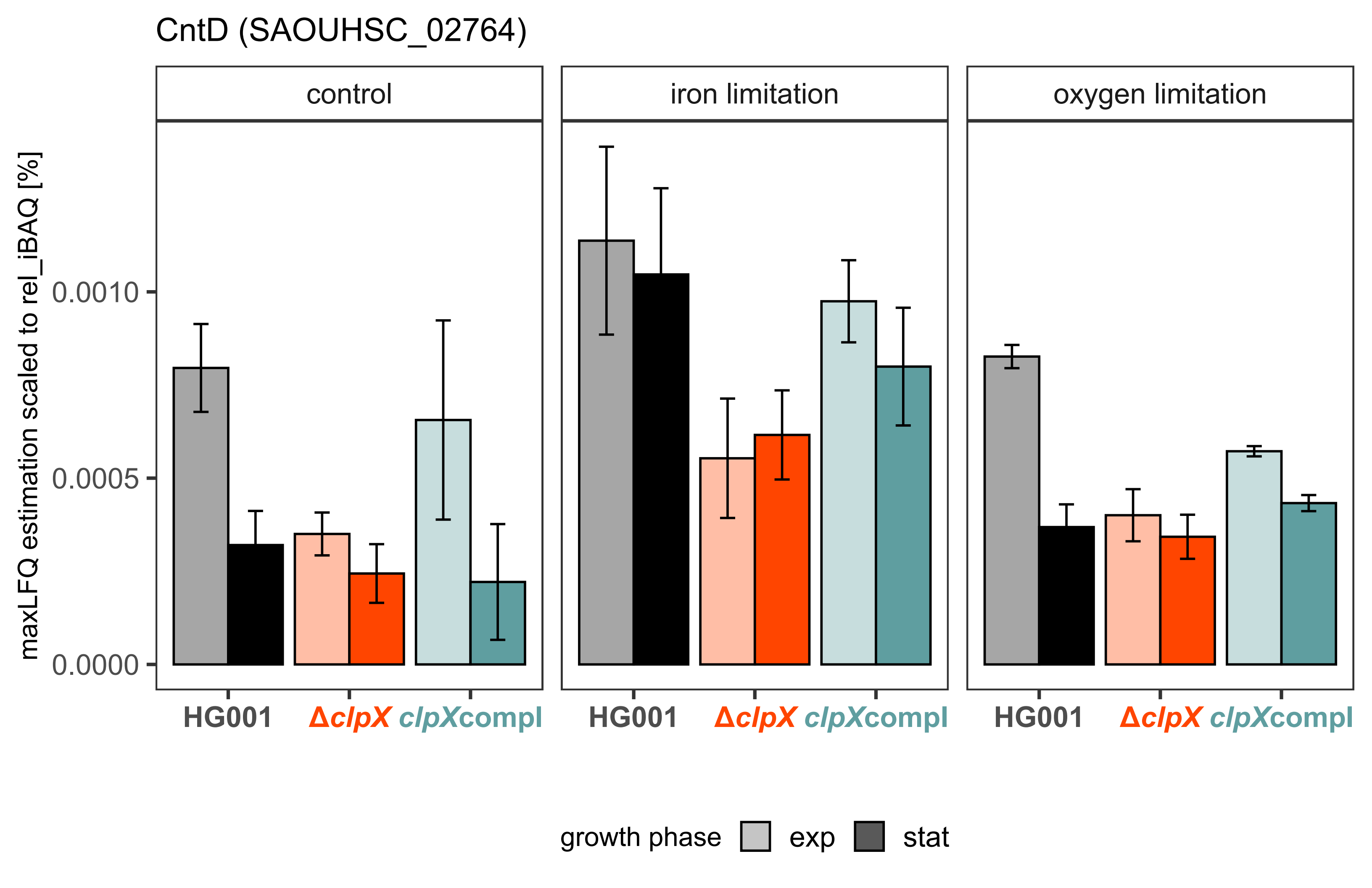
CntD (SAOUHSC_02764)
control
iron limitation
oxygen limitation
maxLFQ estimation scaled to rel_iBAQ [%]
0.0010
0.0005
0.0000
HG001
ΔclpX
clpXcompl
growth phase
exp
stat

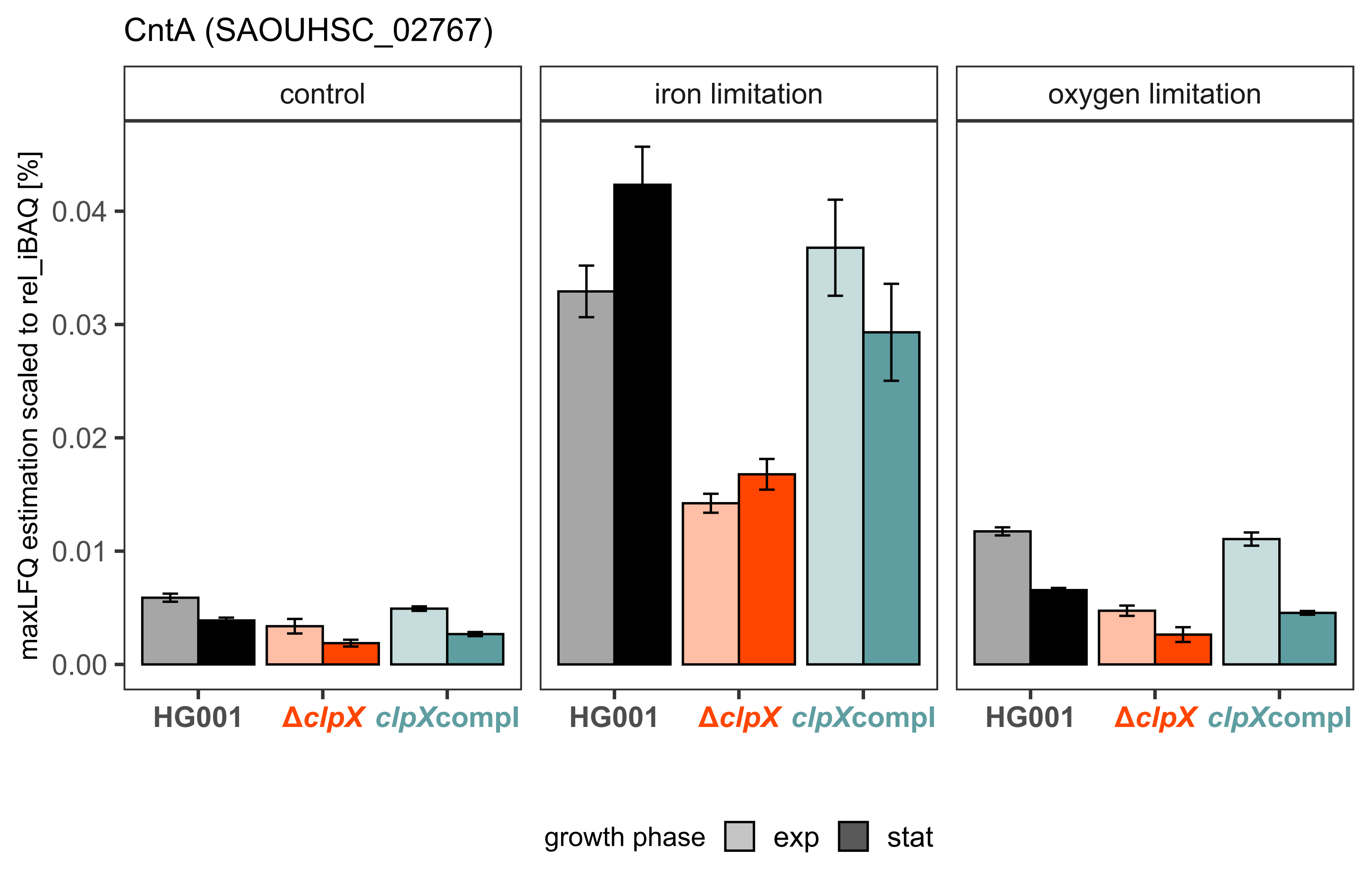
CntA (SAOUHSC_02767)
control
iron limitation
oxygen limitation
maxLFQ estimation scaled to rel_iBAQ [%]
0.04
0.03
0.02
0.01
0.00
HG001
ΔclpX
clpXcompl
growth phase
exp
stat

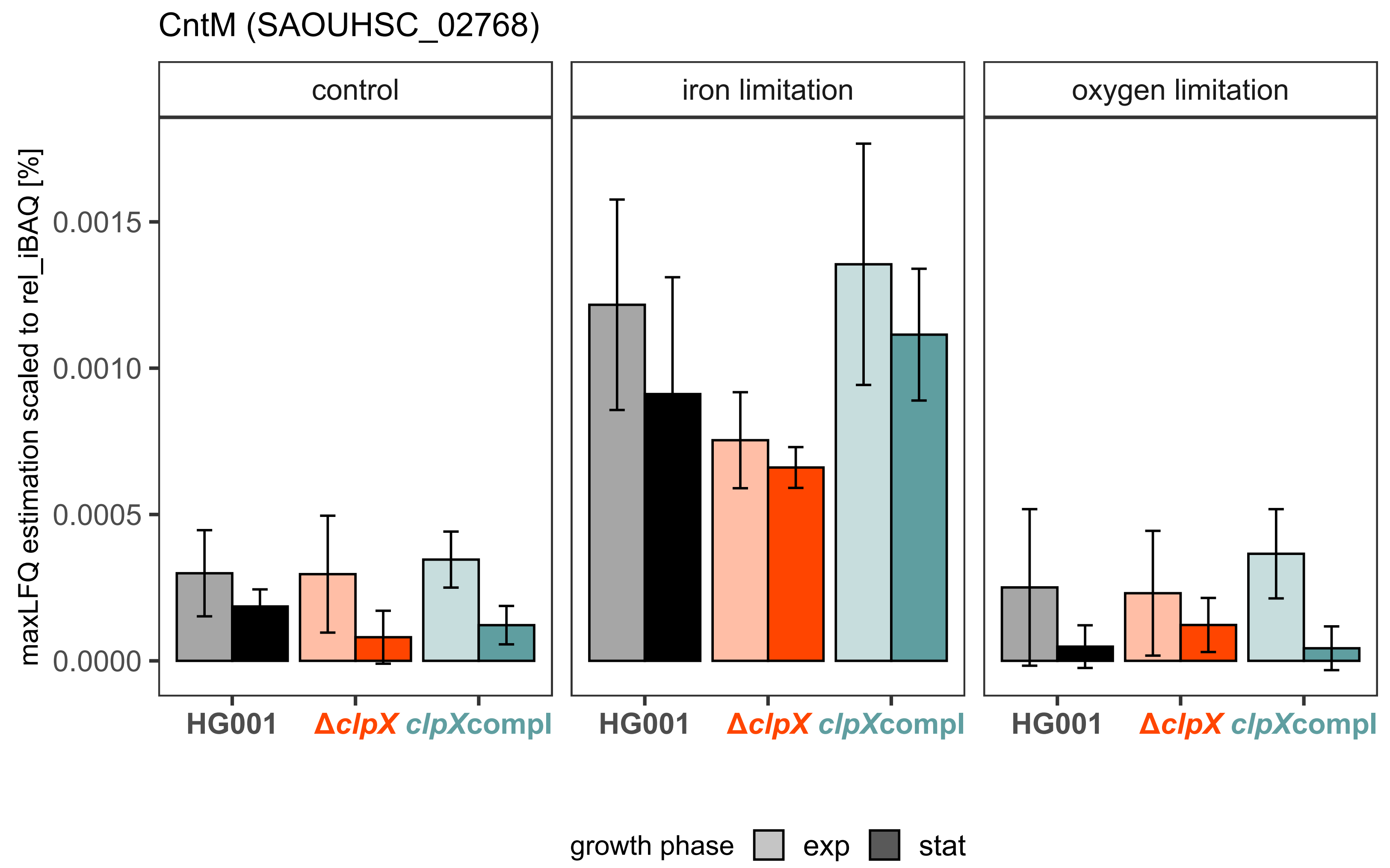

CntM (SAOUHSC_02768)
control
iron limitation
oxygen limitation
maxLFQ estimation scaled to rel_iBAQ [%]
0.0015
0.0010
0.0005
0.0000
HG001
ΔclpX
clpXcompl
growth phase
exp
stat

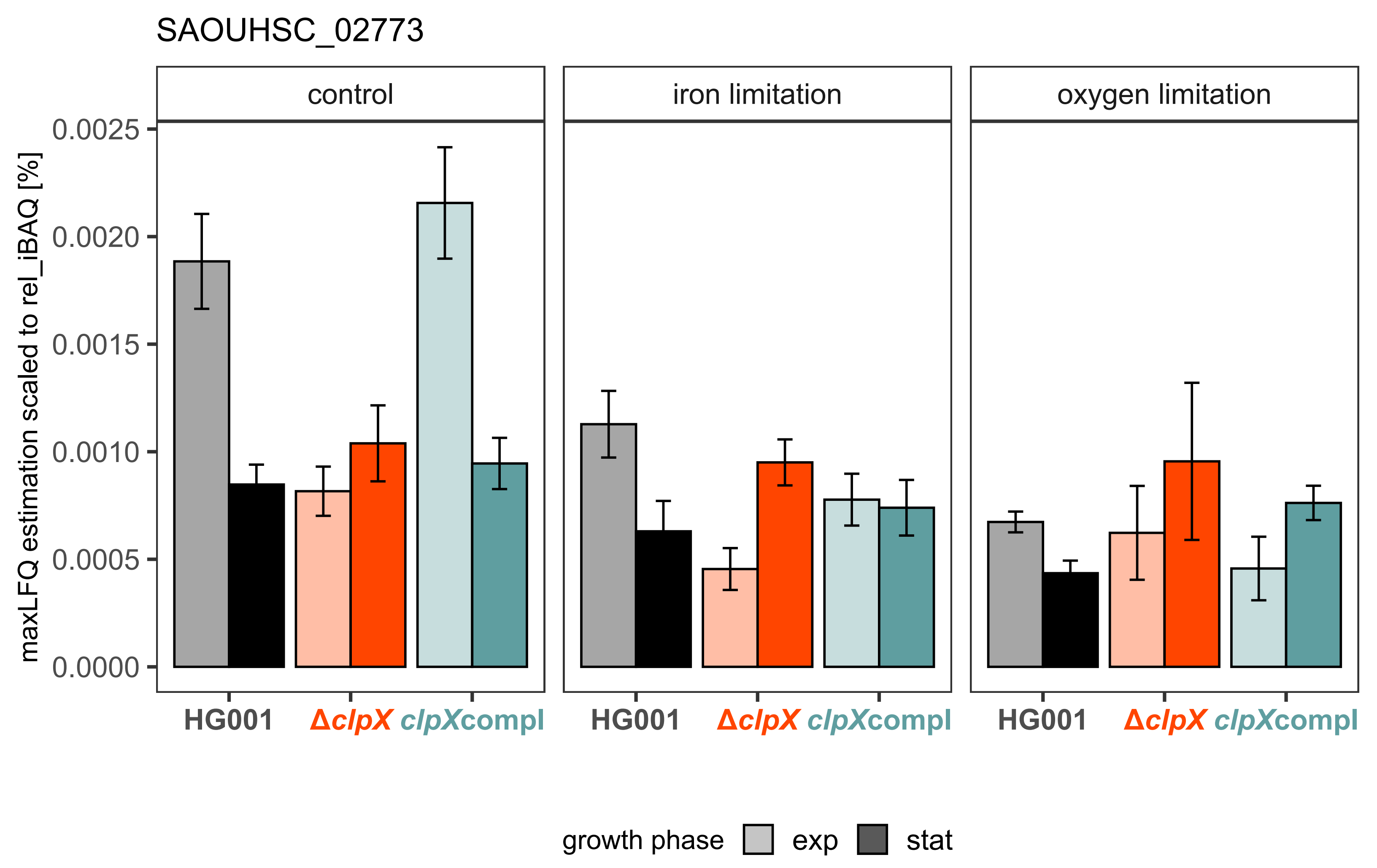
SAOUHSC_02773
control
iron limitation
oxygen limitation
maxLFQ estimation scaled to rel_iBAQ [%]
0.0025
0.0020
0.0015
0.0010
0.0005
0.0000
HG001
ΔclpX
clpXcompl
growth phase
exp
stat

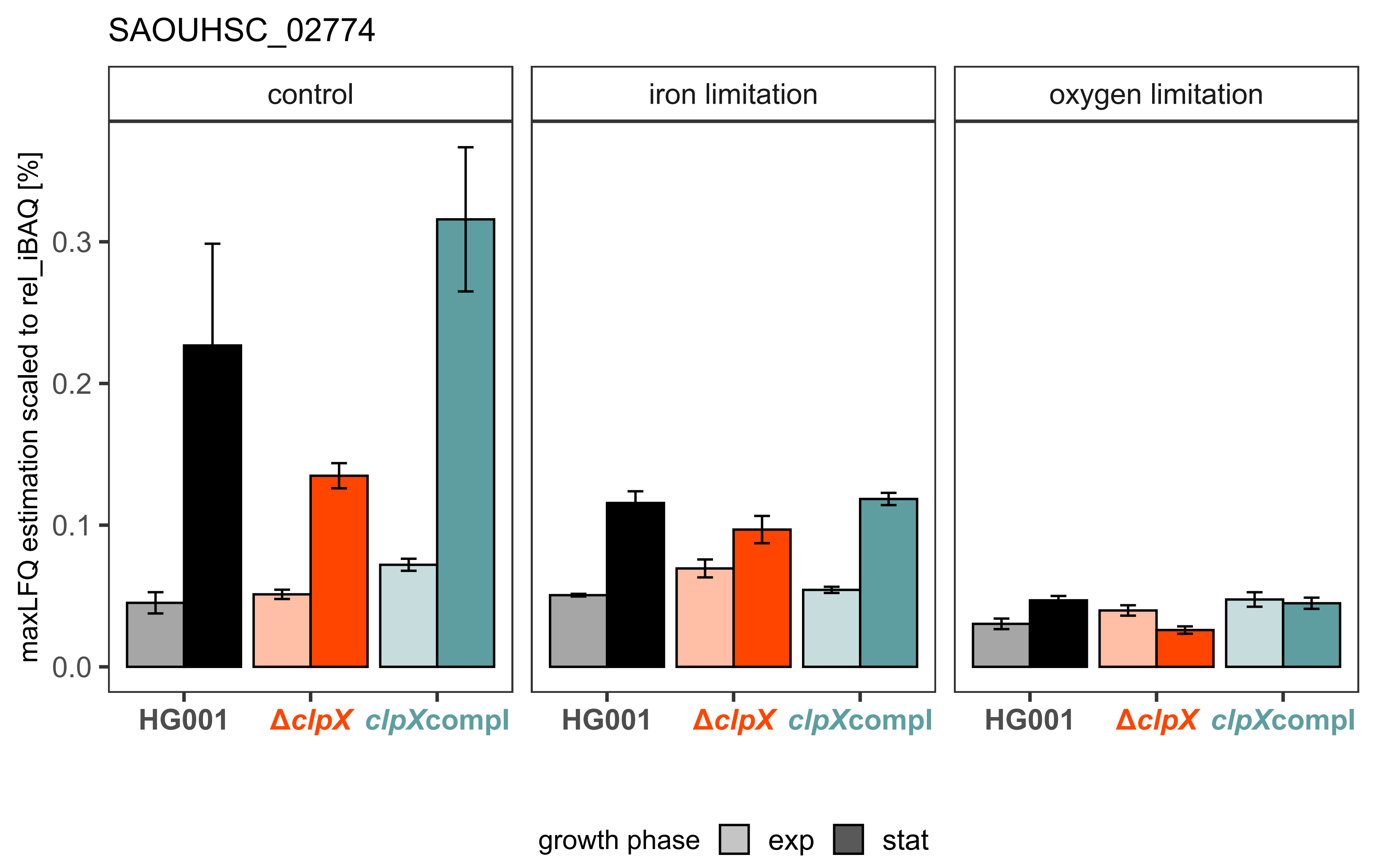

SAOUHSC_02774
control
iron limitation
oxygen limitation
maxLFQ estimation scaled to rel_iBAQ [%]
0.0
0.1
0.2
0.3
HG001
ΔclpX
clpXcompl
growth phase
exp
stat

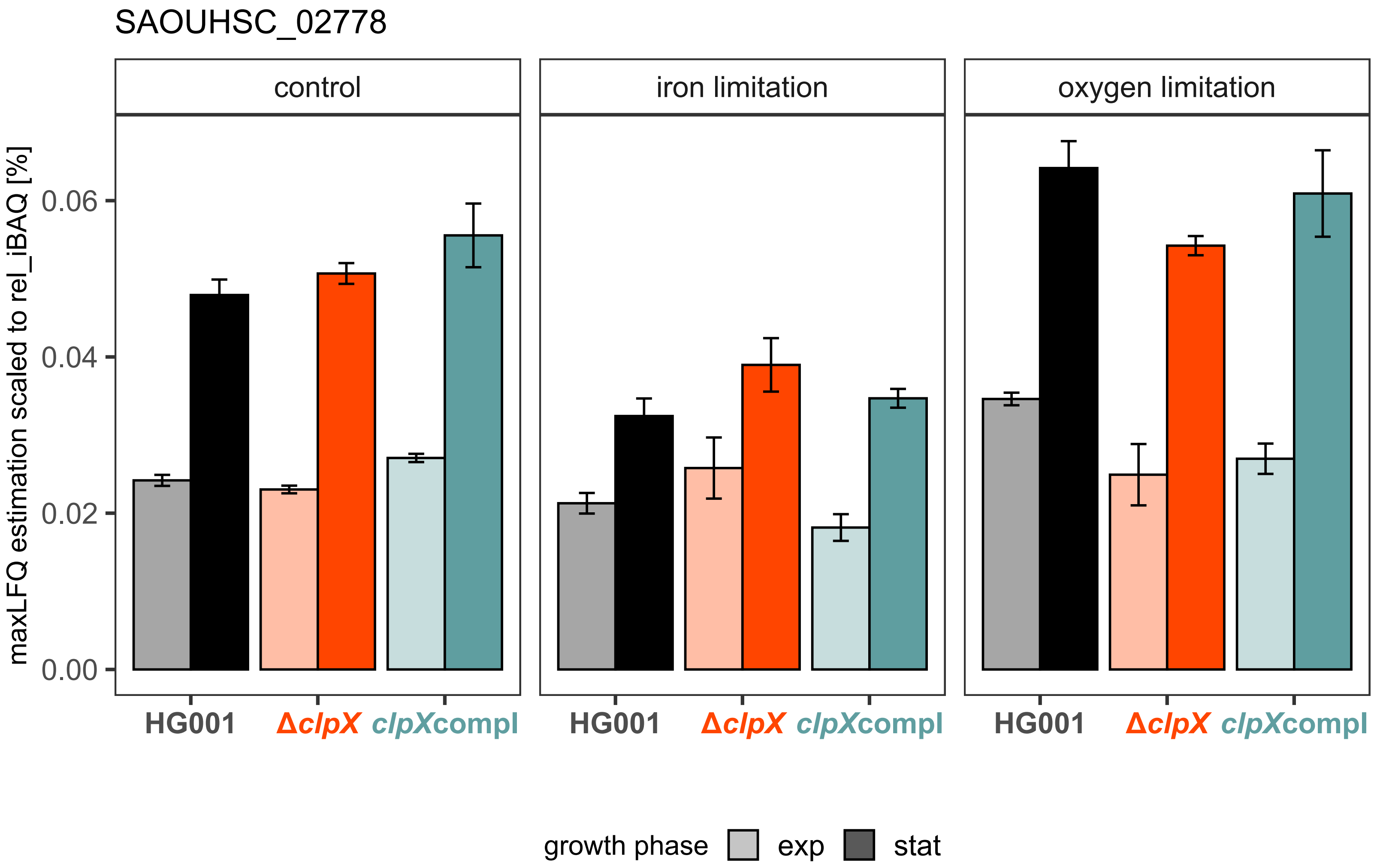

SAOUHSC_02778
control
iron limitation
oxygen limitation
maxLFQ estimation scaled to rel_iBAQ [%]
0.06
0.04
0.02
0.00
HG001
ΔclpX
clpXcompl
growth phase
exp
stat

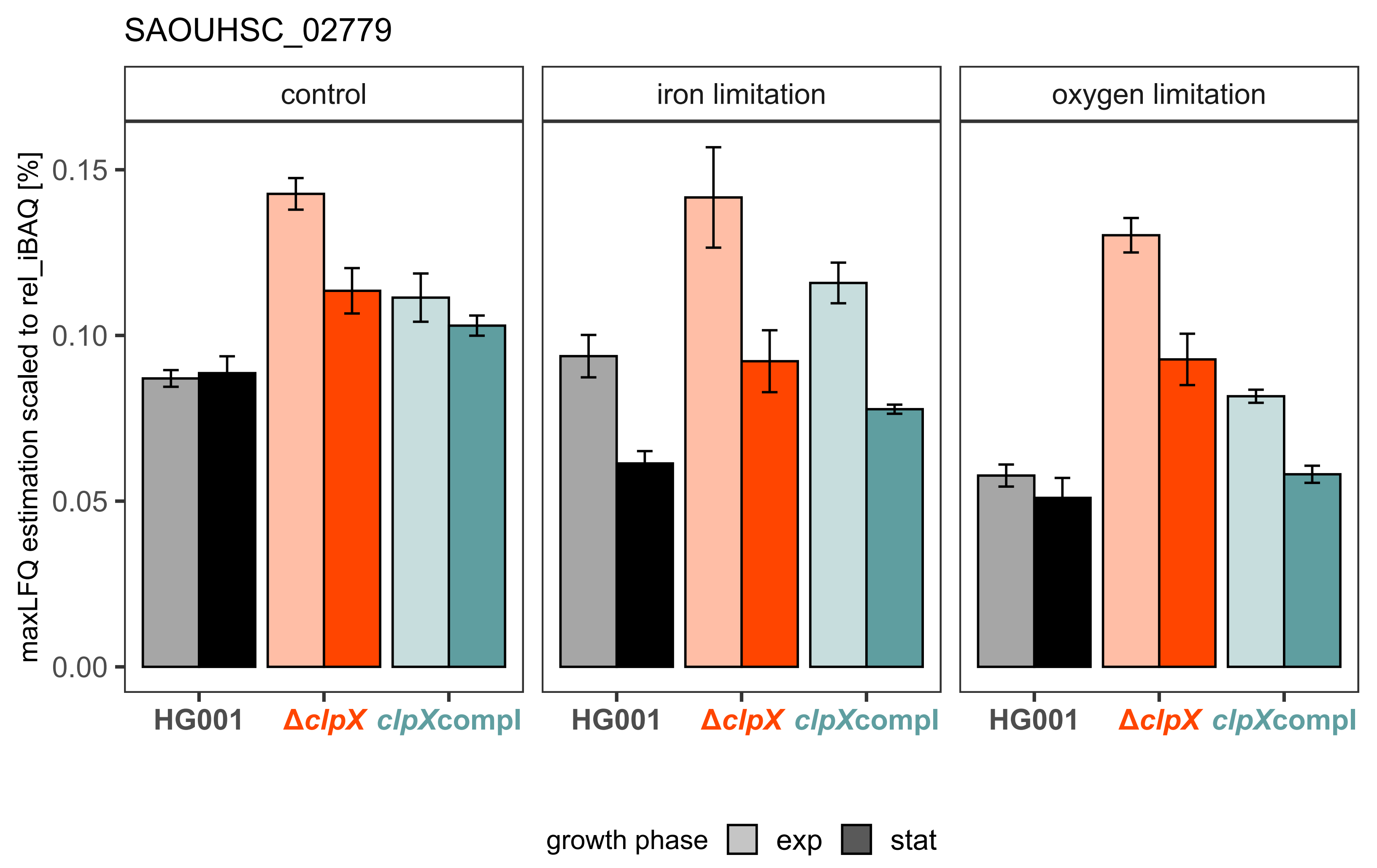

SAOUHSC_02779
control
iron limitation
oxygen limitation
maxLFQ estimation scaled to rel_iBAQ [%]
0.15
0.10
0.05
0.00
HG001
ΔclpX
clpXcompl
growth phase
exp
stat

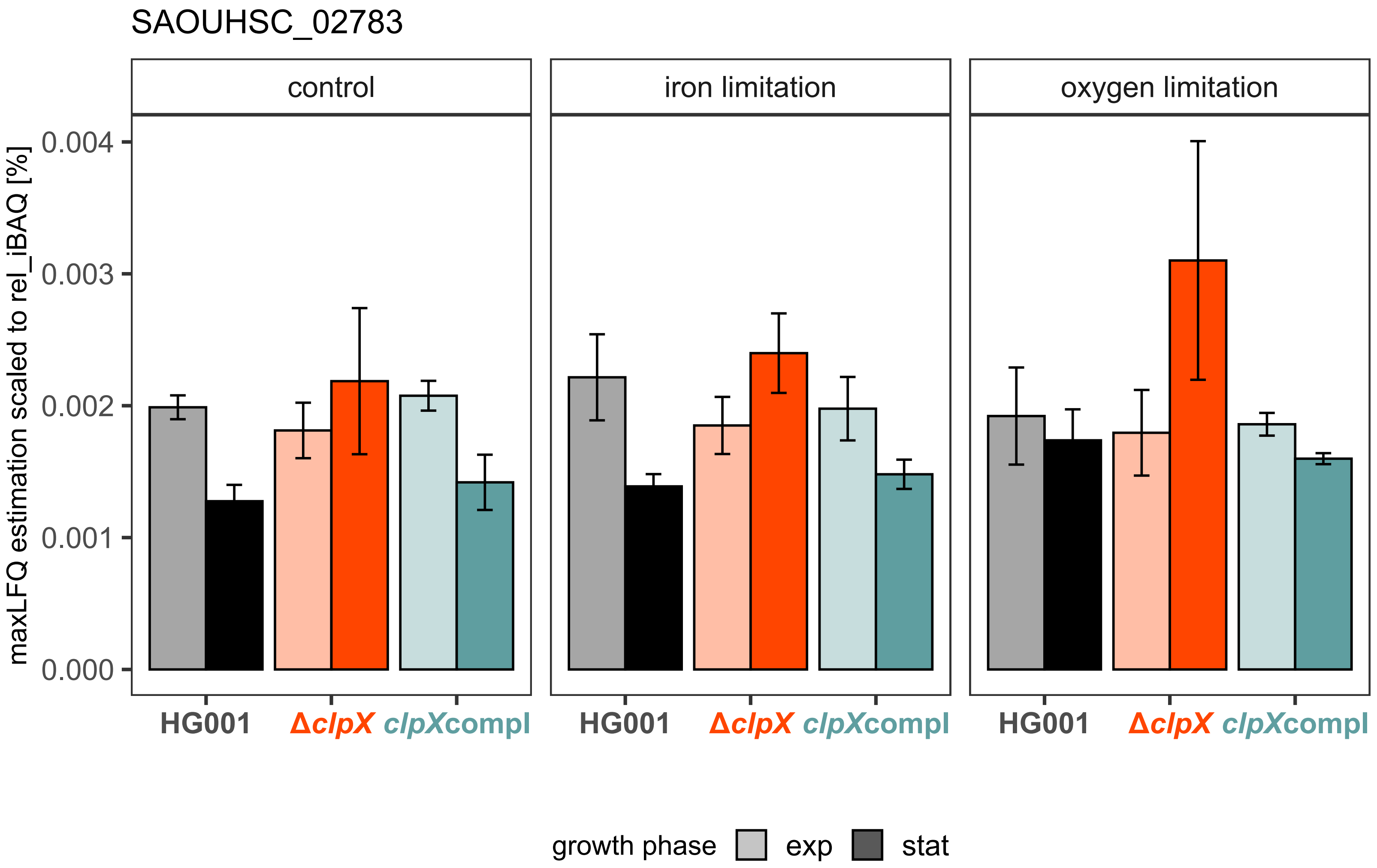
SAOUHSC_02783
control
iron limitation
oxygen limitation
maxLFQ estimation scaled to rel_iBAQ [%]
0.004
0.003
0.002
0.001
0.000
HG001
ΔclpX
clpXcompl
growth phase
exp
stat

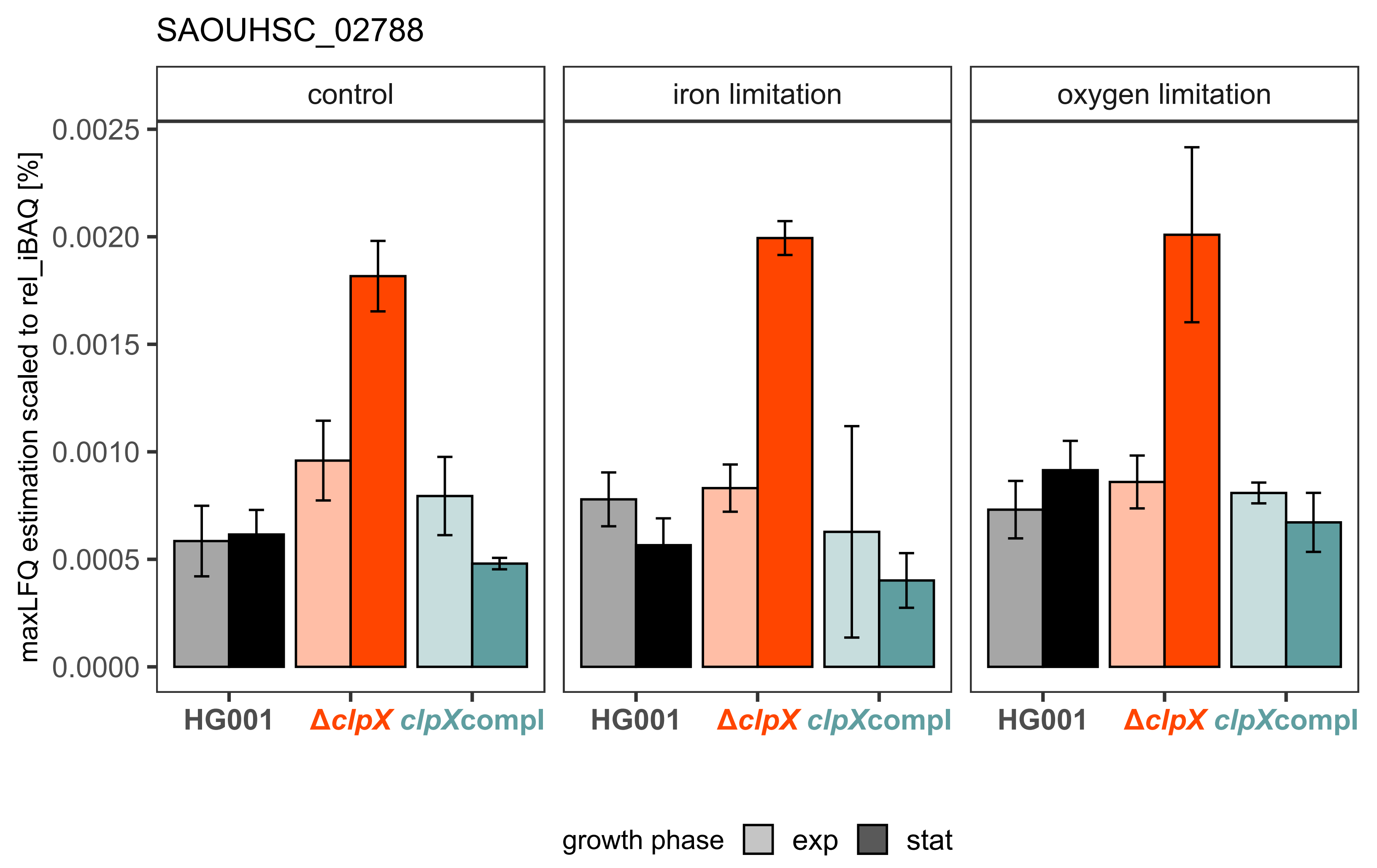

SAOUHSC_02788
control
iron limitation
oxygen limitation
maxLFQ estimation scaled to rel_iBAQ [%]
0.0025
0.0020
0.0015
0.0010
0.0005
0.0000
HG001
ΔclpX
clpXcompl
growth phase
exp
stat

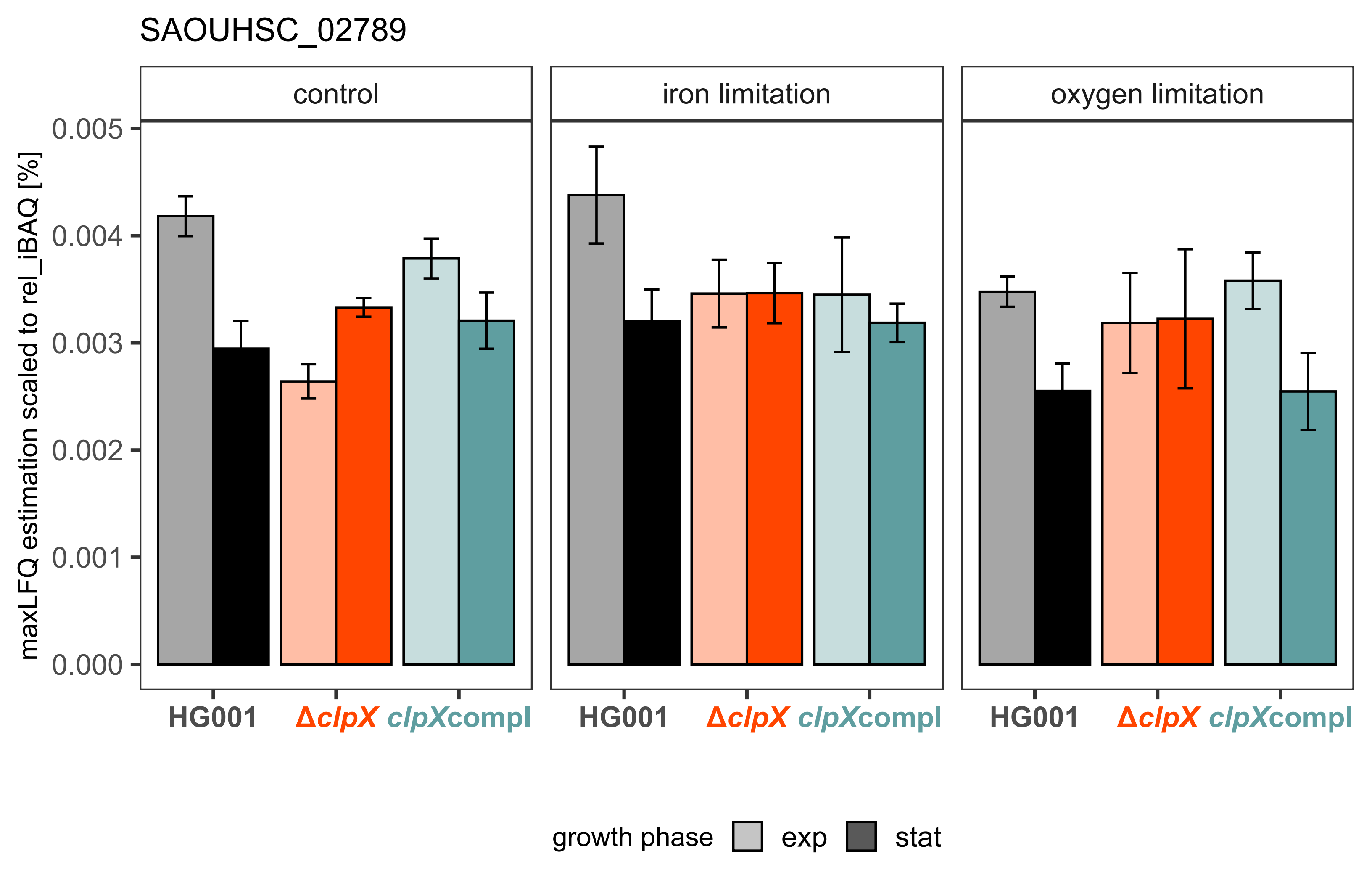
SAOUHSC_02789
control
iron limitation
oxygen limitation
0.005
0.004
0.003
0.002
0.001
0.000
maxLFQ estimation scaled to rel_iBAQ [%]
HG001
ΔclpX
clpXcompl
growth phase
exp
stat

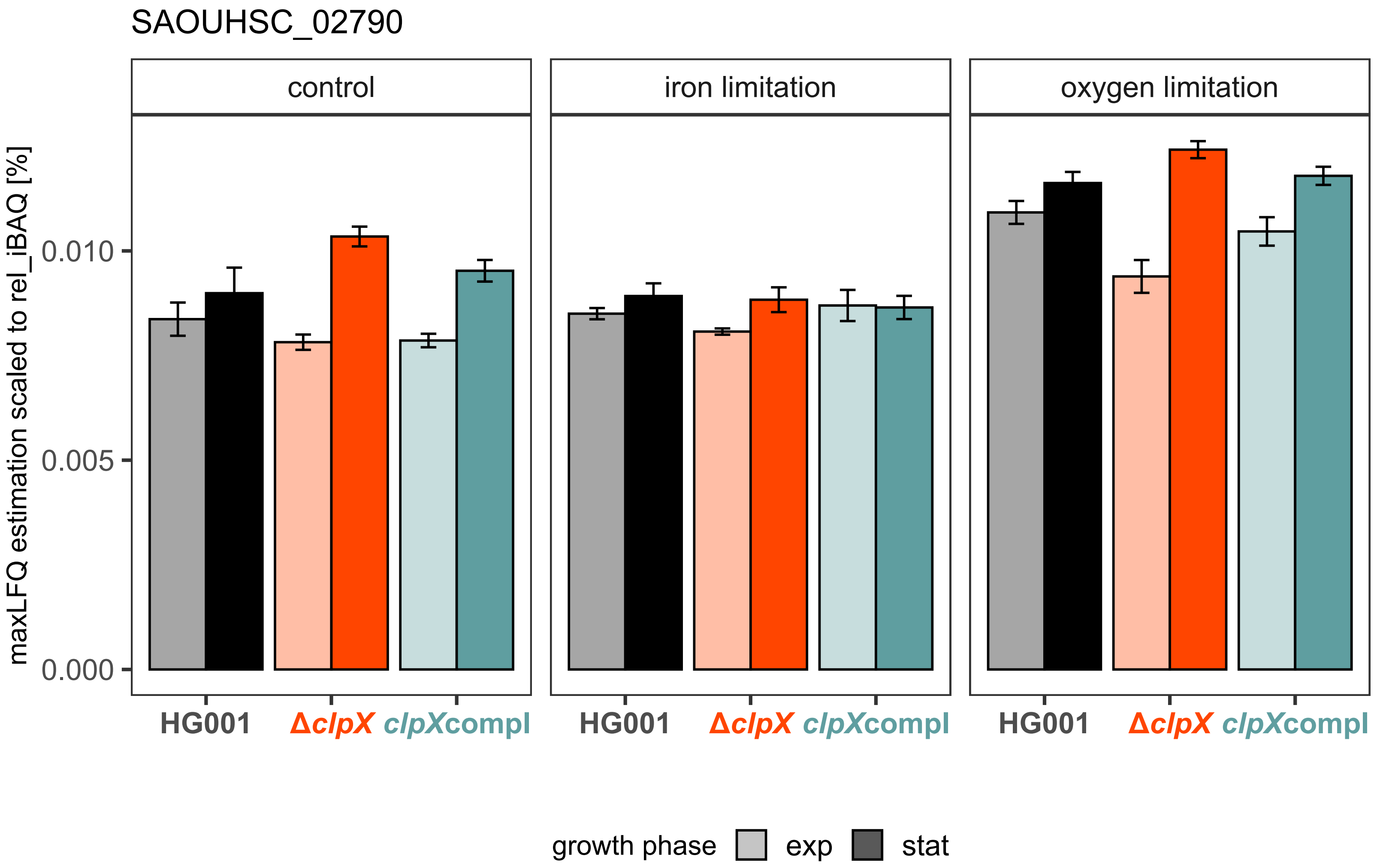
SAOUHSC_02790
control
iron limitation
oxygen limitation
maxLFQ estimation scaled to rel_iBAQ [%]
0.010
0.005
0.000
HG001
ΔclpX
clpXcompl
growth phase
exp
stat

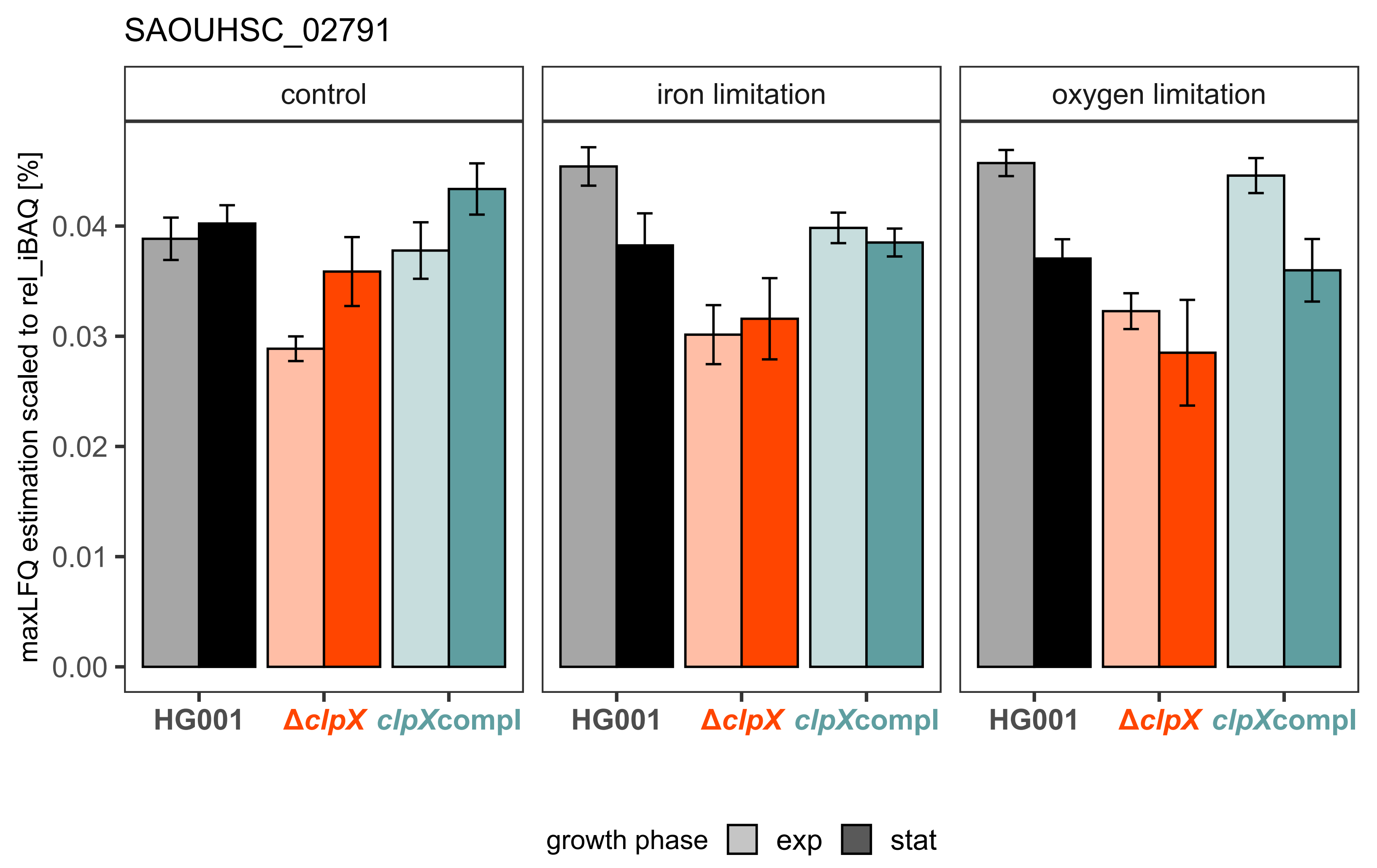
SAOUHSC_02791
control
iron limitation
oxygen limitation
maxLFQ estimation scaled to rel_iBAQ [%]
0.00
0.01
0.02
0.03
0.04
HG001
ΔclpX
clpXcompl
growth phase
exp
stat

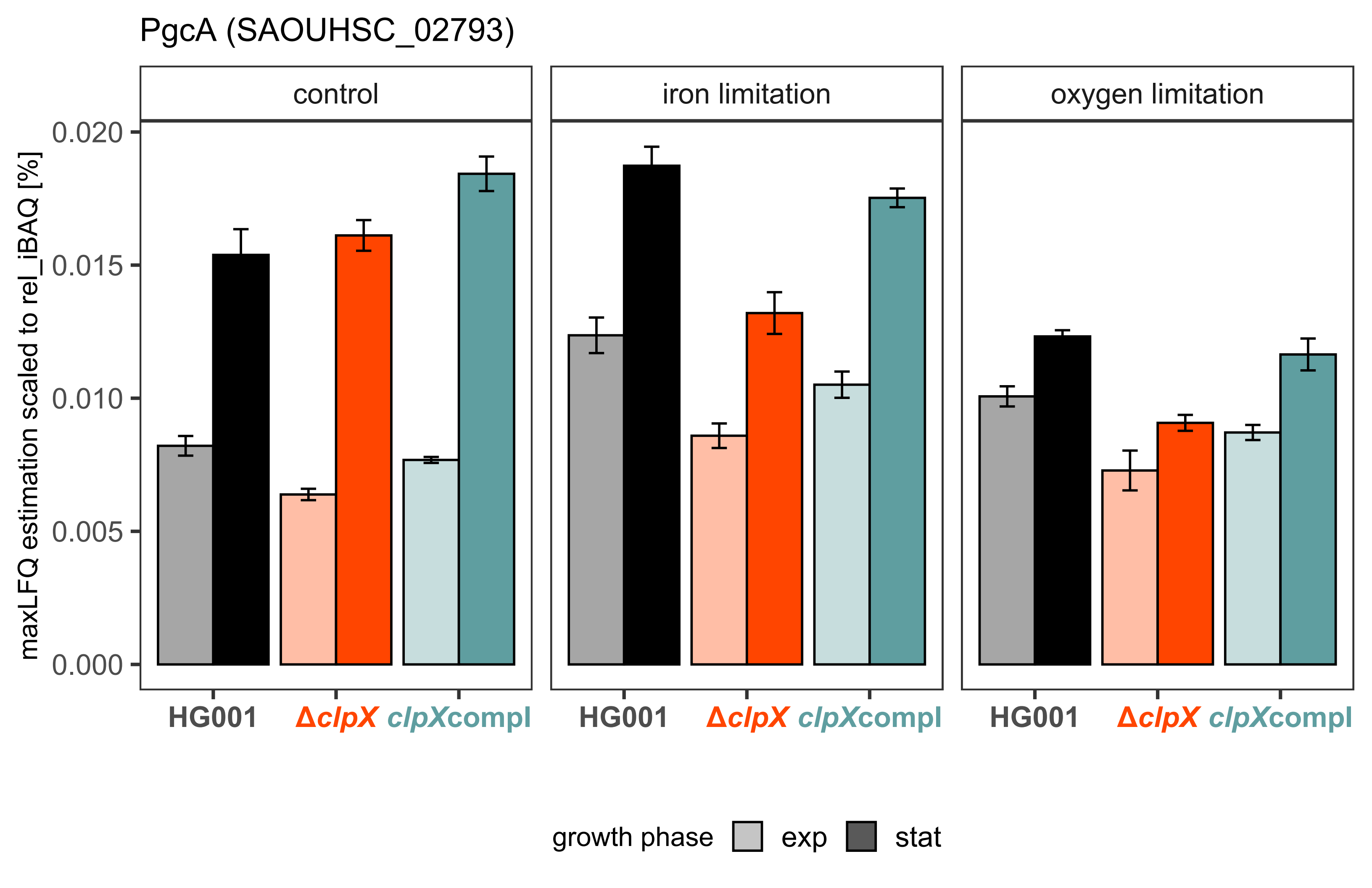

PgcA (SAOUHSC_02793)
control
iron limitation
oxygen limitation
maxLFQ estimation scaled to rel_iBAQ [%]
0.020
0.015
0.010
0.005
0.000
HG001
ΔclpX
clpXcompl
growth phase
exp
stat

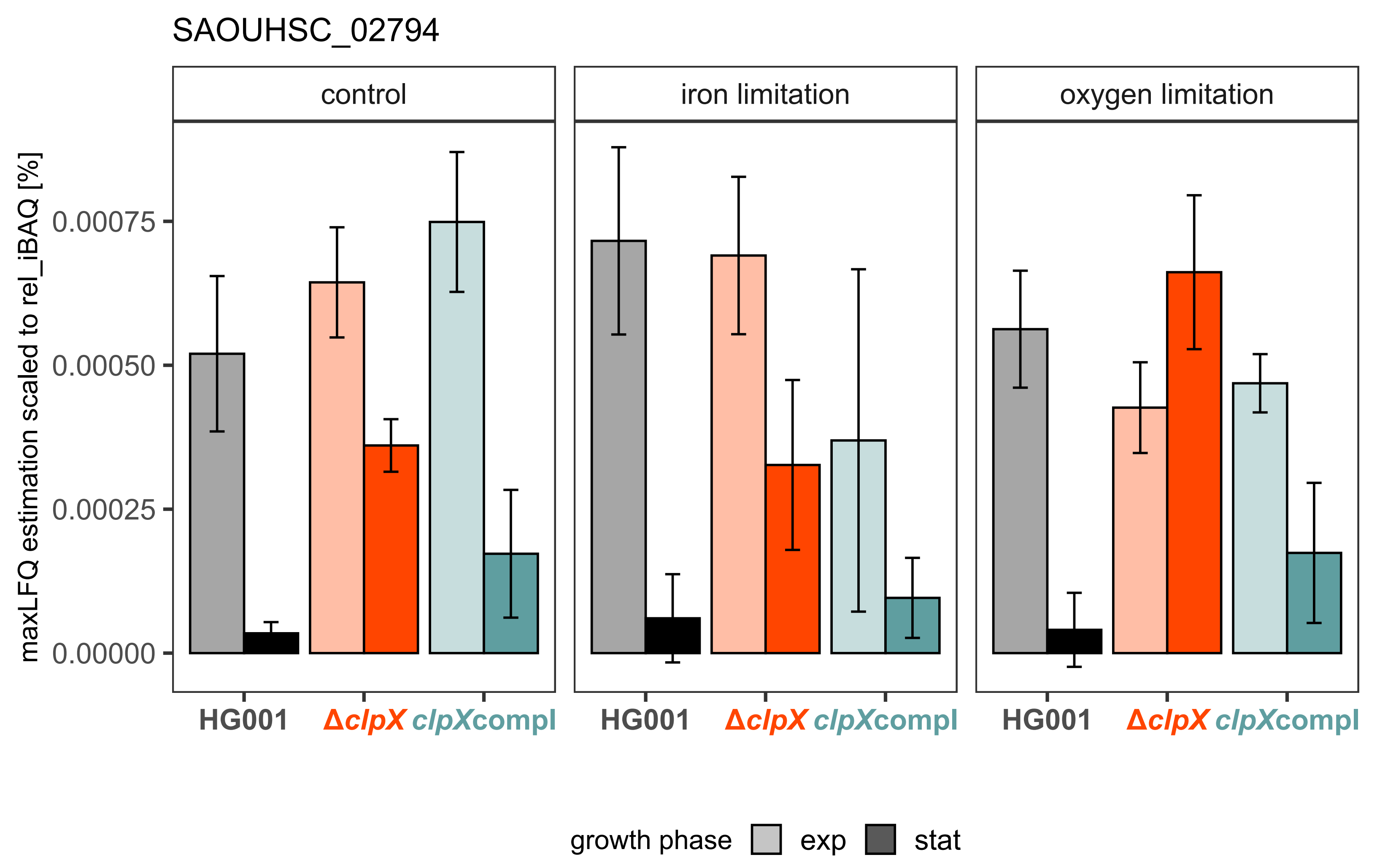

SAOUHSC_02794
control
iron limitation
oxygen limitation
maxLFQ estimation scaled to rel_iBAQ [%]
0.00075
0.00050
0.00025
0.00000
HG001
ΔclpX
clpXcompl
growth phase
exp
stat

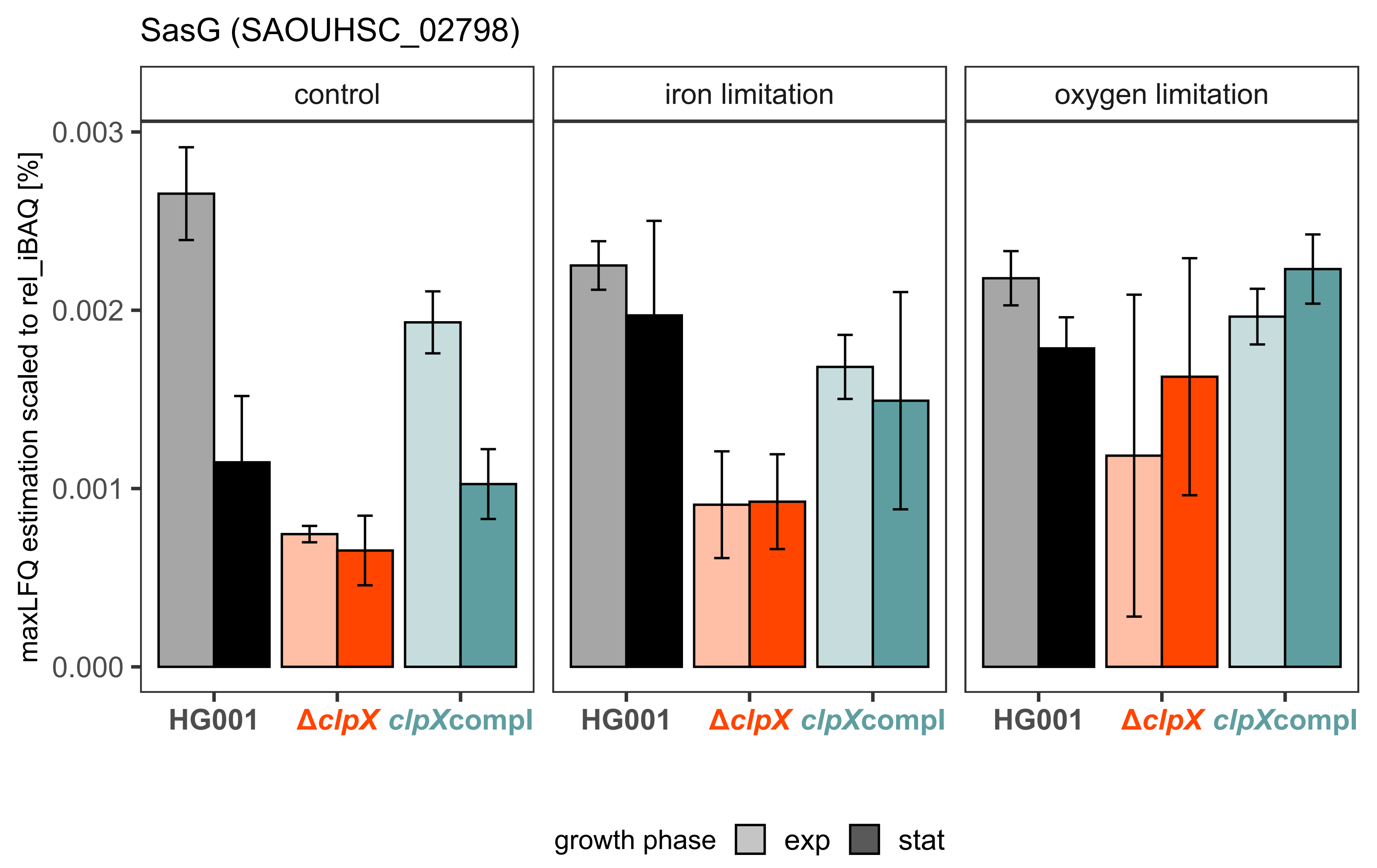

SasG (SAOUHSC_02798)
control
iron limitation
oxygen limitation
maxLFQ estimation scaled to rel_iBAQ [%]
0.003
0.002
0.001
0.000
HG001
ΔclpX
clpXcompl
growth phase
exp
stat

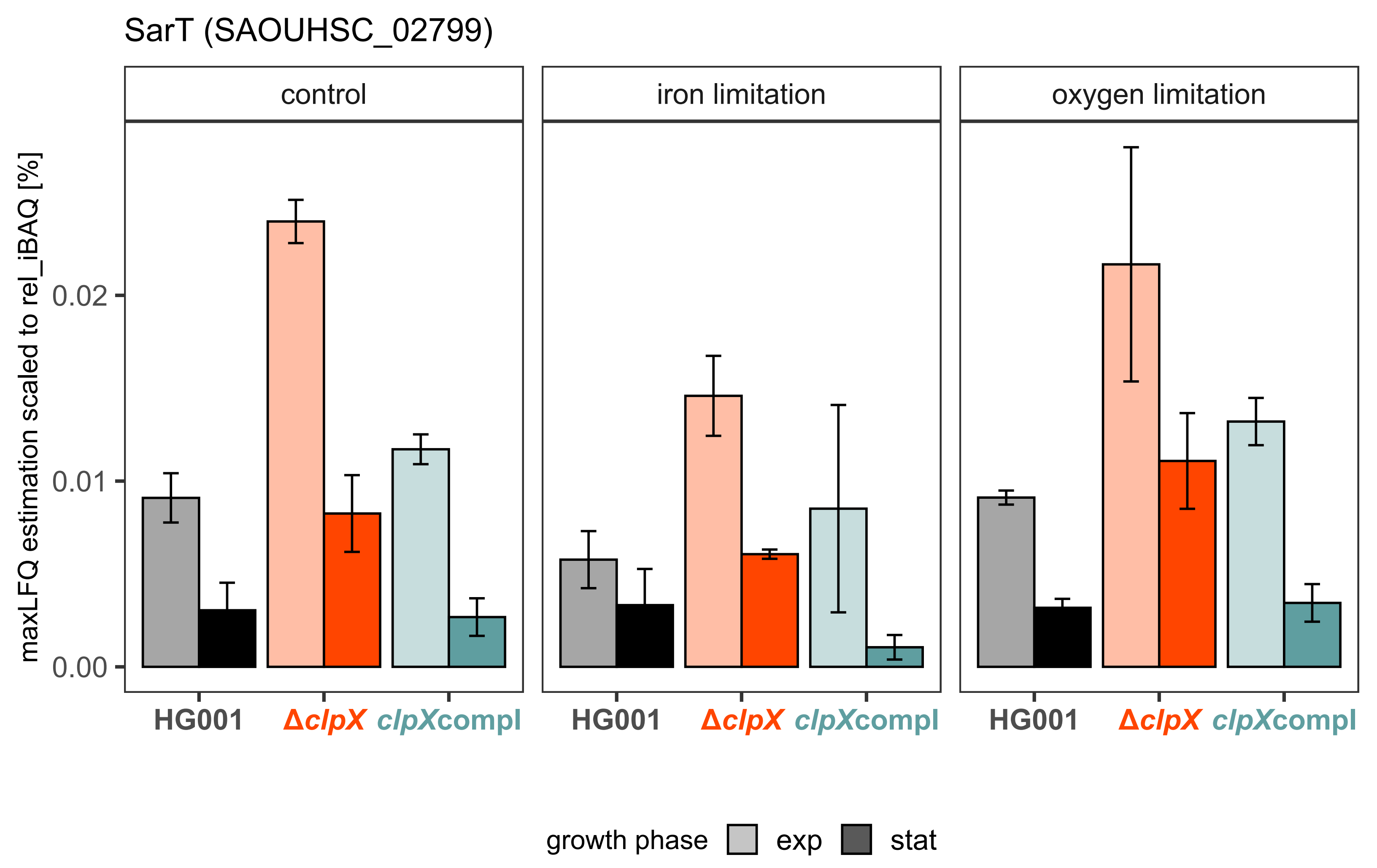
SarT (SAOUHSC_02799)
control
iron limitation
oxygen limitation
maxLFQ estimation scaled to rel_iBAQ [%]
0.00
0.01
0.02
HG001
ΔclpX
clpXcompl
growth phase
exp
stat

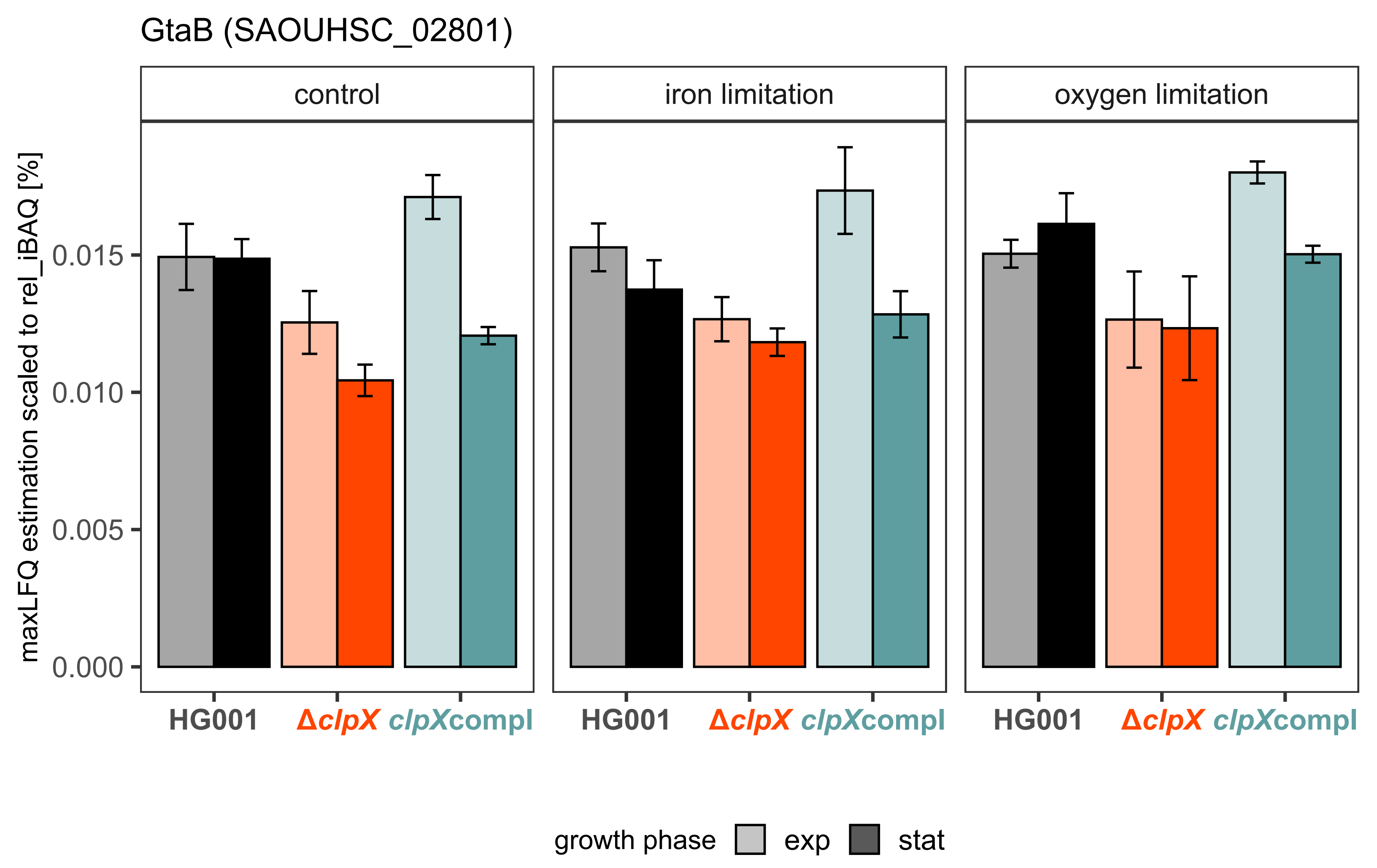
GtaB (SAOUHSC_02801)
control
iron limitation
oxygen limitation
maxLFQ estimation scaled to rel_iBAQ [%]
0.015
0.010
0.005
0.000
HG001
ΔclpX
clpXcompl
growth phase
exp
stat

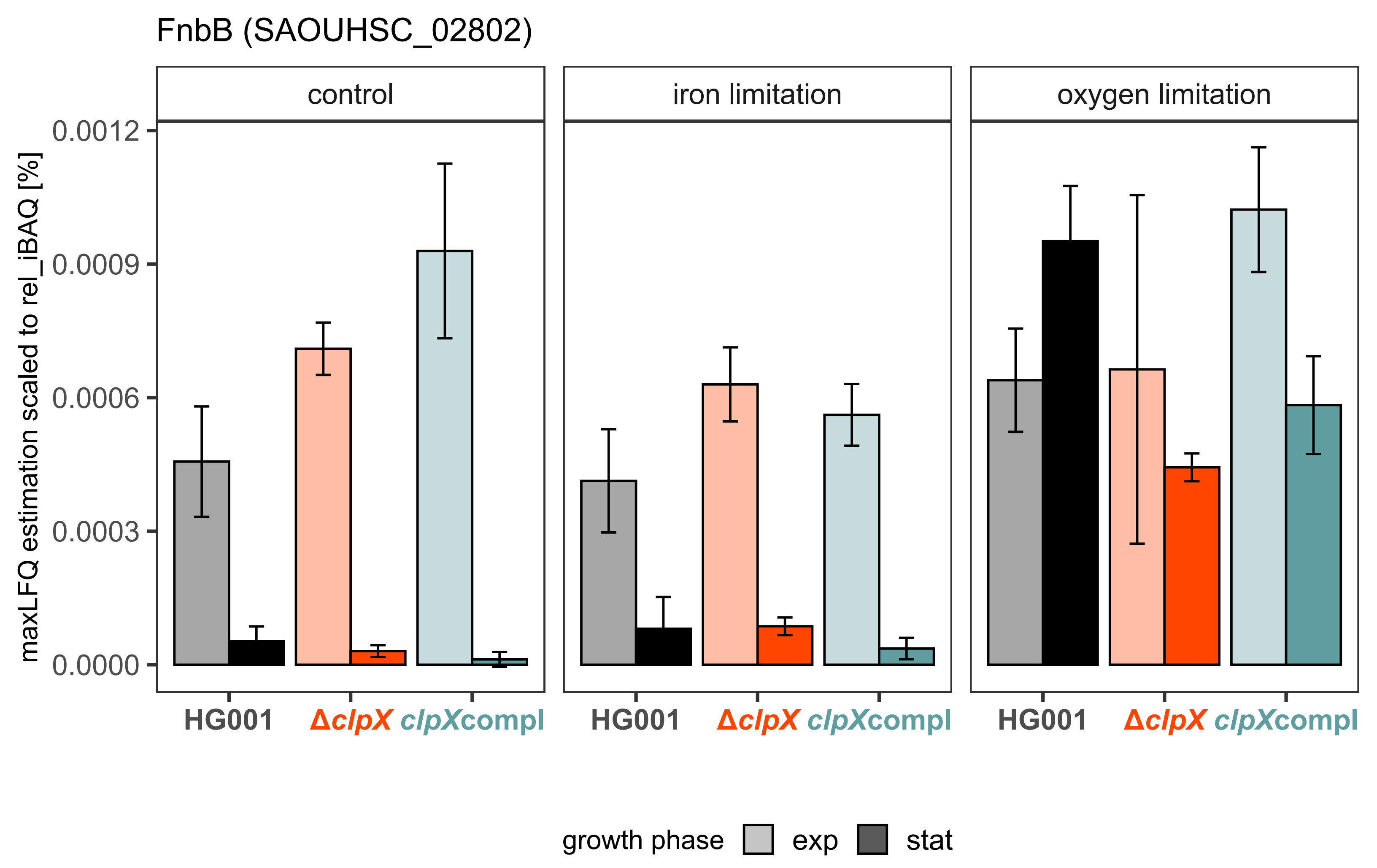

FnbB (SAOUHSC_02802)
control
iron limitation
oxygen limitation
maxLFQ estimation scaled to rel_iBAQ [%]
0.0012
0.0009
0.0006
0.0003
0.0000
HG001
ΔclpX
clpXcompl
growth phase
exp
stat

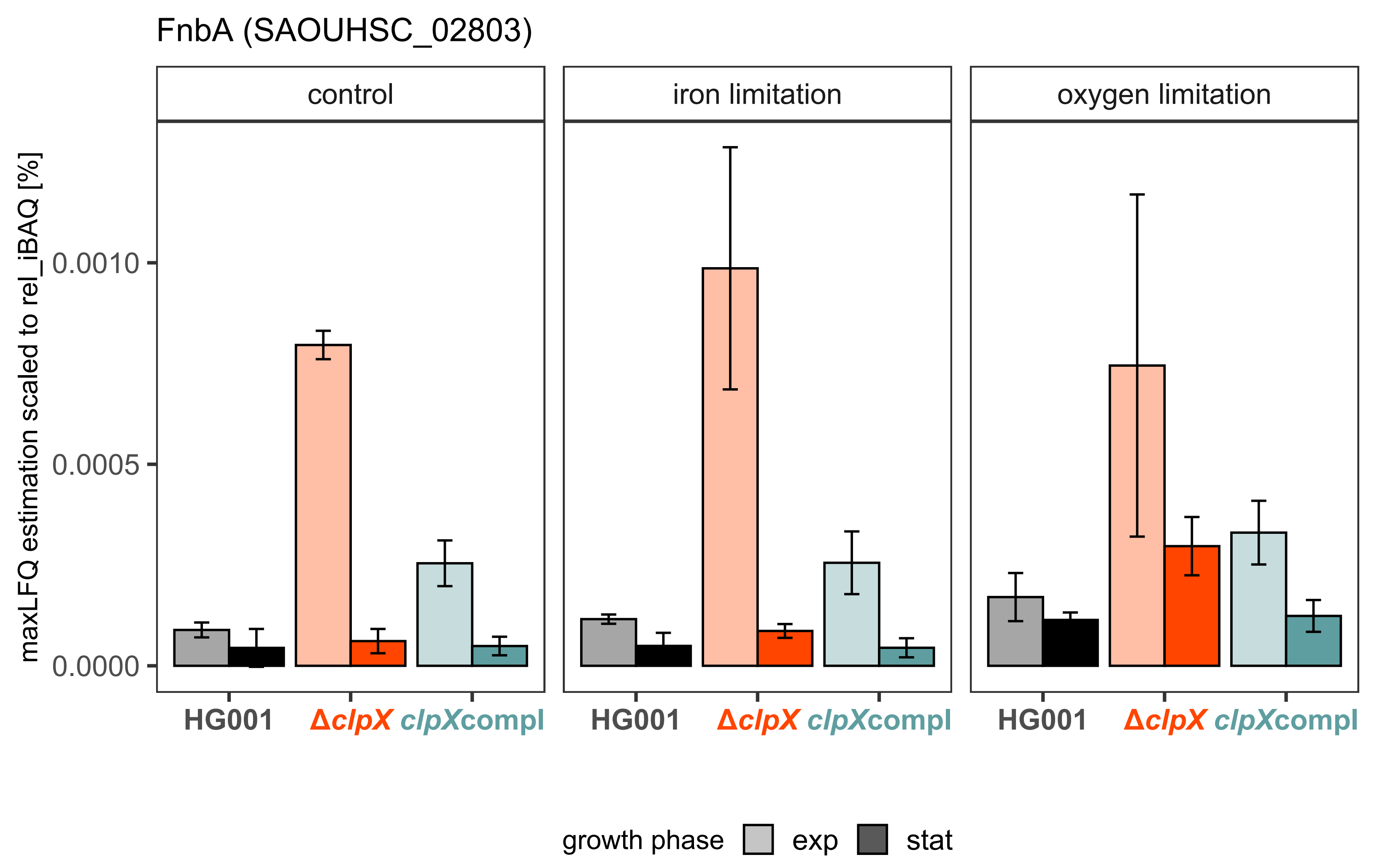

FnbA (SAOUHSC_02803)
control
iron limitation
oxygen limitation
maxLFQ estimation scaled to rel_iBAQ [%]
0.0010
0.0005
0.0000
HG001
ΔclpX
clpXcompl
growth phase
exp
stat

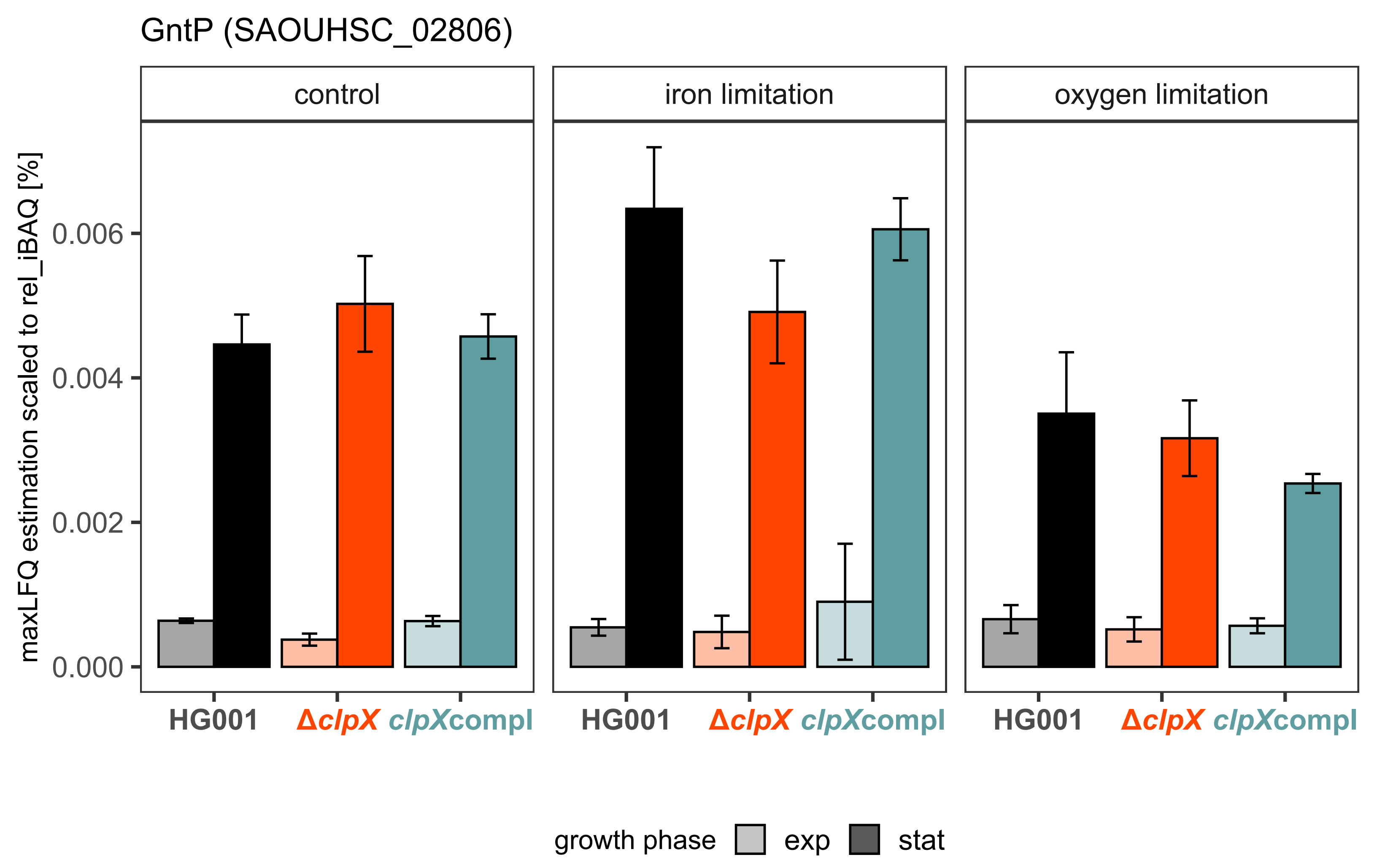
GntP (SAOUHSC_02806)
control
iron limitation
oxygen limitation
maxLFQ estimation scaled to rel_iBAQ [%]
0.006
0.004
0.002
0.000
HG001
ΔclpX
clpXcompl
growth phase
exp
stat

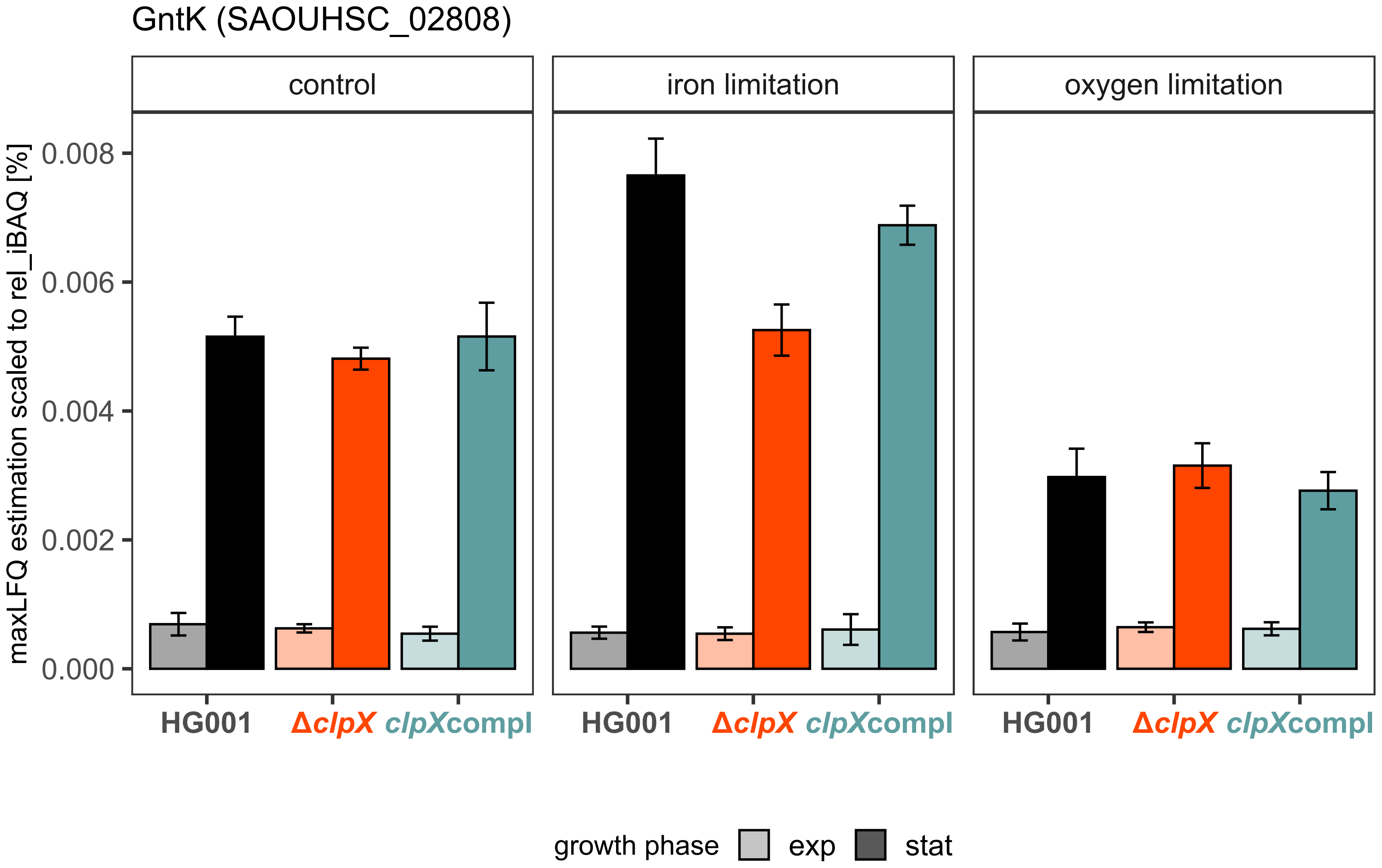
GntK (SAOUHSC_02808)
control
iron limitation
oxygen limitation
maxLFQ estimation scaled to rel_iBAQ [%]
0.008
0.006
0.004
0.002
0.000
HG001
ΔclpX
clpXcompl
growth phase
exp
stat

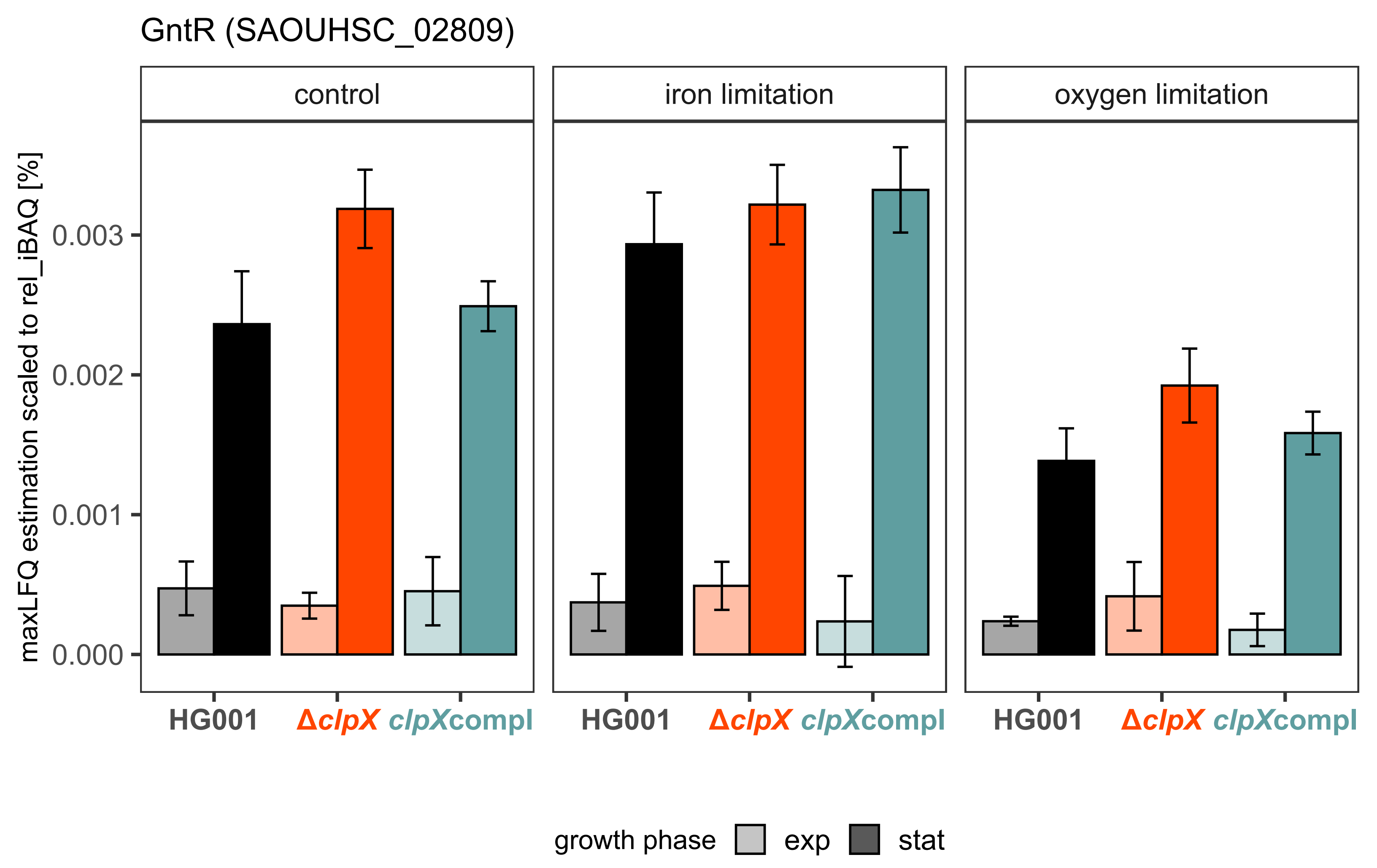
GntR (SAOUHSC_02809)
control
iron limitation
oxygen limitation
maxLFQ estimation scaled to rel_iBAQ [%]
0.000
0.001
0.002
0.003
HG001
ΔclpX
clpXcompl
growth phase
exp
stat

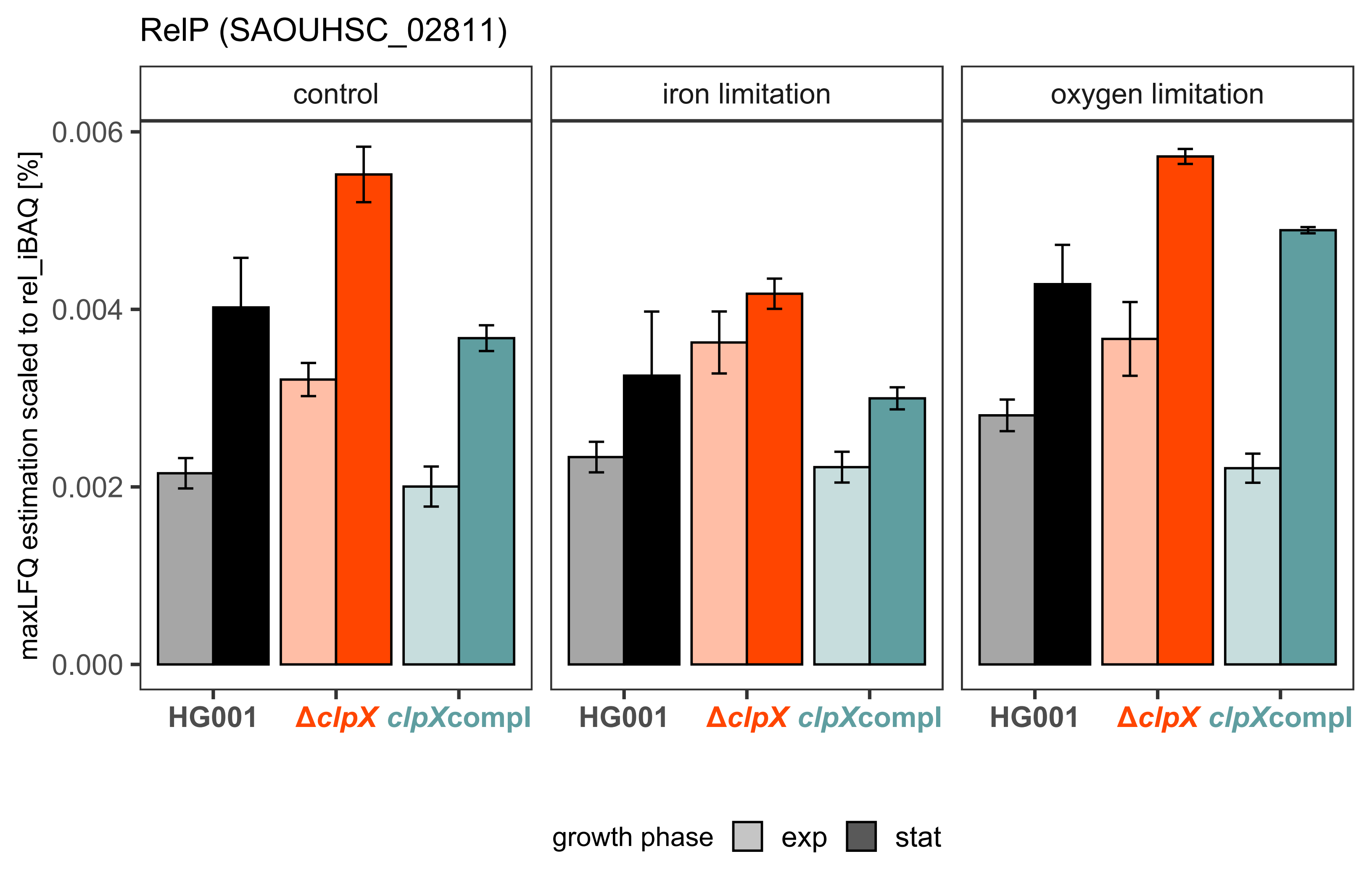
RelP (SAOUHSC_02811)
control
iron limitation
oxygen limitation
maxLFQ estimation scaled to rel_iBAQ [%]
0.006
0.004
0.002
0.000
HG001
ΔclpX
clpXcompl
growth phase
exp
stat

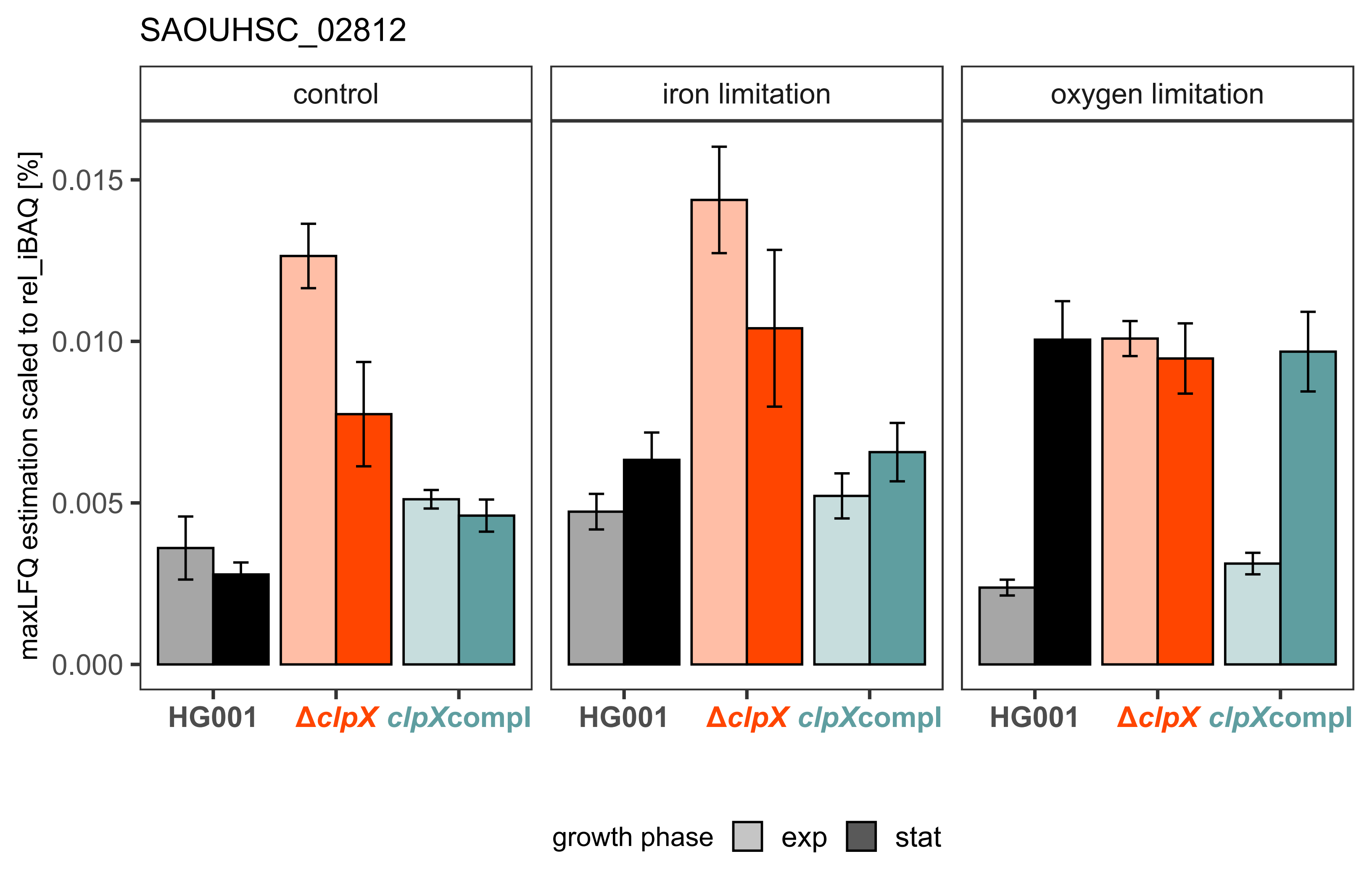

SAOUHSC_02812
control
iron limitation
oxygen limitation
maxLFQ estimation scaled to rel_iBAQ [%]
0.015
0.010
0.005
0.000
HG001
ΔclpX
clpXcompl
growth phase
exp
stat

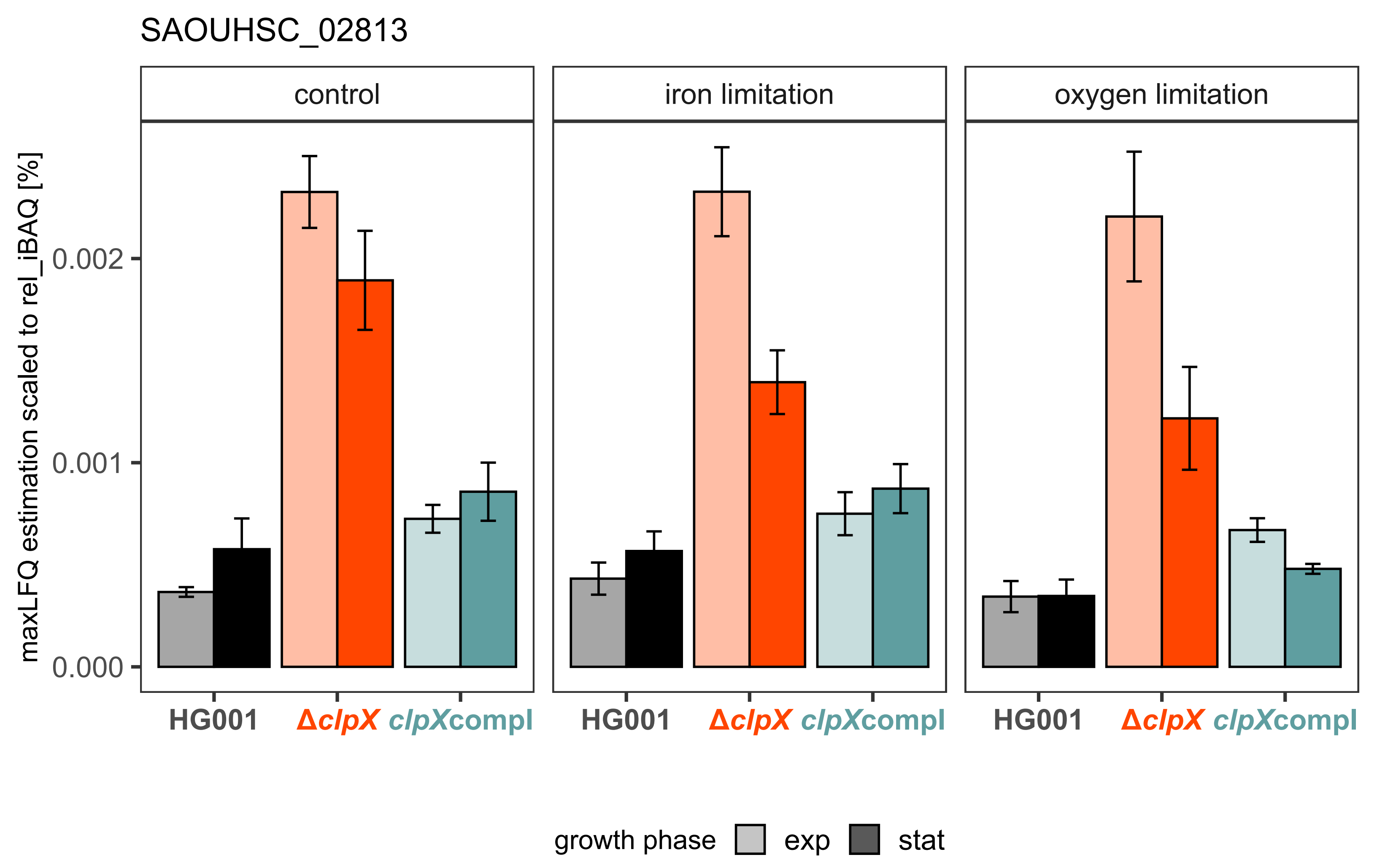
SAOUHSC_02813
control
iron limitation
oxygen limitation
maxLFQ estimation scaled to rel_iBAQ [%]
0.002
0.001
0.000
HG001
ΔclpX
clpXcompl
growth phase
exp
stat

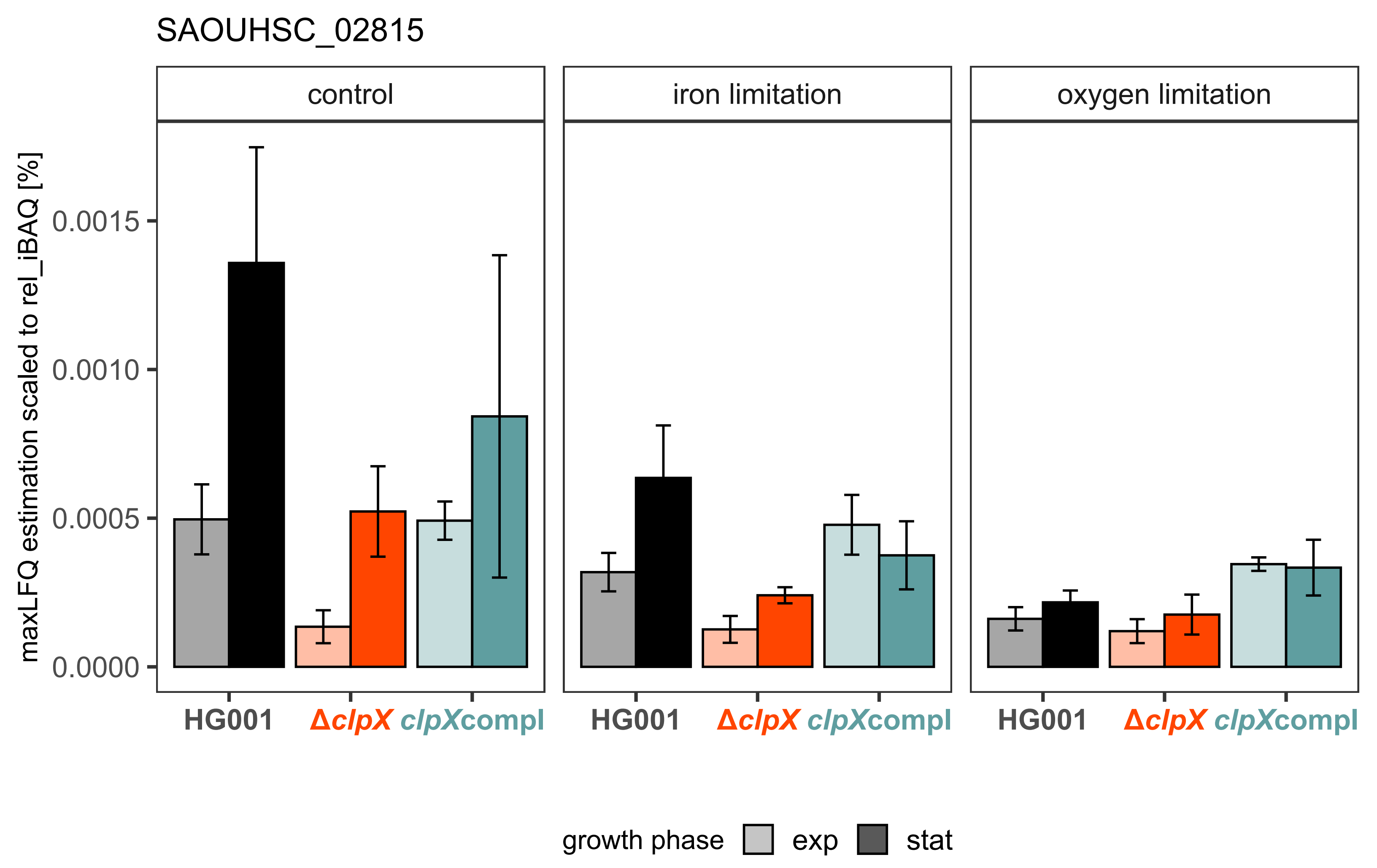

SAOUHSC_02815
control
iron limitation
oxygen limitation
maxLFQ estimation scaled to rel_iBAQ [%]
0.0015
0.0010
0.0005
0.0000
HG001
ΔclpX
clpXcompl
growth phase
exp
stat

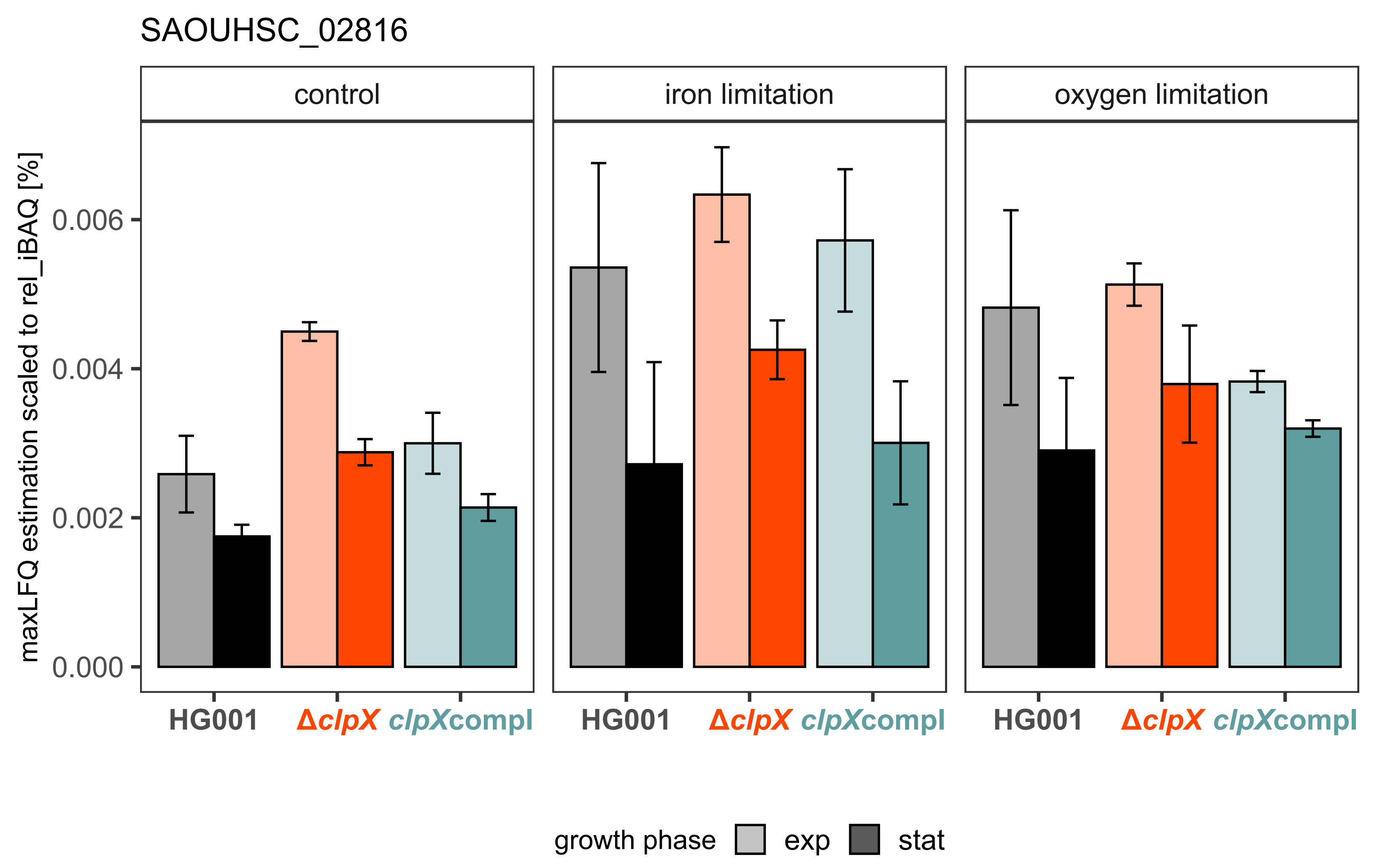

SAOUHSC_02816
control
iron limitation
oxygen limitation
maxLFQ estimation scaled to rel_iBAQ [%]
0.000
0.002
0.004
0.006
HG001
ΔclpX
clpXcompl
growth phase
exp
stat

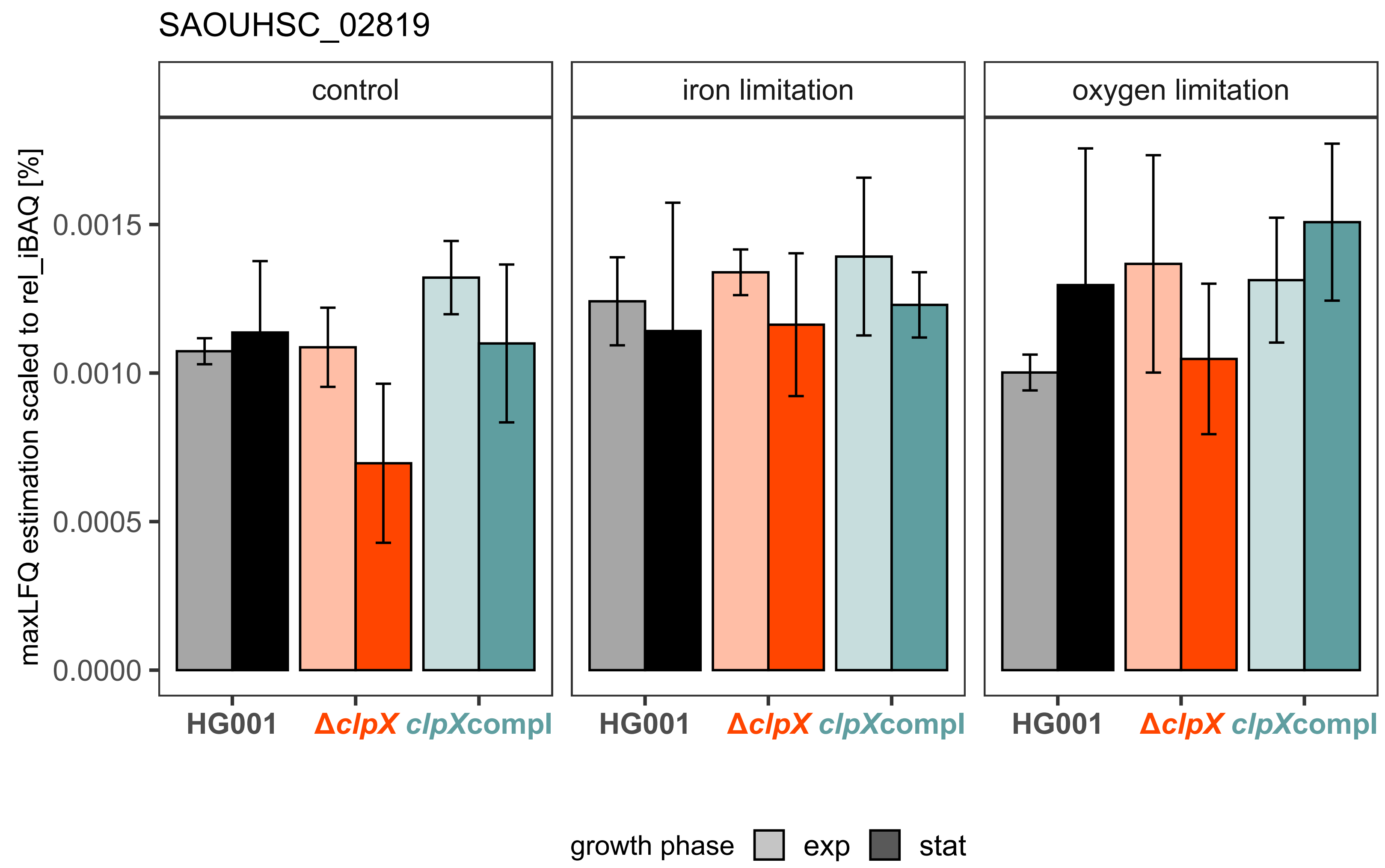
SAOUHSC_02819
control
iron limitation
oxygen limitation
maxLFQ estimation scaled to rel_iBAQ [%]
0.0015
0.0010
0.0005
0.0000
HG001
ΔclpX
clpXcompl
growth phase
exp
stat

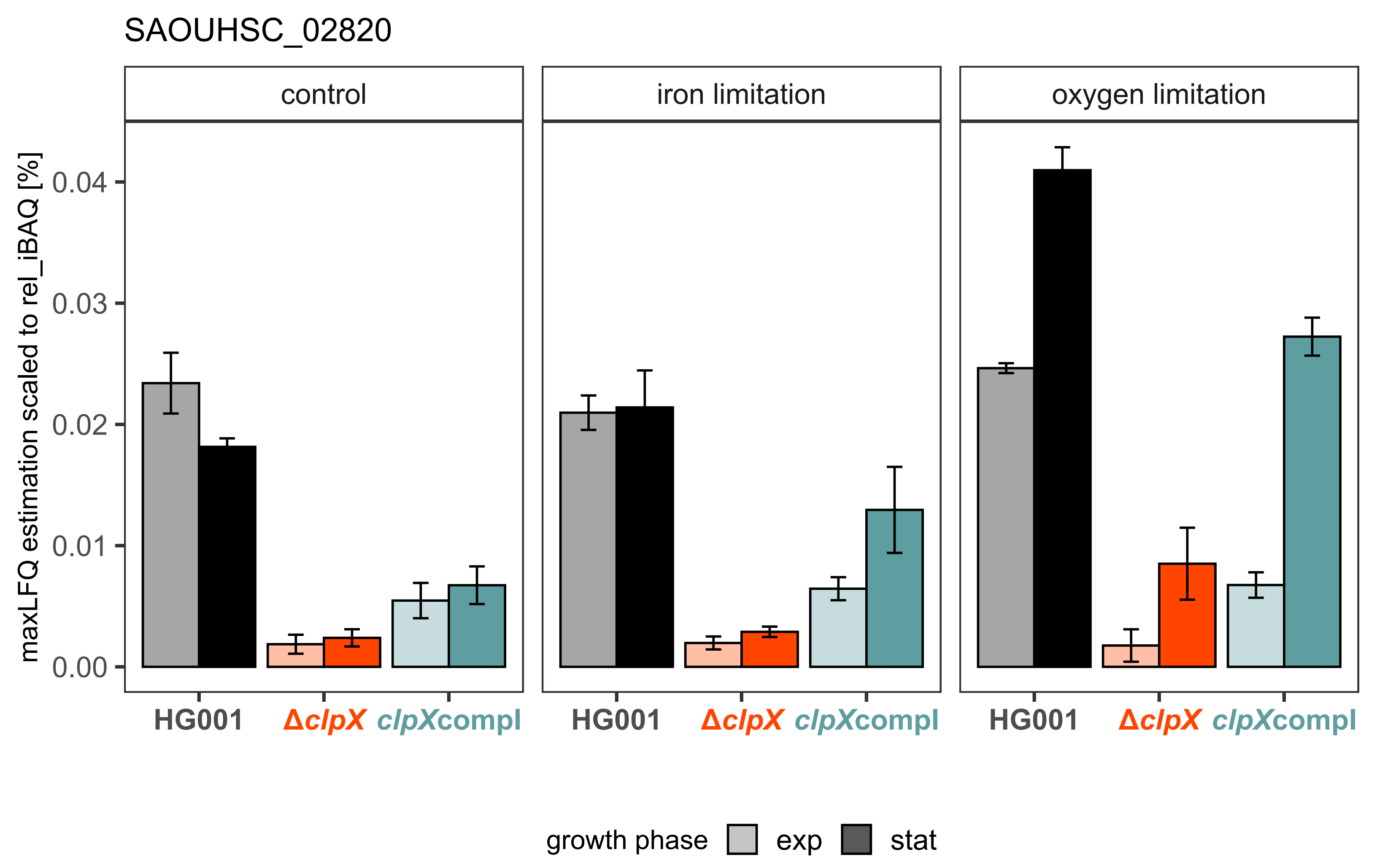
SAOUHSC_02820
control
iron limitation
oxygen limitation
maxLFQ estimation scaled to rel_iBAQ [%]
0.04
0.03
0.02
0.01
0.00
HG001
ΔclpX
clpXcompl
growth phase
exp
stat

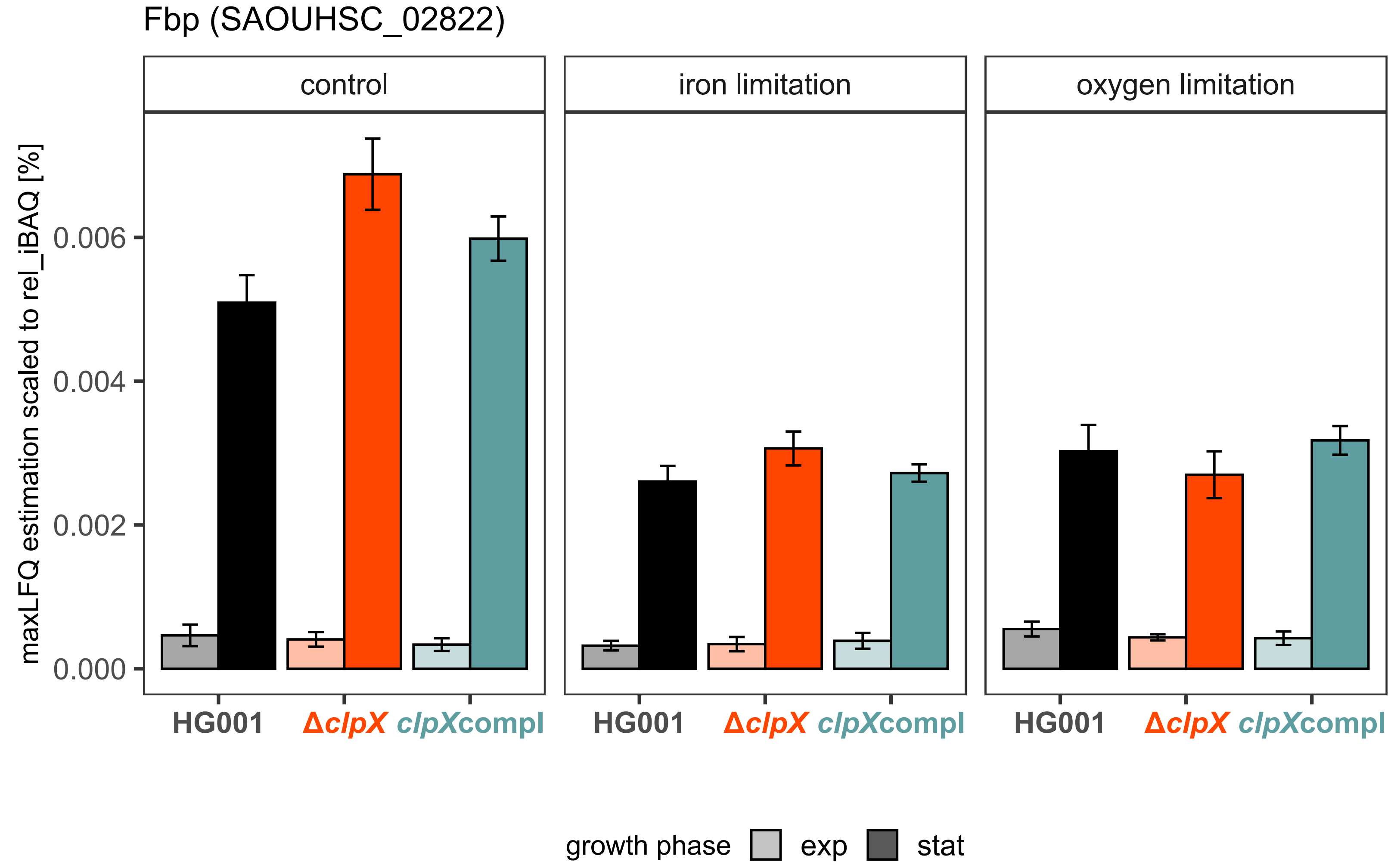

Fbp (SAOUHSC_02822)
control
iron limitation
oxygen limitation
maxLFQ estimation scaled to rel_iBAQ [%]
0.006
0.004
0.002
0.000
HG001
ΔclpX
clpXcompl
growth phase
exp
stat

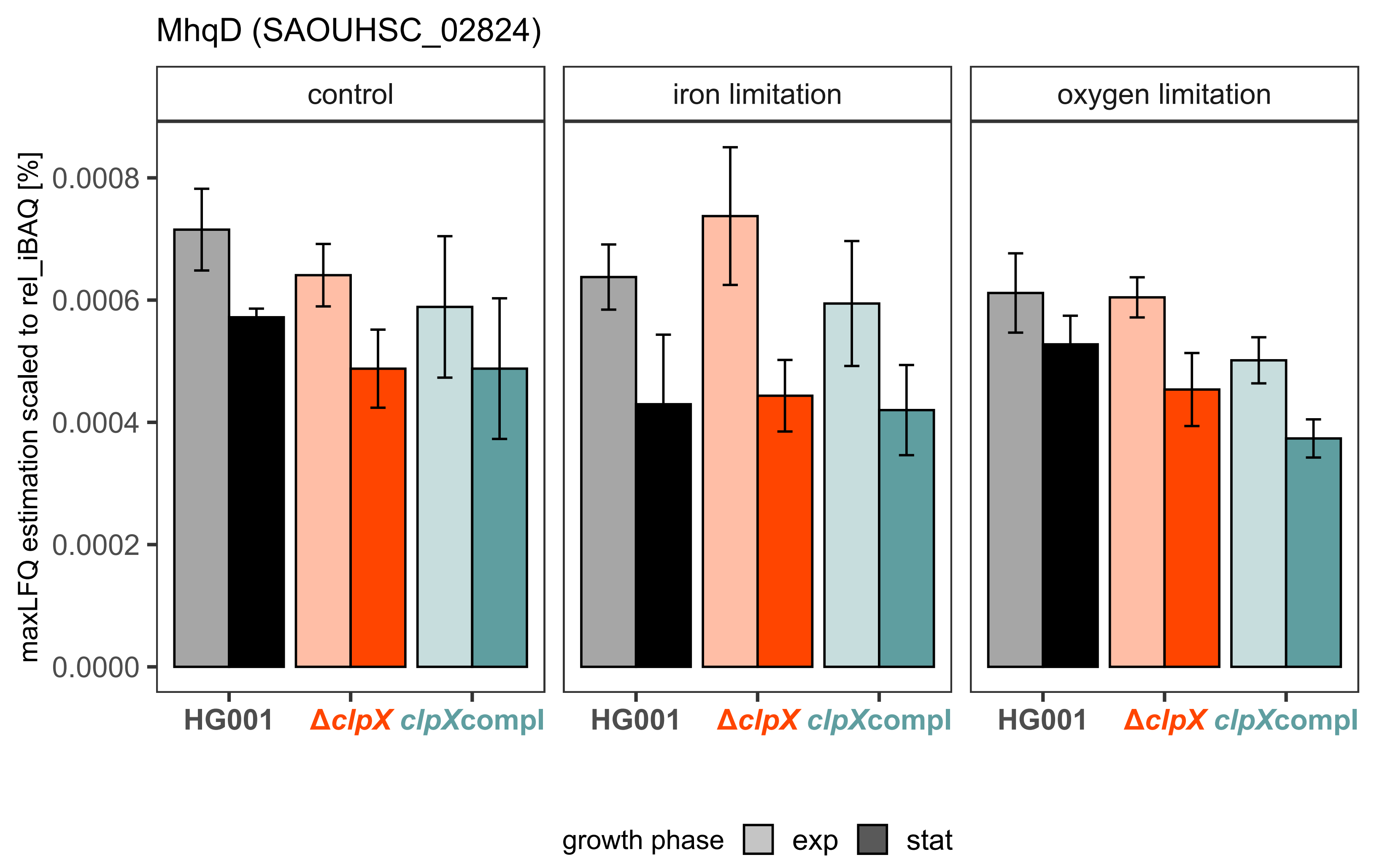
MhqD (SAOUHSC_02824)
control
iron limitation
oxygen limitation
maxLFQ estimation scaled to rel_iBAQ [%]
0.0008
0.0006
0.0004
0.0002
0.0000
HG001
ΔclpX
clpXcompl
growth phase
exp
stat

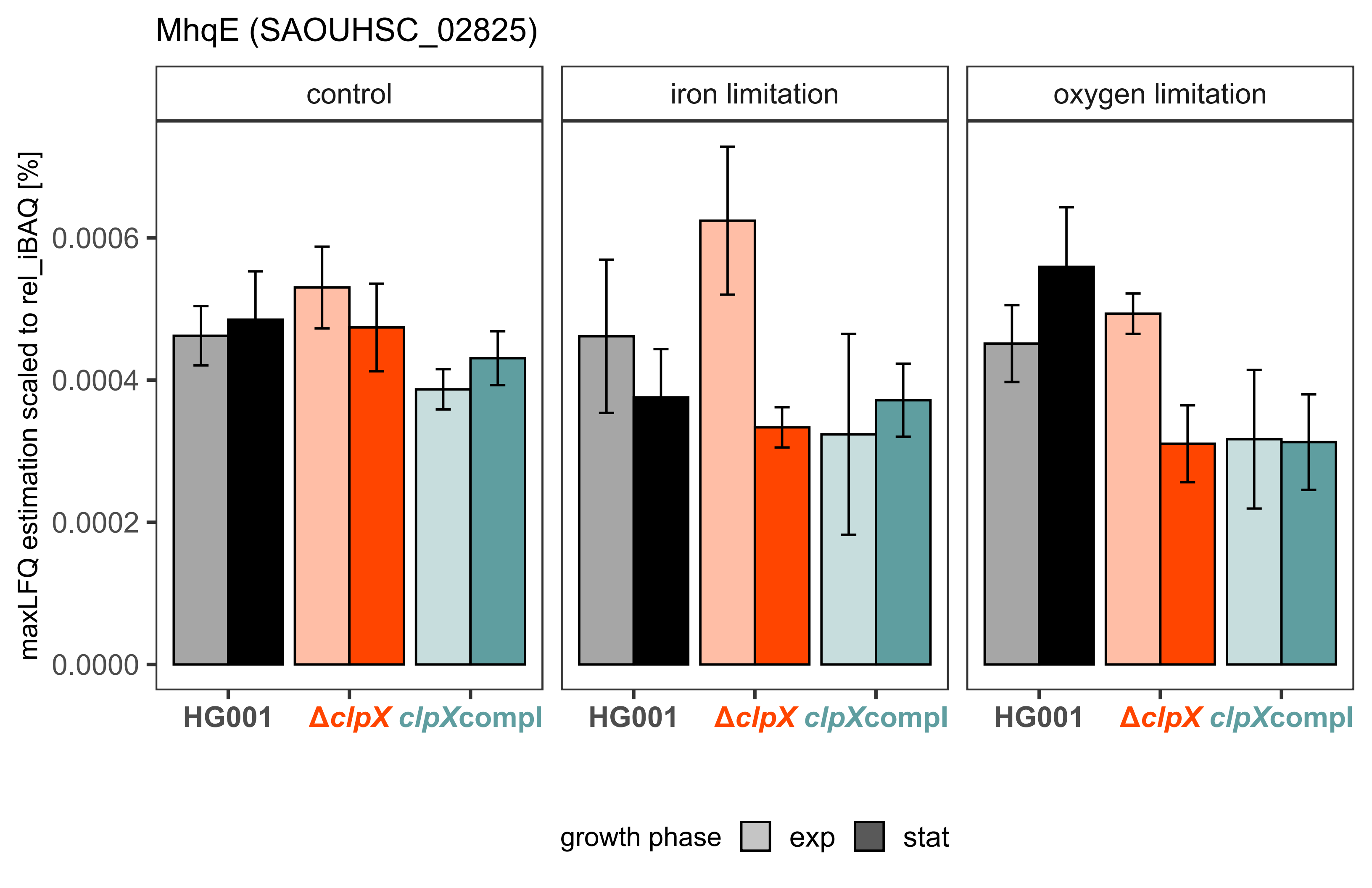
MhqE (SAOUHSC_02825)
control
iron limitation
oxygen limitation
maxLFQ estimation scaled to rel_iBAQ [%]
0.0000
0.0002
0.0004
0.0006
HG001
ΔclpX
clpXcompl
growth phase
exp
stat

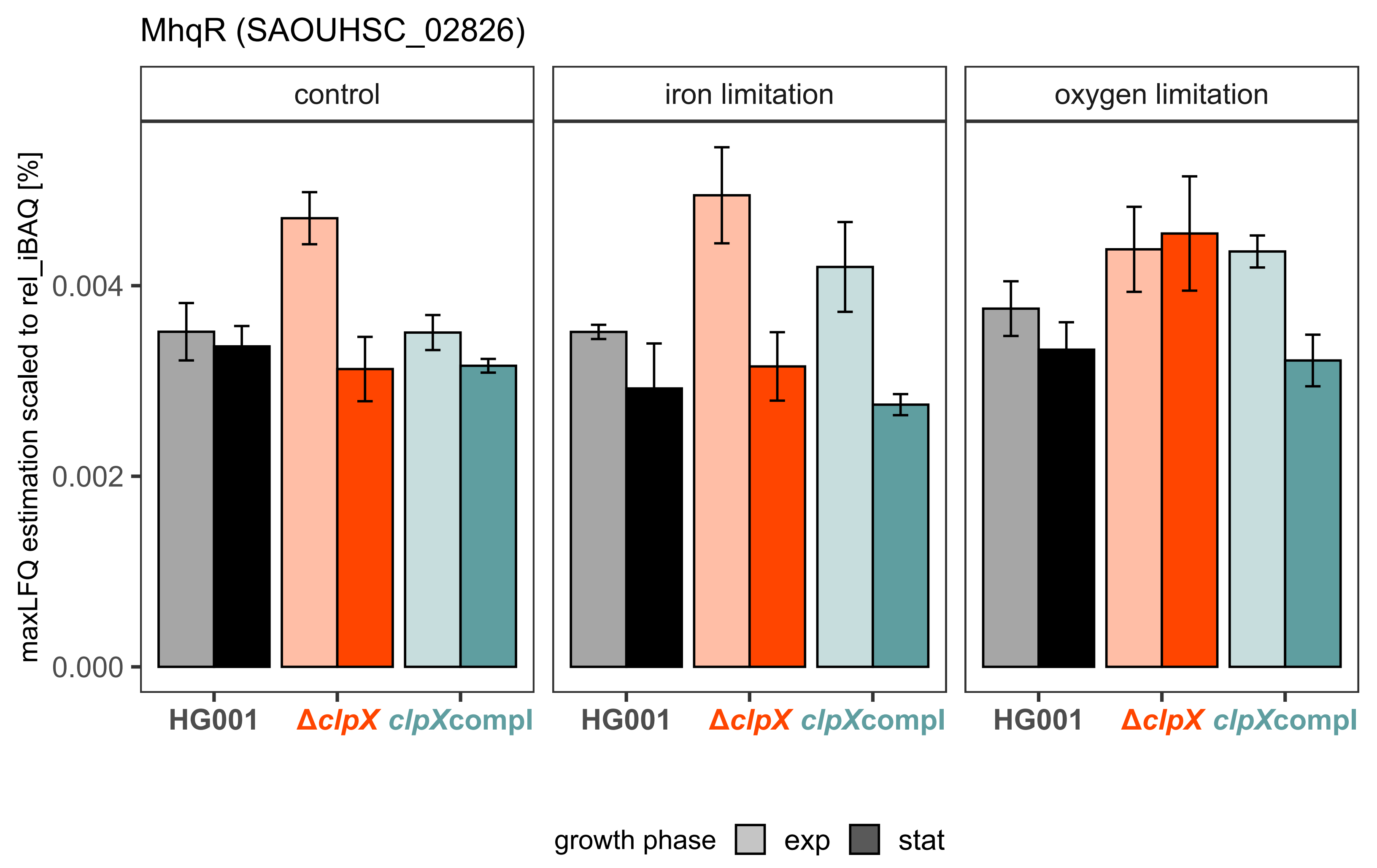

MhqR (SAOUHSC_02826)
control
iron limitation
oxygen limitation
maxLFQ estimation scaled to rel_iBAQ [%]
0.004
0.002
0.000
HG001
ΔclpX
clpXcompl
growth phase
exp
stat

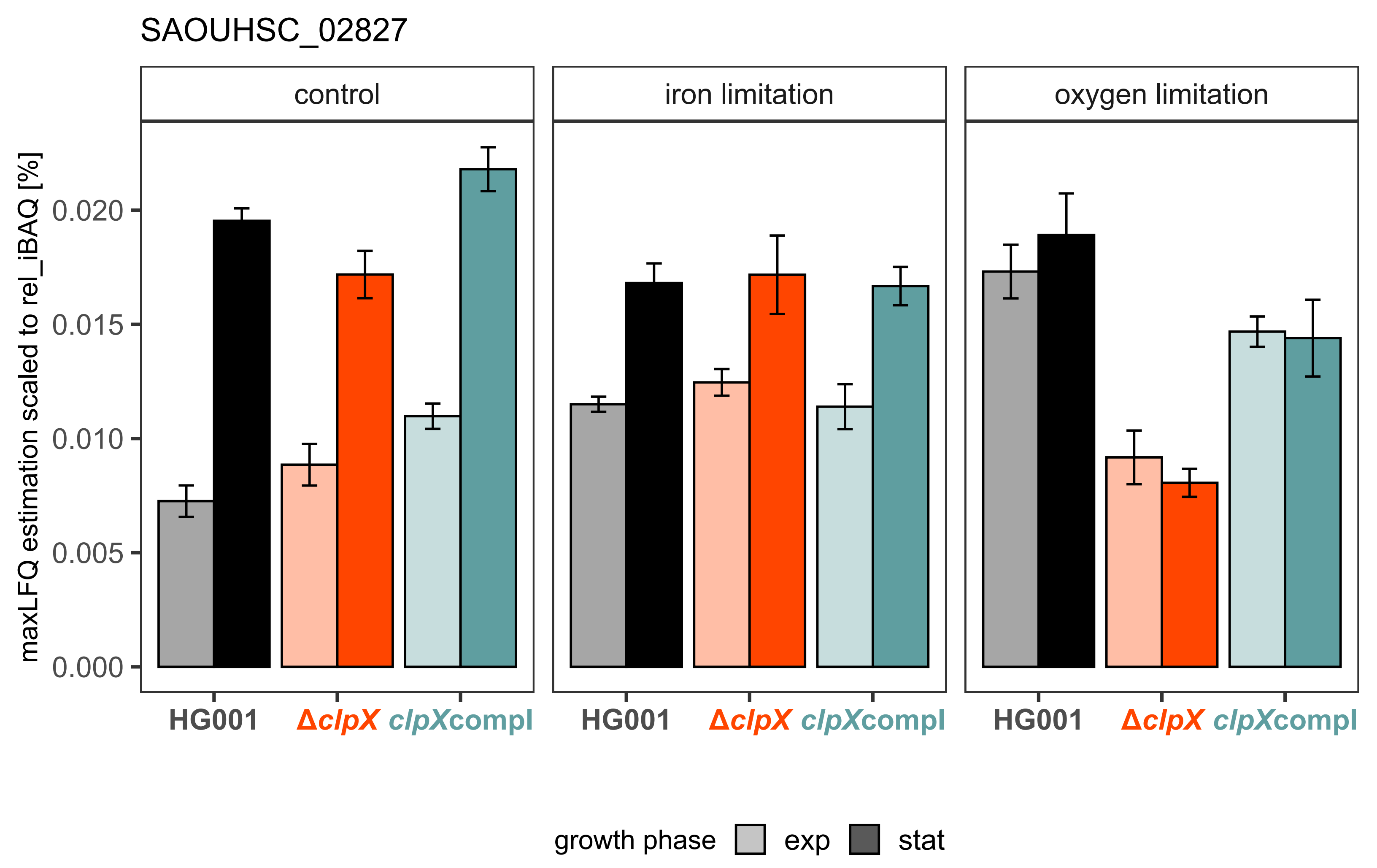

SAOUHSC_02827
control
iron limitation
oxygen limitation
maxLFQ estimation scaled to rel_iBAQ [%]
0.000
0.005
0.010
0.015
0.020
HG001
ΔclpX
clpXcompl
growth phase
exp
stat

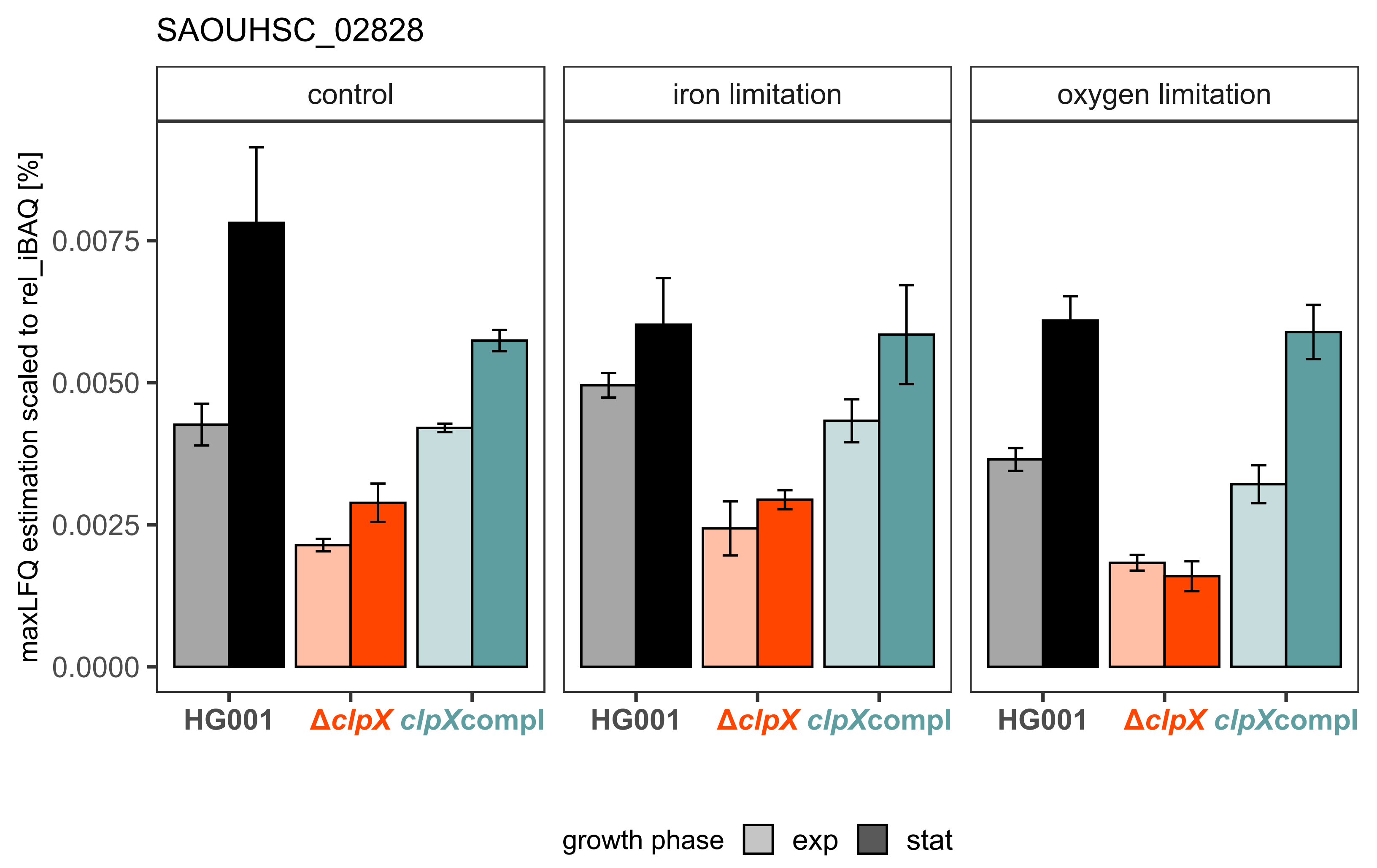
SAOUHSC_02828
control
iron limitation
oxygen limitation
maxLFQ estimation scaled to rel_iBAQ [%]
0.0075
0.0050
0.0025
0.0000
HG001
ΔclpX
clpXcompl
growth phase
exp
stat

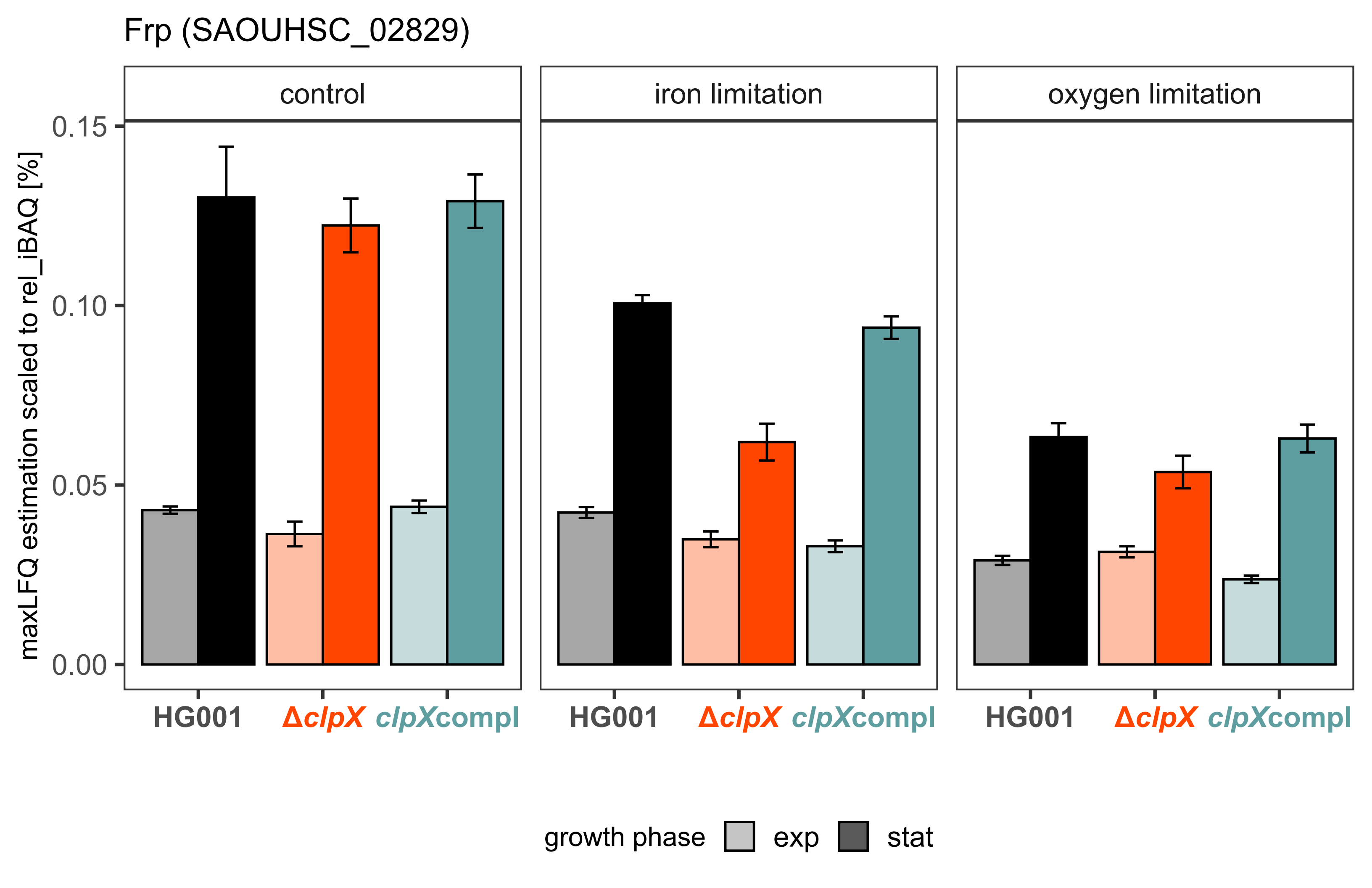
Frp (SAOUHSC_02829)
control
iron limitation
oxygen limitation
maxLFQ estimation scaled to rel_iBAQ [%]
0.15
0.10
0.05
0.00
HG001
ΔclpX
clpXcompl
growth phase
exp
stat

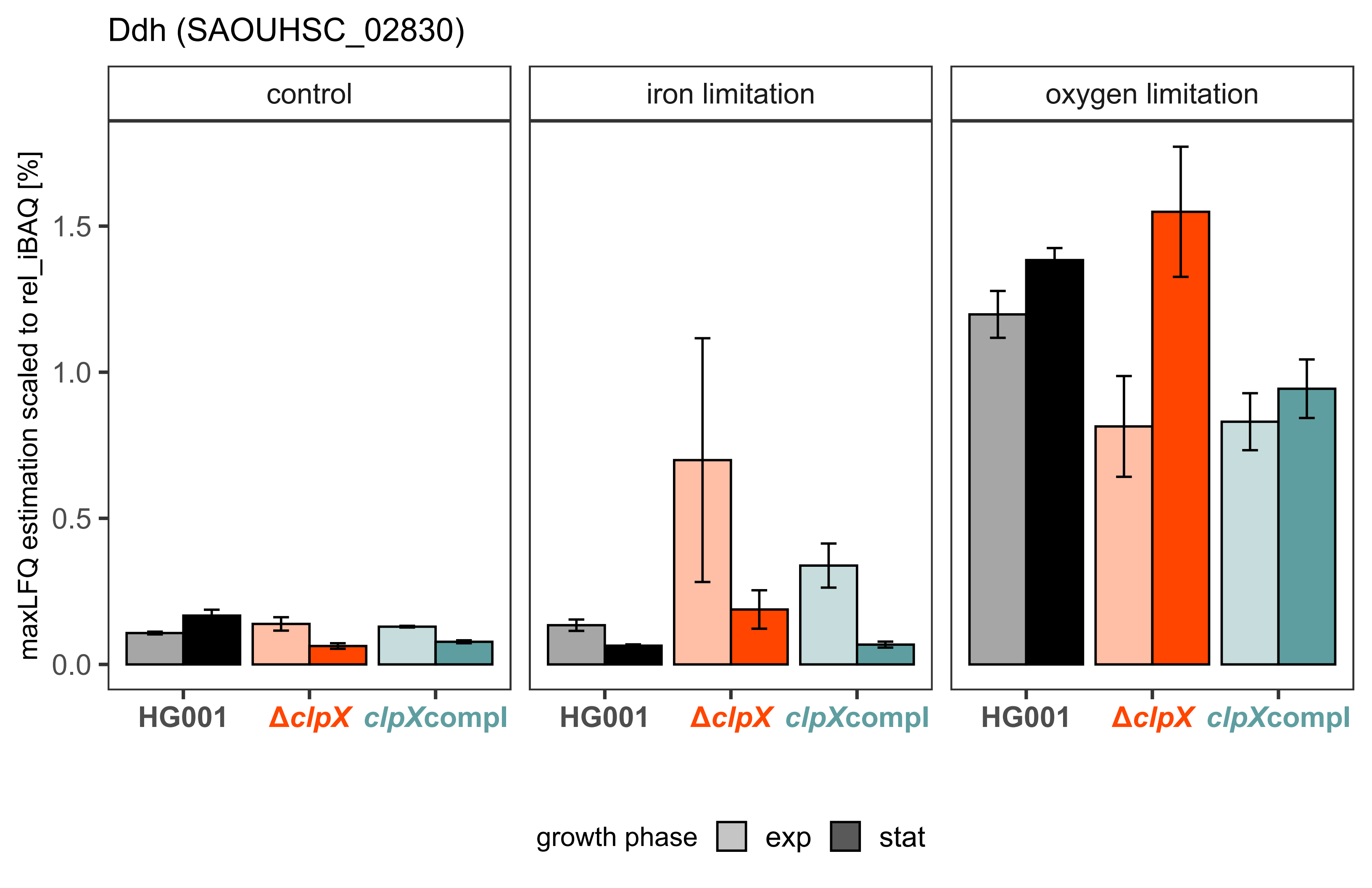
Ddh (SAOUHSC_02830)
control
iron limitation
oxygen limitation
maxLFQ estimation scaled to rel_iBAQ [%]
0.0
0.5
1.0
1.5
HG001
ΔclpX
clpXcompl
growth phase
exp
stat

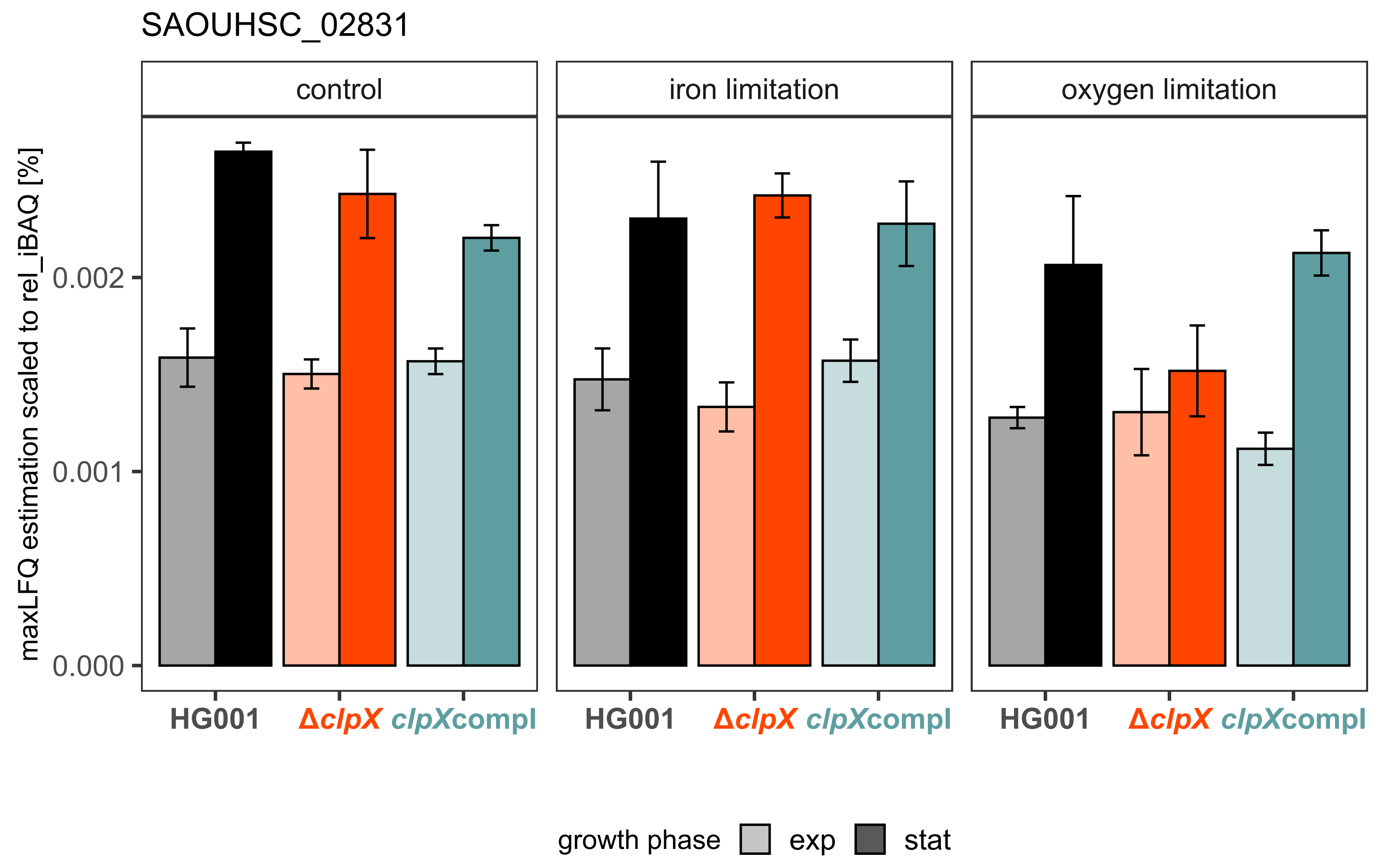
SAOUHSC_02831
control
iron limitation
oxygen limitation
maxLFQ estimation scaled to rel_iBAQ [%]
0.002
0.001
0.000
HG001
ΔclpX
clpXcompl
growth phase
exp
stat

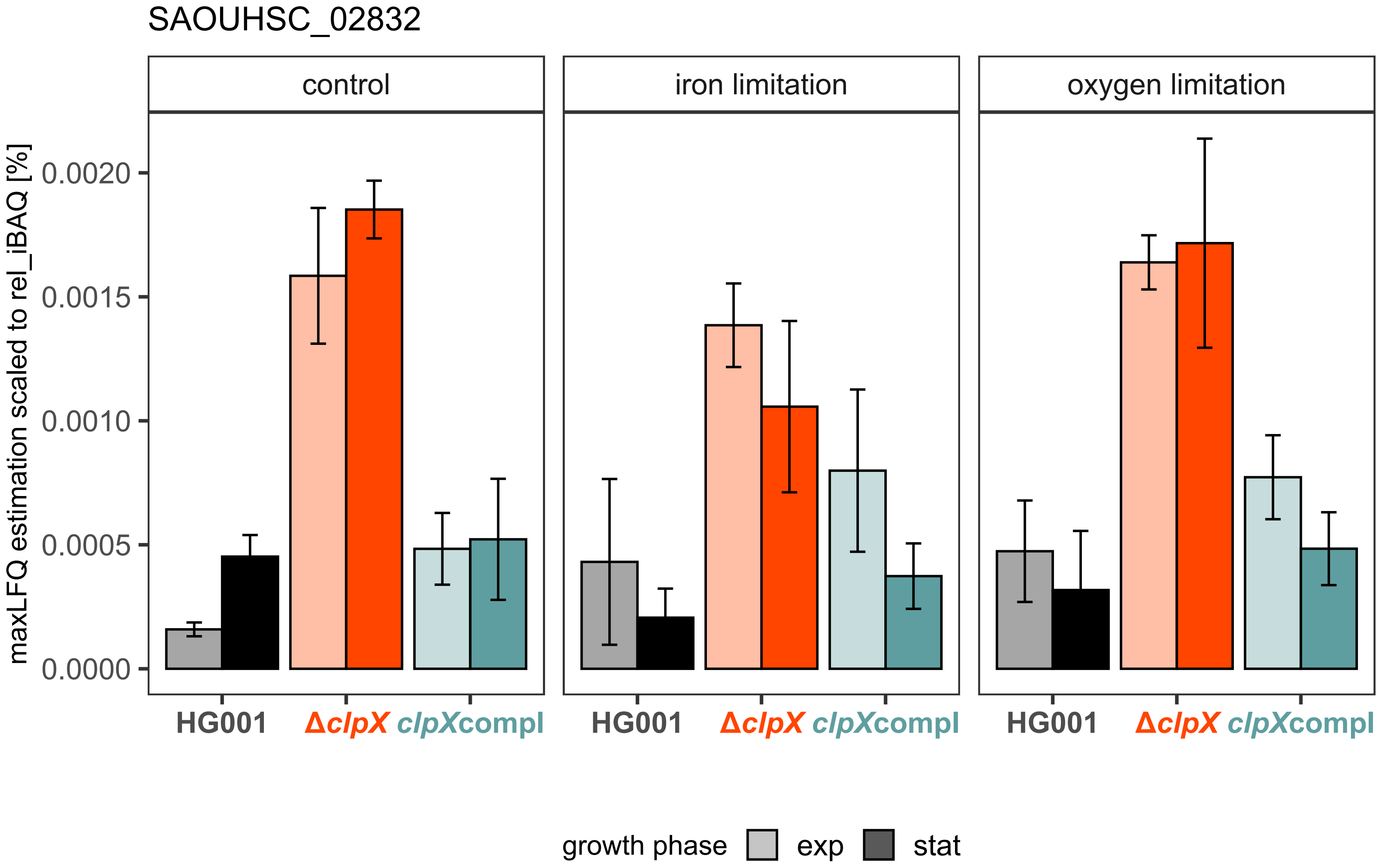
SAOUHSC_02832
control
iron limitation
oxygen limitation
maxLFQ estimation scaled to rel_iBAQ [%]
0.0020
0.0015
0.0010
0.0005
0.0000
HG001
ΔclpX
clpXcompl
growth phase
exp
stat

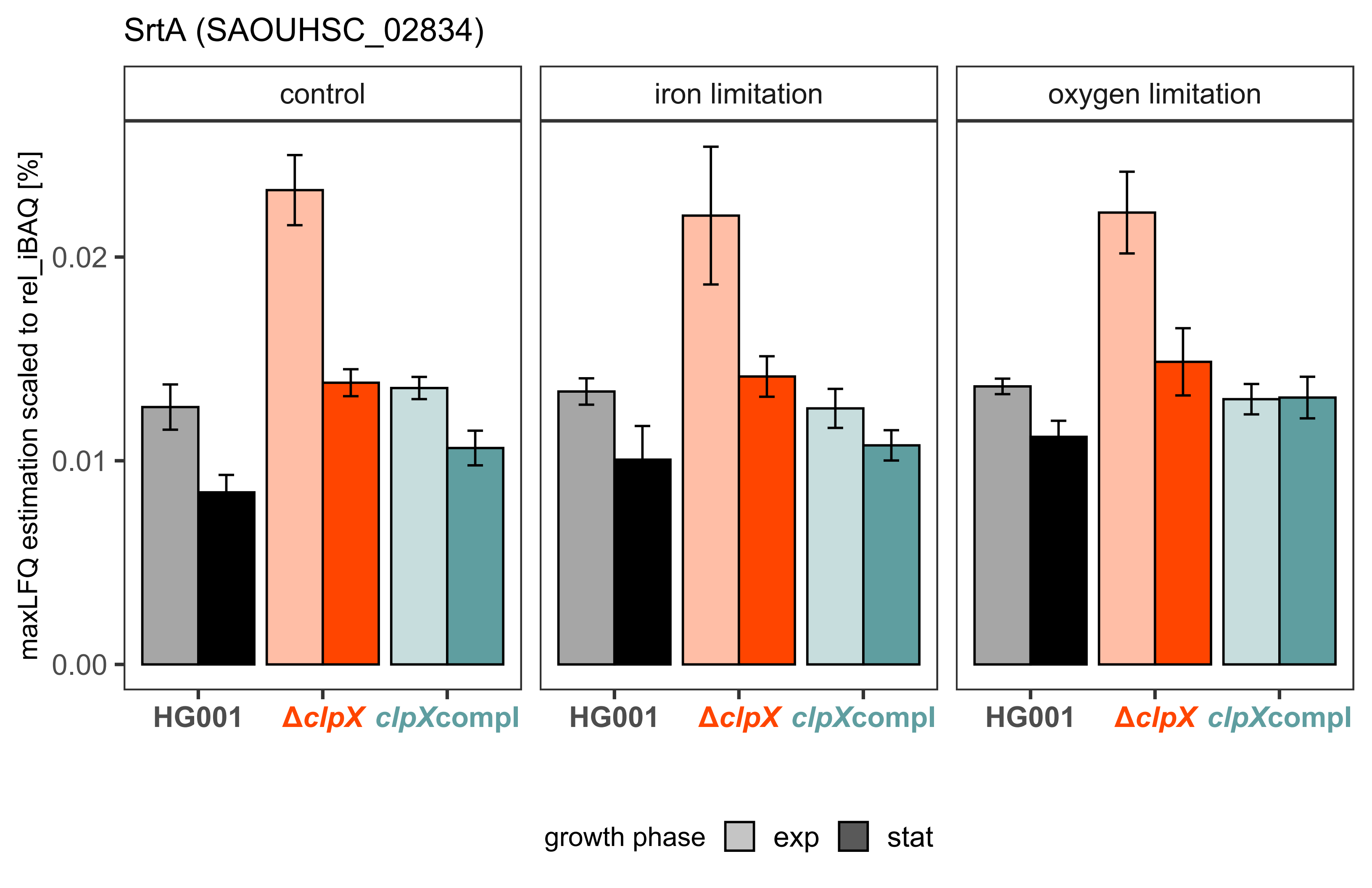

SrtA (SAOUHSC_02834)
control
iron limitation
oxygen limitation
maxLFQ estimation scaled to rel_iBAQ [%]
0.00
0.01
0.02
HG001
ΔclpX
clpXcompl
growth phase
exp
stat

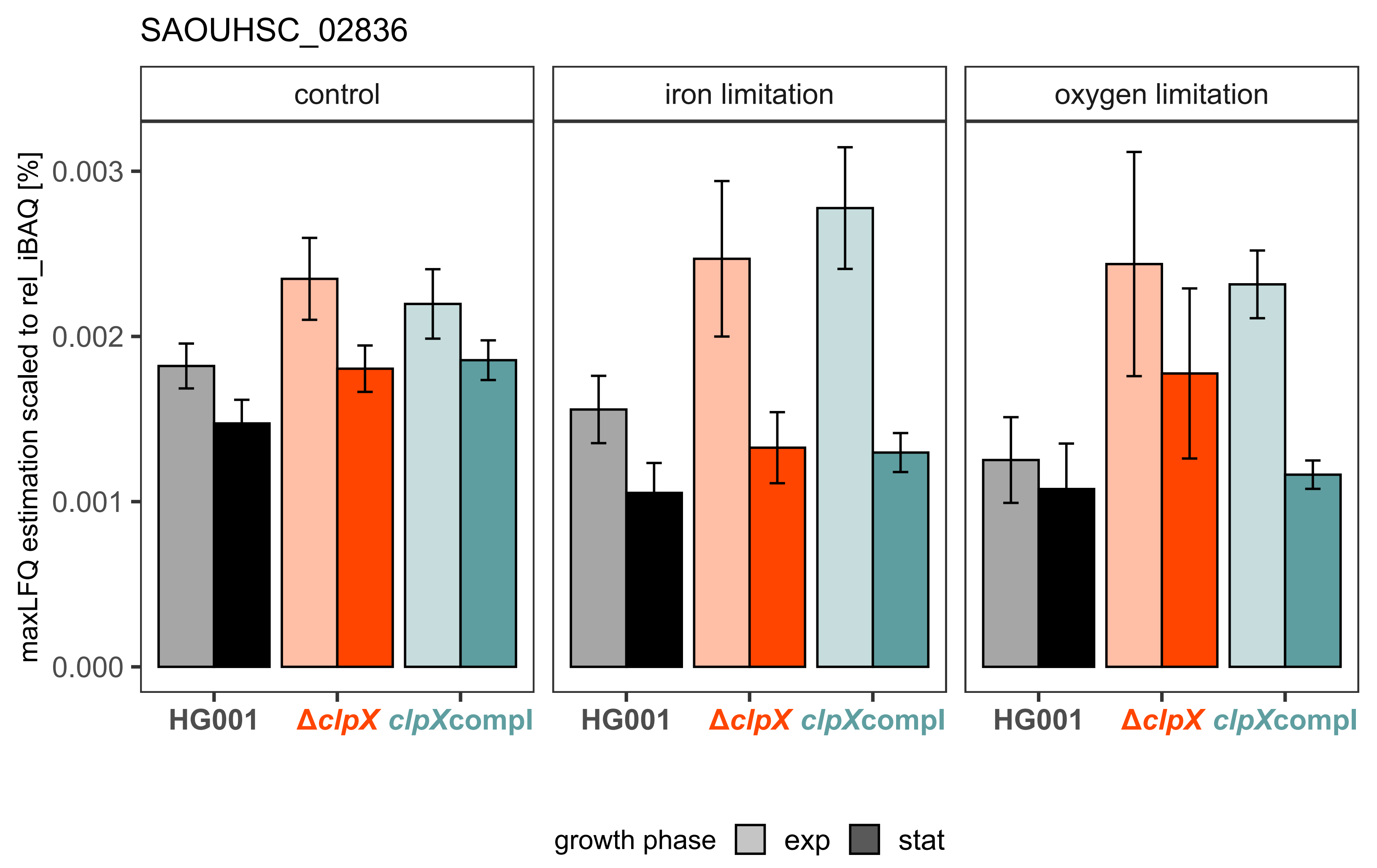
SAOUHSC_02836
control
iron limitation
oxygen limitation
maxLFQ estimation scaled to rel_iBAQ [%]
0.003
0.002
0.001
0.000
HG001
ΔclpX
clpXcompl
growth phase
exp
stat

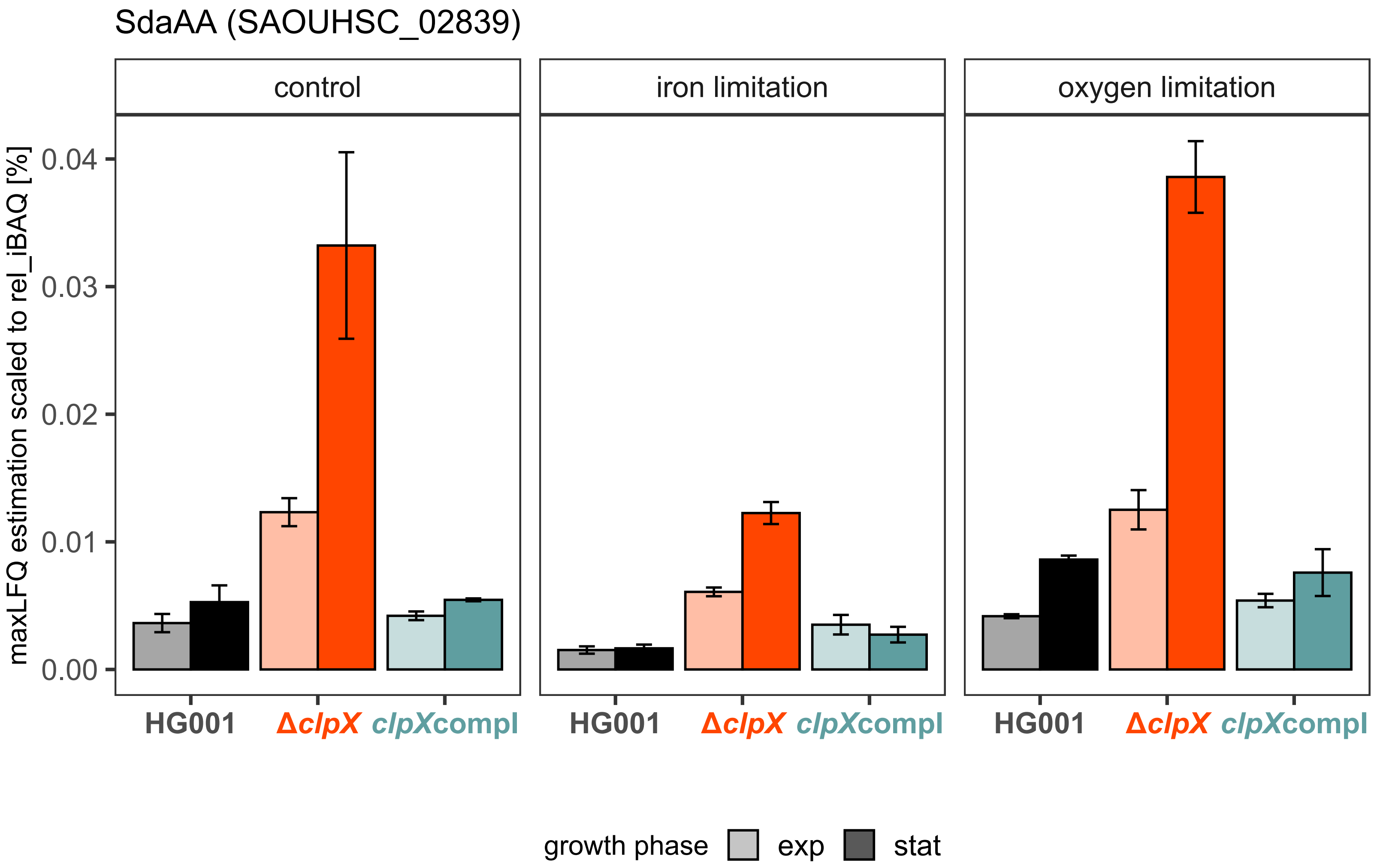

SdaAA (SAOUHSC_02839)
control
iron limitation
oxygen limitation
maxLFQ estimation scaled to rel_iBAQ [%]
0.04
0.03
0.02
0.01
0.00
HG001
ΔclpX
clpXcompl
growth phase
exp
stat

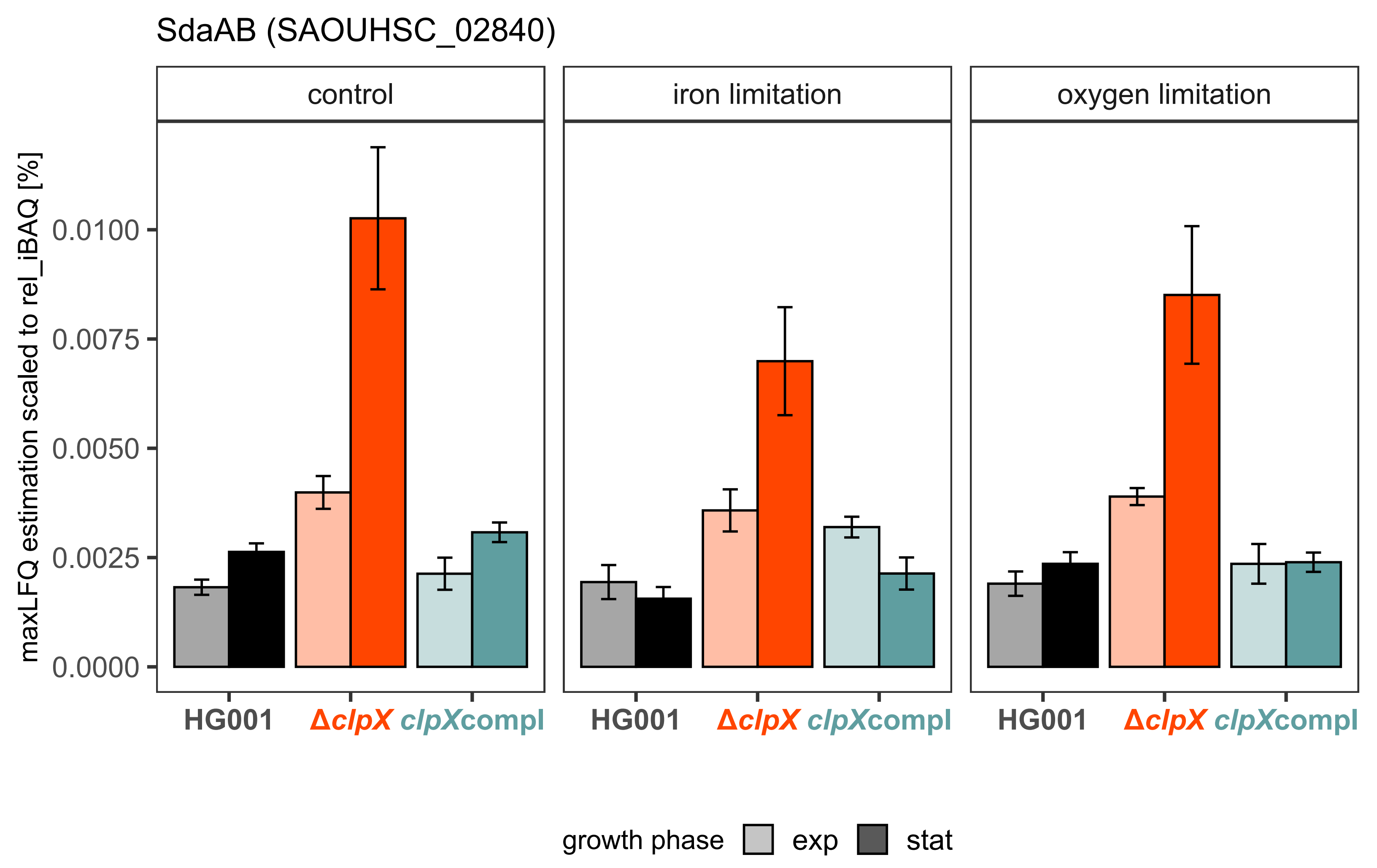

SdaAB (SAOUHSC_02840)
control
iron limitation
oxygen limitation
maxLFQ estimation scaled to rel_iBAQ [%]
0.0100
0.0075
0.0050
0.0025
0.0000
HG001
ΔclpX
clpXcompl
growth phase
exp
stat

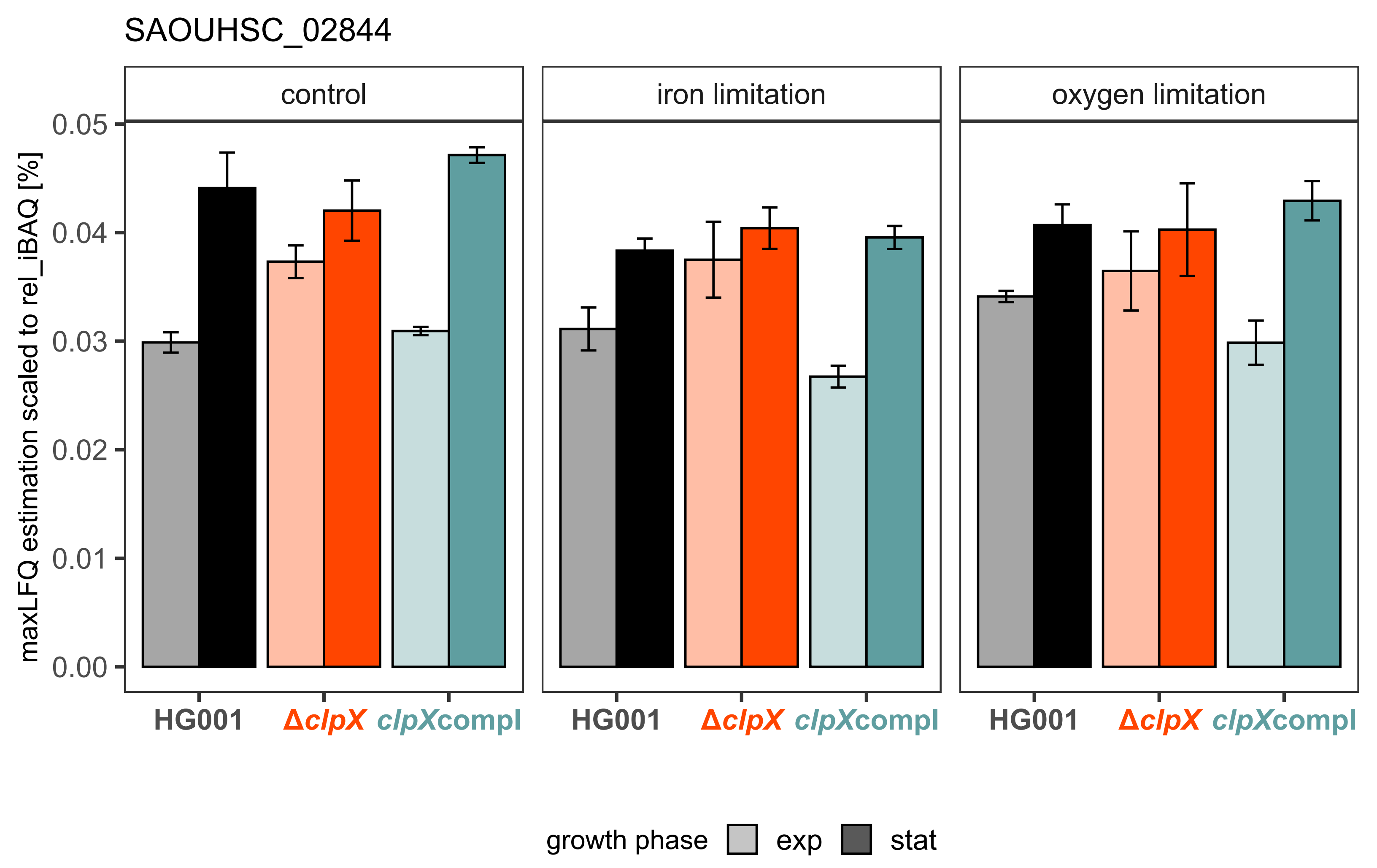
SAOUHSC_02844
control
iron limitation
oxygen limitation
maxLFQ estimation scaled to rel_iBAQ [%]
0.05
0.04
0.03
0.02
0.01
0.00
HG001
ΔclpX
clpXcompl
growth phase
exp
stat

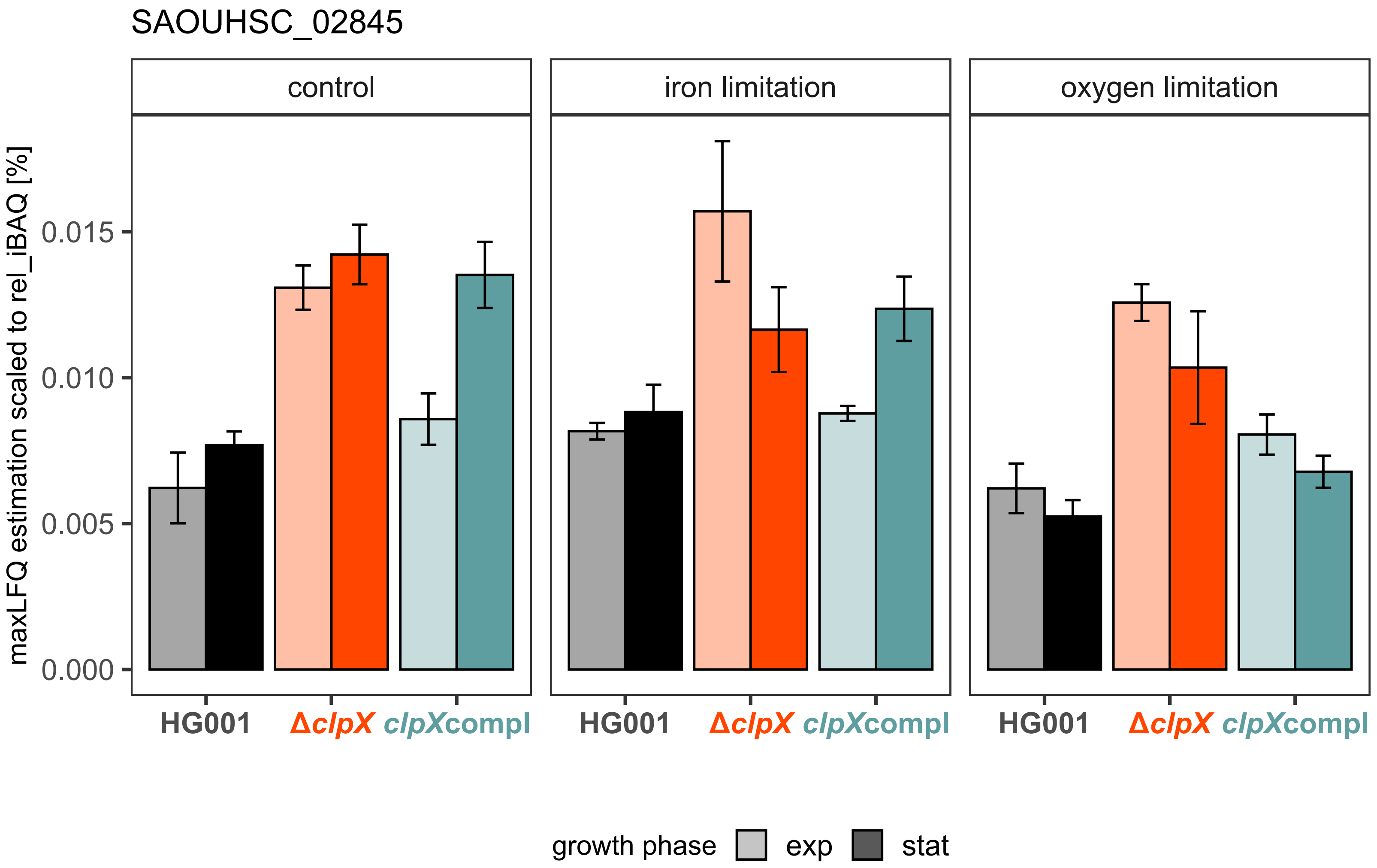
SAOUHSC_02845
control
iron limitation
oxygen limitation
maxLFQ estimation scaled to rel_iBAQ [%]
0.015
0.010
0.005
0.000
HG001
ΔclpX
clpXcompl
growth phase
exp
stat

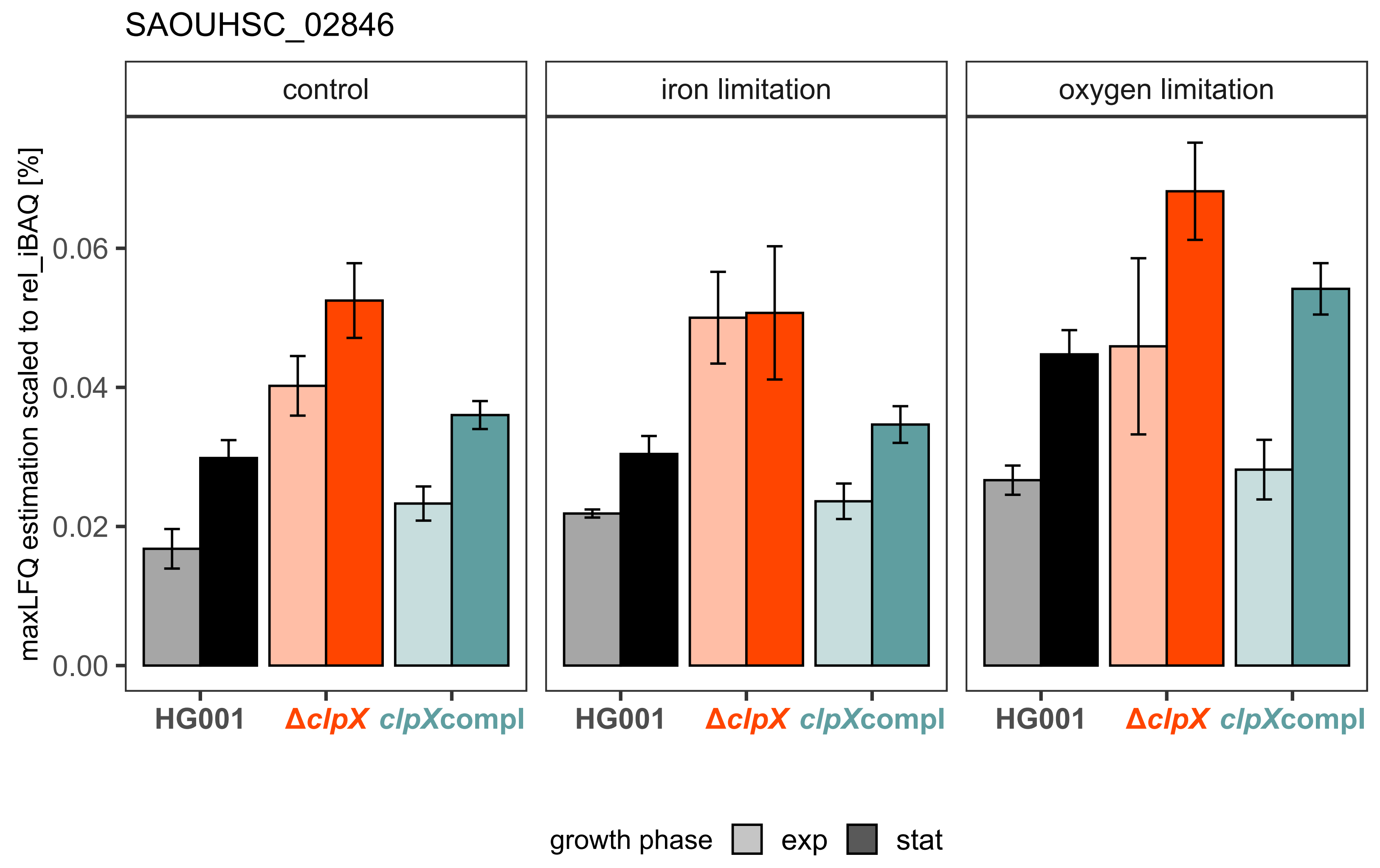
SAOUHSC_02846
control
iron limitation
oxygen limitation
maxLFQ estimation scaled to rel_iBAQ [%]
0.00
0.02
0.04
0.06
HG001
ΔclpX
clpXcompl
growth phase
exp
stat

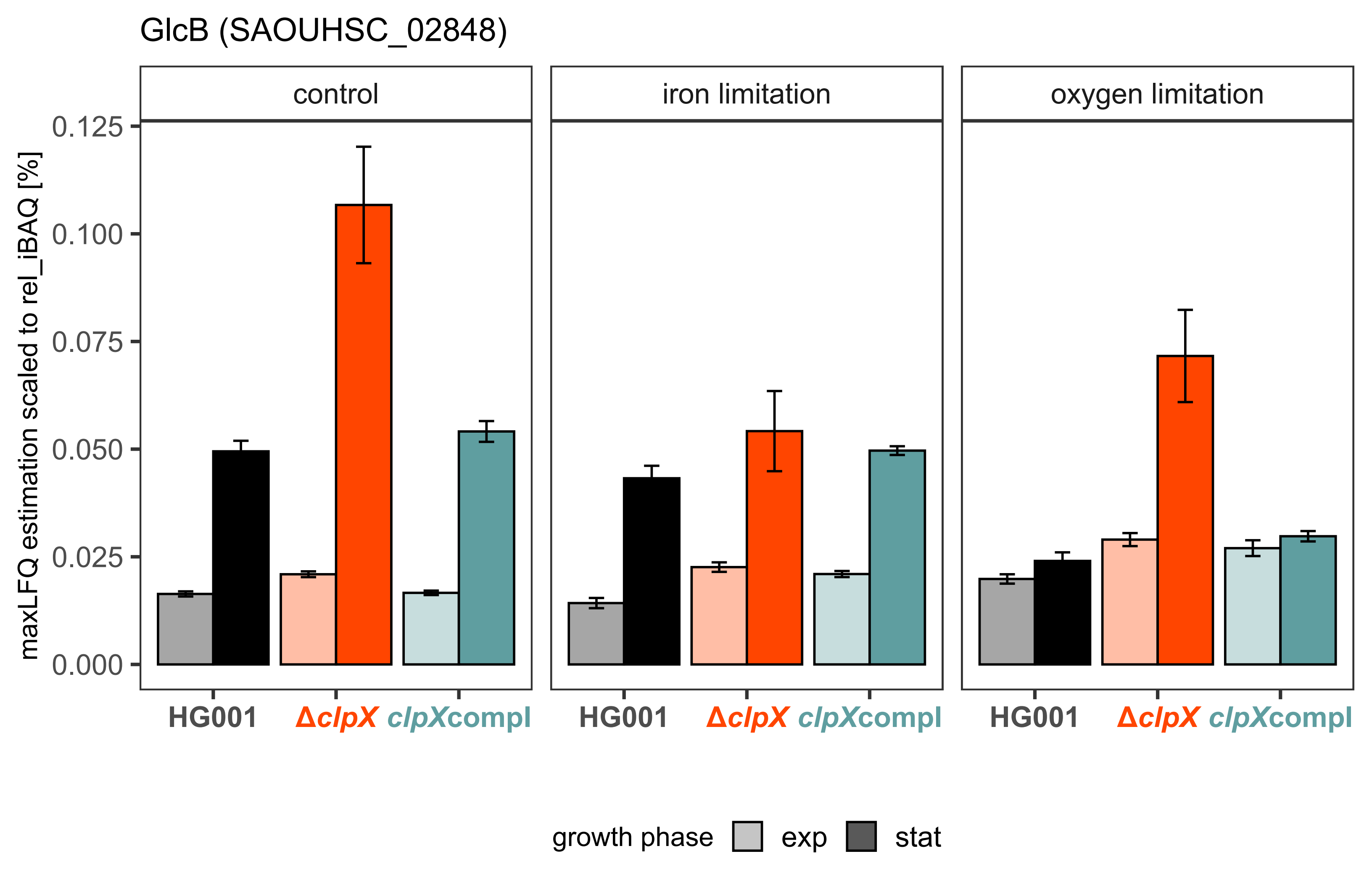

GlcB (SAOUHSC_02848)
control
iron limitation
oxygen limitation
maxLFQ estimation scaled to rel_iBAQ [%]
0.125
0.100
0.075
0.050
0.025
0.000
HG001
ΔclpX
clpXcompl
growth phase
exp
stat

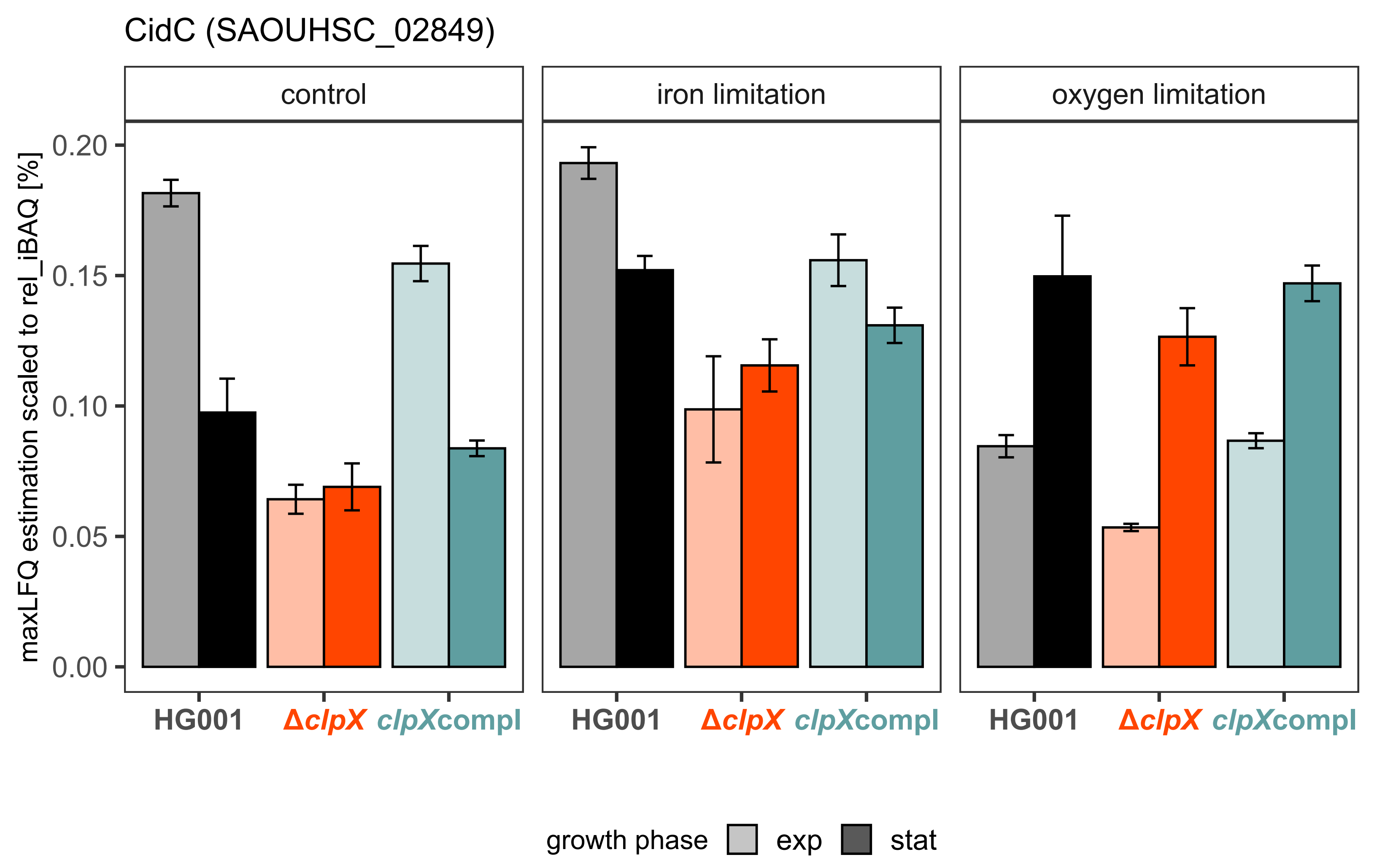

CidC (SAOUHSC_02849)
control
iron limitation
oxygen limitation
maxLFQ estimation scaled to rel_iBAQ [%]
0.20
0.15
0.10
0.05
0.00
HG001
ΔclpX
clpXcompl
growth phase
exp
stat

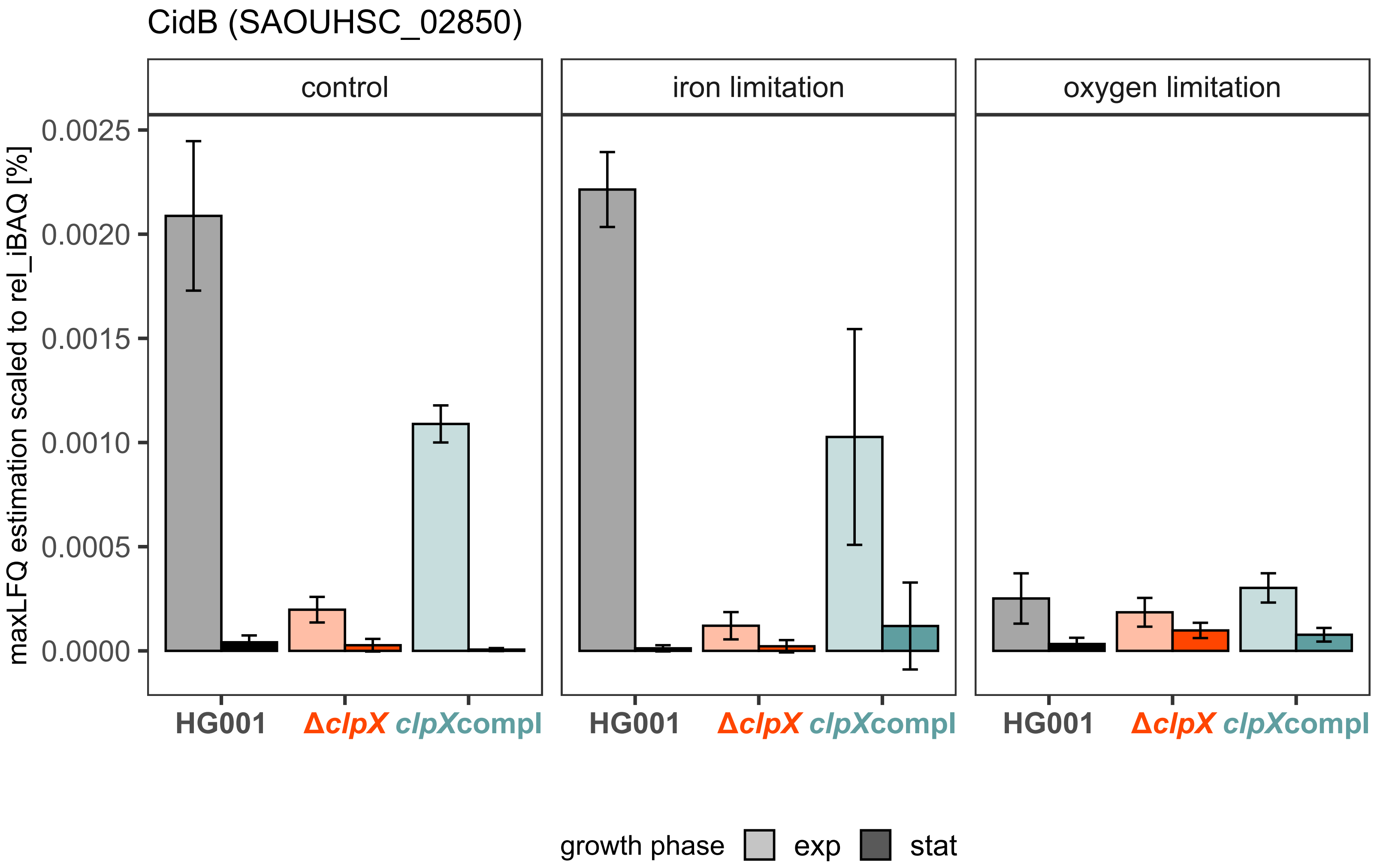

CidB (SAOUHSC_02850)
control
iron limitation
oxygen limitation
maxLFQ estimation scaled to rel_iBAQ [%]
0.0025
0.0020
0.0015
0.0010
0.0005
0.0000
HG001
ΔclpX
clpXcompl
growth phase
exp
stat

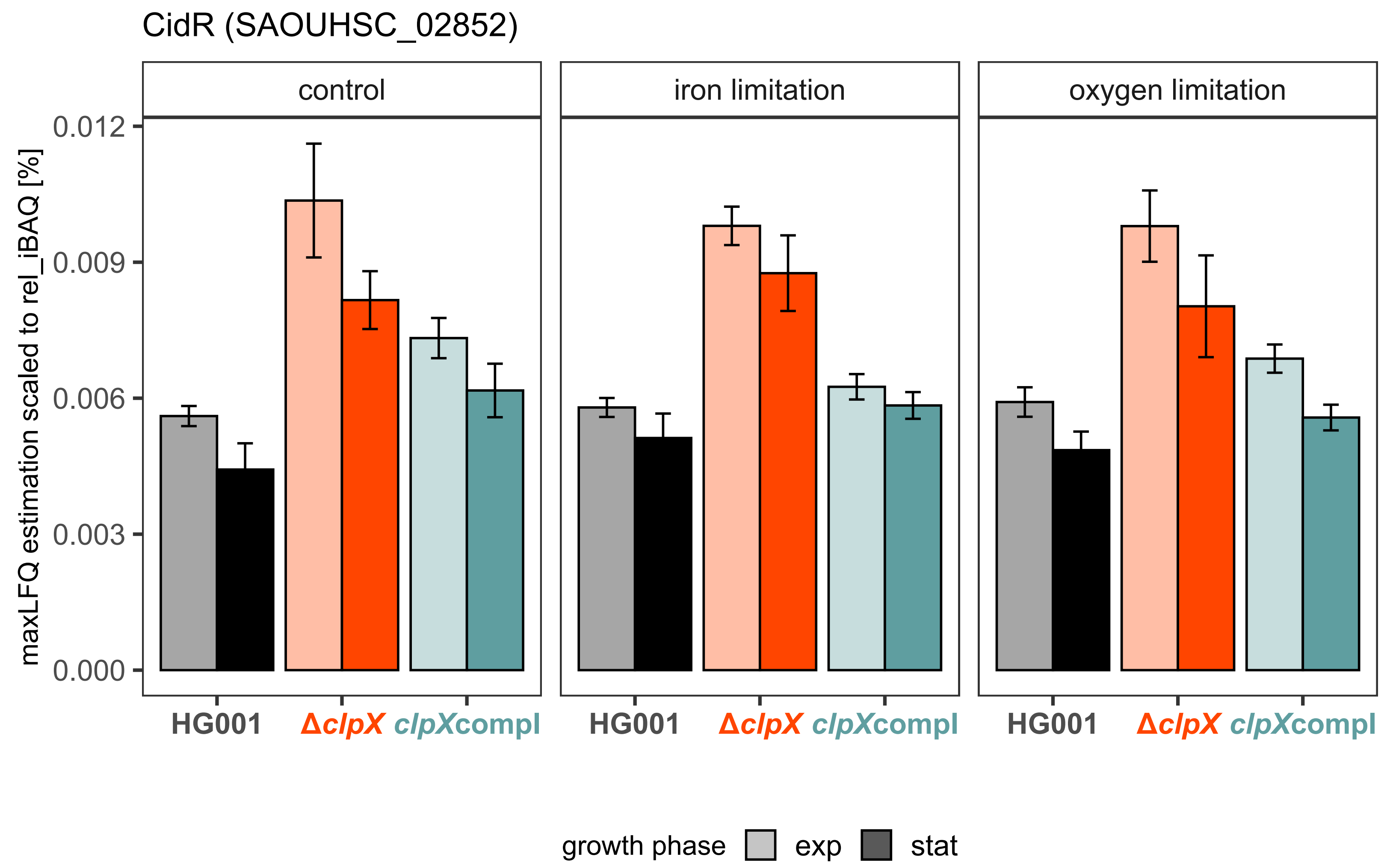
CidR (SAOUHSC_02852)
control
iron limitation
oxygen limitation
maxLFQ estimation scaled to rel_iBAQ [%]
0.012
0.009
0.006
0.003
0.000
HG001
ΔclpX
clpXcompl
growth phase
exp
stat

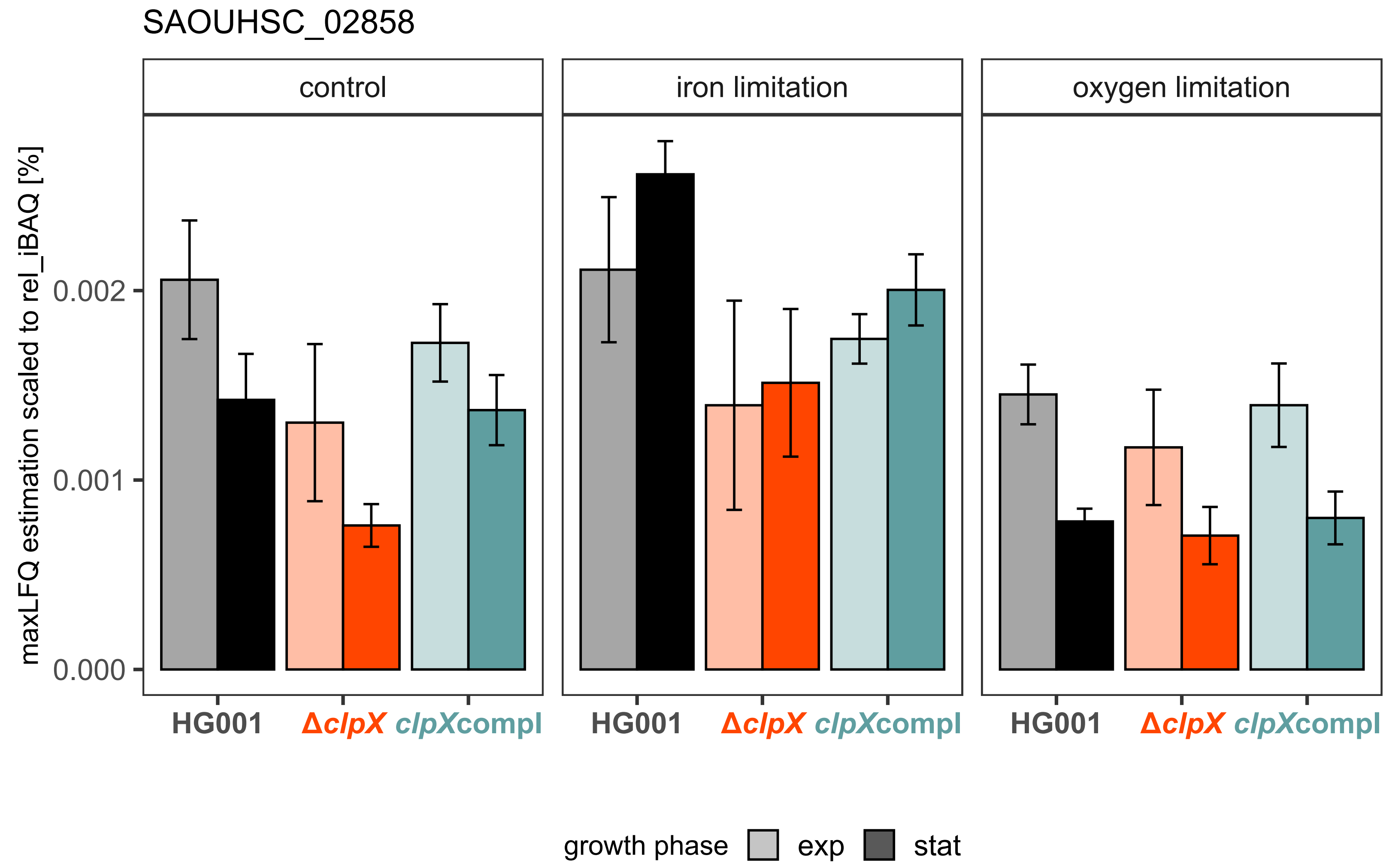

SAOUHSC_02858
control
iron limitation
oxygen limitation
maxLFQ estimation scaled to rel_iBAQ [%]
0.002
0.001
0.000
HG001
ΔclpX
clpXcompl
growth phase
exp
stat

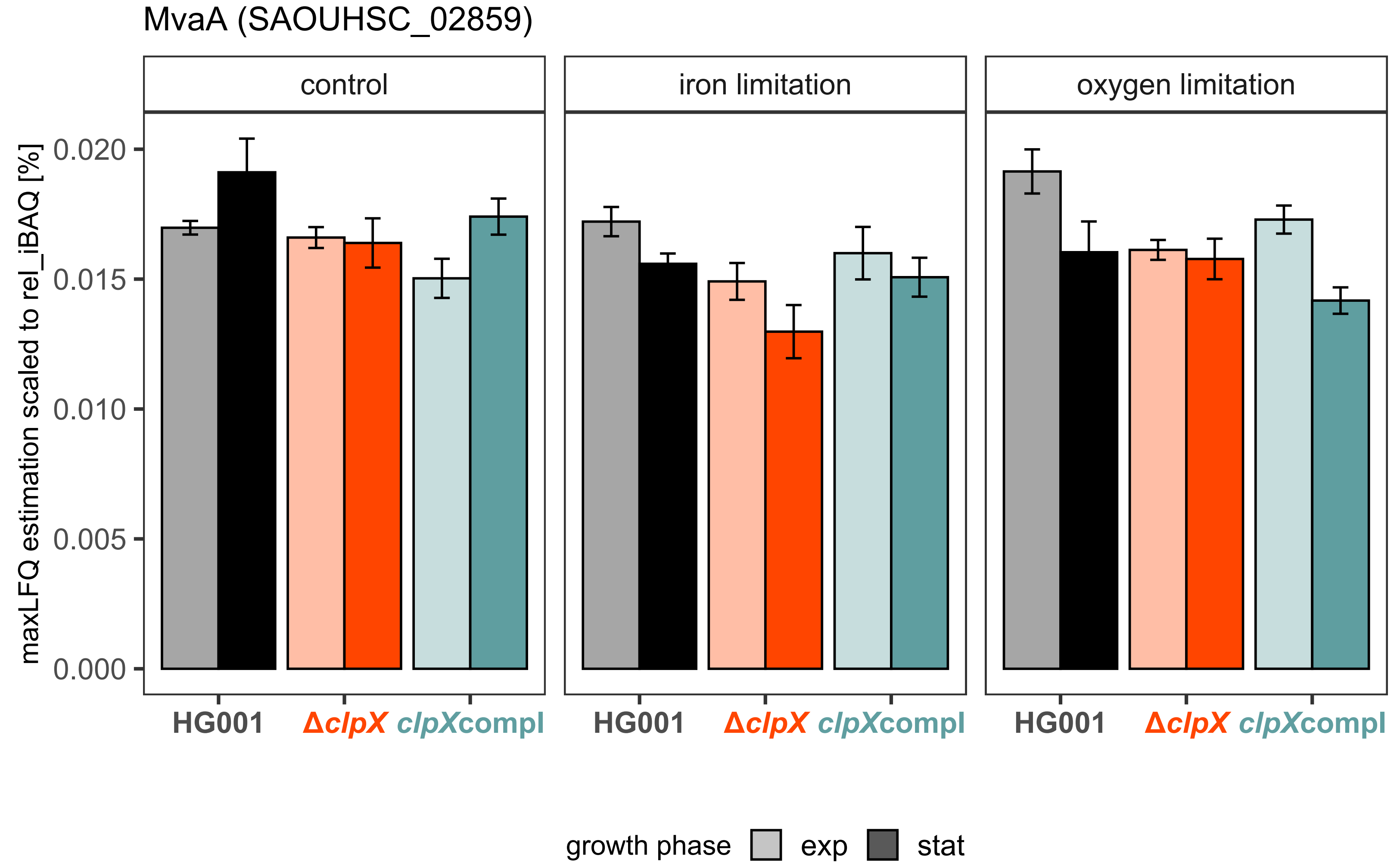

MvaA (SAOUHSC_02859)
control
iron limitation
oxygen limitation
maxLFQ estimation scaled to rel_iBAQ [%]
0.020
0.015
0.010
0.005
0.000
HG001
ΔclpX
clpXcompl
growth phase
exp
stat

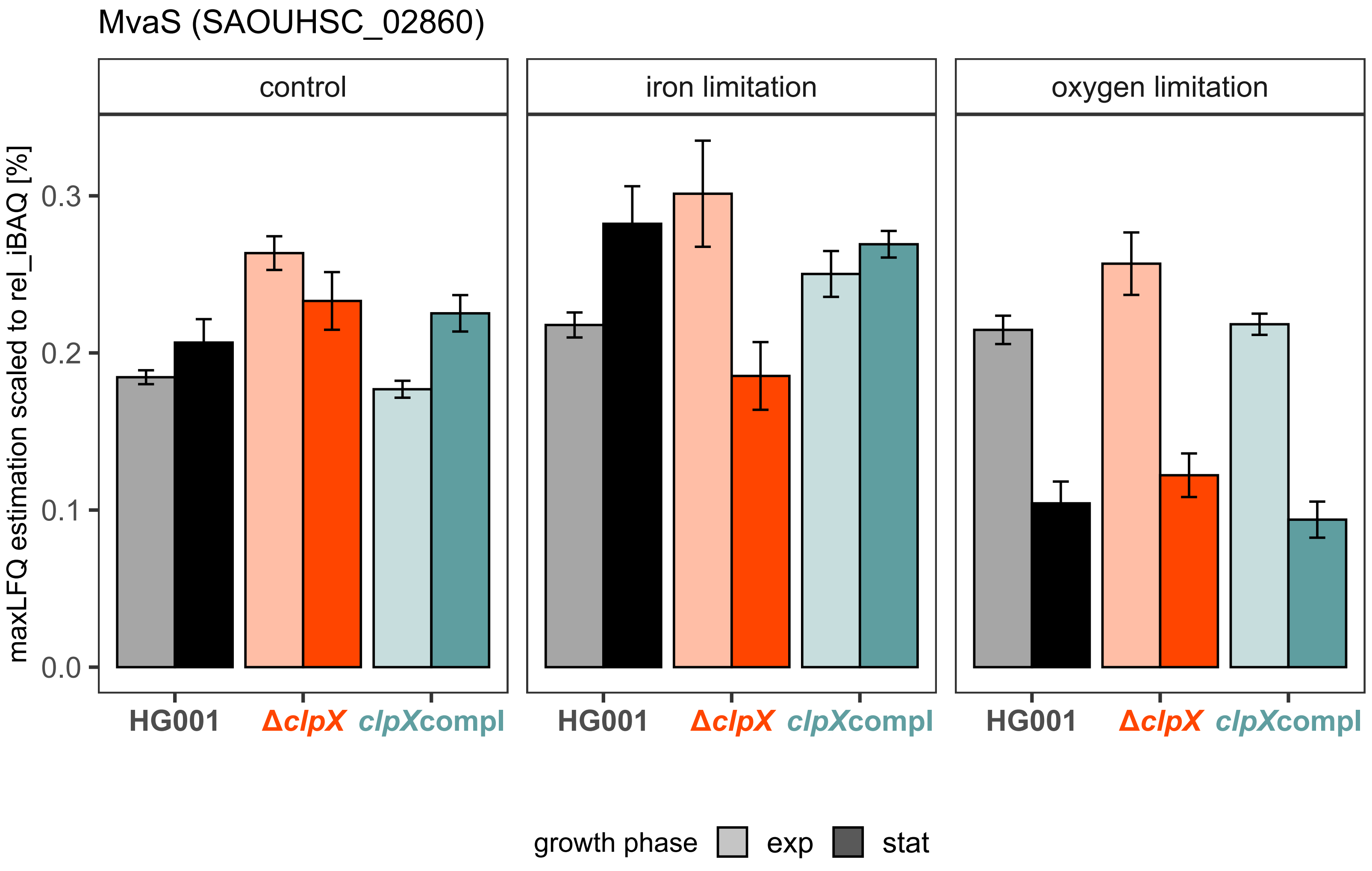
MvaS (SAOUHSC_02860)
control
iron limitation
oxygen limitation
maxLFQ estimation scaled to rel_iBAQ [%]
0.0
0.1
0.2
0.3
HG001
ΔclpX
clpXcompl
growth phase
exp
stat

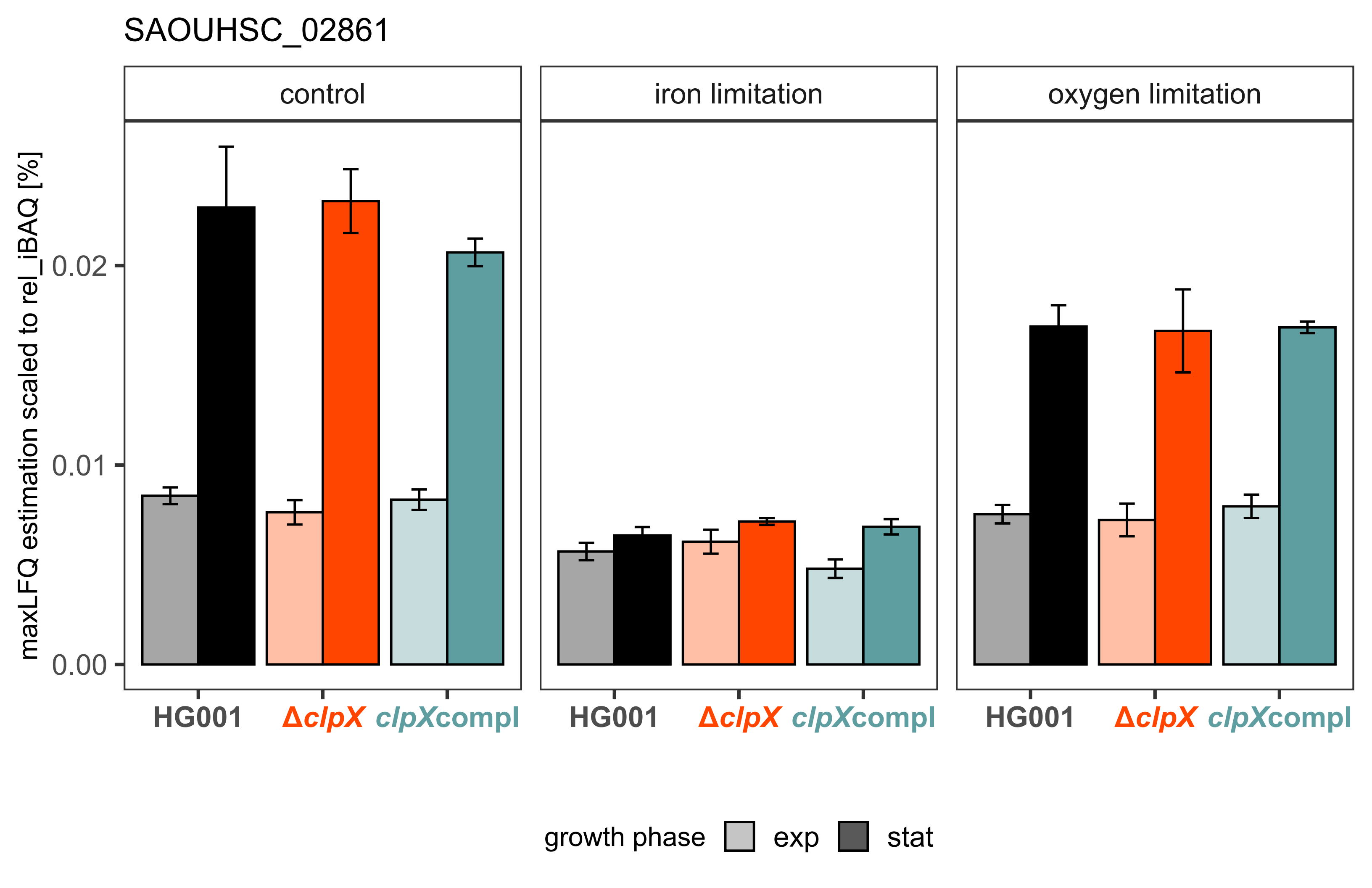
SAOUHSC_02861
control
iron limitation
oxygen limitation
maxLFQ estimation scaled to rel_iBAQ [%]
0.00
0.01
0.02
HG001
ΔclpX
clpXcompl
growth phase
exp
stat

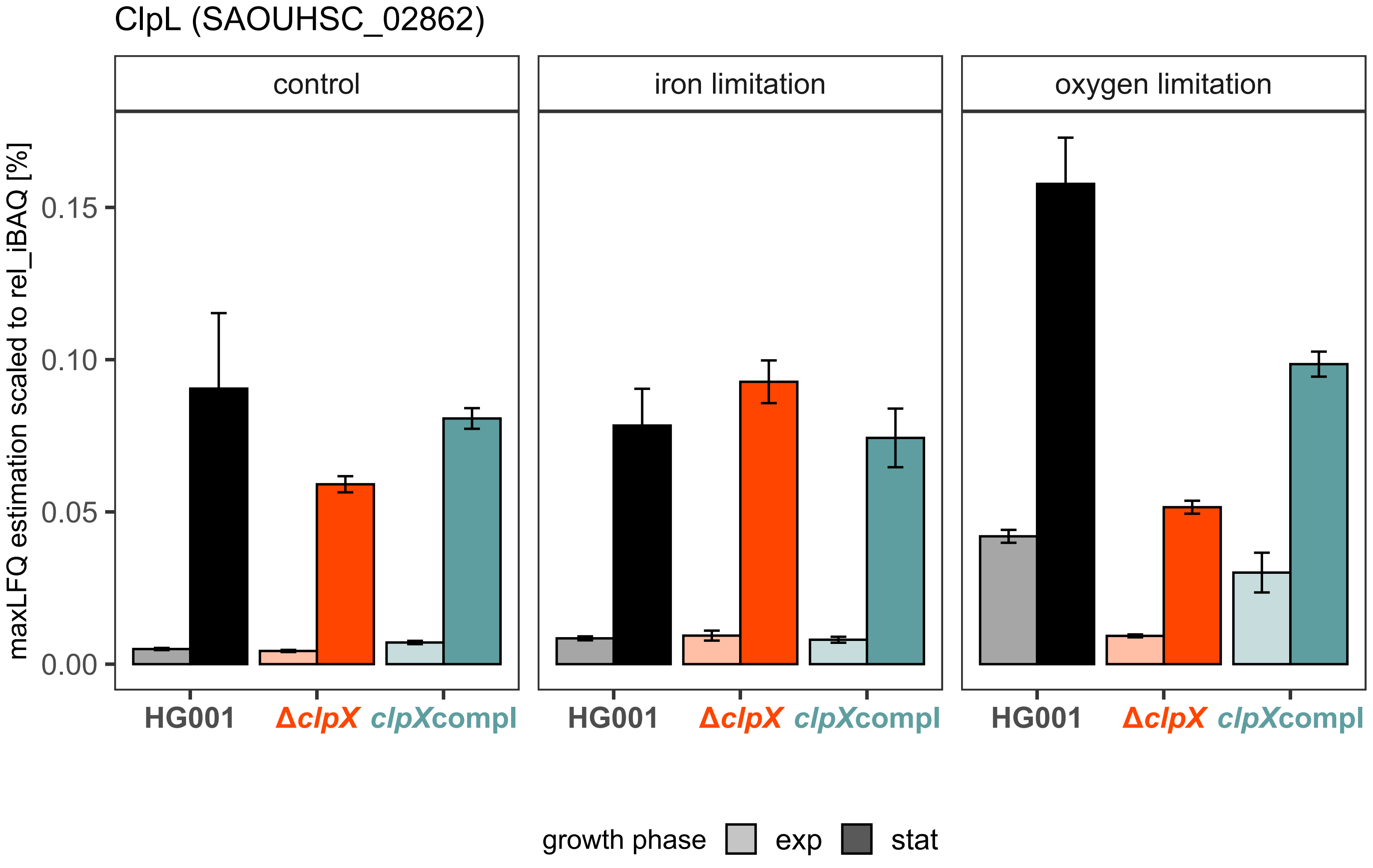

ClpL (SAOUHSC_02862)
control
iron limitation
oxygen limitation
maxLFQ estimation scaled to rel_iBAQ [%]
0.00
0.05
0.10
0.15
HG001
ΔclpX
clpXcompl
growth phase
exp
stat

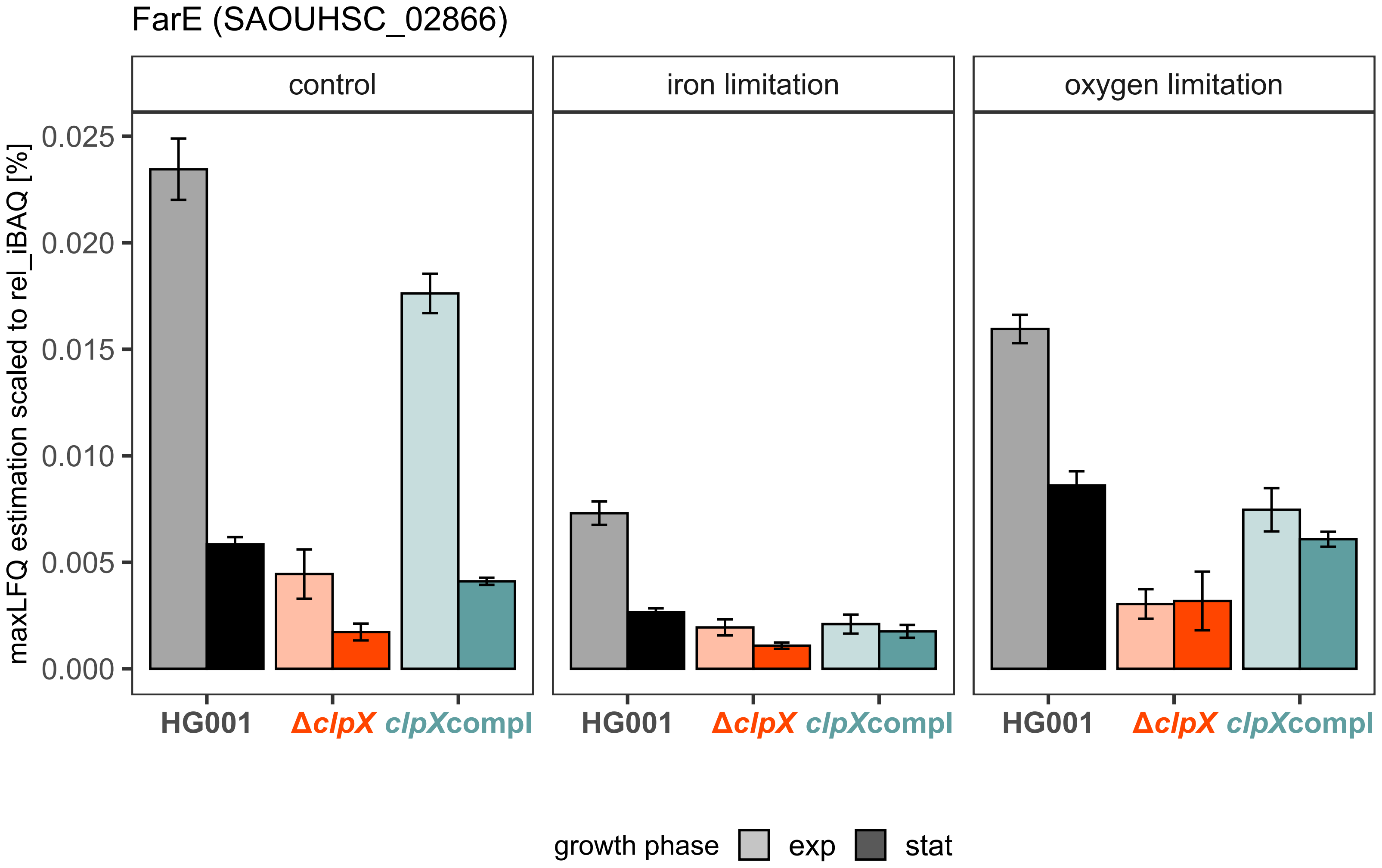
FarE (SAOUHSC_02866)
control
iron limitation
oxygen limitation
maxLFQ estimation scaled to rel_iBAQ [%]
0.025
0.020
0.015
0.010
0.005
0.000
HG001
ΔclpX
clpXcompl
growth phase
exp
stat

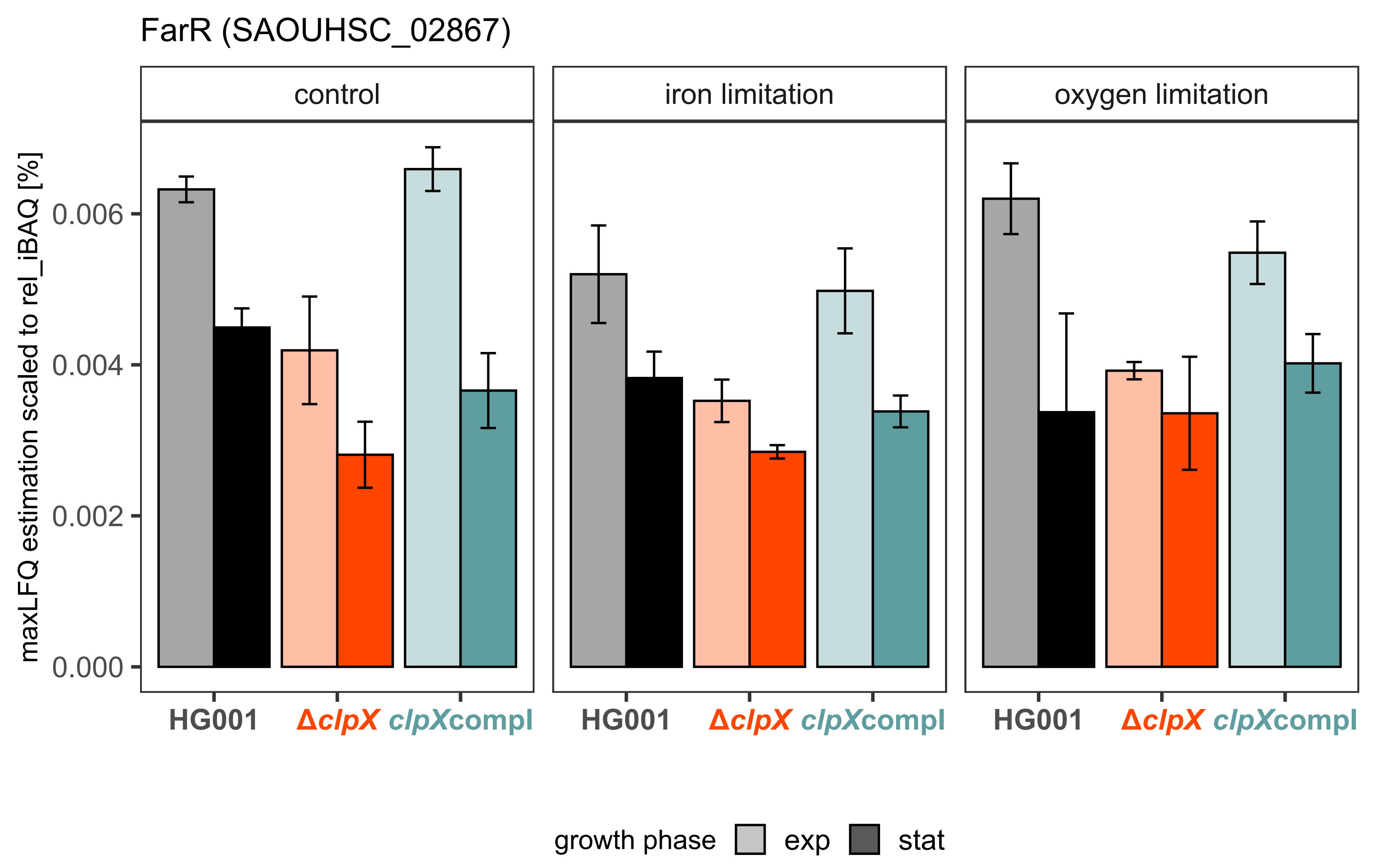
FarR (SAOUHSC_02867)
control
iron limitation
oxygen limitation
maxLFQ estimation scaled to rel_iBAQ [%]
0.006
0.004
0.002
0.000
HG001
ΔclpX
clpXcompl
growth phase
exp
stat

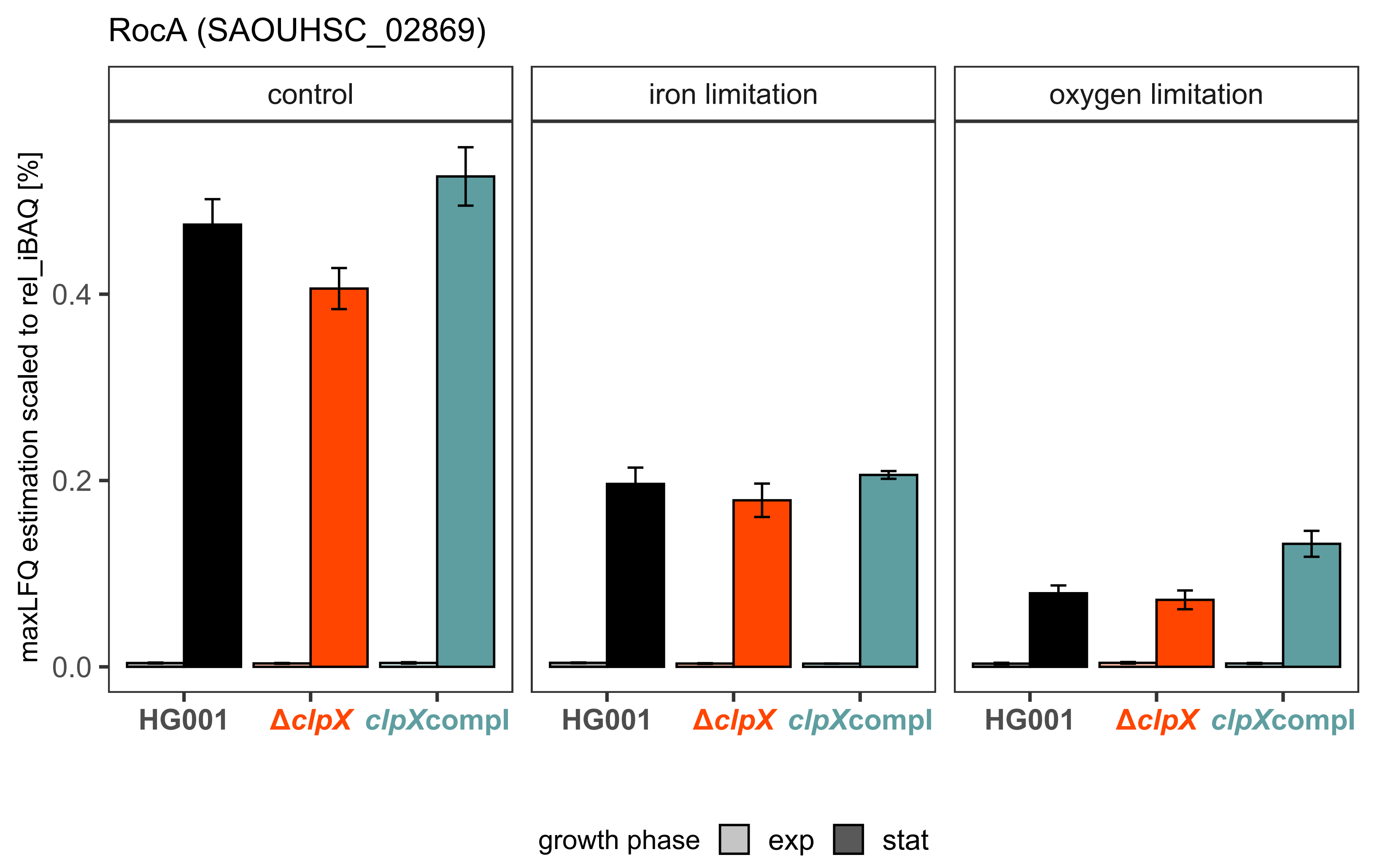
RocA (SAOUHSC_02869)
control
iron limitation
oxygen limitation
maxLFQ estimation scaled to rel_iBAQ [%]
0.4
0.2
0.0
HG001
ΔclpX
clpXcompl
growth phase
exp
stat

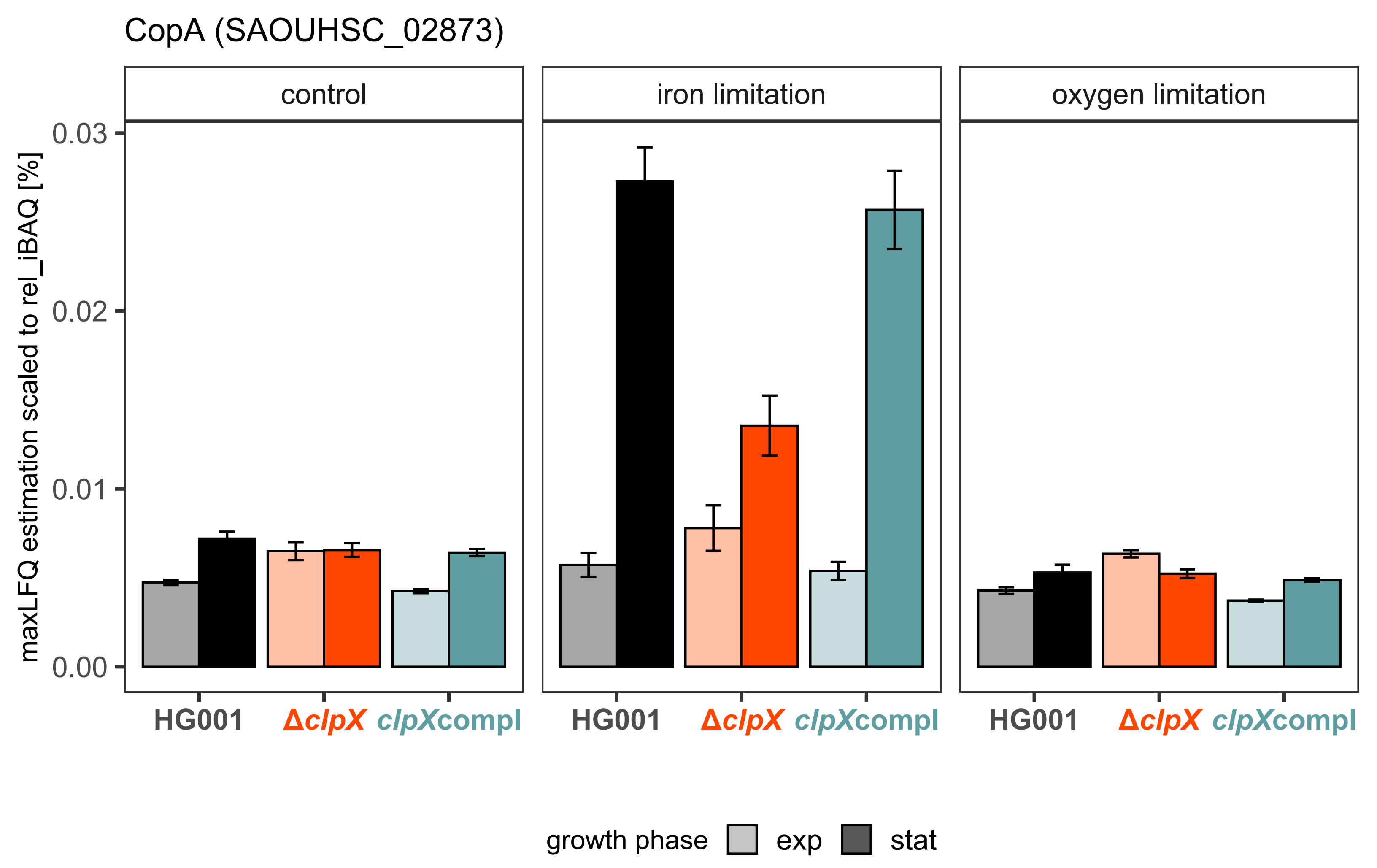
CopA (SAOUHSC_02873)
control
iron limitation
oxygen limitation
maxLFQ estimation scaled to rel_iBAQ [%]
0.03
0.02
0.01
0.00
HG001
ΔclpX
clpXcompl
growth phase
exp
stat

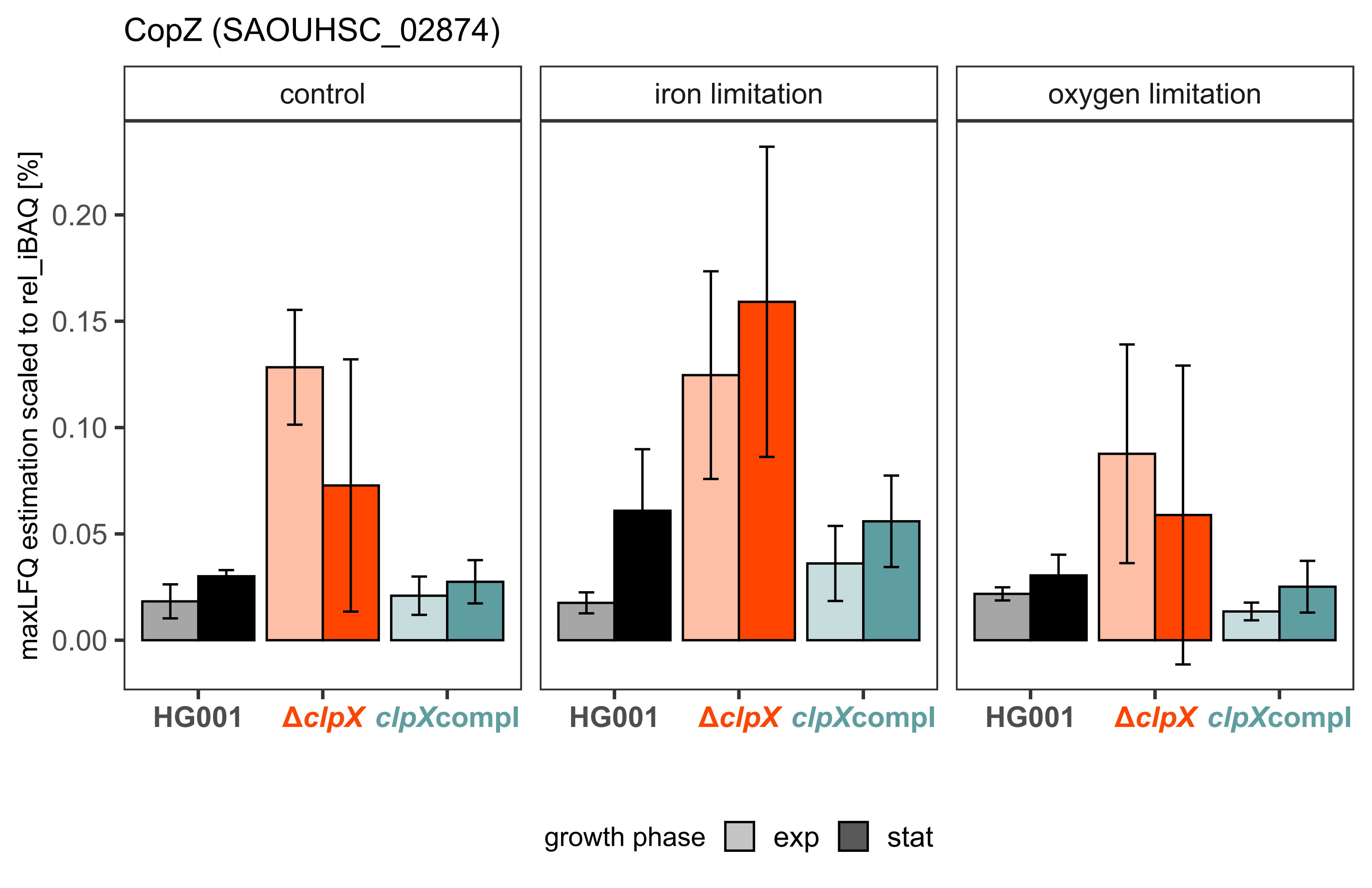

CopZ (SAOUHSC_02874)
control
iron limitation
oxygen limitation
maxLFQ estimation scaled to rel_iBAQ [%]
0.20
0.15
0.10
0.05
0.00
HG001
ΔclpX
clpXcompl
growth phase
exp
stat

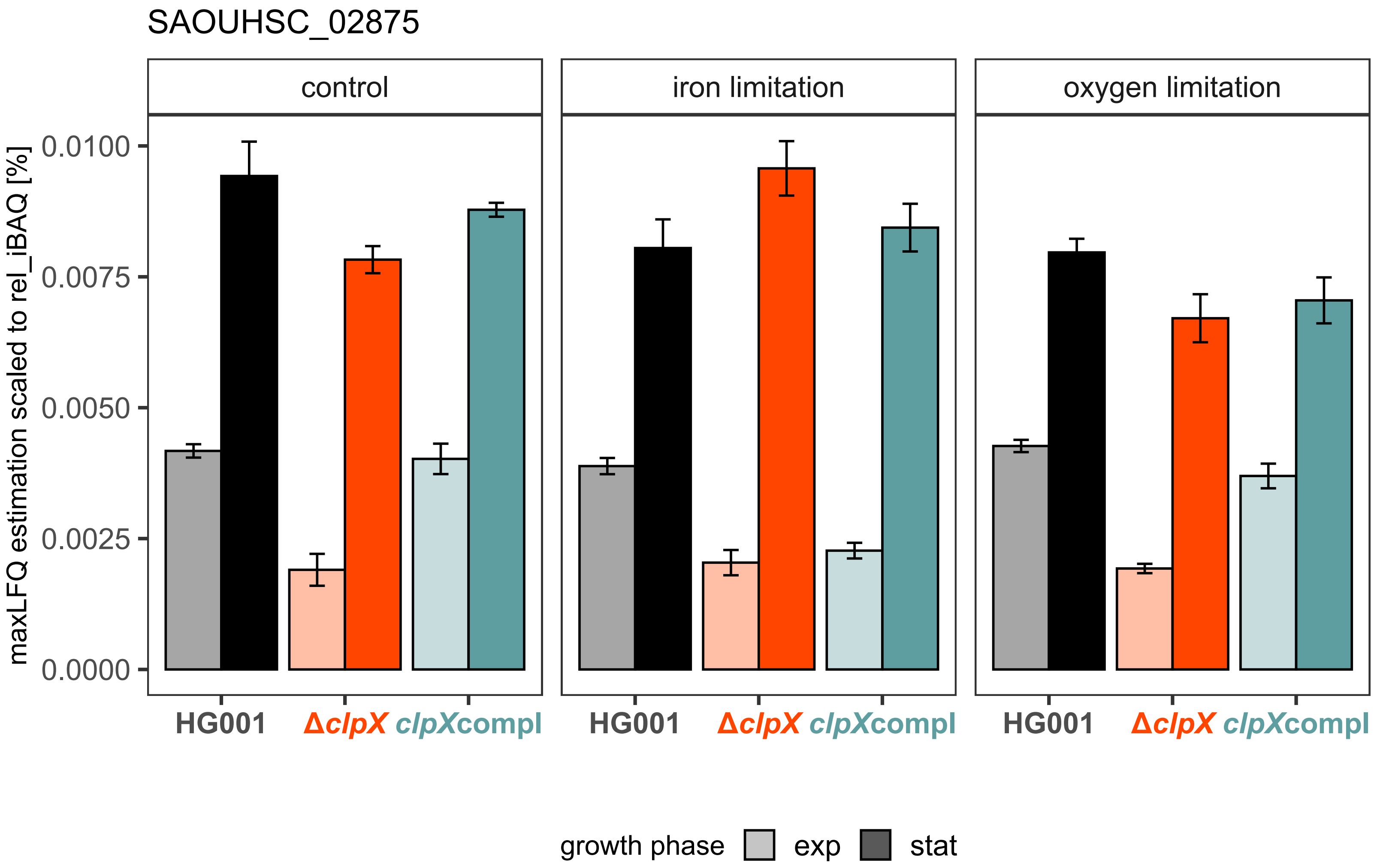
SAOUHSC_02875
control
iron limitation
oxygen limitation
maxLFQ estimation scaled to rel_iBAQ [%]
0.0100
0.0075
0.0050
0.0025
0.0000
HG001
ΔclpX
clpXcompl
growth phase
exp
stat

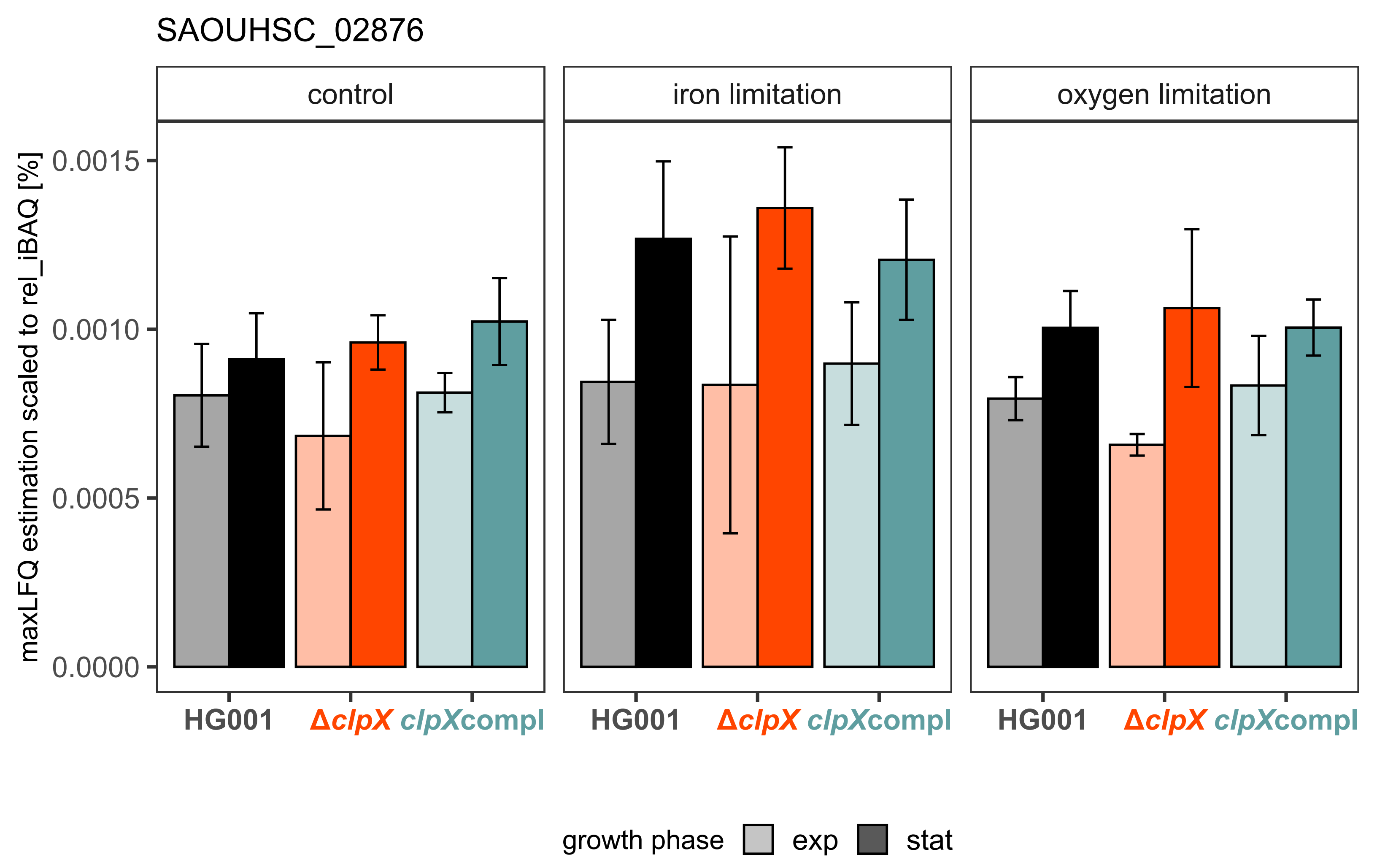

SAOUHSC_02876
control
iron limitation
oxygen limitation
maxLFQ estimation scaled to rel_iBAQ [%]
0.0015
0.0010
0.0005
0.0000
HG001
ΔclpX
clpXcompl
growth phase
exp
stat

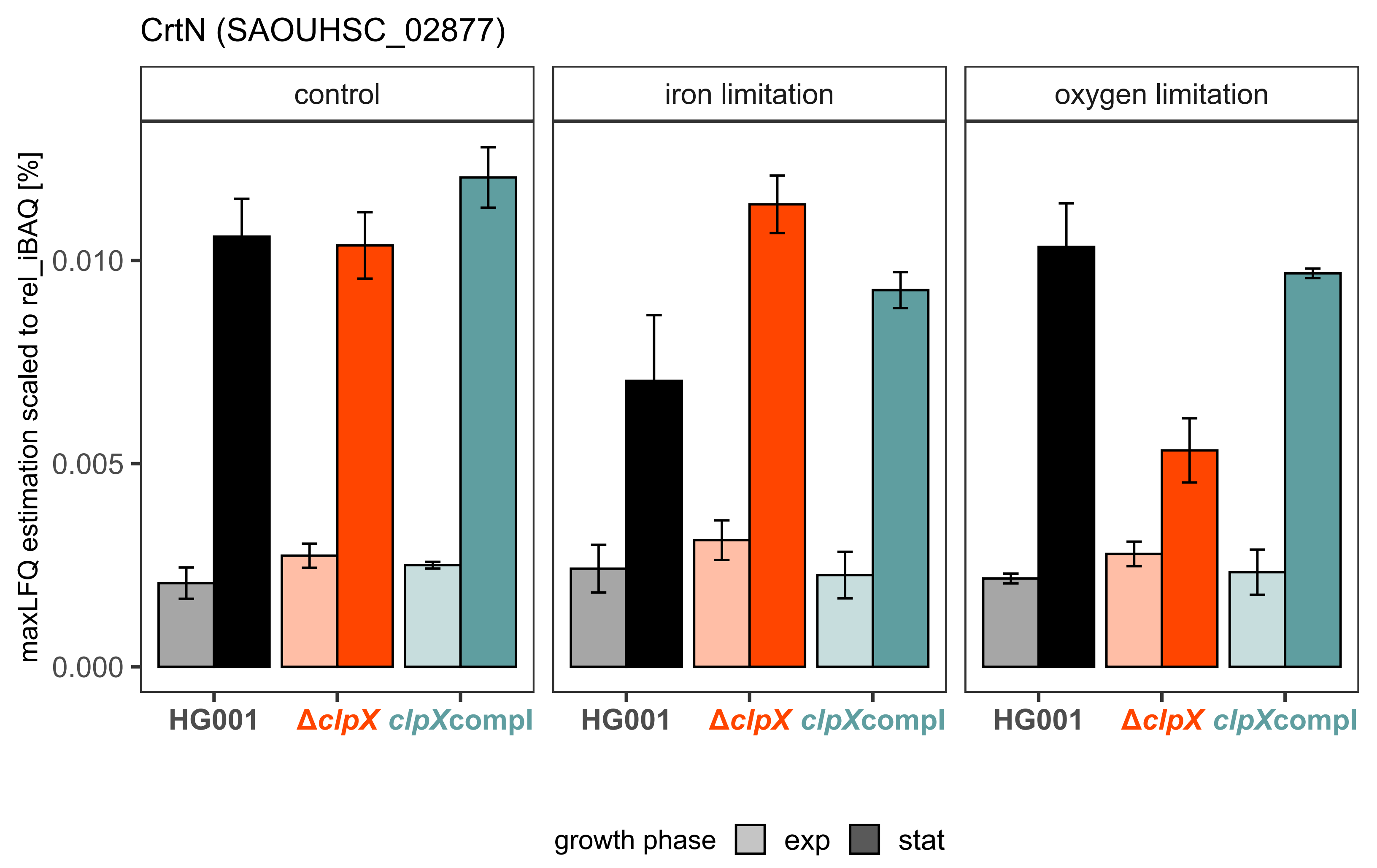
CrtN (SAOUHSC_02877)
control
iron limitation
oxygen limitation
maxLFQ estimation scaled to rel_iBAQ [%]
0.010
0.005
0.000
HG001
ΔclpX
clpXcompl
growth phase
exp
stat

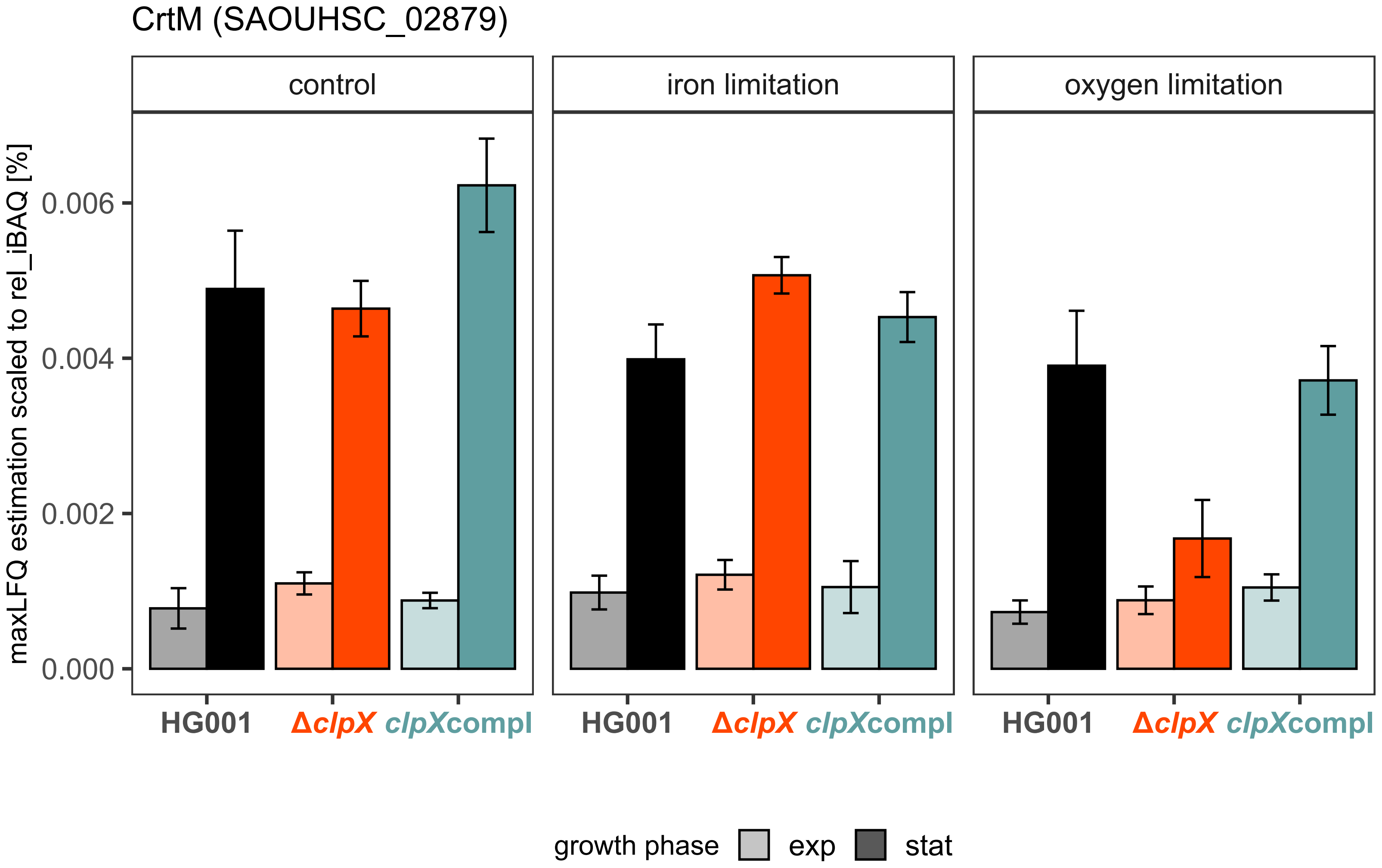

CrtM (SAOUHSC_02879)
control
iron limitation
oxygen limitation
maxLFQ estimation scaled to rel_iBAQ [%]
0.006
0.004
0.002
0.000
HG001
ΔclpX
clpXcompl
growth phase
exp
stat

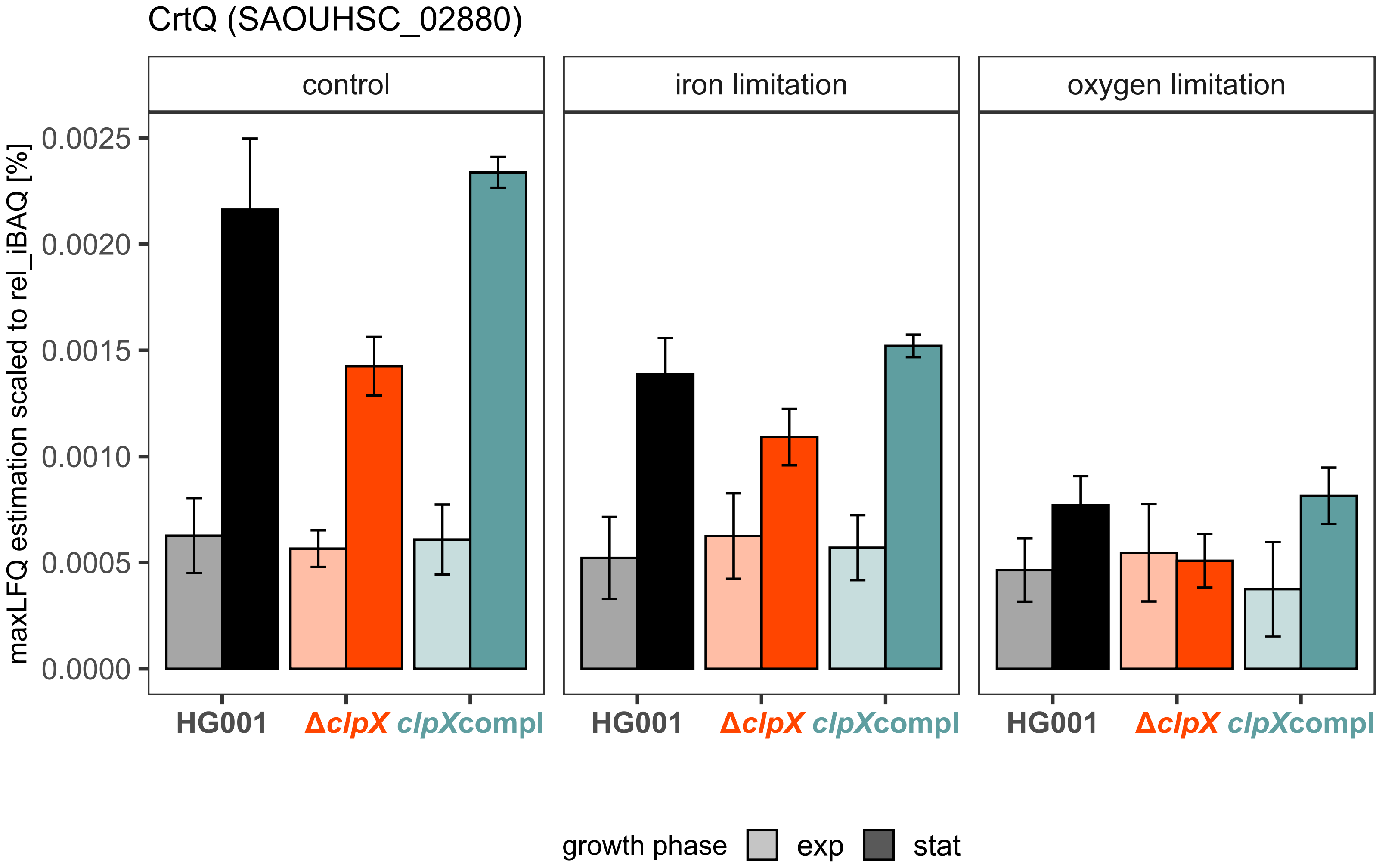
CrtQ (SAOUHSC_02880)
control
iron limitation
oxygen limitation
maxLFQ estimation scaled to rel_iBAQ [%]
0.0025
0.0020
0.0015
0.0010
0.0005
0.0000
HG001
ΔclpX
clpXcompl
growth phase
exp
stat

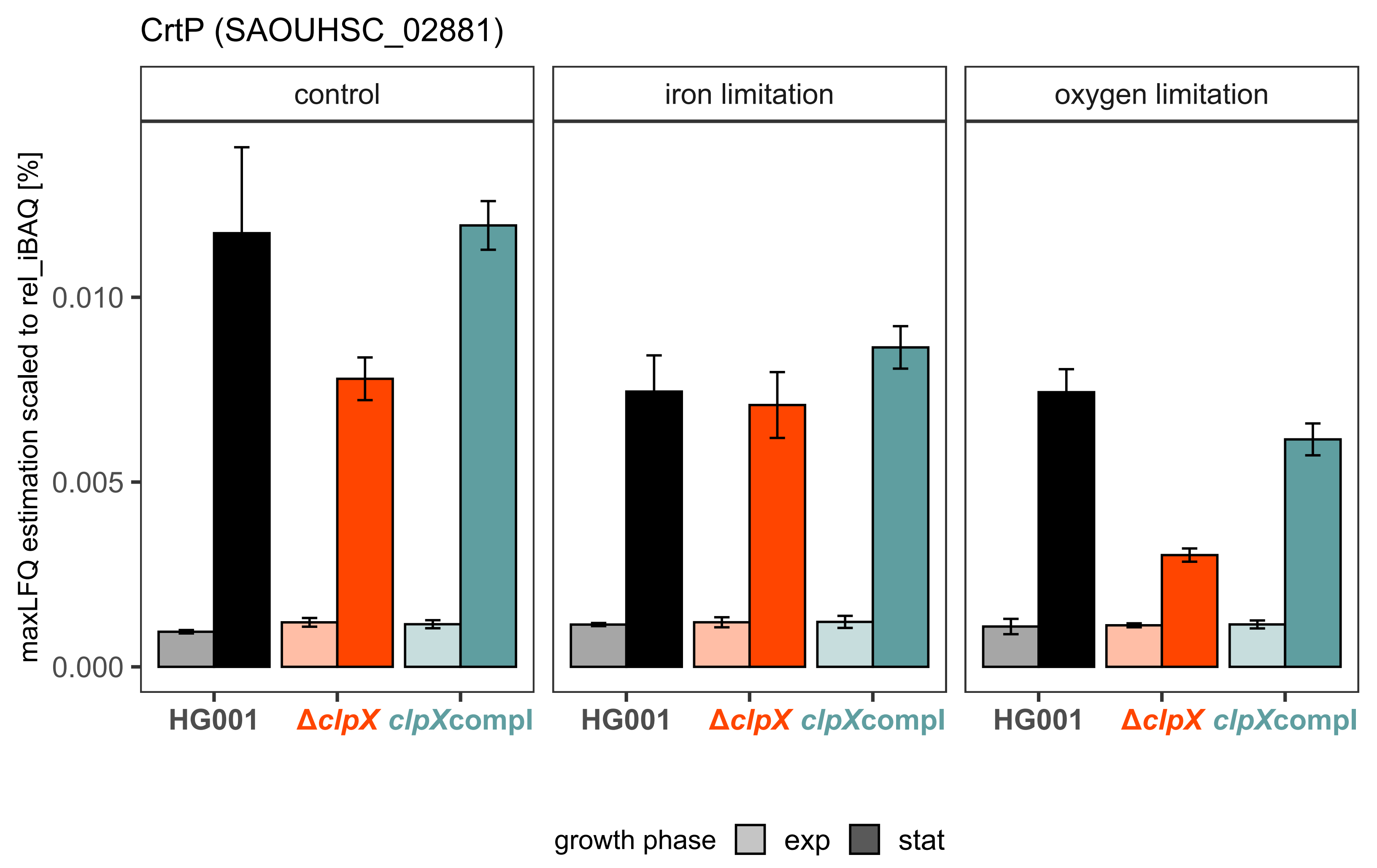

CrtP (SAOUHSC_02881)
control
iron limitation
oxygen limitation
maxLFQ estimation scaled to rel_iBAQ [%]
0.010
0.005
0.000
HG001
ΔclpX
clpXcompl
growth phase
exp
stat

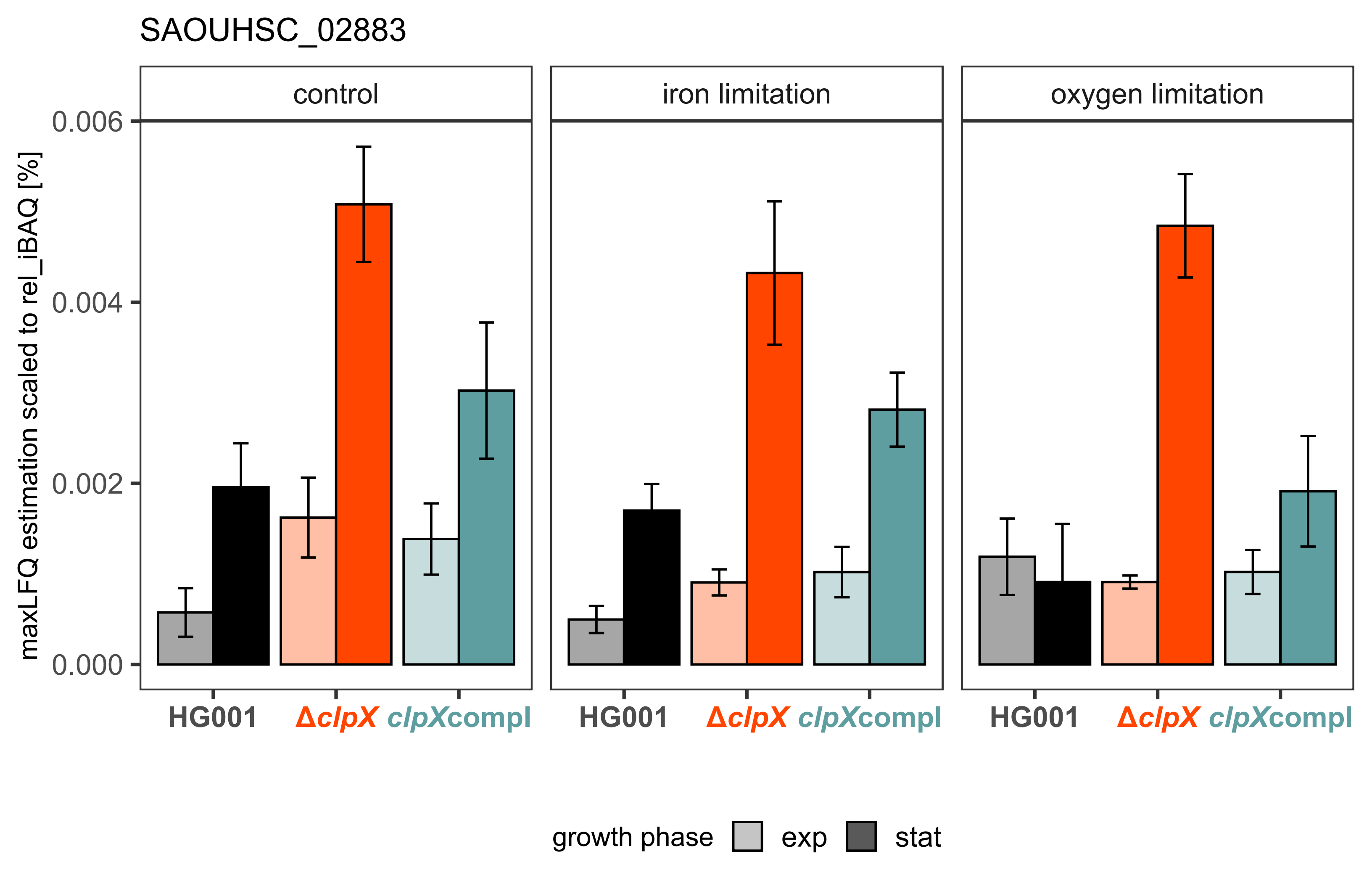

SAOUHSC_02883
control
iron limitation
oxygen limitation
0.006
0.004
0.002
0.000
maxLFQ estimation scaled to rel_iBAQ [%]
HG001
ΔclpX
clpXcompl
growth phase
exp
stat

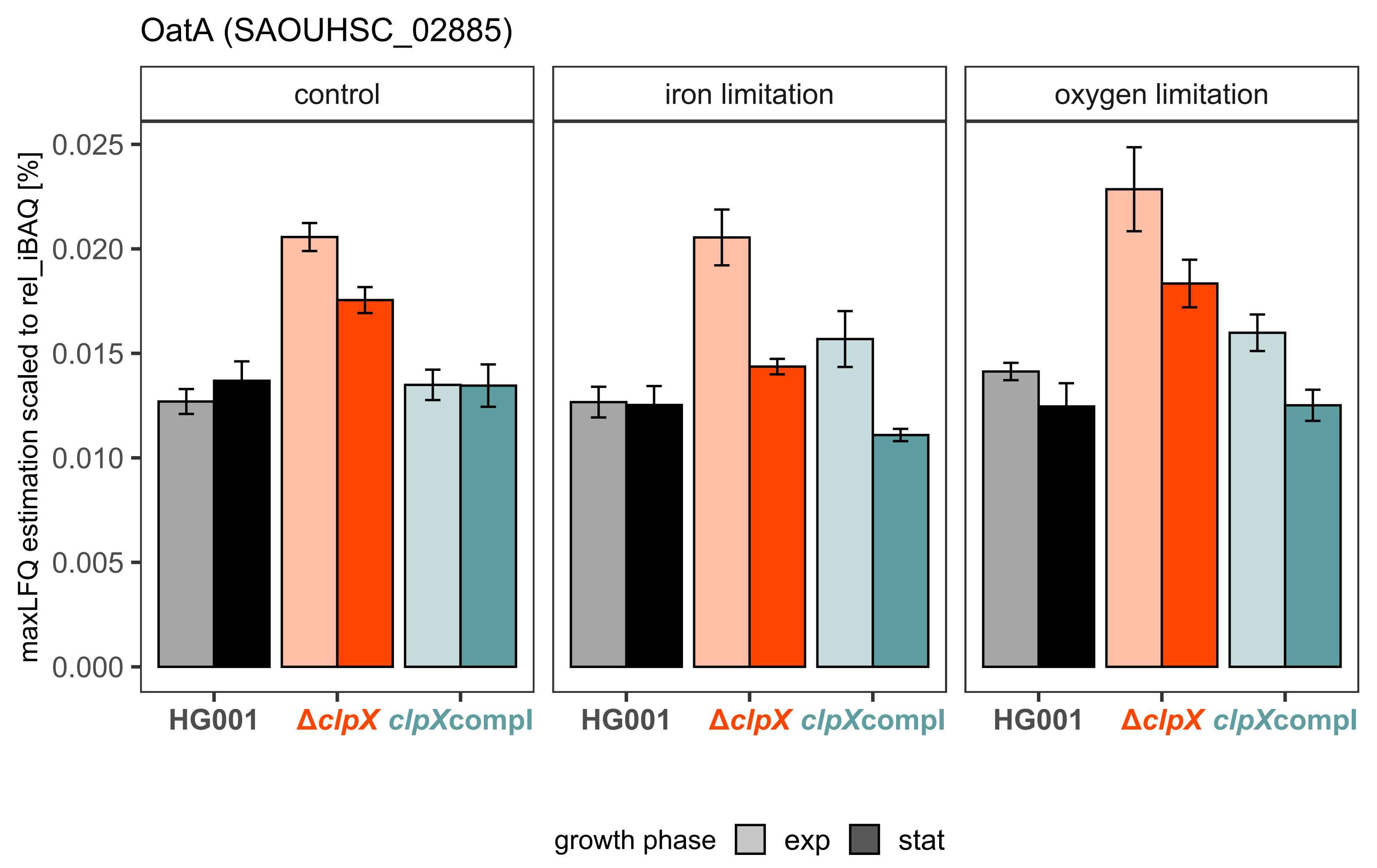

OatA (SAOUHSC_02885)
control
iron limitation
oxygen limitation
maxLFQ estimation scaled to rel_iBAQ [%]
0.025
0.020
0.015
0.010
0.005
0.000
HG001
ΔclpX
clpXcompl
growth phase
exp
stat

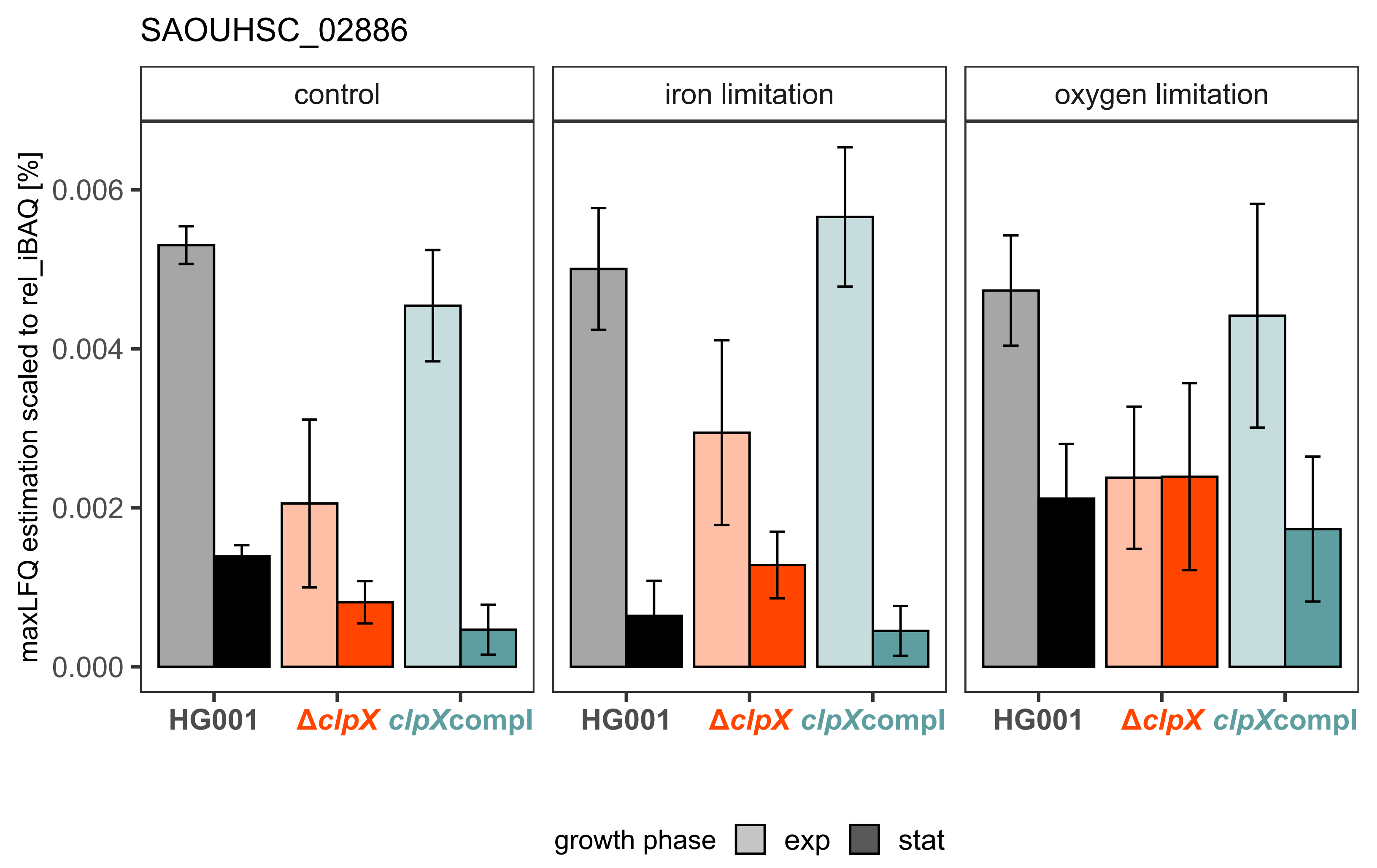
SAOUHSC_02886
control
iron limitation
oxygen limitation
maxLFQ estimation scaled to rel_iBAQ [%]
0.006
0.004
0.002
0.000
HG001
ΔclpX
clpXcompl
growth phase
exp
stat

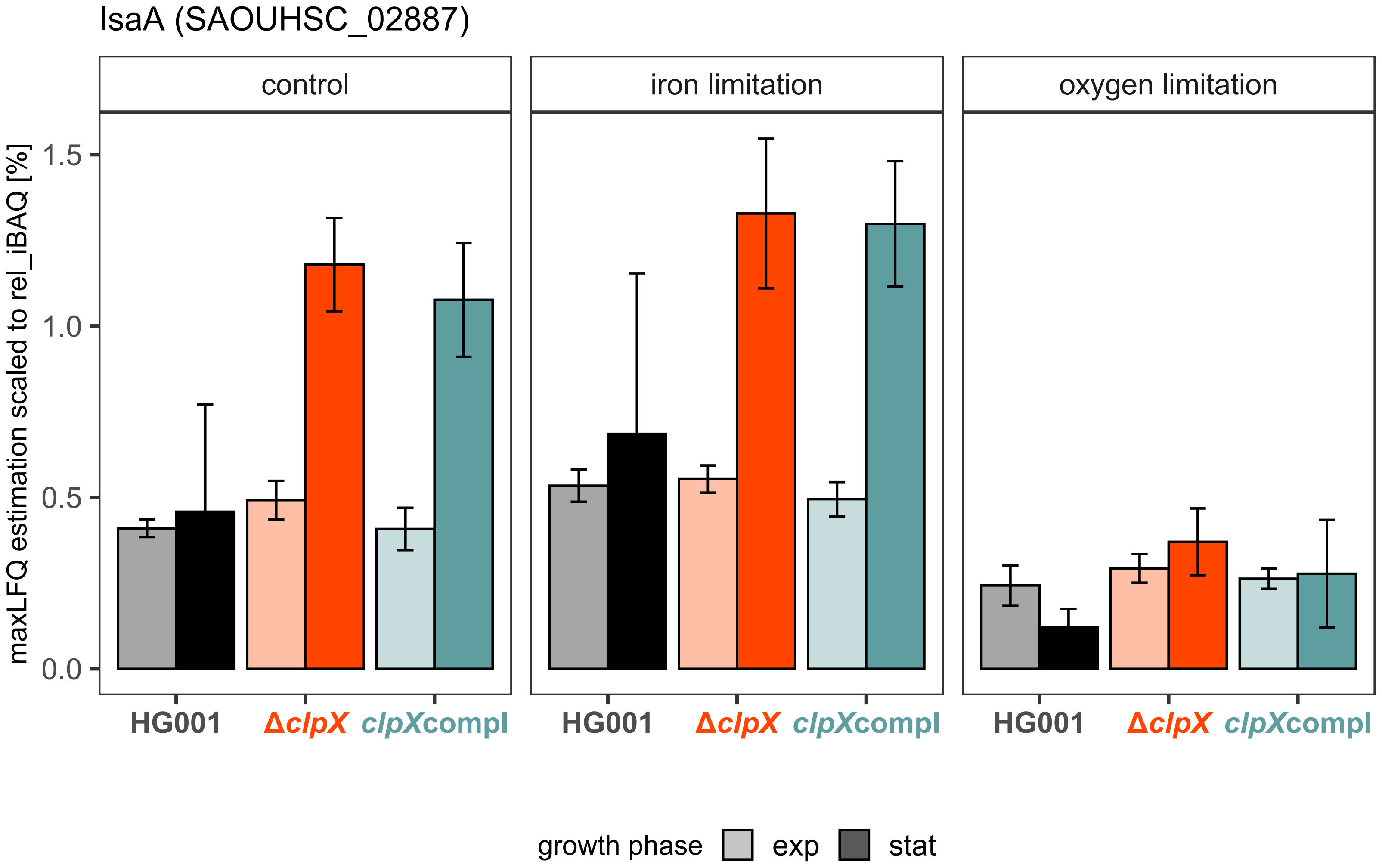
IsaA (SAOUHSC_02887)
control
iron limitation
oxygen limitation
maxLFQ estimation scaled to rel_iBAQ [%]
0.0
0.5
1.0
1.5
HG001
ΔclpX
clpXcompl
growth phase
exp
stat

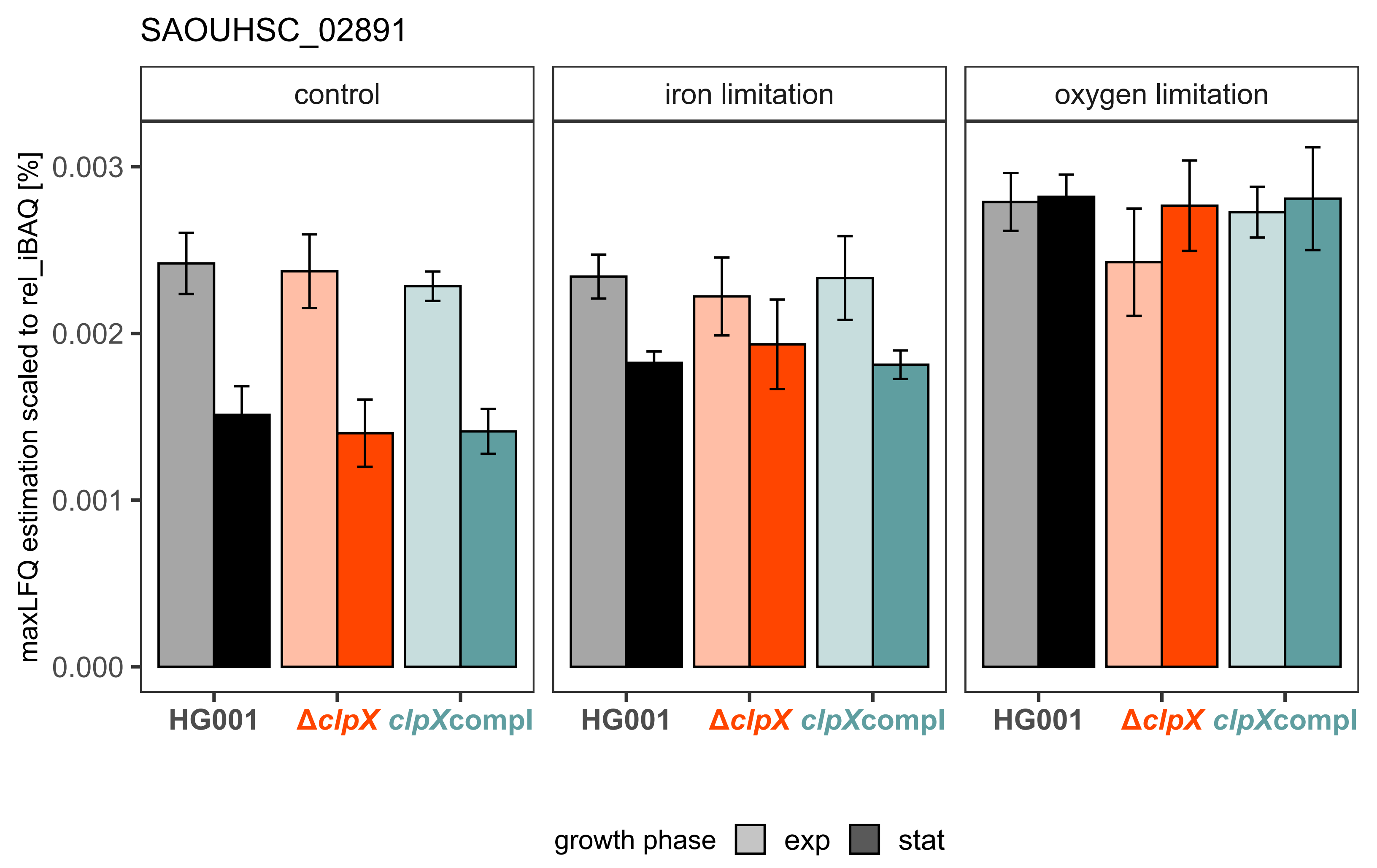
SAOUHSC_02891
control
iron limitation
oxygen limitation
maxLFQ estimation scaled to rel_iBAQ [%]
0.003
0.002
0.001
0.000
HG001
ΔclpX
clpXcompl
growth phase
exp
stat

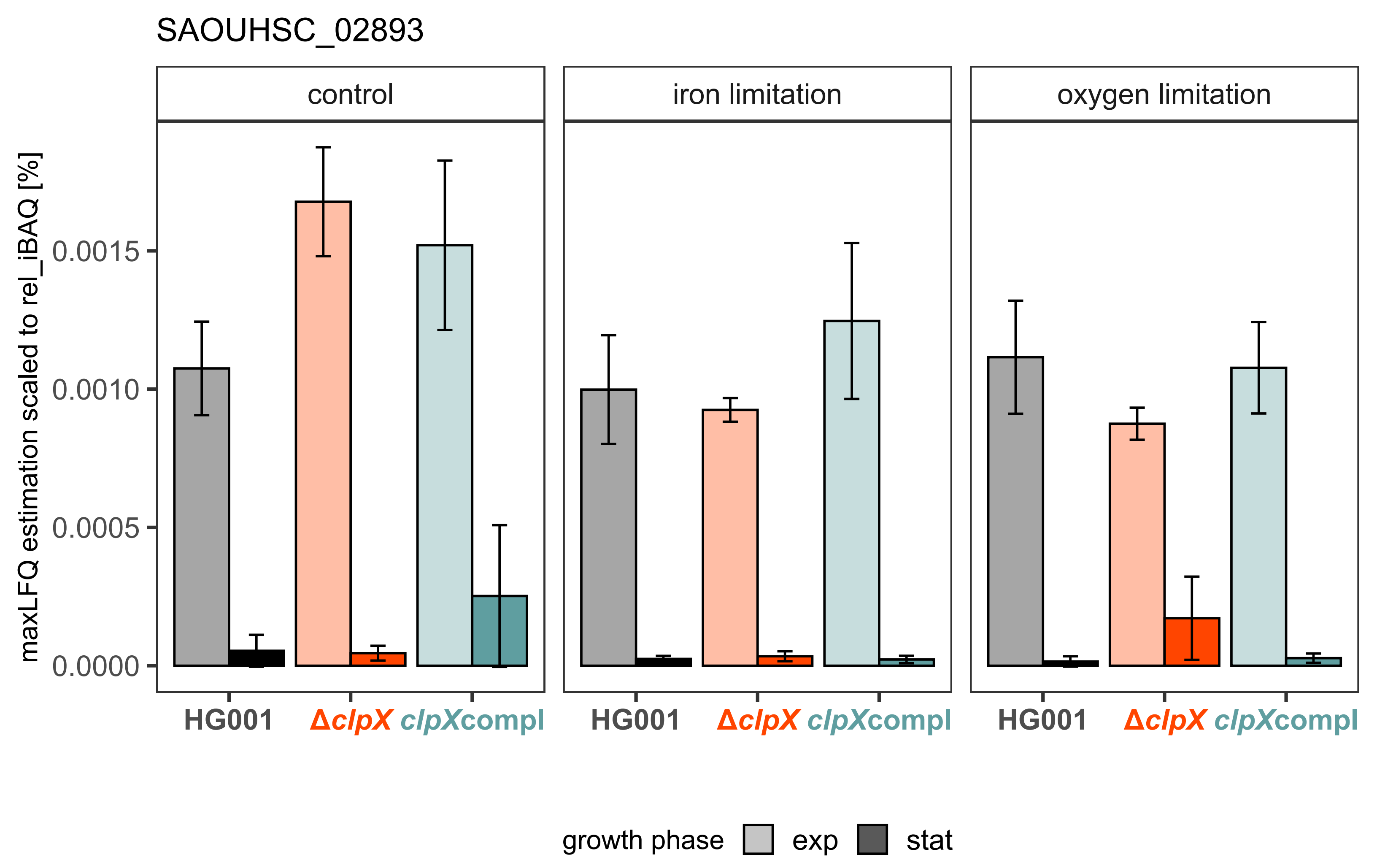

SAOUHSC_02893
control
iron limitation
oxygen limitation
maxLFQ estimation scaled to rel_iBAQ [%]
0.0015
0.0010
0.0005
0.0000
HG001
ΔclpX
clpXcompl
growth phase
exp
stat

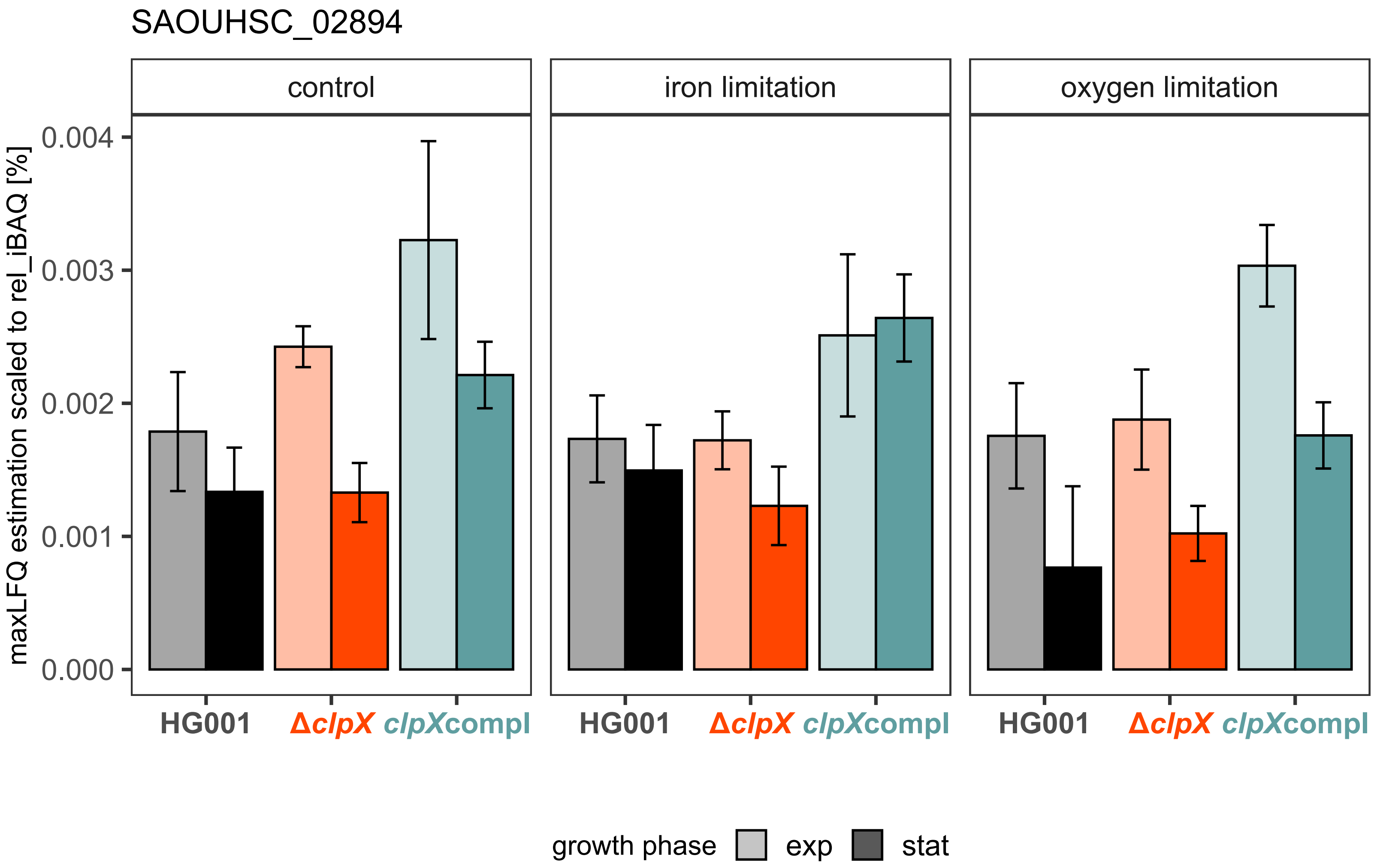
SAOUHSC_02894
control
iron limitation
oxygen limitation
maxLFQ estimation scaled to rel_iBAQ [%]
0.004
0.003
0.002
0.001
0.000
HG001
ΔclpX
clpXcompl
growth phase
exp
stat

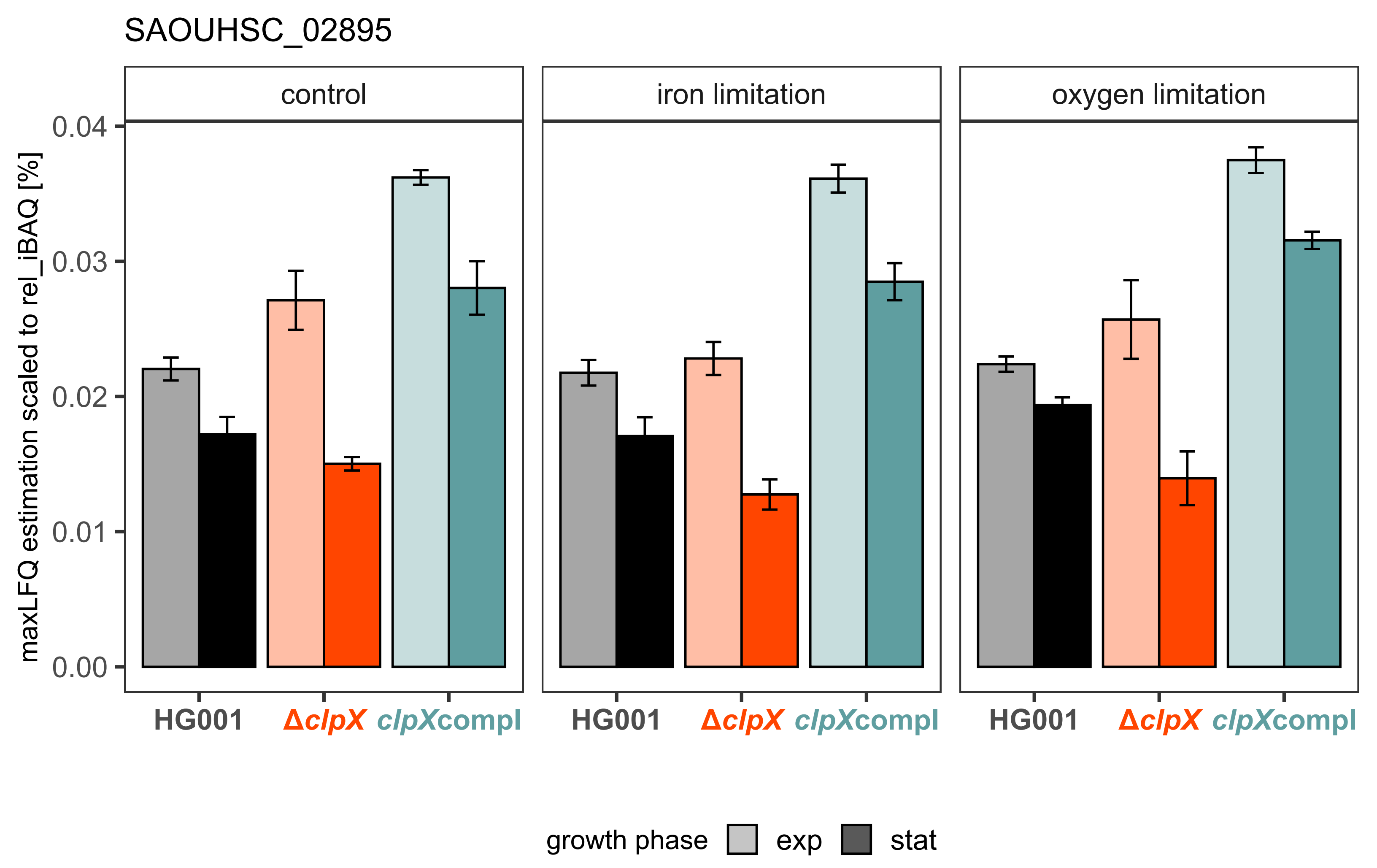
SAOUHSC_02895
control
iron limitation
oxygen limitation
maxLFQ estimation scaled to rel_iBAQ [%]
0.04
0.03
0.02
0.01
0.00
HG001
ΔclpX
clpXcompl
growth phase
exp
stat

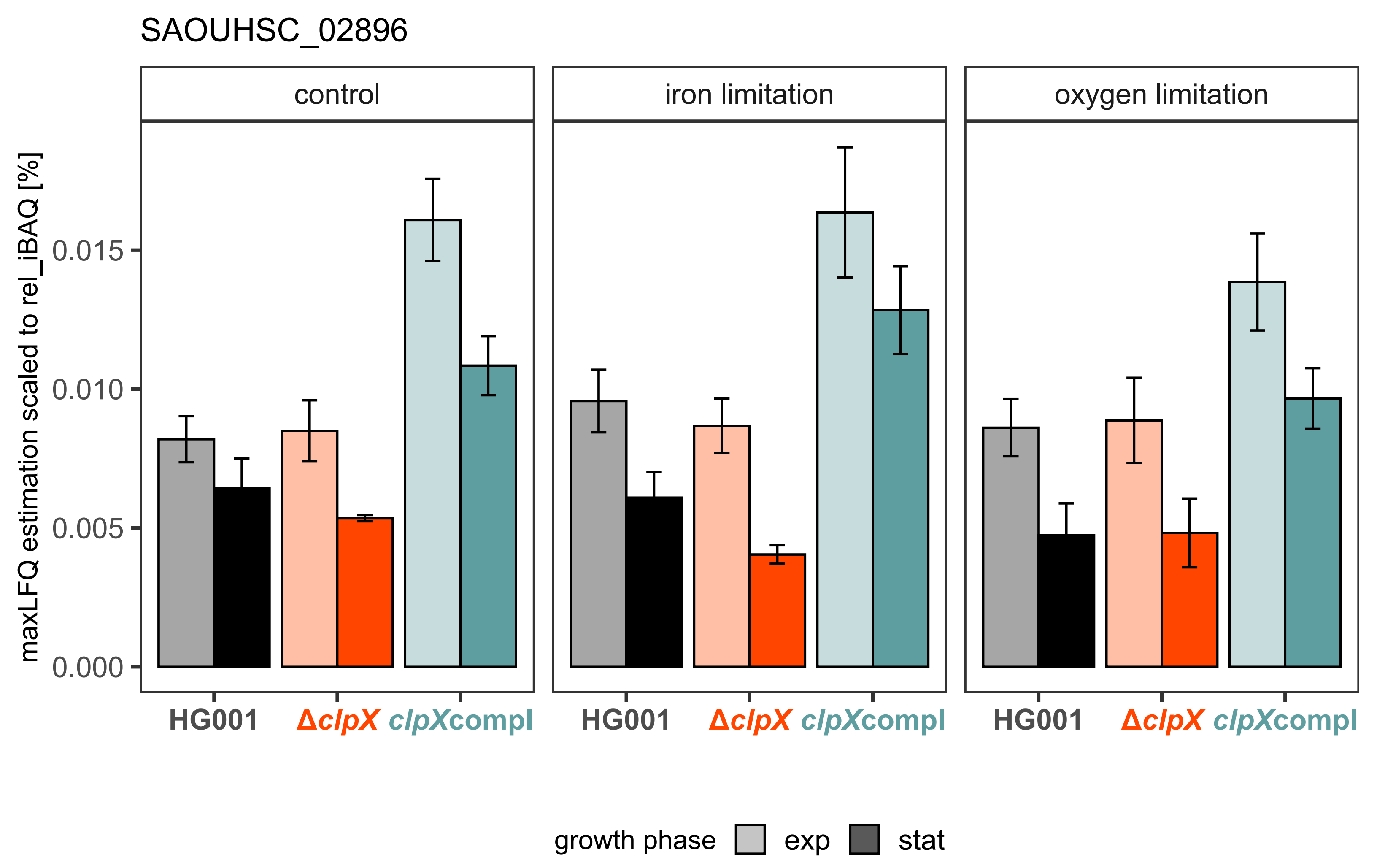

SAOUHSC_02896
control
iron limitation
oxygen limitation
maxLFQ estimation scaled to rel_iBAQ [%]
0.000
0.005
0.010
0.015
HG001
ΔclpX
clpXcompl
growth phase
exp
stat

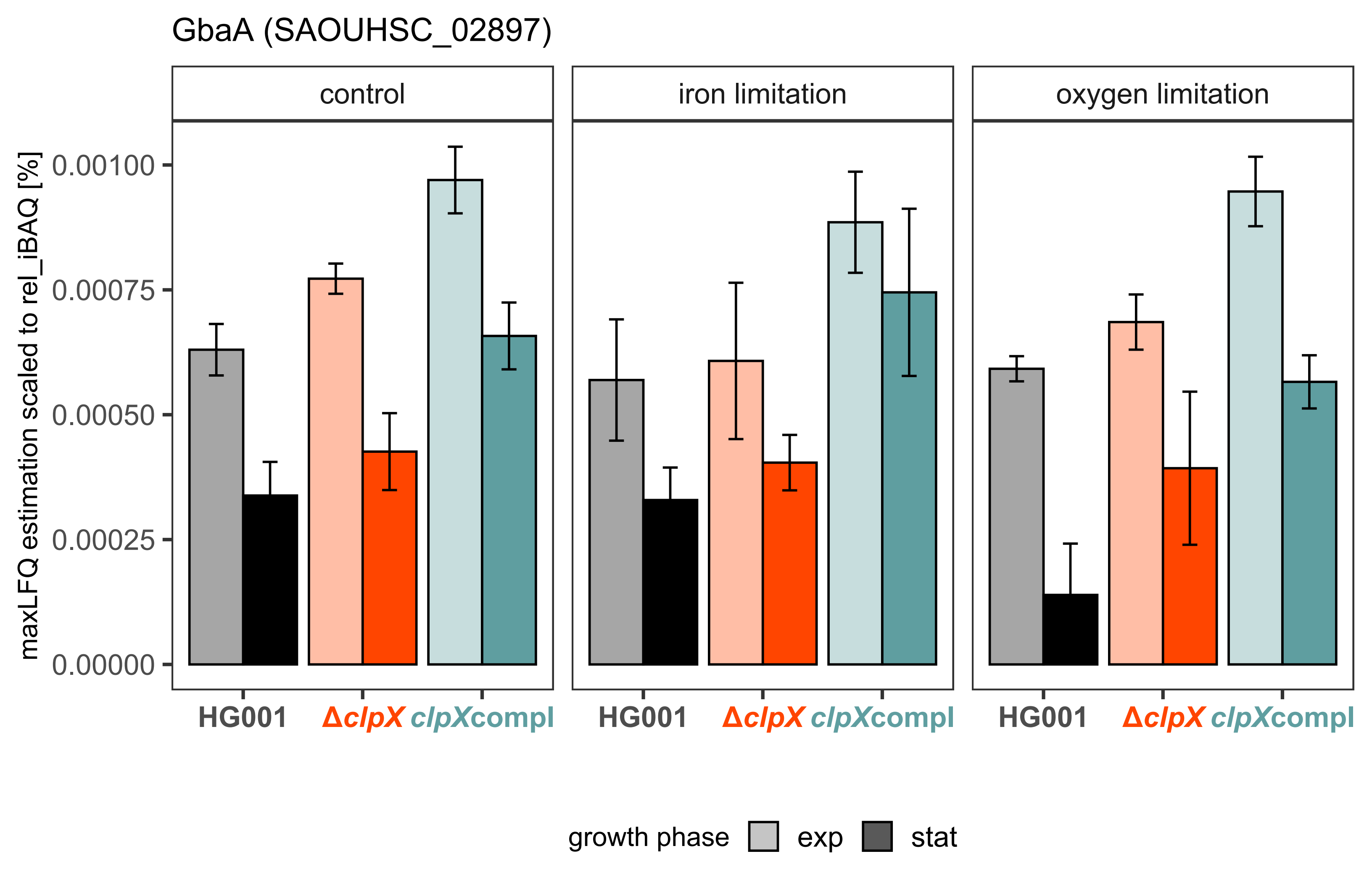

GbaA (SAOUHSC_02897)
control
iron limitation
oxygen limitation
maxLFQ estimation scaled to rel_iBAQ [%]
0.00100
0.00075
0.00050
0.00025
0.00000
HG001
ΔclpX
clpXcompl
growth phase
exp
stat

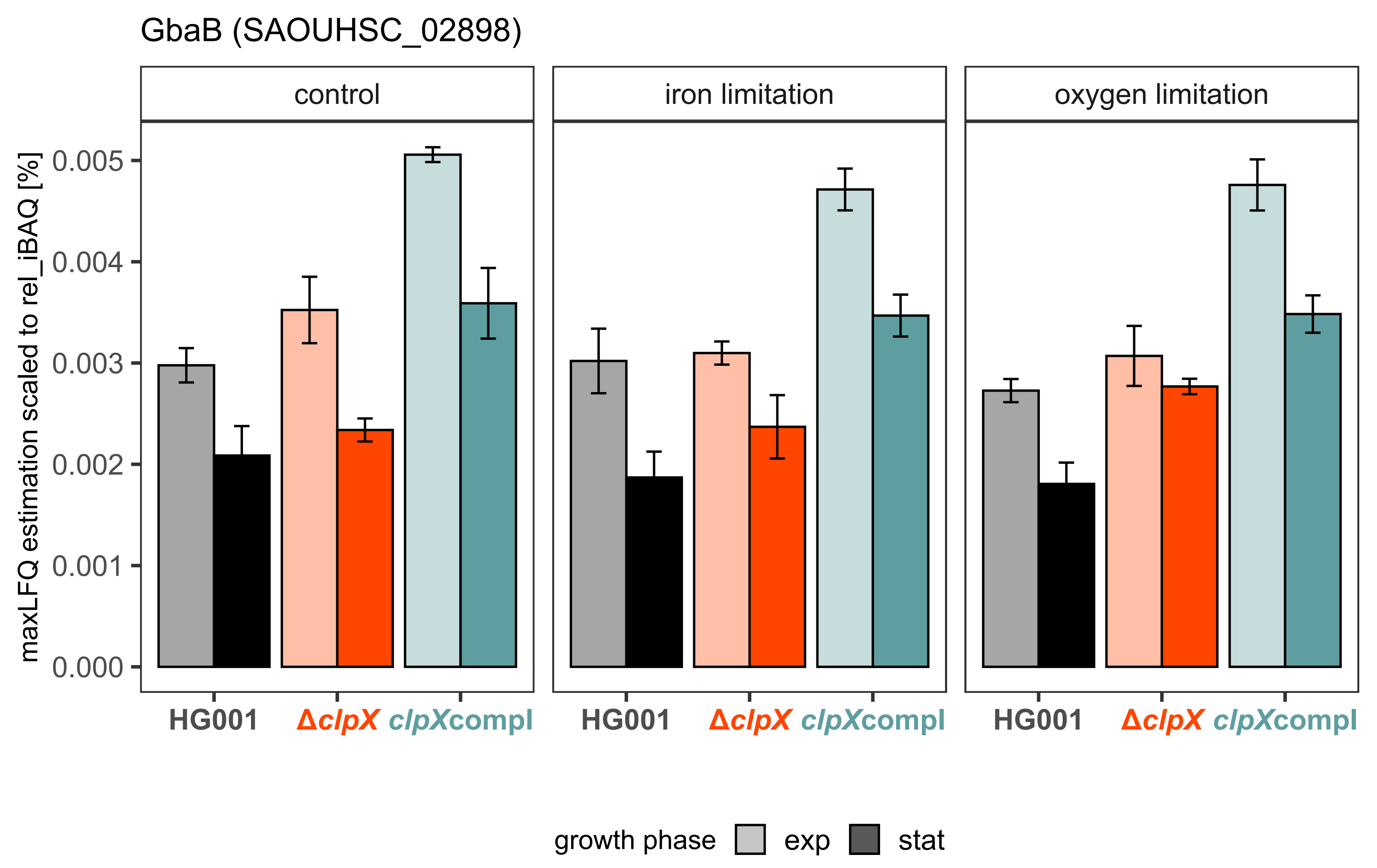
GbaB (SAOUHSC_02898)
control
iron limitation
oxygen limitation
maxLFQ estimation scaled to rel_iBAQ [%]
0.005
0.004
0.003
0.002
0.001
0.000
HG001
ΔclpX
clpXcompl
growth phase
exp
stat

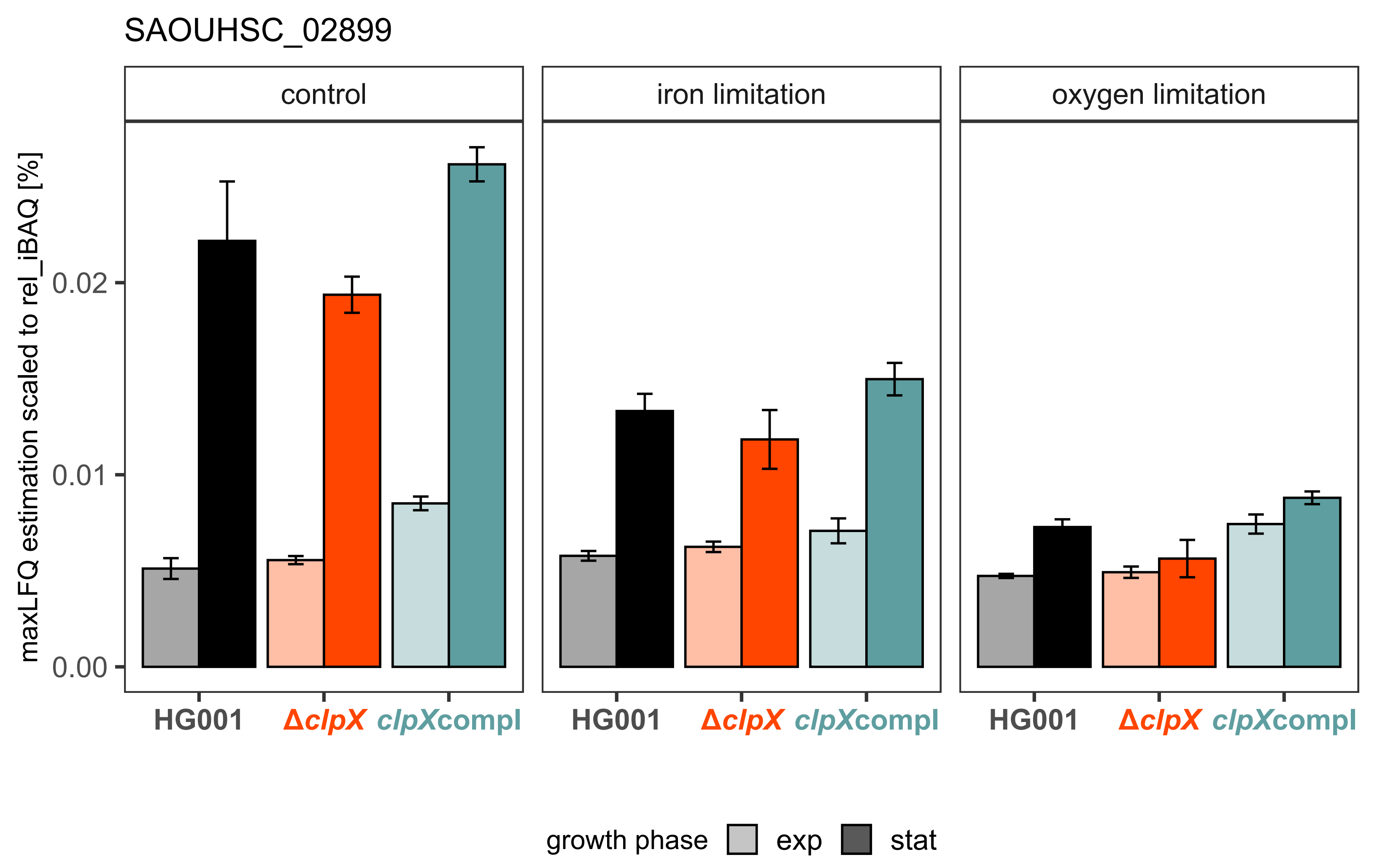

SAOUHSC_02899
control
iron limitation
oxygen limitation
maxLFQ estimation scaled to rel_iBAQ [%]
0.02
0.01
0.00
HG001
ΔclpX
clpXcompl
growth phase
exp
stat

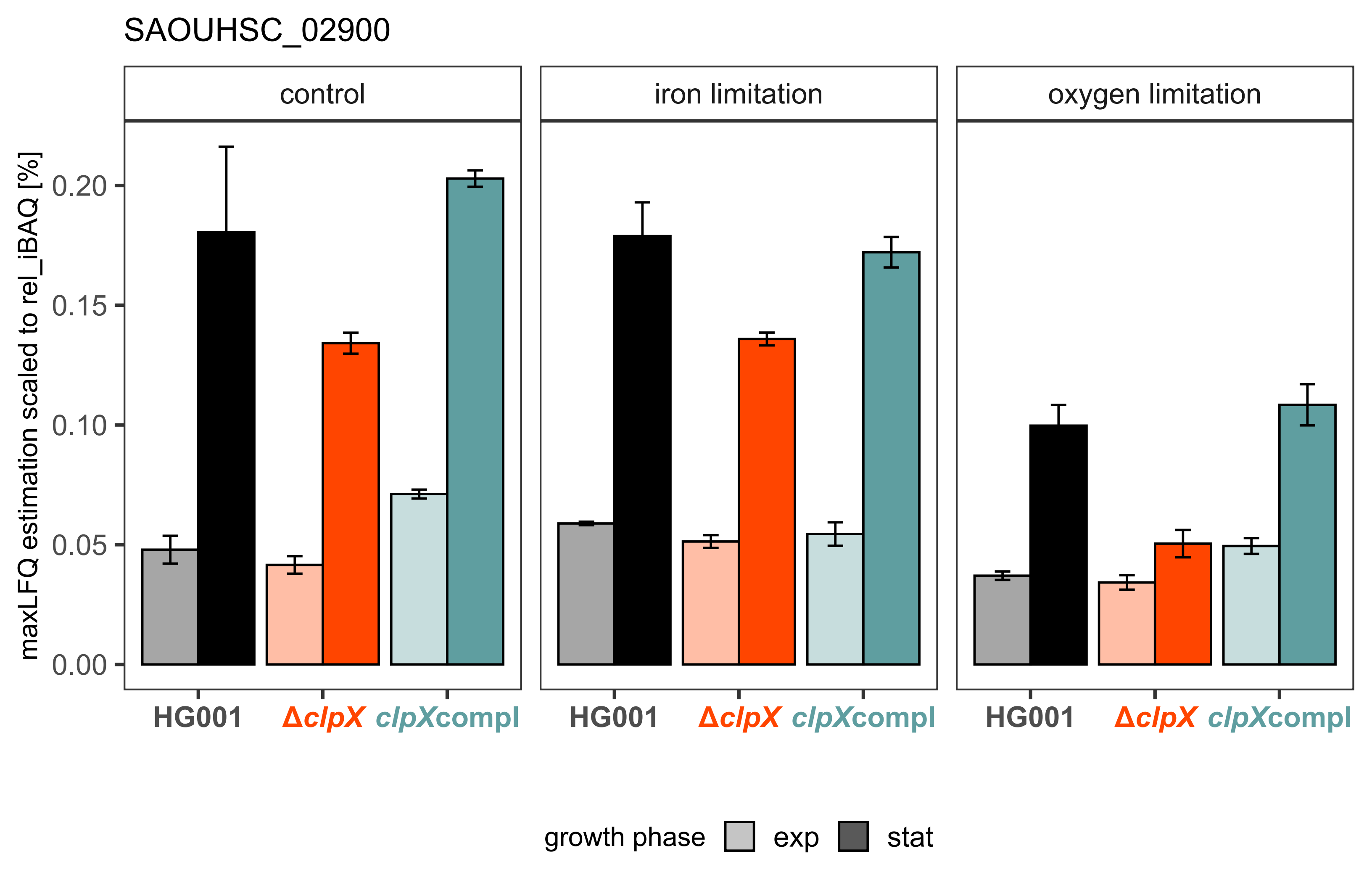
SAOUHSC_02900
control
iron limitation
oxygen limitation
maxLFQ estimation scaled to rel_iBAQ [%]
0.20
0.15
0.10
0.05
0.00
HG001
ΔclpX
clpXcompl
growth phase
exp
stat

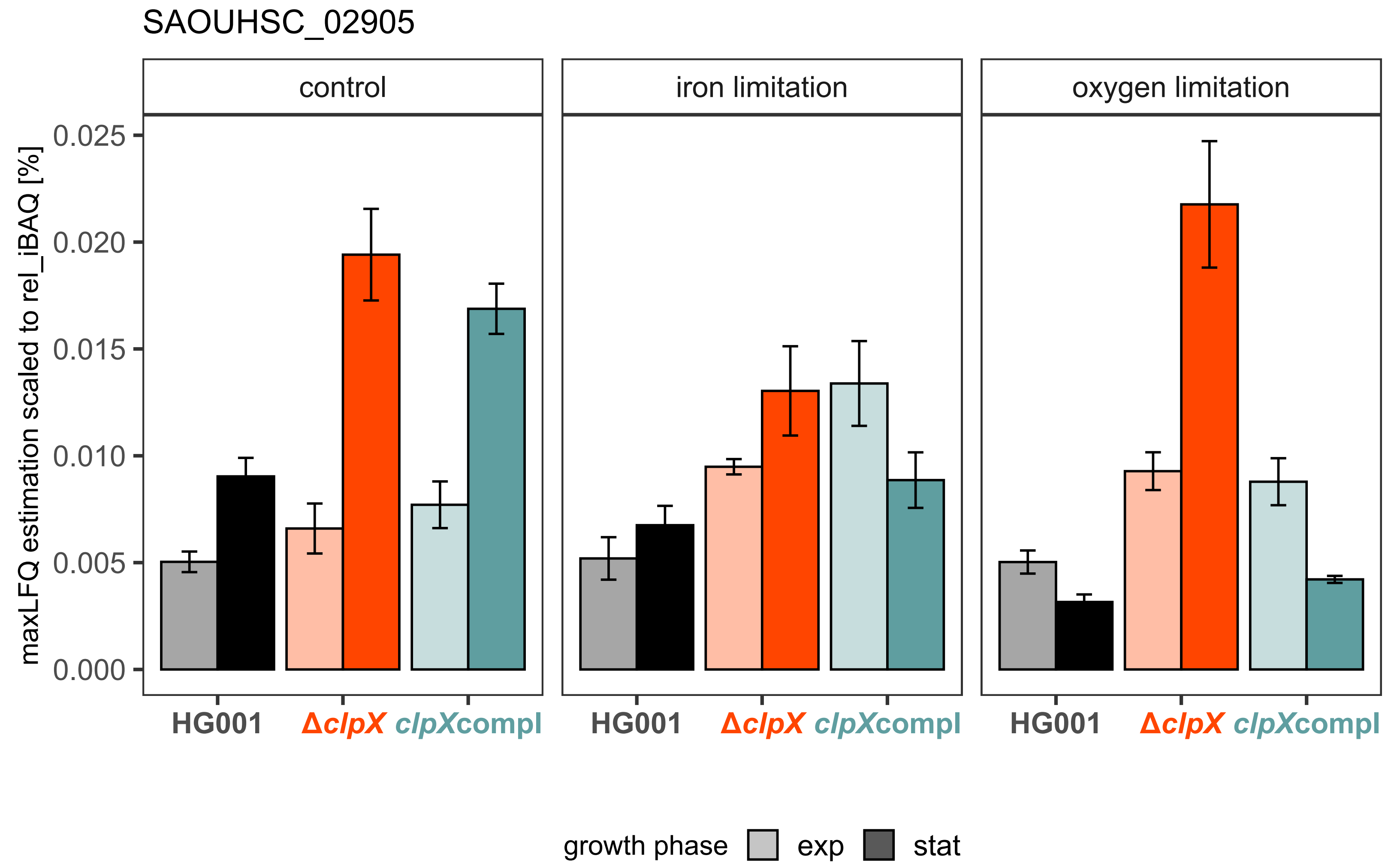
SAOUHSC_02905
control
iron limitation
oxygen limitation
maxLFQ estimation scaled to rel_iBAQ [%]
0.025
0.020
0.015
0.010
0.005
0.000
HG001
ΔclpX
clpXcompl
growth phase
exp
stat

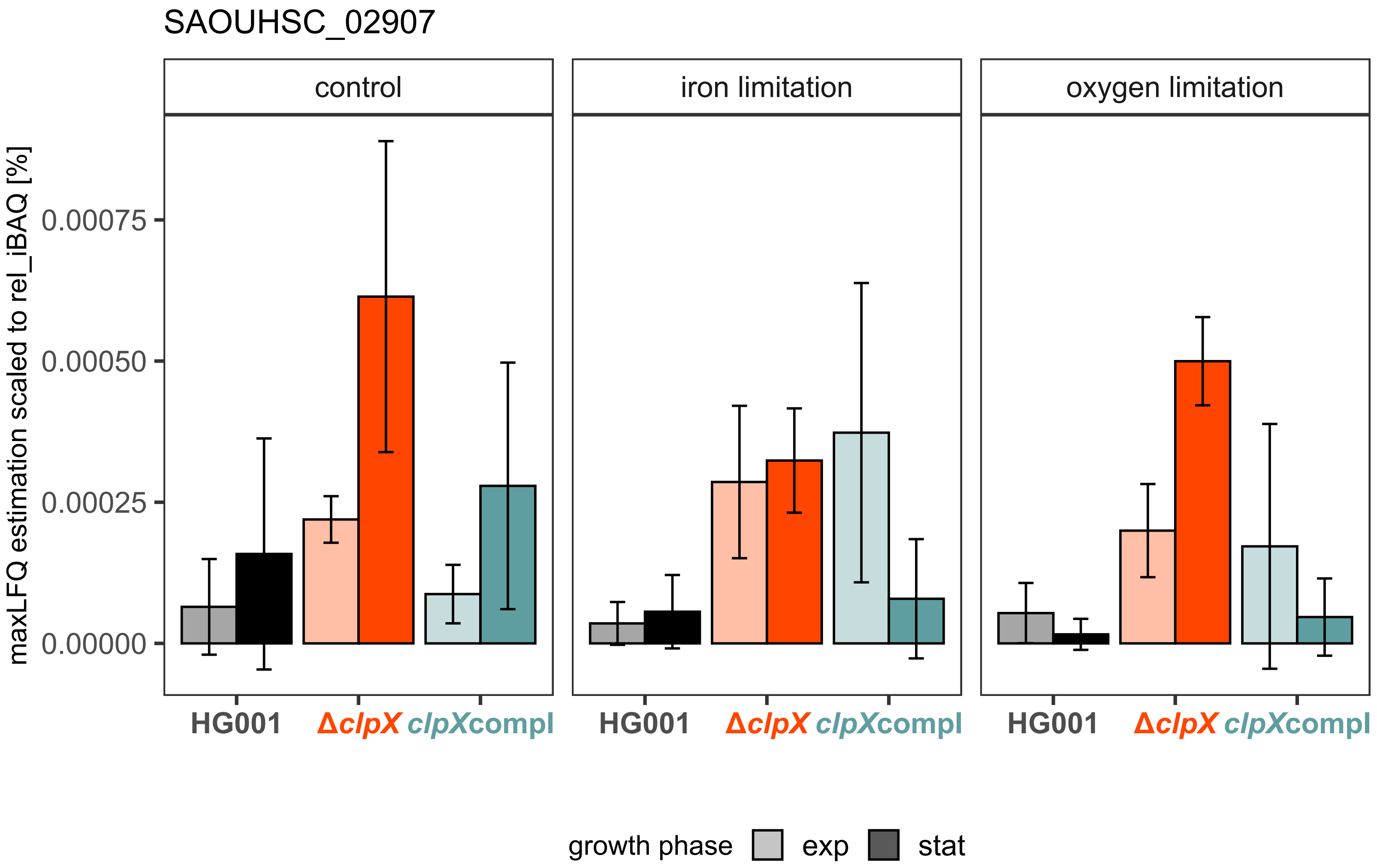

SAOUHSC_02907
control
iron limitation
oxygen limitation
maxLFQ estimation scaled to rel_iBAQ [%]
0.00075
0.00050
0.00025
0.00000
HG001
ΔclpX
clpXcompl
growth phase
exp
stat

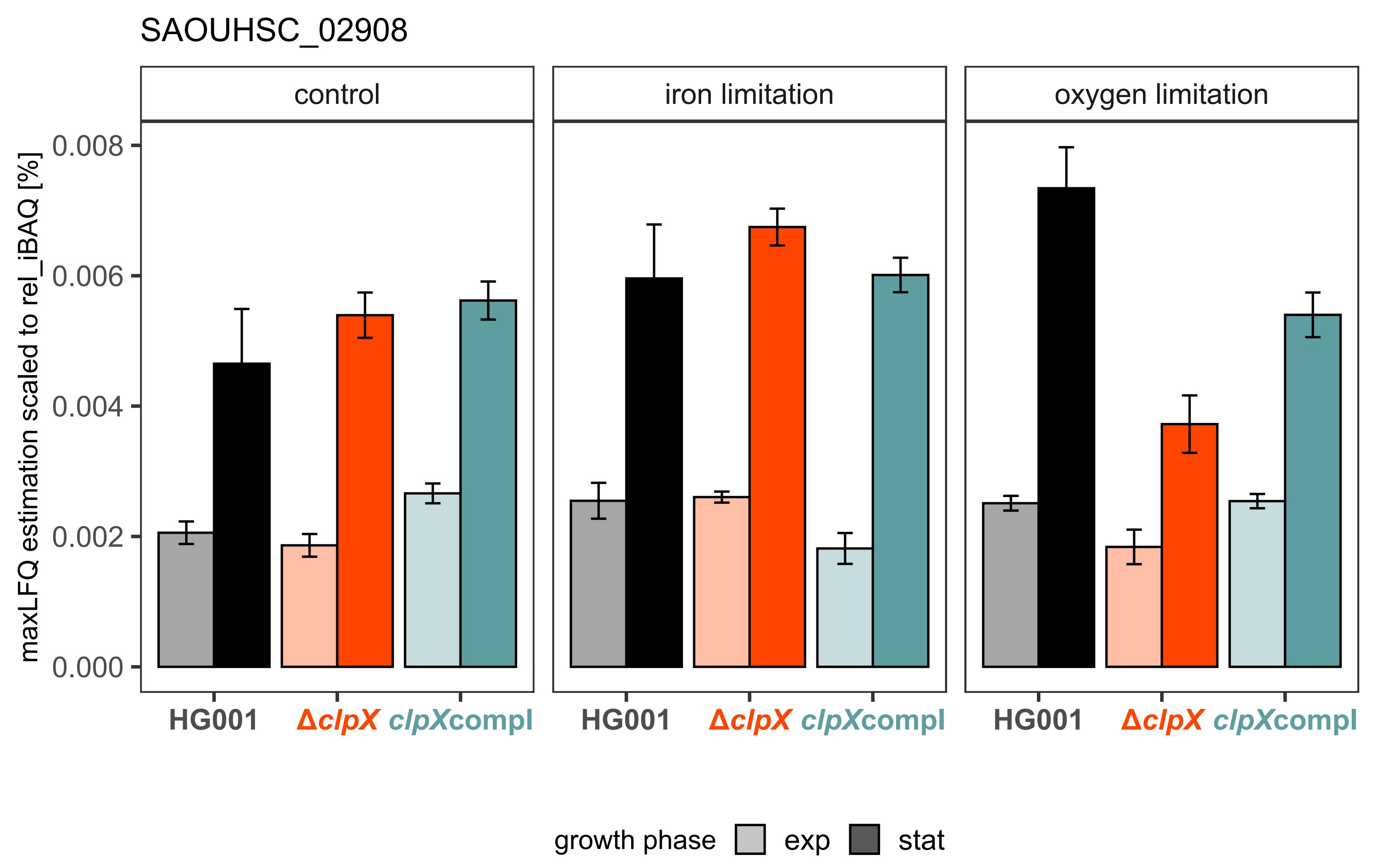
SAOUHSC_02908
control
iron limitation
oxygen limitation
maxLFQ estimation scaled to rel_iBAQ [%]
0.008
0.006
0.004
0.002
0.000
HG001
ΔclpX
clpXcompl
growth phase
exp
stat

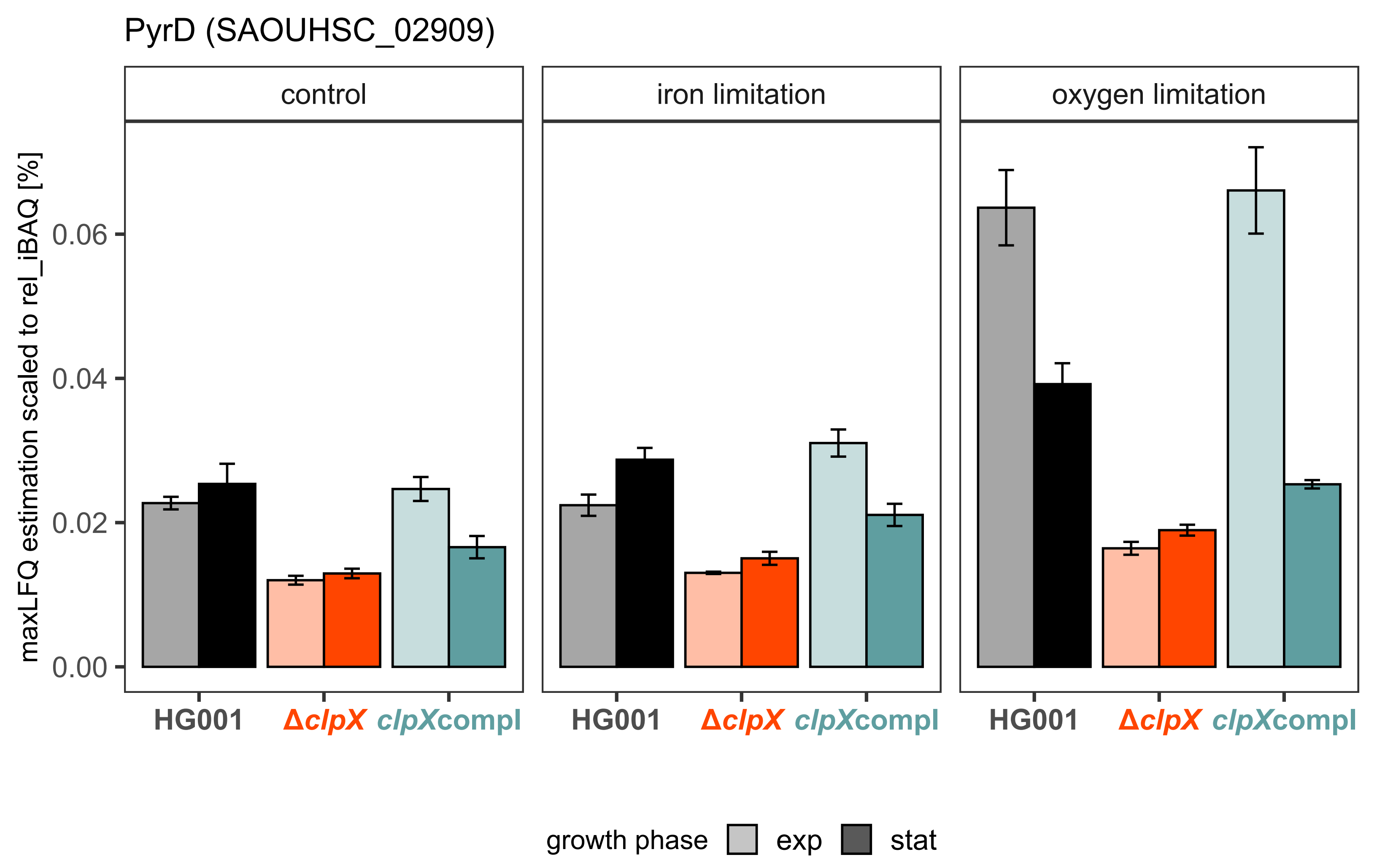

PyrD (SAOUHSC_02909)
control
iron limitation
oxygen limitation
maxLFQ estimation scaled to rel_iBAQ [%]
0.06
0.04
0.02
0.00
HG001
ΔclpX
clpXcompl
growth phase
exp
stat

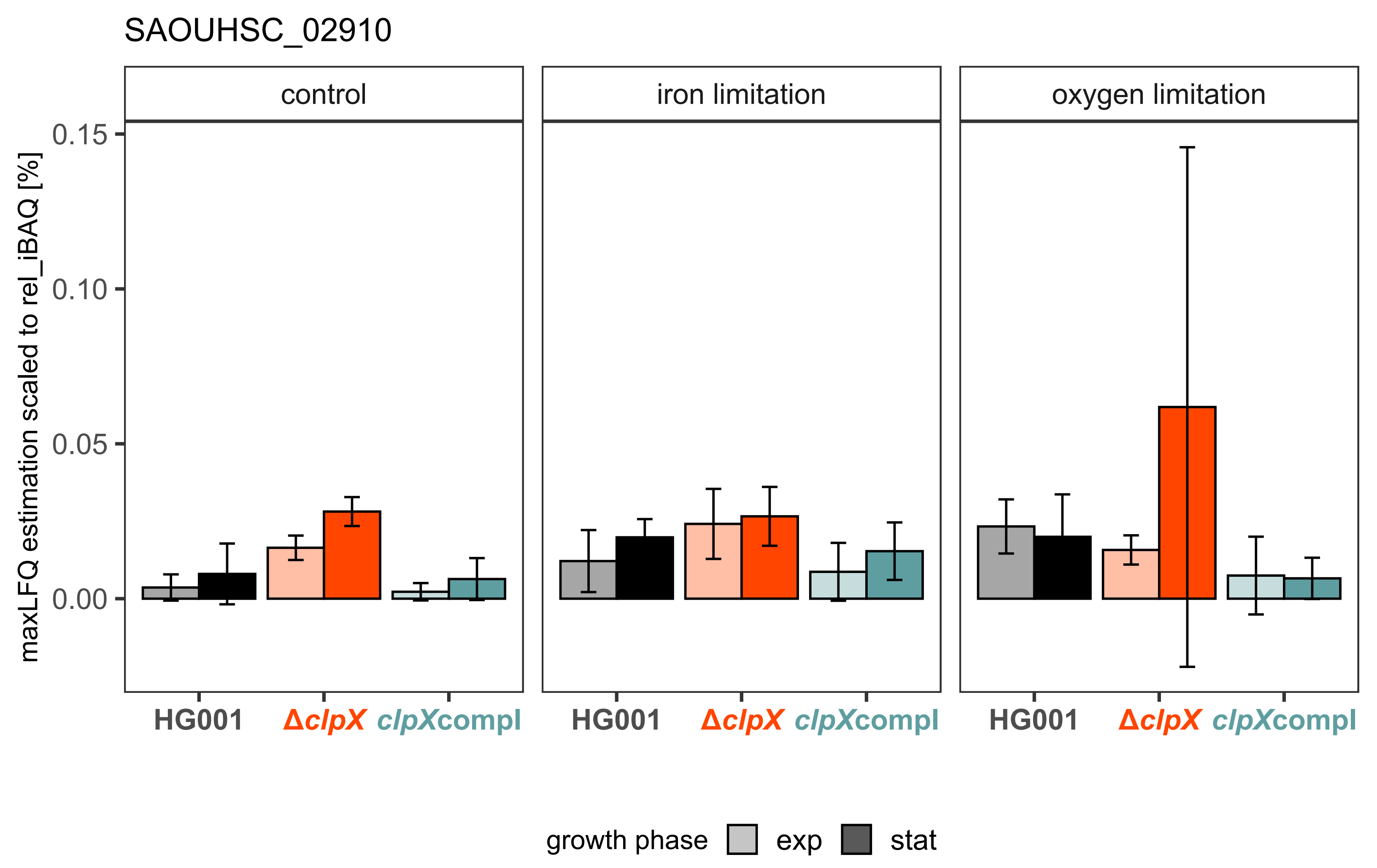

SAOUHSC_02910
control
iron limitation
oxygen limitation
maxLFQ estimation scaled to rel_iBAQ [%]
0.15
0.10
0.05
0.00
HG001
ΔclpX
clpXcompl
growth phase
exp
stat

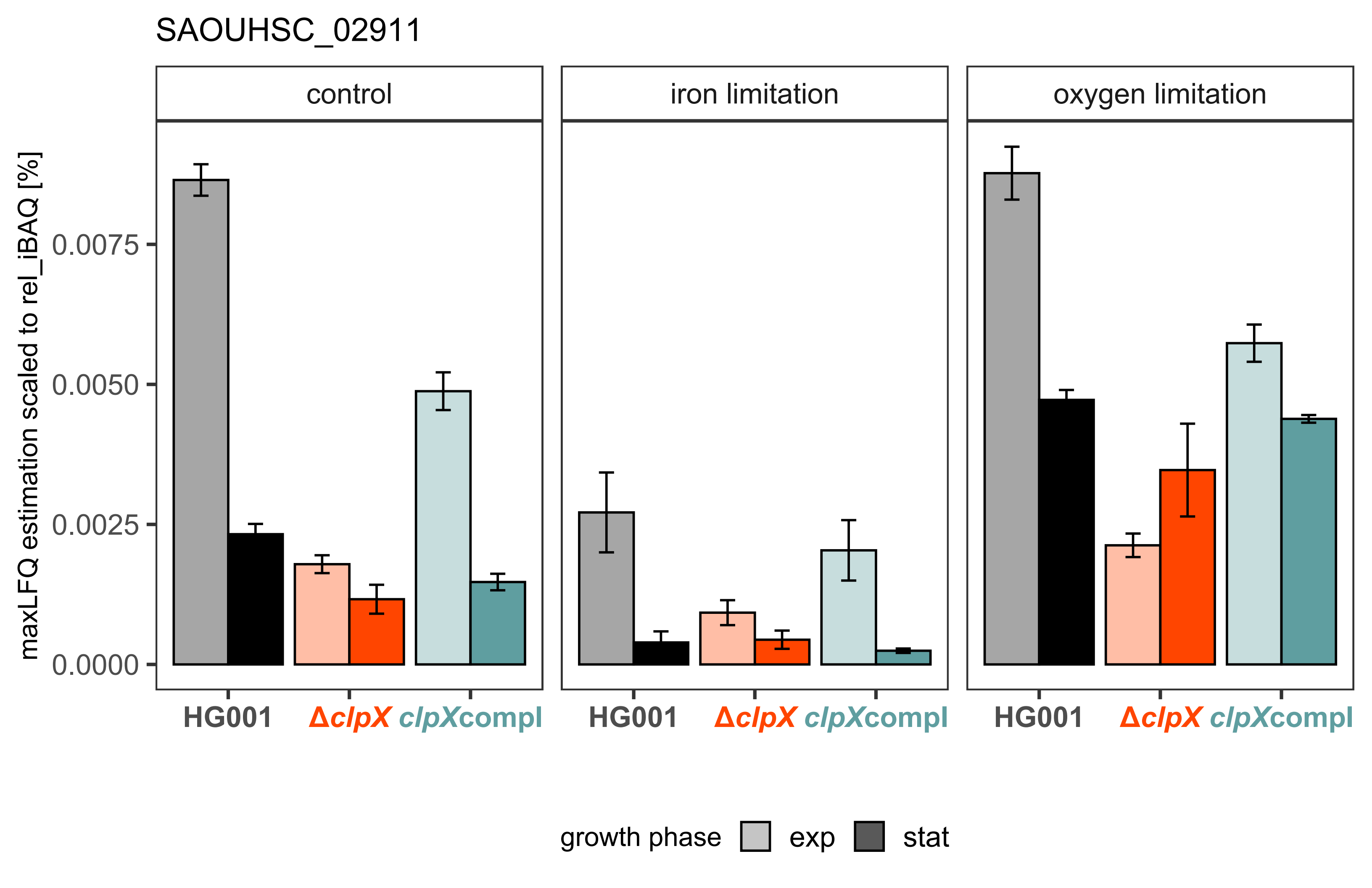
SAOUHSC_02911
control
iron limitation
oxygen limitation
maxLFQ estimation scaled to rel_iBAQ [%]
0.0075
0.0050
0.0025
0.0000
HG001
ΔclpX
clpXcompl
growth phase
exp
stat

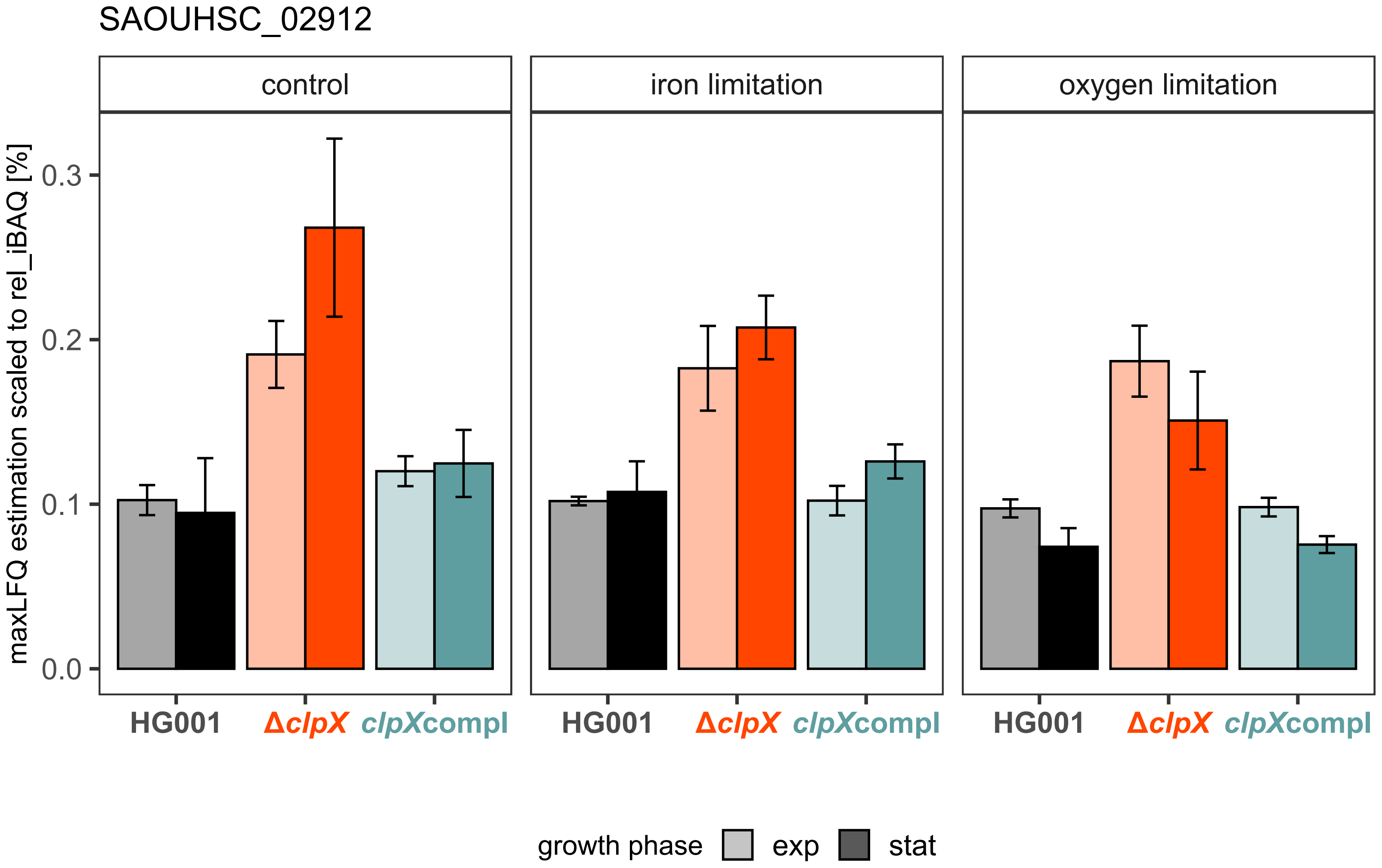
SAOUHSC_02912
control
iron limitation
oxygen limitation
maxLFQ estimation scaled to rel_iBAQ [%]
0.0
0.1
0.2
0.3
HG001
ΔclpX
clpXcompl
growth phase
exp
stat

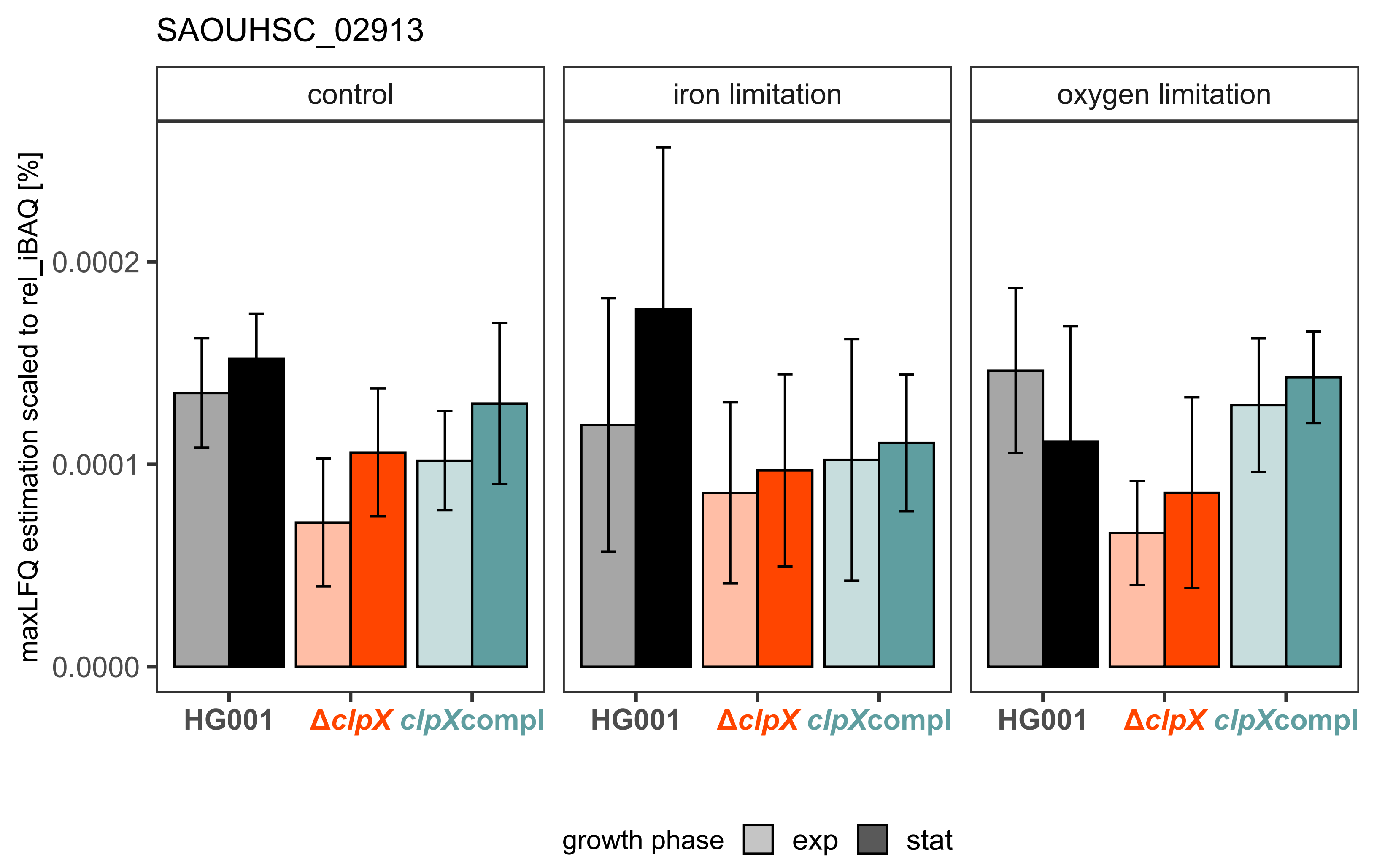
SAOUHSC_02913
control
iron limitation
oxygen limitation
maxLFQ estimation scaled to rel_iBAQ [%]
0.0002
0.0001
0.0000
HG001
ΔclpX
clpXcompl
growth phase
exp
stat

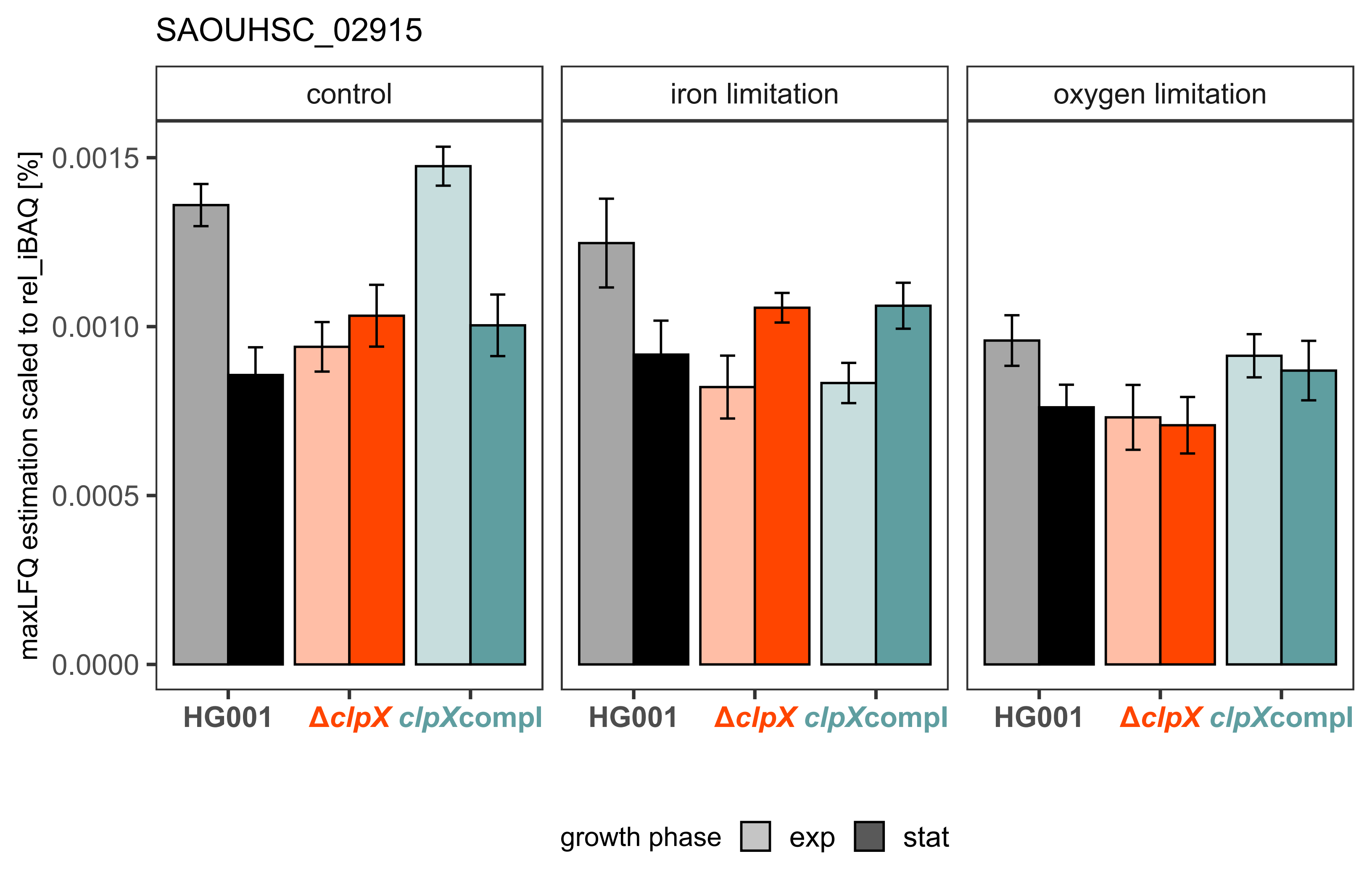
SAOUHSC_02915
control
iron limitation
oxygen limitation
maxLFQ estimation scaled to rel_iBAQ [%]
0.0015
0.0010
0.0005
0.0000
HG001
ΔclpX
clpXcompl
growth phase
exp
stat

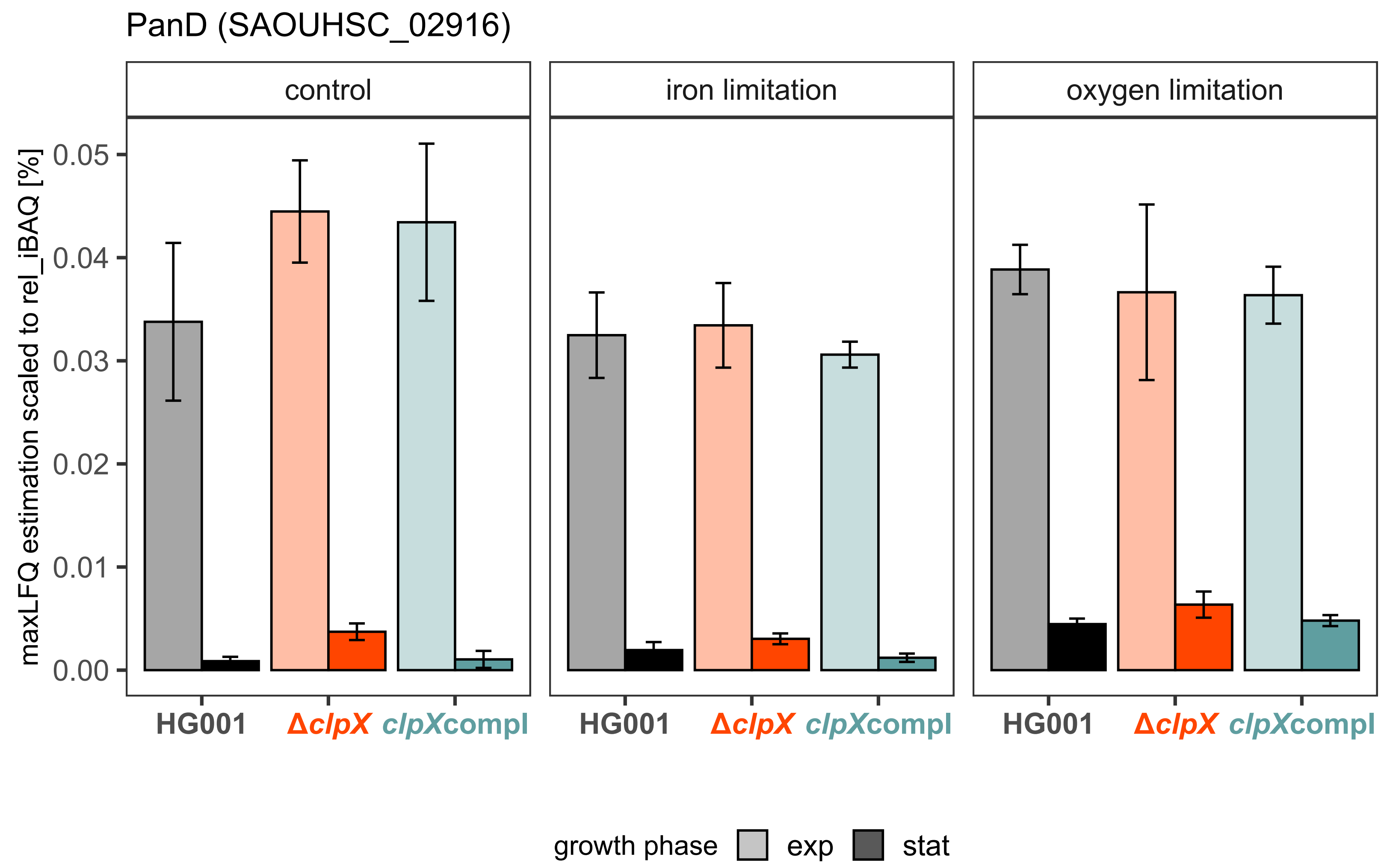
PanD (SAOUHSC_02916)
control
iron limitation
oxygen limitation
maxLFQ estimation scaled to rel_iBAQ [%]
0.05
0.04
0.03
0.02
0.01
0.00
HG001
ΔclpX
clpXcompl
growth phase
exp
stat

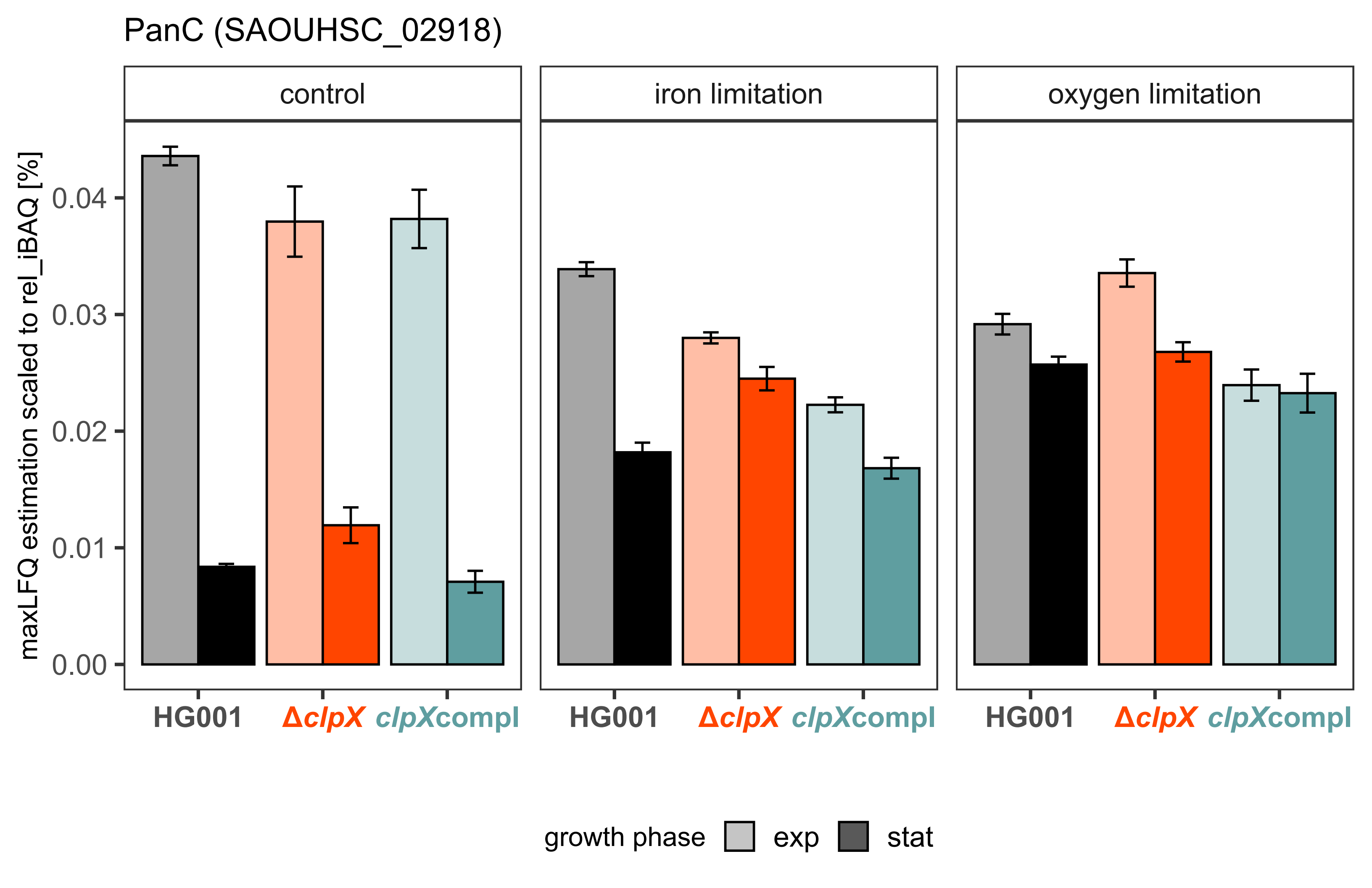

PanC (SAOUHSC_02918)
control
iron limitation
oxygen limitation
maxLFQ estimation scaled to rel_iBAQ [%]
0.00
0.01
0.02
0.03
0.04
HG001
ΔclpX
clpXcompl
growth phase
exp
stat

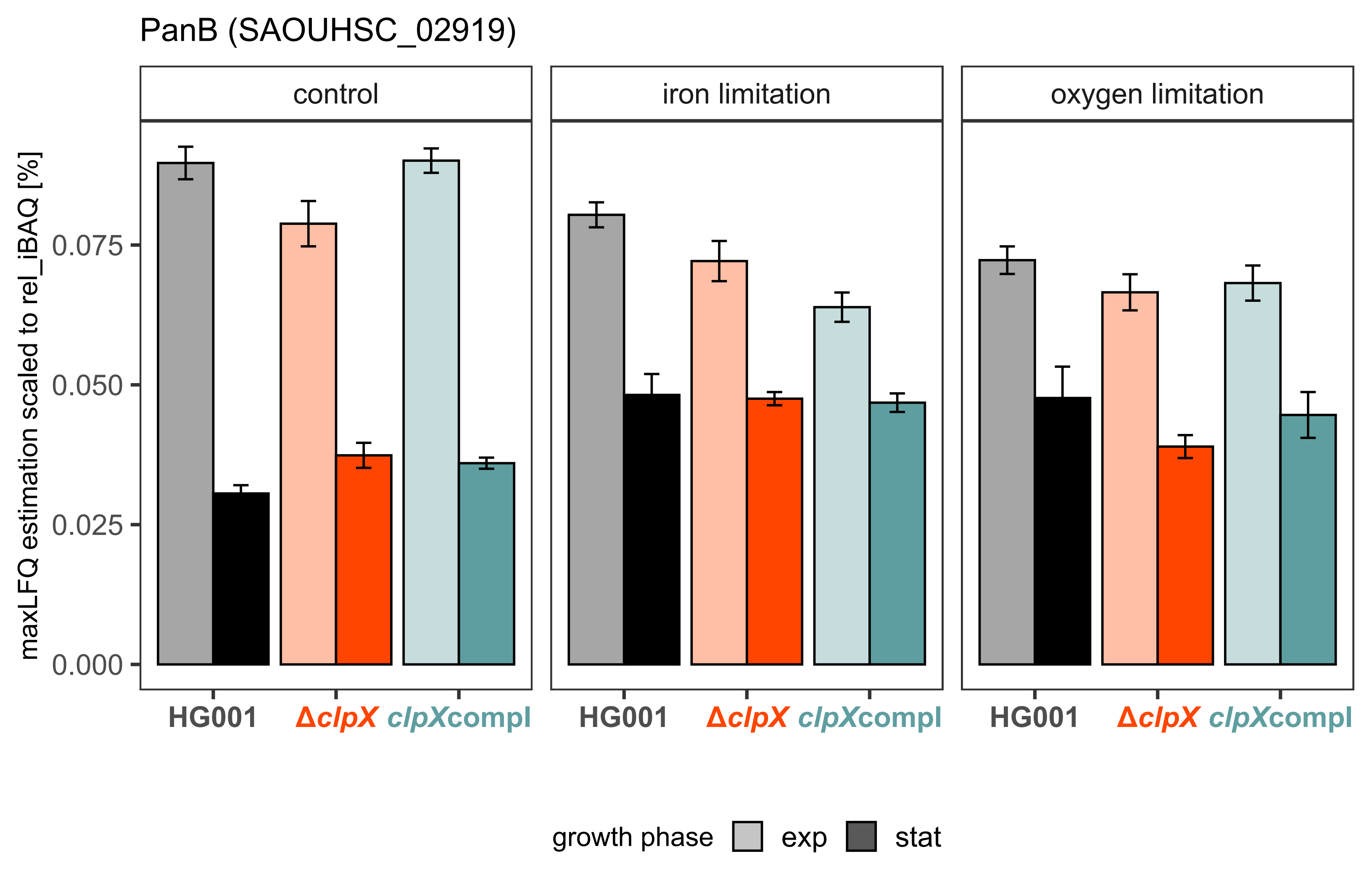
PanB (SAOUHSC_02919)
control
iron limitation
oxygen limitation
maxLFQ estimation scaled to rel_iBAQ [%]
0.075
0.050
0.025
0.000
HG001
ΔclpX
clpXcompl
growth phase
exp
stat

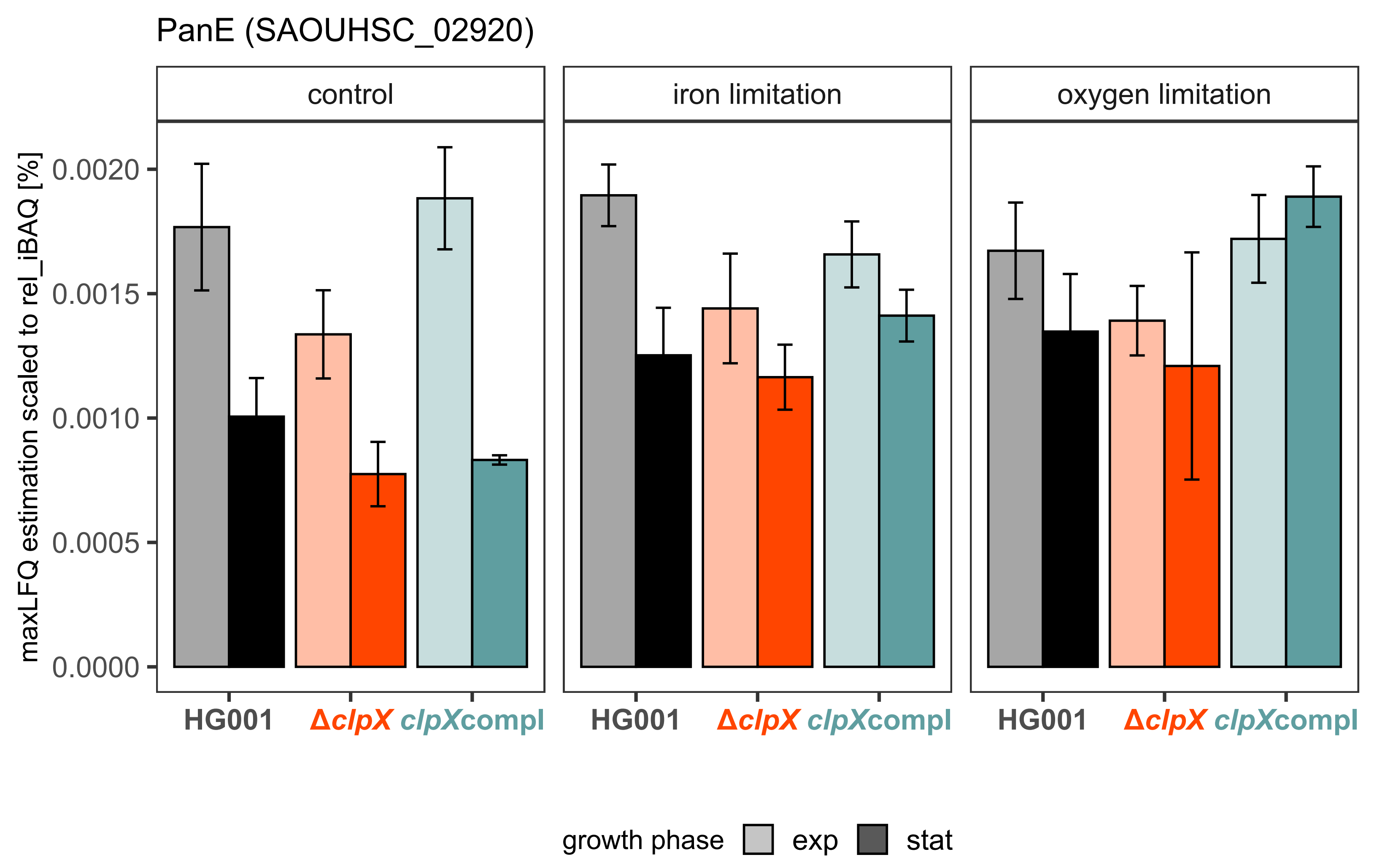

PanE (SAOUHSC_02920)
control
iron limitation
oxygen limitation
maxLFQ estimation scaled to rel_iBAQ [%]
0.0020
0.0015
0.0010
0.0005
0.0000
HG001
ΔclpX
clpXcompl
growth phase
exp
stat

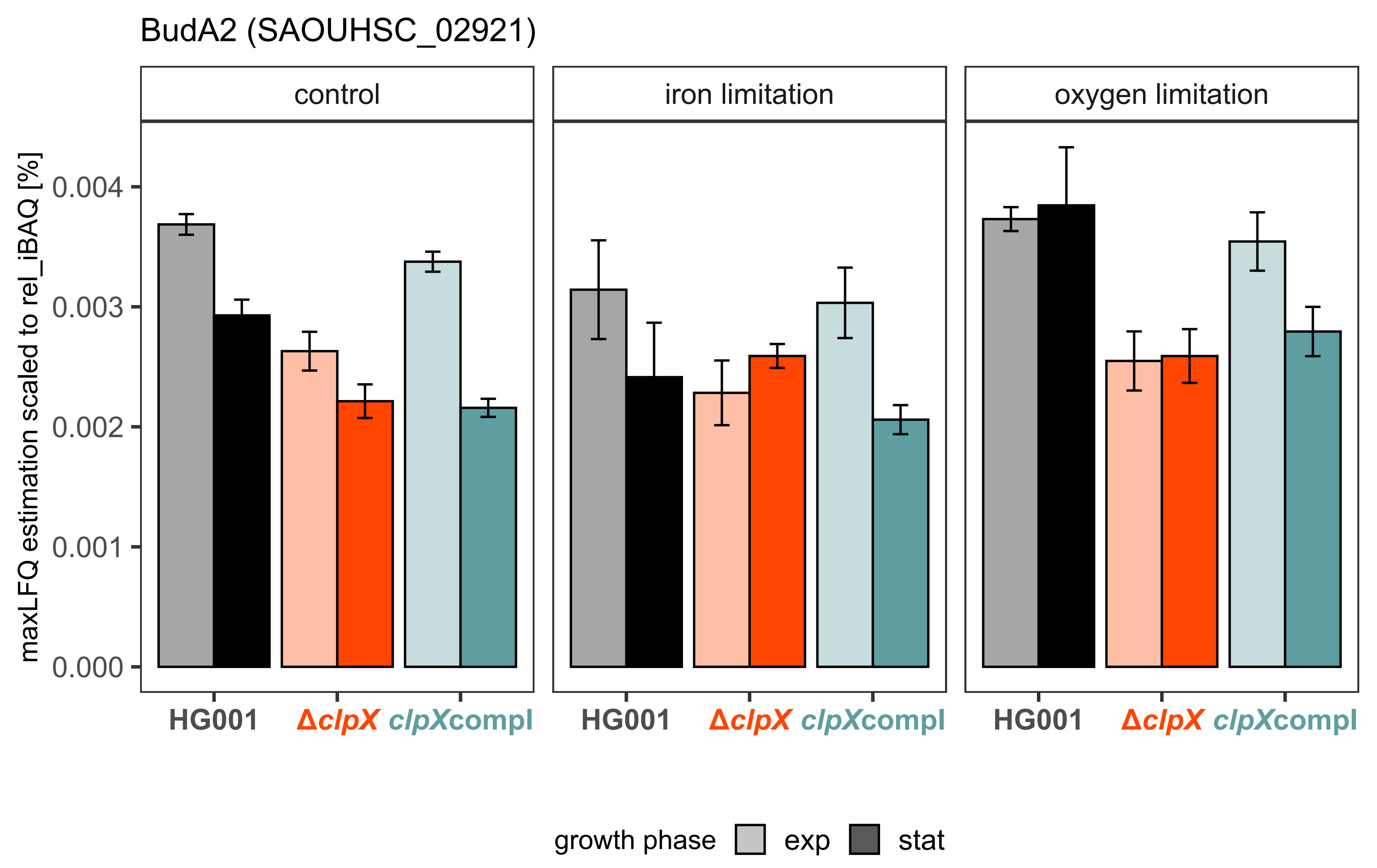

BudA2 (SAOUHSC_02921)
control
iron limitation
oxygen limitation
maxLFQ estimation scaled to rel_iBAQ [%]
0.004
0.003
0.002
0.001
0.000
HG001
ΔclpX
clpXcompl
growth phase
exp
stat

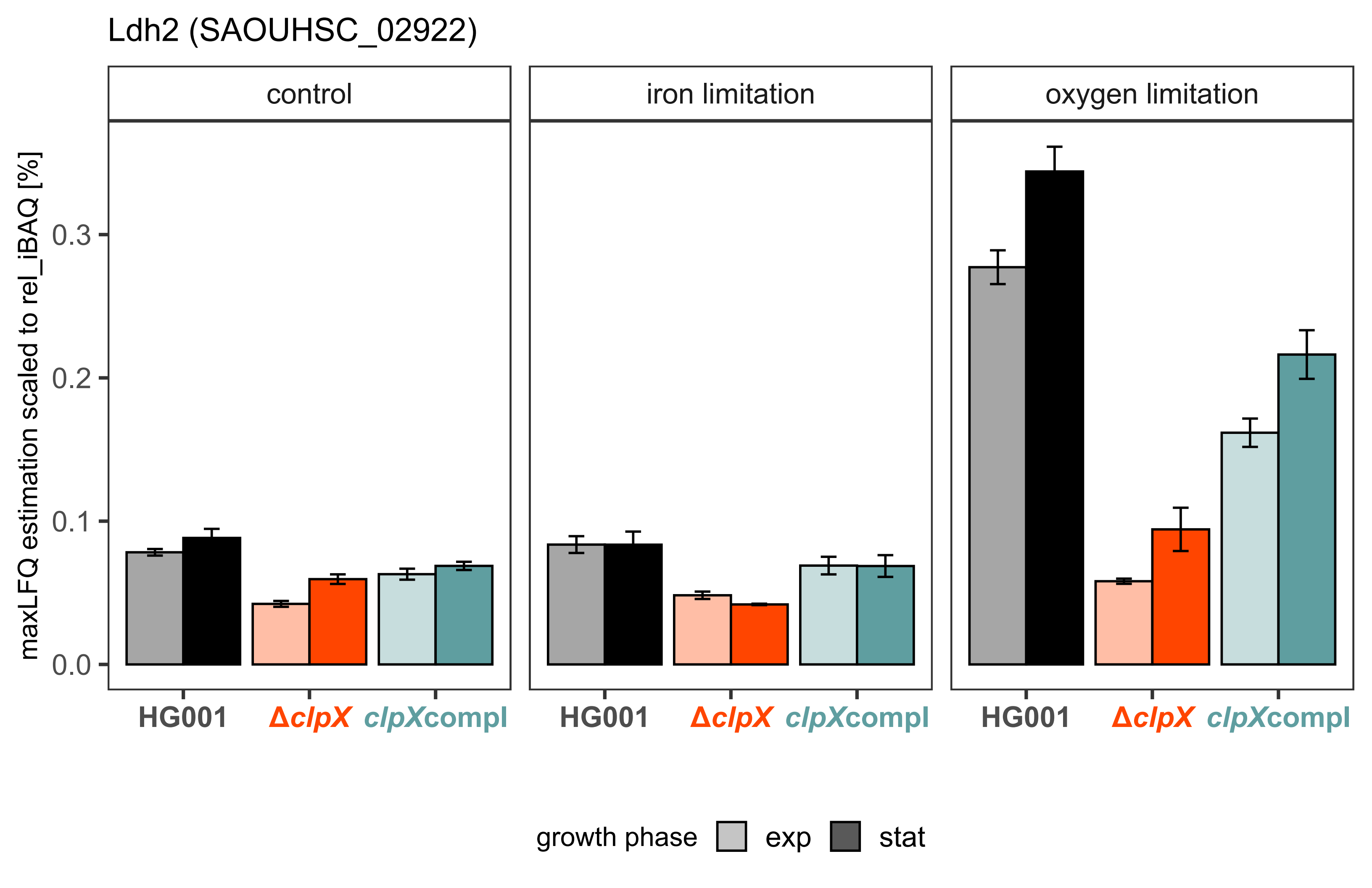

Ldh2 (SAOUHSC_02922)
control
iron limitation
oxygen limitation
maxLFQ estimation scaled to rel_iBAQ [%]
0.0
0.1
0.2
0.3
HG001
ΔclpX
clpXcompl
growth phase
exp
stat

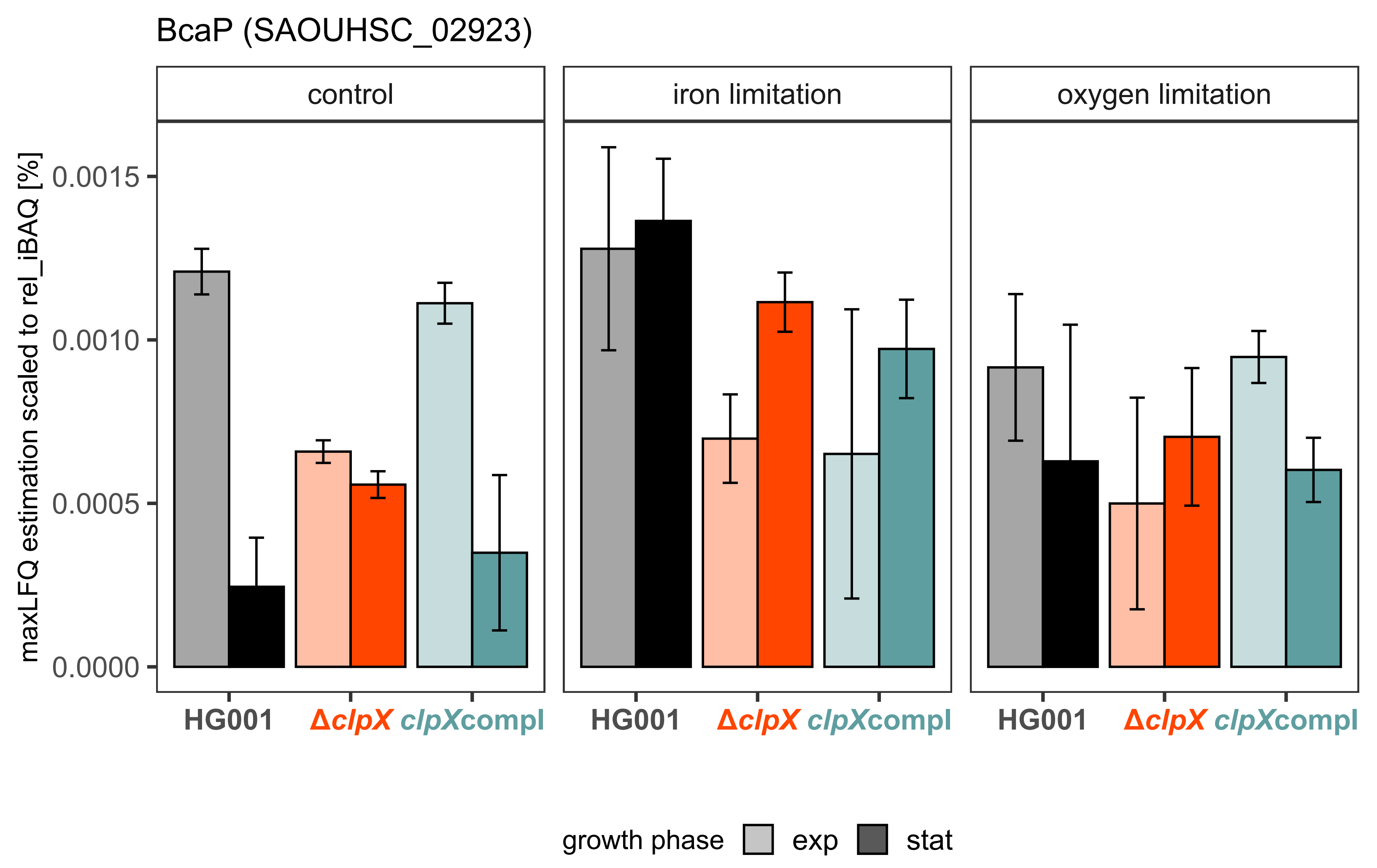
BcaP (SAOUHSC_02923)
control
iron limitation
oxygen limitation
maxLFQ estimation scaled to rel_iBAQ [%]
0.0015
0.0010
0.0005
0.0000
HG001
ΔclpX
clpXcompl
growth phase
exp
stat

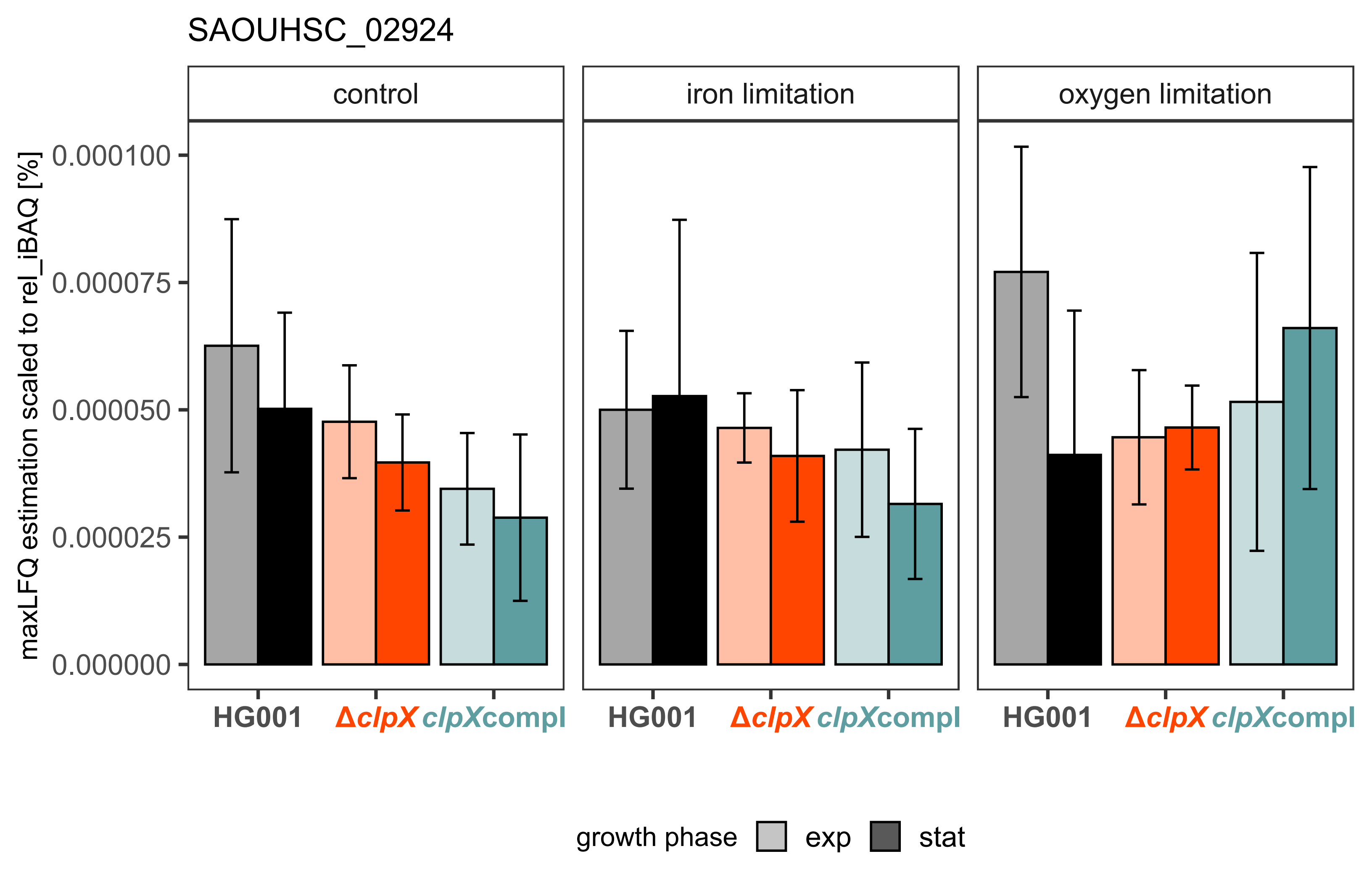
SAOUHSC_02924
control
iron limitation
oxygen limitation
maxLFQ estimation scaled to rel_iBAQ [%]
0.000100
0.000075
0.000050
0.000025
0.000000
HG001
ΔclpX
clpXcompl
growth phase
exp
stat

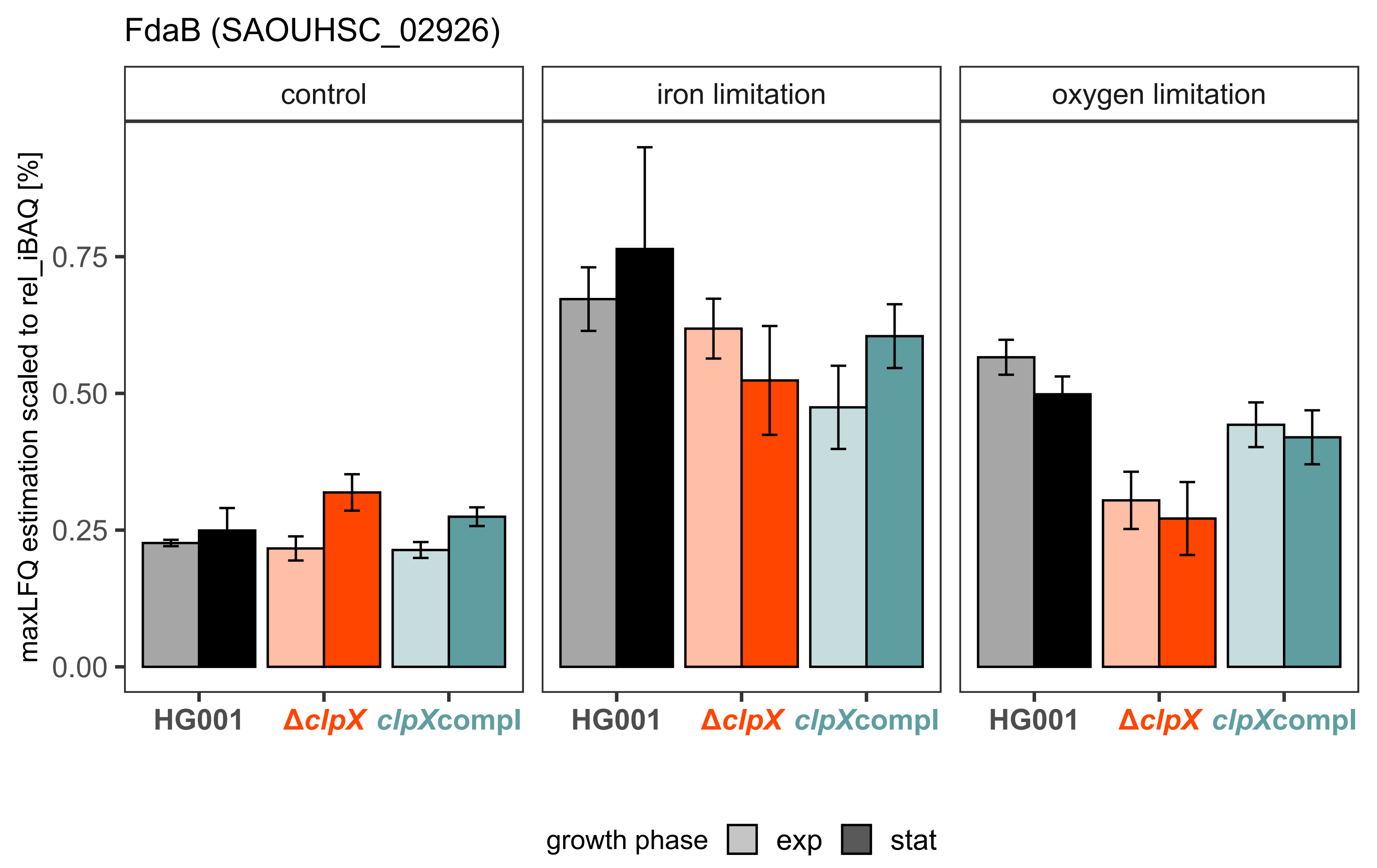

FdaB (SAOUHSC_02926)
control
iron limitation
oxygen limitation
maxLFQ estimation scaled to rel_iBAQ [%]
0.00
0.25
0.50
0.75
HG001
ΔclpX
clpXcompl
growth phase
exp
stat

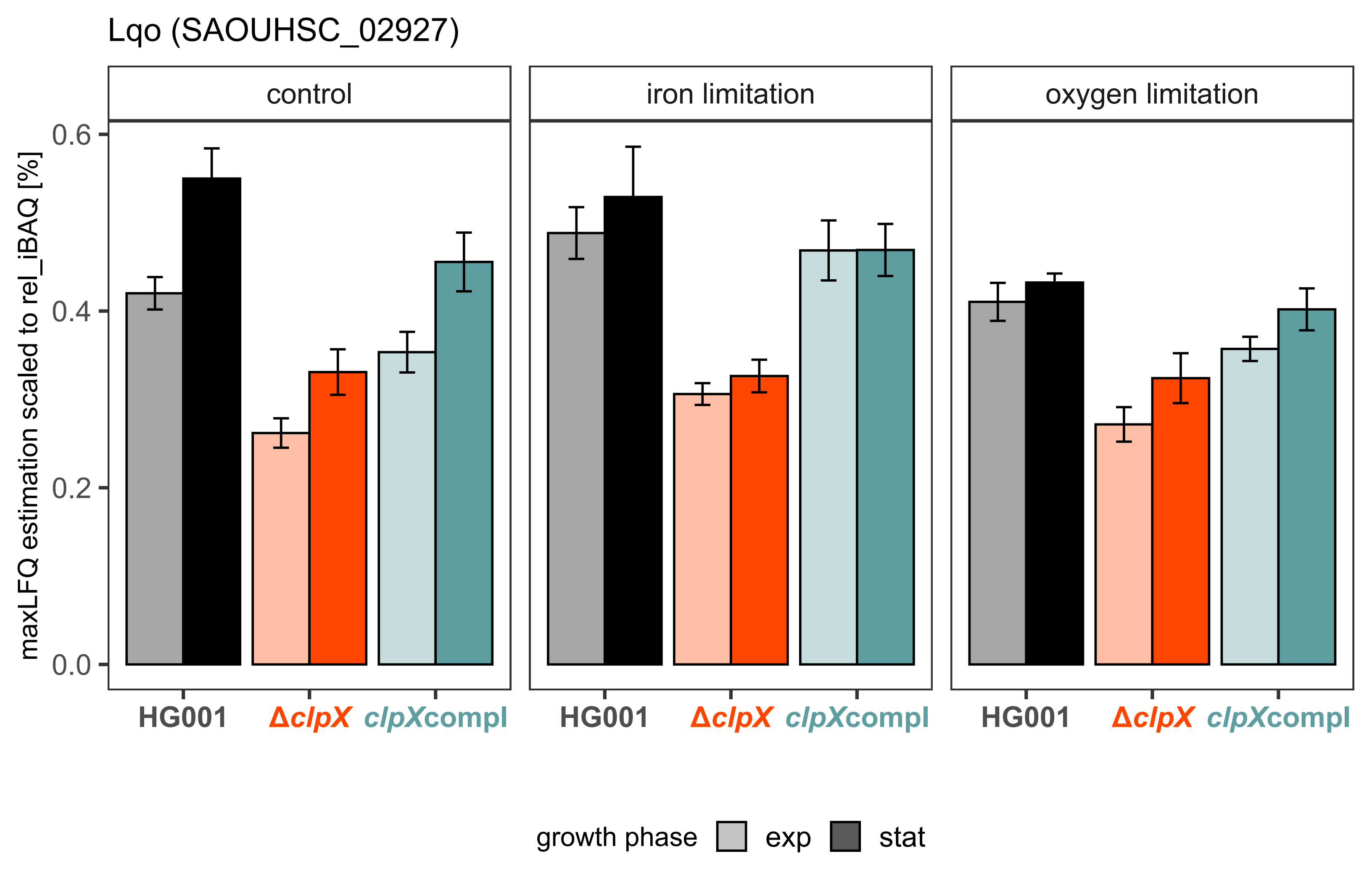

Lqo (SAOUHSC_02927)
control
iron limitation
oxygen limitation
maxLFQ estimation scaled to rel_iBAQ [%]
0.0
0.2
0.4
0.6
HG001
ΔclpX
clpXcompl
growth phase
exp
stat

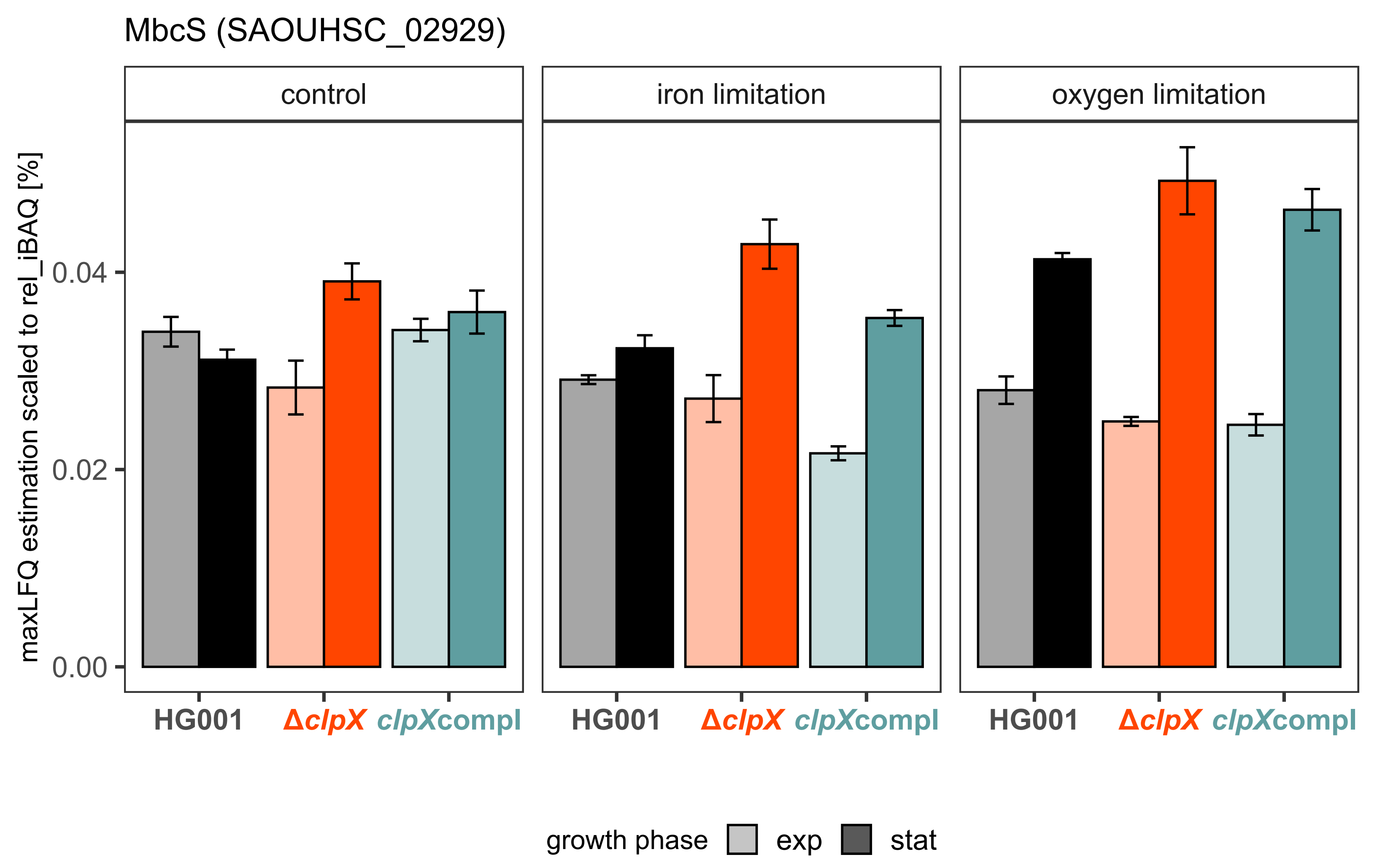

MbcS (SAOUHSC_02929)
control
iron limitation
oxygen limitation
maxLFQ estimation scaled to rel_iBAQ [%]
0.00
0.02
0.04
HG001
ΔclpX
clpXcompl
growth phase
exp
stat

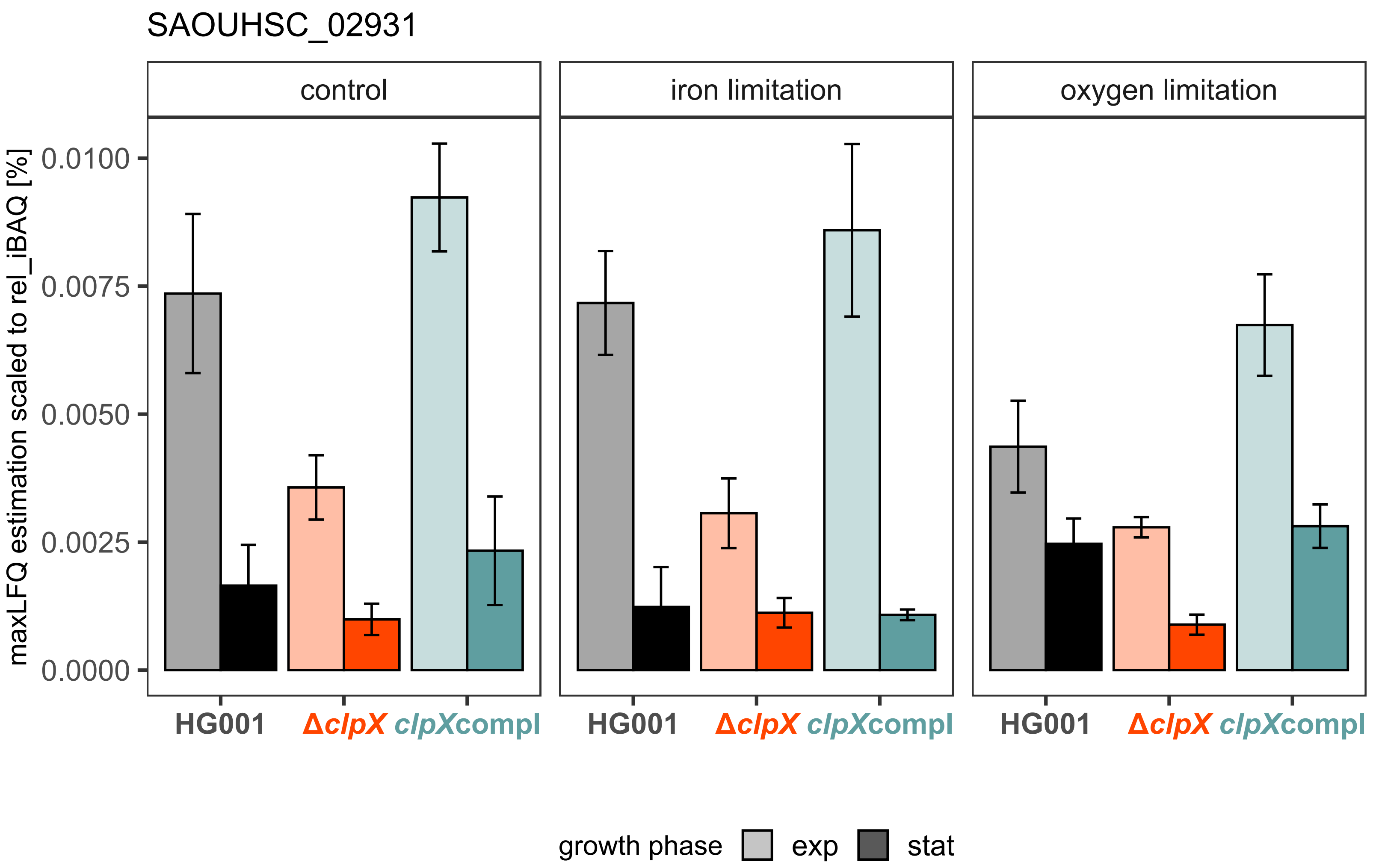

SAOUHSC_02931
control
iron limitation
oxygen limitation
maxLFQ estimation scaled to rel_iBAQ [%]
0.0100
0.0075
0.0050
0.0025
0.0000
HG001
ΔclpX
clpXcompl
growth phase
exp
stat

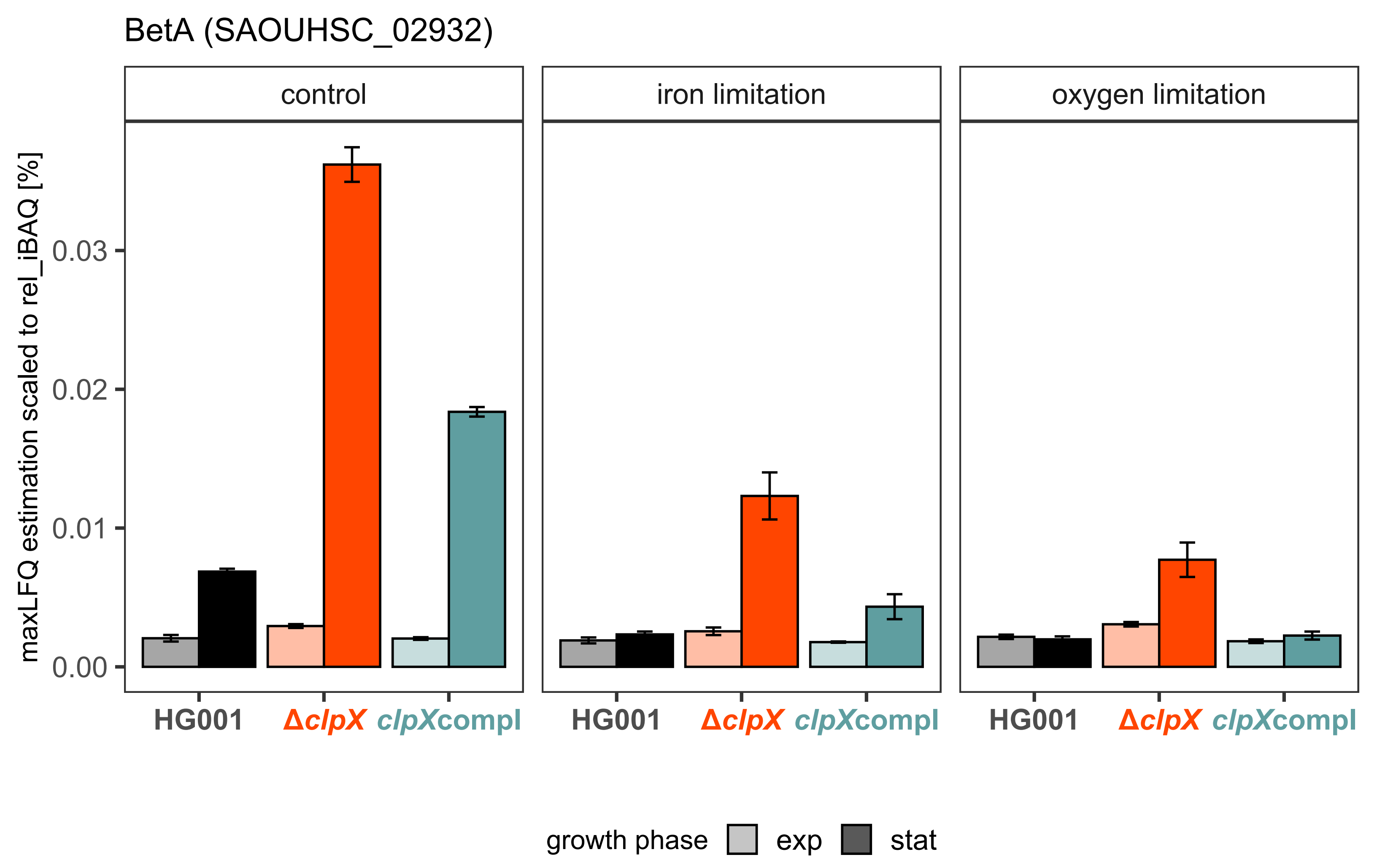

BetA (SAOUHSC_02932)
control
iron limitation
oxygen limitation
maxLFQ estimation scaled to rel_iBAQ [%]
0.03
0.02
0.01
0.00
HG001
ΔclpX
clpXcompl
growth phase
exp
stat

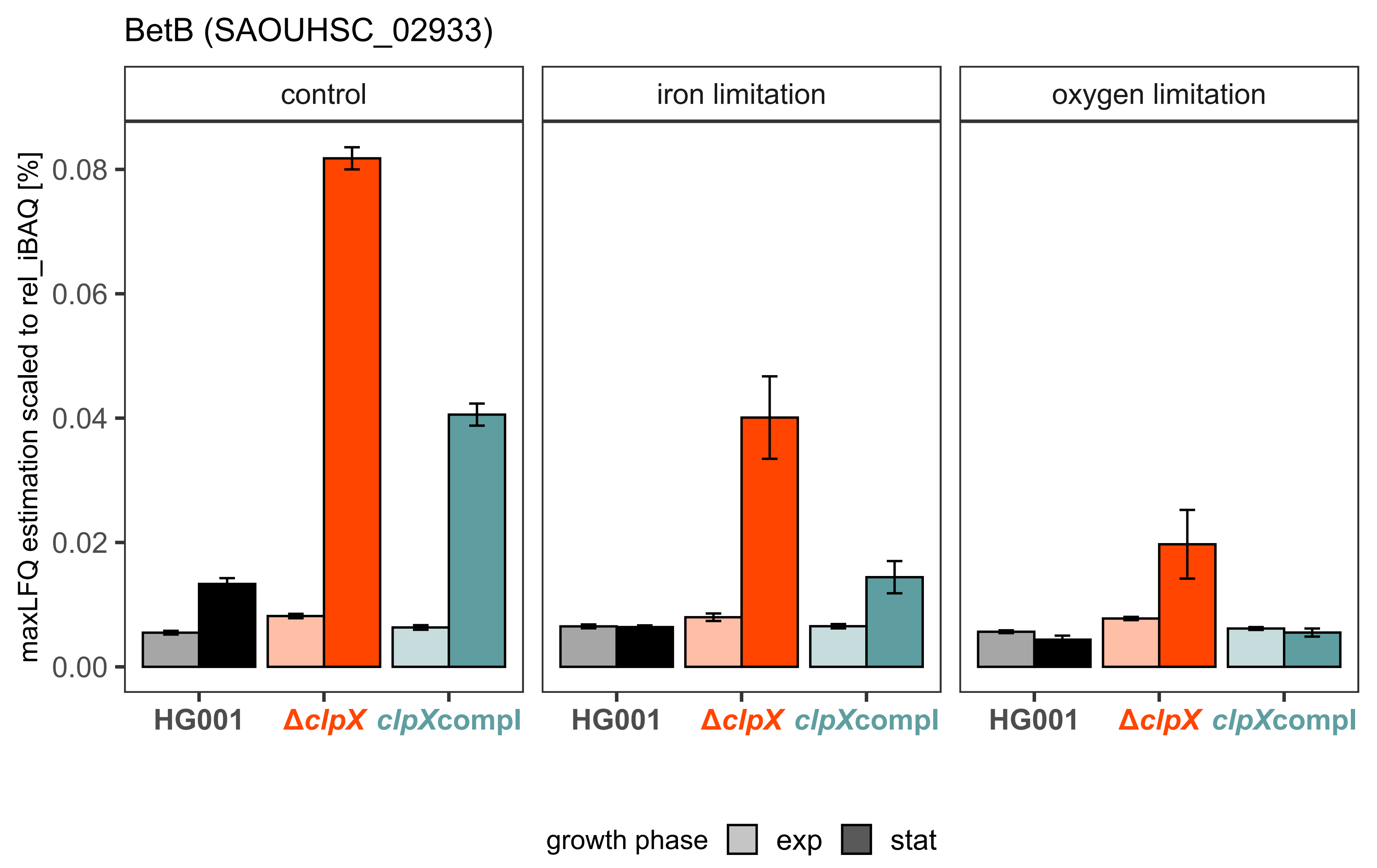

BetB (SAOUHSC_02933)
control
iron limitation
oxygen limitation
maxLFQ estimation scaled to rel_iBAQ [%]
0.08
0.06
0.04
0.02
0.00
HG001
ΔclpX
clpXcompl
growth phase
exp
stat

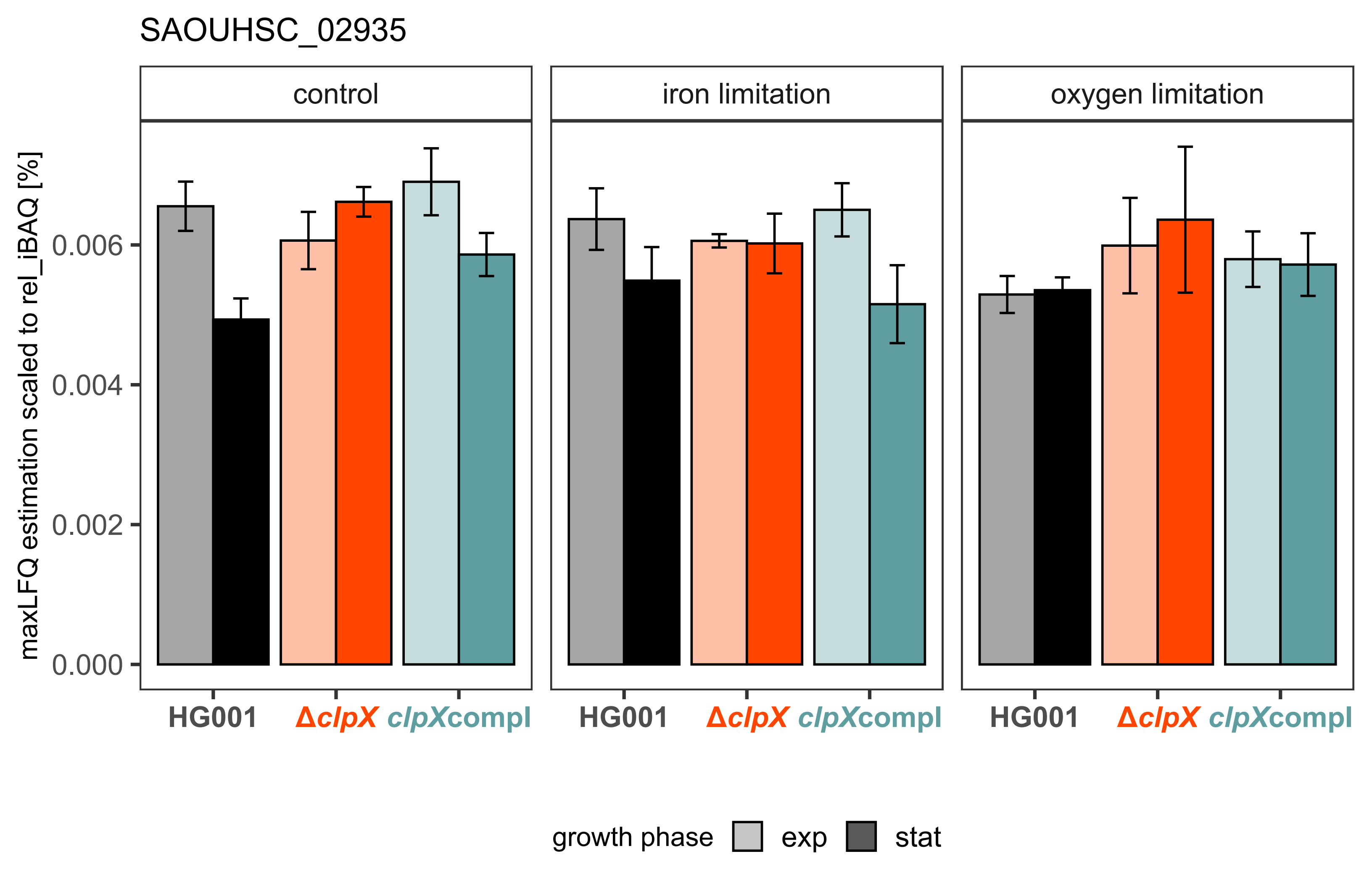
SAOUHSC_02935
control
iron limitation
oxygen limitation
maxLFQ estimation scaled to rel_iBAQ [%]
0.000
0.002
0.004
0.006
HG001
ΔclpX
clpXcompl
growth phase
exp
stat

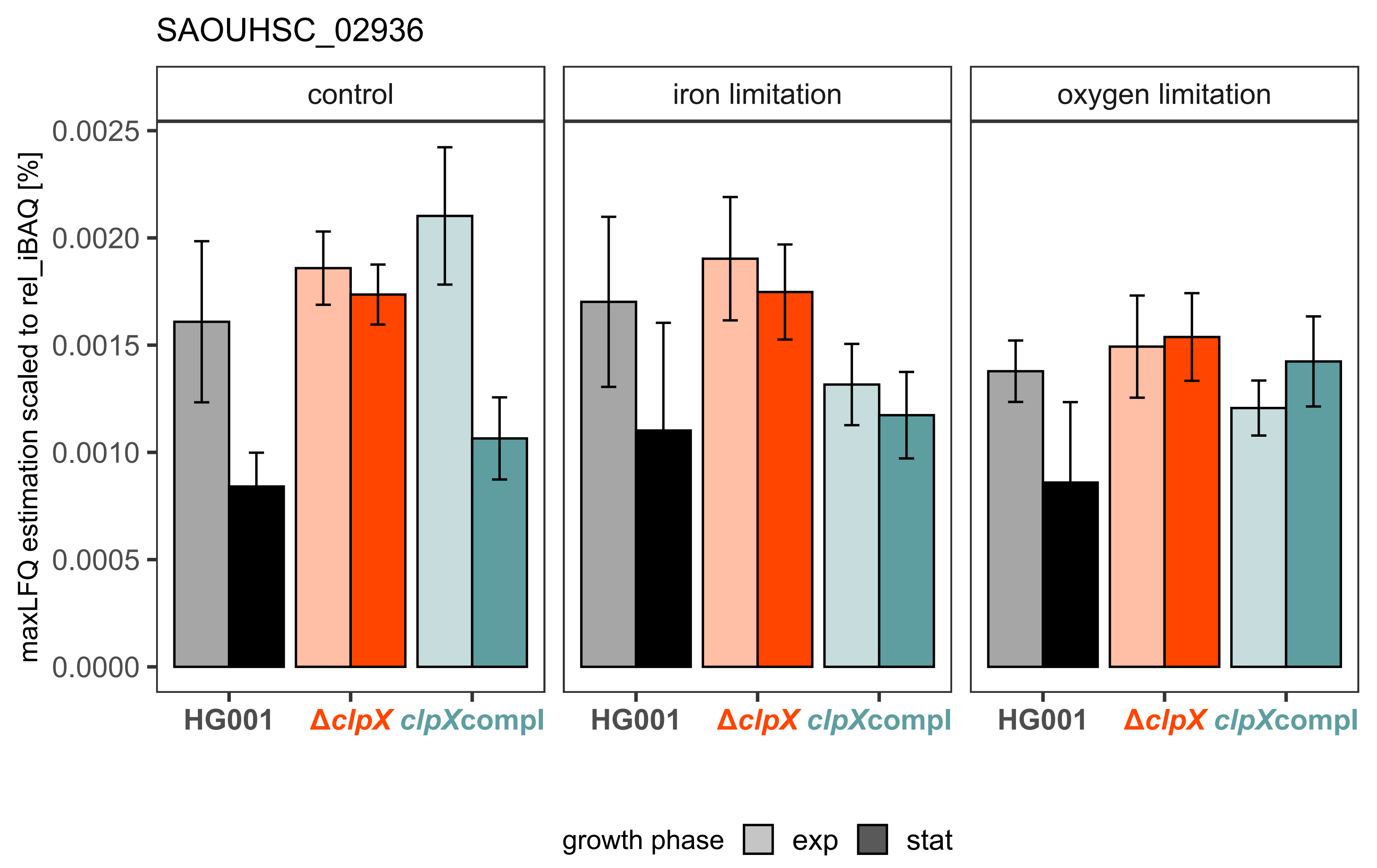
SAOUHSC_02936
control
iron limitation
oxygen limitation
maxLFQ estimation scaled to rel_iBAQ [%]
0.0025
0.0020
0.0015
0.0010
0.0005
0.0000
HG001
ΔclpX
clpXcompl
growth phase
exp
stat

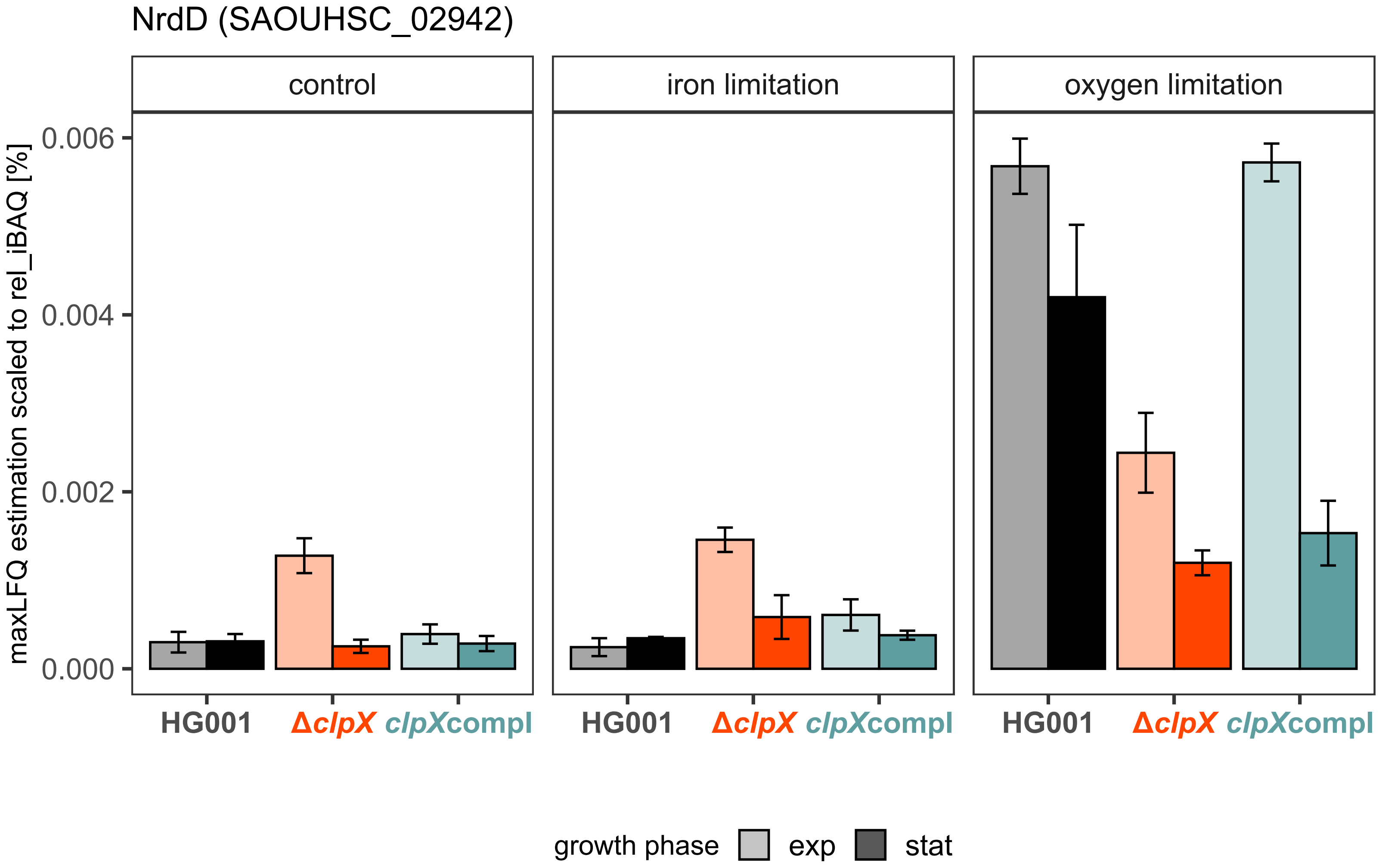

NrdD (SAOUHSC_02942)
control
iron limitation
oxygen limitation
maxLFQ estimation scaled to rel_iBAQ [%]
0.006
0.004
0.002
0.000
HG001
ΔclpX
clpXcompl
growth phase
exp
stat

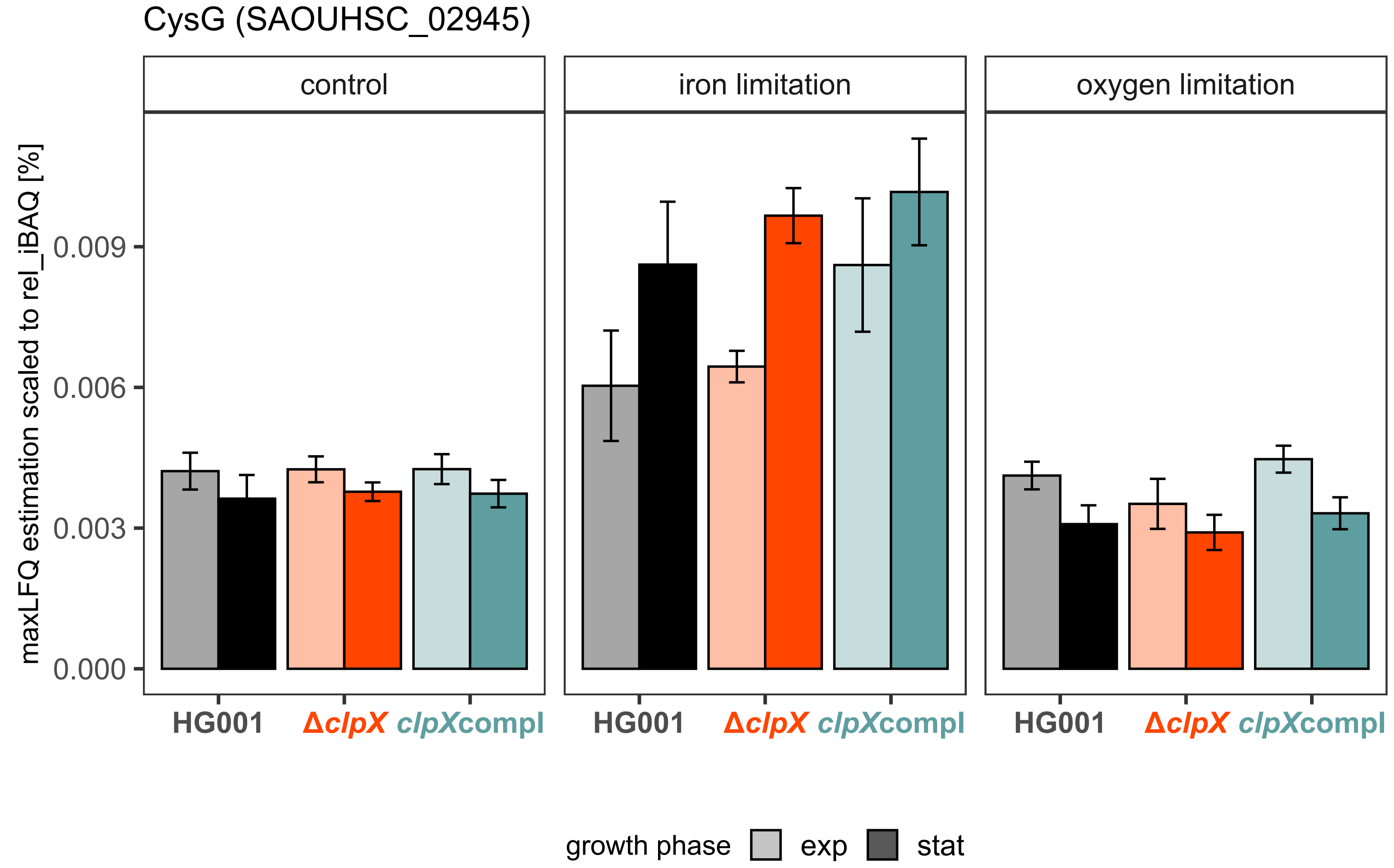
CysG (SAOUHSC_02945)
control
iron limitation
oxygen limitation
maxLFQ estimation scaled to rel_iBAQ [%]
0.009
0.006
0.003
0.000
HG001
ΔclpX
clpXcompl
growth phase
exp
stat

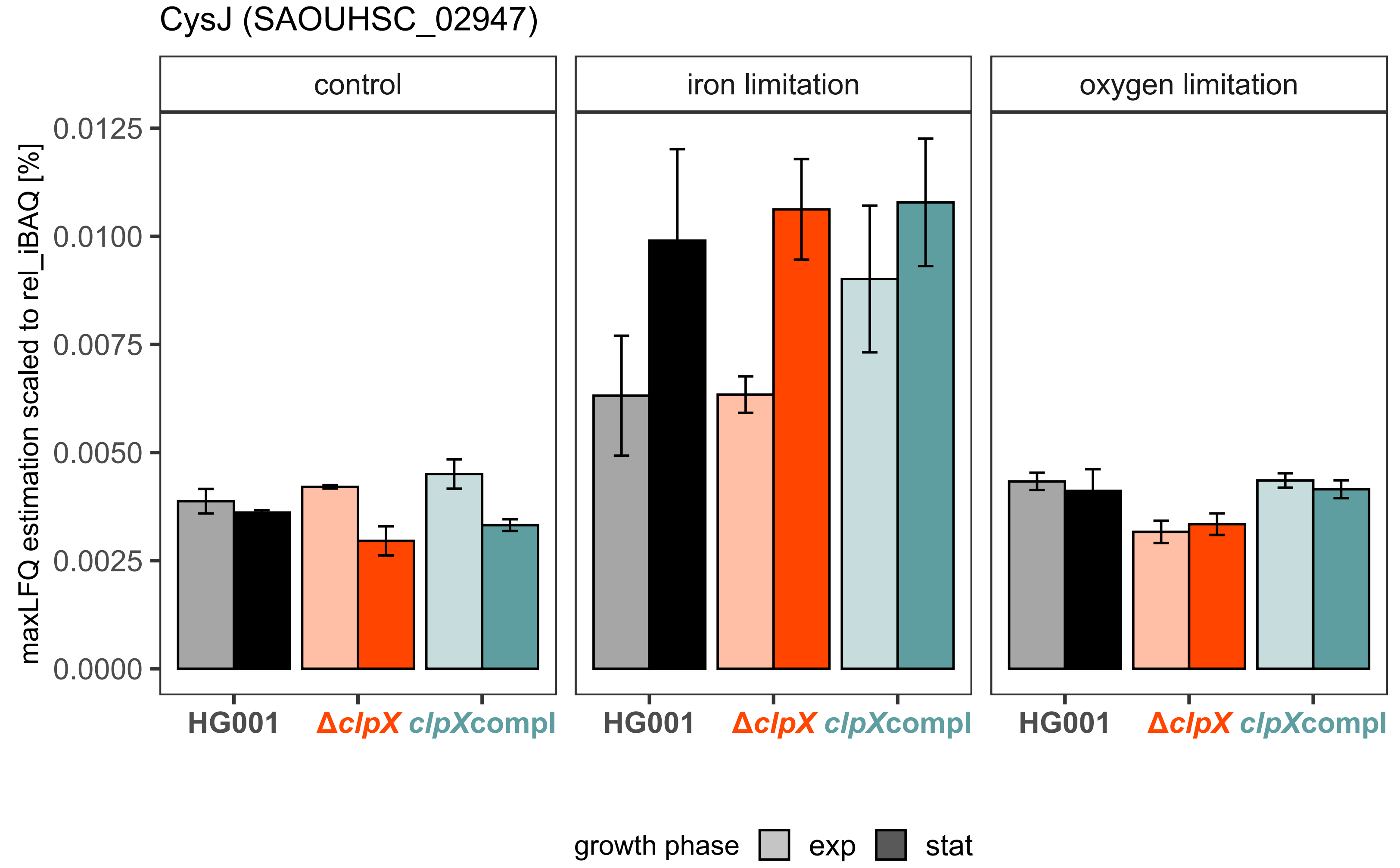

CysJ (SAOUHSC_02947)
control
iron limitation
oxygen limitation
maxLFQ estimation scaled to rel_iBAQ [%]
0.0125
0.0100
0.0075
0.0050
0.0025
0.0000
HG001
ΔclpX
clpXcompl
growth phase
exp
stat

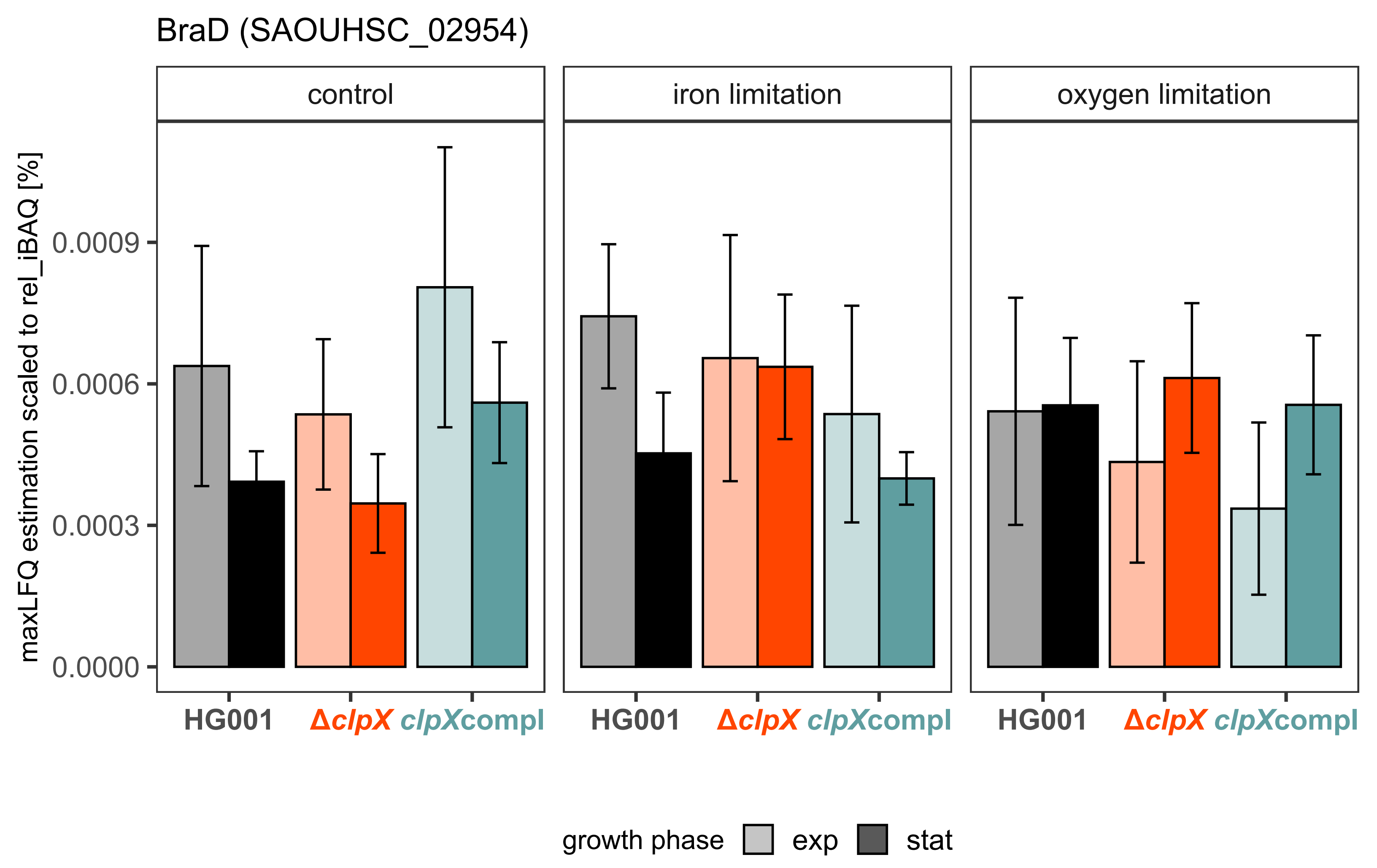
BraD (SAOUHSC_02954)
control
iron limitation
oxygen limitation
maxLFQ estimation scaled to rel_iBAQ [%]
0.0009
0.0006
0.0003
0.0000
HG001
ΔclpX
clpXcompl
growth phase
exp
stat

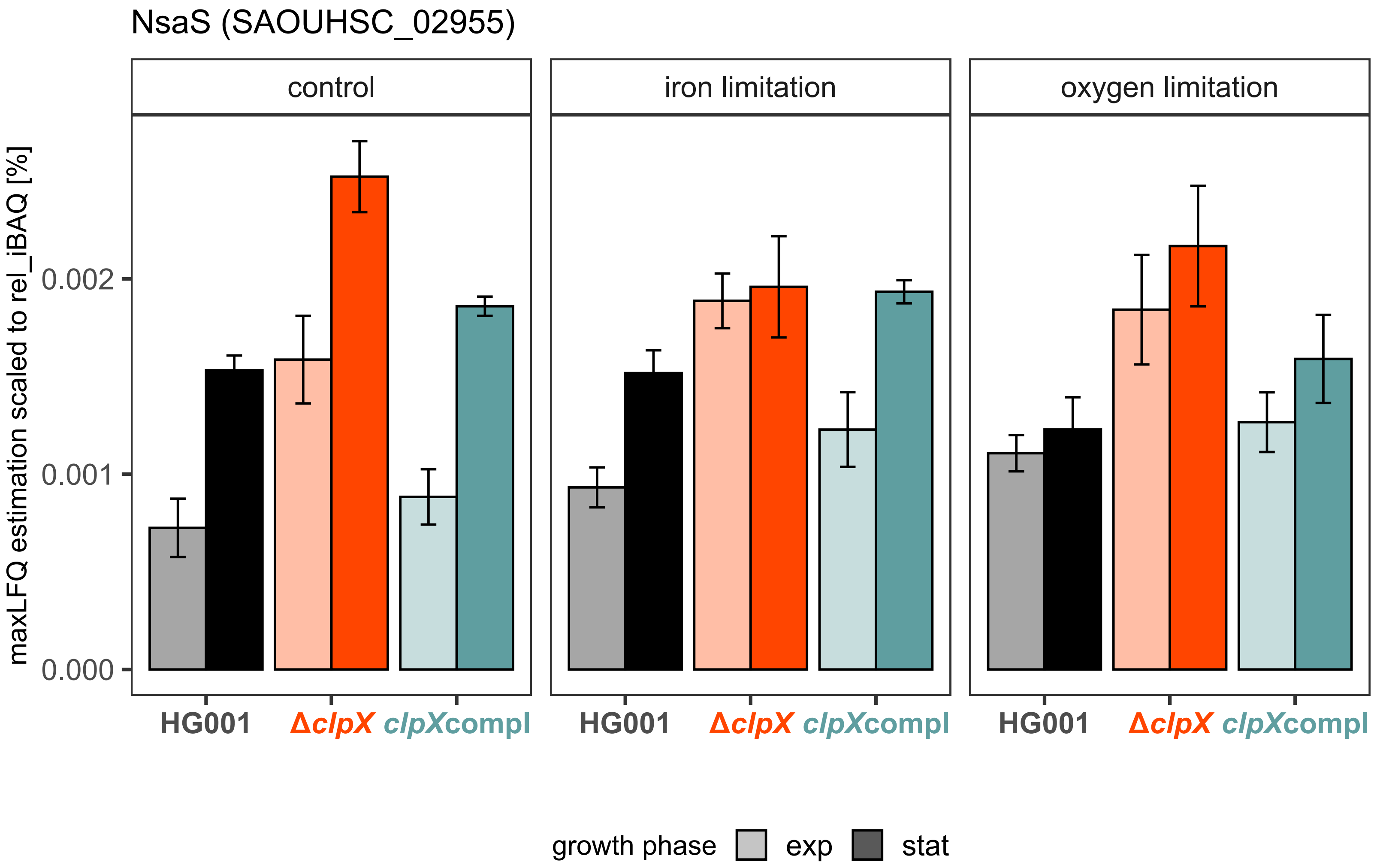
NsaS (SAOUHSC_02955)
control
iron limitation
oxygen limitation
maxLFQ estimation scaled to rel_iBAQ [%]
0.000
0.001
0.002
HG001
ΔclpX
clpXcompl
growth phase
exp
stat

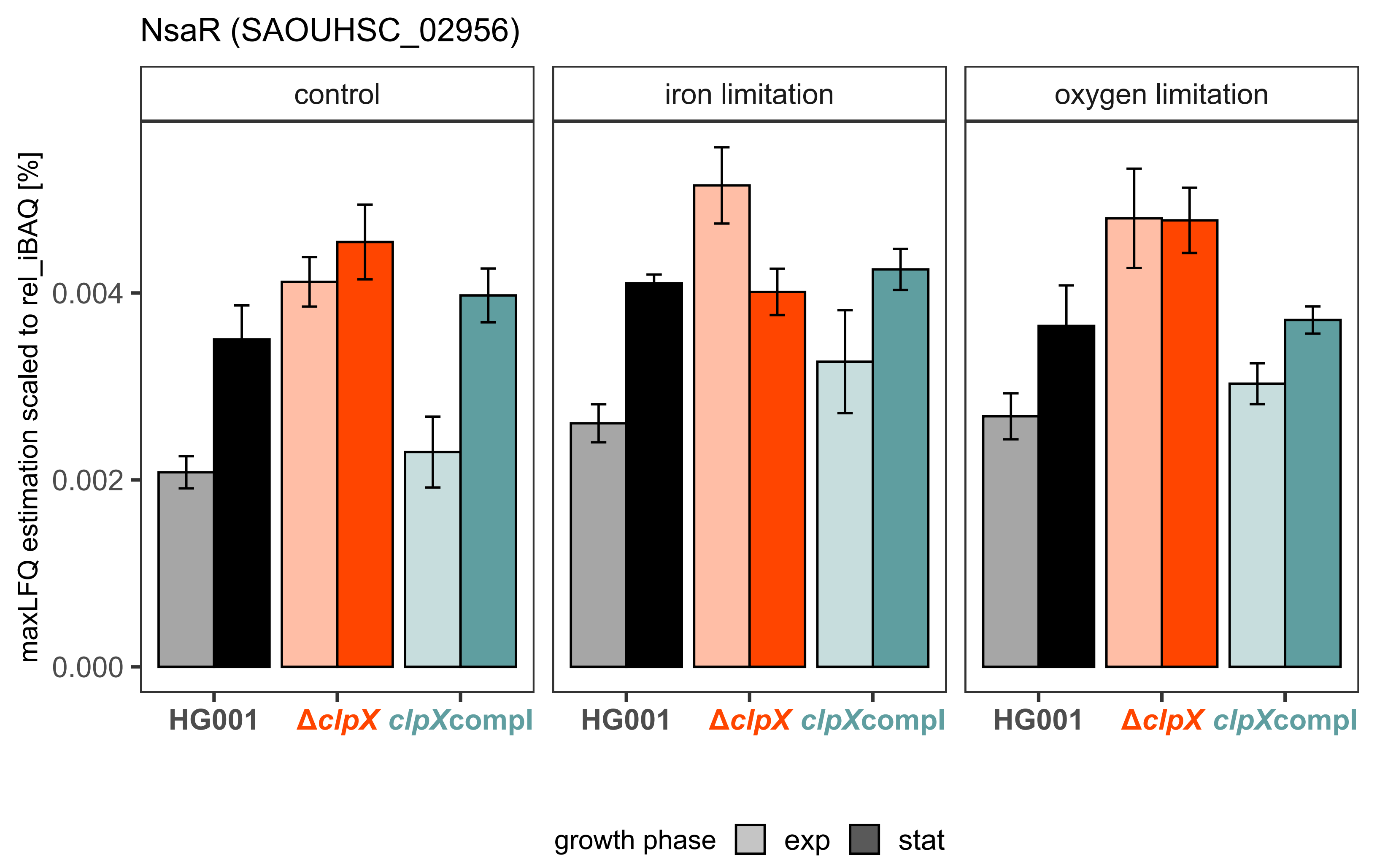

NsaR (SAOUHSC_02956)
control
iron limitation
oxygen limitation
maxLFQ estimation scaled to rel_iBAQ [%]
0.004
0.002
0.000
HG001
ΔclpX
clpXcompl
growth phase
exp
stat

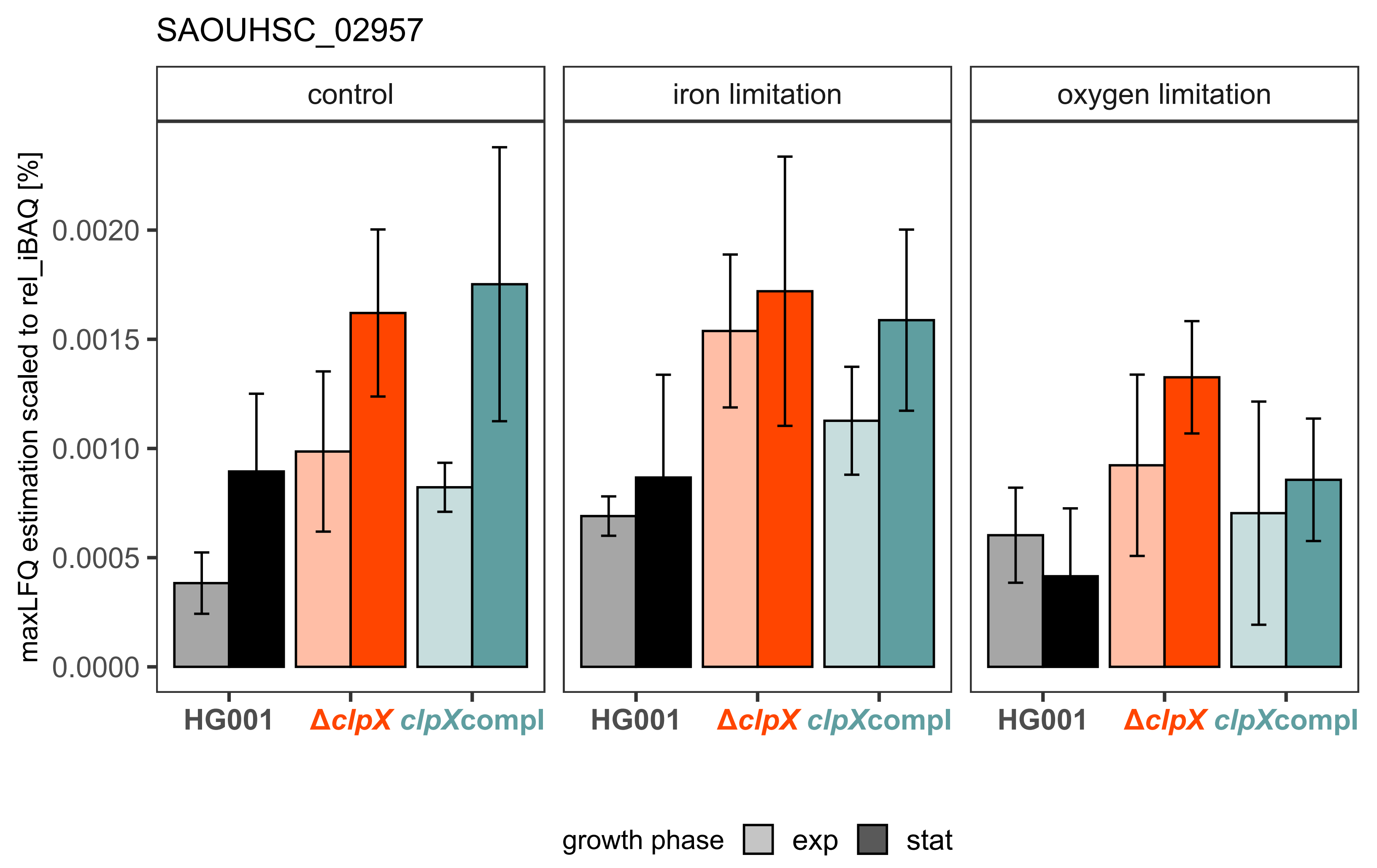
SAOUHSC_02957
control
iron limitation
oxygen limitation
maxLFQ estimation scaled to rel_iBAQ [%]
0.0020
0.0015
0.0010
0.0005
0.0000
HG001
ΔclpX
clpXcompl
growth phase
exp
stat

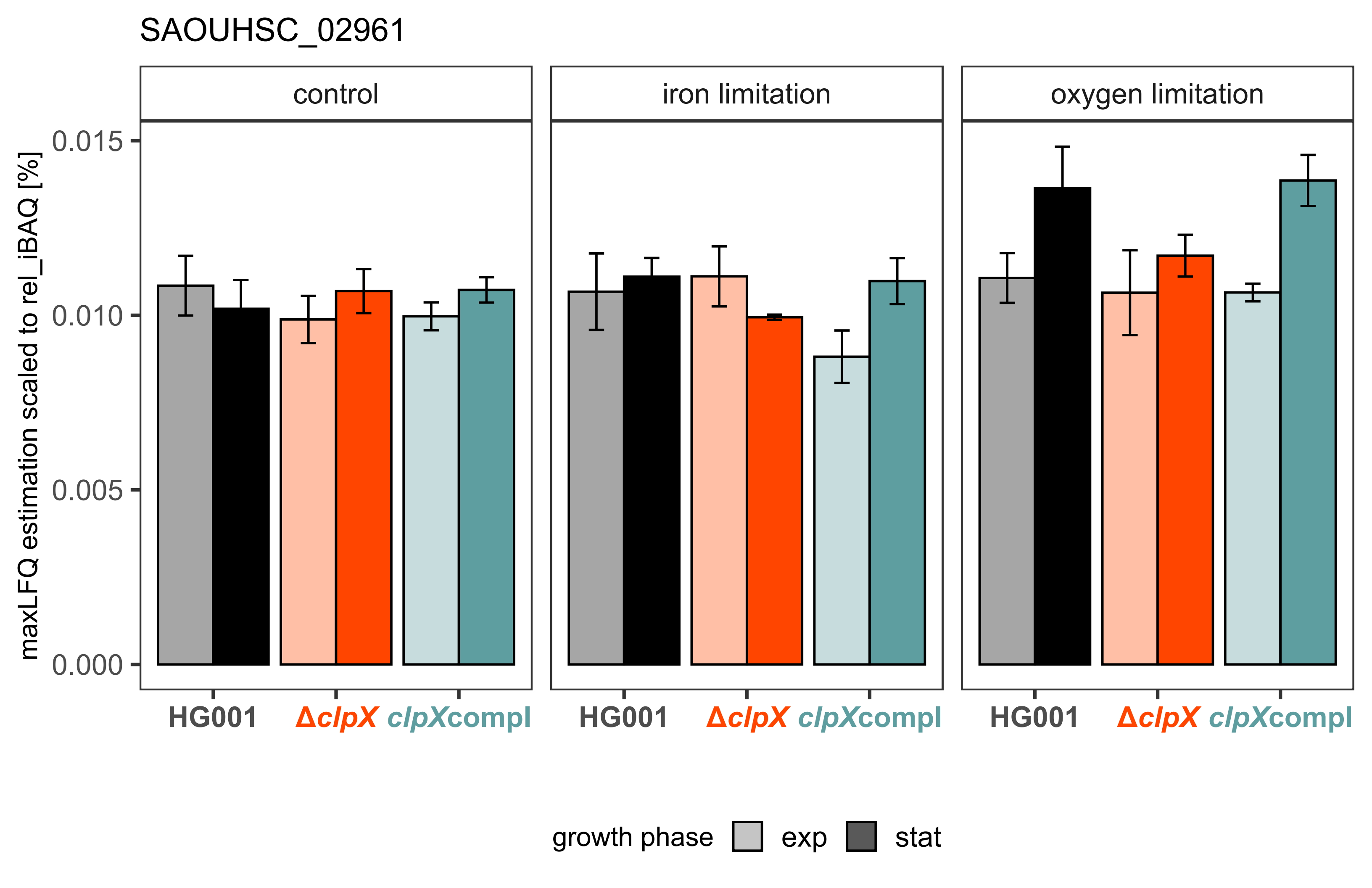
SAOUHSC_02961
control
iron limitation
oxygen limitation
maxLFQ estimation scaled to rel_iBAQ [%]
0.015
0.010
0.005
0.000
HG001
ΔclpX
clpXcompl
growth phase
exp
stat

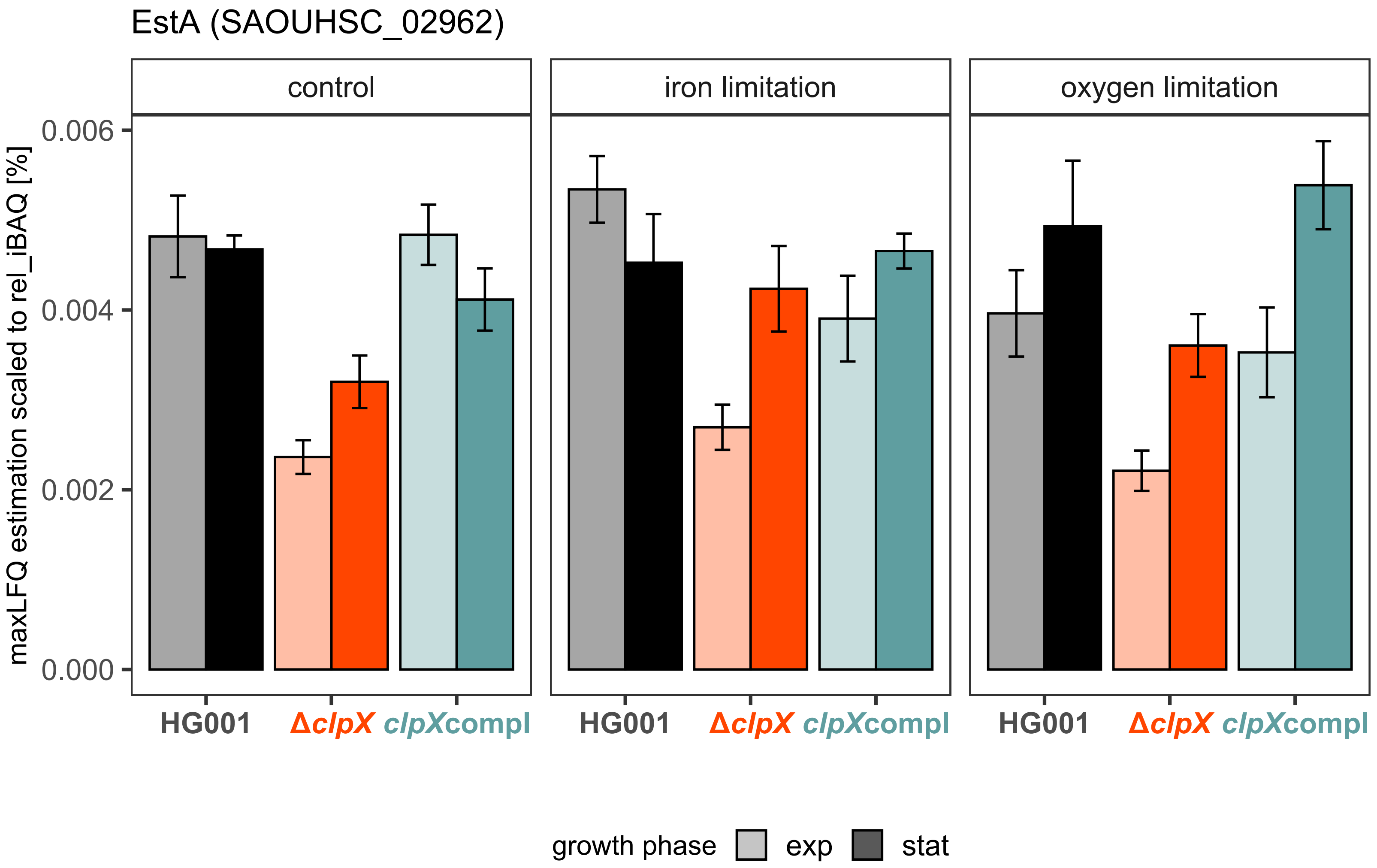
EstA (SAOUHSC_02962)
control
iron limitation
oxygen limitation
maxLFQ estimation scaled to rel_iBAQ [%]
0.006
0.004
0.002
0.000
HG001
ΔclpX
clpXcompl
growth phase
exp
stat

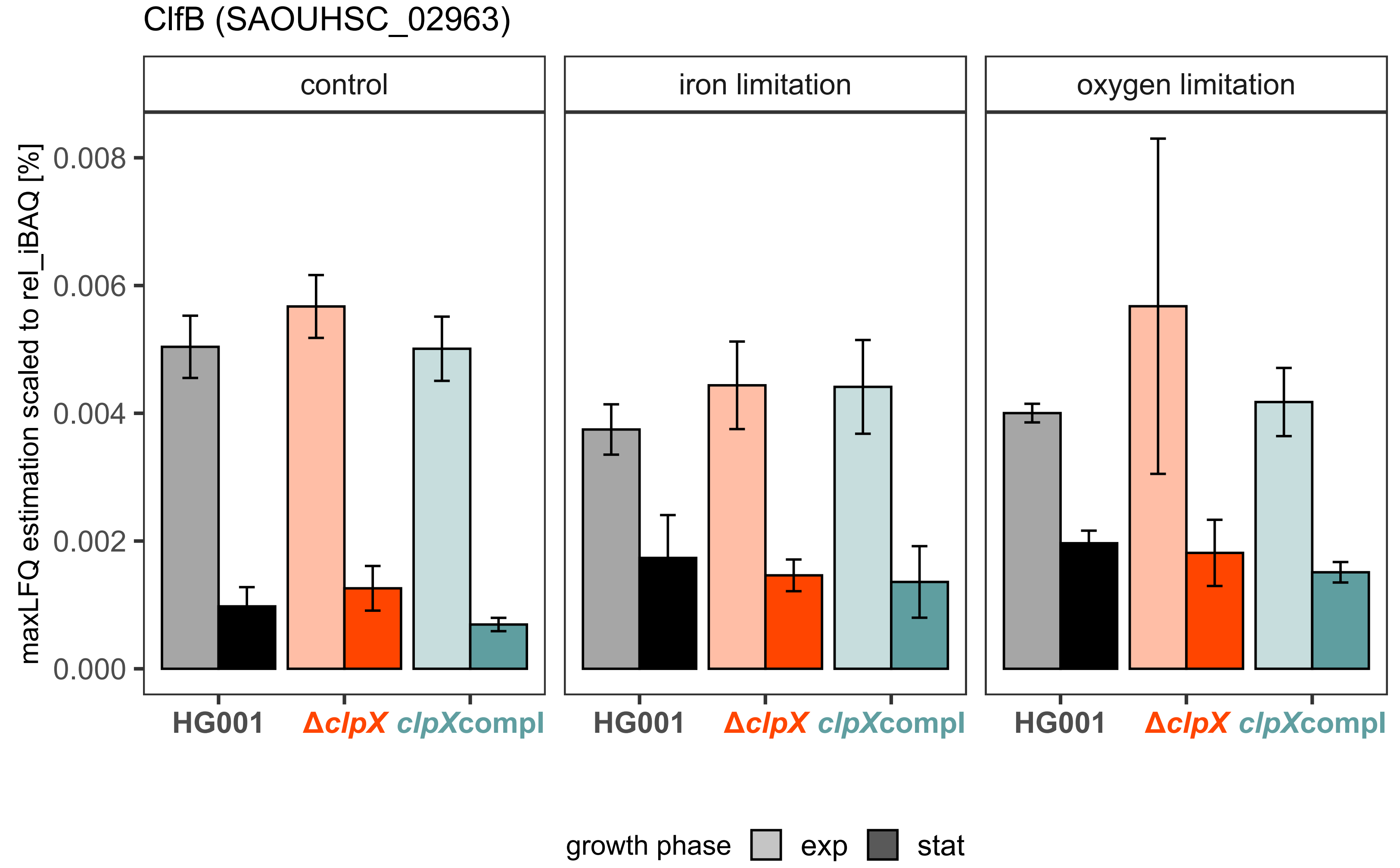

ClfB (SAOUHSC_02963)
control
iron limitation
oxygen limitation
maxLFQ estimation scaled to rel_iBAQ [%]
0.008
0.006
0.004
0.002
0.000
HG001
ΔclpX
clpXcompl
growth phase
exp
stat

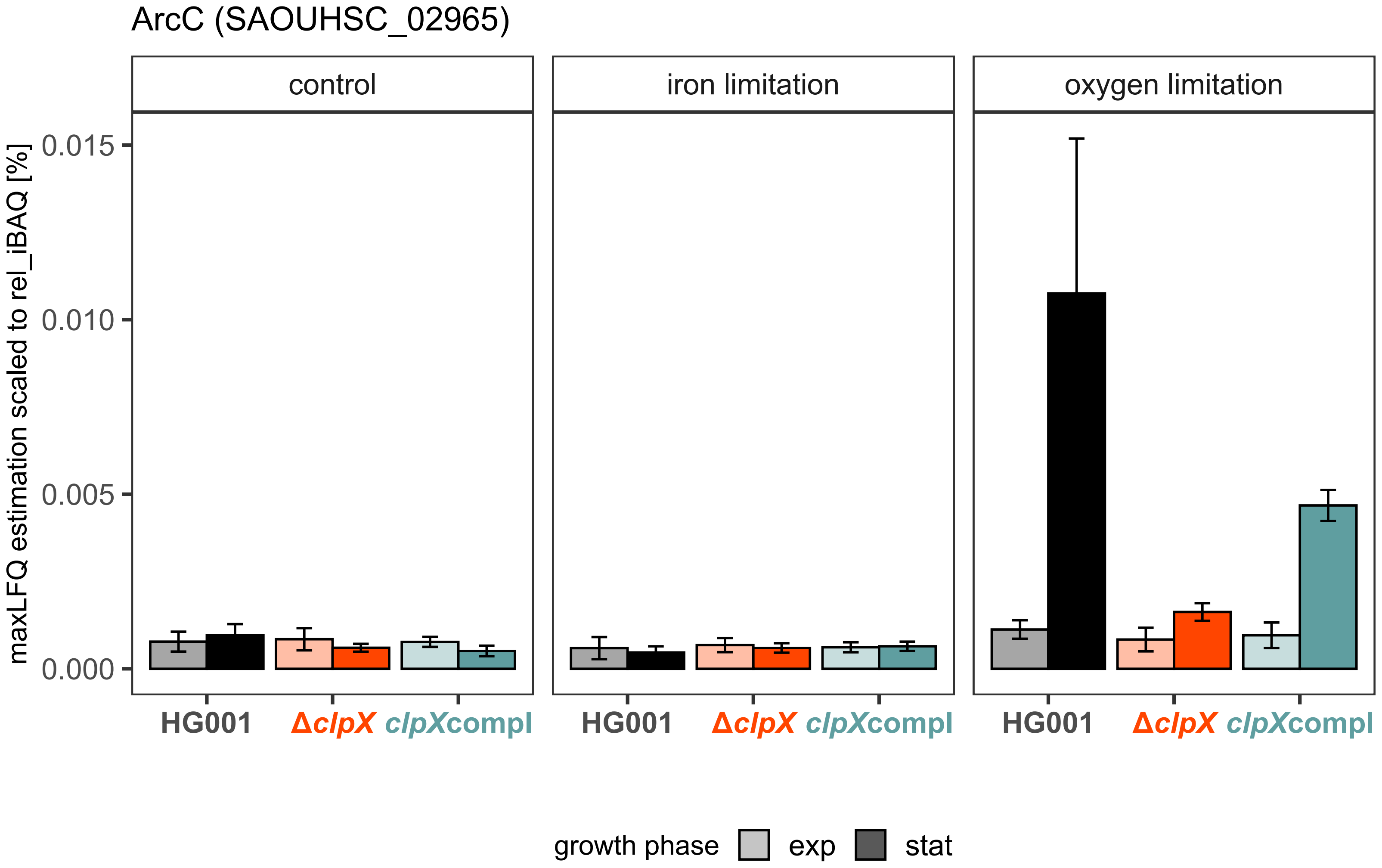

ArcC (SAOUHSC_02965)
control
iron limitation
oxygen limitation
maxLFQ estimation scaled to rel_iBAQ [%]
0.015
0.010
0.005
0.000
HG001
ΔclpX
clpXcompl
growth phase
exp
stat

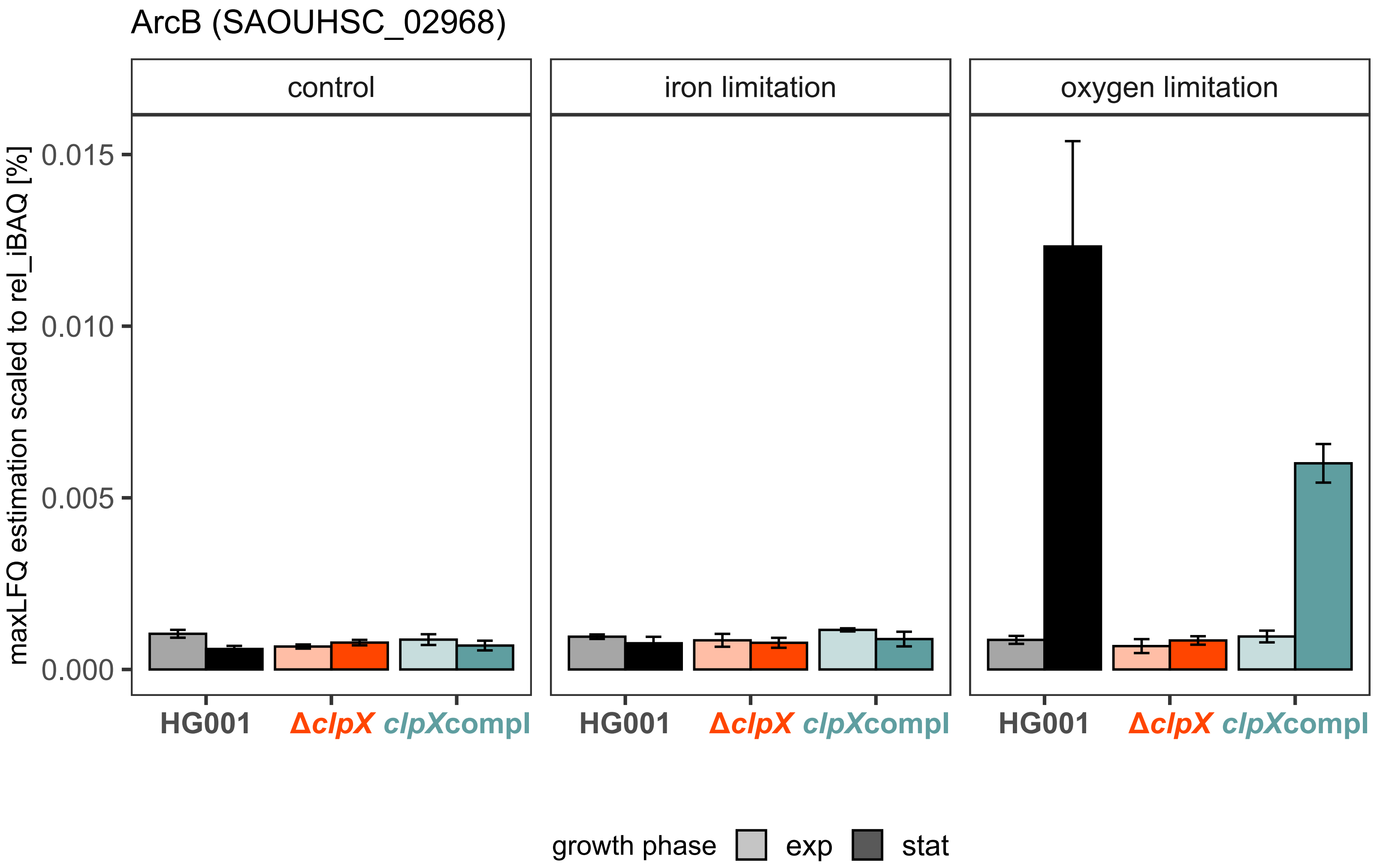

ArcB (SAOUHSC_02968)
control
iron limitation
oxygen limitation
maxLFQ estimation scaled to rel_iBAQ [%]
0.015
0.010
0.005
0.000
HG001
ΔclpX
clpXcompl
growth phase
exp
stat

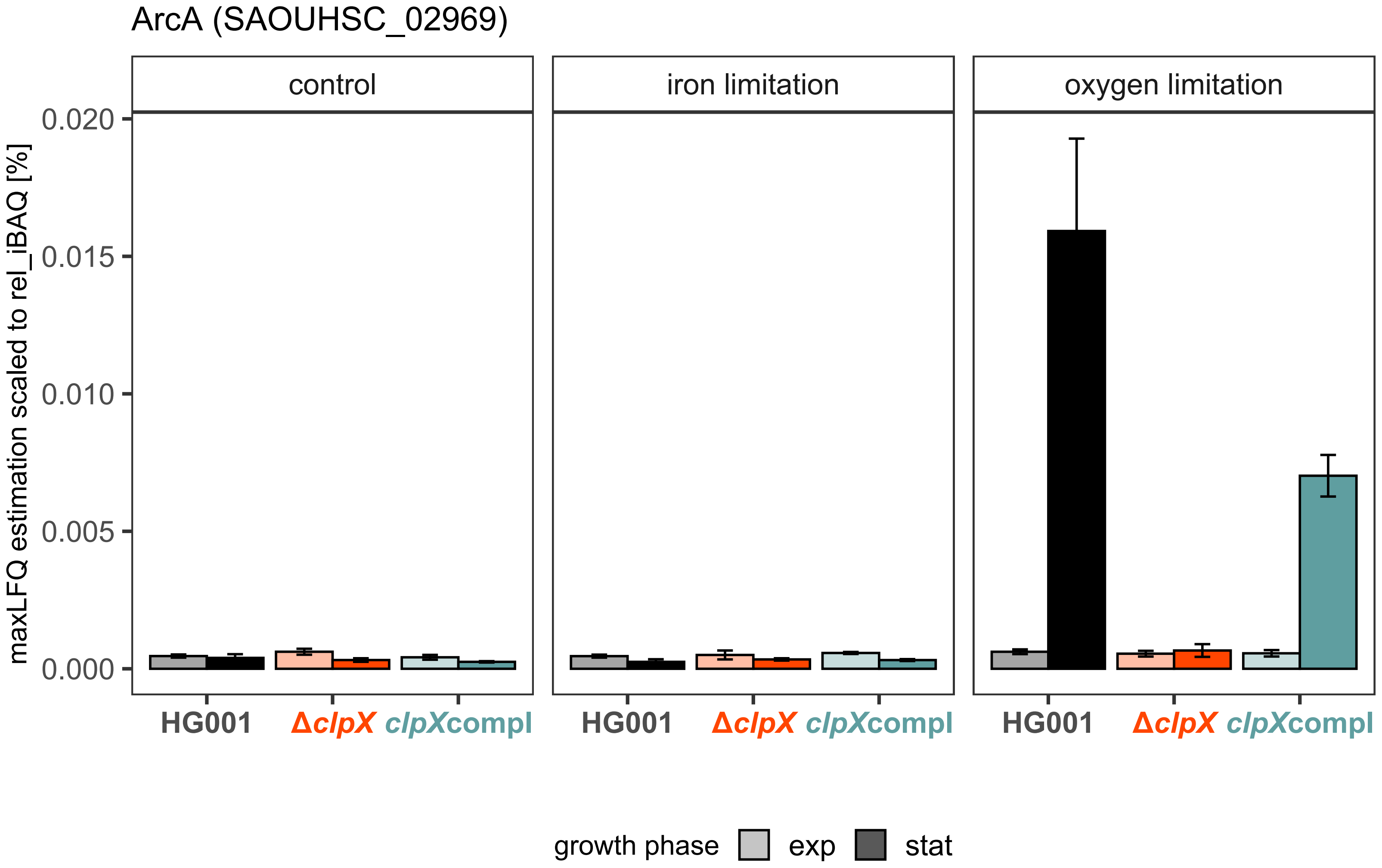

ArcA (SAOUHSC_02969)
control
iron limitation
oxygen limitation
maxLFQ estimation scaled to rel_iBAQ [%]
0.020
0.015
0.010
0.005
0.000
HG001
ΔclpX
clpXcompl
growth phase
exp
stat

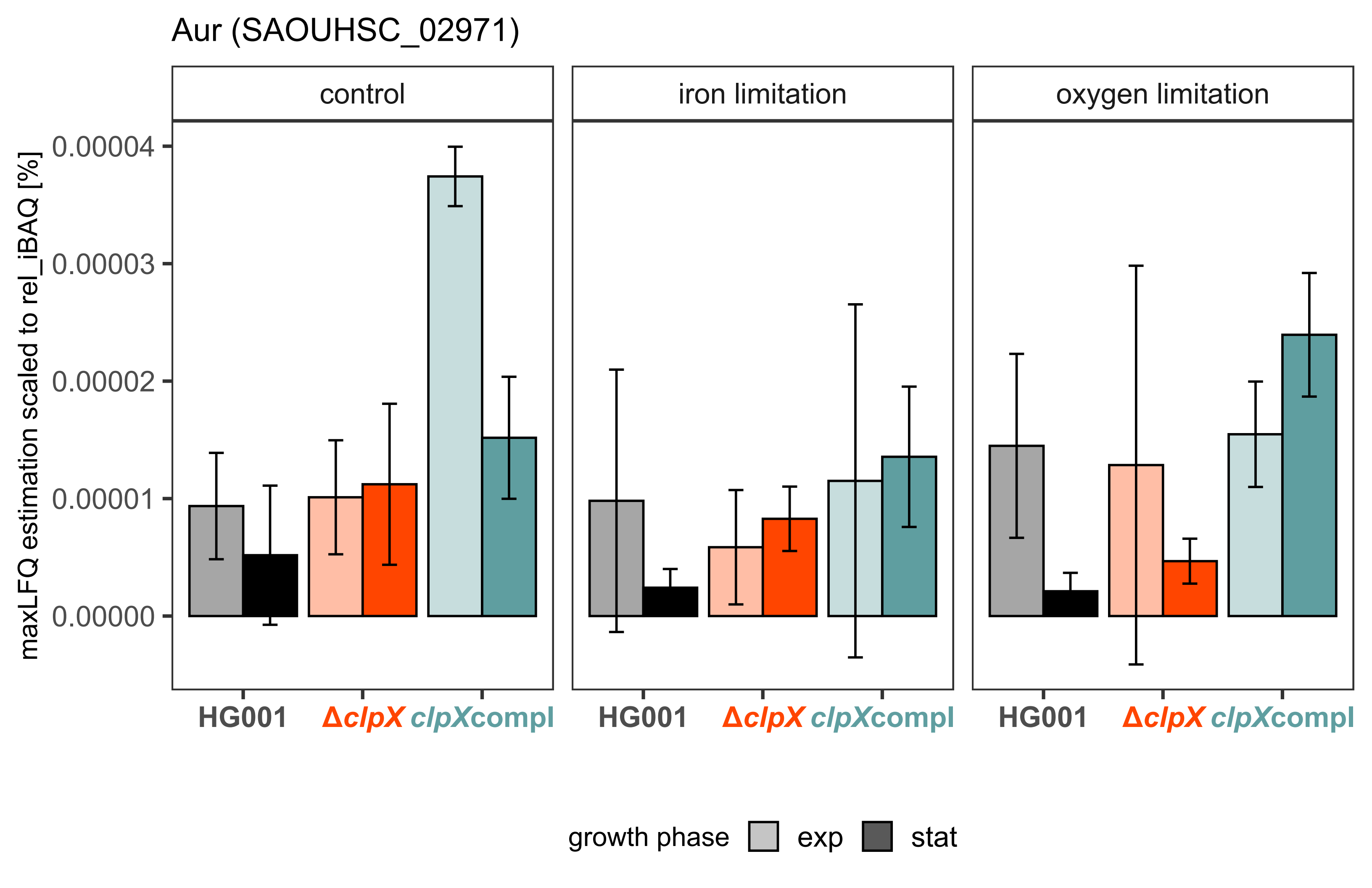
Aur (SAOUHSC_02971)
control
iron limitation
oxygen limitation
maxLFQ estimation scaled to rel_iBAQ [%]
0.00004
0.00003
0.00002
0.00001
0.00000
HG001
ΔclpX
clpXcompl
growth phase
exp
stat

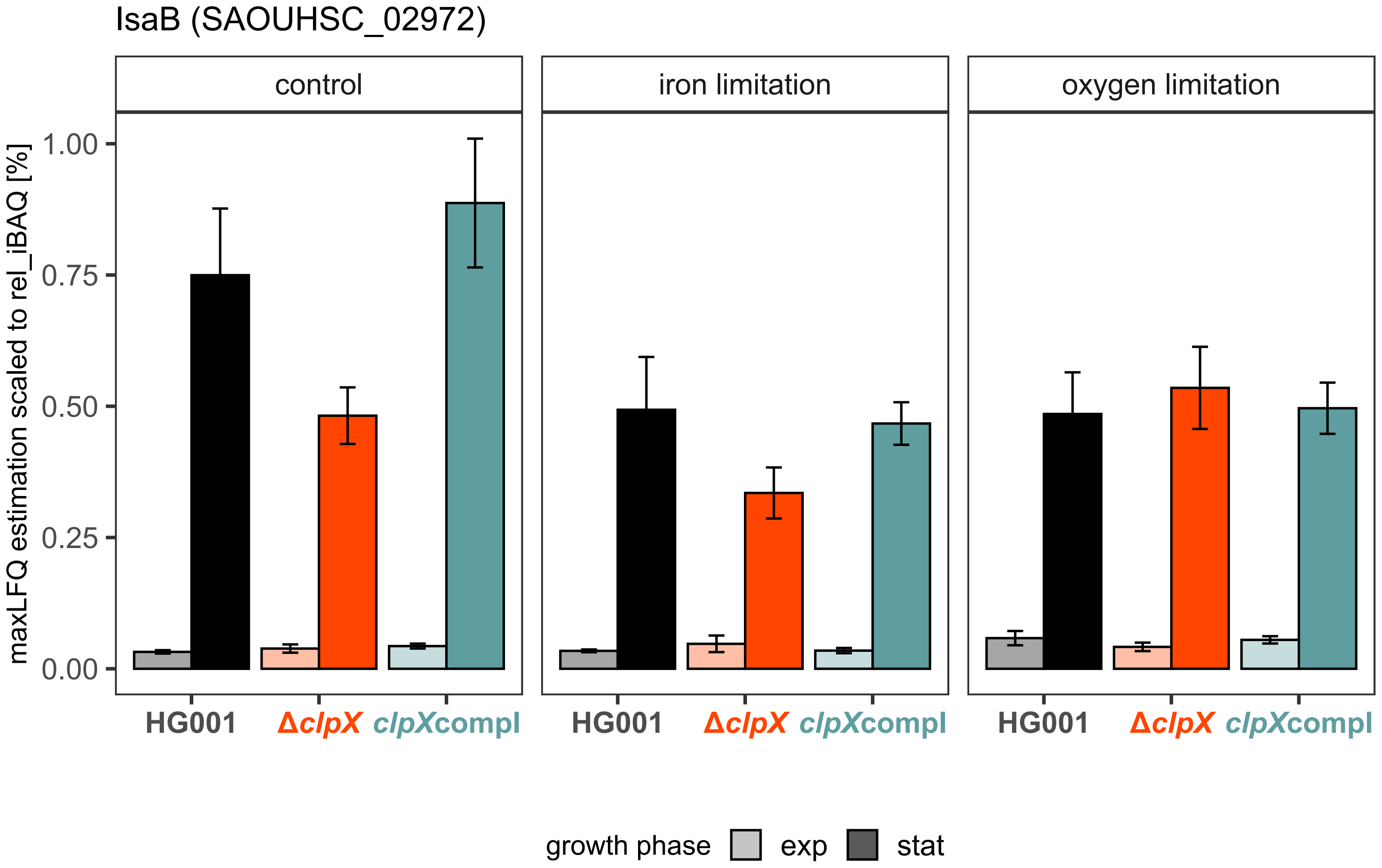
IsaB (SAOUHSC_02972)
control
iron limitation
oxygen limitation
maxLFQ estimation scaled to rel_iBAQ [%]
1.00
0.75
0.50
0.25
0.00
HG001
ΔclpX
clpXcompl
growth phase
exp
stat

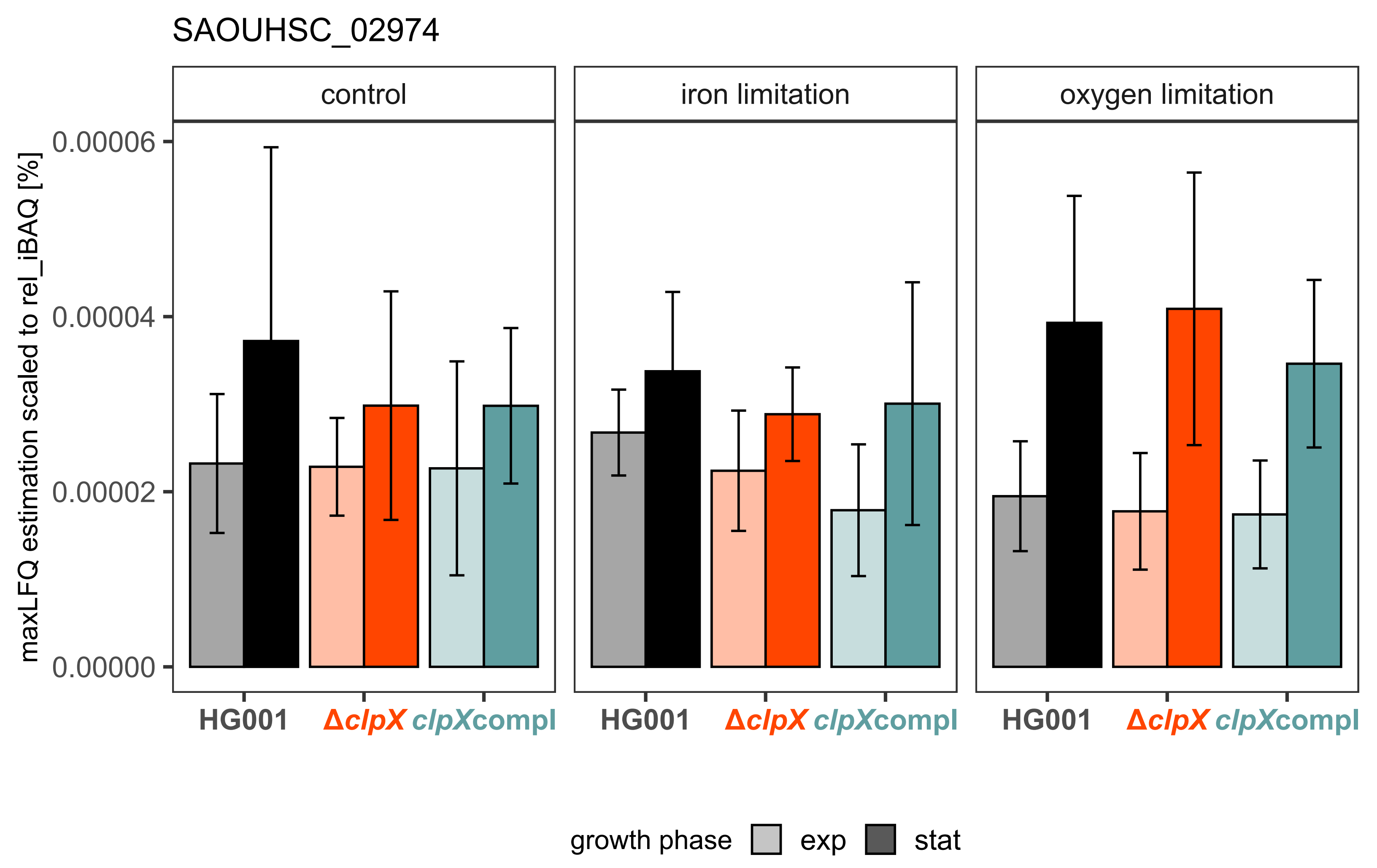

SAOUHSC_02974
control
iron limitation
oxygen limitation
maxLFQ estimation scaled to rel_iBAQ [%]
0.00006
0.00004
0.00002
0.00000
HG001
ΔclpX
clpXcompl
HG001
ΔclpX
clpXcompl
HG001
ΔclpX
clpXcompl
growth phase
exp
stat

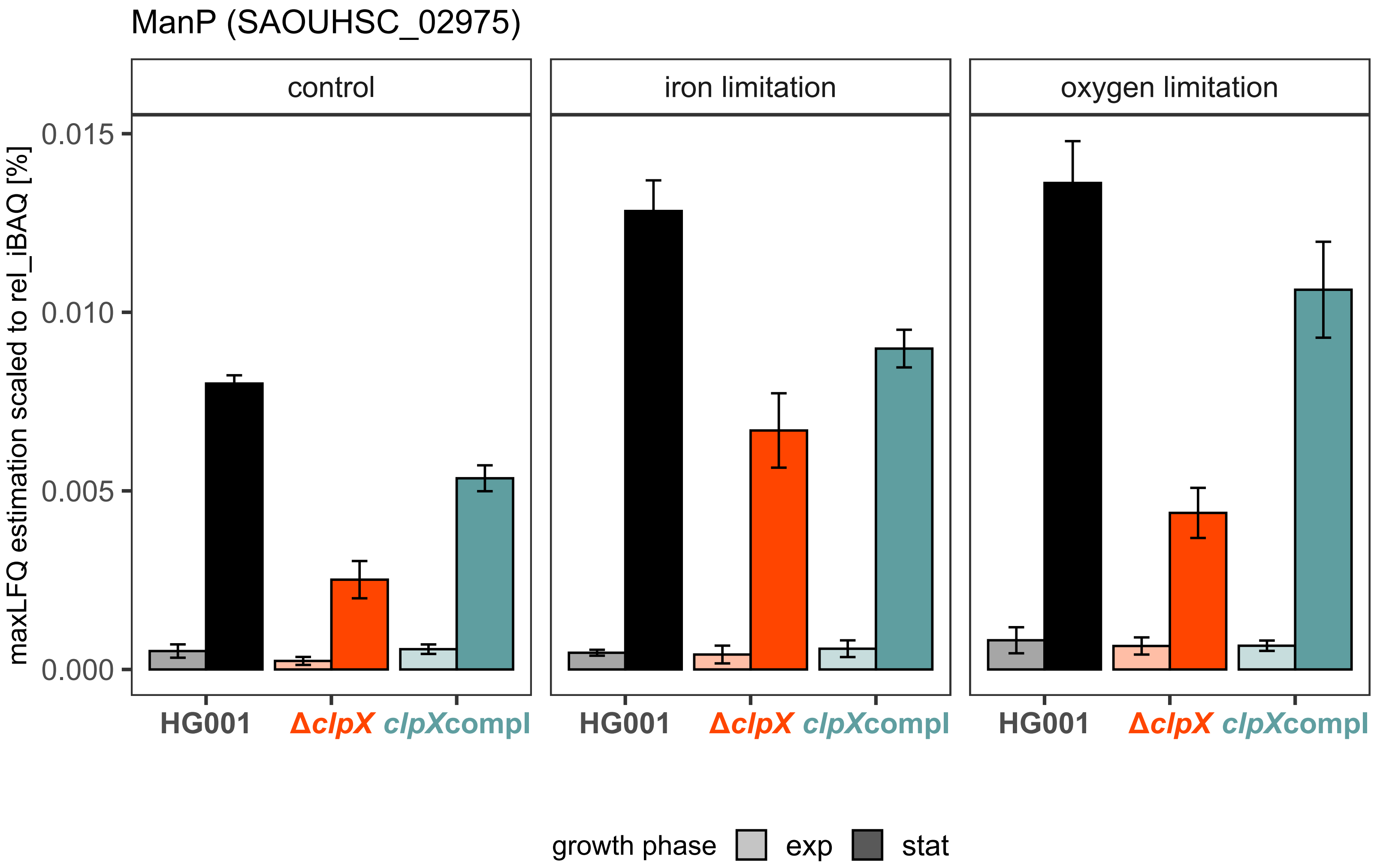

ManP (SAOUHSC_02975)
control
iron limitation
oxygen limitation
maxLFQ estimation scaled to rel_iBAQ [%]
0.015
0.010
0.005
0.000
HG001
ΔclpX
clpXcompl
growth phase
exp
stat

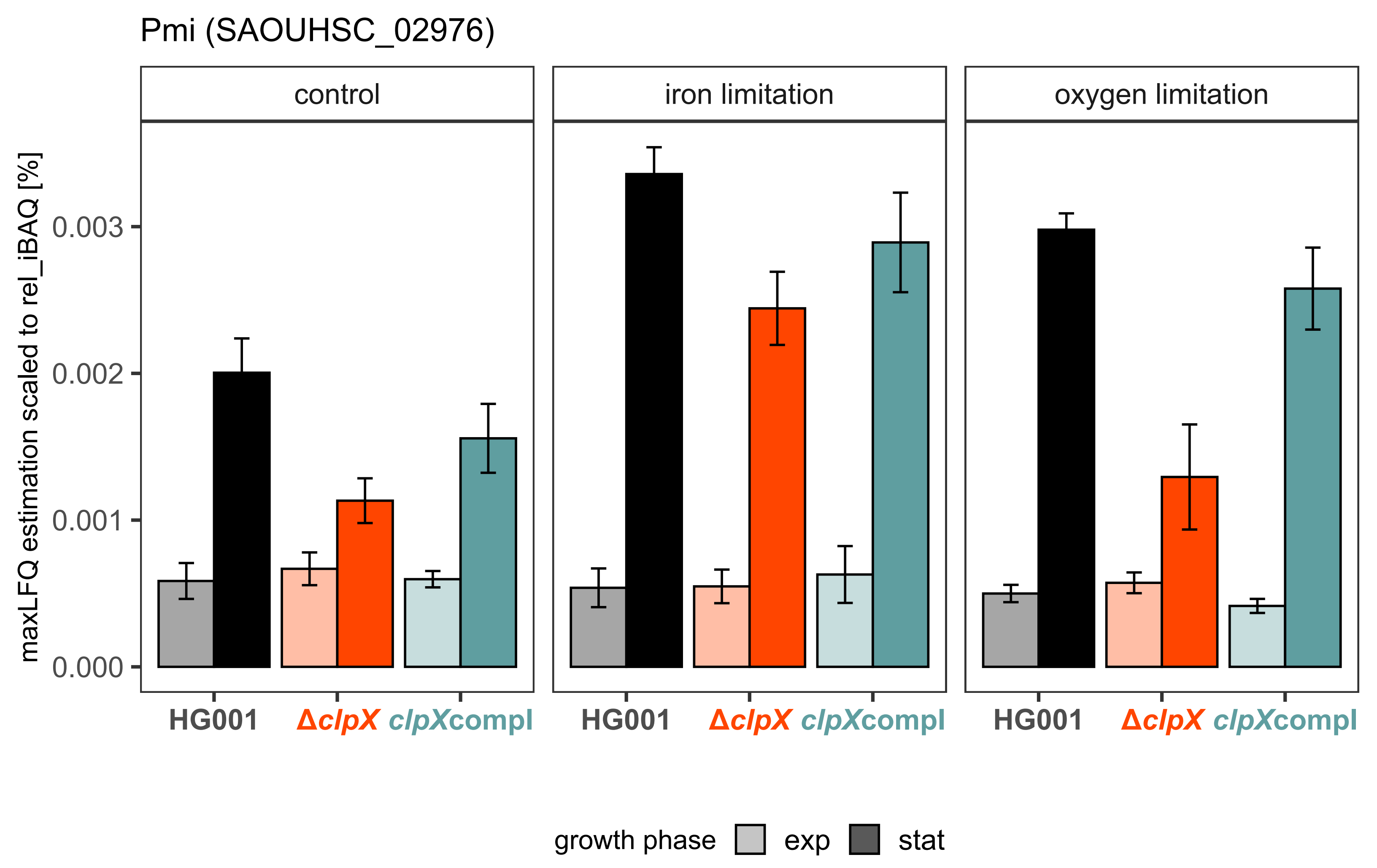

Pmi (SAOUHSC_02976)
control
iron limitation
oxygen limitation
maxLFQ estimation scaled to rel_iBAQ [%]
0.003
0.002
0.001
0.000
HG001
ΔclpX
clpXcompl
growth phase
exp
stat

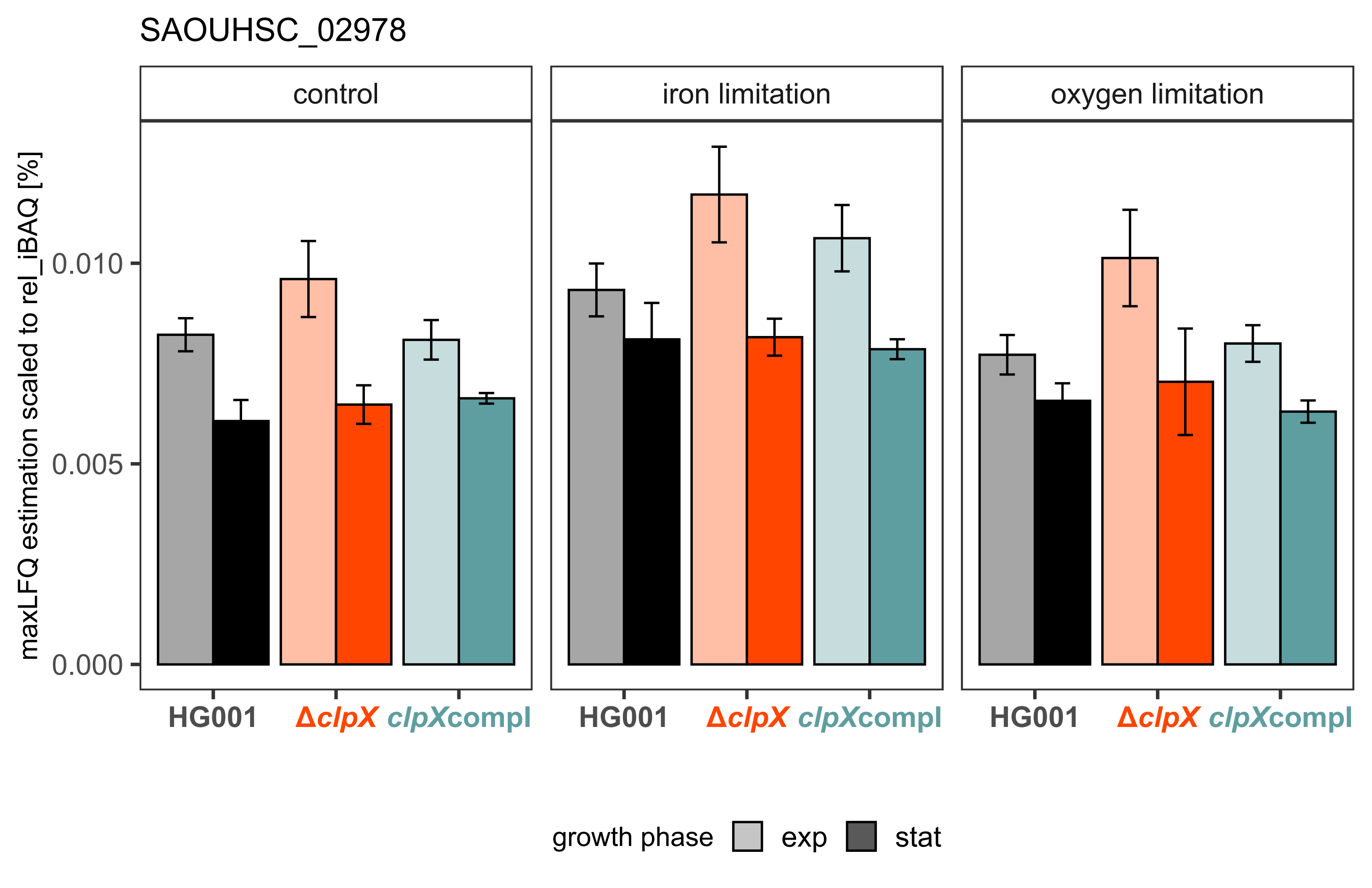

SAOUHSC_02978
control
iron limitation
oxygen limitation
maxLFQ estimation scaled to rel_iBAQ [%]
0.010
0.005
0.000
HG001
ΔclpX
clpXcompl
growth phase
exp
stat

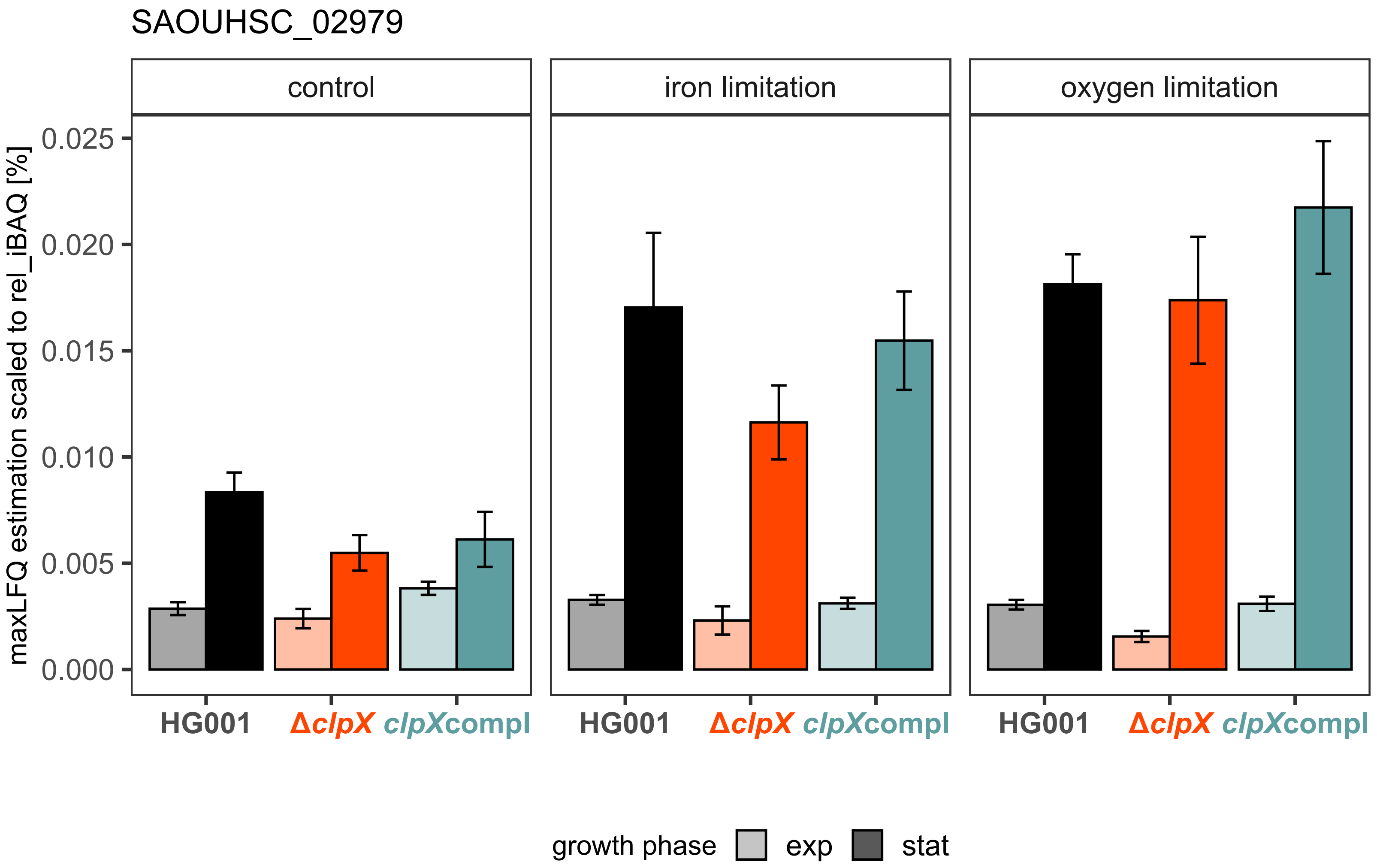
SAOUHSC_02979
control
iron limitation
oxygen limitation
maxLFQ estimation scaled to rel_iBAQ [%]
0.025
0.020
0.015
0.010
0.005
0.000
HG001
ΔclpX
clpXcompl
growth phase
exp
stat

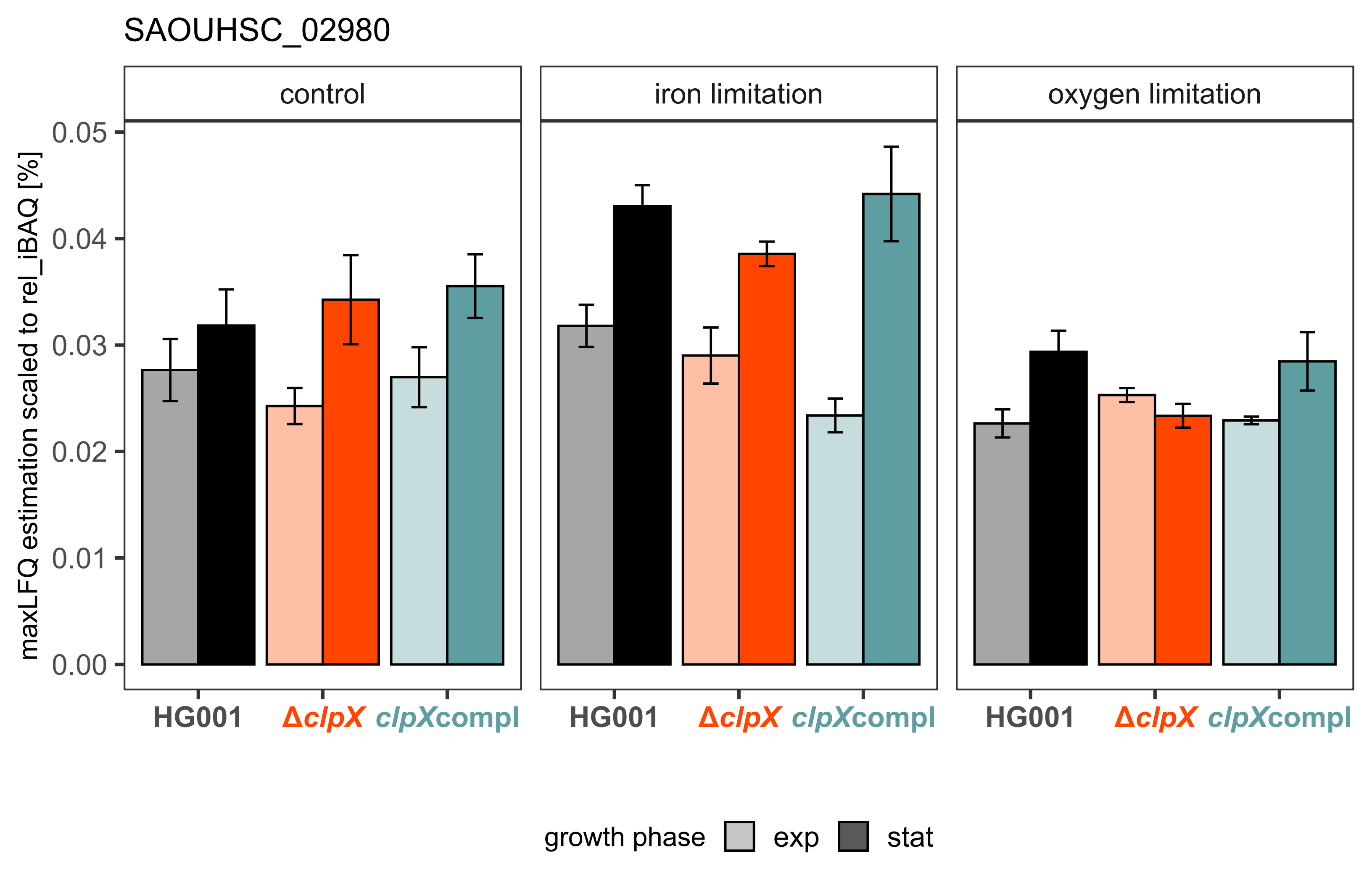
SAOUHSC_02980
control
iron limitation
oxygen limitation
maxLFQ estimation scaled to rel_iBAQ [%]
0.05
0.04
0.03
0.02
0.01
0.00
HG001
ΔclpX
clpXcompl
growth phase
exp
stat

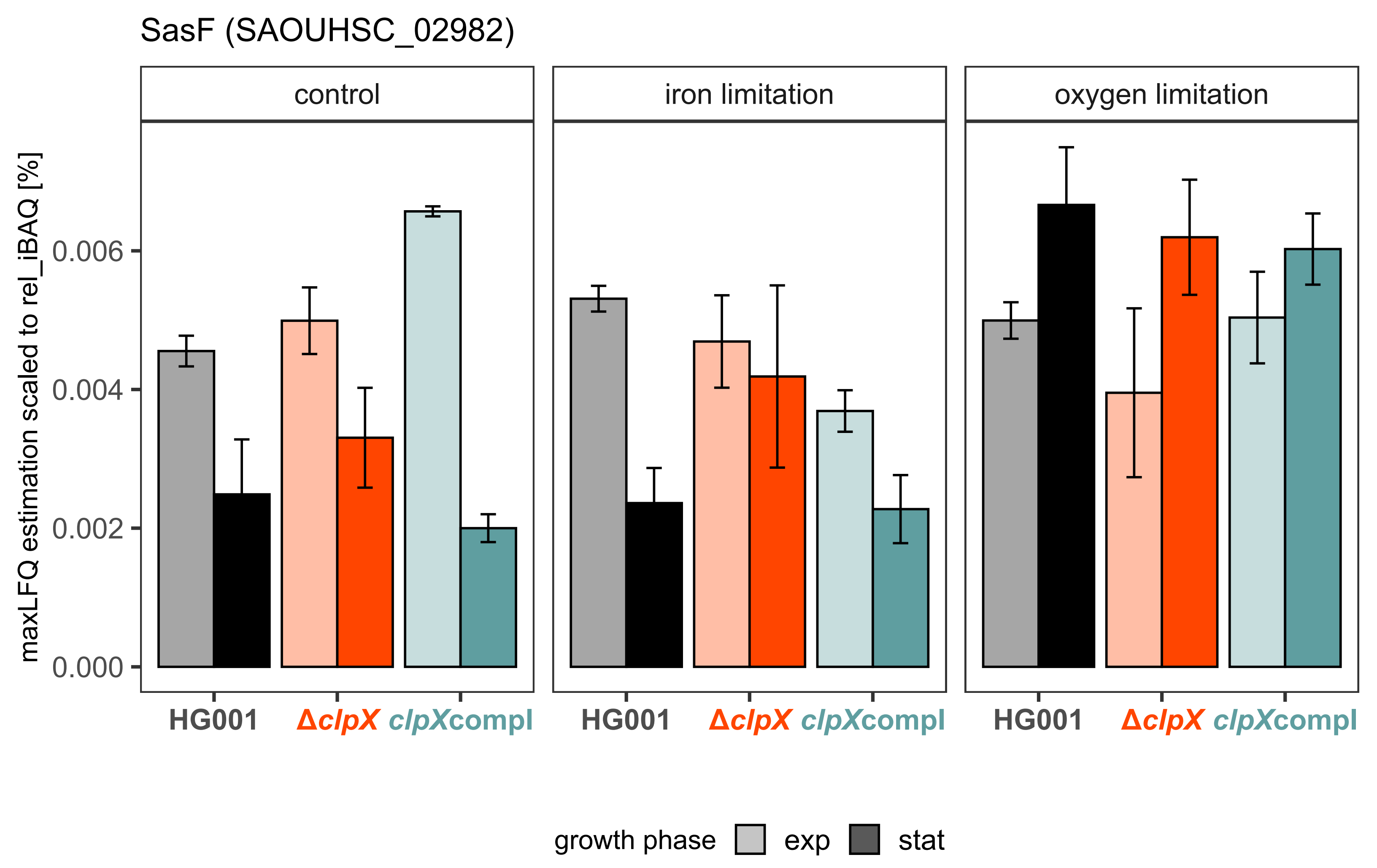
SasF (SAOUHSC_02982)
control
iron limitation
oxygen limitation
maxLFQ estimation scaled to rel_iBAQ [%]
0.006
0.004
0.002
0.000
HG001
ΔclpX
clpXcompl
growth phase
exp
stat

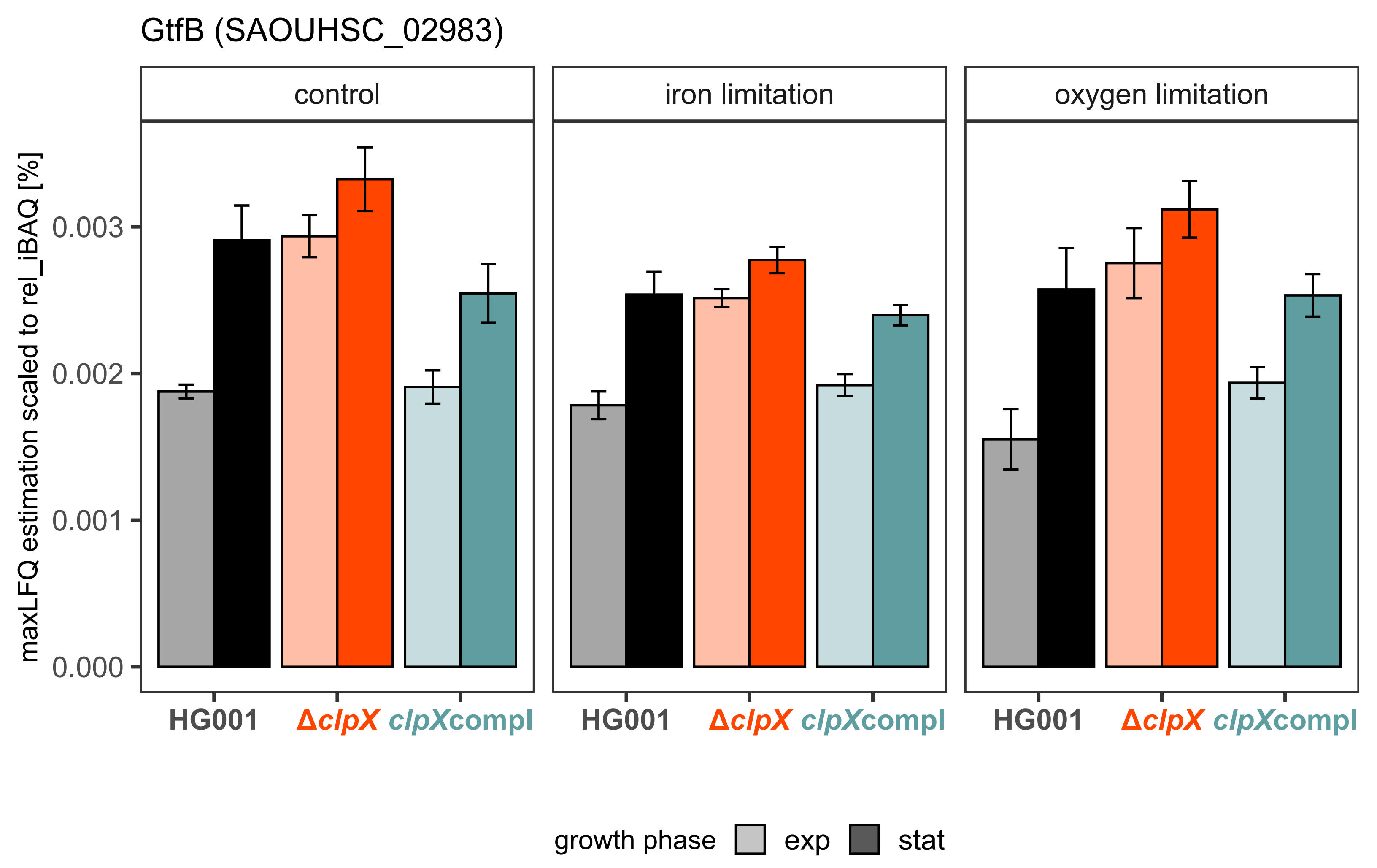
GtfB (SAOUHSC_02983)
control
iron limitation
oxygen limitation
maxLFQ estimation scaled to rel_iBAQ [%]
0.000
0.001
0.002
0.003
HG001
ΔclpX
clpXcompl
growth phase
exp
stat

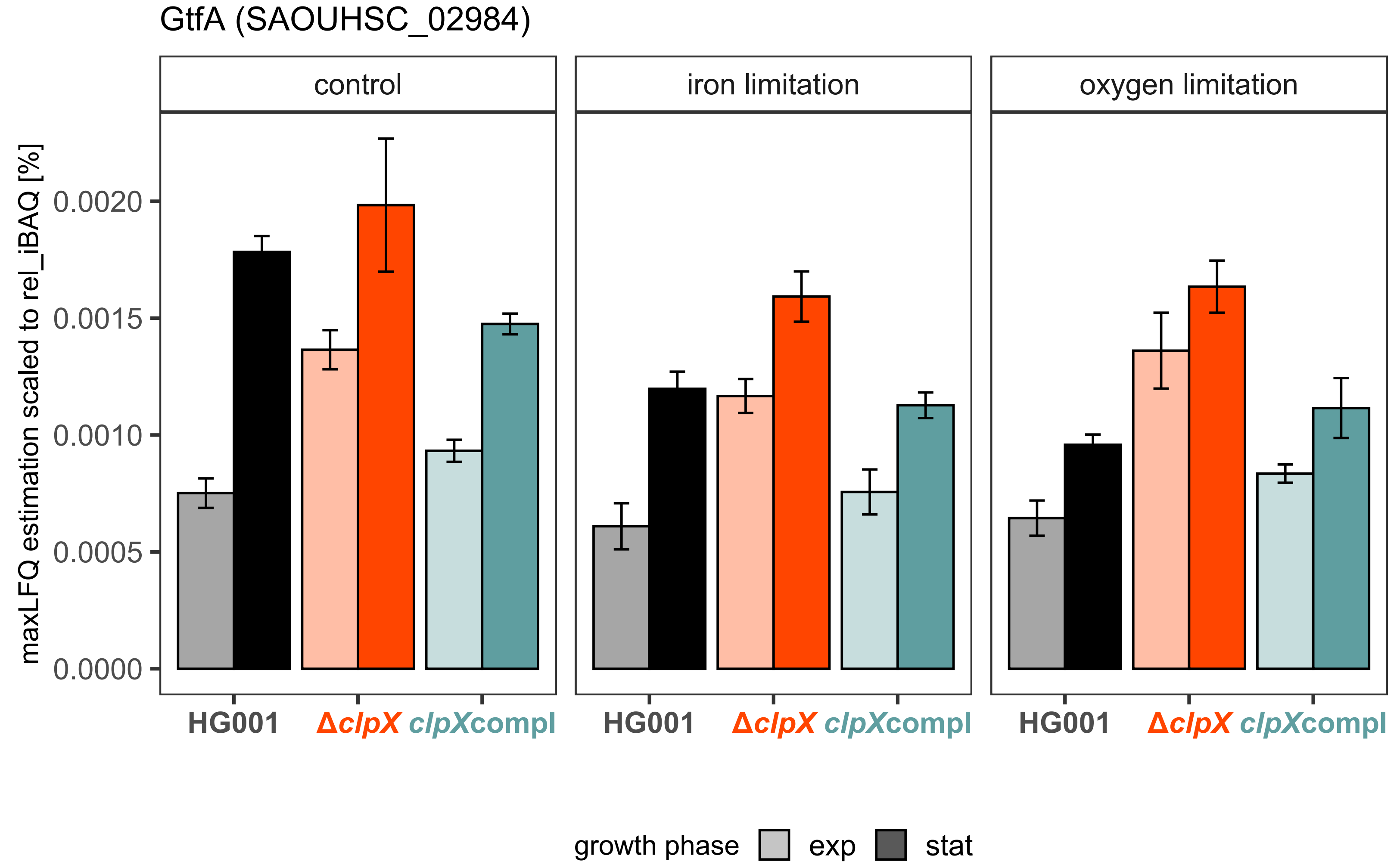
GtfA (SAOUHSC_02984)
control
iron limitation
oxygen limitation
maxLFQ estimation scaled to rel_iBAQ [%]
0.0020
0.0015
0.0010
0.0005
0.0000
HG001
ΔclpX
clpXcompl
growth phase
exp
stat

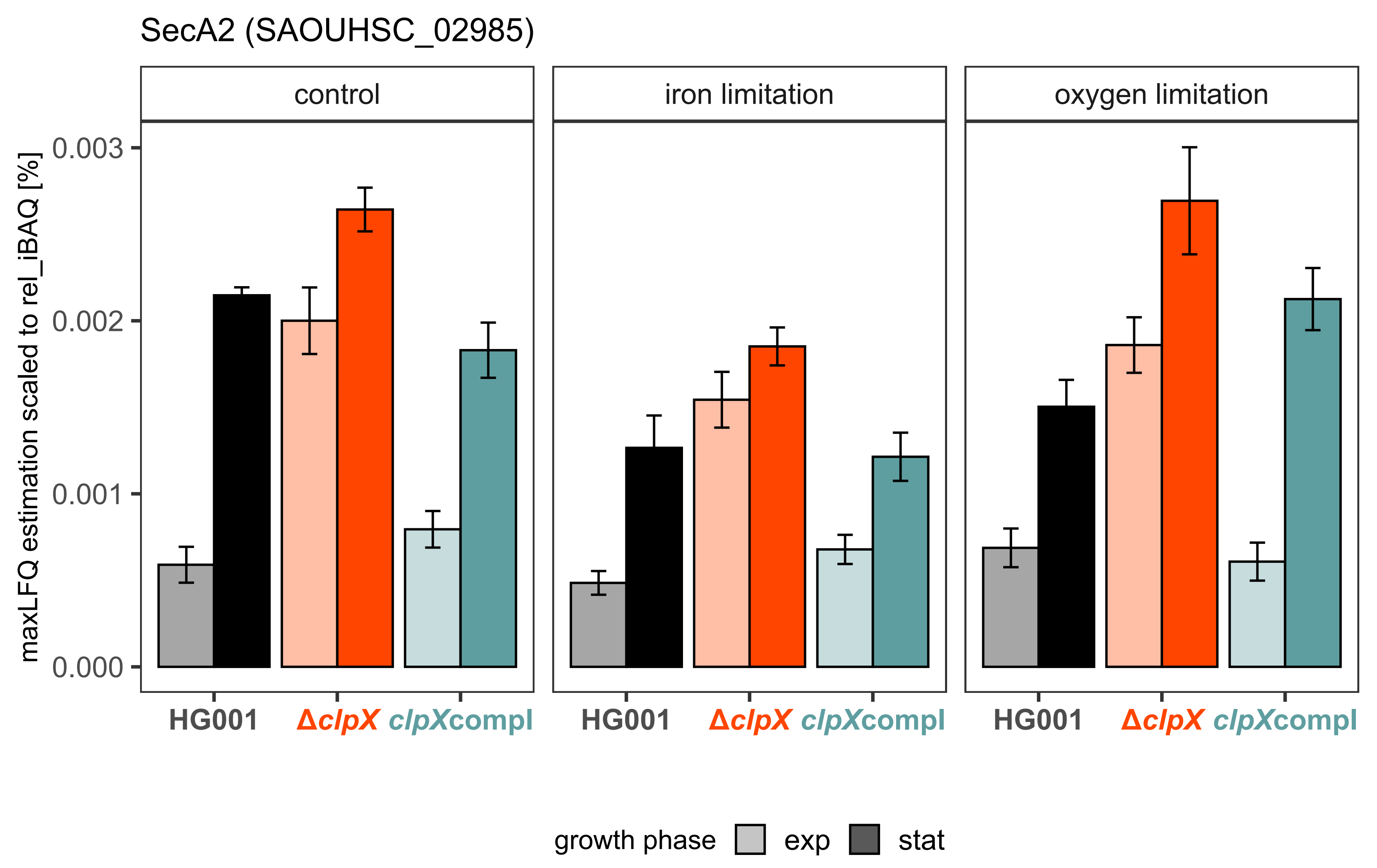
SecA2 (SAOUHSC_02985)
control
iron limitation
oxygen limitation
maxLFQ estimation scaled to rel_iBAQ [%]
0.003
0.002
0.001
0.000
HG001
ΔclpX
clpXcompl
growth phase
exp
stat

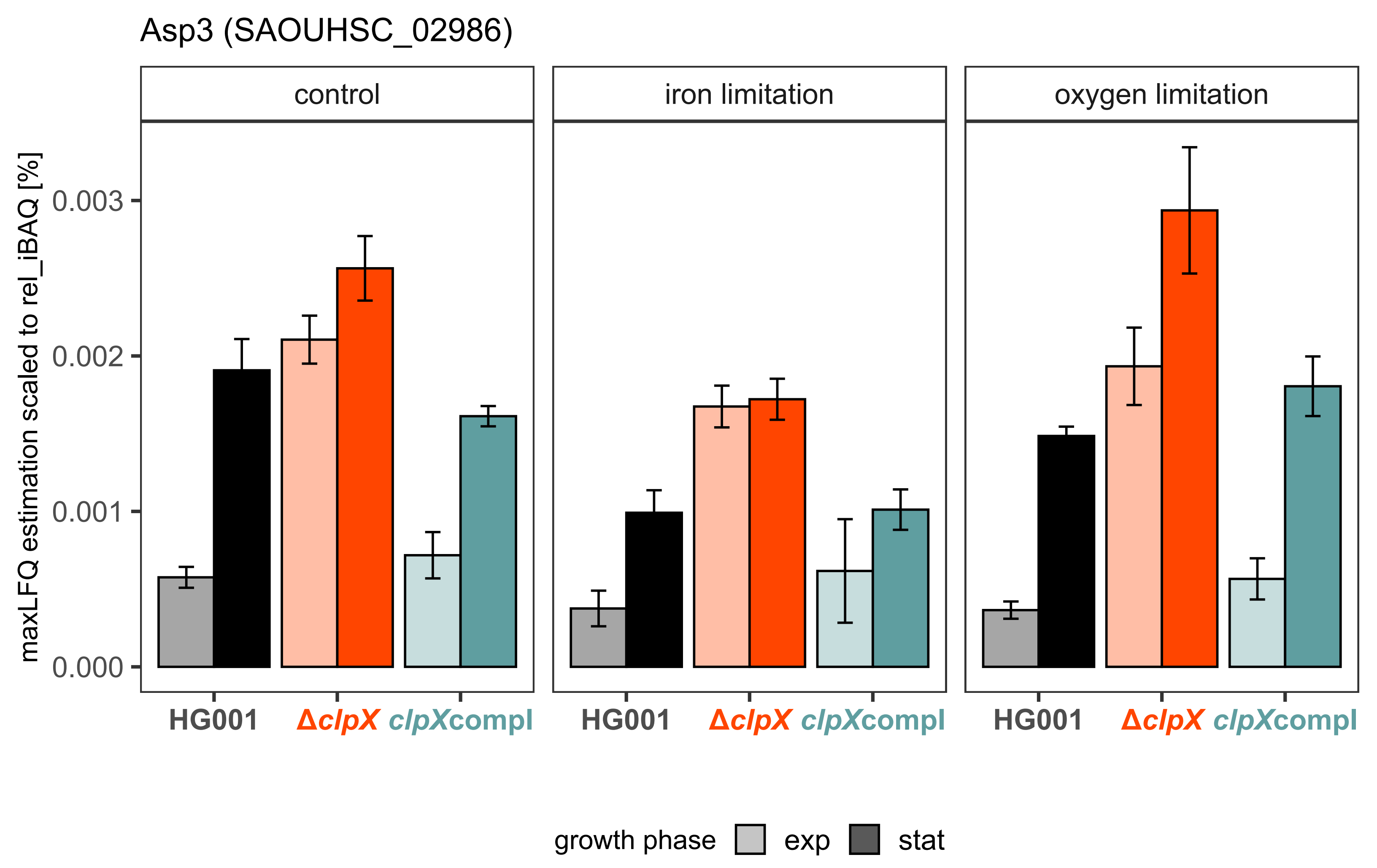

Asp3 (SAOUHSC_02986)
control
iron limitation
oxygen limitation
maxLFQ estimation scaled to rel_iBAQ [%]
0.003
0.002
0.001
0.000
HG001
ΔclpX
clpXcompl
growth phase
exp
stat

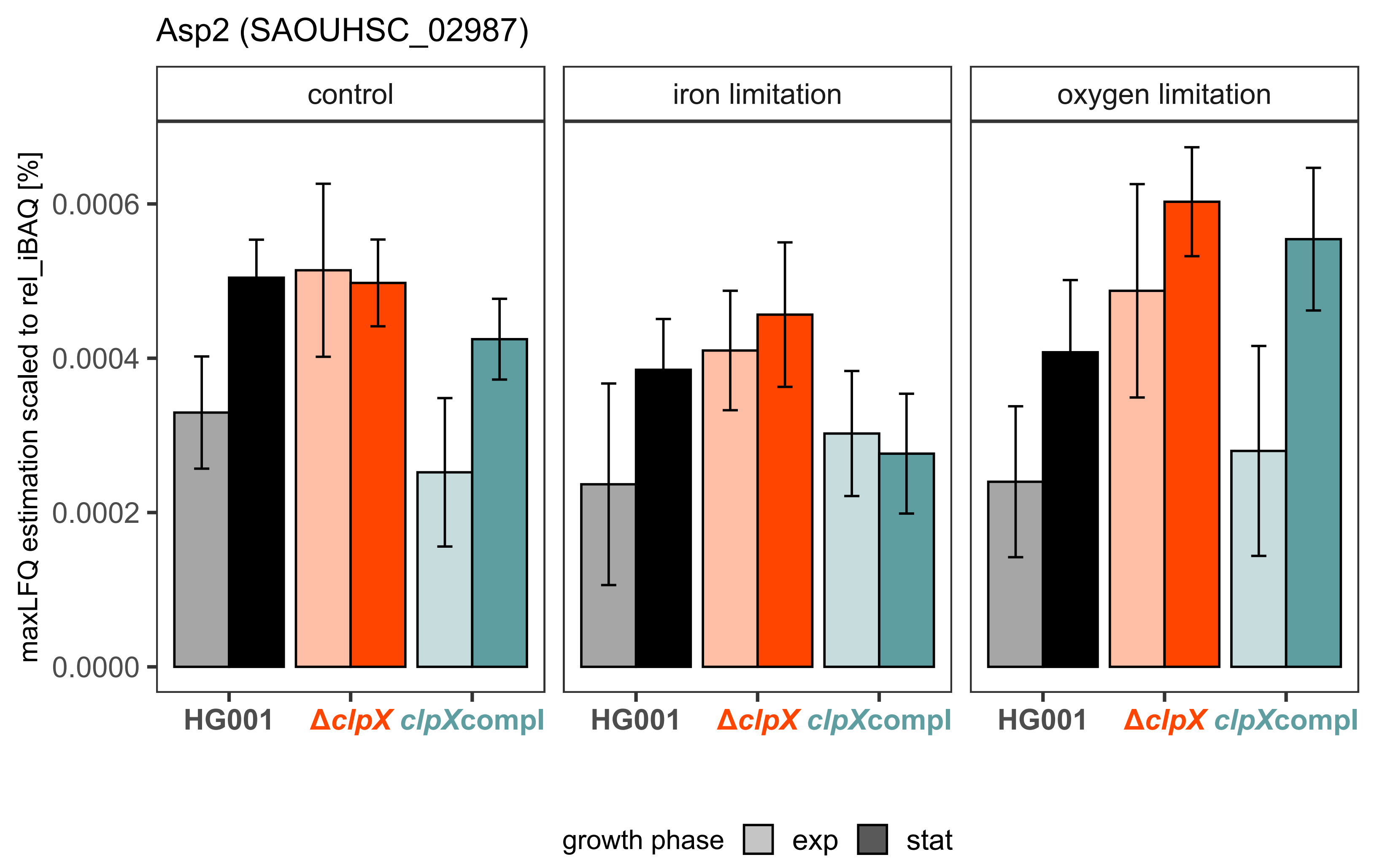

Asp2 (SAOUHSC_02987)
control
iron limitation
oxygen limitation
maxLFQ estimation scaled to rel_iBAQ [%]
0.0006
0.0004
0.0002
0.0000
HG001
ΔclpX
clpXcompl
growth phase
exp
stat

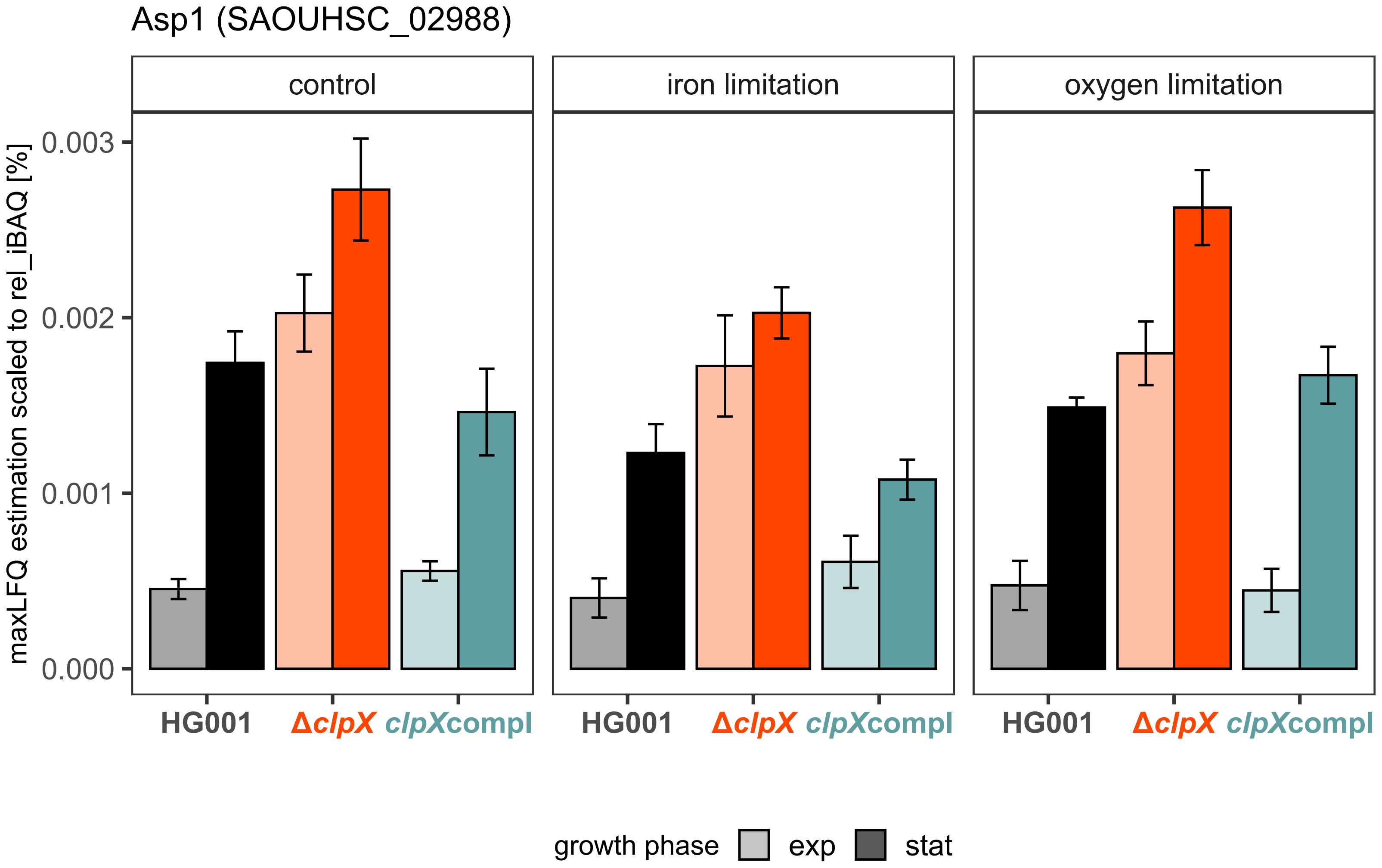

Asp1 (SAOUHSC_02988)
control
iron limitation
oxygen limitation
maxLFQ estimation scaled to rel_iBAQ [%]
0.003
0.002
0.001
0.000
HG001
ΔclpX
clpXcompl
growth phase
exp
stat

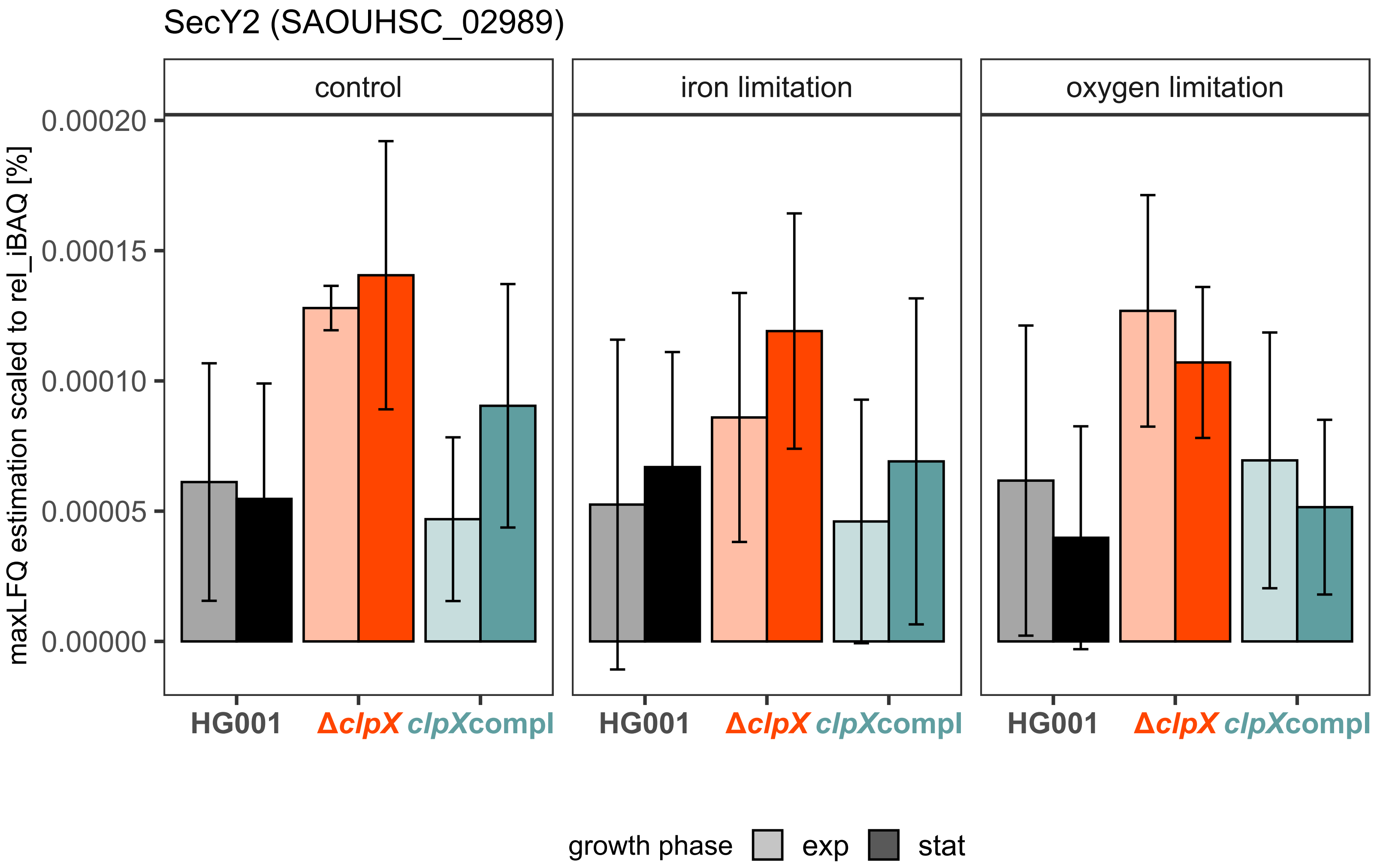
SecY2 (SAOUHSC_02989)
control
iron limitation
oxygen limitation
0.00020
0.00015
0.00010
0.00005
0.00000
maxLFQ estimation scaled to rel_iBAQ [%]
HG001
ΔclpX
clpXcompl
growth phase
exp
stat

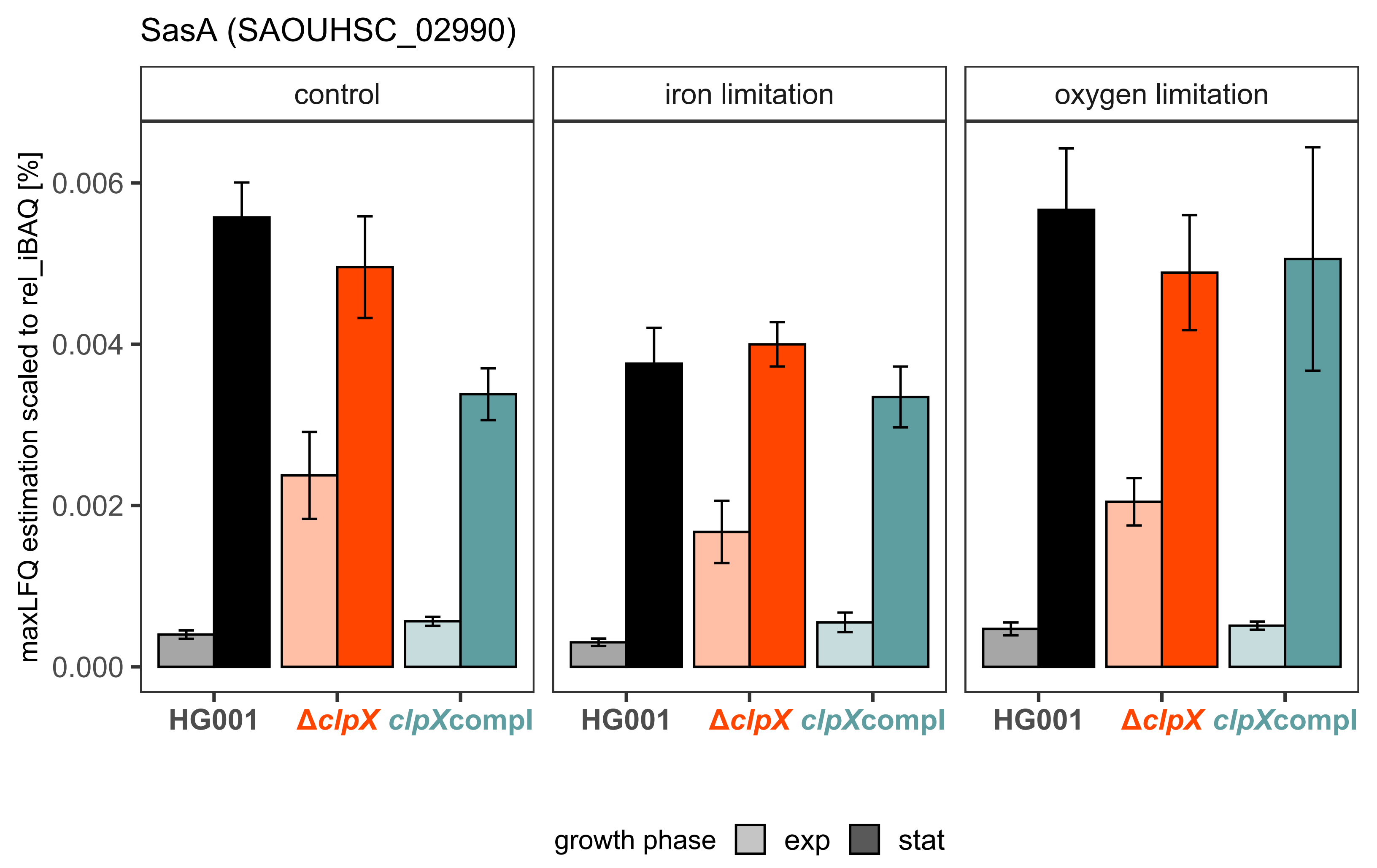
SasA (SAOUHSC_02990)
control
iron limitation
oxygen limitation
maxLFQ estimation scaled to rel_iBAQ [%]
0.006
0.004
0.002
0.000
HG001
ΔclpX
clpXcompl
growth phase
exp
stat

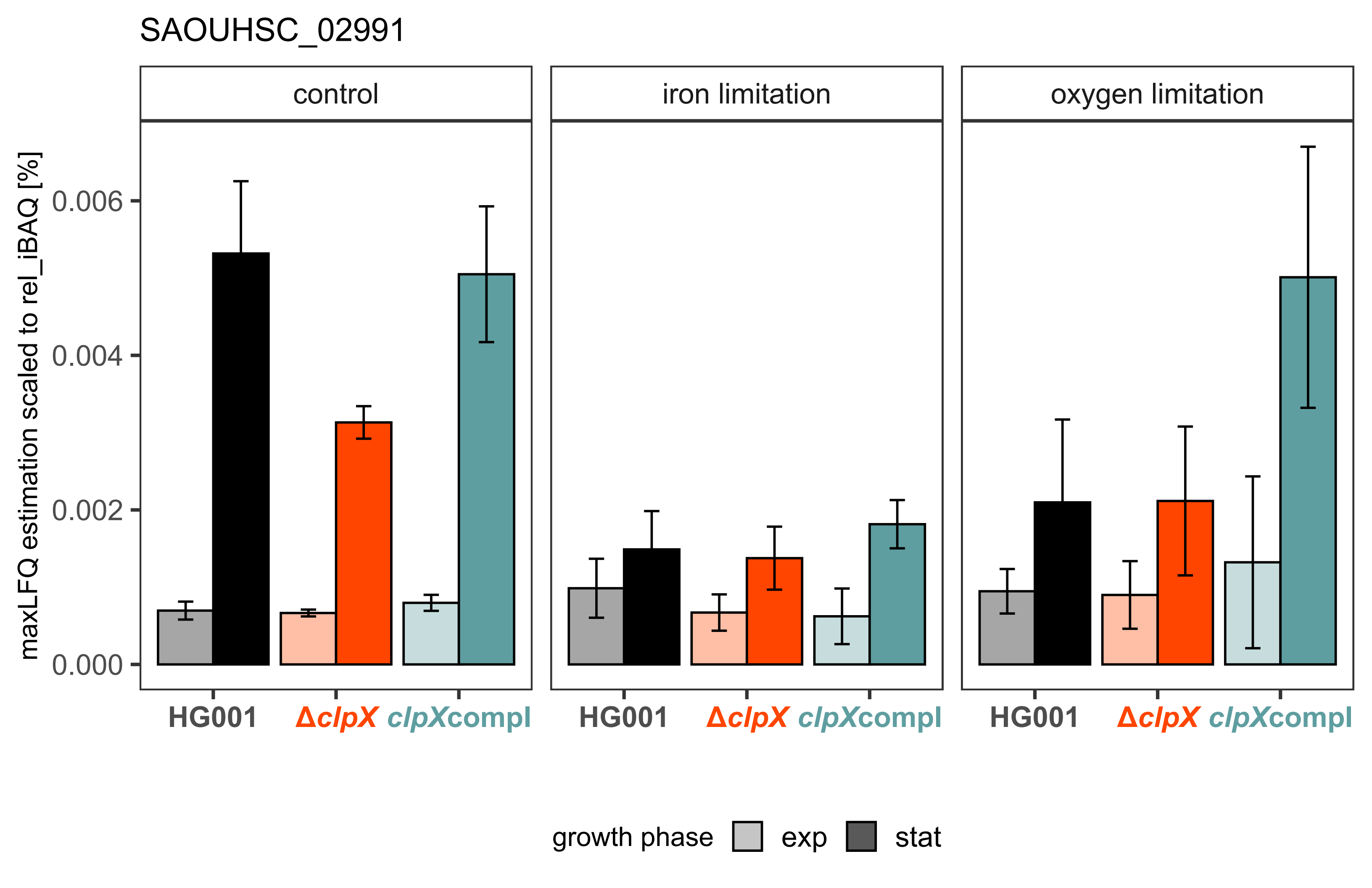

SAOUHSC_02991
control
iron limitation
oxygen limitation
maxLFQ estimation scaled to rel_iBAQ [%]
0.006
0.004
0.002
0.000
HG001
ΔclpX
clpXcompl
growth phase
exp
stat

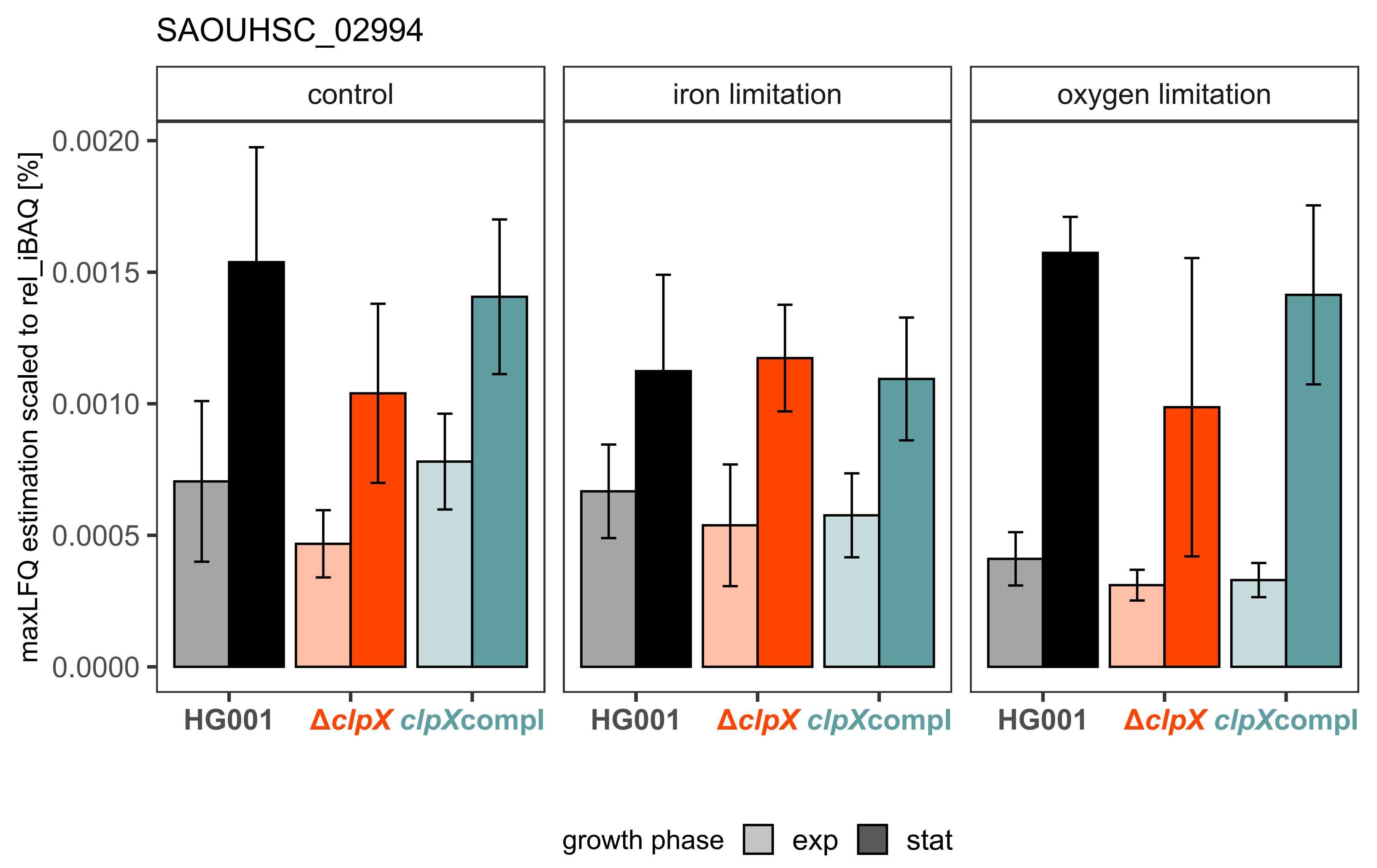
SAOUHSC_02994
control
iron limitation
oxygen limitation
maxLFQ estimation scaled to rel_iBAQ [%]
0.0020
0.0015
0.0010
0.0005
0.0000
HG001
ΔclpX
clpXcompl
growth phase
exp
stat

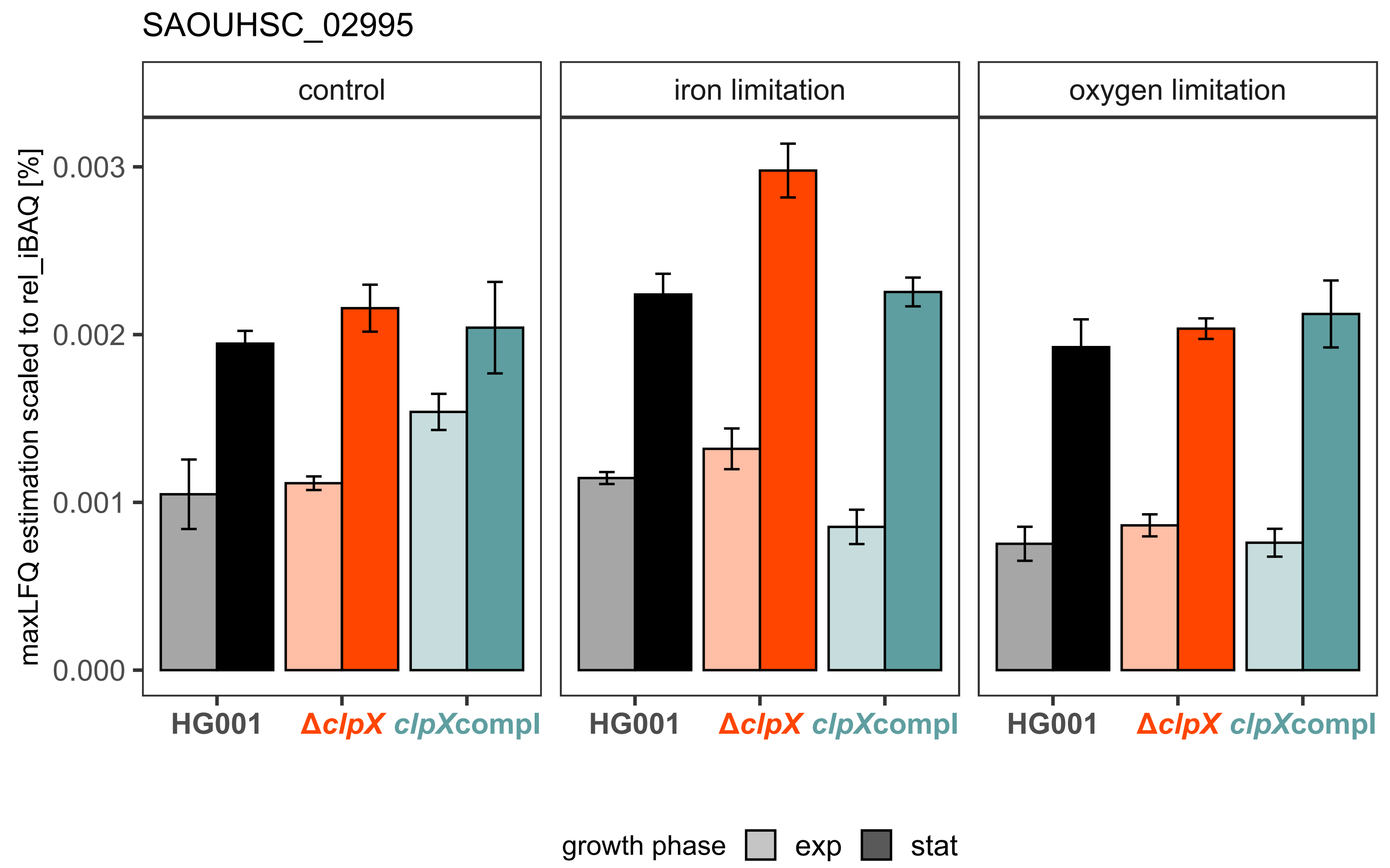
SAOUHSC_02995
control
iron limitation
oxygen limitation
maxLFQ estimation scaled to rel_iBAQ [%]
0.003
0.002
0.001
0.000
HG001
ΔclpX
clpXcompl
growth phase
exp
stat

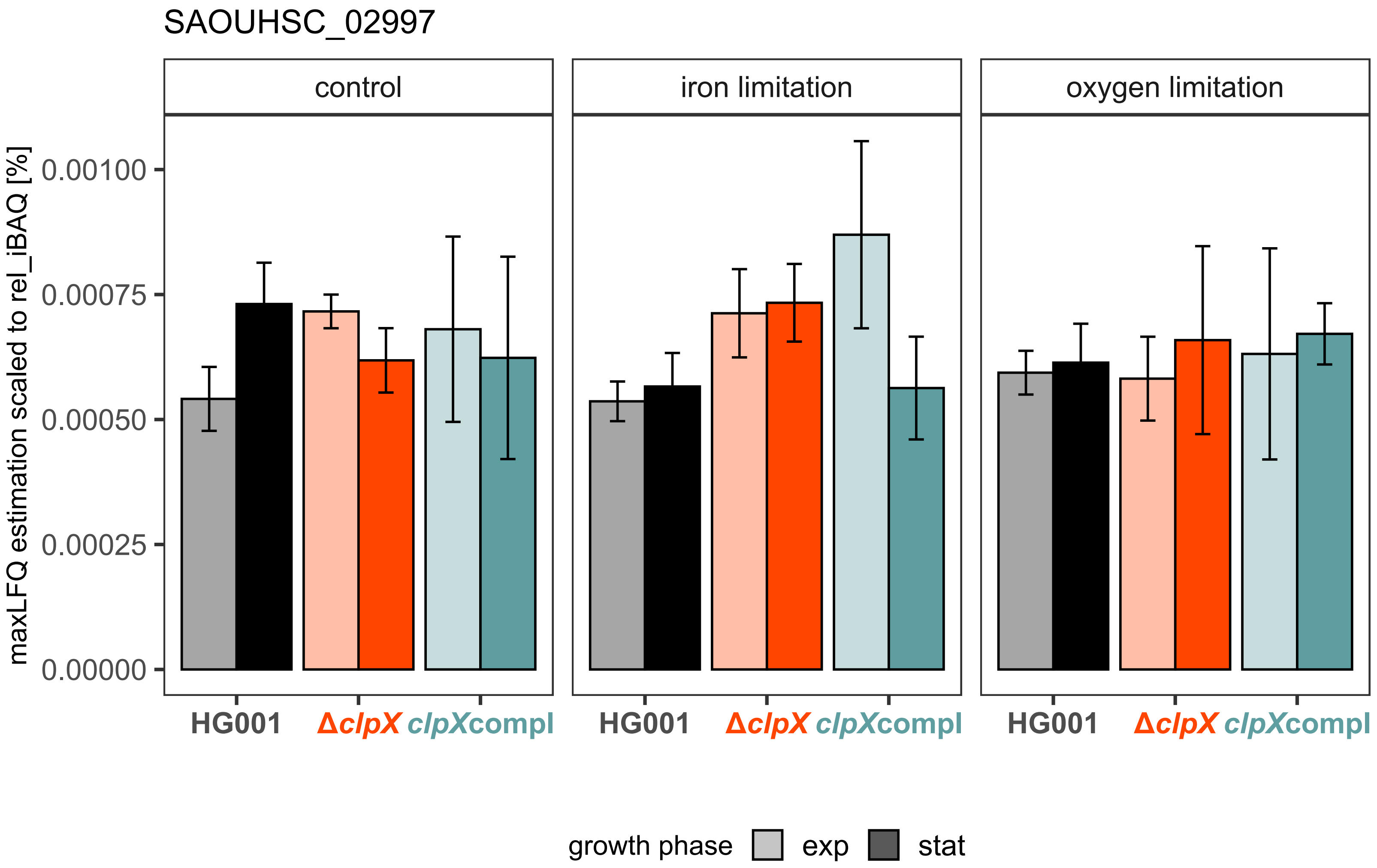
SAOUHSC_02997
control
iron limitation
oxygen limitation
maxLFQ estimation scaled to rel_iBAQ [%]
0.00100
0.00075
0.00050
0.00025
0.00000
HG001
ΔclpX
clpXcompl
growth phase
exp
stat

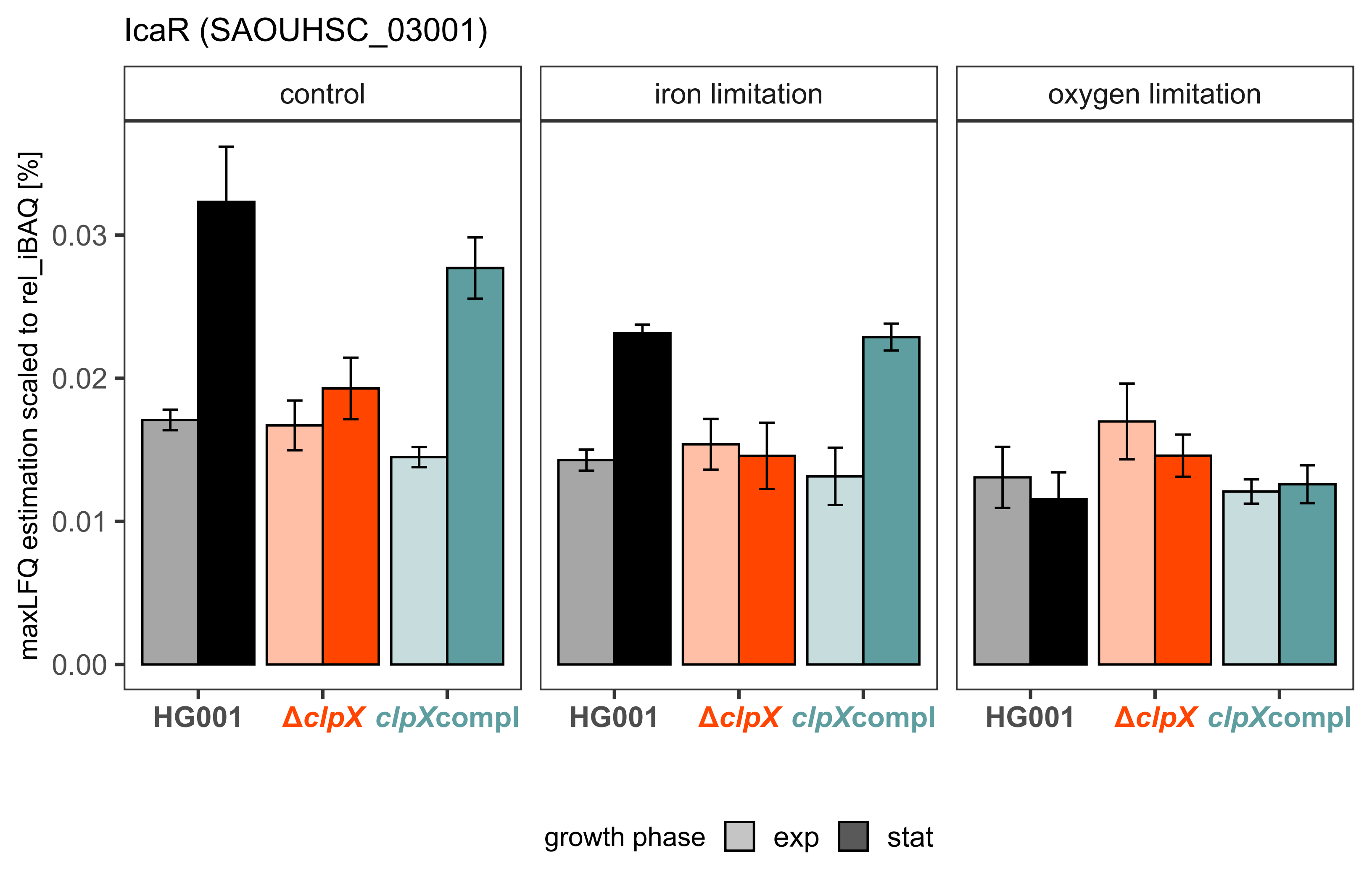

IcaR (SAOUHSC_03001)
control
iron limitation
oxygen limitation
maxLFQ estimation scaled to rel_iBAQ [%]
0.00
0.01
0.02
0.03
HG001
ΔclpX
clpXcompl
growth phase
exp
stat

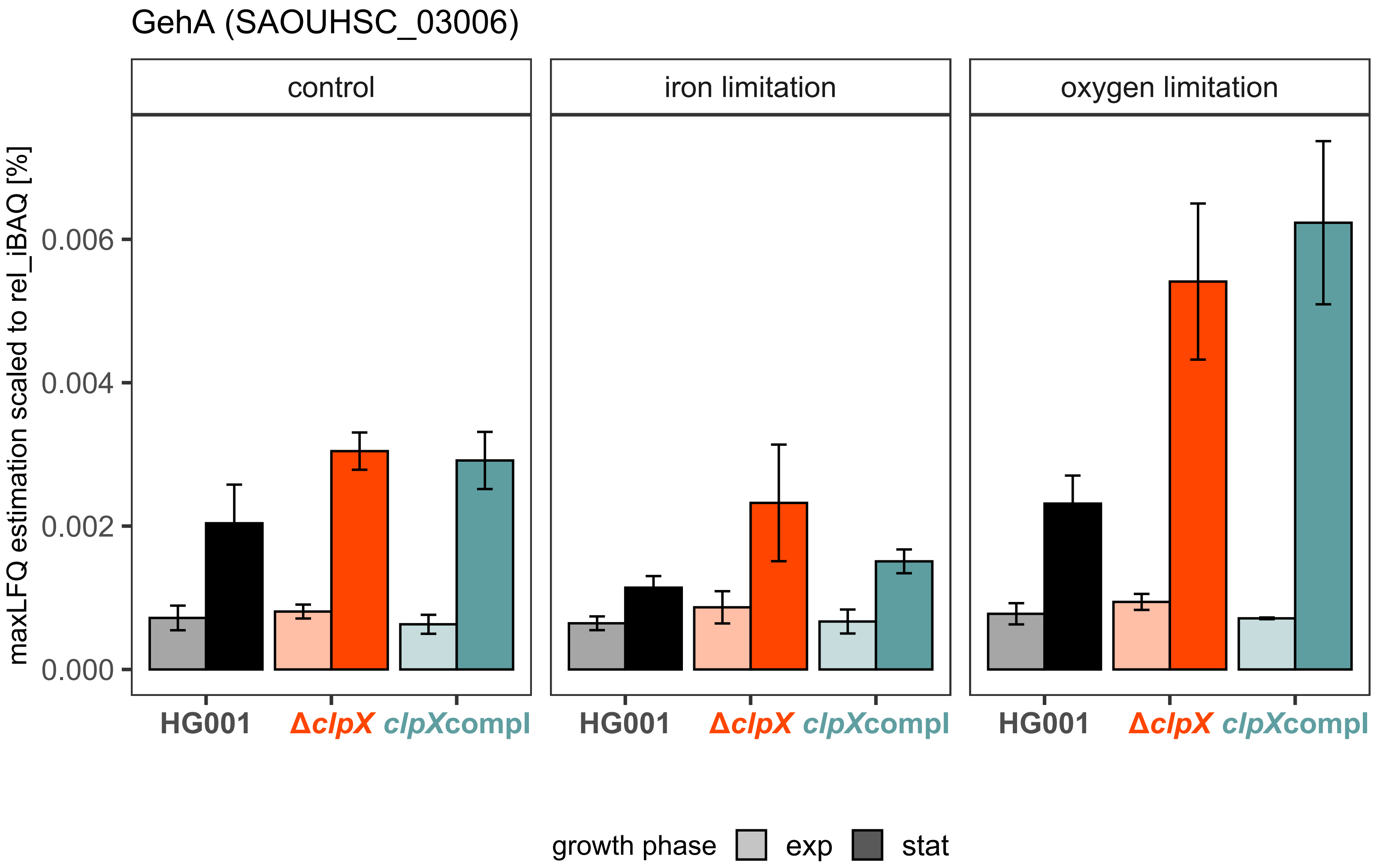

GehA (SAOUHSC_03006)
control
iron limitation
oxygen limitation
maxLFQ estimation scaled to rel_iBAQ [%]
0.006
0.004
0.002
0.000
HG001
ΔclpX
clpXcompl
growth phase
exp
stat

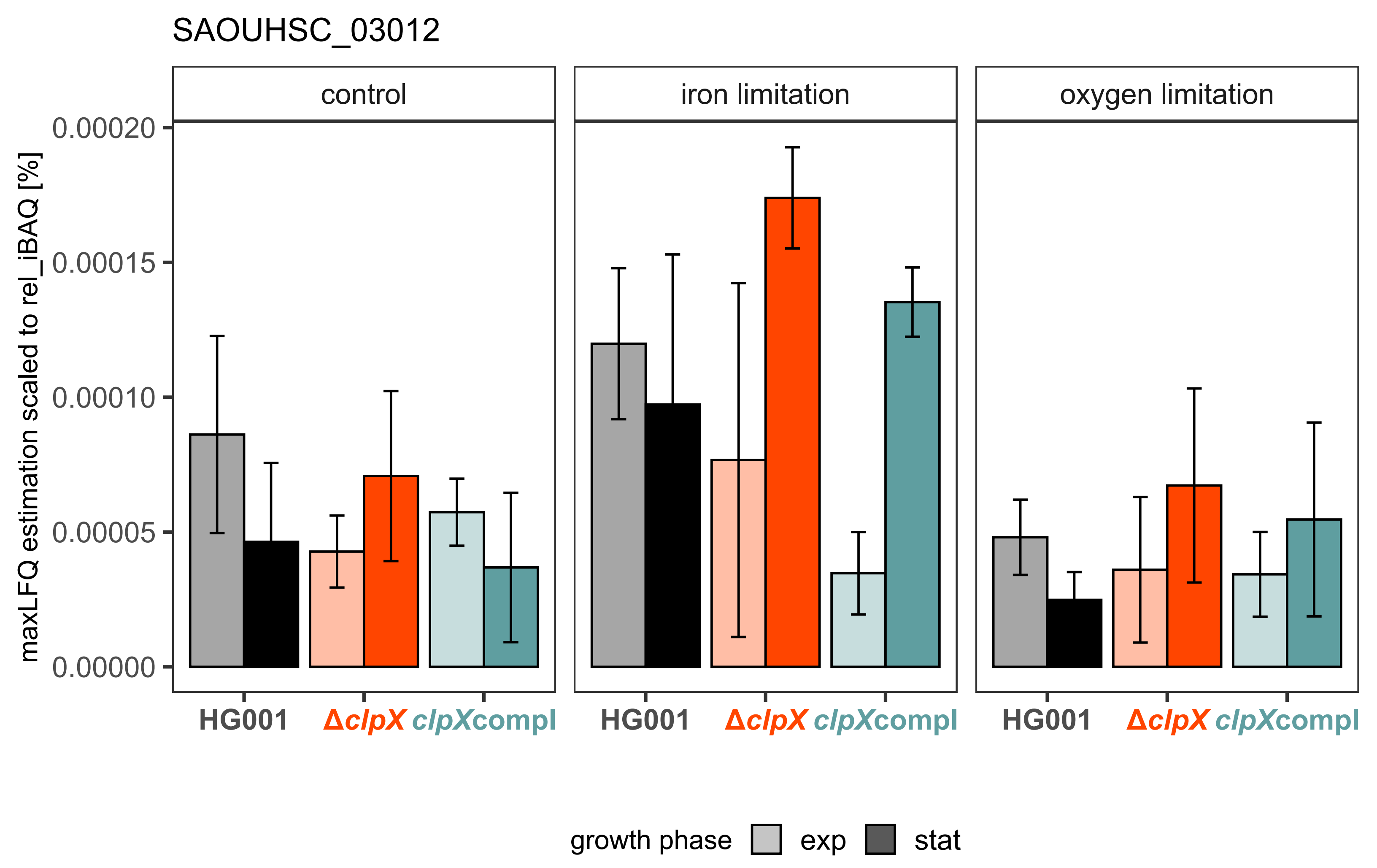
SAOUHSC_03012
control
iron limitation
oxygen limitation
maxLFQ estimation scaled to rel_iBAQ [%]
0.00020
0.00015
0.00010
0.00005
0.00000
HG001
ΔclpX
clpXcompl
growth phase
exp
stat

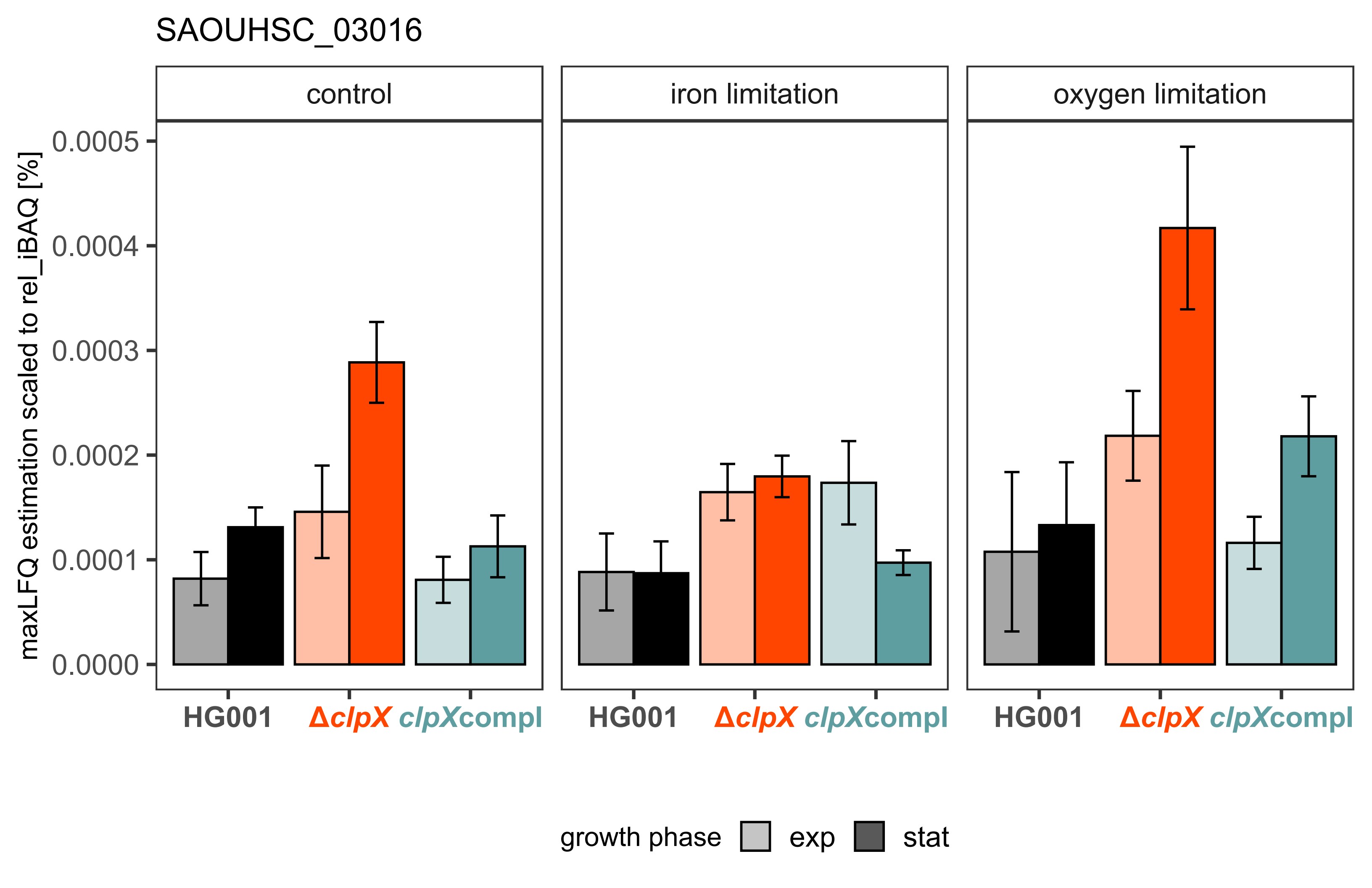
SAOUHSC_03016
control
iron limitation
oxygen limitation
maxLFQ estimation scaled to rel_iBAQ [%]
0.0005
0.0004
0.0003
0.0002
0.0001
0.0000
HG001
ΔclpX
clpXcompl
growth phase
exp
stat

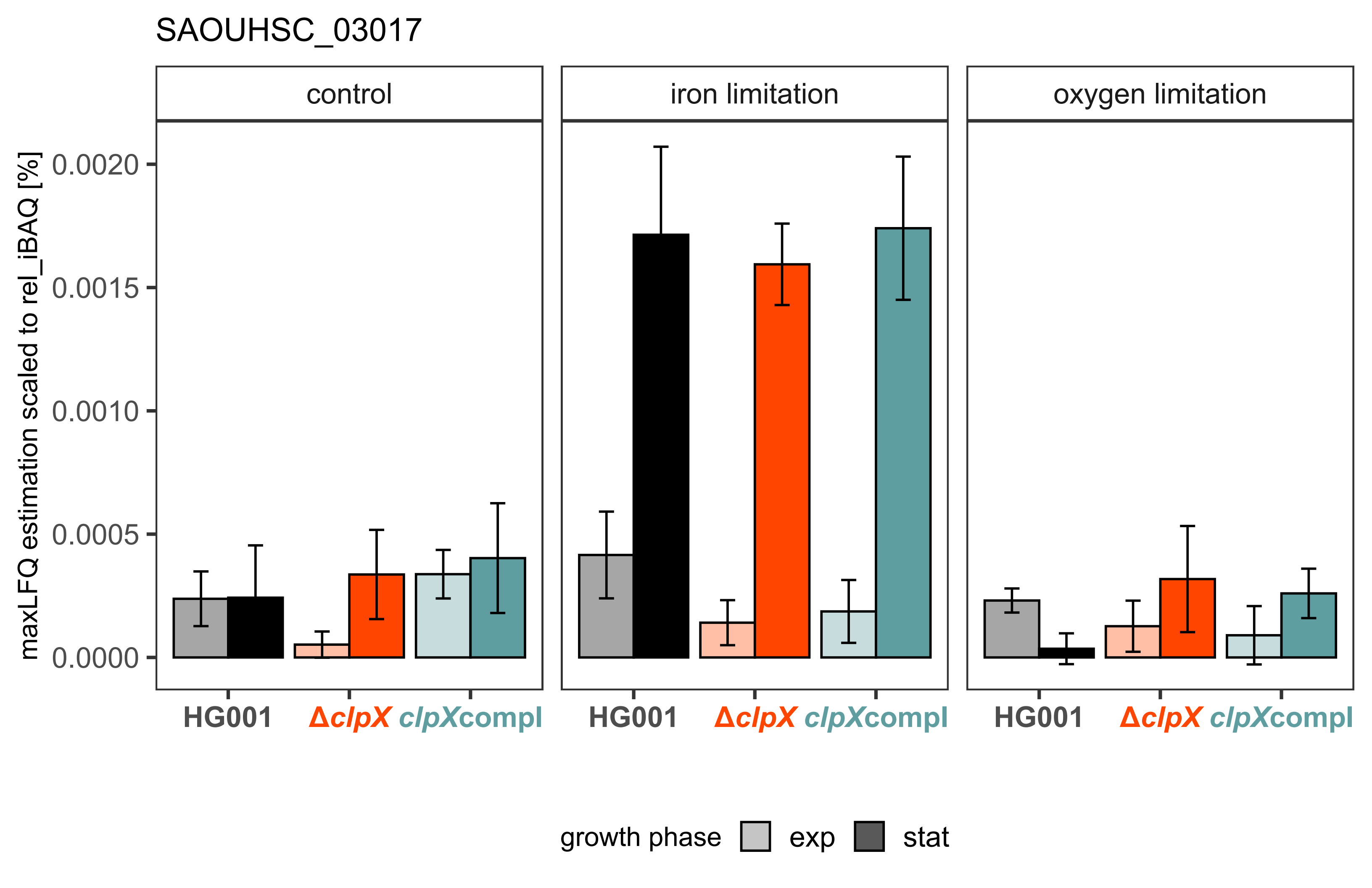

SAOUHSC_03017
control
iron limitation
oxygen limitation
maxLFQ estimation scaled to rel_iBAQ [%]
0.0020
0.0015
0.0010
0.0005
0.0000
HG001
ΔclpX
clpXcompl
growth phase
exp
stat

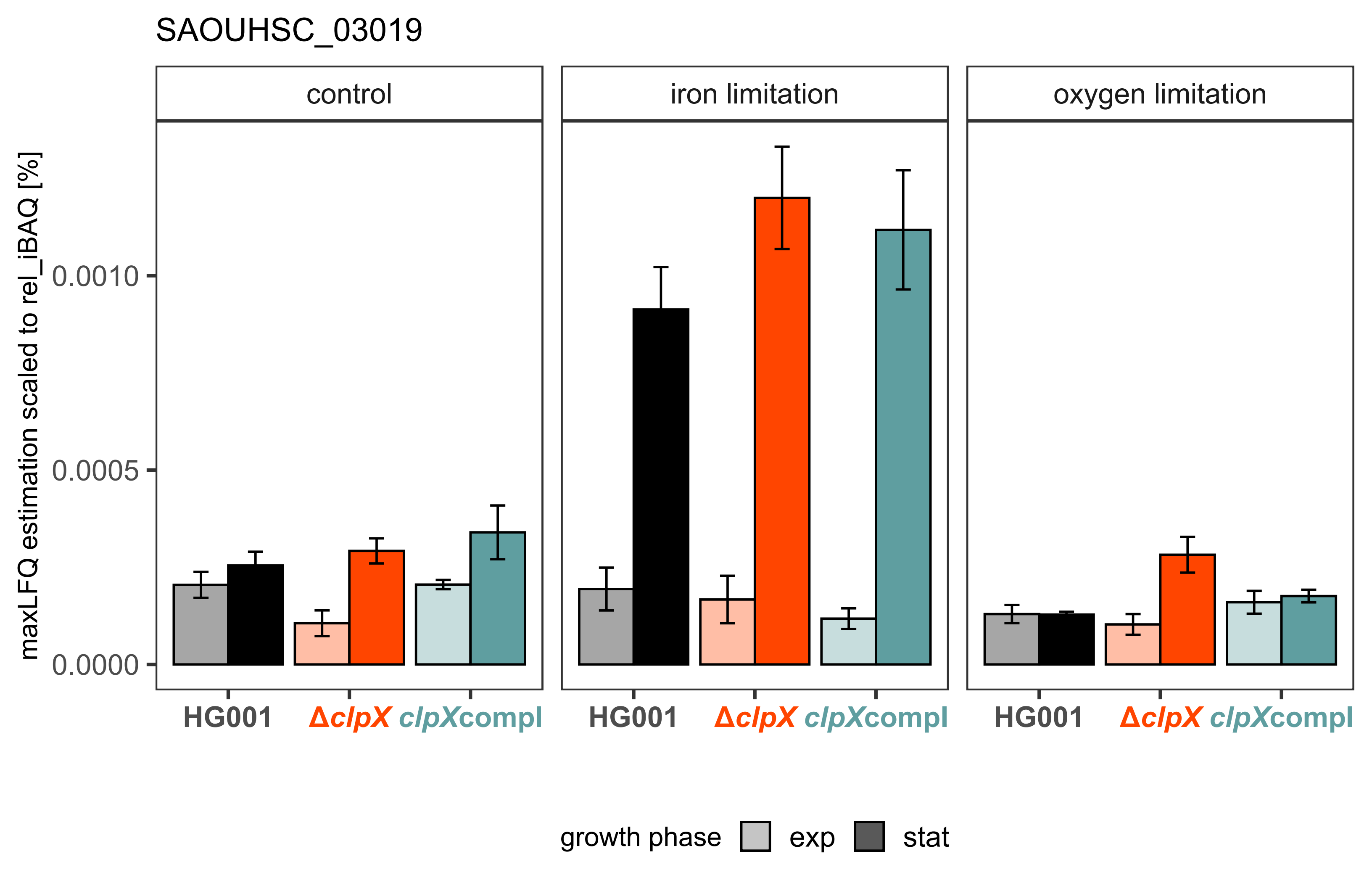

SAOUHSC_03019
control
iron limitation
oxygen limitation
maxLFQ estimation scaled to rel_iBAQ [%]
0.0010
0.0005
0.0000
HG001
ΔclpX
clpXcompl
growth phase
exp
stat

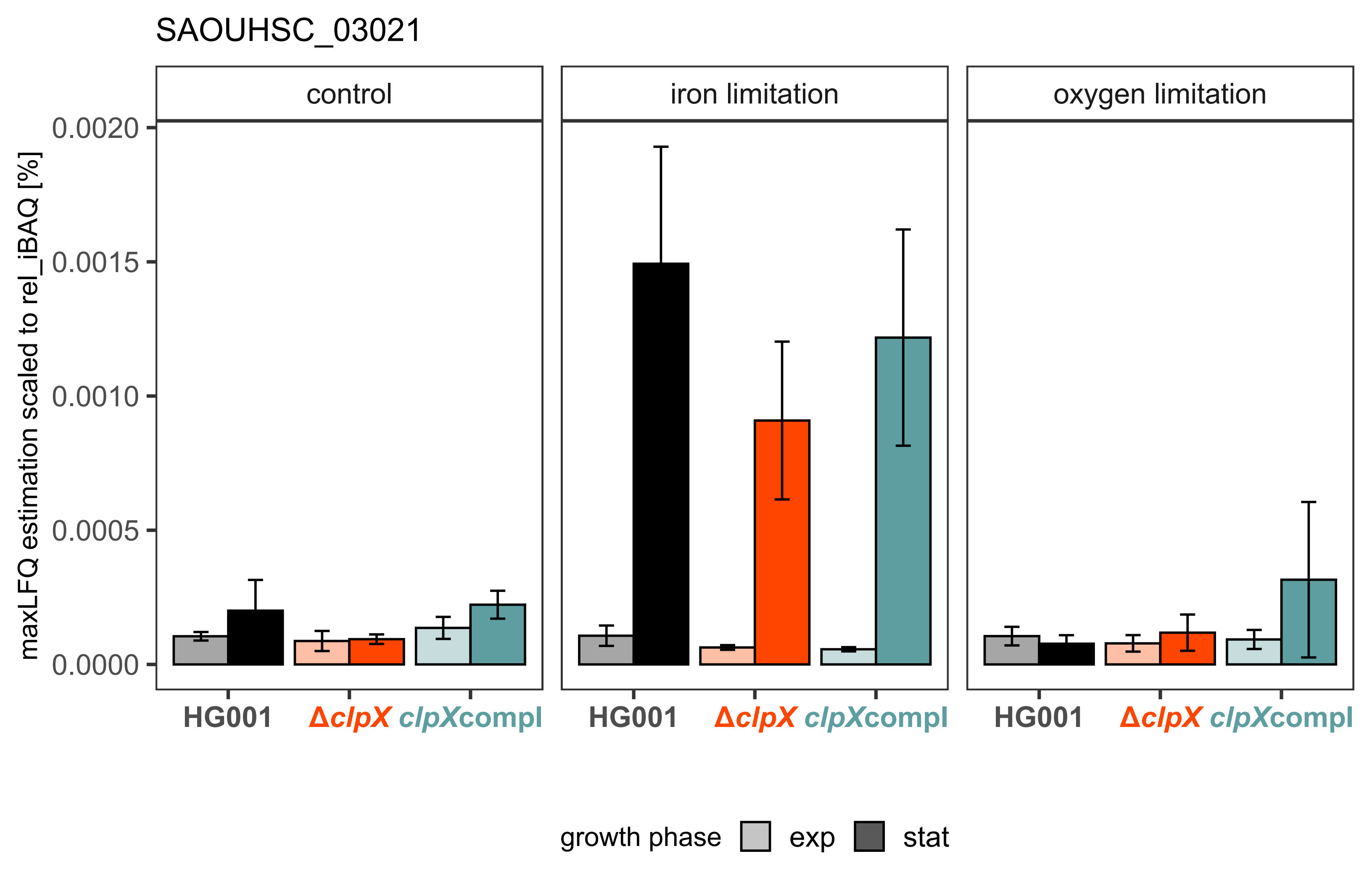

SAOUHSC_03021
control
iron limitation
oxygen limitation
maxLFQ estimation scaled to rel_iBAQ [%]
0.0020
0.0015
0.0010
0.0005
0.0000
HG001
ΔclpX
clpXcompl
growth phase
exp
stat

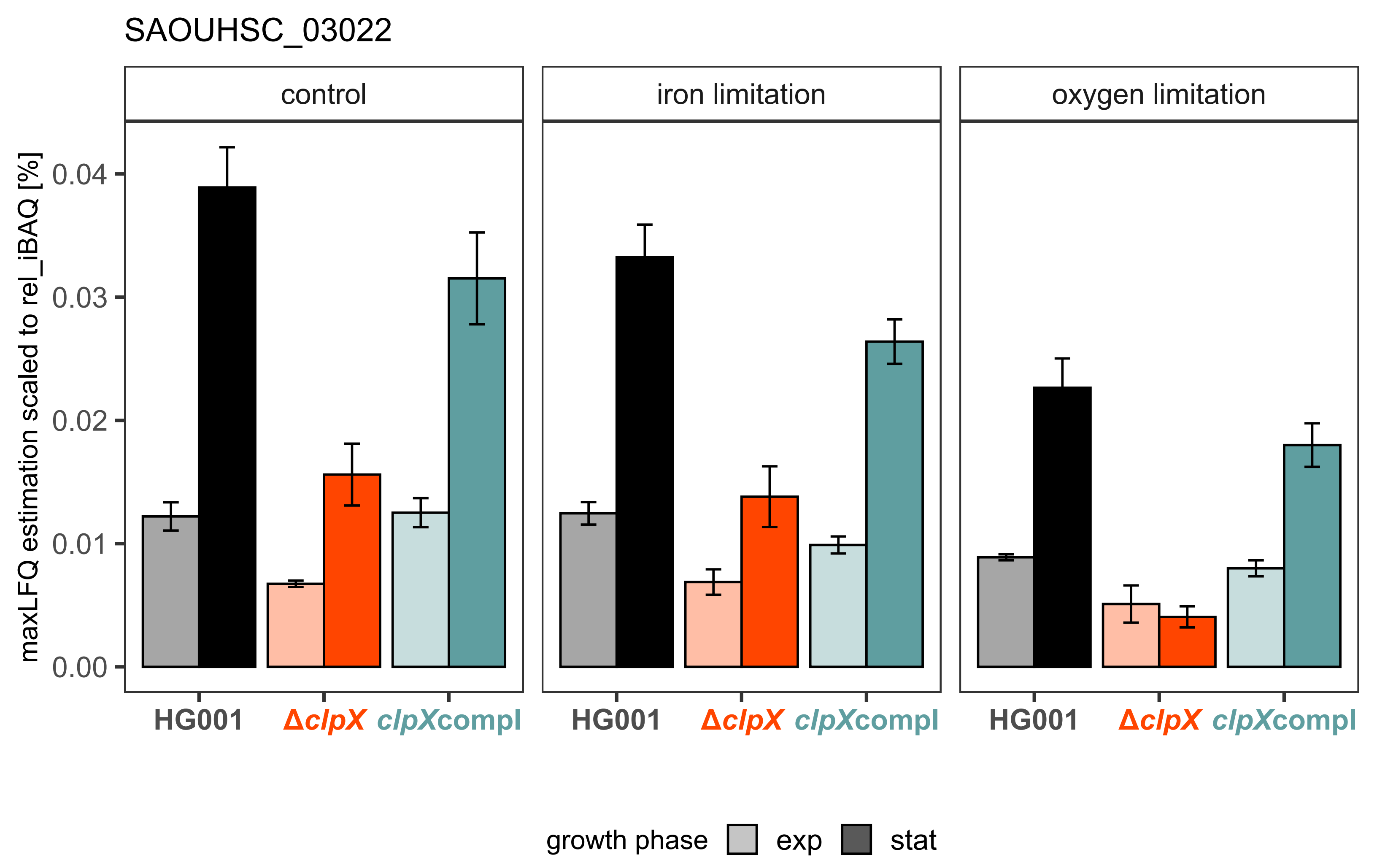

SAOUHSC_03022
control
iron limitation
oxygen limitation
maxLFQ estimation scaled to rel_iBAQ [%]
0.00
0.01
0.02
0.03
0.04
HG001
ΔclpX
clpXcompl
growth phase
exp
stat

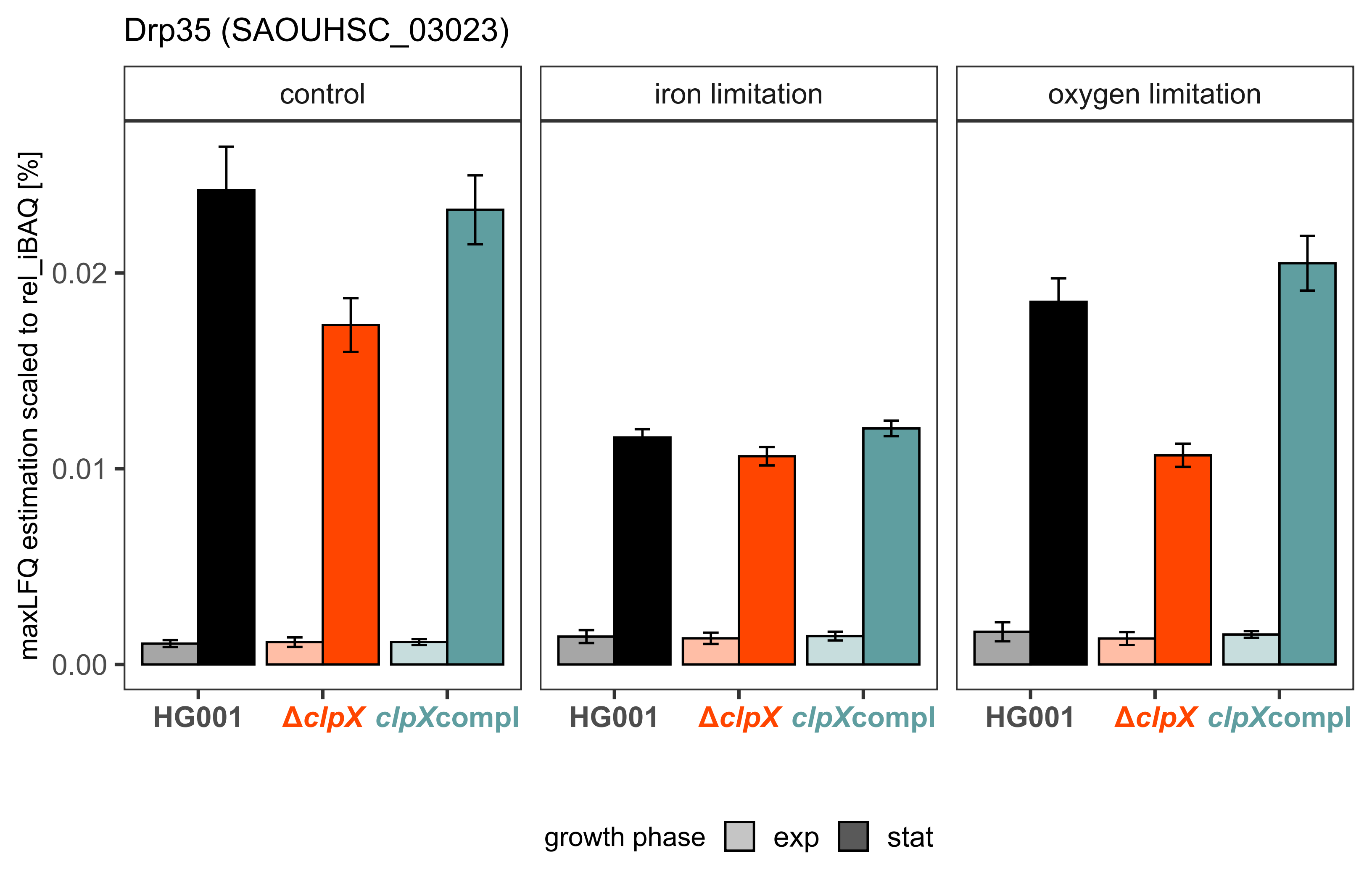

Drp35 (SAOUHSC_03023)
control
iron limitation
oxygen limitation
maxLFQ estimation scaled to rel_iBAQ [%]
0.00
0.01
0.02
HG001
ΔclpX
clpXcompl
growth phase
exp
stat

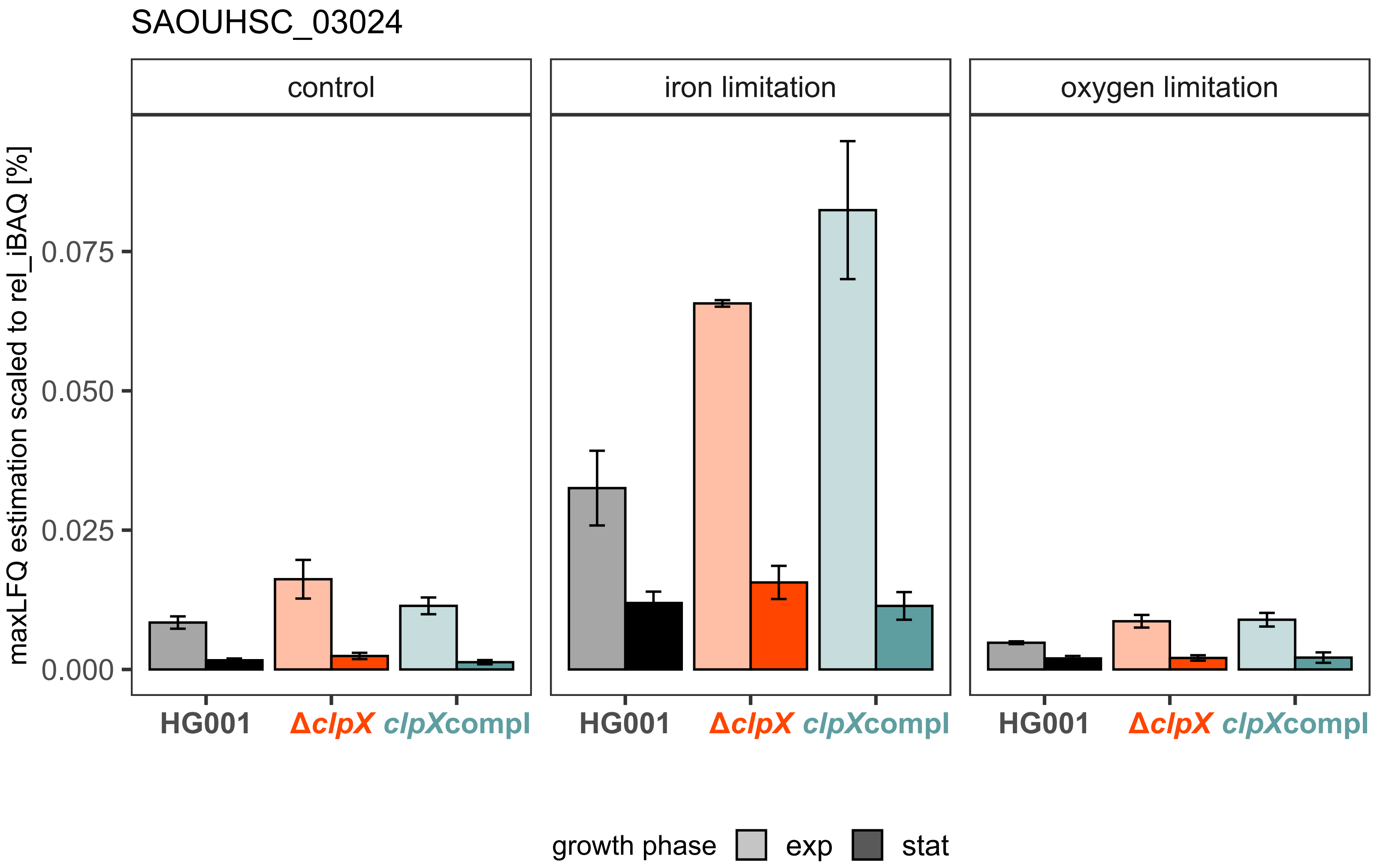

SAOUHSC_03024
control
iron limitation
oxygen limitation
maxLFQ estimation scaled to rel_iBAQ [%]
0.000
0.025
0.050
0.075
HG001
ΔclpX
clpXcompl
growth phase
exp
stat

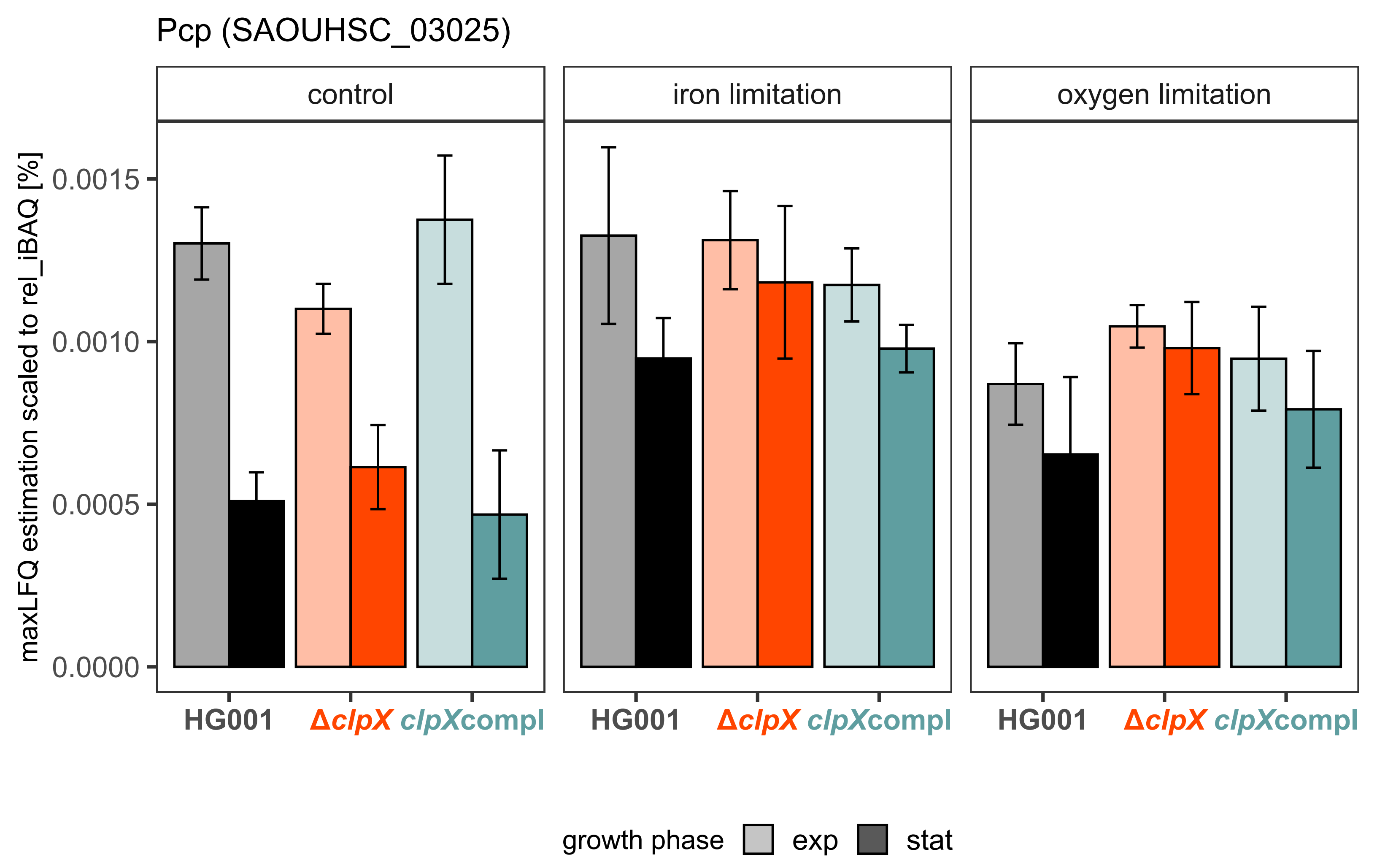

Pcp (SAOUHSC_03025)
control
iron limitation
oxygen limitation
maxLFQ estimation scaled to rel_iBAQ [%]
0.0015
0.0010
0.0005
0.0000
HG001
ΔclpX
clpXcompl
growth phase
exp
stat

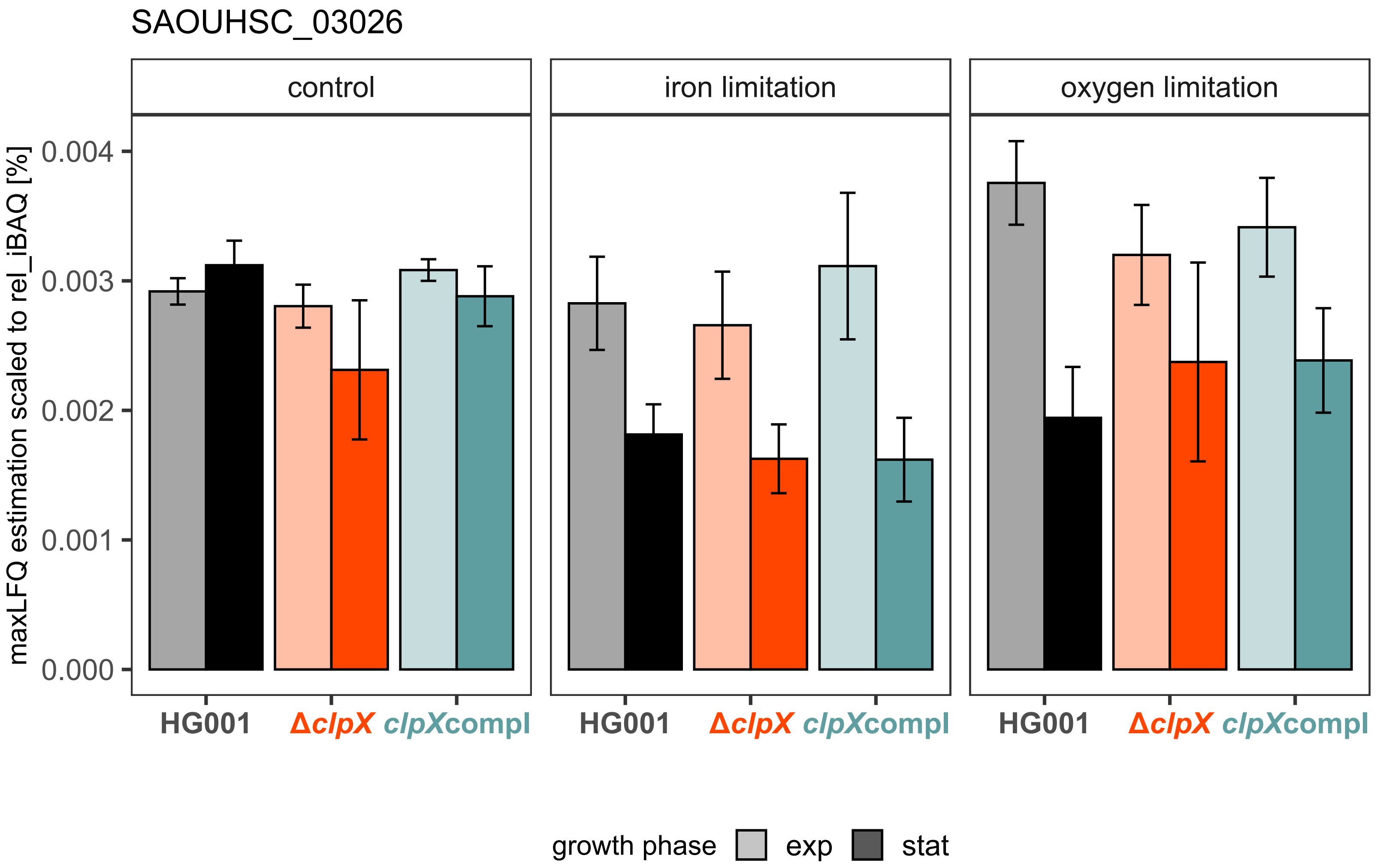
SAOUHSC_03026
control
iron limitation
oxygen limitation
maxLFQ estimation scaled to rel_iBAQ [%]
0.004
0.003
0.002
0.001
0.000
HG001
ΔclpX
clpXcompl
growth phase
exp
stat

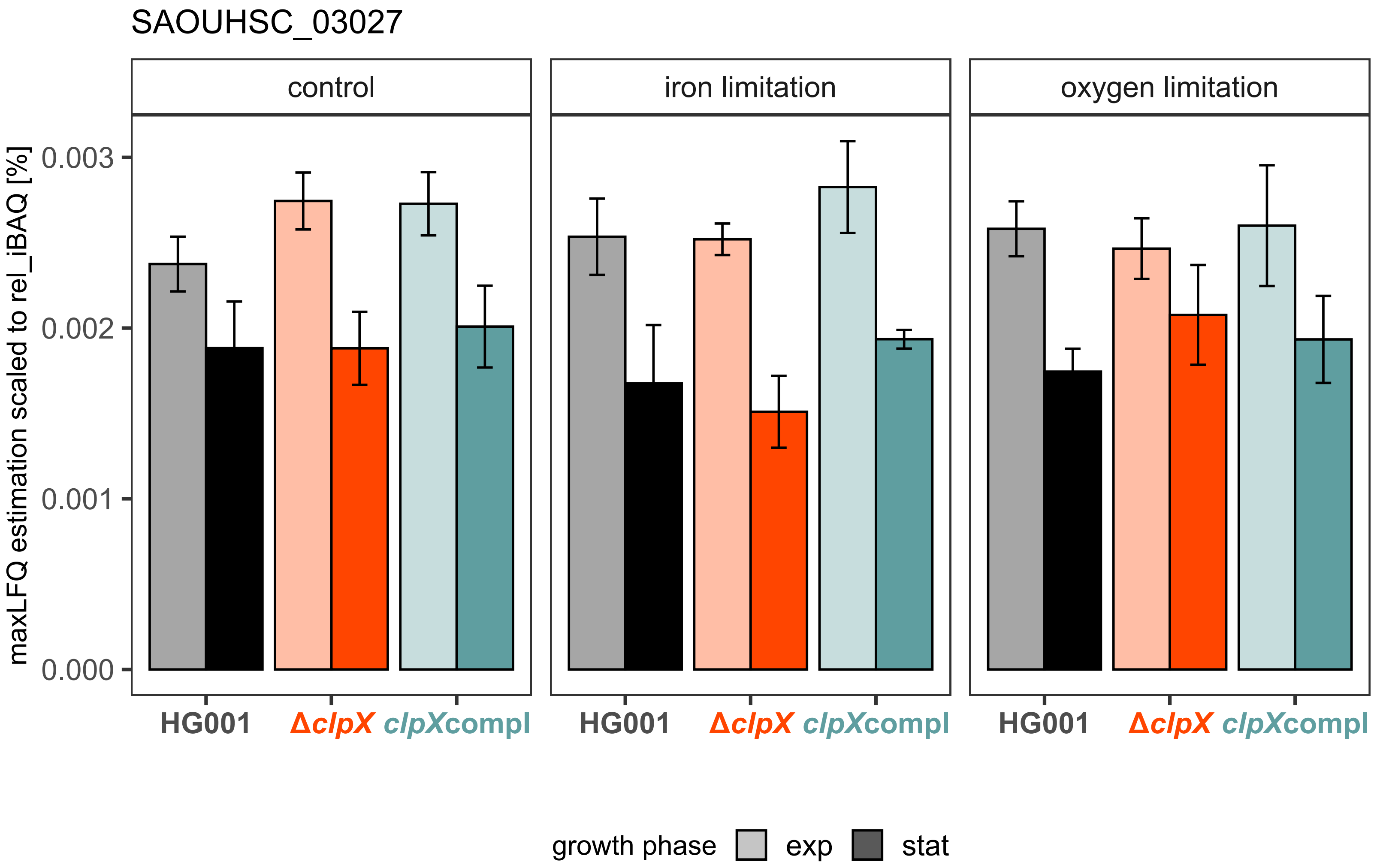

SAOUHSC_03027
control
iron limitation
oxygen limitation
maxLFQ estimation scaled to rel_iBAQ [%]
0.003
0.002
0.001
0.000
HG001
ΔclpX
clpXcompl
growth phase
exp
stat

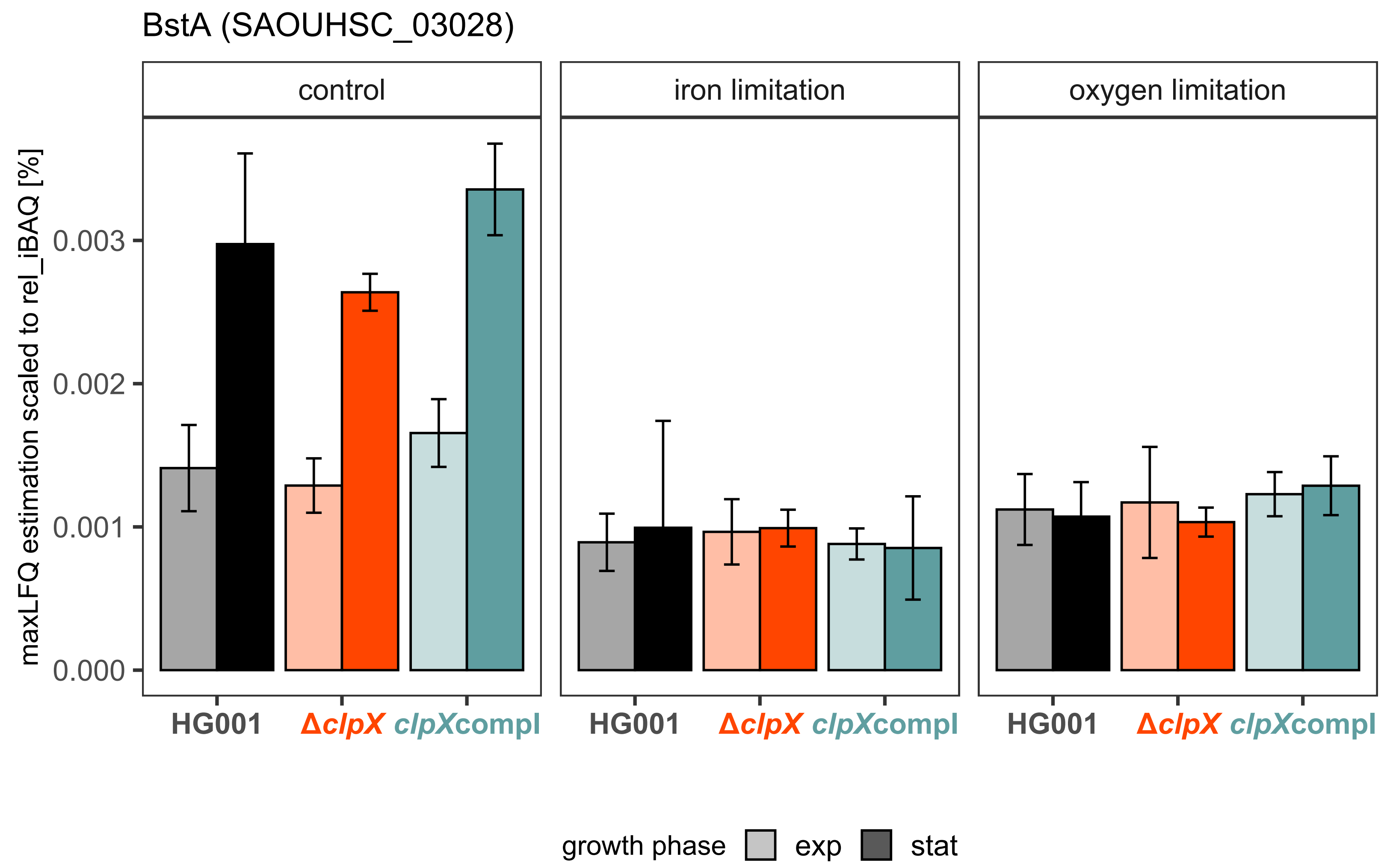

BstA (SAOUHSC_03028)
control
iron limitation
oxygen limitation
maxLFQ estimation scaled to rel_iBAQ [%]
0.003
0.002
0.001
0.000
HG001
ΔclpX
clpXcompl
growth phase
exp
stat

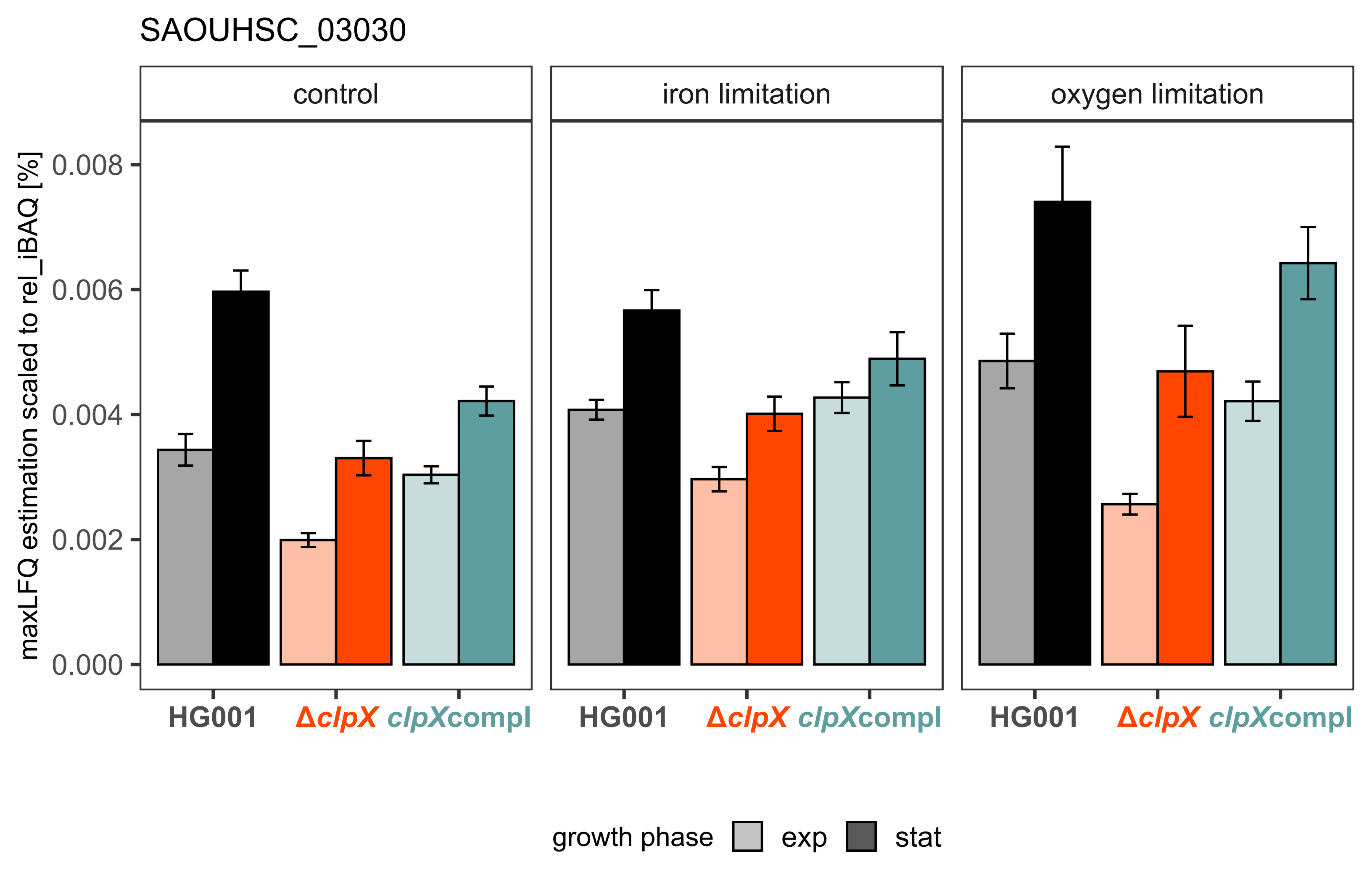

SAOUHSC_03030
control
iron limitation
oxygen limitation
maxLFQ estimation scaled to rel_iBAQ [%]
0.008
0.006
0.004
0.002
0.000
HG001
ΔclpX
clpXcompl
growth phase
exp
stat

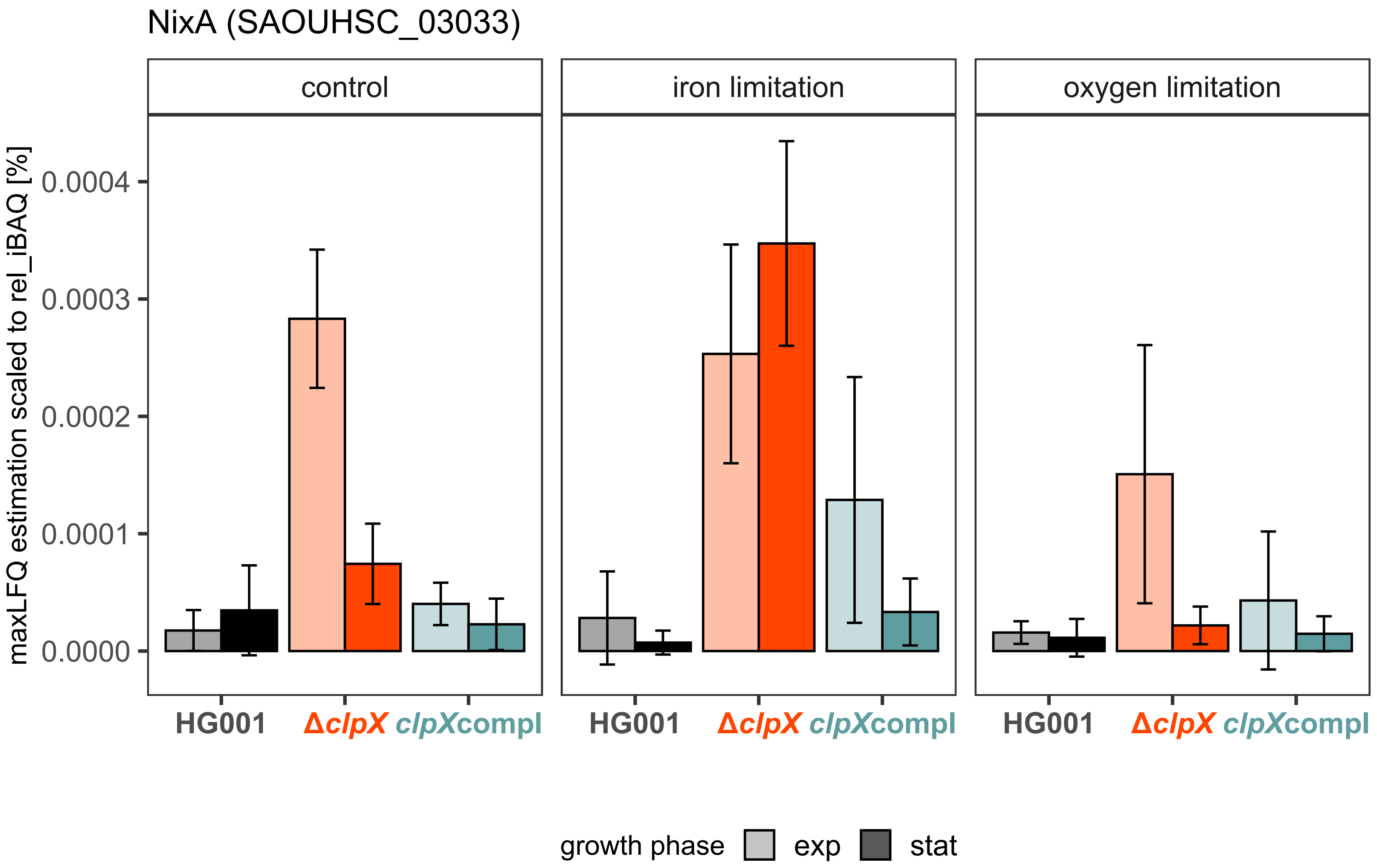
NixA (SAOUHSC_03033)
control
iron limitation
oxygen limitation
maxLFQ estimation scaled to rel_iBAQ [%]
0.0004
0.0003
0.0002
0.0001
0.0000
HG001
ΔclpX
clpXcompl
growth phase
exp
stat

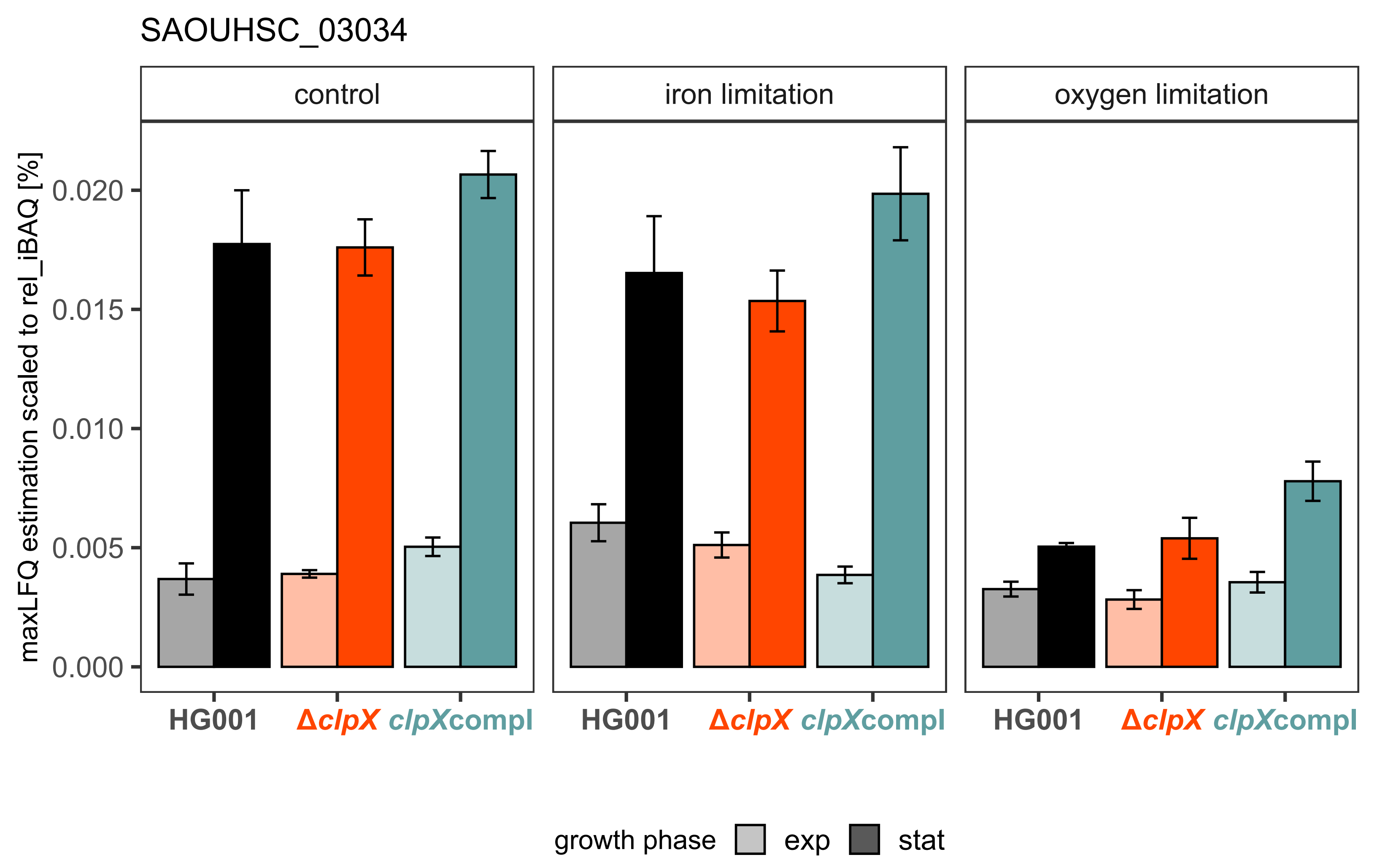
SAOUHSC_03034
control
iron limitation
oxygen limitation
maxLFQ estimation scaled to rel_iBAQ [%]
0.020
0.015
0.010
0.005
0.000
HG001
ΔclpX
clpXcompl
growth phase
exp
stat

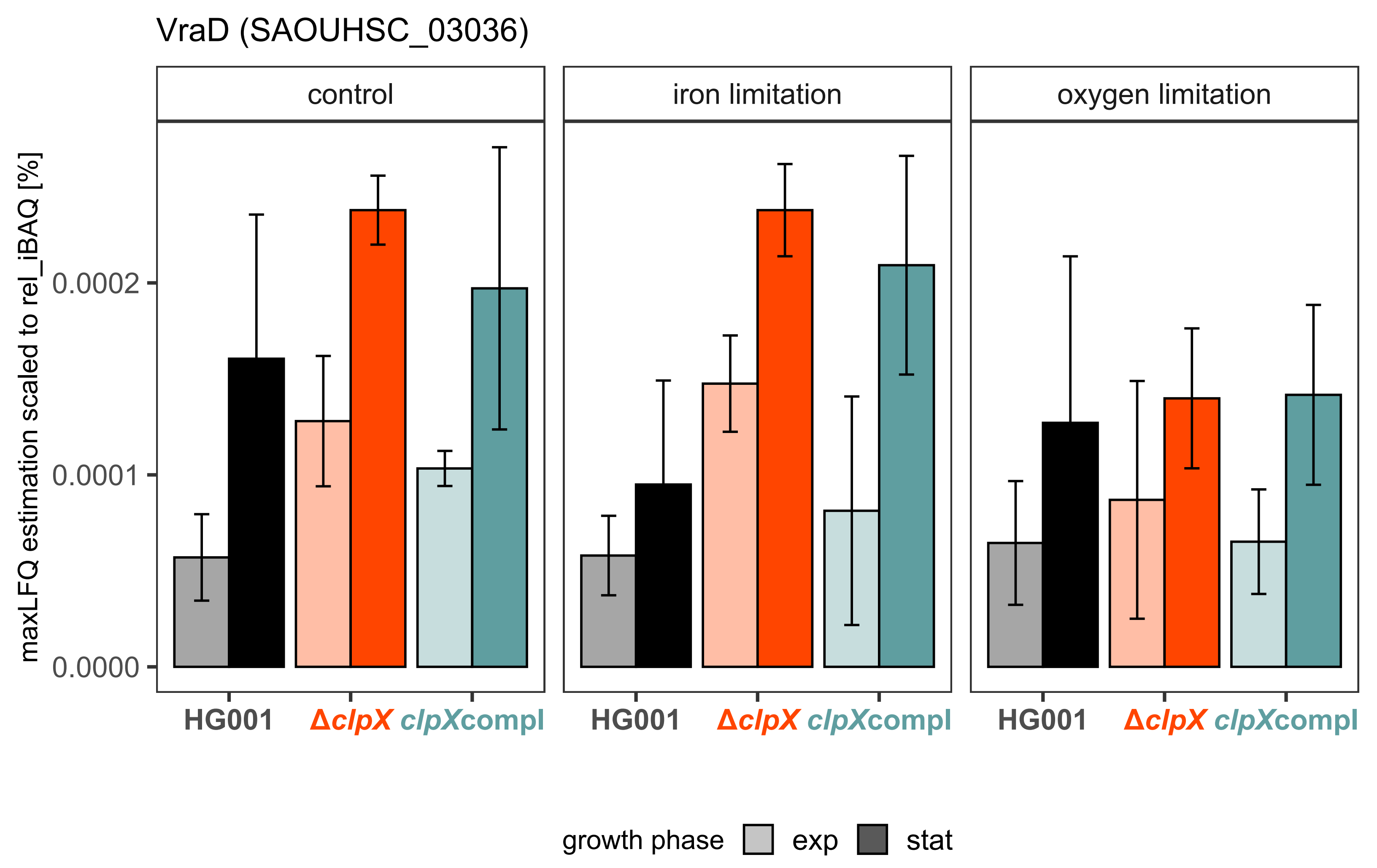
VraD (SAOUHSC_03036)
control
iron limitation
oxygen limitation
maxLFQ estimation scaled to rel_iBAQ [%]
0.0002
0.0001
0.0000
HG001
ΔclpX
clpXcompl
growth phase
exp
stat

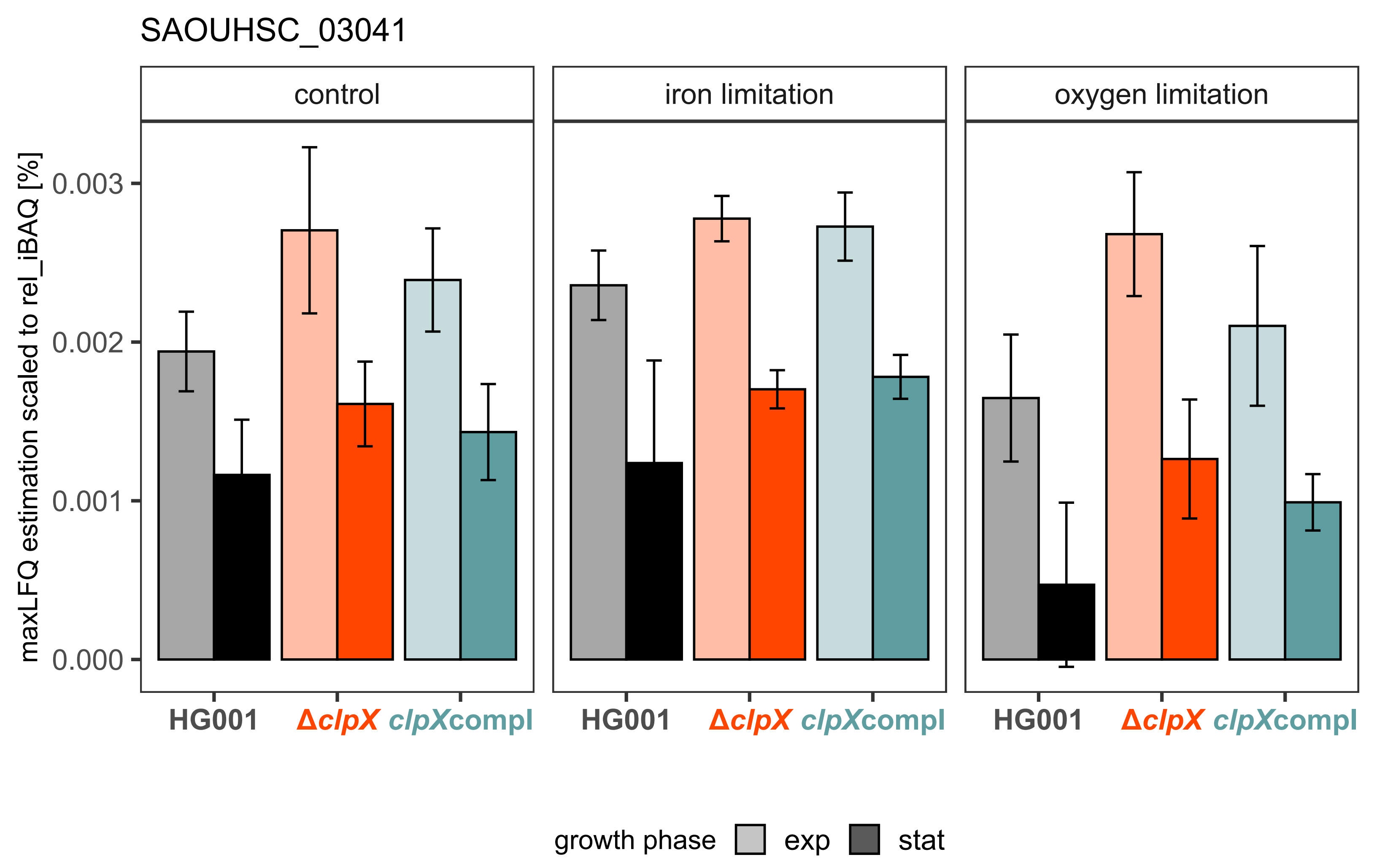
SAOUHSC_03041
control
iron limitation
oxygen limitation
maxLFQ estimation scaled to rel_iBAQ [%]
0.003
0.002
0.001
0.000
HG001
ΔclpX
clpXcompl
growth phase
exp
stat

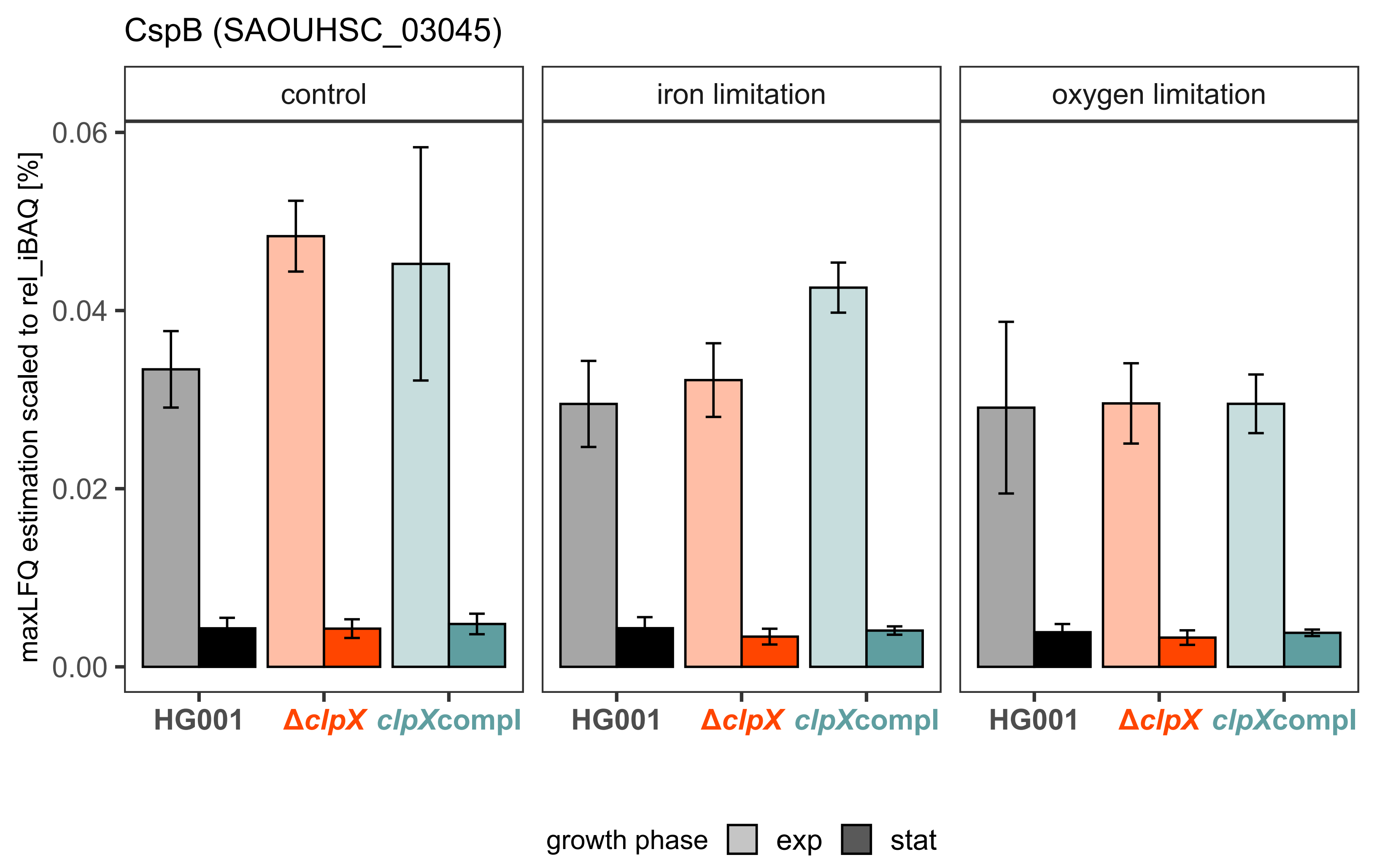
CspB (SAOUHSC_03045)
control
iron limitation
oxygen limitation
maxLFQ estimation scaled to rel_iBAQ [%]
0.06
0.04
0.02
0.00
HG001
ΔclpX
clpXcompl
growth phase
exp
stat

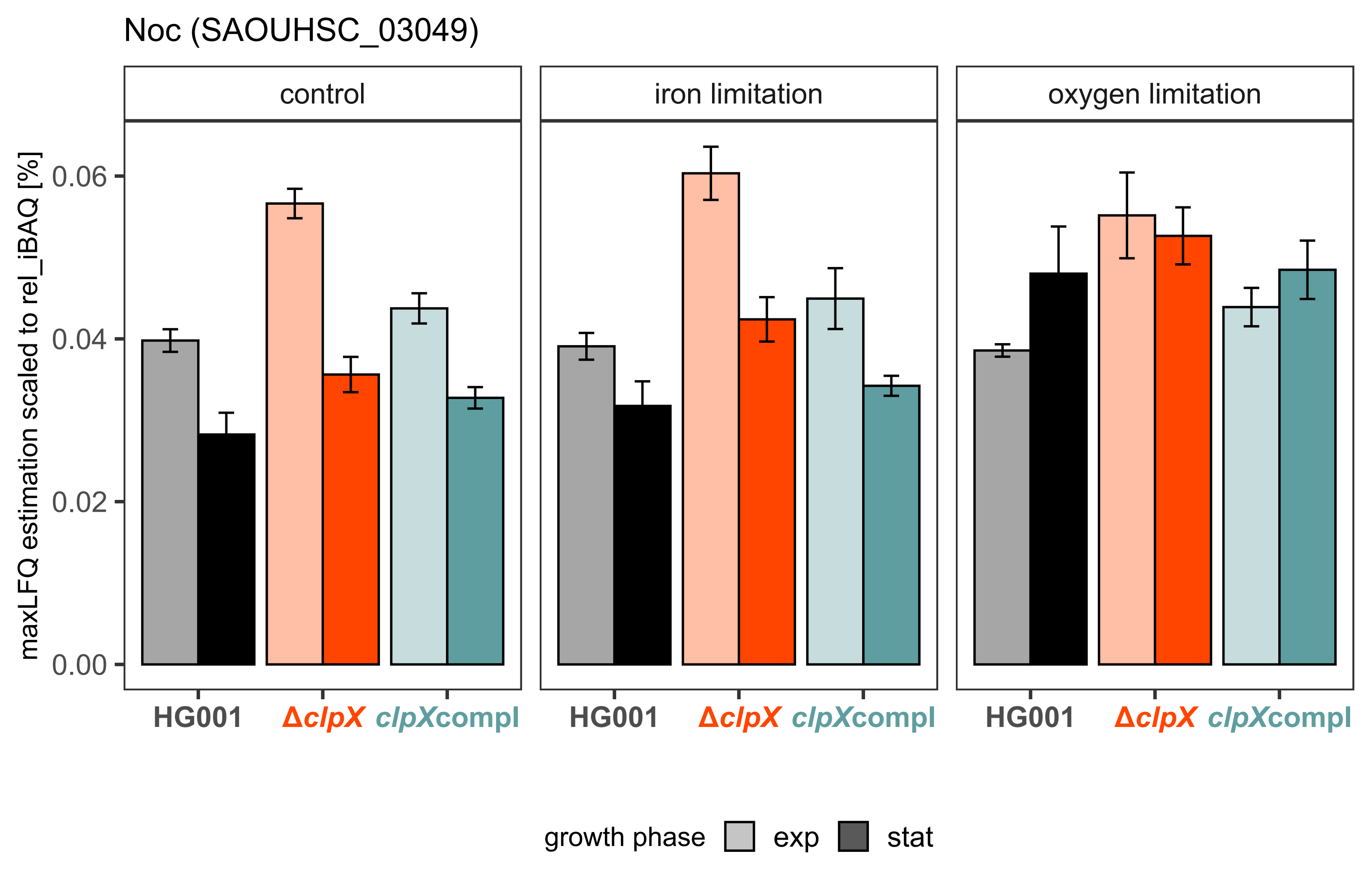

Noc (SAOUHSC_03049)
control
iron limitation
oxygen limitation
maxLFQ estimation scaled to rel_iBAQ [%]
0.06
0.04
0.02
0.00
HG001
ΔclpX
clpXcompl
growth phase
exp
stat

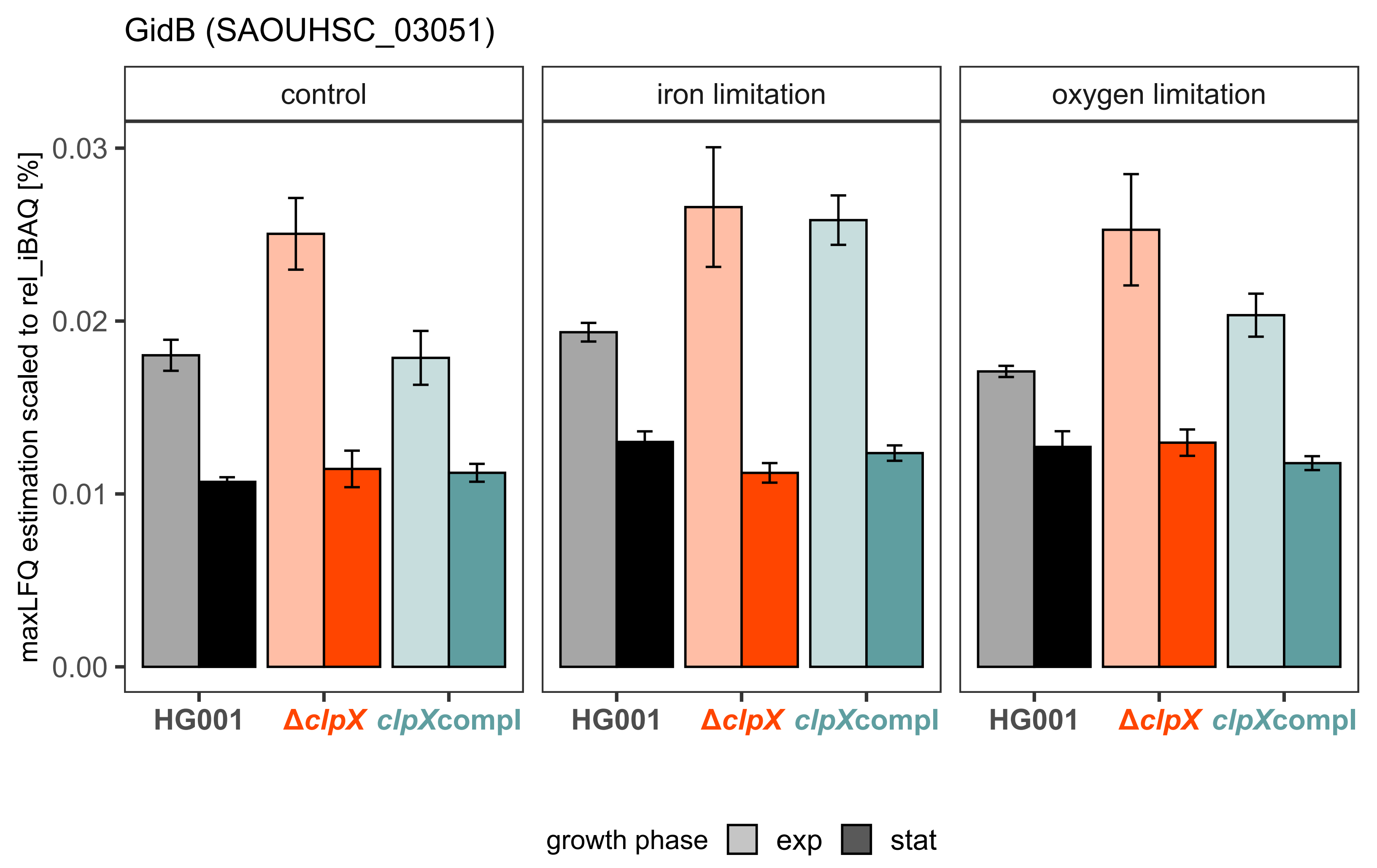
GidB (SAOUHSC_03051)
control
iron limitation
oxygen limitation
maxLFQ estimation scaled to rel_iBAQ [%]
0.03
0.02
0.01
0.00
HG001
ΔclpX
clpXcompl
HG001
ΔclpX
clpXcompl
HG001
ΔclpX
clpXcompl
growth phase
exp
stat

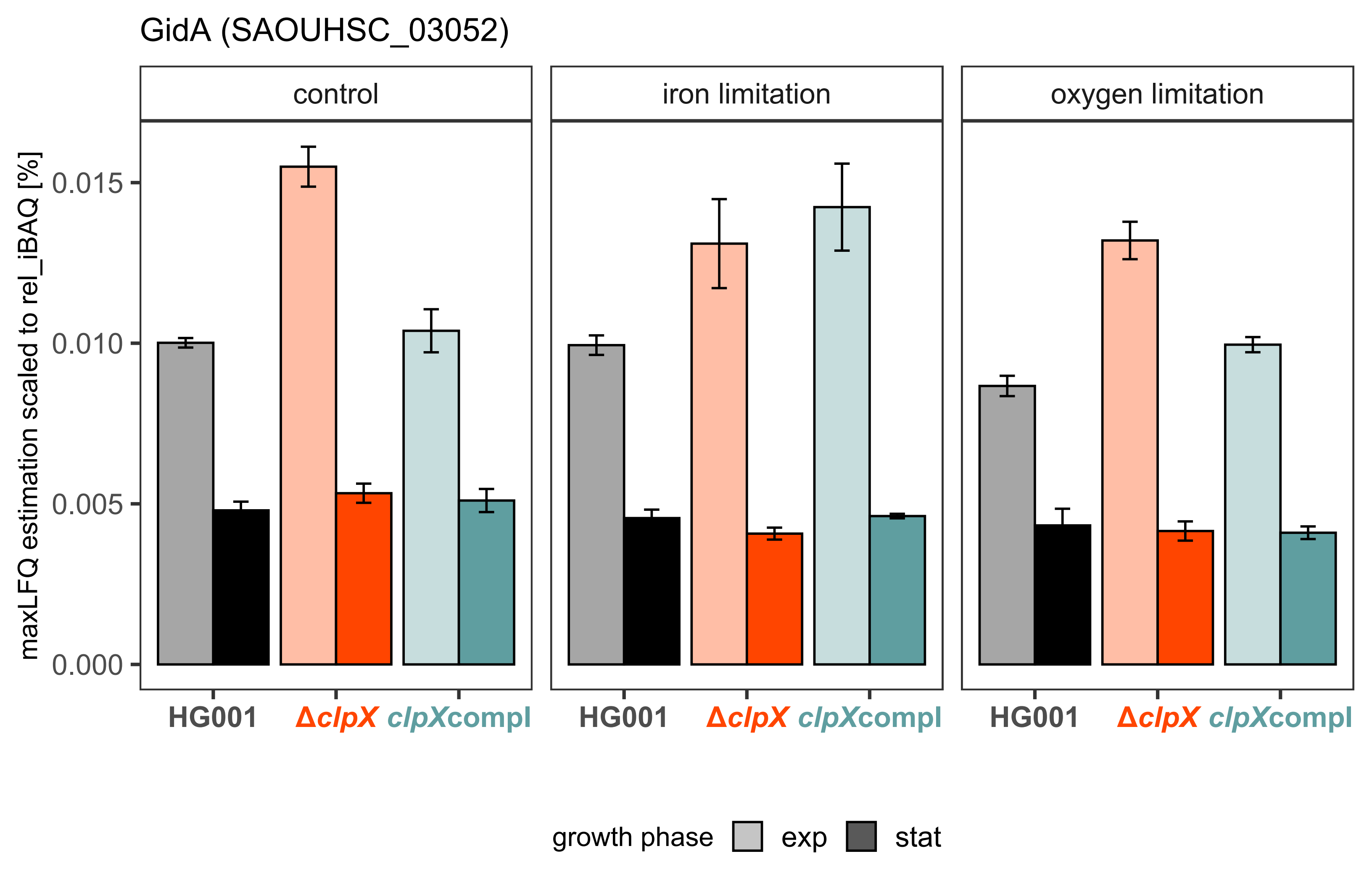
GidA (SAOUHSC_03052)
control
iron limitation
oxygen limitation
maxLFQ estimation scaled to rel_iBAQ [%]
0.015
0.010
0.005
0.000
HG001
ΔclpX
clpXcompl
growth phase
exp
stat

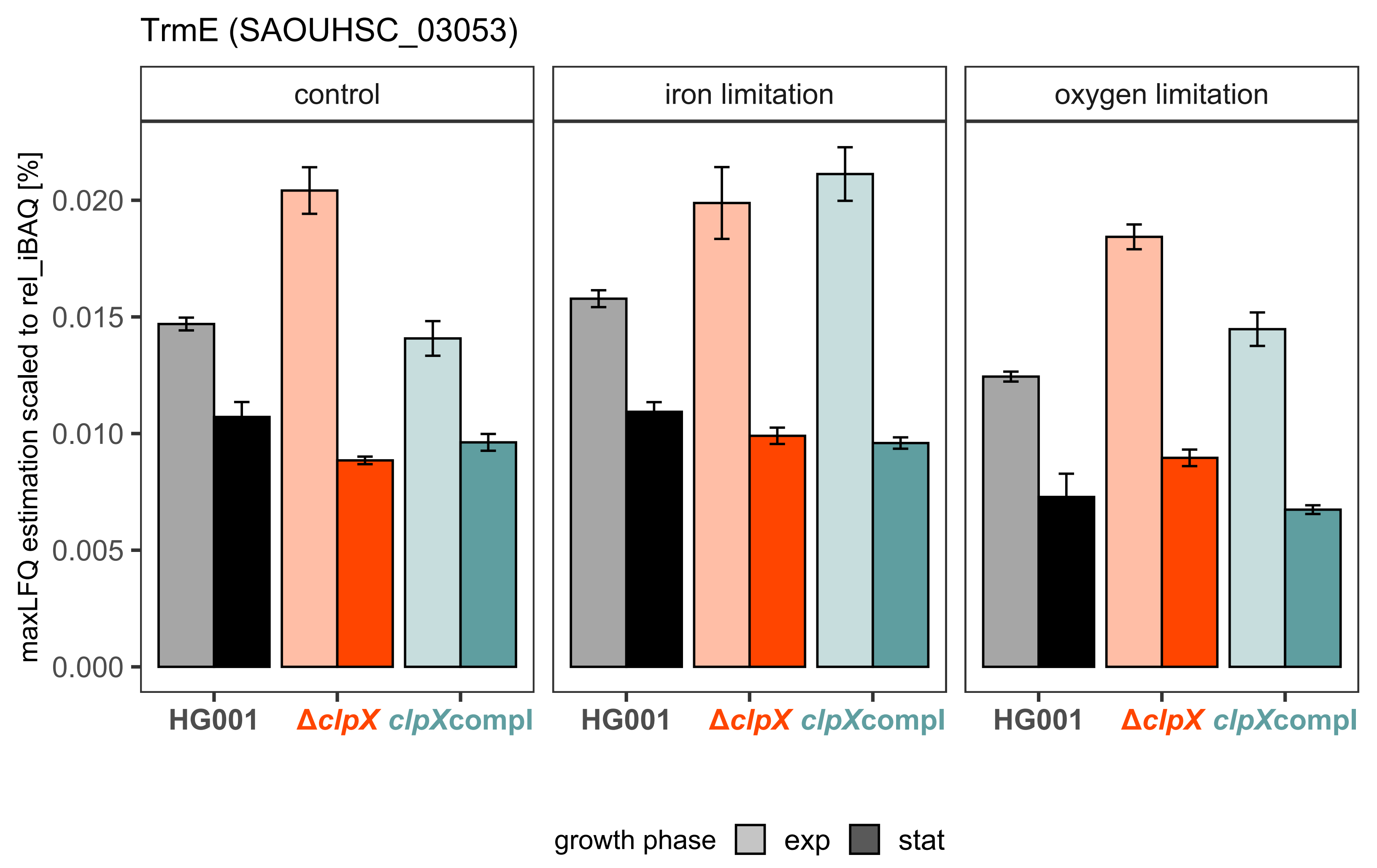

TrmE (SAOUHSC_03053)
control
iron limitation
oxygen limitation
maxLFQ estimation scaled to rel_iBAQ [%]
0.020
0.015
0.010
0.005
0.000
HG001
ΔclpX
clpXcompl
growth phase
exp
stat

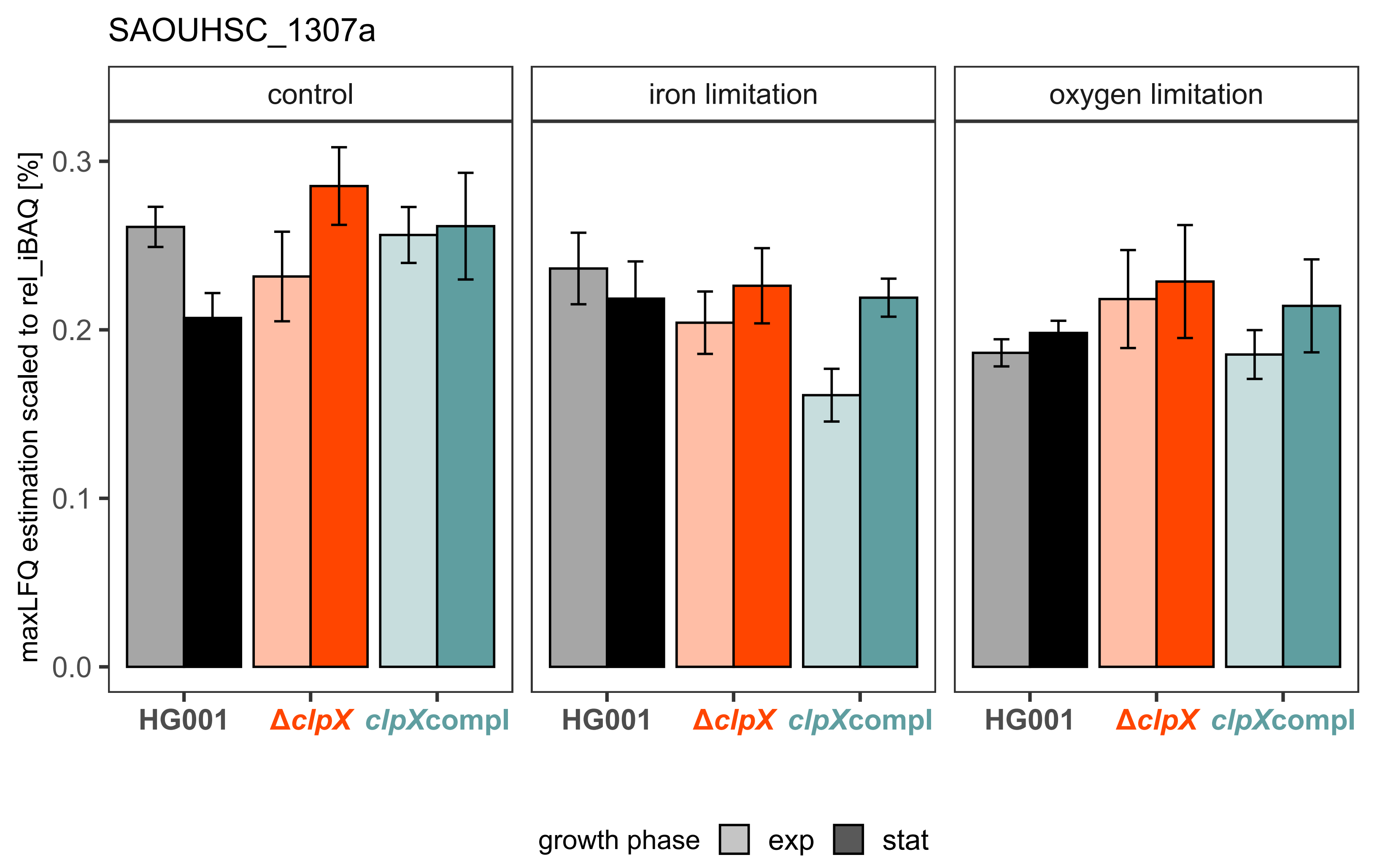
SAOUHSC_1307a
control
iron limitation
oxygen limitation
maxLFQ estimation scaled to rel_iBAQ [%]
0.0
0.1
0.2
0.3
HG001
ΔclpX
clpXcompl
growth phase
exp
stat

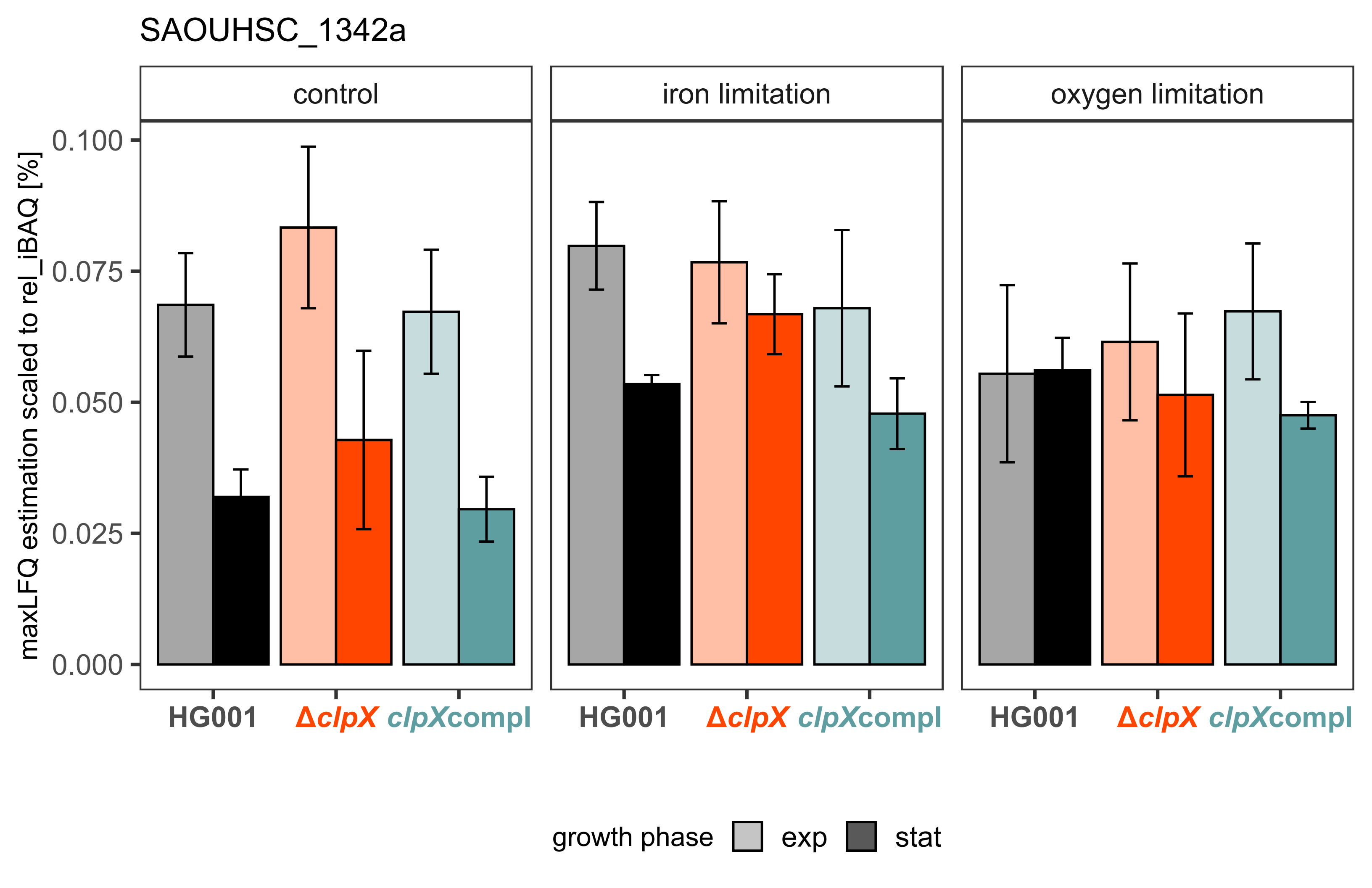
SAOUHSC_1342a
control
iron limitation
oxygen limitation
maxLFQ estimation scaled to rel_iBAQ [%]
0.100
0.075
0.050
0.025
0.000
HG001
ΔclpX
clpXcompl
growth phase
exp
stat

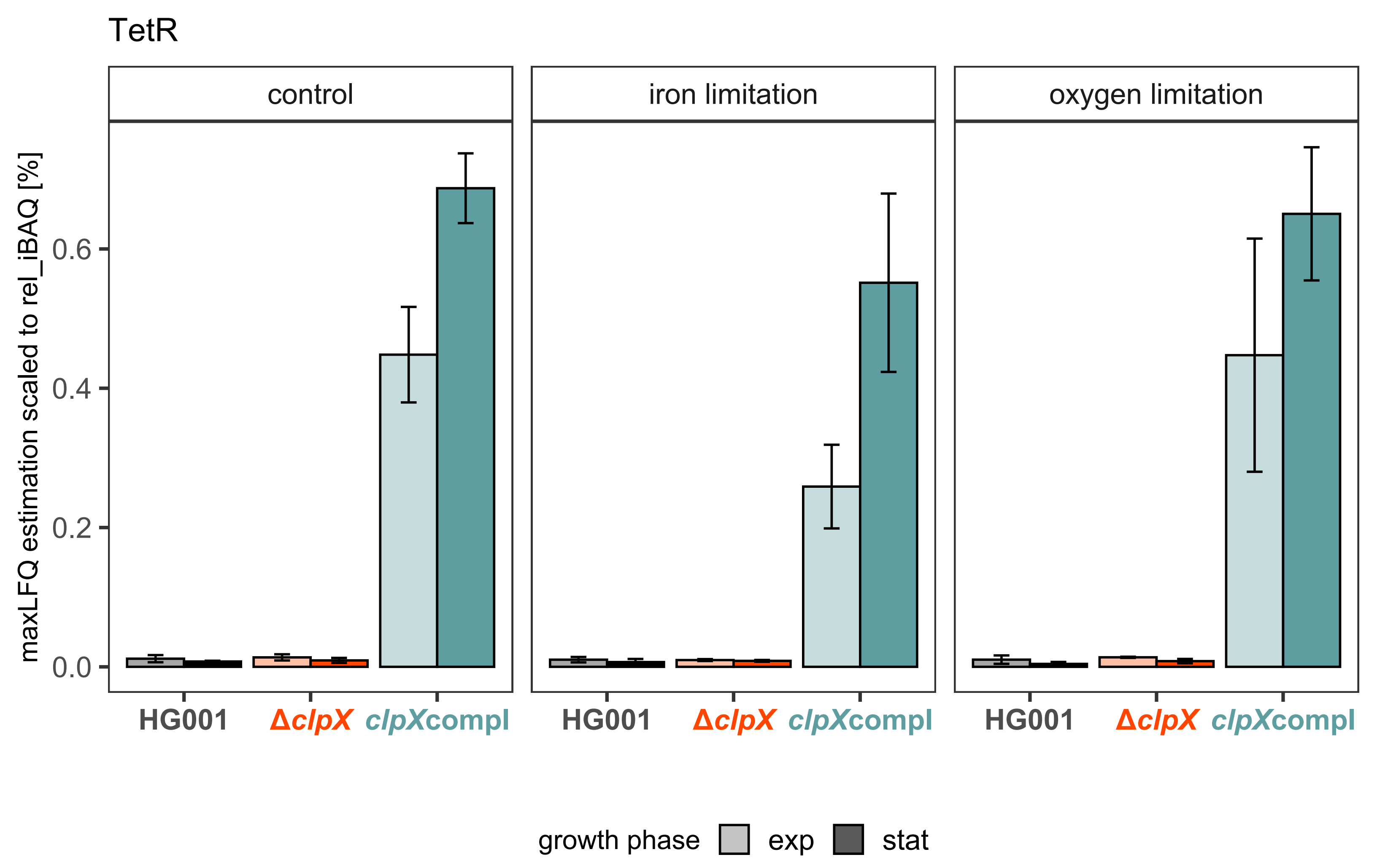

TetR
control
iron limitation
oxygen limitation
maxLFQ estimation scaled to rel_iBAQ [%]
0.0
0.2
0.4
0.6
HG001
ΔclpX
clpXcompl
growth phase
exp
stat
